# Metabolic capacity is maintained despite shifts in microbial diversity in estuary sediments

**DOI:** 10.1093/ismeco/ycaf182

**Published:** 2025-10-11

**Authors:** Marguerite V Langwig, Sunny Lyn Sneed, Anna Rasmussen, Kiley W Seitz, Jessica A Lee, Karthik Anantharaman, Valerie De Anda, Christopher A Francis, Brett J Baker

**Affiliations:** Department of Marine Science, University of Texas at Austin, Marine Science Institute, Port Aransas, TX, USA; Department of Freshwater and Marine Sciences & Department of Bacteriology, University of Wisconsin-Madison, Madison, Wisconsin, USA; Department of Biological Sciences, Rensselaer Polytechnic Institute, Troy, NY, USA; Department of Microbiology and Immunology, Brody School of Medicine, East Carolina University, Greenville, USA; Department of Earth System Science, Stanford University, Stanford, California, USA; Department of Marine Science, University of Texas at Austin, Marine Science Institute, Port Aransas, TX, USA; Current Address: EMBL Heidelberg, Meyerhofstraße 1, 69117 Heidelberg, Germany; Department of Earth System Science, Stanford University, Stanford, California, USA; NASA Ames Research Center, Moffett Field, CA, USA; Department of Bacteriology, University of Wisconsin- Madison, WI, USA; Department of Marine Science, University of Texas at Austin, Marine Science Institute, Port Aransas, TX, USA; Department of Integrative Biology, University of Texas at Austin, Vienna, TX, USA; Department of Functional and Evolutionary Ecology, Microbial Oceanography Unit, University of Vienna, Djerassiplatz 1, 1030 Vienna, Austria; Department of Earth System Science, Stanford University, Stanford, California, USA; Department of Marine Science, University of Texas at Austin, Marine Science Institute, Port Aransas, TX, USA; Department of Integrative Biology, University of Texas at Austin, Vienna, TX, USA

**Keywords:** metagenomics, estuarine microorganisms, functional redundancy, nitrogen and sulfur cycling

## Abstract

Estuaries are highly productive ecosystems where microbial communities drive nutrient and carbon cycling, supporting complex food webs. With intensifying anthropogenic pressures, it is critical to understand the capacity of these communities to maintain essential functions under environmental change. Here, we examined the metabolic functions and redundancy in the microbial community of San Francisco Bay (SFB) sediments, providing the first large-scale, genome-resolved, and spatiotemporally resolved characterization of the estuary. Salinity, iron, phosphorus, sulfur, and total sediment nitrogen were significantly correlated with microbial community composition, suggesting these factors play a key role in structuring SFB communities. In support of this, we identified broad capabilities for iron cycling and key uncultured players that contribute to denitrification, nitrification, and complete nitrification (comammox). We also identified widespread capabilities for sulfur cycling, including understudied lineages capable of rDsr-mediated sulfur oxidation. SFB MAGs exhibited partitioning of multistep metabolisms, or metabolic handoffs, and the rare biosphere broadly encoded key nitrogen and sulfur cycling genes. Despite shifts in community composition across sites and fluctuations in environmental parameters, key nitrogen and sulfur metabolisms were maintained throughout the estuary, especially in nitrate reduction, nitrite reduction, and the Dsr/Sox pathway. The presence of multiple microbial taxa with similar functional roles (functional redundancy) may provide an ecosystem buffer, suggesting these functions could better recover from disturbances and ultimately contribute to the long-term health and sustainability of these vital coastal habitats.

## Introduction

Estuaries exist at the boundary of land and sea, where they experience large spatiotemporal gradients. These dynamic ecosystems host diverse microbial communities that drive the chemical state and availability of essential elements such as nitrogen, sulfur, and phosphorus [1], and support complex food webs. Estuarine microorganisms and the ecosystems they support are under increasing threat from anthropogenic impacts [2]. Climate change, eutrophication, hypoxia, and seawater acidification are increasing in severity globally, resulting in intense perturbations and deterioration of estuarine environments. The negative impact of these changes is evident in increasingly poor estuarine water quality, declines in fisheries, and harmful phytoplankton blooms, but is less clear for the microscopic community. Open questions remain in understanding how microorganisms are responding and how resilient they will be to rapid change.

San Francisco Bay (SFB) is an ecologically, culturally, and economically vital estuary that has a watershed encompassing 40% of California [3]. Like estuaries globally, SFB water quality is increasingly threatened by human activity and climate change [4]. In response, conservation and management strategies have been established to study the estuary, including a 50-year monitoring project by the U.S. Geological Survey [5]. These long-term datasets have shown that SFB is characterized by high turbidity, intermittent stratification, high nutrient loading, well-oxygenated bottom waters, and some of the highest nitrogen (N) and phosphorus concentrations among estuaries globally [6]. Despite these characteristics, SFB often exhibits low primary productivity and resistance to eutrophication, in contrast to many other well studied estuaries [6]. This resistance has been attributed to the high turbidity of SFB, which limits light penetration and restricts algal growth. However, recent increases in phytoplankton blooms suggest SFB may be undergoing a breakdown in the historical resistance of the estuary to eutrophication [7]. This shift highlights an urgent need to better understand nutrient cycling in SFB, particularly the microorganisms that mediate sources and losses of N compounds and other biogeochemical processes.

Due to the high N concentrations throughout the estuary from agricultural and wastewater inputs [6], prior SFB microbial studies have focused on specific N-cycling community members such as denitrifiers and nitrifiers using qPCR, amplicon, metagenomic, and metatranscriptomic sequencing. This has revealed variable nitrification activity [8] and seasonal AOA blooms in the South SFB water column, with high nitrification rates and nitrite accumulation [9, 10]. 16S rRNA gene analyses have shown that salinity is the key driver of microbial community structure in North SFB and seasonality dominates in South SFB [11]. In SFB sediments, dramatic shifts in AOA and AOB communities have been observed spatially [12]. In addition, *nirS* was found to be highly diverse [13] and abundant throughout SFB, and generally more abundant than *nirK* [14, 15].

Although 16S rRNA and functional gene studies provide insight into community dynamics [16], they cannot capture complex metabolic capabilities. Genome-resolved metagenomics provides data about the metabolic potential of whole communities and can reveal important ecological phenomena, such as whether microbial communities are functionally redundant. Functional redundancy occurs when taxonomically distinct organisms coexist that share the same function [17, 18], and is thought to promote ecosystem stability and resilience [19, 20]. Functional redundancy can be “strict” when organisms with the same function fully replace each other or “partial” when niche differences allow coexistence, driven by mechanisms such as differing growth kinetics or viral predation pressure [17]. Genome-resolved metagenomics has also shown the existence of metabolic “handoffs”, where most microbes encode genes for fragmented biogeochemical pathways and rely on substrate exchange for growth [21, 22]. Further, these methods can reveal the metabolisms of rare biosphere microorganisms, which can act as microbial seed banks and provide reservoirs of different ecological functions [23, 24]. Understanding the SFB sediment microbial community, metabolic shifts, and potential functional redundancy across spatiotemporal scales is essential for advancing our understanding of the response of microorganisms to anthropogenic influence and climate change. To help address this, we report the first large-scale, genome-resolved characterization of a spatiotemporal dataset from SFB sediments to elucidate shifts and “handoffs” in metabolic processes that could impact ecosystem functioning.

## Materials and methods

### Sampling

Sampling was conducted aboard the R/V *Polaris* with the US Geological Survey (Menlo Park, CA) at five USGS sites (4.1, 8.1, 13, 21, and 24) across four seasons (July, October, January, and May) between July 2011 and May 2012 [15]. Sediment samples and bottom water were collected by overboard Van Veen grab. Surface cores were collected using sterile 6-cc cutoff syringes, placed immediately on dry ice, and stored at −80°C until processing. Sediment and water column chemical measurements, as well as DNA extractions, were performed as described previously [15]. Dates sampled and geochemical measurements are shown in Supplementary Table 1.

### Metagenomic sequencing and assembly

Illumina library preparation, sequencing, and assembly was completed by the Joint Genome Institute (JGI) under project ID Gs0131968 (processing pipeline jgi_mga_meta_rqc.py v2.1.0). Briefly, each of 20 samples was sequenced on an Illumina HiSeq-2000 using paired-end sequencing. Raw reads were quality filtered and read corrected using bbtools (https://jgi.doe.gov/data-and-tools/bbtools/) and assembled with SPAdes v3.11.1 using the following options: -m 2000--only-assembler -k 33 55 77 99 127--meta -t 32. The entire filtered read set was mapped to the final assembly and coverage information was generated using bbmap v37.76 using default parameters except ambiguous = random.

### Genome binning

Assemblies were indexed using BWA v0.7.17 and mapped using “bwa aln” and “bwa samse.” SAM files were converted to BAM using samtools view v1.7 -bS, and a depth file was created using jgi_summarize_bam_contig_depths. Binning was completed using Metabat v2 with the following command line options: metabat --minSamples 4 --minCVSum 0 -s 500000 --saveCls --unbinned --keep -d -v --minCV 0.1 -m 2000. A second round of binning was performed with CONCOCT via Anvi’o v6.2 using the command: anvi-gen-contigs-database --skip-gene-calling -n name -o contigs.db. For sorted BAMs, a contig profile was created by running the command: anvi-profile -i -c contigs.db --min-contig-length 2000 -M 2000. Profiles were merged with the following command line options: anvi-merge -p -c. The binning results were generated with: anvi-summarize -p -c -C CONCOCT. DasTool v1.1.2 was used to generate nonredundant metagenome-assembled genomes (MAGs) via the command: DAS_Tool.sh -i metabat_contigs_list.tsv,anvio_contigs_list.tsv -l Anvio,Metabat -c contigs.fasta --write_bins 1. MAGs were quality checked with CheckM v1.0.18 and MAGs >50% complete and <10% contamination were used for further analyses. Mmgenome (https://github.com/MadsAlbertsen/mmgenome) was used to remove contaminants and retain additional MAGs for downstream analysis, producing 639 MAGs. Genome size was calculated using Seqkit v2.3.0 with the stats command (Supplementary Table 2).

### Metagenome-assembled genome taxonomy

**Figure 1:**
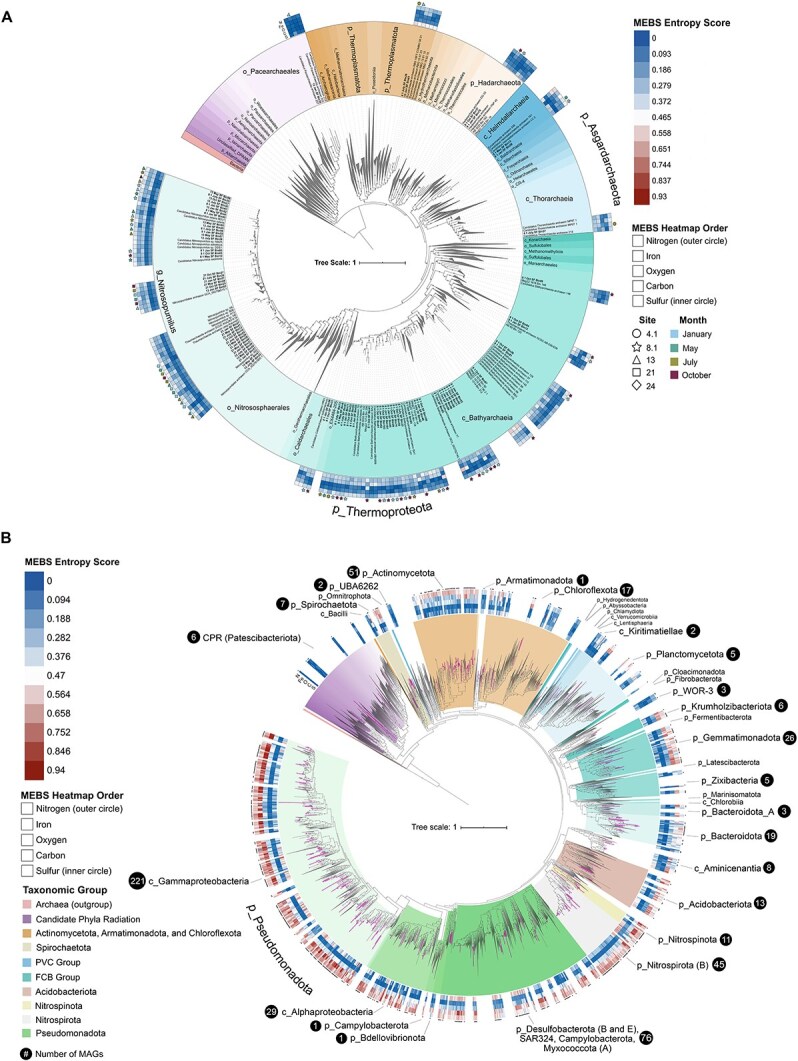
Diversity of 639 archaeal and bacterial MAGs reconstructed from SFB using 37 marker genes. (A) 81 archaea reconstructed from SFB are labeled with colored shapes in the outer ring. This tree was constructed with 3617 reference archaea and bacteria genomes. The heatmap of both phylogenies shows entropy scores for nitrogen (the outermost heatmap ring), iron, oxygen, carbon (primarily methane-related), and sulfur (the innermost heatmap ring). Heatmap colors and values shown in the legend and outer ring indicate a lower or higher number of proteins present in each genome for a cycle. Symbols shown outside of the heatmap indicate the site from which the genome was reconstructed (indicated by shape) and the month (shown with the color of the shape). (B) 558 bacteria reconstructed from SFB are shown with black dots in the outer ring, purple branches, and black circles showing the number of MAGs in a taxonomic group. The phylogeny was constructed with 4186 reference bacteria genomes. Different taxonomic groups are indicated with differing background colors and are shown in the figure legend under “Taxonomic Group”. The phylogenies were stylized in iTOL and visualized using Inkscape.

Phylosift v.0.1 was used to extract 37 single-copy marker genes from the MAGs. The alignment of archaeal MAGs was combined with a 37 marker alignment from a reference dataset of 3698 archaea and bacteria outgroup genomes published elsewhere [25]. The bacterial alignment was combined with a previously published alignment of 4186 bacteria and archaea outgroup genomes (Fig. 1B) [26]. Alignments were generated with MAFFT v7.471 using default parameters, and masked (>50% gaps removed) in Geneious v8.1.9. 45 bacteria MAGs were removed from the alignment due to poor quality (<40% of sequence length). The archaeal phylogeny was constructed using IQ-TREE v1.6.1 with the following parameters: -m LG + F + R10 -bb 1000 -bnni. This model was chosen based on a previous publication examining archaeal phylogeny [25]. Ultrafast bootstrapping option –bb 1000 and –bnni reduce the impact of severe model violations. The bacterial phylogeny was constructed using RAxML v8.2.11 with the following parameters: raxmlHPC-PTHREADS-AVX -f a -m PROTGAMMAAUTO -x 12 345 -p 12345 -N autoMRE. Taxonomic classifications for all MAGs are provided in Supplementary Table 2, predicted using GTDB-tk v2.4.1, r226.

### Metagenome-assembled genome abundance

**Figure 2:**
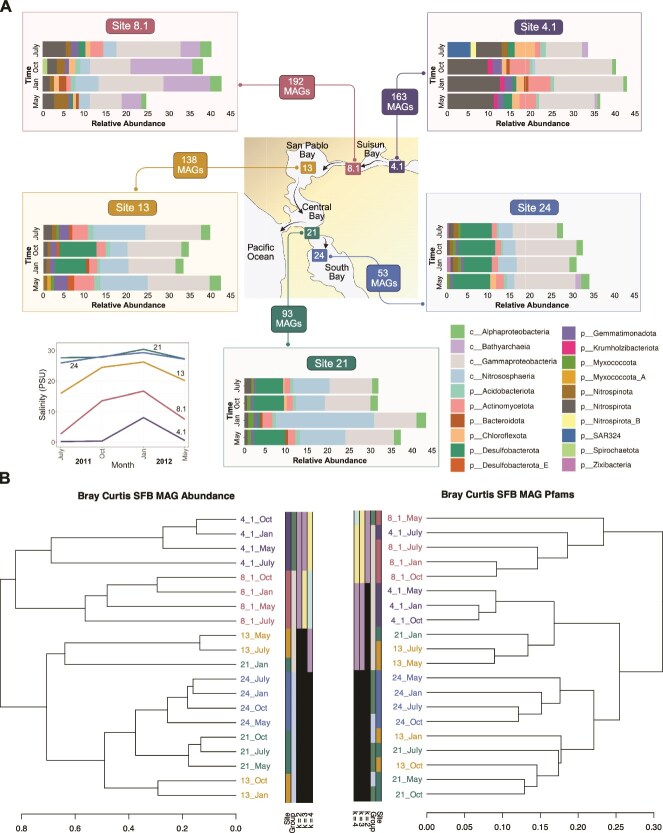
SFB sampling sites and the relative abundance of 639 MAGs reconstructed in this study. (A) The central map shows the five sampling sites. The number of MAGs reconstructed for each site is shown in colored boxes. Percent relative abundances, (calculated using CoverM) are shown in the stacked bar plots. Each color in the stacked bar plot corresponds to a microbial class or phyla, labeled in the bottom right legend. Class designation was chosen for Pseudomonadota and Thermoproteota to better show relative abundance changes within these taxa. Salinity is shown in the bottom left line graph, where salinity in PSU is shown on the y axis, time is shown on the x axis, and colored, labeled lines designate the five sites. (B) Hierarchical clustering results of the relative abundance of microbial MAGs (left dendrogram) and Pfam presence/absence (right dendrogram). The dendrograms were generated using a Bray–Curtis dissimilarity index and the agglomeration method Ward D (see Materials and Methods). The abundance plots, line graph, and dendrograms were generated in R and modified using Biorender and Inkscape.

MAG abundance was calculated using CoverM v0.2.0-alpha7 with method “genome” in default mode [27]. A custom R script was used to sum abundance values by taxa and visualize the top 10 abundant taxa per sample (Fig. 2A, Abundance_Barplot.R). The CoverM output was used to calculate the percent of reads mapped and unmapped to SFB MAGs (Supplementary Fig. 1, Reads_Barplot.R). Rare community members were identified by filtering CoverM output for MAGs that had an average relative abundance <0.1% in all months of the site where they were reconstructed (Supplementary Fig. 2).

### Non-metric multidimensional scaling

MAG relative abundance data was used to generate an non-metric multidimensional scaling (NMDS) ordination in R with the metaMDS function (Supplementary Fig. 3, package vegan, Bray–Curtis dissimilarity) [28]. Environmental variables were examined for correlation and removed if they were highly correlated (*r* > 0.7, Supplementary Table 3) and then scaled and centered using the method “standardize” in decostand. This resulted in a set of 10 environmental variables that were fit to the NMDS axes using the envfit function. Because Fe had a high correlation coefficient with salinity (*r* > 0.7), Fe was tested separately (NMDS.R).

### Hierarchical clustering

MAG relative abundance and Pfam annotations (see below) were used as input for hierarchical clustering in R (Fig. 2B) [29]. The dissimilarity matrices were generated using Bray Curtis and clustered using the method “ward.D.” Clusterwise stability was assessed using clusterboot with 1000 bootstraps (Clustering.R).

### Annotations and metabolism

**Figure 3:**
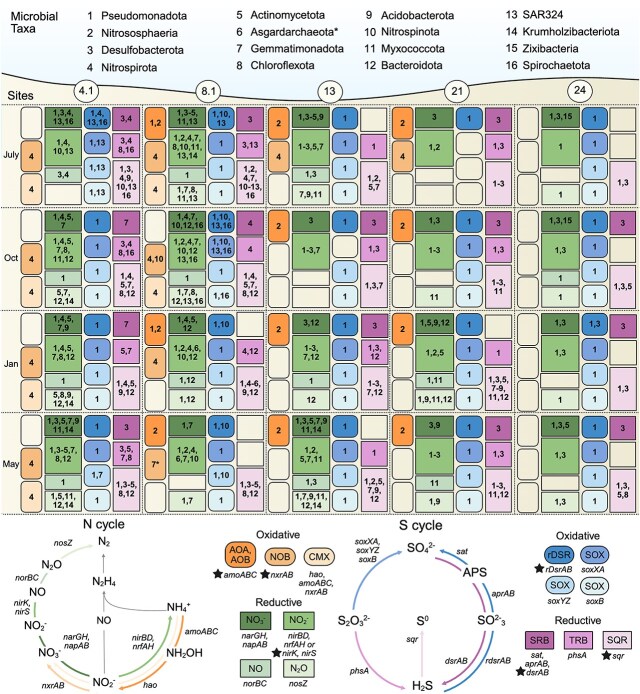
Functional redundancy of nitrogen and sulfur metabolisms in SFB microorganisms. Selected nitrogen (N) and sulfur (S) cycling genes present in 639 SFB bacterial and archaeal MAGs. Presence is indicated with colored shapes, and the number inside the shape shows the microbial taxa that encode the corresponding gene or pathway (shown in the top of the figure). Asterisks show putative Nxr-encoding phyla that lack experimental evidence. Stars next to gene names signify a gene phylogeny was constructed. Oxidative genes and pathways are in rounded shapes while reductive are in sharp edges. The panels are ordered from left to right according to geographic location and top to bottom by sampling date, starting in July of 2011 and ending in May of 2012. Asgardarchaeota are asterisked because they are not abundant but were included for their gene content. Abbreviations: AOA, (AOB) ammonia oxidizing archaea, ammonia oxidizing bacteria; (NOB) nitrite oxidizing bacteria; (CMX) comammox; (rDSR) reverse dissimilatory sulfite reductase; (SOX) sulfur oxidizing; (SRB) sulfate reducing bacteria; (TRB) thiosulfate reducing bacteria; (SQR) sulfide:quinone oxidoreductase. The nitrogen cycling diagram was adapted from Soler-Jofra, Pérez, and Loosdrecht, 2021 [84]. This figure was generated using Biorender.

Gene annotations were completed by IMG/MER for all assemblies using the IMG Annotation Pipeline v4.15.2 (Supplementary Table 4) [30]. Annotations include KAAS (KEGG Automatic Annotation Server), Pfam, and COG databases. All MAGs were annotated using MEBS, which calculates entropy-based scores from genomic data to determine the potential for metabolic machinery related to sulfur, nitrogen, iron, carbon, and oxygen cycling [31]. If MAGs contain proteins related to these cycles, they have an increased entropy score and are inferred to have metabolic machinery for these pathways. MEBS was run with the following parameters: -type genomic -comp. MAGs were also annotated using METABOLIC v4.0 with the following command line options: perl METABOLIC-C.pl -in-gn -r (Supplementary Table 5), as well asFeGenie v1.2 to categorize iron genes and operons (Supplementary Table 6) [32]. These annotations were used to analyze gene presence/absence, illustrated in Fig. 3 using Biorender.

### Measures of functional redundancy

To assign a quantitative measure of functional redundancy to the traits examined here, we applied the metric Contribution Evenness (CE) through the R package funfunfun (Supplementary Fig. 4, Supplementary Table 7) [33]. This measure determines how evenly species contribute to a trait, where more species contributing to a trait equates to higher functional redundancy. We used a diversity order of q = 0.5, which signifies the weight of species abundance in calculating CE. We also calculated the fraction of microorganisms that encoded genes for a specific function by dividing the number of MAGs with genes for a trait by the total number of MAGs, 639. These analyses were completed for 14 nitrogen and sulfur cycling traits in R (MetadataPlotting.R).

### Nitrogen gene trees

#### NirK

NirK sequences were identified in MAGs (122 NirK) using IMG/MER annotations of K00368 (Supplementary Fig. 5). Reference NirK (230 NirK) were obtained using the NCBI nonredundant database and UniProt [34]. LG + F + R7 was chosen as the best fit model. NirK sequences were aligned using MAFFT v7.310 (default settings and --anysymbol) and trimmed and masked in Geneious v8.1.9. Phylogenetic trees were constructed using IQ-TREE v1.6.1 with ultrafast bootstrapping option --bb 1000 and --bnni to reduce the impact of severe model violations. All gene alignments and phylogenies described below were run with the same command line options and processed using the same software unless otherwise specified. The results of gene phylogenies were used to determine directionality or gene category in Supplementary Table 8.

#### AmoA

AmoA was identified in 14 SFB bacteria and 26 SFB archaea MAGs using IMG/MER annotations of K10944 (Supplementary Fig. 6). Additional AmoAs were downloaded from Pfam (PF02461) [35, 36], resulting in 357 AmoA references. LG + F + R6 was chosen as the best-fit model. To generate the archaeal AmoA phylogeny, 78 archaeal AmoA references were obtained following methods in Rasmussen and Francis (2022) [9]. Archaeal references were aligned using MAFFTv7.450 with default options and manually trimmed in Geneious to 649 nt. SFB AmoA were added to the alignment using ClustalOmega v1.2.3 using fast clustering (mBed) and command line option –auto. A phylogenetic tree was constructed using RAxML v8.2.11 [37] with 1000 bootstrap replicates and the following command line options: -f a × 1.

#### NarG/NxrA

**Figure 4:**
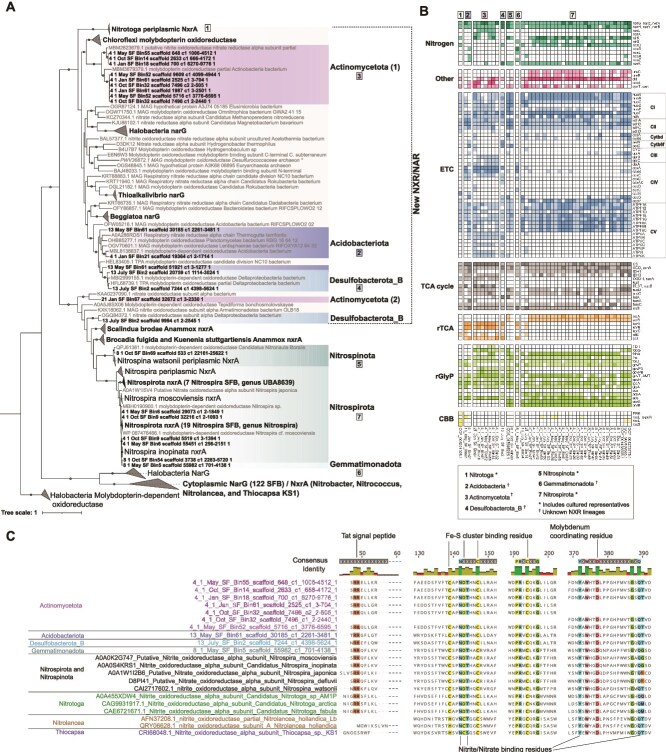
Phylogeny and metabolic potential of NarG/NxrA-encoding SFB MAGs. (A) 153 periplasmic NarG/NxrA proteins identified in SFB MAGs, as well as 255 reference NarG/NxrA. NxrA reference sequences and the phylogeny layout are based on Kitzinger *et al.* 2018 [38]. Cytoplasmic NarG/NxrA are collapsed to highlight periplasmic sequences. The phylogeny was stylized using iTOL. (B) Key marker genes present in NarG/NxrA-encoding phyla from SFB MAGs. Marker gene category is shown on the left and gene names are shown on the right. The heatmap was generated in R. (C) Partial alignment of important residues in 10 sequences from NarG/NxrA-encoding phyla and references. Positions that are highlighted and shown in the consensus identity are conserved in 100% of sequences. The alignment layout is adapted from Boddicker and Mosier, 2018 [47]. The alignment was visualized using Geneious. The figure was modified using Inkscape.

Putative NarG/NxrA sequences were identified in SFB MAGs using IMG/MER annotations of K00370. References were downloaded from UniProt [34] using 1263 KEGG accessions, the NCBI nonredundant database, and Kitzinger *et al.* (2018) [38], resulting in 255 references (Fig. 4A). LG + R10 was chosen as the best-fit model. MAGs encoding phylogenetically unique NarG/NxrA were further analyzed for their gene content with five reference genomes downloaded from NCBI and annotated using eggNOG-mapper v2 (Fig. 4B, Supplementary Table 9, NxrHeatmap.R) [39]. Gene alignments of the NarG/NxrA sequences were visualized in Geneious and modified using Inkscape (Fig. 4C).

#### NarG/NxrA protein modeling

Protein structure of putative Nxr sequences was determined using SWISS-MODEL [40] (Supplementary Data S1) and theFoldSeek online portal [41]. Nar/Nxr fasta sequences were searched against the AlphaFold/UniProt50 v4 database to accurately predict the protein structure of NxrA from cultured organisms (Supplementary Data S2).

### Sulfur gene trees

#### Dsr

DIAMOND was used to identify dissimilatory sulfite reductase subunit A (DsrA) and subunit B (DsrB) in SFB MAGs (≥40% identity, ≥105 base pairs) and aligned with references (197 MAG DsrA, 181 MAG DsrB, Supplementary Fig. 7). For both phylogenies, LG + R10 was chosen as the best-fit model. The phylogenies were visualized in iTOL v5 and Inkscape.

#### Sqr

Sulfide:quinone oxidoreductases (Sqrs) were identified in SFB MAGs using IMG/MER annotations of K17218 and reference Sqrs were obtained from a publicly available database [42]. The final alignment and tree consist of 251 SFB Sqrs and 333 references (Supplementary Fig. 8).

### Rubisco gene tree

MAG Rubisco sequences were identified via the IMG annotation pipeline (K01601). DIAMOND was used to identify closely related references from the NCBI nonredundant database and combined with publicly available Rubisco references [43, 44]. LG + F + R10 was chosen as the best fit model (Supplementary Fig. 9).

## Results

A total of 639 MAGs were recovered from 20 shallow SFB sediment samples at five sites spanning four seasons (Supplementary Table 1) [15]. This sampling scheme captured microbial communities along the major salinity gradient of SFB. To determine the identity of the MAGs, we completed phylogenomic analyses using ribosomal marker proteins and taxonomic assignment using Genome Taxonomy Database (Release 226, Fig. 1, Supplementary Table 2) [45]. This revealed 81 archaeal (Fig. 1A) and 558 bacterial MAGs (Fig. 1B). Nearly half of the archaeal community were Nitrososphaeria (referred to as Thaumarchaeota in text, 37 MAGs) and Bathyarchaeia (34 MAGs), while bacteria were dominated by Gammaproteobacteria (221 MAGs), Desulfobacterota (55 MAGs), and Actinomycetota (51 MAGs). Relative abundance showed that these were some of the most abundant microbial groups across SFB sites (Fig. 2A). In addition to dominant taxa, we identified 161 MAGs that had an average relative abundance <0.1% at a site across seasons and thus may be considered part of the rare biosphere (Supplementary Fig. 2). These MAGs encompassed 27 phyla (of 31 in SFB), including three archaeal and 24 bacterial phyla.

SFB environmental variables (Supplementary Table 1) were correlated to microbial community structure by fitting the environmental variables to a NMDS ordination of microbial relative abundance. We identified salinity, phosphorus, sulfur, and total sediment nitrogen content as significantly correlated with the ordination (*P* < .03, nonsignificant variables included nitrate, ammonium, C:N, magnesium, and lead, stress = 0.06, Supplementary Fig. 3, Supplementary Table 3). Due to high collinearity of Fe and salinity (*r* = −0.83), we evaluated these variables separately. When Fe was excluded, salinity was a significant explanatory variable (r^2^ = 0.89, *P* = .001), and when salinity was removed, Fe was also significant (r^2^ = 0.79, *P =* .001). Given their collinearity, it is difficult to separate their independent effects, but these results and those described below suggest both variables are important in shaping SFB community composition.

To assess the metabolic capabilities of SFB microorganisms, we applied entropy-based scoring via MEBS (see Materials and Methods). This analysis aligned with the NMDS results, where SFB MAGs had high entropy scores for N, S, and iron (Fe) cycling, indicating widespread metabolic capacity for these pathways (Fig. 1A and B, Supplementary Tables 4–6). High Fe scores were primarily driven by the widespread presence of iron acquisition genes such as siderophores (Supplementary Table 6), while high N and S scores were due to the presence of dissimilatory N and S genes across the estuary (Supplementary Table 4 and 5).

### Taxonomic but not metabolic differentiation across the San Francisco Bay salinity gradient

To assess the structure of SFB at both a species and functional level, we performed hierarchical clustering of MAG relative abundance and protein content, based on Pfam presence/absence. The abundance-based clustering showed that the two most freshwater-influenced sites (4.1 and 8.1) formed distinct clusters (Fig. 2B, left dendrogram), while the marine-influenced sites (13, 21, and 24) were intermixed and did not cluster separately. No clusters were season specific. This suggests that community composition was more similar among marine-influenced sites, and less similar among freshwater-influenced sites. In contrast, clustering based on metabolic potential (pfam annotations) did not recover any clear grouping by site or season, indicating that metabolisms were broadly similar across samples (Fig. 2B, right dendrogram). NMDS analyses were consistent with these results, where the NMDS showed no observable patterns based on season, sites 4.1 and 8.1 broadly clustered separately from other sites, and sites 13–24 were closer in ordination space. These results provide evidence for functional redundancy. Although community composition varied spatially, metabolic potential remained relatively consistent.

To quantitatively assess SFB functional redundancy, we used the CE metric (see Materials and Methods) [33] to measure functional redundancy in 14 dissimilatory nitrogen and sulfur pathways (Supplementary Fig. 4, Supplementary Table 7). Nitrite reduction had the highest average functional redundancy (0.40 $\pm$ 0.05). Nitrite reduction is the second step of denitrification (*nirS, nirK*) or DNRA (*nirBD*, *nrfAH*) [14, 46] and the high functional redundancy of this pathway was reflected in the widespread presence of these genes in the estuary (*nirS*: 32.6% of MAGs, *nirK*: 18.6%, *nirBD*: 17%, and *nrfAH*: 4.5%, Supplementary Fig. 5). Second to nitrite reduction, nitrate reduction, or the first step of denitrification and DNRA, was most functionally redundant (0.22 $\pm$ 0.03), followed by steps of Sox-mediated sulfur oxidation, and sulfur oxidation catalyzed by reverse dissimilatory-sulfite reductase (rDsr, Supplementary Fig. 7). The least functionally redundant pathway we identified was nitrogen fixation (0.02 $\pm$ 0.03), consistent with the limited distribution of *nifHDK* genes in SFB and prior findings in pelagic marine environment [33].

### Metabolic handoffs and functional redundancy in denitrification and DNRA

Denitrification and DNRA are important nitrogen cycling pathways in SFB that act as a significant mechanism of nitrogen loss [14] and a source of biologically available ammonium, respectively [46]. The first step in these pathways, *narGH* or *napAB*-mediated nitrate reduction, had relatively high functional redundancy in SFB and was present in 32% of MAGs (Supplementary Table 7). Nitrite reduction genes for the second and final step of DNRA were at most present in 17% of MAGs (*nirBD*), and nitrite reduction genes for the second step of denitrification were widespread (*nirK* and *nirS*). Finally, the penultimate step of denitrification had relatively low functional redundancy and was present in fewer community members (*norBC*: 8% of MAGs), while the final step of denitrification (*nosZ*-mediated nitrous oxide reduction) was the third most functionally redundant nitrogen pathway of the eight analyzed (0.12 $\pm$ 0.01) and present in 16% of MAGs.

The variable redundancy and proportion of community members encoding genes for denitrification and DNRA highlights that, like other ecosystems, these nitrogen cycling pathways were often incomplete within a single SFB organism. Among SFB MAGs estimated to be ≥75% complete, 78.9% of SFB taxa encoded genes for one or more steps of denitrification or DNRA. Alphaproteobacteria, Gammaproteobacteria, and Desulfobacterota frequently encoded partial steps of these pathways, often in specific taxonomic orders. For example, abundant station 13 Desulfobacterota MAGs (12 MAGs, six classes) in classes Desulfobulbia and Desulfuromonadia uniquely fixed nitrogen. At the same site, Desulfobulbia and Deferrimicrobia were the only community members capable of *napAB*-mediated nitrate reduction, while Syntrophobacteria and Desulfuromonadia were capable of *narGH*-mediated nitrate reduction. Some N cyclers were predicted to have unique gene content, where one site 8.1 Gemmatimonadota (8_1_May_SF_Bin5, 57.5% completeness, 5% contamination) encoded a nitrate reductase/nitrite oxidoreductase closely related to Nitrospirota *nxrA* (Fig. 4A; Supplementary Text and Supplementary Table 8 and 9). In addition, several phyla not known to oxidize nitrite (Acidobacteriota, Actinomycetota, and Desulfobacterota) encoded nitrate reductase/nitrite oxidoreductases that were more closely related to *nxrA* in species of the cultured, nitrite oxidizing *Nitrotoga* genus. Although these MAGs encoded the beta subunit of nitrate reductase/nitrite oxidoreductase and the conserved residues in these genes (Fig. 4B and C), experimental evidence is needed to determine the direction of these enzymes. The SFB MAGs did not encode Rubisco for carbon fixation via the Calvin-Benson-Bassham (Supplementary Fig. 9, Supplementary Table 8) cycle as observed in *Nitrotoga* [47], though they did have highly complete pathways for the reductive glycine pathway (rGlyP), a recently proposed seventh CO_2_ fixation pathway [48].

### Coexistence of microorganisms driving ammonia oxidation

Ammonia oxidation is the first and rate-limiting step of nitrification, which is crucial for the removal of nitrogen in an ecosystem [12]. In SFB MAGs, ammonia oxidation was encoded in 8.6% of the community and relative to other nitrogen pathways, had moderately low functional redundancy (11th lowest of 14 CE scores, Supplementary Table 7). Despite this, we observed shifts in the organisms catalyzing this pathway. At the head of the estuary (stations 4.1 and 8.1), we observed a predominance of comammox Nitrospirota, encoding *amoABC*, *hao*, and *nxrAB* genes (Fig. 3, Supplementary Fig. 6 and 10, Supplementary Table 4 and 5). Comammox are capable of the complete oxidation of ammonia to nitrate (both steps of nitrification), which has historically been thought of as a two-step process catalyzed by ammonia oxidizers and nitrite oxidizers [49]. At site 4.1, nitrite-oxidizing Nitrospirota were identified in the same months as comammox Nitrospira and at site 8.1, three comammox Nitrospirota MAGs appeared to coexist with AOA *Nitrosopumilus* (Thaumarchaeota), AOB *Nitrosomonas*, and nitrite-oxidizing bacteria (NOB) Nitrospirota and Nitrospinota. With closer proximity to the ocean, USGS stations 13 and 21 had a higher salinity compared to 4.1 and 8.1 (Fig. 2A) and were dominated by AmoABC-encoding Thaumarchaeota within the *Nitrosopumilaceae* family (12/14 station 13 MAGs, 11/14 station 21 MAGs). Several of these MAGs were closely related to *Nitrosopumilus salaria* BD31, an AOA originally isolated from sediments of San Pablo Bay [50].

### Functional redundancy and unique capabilities in San Francisco Bay sulfur cyclers

In support of high sulfur cycling entropy scores and the correlation between sulfur and community structure, we found that the Dsr/Sox pathway for sulfur compound oxidation was widespread and functionally redundant throughout the estuary (Fig. 3, Supplementary Table 7). All steps of Sox-mediated sulfur oxidation were present in 21%–26% of MAGs apart from *soxCD*, which only occurred in ~9% of MAGs. In addition, *rdsr* genes were present in 21% of MAGs. The absence of *soxCD* in combination with the more widespread presence of all other components of the Sox pathway and *rdsr* suggests SFB organisms largely utilized the Dsr/Sox pathway for sulfur compound oxidation [51, 52]. In this modified, truncated Sox pathway, reduced sulfur compounds have been found to be oxidized with elemental sulfur as an intermediate and then further oxidized via *rdsr*. Alphaproteobacteria, Gammaproteobacteria, Nitrospinota, SAR324, and Spirochaetota encoded genes for *rdsrAB*-mediated sulfur oxidation, and these genes were also present in one Desulfobacterota and Nitrospirota MAG (Supplementary Table 8). Genes for Sox-mediated sulfur oxidation were widely present in Alphaproteobacteria, Gammaproteobacteria, and SAR324. Relative to the other dissimilatory pathways examined, reductive dissimilatory sulfate reduction was less functionally redundant, present in 6.4% of MAGs. Ninety-seven MAGs also encoded type I, IV, or VI SQRs (Supplementary Fig. 8, Supplementary Table 8), associated with sulfide-dependent respiration and photosynthesis (type I) or growth on sulfide as the sole electron source (types IV and VI) [53].

Some SFB MAGs had unique sulfur cycling abilities. At site 8.1, Spirochaetota MAGs were identified encoding *rdsrAB* for sulfur oxidation (Supplementary Table 8). To our knowledge, rDsr has not been identified in Spirochaetota, though reductive DsrAB was recently identified in this phylum [54] and sulfur oxidation has been shown in *Spirochaeta perfilievii* [55]. One *rdsrAB*-encoding MAG in Spirochaetota class Leptospirae (8_1_Oct_SF_Bin1) also encoded *sat*, *aprAB*, *dsrC*, *dsrEFH*, and *qmoABC*, and lacked *dsrD*, which aligns with previously outlined genomic-based evidence for sulfur oxidation [56]. In total, five Leptospirae MAGs contained *rdsrAB* genes on contigs up to 46 kb in length (62.5%–93.8% completeness, 0.0%–8.8% contamination) and had neighboring proteins with homology to other Leptospirae. Therefore, we did not find support that this was the result of binning contamination. Three *rdsr*-encoding Spirochaetota MAGs from the same classes were identified at site 4.1 in July, indicating these organisms may prefer low-mid salinity environments. Two SFB organisms also encoded both a reductive *dsrA* and oxidative *rdsrA* (1 Desulfobacterota MAG and 1 Nitrospirota MAG). Both Dsr-dependent and sulfur-oxidizing and -reducing genes have previously been identified in Nitrospirota [54] and Desulfobacterota [57] but organisms that encode genes for both pathways have not been well described [58].

### Gammaproteobacteria are broadly distributed across the estuarine salinity gradient

In evaluating functional redundancy, weight is often given to abundant taxa, as these organisms are predicted to have a greater impact on community functions [59, 60]. In SFB, Gammaproteobacteria were highly abundant at all sites and were the microbial class for which we recovered the most MAGs (221/639; Fig. 2A). Woeseiales Gammaproteobacteria were uniquely widespread, as the only microbial order present at all sites. In support of the high Fe scores we observed, Gammaproteobacteria and Woeseiales were predicted to broadly encode the capacity for Fe reduction (45 MAGs) and Fe oxidation (29 MAGs), including MtrCAB for Fe reduction and the candidate iron oxidase Cyc2 for Fe oxidation (Supplementary Table 5 and 6). Gammaproteobacteria MAGs contained a variety of S-cycling metabolic pathways that were maintained over space and time, including *rdsr* (Supplementary Fig. 7, Supplementary Table 8) and the truncated Sox system for sulfur compound oxidation (Fig. 3). In support of high N scores, 41% of SFB Gammaproteobacteria encoded *nirS* for nitrite reduction to NO. In contrast, *nirK* was only present in 6% of Gammaproteobacteria MAGs (Supplementary Fig. 5). These findings are consistent with a previous qPCR-based study in SFB on the same samples [15]. Uncultured, site 8.1 Gammaproteobacteria (orders UBA6429 and AKS1) were some of the few predicted complete denitrifiers in SFB (plus two Bacteroidota from the same site). These complete denitrifiers encoded *rdsr* and the truncated Sox system for sulfur oxidation. In support of this, the closest cultured representative to these MAGs (*Sulfuriflexus mobilis* and *Thiohalophilus thiocyanatoxydans*) are known to be capable of nitrogen oxide reduction paired to thiosulfate and sulfur oxidation [61, 62]. The distribution and metabolic capacity of SFB Gammaproteobacteria, especially order Woeseiales, suggest these taxa are “generalist” microorganisms that have broad environmental tolerances [63].

### The role of rare taxa in sulfur and nitrogen cycling

Rare taxa have been described as contributing to an “insurance” pool of genetic resources, serving as reservoirs of genetic and functional diversity [23, 64]. We found evidence for the role of rare community members contributing to nitrogen and sulfur cycling in SFB. Of the 162 MAGs within the rare biosphere (<0.1% average abundance) ~80% encoded genes for the examined dissimilatory nitrogen cycling pathways, while ~60% encoded genes for the dissimilatory sulfur pathways (Supplementary Table 7). Reflecting overall community patterns, most rare taxa encoded genes for nitrite reduction (58.4%), followed by nitrate reduction (47.8%), the partial Sox pathway (*soxYZ*: 30.4%, *soxAX*: 28.6%, *soxB*: 25.5%), and *rdsr* (26.7%). An example of a rare community member included a single representative of the Tectomicrobia phylum (21_Jan_SF_Bin1, family Entotheonellaceae), which encoded genes for nitrite reduction (*nirK* and *nirBD*) and NO reduction to N_2_O (*norBC*). Few other site 21 MAGs encoded *norBC*, apart from one Gammaproteobacteria and three Myxococcota MAGs. In addition, the Tectomicrobia organism encoded *dmsAB* for DMSO reduction, and as previous studies have found [65], had a large genome size (7.13 MB, the largest of all MAGs in this dataset, Supplementary Table 2).

Few Heimdallarchaeota (LC-2 group) were recovered in SFB, and one of the two Heimdall MAGs identified was predicted to be part of the rare community based on relative abundance. In these Heimdallarchaea, we identified putative Cu-containing nitrite reductases (*nirK* in 8_1_Jan_SF_Bin58 and 8_1_May_SF_Bin13). Phylogenetic and coverage analyses of *nirK* provide support for these annotations (coverage >8.5), where the MAG nitrite reductases were related to a “copper-containing nitrite reductase precursor” in a Heimdallarchaeota archaeon in group LC-3 (OLS27132.1; Supplementary Fig. 5 and Supplementary Text) [66]. These MAGs had completeness estimates of 72.7% and 92.5%, and contamination estimates of 4.5% and 9%. Although nitrite reductases have recently been reported in Heimdallarchaea, they are not well studied and require further validation to determine whether these annotations are the result of false positives due to NirK homology to multicopper oxidases [67]. Overall, low abundance and rare microorganisms in SFB have the capacity to contribute to important nitrogen and sulfur metabolisms in the estuary.

## Discussion

Here, we recovered the first large-scale, genome-resolved dataset of SFB microorganisms and one of the largest metagenomic reconstructions from an estuary globally [68–70]. We characterized 639 MAGs spatially across the five sites and temporally at four time points (seasons). This sampling scheme allowed us to examine shifts in biodiversity and function over space and time. We found that the SFB community is shaped by salinity, iron, phosphorus, sulfur, and total sediment nitrogen. SFB microbes maintained functional redundancy, where different taxa replaced each other or coexisted to maintain steps of denitrification, DNRA, ammonia oxidation, and sulfur compound oxidation throughout the estuary.

The high functional redundancy we observed in *nirS-* and *nirK*-mediated nitrite reduction aligned with prior work, where these genes were found to be widespread in SFB [14, 15]. Nitrate reduction and the Dsr/Sox pathway also exhibited high redundancy, though the genomic potential for these pathways has never previously been characterized in the estuary. However, rate measurements taken in South SFB have indicated that denitrification may remove ~14% of N and DNRA may account for ~10% of nitrate reduction, underscoring the importance of these processes for the fate of N in SFB [7]. In support of redundancy in sulfur compound oxidation, studies in other estuaries have observed widespread S oxidizing Gammaproteobacteria [71]. Also, our observation that very few genomes contained nitrogenase genes suggests nitrogen fixation is not an important source of N for surface sediment microbial communities in SFB, likely because SFB is a nitrogen-rich estuary (Supplementary Table 1) and has one of the highest N loads of estuaries globally [7]. The metabolic handoffs we observed in some N and S pathways may contribute to their resilience, where the modularity provided by handoffs creates opportunities for several taxa to complete a part of a reaction. However, the connections between functional redundancy and metabolic handoffs, as well as the role they play in ecosystem stability, warrant further exploration. Overall, the high redundancy observed in SFB nitrite and nitrate reduction and the Dsr/Sox pathway lead us to predict that these functions may have high resistance to environmental disturbance, while lower redundancy metabolisms like N fixation may be more vulnerable to perturbation.

In studies of estuarine microorganisms globally, some have found evidence for functional redundancy in N and S cycling [72], while others have identified significant changes in the spatial patterns of these metabolisms [73, 74]. In addition, some estuarine studies with similar sampling resolution as the dataset recovered here have identified pronounced seasonal changes in microbial community composition [72], while others have not [73]. In other environments, evidence for microbial functional redundancy in N and S cycling has been observed in diverse ecosystems such as subseafloor fluids [75], glaciers [76], and the global pelagic ocean [77]. Thus, the environmental characteristics that lead to the occurrence of functional redundancy versus its absence, as well as distinct patterns in community composition, are unclear. Furthermore, additional studies are needed to determine whether functional redundancy results in resistance to environmental disturbance. A meta-analysis of functional redundancy in diverse ecosystems has suggested that functional redundancy is positively correlated with ecological stability and resilience [19]. Experimental evidence has indicated that in soils, nitrogen cycling microbial communities are resilient to repeated freeze–thaw cycle disturbance, but exhibit changes in response to heat disturbance, and only short-term resilience to anoxia [78]. Additional work is needed to understand the practical outcomes of redundancy in functional traits.

When examining the functional trait space of a community, abundant and generalist species are often thought to contribute to a higher occurrence of functional redundancy. In SFB, Gammaproteobacteria were the only class present and abundant throughout the entire estuary over space and time, encoding a diversity of N and S cycling genes. This reached the family level, where Woeseiaceae was identified in all samples, and these findings align with another recent metagenomics-based study, which classified this group as habitat generalists due to their distribution and metabolic flexibility [79]. However, rare taxa have also been identified as important contributors to functional biodiversity and community resilience in diverse environments [80–82] for many key functions [64]. The widespread presence of dissimilatory N and S cycling genes in the SFB rare microbial community adds to increasing recognition that rare taxa are important contributors to functional redundancy.

This study provides crucial baseline data of estuarine microbial communities on a spatiotemporal scale as well as hypotheses about redundant and potentially resilient ecological functions. These data are critical in the face of climate change and human influence; however, further work is needed to fully understand the implications of our findings. Future studies with repeat sampling of the estuary would help determine whether the patterns we observed here are consistently recovered. Greater sampling resolution is needed over time, and metatranscriptomes as well as activity measurements are needed to understand how redundant functions are differentially expressed, and under what environmental conditions. We also observed that several taxa with the same function coexisted at the same site, such as comammox Nitrospirota that co-occurred with AOB and NOB. This phenomenon is known to be facilitated by niche-separating physiological features such as differences in substrate affinities or energetic efficiencies of a reaction [83]. Future research should aim to understand the mechanisms that allow overlap in diverse organisms with redundant N and S cycling pathways. Furthermore, mesocosm studies simulating disturbance would allow us to test how redundancy is affected by the magnitude, frequency, and chronology of a disturbance, which are factors known to affect the ability of a community to maintain resilience [78]. Future work should aim to reveal how anthropogenic disturbance will affect the ability of microbial communities to maintain functions, to help determine whether the functional redundancy observed here truly promotes a more stable and resilient ecosystem.

## Supplementary Material

SupplementaryTable1_ycaf182

SupplementaryTable2_ycaf182

SupplementaryTable3_ycaf182

SupplementaryTable4_ycaf182

SupplementaryTable5_ycaf182

SupplementaryTable6_ycaf182

SupplementaryTable7_ycaf182

SupplementaryTable8_ycaf182

SupplementaryTable9_ycaf182

Supplementary_Data_1_ycaf182

Supplementary_Data_2_ycaf182

Supplementary_Information_ycaf182

## Data Availability

Raw data and annotations are provided in IMG/MER under project ID Gs0131968. Metagenome-assembled genomes are available under BioProject ID PRJNA865744. All scripts used in this work and cited in the Materials and Methods are publicly available at https://github.com/mlangwig/SanFranciscoBay.
