# Supplementary material for: Metabolic capacity is maintained despite shifts in microbial diversity in estuary sediments
Source: ISME Commun. 2025 Oct 11;5(1):ycaf182. doi: 10.1093/ismeco/ycaf182 (PMC12687941; doi:10.1093/ismeco/ycaf182)
Supplement: Supplementary_Data_1_ycaf182 [file supplementary_data_1_ycaf182.zip › SWISS-MODEL/8_1_May_SF_Bin5_scaffold_55982_c1/templates.html]

Untitled Project | Templates


**Export Alignment**
  
FASTA format
Clustal Format
PNG Image

**Secondary Structure**
  
None
DSSP
PSIPRED
SSpro

**Colour Scheme** 


Fade Mismatches
Enhance Mismatches

Confidencegradient
Confidenceclass
Indels
Chain
Unique Chain
Rainbow
2° Structure
Clustal
Hydrophobic
Size
Charged
Polar
Proline
Ser/Thr
Cysteine
Aliphatic
Aromatic
No Colour

Use QMEANBrane values

|  |  |  |  |
| --- | --- | --- | --- |
| Background |  |  |  |

**3D Viewer**  
NGL
PV

FASTA
Multi FASTA
ClustalW
PNG


SWISS-MODEL

### Untitled Project

### Created: March 29, 2023, 4:10 a.m. at 04:10

- Templates
- Models

Models | Name | Description | GMQE | QSQE | Seq Id | Coverage | Range | Method | Resolution | Oligo-state | Ligands | Found by | Seq Similarity ||  | 7b04.1.B | Nitrite oxidoreductase subunit A  *Structure of Nitrite oxidoreductase (Nxr) from the anammox bacterium Kuenenia stuttgartiensis.* | 0.88 | 0.00 | 58.37 | 1.00 | 1-1143 | X-ray | 2.97 | monomer | 4 x SF4, 1 x F3S, 2 x MD1, 1 x MO, 1 x HEM, 2 x CA | BLAST | 0.49 |
| ``` target    MFLSRRQFLKVSVGTVAAVAVADKVLALTALQPVIEVGNPLGDYPDRSWERVYHDQYRYDSSFTWVCSPNDTHACRVRAF 7b04.1    MKLTRRAFLQVAGATGATLTLAKNAMAFRLLKPAVVVDNPLDTYPDRRWESVYRDQYQYDRTFTYCCSPNDTHACRIRAF  target    VRNGVVMRVEQNYDHQTYEDLYGNRGTFAHNPRMCLKGFTFHRRVYGPYRLKGPLMRKGWKQWMDDNAPELTAETKRKYK 7b04.1    VRNNVMMRVEQNYDHQNYSDLYGNKATRNWNPRMCLKGYTFHRRVYGPYRLRYPLIRKGWKRWADDGFPELTPENKTKYM  target    FDSRFLDDMLRVSWDTAFTYAAKAMITIATRYSGEAGARRLREQGYAPEMIEMMKGAGTRCFKHRAGMPVLGIIGKMGNT 7b04.1    FDNRGNDELLRASWDEAFTYASKGIIHITKKYSGPEGAQKLIDQGYPKEMVDRMQGAGTRTFKGRGGMGLLGVIGKYGMY  target    RMNGGINALLDTWIRKVSPDQAQGGRYWSNYTWHGDQNPAHPFWSGVQGSDIDLSDMRFSKLNTSWGKNFVENKMPEAHW 7b04.1    RFNNCL-AIVDAHNRGVGPDQALGGRNWSNYTWHGDQAPGHPFSHGLQTSDVDMNDVRFSKLLIQTGKNLIENKMPEAHW  target    KLECIERGARVVVITPEYNPTAYRADYWMPLRPESDGALFLGAMKIIIDENMHDIDFLKSFTDAPILVRTDTLQYLDPRD 7b04.1    VTEVMERGGKIVVITPEYSPSAQKADYWIPIRNNTDTALFLGITKILIDNKWYDADYVKKFTDFPLLIRTDTLKRVSPKD  target    VIADYKFPDFSKSYSGRIQSLKPEQIQRLGGMMVWDLNKKQVVPLHREQVGWHYTNSGIDAALTGTYRVKLLNGREIDAM 7b04.1    IIPNYKLQDISDGPSYHIQGLKDEQREIIGDFVVWDAKSKGPKAITRDDVGETLVKKGIDPVLEGSFKLKTIDGKEIEVM  target    PIWQMYMVHFQDYDLDTVHQITRTPKDLIVRWARDSGTIKPAAIHNGEGTCHYFHQTANARGAAMVLIITGNVGKFGTGQ 7b04.1    TLLEMYKIHLRDYDIDSVVSMTNSPKDLIERLAKDIATIKPVAIHYGEGVNHYFHATLMNRSYYLPVMLTGNVGYFGSGS  target    HTWAGNYKAGTWTATPWSGAGLSVHTGEDPFNITLDPNAHGKEIKTRSYYYGEEVGYWNHGDTALIVNTPKYGRKVFTGK 7b04.1    HTWAGNYKAGNFQASKWSGPGFYGWVAEDVFKPNLDPYASAKDLNIKGRALDEEVAYWNHSERPLIVNTPKYGRKVFTGK  target    THMPTPSKFRWVVNVNVVNNAKHHYDMVRNVDPNIECLITQDIEMTSDINHADIAFAANSWMEFTYPEMTVTVSNPWVQI 7b04.1    THMPSPTKVLWFTNVNLINNAKHVYQMLKNVNPNIEQIMSTDIEITGSIEYADFAFPANSWVEFQEFEITNSCSNPFIQI  target    W-KGGIRPLYDTRNDLDTFAGVAAKLSDMTGDKRMRDYFAMVYQNRVDVYVQRMLDASSTFYGYSADVMLKSEKG----W 7b04.1    WGKTGITPVYESKDDVKILAGMASKLGELLRDKRFEDNWKFAIEGRASVYINRLLDGSTTMKGYTCEDILNGKYGEPGVA  target    MVMVRTYPRHPFWEETNESKPMWTRSGRYENYRIEPEAIEYGENFISHREGPEATPYLPNAIFTTNPYVRPDDYGIPITA 7b04.1    MLLFRTYPRHPFWEQVHESLPFYTPTGRLQAYNDEPEIIEYGENFIVHREGPEATPYLPNAIVSTNPYIRPDDYGIPENA  target    QHHDDKTVRNIKLSWHEIKRHSNPLWEKGYQFYCVTPKTRHRVHSQWSVNDWVQIYESNFGDPYRMDKRTPGVGEHQIHI 7b04.1    EYWEDRTVRNIKKSWEETKKTKNFLWEKGYHFYCVTPKSRHTVHSQWAVTDWNFIWNNNFGDPYRMDKRMPGVGEHQIHI  target    NPQAAKDRGINDGDYVYVDGNPVDRPYRGWKPSDPYYKVARLMIRAKYNPAYPYHVTMAKHAPFVATAKSVKGHETRPDG 7b04.1    HPQAARDLGIEDGDYVYVDANPADRPYEGWKPNDSFYKVSRLMLRAKYNPAYPYNCTMMKHSAWISSDKTVQAHETRPDG  target    RAIAIDTGYQSNFRYGAQQSFTRNWLMPMHQTDSLPGKHAVAWKFKWGYQVDHHAINTVPKECLIRITKAEDGGIGARGP 7b04.1    RALS-PSGYQSSFRYGSQQSITRDWSMPMHQLDSLFHKAKIGMKFIFGFEADNHCINTVPKETLVKITKAENGGMGGKGV  target    WEPVRTGFTPGQENEFMIKWLKGEHIKIKV 7b04.1    WDPVKTGYTAGNENDFMKKFLNGELIKV-- ``` | | | | | | | | | | | | | | | | | | | | | | | | | | | | | | | | | | | | | | | | | | | | | | | | | |
|  | 7b04.2.B | Nitrite oxidoreductase subunit A  *Structure of Nitrite oxidoreductase (Nxr) from the anammox bacterium Kuenenia stuttgartiensis.* | 0.86 | 0.00 | 58.37 | 1.00 | 1-1143 | X-ray | 2.97 | monomer | 4 x SF4, 1 x F3S, 2 x MD1, 1 x MO, 1 x HEM, 2 x CA | BLAST | 0.49 |
| ``` target    MFLSRRQFLKVSVGTVAAVAVADKVLALTALQPVIEVGNPLGDYPDRSWERVYHDQYRYDSSFTWVCSPNDTHACRVRAF 7b04.2    MKLTRRAFLQVAGATGATLTLAKNAMAFRLLKPAVVVDNPLDTYPDRRWESVYRDQYQYDRTFTYCCSPNDTHACRIRAF  target    VRNGVVMRVEQNYDHQTYEDLYGNRGTFAHNPRMCLKGFTFHRRVYGPYRLKGPLMRKGWKQWMDDNAPELTAETKRKYK 7b04.2    VRNNVMMRVEQNYDHQNYSDLYGNKATRNWNPRMCLKGYTFHRRVYGPYRLRYPLIRKGWKRWADDGFPELTPENKTKYM  target    FDSRFLDDMLRVSWDTAFTYAAKAMITIATRYSGEAGARRLREQGYAPEMIEMMKGAGTRCFKHRAGMPVLGIIGKMGNT 7b04.2    FDNRGNDELLRASWDEAFTYASKGIIHITKKYSGPEGAQKLIDQGYPKEMVDRMQGAGTRTFKGRGGMGLLGVIGKYGMY  target    RMNGGINALLDTWIRKVSPDQAQGGRYWSNYTWHGDQNPAHPFWSGVQGSDIDLSDMRFSKLNTSWGKNFVENKMPEAHW 7b04.2    RFNNCL-AIVDAHNRGVGPDQALGGRNWSNYTWHGDQAPGHPFSHGLQTSDVDMNDVRFSKLLIQTGKNLIENKMPEAHW  target    KLECIERGARVVVITPEYNPTAYRADYWMPLRPESDGALFLGAMKIIIDENMHDIDFLKSFTDAPILVRTDTLQYLDPRD 7b04.2    VTEVMERGGKIVVITPEYSPSAQKADYWIPIRNNTDTALFLGITKILIDNKWYDADYVKKFTDFPLLIRTDTLKRVSPKD  target    VIADYKFPDFSKSYSGRIQSLKPEQIQRLGGMMVWDLNKKQVVPLHREQVGWHYTNSGIDAALTGTYRVKLLNGREIDAM 7b04.2    IIPNYKLQDISDGPSYHIQGLKDEQREIIGDFVVWDAKSKGPKAITRDDVGETLVKKGIDPVLEGSFKLKTIDGKEIEVM  target    PIWQMYMVHFQDYDLDTVHQITRTPKDLIVRWARDSGTIKPAAIHNGEGTCHYFHQTANARGAAMVLIITGNVGKFGTGQ 7b04.2    TLLEMYKIHLRDYDIDSVVSMTNSPKDLIERLAKDIATIKPVAIHYGEGVNHYFHATLMNRSYYLPVMLTGNVGYFGSGS  target    HTWAGNYKAGTWTATPWSGAGLSVHTGEDPFNITLDPNAHGKEIKTRSYYYGEEVGYWNHGDTALIVNTPKYGRKVFTGK 7b04.2    HTWAGNYKAGNFQASKWSGPGFYGWVAEDVFKPNLDPYASAKDLNIKGRALDEEVAYWNHSERPLIVNTPKYGRKVFTGK  target    THMPTPSKFRWVVNVNVVNNAKHHYDMVRNVDPNIECLITQDIEMTSDINHADIAFAANSWMEFTYPEMTVTVSNPWVQI 7b04.2    THMPSPTKVLWFTNVNLINNAKHVYQMLKNVNPNIEQIMSTDIEITGSIEYADFAFPANSWVEFQEFEITNSCSNPFIQI  target    W-KGGIRPLYDTRNDLDTFAGVAAKLSDMTGDKRMRDYFAMVYQNRVDVYVQRMLDASSTFYGYSADVMLKSEKG----W 7b04.2    WGKTGITPVYESKDDVKILAGMASKLGELLRDKRFEDNWKFAIEGRASVYINRLLDGSTTMKGYTCEDILNGKYGEPGVA  target    MVMVRTYPRHPFWEETNESKPMWTRSGRYENYRIEPEAIEYGENFISHREGPEATPYLPNAIFTTNPYVRPDDYGIPITA 7b04.2    MLLFRTYPRHPFWEQVHESLPFYTPTGRLQAYNDEPEIIEYGENFIVHREGPEATPYLPNAIVSTNPYIRPDDYGIPENA  target    QHHDDKTVRNIKLSWHEIKRHSNPLWEKGYQFYCVTPKTRHRVHSQWSVNDWVQIYESNFGDPYRMDKRTPGVGEHQIHI 7b04.2    EYWEDRTVRNIKKSWEETKKTKNFLWEKGYHFYCVTPKSRHTVHSQWAVTDWNFIWNNNFGDPYRMDKRMPGVGEHQIHI  target    NPQAAKDRGINDGDYVYVDGNPVDRPYRGWKPSDPYYKVARLMIRAKYNPAYPYHVTMAKHAPFVATAKSVKGHETRPDG 7b04.2    HPQAARDLGIEDGDYVYVDANPADRPYEGWKPNDSFYKVSRLMLRAKYNPAYPYNCTMMKHSAWISSDKTVQAHETRPDG  target    RAIAIDTGYQSNFRYGAQQSFTRNWLMPMHQTDSLPGKHAVAWKFKWGYQVDHHAINTVPKECLIRITKAEDGGIGARGP 7b04.2    RALS-PSGYQSSFRYGSQQSITRDWSMPMHQLDSLFHKAKIGMKFIFGFEADNHCINTVPKETLVKITKAENGGMGGKGV  target    WEPVRTGFTPGQENEFMIKWLKGEHIKIKV 7b04.2    WDPVKTGYTAGNENDFMKKFLNGELIKV-- ``` | | | | | | | | | | | | | | | | | | | | | | | | | | | | | | | | | | | | | | | | | | | | | | | | | |
| ✓ | 7b04.1.B | Nitrite oxidoreductase subunit A  *Structure of Nitrite oxidoreductase (Nxr) from the anammox bacterium Kuenenia stuttgartiensis.* | 0.88 | 0.00 | 57.58 | 1.00 | 1-1144 | X-ray | 2.97 | monomer | 4 x SF4, 1 x F3S, 2 x MD1, 1 x MO, 1 x HEM, 2 x CA | HHblits | 0.48 |
| ``` target    MFLSRRQFLKVSVGTVAAVAVADKVLALTALQPVIEVGNPLGDYPDRSWERVYHDQYRYDSSFTWVCSPNDTHACRVRAF 7b04.1    MKLTRRAFLQVAGATGATLTLAKNAMAFRLLKPAVVVDNPLDTYPDRRWESVYRDQYQYDRTFTYCCSPNDTHACRIRAF  target    VRNGVVMRVEQNYDHQTYEDLYGNRGTFAHNPRMCLKGFTFHRRVYGPYRLKGPLMRKGWKQWMDDNAPELTAETKRKYK 7b04.1    VRNNVMMRVEQNYDHQNYSDLYGNKATRNWNPRMCLKGYTFHRRVYGPYRLRYPLIRKGWKRWADDGFPELTPENKTKYM  target    FDSRFLDDMLRVSWDTAFTYAAKAMITIATRY-SGEAGARRLREQGYAPEMIEMMKGAGTRCFKHRAGMPVLGIIGKMGN 7b04.1    FDNRGNDELLRASWDEAFTYASKGIIHITKKYSGPEGAQK-LIDQGYPKEMVDRMQGAGTRTFKGRGGMGLLGVIGKYGM  target    TRMNGGINALLDTWIRKVSPDQAQGGRYWSNYTWHGDQNPAHPFWSGVQGSDIDLSDMRFSKLNTSWGKNFVENKMPEAH 7b04.1    YRFNNC-LAIVDAHNRGVGPDQALGGRNWSNYTWHGDQAPGHPFSHGLQTSDVDMNDVRFSKLLIQTGKNLIENKMPEAH  target    WKLECIERGARVVVITPEYNPTAYRADYWMPLRPESDGALFLGAMKIIIDENMHDIDFLKSFTDAPILVRTDTLQYLDPR 7b04.1    WVTEVMERGGKIVVITPEYSPSAQKADYWIPIRNNTDTALFLGITKILIDNKWYDADYVKKFTDFPLLIRTDTLKRVSPK  target    DVIADYKFPDFSKSYSGRIQSLKPEQIQRLGGMMVWDLNKKQVVPLHREQVGWHYTNSGIDAALTGTYRVKLLNGREIDA 7b04.1    DIIPNYKLQDISDGPSYHIQGLKDEQREIIGDFVVWDAKSKGPKAITRDDVGETLVKKGIDPVLEGSFKLKTIDGKEIEV  target    MPIWQMYMVHFQDYDLDTVHQITRTPKDLIVRWARDSGTIKPAAIHNGEGTCHYFHQTANARGAAMVLIITGNVGKFGTG 7b04.1    MTLLEMYKIHLRDYDIDSVVSMTNSPKDLIERLAKDIATIKPVAIHYGEGVNHYFHATLMNRSYYLPVMLTGNVGYFGSG  target    QHTWAGNYKAGTWTATPWSGAGLSVHTGEDPFNITLDPNAHGKEIKTRSYYYGEEVGYWNHGDTALIVNTPKYGRKVFTG 7b04.1    SHTWAGNYKAGNFQASKWSGPGFYGWVAEDVFKPNLDPYASAKDLNIKGRALDEEVAYWNHSERPLIVNTPKYGRKVFTG  target    KTHMPTPSKFRWVVNVNVVNNAKHHYDMVRNVDPNIECLITQDIEMTSDINHADIAFAANSWMEFTYPEMTVTVSNPWVQ 7b04.1    KTHMPSPTKVLWFTNVNLINNAKHVYQMLKNVNPNIEQIMSTDIEITGSIEYADFAFPANSWVEFQEFEITNSCSNPFIQ  target    IWK-GGIRPLYDTRNDLDTFAGVAAKLSDMTGDKRMRDYFAMVYQNRVDVYVQRMLDASSTFYGYSADVMLKSE---KGW 7b04.1    IWGKTGITPVYESKDDVKILAGMASKLGELLRDKRFEDNWKFAIEGRASVYINRLLDGSTTMKGYTCEDILNGKYGEPGV  target    MV-MVRTYPRHPFWEETNESKPMWTRSGRYENYRIEPEAIEYGENFISHREGPEATPYLPNAIFTTNPYVRPDDYGIPIT 7b04.1    AMLLFRTYPRHPFWEQVHESLPFYTPTGRLQAYNDEPEIIEYGENFIVHREGPEATPYLPNAIVSTNPYIRPDDYGIPEN  target    AQHHDDKTVRNIKLSWHEIKRHSNPLWEKGYQFYCVTPKTRHRVHSQWSVNDWVQIYESNFGDPYRMDKRTPGVGEHQIH 7b04.1    AEYWEDRTVRNIKKSWEETKKTKNFLWEKGYHFYCVTPKSRHTVHSQWAVTDWNFIWNNNFGDPYRMDKRMPGVGEHQIH  target    INPQAAKDRGINDGDYVYVDGNPVDRPYRGWKPSDPYYKVARLMIRAKYNPAYPYHVTMAKHAPFVATAKSVKGHETRPD 7b04.1    IHPQAARDLGIEDGDYVYVDANPADRPYEGWKPNDSFYKVSRLMLRAKYNPAYPYNCTMMKHSAWISSDKTVQAHETRP-  target    GRAIAIDTGYQSNFRYGAQQSFTRNWLMPMHQTDSLPGKHAVAWKFKWGYQVDHHAINTVPKECLIRITKAEDGGIGARG 7b04.1    DGRALSPSGYQSSFRYGSQQSITRDWSMPMHQLDSLFHKAKIGMKFIFGFEADNHCINTVPKETLVKITKAENGGMGGKG  target    PWEPVRTGFTPGQENEFMIKWLKGEHIKIKV 7b04.1    VWDPVKTGYTAGNENDFMKKFLNGELIKVD- ``` | | | | | | | | | | | | | | | | | | | | | | | | | | | | | | | | | | | | | | | | | | | | | | | | | |
|  | 7b04.2.B | Nitrite oxidoreductase subunit A  *Structure of Nitrite oxidoreductase (Nxr) from the anammox bacterium Kuenenia stuttgartiensis.* | 0.86 | 0.00 | 57.58 | 1.00 | 1-1144 | X-ray | 2.97 | monomer | 4 x SF4, 1 x F3S, 2 x MD1, 1 x MO, 1 x HEM, 2 x CA | HHblits | 0.48 |
| ``` target    MFLSRRQFLKVSVGTVAAVAVADKVLALTALQPVIEVGNPLGDYPDRSWERVYHDQYRYDSSFTWVCSPNDTHACRVRAF 7b04.2    MKLTRRAFLQVAGATGATLTLAKNAMAFRLLKPAVVVDNPLDTYPDRRWESVYRDQYQYDRTFTYCCSPNDTHACRIRAF  target    VRNGVVMRVEQNYDHQTYEDLYGNRGTFAHNPRMCLKGFTFHRRVYGPYRLKGPLMRKGWKQWMDDNAPELTAETKRKYK 7b04.2    VRNNVMMRVEQNYDHQNYSDLYGNKATRNWNPRMCLKGYTFHRRVYGPYRLRYPLIRKGWKRWADDGFPELTPENKTKYM  target    FDSRFLDDMLRVSWDTAFTYAAKAMITIATRY-SGEAGARRLREQGYAPEMIEMMKGAGTRCFKHRAGMPVLGIIGKMGN 7b04.2    FDNRGNDELLRASWDEAFTYASKGIIHITKKYSGPEGAQK-LIDQGYPKEMVDRMQGAGTRTFKGRGGMGLLGVIGKYGM  target    TRMNGGINALLDTWIRKVSPDQAQGGRYWSNYTWHGDQNPAHPFWSGVQGSDIDLSDMRFSKLNTSWGKNFVENKMPEAH 7b04.2    YRFNNC-LAIVDAHNRGVGPDQALGGRNWSNYTWHGDQAPGHPFSHGLQTSDVDMNDVRFSKLLIQTGKNLIENKMPEAH  target    WKLECIERGARVVVITPEYNPTAYRADYWMPLRPESDGALFLGAMKIIIDENMHDIDFLKSFTDAPILVRTDTLQYLDPR 7b04.2    WVTEVMERGGKIVVITPEYSPSAQKADYWIPIRNNTDTALFLGITKILIDNKWYDADYVKKFTDFPLLIRTDTLKRVSPK  target    DVIADYKFPDFSKSYSGRIQSLKPEQIQRLGGMMVWDLNKKQVVPLHREQVGWHYTNSGIDAALTGTYRVKLLNGREIDA 7b04.2    DIIPNYKLQDISDGPSYHIQGLKDEQREIIGDFVVWDAKSKGPKAITRDDVGETLVKKGIDPVLEGSFKLKTIDGKEIEV  target    MPIWQMYMVHFQDYDLDTVHQITRTPKDLIVRWARDSGTIKPAAIHNGEGTCHYFHQTANARGAAMVLIITGNVGKFGTG 7b04.2    MTLLEMYKIHLRDYDIDSVVSMTNSPKDLIERLAKDIATIKPVAIHYGEGVNHYFHATLMNRSYYLPVMLTGNVGYFGSG  target    QHTWAGNYKAGTWTATPWSGAGLSVHTGEDPFNITLDPNAHGKEIKTRSYYYGEEVGYWNHGDTALIVNTPKYGRKVFTG 7b04.2    SHTWAGNYKAGNFQASKWSGPGFYGWVAEDVFKPNLDPYASAKDLNIKGRALDEEVAYWNHSERPLIVNTPKYGRKVFTG  target    KTHMPTPSKFRWVVNVNVVNNAKHHYDMVRNVDPNIECLITQDIEMTSDINHADIAFAANSWMEFTYPEMTVTVSNPWVQ 7b04.2    KTHMPSPTKVLWFTNVNLINNAKHVYQMLKNVNPNIEQIMSTDIEITGSIEYADFAFPANSWVEFQEFEITNSCSNPFIQ  target    IWK-GGIRPLYDTRNDLDTFAGVAAKLSDMTGDKRMRDYFAMVYQNRVDVYVQRMLDASSTFYGYSADVMLKSE---KGW 7b04.2    IWGKTGITPVYESKDDVKILAGMASKLGELLRDKRFEDNWKFAIEGRASVYINRLLDGSTTMKGYTCEDILNGKYGEPGV  target    MV-MVRTYPRHPFWEETNESKPMWTRSGRYENYRIEPEAIEYGENFISHREGPEATPYLPNAIFTTNPYVRPDDYGIPIT 7b04.2    AMLLFRTYPRHPFWEQVHESLPFYTPTGRLQAYNDEPEIIEYGENFIVHREGPEATPYLPNAIVSTNPYIRPDDYGIPEN  target    AQHHDDKTVRNIKLSWHEIKRHSNPLWEKGYQFYCVTPKTRHRVHSQWSVNDWVQIYESNFGDPYRMDKRTPGVGEHQIH 7b04.2    AEYWEDRTVRNIKKSWEETKKTKNFLWEKGYHFYCVTPKSRHTVHSQWAVTDWNFIWNNNFGDPYRMDKRMPGVGEHQIH  target    INPQAAKDRGINDGDYVYVDGNPVDRPYRGWKPSDPYYKVARLMIRAKYNPAYPYHVTMAKHAPFVATAKSVKGHETRPD 7b04.2    IHPQAARDLGIEDGDYVYVDANPADRPYEGWKPNDSFYKVSRLMLRAKYNPAYPYNCTMMKHSAWISSDKTVQAHETRP-  target    GRAIAIDTGYQSNFRYGAQQSFTRNWLMPMHQTDSLPGKHAVAWKFKWGYQVDHHAINTVPKECLIRITKAEDGGIGARG 7b04.2    DGRALSPSGYQSSFRYGSQQSITRDWSMPMHQLDSLFHKAKIGMKFIFGFEADNHCINTVPKETLVKITKAENGGMGGKG  target    PWEPVRTGFTPGQENEFMIKWLKGEHIKIKV 7b04.2    VWDPVKTGYTAGNENDFMKKFLNGELIKVD- ``` | | | | | | | | | | | | | | | | | | | | | | | | | | | | | | | | | | | | | | | | | | | | | | | | | |
| ✓ | 3ir5.1.A | Respiratory nitrate reductase 1 alpha chain  *Crystal structure of NarGHI mutant NarG-H49C* | 0.41 | 0.00 | 20.97 | 0.81 | 2-1111 | X-ray | 2.30 | monomer | 2 x MD1, 1 x 6MO, 4 x SF4, 1 x AGA, 1 x F3S, 2 x HEM | HHblits | 0.31 |
| ``` target    MFLSRRQFLKVSVGTVAAVAVADKVLALTALQPVIEVGNPLGDYPDRSWERVYHDQYRYDSSFTWVCSPNDTHACRVRAF 3ir5.1    -FLDRFRYFKQKGETFADGH--------GQL-----------LNTNRDWEDGYRQRWQHDKIVRSTCGVNCTGSCSWKIY  target    VRNGVVMRVEQNYDHQTYEDLYGNRGTFAHNPRMCLKGFTFHRRVYGPYRLKGPLMRKGWK-QWMDDNA----P-----E 3ir5.1    VKNGLVTWETQQTDYPRT-----RPDLPNHEPRGCPRGASYSWYLYSANRLKYPMMRKRLMKMWREAKALHSDPVEAWAS  target    -LTAETKRKYKFDSRFLDDMLRVSWDTAFTYAAKAMITIATRYSGEAGARRLREQGYAPEMIEMMKGAGTRCF-KHRAGM 3ir5.1    IIEDADKAKSFKQARGRGGFVRSSWQEVNELIAASNVYTIKNYGPDRVAGF--------------SPIPAMSMVSYASGA  target    PVLGIIGKMGNTRMNGGINALLDTWIRKVSPDQAQGGRYWSNYTWHGDQNPAHPFWSGVQGSDIDLSDMRFSKLNTSWGK 3ir5.1    RYLS---LIGGT--------------------------CLSFYDWYCDLPPASPQTWGEQTDVPESADWYNSSYIIAWGS  target    NFVENKMPEAHWKLECIERGARVVVITPEYNPTAYRADYWMPLRPESDGALFLGAMKIIIDENM------HDIDFLKSFT 3ir5.1    NVPQTRTPDAHFFTEVRYKGTKTVAVTPDYAEIAKLCDLWLAPKQGTDAAMALAMGHVMLREFHLDNPSQYFTDYVRRYT  target    DAPILVRTD-------TLQYLDPRDVIADYKFPDFSKSYSGRIQSLKPEQIQRLGGMMVWDLNKKQVVPLHREQV----- 3ir5.1    DMPMLVMLEERDGYYAAGRMLRAADLVDALGQEN-----------------NPEWKTVAFNT-NGEMVAPNGSIGFRWGE  target    --GWHYT----------------------------------------NSGIDAALTG---TYRVKLLNGREIDAMPIWQM 3ir5.1    KGKWNLEQRDGKTGEETELQLSLLGSQDEIAEVGFPYFGGDGTEHFNKVELENVLLHKLPVKRLQLADGSTALVTTVYDL  target    YM------------------VHFQDYDLDTVHQITRTPKDLIVRWARDSGTI-----KPAAIHNGEGTCHYFHQTANARG 3ir5.1    TLANYGLERGLNDVNCATSYDDVKAYTPAWAEQITGVSRSQIIRIAREFADNADKTHGRSMIIVGAGLNHWYHLDMNYRG  target    AAMVLIITGNVGKFGTGQHTWAGNYKAGTWTATPWSGAGLSV----HT---GEDPF--------------N-ITLDPNAH 3ir5.1    LINMLIFCGCVGQSGGGWAHYVGQEKLRPQ--TGWQPLAFALDWQRPARHMNSTSYFYNHSSQWRYETVTAEELLSPMAD  target    GKEIKTR---SYYYGEEVGYWNHG----DTALI---------VNTPKY-GRKVFTGKT--------HMPTPSKFRWVVNV 3ir5.1    KSRYTGHLIDFNVRAERMGWLPSAPQLGTNPLTIAGEAEKAGMNPVDYTVKSLKEGSIRFAAEQPENGKNHPRNLFIWRS  target    NVVNNAKHHYD-MV------------------------------RNVDPNIECLITQDIEMTSDINHADIAFAANSWMEF 3ir5.1    NLLGSSGKGHEFMLKYLLGTEHGIQGKDLGQQGGVKPEEVDWQDNGLEGKLDLVVTLDFRLSSTCLYSDIILPTATWYEK  target    TYPEMTVTVSNPWVQIWKGGIRPLYDTRNDLDTFAGVAAKLSDMTGD----K--------------------RMRDYFAM 3ir5.1    DDM--NTSDMHPFIHPLSAAVDPAWEAKSDWEIYKAIAKKFSEVCVGHLGKETDIVTLPIQHDSAAELAQPLDVKDWKKG  target    V------------------YQNR---------------------------VDVYVQR----------------------- 3ir5.1    ECDLIPGKTAPHIMVVERDYPATYERFTSIGPLMEKIGNGGKGIAWNTQSEMDLLRKLNYTKAEGPAKGQPMLNTAIDAA  target    --MLDASSTFYGY----SADVMLKS------------EKGWMVMVR--------------------TYPRHPFWEETNES 3ir5.1    EMILTLAPETNGQVAVKAWAALSEFTGRDHTHLALNKEDEKIRFRDIQAQPRKIISSPTWSGLEDEHVSYNAGYTNVHEL  target    KPMWTRSGRYENYRIEPEAIEYGENFISHREGPEATPYLPNAIFTTNPYVRPDDYGIPITAQHHDDKTVRNIKLSWHEIK 3ir5.1    IPWRTLSGRQQLYQDHQWMRDFGESLLVYRPPIDTRSV-----------------------------------KEVIGQ-  target    RHSNPLWEKGYQFYCVTPKTRHRVHSQWSVNDWVQIYESNFGDPYRMDKRTPGVGEHQIHINPQAAKDRGINDGDYVYVD 3ir5.1    ---KSNGNQEKALNFLTPHQKWGIHSTYSDNLLMLTLG---------------RGGPVVWLSEADAKDLGIADNDWIEVF  target    GNPVDRPYRGWKPSDPYYKVARLMIRAKYNPAYPYHVTMAKHAPFVATAKSVKGHETRPDGRAIAIDTGYQSNFRYGAQQ 3ir5.1    NS-----------------NGALTARAVVSQRVPAGMTMMYHAQERIVNL--------P--GSE------ITQQRGGIHN  target    SFTRNWLMPMHQTDSLPGKHAVAWKFKWGYQVDHHAINTVPKECLIRITKAEDGGIGARGPWEPVRTGFTPGQENEFMIK 3ir5.1    SVTRITPKPTHMIGGYAHLA---------YGFNYYGTVGSNRDEFVVVRKMKNIDWL-----------------------  target    WLKGEHIKIKV 3ir5.1    ----------- ``` | | | | | | | | | | | | | | | | | | | | | | | | | | | | | | | | | | | | | | | | | | | | | | | | | |
|  | 3ir7.1.A | Respiratory nitrate reductase 1 alpha chain  *Crystal structure of NarGHI mutant NarG-R94S* | 0.41 | 0.00 | 20.86 | 0.81 | 2-1111 | X-ray | 2.50 | monomer | 2 x MD1, 4 x SF4, 1 x 6MO, 1 x AGA, 1 x F3S, 2 x HEM | HHblits | 0.31 |
| ``` target    MFLSRRQFLKVSVGTVAAVAVADKVLALTALQPVIEVGNPLGDYPDRSWERVYHDQYRYDSSFTWVCSPNDTHACRVRAF 3ir7.1    -FLDRFRYFKQKGETFADGHG--------QL-----------LNTNRDWEDGYRQRWQHDKIVRSTHGVNCTGSCSWKIY  target    VRNGVVMRVEQNYDHQTYEDLYGNRGTFAHNPRMCLKGFTFHRRVYGPYRLKGPLMRKGWK-QWMDDNA----PE----- 3ir7.1    VKNGLVTWETQQTDYPRT-----RPDLPNHEPRGCPSGASYSWYLYSANRLKYPMMRKRLMKMWREAKALHSDPVEAWAS  target    -LTAETKRKYKFDSRFLDDMLRVSWDTAFTYAAKAMITIATRYSGEAGARRLREQGYAPEMIEMMKGAGTRCF-KHRAGM 3ir7.1    IIEDADKAKSFKQARGRGGFVRSSWQEVNELIAASNVYTIKNYGPDRVAGF--------------SPIPAMSMVSYASGA  target    PVLGIIGKMGNTRMNGGINALLDTWIRKVSPDQAQGGRYWSNYTWHGDQNPAHPFWSGVQGSDIDLSDMRFSKLNTSWGK 3ir7.1    RYLS---LIGGT--------------------------CLSFYDWYCDLPPASPQTWGEQTDVPESADWYNSSYIIAWGS  target    NFVENKMPEAHWKLECIERGARVVVITPEYNPTAYRADYWMPLRPESDGALFLGAMKIIIDENM------HDIDFLKSFT 3ir7.1    NVPQTRTPDAHFFTEVRYKGTKTVAVTPDYAEIAKLCDLWLAPKQGTDAAMALAMGHVMLREFHLDNPSQYFTDYVRRYT  target    DAPILVRTDT-------LQYLDPRDVIADYKFPDFSKSYSGRIQSLKPEQIQRLGGMMVWDLNKKQVVPLHREQ------ 3ir7.1    DMPMLVMLEERDGYYAAGRMLRAADLVDALG-QE----------------NNPEWKTVAFNT-NGEMVAPNGSIGFRWGE  target    -VGWHYTN----------------S------------------------GIDAALTG---TYRVKLLNGREIDAMPIWQM 3ir7.1    KGKWNLEQRDGKTGEETELQLSLLGSQDEIAEVGFPYFGGDGTEHFNKVELENVLLHKLPVKRLQLADGSTALVTTVYDL  target    YM------------------VHFQDYDLDTVHQITRTPKDLIVRWARDSGTI-----KPAAIHNGEGTCHYFHQTANARG 3ir7.1    TLANYGLERGLNDVNCATSYDDVKAYTPAWAEQITGVSRSQIIRIAREFADNADKTHGRSMIIVGAGLNHWYHLDMNYRG  target    AAMVLIITGNVGKFGTGQHTWAGNYKAGTWTATPWSGAGLSV----HT---GEDPF--------------N-ITLDPNAH 3ir7.1    LINMLIFCGCVGQSGGGWAHYVGQEKLRPQ--TGWQPLAFALDWQRPARHMNSTSYFYNHSSQWRYETVTAEELLSPMAD  target    GKEIKTR---SYYYGEEVGYWNH----GDTALI---------VNTPKY-GRKVFTGKT--------HMPTPSKFRWVVNV 3ir7.1    KSRYTGHLIDFNVRAERMGWLPSAPQLGTNPLTIAGEAEKAGMNPVDYTVKSLKEGSIRFAAEQPENGKNHPRNLFIWRS  target    NVVNNAKHHYD-MV------------------------------RNVDPNIECLITQDIEMTSDINHADIAFAANSWMEF 3ir7.1    NLLGSSGKGHEFMLKYLLGTEHGIQGKDLGQQGGVKPEEVDWQDNGLEGKLDLVVTLDFRLSSTCLYSDIILPTATWYEK  target    TYPEMTVTVSNPWVQIWKGGIRPLYDTRNDLDTFAGVAAKLSDMTGD----K--------------------RMRDYFAM 3ir7.1    DDM--NTSDMHPFIHPLSAAVDPAWEAKSDWEIYKAIAKKFSEVCVGHLGKETDIVTLPIQHDSAAELAQPLDVKDWKKG  target    ------------------VYQN---------------------------RVDVYVQRM---------------------- 3ir7.1    ECDLIPGKTAPHIMVVERDYPATYERFTSIGPLMEKIGNGGKGIAWNTQSEMDLLRKLNYTKAEGPAKGQPMLNTAIDAA  target    ---LDASSTFYG----YSADVMLKS------------EKGWMVMV--------------------RTYPRHPFWEETNES 3ir7.1    EMILTLAPETNGQVAVKAWAALSEFTGRDHTHLALNKEDEKIRFRDIQAQPRKIISSPTWSGLEDEHVSYNAGYTNVHEL  target    KPMWTRSGRYENYRIEPEAIEYGENFISHREGPEATPYLPNAIFTTNPYVRPDDYGIPITAQHHDDKTVRNIKLSWHEIK 3ir7.1    IPWRTLSGRQQLYQDHQWMRDFGESLLVYRPPIDTRSV-------------------K----------------EVIG--  target    RHSNPLWEKGYQFYCVTPKTRHRVHSQWSVNDWVQIYESNFGDPYRMDKRTPGVGEHQIHINPQAAKDRGINDGDYVYVD 3ir7.1    --QKSNGNQEKALNFLTPHQKWGIHSTYSDNLLMLTLG---------------RGGPVVWLSEADAKDLGIADNDWIEVF  target    GNPVDRPYRGWKPSDPYYKVARLMIRAKYNPAYPYHVTMAKHAPFVATAKSVKGHETRPDGRAIAIDTGYQSNFRYGAQQ 3ir7.1    NS-----------------NGALTARAVVSQRVPAGMTMMYHAQERIVN----------------LPGSEITQQRGGIHN  target    SFTRNWLMPMHQTDSLPGKHAVAWKFKWGYQVDHHAINTVPKECLIRITKAEDGGIGARGPWEPVRTGFTPGQENEFMIK 3ir7.1    SVTRITPKPTHMIGGYAHLA---------YGFNYYGTVGSNRDEFVVVRKMKNIDWL-----------------------  target    WLKGEHIKIKV 3ir7.1    ----------- ``` | | | | | | | | | | | | | | | | | | | | | | | | | | | | | | | | | | | | | | | | | | | | | | | | | |
|  | 1q16.1.A | Respiratory nitrate reductase 1 alpha chain  *Crystal structure of Nitrate Reductase A, NarGHI, from Escherichia coli* | 0.40 | 0.00 | 20.78 | 0.81 | 2-1111 | X-ray | 1.90 | monomer | 2 x MD1, 1 x 6MO, 2 x HEM, 4 x SF4, 1 x F3S, 1 x AGA, 1 x 3PH | HHblits | 0.31 |
| ``` target    MFLSRRQFLKVSVGTVAAVAVADKVLALTALQPVIEVGNPLGDYPDRSWERVYHDQYRYDSSFTWVCSPNDTHACRVRAF 1q16.1    -FLDRFRYFKQKGETFADGHG--------QL-----------LNTNRDWEDGYRQRWQHDKIVRSTHGVNCTGSCSWKIY  target    VRNGVVMRVEQNYDHQTYEDLYGNRGTFAHNPRMCLKGFTFHRRVYGPYRLKGPLMRKGWK-QWMDDN----APE----- 1q16.1    VKNGLVTWETQQTDYPRT-----RPDLPNHEPRGCPRGASYSWYLYSANRLKYPMMRKRLMKMWREAKALHSDPVEAWAS  target    -LTAETKRKYKFDSRFLDDMLRVSWDTAFTYAAKAMITIATRYSGEAGARRLREQGYAPEMIEMMKGAGTRCF-KHRAGM 1q16.1    IIEDADKAKSFKQARGRGGFVRSSWQEVNELIAASNVYTIKNYGPDRVAGF--------------SPIPAMSMVSYASGA  target    PVLGIIGKMGNTRMNGGINALLDTWIRKVSPDQAQGGRYWSNYTWHGDQNPAHPFWSGVQGSDIDLSDMRFSKLNTSWGK 1q16.1    RYL---SLIGGT--------------------------CLSFYDWYCDLPPASPQTWGEQTDVPESADWYNSSYIIAWGS  target    NFVENKMPEAHWKLECIERGARVVVITPEYNPTAYRADYWMPLRPESDGALFLGAMKIIIDENM------HDIDFLKSFT 1q16.1    NVPQTRTPDAHFFTEVRYKGTKTVAVTPDYAEIAKLCDLWLAPKQGTDAAMALAMGHVMLREFHLDNPSQYFTDYVRRYT  target    DAPILVRTDT-------LQYLDPRDVIADYKFPDFSKSYSGRIQSLKPEQIQRLGGMMVWDLNKKQVVPLHREQV----- 1q16.1    DMPMLVMLEERDGYYAAGRMLRAADLVDALG-QE----------------NNPEWKTVAFNT-NGEMVAPNGSIGFRWGE  target    --GWHYTN----------------SGI-------D-------------AALT-------GTYRVKLLNGREIDAMPIWQM 1q16.1    KGKWNLEQRDGKTGEETELQLSLLGSQDEIAEVGFPYFGGDGTEHFNKVELENVLLHKLPVKRLQLADGSTALVTTVYDL  target    YM------------------VHFQDYDLDTVHQITRTPKDLIVRWARDSGTI-----KPAAIHNGEGTCHYFHQTANARG 1q16.1    TLANYGLERGLNDVNCATSYDDVKAYTPAWAEQITGVSRSQIIRIAREFADNADKTHGRSMIIVGAGLNHWYHLDMNYRG  target    AAMVLIITGNVGKFGTGQHTWAGNYKA-GTWTATPWSGAGLSVH----T---GED--------PF------N-ITLDPNA 1q16.1    LINMLIFCGCVGQSGGGWAHYVGQEKLRPQT---GWQPLAFALDWQRPARHMNSTSYFYNHSSQWRYETVTAEELLSPMA  target    HGKEIKTRS--Y-YYGEEVGYWNH----GDTALI---------VNTPKY-GRKVFTGKT--------HMPTPSKFRWVVN 1q16.1    DKSRYTGHLIDFNVRAERMGWLPSAPQLGTNPLTIAGEAEKAGMNPVDYTVKSLKEGSIRFAAEQPENGKNHPRNLFIWR  target    VNVVNNAKHHYD-MV------------------------------RNVDPNIECLITQDIEMTSDINHADIAFAANSWME 1q16.1    SNLLGSSGKGHEFMLKYLLGTEHGIQGKDLGQQGGVKPEEVDWQDNGLEGKLDLVVTLDFRLSSTCLYSDIILPTATWYE  target    FTYPEMTVTVSNPWVQIWKGGIRPLYDTRNDLDTFAGVAAKLSDMTGD----K--------------------RMRDYFA 1q16.1    KDDM--NTSDMHPFIHPLSAAVDPAWEAKSDWEIYKAIAKKFSEVCVGHLGKETDIVTLPIQHDSAAELAQPLDVKDWKK  target    MV------------------YQN---------------------------RVDVYVQR---------------------- 1q16.1    GECDLIPGKTAPHIMVVERDYPATYERFTSIGPLMEKIGNGGKGIAWNTQSEMDLLRKLNYTKAEGPAKGQPMLNTAIDA  target    ---MLDASSTFYGY----SADVMLKS------------EKGWMVMV--------------------RTYPRHPFWEETNE 1q16.1    AEMILTLAPETNGQVAVKAWAALSEFTGRDHTHLALNKEDEKIRFRDIQAQPRKIISSPTWSGLEDEHVSYNAGYTNVHE  target    SKPMWTRSGRYENYRIEPEAIEYGENFISHREGPEATPYLPNAIFTTNPYVRPDDYGIPITAQHHDDKTVRNIKLSWHEI 1q16.1    LIPWRTLSGRQQLYQDHQWMRDFGESLLVYRPPIDTRSV-------------------K----------------EVIG-  target    KRHSNPLWEKGYQFYCVTPKTRHRVHSQWSVNDWVQIYESNFGDPYRMDKRTPGVGEHQIHINPQAAKDRGINDGDYVYV 1q16.1    ---QKSNGNQEKALNFLTPHQKWGIHSTYSDNLLMLTLG---------------RGGPVVWLSEADAKDLGIADNDWIEV  target    DGNPVDRPYRGWKPSDPYYKVARLMIRAKYNPAYPYHVTMAKHAPFVATAKSVKGHETRPDGRAIAIDTGYQSNFRYGAQ 1q16.1    FNS-----------------NGALTARAVVSQRVPAGMTMMYHAQERIVNL--------P--G------SEITQQRGGIH  target    QSFTRNWLMPMHQTDSLPGKHAVAWKFKWGYQVDHHAINTVPKECLIRITKAEDGGIGARGPWEPVRTGFTPGQENEFMI 1q16.1    NSVTRITPKPTHMIGGYAHLA---------YGFNYYGTVGSNRDEFVVVRKMKNIDWL----------------------  target    KWLKGEHIKIKV 1q16.1    ------------ ``` | | | | | | | | | | | | | | | | | | | | | | | | | | | | | | | | | | | | | | | | | | | | | | | | | |
| ✓ | 1r27.4.A | Respiratory nitrate reductase 1 alpha chain  *Crystal Structure of NarGH complex* | 0.41 | 0.25 | 20.78 | 0.81 | 2-1111 | X-ray | 2.00 | homo-dimer | 4 x MO, 16 x SF4, 8 x MGD, 4 x F3S | HHblits | 0.31 |
| ``` target    MFLSRRQFLKVSVGTVAAVAVADKVLALTALQPVIEVGNPLGDYPDRSWERVYHDQYRYDSSFTWVCSPNDTHACRVRAF 1r27.4    -FLDRFRYFKQKGETFADGHG--------QL-----------LNTNRDWEDGYRQRWQHDKIVRSTHGVNCTGSCSWKIY  target    VRNGVVMRVEQNYDHQTYEDLYGNRGTFAHNPRMCLKGFTFHRRVYGPYRLKGPLMRKGWK-QWMDDN----APEL---- 1r27.4    VKNGLVTWETQQTDYPRT-----RPDLPNHEPRGCPRGASYSWYLYSANRLKYPMMRKRLMKMWREAKALHSDPVEAWAS  target    --TAETKRKYKFDSRFLDDMLRVSWDTAFTYAAKAMITIATRYSGEAGARRLREQGYAPEMIEMMKGAGTRCF-KHRAGM 1r27.4    IIEDADKAKSFKQARGRGGFVRSSWQEVNELIAASNVYTIKNYGPDRVAGFS--------------PIPAMSMVSYASGA  target    PVLGIIGKMGNTRMNGGINALLDTWIRKVSPDQAQGGRYWSNYTWHGDQNPAHPFWSGVQGSDIDLSDMRFSKLNTSWGK 1r27.4    RYL---SLIGGT--------------------------CLSFYDWYCDLPPASPQTWGEQTDVPESADWYNSSYIIAWGS  target    NFVENKMPEAHWKLECIERGARVVVITPEYNPTAYRADYWMPLRPESDGALFLGAMKIIIDENM------HDIDFLKSFT 1r27.4    NVPQTRTPDAHFFTEVRYKGTKTVAVTPDYAEIAKLCDLWLAPKQGTDAAMALAMGHVMLREFHLDNPSQYFTDYVRRYT  target    DAPILVRTDT-------LQYLDPRDVIADYKFPDFSKSYSGRIQSLKPEQIQRLGGMMVWDLNKKQVVPLHREQV----- 1r27.4    DMPMLVMLEERDGYYAAGRMLRAADLVDALGQEN-----------------NPEWKTVAFNT-NGEMVAPNGSIGFRWGE  target    --GWHYTN----------------SGI-------D-------------AALT-------GTYRVKLLNGREIDAMPIWQM 1r27.4    KGKWNLEQRDGKTGEETELQLSLLGSQDEIAEVGFPYFGGDGTEHFNKVELENVLLHKLPVKRLQLADGSTALVTTVYDL  target    YM------------------VHFQDYDLDTVHQITRTPKDLIVRWARDSGTI-----KPAAIHNGEGTCHYFHQTANARG 1r27.4    TLANYGLERGLNDVNCATSYDDVKAYTPAWAEQITGVSRSQIIRIAREFADNADKTHGRSMIIVGAGLNHWYHLDMNYRG  target    AAMVLIITGNVGKFGTGQHTWAGNYKA-GTWTATPWSGAGLSV----HT---GEDPF--------------N-ITLDPNA 1r27.4    LINMLIFCGCVGQSGGGWAHYVGQEKLRPQT---GWQPLAFALDWQRPARHMNSTSYFYNHSSQWRYETVTAEELLSPMA  target    HGKEIKTR---SYYYGEEVGYWNH----GDTALI---------VNTPKYG-RKVFTGKT--------HMPTPSKFRWVVN 1r27.4    DKSRYTGHLIDFNVRAERMGWLPSAPQLGTNPLTIAGEAEKAGMNPVDYTVKSLKEGSIRFAAEQPENGKNHPRNLFIWR  target    VNVVNNAKHHYD-MV------------------------------RNVDPNIECLITQDIEMTSDINHADIAFAANSWME 1r27.4    SNLLGSSGKGHEFMLKYLLGTEHGIQGKDLGQQGGVKPEEVDWQDNGLEGKLDLVVTLDFRLSSTCLYSDIILPTATWYE  target    FTYPEMTVTVSNPWVQIWKGGIRPLYDTRNDLDTFAGVAAKLSDMTGD----K--------------------RMRDYFA 1r27.4    KDDM--NTSDMHPFIHPLSAAVDPAWEAKSDWEIYKAIAKKFSEVCVGHLGKETDIVTLPIQHDSAAELAQPLDVKDWKK  target    MV------------------YQN---------------------------RVDVYVQR---------------------- 1r27.4    GECDLIPGKTAPHIMVVERDYPATYERFTSIGPLMEKIGNGGKGIAWNTQSEMDLLRKLNYTKAEGPAKGQPMLNTAIDA  target    ---MLDASSTFYGY----SADVMLKSE------------KGWMVMV--------------------RTYPRHPFWEETNE 1r27.4    AEMILTLAPETNGQVAVKAWAALSEFTGRDHTHLALNKEDEKIRFRDIQAQPRKIISSPTWSGLEDEHVSYNAGYTNVHE  target    SKPMWTRSGRYENYRIEPEAIEYGENFISHREGPEATPYLPNAIFTTNPYVRPDDYGIPITAQHHDDKTVRNIKLSWHEI 1r27.4    LIPWRTLSGRQQLYQDHQWMRDFGESLLVYRPPIDTRSV-------------KEVIG-----------------------  target    KRHSNPLWEKGYQFYCVTPKTRHRVHSQWSVNDWVQIYESNFGDPYRMDKRTPGVGEHQIHINPQAAKDRGINDGDYVYV 1r27.4    ---QKSNGNQEKALNFLTPHQKWGIHSTYSDNLLMLTLG---------------RGGPVVWLSEADAKDLGIADNDWIEV  target    DGNPVDRPYRGWKPSDPYYKVARLMIRAKYNPAYPYHVTMAKHAPFVATAKSVKGHETRPDGRAIAIDTGYQSNFRYGAQ 1r27.4    FNS-----------------NGALTARAVVSQRVPAGMTMMYHAQERIVNL--------P--------GSEITQQRGGIH  target    QSFTRNWLMPMHQTDSLPGKHAVAWKFKWGYQVDHHAINTVPKECLIRITKAEDGGIGARGPWEPVRTGFTPGQENEFMI 1r27.4    NSVTRITPKPTHMIGGYAHLA---------YGFNYYGTVGSNRDEFVVVRKMKNIDWL----------------------  target    KWLKGEHIKIKV 1r27.4    ------------ ``` | | | | | | | | | | | | | | | | | | | | | | | | | | | | | | | | | | | | | | | | | | | | | | | | | |
|  | 3ir6.1.A | Respiratory nitrate reductase 1 alpha chain  *Crystal structure of NarGHI mutant NarG-H49S* | 0.39 | 0.00 | 20.85 | 0.80 | 2-1108 | X-ray | 2.80 | monomer | 2 x GDP, 1 x AGA, 3 x SF4, 1 x F3S, 2 x HEM | HHblits | 0.31 |
| ``` target    MFLSRRQFLKVSVGTVAAVAVADKVLALTALQPVIEVGNPLGDYPDRSWERVYHDQYRYDSSFTWVCSPNDTHACRVRAF 3ir6.1    -FLDRFRYFKQKGETFADGHG--------QL-----------LNTNRDWEDGYRQRWQHDKIVRSTSGVNCTGSCSWKIY  target    VRNGVVMRVEQNYDHQTYEDLYGNRGTFAHNPRMCLKGFTFHRRVYGPYRLKGPLMRKGWK-QWMDDN----APE----- 3ir6.1    VKNGLVTWETQQTDYPRT-----RPDLPNHEPRGCPRGASYSWYLYSANRLKYPMMRKRLMKMWREAKALHSDPVEAWAS  target    -LTAETKRKYKFDSRFLDDMLRVSWDTAFTYAAKAMITIATRYSGEAGARRLREQGYAPEMIEMMKGAGTRCF-KHRAGM 3ir6.1    IIEDADKAKSFKQARGRGGFVRSSWQEVNELIAASNVYTIKNYGPDRVAGF--------------SPIPAMSMVSYASGA  target    PVLGIIGKMGNTRMNGGINALLDTWIRKVSPDQAQGGRYWSNYTWHGDQNPAHPFWSGVQGSDIDLSDMRFSKLNTSWGK 3ir6.1    RYL---SLIGGTC--------------------------LSFYDWYCDLPPASPQTWGEQTDVPESADWYNSSYIIAWGS  target    NFVENKMPEAHWKLECIERGARVVVITPEYNPTAYRADYWMPLRPESDGALFLGAMKIIIDENM------HDIDFLKSFT 3ir6.1    NVPQTRTPDAHFFTEVRYKGTKTVAVTPDYAEIAKLCDLWLAPKQGTDAAMALAMGHVMLREFHLDNPSQYFTDYVRRYT  target    DAPILVRTD-------TLQYLDPRDVIADYKFPDFSKSYSGRIQSLKPEQIQRLGGMMVWDLNKKQVVPLHREQV----- 3ir6.1    DMPMLVMLEERDGYYAAGRMLRAADLVDALG-QE----------------NNPEWKTVAFNT-NGEMVAPNGSIGFRWGE  target    --GWHYT----------------NSGI-------DA-------------ALT----G---TYRVKLLNGREIDAMPIWQM 3ir6.1    KGKWNLEQRDGKTGEETELQLSLLGSQDEIAEVGFPYFGGDGTEHFNKVELENVLLHKLPVKRLQLADGSTALVTTVYDL  target    YM------------------VHFQDYDLDTVHQITRTPKDLIVRWARDSGTI-----KPAAIHNGEGTCHYFHQTANARG 3ir6.1    TLANYGLERGLNDVNCATSYDDVKAYTPAWAEQITGVSRSQIIRIAREFADNADKTHGRSMIIVGAGLNHWYHLDMNYRG  target    AAMVLIITGNVGKFGTGQHTWAGNYKA-GTWTATPWSGAGLSV----HT---GEDPF--------------N-ITLDPNA 3ir6.1    LINMLIFCGCVGQSGGGWAHYVGQEKLRPQT---GWQPLAFALDWQRPARHMNSTSYFYNHSSQWRYETVTAEELLSPMA  target    HGKEIKTRS---YYYGEEVGYWNH----GDTALI---------VNTPKY-GRKVFTGKT--------HMPTPSKFRWVVN 3ir6.1    DKSRYTGHLIDFNVRAERMGWLPSAPQLGTNPLTIAGEAEKAGMNPVDYTVKSLKEGSIRFAAEQPENGKNHPRNLFIWR  target    VNVVNNAKHHYD-M------------------------------VRNVDPNIECLITQDIEMTSDINHADIAFAANSWME 3ir6.1    SNLLGSSGKGHEFMLKYLLGTEHGIQGKDLGQQGGVKPEEVDWQDNGLEGKLDLVVTLDFRLSSTCLYSDIILPTATWYE  target    FTYPEMTVTVSNPWVQIWKGGIRPLYDTRNDLDTFAGVAAKLSDMTGD----K--------------------RMRDYFA 3ir6.1    KDDM--NTSDMHPFIHPLSAAVDPAWEAKSDWEIYKAIAKKFSEVCVGHLGKETDIVTLPIQHDSAAELAQPLDVKDWKK  target    MV------------------YQN---------------------------RVDVYVQRM--------------------- 3ir6.1    GECDLIPGKTAPHIMVVERDYPATYERFTSIGPLMEKIGNGGKGIAWNTQSEMDLLRKLNYTKAEGPAKGQPMLNTAIDA  target    ----LDASSTFYGY----SADVMLKS------------EKGWMVMV--------------------RTYPRHPFWEETNE 3ir6.1    AEMILTLAPETNGQVAVKAWAALSEFTGRDHTHLALNKEDEKIRFRDIQAQPRKIISSPTWSGLEDEHVSYNAGYTNVHE  target    SKPMWTRSGRYENYRIEPEAIEYGENFISHREGPEATPYLPNAIFTTNPYVRPDDYGIPITAQHHDDKTVRNIKLSWHEI 3ir6.1    LIPWRTLSGRQQLYQDHQWMRDFGESLLVYRPPIDTRSV-------------K----------------------EVIG-  target    KRHSNPLWEKGYQFYCVTPKTRHRVHSQWSVNDWVQIYESNFGDPYRMDKRTPGVGEHQIHINPQAAKDRGINDGDYVYV 3ir6.1    ---QKSNGNQEKALNFLTPHQKWGIHSTYSDNLLMLTLG---------------RGGPVVWLSEADAKDLGIADNDWIEV  target    DGNPVDRPYRGWKPSDPYYKVARLMIRAKYNPAYPYHVTMAKHAPFVATAKSVKGHETRPDGRAIAIDTGYQSNFRYGAQ 3ir6.1    FNS-----------------NGALTARAVVSQRVPAGMTMMYHAQERIVNL--------P--G------SEITQQRGGIH  target    QSFTRNWLMPMHQTDSLPGKHAVAWKFKWGYQVDHHAINTVPKECLIRITKAEDGGIGARGPWEPVRTGFTPGQENEFMI 3ir6.1    NSVTRITPKPTHMIGGYAHLA---------YGFNYYGTVGSNRDEFVVVRKMKNI-------------------------  target    KWLKGEHIKIKV 3ir6.1    ------------ ``` | | | | | | | | | | | | | | | | | | | | | | | | | | | | | | | | | | | | | | | | | | | | | | | | | |
|  | 3egw.1.A | Respiratory nitrate reductase 1 alpha chain  *The crystal structure of the NarGHI mutant NarH - C16A* | 0.41 | 0.17 | 20.85 | 0.78 | 45-1108 | X-ray | 1.90 | homo-dimer | 2 x MD1, 2 x MGD, 2 x 6MO, 6 x SF4, 4 x F3S, 2 x 3PH, 4 x HEM, 2 x AGA | HHblits | 0.31 |
| ``` target    MFLSRRQFLKVSVGTVAAVAVADKVLALTALQPVIEVGNPLGDYPDRSWERVYHDQYRYDSSFTWVCSPNDTHACRVRAF 3egw.1    --------------------------------------------TNRDWEDGYRQRWQHDKIVRSTHGVNCTGSCSWKIY  target    VRNGVVMRVEQNYDHQTYEDLYGNRGTFAHNPRMCLKGFTFHRRVYGPYRLKGPLMRKGWK-QWMDDNA----PE----- 3egw.1    VKNGLVTWETQQTDYPRT-----RPDLPNHEPRGCPRGASYSWYLYSANRLKYPMMRKRLMKMWREAKALHSDPVEAWAS  target    -LTAETKRKYKFDSRFLDDMLRVSWDTAFTYAAKAMITIATRYSGEAGARRLREQGYAPEMIEMMKGAGTRCF-KHRAGM 3egw.1    IIEDADKAKSFKQARGRGGFVRSSWQEVNELIAASNVYTIKNYGPDRVAGF--------------SPIPAMSMVSYASGA  target    PVLGIIGKMGNTRMNGGINALLDTWIRKVSPDQAQGGRYWSNYTWHGDQNPAHPFWSGVQGSDIDLSDMRFSKLNTSWGK 3egw.1    RYL---SLIGGTC--------------------------LSFYDWYCDLPPASPQTWGEQTDVPESADWYNSSYIIAWGS  target    NFVENKMPEAHWKLECIERGARVVVITPEYNPTAYRADYWMPLRPESDGALFLGAMKIIIDENM------HDIDFLKSFT 3egw.1    NVPQTRTPDAHFFTEVRYKGTKTVAVTPDYAEIAKLCDLWLAPKQGTDAAMALAMGHVMLREFHLDNPSQYFTDYVRRYT  target    DAPILVRTDT-------LQYLDPRDVIADYKFPDFSKSYSGRIQSLKPEQIQRLGGMMVWDLNKKQVVPLHREQV----- 3egw.1    DMPMLVMLEERDGYYAAGRMLRAADLVAALGQE-----------------NNPEWKTVAFNT-NGEMVAPNGSIGFRWGE  target    --GWHY----------------------------------------TNSGIDAALTG---TYRVKLLNGREIDAMPIWQM 3egw.1    KGKWNLEQRDGKTGEETELQLSLLGSQDEIAEVGFPYFGGDGTEHFNKVELENVLLHKLPVKRLQLADGSTALVTTVYDL  target    YM------------------VHFQDYDLDTVHQITRTPKDLIVRWARDSGTI-----KPAAIHNGEGTCHYFHQTANARG 3egw.1    TLANYGLERGLNDVNCATSYDDVKAYTPAWAEQITGVSRSQIIRIAREFADNADKTHGRSMIIVGAGLNHWYHLDMNYRG  target    AAMVLIITGNVGKFGTGQHTWAGNYKA-GTWTATPWSGAGLSV----HT---GEDPF--------------N-ITLDPNA 3egw.1    LINMLIFCGCVGQSGGGWAHYVGQEKLRPQT---GWQPLAFALDWQRPARHMNSTSYFYNHSSQWRYETVTAEELLSPMA  target    HGKEIKTR---SYYYGEEVGYWNHG----DTALI---------VNTPKY-GRKVFTGKT--------HMPTPSKFRWVVN 3egw.1    DKSRYTGHLIDFNVRAERMGWLPSAPQLGTNPLTIAGEAEKAGMNPVDYTVKSLKEGSIRFAAEQPENGKNHPRNLFIWR  target    VNVVNNAKHHYD-M------------------------------VRNVDPNIECLITQDIEMTSDINHADIAFAANSWME 3egw.1    SNLLGSSGKGHEFMLKYLLGTEHGIQGKDLGQQGGVKPEEVDWQDNGLEGKLDLVVTLDFRLSSTCLYSDIILPTATWYE  target    FTYPEMTVTVSNPWVQIWKGGIRPLYDTRNDLDTFAGVAAKLSDMTGD---K---------------------RMRDYFA 3egw.1    KDDM--NTSDMHPFIHPLSAAVDPAWEAKSDWEIYKAIAKKFSEVCVGHLGKETDIVTLPIQHDSAAELAQPLDVKDWKK  target    MV------------------YQN---------------------------RVDVYVQRM--------------------- 3egw.1    GECDLIPGKTAPHIMVVERDYPATYERFTSIGPLMEKIGNGGKGIAWNTQSEMDLLRKLNYTKAEGPAKGQPMLNTAIDA  target    ----LDASSTFYGY----SADVMLKS------------EKGWMVMVR--------------------TYPRHPFWEETNE 3egw.1    AEMILTLAPETNGQVAVKAWAALSEFTGRDHTHLALNKEDEKIRFRDIQAQPRKIISSPTWSGLEDEHVSYNAGYTNVHE  target    SKPMWTRSGRYENYRIEPEAIEYGENFISHREGPEATPYLPNAIFTTNPYVRPDDYGIPITAQHHDDKTVRNIKLSWHEI 3egw.1    LIPWRTLSGRQQLYQDHQWMRDFGESLLVYRPPIDTRSV-------------------K----------------EVIG-  target    KRHSNPLWEKGYQFYCVTPKTRHRVHSQWSVNDWVQIYESNFGDPYRMDKRTPGVGEHQIHINPQAAKDRGINDGDYVYV 3egw.1    ---QKSNGNQEKALNFLTPHQKWGIHSTYSDNLLMLTLG---------------RGGPVVWLSEADAKDLGIADNDWIEV  target    DGNPVDRPYRGWKPSDPYYKVARLMIRAKYNPAYPYHVTMAKHAPFVATAKSVKGHETRPDGRAIAIDTGYQSNFRYGAQ 3egw.1    FNS-----------------NGALTARAVVSQRVPAGMTMMYHAQERIVNL--------P--------GSEITQQRGGIH  target    QSFTRNWLMPMHQTDSLPGKHAVAWKFKWGYQVDHHAINTVPKECLIRITKAEDGGIGARGPWEPVRTGFTPGQENEFMI 3egw.1    NSVTRITPKPTHMIGGYAHLA---------YGFNYYGTVGSNRDEFVVVRKMKNI-------------------------  target    KWLKGEHIKIKV 3egw.1    ------------ ``` | | | | | | | | | | | | | | | | | | | | | | | | | | | | | | | | | | | | | | | | | | | | | | | | | |
|  | 5e7o.1.A | DMSO reductase family type II enzyme, molybdopterin subunit  *Crystal structure of the perchlorate reductase PcrAB mutant W461E of PcrA from Azospira suillum PS* | 0.43 |  | 23.23 | 0.73 | 44-1108 | X-ray | 2.40 | hetero-oligomer | 4 x SF4, 1 x MO, 1 x MGD, 1 x MD1, 1 x F3S | HHblits | 0.32 |
| ``` target    MFLSRRQFLKVSVGTVAAVAVADKVLALTALQPVIEVGNPLGDYPDRSWERVYHDQYRYDSSFTWVCSPNDTHACRVRAF 5e7o.1    -------------------------------------------FEYSGWENFHRTQWSWDKKTRGAHLVNCTGACPHFVY  target    VRNGVVMRVEQNYDHQTYEDLYGNRGTFAHNPRMCLKGFTFHRRVYGPYRLKGPLMRKGWKQWMDDNAPELTAETKRKYK 5e7o.1    SKDGVVMREEQSKDIAP------MPNIPEYNPRGCNKGECGHDYMYGPHRIKYPLIRVGER-------------------  target    FDSRFLDDMLRVSWDTAFTYAAKAMITIATRYSGEAGARRLREQGYAPEMIEMMKGAGTRCFKHRAGMPVLGIIGKMGNT 5e7o.1    ----GEGKWRRATWEEALDMIADKCVDTIKNHAPDCISVYSP----VP---------AVSPVSFSAGHRFAH---YIGAH  target    RMNGGINALLDTWIRKVSPDQAQGGRYWSNYTWHGDQNPAHPFWSGVQGSDIDLSDMRFSKLNTSWGKNFVENKMPEAHW 5e7o.1    A--------------------------HTFYDWYGDHPTGQTQTCGVQGDTCETADWFNSKYIILWGSNPTQTRIPDAHF  target    KLECIERGARVVVITPEYNPTAYRADYWMPLRPESDGALFLGAMKIIIDENMHDIDFLKSFTDAPILVRTDTLQYLDPRD 5e7o.1    LSEAQLNGAKIVSISPDYNSSTIKVDKWIHPQPGTDGALAMAMAHVIIKEKLYDAHSLKEQTDLSYLVRSDTKRFLREAD  target    VIADYKFPDFSKSYSGRIQSLKPEQIQRLGGMMVWDLNKKQVVPLHREQVG------------------W---HYTNSGI 5e7o.1    VVAGG----------------------SKDKFYFWNAKTGKPVIPKGSWGDQPEKKGSPVGFLGRNTFAFPKGYIDLGDL  target    DAALTGTYRVKLLNGREIDAMPIWQMYMVHF-QDYDLDTVHQITRTPKDLIVRWARDSGTIKPAAIHNGEGTCHYFHQTA 5e7o.1    DPALEGKFNMQLLDGKTVEVRPVFEILKSRLMADNTPEKAAKITGVTAKAITELAREFATAKPSMIICGGGTQHWYYSDV  target    NARGAAMVLIITGNVGKFGTGQHTWAGNYKAGTWTATPWSGAGLSVHTGEDPFNITLDPNAHGKEIKTRSYYYGEEV-GY 5e7o.1    LLRAMHLLTALTGTEGTNGGGMNHYIGQEKPA-FVA-GLV---ALAFPEG------VNKQ-RFCQTTIWTYIHAEVNDEI  target    WNHGDTALIVNTPK-YGRKVFTG----KTHMPTPSKFRWVVNVNVVNNAKHHYDMVRNVDPNIECLITQDIEMTSDINHA 5e7o.1    ISSD-----IDTEKYLRDSITTGQMPNMPEQGRDPKVFFVYRGNWLNQAKGQKYVLENLWPKLELIVDINIRMDSTALYS  target    DIAFAANSWMEFTYPEMTVTVSNPWVQIWKGGIRPLYDTRNDLDTFAGVAAKLSDMTGDKRMR-------DYFAMV---Y 5e7o.1    DVVLPSAHWYEKLD--LNVTSEHSYINMTEPAIKPMWESKTDWQIFLALAKRVEMAAKRKKYEKFNDEKFKWVRDLSNLW  target    --------QNRVDVYVQRMLDASSTFYGYSADVMLKSEKG----WMVMVR-TYPRHPFWEETNESKPMWTRSGRYENYRI 5e7o.1    NQMTMDGKLAEDEAAAQYILDNAPQSKGITIQMLREKPQRFKSNWTSPLKEGVPYTPFQYFVVDKKPWPTLTGRQQFYLD  target    EPEAIEYGENFISHREGPEATPYLPNAIFTTNPYVRPDDYGIPITAQHHDDKTVRNIKLSWHEIKRHSNPLWEKGYQFYC 5e7o.1    HDTFFDMGVEL-----------------------------------------------PTYKAP------IDADKYPFRF  target    VTPKTRHRVHSQWSVNDWVQIYESNFGDPYRMDKRTPGVGEHQIHINPQAAKDRGINDGDYVYVDGNPVDRPYRGWKPSD 5e7o.1    NSPHSRHSVHSTFKDNVLMLRL---------------QRGGPSIEMSPLDAKPLGIKDNDWVEAWNN-------------  target    PYYKVARLMIRAKYNPAYPYHVTMAKHAPFVATAKSVKGHETRPDGRAIAIDTGYQSNFRYGAQQSFTRNWLMPMHQTDS 5e7o.1    ----HGKVICRVKIRNGEQRGRVSMWHCPELYMD------------------------LLTGGSQSVCPVRINPTNLVGN  target    LPGKHAVAWKFKWGYQVDHHAINTVPKECLIRITKAEDGGIGARGPWEPVRTGFTPGQENEFMIKWLKGEHIKIKV 5e7o.1    YGHL---------FFRPNYYGPAGSQRDVRVNVKRYIGA------------------------------------- ``` | | | | | | | | | | | | | | | | | | | | | | | | | | | | | | | | | | | | | | | | | | | | | | | | | |
|  | 4ydd.1.A | DMSO reductase family type II enzyme, molybdopterin subunit  *Crystal structure of the perchlorate reductase PcrAB from Azospira suillum PS* | 0.43 |  | 23.38 | 0.73 | 44-1108 | X-ray | 1.86 | hetero-oligomer | 4 x SF4, 1 x MO, 1 x MGD, 1 x MD1, 1 x F3S | HHblits | 0.32 |
| ``` target    MFLSRRQFLKVSVGTVAAVAVADKVLALTALQPVIEVGNPLGDYPDRSWERVYHDQYRYDSSFTWVCSPNDTHACRVRAF 4ydd.1    -------------------------------------------FEYSGWENFHRTQWSWDKKTRGAHLVNCTGACPHFVY  target    VRNGVVMRVEQNYDHQTYEDLYGNRGTFAHNPRMCLKGFTFHRRVYGPYRLKGPLMRKGWKQWMDDNAPELTAETKRKYK 4ydd.1    SKDGVVMREEQSKDIAP------MPNIPEYNPRGCNKGECGHDYMYGPHRIKYPLIRVGER-------------------  target    FDSRFLDDMLRVSWDTAFTYAAKAMITIATRYSGEAGARRLREQGYAPEMIEMMKGAG-TRCFKHRAGMPVLGIIGKMGN 4ydd.1    ----GEGKWRRATWEEALDMIADKCVDTIKNHAPDCISVY--------------SPVPAVSPVSFSAGHRFAH---YIGA  target    TRMNGGINALLDTWIRKVSPDQAQGGRYWSNYTWHGDQNPAHPFWSGVQGSDIDLSDMRFSKLNTSWGKNFVENKMPEAH 4ydd.1    HAH--------------------------TFYDWYGDHPTGQTQTCGVQGDTCETADWFNSKYIILWGSNPTQTRIPDAH  target    WKLECIERGARVVVITPEYNPTAYRADYWMPLRPESDGALFLGAMKIIIDENMHDIDFLKSFTDAPILVRTDTLQYLDPR 4ydd.1    FLSEAQLNGAKIVSISPDYNSSTIKVDKWIHPQPGTDGALAMAMAHVIIKEKLYDAHSLKEQTDLSYLVRSDTKRFLREA  target    DVIADYKFPDFSKSYSGRIQSLKPEQIQRLGGMMVWDLNKKQVVPLHREQVGW---------------------HYTNSG 4ydd.1    DVVAGG----------------------SKDKFYFWNAKTGKPVIPKGSWGDQPEKKGSPVGFLGRNTFAFPKGYIDLGD  target    IDAALTGTYRVKLLNGREIDAMPIWQMYMVHF-QDYDLDTVHQITRTPKDLIVRWARDSGTIKPAAIHNGEGTCHYFHQT 4ydd.1    LDPALEGKFNMQLLDGKTVEVRPVFEILKSRLMADNTPEKAAKITGVTAKAITELAREFATAKPSMIICGGGTQHWYYSD  target    ANARGAAMVLIITGNVGKFGTGQHTWAGNYKAGTWTATPWSGAGLSVHTGEDPFNITLDPNAHGKEIKTRSYYYGEEV-G 4ydd.1    VLLRAMHLLTALTGTEGTNGGGMNHYIGQWKPAFV-----A--GLV--ALAFPEG--VNKQ-RFCQTTIWTYIHAEVNDE  target    YWNHGDTALIVNTPK-YGRKVFTGK----THMPTPSKFRWVVNVNVVNNAKHHYDMVRNVDPNIECLITQDIEMTSDINH 4ydd.1    IISSD-----IDTEKYLRDSITTGQMPNMPEQGRDPKVFFVYRGNWLNQAKGQKYVLENLWPKLELIVDINIRMDSTALY  target    ADIAFAANSWMEFTYPEMTVTVSNPWVQIWKGGIRPLYDTRNDLDTFAGVAAKLSDMTGDKRMR-------DYFAMV--- 4ydd.1    SDVVLPSAHWYEKLD--LNVTSEHSYINMTEPAIKPMWESKTDWQIFLALAKRVEMAAKRKKYEKFNDEKFKWVRDLSNL  target    Y--------QNRVDVYVQRMLDASSTFYGYSADVMLKSEKG----WMVMVR-TYPRHPFWEETNESKPMWTRSGRYENYR 4ydd.1    WNQMTMDGKLAEDEAAAQYILDNAPQSKGITIQMLREKPQRFKSNWTSPLKEGVPYTPFQYFVVDKKPWPTLTGRQQFYL  target    IEPEAIEYGENFISHREGPEATPYLPNAIFTTNPYVRPDDYGIPITAQHHDDKTVRNIKLSWHEIKRHSNPLWEKGYQFY 4ydd.1    DHDTFFDMGVEL-----------------------------------------------PTYKAP------IDADKYPFR  target    CVTPKTRHRVHSQWSVNDWVQIYESNFGDPYRMDKRTPGVGEHQIHINPQAAKDRGINDGDYVYVDGNPVDRPYRGWKPS 4ydd.1    FNSPHSRHSVHSTFKDNVLMLRL---------------QRGGPSIEMSPLDAKPLGIKDNDWVEAWNN------------  target    DPYYKVARLMIRAKYNPAYPYHVTMAKHAPFVATAKSVKGHETRPDGRAIAIDTGYQSNFRYGAQQSFTRNWLMPMHQTD 4ydd.1    -----HGKVICRVKIRNGEQRGRVSMWHCPELYMD------------------------LLTGGSQSVCPVRINPTNLVG  target    SLPGKHAVAWKFKWGYQVDHHAINTVPKECLIRITKAEDGGIGARGPWEPVRTGFTPGQENEFMIKWLKGEHIKIKV 4ydd.1    NYGHLF---------FRPNYYGPAGSQRDVRVNVKRYIGA------------------------------------- ``` | | | | | | | | | | | | | | | | | | | | | | | | | | | | | | | | | | | | | | | | | | | | | | | | | |
|  | 2ivf.1.A | ETHYLBENZENE DEHYDROGENASE ALPHA-SUBUNIT  *ETHYLBENZENE DEHYDROGENASE FROM AROMATOLEUM AROMATICUM* | 0.40 |  | 23.07 | 0.72 | 1-1020 | X-ray | 1.88 | hetero-oligomer | 1 x MES, 4 x SF4, 1 x MO, 1 x MGD, 1 x MD1, 1 x F3S, 1 x HEM | HHblits | 0.31 |
| ``` target    MFLSRRQFLKVSVGTVAAVAVADKVLAL--TALQPVIEVGNPLGDYPDRSWERVYHDQYRYDSS----FTWVCSPNDTHA 2ivf.1    QDQHRRDFLKRSGAAVLSLSLSSLATGVVPGFLKDAQA---GTKAPGYASWEDIYRKEWKWDKVNWGSHLNICW--PQGS  target    CRVRAFVRNGVVMRVEQNYDHQTYEDLYGNRGTFAHNPRMCLKGFTFHRRVYGPYRLKGPLMRKGWKQWMDDNAPELTAE 2ivf.1    CKFYVYVRNGIVWREEQAAQTPAC-----NVDYVDYNPLGCQKGSAFNNNLYGDERVKYPLKRVGK--------------  target    TKRKYKFDSRFLDDMLRVSWDTAFTYAAKAMITIATRYSGEAGARRLREQGYAPEMIEMMKGAGTRCFKHRAGMPVLGII 2ivf.1    ---------RGEGKWKRVSWDEAAGDIADSIIDSFEAQGSDGFILD------APH-----VHAGSIAW--GAGFRMTYLM  target    GKMGNTRMNGGINALLDTWIRKVSPDQAQGGRYWSNYTWHGDQNPAHPFWSGVQGSDIDLSDMRFSKLNTSWGKNFVENK 2ivf.1    DGV---SPDI---------------NVDIGDTYM-----------GAFHTFGKMHMGYSADNLLDAELIFMTCSNWSYTY  target    MPEAHWKLECIERGARVVVITPEYNPTAYRADYWMPLRPESDGALFLGAMKIIIDENMHDIDFLKSFTDAPILVRTDTLQ 2ivf.1    PSSYHFLSEARYKGAEVVVIAPDFNPTTPAADLHVPVRVGSDAAFWLGLSQVMIDEKLFDRQFVCEQTDLPLLVRMDTGK  target    YLDPRDVIADYKFPDFSKSYSGRIQSLKPEQIQRLGGMMVWDLNKKQVVPLHREQVGWHYTNSGIDAALTGTYRVKLLNG 2ivf.1    FLSAEDVDGG-----------------------EAKQFYFFDEKAGSVRKASRGTL-----KLDFMPALEGTFSARLKNG  target    REIDAMPIWQMYMVHFQDYDLDTVHQITRTPKDLIVRWARDSGTIKPAAIHNGEGTCHYFHQTANARGAAMVLIITGNVG 2ivf.1    KTIQVRTVFEGLREHLKDYTPEKASAKCGVPVSLIRELGRKVAKK-RTCSYIGFSSAKSYHGDLMERSLFLAMALSGNWG  target    KFGTGQHTWAGNYKAG----TWTATPWSGA-G-LSVH-TGEDPFNITLDPNAHGKEIKTRSYYY--GEEVG--------- 2ivf.1    KPGTGAFAWAYSDDNMVYLGVMSKPTAQGGMDELHQMAEGFNKRTLEADPTS-TDEMGNIEFMKVVTSAVGLVPPAMWLY  target    -------YWNH---GDTALIVNTPKYGR-KVFTG-------KTHMPTPSKFRWVVNVNVVNNAKHHYDMV-RNVDPNIEC 2ivf.1    YHVGYDQLWNNKAWTDPALKKSFGAYLDEAKEKGWWTNDHIRPAPDKTPQVYMLLSQNPMRRKRSGAKMFPDVLFPKLKM  target    LITQDIEMTSDINHADIAFAANSWMEFTYPEMTVTVSNPWVQIWKGGIRPLYDTRNDLDTFAGVAAKLSDMTGDK----- 2ivf.1    IFALETRMSSSAMYADIVLPCAWYYEKHEM-TTPCSGNPFFTFVDRSVAPPGECREEWDAIALILKKVGERAAARGLTEF  target    --------RMRDYFAMVY----QNRVDVYVQRMLDASST----FYGYSADVMLKSEKGWMVMVR---------------- 2ivf.1    NDHNGRKRRYDELYKKFTMDGHLLTNEDCLKEMVDINRAVGVFAKDYTYEKFKK--EGQTRFLSMGTGVSRYAHANEVDV  target    TYPRHPFWEETNESKPMWTRSGRYENYRIEPEAIEYGENFISHREGPEATPYLPNAIFTTNPYVRPDDYGIPITAQHHDD 2ivf.1    TKPIYPMRWHFDDKKVFPTHTRRAQFYLDHDWYLEAGESLPT--------------------------------------  target    KTVRNIKLSWHEIKRHSNPLWEKGYQFYCVTPKTRHRVHSQWSVNDWVQIYESNFGDPYRMDKRTPGVGEHQIHINPQAA 2ivf.1    ---------HKDT-----PMVGGDHPFKITGGHPRVSIHSTHLTNSHLSRLH---------------RGQPVVHMNSKDA  target    KDRGINDGDYVYVDGNPVDRPYRGWKPSDPYYKVARLMIRAKYNPAYPYHVTMAKHAPFVATAKSVKGHETRPDGRAIAI 2ivf.1    AELGIKDGDMAKLFND-----------------FADCEIMVRTAPNVQPKQCIVYFWDAH--------------------  target    DTGYQSNFRYGAQQSFTRNWLMPMHQTDSLPGKHAVAWKFKWGYQVDHHAINTVPKECLIRITKAEDGGIGARGPWEPVR 2ivf.1    --------------------------------------------------------------------------------  target    TGFTPGQENEFMIKWLKGEHIKIKV 2ivf.1    ------------------------- ``` | | | | | | | | | | | | | | | | | | | | | | | | | | | | | | | | | | | | | | | | | | | | | | | | | |
|  | 4ydd.1.A | DMSO reductase family type II enzyme, molybdopterin subunit  *Crystal structure of the perchlorate reductase PcrAB from Azospira suillum PS* | 0.34 |  | 28.21 | 0.69 | 42-971 | X-ray | 1.86 | hetero-oligomer | 4 x SF4, 1 x MO, 1 x MGD, 1 x MD1, 1 x F3S | BLAST | 0.34 |
| ``` target    MFLSRRQFLKVSVGTVAAVAVADKVLALTALQPVIEVGNPLGDYPDRSWERVYHDQYRYDSSFTWVCSPNDTHACRVRAF 4ydd.1    -----------------------------------------GAFEYSGWENFHRTQWSWDKKTRGAHLVNCTGACPHFVY  target    VRNGVVMRVEQNYDHQTYEDLYGNRGTFAHNPRMCLKGFTFHRRVYGPYRLKGPLMRKGWKQWMDDNAPELTAETKRKYK 4ydd.1    SKDGVVMREEQSKDIAPMPNIP------EYNPRGCNKGECGHDYMYGPHRIKYPLIRVGER-----------GEGKWR--  target    FDSRFLDDMLRVSWDTAFTYAAKAMITIATRYSGEAGARRLREQGYAPEMIEMMKGAGTRCFKHRAGMPVLGIIGKMGNT 4ydd.1    ----------RATWEEALD-----MI--------------------ADKCVDTIKNHAPDCISVYSPVPAVSPVSFSAGH  target    RMNGGINALLDTWIRKVSPDQAQGGRYWSNYTWHGDQNPAHPFWSGVQGSDIDLSDMRFSKLNTSWGKNFVENKMPEAHW 4ydd.1    RFAHYIGAHAHTF-----------------YDWYGDHPTGQTQTCGVQGDTCETADWFNSKYIILWGSNPTQTRIPDAHF  target    KLECIERGARVVVITPEYNPTAYRADYWMPLRPESDGALFLGAMKIIIDENMHDIDFLKSFTDAPILVRTDTLQYLDPRD 4ydd.1    LSEAQLNGAKIVSISPDYNSSTIKVDKWIHPQPGTDGALAMAMAHVIIKEKLYDAHSLKEQTDLSYLVRSDTKRFLREAD  target    VIADYKFPDFSKSYSGRIQSLKPEQIQRLGGMMVWDLNKKQVVP---LHREQVGW--HYTNSG-IDAALTGTYRVKLLNG 4ydd.1    VVAGGSKDKF---YFWNAKTGKPVIPKGSWG----DQPEKKGSPVGFLGRNTFAFPKGYIDLGDLDPALEGKFNMQLLDG  target    REIDAMPIWQMYMVHFQ-DYDLDTVHQITRTPKDLIVRWARDSGTIKPAAIHNGEGTCHYFHQTANARGAAMVLIITGNV 4ydd.1    KTVEVRPVFEILKSRLMADNTPEKAAKITGVTAKAITELAREFATAKPSMIICGGGTQHWYYSDVLLRAMHLLTALTGTE  target    GKFGTGQHTWAGNYKAGTWTATPWSGAGLSVHTGEDPFNITLDPNAHGKEIKTRSYYYGEEVGYWNHGDTALIVNTPKYG 4ydd.1    GTNGGGMNHYIGQWK-------PAFVAGLVALAFPEGVN-----KQRFCQTTIWTYIHAEV----NDEIISSDIDTEKYL  target    RKVFTGKTHMPTPSKFR-----WVVNVNVVNNAKHHYDMVRNVDPNIECLITQDIEMTSDINHADIAFAANSWMEFTYPE 4ydd.1    RDSITTGQMPNMPEQGRDPKVFFVYRGNWLNQAKGQKYVLENLWPKLELIVDINIRMDSTALYSDVVLPSAHWYEKL--D  target    MTVTVSNPWVQIWKGGIRPLYDTRNDLDTFAGVAAKLSDMTGDKRMRDYFAMVYQNRVDVYVQRMLDASSTFYGYSADVM 4ydd.1    LNVTSEHSYINMTEPAIKPMWESKTDWQIFLALAKRVEMAAKRKK--------YEKFNDEKFKWVRDLSNLWNQMTMDGK  target    LKSEKGWMVMVRTYPRHPFWEETNESKPMWTRSGRYENYRIEPEAIEYGENFISHREGP--EATPYLPNAIFTTNPYVRP 4ydd.1    LAEDEAAAQYI--------LDNAPQSKGITIQMLR-----------EKPQRFKSNWTSPLKEGVPYTPFQYFVVDKKPWP  target    DDYGIPITAQHHDDKTVRNIKLSWHEIKRHSNPLWEKGYQFYCVTPKTRHRVHSQWSVNDWVQIYESNFGDPYRMDKRTP 4ydd.1    TLTGRQQFYLDHD--TFFDMGV---ELPTYKAPIDADKYPFRFNSPHSRHSVHSTFKDN--VLMLRLQRGGP--------  target    GVGEHQIHINPQAAKDRGINDGDYVYVDGNPVDRPYRGWKPSDPYYKVARLMIRAKYNPAYPYHVTMAKHAPFVATAKSV 4ydd.1    -----SIEMSPLDAKPLGIKDNDWV-------------------------------------------------------  target    KGHETRPDGRAIAIDTGYQSNFRYGAQQSFTRNWLMPMHQTDSLPGKHAVAWKFKWGYQVDHHAINTVPKECLIRITKAE 4ydd.1    --------------------------------------------------------------------------------  target    DGGIGARGPWEPVRTGFTPGQENEFMIKWLKGEHIKIKV 4ydd.1    --------------------------------------- ``` | | | | | | | | | | | | | | | | | | | | | | | | | | | | | | | | | | | | | | | | | | | | | | | | | |
|  | 5e7o.1.A | DMSO reductase family type II enzyme, molybdopterin subunit  *Crystal structure of the perchlorate reductase PcrAB mutant W461E of PcrA from Azospira suillum PS* | 0.34 |  | 28.21 | 0.69 | 42-971 | X-ray | 2.40 | hetero-oligomer | 4 x SF4, 1 x MO, 1 x MGD, 1 x MD1, 1 x F3S | BLAST | 0.34 |
| ``` target    MFLSRRQFLKVSVGTVAAVAVADKVLALTALQPVIEVGNPLGDYPDRSWERVYHDQYRYDSSFTWVCSPNDTHACRVRAF 5e7o.1    -----------------------------------------GAFEYSGWENFHRTQWSWDKKTRGAHLVNCTGACPHFVY  target    VRNGVVMRVEQNYDHQTYEDLYGNRGTFAHNPRMCLKGFTFHRRVYGPYRLKGPLMRKGWKQWMDDNAPELTAETKRKYK 5e7o.1    SKDGVVMREEQSKDIAPMPNIP------EYNPRGCNKGECGHDYMYGPHRIKYPLIRVGER-----------GEGKWR--  target    FDSRFLDDMLRVSWDTAFTYAAKAMITIATRYSGEAGARRLREQGYAPEMIEMMKGAGTRCFKHRAGMPVLGIIGKMGNT 5e7o.1    ----------RATWEEALD-----MI--------------------ADKCVDTIKNHAPDCISVYSPVPAVSPVSFSAGH  target    RMNGGINALLDTWIRKVSPDQAQGGRYWSNYTWHGDQNPAHPFWSGVQGSDIDLSDMRFSKLNTSWGKNFVENKMPEAHW 5e7o.1    RFAHYIGAHAHTF-----------------YDWYGDHPTGQTQTCGVQGDTCETADWFNSKYIILWGSNPTQTRIPDAHF  target    KLECIERGARVVVITPEYNPTAYRADYWMPLRPESDGALFLGAMKIIIDENMHDIDFLKSFTDAPILVRTDTLQYLDPRD 5e7o.1    LSEAQLNGAKIVSISPDYNSSTIKVDKWIHPQPGTDGALAMAMAHVIIKEKLYDAHSLKEQTDLSYLVRSDTKRFLREAD  target    VIADYKFPDFSKSYSGRIQSLKPEQIQRLGGMMVWDLNKKQVVP---LHREQVGW--HYTNSG-IDAALTGTYRVKLLNG 5e7o.1    VVAGGSKDKF---YFWNAKTGKPVIPKGSWG----DQPEKKGSPVGFLGRNTFAFPKGYIDLGDLDPALEGKFNMQLLDG  target    REIDAMPIWQMYMVHFQ-DYDLDTVHQITRTPKDLIVRWARDSGTIKPAAIHNGEGTCHYFHQTANARGAAMVLIITGNV 5e7o.1    KTVEVRPVFEILKSRLMADNTPEKAAKITGVTAKAITELAREFATAKPSMIICGGGTQHWYYSDVLLRAMHLLTALTGTE  target    GKFGTGQHTWAGNYKAGTWTATPWSGAGLSVHTGEDPFNITLDPNAHGKEIKTRSYYYGEEVGYWNHGDTALIVNTPKYG 5e7o.1    GTNGGGMNHYIGQEK-------PAFVAGLVALAFPEGVN-----KQRFCQTTIWTYIHAEV----NDEIISSDIDTEKYL  target    RKVFTGKTHMPTPSKFR-----WVVNVNVVNNAKHHYDMVRNVDPNIECLITQDIEMTSDINHADIAFAANSWMEFTYPE 5e7o.1    RDSITTGQMPNMPEQGRDPKVFFVYRGNWLNQAKGQKYVLENLWPKLELIVDINIRMDSTALYSDVVLPSAHWYEKL--D  target    MTVTVSNPWVQIWKGGIRPLYDTRNDLDTFAGVAAKLSDMTGDKRMRDYFAMVYQNRVDVYVQRMLDASSTFYGYSADVM 5e7o.1    LNVTSEHSYINMTEPAIKPMWESKTDWQIFLALAKRVEMAAKRKK--------YEKFNDEKFKWVRDLSNLWNQMTMDGK  target    LKSEKGWMVMVRTYPRHPFWEETNESKPMWTRSGRYENYRIEPEAIEYGENFISHREGP--EATPYLPNAIFTTNPYVRP 5e7o.1    LAEDEAAAQYI--------LDNAPQSKGITIQMLR-----------EKPQRFKSNWTSPLKEGVPYTPFQYFVVDKKPWP  target    DDYGIPITAQHHDDKTVRNIKLSWHEIKRHSNPLWEKGYQFYCVTPKTRHRVHSQWSVNDWVQIYESNFGDPYRMDKRTP 5e7o.1    TLTGRQQFYLDHD--TFFDMGV---ELPTYKAPIDADKYPFRFNSPHSRHSVHSTFKDN--VLMLRLQRGGP--------  target    GVGEHQIHINPQAAKDRGINDGDYVYVDGNPVDRPYRGWKPSDPYYKVARLMIRAKYNPAYPYHVTMAKHAPFVATAKSV 5e7o.1    -----SIEMSPLDAKPLGIKDNDWV-------------------------------------------------------  target    KGHETRPDGRAIAIDTGYQSNFRYGAQQSFTRNWLMPMHQTDSLPGKHAVAWKFKWGYQVDHHAINTVPKECLIRITKAE 5e7o.1    --------------------------------------------------------------------------------  target    DGGIGARGPWEPVRTGFTPGQENEFMIKWLKGEHIKIKV 5e7o.1    --------------------------------------- ``` | | | | | | | | | | | | | | | | | | | | | | | | | | | | | | | | | | | | | | | | | | | | | | | | | |
|  | 7l5i.1.A | Trimethylamine-N-oxide reductase  *Crystal Structure of Haemophilus influenzae MtsZ at pH 7.0* | 0.31 |  | 18.01 | 0.65 | 61-1111 | X-ray | 1.73 | monomer | 2 x MGD, 1 x MO, 1 x O | HHblits | 0.29 |
| ``` target    MFLSRRQFLKVSVGTVAAVAVADKVLALTALQPVIEVGNPLGDYPDRSWERVYHDQYRYDSSFTWVCSPNDTHACRVRAF 7l5i.1    ------------------------------------------------------------KTVVTAAH-----WGSIGVV  target    VRNGVVMRVEQNYDHQTYEDLYGNRGTFAHNPRMCLKGFTFHRRVYGPYRLKGPLMRKGWKQWMDDNAPELTAETKRKYK 7l5i.1    VQDGKVVKSGPAIEPAV-------------PNELQT---VVADQLYSEARVKCPMVRKGFLAN------------PGKSD  target    FDSRFLDDMLRVSWDTAFTYAAKAMITIATRYSGEAGARRLREQGYAPEMIEMMKGAGTRCFKHRAGMPVLGIIGKMGNT 7l5i.1    TTMRGRDEWVRVSWDEALDLVHNQLKRVRDEHGSTGIFAGSY-GWFSC---------GSLH-------------------  target    RMNGGINALLDTWIRKVSPDQAQGGRYWSNYTWHGDQNPAHPFWSGV---QGSDIDL-SDMRFSKLNTSWGKNFVENKMP 7l5i.1    ----ASRTLLQRYMNATGGFVGHKGDYSTG-----AAQVIMPHVLGTIEVYEQQTSWESILESSDIIVLWSANPLTTMRI  target    E--------AHWKLECIERGARVVVITPEYNPTAYR-ADYWMPLRPESDGALFLGAMKIIIDENMHDIDFLKSFTDAPIL 7l5i.1    AWMSTDQKGIEYFKKFQASGKRIICIDPQKSETCQMLNAEWIPVNTATDVPLMLGIAHTLVEQGKHDKDFLKKYTSGYAK  target    VRTDTLQYLDPRDVIADYKFPDFSKSYSGRIQSLKPEQIQRLGGMMVWDLNKKQVVPLHREQVGWHYTNSGIDAALTGTY 7l5i.1    FEE----YLL----------------------------------------------------------------------  target    RVKLLNGREIDAMPIWQMYMVHFQDYDLDTVHQITRTPKDLIVRWARDSGTIKPAAIHNGEGTCHYFHQTANARGAAMVL 7l5i.1    ------------------GKTDGQPKTAEWAAKICGVPAETIKQLAADFAS-KRTMLMGGWGMQRQRHGEQTHWMLVTLA  target    IITGNVGKFGTGQHTWAGNYKAG-TWTATPWSGAGLSVHTGEDPFNITLDPNAHGKEIKTRSYYYGEEVGYWNHGDTALI 7l5i.1    SMLGQIGLPGGGFGLSYHYSNGGVPTATGGIIG-SITASPSGKAGAKTWLDDTSKSAFPL---------ARIAD----VL  target    VNTPKYGRKVFTGKTHMP-TPSKFRWVVNVNVVNNAKHHYDMVRNVDPNIECLITQDIEMTSDINHADIAFAANSWMEFT 7l5i.1    LHPGKKIQYNG---TEITYPDIKAVYWAGGNPFVHHQD-TNTLVKAFQKPDVVIVNEVNWTPTARMADIVLPATTSYERN  target    YPEMTVTVSNPWVQIWKGGIRPLYDTRNDLDTFAGVAAKLSDMTGD---KRMRDYFAMVYQNRVDVYVQRMLDASST--F 7l5i.1    DLTMAGDYSMMSVYPMKQVVPPQFEAKNDYDIFVELAKRAGVEEQYTEGKTEMEWL--------EEFYNAAFSAARANRV  target    YGYSADVMLKSEKGWMV--MV--RTYPRHPFWEETNESKPMWTRSGRYENYRIEPEAIEYGENFISHREGPEATPYLPNA 7l5i.1    AMPRFDKFWAENKPLSFEAGEAAKKWVRYGEFREDPLLNPLGTPSGKIEIFSDVVEKM--NYN------D---CKGHP--  target    IFTTNPYVRPDDYGIPITAQHHDDKTVRNIKLSWHEIKRHSNPLWEKGYQFYCVTPKTRHRVHSQWSVNDWVQIYESNFG 7l5i.1    --------------------------------SWMEPEEFA-GNVTEEYPLALVTPHPYYRLHSQLAHTSLRQKYA----  target    DPYRMDKRTPGVGEHQIHINPQAAKDRGINDGDYVYVDGNPVDRPYRGWKPSDPYYKVARLMIRAKYNPAYPYHVTMAKH 7l5i.1    ----------VNDREPVMIHPEDAAARGIKDGDIVRIHSK-----------------RGQVLAGAAVTENIIKGTVALHE  target    APFVATAKSVKGHETRPDGRAIAIDTGYQSNFRYGAQQSFTRNW-LMPMHQTDSLPGKHAVAWKFKWGYQVDHHAINTVP 7l5i.1    GAWYDPMYLGESE---------------KPLCKNGCANVLTRDEGTSKL-------------------------AQGNSP  target    KECLIRITKAEDGGIGARGPWEPVRTGFTPGQENEFMIKWLKGEHIKIKV 7l5i.1    NTCIVQIEKFIGVAPE---------------------------------- ``` | | | | | | | | | | | | | | | | | | | | | | | | | | | | | | | | | | | | | | | | | | | | | | | | | |
|  | 7l5s.1.A | Trimethylamine-N-oxide reductase  *Crystal Structure of Haemophilus influenzae MtsZ at pH 5.5* | 0.31 |  | 18.01 | 0.65 | 61-1111 | X-ray | 2.09 | monomer | 1 x O, 2 x MGD, 1 x MO | HHblits | 0.29 |
| ``` target    MFLSRRQFLKVSVGTVAAVAVADKVLALTALQPVIEVGNPLGDYPDRSWERVYHDQYRYDSSFTWVCSPNDTHACRVRAF 7l5s.1    ------------------------------------------------------------KTVVTAAH-----WGSIGVV  target    VRNGVVMRVEQNYDHQTYEDLYGNRGTFAHNPRMCLKGFTFHRRVYGPYRLKGPLMRKGWKQWMDDNAPELTAETKRKYK 7l5s.1    VQDGKVVKSGPAIEPAV-------------PNELQT---VVADQLYSEARVKCPMVRKGFLAN------------PGKSD  target    FDSRFLDDMLRVSWDTAFTYAAKAMITIATRYSGEAGARRLREQGYAPEMIEMMKGAGTRCFKHRAGMPVLGIIGKMGNT 7l5s.1    TTMRGRDEWVRVSWDEALDLVHNQLKRVRDEHGSTGIFAGSY-GWFSC---------GSLH-------------------  target    RMNGGINALLDTWIRKVSPDQAQGGRYWSNYTWHGDQNPAHPFWSGV---QGSDIDL-SDMRFSKLNTSWGKNFVENKMP 7l5s.1    ----ASRTLLQRYMNATGGFVGHKGDYSTG-----AAQVIMPHVLGTIEVYEQQTSWESILESSDIIVLWSANPLTTMRI  target    E--------AHWKLECIERGARVVVITPEYNPTAYR-ADYWMPLRPESDGALFLGAMKIIIDENMHDIDFLKSFTDAPIL 7l5s.1    AWMSTDQKGIEYFKKFQASGKRIICIDPQKSETCQMLNAEWIPVNTATDVPLMLGIAHTLVEQGKHDKDFLKKYTSGYAK  target    VRTDTLQYLDPRDVIADYKFPDFSKSYSGRIQSLKPEQIQRLGGMMVWDLNKKQVVPLHREQVGWHYTNSGIDAALTGTY 7l5s.1    FEE----YLL----------------------------------------------------------------------  target    RVKLLNGREIDAMPIWQMYMVHFQDYDLDTVHQITRTPKDLIVRWARDSGTIKPAAIHNGEGTCHYFHQTANARGAAMVL 7l5s.1    ------------------GKTDGQPKTAEWAAKICGVPAETIKQLAADFAS-KRTMLMGGWGMQRQRHGEQTHWMLVTLA  target    IITGNVGKFGTGQHTWAGNYKAG-TWTATPWSGAGLSVHTGEDPFNITLDPNAHGKEIKTRSYYYGEEVGYWNHGDTALI 7l5s.1    SMLGQIGLPGGGFGLSYHYSNGGVPTATGGIIG-SITASPSGKAGAKTWLDDTSKSAFPL---------ARIAD----VL  target    VNTPKYGRKVFTGKTHMP-TPSKFRWVVNVNVVNNAKHHYDMVRNVDPNIECLITQDIEMTSDINHADIAFAANSWMEFT 7l5s.1    LHPGKKIQYNG---TEITYPDIKAVYWAGGNPFVHHQD-TNTLVKAFQKPDVVIVNEVNWTPTARMADIVLPATTSYERN  target    YPEMTVTVSNPWVQIWKGGIRPLYDTRNDLDTFAGVAAKLSDMTGD---KRMRDYFAMVYQNRVDVYVQRMLDASST--F 7l5s.1    DLTMAGDYSMMSVYPMKQVVPPQFEAKNDYDIFVELAKRAGVEEQYTEGKTEMEWL--------EEFYNAAFSAARANRV  target    YGYSADVMLKSEKGWMV--MV--RTYPRHPFWEETNESKPMWTRSGRYENYRIEPEAIEYGENFISHREGPEATPYLPNA 7l5s.1    AMPRFDKFWAENKPLSFEAGEAAKKWVRYGEFREDPLLNPLGTPSGKIEIFSDVVEKM--NYN------D---CKGHP--  target    IFTTNPYVRPDDYGIPITAQHHDDKTVRNIKLSWHEIKRHSNPLWEKGYQFYCVTPKTRHRVHSQWSVNDWVQIYESNFG 7l5s.1    --------------------------------SWMEPEEFA-GNVTEEYPLALVTPHPYYRLHSQLAHTSLRQKYA----  target    DPYRMDKRTPGVGEHQIHINPQAAKDRGINDGDYVYVDGNPVDRPYRGWKPSDPYYKVARLMIRAKYNPAYPYHVTMAKH 7l5s.1    ----------VNDREPVMIHPEDAAARGIKDGDIVRIHSK-----------------RGQVLAGAAVTENIIKGTVALHE  target    APFVATAKSVKGHETRPDGRAIAIDTGYQSNFRYGAQQSFTRNW-LMPMHQTDSLPGKHAVAWKFKWGYQVDHHAINTVP 7l5s.1    GAWYDPMYLGESE---------------KPLCKNGCANVLTRDEGTSKL-------------------------AQGNSP  target    KECLIRITKAEDGGIGARGPWEPVRTGFTPGQENEFMIKWLKGEHIKIKV 7l5s.1    NTCIVQIEKFIGVAPE---------------------------------- ``` | | | | | | | | | | | | | | | | | | | | | | | | | | | | | | | | | | | | | | | | | | | | | | | | | |
|  | 4dmr.1.A | DMSO REDUCTASE  *REDUCED DMSO REDUCTASE FROM RHODOBACTER CAPSULATUS WITH BOUND DMSO SUBSTRATE* | 0.31 |  | 19.65 | 0.64 | 1-1023 | X-ray | 1.90 | monomer | 2 x PGD, 1 x 4MO, 1 x O | HHblits | 0.29 |
| ``` target    MFLSRRQFLKVSVGTVAAVAVADKVLALTALQPVIEVGNPLGDYPDRSWERVYHDQYRYDSSFTWVCSPNDTHACR-VRA 4dmr.1    AELYRRAFLSYSVAPGALGMFGRSLL------A--KG-----ARA----E--------ALA---NGTV-MSGSHWGVFTA  target    FVRNGVVMRVEQNYDHQTYEDLYGNRGTFAHNPRMCLKGFTFHRRVYGPYRLKGPLMRKGWKQWMDDNAPELTAETKRKY 4dmr.1    TVENGRATAFTPWEKDPH----------------PSPMLAGVLDSIYSPTRIKYPMVRREFLEKG-----------V-NA  target    KFDSRFLDDMLRVSWDTAFTYAAKAMITIATRYSGEAGARRLREQGYAPEMIEMMKGAGTRCFKHRAGMPVLGIIGKMGN 4dmr.1    DRSTRGNGDFVRVSWDQALDLVAAEVKRVEETYGPSGVFGG----SYGWK------SPGR--LHNCT-TLLRRMLTLAGG  target    TRMNGGINALLDTWIRKVSPDQAQGGRYWSNYTWHGDQNPAHPFWSGVQGSDIDLSDMRFSKLNTSWGKNFVENKMPEA- 4dmr.1    YVNGAG--------------DYSTGAAQVIM-----PHVVGTLEVYE--QQTAWPVLAENTEVMVFWAADPIKTSQIGWV  target    -------HWKLECIERGARVVVITPEYNPTAYR-ADYWMPLRPESDGALFLGAMKIIIDENMHDIDFLKSFTDAPILVRT 4dmr.1    IPEHGAYPGLEALKAKGTKVIVIDPVRTKTVEFFGAEHITPKPQTDVAIMLGMAHTLVAEDLYDKDFIANYTSGF-----  target    DTLQYLDPRDVIADYKFPDFSKSYSGRIQSLKPEQIQRLGGMMVWDLNKKQVVPLHREQVGWHYTNSGIDAALTGTYRVK 4dmr.1    -----------------DKFLP-------------------------------------------------YLDGET---  target    LLNGREIDAMPIWQMYMVHFQDYDLDTVHQITRTPKDLIVRWARDSGTIKPAAIHNGEGTCHYFHQTANARGAAMVLIIT 4dmr.1    ------------------DSTPKTAEWAEGISGVPAETIKELARLFES-KRTMLAAGWSMQRMHHGEQAHWMLVTLASML  target    GNVGKFGTGQHTWAGNYKAGTWTATPWSGAGLSVHTGEDP-FNITLDPNAHGKEIKTRSYYYGEEVGYWNHGDTALIVNT 4dmr.1    GQIGLPGGGFGLSYHYSGGGT-PS--TSGPAL---AGITDGGAATKGPE----WLAA----SG--ASVIPVARVVDMLEN  target    PKYGRKVFTGKTHMPTPSKFRWVVNVNVVNNAKHHYDMVRNVDPNIECLITQDIEMTSDINHADIAFAANSWMEFTYPEM 4dmr.1    PGAEF-DFNGTRSKFPDVKMAYWVGGNPFVHHQD-RNRMVKAWEKLETFVVHDFQWTPTARHADIVLPATTSYERNDIET  target    TVTVSNPWVQIWKGGIRPLYDTRNDLDTFAGVAAKLSDMTGDKRMRDYFAMVYQNRVDVYVQRMLDASS---TFYGY--- 4dmr.1    IGDYSNTGILAMKKIVEPLYEARSDYDIFAAVAERLGKGA-------EFTE--GKDEMGWIKSFYDDAAKQGKAAGVQMP  target    SADVMLKSEKGWMVMVR----TYPRHPFWEETNESKPMWTRSGRYENYRIEPEAIEYGENFISHREGPEATPYLPNAIFT 4dmr.1    AFDAFWA--EGIVEFPVTDGADFVRYASFREDPLLNPLGTPTGLIEIYSKNIEKMGYD---------D--CPAHP-----  target    TNPYVRPDDYGIPITAQHHDDKTVRNIKLSWHEIKRHSNPLWEKGYQFYCVTPKTRHRVHSQWSVNDWVQIYESNFGDPY 4dmr.1    -----------------------------TWMEPLERL-DGPGAKYPLHIAASHPFNRLHSQL-NGTVLREGY-------  target    RMDKRTPGVGEHQIHINPQAAKDRGINDGDYVYVDGNPVDRPYRGWKPSDPYYKVARLMIRAKYNPAYPYHVTMAKHAPF 4dmr.1    ---A---VQGHEPCLMHPDDAAARGIADGDVVRVHND-----------------RGQILTGVKVTDAVMKGVIQIYEGGW  target    VATAKSVKGHETRPDGRAIAIDTGYQSNFRYGAQQSFTRNWLMPMHQTDSLPGKHAVAWKFKWGYQVDHHAINTVPKECL 4dmr.1    YDPS----------------------------------------------------------------------------  target    IRITKAEDGGIGARGPWEPVRTGFTPGQENEFMIKWLKGEHIKIKV 4dmr.1    ---------------------------------------------- ``` | | | | | | | | | | | | | | | | | | | | | | | | | | | | | | | | | | | | | | | | | | | | | | | | | |
|  | 4v4c.1.A | Pyrogallol hydroxytransferase large subunit  *Crystal Structure of Pyrogallol-Phloroglucinol Transhydroxylase from Pelobacter acidigallici* | 0.30 |  | 17.21 | 0.64 | 66-1019 | X-ray | 2.35 | hetero-oligomer | 2 x CA, 2 x MGD, 1 x 4MO, 3 x SF4 | HHblits | 0.29 |
| ``` target    MFLSRRQFLKVSVGTVAAVAVADKVLALTALQPVIEVGNPLGDYPDRSWERVYHDQYRYDSSFTWVCSPNDTHACRVRAF 4v4c.1    -----------------------------------------------------------------RLTN-SSTGGPVFVY  target    VRNGVVMRVEQNYDHQTYE--D-LYGNRG---TFAHNPRMCLKGFTFHRRVYGPYRLKGPLMRKGWKQWMDDNAPELTAE 4v4c.1    VKDGKIIRMTPMDFDDAVDAPSWKIEARGKTFTPPRKTSIAPYTAGFKSMIYSDLRIPYPMKRKSFDPNG----------  target    TKRKYKFDSRFL--------DDMLRVSWDTAFTYAAKAMITIATRYSGEAGARRLREQGYAPEMIEMMKGAGTRCFKHRA 4v4c.1    ---ERNPQLRGAGLSKQDPWSDYERISWDEATDIVVAEINRIKHAYGPSAILSTPSSHHM----------WGNVGYRHST  target    GMPVLGIIGKMGNTRMNGGINALLDTWIRKVSPDQAQGGRYWSNYTWHGDQNPAHPFWSGVQGS-DIDLSDMRFSKLNTS 4v4c.1    YFRF---MNMMGFTYADHNP-------------------DSWEGWHWGGMHMWGFSWRLGNPEQYDLLEDGLKHAEMIVF  target    WGKNFVENKMPEAHW-----KLECIERGARVVVITPEYNPTAY-RADYWMPLRPESDGALFLGAMKIIIDENMHDIDFLK 4v4c.1    WSSDPETNSGIYAGFESNIRRQWLKDLGVDFVFIDPHMNHTARLVADKWFSPKIGTDHALSFAIAYTWLKEDSYDKEYVA  target    SFTDAPILVRTDTLQYLDPRDVIADYKFPDFSKSYSGRIQSLKPEQIQRLGGMMVWDLNKKQVVPLHREQVGWHYTNSGI 4v4c.1    ANAHGF----------------------EEWAD-------------------Y-VL------------------------  target    DAALTGTYRVKLLNGREIDAMPIWQMYMVHFQDYDLDTVHQITRTPKDLIVRWARDSGTIKPAAIHNGE----GTCHYFH 4v4c.1    -------------G-------------KTDGTPKTCEWAEEESGVPACEIRALARQWAKKNTYLAAGGLGGWGGACRASH  target    QTANARGAAMVLIITGNVGKFGTGQHTWAGNYKAG-TWTATPWSGAGLS--VHTGEDPFNIT--------LDPNA----- 4v4c.1    GIEWARGMIALATMQG-MGKPGSNMWSTTQGVPLDYEFYFPGYAEGGISGDCENSAAGFKFAWRMFDGKTTFPSPSNLNT  target    -HGKEIKTRSYY---YGEEVGYWNHGDTALIVNTPKYGRKVFTGKTH---MPTPSKFRWVVNVNVVNNAKHHYDMVRNVD 4v4c.1    SAGQHIPRLKIPECIMGGKFQWSGKG-----FAGGDISH-QLHQYEYPAPGYSKIKMFWKYGGPHLGTMTAT-NRYAKMY  target    --PNIECLITQDIEMTSDINHADIAFAANSWMEFTYPEMTVTV------------SNPWVQIWKGGIRPLYDTRNDLDTF 4v4c.1    THDSLEFVVSQSIWFEGEVPFADIILPACTNFERWDIS-EFANCSGYIPDNYQLCNHRVISLQAKCIEPVGESMSDYEIY  target    AGVAAKLSDMTGDKRMRDYFAMVYQNRVDVYVQRMLDASSTFYGYSADVMLKSEKGWMVMVRTY------P--------- 4v4c.1    RLFAKKLNIE-------EMFSE--GKDELAWCEQYFNATDMPKYMTWDEFFK--KGYFVVPDNPNRKKTVALRWFAEGRE  target    -RHPFWEE----TNESKPMWTRSGRYENYRIEPEAIE-YGENFISHREGPEATPYLPNAIFTTNPYVRPDDYGIPITAQH 4v4c.1    KDTPDWGPRLNNQVCRKGLQTTTGKVEFIATSLKNFEEQG--YI-----DEHRPSMHT----------------------  target    HDDKTVRNIKLSWHEIKRHSNPLWEKGYQFYCVTPKTRHRVHSQWSV-NDWVQIYESNFGDPYRMDKRTPGVGEHQIHIN 4v4c.1    --------YVPAWESQKH--SP-LAVKYPLGMLSPHPRFSMHTMGDGKNSYMNYIKDHRVEV----D---GYKYWIMRVN  target    PQAAKDRGINDGDYVYVDGNPVDRPYRGWKPSDPYYKVARLMIRAKYNPAYPYHVTMAKHAPFVATAKSVKGHETRPDGR 4v4c.1    SIDAEARGIKNGDLIRAYND-----------------RGSVILAAQVTECLQPGTVHSYESCA-----------------  target    AIAIDTGYQSNFRYGAQQSFTRNWLMPMHQTDSLPGKHAVAWKFKWGYQVDHHAINTVPKECLIRITKAEDGGIGARGPW 4v4c.1    --------------------------------------------------------------------------------  target    EPVRTGFTPGQENEFMIKWLKGEHIKIKV 4v4c.1    ----------------------------- ``` | | | | | | | | | | | | | | | | | | | | | | | | | | | | | | | | | | | | | | | | | | | | | | | | | |
|  | 1e18.1.A | DMSO REDUCTASE.  *TUNGSTEN-SUSBSTITUTED DMSO REDUCTASE FROM RHODOBACTER CAPSULATUS* | 0.31 |  | 19.65 | 0.64 | 1-1023 | X-ray | 2.00 | monomer | 2 x PGD, 1 x 6WO | HHblits | 0.29 |
| ``` target    MFLSRRQFLKVSVGTVAAVAVADKVLALTALQPVIEVGNPLGDYPDRSWERVYHDQYRYDSSFTWVCSPNDTHACR-VRA 1e18.1    AELYRRAFLSYSVAPGALGMFGRSLL------AK-------GARA--E----------ALA---NGTV-MSGSHWGVFTA  target    FVRNGVVMRVEQNYDHQTYEDLYGNRGTFAHNPRMCLKGFTFHRRVYGPYRLKGPLMRKGWKQWMDDNAPELTAETKRKY 1e18.1    TVENGRATAFTPWEKDPH----------------PSPMLAGVLDSIYSPTRIKYPMVRREFLEKGV------------NA  target    KFDSRFLDDMLRVSWDTAFTYAAKAMITIATRYSGEAGARRLREQGYAPEMIEMMKGAGTRCFKHRAGMPVLGIIGKMGN 1e18.1    DRSTRGNGDFVRVSWDQALDLVAAEVKRVEETYGPQGVFGG----SYGWK------SPGR--LHNCT-TLLRRMLTLAGG  target    TRMNGGINALLDTWIRKVSPDQAQGGRYWSNYTWHGDQNPAHPFWSGVQGSDIDLSDMRFSKLNTSWGKNFVENKMPEA- 1e18.1    YVNGAG--------------DYSTGAAQVIM-----PHVVGTLEVYE--QQTAWPVLAENTEVMVFWAADPIKTSQIGWV  target    -------HWKLECIERGARVVVITPEYNPTAYR-ADYWMPLRPESDGALFLGAMKIIIDENMHDIDFLKSFTDAPILVRT 1e18.1    IPEHGAYPGLEALKAKGTKVIVIDPVRTKTVEFFGAEHITPKPQTDVAIMLGMAHTLVAEDLYDKDFIANYTSGF-----  target    DTLQYLDPRDVIADYKFPDFSKSYSGRIQSLKPEQIQRLGGMMVWDLNKKQVVPLHREQVGWHYTNSGIDAALTGTYRVK 1e18.1    -----------------DKFLP-------------------------------------------------YLDGET---  target    LLNGREIDAMPIWQMYMVHFQDYDLDTVHQITRTPKDLIVRWARDSGTIKPAAIHNGEGTCHYFHQTANARGAAMVLIIT 1e18.1    ------------------DSTPKTAEWAEGISGVPAETIKELARLFES-KRTMLAAGWSMQRMHHGEQAHWMLVTLASML  target    GNVGKFGTGQHTWAGNYKAGTWTATPWSGAGLSVHTGEDP-FNITLDPNAHGKEIKTRSYYYGEEVGYWNHGDTALIVNT 1e18.1    GQIGLPGGGFGLSYHYSGGGT-PS--TSGPAL---AGITDGGAATKGPE-----WLA---ASG--ASVIPVARVVDMLEN  target    PKYGRKVFTGKTHMPTPSKFRWVVNVNVVNNAKHHYDMVRNVDPNIECLITQDIEMTSDINHADIAFAANSWMEFTYPEM 1e18.1    PGAEF-DFNGTRSKFPDVKMAYWVGGNPFVHHQD-RNRMVKAWEKLETFVVHDFQWTPTARHADIVLPATTSYERNDIET  target    TVTVSNPWVQIWKGGIRPLYDTRNDLDTFAGVAAKLSDMTGDKRMRDYFAMVYQNRVDVYVQRMLDASS---TFYGY--- 1e18.1    IGDYSNTGILAMKKIVEPLYEARSDYDIFAAVAERLGKGK-------EFTE--GKDEMGWIKSFYDDAAKQGKAAGVEMP  target    SADVMLKSEKGWMVMVRT--YPR--HPFWEETNESKPMWTRSGRYENYRIEPEAIEYGENFISHREGPEATPYLPNAIFT 1e18.1    AFDAFWA--EGIVEFPVTDGADFVRYASFREDPLLNPLGTPTGLIEIYSKNIEKMGYD-----------DCPAHP-----  target    TNPYVRPDDYGIPITAQHHDDKTVRNIKLSWHEIKRHSNPLWEKGYQFYCVTPKTRHRVHSQWSVNDWVQIYESNFGDPY 1e18.1    -----------------------------TWMEPLERL-DGPGAKYPLHIAASHPFNRLHSQLN-GTVLREGY-------  target    RMDKRTPGVGEHQIHINPQAAKDRGINDGDYVYVDGNPVDRPYRGWKPSDPYYKVARLMIRAKYNPAYPYHVTMAKHAPF 1e18.1    ---A---VQGHEPCLMHPDDAAARGIADGDVVRVHND-----------------RGQILTGVKVTDAVMKGVIQIYEGGW  target    VATAKSVKGHETRPDGRAIAIDTGYQSNFRYGAQQSFTRNWLMPMHQTDSLPGKHAVAWKFKWGYQVDHHAINTVPKECL 1e18.1    YDPS----------------------------------------------------------------------------  target    IRITKAEDGGIGARGPWEPVRTGFTPGQENEFMIKWLKGEHIKIKV 1e18.1    ---------------------------------------------- ``` | | | | | | | | | | | | | | | | | | | | | | | | | | | | | | | | | | | | | | | | | | | | | | | | | |
|  | 1e60.1.A | Dimethyl sulfoxide/trimethylamine N-oxide reductase  *OXIDIZED DMSO REDUCTASE EXPOSED TO HEPES - Structure II BUFFER* | 0.31 |  | 19.51 | 0.64 | 1-1023 | X-ray | 2.00 | monomer | 2 x PGD, 1 x 2MO | HHblits | 0.28 |
| ``` target    MFLSRRQFLKVSVGTVAAVAVADKVLALTALQPVIEVGNPLGDYPDRSWERVYHDQYRYDSSFTWVCSPNDTHACR-VRA 1e60.1    AELYRRAFLSYSVAPGALGMFGRSLL-----AK---G-----ARA--E----------AL---ANGTV-MSGSHWGVFTA  target    FVRNGVVMRVEQNYDHQTYEDLYGNRGTFAHNPRMCLKGFTFHRRVYGPYRLKGPLMRKGWKQWMDDNAPELTAETKRKY 1e60.1    TVENGRATAFTPWEKDPH----------------PSPMLAGVLDSIYSPTRIKYPMVRREFLEKGV------------NA  target    KFDSRFLDDMLRVSWDTAFTYAAKAMITIATRYSGEAGARRLREQGYAPEMIEMMKGAGTRCFKHRAGMPVLGIIGKMGN 1e60.1    DRSTRGNGDFVRVSWDQALDLVAAEVKRVEETYGPEGVFGG----SYGW------KSPGR--LHNCT-TLLRRMLTLAGG  target    TRMNGGINALLDTWIRKVSPDQAQGGRYWSNYTWHGDQNPAHPFWSGVQGSDIDLSDMRFSKLNTSWGKNFVENKMPEA- 1e60.1    YVNGAG--------------DYSTGAAQVIMPHVV-----GTLEVYE--QQTAWPVLAENTEVMVFWAADPIKTSQIGWV  target    -------HWKLECIERGARVVVITPEYNPTAYR-ADYWMPLRPESDGALFLGAMKIIIDENMHDIDFLKSFTDAPILVRT 1e60.1    IPEHGAYPGLEALKAKGTKVIVIDPVRTKTVEFFGAEHITPKPQTDVAIMLGMAHTLVAEDLYDKDFIANYTSGF-----  target    DTLQYLDPRDVIADYKFPDFSKSYSGRIQSLKPEQIQRLGGMMVWDLNKKQVVPLHREQVGWHYTNSGIDAALTGTYRVK 1e60.1    -----------------DKFLP-------------------------------------------------YLDGET---  target    LLNGREIDAMPIWQMYMVHFQDYDLDTVHQITRTPKDLIVRWARDSGTIKPAAIHNGEGTCHYFHQTANARGAAMVLIIT 1e60.1    ------------------DSTPKTAEWAEGISGVPAETIKELARLFES-KRTMLAAGWSMQRMHHGEQAHWMLVTLASML  target    GNVGKFGTGQHTWAGNYKAGTWTATPWSGAGLSVHTGE-DPFNITLDPNAHGKEIKTRSYYYGEEVGYWNHGDTALIVNT 1e60.1    GQIGLPGGGFGLSYHYSGGG-TPS--TSGPAL---AGITDGGAATKGPEW----LA----ASG--ASVIPVARVVDMLEN  target    PKYGRKVFTGKTHMPTPSKFRWVVNVNVVNNAKHHYDMVRNVDPNIECLITQDIEMTSDINHADIAFAANSWMEFTYPEM 1e60.1    PGAEFD-FNGTRSKFPDVKMAYWVGGNPFVHHQD-RNRMVKAWEKLETFVVHDFQWTPTARHADIVLPATTSYERNDIET  target    TVTVSNPWVQIWKGGIRPLYDTRNDLDTFAGVAAKLSDMTGDKRMRDYFAMVYQNRVDVYVQRMLDASS---TFYGY--- 1e60.1    IGDYSNTGILAMKKIVEPLYEARSDYDIFAAVAERLGKGAE---FTE------GKDEMGWIKSFYDDAAKQGKAAGVEMP  target    SADVMLKSEKGWMVMVRT--YPR--HPFWEETNESKPMWTRSGRYENYRIEPEAIEYGENFISHREGPEATPYLPNAIFT 1e60.1    AFDAFWA--EGIVEFPVTDGADFVRYASFREDPLLNPLGTPTGLIEIYSKNIEKMGYD-----------DCPAH------  target    TNPYVRPDDYGIPITAQHHDDKTVRNIKLSWHEIKRHSNPLWEKGYQFYCVTPKTRHRVHSQWSVNDWVQIYESNFGDPY 1e60.1    ----------------------------PTWMEPLERL-DGPGAKYPLHIAASHPFNRLHSQLN-GTVLREGY-------  target    RMDKRTPGVGEHQIHINPQAAKDRGINDGDYVYVDGNPVDRPYRGWKPSDPYYKVARLMIRAKYNPAYPYHVTMAKHAPF 1e60.1    ---A---VQGHEPCLMHPDDAAARGIADGDVVRVHND-----------------RGQILTGVKVTDAVMKGVIQIYEGGW  target    VATAKSVKGHETRPDGRAIAIDTGYQSNFRYGAQQSFTRNWLMPMHQTDSLPGKHAVAWKFKWGYQVDHHAINTVPKECL 1e60.1    YDPS----------------------------------------------------------------------------  target    IRITKAEDGGIGARGPWEPVRTGFTPGQENEFMIKWLKGEHIKIKV 1e60.1    ---------------------------------------------- ``` | | | | | | | | | | | | | | | | | | | | | | | | | | | | | | | | | | | | | | | | | | | | | | | | | |
|  | 1e5v.2.A | Dimethyl sulfoxide/trimethylamine N-oxide reductase  *OXIDIZED DMSO REDUCTASE EXPOSED TO HEPES BUFFER* | 0.31 |  | 19.67 | 0.64 | 1-1022 | X-ray | 2.40 | monomer | 2 x PGD, 1 x 2MO | HHblits | 0.28 |
| ``` target    MFLSRRQFLKVSVGTVAAVAVADKVLALTALQPVIEVGNPLGDYPDRSWERVYHDQYRYDSSFTWVCSPNDTHACR-VRA 1e5v.2    AELYRRAFLSYSVAPGALGMFGRSLL------A--KG-----ARA----E--------ALA---NGTV-MSGSHWGVFTA  target    FVRNGVVMRVEQNYDHQTYEDLYGNRGTFAHNPRMCLKGFTFHRRVYGPYRLKGPLMRKGWKQWMDDNAPELTAETKRKY 1e5v.2    TVENGRATAFTPWEKDPH----------------PSPMLAGVLDSIYSPTRIKYPMVRREFLEKGV------------NA  target    KFDSRFLDDMLRVSWDTAFTYAAKAMITIATRYSGEAGARRLREQGYAPEMIEMMKGAGTRCFKHRAGMPVLGIIGKMGN 1e5v.2    DRSTRGNGDFVRVSWDQALDLVAAEVKRVEETYGPEGVFGG----SYGW------KSPGR--LHNCT-TLLRRMLTLAGG  target    TRMNGGINALLDTWIRKVSPDQAQGGRYWSNYTWHGDQNPAHPFWSGVQGSDIDLSDMRFSKLNTSWGKNFVENKMPEA- 1e5v.2    YVNGAG--------------DYSTGAAQV-----IMPHVVGTLEVYEQ--QTAWPVLAENTEVMVFWAADPIKTSQIGWV  target    -------HWKLECIERGARVVVITPEYNPTAYR-ADYWMPLRPESDGALFLGAMKIIIDENMHDIDFLKSFTDAPILVRT 1e5v.2    IPEHGAYPGLEALKAKGTKVIVIDPVRTKTVEFFGAEHITPKPQTDVAIMLGMAHTLVAEDLYDKDFIANYTSGF-----  target    DTLQYLDPRDVIADYKFPDFSKSYSGRIQSLKPEQIQRLGGMMVWDLNKKQVVPLHREQVGWHYTNSGIDAALTGTYRVK 1e5v.2    -----------------DKFLP-------------------------------------------------YLDGET---  target    LLNGREIDAMPIWQMYMVHFQDYDLDTVHQITRTPKDLIVRWARDSGTIKPAAIHNGEGTCHYFHQTANARGAAMVLIIT 1e5v.2    ------------------DSTPKTAEWAEGISGVPAETIKELARLFES-KRTMLAAGWSMQRMHHGEQAHWMLVTLASML  target    GNVGKFGTGQHTWAGNYKAGTWTATPWSGAGLSVHTGEDP-FNITLDPNAHGKEIKTRSYYYGEEVGYWNHGDTALIVNT 1e5v.2    GQIGLPGGGFGLSYHYSGGGTP-S--TSGPAL---AGITDGGAATKGPEW-LAASGA------SVIPVARV--VDM-LEN  target    PKYGRKVFTGKTHMPTPSKFRWVVNVNVVNNAKHHYDMVRNVDPNIECLITQDIEMTSDINHADIAFAANSWMEFTYPEM 1e5v.2    PGAE-FDFNGTRSKFPDVKMAYWVGGNPFVHHQD-RNRMVKAWEKLETFVVHDFQWTPTARHADIVLPATTSYERNDIET  target    TVTVSNPWVQIWKGGIRPLYDTRNDLDTFAGVAAKLSDMTGDKRMRDYFAMVYQNRVDVYVQRMLDASS---TFYGY--- 1e5v.2    IGDYSNTGILAMKKIVEPLYEARSDYDIFAAVAERLGKGA-------EFTE--GKDEMGWIKSFYDDAAKQGKAAGVQMP  target    SADVMLKSEKGWMVMVR----TYPRHPFWEETNESKPMWTRSGRYENYRIEPEAIEYGENFISHREGPEATPYLPNAIFT 1e5v.2    AFDAFWA--EGIVEFPVTDGADFVRYASFREDPLLNPLGTPTGLIEIYSKNIEKMGYD-----------DCPAH------  target    TNPYVRPDDYGIPITAQHHDDKTVRNIKLSWHEIKRHSNPLWEKGYQFYCVTPKTRHRVHSQWSVNDWVQIYESNFGDPY 1e5v.2    ----------------------------PTWMEPLERL-DGPGAKYPLHIAASHPFNRLHSQL-NGTVLREGY-------  target    RMDKRTPGVGEHQIHINPQAAKDRGINDGDYVYVDGNPVDRPYRGWKPSDPYYKVARLMIRAKYNPAYPYHVTMAKHAPF 1e5v.2    ---A---VQGHEPCLMHPDDAAARGIADGDVVRVHND-----------------RGQILTGVKVTDAVMKGVIQIYEGGW  target    VATAKSVKGHETRPDGRAIAIDTGYQSNFRYGAQQSFTRNWLMPMHQTDSLPGKHAVAWKFKWGYQVDHHAINTVPKECL 1e5v.2    YDP-----------------------------------------------------------------------------  target    IRITKAEDGGIGARGPWEPVRTGFTPGQENEFMIKWLKGEHIKIKV 1e5v.2    ---------------------------------------------- ``` | | | | | | | | | | | | | | | | | | | | | | | | | | | | | | | | | | | | | | | | | | | | | | | | | |
|  | 1tmo.1.A | TRIMETHYLAMINE N-OXIDE REDUCTASE  *TRIMETHYLAMINE N-OXIDE REDUCTASE FROM SHEWANELLA MASSILIA* | 0.31 |  | 18.13 | 0.64 | 75-1109 | X-ray | 2.50 | monomer | 2 x 2MD, 1 x 2MO | HHblits | 0.29 |
| ``` target    MFLSRRQFLKVSVGTVAAVAVADKVLALTALQPVIEVGNPLGDYPDRSWERVYHDQYRYDSSFTWVCSPNDTHACRVRAF 1tmo.1    --------------------------------------------------------------------------GAFKMK  target    VRNGVVMRVEQNYDHQTYEDLYGNRGTFAHNPRMCLKGFTFHRRVYGPYRLKGPLMRKGWKQWMDDNAPELTAETKRKYK 1tmo.1    RKNGVIAEVKPFDLDKY------P--------TDMING--IRGMVYNPSRVRYPMVRLDFLLKG------------HKSN  target    FDSRFLDDMLRVSWDTAFTYAAKAMITIATRYSGEAGARRLREQGYAPEMIEMMKGAGTRCFKHRAGMPVLGIIGKMGNT 1tmo.1    THQRGDFRFVRVTWDKALTLFKHSLDEVQTQYGPSGLHAGQT----GWR------ATGQLHSS---TSHMQRAVGMHGNY  target    RMNGGINALLDTWIRKVSPDQAQGGRYW-SNYTWHGDQNPAHPFWSGVQGSDIDLSDMRFSKLNTSWGKNFVENKMP--- 1tmo.1    ---------VKKI-G----DYSTGAGQTILPYVL------GSTEVYA--QGTSWPLILEHSDTIVLWSNDPYKNLQVGWN  target    -----EAH---WKLECIE-RGARVVVITPEYNPTAYR-ADYWMPLRPESDGALFLGAMKIIIDENMHDIDFLKSFTDAPI 1tmo.1    AETHESFAYLAQLKEKVKQGKIRVISIDPVVTKTQAYLGCEQLYVNPQTDVTLMLAIAHEMISKKLYDDKFIQGYSLGF-  target    LVRTDTLQYLDPRDVIADYKFPDFSKSYSGRIQSLKPEQIQRLGGMMVWDLNKKQVVPLHREQVGWHYTNSGIDAALTGT 1tmo.1    ---------------------EEF--------------------------------------------------------  target    YRVKLLNGREIDAMPIWQMYMVHF------QDYDLDTVHQITRTPKDLIVRWARDSGTIKPAAIHNGEGTCHYFHQTANA 1tmo.1    --------------------VPYVMGTKDGVAKTPEWAAPICGVEAHVIRDLAKTLVKGR-TQFMMGWCIQRQQHGEQPY  target    RGAAMVLIITGNVGKFGTGQHTWAGNYKAGTWTATPWSGAGLSVHTGEDPFNITLDPNAHGKEIKTRSYYYGEEVGYWNH 1tmo.1    WMAAVLATMIGQIGLPGGGISYGHHYSSIGV-P---SS--GAAA-PGAFPR--NLDENQKP-LFDSSD-FKG-ASSTI--  target    GDTALIVNTPKYGRKVFT--------GKTHMPTPSKFRWVVNVNVVNNAKHHYDMVRNVDPNIECLITQDIEMTSDINHA 1tmo.1    -------PVARWIDAILEPGKTIDANGSKVVYPDIKMMIFSGNNPWNHHQD-RNRMKQAFHKLECVVTVDVNWTATCRFS  target    DIAFAANSWMEFTYPEMTVTVSNPWVQIWKGGIRPLYDTRNDLDTFAGVAAKLSDMTGDKRMRDYFAMVYQNRVDVYVQR 1tmo.1    DIVLPACTTYERNDIDVYGAYANRGILAMQKMVEPLFDSLSDFEIFTRFAAVLGKEK------EYTR---NMGEMEWLET  target    MLDASS-----TFYGYSADVMLKSEKGWMVMVRT--YPRHPFWEETNESKPMWTRSGRYENYRIEPEAIEYGENFISHRE 1tmo.1    LYNECKAANAGKFEMPDFATFWK--QGYVHFGDGEVWTRHADFRNDPEINPLGTPSGLIEIFSRKIDQFGYD--------  target    GPEATPYLPNAIFTTNPYVRPDDYGIPITAQHHDDKTVRNIKLSWHEIKRH--SNPLWEKGYQFYCVTPKTRHRVHSQWS 1tmo.1    -D--CKGHPT----------------------------------WMEKTERSHGGP-GSDKHPIWLQSCHPDKRLHSQMC  target    VNDWVQIYESNFGDPYRMDKRTPGVGEHQIHINPQAAKDRGINDGDYVYVDGNPVDRPYRGWKPSDPYYKVARLMIRAKY 1tmo.1    ESREYRETY----------A---VNGREPVYISPVDAKARGIKDGDIVRVFND-----------------RGQLLAGAVV  target    NPAYPYHVTMAKHAPFVATAKSVKGHETRPDGRAIAIDTGYQS-NFR-YGAQQSFTRNW-LMPMHQTDSLPGKHAVAWKF 1tmo.1    SDNFPKGIVRIHEGAWYGPVGKDG-----------STEGGAEVGALCSYGDPNTLTLDIGTSKL----------------  target    KWGYQVDHHAINTVPKECLIRITKAEDGGIGARGPWEPVRTGFTPGQENEFMIKWLKGEHIKIKV 1tmo.1    ---------AQACSAYTCLVEFEKYQGKV------------------------------------ ``` | | | | | | | | | | | | | | | | | | | | | | | | | | | | | | | | | | | | | | | | | | | | | | | | | |
|  | 6cz7.1.A | ArrA  *The arsenate respiratory reductase (Arr) complex from Shewanella sp. ANA-3* | 0.31 |  | 15.95 | 0.63 | 62-1017 | X-ray | 1.62 | hetero-1-1-mer | 5 x SF4, 2 x MGD, 1 x MO, 1 x PG5 | HHblits | 0.28 |
| ``` target    MFLSRRQFLKVSVGTVAAVAVADKVLALTALQPVIEVGNPLGDYPDRSWERVYHDQYRYDSSFTWVCSPNDTHACRVRAF 6cz7.1    -------------------------------------------------------------WLATTCQ-GCTSWCAKQIY  target    VRNGVVMRVEQNYDHQTYEDLYGNRGTFAHNPRMCLKGFTFHRRVYGPYRLKGPLMRKGWKQWMDDNAPELTAETKRKYK 6cz7.1    VMDGRALKVRGNPNSGV------------HGMSSCPRQHLSLQQVYDPDRLRTPMMRTNPKKG-----------------  target    FDSRFLDDMLRVSWDTAFTYAAKAMITIATRYSGEAGARRLREQGYAPEMIEMMKGAGTRCFKHRAGMPVLGIIGKMGNT 6cz7.1    --RDQDPKFVPISWDKALDMLADKIIALRVANEPHKYAL--LRGRYSHIN----------DL---LYKKM---TNLIGSP  target    RMNGGINALLDTWIRKVSPDQAQGGRYWSNYTWHGDQNPAHPFWSGVQGSDIDLSDMRFSKLNTSWGKNFVENKMPEAHW 6cz7.1    NNISHS-------------SVCAEAHK-----------MGPYYLDG--NWGYNQYDVKNAKFILSFGADPIASNRQVSFY  target    KL--ECIERGARVVVITPEYNPTAYRADYWMPLRPESDGALFLGAMKIIIDENMHDIDFLKSFTDAPILVRTDTLQYLDP 6cz7.1    SQTWGDSLDHAKVVVVDPRLSASAAKAHKWIPIEPGQDSVLALAIAHVALVEGVWHKPFVGDFIEGKNLFKAGKT-V---  target    RDVIADYKFPDFSKSYSGRIQSLKPEQIQRLGGMMVWDLNKKQVVPLHREQVGWHYTNSGIDAALTGTYRVKLLNGREID 6cz7.1    -------SVE---------------------------------------------------------SFK-------ETH  target    AMPIWQMYMVHFQDYDLDTVHQITRTPKDLIVRWARDSGTIKPAA-IHNGEGTCHYFHQTANARGAAMVLIITGNVGKFG 6cz7.1    TYGLVEWWNQALKDYTPEWASKITGIDPKTIIAIAKDMGAAAPAVQVWTSRGAVMQARGTYTSISCHALNGLFGGIDSKG  target    TGQHTWAGNYKAGTWTATPWSGAGLSVHTGEDPFNITLDPNAHGKEIKTRSYYYGEEVGYWNHGDTALIVNTPKYGRKVF 6cz7.1    GLFPGNKTPLL-KEYPE---A-KAYMDEI--AAKG--VKKEK-IDQRGRLEF-----PALAKGKSGGG-VITANAANGIR  target    TGKTHMPTPSKFRWVVNVNVVNNAKHHYDMVRNVDPNIECLITQDIEMTSDINHADIAFAANS-WMEFTYPEMTVTVSNP 6cz7.1    N---QDPYEIKVMLAYFNNFNFSNPE-GQRWDEALSKVDFMAHITTNVSEFSWFADVLLPSSHHMFEKWGVLDSIGNGVA  target    WVQIWKGGIRPLYDTRND-LDTFAGVAAKLSDMTGDKRMRDYFAMVY-----------QNRVDVYVQRMLDASS------ 6cz7.1    QISIQQPSIKRLWDTRIDESEIPYMLAKKLADKG----FDAPWRYINEQIVDPETGKPAADEAEFAKLMVRYLTAPLWKE  target    -----TFYGYSADVMLKSEKGWMVMVRTYPRHPFWEETNESKPMWTRSGRYENYRIEPEAIEYGENFISHREGPEATPYL 6cz7.1    DASKYGDKLSSWDEFVQ--KGVWNSS----PYKL---EARWGKFKTETTKFEFYSKTLEKA-----LQSHADK---HKVS  target    PNAIFTTNPYVRPDDYGIPITAQHHDDKTVRNIKLSWHEIKRHSNPLWEKGYQFYCVTPKTRHRVHSQWSVNDWVQIYES 6cz7.1    IDEVMKACDYQA---RG------------HLAFIPHYEEPYR--FG-DESEFPLLLVDQKSRLNKEGRTANSPWYYEFKD  target    NFGDPYRMDKRTPGVGEHQIHINPQAAKDRGINDGDYVYVDGNPVDRPYRGWKPSDPYYKVARLMIRAKYNPAYPYHVTM 6cz7.1    V----DP-GD---VANEDVAKFNPIDGKKFGLKDGDEIRITSP-----------------VGMLTCKAKLWEGVRPGTVA  target    AKHAPFVATAKSVKGHETRPDGRAIAIDTGYQSNFRYGAQQSFTRNWLMPMHQTDSLPGKHAVAWKFKWGYQVDHHAINT 6cz7.1    KCFG----------------------------------------------------------------------------  target    VPKECLIRITKAEDGGIGARGPWEPVRTGFTPGQENEFMIKWLKGEHIKIKV 6cz7.1    ---------------------------------------------------- ``` | | | | | | | | | | | | | | | | | | | | | | | | | | | | | | | | | | | | | | | | | | | | | | | | | |
|  | 2vpz.1.A | THIOSULFATE REDUCTASE  *POLYSULFIDE REDUCTASE NATIVE STRUCTURE* | 0.32 | 0.00 | 20.23 | 0.61 | 3-1021 | X-ray | 2.40 | monomer | 10 x SF4, 4 x MGD, 2 x MO | HHblits | 0.30 |
| ``` target    MFLSRRQFLKVSVGTVAAVAVADKVLALTALQPVIEVGNPLGDYPDRSWERVYHDQYRYDSSFTWVCSPNDTHACRVRAF 2vpz.1    --MQRREFLKLSALGVGAMALRGSG-PAK--------------ALKAPWYA------QEVKSVYQICE-GCFWRCGIVAH  target    VRNGVVMRVEQNYDHQTYEDLYGNRGTFAHNPRMCLKGFTFHRRVYGPYRLKGPLMRKGWKQWMDDNAPELTAETKRKYK 2vpz.1    AVGNRVYKVEGYEANPK------------SRGRLCPRGQGAPQTTYDPDRLKRPLIRVEGS-------------------  target    FDSRFLDDMLRVSWDTAFTYAAKAMITIATRYSGEAGARRLREQGYAPEMIEMMKGAGTRCFKHRAGMPVLGIIGKMGNT 2vpz.1    --QRGEGKYRVATWEEALDHIAKKMLEIREKYGPEAIAFFG--H--G---------TGDYWFVDF----LPAA---WGSP  target    RMNGGINALLDTWIRKVSPDQAQGGRYWSNYTWHGDQNPAHPFWSGVQGSDIDLSDMRFSKLNTSWGKNFVENKM-PEAH 2vpz.1    NAAKPSVS------------LCTAPR-----------EVASQWVFGRPIGGHEPIDWENARYIVLIGHHIGEDTHNTQLQ  target    WKLECIERGARVVVITPEYNPTAYRADYWMPLRPESDGALFLGAMKIIIDENMHDIDFLKSFTDAPILVRTDTLQYLDPR 2vpz.1    DFALALKNGAKVVVVDPRFSTAAAKAHRWLPIKPGTDTALLLAWIHVLIYEDLYDKEYVAKYTVGF--------------  target    DVIADYKFPDFSKSYSGRIQSLKPEQIQRLGGMMVWDLNKKQVVPLHREQVGWHYTNSGIDAALTGTYRVKLLNGREIDA 2vpz.1    --------E-----------------------------------------------------------------------  target    MPIWQMYMVHFQDYDLDTVHQITRTPKDLIVRWARDSGTIKPAAIHNGEGT-CHYFHQTANARGAAMVLIITGNVGKFGT 2vpz.1    -----ELKAHVKDFTPEWAEKHTEIPAQVIREVAREMAAHKPRAVLPPTRHNVWYGDDTYRVMALLYVNVLLGNYGRPGG  target    GQHTWAGNYKAGTWTATPWSGAGLSVHTGE-DPFNITLDPNAHGKEIKTRSYYYGEEVGYWNHGDTALIVNTPKYGRKVF 2vpz.1    FYIAQSPYLEK--YPLPPLPL-E-PAAGGCSGPSGGDHEPEGFKPRAD--------KGKFFARS-----TAIQELIEPMI  target    TGKTHMPTPSKFRWVVNVNVVNNAKHHYDMVRNVDPNIECLITQDIEMTSDINHADIAFAANSWMEFTYPEMTVTVSNPW 2vpz.1    TGE---PYPIKGLFAYGINLFHSIPNV-PRTKEALKNLDLYVAIDVLPQEHVMWADVILPEATYLERYDDFVLVAHKTPF  target    VQIWKGGIRPLYDTRNDLDTFAGVAAKLSDMTGDKRMRDYFAMVYQNRVDVYVQRMLDASSTFYGYSADVMLKSEKGWMV 2vpz.1    IQLRTPAHEPLFDTKPGWWIARELGLRLGLE-------QYFP---WKTIEEYLETRLQSL----GLDLETMKG--MGTLV  target    MVRTYPRHPFWEETNESKPMWTRSGRYENYRIEPEAIEYGENFISHREGPEATPYLPNAIFTTNPYVRPDDYGIPITAQH 2vpz.1    Q-RGKPWLEDWE-KEGRLPFGTASGKIELYCQRFKEAGH---------Q----P-LP-----------------------  target    HDDKTVRNIKLSWHEIKRHSNPLWEKGYQFYCVTPKTRHRVHSQWSVNDWVQIYESNFGDPYRMDKRTPGVGEHQIHINP 2vpz.1    -----------VFTPPE------EPPEGFYRLLYGRSPVHTFARTQNNWVLMEM----------------DPENEVWIHK  target    QAAKDRGINDGDYVYVDGNPVDRPYRGWKPSDPYYKVARLM--IRAKYNPAYPYHVTMAKHAPFVATAKSVKGHETRPDG 2vpz.1    EEAKRLGLKEGDYVMLVNQ-----------------DGVKEGPVRVKPTARIRKDCVYIVHGFGHK--------------  target    RAIAIDTGYQSNFRYGAQQSFTRNWLMPMHQTDSLPGKHAVAWKFKWGYQVDHHAINTVPKECLIRITKAEDGGIGARGP 2vpz.1    --------------------------------------------------------------------------------  target    WEPVRTGFTPGQENEFMIKWLKGEHIKIKV 2vpz.1    ------------------------------ ``` | | | | | | | | | | | | | | | | | | | | | | | | | | | | | | | | | | | | | | | | | | | | | | | | | |
|  | 2vpx.1.D | THIOSULFATE REDUCTASE  *POLYSULFIDE REDUCTASE WITH BOUND QUINONE (UQ1)* | 0.31 | 0.00 | 20.23 | 0.61 | 3-1021 | X-ray | 3.10 | monomer | 10 x SF4, 4 x MGD, 2 x MO, 2 x UQ1 | HHblits | 0.30 |
| ``` target    MFLSRRQFLKVSVGTVAAVAVADKVLALTALQPVIEVGNPLGDYPDRSWERVYHDQYRYDSSFTWVCSPNDTHACRVRAF 2vpx.1    --MQRREFLKLSALGVGAMALRGSG-PAK--------------ALKAPWYA------QEVKSVYQICE-GCFWRCGIVAH  target    VRNGVVMRVEQNYDHQTYEDLYGNRGTFAHNPRMCLKGFTFHRRVYGPYRLKGPLMRKGWKQWMDDNAPELTAETKRKYK 2vpx.1    AVGNRVYKVEGYEANPK------------SRGRLCPRGQGAPQTTYDPDRLKRPLIRVEGS-------------------  target    FDSRFLDDMLRVSWDTAFTYAAKAMITIATRYSGEAGARRLREQGYAPEMIEMMKGAGTRCFKHRAGMPVLGIIGKMGNT 2vpx.1    --QRGEGKYRVATWEEALDHIAKKMLEIREKYGPEAIAFFG--H--G---------TGDYWFVDF----LPAA---WGSP  target    RMNGGINALLDTWIRKVSPDQAQGGRYWSNYTWHGDQNPAHPFWSGVQGSDIDLSDMRFSKLNTSWGKNFVENKM-PEAH 2vpx.1    NAAKPSVS------------LCTAPR-----------EVASQWVFGRPIGGHEPIDWENARYIVLIGHHIGEDTHNTQLQ  target    WKLECIERGARVVVITPEYNPTAYRADYWMPLRPESDGALFLGAMKIIIDENMHDIDFLKSFTDAPILVRTDTLQYLDPR 2vpx.1    DFALALKNGAKVVVVDPRFSTAAAKAHRWLPIKPGTDTALLLAWIHVLIYEDLYDKEYVAKYTVGF--------------  target    DVIADYKFPDFSKSYSGRIQSLKPEQIQRLGGMMVWDLNKKQVVPLHREQVGWHYTNSGIDAALTGTYRVKLLNGREIDA 2vpx.1    --------E-----------------------------------------------------------------------  target    MPIWQMYMVHFQDYDLDTVHQITRTPKDLIVRWARDSGTIKPAAIHNGEGT-CHYFHQTANARGAAMVLIITGNVGKFGT 2vpx.1    -----ELKAHVKDFTPEWAEKHTEIPAQVIREVAREMAAHKPRAVLPPTRHNVWYGDDTYRVMALLYVNVLLGNYGRPGG  target    GQHTWAGNYKAGTWTATPWSGAGLSVHTGE-DPFNITLDPNAHGKEIKTRSYYYGEEVGYWNHGDTALIVNTPKYGRKVF 2vpx.1    FYIAQSPYLEK--YPLPPLPL-E-PAAGGCSGPSGGDHEPEGFKPRAD--------KGKFFARS-----TAIQELIEPMI  target    TGKTHMPTPSKFRWVVNVNVVNNAKHHYDMVRNVDPNIECLITQDIEMTSDINHADIAFAANSWMEFTYPEMTVTVSNPW 2vpx.1    TGE---PYPIKGLFAYGINLFHSIPNV-PRTKEALKNLDLYVAIDVLPQEHVMWADVILPEATYLERYDDFVLVAHKTPF  target    VQIWKGGIRPLYDTRNDLDTFAGVAAKLSDMTGDKRMRDYFAMVYQNRVDVYVQRMLDASSTFYGYSADVMLKSEKGWMV 2vpx.1    IQLRTPAHEPLFDTKPGWWIARELGLRLGLE-------QYFP---WKTIEEYLETRLQSL----GLDLETMKG--MGTLV  target    MVRTYPRHPFWEETNESKPMWTRSGRYENYRIEPEAIEYGENFISHREGPEATPYLPNAIFTTNPYVRPDDYGIPITAQH 2vpx.1    Q-RGKPWLEDWE-KEGRLPFGTASGKIELYCQRFKEAGH---------Q----P-LP-----------------------  target    HDDKTVRNIKLSWHEIKRHSNPLWEKGYQFYCVTPKTRHRVHSQWSVNDWVQIYESNFGDPYRMDKRTPGVGEHQIHINP 2vpx.1    -----------VFTPPE------EPPEGFYRLLYGRSPVHTFARTQNNWVLMEM----------------DPENEVWIHK  target    QAAKDRGINDGDYVYVDGNPVDRPYRGWKPSDPYYKVARLM--IRAKYNPAYPYHVTMAKHAPFVATAKSVKGHETRPDG 2vpx.1    EEAKRLGLKEGDYVMLVNQ-----------------DGVKEGPVRVKPTARIRKDCVYIVHGFGHK--------------  target    RAIAIDTGYQSNFRYGAQQSFTRNWLMPMHQTDSLPGKHAVAWKFKWGYQVDHHAINTVPKECLIRITKAEDGGIGARGP 2vpx.1    --------------------------------------------------------------------------------  target    WEPVRTGFTPGQENEFMIKWLKGEHIKIKV 2vpx.1    ------------------------------ ``` | | | | | | | | | | | | | | | | | | | | | | | | | | | | | | | | | | | | | | | | | | | | | | | | | |
|  | 1dms.1.A | DMSO REDUCTASE  *STRUCTURE OF DMSO REDUCTASE* | 0.29 |  | 19.54 | 0.61 | 66-1023 | X-ray | 1.88 | monomer | 2 x PGD, 1 x 2MO | HHblits | 0.29 |
| ``` target    MFLSRRQFLKVSVGTVAAVAVADKVLALTALQPVIEVGNPLGDYPDRSWERVYHDQYRYDSSFTWVCSPNDTHACRVRAF 1dms.1    -----------------------------------------------------------------GTVMSGSHWGVFTAT  target    VRNGVVMRVEQNYDHQTYEDLYGNRGTFAHNPRMCLKGFTFHRRVYGPYRLKGPLMRKGWKQWMDDNAPELTAETKRKYK 1dms.1    VENGRATAFTPWEKDPH----------------PTPMLEGVLDSIYSPTRIKYPMVRREFLEKGV------------NAD  target    FDSRFLDDMLRVSWDTAFTYAAKAMITIATRYSGEAGARRLREQGYAPEMIEMMKGAGTRCFKHRAGMPVLGIIGKMGNT 1dms.1    RSTRGNGDFVRVSWDQALDLVAAEVKRVEETYGPQGVFGG----SYGWK------SPGRLHNCTTLLRRM---LTLAGGY  target    RMNGGINALLDTWIRKVSPDQAQGGRYWSNYTWHGDQNPAHPFWSGVQGSDIDLSDMRFSKLNTSWGKNFVENKMPEA-- 1dms.1    VNGAG--------------DYSTGAAQVIMPHVVGT-----LEVYE--QQTAWPVLAENTEVMVFWAADPIKTSQIGWVI  target    ------HWKLECIERGARVVVITPEYNPTAYR-ADYWMPLRPESDGALFLGAMKIIIDENMHDIDFLKSFTDAPILVRTD 1dms.1    PEHGAYPGLEALKAKGTKVIVIDPVRTKTVEFFGADHVTPKPQTDVAIMLGMAHTLVAEDLYDKDFIANYTSGF------  target    TLQYLDPRDVIADYKFPDFSKSYSGRIQSLKPEQIQRLGGMMVWDLNKKQVVPLHREQVGWHYTNSGIDAALTGTYRVKL 1dms.1    ----------------DKFLP-------------------------------------------------YLMGE-----  target    LNGREIDAMPIWQMYMVHFQDYDLDTVHQITRTPKDLIVRWARDSGTIKPAAIHNGEGTCHYFHQTANARGAAMVLIITG 1dms.1    ----------------TDSTPKTAEWASDISGVPAETIKELARLFKS-KRTMLAAGWSMQRMHHGEQAHWMLVTLASMLG  target    NVGKFGTGQHTWAGNYKAGTWTATPWSGAGLSVHTGE-DPFNITLDPNAHGKEIKTRSYYYGEEVGYWNHGD-TALIVNT 1dms.1    QIGLPGGGFGLSYHYSGGGTPSS---SGPAL---SGITDGGAATKGPE----WLAA----SG--ASVIPVARVVDMLENP  target    PKYGRKVFTGKTHMPTPSKFRWVVNVNVVNNAKHHYDMVRNVDPNIECLITQDIEMTSDINHADIAFAANSWMEFTYPEM 1dms.1    GAEFDF--NGTRSKFPDVKMAYWVGGNPFVHHQD-RNRMVKAWEKLETFIVHDFQWTPTARHADIVLPATTSYERNDIET  target    TVTVSNPWVQIWKGGIRPLYDTRNDLDTFAGVAAKLSDMTGDKRMRDYFAMVYQNRVDVYVQRMLDASS---TFYGY--- 1dms.1    IGDYSNTGILAMKKIVEPLYEARSDYDIFAAVAERLGKGKE------F-TE--GKDEMGWIKSFYDDAAKQGKAGGVEMP  target    SADVMLKSEKGWMVMVRT--YPRHP--FWEETNESKPMWTRSGRYENYRIEPEAIEYGENFISHREGPEATPYLPNAIFT 1dms.1    AFDAFWA--EGIVEFPVTDGADFVRYASFREDPLLNPLGTPTGLIEIYSKNIEKMGYD---------D--CPAHP-----  target    TNPYVRPDDYGIPITAQHHDDKTVRNIKLSWHEIKRHSNPLWEKGYQFYCVTPKTRHRVHSQWSVNDWVQIYESNFGDPY 1dms.1    -----------------------------TWMEPLER-LDGPGAKYPLHIAASHPFNRLHSQLNG-TVLREGY-------  target    RMDKRTPGVGEHQIHINPQAAKDRGINDGDYVYVDGNPVDRPYRGWKPSDPYYKVARLMIRAKYNPAYPYHVTMAKHAPF 1dms.1    ---A---VQGHEPCLMHPDDAAARGIADGDVVRVHND-----------------RGQILTGVKVTDAVMKGVIQIYEGGW  target    VATAKSVKGHETRPDGRAIAIDTGYQSNFRYGAQQSFTRNWLMPMHQTDSLPGKHAVAWKFKWGYQVDHHAINTVPKECL 1dms.1    YDPS----------------------------------------------------------------------------  target    IRITKAEDGGIGARGPWEPVRTGFTPGQENEFMIKWLKGEHIKIKV 1dms.1    ---------------------------------------------- ``` | | | | | | | | | | | | | | | | | | | | | | | | | | | | | | | | | | | | | | | | | | | | | | | | | |
|  | 1eu1.1.A | DIMETHYL SULFOXIDE REDUCTASE  *THE CRYSTAL STRUCTURE OF RHODOBACTER SPHAEROIDES DIMETHYLSULFOXIDE REDUCTASE REVEALS TWO DISTINCT MOLYBDENUM COORDINATION ENVIRONMENTS.* | 0.29 |  | 19.74 | 0.60 | 70-1020 | X-ray | 1.30 | monomer | 3 x GLC, 1 x CD, 2 x MGD, 1 x 6MO, 2 x O | HHblits | 0.29 |
| ``` target    MFLSRRQFLKVSVGTVAAVAVADKVLALTALQPVIEVGNPLGDYPDRSWERVYHDQYRYDSSFTWVCSPNDTHACRVRAF 1eu1.1    ---------------------------------------------------------------------GCHWGV-FKAR  target    VRNGVVMRVEQNYDHQTYEDLYGNRGTFAHNPRMCLKGFTFHRRVYGPYRLKGPLMRKGW-KQWMDDNAPELTAETKRKY 1eu1.1    VENGRAVAFEPWDKDPA------P------SHQLPG----VLDSIYSPTRIKYPMVRREFLEKGVNA-------------  target    KFDSRFLDDMLRVSWDTAFTYAAKAMITIATRYSGEAGARRLREQGYAPEMIEMMKGAGTRCFKHRAGMPVLGIIGKMGN 1eu1.1    DRSTRGNGDFVRVTWDEALDLVARELKRVQESYGPTGTFGGSYG-WKSPGRLHNC-----QVLMR----RA---LNLAGG  target    TRMNGGINALLDTWIRKVSPDQAQGGR-YWSNYTWHGDQNPAHPFWSGVQGSDIDLSDMRFSKLNTSWGKNFVENKMPEA 1eu1.1    FVNSSG------D--------YSTAAAQIIMPHVM-GT-----LEVYE--QQTAWPVVVENTDLMVFWAADPMKTNEIGW  target    --------HWKLECIERGARVVVITPEYNPTAYRAD-YWMPLRPESDGALFLGAMKIIIDENMHDIDFLKSFTDAPILVR 1eu1.1    VIPDHGAYAGMKALKEKGTRVICINPVRTETADYFGADVVSPRPQTDVALMLGMAHTLYSEDLHDKDFLENCTTGFDLFA  target    TDTLQYLDPRDVIADYKFPDFSKSYSGRIQSLKPEQIQRLGGMMVWDLNKKQVVPLHREQVGWHYTNSGIDAALTGTYRV 1eu1.1    A----Y-------------------------------------------------------------------LTGE---  target    KLLNGREIDAMPIWQMYMVHFQDYDLDTVHQITRTPKDLIVRWARDSGTIKPAAIHNGEGTCHYFHQTANARGAAMVLII 1eu1.1    ------------------SDGTPKTAEWAAEICGLPAEQIRELARSFVAGR-TMLAAGWSIQRMHHGEQAHWMLVTLASM  target    TGNVGKFGTGQHTWAGNYKAGTWTATPWSGAGLSVHTGEDPFNITLDPNAHGKEIKTRSYYYGEEVGYWNHGDTALIVNT 1eu1.1    IGQIGLPGGGFGLSYHYSNGGSP-TSDGPAL-GGISDGGKAV----EGAAWLSESGA------TS--IPCARVVDMLLNP  target    PKYGRKVFTGKTHMPTPSKFRWVVNVNVVNNAKHHYDMVRNVDPNIECLITQDIEMTSDINHADIAFAANSWMEFTYPEM 1eu1.1    GGEFQ--FNGATATYPDVKLAYWAGGNPFAHHQD-RNRMLKAWEKLETFIVQDFQWTATARHADIVLPATTSYERNDIES  target    TVTVSNPWVQIWKGGIRPLYDTRNDLDTFAGVAAKLSDMTGD---KRMRDYFAMVYQNRVDVYVQRMLDAS--STFYGYS 1eu1.1    VGDYSNRAILAMKKVVDPLYEARSDYDIFAALAERLGKGAEFTEGRDEMGWISS--------FYEAAVKQAEFKNVAMPS  target    ADVMLKSEKGWMVMVRT--YPRHPF--WEETNESKPMWTRSGRYENYRIEPEAIEYGENFISHREGPEATPYLPNAIFTT 1eu1.1    FEDFWS--EGIVEFPITEGANFVRYADFREDPLFNPLGTPSGLIEIYSKNIEKMGYD---------D--CPAHP------  target    NPYVRPDDYGIPITAQHHDDKTVRNIKLSWHEIKRHSNPLWEKGYQFYCVTPKTRHRVHSQWSVNDWVQIYESNFGDPYR 1eu1.1    ----------------------------TWMEPAERL-GGAGAKYPLHVVASHPKSRLHSQLNGT-SLRDLY--------  target    MDKRTPGVGEHQIHINPQAAKDRGINDGDYVYVDGNPVDRPYRGWKPSDPYYKVARLMIRAKYNPAYPYHVTMAKHAPFV 1eu1.1    --A---VAGHEPCLINPADAAARGIADGDVLRVFND-----------------RGQILVGAKVSDAVMPGAIQIYEGGWY  target    ATAKSVKGHETRPDGRAIAIDTGYQSNFRYGAQQSFTRNWLMPMHQTDSLPGKHAVAWKFKWGYQVDHHAINTVPKECLI 1eu1.1    --------------------------------------------------------------------------------  target    RITKAEDGGIGARGPWEPVRTGFTPGQENEFMIKWLKGEHIKIKV 1eu1.1    --------------------------------------------- ``` | | | | | | | | | | | | | | | | | | | | | | | | | | | | | | | | | | | | | | | | | | | | | | | | | |
|  | 7qv7.1.L | Hydrogen dependent carbon dioxide reductase subunit FdhF  *Cryo-EM structure of Hydrogen-dependent CO2 reductase.* | 0.29 |  | 21.42 | 0.58 | 63-1019 | EM | 0.00 | hetero-2-6-6-2-mer | 52 x SF4, 6 x 402 | HHblits | 0.30 |
| ``` target    MFLSRRQFLKVSVGTVAAVAVADKVLALTALQPVIEVGNPLGDYPDRSWERVYHDQYRYDSSFTWVCSPNDTHACRVRAF 7qv7.1    --------------------------------------------------------------VLTTCP-YCGTGCGLYLK  target    VRNGVVMRVEQNYDHQTYEDLYGNRGTFAHNPRMCLKGFTFHRRVYGPYRLKGPLMRKGWKQWMDDNAPELTAETKRKYK 7qv7.1    VENEKIVGVEPDKLHPV------------NQGELCIKGYYGYKYVHDPRRLTSPLIKKN---------------------  target    FDSRFLDDMLRVSWDTAFTYAAKAMITIATRYSGEAGARRLREQGYAPEMIEMMKGAGTRCFKHRAGMPVLGIIGKMGNT 7qv7.1    ------GKFVPVSWDEALNFIANGLKKIKSEYGSDAFAMFCSAR-ATNEDNYAA-------------QKFARA--VIGIN  target    RMNGGINALLDTWIRKVSPDQAQGGRYWSNYTWHGDQNPAHPFWSGVQGSDIDLSDMR-FSKLNTSWGKNFVENKMPEAH 7qv7.1    NVDH-------------------CARLCH-----APTVAGLAMTLGSGAMTNSIPEISTYSDVIFIIGSNTAECHPLIAA  target    WKLECIERGARVVVITPEYNPTAYRADYWMPLRPESDGALFLGAMKIIIDENMHDIDFLKSFTDAPILVRTDTLQYLDPR 7qv7.1    HVIKAKERGAKLIVADPRMNAMVHKADIWLRVPSGYNIPLINGMIHIIIKEGLVKTDFVKNHAVGF--------------  target    DVIADYKFPDFSKSYSGRIQSLKPEQIQRLGGMMVWDLNKKQVVPLHREQVGWHYTNSGIDAALTGTYRVKLLNGREIDA 7qv7.1    --------------------------------------------------------------------------------  target    MPIWQMYMVHFQDYDLDTVHQITRTPKDLIVRWARDSGTIKPAAIHNGEGTCHYFHQTANARGAAMVLIITGNVGKFGTG 7qv7.1    ----EEMAKAVEKYTPEYVEELTGIPKKDLIKAARFYGQAQAAAILYSMGVTQFSHGTGNVVSLANLAVITGNLGRPGAG  target    QHTWAGNYK-AGTWTATPWSGAGLSVHTGEDPFNITLDPNAHGKEIKTRSYYYGEEVGYWNHGDT-ALIVNTPKYGRKVF 7qv7.1    ICPLRGQNNVQGACDV----GALPNVLPGYLD----VTKEQNRERFE----------KVWGVKLPSNIGLRVTEVPDAIL  target    TGKTHMPTPSKFRWVVNVNVVNNAKHHYDMVRNVDPNIECLITQDIEMTSDINHADIAFAANSWMEFTYPEMTVTVSNPW 7qv7.1    ------NKRVRALYIFGENPIMSDPD-SDHLRHALEHLDLLIVQDIFLTETARLAHVVLPAACWAEKDG---TFTNTERR  target    VQIWKGGIRPLYDTRNDLDTFAGVAAKLSDMTGDKRMRDYFAMVYQNRVDVYVQRMLDASS-TFYGYSADVMLKSEKGWM 7qv7.1    VQRVRKAVEAPGEAKPDWWIFSQIAERMGYT-------GM----QYNNVQEIWDEVRKIVPEKFGGISYARLEK-EKGLA  target    VMVRTYPRHPFWEETNESKPMWTRSGRYENYRIEPEAIEYGENFISHREGPEATPYLPNAIFTTNPYVRPDDYGIPITAQ 7qv7.1    WPCP-TEDHTGTPILYLGGKFATPSGKAQMYPVIFYPNT-----CICDEGAEKQDF------------NH----VI----  target    HHDDKTVRNIKLSWHEIKRHSNPLWEKGYQFYCVTPKTRHRVHSQW--SVNDWVQIYESNFGDPYRMDKRTPGVGEHQIH 7qv7.1    -----------VGS------IAELPDEEYPFTLTTGRRVYHYHTATMTRKSPVIDQI----------------APQELVE  target    INPQAAKDRGINDGDYVYVDGNPVDRPYRGWKPSDPYYKVARLMIRAKYNPAYPYHVTMAKHAPFVATAKSVKGHETRPD 7qv7.1    INPQDATRLGINDGDFLRVSTR-----------------RGYVATRAWVTERVPKGTIFMTFHYW---------------  target    GRAIAIDTGYQSNFRYGAQQSFTRNWLMPMHQTDSLPGKHAVAWKFKWGYQVDHHAINTVPKECLIRITKAEDGGIGARG 7qv7.1    --------------------------------------------------------------------------------  target    PWEPVRTGFTPGQENEFMIKWLKGEHIKIKV 7qv7.1    ------------------------------- ``` | | | | | | | | | | | | | | | | | | | | | | | | | | | | | | | | | | | | | | | | | | | | | | | | | |
|  | 7qv7.1.O | Hydrogen dependent carbon dioxide reductase subunit FdhF  *Cryo-EM structure of Hydrogen-dependent CO2 reductase.* | 0.28 |  | 21.42 | 0.58 | 63-1019 | EM | 0.00 | hetero-2-6-6-2-mer | 52 x SF4, 6 x 402 | HHblits | 0.30 |
| ``` target    MFLSRRQFLKVSVGTVAAVAVADKVLALTALQPVIEVGNPLGDYPDRSWERVYHDQYRYDSSFTWVCSPNDTHACRVRAF 7qv7.1    --------------------------------------------------------------VLTTCP-YCGTGCGLYLK  target    VRNGVVMRVEQNYDHQTYEDLYGNRGTFAHNPRMCLKGFTFHRRVYGPYRLKGPLMRKGWKQWMDDNAPELTAETKRKYK 7qv7.1    VENEKIVGVEPDKLHPV------------NQGELCIKGYYGYKYVHDPRRLTSPLIKKN---------------------  target    FDSRFLDDMLRVSWDTAFTYAAKAMITIATRYSGEAGARRLREQGYAPEMIEMMKGAGTRCFKHRAGMPVLGIIGKMGNT 7qv7.1    ------GKFVPVSWDEALNFIANGLKKIKSEYGSDAFAMFCSAR-ATNEDNYAA-------------QKFARA--VIGIN  target    RMNGGINALLDTWIRKVSPDQAQGGRYWSNYTWHGDQNPAHPFWSGVQGSDIDLSDMR-FSKLNTSWGKNFVENKMPEAH 7qv7.1    NVDH-------------------CARLCH-----APTVAGLAMTLGSGAMTNSIPEISTYSDVIFIIGSNTAECHPLIAA  target    WKLECIERGARVVVITPEYNPTAYRADYWMPLRPESDGALFLGAMKIIIDENMHDIDFLKSFTDAPILVRTDTLQYLDPR 7qv7.1    HVIKAKERGAKLIVADPRMNAMVHKADIWLRVPSGYNIPLINGMIHIIIKEGLVKTDFVKNHAVGF--------------  target    DVIADYKFPDFSKSYSGRIQSLKPEQIQRLGGMMVWDLNKKQVVPLHREQVGWHYTNSGIDAALTGTYRVKLLNGREIDA 7qv7.1    --------------------------------------------------------------------------------  target    MPIWQMYMVHFQDYDLDTVHQITRTPKDLIVRWARDSGTIKPAAIHNGEGTCHYFHQTANARGAAMVLIITGNVGKFGTG 7qv7.1    ----EEMAKAVEKYTPEYVEELTGIPKKDLIKAARFYGQAQAAAILYSMGVTQFSHGTGNVVSLANLAVITGNLGRPGAG  target    QHTWAGNYK-AGTWTATPWSGAGLSVHTGEDPFNITLDPNAHGKEIKTRSYYYGEEVGYWNHGDT-ALIVNTPKYGRKVF 7qv7.1    ICPLRGQNNVQGACDV----GALPNVLPGYLD----VTKEQNRERFE----------KVWGVKLPSNIGLRVTEVPDAIL  target    TGKTHMPTPSKFRWVVNVNVVNNAKHHYDMVRNVDPNIECLITQDIEMTSDINHADIAFAANSWMEFTYPEMTVTVSNPW 7qv7.1    ------NKRVRALYIFGENPIMSDPD-SDHLRHALEHLDLLIVQDIFLTETARLAHVVLPAACWAEKDG---TFTNTERR  target    VQIWKGGIRPLYDTRNDLDTFAGVAAKLSDMTGDKRMRDYFAMVYQNRVDVYVQRMLDASS-TFYGYSADVMLKSEKGWM 7qv7.1    VQRVRKAVEAPGEAKPDWWIFSQIAERMGYT-------GM----QYNNVQEIWDEVRKIVPEKFGGISYARLEK-EKGLA  target    VMVRTYPRHPFWEETNESKPMWTRSGRYENYRIEPEAIEYGENFISHREGPEATPYLPNAIFTTNPYVRPDDYGIPITAQ 7qv7.1    WPCP-TEDHTGTPILYLGGKFATPSGKAQMYPVIFYPNT-----CICDEGAEKQDF------------NH----VI----  target    HHDDKTVRNIKLSWHEIKRHSNPLWEKGYQFYCVTPKTRHRVHSQW--SVNDWVQIYESNFGDPYRMDKRTPGVGEHQIH 7qv7.1    -----------VGS------IAELPDEEYPFTLTTGRRVYHYHTATMTRKSPVIDQI----------------APQELVE  target    INPQAAKDRGINDGDYVYVDGNPVDRPYRGWKPSDPYYKVARLMIRAKYNPAYPYHVTMAKHAPFVATAKSVKGHETRPD 7qv7.1    INPQDATRLGINDGDFLRVSTR-----------------RGYVATRAWVTERVPKGTIFMTFHYW---------------  target    GRAIAIDTGYQSNFRYGAQQSFTRNWLMPMHQTDSLPGKHAVAWKFKWGYQVDHHAINTVPKECLIRITKAEDGGIGARG 7qv7.1    --------------------------------------------------------------------------------  target    PWEPVRTGFTPGQENEFMIKWLKGEHIKIKV 7qv7.1    ------------------------------- ``` | | | | | | | | | | | | | | | | | | | | | | | | | | | | | | | | | | | | | | | | | | | | | | | | | |
|  | 2e7z.1.A | Acetylene hydratase Ahy  *Acetylene Hydratase from Pelobacter acetylenicus* | 0.30 | 0.00 | 19.55 | 0.58 | 65-1021 | X-ray | 1.26 | monomer | 1 x SF4, 2 x MGD, 1 x W | HHblits | 0.30 |
| ``` target    MFLSRRQFLKVSVGTVAAVAVADKVLALTALQPVIEVGNPLGDYPDRSWERVYHDQYRYDSSFTWVCSPNDTHACRVRAF 2e7z.1    ----------------------------------------------------------------VVCQ-SCDINCVVEAE  target    VR-NGVVMRVEQNYDHQTYEDLYGNRGTFAHNPRMCLKGFTFHRRVYGPYRLKGPLMRKGWKQWMDDNAPELTAETKRKY 2e7z.1    VKADGKIQTKSISEPHPTT-----------PPNSICMKSVNADTIRTHKDRVLYPLKNVGSK------------------  target    KFDSRFLDDMLRVSWDTAFTYAAKAMITIATRYSGEAGARRLREQGYAPEMIEMMKGAGTRCFKHRAGMPVLGIIGKMGN 2e7z.1    ----RGEQRWERISWDQALDEIAEKLKKIIAKYGPESLGVSQ-TEINQQSEYGTL-------------RRF---MNLLGS  target    TRMNGGINALLDTWIRKVSPDQAQGGRYWSNYTWHGDQNPAHPFWSGVQGSDIDLSDMRFSKLNTSWGKNFVENKMPE-A 2e7z.1    PNWTSAM-------------YMCIG-----------NTAGVHRVTHG----SYSFASFADSNCLLFIGKNLSNHNWVSQF  target    HWKLECIERGARVVVITPEYNPTAYRADYWMPLRPESDGALFLGAMKIIIDENMHDIDFLKSFTDAPILVRTDTLQYLDP 2e7z.1    NDLKAALKRGCKLIVLDPRRTKVAEMADIWLPLRYGTDAALFLGMINVIINEQLYDKEFVENWCVGF-------------  target    RDVIADYKFPDFSKSYSGRIQSLKPEQIQRLGGMMVWDLNKKQVVPLHREQVGWHYTNSGIDAALTGTYRVKLLNGREID 2e7z.1    --------------------------------------------------------------------------------  target    AMPIWQMYMVHFQDYDLDTVHQITRTPKDLIVRWARDSGTIKPAAIHNGEGTCHYFHQTANARGAAMVLIITGNVGKFGT 2e7z.1    -----EELKERVQEYPLDKVAEITGCDAGEIRKAAVMFATESPASIPWAVSTDMQKNSCSAIRAQCILRAIVGSFVN-GA  target    GQHTWAGNYKAGTWTATPWSGAGLSV-----HTGEDPFNITLDPNAHGKEIKTRSYYYGEEVGYWNHGDTALIVNTPKYG 2e7z.1    EILGAPHSDLVPISKI-QMH-EALPEEKKKLQLGTETYPFLTYTG-MSALEEPSERVYGV--KYFHN-MGAFMANPTALF  target    RKVFTGKTHMPTPSKFRWVVNVNVVNNAKHHYDMVRNVDPNIECLITQDIEMTSDINHADIAFAANSWMEFTYPEMTVTV 2e7z.1    TAMATE---KPYPVKAFFALASNALMGYANQ-QNALKGLMNQDLVVCYDQFMTPTAQLADYVLPGDHWLERPVVQPN-WE  target    SNPWVQIWKGGIRPLYDTRNDLDTFAGVAAKLSDMTGDKRMRDYFAMVYQNRVDVYVQRMLDASSTFYGYSADVMLKSEK 2e7z.1    GIPFGNTSQQVVEPAGEAKDEYYFIRELAVRMGLE-------EHFP---WKDRLELINYRISP----TGMEWEEYQKQ-Y  target    GWMVMVRTYPRHPFWEETNESKPMWTRSGRYENYRIEPEAIEYGENFISHREGPEATPYLPNAIFTTNPYVRPDDYGIPI 2e7z.1    TYMS--KL-P--DYF--GPEGVGVATPSGKVELYSSVFEKLGYD-P-------------LPY------------------  target    TAQHHDDKTVRNIKLSWHEIKR--HSNPLWEKGYQFYCVTPKTR-HRVHSQWSVNDWVQIYESNFGDPYRMDKRTPGVGE 2e7z.1    ----------------YHEPLQTEISDPELAKEYPLILFAGLREDSNFQSCYHQPGILRDA----------------EPD  target    HQIHINPQAAKDRGINDGDYVYVDGNPVDRPYRGWKPSDPYYKVARLMIRAKYNPAYPYHVTMAKHAPFVATAKSVKGHE 2e7z.1    PVALLHPKTAQSLGLPSGEWIWVETT-----------------HGRLKLLLKHDGAQPEGTIRIPHGRWCP---------  target    TRPDGRAIAIDTGYQSNFRYGAQQSFTRNWLMPMHQTDSLPGKHAVAWKFKWGYQVDHHAINTVPKECLIRITKAEDGGI 2e7z.1    --------------------------------------------------------------------------------  target    GARGPWEPVRTGFTPGQENEFMIKWLKGEHIKIKV 2e7z.1    ----------------------------------- ``` | | | | | | | | | | | | | | | | | | | | | | | | | | | | | | | | | | | | | | | | | | | | | | | | | |
|  | 4aay.1.A | AROA  *Crystal Structure of the arsenite oxidase protein complex from Rhizobium species strain NT-26* | 0.25 |  | 15.04 | 0.59 | 63-1021 | X-ray | 2.70 | hetero-oligomer | 4 x MGD, 2 x O, 2 x 4MO, 2 x F3S, 2 x FES | HHblits | 0.27 |
| ``` target    MFLSRRQFLKVSVGTVAAVAVADKVLALTALQPVIEVGNPLGDYPDRSWERVYHDQYRYDSSFTWVCSPNDTHACRVRAF 4aay.1    --------------------------------------------------------------HNVTCH-FCIVGCGYHAY  target    V-----------------------------------------RNGVVMRVEQNYDHQTYEDLYGNRGTFAHNPRMCLKGF 4aay.1    TWPINKQGGTDPQNNIFGVDLSEQQQAESDAWYSPSMYNVVKQDGRDVHVVIKPDHEC----------VVNSGLGSVRGA  target    TFHRRVY------GPYRLKGPLMRKGWKQWMDDNAPELTAETKRKYKFDSRFLDDMLRVSWDTAFTYAAKAMITIATRYS 4aay.1    RMAETSFSEARNTQQQRLTDPLVWRY---------------------------GQMQPTSWDDALDLVARVTAKIVKEKG  target    GEAGARRLREQGYAPEMIEMMKGAG-TRCFKHRAGMPVLGIIGKMGNTRMNGGINALLDTWIRKVSPDQAQGGRYWSNYT 4aay.1    EDALIVSA----FD------HGGAGGGYENTWGT-GKLY--FEAMKVKNIRIH-------------NRPAYNSEV-----  target    WHGDQNPAHPFWSGVQGSDIDLSDMRFSKLNTSWGKNFVENKMPEA--HWKL---------------ECIERGARVVVIT 4aay.1    ------HGT-RDMGVGELNNCYEDAELADTIVAVGTNALETQTNYFLNHWIPNLRGESLGKKKELMPEEPHEAGRIIIVD  target    PEYNPTAY------RAD--YWMPLRPESDGALFLGAMKIIIDENMHDIDFLKSFTDAPILVRTDTLQYLDPRDVIADYKF 4aay.1    PRRTVTVNACEQTAGADNVLHLAINSGTDLALFNALFTYIADKGWVDRDFIDKSTLREGTARPP---L------------  target    PDFSKSYSGRIQSLKPEQIQRLGGMMVWDLNKKQVVPLHREQVGWHYTNSGIDAALTGTYRVKLLNGREIDAMPIWQMYM 4aay.1    ---------------------------YPAR-G-----------VS-------------------E----ANPGHLSSFE  target    VHFQ--DYDLDTVHQITRTPKDLIVRWARDSGTIK------PAAIHNGEGTCHYFHQTANARGAAMVLIITGNVGKFGTG 4aay.1    DAVEGCRMSIEEAAEITGLDAAQIIKAAEWIGMPKEGGKRRRVMFGYEKGLIWGNDNYRTNGALVNLALATGNIGRPGGG  target    QHTWAGNYKAGTWTATPWSGAGLSVHTGEDPFNITLDPNAHGKEIKTRSYYYGEEVGYWNHGDTALIVNTPKYGRKVFTG 4aay.1    VVRLGGHQEG--YV-----RP--SDAHVGRP-------AAYVDQLLIG--GQGGVHHIWGC---------DHYKT-----  target    KTHMPTPSKFRWVVNVNVVNN------AK---HHYDMVRNVDPNI-ECLITQDIEMTSDINHADIAFAANSWMEFTYPEM 4aay.1    -TLNAHEFKRVYKKRTDMVKDAMSAAPYGDREAMVNAIVDAINQGGLFAVNVDIIPTKIGEACHVILPAATSGEMN---L  target    TVTVSNPWVQIWKGGIRPLYDTRNDLDTFAGVAAKLSDMTGDK---RMRDYFAMVYQNRVDVYV-QRMLDASSTFYGYSA 4aay.1    TSMNGERRMRLTERYMDPPGQSMPDCLIAARLANTMERVLTEMGDVGYAAQFKGFDWQTEEDAFMDGYNKNAHGGEFVTY  target    DVMLKSE-KGWMVMVRTY--PRHPFWEETNESKPMWTRSGRYENYRIEPEAIEYGENFISHREGPEATPYLPNAIFTTNP 4aay.1    ERLSAMGTNGFQEPATGFTDGKIEGTQRLYTDGVFSTDDGKARFMDAPWRGL---------------QA--PG-------  target    YVRPDDYGIPITAQHHDDKTVRNIKLSWHEIKRHSNPLWEKGYQFYCVTPKTRHRVHSQWS--VNDWVQIYESNFGDPYR 4aay.1    -----------------------------------KQQQKDSHKYLINNGRANVVWQSAYLDQENDFVMDR---------  target    MDKRTPGVGEHQIHINPQAAKDRGINDGDYVYVDGNPVDRPYRGWKPSDPYYKVARLMIRAKYNPAYPYHVTMAKHAPFV 4aay.1    -------FPYPFIEMNPEDMAEAGLKEGDLVEIYND-----------------AGATQAMAYPTPTARRGETFMLFGFPT  target    ATAKSVKGHETRPDGRAIAIDTGYQSNFRYGAQQSFTRNWLMPMHQTDSLPGKHAVAWKFKWGYQVDHHAINTVPKECLI 4aay.1    G-------------------------------------------------------------------------------  target    RITKAEDGGIGARGPWEPVRTGFTPGQENEFMIKWLKGEHIKIKV 4aay.1    --------------------------------------------- ``` | | | | | | | | | | | | | | | | | | | | | | | | | | | | | | | | | | | | | | | | | | | | | | | | | |
|  | 5nqd.1.A | AroA  *Arsenite oxidase AioAB from Rhizobium sp. str. NT-26 mutant AioBF108A* | 0.25 |  | 14.60 | 0.59 | 63-1022 | X-ray | 2.20 | hetero-2-2-mer | 4 x MGD, 2 x O, 2 x 4MO, 2 x F3S, 2 x FES | HHblits | 0.27 |
| ``` target    MFLSRRQFLKVSVGTVAAVAVADKVLALTALQPVIEVGNPLGDYPDRSWERVYHDQYRYDSSFTWVCSPNDTHACRVRAF 5nqd.1    --------------------------------------------------------------HNVTCH-FCIVGCGYHAY  target    V-----------------------------------------RNGVVMRVEQNYDHQTYEDLYGNRGTFAHNPRMCLKGF 5nqd.1    TWPINKQGGTDPQNNIFGVDLSEQQQAESDAWYSPSMYNVVKQDGRDVHVVIKPDHEC----------VVNSGLGSVRGA  target    TFHRRVY------GPYRLKGPLMRKGWKQWMDDNAPELTAETKRKYKFDSRFLDDMLRVSWDTAFTYAAKAMITIATRYS 5nqd.1    RMAETSFSEARNTQQQRLTDPLVWRY---------------------------GQMQPTSWDDALDLVARVTAKIVKEKG  target    GEAGARRLREQGYAPEMIEMMKGAGTRCFKHRAGMPVLGIIGKMGNTRMNGGINALLDTWIRKVSPDQAQGGRYWSNYTW 5nqd.1    EDALIVSA----FDH-----GGAGGGYENTWGTG-KLY--FEAMKVKNIRIH-------------NRPAYNSEV------  target    HGDQNPAHPFWSGVQGSDIDLSDMRFSKLNTSWGKNFVENKMPEA--HWKL---------------ECIERGARVVVITP 5nqd.1    -----HGT-RDMGVGELNNCYEDAELADTIVAVGTNALETQTNYFLNHWIPNLRGESLGKKKELMPEEPHEAGRIIIVDP  target    EYNPTAY------RAD--YWMPLRPESDGALFLGAMKIIIDENMHDIDFLKSFTDAPILVRTDTLQYLDPRDVIADYKFP 5nqd.1    RRTVTVNACEQTAGADNVLHLAINSGTDLALFNALFTYIADKGWVDRDFIDKSTLREGTARPP---L-------------  target    DFSKSYSGRIQSLKPEQIQRLGGMMVWDLNKKQVVPLHREQVGWHYTNSGIDAALTGTYRVKLLNGREIDAMPIWQMYMV 5nqd.1    --------------------------YPA-RG-----------V-----------------------SEANPGHLSSFED  target    HFQ--DYDLDTVHQITRTPKDLIVRWARDSGTIK------PAAIHNGEGTCHYFHQTANARGAAMVLIITGNVGKFGTGQ 5nqd.1    AVEGCRMSIEEAAEITGLDAAQIIKAAEWIGMPKEGGKRRRVMFGYEKGLIWGNDNYRTNGALVNLALATGNIGRPGGGV  target    HTWAGNYKAGTWTATPWSGAGLSVHTGEDP--FNITLDPNAHGKEIKTRSYYYGEEVGYWNHGDTALIVNTPKYGRKVFT 5nqd.1    VRLGGHQEG--YVR-----PS--DAHVGRPAAYVDQLLIGGQGG-----------VHHIWGCD---------HYKTTL--  target    GKTHMPTPSKFRWVVNVNVVN------NAK---HHYDMVRNVDPNI-ECLITQDIEMTSDINHADIAFAANSWMEFTYPE 5nqd.1    ----NAHEFKRVYKKRTDMVKDAMSAAPYGDREAMVNAIVDAINQGGLFAVNVDIIPTKIGEACHVILPAATSGEMN---  target    MTVTVSNPWVQIWKGGIRPLYDTRNDLDTFAGVAAKLSDMTGD---KRMRDYFAMVYQNRVDVYVQR-MLDASSTFYGYS 5nqd.1    LTSMNGERRMRLTERYMDPPGQSMPDCLIAARLANTMERVLTEMGDVGYAAQFKGFDWQTEEDAFMDGYNKNAHGGEFVT  target    ADVMLKSE-KGWMVMVRTY--PRHPFWEETNESKPMWTRSGRYENYRIEPEAIEYGENFISHREGPEATPYLPNAIFTTN 5nqd.1    YERLSAMGTNGFQEPATGFTDGKIEGTQRLYTDGVFSTDDGKARFMDAPWRGL------------Q--AP---G------  target    PYVRPDDYGIPITAQHHDDKTVRNIKLSWHEIKRHSNPLWEKGYQFYCVTPKTRHRVHSQWS--VNDWVQIYESNFGDPY 5nqd.1    ------------------------------------KQQQKDSHKYLINNGRANVVWQSAYLDQENDFVMDR--------  target    RMDKRTPGVGEHQIHINPQAAKDRGINDGDYVYVDGNPVDRPYRGWKPSDPYYKVARLMIRAKYNPAYPYHVTMAKHAPF 5nqd.1    --------FPYPFIEMNPEDMAEAGLKEGDLVEIYND-----------------AGATQAMAYPTPTARRGETFMLFGFP  target    VATAKSVKGHETRPDGRAIAIDTGYQSNFRYGAQQSFTRNWLMPMHQTDSLPGKHAVAWKFKWGYQVDHHAINTVPKECL 5nqd.1    TGV-----------------------------------------------------------------------------  target    IRITKAEDGGIGARGPWEPVRTGFTPGQENEFMIKWLKGEHIKIKV 5nqd.1    ---------------------------------------------- ``` | | | | | | | | | | | | | | | | | | | | | | | | | | | | | | | | | | | | | | | | | | | | | | | | | |
|  | 2v3v.1.A | PERIPLASMIC NITRATE REDUCTASE  *A NEW CATALYTIC MECHANISM OF PERIPLASMIC NITRATE REDUCTASE FROM DESULFOVIBRIO DESULFURICANS ATCC 27774 FROM CRYSTALLOGRAPHIC AND EPR DATA AND BASED ON DETAILED ANALYSIS OF THE SIXTH LIGAND* | 0.26 |  | 15.67 | 0.59 | 62-1019 | X-ray | 1.99 | monomer | 1 x SF4, 1 x MO, 2 x MGD, 4 x LCP | HHblits | 0.27 |
| ``` target    MFLSRRQFLKVSVGTVAAVAVADKVLALTALQPVIEVGNPLGDYPDRSWERVYHDQYRYDSSFTWVCSPNDTHACRVRAF 2v3v.1    -------------------------------------------------------------WVKGVCR-YCGTGCGVLVG  target    VRNGVVMRVEQNYDHQTYEDLYGNRGTFAHNPRMCLKGFTFHRRVYGPYRLKGPLMRKGWKQWMDDNAPELTAETKRKYK 2v3v.1    VKDGKAVAIQGDPNNH-------N------AGLLCLKGSLLIPVLNSKERVTQPLVRRHKG-------------------  target    FDSRFLDDMLRVSWDTAFTYAAKAMITIATRYSGEAGARRLREQGYAPEMIEMMKGAGTRCFKHRAGMPVLGIIGKMGNT 2v3v.1    ------GKLEPVSWDEALDLMASRFRSSIDMYGPNSVAWYGSGQ-CLTEES----------Y--VA-NKIF--KGGFGTN  target    RMNGGINALLDTWIRKVSPDQAQGGRYWSNYTWHGDQNPAHPFWSGVQGSDIDLSDMRFSKLNTSWGKNFVENKMPEAHW 2v3v.1    NVDGNP-------------RLCMAS-----------AVGGYVTSFGKDEPMGTYADIDQATCFFIIGSNTSEAHPVLFRR  target    KLECI--ERGARVVVITPEYNPTAYRADYWMPLRPESDGALFLGAMKIIIDENMHDIDFLKSFTDAPILVRTDTLQYLDP 2v3v.1    IARRKQVEPGVKIIVADPRRTNTSRIADMHVAFRPGTDLAFMHSMAWVIINEELDNPRFWQRYVNFM-----DA------  target    RDVIADYKFPDFSKSYSGRIQSLKPEQIQRLGGMMVWDLNKKQVVPLHREQVGWHYTNSGIDAALTGTYRVKLLNGREID 2v3v.1    ------------------------------------------------------------------------------EG  target    AMPIWQMYMVHFQDYDLDTVHQITRTPKDLIVRWARDSGTIKPAAIHNGEGTCHYFHQTANARGAAMVLIITGNVGKFGT 2v3v.1    KPSDFEGYKAFLENYRPEKVAEICRVPVEQIYGAARAFAESAATMSLWCMGINQRVQGVFANNLIHNLHLITGQICRPGA  target    GQHTWAGNYK-AGTWTATPWSGAGLSVHTGEDPFNITLDPNAHGKEIKTRSYYYGEEVGYWNHGDTALIVNTPKYGRKVF 2v3v.1    TSFSLTGQPNACGGVRDGGAL-SH--LLPAGRAIP--NAKH--RAEME----------KLWGLPEGRIAPEPGYHTVALF  target    TGKTHMPTPSKFRWVVNVNVVNNAKHHYDMVRNVDPNI-ECLITQDIEMT-SDINHADIAFAANSWMEFTYPEMTVTVSN 2v3v.1    --EALGRGDVKCMIICETNPAHTLPNL-NKVHKAMSHPESFIVCIEAFPDAVTLEYADLVLPPAFWCERDG---VYGCGE  target    PWVQIWKGGIRPLYDTRNDLDTFAGVAAKLSDMTGDKRMRDYFAMVYQNRVDVYVQRMLDAS----STFYGYSADVMLKS 2v3v.1    RRYSLTEKAVDPPGQCRPTVNTLVEFARRAGVDPQ------LVN--F-RNAEDVWNEWRMVSKGTTYDFWGMTRERLRK-  target    EKGWMVMVR--TYPR--HPFWEETNESKPMWTRSGRYENYRIEPEAIEYGENFISHREGPEATPYLPNAIFTTNPYVRPD 2v3v.1    ESGLIWPCPSEDHPGTSLRYVRGQDPCVPA-DHPDRFFFYGKPDGRA---------------------------------  target    DYGIPITAQHHDDKTVRNIKLSWHEIKRHSNPLWEKGYQFYCVTPKTRHRVHSQW--SVNDWVQIYESNFGDPYRMDKRT 2v3v.1    --------------------VIWMRPAKGAAEEPDAEYPLYLTSMRVIDHWHTATMTGKVPELQKA--------------  target    PGVGEHQIHINPQAAKDRGINDGDYVYVDGNPVDRPYRGWKPSDPYYKVARLMIRAKYNPAYPYHVTMAKHAPFVATAKS 2v3v.1    --NPIAFVEINEEDAARTGIKHGDSVIVETR-----------------RDAMELPARVSDVCRPGLIAVPFFDP------  target    VKGHETRPDGRAIAIDTGYQSNFRYGAQQSFTRNWLMPMHQTDSLPGKHAVAWKFKWGYQVDHHAINTVPKECLIRITKA 2v3v.1    --------------------------------------------------------------------------------  target    EDGGIGARGPWEPVRTGFTPGQENEFMIKWLKGEHIKIKV 2v3v.1    ---------------------------------------- ``` | | | | | | | | | | | | | | | | | | | | | | | | | | | | | | | | | | | | | | | | | | | | | | | | | |
|  | 2v45.1.A | PERIPLASMIC NITRATE REDUCTASE  *A NEW CATALYTIC MECHANISM OF PERIPLASMIC NITRATE REDUCTASE FROM DESULFOVIBRIO DESULFURICANS ATCC 27774 FROM CRYSTALLOGRAPHIC AND EPR DATA AND BASED ON DETAILED ANALYSIS OF THE SIXTH LIGAND* | 0.26 |  | 16.02 | 0.58 | 63-1019 | X-ray | 2.40 | monomer | 1 x SF4, 1 x MO, 2 x MGD, 1 x LCP | HHblits | 0.27 |
| ``` target    MFLSRRQFLKVSVGTVAAVAVADKVLALTALQPVIEVGNPLGDYPDRSWERVYHDQYRYDSSFTWVCSPNDTHACRVRAF 2v45.1    --------------------------------------------------------------VKGVCR-YCGTGCGVLVG  target    VRNGVVMRVEQNYDHQTYEDLYGNRGTFAHNPRMCLKGFTFHRRVYGPYRLKGPLMRKGWKQWMDDNAPELTAETKRKYK 2v45.1    VKDGKAVAIQGNPNNH-------N------AGLLCLKGSLLIPVLNSKERVTQPLVRRHK--------------------  target    FDSRFLDDMLRVSWDTAFTYAAKAMITIATRYSGEAGARRLREQGYAPEMIEMMKGAGTRCFKHRAGMPVLGIIGKMGNT 2v45.1    -----GGKLEPVSWDEALDLMASRFRSSIDMYGPNSVAWYGSG-QCLTEESYVAN-------------KIF--KGGFGTN  target    RMNGGINALLDTWIRKVSPDQAQGGRYWSNYTWHGDQNPAHPFWSGVQGSDIDLSDMRFSKLNTSWGKNFVENKMPEAHW 2v45.1    NVDGNP-------------RLCMA-----------SAVGGYVTSFGKDEPMGTYADIDQATCFFIIGSNTSEAHPVLFRR  target    KLECI--ERGARVVVITPEYNPTAYRADYWMPLRPESDGALFLGAMKIIIDENMHDIDFLKSFTDAPILVRTDTLQYLDP 2v45.1    IARRKQVEPGVKIIVADPRRTNTSRIADMHVAFRPGTDLAFMHSMAWVIINEELDNPRFWQRYVNFMD---A--------  target    RDVIADYKFPDFSKSYSGRIQSLKPEQIQRLGGMMVWDLNKKQVVPLHREQVGWHYTNSGIDAALTGTYRVKLLNGREID 2v45.1    ------------------------------------------------------------------------------EG  target    AMPIWQMYMVHFQDYDLDTVHQITRTPKDLIVRWARDSGTIKPAAIHNGEGTCHYFHQTANARGAAMVLIITGNVGKFGT 2v45.1    KPSDFEGYKAFLENYRPEKVAEICRVPVEQIYGAARAFAESAATMSLWCMGINQRVQGVFANNLIHNLHLITGQICRPGA  target    GQHTWAGNYK-AGTWTATPWSGAGLSVHTGEDPFNITLDPNAH-GKEIKTRSYYYGEEVGYWNHGDTALIVNTPKYGRKV 2v45.1    TSFSLTGQPNACGGVRD---GGALSHLLPAGRA-----IPNAKHRAEME----------KLWGLPEGRIAPEPGYHTVAL  target    FTGKTHMPTPSKFRWVVNVNVVNNAKHHYDMVRNVDPNIE-CLITQDIEMT-SDINHADIAFAANSWMEFTYPEMTVTVS 2v45.1    F--EALGRGDVKCMIICETNPAHTLPNL-NKVHKAMSHPESFIVCIEAFPDAVTLEYADLVLPPAFWCERDG---VYGCG  target    NPWVQIWKGGIRPLYDTRNDLDTFAGVAAKLSDMTGDKRMRDYFAMVYQNRVDVYVQRMLDA----SSTFYGYSADVMLK 2v45.1    ERRYSLTEKAVDPPGQCRPTVNTLVEFARRAGVDPQ------LVNF---RNAEDVWNEWRMVSKGTTYDFWGMTRERLRK  target    SEKGWMVMV--RTYPR--HPFWEETNESKPMWTRSGRYENYRIEPEAIEYGENFISHREGPEATPYLPNAIFTTNPYVRP 2v45.1    -ESGLIWPCPSEDHPGTSLRYVRG-QDPCVPADHPDRFFFYGKPDGRAV-------------------------------  target    DDYGIPITAQHHDDKTVRNIKLSWHEIKRHSNPLWEKGYQFYCVTPKTRHRVHSQW--SVNDWVQIYESNFGDPYRMDKR 2v45.1    ----------------------IWMRPAKGAAEEPDAEYPLYLTSMRVIDHWHTATMTGKVPELQKA-------------  target    TPGVGEHQIHINPQAAKDRGINDGDYVYVDGNPVDRPYRGWKPSDPYYKVARLMIRAKYNPAYPYHVTMAKHAPFVATAK 2v45.1    ---NPIAFVEINEEDAARTGIKHGDSVIVETR-----------------RDAMELPARVSDVCRPGLIAVPFFDP-----  target    SVKGHETRPDGRAIAIDTGYQSNFRYGAQQSFTRNWLMPMHQTDSLPGKHAVAWKFKWGYQVDHHAINTVPKECLIRITK 2v45.1    --------------------------------------------------------------------------------  target    AEDGGIGARGPWEPVRTGFTPGQENEFMIKWLKGEHIKIKV 2v45.1    ----------------------------------------- ``` | | | | | | | | | | | | | | | | | | | | | | | | | | | | | | | | | | | | | | | | | | | | | | | | | |
|  | 1aa6.1.A | FORMATE DEHYDROGENASE H  *REDUCED FORM OF FORMATE DEHYDROGENASE H FROM E. COLI* | 0.30 |  | 20.09 | 0.57 | 63-1020 | X-ray | 2.30 | monomer | 1 x SF4, 2 x MGD, 1 x 4MO | HHblits | 0.30 |
| ``` target    MFLSRRQFLKVSVGTVAAVAVADKVLALTALQPVIEVGNPLGDYPDRSWERVYHDQYRYDSSFTWVCSPNDTHACRVRAF 1aa6.1    --------------------------------------------------------------VVTVCP-YCASGCKINLV  target    VRNGVVMRVEQNYDHQTYEDLYGNRGTFAHNPRMCLKGFTFHRRVYGP----YRLKGPLMRKGWKQWMDDNAPELTAETK 1aa6.1    VDNGKIVRAEAAQG-KT------------NQGTLCLKGYYGWDFINDTQILTPRLKTPMIRRQR----------------  target    RKYKFDSRFLDDMLRVSWDTAFTYAAKAMITIATRYSGEAGARRLREQGYAPEMIEMMKGAGTRCFKHRAGMPVLGIIGK 1aa6.1    ---------GGKLEPVSWDEALNYVAERLSAIKEKYGPDAIQTTGSSRGTGNETNYVM-------------QKFARA--V  target    MGNTRMNGGINALLDTWIRKVSPDQAQGGRYWSNYTWHGDQNPAHPFWSGVQGSDIDLSDMRFSKLNTSWGKNFVENKMP 1aa6.1    IGTNNVDC-C------------ARVUHGPSV-----------AGLHQSVGNGAMSNAINEIDNTDLVFVFGYNPADSHPI  target    EAHWKLECIERGARVVVITPEYNPTAYRADYWMPLRPESDGALFLGAMKIIIDENMHDIDFLKSFTDAPILVRTDTLQYL 1aa6.1    VANHVINAKRNGAKIIVCDPRKIETARIADMHIALKNGSNIALLNAMGHVIIEENLYDKAFVASRTEGF-----------  target    DPRDVIADYKFPDFSKSYSGRIQSLKPEQIQRLGGMMVWDLNKKQVVPLHREQVGWHYTNSGIDAALTGTYRVKLLNGRE 1aa6.1    -----------E--------------------------------------------------------------------  target    IDAMPIWQMYMVHFQDYDLDTVHQITRTPKDLIVRWARDSGTIKPAAIHNGEGTCHYFHQTANARGAAMVLIITGNVGKF 1aa6.1    --------EYRKIVEGYTPESVEDITGVSASEIRQAARMYAQAKSAAILWGMGVTQFYQGVETVRSLTSLAMLTGNLGKP  target    GTGQHTWAGNYK-AGTWTATPWSGAGLSVHTGEDPFNITLDPNAHGKEIKTRSYYYGEEVGYWNHGDTALIVNTPKYGRK 1aa6.1    HAGVNPVRGQNNVQGACDMGAL----PDTYPGY---QYVKDPAN-REKFAK-------AWGVESLPAHT-GYRISELPH-  target    VFTGKTHMPTPSKFRWVVNVNVVNNAKHHYDMVRNVDPNIECLITQDIEMTSDINHADIAFAANSWMEFTYPEMTVTVSN 1aa6.1    -----RAAHGEVRAAYIMGEDPLQTDAEL-SAVRKAFEDLELVIVQDIFMTKTASAADVILPSTSWGEHEG---VFTAAD  target    PWVQIWKGGIRPLYDTRNDLDTFAGVAAKLSDMTGDKRMRDYFAMVYQNRVDVYVQRMLDASSTFYGYSADVMLKSEKGW 1aa6.1    RGFQRFFKAVEPKWDLKTDWQIISEIATRMGYPMH------Y------NNTQEIWDELRHLCPDFYGATYEKMGE--LGF  target    MVMVRTYPRH-PFWEETNESKPMWTRSGRYENYRIEPEAIEYGENFISHREGPEATPYLPNAIFTTNPYVRPDDYGIPIT 1aa6.1    IQWPCRDTSDADQGTSYLFKEKFDTPNGLAQFFTCDWVA-----------------P---I-------------------  target    AQHHDDKTVRNIKLSWHEIKRHSNPLWEKGYQFYCVTPKTR--HRVHSQWSVNDWVQIYESNFGDPYRMDKRTPGVGEHQ 1aa6.1    ------------------------DKLTDEYPMVLSTVREVGHYSCRSMTGNCAALAALA---------------DEPGY  target    IHINPQAAKDRGINDGDYVYVDGNPVDRPYRGWKPSDPYYKVARLMIRAKYNPAYPYHVTMAKHAPFVATAKSVKGHETR 1aa6.1    AQINTEDAKRLGIEDEALVWVHSR-----------------KGKIITRAQVSDRPNKGAIYMTYQWWI------------  target    PDGRAIAIDTGYQSNFRYGAQQSFTRNWLMPMHQTDSLPGKHAVAWKFKWGYQVDHHAINTVPKECLIRITKAEDGGIGA 1aa6.1    --------------------------------------------------------------------------------  target    RGPWEPVRTGFTPGQENEFMIKWLKGEHIKIKV 1aa6.1    --------------------------------- ``` | | | | | | | | | | | | | | | | | | | | | | | | | | | | | | | | | | | | | | | | | | | | | | | | | |
|  | 1fdo.1.A | FORMATE DEHYDROGENASE H  *OXIDIZED FORM OF FORMATE DEHYDROGENASE H FROM E. COLI* | 0.30 |  | 20.09 | 0.57 | 63-1020 | X-ray | 2.80 | monomer | 1 x SF4, 2 x MGD, 1 x 6MO | HHblits | 0.30 |
| ``` target    MFLSRRQFLKVSVGTVAAVAVADKVLALTALQPVIEVGNPLGDYPDRSWERVYHDQYRYDSSFTWVCSPNDTHACRVRAF 1fdo.1    --------------------------------------------------------------VVTVCP-YCASGCKINLV  target    VRNGVVMRVEQNYDHQTYEDLYGNRGTFAHNPRMCLKGFTFHRRVYGP----YRLKGPLMRKGWKQWMDDNAPELTAETK 1fdo.1    VDNGKIVRAEAAQG-KT------------NQGTLCLKGYYGWDFINDTQILTPRLKTPMIRRQR----------------  target    RKYKFDSRFLDDMLRVSWDTAFTYAAKAMITIATRYSGEAGARRLREQGYAPEMIEMMKGAGTRCFKHRAGMPVLGIIGK 1fdo.1    ---------GGKLEPVSWDEALNYVAERLSAIKEKYGPDAIQTTGSSRGTGNETNYVM-------------QKFARA--V  target    MGNTRMNGGINALLDTWIRKVSPDQAQGGRYWSNYTWHGDQNPAHPFWSGVQGSDIDLSDMRFSKLNTSWGKNFVENKMP 1fdo.1    IGTNNVDC-C------------ARVUHGPSV-----------AGLHQSVGNGAMSNAINEIDNTDLVFVFGYNPADSHPI  target    EAHWKLECIERGARVVVITPEYNPTAYRADYWMPLRPESDGALFLGAMKIIIDENMHDIDFLKSFTDAPILVRTDTLQYL 1fdo.1    VANHVINAKRNGAKIIVCDPRKIETARIADMHIALKNGSNIALLNAMGHVIIEENLYDKAFVASRTEGF-----------  target    DPRDVIADYKFPDFSKSYSGRIQSLKPEQIQRLGGMMVWDLNKKQVVPLHREQVGWHYTNSGIDAALTGTYRVKLLNGRE 1fdo.1    -----------E--------------------------------------------------------------------  target    IDAMPIWQMYMVHFQDYDLDTVHQITRTPKDLIVRWARDSGTIKPAAIHNGEGTCHYFHQTANARGAAMVLIITGNVGKF 1fdo.1    --------EYRKIVEGYTPESVEDITGVSASEIRQAARMYAQAKSAAILWGMGVTQFYQGVETVRSLTSLAMLTGNLGKP  target    GTGQHTWAGNYK-AGTWTATPWSGAGLSVHTGEDPFNITLDPNAHGKEIKTRSYYYGEEVGYWNHGDTALIVNTPKYGRK 1fdo.1    HAGVNPVRGQNNVQGACDMGAL----PDTYPGY---QYVKDPAN-REKFAK-------AWGVESLPAHT-GYRISELPH-  target    VFTGKTHMPTPSKFRWVVNVNVVNNAKHHYDMVRNVDPNIECLITQDIEMTSDINHADIAFAANSWMEFTYPEMTVTVSN 1fdo.1    -----RAAHGEVRAAYIMGEDPLQTDAEL-SAVRKAFEDLELVIVQDIFMTKTASAADVILPSTSWGEHEG---VFTAAD  target    PWVQIWKGGIRPLYDTRNDLDTFAGVAAKLSDMTGDKRMRDYFAMVYQNRVDVYVQRMLDASSTFYGYSADVMLKSEKGW 1fdo.1    RGFQRFFKAVEPKWDLKTDWQIISEIATRMGYPMH------Y------NNTQEIWDELRHLCPDFYGATYEKMGE--LGF  target    MVMVRTYPRH-PFWEETNESKPMWTRSGRYENYRIEPEAIEYGENFISHREGPEATPYLPNAIFTTNPYVRPDDYGIPIT 1fdo.1    IQWPCRDTSDADQGTSYLFKEKFDTPNGLAQFFTCDWVA-----------------P---I-------------------  target    AQHHDDKTVRNIKLSWHEIKRHSNPLWEKGYQFYCVTPKTR--HRVHSQWSVNDWVQIYESNFGDPYRMDKRTPGVGEHQ 1fdo.1    ------------------------DKLTDEYPMVLSTVREVGHYSCRSMTGNCAALAALA---------------DEPGY  target    IHINPQAAKDRGINDGDYVYVDGNPVDRPYRGWKPSDPYYKVARLMIRAKYNPAYPYHVTMAKHAPFVATAKSVKGHETR 1fdo.1    AQINTEDAKRLGIEDEALVWVHSR-----------------KGKIITRAQVSDRPNKGAIYMTYQWWI------------  target    PDGRAIAIDTGYQSNFRYGAQQSFTRNWLMPMHQTDSLPGKHAVAWKFKWGYQVDHHAINTVPKECLIRITKAEDGGIGA 1fdo.1    --------------------------------------------------------------------------------  target    RGPWEPVRTGFTPGQENEFMIKWLKGEHIKIKV 1fdo.1    --------------------------------- ``` | | | | | | | | | | | | | | | | | | | | | | | | | | | | | | | | | | | | | | | | | | | | | | | | | |
|  | 2iv2.1.A | Formate dehydrogenase H  *Reinterpretation of reduced form of formate dehydrogenase H from E. coli* | 0.30 |  | 20.09 | 0.57 | 63-1020 | X-ray | 2.27 | monomer | 1 x SF4, 1 x 2MD, 1 x MGD | HHblits | 0.30 |
| ``` target    MFLSRRQFLKVSVGTVAAVAVADKVLALTALQPVIEVGNPLGDYPDRSWERVYHDQYRYDSSFTWVCSPNDTHACRVRAF 2iv2.1    --------------------------------------------------------------VVTVCP-YCASGCKINLV  target    VRNGVVMRVEQNYDHQTYEDLYGNRGTFAHNPRMCLKGFTFHRRVYGP----YRLKGPLMRKGWKQWMDDNAPELTAETK 2iv2.1    VDNGKIVRAEAAQG-KT------------NQGTLCLKGYYGWDFINDTQILTPRLKTPMIRRQR----------------  target    RKYKFDSRFLDDMLRVSWDTAFTYAAKAMITIATRYSGEAGARRLREQGYAPEMIEMMKGAGTRCFKHRAGMPVLGIIGK 2iv2.1    ---------GGKLEPVSWDEALNYVAERLSAIKEKYGPDAIQTTGSSRGTGNETNYVM-------------QKFARA--V  target    MGNTRMNGGINALLDTWIRKVSPDQAQGGRYWSNYTWHGDQNPAHPFWSGVQGSDIDLSDMRFSKLNTSWGKNFVENKMP 2iv2.1    IGTNNVDC-C------------ARVUHGPSV-----------AGLHQSVGNGAMSNAINEIDNTDLVFVFGYNPADSHPI  target    EAHWKLECIERGARVVVITPEYNPTAYRADYWMPLRPESDGALFLGAMKIIIDENMHDIDFLKSFTDAPILVRTDTLQYL 2iv2.1    VANHVINAKRNGAKIIVCDPRKIETARIADMHIALKNGSNIALLNAMGHVIIEENLYDKAFVASRTEGF-----------  target    DPRDVIADYKFPDFSKSYSGRIQSLKPEQIQRLGGMMVWDLNKKQVVPLHREQVGWHYTNSGIDAALTGTYRVKLLNGRE 2iv2.1    -----------E--------------------------------------------------------------------  target    IDAMPIWQMYMVHFQDYDLDTVHQITRTPKDLIVRWARDSGTIKPAAIHNGEGTCHYFHQTANARGAAMVLIITGNVGKF 2iv2.1    --------EYRKIVEGYTPESVEDITGVSASEIRQAARMYAQAKSAAILWGMGVTQFYQGVETVRSLTSLAMLTGNLGKP  target    GTGQHTWAGNYK-AGTWTATPWSGAGLSVHTGEDPFNITLDPNAHGKEIKTRSYYYGEEVGYWNHGDTALIVNTPKYGRK 2iv2.1    HAGVNPVRGQNNVQGACDMGAL----PDTYPGY---QYVKDPAN-REKFAK-------AWGVESLPAHT-GYRISELPH-  target    VFTGKTHMPTPSKFRWVVNVNVVNNAKHHYDMVRNVDPNIECLITQDIEMTSDINHADIAFAANSWMEFTYPEMTVTVSN 2iv2.1    -----RAAHGEVRAAYIMGEDPLQTDAEL-SAVRKAFEDLELVIVQDIFMTKTASAADVILPSTSWGEHEG---VFTAAD  target    PWVQIWKGGIRPLYDTRNDLDTFAGVAAKLSDMTGDKRMRDYFAMVYQNRVDVYVQRMLDASSTFYGYSADVMLKSEKGW 2iv2.1    RGFQRFFKAVEPKWDLKTDWQIISEIATRMGYPMH------Y------NNTQEIWDELRHLCPDFYGATYEKMGE--LGF  target    MVMVRTYPRH-PFWEETNESKPMWTRSGRYENYRIEPEAIEYGENFISHREGPEATPYLPNAIFTTNPYVRPDDYGIPIT 2iv2.1    IQWPCRDTSDADQGTSYLFKEKFDTPNGLAQFFTCDWVA-----------------P---I-------------------  target    AQHHDDKTVRNIKLSWHEIKRHSNPLWEKGYQFYCVTPKTR--HRVHSQWSVNDWVQIYESNFGDPYRMDKRTPGVGEHQ 2iv2.1    ------------------------DKLTDEYPMVLSTVREVGHYSCRSMTGNCAALAALA---------------DEPGY  target    IHINPQAAKDRGINDGDYVYVDGNPVDRPYRGWKPSDPYYKVARLMIRAKYNPAYPYHVTMAKHAPFVATAKSVKGHETR 2iv2.1    AQINTEDAKRLGIEDEALVWVHSR-----------------KGKIITRAQVSDRPNKGAIYMTYQWWI------------  target    PDGRAIAIDTGYQSNFRYGAQQSFTRNWLMPMHQTDSLPGKHAVAWKFKWGYQVDHHAINTVPKECLIRITKAEDGGIGA 2iv2.1    --------------------------------------------------------------------------------  target    RGPWEPVRTGFTPGQENEFMIKWLKGEHIKIKV 2iv2.1    --------------------------------- ``` | | | | | | | | | | | | | | | | | | | | | | | | | | | | | | | | | | | | | | | | | | | | | | | | | |
|  | 7z0t.1.G | Formate dehydrogenase H  *Structure of the Escherichia coli formate hydrogenlyase complex (aerobic preparation, composite structure)* | 0.30 |  | 20.09 | 0.57 | 63-1020 | EM | 0.00 | hetero-1-1-1-1-1-1-… | 1 x NI, 1 x FCO, 8 x SF4, 1 x FE, 2 x MGD, 1 x 6MO | HHblits | 0.30 |
| ``` target    MFLSRRQFLKVSVGTVAAVAVADKVLALTALQPVIEVGNPLGDYPDRSWERVYHDQYRYDSSFTWVCSPNDTHACRVRAF 7z0t.1    --------------------------------------------------------------VVTVCP-YCASGCKINLV  target    VRNGVVMRVEQNYDHQTYEDLYGNRGTFAHNPRMCLKGFTFHRRVYGP----YRLKGPLMRKGWKQWMDDNAPELTAETK 7z0t.1    VDNGKIVRAEAAQG-KT------------NQGTLCLKGYYGWDFINDTQILTPRLKTPMIRRQR----------------  target    RKYKFDSRFLDDMLRVSWDTAFTYAAKAMITIATRYSGEAGARRLREQGYAPEMIEMMKGAGTRCFKHRAGMPVLGIIGK 7z0t.1    ---------GGKLEPVSWDEALNYVAERLSAIKEKYGPDAIQTTGSSRGTGNETNYVM-------------QKFARA--V  target    MGNTRMNGGINALLDTWIRKVSPDQAQGGRYWSNYTWHGDQNPAHPFWSGVQGSDIDLSDMRFSKLNTSWGKNFVENKMP 7z0t.1    IGTNNVDC-C------------ARVUHGPSV-----------AGLHQSVGNGAMSNAINEIDNTDLVFVFGYNPADSHPI  target    EAHWKLECIERGARVVVITPEYNPTAYRADYWMPLRPESDGALFLGAMKIIIDENMHDIDFLKSFTDAPILVRTDTLQYL 7z0t.1    VANHVINAKRNGAKIIVCDPRKIETARIADMHIALKNGSNIALLNAMGHVIIEENLYDKAFVASRTEGF-----------  target    DPRDVIADYKFPDFSKSYSGRIQSLKPEQIQRLGGMMVWDLNKKQVVPLHREQVGWHYTNSGIDAALTGTYRVKLLNGRE 7z0t.1    -----------E--------------------------------------------------------------------  target    IDAMPIWQMYMVHFQDYDLDTVHQITRTPKDLIVRWARDSGTIKPAAIHNGEGTCHYFHQTANARGAAMVLIITGNVGKF 7z0t.1    --------EYRKIVEGYTPESVEDITGVSASEIRQAARMYAQAKSAAILWGMGVTQFYQGVETVRSLTSLAMLTGNLGKP  target    GTGQHTWAGNYK-AGTWTATPWSGAGLSVHTGEDPFNITLDPNAHGKEIKTRSYYYGEEVGYWNHGDTALIVNTPKYGRK 7z0t.1    HAGVNPVRGQNNVQGACDMGAL----PDTYPGY---QYVKDPAN-REKFAK-------AWGVESLPAHT-GYRISELPH-  target    VFTGKTHMPTPSKFRWVVNVNVVNNAKHHYDMVRNVDPNIECLITQDIEMTSDINHADIAFAANSWMEFTYPEMTVTVSN 7z0t.1    -----RAAHGEVRAAYIMGEDPLQTDAEL-SAVRKAFEDLELVIVQDIFMTKTASAADVILPSTSWGEHEG---VFTAAD  target    PWVQIWKGGIRPLYDTRNDLDTFAGVAAKLSDMTGDKRMRDYFAMVYQNRVDVYVQRMLDASSTFYGYSADVMLKSEKGW 7z0t.1    RGFQRFFKAVEPKWDLKTDWQIISEIATRMGYPMH------Y------NNTQEIWDELRHLCPDFYGATYEKMGE--LGF  target    MVMVRTYPRH-PFWEETNESKPMWTRSGRYENYRIEPEAIEYGENFISHREGPEATPYLPNAIFTTNPYVRPDDYGIPIT 7z0t.1    IQWPCRDTSDADQGTSYLFKEKFDTPNGLAQFFTCDWVA-----------------P---I-------------------  target    AQHHDDKTVRNIKLSWHEIKRHSNPLWEKGYQFYCVTPKTR--HRVHSQWSVNDWVQIYESNFGDPYRMDKRTPGVGEHQ 7z0t.1    ------------------------DKLTDEYPMVLSTVREVGHYSCRSMTGNCAALAALA---------------DEPGY  target    IHINPQAAKDRGINDGDYVYVDGNPVDRPYRGWKPSDPYYKVARLMIRAKYNPAYPYHVTMAKHAPFVATAKSVKGHETR 7z0t.1    AQINTEDAKRLGIEDEALVWVHSR-----------------KGKIITRAQVSDRPNKGAIYMTYQWWI------------  target    PDGRAIAIDTGYQSNFRYGAQQSFTRNWLMPMHQTDSLPGKHAVAWKFKWGYQVDHHAINTVPKECLIRITKAEDGGIGA 7z0t.1    --------------------------------------------------------------------------------  target    RGPWEPVRTGFTPGQENEFMIKWLKGEHIKIKV 7z0t.1    --------------------------------- ``` | | | | | | | | | | | | | | | | | | | | | | | | | | | | | | | | | | | | | | | | | | | | | | | | | |
|  | 7vw6.1.A | Formate dehydrogenase  *Cryo-EM Structure of Formate Dehydrogenase 1 from Methylorubrum extorquens AM1* | 0.30 |  | 17.71 | 0.57 | 60-1018 | EM | 0.00 | hetero-1-1-mer | 4 x SF4, 2 x FES, 2 x MGD, 1 x W, 1 x FMN | HHblits | 0.29 |
| ``` target    MFLSRRQFLKVSVGTVAAVAVADKVLALTALQPVIEVGNPLGDYPDRSWERVYHDQYRYDSSFTWVCSPNDTHACRVRAF 7vw6.1    -----------------------------------------------------------DREVKSLCP-YCGVGCQVSYK  target    VRNGVVMRVEQNYDHQTYEDLYGNRGTFAHNPRMCLKGFTFHRRVYGPYRLKGPLMRKGWKQWMDDNAPELTAETKRKYK 7vw6.1    VKDERIVYAEGV-NGPA------------NQNRLCVKGRFGFDYVHHPHRLTVPLIRLENVPKD-----------ANDQV  target    FDSRFLDDMLRVSWDTAFTYAAKAMITIATRYSGEAGARRLREQGYAPEMIEMMKGAGTRCFKHRAGMPVLGIIGKMGNT 7vw6.1    DPANPWTHFREATWEEALDRAAGGLKAIRDTNGRKALAGFGSAK-GSNEEA-----------------------------  target    RMNGGINALLDTWIRKVS--PDQAQGGRYWSNYTWHGDQNPAHPFWSGVQGSDIDLSDMRFSKLNTSWGKNFVENKMPEA 7vw6.1    -------YLFQKLVRLGFGTNNVDHCTRLC-----HASSVAALMEGLNSGAVTAPFSAALDAEVIVVIGANPTVNHPVAA  target    HWKLECI-ERGARVVVITPEYNPTAYRADYWMPLRPESDGALFLGAMKIIIDENMHDIDFLKSFTDAPILVRTDTLQYLD 7vw6.1    TFLKNAVKQRGAKLIIMDPRRQTLSRHAYRHLAFRPGSDVAMLNAMLNVIVTEGLYDEQYIAGYTENF------------  target    PRDVIADYKFPDFSKSYSGRIQSLKPEQIQRLGGMMVWDLNKKQVVPLHREQVGWHYTNSGIDAALTGTYRVKLLNGREI 7vw6.1    --------------------------------------------------------------------------------  target    DAMPIWQMYMVHFQDYDLDTVHQITRTPKDLIVRWARDSGTIKPAAIHNGEGTCHYFHQTANARGAAMVLIITGNVGKFG 7vw6.1    ------EALREKIVDFTPEKMASVCGIDAETLREVARLYARAKSSLIFWGMGVSQHVHGTDNSRCLIALALITGQIGRPG  target    TGQHTWAGNYK-AGTWTATPWSGAGLSVHTGEDPFNITLDPNAHGKEIKTRSYYYGEEVGYWNHGDTA-LIVNTPKYGRK 7vw6.1    TGLHPLRGQNNVQGASD----AGLIPMVYPDYQSV----EKDAVRELF----------EEFWGQSLDPQKGLTVVEIMRA  target    VFTGKTHMPTPSKFRWVVNVNVVNNAKHHYDMVRNVDPNIECLITQDIEMTSDINHADIAFAANSWMEFTYPEMTVTVSN 7vw6.1    I------HAGEIRGMFVEGENPAMSDPD-LNHARHALAMLDHLVVQDLFLTETAFHADVVLPASAFAEKA---GTFTNTD  target    PWVQIWKGGIRPLYDTRNDLDTFAGVAAKLSDMTGDKRMRDYFAMVYQNRVDVYVQRMLDASSTFYGYSADVMLKSEKGW 7vw6.1    RRVQIAQPVVAPPGDARQDWWIIQELARRLDLDWN-----------Y-GGPADIFAEMAQVMPSLNNITWERLER--EGA  target    MVMVRTYPRHPFWEETNESKPMWTRSGRYENYRIEPEAIEYGENFISHREGPEATPYLPNAIFTTNPYVRPDDYGIPITA 7vw6.1    VTYPVDAPDQPGNEI-IFYAGFPTESGRAKIVPAAIVP-----------------P------------------------  target    QHHDDKTVRNIKLSWHEIKRHSNPLWEKGYQFYCVTPKTRHRVHS--QWSVNDWVQIYESNFGDPYRMDKRTPGVGEHQI 7vw6.1    ----------------------DEVPDDEFPMVLSTGRVLEHWHTGSMTRRAGVLDAL----------------EPEAVA  target    HINPQAAKDRGINDGDYVYVDGNPVDRPYRGWKPSDPYYKVARLMIRAKYNPAYPYHVTMAKHAPFVATAKSVKGHETRP 7vw6.1    FMAPKELYRLGLRPGGSMRLETR-----------------RGAVVLKVRSDRDVPIGMIFMPFCY---------------  target    DGRAIAIDTGYQSNFRYGAQQSFTRNWLMPMHQTDSLPGKHAVAWKFKWGYQVDHHAINTVPKECLIRITKAEDGGIGAR 7vw6.1    --------------------------------------------------------------------------------  target    GPWEPVRTGFTPGQENEFMIKWLKGEHIKIKV 7vw6.1    -------------------------------- ``` | | | | | | | | | | | | | | | | | | | | | | | | | | | | | | | | | | | | | | | | | | | | | | | | | |
|  | 7e5z.1.A | Formate dehydrogenase  *Dehydrogenase holoenzyme* | 0.27 |  | 18.04 | 0.57 | 60-1019 | EM | 0.00 | hetero-1-1-mer | 1 x W, 2 x MGD, 2 x FES, 4 x SF4, 1 x FMN | HHblits | 0.29 |
| ``` target    MFLSRRQFLKVSVGTVAAVAVADKVLALTALQPVIEVGNPLGDYPDRSWERVYHDQYRYDSSFTWVCSPNDTHACRVRAF 7e5z.1    -----------------------------------------------------------DREVKSLCP-YCGVGCQVSYK  target    VRNGVVMRVEQNYDHQTYEDLYGNRGTFAHNPRMCLKGFTFHRRVYGPYRLKGPLMRKGWKQWMDDNAPELTAETKRKYK 7e5z.1    VKDERIVYAEGV-NGPA------------NQNRLCVKGRFGFDYVHHPHRLTVPLIRLENVPKD-----------ANDQV  target    FDSRFLDDMLRVSWDTAFTYAAKAMITIATRYSGEAGARRLREQGYAPEMIEMMKGAGTRCFKHRAGMPVLGIIGKMGNT 7e5z.1    DPANPWTHFREATWEEALDRAAGGLKAIRDTNGRKALAGFGSAK-GSNEEA-----------------------------  target    RMNGGINALLDTWIRKVS--PDQAQGGRYWSNYTWHGDQNPAHPFWSGVQGSDIDLSDMRFSKLNTSWGKNFVENKMPEA 7e5z.1    -------YLFQKLVRLGFGTNNVDHCTRLC-----HASSVAALMEGLNSGAVTAPFSAALDAEVIVVIGANPTVNHPVAA  target    HWKLECI-ERGARVVVITPEYNPTAYRADYWMPLRPESDGALFLGAMKIIIDENMHDIDFLKSFTDAPILVRTDTLQYLD 7e5z.1    TFLKNAVKQRGAKLIIMDPRRQTLSRHAYRHLAFRPGSDVAMLNAMLNVIVTEGLYDEQYIAGYTENF------------  target    PRDVIADYKFPDFSKSYSGRIQSLKPEQIQRLGGMMVWDLNKKQVVPLHREQVGWHYTNSGIDAALTGTYRVKLLNGREI 7e5z.1    --------------------------------------------------------------------------------  target    DAMPIWQMYMVHFQDYDLDTVHQITRTPKDLIVRWARDSGTIKPAAIHNGEGTCHYFHQTANARGAAMVLIITGNVGKFG 7e5z.1    ------EALREKIVDFTPEKMASVCGIDAETLREVARLYARAKSSLIFWGMGVSQHVHGTDNSRCLIALALITGQIGRPG  target    TGQHTWAGNYK-AGTWTATPWSGAGLS--VHTGEDPFNITLDPNAHGKEIKTRSYYYGEEVGYWNHGDTA-LIVNTPKYG 7e5z.1    TGLHPLRGQNNVQGASD------AGLIPMVYPDYQSV----EKDAVRELF----------EEFWGQSLDPQKGLTVVEIM  target    RKVFTGKTHMPTPSKFRWVVNVNVVNNAKHHYDMVRNVDPNIECLITQDIEMTSDINHADIAFAANSWMEFTYPEMTVTV 7e5z.1    RAIH------AGEIRGMFVEGENPAMSDPD-LNHARHALAMLDHLVVQDLFLTETAFHADVVLPASAFAEKA---GTFTN  target    SNPWVQIWKGGIRPLYDTRNDLDTFAGVAAKLSDMTGDKRMRDYFAMVYQNRVDVYVQRMLDASSTFYGYSADVMLKSEK 7e5z.1    TDRRVQIAQPVVAPPGDARQDWWIIQELARRLDLDWN-----------Y-GGPADIFAEMAQVMPSLNNITWERLER--E  target    GWMVMVRTYPRHPFWEETNESKPMWTRSGRYENYRIEPEAIEYGENFISHREGPEATPYLPNAIFTTNPYVRPDDYGIPI 7e5z.1    GAVTYPVDAPDQPGNEI-IFYAGFPTESGRAKIVPAAIVP-----------------P----------------------  target    TAQHHDDKTVRNIKLSWHEIKRHSNPLWEKGYQFYCVTPKTRHRVHS--QWSVNDWVQIYESNFGDPYRMDKRTPGVGEH 7e5z.1    --------------------D----EVPDDEFPMVLSTGRVLEHWHTGSMTRRAGVLDAL----------------EPEA  target    QIHINPQAAKDRGINDGDYVYVDGNPVDRPYRGWKPSDPYYKVARLMIRAKYNPAYPYHVTMAKHAPFVATAKSVKGHET 7e5z.1    VAFMAPKELYRLGLRPGGSMRLETR-----------------RGAVVLKVRSDRDVPIGMIFMPFCYA------------  target    RPDGRAIAIDTGYQSNFRYGAQQSFTRNWLMPMHQTDSLPGKHAVAWKFKWGYQVDHHAINTVPKECLIRITKAEDGGIG 7e5z.1    --------------------------------------------------------------------------------  target    ARGPWEPVRTGFTPGQENEFMIKWLKGEHIKIKV 7e5z.1    ---------------------------------- ``` | | | | | | | | | | | | | | | | | | | | | | | | | | | | | | | | | | | | | | | | | | | | | | | | | |
|  | 1g8k.1.A | ARSENITE OXIDASE  *CRYSTAL STRUCTURE ANALYSIS OF ARSENITE OXIDASE FROM ALCALIGENES FAECALIS* | 0.26 |  | 16.52 | 0.58 | 63-1020 | X-ray | 1.64 | hetero-1-1-mer | 3 x HG, 2 x CA, 2 x MGD, 1 x O, 1 x 4MO, 1 x F3S, 1 x FES | HHblits | 0.27 |
| ``` target    MFLSRRQFLKVSVGTVAAVAVADKVLALTALQPVIEVGNPLGDYPDRSWERVYHDQYRYDSSFTWVCSPNDTHACRVRAF 1g8k.1    --------------------------------------------------------------TNMTCHF-CIVGCGYHVY  target    VRN-----GV--------------------------------------VMRVEQNYDHQTYEDLYGNRGTFAHNPRMCLK 1g8k.1    KWPELEEGGRAPEQNALGLDFRKQLPPLAVTLTPAMTNVVTEHDGARYDIMVVPDKACVV------------NSGLSSTR  target    GFTFHRRVYGP-----YRLKGPLMRKGWKQWMDDNAPELTAETKRKYKFDSRFLDDMLRVSWDTAFTYAAKAMITIATRY 1g8k.1    GGKMASYMYTPTGDGKERLSAPRLYAA---------------------------DEWVDTTWDHAMALYAGLIKKTLDKD  target    SGEAGARRLREQGYAPEMIEMMKGAGTRCFKHRAGMPVLGIIGKMGNTRMNGGINALLDTWIRKVSPDQAQGGRYWSNYT 1g8k.1    GPQGVFFSCF----DH------GGAGG-GFENTWGTGK-LMFSAIQTPMVRIH------------------------NRP  target    WHGDQNPAHPFWSGVQGSDIDLSDMRFSKLNTSWGKNFVENKMPEA--HWK---------------LECIERGARVVVIT 1g8k.1    AYNSECHA-TREMGIGELNNAYEDAQLADVIWSIGNNPYESQTNYFLNHWLPNLQGATTSKKKERFPNENFPQARIIFVD  target    PEYNPTAYRA--------DYWMPLRPESDGALFLGAMKIIIDENMHDIDFLKSFTDAPILVRTDTLQYLDPRDVIADYKF 1g8k.1    PRETPSVAIARHVAGNDRVLHLAIEPGTDTALFNGLFTYVVEQGWIDKPFIEAHTKGF----------------------  target    PDFSKSYSGRIQSLKPEQIQRLGGMMVWDLNKKQVVPLHREQVGWHYTNSGIDAALTGTYRVKLLNGREIDAMPIWQMYM 1g8k.1    DD----------------------------------------------------------------------------AV  target    VHFQDYDLDTVHQITRTPKDLIVRWARDSGTIK------PAAIHNGEGTCHYFHQTANARGAAMVLIITGNVGKFGTGQH 1g8k.1    K-TNRLSLDECSNITGVPVDMLKRAAEWSYKPKASGQAPRTMHAYEKGIIWGNDNYVIQSALLDLVIATHNVGRRGTGCV  target    TWAGNYKAGTWTATPWSGAGLSVHTGEDPFNITLDPNAHGKEIKTRSYYYGEEVGYWNHGDTALIVNTPKYGRKVFTGKT 1g8k.1    RMGGHQE-G-YTR--------PPYPGDKKIY--IDQELIKGKGRI-MTWWGCNN-FQTS------NNAQALREAILQ---  target    HMPTPSKFRWVVNVNVVNNAKHHYDMVRNVDPN-IECLITQDIEMTSDINHADIAFAANSWMEFTYPEMTVTVSNPWVQI 1g8k.1    RSAIVKQAMQKARGATTEE---MVDVIYEATQNGGLFVTSINLYPTKLAEAAHLMLPAAHPGEMNL---TSMNGERRIRL  target    WKGGIRPLYDTRNDLDTFAGVAAKLSDMTGDK---RMRDYFAMVYQNRVDVYVQRMLDASS--------TF-----YGYS 1g8k.1    SEKFMDPPGTAMADCLIAARIANALRDMYQKDGKAEMAAQFEGFDWKTEEDAFNDGFRRAGQPGAPAIDSQGGSTGHLVT  target    ADVMLKSE-KGWMVMVRTY---PRHPFWEETNESKPMWTRSGRYENYRIEPEAIEYGENFISHREGPEATPYLPNAIFTT 1g8k.1    YDRLRKSGNNGVQLPVVSWDESKGLVGTEMLYTEGKFDTDDGKAHFKPAPWNG----------------L---PA-----  target    NPYVRPDDYGIPITAQHHDDKTVRNIKLSWHEIKRHSNPLWEKGYQFYCVTPKTRHRVHSQWS--VNDWVQIYESNFGDP 1g8k.1    -----------------------------TVQ------Q-QKDKYRFWLNNGRNNEVWQTAYHDQYNSLMQER-------  target    YRMDKRTPGVGEHQIHINPQAAKDRGINDGDYVYVDGNPVDRPYRGWKPSDPYYKVARLMIRAKYNPAYPYHVTMAKHAP 1g8k.1    ---------YPMAYIEMNPDDCKQLDVTGGDIVEVYND-----------------FGSTFAMVYPVAEIKRGQTFMLFGY  target    FVATAKSVKGHETRPDGRAIAIDTGYQSNFRYGAQQSFTRNWLMPMHQTDSLPGKHAVAWKFKWGYQVDHHAINTVPKEC 1g8k.1    VN------------------------------------------------------------------------------  target    LIRITKAEDGGIGARGPWEPVRTGFTPGQENEFMIKWLKGEHIKIKV 1g8k.1    ----------------------------------------------- ``` | | | | | | | | | | | | | | | | | | | | | | | | | | | | | | | | | | | | | | | | | | | | | | | | | |
|  | 1g8j.1.A | ARSENITE OXIDASE  *CRYSTAL STRUCTURE ANALYSIS OF ARSENITE OXIDASE FROM ALCALIGENES FAECALIS* | 0.26 |  | 16.54 | 0.58 | 63-1019 | X-ray | 2.03 | hetero-oligomer | 2 x MGD, 1 x O, 1 x 4MO, 1 x F3S, 1 x FES | HHblits | 0.28 |
| ``` target    MFLSRRQFLKVSVGTVAAVAVADKVLALTALQPVIEVGNPLGDYPDRSWERVYHDQYRYDSSFTWVCSPNDTHACRVRAF 1g8j.1    --------------------------------------------------------------TNMTCH-FCIVGCGYHVY  target    VRN-----GV--------------------------------------VMRVEQNYDHQTYEDLYGNRGTFAHNPRMCLK 1g8j.1    KWPELEEGGRAPEQNALGLDFRKQLPPLASTLTPAMTNVVTEHDGARYDIMVVPDKACVV------------NSGLSSTR  target    GFTFHRRVYGP-----YRLKGPLMRKGWKQWMDDNAPELTAETKRKYKFDSRFLDDMLRVSWDTAFTYAAKAMITIATRY 1g8j.1    GGKMASYMYTPTGDGKERLSAPRLYAA---------------------------DEWVDTTWDHAMALYAGLIKKTLDSD  target    SGEAGARRLREQGYAPEMIEMMKGAGTRCFKHRAGMPVLGIIGKMGNTRMNGGINALLDTWIRKVSPDQAQGGRYWSNYT 1g8j.1    GPQGVFFSCFD--H--------GGAG-GGFENTWGTG-KLMFSAIQTPMVRIH------------------NR---PAYN  target    WHGDQNPAHPFWSGVQGSDIDLSDMRFSKLNTSWGKNFVENKMPEA--HWK---------------LECIERGARVVVIT 1g8j.1    ---SECHAT-REMGIGELNNAYEDAQLADVIWSIGNNPYESQTNYFLNHWLPNLQGATTSKKKERFPNENFPQARIIFVD  target    PEYNPTAYRA--------DYWMPLRPESDGALFLGAMKIIIDENMHDIDFLKSFTDAPILVRTDTLQYLDPRDVIADYKF 1g8j.1    PRETPSVAIARHVAGNDRVLHLAIEPGTDTALFNGLFTYVVEQGWIDKPFIEAHTKGF----------------------  target    PDFSKSYSGRIQSLKPEQIQRLGGMMVWDLNKKQVVPLHREQVGWHYTNSGIDAALTGTYRVKLLNGREIDAMPIWQMYM 1g8j.1    DD----------------------------------------------------------------------------AV  target    VHFQDYDLDTVHQITRTPKDLIVRWARDSGTI------KPAAIHNGEGTCHYFHQTANARGAAMVLIITGNVGKFGTGQH 1g8j.1    K-TNRLSLDECSNITGVPVDMLKRAAEWSYKPKASGQAPRTMHAYEKGIIWGNDNYVIQSALLDLVIATHNVGRRGTGCV  target    TWAGNYKAGTWTATPWSGAGLSVHTGEDPFNITLDPNAHGKEIKTRSYYYGEEVGYWNHGDTALIVNTPKYGRKVFTGKT 1g8j.1    RMGGHQE-G-YTR--------PPYPGDKKIY--IDQELIKGKGRI-MTWWGCNN-FQT------SNNAQALREAILQR--  target    HMPTPSKFRWVVNVNVVNNAKHHYDMVRNVDPNI-ECLITQDIEMTSDINHADIAFAANSWMEFTYPEMTVTVSNPWVQI 1g8j.1    -SAIVKQAMQKARGATTEE---MVDVIYEATQNGGLFVTSINLYPTKLAEAAHLMLPAAHPGEMNL---TSMNGERRIRL  target    WKGGIRPLYDTRNDLDTFAGVAAKLSDMTGDKR---MRDYFAMVYQNRVDVYVQRMLDASS--------TF-----YGYS 1g8j.1    SEKFMDPPGTAMADCLIAARIANALRDMYQKDGKAEMAAQFEGFDWKTEEDAFNDGFRRAGQPGAPAIDSQGGSTGHLVT  target    ADVMLKSE-KGWMVMVRTYP---RHPFWEETNESKPMWTRSGRYENYRIEPEAIEYGENFISHREGPEATPYLPNAIFTT 1g8j.1    YDRLRKSGNNGVQLPVVSWDESKGLVGTEMLYTEGKFDTDDGKAHFKPAPWNG-------------------LPA-----  target    NPYVRPDDYGIPITAQHHDDKTVRNIKLSWHEIKRHSNPLWEKGYQFYCVTPKTRHRVHSQWS--VNDWVQIYESNFGDP 1g8j.1    -----------------------------TVQ------Q-QKDKYRFWLNNGRNNEVWQTAYHDQYNSLMQER-------  target    YRMDKRTPGVGEHQIHINPQAAKDRGINDGDYVYVDGNPVDRPYRGWKPSDPYYKVARLMIRAKYNPAYPYHVTMAKHAP 1g8j.1    ---------YPMAYIEMNPDDCKQLDVTGGDIVEVYND-----------------FGSTFAMVYPVAEIKRGQTFMLFGY  target    FVATAKSVKGHETRPDGRAIAIDTGYQSNFRYGAQQSFTRNWLMPMHQTDSLPGKHAVAWKFKWGYQVDHHAINTVPKEC 1g8j.1    V-------------------------------------------------------------------------------  target    LIRITKAEDGGIGARGPWEPVRTGFTPGQENEFMIKWLKGEHIKIKV 1g8j.1    ----------------------------------------------- ``` | | | | | | | | | | | | | | | | | | | | | | | | | | | | | | | | | | | | | | | | | | | | | | | | | |
|  | 7bkb.1.F | Formate dehydrogenase  *Formate dehydrogenase - heterodisulfide reductase - formylmethanofuran dehydrogenase complex from Methanospirillum hungatei (hexameric, composite structure)* | 0.27 |  | 20.13 | 0.56 | 63-1019 | EM | 0.00 | hetero-2-2-2-2-2-2-… | 48 x SF4, 4 x FAD, 2 x FES, 4 x 9S8, 4 x ZN, 2 x MO, 4 x MGD | HHblits | 0.30 |
| ``` target    MFLSRRQFLKVSVGTVAAVAVADKVLALTALQPVIEVGNPLGDYPDRSWERVYHDQYRYDSSFTWVCSPNDTHACRVRAF 7bkb.1    --------------------------------------------------------------VATTCP-YCGVGCTLNLV  target    VRNGVVMRVEQNYDHQTYEDLYGNRGTFAHNPRMCLKGFTFHRRVYGPYRLKGPLMRKGWKQWMDDNAPELTAETKRKYK 7bkb.1    VSNGKVVGVEPNQRSPI------------NEGKLCPKGVTCWEHIHSPDRLTTPLIKKD---------------------  target    FDSRFLDDMLRVSWDTAFTYAAKAMITIATRYSGEAGARRLREQGYAPEMIEMMKGAGTRCFKHRAGMPVLGIIGKMGNT 7bkb.1    ------GKFIEASWDEALDLVAKNLKVIYDKHGPKGLGFQTSCR-TVNEDC-----------------------------  target    RMNGGINALLDTWIRKVSPDQAQGGRYWSNYTW--HGDQNPAHPFWSGVQGSDIDLSDMRFSKLNTSWGKNFVENKMPEA 7bkb.1    -------YIFQKFARVG-----FKTNNVDNCARICHGPSVAGLSLSFGSGAATNGFEDALNADLILIWGSNAVEAHPLAG  target    HWKLECIERGARVVVITPEYNPTAYRADYWMPLRPESDGALFLGAMKIIIDENMHDIDFLKSFTDAPILVRTDTLQYLDP 7bkb.1    RRIAQAKKKGIQIIAVDPRYTMTARLADTYVRFNPSTHIALANSMMYWIIKEGLEDKKFIQDRVNGF-------------  target    RDVIADYKFPDFSKSYSGRIQSLKPEQIQRLGGMMVWDLNKKQVVPLHREQVGWHYTNSGIDAALTGTYRVKLLNGREID 7bkb.1    ---------ED---------------------------------------------------------------------  target    AMPIWQMYMVHFQDYDLDTVHQITRTPKDLIVRWARDSGTIKPAAIHNGEGTCHYFHQTANARGAAMVLIITGNVGKFGT 7bkb.1    -------LKKTVENY--ADAEAIHGVPLDVVKDIAFRYAKAKNAVIIYCLGITELTTGTDNVRSMGNLALLTGNVGREGV  target    GQHTWAGNYK-AGTWTATPWSGAGLSVHTGEDPFNITLDPNAHGKEIKTRSYYYGEEVGYWNHGDTALIVNTPKYGRKVF 7bkb.1    GVNPLRGQNNVQGACDM----GAYPNVYSGYQK----CEVAENRAKME----------KAWSVTNLP-----DWYGATLT  target    TGKTHMPTPSKFRWVVNVNVVNNAKHHYDMVRNVDPNIECLITQDIEMTSDINHADIAFAANSWMEFTYPEMTVTVSNPW 7bkb.1    EQINQCGDEIKGMYILGLNPVVTYPS-SNHVKAQLEKLDFLVVQDIFFTETCQYADVILPGACFAEKDG---TFTSGERR  target    VQIWKGGIRPLYDTRNDLDTFAGVAAKLSDMTGDKRMRDYFAMVYQNRVDVYVQRMLDASSTFYGYSADVMLKSEKGWMV 7bkb.1    INRVRKAVNPPGQAKEDIHIISELAAKMGFK-------G-FE---LPTAKDVWDDMRAVTPSMFGATYEKLER-PEGICW  target    MV--RTYPRHPFWEETNESKPMWTRSGRYENYRIEPEAIEYGENFISHREGPEATPYLPNAIFTTNPYVRPDDYGIPITA 7bkb.1    PCPTEEHPGTPIL----HREKFATADGKGNLFGIDYRP-----------------P------------------------  target    QHHDDKTVRNIKLSWHEIKRHSNPLWEKGYQFYCVTPKTRHRVHSQWSV--NDWVQIYESNFGDPYRMDKRTPGVGEHQI 7bkb.1    ------------------AE--VA--DAEYPFTLMTGRLIFHYHSRTQTDRAADLHR----------------EVPESYA  target    HINPQAAKDRGINDGDYVYVDGNPVDRPYRGWKPSDPYYKVARLMIRAKYNPAYPYHVTMAKHAPFVATAKSVKGHETRP 7bkb.1    QINIEDARRLGIKNNEYIKLKSR-----------------RGETTTLARVTDEVAPGVVYMTMHFA--------------  target    DGRAIAIDTGYQSNFRYGAQQSFTRNWLMPMHQTDSLPGKHAVAWKFKWGYQVDHHAINTVPKECLIRITKAEDGGIGAR 7bkb.1    --------------------------------------------------------------------------------  target    GPWEPVRTGFTPGQENEFMIKWLKGEHIKIKV 7bkb.1    -------------------------------- ``` | | | | | | | | | | | | | | | | | | | | | | | | | | | | | | | | | | | | | | | | | | | | | | | | | |
|  | 6tg9.1.A | Formate dehydrogenase subunit alpha  *Cryo-EM Structure of NADH reduced form of NAD+-dependent Formate Dehydrogenase from Rhodobacter capsulatus* | 0.27 |  | 15.66 | 0.56 | 61-1019 | EM | 3.24 | hetero-2-2-2-2-mer | 4 x MGD, 2 x 6MO, 4 x FES, 10 x SF4, 2 x H2S, 2 x FMN, 2 x NAI | HHblits | 0.28 |
| ``` target    MFLSRRQFLKVSVGTVAAVAVADKVLALTALQPVIEVGNPLGDYPDRSWERVYHDQYRYDSSFTWVCSPNDTHACRVRAF 6tg9.1    ------------------------------------------------------------RKVVTTCA-YCGVGCSFEAH  target    VRNGVVMRVEQNYDHQTYEDLYGNRGTFAHNPRMCLKGFTFHRRVYGPYRLKGPLMRKGWKQWMDDNAPELTAETKRKYK 6tg9.1    MLGDQLVRMVPWKGGAA------------NRGHSCVKGRFAYGYATHQDRILKPMIRDKI--------------------  target    FDSRFLDDMLRVSWDTAFTYAAKAMITIATRYSGEAGARRLREQGYAPEMIEMMKGAGTRCFKHRAGMPVLGIIGKMGNT 6tg9.1    -----TDPWREVNWTEALDFTATRLRALRDSHGADALGVITSSR-CTNEETYLVQ-------------KLARA--VFGTN  target    RMNGGINALLDTWIRKVSPDQAQGGRYWSNYTWHGDQNPAHPFWSGVQGSDIDLSDMRFSKLNTSWGKNFVENKMPEAHW 6tg9.1    NTD-TCAR------------VCHSPTG-----------YGLKQTFGTSAGTQDFDSVEETDLALVIGANPTDGHPVFASR  target    KLECIERGARVVVITPEYNPTA----YRADYWMPLRPESDGALFLGAMKIIIDENMHDIDFLKSFTDAPILVRTDTLQYL 6tg9.1    LRKRLRAGAKLIVVDPRRIDLLNTPHRGEAWHLQLKPGTNVAVMTAMAHVIVTEQIFDKRFIGDRCDWD-----------  target    DPRDVIADYKFPDFSKSYSGRIQSLKPEQIQRLGGMMVWDLNKKQVVPLHREQVGWHYTNSGIDAALTGTYRVKLLNGRE 6tg9.1    ---------EWAD-------------------------------------------------------------------  target    IDAMPIWQMYMVHF--QDYDLDTVHQITRTPKDLIVRWARDSGTIKPAAIHNGEGTCHYFHQTANARGAAMVLIITGNVG 6tg9.1    ---------YAEFVANPEYAPEAVESLTGVPAGLLRQAARAYAAAPNAAIYYGLGVTEHSQGSTTVIAIANLAMMTGNIG  target    KFGTGQHTWAGNYK-AGTWTATPWSGAGLSVHTGEDPFNITLDPNAHGKEIKTRSYYYGEEVGYWNHGDTALIVNTPKYG 6tg9.1    RPGVGVNPLRGQNNVQGSCD----MGSFPHEFPGYRH----VSDDATRGLFE---RTWGVTLSSEP----G--LRIPNML  target    RKVFTGKTHMPTPSKFRWVVNVNVVNNAKHHYDMVRNVDPNIECLITQDIEMTSDINHADIAFAANSWMEFTYPEMTVTV 6tg9.1    DAA------VEGRFKALYVQGEDILQSDPD-TRHVSAGLAAMDLVIVHDLFLNETANYAHVFLPGSTFLEKD---GTFTN  target    SNPWVQIWKGGIRPLYDTRNDLDTFAGVAAKLSDMTGDKRMRDYFAMVYQNRVDVYVQRMLDASSTFYGYSADVMLKSEK 6tg9.1    AERRINRVRRVMAPKA-GFADWEVTQMLANALGAG-------WH----Y-THPSEIMAEIAATTPGFAAVTYEMLDA--R  target    GWMVMVRTYPRHPFWEETNESKPMWTRSGRYENYRIEPEAIEYGENFISHREGPEATPYLPNAIFTTNPYVRPDDYGIPI 6tg9.1    GSVQWPC----N---E----KAPEGSPIMHVEGFVRGK-----G--------RF--IR----------------------  target    TAQHHDDKTVRNIKLSWHEIKRHSNPLWEKGYQFYCVTPKTRHRVHSQWSVNDWVQIYESNFGDPYRMDKRTPGVGEHQI 6tg9.1    --------------TAYLPTD----EKTGPRFPLLLTTGRILSQYNVGAQTRRTENT------------V---WHGEDRL  target    HINPQAAKDRGINDGDYVYVDGNPVDRPYRGWKPSDPYYKVARLMIRAKYNPAYPYHVTMAKHAPFVATAKSVKGHETRP 6tg9.1    EIHPTDAETRGIRDGDWVRLASR-----------------AGETTLRATVTDRVSPGVVYTTFHHP--------------  target    DGRAIAIDTGYQSNFRYGAQQSFTRNWLMPMHQTDSLPGKHAVAWKFKWGYQVDHHAINTVPKECLIRITKAEDGGIGAR 6tg9.1    --------------------------------------------------------------------------------  target    GPWEPVRTGFTPGQENEFMIKWLKGEHIKIKV 6tg9.1    -------------------------------- ``` | | | | | | | | | | | | | | | | | | | | | | | | | | | | | | | | | | | | | | | | | | | | | | | | | |
| ✓ | 6sdv.1.A | Formate dehydrogenase, alpha subunit, selenocysteine-containing,Formate dehydrogenase, alpha subunit, selenocysteine-containing,W-formate dehydrogenase - alpha subunit  *W-formate dehydrogenase from Desulfovibrio vulgaris - Formate reduced form* | 0.28 | 0.00 | 20.13 | 0.53 | 1-747 | X-ray | 1.90 | monomer | 2 x MGD, 4 x SF4, 1 x W, 1 x H2S | HHblits | 0.30 |
| ``` target    MFLSRRQFLKVSVGTVAAVAVADKVLALTALQPVIEVGNPLGDYPDRSWERVYHDQYRYDSSFTWVCSPNDTHACRVRAF 6sdv.1    MTVTRRHFLKLSAGAAVAGAFTGLGLSL---APTVA---RAEL--QK---------LQWAKQTTSICC-YCAVGCGLIVH  target    VR---NGVVMRVEQNYDHQTYEDLYGNRGTFAHNPRMCLKGFTFHRRVYGPYRLKGPLMRKGWKQWMDDNAPELTAETKR 6sdv.1    TAKDGQGRAVNVEGDPDHPI------------NEGSLCPKGASIFQLGENDQRGTQPLYRAPFS----------------  target    KYKFDSRFLDDMLRVSWDTAFTYAAKAMITIATRYSGEAGARRLREQGYAPEMIEMMKGAGTRCFKHRAGMPVLGIIGKM 6sdv.1    ---------DTWKPVTWDFALTEIAKRIKKTRDASFTEKNAAGDLV--NRTEAIASFGSAAM------------------  target    GNTRMNGGINALLDTWIRKVSPD-QAQGGRYWSNYTWHGDQNPAHPFWSGVQGSDIDLSDMRFSKLNTSWGKNFVENKMP 6sdv.1    -----DNEECWAYGNILRSLGLVYIEHQARIU-----HSPTVPALAESFGRGAMTNHWNDLANSDCILIMGSNAAENHPI  target    EAHWKLECIERGARVVVITPEYNPTAYRADYWMPLRPESDGALFLGAMKIIIDENMHDIDFLKSFTDAPILVRTDTLQYL 6sdv.1    AFKWVLRAKDKGATLIHVDPRFTRTSARCDVYAPIRSGADIPFLGGLIKYILDNKLYFTDYVREYTNASLIVGEKFSF--  target    DPRDVIADYKFPDFSKSYSGRIQSLKPEQIQRLGGMMVWDLNKKQVVPLHREQVGWHYTNSGIDAALTGTYRVKLLNGRE 6sdv.1    --KDG----LFSGYD----A--------------ANKKYDKSM----------WAFELDA-------NG---VPKRDPAL  target    IDAMPIWQMYMVHFQDYDLDTVHQITRTPKDLIVRWARDSGTI----KPAAIHNGEGTCHYFHQTANARGAAMVLIITGN 6sdv.1    KHPRCVINLLKKHYERYNLDKVAAITGTSKEQLQQVYKAYAATGKPDKAGTIMYAMGWTQHSVGVQNIRAMAMIQLLLGN  target    VGKFGTGQHTWAGNYK-AGTWTATPWSGAGLSVHTGEDPFNIT-------LDPNAHGKEIKTRSYYYGEEVGYW------ 6sdv.1    IGVAGGGVNALRGESNVQGSTDQ----GLLAHIWPGYNPVPNSKAATLELYNAATPQSKDPMSVNWWQNRPKYVASYLKA  target    --NHGDT---ALIVN---T-----PKYGRKVFTGKTHMPTPSKFRWVVNVNVVNNAKHHYDMVRNVDPNIECLITQDIEM 6sdv.1    LYPDEEPAAAYDYLPRIDAGRKLTDYFWLNIFE--KMDKGEFKGLFAWGMNPACGGANA-NKNRKAMGKLEWLVNVNLFE  target    TSDINH--------AD-----IAFAANSWMEFTYPEMTVTVSNPWVQIWKGGIRPLYDTRNDLDTFAGVAAKLSDMTGDK 6sdv.1    NETSSFWKGPGMNPAEIGTEVFFLPCCVSIEKEGS---VANSGRWMQWRYRGPKPYAETKPDGDIMLDMFKKVRE-----  target    RMRDYFAMVYQNRVDVYVQRMLDASSTFYGYSADVMLKSEKGWMVMVRTYPRHPFWEETNESKPMWTRSGRYENYRIEPE 6sdv.1    --------------------------------------------------------------------------------  target    AIEYGENFISHREGPEATPYLPNAIFTTNPYVRPDDYGIPITAQHHDDKTVRNIKLSWHEIKRHSNPLWEKGYQFYCVTP 6sdv.1    --------------------------------------------------------------------------------  target    KTRHRVHSQWSVNDWVQIYESNFGDPYRMDKRTPGVGEHQIHINPQAAKDRGINDGDYVYVDGNPVDRPYRGWKPSDPYY 6sdv.1    --------------------------------------------------------------------------------  target    KVARLMIRAKYNPAYPYHVTMAKHAPFVATAKSVKGHETRPDGRAIAIDTGYQSNFRYGAQQSFTRNWLMPMHQTDSLPG 6sdv.1    --------------------------------------------------------------------------------  target    KHAVAWKFKWGYQVDHHAINTVPKECLIRITKAEDGGIGARGPWEPVRTGFTPGQENEFMIKWLKGEHIKIKV 6sdv.1    ------------------------------------------------------------------------- ``` | | | | | | | | | | | | | | | | | | | | | | | | | | | | | | | | | | | | | | | | | | | | | | | | | |
|  | 6sdr.1.A | Formate dehydrogenase, alpha subunit, selenocysteine-containing  *W-formate dehydrogenase from Desulfovibrio vulgaris - Oxidized form* | 0.28 |  | 20.56 | 0.53 | 1-747 | X-ray | 2.10 | hetero-1-1-mer | 2 x MGD, 4 x SF4, 1 x H2S, 1 x W | HHblits | 0.30 |
| ``` target    MFLSRRQFLKVSVGTVAAVAVADKVLALTALQPVIEVGNPLGDYPDRSWERVYHDQYRYDSSFTWVCSPNDTHACRVRAF 6sdr.1    MTVTRRHFLKLSAGAAVAGAFTGLGLSL---APTVA---RAEL-Q-K---------LQWAKQTTSICC-YCAVGCGLIVH  target    VR---NGVVMRVEQNYDHQTYEDLYGNRGTFAHNPRMCLKGFTFHRRVYGPYRLKGPLMRKGWKQWMDDNAPELTAETKR 6sdr.1    TAKDGQGRAVNVEGDPDHPI------------NEGSLCPKGASIFQLGENDQRGTQPLYRAPFS----------------  target    KYKFDSRFLDDMLRVSWDTAFTYAAKAMITIATRYSGEAGARRLREQGYAPEMIEMMKGAGTRCFKHRAGMPVLGIIGKM 6sdr.1    ---------DTWKPVTWDFALTEIAKRIKKTRDASFTEKNAAGDL--VNRTEAIASFGSAAM------------------  target    GNTRMNGGINALLDTWIRKVSPDQAQGGRYWSNY--TWHGDQNPAHPFWSGVQGSDIDLSDMRFSKLNTSWGKNFVENKM 6sdr.1    -----DNEECWAYGNILRSL------GLVYIEHQARIUHSPTVPALAESFGRGAMTNHWNDLANSDCILIMGSNAAENHP  target    PEAHWKLECIERGARVVVITPEYNPTAYRADYWMPLRPESDGALFLGAMKIIIDENMHDIDFLKSFTDAPILVRTDTLQY 6sdr.1    IAFKWVLRAKDKGATLIHVDPRFTRTSARCDVYAPIRSGADIPFLGGLIKYILDNKLYFTDYVREYTNASLIVGEKFS--  target    LDPRDVIADYKFPDFSKSYSGRIQSLKPEQIQRLGGMMVWDLNKKQVVPLHREQVGWHYTNSGIDAALTGTYRVKLLNGR 6sdr.1    --FKDG-----------------------------LFSGYDAANKK---YDKSM--WAF-----ELDANGV---PKRDPA  target    EIDAMPIWQMYMVHFQDYDLDTVHQITRTPKDLIVRWARDSGTI----KPAAIHNGEGTCHYFHQTANARGAAMVLIITG 6sdr.1    LKHPRCVINLLKKHYERYNLDKVAAITGTSKEQLQQVYKAYAATGKPDKAGTIMYAMGWTQHSVGVQNIRAMAMIQLLLG  target    NVGKFGTGQHTWAGNYKA-GTWTATPWSGAGLSVHTGEDPFNITLDPN--AHGKEIKTRSYYYGEEVGYWNHG------- 6sdr.1    NIGVAGGGVNALRGESNVQGSTDQGLL----AHIWPGYNPVPNSKAATLELYNAATPQSKDPM--SVNWWQNRPKYVASY  target    --------DT---ALIVNTPKYG--------RKVFTGKTHMPTPSKFRWVVNVNVVNNAKHHYDMVRNVDPNIECLITQD 6sdr.1    LKALYPDEEPAAAYDYLPRIDAGRKLTDYFWLNIFE--KMDKGEFKGLFAWGMNPACGGANA-NKNRKAMGKLEWLVNVN  target    IEMTSDINH--------AD-----IAFAANSWMEFTYPEMTVTVSNPWVQIWKGGIRPLYDTRNDLDTFAGVAAKLSDMT 6sdr.1    LFENETSSFWKGPGMNPAEIGTEVFFLPCCVSIEKEGS---VANSGRWMQWRYRGPKPYAETKPDGDIMLDMFKKVRE--  target    GDKRMRDYFAMVYQNRVDVYVQRMLDASSTFYGYSADVMLKSEKGWMVMVRTYPRHPFWEETNESKPMWTRSGRYENYRI 6sdr.1    --------------------------------------------------------------------------------  target    EPEAIEYGENFISHREGPEATPYLPNAIFTTNPYVRPDDYGIPITAQHHDDKTVRNIKLSWHEIKRHSNPLWEKGYQFYC 6sdr.1    --------------------------------------------------------------------------------  target    VTPKTRHRVHSQWSVNDWVQIYESNFGDPYRMDKRTPGVGEHQIHINPQAAKDRGINDGDYVYVDGNPVDRPYRGWKPSD 6sdr.1    --------------------------------------------------------------------------------  target    PYYKVARLMIRAKYNPAYPYHVTMAKHAPFVATAKSVKGHETRPDGRAIAIDTGYQSNFRYGAQQSFTRNWLMPMHQTDS 6sdr.1    --------------------------------------------------------------------------------  target    LPGKHAVAWKFKWGYQVDHHAINTVPKECLIRITKAEDGGIGARGPWEPVRTGFTPGQENEFMIKWLKGEHIKIKV 6sdr.1    ---------------------------------------------------------------------------- ``` | | | | | | | | | | | | | | | | | | | | | | | | | | | | | | | | | | | | | | | | | | | | | | | | | |
|  | 1kqf.1.A | FORMATE DEHYDROGENASE, NITRATE-INDUCIBLE, MAJOR SUBUNIT  *FORMATE DEHYDROGENASE N FROM E. COLI* | 0.27 |  | 20.03 | 0.52 | 1-747 | X-ray | 1.60 | hetero-oligomer | 3 x 6MO, 15 x SF4, 6 x MGD, 6 x HEM, 3 x CDL | HHblits | 0.30 |
| ``` target    MFLSRRQFLKVSVGTVAAVAVADKVLALTALQPVIEVGNPLGDYPDRSWERVYHDQYRYDSSFTWVCSPNDTHACRVRAF 1kqf.1    MDVSRRQFFKICAGGMAGTTVAALGFA-----PKQALA------QARNYKL------LRAKEIRNTCT-YCSVGCGLLMY  target    VRNG-------VVMRVEQNYDHQTYEDLYGNRGTFAHNPRMCLKGFTFHRRVYGPYRLKGPLMRKGWKQWMDDNAPELTA 1kqf.1    SLGDGAKNAREAIYHIEGDPDHPV------S------RGALCPKGAGLLDYVNSENRLRYPEYRAPG-------------  target    ETKRKYKFDSRFLDDMLRVSWDTAFTYAAKAMITIATRYSGEAGARRLREQGYAPE-----MIEMMKGAGTRCFKHRAGM 1kqf.1    ------------SDKWQRISWEEAFSRIAKLMKADRDANFIEKNE-------QGVTVNRWLSTGMLCASGASN-------  target    PVLGIIGKMGNTRMNGGINALLDTWIRKVSPDQAQGGRYWSNYT--WHGDQNPAHPFWSGVQGSDIDLSDMRFSKLNTSW 1kqf.1    ----------------ETGMLTQKFARSL------GMLAVDNQARVUHGPTVASLAPTFGRGAMTNHWVDIKNANVVMVM  target    GKNFVENKMPEAHWKLECI-ERGARVVVITPEYNPTAYRADYWMPLRPESDGALFLGAMKIIIDENMHDIDFLKSFTDAP 1kqf.1    GGNAAEAHPVGFRWAMEAKNNNDATLIVVDPRFTRTASVADIYAPIRSGTDITFLSGVLRYLIENNKINAEYVKHYTNAS  target    ILVRTDTLQYLDPRDVIADYKFPDFSKSYSGRIQSLKPEQIQRLGGMMVWDLNKKQVVPLHREQVGWHYTNSGIDAALTG 1kqf.1    LLVRDDFA-FEDGLFSG----YDAEK--------------R--QYDKSSWNYQLD----------------------ENG  target    TYRVKLLNGREIDAMPIWQMYMVHFQDYDLDTVHQITRTPKDLIVRWARDSGTI----KPAAIHNGEGTCHYFHQTANAR 1kqf.1    Y---AKRDETLTHPRCVWNLLKEHVSRYTPDVVENICGTPKADFLKVCEVLASTSAPDRTTTFLYALGWTQHTVGAQNIR  target    GAAMVLIITGNVGKFGTGQHTWAGNYKA-GTWTATPWSGAGLSVHTGEDPFNIT--L---------DPNAH-GKE----- 1kqf.1    TMAMIQLLLGNMGMAGGGVNALRGHSNIQGLTDL-GLLSTS---LPGYLTLPSEKQVDLQSYLEANTPKATLADQVNYWS  target    -IKTR-----SYYYGEE---VGYWNHGDTALIVNTPKYGRKVFTGKTHMPTPSKFRWVVNVNVVNNAKHHYDMVRNVDPN 1kqf.1    NYPKFFVSLMKSFYGDAAQKENNWGYDWLPKWDQTYDVIK-YF--NMMDEGKVTGYFCQGFNPVASFPDK-NKVVSCLSK  target    IECLITQDIEMTSDINHAD-----------------IAFAANSWMEFTYPEMTVTVSNPWVQIWKGGIRPLYDTRNDLDT 1kqf.1    LKYMVVIDPLVTETSTFWQNHGESNDVDPASIQTEVFRLPSTCFAEEDG---SIANSGRWLQWHWKGQDAPGEARNDGEI  target    FAGVAAKLSDMTGDKRMRDYFAMVYQNRVDVYVQRMLDASSTFYGYSADVMLKSEKGWMVMVRTYPRHPFWEETNESKPM 1kqf.1    LAGIYHHLRE----------------------------------------------------------------------  target    WTRSGRYENYRIEPEAIEYGENFISHREGPEATPYLPNAIFTTNPYVRPDDYGIPITAQHHDDKTVRNIKLSWHEIKRHS 1kqf.1    --------------------------------------------------------------------------------  target    NPLWEKGYQFYCVTPKTRHRVHSQWSVNDWVQIYESNFGDPYRMDKRTPGVGEHQIHINPQAAKDRGINDGDYVYVDGNP 1kqf.1    --------------------------------------------------------------------------------  target    VDRPYRGWKPSDPYYKVARLMIRAKYNPAYPYHVTMAKHAPFVATAKSVKGHETRPDGRAIAIDTGYQSNFRYGAQQSFT 1kqf.1    --------------------------------------------------------------------------------  target    RNWLMPMHQTDSLPGKHAVAWKFKWGYQVDHHAINTVPKECLIRITKAEDGGIGARGPWEPVRTGFTPGQENEFMIKWLK 1kqf.1    --------------------------------------------------------------------------------  target    GEHIKIKV 1kqf.1    -------- ``` | | | | | | | | | | | | | | | | | | | | | | | | | | | | | | | | | | | | | | | | | | | | | | | | | |
|  | 6f0k.1.B | Fe-S-cluster-containing hydrogenase  *Alternative complex III* | 0.21 |  | 16.44 | 0.52 | 2-1023 | EM | 0.00 | hetero-1-1-1-1-1-1-… | 6 x HEC, 1 x F3S, 3 x SF4 | HHblits | 0.27 |
| ``` target    MFLSRRQFLKVSVGTVAAVAVADKVLALTALQPVIEVGNPLGDYPDRSWERVYHDQYRYDSSFTWVCSPNDTHACRVRAF 6f0k.1    -GTSRRQFLQIMGASMALAGLTACRR------PV-EKILP---YV-RQPEEI----IPGIPLYYATAMPFRGSVRPLLVE  target    VRNGVVMRVEQNYDHQTYEDLYGNRGTFAHNPRMCLKGFTFHRRVYGPYRLKGPLMRKGWKQWMDDNAPELTAETKRKYK 6f0k.1    SHEGRPTKIEGNPDHPL------S------RGATGVFEQASLLNLYDPDRSQQVLRK-G---------------------  target    FDSRFLDDMLRVSWDTAFTYAAKAMITIATRYSGEAGARRLREQGYAPEMIEMMKGAGTRCFKHRAGMPVLGIIGKMGNT 6f0k.1    ---------EPASWGDFVQFARSLAA----EAGTKRLA--------------VLCEPSSSP-------------------  target    RMNGGINALLDTWIRKVSPDQAQGGRYWSNYTWHGDQN--PAHPFWSGVQGSDIDLSDMRFSKLNTSWGKNFVENK-MPE 6f0k.1    ----TLAALRRELERRY------AQVRWVTYRPEGDDHEALGLQQAFGR--PVRARYRFSEARVIVSLDADFLGPTDRNF  target    AH---------WKLECIERGARVVVITPEYNPTAYRADYWMPLRPESDGALFLGAMKIIIDENMHDIDFLKSFTDAPILV 6f0k.1    VENTREFAASRRMERPEDEISRLYVIESTYTVTGGMADHRLRLRAGDIPAFAAALAAELGVGELREA-------------  target    RTDTLQYLDPRDVIADYKFPDFSKSYSGRIQSLKPEQIQRLGGMMVWDLNKKQVVPLHREQVGWHYTNSGIDAALTGTYR 6f0k.1    --------------------------------------------------------------------------------  target    VKLLNGREIDAMPIWQMYMVHFQDYDLDTVHQITRTPKDLIVRWARDSGTIKPAAIHNGEGTCHYFHQTANARGAAMVLI 6f0k.1    -----------------------------GARFAGH--PYVVEIARDLRAAGARGVVLAGET--QPPA--VHALCAVIND  target    ITGNVGKFGTGQHTWAGNYKAGTWTATPWSGAGLSVHTGEDPFNITLDPNAHGKEIKTRSYYYGEEVGYWNHGDTALIVN 6f0k.1    LLGSLGRTVILHA----------LD----------E-P---AT----AQ---HAAL------------------------  target    TPKYGRKVFTGKTHMPTPSKFRWVVNVNVVNNAKHHYDMVRNVDPNIECLITQDIEMTSDINHADIAFAANSWMEFTYPE 6f0k.1    -AELVQAMQ------AGAVDALLLLNVNPVYDAPAA-LGFAEALAQVPEVIHLGLHVDETARRSTWHLPSTHYLEAWGDG  target    MTVTVSNPWVQIWKGGIRPLYDT-RNDLDTFAGVAAKLSDMTGDKRMRDYFAMVYQNRVDVYVQRMLDASSTFYGYSADV 6f0k.1    ---RAYDGTLSVIQPLIAPLYEAAHSPLEVLALLATGEEQS-----AYDL--------VRNTWRRLLAGRGAFE-QAWQR  target    MLKSEKGWMVMVRTYPRHPFWEETNESKPMWTRSGRYENYRIEPEAIEYGENFISHREGPEATPYLPNAIFTTNPYVRPD 6f0k.1    VLH--DGFL------------P----DSGYPTVSLRPNR------------------------QALA-------------  target    DYGIPITAQHHDDKTVRNIKLSWHEIKRHSNPLWEKGYQFYCVTPKTRHRVHSQWSVNDWVQIYESNFGDPYRMDKRTPG 6f0k.1    ---------------------DW--------PQ-AAEGGLEVVFRLDPTVLDGSFANNAWAQELP----DP--ITKI---  target    VGEHQIHINPQAAKDRGIND--------GDYVYVDGNPVDRPYRGWKPSDPYYKVARLMIRAKYNPAYPYHVTMAKHAPF 6f0k.1    VWDNVAILSPKTAAALGVKAEYHKGVYIADVIELSLD-----------------GRAVELPVWVLPGHPDDSITVYLGYG  target    VATAKSVKGHETRPDGRAIAIDTGYQSNFRYGAQQSFTRNWLMPMHQTDSLPGKHAVAWKFKWGYQVDHHAINTVPKECL 6f0k.1    REIT----------------------------------------------------------------------------  target    IRITKAEDGGIGARGPWEPVRTGFTPGQENEFMIKWLKGEHIKIKV 6f0k.1    ---------------------------------------------- ``` | | | | | | | | | | | | | | | | | | | | | | | | | | | | | | | | | | | | | | | | | | | | | | | | | |
|  | 8bqg.1.A | Formate dehydrogenase, alpha subunit, selenocysteine-containing  *W-formate dehydrogenase from Desulfovibrio vulgaris - Soaking with Formate 1 min* | 0.26 | 0.00 | 19.54 | 0.50 | 42-747 | X-ray | 1.95 | monomer | 2 x MGD, 4 x SF4, 1 x H2S, 1 x W | HHblits | 0.30 |
| ``` target    MFLSRRQFLKVSVGTVAAVAVADKVLALTALQPVIEVGNPLGDYPDRSWERVYHDQYRYDSSFTWVCSPNDTHACRVRAF 8bqg.1    -----------------------------------------ELQ--K---------LQWAKQTTSICC-YCAVGCGLIVH  target    VR---NGVVMRVEQNYDHQTYEDLYGNRGTFAHNPRMCLKGFTFHRRVYGPYRLKGPLMRKGWKQWMDDNAPELTAETKR 8bqg.1    TAKDGQGRAVNVEGDPDHPI------------NEGSLCPKGASIFQLGENDQRGTQPLYRAPF-----------------  target    KYKFDSRFLDDMLRVSWDTAFTYAAKAMITIATRYSGEAGARRLREQGYAPEMIEMMKGAGTRCFKHRAGMPVLGIIGKM 8bqg.1    --------SDTWKPVTWDFALTEIAKRIKKTRDASFTEKNAAGDL--VNRTEAIASFGSAAMD-----------------  target    GNTRMNGGINALLDTWIRKVSP-DQAQGGRYWSNYTWHGDQNPAHPFWSGVQGSDIDLSDMRFSKLNTSWGKNFVENKMP 8bqg.1    ------NEECWAYGNILRSLGLVYIEHQARIU-----HSPTVPALAESFGRGAMTNHWNDLANSDCILIMGSNAAENHPI  target    EAHWKLECIERGARVVVITPEYNPTAYRADYWMPLRPESDGALFLGAMKIIIDENMHDIDFLKSFTDAPILVRTDTLQYL 8bqg.1    AFKWVLRAKDKGATLIHVDPRFTRTSARCDVYAPIRSGADIPFLGGLIKYILDNKLYFTDYVREYTNASLIVGEKFS---  target    DPRDVIADYKFPDFSKSYSGRIQSLKPEQIQRLGGMMVWDLNKKQVVPLHREQVGWHYTNSGIDAALTGTYRVKLLNGRE 8bqg.1    -FKD----GLFSG-------------------------YDAANKK---YDKSM--WAF-----ELDANG---VPKRDPAL  target    IDAMPIWQMYMVHFQDYDLDTVHQITRTPKDLIVRWARDSGTI----KPAAIHNGEGTCHYFHQTANARGAAMVLIITGN 8bqg.1    KHPRCVINLLKKHYERYNLDKVAAITGTSKEQLQQVYKAYAATGKPDKAGTIMYAMGWTQHSVGVQNIRAMAMIQLLLGN  target    VGKFGTGQHTWAGNYK-AGTWTATPWSGAGLSVHTGEDPFNIT-------L---DPNAHGKEIKTRSYYYGEEVG----- 8bqg.1    IGVAGGGVNALRGESNVQGSTDQGLL----AHIWPGYNPVPNSKAATLELYNAATPQS-KDPMSV-N-WWQNRPKYVASY  target    ---YWNHGDT---ALIV---NTP-K----YGRKVFTGKTHMPTPSKFRWVVNVNVVNNAKHHYDMVRNVDPNIECLITQD 8bqg.1    LKALYPDEEPAAAYDYLPRIDAGRKLTDYFWLNIFE--KMDKGEFKGLFAWGMNPACGGANA-NKNRKAMGKLEWLVNVN  target    IEMTSDINH--------AD-----IAFAANSWMEFTYPEMTVTVSNPWVQIWKGGIRPLYDTRNDLDTFAGVAAKLSDMT 8bqg.1    LFENETSSFWKGPGMNPAEIGTEVFFLPCCVSIEKEGS---VANSGRWMQWRYRGPKPYAETKPDGDIMLDMFKKVRE--  target    GDKRMRDYFAMVYQNRVDVYVQRMLDASSTFYGYSADVMLKSEKGWMVMVRTYPRHPFWEETNESKPMWTRSGRYENYRI 8bqg.1    --------------------------------------------------------------------------------  target    EPEAIEYGENFISHREGPEATPYLPNAIFTTNPYVRPDDYGIPITAQHHDDKTVRNIKLSWHEIKRHSNPLWEKGYQFYC 8bqg.1    --------------------------------------------------------------------------------  target    VTPKTRHRVHSQWSVNDWVQIYESNFGDPYRMDKRTPGVGEHQIHINPQAAKDRGINDGDYVYVDGNPVDRPYRGWKPSD 8bqg.1    --------------------------------------------------------------------------------  target    PYYKVARLMIRAKYNPAYPYHVTMAKHAPFVATAKSVKGHETRPDGRAIAIDTGYQSNFRYGAQQSFTRNWLMPMHQTDS 8bqg.1    --------------------------------------------------------------------------------  target    LPGKHAVAWKFKWGYQVDHHAINTVPKECLIRITKAEDGGIGARGPWEPVRTGFTPGQENEFMIKWLKGEHIKIKV 8bqg.1    ---------------------------------------------------------------------------- ``` | | | | | | | | | | | | | | | | | | | | | | | | | | | | | | | | | | | | | | | | | | | | | | | | | |
|  | 3ir5.1.A | Respiratory nitrate reductase 1 alpha chain  *Crystal structure of NarGHI mutant NarG-H49C* | 0.22 | 0.00 | 27.39 | 0.47 | 46-639 | X-ray | 2.30 | monomer | 2 x MD1, 1 x 6MO, 4 x SF4, 1 x AGA, 1 x F3S, 2 x HEM | BLAST | 0.34 |
| ``` target    MFLSRRQFLKVSVGTVAAVAVADKVLALTALQPVIEVGNPLGDYPDRSWERVYHDQYRYDSSFTWVCSPNDTHACRVRAF 3ir5.1    ---------------------------------------------NRDWEDGYRQRWQHDKIVRSTCGVNCTGSCSWKIY  target    VRNGVVMRVEQNYDH-QTYEDLYGNRGTFAHNPRMCLKGFTFHRRVYGPYRLKGPLMRKGW-KQWMDDNA---------P 3ir5.1    VKNGLVTWETQQTDYPRTRPDLPN------HEPRGCPRGASYSWYLYSANRLKYPMMRKRLMKMWREAKALHSDPVEAWA  target    ELTAETKRKYKF-DSRFLDDMLRVSWDTAFTYAAKAMITIATRYSGEAGARRLREQGYAPEMIEMMKGAGTRCFKHRAGM 3ir5.1    SIIEDADKAKSFKQARGRGGFVRSSWQEVNELIAASNVYTIKNYGPD------RVAGFSP-------------------I  target    PVLGIIGKMGNTRMNGGINALLDTWIRKVSPDQAQGGRYWSNYTWHGDQNPAHPFWSGVQGSDIDLSDMRFSKLNTSWGK 3ir5.1    PAMSMVSYASGARYLSLI-----------------GGTCLSFYDWYCDLPPASPQTWGEQTDVPESADWYNSSYIIAWGS  target    NFVENKMPEAHWKLECIERGARVVVITPEYNPTAYRADYWMPLRPESDGALFLGAMKIIIDENMHD------IDFLKSFT 3ir5.1    NVPQTRTPDAHFFTEVRYKGTKTVAVTPDYAEIAKLCDLWLAPKQGTDAAMALAMGHVMLREFHLDNPSQYFTDYVRRYT  target    DAPILVRTD----------TLQYLDPRDVIADYKFPDFSK---SYSGRIQSLKPEQIQRLGGMMVWDLNKK--------- 3ir5.1    DMPMLVMLEERDGYYAAGRMLRAADLVDALGQENNPEWKTVAFNTNGEMVAPNGSIGFRWGEKGKWNLEQRDGKTGEETE  target    -QVVPLHRE----QVGW---------HYTNSGIDAALTGTYRVKLLNGREIDAMPIWQMYMVHFQDYDLDT--------- 3ir5.1    LQLSLLGSQDEIAEVGFPYFGGDGTEHFNKVELENVLLHKLPVKRLQLADGSTALVTTVYDLTLANYGLERGLNDVNCAT  target    ------------VHQITRTPKDLIVRWARD-----SGTIKPAAIHNGEGTCHYFHQTANARGAAMVLIITGNVGKFGTGQ 3ir5.1    SYDDVKAYTPAWAEQITGVSRSQIIRIAREFADNADKTHGRSMIIVGAGLNHWYHLDMNYRGLINMLIFCGCVGQSGGGW  target    HTWAGNYKAGTWTATPWSGAGLSVHTGEDPFNITLDPNAHGKEIKTRSYYYGEEVGYWNHGDTALIVNTPKYGRKVFTGK 3ir5.1    AHYVGQEK-------------LRPQTGWQPLAFALDWQRPARHMNSTSYFYNHSSQWRYETVTAEELLSPMADKSRYTG-  target    THMPTPSKFRWVVNVNVVNNAKHHYDMVRNVDPNIECLITQDIEMTSDINHADIAFAANSWMEFTYPEMTVTVSNPWVQI 3ir5.1    --------------------------------------------------------------------------------  target    WKGGIRPLYDTRNDLDTFAGVAAKLSDMTGDKRMRDYFAMVYQNRVDVYVQRMLDASSTFYGYSADVMLKSEKGWMVMVR 3ir5.1    --------------------------------------------------------------------------------  target    TYPRHPFWEETNESKPMWTRSGRYENYRIEPEAIEYGENFISHREGPEATPYLPNAIFTTNPYVRPDDYGIPITAQHHDD 3ir5.1    --------------------------------------------------------------------------------  target    KTVRNIKLSWHEIKRHSNPLWEKGYQFYCVTPKTRHRVHSQWSVNDWVQIYESNFGDPYRMDKRTPGVGEHQIHINPQAA 3ir5.1    --------------------------------------------------------------------------------  target    KDRGINDGDYVYVDGNPVDRPYRGWKPSDPYYKVARLMIRAKYNPAYPYHVTMAKHAPFVATAKSVKGHETRPDGRAIAI 3ir5.1    --------------------------------------------------------------------------------  target    DTGYQSNFRYGAQQSFTRNWLMPMHQTDSLPGKHAVAWKFKWGYQVDHHAINTVPKECLIRITKAEDGGIGARGPWEPVR 3ir5.1    --------------------------------------------------------------------------------  target    TGFTPGQENEFMIKWLKGEHIKIKV 3ir5.1    ------------------------- ``` | | | | | | | | | | | | | | | | | | | | | | | | | | | | | | | | | | | | | | | | | | | | | | | | | |
|  | 3ir6.1.A | Respiratory nitrate reductase 1 alpha chain  *Crystal structure of NarGHI mutant NarG-H49S* | 0.21 | 0.00 | 27.20 | 0.47 | 46-639 | X-ray | 2.80 | monomer | 2 x GDP, 1 x AGA, 3 x SF4, 1 x F3S, 2 x HEM | BLAST | 0.34 |
| ``` target    MFLSRRQFLKVSVGTVAAVAVADKVLALTALQPVIEVGNPLGDYPDRSWERVYHDQYRYDSSFTWVCSPNDTHACRVRAF 3ir6.1    ---------------------------------------------NRDWEDGYRQRWQHDKIVRSTSGVNCTGSCSWKIY  target    VRNGVVMRVEQNYDH-QTYEDLYGNRGTFAHNPRMCLKGFTFHRRVYGPYRLKGPLMRKGW-KQWMDDNA---------P 3ir6.1    VKNGLVTWETQQTDYPRTRPDLPN------HEPRGCPRGASYSWYLYSANRLKYPMMRKRLMKMWREAKALHSDPVEAWA  target    ELTAETKRKYKF-DSRFLDDMLRVSWDTAFTYAAKAMITIATRYSGEAGARRLREQGYAPEMIEMMKGAGTRCFKHRAGM 3ir6.1    SIIEDADKAKSFKQARGRGGFVRSSWQEVNELIAASNVYTIKNYGPD------RVAGFSP-------------------I  target    PVLGIIGKMGNTRMNGGINALLDTWIRKVSPDQAQGGRYWSNYTWHGDQNPAHPFWSGVQGSDIDLSDMRFSKLNTSWGK 3ir6.1    PAMSMVSYASGARYLSLI-----------------GGTCLSFYDWYCDLPPASPQTWGEQTDVPESADWYNSSYIIAWGS  target    NFVENKMPEAHWKLECIERGARVVVITPEYNPTAYRADYWMPLRPESDGALFLGAMKIIIDENMHD------IDFLKSFT 3ir6.1    NVPQTRTPDAHFFTEVRYKGTKTVAVTPDYAEIAKLCDLWLAPKQGTDAAMALAMGHVMLREFHLDNPSQYFTDYVRRYT  target    DAPILVRTD----------TLQYLDPRDVIADYKFPDFSK---SYSGRIQSLKPEQIQRLGGMMVWDLNKK--------- 3ir6.1    DMPMLVMLEERDGYYAAGRMLRAADLVDALGQENNPEWKTVAFNTNGEMVAPNGSIGFRWGEKGKWNLEQRDGKTGEETE  target    -QVVPLHRE----QVGW---------HYTNSGIDAALTGTYRVKLLNGREIDAMPIWQMYMVHFQDYDLDT--------- 3ir6.1    LQLSLLGSQDEIAEVGFPYFGGDGTEHFNKVELENVLLHKLPVKRLQLADGSTALVTTVYDLTLANYGLERGLNDVNCAT  target    ------------VHQITRTPKDLIVRWARD-----SGTIKPAAIHNGEGTCHYFHQTANARGAAMVLIITGNVGKFGTGQ 3ir6.1    SYDDVKAYTPAWAEQITGVSRSQIIRIAREFADNADKTHGRSMIIVGAGLNHWYHLDMNYRGLINMLIFCGCVGQSGGGW  target    HTWAGNYKAGTWTATPWSGAGLSVHTGEDPFNITLDPNAHGKEIKTRSYYYGEEVGYWNHGDTALIVNTPKYGRKVFTGK 3ir6.1    AHYVGQEK-------------LRPQTGWQPLAFALDWQRPARHMNSTSYFYNHSSQWRYETVTAEELLSPMADKSRYTG-  target    THMPTPSKFRWVVNVNVVNNAKHHYDMVRNVDPNIECLITQDIEMTSDINHADIAFAANSWMEFTYPEMTVTVSNPWVQI 3ir6.1    --------------------------------------------------------------------------------  target    WKGGIRPLYDTRNDLDTFAGVAAKLSDMTGDKRMRDYFAMVYQNRVDVYVQRMLDASSTFYGYSADVMLKSEKGWMVMVR 3ir6.1    --------------------------------------------------------------------------------  target    TYPRHPFWEETNESKPMWTRSGRYENYRIEPEAIEYGENFISHREGPEATPYLPNAIFTTNPYVRPDDYGIPITAQHHDD 3ir6.1    --------------------------------------------------------------------------------  target    KTVRNIKLSWHEIKRHSNPLWEKGYQFYCVTPKTRHRVHSQWSVNDWVQIYESNFGDPYRMDKRTPGVGEHQIHINPQAA 3ir6.1    --------------------------------------------------------------------------------  target    KDRGINDGDYVYVDGNPVDRPYRGWKPSDPYYKVARLMIRAKYNPAYPYHVTMAKHAPFVATAKSVKGHETRPDGRAIAI 3ir6.1    --------------------------------------------------------------------------------  target    DTGYQSNFRYGAQQSFTRNWLMPMHQTDSLPGKHAVAWKFKWGYQVDHHAINTVPKECLIRITKAEDGGIGARGPWEPVR 3ir6.1    --------------------------------------------------------------------------------  target    TGFTPGQENEFMIKWLKGEHIKIKV 3ir6.1    ------------------------- ``` | | | | | | | | | | | | | | | | | | | | | | | | | | | | | | | | | | | | | | | | | | | | | | | | | |
| ✓ | 1r27.4.A | Respiratory nitrate reductase 1 alpha chain  *Crystal Structure of NarGH complex* | 0.22 | 0.07 | 27.20 | 0.47 | 46-639 | X-ray | 2.00 | homo-dimer | 4 x MO, 16 x SF4, 8 x MGD, 4 x F3S | BLAST | 0.34 |
| ``` target    MFLSRRQFLKVSVGTVAAVAVADKVLALTALQPVIEVGNPLGDYPDRSWERVYHDQYRYDSSFTWVCSPNDTHACRVRAF 1r27.4    ---------------------------------------------NRDWEDGYRQRWQHDKIVRSTHGVNCTGSCSWKIY  target    VRNGVVMRVEQNYDH-QTYEDLYGNRGTFAHNPRMCLKGFTFHRRVYGPYRLKGPLMRKGW-KQWMDDNA---------P 1r27.4    VKNGLVTWETQQTDYPRTRPDLPN------HEPRGCPRGASYSWYLYSANRLKYPMMRKRLMKMWREAKALHSDPVEAWA  target    ELTAETKRKYKF-DSRFLDDMLRVSWDTAFTYAAKAMITIATRYSGEAGARRLREQGYAPEMIEMMKGAGTRCFKHRAGM 1r27.4    SIIEDADKAKSFKQARGRGGFVRSSWQEVNELIAASNVYTIKNYGPD------RVAGFSP-------------------I  target    PVLGIIGKMGNTRMNGGINALLDTWIRKVSPDQAQGGRYWSNYTWHGDQNPAHPFWSGVQGSDIDLSDMRFSKLNTSWGK 1r27.4    PAMSMVSYASGARYLSLI-----------------GGTCLSFYDWYCDLPPASPQTWGEQTDVPESADWYNSSYIIAWGS  target    NFVENKMPEAHWKLECIERGARVVVITPEYNPTAYRADYWMPLRPESDGALFLGAMKIIIDENMHD------IDFLKSFT 1r27.4    NVPQTRTPDAHFFTEVRYKGTKTVAVTPDYAEIAKLCDLWLAPKQGTDAAMALAMGHVMLREFHLDNPSQYFTDYVRRYT  target    DAPILVRTD----------TLQYLDPRDVIADYKFPDFSK---SYSGRIQSLKPEQIQRLGGMMVWDLNKK--------- 1r27.4    DMPMLVMLEERDGYYAAGRMLRAADLVDALGQENNPEWKTVAFNTNGEMVAPNGSIGFRWGEKGKWNLEQRDGKTGEETE  target    -QVVPLHRE----QVGW---------HYTNSGIDAALTGTYRVKLLNGREIDAMPIWQMYMVHFQDYDLDT--------- 1r27.4    LQLSLLGSQDEIAEVGFPYFGGDGTEHFNKVELENVLLHKLPVKRLQLADGSTALVTTVYDLTLANYGLERGLNDVNCAT  target    ------------VHQITRTPKDLIVRWARD-----SGTIKPAAIHNGEGTCHYFHQTANARGAAMVLIITGNVGKFGTGQ 1r27.4    SYDDVKAYTPAWAEQITGVSRSQIIRIAREFADNADKTHGRSMIIVGAGLNHWYHLDMNYRGLINMLIFCGCVGQSGGGW  target    HTWAGNYKAGTWTATPWSGAGLSVHTGEDPFNITLDPNAHGKEIKTRSYYYGEEVGYWNHGDTALIVNTPKYGRKVFTGK 1r27.4    AHYVGQEK-------------LRPQTGWQPLAFALDWQRPARHMNSTSYFYNHSSQWRYETVTAEELLSPMADKSRYTG-  target    THMPTPSKFRWVVNVNVVNNAKHHYDMVRNVDPNIECLITQDIEMTSDINHADIAFAANSWMEFTYPEMTVTVSNPWVQI 1r27.4    --------------------------------------------------------------------------------  target    WKGGIRPLYDTRNDLDTFAGVAAKLSDMTGDKRMRDYFAMVYQNRVDVYVQRMLDASSTFYGYSADVMLKSEKGWMVMVR 1r27.4    --------------------------------------------------------------------------------  target    TYPRHPFWEETNESKPMWTRSGRYENYRIEPEAIEYGENFISHREGPEATPYLPNAIFTTNPYVRPDDYGIPITAQHHDD 1r27.4    --------------------------------------------------------------------------------  target    KTVRNIKLSWHEIKRHSNPLWEKGYQFYCVTPKTRHRVHSQWSVNDWVQIYESNFGDPYRMDKRTPGVGEHQIHINPQAA 1r27.4    --------------------------------------------------------------------------------  target    KDRGINDGDYVYVDGNPVDRPYRGWKPSDPYYKVARLMIRAKYNPAYPYHVTMAKHAPFVATAKSVKGHETRPDGRAIAI 1r27.4    --------------------------------------------------------------------------------  target    DTGYQSNFRYGAQQSFTRNWLMPMHQTDSLPGKHAVAWKFKWGYQVDHHAINTVPKECLIRITKAEDGGIGARGPWEPVR 1r27.4    --------------------------------------------------------------------------------  target    TGFTPGQENEFMIKWLKGEHIKIKV 1r27.4    ------------------------- ``` | | | | | | | | | | | | | | | | | | | | | | | | | | | | | | | | | | | | | | | | | | | | | | | | | |
|  | 1q16.1.A | Respiratory nitrate reductase 1 alpha chain  *Crystal structure of Nitrate Reductase A, NarGHI, from Escherichia coli* | 0.22 | 0.00 | 27.20 | 0.47 | 46-639 | X-ray | 1.90 | monomer | 2 x MD1, 1 x 6MO, 2 x HEM, 4 x SF4, 1 x F3S, 1 x AGA, 1 x 3PH | BLAST | 0.34 |
| ``` target    MFLSRRQFLKVSVGTVAAVAVADKVLALTALQPVIEVGNPLGDYPDRSWERVYHDQYRYDSSFTWVCSPNDTHACRVRAF 1q16.1    ---------------------------------------------NRDWEDGYRQRWQHDKIVRSTHGVNCTGSCSWKIY  target    VRNGVVMRVEQNYDH-QTYEDLYGNRGTFAHNPRMCLKGFTFHRRVYGPYRLKGPLMRKGW-KQWMDDNA---------P 1q16.1    VKNGLVTWETQQTDYPRTRPDLPN------HEPRGCPRGASYSWYLYSANRLKYPMMRKRLMKMWREAKALHSDPVEAWA  target    ELTAETKRKYKF-DSRFLDDMLRVSWDTAFTYAAKAMITIATRYSGEAGARRLREQGYAPEMIEMMKGAGTRCFKHRAGM 1q16.1    SIIEDADKAKSFKQARGRGGFVRSSWQEVNELIAASNVYTIKNYGPD------RVAGFSP-------------------I  target    PVLGIIGKMGNTRMNGGINALLDTWIRKVSPDQAQGGRYWSNYTWHGDQNPAHPFWSGVQGSDIDLSDMRFSKLNTSWGK 1q16.1    PAMSMVSYASGARYLSLI-----------------GGTCLSFYDWYCDLPPASPQTWGEQTDVPESADWYNSSYIIAWGS  target    NFVENKMPEAHWKLECIERGARVVVITPEYNPTAYRADYWMPLRPESDGALFLGAMKIIIDENMHD------IDFLKSFT 1q16.1    NVPQTRTPDAHFFTEVRYKGTKTVAVTPDYAEIAKLCDLWLAPKQGTDAAMALAMGHVMLREFHLDNPSQYFTDYVRRYT  target    DAPILVRTD----------TLQYLDPRDVIADYKFPDFSK---SYSGRIQSLKPEQIQRLGGMMVWDLNKK--------- 1q16.1    DMPMLVMLEERDGYYAAGRMLRAADLVDALGQENNPEWKTVAFNTNGEMVAPNGSIGFRWGEKGKWNLEQRDGKTGEETE  target    -QVVPLHRE----QVGW---------HYTNSGIDAALTGTYRVKLLNGREIDAMPIWQMYMVHFQDYDLDT--------- 1q16.1    LQLSLLGSQDEIAEVGFPYFGGDGTEHFNKVELENVLLHKLPVKRLQLADGSTALVTTVYDLTLANYGLERGLNDVNCAT  target    ------------VHQITRTPKDLIVRWARD-----SGTIKPAAIHNGEGTCHYFHQTANARGAAMVLIITGNVGKFGTGQ 1q16.1    SYDDVKAYTPAWAEQITGVSRSQIIRIAREFADNADKTHGRSMIIVGAGLNHWYHLDMNYRGLINMLIFCGCVGQSGGGW  target    HTWAGNYKAGTWTATPWSGAGLSVHTGEDPFNITLDPNAHGKEIKTRSYYYGEEVGYWNHGDTALIVNTPKYGRKVFTGK 1q16.1    AHYVGQEK-------------LRPQTGWQPLAFALDWQRPARHMNSTSYFYNHSSQWRYETVTAEELLSPMADKSRYTG-  target    THMPTPSKFRWVVNVNVVNNAKHHYDMVRNVDPNIECLITQDIEMTSDINHADIAFAANSWMEFTYPEMTVTVSNPWVQI 1q16.1    --------------------------------------------------------------------------------  target    WKGGIRPLYDTRNDLDTFAGVAAKLSDMTGDKRMRDYFAMVYQNRVDVYVQRMLDASSTFYGYSADVMLKSEKGWMVMVR 1q16.1    --------------------------------------------------------------------------------  target    TYPRHPFWEETNESKPMWTRSGRYENYRIEPEAIEYGENFISHREGPEATPYLPNAIFTTNPYVRPDDYGIPITAQHHDD 1q16.1    --------------------------------------------------------------------------------  target    KTVRNIKLSWHEIKRHSNPLWEKGYQFYCVTPKTRHRVHSQWSVNDWVQIYESNFGDPYRMDKRTPGVGEHQIHINPQAA 1q16.1    --------------------------------------------------------------------------------  target    KDRGINDGDYVYVDGNPVDRPYRGWKPSDPYYKVARLMIRAKYNPAYPYHVTMAKHAPFVATAKSVKGHETRPDGRAIAI 1q16.1    --------------------------------------------------------------------------------  target    DTGYQSNFRYGAQQSFTRNWLMPMHQTDSLPGKHAVAWKFKWGYQVDHHAINTVPKECLIRITKAEDGGIGARGPWEPVR 1q16.1    --------------------------------------------------------------------------------  target    TGFTPGQENEFMIKWLKGEHIKIKV 1q16.1    ------------------------- ``` | | | | | | | | | | | | | | | | | | | | | | | | | | | | | | | | | | | | | | | | | | | | | | | | | |
|  | 3ir7.1.A | Respiratory nitrate reductase 1 alpha chain  *Crystal structure of NarGHI mutant NarG-R94S* | 0.22 | 0.00 | 27.20 | 0.47 | 46-639 | X-ray | 2.50 | monomer | 2 x MD1, 4 x SF4, 1 x 6MO, 1 x AGA, 1 x F3S, 2 x HEM | BLAST | 0.34 |
| ``` target    MFLSRRQFLKVSVGTVAAVAVADKVLALTALQPVIEVGNPLGDYPDRSWERVYHDQYRYDSSFTWVCSPNDTHACRVRAF 3ir7.1    ---------------------------------------------NRDWEDGYRQRWQHDKIVRSTHGVNCTGSCSWKIY  target    VRNGVVMRVEQNYDH-QTYEDLYGNRGTFAHNPRMCLKGFTFHRRVYGPYRLKGPLMRKGW-KQWMDDNA---------P 3ir7.1    VKNGLVTWETQQTDYPRTRPDLPN------HEPRGCPSGASYSWYLYSANRLKYPMMRKRLMKMWREAKALHSDPVEAWA  target    ELTAETKRKYKF-DSRFLDDMLRVSWDTAFTYAAKAMITIATRYSGEAGARRLREQGYAPEMIEMMKGAGTRCFKHRAGM 3ir7.1    SIIEDADKAKSFKQARGRGGFVRSSWQEVNELIAASNVYTIKNYGPD------RVAGFSP-------------------I  target    PVLGIIGKMGNTRMNGGINALLDTWIRKVSPDQAQGGRYWSNYTWHGDQNPAHPFWSGVQGSDIDLSDMRFSKLNTSWGK 3ir7.1    PAMSMVSYASGARYLSLI-----------------GGTCLSFYDWYCDLPPASPQTWGEQTDVPESADWYNSSYIIAWGS  target    NFVENKMPEAHWKLECIERGARVVVITPEYNPTAYRADYWMPLRPESDGALFLGAMKIIIDENMHD------IDFLKSFT 3ir7.1    NVPQTRTPDAHFFTEVRYKGTKTVAVTPDYAEIAKLCDLWLAPKQGTDAAMALAMGHVMLREFHLDNPSQYFTDYVRRYT  target    DAPILVRTD----------TLQYLDPRDVIADYKFPDFSK---SYSGRIQSLKPEQIQRLGGMMVWDLNKK--------- 3ir7.1    DMPMLVMLEERDGYYAAGRMLRAADLVDALGQENNPEWKTVAFNTNGEMVAPNGSIGFRWGEKGKWNLEQRDGKTGEETE  target    -QVVPLHRE----QVGW---------HYTNSGIDAALTGTYRVKLLNGREIDAMPIWQMYMVHFQDYDLDT--------- 3ir7.1    LQLSLLGSQDEIAEVGFPYFGGDGTEHFNKVELENVLLHKLPVKRLQLADGSTALVTTVYDLTLANYGLERGLNDVNCAT  target    ------------VHQITRTPKDLIVRWARD-----SGTIKPAAIHNGEGTCHYFHQTANARGAAMVLIITGNVGKFGTGQ 3ir7.1    SYDDVKAYTPAWAEQITGVSRSQIIRIAREFADNADKTHGRSMIIVGAGLNHWYHLDMNYRGLINMLIFCGCVGQSGGGW  target    HTWAGNYKAGTWTATPWSGAGLSVHTGEDPFNITLDPNAHGKEIKTRSYYYGEEVGYWNHGDTALIVNTPKYGRKVFTGK 3ir7.1    AHYVGQEK-------------LRPQTGWQPLAFALDWQRPARHMNSTSYFYNHSSQWRYETVTAEELLSPMADKSRYTG-  target    THMPTPSKFRWVVNVNVVNNAKHHYDMVRNVDPNIECLITQDIEMTSDINHADIAFAANSWMEFTYPEMTVTVSNPWVQI 3ir7.1    --------------------------------------------------------------------------------  target    WKGGIRPLYDTRNDLDTFAGVAAKLSDMTGDKRMRDYFAMVYQNRVDVYVQRMLDASSTFYGYSADVMLKSEKGWMVMVR 3ir7.1    --------------------------------------------------------------------------------  target    TYPRHPFWEETNESKPMWTRSGRYENYRIEPEAIEYGENFISHREGPEATPYLPNAIFTTNPYVRPDDYGIPITAQHHDD 3ir7.1    --------------------------------------------------------------------------------  target    KTVRNIKLSWHEIKRHSNPLWEKGYQFYCVTPKTRHRVHSQWSVNDWVQIYESNFGDPYRMDKRTPGVGEHQIHINPQAA 3ir7.1    --------------------------------------------------------------------------------  target    KDRGINDGDYVYVDGNPVDRPYRGWKPSDPYYKVARLMIRAKYNPAYPYHVTMAKHAPFVATAKSVKGHETRPDGRAIAI 3ir7.1    --------------------------------------------------------------------------------  target    DTGYQSNFRYGAQQSFTRNWLMPMHQTDSLPGKHAVAWKFKWGYQVDHHAINTVPKECLIRITKAEDGGIGARGPWEPVR 3ir7.1    --------------------------------------------------------------------------------  target    TGFTPGQENEFMIKWLKGEHIKIKV 3ir7.1    ------------------------- ``` | | | | | | | | | | | | | | | | | | | | | | | | | | | | | | | | | | | | | | | | | | | | | | | | | |
|  | 3egw.1.A | Respiratory nitrate reductase 1 alpha chain  *The crystal structure of the NarGHI mutant NarH - C16A* | 0.22 | 0.07 | 27.02 | 0.47 | 46-639 | X-ray | 1.90 | homo-dimer | 2 x MD1, 2 x MGD, 2 x 6MO, 6 x SF4, 4 x F3S, 2 x 3PH, 4 x HEM, 2 x AGA | BLAST | 0.34 |
| ``` target    MFLSRRQFLKVSVGTVAAVAVADKVLALTALQPVIEVGNPLGDYPDRSWERVYHDQYRYDSSFTWVCSPNDTHACRVRAF 3egw.1    ---------------------------------------------NRDWEDGYRQRWQHDKIVRSTHGVNCTGSCSWKIY  target    VRNGVVMRVEQNYDH-QTYEDLYGNRGTFAHNPRMCLKGFTFHRRVYGPYRLKGPLMRKGW-KQWMDDNA---------P 3egw.1    VKNGLVTWETQQTDYPRTRPDLPN------HEPRGCPRGASYSWYLYSANRLKYPMMRKRLMKMWREAKALHSDPVEAWA  target    ELTAETKRKYKF-DSRFLDDMLRVSWDTAFTYAAKAMITIATRYSGEAGARRLREQGYAPEMIEMMKGAGTRCFKHRAGM 3egw.1    SIIEDADKAKSFKQARGRGGFVRSSWQEVNELIAASNVYTIKNYGPD------RVAGFSP-------------------I  target    PVLGIIGKMGNTRMNGGINALLDTWIRKVSPDQAQGGRYWSNYTWHGDQNPAHPFWSGVQGSDIDLSDMRFSKLNTSWGK 3egw.1    PAMSMVSYASGARYLSLI-----------------GGTCLSFYDWYCDLPPASPQTWGEQTDVPESADWYNSSYIIAWGS  target    NFVENKMPEAHWKLECIERGARVVVITPEYNPTAYRADYWMPLRPESDGALFLGAMKIIIDENMHD------IDFLKSFT 3egw.1    NVPQTRTPDAHFFTEVRYKGTKTVAVTPDYAEIAKLCDLWLAPKQGTDAAMALAMGHVMLREFHLDNPSQYFTDYVRRYT  target    DAPILVRTD----------TLQYLDPRDVIADYKFPDFSK---SYSGRIQSLKPEQIQRLGGMMVWDLNKK--------- 3egw.1    DMPMLVMLEERDGYYAAGRMLRAADLVAALGQENNPEWKTVAFNTNGEMVAPNGSIGFRWGEKGKWNLEQRDGKTGEETE  target    -QVVPLHRE----QVGW---------HYTNSGIDAALTGTYRVKLLNGREIDAMPIWQMYMVHFQDYDLDT--------- 3egw.1    LQLSLLGSQDEIAEVGFPYFGGDGTEHFNKVELENVLLHKLPVKRLQLADGSTALVTTVYDLTLANYGLERGLNDVNCAT  target    ------------VHQITRTPKDLIVRWARD-----SGTIKPAAIHNGEGTCHYFHQTANARGAAMVLIITGNVGKFGTGQ 3egw.1    SYDDVKAYTPAWAEQITGVSRSQIIRIAREFADNADKTHGRSMIIVGAGLNHWYHLDMNYRGLINMLIFCGCVGQSGGGW  target    HTWAGNYKAGTWTATPWSGAGLSVHTGEDPFNITLDPNAHGKEIKTRSYYYGEEVGYWNHGDTALIVNTPKYGRKVFTGK 3egw.1    AHYVGQEK-------------LRPQTGWQPLAFALDWQRPARHMNSTSYFYNHSSQWRYETVTAEELLSPMADKSRYTG-  target    THMPTPSKFRWVVNVNVVNNAKHHYDMVRNVDPNIECLITQDIEMTSDINHADIAFAANSWMEFTYPEMTVTVSNPWVQI 3egw.1    --------------------------------------------------------------------------------  target    WKGGIRPLYDTRNDLDTFAGVAAKLSDMTGDKRMRDYFAMVYQNRVDVYVQRMLDASSTFYGYSADVMLKSEKGWMVMVR 3egw.1    --------------------------------------------------------------------------------  target    TYPRHPFWEETNESKPMWTRSGRYENYRIEPEAIEYGENFISHREGPEATPYLPNAIFTTNPYVRPDDYGIPITAQHHDD 3egw.1    --------------------------------------------------------------------------------  target    KTVRNIKLSWHEIKRHSNPLWEKGYQFYCVTPKTRHRVHSQWSVNDWVQIYESNFGDPYRMDKRTPGVGEHQIHINPQAA 3egw.1    --------------------------------------------------------------------------------  target    KDRGINDGDYVYVDGNPVDRPYRGWKPSDPYYKVARLMIRAKYNPAYPYHVTMAKHAPFVATAKSVKGHETRPDGRAIAI 3egw.1    --------------------------------------------------------------------------------  target    DTGYQSNFRYGAQQSFTRNWLMPMHQTDSLPGKHAVAWKFKWGYQVDHHAINTVPKECLIRITKAEDGGIGARGPWEPVR 3egw.1    --------------------------------------------------------------------------------  target    TGFTPGQENEFMIKWLKGEHIKIKV 3egw.1    ------------------------- ``` | | | | | | | | | | | | | | | | | | | | | | | | | | | | | | | | | | | | | | | | | | | | | | | | | |
|  | 7p63.1.C | NADH-quinone oxidoreductase  *Complex I from E. coli, DDM/LMNG-purified, under Turnover at pH 6, Closed state* | 0.22 |  | 13.24 | 0.51 | 61-1020 | EM | 0.00 | hetero-1-1-1-1-1-1-… | 7 x SF4, 1 x FMN, 1 x NAI, 2 x FES, 1 x CA, 1 x DCQ, 4 x LFA, 8 x 3PE | HHblits | 0.26 |
| ``` target    MFLSRRQFLKVSVGTVAAVAVADKVLALTALQPVIEVGNPLGDYPDRSWERVYHDQYRYDSSFTWVCSPNDTHACRVRAF 7p63.1    ------------------------------------------------------------QFAPSICQ-QCSIGCNISPG  target    VRNGVVMRVEQNYDHQTYEDLYGNRGTFAHNPRMCLKGFTFHRRVYGPYRLKGPLMRKGWKQWMDDNAPELTAETKRKYK 7p63.1    ERYGELRRIENRYNGTV------------NHYFLCDRGRFGYGYVNLKDRPRQPVQRRG---------------------  target    FDSRFLDDMLRVSWDTAFTYAAKAMITIATRYSGEAGARRLREQGYAPEMIEMMKGAGTRCFKHRAGMPVLGIIGKMGNT 7p63.1    ------DDFITLNAEQAMQGAADILRQSKKVIGIGSP-------RASVESNFALR----------------EL---VGEE  target    RMNGGINALLDTWIRKVSPDQAQGGRYWSNYTWHGDQNPAHPFWSGVQGSDIDLSDMRFSKLNTSWGKNFVENKMPEAHW 7p63.1    NFYTGIA-------------HGEQERLQ----------LALKVLREGGIYTPALREIESYDAVLVLGEDVTQTGARVALA  target    KLECIERGAR--------------------------VVVITPEYNPTAYRADYWMPLRPESDGALFLGAMKIIIDENMHD 7p63.1    VRQAVKGKAREMAAAQKVADWQIAAILNIGQRAKHPLFVTNVDDTRLDDIAAWTYRAPVEDQARLGFAIAHALDNSAP--  target    IDFLKSFTDAPILVRTDTLQYLDPRDVIADYKFPDFSKSYSGRIQSLKPEQIQRLGGMMVWDLNKKQVVPLHREQVGWHY 7p63.1    --------------------------------------------------------------------------------  target    TNSGIDAALTGTYRVKLLNGREIDAMPIWQMYMVHFQDYDLDTVHQITRTPKDLIVRWARDSGTIKPAAIHNGEGTCHYF 7p63.1    ----------------------------------AVDGIEPE--------LQSKIDVIVQALAGAKKPLIISGTNAG---  target    HQTANARGAAMVLIITGNVGKFGTGQHTWAGNYKAGTWTATPWSGAGLSVHTGEDPFNITLDPNAHGKEIKTRSYYYGEE 7p63.1    -SLEVIQAAANVAKALKGRGADVGITMIARSVNSM-----------GLG------IM-----G---GGS-----------  target    VGYWNHGDTALIVNTPKYGRKVFTGKTHMPTPSKFRWVVNVNVVNNAKHHYDMVRNVDPNIECLITQDIEMTSDINHADI 7p63.1    --------------LEEALTEL------ETGRADAVVVLE-NDLHRHAS-ATRVNAALAKAPLVMVVDHQRTAIMENAHL  target    AFAANSWMEFTYPEMTVTVSNPWVQIWKGGIRPLY-----DTRNDLDTFAGVAAKLSDMTGDKRMRDYFAMVYQNRVDVY 7p63.1    VLSAASFAESDG---TVINNEGRAQRFFQVYDPAYYDSKTVMLESWRWLHSLHSTLLSRE--------VDW---TQLDHV  target    VQRMLDASSTFYGYSADVML-----------K------------SEKGWMVMVR-------------------------T 7p63.1    IDAVVAKIPELAGIKDAAPDATFRIRGQKLAREPHRYSGRTAMRANISVHEPRQPQDIDTMFTFSMEGNNQPTAHRSQVP  target    YPRHPFWE-ETNESKPMWTRSGRYENYRIEPEAIEYGENFISHREGPEATPYLPNAIFTTNPYVRPDDYGIPITAQHHDD 7p63.1    FAWAPGWNSPQAWNKFQDEVGGKLRFGDPGVRLFETSE---------NGLD-----------YF----------------  target    KTVRNIKLSWHEIKRHSNPLWEKGYQFYCVTPKTRHRVHSQWSVNDWVQIYESNFGDPYRMDKRTPGVGEHQIHINPQAA 7p63.1    ----------TSVPA---RFQPQDGKWRIAPYYHLFGSDELSQRAPVFQSR----------------MPQPYIKLNPADA  target    KDRGINDGDYVYVDGNPVDRPYRGWKPSDPYYKVARLMIRAKYNPAYPYHVTMAKHAPFVATAKSVKGHETRPDGRAIAI 7p63.1    AKLGVNAGTRVSFSYD-----------------GNTVTLPVEIAEGLTAGQVGLPMGMSG--------------------  target    DTGYQSNFRYGAQQSFTRNWLMPMHQTDSLPGKHAVAWKFKWGYQVDHHAINTVPKECLIRITKAEDGGIGARGPWEPVR 7p63.1    --------------------------------------------------------------------------------  target    TGFTPGQENEFMIKWLKGEHIKIKV 7p63.1    ------------------------- ``` | | | | | | | | | | | | | | | | | | | | | | | | | | | | | | | | | | | | | | | | | | | | | | | | | |
|  | 1h0h.1.A | FORMATE DEHYDROGENASE SUBUNIT ALPHA  *Tungsten containing Formate Dehydrogenase from Desulfovibrio Gigas* | 0.27 |  | 18.09 | 0.49 | 63-747 | X-ray | 1.80 | hetero-1-1-mer | 1 x W, 1 x 2MD, 1 x MGD, 4 x SF4, 1 x CA | HHblits | 0.29 |
| ``` target    MFLSRRQFLKVSVGTVAAVAVADKVLALTALQPVIEVGNPLGDYPDRSWERVYHDQYRYDSSFTWVCSPNDTHACRVRAF 1h0h.1    --------------------------------------------------------------TTSVCC-YCSVGCGLIVH  target    V--RNGVVMRVEQNYDHQTYEDLYGNRGTFAHNPRMCLKGFTFHRRVYGPYRLKGPLMRKGWKQWMDDNAPELTAETKRK 1h0h.1    TDKKTNRAINVEGDPDHPI------------NEGSLCAKGASTWQLAENERRPANPLYRAP-------------------  target    YKFDSRFLDDMLRVSWDTAFTYAAKAMITIATRYSGEAGARRLREQGYAPEMIEMMKGAGTRCFKHRAGMPVLGIIGKMG 1h0h.1    ------GSDQWEEKSWDWMLDTIAERVAKTREATFVTKNAKGQV--VNRCDGIASVGSAA--------------------  target    NTRMNGGINALLDTWIRKVSPDQAQGGRYWSNYT--WHGDQNPAHPFWSGVQGSDIDLSDMRFSKLNTSWGKNFVENKMP 1h0h.1    ---MDNEECWIYQAWLRSL------GLFYIEHQARIUHSATVAALAESYGRGAMTNHWIDLKNSDVILMMGSNPAENHPI  target    EAHWKLECIERGARVVVITPEYNPTAYRADYWMPLRPESDGALFLGAMKIIIDENMHDIDFLKSFTDAPILVRTDTLQYL 1h0h.1    SFKWVMRAKDKGATLIHVDPRYTRTSTKCDLYAPLRSGSDIAFLNGMTKYILEKELYFKDYVVNYTNASFIVGEGFA---  target    DPRDVIADYKFPDFSKSYSGRIQSLKPEQIQRLGGMMVWDLNKKQVVPLHREQVGWHYTNSGIDAALTGTYRVKLLNGRE 1h0h.1    FEEG------------------------------LFAGYNKETRKYDKSKW----------GFERDENGNP---KRDETL  target    IDAMPIWQMYMVHFQDYDLDTVHQITRTPKDLIVRWARDSGTI----KPAAIHNGEGTCHYFHQTANARGAAMVLIITGN 1h0h.1    KHPRCVFQIMKKHYERYDLDKISAICGTPKELILKVYDAYCATGKPDKAGTIMYAMGWTQHTVGVQNIRAMSINQLLLGN  target    VGKFGTGQHTWAGNYK-AGTWTATPWSG--AGLSV-----HTGEDPFNITLDPNAHGKEIKTRSYYYGE-----EVGYWN 1h0h.1    IGVAGGGVNALRGEANVQGSTDHGLLMHIYPGYLGTARASIPTYEEYTKKFTPVSKDPQSANWWSNFPKYSASYIKSMWP  target    HGDTALIV---NT----PKYG-RKVFTGKTHMPTPSKFRWVVNVNVVNNAKHHYDMVRNVDPNIECLITQDIEMTSDINH 1h0h.1    DADLNEAYGYLPKGEDGKDYSWLTLFD--DMFQGKIKGFFAWGQNPACSGANS-NKTREALTKLDWMVNVNIFDNETGSF  target    A-------------DIAFAANSWMEFTYPEMTVTVSNPWVQIWKGGIRPLYDTRNDLDTFAGVAAKLSDMTGDKRMRDYF 1h0h.1    WRGPDMDPKKIKTEVFFLPCAVAIEKEG---SISNSGRWMQWRYVGPEPRKNAIPDGDLIVELAKRVQK-----------  target    AMVYQNRVDVYVQRMLDASSTFYGYSADVMLKSEKGWMVMVRTYPRHPFWEETNESKPMWTRSGRYENYRIEPEAIEYGE 1h0h.1    --------------------------------------------------------------------------------  target    NFISHREGPEATPYLPNAIFTTNPYVRPDDYGIPITAQHHDDKTVRNIKLSWHEIKRHSNPLWEKGYQFYCVTPKTRHRV 1h0h.1    --------------------------------------------------------------------------------  target    HSQWSVNDWVQIYESNFGDPYRMDKRTPGVGEHQIHINPQAAKDRGINDGDYVYVDGNPVDRPYRGWKPSDPYYKVARLM 1h0h.1    --------------------------------------------------------------------------------  target    IRAKYNPAYPYHVTMAKHAPFVATAKSVKGHETRPDGRAIAIDTGYQSNFRYGAQQSFTRNWLMPMHQTDSLPGKHAVAW 1h0h.1    --------------------------------------------------------------------------------  target    KFKWGYQVDHHAINTVPKECLIRITKAEDGGIGARGPWEPVRTGFTPGQENEFMIKWLKGEHIKIKV 1h0h.1    ------------------------------------------------------------------- ``` | | | | | | | | | | | | | | | | | | | | | | | | | | | | | | | | | | | | | | | | | | | | | | | | | |
|  | 7p61.1.C | NADH-quinone oxidoreductase  *Complex I from E. coli, DDM-purified, with NADH, Resting state* | 0.22 |  | 13.48 | 0.51 | 62-1020 | EM | 0.00 | hetero-1-1-1-1-1-1-… | 7 x SF4, 1 x FMN, 1 x NAI, 2 x FES, 1 x CA, 2 x 3PE, 1 x UQ8 | HHblits | 0.26 |
| ``` target    MFLSRRQFLKVSVGTVAAVAVADKVLALTALQPVIEVGNPLGDYPDRSWERVYHDQYRYDSSFTWVCSPNDTHACRVRAF 7p61.1    -------------------------------------------------------------FAPSICQ-QCSIGCNISPG  target    VRNGVVMRVEQNYDHQTYEDLYGNRGTFAHNPRMCLKGFTFHRRVYGPYRLKGPLMRKGWKQWMDDNAPELTAETKRKYK 7p61.1    ERYGELRRIENRYNGTV------------NHYFLCDRGRFGYGYVNLKDRPRQPVQRRG---------------------  target    FDSRFLDDMLRVSWDTAFTYAAKAMITIATRYSGEAGARRLREQGYAPEMIEMMKGAGTRCFKHRAGMPVLGIIGKMGNT 7p61.1    ------DDFITLNAEQAMQGAADILRQSKKVIGIGSP-------RASVESN-----------------------------  target    RMNGGINALLDTWIRKVSPD--QAQGGRYWSNYTWHGDQNPAHPFWSGVQGSDIDLSDMRFSKLNTSWGKNFVENKMPEA 7p61.1    -------FALRELVGEENFYTGIAHGE--------QERLQLALKVLREGGIYTPALREIESYDAVLVLGEDVTQTGARVA  target    HWKLECIERGAR--------------------------VVVITPEYNPTAYRADYWMPLRPESDGALFLGAMKIIIDENM 7p61.1    LAVRQAVKGKAREMAAAQKVADWQIAAILNIGQRAKHPLFVTNVDDTRLDDIAAWTYRAPVEDQARLGFAIAHALDNSAP  target    HDIDFLKSFTDAPILVRTDTLQYLDPRDVIADYKFPDFSKSYSGRIQSLKPEQIQRLGGMMVWDLNKKQVVPLHREQVGW 7p61.1    --------------------------------------------------------------------------------  target    HYTNSGIDAALTGTYRVKLLNGREIDAMPIWQMYMVHFQDYDLDTVHQITRTPKDLIVRWARDSGTIKPAAIHNGEGTCH 7p61.1    ------------------------------------AVDGIEPE--------LQSKIDVIVQALAGAKKPLIISGTNAG-  target    YFHQTANARGAAMVLIITGNVGKFGTGQHTWAGNYKAGTWTATPWSGAGLSVHTGEDPFNITLDPNAHGKEIKTRSYYYG 7p61.1    ---SLEVIQAAANVAKALKGRGADVGITMIARSVNSM-----------GLG------IM-----G---GGS---------  target    EEVGYWNHGDTALIVNTPKYGRKVFTGKTHMPTPSKFRWVVNVNVVNNAKHHYDMVRNVDPNIECLITQDIEMTSDINHA 7p61.1    ----------------LEEALTEL------ETGRADAVVVLE-NDLHRHAS-ATRVNAALAKAPLVMVVDHQRTAIMENA  target    DIAFAANSWMEFTYPEMTVTVSNPWVQIWKGGIRPLY-----DTRNDLDTFAGVAAKLSDMTGDKRMRDYFAMVYQNRVD 7p61.1    HLVLSAASFAESDG---TVINNEGRAQRFFQVYDPAYYDSKTVMLESWRWLHSLHSTLLSREV-----D-----W-TQLD  target    VYVQRMLDASSTFYGYSADVM-----------LK------------SEKGWMV---------MV-----------RTYPR 7p61.1    HVIDAVVAKIPELAGIKDAAPDATFRIRGQKLAREPHRYSGRTAMRANISVHEPRQPQDIDTMFTFSMEGNNQPTAHRSQ  target    HPF-----W-EETNESKPMWTRSGRYENYRIEPEAIEYGENFISHREGPEATPYLPNAIFTTNPYVRPDDYGIPITAQHH 7p61.1    VPFAWAPGWNSPQAWNKFQDEVGGKLRFGDPGVRLFETS---------ENGLDY--------------------------  target    DDKTVRNIKLSWHEIKRHSNPLWEKGYQFYCVTPKTRHRVHSQWSVNDWVQIYESNFGDPYRMDKRTPGVGEHQIHINPQ 7p61.1    -----------FTSVPA--R-FQPQDGKWRIAPYYHLFGSDELSQRAPVFQSR----------------MPQPYIKLNPA  target    AAKDRGINDGDYVYVDGNPVDRPYRGWKPSDPYYKVARLMIRAKYNPAYPYHVTMAKHAPFVATAKSVKGHETRPDGRAI 7p61.1    DAAKLGVNAGTRVSFSYD-----------------GNTVTLPVEIAEGLTAGQVGLPMGMSG------------------  target    AIDTGYQSNFRYGAQQSFTRNWLMPMHQTDSLPGKHAVAWKFKWGYQVDHHAINTVPKECLIRITKAEDGGIGARGPWEP 7p61.1    --------------------------------------------------------------------------------  target    VRTGFTPGQENEFMIKWLKGEHIKIKV 7p61.1    --------------------------- ``` | | | | | | | | | | | | | | | | | | | | | | | | | | | | | | | | | | | | | | | | | | | | | | | | | |
|  | 7nz1.1.E | NADH-quinone oxidoreductase subunit G  *Respiratory complex I from Escherichia coli - focused refinement of cytoplasmic arm* | 0.22 |  | 13.33 | 0.51 | 62-1020 | EM | 0.00 | hetero-1-1-1-1-1-1-… | 7 x SF4, 2 x FES, 1 x FMN, 1 x CA | HHblits | 0.25 |
| ``` target    MFLSRRQFLKVSVGTVAAVAVADKVLALTALQPVIEVGNPLGDYPDRSWERVYHDQYRYDSSFTWVCSPNDTHACRVRAF 7nz1.1    -------------------------------------------------------------FAPSICQ-QCSIGCNISPG  target    VRNGVVMRVEQNYDHQTYEDLYGNRGTFAHNPRMCLKGFTFHRRVYGPYRLKGPLMRKGWKQWMDDNAPELTAETKRKYK 7nz1.1    ERYGELRRIENRYNGTV------N------HYFLCDRGRFGYGYVNLKDRPRQPVQRRG---------------------  target    FDSRFLDDMLRVSWDTAFTYAAKAMITIATRYSGEAGARRLREQGYAPEMIEMMKGAGTRCFKHRAGMPVLGIIGKMGNT 7nz1.1    ------DDFITLNAEQAMQGAADILRQSKKVIGIGSP-------RASVESNF----------------------------  target    RMNGGINALLDTWIRKVSPD--QAQGGRYWSNYTWHGDQNPAHPFWSGVQGSDIDLSDMRFSKLNTSWGKNFVENKMPEA 7nz1.1    --------ALRELVGEENFYTGIAHGE----Q----ERLQLALKVLREGGIYTPALREIESYDAVLVLGEDVTQTGARVA  target    HWKLECIERGAR--------------------------VVVITPEYNPTAYRADYWMPLRPESDGALFLGAMKIIIDENM 7nz1.1    LAVRQAVKGKAREMAAAQKVADWQIAAILNIGQRAKHPLFVTNVDDTRLDDIAAWTYRAPVEDQARLGFAIAHALDNSAP  target    HDIDFLKSFTDAPILVRTDTLQYLDPRDVIADYKFPDFSKSYSGRIQSLKPEQIQRLGGMMVWDLNKKQVVPLHREQVGW 7nz1.1    --------------------------------------------------------------------------------  target    HYTNSGIDAALTGTYRVKLLNGREIDAMPIWQMYMVHFQDYDLDTVHQITRTPKDLIVRWARDSGTIKPAAIHNGEGTCH 7nz1.1    ------------------------------------AVDGIEPE--------LQSKIDVIVQALAGAKKPLIISGTNAG-  target    YFHQTANARGAAMVLIITGNVGKFGTGQHTWAGNYKAGTWTATPWSGAGLSVHTGEDPFNITLDPNAHGKEIKTRSYYYG 7nz1.1    ---SLEVIQAAANVAKALKGRGADVGITMI-ARSVNS----------MGLG------IM----G----GGS---------  target    EEVGYWNHGDTALIVNTPKYGRKVFTGKTHMPTPSKFRWVVNVNVVNNAKHHYDMVRNVDPNIECLITQDIEMTSDINHA 7nz1.1    ----------------LEEALTEL------ETGRADAVVVLE-NDLHRHAS-AIRVNAALAKAPLVMVVDHQRTAIMENA  target    DIAFAANSWMEFTYPEMTVTVSNPWVQIWKGGIRPLY-----DTRNDLDTFAGVAAKLSDMTGDKRMRDYFAMVYQNRVD 7nz1.1    HLVLSAASFAESDG---TVINNEGRAQRFFQVYDPAYYDSKTVMLESWRWLHSLHSTLLSRE--------VD--W-TQLD  target    VYVQRMLDASSTFYGYSADV------------------------MLKSEKGWMVM---------V--------------R 7nz1.1    HVIDAVVAKIPELAGIKDAAPDATFRIRGQKLAREPHRYSGRTAMRA-NISVHEPRQPQDIDTMFTFSMEGNNQPTAHRS  target    --TYPRHPFWE-ETNESKPMWTRSGRYENYRIEPEAIEYGENFISHREGPEATPYLPNAIFTTNPYVRPDDYGIPITAQH 7nz1.1    QVPFAWAPGWNSPQAWNKFQDEVGGKLRFGDPGVRLFETS---------ENGLD---Y----------------------  target    HDDKTVRNIKLSWHEIKRHSNPLWEKGYQFYCVTPKTRHRVHSQWSVNDWVQIYESNFGDPYRMDKRTPGVGEHQIHINP 7nz1.1    ------------FTSVPA---RFQPQDGKWRIAPYYHLFGSDELSQRAPVFQSR----------------MPQPYIKLNP  target    QAAKDRGINDGDYVYVDGNPVDRPYRGWKPSDPYYKVARLMIRAKYNPAYPYHVTMAKHAPFVATAKSVKGHETRPDGRA 7nz1.1    ADAAKLGVNAGTRVSFSYD-----------------GNTVTLPVEIAEGLTAGQVGLPMGMSG-----------------  target    IAIDTGYQSNFRYGAQQSFTRNWLMPMHQTDSLPGKHAVAWKFKWGYQVDHHAINTVPKECLIRITKAEDGGIGARGPWE 7nz1.1    --------------------------------------------------------------------------------  target    PVRTGFTPGQENEFMIKWLKGEHIKIKV 7nz1.1    ---------------------------- ``` | | | | | | | | | | | | | | | | | | | | | | | | | | | | | | | | | | | | | | | | | | | | | | | | | |
|  | 6lod.1.B | Fe-S-cluster-containing hydrogenase components 1-like protein  *Cryo-EM structure of the air-oxidized photosynthetic alternative complex III from Roseiflexus castenholzii* | 0.22 |  | 14.65 | 0.48 | 65-1025 | EM | 0.00 | hetero-1-1-1-1-1-1-… | 6 x HEC, 2 x EL6, 3 x SF4, 1 x F3S | HHblits | 0.27 |
| ``` target    MFLSRRQFLKVSVGTVAAVAVADKVLALTALQPVIEVGNPLGDYPDRSWERVYHDQYRYDSSFTWVCSPNDTHACRVRAF 6lod.1    ----------------------------------------------------------------TAVT-FAGFGVGLLVE  target    VRNGVVMRVEQNYDHQTYEDLYGNRGTFAHNPRMCLKGFTFHRRVYGPYRLKGPLMRKGWKQWMDDNAPELTAETKRKYK 6lod.1    SHEGRPTKIEGNPDHPA------S------LGSTDLITQAMILTMYDPDRSQAPTNA-----------------------  target    FDSRFLDDMLRVSWDTAFTYAAKAMITIATRYSGEAGARRLREQGYAPEMIEMMKGAGTRCFKHRAGMPVLGIIGKMGNT 6lod.1    --------GQETTWDAFVAAATAAMQAQTAKQGAGLR--VLSGS------------LTSPTL------------------  target    RMNGGINALLDTWIRKVSPDQAQGGRYWSNYTWHGDQ--NPAHPFWSGVQGSDIDLSDMRFSKLNTSWGKNFVENKMPEA 6lod.1    --IAQKQQLLTQFPQ----------AKWYEYEPVGRDNANAGARLAFGA--DVHTIYRLDTAKVIVGFDADFTAPSPTGV  target    H---WKLECI------ERGARVVVITPEYNPTAYRADYWMPLRPESDGALFLGAMKIIIDENMHDIDFLKSFTDAPILVR 6lod.1    RMARQLADGRRIRKGTKEVNRLYLAESTPSITGLLADHRLPVRSSQIEHLVRALATLVGVPNVAA---------------  target    TDTLQYLDPRDVIADYKFPDFSKSYSGRIQSLKPEQIQRLGGMMVWDLNKKQVVPLHREQVGWHYTNSGIDAALTGTYRV 6lod.1    --------------------------------------------------------------------------------  target    KLLNGREIDAMPIWQMYMVHFQDYDLDTVHQITRTPKDLIVRWARDSGTIKPAAIHNGEGTCHYFHQTANARGAAMVLII 6lod.1    ----------------------------GAPLSDTEKKWVEAAAKDLQANRGACVVLV-GESQPP---VVHALGHAINAQ  target    TGNVGKFGTGQHTWAGNYKAGTWTATPWSGAGLSVHTGEDPFNITLDPNAHGKEIKTRSYYYGEEVGYWNHGDTALIVNT 6lod.1    LGNVGST---VVYTE-----------P---------VE-------DDPSGGIAA-------------------------L  target    PKYGRKVFTGKTHMPTPSKFRWVVNVNVVNNAKHHYDMVRNVDPNIECLITQDIEMTSDINHADIAFAANSWMEFTYPEM 6lod.1    SALTQEM------NAGTVEVLLMIESNPVYNAPAD-IPFAEALAKVPLSMHVGLYRDETAQQSVWHINGAHFLEAWGD--  target    TVTVSNPWVQIWKGGIRPLYDTRNDLDTFAGVAAKLSDMTGDKRMRDYFAMVYQNRVDVYVQRMLDASSTFYGYSADVML 6lod.1    -VRAFDGTTTIVQPLIAPLYNGKSAIEVLNVLLGKPQETG----Y-QT--------LTAYWQTQDASG-N-FRVFWNTAL  target    KSEKGWMVMVRTYPRHPFWEETNESKPMWTRSGRYENYRIEPEAIEYGENFISHREGPEATPYLPNAIFTTNPYVRPDDY 6lod.1    H--DGVITAT----------------Q--ARSRQVTLQ--------QG--F---------AD-----------A------  target    GIPITAQHHDDKTVRNIKLSWHEIKRHSNPLWEKGYQFYCVTPKTRHRVHSQWSVNDWVQIYESNFGDPYRMDKRTPGVG 6lod.1    ---------------------A------PPAPTQGLEIVFRPD--PSLWDGAFANNAWLQETP----KPY--TKL---TW  target    EHQIHINPQAAKDRGINDGDYVYVDGNPVDRPYRGWKPSDPYYKVARLMIRAKYNPAYPYHVTMAKHAPFVATAKSVKGH 6lod.1    DNVALMSVRTANALGLKNGDVVRLTYQ-----------------GRSVDAPVWVQPGHADDSVTVHFGFGRTAAGR----  target    ETRPDGRAIAIDTGYQSNFRYGAQQSFTRNWLMPMHQTDSLPGKHAVAWKFKWGYQVDHHAINTVPKECLIRITKAEDGG 6lod.1    --------------------------------------------------------------------------------  target    IGARGPWEPVRTGFTPGQENEFMIKWLKGEHIKIKV 6lod.1    ------------------------------------ ``` | | | | | | | | | | | | | | | | | | | | | | | | | | | | | | | | | | | | | | | | | | | | | | | | | |
|  | 6btm.1.B | Alternative Complex III subunit B  *Structure of Alternative Complex III from Flavobacterium johnsoniae (Wild Type)* | 0.22 |  | 13.15 | 0.47 | 65-1024 | EM | 3.40 | hetero-1-1-1-1-1-1-… | 6 x HEC, 1 x F3S, 1 x SF4, 2 x E87 | HHblits | 0.26 |
| ``` target    MFLSRRQFLKVSVGTVAAVAVADKVLALTALQPVIEVGNPLGDYPDRSWERVYHDQYRYDSSFTWVCSPNDTHACRVRAF 6btm.1    ----------------------------------------------------------------TTVF-DGFDFANLLVK  target    VRNGVVMRVEQNYDHQTYEDLYGNRGTFAHNPRMCLKGFTFHRRVYGPYRLKGPLMRKGWKQWMDDNAPELTAETKRKYK 6btm.1    TREGRPIKIENNTIAGA------K-------FSANARIHASILGLYDSMRLKEPKLDG----------------------  target    FDSRFLDDMLRVSWDTAFTYAAKAMITIATRYSGEAGARRLREQGYAPEMIEMMKGAGTRCFKHRAGMPVLGIIGKMGNT 6btm.1    ---------KNSSWSAVDLKIKSSLADAKAK-GGQV---------------VLLTNTLA---------------------  target    RMNGGINALLDTWIRKVSPDQAQGGRYWSNYTWHGDQ--NPAHPFWSGVQGSDIDLSDMRFSKLNTSWGKNFVENKMPEA 6btm.1    --SPTTEKLIGEFIAKN------PNAKHVVYDAVSSSDALDAFETVYGE--RALVDYDFSKASLIVSVGADFLGDWQGGG  target    H--WKLECIE----RGARVVVITPEYNPTAYRADYWMPLRPESDGALFLGAMKIIIDENMHDIDFLKSFTDAPILVRTDT 6btm.1    YDAGYAKGRIPQNGKMSRHFQFESNMTLSGAAADKRVPMTTADQKQALVQIYNIVVGASVPV-------S----------  target    LQYLDPRDVIADYKFPDFSKSYSGRIQSLKPEQIQRLGGMMVWDLNKKQVVPLHREQVGWHYTNSGIDAALTGTYRVKLL 6btm.1    --------------------------------------------------------------------------------  target    NGREIDAMPIWQMYMVHFQDYDLDTVHQITRTPKDLIVRWARDSGTIKPA-AIHNGEGTCHYFHQTANARGAAMVLIITG 6btm.1    ----------------------------LDAKFKAEVVKAAQQLKAAGTKGILVSGIED------KNAQLLVLAINQALA  target    NVGKFGTGQHTWAGNYKAGTWTATPWSGAGLSVHTGEDPFNITLDPNAHGKEIKTRSYYYGEEVGYWNHGDTALIVNTPK 6btm.1    SEAFSTAGTRQ---------------------IRKG---------S---NAVVA------------------Q-------  target    YGRKVFTGKTHMPTPSKFRWVVNVNVVNNAKHHYDMVRNVDPNIECLITQDIEMTSDINHADIAFAANSWMEFTYPEMTV 6btm.1    LIKD------MNAGSVHTLIMSGVNPVYTLADS-ASFVSGLKKVKTSVAFSLKEDETAAVSTIAAAAPHYLESWGDVE--  target    TVSNPWVQIWKGGIRPLYDTRNDLDTFAGVAAKLSDMTGDKRMRDYFAMVYQNRVDVYVQRMLDASSTFYGYSADVMLKS 6btm.1    -ITKGTYSLTQPTIRPIFDTKQFQDVLLSVNGTPG------NFYDYLKANSGAI--------I------AGSSWNKVLH-  target    EKGWMVMVRTYPRHPFWEETNESKPMWTRSGRYENYRIEPEAIEYGENFISHREGPEATPYLPNAIFTTNPYVRPDDYGI 6btm.1    -DGIFVVG----------------SAALAGGSYDFAGA-----------------A--SL--------------------  target    PITAQHHDDKTVRNIKLSWHEIKRHSNPLWEKGYQFYCVTPKTRHRVHSQWSVNDWVQIYESNFGDPYRMDKRTPGVGEH 6btm.1    -------------------------LSK-AKSSGELELVLYTKTGMGDGQHANNPWLQEFP----DPIT--R---VSWDN  target    QIHINPQAAKDRGIND---------GDYVYVDGNPVDRPYRGWKPSDPYYKVAR--LMIRAKYNPAYPYHVTMAKHAPFV 6btm.1    YVTVSNADAKKFNLSNEIVANGGLNGSYATITTA-----------------DGNKLENVPVIVQPGQAVGTVGLAVGYGR  target    ATAKSVKGHETRPDGRAIAIDTGYQSNFRYGAQQSFTRNWLMPMHQTDSLPGKHAVAWKFKWGYQVDHHAINTVPKECLI 6btm.1    KAAL----------------------------------------------------------------------------  target    RITKAEDGGIGARGPWEPVRTGFTPGQENEFMIKWLKGEHIKIKV 6btm.1    --------------------------------------------- ``` | | | | | | | | | | | | | | | | | | | | | | | | | | | | | | | | | | | | | | | | | | | | | | | | | |
|  | 8e9g.1.G | NADH-quinone oxidoreductase subunit G  *Mycobacterial respiratory complex I with both quinone positions modelled* | 0.19 |  | 13.89 | 0.47 | 62-1024 | EM | 0.00 | hetero-1-1-1-1-1-1-… |  | HHblits | 0.26 |
| ``` target    MFLSRRQFLKVSVGTVAAVAVADKVLALTALQPVIEVGNPLGDYPDRSWERVYHDQYRYDSSFTWVCSPNDTHACRVRAF 8e9g.1    -------------------------------------------------------------SSPSVCEH-CASGCAQRTD  target    VRNGVVMRVEQNYDHQTYEDLYGNRGTFAHNPRMCLKGFTFHRRVYGPYRLKGPLMRKGWKQWMDDNAPELTAETKRKYK 8e9g.1    HRRGKVLRRLAGDEPEV------------NEEWNCDKGRWAFTYATVGDRITTPMLRDG---------------------  target    FDSRFLDDMLRVSWDTAFTYAAKAMITIATRYSGEAGARRLREQGYAPEMIEMMKGAGTRCFKHRAGMPVLGIIGKMGNT 8e9g.1    ------GVLRPASWSEALTVAAAGLLTAAGS-----T--------------GVLVGGRCT--------------------  target    RMNGGINALLDTWIRKVSPD--QAQGGRYWSNYTWHGDQNPAHPFWSGVQGSDIDLSDMRFSKLNTSWGKNFVENKMPEA 8e9g.1    ---VEDAYAYAKFARMVLNTNDVDFRARPHSAE----EAEFLAAH-VAGQTMGLRYAELENAPTVLLAGFEPEEESPIVF  target    HWKLEC-IERGARVVVITPEYNP-TAYRADYWMPLRPESDGALFLGAMKIIIDENMHDIDFLKSFTDAPILVRTDTLQYL 8e9g.1    LRLRKGVRKNGVQVVAVAPWASRGLTKLAGTVVPTVPGDEPAALDGMHD---------DDRLRRP---------------  target    DPRDVIADYKFPDFSKSYSGRIQSLKPEQIQRLGGMMVWDLNKKQVVPLHREQVGWHYTNSGIDAALTGTYRVKLLNGRE 8e9g.1    --------------------------------------------------------------------------------  target    IDAMPIWQMYMVHFQDYDLDTVHQITRTPKDLIVRWARDSGTIKPAAIHNGEGTCHYFHQTANARGAAMVLIITGNVGKF 8e9g.1    ----------------GAVILVGERLATSPGALSAAVRLAAATGARLAWIP----RRAGERGAIEAGALPNLLPGGRPVD  target    GTGQHTWAGNYKAGTWTATPWSGAGLSVHTGEDPFNITLDPNAHGKEIKTRSYYYGEEVGYWNHGDTALIVNTPKYGRKV 8e9g.1    DADAR--------AEVA-RAWY--------------ISALPEAPG-------------------------RDTAAILSTA  target    FTGKTHMPTPSKFRWVVNVNVVNNAKHHYDMVRNVDPNIECLITQDIEMTSDINHADIAFAANSWMEFTYPEMTVTVSNP 8e9g.1    ------ASGHLAALLVGG-VELGDLPD-PELAVAAVRTTPFVVSLELRESAVTELADVVFPVAPVVEKAG---SFLNWEG  target    WVQIWKGGIRPLYDTRNDLDTFAGVAAKLSDMTGDKRMRDYFAMVYQNRVDVYVQRMLDASSTFYGYSADVMLKSEKGWM 8e9g.1    RPRPFAPSLK--TNAIPDLRVLHYLADEIGVDL-------A----LP-TAEAADA---------------ELAQ-LGTW-  target    VMVRTYPRHPFWEETNESKPMWTRSGRYENYRIEPEAIEYGENFISHREGPEATPYLPNAIFTTNPYVRPDDYGIPITAQ 8e9g.1    ------------G----GARPPA-------------------------------PTAPP-------T--------A----  target    HHDDKTVRNIKLSWHEIKRHSNPLWEKGYQFYCVTPKTRHRVHSQWSVNDWVQIYESNFGDPYRMDKRTPGVGEHQIHIN 8e9g.1    ---------------------RP-EAGSGQAVLASWRMLLDAGRLQDGEPHLAGT----------------AVRPVARMS  target    PQAAKDRGINDGDYVYVDGNPVDRPYRGWKPSDPYYKVARLMIRAKYNPAYPYHVTMAKHAPFVATAKSVKGHETRPDGR 8e9g.1    AATAAGIGASDGAPVTVSTE-----------------RGAVTLPLAVTD-MPDGVVWLPMNSPGSAVH------------  target    AIAIDTGYQSNFRYGAQQSFTRNWLMPMHQTDSLPGKHAVAWKFKWGYQVDHHAINTVPKECLIRITKAEDGGIGARGPW 8e9g.1    --------------------------------------------------------------------------------  target    EPVRTGFTPGQENEFMIKWLKGEHIKIKV 8e9g.1    ----------------------------- ``` | | | | | | | | | | | | | | | | | | | | | | | | | | | | | | | | | | | | | | | | | | | | | | | | | |
|  | 3o5a.1.A | Periplasmic nitrate reductase  *Crystal Structure of partially reduced Periplasmic Nitrate Reductase from Cupriavidus necator using Ionic Liquids* | 0.23 |  | 16.38 | 0.46 | 63-746 | X-ray | 1.72 | hetero-oligomer | 1 x SF4, 1 x MOS, 2 x MGD, 2 x HEC | HHblits | 0.27 |
| ``` target    MFLSRRQFLKVSVGTVAAVAVADKVLALTALQPVIEVGNPLGDYPDRSWERVYHDQYRYDSSFTWVCSPNDTHACRVRAF 3o5a.1    --------------------------------------------------------------SKAPCR-FCGTGCGVTVA  target    VRNGVVMRVEQNYDHQTYEDLYGNRGTFAHNPRMCLKGFTFHRRVYGPYRLKGPLMRKGWKQWMDDNAPELTAETKRKYK 3o5a.1    VKDNKVVATQGDPQAEV------------NKGLNCVKGYFLSKIMYGQDRLTRPLMRMKNGK------------------  target    FDSRFLDDMLRVSWDTAFTYAAKAMITIATRYSGEAGARRLREQGYAPEMIEMMKGAGTRCFKHRAGMPVLGIIGKMGNT 3o5a.1    --YDKNGDFAPVTWDQAFDEMERQFKRVLKEKGPTAVGMFG-SGQWTVWE----------GYA---AAKLYK--AGFRSN  target    RMNGGINALLDTWIRKVSPDQAQGGRYWSNYTWHGDQNPAHPFWSGVQGSDIDLSDMRFSKLNTSWGKNFVENKMPEAHW 3o5a.1    NIDPNA-------------RHCMAS-----------AAAGFMRTFGMDEPMGCYDDFEAADAFVLWGSNMAEMHPILWTR  target    KLECI--ERGARVVVITPEYNPTAYRADYWMPLRPESDGALFLGAMKIIIDENMHDIDFLKSFTDAPILVRTDTLQYLDP 3o5a.1    VTDRRLSHPKTRVVVLSTFTHRCFDLADIGIIFKPQTDLAMLNYIANYIIRNNKVNKDFVNKHTVFKEGVTDIGYGLRPD  target    RDVIADYKFPDFSKSYSGRIQSLKPEQIQRLGGMMVWDLNKKQVVPLHREQVGWHYTNSGIDAALTGTYRVKLLNGREID 3o5a.1    HPLQKA----------------------------------AK------------------------------NASDPGAA  target    AMPIWQMYMVHFQDYDLDTVHQITRTPKDLIVRWARDSGTIK-PAAIHNGEGTCHYFHQTANARGAAMVLIITGNVGKFG 3o5a.1    KVITFDEFAKFVSKYDADYVSKLSAVPKAKLDQLAELYADPNIKVMSLWTMGFNQHTRGTWANNMVYNLHLLTGKIATPG  target    TGQHTWAGNYKA-GTWTATPWSGAGLSVHTGEDPFNITLDPNAHGKEIKTRSYYYGEEVGYWNHGDTALIVNTPKYGRKV 3o5a.1    NSPFSLTGQPSACGTAR--EV-GTFSHRLPADMVV---TNPKH-REEAER----------IWKLPPGT-IPDKPGY-DAV  target    FTGKTHMPTPSKFRWVVNVNVVNNAKH-HYDMVRNVDPNIECLITQDIEMTSDINHADIAFAANSWMEFTYPEMTVTVSN 3o5a.1    LQNRMLKDGKLNAYWVQVNNNMQAAANLMEEGLPGYRNPANFIVVSDAYPTVTALAADLVLPSAMWVEKEG---AYGNAE  target    PWVQIWKGGIRPLYDTRNDLDTFAGVAAKLSDMTGDKRMRDYFAMVYQNRVDVYVQRMLDASSTFYGYSADVMLKSEKGW 3o5a.1    RRTQFWHQLVDAPGEARSDLWQLVEFAKRFK-------------------------------------------------  target    MVMVRTYPRHPFWEETNESKPMWTRSGRYENYRIEPEAIEYGENFISHREGPEATPYLPNAIFTTNPYVRPDDYGIPITA 3o5a.1    --------------------------------------------------------------------------------  target    QHHDDKTVRNIKLSWHEIKRHSNPLWEKGYQFYCVTPKTRHRVHSQWSVNDWVQIYESNFGDPYRMDKRTPGVGEHQIHI 3o5a.1    --------------------------------------------------------------------------------  target    NPQAAKDRGINDGDYVYVDGNPVDRPYRGWKPSDPYYKVARLMIRAKYNPAYPYHVTMAKHAPFVATAKSVKGHETRPDG 3o5a.1    --------------------------------------------------------------------------------  target    RAIAIDTGYQSNFRYGAQQSFTRNWLMPMHQTDSLPGKHAVAWKFKWGYQVDHHAINTVPKECLIRITKAEDGGIGARGP 3o5a.1    --------------------------------------------------------------------------------  target    WEPVRTGFTPGQENEFMIKWLKGEHIKIKV 3o5a.1    ------------------------------ ``` | | | | | | | | | | | | | | | | | | | | | | | | | | | | | | | | | | | | | | | | | | | | | | | | | |
|  | 1ogy.1.A | PERIPLASMIC NITRATE REDUCTASE  *Crystal structure of the heterodimeric nitrate reductase from Rhodobacter sphaeroides* | 0.23 |  | 16.25 | 0.46 | 63-746 | X-ray | 3.20 | hetero-1-1-mer | 1 x SF4, 1 x MO, 2 x MGD, 2 x HEC | HHblits | 0.27 |
| ``` target    MFLSRRQFLKVSVGTVAAVAVADKVLALTALQPVIEVGNPLGDYPDRSWERVYHDQYRYDSSFTWVCSPNDTHACRVRAF 1ogy.1    --------------------------------------------------------------SKAPCR-FCGTGCGVMVG  target    VRNGVVMRVEQNYDHQTYEDLYGNRGTFAHNPRMCLKGFTFHRRVYGPYRLKGPLMRKGWKQWMDDNAPELTAETKRKYK 1ogy.1    TRDGQVVATHGDTQAEV------------NRGLNCVKGYFLSKIMYGEDRLTTPLLRMKDGV------------------  target    FDSRFLDDMLRVSWDTAFTYAAKAMITIATRYSGEAGARRLREQGYAPEMIEMMKGAGTRCFKHRAGMPVLGIIGK-MGN 1ogy.1    --YHKEGEFAPVSWDEAFDVMAAQAKLVLKEKAPEAVG--------------MFGSGQWTIWE---GYAASKLMRAGFRS  target    TRMNGGINALLDTWIRKVSPDQAQGGRYWSNYTWHGDQNPAHPFWSGVQGSDIDLSDMRFSKLNTSWGKNFVENKMPEAH 1ogy.1    NNLDP-------------------NARH-----CMASAATAFMRTFGMDEPMGCYDDFEAADAFVLWGSNMAEMHPILWS  target    WKLECI--ERGARVVVITPEYNPTAYRADYWMPLRPESDGALFLGAMKIIIDENMHDIDFLKSFTDAPILVRTDTLQYLD 1ogy.1    RLTDRRLSHEHVRVAVLSTFTHRSSDLSDTPIIFRPGTDRAILNYIAHHIISTGRVNRDFVDRHTNFALGATDIGY-GLR  target    PRDVIADYKFPDFSKSYSGRIQSLKPEQIQRLGGMMVWDLNKKQVVPLHREQVGWHYTNSGIDAALTGTYRVKLLNGREI 1ogy.1    PEH-------------------------------QLQLAA--------------------------------KGAADAGA  target    DAMPIWQMYMVHFQDYDLDTVHQITRTPKDLIVRWARDSGTIKP-AAIHNGEGTCHYFHQTANARGAAMVLIITGNVGKF 1ogy.1    MTPTDFETFAALVSEYTLEKAAEISGVEPALLEELAELYADPDRKWMSLWTMGFNQHVRGVWANHMVYNLHLLTGKISEP  target    GTGQHTWAGNYKA-GTWTATPWSGAGLSVHTGEDPFNITL-DPNAHGKEIKTRSYYYGEEVGYWNHGDTALIVNTPKYGR 1ogy.1    GNSPFSLTGQPFACGTA-----REVG--TFAHRLPADMVVTNPEH-RAHAEEI-WKL--PAGLLPDW-----V--GAHAV  target    KVFTGKTHMPTPSKFRWVVNVNVVNNAKHHY-DMVRNVDPNIECLITQDIEMTSDINHADIAFAANSWMEFTYPEMTVTV 1ogy.1    EQDR--KLHDGEINFYWVQVNNNMQAAPNIDQETYPGYRNPENFIVVSDAYPTVTGRAADLVLPAAMWVEKEG---AYGN  target    SNPWVQIWKGGIRPLYDTRNDLDTFAGVAAKLSDMTGDKRMRDYFAMVYQNRVDVYVQRMLDASSTFYGYSADVMLKSEK 1ogy.1    AERRTHFWHQLVEAPGEARSDLWQLMEFSKRFT-----------------------------------------------  target    GWMVMVRTYPRHPFWEETNESKPMWTRSGRYENYRIEPEAIEYGENFISHREGPEATPYLPNAIFTTNPYVRPDDYGIPI 1ogy.1    --------------------------------------------------------------------------------  target    TAQHHDDKTVRNIKLSWHEIKRHSNPLWEKGYQFYCVTPKTRHRVHSQWSVNDWVQIYESNFGDPYRMDKRTPGVGEHQI 1ogy.1    --------------------------------------------------------------------------------  target    HINPQAAKDRGINDGDYVYVDGNPVDRPYRGWKPSDPYYKVARLMIRAKYNPAYPYHVTMAKHAPFVATAKSVKGHETRP 1ogy.1    --------------------------------------------------------------------------------  target    DGRAIAIDTGYQSNFRYGAQQSFTRNWLMPMHQTDSLPGKHAVAWKFKWGYQVDHHAINTVPKECLIRITKAEDGGIGAR 1ogy.1    --------------------------------------------------------------------------------  target    GPWEPVRTGFTPGQENEFMIKWLKGEHIKIKV 1ogy.1    -------------------------------- ``` | | | | | | | | | | | | | | | | | | | | | | | | | | | | | | | | | | | | | | | | | | | | | | | | | |
|  | 2nya.1.A | Periplasmic nitrate reductase  *Crystal structure of the periplasmic nitrate reductase (NAP) from Escherichia coli* | 0.23 |  | 15.33 | 0.46 | 65-747 | X-ray | 2.50 | monomer | 1 x SF4, 1 x 6MO, 2 x MGD | HHblits | 0.27 |
| ``` target    MFLSRRQFLKVSVGTVAAVAVADKVLALTALQPVIEVGNPLGDYPDRSWERVYHDQYRYDSSFTWVCSPNDTHACRVRAF 2nya.1    ----------------------------------------------------------------APCR-FCGTGCGVLVG  target    VRNGVVMRVEQNYDHQTYEDLYGNRGTFAHNPRMCLKGFTFHRRVYGPYRLKGPLMRKGWKQWMDDNAPELTAETKRKYK 2nya.1    TQQGRVVACQGDPDAPV------N------RGLNCIKGYFLPKIMYGKDRLTQPLLRMKNGK------------------  target    FDSRFLDDMLRVSWDTAFTYAAKAMITIATRYSGEAGARRLREQGYAPEMIEMMKGAGTRCFKHRAGMPVLGIIG-KMGN 2nya.1    --YDKEGEFTPITWDQAFDVMEEKFKTALKEKGPESIGMFGS-GQWTIWEGYA----------------ASKLFKAGFRS  target    TRMNGGINALLDTWIRKVSPDQAQGGRYWSNYTWHGDQNPAHPFWSGVQGSDIDLSDMRFSKLNTSWGKNFVENKMPEAH 2nya.1    NNIDP-------------------NARHCMA-----SAVVGFMRTFGMDEPMGCYDDIEQADAFVLWGANMAEMHPILWS  target    WKLECI--ERGARVVVITPEYNPTAYRADYWMPLRPESDGALFLGAMKIIIDENMHDIDFLKSFTDAPILVRTDTLQYLD 2nya.1    RITNRRLSNQNVTVAVLSTYQHRSFELADNGIIFTPQSDLVILNYIANYIIQNNAINQDFFSKHVNLRKGATDIG-YGLR  target    PRDVIADYKFPDFSKSYSGRIQSLKPEQIQRLGGMMVWDLNKKQVVPLHREQVGWHYTNSGIDAALTGTYRVKLLNGREI 2nya.1    PTHPLE--------------------------------KA--------------------AKN------------PGSDA  target    DAMPIWQMYMVHFQDYDLDTVHQITRTPKDLIVRWARDSGTIKPAA-IHNGEGTCHYFHQTANARGAAMVLIITGNVGKF 2nya.1    SEPMSFEDYKAFVAEYTLEKTAEMTGVPKDQLEQLAQLYADPNKKVISYWTMGFNQHTRGVWANNLVYNLHLLTGKISQP  target    GTGQHTWAGNYKA-GTWTATPWSGAGLSVHTGEDPFNITLDPNAHGKEIKTRSYYYGEEVGYWNHGDTALIVNTPKYGRK 2nya.1    GCGPFSLTGQPSACGTAR-----EVG--TFAHRLPADMVVTNEKHRDICE----------KKWNIPSGTIPAKIGLHAVA  target    VFTGKTHMPTPSKFRWVVNVNVVNNAKHHY-DMVRNVDPNIECLITQDIEMTSDINHADIAFAANSWMEFTYPEMTVTVS 2nya.1    Q--DRALKDGKLNVYWTMCTNNMQAGPNINEERMPGWRDPRNFIIVSDPYPTVSALAADLILPTAMWVEKEG---AYGNA  target    NPWVQIWKGGIRPLYDTRNDLDTFAGVAAKLSDMTGDKRMRDYFAMVYQNRVDVYVQRMLDASSTFYGYSADVMLKSEKG 2nya.1    ERRTQFWRQQVQAPGEAKSDLWQLVQFSRRFKT-----------------------------------------------  target    WMVMVRTYPRHPFWEETNESKPMWTRSGRYENYRIEPEAIEYGENFISHREGPEATPYLPNAIFTTNPYVRPDDYGIPIT 2nya.1    --------------------------------------------------------------------------------  target    AQHHDDKTVRNIKLSWHEIKRHSNPLWEKGYQFYCVTPKTRHRVHSQWSVNDWVQIYESNFGDPYRMDKRTPGVGEHQIH 2nya.1    --------------------------------------------------------------------------------  target    INPQAAKDRGINDGDYVYVDGNPVDRPYRGWKPSDPYYKVARLMIRAKYNPAYPYHVTMAKHAPFVATAKSVKGHETRPD 2nya.1    --------------------------------------------------------------------------------  target    GRAIAIDTGYQSNFRYGAQQSFTRNWLMPMHQTDSLPGKHAVAWKFKWGYQVDHHAINTVPKECLIRITKAEDGGIGARG 2nya.1    --------------------------------------------------------------------------------  target    PWEPVRTGFTPGQENEFMIKWLKGEHIKIKV 2nya.1    ------------------------------- ``` | | | | | | | | | | | | | | | | | | | | | | | | | | | | | | | | | | | | | | | | | | | | | | | | | |
|  | 3m9s.1.C | NADH-quinone oxidoreductase subunit 3  *Crystal structure of respiratory complex I from Thermus thermophilus* | 0.20 | 0.00 | 14.74 | 0.44 | 62-1033 | X-ray | 4.50 | monomer | 7 x SF4, 2 x FES, 1 x FMN | HHblits | 0.26 |
| ``` target    MFLSRRQFLKVSVGTVAAVAVADKVLALTALQPVIEVGNPLGDYPDRSWERVYHDQYRYDSSFTWVCSPNDTHACRVRAF 3m9s.1    -------------------------------------------------------------ETPTTCAL-CPVGCGITAD  target    VRNGVVMRVEQNYDHQTYEDLYGNRGTFAHNPRMCLKGFTFHRRVYGPYRLKGPLMRKGWKQWMDDNAPELTAETKRKYK 3m9s.1    TRSGELLRIRAREVPEV------------NEIWICDAGRFGHEW-ADQNRLKTPLVRKE---------------------  target    FDSRFLDDMLRVSWDTAFTYAAKAMITIATRYSGEAGARRLREQGYAPEMIEMMKGAGTRCFKHRAGMPVLGIIGKMGNT 3m9s.1    ------GRLVEATWEEAFLALKEGLKEARG----EEV--------------GLYLAHDA---------------------  target    RMNGGINALLDTWIRKVSPDQAQGGRYWSNYTWHGDQNPAHPFWSGVQGSDIDLSDMRFSKLNTSWGKNFVENKMPEAHW 3m9s.1    --TLEEGLLASELAKAL------KTPHLDFQGRTAAP-------A-SLFPPASLEDLLQADFALVLGD-PTEEAPILHLR  target    KLE-------------------------CIERGARVVVITPEYNPTAYRADYWMPLRPESDGALFLGAMKIIIDENMHDI 3m9s.1    LSEFVRDLKPPHRYNHGTPFADLQIKERMPRRTDKMALFAPYRAPLMKWAAIHEVHRPGEEREILLALLGDKE-------  target    DFLKSFTDAPILVRTDTLQYLDPRDVIADYKFPDFSKSYSGRIQSLKPEQIQRLGGMMVWDLNKKQVVPLHREQVGWHYT 3m9s.1    --------------------------------------------------------------------------------  target    NSGIDAALTGTYRVKLLNGREIDAMPIWQMYMVHFQDYDLDTVHQITRTPKDLIVRWARDSGTIKPAAIHNGEGTCHYFH 3m9s.1    -------------------------------------------------GSEMVAKAKEAWEKAKNPVLILGAGVLQDTV  target    QTANARGAAMVLIITGNVGKFGTGQHTWAGNYKAGTWTATPWSGAGLSVHTGEDPFNITLDPNAHGKEIKTRSYYYGEEV 3m9s.1    AAERARLL------A---ERKGAKVLAMTPAANA----------RGL------EAMG--VLPGAKGA-------------  target    GYWNHGDTALIVNTPKYGRKVFTGKTHMPTPSKFRWVVNVNVVNNAKHHYDMVRNVDPNIECLITQDIEMTSDI-NHADI 3m9s.1    -S---------W-----------D---EPGALYAY--YGFVP----------PEEALKGKRFVVMHLSHLHPLAERYAHV  target    AFAANSWMEFTYPEMTVTVSNPWVQIWKGGIRPLYDTRNDLDTFAGVAAKLSDMTGDKRMRDYFAMVYQNRVDVYVQRML 3m9s.1    VLPAPTFYEKRG---HLVNLEGRVLPLSPAPIENGEAEGALQVLALLAEALGVRP------PF-----R-LHLEAQ----  target    DASSTFYGYSADVMLKSEKGWMVMVRTYPRHPFWEETNESKPMWTRSGRYENYRIEPEAIEYGENFISHREGPEATPYLP 3m9s.1    -----------KALK------------------------ARKVPEAMGRLSFRLKELR----------------------  target    NAIFTTNPYVRPDDYGIPITAQHHDDKTVRNIKLSWHEIKRHSNPLWEKGYQFYCVTPKTRHRVHSQWSVNDWVQIYESN 3m9s.1    --------------------------------------------P-KERKGAFYLRPTMWKAH-----QAVGKAQE----  target    FGDPYRMDKRTPGVGEHQIHINPQAAKDRGINDGDYVYVDGNPVDRPYRGWKPSDPYYKVARLMIRAKYNPAYPYHVTMA 3m9s.1    -------------AARAELWAHPETARAEALPEGAQVAVETP-----------------FGRVEARVVHREDVPKGHLYL  target    KHAPFVATAKSVKGHETRPDGRAIAIDTGYQSNFRYGAQQSFTRNWLMPMHQTDSLPGKHAVAWKFKWGYQVDHHAINTV 3m9s.1    SALGPAAG-LRVEGRVLVP-------------------------------------------------------------  target    PKECLIRITKAEDGGIGARGPWEPVRTGFTPGQENEFMIKWLKGEHIKIKV 3m9s.1    --------------------------------------------------- ``` | | | | | | | | | | | | | | | | | | | | | | | | | | | | | | | | | | | | | | | | | | | | | | | | | |
|  | 2fug.2.C | NADH-quinone oxidoreductase chain 3  *Crystal structure of the hydrophilic domain of respiratory complex I from Thermus thermophilus* | 0.19 | 0.00 | 14.74 | 0.44 | 62-1033 | X-ray | 3.30 | monomer | 7 x SF4, 2 x FES, 1 x FMN | HHblits | 0.26 |
| ``` target    MFLSRRQFLKVSVGTVAAVAVADKVLALTALQPVIEVGNPLGDYPDRSWERVYHDQYRYDSSFTWVCSPNDTHACRVRAF 2fug.2    -------------------------------------------------------------ETPTTCAL-CPVGCGITAD  target    VRNGVVMRVEQNYDHQTYEDLYGNRGTFAHNPRMCLKGFTFHRRVYGPYRLKGPLMRKGWKQWMDDNAPELTAETKRKYK 2fug.2    TRSGELLRIRAREVPEV------------NEIWICDAGRFGHEW-ADQNRLKTPLVRKE---------------------  target    FDSRFLDDMLRVSWDTAFTYAAKAMITIATRYSGEAGARRLREQGYAPEMIEMMKGAGTRCFKHRAGMPVLGIIGKMGNT 2fug.2    ------GRLVEATWEEAFLALKEGLKEARG----EEV--------------GLYLAHDA---------------------  target    RMNGGINALLDTWIRKVSPDQAQGGRYWSNYTWHGDQNPAHPFWSGVQGSDIDLSDMRFSKLNTSWGKNFVENKMPEAHW 2fug.2    --TLEEGLLASELAKAL------KTPHLDFQGRTAAP-------A-SLFPPASLEDLLQADFALVLGD-PTEEAPILHLR  target    KLE-------------------------CIERGARVVVITPEYNPTAYRADYWMPLRPESDGALFLGAMKIIIDENMHDI 2fug.2    LSEFVRDLKPPHRYNHGTPFADLQIKERMPRRTDKMALFAPYRAPLMKWAAIHEVHRPGEEREILLALLGDKE-------  target    DFLKSFTDAPILVRTDTLQYLDPRDVIADYKFPDFSKSYSGRIQSLKPEQIQRLGGMMVWDLNKKQVVPLHREQVGWHYT 2fug.2    --------------------------------------------------------------------------------  target    NSGIDAALTGTYRVKLLNGREIDAMPIWQMYMVHFQDYDLDTVHQITRTPKDLIVRWARDSGTIKPAAIHNGEGTCHYFH 2fug.2    -------------------------------------------------GSEMVAKAKEAWEKAKNPVLILGAGVLQDTV  target    QTANARGAAMVLIITGNVGKFGTGQHTWAGNYKAGTWTATPWSGAGLSVHTGEDPFNITLDPNAHGKEIKTRSYYYGEEV 2fug.2    AAERARLL------A---ERKGAKVLAMTPAANA----------RGL------EAMG--VLPGAKGA-------------  target    GYWNHGDTALIVNTPKYGRKVFTGKTHMPTPSKFRWVVNVNVVNNAKHHYDMVRNVDPNIECLITQDIEMTSDI-NHADI 2fug.2    -S---------W-----------D---EPGALYAY--YGFVP----------PEEALKGKRFVVMHLSHLHPLAERYAHV  target    AFAANSWMEFTYPEMTVTVSNPWVQIWKGGIRPLYDTRNDLDTFAGVAAKLSDMTGDKRMRDYFAMVYQNRVDVYVQRML 2fug.2    VLPAPTFYEKRG---HLVNLEGRVLPLSPAPIENGEAEGALQVLALLAEALGVRP------PF-----R-LHLEAQ----  target    DASSTFYGYSADVMLKSEKGWMVMVRTYPRHPFWEETNESKPMWTRSGRYENYRIEPEAIEYGENFISHREGPEATPYLP 2fug.2    -----------KALK------------------------ARKVPEAMGRLSFRLKELR----------------------  target    NAIFTTNPYVRPDDYGIPITAQHHDDKTVRNIKLSWHEIKRHSNPLWEKGYQFYCVTPKTRHRVHSQWSVNDWVQIYESN 2fug.2    --------------------------------------------P-KERKGAFYLRPTMWKAH-----QAVGKAQE----  target    FGDPYRMDKRTPGVGEHQIHINPQAAKDRGINDGDYVYVDGNPVDRPYRGWKPSDPYYKVARLMIRAKYNPAYPYHVTMA 2fug.2    -------------AARAELWAHPETARAEALPEGAQVAVETP-----------------FGRVEARVVHREDVPKGHLYL  target    KHAPFVATAKSVKGHETRPDGRAIAIDTGYQSNFRYGAQQSFTRNWLMPMHQTDSLPGKHAVAWKFKWGYQVDHHAINTV 2fug.2    SALGPAAG-LRVEGRVLVP-------------------------------------------------------------  target    PKECLIRITKAEDGGIGARGPWEPVRTGFTPGQENEFMIKWLKGEHIKIKV 2fug.2    --------------------------------------------------- ``` | | | | | | | | | | | | | | | | | | | | | | | | | | | | | | | | | | | | | | | | | | | | | | | | | |
|  | 6zjl.1.C | NADH-quinone oxidoreductase subunit 3  *Respiratory complex I from Thermus thermophilus, NAD+ dataset, major state* | 0.21 | 0.00 | 14.74 | 0.44 | 62-1033 | EM | 0.00 | monomer | 7 x SF4, 1 x FMN, 2 x FES | HHblits | 0.26 |
| ``` target    MFLSRRQFLKVSVGTVAAVAVADKVLALTALQPVIEVGNPLGDYPDRSWERVYHDQYRYDSSFTWVCSPNDTHACRVRAF 6zjl.1    -------------------------------------------------------------ETPTTCAL-CPVGCGITAD  target    VRNGVVMRVEQNYDHQTYEDLYGNRGTFAHNPRMCLKGFTFHRRVYGPYRLKGPLMRKGWKQWMDDNAPELTAETKRKYK 6zjl.1    TRSGELLRIRAREVPEV------------NEIWICDAGRFGHEW-ADQNRLKTPLVRKE---------------------  target    FDSRFLDDMLRVSWDTAFTYAAKAMITIATRYSGEAGARRLREQGYAPEMIEMMKGAGTRCFKHRAGMPVLGIIGKMGNT 6zjl.1    ------GRLVEATWEEAFLALKEGLKEARG----EEV--------------GLYLAHDA---------------------  target    RMNGGINALLDTWIRKVSPDQAQGGRYWSNYTWHGDQNPAHPFWSGVQGSDIDLSDMRFSKLNTSWGKNFVENKMPEAHW 6zjl.1    --TLEEGLLASELAKAL------KTPHLDFQGRTAAP-------A-SLFPPASLEDLLQADFALVLGD-PTEEAPILHLR  target    KLE-------------------------CIERGARVVVITPEYNPTAYRADYWMPLRPESDGALFLGAMKIIIDENMHDI 6zjl.1    LSEFVRDLKPPHRYNHGTPFADLQIKERMPRRTDKMALFAPYRAPLMKWAAIHEVHRPGEEREILLALLGDKE-------  target    DFLKSFTDAPILVRTDTLQYLDPRDVIADYKFPDFSKSYSGRIQSLKPEQIQRLGGMMVWDLNKKQVVPLHREQVGWHYT 6zjl.1    --------------------------------------------------------------------------------  target    NSGIDAALTGTYRVKLLNGREIDAMPIWQMYMVHFQDYDLDTVHQITRTPKDLIVRWARDSGTIKPAAIHNGEGTCHYFH 6zjl.1    -------------------------------------------------GSEMVAKAKEAWEKAKNPVLILGAGVLQDTV  target    QTANARGAAMVLIITGNVGKFGTGQHTWAGNYKAGTWTATPWSGAGLSVHTGEDPFNITLDPNAHGKEIKTRSYYYGEEV 6zjl.1    AAERARLL------A---ERKGAKVLAMTPAANA----------RGL------EAMG--VLPGAKGA-------------  target    GYWNHGDTALIVNTPKYGRKVFTGKTHMPTPSKFRWVVNVNVVNNAKHHYDMVRNVDPNIECLITQDIEMTSDI-NHADI 6zjl.1    -S---------W-----------D---EPGALYAY--YGFVP----------PEEALKGKRFVVMHLSHLHPLAERYAHV  target    AFAANSWMEFTYPEMTVTVSNPWVQIWKGGIRPLYDTRNDLDTFAGVAAKLSDMTGDKRMRDYFAMVYQNRVDVYVQRML 6zjl.1    VLPAPTFYEKRG---HLVNLEGRVLPLSPAPIENGEAEGALQVLALLAEALGVRP------PF-----R-LHLEAQ----  target    DASSTFYGYSADVMLKSEKGWMVMVRTYPRHPFWEETNESKPMWTRSGRYENYRIEPEAIEYGENFISHREGPEATPYLP 6zjl.1    -----------KALK------------------------ARKVPEAMGRLSFRLKELR----------------------  target    NAIFTTNPYVRPDDYGIPITAQHHDDKTVRNIKLSWHEIKRHSNPLWEKGYQFYCVTPKTRHRVHSQWSVNDWVQIYESN 6zjl.1    --------------------------------------------P-KERKGAFYLRPTMWKAH-----QAVGKAQE----  target    FGDPYRMDKRTPGVGEHQIHINPQAAKDRGINDGDYVYVDGNPVDRPYRGWKPSDPYYKVARLMIRAKYNPAYPYHVTMA 6zjl.1    -------------AARAELWAHPETARAEALPEGAQVAVETP-----------------FGRVEARVVHREDVPKGHLYL  target    KHAPFVATAKSVKGHETRPDGRAIAIDTGYQSNFRYGAQQSFTRNWLMPMHQTDSLPGKHAVAWKFKWGYQVDHHAINTV 6zjl.1    SALGPAAG-LRVEGRVLVP-------------------------------------------------------------  target    PKECLIRITKAEDGGIGARGPWEPVRTGFTPGQENEFMIKWLKGEHIKIKV 6zjl.1    --------------------------------------------------- ``` | | | | | | | | | | | | | | | | | | | | | | | | | | | | | | | | | | | | | | | | | | | | | | | | | |
|  | 6q8o.1.C | NADH-quinone oxidoreductase subunit 3  *Respiratory complex I from Thermus thermophilus with bound Piericidin A* | 0.21 | 0.00 | 14.74 | 0.44 | 62-1033 | X-ray | 3.61 | monomer | 7 x SF4, 1 x FMN, 2 x FES, 1 x HQH | HHblits | 0.26 |
| ``` target    MFLSRRQFLKVSVGTVAAVAVADKVLALTALQPVIEVGNPLGDYPDRSWERVYHDQYRYDSSFTWVCSPNDTHACRVRAF 6q8o.1    -------------------------------------------------------------ETPTTCAL-CPVGCGITAD  target    VRNGVVMRVEQNYDHQTYEDLYGNRGTFAHNPRMCLKGFTFHRRVYGPYRLKGPLMRKGWKQWMDDNAPELTAETKRKYK 6q8o.1    TRSGELLRIRAREVPEV------------NEIWICDAGRFGHEW-ADQNRLKTPLVRKE---------------------  target    FDSRFLDDMLRVSWDTAFTYAAKAMITIATRYSGEAGARRLREQGYAPEMIEMMKGAGTRCFKHRAGMPVLGIIGKMGNT 6q8o.1    ------GRLVEATWEEAFLALKEGLKEARG----EEV--------------GLYLAHDA---------------------  target    RMNGGINALLDTWIRKVSPDQAQGGRYWSNYTWHGDQNPAHPFWSGVQGSDIDLSDMRFSKLNTSWGKNFVENKMPEAHW 6q8o.1    --TLEEGLLASELAKAL------KTPHLDFQGRTAAP-------A-SLFPPASLEDLLQADFALVLGD-PTEEAPILHLR  target    KLE-------------------------CIERGARVVVITPEYNPTAYRADYWMPLRPESDGALFLGAMKIIIDENMHDI 6q8o.1    LSEFVRDLKPPHRYNHGTPFADLQIKERMPRRTDKMALFAPYRAPLMKWAAIHEVHRPGEEREILLALLGDKE-------  target    DFLKSFTDAPILVRTDTLQYLDPRDVIADYKFPDFSKSYSGRIQSLKPEQIQRLGGMMVWDLNKKQVVPLHREQVGWHYT 6q8o.1    --------------------------------------------------------------------------------  target    NSGIDAALTGTYRVKLLNGREIDAMPIWQMYMVHFQDYDLDTVHQITRTPKDLIVRWARDSGTIKPAAIHNGEGTCHYFH 6q8o.1    -------------------------------------------------GSEMVAKAKEAWEKAKNPVLILGAGVLQDTV  target    QTANARGAAMVLIITGNVGKFGTGQHTWAGNYKAGTWTATPWSGAGLSVHTGEDPFNITLDPNAHGKEIKTRSYYYGEEV 6q8o.1    AAERARLL------A---ERKGAKVLAMTPAANA----------RGL------EAMG--VLPGAKGA-------------  target    GYWNHGDTALIVNTPKYGRKVFTGKTHMPTPSKFRWVVNVNVVNNAKHHYDMVRNVDPNIECLITQDIEMTSDI-NHADI 6q8o.1    -S---------W-----------D---EPGALYAY--YGFVP----------PEEALKGKRFVVMHLSHLHPLAERYAHV  target    AFAANSWMEFTYPEMTVTVSNPWVQIWKGGIRPLYDTRNDLDTFAGVAAKLSDMTGDKRMRDYFAMVYQNRVDVYVQRML 6q8o.1    VLPAPTFYEKRG---HLVNLEGRVLPLSPAPIENGEAEGALQVLALLAEALGVRP------PF-----R-LHLEAQ----  target    DASSTFYGYSADVMLKSEKGWMVMVRTYPRHPFWEETNESKPMWTRSGRYENYRIEPEAIEYGENFISHREGPEATPYLP 6q8o.1    -----------KALK------------------------ARKVPEAMGRLSFRLKELR----------------------  target    NAIFTTNPYVRPDDYGIPITAQHHDDKTVRNIKLSWHEIKRHSNPLWEKGYQFYCVTPKTRHRVHSQWSVNDWVQIYESN 6q8o.1    --------------------------------------------P-KERKGAFYLRPTMWKAH-----QAVGKAQE----  target    FGDPYRMDKRTPGVGEHQIHINPQAAKDRGINDGDYVYVDGNPVDRPYRGWKPSDPYYKVARLMIRAKYNPAYPYHVTMA 6q8o.1    -------------AARAELWAHPETARAEALPEGAQVAVETP-----------------FGRVEARVVHREDVPKGHLYL  target    KHAPFVATAKSVKGHETRPDGRAIAIDTGYQSNFRYGAQQSFTRNWLMPMHQTDSLPGKHAVAWKFKWGYQVDHHAINTV 6q8o.1    SALGPAAG-LRVEGRVLVP-------------------------------------------------------------  target    PKECLIRITKAEDGGIGARGPWEPVRTGFTPGQENEFMIKWLKGEHIKIKV 6q8o.1    --------------------------------------------------- ``` | | | | | | | | | | | | | | | | | | | | | | | | | | | | | | | | | | | | | | | | | | | | | | | | | |
|  | 6zjy.1.C | NADH-quinone oxidoreductase subunit 3  *Respiratory complex I from Thermus thermophilus, NAD+ dataset, minor state* | 0.20 | 0.00 | 14.74 | 0.44 | 62-1033 | EM | 0.00 | monomer | 7 x SF4, 2 x FES | HHblits | 0.26 |
| ``` target    MFLSRRQFLKVSVGTVAAVAVADKVLALTALQPVIEVGNPLGDYPDRSWERVYHDQYRYDSSFTWVCSPNDTHACRVRAF 6zjy.1    -------------------------------------------------------------ETPTTCAL-CPVGCGITAD  target    VRNGVVMRVEQNYDHQTYEDLYGNRGTFAHNPRMCLKGFTFHRRVYGPYRLKGPLMRKGWKQWMDDNAPELTAETKRKYK 6zjy.1    TRSGELLRIRAREVPEV------------NEIWICDAGRFGHEW-ADQNRLKTPLVRKE---------------------  target    FDSRFLDDMLRVSWDTAFTYAAKAMITIATRYSGEAGARRLREQGYAPEMIEMMKGAGTRCFKHRAGMPVLGIIGKMGNT 6zjy.1    ------GRLVEATWEEAFLALKEGLKEARG----EEV--------------GLYLAHDA---------------------  target    RMNGGINALLDTWIRKVSPDQAQGGRYWSNYTWHGDQNPAHPFWSGVQGSDIDLSDMRFSKLNTSWGKNFVENKMPEAHW 6zjy.1    --TLEEGLLASELAKAL------KTPHLDFQGRTAAP-------A-SLFPPASLEDLLQADFALVLGD-PTEEAPILHLR  target    KLE-------------------------CIERGARVVVITPEYNPTAYRADYWMPLRPESDGALFLGAMKIIIDENMHDI 6zjy.1    LSEFVRDLKPPHRYNHGTPFADLQIKERMPRRTDKMALFAPYRAPLMKWAAIHEVHRPGEEREILLALLGDKE-------  target    DFLKSFTDAPILVRTDTLQYLDPRDVIADYKFPDFSKSYSGRIQSLKPEQIQRLGGMMVWDLNKKQVVPLHREQVGWHYT 6zjy.1    --------------------------------------------------------------------------------  target    NSGIDAALTGTYRVKLLNGREIDAMPIWQMYMVHFQDYDLDTVHQITRTPKDLIVRWARDSGTIKPAAIHNGEGTCHYFH 6zjy.1    -------------------------------------------------GSEMVAKAKEAWEKAKNPVLILGAGVLQDTV  target    QTANARGAAMVLIITGNVGKFGTGQHTWAGNYKAGTWTATPWSGAGLSVHTGEDPFNITLDPNAHGKEIKTRSYYYGEEV 6zjy.1    AAERARLL------A---ERKGAKVLAMTPAANA----------RGL------EAMG--VLPGAKGA-------------  target    GYWNHGDTALIVNTPKYGRKVFTGKTHMPTPSKFRWVVNVNVVNNAKHHYDMVRNVDPNIECLITQDIEMTSDI-NHADI 6zjy.1    -S---------W-----------D---EPGALYAY--YGFVP----------PEEALKGKRFVVMHLSHLHPLAERYAHV  target    AFAANSWMEFTYPEMTVTVSNPWVQIWKGGIRPLYDTRNDLDTFAGVAAKLSDMTGDKRMRDYFAMVYQNRVDVYVQRML 6zjy.1    VLPAPTFYEKRG---HLVNLEGRVLPLSPAPIENGEAEGALQVLALLAEALGVRP------PF-----R-LHLEAQ----  target    DASSTFYGYSADVMLKSEKGWMVMVRTYPRHPFWEETNESKPMWTRSGRYENYRIEPEAIEYGENFISHREGPEATPYLP 6zjy.1    -----------KALK------------------------ARKVPEAMGRLSFRLKELR----------------------  target    NAIFTTNPYVRPDDYGIPITAQHHDDKTVRNIKLSWHEIKRHSNPLWEKGYQFYCVTPKTRHRVHSQWSVNDWVQIYESN 6zjy.1    --------------------------------------------P-KERKGAFYLRPTMWKAH-----QAVGKAQE----  target    FGDPYRMDKRTPGVGEHQIHINPQAAKDRGINDGDYVYVDGNPVDRPYRGWKPSDPYYKVARLMIRAKYNPAYPYHVTMA 6zjy.1    -------------AARAELWAHPETARAEALPEGAQVAVETP-----------------FGRVEARVVHREDVPKGHLYL  target    KHAPFVATAKSVKGHETRPDGRAIAIDTGYQSNFRYGAQQSFTRNWLMPMHQTDSLPGKHAVAWKFKWGYQVDHHAINTV 6zjy.1    SALGPAAG-LRVEGRVLVP-------------------------------------------------------------  target    PKECLIRITKAEDGGIGARGPWEPVRTGFTPGQENEFMIKWLKGEHIKIKV 6zjy.1    --------------------------------------------------- ``` | | | | | | | | | | | | | | | | | | | | | | | | | | | | | | | | | | | | | | | | | | | | | | | | | |
|  | 6zjn.1.C | NADH-quinone oxidoreductase subunit 3  *Respiratory complex I from Thermus thermophilus, NADH dataset, minor state* | 0.21 | 0.00 | 14.74 | 0.44 | 62-1033 | EM | 0.00 | monomer | 7 x SF4, 2 x FES | HHblits | 0.26 |
| ``` target    MFLSRRQFLKVSVGTVAAVAVADKVLALTALQPVIEVGNPLGDYPDRSWERVYHDQYRYDSSFTWVCSPNDTHACRVRAF 6zjn.1    -------------------------------------------------------------ETPTTCAL-CPVGCGITAD  target    VRNGVVMRVEQNYDHQTYEDLYGNRGTFAHNPRMCLKGFTFHRRVYGPYRLKGPLMRKGWKQWMDDNAPELTAETKRKYK 6zjn.1    TRSGELLRIRAREVPEV------------NEIWICDAGRFGHEW-ADQNRLKTPLVRKE---------------------  target    FDSRFLDDMLRVSWDTAFTYAAKAMITIATRYSGEAGARRLREQGYAPEMIEMMKGAGTRCFKHRAGMPVLGIIGKMGNT 6zjn.1    ------GRLVEATWEEAFLALKEGLKEARG----EEV--------------GLYLAHDA---------------------  target    RMNGGINALLDTWIRKVSPDQAQGGRYWSNYTWHGDQNPAHPFWSGVQGSDIDLSDMRFSKLNTSWGKNFVENKMPEAHW 6zjn.1    --TLEEGLLASELAKAL------KTPHLDFQGRTAAP-------A-SLFPPASLEDLLQADFALVLGD-PTEEAPILHLR  target    KLE-------------------------CIERGARVVVITPEYNPTAYRADYWMPLRPESDGALFLGAMKIIIDENMHDI 6zjn.1    LSEFVRDLKPPHRYNHGTPFADLQIKERMPRRTDKMALFAPYRAPLMKWAAIHEVHRPGEEREILLALLGDKE-------  target    DFLKSFTDAPILVRTDTLQYLDPRDVIADYKFPDFSKSYSGRIQSLKPEQIQRLGGMMVWDLNKKQVVPLHREQVGWHYT 6zjn.1    --------------------------------------------------------------------------------  target    NSGIDAALTGTYRVKLLNGREIDAMPIWQMYMVHFQDYDLDTVHQITRTPKDLIVRWARDSGTIKPAAIHNGEGTCHYFH 6zjn.1    -------------------------------------------------GSEMVAKAKEAWEKAKNPVLILGAGVLQDTV  target    QTANARGAAMVLIITGNVGKFGTGQHTWAGNYKAGTWTATPWSGAGLSVHTGEDPFNITLDPNAHGKEIKTRSYYYGEEV 6zjn.1    AAERARLL------A---ERKGAKVLAMTPAANA----------RGL------EAMG--VLPGAKGA-------------  target    GYWNHGDTALIVNTPKYGRKVFTGKTHMPTPSKFRWVVNVNVVNNAKHHYDMVRNVDPNIECLITQDIEMTSDI-NHADI 6zjn.1    -S---------W-----------D---EPGALYAY--YGFVP----------PEEALKGKRFVVMHLSHLHPLAERYAHV  target    AFAANSWMEFTYPEMTVTVSNPWVQIWKGGIRPLYDTRNDLDTFAGVAAKLSDMTGDKRMRDYFAMVYQNRVDVYVQRML 6zjn.1    VLPAPTFYEKRG---HLVNLEGRVLPLSPAPIENGEAEGALQVLALLAEALGVRP------PF-----R-LHLEAQ----  target    DASSTFYGYSADVMLKSEKGWMVMVRTYPRHPFWEETNESKPMWTRSGRYENYRIEPEAIEYGENFISHREGPEATPYLP 6zjn.1    -----------KALK------------------------ARKVPEAMGRLSFRLKELR----------------------  target    NAIFTTNPYVRPDDYGIPITAQHHDDKTVRNIKLSWHEIKRHSNPLWEKGYQFYCVTPKTRHRVHSQWSVNDWVQIYESN 6zjn.1    --------------------------------------------P-KERKGAFYLRPTMWKAH-----QAVGKAQE----  target    FGDPYRMDKRTPGVGEHQIHINPQAAKDRGINDGDYVYVDGNPVDRPYRGWKPSDPYYKVARLMIRAKYNPAYPYHVTMA 6zjn.1    -------------AARAELWAHPETARAEALPEGAQVAVETP-----------------FGRVEARVVHREDVPKGHLYL  target    KHAPFVATAKSVKGHETRPDGRAIAIDTGYQSNFRYGAQQSFTRNWLMPMHQTDSLPGKHAVAWKFKWGYQVDHHAINTV 6zjn.1    SALGPAAG-LRVEGRVLVP-------------------------------------------------------------  target    PKECLIRITKAEDGGIGARGPWEPVRTGFTPGQENEFMIKWLKGEHIKIKV 6zjn.1    --------------------------------------------------- ``` | | | | | | | | | | | | | | | | | | | | | | | | | | | | | | | | | | | | | | | | | | | | | | | | | |
|  | 6ziy.1.C | NADH-quinone oxidoreductase subunit 3  *Respiratory complex I from Thermus thermophilus, NADH dataset, major state* | 0.21 | 0.00 | 14.74 | 0.44 | 62-1033 | EM | 0.00 | monomer | 7 x SF4, 1 x FMN, 1 x NAI, 2 x FES | HHblits | 0.26 |
| ``` target    MFLSRRQFLKVSVGTVAAVAVADKVLALTALQPVIEVGNPLGDYPDRSWERVYHDQYRYDSSFTWVCSPNDTHACRVRAF 6ziy.1    -------------------------------------------------------------ETPTTCAL-CPVGCGITAD  target    VRNGVVMRVEQNYDHQTYEDLYGNRGTFAHNPRMCLKGFTFHRRVYGPYRLKGPLMRKGWKQWMDDNAPELTAETKRKYK 6ziy.1    TRSGELLRIRAREVPEV------------NEIWICDAGRFGHEW-ADQNRLKTPLVRKE---------------------  target    FDSRFLDDMLRVSWDTAFTYAAKAMITIATRYSGEAGARRLREQGYAPEMIEMMKGAGTRCFKHRAGMPVLGIIGKMGNT 6ziy.1    ------GRLVEATWEEAFLALKEGLKEARG----EEV--------------GLYLAHDA---------------------  target    RMNGGINALLDTWIRKVSPDQAQGGRYWSNYTWHGDQNPAHPFWSGVQGSDIDLSDMRFSKLNTSWGKNFVENKMPEAHW 6ziy.1    --TLEEGLLASELAKAL------KTPHLDFQGRTAAP-------A-SLFPPASLEDLLQADFALVLGD-PTEEAPILHLR  target    KLE-------------------------CIERGARVVVITPEYNPTAYRADYWMPLRPESDGALFLGAMKIIIDENMHDI 6ziy.1    LSEFVRDLKPPHRYNHGTPFADLQIKERMPRRTDKMALFAPYRAPLMKWAAIHEVHRPGEEREILLALLGDKE-------  target    DFLKSFTDAPILVRTDTLQYLDPRDVIADYKFPDFSKSYSGRIQSLKPEQIQRLGGMMVWDLNKKQVVPLHREQVGWHYT 6ziy.1    --------------------------------------------------------------------------------  target    NSGIDAALTGTYRVKLLNGREIDAMPIWQMYMVHFQDYDLDTVHQITRTPKDLIVRWARDSGTIKPAAIHNGEGTCHYFH 6ziy.1    -------------------------------------------------GSEMVAKAKEAWEKAKNPVLILGAGVLQDTV  target    QTANARGAAMVLIITGNVGKFGTGQHTWAGNYKAGTWTATPWSGAGLSVHTGEDPFNITLDPNAHGKEIKTRSYYYGEEV 6ziy.1    AAERARLL------A---ERKGAKVLAMTPAANA----------RGL------EAMG--VLPGAKGA-------------  target    GYWNHGDTALIVNTPKYGRKVFTGKTHMPTPSKFRWVVNVNVVNNAKHHYDMVRNVDPNIECLITQDIEMTSDI-NHADI 6ziy.1    -S---------W-----------D---EPGALYAY--YGFVP----------PEEALKGKRFVVMHLSHLHPLAERYAHV  target    AFAANSWMEFTYPEMTVTVSNPWVQIWKGGIRPLYDTRNDLDTFAGVAAKLSDMTGDKRMRDYFAMVYQNRVDVYVQRML 6ziy.1    VLPAPTFYEKRG---HLVNLEGRVLPLSPAPIENGEAEGALQVLALLAEALGVRP------PF-----R-LHLEAQ----  target    DASSTFYGYSADVMLKSEKGWMVMVRTYPRHPFWEETNESKPMWTRSGRYENYRIEPEAIEYGENFISHREGPEATPYLP 6ziy.1    -----------KALK------------------------ARKVPEAMGRLSFRLKELR----------------------  target    NAIFTTNPYVRPDDYGIPITAQHHDDKTVRNIKLSWHEIKRHSNPLWEKGYQFYCVTPKTRHRVHSQWSVNDWVQIYESN 6ziy.1    --------------------------------------------P-KERKGAFYLRPTMWKAH-----QAVGKAQE----  target    FGDPYRMDKRTPGVGEHQIHINPQAAKDRGINDGDYVYVDGNPVDRPYRGWKPSDPYYKVARLMIRAKYNPAYPYHVTMA 6ziy.1    -------------AARAELWAHPETARAEALPEGAQVAVETP-----------------FGRVEARVVHREDVPKGHLYL  target    KHAPFVATAKSVKGHETRPDGRAIAIDTGYQSNFRYGAQQSFTRNWLMPMHQTDSLPGKHAVAWKFKWGYQVDHHAINTV 6ziy.1    SALGPAAG-LRVEGRVLVP-------------------------------------------------------------  target    PKECLIRITKAEDGGIGARGPWEPVRTGFTPGQENEFMIKWLKGEHIKIKV 6ziy.1    --------------------------------------------------- ``` | | | | | | | | | | | | | | | | | | | | | | | | | | | | | | | | | | | | | | | | | | | | | | | | | |
|  | 2ivf.1.A | ETHYLBENZENE DEHYDROGENASE ALPHA-SUBUNIT  *ETHYLBENZENE DEHYDROGENASE FROM AROMATOLEUM AROMATICUM* | 0.19 |  | 32.93 | 0.36 | 48-564 | X-ray | 1.88 | hetero-oligomer | 1 x MES, 4 x SF4, 1 x MO, 1 x MGD, 1 x MD1, 1 x F3S, 1 x HEM | BLAST | 0.36 |
| ``` target    MFLSRRQFLKVSVGTVAAVAVADKVLALTALQPVIEVGNPLGDYPDRSWERVYHDQYRYD----SSFTWVCSPNDTHACR 2ivf.1    -----------------------------------------------SWEDIYRKEWKWDKVNWGSHLNICWPQGS--CK  target    VRAFVRNGVVMRVEQNYDHQTYEDLYGNRGTFAHNPRMCLKGFTFHRRVYGPYRLKGPLMRKGWKQWMDDNAPELTAETK 2ivf.1    FYVYVRNGIVWREEQ-----AAQTPACNVDYVDYNPLGCQKGSAFNNNLYGDERVKYPLKRVGKR-----------GEGK  target    RKYKFDSRFLDDMLRVSWDTAFTYAAKAMITIATRYSGEAGARRLREQGYAPEMIEMMKGAGTRCFKHRAGMPVLGIIGK 2ivf.1    WK------------RVSWDEA---------------AGDI----------ADSIIDSFEAQGSDGFILDAPHVHAGSIAW  target    MGNTRMNGGINALLDTWIRKVSPDQAQ--GGRYWSNYTWHGDQNPAHPFWSGVQGSDIDLSDMRFSKLNTSWGKNFVENK 2ivf.1    GAGFRMT----YLMDG----VSPDINVDIGDTYMGAFHTFGKM---HMGYSADNLLDAELIFMTCSNWSYTYPSSY----  target    MPEAHWKLECIERGARVVVITPEYNPTAYRADYWMPLRPESDGALFLGAMKIIIDENMHDIDFLKSFTDAPILVRTDTLQ 2ivf.1    ----HFLSEARYKGAEVVVIAPDFNPTTPAADLHVPVRVGSDAAFWLGLSQVMIDEKLFDRQFVCEQTDLPLLVRMDTGK  target    YLDPRDVI-ADYKFPDFSKSYSGRIQSLKPEQIQRLGGMMVWDLNKKQVVPLHREQVGWHYTNSGIDAALTGTYRVKLLN 2ivf.1    FLSAEDVDGGEAKQFYFFDEKAGSVRKASRGTL------------KLDFMP-----------------ALEGTFSARLKN  target    GREIDAMPIWQMYMVHFQDYDLDTVHQITRTPKDLIVRWARDSGTIKPAAIHNGEGTCHYFHQTANARGAAMVLIITGNV 2ivf.1    GKTIQVRTVFEGLREHLKDYTPEKASAKCGVPVSLIRELGRKVAK-KRTCSYIGFSSAKSYHGDLMERSLFLAMALSGNW  target    GKFGTGQHTWAGNYKAGTWTATPWSGAGLSVHTGEDPFNITLDPNAHGKEIKTRSYYYGEEVGYWNHGDTALIVNTPKYG 2ivf.1    GKPGTGAFAWA---------------------------------------------------------------------  target    RKVFTGKTHMPTPSKFRWVVNVNVVNNAKHHYDMVRNVDPNIECLITQDIEMTSDINHADIAFAANSWMEFTYPEMTVTV 2ivf.1    --------------------------------------------------------------------------------  target    SNPWVQIWKGGIRPLYDTRNDLDTFAGVAAKLSDMTGDKRMRDYFAMVYQNRVDVYVQRMLDASSTFYGYSADVMLKSEK 2ivf.1    --------------------------------------------------------------------------------  target    GWMVMVRTYPRHPFWEETNESKPMWTRSGRYENYRIEPEAIEYGENFISHREGPEATPYLPNAIFTTNPYVRPDDYGIPI 2ivf.1    --------------------------------------------------------------------------------  target    TAQHHDDKTVRNIKLSWHEIKRHSNPLWEKGYQFYCVTPKTRHRVHSQWSVNDWVQIYESNFGDPYRMDKRTPGVGEHQI 2ivf.1    --------------------------------------------------------------------------------  target    HINPQAAKDRGINDGDYVYVDGNPVDRPYRGWKPSDPYYKVARLMIRAKYNPAYPYHVTMAKHAPFVATAKSVKGHETRP 2ivf.1    --------------------------------------------------------------------------------  target    DGRAIAIDTGYQSNFRYGAQQSFTRNWLMPMHQTDSLPGKHAVAWKFKWGYQVDHHAINTVPKECLIRITKAEDGGIGAR 2ivf.1    --------------------------------------------------------------------------------  target    GPWEPVRTGFTPGQENEFMIKWLKGEHIKIKV 2ivf.1    -------------------------------- ``` | | | | | | | | | | | | | | | | | | | | | | | | | | | | | | | | | | | | | | | | | | | | | | | | | |
|  | 7t2r.1.A | NiFe hydrogenase subunit A  *Structure of electron bifurcating Ni-Fe hydrogenase complex HydABCSL in FMN-free apo state* | 0.18 |  | 14.09 | 0.38 | 61-746 | EM | 0.00 | hetero-2-2-2-2-2-mer | 6 x FES, 12 x SF4, 2 x 3NI, 2 x FCO | HHblits | 0.27 |
| ``` target    MFLSRRQFLKVSVGTVAAVAVADKVLALTALQPVIEVGNPLGDYPDRSWERVYHDQYRYDSSFTWVCSPNDTHACRVRAF 7t2r.1    ------------------------------------------------------------AVVESVCP-LCAVGCKIKTY  target    VRNGVVMRVEQNYDHQTYEDLYGNRGTFAHNPRMCLKGFTFHRRVYGPYRLKGPLMRKGWKQWMDDNAPELTAETKRKYK 7t2r.1    VRTGSIVRVEGTGVEEP------------DGGQLCHMGRWWLPESTERERVTVPLIREG---------------------  target    FDSRFLDDMLRVSWDTAFTYAAKAMITIATRYSGEAGARRLREQGYAPEMIEMMKGAGTRCFKHRAGMPVLGIIGKMGNT 7t2r.1    ------ASYREATWEEALALASAEFKKAYDQEKAGAIL----SSLCTDEELTLFSAL-------------FR--NALKMK  target    RMNGGINALLDTWIRKVSPDQAQGGRYWSNYTWHGDQNPAHPF-WSGVQGSDIDLSDMRFSKLNTSWGKNFVENKMPEAH 7t2r.1    HIDT--------FDG----DII--RGFFKGF---------MPFREQGV-RPFTAAHHILDSDLIITMFADPQKEAPVVAS  target    WKLECI-ERGARVVVITPEYNPTAYRADYWMPLRPESDGALFLGAMKIIIDENMHDIDFLK---SFTDAPILVRTDTLQY 7t2r.1    YIRVACLHRNAKLMNLSYGPSPFPGLVDLDIRLPEGQAVPKALSNLAEIIGKISLGPSDMASFGEFEAG-----------  target    LDPRDVIADYKFPDFSKSYSGRIQSLKPEQIQRLGGMMVWDLNKKQVVPLHREQVGWHYTNSGIDAALTGTYRVKLLNGR 7t2r.1    --------------------------------------------------------------------------------  target    EIDAMPIWQMYMVHFQD--YDLDTVHQITRTPKDLIVRWARDSGTIKPAAIHNGEGTCHYFHQTANARGAAMVLIITGNV 7t2r.1    ----------AGKALSSYRESIEESARAMGLDPKIAEEVALMLISARRPIFIIGGRA---TKSHELVTAACNLAVASKAF  target    GKFGTGQHTWAGNYKAGTWTATPWSGAGLSVHTGEDPFNITLDPNAHGKEIKTRSYYYGEEVGYWNHGDTALIVNTPKYG 7t2r.1    FEDGLGVVPLLVSANS----------------LGA-------------RNTVV-------S-------------ENP-W-  target    RKVFTGKTHMPTPSKFRWVVNVNVVNNAKHHYDMVRNVDPNIECLITQDIEMT-SDINHADIAFAANSWMEFTYPEMTVT 7t2r.1    ----LG----RERRDFLYVFSTAMVPE----EEEILAAISATRFVVVQTPFKVRPLVNLADILLPAPAWYERS---GHFC  target    VSNPWVQIWKGGIRPLYDTRNDLDTFAGVAAKLSDMTGDKRMRDYFAMVYQNRVDVYVQRMLDASSTFYGYSADVMLKSE 7t2r.1    TIEGERRKLNTIVPPKGEIKSLHYVMDEFAKKLG----------------------------------------------  target    KGWMVMVRTYPRHPFWEETNESKPMWTRSGRYENYRIEPEAIEYGENFISHREGPEATPYLPNAIFTTNPYVRPDDYGIP 7t2r.1    --------------------------------------------------------------------------------  target    ITAQHHDDKTVRNIKLSWHEIKRHSNPLWEKGYQFYCVTPKTRHRVHSQWSVNDWVQIYESNFGDPYRMDKRTPGVGEHQ 7t2r.1    --------------------------------------------------------------------------------  target    IHINPQAAKDRGINDGDYVYVDGNPVDRPYRGWKPSDPYYKVARLMIRAKYNPAYPYHVTMAKHAPFVATAKSVKGHETR 7t2r.1    --------------------------------------------------------------------------------  target    PDGRAIAIDTGYQSNFRYGAQQSFTRNWLMPMHQTDSLPGKHAVAWKFKWGYQVDHHAINTVPKECLIRITKAEDGGIGA 7t2r.1    --------------------------------------------------------------------------------  target    RGPWEPVRTGFTPGQENEFMIKWLKGEHIKIKV 7t2r.1    --------------------------------- ``` | | | | | | | | | | | | | | | | | | | | | | | | | | | | | | | | | | | | | | | | | | | | | | | | | |
|  | 7t30.1.A | NiFe hydrogenase subunit A  *Structure of electron bifurcating Ni-Fe hydrogenase complex HydABCSL in FMN/NAD(H) bound state* | 0.17 |  | 14.09 | 0.38 | 61-746 | EM | 0.00 | hetero-2-2-2-2-2-mer | 4 x FES, 12 x SF4, 2 x NAD, 2 x FMN, 2 x 3NI, 2 x FCO | HHblits | 0.27 |
| ``` target    MFLSRRQFLKVSVGTVAAVAVADKVLALTALQPVIEVGNPLGDYPDRSWERVYHDQYRYDSSFTWVCSPNDTHACRVRAF 7t30.1    ------------------------------------------------------------AVVESVCP-LCAVGCKIKTY  target    VRNGVVMRVEQNYDHQTYEDLYGNRGTFAHNPRMCLKGFTFHRRVYGPYRLKGPLMRKGWKQWMDDNAPELTAETKRKYK 7t30.1    VRTGSIVRVEGTGVEEP------------DGGQLCHMGRWWLPESTERERVTVPLIREG---------------------  target    FDSRFLDDMLRVSWDTAFTYAAKAMITIATRYSGEAGARRLREQGYAPEMIEMMKGAGTRCFKHRAGMPVLGIIGKMGNT 7t30.1    ------ASYREATWEEALALASAEFKKAYDQEKAGAIL----SSLCTDEELTLFSAL-------------FR--NALKMK  target    RMNGGINALLDTWIRKVSPDQAQGGRYWSNYTWHGDQNPAHPF-WSGVQGSDIDLSDMRFSKLNTSWGKNFVENKMPEAH 7t30.1    HIDT--------FDG----DII--RGFFKGF---------MPFREQGV-RPFTAAHHILDSDLIITMFADPQKEAPVVAS  target    WKLECI-ERGARVVVITPEYNPTAYRADYWMPLRPESDGALFLGAMKIIIDENMHDIDFLK---SFTDAPILVRTDTLQY 7t30.1    YIRVACLHRNAKLMNLSYGPSPFPGLVDLDIRLPEGQAVPKALSNLAEIIGKISLGPSDMASFGEFEAG-----------  target    LDPRDVIADYKFPDFSKSYSGRIQSLKPEQIQRLGGMMVWDLNKKQVVPLHREQVGWHYTNSGIDAALTGTYRVKLLNGR 7t30.1    --------------------------------------------------------------------------------  target    EIDAMPIWQMYMVHFQD--YDLDTVHQITRTPKDLIVRWARDSGTIKPAAIHNGEGTCHYFHQTANARGAAMVLIITGNV 7t30.1    ----------AGKALSSYRESIEESARAMGLDPKIAEEVALMLISARRPIFIIGGRA---TKSHELVTAACNLAVASKAF  target    GKFGTGQHTWAGNYKAGTWTATPWSGAGLSVHTGEDPFNITLDPNAHGKEIKTRSYYYGEEVGYWNHGDTALIVNTPKYG 7t30.1    FEDGLGVVPLLVSANS----------------LGA-------------RNTVV-------S-------------ENP-W-  target    RKVFTGKTHMPTPSKFRWVVNVNVVNNAKHHYDMVRNVDPNIECLITQDIEMT-SDINHADIAFAANSWMEFTYPEMTVT 7t30.1    ----LG----RERRDFLYVFSTAMVPE----EEEILAAISATRFVVVQTPFKVRPLVNLADILLPAPAWYERS---GHFC  target    VSNPWVQIWKGGIRPLYDTRNDLDTFAGVAAKLSDMTGDKRMRDYFAMVYQNRVDVYVQRMLDASSTFYGYSADVMLKSE 7t30.1    TIEGERRKLNTIVPPKGEIKSLHYVMDEFAKKLG----------------------------------------------  target    KGWMVMVRTYPRHPFWEETNESKPMWTRSGRYENYRIEPEAIEYGENFISHREGPEATPYLPNAIFTTNPYVRPDDYGIP 7t30.1    --------------------------------------------------------------------------------  target    ITAQHHDDKTVRNIKLSWHEIKRHSNPLWEKGYQFYCVTPKTRHRVHSQWSVNDWVQIYESNFGDPYRMDKRTPGVGEHQ 7t30.1    --------------------------------------------------------------------------------  target    IHINPQAAKDRGINDGDYVYVDGNPVDRPYRGWKPSDPYYKVARLMIRAKYNPAYPYHVTMAKHAPFVATAKSVKGHETR 7t30.1    --------------------------------------------------------------------------------  target    PDGRAIAIDTGYQSNFRYGAQQSFTRNWLMPMHQTDSLPGKHAVAWKFKWGYQVDHHAINTVPKECLIRITKAEDGGIGA 7t30.1    --------------------------------------------------------------------------------  target    RGPWEPVRTGFTPGQENEFMIKWLKGEHIKIKV 7t30.1    --------------------------------- ``` | | | | | | | | | | | | | | | | | | | | | | | | | | | | | | | | | | | | | | | | | | | | | | | | | |
|  | 5t5i.1.B | Tungsten formylmethanofuran dehydrogenase subunit B  *TUNGSTEN-CONTAINING FORMYLMETHANOFURAN DEHYDROGENASE FROM METHANOTHERMOBACTER WOLFEII, ORTHORHOMBIC FORM AT 1.9 A* | 0.16 |  | 16.22 | 0.36 | 63-748 | X-ray | 1.90 | hetero-oligomer | 4 x ZN, 2 x MG, 18 x K, 22 x SF4, 2 x W, 4 x MGD, 2 x H2S, 2 x CA | HHblits | 0.27 |
| ``` target    MFLSRRQFLKVSVGTVAAVAVADKVLALTALQPVIEVGNPLGDYPDRSWERVYHDQYRYDSSFTWVCSPNDTHACR-VRA 5t5i.1    --------------------------------------------------------------KNVVCPF-CGTLCDDIIC  target    FVRNGVVMRVEQNYDHQTYEDLYGNRGTFAHNPRMCLKGFTFHRRVYGPYRLKGPLMRKGWKQWMDDNAPELTAETKRKY 5t5i.1    KVEGNEIVGT----------------------INACRIGHSKFVHAEGAMRYKKPLIRKN--------------------  target    KFDSRFLDDMLRVSWDTAFTYAAKAMITIATRYSGEAGARRLREQGYAPEMIEMMKGAGTRCFKHRAGMPVLGIIGKMGN 5t5i.1    -------GEFVEVSYDEAIDKAAKILAESKRP----L--------MYGWSCT----ECEAQAV----GV---ELAEEAGA  target    TRMNGGINALLDTWIRKVSPDQAQGGRYWSNYTWHGDQNPAHPFWSGVQGSDIDLSDM-RFSKLNTSWGKNFVENKMPEA 5t5i.1    VIDNTA--------------SVCHGPSVL-----------ALQ-DVG--YPICTFGEVKNRADVVVYWGCNPMHAHPRHM  target    HW-------KLECIERGARVVVITPEYNPTAYRADYWMPLRPESDGALFLGAMKIIIDENMHDIDFLKSFTDAPILVRTD 5t5i.1    SRNVFARGFFRERGRSDRTLIVVDPRKTDSAKLADIHLQLDFDRDYELLDAMRACLLGHEI-------------------  target    TLQYLDPRDVIADYKFPDFSKSYSGRIQSLKPEQIQRLGGMMVWDLNKKQVVPLHREQVGWHYTNSGIDAALTGTYRVKL 5t5i.1    --------------------------------------------------------------------------------  target    LNGREIDAMPIWQMYMVHFQDYDLDTVHQITRTPKDLIVRWARDSGTIKPAAIHNGEGTCHYFHQTANARGAAMVLIITG 5t5i.1    -------------------------LYDEVAGVPREQIEEAVEVLKNAQFGILFFGMGITHSRGKHRNIDTAIMMVQDLN  target    NVGKFGTGQHTWAGNYK-AGTWTATPWSGAGLSVHTGEDPFNITLDPNAHGKEIKTRSYYYGEEVGYWNHGDTALIVNTP 5t5i.1    DY--AKWTLIPMRGHYNVTGFNQV------C--TWESGYPYCVDFSG---------------GEPRYNP----G-ETG--  target    KYGRKVFTGKTHMPTPSKFRWVVNVNVVNNAKHHYDMVRNVDPNIECLITQDIEMTSDINHADIAFAANS-WMEFTYPEM 5t5i.1    -ANDL------LQNREADAMMVIASDPGAHFPQ---RALERMAEIP-VIAIEPHRTPTTEMADIIIPPAIVGMEAEG---  target    TVTVSNPWVQIWKGGIRPLYDTRNDLDTFAGVAAKLSDMTGDKRMRDYFAMVYQNRVDVYVQRMLDASSTFYGYSADVML 5t5i.1    TAYRMEGVPIRMKKVVDS--DLLSDREILERLLEKVREY-----------------------------------------  target    KSEKGWMVMVRTYPRHPFWEETNESKPMWTRSGRYENYRIEPEAIEYGENFISHREGPEATPYLPNAIFTTNPYVRPDDY 5t5i.1    --------------------------------------------------------------------------------  target    GIPITAQHHDDKTVRNIKLSWHEIKRHSNPLWEKGYQFYCVTPKTRHRVHSQWSVNDWVQIYESNFGDPYRMDKRTPGVG 5t5i.1    --------------------------------------------------------------------------------  target    EHQIHINPQAAKDRGINDGDYVYVDGNPVDRPYRGWKPSDPYYKVARLMIRAKYNPAYPYHVTMAKHAPFVATAKSVKGH 5t5i.1    --------------------------------------------------------------------------------  target    ETRPDGRAIAIDTGYQSNFRYGAQQSFTRNWLMPMHQTDSLPGKHAVAWKFKWGYQVDHHAINTVPKECLIRITKAEDGG 5t5i.1    --------------------------------------------------------------------------------  target    IGARGPWEPVRTGFTPGQENEFMIKWLKGEHIKIKV 5t5i.1    ------------------------------------ ``` | | | | | | | | | | | | | | | | | | | | | | | | | | | | | | | | | | | | | | | | | | | | | | | | | |
|  | 7bkb.1.L | Formylmethanofuran dehydrogenase, subunit B  *Formate dehydrogenase - heterodisulfide reductase - formylmethanofuran dehydrogenase complex from Methanospirillum hungatei (hexameric, composite structure)* | 0.15 |  | 14.46 | 0.36 | 63-747 | EM | 0.00 | hetero-2-2-2-2-2-2-… | 48 x SF4, 4 x FAD, 2 x FES, 4 x 9S8, 4 x ZN, 2 x MO, 4 x MGD | HHblits | 0.26 |
| ``` target    MFLSRRQFLKVSVGTVAAVAVADKVLALTALQPVIEVGNPLGDYPDRSWERVYHDQYRYDSSFTWVCSPNDTHACR-VRA 7bkb.1    --------------------------------------------------------------ENVGCPY-CGCSCDDVRI  target    FVRNGVVMRVEQNYDHQTYEDLYGNRGTFAHNPRMCLKGFTFHRRVYGPYRLKGPLMRKGWKQWMDDNAPELTAETKRKY 7bkb.1    TVSDDG--------KDIL------------EVENVCAIGTEIFKHGCSKDRIRLPRMRQPD-------------------  target    KFDSRFLDDMLRVSWDTAFTYAAKAMITIATRYSGEAGARRLREQGYAPEMIEMMKGAGTRCFKHRAGMPVLGIIGKMGN 7bkb.1    -------GSMKDISYEEAIDWTARHLLKAKKP----LM--------YG------FGSTNCE-------------------  target    TRMNGGINALLDTWIRKVSPDQAQGGRYWSNYTWHGDQNPAHPFWSGVQGSDIDLSDM-RFSKLNTSWGKNFVENKMPEA 7bkb.1    ------GQAAAARVMEIAGGMLDNCAT----ICHGPSF-LA---IFDNGYPSCTLGEVKNRADVIVYWGSNPAHAHPRHM  target    HWK--------LECIERGARVVVITPEYNPTAYRADYWMPLRPESDGALFLGAMKIIIDENMHDIDFLKSFTDAPILVRT 7bkb.1    SRYSIFPRGFFTGKGQKKRTVIVIDPRFTDTANVADYHLQVKQGHDYELFNAFRMVIHGHGK------------------  target    DTLQYLDPRDVIADYKFPDFSKSYSGRIQSLKPEQIQRLGGMMVWDLNKKQVVPLHREQVGWHYTNSGIDAALTGTYRVK 7bkb.1    --------------------------------------------------------------------------------  target    LLNGREIDAMPIWQMYMVHFQDYDLDTVHQITRTPKDLIVRWARDSGTIKPAAIHNGEGTCHYFHQTANARGAA------ 7bkb.1    -------------------------DLPDEVAGIKKETILEVAEIMKNARFGTTFFGMGLTHTDGRNHNIDIAISLTRDL  target    ------MVLIITGNVGKFGTGQHTWAGNYKAGTWTATPWSGAGLSVHTGEDPFNITLDPNAHGKEIKTRSYYYGEEVGYW 7bkb.1    NKISKWTIMAMRGHYNIAGPGVVWSWT---F-----------GF---PYCLDL----TKQ---N-HA-----------HM  target    NHGDTALIVNTPKYGRKVFTGKTHMPTPSKFRWVVNVNVVNNAKHHYDMVRNVDPNIECLITQDIEMTSDINHADIAFAA 7bkb.1    NPG-------ETSSVDM------AMRDEVDMFINIGTDAAAHFPIP---AVKQLKKHPW-VTIDPSINMASEISDLHIPV  target    NSW-MEFTYPEMTVTVSNPWVQIWKGGIRPLYDTRNDLDTFAGVAAKLSDMTGDKRMRDYFAMVYQNRVDVYVQRMLDAS 7bkb.1    CICGVDVGG---IVYRMDNVPIQFRKVIEPPEGVMDDETLLNKIADRMEE------------------------------  target    STFYGYSADVMLKSEKGWMVMVRTYPRHPFWEETNESKPMWTRSGRYENYRIEPEAIEYGENFISHREGPEATPYLPNAI 7bkb.1    --------------------------------------------------------------------------------  target    FTTNPYVRPDDYGIPITAQHHDDKTVRNIKLSWHEIKRHSNPLWEKGYQFYCVTPKTRHRVHSQWSVNDWVQIYESNFGD 7bkb.1    --------------------------------------------------------------------------------  target    PYRMDKRTPGVGEHQIHINPQAAKDRGINDGDYVYVDGNPVDRPYRGWKPSDPYYKVARLMIRAKYNPAYPYHVTMAKHA 7bkb.1    --------------------------------------------------------------------------------  target    PFVATAKSVKGHETRPDGRAIAIDTGYQSNFRYGAQQSFTRNWLMPMHQTDSLPGKHAVAWKFKWGYQVDHHAINTVPKE 7bkb.1    --------------------------------------------------------------------------------  target    CLIRITKAEDGGIGARGPWEPVRTGFTPGQENEFMIKWLKGEHIKIKV 7bkb.1    ------------------------------------------------ ``` | | | | | | | | | | | | | | | | | | | | | | | | | | | | | | | | | | | | | | | | | | | | | | | | | |
|  | 6yj4.1.G | Subunit NUAM of NADH:Ubiquinone Oxidoreductase (Complex I)  *Structure of Yarrowia lipolytica complex I at 2.7 A* | 0.16 |  | 16.14 | 0.33 | 63-746 | EM | 0.00 | hetero-1-1-1-1-1-1-… | 18 x 3PE, 6 x SF4, 5 x LMT, 8 x PLC, 2 x FES, 1 x FMN, 6 x CDL, 1 x NDP, 1 x ZN, 2 x EHZ | HHblits | 0.27 |
| ``` target    MFLSRRQFLKVSVGTVAAVAVADKVLALTALQPVIEVGNPLGDYPDRSWERVYHDQYRYDSSFTWVCSPNDTHACRVRAF 6yj4.1    --------------------------------------------------------------TESIDV-MDAVGSNIRID  target    VRNGVVMRVEQNYDHQTYEDLYGNRGTFAHNPRMCLKGFTFHRRVYGPYRLKGPLMRKGWKQWMDDNAPELTAETKRKYK 6yj4.1    SKGVEVMRVIPRVHEDV------------NEEWINDKSRFACDGLK-TQRLTTPLIRVG---------------------  target    FDSRFLDDMLRVSWDTAFTYAAKAMITIATRYSGEAGARRLREQGYAPEMIEMMKGAGTRCFKHRAGMPVLGIIGKMGNT 6yj4.1    ------DKFVNATWDDALSTIAKAYQQKAP--KGDEFK--------------AVAGALVEVESMVA---LKDMTNALGSE  target    RMNGGINALLDTWIRKVSPDQAQGGRYWSNYTWHGDQNPAHPFWSGVQGSDIDLSDMRFSKLNTSWGKNFVENKMPEAHW 6yj4.1    NTTTDT-------------PNGNS-----------APAHGITFRSNY-LFNSSIAGIEDADAILLVGTNPRREAAVMNAR  target    KLECI-ERGARVVVITPEYNPTAYRADYWMPLRPESDGALFLGAMKIIIDENMHDIDFLKSFTDAPILVRTDTLQYLDPR 6yj4.1    IRKAWLRQELEIASVGPTLDATFDVAEL----------------------------------GNTH--------------  target    DVIADYKFPDFSKSYSGRIQSLKPEQIQRLGGMMVWDLNKKQVVPLHREQVGWHYTNSGIDAALTGTYRVKLLNGREIDA 6yj4.1    --------AD----------------------------------------------------------------------  target    MPIWQMYMVHFQDYDLDTVHQITRTPKDLIVRWARDSGTIKPAAIHNGEGTCHYFHQTANARGAAMVLIITGNV-GKFGT 6yj4.1    ------LEKALS------------------GEFGEVLKNAKNPLIIVGSGITDREDAGAFFNTIGKFVESTPSVLNENWN  target    GQHTWAGNYKAGTWTATPWSGAGLSVHTGEDPFNITLDPNAHGKEIKTRSYYYGEEVGYWNHGDTALIVNTPKYGRKVFT 6yj4.1    GYNVLQRSAS-------R---------AG-------------AYDIG-----------FTP---------S----D----  target    GKTHMPTPSKFRWVVNVNVVNNAKHHYDMVRNVDPNIECLITQDIEMTSDINHADIAFAANSWMEFTYPEMTVTVSNPWV 6yj4.1    --EASKTTPKMVWLLGADEVAAS---------DIPADAFVVYQGHNGDVGAQFADVVLPGAAYTEKA---GTYVNTEGRS  target    QIWKGGIRPLYDTRNDLDTFAGVAAKLSDMTGDKRMRDYFAMVYQNRVDVYVQRMLDASSTFYGYSADVMLKSEKGWMVM 6yj4.1    QISRAATGPPGGAREDWKILRAVSEYLG----------------------------------------------------  target    VRTYPRHPFWEETNESKPMWTRSGRYENYRIEPEAIEYGENFISHREGPEATPYLPNAIFTTNPYVRPDDYGIPITAQHH 6yj4.1    --------------------------------------------------------------------------------  target    DDKTVRNIKLSWHEIKRHSNPLWEKGYQFYCVTPKTRHRVHSQWSVNDWVQIYESNFGDPYRMDKRTPGVGEHQIHINPQ 6yj4.1    --------------------------------------------------------------------------------  target    AAKDRGINDGDYVYVDGNPVDRPYRGWKPSDPYYKVARLMIRAKYNPAYPYHVTMAKHAPFVATAKSVKGHETRPDGRAI 6yj4.1    --------------------------------------------------------------------------------  target    AIDTGYQSNFRYGAQQSFTRNWLMPMHQTDSLPGKHAVAWKFKWGYQVDHHAINTVPKECLIRITKAEDGGIGARGPWEP 6yj4.1    --------------------------------------------------------------------------------  target    VRTGFTPGQENEFMIKWLKGEHIKIKV 6yj4.1    --------------------------- ``` | | | | | | | | | | | | | | | | | | | | | | | | | | | | | | | | | | | | | | | | | | | | | | | | | |
|  | 6rfs.1.A | Subunit NUAM of NADH:Ubiquinone Oxidoreductase (Complex I)  *Cryo-EM structure of a respiratory complex I mutant lacking NDUFS4* | 0.15 |  | 16.14 | 0.33 | 63-746 | EM | 4.04 | hetero-1-1-1-1-1-1-… | 6 x SF4, 2 x FES, 1 x FMN, 1 x NDP, 1 x ZN, 1 x ZMP | HHblits | 0.27 |
| ``` target    MFLSRRQFLKVSVGTVAAVAVADKVLALTALQPVIEVGNPLGDYPDRSWERVYHDQYRYDSSFTWVCSPNDTHACRVRAF 6rfs.1    --------------------------------------------------------------TESIDV-MDAVGSNIRID  target    VRNGVVMRVEQNYDHQTYEDLYGNRGTFAHNPRMCLKGFTFHRRVYGPYRLKGPLMRKGWKQWMDDNAPELTAETKRKYK 6rfs.1    SKGVEVMRVIPRVHEDV------------NEEWINDKSRFACDGLK-TQRLTTPLIRVG---------------------  target    FDSRFLDDMLRVSWDTAFTYAAKAMITIATRYSGEAGARRLREQGYAPEMIEMMKGAGTRCFKHRAGMPVLGIIGKMGNT 6rfs.1    ------DKFVNATWDDALSTIAKAYQQKAP--KGDEFK--------------AVAGALVEVESMVA---LKDMTNALGSE  target    RMNGGINALLDTWIRKVSPDQAQGGRYWSNYTWHGDQNPAHPFWSGVQGSDIDLSDMRFSKLNTSWGKNFVENKMPEAHW 6rfs.1    NTTTDT-------------PNGNS-----------APAHGITFRSNY-LFNSSIAGIEDADAILLVGTNPRREAAVMNAR  target    KLECI-ERGARVVVITPEYNPTAYRADYWMPLRPESDGALFLGAMKIIIDENMHDIDFLKSFTDAPILVRTDTLQYLDPR 6rfs.1    IRKAWLRQELEIASVGPTLDATFDVAEL----------------------------------GNTH--------------  target    DVIADYKFPDFSKSYSGRIQSLKPEQIQRLGGMMVWDLNKKQVVPLHREQVGWHYTNSGIDAALTGTYRVKLLNGREIDA 6rfs.1    --------AD----------------------------------------------------------------------  target    MPIWQMYMVHFQDYDLDTVHQITRTPKDLIVRWARDSGTIKPAAIHNGEGTCHYFHQTANARGAAMVLIITGNV-GKFGT 6rfs.1    ------LEKALS------------------GEFGEVLKNAKNPLIIVGSGITDREDAGAFFNTIGKFVESTPSVLNENWN  target    GQHTWAGNYKAGTWTATPWSGAGLSVHTGEDPFNITLDPNAHGKEIKTRSYYYGEEVGYWNHGDTALIVNTPKYGRKVFT 6rfs.1    GYNVLQRSAS-------R---------AG-------------AYDIG-----------FTP---------S----D----  target    GKTHMPTPSKFRWVVNVNVVNNAKHHYDMVRNVDPNIECLITQDIEMTSDINHADIAFAANSWMEFTYPEMTVTVSNPWV 6rfs.1    --EASKTTPKMVWLLGADEVAAS---------DIPADAFVVYQGHNGDVGAQFADVVLPGAAYTEKA---GTYVNTEGRS  target    QIWKGGIRPLYDTRNDLDTFAGVAAKLSDMTGDKRMRDYFAMVYQNRVDVYVQRMLDASSTFYGYSADVMLKSEKGWMVM 6rfs.1    QISRAATGPPGGAREDWKILRAVSEYLG----------------------------------------------------  target    VRTYPRHPFWEETNESKPMWTRSGRYENYRIEPEAIEYGENFISHREGPEATPYLPNAIFTTNPYVRPDDYGIPITAQHH 6rfs.1    --------------------------------------------------------------------------------  target    DDKTVRNIKLSWHEIKRHSNPLWEKGYQFYCVTPKTRHRVHSQWSVNDWVQIYESNFGDPYRMDKRTPGVGEHQIHINPQ 6rfs.1    --------------------------------------------------------------------------------  target    AAKDRGINDGDYVYVDGNPVDRPYRGWKPSDPYYKVARLMIRAKYNPAYPYHVTMAKHAPFVATAKSVKGHETRPDGRAI 6rfs.1    --------------------------------------------------------------------------------  target    AIDTGYQSNFRYGAQQSFTRNWLMPMHQTDSLPGKHAVAWKFKWGYQVDHHAINTVPKECLIRITKAEDGGIGARGPWEP 6rfs.1    --------------------------------------------------------------------------------  target    VRTGFTPGQENEFMIKWLKGEHIKIKV 6rfs.1    --------------------------- ``` | | | | | | | | | | | | | | | | | | | | | | | | | | | | | | | | | | | | | | | | | | | | | | | | | |
|  | 6rfq.1.A | Subunit NUAM of NADH:Ubiquinone Oxidoreductase (Complex I)  *Cryo-EM structure of a respiratory complex I assembly intermediate with NDUFAF2* | 0.15 |  | 16.14 | 0.33 | 63-746 | EM | 3.30 | hetero-1-1-1-1-1-1-… | 6 x SF4, 2 x FES, 1 x FMN, 1 x NDP, 10 x 3PE, 2 x LMN, 4 x CDL, 2 x ZMP, 4 x PLC, 3 x T7X, 1 x CPL | HHblits | 0.27 |
| ``` target    MFLSRRQFLKVSVGTVAAVAVADKVLALTALQPVIEVGNPLGDYPDRSWERVYHDQYRYDSSFTWVCSPNDTHACRVRAF 6rfq.1    --------------------------------------------------------------TESIDV-MDAVGSNIRID  target    VRNGVVMRVEQNYDHQTYEDLYGNRGTFAHNPRMCLKGFTFHRRVYGPYRLKGPLMRKGWKQWMDDNAPELTAETKRKYK 6rfq.1    SKGVEVMRVIPRVHEDV------------NEEWINDKSRFACDGLK-TQRLTTPLIRVG---------------------  target    FDSRFLDDMLRVSWDTAFTYAAKAMITIATRYSGEAGARRLREQGYAPEMIEMMKGAGTRCFKHRAGMPVLGIIGKMGNT 6rfq.1    ------DKFVNATWDDALSTIAKAYQQKAP--KGDEFK--------------AVAGALVEVESMVA---LKDMTNALGSE  target    RMNGGINALLDTWIRKVSPDQAQGGRYWSNYTWHGDQNPAHPFWSGVQGSDIDLSDMRFSKLNTSWGKNFVENKMPEAHW 6rfq.1    NTTTDT-------------PNGNS-----------APAHGITFRSNY-LFNSSIAGIEDADAILLVGTNPRREAAVMNAR  target    KLECI-ERGARVVVITPEYNPTAYRADYWMPLRPESDGALFLGAMKIIIDENMHDIDFLKSFTDAPILVRTDTLQYLDPR 6rfq.1    IRKAWLRQELEIASVGPTLDATFDVAEL----------------------------------GNTH--------------  target    DVIADYKFPDFSKSYSGRIQSLKPEQIQRLGGMMVWDLNKKQVVPLHREQVGWHYTNSGIDAALTGTYRVKLLNGREIDA 6rfq.1    --------AD----------------------------------------------------------------------  target    MPIWQMYMVHFQDYDLDTVHQITRTPKDLIVRWARDSGTIKPAAIHNGEGTCHYFHQTANARGAAMVLIITGNV-GKFGT 6rfq.1    ------LEKALS------------------GEFGEVLKNAKNPLIIVGSGITDREDAGAFFNTIGKFVESTPSVLNENWN  target    GQHTWAGNYKAGTWTATPWSGAGLSVHTGEDPFNITLDPNAHGKEIKTRSYYYGEEVGYWNHGDTALIVNTPKYGRKVFT 6rfq.1    GYNVLQRSAS-------R---------AG-------------AYDIG-----------FTP---------S----D----  target    GKTHMPTPSKFRWVVNVNVVNNAKHHYDMVRNVDPNIECLITQDIEMTSDINHADIAFAANSWMEFTYPEMTVTVSNPWV 6rfq.1    --EASKTTPKMVWLLGADEVAAS---------DIPADAFVVYQGHNGDVGAQFADVVLPGAAYTEKA---GTYVNTEGRS  target    QIWKGGIRPLYDTRNDLDTFAGVAAKLSDMTGDKRMRDYFAMVYQNRVDVYVQRMLDASSTFYGYSADVMLKSEKGWMVM 6rfq.1    QISRAATGPPGGAREDWKILRAVSEYLG----------------------------------------------------  target    VRTYPRHPFWEETNESKPMWTRSGRYENYRIEPEAIEYGENFISHREGPEATPYLPNAIFTTNPYVRPDDYGIPITAQHH 6rfq.1    --------------------------------------------------------------------------------  target    DDKTVRNIKLSWHEIKRHSNPLWEKGYQFYCVTPKTRHRVHSQWSVNDWVQIYESNFGDPYRMDKRTPGVGEHQIHINPQ 6rfq.1    --------------------------------------------------------------------------------  target    AAKDRGINDGDYVYVDGNPVDRPYRGWKPSDPYYKVARLMIRAKYNPAYPYHVTMAKHAPFVATAKSVKGHETRPDGRAI 6rfq.1    --------------------------------------------------------------------------------  target    AIDTGYQSNFRYGAQQSFTRNWLMPMHQTDSLPGKHAVAWKFKWGYQVDHHAINTVPKECLIRITKAEDGGIGARGPWEP 6rfq.1    --------------------------------------------------------------------------------  target    VRTGFTPGQENEFMIKWLKGEHIKIKV 6rfq.1    --------------------------- ``` | | | | | | | | | | | | | | | | | | | | | | | | | | | | | | | | | | | | | | | | | | | | | | | | | |
|  | 6gcs.1.A | 75-KDA PROTEIN (NUAM)  *Cryo-EM structure of respiratory complex I from Yarrowia lipolytica* | 0.15 |  | 16.14 | 0.33 | 63-746 | EM | 4.32 | hetero-1-1-1-1-1-1-… | 6 x SF4, 2 x FES, 1 x FMN, 1 x NDP, 1 x ZN, 1 x ZMP, 1 x CDL, 3 x 3PE | HHblits | 0.27 |
| ``` target    MFLSRRQFLKVSVGTVAAVAVADKVLALTALQPVIEVGNPLGDYPDRSWERVYHDQYRYDSSFTWVCSPNDTHACRVRAF 6gcs.1    --------------------------------------------------------------TESIDV-MDAVGSNIRID  target    VRNGVVMRVEQNYDHQTYEDLYGNRGTFAHNPRMCLKGFTFHRRVYGPYRLKGPLMRKGWKQWMDDNAPELTAETKRKYK 6gcs.1    SKGVEVMRVIPRVHEDV------------NEEWINDKSRFACDGLK-TQRLTTPLIRVG---------------------  target    FDSRFLDDMLRVSWDTAFTYAAKAMITIATRYSGEAGARRLREQGYAPEMIEMMKGAGTRCFKHRAGMPVLGIIGKMGNT 6gcs.1    ------DKFVNATWDDALSTIAKAYQQKAP--KGDEFK--------------AVAGALVEVESMVA---LKDMTNALGSE  target    RMNGGINALLDTWIRKVSPDQAQGGRYWSNYTWHGDQNPAHPFWSGVQGSDIDLSDMRFSKLNTSWGKNFVENKMPEAHW 6gcs.1    NTTTDT-------------PNGNS-----------APAHGITFRSNY-LFNSSIAGIEDADAILLVGTNPRREAAVMNAR  target    KLECI-ERGARVVVITPEYNPTAYRADYWMPLRPESDGALFLGAMKIIIDENMHDIDFLKSFTDAPILVRTDTLQYLDPR 6gcs.1    IRKAWLRQELEIASVGPTLDATFDVAEL----------------------------------GNTH--------------  target    DVIADYKFPDFSKSYSGRIQSLKPEQIQRLGGMMVWDLNKKQVVPLHREQVGWHYTNSGIDAALTGTYRVKLLNGREIDA 6gcs.1    --------AD----------------------------------------------------------------------  target    MPIWQMYMVHFQDYDLDTVHQITRTPKDLIVRWARDSGTIKPAAIHNGEGTCHYFHQTANARGAAMVLIITGNV-GKFGT 6gcs.1    ------LEKALS------------------GEFGEVLKNAKNPLIIVGSGITDREDAGAFFNTIGKFVESTPSVLNENWN  target    GQHTWAGNYKAGTWTATPWSGAGLSVHTGEDPFNITLDPNAHGKEIKTRSYYYGEEVGYWNHGDTALIVNTPKYGRKVFT 6gcs.1    GYNVLQRSAS-------R---------AG-------------AYDIG-----------FTP---------S----D----  target    GKTHMPTPSKFRWVVNVNVVNNAKHHYDMVRNVDPNIECLITQDIEMTSDINHADIAFAANSWMEFTYPEMTVTVSNPWV 6gcs.1    --EASKTTPKMVWLLGADEVAAS---------DIPADAFVVYQGHNGDVGAQFADVVLPGAAYTEKA---GTYVNTEGRS  target    QIWKGGIRPLYDTRNDLDTFAGVAAKLSDMTGDKRMRDYFAMVYQNRVDVYVQRMLDASSTFYGYSADVMLKSEKGWMVM 6gcs.1    QISRAATGPPGGAREDWKILRAVSEYLG----------------------------------------------------  target    VRTYPRHPFWEETNESKPMWTRSGRYENYRIEPEAIEYGENFISHREGPEATPYLPNAIFTTNPYVRPDDYGIPITAQHH 6gcs.1    --------------------------------------------------------------------------------  target    DDKTVRNIKLSWHEIKRHSNPLWEKGYQFYCVTPKTRHRVHSQWSVNDWVQIYESNFGDPYRMDKRTPGVGEHQIHINPQ 6gcs.1    --------------------------------------------------------------------------------  target    AAKDRGINDGDYVYVDGNPVDRPYRGWKPSDPYYKVARLMIRAKYNPAYPYHVTMAKHAPFVATAKSVKGHETRPDGRAI 6gcs.1    --------------------------------------------------------------------------------  target    AIDTGYQSNFRYGAQQSFTRNWLMPMHQTDSLPGKHAVAWKFKWGYQVDHHAINTVPKECLIRITKAEDGGIGARGPWEP 6gcs.1    --------------------------------------------------------------------------------  target    VRTGFTPGQENEFMIKWLKGEHIKIKV 6gcs.1    --------------------------- ``` | | | | | | | | | | | | | | | | | | | | | | | | | | | | | | | | | | | | | | | | | | | | | | | | | |
|  | 6zr2.1.G | NADH-ubiquinone oxidoreductase 75 kDa subunit, mitochondrial  *Cryo-EM structure of respiratory complex I in the active state from Mus musculus at 3.1 A* | 0.15 |  | 12.76 | 0.34 | 62-746 | EM | 3.10 | hetero-1-1-1-1-1-1-… | 6 x SF4, 4 x PC1, 2 x FES, 1 x FMN, 9 x 3PE, 7 x CDL, 1 x ATP, 1 x NDP, 1 x ZN, 2 x EHZ | HHblits | 0.25 |
| ``` target    MFLSRRQFLKVSVGTVAAVAVADKVLALTALQPVIEVGNPLGDYPDRSWERVYHDQYRYDSSFTWVCSPNDTHACRVRAF 6zr2.1    -------------------------------------------------------------KTESIDV-MDAVGSNIVVS  target    VRNGVVMRVEQNYDHQTYEDLYGNRGTFAHNPRMCLKGFTFHRRVYGPYRLKGPLMRKGWKQWMDDNAPELTAETKRKYK 6zr2.1    TRTGEVMRILPRMHEDI------------NEEWISDKTRFAYDGLK-RQRLTEPMVRNEK--------------------  target    FDSRFLDDMLRVSWDTAFTYAAKAMITIATRYSGEAGARRLREQGYAPEMIEMMKGAGTRCFKHRAGMPVLGIIGKMGNT 6zr2.1    ------GLLTYTSWEDALSRVAGMLQNF----EGNAVA--------------AIAGGLVD--------------------  target    RMNGGINALLDTWIRKVSPDQAQGGRYWSNYTWHGDQNPAHPFWSGVQGSDIDLSDMRFSKLNTSWGKNFVENKMPEAHW 6zr2.1    ---AEALVALKDLLNKVDSD-----NLCTEEI-FPTEGAGTDLRS-NYLLNTTIAGVEEADVVLLVGTNPRFEAPLFNAR  target    KLECI-ERGARVVVITPEYNPTAYRADYWMPLRPESDGALFLGAMKIIIDENMHDIDFLKSFTDAPILVRTDTLQYLDPR 6zr2.1    IRKSWLHNDLKVALIGSPVDLTYRYDHLGDSP------------------------KILQDIASG---------------  target    DVIADYKFPDFSKSYSGRIQSLKPEQIQRLGGMMVWDLNKKQVVPLHREQVGWHYTNSGIDAALTGTYRVKLLNGREIDA 6zr2.1    --------------------------------------------------------------------------------  target    MPIWQMYMVHFQDYDLDTVHQITRTPKDLIVRWARDSGTIKPAAIHNGEGTCHYFHQTANARGAAMVLIITGNVGKFGTG 6zr2.1    -----------------------------RHSFCEVLKDAKKPMVVLGSSALQRDDGAAILVAVSNMVQKIRVTTGVAAE  target    QHTWAGNYKAGTWTATPWSGAGLSVHTGEDPFNITLDPNAHGKEIKTRSYYYGEEVGYWNHGDTALIVNTPKYGRKVFTG 6zr2.1    WKV---------MN--ILH-------RIASQVA--------ALDL-----------GYKP------------GVEAI---  target    KTHMPTPSKFRWVVNVNVVNNAKHHYDMVRNVDPNIECLITQDIEMTSDINHADIAFAANSWMEFTYPEMTVTVSNPWVQ 6zr2.1    ---RKNPPKMLFLLGADGGC-------ITRQDLPKDCFIVYQGHHGDVGAPMADVILPGAAYTEKS---ATYVNTEGRAQ  target    IWKGGIRPLYDTRNDLDTFAGVAAKLSDMTGDKRMRDYFAMVYQNRVDVYVQRMLDASSTFYGYSADVMLKSEKGWMVMV 6zr2.1    QTKVAVTPPGLAREDWKIIRALSEIAG-----------------------------------------------------  target    RTYPRHPFWEETNESKPMWTRSGRYENYRIEPEAIEYGENFISHREGPEATPYLPNAIFTTNPYVRPDDYGIPITAQHHD 6zr2.1    --------------------------------------------------------------------------------  target    DKTVRNIKLSWHEIKRHSNPLWEKGYQFYCVTPKTRHRVHSQWSVNDWVQIYESNFGDPYRMDKRTPGVGEHQIHINPQA 6zr2.1    --------------------------------------------------------------------------------  target    AKDRGINDGDYVYVDGNPVDRPYRGWKPSDPYYKVARLMIRAKYNPAYPYHVTMAKHAPFVATAKSVKGHETRPDGRAIA 6zr2.1    --------------------------------------------------------------------------------  target    IDTGYQSNFRYGAQQSFTRNWLMPMHQTDSLPGKHAVAWKFKWGYQVDHHAINTVPKECLIRITKAEDGGIGARGPWEPV 6zr2.1    --------------------------------------------------------------------------------  target    RTGFTPGQENEFMIKWLKGEHIKIKV 6zr2.1    -------------------------- ``` | | | | | | | | | | | | | | | | | | | | | | | | | | | | | | | | | | | | | | | | | | | | | | | | | |
|  | 6g72.1.G | NADH-ubiquinone oxidoreductase 75 kDa subunit, mitochondrial  *Mouse mitochondrial complex I in the deactive state* | 0.15 |  | 12.76 | 0.34 | 62-746 | EM | 0.00 | hetero-1-1-1-1-1-1-… | 6 x SF4, 2 x FES, 1 x FMN, 1 x ADP, 1 x NDP, 1 x ZN, 2 x EHZ | HHblits | 0.25 |
| ``` target    MFLSRRQFLKVSVGTVAAVAVADKVLALTALQPVIEVGNPLGDYPDRSWERVYHDQYRYDSSFTWVCSPNDTHACRVRAF 6g72.1    -------------------------------------------------------------KTESIDV-MDAVGSNIVVS  target    VRNGVVMRVEQNYDHQTYEDLYGNRGTFAHNPRMCLKGFTFHRRVYGPYRLKGPLMRKGWKQWMDDNAPELTAETKRKYK 6g72.1    TRTGEVMRILPRMHEDI------------NEEWISDKTRFAYDGLK-RQRLTEPMVRNEK--------------------  target    FDSRFLDDMLRVSWDTAFTYAAKAMITIATRYSGEAGARRLREQGYAPEMIEMMKGAGTRCFKHRAGMPVLGIIGKMGNT 6g72.1    ------GLLTYTSWEDALSRVAGMLQNF----EGNAVA--------------AIAGGLVD--------------------  target    RMNGGINALLDTWIRKVSPDQAQGGRYWSNYTWHGDQNPAHPFWSGVQGSDIDLSDMRFSKLNTSWGKNFVENKMPEAHW 6g72.1    ---AEALVALKDLLNKVDSD-----NLCTEEI-FPTEGAGTDLRS-NYLLNTTIAGVEEADVVLLVGTNPRFEAPLFNAR  target    KLECI-ERGARVVVITPEYNPTAYRADYWMPLRPESDGALFLGAMKIIIDENMHDIDFLKSFTDAPILVRTDTLQYLDPR 6g72.1    IRKSWLHNDLKVALIGSPVDLTYRYDHLGDSP------------------------KILQDIASG---------------  target    DVIADYKFPDFSKSYSGRIQSLKPEQIQRLGGMMVWDLNKKQVVPLHREQVGWHYTNSGIDAALTGTYRVKLLNGREIDA 6g72.1    --------------------------------------------------------------------------------  target    MPIWQMYMVHFQDYDLDTVHQITRTPKDLIVRWARDSGTIKPAAIHNGEGTCHYFHQTANARGAAMVLIITGNVGKFGTG 6g72.1    -----------------------------RHSFCEVLKDAKKPMVVLGSSALQRDDGAAILVAVSNMVQKIRVTTGVAAE  target    QHTWAGNYKAGTWTATPWSGAGLSVHTGEDPFNITLDPNAHGKEIKTRSYYYGEEVGYWNHGDTALIVNTPKYGRKVFTG 6g72.1    WKV---------MN--ILH-------RIASQVA--------ALDL-----------GYKP------------GVEAI---  target    KTHMPTPSKFRWVVNVNVVNNAKHHYDMVRNVDPNIECLITQDIEMTSDINHADIAFAANSWMEFTYPEMTVTVSNPWVQ 6g72.1    ---RKNPPKMLFLLGADGGC-------ITRQDLPKDCFIVYQGHHGDVGAPMADVILPGAAYTEKS---ATYVNTEGRAQ  target    IWKGGIRPLYDTRNDLDTFAGVAAKLSDMTGDKRMRDYFAMVYQNRVDVYVQRMLDASSTFYGYSADVMLKSEKGWMVMV 6g72.1    QTKVAVTPPGLAREDWKIIRALSEIAG-----------------------------------------------------  target    RTYPRHPFWEETNESKPMWTRSGRYENYRIEPEAIEYGENFISHREGPEATPYLPNAIFTTNPYVRPDDYGIPITAQHHD 6g72.1    --------------------------------------------------------------------------------  target    DKTVRNIKLSWHEIKRHSNPLWEKGYQFYCVTPKTRHRVHSQWSVNDWVQIYESNFGDPYRMDKRTPGVGEHQIHINPQA 6g72.1    --------------------------------------------------------------------------------  target    AKDRGINDGDYVYVDGNPVDRPYRGWKPSDPYYKVARLMIRAKYNPAYPYHVTMAKHAPFVATAKSVKGHETRPDGRAIA 6g72.1    --------------------------------------------------------------------------------  target    IDTGYQSNFRYGAQQSFTRNWLMPMHQTDSLPGKHAVAWKFKWGYQVDHHAINTVPKECLIRITKAEDGGIGARGPWEPV 6g72.1    --------------------------------------------------------------------------------  target    RTGFTPGQENEFMIKWLKGEHIKIKV 6g72.1    -------------------------- ``` | | | | | | | | | | | | | | | | | | | | | | | | | | | | | | | | | | | | | | | | | | | | | | | | | |
|  | 7ak6.1.G | NADH-ubiquinone oxidoreductase 75 kDa subunit, mitochondrial  *Cryo-EM structure of ND6-P25L mutant respiratory complex I from Mus musculus at 3.8 A* | 0.15 |  | 12.76 | 0.34 | 62-746 | EM | 0.00 | hetero-1-1-1-1-1-1-… | 6 x SF4, 1 x PC1, 2 x FES, 1 x FMN, 4 x 3PE, 2 x CDL, 1 x ATP, 1 x NDP, 1 x ZN, 2 x EHZ | HHblits | 0.25 |
| ``` target    MFLSRRQFLKVSVGTVAAVAVADKVLALTALQPVIEVGNPLGDYPDRSWERVYHDQYRYDSSFTWVCSPNDTHACRVRAF 7ak6.1    -------------------------------------------------------------KTESIDV-MDAVGSNIVVS  target    VRNGVVMRVEQNYDHQTYEDLYGNRGTFAHNPRMCLKGFTFHRRVYGPYRLKGPLMRKGWKQWMDDNAPELTAETKRKYK 7ak6.1    TRTGEVMRILPRMHEDI------------NEEWISDKTRFAYDGLK-RQRLTEPMVRNEK--------------------  target    FDSRFLDDMLRVSWDTAFTYAAKAMITIATRYSGEAGARRLREQGYAPEMIEMMKGAGTRCFKHRAGMPVLGIIGKMGNT 7ak6.1    ------GLLTYTSWEDALSRVAGMLQNF----EGNAVA--------------AIAGGLVD--------------------  target    RMNGGINALLDTWIRKVSPDQAQGGRYWSNYTWHGDQNPAHPFWSGVQGSDIDLSDMRFSKLNTSWGKNFVENKMPEAHW 7ak6.1    ---AEALVALKDLLNKVDSD-----NLCTEEI-FPTEGAGTDLRS-NYLLNTTIAGVEEADVVLLVGTNPRFEAPLFNAR  target    KLECI-ERGARVVVITPEYNPTAYRADYWMPLRPESDGALFLGAMKIIIDENMHDIDFLKSFTDAPILVRTDTLQYLDPR 7ak6.1    IRKSWLHNDLKVALIGSPVDLTYRYDHLGDSP------------------------KILQDIASG---------------  target    DVIADYKFPDFSKSYSGRIQSLKPEQIQRLGGMMVWDLNKKQVVPLHREQVGWHYTNSGIDAALTGTYRVKLLNGREIDA 7ak6.1    --------------------------------------------------------------------------------  target    MPIWQMYMVHFQDYDLDTVHQITRTPKDLIVRWARDSGTIKPAAIHNGEGTCHYFHQTANARGAAMVLIITGNVGKFGTG 7ak6.1    -----------------------------RHSFCEVLKDAKKPMVVLGSSALQRDDGAAILVAVSNMVQKIRVTTGVAAE  target    QHTWAGNYKAGTWTATPWSGAGLSVHTGEDPFNITLDPNAHGKEIKTRSYYYGEEVGYWNHGDTALIVNTPKYGRKVFTG 7ak6.1    WKV---------MN--ILH-------RIASQVA--------ALDL-----------GYKP------------GVEAI---  target    KTHMPTPSKFRWVVNVNVVNNAKHHYDMVRNVDPNIECLITQDIEMTSDINHADIAFAANSWMEFTYPEMTVTVSNPWVQ 7ak6.1    ---RKNPPKMLFLLGADGGC-------ITRQDLPKDCFIVYQGHHGDVGAPMADVILPGAAYTEKS---ATYVNTEGRAQ  target    IWKGGIRPLYDTRNDLDTFAGVAAKLSDMTGDKRMRDYFAMVYQNRVDVYVQRMLDASSTFYGYSADVMLKSEKGWMVMV 7ak6.1    QTKVAVTPPGLAREDWKIIRALSEIAG-----------------------------------------------------  target    RTYPRHPFWEETNESKPMWTRSGRYENYRIEPEAIEYGENFISHREGPEATPYLPNAIFTTNPYVRPDDYGIPITAQHHD 7ak6.1    --------------------------------------------------------------------------------  target    DKTVRNIKLSWHEIKRHSNPLWEKGYQFYCVTPKTRHRVHSQWSVNDWVQIYESNFGDPYRMDKRTPGVGEHQIHINPQA 7ak6.1    --------------------------------------------------------------------------------  target    AKDRGINDGDYVYVDGNPVDRPYRGWKPSDPYYKVARLMIRAKYNPAYPYHVTMAKHAPFVATAKSVKGHETRPDGRAIA 7ak6.1    --------------------------------------------------------------------------------  target    IDTGYQSNFRYGAQQSFTRNWLMPMHQTDSLPGKHAVAWKFKWGYQVDHHAINTVPKECLIRITKAEDGGIGARGPWEPV 7ak6.1    --------------------------------------------------------------------------------  target    RTGFTPGQENEFMIKWLKGEHIKIKV 7ak6.1    -------------------------- ``` | | | | | | | | | | | | | | | | | | | | | | | | | | | | | | | | | | | | | | | | | | | | | | | | | |
|  | 7ak5.1.G | NADH-ubiquinone oxidoreductase 75 kDa subunit, mitochondrial  *Cryo-EM structure of respiratory complex I in the deactive state from Mus musculus at 3.2 A* | 0.16 |  | 12.76 | 0.34 | 62-746 | EM | 0.00 | hetero-1-1-1-1-1-1-… | 6 x SF4, 2 x PC1, 2 x FES, 1 x FMN, 8 x 3PE, 4 x CDL, 1 x ATP, 1 x NDP, 1 x ZN, 2 x EHZ | HHblits | 0.25 |
| ``` target    MFLSRRQFLKVSVGTVAAVAVADKVLALTALQPVIEVGNPLGDYPDRSWERVYHDQYRYDSSFTWVCSPNDTHACRVRAF 7ak5.1    -------------------------------------------------------------KTESIDV-MDAVGSNIVVS  target    VRNGVVMRVEQNYDHQTYEDLYGNRGTFAHNPRMCLKGFTFHRRVYGPYRLKGPLMRKGWKQWMDDNAPELTAETKRKYK 7ak5.1    TRTGEVMRILPRMHEDI------------NEEWISDKTRFAYDGLK-RQRLTEPMVRNEK--------------------  target    FDSRFLDDMLRVSWDTAFTYAAKAMITIATRYSGEAGARRLREQGYAPEMIEMMKGAGTRCFKHRAGMPVLGIIGKMGNT 7ak5.1    ------GLLTYTSWEDALSRVAGMLQNF----EGNAVA--------------AIAGGLVD--------------------  target    RMNGGINALLDTWIRKVSPDQAQGGRYWSNYTWHGDQNPAHPFWSGVQGSDIDLSDMRFSKLNTSWGKNFVENKMPEAHW 7ak5.1    ---AEALVALKDLLNKVDSD-----NLCTEE-IFPTEGAGTDLRS-NYLLNTTIAGVEEADVVLLVGTNPRFEAPLFNAR  target    KLECI-ERGARVVVITPEYNPTAYRADYWMPLRPESDGALFLGAMKIIIDENMHDIDFLKSFTDAPILVRTDTLQYLDPR 7ak5.1    IRKSWLHNDLKVALIGSPVDLTYRYDHLGDSP------------------------KILQDIASG---------------  target    DVIADYKFPDFSKSYSGRIQSLKPEQIQRLGGMMVWDLNKKQVVPLHREQVGWHYTNSGIDAALTGTYRVKLLNGREIDA 7ak5.1    --------------------------------------------------------------------------------  target    MPIWQMYMVHFQDYDLDTVHQITRTPKDLIVRWARDSGTIKPAAIHNGEGTCHYFHQTANARGAAMVLIITGNVGKFGTG 7ak5.1    -----------------------------RHSFCEVLKDAKKPMVVLGSSALQRDDGAAILVAVSNMVQKIRVTTGVAAE  target    QHTWAGNYKAGTWTATPWSGAGLSVHTGEDPFNITLDPNAHGKEIKTRSYYYGEEVGYWNHGDTALIVNTPKYGRKVFTG 7ak5.1    WK---V------MN--ILH-------RIASQVA--------ALDL-----------GYK-----P------GV-EAI---  target    KTHMPTPSKFRWVVNVNVVNNAKHHYDMVRNVDPNIECLITQDIEMTSDINHADIAFAANSWMEFTYPEMTVTVSNPWVQ 7ak5.1    ---RKNPPKMLFLLGADGGC-------ITRQDLPKDCFIVYQGHHGDVGAPMADVILPGAAYTEKS---ATYVNTEGRAQ  target    IWKGGIRPLYDTRNDLDTFAGVAAKLSDMTGDKRMRDYFAMVYQNRVDVYVQRMLDASSTFYGYSADVMLKSEKGWMVMV 7ak5.1    QTKVAVTPPGLAREDWKIIRALSEIAG-----------------------------------------------------  target    RTYPRHPFWEETNESKPMWTRSGRYENYRIEPEAIEYGENFISHREGPEATPYLPNAIFTTNPYVRPDDYGIPITAQHHD 7ak5.1    --------------------------------------------------------------------------------  target    DKTVRNIKLSWHEIKRHSNPLWEKGYQFYCVTPKTRHRVHSQWSVNDWVQIYESNFGDPYRMDKRTPGVGEHQIHINPQA 7ak5.1    --------------------------------------------------------------------------------  target    AKDRGINDGDYVYVDGNPVDRPYRGWKPSDPYYKVARLMIRAKYNPAYPYHVTMAKHAPFVATAKSVKGHETRPDGRAIA 7ak5.1    --------------------------------------------------------------------------------  target    IDTGYQSNFRYGAQQSFTRNWLMPMHQTDSLPGKHAVAWKFKWGYQVDHHAINTVPKECLIRITKAEDGGIGARGPWEPV 7ak5.1    --------------------------------------------------------------------------------  target    RTGFTPGQENEFMIKWLKGEHIKIKV 7ak5.1    -------------------------- ``` | | | | | | | | | | | | | | | | | | | | | | | | | | | | | | | | | | | | | | | | | | | | | | | | | |
|  | 7dgr.10.A | NADH-ubiquinone oxidoreductase 75 kDa subunit, mitochondrial  *Activity optimized supercomplex state2* | 0.13 | 0.00 | 12.01 | 0.33 | 62-746 | EM | 0.00 | monomer |  | HHblits | 0.25 |
| ``` target    MFLSRRQFLKVSVGTVAAVAVADKVLALTALQPVIEVGNPLGDYPDRSWERVYHDQYRYDSSFTWVCSPNDTHACRVRAF 7dgr.10   -------------------------------------------------------------KTESIDV-MDAVGSNIVVS  target    VRNGVVMRVEQNYDHQTYEDLYGNRGTFAHNPRMCLKGFTFHRRVYGPYRLKGPLMRKGWKQWMDDNAPELTAETKRKYK 7dgr.10   TRTGEVMRILPRMHEDI------------NEEWISDKTRFAYDGLK-RQRLTEPMVRNEK--------------------  target    FDSRFLDDMLRVSWDTAFTYAAKAMITIATRYSGEAGARRLREQGYAPEMIEMMKGAGTRCFKHRAGMPVLGIIGKMGNT 7dgr.10   ------GLLTHTTWEDALSRVAGMLQSF----QGNDVA--------------AIAGGLVD--------------------  target    RMNGGINALLDTWIRKVSPD-QAQGGRYWSNYTWHGDQNPAHPFWSGVQGSDIDLSDMRFSKLNTSWGKNFVENKMPEAH 7dgr.10   ---AEALIALKDLLNRVDSDTLCTEEV----FP---TAGAGTDL-RSNYLLNTTIAGVEEADVVLLVGTNPRFEAPLFNA  target    WKLECIE-RGARVVVITPEYNPTAYRADYWMPLRPESDGALFLGAMKIIIDENMHDIDFLKSFTDAPILVRTDTLQYLDP 7dgr.10   RIRKSWLHNDLKVALIGSPVDLTYRYDHLGDSPKILQDIA------------------------SG--------------  target    RDVIADYKFPDFSKSYSGRIQSLKPEQIQRLGGMMVWDLNKKQVVPLHREQVGWHYTNSGIDAALTGTYRVKLLNGREID 7dgr.10   --------------------------------------------------------------------------------  target    AMPIWQMYMVHFQDYDLDTVHQITRTPKDLIVRWARDSGTIKPAAIHNGEGTCHYFHQTANARGAAMVLIITGNVGKFGT 7dgr.10   ------------------------------SHPFSQVLQEAKKPMVILGSSALQRNDGAAILAAVSNIAQKIRTSSGVTG  target    GQHTWAGNYKAGTWTATPWSGAGLSVHTGEDPFNITLDPNAHGKEIKTRSYYYGEEVGYWNHGDTALIVNTPKYGRKVFT 7dgr.10   DWKVMN--I----L-----H-------RIASQV--------AALDL-----------GYKP------------GVEAI--  target    GKTHMPTPSKFRWVVNVNVVNNAKHHYDMVRNVDPNIECLITQDIEMTSDINHADIAFAANSWMEFTYPEMTVTVSNPWV 7dgr.10   ----QKNPPKMLFLLGADGGC-------ITRQDLPKDCFIVYQGHHGDVGAPIADVILPGAAYTEKS---ATYVNTEGRA  target    QIWKGGIRPLYDTRNDLDTFAGVAAKLSDMTGDKRMRDYFAMVYQNRVDVYVQRMLDASSTFYGYSADVMLKSEKGWMVM 7dgr.10   QQTKVAVTPPGLAREDWKIIRALSEIAG----------------------------------------------------  target    VRTYPRHPFWEETNESKPMWTRSGRYENYRIEPEAIEYGENFISHREGPEATPYLPNAIFTTNPYVRPDDYGIPITAQHH 7dgr.10   --------------------------------------------------------------------------------  target    DDKTVRNIKLSWHEIKRHSNPLWEKGYQFYCVTPKTRHRVHSQWSVNDWVQIYESNFGDPYRMDKRTPGVGEHQIHINPQ 7dgr.10   --------------------------------------------------------------------------------  target    AAKDRGINDGDYVYVDGNPVDRPYRGWKPSDPYYKVARLMIRAKYNPAYPYHVTMAKHAPFVATAKSVKGHETRPDGRAI 7dgr.10   --------------------------------------------------------------------------------  target    AIDTGYQSNFRYGAQQSFTRNWLMPMHQTDSLPGKHAVAWKFKWGYQVDHHAINTVPKECLIRITKAEDGGIGARGPWEP 7dgr.10   --------------------------------------------------------------------------------  target    VRTGFTPGQENEFMIKWLKGEHIKIKV 7dgr.10   --------------------------- ``` | | | | | | | | | | | | | | | | | | | | | | | | | | | | | | | | | | | | | | | | | | | | | | | | | |
|  | 5o31.1.8 | NADH-ubiquinone oxidoreductase 75 kDa subunit, mitochondrial  *Mitochondrial complex I in the deactive state* | 0.14 | 0.00 | 12.01 | 0.33 | 62-746 | EM | 4.13 | monomer | 6 x SF4, 2 x FES, 1 x FMN, 1 x NAP, 1 x ZN | HHblits | 0.25 |
| ``` target    MFLSRRQFLKVSVGTVAAVAVADKVLALTALQPVIEVGNPLGDYPDRSWERVYHDQYRYDSSFTWVCSPNDTHACRVRAF 5o31.1    -------------------------------------------------------------KTESIDV-MDAVGSNIVVS  target    VRNGVVMRVEQNYDHQTYEDLYGNRGTFAHNPRMCLKGFTFHRRVYGPYRLKGPLMRKGWKQWMDDNAPELTAETKRKYK 5o31.1    TRTGEVMRILPRMHEDI------------NEEWISDKTRFAYDGLK-RQRLTEPMVRNEK--------------------  target    FDSRFLDDMLRVSWDTAFTYAAKAMITIATRYSGEAGARRLREQGYAPEMIEMMKGAGTRCFKHRAGMPVLGIIGKMGNT 5o31.1    ------GLLTHTTWEDALSRVAGMLQSF----QGNDVA--------------AIAGGLVD--------------------  target    RMNGGINALLDTWIRKVSPD-QAQGGRYWSNYTWHGDQNPAHPFWSGVQGSDIDLSDMRFSKLNTSWGKNFVENKMPEAH 5o31.1    ---AEALIALKDLLNRVDSDTLCTEEV----FP---TAGAGTDL-RSNYLLNTTIAGVEEADVVLLVGTNPRFEAPLFNA  target    WKLECIE-RGARVVVITPEYNPTAYRADYWMPLRPESDGALFLGAMKIIIDENMHDIDFLKSFTDAPILVRTDTLQYLDP 5o31.1    RIRKSWLHNDLKVALIGSPVDLTYRYDHLGDSPKILQDIA------------------------SG--------------  target    RDVIADYKFPDFSKSYSGRIQSLKPEQIQRLGGMMVWDLNKKQVVPLHREQVGWHYTNSGIDAALTGTYRVKLLNGREID 5o31.1    --------------------------------------------------------------------------------  target    AMPIWQMYMVHFQDYDLDTVHQITRTPKDLIVRWARDSGTIKPAAIHNGEGTCHYFHQTANARGAAMVLIITGNVGKFGT 5o31.1    ------------------------------SHPFSQVLQEAKKPMVILGSSALQRNDGAAILAAVSNIAQKIRTSSGVTG  target    GQHTWAGNYKAGTWTATPWSGAGLSVHTGEDPFNITLDPNAHGKEIKTRSYYYGEEVGYWNHGDTALIVNTPKYGRKVFT 5o31.1    DWKVMN--I----L-----H-------RIASQV--------AALDL-----------GYKP------------GVEAI--  target    GKTHMPTPSKFRWVVNVNVVNNAKHHYDMVRNVDPNIECLITQDIEMTSDINHADIAFAANSWMEFTYPEMTVTVSNPWV 5o31.1    ----QKNPPKMLFLLGADGGC-------ITRQDLPKDCFIVYQGHHGDVGAPIADVILPGAAYTEKS---ATYVNTEGRA  target    QIWKGGIRPLYDTRNDLDTFAGVAAKLSDMTGDKRMRDYFAMVYQNRVDVYVQRMLDASSTFYGYSADVMLKSEKGWMVM 5o31.1    QQTKVAVTPPGLAREDWKIIRALSEIAG----------------------------------------------------  target    VRTYPRHPFWEETNESKPMWTRSGRYENYRIEPEAIEYGENFISHREGPEATPYLPNAIFTTNPYVRPDDYGIPITAQHH 5o31.1    --------------------------------------------------------------------------------  target    DDKTVRNIKLSWHEIKRHSNPLWEKGYQFYCVTPKTRHRVHSQWSVNDWVQIYESNFGDPYRMDKRTPGVGEHQIHINPQ 5o31.1    --------------------------------------------------------------------------------  target    AAKDRGINDGDYVYVDGNPVDRPYRGWKPSDPYYKVARLMIRAKYNPAYPYHVTMAKHAPFVATAKSVKGHETRPDGRAI 5o31.1    --------------------------------------------------------------------------------  target    AIDTGYQSNFRYGAQQSFTRNWLMPMHQTDSLPGKHAVAWKFKWGYQVDHHAINTVPKECLIRITKAEDGGIGARGPWEP 5o31.1    --------------------------------------------------------------------------------  target    VRTGFTPGQENEFMIKWLKGEHIKIKV 5o31.1    --------------------------- ``` | | | | | | | | | | | | | | | | | | | | | | | | | | | | | | | | | | | | | | | | | | | | | | | | | |
|  | 7qsd.1.G | NADH-ubiquinone oxidoreductase 75 kDa subunit, mitochondrial  *Bovine complex I in the active state at 3.1 A* | 0.15 | 0.00 | 11.75 | 0.33 | 62-746 | EM | 0.00 | monomer | 5 x PC1, 13 x 3PE, 6 x SF4, 2 x FES, 1 x FMN, 4 x CDL, 3 x LMT, 1 x GTP, 1 x MG, 1 x NDP, 1 x ZN, 2 x EHZ | HHblits | 0.25 |
| ``` target    MFLSRRQFLKVSVGTVAAVAVADKVLALTALQPVIEVGNPLGDYPDRSWERVYHDQYRYDSSFTWVCSPNDTHACRVRAF 7qsd.1    -------------------------------------------------------------KTESIDV-MDAVGSNIVVS  target    VRNGVVMRVEQNYDHQTYEDLYGNRGTFAHNPRMCLKGFTFHRRVYGPYRLKGPLMRKGWKQWMDDNAPELTAETKRKYK 7qsd.1    TRTGEVMRILPRMHEDI------------NEEWISDKTRFAYDGLK-RQRLTEPMVRNEK--------------------  target    FDSRFLDDMLRVSWDTAFTYAAKAMITIATRYSGEAGARRLREQGYAPEMIEMMKGAGTRCFKHRAGMPVLGIIGKMGNT 7qsd.1    ------GLLTHTTWEDALSRVAGMLQSF----QGNDVA--------------AIAGGLVD--------------------  target    RMNGGINALLDTWIRKVSPD-QAQGGRYWSNYTWHGDQNPAHPFWSGVQGSDIDLSDMRFSKLNTSWGKNFVENKMPEAH 7qsd.1    ---AEALIALKDLLNRVDSDTLCTEEVFP-------TAGAGTDL-RSNYLLNTTIAGVEEADVVLLVGTNPRFEAPLFNA  target    WKLECIE-RGARVVVITPEYNPTAYRADYWMPLRPESDGALFLGAMKIIIDENMHDIDFLKSFTDAPILVRTDTLQYLDP 7qsd.1    RIRKSWLHNDLKVALIGSPVDLTYRYDHLGDSPKILQD------------------------IASG--------------  target    RDVIADYKFPDFSKSYSGRIQSLKPEQIQRLGGMMVWDLNKKQVVPLHREQVGWHYTNSGIDAALTGTYRVKLLNGREID 7qsd.1    --------------------------------------------------------------------------------  target    AMPIWQMYMVHFQDYDLDTVHQITRTPKDLIVRWARDSGTIKPAAIHNGEGTCHYFHQTANARGAAMVLIITGNVGKFGT 7qsd.1    ------------------------------SHPFSQVLQEAKKPMVILGSSALQRNDGAAILAAVSNIAQKIRTSSGVTG  target    GQHTWAGNYKAGTWTATPWSGAGLSVHTGEDPFNITLDPNAHGKEIKTRSYYYGEEVGYWNHGDTALIVNTPKYGRKVFT 7qsd.1    DWKVM---N----I----LH-------RIASQV---------AA-L---------DLGYKP------------GVEAI--  target    GKTHMPTPSKFRWVVNVNVVNNAKHHYDMVRNVDPNIECLITQDIEMTSDINHADIAFAANSWMEFTYPEMTVTVSNPWV 7qsd.1    ----QKNPPKMLFLLGADGGC-------ITRQDLPKDCFIVYQGHHGDVGAPIADVILPGAAYTEKS---ATYVNTEGRA  target    QIWKGGIRPLYDTRNDLDTFAGVAAKLSDMTGDKRMRDYFAMVYQNRVDVYVQRMLDASSTFYGYSADVMLKSEKGWMVM 7qsd.1    QQTKVAVTPPGLAREDWKIIRALSEIAG----------------------------------------------------  target    VRTYPRHPFWEETNESKPMWTRSGRYENYRIEPEAIEYGENFISHREGPEATPYLPNAIFTTNPYVRPDDYGIPITAQHH 7qsd.1    --------------------------------------------------------------------------------  target    DDKTVRNIKLSWHEIKRHSNPLWEKGYQFYCVTPKTRHRVHSQWSVNDWVQIYESNFGDPYRMDKRTPGVGEHQIHINPQ 7qsd.1    --------------------------------------------------------------------------------  target    AAKDRGINDGDYVYVDGNPVDRPYRGWKPSDPYYKVARLMIRAKYNPAYPYHVTMAKHAPFVATAKSVKGHETRPDGRAI 7qsd.1    --------------------------------------------------------------------------------  target    AIDTGYQSNFRYGAQQSFTRNWLMPMHQTDSLPGKHAVAWKFKWGYQVDHHAINTVPKECLIRITKAEDGGIGARGPWEP 7qsd.1    --------------------------------------------------------------------------------  target    VRTGFTPGQENEFMIKWLKGEHIKIKV 7qsd.1    --------------------------- ``` | | | | | | | | | | | | | | | | | | | | | | | | | | | | | | | | | | | | | | | | | | | | | | | | | |
|  | 6zk9.1.C | NADH:ubiquinone oxidoreductase core subunit S1  *Peripheral domain of open complex I during turnover* | 0.16 | 0.00 | 11.75 | 0.33 | 62-746 | EM | 0.00 | monomer | 6 x SF4, 1 x FMN, 1 x NAI, 2 x FES, 1 x K, 2 x PC1, 2 x 3PE, 1 x ZN, 1 x NDP, 1 x ZMP, 1 x CDL | HHblits | 0.25 |
| ``` target    MFLSRRQFLKVSVGTVAAVAVADKVLALTALQPVIEVGNPLGDYPDRSWERVYHDQYRYDSSFTWVCSPNDTHACRVRAF 6zk9.1    -------------------------------------------------------------KTESIDV-MDAVGSNIVVS  target    VRNGVVMRVEQNYDHQTYEDLYGNRGTFAHNPRMCLKGFTFHRRVYGPYRLKGPLMRKGWKQWMDDNAPELTAETKRKYK 6zk9.1    TRTGEVMRILPRMHEDI------------NEEWISDKTRFAYDGLK-RQRLTEPMVRNEK--------------------  target    FDSRFLDDMLRVSWDTAFTYAAKAMITIATRYSGEAGARRLREQGYAPEMIEMMKGAGTRCFKHRAGMPVLGIIGKMGNT 6zk9.1    ------GLLTHTTWEDALSRVAGMLQSC----QGNDVA--------------AIAGGLVD--------------------  target    RMNGGINALLDTWIRKVSPD-QAQGGRYWSNYTWHGDQNPAHPFWSGVQGSDIDLSDMRFSKLNTSWGKNFVENKMPEAH 6zk9.1    ---AEALIALKDLLNRVDSDTLCTEEVFP-------TAGAGTDL-RSNYLLNTTIAGVEEADVVLLVGTNPRFEAPLFNA  target    WKLECIE-RGARVVVITPEYNPTAYRADYWMPLRPESDGALFLGAMKIIIDENMHDIDFLKSFTDAPILVRTDTLQYLDP 6zk9.1    RIRKSWLHNDLKVALIGSPVDLTYRYDHLGD------SP------------------KILQDIASG--------------  target    RDVIADYKFPDFSKSYSGRIQSLKPEQIQRLGGMMVWDLNKKQVVPLHREQVGWHYTNSGIDAALTGTYRVKLLNGREID 6zk9.1    --------------------------------------------------------------------------------  target    AMPIWQMYMVHFQDYDLDTVHQITRTPKDLIVRWARDSGTIKPAAIHNGEGTCHYFHQTANARGAAMVLIITGNVGKFGT 6zk9.1    ------------------------------SHPFSQVLQEAKKPMVVLGSSALQRNDGAAILAAVSNIAQKIRTSSGVTG  target    GQHTWAGNYKAGTWTATPWSGAGLSVHTGEDPFNITLDPNAHGKEIKTRSYYYGEEVGYWNHGDTALIVNTPKYGRKVFT 6zk9.1    DWKVMN--I----L-----H-------RIASQV--------AALDL-----------GYKP------------GVEAIR-  target    GKTHMPTPSKFRWVVNVNVVNNAKHHYDMVRNVDPNIECLITQDIEMTSDINHADIAFAANSWMEFTYPEMTVTVSNPWV 6zk9.1    -----KNPPKMLFLLGADGGC-------VTRQDLPKDCFIVYQGHHGDVGAPIADVILPGAAYTEKS---ATYVNTEGRA  target    QIWKGGIRPLYDTRNDLDTFAGVAAKLSDMTGDKRMRDYFAMVYQNRVDVYVQRMLDASSTFYGYSADVMLKSEKGWMVM 6zk9.1    QQTKVAVMPPGLAREDWKIIRALSEIAG----------------------------------------------------  target    VRTYPRHPFWEETNESKPMWTRSGRYENYRIEPEAIEYGENFISHREGPEATPYLPNAIFTTNPYVRPDDYGIPITAQHH 6zk9.1    --------------------------------------------------------------------------------  target    DDKTVRNIKLSWHEIKRHSNPLWEKGYQFYCVTPKTRHRVHSQWSVNDWVQIYESNFGDPYRMDKRTPGVGEHQIHINPQ 6zk9.1    --------------------------------------------------------------------------------  target    AAKDRGINDGDYVYVDGNPVDRPYRGWKPSDPYYKVARLMIRAKYNPAYPYHVTMAKHAPFVATAKSVKGHETRPDGRAI 6zk9.1    --------------------------------------------------------------------------------  target    AIDTGYQSNFRYGAQQSFTRNWLMPMHQTDSLPGKHAVAWKFKWGYQVDHHAINTVPKECLIRITKAEDGGIGARGPWEP 6zk9.1    --------------------------------------------------------------------------------  target    VRTGFTPGQENEFMIKWLKGEHIKIKV 6zk9.1    --------------------------- ``` | | | | | | | | | | | | | | | | | | | | | | | | | | | | | | | | | | | | | | | | | | | | | | | | | |
|  | 7arc.1.F | 75 kDa  *Cryo-EM structure of Polytomella Complex-I (peripheral arm)* | 0.15 |  | 13.26 | 0.33 | 62-746 | EM | 0.00 | hetero-1-1-1-1-1-1-… | 6 x SF4, 2 x FES, 1 x FMN, 1 x NDP, 1 x ZN, 1 x 8Q1 | HHblits | 0.26 |
| ``` target    MFLSRRQFLKVSVGTVAAVAVADKVLALTALQPVIEVGNPLGDYPDRSWERVYHDQYRYDSSFTWVCSPNDTHACRVRAF 7arc.1    -------------------------------------------------------------GTETIDV-SDALGSNIKVD  target    VRNGVVMRVEQNYDHQTYEDLYGNRGTFAHNPRMCLKGFTFHRRVYGPYRLKGPLMRKGWKQWMDDNAPELTAETKRKYK 7arc.1    CRGTEVMRITPRLNDAI------------NEEWLSDKGRFQYDGLK-RQRLNTPLVKGA---------------------  target    FDSRFLDDMLRVSWDTAFTYAAKAMITIATRYSGEAGARRLREQGYAPEMIEMMKGAGTRCFKHRAGMPVLGIIGKMGNT 7arc.1    ------KGLENATWSAAFDAIRTAIAGAK----GNEL--------------KAIAGKLADAESMI---ALKDLFNKLGSG  target    RMNGGINALLDTWIRKVSPDQAQGGRYWSNYTWHGDQNPAHPFWSGVQGSDIDLSDMRFSKLNTSWGKNFVENKMPEAHW 7arc.1    NLI-------------------HEDGS---ATL-------SADVRSSYIANTTIASIEKADVILLVGTNPRFESPVFNAR  target    KLECIERGARVVVITPEYNPTAYRADYWMPLRPESDGALFLGAMKIIIDENMHDIDFLKSFTDAPILVRTDTLQYLDPRD 7arc.1    LRKVFLDGAKVGLVGEKVDLT------YAYQHLGADVAALESLASGK---------------------------------  target    VIADYKFPDFSKSYSGRIQSLKPEQIQRLGGMMVWDLNKKQVVPLHREQVGWHYTNSGIDAALTGTYRVKLLNGREIDAM 7arc.1    --------------------------------------------------------------------------------  target    PIWQMYMVHFQDYDLDTVHQITRTPKDLIVRWARDSGTIKPAAIHNGEGTCHYFHQTANARGAAMVLIITGNVGKFGTGQ 7arc.1    -----------------------------GAFFEALKGAKNPVVIVGSSVLRRDDREAVLKTVNDLVDAAGVVKEGWNGF  target    HTWAGNYKAGTWTATPWSGAGLSVHTGEDPFNITLDPNAHGKEIKTRSYYYGEEVGYWNHGDTALIVNTPKYGRKVFTGK 7arc.1    NVLHDNASR----------------VAA--L-----------DIG-----------FVPSA-----S----------A--  target    THMPTPSKFRWVVNVNVVNNAKHHYDMVRNVDPNIECLITQDIEMTSDINHADIAFAANSWMEFTYPEMTVTVSNPWVQI 7arc.1    RTNPVPAKVVYLLGSDDFKD---------EEIPADAFVIYQGHHGDKGAARANVVLPGAAYTEKAS---LFANTEGRVQT  target    WKGGIRPLYDTRNDLDTFAGVAAKLSDMTGDKRMRDYFAMVYQNRVDVYVQRMLDASSTFYGYSADVMLKSEKGWMVMVR 7arc.1    TRTAVPVLGDAREDWKIIRALSEVVG------------------------------------------------------  target    TYPRHPFWEETNESKPMWTRSGRYENYRIEPEAIEYGENFISHREGPEATPYLPNAIFTTNPYVRPDDYGIPITAQHHDD 7arc.1    --------------------------------------------------------------------------------  target    KTVRNIKLSWHEIKRHSNPLWEKGYQFYCVTPKTRHRVHSQWSVNDWVQIYESNFGDPYRMDKRTPGVGEHQIHINPQAA 7arc.1    --------------------------------------------------------------------------------  target    KDRGINDGDYVYVDGNPVDRPYRGWKPSDPYYKVARLMIRAKYNPAYPYHVTMAKHAPFVATAKSVKGHETRPDGRAIAI 7arc.1    --------------------------------------------------------------------------------  target    DTGYQSNFRYGAQQSFTRNWLMPMHQTDSLPGKHAVAWKFKWGYQVDHHAINTVPKECLIRITKAEDGGIGARGPWEPVR 7arc.1    --------------------------------------------------------------------------------  target    TGFTPGQENEFMIKWLKGEHIKIKV 7arc.1    ------------------------- ``` | | | | | | | | | | | | | | | | | | | | | | | | | | | | | | | | | | | | | | | | | | | | | | | | | |
|  | 7zm7.1.I | NADH-ubiquinone oxidoreductase-like protein  *CryoEM structure of mitochondrial complex I from Chaetomium thermophilum (inhibited by DDM)* | 0.15 |  | 15.04 | 0.33 | 62-746 | EM | 0.00 | hetero-1-1-1-1-1-1-… | 4 x PC1, 14 x LMT, 5 x CDL, 8 x 3PE, 2 x FES, 6 x SF4, 1 x FMN, 1 x NDP, 1 x ZN, 2 x ZMP | HHblits | 0.26 |
| ``` target    MFLSRRQFLKVSVGTVAAVAVADKVLALTALQPVIEVGNPLGDYPDRSWERVYHDQYRYDSSFTWVCSPNDTHACRVRAF 7zm7.1    -------------------------------------------------------------RTESIDV-LDGLGSNIRVD  target    VRNGVVMRVEQNYDHQTYEDLYGNRGTFAHNPRMCLKGFTFHRRVYGPYRLKGPLMRKGWKQWMDDNAPELTAETKRKYK 7zm7.1    SRGLEVMRILPRLNDDV------------NEEWINDKTRFACDGLK-TQRLTMPLVRRD---------------------  target    FDSRFLDDMLRVSWDTAFTYAAKAMITIATRYSGEAGARRLREQGYAPEMIEMMKGAGTRCFKHRAGMPVLGIIGKMGNT 7zm7.1    ------GKFEPATWEQALTEIAHAYQTLAPKENEFKV----------------IAGQLVEV-------------------  target    RMNGGINALLDTWIRKVSPDQAQGGRYWSNYTWHGDQNPAHPFWSGV-QGSDIDLSDMRFSKLNTSWGKNFVENKMPEAH 7zm7.1    ----ESLVAMKDLANRL------GSENLALDFPGGSQPLAHGVDIRSNYLFNSKIWGIEEADAILLVGTNPRHEAAVLNA  target    WKLECI-ERGARVVVITPEYNPTAYRADYWMPLRPESDGALFLGAMKIIIDENMHDIDFLKSFTDAPILVRTDTLQYLDP 7zm7.1    RIRKQWLRSDLEIAAVGQPWESTFDYEH------LGTDLAALKNALSGPF------------------------------  target    RDVIADYKFPDFSKSYSGRIQSLKPEQIQRLGGMMVWDLNKKQVVPLHREQVGWHYTNSGIDAALTGTYRVKLLNGREID 7zm7.1    --------------------------------------------------------------------------------  target    AMPIWQMYMVHFQDYDLDTVHQITRTPKDLIVRWARDSGTIKPAAIHNGEGTCHYFHQTANARGAAMVLIITGN--VGKF 7zm7.1    ----------------------------------GEKLKKAKRPMIIVGSGVTEHPDAKAFYETVWSFVEKNASNFLTEE  target    GTGQHTWAGNYKAGTWTATPWSGAGLSVHTGEDPFNITLDPNAHGKEIKTRSYYYGEEVGYWNHGDTALIVNTPKYGRKV 7zm7.1    WCGYNVLQRAA---------------------SR---------AGA-FE---------VGFVVP------------SPE-  target    FTGKTHMPTPSKFRWVVNVNVVNNAKHHYDMVRNVDPNIECLITQDIEMTSDINHADIAFAANSWMEFTYPEMTVTVSNP 7zm7.1    -----VAATKPKFVWLLGADEFDP---------ADVPKDAFIVYQGHHGDRGAEIADIVLPGAAYTEKA---GTYVNTEG  target    WVQIWKGGIRPLYDTRNDLDTFAGVAAKLSDMTGDKRMRDYFAMVYQNRVDVYVQRMLDASSTFYGYSADVMLKSEKGWM 7zm7.1    RVQMTRAATGLPGAARTDWKIIRAVSEFLG--------------------------------------------------  target    VMVRTYPRHPFWEETNESKPMWTRSGRYENYRIEPEAIEYGENFISHREGPEATPYLPNAIFTTNPYVRPDDYGIPITAQ 7zm7.1    --------------------------------------------------------------------------------  target    HHDDKTVRNIKLSWHEIKRHSNPLWEKGYQFYCVTPKTRHRVHSQWSVNDWVQIYESNFGDPYRMDKRTPGVGEHQIHIN 7zm7.1    --------------------------------------------------------------------------------  target    PQAAKDRGINDGDYVYVDGNPVDRPYRGWKPSDPYYKVARLMIRAKYNPAYPYHVTMAKHAPFVATAKSVKGHETRPDGR 7zm7.1    --------------------------------------------------------------------------------  target    AIAIDTGYQSNFRYGAQQSFTRNWLMPMHQTDSLPGKHAVAWKFKWGYQVDHHAINTVPKECLIRITKAEDGGIGARGPW 7zm7.1    --------------------------------------------------------------------------------  target    EPVRTGFTPGQENEFMIKWLKGEHIKIKV 7zm7.1    ----------------------------- ``` | | | | | | | | | | | | | | | | | | | | | | | | | | | | | | | | | | | | | | | | | | | | | | | | | |
|  | 7zd6.1.4 | NADH-ubiquinone oxidoreductase 75 kDa subunit, mitochondrial  *Complex I from Ovis aries, at pH7.4, Open state* | 0.15 | 0.00 | 11.75 | 0.33 | 62-746 | EM | 0.00 | monomer | 6 x PC1, 14 x 3PE, 1 x DCQ, 2 x ZMP, 1 x AMP, 1 x MYR, 6 x SF4, 1 x FMN, 1 x NAI, 2 x FES, 1 x K, 1 x ZN, 1 x NDP | HHblits | 0.25 |
| ``` target    MFLSRRQFLKVSVGTVAAVAVADKVLALTALQPVIEVGNPLGDYPDRSWERVYHDQYRYDSSFTWVCSPNDTHACRVRAF 7zd6.1    -------------------------------------------------------------KTESIDV-MDAVGSNIVVS  target    VRNGVVMRVEQNYDHQTYEDLYGNRGTFAHNPRMCLKGFTFHRRVYGPYRLKGPLMRKGWKQWMDDNAPELTAETKRKYK 7zd6.1    TRTGEVMRILPRMHEDI------------NEEWISDKTRFAYDGLK-RQRLTEPMVRNEK--------------------  target    FDSRFLDDMLRVSWDTAFTYAAKAMITIATRYSGEAGARRLREQGYAPEMIEMMKGAGTRCFKHRAGMPVLGIIGKMGNT 7zd6.1    ------GLLTHTTWEDALSRVAGMLQSC----QGNDVA--------------AIAGGLVD--------------------  target    RMNGGINALLDTWIRKVSPD-QAQGGRYWSNYTWHGDQNPAHPFWSGVQGSDIDLSDMRFSKLNTSWGKNFVENKMPEAH 7zd6.1    ---AEALIALKDLLNRVDSDTLCTEEVFP-------TAGAGTDL-RSNYLLNTTIAGVEEADVVLLVGTNPRFEAPLFNA  target    WKLECIE-RGARVVVITPEYNPTAYRADYWMPLRPESDGALFLGAMKIIIDENMHDIDFLKSFTDAPILVRTDTLQYLDP 7zd6.1    RIRKSWLHNDLKVALIGSPVDLTYRYDHLGD------SP------------------KILQDIASG--------------  target    RDVIADYKFPDFSKSYSGRIQSLKPEQIQRLGGMMVWDLNKKQVVPLHREQVGWHYTNSGIDAALTGTYRVKLLNGREID 7zd6.1    --------------------------------------------------------------------------------  target    AMPIWQMYMVHFQDYDLDTVHQITRTPKDLIVRWARDSGTIKPAAIHNGEGTCHYFHQTANARGAAMVLIITGNVGKFGT 7zd6.1    ------------------------------SHPFSQVLQEAKKPMVVLGSSALQRNDGAAILAAVSNIAQKIRTSSGVTG  target    GQHTWAGNYKAGTWTATPWSGAGLSVHTGEDPFNITLDPNAHGKEIKTRSYYYGEEVGYWNHGDTALIVNTPKYGRKVFT 7zd6.1    DWKVM---N----I----LH-------RIASQV--------AALDL-----------GYKP------------GVEAIR-  target    GKTHMPTPSKFRWVVNVNVVNNAKHHYDMVRNVDPNIECLITQDIEMTSDINHADIAFAANSWMEFTYPEMTVTVSNPWV 7zd6.1    -----KNPPKMLFLLGADGGC-------VTRQDLPKDCFIVYQGHHGDVGAPIADVILPGAAYTEKS---ATYVNTEGRA  target    QIWKGGIRPLYDTRNDLDTFAGVAAKLSDMTGDKRMRDYFAMVYQNRVDVYVQRMLDASSTFYGYSADVMLKSEKGWMVM 7zd6.1    QQTKVAVMPPGLAREDWKIIRALSEIAG----------------------------------------------------  target    VRTYPRHPFWEETNESKPMWTRSGRYENYRIEPEAIEYGENFISHREGPEATPYLPNAIFTTNPYVRPDDYGIPITAQHH 7zd6.1    --------------------------------------------------------------------------------  target    DDKTVRNIKLSWHEIKRHSNPLWEKGYQFYCVTPKTRHRVHSQWSVNDWVQIYESNFGDPYRMDKRTPGVGEHQIHINPQ 7zd6.1    --------------------------------------------------------------------------------  target    AAKDRGINDGDYVYVDGNPVDRPYRGWKPSDPYYKVARLMIRAKYNPAYPYHVTMAKHAPFVATAKSVKGHETRPDGRAI 7zd6.1    --------------------------------------------------------------------------------  target    AIDTGYQSNFRYGAQQSFTRNWLMPMHQTDSLPGKHAVAWKFKWGYQVDHHAINTVPKECLIRITKAEDGGIGARGPWEP 7zd6.1    --------------------------------------------------------------------------------  target    VRTGFTPGQENEFMIKWLKGEHIKIKV 7zd6.1    --------------------------- ``` | | | | | | | | | | | | | | | | | | | | | | | | | | | | | | | | | | | | | | | | | | | | | | | | | |
|  | 5gpn.24.A | NADH-ubiquinone oxidoreductase 75 kDa subunit  *Architecture of mammalian respirasome* | 0.14 |  | 12.01 | 0.33 | 63-746 | EM | 0.00 | monomer |  | HHblits | 0.25 |
| ``` target    MFLSRRQFLKVSVGTVAAVAVADKVLALTALQPVIEVGNPLGDYPDRSWERVYHDQYRYDSSFTWVCSPNDTHACRVRAF 5gpn.24   --------------------------------------------------------------TESIDV-MDAVGSNIVVS  target    VRNGVVMRVEQNYDHQTYEDLYGNRGTFAHNPRMCLKGFTFHRRVYGPYRLKGPLMRKGWKQWMDDNAPELTAETKRKYK 5gpn.24   TRTGEVMRILPRMHEDI------------NEEWISDKTRFAYDGLK-RQRLTQPMIRNEK--------------------  target    FDSRFLDDMLRVSWDTAFTYAAKAMITIATRYSGEAGARRLREQGYAPEMIEMMKGAGTRCFKHRAGMPVLGIIGKMGNT 5gpn.24   ------GLLTYTTWEDALSRVAGMLQSF----QGNDVA--------------AIAGGLVDA-------------------  target    RMNGGINALLDTWIRKVSPDQAQGGRYWSNYTWHGDQNPAHPFWSGVQGSDIDLSDMRFSKLNTSWGKNFVENKMPEAHW 5gpn.24   ----EALVALKDLLNRVD------SDSLCTEEVFPTAGAGTDL-RSNYLLNTTIAGVEEADVILLVGTNPRFEAPLFNAR  target    KLECIE-RGARVVVITPEYNPTAYRADYWMPLRPESDGALFLGAMKIIIDENMHDIDFLKSFTDAPILVRTDTLQYLDPR 5gpn.24   IRKSWLHNDLKVALIGSPVDLTYRYDHLGDSPK------------------------ILQDIASG---------------  target    DVIADYKFPDFSKSYSGRIQSLKPEQIQRLGGMMVWDLNKKQVVPLHREQVGWHYTNSGIDAALTGTYRVKLLNGREIDA 5gpn.24   --------------------------------------------------------------------------------  target    MPIWQMYMVHFQDYDLDTVHQITRTPKDLIVRWARDSGTIKPAAIHNGEGTCHYFHQTANARGAAMVLIITGNVGKFGTG 5gpn.24   -----------------------------NHPFSQILKEAKKPMVVLGSSALQRSDGTAILAAVSNIAQNIRLSSGVTGD  target    QHTWAGNYKAGTWTATPWSGAGLSVHTGEDPFNITLDPNAHGKEIKTRSYYYGEEVGYWNHGDTALIVNTPKYGRKVFTG 5gpn.24   WKVM---N----I----LH-------RIASQV--------AALDL-----------GYK-----P-------GVEAI---  target    KTHMPTPSKFRWVVNVNVVNNAKHHYDMVRNVDPNIECLITQDIEMTSDINHADIAFAANSWMEFTYPEMTVTVSNPWVQ 5gpn.24   ---RKNPPKVLFLLGADGGC-------ITRQDLPKDCFIIYQGHHGDVGAPMADVILPGAAYTEKS---ATYVNTEGRAQ  target    IWKGGIRPLYDTRNDLDTFAGVAAKLSDMTGDKRMRDYFAMVYQNRVDVYVQRMLDASSTFYGYSADVMLKSEKGWMVMV 5gpn.24   QTKVAVTPPGLAREDWKIIRALSEIAG-----------------------------------------------------  target    RTYPRHPFWEETNESKPMWTRSGRYENYRIEPEAIEYGENFISHREGPEATPYLPNAIFTTNPYVRPDDYGIPITAQHHD 5gpn.24   --------------------------------------------------------------------------------  target    DKTVRNIKLSWHEIKRHSNPLWEKGYQFYCVTPKTRHRVHSQWSVNDWVQIYESNFGDPYRMDKRTPGVGEHQIHINPQA 5gpn.24   --------------------------------------------------------------------------------  target    AKDRGINDGDYVYVDGNPVDRPYRGWKPSDPYYKVARLMIRAKYNPAYPYHVTMAKHAPFVATAKSVKGHETRPDGRAIA 5gpn.24   --------------------------------------------------------------------------------  target    IDTGYQSNFRYGAQQSFTRNWLMPMHQTDSLPGKHAVAWKFKWGYQVDHHAINTVPKECLIRITKAEDGGIGARGPWEPV 5gpn.24   --------------------------------------------------------------------------------  target    RTGFTPGQENEFMIKWLKGEHIKIKV 5gpn.24   -------------------------- ``` | | | | | | | | | | | | | | | | | | | | | | | | | | | | | | | | | | | | | | | | | | | | | | | | | |
|  | 6qcf.1.C | NADH:ubiquinone oxidoreductase core subunit S1  *Ovine respiratory complex I FRC open class 6* | 0.15 | 0.00 | 11.78 | 0.33 | 63-746 | EM | 0.00 | monomer | 6 x SF4, 1 x FMN, 2 x FES, 1 x ZN, 1 x NDP, 2 x ZMP | HHblits | 0.25 |
| ``` target    MFLSRRQFLKVSVGTVAAVAVADKVLALTALQPVIEVGNPLGDYPDRSWERVYHDQYRYDSSFTWVCSPNDTHACRVRAF 6qcf.1    --------------------------------------------------------------TESIDV-MDAVGSNIVVS  target    VRNGVVMRVEQNYDHQTYEDLYGNRGTFAHNPRMCLKGFTFHRRVYGPYRLKGPLMRKGWKQWMDDNAPELTAETKRKYK 6qcf.1    TRTGEVMRILPRMHEDI------------NEEWISDKTRFAYDGLK-RQRLTEPMVRNEK--------------------  target    FDSRFLDDMLRVSWDTAFTYAAKAMITIATRYSGEAGARRLREQGYAPEMIEMMKGAGTRCFKHRAGMPVLGIIGKMGNT 6qcf.1    ------GLLTHTTWEDALSRVAGMLQSC----QGNDVA--------------AIAGGLVD--------------------  target    RMNGGINALLDTWIRKVSPD-QAQGGRYWSNYTWHGDQNPAHPFWSGVQGSDIDLSDMRFSKLNTSWGKNFVENKMPEAH 6qcf.1    ---AEALIALKDLLNRVDSDTLCTEEVFP-TA----G--AGTDL-RSNYLLNTTIAGVEEADVVLLVGTNPRFEAPLFNA  target    WKLECIE-RGARVVVITPEYNPTAYRADYWMPLRPESDGALFLGAMKIIIDENMHDIDFLKSFTDAPILVRTDTLQYLDP 6qcf.1    RIRKSWLHNDLKVALIGSPVDLTYRYDHLGDSP------------------------KILQDIASG--------------  target    RDVIADYKFPDFSKSYSGRIQSLKPEQIQRLGGMMVWDLNKKQVVPLHREQVGWHYTNSGIDAALTGTYRVKLLNGREID 6qcf.1    --------------------------------------------------------------------------------  target    AMPIWQMYMVHFQDYDLDTVHQITRTPKDLIVRWARDSGTIKPAAIHNGEGTCHYFHQTANARGAAMVLIITGNVGKFGT 6qcf.1    ------------------------------SHPFSQVLQEAKKPMVVLGSSALQRNDGAAILAAVSNIAQKIRTSSGVTG  target    GQHTWAGNYKAGTWTATPWSGAGLSVHTGEDPFNITLDPNAHGKEIKTRSYYYGEEVGYWNHGDTALIVNTPKYGRKVFT 6qcf.1    DWKVMN--I----L-----H-------RIASQV---------AA-L---------DLGYKP------------GVEAI--  target    GKTHMPTPSKFRWVVNVNVVNNAKHHYDMVRNVDPNIECLITQDIEMTSDINHADIAFAANSWMEFTYPEMTVTVSNPWV 6qcf.1    ----RKNPPKMLFLLGADGGC-------VTRQDLPKDCFIVYQGHHGDVGAPIADVILPGAAYTEKS---ATYVNTEGRA  target    QIWKGGIRPLYDTRNDLDTFAGVAAKLSDMTGDKRMRDYFAMVYQNRVDVYVQRMLDASSTFYGYSADVMLKSEKGWMVM 6qcf.1    QQTKVAVMPPGLAREDWKIIRALSEIAG----------------------------------------------------  target    VRTYPRHPFWEETNESKPMWTRSGRYENYRIEPEAIEYGENFISHREGPEATPYLPNAIFTTNPYVRPDDYGIPITAQHH 6qcf.1    --------------------------------------------------------------------------------  target    DDKTVRNIKLSWHEIKRHSNPLWEKGYQFYCVTPKTRHRVHSQWSVNDWVQIYESNFGDPYRMDKRTPGVGEHQIHINPQ 6qcf.1    --------------------------------------------------------------------------------  target    AAKDRGINDGDYVYVDGNPVDRPYRGWKPSDPYYKVARLMIRAKYNPAYPYHVTMAKHAPFVATAKSVKGHETRPDGRAI 6qcf.1    --------------------------------------------------------------------------------  target    AIDTGYQSNFRYGAQQSFTRNWLMPMHQTDSLPGKHAVAWKFKWGYQVDHHAINTVPKECLIRITKAEDGGIGARGPWEP 6qcf.1    --------------------------------------------------------------------------------  target    VRTGFTPGQENEFMIKWLKGEHIKIKV 6qcf.1    --------------------------- ``` | | | | | | | | | | | | | | | | | | | | | | | | | | | | | | | | | | | | | | | | | | | | | | | | | |
|  | 6qc5.1.C | NADH:ubiquinone oxidoreductase core subunit S1  *Ovine respiratory complex I FRC closed class 1* | 0.15 | 0.00 | 11.78 | 0.33 | 63-746 | EM | 0.00 | monomer | 6 x SF4, 1 x FMN, 2 x FES, 2 x 3PE, 1 x ZN, 1 x NDP, 2 x ZMP, 1 x PC1 | HHblits | 0.25 |
| ``` target    MFLSRRQFLKVSVGTVAAVAVADKVLALTALQPVIEVGNPLGDYPDRSWERVYHDQYRYDSSFTWVCSPNDTHACRVRAF 6qc5.1    --------------------------------------------------------------TESIDV-MDAVGSNIVVS  target    VRNGVVMRVEQNYDHQTYEDLYGNRGTFAHNPRMCLKGFTFHRRVYGPYRLKGPLMRKGWKQWMDDNAPELTAETKRKYK 6qc5.1    TRTGEVMRILPRMHEDI------------NEEWISDKTRFAYDGLK-RQRLTEPMVRNEK--------------------  target    FDSRFLDDMLRVSWDTAFTYAAKAMITIATRYSGEAGARRLREQGYAPEMIEMMKGAGTRCFKHRAGMPVLGIIGKMGNT 6qc5.1    ------GLLTHTTWEDALSRVAGMLQSC----QGNDVA--------------AIAGGLVD--------------------  target    RMNGGINALLDTWIRKVSPD-QAQGGRYWSNYTWHGDQNPAHPFWSGVQGSDIDLSDMRFSKLNTSWGKNFVENKMPEAH 6qc5.1    ---AEALIALKDLLNRVDSDTLCTEEVFP-TA----G--AGTDL-RSNYLLNTTIAGVEEADVVLLVGTNPRFEAPLFNA  target    WKLECIE-RGARVVVITPEYNPTAYRADYWMPLRPESDGALFLGAMKIIIDENMHDIDFLKSFTDAPILVRTDTLQYLDP 6qc5.1    RIRKSWLHNDLKVALIGSPVDLTYRYDHLGDSP------------------------KILQDIASG--------------  target    RDVIADYKFPDFSKSYSGRIQSLKPEQIQRLGGMMVWDLNKKQVVPLHREQVGWHYTNSGIDAALTGTYRVKLLNGREID 6qc5.1    --------------------------------------------------------------------------------  target    AMPIWQMYMVHFQDYDLDTVHQITRTPKDLIVRWARDSGTIKPAAIHNGEGTCHYFHQTANARGAAMVLIITGNVGKFGT 6qc5.1    ------------------------------SHPFSQVLQEAKKPMVVLGSSALQRNDGAAILAAVSNIAQKIRTSSGVTG  target    GQHTWAGNYKAGTWTATPWSGAGLSVHTGEDPFNITLDPNAHGKEIKTRSYYYGEEVGYWNHGDTALIVNTPKYGRKVFT 6qc5.1    DWKVMN--I----L-----H-------RIASQV---------AA-L---------DLGYKP------------GVEAI--  target    GKTHMPTPSKFRWVVNVNVVNNAKHHYDMVRNVDPNIECLITQDIEMTSDINHADIAFAANSWMEFTYPEMTVTVSNPWV 6qc5.1    ----RKNPPKMLFLLGADGGC-------VTRQDLPKDCFIVYQGHHGDVGAPIADVILPGAAYTEKS---ATYVNTEGRA  target    QIWKGGIRPLYDTRNDLDTFAGVAAKLSDMTGDKRMRDYFAMVYQNRVDVYVQRMLDASSTFYGYSADVMLKSEKGWMVM 6qc5.1    QQTKVAVMPPGLAREDWKIIRALSEIAG----------------------------------------------------  target    VRTYPRHPFWEETNESKPMWTRSGRYENYRIEPEAIEYGENFISHREGPEATPYLPNAIFTTNPYVRPDDYGIPITAQHH 6qc5.1    --------------------------------------------------------------------------------  target    DDKTVRNIKLSWHEIKRHSNPLWEKGYQFYCVTPKTRHRVHSQWSVNDWVQIYESNFGDPYRMDKRTPGVGEHQIHINPQ 6qc5.1    --------------------------------------------------------------------------------  target    AAKDRGINDGDYVYVDGNPVDRPYRGWKPSDPYYKVARLMIRAKYNPAYPYHVTMAKHAPFVATAKSVKGHETRPDGRAI 6qc5.1    --------------------------------------------------------------------------------  target    AIDTGYQSNFRYGAQQSFTRNWLMPMHQTDSLPGKHAVAWKFKWGYQVDHHAINTVPKECLIRITKAEDGGIGARGPWEP 6qc5.1    --------------------------------------------------------------------------------  target    VRTGFTPGQENEFMIKWLKGEHIKIKV 6qc5.1    --------------------------- ``` | | | | | | | | | | | | | | | | | | | | | | | | | | | | | | | | | | | | | | | | | | | | | | | | | |
|  | 5xtb.1.L | NADH-ubiquinone oxidoreductase 75 kDa subunit, mitochondrial  *Cryo-EM structure of human respiratory complex I matrix arm* | 0.15 |  | 12.01 | 0.33 | 62-746 | EM | 0.00 | hetero-1-1-1-1-1-1-… | 6 x SF4, 1 x FMN, 1 x 8Q1, 1 x NDP, 2 x FES | HHblits | 0.25 |
| ``` target    MFLSRRQFLKVSVGTVAAVAVADKVLALTALQPVIEVGNPLGDYPDRSWERVYHDQYRYDSSFTWVCSPNDTHACRVRAF 5xtb.1    -------------------------------------------------------------KTESIDV-MDAVGSNIVVS  target    VRNGVVMRVEQNYDHQTYEDLYGNRGTFAHNPRMCLKGFTFHRRVYGPYRLKGPLMRKGWKQWMDDNAPELTAETKRKYK 5xtb.1    TRTGEVMRILPRMHEDI------------NEEWISDKTRFAYDGLK-RQRLTEPMVRNEK--------------------  target    FDSRFLDDMLRVSWDTAFTYAAKAMITIATRYSGEAGARRLREQGYAPEMIEMMKGAGTRCFKHRAGMPVLGIIGKMGNT 5xtb.1    ------GLLTYTSWEDALSRVAGMLQSF----QGKDVA--------------AIAGGLVDA-------------------  target    RMNGGINALLDTWIRKVSPD-QAQGGRYWSNYTWHGDQNPAHPFWSGVQGSDIDLSDMRFSKLNTSWGKNFVENKMPEAH 5xtb.1    ----EALVALKDLLNRVDSDTLCTEEVFP-T------AGAGTDL-RSNYLLNTTIAGVEEADVVLLVGTNPRFEAPLFNA  target    WKLECIE-RGARVVVITPEYNPTAYRADYWMPLRPESDGALFLGAMKIIIDENMHDIDFLKSFTDAPILVRTDTLQYLDP 5xtb.1    RIRKSWLHNDLKVALIGSPVDLTYTYD--H----LGDSPKILQDIA----------------------------------  target    RDVIADYKFPDFSKSYSGRIQSLKPEQIQRLGGMMVWDLNKKQVVPLHREQVGWHYTNSGIDAALTGTYRVKLLNGREID 5xtb.1    --------------------------------------------------------------------------------  target    AMPIWQMYMVHFQDYDLDTVHQITRTPKDLIVRWARDSGTIKPAAIHNGEGTCHYFHQTANARGAAMVLIITGNVGKFGT 5xtb.1    ----------------------------SGSHPFSQVLKEAKKPMVVLGSSALQRNDGAAILAAVSSIAQKIRMTSGVTG  target    GQHTWAGNYKAGTWTATPWSGAGLSVHTGEDPFNITLDPNAHGKEIKTRSYYYGEEVGYWNHGDTALIVNTPKYGRKVFT 5xtb.1    DWKVMNI------L-----H-------RIASQVA--------ALDL-----------GYKP------------GVEAIR-  target    GKTHMPTPSKFRWVVNVNVVNNAKHHYDMVRNVDPNIECLITQDIEMTSDINHADIAFAANSWMEFTYPEMTVTVSNPWV 5xtb.1    -----KNPPKVLFLLGADGGC-------ITRQDLPKDCFIIYQGHHGDVGAPIADVILPGAAYTEKS---ATYVNTEGRA  target    QIWKGGIRPLYDTRNDLDTFAGVAAKLSDMTGDKRMRDYFAMVYQNRVDVYVQRMLDASSTFYGYSADVMLKSEKGWMVM 5xtb.1    QQTKVAVTPPGLAREDWKIIRALSEIAG----------------------------------------------------  target    VRTYPRHPFWEETNESKPMWTRSGRYENYRIEPEAIEYGENFISHREGPEATPYLPNAIFTTNPYVRPDDYGIPITAQHH 5xtb.1    --------------------------------------------------------------------------------  target    DDKTVRNIKLSWHEIKRHSNPLWEKGYQFYCVTPKTRHRVHSQWSVNDWVQIYESNFGDPYRMDKRTPGVGEHQIHINPQ 5xtb.1    --------------------------------------------------------------------------------  target    AAKDRGINDGDYVYVDGNPVDRPYRGWKPSDPYYKVARLMIRAKYNPAYPYHVTMAKHAPFVATAKSVKGHETRPDGRAI 5xtb.1    --------------------------------------------------------------------------------  target    AIDTGYQSNFRYGAQQSFTRNWLMPMHQTDSLPGKHAVAWKFKWGYQVDHHAINTVPKECLIRITKAEDGGIGARGPWEP 5xtb.1    --------------------------------------------------------------------------------  target    VRTGFTPGQENEFMIKWLKGEHIKIKV 5xtb.1    --------------------------- ``` | | | | | | | | | | | | | | | | | | | | | | | | | | | | | | | | | | | | | | | | | | | | | | | | | |
|  | 7v2c.1.L | NADH-ubiquinone oxidoreductase 75 kDa subunit, mitochondrial  *Active state complex I from Q10 dataset* | 0.15 |  | 12.30 | 0.33 | 63-746 | EM | 0.00 | hetero-1-1-1-1-1-2-… | 6 x SF4, 1 x FMN, 10 x PEE, 8 x PLX, 2 x 8Q1, 1 x NDP, 2 x UQ, 11 x CDL, 2 x FES, 1 x MG, 1 x ZN, 1 x ADP | HHblits | 0.25 |
| ``` target    MFLSRRQFLKVSVGTVAAVAVADKVLALTALQPVIEVGNPLGDYPDRSWERVYHDQYRYDSSFTWVCSPNDTHACRVRAF 7v2c.1    --------------------------------------------------------------TESIDV-MDAVGSNIVVS  target    VRNGVVMRVEQNYDHQTYEDLYGNRGTFAHNPRMCLKGFTFHRRVYGPYRLKGPLMRKGWKQWMDDNAPELTAETKRKYK 7v2c.1    TRTGEVMRILPRMHEDI------------NEEWISDKTRFAYDGLK-RQRLTQPMIRNEK--------------------  target    FDSRFLDDMLRVSWDTAFTYAAKAMITIATRYSGEAGARRLREQGYAPEMIEMMKGAGTRCFKHRAGMPVLGIIGKMGNT 7v2c.1    ------GLLTYTTWEDALSRVAGMLQSF----QGNDVA--------------AIAGGLVDA-------------------  target    RMNGGINALLDTWIRKVSPD-QAQGGRYWSNYTWHGDQNPAHPFWSGVQGSDIDLSDMRFSKLNTSWGKNFVENKMPEAH 7v2c.1    ----EALVALKDLLNRVDSDSLCTEEV----FPT-AG--AGTDL-RSNYLLNTTIAGVEEADVILLVGTNPRFEAPLFNA  target    WKLECIE-RGARVVVITPEYNPTAYRADYWMPLRPESDGALFLGAMKIIIDENMHDIDFLKSFTDAPILVRTDTLQYLDP 7v2c.1    RIRKSWLHNDLKVALIGSPVDLTYRYDHLGDSPK------------------------ILQDIASG--------------  target    RDVIADYKFPDFSKSYSGRIQSLKPEQIQRLGGMMVWDLNKKQVVPLHREQVGWHYTNSGIDAALTGTYRVKLLNGREID 7v2c.1    --------------------------------------------------------------------------------  target    AMPIWQMYMVHFQDYDLDTVHQITRTPKDLIVRWARDSGTIKPAAIHNGEGTCHYFHQTANARGAAMVLIITGNVGKFGT 7v2c.1    ------------------------------NHPFSQILKEAKKPMVVLGSSALQRSDGTAILAAVSNIAQNIRLSSGVTG  target    GQHTWAGNYKAGTWTATPWSGAGLSVHTGEDPFNITLDPNAHGKEIKTRSYYYGEEVGYWNHGDTALIVNTPKYGRKVFT 7v2c.1    DWK---VMN----I----LH-------RIASQVA--------ALDL-----------GYKP------------GVEAIR-  target    GKTHMPTPSKFRWVVNVNVVNNAKHHYDMVRNVDPNIECLITQDIEMTSDINHADIAFAANSWMEFTYPEMTVTVSNPWV 7v2c.1    -----KNPPKVLFLLGADGGC-------ITRQDLPKDCFIIYQGHHGDVGAPMADVILPGAAYTEKS---ATYVNTEGRA  target    QIWKGGIRPLYDTRNDLDTFAGVAAKLSDMTGDKRMRDYFAMVYQNRVDVYVQRMLDASSTFYGYSADVMLKSEKGWMVM 7v2c.1    QQTKVAVTPPGLAREDWKIIRALSEIAG----------------------------------------------------  target    VRTYPRHPFWEETNESKPMWTRSGRYENYRIEPEAIEYGENFISHREGPEATPYLPNAIFTTNPYVRPDDYGIPITAQHH 7v2c.1    --------------------------------------------------------------------------------  target    DDKTVRNIKLSWHEIKRHSNPLWEKGYQFYCVTPKTRHRVHSQWSVNDWVQIYESNFGDPYRMDKRTPGVGEHQIHINPQ 7v2c.1    --------------------------------------------------------------------------------  target    AAKDRGINDGDYVYVDGNPVDRPYRGWKPSDPYYKVARLMIRAKYNPAYPYHVTMAKHAPFVATAKSVKGHETRPDGRAI 7v2c.1    --------------------------------------------------------------------------------  target    AIDTGYQSNFRYGAQQSFTRNWLMPMHQTDSLPGKHAVAWKFKWGYQVDHHAINTVPKECLIRITKAEDGGIGARGPWEP 7v2c.1    --------------------------------------------------------------------------------  target    VRTGFTPGQENEFMIKWLKGEHIKIKV 7v2c.1    --------------------------- ``` | | | | | | | | | | | | | | | | | | | | | | | | | | | | | | | | | | | | | | | | | | | | | | | | | |
|  | 7vxu.1.L | NADH-ubiquinone oxidoreductase 75 kDa subunit, mitochondrial  *Matrix arm of deactive state CI from Q10 dataset* | 0.15 |  | 11.75 | 0.33 | 63-746 | EM | 0.00 | hetero-1-1-1-1-1-1-… | 6 x SF4, 1 x FMN, 1 x PEE, 1 x PLX, 1 x 8Q1, 1 x NDP, 2 x FES, 1 x MG, 1 x CDL, 1 x ZN | HHblits | 0.25 |
| ``` target    MFLSRRQFLKVSVGTVAAVAVADKVLALTALQPVIEVGNPLGDYPDRSWERVYHDQYRYDSSFTWVCSPNDTHACRVRAF 7vxu.1    --------------------------------------------------------------TESIDV-MDAVGSNIVVS  target    VRNGVVMRVEQNYDHQTYEDLYGNRGTFAHNPRMCLKGFTFHRRVYGPYRLKGPLMRKGWKQWMDDNAPELTAETKRKYK 7vxu.1    TRTGEVMRILPRMHEDI------------NEEWISDKTRFAYDGLK-RQRLTQPMIRNEK--------------------  target    FDSRFLDDMLRVSWDTAFTYAAKAMITIATRYSGEAGARRLREQGYAPEMIEMMKGAGTRCFKHRAGMPVLGIIGKMGNT 7vxu.1    ------GLLTYTTWEDALSRVAGMLQSF----QGNDV--------------AAIAGGLVDA-------------------  target    RMNGGINALLDTWIRKVSPDQAQGGRYWSNYTWHGDQNPAHPFWSGVQGSDIDLSDMRFSKLNTSWGKNFVENKMPEAHW 7vxu.1    ----EALVALKDLLNRVD------SDSLCTEEVFPTAGAGTDL-RSNYLLNTTIAGVEEADVILLVGTNPRFEAPLFNAR  target    KLECIE-RGARVVVITPEYNPTAYRADYWMPLRPESDGALFLGAMKIIIDENMHDIDFLKSFTDAPILVRTDTLQYLDPR 7vxu.1    IRKSWLHNDLKVALIGSPVDLTYRYDHLGDSPK------------------------ILQDIASG---------------  target    DVIADYKFPDFSKSYSGRIQSLKPEQIQRLGGMMVWDLNKKQVVPLHREQVGWHYTNSGIDAALTGTYRVKLLNGREIDA 7vxu.1    --------------------------------------------------------------------------------  target    MPIWQMYMVHFQDYDLDTVHQITRTPKDLIVRWARDSGTIKPAAIHNGEGTCHYFHQTANARGAAMVLIITGNVGKFGTG 7vxu.1    -----------------------------NHPFSQILKEAKKPMVVLGSSALQRSDGTAILAAVSNIAQNIRLSSGVTGD  target    QHTWAGNYKAGTWTATPWSGAGLSVHTGEDPFNITLDPNAHGKEIKTRSYYYGEEVGYWNHGDTALIVNTPKYGRKVFTG 7vxu.1    WKVM---N----I----LH-------RIASQV--------AALDL-----------GYKP------------GVEAIR--  target    KTHMPTPSKFRWVVNVNVVNNAKHHYDMVRNVDPNIECLITQDIEMTSDINHADIAFAANSWMEFTYPEMTVTVSNPWVQ 7vxu.1    ----KNPPKVLFLLGADGGC-------ITRQDLPKDCFIIYQGHHGDVGAPMADVILPGAAYTEKS---ATYVNTEGRAQ  target    IWKGGIRPLYDTRNDLDTFAGVAAKLSDMTGDKRMRDYFAMVYQNRVDVYVQRMLDASSTFYGYSADVMLKSEKGWMVMV 7vxu.1    QTKVAVTPPGLAREDWKIIRALSEIAG-----------------------------------------------------  target    RTYPRHPFWEETNESKPMWTRSGRYENYRIEPEAIEYGENFISHREGPEATPYLPNAIFTTNPYVRPDDYGIPITAQHHD 7vxu.1    --------------------------------------------------------------------------------  target    DKTVRNIKLSWHEIKRHSNPLWEKGYQFYCVTPKTRHRVHSQWSVNDWVQIYESNFGDPYRMDKRTPGVGEHQIHINPQA 7vxu.1    --------------------------------------------------------------------------------  target    AKDRGINDGDYVYVDGNPVDRPYRGWKPSDPYYKVARLMIRAKYNPAYPYHVTMAKHAPFVATAKSVKGHETRPDGRAIA 7vxu.1    --------------------------------------------------------------------------------  target    IDTGYQSNFRYGAQQSFTRNWLMPMHQTDSLPGKHAVAWKFKWGYQVDHHAINTVPKECLIRITKAEDGGIGARGPWEPV 7vxu.1    --------------------------------------------------------------------------------  target    RTGFTPGQENEFMIKWLKGEHIKIKV 7vxu.1    -------------------------- ``` | | | | | | | | | | | | | | | | | | | | | | | | | | | | | | | | | | | | | | | | | | | | | | | | | |
|  | 7tgh.58.A | NADH-ubiquinone oxidoreductase 75 kDa subunit  *Cryo-EM structure of respiratory super-complex CI+III2 from Tetrahymena thermophila* | 0.16 |  | 12.70 | 0.33 | 62-746 | EM | 0.00 | monomer |  | HHblits | 0.26 |
| ``` target    MFLSRRQFLKVSVGTVAAVAVADKVLALTALQPVIEVGNPLGDYPDRSWERVYHDQYRYDSSFTWVCSPNDTHACRVRAF 7tgh.58   -------------------------------------------------------------SFYTSDV-FDTLGSAIQVD  target    VRNGVVMRVEQNYDHQTYEDLYGNRGTFAHNPRMCLKGFTFHRRVYGPYRLKGPLMRKGWKQWMDDNAPELTAETKRKYK 7tgh.58   TRGPEIMRVLPRIHEEI------------NEEWISDKTRHAFDGLK-RQRINSPMKRSKD--------------------  target    FDSRFLDDMLRVSWDTAFTYAAKAMITIATRYSGEAGARRLREQGYAPEMIEMMKGAGTRCFKHRAGMPVLGIIGKMGNT 7tgh.58   ------GNYEDIFWEEAIQTISKKCLNTPS----DQIG--------------AIIGEFADI-------------------  target    RMNGGINALLDTWIRKVSPDQAQGGRYWSNYTWHGDQNPAHPFWSGVQGSDIDLSDMRFSKLNTSWGKNFVENKMPEAHW 7tgh.58   ----ESITALKDFLNRL------DVDNFE-VRQHGNLKVSPDF-RANYLMNSKITGVEDADVLLLVGCNPRYEAPVLNAR  target    KLECIERGARVVVITPEYNPTAYRADYWMPLRPESDGALFLGAMKIIIDENMHDIDFLKSFTDAPILVRTDTLQYLDPRD 7tgh.58   ILKSTRKNLKVFNIGTNQDLN--YKNVHL----GNSTKVL----------------------------------------  target    VIADYKFPDFSKSYSGRIQSLKPEQIQRLGGMMVWDLNKKQVVPLHREQVGWHYTNSGIDAALTGTYRVKLLNGREIDAM 7tgh.58   --------------------------------------------------------------------------------  target    PIWQMYMVHFQDYDLDTVHQITRTPKDLIVRWARDSGTIKPAAIHNGEGTCHYFHQTANARGAAMVLIITGNVGKFGTGQ 7tgh.58   ----------------------KEIADGTHPFAERLKKAKLPMIMVGASALEREDGAELYNTLKVISNKTGVISEEKSWN  target    HTWAGNYKAGTWTATPWSGAGLSVHTGEDPFNITLDPNAHGKEIKTRSYYYGEEVGYWNHGDTALIVNTPKYGRKVFTGK 7tgh.58   GFNIL------------HK-------EMGRI---------NA-LE---------LGI----------NP-----------  target    THMPTPSKFRWVVNVNVVNNAKHHYDMVRNVDPNIECLITQDIEMTSDINHADIAFAANSWMEFTYPEMTVTVSNPWVQI 7tgh.58   TSVNKNAKLVFILGADNNLRP--------EDIPADAFVVYFGTHGDEGAYYADIILPTAAYTEKNA---TWVNTEGRVQQ  target    WKGGIRPLYDTRNDLDTFAGVAAKLSDMTGDKRMRDYFAMVYQNRVDVYVQRMLDASSTFYGYSADVMLKSEKGWMVMVR 7tgh.58   GRLVVMPPGDAREDWQIIRALSEEAG------------------------------------------------------  target    TYPRHPFWEETNESKPMWTRSGRYENYRIEPEAIEYGENFISHREGPEATPYLPNAIFTTNPYVRPDDYGIPITAQHHDD 7tgh.58   --------------------------------------------------------------------------------  target    KTVRNIKLSWHEIKRHSNPLWEKGYQFYCVTPKTRHRVHSQWSVNDWVQIYESNFGDPYRMDKRTPGVGEHQIHINPQAA 7tgh.58   --------------------------------------------------------------------------------  target    KDRGINDGDYVYVDGNPVDRPYRGWKPSDPYYKVARLMIRAKYNPAYPYHVTMAKHAPFVATAKSVKGHETRPDGRAIAI 7tgh.58   --------------------------------------------------------------------------------  target    DTGYQSNFRYGAQQSFTRNWLMPMHQTDSLPGKHAVAWKFKWGYQVDHHAINTVPKECLIRITKAEDGGIGARGPWEPVR 7tgh.58   --------------------------------------------------------------------------------  target    TGFTPGQENEFMIKWLKGEHIKIKV 7tgh.58   ------------------------- ``` | | | | | | | | | | | | | | | | | | | | | | | | | | | | | | | | | | | | | | | | | | | | | | | | | |
|  | 7ar7.1.G | NADH dehydrogenase [ubiquinone] iron-sulfur protein 1, mitochondrial  *Cryo-EM structure of Arabidopsis thaliana complex-I (open conformation)* | 0.15 |  | 13.07 | 0.33 | 63-746 | EM | 0.00 | hetero-1-1-1-1-1-1-… | 6 x SF4, 2 x FES, 1 x FMN, 1 x UQ9, 3 x PTY, 2 x PC7, 1 x LMN, 1 x NDP, 2 x ZN, 2 x 8Q1, 1 x PGT, 1 x PSF, 1 x T7X | HHblits | 0.26 |
| ``` target    MFLSRRQFLKVSVGTVAAVAVADKVLALTALQPVIEVGNPLGDYPDRSWERVYHDQYRYDSSFTWVCSPNDTHACRVRAF 7ar7.1    --------------------------------------------------------------TETIDV-SDAVGSNIRVD  target    VRNGVVMRVEQNYDHQTYEDLYGNRGTFAHNPRMCLKGFTFHRRVYGPYRLKGPLMRKGWKQWMDDNAPELTAETKRKYK 7ar7.1    SRGPEVMRIIPRLNEDI------------NEEWISDKTRFCYDGLK-RQRLSDPMIRDSD--------------------  target    FDSRFLDDMLRVSWDTAFTYAAKAMITIATRYSGEAGARRLREQGYAPEMIEMMKGAGTRCFKHRAGMPVLGIIGKMGNT 7ar7.1    ------GRFKAVSWRDALAVVGDIIHQV----KPDEI--------------VGVAGQLSDA-------------------  target    RMNGGINALLDTWIRKVSPDQAQGGRYWSNYTWHGDQNPAHPFWSGVQGSDIDLSDMRFSKLNTSWGKNFVENKMPEAHW 7ar7.1    ----ESMMVLKDFVNRM------GSDNV---WCEGTAAGVDADLRYSYLMNTSISGLENADLFLLIGTQPRVEAAMVNAR  target    KLECI-ERGARVVVITPEYNPTAYRADYWMPLRPESDGALFLGAMKIIIDENMHDIDFLKSFTDAPILVRTDTLQYLDPR 7ar7.1    ICKTVRASNAKVGYVGPPAEFN--YDCKHLGTGPDTLKEI----------------------------------------  target    DVIADYKFPDFSKSYSGRIQSLKPEQIQRLGGMMVWDLNKKQVVPLHREQVGWHYTNSGIDAALTGTYRVKLLNGREIDA 7ar7.1    --------------------------------------------------------------------------------  target    MPIWQMYMVHFQDYDLDTVHQITRTPKDLIVRWARDSGTIKPAAIHNGEGTCHYFHQTANARGAAMVLIITGNVGKFGTG 7ar7.1    --------------------------AEGRHPFCTALKNAKNPAIIVGAGLFNRTDKNAILSSVESIAQANNVVRPDWNG  target    QHTWAGNYKAGTWTATPWSGAGLSVHTGEDPFNITLDPNAHGKEIKTRSYYYGEEVGYWNHGDTALIVNTPKYGRKVFTG 7ar7.1    LNFLLQYAAQ----------------AA-------------ALDL-----------GLIQ--------------Q-SAK-  target    KTHMPTPSKFRWVVNVNVVNNAKHHYDMVRNVDPNIECLITQDIEMTSDINHADIAFAANSWMEFTYPEMTVTVSNPWVQ 7ar7.1    ---ALESAKFVYLMGADDVN---------VDKIPKDAFVVYQGHHGDKAVYRANVILPASAFTEKE---GTYENTEGFTQ  target    IWKGGIRPLYDTRNDLDTFAGVAAKLSDMTGDKRMRDYFAMVYQNRVDVYVQRMLDASSTFYGYSADVMLKSEKGWMVMV 7ar7.1    QTVPAVPTVGDARDDWKIVRALSEVSG-----------------------------------------------------  target    RTYPRHPFWEETNESKPMWTRSGRYENYRIEPEAIEYGENFISHREGPEATPYLPNAIFTTNPYVRPDDYGIPITAQHHD 7ar7.1    --------------------------------------------------------------------------------  target    DKTVRNIKLSWHEIKRHSNPLWEKGYQFYCVTPKTRHRVHSQWSVNDWVQIYESNFGDPYRMDKRTPGVGEHQIHINPQA 7ar7.1    --------------------------------------------------------------------------------  target    AKDRGINDGDYVYVDGNPVDRPYRGWKPSDPYYKVARLMIRAKYNPAYPYHVTMAKHAPFVATAKSVKGHETRPDGRAIA 7ar7.1    --------------------------------------------------------------------------------  target    IDTGYQSNFRYGAQQSFTRNWLMPMHQTDSLPGKHAVAWKFKWGYQVDHHAINTVPKECLIRITKAEDGGIGARGPWEPV 7ar7.1    --------------------------------------------------------------------------------  target    RTGFTPGQENEFMIKWLKGEHIKIKV 7ar7.1    -------------------------- ``` | | | | | | | | | | | | | | | | | | | | | | | | | | | | | | | | | | | | | | | | | | | | | | | | | |
|  | 7aqr.1.F | NADH dehydrogenase [ubiquinone] iron-sulfur protein 1, mitochondrial  *Cryo-EM structure of Arabidopsis thaliana Complex-I (peripheral arm)* | 0.15 |  | 12.80 | 0.33 | 63-746 | EM | 0.00 | hetero-1-1-1-1-1-1-… | 6 x SF4, 2 x FES, 1 x FMN, 1 x NDP, 1 x ZN, 1 x 8Q1 | HHblits | 0.26 |
| ``` target    MFLSRRQFLKVSVGTVAAVAVADKVLALTALQPVIEVGNPLGDYPDRSWERVYHDQYRYDSSFTWVCSPNDTHACRVRAF 7aqr.1    --------------------------------------------------------------TETIDV-SDAVGSNIRVD  target    VRNGVVMRVEQNYDHQTYEDLYGNRGTFAHNPRMCLKGFTFHRRVYGPYRLKGPLMRKGWKQWMDDNAPELTAETKRKYK 7aqr.1    SRGPEVMRIIPRLNEDI------------NEEWISDKTRFCYDGLK-RQRLSDPMIRDSD--------------------  target    FDSRFLDDMLRVSWDTAFTYAAKAMITIATRYSGEAGARRLREQGYAPEMIEMMKGAGTRCFKHRAGMPVLGIIGKMGNT 7aqr.1    ------GRFKAVSWRDALAVVGDIIHQV----KPDEIV--------------GVAGQLSDA-------------------  target    RMNGGINALLDTWIRKVSPDQAQGGRYWSNYTWHGDQNPAHPFWSGVQGSDIDLSDMRFSKLNTSWGKNFVENKMPEAHW 7aqr.1    ----ESMMVLKDFVNRM------GSDNV---WCEGTAAGVDADLRYSYLMNTSISGLENADLFLLIGTQPRVEAAMVNAR  target    KLECI-ERGARVVVITPEYNPTAYRADYWMPLRPESDGALFLGAMKIIIDENMHDIDFLKSFTDAPILVRTDTLQYLDPR 7aqr.1    ICKTVRASNAKVGYVGPPAEFN--YDCKHLGTGPDTLKEI----------------------------------------  target    DVIADYKFPDFSKSYSGRIQSLKPEQIQRLGGMMVWDLNKKQVVPLHREQVGWHYTNSGIDAALTGTYRVKLLNGREIDA 7aqr.1    --------------------------------------------------------------------------------  target    MPIWQMYMVHFQDYDLDTVHQITRTPKDLIVRWARDSGTIKPAAIHNGEGTCHYFHQTANARGAAMVLIITGNVGKFGTG 7aqr.1    --------------------------AEGRHPFCTALKNAKNPAIIVGAGLFNRTDKNAILSSVESIAQANNVVRPDWNG  target    QHTWAGNYKAGTWTATPWSGAGLSVHTGEDPFNITLDPNAHGKEIKTRSYYYGEEVGYWNHGDTALIVNTPKYGRKVFTG 7aqr.1    LNFLLQYAAQ----------------AA-------------ALDLG-----------LIQ--------------Q-SAK-  target    KTHMPTPSKFRWVVNVNVVNNAKHHYDMVRNVDPNIECLITQDIEMTSDINHADIAFAANSWMEFTYPEMTVTVSNPWVQ 7aqr.1    ---ALESAKFVYLMGADDVN---------VDKIPKDAFVVYQGHHGDKAVYRANVILPASAFTEKE---GTYENTEGFTQ  target    IWKGGIRPLYDTRNDLDTFAGVAAKLSDMTGDKRMRDYFAMVYQNRVDVYVQRMLDASSTFYGYSADVMLKSEKGWMVMV 7aqr.1    QTVPAVPTVGDARDDWKIVRALSEVSG-----------------------------------------------------  target    RTYPRHPFWEETNESKPMWTRSGRYENYRIEPEAIEYGENFISHREGPEATPYLPNAIFTTNPYVRPDDYGIPITAQHHD 7aqr.1    --------------------------------------------------------------------------------  target    DKTVRNIKLSWHEIKRHSNPLWEKGYQFYCVTPKTRHRVHSQWSVNDWVQIYESNFGDPYRMDKRTPGVGEHQIHINPQA 7aqr.1    --------------------------------------------------------------------------------  target    AKDRGINDGDYVYVDGNPVDRPYRGWKPSDPYYKVARLMIRAKYNPAYPYHVTMAKHAPFVATAKSVKGHETRPDGRAIA 7aqr.1    --------------------------------------------------------------------------------  target    IDTGYQSNFRYGAQQSFTRNWLMPMHQTDSLPGKHAVAWKFKWGYQVDHHAINTVPKECLIRITKAEDGGIGARGPWEPV 7aqr.1    --------------------------------------------------------------------------------  target    RTGFTPGQENEFMIKWLKGEHIKIKV 7aqr.1    -------------------------- ``` | | | | | | | | | | | | | | | | | | | | | | | | | | | | | | | | | | | | | | | | | | | | | | | | | |
|  | 7a23.1.O | 75kDa  *Plant mitochondrial respiratory complex I* | 0.15 |  | 12.80 | 0.33 | 63-746 | EM | 0.00 | hetero-1-1-1-1-1-1-… | 6 x SF4, 1 x FMN, 2 x T7X, 3 x CDL, 1 x U10, 1 x PEV, 2 x FES, 1 x NDP, 2 x ZN | HHblits | 0.26 |
| ``` target    MFLSRRQFLKVSVGTVAAVAVADKVLALTALQPVIEVGNPLGDYPDRSWERVYHDQYRYDSSFTWVCSPNDTHACRVRAF 7a23.1    --------------------------------------------------------------TETIDV-SDAVGSNIRVD  target    VRNGVVMRVEQNYDHQTYEDLYGNRGTFAHNPRMCLKGFTFHRRVYGPYRLKGPLMRKGWKQWMDDNAPELTAETKRKYK 7a23.1    SRGPEVMRIIPRLNEDI------------NEEWISDKTRFCYDGLK-RQRLSDPMIRDSD--------------------  target    FDSRFLDDMLRVSWDTAFTYAAKAMITIATRYSGEAGARRLREQGYAPEMIEMMKGAGTRCFKHRAGMPVLGIIGKMGNT 7a23.1    ------GRFKAVSWRDALAVVGDIIHQV----KPDEIV--------------GVAGQLSDA-------------------  target    RMNGGINALLDTWIRKVSPDQAQGGRYWSNYTWHGDQNPAHPFWSGVQGSDIDLSDMRFSKLNTSWGKNFVENKMPEAHW 7a23.1    ----ESMMVLKDFVNRM------GSDNV---WCEGTAAGVDADLRYSYLMNTSISGLENADLFLLIGTQPRVEAAMVNAR  target    KLECI-ERGARVVVITPEYNPTAYRADYWMPLRPESDGALFLGAMKIIIDENMHDIDFLKSFTDAPILVRTDTLQYLDPR 7a23.1    ICKTVRASNAKVGYVGPPAEFN--YDCKHLGTGPDTLKEI----------------------------------------  target    DVIADYKFPDFSKSYSGRIQSLKPEQIQRLGGMMVWDLNKKQVVPLHREQVGWHYTNSGIDAALTGTYRVKLLNGREIDA 7a23.1    --------------------------------------------------------------------------------  target    MPIWQMYMVHFQDYDLDTVHQITRTPKDLIVRWARDSGTIKPAAIHNGEGTCHYFHQTANARGAAMVLIITGNVGKFGTG 7a23.1    --------------------------AEGRHPFCTALKNAKNPAIIVGAGLFNRTDKNAILSSVESIAQANNVVRPDWNG  target    QHTWAGNYKAGTWTATPWSGAGLSVHTGEDPFNITLDPNAHGKEIKTRSYYYGEEVGYWNHGDTALIVNTPKYGRKVFTG 7a23.1    LNFLLQYAAQ----------------AA-------------ALDLG-----------LIQ--------------Q-SAK-  target    KTHMPTPSKFRWVVNVNVVNNAKHHYDMVRNVDPNIECLITQDIEMTSDINHADIAFAANSWMEFTYPEMTVTVSNPWVQ 7a23.1    ---ALESAKFVYLMGADDVN---------VDKIPKDAFVVYQGHHGDKAVYRANVILPASAFTEKE---GTYENTEGFTQ  target    IWKGGIRPLYDTRNDLDTFAGVAAKLSDMTGDKRMRDYFAMVYQNRVDVYVQRMLDASSTFYGYSADVMLKSEKGWMVMV 7a23.1    QTVPAVPTVGDARDDWKIVRALSEVSG-----------------------------------------------------  target    RTYPRHPFWEETNESKPMWTRSGRYENYRIEPEAIEYGENFISHREGPEATPYLPNAIFTTNPYVRPDDYGIPITAQHHD 7a23.1    --------------------------------------------------------------------------------  target    DKTVRNIKLSWHEIKRHSNPLWEKGYQFYCVTPKTRHRVHSQWSVNDWVQIYESNFGDPYRMDKRTPGVGEHQIHINPQA 7a23.1    --------------------------------------------------------------------------------  target    AKDRGINDGDYVYVDGNPVDRPYRGWKPSDPYYKVARLMIRAKYNPAYPYHVTMAKHAPFVATAKSVKGHETRPDGRAIA 7a23.1    --------------------------------------------------------------------------------  target    IDTGYQSNFRYGAQQSFTRNWLMPMHQTDSLPGKHAVAWKFKWGYQVDHHAINTVPKECLIRITKAEDGGIGARGPWEPV 7a23.1    --------------------------------------------------------------------------------  target    RTGFTPGQENEFMIKWLKGEHIKIKV 7a23.1    -------------------------- ``` | | | | | | | | | | | | | | | | | | | | | | | | | | | | | | | | | | | | | | | | | | | | | | | | | |
|  | 7ar8.1.G | NADH dehydrogenase [ubiquinone] iron-sulfur protein 1, mitochondrial  *Cryo-EM structure of Arabidopsis thaliana complex-I (closed conformation)* | 0.15 |  | 12.80 | 0.33 | 63-746 | EM | 0.00 | hetero-1-1-1-1-1-1-… | 6 x SF4, 2 x FES, 1 x FMN, 1 x UQ9, 3 x PTY, 2 x PC7, 1 x PGT, 1 x FE, 1 x NDP, 2 x ZN, 2 x 8Q1, 1 x LMN, 1 x PSF, 1 x T7X | HHblits | 0.26 |
| ``` target    MFLSRRQFLKVSVGTVAAVAVADKVLALTALQPVIEVGNPLGDYPDRSWERVYHDQYRYDSSFTWVCSPNDTHACRVRAF 7ar8.1    --------------------------------------------------------------TETIDV-SDAVGSNIRVD  target    VRNGVVMRVEQNYDHQTYEDLYGNRGTFAHNPRMCLKGFTFHRRVYGPYRLKGPLMRKGWKQWMDDNAPELTAETKRKYK 7ar8.1    SRGPEVMRIIPRLNEDI------------NEEWISDKTRFCYDGLK-RQRLSDPMIRDSD--------------------  target    FDSRFLDDMLRVSWDTAFTYAAKAMITIATRYSGEAGARRLREQGYAPEMIEMMKGAGTRCFKHRAGMPVLGIIGKMGNT 7ar8.1    ------GRFKAVSWRDALAVVGDIIHQV----KPDEIV--------------GVAGQLSDA-------------------  target    RMNGGINALLDTWIRKVSPDQAQGGRYWSNYTWHGDQNPAHPFWSGVQGSDIDLSDMRFSKLNTSWGKNFVENKMPEAHW 7ar8.1    ----ESMMVLKDFVNRM------GSDNV---WCEGTAAGVDADLRYSYLMNTSISGLENADLFLLIGTQPRVEAAMVNAR  target    KLECI-ERGARVVVITPEYNPTAYRADYWMPLRPESDGALFLGAMKIIIDENMHDIDFLKSFTDAPILVRTDTLQYLDPR 7ar8.1    ICKTVRASNAKVGYVGPPAEFN--YDCKHLGTGPDTLKEI----------------------------------------  target    DVIADYKFPDFSKSYSGRIQSLKPEQIQRLGGMMVWDLNKKQVVPLHREQVGWHYTNSGIDAALTGTYRVKLLNGREIDA 7ar8.1    --------------------------------------------------------------------------------  target    MPIWQMYMVHFQDYDLDTVHQITRTPKDLIVRWARDSGTIKPAAIHNGEGTCHYFHQTANARGAAMVLIITGNVGKFGTG 7ar8.1    --------------------------AEGRHPFCTALKNAKNPAIIVGAGLFNRTDKNAILSSVESIAQANNVVRPDWNG  target    QHTWAGNYKAGTWTATPWSGAGLSVHTGEDPFNITLDPNAHGKEIKTRSYYYGEEVGYWNHGDTALIVNTPKYGRKVFTG 7ar8.1    LNFLLQYAAQ----------------AA-------------ALDLG-----------LIQ--------------Q-SAK-  target    KTHMPTPSKFRWVVNVNVVNNAKHHYDMVRNVDPNIECLITQDIEMTSDINHADIAFAANSWMEFTYPEMTVTVSNPWVQ 7ar8.1    ---ALESAKFVYLMGADDVN---------VDKIPKDAFVVYQGHHGDKAVYRANVILPASAFTEKE---GTYENTEGFTQ  target    IWKGGIRPLYDTRNDLDTFAGVAAKLSDMTGDKRMRDYFAMVYQNRVDVYVQRMLDASSTFYGYSADVMLKSEKGWMVMV 7ar8.1    QTVPAVPTVGDARDDWKIVRALSEVSG-----------------------------------------------------  target    RTYPRHPFWEETNESKPMWTRSGRYENYRIEPEAIEYGENFISHREGPEATPYLPNAIFTTNPYVRPDDYGIPITAQHHD 7ar8.1    --------------------------------------------------------------------------------  target    DKTVRNIKLSWHEIKRHSNPLWEKGYQFYCVTPKTRHRVHSQWSVNDWVQIYESNFGDPYRMDKRTPGVGEHQIHINPQA 7ar8.1    --------------------------------------------------------------------------------  target    AKDRGINDGDYVYVDGNPVDRPYRGWKPSDPYYKVARLMIRAKYNPAYPYHVTMAKHAPFVATAKSVKGHETRPDGRAIA 7ar8.1    --------------------------------------------------------------------------------  target    IDTGYQSNFRYGAQQSFTRNWLMPMHQTDSLPGKHAVAWKFKWGYQVDHHAINTVPKECLIRITKAEDGGIGARGPWEPV 7ar8.1    --------------------------------------------------------------------------------  target    RTGFTPGQENEFMIKWLKGEHIKIKV 7ar8.1    -------------------------- ``` | | | | | | | | | | | | | | | | | | | | | | | | | | | | | | | | | | | | | | | | | | | | | | | | | |
|  | 6x89.1.H | NADH dehydrogenase [ubiquinone] iron-sulfur protein 1, mitochondrial  *Vigna radiata mitochondrial complex I\** | 0.15 |  | 12.53 | 0.33 | 63-746 | EM | 0.00 | hetero-1-1-1-1-1-1-… | 1 x NAP, 6 x PC1, 6 x SF4, 2 x FES, 2 x ZN, 1 x FMN | HHblits | 0.26 |
| ``` target    MFLSRRQFLKVSVGTVAAVAVADKVLALTALQPVIEVGNPLGDYPDRSWERVYHDQYRYDSSFTWVCSPNDTHACRVRAF 6x89.1    --------------------------------------------------------------TETIDV-TDAVGSNIRID  target    VRNGVVMRVEQNYDHQTYEDLYGNRGTFAHNPRMCLKGFTFHRRVYGPYRLKGPLMRKGWKQWMDDNAPELTAETKRKYK 6x89.1    SRGPEVMRIVPRLNEDI------------NEEWISDKTRFCYDGLK-RQRLNDPMIRGPD--------------------  target    FDSRFLDDMLRVSWDTAFTYAAKAMITIATRYSGEAGARRLREQGYAPEMIEMMKGAGTRCFKHRAGMPVLGIIGKMGNT 6x89.1    ------GRFKAVNWRDALSVIADIAHQV----KPEEIV--------------GVAGKLSDA-------------------  target    RMNGGINALLDTWIRKVSPDQAQGGRYWSNYTWHGDQNPAHPFWSGVQGSDIDLSDMRFSKLNTSWGKNFVENKMPEAHW 6x89.1    ----ESMIALKDFLNRM------GSNDVWG---EGIGVNTNADFRSGYIMNTSIAGLEKADVFLLVGTQPRVEAAMVNAR  target    KLECIE-RGARVVVITPEYNPTAYRADYWMPLRPESDGALFLGAMKIIIDENMHDIDFLKSFTDAPILVRTDTLQYLDPR 6x89.1    IRKTVRSNQAKVGYIGPATDFN--YDHKHLGTDPQTLVEIAE----------------------G---------------  target    DVIADYKFPDFSKSYSGRIQSLKPEQIQRLGGMMVWDLNKKQVVPLHREQVGWHYTNSGIDAALTGTYRVKLLNGREIDA 6x89.1    --------------------------------------------------------------------------------  target    MPIWQMYMVHFQDYDLDTVHQITRTPKDLIVRWARDSGTIKPAAIHNGEGTCHYFHQTANARGAAMVLIITGNVGKFGTG 6x89.1    -----------------------------RHPFFKTLSDAKNPVIIVGAGVFERKDQDAIFAAVETIAQKANVVRPDWNG  target    QHTWAGNYKAGTWTATPWSGAGLSVHTGEDPFNITLDPNAHGKEIKTRSYYYGEEVGYWNHGDTALIVNTPKYGRKVFTG 6x89.1    LNVLLLHAAQ----------------AA-------------ALDLG-----------LVPQ-----------S-----E-  target    KTHMPTPSKFRWVVNVNVVNNAKHHYDMVRNVDPNIECLITQDIEMTSDINHADIAFAANSWMEFTYPEMTVTVSNPWVQ 6x89.1    --KSLESAKFVYLMGADDVNL---------DKIPDDAFVVYQGHHGDKSVYRANVILPTAAFSEKE---GTYQNTEGCTQ  target    IWKGGIRPLYDTRNDLDTFAGVAAKLSDMTGDKRMRDYFAMVYQNRVDVYVQRMLDASSTFYGYSADVMLKSEKGWMVMV 6x89.1    QTLPAVPTVGDSRDDWKIIRALSEVAG-----------------------------------------------------  target    RTYPRHPFWEETNESKPMWTRSGRYENYRIEPEAIEYGENFISHREGPEATPYLPNAIFTTNPYVRPDDYGIPITAQHHD 6x89.1    --------------------------------------------------------------------------------  target    DKTVRNIKLSWHEIKRHSNPLWEKGYQFYCVTPKTRHRVHSQWSVNDWVQIYESNFGDPYRMDKRTPGVGEHQIHINPQA 6x89.1    --------------------------------------------------------------------------------  target    AKDRGINDGDYVYVDGNPVDRPYRGWKPSDPYYKVARLMIRAKYNPAYPYHVTMAKHAPFVATAKSVKGHETRPDGRAIA 6x89.1    --------------------------------------------------------------------------------  target    IDTGYQSNFRYGAQQSFTRNWLMPMHQTDSLPGKHAVAWKFKWGYQVDHHAINTVPKECLIRITKAEDGGIGARGPWEPV 6x89.1    --------------------------------------------------------------------------------  target    RTGFTPGQENEFMIKWLKGEHIKIKV 6x89.1    -------------------------- ``` | | | | | | | | | | | | | | | | | | | | | | | | | | | | | | | | | | | | | | | | | | | | | | | | | |
|  | 8e73.55.A | NDUS1  *Vigna radiata supercomplex I+III2 (full bridge)* | 0.16 |  | 12.53 | 0.33 | 63-746 | EM | 0.00 | monomer |  | HHblits | 0.26 |
| ``` target    MFLSRRQFLKVSVGTVAAVAVADKVLALTALQPVIEVGNPLGDYPDRSWERVYHDQYRYDSSFTWVCSPNDTHACRVRAF 8e73.55   --------------------------------------------------------------TETIDV-TDAVGSNIRID  target    VRNGVVMRVEQNYDHQTYEDLYGNRGTFAHNPRMCLKGFTFHRRVYGPYRLKGPLMRKGWKQWMDDNAPELTAETKRKYK 8e73.55   SRGPEVMRIVPRLNEDI------------NEEWISDKTRFCYDGLK-RQRLNDPMIRGPD--------------------  target    FDSRFLDDMLRVSWDTAFTYAAKAMITIATRYSGEAGARRLREQGYAPEMIEMMKGAGTRCFKHRAGMPVLGIIGKMGNT 8e73.55   ------GRFKAVNWRDALSVIADIAHQV----KPEEIV--------------GVAGKLSDA-------------------  target    RMNGGINALLDTWIRKVSPDQAQGGRYWSNYTWHGDQNPAHPFWSGVQGSDIDLSDMRFSKLNTSWGKNFVENKMPEAHW 8e73.55   ----ESMIALKDFLNRM------GSNDVWG---EGIGVNTNADFRSGYIMNTSIAGLEKADVFLLVGTQPRVEAAMVNAR  target    KLECIE-RGARVVVITPEYNPTAYRADYWMPLRPESDGALFLGAMKIIIDENMHDIDFLKSFTDAPILVRTDTLQYLDPR 8e73.55   IRKTVRSNQAKVGYIGPATDFN--YDHKHLGTDPQTLVEIAE----------------------G---------------  target    DVIADYKFPDFSKSYSGRIQSLKPEQIQRLGGMMVWDLNKKQVVPLHREQVGWHYTNSGIDAALTGTYRVKLLNGREIDA 8e73.55   --------------------------------------------------------------------------------  target    MPIWQMYMVHFQDYDLDTVHQITRTPKDLIVRWARDSGTIKPAAIHNGEGTCHYFHQTANARGAAMVLIITGNVGKFGTG 8e73.55   -----------------------------RHPFFKTLSDAKNPVIIVGAGVFERKDQDAIFAAVETIAQKANVVRPDWNG  target    QHTWAGNYKAGTWTATPWSGAGLSVHTGEDPFNITLDPNAHGKEIKTRSYYYGEEVGYWNHGDTALIVNTPKYGRKVFTG 8e73.55   LNVLLLHAAQ----------------AA-------------ALDLG-----------LVPQ-----------S-----E-  target    KTHMPTPSKFRWVVNVNVVNNAKHHYDMVRNVDPNIECLITQDIEMTSDINHADIAFAANSWMEFTYPEMTVTVSNPWVQ 8e73.55   --KSLESAKFVYLMGADDVNL---------DKIPDDAFVVYQGHHGDKSVYRANVILPTAAFSEKE---GTYQNTEGCTQ  target    IWKGGIRPLYDTRNDLDTFAGVAAKLSDMTGDKRMRDYFAMVYQNRVDVYVQRMLDASSTFYGYSADVMLKSEKGWMVMV 8e73.55   QTLPAVPTVGDSRDDWKIIRALSEVAG-----------------------------------------------------  target    RTYPRHPFWEETNESKPMWTRSGRYENYRIEPEAIEYGENFISHREGPEATPYLPNAIFTTNPYVRPDDYGIPITAQHHD 8e73.55   --------------------------------------------------------------------------------  target    DKTVRNIKLSWHEIKRHSNPLWEKGYQFYCVTPKTRHRVHSQWSVNDWVQIYESNFGDPYRMDKRTPGVGEHQIHINPQA 8e73.55   --------------------------------------------------------------------------------  target    AKDRGINDGDYVYVDGNPVDRPYRGWKPSDPYYKVARLMIRAKYNPAYPYHVTMAKHAPFVATAKSVKGHETRPDGRAIA 8e73.55   --------------------------------------------------------------------------------  target    IDTGYQSNFRYGAQQSFTRNWLMPMHQTDSLPGKHAVAWKFKWGYQVDHHAINTVPKECLIRITKAEDGGIGARGPWEPV 8e73.55   --------------------------------------------------------------------------------  target    RTGFTPGQENEFMIKWLKGEHIKIKV 8e73.55   -------------------------- ``` | | | | | | | | | | | | | | | | | | | | | | | | | | | | | | | | | | | | | | | | | | | | | | | | | |
|  | 8b9z.1.G | NADH-ubiquinone oxidoreductase 75 kDa subunit, mitochondrial  *Drosophila melanogaster complex I in the Active state (Dm1)* | 0.15 |  | 12.53 | 0.33 | 62-746 | EM | 3.28 | hetero-1-1-1-1-1-1-… | 3 x PC1, 16 x 3PE, 6 x SF4, 4 x CDL, 2 x FES, 1 x FMN, 1 x UQ9, 1 x DGT, 1 x NDP, 1 x ZN, 2 x EHZ | HHblits | 0.26 |
| ``` target    MFLSRRQFLKVSVGTVAAVAVADKVLALTALQPVIEVGNPLGDYPDRSWERVYHDQYRYDSSFTWVCSPNDTHACRVRAF 8b9z.1    -------------------------------------------------------------KVSSIDV-LDAVGSNIVVS  target    VRNGVVMRVEQNYDHQTYEDLYGNRGTFAHNPRMCLKGFTFHRRVYGPYRLKGPLMRKGWKQWMDDNAPELTAETKRKYK 8b9z.1    TRTNEVLRILPRENEDV------------NEEWLADKSRFACDGLK-RQRLVAPMVRMPN--------------------  target    FDSRFLDDMLRVSWDTAFTYAAKAMITIATRYSGEAGARRLREQGYAPEMIEMMKGAGTRCFKHRAGMPVLGIIGKMGNT 8b9z.1    ------GELQAVEWEGALIAVAKAIKAAGG-----QI--------------AGISGQLAD--------------------  target    RMNGGINALLDTWIRKVSPDQAQGGRYWSNYTWHGDQNPAHPFWSGVQGSDIDLSDMRFSKLNTSWGKNFVENKMPEAHW 8b9z.1    ---LEAQVALKDLLNRL------GSEVVATEQGFIAGGTD---NRANYLLNSTIAGLEEADAVLLVGTNPRYEAPLVNTR  target    KLECI-ERGARVVVITPEYNPTAYRADYWMPLRPESDGALFLGAMKIIIDENMHDIDFLKSFTDAPILVRTDTLQYLDPR 8b9z.1    LRKAYVHNELQIASIGPKIDLS------YDHENLGADAALVKDV------------------CSG---------------  target    DVIADYKFPDFSKSYSGRIQSLKPEQIQRLGGMMVWDLNKKQVVPLHREQVGWHYTNSGIDAALTGTYRVKLLNGREIDA 8b9z.1    --------------------------------------------------------------------------------  target    MPIWQMYMVHFQDYDLDTVHQITRTPKDLIVRWARDSGTIKPAAIHNGEGTCHYFHQTANARGAAMVLIITGNVGKFGTG 8b9z.1    -----------------------------AHAFSKVLEGAKKPAIIIGADLLERADGAAIHAT---VAEYCKKLKKPNWN  target    Q-HTWAGNYKAGTWTATPWSGAGLSVHTGEDPFNITLDPNAHGKEIKTRSYYYGEEVGYWNHGDTALIVNTPKYGRKVFT 8b9z.1    PFNVLQTNAA----------------QVGA--L-----------DVG-----------YKAG-----------A-QT---  target    GKTHMPTPSKFRWVVNVNVVNNAKHHYDMVRNVDPNIECLITQDIEMTSDINHADIAFAANSWMEFTYPEMTVTVSNPWV 8b9z.1    ---AVKAQPKVLFLLNADAG-------KVTREQLPKDCFVVYIGSHGDNGASIADAVLPGAAYTEKQ---GIYVNTEGRP  target    QIWKGGIRPLYDTRNDLDTFAGVAAKLSDMTGDKRMRDYFAMVYQNRVDVYVQRMLDASSTFYGYSADVMLKSEKGWMVM 8b9z.1    QQTLPGVSPPGMAREDWKILRALSEVVG----------------------------------------------------  target    VRTYPRHPFWEETNESKPMWTRSGRYENYRIEPEAIEYGENFISHREGPEATPYLPNAIFTTNPYVRPDDYGIPITAQHH 8b9z.1    --------------------------------------------------------------------------------  target    DDKTVRNIKLSWHEIKRHSNPLWEKGYQFYCVTPKTRHRVHSQWSVNDWVQIYESNFGDPYRMDKRTPGVGEHQIHINPQ 8b9z.1    --------------------------------------------------------------------------------  target    AAKDRGINDGDYVYVDGNPVDRPYRGWKPSDPYYKVARLMIRAKYNPAYPYHVTMAKHAPFVATAKSVKGHETRPDGRAI 8b9z.1    --------------------------------------------------------------------------------  target    AIDTGYQSNFRYGAQQSFTRNWLMPMHQTDSLPGKHAVAWKFKWGYQVDHHAINTVPKECLIRITKAEDGGIGARGPWEP 8b9z.1    --------------------------------------------------------------------------------  target    VRTGFTPGQENEFMIKWLKGEHIKIKV 8b9z.1    --------------------------- ``` | | | | | | | | | | | | | | | | | | | | | | | | | | | | | | | | | | | | | | | | | | | | | | | | | |
|  | 8ba0.1.G | NADH-ubiquinone oxidoreductase 75 kDa subunit, mitochondrial  *Drosophila melanogaster complex I in the Twisted state (Dm2)* | 0.15 |  | 12.53 | 0.33 | 62-746 | EM | 3.68 | hetero-1-1-1-1-1-1-… | 6 x SF4, 6 x 3PE, 2 x FES, 1 x FMN, 2 x CDL, 1 x DGT, 1 x NDP, 1 x ZN, 2 x EHZ | HHblits | 0.26 |
| ``` target    MFLSRRQFLKVSVGTVAAVAVADKVLALTALQPVIEVGNPLGDYPDRSWERVYHDQYRYDSSFTWVCSPNDTHACRVRAF 8ba0.1    -------------------------------------------------------------KVSSIDV-LDAVGSNIVVS  target    VRNGVVMRVEQNYDHQTYEDLYGNRGTFAHNPRMCLKGFTFHRRVYGPYRLKGPLMRKGWKQWMDDNAPELTAETKRKYK 8ba0.1    TRTNEVLRILPRENEDV------------NEEWLADKSRFACDGLK-RQRLVAPMVRMPN--------------------  target    FDSRFLDDMLRVSWDTAFTYAAKAMITIATRYSGEAGARRLREQGYAPEMIEMMKGAGTRCFKHRAGMPVLGIIGKMGNT 8ba0.1    ------GELQAVEWEGALIAVAKAIKAAGG-----QI--------------AGISGQLAD--------------------  target    RMNGGINALLDTWIRKVSPDQAQGGRYWSNYTWHGDQNPAHPFWSGVQGSDIDLSDMRFSKLNTSWGKNFVENKMPEAHW 8ba0.1    ---LEAQVALKDLLNRL------GSEVVATEQGFIAGGTD---NRANYLLNSTIAGLEEADAVLLVGTNPRYEAPLVNTR  target    KLECI-ERGARVVVITPEYNPTAYRADYWMPLRPESDGALFLGAMKIIIDENMHDIDFLKSFTDAPILVRTDTLQYLDPR 8ba0.1    LRKAYVHNELQIASIGPKIDLS------YDHENLGADAALVKDV------------------CSG---------------  target    DVIADYKFPDFSKSYSGRIQSLKPEQIQRLGGMMVWDLNKKQVVPLHREQVGWHYTNSGIDAALTGTYRVKLLNGREIDA 8ba0.1    --------------------------------------------------------------------------------  target    MPIWQMYMVHFQDYDLDTVHQITRTPKDLIVRWARDSGTIKPAAIHNGEGTCHYFHQTANARGAAMVLIITGNVGKFGTG 8ba0.1    -----------------------------AHAFSKVLEGAKKPAIIIGADLLERADGAAIHAT---VAEYCKKLKKPNWN  target    Q-HTWAGNYKAGTWTATPWSGAGLSVHTGEDPFNITLDPNAHGKEIKTRSYYYGEEVGYWNHGDTALIVNTPKYGRKVFT 8ba0.1    PFNVLQTNAA----------------QVGA--L-----------DVG-----------YKAG-----------A-QT---  target    GKTHMPTPSKFRWVVNVNVVNNAKHHYDMVRNVDPNIECLITQDIEMTSDINHADIAFAANSWMEFTYPEMTVTVSNPWV 8ba0.1    ---AVKAQPKVLFLLNADAG-------KVTREQLPKDCFVVYIGSHGDNGASIADAVLPGAAYTEKQ---GIYVNTEGRP  target    QIWKGGIRPLYDTRNDLDTFAGVAAKLSDMTGDKRMRDYFAMVYQNRVDVYVQRMLDASSTFYGYSADVMLKSEKGWMVM 8ba0.1    QQTLPGVSPPGMAREDWKILRALSEVVG----------------------------------------------------  target    VRTYPRHPFWEETNESKPMWTRSGRYENYRIEPEAIEYGENFISHREGPEATPYLPNAIFTTNPYVRPDDYGIPITAQHH 8ba0.1    --------------------------------------------------------------------------------  target    DDKTVRNIKLSWHEIKRHSNPLWEKGYQFYCVTPKTRHRVHSQWSVNDWVQIYESNFGDPYRMDKRTPGVGEHQIHINPQ 8ba0.1    --------------------------------------------------------------------------------  target    AAKDRGINDGDYVYVDGNPVDRPYRGWKPSDPYYKVARLMIRAKYNPAYPYHVTMAKHAPFVATAKSVKGHETRPDGRAI 8ba0.1    --------------------------------------------------------------------------------  target    AIDTGYQSNFRYGAQQSFTRNWLMPMHQTDSLPGKHAVAWKFKWGYQVDHHAINTVPKECLIRITKAEDGGIGARGPWEP 8ba0.1    --------------------------------------------------------------------------------  target    VRTGFTPGQENEFMIKWLKGEHIKIKV 8ba0.1    --------------------------- ``` | | | | | | | | | | | | | | | | | | | | | | | | | | | | | | | | | | | | | | | | | | | | | | | | | |
|  | 1eu1.1.A | DIMETHYL SULFOXIDE REDUCTASE  *THE CRYSTAL STRUCTURE OF RHODOBACTER SPHAEROIDES DIMETHYLSULFOXIDE REDUCTASE REVEALS TWO DISTINCT MOLYBDENUM COORDINATION ENVIRONMENTS.* | 0.08 |  | 29.84 | 0.22 | 663-973 | X-ray | 1.30 | monomer | 3 x GLC, 1 x CD, 2 x MGD, 1 x 6MO, 2 x O | BLAST | 0.34 |
| ``` target    MFLSRRQFLKVSVGTVAAVAVADKVLALTALQPVIEVGNPLGDYPDRSWERVYHDQYRYDSSFTWVCSPNDTHACRVRAF 1eu1.1    --------------------------------------------------------------------------------  target    VRNGVVMRVEQNYDHQTYEDLYGNRGTFAHNPRMCLKGFTFHRRVYGPYRLKGPLMRKGWKQWMDDNAPELTAETKRKYK 1eu1.1    --------------------------------------------------------------------------------  target    FDSRFLDDMLRVSWDTAFTYAAKAMITIATRYSGEAGARRLREQGYAPEMIEMMKGAGTRCFKHRAGMPVLGIIGKMGNT 1eu1.1    --------------------------------------------------------------------------------  target    RMNGGINALLDTWIRKVSPDQAQGGRYWSNYTWHGDQNPAHPFWSGVQGSDIDLSDMRFSKLNTSWGKNFVENKMPEAHW 1eu1.1    --------------------------------------------------------------------------------  target    KLECIERGARVVVITPEYNPTAYRADYWMPLRPESDGALFLGAMKIIIDENMHDIDFLKSFTDAPILVRTDTLQYLDPRD 1eu1.1    --------------------------------------------------------------------------------  target    VIADYKFPDFSKSYSGRIQSLKPEQIQRLGGMMVWDLNKKQVVPLHREQVGWHYTNSGIDAALTGTYRVKLLNGREIDAM 1eu1.1    --------------------------------------------------------------------------------  target    PIWQMYMVHFQDYDLDTVHQITRTPKDLIVRWARDSGTIKPAAIHNGEGTCHYFHQTANARGAAMVLIITGNVGKFGTGQ 1eu1.1    --------------------------------------------------------------------------------  target    HTWAGNYKAGTWTATPWSGAGLSVHTGEDPFNITLDPNAHGKEIKTRSYYYGEEVGYWNHGDTALIVNTPKYGRKVFTGK 1eu1.1    --------------------------------------------------------------------------------  target    THMPTPSKFRWVVNVNVVNNAKHHYDMVRNVDP--NIECLITQDIEMTSDINHADIAFAANSWMEFTYPEMTVTVSNPWV 1eu1.1    ----------------------HHQDRNRMLKAWEKLETFIVQDFQWTATARHADIVLPATTSYERNDIESVGDYSNRAI  target    QIWKGGIRPLYDTRNDLDTFAGVAAKLSDMTGDKRMRDYFAMVYQNRVDVYVQRMLDASSTFYGYSADVMLKSEKGWMVM 1eu1.1    LAMKKVVDPLYEARSDYDIFAALAERLGKGAEFTEGRDEMGWI----------------SSFY----EAAVKQAEFKNVA  target    VRTYPRHPFWEETNESKPMWTRSG--RYENYRIEPEAIEYGENFISHREGPEATPYLPNAIFTTNPYVRPDDYGIPITAQ 1eu1.1    MPSF--EDFWSEGIVEFPITEGANFVRYADFREDP------------LFNPLGTPSGLIEIYSKN-----------IEKM  target    HHDDKTVRNIKLSWHEIKRHSNPLWEKGYQFYCVTPKTRHRVHSQWSVNDWVQIYESNFGDPYRMDKRTPGVGEHQIHIN 1eu1.1    GYDDCPAHP---TWMEPAERLGGAGAK-YPLHVVASHPKSRLHSQLNGTSLRDLYAV--------------AGHEPCLIN  target    PQAAKDRGINDGDYVYVDGNPVDRPYRGWKPSDPYYKVARLMIRAKYNPAYPYHVTMAKHAPFVATAKSVKGHETRPDGR 1eu1.1    PADAAARGIADGDVLRV---------------------------------------------------------------  target    AIAIDTGYQSNFRYGAQQSFTRNWLMPMHQTDSLPGKHAVAWKFKWGYQVDHHAINTVPKECLIRITKAEDGGIGARGPW 1eu1.1    --------------------------------------------------------------------------------  target    EPVRTGFTPGQENEFMIKWLKGEHIKIKV 1eu1.1    ----------------------------- ``` | | | | | | | | | | | | | | | | | | | | | | | | | | | | | | | | | | | | | | | | | | | | | | | | | |
|  | 1aa6.1.A | FORMATE DEHYDROGENASE H  *REDUCED FORM OF FORMATE DEHYDROGENASE H FROM E. COLI* | 0.09 |  | 33.04 | 0.20 | 66-383 | X-ray | 2.30 | monomer | 1 x SF4, 2 x MGD, 1 x 4MO | BLAST | 0.36 |
| ``` target    MFLSRRQFLKVSVGTVAAVAVADKVLALTALQPVIEVGNPLGDYPDRSWERVYHDQYRYDSSFTWVCSPNDTHACRVRAF 1aa6.1    -----------------------------------------------------------------VC-PYCASGCKINLV  target    VRNGVVMRVEQNYDHQTYEDLYGNRGTFAHNPRMCLKG-----FTFHRRVYGPYRLKGPLMRKGWKQWMDDNAPELTAET 1aa6.1    VDNGKIVRAEAAQGKT-------NQGT------LCLKGYYGWDFINDTQILTP-RLKTPMIRR-----------------  target    KRKYKFDSRFLDDMLRVSWDTAFTYAAKAMITIATRYSGEAGARRLREQGYAPEMIEMMKG-----AGTR----CFKHRA 1aa6.1    QRGGKLEP--------VSWDEALNYVAERLSAIKEKYGPDAIQTTGSSRGTGNETNYVMQKFARAVIGTNNVDCCARVUH  target    GMPVLGIIGKMGNTRMNGGINALLDTWIRKVSPDQAQGGRYWSNYTWHGDQNPAHPFWSGVQGSDIDLSDMRFSKLNTSW 1aa6.1    GPSVAGLHQSVGNGAMSNAIN-------------------------------------------EIDNTDLVFV-----F  target    GKNFVENKMPEAHWKLECIERGARVVVITPEYNPTAYRADYWMPLRPESDGALFLGAMKIIIDENMHDIDFLKSFTDAPI 1aa6.1    GYNPADSHPIVANHVINAKRNGAKIIVCDPRKIETARIADMHIALKNGSNIALLNAMGHVIIEENLYDKAFVASRTE---  target    LVRTDTLQYLDPRDVIADYKFPDFSKSYSGRIQSLKPEQIQRLGGMMVWDLNKKQVVPLHREQVGWHYTNSGIDAALTGT 1aa6.1    --------------------------------------------------------------------------------  target    YRVKLLNGREIDAMPIWQMYMVHFQDYDLDTVHQITRTPKDLIVRWARDSGTIKPAAIHNGEGTCHYFHQTANARGAAMV 1aa6.1    --------------------------------------------------------------------------------  target    LIITGNVGKFGTGQHTWAGNYKAGTWTATPWSGAGLSVHTGEDPFNITLDPNAHGKEIKTRSYYYGEEVGYWNHGDTALI 1aa6.1    --------------------------------------------------------------------------------  target    VNTPKYGRKVFTGKTHMPTPSKFRWVVNVNVVNNAKHHYDMVRNVDPNIECLITQDIEMTSDINHADIAFAANSWMEFTY 1aa6.1    --------------------------------------------------------------------------------  target    PEMTVTVSNPWVQIWKGGIRPLYDTRNDLDTFAGVAAKLSDMTGDKRMRDYFAMVYQNRVDVYVQRMLDASSTFYGYSAD 1aa6.1    --------------------------------------------------------------------------------  target    VMLKSEKGWMVMVRTYPRHPFWEETNESKPMWTRSGRYENYRIEPEAIEYGENFISHREGPEATPYLPNAIFTTNPYVRP 1aa6.1    --------------------------------------------------------------------------------  target    DDYGIPITAQHHDDKTVRNIKLSWHEIKRHSNPLWEKGYQFYCVTPKTRHRVHSQWSVNDWVQIYESNFGDPYRMDKRTP 1aa6.1    --------------------------------------------------------------------------------  target    GVGEHQIHINPQAAKDRGINDGDYVYVDGNPVDRPYRGWKPSDPYYKVARLMIRAKYNPAYPYHVTMAKHAPFVATAKSV 1aa6.1    --------------------------------------------------------------------------------  target    KGHETRPDGRAIAIDTGYQSNFRYGAQQSFTRNWLMPMHQTDSLPGKHAVAWKFKWGYQVDHHAINTVPKECLIRITKAE 1aa6.1    --------------------------------------------------------------------------------  target    DGGIGARGPWEPVRTGFTPGQENEFMIKWLKGEHIKIKV 1aa6.1    --------------------------------------- ``` | | | | | | | | | | | | | | | | | | | | | | | | | | | | | | | | | | | | | | | | | | | | | | | | | |
|  | 1fdo.1.A | FORMATE DEHYDROGENASE H  *OXIDIZED FORM OF FORMATE DEHYDROGENASE H FROM E. COLI* | 0.09 |  | 33.04 | 0.20 | 66-383 | X-ray | 2.80 | monomer | 1 x SF4, 2 x MGD, 1 x 6MO | BLAST | 0.36 |
| ``` target    MFLSRRQFLKVSVGTVAAVAVADKVLALTALQPVIEVGNPLGDYPDRSWERVYHDQYRYDSSFTWVCSPNDTHACRVRAF 1fdo.1    -----------------------------------------------------------------VC-PYCASGCKINLV  target    VRNGVVMRVEQNYDHQTYEDLYGNRGTFAHNPRMCLKG-----FTFHRRVYGPYRLKGPLMRKGWKQWMDDNAPELTAET 1fdo.1    VDNGKIVRAEAAQGKT-------NQGT------LCLKGYYGWDFINDTQILTP-RLKTPMIRR-----------------  target    KRKYKFDSRFLDDMLRVSWDTAFTYAAKAMITIATRYSGEAGARRLREQGYAPEMIEMMKG-----AGTR----CFKHRA 1fdo.1    QRGGKLEP--------VSWDEALNYVAERLSAIKEKYGPDAIQTTGSSRGTGNETNYVMQKFARAVIGTNNVDCCARVUH  target    GMPVLGIIGKMGNTRMNGGINALLDTWIRKVSPDQAQGGRYWSNYTWHGDQNPAHPFWSGVQGSDIDLSDMRFSKLNTSW 1fdo.1    GPSVAGLHQSVGNGAMSNAIN-------------------------------------------EIDNTDLVFV-----F  target    GKNFVENKMPEAHWKLECIERGARVVVITPEYNPTAYRADYWMPLRPESDGALFLGAMKIIIDENMHDIDFLKSFTDAPI 1fdo.1    GYNPADSHPIVANHVINAKRNGAKIIVCDPRKIETARIADMHIALKNGSNIALLNAMGHVIIEENLYDKAFVASRTE---  target    LVRTDTLQYLDPRDVIADYKFPDFSKSYSGRIQSLKPEQIQRLGGMMVWDLNKKQVVPLHREQVGWHYTNSGIDAALTGT 1fdo.1    --------------------------------------------------------------------------------  target    YRVKLLNGREIDAMPIWQMYMVHFQDYDLDTVHQITRTPKDLIVRWARDSGTIKPAAIHNGEGTCHYFHQTANARGAAMV 1fdo.1    --------------------------------------------------------------------------------  target    LIITGNVGKFGTGQHTWAGNYKAGTWTATPWSGAGLSVHTGEDPFNITLDPNAHGKEIKTRSYYYGEEVGYWNHGDTALI 1fdo.1    --------------------------------------------------------------------------------  target    VNTPKYGRKVFTGKTHMPTPSKFRWVVNVNVVNNAKHHYDMVRNVDPNIECLITQDIEMTSDINHADIAFAANSWMEFTY 1fdo.1    --------------------------------------------------------------------------------  target    PEMTVTVSNPWVQIWKGGIRPLYDTRNDLDTFAGVAAKLSDMTGDKRMRDYFAMVYQNRVDVYVQRMLDASSTFYGYSAD 1fdo.1    --------------------------------------------------------------------------------  target    VMLKSEKGWMVMVRTYPRHPFWEETNESKPMWTRSGRYENYRIEPEAIEYGENFISHREGPEATPYLPNAIFTTNPYVRP 1fdo.1    --------------------------------------------------------------------------------  target    DDYGIPITAQHHDDKTVRNIKLSWHEIKRHSNPLWEKGYQFYCVTPKTRHRVHSQWSVNDWVQIYESNFGDPYRMDKRTP 1fdo.1    --------------------------------------------------------------------------------  target    GVGEHQIHINPQAAKDRGINDGDYVYVDGNPVDRPYRGWKPSDPYYKVARLMIRAKYNPAYPYHVTMAKHAPFVATAKSV 1fdo.1    --------------------------------------------------------------------------------  target    KGHETRPDGRAIAIDTGYQSNFRYGAQQSFTRNWLMPMHQTDSLPGKHAVAWKFKWGYQVDHHAINTVPKECLIRITKAE 1fdo.1    --------------------------------------------------------------------------------  target    DGGIGARGPWEPVRTGFTPGQENEFMIKWLKGEHIKIKV 1fdo.1    --------------------------------------- ``` | | | | | | | | | | | | | | | | | | | | | | | | | | | | | | | | | | | | | | | | | | | | | | | | | |
|  | 2iv2.1.A | Formate dehydrogenase H  *Reinterpretation of reduced form of formate dehydrogenase H from E. coli* | 0.09 |  | 33.04 | 0.20 | 66-383 | X-ray | 2.27 | monomer | 1 x SF4, 1 x 2MD, 1 x MGD | BLAST | 0.36 |
| ``` target    MFLSRRQFLKVSVGTVAAVAVADKVLALTALQPVIEVGNPLGDYPDRSWERVYHDQYRYDSSFTWVCSPNDTHACRVRAF 2iv2.1    -----------------------------------------------------------------VC-PYCASGCKINLV  target    VRNGVVMRVEQNYDHQTYEDLYGNRGTFAHNPRMCLKG-----FTFHRRVYGPYRLKGPLMRKGWKQWMDDNAPELTAET 2iv2.1    VDNGKIVRAEAAQGKT-------NQGT------LCLKGYYGWDFINDTQILTP-RLKTPMIRR-----------------  target    KRKYKFDSRFLDDMLRVSWDTAFTYAAKAMITIATRYSGEAGARRLREQGYAPEMIEMMKG-----AGTR----CFKHRA 2iv2.1    QRGGKLEP--------VSWDEALNYVAERLSAIKEKYGPDAIQTTGSSRGTGNETNYVMQKFARAVIGTNNVDCCARVUH  target    GMPVLGIIGKMGNTRMNGGINALLDTWIRKVSPDQAQGGRYWSNYTWHGDQNPAHPFWSGVQGSDIDLSDMRFSKLNTSW 2iv2.1    GPSVAGLHQSVGNGAMSNAIN-------------------------------------------EIDNTDLVFV-----F  target    GKNFVENKMPEAHWKLECIERGARVVVITPEYNPTAYRADYWMPLRPESDGALFLGAMKIIIDENMHDIDFLKSFTDAPI 2iv2.1    GYNPADSHPIVANHVINAKRNGAKIIVCDPRKIETARIADMHIALKNGSNIALLNAMGHVIIEENLYDKAFVASRTE---  target    LVRTDTLQYLDPRDVIADYKFPDFSKSYSGRIQSLKPEQIQRLGGMMVWDLNKKQVVPLHREQVGWHYTNSGIDAALTGT 2iv2.1    --------------------------------------------------------------------------------  target    YRVKLLNGREIDAMPIWQMYMVHFQDYDLDTVHQITRTPKDLIVRWARDSGTIKPAAIHNGEGTCHYFHQTANARGAAMV 2iv2.1    --------------------------------------------------------------------------------  target    LIITGNVGKFGTGQHTWAGNYKAGTWTATPWSGAGLSVHTGEDPFNITLDPNAHGKEIKTRSYYYGEEVGYWNHGDTALI 2iv2.1    --------------------------------------------------------------------------------  target    VNTPKYGRKVFTGKTHMPTPSKFRWVVNVNVVNNAKHHYDMVRNVDPNIECLITQDIEMTSDINHADIAFAANSWMEFTY 2iv2.1    --------------------------------------------------------------------------------  target    PEMTVTVSNPWVQIWKGGIRPLYDTRNDLDTFAGVAAKLSDMTGDKRMRDYFAMVYQNRVDVYVQRMLDASSTFYGYSAD 2iv2.1    --------------------------------------------------------------------------------  target    VMLKSEKGWMVMVRTYPRHPFWEETNESKPMWTRSGRYENYRIEPEAIEYGENFISHREGPEATPYLPNAIFTTNPYVRP 2iv2.1    --------------------------------------------------------------------------------  target    DDYGIPITAQHHDDKTVRNIKLSWHEIKRHSNPLWEKGYQFYCVTPKTRHRVHSQWSVNDWVQIYESNFGDPYRMDKRTP 2iv2.1    --------------------------------------------------------------------------------  target    GVGEHQIHINPQAAKDRGINDGDYVYVDGNPVDRPYRGWKPSDPYYKVARLMIRAKYNPAYPYHVTMAKHAPFVATAKSV 2iv2.1    --------------------------------------------------------------------------------  target    KGHETRPDGRAIAIDTGYQSNFRYGAQQSFTRNWLMPMHQTDSLPGKHAVAWKFKWGYQVDHHAINTVPKECLIRITKAE 2iv2.1    --------------------------------------------------------------------------------  target    DGGIGARGPWEPVRTGFTPGQENEFMIKWLKGEHIKIKV 2iv2.1    --------------------------------------- ``` | | | | | | | | | | | | | | | | | | | | | | | | | | | | | | | | | | | | | | | | | | | | | | | | | |
|  | 7z0t.1.G | Formate dehydrogenase H  *Structure of the Escherichia coli formate hydrogenlyase complex (aerobic preparation, composite structure)* | 0.09 |  | 33.04 | 0.20 | 66-383 | EM | 0.00 | hetero-1-1-1-1-1-1-… | 1 x NI, 1 x FCO, 8 x SF4, 1 x FE, 2 x MGD, 1 x 6MO | BLAST | 0.36 |
| ``` target    MFLSRRQFLKVSVGTVAAVAVADKVLALTALQPVIEVGNPLGDYPDRSWERVYHDQYRYDSSFTWVCSPNDTHACRVRAF 7z0t.1    -----------------------------------------------------------------VC-PYCASGCKINLV  target    VRNGVVMRVEQNYDHQTYEDLYGNRGTFAHNPRMCLKG-----FTFHRRVYGPYRLKGPLMRKGWKQWMDDNAPELTAET 7z0t.1    VDNGKIVRAEAAQGKT-------NQGT------LCLKGYYGWDFINDTQILTP-RLKTPMIRR-----------------  target    KRKYKFDSRFLDDMLRVSWDTAFTYAAKAMITIATRYSGEAGARRLREQGYAPEMIEMMKG-----AGTR----CFKHRA 7z0t.1    QRGGKLEP--------VSWDEALNYVAERLSAIKEKYGPDAIQTTGSSRGTGNETNYVMQKFARAVIGTNNVDCCARVUH  target    GMPVLGIIGKMGNTRMNGGINALLDTWIRKVSPDQAQGGRYWSNYTWHGDQNPAHPFWSGVQGSDIDLSDMRFSKLNTSW 7z0t.1    GPSVAGLHQSVGNGAMSNAIN-------------------------------------------EIDNTDLVFV-----F  target    GKNFVENKMPEAHWKLECIERGARVVVITPEYNPTAYRADYWMPLRPESDGALFLGAMKIIIDENMHDIDFLKSFTDAPI 7z0t.1    GYNPADSHPIVANHVINAKRNGAKIIVCDPRKIETARIADMHIALKNGSNIALLNAMGHVIIEENLYDKAFVASRTE---  target    LVRTDTLQYLDPRDVIADYKFPDFSKSYSGRIQSLKPEQIQRLGGMMVWDLNKKQVVPLHREQVGWHYTNSGIDAALTGT 7z0t.1    --------------------------------------------------------------------------------  target    YRVKLLNGREIDAMPIWQMYMVHFQDYDLDTVHQITRTPKDLIVRWARDSGTIKPAAIHNGEGTCHYFHQTANARGAAMV 7z0t.1    --------------------------------------------------------------------------------  target    LIITGNVGKFGTGQHTWAGNYKAGTWTATPWSGAGLSVHTGEDPFNITLDPNAHGKEIKTRSYYYGEEVGYWNHGDTALI 7z0t.1    --------------------------------------------------------------------------------  target    VNTPKYGRKVFTGKTHMPTPSKFRWVVNVNVVNNAKHHYDMVRNVDPNIECLITQDIEMTSDINHADIAFAANSWMEFTY 7z0t.1    --------------------------------------------------------------------------------  target    PEMTVTVSNPWVQIWKGGIRPLYDTRNDLDTFAGVAAKLSDMTGDKRMRDYFAMVYQNRVDVYVQRMLDASSTFYGYSAD 7z0t.1    --------------------------------------------------------------------------------  target    VMLKSEKGWMVMVRTYPRHPFWEETNESKPMWTRSGRYENYRIEPEAIEYGENFISHREGPEATPYLPNAIFTTNPYVRP 7z0t.1    --------------------------------------------------------------------------------  target    DDYGIPITAQHHDDKTVRNIKLSWHEIKRHSNPLWEKGYQFYCVTPKTRHRVHSQWSVNDWVQIYESNFGDPYRMDKRTP 7z0t.1    --------------------------------------------------------------------------------  target    GVGEHQIHINPQAAKDRGINDGDYVYVDGNPVDRPYRGWKPSDPYYKVARLMIRAKYNPAYPYHVTMAKHAPFVATAKSV 7z0t.1    --------------------------------------------------------------------------------  target    KGHETRPDGRAIAIDTGYQSNFRYGAQQSFTRNWLMPMHQTDSLPGKHAVAWKFKWGYQVDHHAINTVPKECLIRITKAE 7z0t.1    --------------------------------------------------------------------------------  target    DGGIGARGPWEPVRTGFTPGQENEFMIKWLKGEHIKIKV 7z0t.1    --------------------------------------- ``` | | | | | | | | | | | | | | | | | | | | | | | | | | | | | | | | | | | | | | | | | | | | | | | | | |
|  | 1kqf.1.A | FORMATE DEHYDROGENASE, NITRATE-INDUCIBLE, MAJOR SUBUNIT  *FORMATE DEHYDROGENASE N FROM E. COLI* | 0.09 |  | 26.79 | 0.20 | 296-565 | X-ray | 1.60 | hetero-oligomer | 3 x 6MO, 15 x SF4, 6 x MGD, 6 x HEM, 3 x CDL | BLAST | 0.34 |
| ``` target    MFLSRRQFLKVSVGTVAAVAVADKVLALTALQPVIEVGNPLGDYPDRSWERVYHDQYRYDSSFTWVCSPNDTHACRVRAF 1kqf.1    --------------------------------------------------------------------------------  target    VRNGVVMRVEQNYDHQTYEDLYGNRGTFAHNPRMCLKGFTFHRRVYGPYRLKGPLMRKGWKQWMDDNAPELTAETKRKYK 1kqf.1    --------------------------------------------------------------------------------  target    FDSRFLDDMLRVSWDTAFTYAAKAMITIATRYSGEAGARRLREQGYAPEMIEMMKGAGTRCFKHRAGMPVLGIIGKMGNT 1kqf.1    --------------------------------------------------------------------------------  target    RMNGGINALLDTWIRKVSPDQAQGGRYWSNYTWHGDQNPAHPFWSGVQGSDIDLSDMRFSKLNTSWGKNFVENKMPEAHW 1kqf.1    -------------------------------------------------------DIKNANVVMVMGGNAAEAHPVGFRW  target    KLECIERG-ARVVVITPEYNPTAYRADYWMPLRPESDGALFLGAMKIIIDENMHDIDFLKSFTDAPILVRTDTLQYLDPR 1kqf.1    AMEAKNNNDATLIVVDPRFTRTASVADIYAPIRSGTDITFLSGVLRYLIENNKINAEYVKHYTNASLLVRD---------  target    DVIADYKFPDFSKSYSGRIQSLKPEQIQRLGGMMVWDLNKKQVVPLHREQVGWHYTNSGIDAALTGTYRVKLLNGREIDA 1kqf.1    ----DFAFED--GLFSG------------------YDAEKRQY-----DKSSWNYQ---LDENGYAKRDETLTHPR----  target    MPIWQMYMVHFQDYDLDTVHQITRTPKDLIVR----WARDSGTIKPAAIHNGEGTCHYFHQTANARGAAMVLIITGNVGK 1kqf.1    -CVWNLLKEHVSRYTPDVVENICGTPKADFLKVCEVLASTSAPDRTTTFLYALGWTQHTVGAQNIRTMAMIQLLLGNMGM  target    FGTGQHTWAGNYKAGTWTATPWSGAGLSVHTGEDPFNITLDPNAHGKEIKTRSYYYGEEVGYWNHGDTALIVNTPKYGRK 1kqf.1    AGGGVNALRG----------------------------------------------------------------------  target    VFTGKTHMPTPSKFRWVVNVNVVNNAKHHYDMVRNVDPNIECLITQDIEMTSDINHADIAFAANSWMEFTYPEMTVTVSN 1kqf.1    --------------------------------------------------------------------------------  target    PWVQIWKGGIRPLYDTRNDLDTFAGVAAKLSDMTGDKRMRDYFAMVYQNRVDVYVQRMLDASSTFYGYSADVMLKSEKGW 1kqf.1    --------------------------------------------------------------------------------  target    MVMVRTYPRHPFWEETNESKPMWTRSGRYENYRIEPEAIEYGENFISHREGPEATPYLPNAIFTTNPYVRPDDYGIPITA 1kqf.1    --------------------------------------------------------------------------------  target    QHHDDKTVRNIKLSWHEIKRHSNPLWEKGYQFYCVTPKTRHRVHSQWSVNDWVQIYESNFGDPYRMDKRTPGVGEHQIHI 1kqf.1    --------------------------------------------------------------------------------  target    NPQAAKDRGINDGDYVYVDGNPVDRPYRGWKPSDPYYKVARLMIRAKYNPAYPYHVTMAKHAPFVATAKSVKGHETRPDG 1kqf.1    --------------------------------------------------------------------------------  target    RAIAIDTGYQSNFRYGAQQSFTRNWLMPMHQTDSLPGKHAVAWKFKWGYQVDHHAINTVPKECLIRITKAEDGGIGARGP 1kqf.1    --------------------------------------------------------------------------------  target    WEPVRTGFTPGQENEFMIKWLKGEHIKIKV 1kqf.1    ------------------------------ ``` | | | | | | | | | | | | | | | | | | | | | | | | | | | | | | | | | | | | | | | | | | | | | | | | | |
|  | 8bqg.1.A | Formate dehydrogenase, alpha subunit, selenocysteine-containing  *W-formate dehydrogenase from Desulfovibrio vulgaris - Soaking with Formate 1 min* | 0.03 | 0.00 | 26.60 | 0.08 | 295-388 | X-ray | 1.95 | monomer | 2 x MGD, 4 x SF4, 1 x H2S, 1 x W | BLAST | 0.35 |
| ``` target    MFLSRRQFLKVSVGTVAAVAVADKVLALTALQPVIEVGNPLGDYPDRSWERVYHDQYRYDSSFTWVCSPNDTHACRVRAF 8bqg.1    --------------------------------------------------------------------------------  target    VRNGVVMRVEQNYDHQTYEDLYGNRGTFAHNPRMCLKGFTFHRRVYGPYRLKGPLMRKGWKQWMDDNAPELTAETKRKYK 8bqg.1    --------------------------------------------------------------------------------  target    FDSRFLDDMLRVSWDTAFTYAAKAMITIATRYSGEAGARRLREQGYAPEMIEMMKGAGTRCFKHRAGMPVLGIIGKMGNT 8bqg.1    --------------------------------------------------------------------------------  target    RMNGGINALLDTWIRKVSPDQAQGGRYWSNYTWHGDQNPAHPFWSGVQGSDIDLSDMRFSKLNTSWGKNFVENKMPEAHW 8bqg.1    ------------------------------------------------------NDLANSDCILIMGSNAAENHPIAFKW  target    KLECIERGARVVVITPEYNPTAYRADYWMPLRPESDGALFLGAMKIIIDENMHDIDFLKSFTDAPILVRTDTLQYLDPRD 8bqg.1    VLRAKDKGATLIHVDPRFTRTSARCDVYAPIRSGADIPFLGGLIKYILDNKLYFTDYVREYTNASLIV------------  target    VIADYKFPDFSKSYSGRIQSLKPEQIQRLGGMMVWDLNKKQVVPLHREQVGWHYTNSGIDAALTGTYRVKLLNGREIDAM 8bqg.1    --------------------------------------------------------------------------------  target    PIWQMYMVHFQDYDLDTVHQITRTPKDLIVRWARDSGTIKPAAIHNGEGTCHYFHQTANARGAAMVLIITGNVGKFGTGQ 8bqg.1    --------------------------------------------------------------------------------  target    HTWAGNYKAGTWTATPWSGAGLSVHTGEDPFNITLDPNAHGKEIKTRSYYYGEEVGYWNHGDTALIVNTPKYGRKVFTGK 8bqg.1    --------------------------------------------------------------------------------  target    THMPTPSKFRWVVNVNVVNNAKHHYDMVRNVDPNIECLITQDIEMTSDINHADIAFAANSWMEFTYPEMTVTVSNPWVQI 8bqg.1    --------------------------------------------------------------------------------  target    WKGGIRPLYDTRNDLDTFAGVAAKLSDMTGDKRMRDYFAMVYQNRVDVYVQRMLDASSTFYGYSADVMLKSEKGWMVMVR 8bqg.1    --------------------------------------------------------------------------------  target    TYPRHPFWEETNESKPMWTRSGRYENYRIEPEAIEYGENFISHREGPEATPYLPNAIFTTNPYVRPDDYGIPITAQHHDD 8bqg.1    --------------------------------------------------------------------------------  target    KTVRNIKLSWHEIKRHSNPLWEKGYQFYCVTPKTRHRVHSQWSVNDWVQIYESNFGDPYRMDKRTPGVGEHQIHINPQAA 8bqg.1    --------------------------------------------------------------------------------  target    KDRGINDGDYVYVDGNPVDRPYRGWKPSDPYYKVARLMIRAKYNPAYPYHVTMAKHAPFVATAKSVKGHETRPDGRAIAI 8bqg.1    --------------------------------------------------------------------------------  target    DTGYQSNFRYGAQQSFTRNWLMPMHQTDSLPGKHAVAWKFKWGYQVDHHAINTVPKECLIRITKAEDGGIGARGPWEPVR 8bqg.1    --------------------------------------------------------------------------------  target    TGFTPGQENEFMIKWLKGEHIKIKV 8bqg.1    ------------------------- ``` | | | | | | | | | | | | | | | | | | | | | | | | | | | | | | | | | | | | | | | | | | | | | | | | | |
|  | 6sdr.1.A | Formate dehydrogenase, alpha subunit, selenocysteine-containing  *W-formate dehydrogenase from Desulfovibrio vulgaris - Oxidized form* | 0.03 | 0.00 | 26.60 | 0.08 | 295-388 | X-ray | 2.10 | monomer | 2 x MGD, 4 x SF4, 1 x H2S, 1 x W | BLAST | 0.35 |
| ``` target    MFLSRRQFLKVSVGTVAAVAVADKVLALTALQPVIEVGNPLGDYPDRSWERVYHDQYRYDSSFTWVCSPNDTHACRVRAF 6sdr.1    --------------------------------------------------------------------------------  target    VRNGVVMRVEQNYDHQTYEDLYGNRGTFAHNPRMCLKGFTFHRRVYGPYRLKGPLMRKGWKQWMDDNAPELTAETKRKYK 6sdr.1    --------------------------------------------------------------------------------  target    FDSRFLDDMLRVSWDTAFTYAAKAMITIATRYSGEAGARRLREQGYAPEMIEMMKGAGTRCFKHRAGMPVLGIIGKMGNT 6sdr.1    --------------------------------------------------------------------------------  target    RMNGGINALLDTWIRKVSPDQAQGGRYWSNYTWHGDQNPAHPFWSGVQGSDIDLSDMRFSKLNTSWGKNFVENKMPEAHW 6sdr.1    ------------------------------------------------------NDLANSDCILIMGSNAAENHPIAFKW  target    KLECIERGARVVVITPEYNPTAYRADYWMPLRPESDGALFLGAMKIIIDENMHDIDFLKSFTDAPILVRTDTLQYLDPRD 6sdr.1    VLRAKDKGATLIHVDPRFTRTSARCDVYAPIRSGADIPFLGGLIKYILDNKLYFTDYVREYTNASLIV------------  target    VIADYKFPDFSKSYSGRIQSLKPEQIQRLGGMMVWDLNKKQVVPLHREQVGWHYTNSGIDAALTGTYRVKLLNGREIDAM 6sdr.1    --------------------------------------------------------------------------------  target    PIWQMYMVHFQDYDLDTVHQITRTPKDLIVRWARDSGTIKPAAIHNGEGTCHYFHQTANARGAAMVLIITGNVGKFGTGQ 6sdr.1    --------------------------------------------------------------------------------  target    HTWAGNYKAGTWTATPWSGAGLSVHTGEDPFNITLDPNAHGKEIKTRSYYYGEEVGYWNHGDTALIVNTPKYGRKVFTGK 6sdr.1    --------------------------------------------------------------------------------  target    THMPTPSKFRWVVNVNVVNNAKHHYDMVRNVDPNIECLITQDIEMTSDINHADIAFAANSWMEFTYPEMTVTVSNPWVQI 6sdr.1    --------------------------------------------------------------------------------  target    WKGGIRPLYDTRNDLDTFAGVAAKLSDMTGDKRMRDYFAMVYQNRVDVYVQRMLDASSTFYGYSADVMLKSEKGWMVMVR 6sdr.1    --------------------------------------------------------------------------------  target    TYPRHPFWEETNESKPMWTRSGRYENYRIEPEAIEYGENFISHREGPEATPYLPNAIFTTNPYVRPDDYGIPITAQHHDD 6sdr.1    --------------------------------------------------------------------------------  target    KTVRNIKLSWHEIKRHSNPLWEKGYQFYCVTPKTRHRVHSQWSVNDWVQIYESNFGDPYRMDKRTPGVGEHQIHINPQAA 6sdr.1    --------------------------------------------------------------------------------  target    KDRGINDGDYVYVDGNPVDRPYRGWKPSDPYYKVARLMIRAKYNPAYPYHVTMAKHAPFVATAKSVKGHETRPDGRAIAI 6sdr.1    --------------------------------------------------------------------------------  target    DTGYQSNFRYGAQQSFTRNWLMPMHQTDSLPGKHAVAWKFKWGYQVDHHAINTVPKECLIRITKAEDGGIGARGPWEPVR 6sdr.1    --------------------------------------------------------------------------------  target    TGFTPGQENEFMIKWLKGEHIKIKV 6sdr.1    ------------------------- ``` | | | | | | | | | | | | | | | | | | | | | | | | | | | | | | | | | | | | | | | | | | | | | | | | | |
|  | 6sdv.1.A | Formate dehydrogenase, alpha subunit, selenocysteine-containing,Formate dehydrogenase, alpha subunit, selenocysteine-containing,W-formate dehydrogenase - alpha subunit  *W-formate dehydrogenase from Desulfovibrio vulgaris - Formate reduced form* | 0.03 | 0.00 | 26.60 | 0.08 | 295-388 | X-ray | 1.90 | monomer | 2 x MGD, 4 x SF4, 1 x W, 1 x H2S | BLAST | 0.35 |
| ``` target    MFLSRRQFLKVSVGTVAAVAVADKVLALTALQPVIEVGNPLGDYPDRSWERVYHDQYRYDSSFTWVCSPNDTHACRVRAF 6sdv.1    --------------------------------------------------------------------------------  target    VRNGVVMRVEQNYDHQTYEDLYGNRGTFAHNPRMCLKGFTFHRRVYGPYRLKGPLMRKGWKQWMDDNAPELTAETKRKYK 6sdv.1    --------------------------------------------------------------------------------  target    FDSRFLDDMLRVSWDTAFTYAAKAMITIATRYSGEAGARRLREQGYAPEMIEMMKGAGTRCFKHRAGMPVLGIIGKMGNT 6sdv.1    --------------------------------------------------------------------------------  target    RMNGGINALLDTWIRKVSPDQAQGGRYWSNYTWHGDQNPAHPFWSGVQGSDIDLSDMRFSKLNTSWGKNFVENKMPEAHW 6sdv.1    ------------------------------------------------------NDLANSDCILIMGSNAAENHPIAFKW  target    KLECIERGARVVVITPEYNPTAYRADYWMPLRPESDGALFLGAMKIIIDENMHDIDFLKSFTDAPILVRTDTLQYLDPRD 6sdv.1    VLRAKDKGATLIHVDPRFTRTSARCDVYAPIRSGADIPFLGGLIKYILDNKLYFTDYVREYTNASLIV------------  target    VIADYKFPDFSKSYSGRIQSLKPEQIQRLGGMMVWDLNKKQVVPLHREQVGWHYTNSGIDAALTGTYRVKLLNGREIDAM 6sdv.1    --------------------------------------------------------------------------------  target    PIWQMYMVHFQDYDLDTVHQITRTPKDLIVRWARDSGTIKPAAIHNGEGTCHYFHQTANARGAAMVLIITGNVGKFGTGQ 6sdv.1    --------------------------------------------------------------------------------  target    HTWAGNYKAGTWTATPWSGAGLSVHTGEDPFNITLDPNAHGKEIKTRSYYYGEEVGYWNHGDTALIVNTPKYGRKVFTGK 6sdv.1    --------------------------------------------------------------------------------  target    THMPTPSKFRWVVNVNVVNNAKHHYDMVRNVDPNIECLITQDIEMTSDINHADIAFAANSWMEFTYPEMTVTVSNPWVQI 6sdv.1    --------------------------------------------------------------------------------  target    WKGGIRPLYDTRNDLDTFAGVAAKLSDMTGDKRMRDYFAMVYQNRVDVYVQRMLDASSTFYGYSADVMLKSEKGWMVMVR 6sdv.1    --------------------------------------------------------------------------------  target    TYPRHPFWEETNESKPMWTRSGRYENYRIEPEAIEYGENFISHREGPEATPYLPNAIFTTNPYVRPDDYGIPITAQHHDD 6sdv.1    --------------------------------------------------------------------------------  target    KTVRNIKLSWHEIKRHSNPLWEKGYQFYCVTPKTRHRVHSQWSVNDWVQIYESNFGDPYRMDKRTPGVGEHQIHINPQAA 6sdv.1    --------------------------------------------------------------------------------  target    KDRGINDGDYVYVDGNPVDRPYRGWKPSDPYYKVARLMIRAKYNPAYPYHVTMAKHAPFVATAKSVKGHETRPDGRAIAI 6sdv.1    --------------------------------------------------------------------------------  target    DTGYQSNFRYGAQQSFTRNWLMPMHQTDSLPGKHAVAWKFKWGYQVDHHAINTVPKECLIRITKAEDGGIGARGPWEPVR 6sdv.1    --------------------------------------------------------------------------------  target    TGFTPGQENEFMIKWLKGEHIKIKV 6sdv.1    ------------------------- ``` | | | | | | | | | | | | | | | | | | | | | | | | | | | | | | | | | | | | | | | | | | | | | | | | | |
|  | 1h0h.1.A | FORMATE DEHYDROGENASE SUBUNIT ALPHA  *Tungsten containing Formate Dehydrogenase from Desulfovibrio Gigas* | 0.03 |  | 27.96 | 0.08 | 296-388 | X-ray | 1.80 | hetero-1-1-mer | 1 x W, 1 x 2MD, 1 x MGD, 4 x SF4, 1 x CA | BLAST | 0.36 |
| ``` target    MFLSRRQFLKVSVGTVAAVAVADKVLALTALQPVIEVGNPLGDYPDRSWERVYHDQYRYDSSFTWVCSPNDTHACRVRAF 1h0h.1    --------------------------------------------------------------------------------  target    VRNGVVMRVEQNYDHQTYEDLYGNRGTFAHNPRMCLKGFTFHRRVYGPYRLKGPLMRKGWKQWMDDNAPELTAETKRKYK 1h0h.1    --------------------------------------------------------------------------------  target    FDSRFLDDMLRVSWDTAFTYAAKAMITIATRYSGEAGARRLREQGYAPEMIEMMKGAGTRCFKHRAGMPVLGIIGKMGNT 1h0h.1    --------------------------------------------------------------------------------  target    RMNGGINALLDTWIRKVSPDQAQGGRYWSNYTWHGDQNPAHPFWSGVQGSDIDLSDMRFSKLNTSWGKNFVENKMPEAHW 1h0h.1    -------------------------------------------------------DLKNSDVILMMGSNPAENHPISFKW  target    KLECIERGARVVVITPEYNPTAYRADYWMPLRPESDGALFLGAMKIIIDENMHDIDFLKSFTDAPILVRTDTLQYLDPRD 1h0h.1    VMRAKDKGATLIHVDPRYTRTSTKCDLYAPLRSGSDIAFLNGMTKYILEKELYFKDYVVNYTNASFIV------------  target    VIADYKFPDFSKSYSGRIQSLKPEQIQRLGGMMVWDLNKKQVVPLHREQVGWHYTNSGIDAALTGTYRVKLLNGREIDAM 1h0h.1    --------------------------------------------------------------------------------  target    PIWQMYMVHFQDYDLDTVHQITRTPKDLIVRWARDSGTIKPAAIHNGEGTCHYFHQTANARGAAMVLIITGNVGKFGTGQ 1h0h.1    --------------------------------------------------------------------------------  target    HTWAGNYKAGTWTATPWSGAGLSVHTGEDPFNITLDPNAHGKEIKTRSYYYGEEVGYWNHGDTALIVNTPKYGRKVFTGK 1h0h.1    --------------------------------------------------------------------------------  target    THMPTPSKFRWVVNVNVVNNAKHHYDMVRNVDPNIECLITQDIEMTSDINHADIAFAANSWMEFTYPEMTVTVSNPWVQI 1h0h.1    --------------------------------------------------------------------------------  target    WKGGIRPLYDTRNDLDTFAGVAAKLSDMTGDKRMRDYFAMVYQNRVDVYVQRMLDASSTFYGYSADVMLKSEKGWMVMVR 1h0h.1    --------------------------------------------------------------------------------  target    TYPRHPFWEETNESKPMWTRSGRYENYRIEPEAIEYGENFISHREGPEATPYLPNAIFTTNPYVRPDDYGIPITAQHHDD 1h0h.1    --------------------------------------------------------------------------------  target    KTVRNIKLSWHEIKRHSNPLWEKGYQFYCVTPKTRHRVHSQWSVNDWVQIYESNFGDPYRMDKRTPGVGEHQIHINPQAA 1h0h.1    --------------------------------------------------------------------------------  target    KDRGINDGDYVYVDGNPVDRPYRGWKPSDPYYKVARLMIRAKYNPAYPYHVTMAKHAPFVATAKSVKGHETRPDGRAIAI 1h0h.1    --------------------------------------------------------------------------------  target    DTGYQSNFRYGAQQSFTRNWLMPMHQTDSLPGKHAVAWKFKWGYQVDHHAINTVPKECLIRITKAEDGGIGARGPWEPVR 1h0h.1    --------------------------------------------------------------------------------  target    TGFTPGQENEFMIKWLKGEHIKIKV 1h0h.1    ------------------------- ``` | | | | | | | | | | | | | | | | | | | | | | | | | | | | | | | | | | | | | | | | | | | | | | | | | |
|  | 7q5y.1.A | NADH dehydrogenase I chain G  *Structure of NADH:ubichinon oxidoreductase (complex I) of the hyperthermophilic eubacterium Aquifex aeolicus* | 0.03 |  | 12.90 | 0.08 | 646-747 | X-ray | 2.70 | hetero-1-1-1-1-1-1-… | 8 x SF4, 2 x FES, 1 x FMN | HHblits | 0.26 |
| ``` target    MFLSRRQFLKVSVGTVAAVAVADKVLALTALQPVIEVGNPLGDYPDRSWERVYHDQYRYDSSFTWVCSPNDTHACRVRAF 7q5y.1    --------------------------------------------------------------------------------  target    VRNGVVMRVEQNYDHQTYEDLYGNRGTFAHNPRMCLKGFTFHRRVYGPYRLKGPLMRKGWKQWMDDNAPELTAETKRKYK 7q5y.1    --------------------------------------------------------------------------------  target    FDSRFLDDMLRVSWDTAFTYAAKAMITIATRYSGEAGARRLREQGYAPEMIEMMKGAGTRCFKHRAGMPVLGIIGKMGNT 7q5y.1    --------------------------------------------------------------------------------  target    RMNGGINALLDTWIRKVSPDQAQGGRYWSNYTWHGDQNPAHPFWSGVQGSDIDLSDMRFSKLNTSWGKNFVENKMPEAHW 7q5y.1    --------------------------------------------------------------------------------  target    KLECIERGARVVVITPEYNPTAYRADYWMPLRPESDGALFLGAMKIIIDENMHDIDFLKSFTDAPILVRTDTLQYLDPRD 7q5y.1    --------------------------------------------------------------------------------  target    VIADYKFPDFSKSYSGRIQSLKPEQIQRLGGMMVWDLNKKQVVPLHREQVGWHYTNSGIDAALTGTYRVKLLNGREIDAM 7q5y.1    --------------------------------------------------------------------------------  target    PIWQMYMVHFQDYDLDTVHQITRTPKDLIVRWARDSGTIKPAAIHNGEGTCHYFHQTANARGAAMVLIITGNVGKFGTGQ 7q5y.1    --------------------------------------------------------------------------------  target    HTWAGNYKAGTWTATPWSGAGLSVHTGEDPFNITLDPNAHGKEIKTRSYYYGEEVGYWNHGDTALIVNTPKYGRKVFTGK 7q5y.1    --------------------------------------------------------------------------------  target    THMPTPSKFRWVVNVNVVNNAKHHYDMVRNVDPNIECLITQDIEMTSDINHADIAFAANSWMEFTYPEMTVTVSNPWVQI 7q5y.1    -----DIENLIIFGEDILEFYED--KVFEELKEKLEHLVVVSPYEDGLSEYAHIKIPMSLMGEN---EGTYKTFFGEVKG  target    WKGGIRPLYDTRNDLDTFAGVAAKLSDMTGDKRMRDYFAMVYQNRVDVYVQRMLDASSTFYGYSADVMLKSEKGWMVMVR 7q5y.1    KK--FLP--WAFDDLAFWKYLGENFKE-----------------------------------------------------  target    TYPRHPFWEETNESKPMWTRSGRYENYRIEPEAIEYGENFISHREGPEATPYLPNAIFTTNPYVRPDDYGIPITAQHHDD 7q5y.1    --------------------------------------------------------------------------------  target    KTVRNIKLSWHEIKRHSNPLWEKGYQFYCVTPKTRHRVHSQWSVNDWVQIYESNFGDPYRMDKRTPGVGEHQIHINPQAA 7q5y.1    --------------------------------------------------------------------------------  target    KDRGINDGDYVYVDGNPVDRPYRGWKPSDPYYKVARLMIRAKYNPAYPYHVTMAKHAPFVATAKSVKGHETRPDGRAIAI 7q5y.1    --------------------------------------------------------------------------------  target    DTGYQSNFRYGAQQSFTRNWLMPMHQTDSLPGKHAVAWKFKWGYQVDHHAINTVPKECLIRITKAEDGGIGARGPWEPVR 7q5y.1    --------------------------------------------------------------------------------  target    TGFTPGQENEFMIKWLKGEHIKIKV 7q5y.1    ------------------------- ``` | | | | | | | | | | | | | | | | | | | | | | | | | | | | | | | | | | | | | | | | | | | | | | | | | |
|  | 6s6y.1.B | Tungsten-containing formylmethanofuran dehydrogenase, subunit B  *X-ray crystal structure of the formyltransferase/hydrolase complex (FhcABCD) from Methylorubrum extorquens in complex with methylofuran* | 0.03 |  | 5.43 | 0.08 | 645-747 | X-ray | 3.10 | hetero-2-2-2-2-mer | 1 x MFN, 4 x ZN, 4 x CA, 4 x K, 3 x DGL, 2 x GLU, 1 x IAS | HHblits | 0.23 |
| ``` target    MFLSRRQFLKVSVGTVAAVAVADKVLALTALQPVIEVGNPLGDYPDRSWERVYHDQYRYDSSFTWVCSPNDTHACRVRAF 6s6y.1    --------------------------------------------------------------------------------  target    VRNGVVMRVEQNYDHQTYEDLYGNRGTFAHNPRMCLKGFTFHRRVYGPYRLKGPLMRKGWKQWMDDNAPELTAETKRKYK 6s6y.1    --------------------------------------------------------------------------------  target    FDSRFLDDMLRVSWDTAFTYAAKAMITIATRYSGEAGARRLREQGYAPEMIEMMKGAGTRCFKHRAGMPVLGIIGKMGNT 6s6y.1    --------------------------------------------------------------------------------  target    RMNGGINALLDTWIRKVSPDQAQGGRYWSNYTWHGDQNPAHPFWSGVQGSDIDLSDMRFSKLNTSWGKNFVENKMPEAHW 6s6y.1    --------------------------------------------------------------------------------  target    KLECIERGARVVVITPEYNPTAYRADYWMPLRPESDGALFLGAMKIIIDENMHDIDFLKSFTDAPILVRTDTLQYLDPRD 6s6y.1    --------------------------------------------------------------------------------  target    VIADYKFPDFSKSYSGRIQSLKPEQIQRLGGMMVWDLNKKQVVPLHREQVGWHYTNSGIDAALTGTYRVKLLNGREIDAM 6s6y.1    --------------------------------------------------------------------------------  target    PIWQMYMVHFQDYDLDTVHQITRTPKDLIVRWARDSGTIKPAAIHNGEGTCHYFHQTANARGAAMVLIITGNVGKFGTGQ 6s6y.1    --------------------------------------------------------------------------------  target    HTWAGNYKAGTWTATPWSGAGLSVHTGEDPFNITLDPNAHGKEIKTRSYYYGEEVGYWNHGDTALIVNTPKYGRKVFTGK 6s6y.1    --------------------------------------------------------------------------------  target    THMPTPSKFRWVVNVNVVNNAKHHYDMVRNVDPNIECLITQD-IEMTSDINHADIAFAANSW-MEFTYPEMTVTVSNPWV 6s6y.1    ----GEADAALWLASLPAP--------RPAWLGSLPTIAIVGEGSQEAAGETAEVVITVGVPGQSVG---GALWNDRRGV  target    QIWKGGIRPL---YDTRNDLDTFAGVAAKLSDMTGDKRMRDYFAMVYQNRVDVYVQRMLDASSTFYGYSADVMLKSEKGW 6s6y.1    IAYAEASDPAKTPAETETAAGVLTRIRDRLIE------------------------------------------------  target    MVMVRTYPRHPFWEETNESKPMWTRSGRYENYRIEPEAIEYGENFISHREGPEATPYLPNAIFTTNPYVRPDDYGIPITA 6s6y.1    --------------------------------------------------------------------------------  target    QHHDDKTVRNIKLSWHEIKRHSNPLWEKGYQFYCVTPKTRHRVHSQWSVNDWVQIYESNFGDPYRMDKRTPGVGEHQIHI 6s6y.1    --------------------------------------------------------------------------------  target    NPQAAKDRGINDGDYVYVDGNPVDRPYRGWKPSDPYYKVARLMIRAKYNPAYPYHVTMAKHAPFVATAKSVKGHETRPDG 6s6y.1    --------------------------------------------------------------------------------  target    RAIAIDTGYQSNFRYGAQQSFTRNWLMPMHQTDSLPGKHAVAWKFKWGYQVDHHAINTVPKECLIRITKAEDGGIGARGP 6s6y.1    --------------------------------------------------------------------------------  target    WEPVRTGFTPGQENEFMIKWLKGEHIKIKV 6s6y.1    ------------------------------ ``` | | | | | | | | | | | | | | | | | | | | | | | | | | | | | | | | | | | | | | | | | | | | | | | | | |
|  | 1kqf.1.A | FORMATE DEHYDROGENASE, NITRATE-INDUCIBLE, MAJOR SUBUNIT  *FORMATE DEHYDROGENASE N FROM E. COLI* | 0.02 |  | 16.47 | 0.07 | 902-1019 | X-ray | 1.60 | hetero-oligomer | 3 x 6MO, 15 x SF4, 6 x MGD, 6 x HEM, 3 x CDL | HHblits | 0.27 |
| ``` target    MFLSRRQFLKVSVGTVAAVAVADKVLALTALQPVIEVGNPLGDYPDRSWERVYHDQYRYDSSFTWVCSPNDTHACRVRAF 1kqf.1    --------------------------------------------------------------------------------  target    VRNGVVMRVEQNYDHQTYEDLYGNRGTFAHNPRMCLKGFTFHRRVYGPYRLKGPLMRKGWKQWMDDNAPELTAETKRKYK 1kqf.1    --------------------------------------------------------------------------------  target    FDSRFLDDMLRVSWDTAFTYAAKAMITIATRYSGEAGARRLREQGYAPEMIEMMKGAGTRCFKHRAGMPVLGIIGKMGNT 1kqf.1    --------------------------------------------------------------------------------  target    RMNGGINALLDTWIRKVSPDQAQGGRYWSNYTWHGDQNPAHPFWSGVQGSDIDLSDMRFSKLNTSWGKNFVENKMPEAHW 1kqf.1    --------------------------------------------------------------------------------  target    KLECIERGARVVVITPEYNPTAYRADYWMPLRPESDGALFLGAMKIIIDENMHDIDFLKSFTDAPILVRTDTLQYLDPRD 1kqf.1    --------------------------------------------------------------------------------  target    VIADYKFPDFSKSYSGRIQSLKPEQIQRLGGMMVWDLNKKQVVPLHREQVGWHYTNSGIDAALTGTYRVKLLNGREIDAM 1kqf.1    --------------------------------------------------------------------------------  target    PIWQMYMVHFQDYDLDTVHQITRTPKDLIVRWARDSGTIKPAAIHNGEGTCHYFHQTANARGAAMVLIITGNVGKFGTGQ 1kqf.1    --------------------------------------------------------------------------------  target    HTWAGNYKAGTWTATPWSGAGLSVHTGEDPFNITLDPNAHGKEIKTRSYYYGEEVGYWNHGDTALIVNTPKYGRKVFTGK 1kqf.1    --------------------------------------------------------------------------------  target    THMPTPSKFRWVVNVNVVNNAKHHYDMVRNVDPNIECLITQDIEMTSDINHADIAFAANSWMEFTYPEMTVTVSNPWVQI 1kqf.1    --------------------------------------------------------------------------------  target    WKGGIRPLYDTRNDLDTFAGVAAKLSDMTGDKRMRDYFAMVYQNRVDVYVQRMLDASSTFYGYSADVMLKSEKGWMVMVR 1kqf.1    --------------------------------------------------------------------------------  target    TYPRHPFWEETNESKPMWTRSGRYENYRIEPEAIEYGENFISHREGPEATPYLPNAIFTTNPYVRPDDYGIPITAQHHDD 1kqf.1    --------------------------------------------------------------------------------  target    KTVRNIKLSWHEIKRHSNPLWEKGYQFYCVTPKTRHRVHSQWSVNDWVQIYESNFGDPYRMDKRTPGVGEHQIHINPQAA 1kqf.1    ---------------------KEQFPYVGTTYRLTEHFHTWTKHALLNAIA----------------QPEQFVEISETLA  target    KDRGINDGDYVYVDGNPVDRPYRGWKPSDPYYKVARLMIRAKYNPAYPY--------HVTMAKHAPFVATAKSVKGHETR 1kqf.1    AAKGINNGDRVTVSSK-----------------RGFIRAVAVVTRRLKPLNVNGQQVETVGIPIHWG-------------  target    PDGRAIAIDTGYQSNFRYGAQQSFTRNWLMPMHQTDSLPGKHAVAWKFKWGYQVDHHAINTVPKECLIRITKAEDGGIGA 1kqf.1    --------------------------------------------------------------------------------  target    RGPWEPVRTGFTPGQENEFMIKWLKGEHIKIKV 1kqf.1    --------------------------------- ``` | | | | | | | | | | | | | | | | | | | | | | | | | | | | | | | | | | | | | | | | | | | | | | | | | |
|  | 1ogy.1.A | PERIPLASMIC NITRATE REDUCTASE  *Crystal structure of the heterodimeric nitrate reductase from Rhodobacter sphaeroides* | 0.02 |  | 19.51 | 0.07 | 903-1017 | X-ray | 3.20 | hetero-1-1-mer | 1 x SF4, 1 x MO, 2 x MGD, 2 x HEC | HHblits | 0.29 |
| ``` target    MFLSRRQFLKVSVGTVAAVAVADKVLALTALQPVIEVGNPLGDYPDRSWERVYHDQYRYDSSFTWVCSPNDTHACRVRAF 1ogy.1    --------------------------------------------------------------------------------  target    VRNGVVMRVEQNYDHQTYEDLYGNRGTFAHNPRMCLKGFTFHRRVYGPYRLKGPLMRKGWKQWMDDNAPELTAETKRKYK 1ogy.1    --------------------------------------------------------------------------------  target    FDSRFLDDMLRVSWDTAFTYAAKAMITIATRYSGEAGARRLREQGYAPEMIEMMKGAGTRCFKHRAGMPVLGIIGKMGNT 1ogy.1    --------------------------------------------------------------------------------  target    RMNGGINALLDTWIRKVSPDQAQGGRYWSNYTWHGDQNPAHPFWSGVQGSDIDLSDMRFSKLNTSWGKNFVENKMPEAHW 1ogy.1    --------------------------------------------------------------------------------  target    KLECIERGARVVVITPEYNPTAYRADYWMPLRPESDGALFLGAMKIIIDENMHDIDFLKSFTDAPILVRTDTLQYLDPRD 1ogy.1    --------------------------------------------------------------------------------  target    VIADYKFPDFSKSYSGRIQSLKPEQIQRLGGMMVWDLNKKQVVPLHREQVGWHYTNSGIDAALTGTYRVKLLNGREIDAM 1ogy.1    --------------------------------------------------------------------------------  target    PIWQMYMVHFQDYDLDTVHQITRTPKDLIVRWARDSGTIKPAAIHNGEGTCHYFHQTANARGAAMVLIITGNVGKFGTGQ 1ogy.1    --------------------------------------------------------------------------------  target    HTWAGNYKAGTWTATPWSGAGLSVHTGEDPFNITLDPNAHGKEIKTRSYYYGEEVGYWNHGDTALIVNTPKYGRKVFTGK 1ogy.1    --------------------------------------------------------------------------------  target    THMPTPSKFRWVVNVNVVNNAKHHYDMVRNVDPNIECLITQDIEMTSDINHADIAFAANSWMEFTYPEMTVTVSNPWVQI 1ogy.1    --------------------------------------------------------------------------------  target    WKGGIRPLYDTRNDLDTFAGVAAKLSDMTGDKRMRDYFAMVYQNRVDVYVQRMLDASSTFYGYSADVMLKSEKGWMVMVR 1ogy.1    --------------------------------------------------------------------------------  target    TYPRHPFWEETNESKPMWTRSGRYENYRIEPEAIEYGENFISHREGPEATPYLPNAIFTTNPYVRPDDYGIPITAQHHDD 1ogy.1    --------------------------------------------------------------------------------  target    KTVRNIKLSWHEIKRHSNPLWEKGYQFYCVTPKTRHRVHSQWSV--NDWVQIYESNFGDPYRMDKRTPGVGEHQIHINPQ 1ogy.1    ----------------------EEFGFWLVTGRVLEHWHSGSMTLRWPELYKA----------------FPGAVCFMHPE  target    AAKDRGINDGDYVYVDGNPVDRPYRGWKPSDPYYKVARLMIRAKY--NPAYPYHVTMAKHAPFVATAKSVKGHETRPDGR 1ogy.1    DARSRGLNRGSEVRVISR-----------------RGEIRTRLETRGRNRMPRGVVFVPWF-------------------  target    AIAIDTGYQSNFRYGAQQSFTRNWLMPMHQTDSLPGKHAVAWKFKWGYQVDHHAINTVPKECLIRITKAEDGGIGARGPW 1ogy.1    --------------------------------------------------------------------------------  target    EPVRTGFTPGQENEFMIKWLKGEHIKIKV 1ogy.1    ----------------------------- ``` | | | | | | | | | | | | | | | | | | | | | | | | | | | | | | | | | | | | | | | | | | | | | | | | | |
|  | 5t5i.1.D | Tungsten formylmethanofuran dehydrogenase subunit fwdD  *TUNGSTEN-CONTAINING FORMYLMETHANOFURAN DEHYDROGENASE FROM METHANOTHERMOBACTER WOLFEII, ORTHORHOMBIC FORM AT 1.9 A* | 0.01 |  | 20.99 | 0.07 | 906-1019 | X-ray | 1.90 | hetero-oligomer | 4 x ZN, 2 x MG, 18 x K, 22 x SF4, 2 x W, 4 x MGD, 2 x H2S, 2 x CA | HHblits | 0.30 |
| ``` target    MFLSRRQFLKVSVGTVAAVAVADKVLALTALQPVIEVGNPLGDYPDRSWERVYHDQYRYDSSFTWVCSPNDTHACRVRAF 5t5i.1    --------------------------------------------------------------------------------  target    VRNGVVMRVEQNYDHQTYEDLYGNRGTFAHNPRMCLKGFTFHRRVYGPYRLKGPLMRKGWKQWMDDNAPELTAETKRKYK 5t5i.1    --------------------------------------------------------------------------------  target    FDSRFLDDMLRVSWDTAFTYAAKAMITIATRYSGEAGARRLREQGYAPEMIEMMKGAGTRCFKHRAGMPVLGIIGKMGNT 5t5i.1    --------------------------------------------------------------------------------  target    RMNGGINALLDTWIRKVSPDQAQGGRYWSNYTWHGDQNPAHPFWSGVQGSDIDLSDMRFSKLNTSWGKNFVENKMPEAHW 5t5i.1    --------------------------------------------------------------------------------  target    KLECIERGARVVVITPEYNPTAYRADYWMPLRPESDGALFLGAMKIIIDENMHDIDFLKSFTDAPILVRTDTLQYLDPRD 5t5i.1    --------------------------------------------------------------------------------  target    VIADYKFPDFSKSYSGRIQSLKPEQIQRLGGMMVWDLNKKQVVPLHREQVGWHYTNSGIDAALTGTYRVKLLNGREIDAM 5t5i.1    --------------------------------------------------------------------------------  target    PIWQMYMVHFQDYDLDTVHQITRTPKDLIVRWARDSGTIKPAAIHNGEGTCHYFHQTANARGAAMVLIITGNVGKFGTGQ 5t5i.1    --------------------------------------------------------------------------------  target    HTWAGNYKAGTWTATPWSGAGLSVHTGEDPFNITLDPNAHGKEIKTRSYYYGEEVGYWNHGDTALIVNTPKYGRKVFTGK 5t5i.1    --------------------------------------------------------------------------------  target    THMPTPSKFRWVVNVNVVNNAKHHYDMVRNVDPNIECLITQDIEMTSDINHADIAFAANSWMEFTYPEMTVTVSNPWVQI 5t5i.1    --------------------------------------------------------------------------------  target    WKGGIRPLYDTRNDLDTFAGVAAKLSDMTGDKRMRDYFAMVYQNRVDVYVQRMLDASSTFYGYSADVMLKSEKGWMVMVR 5t5i.1    --------------------------------------------------------------------------------  target    TYPRHPFWEETNESKPMWTRSGRYENYRIEPEAIEYGENFISHREGPEATPYLPNAIFTTNPYVRPDDYGIPITAQHHDD 5t5i.1    --------------------------------------------------------------------------------  target    KTVRNIKLSWHEIKRHSNPLWEKGYQFYCVTPKTRHRVHSQWSVNDWVQIYESNFGDPYRMDKRTPGVGEHQIHINPQAA 5t5i.1    -------------------------RVILNTGRTIWQGQAIESGKDLKMYV----------------DAAAIIQMNPEMM  target    KDRGINDGDYVYVDGNPVDRPYRGWKPSDPYYKVARLMIRAK-YNPAYPYHVTMAKHAPFVATAKSVKGHETRPDGRAIA 5t5i.1    KQLGIAEGDNVKVISE-----------------YGDVVVKAVEAKEPLPEGMVYIPMGPW--------------------  target    IDTGYQSNFRYGAQQSFTRNWLMPMHQTDSLPGKHAVAWKFKWGYQVDHHAINTVPKECLIRITKAEDGGIGARGPWEPV 5t5i.1    --------------------------------------------------------------------------------  target    RTGFTPGQENEFMIKWLKGEHIKIKV 5t5i.1    -------------------------- ``` | | | | | | | | | | | | | | | | | | | | | | | | | | | | | | | | | | | | | | | | | | | | | | | | | |
|  | 3o5a.1.A | Periplasmic nitrate reductase  *Crystal Structure of partially reduced Periplasmic Nitrate Reductase from Cupriavidus necator using Ionic Liquids* | 0.02 |  | 20.99 | 0.07 | 903-1016 | X-ray | 1.72 | hetero-oligomer | 1 x SF4, 1 x MOS, 2 x MGD, 2 x HEC | HHblits | 0.29 |
| ``` target    MFLSRRQFLKVSVGTVAAVAVADKVLALTALQPVIEVGNPLGDYPDRSWERVYHDQYRYDSSFTWVCSPNDTHACRVRAF 3o5a.1    --------------------------------------------------------------------------------  target    VRNGVVMRVEQNYDHQTYEDLYGNRGTFAHNPRMCLKGFTFHRRVYGPYRLKGPLMRKGWKQWMDDNAPELTAETKRKYK 3o5a.1    --------------------------------------------------------------------------------  target    FDSRFLDDMLRVSWDTAFTYAAKAMITIATRYSGEAGARRLREQGYAPEMIEMMKGAGTRCFKHRAGMPVLGIIGKMGNT 3o5a.1    --------------------------------------------------------------------------------  target    RMNGGINALLDTWIRKVSPDQAQGGRYWSNYTWHGDQNPAHPFWSGVQGSDIDLSDMRFSKLNTSWGKNFVENKMPEAHW 3o5a.1    --------------------------------------------------------------------------------  target    KLECIERGARVVVITPEYNPTAYRADYWMPLRPESDGALFLGAMKIIIDENMHDIDFLKSFTDAPILVRTDTLQYLDPRD 3o5a.1    --------------------------------------------------------------------------------  target    VIADYKFPDFSKSYSGRIQSLKPEQIQRLGGMMVWDLNKKQVVPLHREQVGWHYTNSGIDAALTGTYRVKLLNGREIDAM 3o5a.1    --------------------------------------------------------------------------------  target    PIWQMYMVHFQDYDLDTVHQITRTPKDLIVRWARDSGTIKPAAIHNGEGTCHYFHQTANARGAAMVLIITGNVGKFGTGQ 3o5a.1    --------------------------------------------------------------------------------  target    HTWAGNYKAGTWTATPWSGAGLSVHTGEDPFNITLDPNAHGKEIKTRSYYYGEEVGYWNHGDTALIVNTPKYGRKVFTGK 3o5a.1    --------------------------------------------------------------------------------  target    THMPTPSKFRWVVNVNVVNNAKHHYDMVRNVDPNIECLITQDIEMTSDINHADIAFAANSWMEFTYPEMTVTVSNPWVQI 3o5a.1    --------------------------------------------------------------------------------  target    WKGGIRPLYDTRNDLDTFAGVAAKLSDMTGDKRMRDYFAMVYQNRVDVYVQRMLDASSTFYGYSADVMLKSEKGWMVMVR 3o5a.1    --------------------------------------------------------------------------------  target    TYPRHPFWEETNESKPMWTRSGRYENYRIEPEAIEYGENFISHREGPEATPYLPNAIFTTNPYVRPDDYGIPITAQHHDD 3o5a.1    --------------------------------------------------------------------------------  target    KTVRNIKLSWHEIKRHSNPLWEKGYQFYCVTPKTRHRVHSQ--WSVNDWVQIYESNFGDPYRMDKRTPGVGEHQIHINPQ 3o5a.1    ----------------------KEYPYWLVTGRVLEHWHSGSMTRRVPELYRS----------------FPNAVVFMHPE  target    AAKDRGINDGDYVYVDGNPVDRPYRGWKPSDPYYKVARLMIRAKYN--PAYPYHVTMAKHAPFVATAKSVKGHETRPDGR 3o5a.1    DAKALGLRRGVEVEVVSR-----------------RGRMRSRIETRGRDAPPRGLVFVPW--------------------  target    AIAIDTGYQSNFRYGAQQSFTRNWLMPMHQTDSLPGKHAVAWKFKWGYQVDHHAINTVPKECLIRITKAEDGGIGARGPW 3o5a.1    --------------------------------------------------------------------------------  target    EPVRTGFTPGQENEFMIKWLKGEHIKIKV 3o5a.1    ----------------------------- ``` | | | | | | | | | | | | | | | | | | | | | | | | | | | | | | | | | | | | | | | | | | | | | | | | | |
|  | 2nya.1.A | Periplasmic nitrate reductase  *Crystal structure of the periplasmic nitrate reductase (NAP) from Escherichia coli* | 0.02 |  | 18.29 | 0.07 | 903-1017 | X-ray | 2.50 | monomer | 1 x SF4, 1 x 6MO, 2 x MGD | HHblits | 0.28 |
| ``` target    MFLSRRQFLKVSVGTVAAVAVADKVLALTALQPVIEVGNPLGDYPDRSWERVYHDQYRYDSSFTWVCSPNDTHACRVRAF 2nya.1    --------------------------------------------------------------------------------  target    VRNGVVMRVEQNYDHQTYEDLYGNRGTFAHNPRMCLKGFTFHRRVYGPYRLKGPLMRKGWKQWMDDNAPELTAETKRKYK 2nya.1    --------------------------------------------------------------------------------  target    FDSRFLDDMLRVSWDTAFTYAAKAMITIATRYSGEAGARRLREQGYAPEMIEMMKGAGTRCFKHRAGMPVLGIIGKMGNT 2nya.1    --------------------------------------------------------------------------------  target    RMNGGINALLDTWIRKVSPDQAQGGRYWSNYTWHGDQNPAHPFWSGVQGSDIDLSDMRFSKLNTSWGKNFVENKMPEAHW 2nya.1    --------------------------------------------------------------------------------  target    KLECIERGARVVVITPEYNPTAYRADYWMPLRPESDGALFLGAMKIIIDENMHDIDFLKSFTDAPILVRTDTLQYLDPRD 2nya.1    --------------------------------------------------------------------------------  target    VIADYKFPDFSKSYSGRIQSLKPEQIQRLGGMMVWDLNKKQVVPLHREQVGWHYTNSGIDAALTGTYRVKLLNGREIDAM 2nya.1    --------------------------------------------------------------------------------  target    PIWQMYMVHFQDYDLDTVHQITRTPKDLIVRWARDSGTIKPAAIHNGEGTCHYFHQTANARGAAMVLIITGNVGKFGTGQ 2nya.1    --------------------------------------------------------------------------------  target    HTWAGNYKAGTWTATPWSGAGLSVHTGEDPFNITLDPNAHGKEIKTRSYYYGEEVGYWNHGDTALIVNTPKYGRKVFTGK 2nya.1    --------------------------------------------------------------------------------  target    THMPTPSKFRWVVNVNVVNNAKHHYDMVRNVDPNIECLITQDIEMTSDINHADIAFAANSWMEFTYPEMTVTVSNPWVQI 2nya.1    --------------------------------------------------------------------------------  target    WKGGIRPLYDTRNDLDTFAGVAAKLSDMTGDKRMRDYFAMVYQNRVDVYVQRMLDASSTFYGYSADVMLKSEKGWMVMVR 2nya.1    --------------------------------------------------------------------------------  target    TYPRHPFWEETNESKPMWTRSGRYENYRIEPEAIEYGENFISHREGPEATPYLPNAIFTTNPYVRPDDYGIPITAQHHDD 2nya.1    --------------------------------------------------------------------------------  target    KTVRNIKLSWHEIKRHSNPLWEKGYQFYCVTPKTRHRVH--SQWSVNDWVQIYESNFGDPYRMDKRTPGVGEHQIHINPQ 2nya.1    ----------------------EEYDLWLSTGRVLEHWHTGSMTRRVPELHRA----------------FPEAVLFIHPL  target    AAKDRGINDGDYVYVDGNPVDRPYRGWKPSDPYYKVARLMIRAKYNPAY--PYHVTMAKHAPFVATAKSVKGHETRPDGR 2nya.1    DAKARDLRRGDKVKVVSR-----------------RGEVISIVETRGRNRPPQGLVYMPFF-------------------  target    AIAIDTGYQSNFRYGAQQSFTRNWLMPMHQTDSLPGKHAVAWKFKWGYQVDHHAINTVPKECLIRITKAEDGGIGARGPW 2nya.1    --------------------------------------------------------------------------------  target    EPVRTGFTPGQENEFMIKWLKGEHIKIKV 2nya.1    ----------------------------- ``` | | | | | | | | | | | | | | | | | | | | | | | | | | | | | | | | | | | | | | | | | | | | | | | | | |
|  | 7bkb.1.J | Formylmethanofuran dehydrogenase, subunit D  *Formate dehydrogenase - heterodisulfide reductase - formylmethanofuran dehydrogenase complex from Methanospirillum hungatei (hexameric, composite structure)* | 0.01 |  | 13.10 | 0.07 | 903-1019 | EM | 0.00 | hetero-2-2-2-2-2-2-… | 48 x SF4, 4 x FAD, 2 x FES, 4 x 9S8, 4 x ZN, 2 x MO, 4 x MGD | HHblits | 0.26 |
| ``` target    MFLSRRQFLKVSVGTVAAVAVADKVLALTALQPVIEVGNPLGDYPDRSWERVYHDQYRYDSSFTWVCSPNDTHACRVRAF 7bkb.1    --------------------------------------------------------------------------------  target    VRNGVVMRVEQNYDHQTYEDLYGNRGTFAHNPRMCLKGFTFHRRVYGPYRLKGPLMRKGWKQWMDDNAPELTAETKRKYK 7bkb.1    --------------------------------------------------------------------------------  target    FDSRFLDDMLRVSWDTAFTYAAKAMITIATRYSGEAGARRLREQGYAPEMIEMMKGAGTRCFKHRAGMPVLGIIGKMGNT 7bkb.1    --------------------------------------------------------------------------------  target    RMNGGINALLDTWIRKVSPDQAQGGRYWSNYTWHGDQNPAHPFWSGVQGSDIDLSDMRFSKLNTSWGKNFVENKMPEAHW 7bkb.1    --------------------------------------------------------------------------------  target    KLECIERGARVVVITPEYNPTAYRADYWMPLRPESDGALFLGAMKIIIDENMHDIDFLKSFTDAPILVRTDTLQYLDPRD 7bkb.1    --------------------------------------------------------------------------------  target    VIADYKFPDFSKSYSGRIQSLKPEQIQRLGGMMVWDLNKKQVVPLHREQVGWHYTNSGIDAALTGTYRVKLLNGREIDAM 7bkb.1    --------------------------------------------------------------------------------  target    PIWQMYMVHFQDYDLDTVHQITRTPKDLIVRWARDSGTIKPAAIHNGEGTCHYFHQTANARGAAMVLIITGNVGKFGTGQ 7bkb.1    --------------------------------------------------------------------------------  target    HTWAGNYKAGTWTATPWSGAGLSVHTGEDPFNITLDPNAHGKEIKTRSYYYGEEVGYWNHGDTALIVNTPKYGRKVFTGK 7bkb.1    --------------------------------------------------------------------------------  target    THMPTPSKFRWVVNVNVVNNAKHHYDMVRNVDPNIECLITQDIEMTSDINHADIAFAANSWMEFTYPEMTVTVSNPWVQI 7bkb.1    --------------------------------------------------------------------------------  target    WKGGIRPLYDTRNDLDTFAGVAAKLSDMTGDKRMRDYFAMVYQNRVDVYVQRMLDASSTFYGYSADVMLKSEKGWMVMVR 7bkb.1    --------------------------------------------------------------------------------  target    TYPRHPFWEETNESKPMWTRSGRYENYRIEPEAIEYGENFISHREGPEATPYLPNAIFTTNPYVRPDDYGIPITAQHHDD 7bkb.1    --------------------------------------------------------------------------------  target    KTVRNIKLSWHEIKRHSNPLWEKGYQFYCVTPKTRHRVHSQWSVNDWVQIYESNFGDPYRMDKRTPGVGEHQIHINPQAA 7bkb.1    ----------------------AKKTLNMITQRAVEEGIAMEI-GKTSRQY---------------FDACSIIEMNEQDM  target    KDRGINDGDYVYVDGNPVDRPYRGWKPSDPYYKVARLMIRAKYN-PAYPYHVTMAKHAPFVATAKSVKGHETRPDGRAIA 7bkb.1    KELGIMKNTNVRVKSE-----------------SGEVVVKAVVGRQTCYPGLCHIRQGVW--------------------  target    IDTGYQSNFRYGAQQSFTRNWLMPMHQTDSLPGKHAVAWKFKWGYQVDHHAINTVPKECLIRITKAEDGGIGARGPWEPV 7bkb.1    --------------------------------------------------------------------------------  target    RTGFTPGQENEFMIKWLKGEHIKIKV 7bkb.1    -------------------------- ``` | | | | | | | | | | | | | | | | | | | | | | | | | | | | | | | | | | | | | | | | | | | | | | | | | |
|  | 2ki8.1.A | Tungsten formylmethanofuran dehydrogenase, subunit D (FwdD-2)  *Solution NMR structure of tungsten formylmethanofuran dehydrogenase subunit D from Archaeoglobus fulgidus, Northeast Structural Genomics Consortium target AtT7* | 0.02 |  | 15.00 | 0.07 | 904-1018 | NMR | 0.00 | monomer |  | HHblits | 0.28 |
| ``` target    MFLSRRQFLKVSVGTVAAVAVADKVLALTALQPVIEVGNPLGDYPDRSWERVYHDQYRYDSSFTWVCSPNDTHACRVRAF 2ki8.1    --------------------------------------------------------------------------------  target    VRNGVVMRVEQNYDHQTYEDLYGNRGTFAHNPRMCLKGFTFHRRVYGPYRLKGPLMRKGWKQWMDDNAPELTAETKRKYK 2ki8.1    --------------------------------------------------------------------------------  target    FDSRFLDDMLRVSWDTAFTYAAKAMITIATRYSGEAGARRLREQGYAPEMIEMMKGAGTRCFKHRAGMPVLGIIGKMGNT 2ki8.1    --------------------------------------------------------------------------------  target    RMNGGINALLDTWIRKVSPDQAQGGRYWSNYTWHGDQNPAHPFWSGVQGSDIDLSDMRFSKLNTSWGKNFVENKMPEAHW 2ki8.1    --------------------------------------------------------------------------------  target    KLECIERGARVVVITPEYNPTAYRADYWMPLRPESDGALFLGAMKIIIDENMHDIDFLKSFTDAPILVRTDTLQYLDPRD 2ki8.1    --------------------------------------------------------------------------------  target    VIADYKFPDFSKSYSGRIQSLKPEQIQRLGGMMVWDLNKKQVVPLHREQVGWHYTNSGIDAALTGTYRVKLLNGREIDAM 2ki8.1    --------------------------------------------------------------------------------  target    PIWQMYMVHFQDYDLDTVHQITRTPKDLIVRWARDSGTIKPAAIHNGEGTCHYFHQTANARGAAMVLIITGNVGKFGTGQ 2ki8.1    --------------------------------------------------------------------------------  target    HTWAGNYKAGTWTATPWSGAGLSVHTGEDPFNITLDPNAHGKEIKTRSYYYGEEVGYWNHGDTALIVNTPKYGRKVFTGK 2ki8.1    --------------------------------------------------------------------------------  target    THMPTPSKFRWVVNVNVVNNAKHHYDMVRNVDPNIECLITQDIEMTSDINHADIAFAANSWMEFTYPEMTVTVSNPWVQI 2ki8.1    --------------------------------------------------------------------------------  target    WKGGIRPLYDTRNDLDTFAGVAAKLSDMTGDKRMRDYFAMVYQNRVDVYVQRMLDASSTFYGYSADVMLKSEKGWMVMVR 2ki8.1    --------------------------------------------------------------------------------  target    TYPRHPFWEETNESKPMWTRSGRYENYRIEPEAIEYGENFISHREGPEATPYLPNAIFTTNPYVRPDDYGIPITAQHHDD 2ki8.1    --------------------------------------------------------------------------------  target    KTVRNIKLSWHEIKRHSNPLWEKGYQFYCVTPKTRHRVHSQWSVNDWVQIYESNFGDPYRMDKRTPGVGEHQIHINPQAA 2ki8.1    -----------------------MLEVEVISGRTLNQGATVE-EKLTEEY----------------FNAVNYAEINEEDW  target    KDRGINDGDYVYVDGNPVDRPYRGWKPSDPYYKVARLMIRAKYNPAYPYHVTMAKHAPFVATAKSVKGHETRPDGRAIAI 2ki8.1    NALGLQEGDRVKVKTE-----------------FGEVVVFAKKG-DVPKGMIFIPMGP----------------------  target    DTGYQSNFRYGAQQSFTRNWLMPMHQTDSLPGKHAVAWKFKWGYQVDHHAINTVPKECLIRITKAEDGGIGARGPWEPVR 2ki8.1    --------------------------------------------------------------------------------  target    TGFTPGQENEFMIKWLKGEHIKIKV 2ki8.1    ------------------------- ``` | | | | | | | | | | | | | | | | | | | | | | | | | | | | | | | | | | | | | | | | | | | | | | | | | |
|  | 1h0h.1.A | FORMATE DEHYDROGENASE SUBUNIT ALPHA  *Tungsten containing Formate Dehydrogenase from Desulfovibrio Gigas* | 0.01 |  | 18.18 | 0.07 | 903-1012 | X-ray | 1.80 | hetero-1-1-mer | 1 x W, 1 x 2MD, 1 x MGD, 4 x SF4, 1 x CA | HHblits | 0.29 |
| ``` target    MFLSRRQFLKVSVGTVAAVAVADKVLALTALQPVIEVGNPLGDYPDRSWERVYHDQYRYDSSFTWVCSPNDTHACRVRAF 1h0h.1    --------------------------------------------------------------------------------  target    VRNGVVMRVEQNYDHQTYEDLYGNRGTFAHNPRMCLKGFTFHRRVYGPYRLKGPLMRKGWKQWMDDNAPELTAETKRKYK 1h0h.1    --------------------------------------------------------------------------------  target    FDSRFLDDMLRVSWDTAFTYAAKAMITIATRYSGEAGARRLREQGYAPEMIEMMKGAGTRCFKHRAGMPVLGIIGKMGNT 1h0h.1    --------------------------------------------------------------------------------  target    RMNGGINALLDTWIRKVSPDQAQGGRYWSNYTWHGDQNPAHPFWSGVQGSDIDLSDMRFSKLNTSWGKNFVENKMPEAHW 1h0h.1    --------------------------------------------------------------------------------  target    KLECIERGARVVVITPEYNPTAYRADYWMPLRPESDGALFLGAMKIIIDENMHDIDFLKSFTDAPILVRTDTLQYLDPRD 1h0h.1    --------------------------------------------------------------------------------  target    VIADYKFPDFSKSYSGRIQSLKPEQIQRLGGMMVWDLNKKQVVPLHREQVGWHYTNSGIDAALTGTYRVKLLNGREIDAM 1h0h.1    --------------------------------------------------------------------------------  target    PIWQMYMVHFQDYDLDTVHQITRTPKDLIVRWARDSGTIKPAAIHNGEGTCHYFHQTANARGAAMVLIITGNVGKFGTGQ 1h0h.1    --------------------------------------------------------------------------------  target    HTWAGNYKAGTWTATPWSGAGLSVHTGEDPFNITLDPNAHGKEIKTRSYYYGEEVGYWNHGDTALIVNTPKYGRKVFTGK 1h0h.1    --------------------------------------------------------------------------------  target    THMPTPSKFRWVVNVNVVNNAKHHYDMVRNVDPNIECLITQDIEMTSDINHADIAFAANSWMEFTYPEMTVTVSNPWVQI 1h0h.1    --------------------------------------------------------------------------------  target    WKGGIRPLYDTRNDLDTFAGVAAKLSDMTGDKRMRDYFAMVYQNRVDVYVQRMLDASSTFYGYSADVMLKSEKGWMVMVR 1h0h.1    --------------------------------------------------------------------------------  target    TYPRHPFWEETNESKPMWTRSGRYENYRIEPEAIEYGENFISHREGPEATPYLPNAIFTTNPYVRPDDYGIPITAQHHDD 1h0h.1    --------------------------------------------------------------------------------  target    KTVRNIKLSWHEIKRHSNPLWEKGYQFYCVTPKTR--HRVHSQWSVNDWVQIYESNFGDPYRMDKRTPGVGEHQIHINPQ 1h0h.1    ----------------------PRYPFICSTYRVTEHWQTGLMTRNTPWLLEA----------------EPQMFCEMSEE  target    AAKDRGINDGDYVYVDGNPVDRPYRGWKPSDPYYKVARLMIRAKYNPAYPYHVTMAKHAPFVATAKSVKGHETRPDGRAI 1h0h.1    LATLRGIKNGDKVILESV-----------------RGKLWAKAIITKRIKPFAI--------------------------  target    AIDTGYQSNFRYGAQQSFTRNWLMPMHQTDSLPGKHAVAWKFKWGYQVDHHAINTVPKECLIRITKAEDGGIGARGPWEP 1h0h.1    --------------------------------------------------------------------------------  target    VRTGFTPGQENEFMIKWLKGEHIKIKV 1h0h.1    --------------------------- ``` | | | | | | | | | | | | | | | | | | | | | | | | | | | | | | | | | | | | | | | | | | | | | | | | | |
|  | 8bqg.1.A | Formate dehydrogenase, alpha subunit, selenocysteine-containing  *W-formate dehydrogenase from Desulfovibrio vulgaris - Soaking with Formate 1 min* | 0.01 |  | 20.51 | 0.07 | 902-1012 | X-ray | 1.95 | hetero-1-1-mer | 2 x MGD, 4 x SF4, 1 x H2S, 1 x W | HHblits | 0.28 |
| ``` target    MFLSRRQFLKVSVGTVAAVAVADKVLALTALQPVIEVGNPLGDYPDRSWERVYHDQYRYDSSFTWVCSPNDTHACRVRAF 8bqg.1    --------------------------------------------------------------------------------  target    VRNGVVMRVEQNYDHQTYEDLYGNRGTFAHNPRMCLKGFTFHRRVYGPYRLKGPLMRKGWKQWMDDNAPELTAETKRKYK 8bqg.1    --------------------------------------------------------------------------------  target    FDSRFLDDMLRVSWDTAFTYAAKAMITIATRYSGEAGARRLREQGYAPEMIEMMKGAGTRCFKHRAGMPVLGIIGKMGNT 8bqg.1    --------------------------------------------------------------------------------  target    RMNGGINALLDTWIRKVSPDQAQGGRYWSNYTWHGDQNPAHPFWSGVQGSDIDLSDMRFSKLNTSWGKNFVENKMPEAHW 8bqg.1    --------------------------------------------------------------------------------  target    KLECIERGARVVVITPEYNPTAYRADYWMPLRPESDGALFLGAMKIIIDENMHDIDFLKSFTDAPILVRTDTLQYLDPRD 8bqg.1    --------------------------------------------------------------------------------  target    VIADYKFPDFSKSYSGRIQSLKPEQIQRLGGMMVWDLNKKQVVPLHREQVGWHYTNSGIDAALTGTYRVKLLNGREIDAM 8bqg.1    --------------------------------------------------------------------------------  target    PIWQMYMVHFQDYDLDTVHQITRTPKDLIVRWARDSGTIKPAAIHNGEGTCHYFHQTANARGAAMVLIITGNVGKFGTGQ 8bqg.1    --------------------------------------------------------------------------------  target    HTWAGNYKAGTWTATPWSGAGLSVHTGEDPFNITLDPNAHGKEIKTRSYYYGEEVGYWNHGDTALIVNTPKYGRKVFTGK 8bqg.1    --------------------------------------------------------------------------------  target    THMPTPSKFRWVVNVNVVNNAKHHYDMVRNVDPNIECLITQDIEMTSDINHADIAFAANSWMEFTYPEMTVTVSNPWVQI 8bqg.1    --------------------------------------------------------------------------------  target    WKGGIRPLYDTRNDLDTFAGVAAKLSDMTGDKRMRDYFAMVYQNRVDVYVQRMLDASSTFYGYSADVMLKSEKGWMVMVR 8bqg.1    --------------------------------------------------------------------------------  target    TYPRHPFWEETNESKPMWTRSGRYENYRIEPEAIEYGENFISHREGPEATPYLPNAIFTTNPYVRPDDYGIPITAQHHDD 8bqg.1    --------------------------------------------------------------------------------  target    KTVRNIKLSWHEIKRHSNPLWEKGYQFYCVTPKTRHRVHS--QWSVNDWVQIYESNFGDPYRMDKRTPGVGEHQIHINPQ 8bqg.1    ---------------------DPRYPFIGTTYRVTEHWQTGLMTRRCAWLVEA----------------EPQIFCEISKE  target    AAKDRGINDGDYVYVDGNPVDRPYRGWKPSDPYYKVARLMIRAKYNPAYPYHVTMAKHAPFVATAKSVKGHETRPDGRAI 8bqg.1    LAKLRGIGNGDTVKVSSL-----------------RGALEAVAIVTERIRPFKI--------------------------  target    AIDTGYQSNFRYGAQQSFTRNWLMPMHQTDSLPGKHAVAWKFKWGYQVDHHAINTVPKECLIRITKAEDGGIGARGPWEP 8bqg.1    --------------------------------------------------------------------------------  target    VRTGFTPGQENEFMIKWLKGEHIKIKV 8bqg.1    --------------------------- ``` | | | | | | | | | | | | | | | | | | | | | | | | | | | | | | | | | | | | | | | | | | | | | | | | | |
|  | 6sdr.1.A | Formate dehydrogenase, alpha subunit, selenocysteine-containing  *W-formate dehydrogenase from Desulfovibrio vulgaris - Oxidized form* | 0.01 |  | 20.78 | 0.07 | 903-1012 | X-ray | 2.10 | hetero-1-1-mer | 2 x MGD, 4 x SF4, 1 x H2S, 1 x W | HHblits | 0.28 |
[truncated: 1,154,971 more chars]
