# Supplementary material for: Metabolic capacity is maintained despite shifts in microbial diversity in estuary sediments
Source: ISME Commun. 2025 Oct 11;5(1):ycaf182. doi: 10.1093/ismeco/ycaf182 (PMC12687941; doi:10.1093/ismeco/ycaf182)
Supplement: Supplementary_Data_1_ycaf182 [file supplementary_data_1_ycaf182.zip › SWISS-MODEL/8_1_May_SF_Bin5_scaffold_55982_c1/report.html]

Untitled Project | Report


|  |  |  |
| --- | --- | --- |
|  |  | SWISS-MODEL Homology Modelling Report |

## Model Building Report

This document lists the results for the homology modelling project "Untitled Project" submitted to SWISS-MODEL workspace
on March 29, 2023, 4:10 a.m..The submitted primary amino acid sequence is given in Table T1.

If you use any results in your research, please cite the relevant publications:

- Waterhouse, A., Bertoni, M., Bienert, S., Studer, G., Tauriello, G., Gumienny, R.,
  Heer, F.T., de Beer, T.A.P., Rempfer, C., Bordoli, L., Lepore, R., Schwede, T.
  SWISS-MODEL: homology modelling of protein structures and complexes.
  Nucleic Acids Res. 46(W1), W296-W303 (2018).
- Bienert, S., Waterhouse, A., de Beer, T.A.P., Tauriello, G., Studer,
  G., Bordoli, L., Schwede, T. The SWISS-MODEL Repository - new features and
  functionality. Nucleic Acids Res. 45, D313-D319 (2017).
- Studer, G., Tauriello, G., Bienert, S.,
  Biasini, M., Johner, N., Schwede, T. ProMod3 - A versatile homology
  modelling toolbox. PLOS Comp. Biol. 17(1), e1008667 (2021).
- Studer, G., Rempfer, C., Waterhouse, A.M.,
  Gumienny, G., Haas, J., Schwede, T. QMEANDisCo - distance constraints
  applied on model quality estimation. Bioinformatics 36, 1765-1771 (2020).
- Bertoni, M., Kiefer, F., Biasini, M., Bordoli, L.,
  Schwede, T. Modeling protein quaternary structure of homo- and
  hetero-oligomers beyond binary interactions by homology. Scientific
  Reports 7 (2017).

## Results

The SWISS-MODEL template library (SMTL version 2023-03-23, PDB release 2023-03-17) was searched with
for evolutionary related structures matching the target sequence in Table T1. For details on the template search, see Materials and Methods. Overall 515 templates were found (Table T2).

## Models

The following models were built (see Materials and Methods "Model Building"):

| Model #01 | File | Built with | Oligo-State | Ligands | GMQE | QMEANDisCo Global |
| --- | --- | --- | --- | --- | --- | --- |
|  | PDB | ProMod3 3.2.1 | monomer | 1 x MO: MOLYBDENUM ATOM; | 0.79 | 0.80 ± 0.05 |

|  |  |  |
| --- | --- | --- |
|  |  |  |

| Template | Seq Identity | Oligo-state | QSQE | Found by | Method | Resolution | Seq Similarity | Range | Coverage | Description |
| --- | --- | --- | --- | --- | --- | --- | --- | --- | --- | --- |
| 7b04.1.B | 57.58 | monomer | 0.00 | HHblits | X-ray | 2.97Å | 0.48 | 28 - 1144 | 1.00 | Nitrite oxidoreductase subunit A |

  

### Included Ligands

| Ligand | Description |
| --- | --- |
| 1 x MO | MOLYBDENUM ATOM |

  

### Excluded ligands

| Ligand Name.Number | Reason for Exclusion | Description |
| --- | --- | --- |
| CA.10 | Binding site not conserved. | CALCIUM ION |
| CA.11 | Binding site not conserved. | CALCIUM ION |
| F3S.4 | Binding site not conserved. | FE3-S4 CLUSTER |
| HEM.9 | Binding site not conserved. | PROTOPORPHYRIN IX CONTAINING FE |
| MD1.5 | Binding site not conserved. | PHOSPHORIC ACID 4-(2-AMINO-4-OXO-3,4,5,6,-TETRAHYDRO-PTERIDIN-6-YL)-2-HYDROXY-3,4-DIMERCAPTO-BUT-3-EN-YL ESTER GUANYLATE ESTER |
| MD1.6 | Binding site not conserved. | PHOSPHORIC ACID 4-(2-AMINO-4-OXO-3,4,5,6,-TETRAHYDRO-PTERIDIN-6-YL)-2-HYDROXY-3,4-DIMERCAPTO-BUT-3-EN-YL ESTER GUANYLATE ESTER |
| SF4.1 | Binding site not conserved. | IRON/SULFUR CLUSTER |
| SF4.2 | Binding site not conserved. | IRON/SULFUR CLUSTER |
| SF4.3 | Binding site not conserved. | IRON/SULFUR CLUSTER |
| SF4.8 | Binding site not conserved. | IRON/SULFUR CLUSTER |

  

```
Target    MFLSRRQFLKVSVGTVAAVAVADKVLALTALQPVIEVGNPLGDYPDRSWERVYHDQYRYDSSFTWVCSPNDTHACRVRAF  
7b04.1.B  MKLTRRAFLQVAGATGATLTLAKNAMAFRLLKPAVVVDNPLDTYPDRRWESVYRDQYQYDRTFTYCCSPNDTHACRIRAF  
  
Target    VRNGVVMRVEQNYDHQTYEDLYGNRGTFAHNPRMCLKGFTFHRRVYGPYRLKGPLMRKGWKQWMDDNAPELTAETKRKYK  
7b04.1.B  VRNNVMMRVEQNYDHQNYSDLYGNKATRNWNPRMCLKGYTFHRRVYGPYRLRYPLIRKGWKRWADDGFPELTPENKTKYM  
  
Target    FDSRFLDDMLRVSWDTAFTYAAKAMITIATRY-SGEAGARRLREQGYAPEMIEMMKGAGTRCFKHRAGMPVLGIIGKMGN  
7b04.1.B  FDNRGNDELLRASWDEAFTYASKGIIHITKKYSGPEGAQK-LIDQGYPKEMVDRMQGAGTRTFKGRGGMGLLGVIGKYGM  
  
Target    TRMNGGINALLDTWIRKVSPDQAQGGRYWSNYTWHGDQNPAHPFWSGVQGSDIDLSDMRFSKLNTSWGKNFVENKMPEAH  
7b04.1.B  YRFNNC-LAIVDAHNRGVGPDQALGGRNWSNYTWHGDQAPGHPFSHGLQTSDVDMNDVRFSKLLIQTGKNLIENKMPEAH  
  
Target    WKLECIERGARVVVITPEYNPTAYRADYWMPLRPESDGALFLGAMKIIIDENMHDIDFLKSFTDAPILVRTDTLQYLDPR  
7b04.1.B  WVTEVMERGGKIVVITPEYSPSAQKADYWIPIRNNTDTALFLGITKILIDNKWYDADYVKKFTDFPLLIRTDTLKRVSPK  
  
Target    DVIADYKFPDFSKSYSGRIQSLKPEQIQRLGGMMVWDLNKKQVVPLHREQVGWHYTNSGIDAALTGTYRVKLLNGREIDA  
7b04.1.B  DIIPNYKLQDISDGPSYHIQGLKDEQREIIGDFVVWDAKSKGPKAITRDDVGETLVKKGIDPVLEGSFKLKTIDGKEIEV  
  
Target    MPIWQMYMVHFQDYDLDTVHQITRTPKDLIVRWARDSGTIKPAAIHNGEGTCHYFHQTANARGAAMVLIITGNVGKFGTG  
7b04.1.B  MTLLEMYKIHLRDYDIDSVVSMTNSPKDLIERLAKDIATIKPVAIHYGEGVNHYFHATLMNRSYYLPVMLTGNVGYFGSG  
  
Target    QHTWAGNYKAGTWTATPWSGAGLSVHTGEDPFNITLDPNAHGKEIKTRSYYYGEEVGYWNHGDTALIVNTPKYGRKVFTG  
7b04.1.B  SHTWAGNYKAGNFQASKWSGPGFYGWVAEDVFKPNLDPYASAKDLNIKGRALDEEVAYWNHSERPLIVNTPKYGRKVFTG  
  
Target    KTHMPTPSKFRWVVNVNVVNNAKHHYDMVRNVDPNIECLITQDIEMTSDINHADIAFAANSWMEFTYPEMTVTVSNPWVQ  
7b04.1.B  KTHMPSPTKVLWFTNVNLINNAKHVYQMLKNVNPNIEQIMSTDIEITGSIEYADFAFPANSWVEFQEFEITNSCSNPFIQ  
  
Target    IWK-GGIRPLYDTRNDLDTFAGVAAKLSDMTGDKRMRDYFAMVYQNRVDVYVQRMLDASSTFYGYSADVMLKSE---KGW  
7b04.1.B  IWGKTGITPVYESKDDVKILAGMASKLGELLRDKRFEDNWKFAIEGRASVYINRLLDGSTTMKGYTCEDILNGKYGEPGV  
  
Target    MV-MVRTYPRHPFWEETNESKPMWTRSGRYENYRIEPEAIEYGENFISHREGPEATPYLPNAIFTTNPYVRPDDYGIPIT  
7b04.1.B  AMLLFRTYPRHPFWEQVHESLPFYTPTGRLQAYNDEPEIIEYGENFIVHREGPEATPYLPNAIVSTNPYIRPDDYGIPEN  
  
Target    AQHHDDKTVRNIKLSWHEIKRHSNPLWEKGYQFYCVTPKTRHRVHSQWSVNDWVQIYESNFGDPYRMDKRTPGVGEHQIH  
7b04.1.B  AEYWEDRTVRNIKKSWEETKKTKNFLWEKGYHFYCVTPKSRHTVHSQWAVTDWNFIWNNNFGDPYRMDKRMPGVGEHQIH  
  
Target    INPQAAKDRGINDGDYVYVDGNPVDRPYRGWKPSDPYYKVARLMIRAKYNPAYPYHVTMAKHAPFVATAKSVKGHETRPD  
7b04.1.B  IHPQAARDLGIEDGDYVYVDANPADRPYEGWKPNDSFYKVSRLMLRAKYNPAYPYNCTMMKHSAWISSDKTVQAHETRP-  
  
Target    GRAIAIDTGYQSNFRYGAQQSFTRNWLMPMHQTDSLPGKHAVAWKFKWGYQVDHHAINTVPKECLIRITKAEDGGIGARG  
7b04.1.B  DGRALSPSGYQSSFRYGSQQSITRDWSMPMHQLDSLFHKAKIGMKFIFGFEADNHCINTVPKETLVKITKAENGGMGGKG  
  
Target    PWEPVRTGFTPGQENEFMIKWLKGEHIKIKV  
7b04.1.B  VWDPVKTGYTAGNENDFMKKFLNGELIKVD-
```

  


---

  

| Model #03 | File | Built with | Oligo-State | Ligands | GMQE | QMEANDisCo Global |
| --- | --- | --- | --- | --- | --- | --- |
|  | PDB | ProMod3 3.2.1 | monomer | 1 x 6MO: MOLYBDENUM(VI) ION; | 0.40 | 0.49 ± 0.05 |

|  |  |  |
| --- | --- | --- |
|  |  |  |

| Template | Seq Identity | Oligo-state | QSQE | Found by | Method | Resolution | Seq Similarity | Range | Coverage | Description |
| --- | --- | --- | --- | --- | --- | --- | --- | --- | --- | --- |
| 3ir5.1.A | 20.97 | monomer | 0.00 | HHblits | X-ray | 2.30Å | 0.31 | 2 - 1111 | 0.81 | Respiratory nitrate reductase 1 alpha chain |

  

### Included Ligands

| Ligand | Description |
| --- | --- |
| 1 x 6MO | MOLYBDENUM(VI) ION |

  

### Excluded ligands

| Ligand Name.Number | Reason for Exclusion | Description |
| --- | --- | --- |
| AGA.5 | Binding site not conserved. | (1S)-2-{[{[(2S)-2,3-DIHYDROXYPROPYL]OXY}(HYDROXY)PHOSPHORYL]OXY}-1-[(PENTANOYLOXY)METHYL]ETHYL OCTANOATE |
| F3S.9 | Binding site not conserved. | FE3-S4 CLUSTER |
| HEM.10 | Binding site not conserved. | PROTOPORPHYRIN IX CONTAINING FE |
| HEM.11 | Binding site not conserved. | PROTOPORPHYRIN IX CONTAINING FE |
| MD1.1 | Binding site not conserved. | PHOSPHORIC ACID 4-(2-AMINO-4-OXO-3,4,5,6,-TETRAHYDRO-PTERIDIN-6-YL)-2-HYDROXY-3,4-DIMERCAPTO-BUT-3-EN-YL ESTER GUANYLATE ESTER |
| MD1.2 | Binding site not conserved. | PHOSPHORIC ACID 4-(2-AMINO-4-OXO-3,4,5,6,-TETRAHYDRO-PTERIDIN-6-YL)-2-HYDROXY-3,4-DIMERCAPTO-BUT-3-EN-YL ESTER GUANYLATE ESTER |
| SF4.4 | Binding site not conserved. | IRON/SULFUR CLUSTER |
| SF4.6 | Binding site not conserved. | IRON/SULFUR CLUSTER |
| SF4.7 | Binding site not conserved. | IRON/SULFUR CLUSTER |
| SF4.8 | Binding site not conserved. | IRON/SULFUR CLUSTER |

  

```
Target    MFLSRRQFLKVSVGTVAAVAVADKVLALTALQPVIEVGNPLGDYPDRSWERVYHDQYRYDSSFTWVCSPNDTHACRVRAF  
3ir5.1.A  -FLDRFRYFKQKGETFADGH--------GQL-----------LNTNRDWEDGYRQRWQHDKIVRSTCGVNCTGSCSWKIY  
  
Target    VRNGVVMRVEQNYDHQTYEDLYGNRGTFAHNPRMCLKGFTFHRRVYGPYRLKGPLMRKGWK-QWMDDNA----P-----E  
3ir5.1.A  VKNGLVTWETQQTDYPRT-----RPDLPNHEPRGCPRGASYSWYLYSANRLKYPMMRKRLMKMWREAKALHSDPVEAWAS  
  
Target    -LTAETKRKYKFDSRFLDDMLRVSWDTAFTYAAKAMITIATRYSGEAGARRLREQGYAPEMIEMMKGAGTRCF-KHRAGM  
3ir5.1.A  IIEDADKAKSFKQARGRGGFVRSSWQEVNELIAASNVYTIKNYGPDRVAGF--------------SPIPAMSMVSYASGA  
  
Target    PVLGIIGKMGNTRMNGGINALLDTWIRKVSPDQAQGGRYWSNYTWHGDQNPAHPFWSGVQGSDIDLSDMRFSKLNTSWGK  
3ir5.1.A  RYLS---LIGGT--------------------------CLSFYDWYCDLPPASPQTWGEQTDVPESADWYNSSYIIAWGS  
  
Target    NFVENKMPEAHWKLECIERGARVVVITPEYNPTAYRADYWMPLRPESDGALFLGAMKIIIDENM------HDIDFLKSFT  
3ir5.1.A  NVPQTRTPDAHFFTEVRYKGTKTVAVTPDYAEIAKLCDLWLAPKQGTDAAMALAMGHVMLREFHLDNPSQYFTDYVRRYT  
  
Target    DAPILVRTD-------TLQYLDPRDVIADYKFPDFSKSYSGRIQSLKPEQIQRLGGMMVWDLNKKQVVPLHREQV-----  
3ir5.1.A  DMPMLVMLEERDGYYAAGRMLRAADLVDALGQEN-----------------NPEWKTVAFNT-NGEMVAPNGSIGFRWGE  
  
Target    --GWHYT----------------------------------------NSGIDAALTG---TYRVKLLNGREIDAMPIWQM  
3ir5.1.A  KGKWNLEQRDGKTGEETELQLSLLGSQDEIAEVGFPYFGGDGTEHFNKVELENVLLHKLPVKRLQLADGSTALVTTVYDL  
  
Target    YM------------------VHFQDYDLDTVHQITRTPKDLIVRWARDSGTI-----KPAAIHNGEGTCHYFHQTANARG  
3ir5.1.A  TLANYGLERGLNDVNCATSYDDVKAYTPAWAEQITGVSRSQIIRIAREFADNADKTHGRSMIIVGAGLNHWYHLDMNYRG  
  
Target    AAMVLIITGNVGKFGTGQHTWAGNYKAGTWTATPWSGAGLSV----HT---GEDPF--------------N-ITLDPNAH  
3ir5.1.A  LINMLIFCGCVGQSGGGWAHYVGQEKLRPQ--TGWQPLAFALDWQRPARHMNSTSYFYNHSSQWRYETVTAEELLSPMAD  
  
Target    GKEIKTR---SYYYGEEVGYWNHG----DTALI---------VNTPKY-GRKVFTGKT--------HMPTPSKFRWVVNV  
3ir5.1.A  KSRYTGHLIDFNVRAERMGWLPSAPQLGTNPLTIAGEAEKAGMNPVDYTVKSLKEGSIRFAAEQPENGKNHPRNLFIWRS  
  
Target    NVVNNAKHHYD-MV------------------------------RNVDPNIECLITQDIEMTSDINHADIAFAANSWMEF  
3ir5.1.A  NLLGSSGKGHEFMLKYLLGTEHGIQGKDLGQQGGVKPEEVDWQDNGLEGKLDLVVTLDFRLSSTCLYSDIILPTATWYEK  
  
Target    TYPEMTVTVSNPWVQIWKGGIRPLYDTRNDLDTFAGVAAKLSDMTGD----K--------------------RMRDYFAM  
3ir5.1.A  DDM--NTSDMHPFIHPLSAAVDPAWEAKSDWEIYKAIAKKFSEVCVGHLGKETDIVTLPIQHDSAAELAQPLDVKDWKKG  
  
Target    V------------------YQNR---------------------------VDVYVQR-----------------------  
3ir5.1.A  ECDLIPGKTAPHIMVVERDYPATYERFTSIGPLMEKIGNGGKGIAWNTQSEMDLLRKLNYTKAEGPAKGQPMLNTAIDAA  
  
Target    --MLDASSTFYGY----SADVMLKS------------EKGWMVMVR--------------------TYPRHPFWEETNES  
3ir5.1.A  EMILTLAPETNGQVAVKAWAALSEFTGRDHTHLALNKEDEKIRFRDIQAQPRKIISSPTWSGLEDEHVSYNAGYTNVHEL  
  
Target    KPMWTRSGRYENYRIEPEAIEYGENFISHREGPEATPYLPNAIFTTNPYVRPDDYGIPITAQHHDDKTVRNIKLSWHEIK  
3ir5.1.A  IPWRTLSGRQQLYQDHQWMRDFGESLLVYRPPIDTRSV-----------------------------------KEVIGQ-  
  
Target    RHSNPLWEKGYQFYCVTPKTRHRVHSQWSVNDWVQIYESNFGDPYRMDKRTPGVGEHQIHINPQAAKDRGINDGDYVYVD  
3ir5.1.A  ---KSNGNQEKALNFLTPHQKWGIHSTYSDNLLMLTLG---------------RGGPVVWLSEADAKDLGIADNDWIEVF  
  
Target    GNPVDRPYRGWKPSDPYYKVARLMIRAKYNPAYPYHVTMAKHAPFVATAKSVKGHETRPDGRAIAIDTGYQSNFRYGAQQ  
3ir5.1.A  NS-----------------NGALTARAVVSQRVPAGMTMMYHAQERIVNL--------P--GSE------ITQQRGGIHN  
  
Target    SFTRNWLMPMHQTDSLPGKHAVAWKFKWGYQVDHHAINTVPKECLIRITKAEDGGIGARGPWEPVRTGFTPGQENEFMIK  
3ir5.1.A  SVTRITPKPTHMIGGYAHLA---------YGFNYYGTVGSNRDEFVVVRKMKNIDWL-----------------------  
  
Target    WLKGEHIKIKV  
3ir5.1.A  -----------
```

  


---

  

| Model #02 | File | Built with | Oligo-State | Ligands | GMQE | QMEANDisCo Global |
| --- | --- | --- | --- | --- | --- | --- |
|  | PDB | ProMod3 3.2.1 | monomer (matching prediction) | None | 0.37 | 0.48 ± 0.05 |

|  |  |  |
| --- | --- | --- |
|  |  |  |

| Template | Seq Identity | Oligo-state | QSQE | Found by | Method | Resolution | Seq Similarity | Range | Coverage | Description |
| --- | --- | --- | --- | --- | --- | --- | --- | --- | --- | --- |
| 1r27.4.A | 20.78 | homo-dimer | 0.25 | HHblits | X-ray | 2.00Å | 0.31 | 47 - 1111 | 0.81 | Respiratory nitrate reductase 1 alpha chain |

  

### Excluded ligands

| Ligand Name.Number | Reason for Exclusion | Description |
| --- | --- | --- |
| F3S.5 | Binding site not conserved. | FE3-S4 CLUSTER |
| F3S.13 | Binding site not conserved. | FE3-S4 CLUSTER |
| F3S.21 | Binding site not conserved. | FE3-S4 CLUSTER |
| F3S.29 | Binding site not conserved. | FE3-S4 CLUSTER |
| MGD.3 | Binding site not conserved. | 2-AMINO-5,6-DIMERCAPTO-7-METHYL-3,7,8A,9-TETRAHYDRO-8-OXA-1,3,9,10-TETRAAZA-ANTHRACEN-4-ONE GUANOSINE DINUCLEOTIDE |
| MGD.4 | Binding site not conserved. | 2-AMINO-5,6-DIMERCAPTO-7-METHYL-3,7,8A,9-TETRAHYDRO-8-OXA-1,3,9,10-TETRAAZA-ANTHRACEN-4-ONE GUANOSINE DINUCLEOTIDE |
| MGD.11 | Binding site not conserved. | 2-AMINO-5,6-DIMERCAPTO-7-METHYL-3,7,8A,9-TETRAHYDRO-8-OXA-1,3,9,10-TETRAAZA-ANTHRACEN-4-ONE GUANOSINE DINUCLEOTIDE |
| MGD.12 | Binding site not conserved. | 2-AMINO-5,6-DIMERCAPTO-7-METHYL-3,7,8A,9-TETRAHYDRO-8-OXA-1,3,9,10-TETRAAZA-ANTHRACEN-4-ONE GUANOSINE DINUCLEOTIDE |
| MGD.19 | Binding site not conserved. | 2-AMINO-5,6-DIMERCAPTO-7-METHYL-3,7,8A,9-TETRAHYDRO-8-OXA-1,3,9,10-TETRAAZA-ANTHRACEN-4-ONE GUANOSINE DINUCLEOTIDE |
| MGD.20 | Binding site not conserved. | 2-AMINO-5,6-DIMERCAPTO-7-METHYL-3,7,8A,9-TETRAHYDRO-8-OXA-1,3,9,10-TETRAAZA-ANTHRACEN-4-ONE GUANOSINE DINUCLEOTIDE |
| MGD.27 | Binding site not conserved. | 2-AMINO-5,6-DIMERCAPTO-7-METHYL-3,7,8A,9-TETRAHYDRO-8-OXA-1,3,9,10-TETRAAZA-ANTHRACEN-4-ONE GUANOSINE DINUCLEOTIDE |
| MGD.28 | Binding site not conserved. | 2-AMINO-5,6-DIMERCAPTO-7-METHYL-3,7,8A,9-TETRAHYDRO-8-OXA-1,3,9,10-TETRAAZA-ANTHRACEN-4-ONE GUANOSINE DINUCLEOTIDE |
| MO.1 | Not in contact with model. | MOLYBDENUM ATOM |
| MO.9 | Binding site not conserved. | MOLYBDENUM ATOM |
| MO.17 | Binding site not conserved. | MOLYBDENUM ATOM |
| MO.25 | Binding site not conserved. | MOLYBDENUM ATOM |
| SF4.2 | Binding site not conserved. | IRON/SULFUR CLUSTER |
| SF4.6 | Binding site not conserved. | IRON/SULFUR CLUSTER |
| SF4.7 | Binding site not conserved. | IRON/SULFUR CLUSTER |
| SF4.8 | Binding site not conserved. | IRON/SULFUR CLUSTER |
| SF4.10 | Binding site not conserved. | IRON/SULFUR CLUSTER |
| SF4.14 | Binding site not conserved. | IRON/SULFUR CLUSTER |
| SF4.15 | Binding site not conserved. | IRON/SULFUR CLUSTER |
| SF4.16 | Binding site not conserved. | IRON/SULFUR CLUSTER |
| SF4.18 | Binding site not conserved. | IRON/SULFUR CLUSTER |
| SF4.22 | Binding site not conserved. | IRON/SULFUR CLUSTER |
| SF4.23 | Binding site not conserved. | IRON/SULFUR CLUSTER |
| SF4.24 | Binding site not conserved. | IRON/SULFUR CLUSTER |
| SF4.26 | Binding site not conserved. | IRON/SULFUR CLUSTER |
| SF4.30 | Binding site not conserved. | IRON/SULFUR CLUSTER |
| SF4.31 | Binding site not conserved. | IRON/SULFUR CLUSTER |
| SF4.32 | Binding site not conserved. | IRON/SULFUR CLUSTER |

  

```
Target    MFLSRRQFLKVSVGTVAAVAVADKVLALTALQPVIEVGNPLGDYPDRSWERVYHDQYRYDSSFTWVCSPNDTHACRVRAF  
1r27.4.A  -FLDRFRYFKQKGETFADGHG--------QL-----------LNTNRDWEDGYRQRWQHDKIVRSTHGVNCTGSCSWKIY  
  
Target    VRNGVVMRVEQNYDHQTYEDLYGNRGTFAHNPRMCLKGFTFHRRVYGPYRLKGPLMRKGWK-QWMDDN----APEL----  
1r27.4.A  VKNGLVTWETQQTDYPRT-----RPDLPNHEPRGCPRGASYSWYLYSANRLKYPMMRKRLMKMWREAKALHSDPVEAWAS  
  
Target    --TAETKRKYKFDSRFLDDMLRVSWDTAFTYAAKAMITIATRYSGEAGARRLREQGYAPEMIEMMKGAGTRCF-KHRAGM  
1r27.4.A  IIEDADKAKSFKQARGRGGFVRSSWQEVNELIAASNVYTIKNYGPDRVAGFS--------------PIPAMSMVSYASGA  
  
Target    PVLGIIGKMGNTRMNGGINALLDTWIRKVSPDQAQGGRYWSNYTWHGDQNPAHPFWSGVQGSDIDLSDMRFSKLNTSWGK  
1r27.4.A  RYL---SLIGGT--------------------------CLSFYDWYCDLPPASPQTWGEQTDVPESADWYNSSYIIAWGS  
  
Target    NFVENKMPEAHWKLECIERGARVVVITPEYNPTAYRADYWMPLRPESDGALFLGAMKIIIDENM------HDIDFLKSFT  
1r27.4.A  NVPQTRTPDAHFFTEVRYKGTKTVAVTPDYAEIAKLCDLWLAPKQGTDAAMALAMGHVMLREFHLDNPSQYFTDYVRRYT  
  
Target    DAPILVRTDT-------LQYLDPRDVIADYKFPDFSKSYSGRIQSLKPEQIQRLGGMMVWDLNKKQVVPLHREQV-----  
1r27.4.A  DMPMLVMLEERDGYYAAGRMLRAADLVDALGQEN-----------------NPEWKTVAFNT-NGEMVAPNGSIGFRWGE  
  
Target    --GWHYTN----------------SGI-------D-------------AALT-------GTYRVKLLNGREIDAMPIWQM  
1r27.4.A  KGKWNLEQRDGKTGEETELQLSLLGSQDEIAEVGFPYFGGDGTEHFNKVELENVLLHKLPVKRLQLADGSTALVTTVYDL  
  
Target    YM------------------VHFQDYDLDTVHQITRTPKDLIVRWARDSGTI-----KPAAIHNGEGTCHYFHQTANARG  
1r27.4.A  TLANYGLERGLNDVNCATSYDDVKAYTPAWAEQITGVSRSQIIRIAREFADNADKTHGRSMIIVGAGLNHWYHLDMNYRG  
  
Target    AAMVLIITGNVGKFGTGQHTWAGNYKA-GTWTATPWSGAGLSV----HT---GEDPF--------------N-ITLDPNA  
1r27.4.A  LINMLIFCGCVGQSGGGWAHYVGQEKLRPQT---GWQPLAFALDWQRPARHMNSTSYFYNHSSQWRYETVTAEELLSPMA  
  
Target    HGKEIKTR---SYYYGEEVGYWNH----GDTALI---------VNTPKYG-RKVFTGKT--------HMPTPSKFRWVVN  
1r27.4.A  DKSRYTGHLIDFNVRAERMGWLPSAPQLGTNPLTIAGEAEKAGMNPVDYTVKSLKEGSIRFAAEQPENGKNHPRNLFIWR  
  
Target    VNVVNNAKHHYD-MV------------------------------RNVDPNIECLITQDIEMTSDINHADIAFAANSWME  
1r27.4.A  SNLLGSSGKGHEFMLKYLLGTEHGIQGKDLGQQGGVKPEEVDWQDNGLEGKLDLVVTLDFRLSSTCLYSDIILPTATWYE  
  
Target    FTYPEMTVTVSNPWVQIWKGGIRPLYDTRNDLDTFAGVAAKLSDMTGD----K--------------------RMRDYFA  
1r27.4.A  KDDM--NTSDMHPFIHPLSAAVDPAWEAKSDWEIYKAIAKKFSEVCVGHLGKETDIVTLPIQHDSAAELAQPLDVKDWKK  
  
Target    MV------------------YQN---------------------------RVDVYVQR----------------------  
1r27.4.A  GECDLIPGKTAPHIMVVERDYPATYERFTSIGPLMEKIGNGGKGIAWNTQSEMDLLRKLNYTKAEGPAKGQPMLNTAIDA  
  
Target    ---MLDASSTFYGY----SADVMLKSE------------KGWMVMV--------------------RTYPRHPFWEETNE  
1r27.4.A  AEMILTLAPETNGQVAVKAWAALSEFTGRDHTHLALNKEDEKIRFRDIQAQPRKIISSPTWSGLEDEHVSYNAGYTNVHE  
  
Target    SKPMWTRSGRYENYRIEPEAIEYGENFISHREGPEATPYLPNAIFTTNPYVRPDDYGIPITAQHHDDKTVRNIKLSWHEI  
1r27.4.A  LIPWRTLSGRQQLYQDHQWMRDFGESLLVYRPPIDTRSV-------------KEVIG-----------------------  
  
Target    KRHSNPLWEKGYQFYCVTPKTRHRVHSQWSVNDWVQIYESNFGDPYRMDKRTPGVGEHQIHINPQAAKDRGINDGDYVYV  
1r27.4.A  ---QKSNGNQEKALNFLTPHQKWGIHSTYSDNLLMLTLG---------------RGGPVVWLSEADAKDLGIADNDWIEV  
  
Target    DGNPVDRPYRGWKPSDPYYKVARLMIRAKYNPAYPYHVTMAKHAPFVATAKSVKGHETRPDGRAIAIDTGYQSNFRYGAQ  
1r27.4.A  FNS-----------------NGALTARAVVSQRVPAGMTMMYHAQERIVNL--------P--------GSEITQQRGGIH  
  
Target    QSFTRNWLMPMHQTDSLPGKHAVAWKFKWGYQVDHHAINTVPKECLIRITKAEDGGIGARGPWEPVRTGFTPGQENEFMI  
1r27.4.A  NSVTRITPKPTHMIGGYAHLA---------YGFNYYGTVGSNRDEFVVVRKMKNIDWL----------------------  
  
Target    KWLKGEHIKIKV  
1r27.4.A  ------------
```

  


---

  

| Model #04 | File | Built with | Oligo-State | Ligands | GMQE | QMEANDisCo Global |
| --- | --- | --- | --- | --- | --- | --- |
|  | PDB | ProMod3 3.2.1 | monomer | None | 0.32 | 0.48 ± 0.05 |

|  |  |  |
| --- | --- | --- |
|  |  |  |

| Template | Seq Identity | Oligo-state | QSQE | Found by | Method | Resolution | Seq Similarity | Range | Coverage | Description |
| --- | --- | --- | --- | --- | --- | --- | --- | --- | --- | --- |
| 6sdv.1.A | 20.13 | monomer | 0.00 | HHblits | X-ray | 1.90Å | 0.30 | 42 - 747 | 0.53 | Formate dehydrogenase, alpha subunit, selenocysteine-containing,Formate dehydrogenase, alpha subunit, selenocysteine-containing,W-formate dehydrogenase - alpha subunit |

  

### Excluded ligands

| Ligand Name.Number | Reason for Exclusion | Description |
| --- | --- | --- |
| GOL.6 | Not biologically relevant. | GLYCEROL |
| GOL.7 | Not biologically relevant. | GLYCEROL |
| GOL.8 | Not biologically relevant. | GLYCEROL |
| GOL.9 | Not biologically relevant. | GLYCEROL |
| GOL.10 | Not biologically relevant. | GLYCEROL |
| GOL.11 | Not biologically relevant. | GLYCEROL |
| GOL.12 | Not biologically relevant. | GLYCEROL |
| GOL.13 | Not biologically relevant. | GLYCEROL |
| GOL.14 | Not biologically relevant. | GLYCEROL |
| GOL.15 | Not biologically relevant. | GLYCEROL |
| H2S.5 | Binding site not conserved. | HYDROSULFURIC ACID |
| MGD.1 | Binding site not conserved. | 2-AMINO-5,6-DIMERCAPTO-7-METHYL-3,7,8A,9-TETRAHYDRO-8-OXA-1,3,9,10-TETRAAZA-ANTHRACEN-4-ONE GUANOSINE DINUCLEOTIDE |
| MGD.2 | Binding site not conserved. | 2-AMINO-5,6-DIMERCAPTO-7-METHYL-3,7,8A,9-TETRAHYDRO-8-OXA-1,3,9,10-TETRAAZA-ANTHRACEN-4-ONE GUANOSINE DINUCLEOTIDE |
| NO3.16 | Not biologically relevant. | NITRATE ION |
| NO3.17 | Not biologically relevant. | NITRATE ION |
| PEG.18 | Not biologically relevant. | DI(HYDROXYETHYL)ETHER |
| SF4.3 | Binding site not conserved. | IRON/SULFUR CLUSTER |
| SF4.19 | Binding site not conserved. | IRON/SULFUR CLUSTER |
| SF4.20 | Binding site not conserved. | IRON/SULFUR CLUSTER |
| SF4.21 | Binding site not conserved. | IRON/SULFUR CLUSTER |
| W.4 | Not in contact with model. | TUNGSTEN ION |

  

```
Target    MFLSRRQFLKVSVGTVAAVAVADKVLALTALQPVIEVGNPLGDYPDRSWERVYHDQYRYDSSFTWVCSPNDTHACRVRAF  
6sdv.1.A  MTVTRRHFLKLSAGAAVAGAFTGLGLSL---APTVA---RAEL--QK---------LQWAKQTTSICC-YCAVGCGLIVH  
  
Target    VR---NGVVMRVEQNYDHQTYEDLYGNRGTFAHNPRMCLKGFTFHRRVYGPYRLKGPLMRKGWKQWMDDNAPELTAETKR  
6sdv.1.A  TAKDGQGRAVNVEGDPDHPI------------NEGSLCPKGASIFQLGENDQRGTQPLYRAPFS----------------  
  
Target    KYKFDSRFLDDMLRVSWDTAFTYAAKAMITIATRYSGEAGARRLREQGYAPEMIEMMKGAGTRCFKHRAGMPVLGIIGKM  
6sdv.1.A  ---------DTWKPVTWDFALTEIAKRIKKTRDASFTEKNAAGDLV--NRTEAIASFGSAAM------------------  
  
Target    GNTRMNGGINALLDTWIRKVSPD-QAQGGRYWSNYTWHGDQNPAHPFWSGVQGSDIDLSDMRFSKLNTSWGKNFVENKMP  
6sdv.1.A  -----DNEECWAYGNILRSLGLVYIEHQARIU-----HSPTVPALAESFGRGAMTNHWNDLANSDCILIMGSNAAENHPI  
  
Target    EAHWKLECIERGARVVVITPEYNPTAYRADYWMPLRPESDGALFLGAMKIIIDENMHDIDFLKSFTDAPILVRTDTLQYL  
6sdv.1.A  AFKWVLRAKDKGATLIHVDPRFTRTSARCDVYAPIRSGADIPFLGGLIKYILDNKLYFTDYVREYTNASLIVGEKFSF--  
  
Target    DPRDVIADYKFPDFSKSYSGRIQSLKPEQIQRLGGMMVWDLNKKQVVPLHREQVGWHYTNSGIDAALTGTYRVKLLNGRE  
6sdv.1.A  --KDG----LFSGYD----A--------------ANKKYDKSM----------WAFELDA-------NG---VPKRDPAL  
  
Target    IDAMPIWQMYMVHFQDYDLDTVHQITRTPKDLIVRWARDSGTI----KPAAIHNGEGTCHYFHQTANARGAAMVLIITGN  
6sdv.1.A  KHPRCVINLLKKHYERYNLDKVAAITGTSKEQLQQVYKAYAATGKPDKAGTIMYAMGWTQHSVGVQNIRAMAMIQLLLGN  
  
Target    VGKFGTGQHTWAGNYK-AGTWTATPWSGAGLSVHTGEDPFNIT-------LDPNAHGKEIKTRSYYYGEEVGYW------  
6sdv.1.A  IGVAGGGVNALRGESNVQGSTDQ----GLLAHIWPGYNPVPNSKAATLELYNAATPQSKDPMSVNWWQNRPKYVASYLKA  
  
Target    --NHGDT---ALIVN---T-----PKYGRKVFTGKTHMPTPSKFRWVVNVNVVNNAKHHYDMVRNVDPNIECLITQDIEM  
6sdv.1.A  LYPDEEPAAAYDYLPRIDAGRKLTDYFWLNIFE--KMDKGEFKGLFAWGMNPACGGANA-NKNRKAMGKLEWLVNVNLFE  
  
Target    TSDINH--------AD-----IAFAANSWMEFTYPEMTVTVSNPWVQIWKGGIRPLYDTRNDLDTFAGVAAKLSDMTGDK  
6sdv.1.A  NETSSFWKGPGMNPAEIGTEVFFLPCCVSIEKEGS---VANSGRWMQWRYRGPKPYAETKPDGDIMLDMFKKVRE-----  
  
Target    RMRDYFAMVYQNRVDVYVQRMLDASSTFYGYSADVMLKSEKGWMVMVRTYPRHPFWEETNESKPMWTRSGRYENYRIEPE  
6sdv.1.A  --------------------------------------------------------------------------------  
  
Target    AIEYGENFISHREGPEATPYLPNAIFTTNPYVRPDDYGIPITAQHHDDKTVRNIKLSWHEIKRHSNPLWEKGYQFYCVTP  
6sdv.1.A  --------------------------------------------------------------------------------  
  
Target    KTRHRVHSQWSVNDWVQIYESNFGDPYRMDKRTPGVGEHQIHINPQAAKDRGINDGDYVYVDGNPVDRPYRGWKPSDPYY  
6sdv.1.A  --------------------------------------------------------------------------------  
  
Target    KVARLMIRAKYNPAYPYHVTMAKHAPFVATAKSVKGHETRPDGRAIAIDTGYQSNFRYGAQQSFTRNWLMPMHQTDSLPG  
6sdv.1.A  --------------------------------------------------------------------------------  
  
Target    KHAVAWKFKWGYQVDHHAINTVPKECLIRITKAEDGGIGARGPWEPVRTGFTPGQENEFMIKWLKGEHIKIKV  
6sdv.1.A  -------------------------------------------------------------------------
```

  


---

  

| Model #05 | File | Built with | Oligo-State | Ligands | GMQE | QMEANDisCo Global |
| --- | --- | --- | --- | --- | --- | --- |
|  | PDB | ProMod3 3.2.1 | monomer (matching prediction) | 1 x MO: MOLYBDENUM ATOM; | 0.21 | 0.44 ± 0.05 |

|  |  |  |
| --- | --- | --- |
|  |  |  |

| Template | Seq Identity | Oligo-state | QSQE | Found by | Method | Resolution | Seq Similarity | Range | Coverage | Description |
| --- | --- | --- | --- | --- | --- | --- | --- | --- | --- | --- |
| 1r27.4.A | 27.20 | homo-dimer | 0.07 | BLAST | X-ray | 2.00Å | 0.34 | 47 - 639 | 0.47 | Respiratory nitrate reductase 1 alpha chain |

  

### Included Ligands

| Ligand | Description |
| --- | --- |
| 1 x MO | MOLYBDENUM ATOM |

  

### Excluded ligands

| Ligand Name.Number | Reason for Exclusion | Description |
| --- | --- | --- |
| F3S.5 | Binding site not conserved. | FE3-S4 CLUSTER |
| F3S.13 | Binding site not conserved. | FE3-S4 CLUSTER |
| F3S.21 | Binding site not conserved. | FE3-S4 CLUSTER |
| F3S.29 | Binding site not conserved. | FE3-S4 CLUSTER |
| MGD.3 | Binding site not conserved. | 2-AMINO-5,6-DIMERCAPTO-7-METHYL-3,7,8A,9-TETRAHYDRO-8-OXA-1,3,9,10-TETRAAZA-ANTHRACEN-4-ONE GUANOSINE DINUCLEOTIDE |
| MGD.4 | Binding site not conserved. | 2-AMINO-5,6-DIMERCAPTO-7-METHYL-3,7,8A,9-TETRAHYDRO-8-OXA-1,3,9,10-TETRAAZA-ANTHRACEN-4-ONE GUANOSINE DINUCLEOTIDE |
| MGD.11 | Binding site not conserved. | 2-AMINO-5,6-DIMERCAPTO-7-METHYL-3,7,8A,9-TETRAHYDRO-8-OXA-1,3,9,10-TETRAAZA-ANTHRACEN-4-ONE GUANOSINE DINUCLEOTIDE |
| MGD.12 | Binding site not conserved. | 2-AMINO-5,6-DIMERCAPTO-7-METHYL-3,7,8A,9-TETRAHYDRO-8-OXA-1,3,9,10-TETRAAZA-ANTHRACEN-4-ONE GUANOSINE DINUCLEOTIDE |
| MGD.19 | Binding site not conserved. | 2-AMINO-5,6-DIMERCAPTO-7-METHYL-3,7,8A,9-TETRAHYDRO-8-OXA-1,3,9,10-TETRAAZA-ANTHRACEN-4-ONE GUANOSINE DINUCLEOTIDE |
| MGD.20 | Binding site not conserved. | 2-AMINO-5,6-DIMERCAPTO-7-METHYL-3,7,8A,9-TETRAHYDRO-8-OXA-1,3,9,10-TETRAAZA-ANTHRACEN-4-ONE GUANOSINE DINUCLEOTIDE |
| MGD.27 | Binding site not conserved. | 2-AMINO-5,6-DIMERCAPTO-7-METHYL-3,7,8A,9-TETRAHYDRO-8-OXA-1,3,9,10-TETRAAZA-ANTHRACEN-4-ONE GUANOSINE DINUCLEOTIDE |
| MGD.28 | Binding site not conserved. | 2-AMINO-5,6-DIMERCAPTO-7-METHYL-3,7,8A,9-TETRAHYDRO-8-OXA-1,3,9,10-TETRAAZA-ANTHRACEN-4-ONE GUANOSINE DINUCLEOTIDE |
| MO.9 | Binding site not conserved. | MOLYBDENUM ATOM |
| MO.17 | Binding site not conserved. | MOLYBDENUM ATOM |
| MO.25 | Binding site not conserved. | MOLYBDENUM ATOM |
| SF4.2 | Binding site not conserved. | IRON/SULFUR CLUSTER |
| SF4.6 | Binding site not conserved. | IRON/SULFUR CLUSTER |
| SF4.7 | Binding site not conserved. | IRON/SULFUR CLUSTER |
| SF4.8 | Binding site not conserved. | IRON/SULFUR CLUSTER |
| SF4.10 | Binding site not conserved. | IRON/SULFUR CLUSTER |
| SF4.14 | Binding site not conserved. | IRON/SULFUR CLUSTER |
| SF4.15 | Binding site not conserved. | IRON/SULFUR CLUSTER |
| SF4.16 | Binding site not conserved. | IRON/SULFUR CLUSTER |
| SF4.18 | Binding site not conserved. | IRON/SULFUR CLUSTER |
| SF4.22 | Binding site not conserved. | IRON/SULFUR CLUSTER |
| SF4.23 | Binding site not conserved. | IRON/SULFUR CLUSTER |
| SF4.24 | Binding site not conserved. | IRON/SULFUR CLUSTER |
| SF4.26 | Binding site not conserved. | IRON/SULFUR CLUSTER |
| SF4.30 | Binding site not conserved. | IRON/SULFUR CLUSTER |
| SF4.31 | Binding site not conserved. | IRON/SULFUR CLUSTER |
| SF4.32 | Binding site not conserved. | IRON/SULFUR CLUSTER |

  

```
Target    MFLSRRQFLKVSVGTVAAVAVADKVLALTALQPVIEVGNPLGDYPDRSWERVYHDQYRYDSSFTWVCSPNDTHACRVRAF  
1r27.4.A  ---------------------------------------------NRDWEDGYRQRWQHDKIVRSTHGVNCTGSCSWKIY  
  
Target    VRNGVVMRVEQNYDH-QTYEDLYGNRGTFAHNPRMCLKGFTFHRRVYGPYRLKGPLMRKGW-KQWMDDNA---------P  
1r27.4.A  VKNGLVTWETQQTDYPRTRPDLPN------HEPRGCPRGASYSWYLYSANRLKYPMMRKRLMKMWREAKALHSDPVEAWA  
  
Target    ELTAETKRKYKF-DSRFLDDMLRVSWDTAFTYAAKAMITIATRYSGEAGARRLREQGYAPEMIEMMKGAGTRCFKHRAGM  
1r27.4.A  SIIEDADKAKSFKQARGRGGFVRSSWQEVNELIAASNVYTIKNYGPD------RVAGFSP-------------------I  
  
Target    PVLGIIGKMGNTRMNGGINALLDTWIRKVSPDQAQGGRYWSNYTWHGDQNPAHPFWSGVQGSDIDLSDMRFSKLNTSWGK  
1r27.4.A  PAMSMVSYASGARYLSLI-----------------GGTCLSFYDWYCDLPPASPQTWGEQTDVPESADWYNSSYIIAWGS  
  
Target    NFVENKMPEAHWKLECIERGARVVVITPEYNPTAYRADYWMPLRPESDGALFLGAMKIIIDENMHD------IDFLKSFT  
1r27.4.A  NVPQTRTPDAHFFTEVRYKGTKTVAVTPDYAEIAKLCDLWLAPKQGTDAAMALAMGHVMLREFHLDNPSQYFTDYVRRYT  
  
Target    DAPILVRTD----------TLQYLDPRDVIADYKFPDFSK---SYSGRIQSLKPEQIQRLGGMMVWDLNKK---------  
1r27.4.A  DMPMLVMLEERDGYYAAGRMLRAADLVDALGQENNPEWKTVAFNTNGEMVAPNGSIGFRWGEKGKWNLEQRDGKTGEETE  
  
Target    -QVVPLHRE----QVGW---------HYTNSGIDAALTGTYRVKLLNGREIDAMPIWQMYMVHFQDYDLDT---------  
1r27.4.A  LQLSLLGSQDEIAEVGFPYFGGDGTEHFNKVELENVLLHKLPVKRLQLADGSTALVTTVYDLTLANYGLERGLNDVNCAT  
  
Target    ------------VHQITRTPKDLIVRWARD-----SGTIKPAAIHNGEGTCHYFHQTANARGAAMVLIITGNVGKFGTGQ  
1r27.4.A  SYDDVKAYTPAWAEQITGVSRSQIIRIAREFADNADKTHGRSMIIVGAGLNHWYHLDMNYRGLINMLIFCGCVGQSGGGW  
  
Target    HTWAGNYKAGTWTATPWSGAGLSVHTGEDPFNITLDPNAHGKEIKTRSYYYGEEVGYWNHGDTALIVNTPKYGRKVFTGK  
1r27.4.A  AHYVGQEK-------------LRPQTGWQPLAFALDWQRPARHMNSTSYFYNHSSQWRYETVTAEELLSPMADKSRYTG-  
  
Target    THMPTPSKFRWVVNVNVVNNAKHHYDMVRNVDPNIECLITQDIEMTSDINHADIAFAANSWMEFTYPEMTVTVSNPWVQI  
1r27.4.A  --------------------------------------------------------------------------------  
  
Target    WKGGIRPLYDTRNDLDTFAGVAAKLSDMTGDKRMRDYFAMVYQNRVDVYVQRMLDASSTFYGYSADVMLKSEKGWMVMVR  
1r27.4.A  --------------------------------------------------------------------------------  
  
Target    TYPRHPFWEETNESKPMWTRSGRYENYRIEPEAIEYGENFISHREGPEATPYLPNAIFTTNPYVRPDDYGIPITAQHHDD  
1r27.4.A  --------------------------------------------------------------------------------  
  
Target    KTVRNIKLSWHEIKRHSNPLWEKGYQFYCVTPKTRHRVHSQWSVNDWVQIYESNFGDPYRMDKRTPGVGEHQIHINPQAA  
1r27.4.A  --------------------------------------------------------------------------------  
  
Target    KDRGINDGDYVYVDGNPVDRPYRGWKPSDPYYKVARLMIRAKYNPAYPYHVTMAKHAPFVATAKSVKGHETRPDGRAIAI  
1r27.4.A  --------------------------------------------------------------------------------  
  
Target    DTGYQSNFRYGAQQSFTRNWLMPMHQTDSLPGKHAVAWKFKWGYQVDHHAINTVPKECLIRITKAEDGGIGARGPWEPVR  
1r27.4.A  --------------------------------------------------------------------------------  
  
Target    TGFTPGQENEFMIKWLKGEHIKIKV  
1r27.4.A  -------------------------
```

  


---

  

## Materials and Methods

## Template Search

Template search with
has been performed against the SWISS-MODEL template library (SMTL, last update: 2023-03-23, last included PDB release: 2023-03-17).

## Template Selection

For each identified template, the template's quality has been predicted from features of the target-template alignment.
The templates with the highest quality have then been selected for model building.

## Model Building

Models are built based on the target-template alignment using ProMod3 (Studer et al.). Coordinates which are conserved between the target and the template are copied from the template to the model. Insertions and deletions are remodelled using a fragment library. Side chains are then rebuilt. Finally, the geometry of the resulting model is regularized by using a force field.

## Model Quality Estimation

The global and per-residue model quality has been assessed using the QMEAN scoring function (Studer et al.).

## Ligand Modelling

Ligands present in the template structure are transferred by homology to the model when the following criteria are met: (a) The ligands are annotated as biologically relevant in the template library, (b) the ligand is in contact with the model, (c) the ligand is not clashing with the protein, (d) the residues in contact with the ligand are conserved between the target and the template. If any of these four criteria is not satisfied, a certain ligand will not be included in the model. The model summary includes information on why and which ligand has not been included.

## Oligomeric State Conservation

The quaternary structure annotation of the template is used to model the target sequence in its oligomeric form. The method (Bertoni et al.) is based on a supervised machine learning algorithm, Support Vector Machines (SVM), which combines interface conservation, structural clustering, and other template features to provide a quaternary structure quality estimate (QSQE). The QSQE score is a number between 0 and 1, reflecting the expected accuracy of the interchain contacts for a model built based a given alignment and template. Higher numbers indicate higher reliability. This complements the GMQE score which estimates the accuracy of the tertiary structure of the resulting model.

## References

- **BLAST**  
  Camacho, C., Coulouris, G., Avagyan, V., Ma, N., Papadopoulos, J.,
  Bealer, K., Madden, T.L. BLAST+: architecture and applications. BMC
  Bioinformatics 10, 421-430 (2009).
- **HHblits**  
  Steinegger, M., Meier, M., Mirdita, M., Vöhringer, H., Haunsberger,
  S. J., Söding, J. HH-suite3 for fast remote homology detection and
  deep protein annotation. BMC Bioinformatics 20, 473 (2019).

## Table T1:

Primary amino acid sequence for which templates were searched and models were built.

MFLSRRQFLKVSVGTVAAVAVADKVLALTALQPVIEVGNPLGDYPDRSWERVYHDQYRYDSSFTWVCSPNDTHACRVRAFVRNGVVMRVEQNYDHQTYED  
LYGNRGTFAHNPRMCLKGFTFHRRVYGPYRLKGPLMRKGWKQWMDDNAPELTAETKRKYKFDSRFLDDMLRVSWDTAFTYAAKAMITIATRYSGEAGARR  
LREQGYAPEMIEMMKGAGTRCFKHRAGMPVLGIIGKMGNTRMNGGINALLDTWIRKVSPDQAQGGRYWSNYTWHGDQNPAHPFWSGVQGSDIDLSDMRFS  
KLNTSWGKNFVENKMPEAHWKLECIERGARVVVITPEYNPTAYRADYWMPLRPESDGALFLGAMKIIIDENMHDIDFLKSFTDAPILVRTDTLQYLDPRD  
VIADYKFPDFSKSYSGRIQSLKPEQIQRLGGMMVWDLNKKQVVPLHREQVGWHYTNSGIDAALTGTYRVKLLNGREIDAMPIWQMYMVHFQDYDLDTVHQ  
ITRTPKDLIVRWARDSGTIKPAAIHNGEGTCHYFHQTANARGAAMVLIITGNVGKFGTGQHTWAGNYKAGTWTATPWSGAGLSVHTGEDPFNITLDPNAH  
GKEIKTRSYYYGEEVGYWNHGDTALIVNTPKYGRKVFTGKTHMPTPSKFRWVVNVNVVNNAKHHYDMVRNVDPNIECLITQDIEMTSDINHADIAFAANS  
WMEFTYPEMTVTVSNPWVQIWKGGIRPLYDTRNDLDTFAGVAAKLSDMTGDKRMRDYFAMVYQNRVDVYVQRMLDASSTFYGYSADVMLKSEKGWMVMVR  
TYPRHPFWEETNESKPMWTRSGRYENYRIEPEAIEYGENFISHREGPEATPYLPNAIFTTNPYVRPDDYGIPITAQHHDDKTVRNIKLSWHEIKRHSNPL  
WEKGYQFYCVTPKTRHRVHSQWSVNDWVQIYESNFGDPYRMDKRTPGVGEHQIHINPQAAKDRGINDGDYVYVDGNPVDRPYRGWKPSDPYYKVARLMIR  
AKYNPAYPYHVTMAKHAPFVATAKSVKGHETRPDGRAIAIDTGYQSNFRYGAQQSFTRNWLMPMHQTDSLPGKHAVAWKFKWGYQVDHHAINTVPKECLI  
RITKAEDGGIGARGPWEPVRTGFTPGQENEFMIKWLKGEHIKIKV

## Table T2:

| Template | Seq Identity | Oligo-state | QSQE | Found by | Method | Resolution | Seq Similarity | Coverage | Description |
| --- | --- | --- | --- | --- | --- | --- | --- | --- | --- |
| 7b04.1.B | 57.58 | monomer | - | HHblits | X-ray | 2.97Å | 0.48 | 1.00 | Nitrite oxidoreductase subunit A |
| 7b04.1.B | 58.37 | monomer | - | BLAST | X-ray | 2.97Å | 0.49 | 1.00 | Nitrite oxidoreductase subunit A |
| 7b04.2.B | 58.37 | monomer | - | BLAST | X-ray | 2.97Å | 0.49 | 1.00 | Nitrite oxidoreductase subunit A |
| 7b04.2.B | 57.58 | monomer | - | HHblits | X-ray | 2.97Å | 0.48 | 1.00 | Nitrite oxidoreductase subunit A |
| 1r27.4.A | 20.78 | homo-dimer | 0.25 | HHblits | X-ray | 2.00Å | 0.31 | 0.81 | Respiratory nitrate reductase 1 alpha chain |
| 3ir5.1.A | 20.97 | monomer | - | HHblits | X-ray | 2.30Å | 0.31 | 0.81 | Respiratory nitrate reductase 1 alpha chain |
| 3egw.1.A | 20.85 | homo-dimer | 0.17 | HHblits | X-ray | 1.90Å | 0.31 | 0.78 | Respiratory nitrate reductase 1 alpha chain |
| 3ir7.1.A | 20.86 | monomer | - | HHblits | X-ray | 2.50Å | 0.31 | 0.81 | Respiratory nitrate reductase 1 alpha chain |
| 1q16.1.A | 20.78 | monomer | - | HHblits | X-ray | 1.90Å | 0.31 | 0.81 | Respiratory nitrate reductase 1 alpha chain |
| 3ir6.1.A | 20.85 | monomer | - | HHblits | X-ray | 2.80Å | 0.31 | 0.80 | Respiratory nitrate reductase 1 alpha chain |
| 2vpz.1.A | 20.23 | monomer | - | HHblits | X-ray | 2.40Å | 0.30 | 0.61 | THIOSULFATE REDUCTASE |
| 2vpx.1.D | 20.23 | monomer | - | HHblits | X-ray | 3.10Å | 0.30 | 0.61 | THIOSULFATE REDUCTASE |
| 2e7z.1.A | 19.55 | monomer | - | HHblits | X-ray | 1.26Å | 0.30 | 0.58 | Acetylene hydratase Ahy |
| 6sdv.1.A | 20.13 | monomer | - | HHblits | X-ray | 1.90Å | 0.30 | 0.53 | Formate dehydrogenase, alpha subunit, selenocysteine-containing,Formate dehydrogenase, alpha subunit, selenocysteine-containing,W-formate dehydrogenase - alpha subunit |
| 8bqg.1.A | 19.54 | monomer | - | HHblits | X-ray | 1.95Å | 0.30 | 0.50 | Formate dehydrogenase, alpha subunit, selenocysteine-containing |
| 1r27.4.A | 27.20 | homo-dimer | 0.07 | BLAST | X-ray | 2.00Å | 0.34 | 0.47 | Respiratory nitrate reductase 1 alpha chain |
| 3ir5.1.A | 27.39 | monomer | - | BLAST | X-ray | 2.30Å | 0.34 | 0.47 | Respiratory nitrate reductase 1 alpha chain |
| 3ir7.1.A | 27.20 | monomer | - | BLAST | X-ray | 2.50Å | 0.34 | 0.47 | Respiratory nitrate reductase 1 alpha chain |
| 3egw.1.A | 27.02 | homo-dimer | 0.07 | BLAST | X-ray | 1.90Å | 0.34 | 0.47 | Respiratory nitrate reductase 1 alpha chain |
| 1q16.1.A | 27.20 | monomer | - | BLAST | X-ray | 1.90Å | 0.34 | 0.47 | Respiratory nitrate reductase 1 alpha chain |
| 3ir6.1.A | 27.20 | monomer | - | BLAST | X-ray | 2.80Å | 0.34 | 0.47 | Respiratory nitrate reductase 1 alpha chain |
| 6q8o.1.C | 14.74 | monomer | - | HHblits | X-ray | 3.61Å | 0.26 | 0.44 | NADH-quinone oxidoreductase subunit 3 |
| 6ziy.1.C | 14.74 | monomer | - | HHblits | EM | NA | 0.26 | 0.44 | NADH-quinone oxidoreductase subunit 3 |
| 6zjl.1.C | 14.74 | monomer | - | HHblits | EM | NA | 0.26 | 0.44 | NADH-quinone oxidoreductase subunit 3 |
| 6zjn.1.C | 14.74 | monomer | - | HHblits | EM | NA | 0.26 | 0.44 | NADH-quinone oxidoreductase subunit 3 |
| 3m9s.1.C | 14.74 | monomer | - | HHblits | X-ray | 4.50Å | 0.26 | 0.44 | NADH-quinone oxidoreductase subunit 3 |
| 6zjy.1.C | 14.74 | monomer | - | HHblits | EM | NA | 0.26 | 0.44 | NADH-quinone oxidoreductase subunit 3 |
| 2fug.2.C | 14.74 | monomer | - | HHblits | X-ray | 3.30Å | 0.26 | 0.44 | NADH-quinone oxidoreductase chain 3 |
| 6zk9.1.C | 11.75 | monomer | - | HHblits | EM | NA | 0.25 | 0.33 | NADH:ubiquinone oxidoreductase core subunit S1 |
| 7zd6.1.4 | 11.75 | monomer | - | HHblits | EM | NA | 0.25 | 0.33 | NADH-ubiquinone oxidoreductase 75 kDa subunit, mitochondrial |
| 6qcf.1.C | 11.78 | monomer | - | HHblits | EM | NA | 0.25 | 0.33 | NADH:ubiquinone oxidoreductase core subunit S1 |
| 7qsd.1.G | 11.75 | monomer | - | HHblits | EM | NA | 0.25 | 0.33 | NADH-ubiquinone oxidoreductase 75 kDa subunit, mitochondrial |
| 6qc5.1.C | 11.78 | monomer | - | HHblits | EM | NA | 0.25 | 0.33 | NADH:ubiquinone oxidoreductase core subunit S1 |
| 5o31.1.8 | 12.01 | monomer | - | HHblits | EM | 4.13Å | 0.25 | 0.33 | NADH-ubiquinone oxidoreductase 75 kDa subunit, mitochondrial |
| 7dgr.10.A | 12.01 | monomer | - | HHblits | EM | NA | 0.25 | 0.33 | NADH-ubiquinone oxidoreductase 75 kDa subunit, mitochondrial |
| 6sdr.1.A | 26.60 | monomer | - | BLAST | X-ray | 2.10Å | 0.35 | 0.08 | Formate dehydrogenase, alpha subunit, selenocysteine-containing |
| 8bqg.1.A | 26.60 | monomer | - | BLAST | X-ray | 1.95Å | 0.35 | 0.08 | Formate dehydrogenase, alpha subunit, selenocysteine-containing |
| 6sdv.1.A | 26.60 | monomer | - | BLAST | X-ray | 1.90Å | 0.35 | 0.08 | Formate dehydrogenase, alpha subunit, selenocysteine-containing,Formate dehydrogenase, alpha subunit, selenocysteine-containing,W-formate dehydrogenase - alpha subunit |
| 2vpx.1.D | 32.20 | monomer | - | BLAST | X-ray | 3.10Å | 0.40 | 0.05 | THIOSULFATE REDUCTASE |
| 2e7z.1.A | 37.93 | monomer | - | BLAST | X-ray | 1.26Å | 0.41 | 0.05 | Acetylene hydratase Ahy |
| 2vpz.1.A | 32.20 | monomer | - | BLAST | X-ray | 2.40Å | 0.40 | 0.05 | THIOSULFATE REDUCTASE |
| 1m2j.1.A | 24.53 | monomer | - | HHblits | X-ray | 1.70Å | 0.32 | 0.05 | Silent Information Regulator 2 |
| 3k35.1.A | 20.37 | monomer | - | HHblits | X-ray | 2.00Å | 0.30 | 0.05 | NAD-dependent deacetylase sirtuin-6 |
| 1ici.1.A | 23.64 | monomer | - | HHblits | X-ray | 2.10Å | 0.31 | 0.05 | TRANSCRIPTIONAL REGULATORY PROTEIN, SIR2 FAMILY |
| 1m2h.1.A | 24.53 | monomer | - | HHblits | X-ray | 1.80Å | 0.32 | 0.05 | Silent Information Regulator 2 |
| 4twi.1.A | 24.07 | monomer | - | HHblits | X-ray | 1.79Å | 0.32 | 0.05 | NAD-dependent protein deacylase 1 |
| 1m2k.1.A | 24.53 | monomer | - | HHblits | X-ray | 1.47Å | 0.32 | 0.05 | Silent Information Regulator 2 |
| 1m2g.1.A | 24.53 | monomer | - | HHblits | X-ray | 1.70Å | 0.32 | 0.05 | Silent Information Regulator 2 |
| 6xvg.3.A | 20.37 | monomer | - | HHblits | X-ray | 2.10Å | 0.30 | 0.05 | NAD-dependent protein deacetylase sirtuin-6 |
| 3riy.2.A | 20.00 | monomer | - | HHblits | X-ray | 1.55Å | 0.29 | 0.05 | NAD-dependent deacetylase sirtuin-5 |

  
The table above shows the top 50 filtered templates. A further 346 templates were found which were considered to be less suitable for modelling than the filtered list.  
1aa6.1.A, 1be3.1.E, 1bgy.1.P, 1cz4.1.A, 1cz5.1.A, 1dms.1.A, 1e18.1.A, 1e5v.2.A, 1e60.1.A, 1eu1.1.A, 1fdo.1.A, 1g8j.1.A, 1g8k.1.A, 1h0h.1.A, 1jeo.1.A, 1kb9.1.E, 1kqf.1.A, 1l0l.1.E, 1l0n.1.E, 1m2n.1.A, 1m2n.1.B, 1m3s.1.A, 1m3s.1.B, 1ma3.1.A, 1ntk.1.E, 1ntm.1.E, 1ogy.1.A, 1q16.1.A, 1q90.1.E, 1r27.4.A, 1s5p.1.A, 1s7g.1.A, 1s7g.1.B, 1s7g.1.C, 1s7g.1.D, 1s7g.1.E, 1sqb.1.E, 1sqp.1.P, 1sqq.1.P, 1sqv.1.E, 1tk9.1.A, 1tmo.1.A, 1vf5.1.D, 1vf5.1.L, 1vim.1.A, 1vim.1.D, 1viv.1.A, 1wlf.1.A, 1x94.1.A, 1x94.1.B, 1yc5.1.A, 1zrt.1.C, 1zrt.1.F, 2b4y.1.A, 2b4y.3.A, 2d2c.1.D, 2d2c.1.L, 2e75.1.D, 2e76.1.D, 2e7z.1.A, 2fyn.1.C, 2fyn.2.C, 2fyu.1.E, 2h2i.1.A, 2h4h.1.A, 2h59.1.B, 2i2w.1.A, 2i2w.2.B, 2iv2.1.A, 2ivf.1.A, 2ki8.1.A, 2nya.1.A, 2nyr.1.A, 2nyr.1.B, 2pjh.1.B, 2pq4.1.B, 2qjk.1.C, 2qjp.1.C, 2qjy.3.F, 2v3v.1.A, 2v45.1.A, 2vpx.1.D, 2vpz.1.A, 2x3y.1.A, 2xbl.1.A, 2ybb.1.b, 3cf4.1.B, 3cwb.1.E, 3egw.1.A, 3etn.1.A, 3fxa.1.A, 3h1h.1.E, 3h1h.1.O, 3h1i.1.E, 3hu2.1.A, 3ir5.1.A, 3ir6.1.A, 3ir7.1.A, 3jr3.1.A, 3jwp.1.A, 3l75.1.E, 3l75.1.O, 3o27.1.A, 3o27.1.B, 3o5a.1.A, 3pki.1.A, 3qc8.1.A, 3qq7.1.A, 3qq8.1.A, 3qwz.1.A, 3sho.1.A, 3sho.1.C, 3tiw.1.A, 3tiw.2.A, 3u31.1.A, 3zg6.1.A, 4aay.1.A, 4bv2.3.A, 4dmr.1.A, 4g1c.1.A, 4g1c.2.A, 4ga5.1.A, 4ga6.1.A, 4h44.1.D, 4hda.1.A, 4hda.2.A, 4kdi.1.A, 4kdi.2.A, 4kdl.1.A, 4ogq.1.L, 4pv1.1.L, 4rv0.1.A, 4twj.1.A, 4u3f.1.E, 4utn.1.A, 4utn.2.A, 4v4c.1.A, 4ydd.1.A, 5b6c.1.A, 5bwl.1.A, 5cuo.1.A, 5cup.1.A, 5e7o.1.A, 5e7p.1.A, 5epp.1.A, 5g4f.1.A, 5g4f.1.B, 5g4f.1.C, 5g4f.1.D, 5g4f.1.E, 5g4f.1.F, 5g4g.1.A, 5glf.1.A, 5glf.2.A, 5glf.3.A, 5glf.4.A, 5gpn.24.A, 5kkz.1.C, 5kli.1.C, 5klv.1.P, 5ltz.1.A, 5lu6.1.A, 5lu7.1.A, 5mf6.1.A, 5nqd.1.A, 5o31.1.8, 5oj7.1.A, 5ojn.1.A, 5okd.1.E, 5t5i.1.B, 5t5i.1.D, 5x16.1.A, 5x4l.1.A, 5x4l.2.A, 5xhs.1.A, 5xtb.1.L, 5xte.1.C, 5y2f.1.A, 6aco.1.A, 6acp.1.A, 6btm.1.B, 6cz7.1.A, 6enx.1.A, 6eo0.1.A, 6eqs.3.A, 6f0k.1.B, 6fky.1.A, 6fky.2.A, 6flg.1.A, 6g72.1.G, 6gcs.1.A, 6giq.1.E, 6hd3.1.A, 6hu9.1.E, 6ljk.1.A, 6ljm.1.A, 6lod.1.B, 6nhg.1.E, 6nin.1.C, 6qc5.1.C, 6qcf.1.C, 6rfq.1.A, 6rfs.1.A, 6rqf.1.D, 6rqf.1.L, 6rxj.1.A, 6rxm.1.A, 6rxm.2.A, 6rxm.3.A, 6rxm.4.A, 6rxm.5.A, 6rxm.6.A, 6rxo.1.A, 6rxo.2.A, 6rxp.2.A, 6rxq.4.A, 6rxs.1.A, 6s6y.1.B, 6sdr.1.A, 6sdv.1.A, 6t0b.1.E, 6t0b.1.O, 6t15.1.E, 6t15.1.O, 6tg9.1.A, 6x89.1.H, 6xkt.1.E, 6xkz.1.F, 6yj4.1.G, 6ymx.1.Q, 6ymx.1.Z, 6zk9.1.C, 6zr2.1.G, 7a23.1.O, 7ak5.1.G, 7ak6.1.G, 7aqr.1.F, 7ar7.1.G, 7ar8.1.G, 7arc.1.F, 7b04.1.B, 7b04.2.B, 7bkb.1.F, 7bkb.1.J, 7bkb.1.L, 7cl0.1.A, 7dbo.1.A, 7dbo.2.A, 7dg7.1.A, 7dg9.1.A, 7dgr.10.A, 7dgr.60.A, 7dgs.50.A, 7dgs.60.A, 7di0.1.A, 7di0.2.A, 7di0.3.A, 7di1.1.A, 7du6.1.A, 7du7.1.A, 7dvc.1.A, 7dvc.5.A, 7dvf.1.A, 7dvh.1.A, 7dvh.2.A, 7dvh.4.A, 7dww.1.A, 7dww.2.A, 7dxr.1.A, 7dxr.1.B, 7dxr.2.B, 7dxs.1.A, 7dxs.1.B, 7dxs.2.A, 7dxs.2.B, 7dxt.1.A, 7dxu.1.A, 7dxu.1.B, 7dxu.2.B, 7dxv.1.A, 7dxv.1.B, 7dxw.1.A, 7dxx.1.A, 7dxx.1.B, 7dxy.1.A, 7dxz.1.A, 7dxz.2.A, 7dxz.2.B, 7dxz.3.A, 7dyc.1.A, 7dyc.2.A, 7dyc.3.A, 7e1v.1.P, 7e5z.1.A, 7en5.1.A, 7en6.1.A, 7en6.1.B, 7en6.1.C, 7en6.1.D, 7jrg.1.E, 7l5i.1.A, 7l5s.1.A, 7nz1.1.E, 7p61.1.C, 7p63.1.C, 7q5y.1.A, 7qrm.1.D, 7qsd.1.G, 7qv7.1.L, 7qv7.1.O, 7r0w.1.L, 7r0w.1.Q, 7rja.1.H, 7rjb.1.I, 7t2r.1.A, 7t30.1.A, 7tce.2.F, 7tgh.58.A, 7tlj.1.C, 7tz6.1.E, 7tz6.1.P, 7v2c.1.L, 7vw6.1.A, 7vxu.1.L, 7wbb.1.A, 7wbb.1.B, 7wbb.1.C, 7wbb.1.D, 7wbb.1.E, 7wbb.1.G, 7z0t.1.G, 7zd6.1.4, 7zm7.1.I, 7zxy.1.D, 7zxy.1.L, 8asi.1.A, 8asi.1.E, 8asj.1.E, 8b9z.1.G, 8ba0.1.G, 8bel.1.B, 8bel.1.I, 8bpx.51.A, 8bqg.1.A, 8e73.55.A, 8e9g.1.G

Swiss Institute of Bioinformatics
Contact Us
