# Supplementary material for: Metabolic capacity is maintained despite shifts in microbial diversity in estuary sediments
Source: ISME Commun. 2025 Oct 11;5(1):ycaf182. doi: 10.1093/ismeco/ycaf182 (PMC12687941; doi:10.1093/ismeco/ycaf182)
Supplement: Supplementary_Data_1_ycaf182 [file supplementary_data_1_ycaf182.zip › SWISS-MODEL/13_July_SF_Bin2_scaffold_9994_c1_22545_1/templates.html]

13\_July\_SF\_Bin2\_scaffold\_9994\_c1\_2-2545\_1 | Templates


**Export Alignment**
  
FASTA format
Clustal Format
PNG Image

**Secondary Structure**
  
None
DSSP
PSIPRED
SSpro

**Colour Scheme** 


Fade Mismatches
Enhance Mismatches

Confidencegradient
Confidenceclass
Indels
Chain
Unique Chain
Rainbow
2° Structure
Clustal
Hydrophobic
Size
Charged
Polar
Proline
Ser/Thr
Cysteine
Aliphatic
Aromatic
No Colour

Use QMEANBrane values

|  |  |  |  |
| --- | --- | --- | --- |
| Background |  |  |  |

**3D Viewer**  
NGL
PV

FASTA
Multi FASTA
ClustalW
PNG


SWISS-MODEL

### 13\_July\_SF\_Bin2\_scaffold\_9994\_c1\_2-2545\_1

### Created: March 29, 2023, 7:37 p.m. at 19:37

- Templates
- Models

Models | Name | Description | GMQE | QSQE | Seq Id | Coverage | Range | Method | Resolution | Oligo-state | Ligands | Found by | Seq Similarity || ✓ | 7b04.1.B | Nitrite oxidoreductase subunit A  *Structure of Nitrite oxidoreductase (Nxr) from the anammox bacterium Kuenenia stuttgartiensis.* | 0.92 | 0.00 | 65.28 | 1.00 | 1-845 | X-ray | 2.97 | monomer | 4 x SF4, 1 x F3S, 2 x MD1, 1 x MO, 1 x HEM, 2 x CA | HHblits | 0.51 |
| ``` target    IMDGKNLVENKLTDSHWFIECMERGAKIVVIAPEYGPPSTKADYWIPIRPQTDAALWLGITRLMIEKKWYDETFVKGFTD 7b04.1    IQTGKNLIENKMPEAHWVTEVMERGGKIVVITPEYSPSAQKADYWIPIRNNTDTALFLGITKILIDNKWYDADYVKKFTD  target    FPLLVRTDTLQRLRAHEVFPQYKTSLSADGPSMKIQGLSAEQHAKLGDFVVWDGKTNAPAAITRDDVGATITKKGIDPVL 7b04.1    FPLLIRTDTLKRVSPKDIIPNYKLQDISDGPSYHIQGLKDEQREIIGDFVVWDAKSKGPKAITRDDVGETLVKKGIDPVL  target    AGSFKVKLVDGKEVEVATLWTLYQDHLKDYDLDTVVEITQAPKEMIEQLAQDIATMKPVAIHQGEGINHWFHATEMNRAA 7b04.1    EGSFKLKTIDGKEIEVMTLLEMYKIHLRDYDIDSVVSMTNSPKDLIERLAKDIATIKPVAIHYGEGVNHYFHATLMNRSY  target    YLPLMLTGNIGRPGAGCQTWAGNYKAALFQGSPWTGPGFKGWVAEDPFDINLNPKAHGKEIHAHAYTKDEEPAYWNHGDL 7b04.1    YLPVMLTGNVGYFGSGSHTWAGNYKAGNFQASKWSGPGFYGWVAEDVFKPNLDPYASAKDLNIKGRALDEEVAYWNHSER  target    ALIVDTPKFGRKNFTGKTHMPTPTKALIFNNVNLINNAKWAYGMIKNVNPNVEMIVSMDIQMTASIEYADLALPANSWLE 7b04.1    PLIVNTPKYGRKVFTGKTHMPSPTKVLWFTNVNLINNAKHVYQMLKNVNPNIEQIMSTDIEITGSIEYADFAFPANSWVE  target    FEGLEITASCSNPFLQIWK-GGIPPVFDSRDDLDILAGIANALADVTGEKRFRDYFAFAAADKRGIYIQRLLDTCTTTAG 7b04.1    FQEFEITNSCSNPFIQIWGKTGITPVYESKDDVKILAGMASKLGELLRDKRFEDNWKFAIEGRASVYINRLLDGSTTMKG  target    YKLADIMAGKYGPPGGCLLNFRTYPRIPFYEQVHDSEPFHTDTGRMHAYADVPEAIEYGENFIVHREGPEATPYLPNVIV 7b04.1    YTCEDILNGKYGEPGVAMLLFRTYPRHPFWEQVHESLPFYTPTGRLQAYNDEPEIIEYGENFIVHREGPEATPYLPNAIV  target    SSNPHIRPEDYGIAADAEHWDDRTIRNIKMPWSKVKETKNFLWEKGFQFYCLTPKTRHRVHSGWSNVDWHMLMDSNFGDP 7b04.1    STNPYIRPDDYGIPENAEYWEDRTVRNIKKSWEETKKTKNFLWEKGYHFYCVTPKSRHTVHSQWAVTDWNFIWNNNFGDP  target    YRLDKRAPCVGEHQLHINPQAARDLNINDGDYVYVDANPADRPYLGAKPDDPFYRVSRCMLRVTYNRAYPYNIVMMKHAP 7b04.1    YRMDKRMPGVGEHQIHIHPQAARDLGIEDGDYVYVDANPADRPYEGWKPNDSFYKVSRLMLRAKYNPAYPYNCTMMKHSA  target    FIATEKSVKAHETRPDGRALSANTGYQANLRYGSQQSVTRNWHMPMHQTDTLFHKSKVFMGFIFGGEADNHAVNTVPKET 7b04.1    WISSDKTVQAHETRPDGRALSP-SGYQSSFRYGSQQSITRDWSMPMHQLDSLFHKAKIGMKFIFGFEADNHCINTVPKET  target    LVRVTKAEDGGMGGKGIWQPATTGFSPDNESDFMKKYLAGELTKVKT 7b04.1    LVKITKAENGGMGGKGVWDPVKTGYTAGNENDFMKKFLNGELIKVD- ``` | | | | | | | | | | | | | | | | | | | | | | | | | | | | | | | | | | | | | | | | | | | | | | | | | |
|  | 7b04.2.B | Nitrite oxidoreductase subunit A  *Structure of Nitrite oxidoreductase (Nxr) from the anammox bacterium Kuenenia stuttgartiensis.* | 0.90 | 0.00 | 65.28 | 1.00 | 1-845 | X-ray | 2.97 | monomer | 4 x SF4, 1 x F3S, 2 x MD1, 1 x MO, 1 x HEM, 2 x CA | HHblits | 0.51 |
| ``` target    IMDGKNLVENKLTDSHWFIECMERGAKIVVIAPEYGPPSTKADYWIPIRPQTDAALWLGITRLMIEKKWYDETFVKGFTD 7b04.2    IQTGKNLIENKMPEAHWVTEVMERGGKIVVITPEYSPSAQKADYWIPIRNNTDTALFLGITKILIDNKWYDADYVKKFTD  target    FPLLVRTDTLQRLRAHEVFPQYKTSLSADGPSMKIQGLSAEQHAKLGDFVVWDGKTNAPAAITRDDVGATITKKGIDPVL 7b04.2    FPLLIRTDTLKRVSPKDIIPNYKLQDISDGPSYHIQGLKDEQREIIGDFVVWDAKSKGPKAITRDDVGETLVKKGIDPVL  target    AGSFKVKLVDGKEVEVATLWTLYQDHLKDYDLDTVVEITQAPKEMIEQLAQDIATMKPVAIHQGEGINHWFHATEMNRAA 7b04.2    EGSFKLKTIDGKEIEVMTLLEMYKIHLRDYDIDSVVSMTNSPKDLIERLAKDIATIKPVAIHYGEGVNHYFHATLMNRSY  target    YLPLMLTGNIGRPGAGCQTWAGNYKAALFQGSPWTGPGFKGWVAEDPFDINLNPKAHGKEIHAHAYTKDEEPAYWNHGDL 7b04.2    YLPVMLTGNVGYFGSGSHTWAGNYKAGNFQASKWSGPGFYGWVAEDVFKPNLDPYASAKDLNIKGRALDEEVAYWNHSER  target    ALIVDTPKFGRKNFTGKTHMPTPTKALIFNNVNLINNAKWAYGMIKNVNPNVEMIVSMDIQMTASIEYADLALPANSWLE 7b04.2    PLIVNTPKYGRKVFTGKTHMPSPTKVLWFTNVNLINNAKHVYQMLKNVNPNIEQIMSTDIEITGSIEYADFAFPANSWVE  target    FEGLEITASCSNPFLQIWK-GGIPPVFDSRDDLDILAGIANALADVTGEKRFRDYFAFAAADKRGIYIQRLLDTCTTTAG 7b04.2    FQEFEITNSCSNPFIQIWGKTGITPVYESKDDVKILAGMASKLGELLRDKRFEDNWKFAIEGRASVYINRLLDGSTTMKG  target    YKLADIMAGKYGPPGGCLLNFRTYPRIPFYEQVHDSEPFHTDTGRMHAYADVPEAIEYGENFIVHREGPEATPYLPNVIV 7b04.2    YTCEDILNGKYGEPGVAMLLFRTYPRHPFWEQVHESLPFYTPTGRLQAYNDEPEIIEYGENFIVHREGPEATPYLPNAIV  target    SSNPHIRPEDYGIAADAEHWDDRTIRNIKMPWSKVKETKNFLWEKGFQFYCLTPKTRHRVHSGWSNVDWHMLMDSNFGDP 7b04.2    STNPYIRPDDYGIPENAEYWEDRTVRNIKKSWEETKKTKNFLWEKGYHFYCVTPKSRHTVHSQWAVTDWNFIWNNNFGDP  target    YRLDKRAPCVGEHQLHINPQAARDLNINDGDYVYVDANPADRPYLGAKPDDPFYRVSRCMLRVTYNRAYPYNIVMMKHAP 7b04.2    YRMDKRMPGVGEHQIHIHPQAARDLGIEDGDYVYVDANPADRPYEGWKPNDSFYKVSRLMLRAKYNPAYPYNCTMMKHSA  target    FIATEKSVKAHETRPDGRALSANTGYQANLRYGSQQSVTRNWHMPMHQTDTLFHKSKVFMGFIFGGEADNHAVNTVPKET 7b04.2    WISSDKTVQAHETRPDGRALSP-SGYQSSFRYGSQQSITRDWSMPMHQLDSLFHKAKIGMKFIFGFEADNHCINTVPKET  target    LVRVTKAEDGGMGGKGIWQPATTGFSPDNESDFMKKYLAGELTKVKT 7b04.2    LVKITKAENGGMGGKGVWDPVKTGYTAGNENDFMKKFLNGELIKVD- ``` | | | | | | | | | | | | | | | | | | | | | | | | | | | | | | | | | | | | | | | | | | | | | | | | | |
|  | 7b04.1.B | Nitrite oxidoreductase subunit A  *Structure of Nitrite oxidoreductase (Nxr) from the anammox bacterium Kuenenia stuttgartiensis.* | 0.92 | 0.00 | 65.48 | 1.00 | 1-844 | X-ray | 2.97 | monomer | 4 x SF4, 1 x F3S, 2 x MD1, 1 x MO, 1 x HEM, 2 x CA | BLAST | 0.51 |
| ``` target    IMDGKNLVENKLTDSHWFIECMERGAKIVVIAPEYGPPSTKADYWIPIRPQTDAALWLGITRLMIEKKWYDETFVKGFTD 7b04.1    IQTGKNLIENKMPEAHWVTEVMERGGKIVVITPEYSPSAQKADYWIPIRNNTDTALFLGITKILIDNKWYDADYVKKFTD  target    FPLLVRTDTLQRLRAHEVFPQYKTSLSADGPSMKIQGLSAEQHAKLGDFVVWDGKTNAPAAITRDDVGATITKKGIDPVL 7b04.1    FPLLIRTDTLKRVSPKDIIPNYKLQDISDGPSYHIQGLKDEQREIIGDFVVWDAKSKGPKAITRDDVGETLVKKGIDPVL  target    AGSFKVKLVDGKEVEVATLWTLYQDHLKDYDLDTVVEITQAPKEMIEQLAQDIATMKPVAIHQGEGINHWFHATEMNRAA 7b04.1    EGSFKLKTIDGKEIEVMTLLEMYKIHLRDYDIDSVVSMTNSPKDLIERLAKDIATIKPVAIHYGEGVNHYFHATLMNRSY  target    YLPLMLTGNIGRPGAGCQTWAGNYKAALFQGSPWTGPGFKGWVAEDPFDINLNPKAHGKEIHAHAYTKDEEPAYWNHGDL 7b04.1    YLPVMLTGNVGYFGSGSHTWAGNYKAGNFQASKWSGPGFYGWVAEDVFKPNLDPYASAKDLNIKGRALDEEVAYWNHSER  target    ALIVDTPKFGRKNFTGKTHMPTPTKALIFNNVNLINNAKWAYGMIKNVNPNVEMIVSMDIQMTASIEYADLALPANSWLE 7b04.1    PLIVNTPKYGRKVFTGKTHMPSPTKVLWFTNVNLINNAKHVYQMLKNVNPNIEQIMSTDIEITGSIEYADFAFPANSWVE  target    FEGLEITASCSNPFLQIW-KGGIPPVFDSRDDLDILAGIANALADVTGEKRFRDYFAFAAADKRGIYIQRLLDTCTTTAG 7b04.1    FQEFEITNSCSNPFIQIWGKTGITPVYESKDDVKILAGMASKLGELLRDKRFEDNWKFAIEGRASVYINRLLDGSTTMKG  target    YKLADIMAGKYGPPGGCLLNFRTYPRIPFYEQVHDSEPFHTDTGRMHAYADVPEAIEYGENFIVHREGPEATPYLPNVIV 7b04.1    YTCEDILNGKYGEPGVAMLLFRTYPRHPFWEQVHESLPFYTPTGRLQAYNDEPEIIEYGENFIVHREGPEATPYLPNAIV  target    SSNPHIRPEDYGIAADAEHWDDRTIRNIKMPWSKVKETKNFLWEKGFQFYCLTPKTRHRVHSGWSNVDWHMLMDSNFGDP 7b04.1    STNPYIRPDDYGIPENAEYWEDRTVRNIKKSWEETKKTKNFLWEKGYHFYCVTPKSRHTVHSQWAVTDWNFIWNNNFGDP  target    YRLDKRAPCVGEHQLHINPQAARDLNINDGDYVYVDANPADRPYLGAKPDDPFYRVSRCMLRVTYNRAYPYNIVMMKHAP 7b04.1    YRMDKRMPGVGEHQIHIHPQAARDLGIEDGDYVYVDANPADRPYEGWKPNDSFYKVSRLMLRAKYNPAYPYNCTMMKHSA  target    FIATEKSVKAHETRPDGRALSANTGYQANLRYGSQQSVTRNWHMPMHQTDTLFHKSKVFMGFIFGGEADNHAVNTVPKET 7b04.1    WISSDKTVQAHETRPDGRALSP-SGYQSSFRYGSQQSITRDWSMPMHQLDSLFHKAKIGMKFIFGFEADNHCINTVPKET  target    LVRVTKAEDGGMGGKGIWQPATTGFSPDNESDFMKKYLAGELTKVKT 7b04.1    LVKITKAENGGMGGKGVWDPVKTGYTAGNENDFMKKFLNGELIKV-- ``` | | | | | | | | | | | | | | | | | | | | | | | | | | | | | | | | | | | | | | | | | | | | | | | | | |
|  | 7b04.2.B | Nitrite oxidoreductase subunit A  *Structure of Nitrite oxidoreductase (Nxr) from the anammox bacterium Kuenenia stuttgartiensis.* | 0.90 | 0.00 | 65.48 | 1.00 | 1-844 | X-ray | 2.97 | monomer | 4 x SF4, 1 x F3S, 2 x MD1, 1 x MO, 1 x HEM, 2 x CA | BLAST | 0.51 |
| ``` target    IMDGKNLVENKLTDSHWFIECMERGAKIVVIAPEYGPPSTKADYWIPIRPQTDAALWLGITRLMIEKKWYDETFVKGFTD 7b04.2    IQTGKNLIENKMPEAHWVTEVMERGGKIVVITPEYSPSAQKADYWIPIRNNTDTALFLGITKILIDNKWYDADYVKKFTD  target    FPLLVRTDTLQRLRAHEVFPQYKTSLSADGPSMKIQGLSAEQHAKLGDFVVWDGKTNAPAAITRDDVGATITKKGIDPVL 7b04.2    FPLLIRTDTLKRVSPKDIIPNYKLQDISDGPSYHIQGLKDEQREIIGDFVVWDAKSKGPKAITRDDVGETLVKKGIDPVL  target    AGSFKVKLVDGKEVEVATLWTLYQDHLKDYDLDTVVEITQAPKEMIEQLAQDIATMKPVAIHQGEGINHWFHATEMNRAA 7b04.2    EGSFKLKTIDGKEIEVMTLLEMYKIHLRDYDIDSVVSMTNSPKDLIERLAKDIATIKPVAIHYGEGVNHYFHATLMNRSY  target    YLPLMLTGNIGRPGAGCQTWAGNYKAALFQGSPWTGPGFKGWVAEDPFDINLNPKAHGKEIHAHAYTKDEEPAYWNHGDL 7b04.2    YLPVMLTGNVGYFGSGSHTWAGNYKAGNFQASKWSGPGFYGWVAEDVFKPNLDPYASAKDLNIKGRALDEEVAYWNHSER  target    ALIVDTPKFGRKNFTGKTHMPTPTKALIFNNVNLINNAKWAYGMIKNVNPNVEMIVSMDIQMTASIEYADLALPANSWLE 7b04.2    PLIVNTPKYGRKVFTGKTHMPSPTKVLWFTNVNLINNAKHVYQMLKNVNPNIEQIMSTDIEITGSIEYADFAFPANSWVE  target    FEGLEITASCSNPFLQIW-KGGIPPVFDSRDDLDILAGIANALADVTGEKRFRDYFAFAAADKRGIYIQRLLDTCTTTAG 7b04.2    FQEFEITNSCSNPFIQIWGKTGITPVYESKDDVKILAGMASKLGELLRDKRFEDNWKFAIEGRASVYINRLLDGSTTMKG  target    YKLADIMAGKYGPPGGCLLNFRTYPRIPFYEQVHDSEPFHTDTGRMHAYADVPEAIEYGENFIVHREGPEATPYLPNVIV 7b04.2    YTCEDILNGKYGEPGVAMLLFRTYPRHPFWEQVHESLPFYTPTGRLQAYNDEPEIIEYGENFIVHREGPEATPYLPNAIV  target    SSNPHIRPEDYGIAADAEHWDDRTIRNIKMPWSKVKETKNFLWEKGFQFYCLTPKTRHRVHSGWSNVDWHMLMDSNFGDP 7b04.2    STNPYIRPDDYGIPENAEYWEDRTVRNIKKSWEETKKTKNFLWEKGYHFYCVTPKSRHTVHSQWAVTDWNFIWNNNFGDP  target    YRLDKRAPCVGEHQLHINPQAARDLNINDGDYVYVDANPADRPYLGAKPDDPFYRVSRCMLRVTYNRAYPYNIVMMKHAP 7b04.2    YRMDKRMPGVGEHQIHIHPQAARDLGIEDGDYVYVDANPADRPYEGWKPNDSFYKVSRLMLRAKYNPAYPYNCTMMKHSA  target    FIATEKSVKAHETRPDGRALSANTGYQANLRYGSQQSVTRNWHMPMHQTDTLFHKSKVFMGFIFGGEADNHAVNTVPKET 7b04.2    WISSDKTVQAHETRPDGRALSP-SGYQSSFRYGSQQSITRDWSMPMHQLDSLFHKAKIGMKFIFGFEADNHCINTVPKET  target    LVRVTKAEDGGMGGKGIWQPATTGFSPDNESDFMKKYLAGELTKVKT 7b04.2    LVKITKAENGGMGGKGVWDPVKTGYTAGNENDFMKKFLNGELIKV-- ``` | | | | | | | | | | | | | | | | | | | | | | | | | | | | | | | | | | | | | | | | | | | | | | | | | |
| ✓ | 1r27.4.A | Respiratory nitrate reductase 1 alpha chain  *Crystal Structure of NarGH complex* | 0.43 |  | 22.77 | 0.82 | 1-812 | X-ray | 2.00 | homo-dimer | 4 x MO, 16 x SF4, 8 x MGD, 4 x F3S | HHblits | 0.32 |
| ``` target    IMDGKNLVENKLTDSHWFIECMERGAKIVVIAPEYGPPSTKADYWIPIRPQTDAALWLGITRLMIEKKW------YDETF 1r27.4    IAWGSNVPQTRTPDAHFFTEVRYKGTKTVAVTPDYAEIAKLCDLWLAPKQGTDAAMALAMGHVMLREFHLDNPSQYFTDY  target    VKGFTDFPLLVRTDT-------LQRLRAHEVFPQYKTSLSADGPSMKIQGLSAEQHAKLGDFVVWDGKTNAPAAITRDDV 1r27.4    VRRYTDMPMLVMLEERDGYYAAGRMLRAADLVDALG-----------------QENNPEWKTVAFNT-NGEMVAPNGS-I  target    GA------TITK------------------KGID--------------------PVLA-------GSFKVKLVDGKEVEV 1r27.4    GFRWGEKGKWNLEQRDGKTGEETELQLSLLGSQDEIAEVGFPYFGGDGTEHFNKVELENVLLHKLPVKRLQLADGSTALV  target    ATLWTLYQ------------------DHLKDYDLDTVVEITQAPKEMIEQLAQDIATM-----KPVAIHQGEGINHWFHA 1r27.4    TTVYDLTLANYGLERGLNDVNCATSYDDVKAYTPAWAEQITGVSRSQIIRIAREFADNADKTHGRSMIIVGAGLNHWYHL  target    TEMNRAAYLPLMLTGNIGRPGAGCQTWAGNYKAALFQG-------SPWTGPGF------------KGWVAEDPFDIN-LN 1r27.4    DMNYRGLINMLIFCGCVGQSGGGWAHYVGQEKLRPQTGWQPLAFALDWQRPARHMNSTSYFYNHSSQWRYETVTAEELLS  target    PKAHGKEIHA-------HAYTKDEEPAYWNHGDLALIV---------DTPKFG-RKNFTGK--------THMPTPTKALI 1r27.4    PMADKSRYTGHLIDFNVRAERMGWLPSAPQLGTNPLTIAGEAEKAGMNPVDYTVKSLKEGSIRFAAEQPENGKNHPRNLF  target    FNNVNLINNAKWAYG-M------------------------------IKNVNPNVEMIVSMDIQMTASIEYADLALPANS 1r27.4    IWRSNLLGSSGKGHEFMLKYLLGTEHGIQGKDLGQQGGVKPEEVDWQDNGLEGKLDLVVTLDFRLSSTCLYSDIILPTAT  target    WLEFEGLEITASCSNPFLQIWKGGIPPVFDSRDDLDILAGIANALADVTGEK------------------------RFRD 1r27.4    WYEKDDMNT--SDMHPFIHPLSAAVDPAWEAKSDWEIYKAIAKKFSEVCVGHLGKETDIVTLPIQHDSAAELAQPLDVKD  target    YFA---------------------------FAAA------------------DKRGIYIQRL------------------ 1r27.4    WKKGECDLIPGKTAPHIMVVERDYPATYERFTSIGPLMEKIGNGGKGIAWNTQSEMDLLRKLNYTKAEGPAKGQPMLNTA  target    -------LDTCTTTAGY----KLADIMAGKY-G-------PPGGCLLNF---------------------RTYPRIPFYE 1r27.4    IDAAEMILTLAPETNGQVAVKAWAALSEFTGRDHTHLALNKEDE-KIRFRDIQAQPRKIISSPTWSGLEDEHVSYNAGYT  target    QVHDSEPFHTDTGRMHAYADVPEAIEYGENFIVHREGPEATPYLPNVIVSSNPHIRPEDYGIAADAEHWDDRTIRNIKMP 1r27.4    NVHELIPWRTLSGRQQLYQDHQWMRDFGESLLVYRPPIDTRSVK-------------EVIG-------------------  target    WSKVKETKNFLWEKGFQFYCLTPKTRHRVHSGWSNVDWHMLMDSNFGDPYRLDKRAPCVGEHQLHINPQAARDLNINDGD 1r27.4    ------QK-SNGNQEKALNFLTPHQKWGIHSTYSDNLLMLTLG---------------RGGPVVWLSEADAKDLGIADND  target    YVYVDANPADRPYLGAKPDDPFYRVSRCMLRVTYNRAYPYNIVMMKHAPFIATEKSVKAHETRPDGRALSANTGYQANLR 1r27.4    WIEVFNSN-----------------GALTARAVVSQRVPAGMTMMYHAQE----RIVNLPG--------SEITQ----QR  target    YGSQQSVTRNWHMPMHQTDTLFHKSKVFMGFIFGGEADNHAVNTVPKETLVRVTKAEDGGMGGKGIWQPATTGFSPDNES 1r27.4    GGIHNSVTRITPKPTHMIGGYAHLAYG-----F----NYYGTVGSNRDEFVVVRKMKNIDWL------------------  target    DFMKKYLAGELTKVKT 1r27.4    ---------------- ``` | | | | | | | | | | | | | | | | | | | | | | | | | | | | | | | | | | | | | | | | | | | | | | | | | |
|  | 3ir5.1.A | Respiratory nitrate reductase 1 alpha chain  *Crystal structure of NarGHI mutant NarG-H49C* | 0.42 | 0.00 | 22.25 | 0.82 | 1-812 | X-ray | 2.30 | monomer | 2 x MD1, 1 x 6MO, 4 x SF4, 1 x AGA, 1 x F3S, 2 x HEM | HHblits | 0.32 |
| ``` target    IMDGKNLVENKLTDSHWFIECMERGAKIVVIAPEYGPPSTKADYWIPIRPQTDAALWLGITRLMIEKKW------YDETF 3ir5.1    IAWGSNVPQTRTPDAHFFTEVRYKGTKTVAVTPDYAEIAKLCDLWLAPKQGTDAAMALAMGHVMLREFHLDNPSQYFTDY  target    VKGFTDFPLLVRTD-------TLQRLRAHEVFPQYKTSLSADGPSMKIQGLSAEQHAKLGDFVVWDGKTNAPAAITRDDV 3ir5.1    VRRYTDMPMLVMLEERDGYYAAGRMLRAADLVDALG-----------------QENNPEWKTVAFN-TNGEMVAPNGSI-  target    GA------TI------------------------------------------TKKGIDPVLAG---SFKVKLVDGKEVEV 3ir5.1    GFRWGEKGKWNLEQRDGKTGEETELQLSLLGSQDEIAEVGFPYFGGDGTEHFNKVELENVLLHKLPVKRLQLADGSTALV  target    ATLWTLYQ------------------DHLKDYDLDTVVEITQAPKEMIEQLAQDIATM-----KPVAIHQGEGINHWFHA 3ir5.1    TTVYDLTLANYGLERGLNDVNCATSYDDVKAYTPAWAEQITGVSRSQIIRIAREFADNADKTHGRSMIIVGAGLNHWYHL  target    TEMNRAAYLPLMLTGNIGRPGAGCQTWAGNYKAALFQG-------SPWTGPGF------------KGWVAEDPFDIN-LN 3ir5.1    DMNYRGLINMLIFCGCVGQSGGGWAHYVGQEKLRPQTGWQPLAFALDWQRPARHMNSTSYFYNHSSQWRYETVTAEELLS  target    PKAHGKEIH-----------AHAYTKDEEPAYWNHGDLALI-------VDTPKFG-RKNFTGK--------THMPTPTKA 3ir5.1    PMADKSRYTGHLIDFNVRAERMGWL-PSAPQL-GTNPLTIAGEAEKAGMNPVDYTVKSLKEGSIRFAAEQPENGKNHPRN  target    LIFNNVNLINNAKWAYG-M------------------------------IKNVNPNVEMIVSMDIQMTASIEYADLALPA 3ir5.1    LFIWRSNLLGSSGKGHEFMLKYLLGTEHGIQGKDLGQQGGVKPEEVDWQDNGLEGKLDLVVTLDFRLSSTCLYSDIILPT  target    NSWLEFEGLEITASCSNPFLQIWKGGIPPVFDSRDDLDILAGIANALADVTGEK--R----------------------F 3ir5.1    ATWYEKDDMNT--SDMHPFIHPLSAAVDPAWEAKSDWEIYKAIAKKFSEVCVGHLGKETDIVTLPIQHDSAAELAQPLDV  target    RDYFA---------------------------FAAAD------------------KRGIYIQRL---------------- 3ir5.1    KDWKKGECDLIPGKTAPHIMVVERDYPATYERFTSIGPLMEKIGNGGKGIAWNTQSEMDLLRKLNYTKAEGPAKGQPMLN  target    ---------LDTCTTTAGY----KLADIMAGK--------YGPPGGCLLNF---------------------RTYPRIPF 3ir5.1    TAIDAAEMILTLAPETNGQVAVKAWAALSEFTGRDHTHLALNKEDE-KIRFRDIQAQPRKIISSPTWSGLEDEHVSYNAG  target    YEQVHDSEPFHTDTGRMHAYADVPEAIEYGENFIVHREGPEATPYLPNVIVSSNPHIRPEDYGIAADAEHWDDRTIRNIK 3ir5.1    YTNVHELIPWRTLSGRQQLYQDHQWMRDFGESLLVYRPPIDTRSVKE---------------------------------  target    MPWSKVKETKNFLWEKGFQFYCLTPKTRHRVHSGWSNVDWHMLMDSNFGDPYRLDKRAPCVGEHQLHINPQAARDLNIND 3ir5.1    --VIG---QK-SNGNQEKALNFLTPHQKWGIHSTYSDNLLMLTLG---------------RGGPVVWLSEADAKDLGIAD  target    GDYVYVDANPADRPYLGAKPDDPFYRVSRCMLRVTYNRAYPYNIVMMKHAPFIATEKSVKAHETRPDGRALSANTGYQAN 3ir5.1    NDWIEVFNSN-----------------GALTARAVVSQRVPAGMTMMYHAQE----RIVNLPGS--------EITQ----  target    LRYGSQQSVTRNWHMPMHQTDTLFHKSKVFMGFIFGGEADNHAVNTVPKETLVRVTKAEDGGMGGKGIWQPATTGFSPDN 3ir5.1    QRGGIHNSVTRITPKPTHMIGGYAHLAYG-----F----NYYGTVGSNRDEFVVVRKMKNIDWL----------------  target    ESDFMKKYLAGELTKVKT 3ir5.1    ------------------ ``` | | | | | | | | | | | | | | | | | | | | | | | | | | | | | | | | | | | | | | | | | | | | | | | | | |
|  | 3ir7.1.A | Respiratory nitrate reductase 1 alpha chain  *Crystal structure of NarGHI mutant NarG-R94S* | 0.41 | 0.00 | 22.66 | 0.82 | 1-812 | X-ray | 2.50 | monomer | 2 x MD1, 4 x SF4, 1 x 6MO, 1 x AGA, 1 x F3S, 2 x HEM | HHblits | 0.32 |
| ``` target    IMDGKNLVENKLTDSHWFIECMERGAKIVVIAPEYGPPSTKADYWIPIRPQTDAALWLGITRLMIEKKW------YDETF 3ir7.1    IAWGSNVPQTRTPDAHFFTEVRYKGTKTVAVTPDYAEIAKLCDLWLAPKQGTDAAMALAMGHVMLREFHLDNPSQYFTDY  target    VKGFTDFPLLVRTDT-------LQRLRAHEVFPQYKTSLSADGPSMKIQGLSAEQHAKLGDFVVWDGKTNAPAAITRDDV 3ir7.1    VRRYTDMPMLVMLEERDGYYAAGRMLRAADLVDALG-----------------QENNPEWKTVAFNT-NGEMVAPNGS-I  target    GAT------I------------------------------------------TKKGIDPVLAG---SFKVKLVDGKEVEV 3ir7.1    GFRWGEKGKWNLEQRDGKTGEETELQLSLLGSQDEIAEVGFPYFGGDGTEHFNKVELENVLLHKLPVKRLQLADGSTALV  target    ATLWTLYQ------------------DHLKDYDLDTVVEITQAPKEMIEQLAQDIATM-----KPVAIHQGEGINHWFHA 3ir7.1    TTVYDLTLANYGLERGLNDVNCATSYDDVKAYTPAWAEQITGVSRSQIIRIAREFADNADKTHGRSMIIVGAGLNHWYHL  target    TEMNRAAYLPLMLTGNIGRPGAGCQTWAGNYKAALFQG-------SPWTGPGF------------KGWVAEDPFDIN-LN 3ir7.1    DMNYRGLINMLIFCGCVGQSGGGWAHYVGQEKLRPQTGWQPLAFALDWQRPARHMNSTSYFYNHSSQWRYETVTAEELLS  target    PKAHGKEI-------HAHAYTKDEEPAYWNHGD--L--------------ALIVDTPKFGRKN--FT-GKTHMPTPTKAL 3ir7.1    PMADKSRYTGHLIDFNVRAERMGWLPSAPQLGTNPLTIAGEAEKAGMNPVDYTVKSLKEGSIRFAAEQPENG-KNHPRNL  target    IFNNVNLINNAKWAYG-M------------------------------IKNVNPNVEMIVSMDIQMTASIEYADLALPAN 3ir7.1    FIWRSNLLGSSGKGHEFMLKYLLGTEHGIQGKDLGQQGGVKPEEVDWQDNGLEGKLDLVVTLDFRLSSTCLYSDIILPTA  target    SWLEFEGLEITASCSNPFLQIWKGGIPPVFDSRDDLDILAGIANALADVTGEK------------------------RFR 3ir7.1    TWYEKDDMNT--SDMHPFIHPLSAAVDPAWEAKSDWEIYKAIAKKFSEVCVGHLGKETDIVTLPIQHDSAAELAQPLDVK  target    DYFAFA--------------AADK-------------------------------RGIYIQRL----------------- 3ir7.1    DWKKGECDLIPGKTAPHIMVVERDYPATYERFTSIGPLMEKIGNGGKGIAWNTQSEMDLLRKLNYTKAEGPAKGQPMLNT  target    --------LDTCTTTAGY----KLADIMAGKY--------GPPGGCLLNF---------------------RTYPRIPFY 3ir7.1    AIDAAEMILTLAPETNGQVAVKAWAALSEFTGRDHTHLALNKEDE-KIRFRDIQAQPRKIISSPTWSGLEDEHVSYNAGY  target    EQVHDSEPFHTDTGRMHAYADVPEAIEYGENFIVHREGPEATPYLPNVIVSSNPHIRPEDYGIAADAEHWDDRTIRNIKM 3ir7.1    TNVHELIPWRTLSGRQQLYQDHQWMRDFGESLLVYRPPIDTRSV-KE------------VI-------------------  target    PWSKVKETKNFLWEKGFQFYCLTPKTRHRVHSGWSNVDWHMLMDSNFGDPYRLDKRAPCVGEHQLHINPQAARDLNINDG 3ir7.1    ------GQK-SNGNQEKALNFLTPHQKWGIHSTYSDNLLMLTLG---------------RGGPVVWLSEADAKDLGIADN  target    DYVYVDANPADRPYLGAKPDDPFYRVSRCMLRVTYNRAYPYNIVMMKHAPFIATEKSVKAHETRPDGRALSANTGYQANL 3ir7.1    DWIEVFNSN-----------------GALTARAVVSQRVPAGMTMMYHAQE----RIVNLPGS--------EIT----QQ  target    RYGSQQSVTRNWHMPMHQTDTLFHKSKVFMGFIFGGEADNHAVNTVPKETLVRVTKAEDGGMGGKGIWQPATTGFSPDNE 3ir7.1    RGGIHNSVTRITPKPTHMIGGYAHLAYG-----F----NYYGTVGSNRDEFVVVRKMKNIDWL-----------------  target    SDFMKKYLAGELTKVKT 3ir7.1    ----------------- ``` | | | | | | | | | | | | | | | | | | | | | | | | | | | | | | | | | | | | | | | | | | | | | | | | | |
|  | 1q16.1.A | Respiratory nitrate reductase 1 alpha chain  *Crystal structure of Nitrate Reductase A, NarGHI, from Escherichia coli* | 0.41 | 0.00 | 22.25 | 0.82 | 1-812 | X-ray | 1.90 | monomer | 2 x MD1, 1 x 6MO, 2 x HEM, 4 x SF4, 1 x F3S, 1 x AGA, 1 x 3PH | HHblits | 0.32 |
| ``` target    IMDGKNLVENKLTDSHWFIECMERGAKIVVIAPEYGPPSTKADYWIPIRPQTDAALWLGITRLMIEKKW------YDETF 1q16.1    IAWGSNVPQTRTPDAHFFTEVRYKGTKTVAVTPDYAEIAKLCDLWLAPKQGTDAAMALAMGHVMLREFHLDNPSQYFTDY  target    VKGFTDFPLLVRTDT-------LQRLRAHEVFPQYKTSLSADGPSMKIQGLSAEQHAKLGDFVVWDGKTNAPAAITRDDV 1q16.1    VRRYTDMPMLVMLEERDGYYAAGRMLRAADLVDALG-----------------QENNPEWKTVAFNT-NGEMVAPNGS-I  target    GA------TITK------------------KGID--------------------PVLA-------GSFKVKLVDGKEVEV 1q16.1    GFRWGEKGKWNLEQRDGKTGEETELQLSLLGSQDEIAEVGFPYFGGDGTEHFNKVELENVLLHKLPVKRLQLADGSTALV  target    ATLWTLYQ------------------DHLKDYDLDTVVEITQAPKEMIEQLAQDIATM-----KPVAIHQGEGINHWFHA 1q16.1    TTVYDLTLANYGLERGLNDVNCATSYDDVKAYTPAWAEQITGVSRSQIIRIAREFADNADKTHGRSMIIVGAGLNHWYHL  target    TEMNRAAYLPLMLTGNIGRPGAGCQTWAGNYKAALFQG-------SPWTGPGF------------KGWVAEDPFDIN-LN 1q16.1    DMNYRGLINMLIFCGCVGQSGGGWAHYVGQEKLRPQTGWQPLAFALDWQRPARHMNSTSYFYNHSSQWRYETVTAEELLS  target    PKAHGKEI-----------HAHAYTKDEEPAYWNHGDLALI-------VDTPKFG-RKNFTGK--------THMPTPTKA 1q16.1    PMADKSRYTGHLIDFNVRAERMGWL-PSAPQL-GTNPLTIAGEAEKAGMNPVDYTVKSLKEGSIRFAAEQPENGKNHPRN  target    LIFNNVNLINNAKWAYG-M------------------------------IKNVNPNVEMIVSMDIQMTASIEYADLALPA 1q16.1    LFIWRSNLLGSSGKGHEFMLKYLLGTEHGIQGKDLGQQGGVKPEEVDWQDNGLEGKLDLVVTLDFRLSSTCLYSDIILPT  target    NSWLEFEGLEITASCSNPFLQIWKGGIPPVFDSRDDLDILAGIANALADVTGEK------------------------RF 1q16.1    ATWYEKDDMNT--SDMHPFIHPLSAAVDPAWEAKSDWEIYKAIAKKFSEVCVGHLGKETDIVTLPIQHDSAAELAQPLDV  target    RDYFAFA--------------AADK-------------------------------RGIYIQRL---------------- 1q16.1    KDWKKGECDLIPGKTAPHIMVVERDYPATYERFTSIGPLMEKIGNGGKGIAWNTQSEMDLLRKLNYTKAEGPAKGQPMLN  target    ---------LDTCTTTAGY----KLADIMAGKY-G-------PPGGCLLNF---------------------RTYPRIPF 1q16.1    TAIDAAEMILTLAPETNGQVAVKAWAALSEFTGRDHTHLALNKEDE-KIRFRDIQAQPRKIISSPTWSGLEDEHVSYNAG  target    YEQVHDSEPFHTDTGRMHAYADVPEAIEYGENFIVHREGPEATPYLPNVIVSSNPHIRPEDYGIAADAEHWDDRTIRNIK 1q16.1    YTNVHELIPWRTLSGRQQLYQDHQWMRDFGESLLVYRPPIDTRSV-KE------------VIG-----------------  target    MPWSKVKETKNFLWEKGFQFYCLTPKTRHRVHSGWSNVDWHMLMDSNFGDPYRLDKRAPCVGEHQLHINPQAARDLNIND 1q16.1    --------QK-SNGNQEKALNFLTPHQKWGIHSTYSDNLLMLTLG---------------RGGPVVWLSEADAKDLGIAD  target    GDYVYVDANPADRPYLGAKPDDPFYRVSRCMLRVTYNRAYPYNIVMMKHAPFIATEKSVKAHETRPDGRALSANTGYQAN 1q16.1    NDWIEVFNSN-----------------GALTARAVVSQRVPAGMTMMYHAQE----RIVNLPG--------SEITQ----  target    LRYGSQQSVTRNWHMPMHQTDTLFHKSKVFMGFIFGGEADNHAVNTVPKETLVRVTKAEDGGMGGKGIWQPATTGFSPDN 1q16.1    QRGGIHNSVTRITPKPTHMIGGYAHLAYG-----F----NYYGTVGSNRDEFVVVRKMKNIDWL----------------  target    ESDFMKKYLAGELTKVKT 1q16.1    ------------------ ``` | | | | | | | | | | | | | | | | | | | | | | | | | | | | | | | | | | | | | | | | | | | | | | | | | |
|  | 3egw.1.A | Respiratory nitrate reductase 1 alpha chain  *The crystal structure of the NarGHI mutant NarH - C16A* | 0.42 | 0.08 | 22.35 | 0.81 | 1-809 | X-ray | 1.90 | homo-dimer | 2 x MD1, 2 x MGD, 2 x 6MO, 6 x SF4, 4 x F3S, 2 x 3PH, 4 x HEM, 2 x AGA | HHblits | 0.32 |
| ``` target    IMDGKNLVENKLTDSHWFIECMERGAKIVVIAPEYGPPSTKADYWIPIRPQTDAALWLGITRLMIEKKW------YDETF 3egw.1    IAWGSNVPQTRTPDAHFFTEVRYKGTKTVAVTPDYAEIAKLCDLWLAPKQGTDAAMALAMGHVMLREFHLDNPSQYFTDY  target    VKGFTDFPLLVRTD-------TLQRLRAHEVFPQYKTSLSADGPSMKIQGLSAEQHAKLGDFVVWDGKTNAPAAITRDDV 3egw.1    VRRYTDMPMLVMLEERDGYYAAGRMLRAADLVAALG-----------------QENNPEWKTVAFNT-NGEMVAPNGSI-  target    GA------T------------------------------------------ITKKGIDPVLAG---SFKVKLVDGKEVEV 3egw.1    GFRWGEKGKWNLEQRDGKTGEETELQLSLLGSQDEIAEVGFPYFGGDGTEHFNKVELENVLLHKLPVKRLQLADGSTALV  target    ATLWTLYQ------------------DHLKDYDLDTVVEITQAPKEMIEQLAQDIATM-----KPVAIHQGEGINHWFHA 3egw.1    TTVYDLTLANYGLERGLNDVNCATSYDDVKAYTPAWAEQITGVSRSQIIRIAREFADNADKTHGRSMIIVGAGLNHWYHL  target    TEMNRAAYLPLMLTGNIGRPGAGCQTWAGNYKAALFQGS-------PWTGPG------------FKGWVAEDPFDIN-LN 3egw.1    DMNYRGLINMLIFCGCVGQSGGGWAHYVGQEKLRPQTGWQPLAFALDWQRPARHMNSTSYFYNHSSQWRYETVTAEELLS  target    PKAHGKEIH-----------AHAYTKDEEPAYWNHGDLALIVD-------TPKFG-RKNFTGK--------THMPTPTKA 3egw.1    PMADKSRYTGHLIDFNVRAERMGWL-PSAPQL-GTNPLTIAGEAEKAGMNPVDYTVKSLKEGSIRFAAEQPENGKNHPRN  target    LIFNNVNLINNAKWAYG-M------------------------------IKNVNPNVEMIVSMDIQMTASIEYADLALPA 3egw.1    LFIWRSNLLGSSGKGHEFMLKYLLGTEHGIQGKDLGQQGGVKPEEVDWQDNGLEGKLDLVVTLDFRLSSTCLYSDIILPT  target    NSWLEFEGLEITASCSNPFLQIWKGGIPPVFDSRDDLDILAGIANALADVTGEK--R----------------------F 3egw.1    ATWYEKDDMNT--SDMHPFIHPLSAAVDPAWEAKSDWEIYKAIAKKFSEVCVGHLGKETDIVTLPIQHDSAAELAQPLDV  target    RDYFA---------------------------FAAA------------------DKRGIYIQRLL--------------- 3egw.1    KDWKKGECDLIPGKTAPHIMVVERDYPATYERFTSIGPLMEKIGNGGKGIAWNTQSEMDLLRKLNYTKAEGPAKGQPMLN  target    ----------DTCTTTAGY----KLADIMAGKY--------GPPGGCLLNF---------------------RTYPRIPF 3egw.1    TAIDAAEMILTLAPETNGQVAVKAWAALSEFTGRDHTHLALNKEDE-KIRFRDIQAQPRKIISSPTWSGLEDEHVSYNAG  target    YEQVHDSEPFHTDTGRMHAYADVPEAIEYGENFIVHREGPEATPYLPNVIVSSNPHIRPEDYGIAADAEHWDDRTIRNIK 3egw.1    YTNVHELIPWRTLSGRQQLYQDHQWMRDFGESLLVYRPPIDTRSVKE---------------------------------  target    MPWSKVKETKNFLWEKGFQFYCLTPKTRHRVHSGWSNVDWHMLMDSNFGDPYRLDKRAPCVGEHQLHINPQAARDLNIND 3egw.1    --VIG---QK-SNGNQEKALNFLTPHQKWGIHSTYSDNLLMLTLG---------------RGGPVVWLSEADAKDLGIAD  target    GDYVYVDANPADRPYLGAKPDDPFYRVSRCMLRVTYNRAYPYNIVMMKHAPFIATEKSVKAHETRPDGRALSANTGYQAN 3egw.1    NDWIEVFNSN-----------------GALTARAVVSQRVPAGMTMMYHAQE----RIVNLPG--------SEITQ----  target    LRYGSQQSVTRNWHMPMHQTDTLFHKSKVFMGFIFGGEADNHAVNTVPKETLVRVTKAEDGGMGGKGIWQPATTGFSPDN 3egw.1    QRGGIHNSVTRITPKPTHMIGGYAHLAYG-----F----NYYGTVGSNRDEFVVVRKMKNI-------------------  target    ESDFMKKYLAGELTKVKT 3egw.1    ------------------ ``` | | | | | | | | | | | | | | | | | | | | | | | | | | | | | | | | | | | | | | | | | | | | | | | | | |
|  | 3ir6.1.A | Respiratory nitrate reductase 1 alpha chain  *Crystal structure of NarGHI mutant NarG-H49S* | 0.41 | 0.00 | 22.53 | 0.81 | 1-808 | X-ray | 2.80 | monomer | 2 x GDP, 1 x AGA, 3 x SF4, 1 x F3S, 2 x HEM | HHblits | 0.32 |
| ``` target    IMDGKNLVENKLTDSHWFIECMERGAKIVVIAPEYGPPSTKADYWIPIRPQTDAALWLGITRLMIEKKW------YDETF 3ir6.1    IAWGSNVPQTRTPDAHFFTEVRYKGTKTVAVTPDYAEIAKLCDLWLAPKQGTDAAMALAMGHVMLREFHLDNPSQYFTDY  target    VKGFTDFPLLVRTD-------TLQRLRAHEVFPQYKTSLSADGPSMKIQGLSAEQHAKLGDFVVWDGKTNAPAAITRDDV 3ir6.1    VRRYTDMPMLVMLEERDGYYAAGRMLRAADLVDAL-----------------GQENNPEWKTVAFNT-NGEMVAPNGSI-  target    GA------TIT------------------KKGID-------P-------------VLA----G---SFKVKLVDGKEVEV 3ir6.1    GFRWGEKGKWNLEQRDGKTGEETELQLSLLGSQDEIAEVGFPYFGGDGTEHFNKVELENVLLHKLPVKRLQLADGSTALV  target    ATLWTLYQ------------------DHLKDYDLDTVVEITQAPKEMIEQLAQDIATM-----KPVAIHQGEGINHWFHA 3ir6.1    TTVYDLTLANYGLERGLNDVNCATSYDDVKAYTPAWAEQITGVSRSQIIRIAREFADNADKTHGRSMIIVGAGLNHWYHL  target    TEMNRAAYLPLMLTGNIGRPGAGCQTWAGNYKAALFQGS-------PWTGPGF------------KGWVAEDPFDIN-LN 3ir6.1    DMNYRGLINMLIFCGCVGQSGGGWAHYVGQEKLRPQTGWQPLAFALDWQRPARHMNSTSYFYNHSSQWRYETVTAEELLS  target    PKAHGKEI-----------HAHAYTKDEEPAYWNHGDLALIVD-------TPKFG-RKNFTGK--------THMPTPTKA 3ir6.1    PMADKSRYTGHLIDFNVRAERMGWL-PSAPQL-GTNPLTIAGEAEKAGMNPVDYTVKSLKEGSIRFAAEQPENGKNHPRN  target    LIFNNVNLINNAKWAYG-M------------------------------IKNVNPNVEMIVSMDIQMTASIEYADLALPA 3ir6.1    LFIWRSNLLGSSGKGHEFMLKYLLGTEHGIQGKDLGQQGGVKPEEVDWQDNGLEGKLDLVVTLDFRLSSTCLYSDIILPT  target    NSWLEFEGLEITASCSNPFLQIWKGGIPPVFDSRDDLDILAGIANALADVTGEK--R----------------------F 3ir6.1    ATWYEKDDMNT--SDMHPFIHPLSAAVDPAWEAKSDWEIYKAIAKKFSEVCVGHLGKETDIVTLPIQHDSAAELAQPLDV  target    RDYFA---------------------------FAAA------------------DKRGIYIQRLL--------------- 3ir6.1    KDWKKGECDLIPGKTAPHIMVVERDYPATYERFTSIGPLMEKIGNGGKGIAWNTQSEMDLLRKLNYTKAEGPAKGQPMLN  target    ----------DTCTTTAGY----KLADIMAGKY-G-------PPGGCLLNF---------------------RTYPRIPF 3ir6.1    TAIDAAEMILTLAPETNGQVAVKAWAALSEFTGRDHTHLALNKEDE-KIRFRDIQAQPRKIISSPTWSGLEDEHVSYNAG  target    YEQVHDSEPFHTDTGRMHAYADVPEAIEYGENFIVHREGPEATPYLPNVIVSSNPHIRPEDYGIAADAEHWDDRTIRNIK 3ir6.1    YTNVHELIPWRTLSGRQQLYQDHQWMRDFGESLLVYRPPIDTRSV-K------------EVI------------------  target    MPWSKVKETKNFLWEKGFQFYCLTPKTRHRVHSGWSNVDWHMLMDSNFGDPYRLDKRAPCVGEHQLHINPQAARDLNIND 3ir6.1    -------GQK-SNGNQEKALNFLTPHQKWGIHSTYSDNLLMLTLG---------------RGGPVVWLSEADAKDLGIAD  target    GDYVYVDANPADRPYLGAKPDDPFYRVSRCMLRVTYNRAYPYNIVMMKHAPFIATEKSVKAHETRPDGRALSANTGYQAN 3ir6.1    NDWIEVFNSN-----------------GALTARAVVSQRVPAGMTMMYHAQE----RIVNLPG--------SEITQ----  target    LRYGSQQSVTRNWHMPMHQTDTLFHKSKVFMGFIFGGEADNHAVNTVPKETLVRVTKAEDGGMGGKGIWQPATTGFSPDN 3ir6.1    QRGGIHNSVTRITPKPTHMIGGYAHLAYG-----F----NYYGTVGSNRDEFVVVRKMKN--------------------  target    ESDFMKKYLAGELTKVKT 3ir6.1    ------------------ ``` | | | | | | | | | | | | | | | | | | | | | | | | | | | | | | | | | | | | | | | | | | | | | | | | | |
|  | 5e7o.1.A | DMSO reductase family type II enzyme, molybdopterin subunit  *Crystal structure of the perchlorate reductase PcrAB mutant W461E of PcrA from Azospira suillum PS* | 0.47 |  | 25.69 | 0.77 | 1-810 | X-ray | 2.40 | hetero-oligomer | 4 x SF4, 1 x MO, 1 x MGD, 1 x MD1, 1 x F3S | HHblits | 0.33 |
| ``` target    IMDGKNLVENKLTDSHWFIECMERGAKIVVIAPEYGPPSTKADYWIPIRPQTDAALWLGITRLMIEKKWYDETFVKGFTD 5e7o.1    ILWGSNPTQTRIPDAHFLSEAQLNGAKIVSISPDYNSSTIKVDKWIHPQPGTDGALAMAMAHVIIKEKLYDAHSLKEQTD  target    FPLLVRTDTLQRLRAHEVFPQYKTSLSADGPSMKIQGLSAEQHAKLGDFVVWDGKTNAPAAITRDDVG------------ 5e7o.1    LSYLVRSDTKRFLREADVVAG----------------------GSKDKFYFWNAKTGKPVIPKGSWGDQPEKKGSPVGFL  target    ---------ATITKKGIDPVLAGSFKVKLVDGKEVEVATLWTLYQDHL-KDYDLDTVVEITQAPKEMIEQLAQDIATMKP 5e7o.1    GRNTFAFPKGYIDLGDLDPALEGKFNMQLLDGKTVEVRPVFEILKSRLMADNTPEKAAKITGVTAKAITELAREFATAKP  target    VAIHQGEGINHWFHATEMNRAAYLPLMLTGNIGRPGAGCQTWAGNYKAALFQGSPWTGPGFKGWVAEDPFDINLNPKAHG 5e7o.1    SMIICGGGTQHWYYSDVLLRAMHLLTALTGTEGTNGGGMNHYIGQEKPAFVAGLV--ALAF-------PEGV--NKQRFC  target    KEIHAHAYTKDEEPA-YW--NHGDLALIVDTPKFGRKNFTGKTHMPTPTKALIFNNVNLINNAKWAYGMIKNVNPNVEMI 5e7o.1    Q-TTIWTYIHAEVNDEIISSDIDTEKYLRDSITTG--QMPNMPEQGRDPKVFFVYRGNWLNQAKGQKYVLENLWPKLELI  target    VSMDIQMTASIEYADLALPANSWLEFEGLEITASCSNPFLQIWKGGIPPVFDSRDDLDILAGIANALADVTGEKRFRDY- 5e7o.1    VDINIRMDSTALYSDVVLPSAHWYEKLDLNV--TSEHSYINMTEPAIKPMWESKTDWQIFLALAKRVEMAAKRKKYEKFN  target    ---FAFAA-----------AD---KRGIYIQRLLDTCTTTAGYKLADIMAGKYGPPGGCLLNF-RTYPRIPFYEQVHDSE 5e7o.1    DEKFKWVRDLSNLWNQMTMDGKLAEDEAAAQYILDNAPQSKGITIQMLREKPQRFKSNWTSPLKEGVPYTPFQYFVVDKK  target    PFHTDTGRMHAYADVPEAIEYGENFIVHREGPEATPYLPNVIVSSNPHIRPEDYGIAADAEHWDDRTIRNIKMPWSKVKE 5e7o.1    PWPTLTGRQQFYLDHDTFFDMGVELPTYKAPI------------------------------------------------  target    TKNFLWEKGFQFYCLTPKTRHRVHSGWSNVDWHMLMDSNFGDPYRLDKRAPCVGEHQLHINPQAARDLNINDGDYVYVDA 5e7o.1    -----DADKYPFRFNSPHSRHSVHSTFKDNVLMLRL------------Q---RGGPSIEMSPLDAKPLGIKDNDWVEAWN  target    NPADRPYLGAKPDDPFYRVSRCMLRVTYNRAYPYNIVMMKHAPFIATEKSVKAHETRPDGRALSANTGYQANLRYGSQQS 5e7o.1    NH-----------------GKVICRVKIRNGEQRGRVSMWHCPE----L-------------------YMDLL-TGGSQS  target    VTRNWHMPMHQTDTLFHKSKVFMGFIFGGEADNHAVNTVPKETLVRVTKAEDGGMGGKGIWQPATTGFSPDNESDFMKKY 5e7o.1    VCPVRINPTNLVGNYGHLFFR-----P----NYYGPAGSQRDVRVNVKRYIGAT--------------------------  target    LAGELTKVKT 5e7o.1    ---------- ``` | | | | | | | | | | | | | | | | | | | | | | | | | | | | | | | | | | | | | | | | | | | | | | | | | |
|  | 4ydd.1.A | DMSO reductase family type II enzyme, molybdopterin subunit  *Crystal structure of the perchlorate reductase PcrAB from Azospira suillum PS* | 0.47 |  | 25.73 | 0.77 | 1-809 | X-ray | 1.86 | hetero-oligomer | 4 x SF4, 1 x MO, 1 x MGD, 1 x MD1, 1 x F3S | HHblits | 0.33 |
| ``` target    IMDGKNLVENKLTDSHWFIECMERGAKIVVIAPEYGPPSTKADYWIPIRPQTDAALWLGITRLMIEKKWYDETFVKGFTD 4ydd.1    ILWGSNPTQTRIPDAHFLSEAQLNGAKIVSISPDYNSSTIKVDKWIHPQPGTDGALAMAMAHVIIKEKLYDAHSLKEQTD  target    FPLLVRTDTLQRLRAHEVFPQYKTSLSADGPSMKIQGLSAEQHAKLGDFVVWDGKTNAPAAITRDDV------------- 4ydd.1    LSYLVRSDTKRFLREADVVAG----------------------GSKDKFYFWNAKTGKPVIPKGSWGDQPEKKGSPVGFL  target    --------GATITKKGIDPVLAGSFKVKLVDGKEVEVATLWTLYQDHL-KDYDLDTVVEITQAPKEMIEQLAQDIATMKP 4ydd.1    GRNTFAFPKGYIDLGDLDPALEGKFNMQLLDGKTVEVRPVFEILKSRLMADNTPEKAAKITGVTAKAITELAREFATAKP  target    VAIHQGEGINHWFHATEMNRAAYLPLMLTGNIGRPGAGCQTWAGNYKAALFQGSPWTGPGFKGWVAEDPFDINLNPKAHG 4ydd.1    SMIICGGGTQHWYYSDVLLRAMHLLTALTGTEGTNGGGMNHYIGQWKPAFVAGLV--ALAF-------PEGV--NKQRFC  target    KEIHAHAYTKDEEPAY-W--NHGDLALIVDTPKFGRKNFTGKTHMPTPTKALIFNNVNLINNAKWAYGMIKNVNPNVEMI 4ydd.1    Q-TTIWTYIHAEVNDEIISSDIDTEKYLRDSITTG--QMPNMPEQGRDPKVFFVYRGNWLNQAKGQKYVLENLWPKLELI  target    VSMDIQMTASIEYADLALPANSWLEFEGLEITASCSNPFLQIWKGGIPPVFDSRDDLDILAGIANALADVTGEKRFR--- 4ydd.1    VDINIRMDSTALYSDVVLPSAHWYEKLDLNV--TSEHSYINMTEPAIKPMWESKTDWQIFLALAKRVEMAAKRKKYEKFN  target    --------DYFAFAA----AD---KRGIYIQRLLDTCTTTAGYKLADIMAGKYGPPGGCLLNF-RTYPRIPFYEQVHDSE 4ydd.1    DEKFKWVRDLSNLWNQMTMDGKLAEDEAAAQYILDNAPQSKGITIQMLREKPQRFKSNWTSPLKEGVPYTPFQYFVVDKK  target    PFHTDTGRMHAYADVPEAIEYGENFIVHREGPEATPYLPNVIVSSNPHIRPEDYGIAADAEHWDDRTIRNIKMPWSKVKE 4ydd.1    PWPTLTGRQQFYLDHDTFFDMGVELPTYKAPI------------------------------------------------  target    TKNFLWEKGFQFYCLTPKTRHRVHSGWSNVDWHMLMDSNFGDPYRLDKRAPCVGEHQLHINPQAARDLNINDGDYVYVDA 4ydd.1    -----DADKYPFRFNSPHSRHSVHSTFKDNVLMLRL------------Q---RGGPSIEMSPLDAKPLGIKDNDWVEAWN  target    NPADRPYLGAKPDDPFYRVSRCMLRVTYNRAYPYNIVMMKHAPFIATEKSVKAHETRPDGRALSANTGYQANLRYGSQQS 4ydd.1    NH-----------------GKVICRVKIRNGEQRGRVSMWHCPE----L-------------------YMD-LLTGGSQS  target    VTRNWHMPMHQTDTLFHKSKVFMGFIFGGEADNHAVNTVPKETLVRVTKAEDGGMGGKGIWQPATTGFSPDNESDFMKKY 4ydd.1    VCPVRINPTNLVGNYGHLFFR-----P----NYYGPAGSQRDVRVNVKRYIGA---------------------------  target    LAGELTKVKT 4ydd.1    ---------- ``` | | | | | | | | | | | | | | | | | | | | | | | | | | | | | | | | | | | | | | | | | | | | | | | | | |
|  | 2ivf.1.A | ETHYLBENZENE DEHYDROGENASE ALPHA-SUBUNIT  *ETHYLBENZENE DEHYDROGENASE FROM AROMATOLEUM AROMATICUM* | 0.45 |  | 23.36 | 0.77 | 1-806 | X-ray | 1.88 | hetero-oligomer | 1 x MES, 4 x SF4, 1 x MO, 1 x MGD, 1 x MD1, 1 x F3S, 1 x HEM | HHblits | 0.32 |
| ``` target    IMDGKNLVENKLTDSHWFIECMERGAKIVVIAPEYGPPSTKADYWIPIRPQTDAALWLGITRLMIEKKWYDETFVKGFTD 2ivf.1    FMTCSNWSYTYPSSYHFLSEARYKGAEVVVIAPDFNPTTPAADLHVPVRVGSDAAFWLGLSQVMIDEKLFDRQFVCEQTD  target    FPLLVRTDTLQRLRAHEVFPQYKTSLSADGPSMKIQGLSAEQHAKLGDFVVWDGKTNAPAAITRDDVGATITKKGIDPVL 2ivf.1    LPLLVRMDTGKFLSAEDVDG-----------------------GEAKQFYFFDEKAGSVRKASRGTLK-----LDFMPAL  target    AGSFKVKLVDGKEVEVATLWTLYQDHLKDYDLDTVVEITQAPKEMIEQLAQDIATMKPVAIHQGEGINHWFHATEMNRAA 2ivf.1    EGTFSARLKNGKTIQVRTVFEGLREHLKDYTPEKASAKCGVPVSLIRELGRKVAKK-RTCSYIGFSSAKSYHGDLMERSL  target    YLPLMLTGNIGRPGAGCQTWAGNYKAALF---QGSPWTGPGFK--GWVAEDPFD---INLNPKAHGKEIHAH----AYT- 2ivf.1    FLAMALSGNWGKPGTGAFAWAYSDDNMVYLGVMSKPTAQGGMDELHQMA-EGFNKRTLEADPTS-TDEMGNIEFMKVVTS  target    -KDEEPA------------YWNH-----GDL----ALIVDTPKFGRKNFT---GKTHMPTPTKALIFNNVNLINNAKWAY 2ivf.1    AVGLVPPAMWLYYHVGYDQLWNNKAWTDPALKKSFGAYLDEAKE-KGWWTNDHIRPAPDKTPQVYMLLSQNPMRRKRSGA  target    GMI-KNVNPNVEMIVSMDIQMTASIEYADLALPANSWLEFEGLEITASCSNPFLQIWKGGIPPVFDSRDDLDILAGIANA 2ivf.1    KMFPDVLFPKLKMIFALETRMSSSAMYADIVLPCAWYYEKHEMTTP-CSGNPFFTFVDRSVAPPGECREEWDAIALILKK  target    LADVTGEKR-------------FRDYFA-FAAA---DKRGIYIQRLLDTCTT----TAGYKLADIMAGKYGPP---GGCL 2ivf.1    VGERAAARGLTEFNDHNGRKRRYDELYKKFTMDGHLLTNEDCLKEMVDINRAVGVFAKDYTYEKFKKEGQTRFLSMGTGV  target    LNFR-------TYPRIPFYEQVHDSEPFHTDTGRMHAYADVPEAIEYGENFIVHREGPEATPYLPNVIVSSNPHIRPEDY 2ivf.1    SRYAHANEVDVTKPIYPMRWHFDDKKVFPTHTRRAQFYLDHDWYLEAGESLPTHKDTPM---------------------  target    GIAADAEHWDDRTIRNIKMPWSKVKETKNFLWEKGFQFYCLTPKTRHRVHSGWSNVDWHMLMDSNFGDPYRLDKRAPCVG 2ivf.1    -------------------------------VGGDHPFKITGGHPRVSIHSTHLTNSHLSRLH---------------RG  target    EHQLHINPQAARDLNINDGDYVYVDANPADRPYLGAKPDDPFYRVSRCMLRVTYNRAYPYNIVMMKHAPFIATEKSVKAH 2ivf.1    QPVVHMNSKDAAELGIKDGDMAKLFNDF-----------------ADCEIMVRTAPNVQPKQCIVYFWDA----------  target    ETRPDGRALSANTGYQANLRYGSQQSVTRNWHMPMHQTDTLFHKSKVFMGFIFGGEADNHAVNT-VPKETLVRVTKAEDG 2ivf.1    --------------HQYKGWK-PYDILLIGMPKPLHLAGGYEQFRYY-----F----MNGSPAPVTDRGVRVSIKKA---  target    GMGGKGIWQPATTGFSPDNESDFMKKYLAGELTKVKT 2ivf.1    ------------------------------------- ``` | | | | | | | | | | | | | | | | | | | | | | | | | | | | | | | | | | | | | | | | | | | | | | | | | |
|  | 1kqf.1.A | FORMATE DEHYDROGENASE, NITRATE-INDUCIBLE, MAJOR SUBUNIT  *FORMATE DEHYDROGENASE N FROM E. COLI* | 0.32 |  | 18.56 | 0.72 | 1-719 | X-ray | 1.60 | hetero-oligomer | 3 x 6MO, 15 x SF4, 6 x MGD, 6 x HEM, 3 x CDL | HHblits | 0.28 |
| ``` target    IMDGKNLVENKLTDSHWFIECME-RGAKIVVIAPEYGPPSTKADYWIPIRPQTDAALWLGITRLMIEKKWYDETFVKGFT 1kqf.1    MVMGGNAAEAHPVGFRWAMEAKNNNDATLIVVDPRFTRTASVADIYAPIRSGTDITFLSGVLRYLIENNKINAEYVKHYT  target    DFPLLVRTDTLQRLRAHEVFPQYKTSLSADGPSMKIQGLSAEQHAKLGDFVVWDGKTNAPAAITRDDVGATITKKGIDPV 1kqf.1    NASLLVRDDFA-FEDGLFS--GYD----------------AEK--RQYDKSSWNYQLDE---------------------  target    LAGSFKVKLVDGKEVEVATLWTLYQDHLKDYDLDTVVEITQAPKEMIEQLAQDIATM----KPVAIHQGEGINHWFHATE 1kqf.1    -NGY---AKRDETLTHPRCVWNLLKEHVSRYTPDVVENICGTPKADFLKVCEVLASTSAPDRTTTFLYALGWTQHTVGAQ  target    MNRAAYLPLMLTGNIGRPGAGCQTWAGNYKAALF--QGS-PWTGPGFKGWVAED-PFDINLNPK--AHGKEIHAHAYTKD 1kqf.1    NIRTMAMIQLLLGNMGMAGGGVNALRGHSNIQGLTDLGLLSTSLPGYLTLPSEKQVDLQSYLEANTPKATLADQVNY-WS  target    EEPAYWNHGDLALIVD-------------TPKF--GRKN-FTGKTHMPTPTKALIFNNVNLINNAKWAYGMIKNVNPNVE 1kqf.1    NYPKFFV-SLMKSFYGDAAQKENNWGYDWLPKWDQTYDVIKYFNMMDEGKVTGYFCQGFNPVASFPDKNKVV-SCLSKLK  target    MIVSMDIQMTASIEYAD-----------------LALPANSWLEFEGLEITASCSNPFLQIWKGGIPPVFDSRDDLDILA 1kqf.1    YMVVIDPLVTETSTFWQNHGESNDVDPASIQTEVFRLPSTCFAEEDGSIA---NSGRWLQWHWKGQDAPGEARNDGEILA  target    GIANALADVTGE---KRFRDY----FAFAAADK----------RGIYIQRLLDTCTT------TAGYKLADIMAGKYGPP 1kqf.1    GIYHHLRELYQSEGGKGVEPLMKMSWNYKQPHEPQSDEVAKENNGYALEDLYDANGVLIAKKGQLLSSFAHLRDDGTTAS  target    G--------------------------------GCLLN------F---------RT--YPRIPF----------YEQVHD 1kqf.1    SCWIYTGSWTEQGNQMANRDNSDPSGLGNTLGWAWAWPLNRRVLYNRASADINGKPWDPKRMLIQWNGSKWTGNDIPDFG  target    SEPFHTDTGRMHAYADVPEAIE----YG-ENFIVHREGPEATPY-LPNVIVS-SNPHIRPEDYGIAADAEHWDDRTIRNI 1kqf.1    NAAPGTPTGPFIMQPEGMGRLFAINKMAEGPFPEHYEPIETPLGTNPLHPNVVSNPVVR-----------LYEQ------  target    KMPWSKVKETKNFLWEKGFQFYCLTPKTRHRVHSGWSNVDWHMLMDSNFGDPYRLDKRAPCVGEHQLHINPQAARDLNIN 1kqf.1    ------DAL--RMGKKEQFPYVGTTYRLTEHFHTWTKHALLNAI-------------AQ---PEQFVEISETLAAAKGIN  target    DGDYVYVDANPADRPYLGAKPDDPFYRVSRCMLRVTYNRAYPY--------NIVMMKHAPFIATEKSVKAHETRPDGRAL 1kqf.1    NGDRVTVSSK-----------------RGFIRAVAVVTRRLKPLNVNGQQVETVGIPIHW--------------------  target    SANTGYQANLRYGSQQSVTRNWHMPMHQTDTLFHKSKVFMGFIFGGEADNHAVNTVPKETLVRVTKAEDGGMGGKGIWQP 1kqf.1    --------------------------------------------------------------------------------  target    ATTGFSPDNESDFMKKYLAGELTKVKT 1kqf.1    --------------------------- ``` | | | | | | | | | | | | | | | | | | | | | | | | | | | | | | | | | | | | | | | | | | | | | | | | | |
|  | 7l5i.1.A | Trimethylamine-N-oxide reductase  *Crystal Structure of Haemophilus influenzae MtsZ at pH 7.0* | 0.37 |  | 18.52 | 0.67 | 1-812 | X-ray | 1.73 | monomer | 2 x MGD, 1 x MO, 1 x O | HHblits | 0.30 |
| ``` target    IMDGKNLVENKLTD--------SHWFIECMERGAKIVVIAPEYGPPSTK-ADYWIPIRPQTDAALWLGITRLMIEKKWYD 7l5i.1    VLWSANPLTTMRIAWMSTDQKGIEYFKKFQASGKRIICIDPQKSETCQMLNAEWIPVNTATDVPLMLGIAHTLVEQGKHD  target    ETFVKGFTDFPLLVRTDTLQRLRAHEVFPQYKTSLSADGPSMKIQGLSAEQHAKLGDFVVWDGKTNAPAAITRDDVGATI 7l5i.1    KDFLKKYTSGYA--------------------------------------------------------------------  target    TKKGIDPVLAGSFKVKLVDGKEVEVATLWTLYQDH------LKDYDLDTVVEITQAPKEMIEQLAQDIATMKPVAIHQGE 7l5i.1    ------------------------------KFEEYLLGKTDGQPKTAEWAAKICGVPAETIKQLAADFAS-KRTMLMGGW  target    GINHWFHATEMNRAAYLPLMLTGNIGRPGAGCQTWAGNYKAALFQGSPWTGPGFKGWVAEDPFDINLNPKAHGKEIHAHA 7l5i.1    GMQRQRHGEQTHWMLVTLASMLGQIGLPGGGFGLSYHYSNGG---V-PTATGGIIGSITASPSG---KAGA--KTWLD-D  target    YTKDEEPAYWNHGDLALIVDTPKFGRKNFTGKTHMPTPTKALIFNNVNLINNAKWAYGMIKNVNPNVEMIVSMDIQMTAS 7l5i.1    TSKSAFPLAR----IADVLLHPGKKIQY-NGTEITYPDIKAVYWAGGNPFVHHQDTNTLV-KAFQKPDVVIVNEVNWTPT  target    IEYADLALPANSWLEFEGLEITASCSNPFLQIWKGGIPPVFDSRDDLDILAGIANALADVTGEKRFRDYFAFAAADKRGI 7l5i.1    ARMADIVLPATTSYERNDLTMAGDYSMMSVYPMKQVVPPQFEAKNDYDIFVELAKRAGVEEQ------YTEGK---TEME  target    YIQRLLDTCTTT---AG---YKLADIMAGK--YGPPGG-CLLNFRTYPRIPFYEQVHDSEPFHTDTGRMHAYADVPEAIE 7l5i.1    WLEEFYNAAFSAARANRVAMPRFDKFWAENKPLSFEAGEAAKKWVRY--GEF-REDPLLNPLGTPSGKIEIFSDVVEKMN  target    YG--ENFIVHREGPEATPYLPNVIVSSNPHIRPEDYGIAADAEHWDDRTIRNIKMPWSKVKETKNFLWEKGFQFYCLTPK 7l5i.1    YNDCKGHPSWMEPEE-----------------------------------------------FA-GNVTEEYPLALVTPH  target    TRHRVHSGWSNVDWHMLMDSNFGDPYRLDKRAPCVGEHQLHINPQAARDLNINDGDYVYVDANPADRPYLGAKPDDPFYR 7l5i.1    PYYRLHSQLAHTSLRQKYA-----------VN---DREPVMIHPEDAAARGIKDGDIVRIHSKR----------------  target    VSRCMLRVTYNRAYPYNIVMMKHAPFIATEKSVKAHETRPDGRALSANTGYQANLRYGSQQSVTRNWHMPMHQTDTLFHK 7l5i.1    -GQVLAGAAVTENIIKGTVALHEGAWYDPM---YLGE------------SEKPLCKNGCANVLTRDEG-TSKLA------  target    SKVFMGFIFGGEADNHAVNTVPKETLVRVTKAEDGGMGGKGIWQPATTGFSPDNESDFMKKYLAGELTKVKT 7l5i.1    -----------------QGNSPNTCIVQIEKFIGVAPE---------------------------------- ``` | | | | | | | | | | | | | | | | | | | | | | | | | | | | | | | | | | | | | | | | | | | | | | | | | |
|  | 7l5s.1.A | Trimethylamine-N-oxide reductase  *Crystal Structure of Haemophilus influenzae MtsZ at pH 5.5* | 0.37 |  | 18.52 | 0.67 | 1-812 | X-ray | 2.09 | monomer | 1 x O, 2 x MGD, 1 x MO | HHblits | 0.30 |
| ``` target    IMDGKNLVENKLTD--------SHWFIECMERGAKIVVIAPEYGPPSTK-ADYWIPIRPQTDAALWLGITRLMIEKKWYD 7l5s.1    VLWSANPLTTMRIAWMSTDQKGIEYFKKFQASGKRIICIDPQKSETCQMLNAEWIPVNTATDVPLMLGIAHTLVEQGKHD  target    ETFVKGFTDFPLLVRTDTLQRLRAHEVFPQYKTSLSADGPSMKIQGLSAEQHAKLGDFVVWDGKTNAPAAITRDDVGATI 7l5s.1    KDFLKKYTSGYA--------------------------------------------------------------------  target    TKKGIDPVLAGSFKVKLVDGKEVEVATLWTLYQDH------LKDYDLDTVVEITQAPKEMIEQLAQDIATMKPVAIHQGE 7l5s.1    ------------------------------KFEEYLLGKTDGQPKTAEWAAKICGVPAETIKQLAADFAS-KRTMLMGGW  target    GINHWFHATEMNRAAYLPLMLTGNIGRPGAGCQTWAGNYKAALFQGSPWTGPGFKGWVAEDPFDINLNPKAHGKEIHAHA 7l5s.1    GMQRQRHGEQTHWMLVTLASMLGQIGLPGGGFGLSYHYSNGG---V-PTATGGIIGSITASPSG---KAGA--KTWLD-D  target    YTKDEEPAYWNHGDLALIVDTPKFGRKNFTGKTHMPTPTKALIFNNVNLINNAKWAYGMIKNVNPNVEMIVSMDIQMTAS 7l5s.1    TSKSAFPLAR----IADVLLHPGKKIQY-NGTEITYPDIKAVYWAGGNPFVHHQDTNTLV-KAFQKPDVVIVNEVNWTPT  target    IEYADLALPANSWLEFEGLEITASCSNPFLQIWKGGIPPVFDSRDDLDILAGIANALADVTGEKRFRDYFAFAAADKRGI 7l5s.1    ARMADIVLPATTSYERNDLTMAGDYSMMSVYPMKQVVPPQFEAKNDYDIFVELAKRAGVEEQ------YTEGK---TEME  target    YIQRLLDTCTTT---AG---YKLADIMAGK--YGPPGG-CLLNFRTYPRIPFYEQVHDSEPFHTDTGRMHAYADVPEAIE 7l5s.1    WLEEFYNAAFSAARANRVAMPRFDKFWAENKPLSFEAGEAAKKWVRY--GEF-REDPLLNPLGTPSGKIEIFSDVVEKMN  target    YG--ENFIVHREGPEATPYLPNVIVSSNPHIRPEDYGIAADAEHWDDRTIRNIKMPWSKVKETKNFLWEKGFQFYCLTPK 7l5s.1    YNDCKGHPSWMEPEE-----------------------------------------------FA-GNVTEEYPLALVTPH  target    TRHRVHSGWSNVDWHMLMDSNFGDPYRLDKRAPCVGEHQLHINPQAARDLNINDGDYVYVDANPADRPYLGAKPDDPFYR 7l5s.1    PYYRLHSQLAHTSLRQKYA-----------VN---DREPVMIHPEDAAARGIKDGDIVRIHSKR----------------  target    VSRCMLRVTYNRAYPYNIVMMKHAPFIATEKSVKAHETRPDGRALSANTGYQANLRYGSQQSVTRNWHMPMHQTDTLFHK 7l5s.1    -GQVLAGAAVTENIIKGTVALHEGAWYDPM---YLGE------------SEKPLCKNGCANVLTRDEG-TSKLA------  target    SKVFMGFIFGGEADNHAVNTVPKETLVRVTKAEDGGMGGKGIWQPATTGFSPDNESDFMKKYLAGELTKVKT 7l5s.1    -----------------QGNSPNTCIVQIEKFIGVAPE---------------------------------- ``` | | | | | | | | | | | | | | | | | | | | | | | | | | | | | | | | | | | | | | | | | | | | | | | | | |
|  | 2nya.1.A | Periplasmic nitrate reductase  *Crystal structure of the periplasmic nitrate reductase (NAP) from Escherichia coli* | 0.30 |  | 16.99 | 0.66 | 1-719 | X-ray | 2.50 | monomer | 1 x SF4, 1 x 6MO, 2 x MGD | HHblits | 0.28 |
| ``` target    IMDGKNLVENKLTDSHWFIECM--ERGAKIVVIAPEYGPPSTKADYWIPIRPQTDAALWLGITRLMIEKKWYDETFVKGF 2nya.1    VLWGANMAEMHPILWSRITNRRLSNQNVTVAVLSTYQHRSFELADNGIIFTPQSDLVILNYIANYIIQNNAINQDFFSKH  target    TDFPLLVRTDTLQRLRAHEVFPQYKTSLSADGPSMKIQGLSAEQHAKLGDFVVWDGKTNAPAAITRDDVGATITKKGIDP 2nya.1    VNLRKGATDIG-YGLRPTHPLE--------------------------------KA--------------------AKN-  target    VLAGSFKVKLVDGKEVEVATLWTLYQDHLKDYDLDTVVEITQAPKEMIEQLAQDIATMKP-VAIHQGEGINHWFHATEMN 2nya.1    -----------PGSDASEPMSFEDYKAFVAEYTLEKTAEMTGVPKDQLEQLAQLYADPNKKVISYWTMGFNQHTRGVWAN  target    RAAYLPLMLTGNIGRPGAGCQTWAGNYKAALFQGSPWTGPGFKGWVAEDPFDINLNPKAHGKEIHAHAYTKDEEPAYWNH 2nya.1    NLVYNLHLLTGKISQPGCGPFSLTGQPSAC---GTAREVGTFAHR---LPADMVVTNEKHRDIC-EKKWN-------IPS  target    GDLALIVDTPKFGRKNFTGKTHMPTPTKALIFNNVNLINNAKWAYG-MIKNVNPNVEMIVSMDIQMTASIEYADLALPAN 2nya.1    GTIPA---KIGLHAVA-QDRALKDGKLNVYWTMCTNNMQAGPNINEERMPGWRDPRNFIIVSDPYPTVSALAADLILPTA  target    SWLEFEGLEITASCSNPFLQIWKGGIPPVFDSRDDLDILAGIANALADVTGEK-RFRDYFAFAAADKRGIYIQRLLDTCT 2nya.1    MWVEKEGAY---GNAERRTQFWRQQVQAPGEAKSDLWQLVQFSRRFKTEEVWPEDLLAKKPELRG---KTLYEVLYAT-P  target    TTAGYKLADIMAGKYGP---PGGCLL------NFR------TYPRIPFYE-QVHDSEPFHTDTGRMHAYA--D-VPEAIE 2nya.1    EVSKFPVSELAEDQLNDESRELGFYLQKGLFEEYAWFGRGHGHDLAPFDDYHKARGLRWPVVNGKETQWRYSEGNDPYVK  target    YGENFIVHREGPEATPYLPNVIVSSNPHIRPEDYGIAADAEHWDDRTIRNIKMPWSKVKETKNFLWEKGFQFYCLTPKTR 2nya.1    AGEGYKFYGKP----------------DGKAVIF-----ALPFEPA--------A--------EAPDEEYDLWLSTGRVL  target    HRVH--SGWSNVDWHMLMDSNFGDPYRLDKRAPCVGEHQLHINPQAARDLNINDGDYVYVDANPADRPYLGAKPDDPFYR 2nya.1    EHWHTGSMTRRVPELHRAF----------------PEAVLFIHPLDAKARDLRRGDKVKVVSRR----------------  target    VSRCMLRVTYNRA--YPYNIVMMKHAPFIATEKSVKAHETRPDGRALSANTGYQANLRYGSQQSVTRNWHMPMHQTDTLF 2nya.1    -GEVISIVETRGRNRPPQGLVYMPFFD-----------------------------------------------------  target    HKSKVFMGFIFGGEADNHAVNTVPKETLVRVTKAEDGGMGGKGIWQPATTGFSPDNESDFMKKYLAGELTKVKT 2nya.1    -------------------------------------------------------------------------- ``` | | | | | | | | | | | | | | | | | | | | | | | | | | | | | | | | | | | | | | | | | | | | | | | | | |
|  | 6cz7.1.A | ArrA  *The arsenate respiratory reductase (Arr) complex from Shewanella sp. ANA-3* | 0.33 |  | 17.03 | 0.65 | 1-719 | X-ray | 1.62 | hetero-1-1-mer | 5 x SF4, 2 x MGD, 1 x MO, 1 x PG5 | HHblits | 0.28 |
| ``` target    IMDGKNLVENKLTDSHWFI--ECMERGAKIVVIAPEYGPPSTKADYWIPIRPQTDAALWLGITRLMIEKKWYDETFVKGF 6cz7.1    LSFGADPIASNRQVSFYSQTWGDSLDHAKVVVVDPRLSASAAKAHKWIPIEPGQDSVLALAIAHVALVEGVWHKPFVGDF  target    TDFPLLVRTDTLQRLRAHEVFPQYKTSLSADGPSMKIQGLSAEQHAKLGDFVVWDGKTNAPAAITRDDVGATITKKGIDP 6cz7.1    IEGKNLFKAG--KTVS-------------------------------VESF-----------------------------  target    VLAGSFKVKLVDGKEVEVATLWTLYQDHLKDYDLDTVVEITQAPKEMIEQLAQDIATMKPVA-IHQGEGINHWFHATEMN 6cz7.1    ------KE-------THTYGLVEWWNQALKDYTPEWASKITGIDPKTIIAIAKDMGAAAPAVQVWTSRGAVMQARGTYTS  target    RAAYLPLMLTGNIGRPGAGCQTWAGNYKAALFQGSPWTGPGFKGWVAEDPFDINLNPKAHGKEIHAHAYTKDEEPAYWNH 6cz7.1    ISCHALNGLFGGIDSKGGLFPGNKTPL----LKEY----PEAKAYMDEIA-AKGVKKE----KIDQ--RGRLEFPALAKG  target    GDLALIVDTPKFGRKNFTGKTHMPTPTKALIFNNVNLINNAKWAYGMIKNVNPNVEMIVSMDIQMTASIEYADLALPANS 6cz7.1    KSGGGVIT-ANAANG---IRNQDPYEIKVMLAYFNNFNFSNPEGQRWD-EALSKVDFMAHITTNVSEFSWFADVLLPSSH  target    -WLEFEGLEITASCSNPFLQIWKGGIPPVFDSRDD-LDILAGIANALADVTGEKRFRDYFAFAAA-----------DKRG 6cz7.1    HMFEKWGVLDSIGNGVAQISIQQPSIKRLWDTRIDESEIPYMLAKKLADKG----FDAPWRYINEQIVDPETGKPAADEA  target    IYIQRLLDTCT-----------TTAGYKLADIMAGKYGPPGGCLLNFRTYPRIPFYEQVHDSEPFHTDTGRMHAYADVPE 6cz7.1    EFAKLMVRYLTAPLWKEDASKYGDKLSSWDEFVQKGVWN-------SSPY------KLEARWGKFKTETTKFEFYSKTLE  target    AIEYGENFIVHREG----PEATPYLPNVIVSSNPHIRPEDYGIAADAEHWDDRTIRNIKMPWSKVKETKNFLWEKGFQFY 6cz7.1    KA-----LQSHADKHKVSIDEVMKACDY----QAR------GHLAFIPHYEE------------PYR---FGDESEFPLL  target    CLTPKTRHRVHSGWSNVDWHMLMDSNFGDPYRLDKRAPCVGEHQLHINPQAARDLNINDGDYVYVDANPADRPYLGAKPD 6cz7.1    LVDQKSRLNKEGRTANSPWYYEFKDV-----DPGDV---ANEDVAKFNPIDGKKFGLKDGDEIRITSPV-----------  target    DPFYRVSRCMLRVTYNRAYPYNIVMMKHAPFIATEKSVKAHETRPDGRALSANTGYQANLRYGSQQSVTRNWHMPMHQTD 6cz7.1    ------GMLTCKAKLWEGVRPGTVAKCFGQ--------------------------------------------------  target    TLFHKSKVFMGFIFGGEADNHAVNTVPKETLVRVTKAEDGGMGGKGIWQPATTGFSPDNESDFMKKYLAGELTKVKT 6cz7.1    ----------------------------------------------------------------------------- ``` | | | | | | | | | | | | | | | | | | | | | | | | | | | | | | | | | | | | | | | | | | | | | | | | | |
|  | 1ogy.1.A | PERIPLASMIC NITRATE REDUCTASE  *Crystal structure of the heterodimeric nitrate reductase from Rhodobacter sphaeroides* | 0.29 |  | 19.93 | 0.64 | 1-719 | X-ray | 3.20 | hetero-1-1-mer | 1 x SF4, 1 x MO, 2 x MGD, 2 x HEC | HHblits | 0.28 |
| ``` target    IMDGKNLVENKLTDSHWFIECM--ERGAKIVVIAPEYGPPSTKADYWIPIRPQTDAALWLGITRLMIEKKWYDETFVKGF 1ogy.1    VLWGSNMAEMHPILWSRLTDRRLSHEHVRVAVLSTFTHRSSDLSDTPIIFRPGTDRAILNYIAHHIISTGRVNRDFVDRH  target    TDFPLLVRTDTLQRLRAHEVFPQYKTSLSADGPSMKIQGLSAEQHAKLGDFVVWDGKTNAPAAITRDDVGATITKKGIDP 1ogy.1    TNFALGATDIG-YGLRPEH-------------------------------QLQLAAK-------------------G---  target    VLAGSFKVKLVDGKEVEVATLWTLYQDHLKDYDLDTVVEITQAPKEMIEQLAQDIATMK-PVAIHQGEGINHWFHATEMN 1ogy.1    ----------AADAGAMTPTDFETFAALVSEYTLEKAAEISGVEPALLEELAELYADPDRKWMSLWTMGFNQHVRGVWAN  target    RAAYLPLMLTGNIGRPGAGCQTWAGNYKAALFQGSPWTGPGFKGWVAEDPFDINL-NPKAHGKEIHAHAYTKDEEPAYWN 1ogy.1    HMVYNLHLLTGKISEPGNSPFSLTGQPFAC---GTAREVGTFAHRL---PADMVVTNPEH-RA----------HAEEIWK  target    H--GDLALIVDTPKFGRKNFTGKTHMPTPTKALIFNNVNLINNAKWAY-GMIKNVNPNVEMIVSMDIQMTASIEYADLAL 1ogy.1    LPAGLLPDWVGA--HAVEQD--RKLHDGEINFYWVQVNNNMQAAPNIDQETYPGYRNPENFIVVSDAYPTVTGRAADLVL  target    PANSWLEFEGLEITASCSNPFLQIWKGGIPPVFDSRDDLDILAGIANALADVTGEKRFRDYFAFAAADKRGI-YIQRLLD 1ogy.1    PAAMWVEKEGAYG---NAERRTHFWHQLVEAPGEARSDLWQLMEFSKRFTTDEVW--PEEILSAAPA-YRGKTLFEVLFA  target    TCTTTAGY------------------------KLADIMAGKYG----PP--------GGCLLNFRTYPRIPFYEQVHDSE 1ogy.1    N-GSVDRFPASDVNPDHANHEAALFGFYPQKGLFEEYAAFGRGHGHDLAPFDTYHEVRGLHWPVVEGE-ETRWRYREGFD  target    PFHTDTGRMHAYADVPEAIEYGENFIVHREGPEATPYLPNVIVSSNPHIRPEDYGIAADAEHWDDRTIRNIKMPWSKVKE 1ogy.1    PYVKPGEGLRFYGKPDGRAVI------L-----GVPYEPP--------------------------------------AE  target    TKNFLWEKGFQFYCLTPKTRHRVHSGWSNVDWHMLMDSNFGDPYRLDKRAPCVGEHQLHINPQAARDLNINDGDYVYVDA 1ogy.1    ----SPDEEFGFWLVTGRVLEHWHSGSMTLRWPE-----------LYKAF---PGAVCFMHPEDARSRGLNRGSEVRVIS  target    NPADRPYLGAKPDDPFYRVSRCMLRVTY--NRAYPYNIVMMKHAPFIATEKSVKAHETRPDGRALSANTGYQANLRYGSQ 1ogy.1    RR-----------------GEIRTRLETRGRNRMPRGVVFVPWFD-----------------------------------  target    QSVTRNWHMPMHQTDTLFHKSKVFMGFIFGGEADNHAVNTVPKETLVRVTKAEDGGMGGKGIWQPATTGFSPDNESDFMK 1ogy.1    --------------------------------------------------------------------------------  target    KYLAGELTKVKT 1ogy.1    ------------ ``` | | | | | | | | | | | | | | | | | | | | | | | | | | | | | | | | | | | | | | | | | | | | | | | | | |
|  | 4v4c.1.A | Pyrogallol hydroxytransferase large subunit  *Crystal Structure of Pyrogallol-Phloroglucinol Transhydroxylase from Pelobacter acidigallici* | 0.32 |  | 15.53 | 0.64 | 1-719 | X-ray | 2.35 | hetero-oligomer | 2 x CA, 2 x MGD, 1 x 4MO, 3 x SF4 | HHblits | 0.27 |
| ``` target    IMDGKNLVENKLTDSHW-----FIECMERGAKIVVIAPEYGPPST-KADYWIPIRPQTDAALWLGITRLMIEKKWYDETF 4v4c.1    VFWSSDPETNSGIYAGFESNIRRQWLKDLGVDFVFIDPHMNHTARLVADKWFSPKIGTDHALSFAIAYTWLKEDSYDKEY  target    VKGFTDFPLLVRTDTLQRLRAHEVFPQYKTSLSADGPSMKIQGLSAEQHAKLGDFVVWDGKTNAPAAITRDDVGATITKK 4v4c.1    VAANAHGF------------------------------------------------------------------------  target    GIDPVLAGSFKVKLVDGKEVEVATLWTLYQDHL------KDYDLDTVVEITQAPKEMIEQLAQDIATMKPVAIHQGE--- 4v4c.1    --------------------------EEWADYVLGKTDGTPKTCEWAEEESGVPACEIRALARQWAKKNTYLAAGGLGGW  target    -GINHWFHATEMNRAAYLPLMLTGNIGRPGAGCQTWAGNYKAALFQGSPWTGPGFKG--W---VAE--DPFDINLNPKAH 4v4c.1    GGACRASHGIEWARGMIALATMQG-MGKPGSNMWSTTQGVPLD----YEFYFPGYAEGGISGDCENSAAGFKFAWRMFDG  target    GKEIHA-HAYTKDEEPAYWNHGDLALIVDT-------PKFG----RKNFTGKTH---MPTPTKALIFNNVNLINNAKWAY 4v4c.1    KTTFPSPSNLNTS-AGQHIPRLKIPECIMGGKFQWSGKGFAGGDISHQLHQYEYPAPGYSKIKMFWKYGGPHLGTMTATN  target    GMIKNVN--PNVEMIVSMDIQMTASIEYADLALPANSWLEFEGLEITAS-----------CSNPFLQIWKGGIPPVFDSR 4v4c.1    RYA-KMYTHDSLEFVVSQSIWFEGEVPFADIILPACTNFERWDISEFANCSGYIPDNYQLCNHRVISLQAKCIEPVGESM  target    DDLDILAGIANALADVTGEKRFRDYFAFAAADKRGIYIQRLLDTCTTTAGYKLADIMAGKYGP-PG-------GCLLNFR 4v4c.1    SDYEIYRLFAKKLNIEE-------MFSEG--KDELAWCEQYFNATDMPKYMTWDEFFKKGYFVVPDNPNRKKTVALRWFA  target    -----TYPRIPF-YEQVHDSEPFHTDTGRMHAYADVPEAIE-YGENFIVHREGPEATPYLPNVIVSSNPHIRPEDYGIAA 4v4c.1    EGREKDTPDWGPRLNNQVCRKGLQTTTGKVEFIATSLKNFEEQG-----YID--EHRPSMHTYV----------------  target    DAEHWDDRTIRNIKMPWSKVKETKNFLWEKGFQFYCLTPKTRHRVHSGWSNV-DWHMLMDSNFGDPYRLDKRAPCVGEHQ 4v4c.1    --PAWESQ------------KH--S-PLAVKYPLGMLSPHPRFSMHTMGDGKNSYMNYIKD----HRVEVDG---YKYWI  target    LHINPQAARDLNINDGDYVYVDANPADRPYLGAKPDDPFYRVSRCMLRVTYNRAYPYNIVMMKHAPFIATEKSVKAHETR 4v4c.1    MRVNSIDAEARGIKNGDLIRAYND-----------------RGSVILAAQVTECLQPGTVHSYESC--------------  target    PDGRALSANTGYQANLRYGSQQSVTRNWHMPMHQTDTLFHKSKVFMGFIFGGEADNHAVNTVPKETLVRVTKAEDGGMGG 4v4c.1    --------------------------------------------------------------------------------  target    KGIWQPATTGFSPDNESDFMKKYLAGELTKVKT 4v4c.1    --------------------------------- ``` | | | | | | | | | | | | | | | | | | | | | | | | | | | | | | | | | | | | | | | | | | | | | | | | | |
|  | 1e5v.2.A | Dimethyl sulfoxide/trimethylamine N-oxide reductase  *OXIDIZED DMSO REDUCTASE EXPOSED TO HEPES BUFFER* | 0.33 | 0.00 | 19.42 | 0.61 | 1-720 | X-ray | 2.40 | monomer | 2 x PGD, 1 x 2MO | HHblits | 0.29 |
| ``` target    IMDGKNLVENKLTD--------SHWFIECMERGAKIVVIAPEYGPPSTK-ADYWIPIRPQTDAALWLGITRLMIEKKWYD 1e5v.2    VFWAADPIKTSQIGWVIPEHGAYPGLEALKAKGTKVIVIDPVRTKTVEFFGAEHITPKPQTDVAIMLGMAHTLVAEDLYD  target    ETFVKGFTDFPLLVRTDTLQRLRAHEVFPQYKTSLSADGPSMKIQGLSAEQHAKLGDFVVWDGKTNAPAAITRDDVGATI 1e5v.2    KDFIANYTSGF-----D------------KFL------------------------------------------------  target    TKKGIDPVLAGSFKVKLVDGKEVEVATLWTLYQDHLKDYDLDTVVEITQAPKEMIEQLAQDIATMKPVAIHQGEGINHWF 1e5v.2    ------PYLDGET---------------------DSTPKTAEWAEGISGVPAETIKELARLFES-KRTMLAAGWSMQRMH  target    HATEMNRAAYLPLMLTGNIGRPGAGCQTWAGNYKAALFQGSPWTGPGFKGWVAEDPFDINLNPKAHGKEIHAHAYTKDEE 1e5v.2    HGEQAHWMLVTLASMLGQIGLPGGGFGLSYHYSGGG---TPSTSGPALAGITDGGA--ATKGPEW----LAAS--GASVI  target    PAYWNHGDLALIVDTPKFGRKNFTGKTHMPTPTKALIFNNVNLINNAKWAYGMIKNVNPNVEMIVSMDIQMTASIEYADL 1e5v.2    PV----ARVVDMLENPGAE-FDFNGTRSKFPDVKMAYWVGGNPFVHHQDRNRM-VKAWEKLETFVVHDFQWTPTARHADI  target    ALPANSWLEFEGLEITASCSNPFLQIWKGGIPPVFDSRDDLDILAGIANALADVTGEKRFRDYFAFAAADKRGIYIQRLL 1e5v.2    VLPATTSYERNDIETIGDYSNTGILAMKKIVEPLYEARSDYDIFAAVAERLGKGAE---FT---EGK---DEMGWIKSFY  target    DTCT---TTAGY---KLADIMAGKYGPPGGCLLNFRTYPRIPF--YEQVHDSEPFHTDTGRMHAYADVPEAIEYGENFIV 1e5v.2    DDAAKQGKAAGVQMPAFDAFWAEGIVEFPV----TDGADFVRYASFREDPLLNPLGTPTGLIEIYSKNIEKMGYDD----  target    HREGPEATPYLPNVIVSSNPHIRPEDYGIAADAEHWDDRTIRNIKMPWSKVKETKNFLWEKGFQFYCLTPKTRHRVHSGW 1e5v.2    -------CPAHPTWME---P---------------LE----------------RL-DGPGAKYPLHIAASHPFNRLHSQL  target    SNVDWHMLMDSNFGDPYRLDKRAPCVGEHQLHINPQAARDLNINDGDYVYVDANPADRPYLGAKPDDPFYRVSRCMLRVT 1e5v.2    NG-TVLREGY----------AVQ---GHEPCLMHPDDAAARGIADGDVVRVHND-----------------RGQILTGVK  target    YNRAYPYNIVMMKHAPFIATEKSVKAHETRPDGRALSANTGYQANLRYGSQQSVTRNWHMPMHQTDTLFHKSKVFMGFIF 1e5v.2    VTDAVMKGVIQIYEGGW---------------------------------------------------------------  target    GGEADNHAVNTVPKETLVRVTKAEDGGMGGKGIWQPATTGFSPDNESDFMKKYLAGELTKVKT 1e5v.2    --------------------------------------------------------------- ``` | | | | | | | | | | | | | | | | | | | | | | | | | | | | | | | | | | | | | | | | | | | | | | | | | |
|  | 1e60.1.A | Dimethyl sulfoxide/trimethylamine N-oxide reductase  *OXIDIZED DMSO REDUCTASE EXPOSED TO HEPES - Structure II BUFFER* | 0.34 | 0.00 | 19.42 | 0.61 | 1-720 | X-ray | 2.00 | monomer | 2 x PGD, 1 x 2MO | HHblits | 0.29 |
| ``` target    IMDGKNLVENKLTDS--------HWFIECMERGAKIVVIAPEYGPPSTK-ADYWIPIRPQTDAALWLGITRLMIEKKWYD 1e60.1    VFWAADPIKTSQIGWVIPEHGAYPGLEALKAKGTKVIVIDPVRTKTVEFFGAEHITPKPQTDVAIMLGMAHTLVAEDLYD  target    ETFVKGFTDFPLLVRTDTLQRLRAHEVFPQYKTSLSADGPSMKIQGLSAEQHAKLGDFVVWDGKTNAPAAITRDDVGATI 1e60.1    KDFIANYTSGF-----D------------KFL------------------------------------------------  target    TKKGIDPVLAGSFKVKLVDGKEVEVATLWTLYQDHLKDYDLDTVVEITQAPKEMIEQLAQDIATMKPVAIHQGEGINHWF 1e60.1    ------PYLDGE---------------------TDSTPKTAEWAEGISGVPAETIKELARLFES-KRTMLAAGWSMQRMH  target    HATEMNRAAYLPLMLTGNIGRPGAGCQTWAGNYKAALFQGSPWTGPGFKGWVAEDPFDINLNPKAHGKEIHAHAYTKDEE 1e60.1    HGEQAHWMLVTLASMLGQIGLPGGGFGLSYHYSGGG---TPSTSGPALAGITD--GGAATKGPEW----LAAS--GASVI  target    PAYWNHGDLALIVDTPKFGRKNFTGKTHMPTPTKALIFNNVNLINNAKWAYGMIKNVNPNVEMIVSMDIQMTASIEYADL 1e60.1    PVA----RVVDMLENPGAE-FDFNGTRSKFPDVKMAYWVGGNPFVHHQDRNRM-VKAWEKLETFVVHDFQWTPTARHADI  target    ALPANSWLEFEGLEITASCSNPFLQIWKGGIPPVFDSRDDLDILAGIANALADVTGEKRFRDYFAFAAADKRGIYIQRLL 1e60.1    VLPATTSYERNDIETIGDYSNTGILAMKKIVEPLYEARSDYDIFAAVAERLGKGAE---FT---E-G--KDEMGWIKSFY  target    DTCT---TTAGY---KLADIMAGKYGPPGGCLLNFRTYPRIPFYE--QVHDSEPFHTDTGRMHAYADVPEAIEYGENFIV 1e60.1    DDAAKQGKAAGVEMPAFDAFWAEGIVEFPV----TDGADFVRYASFREDPLLNPLGTPTGLIEIYSKNIEKMGYDD----  target    HREGPEATPYLPNVIVSSNPHIRPEDYGIAADAEHWDDRTIRNIKMPWSKVKETKNFLWEKGFQFYCLTPKTRHRVHSGW 1e60.1    -------CPAHPTWME---P---------------LE----------------RL-DGPGAKYPLHIAASHPFNRLHSQL  target    SNVDWHMLMDSNFGDPYRLDKRAPCVGEHQLHINPQAARDLNINDGDYVYVDANPADRPYLGAKPDDPFYRVSRCMLRVT 1e60.1    NG-TVLREGY----------AVQ---GHEPCLMHPDDAAARGIADGDVVRVHNDR-----------------GQILTGVK  target    YNRAYPYNIVMMKHAPFIATEKSVKAHETRPDGRALSANTGYQANLRYGSQQSVTRNWHMPMHQTDTLFHKSKVFMGFIF 1e60.1    VTDAVMKGVIQIYEGGW---------------------------------------------------------------  target    GGEADNHAVNTVPKETLVRVTKAEDGGMGGKGIWQPATTGFSPDNESDFMKKYLAGELTKVKT 1e60.1    --------------------------------------------------------------- ``` | | | | | | | | | | | | | | | | | | | | | | | | | | | | | | | | | | | | | | | | | | | | | | | | | |
|  | 1e18.1.A | DMSO REDUCTASE.  *TUNGSTEN-SUSBSTITUTED DMSO REDUCTASE FROM RHODOBACTER CAPSULATUS* | 0.33 | 0.00 | 19.61 | 0.61 | 1-720 | X-ray | 2.00 | monomer | 2 x PGD, 1 x 6WO | HHblits | 0.29 |
| ``` target    IMDGKNLVENKLTDS--------HWFIECMERGAKIVVIAPEYGPPSTK-ADYWIPIRPQTDAALWLGITRLMIEKKWYD 1e18.1    VFWAADPIKTSQIGWVIPEHGAYPGLEALKAKGTKVIVIDPVRTKTVEFFGAEHITPKPQTDVAIMLGMAHTLVAEDLYD  target    ETFVKGFTDFPLLVRTDTLQRLRAHEVFPQYKTSLSADGPSMKIQGLSAEQHAKLGDFVVWDGKTNAPAAITRDDVGATI 1e18.1    KDFIANYTSGF---------------------------------------------------------------------  target    TKKGIDPVLAGSFKVKLVDGKEVEVATLWTLYQDH------LKDYDLDTVVEITQAPKEMIEQLAQDIATMKPVAIHQGE 1e18.1    -----------------------------DKFLPYLDGETDSTPKTAEWAEGISGVPAETIKELARLFES-KRTMLAAGW  target    GINHWFHATEMNRAAYLPLMLTGNIGRPGAGCQTWAGNYKAALFQGSPWTGPGFKGWVAEDPFDINLNPKAHGKEIHAHA 1e18.1    SMQRMHHGEQAHWMLVTLASMLGQIGLPGGGFGLSYHYSGGG---TPSTSGPALAGITD--GGAATKGPEW----LAAS-  target    YTKDEEPAYWNHGDLALIVDTPKFGRKNFTGKTHMPTPTKALIFNNVNLINNAKWAYGMIKNVNPNVEMIVSMDIQMTAS 1e18.1    -GASVIPV----ARVVDMLENPGAE-FDFNGTRSKFPDVKMAYWVGGNPFVHHQDRNRM-VKAWEKLETFVVHDFQWTPT  target    IEYADLALPANSWLEFEGLEITASCSNPFLQIWKGGIPPVFDSRDDLDILAGIANALADVTGEKRFRDYFAFAAADKRGI 1e18.1    ARHADIVLPATTSYERNDIETIGDYSNTGILAMKKIVEPLYEARSDYDIFAAVAERLGKGKE------FTE-G--KDEMG  target    YIQRLLDTCT---TTAGY---KLADIMAGKYGP-PGGCLLNFRTYPRIPFYEQVHDSEPFHTDTGRMHAYADVPEAIEYG 1e18.1    WIKSFYDDAAKQGKAAGVEMPAFDAFWAEGIVEFPVTDGADFVRY--ASFRE-DPLLNPLGTPTGLIEIYSKNIEKMGYD  target    ENFIVHREGPEATPYLPNVIVSSNPHIRPEDYGIAADAEHWDDRTIRNIKMPWSKVKETKNFLWEKGFQFYCLTPKTRHR 1e18.1    D-----------CPAHPTWME---P---------------LE----------------RL-DGPGAKYPLHIAASHPFNR  target    VHSGWSNVDWHMLMDSNFGDPYRLDKRAPCVGEHQLHINPQAARDLNINDGDYVYVDANPADRPYLGAKPDDPFYRVSRC 1e18.1    LHSQLNG-TVLREGY----------AV---QGHEPCLMHPDDAAARGIADGDVVRVHND-----------------RGQI  target    MLRVTYNRAYPYNIVMMKHAPFIATEKSVKAHETRPDGRALSANTGYQANLRYGSQQSVTRNWHMPMHQTDTLFHKSKVF 1e18.1    LTGVKVTDAVMKGVIQIYEGGW----------------------------------------------------------  target    MGFIFGGEADNHAVNTVPKETLVRVTKAEDGGMGGKGIWQPATTGFSPDNESDFMKKYLAGELTKVKT 1e18.1    -------------------------------------------------------------------- ``` | | | | | | | | | | | | | | | | | | | | | | | | | | | | | | | | | | | | | | | | | | | | | | | | | |
|  | 1tmo.1.A | TRIMETHYLAMINE N-OXIDE REDUCTASE  *TRIMETHYLAMINE N-OXIDE REDUCTASE FROM SHEWANELLA MASSILIA* | 0.33 |  | 18.41 | 0.61 | 1-720 | X-ray | 2.50 | monomer | 2 x 2MD, 1 x 2MO | HHblits | 0.29 |
| ``` target    IMDGKNLVENKLT--------DS---HWFIECME-RGAKIVVIAPEYGPPSTK-ADYWIPIRPQTDAALWLGITRLMIEK 1tmo.1    VLWSNDPYKNLQVGWNAETHESFAYLAQLKEKVKQGKIRVISIDPVVTKTQAYLGCEQLYVNPQTDVTLMLAIAHEMISK  target    KWYDETFVKGFTDFPLLVRTDTLQRLRAHEVFPQYKTSLSADGPSMKIQGLSAEQHAKLGDFVVWDGKTNAPAAITRDDV 1tmo.1    KLYDDKFIQGYSLG------------------------------------------------------------------  target    GATITKKGIDPVLAGSFKVKLVDGKEVEVATLWTLYQDHLK------DYDLDTVVEITQAPKEMIEQLAQDIATMKPVAI 1tmo.1    --------------------------------FEEFVPYVMGTKDGVAKTPEWAAPICGVEAHVIRDLAKTLVKGR-TQF  target    HQGEGINHWFHATEMNRAAYLPLMLTGNIGRPGAGCQTWAGNYKA-ALFQGSPWTGPGFKGWVAEDPFDINLNPKAHGKE 1tmo.1    MMGWCIQRQQHGEQPYWMAAVLATMIGQIGLPGGGISYGHHYSSIGVPSSGAA-----APGAFP-RNLDENQKPLFDSSD  target    IHAHAYTKDEEPAYWNHGDLALIVDTPKFGRKNFTGKTHMPTPTKALIFNNVNLINNAKWAYGMIKNVNPNVEMIVSMDI 1tmo.1    FKG---ASSTIP----VARWIDAILEPGKTID-ANGSKVVYPDIKMMIFSGNNPWNHHQDRNRMK-QAFHKLECVVTVDV  target    QMTASIEYADLALPANSWLEFEGLEITASCSNPFLQIWKGGIPPVFDSRDDLDILAGIANALADVTGEKRFRDYFAFAAA 1tmo.1    NWTATCRFSDIVLPACTTYERNDIDVYGAYANRGILAMQKMVEPLFDSLSDFEIFTRFAAVLGKEKE------YTRNM--  target    DKRGIYIQRLLDTCTT-----TAGYKLADIMAGKYGP-PGGCLLNFRTYPRIPFYEQVHDSEPFHTDTGRMHAYADVPEA 1tmo.1    -GEMEWLETLYNECKAANAGKFEMPDFATFWKQGYVHFGDGEV-----WTRHADFRNDPEINPLGTPSGLIEIFSRKIDQ  target    IEYGE--NFIVHREGPEATPYLPNVIVSSNPHIRPEDYGIAADAEHWDDRTIRNIKMPWSKVKETKNFLWEKGFQFYCLT 1tmo.1    FGYDDCKGHPTWMEKTER------------S---------------H-------------------GGPGSDKHPIWLQS  target    PKTRHRVHSGWSNVDWHMLMDSNFGDPYRLDKRAPCVGEHQLHINPQAARDLNINDGDYVYVDANPADRPYLGAKPDDPF 1tmo.1    CHPDKRLHSQMCESREYRETY----------AV---NGREPVYISPVDAKARGIKDGDIVRVFNDR--------------  target    YRVSRCMLRVTYNRAYPYNIVMMKHAPFIATEKSVKAHETRPDGRALSANTGYQANLRYGSQQSVTRNWHMPMHQTDTLF 1tmo.1    ---GQLLAGAVVSDNFPKGIVRIHEGAW----------------------------------------------------  target    HKSKVFMGFIFGGEADNHAVNTVPKETLVRVTKAEDGGMGGKGIWQPATTGFSPDNESDFMKKYLAGELTKVKT 1tmo.1    -------------------------------------------------------------------------- ``` | | | | | | | | | | | | | | | | | | | | | | | | | | | | | | | | | | | | | | | | | | | | | | | | | |
|  | 4dmr.1.A | DMSO REDUCTASE  *REDUCED DMSO REDUCTASE FROM RHODOBACTER CAPSULATUS WITH BOUND DMSO SUBSTRATE* | 0.33 | 0.00 | 18.87 | 0.61 | 1-720 | X-ray | 1.90 | monomer | 2 x PGD, 1 x 4MO, 1 x O | HHblits | 0.29 |
| ``` target    IMDGKNLVENKLTDS--------HWFIECMERGAKIVVIAPEYGPPSTK-ADYWIPIRPQTDAALWLGITRLMIEKKWYD 4dmr.1    VFWAADPIKTSQIGWVIPEHGAYPGLEALKAKGTKVIVIDPVRTKTVEFFGAEHITPKPQTDVAIMLGMAHTLVAEDLYD  target    ETFVKGFTDFPLLVRTDTLQRLRAHEVFPQYKTSLSADGPSMKIQGLSAEQHAKLGDFVVWDGKTNAPAAITRDDVGATI 4dmr.1    KDFIANYTSGF---------------------------------------------------------------------  target    TKKGIDPVLAGSFKVKLVDGKEVEVATLWTLYQDH------LKDYDLDTVVEITQAPKEMIEQLAQDIATMKPVAIHQGE 4dmr.1    -----------------------------DKFLPYLDGETDSTPKTAEWAEGISGVPAETIKELARLFES-KRTMLAAGW  target    GINHWFHATEMNRAAYLPLMLTGNIGRPGAGCQTWAGNYKAALFQGSPWTGPGFKGWVAEDPFDINLNPKAHGKEIHAHA 4dmr.1    SMQRMHHGEQAHWMLVTLASMLGQIGLPGGGFGLSYHYSGGG---TPSTSGPALAGITD--GGAATKGPEW----LAAS-  target    YTKDEEPAYWNHGDLALIVDTPKFGRKNFTGKTHMPTPTKALIFNNVNLINNAKWAYGMIKNVNPNVEMIVSMDIQMTAS 4dmr.1    -GASVIPV----ARVVDMLENPGAE-FDFNGTRSKFPDVKMAYWVGGNPFVHHQDRNRM-VKAWEKLETFVVHDFQWTPT  target    IEYADLALPANSWLEFEGLEITASCSNPFLQIWKGGIPPVFDSRDDLDILAGIANALADVTGEKRFRDYFAFAAADKRGI 4dmr.1    ARHADIVLPATTSYERNDIETIGDYSNTGILAMKKIVEPLYEARSDYDIFAAVAERLGKGAE---FT---E-G--KDEMG  target    YIQRLLDTCT---TTAGY---KLADIMAGKYGPPGGCLLNFRTYPRIPF--YEQVHDSEPFHTDTGRMHAYADVPEAIEY 4dmr.1    WIKSFYDDAAKQGKAAGVQMPAFDAFWAEGIVEFP---V-TDGADFVRYASFREDPLLNPLGTPTGLIEIYSKNIEKMGY  target    GENFIVHREGPEATPYLPNVIVSSNPHIRPEDYGIAADAEHWDDRTIRNIKMPWSKVKETKNFLWEKGFQFYCLTPKTRH 4dmr.1    DD-----------CPAHPTWME---P---------------LER----------------L-DGPGAKYPLHIAASHPFN  target    RVHSGWSNVDWHMLMDSNFGDPYRLDKRAPCVGEHQLHINPQAARDLNINDGDYVYVDANPADRPYLGAKPDDPFYRVSR 4dmr.1    RLHSQLNG-TVLREGY----------AVQ---GHEPCLMHPDDAAARGIADGDVVRVHND-----------------RGQ  target    CMLRVTYNRAYPYNIVMMKHAPFIATEKSVKAHETRPDGRALSANTGYQANLRYGSQQSVTRNWHMPMHQTDTLFHKSKV 4dmr.1    ILTGVKVTDAVMKGVIQIYEGGW---------------------------------------------------------  target    FMGFIFGGEADNHAVNTVPKETLVRVTKAEDGGMGGKGIWQPATTGFSPDNESDFMKKYLAGELTKVKT 4dmr.1    --------------------------------------------------------------------- ``` | | | | | | | | | | | | | | | | | | | | | | | | | | | | | | | | | | | | | | | | | | | | | | | | | |
|  | 1eu1.1.A | DIMETHYL SULFOXIDE REDUCTASE  *THE CRYSTAL STRUCTURE OF RHODOBACTER SPHAEROIDES DIMETHYLSULFOXIDE REDUCTASE REVEALS TWO DISTINCT MOLYBDENUM COORDINATION ENVIRONMENTS.* | 0.33 |  | 19.10 | 0.61 | 1-720 | X-ray | 1.30 | monomer | 3 x GLC, 1 x CD, 2 x MGD, 1 x 6MO, 2 x O | HHblits | 0.29 |
| ``` target    IMDGKNLVENKLTD--------SHWFIECMERGAKIVVIAPEYGPPSTKAD-YWIPIRPQTDAALWLGITRLMIEKKWYD 1eu1.1    VFWAADPMKTNEIGWVIPDHGAYAGMKALKEKGTRVICINPVRTETADYFGADVVSPRPQTDVALMLGMAHTLYSEDLHD  target    ETFVKGFTDFPLLVRTDTLQRLRAHEVFPQYKTSLSADGPSMKIQGLSAEQHAKLGDFVVWDGKTNAPAAITRDDVGATI 1eu1.1    KDFLENCTTGF---------------------------------------------------------------------  target    TKKGIDPVLAGSFKVKLVDGKEVEVATLWTLYQDHL------KDYDLDTVVEITQAPKEMIEQLAQDIATMKPVAIHQGE 1eu1.1    -----------------------------DLFAAYLTGESDGTPKTAEWAAEICGLPAEQIRELARSFVAGR-TMLAAGW  target    GINHWFHATEMNRAAYLPLMLTGNIGRPGAGCQTWAGNYKAALFQGSPWTGPGFKGWVAEDPFDINLNPKAHGKEIHAHA 1eu1.1    SIQRMHHGEQAHWMLVTLASMIGQIGLPGGGFGLSYHYSNGGS--P-TSDGPALGGISD--G-GKAVEGAAW---LSES-  target    YTKDEEPAYWNHGDLALIVDTPKFGRKNFTGKTHMPTPTKALIFNNVNLINNAKWAYGMIKNVNPNVEMIVSMDIQMTAS 1eu1.1    -GATSIPC----ARVVDMLLNPGGE-FQFNGATATYPDVKLAYWAGGNPFAHHQDRNRML-KAWEKLETFIVQDFQWTAT  target    IEYADLALPANSWLEFEGLEITASCSNPFLQIWKGGIPPVFDSRDDLDILAGIANALADVTGEKRFRDYFAFAAADKRGI 1eu1.1    ARHADIVLPATTSYERNDIESVGDYSNRAILAMKKVVDPLYEARSDYDIFAALAERLGKGAE---FT---EGR---DEMG  target    YIQRLLDTC------TTTAGYKLADIMAGKYGPPGGCLLNF-RTYPRIPFYEQV--HDSEPFHTDTGRMHAYADVPEAIE 1eu1.1    WISSFYEAAVKQAEFKNVAMPSFEDFWSEGIVE-----FPITEGANFVRYADFREDPLFNPLGTPSGLIEIYSKNIEKMG  target    YGENFIVHREGPEATPYLPNVIVSSNPHIRPEDYGIAADAEHWDDRTIRNIKMPWSKVKETKNFLWEKGFQFYCLTPKTR 1eu1.1    YDD-C----------PAHPTWME---P-------------------------------AERL-GGAGAKYPLHVVASHPK  target    HRVHSGWSNVDWHMLMDSNFGDPYRLDKRAPCVGEHQLHINPQAARDLNINDGDYVYVDANPADRPYLGAKPDDPFYRVS 1eu1.1    SRLHSQLNGT-SLRDL----------YAVA---GHEPCLINPADAAARGIADGDVLRVFNDR-----------------G  target    RCMLRVTYNRAYPYNIVMMKHAPFIATEKSVKAHETRPDGRALSANTGYQANLRYGSQQSVTRNWHMPMHQTDTLFHKSK 1eu1.1    QILVGAKVSDAVMPGAIQIYEGGW--------------------------------------------------------  target    VFMGFIFGGEADNHAVNTVPKETLVRVTKAEDGGMGGKGIWQPATTGFSPDNESDFMKKYLAGELTKVKT 1eu1.1    ---------------------------------------------------------------------- ``` | | | | | | | | | | | | | | | | | | | | | | | | | | | | | | | | | | | | | | | | | | | | | | | | | |
|  | 1dms.1.A | DMSO REDUCTASE  *STRUCTURE OF DMSO REDUCTASE* | 0.32 | 0.00 | 18.13 | 0.61 | 1-720 | X-ray | 1.88 | monomer | 2 x PGD, 1 x 2MO | HHblits | 0.29 |
| ``` target    IMDGKNLVENKLTDS--------HWFIECMERGAKIVVIAPEYGPPSTK-ADYWIPIRPQTDAALWLGITRLMIEKKWYD 1dms.1    VFWAADPIKTSQIGWVIPEHGAYPGLEALKAKGTKVIVIDPVRTKTVEFFGADHVTPKPQTDVAIMLGMAHTLVAEDLYD  target    ETFVKGFTDFPLLVRTDTLQRLRAHEVFPQYKTSLSADGPSMKIQGLSAEQHAKLGDFVVWDGKTNAPAAITRDDVGATI 1dms.1    KDFIANYTSGF---------------------------------------------------------------------  target    TKKGIDPVLAGSFKVKLVDGKEVEVATLWTLYQDH------LKDYDLDTVVEITQAPKEMIEQLAQDIATMKPVAIHQGE 1dms.1    -----------------------------DKFLPYLMGETDSTPKTAEWASDISGVPAETIKELARLFKS-KRTMLAAGW  target    GINHWFHATEMNRAAYLPLMLTGNIGRPGAGCQTWAGNYKAALFQGSPWTGPGFKGWVAEDPFDINLNPKAHGKEIHAHA 1dms.1    SMQRMHHGEQAHWMLVTLASMLGQIGLPGGGFGLSYHYSGGGT---PSSSGPALSGITD--GGAATKGPEW----LAAS-  target    YTKDEEPAYWNHGDLALIVDTPKFGRKNFTGKTHMPTPTKALIFNNVNLINNAKWAYGMIKNVNPNVEMIVSMDIQMTAS 1dms.1    -GASVIPV----ARVVDMLENPGAE-FDFNGTRSKFPDVKMAYWVGGNPFVHHQDRNRM-VKAWEKLETFIVHDFQWTPT  target    IEYADLALPANSWLEFEGLEITASCSNPFLQIWKGGIPPVFDSRDDLDILAGIANALADVTGEKRFRDYFAFAAADKRGI 1dms.1    ARHADIVLPATTSYERNDIETIGDYSNTGILAMKKIVEPLYEARSDYDIFAAVAERLGKGKE------FTEGK---DEMG  target    YIQRLLDTCT---TTAGY---KLADIMAGKYGPPGGCLLNF-RTYPRIPFYEQ--VHDSEPFHTDTGRMHAYADVPEAIE 1dms.1    WIKSFYDDAAKQGKAGGVEMPAFDAFWAEGIVE-----FPVTDGADFVRYASFREDPLLNPLGTPTGLIEIYSKNIEKMG  target    YGENFIVHREGPEATPYLPNVIVSSNPHIRPEDYGIAADAEHWDDRTIRNIKMPWSKVKETKNFLWEKGFQFYCLTPKTR 1dms.1    YDD-----------CPAHPTWME---P---------------LE----------------RL-DGPGAKYPLHIAASHPF  target    HRVHSGWSNVDWHMLMDSNFGDPYRLDKRAPCVGEHQLHINPQAARDLNINDGDYVYVDANPADRPYLGAKPDDPFYRVS 1dms.1    NRLHSQLNG-TVLREGY----------AV---QGHEPCLMHPDDAAARGIADGDVVRVHND-----------------RG  target    RCMLRVTYNRAYPYNIVMMKHAPFIATEKSVKAHETRPDGRALSANTGYQANLRYGSQQSVTRNWHMPMHQTDTLFHKSK 1dms.1    QILTGVKVTDAVMKGVIQIYEGGW--------------------------------------------------------  target    VFMGFIFGGEADNHAVNTVPKETLVRVTKAEDGGMGGKGIWQPATTGFSPDNESDFMKKYLAGELTKVKT 1dms.1    ---------------------------------------------------------------------- ``` | | | | | | | | | | | | | | | | | | | | | | | | | | | | | | | | | | | | | | | | | | | | | | | | | |
|  | 2e7z.1.A | Acetylene hydratase Ahy  *Acetylene Hydratase from Pelobacter acetylenicus* | 0.32 |  | 19.12 | 0.59 | 1-720 | X-ray | 1.26 | monomer | 1 x SF4, 2 x MGD, 1 x W | HHblits | 0.30 |
| ``` target    IMDGKNLVENKLTD-SHWFIECMERGAKIVVIAPEYGPPSTKADYWIPIRPQTDAALWLGITRLMIEKKWYDETFVKGFT 2e7z.1    LFIGKNLSNHNWVSQFNDLKAALKRGCKLIVLDPRRTKVAEMADIWLPLRYGTDAALFLGMINVIINEQLYDKEFVENWC  target    DFPLLVRTDTLQRLRAHEVFPQYKTSLSADGPSMKIQGLSAEQHAKLGDFVVWDGKTNAPAAITRDDVGATITKKGIDPV 2e7z.1    VG------------------------------------------------------------------------------  target    LAGSFKVKLVDGKEVEVATLWTLYQDHLKDYDLDTVVEITQAPKEMIEQLAQDIATMKPVAIHQGEGINHWFHATEMNRA 2e7z.1    --------------------FEELKERVQEYPLDKVAEITGCDAGEIRKAAVMFATESPASIPWAVSTDMQKNSCSAIRA  target    AYLPLMLTGNIGRPGAGCQTWAGNYKAALFQGSP-W-TGPGFKGW--VAEDPFDINLNPKAHGKEIHAHAYTKDEEPAYW 2e7z.1    QCILRAIVGSFVN-GAEILGAPHSD-LVPISKIQMHEALPEEKKKLQLGTETYPFLTYTGM--SALE------EPSERVY  target    NHG---DLALIVDTPKFGRKNFT-GKTHMPTPTKALIFNNVNLINNAKWAYGMIKNVNPNVEMIVSMDIQMTASIEYADL 2e7z.1    GVKYFHNMGAFMANPT---ALFTAMATEKPYPVKAFFALASNALMGYANQQNA-LKGLMNQDLVVCYDQFMTPTAQLADY  target    ALPANSWLEFEGLEITASCSNPFLQIWKGGIPPVFDSRDDLDILAGIANALADVTGEKRFRDYFAFAAADKRGIYIQRLL 2e7z.1    VLPGDHWLERPVVQPNW-EGIPFGNTSQQVVEPAGEAKDEYYFIRELAVRMGLEE-------HFPWKD---RLELINYRI  target    DTCTTTAGYKLADIMAGKYGPPGGCLLNFRTYPRIPFYEQVHDSEPFHTDTGRMHAYADVPEAIEYGENFIVHREGPEAT 2e7z.1    S----PTGMEWEEYQKQYTYM-----SKLPDY--F-----GPEGVGVATPSGKVELYSSVFEKLGYD-PLPYYHEPLQT-  target    PYLPNVIVSSNPHIRPEDYGIAADAEHWDDRTIRNIKMPWSKVKETKNFLWEKGFQFYCLTPKT-RHRVHSGWSNVDWHM 2e7z.1    E-----------IS---------------------------------DPELAKEYPLILFAGLREDSNFQSCYHQPGILR  target    LMDSNFGDPYRLDKRAPCVGEHQLHINPQAARDLNINDGDYVYVDANPADRPYLGAKPDDPFYRVSRCMLRVTYNRAYPY 2e7z.1    DA-------------E---PDPVALLHPKTAQSLGLPSGEWIWVETTH-----------------GRLKLLLKHDGAQPE  target    NIVMMKHAPFIATEKSVKAHETRPDGRALSANTGYQANLRYGSQQSVTRNWHMPMHQTDTLFHKSKVFMGFIFGGEADNH 2e7z.1    GTIRIPHGRW----------------------------------------------------------------------  target    AVNTVPKETLVRVTKAEDGGMGGKGIWQPATTGFSPDNESDFMKKYLAGELTKVKT 2e7z.1    -------------------------------------------------------- ``` | | | | | | | | | | | | | | | | | | | | | | | | | | | | | | | | | | | | | | | | | | | | | | | | | |
|  | 7qv7.1.L | Hydrogen dependent carbon dioxide reductase subunit FdhF  *Cryo-EM structure of Hydrogen-dependent CO2 reductase.* | 0.30 |  | 19.48 | 0.59 | 1-720 | EM | 0.00 | hetero-2-6-6-2-mer | 52 x SF4, 6 x 402 | HHblits | 0.29 |
| ``` target    IMDGKNLVENKLTDSHWFIECMERGAKIVVIAPEYGPPSTKADYWIPIRPQTDAALWLGITRLMIEKKWYDETFVKGFTD 7qv7.1    FIIGSNTAECHPLIAAHVIKAKERGAKLIVADPRMNAMVHKADIWLRVPSGYNIPLINGMIHIIIKEGLVKTDFVKNHAV  target    FPLLVRTDTLQRLRAHEVFPQYKTSLSADGPSMKIQGLSAEQHAKLGDFVVWDGKTNAPAAITRDDVGATITKKGIDPVL 7qv7.1    G-------------------------------------------------------------------------------  target    AGSFKVKLVDGKEVEVATLWTLYQDHLKDYDLDTVVEITQAPKEMIEQLAQDIATMKPVAIHQGEGINHWFHATEMNRAA 7qv7.1    -------------------FEEMAKAVEKYTPEYVEELTGIPKKDLIKAARFYGQAQAAAILYSMGVTQFSHGTGNVVSL  target    YLPLMLTGNIGRPGAGCQTWAGNYKAALFQGSPWTGPGFKGWVAEDPFDINLNPKAHGKEIHAHAYTKDEEPAYWNHGDL 7qv7.1    ANLAVITGNLGRPGAGICPLRGQNNV---QGACDVG-ALPNVL---PGYLDVTKEQNR----------ERFEKVWGVK-L  target    ALIVDTPKFG-RKNFTGKTHMPTPTKALIFNNVNLINNAKWAYGMIKNVNPNVEMIVSMDIQMTASIEYADLALPANSWL 7qv7.1    P---SNIGLRVTEVP--DAILNKRVRALYIFGENPIMSDPDSDHL-RHALEHLDLLIVQDIFLTETARLAHVVLPAACWA  target    EFEGLEITASCSNPFLQIWKGGIPPVFDSRDDLDILAGIANALADVTGEKRFRDYFAFAAADKRGIYIQRLLDTCT-TTA 7qv7.1    EKDGTF---TNTERRVQRVRKAVEAPGEAKPDWWIFSQIAERMGYTG--M------QYNNV---QEIWDEVRKIVPEKFG  target    GYKLADIMAGKYGPPGGCLLNFRTYPRIPFYEQVHDSEPFHTDTGRMHAYADVPEAIEYGENFIVHREGPEATPYLPNVI 7qv7.1    GISYARLEKEKGL-----AWPCP-TEDHTGTPILYLGGKFATPSGKAQMYPVIFYPN-----TCICDEGAEKQDF-N---  target    VSSNPHIRPEDYGIAADAEHWDDRTIRNIKMPWSKVKETKNFLWEKGFQFYCLTPKTRHRVHSGW--SNVDWHMLMDSNF 7qv7.1    ----HV--IV---------------------GS------IAELPDEEYPFTLTTGRRVYHYHTATMTRKSPVIDQ-----  target    GDPYRLDKRAPCVGEHQLHINPQAARDLNINDGDYVYVDANPADRPYLGAKPDDPFYRVSRCMLRVTYNRAYPYNIVMMK 7qv7.1    --------IA---PQELVEINPQDATRLGINDGDFLRVSTRR-----------------GYVATRAWVTERVPKGTIFMT  target    HAPFIATEKSVKAHETRPDGRALSANTGYQANLRYGSQQSVTRNWHMPMHQTDTLFHKSKVFMGFIFGGEADNHAVNTVP 7qv7.1    FHYW----------------------------------------------------------------------------  target    KETLVRVTKAEDGGMGGKGIWQPATTGFSPDNESDFMKKYLAGELTKVKT 7qv7.1    -------------------------------------------------- ``` | | | | | | | | | | | | | | | | | | | | | | | | | | | | | | | | | | | | | | | | | | | | | | | | | |
|  | 7qv7.1.O | Hydrogen dependent carbon dioxide reductase subunit FdhF  *Cryo-EM structure of Hydrogen-dependent CO2 reductase.* | 0.29 |  | 19.48 | 0.59 | 1-720 | EM | 0.00 | hetero-2-6-6-2-mer | 52 x SF4, 6 x 402 | HHblits | 0.29 |
| ``` target    IMDGKNLVENKLTDSHWFIECMERGAKIVVIAPEYGPPSTKADYWIPIRPQTDAALWLGITRLMIEKKWYDETFVKGFTD 7qv7.1    FIIGSNTAECHPLIAAHVIKAKERGAKLIVADPRMNAMVHKADIWLRVPSGYNIPLINGMIHIIIKEGLVKTDFVKNHAV  target    FPLLVRTDTLQRLRAHEVFPQYKTSLSADGPSMKIQGLSAEQHAKLGDFVVWDGKTNAPAAITRDDVGATITKKGIDPVL 7qv7.1    G-------------------------------------------------------------------------------  target    AGSFKVKLVDGKEVEVATLWTLYQDHLKDYDLDTVVEITQAPKEMIEQLAQDIATMKPVAIHQGEGINHWFHATEMNRAA 7qv7.1    -------------------FEEMAKAVEKYTPEYVEELTGIPKKDLIKAARFYGQAQAAAILYSMGVTQFSHGTGNVVSL  target    YLPLMLTGNIGRPGAGCQTWAGNYKAALFQGSPWTGPGFKGWVAEDPFDINLNPKAHGKEIHAHAYTKDEEPAYWNHGDL 7qv7.1    ANLAVITGNLGRPGAGICPLRGQNNV---QGACDVG-ALPNVL---PGYLDVTKEQNR----------ERFEKVWGVK-L  target    ALIVDTPKFG-RKNFTGKTHMPTPTKALIFNNVNLINNAKWAYGMIKNVNPNVEMIVSMDIQMTASIEYADLALPANSWL 7qv7.1    P---SNIGLRVTEVP--DAILNKRVRALYIFGENPIMSDPDSDHL-RHALEHLDLLIVQDIFLTETARLAHVVLPAACWA  target    EFEGLEITASCSNPFLQIWKGGIPPVFDSRDDLDILAGIANALADVTGEKRFRDYFAFAAADKRGIYIQRLLDTCT-TTA 7qv7.1    EKDGTF---TNTERRVQRVRKAVEAPGEAKPDWWIFSQIAERMGYTG--M------QYNNV---QEIWDEVRKIVPEKFG  target    GYKLADIMAGKYGPPGGCLLNFRTYPRIPFYEQVHDSEPFHTDTGRMHAYADVPEAIEYGENFIVHREGPEATPYLPNVI 7qv7.1    GISYARLEKEKGL-----AWPCP-TEDHTGTPILYLGGKFATPSGKAQMYPVIFYPN-----TCICDEGAEKQDF-N---  target    VSSNPHIRPEDYGIAADAEHWDDRTIRNIKMPWSKVKETKNFLWEKGFQFYCLTPKTRHRVHSGW--SNVDWHMLMDSNF 7qv7.1    ----HV--IV---------------------GS------IAELPDEEYPFTLTTGRRVYHYHTATMTRKSPVIDQ-----  target    GDPYRLDKRAPCVGEHQLHINPQAARDLNINDGDYVYVDANPADRPYLGAKPDDPFYRVSRCMLRVTYNRAYPYNIVMMK 7qv7.1    --------IA---PQELVEINPQDATRLGINDGDFLRVSTRR-----------------GYVATRAWVTERVPKGTIFMT  target    HAPFIATEKSVKAHETRPDGRALSANTGYQANLRYGSQQSVTRNWHMPMHQTDTLFHKSKVFMGFIFGGEADNHAVNTVP 7qv7.1    FHYW----------------------------------------------------------------------------  target    KETLVRVTKAEDGGMGGKGIWQPATTGFSPDNESDFMKKYLAGELTKVKT 7qv7.1    -------------------------------------------------- ``` | | | | | | | | | | | | | | | | | | | | | | | | | | | | | | | | | | | | | | | | | | | | | | | | | |
|  | 2vpz.1.A | THIOSULFATE REDUCTASE  *POLYSULFIDE REDUCTASE NATIVE STRUCTURE* | 0.33 |  | 21.91 | 0.58 | 1-720 | X-ray | 2.40 | hetero-oligomer | 10 x SF4, 4 x MGD, 2 x MO | HHblits | 0.31 |
| ``` target    IMDGKNLVEN-KLTDSHWFIECMERGAKIVVIAPEYGPPSTKADYWIPIRPQTDAALWLGITRLMIEKKWYDETFVKGFT 2vpz.1    VLIGHHIGEDTHNTQLQDFALALKNGAKVVVVDPRFSTAAAKAHRWLPIKPGTDTALLLAWIHVLIYEDLYDKEYVAKYT  target    DFPLLVRTDTLQRLRAHEVFPQYKTSLSADGPSMKIQGLSAEQHAKLGDFVVWDGKTNAPAAITRDDVGATITKKGIDPV 2vpz.1    VG------------------------------------------------------------------------------  target    LAGSFKVKLVDGKEVEVATLWTLYQDHLKDYDLDTVVEITQAPKEMIEQLAQDIATMKPVAIHQGEGINH-WFHATEMNR 2vpz.1    --------------------FEELKAHVKDFTPEWAEKHTEIPAQVIREVAREMAAHKPRAVLPPTRHNVWYGDDTYRVM  target    AAYLPLMLTGNIGRPGAGCQTWAGNYKAALFQGSPWTGPGFKGWVAEDPFDINLNPKAHGKEIHAHAYTKDEEPAYWNHG 2vpz.1    ALLYVNVLLGNYGRPGGFYIAQSPYLEKYPLPPLPL-EPAAGGCS--GPSGGDHEPEGFKPRA-------DK-GKFFARS  target    DLALIVDTPKFGRKNFTGKTHMPTPTKALIFNNVNLINNAKWAYGMIKNVNPNVEMIVSMDIQMTASIEYADLALPANSW 2vpz.1    -----TAIQELIEPMIT---GEPYPIKGLFAYGINLFHSIPNVPRT-KEALKNLDLYVAIDVLPQEHVMWADVILPEATY  target    LEFEGLEITASCSNPFLQIWKGGIPPVFDSRDDLDILAGIANALADVTGEKRFRDYFAFAAADKRGIYIQRLLDTCTTTA 2vpz.1    LERYDDFVLVAHKTPFIQLRTPAHEPLFDTKPGWWIARELGLRLGLE-------QYFPWKT---IEEYLETRLQS----L  target    GYKLADIMAGKYGPPGGCLLNFRTYPRIPFYEQVHDSEPFHTDTGRMHAYADVPEAIEYGENFIVHREGPEATPYLPNVI 2vpz.1    GLDLETMKGMGTLVQR-------GKPWLEDWE-KEGRLPFGTASGKIELYCQRFKEAGH-QPLPVFTPP-----------  target    VSSNPHIRPEDYGIAADAEHWDDRTIRNIKMPWSKVKETKNFLWEKGFQFYCLTPKTRHRVHSGWSNVDWHMLMDSNFGD 2vpz.1    ------------------------------------------EEPPEGFYRLLYGRSPVHTFARTQNNWVLMEM------  target    PYRLDKRAPCVGEHQLHINPQAARDLNINDGDYVYVDANPADRPYLGAKPDDPFYRVSRCM--LRVTYNRAYPYNIVMMK 2vpz.1    -------D---PENEVWIHKEEAKRLGLKEGDYVMLVNQD-----------------GVKEGPVRVKPTARIRKDCVYIV  target    HAPFIATEKSVKAHETRPDGRALSANTGYQANLRYGSQQSVTRNWHMPMHQTDTLFHKSKVFMGFIFGGEADNHAVNTVP 2vpz.1    HGFG----------------------------------------------------------------------------  target    KETLVRVTKAEDGGMGGKGIWQPATTGFSPDNESDFMKKYLAGELTKVKT 2vpz.1    -------------------------------------------------- ``` | | | | | | | | | | | | | | | | | | | | | | | | | | | | | | | | | | | | | | | | | | | | | | | | | |
|  | 2vpx.1.D | THIOSULFATE REDUCTASE  *POLYSULFIDE REDUCTASE WITH BOUND QUINONE (UQ1)* | 0.32 |  | 21.91 | 0.58 | 1-720 | X-ray | 3.10 | hetero-oligomer | 10 x SF4, 4 x MGD, 2 x MO, 2 x UQ1 | HHblits | 0.31 |
| ``` target    IMDGKNLVEN-KLTDSHWFIECMERGAKIVVIAPEYGPPSTKADYWIPIRPQTDAALWLGITRLMIEKKWYDETFVKGFT 2vpx.1    VLIGHHIGEDTHNTQLQDFALALKNGAKVVVVDPRFSTAAAKAHRWLPIKPGTDTALLLAWIHVLIYEDLYDKEYVAKYT  target    DFPLLVRTDTLQRLRAHEVFPQYKTSLSADGPSMKIQGLSAEQHAKLGDFVVWDGKTNAPAAITRDDVGATITKKGIDPV 2vpx.1    VG------------------------------------------------------------------------------  target    LAGSFKVKLVDGKEVEVATLWTLYQDHLKDYDLDTVVEITQAPKEMIEQLAQDIATMKPVAIHQGEGINH-WFHATEMNR 2vpx.1    --------------------FEELKAHVKDFTPEWAEKHTEIPAQVIREVAREMAAHKPRAVLPPTRHNVWYGDDTYRVM  target    AAYLPLMLTGNIGRPGAGCQTWAGNYKAALFQGSPWTGPGFKGWVAEDPFDINLNPKAHGKEIHAHAYTKDEEPAYWNHG 2vpx.1    ALLYVNVLLGNYGRPGGFYIAQSPYLEKYPLPPLPL-EPAAGGCS--GPSGGDHEPEGFKPRA-------DK-GKFFARS  target    DLALIVDTPKFGRKNFTGKTHMPTPTKALIFNNVNLINNAKWAYGMIKNVNPNVEMIVSMDIQMTASIEYADLALPANSW 2vpx.1    -----TAIQELIEPMIT---GEPYPIKGLFAYGINLFHSIPNVPRT-KEALKNLDLYVAIDVLPQEHVMWADVILPEATY  target    LEFEGLEITASCSNPFLQIWKGGIPPVFDSRDDLDILAGIANALADVTGEKRFRDYFAFAAADKRGIYIQRLLDTCTTTA 2vpx.1    LERYDDFVLVAHKTPFIQLRTPAHEPLFDTKPGWWIARELGLRLGLE-------QYFPWKT---IEEYLETRLQS----L  target    GYKLADIMAGKYGPPGGCLLNFRTYPRIPFYEQVHDSEPFHTDTGRMHAYADVPEAIEYGENFIVHREGPEATPYLPNVI 2vpx.1    GLDLETMKGMGTLVQR-------GKPWLEDWE-KEGRLPFGTASGKIELYCQRFKEAGH-QPLPVFTPP-----------  target    VSSNPHIRPEDYGIAADAEHWDDRTIRNIKMPWSKVKETKNFLWEKGFQFYCLTPKTRHRVHSGWSNVDWHMLMDSNFGD 2vpx.1    ------------------------------------------EEPPEGFYRLLYGRSPVHTFARTQNNWVLMEM------  target    PYRLDKRAPCVGEHQLHINPQAARDLNINDGDYVYVDANPADRPYLGAKPDDPFYRVSRCM--LRVTYNRAYPYNIVMMK 2vpx.1    -------D---PENEVWIHKEEAKRLGLKEGDYVMLVNQD-----------------GVKEGPVRVKPTARIRKDCVYIV  target    HAPFIATEKSVKAHETRPDGRALSANTGYQANLRYGSQQSVTRNWHMPMHQTDTLFHKSKVFMGFIFGGEADNHAVNTVP 2vpx.1    HGFG----------------------------------------------------------------------------  target    KETLVRVTKAEDGGMGGKGIWQPATTGFSPDNESDFMKKYLAGELTKVKT 2vpx.1    -------------------------------------------------- ``` | | | | | | | | | | | | | | | | | | | | | | | | | | | | | | | | | | | | | | | | | | | | | | | | | |
|  | 5nqd.1.A | AroA  *Arsenite oxidase AioAB from Rhizobium sp. str. NT-26 mutant AioBF108A* | 0.27 |  | 17.09 | 0.60 | 1-720 | X-ray | 2.20 | hetero-2-2-mer | 4 x MGD, 2 x O, 2 x 4MO, 2 x F3S, 2 x FES | HHblits | 0.28 |
| ``` target    IMDGKNLVENKLTDS--HWFI---------------ECMERGAKIVVIAPEYGPPST------KAD--YWIPIRPQTDAA 5nqd.1    VAVGTNALETQTNYFLNHWIPNLRGESLGKKKELMPEEPHEAGRIIIVDPRRTVTVNACEQTAGADNVLHLAINSGTDLA  target    LWLGITRLMIEKKWYDETFVKGFTDFPLLVRTDTLQRLRAHEVFPQYKTSLSADGPSMKIQGLSAEQHAKLGDFVVWDGK 5nqd.1    LFNALFTYIADKGWVDRDFIDKSTLREGTARP----------------------------------------P--LYPAR  target    TNAPAAITRDDVGATITKKGIDPVLAGSFKVKLVDGKEVEVATLWTLYQDHLK--DYDLDTVVEITQAPKEMIEQLAQDI 5nqd.1    -GV----------------------------------SEANPGHLSSFEDAVEGCRMSIEEAAEITGLDAAQIIKAAEWI  target    ATMK------PVAIHQGEGINHWFHATEMNRAAYLPLMLTGNIGRPGAGCQTWAGNYKAALFQGSPWTGPGFKGWVAEDP 5nqd.1    GMPKEGGKRRRVMFGYEKGLIWGNDNYRTNGALVNLALATGNIGRPGGGVVRLGGHQEGYV-------RPSDAH--VGRP  target    FDINLNPKAHGKEIHAHAYTKDEEPAYWNHGDLALIVDTPKFGRKNFTGKTHMPTPTKALIFNNVNLINN------AKWA 5nqd.1    -------AAYVDQLLIG--GQGGVHHIWGCDHYK---------------TTLNAHEFKRVYKKRTDMVKDAMSAAPYGDR  target    ---YGMIKNVNPNV-EMIVSMDIQMTASIEYADLALPANSWLEFEGLEITASCSNPFLQIWKGGIPPVFDSRDDLDILAG 5nqd.1    EAMVNAIVDAINQGGLFAVNVDIIPTKIGEACHVILPAATSGEMNLTS---MNGERRMRLTERYMDPPGQSMPDCLIAAR  target    IANALADVTGEK---RFRDY---FAFAAADKRGIYIQRLLDTCTTTAGYKLADIMAGK---YGPPGGCLLNFRTYPRIPF 5nqd.1    LANTMERVLTEMGDVGYAAQFKGFDWQTE--EDAFMDGYNKNAHGGEFVTYERLSAMGTNGFQEPATGFTD--GK--IEG  target    YEQVHDSEPFHTDTGRMHAYADVPEAIEYGENFIVHREGPEATPYLPNVIVSSNPHIRPEDYGIAADAEHWDDRTIRNIK 5nqd.1    TQRLYTDGVFSTDDGKARFMDAPWRG---------LQA-----PGKQ---------------------------------  target    MPWSKVKETKNFLWEKGFQFYCLTPKTRHRVHSGWS--NVDWHMLMDSNFGDPYRLDKRAPCVGEHQLHINPQAARDLNI 5nqd.1    ------------QQKDSHKYLINNGRANVVWQSAYLDQENDFVMD-------------RF---PYPFIEMNPEDMAEAGL  target    NDGDYVYVDANPADRPYLGAKPDDPFYRVSRCMLRVTYNRAYPYNIVMMKHAPFIATEKSVKAHETRPDGRALSANTGYQ 5nqd.1    KEGDLVEIYNDA-----------------GATQAMAYPTPTARRGETFMLFGFP--------------------------  target    ANLRYGSQQSVTRNWHMPMHQTDTLFHKSKVFMGFIFGGEADNHAVNTVPKETLVRVTKAEDGGMGGKGIWQPATTGFSP 5nqd.1    --------------------------------------------------------------------------------  target    DNESDFMKKYLAGELTKVKT 5nqd.1    -------------------- ``` | | | | | | | | | | | | | | | | | | | | | | | | | | | | | | | | | | | | | | | | | | | | | | | | | |
|  | 4aay.1.A | AROA  *Crystal Structure of the arsenite oxidase protein complex from Rhizobium species strain NT-26* | 0.26 |  | 16.86 | 0.60 | 1-720 | X-ray | 2.70 | hetero-oligomer | 4 x MGD, 2 x O, 2 x 4MO, 2 x F3S, 2 x FES | HHblits | 0.28 |
| ``` target    IMDGKNLVENKLTDS--HWFI---------------ECMERGAKIVVIAPEYGPPST------KAD--YWIPIRPQTDAA 4aay.1    VAVGTNALETQTNYFLNHWIPNLRGESLGKKKELMPEEPHEAGRIIIVDPRRTVTVNACEQTAGADNVLHLAINSGTDLA  target    LWLGITRLMIEKKWYDETFVKGFTDFPLLVRTDTLQRLRAHEVFPQYKTSLSADGPSMKIQGLSAEQHAKLGDFVVWDGK 4aay.1    LFNALFTYIADKGWVDRDFIDKSTLREGTAR----------------------------------------PP--LYPAR  target    TNAPAAITRDDVGATITKKGIDPVLAGSFKVKLVDGKEVEVATLWTLYQDHLK--DYDLDTVVEITQAPKEMIEQLAQDI 4aay.1    -GVS---------------EAN-------------------PGHLSSFEDAVEGCRMSIEEAAEITGLDAAQIIKAAEWI  target    ATMK------PVAIHQGEGINHWFHATEMNRAAYLPLMLTGNIGRPGAGCQTWAGNYKAALFQGSPWTGPGFKGWVAEDP 4aay.1    GMPKEGGKRRRVMFGYEKGLIWGNDNYRTNGALVNLALATGNIGRPGGGVVRLGGHQEGYV-------RPSDAHV--GRP  target    FDINLNPKAHGKEIHAHAYTKDEEPAYWNHGDLALIVDTPKFGRKNFTGKTHMPTPTKALIFNNVNLINN------AKWA 4aay.1    -------AAYVDQLLIG--GQGGVHHIWG---------CDHY------KTTLNAHEFKRVYKKRTDMVKDAMSAAPYGDR  target    ---YGMIKNVNPNV-EMIVSMDIQMTASIEYADLALPANSWLEFEGLEITASCSNPFLQIWKGGIPPVFDSRDDLDILAG 4aay.1    EAMVNAIVDAINQGGLFAVNVDIIPTKIGEACHVILPAATSGEMNLTS---MNGERRMRLTERYMDPPGQSMPDCLIAAR  target    IANALADVTGEKR---FRDY---FAFAAADKRGIYIQRLLDTCTTTAGYKLADIMAGKYGPPGGCLLNFRT--YPRIPFY 4aay.1    LANTMERVLTEMGDVGYAAQFKGFDWQTE--EDAFMDGYNKNAHGGEFVTYERLSAMGTN---GFQEPATGFTDGKIEGT  target    EQVHDSEPFHTDTGRMHAYADVPEAIEYGENFIVHREGPEATPYLPNVIVSSNPHIRPEDYGIAADAEHWDDRTIRNIKM 4aay.1    QRLYTDGVFSTDDGKARFMDAPWRG---------LQAPG-----KQ----------------------------------  target    PWSKVKETKNFLWEKGFQFYCLTPKTRHRVHSGWSN--VDWHMLMDSNFGDPYRLDKRAPCVGEHQLHINPQAARDLNIN 4aay.1    -----------QQKDSHKYLINNGRANVVWQSAYLDQENDFVMD-------------RF---PYPFIEMNPEDMAEAGLK  target    DGDYVYVDANPADRPYLGAKPDDPFYRVSRCMLRVTYNRAYPYNIVMMKHAPFIATEKSVKAHETRPDGRALSANTGYQA 4aay.1    EGDLVEIYNDA-----------------GATQAMAYPTPTARRGETFMLFGFP---------------------------  target    NLRYGSQQSVTRNWHMPMHQTDTLFHKSKVFMGFIFGGEADNHAVNTVPKETLVRVTKAEDGGMGGKGIWQPATTGFSPD 4aay.1    --------------------------------------------------------------------------------  target    NESDFMKKYLAGELTKVKT 4aay.1    ------------------- ``` | | | | | | | | | | | | | | | | | | | | | | | | | | | | | | | | | | | | | | | | | | | | | | | | | |
|  | 2v45.1.A | PERIPLASMIC NITRATE REDUCTASE  *A NEW CATALYTIC MECHANISM OF PERIPLASMIC NITRATE REDUCTASE FROM DESULFOVIBRIO DESULFURICANS ATCC 27774 FROM CRYSTALLOGRAPHIC AND EPR DATA AND BASED ON DETAILED ANALYSIS OF THE SIXTH LIGAND* | 0.29 |  | 18.70 | 0.60 | 1-719 | X-ray | 2.40 | monomer | 1 x SF4, 1 x MO, 2 x MGD, 1 x LCP | HHblits | 0.28 |
| ``` target    IMDGKNLVENKLTDSHWFIECM--ERGAKIVVIAPEYGPPSTKADYWIPIRPQTDAALWLGITRLMIEKKWYDETFVKGF 2v45.1    FIIGSNTSEAHPVLFRRIARRKQVEPGVKIIVADPRRTNTSRIADMHVAFRPGTDLAFMHSMAWVIINEELDNPRFWQRY  target    TDFPLLVRTDTLQRLRAHEVFPQYKTSLSADGPSMKIQGLSAEQHAKLGDFVVWDGKTNAPAAITRDDVGATITKKGIDP 2v45.1    VNFM-----DA---------------------------------------------------------------------  target    VLAGSFKVKLVDGKEVEVATLWTLYQDHLKDYDLDTVVEITQAPKEMIEQLAQDIATMKPVAIHQGEGINHWFHATEMNR 2v45.1    -----------E----GKPSDFEGYKAFLENYRPEKVAEICRVPVEQIYGAARAFAESAATMSLWCMGINQRVQGVFANN  target    AAYLPLMLTGNIGRPGAGCQTWAGNYKAALFQGSPWTGPGFKGWVAEDPFDINLNPKAHGKEIHAHAYTKDEEPAYWNHG 2v45.1    LIHNLHLITGQICRPGATSFSLTGQPNA---CGGVRDGGALSHLL---PAGR-AIPNAKHR---------AEMEKLWGLP  target    DLALIVDTPKFG-RKNFTGKTHMPTPTKALIFNNVNLINNAKWAYGMIKNVNPNV-EMIVSMDIQMT-ASIEYADLALPA 2v45.1    EGR-IAPEPGYHTVALF--EALGRGDVKCMIICETNPAHTLPNLNKVH-KAMSHPESFIVCIEAFPDAVTLEYADLVLPP  target    NSWLEFEGLEITASCSNPFLQIWKGGIPPVFDSRDDLDILAGIANALADVTGEKRFRDYFAFAAADKRGIYIQRLLDTC- 2v45.1    AFWCERDGVY---GCGERRYSLTEKAVDPPGQCRPTVNTLVEFARRAGVDPQ------LVNFRNAE---DVWNEWRMVSK  target    ---TTTAGYKLADIMAGK-YGPPGG-CLLNFRTYPRIPFYEQVHDSEPFHTDTGRMHAYADVPEAIEYGENFIVHREGPE 2v45.1    GTTYDFWGMTRERLRKESGLIWPCPSEDHPGTSL---RYVR-GQDPCVPADHPDRFFFYGKPDGR------AVIWMRPAK  target    ATPYLPNVIVSSNPHIRPEDYGIAADAEHWDDRTIRNIKMPWSKVKETKNFLWEKGFQFYCLTPKTRHRVHSGWSN--VD 2v45.1    G-----A------------------------------------------AEEPDAEYPLYLTSMRVIDHWHTATMTGKVP  target    WHMLMDSNFGDPYRLDKRAPCVGEHQLHINPQAARDLNINDGDYVYVDANPADRPYLGAKPDDPFYRVSRCMLRVTYNRA 2v45.1    ELQ-------------KAN---PIAFVEINEEDAARTGIKHGDSVIVETRR-----------------DAMELPARVSDV  target    YPYNIVMMKHAPFIATEKSVKAHETRPDGRALSANTGYQANLRYGSQQSVTRNWHMPMHQTDTLFHKSKVFMGFIFGGEA 2v45.1    CRPGLIAVPFFD--------------------------------------------------------------------  target    DNHAVNTVPKETLVRVTKAEDGGMGGKGIWQPATTGFSPDNESDFMKKYLAGELTKVKT 2v45.1    ----------------------------------------------------------- ``` | | | | | | | | | | | | | | | | | | | | | | | | | | | | | | | | | | | | | | | | | | | | | | | | | |
|  | 2v3v.1.A | PERIPLASMIC NITRATE REDUCTASE  *A NEW CATALYTIC MECHANISM OF PERIPLASMIC NITRATE REDUCTASE FROM DESULFOVIBRIO DESULFURICANS ATCC 27774 FROM CRYSTALLOGRAPHIC AND EPR DATA AND BASED ON DETAILED ANALYSIS OF THE SIXTH LIGAND* | 0.29 |  | 18.25 | 0.60 | 1-720 | X-ray | 1.99 | monomer | 1 x SF4, 1 x MO, 2 x MGD, 4 x LCP | HHblits | 0.28 |
| ``` target    IMDGKNLVENKLTDSHWFIECM--ERGAKIVVIAPEYGPPSTKADYWIPIRPQTDAALWLGITRLMIEKKWYDETFVKGF 2v3v.1    FIIGSNTSEAHPVLFRRIARRKQVEPGVKIIVADPRRTNTSRIADMHVAFRPGTDLAFMHSMAWVIINEELDNPRFWQRY  target    TDFPLLVRTDTLQRLRAHEVFPQYKTSLSADGPSMKIQGLSAEQHAKLGDFVVWDGKTNAPAAITRDDVGATITKKGIDP 2v3v.1    VNFM-----DA---------------------------------------------------------------------  target    VLAGSFKVKLVDGKEVEVATLWTLYQDHLKDYDLDTVVEITQAPKEMIEQLAQDIATMKPVAIHQGEGINHWFHATEMNR 2v3v.1    -----------E----GKPSDFEGYKAFLENYRPEKVAEICRVPVEQIYGAARAFAESAATMSLWCMGINQRVQGVFANN  target    AAYLPLMLTGNIGRPGAGCQTWAGNYKAALFQGSPWTGPGFKGWVAEDPFDINLNPKAHGKEIHAHAYTKDEEPAYWNHG 2v3v.1    LIHNLHLITGQICRPGATSFSLTGQPNA---CGGVRDGGALSHLLP-AGRA---IPNAKHR---------AEMEKLWGLP  target    DLALIVDTPKFGRKNFTGKTHMPTPTKALIFNNVNLINNAKWAYGMIKNVNPNV-EMIVSMDIQMT-ASIEYADLALPAN 2v3v.1    EGR-IAPEPGYHTVAL-FEALGRGDVKCMIICETNPAHTLPNLNKVH-KAMSHPESFIVCIEAFPDAVTLEYADLVLPPA  target    SWLEFEGLEITASCSNPFLQIWKGGIPPVFDSRDDLDILAGIANALADVTGEKRFRDYFAFAAADKRGIYIQRLLDTCT- 2v3v.1    FWCERDGVY---GCGERRYSLTEKAVDPPGQCRPTVNTLVEFARRAGVDPQ------LVNFRNA---EDVWNEWRMVSKG  target    ---TTAGYKLADIMAGK-YGPPGG-CLLNFRTYPRIPF------YEQVHDSEPFHTDTGRMHAYADVPEAIEYGENFIVH 2v3v.1    TTYDFWGMTRERLRKESGLIWPCPSEDHPGTSLRYVRGQDPCVPADHPDRFFFYGKPDGRAVIWMRPAK-----------  target    REGPEATPYLPNVIVSSNPHIRPEDYGIAADAEHWDDRTIRNIKMPWSKVKETKNFLWEKGFQFYCLTPKTRHRVHSGWS 2v3v.1    -----G---AA--------------------------------------------EEPDAEYPLYLTSMRVIDHWHTATM  target    N--VDWHMLMDSNFGDPYRLDKRAPCVGEHQLHINPQAARDLNINDGDYVYVDANPADRPYLGAKPDDPFYRVSRCMLRV 2v3v.1    TGKVPELQ-------------KAN---PIAFVEINEEDAARTGIKHGDSVIVETRR-----------------DAMELPA  target    TYNRAYPYNIVMMKHAPFIATEKSVKAHETRPDGRALSANTGYQANLRYGSQQSVTRNWHMPMHQTDTLFHKSKVFMGFI 2v3v.1    RVSDVCRPGLIAVPFFDP--------------------------------------------------------------  target    FGGEADNHAVNTVPKETLVRVTKAEDGGMGGKGIWQPATTGFSPDNESDFMKKYLAGELTKVKT 2v3v.1    ---------------------------------------------------------------- ``` | | | | | | | | | | | | | | | | | | | | | | | | | | | | | | | | | | | | | | | | | | | | | | | | | |
|  | 1g8k.1.A | ARSENITE OXIDASE  *CRYSTAL STRUCTURE ANALYSIS OF ARSENITE OXIDASE FROM ALCALIGENES FAECALIS* | 0.28 |  | 17.61 | 0.58 | 1-720 | X-ray | 1.64 | hetero-1-1-mer | 3 x HG, 2 x CA, 2 x MGD, 1 x O, 1 x 4MO, 1 x F3S, 1 x FES | HHblits | 0.28 |
| ``` target    IMDGKNLVENKLTD--SHWF---------------IECMERGAKIVVIAPEYGPPSTKA--------DYWIPIRPQTDAA 1g8k.1    WSIGNNPYESQTNYFLNHWLPNLQGATTSKKKERFPNENFPQARIIFVDPRETPSVAIARHVAGNDRVLHLAIEPGTDTA  target    LWLGITRLMIEKKWYDETFVKGFTDFPLLVRTDTLQRLRAHEVFPQYKTSLSADGPSMKIQGLSAEQHAKLGDFVVWDGK 1g8k.1    LFNGLFTYVVEQGWIDKPFIEAHTKG------------------------------------------------------  target    TNAPAAITRDDVGATITKKGIDPVLAGSFKVKLVDGKEVEVATLWTLYQDHLKDYDLDTVVEITQAPKEMIEQLAQDIAT 1g8k.1    --------------------------------------------FDDAVK-TNRLSLDECSNITGVPVDMLKRAAEWSYK  target    M------KPVAIHQGEGINHWFHATEMNRAAYLPLMLTGNIGRPGAGCQTWAGNYKAALFQGSPWTGPGFKGWVAEDPFD 1g8k.1    PKASGQAPRTMHAYEKGIIWGNDNYVIQSALLDLVIATHNVGRRGTGCVRMGGHQEGYT-------RPPYPGDK---KI-  target    INLNPKAHGKEIHAHAYTKDEEPAYWNHGDLALIVDTPKFG--RKNFTGKTHMPTPTKALIFNNVNLINNAKWAYGMIKN 1g8k.1    -YID-----QELIKGK---GRIMTWWGC-NN--FQTSNNAQALREAI--LQRSAIVKQAMQKARGATTEEM--V-DVIYE  target    VNPN-VEMIVSMDIQMTASIEYADLALPANSWLEFEGLEITASCSNPFLQIWKGGIPPVFDSRDDLDILAGIANALADVT 1g8k.1    ATQNGGLFVTSINLYPTKLAEAAHLMLPAAHPGEMNLTS---MNGERRIRLSEKFMDPPGTAMADCLIAARIANALRDMY  target    GEK---RFRDYFAFAAADKRGIYIQRLLDTCTT-------------TAGYKLADIMAGKYGPPGGCLLNFRTY---PRIP 1g8k.1    QKDGKAEMAAQFEGFDWKTEEDAFNDGFRRAGQPGAPAIDSQGGSTGHLVTYDRLRKSGNN---GVQLPVVSWDESKGLV  target    FYEQVHDSEPFHTDTGRMHAYADVPEAIEYGENFIVHREGPEATPYLPNVIVSSNPHIRPEDYGIAADAEHWDDRTIRNI 1g8k.1    GTEMLYTEGKFDTDDGKAHFKPAPWN------GLPATVQ-----------------------------------------  target    KMPWSKVKETKNFLWEKGFQFYCLTPKTRHRVHSGWSN--VDWHMLMDSNFGDPYRLDKRAPCVGEHQLHINPQAARDLN 1g8k.1    -------------QQKDKYRFWLNNGRNNEVWQTAYHDQYNSLMQ-------------ERY---PMAYIEMNPDDCKQLD  target    INDGDYVYVDANPADRPYLGAKPDDPFYRVSRCMLRVTYNRAYPYNIVMMKHAPFIATEKSVKAHETRPDGRALSANTGY 1g8k.1    VTGGDIVEVYNDF-----------------GSTFAMVYPVAEIKRGQTFMLFGYV-------------------------  target    QANLRYGSQQSVTRNWHMPMHQTDTLFHKSKVFMGFIFGGEADNHAVNTVPKETLVRVTKAEDGGMGGKGIWQPATTGFS 1g8k.1    --------------------------------------------------------------------------------  target    PDNESDFMKKYLAGELTKVKT 1g8k.1    --------------------- ``` | | | | | | | | | | | | | | | | | | | | | | | | | | | | | | | | | | | | | | | | | | | | | | | | | |
|  | 1g8j.1.A | ARSENITE OXIDASE  *CRYSTAL STRUCTURE ANALYSIS OF ARSENITE OXIDASE FROM ALCALIGENES FAECALIS* | 0.28 |  | 17.92 | 0.58 | 1-720 | X-ray | 2.03 | hetero-oligomer | 2 x MGD, 1 x O, 1 x 4MO, 1 x F3S, 1 x FES | HHblits | 0.28 |
| ``` target    IMDGKNLVENKLTDS--HWF---------------IECMERGAKIVVIAPEYGPPSTKA--------DYWIPIRPQTDAA 1g8j.1    WSIGNNPYESQTNYFLNHWLPNLQGATTSKKKERFPNENFPQARIIFVDPRETPSVAIARHVAGNDRVLHLAIEPGTDTA  target    LWLGITRLMIEKKWYDETFVKGFTDFPLLVRTDTLQRLRAHEVFPQYKTSLSADGPSMKIQGLSAEQHAKLGDFVVWDGK 1g8j.1    LFNGLFTYVVEQGWIDKPFIEAHTKG------------------------------------------------------  target    TNAPAAITRDDVGATITKKGIDPVLAGSFKVKLVDGKEVEVATLWTLYQDHLKDYDLDTVVEITQAPKEMIEQLAQDIAT 1g8j.1    --------------------------------------------FDDAVK-TNRLSLDECSNITGVPVDMLKRAAEWSYK  target    M------KPVAIHQGEGINHWFHATEMNRAAYLPLMLTGNIGRPGAGCQTWAGNYKAALFQGSPWTGPGFKGWVAEDPFD 1g8j.1    PKASGQAPRTMHAYEKGIIWGNDNYVIQSALLDLVIATHNVGRRGTGCVRMGGHQEGY-------TRPPYPGDK---KIY  target    INLNPKAHGKEIHAHAYTKDEEPAYWNHGDLALIVDTPKFG--RKNFTGKTHMPTPTKALIFNNVNLINNAKWAYGMIKN 1g8j.1    --ID-Q----ELIKGK---GRIMTWWGCNNF---QTSNNAQALREAI--LQRSAIVKQAMQKARGATTEEMV---DVIYE  target    VNPNV-EMIVSMDIQMTASIEYADLALPANSWLEFEGLEITASCSNPFLQIWKGGIPPVFDSRDDLDILAGIANALADVT 1g8j.1    ATQNGGLFVTSINLYPTKLAEAAHLMLPAAHPGEMNLTS---MNGERRIRLSEKFMDPPGTAMADCLIAARIANALRDMY  target    GEKR---FRDY---FAFAAADKRGIYIQRLLDTCTT-------------TAGYKLADIMAGKYGPPGGCLLNFRTY---P 1g8j.1    QKDGKAEMAAQFEGFDWKT---EEDAFNDGFRRAGQPGAPAIDSQGGSTGHLVTYDRLRKSGNN---GVQLPVVSWDESK  target    RIPFYEQVHDSEPFHTDTGRMHAYADVPEAIEYGENFIVHREGPEATPYLPNVIVSSNPHIRPEDYGIAADAEHWDDRTI 1g8j.1    GLVGTEMLYTEGKFDTDDGKAHFKPAPWN------GLPATVQ--------------------------------------  target    RNIKMPWSKVKETKNFLWEKGFQFYCLTPKTRHRVHSGWSN--VDWHMLMDSNFGDPYRLDKRAPCVGEHQLHINPQAAR 1g8j.1    ----------------QQKDKYRFWLNNGRNNEVWQTAYHDQYNSLMQ-------------ERY---PMAYIEMNPDDCK  target    DLNINDGDYVYVDANPADRPYLGAKPDDPFYRVSRCMLRVTYNRAYPYNIVMMKHAPFIATEKSVKAHETRPDGRALSAN 1g8j.1    QLDVTGGDIVEVYNDF-----------------GSTFAMVYPVAEIKRGQTFMLFGYV----------------------  target    TGYQANLRYGSQQSVTRNWHMPMHQTDTLFHKSKVFMGFIFGGEADNHAVNTVPKETLVRVTKAEDGGMGGKGIWQPATT 1g8j.1    --------------------------------------------------------------------------------  target    GFSPDNESDFMKKYLAGELTKVKT 1g8j.1    ------------------------ ``` | | | | | | | | | | | | | | | | | | | | | | | | | | | | | | | | | | | | | | | | | | | | | | | | | |
|  | 1aa6.1.A | FORMATE DEHYDROGENASE H  *REDUCED FORM OF FORMATE DEHYDROGENASE H FROM E. COLI* | 0.35 |  | 19.59 | 0.57 | 1-720 | X-ray | 2.30 | monomer | 1 x SF4, 2 x MGD, 1 x 4MO | HHblits | 0.29 |
| ``` target    IMDGKNLVENKLTDSHWFIECMERGAKIVVIAPEYGPPSTKADYWIPIRPQTDAALWLGITRLMIEKKWYDETFVKGFTD 1aa6.1    FVFGYNPADSHPIVANHVINAKRNGAKIIVCDPRKIETARIADMHIALKNGSNIALLNAMGHVIIEENLYDKAFVASRTE  target    FPLLVRTDTLQRLRAHEVFPQYKTSLSADGPSMKIQGLSAEQHAKLGDFVVWDGKTNAPAAITRDDVGATITKKGIDPVL 1aa6.1    G-------------------------------------------------------------------------------  target    AGSFKVKLVDGKEVEVATLWTLYQDHLKDYDLDTVVEITQAPKEMIEQLAQDIATMKPVAIHQGEGINHWFHATEMNRAA 1aa6.1    -------------------FEEYRKIVEGYTPESVEDITGVSASEIRQAARMYAQAKSAAILWGMGVTQFYQGVETVRSL  target    YLPLMLTGNIGRPGAGCQTWAGNYKAALFQGSPWTGPGFKGWVAEDPFDINLNPKAHGKEIHAHAYTKDEEPAYWNHGDL 1aa6.1    TSLAMLTGNLGKPHAGVNPVRGQNNV---QGAC-DMGALPDTYP--GYQYVKDPANR-E----------KFAKAWGVESL  target    ALIVDTPKFGRKNFTGKTHMPTPTKALIFNNVNLINNAKWAYGMIKNVNPNVEMIVSMDIQMTASIEYADLALPANSWLE 1aa6.1    P---AHTGYRISEL-PHRAAHGEVRAAYIMGEDPLQTDAELSAVR-KAFEDLELVIVQDIFMTKTASAADVILPSTSWGE  target    FEGLEITASCSNPFLQIWKGGIPPVFDSRDDLDILAGIANALADVTGEKRFRDYFAFAAADKRGIYIQRLLDTCTTTAGY 1aa6.1    HEGVFT---AADRGFQRFFKAVEPKWDLKTDWQIISEIATRMGYPMHYNNTQ------------EIWDELRHLCPDFYGA  target    KLADIMAGKYGPPGGCLLNFRTYPRIPFYEQVHDSEPFHTDTGRMHAYADVPEAIEYGENFIVHREGPEATPYLPNVIVS 1aa6.1    TYEKMGELGFIQW-----PCRDTSDADQGTSYLFKEKFDTPNGLAQFFTCDWV------------API------D-----  target    SNPHIRPEDYGIAADAEHWDDRTIRNIKMPWSKVKETKNFLWEKGFQFYCLTPKTR--HRVHSGWSNVDWHMLMDSNFGD 1aa6.1    ----------------------------------------KLTDEYPMVLSTVREVGHYSCRSMTGNCAALAALAD----  target    PYRLDKRAPCVGEHQLHINPQAARDLNINDGDYVYVDANPADRPYLGAKPDDPFYRVSRCMLRVTYNRAYPYNIVMMKHA 1aa6.1    -----------EPGYAQINTEDAKRLGIEDEALVWVHSRK-----------------GKIITRAQVSDRPNKGAIYMTYQ  target    PFIATEKSVKAHETRPDGRALSANTGYQANLRYGSQQSVTRNWHMPMHQTDTLFHKSKVFMGFIFGGEADNHAVNTVPKE 1aa6.1    WW------------------------------------------------------------------------------  target    TLVRVTKAEDGGMGGKGIWQPATTGFSPDNESDFMKKYLAGELTKVKT 1aa6.1    ------------------------------------------------ ``` | | | | | | | | | | | | | | | | | | | | | | | | | | | | | | | | | | | | | | | | | | | | | | | | | |
|  | 1fdo.1.A | FORMATE DEHYDROGENASE H  *OXIDIZED FORM OF FORMATE DEHYDROGENASE H FROM E. COLI* | 0.34 |  | 19.59 | 0.57 | 1-720 | X-ray | 2.80 | monomer | 1 x SF4, 2 x MGD, 1 x 6MO | HHblits | 0.29 |
| ``` target    IMDGKNLVENKLTDSHWFIECMERGAKIVVIAPEYGPPSTKADYWIPIRPQTDAALWLGITRLMIEKKWYDETFVKGFTD 1fdo.1    FVFGYNPADSHPIVANHVINAKRNGAKIIVCDPRKIETARIADMHIALKNGSNIALLNAMGHVIIEENLYDKAFVASRTE  target    FPLLVRTDTLQRLRAHEVFPQYKTSLSADGPSMKIQGLSAEQHAKLGDFVVWDGKTNAPAAITRDDVGATITKKGIDPVL 1fdo.1    G-------------------------------------------------------------------------------  target    AGSFKVKLVDGKEVEVATLWTLYQDHLKDYDLDTVVEITQAPKEMIEQLAQDIATMKPVAIHQGEGINHWFHATEMNRAA 1fdo.1    -------------------FEEYRKIVEGYTPESVEDITGVSASEIRQAARMYAQAKSAAILWGMGVTQFYQGVETVRSL  target    YLPLMLTGNIGRPGAGCQTWAGNYKAALFQGSPWTGPGFKGWVAEDPFDINLNPKAHGKEIHAHAYTKDEEPAYWNHGDL 1fdo.1    TSLAMLTGNLGKPHAGVNPVRGQNNV---QGAC-DMGALPDTYP--GYQYVKDPANR-E----------KFAKAWGVESL  target    ALIVDTPKFGRKNFTGKTHMPTPTKALIFNNVNLINNAKWAYGMIKNVNPNVEMIVSMDIQMTASIEYADLALPANSWLE 1fdo.1    P---AHTGYRISEL-PHRAAHGEVRAAYIMGEDPLQTDAELSAVR-KAFEDLELVIVQDIFMTKTASAADVILPSTSWGE  target    FEGLEITASCSNPFLQIWKGGIPPVFDSRDDLDILAGIANALADVTGEKRFRDYFAFAAADKRGIYIQRLLDTCTTTAGY 1fdo.1    HEGVFT---AADRGFQRFFKAVEPKWDLKTDWQIISEIATRMGYPMHYNNTQ------------EIWDELRHLCPDFYGA  target    KLADIMAGKYGPPGGCLLNFRTYPRIPFYEQVHDSEPFHTDTGRMHAYADVPEAIEYGENFIVHREGPEATPYLPNVIVS 1fdo.1    TYEKMGELGFIQW-----PCRDTSDADQGTSYLFKEKFDTPNGLAQFFTCDWV------------API------D-----  target    SNPHIRPEDYGIAADAEHWDDRTIRNIKMPWSKVKETKNFLWEKGFQFYCLTPKTR--HRVHSGWSNVDWHMLMDSNFGD 1fdo.1    ----------------------------------------KLTDEYPMVLSTVREVGHYSCRSMTGNCAALAALAD----  target    PYRLDKRAPCVGEHQLHINPQAARDLNINDGDYVYVDANPADRPYLGAKPDDPFYRVSRCMLRVTYNRAYPYNIVMMKHA 1fdo.1    -----------EPGYAQINTEDAKRLGIEDEALVWVHSRK-----------------GKIITRAQVSDRPNKGAIYMTYQ  target    PFIATEKSVKAHETRPDGRALSANTGYQANLRYGSQQSVTRNWHMPMHQTDTLFHKSKVFMGFIFGGEADNHAVNTVPKE 1fdo.1    WW------------------------------------------------------------------------------  target    TLVRVTKAEDGGMGGKGIWQPATTGFSPDNESDFMKKYLAGELTKVKT 1fdo.1    ------------------------------------------------ ``` | | | | | | | | | | | | | | | | | | | | | | | | | | | | | | | | | | | | | | | | | | | | | | | | | |
|  | 2iv2.1.A | Formate dehydrogenase H  *Reinterpretation of reduced form of formate dehydrogenase H from E. coli* | 0.34 |  | 19.59 | 0.57 | 1-720 | X-ray | 2.27 | monomer | 1 x SF4, 1 x 2MD, 1 x MGD | HHblits | 0.29 |
| ``` target    IMDGKNLVENKLTDSHWFIECMERGAKIVVIAPEYGPPSTKADYWIPIRPQTDAALWLGITRLMIEKKWYDETFVKGFTD 2iv2.1    FVFGYNPADSHPIVANHVINAKRNGAKIIVCDPRKIETARIADMHIALKNGSNIALLNAMGHVIIEENLYDKAFVASRTE  target    FPLLVRTDTLQRLRAHEVFPQYKTSLSADGPSMKIQGLSAEQHAKLGDFVVWDGKTNAPAAITRDDVGATITKKGIDPVL 2iv2.1    G-------------------------------------------------------------------------------  target    AGSFKVKLVDGKEVEVATLWTLYQDHLKDYDLDTVVEITQAPKEMIEQLAQDIATMKPVAIHQGEGINHWFHATEMNRAA 2iv2.1    -------------------FEEYRKIVEGYTPESVEDITGVSASEIRQAARMYAQAKSAAILWGMGVTQFYQGVETVRSL  target    YLPLMLTGNIGRPGAGCQTWAGNYKAALFQGSPWTGPGFKGWVAEDPFDINLNPKAHGKEIHAHAYTKDEEPAYWNHGDL 2iv2.1    TSLAMLTGNLGKPHAGVNPVRGQNNV---QGAC-DMGALPDTYP--GYQYVKDPANR-E----------KFAKAWGVESL  target    ALIVDTPKFGRKNFTGKTHMPTPTKALIFNNVNLINNAKWAYGMIKNVNPNVEMIVSMDIQMTASIEYADLALPANSWLE 2iv2.1    P---AHTGYRISEL-PHRAAHGEVRAAYIMGEDPLQTDAELSAVR-KAFEDLELVIVQDIFMTKTASAADVILPSTSWGE  target    FEGLEITASCSNPFLQIWKGGIPPVFDSRDDLDILAGIANALADVTGEKRFRDYFAFAAADKRGIYIQRLLDTCTTTAGY 2iv2.1    HEGVFT---AADRGFQRFFKAVEPKWDLKTDWQIISEIATRMGYPMHYNNTQ------------EIWDELRHLCPDFYGA  target    KLADIMAGKYGPPGGCLLNFRTYPRIPFYEQVHDSEPFHTDTGRMHAYADVPEAIEYGENFIVHREGPEATPYLPNVIVS 2iv2.1    TYEKMGELGFIQW-----PCRDTSDADQGTSYLFKEKFDTPNGLAQFFTCDWV------------API------D-----  target    SNPHIRPEDYGIAADAEHWDDRTIRNIKMPWSKVKETKNFLWEKGFQFYCLTPKTR--HRVHSGWSNVDWHMLMDSNFGD 2iv2.1    ----------------------------------------KLTDEYPMVLSTVREVGHYSCRSMTGNCAALAALAD----  target    PYRLDKRAPCVGEHQLHINPQAARDLNINDGDYVYVDANPADRPYLGAKPDDPFYRVSRCMLRVTYNRAYPYNIVMMKHA 2iv2.1    -----------EPGYAQINTEDAKRLGIEDEALVWVHSRK-----------------GKIITRAQVSDRPNKGAIYMTYQ  target    PFIATEKSVKAHETRPDGRALSANTGYQANLRYGSQQSVTRNWHMPMHQTDTLFHKSKVFMGFIFGGEADNHAVNTVPKE 2iv2.1    WW------------------------------------------------------------------------------  target    TLVRVTKAEDGGMGGKGIWQPATTGFSPDNESDFMKKYLAGELTKVKT 2iv2.1    ------------------------------------------------ ``` | | | | | | | | | | | | | | | | | | | | | | | | | | | | | | | | | | | | | | | | | | | | | | | | | |
|  | 7z0t.1.G | Formate dehydrogenase H  *Structure of the Escherichia coli formate hydrogenlyase complex (aerobic preparation, composite structure)* | 0.34 |  | 19.59 | 0.57 | 1-720 | EM | 0.00 | hetero-1-1-1-1-1-1-… | 1 x NI, 1 x FCO, 8 x SF4, 1 x FE, 2 x MGD, 1 x 6MO | HHblits | 0.29 |
| ``` target    IMDGKNLVENKLTDSHWFIECMERGAKIVVIAPEYGPPSTKADYWIPIRPQTDAALWLGITRLMIEKKWYDETFVKGFTD 7z0t.1    FVFGYNPADSHPIVANHVINAKRNGAKIIVCDPRKIETARIADMHIALKNGSNIALLNAMGHVIIEENLYDKAFVASRTE  target    FPLLVRTDTLQRLRAHEVFPQYKTSLSADGPSMKIQGLSAEQHAKLGDFVVWDGKTNAPAAITRDDVGATITKKGIDPVL 7z0t.1    G-------------------------------------------------------------------------------  target    AGSFKVKLVDGKEVEVATLWTLYQDHLKDYDLDTVVEITQAPKEMIEQLAQDIATMKPVAIHQGEGINHWFHATEMNRAA 7z0t.1    -------------------FEEYRKIVEGYTPESVEDITGVSASEIRQAARMYAQAKSAAILWGMGVTQFYQGVETVRSL  target    YLPLMLTGNIGRPGAGCQTWAGNYKAALFQGSPWTGPGFKGWVAEDPFDINLNPKAHGKEIHAHAYTKDEEPAYWNHGDL 7z0t.1    TSLAMLTGNLGKPHAGVNPVRGQNNV---QGAC-DMGALPDTYP--GYQYVKDPANR-E----------KFAKAWGVESL  target    ALIVDTPKFGRKNFTGKTHMPTPTKALIFNNVNLINNAKWAYGMIKNVNPNVEMIVSMDIQMTASIEYADLALPANSWLE 7z0t.1    P---AHTGYRISEL-PHRAAHGEVRAAYIMGEDPLQTDAELSAVR-KAFEDLELVIVQDIFMTKTASAADVILPSTSWGE  target    FEGLEITASCSNPFLQIWKGGIPPVFDSRDDLDILAGIANALADVTGEKRFRDYFAFAAADKRGIYIQRLLDTCTTTAGY 7z0t.1    HEGVFT---AADRGFQRFFKAVEPKWDLKTDWQIISEIATRMGYPMHYNNTQ------------EIWDELRHLCPDFYGA  target    KLADIMAGKYGPPGGCLLNFRTYPRIPFYEQVHDSEPFHTDTGRMHAYADVPEAIEYGENFIVHREGPEATPYLPNVIVS 7z0t.1    TYEKMGELGFIQW-----PCRDTSDADQGTSYLFKEKFDTPNGLAQFFTCDWV------------API------D-----  target    SNPHIRPEDYGIAADAEHWDDRTIRNIKMPWSKVKETKNFLWEKGFQFYCLTPKTR--HRVHSGWSNVDWHMLMDSNFGD 7z0t.1    ----------------------------------------KLTDEYPMVLSTVREVGHYSCRSMTGNCAALAALAD----  target    PYRLDKRAPCVGEHQLHINPQAARDLNINDGDYVYVDANPADRPYLGAKPDDPFYRVSRCMLRVTYNRAYPYNIVMMKHA 7z0t.1    -----------EPGYAQINTEDAKRLGIEDEALVWVHSRK-----------------GKIITRAQVSDRPNKGAIYMTYQ  target    PFIATEKSVKAHETRPDGRALSANTGYQANLRYGSQQSVTRNWHMPMHQTDTLFHKSKVFMGFIFGGEADNHAVNTVPKE 7z0t.1    WW------------------------------------------------------------------------------  target    TLVRVTKAEDGGMGGKGIWQPATTGFSPDNESDFMKKYLAGELTKVKT 7z0t.1    ------------------------------------------------ ``` | | | | | | | | | | | | | | | | | | | | | | | | | | | | | | | | | | | | | | | | | | | | | | | | | |
|  | 6tg9.1.A | Formate dehydrogenase subunit alpha  *Cryo-EM Structure of NADH reduced form of NAD+-dependent Formate Dehydrogenase from Rhodobacter capsulatus* | 0.30 |  | 19.42 | 0.57 | 1-719 | EM | 3.24 | hetero-2-2-2-2-mer | 4 x MGD, 2 x 6MO, 4 x FES, 10 x SF4, 2 x H2S, 2 x FMN, 2 x NAI | HHblits | 0.30 |
| ``` target    IMDGKNLVENKLTDSHWFIECMERGAKIVVIAPEYGPPS----TKADYWIPIRPQTDAALWLGITRLMIEKKWYDETFVK 6tg9.1    LVIGANPTDGHPVFASRLRKRLRAGAKLIVVDPRRIDLLNTPHRGEAWHLQLKPGTNVAVMTAMAHVIVTEQIFDKRFIG  target    GFTDFPLLVRTDTLQRLRAHEVFPQYKTSLSADGPSMKIQGLSAEQHAKLGDFVVWDGKTNAPAAITRDDVGATITKKGI 6tg9.1    DRCDWD------E-------------------------------------------------------------------  target    DPVLAGSFKVKLVDGKEVEVATLWTLYQDHL--KDYDLDTVVEITQAPKEMIEQLAQDIATMKPVAIHQGEGINHWFHAT 6tg9.1    -----------------------WADYAEFVANPEYAPEAVESLTGVPAGLLRQAARAYAAAPNAAIYYGLGVTEHSQGS  target    EMNRAAYLPLMLTGNIGRPGAGCQTWAGNYKAALFQGSPWTGPGFKGWVAEDPFDINLNPKAHGKEIHAHAYTKDEEPAY 6tg9.1    TTVIAIANLAMMTGNIGRPGVGVNPLRGQNNV---QGSCDMG-SFPHEF---PGYRHVSDDATRG----------LFERT  target    WNHGDLALIVDTPKFG-RKNFTGKTHMPTPTKALIFNNVNLINNAKWAYGMIKNVNPNVEMIVSMDIQMTASIEYADLAL 6tg9.1    WGVT-LS---SEPGLRIPNMLD--AAVEGRFKALYVQGEDILQSDPDTRHV-SAGLAAMDLVIVHDLFLNETANYAHVFL  target    PANSWLEFEGLEITASCSNPFLQIWKGGIPPVFDSRDDLDILAGIANALADVTGEKRFRDYFAFAAADKRGIYIQRLLDT 6tg9.1    PGSTFLEKDGTF---TNAERRINRVRRVMAPKA-GFADWEVTQMLANALGAGWH---------YTHP---SEIMAEIAAT  target    CTTTAGYKLADIMAGKYGPPGGCLLNFRTYPRIPFYEQVHDSEPFHTDTGRMHAYADVPEAIEYGENFIVHREGPEATPY 6tg9.1    TPGFAAVTYEMLDARGSVQ-----WPCN--------E----KAPEGSPIMHVEGFVRGK-----GR----FIR----TAY  target    LPNVIVSSNPHIRPEDYGIAADAEHWDDRTIRNIKMPWSKVKETKNFLWEKGFQFYCLTPKTRHRVHSGWSNVDWHMLMD 6tg9.1    LPT------D------------------------------------EKTGPRFPLLLTTGRILSQYNVGAQTRRTEN---  target    SNFGDPYRLDKRAPCVGEHQLHINPQAARDLNINDGDYVYVDANPADRPYLGAKPDDPFYRVSRCMLRVTYNRAYPYNIV 6tg9.1    --------T-VW---HGEDRLEIHPTDAETRGIRDGDWVRLASRA-----------------GETTLRATVTDRVSPGVV  target    MMKHAPFIATEKSVKAHETRPDGRALSANTGYQANLRYGSQQSVTRNWHMPMHQTDTLFHKSKVFMGFIFGGEADNHAVN 6tg9.1    YTTFHH--------------------------------------------------------------------------  target    TVPKETLVRVTKAEDGGMGGKGIWQPATTGFSPDNESDFMKKYLAGELTKVKT 6tg9.1    ----------------------------------------------------- ``` | | | | | | | | | | | | | | | | | | | | | | | | | | | | | | | | | | | | | | | | | | | | | | | | | |
|  | 7bkb.1.F | Formate dehydrogenase  *Formate dehydrogenase - heterodisulfide reductase - formylmethanofuran dehydrogenase complex from Methanospirillum hungatei (hexameric, composite structure)* | 0.29 |  | 18.09 | 0.57 | 1-719 | EM | 0.00 | hetero-2-2-2-2-2-2-… | 48 x SF4, 4 x FAD, 2 x FES, 4 x 9S8, 4 x ZN, 2 x MO, 4 x MGD | HHblits | 0.29 |
| ``` target    IMDGKNLVENKLTDSHWFIECMERGAKIVVIAPEYGPPSTKADYWIPIRPQTDAALWLGITRLMIEKKWYDETFVKGFTD 7bkb.1    LIWGSNAVEAHPLAGRRIAQAKKKGIQIIAVDPRYTMTARLADTYVRFNPSTHIALANSMMYWIIKEGLEDKKFIQDRVN  target    FPLLVRTDTLQRLRAHEVFPQYKTSLSADGPSMKIQGLSAEQHAKLGDFVVWDGKTNAPAAITRDDVGATITKKGIDPVL 7bkb.1    G-------------------------------------------------------------------------------  target    AGSFKVKLVDGKEVEVATLWTLYQDHLKDYDLDTVVEITQAPKEMIEQLAQDIATMKPVAIHQGEGINHWFHATEMNRAA 7bkb.1    -------------------FEDLKKTVENY--ADAEAIHGVPLDVVKDIAFRYAKAKNAVIIYCLGITELTTGTDNVRSM  target    YLPLMLTGNIGRPGAGCQTWAGNYKAALFQGSPWTGPGFKGWVAEDPFDINLNPKAHGKEIHAHAYTKDEEPAYWNHGDL 7bkb.1    GNLALLTGNVGREGVGVNPLRGQNNV---QGACDMG-AYPNVYS--GYQ-KCEVAENRA----------KMEKAWSVTNL  target    ALIVDTPKFGRKNFTGKTHMPTPTKALIFNNVNLINNAKWAYGMIKNVNPNVEMIVSMDIQMTASIEYADLALPANSWLE 7bkb.1    PDW-----YGATLTEQINQCGDEIKGMYILGLNPVVTYPSSNHV-KAQLEKLDFLVVQDIFFTETCQYADVILPGACFAE  target    FEGLEITASCSNPFLQIWKGGIPPVFDSRDDLDILAGIANALADVTGEKRFRDYFAFAAADKRGIYIQRLLDTCTTTAGY 7bkb.1    KDGTF---TSGERRINRVRKAVNPPGQAKEDIHIISELAAKMGFKG--------FELPTA---KDVWDDMRAVTPSMFGA  target    KLADIMAGKYGPPGGCLLNFRTYPRIPFYEQVHDSEPFHTDTGRMHAYADVPEAIEYGENFIVHREGPEATPYLPNVIVS 7bkb.1    TYEKLERPEGIC---WPCPTEEHPGTPIL----HREKFATADGKGNLFGID------------YRPPAE-----------  target    SNPHIRPEDYGIAADAEHWDDRTIRNIKMPWSKVKETKNFLWEKGFQFYCLTPKTRHRVHSGWSNVDWHMLMDSNFGDPY 7bkb.1    ----------------------------------------VADAEYPFTLMTGRLIFHYHSRTQTDR-AA----------  target    RLDKRAPCVGEHQLHINPQAARDLNINDGDYVYVDANPADRPYLGAKPDDPFYRVSRCMLRVTYNRAYPYNIVMMKHAPF 7bkb.1    DLHREV---PESYAQINIEDARRLGIKNNEYIKLKSRR-----------------GETTTLARVTDEVAPGVVYMTMHF-  target    IATEKSVKAHETRPDGRALSANTGYQANLRYGSQQSVTRNWHMPMHQTDTLFHKSKVFMGFIFGGEADNHAVNTVPKETL 7bkb.1    --------------------------------------------------------------------------------  target    VRVTKAEDGGMGGKGIWQPATTGFSPDNESDFMKKYLAGELTKVKT 7bkb.1    ---------------------------------------------- ``` | | | | | | | | | | | | | | | | | | | | | | | | | | | | | | | | | | | | | | | | | | | | | | | | | |
|  | 7vw6.1.A | Formate dehydrogenase  *Cryo-EM Structure of Formate Dehydrogenase 1 from Methylorubrum extorquens AM1* | 0.32 |  | 18.16 | 0.57 | 1-719 | EM | 0.00 | hetero-1-1-mer | 4 x SF4, 2 x FES, 2 x MGD, 1 x W, 1 x FMN | HHblits | 0.30 |
| ``` target    IMDGKNLVENKLTDSHWFIECM-ERGAKIVVIAPEYGPPSTKADYWIPIRPQTDAALWLGITRLMIEKKWYDETFVKGFT 7vw6.1    VVIGANPTVNHPVAATFLKNAVKQRGAKLIIMDPRRQTLSRHAYRHLAFRPGSDVAMLNAMLNVIVTEGLYDEQYIAGYT  target    DFPLLVRTDTLQRLRAHEVFPQYKTSLSADGPSMKIQGLSAEQHAKLGDFVVWDGKTNAPAAITRDDVGATITKKGIDPV 7vw6.1    EN------------------------------------------------------------------------------  target    LAGSFKVKLVDGKEVEVATLWTLYQDHLKDYDLDTVVEITQAPKEMIEQLAQDIATMKPVAIHQGEGINHWFHATEMNRA 7vw6.1    --------------------FEALREKIVDFTPEKMASVCGIDAETLREVARLYARAKSSLIFWGMGVSQHVHGTDNSRC  target    AYLPLMLTGNIGRPGAGCQTWAGNYKAALFQGSPWTGPGFKGWVAE-DPFDINLNPKAHGKEIHAHAYTKDEEPAYWNHG 7vw6.1    LIALALITGQIGRPGTGLHPLRGQNNV---QGASD-----AGLIPMVYPDYQSVEKDAVRE----------LFEEFWGQS  target    DLALIVDTPKFGRKNFTGKTHMPTPTKALIFNNVNLINNAKWAYGMIKNVNPNVEMIVSMDIQMTASIEYADLALPANSW 7vw6.1    -LD---PQKGLTVVEI-MRAIHAGEIRGMFVEGENPAMSDPDLNHA-RHALAMLDHLVVQDLFLTETAFHADVVLPASAF  target    LEFEGLEITASCSNPFLQIWKGGIPPVFDSRDDLDILAGIANALADVTGEKRFRDYFAFAAADKRGIYIQRLLDTCTTTA 7vw6.1    AEKAGTF---TNTDRRVQIAQPVVAPPGDARQDWWIIQELARRLDLDWNYGGPA------------DIFAEMAQVMPSLN  target    GYKLADIMAGKYGPPGGCLLNFRTYPRIPFYEQVHDSEPFHTDTGRMHAYADVPEAIEYGENFIVHREGPEATPYLPNVI 7vw6.1    NITWERLEREGAVT-----YPVDA-PDQPGNE-IIFYAGFPTESGRAKIVPAAIV-------------PPD-----E---  target    VSSNPHIRPEDYGIAADAEHWDDRTIRNIKMPWSKVKETKNFLWEKGFQFYCLTPKTRHRVHSG--WSNVDWHMLMDSNF 7vw6.1    ------------------------------------------VPDDEFPMVLSTGRVLEHWHTGSMTRRAGVLDA-----  target    GDPYRLDKRAPCVGEHQLHINPQAARDLNINDGDYVYVDANPADRPYLGAKPDDPFYRVSRCMLRVTYNRAYPYNIVMMK 7vw6.1    --------LE---PEAVAFMAPKELYRLGLRPGGSMRLETRR-----------------GAVVLKVRSDRDVPIGMIFMP  target    HAPFIATEKSVKAHETRPDGRALSANTGYQANLRYGSQQSVTRNWHMPMHQTDTLFHKSKVFMGFIFGGEADNHAVNTVP 7vw6.1    FCY-----------------------------------------------------------------------------  target    KETLVRVTKAEDGGMGGKGIWQPATTGFSPDNESDFMKKYLAGELTKVKT 7vw6.1    -------------------------------------------------- ``` | | | | | | | | | | | | | | | | | | | | | | | | | | | | | | | | | | | | | | | | | | | | | | | | | |
|  | 7e5z.1.A | Formate dehydrogenase  *Dehydrogenase holoenzyme* | 0.27 |  | 17.82 | 0.56 | 1-720 | EM | 0.00 | hetero-1-1-mer | 1 x W, 2 x MGD, 2 x FES, 4 x SF4, 1 x FMN | HHblits | 0.29 |
| ``` target    IMDGKNLVENKLTDSHWFIEC-MERGAKIVVIAPEYGPPSTKADYWIPIRPQTDAALWLGITRLMIEKKWYDETFVKGFT 7e5z.1    VVIGANPTVNHPVAATFLKNAVKQRGAKLIIMDPRRQTLSRHAYRHLAFRPGSDVAMLNAMLNVIVTEGLYDEQYIAGYT  target    DFPLLVRTDTLQRLRAHEVFPQYKTSLSADGPSMKIQGLSAEQHAKLGDFVVWDGKTNAPAAITRDDVGATITKKGIDPV 7e5z.1    EN------------------------------------------------------------------------------  target    LAGSFKVKLVDGKEVEVATLWTLYQDHLKDYDLDTVVEITQAPKEMIEQLAQDIATMKPVAIHQGEGINHWFHATEMNRA 7e5z.1    --------------------FEALREKIVDFTPEKMASVCGIDAETLREVARLYARAKSSLIFWGMGVSQHVHGTDNSRC  target    AYLPLMLTGNIGRPGAGCQTWAGNYKAA--LFQGS-PWTGPGFKGWVAEDPFDINLNPKAHGKEIHAHAYTKDEEPAYWN 7e5z.1    LIALALITGQIGRPGTGLHPLRGQNNVQGASDAGLIPMVYPDYQS----------VEKDAVRE----------LFEEFWG  target    HGDLALIVDTPKFG-RKNFTGKTHMPTPTKALIFNNVNLINNAKWAYGMIKNVNPNVEMIVSMDIQMTASIEYADLALPA 7e5z.1    QS-LD---PQKGLTVVEIM--RAIHAGEIRGMFVEGENPAMSDPDLNHA-RHALAMLDHLVVQDLFLTETAFHADVVLPA  target    NSWLEFEGLEITASCSNPFLQIWKGGIPPVFDSRDDLDILAGIANALADVTGEKRFRDYFAFAAADKRGIYIQRLLDTCT 7e5z.1    SAFAEKAGTF---TNTDRRVQIAQPVVAPPGDARQDWWIIQELARRLDLDWNYGGPA------------DIFAEMAQVMP  target    TTAGYKLADIMAGKYGPPGGCLLNFRTYPRIPFYEQVHDSEPFHTDTGRMHAYADVPEAIEYGENFIVHREGPEATPYLP 7e5z.1    SLNNITWERLEREGAVT-----YPVDA-PDQPGNE-IIFYAGFPTESGRAKIVPAAIV------------PPD------E  target    NVIVSSNPHIRPEDYGIAADAEHWDDRTIRNIKMPWSKVKETKNFLWEKGFQFYCLTPKTRHRVHSG--WSNVDWHMLMD 7e5z.1    ---------------------------------------------VPDDEFPMVLSTGRVLEHWHTGSMTRRAGVLDA--  target    SNFGDPYRLDKRAPCVGEHQLHINPQAARDLNINDGDYVYVDANPADRPYLGAKPDDPFYRVSRCMLRVTYNRAYPYNIV 7e5z.1    -----------LE---PEAVAFMAPKELYRLGLRPGGSMRLETRR-----------------GAVVLKVRSDRDVPIGMI  target    MMKHAPFIATEKSVKAHETRPDGRALSANTGYQANLRYGSQQSVTRNWHMPMHQTDTLFHKSKVFMGFIFGGEADNHAVN 7e5z.1    FMPFCYA-------------------------------------------------------------------------  target    TVPKETLVRVTKAEDGGMGGKGIWQPATTGFSPDNESDFMKKYLAGELTKVKT 7e5z.1    ----------------------------------------------------- ``` | | | | | | | | | | | | | | | | | | | | | | | | | | | | | | | | | | | | | | | | | | | | | | | | | |
|  | 4ydd.1.A | DMSO reductase family type II enzyme, molybdopterin subunit  *Crystal structure of the perchlorate reductase PcrAB from Azospira suillum PS* | 0.28 |  | 30.12 | 0.49 | 1-442 | X-ray | 1.86 | hetero-oligomer | 4 x SF4, 1 x MO, 1 x MGD, 1 x MD1, 1 x F3S | BLAST | 0.35 |
| ``` target    IMDGKNLVENKLTDSHWFIECMERGAKIVVIAPEYGPPSTKADYWIPIRPQTDAALWLGITRLMIEKKWYDETFVKGFTD 4ydd.1    ILWGSNPTQTRIPDAHFLSEAQLNGAKIVSISPDYNSSTIKVDKWIHPQPGTDGALAMAMAHVIIKEKLYDAHSLKEQTD  target    FPLLVRTDTLQRLRAHEVFP-----QYKTSLSADGPSMKIQGLSAEQHAKLGDFVVWDGKTNAPAAITRDDVGATITKKG 4ydd.1    LSYLVRSDTKRFLREADVVAGGSKDKFYFWNAKTGKPVIPKGSWGDQPEKKGSPVGFLGRNTFAFPKGYIDLG------D  target    IDPVLAGSFKVKLVDGKEVEVATLWTLYQDHL-KDYDLDTVVEITQAPKEMIEQLAQDIATMKPVAIHQGEGINHWFHAT 4ydd.1    LDPALEGKFNMQLLDGKTVEVRPVFEILKSRLMADNTPEKAAKITGVTAKAITELAREFATAKPSMIICGGGTQHWYYSD  target    EMNRAAYLPLMLTGNIGRPGAGCQTWAGNYKAALFQGSPWTGPGFKGWVAEDPFDINLNPKAHGKE---IHAHAYTKDEE 4ydd.1    VLLRAMHLLTALTGTEGTNGGGMNHYIGQWKPAFVAG-----------LVALAFPEGVNKQRFCQTTIWTYIHAEVNDE-  target    PAYWNHGDLALIVDTPKFGRKNFTGKTHMPTPT-----KALIFNNVNLINNAKWAYGMIKNVNPNVEMIVSMDIQMTASI 4ydd.1    -------IISSDIDTEKYLRDSITTGQMPNMPEQGRDPKVFFVYRGNWLNQAKGQKYVLENLWPKLELIVDINIRMDSTA  target    EYADLALPANSWLEFEGLEITASCSNPFLQIWKGGIPPVFDSRDDLDILAGIANALADVTGEKRFRDYFAFAAADKRGIY 4ydd.1    LYSDVVLPSAHW--YEKLDLNVTSEHSYINMTEPAIKPMWESKTDWQIFLALAKRV------------------------  target    IQRLLDTCTTTAGYKLADIMAGKYGPPGGCLLNFRTYPRIPFYEQVHDSEPFHTDTGRMHAYADVPEAIEYGENFIVHRE 4ydd.1    --------------------------------------------------------------------------------  target    GPEATPYLPNVIVSSNPHIRPEDYGIAADAEHWDDRTIRNIKMPWSKVKETKNFLWEKGFQFYCLTPKTRHRVHSGWSNV 4ydd.1    --------------------------------------------------------------------------------  target    DWHMLMDSNFGDPYRLDKRAPCVGEHQLHINPQAARDLNINDGDYVYVDANPADRPYLGAKPDDPFYRVSRCMLRVTYNR 4ydd.1    --------------------------------------------------------------------------------  target    AYPYNIVMMKHAPFIATEKSVKAHETRPDGRALSANTGYQANLRYGSQQSVTRNWHMPMHQTDTLFHKSKVFMGFIFGGE 4ydd.1    --------------------------------------------------------------------------------  target    ADNHAVNTVPKETLVRVTKAEDGGMGGKGIWQPATTGFSPDNESDFMKKYLAGELTKVKT 4ydd.1    ------------------------------------------------------------ ``` | | | | | | | | | | | | | | | | | | | | | | | | | | | | | | | | | | | | | | | | | | | | | | | | | |
|  | 5e7o.1.A | DMSO reductase family type II enzyme, molybdopterin subunit  *Crystal structure of the perchlorate reductase PcrAB mutant W461E of PcrA from Azospira suillum PS* | 0.28 |  | 30.12 | 0.49 | 1-442 | X-ray | 2.40 | hetero-oligomer | 4 x SF4, 1 x MO, 1 x MGD, 1 x MD1, 1 x F3S | BLAST | 0.35 |
| ``` target    IMDGKNLVENKLTDSHWFIECMERGAKIVVIAPEYGPPSTKADYWIPIRPQTDAALWLGITRLMIEKKWYDETFVKGFTD 5e7o.1    ILWGSNPTQTRIPDAHFLSEAQLNGAKIVSISPDYNSSTIKVDKWIHPQPGTDGALAMAMAHVIIKEKLYDAHSLKEQTD  target    FPLLVRTDTLQRLRAHEVFP-----QYKTSLSADGPSMKIQGLSAEQHAKLGDFVVWDGKTNAPAAITRDDVGATITKKG 5e7o.1    LSYLVRSDTKRFLREADVVAGGSKDKFYFWNAKTGKPVIPKGSWGDQPEKKGSPVGFLGRNTFAFPKGYIDLG------D  target    IDPVLAGSFKVKLVDGKEVEVATLWTLYQDHL-KDYDLDTVVEITQAPKEMIEQLAQDIATMKPVAIHQGEGINHWFHAT 5e7o.1    LDPALEGKFNMQLLDGKTVEVRPVFEILKSRLMADNTPEKAAKITGVTAKAITELAREFATAKPSMIICGGGTQHWYYSD  target    EMNRAAYLPLMLTGNIGRPGAGCQTWAGNYKAALFQGSPWTGPGFKGWVAEDPFDINLNPKAHGKE---IHAHAYTKDEE 5e7o.1    VLLRAMHLLTALTGTEGTNGGGMNHYIGQEK-----------PAFVAGLVALAFPEGVNKQRFCQTTIWTYIHAEVNDE-  target    PAYWNHGDLALIVDTPKFGRKNFTGKTHMPTPT-----KALIFNNVNLINNAKWAYGMIKNVNPNVEMIVSMDIQMTASI 5e7o.1    -------IISSDIDTEKYLRDSITTGQMPNMPEQGRDPKVFFVYRGNWLNQAKGQKYVLENLWPKLELIVDINIRMDSTA  target    EYADLALPANSWLEFEGLEITASCSNPFLQIWKGGIPPVFDSRDDLDILAGIANALADVTGEKRFRDYFAFAAADKRGIY 5e7o.1    LYSDVVLPSAHW--YEKLDLNVTSEHSYINMTEPAIKPMWESKTDWQIFLALAKRV------------------------  target    IQRLLDTCTTTAGYKLADIMAGKYGPPGGCLLNFRTYPRIPFYEQVHDSEPFHTDTGRMHAYADVPEAIEYGENFIVHRE 5e7o.1    --------------------------------------------------------------------------------  target    GPEATPYLPNVIVSSNPHIRPEDYGIAADAEHWDDRTIRNIKMPWSKVKETKNFLWEKGFQFYCLTPKTRHRVHSGWSNV 5e7o.1    --------------------------------------------------------------------------------  target    DWHMLMDSNFGDPYRLDKRAPCVGEHQLHINPQAARDLNINDGDYVYVDANPADRPYLGAKPDDPFYRVSRCMLRVTYNR 5e7o.1    --------------------------------------------------------------------------------  target    AYPYNIVMMKHAPFIATEKSVKAHETRPDGRALSANTGYQANLRYGSQQSVTRNWHMPMHQTDTLFHKSKVFMGFIFGGE 5e7o.1    --------------------------------------------------------------------------------  target    ADNHAVNTVPKETLVRVTKAEDGGMGGKGIWQPATTGFSPDNESDFMKKYLAGELTKVKT 5e7o.1    ------------------------------------------------------------ ``` | | | | | | | | | | | | | | | | | | | | | | | | | | | | | | | | | | | | | | | | | | | | | | | | | |
|  | 7nz1.1.E | NADH-quinone oxidoreductase subunit G  *Respiratory complex I from Escherichia coli - focused refinement of cytoplasmic arm* | 0.24 |  | 14.68 | 0.52 | 1-720 | EM | 0.00 | hetero-1-1-1-1-1-1-… | 7 x SF4, 2 x FES, 1 x FMN, 1 x CA | HHblits | 0.26 |
| ``` target    IMDGKNLVENKLTDSHWFIECMERGAK--------------------------IVVIAPEYGPPSTKADYWIPIRPQTDA 7nz1.1    LVLGEDVTQTGARVALAVRQAVKGKAREMAAAQKVADWQIAAILNIGQRAKHPLFVTNVDDTRLDDIAAWTYRAPVEDQA  target    ALWLGITRLMIEKKWYDETFVKGFTDFPLLVRTDTLQRLRAHEVFPQYKTSLSADGPSMKIQGLSAEQHAKLGDFVVWDG 7nz1.1    RLGFAIAHALDNSAP-----------------------------------------------------------------  target    KTNAPAAITRDDVGATITKKGIDPVLAGSFKVKLVDGKEVEVATLWTLYQDHLKDYDLDTVVEITQAPKEMIEQLAQDIA 7nz1.1    ---------------------------------------------------AVDGIEPEL--------QSKIDVIVQALA  target    TMKPVAIHQGEGINHWFHATEMNRAAYLPLMLTGNIGRPGAGCQTWAGNYKAALFQGSPWTGPGFKGWVAEDPFDINLNP 7nz1.1    GAKKPLIISGTNAG----SLEVIQAAANVAKALKGRGADVGITMIA-RSV---NSMG-------L---G---IM------  target    KAHGKEIHAHAYTKDEEPAYWNHGDLALIVDTPKFGRKNFTGKTHMPTPTKALIFNNVNLINNAKWAYGMIKNVNPNVEM 7nz1.1    ---G-----------G-------GSLEEALTE------------LETGRADAVVVLE-NDLHRHASAI-RVNAALAKAPL  target    IVSMDIQMTASIEYADLALPANSWLEFEGLEITASCSNPFLQIWKGGIPPVF-----DSRDDLDILAGIANALADVT-GE 7nz1.1    VMVVDHQRTAIMENAHLVLSAASFAESDGTVI---NNEGRAQRFFQVYDPAYYDSKTVMLESWRWLHSLHSTLLSREVDW  target    KRFRDYFAFAAADKRGIYIQRLLDTCTTTAGYKLA------------------------DIMAGK-YGPP---GG--CLL 7nz1.1    TQLDHVID------------AVVAKIPELAGIKDAAPDATFRIRGQKLAREPHRYSGRTAMRANISVHEPRQPQDIDTMF  target    NF--------RTY-PRIPFY-----E-QVHDSEPFHTDTGRMHAYADVPEAIEYGE-NFIVHREGPEATPYLPNVIVSSN 7nz1.1    TFSMEGNNQPTAHRSQVPFAWAPGWNSPQAWNKFQDEVGGKLRFGDPGVRLFETSENGLDYFTSV---------------  target    PHIRPEDYGIAADAEHWDDRTIRNIKMPWSKVKETKNFLWEKGFQFYCLTPKTRHRVHSGWSNVDWHMLMDSNFGDPYRL 7nz1.1    ----PA-------------------------------RFQPQDGKWRIAPYYHLFGSDELSQRAPVFQSRM---------  target    DKRAPCVGEHQLHINPQAARDLNINDGDYVYVDANPADRPYLGAKPDDPFYRVSRCMLRVTYNRAYPYNIVMMKHAPFIA 7nz1.1    -------PQPYIKLNPADAAKLGVNAGTRVSFSYD-----------------GNTVTLPVEIAEGLTAGQVGLPMGMS--  target    TEKSVKAHETRPDGRALSANTGYQANLRYGSQQSVTRNWHMPMHQTDTLFHKSKVFMGFIFGGEADNHAVNTVPKETLVR 7nz1.1    --------------------------------------------------------------------------------  target    VTKAEDGGMGGKGIWQPATTGFSPDNESDFMKKYLAGELTKVKT 7nz1.1    -------------------------------------------- ``` | | | | | | | | | | | | | | | | | | | | | | | | | | | | | | | | | | | | | | | | | | | | | | | | | |
|  | 7p61.1.C | NADH-quinone oxidoreductase  *Complex I from E. coli, DDM-purified, with NADH, Resting state* | 0.23 |  | 14.19 | 0.52 | 1-720 | EM | 0.00 | hetero-1-1-1-1-1-1-… | 7 x SF4, 1 x FMN, 1 x NAI, 2 x FES, 1 x CA, 2 x 3PE, 1 x UQ8 | HHblits | 0.26 |
| ``` target    IMDGKNLVENKLTDSHWFIECMERGAK--------------------------IVVIAPEYGPPSTKADYWIPIRPQTDA 7p61.1    LVLGEDVTQTGARVALAVRQAVKGKAREMAAAQKVADWQIAAILNIGQRAKHPLFVTNVDDTRLDDIAAWTYRAPVEDQA  target    ALWLGITRLMIEKKWYDETFVKGFTDFPLLVRTDTLQRLRAHEVFPQYKTSLSADGPSMKIQGLSAEQHAKLGDFVVWDG 7p61.1    RLGFAIAHALDNSAP-----------------------------------------------------------------  target    KTNAPAAITRDDVGATITKKGIDPVLAGSFKVKLVDGKEVEVATLWTLYQDHLKDYDLDTVVEITQAPKEMIEQLAQDIA 7p61.1    ---------------------------------------------------AVDGIEPEL--------QSKIDVIVQALA  target    TMKPVAIHQGEGINHWFHATEMNRAAYLPLMLTGNIGRPGAGCQTWAGNYKAALFQGSPWTGPGFKGWVAEDPFDINLNP 7p61.1    GAKKPLIISGTNAG----SLEVIQAAANVAKALKGRGADVGITMIA-RSVN---SMGL----------G---IM------  target    KAHGKEIHAHAYTKDEEPAYWNHGDLALIVDTPKFGRKNFTGKTHMPTPTKALIFNNVNLINNAKWAYGMIKNVNPNVEM 7p61.1    ---G-----------G-------GSLEEALTE------------LETGRADAVVVLE-NDLHRHASAT-RVNAALAKAPL  target    IVSMDIQMTASIEYADLALPANSWLEFEGLEITASCSNPFLQIWKGGIPPVF-----DSRDDLDILAGIANALADVT-GE 7p61.1    VMVVDHQRTAIMENAHLVLSAASFAESDGTVI---NNEGRAQRFFQVYDPAYYDSKTVMLESWRWLHSLHSTLLSREVDW  target    KRFRDYFAFAAADKRGIYIQRLLDTCTTTAGYKLAD-----------IMAG--------------KYGPP---GG--CLL 7p61.1    TQLDHVID------------AVVAKIPELAGIKDAAPDATFRIRGQKLAREPHRYSGRTAMRANISVHEPRQPQDIDTMF  target    N--------------FRTYPRI-PFYEQVHDSEPFHTDTGRMHAYADVPEAIEYGENFIVHREGPEATPYLPNVIVSSNP 7p61.1    TFSMEGNNQPTAHRSQVPFAWAPGWNSPQAWNKFQDEVGGKLRFGDPGVRLFETSE------NG---LDYFTS------V  target    HIRPEDYGIAADAEHWDDRTIRNIKMPWSKVKETKNFLWEKGFQFYCLTPKTRHRVHSGWSNVDWHMLMDSNFGDPYRLD 7p61.1    ---PA-------------------------------RFQPQDGKWRIAPYYHLFGSDELSQRAPVFQSRM----------  target    KRAPCVGEHQLHINPQAARDLNINDGDYVYVDANPADRPYLGAKPDDPFYRVSRCMLRVTYNRAYPYNIVMMKHAPFIAT 7p61.1    ------PQPYIKLNPADAAKLGVNAGTRVSFSYD-----------------GNTVTLPVEIAEGLTAGQVGLPMGMS---  target    EKSVKAHETRPDGRALSANTGYQANLRYGSQQSVTRNWHMPMHQTDTLFHKSKVFMGFIFGGEADNHAVNTVPKETLVRV 7p61.1    --------------------------------------------------------------------------------  target    TKAEDGGMGGKGIWQPATTGFSPDNESDFMKKYLAGELTKVKT 7p61.1    ------------------------------------------- ``` | | | | | | | | | | | | | | | | | | | | | | | | | | | | | | | | | | | | | | | | | | | | | | | | | |
|  | 7p63.1.C | NADH-quinone oxidoreductase  *Complex I from E. coli, DDM/LMNG-purified, under Turnover at pH 6, Closed state* | 0.23 |  | 13.99 | 0.52 | 1-720 | EM | 0.00 | hetero-1-1-1-1-1-1-… | 7 x SF4, 1 x FMN, 1 x NAI, 2 x FES, 1 x CA, 1 x DCQ, 4 x LFA, 8 x 3PE | HHblits | 0.26 |
| ``` target    IMDGKNLVENKLTDSHWFIECMERGAK--------------------------IVVIAPEYGPPSTKADYWIPIRPQTDA 7p63.1    LVLGEDVTQTGARVALAVRQAVKGKAREMAAAQKVADWQIAAILNIGQRAKHPLFVTNVDDTRLDDIAAWTYRAPVEDQA  target    ALWLGITRLMIEKKWYDETFVKGFTDFPLLVRTDTLQRLRAHEVFPQYKTSLSADGPSMKIQGLSAEQHAKLGDFVVWDG 7p63.1    RLGFAIAHALDNSAP-----------------------------------------------------------------  target    KTNAPAAITRDDVGATITKKGIDPVLAGSFKVKLVDGKEVEVATLWTLYQDHLKDYDLDTVVEITQAPKEMIEQLAQDIA 7p63.1    ---------------------------------------------------AVDGIEPEL--------QSKIDVIVQALA  target    TMKPVAIHQGEGINHWFHATEMNRAAYLPLMLTGNIGRPGAGCQTWAGNYKAALFQGSPWTGPGFKGWVAEDPFDINLNP 7p63.1    GAKKPLIISGTNAG----SLEVIQAAANVAKALKGRGADVGITMIA-RSV---NSMGLG-----IM--------------  target    KAHGKEIHAHAYTKDEEPAYWNHGDLALIVDTPKFGRKNFTGKTHMPTPTKALIFNNVNLINNAKWAYGMIKNVNPNVEM 7p63.1    -----------------G----GGSLEEALTE------------LETGRADAVVVLE-NDLHRHASAT-RVNAALAKAPL  target    IVSMDIQMTASIEYADLALPANSWLEFEGLEITASCSNPFLQIWKGGIPPVF-----DSRDDLDILAGIANALADVT-GE 7p63.1    VMVVDHQRTAIMENAHLVLSAASFAESDGTVI---NNEGRAQRFFQVYDPAYYDSKTVMLESWRWLHSLHSTLLSREVDW  target    KRFRDYFAFAAADKRGIYIQRLLDTCTTTAGYKLAD------------------------IMAG-KYGPPGG-----CLL 7p63.1    TQLDHVI------------DAVVAKIPELAGIKDAAPDATFRIRGQKLAREPHRYSGRTAMRANISVHEPRQPQDIDTMF  target    N--------------FRTYPRIPFYEQ-VHDSEPFHTDTGRMHAYADVPEAIEYGE-NFIVHREGPEATPYLPNVIVSSN 7p63.1    TFSMEGNNQPTAHRSQVPFAWAPGWNSPQAWNKFQDEVGGKLRFGDPGVRLFETSENGLDYFTSV---------------  target    PHIRPEDYGIAADAEHWDDRTIRNIKMPWSKVKETKNFLWEKGFQFYCLTPKTRHRVHSGWSNVDWHMLMDSNFGDPYRL 7p63.1    P----A-------------------------------RFQPQDGKWRIAPYYHLFGSDELSQRAPVFQSRM---------  target    DKRAPCVGEHQLHINPQAARDLNINDGDYVYVDANPADRPYLGAKPDDPFYRVSRCMLRVTYNRAYPYNIVMMKHAPFIA 7p63.1    -------PQPYIKLNPADAAKLGVNAGTRVSFSYD-----------------GNTVTLPVEIAEGLTAGQVGLPMGMS--  target    TEKSVKAHETRPDGRALSANTGYQANLRYGSQQSVTRNWHMPMHQTDTLFHKSKVFMGFIFGGEADNHAVNTVPKETLVR 7p63.1    --------------------------------------------------------------------------------  target    VTKAEDGGMGGKGIWQPATTGFSPDNESDFMKKYLAGELTKVKT 7p63.1    -------------------------------------------- ``` | | | | | | | | | | | | | | | | | | | | | | | | | | | | | | | | | | | | | | | | | | | | | | | | | |
|  | 8bqg.1.A | Formate dehydrogenase, alpha subunit, selenocysteine-containing  *W-formate dehydrogenase from Desulfovibrio vulgaris - Soaking with Formate 1 min* | 0.27 |  | 22.16 | 0.46 | 1-444 | X-ray | 1.95 | hetero-1-1-mer | 2 x MGD, 4 x SF4, 1 x H2S, 1 x W | HHblits | 0.30 |
| ``` target    IMDGKNLVENKLTDSHWFIECMERGAKIVVIAPEYGPPSTKADYWIPIRPQTDAALWLGITRLMIEKKWYDETFVKGFTD 8bqg.1    LIMGSNAAENHPIAFKWVLRAKDKGATLIHVDPRFTRTSARCDVYAPIRSGADIPFLGGLIKYILDNKLYFTDYVREYTN  target    FPLLVRTDTLQRLRAHEVFPQYKTSLSADGPSMKIQGLSAEQHAKLGDFVVWDGKTNAPAAITRDDVGATITKKGIDPVL 8bqg.1    ASLIVGEKFSF---KD------------------------------GLFSGYDAANKKYDKS--------------MWAF  target    AGSF-KVKLVDGKEVEVATLWTLYQDHLKDYDLDTVVEITQAPKEMIEQLAQDIATM----KPVAIHQGEGINHWFHATE 8bqg.1    ELDANGVPKRDPALKHPRCVINLLKKHYERYNLDKVAAITGTSKEQLQQVYKAYAATGKPDKAGTIMYAMGWTQHSVGVQ  target    MNRAAYLPLMLTGNIGRPGAGCQTWAGNYKAA--LFQGSP-WTGPGFKGWVAE-DPFD--IN-LNPKAHGKEIH------ 8bqg.1    NIRAMAMIQLLLGNIGVAGGGVNALRGESNVQGSTDQGLLAHIWPGYNPVPNSKAATLELYNAATPQS-KDPMSVNWWQN  target    ---------AHAYTKDEEPAYWNHGDLALIVDTPKF----GRKNFTGKTHMPTPTKALIFNNVNLINNAKWAYGMIKNVN 8bqg.1    RPKYVASYLKALYP-DEEPAA-AYDYLPRIDAGRKLTDYFWLNIF--EKMDKGEFKGLFAWGMNPACGGANANKNR-KAM  target    PNVEMIVSMDIQMTASIEY--------AD-----LALPANSWLEFEGLEITASCSNPFLQIWKGGIPPVFDSRDDLDILA 8bqg.1    GKLEWLVNVNLFENETSSFWKGPGMNPAEIGTEVFFLPCCVSIEKEGSVA---NSGRWMQWRYRGPKPYAETKPDGDIML  target    GIANALADVTGEKRFRDYFAFAAADKRGIYIQRLLDTCTTTAGYKLADIMAGKYGPPGGCLLNFRTYPRIPFYEQVHDSE 8bqg.1    DMFKKVRE------------------------------------------------------------------------  target    PFHTDTGRMHAYADVPEAIEYGENFIVHREGPEATPYLPNVIVSSNPHIRPEDYGIAADAEHWDDRTIRNIKMPWSKVKE 8bqg.1    --------------------------------------------------------------------------------  target    TKNFLWEKGFQFYCLTPKTRHRVHSGWSNVDWHMLMDSNFGDPYRLDKRAPCVGEHQLHINPQAARDLNINDGDYVYVDA 8bqg.1    --------------------------------------------------------------------------------  target    NPADRPYLGAKPDDPFYRVSRCMLRVTYNRAYPYNIVMMKHAPFIATEKSVKAHETRPDGRALSANTGYQANLRYGSQQS 8bqg.1    --------------------------------------------------------------------------------  target    VTRNWHMPMHQTDTLFHKSKVFMGFIFGGEADNHAVNTVPKETLVRVTKAEDGGMGGKGIWQPATTGFSPDNESDFMKKY 8bqg.1    --------------------------------------------------------------------------------  target    LAGELTKVKT 8bqg.1    ---------- ``` | | | | | | | | | | | | | | | | | | | | | | | | | | | | | | | | | | | | | | | | | | | | | | | | | |
|  | 1h0h.1.A | FORMATE DEHYDROGENASE SUBUNIT ALPHA  *Tungsten containing Formate Dehydrogenase from Desulfovibrio Gigas* | 0.26 |  | 20.05 | 0.46 | 1-444 | X-ray | 1.80 | hetero-1-1-mer | 1 x W, 1 x 2MD, 1 x MGD, 4 x SF4, 1 x CA | HHblits | 0.30 |
| ``` target    IMDGKNLVENKLTDSHWFIECMERGAKIVVIAPEYGPPSTKADYWIPIRPQTDAALWLGITRLMIEKKWYDETFVKGFTD 1h0h.1    LMMGSNPAENHPISFKWVMRAKDKGATLIHVDPRYTRTSTKCDLYAPLRSGSDIAFLNGMTKYILEKELYFKDYVVNYTN  target    FPLLVRTDTLQRLRAHEVFPQYKTSLSADGPSMKIQGLSAEQHAKLGDFVVWDGKTNAPAAITRDDVGATITKKGIDPVL 1h0h.1    ASFIVGEGFA---FE------------------------------EGLFAGYNKETRKYDKSKW----------GFERDE  target    AGSFKVKLVDGKEVEVATLWTLYQDHLKDYDLDTVVEITQAPKEMIEQLAQDIATM----KPVAIHQGEGINHWFHATEM 1h0h.1    NGNP---KRDETLKHPRCVFQIMKKHYERYDLDKISAICGTPKELILKVYDAYCATGKPDKAGTIMYAMGWTQHTVGVQN  target    NRAAYLPLMLTGNIGRPGAGCQTWAGNYKAA--LFQGS-PWTGPGFKGWVA-E----DPFDINLNPKAHGKEIHAHAYTK 1h0h.1    IRAMSINQLLLGNIGVAGGGVNALRGEANVQGSTDHGLLMHIYPGYLGTARASIPTYEEYTKKFTPVSKDP-QS-ANW-W  target    DEEPAYWNHGDLALIVDTPK----------------FGRKNFTGKTHMPTPTKALIFNNVNLINNAKWAYGMIKNVNPNV 1h0h.1    SNFPKYSA-SYIKSMWPDADLNEAYGYLPKGEDGKDYSWLTL-FDDMFQGKIKGFFAWGQNPACSGANSNKTR-EALTKL  target    EMIVSMDIQMTASIEYA-------------DLALPANSWLEFEGLEITASCSNPFLQIWKGGIPPVFDSRDDLDILAGIA 1h0h.1    DWMVNVNIFDNETGSFWRGPDMDPKKIKTEVFFLPCAVAIEKEGSIS---NSGRWMQWRYVGPEPRKNAIPDGDLIVELA  target    NALADVTGEKRFRDYFAFAAADKRGIYIQRLLDTCTTTAGYKLADIMAGKYGPPGGCLLNFRTYPRIPFYEQVHDSEPFH 1h0h.1    KRVQK---------------------------------------------------------------------------  target    TDTGRMHAYADVPEAIEYGENFIVHREGPEATPYLPNVIVSSNPHIRPEDYGIAADAEHWDDRTIRNIKMPWSKVKETKN 1h0h.1    --------------------------------------------------------------------------------  target    FLWEKGFQFYCLTPKTRHRVHSGWSNVDWHMLMDSNFGDPYRLDKRAPCVGEHQLHINPQAARDLNINDGDYVYVDANPA 1h0h.1    --------------------------------------------------------------------------------  target    DRPYLGAKPDDPFYRVSRCMLRVTYNRAYPYNIVMMKHAPFIATEKSVKAHETRPDGRALSANTGYQANLRYGSQQSVTR 1h0h.1    --------------------------------------------------------------------------------  target    NWHMPMHQTDTLFHKSKVFMGFIFGGEADNHAVNTVPKETLVRVTKAEDGGMGGKGIWQPATTGFSPDNESDFMKKYLAG 1h0h.1    --------------------------------------------------------------------------------  target    ELTKVKT 1h0h.1    ------- ``` | | | | | | | | | | | | | | | | | | | | | | | | | | | | | | | | | | | | | | | | | | | | | | | | | |
|  | 6sdr.1.A | Formate dehydrogenase, alpha subunit, selenocysteine-containing  *W-formate dehydrogenase from Desulfovibrio vulgaris - Oxidized form* | 0.27 |  | 20.73 | 0.46 | 1-444 | X-ray | 2.10 | hetero-1-1-mer | 2 x MGD, 4 x SF4, 1 x H2S, 1 x W | HHblits | 0.30 |
| ``` target    IMDGKNLVENKLTDSHWFIECMERGAKIVVIAPEYGPPSTKADYWIPIRPQTDAALWLGITRLMIEKKWYDETFVKGFTD 6sdr.1    LIMGSNAAENHPIAFKWVLRAKDKGATLIHVDPRFTRTSARCDVYAPIRSGADIPFLGGLIKYILDNKLYFTDYVREYTN  target    FPLLVRTDTLQRLRAHEVFPQYKTSLSADGPSMKIQGLSAEQHAKLGDFVVWDGKTNAPAAITRDDVGATITKKGIDPVL 6sdr.1    ASLIVGEKFSF---K------------------------------DGLFSGYDAANKKYDKSM-------W---AFELDA  target    AGSFKVKLVDGKEVEVATLWTLYQDHLKDYDLDTVVEITQAPKEMIEQLAQDIATM----KPVAIHQGEGINHWFHATEM 6sdr.1    NG---VPKRDPALKHPRCVINLLKKHYERYNLDKVAAITGTSKEQLQQVYKAYAATGKPDKAGTIMYAMGWTQHSVGVQN  target    NRAAYLPLMLTGNIGRPGAGCQTWAGNYKAAL--FQGS-PWTGPGFKGWVAEDPFDINLNPKAHGKEIHAHAYTKDEEPA 6sdr.1    IRAMAMIQLLLGNIGVAGGGVNALRGESNVQGSTDQGLLAHIWPGYNPVPNSKAATLEL----YNAATPQSKD--PMSVN  target    YWNH-GD-------------L----ALIVDTPKFGR--------KNFTGKTHMPTPTKALIFNNVNLINNAKWAYGMIKN 6sdr.1    WWQNRPKYVASYLKALYPDEEPAAAYDYLPRIDAGRKLTDYFWLNIF--EKMDKGEFKGLFAWGMNPACGGANANKNR-K  target    VNPNVEMIVSMDIQMTASIEY--------AD-----LALPANSWLEFEGLEITASCSNPFLQIWKGGIPPVFDSRDDLDI 6sdr.1    AMGKLEWLVNVNLFENETSSFWKGPGMNPAEIGTEVFFLPCCVSIEKEGSVA---NSGRWMQWRYRGPKPYAETKPDGDI  target    LAGIANALADVTGEKRFRDYFAFAAADKRGIYIQRLLDTCTTTAGYKLADIMAGKYGPPGGCLLNFRTYPRIPFYEQVHD 6sdr.1    MLDMFKKVRE----------------------------------------------------------------------  target    SEPFHTDTGRMHAYADVPEAIEYGENFIVHREGPEATPYLPNVIVSSNPHIRPEDYGIAADAEHWDDRTIRNIKMPWSKV 6sdr.1    --------------------------------------------------------------------------------  target    KETKNFLWEKGFQFYCLTPKTRHRVHSGWSNVDWHMLMDSNFGDPYRLDKRAPCVGEHQLHINPQAARDLNINDGDYVYV 6sdr.1    --------------------------------------------------------------------------------  target    DANPADRPYLGAKPDDPFYRVSRCMLRVTYNRAYPYNIVMMKHAPFIATEKSVKAHETRPDGRALSANTGYQANLRYGSQ 6sdr.1    --------------------------------------------------------------------------------  target    QSVTRNWHMPMHQTDTLFHKSKVFMGFIFGGEADNHAVNTVPKETLVRVTKAEDGGMGGKGIWQPATTGFSPDNESDFMK 6sdr.1    --------------------------------------------------------------------------------  target    KYLAGELTKVKT 6sdr.1    ------------ ``` | | | | | | | | | | | | | | | | | | | | | | | | | | | | | | | | | | | | | | | | | | | | | | | | | |
|  | 6sdv.1.A | Formate dehydrogenase, alpha subunit, selenocysteine-containing,Formate dehydrogenase, alpha subunit, selenocysteine-containing,W-formate dehydrogenase - alpha subunit  *W-formate dehydrogenase from Desulfovibrio vulgaris - Formate reduced form* | 0.26 |  | 20.47 | 0.46 | 1-444 | X-ray | 1.90 | hetero-1-1-mer | 2 x MGD, 4 x SF4, 1 x W, 1 x H2S | HHblits | 0.30 |
| ``` target    IMDGKNLVENKLTDSHWFIECMERGAKIVVIAPEYGPPSTKADYWIPIRPQTDAALWLGITRLMIEKKWYDETFVKGFTD 6sdv.1    LIMGSNAAENHPIAFKWVLRAKDKGATLIHVDPRFTRTSARCDVYAPIRSGADIPFLGGLIKYILDNKLYFTDYVREYTN  target    FPLLVRTDTLQRLRAHEVFPQYKTSLSADGPSMKIQGLSAEQHAKLGDFVVWDGKTNAPAAITRDDVGATITKKGIDPVL 6sdv.1    ASLIVGEKFSF---KDG------------------------------LFSGYDAANKKYDKSM-WA----F--ELDA---  target    AGSFKVKLVDGKEVEVATLWTLYQDHLKDYDLDTVVEITQAPKEMIEQLAQDIATM----KPVAIHQGEGINHWFHATEM 6sdv.1    NG---VPKRDPALKHPRCVINLLKKHYERYNLDKVAAITGTSKEQLQQVYKAYAATGKPDKAGTIMYAMGWTQHSVGVQN  target    NRAAYLPLMLTGNIGRPGAGCQTWAGNYKA--ALFQGSP-WTGPGFKGWVAEDPFDINLNPKAHGKEIHAHAYTKDEEPA 6sdv.1    IRAMAMIQLLLGNIGVAGGGVNALRGESNVQGSTDQGLLAHIWPGYNPVP--NSKAATLELYN-AA-TPQSKD--PMSVN  target    YWNH-GD-LA----------------LIVDTPKFG--------RKNFTGKTHMPTPTKALIFNNVNLINNAKWAYGMIKN 6sdv.1    WWQNRPKYVASYLKALYPDEEPAAAYDYLPRIDAGRKLTDYFWLNIF--EKMDKGEFKGLFAWGMNPACGGANANKNR-K  target    VNPNVEMIVSMDIQMTASIEY--------AD-----LALPANSWLEFEGLEITASCSNPFLQIWKGGIPPVFDSRDDLDI 6sdv.1    AMGKLEWLVNVNLFENETSSFWKGPGMNPAEIGTEVFFLPCCVSIEKEGSVA---NSGRWMQWRYRGPKPYAETKPDGDI  target    LAGIANALADVTGEKRFRDYFAFAAADKRGIYIQRLLDTCTTTAGYKLADIMAGKYGPPGGCLLNFRTYPRIPFYEQVHD 6sdv.1    MLDMFKKVRE----------------------------------------------------------------------  target    SEPFHTDTGRMHAYADVPEAIEYGENFIVHREGPEATPYLPNVIVSSNPHIRPEDYGIAADAEHWDDRTIRNIKMPWSKV 6sdv.1    --------------------------------------------------------------------------------  target    KETKNFLWEKGFQFYCLTPKTRHRVHSGWSNVDWHMLMDSNFGDPYRLDKRAPCVGEHQLHINPQAARDLNINDGDYVYV 6sdv.1    --------------------------------------------------------------------------------  target    DANPADRPYLGAKPDDPFYRVSRCMLRVTYNRAYPYNIVMMKHAPFIATEKSVKAHETRPDGRALSANTGYQANLRYGSQ 6sdv.1    --------------------------------------------------------------------------------  target    QSVTRNWHMPMHQTDTLFHKSKVFMGFIFGGEADNHAVNTVPKETLVRVTKAEDGGMGGKGIWQPATTGFSPDNESDFMK 6sdv.1    --------------------------------------------------------------------------------  target    KYLAGELTKVKT 6sdv.1    ------------ ``` | | | | | | | | | | | | | | | | | | | | | | | | | | | | | | | | | | | | | | | | | | | | | | | | | |
|  | 6f0k.1.B | Fe-S-cluster-containing hydrogenase  *Alternative complex III* | 0.19 |  | 14.61 | 0.47 | 1-720 | EM | 0.00 | hetero-1-1-1-1-1-1-… | 6 x HEC, 1 x F3S, 3 x SF4 | HHblits | 0.27 |
| ``` target    IMDGKNLVENK-LTD---S------HWFIECMERGAKIVVIAPEYGPPSTKADYWIPIRPQTDAALWLGITRLMIEKKWY 6f0k.1    VSLDADFLGPTDRNFVENTREFAASRRMERPEDEISRLYVIESTYTVTGGMADHRLRLRAGDIPAFAAALAAELGVGELR  target    DETFVKGFTDFPLLVRTDTLQRLRAHEVFPQYKTSLSADGPSMKIQGLSAEQHAKLGDFVVWDGKTNAPAAITRDDVGAT 6f0k.1    E-------------------------------------------------------------------------------  target    ITKKGIDPVLAGSFKVKLVDGKEVEVATLWTLYQDHLKDYDLDTVVEITQAPKEMIEQLAQDIATMKPVAIHQGEGINHW 6f0k.1    -------------------------------------------AGARFAGH--PYVVEIARDLRAAGARGVVLAGET--Q  target    FHATEMNRAAYLPLMLTGNIGRPGAGCQTWAGNYKAALFQGSPWTGPGFKGWVAEDPFDINLNPKAHGKEIHAHAYTKDE 6f0k.1    --PPAVHALCAVINDLLGSLGRTVILHA---------LD------EPAT-------------AQH---------------  target    EPAYWNHGDLALIVDTPKFGRKNFTGKTHMPTPTKALIFNNVNLINNAKWAYGMIKNVNPNVEMIVSMDIQMTASIEYAD 6f0k.1    -------AALAELVQA------------MQAGAVDALLLLNVNPVYDAPAALGFA-EALAQVPEVIHLGLHVDETARRST  target    LALPANSWLEFEGLEITASCSNPFLQIWKGGIPPVFDS-RDDLDILAGIANALADVTGEKRFRDYFAFAAADKRGIYIQR 6f0k.1    WHLPSTHYLEAWGDGRA---YDGTLSVIQPLIAPLYEAAHSPLEVLALLATGEEQS-----AYDLVR--------NTWRR  target    LLDTCTTTAGYKLADIMAGKYGPPGGCLLNFRTYPRIPFYEQVHDSEPFHTDTGRMHAYADVPEAIEYGENFIVHREGPE 6f0k.1    LLAGR-----GAFEQAWQR-VLHD-G------------FL----PDSGYPTVSLRPNR-----------QALADW-----  target    ATPYLPNVIVSSNPHIRPEDYGIAADAEHWDDRTIRNIKMPWSKVKETKNFLWEKGFQFYCLTPKTRHRVHSGWSNVDWH 6f0k.1    -----P----------------------------------------------QAAEGGLEVVFRLDPTVLDGSFANNAWA  target    MLMDSNFGDPYRLDKRAPCVGEHQLHINPQAARDLNIND--------GDYVYVDANPADRPYLGAKPDDPFYRVSRCMLR 6f0k.1    QELPD------PITKIV---WDNVAILSPKTAAALGVKAEYHKGVYIADVIELSLDG-----------------RAVELP  target    VTYNRAYPYNIVMMKHAPFIATEKSVKAHETRPDGRALSANTGYQANLRYGSQQSVTRNWHMPMHQTDTLFHKSKVFMGF 6f0k.1    VWVLPGHPDDSITVYLGYG-------------------------------------------------------------  target    IFGGEADNHAVNTVPKETLVRVTKAEDGGMGGKGIWQPATTGFSPDNESDFMKKYLAGELTKVKT 6f0k.1    ----------------------------------------------------------------- ``` | | | | | | | | | | | | | | | | | | | | | | | | | | | | | | | | | | | | | | | | | | | | | | | | | |
|  | 6lod.1.B | Fe-S-cluster-containing hydrogenase components 1-like protein  *Cryo-EM structure of the air-oxidized photosynthetic alternative complex III from Roseiflexus castenholzii* | 0.21 |  | 11.06 | 0.47 | 1-720 | EM | 0.00 | hetero-1-1-1-1-1-1-… | 6 x HEC, 2 x EL6, 3 x SF4, 1 x F3S | HHblits | 0.26 |
| ``` target    IMDGKNLVENKLTD---SHWFIECM------ERGAKIVVIAPEYGPPSTKADYWIPIRPQTDAALWLGITRLMIEKKWYD 6lod.1    VGFDADFTAPSPTGVRMARQLADGRRIRKGTKEVNRLYLAESTPSITGLLADHRLPVRSSQIEHLVRALATLVGVPNVA-  target    ETFVKGFTDFPLLVRTDTLQRLRAHEVFPQYKTSLSADGPSMKIQGLSAEQHAKLGDFVVWDGKTNAPAAITRDDVGATI 6lod.1    --------------------------------------------------------------------------------  target    TKKGIDPVLAGSFKVKLVDGKEVEVATLWTLYQDHLKDYDLDTVVEITQAPKEMIEQLAQDIATMKPVAIHQGEGINHWF 6lod.1    ------------------------------------------AGAPLSDTEKKWVEAAAKDLQANRGACVVLV-GESQP-  target    HATEMNRAAYLPLMLTGNIGRPGAGCQTWAGNYKAALFQGSPWTGPGFKGWVAEDPFDINLNPKAHGKEIHAHAYTKDEE 6lod.1    --PVVHALGHAINAQLGNVGST---VVYTE------P---VE--D----------------DPSG-G-------------  target    PAYWNHGDLALIVDTPKFGRKNFTGKTHMPTPTKALIFNNVNLINNAKWAYGMIKNVNPNVEMIVSMDIQMTASIEYADL 6lod.1    -----IAALSALTQE------------MNAGTVEVLLMIESNPVYNAPADIPF-AEALAKVPLSMHVGLYRDETAQQSVW  target    ALPANSWLEFEGLEITASCSNPFLQIWKGGIPPVFDSRDDLDILAGIANALADVTGEKRFRDYFAFAAADKRGIYIQRLL 6lod.1    HINGAHFLEAWGDVR---AFDGTTTIVQPLIAPLYNGKSAIEVLNVLLGKPQETG-YQTLTAYWQ------------TQD  target    DTCTTTAGYKLADIMAGKYGPPGGCLLNFRTYPRIPFYEQVHDSEPFHTDTGRMHAYADVPEAIEYGENFIVHREGPEAT 6lod.1    AS-G-NFRVFWNTALHDGVIT-------A---------------TQ--ARSRQVTLQ----------QGFADAAP-----  target    PYLPNVIVSSNPHIRPEDYGIAADAEHWDDRTIRNIKMPWSKVKETKNFLWEKGFQFYCLTPKTRHRVHSGWSNVDWHML 6lod.1    ---P---------------------------------------------APTQGLEIVFRPDP--SLWDGAFANNAWLQE  target    MDSNFGDPYRLDKRAPCVGEHQLHINPQAARDLNINDGDYVYVDANPADRPYLGAKPDDPFYRVSRCMLRVTYNRAYPYN 6lod.1    TPK------PYTKLT---WDNVALMSVRTANALGLKNGDVVRLTYQG-----------------RSVDAPVWVQPGHADD  target    IVMMKHAPFIATEKSVKAHETRPDGRALSANTGYQANLRYGSQQSVTRNWHMPMHQTDTLFHKSKVFMGFIFGGEADNHA 6lod.1    SVTVHFGFG-----------------------------------------------------------------------  target    VNTVPKETLVRVTKAEDGGMGGKGIWQPATTGFSPDNESDFMKKYLAGELTKVKT 6lod.1    ------------------------------------------------------- ``` | | | | | | | | | | | | | | | | | | | | | | | | | | | | | | | | | | | | | | | | | | | | | | | | | |
|  | 3o5a.1.A | Periplasmic nitrate reductase  *Crystal Structure of partially reduced Periplasmic Nitrate Reductase from Cupriavidus necator using Ionic Liquids* | 0.24 |  | 22.01 | 0.42 | 1-444 | X-ray | 1.72 | hetero-oligomer | 1 x SF4, 1 x MOS, 2 x MGD, 2 x HEC | HHblits | 0.29 |
| ``` target    IMDGKNLVENKLTDSHWFIECM--ERGAKIVVIAPEYGPPSTKADYWIPIRPQTDAALWLGITRLMIEKKWYDETFVKGF 3o5a.1    VLWGSNMAEMHPILWTRVTDRRLSHPKTRVVVLSTFTHRCFDLADIGIIFKPQTDLAMLNYIANYIIRNNKVNKDFVNKH  target    TDFPLLVRTDTLQRLRAHEVFPQYKTSLSADGPSMKIQGLSAEQHAKLGDFVVWDGKTNAPAAITRDDVGATITKKGIDP 3o5a.1    TVFKEGVTDIGYGLRPDHPLQK----------------------------------AAK---------------------  target    VLAGSFKVKLVDGKEVEVATLWTLYQDHLKDYDLDTVVEITQAPKEMIEQLAQDIATMK-PVAIHQGEGINHWFHATEMN 3o5a.1    ---------NASDPGAAKVITFDEFAKFVSKYDADYVSKLSAVPKAKLDQLAELYADPNIKVMSLWTMGFNQHTRGTWAN  target    RAAYLPLMLTGNIGRPGAGCQTWAGNYKAALFQGSPWTGPGFKGWVAEDPFDINLNPKAHGKEIHAHAYTKDEEPAYWNH 3o5a.1    NMVYNLHLLTGKIATPGNSPFSLTGQPSAC---GTAREVGTFSHRLPAD--MVVTNPKH-R----------EEAERIWKL  target    GDLALIVDTPKFGRKNFTGKTHMPTPTKALIFNNVNLINNAKWA-YGMIKNVNPNVEMIVSMDIQMTASIEYADLALPAN 3o5a.1    PP-GTIPDKPGYD-AVLQNRMLKDGKLNAYWVQVNNNMQAAANLMEEGLPGYRNPANFIVVSDAYPTVTALAADLVLPSA  target    SWLEFEGLEITASCSNPFLQIWKGGIPPVFDSRDDLDILAGIANALADVTGEKRFRDYFAFAAADKRGIYIQRLLDTCTT 3o5a.1    MWVEKEGAYG---NAERRTQFWHQLVDAPGEARSDLWQLVEFAKRFKV--------------------------------  target    TAGYKLADIMAGKYGPPGGCLLNFRTYPRIPFYEQVHDSEPFHTDTGRMHAYADVPEAIEYGENFIVHREGPEATPYLPN 3o5a.1    --------------------------------------------------------------------------------  target    VIVSSNPHIRPEDYGIAADAEHWDDRTIRNIKMPWSKVKETKNFLWEKGFQFYCLTPKTRHRVHSGWSNVDWHMLMDSNF 3o5a.1    --------------------------------------------------------------------------------  target    GDPYRLDKRAPCVGEHQLHINPQAARDLNINDGDYVYVDANPADRPYLGAKPDDPFYRVSRCMLRVTYNRAYPYNIVMMK 3o5a.1    --------------------------------------------------------------------------------  target    HAPFIATEKSVKAHETRPDGRALSANTGYQANLRYGSQQSVTRNWHMPMHQTDTLFHKSKVFMGFIFGGEADNHAVNTVP 3o5a.1    --------------------------------------------------------------------------------  target    KETLVRVTKAEDGGMGGKGIWQPATTGFSPDNESDFMKKYLAGELTKVKT 3o5a.1    -------------------------------------------------- ``` | | | | | | | | | | | | | | | | | | | | | | | | | | | | | | | | | | | | | | | | | | | | | | | | | |
|  | 3ir5.1.A | Respiratory nitrate reductase 1 alpha chain  *Crystal structure of NarGHI mutant NarG-H49C* | 0.15 | 0.00 | 28.63 | 0.31 | 4-265 | X-ray | 2.30 | monomer | 2 x MD1, 1 x 6MO, 4 x SF4, 1 x AGA, 1 x F3S, 2 x HEM | BLAST | 0.35 |
| ``` target    IMDGKNLVENKLTDSHWFIECMERGAKIVVIAPEYGPPSTKADYWIPIRPQTDAALWLGITRLMIEK------KWYDETF 3ir5.1    ---GSNVPQTRTPDAHFFTEVRYKGTKTVAVTPDYAEIAKLCDLWLAPKQGTDAAMALAMGHVMLREFHLDNPSQYFTDY  target    VKGFTDFPLLVRTDTL-------QRLRAHEVF--------PQYKT-SLSADGPSMKIQGLSAEQHAKLGDFVV--WDGKT 3ir5.1    VRRYTDMPMLVMLEERDGYYAAGRMLRAADLVDALGQENNPEWKTVAFNTNGEMVAPNGSIGFRWGEKGKWNLEQRDGKT  target    NAPAAITRDDVGAT------------------ITKKGIDPVLAGSFKVK---LVDGKEVEVATLWTL------------- 3ir5.1    GEETELQLSLLGSQDEIAEVGFPYFGGDGTEHFNKVELENVLLHKLPVKRLQLADGSTALVTTVYDLTLANYGLERGLND  target    -----YQDHLKDYDLDTVVEITQAPKEMIEQLAQDIA-----TMKPVAIHQGEGINHWFHATEMNRAAYLPLMLTGNIGR 3ir5.1    VNCATSYDDVKAYTPAWAEQITGVSRSQIIRIAREFADNADKTHGRSMIIVGAGLNHWYHLDMNYRGLINMLIFCGCVGQ  target    PGAGCQTWAGNYKAALFQGSPWTGPGFKGWVAEDPFDINLNPKAHGKEIHAHAYTKDEEPAYWNHGDLALIVDTPKFGRK 3ir5.1    SGGGWAHYVGQEK-------------------------------------------------------------------  target    NFTGKTHMPTPTKALIFNNVNLINNAKWAYGMIKNVNPNVEMIVSMDIQMTASIEYADLALPANSWLEFEGLEITASCSN 3ir5.1    --------------------------------------------------------------------------------  target    PFLQIWKGGIPPVFDSRDDLDILAGIANALADVTGEKRFRDYFAFAAADKRGIYIQRLLDTCTTTAGYKLADIMAGKYGP 3ir5.1    --------------------------------------------------------------------------------  target    PGGCLLNFRTYPRIPFYEQVHDSEPFHTDTGRMHAYADVPEAIEYGENFIVHREGPEATPYLPNVIVSSNPHIRPEDYGI 3ir5.1    --------------------------------------------------------------------------------  target    AADAEHWDDRTIRNIKMPWSKVKETKNFLWEKGFQFYCLTPKTRHRVHSGWSNVDWHMLMDSNFGDPYRLDKRAPCVGEH 3ir5.1    --------------------------------------------------------------------------------  target    QLHINPQAARDLNINDGDYVYVDANPADRPYLGAKPDDPFYRVSRCMLRVTYNRAYPYNIVMMKHAPFIATEKSVKAHET 3ir5.1    --------------------------------------------------------------------------------  target    RPDGRALSANTGYQANLRYGSQQSVTRNWHMPMHQTDTLFHKSKVFMGFIFGGEADNHAVNTVPKETLVRVTKAEDGGMG 3ir5.1    --------------------------------------------------------------------------------  target    GKGIWQPATTGFSPDNESDFMKKYLAGELTKVKT 3ir5.1    ---------------------------------- ``` | | | | | | | | | | | | | | | | | | | | | | | | | | | | | | | | | | | | | | | | | | | | | | | | | |
|  | 1r27.4.A | Respiratory nitrate reductase 1 alpha chain  *Crystal Structure of NarGH complex* | 0.15 | 0.00 | 28.63 | 0.31 | 4-265 | X-ray | 2.00 | monomer | 4 x MO, 16 x SF4, 8 x MGD, 4 x F3S | BLAST | 0.35 |
| ``` target    IMDGKNLVENKLTDSHWFIECMERGAKIVVIAPEYGPPSTKADYWIPIRPQTDAALWLGITRLMIEK------KWYDETF 1r27.4    ---GSNVPQTRTPDAHFFTEVRYKGTKTVAVTPDYAEIAKLCDLWLAPKQGTDAAMALAMGHVMLREFHLDNPSQYFTDY  target    VKGFTDFPLLVRTDTL-------QRLRAHEVF--------PQYKT-SLSADGPSMKIQGLSAEQHAKLGDFVV--WDGKT 1r27.4    VRRYTDMPMLVMLEERDGYYAAGRMLRAADLVDALGQENNPEWKTVAFNTNGEMVAPNGSIGFRWGEKGKWNLEQRDGKT  target    NAPAAITRDDVGAT------------------ITKKGIDPVLAGSFKVK---LVDGKEVEVATLWTL------------- 1r27.4    GEETELQLSLLGSQDEIAEVGFPYFGGDGTEHFNKVELENVLLHKLPVKRLQLADGSTALVTTVYDLTLANYGLERGLND  target    -----YQDHLKDYDLDTVVEITQAPKEMIEQLAQDIA-----TMKPVAIHQGEGINHWFHATEMNRAAYLPLMLTGNIGR 1r27.4    VNCATSYDDVKAYTPAWAEQITGVSRSQIIRIAREFADNADKTHGRSMIIVGAGLNHWYHLDMNYRGLINMLIFCGCVGQ  target    PGAGCQTWAGNYKAALFQGSPWTGPGFKGWVAEDPFDINLNPKAHGKEIHAHAYTKDEEPAYWNHGDLALIVDTPKFGRK 1r27.4    SGGGWAHYVGQEK-------------------------------------------------------------------  target    NFTGKTHMPTPTKALIFNNVNLINNAKWAYGMIKNVNPNVEMIVSMDIQMTASIEYADLALPANSWLEFEGLEITASCSN 1r27.4    --------------------------------------------------------------------------------  target    PFLQIWKGGIPPVFDSRDDLDILAGIANALADVTGEKRFRDYFAFAAADKRGIYIQRLLDTCTTTAGYKLADIMAGKYGP 1r27.4    --------------------------------------------------------------------------------  target    PGGCLLNFRTYPRIPFYEQVHDSEPFHTDTGRMHAYADVPEAIEYGENFIVHREGPEATPYLPNVIVSSNPHIRPEDYGI 1r27.4    --------------------------------------------------------------------------------  target    AADAEHWDDRTIRNIKMPWSKVKETKNFLWEKGFQFYCLTPKTRHRVHSGWSNVDWHMLMDSNFGDPYRLDKRAPCVGEH 1r27.4    --------------------------------------------------------------------------------  target    QLHINPQAARDLNINDGDYVYVDANPADRPYLGAKPDDPFYRVSRCMLRVTYNRAYPYNIVMMKHAPFIATEKSVKAHET 1r27.4    --------------------------------------------------------------------------------  target    RPDGRALSANTGYQANLRYGSQQSVTRNWHMPMHQTDTLFHKSKVFMGFIFGGEADNHAVNTVPKETLVRVTKAEDGGMG 1r27.4    --------------------------------------------------------------------------------  target    GKGIWQPATTGFSPDNESDFMKKYLAGELTKVKT 1r27.4    ---------------------------------- ``` | | | | | | | | | | | | | | | | | | | | | | | | | | | | | | | | | | | | | | | | | | | | | | | | | |
|  | 3ir6.1.A | Respiratory nitrate reductase 1 alpha chain  *Crystal structure of NarGHI mutant NarG-H49S* | 0.15 | 0.00 | 28.63 | 0.31 | 4-265 | X-ray | 2.80 | monomer | 2 x GDP, 1 x AGA, 3 x SF4, 1 x F3S, 2 x HEM | BLAST | 0.35 |
| ``` target    IMDGKNLVENKLTDSHWFIECMERGAKIVVIAPEYGPPSTKADYWIPIRPQTDAALWLGITRLMIEK------KWYDETF 3ir6.1    ---GSNVPQTRTPDAHFFTEVRYKGTKTVAVTPDYAEIAKLCDLWLAPKQGTDAAMALAMGHVMLREFHLDNPSQYFTDY  target    VKGFTDFPLLVRTDTL-------QRLRAHEVF--------PQYKT-SLSADGPSMKIQGLSAEQHAKLGDFVV--WDGKT 3ir6.1    VRRYTDMPMLVMLEERDGYYAAGRMLRAADLVDALGQENNPEWKTVAFNTNGEMVAPNGSIGFRWGEKGKWNLEQRDGKT  target    NAPAAITRDDVGAT------------------ITKKGIDPVLAGSFKVK---LVDGKEVEVATLWTL------------- 3ir6.1    GEETELQLSLLGSQDEIAEVGFPYFGGDGTEHFNKVELENVLLHKLPVKRLQLADGSTALVTTVYDLTLANYGLERGLND  target    -----YQDHLKDYDLDTVVEITQAPKEMIEQLAQDIA-----TMKPVAIHQGEGINHWFHATEMNRAAYLPLMLTGNIGR 3ir6.1    VNCATSYDDVKAYTPAWAEQITGVSRSQIIRIAREFADNADKTHGRSMIIVGAGLNHWYHLDMNYRGLINMLIFCGCVGQ  target    PGAGCQTWAGNYKAALFQGSPWTGPGFKGWVAEDPFDINLNPKAHGKEIHAHAYTKDEEPAYWNHGDLALIVDTPKFGRK 3ir6.1    SGGGWAHYVGQEK-------------------------------------------------------------------  target    NFTGKTHMPTPTKALIFNNVNLINNAKWAYGMIKNVNPNVEMIVSMDIQMTASIEYADLALPANSWLEFEGLEITASCSN 3ir6.1    --------------------------------------------------------------------------------  target    PFLQIWKGGIPPVFDSRDDLDILAGIANALADVTGEKRFRDYFAFAAADKRGIYIQRLLDTCTTTAGYKLADIMAGKYGP 3ir6.1    --------------------------------------------------------------------------------  target    PGGCLLNFRTYPRIPFYEQVHDSEPFHTDTGRMHAYADVPEAIEYGENFIVHREGPEATPYLPNVIVSSNPHIRPEDYGI 3ir6.1    --------------------------------------------------------------------------------  target    AADAEHWDDRTIRNIKMPWSKVKETKNFLWEKGFQFYCLTPKTRHRVHSGWSNVDWHMLMDSNFGDPYRLDKRAPCVGEH 3ir6.1    --------------------------------------------------------------------------------  target    QLHINPQAARDLNINDGDYVYVDANPADRPYLGAKPDDPFYRVSRCMLRVTYNRAYPYNIVMMKHAPFIATEKSVKAHET 3ir6.1    --------------------------------------------------------------------------------  target    RPDGRALSANTGYQANLRYGSQQSVTRNWHMPMHQTDTLFHKSKVFMGFIFGGEADNHAVNTVPKETLVRVTKAEDGGMG 3ir6.1    --------------------------------------------------------------------------------  target    GKGIWQPATTGFSPDNESDFMKKYLAGELTKVKT 3ir6.1    ---------------------------------- ``` | | | | | | | | | | | | | | | | | | | | | | | | | | | | | | | | | | | | | | | | | | | | | | | | | |
|  | 1q16.1.A | Respiratory nitrate reductase 1 alpha chain  *Crystal structure of Nitrate Reductase A, NarGHI, from Escherichia coli* | 0.15 | 0.00 | 28.63 | 0.31 | 4-265 | X-ray | 1.90 | monomer | 2 x MD1, 1 x 6MO, 2 x HEM, 4 x SF4, 1 x F3S, 1 x AGA, 1 x 3PH | BLAST | 0.35 |
| ``` target    IMDGKNLVENKLTDSHWFIECMERGAKIVVIAPEYGPPSTKADYWIPIRPQTDAALWLGITRLMIEK------KWYDETF 1q16.1    ---GSNVPQTRTPDAHFFTEVRYKGTKTVAVTPDYAEIAKLCDLWLAPKQGTDAAMALAMGHVMLREFHLDNPSQYFTDY  target    VKGFTDFPLLVRTDTL-------QRLRAHEVF--------PQYKT-SLSADGPSMKIQGLSAEQHAKLGDFVV--WDGKT 1q16.1    VRRYTDMPMLVMLEERDGYYAAGRMLRAADLVDALGQENNPEWKTVAFNTNGEMVAPNGSIGFRWGEKGKWNLEQRDGKT  target    NAPAAITRDDVGAT------------------ITKKGIDPVLAGSFKVK---LVDGKEVEVATLWTL------------- 1q16.1    GEETELQLSLLGSQDEIAEVGFPYFGGDGTEHFNKVELENVLLHKLPVKRLQLADGSTALVTTVYDLTLANYGLERGLND  target    -----YQDHLKDYDLDTVVEITQAPKEMIEQLAQDIA-----TMKPVAIHQGEGINHWFHATEMNRAAYLPLMLTGNIGR 1q16.1    VNCATSYDDVKAYTPAWAEQITGVSRSQIIRIAREFADNADKTHGRSMIIVGAGLNHWYHLDMNYRGLINMLIFCGCVGQ  target    PGAGCQTWAGNYKAALFQGSPWTGPGFKGWVAEDPFDINLNPKAHGKEIHAHAYTKDEEPAYWNHGDLALIVDTPKFGRK 1q16.1    SGGGWAHYVGQEK-------------------------------------------------------------------  target    NFTGKTHMPTPTKALIFNNVNLINNAKWAYGMIKNVNPNVEMIVSMDIQMTASIEYADLALPANSWLEFEGLEITASCSN 1q16.1    --------------------------------------------------------------------------------  target    PFLQIWKGGIPPVFDSRDDLDILAGIANALADVTGEKRFRDYFAFAAADKRGIYIQRLLDTCTTTAGYKLADIMAGKYGP 1q16.1    --------------------------------------------------------------------------------  target    PGGCLLNFRTYPRIPFYEQVHDSEPFHTDTGRMHAYADVPEAIEYGENFIVHREGPEATPYLPNVIVSSNPHIRPEDYGI 1q16.1    --------------------------------------------------------------------------------  target    AADAEHWDDRTIRNIKMPWSKVKETKNFLWEKGFQFYCLTPKTRHRVHSGWSNVDWHMLMDSNFGDPYRLDKRAPCVGEH 1q16.1    --------------------------------------------------------------------------------  target    QLHINPQAARDLNINDGDYVYVDANPADRPYLGAKPDDPFYRVSRCMLRVTYNRAYPYNIVMMKHAPFIATEKSVKAHET 1q16.1    --------------------------------------------------------------------------------  target    RPDGRALSANTGYQANLRYGSQQSVTRNWHMPMHQTDTLFHKSKVFMGFIFGGEADNHAVNTVPKETLVRVTKAEDGGMG 1q16.1    --------------------------------------------------------------------------------  target    GKGIWQPATTGFSPDNESDFMKKYLAGELTKVKT 1q16.1    ---------------------------------- ``` | | | | | | | | | | | | | | | | | | | | | | | | | | | | | | | | | | | | | | | | | | | | | | | | | |
|  | 3ir7.1.A | Respiratory nitrate reductase 1 alpha chain  *Crystal structure of NarGHI mutant NarG-R94S* | 0.15 | 0.00 | 28.63 | 0.31 | 4-265 | X-ray | 2.50 | monomer | 2 x MD1, 4 x SF4, 1 x 6MO, 1 x AGA, 1 x F3S, 2 x HEM | BLAST | 0.35 |
| ``` target    IMDGKNLVENKLTDSHWFIECMERGAKIVVIAPEYGPPSTKADYWIPIRPQTDAALWLGITRLMIEK------KWYDETF 3ir7.1    ---GSNVPQTRTPDAHFFTEVRYKGTKTVAVTPDYAEIAKLCDLWLAPKQGTDAAMALAMGHVMLREFHLDNPSQYFTDY  target    VKGFTDFPLLVRTDTL-------QRLRAHEVF--------PQYKT-SLSADGPSMKIQGLSAEQHAKLGDFVV--WDGKT 3ir7.1    VRRYTDMPMLVMLEERDGYYAAGRMLRAADLVDALGQENNPEWKTVAFNTNGEMVAPNGSIGFRWGEKGKWNLEQRDGKT  target    NAPAAITRDDVGAT------------------ITKKGIDPVLAGSFKVK---LVDGKEVEVATLWTL------------- 3ir7.1    GEETELQLSLLGSQDEIAEVGFPYFGGDGTEHFNKVELENVLLHKLPVKRLQLADGSTALVTTVYDLTLANYGLERGLND  target    -----YQDHLKDYDLDTVVEITQAPKEMIEQLAQDIA-----TMKPVAIHQGEGINHWFHATEMNRAAYLPLMLTGNIGR 3ir7.1    VNCATSYDDVKAYTPAWAEQITGVSRSQIIRIAREFADNADKTHGRSMIIVGAGLNHWYHLDMNYRGLINMLIFCGCVGQ  target    PGAGCQTWAGNYKAALFQGSPWTGPGFKGWVAEDPFDINLNPKAHGKEIHAHAYTKDEEPAYWNHGDLALIVDTPKFGRK 3ir7.1    SGGGWAHYVGQEK-------------------------------------------------------------------  target    NFTGKTHMPTPTKALIFNNVNLINNAKWAYGMIKNVNPNVEMIVSMDIQMTASIEYADLALPANSWLEFEGLEITASCSN 3ir7.1    --------------------------------------------------------------------------------  target    PFLQIWKGGIPPVFDSRDDLDILAGIANALADVTGEKRFRDYFAFAAADKRGIYIQRLLDTCTTTAGYKLADIMAGKYGP 3ir7.1    --------------------------------------------------------------------------------  target    PGGCLLNFRTYPRIPFYEQVHDSEPFHTDTGRMHAYADVPEAIEYGENFIVHREGPEATPYLPNVIVSSNPHIRPEDYGI 3ir7.1    --------------------------------------------------------------------------------  target    AADAEHWDDRTIRNIKMPWSKVKETKNFLWEKGFQFYCLTPKTRHRVHSGWSNVDWHMLMDSNFGDPYRLDKRAPCVGEH 3ir7.1    --------------------------------------------------------------------------------  target    QLHINPQAARDLNINDGDYVYVDANPADRPYLGAKPDDPFYRVSRCMLRVTYNRAYPYNIVMMKHAPFIATEKSVKAHET 3ir7.1    --------------------------------------------------------------------------------  target    RPDGRALSANTGYQANLRYGSQQSVTRNWHMPMHQTDTLFHKSKVFMGFIFGGEADNHAVNTVPKETLVRVTKAEDGGMG 3ir7.1    --------------------------------------------------------------------------------  target    GKGIWQPATTGFSPDNESDFMKKYLAGELTKVKT 3ir7.1    ---------------------------------- ``` | | | | | | | | | | | | | | | | | | | | | | | | | | | | | | | | | | | | | | | | | | | | | | | | | |
|  | 3egw.1.A | Respiratory nitrate reductase 1 alpha chain  *The crystal structure of the NarGHI mutant NarH - C16A* | 0.14 | 0.00 | 28.63 | 0.31 | 4-265 | X-ray | 1.90 | monomer | 2 x MD1, 2 x MGD, 2 x 6MO, 6 x SF4, 4 x F3S, 2 x 3PH, 4 x HEM, 2 x AGA | BLAST | 0.35 |
| ``` target    IMDGKNLVENKLTDSHWFIECMERGAKIVVIAPEYGPPSTKADYWIPIRPQTDAALWLGITRLMIEK------KWYDETF 3egw.1    ---GSNVPQTRTPDAHFFTEVRYKGTKTVAVTPDYAEIAKLCDLWLAPKQGTDAAMALAMGHVMLREFHLDNPSQYFTDY  target    VKGFTDFPLLV---------------RTDTLQRLRAHEVFPQYKT-SLSADGPSMKIQGLSAEQHAKLGDFVV--WDGKT 3egw.1    VRRYTDMPMLVMLEERDGYYAAGRMLRAADLVAALGQENNPEWKTVAFNTNGEMVAPNGSIGFRWGEKGKWNLEQRDGKT  target    NAPAAITRDDVGAT------------------ITKKGIDPVLAGSFKVK---LVDGKEVEVATLWTL------------- 3egw.1    GEETELQLSLLGSQDEIAEVGFPYFGGDGTEHFNKVELENVLLHKLPVKRLQLADGSTALVTTVYDLTLANYGLERGLND  target    -----YQDHLKDYDLDTVVEITQAPKEMIEQLAQDIA-----TMKPVAIHQGEGINHWFHATEMNRAAYLPLMLTGNIGR 3egw.1    VNCATSYDDVKAYTPAWAEQITGVSRSQIIRIAREFADNADKTHGRSMIIVGAGLNHWYHLDMNYRGLINMLIFCGCVGQ  target    PGAGCQTWAGNYKAALFQGSPWTGPGFKGWVAEDPFDINLNPKAHGKEIHAHAYTKDEEPAYWNHGDLALIVDTPKFGRK 3egw.1    SGGGWAHYVGQEK-------------------------------------------------------------------  target    NFTGKTHMPTPTKALIFNNVNLINNAKWAYGMIKNVNPNVEMIVSMDIQMTASIEYADLALPANSWLEFEGLEITASCSN 3egw.1    --------------------------------------------------------------------------------  target    PFLQIWKGGIPPVFDSRDDLDILAGIANALADVTGEKRFRDYFAFAAADKRGIYIQRLLDTCTTTAGYKLADIMAGKYGP 3egw.1    --------------------------------------------------------------------------------  target    PGGCLLNFRTYPRIPFYEQVHDSEPFHTDTGRMHAYADVPEAIEYGENFIVHREGPEATPYLPNVIVSSNPHIRPEDYGI 3egw.1    --------------------------------------------------------------------------------  target    AADAEHWDDRTIRNIKMPWSKVKETKNFLWEKGFQFYCLTPKTRHRVHSGWSNVDWHMLMDSNFGDPYRLDKRAPCVGEH 3egw.1    --------------------------------------------------------------------------------  target    QLHINPQAARDLNINDGDYVYVDANPADRPYLGAKPDDPFYRVSRCMLRVTYNRAYPYNIVMMKHAPFIATEKSVKAHET 3egw.1    --------------------------------------------------------------------------------  target    RPDGRALSANTGYQANLRYGSQQSVTRNWHMPMHQTDTLFHKSKVFMGFIFGGEADNHAVNTVPKETLVRVTKAEDGGMG 3egw.1    --------------------------------------------------------------------------------  target    GKGIWQPATTGFSPDNESDFMKKYLAGELTKVKT 3egw.1    ---------------------------------- ``` | | | | | | | | | | | | | | | | | | | | | | | | | | | | | | | | | | | | | | | | | | | | | | | | | |
|  | 6sdr.1.A | Formate dehydrogenase, alpha subunit, selenocysteine-containing  *W-formate dehydrogenase from Desulfovibrio vulgaris - Oxidized form* | 0.17 |  | 27.69 | 0.31 | 1-312 | X-ray | 2.10 | hetero-1-1-mer | 2 x MGD, 4 x SF4, 1 x H2S, 1 x W | BLAST | 0.34 |
| ``` target    IMDGKNLVENKLTDSHWFIECMERGAKIVVIAPEYGPPSTKADYWIPIRPQTDAALWLGITRLMIEKKWYDETFVKGFTD 6sdr.1    LIMGSNAAENHPIAFKWVLRAKDKGATLIHVDPRFTRTSARCDVYAPIRSGADIPFLGGLIKYILDNKLYFTDYVREYTN  target    FPLLVRTDTLQRLRAHEVFPQYKTSLSADGPSMKIQGLSAEQHAKLGDFVVWDGKTNAPAAITRDDVGATITKKGIDPVL 6sdr.1    ASLIV----------GEKF-SFKDGL--------FSGYDAAN--KKYDKSMWAFELDANGVPKR------------DPAL  target    AGSFKVKLVDGKEVEVATLWTLYQDHLKDYDLDTVVEITQAPKEMIEQLAQDIATM----KPVAIHQGEGINHWFHATEM 6sdr.1    K-------------HPRCVINLLKKHYERYNLDKVAAITGTSKEQLQQVYKAYAATGKPDKAGTIMYAMGWTQHSVGVQN  target    NRAAYLPLMLTGNIGRPGAGCQTWAGNYKAALFQGSPWTGPGFKGWVAEDPFDINLNPKAHGKEIHAHAYTKDEEPAYWN 6sdr.1    IRAMAMIQLLLGNIGVAGGGVNALRGESNV---QGSTDQGLLAHIWPGYNPVP---NSKAATLELYNAATPQSKDP----  target    HGDLALIVDTPKFGRKNFTGKTHMPTPTKALIFNNVNLINNAKWAYGMIKNVNPNVEMIVSMDIQMTASIEYADLALPAN 6sdr.1    --------------------------------------------------------------------------------  target    SWLEFEGLEITASCSNPFLQIWKGGIPPVFDSRDDLDILAGIANALADVTGEKRFRDYFAFAAADKRGIYIQRLLDTCTT 6sdr.1    --------------------------------------------------------------------------------  target    TAGYKLADIMAGKYGPPGGCLLNFRTYPRIPFYEQVHDSEPFHTDTGRMHAYADVPEAIEYGENFIVHREGPEATPYLPN 6sdr.1    --------------------------------------------------------------------------------  target    VIVSSNPHIRPEDYGIAADAEHWDDRTIRNIKMPWSKVKETKNFLWEKGFQFYCLTPKTRHRVHSGWSNVDWHMLMDSNF 6sdr.1    --------------------------------------------------------------------------------  target    GDPYRLDKRAPCVGEHQLHINPQAARDLNINDGDYVYVDANPADRPYLGAKPDDPFYRVSRCMLRVTYNRAYPYNIVMMK 6sdr.1    --------------------------------------------------------------------------------  target    HAPFIATEKSVKAHETRPDGRALSANTGYQANLRYGSQQSVTRNWHMPMHQTDTLFHKSKVFMGFIFGGEADNHAVNTVP 6sdr.1    --------------------------------------------------------------------------------  target    KETLVRVTKAEDGGMGGKGIWQPATTGFSPDNESDFMKKYLAGELTKVKT 6sdr.1    -------------------------------------------------- ``` | | | | | | | | | | | | | | | | | | | | | | | | | | | | | | | | | | | | | | | | | | | | | | | | | |
|  | 8bqg.1.A | Formate dehydrogenase, alpha subunit, selenocysteine-containing  *W-formate dehydrogenase from Desulfovibrio vulgaris - Soaking with Formate 1 min* | 0.17 |  | 27.69 | 0.31 | 1-312 | X-ray | 1.95 | hetero-1-1-mer | 2 x MGD, 4 x SF4, 1 x H2S, 1 x W | BLAST | 0.34 |
| ``` target    IMDGKNLVENKLTDSHWFIECMERGAKIVVIAPEYGPPSTKADYWIPIRPQTDAALWLGITRLMIEKKWYDETFVKGFTD 8bqg.1    LIMGSNAAENHPIAFKWVLRAKDKGATLIHVDPRFTRTSARCDVYAPIRSGADIPFLGGLIKYILDNKLYFTDYVREYTN  target    FPLLVRTDTLQRLRAHEVFPQYKTSLSADGPSMKIQGLSAEQHAKLGDFVVWDGKTNAPAAITRDDVGATITKKGIDPVL 8bqg.1    ASLIV----------GEKF-SFKDGL--------FSGYDAAN--KKYDKSMWAFELDANGVPKR------------DPAL  target    AGSFKVKLVDGKEVEVATLWTLYQDHLKDYDLDTVVEITQAPKEMIEQLAQDIATM----KPVAIHQGEGINHWFHATEM 8bqg.1    K-------------HPRCVINLLKKHYERYNLDKVAAITGTSKEQLQQVYKAYAATGKPDKAGTIMYAMGWTQHSVGVQN  target    NRAAYLPLMLTGNIGRPGAGCQTWAGNYKAALFQGSPWTGPGFKGWVAEDPFDINLNPKAHGKEIHAHAYTKDEEPAYWN 8bqg.1    IRAMAMIQLLLGNIGVAGGGVNALRGESNV---QGSTDQGLLAHIWPGYNPVP---NSKAATLELYNAATPQSKDP----  target    HGDLALIVDTPKFGRKNFTGKTHMPTPTKALIFNNVNLINNAKWAYGMIKNVNPNVEMIVSMDIQMTASIEYADLALPAN 8bqg.1    --------------------------------------------------------------------------------  target    SWLEFEGLEITASCSNPFLQIWKGGIPPVFDSRDDLDILAGIANALADVTGEKRFRDYFAFAAADKRGIYIQRLLDTCTT 8bqg.1    --------------------------------------------------------------------------------  target    TAGYKLADIMAGKYGPPGGCLLNFRTYPRIPFYEQVHDSEPFHTDTGRMHAYADVPEAIEYGENFIVHREGPEATPYLPN 8bqg.1    --------------------------------------------------------------------------------  target    VIVSSNPHIRPEDYGIAADAEHWDDRTIRNIKMPWSKVKETKNFLWEKGFQFYCLTPKTRHRVHSGWSNVDWHMLMDSNF 8bqg.1    --------------------------------------------------------------------------------  target    GDPYRLDKRAPCVGEHQLHINPQAARDLNINDGDYVYVDANPADRPYLGAKPDDPFYRVSRCMLRVTYNRAYPYNIVMMK 8bqg.1    --------------------------------------------------------------------------------  target    HAPFIATEKSVKAHETRPDGRALSANTGYQANLRYGSQQSVTRNWHMPMHQTDTLFHKSKVFMGFIFGGEADNHAVNTVP 8bqg.1    --------------------------------------------------------------------------------  target    KETLVRVTKAEDGGMGGKGIWQPATTGFSPDNESDFMKKYLAGELTKVKT 8bqg.1    -------------------------------------------------- ``` | | | | | | | | | | | | | | | | | | | | | | | | | | | | | | | | | | | | | | | | | | | | | | | | | |
|  | 5t5i.1.B | Tungsten formylmethanofuran dehydrogenase subunit B  *TUNGSTEN-CONTAINING FORMYLMETHANOFURAN DEHYDROGENASE FROM METHANOTHERMOBACTER WOLFEII, ORTHORHOMBIC FORM AT 1.9 A* | 0.15 |  | 14.65 | 0.32 | 1-444 | X-ray | 1.90 | hetero-oligomer | 4 x ZN, 2 x MG, 18 x K, 22 x SF4, 2 x W, 4 x MGD, 2 x H2S, 2 x CA | HHblits | 0.27 |
| ``` target    IMDGKNLVENKLTDSHW-------FIECMERGAKIVVIAPEYGPPSTKADYWIPIRPQTDAALWLGITRLMIEKKWYDET 5t5i.1    VYWGCNPMHAHPRHMSRNVFARGFFRERGRSDRTLIVVDPRKTDSAKLADIHLQLDFDRDYELLDAMRACLLGHEI----  target    FVKGFTDFPLLVRTDTLQRLRAHEVFPQYKTSLSADGPSMKIQGLSAEQHAKLGDFVVWDGKTNAPAAITRDDVGATITK 5t5i.1    --------------------------------------------------------------------------------  target    KGIDPVLAGSFKVKLVDGKEVEVATLWTLYQDHLKDYDLDTVVEITQAPKEMIEQLAQDIATMKPVAIHQGEGINHWFHA 5t5i.1    ----------------------------------------LYDEVAGVPREQIEEAVEVLKNAQFGILFFGMGITHSRGK  target    TEMNRAAYLPLMLTGNIGRPGAGCQTWAGNYKAALFQGSPWTGPGFKGWVAEDPFDINLNPKAHGKEIHAHAYTKDEEPA 5t5i.1    HRNIDTAIMMVQDLNDY--AKWTLIPMRGHYNV---TGFNQVC----TWESGYPYCVDFS---------GG--EPRYNPG  target    YWNHGDLALIVDTPKFGRKNFTGKTHMPTPTKALIFNNVNLINNAKWAYGMIKNVNPNVEMIVSMDIQMTASIEYADLAL 5t5i.1    ET---G--------------AN-DLLQNREADAMMVIASDPGAHFPQR---ALERMAEIP-VIAIEPHRTPTTEMADIII  target    PANS-WLEFEGLEITASCSNPFLQIWKGGIPPVFDSRDDLDILAGIANALADVTGEKRFRDYFAFAAADKRGIYIQRLLD 5t5i.1    PPAIVGMEAEGTAY---RMEGVPIRMKKVVDS--DLLSDREILERLLEKVRE----------------------------  target    TCTTTAGYKLADIMAGKYGPPGGCLLNFRTYPRIPFYEQVHDSEPFHTDTGRMHAYADVPEAIEYGENFIVHREGPEATP 5t5i.1    --------------------------------------------------------------------------------  target    YLPNVIVSSNPHIRPEDYGIAADAEHWDDRTIRNIKMPWSKVKETKNFLWEKGFQFYCLTPKTRHRVHSGWSNVDWHMLM 5t5i.1    --------------------------------------------------------------------------------  target    DSNFGDPYRLDKRAPCVGEHQLHINPQAARDLNINDGDYVYVDANPADRPYLGAKPDDPFYRVSRCMLRVTYNRAYPYNI 5t5i.1    --------------------------------------------------------------------------------  target    VMMKHAPFIATEKSVKAHETRPDGRALSANTGYQANLRYGSQQSVTRNWHMPMHQTDTLFHKSKVFMGFIFGGEADNHAV 5t5i.1    --------------------------------------------------------------------------------  target    NTVPKETLVRVTKAEDGGMGGKGIWQPATTGFSPDNESDFMKKYLAGELTKVKT 5t5i.1    ------------------------------------------------------ ``` | | | | | | | | | | | | | | | | | | | | | | | | | | | | | | | | | | | | | | | | | | | | | | | | | |
|  | 7t2r.1.A | NiFe hydrogenase subunit A  *Structure of electron bifurcating Ni-Fe hydrogenase complex HydABCSL in FMN-free apo state* | 0.15 |  | 14.08 | 0.33 | 1-444 | EM | 0.00 | hetero-2-2-2-2-2-mer | 6 x FES, 12 x SF4, 2 x 3NI, 2 x FCO | HHblits | 0.26 |
| ``` target    IMDGKNLVENKLTDSHWFIECM-ERGAKIVVIAPEYGPPSTKADYWIPIRPQTDAALWLGITRLMIEKKWYDETFVKGFT 7t2r.1    ITMFADPQKEAPVVASYIRVACLHRNAKLMNLSYGPSPFPGLVDLDIRLPEGQAVPKALSNLAEIIGKISLGPSDMASFG  target    DFPLLVRTDTLQRLRAHEVFPQYKTSLSADGPSMKIQGLSAEQHAKLGDFVVWDGKTNAPAAITRDDVGATITKKGIDPV 7t2r.1    EF------------------------------------------------------------------------------  target    LAGSFKVKLVDGKEVEVATLWTLYQDHLKD--YDLDTVVEITQAPKEMIEQLAQDIATMKPVAIHQGEGINHWFHATEMN 7t2r.1    --------------------EAGAGKALSSYRESIEESARAMGLDPKIAEEVALMLISARRPIFIIGGRA---TKSHELV  target    RAAYLPLMLTGNIGRPGAGCQTWAGNYKAALFQGSPWTGPGFKGWVAEDPFDINLNPKAHGKEIHAHAYTKDEEPAYWNH 7t2r.1    TAACNLAVASKAFFEDGLGVVPLLVSANS---LGA----------------------RN----T---V------------  target    GDLALIVDTPKFGRKNFTGKTHMPTPTKALIFNNVNLINNAKWAYGMIKNVNPNVEMIVSMDIQMT-ASIEYADLALPAN 7t2r.1    -----VSENPWL------G----RERRDFLYVFSTAMVPEE---EE-ILAAISATRFVVVQTPFKVRPLVNLADILLPAP  target    SWLEFEGLEITASCSNPFLQIWKGGIPPVFDSRDDLDILAGIANALADVTGEKRFRDYFAFAAADKRGIYIQRLLDTCTT 7t2r.1    AWYERSGHFC---TIEGERRKLNTIVPPKGEIKSLHYVMDEFAKKLGV--------------------------------  target    TAGYKLADIMAGKYGPPGGCLLNFRTYPRIPFYEQVHDSEPFHTDTGRMHAYADVPEAIEYGENFIVHREGPEATPYLPN 7t2r.1    --------------------------------------------------------------------------------  target    VIVSSNPHIRPEDYGIAADAEHWDDRTIRNIKMPWSKVKETKNFLWEKGFQFYCLTPKTRHRVHSGWSNVDWHMLMDSNF 7t2r.1    --------------------------------------------------------------------------------  target    GDPYRLDKRAPCVGEHQLHINPQAARDLNINDGDYVYVDANPADRPYLGAKPDDPFYRVSRCMLRVTYNRAYPYNIVMMK 7t2r.1    --------------------------------------------------------------------------------  target    HAPFIATEKSVKAHETRPDGRALSANTGYQANLRYGSQQSVTRNWHMPMHQTDTLFHKSKVFMGFIFGGEADNHAVNTVP 7t2r.1    --------------------------------------------------------------------------------  target    KETLVRVTKAEDGGMGGKGIWQPATTGFSPDNESDFMKKYLAGELTKVKT 7t2r.1    -------------------------------------------------- ``` | | | | | | | | | | | | | | | | | | | | | | | | | | | | | | | | | | | | | | | | | | | | | | | | | |
|  | 7t30.1.A | NiFe hydrogenase subunit A  *Structure of electron bifurcating Ni-Fe hydrogenase complex HydABCSL in FMN/NAD(H) bound state* | 0.15 |  | 14.08 | 0.33 | 1-444 | EM | 0.00 | hetero-2-2-2-2-2-mer | 4 x FES, 12 x SF4, 2 x NAD, 2 x FMN, 2 x 3NI, 2 x FCO | HHblits | 0.26 |
| ``` target    IMDGKNLVENKLTDSHWFIECM-ERGAKIVVIAPEYGPPSTKADYWIPIRPQTDAALWLGITRLMIEKKWYDETFVKGFT 7t30.1    ITMFADPQKEAPVVASYIRVACLHRNAKLMNLSYGPSPFPGLVDLDIRLPEGQAVPKALSNLAEIIGKISLGPSDMASFG  target    DFPLLVRTDTLQRLRAHEVFPQYKTSLSADGPSMKIQGLSAEQHAKLGDFVVWDGKTNAPAAITRDDVGATITKKGIDPV 7t30.1    EF------------------------------------------------------------------------------  target    LAGSFKVKLVDGKEVEVATLWTLYQDHLKD--YDLDTVVEITQAPKEMIEQLAQDIATMKPVAIHQGEGINHWFHATEMN 7t30.1    --------------------EAGAGKALSSYRESIEESARAMGLDPKIAEEVALMLISARRPIFIIGGRA---TKSHELV  target    RAAYLPLMLTGNIGRPGAGCQTWAGNYKAALFQGSPWTGPGFKGWVAEDPFDINLNPKAHGKEIHAHAYTKDEEPAYWNH 7t30.1    TAACNLAVASKAFFEDGLGVVPLLVSANS---LGA----------------------RN----T---V------------  target    GDLALIVDTPKFGRKNFTGKTHMPTPTKALIFNNVNLINNAKWAYGMIKNVNPNVEMIVSMDIQMT-ASIEYADLALPAN 7t30.1    -----VSENPWL------G----RERRDFLYVFSTAMVPEE---EE-ILAAISATRFVVVQTPFKVRPLVNLADILLPAP  target    SWLEFEGLEITASCSNPFLQIWKGGIPPVFDSRDDLDILAGIANALADVTGEKRFRDYFAFAAADKRGIYIQRLLDTCTT 7t30.1    AWYERSGHFC---TIEGERRKLNTIVPPKGEIKSLHYVMDEFAKKLGV--------------------------------  target    TAGYKLADIMAGKYGPPGGCLLNFRTYPRIPFYEQVHDSEPFHTDTGRMHAYADVPEAIEYGENFIVHREGPEATPYLPN 7t30.1    --------------------------------------------------------------------------------  target    VIVSSNPHIRPEDYGIAADAEHWDDRTIRNIKMPWSKVKETKNFLWEKGFQFYCLTPKTRHRVHSGWSNVDWHMLMDSNF 7t30.1    --------------------------------------------------------------------------------  target    GDPYRLDKRAPCVGEHQLHINPQAARDLNINDGDYVYVDANPADRPYLGAKPDDPFYRVSRCMLRVTYNRAYPYNIVMMK 7t30.1    --------------------------------------------------------------------------------  target    HAPFIATEKSVKAHETRPDGRALSANTGYQANLRYGSQQSVTRNWHMPMHQTDTLFHKSKVFMGFIFGGEADNHAVNTVP 7t30.1    --------------------------------------------------------------------------------  target    KETLVRVTKAEDGGMGGKGIWQPATTGFSPDNESDFMKKYLAGELTKVKT 7t30.1    -------------------------------------------------- ``` | | | | | | | | | | | | | | | | | | | | | | | | | | | | | | | | | | | | | | | | | | | | | | | | | |
|  | 7bkb.1.L | Formylmethanofuran dehydrogenase, subunit B  *Formate dehydrogenase - heterodisulfide reductase - formylmethanofuran dehydrogenase complex from Methanospirillum hungatei (hexameric, composite structure)* | 0.14 |  | 16.92 | 0.31 | 1-445 | EM | 0.00 | hetero-2-2-2-2-2-2-… | 48 x SF4, 4 x FAD, 2 x FES, 4 x 9S8, 4 x ZN, 2 x MO, 4 x MGD | HHblits | 0.28 |
| ``` target    IMDGKNLVENKLTDSHWF--------IECMERGAKIVVIAPEYGPPSTKADYWIPIRPQTDAALWLGITRLMIEKKWYDE 7bkb.1    VYWGSNPAHAHPRHMSRYSIFPRGFFTGKGQKKRTVIVIDPRFTDTANVADYHLQVKQGHDYELFNAFRMVIHGHGK---  target    TFVKGFTDFPLLVRTDTLQRLRAHEVFPQYKTSLSADGPSMKIQGLSAEQHAKLGDFVVWDGKTNAPAAITRDDVGATIT 7bkb.1    --------------------------------------------------------------------------------  target    KKGIDPVLAGSFKVKLVDGKEVEVATLWTLYQDHLKDYDLDTVVEITQAPKEMIEQLAQDIATMKPVAIHQGEGINHWFH 7bkb.1    ----------------------------------------DLPDEVAGIKKETILEVAEIMKNARFGTTFFGMGLTHTDG  target    ATEMNRAAY------------LPLMLTGNIGRPGAGCQTWAGNYKAALFQGSPWTGPGFKGWVAEDPFDINLNPKAHGKE 7bkb.1    RNHNIDIAISLTRDLNKISKWTIMAMRGHYNIAGPGVVWSWTF-------G----FPYCLD------L----TK------  target    IHAHAYTKDEEPAYWNHGDLALIVDTPKFGRKNFTGKTHMPTPTKALIFNNVNLINNAKWAYGMIKNVNPNVEMIVSMDI 7bkb.1    --------QNHAH-MNPG-ETSSVD------------MAMRDEVDMFINIGTDAAAHFPIP---AVKQLKKHPW-VTIDP  target    QMTASIEYADLALPANSW-LEFEGLEITASCSNPFLQIWKGGIPPVFDSRDDLDILAGIANALADVTGEKRFRDYFAFAA 7bkb.1    SINMASEISDLHIPVCICGVDVGGIVY---RMDNVPIQFRKVIEPPEGVMDDETLLNKIADRMEEL--------------  target    ADKRGIYIQRLLDTCTTTAGYKLADIMAGKYGPPGGCLLNFRTYPRIPFYEQVHDSEPFHTDTGRMHAYADVPEAIEYGE 7bkb.1    --------------------------------------------------------------------------------  target    NFIVHREGPEATPYLPNVIVSSNPHIRPEDYGIAADAEHWDDRTIRNIKMPWSKVKETKNFLWEKGFQFYCLTPKTRHRV 7bkb.1    --------------------------------------------------------------------------------  target    HSGWSNVDWHMLMDSNFGDPYRLDKRAPCVGEHQLHINPQAARDLNINDGDYVYVDANPADRPYLGAKPDDPFYRVSRCM 7bkb.1    --------------------------------------------------------------------------------  target    LRVTYNRAYPYNIVMMKHAPFIATEKSVKAHETRPDGRALSANTGYQANLRYGSQQSVTRNWHMPMHQTDTLFHKSKVFM 7bkb.1    --------------------------------------------------------------------------------  target    GFIFGGEADNHAVNTVPKETLVRVTKAEDGGMGGKGIWQPATTGFSPDNESDFMKKYLAGELTKVKT 7bkb.1    ------------------------------------------------------------------- ``` | | | | | | | | | | | | | | | | | | | | | | | | | | | | | | | | | | | | | | | | | | | | | | | | | |
|  | 1aa6.1.A | FORMATE DEHYDROGENASE H  *REDUCED FORM OF FORMATE DEHYDROGENASE H FROM E. COLI* | 0.11 |  | 31.74 | 0.27 | 183-442 | X-ray | 2.30 | monomer | 1 x SF4, 2 x MGD, 1 x 4MO | BLAST | 0.36 |
| ``` target    IMDGKNLVENKLTDSHWFIECMERGAKIVVIAPEYGPPSTKADYWIPIRPQTDAALWLGITRLMIEKKWYDETFVKGFTD 1aa6.1    --------------------------------------------------------------------------------  target    FPLLVRTDTLQRLRAHEVFPQYKTSLSADGPSMKIQGLSAEQHAKLGDFVVWDGKTNAPAAITRDDVGATITKKGIDPVL 1aa6.1    --------------------------------------------------------------------------------  target    AGSFKVKLVDGKEVEVATLWTLYQDHLKDYDLDTVVEITQAPKEMIEQLAQDIATMKPVAIHQGEGINHWFHATEMNRAA 1aa6.1    ----------------------YRKIVEGYTPESVEDITGVSASEIRQAARMYAQAKSAAILWGMGVTQFYQGVETVRSL  target    YLPLMLTGNIGRPGAGCQTWAG--NYKAALFQGS-PWTGPGFKGWVAEDPFDINLNPKAHGKE-IHAH-AYTKDEEPAYW 1aa6.1    TSLAMLTGNLGKPHAGVNPVRGQNNVQGACDMGALPDTYPGYQ--YVKDPANREKFAKAWGVESLPAHTGYRISELPHRA  target    NHGDLALIVDTPKFGRKNFTGKTHMPTPTKALIFNNVNLINNAKWAYGMIKNVNPNVEMIVSMDIQMTASIEYADLALPA 1aa6.1    AHGEV-----------------------RAAYIMGEDPLQTDAELS--AVRKAFEDLELVIVQDIFMTKTASAADVILPS  target    NSWLEFEGLEITASCSNPFLQIWKGGIPPVFDSRDDLDILAGIANALADVTGEKRFRDYFAFAAADKRGIYIQRLLDTCT 1aa6.1    TSWGEHEGVFTAA--DRGFQRFFK-AVEPKWDLKTDWQIISEIATRM---------------------------------  target    TTAGYKLADIMAGKYGPPGGCLLNFRTYPRIPFYEQVHDSEPFHTDTGRMHAYADVPEAIEYGENFIVHREGPEATPYLP 1aa6.1    --------------------------------------------------------------------------------  target    NVIVSSNPHIRPEDYGIAADAEHWDDRTIRNIKMPWSKVKETKNFLWEKGFQFYCLTPKTRHRVHSGWSNVDWHMLMDSN 1aa6.1    --------------------------------------------------------------------------------  target    FGDPYRLDKRAPCVGEHQLHINPQAARDLNINDGDYVYVDANPADRPYLGAKPDDPFYRVSRCMLRVTYNRAYPYNIVMM 1aa6.1    --------------------------------------------------------------------------------  target    KHAPFIATEKSVKAHETRPDGRALSANTGYQANLRYGSQQSVTRNWHMPMHQTDTLFHKSKVFMGFIFGGEADNHAVNTV 1aa6.1    --------------------------------------------------------------------------------  target    PKETLVRVTKAEDGGMGGKGIWQPATTGFSPDNESDFMKKYLAGELTKVKT 1aa6.1    --------------------------------------------------- ``` | | | | | | | | | | | | | | | | | | | | | | | | | | | | | | | | | | | | | | | | | | | | | | | | | |
|  | 1fdo.1.A | FORMATE DEHYDROGENASE H  *OXIDIZED FORM OF FORMATE DEHYDROGENASE H FROM E. COLI* | 0.11 |  | 31.74 | 0.27 | 183-442 | X-ray | 2.80 | monomer | 1 x SF4, 2 x MGD, 1 x 6MO | BLAST | 0.36 |
| ``` target    IMDGKNLVENKLTDSHWFIECMERGAKIVVIAPEYGPPSTKADYWIPIRPQTDAALWLGITRLMIEKKWYDETFVKGFTD 1fdo.1    --------------------------------------------------------------------------------  target    FPLLVRTDTLQRLRAHEVFPQYKTSLSADGPSMKIQGLSAEQHAKLGDFVVWDGKTNAPAAITRDDVGATITKKGIDPVL 1fdo.1    --------------------------------------------------------------------------------  target    AGSFKVKLVDGKEVEVATLWTLYQDHLKDYDLDTVVEITQAPKEMIEQLAQDIATMKPVAIHQGEGINHWFHATEMNRAA 1fdo.1    ----------------------YRKIVEGYTPESVEDITGVSASEIRQAARMYAQAKSAAILWGMGVTQFYQGVETVRSL  target    YLPLMLTGNIGRPGAGCQTWAG--NYKAALFQGS-PWTGPGFKGWVAEDPFDINLNPKAHGKE-IHAH-AYTKDEEPAYW 1fdo.1    TSLAMLTGNLGKPHAGVNPVRGQNNVQGACDMGALPDTYPGYQ--YVKDPANREKFAKAWGVESLPAHTGYRISELPHRA  target    NHGDLALIVDTPKFGRKNFTGKTHMPTPTKALIFNNVNLINNAKWAYGMIKNVNPNVEMIVSMDIQMTASIEYADLALPA 1fdo.1    AHGEV-----------------------RAAYIMGEDPLQTDAELS--AVRKAFEDLELVIVQDIFMTKTASAADVILPS  target    NSWLEFEGLEITASCSNPFLQIWKGGIPPVFDSRDDLDILAGIANALADVTGEKRFRDYFAFAAADKRGIYIQRLLDTCT 1fdo.1    TSWGEHEGVFTAA--DRGFQRFFK-AVEPKWDLKTDWQIISEIATRM---------------------------------  target    TTAGYKLADIMAGKYGPPGGCLLNFRTYPRIPFYEQVHDSEPFHTDTGRMHAYADVPEAIEYGENFIVHREGPEATPYLP 1fdo.1    --------------------------------------------------------------------------------  target    NVIVSSNPHIRPEDYGIAADAEHWDDRTIRNIKMPWSKVKETKNFLWEKGFQFYCLTPKTRHRVHSGWSNVDWHMLMDSN 1fdo.1    --------------------------------------------------------------------------------  target    FGDPYRLDKRAPCVGEHQLHINPQAARDLNINDGDYVYVDANPADRPYLGAKPDDPFYRVSRCMLRVTYNRAYPYNIVMM 1fdo.1    --------------------------------------------------------------------------------  target    KHAPFIATEKSVKAHETRPDGRALSANTGYQANLRYGSQQSVTRNWHMPMHQTDTLFHKSKVFMGFIFGGEADNHAVNTV 1fdo.1    --------------------------------------------------------------------------------  target    PKETLVRVTKAEDGGMGGKGIWQPATTGFSPDNESDFMKKYLAGELTKVKT 1fdo.1    --------------------------------------------------- ``` | | | | | | | | | | | | | | | | | | | | | | | | | | | | | | | | | | | | | | | | | | | | | | | | | |
|  | 2iv2.1.A | Formate dehydrogenase H  *Reinterpretation of reduced form of formate dehydrogenase H from E. coli* | 0.11 |  | 31.74 | 0.27 | 183-442 | X-ray | 2.27 | monomer | 1 x SF4, 1 x 2MD, 1 x MGD | BLAST | 0.36 |
| ``` target    IMDGKNLVENKLTDSHWFIECMERGAKIVVIAPEYGPPSTKADYWIPIRPQTDAALWLGITRLMIEKKWYDETFVKGFTD 2iv2.1    --------------------------------------------------------------------------------  target    FPLLVRTDTLQRLRAHEVFPQYKTSLSADGPSMKIQGLSAEQHAKLGDFVVWDGKTNAPAAITRDDVGATITKKGIDPVL 2iv2.1    --------------------------------------------------------------------------------  target    AGSFKVKLVDGKEVEVATLWTLYQDHLKDYDLDTVVEITQAPKEMIEQLAQDIATMKPVAIHQGEGINHWFHATEMNRAA 2iv2.1    ----------------------YRKIVEGYTPESVEDITGVSASEIRQAARMYAQAKSAAILWGMGVTQFYQGVETVRSL  target    YLPLMLTGNIGRPGAGCQTWAG--NYKAALFQGS-PWTGPGFKGWVAEDPFDINLNPKAHGKE-IHAH-AYTKDEEPAYW 2iv2.1    TSLAMLTGNLGKPHAGVNPVRGQNNVQGACDMGALPDTYPGYQ--YVKDPANREKFAKAWGVESLPAHTGYRISELPHRA  target    NHGDLALIVDTPKFGRKNFTGKTHMPTPTKALIFNNVNLINNAKWAYGMIKNVNPNVEMIVSMDIQMTASIEYADLALPA 2iv2.1    AHGEV-----------------------RAAYIMGEDPLQTDAELS--AVRKAFEDLELVIVQDIFMTKTASAADVILPS  target    NSWLEFEGLEITASCSNPFLQIWKGGIPPVFDSRDDLDILAGIANALADVTGEKRFRDYFAFAAADKRGIYIQRLLDTCT 2iv2.1    TSWGEHEGVFTAA--DRGFQRFFK-AVEPKWDLKTDWQIISEIATRM---------------------------------  target    TTAGYKLADIMAGKYGPPGGCLLNFRTYPRIPFYEQVHDSEPFHTDTGRMHAYADVPEAIEYGENFIVHREGPEATPYLP 2iv2.1    --------------------------------------------------------------------------------  target    NVIVSSNPHIRPEDYGIAADAEHWDDRTIRNIKMPWSKVKETKNFLWEKGFQFYCLTPKTRHRVHSGWSNVDWHMLMDSN 2iv2.1    --------------------------------------------------------------------------------  target    FGDPYRLDKRAPCVGEHQLHINPQAARDLNINDGDYVYVDANPADRPYLGAKPDDPFYRVSRCMLRVTYNRAYPYNIVMM 2iv2.1    --------------------------------------------------------------------------------  target    KHAPFIATEKSVKAHETRPDGRALSANTGYQANLRYGSQQSVTRNWHMPMHQTDTLFHKSKVFMGFIFGGEADNHAVNTV 2iv2.1    --------------------------------------------------------------------------------  target    PKETLVRVTKAEDGGMGGKGIWQPATTGFSPDNESDFMKKYLAGELTKVKT 2iv2.1    --------------------------------------------------- ``` | | | | | | | | | | | | | | | | | | | | | | | | | | | | | | | | | | | | | | | | | | | | | | | | | |
|  | 7z0t.1.G | Formate dehydrogenase H  *Structure of the Escherichia coli formate hydrogenlyase complex (aerobic preparation, composite structure)* | 0.11 |  | 31.74 | 0.27 | 183-442 | EM | 0.00 | hetero-1-1-1-1-1-1-… | 1 x NI, 1 x FCO, 8 x SF4, 1 x FE, 2 x MGD, 1 x 6MO | BLAST | 0.36 |
| ``` target    IMDGKNLVENKLTDSHWFIECMERGAKIVVIAPEYGPPSTKADYWIPIRPQTDAALWLGITRLMIEKKWYDETFVKGFTD 7z0t.1    --------------------------------------------------------------------------------  target    FPLLVRTDTLQRLRAHEVFPQYKTSLSADGPSMKIQGLSAEQHAKLGDFVVWDGKTNAPAAITRDDVGATITKKGIDPVL 7z0t.1    --------------------------------------------------------------------------------  target    AGSFKVKLVDGKEVEVATLWTLYQDHLKDYDLDTVVEITQAPKEMIEQLAQDIATMKPVAIHQGEGINHWFHATEMNRAA 7z0t.1    ----------------------YRKIVEGYTPESVEDITGVSASEIRQAARMYAQAKSAAILWGMGVTQFYQGVETVRSL  target    YLPLMLTGNIGRPGAGCQTWAG--NYKAALFQGS-PWTGPGFKGWVAEDPFDINLNPKAHGKE-IHAH-AYTKDEEPAYW 7z0t.1    TSLAMLTGNLGKPHAGVNPVRGQNNVQGACDMGALPDTYPGYQ--YVKDPANREKFAKAWGVESLPAHTGYRISELPHRA  target    NHGDLALIVDTPKFGRKNFTGKTHMPTPTKALIFNNVNLINNAKWAYGMIKNVNPNVEMIVSMDIQMTASIEYADLALPA 7z0t.1    AHGEV-----------------------RAAYIMGEDPLQTDAELS--AVRKAFEDLELVIVQDIFMTKTASAADVILPS  target    NSWLEFEGLEITASCSNPFLQIWKGGIPPVFDSRDDLDILAGIANALADVTGEKRFRDYFAFAAADKRGIYIQRLLDTCT 7z0t.1    TSWGEHEGVFTAA--DRGFQRFFK-AVEPKWDLKTDWQIISEIATRM---------------------------------  target    TTAGYKLADIMAGKYGPPGGCLLNFRTYPRIPFYEQVHDSEPFHTDTGRMHAYADVPEAIEYGENFIVHREGPEATPYLP 7z0t.1    --------------------------------------------------------------------------------  target    NVIVSSNPHIRPEDYGIAADAEHWDDRTIRNIKMPWSKVKETKNFLWEKGFQFYCLTPKTRHRVHSGWSNVDWHMLMDSN 7z0t.1    --------------------------------------------------------------------------------  target    FGDPYRLDKRAPCVGEHQLHINPQAARDLNINDGDYVYVDANPADRPYLGAKPDDPFYRVSRCMLRVTYNRAYPYNIVMM 7z0t.1    --------------------------------------------------------------------------------  target    KHAPFIATEKSVKAHETRPDGRALSANTGYQANLRYGSQQSVTRNWHMPMHQTDTLFHKSKVFMGFIFGGEADNHAVNTV 7z0t.1    --------------------------------------------------------------------------------  target    PKETLVRVTKAEDGGMGGKGIWQPATTGFSPDNESDFMKKYLAGELTKVKT 7z0t.1    --------------------------------------------------- ``` | | | | | | | | | | | | | | | | | | | | | | | | | | | | | | | | | | | | | | | | | | | | | | | | | |
|  | 2ivf.1.A | ETHYLBENZENE DEHYDROGENASE ALPHA-SUBUNIT  *ETHYLBENZENE DEHYDROGENASE FROM AROMATOLEUM AROMATICUM* | 0.18 |  | 36.41 | 0.26 | 16-261 | X-ray | 1.88 | hetero-oligomer | 1 x MES, 4 x SF4, 1 x MO, 1 x MGD, 1 x MD1, 1 x F3S, 1 x HEM | BLAST | 0.39 |
| ``` target    IMDGKNLVENKLTDSHWFIECMERGAKIVVIAPEYGPPSTKADYWIPIRPQTDAALWLGITRLMIEKKWYDETFVKGFTD 2ivf.1    ---------------HFLSEARYKGAEVVVIAPDFNPTTPAADLHVPVRVGSDAAFWLGLSQVMIDEKLFDRQFVCEQTD  target    FPLLVRTDTLQRLRAHEVFPQYKTSLSADGPSMKIQGLSAEQHAKLGDFVVWDGKTNAPAAITRDDVGATITKKGIDPVL 2ivf.1    LPLLVRMDTGKFLSAEDV----------DGGEAK-------------QFYFFDEKAGSVRKASRGTL-----KLDFMPAL  target    AGSFKVKLVDGKEVEVATLWTLYQDHLKDYDLDTVVEITQAPKEMIEQLAQDIATMKPVAIHQGEGINHWFHATEMNRAA 2ivf.1    EGTFSARLKNGKTIQVRTVFEGLREHLKDYTPEKASAKCGVPVSLIRELGRKVAK-KRTCSYIGFSSAKSYHGDLMERSL  target    YLPLMLTGNIGRPGAGCQTWAGNYKAALFQGSPWTGPGFKGWVAEDPFDINLNPKAHGKEIHAHAYTKDEEPAYWNHGDL 2ivf.1    FLAMALSGNWGKPGTGAFAWA-----------------------------------------------------------  target    ALIVDTPKFGRKNFTGKTHMPTPTKALIFNNVNLINNAKWAYGMIKNVNPNVEMIVSMDIQMTASIEYADLALPANSWLE 2ivf.1    --------------------------------------------------------------------------------  target    FEGLEITASCSNPFLQIWKGGIPPVFDSRDDLDILAGIANALADVTGEKRFRDYFAFAAADKRGIYIQRLLDTCTTTAGY 2ivf.1    --------------------------------------------------------------------------------  target    KLADIMAGKYGPPGGCLLNFRTYPRIPFYEQVHDSEPFHTDTGRMHAYADVPEAIEYGENFIVHREGPEATPYLPNVIVS 2ivf.1    --------------------------------------------------------------------------------  target    SNPHIRPEDYGIAADAEHWDDRTIRNIKMPWSKVKETKNFLWEKGFQFYCLTPKTRHRVHSGWSNVDWHMLMDSNFGDPY 2ivf.1    --------------------------------------------------------------------------------  target    RLDKRAPCVGEHQLHINPQAARDLNINDGDYVYVDANPADRPYLGAKPDDPFYRVSRCMLRVTYNRAYPYNIVMMKHAPF 2ivf.1    --------------------------------------------------------------------------------  target    IATEKSVKAHETRPDGRALSANTGYQANLRYGSQQSVTRNWHMPMHQTDTLFHKSKVFMGFIFGGEADNHAVNTVPKETL 2ivf.1    --------------------------------------------------------------------------------  target    VRVTKAEDGGMGGKGIWQPATTGFSPDNESDFMKKYLAGELTKVKT 2ivf.1    ---------------------------------------------- ``` | | | | | | | | | | | | | | | | | | | | | | | | | | | | | | | | | | | | | | | | | | | | | | | | | |
|  | 6btm.1.B | Alternative Complex III subunit B  *Structure of Alternative Complex III from Flavobacterium johnsoniae (Wild Type)* | 0.13 |  | 12.35 | 0.29 | 1-442 | EM | 3.40 | hetero-1-1-1-1-1-1-… | 6 x HEC, 1 x F3S, 1 x SF4, 2 x E87 | HHblits | 0.26 |
| ``` target    IMDGKNLVENKLTD--SHWFIECME----RGAKIVVIAPEYGPPSTKADYWIPIRPQTDAALWLGITRLMIEKKWYDETF 6btm.1    VSVGADFLGDWQGGGYDAGYAKGRIPQNGKMSRHFQFESNMTLSGAAADKRVPMTTADQKQALVQIYNIVVGASVP----  target    VKGFTDFPLLVRTDTLQRLRAHEVFPQYKTSLSADGPSMKIQGLSAEQHAKLGDFVVWDGKTNAPAAITRDDVGATITKK 6btm.1    --------------------------------------------------------------------------------  target    GIDPVLAGSFKVKLVDGKEVEVATLWTLYQDHLKDYDLDTVVEITQAPKEMIEQLAQDIATMK-PVAIHQGEGINHWFHA 6btm.1    -----------------------------------V------SLDAKFKAEVVKAAQQLKAAGTKGILVSGIED------  target    TEMNRAAYLPLMLTGNIGRPGAGCQTWAGNYKAALFQGSPWTGPGFKGWVAEDPFDINLNPKAHGKEIHAHAYTKDEEPA 6btm.1    KNAQLLVLAINQALASEAFSTAGTRQI--------RKG---------------S-------N---A--------------  target    YWNHGDLALIVDTPKFGRKNFTGKTHMPTPTKALIFNNVNLINNAKWAYGMIKNVNPNVEMIVSMDIQMTASIEYADLAL 6btm.1    -----VVAQLIK------------DMNAGSVHTLIMSGVNPVYTLADSASF-VSGLKKVKTSVAFSLKEDETAAVSTIAA  target    PANSWLEFEGLEITASCSNPFLQIWKGGIPPVFDSRDDLDILAGIANALADVTGEKRFRDYFAFAAADKRGIYIQRLLDT 6btm.1    AAPHYLESWGDVEI---TKGTYSLTQPTIRPIFDTKQFQDVLLSVNGTP-------------------------------  target    CTTTAGYKLADIMAGKYGPPGGCLLNFRTYPRIPFYEQVHDSEPFHTDTGRMHAYADVPEAIEYGENFIVHREGPEATPY 6btm.1    --------------------------------------------------------------------------------  target    LPNVIVSSNPHIRPEDYGIAADAEHWDDRTIRNIKMPWSKVKETKNFLWEKGFQFYCLTPKTRHRVHSGWSNVDWHMLMD 6btm.1    --------------------------------------------------------------------------------  target    SNFGDPYRLDKRAPCVGEHQLHINPQAARDLNINDGDYVYVDANPADRPYLGAKPDDPFYRVSRCMLRVTYNRAYPYNIV 6btm.1    --------------------------------------------------------------------------------  target    MMKHAPFIATEKSVKAHETRPDGRALSANTGYQANLRYGSQQSVTRNWHMPMHQTDTLFHKSKVFMGFIFGGEADNHAVN 6btm.1    --------------------------------------------------------------------------------  target    TVPKETLVRVTKAEDGGMGGKGIWQPATTGFSPDNESDFMKKYLAGELTKVKT 6btm.1    ----------------------------------------------------- ``` | | | | | | | | | | | | | | | | | | | | | | | | | | | | | | | | | | | | | | | | | | | | | | | | | |
|  | 2vpz.1.A | THIOSULFATE REDUCTASE  *POLYSULFIDE REDUCTASE NATIVE STRUCTURE* | 0.10 |  | 26.48 | 0.26 | 186-429 | X-ray | 2.40 | hetero-oligomer | 10 x SF4, 4 x MGD, 2 x MO | BLAST | 0.34 |
| ``` target    IMDGKNLVENKLTDSHWFIECMERGAKIVVIAPEYGPPSTKADYWIPIRPQTDAALWLGITRLMIEKKWYDETFVKGFTD 2vpz.1    --------------------------------------------------------------------------------  target    FPLLVRTDTLQRLRAHEVFPQYKTSLSADGPSMKIQGLSAEQHAKLGDFVVWDGKTNAPAAITRDDVGATITKKGIDPVL 2vpz.1    --------------------------------------------------------------------------------  target    AGSFKVKLVDGKEVEVATLWTLYQDHLKDYDLDTVVEITQAPKEMIEQLAQDIATMKPVAIHQGEGINHWFHATEMNRAA 2vpz.1    -------------------------HVKDFTPEWAEKHTEIPAQVIREVAREMAAHKPRAVLPPTRHNVWYGDDTYRVMA  target    YLPL-MLTGNIGRPGA---GCQTWAGNYKAALFQGSPWTGPGFKGWVAEDPFDINLNPKAHGKEIHAHAYTKDE--EPAY 2vpz.1    LLYVNVLLGNYGRPGGFYIAQSPYLEKYPLPPLPLEPAAG-GCSGPSGGDHEPEGFKPRADKGKFFARSTAIQELIEPM-  target    WNHGDLALIVDTPKFGRKNFTGKTHMPTPTKALIFNNVNLINNAKWAYGMIKNVNPNVEMIVSMDIQMTASIEYADLALP 2vpz.1    -------------------ITGE---PYPIKGLFAYGINLFHSIP-NVPRTKEALKNLDLYVAIDVLPQEHVMWADVILP  target    ANSWLEFEGLEITASCSNPFLQIWKGGIPPVFDSRDDLDILAGIANALADVTGEKRFRDYFAFAAADKRGIYIQRLLDTC 2vpz.1    EATYLERYDDFVLVAHKTPFIQLRTPAHEPLFDTK---------------------------------------------  target    TTTAGYKLADIMAGKYGPPGGCLLNFRTYPRIPFYEQVHDSEPFHTDTGRMHAYADVPEAIEYGENFIVHREGPEATPYL 2vpz.1    --------------------------------------------------------------------------------  target    PNVIVSSNPHIRPEDYGIAADAEHWDDRTIRNIKMPWSKVKETKNFLWEKGFQFYCLTPKTRHRVHSGWSNVDWHMLMDS 2vpz.1    --------------------------------------------------------------------------------  target    NFGDPYRLDKRAPCVGEHQLHINPQAARDLNINDGDYVYVDANPADRPYLGAKPDDPFYRVSRCMLRVTYNRAYPYNIVM 2vpz.1    --------------------------------------------------------------------------------  target    MKHAPFIATEKSVKAHETRPDGRALSANTGYQANLRYGSQQSVTRNWHMPMHQTDTLFHKSKVFMGFIFGGEADNHAVNT 2vpz.1    --------------------------------------------------------------------------------  target    VPKETLVRVTKAEDGGMGGKGIWQPATTGFSPDNESDFMKKYLAGELTKVKT 2vpz.1    ---------------------------------------------------- ``` | | | | | | | | | | | | | | | | | | | | | | | | | | | | | | | | | | | | | | | | | | | | | | | | | |
|  | 2vpx.1.D | THIOSULFATE REDUCTASE  *POLYSULFIDE REDUCTASE WITH BOUND QUINONE (UQ1)* | 0.10 |  | 26.48 | 0.26 | 186-429 | X-ray | 3.10 | hetero-oligomer | 10 x SF4, 4 x MGD, 2 x MO, 2 x UQ1 | BLAST | 0.34 |
| ``` target    IMDGKNLVENKLTDSHWFIECMERGAKIVVIAPEYGPPSTKADYWIPIRPQTDAALWLGITRLMIEKKWYDETFVKGFTD 2vpx.1    --------------------------------------------------------------------------------  target    FPLLVRTDTLQRLRAHEVFPQYKTSLSADGPSMKIQGLSAEQHAKLGDFVVWDGKTNAPAAITRDDVGATITKKGIDPVL 2vpx.1    --------------------------------------------------------------------------------  target    AGSFKVKLVDGKEVEVATLWTLYQDHLKDYDLDTVVEITQAPKEMIEQLAQDIATMKPVAIHQGEGINHWFHATEMNRAA 2vpx.1    -------------------------HVKDFTPEWAEKHTEIPAQVIREVAREMAAHKPRAVLPPTRHNVWYGDDTYRVMA  target    YLPL-MLTGNIGRPGA---GCQTWAGNYKAALFQGSPWTGPGFKGWVAEDPFDINLNPKAHGKEIHAHAYTKDE--EPAY 2vpx.1    LLYVNVLLGNYGRPGGFYIAQSPYLEKYPLPPLPLEPAAG-GCSGPSGGDHEPEGFKPRADKGKFFARSTAIQELIEPM-  target    WNHGDLALIVDTPKFGRKNFTGKTHMPTPTKALIFNNVNLINNAKWAYGMIKNVNPNVEMIVSMDIQMTASIEYADLALP 2vpx.1    -------------------ITGE---PYPIKGLFAYGINLFHSIP-NVPRTKEALKNLDLYVAIDVLPQEHVMWADVILP  target    ANSWLEFEGLEITASCSNPFLQIWKGGIPPVFDSRDDLDILAGIANALADVTGEKRFRDYFAFAAADKRGIYIQRLLDTC 2vpx.1    EATYLERYDDFVLVAHKTPFIQLRTPAHEPLFDTK---------------------------------------------  target    TTTAGYKLADIMAGKYGPPGGCLLNFRTYPRIPFYEQVHDSEPFHTDTGRMHAYADVPEAIEYGENFIVHREGPEATPYL 2vpx.1    --------------------------------------------------------------------------------  target    PNVIVSSNPHIRPEDYGIAADAEHWDDRTIRNIKMPWSKVKETKNFLWEKGFQFYCLTPKTRHRVHSGWSNVDWHMLMDS 2vpx.1    --------------------------------------------------------------------------------  target    NFGDPYRLDKRAPCVGEHQLHINPQAARDLNINDGDYVYVDANPADRPYLGAKPDDPFYRVSRCMLRVTYNRAYPYNIVM 2vpx.1    --------------------------------------------------------------------------------  target    MKHAPFIATEKSVKAHETRPDGRALSANTGYQANLRYGSQQSVTRNWHMPMHQTDTLFHKSKVFMGFIFGGEADNHAVNT 2vpx.1    --------------------------------------------------------------------------------  target    VPKETLVRVTKAEDGGMGGKGIWQPATTGFSPDNESDFMKKYLAGELTKVKT 2vpx.1    ---------------------------------------------------- ``` | | | | | | | | | | | | | | | | | | | | | | | | | | | | | | | | | | | | | | | | | | | | | | | | | |
|  | 1kqf.1.A | FORMATE DEHYDROGENASE, NITRATE-INDUCIBLE, MAJOR SUBUNIT  *FORMATE DEHYDROGENASE N FROM E. COLI* | 0.13 |  | 30.66 | 0.25 | 4-262 | X-ray | 1.60 | hetero-oligomer | 3 x 6MO, 15 x SF4, 6 x MGD, 6 x HEM, 3 x CDL | BLAST | 0.35 |
| ``` target    IMDGKNLVENKLTDSHWFIECMERG-AKIVVIAPEYGPPSTKADYWIPIRPQTDAALWLGITRLMIEKKWYDETFVKGFT 1kqf.1    ---GGNAAEAHPVGFRWAMEAKNNNDATLIVVDPRFTRTASVADIYAPIRSGTDITFLSGVLRYLIENNKINAEYVKHYT  target    DFPLLVRTDTLQRLRAHEVFPQYKTSLSADGPSMKIQGLSAEQHAKLGDFVVWDGKTNAPAAITRDDVGATITKKGIDPV 1kqf.1    NASLLVRDDF-----AFE-----------DG---LFSGYDAEKRQY--DKSSWNYQLDENGYAKRDE---TLTHP-----  target    LAGSFKVKLVDGKEVEVATLWTLYQDHLKDYDLDTVVEITQAPK----EMIEQLAQDIATMKPVAIHQGEG-INHWFHAT 1kqf.1    -----------------RCVWNLLKEHVSRYTPDVVENICGTPKADFLKVCEVLASTSAPDRTTTFLYALGWTQHTVGAQ  target    EMNRAAYLPLMLTGNIGRPGAGCQTWAGNYKAALFQGSPWTGPGFKGWVAEDPFDINLNPKAHGKEIHAHAYTKDEEPAY 1kqf.1    NIRTMAMIQLLL-GNMGMAGGGVNALRG----------------------------------------------------  target    WNHGDLALIVDTPKFGRKNFTGKTHMPTPTKALIFNNVNLINNAKWAYGMIKNVNPNVEMIVSMDIQMTASIEYADLALP 1kqf.1    --------------------------------------------------------------------------------  target    ANSWLEFEGLEITASCSNPFLQIWKGGIPPVFDSRDDLDILAGIANALADVTGEKRFRDYFAFAAADKRGIYIQRLLDTC 1kqf.1    --------------------------------------------------------------------------------  target    TTTAGYKLADIMAGKYGPPGGCLLNFRTYPRIPFYEQVHDSEPFHTDTGRMHAYADVPEAIEYGENFIVHREGPEATPYL 1kqf.1    --------------------------------------------------------------------------------  target    PNVIVSSNPHIRPEDYGIAADAEHWDDRTIRNIKMPWSKVKETKNFLWEKGFQFYCLTPKTRHRVHSGWSNVDWHMLMDS 1kqf.1    --------------------------------------------------------------------------------  target    NFGDPYRLDKRAPCVGEHQLHINPQAARDLNINDGDYVYVDANPADRPYLGAKPDDPFYRVSRCMLRVTYNRAYPYNIVM 1kqf.1    --------------------------------------------------------------------------------  target    MKHAPFIATEKSVKAHETRPDGRALSANTGYQANLRYGSQQSVTRNWHMPMHQTDTLFHKSKVFMGFIFGGEADNHAVNT 1kqf.1    --------------------------------------------------------------------------------  target    VPKETLVRVTKAEDGGMGGKGIWQPATTGFSPDNESDFMKKYLAGELTKVKT 1kqf.1    ---------------------------------------------------- ``` | | | | | | | | | | | | | | | | | | | | | | | | | | | | | | | | | | | | | | | | | | | | | | | | | |
|  | 6x89.1.H | NADH dehydrogenase [ubiquinone] iron-sulfur protein 1, mitochondrial  *Vigna radiata mitochondrial complex I\** | 0.12 |  | 16.44 | 0.27 | 1-444 | EM | 0.00 | hetero-1-1-1-1-1-1-… | 1 x NAP, 6 x PC1, 6 x SF4, 2 x FES, 2 x ZN, 1 x FMN | HHblits | 0.27 |
| ``` target    IMDGKNLVENKLTDSHWFIECME-RGAKIVVIAPEYGPPSTKADYWIPIRPQTDAALWLGITRLMIEKKWYDETFVKGFT 6x89.1    LLVGTQPRVEAAMVNARIRKTVRSNQAKVGYIGPATDFN--YDHKHLGTDPQTLVEIAEG--------------------  target    DFPLLVRTDTLQRLRAHEVFPQYKTSLSADGPSMKIQGLSAEQHAKLGDFVVWDGKTNAPAAITRDDVGATITKKGIDPV 6x89.1    --------------------------------------------------------------------------------  target    LAGSFKVKLVDGKEVEVATLWTLYQDHLKDYDLDTVVEITQAPKEMIEQLAQDIATMKPVAIHQGEGINHWFHATEMNRA 6x89.1    ----------------------------------------------RHPFFKTLSDAKNPVIIVGAGVFERKDQDAIFAA  target    AYLPLMLTGNIGRPGAGCQTWAGNYKAALFQGSPWTGPGFKGWVAEDPFDINLNPKAHGKEIHAHAYTKDEEPAYWNHGD 6x89.1    VETIAQKANVVRPDWNGLNVLLLHAAQ---AA---------------ALDLG------------------LVP-------  target    LALIVDTPKFGRKNFTGKTHMPTPTKALIFNNVNLINNAKWAYGMIKNVNPNVEMIVSMDIQMTASIEYADLALPANSWL 6x89.1    --------QS--------EKSLESAKFVYLMGADDVNL---------DKIPDDAFVVYQGHHGDKSVYRANVILPTAAFS  target    EFEGLEITASCSNPFLQIWKGGIPPVFDSRDDLDILAGIANALADVTGEKRFRDYFAFAAADKRGIYIQRLLDTCTTTAG 6x89.1    EKEGTY---QNTEGCTQQTLPAVPTVGDSRDDWKIIRALSEVAGV-----------------------------------  target    YKLADIMAGKYGPPGGCLLNFRTYPRIPFYEQVHDSEPFHTDTGRMHAYADVPEAIEYGENFIVHREGPEATPYLPNVIV 6x89.1    --------------------------------------------------------------------------------  target    SSNPHIRPEDYGIAADAEHWDDRTIRNIKMPWSKVKETKNFLWEKGFQFYCLTPKTRHRVHSGWSNVDWHMLMDSNFGDP 6x89.1    --------------------------------------------------------------------------------  target    YRLDKRAPCVGEHQLHINPQAARDLNINDGDYVYVDANPADRPYLGAKPDDPFYRVSRCMLRVTYNRAYPYNIVMMKHAP 6x89.1    --------------------------------------------------------------------------------  target    FIATEKSVKAHETRPDGRALSANTGYQANLRYGSQQSVTRNWHMPMHQTDTLFHKSKVFMGFIFGGEADNHAVNTVPKET 6x89.1    --------------------------------------------------------------------------------  target    LVRVTKAEDGGMGGKGIWQPATTGFSPDNESDFMKKYLAGELTKVKT 6x89.1    ----------------------------------------------- ``` | | | | | | | | | | | | | | | | | | | | | | | | | | | | | | | | | | | | | | | | | | | | | | | | | |
|  | 8e73.55.A | NDUS1  *Vigna radiata supercomplex I+III2 (full bridge)* | 0.12 |  | 16.44 | 0.27 | 1-444 | EM | 0.00 | monomer |  | HHblits | 0.27 |
| ``` target    IMDGKNLVENKLTDSHWFIECME-RGAKIVVIAPEYGPPSTKADYWIPIRPQTDAALWLGITRLMIEKKWYDETFVKGFT 8e73.55   LLVGTQPRVEAAMVNARIRKTVRSNQAKVGYIGPATDFN--YDHKHLGTDPQTLVEIAEG--------------------  target    DFPLLVRTDTLQRLRAHEVFPQYKTSLSADGPSMKIQGLSAEQHAKLGDFVVWDGKTNAPAAITRDDVGATITKKGIDPV 8e73.55   --------------------------------------------------------------------------------  target    LAGSFKVKLVDGKEVEVATLWTLYQDHLKDYDLDTVVEITQAPKEMIEQLAQDIATMKPVAIHQGEGINHWFHATEMNRA 8e73.55   ----------------------------------------------RHPFFKTLSDAKNPVIIVGAGVFERKDQDAIFAA  target    AYLPLMLTGNIGRPGAGCQTWAGNYKAALFQGSPWTGPGFKGWVAEDPFDINLNPKAHGKEIHAHAYTKDEEPAYWNHGD 8e73.55   VETIAQKANVVRPDWNGLNVLLLHAAQ---AA---------------ALDLG------------------LVP-------  target    LALIVDTPKFGRKNFTGKTHMPTPTKALIFNNVNLINNAKWAYGMIKNVNPNVEMIVSMDIQMTASIEYADLALPANSWL 8e73.55   --------QS--------EKSLESAKFVYLMGADDVNL---------DKIPDDAFVVYQGHHGDKSVYRANVILPTAAFS  target    EFEGLEITASCSNPFLQIWKGGIPPVFDSRDDLDILAGIANALADVTGEKRFRDYFAFAAADKRGIYIQRLLDTCTTTAG 8e73.55   EKEGTY---QNTEGCTQQTLPAVPTVGDSRDDWKIIRALSEVAGV-----------------------------------  target    YKLADIMAGKYGPPGGCLLNFRTYPRIPFYEQVHDSEPFHTDTGRMHAYADVPEAIEYGENFIVHREGPEATPYLPNVIV 8e73.55   --------------------------------------------------------------------------------  target    SSNPHIRPEDYGIAADAEHWDDRTIRNIKMPWSKVKETKNFLWEKGFQFYCLTPKTRHRVHSGWSNVDWHMLMDSNFGDP 8e73.55   --------------------------------------------------------------------------------  target    YRLDKRAPCVGEHQLHINPQAARDLNINDGDYVYVDANPADRPYLGAKPDDPFYRVSRCMLRVTYNRAYPYNIVMMKHAP 8e73.55   --------------------------------------------------------------------------------  target    FIATEKSVKAHETRPDGRALSANTGYQANLRYGSQQSVTRNWHMPMHQTDTLFHKSKVFMGFIFGGEADNHAVNTVPKET 8e73.55   --------------------------------------------------------------------------------  target    LVRVTKAEDGGMGGKGIWQPATTGFSPDNESDFMKKYLAGELTKVKT 8e73.55   ----------------------------------------------- ``` | | | | | | | | | | | | | | | | | | | | | | | | | | | | | | | | | | | | | | | | | | | | | | | | | |
|  | 8e9g.1.G | NADH-quinone oxidoreductase subunit G  *Mycobacterial respiratory complex I with both quinone positions modelled* | 0.13 |  | 11.98 | 0.26 | 342-720 | EM | 0.00 | hetero-1-1-1-1-1-1-… |  | HHblits | 0.25 |
| ``` target    IMDGKNLVENKLTDSHWFIECMERGAKIVVIAPEYGPPSTKADYWIPIRPQTDAALWLGITRLMIEKKWYDETFVKGFTD 8e9g.1    --------------------------------------------------------------------------------  target    FPLLVRTDTLQRLRAHEVFPQYKTSLSADGPSMKIQGLSAEQHAKLGDFVVWDGKTNAPAAITRDDVGATITKKGIDPVL 8e9g.1    --------------------------------------------------------------------------------  target    AGSFKVKLVDGKEVEVATLWTLYQDHLKDYDLDTVVEITQAPKEMIEQLAQDIATMKPVAIHQGEGINHWFHATEMNRAA 8e9g.1    --------------------------------------------------------------------------------  target    YLPLMLTGNIGRPGAGCQTWAGNYKAALFQGSPWTGPGFKGWVAEDPFDINLNPKAHGKEIHAHAYTKDEEPAYWNHGDL 8e9g.1    --------------------------------------------------------------------------------  target    ALIVDTPKFGRKNFTGKTHMPTPTKALIFNNVNLINNAKWAYGMIKNVNPNVEMIVSMDIQMTASIEYADLALPANSWLE 8e9g.1    ---------------------GHLAALLVGG-VELGDLPDPELA-VAAVRTTPFVVSLELRESAVTELADVVFPVAPVVE  target    FEGLEITASCSNPFLQIWKGGIPPVFDSRDDLDILAGIANALADVTGEKRFRDYFAFAAADKRGIYIQRLLDTCTTTAGY 8e9g.1    KAGSFL---NWEGRPRPFAPSLK--TNAIPDLRVLHYLADEIGVDLA-------LP--TA---EAADAE-----------  target    KLADIMAGKYGPPGGCLLNFRTYPRIPFYEQVHDSEPFHTDTGRMHAYADVPEAIEYGENFIVHREGPEATPYLPNVIVS 8e9g.1    ----LAQLG-----------------TWGG-----ARPPAPT----------------------APPT------AR----  target    SNPHIRPEDYGIAADAEHWDDRTIRNIKMPWSKVKETKNFLWEKGFQFYCLTPKTRHRVHSGWSNVDWHMLMDSNFGDPY 8e9g.1    ----------------------------------------PEAGSGQAVLASWRMLLDAGRLQDGEPHLAGTA-------  target    RLDKRAPCVGEHQLHINPQAARDLNINDGDYVYVDANPADRPYLGAKPDDPFYRVSRCMLRVTYNRAYPYNIVMMKHAPF 8e9g.1    ---------VRPVARMSAATAAGIGASDGAPVTVSTER-----------------GAVTLPLAVTD-MPDGVVWLPMNSP  target    IATEKSVKAHETRPDGRALSANTGYQANLRYGSQQSVTRNWHMPMHQTDTLFHKSKVFMGFIFGGEADNHAVNTVPKETL 8e9g.1    --------------------------------------------------------------------------------  target    VRVTKAEDGGMGGKGIWQPATTGFSPDNESDFMKKYLAGELTKVKT 8e9g.1    ---------------------------------------------- ``` | | | | | | | | | | | | | | | | | | | | | | | | | | | | | | | | | | | | | | | | | | | | | | | | | |
|  | 3m9s.1.C | NADH-quinone oxidoreductase subunit 3  *Crystal structure of respiratory complex I from Thermus thermophilus* | 0.11 | 0.00 | 17.10 | 0.23 | 367-723 | X-ray | 4.50 | monomer | 7 x SF4, 2 x FES, 1 x FMN | HHblits | 0.27 |
| ``` target    IMDGKNLVENKLTDSHWFIECMERGAKIVVIAPEYGPPSTKADYWIPIRPQTDAALWLGITRLMIEKKWYDETFVKGFTD 3m9s.1    --------------------------------------------------------------------------------  target    FPLLVRTDTLQRLRAHEVFPQYKTSLSADGPSMKIQGLSAEQHAKLGDFVVWDGKTNAPAAITRDDVGATITKKGIDPVL 3m9s.1    --------------------------------------------------------------------------------  target    AGSFKVKLVDGKEVEVATLWTLYQDHLKDYDLDTVVEITQAPKEMIEQLAQDIATMKPVAIHQGEGINHWFHATEMNRAA 3m9s.1    --------------------------------------------------------------------------------  target    YLPLMLTGNIGRPGAGCQTWAGNYKAALFQGSPWTGPGFKGWVAEDPFDINLNPKAHGKEIHAHAYTKDEEPAYWNHGDL 3m9s.1    --------------------------------------------------------------------------------  target    ALIVDTPKFGRKNFTGKTHMPTPTKALIFNNVNLINNAKWAYGMIKNVNPNVEMIVSMDIQMTASI-EYADLALPANSWL 3m9s.1    ----------------------------------------------EALKGKRFVVMHLSHLHPLAERYAHVVLPAPTFY  target    EFEGLEITASCSNPFLQIWKGGIPPVFDSRDDLDILAGIANALADVTGEKRFRDYFAFAAADKRGIYIQRLLDTCTTTAG 3m9s.1    EKRGHL---VNLEGRVLPLSPAPIENGEAEGALQVLALLAEALGVRPP------F---R-------LHLEA---------  target    YKLADIMAGKYGPPGGCLLNFRTYPRIPFYEQVHDSEPFHTDTGRMHAYADVPEAIEYGENFIVHREGPEATPYLPNVIV 3m9s.1    --QKALK----------------------------ARKVPEAMGRLSFRLKELR---------------------P----  target    SSNPHIRPEDYGIAADAEHWDDRTIRNIKMPWSKVKETKNFLWEKGFQFYCLTPKTRHRVHSGWSNVDWHMLMDSNFGDP 3m9s.1    ------------------------------------------KERKGAFYLRPTMWKA-----HQAVGKAQE--------  target    YRLDKRAPCVGEHQLHINPQAARDLNINDGDYVYVDANPADRPYLGAKPDDPFYRVSRCMLRVTYNRAYPYNIVMMKHAP 3m9s.1    ---------AARAELWAHPETARAEALPEGAQVAVETPF-----------------GRVEARVVHREDVPKGHLYLSALG  target    FIATEKSVKAHETRPDGRALSANTGYQANLRYGSQQSVTRNWHMPMHQTDTLFHKSKVFMGFIFGGEADNHAVNTVPKET 3m9s.1    PAAG----------------------------------------------------------------------------  target    LVRVTKAEDGGMGGKGIWQPATTGFSPDNESDFMKKYLAGELTKVKT 3m9s.1    ----------------------------------------------- ``` | | | | | | | | | | | | | | | | | | | | | | | | | | | | | | | | | | | | | | | | | | | | | | | | | |
| ✓ | 2fug.2.C | NADH-quinone oxidoreductase chain 3  *Crystal structure of the hydrophilic domain of respiratory complex I from Thermus thermophilus* | 0.10 | 0.00 | 17.10 | 0.23 | 367-723 | X-ray | 3.30 | monomer | 7 x SF4, 2 x FES, 1 x FMN | HHblits | 0.27 |
| ``` target    IMDGKNLVENKLTDSHWFIECMERGAKIVVIAPEYGPPSTKADYWIPIRPQTDAALWLGITRLMIEKKWYDETFVKGFTD 2fug.2    --------------------------------------------------------------------------------  target    FPLLVRTDTLQRLRAHEVFPQYKTSLSADGPSMKIQGLSAEQHAKLGDFVVWDGKTNAPAAITRDDVGATITKKGIDPVL 2fug.2    --------------------------------------------------------------------------------  target    AGSFKVKLVDGKEVEVATLWTLYQDHLKDYDLDTVVEITQAPKEMIEQLAQDIATMKPVAIHQGEGINHWFHATEMNRAA 2fug.2    --------------------------------------------------------------------------------  target    YLPLMLTGNIGRPGAGCQTWAGNYKAALFQGSPWTGPGFKGWVAEDPFDINLNPKAHGKEIHAHAYTKDEEPAYWNHGDL 2fug.2    --------------------------------------------------------------------------------  target    ALIVDTPKFGRKNFTGKTHMPTPTKALIFNNVNLINNAKWAYGMIKNVNPNVEMIVSMDIQMTASI-EYADLALPANSWL 2fug.2    ----------------------------------------------EALKGKRFVVMHLSHLHPLAERYAHVVLPAPTFY  target    EFEGLEITASCSNPFLQIWKGGIPPVFDSRDDLDILAGIANALADVTGEKRFRDYFAFAAADKRGIYIQRLLDTCTTTAG 2fug.2    EKRGHL---VNLEGRVLPLSPAPIENGEAEGALQVLALLAEALGVRPP------F---R-------LHLEA---------  target    YKLADIMAGKYGPPGGCLLNFRTYPRIPFYEQVHDSEPFHTDTGRMHAYADVPEAIEYGENFIVHREGPEATPYLPNVIV 2fug.2    --QKALK----------------------------ARKVPEAMGRLSFRLKELR---------------------P----  target    SSNPHIRPEDYGIAADAEHWDDRTIRNIKMPWSKVKETKNFLWEKGFQFYCLTPKTRHRVHSGWSNVDWHMLMDSNFGDP 2fug.2    ------------------------------------------KERKGAFYLRPTMWKA-----HQAVGKAQE--------  target    YRLDKRAPCVGEHQLHINPQAARDLNINDGDYVYVDANPADRPYLGAKPDDPFYRVSRCMLRVTYNRAYPYNIVMMKHAP 2fug.2    ---------AARAELWAHPETARAEALPEGAQVAVETPF-----------------GRVEARVVHREDVPKGHLYLSALG  target    FIATEKSVKAHETRPDGRALSANTGYQANLRYGSQQSVTRNWHMPMHQTDTLFHKSKVFMGFIFGGEADNHAVNTVPKET 2fug.2    PAAG----------------------------------------------------------------------------  target    LVRVTKAEDGGMGGKGIWQPATTGFSPDNESDFMKKYLAGELTKVKT 2fug.2    ----------------------------------------------- ``` | | | | | | | | | | | | | | | | | | | | | | | | | | | | | | | | | | | | | | | | | | | | | | | | | |
|  | 6zjl.1.C | NADH-quinone oxidoreductase subunit 3  *Respiratory complex I from Thermus thermophilus, NAD+ dataset, major state* | 0.11 | 0.00 | 17.10 | 0.23 | 367-723 | EM | 0.00 | monomer | 7 x SF4, 1 x FMN, 2 x FES | HHblits | 0.27 |
| ``` target    IMDGKNLVENKLTDSHWFIECMERGAKIVVIAPEYGPPSTKADYWIPIRPQTDAALWLGITRLMIEKKWYDETFVKGFTD 6zjl.1    --------------------------------------------------------------------------------  target    FPLLVRTDTLQRLRAHEVFPQYKTSLSADGPSMKIQGLSAEQHAKLGDFVVWDGKTNAPAAITRDDVGATITKKGIDPVL 6zjl.1    --------------------------------------------------------------------------------  target    AGSFKVKLVDGKEVEVATLWTLYQDHLKDYDLDTVVEITQAPKEMIEQLAQDIATMKPVAIHQGEGINHWFHATEMNRAA 6zjl.1    --------------------------------------------------------------------------------  target    YLPLMLTGNIGRPGAGCQTWAGNYKAALFQGSPWTGPGFKGWVAEDPFDINLNPKAHGKEIHAHAYTKDEEPAYWNHGDL 6zjl.1    --------------------------------------------------------------------------------  target    ALIVDTPKFGRKNFTGKTHMPTPTKALIFNNVNLINNAKWAYGMIKNVNPNVEMIVSMDIQMTASI-EYADLALPANSWL 6zjl.1    ----------------------------------------------EALKGKRFVVMHLSHLHPLAERYAHVVLPAPTFY  target    EFEGLEITASCSNPFLQIWKGGIPPVFDSRDDLDILAGIANALADVTGEKRFRDYFAFAAADKRGIYIQRLLDTCTTTAG 6zjl.1    EKRGHL---VNLEGRVLPLSPAPIENGEAEGALQVLALLAEALGVRPP------F---R-------LHLEA---------  target    YKLADIMAGKYGPPGGCLLNFRTYPRIPFYEQVHDSEPFHTDTGRMHAYADVPEAIEYGENFIVHREGPEATPYLPNVIV 6zjl.1    --QKALK----------------------------ARKVPEAMGRLSFRLKELR---------------------P----  target    SSNPHIRPEDYGIAADAEHWDDRTIRNIKMPWSKVKETKNFLWEKGFQFYCLTPKTRHRVHSGWSNVDWHMLMDSNFGDP 6zjl.1    ------------------------------------------KERKGAFYLRPTMWKA-----HQAVGKAQE--------  target    YRLDKRAPCVGEHQLHINPQAARDLNINDGDYVYVDANPADRPYLGAKPDDPFYRVSRCMLRVTYNRAYPYNIVMMKHAP 6zjl.1    ---------AARAELWAHPETARAEALPEGAQVAVETPF-----------------GRVEARVVHREDVPKGHLYLSALG  target    FIATEKSVKAHETRPDGRALSANTGYQANLRYGSQQSVTRNWHMPMHQTDTLFHKSKVFMGFIFGGEADNHAVNTVPKET 6zjl.1    PAAG----------------------------------------------------------------------------  target    LVRVTKAEDGGMGGKGIWQPATTGFSPDNESDFMKKYLAGELTKVKT 6zjl.1    ----------------------------------------------- ``` | | | | | | | | | | | | | | | | | | | | | | | | | | | | | | | | | | | | | | | | | | | | | | | | | |
|  | 6q8o.1.C | NADH-quinone oxidoreductase subunit 3  *Respiratory complex I from Thermus thermophilus with bound Piericidin A* | 0.11 | 0.00 | 17.10 | 0.23 | 367-723 | X-ray | 3.61 | monomer | 7 x SF4, 1 x FMN, 2 x FES, 1 x HQH | HHblits | 0.27 |
| ``` target    IMDGKNLVENKLTDSHWFIECMERGAKIVVIAPEYGPPSTKADYWIPIRPQTDAALWLGITRLMIEKKWYDETFVKGFTD 6q8o.1    --------------------------------------------------------------------------------  target    FPLLVRTDTLQRLRAHEVFPQYKTSLSADGPSMKIQGLSAEQHAKLGDFVVWDGKTNAPAAITRDDVGATITKKGIDPVL 6q8o.1    --------------------------------------------------------------------------------  target    AGSFKVKLVDGKEVEVATLWTLYQDHLKDYDLDTVVEITQAPKEMIEQLAQDIATMKPVAIHQGEGINHWFHATEMNRAA 6q8o.1    --------------------------------------------------------------------------------  target    YLPLMLTGNIGRPGAGCQTWAGNYKAALFQGSPWTGPGFKGWVAEDPFDINLNPKAHGKEIHAHAYTKDEEPAYWNHGDL 6q8o.1    --------------------------------------------------------------------------------  target    ALIVDTPKFGRKNFTGKTHMPTPTKALIFNNVNLINNAKWAYGMIKNVNPNVEMIVSMDIQMTASI-EYADLALPANSWL 6q8o.1    ----------------------------------------------EALKGKRFVVMHLSHLHPLAERYAHVVLPAPTFY  target    EFEGLEITASCSNPFLQIWKGGIPPVFDSRDDLDILAGIANALADVTGEKRFRDYFAFAAADKRGIYIQRLLDTCTTTAG 6q8o.1    EKRGHL---VNLEGRVLPLSPAPIENGEAEGALQVLALLAEALGVRPP------F---R-------LHLEA---------  target    YKLADIMAGKYGPPGGCLLNFRTYPRIPFYEQVHDSEPFHTDTGRMHAYADVPEAIEYGENFIVHREGPEATPYLPNVIV 6q8o.1    --QKALK----------------------------ARKVPEAMGRLSFRLKELR---------------------P----  target    SSNPHIRPEDYGIAADAEHWDDRTIRNIKMPWSKVKETKNFLWEKGFQFYCLTPKTRHRVHSGWSNVDWHMLMDSNFGDP 6q8o.1    ------------------------------------------KERKGAFYLRPTMWKA-----HQAVGKAQE--------  target    YRLDKRAPCVGEHQLHINPQAARDLNINDGDYVYVDANPADRPYLGAKPDDPFYRVSRCMLRVTYNRAYPYNIVMMKHAP 6q8o.1    ---------AARAELWAHPETARAEALPEGAQVAVETPF-----------------GRVEARVVHREDVPKGHLYLSALG  target    FIATEKSVKAHETRPDGRALSANTGYQANLRYGSQQSVTRNWHMPMHQTDTLFHKSKVFMGFIFGGEADNHAVNTVPKET 6q8o.1    PAAG----------------------------------------------------------------------------  target    LVRVTKAEDGGMGGKGIWQPATTGFSPDNESDFMKKYLAGELTKVKT 6q8o.1    ----------------------------------------------- ``` | | | | | | | | | | | | | | | | | | | | | | | | | | | | | | | | | | | | | | | | | | | | | | | | | |
|  | 6zjy.1.C | NADH-quinone oxidoreductase subunit 3  *Respiratory complex I from Thermus thermophilus, NAD+ dataset, minor state* | 0.11 | 0.00 | 17.10 | 0.23 | 367-723 | EM | 0.00 | monomer | 7 x SF4, 2 x FES | HHblits | 0.27 |
| ``` target    IMDGKNLVENKLTDSHWFIECMERGAKIVVIAPEYGPPSTKADYWIPIRPQTDAALWLGITRLMIEKKWYDETFVKGFTD 6zjy.1    --------------------------------------------------------------------------------  target    FPLLVRTDTLQRLRAHEVFPQYKTSLSADGPSMKIQGLSAEQHAKLGDFVVWDGKTNAPAAITRDDVGATITKKGIDPVL 6zjy.1    --------------------------------------------------------------------------------  target    AGSFKVKLVDGKEVEVATLWTLYQDHLKDYDLDTVVEITQAPKEMIEQLAQDIATMKPVAIHQGEGINHWFHATEMNRAA 6zjy.1    --------------------------------------------------------------------------------  target    YLPLMLTGNIGRPGAGCQTWAGNYKAALFQGSPWTGPGFKGWVAEDPFDINLNPKAHGKEIHAHAYTKDEEPAYWNHGDL 6zjy.1    --------------------------------------------------------------------------------  target    ALIVDTPKFGRKNFTGKTHMPTPTKALIFNNVNLINNAKWAYGMIKNVNPNVEMIVSMDIQMTASI-EYADLALPANSWL 6zjy.1    ----------------------------------------------EALKGKRFVVMHLSHLHPLAERYAHVVLPAPTFY  target    EFEGLEITASCSNPFLQIWKGGIPPVFDSRDDLDILAGIANALADVTGEKRFRDYFAFAAADKRGIYIQRLLDTCTTTAG 6zjy.1    EKRGHL---VNLEGRVLPLSPAPIENGEAEGALQVLALLAEALGVRPP------F---R-------LHLEA---------  target    YKLADIMAGKYGPPGGCLLNFRTYPRIPFYEQVHDSEPFHTDTGRMHAYADVPEAIEYGENFIVHREGPEATPYLPNVIV 6zjy.1    --QKALK----------------------------ARKVPEAMGRLSFRLKELR---------------------P----  target    SSNPHIRPEDYGIAADAEHWDDRTIRNIKMPWSKVKETKNFLWEKGFQFYCLTPKTRHRVHSGWSNVDWHMLMDSNFGDP 6zjy.1    ------------------------------------------KERKGAFYLRPTMWKA-----HQAVGKAQE--------  target    YRLDKRAPCVGEHQLHINPQAARDLNINDGDYVYVDANPADRPYLGAKPDDPFYRVSRCMLRVTYNRAYPYNIVMMKHAP 6zjy.1    ---------AARAELWAHPETARAEALPEGAQVAVETPF-----------------GRVEARVVHREDVPKGHLYLSALG  target    FIATEKSVKAHETRPDGRALSANTGYQANLRYGSQQSVTRNWHMPMHQTDTLFHKSKVFMGFIFGGEADNHAVNTVPKET 6zjy.1    PAAG----------------------------------------------------------------------------  target    LVRVTKAEDGGMGGKGIWQPATTGFSPDNESDFMKKYLAGELTKVKT 6zjy.1    ----------------------------------------------- ``` | | | | | | | | | | | | | | | | | | | | | | | | | | | | | | | | | | | | | | | | | | | | | | | | | |
|  | 6zjn.1.C | NADH-quinone oxidoreductase subunit 3  *Respiratory complex I from Thermus thermophilus, NADH dataset, minor state* | 0.11 | 0.00 | 17.10 | 0.23 | 367-723 | EM | 0.00 | monomer | 7 x SF4, 2 x FES | HHblits | 0.27 |
| ``` target    IMDGKNLVENKLTDSHWFIECMERGAKIVVIAPEYGPPSTKADYWIPIRPQTDAALWLGITRLMIEKKWYDETFVKGFTD 6zjn.1    --------------------------------------------------------------------------------  target    FPLLVRTDTLQRLRAHEVFPQYKTSLSADGPSMKIQGLSAEQHAKLGDFVVWDGKTNAPAAITRDDVGATITKKGIDPVL 6zjn.1    --------------------------------------------------------------------------------  target    AGSFKVKLVDGKEVEVATLWTLYQDHLKDYDLDTVVEITQAPKEMIEQLAQDIATMKPVAIHQGEGINHWFHATEMNRAA 6zjn.1    --------------------------------------------------------------------------------  target    YLPLMLTGNIGRPGAGCQTWAGNYKAALFQGSPWTGPGFKGWVAEDPFDINLNPKAHGKEIHAHAYTKDEEPAYWNHGDL 6zjn.1    --------------------------------------------------------------------------------  target    ALIVDTPKFGRKNFTGKTHMPTPTKALIFNNVNLINNAKWAYGMIKNVNPNVEMIVSMDIQMTASI-EYADLALPANSWL 6zjn.1    ----------------------------------------------EALKGKRFVVMHLSHLHPLAERYAHVVLPAPTFY  target    EFEGLEITASCSNPFLQIWKGGIPPVFDSRDDLDILAGIANALADVTGEKRFRDYFAFAAADKRGIYIQRLLDTCTTTAG 6zjn.1    EKRGHL---VNLEGRVLPLSPAPIENGEAEGALQVLALLAEALGVRPP------F---R-------LHLEA---------  target    YKLADIMAGKYGPPGGCLLNFRTYPRIPFYEQVHDSEPFHTDTGRMHAYADVPEAIEYGENFIVHREGPEATPYLPNVIV 6zjn.1    --QKALK----------------------------ARKVPEAMGRLSFRLKELR---------------------P----  target    SSNPHIRPEDYGIAADAEHWDDRTIRNIKMPWSKVKETKNFLWEKGFQFYCLTPKTRHRVHSGWSNVDWHMLMDSNFGDP 6zjn.1    ------------------------------------------KERKGAFYLRPTMWKA-----HQAVGKAQE--------  target    YRLDKRAPCVGEHQLHINPQAARDLNINDGDYVYVDANPADRPYLGAKPDDPFYRVSRCMLRVTYNRAYPYNIVMMKHAP 6zjn.1    ---------AARAELWAHPETARAEALPEGAQVAVETPF-----------------GRVEARVVHREDVPKGHLYLSALG  target    FIATEKSVKAHETRPDGRALSANTGYQANLRYGSQQSVTRNWHMPMHQTDTLFHKSKVFMGFIFGGEADNHAVNTVPKET 6zjn.1    PAAG----------------------------------------------------------------------------  target    LVRVTKAEDGGMGGKGIWQPATTGFSPDNESDFMKKYLAGELTKVKT 6zjn.1    ----------------------------------------------- ``` | | | | | | | | | | | | | | | | | | | | | | | | | | | | | | | | | | | | | | | | | | | | | | | | | |
|  | 6ziy.1.C | NADH-quinone oxidoreductase subunit 3  *Respiratory complex I from Thermus thermophilus, NADH dataset, major state* | 0.11 | 0.00 | 17.10 | 0.23 | 367-723 | EM | 0.00 | monomer | 7 x SF4, 1 x FMN, 1 x NAI, 2 x FES | HHblits | 0.27 |
| ``` target    IMDGKNLVENKLTDSHWFIECMERGAKIVVIAPEYGPPSTKADYWIPIRPQTDAALWLGITRLMIEKKWYDETFVKGFTD 6ziy.1    --------------------------------------------------------------------------------  target    FPLLVRTDTLQRLRAHEVFPQYKTSLSADGPSMKIQGLSAEQHAKLGDFVVWDGKTNAPAAITRDDVGATITKKGIDPVL 6ziy.1    --------------------------------------------------------------------------------  target    AGSFKVKLVDGKEVEVATLWTLYQDHLKDYDLDTVVEITQAPKEMIEQLAQDIATMKPVAIHQGEGINHWFHATEMNRAA 6ziy.1    --------------------------------------------------------------------------------  target    YLPLMLTGNIGRPGAGCQTWAGNYKAALFQGSPWTGPGFKGWVAEDPFDINLNPKAHGKEIHAHAYTKDEEPAYWNHGDL 6ziy.1    --------------------------------------------------------------------------------  target    ALIVDTPKFGRKNFTGKTHMPTPTKALIFNNVNLINNAKWAYGMIKNVNPNVEMIVSMDIQMTASI-EYADLALPANSWL 6ziy.1    ----------------------------------------------EALKGKRFVVMHLSHLHPLAERYAHVVLPAPTFY  target    EFEGLEITASCSNPFLQIWKGGIPPVFDSRDDLDILAGIANALADVTGEKRFRDYFAFAAADKRGIYIQRLLDTCTTTAG 6ziy.1    EKRGHL---VNLEGRVLPLSPAPIENGEAEGALQVLALLAEALGVRPP------F---R-------LHLEA---------  target    YKLADIMAGKYGPPGGCLLNFRTYPRIPFYEQVHDSEPFHTDTGRMHAYADVPEAIEYGENFIVHREGPEATPYLPNVIV 6ziy.1    --QKALK----------------------------ARKVPEAMGRLSFRLKELR---------------------P----  target    SSNPHIRPEDYGIAADAEHWDDRTIRNIKMPWSKVKETKNFLWEKGFQFYCLTPKTRHRVHSGWSNVDWHMLMDSNFGDP 6ziy.1    ------------------------------------------KERKGAFYLRPTMWKA-----HQAVGKAQE--------  target    YRLDKRAPCVGEHQLHINPQAARDLNINDGDYVYVDANPADRPYLGAKPDDPFYRVSRCMLRVTYNRAYPYNIVMMKHAP 6ziy.1    ---------AARAELWAHPETARAEALPEGAQVAVETPF-----------------GRVEARVVHREDVPKGHLYLSALG  target    FIATEKSVKAHETRPDGRALSANTGYQANLRYGSQQSVTRNWHMPMHQTDTLFHKSKVFMGFIFGGEADNHAVNTVPKET 6ziy.1    PAAG----------------------------------------------------------------------------  target    LVRVTKAEDGGMGGKGIWQPATTGFSPDNESDFMKKYLAGELTKVKT 6ziy.1    ----------------------------------------------- ``` | | | | | | | | | | | | | | | | | | | | | | | | | | | | | | | | | | | | | | | | | | | | | | | | | |
|  | 2e7z.1.A | Acetylene hydratase Ahy  *Acetylene Hydratase from Pelobacter acetylenicus* | 0.05 |  | 31.58 | 0.18 | 4-174 | X-ray | 1.26 | monomer | 1 x SF4, 2 x MGD, 1 x W | BLAST | 0.36 |
| ``` target    IMDGKNLV-ENKLTDSHWFIECMERGAKIVVIAPEYGPPSTKADYWIPIRPQTDAALWLGITRLMIEKKWYDETFVKGFT 2e7z.1    ---GKNLSNHNWVSQFNDLKAALKRGCKLIVLDPRRTKVAEMADIWLPLRYGTDAALFLGMINVIINEQLYDKEFVENWC  target    DFPLLVRTDTLQRLRAHEVFPQYKTSLSADGPSMKIQGLSAEQHAKLGDFVVWDGKTNAPAAITRD---DVGATITKKGI 2e7z.1    -----VGFEELKE-RVQE-YPLDKVA--------EITGCDAGEIRKAAVMFATESPASIPWAVSTDMQKNSCSAIRAQCI  target    DPVLAGSFKVKLVDGKEVEVATLWTLYQDHLKDYDLDTVVEITQAPKEMIEQLAQDIATMKPVAIHQGEGINHWFHATEM 2e7z.1    LRAIVGSF----VNGAEI--------------------------------------------------------------  target    NRAAYLPLMLTGNIGRPGAGCQTWAGNYKAALFQGSPWTGPGFKGWVAEDPFDINLNPKAHGKEIHAHAYTKDEEPAYWN 2e7z.1    --------------------------------------------------------------------------------  target    HGDLALIVDTPKFGRKNFTGKTHMPTPTKALIFNNVNLINNAKWAYGMIKNVNPNVEMIVSMDIQMTASIEYADLALPAN 2e7z.1    --------------------------------------------------------------------------------  target    SWLEFEGLEITASCSNPFLQIWKGGIPPVFDSRDDLDILAGIANALADVTGEKRFRDYFAFAAADKRGIYIQRLLDTCTT 2e7z.1    --------------------------------------------------------------------------------  target    TAGYKLADIMAGKYGPPGGCLLNFRTYPRIPFYEQVHDSEPFHTDTGRMHAYADVPEAIEYGENFIVHREGPEATPYLPN 2e7z.1    --------------------------------------------------------------------------------  target    VIVSSNPHIRPEDYGIAADAEHWDDRTIRNIKMPWSKVKETKNFLWEKGFQFYCLTPKTRHRVHSGWSNVDWHMLMDSNF 2e7z.1    --------------------------------------------------------------------------------  target    GDPYRLDKRAPCVGEHQLHINPQAARDLNINDGDYVYVDANPADRPYLGAKPDDPFYRVSRCMLRVTYNRAYPYNIVMMK 2e7z.1    --------------------------------------------------------------------------------  target    HAPFIATEKSVKAHETRPDGRALSANTGYQANLRYGSQQSVTRNWHMPMHQTDTLFHKSKVFMGFIFGGEADNHAVNTVP 2e7z.1    --------------------------------------------------------------------------------  target    KETLVRVTKAEDGGMGGKGIWQPATTGFSPDNESDFMKKYLAGELTKVKT 2e7z.1    -------------------------------------------------- ``` | | | | | | | | | | | | | | | | | | | | | | | | | | | | | | | | | | | | | | | | | | | | | | | | | |
|  | 5xtb.1.L | NADH-ubiquinone oxidoreductase 75 kDa subunit, mitochondrial  *Cryo-EM structure of human respiratory complex I matrix arm* | 0.08 | 0.00 | 12.64 | 0.21 | 206-444 | EM | 0.00 | monomer | 6 x SF4, 1 x FMN, 1 x 8Q1, 1 x NDP, 2 x FES | HHblits | 0.26 |
| ``` target    IMDGKNLVENKLTDSHWFIECMERGAKIVVIAPEYGPPSTKADYWIPIRPQTDAALWLGITRLMIEKKWYDETFVKGFTD 5xtb.1    --------------------------------------------------------------------------------  target    FPLLVRTDTLQRLRAHEVFPQYKTSLSADGPSMKIQGLSAEQHAKLGDFVVWDGKTNAPAAITRDDVGATITKKGIDPVL 5xtb.1    --------------------------------------------------------------------------------  target    AGSFKVKLVDGKEVEVATLWTLYQDHLKDYDLDTVVEITQAPKEMIEQLAQDIATMKPVAIHQGEGINHWFHATEMNRAA 5xtb.1    ---------------------------------------------SHPFSQVLKEAKKPMVVLGSSALQRNDGAAILAAV  target    YLPLMLTGNIGRPGAGCQTWAGNYKAALFQGSPWTGPGFKGWVAEDPFDINLNPKAHGKEIHAHAYTKDEEPAYWNHGDL 5xtb.1    SSIAQKIRMTSGVTGDWKVMNILH-----RIA--SQV--------------------AA---------------LDLG--  target    ALIVDTPKFGRKNFTGKTHMPTPTKALIFNNVNLINNAKWAYGMIKNVNPNVEMIVSMDIQMTASIEYADLALPANSWLE 5xtb.1    -Y---KPGV-E------AIRKNPPKVLFLLGADGGCI-------TRQDLPKDCFIIYQGHHGDVGAPIADVILPGAAYTE  target    FEGLEITASCSNPFLQIWKGGIPPVFDSRDDLDILAGIANALADVTGEKRFRDYFAFAAADKRGIYIQRLLDTCTTTAGY 5xtb.1    KSATYV---NTEGRAQQTKVAVTPPGLAREDWKIIRALSEIAGM------------------------------------  target    KLADIMAGKYGPPGGCLLNFRTYPRIPFYEQVHDSEPFHTDTGRMHAYADVPEAIEYGENFIVHREGPEATPYLPNVIVS 5xtb.1    --------------------------------------------------------------------------------  target    SNPHIRPEDYGIAADAEHWDDRTIRNIKMPWSKVKETKNFLWEKGFQFYCLTPKTRHRVHSGWSNVDWHMLMDSNFGDPY 5xtb.1    --------------------------------------------------------------------------------  target    RLDKRAPCVGEHQLHINPQAARDLNINDGDYVYVDANPADRPYLGAKPDDPFYRVSRCMLRVTYNRAYPYNIVMMKHAPF 5xtb.1    --------------------------------------------------------------------------------  target    IATEKSVKAHETRPDGRALSANTGYQANLRYGSQQSVTRNWHMPMHQTDTLFHKSKVFMGFIFGGEADNHAVNTVPKETL 5xtb.1    --------------------------------------------------------------------------------  target    VRVTKAEDGGMGGKGIWQPATTGFSPDNESDFMKKYLAGELTKVKT 5xtb.1    ---------------------------------------------- ``` | | | | | | | | | | | | | | | | | | | | | | | | | | | | | | | | | | | | | | | | | | | | | | | | | |
|  | 7tgh.58.A | NADH-ubiquinone oxidoreductase 75 kDa subunit  *Cryo-EM structure of respiratory super-complex CI+III2 from Tetrahymena thermophila* | 0.09 |  | 15.20 | 0.20 | 204-444 | EM | 0.00 | monomer |  | HHblits | 0.27 |
| ``` target    IMDGKNLVENKLTDSHWFIECMERGAKIVVIAPEYGPPSTKADYWIPIRPQTDAALWLGITRLMIEKKWYDETFVKGFTD 7tgh.58   --------------------------------------------------------------------------------  target    FPLLVRTDTLQRLRAHEVFPQYKTSLSADGPSMKIQGLSAEQHAKLGDFVVWDGKTNAPAAITRDDVGATITKKGIDPVL 7tgh.58   --------------------------------------------------------------------------------  target    AGSFKVKLVDGKEVEVATLWTLYQDHLKDYDLDTVVEITQAPKEMIEQLAQDIATMKPVAIHQGEGINHWFHATEMNRAA 7tgh.58   -------------------------------------------DGTHPFAERLKKAKLPMIMVGASALEREDGAELYNTL  target    YLPLMLTGNIGRPGAGCQTWAGNYKAALFQGSPWTGPGFKGWVAEDPFDINLNPKAHGKEIHAHAYTKDEEPAYWNHGDL 7tgh.58   KVISNKTGVISEEKSWNGFNILH-------KE---M---------GR-------I--N---------------ALE---L  target    ALIVDTPKFGRKNFTGKTHMPTPTKALIFNNVNLINNAKWAYGMIKNVNPNVEMIVSMDIQMTASIEYADLALPANSWLE 7tgh.58   G--I-NP----------TSVNKNAKLVFILGADNNLRP--------EDIPADAFVVYFGTHGDEGAYYADIILPTAAYTE  target    FEGLEITASCSNPFLQIWKGGIPPVFDSRDDLDILAGIANALADVTGEKRFRDYFAFAAADKRGIYIQRLLDTCTTTAGY 7tgh.58   KNATWV---NTEGRVQQGRLVVMPPGDAREDWQIIRALSEEAGV------------------------------------  target    KLADIMAGKYGPPGGCLLNFRTYPRIPFYEQVHDSEPFHTDTGRMHAYADVPEAIEYGENFIVHREGPEATPYLPNVIVS 7tgh.58   --------------------------------------------------------------------------------  target    SNPHIRPEDYGIAADAEHWDDRTIRNIKMPWSKVKETKNFLWEKGFQFYCLTPKTRHRVHSGWSNVDWHMLMDSNFGDPY 7tgh.58   --------------------------------------------------------------------------------  target    RLDKRAPCVGEHQLHINPQAARDLNINDGDYVYVDANPADRPYLGAKPDDPFYRVSRCMLRVTYNRAYPYNIVMMKHAPF 7tgh.58   --------------------------------------------------------------------------------  target    IATEKSVKAHETRPDGRALSANTGYQANLRYGSQQSVTRNWHMPMHQTDTLFHKSKVFMGFIFGGEADNHAVNTVPKETL 7tgh.58   --------------------------------------------------------------------------------  target    VRVTKAEDGGMGGKGIWQPATTGFSPDNESDFMKKYLAGELTKVKT 7tgh.58   ---------------------------------------------- ``` | | | | | | | | | | | | | | | | | | | | | | | | | | | | | | | | | | | | | | | | | | | | | | | | | |
|  | 6yj4.1.G | Subunit NUAM of NADH:Ubiquinone Oxidoreductase (Complex I)  *Structure of Yarrowia lipolytica complex I at 2.7 A* | 0.08 |  | 18.07 | 0.20 | 208-444 | EM | 0.00 | hetero-1-1-1-1-1-1-… | 18 x 3PE, 6 x SF4, 5 x LMT, 8 x PLC, 2 x FES, 1 x FMN, 6 x CDL, 1 x NDP, 1 x ZN, 2 x EHZ | HHblits | 0.29 |
| ``` target    IMDGKNLVENKLTDSHWFIECMERGAKIVVIAPEYGPPSTKADYWIPIRPQTDAALWLGITRLMIEKKWYDETFVKGFTD 6yj4.1    --------------------------------------------------------------------------------  target    FPLLVRTDTLQRLRAHEVFPQYKTSLSADGPSMKIQGLSAEQHAKLGDFVVWDGKTNAPAAITRDDVGATITKKGIDPVL 6yj4.1    --------------------------------------------------------------------------------  target    AGSFKVKLVDGKEVEVATLWTLYQDHLKDYDLDTVVEITQAPKEMIEQLAQDIATMKPVAIHQGEGINHWFHATEMNRAA 6yj4.1    -----------------------------------------------EFGEVLKNAKNPLIIVGSGITDREDAGAFFNTI  target    YLPLMLTGN-IGRPGAGCQTWAGNYKAALFQGSPWTGPGFKGWVAEDPFDINLNPKAHGKEIHAHAYTKDEEPAYWNHGD 6yj4.1    GKFVESTPSVLNENWNGYNVLQRSAS----------R---AG-----AYD------------------------------  target    LALIVDTPKFGRKNFTGKTHMPTPTKALIFNNVNLINNAKWAYGMIKNVNPNVEMIVSMDIQMTASIEYADLALPANSWL 6yj4.1    IGF---TPS--------DEASKTTPKMVWLLGADEVAASD---------IPADAFVVYQGHNGDVGAQFADVVLPGAAYT  target    EFEGLEITASCSNPFLQIWKGGIPPVFDSRDDLDILAGIANALADVTGEKRFRDYFAFAAADKRGIYIQRLLDTCTTTAG 6yj4.1    EKAGTY---VNTEGRSQISRAATGPPGGAREDWKILRAVSEYLGV-----------------------------------  target    YKLADIMAGKYGPPGGCLLNFRTYPRIPFYEQVHDSEPFHTDTGRMHAYADVPEAIEYGENFIVHREGPEATPYLPNVIV 6yj4.1    --------------------------------------------------------------------------------  target    SSNPHIRPEDYGIAADAEHWDDRTIRNIKMPWSKVKETKNFLWEKGFQFYCLTPKTRHRVHSGWSNVDWHMLMDSNFGDP 6yj4.1    --------------------------------------------------------------------------------  target    YRLDKRAPCVGEHQLHINPQAARDLNINDGDYVYVDANPADRPYLGAKPDDPFYRVSRCMLRVTYNRAYPYNIVMMKHAP 6yj4.1    --------------------------------------------------------------------------------  target    FIATEKSVKAHETRPDGRALSANTGYQANLRYGSQQSVTRNWHMPMHQTDTLFHKSKVFMGFIFGGEADNHAVNTVPKET 6yj4.1    --------------------------------------------------------------------------------  target    LVRVTKAEDGGMGGKGIWQPATTGFSPDNESDFMKKYLAGELTKVKT 6yj4.1    ----------------------------------------------- ``` | | | | | | | | | | | | | | | | | | | | | | | | | | | | | | | | | | | | | | | | | | | | | | | | | |
|  | 6rfs.1.A | Subunit NUAM of NADH:Ubiquinone Oxidoreductase (Complex I)  *Cryo-EM structure of a respiratory complex I mutant lacking NDUFS4* | 0.08 |  | 18.07 | 0.20 | 208-444 | EM | 4.04 | hetero-1-1-1-1-1-1-… | 6 x SF4, 2 x FES, 1 x FMN, 1 x NDP, 1 x ZN, 1 x ZMP | HHblits | 0.29 |
| ``` target    IMDGKNLVENKLTDSHWFIECMERGAKIVVIAPEYGPPSTKADYWIPIRPQTDAALWLGITRLMIEKKWYDETFVKGFTD 6rfs.1    --------------------------------------------------------------------------------  target    FPLLVRTDTLQRLRAHEVFPQYKTSLSADGPSMKIQGLSAEQHAKLGDFVVWDGKTNAPAAITRDDVGATITKKGIDPVL 6rfs.1    --------------------------------------------------------------------------------  target    AGSFKVKLVDGKEVEVATLWTLYQDHLKDYDLDTVVEITQAPKEMIEQLAQDIATMKPVAIHQGEGINHWFHATEMNRAA 6rfs.1    -----------------------------------------------EFGEVLKNAKNPLIIVGSGITDREDAGAFFNTI  target    YLPLMLTGN-IGRPGAGCQTWAGNYKAALFQGSPWTGPGFKGWVAEDPFDINLNPKAHGKEIHAHAYTKDEEPAYWNHGD 6rfs.1    GKFVESTPSVLNENWNGYNVLQRSAS----------R---AG-----AYD------------------------------  target    LALIVDTPKFGRKNFTGKTHMPTPTKALIFNNVNLINNAKWAYGMIKNVNPNVEMIVSMDIQMTASIEYADLALPANSWL 6rfs.1    IGF---TPS--------DEASKTTPKMVWLLGADEVAASD---------IPADAFVVYQGHNGDVGAQFADVVLPGAAYT  target    EFEGLEITASCSNPFLQIWKGGIPPVFDSRDDLDILAGIANALADVTGEKRFRDYFAFAAADKRGIYIQRLLDTCTTTAG 6rfs.1    EKAGTY---VNTEGRSQISRAATGPPGGAREDWKILRAVSEYLGV-----------------------------------  target    YKLADIMAGKYGPPGGCLLNFRTYPRIPFYEQVHDSEPFHTDTGRMHAYADVPEAIEYGENFIVHREGPEATPYLPNVIV 6rfs.1    --------------------------------------------------------------------------------  target    SSNPHIRPEDYGIAADAEHWDDRTIRNIKMPWSKVKETKNFLWEKGFQFYCLTPKTRHRVHSGWSNVDWHMLMDSNFGDP 6rfs.1    --------------------------------------------------------------------------------  target    YRLDKRAPCVGEHQLHINPQAARDLNINDGDYVYVDANPADRPYLGAKPDDPFYRVSRCMLRVTYNRAYPYNIVMMKHAP 6rfs.1    --------------------------------------------------------------------------------  target    FIATEKSVKAHETRPDGRALSANTGYQANLRYGSQQSVTRNWHMPMHQTDTLFHKSKVFMGFIFGGEADNHAVNTVPKET 6rfs.1    --------------------------------------------------------------------------------  target    LVRVTKAEDGGMGGKGIWQPATTGFSPDNESDFMKKYLAGELTKVKT 6rfs.1    ----------------------------------------------- ``` | | | | | | | | | | | | | | | | | | | | | | | | | | | | | | | | | | | | | | | | | | | | | | | | | |
|  | 6rfq.1.A | Subunit NUAM of NADH:Ubiquinone Oxidoreductase (Complex I)  *Cryo-EM structure of a respiratory complex I assembly intermediate with NDUFAF2* | 0.08 |  | 18.07 | 0.20 | 208-444 | EM | 3.30 | hetero-1-1-1-1-1-1-… | 6 x SF4, 2 x FES, 1 x FMN, 1 x NDP, 10 x 3PE, 2 x LMN, 4 x CDL, 2 x ZMP, 4 x PLC, 3 x T7X, 1 x CPL | HHblits | 0.29 |
| ``` target    IMDGKNLVENKLTDSHWFIECMERGAKIVVIAPEYGPPSTKADYWIPIRPQTDAALWLGITRLMIEKKWYDETFVKGFTD 6rfq.1    --------------------------------------------------------------------------------  target    FPLLVRTDTLQRLRAHEVFPQYKTSLSADGPSMKIQGLSAEQHAKLGDFVVWDGKTNAPAAITRDDVGATITKKGIDPVL 6rfq.1    --------------------------------------------------------------------------------  target    AGSFKVKLVDGKEVEVATLWTLYQDHLKDYDLDTVVEITQAPKEMIEQLAQDIATMKPVAIHQGEGINHWFHATEMNRAA 6rfq.1    -----------------------------------------------EFGEVLKNAKNPLIIVGSGITDREDAGAFFNTI  target    YLPLMLTGN-IGRPGAGCQTWAGNYKAALFQGSPWTGPGFKGWVAEDPFDINLNPKAHGKEIHAHAYTKDEEPAYWNHGD 6rfq.1    GKFVESTPSVLNENWNGYNVLQRSAS----------R---AG-----AYD------------------------------  target    LALIVDTPKFGRKNFTGKTHMPTPTKALIFNNVNLINNAKWAYGMIKNVNPNVEMIVSMDIQMTASIEYADLALPANSWL 6rfq.1    IGF---TPS--------DEASKTTPKMVWLLGADEVAASD---------IPADAFVVYQGHNGDVGAQFADVVLPGAAYT  target    EFEGLEITASCSNPFLQIWKGGIPPVFDSRDDLDILAGIANALADVTGEKRFRDYFAFAAADKRGIYIQRLLDTCTTTAG 6rfq.1    EKAGTY---VNTEGRSQISRAATGPPGGAREDWKILRAVSEYLGV-----------------------------------  target    YKLADIMAGKYGPPGGCLLNFRTYPRIPFYEQVHDSEPFHTDTGRMHAYADVPEAIEYGENFIVHREGPEATPYLPNVIV 6rfq.1    --------------------------------------------------------------------------------  target    SSNPHIRPEDYGIAADAEHWDDRTIRNIKMPWSKVKETKNFLWEKGFQFYCLTPKTRHRVHSGWSNVDWHMLMDSNFGDP 6rfq.1    --------------------------------------------------------------------------------  target    YRLDKRAPCVGEHQLHINPQAARDLNINDGDYVYVDANPADRPYLGAKPDDPFYRVSRCMLRVTYNRAYPYNIVMMKHAP 6rfq.1    --------------------------------------------------------------------------------  target    FIATEKSVKAHETRPDGRALSANTGYQANLRYGSQQSVTRNWHMPMHQTDTLFHKSKVFMGFIFGGEADNHAVNTVPKET 6rfq.1    --------------------------------------------------------------------------------  target    LVRVTKAEDGGMGGKGIWQPATTGFSPDNESDFMKKYLAGELTKVKT 6rfq.1    ----------------------------------------------- ``` | | | | | | | | | | | | | | | | | | | | | | | | | | | | | | | | | | | | | | | | | | | | | | | | | |
|  | 6gcs.1.A | 75-KDA PROTEIN (NUAM)  *Cryo-EM structure of respiratory complex I from Yarrowia lipolytica* | 0.08 |  | 18.07 | 0.20 | 208-444 | EM | 4.32 | hetero-1-1-1-1-1-1-… | 6 x SF4, 2 x FES, 1 x FMN, 1 x NDP, 1 x ZN, 1 x ZMP, 1 x CDL, 3 x 3PE | HHblits | 0.29 |
| ``` target    IMDGKNLVENKLTDSHWFIECMERGAKIVVIAPEYGPPSTKADYWIPIRPQTDAALWLGITRLMIEKKWYDETFVKGFTD 6gcs.1    --------------------------------------------------------------------------------  target    FPLLVRTDTLQRLRAHEVFPQYKTSLSADGPSMKIQGLSAEQHAKLGDFVVWDGKTNAPAAITRDDVGATITKKGIDPVL 6gcs.1    --------------------------------------------------------------------------------  target    AGSFKVKLVDGKEVEVATLWTLYQDHLKDYDLDTVVEITQAPKEMIEQLAQDIATMKPVAIHQGEGINHWFHATEMNRAA 6gcs.1    -----------------------------------------------EFGEVLKNAKNPLIIVGSGITDREDAGAFFNTI  target    YLPLMLTGN-IGRPGAGCQTWAGNYKAALFQGSPWTGPGFKGWVAEDPFDINLNPKAHGKEIHAHAYTKDEEPAYWNHGD 6gcs.1    GKFVESTPSVLNENWNGYNVLQRSAS----------R---AG-----AYD------------------------------  target    LALIVDTPKFGRKNFTGKTHMPTPTKALIFNNVNLINNAKWAYGMIKNVNPNVEMIVSMDIQMTASIEYADLALPANSWL 6gcs.1    IGF---TPS--------DEASKTTPKMVWLLGADEVAASD---------IPADAFVVYQGHNGDVGAQFADVVLPGAAYT  target    EFEGLEITASCSNPFLQIWKGGIPPVFDSRDDLDILAGIANALADVTGEKRFRDYFAFAAADKRGIYIQRLLDTCTTTAG 6gcs.1    EKAGTY---VNTEGRSQISRAATGPPGGAREDWKILRAVSEYLGV-----------------------------------  target    YKLADIMAGKYGPPGGCLLNFRTYPRIPFYEQVHDSEPFHTDTGRMHAYADVPEAIEYGENFIVHREGPEATPYLPNVIV 6gcs.1    --------------------------------------------------------------------------------  target    SSNPHIRPEDYGIAADAEHWDDRTIRNIKMPWSKVKETKNFLWEKGFQFYCLTPKTRHRVHSGWSNVDWHMLMDSNFGDP 6gcs.1    --------------------------------------------------------------------------------  target    YRLDKRAPCVGEHQLHINPQAARDLNINDGDYVYVDANPADRPYLGAKPDDPFYRVSRCMLRVTYNRAYPYNIVMMKHAP 6gcs.1    --------------------------------------------------------------------------------  target    FIATEKSVKAHETRPDGRALSANTGYQANLRYGSQQSVTRNWHMPMHQTDTLFHKSKVFMGFIFGGEADNHAVNTVPKET 6gcs.1    --------------------------------------------------------------------------------  target    LVRVTKAEDGGMGGKGIWQPATTGFSPDNESDFMKKYLAGELTKVKT 6gcs.1    ----------------------------------------------- ``` | | | | | | | | | | | | | | | | | | | | | | | | | | | | | | | | | | | | | | | | | | | | | | | | | |
|  | 7ak5.1.G | NADH-ubiquinone oxidoreductase 75 kDa subunit, mitochondrial  *Cryo-EM structure of respiratory complex I in the deactive state from Mus musculus at 3.2 A* | 0.09 |  | 13.37 | 0.20 | 208-444 | EM | 0.00 | hetero-1-1-1-1-1-1-… | 6 x SF4, 2 x PC1, 2 x FES, 1 x FMN, 8 x 3PE, 4 x CDL, 1 x ATP, 1 x NDP, 1 x ZN, 2 x EHZ | HHblits | 0.26 |
| ``` target    IMDGKNLVENKLTDSHWFIECMERGAKIVVIAPEYGPPSTKADYWIPIRPQTDAALWLGITRLMIEKKWYDETFVKGFTD 7ak5.1    --------------------------------------------------------------------------------  target    FPLLVRTDTLQRLRAHEVFPQYKTSLSADGPSMKIQGLSAEQHAKLGDFVVWDGKTNAPAAITRDDVGATITKKGIDPVL 7ak5.1    --------------------------------------------------------------------------------  target    AGSFKVKLVDGKEVEVATLWTLYQDHLKDYDLDTVVEITQAPKEMIEQLAQDIATMKPVAIHQGEGINHWFHATEMNRAA 7ak5.1    -----------------------------------------------SFCEVLKDAKKPMVVLGSSALQRDDGAAILVAV  target    YLPLMLTGNIGRPGAGCQTWAGNYKAALFQGSPWTGPGFKGWVAEDPFDINLNPKAHGKEIHAHAYTKDEEPAYWNHGDL 7ak5.1    SNMVQKIRVTTGVAAEWKVMN------ILHRI---A---------SQV---------AA---------------LD---L  target    ALIVDTPKFGRKNFTGKTHMPTPTKALIFNNVNLINNAKWAYGMIKNVNPNVEMIVSMDIQMTASIEYADLALPANSWLE 7ak5.1    GY---KPGV-E------AIRKNPPKMLFLLGADGGCI-------TRQDLPKDCFIVYQGHHGDVGAPMADVILPGAAYTE  target    FEGLEITASCSNPFLQIWKGGIPPVFDSRDDLDILAGIANALADVTGEKRFRDYFAFAAADKRGIYIQRLLDTCTTTAGY 7ak5.1    KSATY---VNTEGRAQQTKVAVTPPGLAREDWKIIRALSEIAGI------------------------------------  target    KLADIMAGKYGPPGGCLLNFRTYPRIPFYEQVHDSEPFHTDTGRMHAYADVPEAIEYGENFIVHREGPEATPYLPNVIVS 7ak5.1    --------------------------------------------------------------------------------  target    SNPHIRPEDYGIAADAEHWDDRTIRNIKMPWSKVKETKNFLWEKGFQFYCLTPKTRHRVHSGWSNVDWHMLMDSNFGDPY 7ak5.1    --------------------------------------------------------------------------------  target    RLDKRAPCVGEHQLHINPQAARDLNINDGDYVYVDANPADRPYLGAKPDDPFYRVSRCMLRVTYNRAYPYNIVMMKHAPF 7ak5.1    --------------------------------------------------------------------------------  target    IATEKSVKAHETRPDGRALSANTGYQANLRYGSQQSVTRNWHMPMHQTDTLFHKSKVFMGFIFGGEADNHAVNTVPKETL 7ak5.1    --------------------------------------------------------------------------------  target    VRVTKAEDGGMGGKGIWQPATTGFSPDNESDFMKKYLAGELTKVKT 7ak5.1    ---------------------------------------------- ``` | | | | | | | | | | | | | | | | | | | | | | | | | | | | | | | | | | | | | | | | | | | | | | | | | |
|  | 7aqr.1.F | NADH dehydrogenase [ubiquinone] iron-sulfur protein 1, mitochondrial  *Cryo-EM structure of Arabidopsis thaliana Complex-I (peripheral arm)* | 0.08 |  | 16.07 | 0.20 | 206-444 | EM | 0.00 | hetero-1-1-1-1-1-1-… | 6 x SF4, 2 x FES, 1 x FMN, 1 x NDP, 1 x ZN, 1 x 8Q1 | HHblits | 0.28 |
| ``` target    IMDGKNLVENKLTDSHWFIECMERGAKIVVIAPEYGPPSTKADYWIPIRPQTDAALWLGITRLMIEKKWYDETFVKGFTD 7aqr.1    --------------------------------------------------------------------------------  target    FPLLVRTDTLQRLRAHEVFPQYKTSLSADGPSMKIQGLSAEQHAKLGDFVVWDGKTNAPAAITRDDVGATITKKGIDPVL 7aqr.1    --------------------------------------------------------------------------------  target    AGSFKVKLVDGKEVEVATLWTLYQDHLKDYDLDTVVEITQAPKEMIEQLAQDIATMKPVAIHQGEGINHWFHATEMNRAA 7aqr.1    ---------------------------------------------RHPFCTALKNAKNPAIIVGAGLFNRTDKNAILSSV  target    YLPLMLTGNIGRPGAGCQTWAGNYKAALFQGSPWTGPGFKGWVAEDPFDINLNPKAHGKEIHAHAYTKDEEPAYWNHGDL 7aqr.1    ESIAQANNVVRPDWNGLNFLLQYAAQ-------------AA-----AL---------------------------DLGLI  target    ALIVDTPKFGRKNFTGKTHMPTPTKALIFNNVNLINNAKWAYGMIKNVNPNVEMIVSMDIQMTASIEYADLALPANSWLE 7aqr.1    QQSA--------------KALESAKFVYLMGADDVNV---------DKIPKDAFVVYQGHHGDKAVYRANVILPASAFTE  target    FEGLEITASCSNPFLQIWKGGIPPVFDSRDDLDILAGIANALADVTGEKRFRDYFAFAAADKRGIYIQRLLDTCTTTAGY 7aqr.1    KEGTY---ENTEGFTQQTVPAVPTVGDARDDWKIVRALSEVSGV------------------------------------  target    KLADIMAGKYGPPGGCLLNFRTYPRIPFYEQVHDSEPFHTDTGRMHAYADVPEAIEYGENFIVHREGPEATPYLPNVIVS 7aqr.1    --------------------------------------------------------------------------------  target    SNPHIRPEDYGIAADAEHWDDRTIRNIKMPWSKVKETKNFLWEKGFQFYCLTPKTRHRVHSGWSNVDWHMLMDSNFGDPY 7aqr.1    --------------------------------------------------------------------------------  target    RLDKRAPCVGEHQLHINPQAARDLNINDGDYVYVDANPADRPYLGAKPDDPFYRVSRCMLRVTYNRAYPYNIVMMKHAPF 7aqr.1    --------------------------------------------------------------------------------  target    IATEKSVKAHETRPDGRALSANTGYQANLRYGSQQSVTRNWHMPMHQTDTLFHKSKVFMGFIFGGEADNHAVNTVPKETL 7aqr.1    --------------------------------------------------------------------------------  target    VRVTKAEDGGMGGKGIWQPATTGFSPDNESDFMKKYLAGELTKVKT 7aqr.1    ---------------------------------------------- ``` | | | | | | | | | | | | | | | | | | | | | | | | | | | | | | | | | | | | | | | | | | | | | | | | | |
|  | 7a23.1.O | 75kDa  *Plant mitochondrial respiratory complex I* | 0.08 |  | 16.07 | 0.20 | 206-444 | EM | 0.00 | hetero-1-1-1-1-1-1-… | 6 x SF4, 1 x FMN, 2 x T7X, 3 x CDL, 1 x U10, 1 x PEV, 2 x FES, 1 x NDP, 2 x ZN | HHblits | 0.28 |
| ``` target    IMDGKNLVENKLTDSHWFIECMERGAKIVVIAPEYGPPSTKADYWIPIRPQTDAALWLGITRLMIEKKWYDETFVKGFTD 7a23.1    --------------------------------------------------------------------------------  target    FPLLVRTDTLQRLRAHEVFPQYKTSLSADGPSMKIQGLSAEQHAKLGDFVVWDGKTNAPAAITRDDVGATITKKGIDPVL 7a23.1    --------------------------------------------------------------------------------  target    AGSFKVKLVDGKEVEVATLWTLYQDHLKDYDLDTVVEITQAPKEMIEQLAQDIATMKPVAIHQGEGINHWFHATEMNRAA 7a23.1    ---------------------------------------------RHPFCTALKNAKNPAIIVGAGLFNRTDKNAILSSV  target    YLPLMLTGNIGRPGAGCQTWAGNYKAALFQGSPWTGPGFKGWVAEDPFDINLNPKAHGKEIHAHAYTKDEEPAYWNHGDL 7a23.1    ESIAQANNVVRPDWNGLNFLLQYAAQ-------------AA-----AL---------------------------DLGLI  target    ALIVDTPKFGRKNFTGKTHMPTPTKALIFNNVNLINNAKWAYGMIKNVNPNVEMIVSMDIQMTASIEYADLALPANSWLE 7a23.1    QQSA--------------KALESAKFVYLMGADDVNV---------DKIPKDAFVVYQGHHGDKAVYRANVILPASAFTE  target    FEGLEITASCSNPFLQIWKGGIPPVFDSRDDLDILAGIANALADVTGEKRFRDYFAFAAADKRGIYIQRLLDTCTTTAGY 7a23.1    KEGTY---ENTEGFTQQTVPAVPTVGDARDDWKIVRALSEVSGV------------------------------------  target    KLADIMAGKYGPPGGCLLNFRTYPRIPFYEQVHDSEPFHTDTGRMHAYADVPEAIEYGENFIVHREGPEATPYLPNVIVS 7a23.1    --------------------------------------------------------------------------------  target    SNPHIRPEDYGIAADAEHWDDRTIRNIKMPWSKVKETKNFLWEKGFQFYCLTPKTRHRVHSGWSNVDWHMLMDSNFGDPY 7a23.1    --------------------------------------------------------------------------------  target    RLDKRAPCVGEHQLHINPQAARDLNINDGDYVYVDANPADRPYLGAKPDDPFYRVSRCMLRVTYNRAYPYNIVMMKHAPF 7a23.1    --------------------------------------------------------------------------------  target    IATEKSVKAHETRPDGRALSANTGYQANLRYGSQQSVTRNWHMPMHQTDTLFHKSKVFMGFIFGGEADNHAVNTVPKETL 7a23.1    --------------------------------------------------------------------------------  target    VRVTKAEDGGMGGKGIWQPATTGFSPDNESDFMKKYLAGELTKVKT 7a23.1    ---------------------------------------------- ``` | | | | | | | | | | | | | | | | | | | | | | | | | | | | | | | | | | | | | | | | | | | | | | | | | |
|  | 7ar8.1.G | NADH dehydrogenase [ubiquinone] iron-sulfur protein 1, mitochondrial  *Cryo-EM structure of Arabidopsis thaliana complex-I (closed conformation)* | 0.08 |  | 16.07 | 0.20 | 206-444 | EM | 0.00 | hetero-1-1-1-1-1-1-… | 6 x SF4, 2 x FES, 1 x FMN, 1 x UQ9, 3 x PTY, 2 x PC7, 1 x PGT, 1 x FE, 1 x NDP, 2 x ZN, 2 x 8Q1, 1 x LMN, 1 x PSF, 1 x T7X | HHblits | 0.28 |
| ``` target    IMDGKNLVENKLTDSHWFIECMERGAKIVVIAPEYGPPSTKADYWIPIRPQTDAALWLGITRLMIEKKWYDETFVKGFTD 7ar8.1    --------------------------------------------------------------------------------  target    FPLLVRTDTLQRLRAHEVFPQYKTSLSADGPSMKIQGLSAEQHAKLGDFVVWDGKTNAPAAITRDDVGATITKKGIDPVL 7ar8.1    --------------------------------------------------------------------------------  target    AGSFKVKLVDGKEVEVATLWTLYQDHLKDYDLDTVVEITQAPKEMIEQLAQDIATMKPVAIHQGEGINHWFHATEMNRAA 7ar8.1    ---------------------------------------------RHPFCTALKNAKNPAIIVGAGLFNRTDKNAILSSV  target    YLPLMLTGNIGRPGAGCQTWAGNYKAALFQGSPWTGPGFKGWVAEDPFDINLNPKAHGKEIHAHAYTKDEEPAYWNHGDL 7ar8.1    ESIAQANNVVRPDWNGLNFLLQYAAQ-------------AA-----AL---------------------------DLGLI  target    ALIVDTPKFGRKNFTGKTHMPTPTKALIFNNVNLINNAKWAYGMIKNVNPNVEMIVSMDIQMTASIEYADLALPANSWLE 7ar8.1    QQSA--------------KALESAKFVYLMGADDVNV---------DKIPKDAFVVYQGHHGDKAVYRANVILPASAFTE  target    FEGLEITASCSNPFLQIWKGGIPPVFDSRDDLDILAGIANALADVTGEKRFRDYFAFAAADKRGIYIQRLLDTCTTTAGY 7ar8.1    KEGTY---ENTEGFTQQTVPAVPTVGDARDDWKIVRALSEVSGV------------------------------------  target    KLADIMAGKYGPPGGCLLNFRTYPRIPFYEQVHDSEPFHTDTGRMHAYADVPEAIEYGENFIVHREGPEATPYLPNVIVS 7ar8.1    --------------------------------------------------------------------------------  target    SNPHIRPEDYGIAADAEHWDDRTIRNIKMPWSKVKETKNFLWEKGFQFYCLTPKTRHRVHSGWSNVDWHMLMDSNFGDPY 7ar8.1    --------------------------------------------------------------------------------  target    RLDKRAPCVGEHQLHINPQAARDLNINDGDYVYVDANPADRPYLGAKPDDPFYRVSRCMLRVTYNRAYPYNIVMMKHAPF 7ar8.1    --------------------------------------------------------------------------------  target    IATEKSVKAHETRPDGRALSANTGYQANLRYGSQQSVTRNWHMPMHQTDTLFHKSKVFMGFIFGGEADNHAVNTVPKETL 7ar8.1    --------------------------------------------------------------------------------  target    VRVTKAEDGGMGGKGIWQPATTGFSPDNESDFMKKYLAGELTKVKT 7ar8.1    ---------------------------------------------- ``` | | | | | | | | | | | | | | | | | | | | | | | | | | | | | | | | | | | | | | | | | | | | | | | | | |
|  | 5gpn.24.A | NADH-ubiquinone oxidoreductase 75 kDa subunit  *Architecture of mammalian respirasome* | 0.08 |  | 13.37 | 0.20 | 208-444 | EM | 0.00 | monomer |  | HHblits | 0.26 |
| ``` target    IMDGKNLVENKLTDSHWFIECMERGAKIVVIAPEYGPPSTKADYWIPIRPQTDAALWLGITRLMIEKKWYDETFVKGFTD 5gpn.24   --------------------------------------------------------------------------------  target    FPLLVRTDTLQRLRAHEVFPQYKTSLSADGPSMKIQGLSAEQHAKLGDFVVWDGKTNAPAAITRDDVGATITKKGIDPVL 5gpn.24   --------------------------------------------------------------------------------  target    AGSFKVKLVDGKEVEVATLWTLYQDHLKDYDLDTVVEITQAPKEMIEQLAQDIATMKPVAIHQGEGINHWFHATEMNRAA 5gpn.24   -----------------------------------------------PFSQILKEAKKPMVVLGSSALQRSDGTAILAAV  target    YLPLMLTGNIGRPGAGCQTWAGNYKAALFQGSPWTGPGFKGWVAEDPFDINLNPKAHGKEIHAHAYTKDEEPAYWNHGDL 5gpn.24   SNIAQNIRLSSGVTGDWKVMN------ILHR----IA---------------S--QVAA---------------LDLG--  target    ALIVDTPKFGRKNFTGKTHMPTPTKALIFNNVNLINNAKWAYGMIKNVNPNVEMIVSMDIQMTASIEYADLALPANSWLE 5gpn.24   -Y---KPGV-E------AIRKNPPKVLFLLGADGGCI-------TRQDLPKDCFIIYQGHHGDVGAPMADVILPGAAYTE  target    FEGLEITASCSNPFLQIWKGGIPPVFDSRDDLDILAGIANALADVTGEKRFRDYFAFAAADKRGIYIQRLLDTCTTTAGY 5gpn.24   KSATY---VNTEGRAQQTKVAVTPPGLAREDWKIIRALSEIAGM------------------------------------  target    KLADIMAGKYGPPGGCLLNFRTYPRIPFYEQVHDSEPFHTDTGRMHAYADVPEAIEYGENFIVHREGPEATPYLPNVIVS 5gpn.24   --------------------------------------------------------------------------------  target    SNPHIRPEDYGIAADAEHWDDRTIRNIKMPWSKVKETKNFLWEKGFQFYCLTPKTRHRVHSGWSNVDWHMLMDSNFGDPY 5gpn.24   --------------------------------------------------------------------------------  target    RLDKRAPCVGEHQLHINPQAARDLNINDGDYVYVDANPADRPYLGAKPDDPFYRVSRCMLRVTYNRAYPYNIVMMKHAPF 5gpn.24   --------------------------------------------------------------------------------  target    IATEKSVKAHETRPDGRALSANTGYQANLRYGSQQSVTRNWHMPMHQTDTLFHKSKVFMGFIFGGEADNHAVNTVPKETL 5gpn.24   --------------------------------------------------------------------------------  target    VRVTKAEDGGMGGKGIWQPATTGFSPDNESDFMKKYLAGELTKVKT 5gpn.24   ---------------------------------------------- ``` | | | | | | | | | | | | | | | | | | | | | | | | | | | | | | | | | | | | | | | | | | | | | | | | | |
|  | 7vxu.1.L | NADH-ubiquinone oxidoreductase 75 kDa subunit, mitochondrial  *Matrix arm of deactive state CI from Q10 dataset* | 0.09 |  | 13.95 | 0.20 | 208-444 | EM | 0.00 | hetero-1-1-1-1-1-1-… | 6 x SF4, 1 x FMN, 1 x PEE, 1 x PLX, 1 x 8Q1, 1 x NDP, 2 x FES, 1 x MG, 1 x CDL, 1 x ZN | HHblits | 0.26 |
| ``` target    IMDGKNLVENKLTDSHWFIECMERGAKIVVIAPEYGPPSTKADYWIPIRPQTDAALWLGITRLMIEKKWYDETFVKGFTD 7vxu.1    --------------------------------------------------------------------------------  target    FPLLVRTDTLQRLRAHEVFPQYKTSLSADGPSMKIQGLSAEQHAKLGDFVVWDGKTNAPAAITRDDVGATITKKGIDPVL 7vxu.1    --------------------------------------------------------------------------------  target    AGSFKVKLVDGKEVEVATLWTLYQDHLKDYDLDTVVEITQAPKEMIEQLAQDIATMKPVAIHQGEGINHWFHATEMNRAA 7vxu.1    -----------------------------------------------PFSQILKEAKKPMVVLGSSALQRSDGTAILAAV  target    YLPLMLTGNIGRPGAGCQTWAGNYKAALFQGSPWTGPGFKGWVAEDPFDINLNPKAHGKEIHAHAYTKDEEPAYWNHGDL 7vxu.1    SNIAQNIRLSSGVTGDWKVMNI-----LHRI----A----------------S--QVA---------------ALDLG--  target    ALIVDTPKFGRKNFTGKTHMPTPTKALIFNNVNLINNAKWAYGMIKNVNPNVEMIVSMDIQMTASIEYADLALPANSWLE 7vxu.1    -Y---KPGV-E------AIRKNPPKVLFLLGADGGCI-------TRQDLPKDCFIIYQGHHGDVGAPMADVILPGAAYTE  target    FEGLEITASCSNPFLQIWKGGIPPVFDSRDDLDILAGIANALADVTGEKRFRDYFAFAAADKRGIYIQRLLDTCTTTAGY 7vxu.1    KSATY---VNTEGRAQQTKVAVTPPGLAREDWKIIRALSEIAGM------------------------------------  target    KLADIMAGKYGPPGGCLLNFRTYPRIPFYEQVHDSEPFHTDTGRMHAYADVPEAIEYGENFIVHREGPEATPYLPNVIVS 7vxu.1    --------------------------------------------------------------------------------  target    SNPHIRPEDYGIAADAEHWDDRTIRNIKMPWSKVKETKNFLWEKGFQFYCLTPKTRHRVHSGWSNVDWHMLMDSNFGDPY 7vxu.1    --------------------------------------------------------------------------------  target    RLDKRAPCVGEHQLHINPQAARDLNINDGDYVYVDANPADRPYLGAKPDDPFYRVSRCMLRVTYNRAYPYNIVMMKHAPF 7vxu.1    --------------------------------------------------------------------------------  target    IATEKSVKAHETRPDGRALSANTGYQANLRYGSQQSVTRNWHMPMHQTDTLFHKSKVFMGFIFGGEADNHAVNTVPKETL 7vxu.1    --------------------------------------------------------------------------------  target    VRVTKAEDGGMGGKGIWQPATTGFSPDNESDFMKKYLAGELTKVKT 7vxu.1    ---------------------------------------------- ``` | | | | | | | | | | | | | | | | | | | | | | | | | | | | | | | | | | | | | | | | | | | | | | | | | |
|  | 6zr2.1.G | NADH-ubiquinone oxidoreductase 75 kDa subunit, mitochondrial  *Cryo-EM structure of respiratory complex I in the active state from Mus musculus at 3.1 A* | 0.09 |  | 13.37 | 0.20 | 208-444 | EM | 3.10 | hetero-1-1-1-1-1-1-… | 6 x SF4, 4 x PC1, 2 x FES, 1 x FMN, 9 x 3PE, 7 x CDL, 1 x ATP, 1 x NDP, 1 x ZN, 2 x EHZ | HHblits | 0.26 |
| ``` target    IMDGKNLVENKLTDSHWFIECMERGAKIVVIAPEYGPPSTKADYWIPIRPQTDAALWLGITRLMIEKKWYDETFVKGFTD 6zr2.1    --------------------------------------------------------------------------------  target    FPLLVRTDTLQRLRAHEVFPQYKTSLSADGPSMKIQGLSAEQHAKLGDFVVWDGKTNAPAAITRDDVGATITKKGIDPVL 6zr2.1    --------------------------------------------------------------------------------  target    AGSFKVKLVDGKEVEVATLWTLYQDHLKDYDLDTVVEITQAPKEMIEQLAQDIATMKPVAIHQGEGINHWFHATEMNRAA 6zr2.1    -----------------------------------------------SFCEVLKDAKKPMVVLGSSALQRDDGAAILVAV  target    YLPLMLTGNIGRPGAGCQTWAGNYKAALFQGSPWTGPGFKGWVAEDPFDINLNPKAHGKEIHAHAYTKDEEPAYWNHGDL 6zr2.1    SNMVQKIRVTTGVAAEWKVMN------ILHRI---A---------SQ---------VA---------------ALD---L  target    ALIVDTPKFGRKNFTGKTHMPTPTKALIFNNVNLINNAKWAYGMIKNVNPNVEMIVSMDIQMTASIEYADLALPANSWLE 6zr2.1    GY---KPGV-E------AIRKNPPKMLFLLGADGGCI-------TRQDLPKDCFIVYQGHHGDVGAPMADVILPGAAYTE  target    FEGLEITASCSNPFLQIWKGGIPPVFDSRDDLDILAGIANALADVTGEKRFRDYFAFAAADKRGIYIQRLLDTCTTTAGY 6zr2.1    KSATY---VNTEGRAQQTKVAVTPPGLAREDWKIIRALSEIAGI------------------------------------  target    KLADIMAGKYGPPGGCLLNFRTYPRIPFYEQVHDSEPFHTDTGRMHAYADVPEAIEYGENFIVHREGPEATPYLPNVIVS 6zr2.1    --------------------------------------------------------------------------------  target    SNPHIRPEDYGIAADAEHWDDRTIRNIKMPWSKVKETKNFLWEKGFQFYCLTPKTRHRVHSGWSNVDWHMLMDSNFGDPY 6zr2.1    --------------------------------------------------------------------------------  target    RLDKRAPCVGEHQLHINPQAARDLNINDGDYVYVDANPADRPYLGAKPDDPFYRVSRCMLRVTYNRAYPYNIVMMKHAPF 6zr2.1    --------------------------------------------------------------------------------  target    IATEKSVKAHETRPDGRALSANTGYQANLRYGSQQSVTRNWHMPMHQTDTLFHKSKVFMGFIFGGEADNHAVNTVPKETL 6zr2.1    --------------------------------------------------------------------------------  target    VRVTKAEDGGMGGKGIWQPATTGFSPDNESDFMKKYLAGELTKVKT 6zr2.1    ---------------------------------------------- ``` | | | | | | | | | | | | | | | | | | | | | | | | | | | | | | | | | | | | | | | | | | | | | | | | | |
|  | 6g72.1.G | NADH-ubiquinone oxidoreductase 75 kDa subunit, mitochondrial  *Mouse mitochondrial complex I in the deactive state* | 0.09 |  | 13.37 | 0.20 | 208-444 | EM | 0.00 | hetero-1-1-1-1-1-1-… | 6 x SF4, 2 x FES, 1 x FMN, 1 x ADP, 1 x NDP, 1 x ZN, 2 x EHZ | HHblits | 0.26 |
| ``` target    IMDGKNLVENKLTDSHWFIECMERGAKIVVIAPEYGPPSTKADYWIPIRPQTDAALWLGITRLMIEKKWYDETFVKGFTD 6g72.1    --------------------------------------------------------------------------------  target    FPLLVRTDTLQRLRAHEVFPQYKTSLSADGPSMKIQGLSAEQHAKLGDFVVWDGKTNAPAAITRDDVGATITKKGIDPVL 6g72.1    --------------------------------------------------------------------------------  target    AGSFKVKLVDGKEVEVATLWTLYQDHLKDYDLDTVVEITQAPKEMIEQLAQDIATMKPVAIHQGEGINHWFHATEMNRAA 6g72.1    -----------------------------------------------SFCEVLKDAKKPMVVLGSSALQRDDGAAILVAV  target    YLPLMLTGNIGRPGAGCQTWAGNYKAALFQGSPWTGPGFKGWVAEDPFDINLNPKAHGKEIHAHAYTKDEEPAYWNHGDL 6g72.1    SNMVQKIRVTTGVAAEWKVMN------ILHRI---A---------SQ---------VA---------------ALD---L  target    ALIVDTPKFGRKNFTGKTHMPTPTKALIFNNVNLINNAKWAYGMIKNVNPNVEMIVSMDIQMTASIEYADLALPANSWLE 6g72.1    GY---KPGV-E------AIRKNPPKMLFLLGADGGCI-------TRQDLPKDCFIVYQGHHGDVGAPMADVILPGAAYTE  target    FEGLEITASCSNPFLQIWKGGIPPVFDSRDDLDILAGIANALADVTGEKRFRDYFAFAAADKRGIYIQRLLDTCTTTAGY 6g72.1    KSATY---VNTEGRAQQTKVAVTPPGLAREDWKIIRALSEIAGI------------------------------------  target    KLADIMAGKYGPPGGCLLNFRTYPRIPFYEQVHDSEPFHTDTGRMHAYADVPEAIEYGENFIVHREGPEATPYLPNVIVS 6g72.1    --------------------------------------------------------------------------------  target    SNPHIRPEDYGIAADAEHWDDRTIRNIKMPWSKVKETKNFLWEKGFQFYCLTPKTRHRVHSGWSNVDWHMLMDSNFGDPY 6g72.1    --------------------------------------------------------------------------------  target    RLDKRAPCVGEHQLHINPQAARDLNINDGDYVYVDANPADRPYLGAKPDDPFYRVSRCMLRVTYNRAYPYNIVMMKHAPF 6g72.1    --------------------------------------------------------------------------------  target    IATEKSVKAHETRPDGRALSANTGYQANLRYGSQQSVTRNWHMPMHQTDTLFHKSKVFMGFIFGGEADNHAVNTVPKETL 6g72.1    --------------------------------------------------------------------------------  target    VRVTKAEDGGMGGKGIWQPATTGFSPDNESDFMKKYLAGELTKVKT 6g72.1    ---------------------------------------------- ``` | | | | | | | | | | | | | | | | | | | | | | | | | | | | | | | | | | | | | | | | | | | | | | | | | |
|  | 7ak6.1.G | NADH-ubiquinone oxidoreductase 75 kDa subunit, mitochondrial  *Cryo-EM structure of ND6-P25L mutant respiratory complex I from Mus musculus at 3.8 A* | 0.09 |  | 13.37 | 0.20 | 208-444 | EM | 0.00 | hetero-1-1-1-1-1-1-… | 6 x SF4, 1 x PC1, 2 x FES, 1 x FMN, 4 x 3PE, 2 x CDL, 1 x ATP, 1 x NDP, 1 x ZN, 2 x EHZ | HHblits | 0.26 |
| ``` target    IMDGKNLVENKLTDSHWFIECMERGAKIVVIAPEYGPPSTKADYWIPIRPQTDAALWLGITRLMIEKKWYDETFVKGFTD 7ak6.1    --------------------------------------------------------------------------------  target    FPLLVRTDTLQRLRAHEVFPQYKTSLSADGPSMKIQGLSAEQHAKLGDFVVWDGKTNAPAAITRDDVGATITKKGIDPVL 7ak6.1    --------------------------------------------------------------------------------  target    AGSFKVKLVDGKEVEVATLWTLYQDHLKDYDLDTVVEITQAPKEMIEQLAQDIATMKPVAIHQGEGINHWFHATEMNRAA 7ak6.1    -----------------------------------------------SFCEVLKDAKKPMVVLGSSALQRDDGAAILVAV  target    YLPLMLTGNIGRPGAGCQTWAGNYKAALFQGSPWTGPGFKGWVAEDPFDINLNPKAHGKEIHAHAYTKDEEPAYWNHGDL 7ak6.1    SNMVQKIRVTTGVAAEWKVMN------ILHRI---A---------SQ---------VA---------------ALD---L  target    ALIVDTPKFGRKNFTGKTHMPTPTKALIFNNVNLINNAKWAYGMIKNVNPNVEMIVSMDIQMTASIEYADLALPANSWLE 7ak6.1    GY---KPGV-E------AIRKNPPKMLFLLGADGGCI-------TRQDLPKDCFIVYQGHHGDVGAPMADVILPGAAYTE  target    FEGLEITASCSNPFLQIWKGGIPPVFDSRDDLDILAGIANALADVTGEKRFRDYFAFAAADKRGIYIQRLLDTCTTTAGY 7ak6.1    KSATY---VNTEGRAQQTKVAVTPPGLAREDWKIIRALSEIAGI------------------------------------  target    KLADIMAGKYGPPGGCLLNFRTYPRIPFYEQVHDSEPFHTDTGRMHAYADVPEAIEYGENFIVHREGPEATPYLPNVIVS 7ak6.1    --------------------------------------------------------------------------------  target    SNPHIRPEDYGIAADAEHWDDRTIRNIKMPWSKVKETKNFLWEKGFQFYCLTPKTRHRVHSGWSNVDWHMLMDSNFGDPY 7ak6.1    --------------------------------------------------------------------------------  target    RLDKRAPCVGEHQLHINPQAARDLNINDGDYVYVDANPADRPYLGAKPDDPFYRVSRCMLRVTYNRAYPYNIVMMKHAPF 7ak6.1    --------------------------------------------------------------------------------  target    IATEKSVKAHETRPDGRALSANTGYQANLRYGSQQSVTRNWHMPMHQTDTLFHKSKVFMGFIFGGEADNHAVNTVPKETL 7ak6.1    --------------------------------------------------------------------------------  target    VRVTKAEDGGMGGKGIWQPATTGFSPDNESDFMKKYLAGELTKVKT 7ak6.1    ---------------------------------------------- ``` | | | | | | | | | | | | | | | | | | | | | | | | | | | | | | | | | | | | | | | | | | | | | | | | | |
|  | 7v2c.1.L | NADH-ubiquinone oxidoreductase 75 kDa subunit, mitochondrial  *Active state complex I from Q10 dataset* | 0.09 |  | 13.37 | 0.20 | 208-444 | EM | 0.00 | hetero-1-1-1-1-1-2-… | 6 x SF4, 1 x FMN, 10 x PEE, 8 x PLX, 2 x 8Q1, 1 x NDP, 2 x UQ, 11 x CDL, 2 x FES, 1 x MG, 1 x ZN, 1 x ADP | HHblits | 0.26 |
| ``` target    IMDGKNLVENKLTDSHWFIECMERGAKIVVIAPEYGPPSTKADYWIPIRPQTDAALWLGITRLMIEKKWYDETFVKGFTD 7v2c.1    --------------------------------------------------------------------------------  target    FPLLVRTDTLQRLRAHEVFPQYKTSLSADGPSMKIQGLSAEQHAKLGDFVVWDGKTNAPAAITRDDVGATITKKGIDPVL 7v2c.1    --------------------------------------------------------------------------------  target    AGSFKVKLVDGKEVEVATLWTLYQDHLKDYDLDTVVEITQAPKEMIEQLAQDIATMKPVAIHQGEGINHWFHATEMNRAA 7v2c.1    -----------------------------------------------PFSQILKEAKKPMVVLGSSALQRSDGTAILAAV  target    YLPLMLTGNIGRPGAGCQTWAGNYKAALFQGSPWTGPGFKGWVAEDPFDINLNPKAHGKEIHAHAYTKDEEPAYWNHGDL 7v2c.1    SNIAQNIRLSSGVTGDWKVMN------ILHR----IA---------------S--QVA---------------ALDLG--  target    ALIVDTPKFGRKNFTGKTHMPTPTKALIFNNVNLINNAKWAYGMIKNVNPNVEMIVSMDIQMTASIEYADLALPANSWLE 7v2c.1    -Y---KPGV-E------AIRKNPPKVLFLLGADGGCI-------TRQDLPKDCFIIYQGHHGDVGAPMADVILPGAAYTE  target    FEGLEITASCSNPFLQIWKGGIPPVFDSRDDLDILAGIANALADVTGEKRFRDYFAFAAADKRGIYIQRLLDTCTTTAGY 7v2c.1    KSATY---VNTEGRAQQTKVAVTPPGLAREDWKIIRALSEIAGM------------------------------------  target    KLADIMAGKYGPPGGCLLNFRTYPRIPFYEQVHDSEPFHTDTGRMHAYADVPEAIEYGENFIVHREGPEATPYLPNVIVS 7v2c.1    --------------------------------------------------------------------------------  target    SNPHIRPEDYGIAADAEHWDDRTIRNIKMPWSKVKETKNFLWEKGFQFYCLTPKTRHRVHSGWSNVDWHMLMDSNFGDPY 7v2c.1    --------------------------------------------------------------------------------  target    RLDKRAPCVGEHQLHINPQAARDLNINDGDYVYVDANPADRPYLGAKPDDPFYRVSRCMLRVTYNRAYPYNIVMMKHAPF 7v2c.1    --------------------------------------------------------------------------------  target    IATEKSVKAHETRPDGRALSANTGYQANLRYGSQQSVTRNWHMPMHQTDTLFHKSKVFMGFIFGGEADNHAVNTVPKETL 7v2c.1    --------------------------------------------------------------------------------  target    VRVTKAEDGGMGGKGIWQPATTGFSPDNESDFMKKYLAGELTKVKT 7v2c.1    ---------------------------------------------- ``` | | | | | | | | | | | | | | | | | | | | | | | | | | | | | | | | | | | | | | | | | | | | | | | | | |
|  | 7ar7.1.G | NADH dehydrogenase [ubiquinone] iron-sulfur protein 1, mitochondrial  *Cryo-EM structure of Arabidopsis thaliana complex-I (open conformation)* | 0.08 |  | 16.07 | 0.20 | 206-444 | EM | 0.00 | hetero-1-1-1-1-1-1-… | 6 x SF4, 2 x FES, 1 x FMN, 1 x UQ9, 3 x PTY, 2 x PC7, 1 x LMN, 1 x NDP, 2 x ZN, 2 x 8Q1, 1 x PGT, 1 x PSF, 1 x T7X | HHblits | 0.27 |
| ``` target    IMDGKNLVENKLTDSHWFIECMERGAKIVVIAPEYGPPSTKADYWIPIRPQTDAALWLGITRLMIEKKWYDETFVKGFTD 7ar7.1    --------------------------------------------------------------------------------  target    FPLLVRTDTLQRLRAHEVFPQYKTSLSADGPSMKIQGLSAEQHAKLGDFVVWDGKTNAPAAITRDDVGATITKKGIDPVL 7ar7.1    --------------------------------------------------------------------------------  target    AGSFKVKLVDGKEVEVATLWTLYQDHLKDYDLDTVVEITQAPKEMIEQLAQDIATMKPVAIHQGEGINHWFHATEMNRAA 7ar7.1    ---------------------------------------------RHPFCTALKNAKNPAIIVGAGLFNRTDKNAILSSV  target    YLPLMLTGNIGRPGAGCQTWAGNYKAALFQGSPWTGPGFKGWVAEDPFDINLNPKAHGKEIHAHAYTKDEEPAYWNHGDL 7ar7.1    ESIAQANNVVRPDWNGLNFLLQYAAQ---A----------A-----AL---------------------------DLGLI  target    ALIVDTPKFGRKNFTGKTHMPTPTKALIFNNVNLINNAKWAYGMIKNVNPNVEMIVSMDIQMTASIEYADLALPANSWLE 7ar7.1    QQS----------A----KALESAKFVYLMGADDVNV---------DKIPKDAFVVYQGHHGDKAVYRANVILPASAFTE  target    FEGLEITASCSNPFLQIWKGGIPPVFDSRDDLDILAGIANALADVTGEKRFRDYFAFAAADKRGIYIQRLLDTCTTTAGY 7ar7.1    KEGTY---ENTEGFTQQTVPAVPTVGDARDDWKIVRALSEVSGV------------------------------------  target    KLADIMAGKYGPPGGCLLNFRTYPRIPFYEQVHDSEPFHTDTGRMHAYADVPEAIEYGENFIVHREGPEATPYLPNVIVS 7ar7.1    --------------------------------------------------------------------------------  target    SNPHIRPEDYGIAADAEHWDDRTIRNIKMPWSKVKETKNFLWEKGFQFYCLTPKTRHRVHSGWSNVDWHMLMDSNFGDPY 7ar7.1    --------------------------------------------------------------------------------  target    RLDKRAPCVGEHQLHINPQAARDLNINDGDYVYVDANPADRPYLGAKPDDPFYRVSRCMLRVTYNRAYPYNIVMMKHAPF 7ar7.1    --------------------------------------------------------------------------------  target    IATEKSVKAHETRPDGRALSANTGYQANLRYGSQQSVTRNWHMPMHQTDTLFHKSKVFMGFIFGGEADNHAVNTVPKETL 7ar7.1    --------------------------------------------------------------------------------  target    VRVTKAEDGGMGGKGIWQPATTGFSPDNESDFMKKYLAGELTKVKT 7ar7.1    ---------------------------------------------- ``` | | | | | | | | | | | | | | | | | | | | | | | | | | | | | | | | | | | | | | | | | | | | | | | | | |
|  | 7zd6.1.4 | NADH-ubiquinone oxidoreductase 75 kDa subunit, mitochondrial  *Complex I from Ovis aries, at pH7.4, Open state* | 0.08 | 0.00 | 14.62 | 0.20 | 209-444 | EM | 0.00 | monomer | 6 x PC1, 14 x 3PE, 1 x DCQ, 2 x ZMP, 1 x AMP, 1 x MYR, 6 x SF4, 1 x FMN, 1 x NAI, 2 x FES, 1 x K, 1 x ZN, 1 x NDP | HHblits | 0.26 |
| ``` target    IMDGKNLVENKLTDSHWFIECMERGAKIVVIAPEYGPPSTKADYWIPIRPQTDAALWLGITRLMIEKKWYDETFVKGFTD 7zd6.1    --------------------------------------------------------------------------------  target    FPLLVRTDTLQRLRAHEVFPQYKTSLSADGPSMKIQGLSAEQHAKLGDFVVWDGKTNAPAAITRDDVGATITKKGIDPVL 7zd6.1    --------------------------------------------------------------------------------  target    AGSFKVKLVDGKEVEVATLWTLYQDHLKDYDLDTVVEITQAPKEMIEQLAQDIATMKPVAIHQGEGINHWFHATEMNRAA 7zd6.1    ------------------------------------------------FSQVLQEAKKPMVVLGSSALQRNDGAAILAAV  target    YLPLMLTGNIGRPGAGCQTWAGNYKAALFQGSPWTGPGFKGWVAEDPFDINLNPKAHGKEIHAHAYTKDEEPAYWNHGDL 7zd6.1    SNIAQKIRTSSGVTGDWKVMNI-----LHRIAS-----QVA-----------------------A---------LD---L  target    ALIVDTPKFGRKNFTGKTHMPTPTKALIFNNVNLINNAKWAYGMIKNVNPNVEMIVSMDIQMTASIEYADLALPANSWLE 7zd6.1    GY---KPGV-E------AIRKNPPKMLFLLGADGGCV-------TRQDLPKDCFIVYQGHHGDVGAPIADVILPGAAYTE  target    FEGLEITASCSNPFLQIWKGGIPPVFDSRDDLDILAGIANALADVTGEKRFRDYFAFAAADKRGIYIQRLLDTCTTTAGY 7zd6.1    KSATY---VNTEGRAQQTKVAVMPPGLAREDWKIIRALSEIAGM------------------------------------  target    KLADIMAGKYGPPGGCLLNFRTYPRIPFYEQVHDSEPFHTDTGRMHAYADVPEAIEYGENFIVHREGPEATPYLPNVIVS 7zd6.1    --------------------------------------------------------------------------------  target    SNPHIRPEDYGIAADAEHWDDRTIRNIKMPWSKVKETKNFLWEKGFQFYCLTPKTRHRVHSGWSNVDWHMLMDSNFGDPY 7zd6.1    --------------------------------------------------------------------------------  target    RLDKRAPCVGEHQLHINPQAARDLNINDGDYVYVDANPADRPYLGAKPDDPFYRVSRCMLRVTYNRAYPYNIVMMKHAPF 7zd6.1    --------------------------------------------------------------------------------  target    IATEKSVKAHETRPDGRALSANTGYQANLRYGSQQSVTRNWHMPMHQTDTLFHKSKVFMGFIFGGEADNHAVNTVPKETL 7zd6.1    --------------------------------------------------------------------------------  target    VRVTKAEDGGMGGKGIWQPATTGFSPDNESDFMKKYLAGELTKVKT 7zd6.1    ---------------------------------------------- ``` | | | | | | | | | | | | | | | | | | | | | | | | | | | | | | | | | | | | | | | | | | | | | | | | | |
|  | 7dgr.10.A | NADH-ubiquinone oxidoreductase 75 kDa subunit, mitochondrial  *Activity optimized supercomplex state2* | 0.08 | 0.00 | 14.04 | 0.20 | 209-444 | EM | 0.00 | monomer |  | HHblits | 0.26 |
| ``` target    IMDGKNLVENKLTDSHWFIECMERGAKIVVIAPEYGPPSTKADYWIPIRPQTDAALWLGITRLMIEKKWYDETFVKGFTD 7dgr.10   --------------------------------------------------------------------------------  target    FPLLVRTDTLQRLRAHEVFPQYKTSLSADGPSMKIQGLSAEQHAKLGDFVVWDGKTNAPAAITRDDVGATITKKGIDPVL 7dgr.10   --------------------------------------------------------------------------------  target    AGSFKVKLVDGKEVEVATLWTLYQDHLKDYDLDTVVEITQAPKEMIEQLAQDIATMKPVAIHQGEGINHWFHATEMNRAA 7dgr.10   ------------------------------------------------FSQVLQEAKKPMVILGSSALQRNDGAAILAAV  target    YLPLMLTGNIGRPGAGCQTWAGNYKAALFQGSPWTGPGFKGWVAEDPFDINLNPKAHGKEIHAHAYTKDEEPAYWNHGDL 7dgr.10   SNIAQKIRTSSGVTGDWKVMNI-----LHRIA-----SQVA-----A---------------------------LD---L  target    ALIVDTPKFGRKNFTGKTHMPTPTKALIFNNVNLINNAKWAYGMIKNVNPNVEMIVSMDIQMTASIEYADLALPANSWLE 7dgr.10   GY---KPGV-E------AIQKNPPKMLFLLGADGGCI-------TRQDLPKDCFIVYQGHHGDVGAPIADVILPGAAYTE  target    FEGLEITASCSNPFLQIWKGGIPPVFDSRDDLDILAGIANALADVTGEKRFRDYFAFAAADKRGIYIQRLLDTCTTTAGY 7dgr.10   KSATY---VNTEGRAQQTKVAVTPPGLAREDWKIIRALSEIAGM------------------------------------  target    KLADIMAGKYGPPGGCLLNFRTYPRIPFYEQVHDSEPFHTDTGRMHAYADVPEAIEYGENFIVHREGPEATPYLPNVIVS 7dgr.10   --------------------------------------------------------------------------------  target    SNPHIRPEDYGIAADAEHWDDRTIRNIKMPWSKVKETKNFLWEKGFQFYCLTPKTRHRVHSGWSNVDWHMLMDSNFGDPY 7dgr.10   --------------------------------------------------------------------------------  target    RLDKRAPCVGEHQLHINPQAARDLNINDGDYVYVDANPADRPYLGAKPDDPFYRVSRCMLRVTYNRAYPYNIVMMKHAPF 7dgr.10   --------------------------------------------------------------------------------  target    IATEKSVKAHETRPDGRALSANTGYQANLRYGSQQSVTRNWHMPMHQTDTLFHKSKVFMGFIFGGEADNHAVNTVPKETL 7dgr.10   --------------------------------------------------------------------------------  target    VRVTKAEDGGMGGKGIWQPATTGFSPDNESDFMKKYLAGELTKVKT 7dgr.10   ---------------------------------------------- ``` | | | | | | | | | | | | | | | | | | | | | | | | | | | | | | | | | | | | | | | | | | | | | | | | | |
|  | 5o31.1.8 | NADH-ubiquinone oxidoreductase 75 kDa subunit, mitochondrial  *Mitochondrial complex I in the deactive state* | 0.08 | 0.00 | 14.04 | 0.20 | 209-444 | EM | 4.13 | monomer | 6 x SF4, 2 x FES, 1 x FMN, 1 x NAP, 1 x ZN | HHblits | 0.26 |
| ``` target    IMDGKNLVENKLTDSHWFIECMERGAKIVVIAPEYGPPSTKADYWIPIRPQTDAALWLGITRLMIEKKWYDETFVKGFTD 5o31.1    --------------------------------------------------------------------------------  target    FPLLVRTDTLQRLRAHEVFPQYKTSLSADGPSMKIQGLSAEQHAKLGDFVVWDGKTNAPAAITRDDVGATITKKGIDPVL 5o31.1    --------------------------------------------------------------------------------  target    AGSFKVKLVDGKEVEVATLWTLYQDHLKDYDLDTVVEITQAPKEMIEQLAQDIATMKPVAIHQGEGINHWFHATEMNRAA 5o31.1    ------------------------------------------------FSQVLQEAKKPMVILGSSALQRNDGAAILAAV  target    YLPLMLTGNIGRPGAGCQTWAGNYKAALFQGSPWTGPGFKGWVAEDPFDINLNPKAHGKEIHAHAYTKDEEPAYWNHGDL 5o31.1    SNIAQKIRTSSGVTGDWKVMNI-----LHRIA-----SQVA-----A---------------------------LD---L  target    ALIVDTPKFGRKNFTGKTHMPTPTKALIFNNVNLINNAKWAYGMIKNVNPNVEMIVSMDIQMTASIEYADLALPANSWLE 5o31.1    GY---KPGV-E------AIQKNPPKMLFLLGADGGCI-------TRQDLPKDCFIVYQGHHGDVGAPIADVILPGAAYTE  target    FEGLEITASCSNPFLQIWKGGIPPVFDSRDDLDILAGIANALADVTGEKRFRDYFAFAAADKRGIYIQRLLDTCTTTAGY 5o31.1    KSATY---VNTEGRAQQTKVAVTPPGLAREDWKIIRALSEIAGM------------------------------------  target    KLADIMAGKYGPPGGCLLNFRTYPRIPFYEQVHDSEPFHTDTGRMHAYADVPEAIEYGENFIVHREGPEATPYLPNVIVS 5o31.1    --------------------------------------------------------------------------------  target    SNPHIRPEDYGIAADAEHWDDRTIRNIKMPWSKVKETKNFLWEKGFQFYCLTPKTRHRVHSGWSNVDWHMLMDSNFGDPY 5o31.1    --------------------------------------------------------------------------------  target    RLDKRAPCVGEHQLHINPQAARDLNINDGDYVYVDANPADRPYLGAKPDDPFYRVSRCMLRVTYNRAYPYNIVMMKHAPF 5o31.1    --------------------------------------------------------------------------------  target    IATEKSVKAHETRPDGRALSANTGYQANLRYGSQQSVTRNWHMPMHQTDTLFHKSKVFMGFIFGGEADNHAVNTVPKETL 5o31.1    --------------------------------------------------------------------------------  target    VRVTKAEDGGMGGKGIWQPATTGFSPDNESDFMKKYLAGELTKVKT 5o31.1    ---------------------------------------------- ``` | | | | | | | | | | | | | | | | | | | | | | | | | | | | | | | | | | | | | | | | | | | | | | | | | |
|  | 6zk9.1.C | NADH:ubiquinone oxidoreductase core subunit S1  *Peripheral domain of open complex I during turnover* | 0.09 | 0.00 | 14.04 | 0.20 | 209-444 | EM | 0.00 | monomer | 6 x SF4, 1 x FMN, 1 x NAI, 2 x FES, 1 x K, 2 x PC1, 2 x 3PE, 1 x ZN, 1 x NDP, 1 x ZMP, 1 x CDL | HHblits | 0.26 |
| ``` target    IMDGKNLVENKLTDSHWFIECMERGAKIVVIAPEYGPPSTKADYWIPIRPQTDAALWLGITRLMIEKKWYDETFVKGFTD 6zk9.1    --------------------------------------------------------------------------------  target    FPLLVRTDTLQRLRAHEVFPQYKTSLSADGPSMKIQGLSAEQHAKLGDFVVWDGKTNAPAAITRDDVGATITKKGIDPVL 6zk9.1    --------------------------------------------------------------------------------  target    AGSFKVKLVDGKEVEVATLWTLYQDHLKDYDLDTVVEITQAPKEMIEQLAQDIATMKPVAIHQGEGINHWFHATEMNRAA 6zk9.1    ------------------------------------------------FSQVLQEAKKPMVVLGSSALQRNDGAAILAAV  target    YLPLMLTGNIGRPGAGCQTWAGNYKAALFQGSPWTGPGFKGWVAEDPFDINLNPKAHGKEIHAHAYTKDEEPAYWNHGDL 6zk9.1    SNIAQKIRTSSGVTGDWKVMNI-----LHRIAS--Q---V-----------------A---------------ALDLG--  target    ALIVDTPKFGRKNFTGKTHMPTPTKALIFNNVNLINNAKWAYGMIKNVNPNVEMIVSMDIQMTASIEYADLALPANSWLE 6zk9.1    -Y---KPGV-E------AIRKNPPKMLFLLGADGGCV-------TRQDLPKDCFIVYQGHHGDVGAPIADVILPGAAYTE  target    FEGLEITASCSNPFLQIWKGGIPPVFDSRDDLDILAGIANALADVTGEKRFRDYFAFAAADKRGIYIQRLLDTCTTTAGY 6zk9.1    KSATY---VNTEGRAQQTKVAVMPPGLAREDWKIIRALSEIAGM------------------------------------  target    KLADIMAGKYGPPGGCLLNFRTYPRIPFYEQVHDSEPFHTDTGRMHAYADVPEAIEYGENFIVHREGPEATPYLPNVIVS 6zk9.1    --------------------------------------------------------------------------------  target    SNPHIRPEDYGIAADAEHWDDRTIRNIKMPWSKVKETKNFLWEKGFQFYCLTPKTRHRVHSGWSNVDWHMLMDSNFGDPY 6zk9.1    --------------------------------------------------------------------------------  target    RLDKRAPCVGEHQLHINPQAARDLNINDGDYVYVDANPADRPYLGAKPDDPFYRVSRCMLRVTYNRAYPYNIVMMKHAPF 6zk9.1    --------------------------------------------------------------------------------  target    IATEKSVKAHETRPDGRALSANTGYQANLRYGSQQSVTRNWHMPMHQTDTLFHKSKVFMGFIFGGEADNHAVNTVPKETL 6zk9.1    --------------------------------------------------------------------------------  target    VRVTKAEDGGMGGKGIWQPATTGFSPDNESDFMKKYLAGELTKVKT 6zk9.1    ---------------------------------------------- ``` | | | | | | | | | | | | | | | | | | | | | | | | | | | | | | | | | | | | | | | | | | | | | | | | | |
|  | 7qsd.1.G | NADH-ubiquinone oxidoreductase 75 kDa subunit, mitochondrial  *Bovine complex I in the active state at 3.1 A* | 0.09 |  | 13.45 | 0.20 | 209-444 | EM | 0.00 | hetero-1-1-1-1-1-1-… | 5 x PC1, 13 x 3PE, 6 x SF4, 2 x FES, 1 x FMN, 4 x CDL, 3 x LMT, 1 x GTP, 1 x MG, 1 x NDP, 1 x ZN, 2 x EHZ | HHblits | 0.25 |
| ``` target    IMDGKNLVENKLTDSHWFIECMERGAKIVVIAPEYGPPSTKADYWIPIRPQTDAALWLGITRLMIEKKWYDETFVKGFTD 7qsd.1    --------------------------------------------------------------------------------  target    FPLLVRTDTLQRLRAHEVFPQYKTSLSADGPSMKIQGLSAEQHAKLGDFVVWDGKTNAPAAITRDDVGATITKKGIDPVL 7qsd.1    --------------------------------------------------------------------------------  target    AGSFKVKLVDGKEVEVATLWTLYQDHLKDYDLDTVVEITQAPKEMIEQLAQDIATMKPVAIHQGEGINHWFHATEMNRAA 7qsd.1    ------------------------------------------------FSQVLQEAKKPMVILGSSALQRNDGAAILAAV  target    YLPLMLTGNIGRPGAGCQTWAGNYKAALFQGSPWTGPGFKGWVAEDPFDINLNPKAHGKEIHAHAYTKDEEPAYWNHGDL 7qsd.1    SNIAQKIRTSSGVTGDWKVMNI------LHR----IASQV-----------------A---------------ALD---L  target    ALIVDTPKFGRKNFTGKTHMPTPTKALIFNNVNLINNAKWAYGMIKNVNPNVEMIVSMDIQMTASIEYADLALPANSWLE 7qsd.1    GY---KPGV-E------AIQKNPPKMLFLLGADGGCI-------TRQDLPKDCFIVYQGHHGDVGAPIADVILPGAAYTE  target    FEGLEITASCSNPFLQIWKGGIPPVFDSRDDLDILAGIANALADVTGEKRFRDYFAFAAADKRGIYIQRLLDTCTTTAGY 7qsd.1    KSATY---VNTEGRAQQTKVAVTPPGLAREDWKIIRALSEIAGM------------------------------------  target    KLADIMAGKYGPPGGCLLNFRTYPRIPFYEQVHDSEPFHTDTGRMHAYADVPEAIEYGENFIVHREGPEATPYLPNVIVS 7qsd.1    --------------------------------------------------------------------------------  target    SNPHIRPEDYGIAADAEHWDDRTIRNIKMPWSKVKETKNFLWEKGFQFYCLTPKTRHRVHSGWSNVDWHMLMDSNFGDPY 7qsd.1    --------------------------------------------------------------------------------  target    RLDKRAPCVGEHQLHINPQAARDLNINDGDYVYVDANPADRPYLGAKPDDPFYRVSRCMLRVTYNRAYPYNIVMMKHAPF 7qsd.1    --------------------------------------------------------------------------------  target    IATEKSVKAHETRPDGRALSANTGYQANLRYGSQQSVTRNWHMPMHQTDTLFHKSKVFMGFIFGGEADNHAVNTVPKETL 7qsd.1    --------------------------------------------------------------------------------  target    VRVTKAEDGGMGGKGIWQPATTGFSPDNESDFMKKYLAGELTKVKT 7qsd.1    ---------------------------------------------- ``` | | | | | | | | | | | | | | | | | | | | | | | | | | | | | | | | | | | | | | | | | | | | | | | | | |
|  | 6qcf.1.C | NADH:ubiquinone oxidoreductase core subunit S1  *Ovine respiratory complex I FRC open class 6* | 0.08 |  | 13.45 | 0.20 | 209-444 | EM | 0.00 | hetero-1-1-1-1-1-1-… | 6 x SF4, 1 x FMN, 2 x FES, 1 x ZN, 1 x NDP, 2 x ZMP | HHblits | 0.25 |
| ``` target    IMDGKNLVENKLTDSHWFIECMERGAKIVVIAPEYGPPSTKADYWIPIRPQTDAALWLGITRLMIEKKWYDETFVKGFTD 6qcf.1    --------------------------------------------------------------------------------  target    FPLLVRTDTLQRLRAHEVFPQYKTSLSADGPSMKIQGLSAEQHAKLGDFVVWDGKTNAPAAITRDDVGATITKKGIDPVL 6qcf.1    --------------------------------------------------------------------------------  target    AGSFKVKLVDGKEVEVATLWTLYQDHLKDYDLDTVVEITQAPKEMIEQLAQDIATMKPVAIHQGEGINHWFHATEMNRAA 6qcf.1    ------------------------------------------------FSQVLQEAKKPMVVLGSSALQRNDGAAILAAV  target    YLPLMLTGNIGRPGAGCQTWAGNYKAALFQGSPWTGPGFKGWVAEDPFDINLNPKAHGKEIHAHAYTKDEEPAYWNHGDL 6qcf.1    SNIAQKIRTSSGVTGDWKVMNI------LHR----IASQV-----------------A---------------ALD---L  target    ALIVDTPKFGRKNFTGKTHMPTPTKALIFNNVNLINNAKWAYGMIKNVNPNVEMIVSMDIQMTASIEYADLALPANSWLE 6qcf.1    GY---KPGV-E------AIRKNPPKMLFLLGADGGCV-------TRQDLPKDCFIVYQGHHGDVGAPIADVILPGAAYTE  target    FEGLEITASCSNPFLQIWKGGIPPVFDSRDDLDILAGIANALADVTGEKRFRDYFAFAAADKRGIYIQRLLDTCTTTAGY 6qcf.1    KSATY---VNTEGRAQQTKVAVMPPGLAREDWKIIRALSEIAGM------------------------------------  target    KLADIMAGKYGPPGGCLLNFRTYPRIPFYEQVHDSEPFHTDTGRMHAYADVPEAIEYGENFIVHREGPEATPYLPNVIVS 6qcf.1    --------------------------------------------------------------------------------  target    SNPHIRPEDYGIAADAEHWDDRTIRNIKMPWSKVKETKNFLWEKGFQFYCLTPKTRHRVHSGWSNVDWHMLMDSNFGDPY 6qcf.1    --------------------------------------------------------------------------------  target    RLDKRAPCVGEHQLHINPQAARDLNINDGDYVYVDANPADRPYLGAKPDDPFYRVSRCMLRVTYNRAYPYNIVMMKHAPF 6qcf.1    --------------------------------------------------------------------------------  target    IATEKSVKAHETRPDGRALSANTGYQANLRYGSQQSVTRNWHMPMHQTDTLFHKSKVFMGFIFGGEADNHAVNTVPKETL 6qcf.1    --------------------------------------------------------------------------------  target    VRVTKAEDGGMGGKGIWQPATTGFSPDNESDFMKKYLAGELTKVKT 6qcf.1    ---------------------------------------------- ``` | | | | | | | | | | | | | | | | | | | | | | | | | | | | | | | | | | | | | | | | | | | | | | | | | |
|  | 6qc5.1.C | NADH:ubiquinone oxidoreductase core subunit S1  *Ovine respiratory complex I FRC closed class 1* | 0.08 |  | 13.45 | 0.20 | 209-444 | EM | 0.00 | hetero-1-1-1-1-1-1-… | 6 x SF4, 1 x FMN, 2 x FES, 2 x 3PE, 1 x ZN, 1 x NDP, 2 x ZMP, 1 x PC1 | HHblits | 0.25 |
| ``` target    IMDGKNLVENKLTDSHWFIECMERGAKIVVIAPEYGPPSTKADYWIPIRPQTDAALWLGITRLMIEKKWYDETFVKGFTD 6qc5.1    --------------------------------------------------------------------------------  target    FPLLVRTDTLQRLRAHEVFPQYKTSLSADGPSMKIQGLSAEQHAKLGDFVVWDGKTNAPAAITRDDVGATITKKGIDPVL 6qc5.1    --------------------------------------------------------------------------------  target    AGSFKVKLVDGKEVEVATLWTLYQDHLKDYDLDTVVEITQAPKEMIEQLAQDIATMKPVAIHQGEGINHWFHATEMNRAA 6qc5.1    ------------------------------------------------FSQVLQEAKKPMVVLGSSALQRNDGAAILAAV  target    YLPLMLTGNIGRPGAGCQTWAGNYKAALFQGSPWTGPGFKGWVAEDPFDINLNPKAHGKEIHAHAYTKDEEPAYWNHGDL 6qc5.1    SNIAQKIRTSSGVTGDWKVMNI------LHR----IASQV-----------------A---------------ALD---L  target    ALIVDTPKFGRKNFTGKTHMPTPTKALIFNNVNLINNAKWAYGMIKNVNPNVEMIVSMDIQMTASIEYADLALPANSWLE 6qc5.1    GY---KPGV-E------AIRKNPPKMLFLLGADGGCV-------TRQDLPKDCFIVYQGHHGDVGAPIADVILPGAAYTE  target    FEGLEITASCSNPFLQIWKGGIPPVFDSRDDLDILAGIANALADVTGEKRFRDYFAFAAADKRGIYIQRLLDTCTTTAGY 6qc5.1    KSATY---VNTEGRAQQTKVAVMPPGLAREDWKIIRALSEIAGM------------------------------------  target    KLADIMAGKYGPPGGCLLNFRTYPRIPFYEQVHDSEPFHTDTGRMHAYADVPEAIEYGENFIVHREGPEATPYLPNVIVS 6qc5.1    --------------------------------------------------------------------------------  target    SNPHIRPEDYGIAADAEHWDDRTIRNIKMPWSKVKETKNFLWEKGFQFYCLTPKTRHRVHSGWSNVDWHMLMDSNFGDPY 6qc5.1    --------------------------------------------------------------------------------  target    RLDKRAPCVGEHQLHINPQAARDLNINDGDYVYVDANPADRPYLGAKPDDPFYRVSRCMLRVTYNRAYPYNIVMMKHAPF 6qc5.1    --------------------------------------------------------------------------------  target    IATEKSVKAHETRPDGRALSANTGYQANLRYGSQQSVTRNWHMPMHQTDTLFHKSKVFMGFIFGGEADNHAVNTVPKETL 6qc5.1    --------------------------------------------------------------------------------  target    VRVTKAEDGGMGGKGIWQPATTGFSPDNESDFMKKYLAGELTKVKT 6qc5.1    ---------------------------------------------- ``` | | | | | | | | | | | | | | | | | | | | | | | | | | | | | | | | | | | | | | | | | | | | | | | | | |
|  | 7arc.1.F | 75 kDa  *Cryo-EM structure of Polytomella Complex-I (peripheral arm)* | 0.08 |  | 14.02 | 0.19 | 212-444 | EM | 0.00 | hetero-1-1-1-1-1-1-… | 6 x SF4, 2 x FES, 1 x FMN, 1 x NDP, 1 x ZN, 1 x 8Q1 | HHblits | 0.28 |
| ``` target    IMDGKNLVENKLTDSHWFIECMERGAKIVVIAPEYGPPSTKADYWIPIRPQTDAALWLGITRLMIEKKWYDETFVKGFTD 7arc.1    --------------------------------------------------------------------------------  target    FPLLVRTDTLQRLRAHEVFPQYKTSLSADGPSMKIQGLSAEQHAKLGDFVVWDGKTNAPAAITRDDVGATITKKGIDPVL 7arc.1    --------------------------------------------------------------------------------  target    AGSFKVKLVDGKEVEVATLWTLYQDHLKDYDLDTVVEITQAPKEMIEQLAQDIATMKPVAIHQGEGINHWFHATEMNRAA 7arc.1    ---------------------------------------------------ALKGAKNPVVIVGSSVLRRDDREAVLKTV  target    YLPLMLTGNIGRPGAGCQTWAGNYKAALFQGSPWTGPGFKGWVAEDPFDINLNPKAHGKEIHAHAYTKDEEPAYWNHGDL 7arc.1    NDLVDAAGVVKEGWNGFNVLHDNASR---VAA---------------LDIG------------------FVPS-------  target    ALIVDTPKFGRKNFTGKTHMPTPTKALIFNNVNLINNAKWAYGMIKNVNPNVEMIVSMDIQMTASIEYADLALPANSWLE 7arc.1    --A--SA----------RTNPVPAKVVYLLGSDDFKD---------EEIPADAFVIYQGHHGDKGAARANVVLPGAAYTE  target    FEGLEITASCSNPFLQIWKGGIPPVFDSRDDLDILAGIANALADVTGEKRFRDYFAFAAADKRGIYIQRLLDTCTTTAGY 7arc.1    KASLF---ANTEGRVQTTRTAVPVLGDAREDWKIIRALSEVVGQ------------------------------------  target    KLADIMAGKYGPPGGCLLNFRTYPRIPFYEQVHDSEPFHTDTGRMHAYADVPEAIEYGENFIVHREGPEATPYLPNVIVS 7arc.1    --------------------------------------------------------------------------------  target    SNPHIRPEDYGIAADAEHWDDRTIRNIKMPWSKVKETKNFLWEKGFQFYCLTPKTRHRVHSGWSNVDWHMLMDSNFGDPY 7arc.1    --------------------------------------------------------------------------------  target    RLDKRAPCVGEHQLHINPQAARDLNINDGDYVYVDANPADRPYLGAKPDDPFYRVSRCMLRVTYNRAYPYNIVMMKHAPF 7arc.1    --------------------------------------------------------------------------------  target    IATEKSVKAHETRPDGRALSANTGYQANLRYGSQQSVTRNWHMPMHQTDTLFHKSKVFMGFIFGGEADNHAVNTVPKETL 7arc.1    --------------------------------------------------------------------------------  target    VRVTKAEDGGMGGKGIWQPATTGFSPDNESDFMKKYLAGELTKVKT 7arc.1    ---------------------------------------------- ``` | | | | | | | | | | | | | | | | | | | | | | | | | | | | | | | | | | | | | | | | | | | | | | | | | |
|  | 7q5y.1.A | NADH dehydrogenase I chain G  *Structure of NADH:ubichinon oxidoreductase (complex I) of the hyperthermophilic eubacterium Aquifex aeolicus* | 0.03 |  | 16.67 | 0.11 | 342-446 | X-ray | 2.70 | hetero-1-1-1-1-1-1-… | 8 x SF4, 2 x FES, 1 x FMN | HHblits | 0.28 |
| ``` target    IMDGKNLVENKLTDSHWFIECMERGAKIVVIAPEYGPPSTKADYWIPIRPQTDAALWLGITRLMIEKKWYDETFVKGFTD 7q5y.1    --------------------------------------------------------------------------------  target    FPLLVRTDTLQRLRAHEVFPQYKTSLSADGPSMKIQGLSAEQHAKLGDFVVWDGKTNAPAAITRDDVGATITKKGIDPVL 7q5y.1    --------------------------------------------------------------------------------  target    AGSFKVKLVDGKEVEVATLWTLYQDHLKDYDLDTVVEITQAPKEMIEQLAQDIATMKPVAIHQGEGINHWFHATEMNRAA 7q5y.1    --------------------------------------------------------------------------------  target    YLPLMLTGNIGRPGAGCQTWAGNYKAALFQGSPWTGPGFKGWVAEDPFDINLNPKAHGKEIHAHAYTKDEEPAYWNHGDL 7q5y.1    --------------------------------------------------------------------------------  target    ALIVDTPKFGRKNFTGKTHMPTPTKALIFNNVNLINNAKWAYGMIKNVNPNVEMIVSMDIQMTASIEYADLALPANSWLE 7q5y.1    ---------------------GDIENLIIFGEDILEFYED--KVFEELKEKLEHLVVVSPYEDGLSEYAHIKIPMSLMGE  target    FEGLEITASCSNPFLQIWKGGIPPVFDSRDDLDILAGIANALADVTGEKRFRDYFAFAAADKRGIYIQRLLDTCTTTAGY 7q5y.1    NEGT---YKTFFGEVKGKK--FLP--WAFDDLAFWKYLGENFKEEK----------------------------------  target    KLADIMAGKYGPPGGCLLNFRTYPRIPFYEQVHDSEPFHTDTGRMHAYADVPEAIEYGENFIVHREGPEATPYLPNVIVS 7q5y.1    --------------------------------------------------------------------------------  target    SNPHIRPEDYGIAADAEHWDDRTIRNIKMPWSKVKETKNFLWEKGFQFYCLTPKTRHRVHSGWSNVDWHMLMDSNFGDPY 7q5y.1    --------------------------------------------------------------------------------  target    RLDKRAPCVGEHQLHINPQAARDLNINDGDYVYVDANPADRPYLGAKPDDPFYRVSRCMLRVTYNRAYPYNIVMMKHAPF 7q5y.1    --------------------------------------------------------------------------------  target    IATEKSVKAHETRPDGRALSANTGYQANLRYGSQQSVTRNWHMPMHQTDTLFHKSKVFMGFIFGGEADNHAVNTVPKETL 7q5y.1    --------------------------------------------------------------------------------  target    VRVTKAEDGGMGGKGIWQPATTGFSPDNESDFMKKYLAGELTKVKT 7q5y.1    ---------------------------------------------- ``` | | | | | | | | | | | | | | | | | | | | | | | | | | | | | | | | | | | | | | | | | | | | | | | | | |
|  | 1h0h.1.A | FORMATE DEHYDROGENASE SUBUNIT ALPHA  *Tungsten containing Formate Dehydrogenase from Desulfovibrio Gigas* | 0.03 |  | 30.59 | 0.10 | 1-85 | X-ray | 1.80 | hetero-1-1-mer | 1 x W, 1 x 2MD, 1 x MGD, 4 x SF4, 1 x CA | BLAST | 0.37 |
| ``` target    IMDGKNLVENKLTDSHWFIECMERGAKIVVIAPEYGPPSTKADYWIPIRPQTDAALWLGITRLMIEKKWYDETFVKGFTD 1h0h.1    LMMGSNPAENHPISFKWVMRAKDKGATLIHVDPRYTRTSTKCDLYAPLRSGSDIAFLNGMTKYILEKELYFKDYVVNYTN  target    FPLLVRTDTLQRLRAHEVFPQYKTSLSADGPSMKIQGLSAEQHAKLGDFVVWDGKTNAPAAITRDDVGATITKKGIDPVL 1h0h.1    ASFIV---------------------------------------------------------------------------  target    AGSFKVKLVDGKEVEVATLWTLYQDHLKDYDLDTVVEITQAPKEMIEQLAQDIATMKPVAIHQGEGINHWFHATEMNRAA 1h0h.1    --------------------------------------------------------------------------------  target    YLPLMLTGNIGRPGAGCQTWAGNYKAALFQGSPWTGPGFKGWVAEDPFDINLNPKAHGKEIHAHAYTKDEEPAYWNHGDL 1h0h.1    --------------------------------------------------------------------------------  target    ALIVDTPKFGRKNFTGKTHMPTPTKALIFNNVNLINNAKWAYGMIKNVNPNVEMIVSMDIQMTASIEYADLALPANSWLE 1h0h.1    --------------------------------------------------------------------------------  target    FEGLEITASCSNPFLQIWKGGIPPVFDSRDDLDILAGIANALADVTGEKRFRDYFAFAAADKRGIYIQRLLDTCTTTAGY 1h0h.1    --------------------------------------------------------------------------------  target    KLADIMAGKYGPPGGCLLNFRTYPRIPFYEQVHDSEPFHTDTGRMHAYADVPEAIEYGENFIVHREGPEATPYLPNVIVS 1h0h.1    --------------------------------------------------------------------------------  target    SNPHIRPEDYGIAADAEHWDDRTIRNIKMPWSKVKETKNFLWEKGFQFYCLTPKTRHRVHSGWSNVDWHMLMDSNFGDPY 1h0h.1    --------------------------------------------------------------------------------  target    RLDKRAPCVGEHQLHINPQAARDLNINDGDYVYVDANPADRPYLGAKPDDPFYRVSRCMLRVTYNRAYPYNIVMMKHAPF 1h0h.1    --------------------------------------------------------------------------------  target    IATEKSVKAHETRPDGRALSANTGYQANLRYGSQQSVTRNWHMPMHQTDTLFHKSKVFMGFIFGGEADNHAVNTVPKETL 1h0h.1    --------------------------------------------------------------------------------  target    VRVTKAEDGGMGGKGIWQPATTGFSPDNESDFMKKYLAGELTKVKT 1h0h.1    ---------------------------------------------- ``` | | | | | | | | | | | | | | | | | | | | | | | | | | | | | | | | | | | | | | | | | | | | | | | | | |
|  | 8b9z.1.G | NADH-ubiquinone oxidoreductase 75 kDa subunit, mitochondrial  *Drosophila melanogaster complex I in the Active state (Dm1)* | 0.04 |  | 19.35 | 0.11 | 342-444 | EM | 3.28 | hetero-1-1-1-1-1-1-… | 3 x PC1, 16 x 3PE, 6 x SF4, 4 x CDL, 2 x FES, 1 x FMN, 1 x UQ9, 1 x DGT, 1 x NDP, 1 x ZN, 2 x EHZ | HHblits | 0.30 |
| ``` target    IMDGKNLVENKLTDSHWFIECMERGAKIVVIAPEYGPPSTKADYWIPIRPQTDAALWLGITRLMIEKKWYDETFVKGFTD 8b9z.1    --------------------------------------------------------------------------------  target    FPLLVRTDTLQRLRAHEVFPQYKTSLSADGPSMKIQGLSAEQHAKLGDFVVWDGKTNAPAAITRDDVGATITKKGIDPVL 8b9z.1    --------------------------------------------------------------------------------  target    AGSFKVKLVDGKEVEVATLWTLYQDHLKDYDLDTVVEITQAPKEMIEQLAQDIATMKPVAIHQGEGINHWFHATEMNRAA 8b9z.1    --------------------------------------------------------------------------------  target    YLPLMLTGNIGRPGAGCQTWAGNYKAALFQGSPWTGPGFKGWVAEDPFDINLNPKAHGKEIHAHAYTKDEEPAYWNHGDL 8b9z.1    --------------------------------------------------------------------------------  target    ALIVDTPKFGRKNFTGKTHMPTPTKALIFNNVNLINNAKWAYGMIKNVNPNVEMIVSMDIQMTASIEYADLALPANSWLE 8b9z.1    ---------------------AQPKVLFLLNADAGK-------VTREQLPKDCFVVYIGSHGDNGASIADAVLPGAAYTE  target    FEGLEITASCSNPFLQIWKGGIPPVFDSRDDLDILAGIANALADVTGEKRFRDYFAFAAADKRGIYIQRLLDTCTTTAGY 8b9z.1    KQGIY---VNTEGRPQQTLPGVSPPGMAREDWKILRALSEVVGK------------------------------------  target    KLADIMAGKYGPPGGCLLNFRTYPRIPFYEQVHDSEPFHTDTGRMHAYADVPEAIEYGENFIVHREGPEATPYLPNVIVS 8b9z.1    --------------------------------------------------------------------------------  target    SNPHIRPEDYGIAADAEHWDDRTIRNIKMPWSKVKETKNFLWEKGFQFYCLTPKTRHRVHSGWSNVDWHMLMDSNFGDPY 8b9z.1    --------------------------------------------------------------------------------  target    RLDKRAPCVGEHQLHINPQAARDLNINDGDYVYVDANPADRPYLGAKPDDPFYRVSRCMLRVTYNRAYPYNIVMMKHAPF 8b9z.1    --------------------------------------------------------------------------------  target    IATEKSVKAHETRPDGRALSANTGYQANLRYGSQQSVTRNWHMPMHQTDTLFHKSKVFMGFIFGGEADNHAVNTVPKETL 8b9z.1    --------------------------------------------------------------------------------  target    VRVTKAEDGGMGGKGIWQPATTGFSPDNESDFMKKYLAGELTKVKT 8b9z.1    ---------------------------------------------- ``` | | | | | | | | | | | | | | | | | | | | | | | | | | | | | | | | | | | | | | | | | | | | | | | | | |
|  | 8ba0.1.G | NADH-ubiquinone oxidoreductase 75 kDa subunit, mitochondrial  *Drosophila melanogaster complex I in the Twisted state (Dm2)* | 0.04 |  | 19.35 | 0.11 | 342-444 | EM | 3.68 | hetero-1-1-1-1-1-1-… | 6 x SF4, 6 x 3PE, 2 x FES, 1 x FMN, 2 x CDL, 1 x DGT, 1 x NDP, 1 x ZN, 2 x EHZ | HHblits | 0.30 |
| ``` target    IMDGKNLVENKLTDSHWFIECMERGAKIVVIAPEYGPPSTKADYWIPIRPQTDAALWLGITRLMIEKKWYDETFVKGFTD 8ba0.1    --------------------------------------------------------------------------------  target    FPLLVRTDTLQRLRAHEVFPQYKTSLSADGPSMKIQGLSAEQHAKLGDFVVWDGKTNAPAAITRDDVGATITKKGIDPVL 8ba0.1    --------------------------------------------------------------------------------  target    AGSFKVKLVDGKEVEVATLWTLYQDHLKDYDLDTVVEITQAPKEMIEQLAQDIATMKPVAIHQGEGINHWFHATEMNRAA 8ba0.1    --------------------------------------------------------------------------------  target    YLPLMLTGNIGRPGAGCQTWAGNYKAALFQGSPWTGPGFKGWVAEDPFDINLNPKAHGKEIHAHAYTKDEEPAYWNHGDL 8ba0.1    --------------------------------------------------------------------------------  target    ALIVDTPKFGRKNFTGKTHMPTPTKALIFNNVNLINNAKWAYGMIKNVNPNVEMIVSMDIQMTASIEYADLALPANSWLE 8ba0.1    ---------------------AQPKVLFLLNADAGK-------VTREQLPKDCFVVYIGSHGDNGASIADAVLPGAAYTE  target    FEGLEITASCSNPFLQIWKGGIPPVFDSRDDLDILAGIANALADVTGEKRFRDYFAFAAADKRGIYIQRLLDTCTTTAGY 8ba0.1    KQGIY---VNTEGRPQQTLPGVSPPGMAREDWKILRALSEVVGK------------------------------------  target    KLADIMAGKYGPPGGCLLNFRTYPRIPFYEQVHDSEPFHTDTGRMHAYADVPEAIEYGENFIVHREGPEATPYLPNVIVS 8ba0.1    --------------------------------------------------------------------------------  target    SNPHIRPEDYGIAADAEHWDDRTIRNIKMPWSKVKETKNFLWEKGFQFYCLTPKTRHRVHSGWSNVDWHMLMDSNFGDPY 8ba0.1    --------------------------------------------------------------------------------  target    RLDKRAPCVGEHQLHINPQAARDLNINDGDYVYVDANPADRPYLGAKPDDPFYRVSRCMLRVTYNRAYPYNIVMMKHAPF 8ba0.1    --------------------------------------------------------------------------------  target    IATEKSVKAHETRPDGRALSANTGYQANLRYGSQQSVTRNWHMPMHQTDTLFHKSKVFMGFIFGGEADNHAVNTVPKETL 8ba0.1    --------------------------------------------------------------------------------  target    VRVTKAEDGGMGGKGIWQPATTGFSPDNESDFMKKYLAGELTKVKT 8ba0.1    ---------------------------------------------- ``` | | | | | | | | | | | | | | | | | | | | | | | | | | | | | | | | | | | | | | | | | | | | | | | | | |
|  | 7zm7.1.I | NADH-ubiquinone oxidoreductase-like protein  *CryoEM structure of mitochondrial complex I from Chaetomium thermophilum (inhibited by DDM)* | 0.04 |  | 18.68 | 0.11 | 342-444 | EM | 0.00 | hetero-1-1-1-1-1-1-… | 4 x PC1, 14 x LMT, 5 x CDL, 8 x 3PE, 2 x FES, 6 x SF4, 1 x FMN, 1 x NDP, 1 x ZN, 2 x ZMP | HHblits | 0.28 |
| ``` target    IMDGKNLVENKLTDSHWFIECMERGAKIVVIAPEYGPPSTKADYWIPIRPQTDAALWLGITRLMIEKKWYDETFVKGFTD 7zm7.1    --------------------------------------------------------------------------------  target    FPLLVRTDTLQRLRAHEVFPQYKTSLSADGPSMKIQGLSAEQHAKLGDFVVWDGKTNAPAAITRDDVGATITKKGIDPVL 7zm7.1    --------------------------------------------------------------------------------  target    AGSFKVKLVDGKEVEVATLWTLYQDHLKDYDLDTVVEITQAPKEMIEQLAQDIATMKPVAIHQGEGINHWFHATEMNRAA 7zm7.1    --------------------------------------------------------------------------------  target    YLPLMLTGNIGRPGAGCQTWAGNYKAALFQGSPWTGPGFKGWVAEDPFDINLNPKAHGKEIHAHAYTKDEEPAYWNHGDL 7zm7.1    --------------------------------------------------------------------------------  target    ALIVDTPKFGRKNFTGKTHMPTPTKALIFNNVNLINNAKWAYGMIKNVNPNVEMIVSMDIQMTASIEYADLALPANSWLE 7zm7.1    ---------------------TKPKFVWLLGADEFDP---------ADVPKDAFIVYQGHHGDRGAEIADIVLPGAAYTE  target    FEGLEITASCSNPFLQIWKGGIPPVFDSRDDLDILAGIANALADVTGEKRFRDYFAFAAADKRGIYIQRLLDTCTTTAGY 7zm7.1    KAGTY---VNTEGRVQMTRAATGLPGAARTDWKIIRAVSEFLGV------------------------------------  target    KLADIMAGKYGPPGGCLLNFRTYPRIPFYEQVHDSEPFHTDTGRMHAYADVPEAIEYGENFIVHREGPEATPYLPNVIVS 7zm7.1    --------------------------------------------------------------------------------  target    SNPHIRPEDYGIAADAEHWDDRTIRNIKMPWSKVKETKNFLWEKGFQFYCLTPKTRHRVHSGWSNVDWHMLMDSNFGDPY 7zm7.1    --------------------------------------------------------------------------------  target    RLDKRAPCVGEHQLHINPQAARDLNINDGDYVYVDANPADRPYLGAKPDDPFYRVSRCMLRVTYNRAYPYNIVMMKHAPF 7zm7.1    --------------------------------------------------------------------------------  target    IATEKSVKAHETRPDGRALSANTGYQANLRYGSQQSVTRNWHMPMHQTDTLFHKSKVFMGFIFGGEADNHAVNTVPKETL 7zm7.1    --------------------------------------------------------------------------------  target    VRVTKAEDGGMGGKGIWQPATTGFSPDNESDFMKKYLAGELTKVKT 7zm7.1    ---------------------------------------------- ``` | | | | | | | | | | | | | | | | | | | | | | | | | | | | | | | | | | | | | | | | | | | | | | | | | |
|  | 6s6y.1.B | Tungsten-containing formylmethanofuran dehydrogenase, subunit B  *X-ray crystal structure of the formyltransferase/hydrolase complex (FhcABCD) from Methylorubrum extorquens in complex with methylofuran* | 0.04 |  | 10.99 | 0.11 | 343-444 | X-ray | 3.10 | hetero-2-2-2-2-mer | 1 x MFN, 4 x ZN, 4 x CA, 4 x K, 3 x DGL, 2 x GLU, 1 x IAS | HHblits | 0.25 |
| ``` target    IMDGKNLVENKLTDSHWFIECMERGAKIVVIAPEYGPPSTKADYWIPIRPQTDAALWLGITRLMIEKKWYDETFVKGFTD 6s6y.1    --------------------------------------------------------------------------------  target    FPLLVRTDTLQRLRAHEVFPQYKTSLSADGPSMKIQGLSAEQHAKLGDFVVWDGKTNAPAAITRDDVGATITKKGIDPVL 6s6y.1    --------------------------------------------------------------------------------  target    AGSFKVKLVDGKEVEVATLWTLYQDHLKDYDLDTVVEITQAPKEMIEQLAQDIATMKPVAIHQGEGINHWFHATEMNRAA 6s6y.1    --------------------------------------------------------------------------------  target    YLPLMLTGNIGRPGAGCQTWAGNYKAALFQGSPWTGPGFKGWVAEDPFDINLNPKAHGKEIHAHAYTKDEEPAYWNHGDL 6s6y.1    --------------------------------------------------------------------------------  target    ALIVDTPKFGRKNFTGKTHMPTPTKALIFNNVNLINNAKWAYGMIKNVNPNVEMIVSMD-IQMTASIEYADLALPANSW- 6s6y.1    ----------------------EADAALWLASLPAPR--------PAWLGSLPTIAIVGEGSQEAAGETAEVVITVGVPG  target    LEFEGLEITASCSNPFLQIWKGGIPPV---FDSRDDLDILAGIANALADVTGEKRFRDYFAFAAADKRGIYIQRLLDTCT 6s6y.1    QSVGGAL---WNDRRGVIAYAEASDPAKTPAETETAAGVLTRIRDRLIE-------------------------------  target    TTAGYKLADIMAGKYGPPGGCLLNFRTYPRIPFYEQVHDSEPFHTDTGRMHAYADVPEAIEYGENFIVHREGPEATPYLP 6s6y.1    --------------------------------------------------------------------------------  target    NVIVSSNPHIRPEDYGIAADAEHWDDRTIRNIKMPWSKVKETKNFLWEKGFQFYCLTPKTRHRVHSGWSNVDWHMLMDSN 6s6y.1    --------------------------------------------------------------------------------  target    FGDPYRLDKRAPCVGEHQLHINPQAARDLNINDGDYVYVDANPADRPYLGAKPDDPFYRVSRCMLRVTYNRAYPYNIVMM 6s6y.1    --------------------------------------------------------------------------------  target    KHAPFIATEKSVKAHETRPDGRALSANTGYQANLRYGSQQSVTRNWHMPMHQTDTLFHKSKVFMGFIFGGEADNHAVNTV 6s6y.1    --------------------------------------------------------------------------------  target    PKETLVRVTKAEDGGMGGKGIWQPATTGFSPDNESDFMKKYLAGELTKVKT 6s6y.1    --------------------------------------------------- ``` | | | | | | | | | | | | | | | | | | | | | | | | | | | | | | | | | | | | | | | | | | | | | | | | | |
|  | 3o5a.1.A | Periplasmic nitrate reductase  *Crystal Structure of partially reduced Periplasmic Nitrate Reductase from Cupriavidus necator using Ionic Liquids* | 0.03 |  | 20.48 | 0.10 | 603-718 | X-ray | 1.72 | hetero-oligomer | 1 x SF4, 1 x MOS, 2 x MGD, 2 x HEC | HHblits | 0.30 |
| ``` target    IMDGKNLVENKLTDSHWFIECMERGAKIVVIAPEYGPPSTKADYWIPIRPQTDAALWLGITRLMIEKKWYDETFVKGFTD 3o5a.1    --------------------------------------------------------------------------------  target    FPLLVRTDTLQRLRAHEVFPQYKTSLSADGPSMKIQGLSAEQHAKLGDFVVWDGKTNAPAAITRDDVGATITKKGIDPVL 3o5a.1    --------------------------------------------------------------------------------  target    AGSFKVKLVDGKEVEVATLWTLYQDHLKDYDLDTVVEITQAPKEMIEQLAQDIATMKPVAIHQGEGINHWFHATEMNRAA 3o5a.1    --------------------------------------------------------------------------------  target    YLPLMLTGNIGRPGAGCQTWAGNYKAALFQGSPWTGPGFKGWVAEDPFDINLNPKAHGKEIHAHAYTKDEEPAYWNHGDL 3o5a.1    --------------------------------------------------------------------------------  target    ALIVDTPKFGRKNFTGKTHMPTPTKALIFNNVNLINNAKWAYGMIKNVNPNVEMIVSMDIQMTASIEYADLALPANSWLE 3o5a.1    --------------------------------------------------------------------------------  target    FEGLEITASCSNPFLQIWKGGIPPVFDSRDDLDILAGIANALADVTGEKRFRDYFAFAAADKRGIYIQRLLDTCTTTAGY 3o5a.1    --------------------------------------------------------------------------------  target    KLADIMAGKYGPPGGCLLNFRTYPRIPFYEQVHDSEPFHTDTGRMHAYADVPEAIEYGENFIVHREGPEATPYLPNVIVS 3o5a.1    --------------------------------------------------------------------------------  target    SNPHIRPEDYGIAADAEHWDDRTIRNIKMPWSKVKETKNFLWEKGFQFYCLTPKTRHRVHSG--WSNVDWHMLMDSNFGD 3o5a.1    ------------------------------------------DKEYPYWLVTGRVLEHWHSGSMTRRVPELYR-------  target    PYRLDKRAPCVGEHQLHINPQAARDLNINDGDYVYVDANPADRPYLGAKPDDPFYRVSRCMLRVTYN--RAYPYNIVMMK 3o5a.1    ------SF---PNAVVFMHPEDAKALGLRRGVEVEVVSRR-----------------GRMRSRIETRGRDAPPRGLVFVP  target    HAPFIATEKSVKAHETRPDGRALSANTGYQANLRYGSQQSVTRNWHMPMHQTDTLFHKSKVFMGFIFGGEADNHAVNTVP 3o5a.1    WF------------------------------------------------------------------------------  target    KETLVRVTKAEDGGMGGKGIWQPATTGFSPDNESDFMKKYLAGELTKVKT 3o5a.1    -------------------------------------------------- ``` | | | | | | | | | | | | | | | | | | | | | | | | | | | | | | | | | | | | | | | | | | | | | | | | | |
|  | 2vpz.1.A | THIOSULFATE REDUCTASE  *POLYSULFIDE REDUCTASE NATIVE STRUCTURE* | 0.03 |  | 35.62 | 0.09 | 4-79 | X-ray | 2.40 | hetero-oligomer | 10 x SF4, 4 x MGD, 2 x MO | BLAST | 0.39 |
| ``` target    IMDGKNLVENKLTDSHWFIECMERGAKIVVIAPEYGPPSTKADYWIPIRPQTDAALWLGITRLMIEKKWYDETFVKGFTD 2vpz.1    ---GEDTHNTQLQD---FALALKNGAKVVVVDPRFSTAAAKAHRWLPIKPGTDTALLLAWIHVLIYEDLYDKEYVAKYT-  target    FPLLVRTDTLQRLRAHEVFPQYKTSLSADGPSMKIQGLSAEQHAKLGDFVVWDGKTNAPAAITRDDVGATITKKGIDPVL 2vpz.1    --------------------------------------------------------------------------------  target    AGSFKVKLVDGKEVEVATLWTLYQDHLKDYDLDTVVEITQAPKEMIEQLAQDIATMKPVAIHQGEGINHWFHATEMNRAA 2vpz.1    --------------------------------------------------------------------------------  target    YLPLMLTGNIGRPGAGCQTWAGNYKAALFQGSPWTGPGFKGWVAEDPFDINLNPKAHGKEIHAHAYTKDEEPAYWNHGDL 2vpz.1    --------------------------------------------------------------------------------  target    ALIVDTPKFGRKNFTGKTHMPTPTKALIFNNVNLINNAKWAYGMIKNVNPNVEMIVSMDIQMTASIEYADLALPANSWLE 2vpz.1    --------------------------------------------------------------------------------  target    FEGLEITASCSNPFLQIWKGGIPPVFDSRDDLDILAGIANALADVTGEKRFRDYFAFAAADKRGIYIQRLLDTCTTTAGY 2vpz.1    --------------------------------------------------------------------------------  target    KLADIMAGKYGPPGGCLLNFRTYPRIPFYEQVHDSEPFHTDTGRMHAYADVPEAIEYGENFIVHREGPEATPYLPNVIVS 2vpz.1    --------------------------------------------------------------------------------  target    SNPHIRPEDYGIAADAEHWDDRTIRNIKMPWSKVKETKNFLWEKGFQFYCLTPKTRHRVHSGWSNVDWHMLMDSNFGDPY 2vpz.1    --------------------------------------------------------------------------------  target    RLDKRAPCVGEHQLHINPQAARDLNINDGDYVYVDANPADRPYLGAKPDDPFYRVSRCMLRVTYNRAYPYNIVMMKHAPF 2vpz.1    --------------------------------------------------------------------------------  target    IATEKSVKAHETRPDGRALSANTGYQANLRYGSQQSVTRNWHMPMHQTDTLFHKSKVFMGFIFGGEADNHAVNTVPKETL 2vpz.1    --------------------------------------------------------------------------------  target    VRVTKAEDGGMGGKGIWQPATTGFSPDNESDFMKKYLAGELTKVKT 2vpz.1    ---------------------------------------------- ``` | | | | | | | | | | | | | | | | | | | | | | | | | | | | | | | | | | | | | | | | | | | | | | | | | |
|  | 2vpx.1.D | THIOSULFATE REDUCTASE  *POLYSULFIDE REDUCTASE WITH BOUND QUINONE (UQ1)* | 0.03 |  | 35.62 | 0.09 | 4-79 | X-ray | 3.10 | hetero-oligomer | 10 x SF4, 4 x MGD, 2 x MO, 2 x UQ1 | BLAST | 0.39 |
| ``` target    IMDGKNLVENKLTDSHWFIECMERGAKIVVIAPEYGPPSTKADYWIPIRPQTDAALWLGITRLMIEKKWYDETFVKGFTD 2vpx.1    ---GEDTHNTQLQD---FALALKNGAKVVVVDPRFSTAAAKAHRWLPIKPGTDTALLLAWIHVLIYEDLYDKEYVAKYT-  target    FPLLVRTDTLQRLRAHEVFPQYKTSLSADGPSMKIQGLSAEQHAKLGDFVVWDGKTNAPAAITRDDVGATITKKGIDPVL 2vpx.1    --------------------------------------------------------------------------------  target    AGSFKVKLVDGKEVEVATLWTLYQDHLKDYDLDTVVEITQAPKEMIEQLAQDIATMKPVAIHQGEGINHWFHATEMNRAA 2vpx.1    --------------------------------------------------------------------------------  target    YLPLMLTGNIGRPGAGCQTWAGNYKAALFQGSPWTGPGFKGWVAEDPFDINLNPKAHGKEIHAHAYTKDEEPAYWNHGDL 2vpx.1    --------------------------------------------------------------------------------  target    ALIVDTPKFGRKNFTGKTHMPTPTKALIFNNVNLINNAKWAYGMIKNVNPNVEMIVSMDIQMTASIEYADLALPANSWLE 2vpx.1    --------------------------------------------------------------------------------  target    FEGLEITASCSNPFLQIWKGGIPPVFDSRDDLDILAGIANALADVTGEKRFRDYFAFAAADKRGIYIQRLLDTCTTTAGY 2vpx.1    --------------------------------------------------------------------------------  target    KLADIMAGKYGPPGGCLLNFRTYPRIPFYEQVHDSEPFHTDTGRMHAYADVPEAIEYGENFIVHREGPEATPYLPNVIVS 2vpx.1    --------------------------------------------------------------------------------  target    SNPHIRPEDYGIAADAEHWDDRTIRNIKMPWSKVKETKNFLWEKGFQFYCLTPKTRHRVHSGWSNVDWHMLMDSNFGDPY 2vpx.1    --------------------------------------------------------------------------------  target    RLDKRAPCVGEHQLHINPQAARDLNINDGDYVYVDANPADRPYLGAKPDDPFYRVSRCMLRVTYNRAYPYNIVMMKHAPF 2vpx.1    --------------------------------------------------------------------------------  target    IATEKSVKAHETRPDGRALSANTGYQANLRYGSQQSVTRNWHMPMHQTDTLFHKSKVFMGFIFGGEADNHAVNTVPKETL 2vpx.1    --------------------------------------------------------------------------------  target    VRVTKAEDGGMGGKGIWQPATTGFSPDNESDFMKKYLAGELTKVKT 2vpx.1    ---------------------------------------------- ``` | | | | | | | | | | | | | | | | | | | | | | | | | | | | | | | | | | | | | | | | | | | | | | | | | |
|  | 5t5i.1.D | Tungsten formylmethanofuran dehydrogenase subunit fwdD  *TUNGSTEN-CONTAINING FORMYLMETHANOFURAN DEHYDROGENASE FROM METHANOTHERMOBACTER WOLFEII, ORTHORHOMBIC FORM AT 1.9 A* | 0.02 |  | 19.75 | 0.10 | 607-720 | X-ray | 1.90 | hetero-oligomer | 4 x ZN, 2 x MG, 18 x K, 22 x SF4, 2 x W, 4 x MGD, 2 x H2S, 2 x CA | HHblits | 0.30 |
| ``` target    IMDGKNLVENKLTDSHWFIECMERGAKIVVIAPEYGPPSTKADYWIPIRPQTDAALWLGITRLMIEKKWYDETFVKGFTD 5t5i.1    --------------------------------------------------------------------------------  target    FPLLVRTDTLQRLRAHEVFPQYKTSLSADGPSMKIQGLSAEQHAKLGDFVVWDGKTNAPAAITRDDVGATITKKGIDPVL 5t5i.1    --------------------------------------------------------------------------------  target    AGSFKVKLVDGKEVEVATLWTLYQDHLKDYDLDTVVEITQAPKEMIEQLAQDIATMKPVAIHQGEGINHWFHATEMNRAA 5t5i.1    --------------------------------------------------------------------------------  target    YLPLMLTGNIGRPGAGCQTWAGNYKAALFQGSPWTGPGFKGWVAEDPFDINLNPKAHGKEIHAHAYTKDEEPAYWNHGDL 5t5i.1    --------------------------------------------------------------------------------  target    ALIVDTPKFGRKNFTGKTHMPTPTKALIFNNVNLINNAKWAYGMIKNVNPNVEMIVSMDIQMTASIEYADLALPANSWLE 5t5i.1    --------------------------------------------------------------------------------  target    FEGLEITASCSNPFLQIWKGGIPPVFDSRDDLDILAGIANALADVTGEKRFRDYFAFAAADKRGIYIQRLLDTCTTTAGY 5t5i.1    --------------------------------------------------------------------------------  target    KLADIMAGKYGPPGGCLLNFRTYPRIPFYEQVHDSEPFHTDTGRMHAYADVPEAIEYGENFIVHREGPEATPYLPNVIVS 5t5i.1    --------------------------------------------------------------------------------  target    SNPHIRPEDYGIAADAEHWDDRTIRNIKMPWSKVKETKNFLWEKGFQFYCLTPKTRHRVHSGWSNVDWHMLMDSNFGDPY 5t5i.1    ----------------------------------------------RVILNTGRTIWQGQAIESGKDLKMY---------  target    RLDKRAPCVGEHQLHINPQAARDLNINDGDYVYVDANPADRPYLGAKPDDPFYRVSRCMLRVT-YNRAYPYNIVMMKHAP 5t5i.1    ----VD---AAAIIQMNPEMMKQLGIAEGDNVKVISEY-----------------GDVVVKAVEAKEPLPEGMVYIPMGP  target    FIATEKSVKAHETRPDGRALSANTGYQANLRYGSQQSVTRNWHMPMHQTDTLFHKSKVFMGFIFGGEADNHAVNTVPKET 5t5i.1    W-------------------------------------------------------------------------------  target    LVRVTKAEDGGMGGKGIWQPATTGFSPDNESDFMKKYLAGELTKVKT 5t5i.1    ----------------------------------------------- ``` | | | | | | | | | | | | | | | | | | | | | | | | | | | | | | | | | | | | | | | | | | | | | | | | | |
|  | 7bkb.1.J | Formylmethanofuran dehydrogenase, subunit D  *Formate dehydrogenase - heterodisulfide reductase - formylmethanofuran dehydrogenase complex from Methanospirillum hungatei (hexameric, composite structure)* | 0.02 |  | 8.33 | 0.10 | 604-720 | EM | 0.00 | hetero-2-2-2-2-2-2-… | 48 x SF4, 4 x FAD, 2 x FES, 4 x 9S8, 4 x ZN, 2 x MO, 4 x MGD | HHblits | 0.25 |
| ``` target    IMDGKNLVENKLTDSHWFIECMERGAKIVVIAPEYGPPSTKADYWIPIRPQTDAALWLGITRLMIEKKWYDETFVKGFTD 7bkb.1    --------------------------------------------------------------------------------  target    FPLLVRTDTLQRLRAHEVFPQYKTSLSADGPSMKIQGLSAEQHAKLGDFVVWDGKTNAPAAITRDDVGATITKKGIDPVL 7bkb.1    --------------------------------------------------------------------------------  target    AGSFKVKLVDGKEVEVATLWTLYQDHLKDYDLDTVVEITQAPKEMIEQLAQDIATMKPVAIHQGEGINHWFHATEMNRAA 7bkb.1    --------------------------------------------------------------------------------  target    YLPLMLTGNIGRPGAGCQTWAGNYKAALFQGSPWTGPGFKGWVAEDPFDINLNPKAHGKEIHAHAYTKDEEPAYWNHGDL 7bkb.1    --------------------------------------------------------------------------------  target    ALIVDTPKFGRKNFTGKTHMPTPTKALIFNNVNLINNAKWAYGMIKNVNPNVEMIVSMDIQMTASIEYADLALPANSWLE 7bkb.1    --------------------------------------------------------------------------------  target    FEGLEITASCSNPFLQIWKGGIPPVFDSRDDLDILAGIANALADVTGEKRFRDYFAFAAADKRGIYIQRLLDTCTTTAGY 7bkb.1    --------------------------------------------------------------------------------  target    KLADIMAGKYGPPGGCLLNFRTYPRIPFYEQVHDSEPFHTDTGRMHAYADVPEAIEYGENFIVHREGPEATPYLPNVIVS 7bkb.1    --------------------------------------------------------------------------------  target    SNPHIRPEDYGIAADAEHWDDRTIRNIKMPWSKVKETKNFLWEKGFQFYCLTPKTRHRVHSGWSNVDWHMLMDSNFGDPY 7bkb.1    -------------------------------------------AKKTLNMITQRAVEEGIAMEIG-KTSRQ---------  target    RLDKRAPCVGEHQLHINPQAARDLNINDGDYVYVDANPADRPYLGAKPDDPFYRVSRCMLRVTYN-RAYPYNIVMMKHAP 7bkb.1    -Y--FD---ACSIIEMNEQDMKELGIMKNTNVRVKSES-----------------GEVVVKAVVGRQTCYPGLCHIRQGV  target    FIATEKSVKAHETRPDGRALSANTGYQANLRYGSQQSVTRNWHMPMHQTDTLFHKSKVFMGFIFGGEADNHAVNTVPKET 7bkb.1    W-------------------------------------------------------------------------------  target    LVRVTKAEDGGMGGKGIWQPATTGFSPDNESDFMKKYLAGELTKVKT 7bkb.1    ----------------------------------------------- ``` | | | | | | | | | | | | | | | | | | | | | | | | | | | | | | | | | | | | | | | | | | | | | | | | | |
|  | 2ki8.1.A | Tungsten formylmethanofuran dehydrogenase, subunit D (FwdD-2)  *Solution NMR structure of tungsten formylmethanofuran dehydrogenase subunit D from Archaeoglobus fulgidus, Northeast Structural Genomics Consortium target AtT7* | 0.03 |  | 12.50 | 0.09 | 605-719 | NMR | 0.00 | monomer |  | HHblits | 0.27 |
| ``` target    IMDGKNLVENKLTDSHWFIECMERGAKIVVIAPEYGPPSTKADYWIPIRPQTDAALWLGITRLMIEKKWYDETFVKGFTD 2ki8.1    --------------------------------------------------------------------------------  target    FPLLVRTDTLQRLRAHEVFPQYKTSLSADGPSMKIQGLSAEQHAKLGDFVVWDGKTNAPAAITRDDVGATITKKGIDPVL 2ki8.1    --------------------------------------------------------------------------------  target    AGSFKVKLVDGKEVEVATLWTLYQDHLKDYDLDTVVEITQAPKEMIEQLAQDIATMKPVAIHQGEGINHWFHATEMNRAA 2ki8.1    --------------------------------------------------------------------------------  target    YLPLMLTGNIGRPGAGCQTWAGNYKAALFQGSPWTGPGFKGWVAEDPFDINLNPKAHGKEIHAHAYTKDEEPAYWNHGDL 2ki8.1    --------------------------------------------------------------------------------  target    ALIVDTPKFGRKNFTGKTHMPTPTKALIFNNVNLINNAKWAYGMIKNVNPNVEMIVSMDIQMTASIEYADLALPANSWLE 2ki8.1    --------------------------------------------------------------------------------  target    FEGLEITASCSNPFLQIWKGGIPPVFDSRDDLDILAGIANALADVTGEKRFRDYFAFAAADKRGIYIQRLLDTCTTTAGY 2ki8.1    --------------------------------------------------------------------------------  target    KLADIMAGKYGPPGGCLLNFRTYPRIPFYEQVHDSEPFHTDTGRMHAYADVPEAIEYGENFIVHREGPEATPYLPNVIVS 2ki8.1    --------------------------------------------------------------------------------  target    SNPHIRPEDYGIAADAEHWDDRTIRNIKMPWSKVKETKNFLWEKGFQFYCLTPKTRHRVHSGWSNVDWHMLMDSNFGDPY 2ki8.1    --------------------------------------------MLEVEVISGRTLNQGATVE-E-KLTE----------  target    RLDKRAPCVGEHQLHINPQAARDLNINDGDYVYVDANPADRPYLGAKPDDPFYRVSRCMLRVTYNRAYPYNIVMMKHAPF 2ki8.1    --EYFN---AVNYAEINEEDWNALGLQEGDRVKVKTEF-----------------GEVVVFAKKG-DVPKGMIFIPMGP-  target    IATEKSVKAHETRPDGRALSANTGYQANLRYGSQQSVTRNWHMPMHQTDTLFHKSKVFMGFIFGGEADNHAVNTVPKETL 2ki8.1    --------------------------------------------------------------------------------  target    VRVTKAEDGGMGGKGIWQPATTGFSPDNESDFMKKYLAGELTKVKT 2ki8.1    ---------------------------------------------- ``` | | | | | | | | | | | | | | | | | | | | | | | | | | | | | | | | | | | | | | | | | | | | | | | | | |
|  | 1h0h.1.A | FORMATE DEHYDROGENASE SUBUNIT ALPHA  *Tungsten containing Formate Dehydrogenase from Desulfovibrio Gigas* | 0.02 |  | 12.82 | 0.09 | 603-713 | X-ray | 1.80 | hetero-1-1-mer | 1 x W, 1 x 2MD, 1 x MGD, 4 x SF4, 1 x CA | HHblits | 0.29 |
| ``` target    IMDGKNLVENKLTDSHWFIECMERGAKIVVIAPEYGPPSTKADYWIPIRPQTDAALWLGITRLMIEKKWYDETFVKGFTD 1h0h.1    --------------------------------------------------------------------------------  target    FPLLVRTDTLQRLRAHEVFPQYKTSLSADGPSMKIQGLSAEQHAKLGDFVVWDGKTNAPAAITRDDVGATITKKGIDPVL 1h0h.1    --------------------------------------------------------------------------------  target    AGSFKVKLVDGKEVEVATLWTLYQDHLKDYDLDTVVEITQAPKEMIEQLAQDIATMKPVAIHQGEGINHWFHATEMNRAA 1h0h.1    --------------------------------------------------------------------------------  target    YLPLMLTGNIGRPGAGCQTWAGNYKAALFQGSPWTGPGFKGWVAEDPFDINLNPKAHGKEIHAHAYTKDEEPAYWNHGDL 1h0h.1    --------------------------------------------------------------------------------  target    ALIVDTPKFGRKNFTGKTHMPTPTKALIFNNVNLINNAKWAYGMIKNVNPNVEMIVSMDIQMTASIEYADLALPANSWLE 1h0h.1    --------------------------------------------------------------------------------  target    FEGLEITASCSNPFLQIWKGGIPPVFDSRDDLDILAGIANALADVTGEKRFRDYFAFAAADKRGIYIQRLLDTCTTTAGY 1h0h.1    --------------------------------------------------------------------------------  target    KLADIMAGKYGPPGGCLLNFRTYPRIPFYEQVHDSEPFHTDTGRMHAYADVPEAIEYGENFIVHREGPEATPYLPNVIVS 1h0h.1    --------------------------------------------------------------------------------  target    SNPHIRPEDYGIAADAEHWDDRTIRNIKMPWSKVKETKNFLWEKGFQFYCLTPKTR--HRVHSGWSNVDWHMLMDSNFGD 1h0h.1    ------------------------------------------DPRYPFICSTYRVTEHWQTGLMTRNTPWLLEAE-----  target    PYRLDKRAPCVGEHQLHINPQAARDLNINDGDYVYVDANPADRPYLGAKPDDPFYRVSRCMLRVTYNRAYPYNIVMMKHA 1h0h.1    -----------PQMFCEMSEELATLRGIKNGDKVILESVR-----------------GKLWAKAIITKRIKPFAI-----  target    PFIATEKSVKAHETRPDGRALSANTGYQANLRYGSQQSVTRNWHMPMHQTDTLFHKSKVFMGFIFGGEADNHAVNTVPKE 1h0h.1    --------------------------------------------------------------------------------  target    TLVRVTKAEDGGMGGKGIWQPATTGFSPDNESDFMKKYLAGELTKVKT 1h0h.1    ------------------------------------------------ ``` | | | | | | | | | | | | | | | | | | | | | | | | | | | | | | | | | | | | | | | | | | | | | | | | | |
|  | 8bqg.1.A | Formate dehydrogenase, alpha subunit, selenocysteine-containing  *W-formate dehydrogenase from Desulfovibrio vulgaris - Soaking with Formate 1 min* | 0.02 |  | 12.82 | 0.09 | 603-713 | X-ray | 1.95 | hetero-1-1-mer | 2 x MGD, 4 x SF4, 1 x H2S, 1 x W | HHblits | 0.26 |
| ``` target    IMDGKNLVENKLTDSHWFIECMERGAKIVVIAPEYGPPSTKADYWIPIRPQTDAALWLGITRLMIEKKWYDETFVKGFTD 8bqg.1    --------------------------------------------------------------------------------  target    FPLLVRTDTLQRLRAHEVFPQYKTSLSADGPSMKIQGLSAEQHAKLGDFVVWDGKTNAPAAITRDDVGATITKKGIDPVL 8bqg.1    --------------------------------------------------------------------------------  target    AGSFKVKLVDGKEVEVATLWTLYQDHLKDYDLDTVVEITQAPKEMIEQLAQDIATMKPVAIHQGEGINHWFHATEMNRAA 8bqg.1    --------------------------------------------------------------------------------  target    YLPLMLTGNIGRPGAGCQTWAGNYKAALFQGSPWTGPGFKGWVAEDPFDINLNPKAHGKEIHAHAYTKDEEPAYWNHGDL 8bqg.1    --------------------------------------------------------------------------------  target    ALIVDTPKFGRKNFTGKTHMPTPTKALIFNNVNLINNAKWAYGMIKNVNPNVEMIVSMDIQMTASIEYADLALPANSWLE 8bqg.1    --------------------------------------------------------------------------------  target    FEGLEITASCSNPFLQIWKGGIPPVFDSRDDLDILAGIANALADVTGEKRFRDYFAFAAADKRGIYIQRLLDTCTTTAGY 8bqg.1    --------------------------------------------------------------------------------  target    KLADIMAGKYGPPGGCLLNFRTYPRIPFYEQVHDSEPFHTDTGRMHAYADVPEAIEYGENFIVHREGPEATPYLPNVIVS 8bqg.1    --------------------------------------------------------------------------------  target    SNPHIRPEDYGIAADAEHWDDRTIRNIKMPWSKVKETKNFLWEKGFQFYCLTPKTRHRVHS--GWSNVDWHMLMDSNFGD 8bqg.1    ------------------------------------------DPRYPFIGTTYRVTEHWQTGLMTRRCAWLVEAE-----  target    PYRLDKRAPCVGEHQLHINPQAARDLNINDGDYVYVDANPADRPYLGAKPDDPFYRVSRCMLRVTYNRAYPYNIVMMKHA 8bqg.1    -----------PQIFCEISKELAKLRGIGNGDTVKVSSLR-----------------GALEAVAIVTERIRPFKI-----  target    PFIATEKSVKAHETRPDGRALSANTGYQANLRYGSQQSVTRNWHMPMHQTDTLFHKSKVFMGFIFGGEADNHAVNTVPKE 8bqg.1    --------------------------------------------------------------------------------  target    TLVRVTKAEDGGMGGKGIWQPATTGFSPDNESDFMKKYLAGELTKVKT 8bqg.1    ------------------------------------------------ ``` | | | | | | | | | | | | | | | | | | | | | | | | | | | | | | | | | | | | | | | | | | | | | | | | | |
|  | 6sdv.1.A | Formate dehydrogenase, alpha subunit, selenocysteine-containing,Formate dehydrogenase, alpha subunit, selenocysteine-containing,W-formate dehydrogenase - alpha subunit  *W-formate dehydrogenase from Desulfovibrio vulgaris - Formate reduced form* | 0.02 |  | 12.82 | 0.09 | 603-713 | X-ray | 1.90 | hetero-1-1-mer | 2 x MGD, 4 x SF4, 1 x W, 1 x H2S | HHblits | 0.26 |
| ``` target    IMDGKNLVENKLTDSHWFIECMERGAKIVVIAPEYGPPSTKADYWIPIRPQTDAALWLGITRLMIEKKWYDETFVKGFTD 6sdv.1    --------------------------------------------------------------------------------  target    FPLLVRTDTLQRLRAHEVFPQYKTSLSADGPSMKIQGLSAEQHAKLGDFVVWDGKTNAPAAITRDDVGATITKKGIDPVL 6sdv.1    --------------------------------------------------------------------------------  target    AGSFKVKLVDGKEVEVATLWTLYQDHLKDYDLDTVVEITQAPKEMIEQLAQDIATMKPVAIHQGEGINHWFHATEMNRAA 6sdv.1    --------------------------------------------------------------------------------  target    YLPLMLTGNIGRPGAGCQTWAGNYKAALFQGSPWTGPGFKGWVAEDPFDINLNPKAHGKEIHAHAYTKDEEPAYWNHGDL 6sdv.1    --------------------------------------------------------------------------------  target    ALIVDTPKFGRKNFTGKTHMPTPTKALIFNNVNLINNAKWAYGMIKNVNPNVEMIVSMDIQMTASIEYADLALPANSWLE 6sdv.1    --------------------------------------------------------------------------------  target    FEGLEITASCSNPFLQIWKGGIPPVFDSRDDLDILAGIANALADVTGEKRFRDYFAFAAADKRGIYIQRLLDTCTTTAGY 6sdv.1    --------------------------------------------------------------------------------  target    KLADIMAGKYGPPGGCLLNFRTYPRIPFYEQVHDSEPFHTDTGRMHAYADVPEAIEYGENFIVHREGPEATPYLPNVIVS 6sdv.1    --------------------------------------------------------------------------------  target    SNPHIRPEDYGIAADAEHWDDRTIRNIKMPWSKVKETKNFLWEKGFQFYCLTPKTRHR--VHSGWSNVDWHMLMDSNFGD 6sdv.1    ------------------------------------------DPRYPFIGTTYRVTEHWQTGLMTRRCAWLVEAE-----  target    PYRLDKRAPCVGEHQLHINPQAARDLNINDGDYVYVDANPADRPYLGAKPDDPFYRVSRCMLRVTYNRAYPYNIVMMKHA 6sdv.1    -----------PQIFCEISKELAKLRGIGNGDTVKVSSLR-----------------GALEAVAIVTERIRPFKI-----  target    PFIATEKSVKAHETRPDGRALSANTGYQANLRYGSQQSVTRNWHMPMHQTDTLFHKSKVFMGFIFGGEADNHAVNTVPKE 6sdv.1    --------------------------------------------------------------------------------  target    TLVRVTKAEDGGMGGKGIWQPATTGFSPDNESDFMKKYLAGELTKVKT 6sdv.1    ------------------------------------------------ ``` | | | | | | | | | | | | | | | | | | | | | | | | | | | | | | | | | | | | | | | | | | | | | | | | | |
|  | 6sdr.1.A | Formate dehydrogenase, alpha subunit, selenocysteine-containing  *W-formate dehydrogenase from Desulfovibrio vulgaris - Oxidized form* | 0.02 |  | 12.82 | 0.09 | 603-713 | X-ray | 2.10 | hetero-1-1-mer | 2 x MGD, 4 x SF4, 1 x H2S, 1 x W | HHblits | 0.26 |
| ``` target    IMDGKNLVENKLTDSHWFIECMERGAKIVVIAPEYGPPSTKADYWIPIRPQTDAALWLGITRLMIEKKWYDETFVKGFTD 6sdr.1    --------------------------------------------------------------------------------  target    FPLLVRTDTLQRLRAHEVFPQYKTSLSADGPSMKIQGLSAEQHAKLGDFVVWDGKTNAPAAITRDDVGATITKKGIDPVL 6sdr.1    --------------------------------------------------------------------------------  target    AGSFKVKLVDGKEVEVATLWTLYQDHLKDYDLDTVVEITQAPKEMIEQLAQDIATMKPVAIHQGEGINHWFHATEMNRAA 6sdr.1    --------------------------------------------------------------------------------  target    YLPLMLTGNIGRPGAGCQTWAGNYKAALFQGSPWTGPGFKGWVAEDPFDINLNPKAHGKEIHAHAYTKDEEPAYWNHGDL 6sdr.1    --------------------------------------------------------------------------------  target    ALIVDTPKFGRKNFTGKTHMPTPTKALIFNNVNLINNAKWAYGMIKNVNPNVEMIVSMDIQMTASIEYADLALPANSWLE 6sdr.1    --------------------------------------------------------------------------------  target    FEGLEITASCSNPFLQIWKGGIPPVFDSRDDLDILAGIANALADVTGEKRFRDYFAFAAADKRGIYIQRLLDTCTTTAGY 6sdr.1    --------------------------------------------------------------------------------  target    KLADIMAGKYGPPGGCLLNFRTYPRIPFYEQVHDSEPFHTDTGRMHAYADVPEAIEYGENFIVHREGPEATPYLPNVIVS 6sdr.1    --------------------------------------------------------------------------------  target    SNPHIRPEDYGIAADAEHWDDRTIRNIKMPWSKVKETKNFLWEKGFQFYCLTPKTRHR--VHSGWSNVDWHMLMDSNFGD 6sdr.1    ------------------------------------------DPRYPFIGTTYRVTEHWQTGLMTRRCAWLVEA------  target    PYRLDKRAPCVGEHQLHINPQAARDLNINDGDYVYVDANPADRPYLGAKPDDPFYRVSRCMLRVTYNRAYPYNIVMMKHA 6sdr.1    -------E---PQIFCEISKELAKLRGIGNGDTVKVSSLR-----------------GALEAVAIVTERIRPFKI-----  target    PFIATEKSVKAHETRPDGRALSANTGYQANLRYGSQQSVTRNWHMPMHQTDTLFHKSKVFMGFIFGGEADNHAVNTVPKE 6sdr.1    --------------------------------------------------------------------------------  target    TLVRVTKAEDGGMGGKGIWQPATTGFSPDNESDFMKKYLAGELTKVKT 6sdr.1    ------------------------------------------------ ``` | | | | | | | | | | | | | | | | | | | | | | | | | | | | | | | | | | | | | | | | | | | | | | | | | |
|  | 2e7z.1.A | Acetylene hydratase Ahy  *Acetylene Hydratase from Pelobacter acetylenicus* | 0.00 |  | 23.64 | 0.07 | 344-398 | X-ray | 1.26 | monomer | 1 x SF4, 2 x MGD, 1 x W | HHblits | 0.31 |
| ``` target    IMDGKNLVENKLTDSHWFIECMERGAKIVVIAPEYGPPSTKADYWIPIRPQTDAALWLGITRLMIEKKWYDETFVKGFTD 2e7z.1    --------------------------------------------------------------------------------  target    FPLLVRTDTLQRLRAHEVFPQYKTSLSADGPSMKIQGLSAEQHAKLGDFVVWDGKTNAPAAITRDDVGATITKKGIDPVL 2e7z.1    --------------------------------------------------------------------------------  target    AGSFKVKLVDGKEVEVATLWTLYQDHLKDYDLDTVVEITQAPKEMIEQLAQDIATMKPVAIHQGEGINHWFHATEMNRAA 2e7z.1    --------------------------------------------------------------------------------  target    YLPLMLTGNIGRPGAGCQTWAGNYKAALFQGSPWTGPGFKGWVAEDPFDINLNPKAHGKEIHAHAYTKDEEPAYWNHGDL 2e7z.1    --------------------------------------------------------------------------------  target    ALIVDTPKFGRKNFTGKTHMPTPTKALIFNNVNLINNAKWA-YGMIKNVNPNVEMIVSMDIQMTASIEYADLALPANSWL 2e7z.1    -----------------------SNCLLFIGKNLSNHNWVSQFNDLKAALKRGCKLIVLDPRRTKVAEMADIWLPLRYG-  target    EFEGLEITASCSNPFLQIWKGGIPPVFDSRDDLDILAGIANALADVTGEKRFRDYFAFAAADKRGIYIQRLLDTCTTTAG 2e7z.1    --------------------------------------------------------------------------------  target    YKLADIMAGKYGPPGGCLLNFRTYPRIPFYEQVHDSEPFHTDTGRMHAYADVPEAIEYGENFIVHREGPEATPYLPNVIV 2e7z.1    --------------------------------------------------------------------------------  target    SSNPHIRPEDYGIAADAEHWDDRTIRNIKMPWSKVKETKNFLWEKGFQFYCLTPKTRHRVHSGWSNVDWHMLMDSNFGDP 2e7z.1    --------------------------------------------------------------------------------  target    YRLDKRAPCVGEHQLHINPQAARDLNINDGDYVYVDANPADRPYLGAKPDDPFYRVSRCMLRVTYNRAYPYNIVMMKHAP 2e7z.1    --------------------------------------------------------------------------------  target    FIATEKSVKAHETRPDGRALSANTGYQANLRYGSQQSVTRNWHMPMHQTDTLFHKSKVFMGFIFGGEADNHAVNTVPKET 2e7z.1    --------------------------------------------------------------------------------  target    LVRVTKAEDGGMGGKGIWQPATTGFSPDNESDFMKKYLAGELTKVKT 2e7z.1    ----------------------------------------------- ``` | | | | | | | | | | | | | | | | | | | | | | | | | | | | | | | | | | | | | | | | | | | | | | | | | |
|  | 1e5v.2.A | Dimethyl sulfoxide/trimethylamine N-oxide reductase  *OXIDIZED DMSO REDUCTASE EXPOSED TO HEPES BUFFER* | 0.00 | 0.00 | 12.73 | 0.07 | 343-397 | X-ray | 2.40 | monomer | 2 x PGD, 1 x 2MO | HHblits | 0.29 |
| ``` target    IMDGKNLVENKLTDSHWFIECMERGAKIVVIAPEYGPPSTKADYWIPIRPQTDAALWLGITRLMIEKKWYDETFVKGFTD 1e5v.2    --------------------------------------------------------------------------------  target    FPLLVRTDTLQRLRAHEVFPQYKTSLSADGPSMKIQGLSAEQHAKLGDFVVWDGKTNAPAAITRDDVGATITKKGIDPVL 1e5v.2    --------------------------------------------------------------------------------  target    AGSFKVKLVDGKEVEVATLWTLYQDHLKDYDLDTVVEITQAPKEMIEQLAQDIATMKPVAIHQGEGINHWFHATEMNRAA 1e5v.2    --------------------------------------------------------------------------------  target    YLPLMLTGNIGRPGAGCQTWAGNYKAALFQGSPWTGPGFKGWVAEDPFDINLNPKAHGKEIHAHAYTKDEEPAYWNHGDL 1e5v.2    --------------------------------------------------------------------------------  target    ALIVDTPKFGRKNFTGKTHMPTPTKALIFNNVNLINNAKWA--------YGMIKNVNPNVEMIVSMDIQMTASIEY-ADL 1e5v.2    ----------------------NTEVMVFWAADPIKTSQIGWVIPEHGAYPGLEALKAKGTKVIVIDPVRTKTVEFFGAE  target    ALPANSWLEFEGLEITASCSNPFLQIWKGGIPPVFDSRDDLDILAGIANALADVTGEKRFRDYFAFAAADKRGIYIQRLL 1e5v.2    HITPKP--------------------------------------------------------------------------  target    DTCTTTAGYKLADIMAGKYGPPGGCLLNFRTYPRIPFYEQVHDSEPFHTDTGRMHAYADVPEAIEYGENFIVHREGPEAT 1e5v.2    --------------------------------------------------------------------------------  target    PYLPNVIVSSNPHIRPEDYGIAADAEHWDDRTIRNIKMPWSKVKETKNFLWEKGFQFYCLTPKTRHRVHSGWSNVDWHML 1e5v.2    --------------------------------------------------------------------------------  target    MDSNFGDPYRLDKRAPCVGEHQLHINPQAARDLNINDGDYVYVDANPADRPYLGAKPDDPFYRVSRCMLRVTYNRAYPYN 1e5v.2    --------------------------------------------------------------------------------  target    IVMMKHAPFIATEKSVKAHETRPDGRALSANTGYQANLRYGSQQSVTRNWHMPMHQTDTLFHKSKVFMGFIFGGEADNHA 1e5v.2    --------------------------------------------------------------------------------  target    VNTVPKETLVRVTKAEDGGMGGKGIWQPATTGFSPDNESDFMKKYLAGELTKVKT 1e5v.2    ------------------------------------------------------- ``` | | | | | | | | | | | | | | | | | | | | | | | | | | | | | | | | | | | | | | | | | | | | | | | | | |
|  | 1e18.1.A | DMSO REDUCTASE.  *TUNGSTEN-SUSBSTITUTED DMSO REDUCTASE FROM RHODOBACTER CAPSULATUS* | 0.00 | 0.00 | 12.73 | 0.07 | 343-397 | X-ray | 2.00 | monomer | 2 x PGD, 1 x 6WO | HHblits | 0.29 |
| ``` target    IMDGKNLVENKLTDSHWFIECMERGAKIVVIAPEYGPPSTKADYWIPIRPQTDAALWLGITRLMIEKKWYDETFVKGFTD 1e18.1    --------------------------------------------------------------------------------  target    FPLLVRTDTLQRLRAHEVFPQYKTSLSADGPSMKIQGLSAEQHAKLGDFVVWDGKTNAPAAITRDDVGATITKKGIDPVL 1e18.1    --------------------------------------------------------------------------------  target    AGSFKVKLVDGKEVEVATLWTLYQDHLKDYDLDTVVEITQAPKEMIEQLAQDIATMKPVAIHQGEGINHWFHATEMNRAA 1e18.1    --------------------------------------------------------------------------------  target    YLPLMLTGNIGRPGAGCQTWAGNYKAALFQGSPWTGPGFKGWVAEDPFDINLNPKAHGKEIHAHAYTKDEEPAYWNHGDL 1e18.1    --------------------------------------------------------------------------------  target    ALIVDTPKFGRKNFTGKTHMPTPTKALIFNNVNLINNAKWA--------YGMIKNVNPNVEMIVSMDIQMTASIEY-ADL 1e18.1    ----------------------NTEVMVFWAADPIKTSQIGWVIPEHGAYPGLEALKAKGTKVIVIDPVRTKTVEFFGAE  target    ALPANSWLEFEGLEITASCSNPFLQIWKGGIPPVFDSRDDLDILAGIANALADVTGEKRFRDYFAFAAADKRGIYIQRLL 1e18.1    HITPKP--------------------------------------------------------------------------  target    DTCTTTAGYKLADIMAGKYGPPGGCLLNFRTYPRIPFYEQVHDSEPFHTDTGRMHAYADVPEAIEYGENFIVHREGPEAT 1e18.1    --------------------------------------------------------------------------------  target    PYLPNVIVSSNPHIRPEDYGIAADAEHWDDRTIRNIKMPWSKVKETKNFLWEKGFQFYCLTPKTRHRVHSGWSNVDWHML 1e18.1    --------------------------------------------------------------------------------  target    MDSNFGDPYRLDKRAPCVGEHQLHINPQAARDLNINDGDYVYVDANPADRPYLGAKPDDPFYRVSRCMLRVTYNRAYPYN 1e18.1    --------------------------------------------------------------------------------  target    IVMMKHAPFIATEKSVKAHETRPDGRALSANTGYQANLRYGSQQSVTRNWHMPMHQTDTLFHKSKVFMGFIFGGEADNHA 1e18.1    --------------------------------------------------------------------------------  target    VNTVPKETLVRVTKAEDGGMGGKGIWQPATTGFSPDNESDFMKKYLAGELTKVKT 1e18.1    ------------------------------------------------------- ``` | | | | | | | | | | | | | | | | | | | | | | | | | | | | | | | | | | | | | | | | | | | | | | | | | |
|  | 1e60.1.A | Dimethyl sulfoxide/trimethylamine N-oxide reductase  *OXIDIZED DMSO REDUCTASE EXPOSED TO HEPES - Structure II BUFFER* | 0.00 | 0.00 | 12.73 | 0.07 | 343-397 | X-ray | 2.00 | monomer | 2 x PGD, 1 x 2MO | HHblits | 0.29 |
| ``` target    IMDGKNLVENKLTDSHWFIECMERGAKIVVIAPEYGPPSTKADYWIPIRPQTDAALWLGITRLMIEKKWYDETFVKGFTD 1e60.1    --------------------------------------------------------------------------------  target    FPLLVRTDTLQRLRAHEVFPQYKTSLSADGPSMKIQGLSAEQHAKLGDFVVWDGKTNAPAAITRDDVGATITKKGIDPVL 1e60.1    --------------------------------------------------------------------------------  target    AGSFKVKLVDGKEVEVATLWTLYQDHLKDYDLDTVVEITQAPKEMIEQLAQDIATMKPVAIHQGEGINHWFHATEMNRAA 1e60.1    --------------------------------------------------------------------------------  target    YLPLMLTGNIGRPGAGCQTWAGNYKAALFQGSPWTGPGFKGWVAEDPFDINLNPKAHGKEIHAHAYTKDEEPAYWNHGDL 1e60.1    --------------------------------------------------------------------------------  target    ALIVDTPKFGRKNFTGKTHMPTPTKALIFNNVNLINNAKWA--------YGMIKNVNPNVEMIVSMDIQMTASIEY-ADL 1e60.1    ----------------------NTEVMVFWAADPIKTSQIGWVIPEHGAYPGLEALKAKGTKVIVIDPVRTKTVEFFGAE  target    ALPANSWLEFEGLEITASCSNPFLQIWKGGIPPVFDSRDDLDILAGIANALADVTGEKRFRDYFAFAAADKRGIYIQRLL 1e60.1    HITPKP--------------------------------------------------------------------------  target    DTCTTTAGYKLADIMAGKYGPPGGCLLNFRTYPRIPFYEQVHDSEPFHTDTGRMHAYADVPEAIEYGENFIVHREGPEAT 1e60.1    --------------------------------------------------------------------------------  target    PYLPNVIVSSNPHIRPEDYGIAADAEHWDDRTIRNIKMPWSKVKETKNFLWEKGFQFYCLTPKTRHRVHSGWSNVDWHML 1e60.1    --------------------------------------------------------------------------------  target    MDSNFGDPYRLDKRAPCVGEHQLHINPQAARDLNINDGDYVYVDANPADRPYLGAKPDDPFYRVSRCMLRVTYNRAYPYN 1e60.1    --------------------------------------------------------------------------------  target    IVMMKHAPFIATEKSVKAHETRPDGRALSANTGYQANLRYGSQQSVTRNWHMPMHQTDTLFHKSKVFMGFIFGGEADNHA 1e60.1    --------------------------------------------------------------------------------  target    VNTVPKETLVRVTKAEDGGMGGKGIWQPATTGFSPDNESDFMKKYLAGELTKVKT 1e60.1    ------------------------------------------------------- ``` | | | | | | | | | | | | | | | | | | | | | | | | | | | | | | | | | | | | | | | | | | | | | | | | | |
|  | 7b04.1.B | Nitrite oxidoreductase subunit A  *Structure of Nitrite oxidoreductase (Nxr) from the anammox bacterium Kuenenia stuttgartiensis.* | 0.01 |  | 25.93 | 0.06 | 343-396 | X-ray | 2.97 | hetero-1-1-1-mer | 4 x SF4, 1 x F3S, 2 x MD1, 1 x MO, 1 x HEM, 2 x CA | HHblits | 0.30 |
| ``` target    IMDGKNLVENKLTDSHWFIECMERGAKIVVIAPEYGPPSTKADYWIPIRPQTDAALWLGITRLMIEKKWYDETFVKGFTD 7b04.1    --------------------------------------------------------------------------------  target    FPLLVRTDTLQRLRAHEVFPQYKTSLSADGPSMKIQGLSAEQHAKLGDFVVWDGKTNAPAAITRDDVGATITKKGIDPVL 7b04.1    --------------------------------------------------------------------------------  target    AGSFKVKLVDGKEVEVATLWTLYQDHLKDYDLDTVVEITQAPKEMIEQLAQDIATMKPVAIHQGEGINHWFHATEMNRAA 7b04.1    --------------------------------------------------------------------------------  target    YLPLMLTGNIGRPGAGCQTWAGNYKAALFQGSPWTGPGFKGWVAEDPFDINLNPKAHGKEIHAHAYTKDEEPAYWNHGDL 7b04.1    --------------------------------------------------------------------------------  target    ALIVDTPKFGRKNFTGKTHMPTPTKALIFNNVNLINNAKWAYGMIKNVNPNVEMIVSMDIQMTASIEYADLALPANSWLE 7b04.1    ----------------------FSKLLIQTGKNLIENKMPEAHWVTEVMERGGKIVVITPEYSPSAQKADYWIPIR----  target    FEGLEITASCSNPFLQIWKGGIPPVFDSRDDLDILAGIANALADVTGEKRFRDYFAFAAADKRGIYIQRLLDTCTTTAGY 7b04.1    --------------------------------------------------------------------------------  target    KLADIMAGKYGPPGGCLLNFRTYPRIPFYEQVHDSEPFHTDTGRMHAYADVPEAIEYGENFIVHREGPEATPYLPNVIVS 7b04.1    --------------------------------------------------------------------------------  target    SNPHIRPEDYGIAADAEHWDDRTIRNIKMPWSKVKETKNFLWEKGFQFYCLTPKTRHRVHSGWSNVDWHMLMDSNFGDPY 7b04.1    --------------------------------------------------------------------------------  target    RLDKRAPCVGEHQLHINPQAARDLNINDGDYVYVDANPADRPYLGAKPDDPFYRVSRCMLRVTYNRAYPYNIVMMKHAPF 7b04.1    --------------------------------------------------------------------------------  target    IATEKSVKAHETRPDGRALSANTGYQANLRYGSQQSVTRNWHMPMHQTDTLFHKSKVFMGFIFGGEADNHAVNTVPKETL 7b04.1    --------------------------------------------------------------------------------  target    VRVTKAEDGGMGGKGIWQPATTGFSPDNESDFMKKYLAGELTKVKT 7b04.1    ---------------------------------------------- ``` | | | | | | | | | | | | | | | | | | | | | | | | | | | | | | | | | | | | | | | | | | | | | | | | | |
|  | 7b04.2.B | Nitrite oxidoreductase subunit A  *Structure of Nitrite oxidoreductase (Nxr) from the anammox bacterium Kuenenia stuttgartiensis.* | 0.00 |  | 25.93 | 0.06 | 343-396 | X-ray | 2.97 | hetero-1-1-1-mer | 4 x SF4, 1 x F3S, 2 x MD1, 1 x MO, 1 x HEM, 2 x CA | HHblits | 0.30 |
| ``` target    IMDGKNLVENKLTDSHWFIECMERGAKIVVIAPEYGPPSTKADYWIPIRPQTDAALWLGITRLMIEKKWYDETFVKGFTD 7b04.2    --------------------------------------------------------------------------------  target    FPLLVRTDTLQRLRAHEVFPQYKTSLSADGPSMKIQGLSAEQHAKLGDFVVWDGKTNAPAAITRDDVGATITKKGIDPVL 7b04.2    --------------------------------------------------------------------------------  target    AGSFKVKLVDGKEVEVATLWTLYQDHLKDYDLDTVVEITQAPKEMIEQLAQDIATMKPVAIHQGEGINHWFHATEMNRAA 7b04.2    --------------------------------------------------------------------------------  target    YLPLMLTGNIGRPGAGCQTWAGNYKAALFQGSPWTGPGFKGWVAEDPFDINLNPKAHGKEIHAHAYTKDEEPAYWNHGDL 7b04.2    --------------------------------------------------------------------------------  target    ALIVDTPKFGRKNFTGKTHMPTPTKALIFNNVNLINNAKWAYGMIKNVNPNVEMIVSMDIQMTASIEYADLALPANSWLE 7b04.2    ----------------------FSKLLIQTGKNLIENKMPEAHWVTEVMERGGKIVVITPEYSPSAQKADYWIPIR----  target    FEGLEITASCSNPFLQIWKGGIPPVFDSRDDLDILAGIANALADVTGEKRFRDYFAFAAADKRGIYIQRLLDTCTTTAGY 7b04.2    --------------------------------------------------------------------------------  target    KLADIMAGKYGPPGGCLLNFRTYPRIPFYEQVHDSEPFHTDTGRMHAYADVPEAIEYGENFIVHREGPEATPYLPNVIVS 7b04.2    --------------------------------------------------------------------------------  target    SNPHIRPEDYGIAADAEHWDDRTIRNIKMPWSKVKETKNFLWEKGFQFYCLTPKTRHRVHSGWSNVDWHMLMDSNFGDPY 7b04.2    --------------------------------------------------------------------------------  target    RLDKRAPCVGEHQLHINPQAARDLNINDGDYVYVDANPADRPYLGAKPDDPFYRVSRCMLRVTYNRAYPYNIVMMKHAPF 7b04.2    --------------------------------------------------------------------------------  target    IATEKSVKAHETRPDGRALSANTGYQANLRYGSQQSVTRNWHMPMHQTDTLFHKSKVFMGFIFGGEADNHAVNTVPKETL 7b04.2    --------------------------------------------------------------------------------  target    VRVTKAEDGGMGGKGIWQPATTGFSPDNESDFMKKYLAGELTKVKT 7b04.2    ---------------------------------------------- ``` | | | | | | | | | | | | | | | | | | | | | | | | | | | | | | | | | | | | | | | | | | | | | | | | | |
|  | 4ga5.1.A | Putative thymidine phosphorylase  *Crystal structure of AMP phosphorylase C-terminal deletion mutant in the apo-form* | 0.02 |  | 16.67 | 0.06 | 650-720 | X-ray | 3.25 | homo-dimer |  | HHblits | 0.29 |
| ``` target    IMDGKNLVENKLTDSHWFIECMERGAKIVVIAPEYGPPSTKADYWIPIRPQTDAALWLGITRLMIEKKWYDETFVKGFTD 4ga5.1    --------------------------------------------------------------------------------  target    FPLLVRTDTLQRLRAHEVFPQYKTSLSADGPSMKIQGLSAEQHAKLGDFVVWDGKTNAPAAITRDDVGATITKKGIDPVL 4ga5.1    --------------------------------------------------------------------------------  target    AGSFKVKLVDGKEVEVATLWTLYQDHLKDYDLDTVVEITQAPKEMIEQLAQDIATMKPVAIHQGEGINHWFHATEMNRAA 4ga5.1    --------------------------------------------------------------------------------  target    YLPLMLTGNIGRPGAGCQTWAGNYKAALFQGSPWTGPGFKGWVAEDPFDINLNPKAHGKEIHAHAYTKDEEPAYWNHGDL 4ga5.1    --------------------------------------------------------------------------------  target    ALIVDTPKFGRKNFTGKTHMPTPTKALIFNNVNLINNAKWAYGMIKNVNPNVEMIVSMDIQMTASIEYADLALPANSWLE 4ga5.1    --------------------------------------------------------------------------------  target    FEGLEITASCSNPFLQIWKGGIPPVFDSRDDLDILAGIANALADVTGEKRFRDYFAFAAADKRGIYIQRLLDTCTTTAGY 4ga5.1    --------------------------------------------------------------------------------  target    KLADIMAGKYGPPGGCLLNFRTYPRIPFYEQVHDSEPFHTDTGRMHAYADVPEAIEYGENFIVHREGPEATPYLPNVIVS 4ga5.1    --------------------------------------------------------------------------------  target    SNPHIRPEDYGIAADAEHWDDRTIRNIKMPWSKVKETKNFLWEKGFQFYCLTPKTRHRVHSGWSNVDWHMLMDSNFGDPY 4ga5.1    --------------------------------------------------------------------------------  target    RLDKRAPCVGEHQLHINPQAARDLNINDGDYVYVDANPADRPYLGAKPDDPFYRVSRCMLRVTYNRAYPYNIVMMKHAPF 4ga5.1    ---------GRYTVLINEEDAKEAKLHPDDLVKIEAGK-----------------KAVYGSVALSNLVGKGEVGISRDVL  target    IATEKSVKAHETRPDGRALSANTGYQANLRYGSQQSVTRNWHMPMHQTDTLFHKSKVFMGFIFGGEADNHAVNTVPKETL 4ga5.1    --------------------------------------------------------------------------------  target    VRVTKAEDGGMGGKGIWQPATTGFSPDNESDFMKKYLAGELTKVKT 4ga5.1    ---------------------------------------------- ``` | | | | | | | | | | | | | | | | | | | | | | | | | | | | | | | | | | | | | | | | | | | | | | | | | |
|  | 4ga6.1.A | Putative thymidine phosphorylase  *Crystal structure of AMP phosphorylase C-terminal deletion mutant in complex with substrates* | 0.02 |  | 16.67 | 0.06 | 650-720 | X-ray | 2.21 | homo-dimer | 2 x AMP | HHblits | 0.29 |
| ``` target    IMDGKNLVENKLTDSHWFIECMERGAKIVVIAPEYGPPSTKADYWIPIRPQTDAALWLGITRLMIEKKWYDETFVKGFTD 4ga6.1    --------------------------------------------------------------------------------  target    FPLLVRTDTLQRLRAHEVFPQYKTSLSADGPSMKIQGLSAEQHAKLGDFVVWDGKTNAPAAITRDDVGATITKKGIDPVL 4ga6.1    --------------------------------------------------------------------------------  target    AGSFKVKLVDGKEVEVATLWTLYQDHLKDYDLDTVVEITQAPKEMIEQLAQDIATMKPVAIHQGEGINHWFHATEMNRAA 4ga6.1    --------------------------------------------------------------------------------  target    YLPLMLTGNIGRPGAGCQTWAGNYKAALFQGSPWTGPGFKGWVAEDPFDINLNPKAHGKEIHAHAYTKDEEPAYWNHGDL 4ga6.1    --------------------------------------------------------------------------------  target    ALIVDTPKFGRKNFTGKTHMPTPTKALIFNNVNLINNAKWAYGMIKNVNPNVEMIVSMDIQMTASIEYADLALPANSWLE 4ga6.1    --------------------------------------------------------------------------------  target    FEGLEITASCSNPFLQIWKGGIPPVFDSRDDLDILAGIANALADVTGEKRFRDYFAFAAADKRGIYIQRLLDTCTTTAGY 4ga6.1    --------------------------------------------------------------------------------  target    KLADIMAGKYGPPGGCLLNFRTYPRIPFYEQVHDSEPFHTDTGRMHAYADVPEAIEYGENFIVHREGPEATPYLPNVIVS 4ga6.1    --------------------------------------------------------------------------------  target    SNPHIRPEDYGIAADAEHWDDRTIRNIKMPWSKVKETKNFLWEKGFQFYCLTPKTRHRVHSGWSNVDWHMLMDSNFGDPY 4ga6.1    --------------------------------------------------------------------------------  target    RLDKRAPCVGEHQLHINPQAARDLNINDGDYVYVDANPADRPYLGAKPDDPFYRVSRCMLRVTYNRAYPYNIVMMKHAPF 4ga6.1    ---------GRYTVLINEEDAKEAKLHPDDLVKIEAGK-----------------KAVYGSVALSNLVGKGEVGISRDVL  target    IATEKSVKAHETRPDGRALSANTGYQANLRYGSQQSVTRNWHMPMHQTDTLFHKSKVFMGFIFGGEADNHAVNTVPKETL 4ga6.1    --------------------------------------------------------------------------------  target    VRVTKAEDGGMGGKGIWQPATTGFSPDNESDFMKKYLAGELTKVKT 4ga6.1    ---------------------------------------------- ``` | | | | | | | | | | | | | | | | | | | | | | | | | | | | | | | | | | | | | | | | | | | | | | | | | |
|  | 7qv7.1.L | Hydrogen dependent carbon dioxide reductase subunit FdhF  *Cryo-EM structure of Hydrogen-dependent CO2 reductase.* | 0.01 |  | 14.55 | 0.07 | 344-398 | EM | 0.00 | hetero-2-6-6-2-mer | 52 x SF4, 6 x 402 | HHblits | 0.28 |
| ``` target    IMDGKNLVENKLTDSHWFIECMERGAKIVVIAPEYGPPSTKADYWIPIRPQTDAALWLGITRLMIEKKWYDETFVKGFTD 7qv7.1    --------------------------------------------------------------------------------  target    FPLLVRTDTLQRLRAHEVFPQYKTSLSADGPSMKIQGLSAEQHAKLGDFVVWDGKTNAPAAITRDDVGATITKKGIDPVL 7qv7.1    --------------------------------------------------------------------------------  target    AGSFKVKLVDGKEVEVATLWTLYQDHLKDYDLDTVVEITQAPKEMIEQLAQDIATMKPVAIHQGEGINHWFHATEMNRAA 7qv7.1    --------------------------------------------------------------------------------  target    YLPLMLTGNIGRPGAGCQTWAGNYKAALFQGSPWTGPGFKGWVAEDPFDINLNPKAHGKEIHAHAYTKDEEPAYWNHGDL 7qv7.1    --------------------------------------------------------------------------------  target    ALIVDTPKFGRKNFTGKTHMPTPTKALIFNNVNLINNAKWAYGMIKNVNPNVEMIVSMDIQMTASIEYADLALPANSWLE 7qv7.1    -----------------------SDVIFIIGSNTAECHPLIAAHVIKAKERGAKLIVADPRMNAMVHKADIWLRVPSG--  target    FEGLEITASCSNPFLQIWKGGIPPVFDSRDDLDILAGIANALADVTGEKRFRDYFAFAAADKRGIYIQRLLDTCTTTAGY 7qv7.1    --------------------------------------------------------------------------------  target    KLADIMAGKYGPPGGCLLNFRTYPRIPFYEQVHDSEPFHTDTGRMHAYADVPEAIEYGENFIVHREGPEATPYLPNVIVS 7qv7.1    --------------------------------------------------------------------------------  target    SNPHIRPEDYGIAADAEHWDDRTIRNIKMPWSKVKETKNFLWEKGFQFYCLTPKTRHRVHSGWSNVDWHMLMDSNFGDPY 7qv7.1    --------------------------------------------------------------------------------  target    RLDKRAPCVGEHQLHINPQAARDLNINDGDYVYVDANPADRPYLGAKPDDPFYRVSRCMLRVTYNRAYPYNIVMMKHAPF 7qv7.1    --------------------------------------------------------------------------------  target    IATEKSVKAHETRPDGRALSANTGYQANLRYGSQQSVTRNWHMPMHQTDTLFHKSKVFMGFIFGGEADNHAVNTVPKETL 7qv7.1    --------------------------------------------------------------------------------  target    VRVTKAEDGGMGGKGIWQPATTGFSPDNESDFMKKYLAGELTKVKT 7qv7.1    ---------------------------------------------- ``` | | | | | | | | | | | | | | | | | | | | | | | | | | | | | | | | | | | | | | | | | | | | | | | | | |
|  | 7qv7.1.O | Hydrogen dependent carbon dioxide reductase subunit FdhF  *Cryo-EM structure of Hydrogen-dependent CO2 reductase.* | 0.01 |  | 14.55 | 0.07 | 344-398 | EM | 0.00 | hetero-2-6-6-2-mer | 52 x SF4, 6 x 402 | HHblits | 0.28 |
| ``` target    IMDGKNLVENKLTDSHWFIECMERGAKIVVIAPEYGPPSTKADYWIPIRPQTDAALWLGITRLMIEKKWYDETFVKGFTD 7qv7.1    --------------------------------------------------------------------------------  target    FPLLVRTDTLQRLRAHEVFPQYKTSLSADGPSMKIQGLSAEQHAKLGDFVVWDGKTNAPAAITRDDVGATITKKGIDPVL 7qv7.1    --------------------------------------------------------------------------------  target    AGSFKVKLVDGKEVEVATLWTLYQDHLKDYDLDTVVEITQAPKEMIEQLAQDIATMKPVAIHQGEGINHWFHATEMNRAA 7qv7.1    --------------------------------------------------------------------------------  target    YLPLMLTGNIGRPGAGCQTWAGNYKAALFQGSPWTGPGFKGWVAEDPFDINLNPKAHGKEIHAHAYTKDEEPAYWNHGDL 7qv7.1    --------------------------------------------------------------------------------  target    ALIVDTPKFGRKNFTGKTHMPTPTKALIFNNVNLINNAKWAYGMIKNVNPNVEMIVSMDIQMTASIEYADLALPANSWLE 7qv7.1    -----------------------SDVIFIIGSNTAECHPLIAAHVIKAKERGAKLIVADPRMNAMVHKADIWLRVPSG--  target    FEGLEITASCSNPFLQIWKGGIPPVFDSRDDLDILAGIANALADVTGEKRFRDYFAFAAADKRGIYIQRLLDTCTTTAGY 7qv7.1    --------------------------------------------------------------------------------  target    KLADIMAGKYGPPGGCLLNFRTYPRIPFYEQVHDSEPFHTDTGRMHAYADVPEAIEYGENFIVHREGPEATPYLPNVIVS 7qv7.1    --------------------------------------------------------------------------------  target    SNPHIRPEDYGIAADAEHWDDRTIRNIKMPWSKVKETKNFLWEKGFQFYCLTPKTRHRVHSGWSNVDWHMLMDSNFGDPY 7qv7.1    --------------------------------------------------------------------------------  target    RLDKRAPCVGEHQLHINPQAARDLNINDGDYVYVDANPADRPYLGAKPDDPFYRVSRCMLRVTYNRAYPYNIVMMKHAPF 7qv7.1    --------------------------------------------------------------------------------  target    IATEKSVKAHETRPDGRALSANTGYQANLRYGSQQSVTRNWHMPMHQTDTLFHKSKVFMGFIFGGEADNHAVNTVPKETL 7qv7.1    --------------------------------------------------------------------------------  target    VRVTKAEDGGMGGKGIWQPATTGFSPDNESDFMKKYLAGELTKVKT 7qv7.1    ---------------------------------------------- ``` | | | | | | | | | | | | | | | | | | | | | | | | | | | | | | | | | | | | | | | | | | | | | | | | | |
|  | 6cz7.1.A | ArrA  *The arsenate respiratory reductase (Arr) complex from Shewanella sp. ANA-3* | 0.01 |  | 14.55 | 0.07 | 344-398 | X-ray | 1.62 | hetero-1-1-mer | 5 x SF4, 2 x MGD, 1 x MO, 1 x PG5 | HHblits | 0.27 |
| ``` target    IMDGKNLVENKLTDSHWFIECMERGAKIVVIAPEYGPPSTKADYWIPIRPQTDAALWLGITRLMIEKKWYDETFVKGFTD 6cz7.1    --------------------------------------------------------------------------------  target    FPLLVRTDTLQRLRAHEVFPQYKTSLSADGPSMKIQGLSAEQHAKLGDFVVWDGKTNAPAAITRDDVGATITKKGIDPVL 6cz7.1    --------------------------------------------------------------------------------  target    AGSFKVKLVDGKEVEVATLWTLYQDHLKDYDLDTVVEITQAPKEMIEQLAQDIATMKPVAIHQGEGINHWFHATEMNRAA 6cz7.1    --------------------------------------------------------------------------------  target    YLPLMLTGNIGRPGAGCQTWAGNYKAALFQGSPWTGPGFKGWVAEDPFDINLNPKAHGKEIHAHAYTKDEEPAYWNHGDL 6cz7.1    --------------------------------------------------------------------------------  target    ALIVDTPKFGRKNFTGKTHMPTPTKALIFNNVNLINNAKWAYGMIKN--VNPNVEMIVSMDIQMTASIEYADLALPANSW 6cz7.1    -----------------------AKFILSFGADPIASNRQVSFYSQTWGDSLDHAKVVVVDPRLSASAAKAHKWIPIEPG  target    LEFEGLEITASCSNPFLQIWKGGIPPVFDSRDDLDILAGIANALADVTGEKRFRDYFAFAAADKRGIYIQRLLDTCTTTA 6cz7.1    --------------------------------------------------------------------------------  target    GYKLADIMAGKYGPPGGCLLNFRTYPRIPFYEQVHDSEPFHTDTGRMHAYADVPEAIEYGENFIVHREGPEATPYLPNVI 6cz7.1    --------------------------------------------------------------------------------  target    VSSNPHIRPEDYGIAADAEHWDDRTIRNIKMPWSKVKETKNFLWEKGFQFYCLTPKTRHRVHSGWSNVDWHMLMDSNFGD 6cz7.1    --------------------------------------------------------------------------------  target    PYRLDKRAPCVGEHQLHINPQAARDLNINDGDYVYVDANPADRPYLGAKPDDPFYRVSRCMLRVTYNRAYPYNIVMMKHA 6cz7.1    --------------------------------------------------------------------------------  target    PFIATEKSVKAHETRPDGRALSANTGYQANLRYGSQQSVTRNWHMPMHQTDTLFHKSKVFMGFIFGGEADNHAVNTVPKE 6cz7.1    --------------------------------------------------------------------------------  target    TLVRVTKAEDGGMGGKGIWQPATTGFSPDNESDFMKKYLAGELTKVKT 6cz7.1    ------------------------------------------------ ``` | | | | | | | | | | | | | | | | | | | | | | | | | | | | | | | | | | | | | | | | | | | | | | | | | |
|  | 7l5i.1.A | Trimethylamine-N-oxide reductase  *Crystal Structure of Haemophilus influenzae MtsZ at pH 7.0* | 0.00 |  | 15.09 | 0.06 | 344-396 | X-ray | 1.73 | monomer | 2 x MGD, 1 x MO, 1 x O | HHblits | 0.30 |
| ``` target    IMDGKNLVENKLTDSHWFIECMERGAKIVVIAPEYGPPSTKADYWIPIRPQTDAALWLGITRLMIEKKWYDETFVKGFTD 7l5i.1    --------------------------------------------------------------------------------  target    FPLLVRTDTLQRLRAHEVFPQYKTSLSADGPSMKIQGLSAEQHAKLGDFVVWDGKTNAPAAITRDDVGATITKKGIDPVL 7l5i.1    --------------------------------------------------------------------------------  target    AGSFKVKLVDGKEVEVATLWTLYQDHLKDYDLDTVVEITQAPKEMIEQLAQDIATMKPVAIHQGEGINHWFHATEMNRAA 7l5i.1    --------------------------------------------------------------------------------  target    YLPLMLTGNIGRPGAGCQTWAGNYKAALFQGSPWTGPGFKGWVAEDPFDINLNPKAHGKEIHAHAYTKDEEPAYWNHGDL 7l5i.1    --------------------------------------------------------------------------------  target    ALIVDTPKFGRKNFTGKTHMPTPTKALIFNNVNLINNAKWA--------YGMIKNVNPNVEMIVSMDIQMTASIEY-ADL 7l5i.1    -----------------------SDIIVLWSANPLTTMRIAWMSTDQKGIEYFKKFQASGKRIICIDPQKSETCQMLNAE  target    ALPANSWLEFEGLEITASCSNPFLQIWKGGIPPVFDSRDDLDILAGIANALADVTGEKRFRDYFAFAAADKRGIYIQRLL 7l5i.1    WIPVN---------------------------------------------------------------------------  target    DTCTTTAGYKLADIMAGKYGPPGGCLLNFRTYPRIPFYEQVHDSEPFHTDTGRMHAYADVPEAIEYGENFIVHREGPEAT 7l5i.1    --------------------------------------------------------------------------------  target    PYLPNVIVSSNPHIRPEDYGIAADAEHWDDRTIRNIKMPWSKVKETKNFLWEKGFQFYCLTPKTRHRVHSGWSNVDWHML 7l5i.1    --------------------------------------------------------------------------------  target    MDSNFGDPYRLDKRAPCVGEHQLHINPQAARDLNINDGDYVYVDANPADRPYLGAKPDDPFYRVSRCMLRVTYNRAYPYN 7l5i.1    --------------------------------------------------------------------------------  target    IVMMKHAPFIATEKSVKAHETRPDGRALSANTGYQANLRYGSQQSVTRNWHMPMHQTDTLFHKSKVFMGFIFGGEADNHA 7l5i.1    --------------------------------------------------------------------------------  target    VNTVPKETLVRVTKAEDGGMGGKGIWQPATTGFSPDNESDFMKKYLAGELTKVKT 7l5i.1    ------------------------------------------------------- ``` | | | | | | | | | | | | | | | | | | | | | | | | | | | | | | | | | | | | | | | | | | | | | | | | | |
|  | 7l5s.1.A | Trimethylamine-N-oxide reductase  *Crystal Structure of Haemophilus influenzae MtsZ at pH 5.5* | 0.00 |  | 15.09 | 0.06 | 344-396 | X-ray | 2.09 | monomer | 1 x O, 2 x MGD, 1 x MO | HHblits | 0.30 |
| ``` target    IMDGKNLVENKLTDSHWFIECMERGAKIVVIAPEYGPPSTKADYWIPIRPQTDAALWLGITRLMIEKKWYDETFVKGFTD 7l5s.1    --------------------------------------------------------------------------------  target    FPLLVRTDTLQRLRAHEVFPQYKTSLSADGPSMKIQGLSAEQHAKLGDFVVWDGKTNAPAAITRDDVGATITKKGIDPVL 7l5s.1    --------------------------------------------------------------------------------  target    AGSFKVKLVDGKEVEVATLWTLYQDHLKDYDLDTVVEITQAPKEMIEQLAQDIATMKPVAIHQGEGINHWFHATEMNRAA 7l5s.1    --------------------------------------------------------------------------------  target    YLPLMLTGNIGRPGAGCQTWAGNYKAALFQGSPWTGPGFKGWVAEDPFDINLNPKAHGKEIHAHAYTKDEEPAYWNHGDL 7l5s.1    --------------------------------------------------------------------------------  target    ALIVDTPKFGRKNFTGKTHMPTPTKALIFNNVNLINNAKWA--------YGMIKNVNPNVEMIVSMDIQMTASIEY-ADL 7l5s.1    -----------------------SDIIVLWSANPLTTMRIAWMSTDQKGIEYFKKFQASGKRIICIDPQKSETCQMLNAE  target    ALPANSWLEFEGLEITASCSNPFLQIWKGGIPPVFDSRDDLDILAGIANALADVTGEKRFRDYFAFAAADKRGIYIQRLL 7l5s.1    WIPVN---------------------------------------------------------------------------  target    DTCTTTAGYKLADIMAGKYGPPGGCLLNFRTYPRIPFYEQVHDSEPFHTDTGRMHAYADVPEAIEYGENFIVHREGPEAT 7l5s.1    --------------------------------------------------------------------------------  target    PYLPNVIVSSNPHIRPEDYGIAADAEHWDDRTIRNIKMPWSKVKETKNFLWEKGFQFYCLTPKTRHRVHSGWSNVDWHML 7l5s.1    --------------------------------------------------------------------------------  target    MDSNFGDPYRLDKRAPCVGEHQLHINPQAARDLNINDGDYVYVDANPADRPYLGAKPDDPFYRVSRCMLRVTYNRAYPYN 7l5s.1    --------------------------------------------------------------------------------  target    IVMMKHAPFIATEKSVKAHETRPDGRALSANTGYQANLRYGSQQSVTRNWHMPMHQTDTLFHKSKVFMGFIFGGEADNHA 7l5s.1    --------------------------------------------------------------------------------  target    VNTVPKETLVRVTKAEDGGMGGKGIWQPATTGFSPDNESDFMKKYLAGELTKVKT 7l5s.1    ------------------------------------------------------- ``` | | | | | | | | | | | | | | | | | | | | | | | | | | | | | | | | | | | | | | | | | | | | | | | | | |
|  | 1aa6.1.A | FORMATE DEHYDROGENASE H  *REDUCED FORM OF FORMATE DEHYDROGENASE H FROM E. COLI* | 0.01 |  | 14.81 | 0.06 | 344-397 | X-ray | 2.30 | monomer | 1 x SF4, 2 x MGD, 1 x 4MO | HHblits | 0.28 |
| ``` target    IMDGKNLVENKLTDSHWFIECMERGAKIVVIAPEYGPPSTKADYWIPIRPQTDAALWLGITRLMIEKKWYDETFVKGFTD 1aa6.1    --------------------------------------------------------------------------------  target    FPLLVRTDTLQRLRAHEVFPQYKTSLSADGPSMKIQGLSAEQHAKLGDFVVWDGKTNAPAAITRDDVGATITKKGIDPVL 1aa6.1    --------------------------------------------------------------------------------  target    AGSFKVKLVDGKEVEVATLWTLYQDHLKDYDLDTVVEITQAPKEMIEQLAQDIATMKPVAIHQGEGINHWFHATEMNRAA 1aa6.1    --------------------------------------------------------------------------------  target    YLPLMLTGNIGRPGAGCQTWAGNYKAALFQGSPWTGPGFKGWVAEDPFDINLNPKAHGKEIHAHAYTKDEEPAYWNHGDL 1aa6.1    --------------------------------------------------------------------------------  target    ALIVDTPKFGRKNFTGKTHMPTPTKALIFNNVNLINNAKWAYGMIKNVNPNVEMIVSMDIQMTASIEYADLALPANSWLE 1aa6.1    -----------------------TDLVFVFGYNPADSHPIVANHVINAKRNGAKIIVCDPRKIETARIADMHIALKN---  target    FEGLEITASCSNPFLQIWKGGIPPVFDSRDDLDILAGIANALADVTGEKRFRDYFAFAAADKRGIYIQRLLDTCTTTAGY 1aa6.1    --------------------------------------------------------------------------------  target    KLADIMAGKYGPPGGCLLNFRTYPRIPFYEQVHDSEPFHTDTGRMHAYADVPEAIEYGENFIVHREGPEATPYLPNVIVS 1aa6.1    --------------------------------------------------------------------------------  target    SNPHIRPEDYGIAADAEHWDDRTIRNIKMPWSKVKETKNFLWEKGFQFYCLTPKTRHRVHSGWSNVDWHMLMDSNFGDPY 1aa6.1    --------------------------------------------------------------------------------  target    RLDKRAPCVGEHQLHINPQAARDLNINDGDYVYVDANPADRPYLGAKPDDPFYRVSRCMLRVTYNRAYPYNIVMMKHAPF 1aa6.1    --------------------------------------------------------------------------------  target    IATEKSVKAHETRPDGRALSANTGYQANLRYGSQQSVTRNWHMPMHQTDTLFHKSKVFMGFIFGGEADNHAVNTVPKETL 1aa6.1    --------------------------------------------------------------------------------  target    VRVTKAEDGGMGGKGIWQPATTGFSPDNESDFMKKYLAGELTKVKT 1aa6.1    ---------------------------------------------- ``` | | | | | | | | | | | | | | | | | | | | | | | | | | | | | | | | | | | | | | | | | | | | | | | | | |
|  | 1fdo.1.A | FORMATE DEHYDROGENASE H  *OXIDIZED FORM OF FORMATE DEHYDROGENASE H FROM E. COLI* | 0.00 |  | 14.81 | 0.06 | 344-397 | X-ray | 2.80 | monomer | 1 x SF4, 2 x MGD, 1 x 6MO | HHblits | 0.28 |
| ``` target    IMDGKNLVENKLTDSHWFIECMERGAKIVVIAPEYGPPSTKADYWIPIRPQTDAALWLGITRLMIEKKWYDETFVKGFTD 1fdo.1    --------------------------------------------------------------------------------  target    FPLLVRTDTLQRLRAHEVFPQYKTSLSADGPSMKIQGLSAEQHAKLGDFVVWDGKTNAPAAITRDDVGATITKKGIDPVL 1fdo.1    --------------------------------------------------------------------------------  target    AGSFKVKLVDGKEVEVATLWTLYQDHLKDYDLDTVVEITQAPKEMIEQLAQDIATMKPVAIHQGEGINHWFHATEMNRAA 1fdo.1    --------------------------------------------------------------------------------  target    YLPLMLTGNIGRPGAGCQTWAGNYKAALFQGSPWTGPGFKGWVAEDPFDINLNPKAHGKEIHAHAYTKDEEPAYWNHGDL 1fdo.1    --------------------------------------------------------------------------------  target    ALIVDTPKFGRKNFTGKTHMPTPTKALIFNNVNLINNAKWAYGMIKNVNPNVEMIVSMDIQMTASIEYADLALPANSWLE 1fdo.1    -----------------------TDLVFVFGYNPADSHPIVANHVINAKRNGAKIIVCDPRKIETARIADMHIALKN---  target    FEGLEITASCSNPFLQIWKGGIPPVFDSRDDLDILAGIANALADVTGEKRFRDYFAFAAADKRGIYIQRLLDTCTTTAGY 1fdo.1    --------------------------------------------------------------------------------  target    KLADIMAGKYGPPGGCLLNFRTYPRIPFYEQVHDSEPFHTDTGRMHAYADVPEAIEYGENFIVHREGPEATPYLPNVIVS 1fdo.1    --------------------------------------------------------------------------------  target    SNPHIRPEDYGIAADAEHWDDRTIRNIKMPWSKVKETKNFLWEKGFQFYCLTPKTRHRVHSGWSNVDWHMLMDSNFGDPY 1fdo.1    --------------------------------------------------------------------------------  target    RLDKRAPCVGEHQLHINPQAARDLNINDGDYVYVDANPADRPYLGAKPDDPFYRVSRCMLRVTYNRAYPYNIVMMKHAPF 1fdo.1    --------------------------------------------------------------------------------  target    IATEKSVKAHETRPDGRALSANTGYQANLRYGSQQSVTRNWHMPMHQTDTLFHKSKVFMGFIFGGEADNHAVNTVPKETL 1fdo.1    --------------------------------------------------------------------------------  target    VRVTKAEDGGMGGKGIWQPATTGFSPDNESDFMKKYLAGELTKVKT 1fdo.1    ---------------------------------------------- ``` | | | | | | | | | | | | | | | | | | | | | | | | | | | | | | | | | | | | | | | | | | | | | | | | | |
|  | 2iv2.1.A | Formate dehydrogenase H  *Reinterpretation of reduced form of formate dehydrogenase H from E. coli* | 0.00 |  | 14.81 | 0.06 | 344-397 | X-ray | 2.27 | monomer | 1 x SF4, 1 x 2MD, 1 x MGD | HHblits | 0.28 |
| ``` target    IMDGKNLVENKLTDSHWFIECMERGAKIVVIAPEYGPPSTKADYWIPIRPQTDAALWLGITRLMIEKKWYDETFVKGFTD 2iv2.1    --------------------------------------------------------------------------------  target    FPLLVRTDTLQRLRAHEVFPQYKTSLSADGPSMKIQGLSAEQHAKLGDFVVWDGKTNAPAAITRDDVGATITKKGIDPVL 2iv2.1    --------------------------------------------------------------------------------  target    AGSFKVKLVDGKEVEVATLWTLYQDHLKDYDLDTVVEITQAPKEMIEQLAQDIATMKPVAIHQGEGINHWFHATEMNRAA 2iv2.1    --------------------------------------------------------------------------------  target    YLPLMLTGNIGRPGAGCQTWAGNYKAALFQGSPWTGPGFKGWVAEDPFDINLNPKAHGKEIHAHAYTKDEEPAYWNHGDL 2iv2.1    --------------------------------------------------------------------------------  target    ALIVDTPKFGRKNFTGKTHMPTPTKALIFNNVNLINNAKWAYGMIKNVNPNVEMIVSMDIQMTASIEYADLALPANSWLE 2iv2.1    -----------------------TDLVFVFGYNPADSHPIVANHVINAKRNGAKIIVCDPRKIETARIADMHIALKN---  target    FEGLEITASCSNPFLQIWKGGIPPVFDSRDDLDILAGIANALADVTGEKRFRDYFAFAAADKRGIYIQRLLDTCTTTAGY 2iv2.1    --------------------------------------------------------------------------------  target    KLADIMAGKYGPPGGCLLNFRTYPRIPFYEQVHDSEPFHTDTGRMHAYADVPEAIEYGENFIVHREGPEATPYLPNVIVS 2iv2.1    --------------------------------------------------------------------------------  target    SNPHIRPEDYGIAADAEHWDDRTIRNIKMPWSKVKETKNFLWEKGFQFYCLTPKTRHRVHSGWSNVDWHMLMDSNFGDPY 2iv2.1    --------------------------------------------------------------------------------  target    RLDKRAPCVGEHQLHINPQAARDLNINDGDYVYVDANPADRPYLGAKPDDPFYRVSRCMLRVTYNRAYPYNIVMMKHAPF 2iv2.1    --------------------------------------------------------------------------------  target    IATEKSVKAHETRPDGRALSANTGYQANLRYGSQQSVTRNWHMPMHQTDTLFHKSKVFMGFIFGGEADNHAVNTVPKETL 2iv2.1    --------------------------------------------------------------------------------  target    VRVTKAEDGGMGGKGIWQPATTGFSPDNESDFMKKYLAGELTKVKT 2iv2.1    ---------------------------------------------- ``` | | | | | | | | | | | | | | | | | | | | | | | | | | | | | | | | | | | | | | | | | | | | | | | | | |
|  | 7z0t.1.G | Formate dehydrogenase H  *Structure of the Escherichia coli formate hydrogenlyase complex (aerobic preparation, composite structure)* | 0.00 |  | 14.81 | 0.06 | 344-397 | EM | 0.00 | hetero-1-1-1-1-1-1-… | 1 x NI, 1 x FCO, 8 x SF4, 1 x FE, 2 x MGD, 1 x 6MO | HHblits | 0.28 |
| ``` target    IMDGKNLVENKLTDSHWFIECMERGAKIVVIAPEYGPPSTKADYWIPIRPQTDAALWLGITRLMIEKKWYDETFVKGFTD 7z0t.1    --------------------------------------------------------------------------------  target    FPLLVRTDTLQRLRAHEVFPQYKTSLSADGPSMKIQGLSAEQHAKLGDFVVWDGKTNAPAAITRDDVGATITKKGIDPVL 7z0t.1    --------------------------------------------------------------------------------  target    AGSFKVKLVDGKEVEVATLWTLYQDHLKDYDLDTVVEITQAPKEMIEQLAQDIATMKPVAIHQGEGINHWFHATEMNRAA 7z0t.1    --------------------------------------------------------------------------------  target    YLPLMLTGNIGRPGAGCQTWAGNYKAALFQGSPWTGPGFKGWVAEDPFDINLNPKAHGKEIHAHAYTKDEEPAYWNHGDL 7z0t.1    --------------------------------------------------------------------------------  target    ALIVDTPKFGRKNFTGKTHMPTPTKALIFNNVNLINNAKWAYGMIKNVNPNVEMIVSMDIQMTASIEYADLALPANSWLE 7z0t.1    -----------------------TDLVFVFGYNPADSHPIVANHVINAKRNGAKIIVCDPRKIETARIADMHIALKN---  target    FEGLEITASCSNPFLQIWKGGIPPVFDSRDDLDILAGIANALADVTGEKRFRDYFAFAAADKRGIYIQRLLDTCTTTAGY 7z0t.1    --------------------------------------------------------------------------------  target    KLADIMAGKYGPPGGCLLNFRTYPRIPFYEQVHDSEPFHTDTGRMHAYADVPEAIEYGENFIVHREGPEATPYLPNVIVS 7z0t.1    --------------------------------------------------------------------------------  target    SNPHIRPEDYGIAADAEHWDDRTIRNIKMPWSKVKETKNFLWEKGFQFYCLTPKTRHRVHSGWSNVDWHMLMDSNFGDPY 7z0t.1    --------------------------------------------------------------------------------  target    RLDKRAPCVGEHQLHINPQAARDLNINDGDYVYVDANPADRPYLGAKPDDPFYRVSRCMLRVTYNRAYPYNIVMMKHAPF 7z0t.1    --------------------------------------------------------------------------------  target    IATEKSVKAHETRPDGRALSANTGYQANLRYGSQQSVTRNWHMPMHQTDTLFHKSKVFMGFIFGGEADNHAVNTVPKETL 7z0t.1    --------------------------------------------------------------------------------  target    VRVTKAEDGGMGGKGIWQPATTGFSPDNESDFMKKYLAGELTKVKT 7z0t.1    ---------------------------------------------- ``` | | | | | | | | | | | | | | | | | | | | | | | | | | | | | | | | | | | | | | | | | | | | | | | | | |
|  | 5t5i.1.B | Tungsten formylmethanofuran dehydrogenase subunit B  *TUNGSTEN-CONTAINING FORMYLMETHANOFURAN DEHYDROGENASE FROM METHANOTHERMOBACTER WOLFEII, ORTHORHOMBIC FORM AT 1.9 A* | 0.00 |  | 12.96 | 0.06 | 344-397 | X-ray | 1.90 | hetero-oligomer | 4 x ZN, 2 x MG, 18 x K, 22 x SF4, 2 x W, 4 x MGD, 2 x H2S, 2 x CA | HHblits | 0.28 |
| ``` target    IMDGKNLVENKLTDSHWFIECMERGAKIVVIAPEYGPPSTKADYWIPIRPQTDAALWLGITRLMIEKKWYDETFVKGFTD 5t5i.1    --------------------------------------------------------------------------------  target    FPLLVRTDTLQRLRAHEVFPQYKTSLSADGPSMKIQGLSAEQHAKLGDFVVWDGKTNAPAAITRDDVGATITKKGIDPVL 5t5i.1    --------------------------------------------------------------------------------  target    AGSFKVKLVDGKEVEVATLWTLYQDHLKDYDLDTVVEITQAPKEMIEQLAQDIATMKPVAIHQGEGINHWFHATEMNRAA 5t5i.1    --------------------------------------------------------------------------------  target    YLPLMLTGNIGRPGAGCQTWAGNYKAALFQGSPWTGPGFKGWVAEDPFDINLNPKAHGKEIHAHAYTKDEEPAYWNHGDL 5t5i.1    --------------------------------------------------------------------------------  target    ALIVDTPKFGRKNFTGKTHMPTPTKALIFNNVNLINNAKWAYG-------MIKNVNPNVEMIVSMDIQMTASIEYADLAL 5t5i.1    -----------------------ADVVVYWGCNPMHAHPRHMSRNVFARGFFRERGRSDRTLIVVDPRKTDSAKLADIHL  target    PANSWLEFEGLEITASCSNPFLQIWKGGIPPVFDSRDDLDILAGIANALADVTGEKRFRDYFAFAAADKRGIYIQRLLDT 5t5i.1    QLDF----------------------------------------------------------------------------  target    CTTTAGYKLADIMAGKYGPPGGCLLNFRTYPRIPFYEQVHDSEPFHTDTGRMHAYADVPEAIEYGENFIVHREGPEATPY 5t5i.1    --------------------------------------------------------------------------------  target    LPNVIVSSNPHIRPEDYGIAADAEHWDDRTIRNIKMPWSKVKETKNFLWEKGFQFYCLTPKTRHRVHSGWSNVDWHMLMD 5t5i.1    --------------------------------------------------------------------------------  target    SNFGDPYRLDKRAPCVGEHQLHINPQAARDLNINDGDYVYVDANPADRPYLGAKPDDPFYRVSRCMLRVTYNRAYPYNIV 5t5i.1    --------------------------------------------------------------------------------  target    MMKHAPFIATEKSVKAHETRPDGRALSANTGYQANLRYGSQQSVTRNWHMPMHQTDTLFHKSKVFMGFIFGGEADNHAVN 5t5i.1    --------------------------------------------------------------------------------  target    TVPKETLVRVTKAEDGGMGGKGIWQPATTGFSPDNESDFMKKYLAGELTKVKT 5t5i.1    ----------------------------------------------------- ``` | | | | | | | | | | | | | | | | | | | | | | | | | | | | | | | | | | | | | | | | | | | | | | | | | |
|  | 4dmr.1.A | DMSO REDUCTASE  *REDUCED DMSO REDUCTASE FROM RHODOBACTER CAPSULATUS WITH BOUND DMSO SUBSTRATE* | 0.00 | 0.00 | 13.21 | 0.06 | 344-396 | X-ray | 1.90 | monomer | 2 x PGD, 1 x 4MO, 1 x O | HHblits | 0.30 |
| ``` target    IMDGKNLVENKLTDSHWFIECMERGAKIVVIAPEYGPPSTKADYWIPIRPQTDAALWLGITRLMIEKKWYDETFVKGFTD 4dmr.1    --------------------------------------------------------------------------------  target    FPLLVRTDTLQRLRAHEVFPQYKTSLSADGPSMKIQGLSAEQHAKLGDFVVWDGKTNAPAAITRDDVGATITKKGIDPVL 4dmr.1    --------------------------------------------------------------------------------  target    AGSFKVKLVDGKEVEVATLWTLYQDHLKDYDLDTVVEITQAPKEMIEQLAQDIATMKPVAIHQGEGINHWFHATEMNRAA 4dmr.1    --------------------------------------------------------------------------------  target    YLPLMLTGNIGRPGAGCQTWAGNYKAALFQGSPWTGPGFKGWVAEDPFDINLNPKAHGKEIHAHAYTKDEEPAYWNHGDL 4dmr.1    --------------------------------------------------------------------------------  target    ALIVDTPKFGRKNFTGKTHMPTPTKALIFNNVNLINNAKWA--------YGMIKNVNPNVEMIVSMDIQMTASIEY-ADL 4dmr.1    -----------------------TEVMVFWAADPIKTSQIGWVIPEHGAYPGLEALKAKGTKVIVIDPVRTKTVEFFGAE  target    ALPANSWLEFEGLEITASCSNPFLQIWKGGIPPVFDSRDDLDILAGIANALADVTGEKRFRDYFAFAAADKRGIYIQRLL 4dmr.1    HITPK---------------------------------------------------------------------------  target    DTCTTTAGYKLADIMAGKYGPPGGCLLNFRTYPRIPFYEQVHDSEPFHTDTGRMHAYADVPEAIEYGENFIVHREGPEAT 4dmr.1    --------------------------------------------------------------------------------  target    PYLPNVIVSSNPHIRPEDYGIAADAEHWDDRTIRNIKMPWSKVKETKNFLWEKGFQFYCLTPKTRHRVHSGWSNVDWHML 4dmr.1    --------------------------------------------------------------------------------  target    MDSNFGDPYRLDKRAPCVGEHQLHINPQAARDLNINDGDYVYVDANPADRPYLGAKPDDPFYRVSRCMLRVTYNRAYPYN 4dmr.1    --------------------------------------------------------------------------------  target    IVMMKHAPFIATEKSVKAHETRPDGRALSANTGYQANLRYGSQQSVTRNWHMPMHQTDTLFHKSKVFMGFIFGGEADNHA 4dmr.1    --------------------------------------------------------------------------------  target    VNTVPKETLVRVTKAEDGGMGGKGIWQPATTGFSPDNESDFMKKYLAGELTKVKT 4dmr.1    ------------------------------------------------------- ``` | | | | | | | | | | | | | | | | | | | | | | | | | | | | | | | | | | | | | | | | | | | | | | | | | |
|  | 1dms.1.A | DMSO REDUCTASE  *STRUCTURE OF DMSO REDUCTASE* | 0.00 | 0.00 | 13.21 | 0.06 | 344-396 | X-ray | 1.88 | monomer | 2 x PGD, 1 x 2MO | HHblits | 0.29 |
| ``` target    IMDGKNLVENKLTDSHWFIECMERGAKIVVIAPEYGPPSTKADYWIPIRPQTDAALWLGITRLMIEKKWYDETFVKGFTD 1dms.1    --------------------------------------------------------------------------------  target    FPLLVRTDTLQRLRAHEVFPQYKTSLSADGPSMKIQGLSAEQHAKLGDFVVWDGKTNAPAAITRDDVGATITKKGIDPVL 1dms.1    --------------------------------------------------------------------------------  target    AGSFKVKLVDGKEVEVATLWTLYQDHLKDYDLDTVVEITQAPKEMIEQLAQDIATMKPVAIHQGEGINHWFHATEMNRAA 1dms.1    --------------------------------------------------------------------------------  target    YLPLMLTGNIGRPGAGCQTWAGNYKAALFQGSPWTGPGFKGWVAEDPFDINLNPKAHGKEIHAHAYTKDEEPAYWNHGDL 1dms.1    --------------------------------------------------------------------------------  target    ALIVDTPKFGRKNFTGKTHMPTPTKALIFNNVNLINNAKWA--------YGMIKNVNPNVEMIVSMDIQMTASIEY-ADL 1dms.1    -----------------------TEVMVFWAADPIKTSQIGWVIPEHGAYPGLEALKAKGTKVIVIDPVRTKTVEFFGAD  target    ALPANSWLEFEGLEITASCSNPFLQIWKGGIPPVFDSRDDLDILAGIANALADVTGEKRFRDYFAFAAADKRGIYIQRLL 1dms.1    HVTPK---------------------------------------------------------------------------  target    DTCTTTAGYKLADIMAGKYGPPGGCLLNFRTYPRIPFYEQVHDSEPFHTDTGRMHAYADVPEAIEYGENFIVHREGPEAT 1dms.1    --------------------------------------------------------------------------------  target    PYLPNVIVSSNPHIRPEDYGIAADAEHWDDRTIRNIKMPWSKVKETKNFLWEKGFQFYCLTPKTRHRVHSGWSNVDWHML 1dms.1    --------------------------------------------------------------------------------  target    MDSNFGDPYRLDKRAPCVGEHQLHINPQAARDLNINDGDYVYVDANPADRPYLGAKPDDPFYRVSRCMLRVTYNRAYPYN 1dms.1    --------------------------------------------------------------------------------  target    IVMMKHAPFIATEKSVKAHETRPDGRALSANTGYQANLRYGSQQSVTRNWHMPMHQTDTLFHKSKVFMGFIFGGEADNHA 1dms.1    --------------------------------------------------------------------------------  target    VNTVPKETLVRVTKAEDGGMGGKGIWQPATTGFSPDNESDFMKKYLAGELTKVKT 1dms.1    ------------------------------------------------------- ``` | | | | | | | | | | | | | | | | | | | | | | | | | | | | | | | | | | | | | | | | | | | | | | | | | |
|  | 1tmo.1.A | TRIMETHYLAMINE N-OXIDE REDUCTASE  *TRIMETHYLAMINE N-OXIDE REDUCTASE FROM SHEWANELLA MASSILIA* | 0.00 |  | 14.81 | 0.06 | 343-396 | X-ray | 2.50 | monomer | 2 x 2MD, 1 x 2MO | HHblits | 0.28 |
| ``` target    IMDGKNLVENKLTDSHWFIECMERGAKIVVIAPEYGPPSTKADYWIPIRPQTDAALWLGITRLMIEKKWYDETFVKGFTD 1tmo.1    --------------------------------------------------------------------------------  target    FPLLVRTDTLQRLRAHEVFPQYKTSLSADGPSMKIQGLSAEQHAKLGDFVVWDGKTNAPAAITRDDVGATITKKGIDPVL 1tmo.1    --------------------------------------------------------------------------------  target    AGSFKVKLVDGKEVEVATLWTLYQDHLKDYDLDTVVEITQAPKEMIEQLAQDIATMKPVAIHQGEGINHWFHATEMNRAA 1tmo.1    --------------------------------------------------------------------------------  target    YLPLMLTGNIGRPGAGCQTWAGNYKAALFQGSPWTGPGFKGWVAEDPFDINLNPKAHGKEIHAHAYTKDEEPAYWNHGDL 1tmo.1    --------------------------------------------------------------------------------  target    ALIVDTPKFGRKNFTGKTHMPTPTKALIFNNVNLINNAKW-----------AYGMIKNVNPN-VEMIVSMDIQMTASIEY 1tmo.1    ----------------------HSDTIVLWSNDPYKNLQVGWNAETHESFAYLAQLKEKVKQGKIRVISIDPVVTKTQAY  target    -ADLALPANSWLEFEGLEITASCSNPFLQIWKGGIPPVFDSRDDLDILAGIANALADVTGEKRFRDYFAFAAADKRGIYI 1tmo.1    LGCEQLYVN-----------------------------------------------------------------------  target    QRLLDTCTTTAGYKLADIMAGKYGPPGGCLLNFRTYPRIPFYEQVHDSEPFHTDTGRMHAYADVPEAIEYGENFIVHREG 1tmo.1    --------------------------------------------------------------------------------  target    PEATPYLPNVIVSSNPHIRPEDYGIAADAEHWDDRTIRNIKMPWSKVKETKNFLWEKGFQFYCLTPKTRHRVHSGWSNVD 1tmo.1    --------------------------------------------------------------------------------  target    WHMLMDSNFGDPYRLDKRAPCVGEHQLHINPQAARDLNINDGDYVYVDANPADRPYLGAKPDDPFYRVSRCMLRVTYNRA 1tmo.1    --------------------------------------------------------------------------------  target    YPYNIVMMKHAPFIATEKSVKAHETRPDGRALSANTGYQANLRYGSQQSVTRNWHMPMHQTDTLFHKSKVFMGFIFGGEA 1tmo.1    --------------------------------------------------------------------------------  target    DNHAVNTVPKETLVRVTKAEDGGMGGKGIWQPATTGFSPDNESDFMKKYLAGELTKVKT 1tmo.1    ----------------------------------------------------------- ``` | | | | | | | | | | | | | | | | | | | | | | | | | | | | | | | | | | | | | | | | | | | | | | | | | |
|  | 5e7o.1.A | DMSO reductase family type II enzyme, molybdopterin subunit  *Crystal structure of the perchlorate reductase PcrAB mutant W461E of PcrA from Azospira suillum PS* | 0.01 |  | 16.36 | 0.07 | 343-397 | X-ray | 2.40 | hetero-oligomer | 4 x SF4, 1 x MO, 1 x MGD, 1 x MD1, 1 x F3S | HHblits | 0.26 |
| ``` target    IMDGKNLVENKLTDSHWFIECMERGAKIVVIAPEYGPPSTKADYWIPIRPQTDAALWLGITRLMIEKKWYDETFVKGFTD 5e7o.1    --------------------------------------------------------------------------------  target    FPLLVRTDTLQRLRAHEVFPQYKTSLSADGPSMKIQGLSAEQHAKLGDFVVWDGKTNAPAAITRDDVGATITKKGIDPVL 5e7o.1    --------------------------------------------------------------------------------  target    AGSFKVKLVDGKEVEVATLWTLYQDHLKDYDLDTVVEITQAPKEMIEQLAQDIATMKPVAIHQGEGINHWFHATEMNRAA 5e7o.1    --------------------------------------------------------------------------------  target    YLPLMLTGNIGRPGAGCQTWAGNYKAALFQGSPWTGPGFKGWVAEDPFDINLNPKAHGKEIHAHAYTKDEEPAYWNHGDL 5e7o.1    --------------------------------------------------------------------------------  target    ALIVDTPKFGRKNFTGKTHMPTPTKALIFNNVNLINNAKWAYGMIKNVNPNVEMIVSMDIQMTASIEYADLALPANSWLE 5e7o.1    ----------------------NSKYIILWGSNPTQTRIPDAHFLSEAQLNGAKIVSISPDYNSSTIKVDKWIHPQP---  target    FEGLEITASCSNPFLQIWKGGIPPVFDSRDDLDILAGIANALADVTGEKRFRDYFAFAAADKRGIYIQRLLDTCTTTAGY 5e7o.1    --------------------------------------------------------------------------------  target    KLADIMAGKYGPPGGCLLNFRTYPRIPFYEQVHDSEPFHTDTGRMHAYADVPEAIEYGENFIVHREGPEATPYLPNVIVS 5e7o.1    --------------------------------------------------------------------------------  target    SNPHIRPEDYGIAADAEHWDDRTIRNIKMPWSKVKETKNFLWEKGFQFYCLTPKTRHRVHSGWSNVDWHMLMDSNFGDPY 5e7o.1    --------------------------------------------------------------------------------  target    RLDKRAPCVGEHQLHINPQAARDLNINDGDYVYVDANPADRPYLGAKPDDPFYRVSRCMLRVTYNRAYPYNIVMMKHAPF 5e7o.1    --------------------------------------------------------------------------------  target    IATEKSVKAHETRPDGRALSANTGYQANLRYGSQQSVTRNWHMPMHQTDTLFHKSKVFMGFIFGGEADNHAVNTVPKETL 5e7o.1    --------------------------------------------------------------------------------  target    VRVTKAEDGGMGGKGIWQPATTGFSPDNESDFMKKYLAGELTKVKT 5e7o.1    ---------------------------------------------- ``` | | | | | | | | | | | | | | | | | | | | | | | | | | | | | | | | | | | | | | | | | | | | | | | | | |
|  | 2ivf.1.A | ETHYLBENZENE DEHYDROGENASE ALPHA-SUBUNIT  *ETHYLBENZENE DEHYDROGENASE FROM AROMATOLEUM AROMATICUM* | 0.01 |  | 12.96 | 0.06 | 344-397 | X-ray | 1.88 | hetero-oligomer | 1 x MES, 4 x SF4, 1 x MO, 1 x MGD, 1 x MD1, 1 x F3S, 1 x HEM | HHblits | 0.27 |
| ``` target    IMDGKNLVENKLTDSHWFIECMERGAKIVVIAPEYGPPSTKADYWIPIRPQTDAALWLGITRLMIEKKWYDETFVKGFTD 2ivf.1    --------------------------------------------------------------------------------  target    FPLLVRTDTLQRLRAHEVFPQYKTSLSADGPSMKIQGLSAEQHAKLGDFVVWDGKTNAPAAITRDDVGATITKKGIDPVL 2ivf.1    --------------------------------------------------------------------------------  target    AGSFKVKLVDGKEVEVATLWTLYQDHLKDYDLDTVVEITQAPKEMIEQLAQDIATMKPVAIHQGEGINHWFHATEMNRAA 2ivf.1    --------------------------------------------------------------------------------  target    YLPLMLTGNIGRPGAGCQTWAGNYKAALFQGSPWTGPGFKGWVAEDPFDINLNPKAHGKEIHAHAYTKDEEPAYWNHGDL 2ivf.1    --------------------------------------------------------------------------------  target    ALIVDTPKFGRKNFTGKTHMPTPTKALIFNNVNLINNAKWAYGMIKNVNPNVEMIVSMDIQMTASIEYADLALPANSWLE 2ivf.1    -----------------------AELIFMTCSNWSYTYPSSYHFLSEARYKGAEVVVIAPDFNPTTPAADLHVPVRV---  target    FEGLEITASCSNPFLQIWKGGIPPVFDSRDDLDILAGIANALADVTGEKRFRDYFAFAAADKRGIYIQRLLDTCTTTAGY 2ivf.1    --------------------------------------------------------------------------------  target    KLADIMAGKYGPPGGCLLNFRTYPRIPFYEQVHDSEPFHTDTGRMHAYADVPEAIEYGENFIVHREGPEATPYLPNVIVS 2ivf.1    --------------------------------------------------------------------------------  target    SNPHIRPEDYGIAADAEHWDDRTIRNIKMPWSKVKETKNFLWEKGFQFYCLTPKTRHRVHSGWSNVDWHMLMDSNFGDPY 2ivf.1    --------------------------------------------------------------------------------  target    RLDKRAPCVGEHQLHINPQAARDLNINDGDYVYVDANPADRPYLGAKPDDPFYRVSRCMLRVTYNRAYPYNIVMMKHAPF 2ivf.1    --------------------------------------------------------------------------------  target    IATEKSVKAHETRPDGRALSANTGYQANLRYGSQQSVTRNWHMPMHQTDTLFHKSKVFMGFIFGGEADNHAVNTVPKETL 2ivf.1    --------------------------------------------------------------------------------  target    VRVTKAEDGGMGGKGIWQPATTGFSPDNESDFMKKYLAGELTKVKT 2ivf.1    ---------------------------------------------- ``` | | | | | | | | | | | | | | | | | | | | | | | | | | | | | | | | | | | | | | | | | | | | | | | | | |
|  | 7bkb.1.F | Formate dehydrogenase  *Formate dehydrogenase - heterodisulfide reductase - formylmethanofuran dehydrogenase complex from Methanospirillum hungatei (hexameric, composite structure)* | 0.01 |  | 16.98 | 0.06 | 344-396 | EM | 0.00 | hetero-2-2-2-2-2-2-… | 48 x SF4, 4 x FAD, 2 x FES, 4 x 9S8, 4 x ZN, 2 x MO, 4 x MGD | HHblits | 0.29 |
| ``` target    IMDGKNLVENKLTDSHWFIECMERGAKIVVIAPEYGPPSTKADYWIPIRPQTDAALWLGITRLMIEKKWYDETFVKGFTD 7bkb.1    --------------------------------------------------------------------------------  target    FPLLVRTDTLQRLRAHEVFPQYKTSLSADGPSMKIQGLSAEQHAKLGDFVVWDGKTNAPAAITRDDVGATITKKGIDPVL 7bkb.1    --------------------------------------------------------------------------------  target    AGSFKVKLVDGKEVEVATLWTLYQDHLKDYDLDTVVEITQAPKEMIEQLAQDIATMKPVAIHQGEGINHWFHATEMNRAA 7bkb.1    --------------------------------------------------------------------------------  target    YLPLMLTGNIGRPGAGCQTWAGNYKAALFQGSPWTGPGFKGWVAEDPFDINLNPKAHGKEIHAHAYTKDEEPAYWNHGDL 7bkb.1    --------------------------------------------------------------------------------  target    ALIVDTPKFGRKNFTGKTHMPTPTKALIFNNVNLINNAKWAYGMIKNVNPNVEMIVSMDIQMTASIEYADLALPANSWLE 7bkb.1    -----------------------ADLILIWGSNAVEAHPLAGRRIAQAKKKGIQIIAVDPRYTMTARLADTYVRFN----  target    FEGLEITASCSNPFLQIWKGGIPPVFDSRDDLDILAGIANALADVTGEKRFRDYFAFAAADKRGIYIQRLLDTCTTTAGY 7bkb.1    --------------------------------------------------------------------------------  target    KLADIMAGKYGPPGGCLLNFRTYPRIPFYEQVHDSEPFHTDTGRMHAYADVPEAIEYGENFIVHREGPEATPYLPNVIVS 7bkb.1    --------------------------------------------------------------------------------  target    SNPHIRPEDYGIAADAEHWDDRTIRNIKMPWSKVKETKNFLWEKGFQFYCLTPKTRHRVHSGWSNVDWHMLMDSNFGDPY 7bkb.1    --------------------------------------------------------------------------------  target    RLDKRAPCVGEHQLHINPQAARDLNINDGDYVYVDANPADRPYLGAKPDDPFYRVSRCMLRVTYNRAYPYNIVMMKHAPF 7bkb.1    --------------------------------------------------------------------------------  target    IATEKSVKAHETRPDGRALSANTGYQANLRYGSQQSVTRNWHMPMHQTDTLFHKSKVFMGFIFGGEADNHAVNTVPKETL 7bkb.1    --------------------------------------------------------------------------------  target    VRVTKAEDGGMGGKGIWQPATTGFSPDNESDFMKKYLAGELTKVKT 7bkb.1    ---------------------------------------------- ``` | | | | | | | | | | | | | | | | | | | | | | | | | | | | | | | | | | | | | | | | | | | | | | | | | |
|  | 1eu1.1.A | DIMETHYL SULFOXIDE REDUCTASE  *THE CRYSTAL STRUCTURE OF RHODOBACTER SPHAEROIDES DIMETHYLSULFOXIDE REDUCTASE REVEALS TWO DISTINCT MOLYBDENUM COORDINATION ENVIRONMENTS.* | 0.00 |  | 11.32 | 0.06 | 344-396 | X-ray | 1.30 | monomer | 3 x GLC, 1 x CD, 2 x MGD, 1 x 6MO, 2 x O | HHblits | 0.28 |
| ``` target    IMDGKNLVENKLTDSHWFIECMERGAKIVVIAPEYGPPSTKADYWIPIRPQTDAALWLGITRLMIEKKWYDETFVKGFTD 1eu1.1    --------------------------------------------------------------------------------  target    FPLLVRTDTLQRLRAHEVFPQYKTSLSADGPSMKIQGLSAEQHAKLGDFVVWDGKTNAPAAITRDDVGATITKKGIDPVL 1eu1.1    --------------------------------------------------------------------------------  target    AGSFKVKLVDGKEVEVATLWTLYQDHLKDYDLDTVVEITQAPKEMIEQLAQDIATMKPVAIHQGEGINHWFHATEMNRAA 1eu1.1    --------------------------------------------------------------------------------  target    YLPLMLTGNIGRPGAGCQTWAGNYKAALFQGSPWTGPGFKGWVAEDPFDINLNPKAHGKEIHAHAYTKDEEPAYWNHGDL 1eu1.1    --------------------------------------------------------------------------------  target    ALIVDTPKFGRKNFTGKTHMPTPTKALIFNNVNLINNAKWA--------YGMIKNVNPNVEMIVSMDIQMTASIEYAD-L 1eu1.1    -----------------------TDLMVFWAADPMKTNEIGWVIPDHGAYAGMKALKEKGTRVICINPVRTETADYFGAD  target    ALPANSWLEFEGLEITASCSNPFLQIWKGGIPPVFDSRDDLDILAGIANALADVTGEKRFRDYFAFAAADKRGIYIQRLL 1eu1.1    VVSPR---------------------------------------------------------------------------  target    DTCTTTAGYKLADIMAGKYGPPGGCLLNFRTYPRIPFYEQVHDSEPFHTDTGRMHAYADVPEAIEYGENFIVHREGPEAT 1eu1.1    --------------------------------------------------------------------------------  target    PYLPNVIVSSNPHIRPEDYGIAADAEHWDDRTIRNIKMPWSKVKETKNFLWEKGFQFYCLTPKTRHRVHSGWSNVDWHML 1eu1.1    --------------------------------------------------------------------------------  target    MDSNFGDPYRLDKRAPCVGEHQLHINPQAARDLNINDGDYVYVDANPADRPYLGAKPDDPFYRVSRCMLRVTYNRAYPYN 1eu1.1    --------------------------------------------------------------------------------  target    IVMMKHAPFIATEKSVKAHETRPDGRALSANTGYQANLRYGSQQSVTRNWHMPMHQTDTLFHKSKVFMGFIFGGEADNHA 1eu1.1    --------------------------------------------------------------------------------  target    VNTVPKETLVRVTKAEDGGMGGKGIWQPATTGFSPDNESDFMKKYLAGELTKVKT 1eu1.1    ------------------------------------------------------- ``` | | | | | | | | | | | | | | | | | | | | | | | | | | | | | | | | | | | | | | | | | | | | | | | | | |
|  | 4ydd.1.A | DMSO reductase family type II enzyme, molybdopterin subunit  *Crystal structure of the perchlorate reductase PcrAB from Azospira suillum PS* | 0.01 |  | 16.67 | 0.06 | 343-396 | X-ray | 1.86 | hetero-oligomer | 4 x SF4, 1 x MO, 1 x MGD, 1 x MD1, 1 x F3S | HHblits | 0.26 |
| ``` target    IMDGKNLVENKLTDSHWFIECMERGAKIVVIAPEYGPPSTKADYWIPIRPQTDAALWLGITRLMIEKKWYDETFVKGFTD 4ydd.1    --------------------------------------------------------------------------------  target    FPLLVRTDTLQRLRAHEVFPQYKTSLSADGPSMKIQGLSAEQHAKLGDFVVWDGKTNAPAAITRDDVGATITKKGIDPVL 4ydd.1    --------------------------------------------------------------------------------  target    AGSFKVKLVDGKEVEVATLWTLYQDHLKDYDLDTVVEITQAPKEMIEQLAQDIATMKPVAIHQGEGINHWFHATEMNRAA 4ydd.1    --------------------------------------------------------------------------------  target    YLPLMLTGNIGRPGAGCQTWAGNYKAALFQGSPWTGPGFKGWVAEDPFDINLNPKAHGKEIHAHAYTKDEEPAYWNHGDL 4ydd.1    --------------------------------------------------------------------------------  target    ALIVDTPKFGRKNFTGKTHMPTPTKALIFNNVNLINNAKWAYGMIKNVNPNVEMIVSMDIQMTASIEYADLALPANSWLE 4ydd.1    ----------------------NSKYIILWGSNPTQTRIPDAHFLSEAQLNGAKIVSISPDYNSSTIKVDKWIHPQ----  target    FEGLEITASCSNPFLQIWKGGIPPVFDSRDDLDILAGIANALADVTGEKRFRDYFAFAAADKRGIYIQRLLDTCTTTAGY 4ydd.1    --------------------------------------------------------------------------------  target    KLADIMAGKYGPPGGCLLNFRTYPRIPFYEQVHDSEPFHTDTGRMHAYADVPEAIEYGENFIVHREGPEATPYLPNVIVS 4ydd.1    --------------------------------------------------------------------------------  target    SNPHIRPEDYGIAADAEHWDDRTIRNIKMPWSKVKETKNFLWEKGFQFYCLTPKTRHRVHSGWSNVDWHMLMDSNFGDPY 4ydd.1    --------------------------------------------------------------------------------  target    RLDKRAPCVGEHQLHINPQAARDLNINDGDYVYVDANPADRPYLGAKPDDPFYRVSRCMLRVTYNRAYPYNIVMMKHAPF 4ydd.1    --------------------------------------------------------------------------------  target    IATEKSVKAHETRPDGRALSANTGYQANLRYGSQQSVTRNWHMPMHQTDTLFHKSKVFMGFIFGGEADNHAVNTVPKETL 4ydd.1    --------------------------------------------------------------------------------  target    VRVTKAEDGGMGGKGIWQPATTGFSPDNESDFMKKYLAGELTKVKT 4ydd.1    ---------------------------------------------- ``` | | | | | | | | | | | | | | | | | | | | | | | | | | | | | | | | | | | | | | | | | | | | | | | | | |
|  | 2nya.1.A | Periplasmic nitrate reductase  *Crystal structure of the periplasmic nitrate reductase (NAP) from Escherichia coli* | 0.01 |  | 15.09 | 0.06 | 344-396 | X-ray | 2.50 | monomer | 1 x SF4, 1 x 6MO, 2 x MGD | HHblits | 0.28 |
| ``` target    IMDGKNLVENKLTDSHWFIECMERGAKIVVIAPEYGPPSTKADYWIPIRPQTDAALWLGITRLMIEKKWYDETFVKGFTD 2nya.1    --------------------------------------------------------------------------------  target    FPLLVRTDTLQRLRAHEVFPQYKTSLSADGPSMKIQGLSAEQHAKLGDFVVWDGKTNAPAAITRDDVGATITKKGIDPVL 2nya.1    --------------------------------------------------------------------------------  target    AGSFKVKLVDGKEVEVATLWTLYQDHLKDYDLDTVVEITQAPKEMIEQLAQDIATMKPVAIHQGEGINHWFHATEMNRAA 2nya.1    --------------------------------------------------------------------------------  target    YLPLMLTGNIGRPGAGCQTWAGNYKAALFQGSPWTGPGFKGWVAEDPFDINLNPKAHGKEIHAHAYTKDEEPAYWNHGDL 2nya.1    --------------------------------------------------------------------------------  target    ALIVDTPKFGRKNFTGKTHMPTPTKALIFNNVNLINNAKWAYGMIKNVN--PNVEMIVSMDIQMTASIEYADLALPANSW 2nya.1    -----------------------ADAFVLWGANMAEMHPILWSRITNRRLSNQNVTVAVLSTYQHRSFELADNGIIFT--  target    LEFEGLEITASCSNPFLQIWKGGIPPVFDSRDDLDILAGIANALADVTGEKRFRDYFAFAAADKRGIYIQRLLDTCTTTA 2nya.1    --------------------------------------------------------------------------------  target    GYKLADIMAGKYGPPGGCLLNFRTYPRIPFYEQVHDSEPFHTDTGRMHAYADVPEAIEYGENFIVHREGPEATPYLPNVI 2nya.1    --------------------------------------------------------------------------------  target    VSSNPHIRPEDYGIAADAEHWDDRTIRNIKMPWSKVKETKNFLWEKGFQFYCLTPKTRHRVHSGWSNVDWHMLMDSNFGD 2nya.1    --------------------------------------------------------------------------------  target    PYRLDKRAPCVGEHQLHINPQAARDLNINDGDYVYVDANPADRPYLGAKPDDPFYRVSRCMLRVTYNRAYPYNIVMMKHA 2nya.1    --------------------------------------------------------------------------------  target    PFIATEKSVKAHETRPDGRALSANTGYQANLRYGSQQSVTRNWHMPMHQTDTLFHKSKVFMGFIFGGEADNHAVNTVPKE 2nya.1    --------------------------------------------------------------------------------  target    TLVRVTKAEDGGMGGKGIWQPATTGFSPDNESDFMKKYLAGELTKVKT 2nya.1    ------------------------------------------------ ``` | | | | | | | | | | | | | | | | | | | | | | | | | | | | | | | | | | | | | | | | | | | | | | | | | |
|  | 2vpz.1.A | THIOSULFATE REDUCTASE  *POLYSULFIDE REDUCTASE NATIVE STRUCTURE* | 0.01 |  | 11.11 | 0.06 | 344-397 | X-ray | 2.40 | hetero-oligomer | 10 x SF4, 4 x MGD, 2 x MO | HHblits | 0.26 |
| ``` target    IMDGKNLVENKLTDSHWFIECMERGAKIVVIAPEYGPPSTKADYWIPIRPQTDAALWLGITRLMIEKKWYDETFVKGFTD 2vpz.1    --------------------------------------------------------------------------------  target    FPLLVRTDTLQRLRAHEVFPQYKTSLSADGPSMKIQGLSAEQHAKLGDFVVWDGKTNAPAAITRDDVGATITKKGIDPVL 2vpz.1    --------------------------------------------------------------------------------  target    AGSFKVKLVDGKEVEVATLWTLYQDHLKDYDLDTVVEITQAPKEMIEQLAQDIATMKPVAIHQGEGINHWFHATEMNRAA 2vpz.1    --------------------------------------------------------------------------------  target    YLPLMLTGNIGRPGAGCQTWAGNYKAALFQGSPWTGPGFKGWVAEDPFDINLNPKAHGKEIHAHAYTKDEEPAYWNHGDL 2vpz.1    --------------------------------------------------------------------------------  target    ALIVDTPKFGRKNFTGKTHMPTPTKALIFNNVNLINN-AKWAYGMIKNVNPNVEMIVSMDIQMTASIEYADLALPANSWL 2vpz.1    -----------------------ARYIVLIGHHIGEDTHNTQLQDFALALKNGAKVVVVDPRFSTAAAKAHRWLPIKP--  target    EFEGLEITASCSNPFLQIWKGGIPPVFDSRDDLDILAGIANALADVTGEKRFRDYFAFAAADKRGIYIQRLLDTCTTTAG 2vpz.1    --------------------------------------------------------------------------------  target    YKLADIMAGKYGPPGGCLLNFRTYPRIPFYEQVHDSEPFHTDTGRMHAYADVPEAIEYGENFIVHREGPEATPYLPNVIV 2vpz.1    --------------------------------------------------------------------------------  target    SSNPHIRPEDYGIAADAEHWDDRTIRNIKMPWSKVKETKNFLWEKGFQFYCLTPKTRHRVHSGWSNVDWHMLMDSNFGDP 2vpz.1    --------------------------------------------------------------------------------  target    YRLDKRAPCVGEHQLHINPQAARDLNINDGDYVYVDANPADRPYLGAKPDDPFYRVSRCMLRVTYNRAYPYNIVMMKHAP 2vpz.1    --------------------------------------------------------------------------------  target    FIATEKSVKAHETRPDGRALSANTGYQANLRYGSQQSVTRNWHMPMHQTDTLFHKSKVFMGFIFGGEADNHAVNTVPKET 2vpz.1    --------------------------------------------------------------------------------  target    LVRVTKAEDGGMGGKGIWQPATTGFSPDNESDFMKKYLAGELTKVKT 2vpz.1    ----------------------------------------------- ``` | | | | | | | | | | | | | | | | | | | | | | | | | | | | | | | | | | | | | | | | | | | | | | | | | |
|  | 2vpx.1.D | THIOSULFATE REDUCTASE  *POLYSULFIDE REDUCTASE WITH BOUND QUINONE (UQ1)* | 0.01 |  | 11.11 | 0.06 | 344-397 | X-ray | 3.10 | hetero-oligomer | 10 x SF4, 4 x MGD, 2 x MO, 2 x UQ1 | HHblits | 0.26 |
| ``` target    IMDGKNLVENKLTDSHWFIECMERGAKIVVIAPEYGPPSTKADYWIPIRPQTDAALWLGITRLMIEKKWYDETFVKGFTD 2vpx.1    --------------------------------------------------------------------------------  target    FPLLVRTDTLQRLRAHEVFPQYKTSLSADGPSMKIQGLSAEQHAKLGDFVVWDGKTNAPAAITRDDVGATITKKGIDPVL 2vpx.1    --------------------------------------------------------------------------------  target    AGSFKVKLVDGKEVEVATLWTLYQDHLKDYDLDTVVEITQAPKEMIEQLAQDIATMKPVAIHQGEGINHWFHATEMNRAA 2vpx.1    --------------------------------------------------------------------------------  target    YLPLMLTGNIGRPGAGCQTWAGNYKAALFQGSPWTGPGFKGWVAEDPFDINLNPKAHGKEIHAHAYTKDEEPAYWNHGDL 2vpx.1    --------------------------------------------------------------------------------  target    ALIVDTPKFGRKNFTGKTHMPTPTKALIFNNVNLINN-AKWAYGMIKNVNPNVEMIVSMDIQMTASIEYADLALPANSWL 2vpx.1    -----------------------ARYIVLIGHHIGEDTHNTQLQDFALALKNGAKVVVVDPRFSTAAAKAHRWLPIKP--  target    EFEGLEITASCSNPFLQIWKGGIPPVFDSRDDLDILAGIANALADVTGEKRFRDYFAFAAADKRGIYIQRLLDTCTTTAG 2vpx.1    --------------------------------------------------------------------------------  target    YKLADIMAGKYGPPGGCLLNFRTYPRIPFYEQVHDSEPFHTDTGRMHAYADVPEAIEYGENFIVHREGPEATPYLPNVIV 2vpx.1    --------------------------------------------------------------------------------  target    SSNPHIRPEDYGIAADAEHWDDRTIRNIKMPWSKVKETKNFLWEKGFQFYCLTPKTRHRVHSGWSNVDWHMLMDSNFGDP 2vpx.1    --------------------------------------------------------------------------------  target    YRLDKRAPCVGEHQLHINPQAARDLNINDGDYVYVDANPADRPYLGAKPDDPFYRVSRCMLRVTYNRAYPYNIVMMKHAP 2vpx.1    --------------------------------------------------------------------------------  target    FIATEKSVKAHETRPDGRALSANTGYQANLRYGSQQSVTRNWHMPMHQTDTLFHKSKVFMGFIFGGEADNHAVNTVPKET 2vpx.1    --------------------------------------------------------------------------------  target    LVRVTKAEDGGMGGKGIWQPATTGFSPDNESDFMKKYLAGELTKVKT 2vpx.1    ----------------------------------------------- ``` | | | | | | | | | | | | | | | | | | | | | | | | | | | | | | | | | | | | | | | | | | | | | | | | | |
|  | 2v45.1.A | PERIPLASMIC NITRATE REDUCTASE  *A NEW CATALYTIC MECHANISM OF PERIPLASMIC NITRATE REDUCTASE FROM DESULFOVIBRIO DESULFURICANS ATCC 27774 FROM CRYSTALLOGRAPHIC AND EPR DATA AND BASED ON DETAILED ANALYSIS OF THE SIXTH LIGAND* | 0.01 |  | 12.96 | 0.06 | 344-397 | X-ray | 2.40 | monomer | 1 x SF4, 1 x MO, 2 x MGD, 1 x LCP | HHblits | 0.26 |
| ``` target    IMDGKNLVENKLTDSHWFIECMERGAKIVVIAPEYGPPSTKADYWIPIRPQTDAALWLGITRLMIEKKWYDETFVKGFTD 2v45.1    --------------------------------------------------------------------------------  target    FPLLVRTDTLQRLRAHEVFPQYKTSLSADGPSMKIQGLSAEQHAKLGDFVVWDGKTNAPAAITRDDVGATITKKGIDPVL 2v45.1    --------------------------------------------------------------------------------  target    AGSFKVKLVDGKEVEVATLWTLYQDHLKDYDLDTVVEITQAPKEMIEQLAQDIATMKPVAIHQGEGINHWFHATEMNRAA 2v45.1    --------------------------------------------------------------------------------  target    YLPLMLTGNIGRPGAGCQTWAGNYKAALFQGSPWTGPGFKGWVAEDPFDINLNPKAHGKEIHAHAYTKDEEPAYWNHGDL 2v45.1    --------------------------------------------------------------------------------  target    ALIVDTPKFGRKNFTGKTHMPTPTKALIFNNVNLINNAKWAYGMIKNVN--PNVEMIVSMDIQMTASIEYADLALPANSW 2v45.1    -----------------------ATCFFIIGSNTSEAHPVLFRRIARRKQVEPGVKIIVADPRRTNTSRIADMHVAFRP-  target    LEFEGLEITASCSNPFLQIWKGGIPPVFDSRDDLDILAGIANALADVTGEKRFRDYFAFAAADKRGIYIQRLLDTCTTTA 2v45.1    --------------------------------------------------------------------------------  target    GYKLADIMAGKYGPPGGCLLNFRTYPRIPFYEQVHDSEPFHTDTGRMHAYADVPEAIEYGENFIVHREGPEATPYLPNVI 2v45.1    --------------------------------------------------------------------------------  target    VSSNPHIRPEDYGIAADAEHWDDRTIRNIKMPWSKVKETKNFLWEKGFQFYCLTPKTRHRVHSGWSNVDWHMLMDSNFGD 2v45.1    --------------------------------------------------------------------------------  target    PYRLDKRAPCVGEHQLHINPQAARDLNINDGDYVYVDANPADRPYLGAKPDDPFYRVSRCMLRVTYNRAYPYNIVMMKHA 2v45.1    --------------------------------------------------------------------------------  target    PFIATEKSVKAHETRPDGRALSANTGYQANLRYGSQQSVTRNWHMPMHQTDTLFHKSKVFMGFIFGGEADNHAVNTVPKE 2v45.1    --------------------------------------------------------------------------------  target    TLVRVTKAEDGGMGGKGIWQPATTGFSPDNESDFMKKYLAGELTKVKT 2v45.1    ------------------------------------------------ ``` | | | | | | | | | | | | | | | | | | | | | | | | | | | | | | | | | | | | | | | | | | | | | | | | | |
|  | 1kqf.1.A | FORMATE DEHYDROGENASE, NITRATE-INDUCIBLE, MAJOR SUBUNIT  *FORMATE DEHYDROGENASE N FROM E. COLI* | 0.00 |  | 13.46 | 0.06 | 344-395 | X-ray | 1.60 | hetero-oligomer | 3 x 6MO, 15 x SF4, 6 x MGD, 6 x HEM, 3 x CDL | HHblits | 0.29 |
| ``` target    IMDGKNLVENKLTDSHWFIECMERGAKIVVIAPEYGPPSTKADYWIPIRPQTDAALWLGITRLMIEKKWYDETFVKGFTD 1kqf.1    --------------------------------------------------------------------------------  target    FPLLVRTDTLQRLRAHEVFPQYKTSLSADGPSMKIQGLSAEQHAKLGDFVVWDGKTNAPAAITRDDVGATITKKGIDPVL 1kqf.1    --------------------------------------------------------------------------------  target    AGSFKVKLVDGKEVEVATLWTLYQDHLKDYDLDTVVEITQAPKEMIEQLAQDIATMKPVAIHQGEGINHWFHATEMNRAA 1kqf.1    --------------------------------------------------------------------------------  target    YLPLMLTGNIGRPGAGCQTWAGNYKAALFQGSPWTGPGFKGWVAEDPFDINLNPKAHGKEIHAHAYTKDEEPAYWNHGDL 1kqf.1    --------------------------------------------------------------------------------  target    ALIVDTPKFGRKNFTGKTHMPTPTKALIFNNVNLINNAKWAYGMIKNVN-PNVEMIVSMDIQMTASIEYADLALPANSWL 1kqf.1    -----------------------ANVVMVMGGNAAEAHPVGFRWAMEAKNNNDATLIVVDPRFTRTASVADIYAPI----  target    EFEGLEITASCSNPFLQIWKGGIPPVFDSRDDLDILAGIANALADVTGEKRFRDYFAFAAADKRGIYIQRLLDTCTTTAG 1kqf.1    --------------------------------------------------------------------------------  target    YKLADIMAGKYGPPGGCLLNFRTYPRIPFYEQVHDSEPFHTDTGRMHAYADVPEAIEYGENFIVHREGPEATPYLPNVIV 1kqf.1    --------------------------------------------------------------------------------  target    SSNPHIRPEDYGIAADAEHWDDRTIRNIKMPWSKVKETKNFLWEKGFQFYCLTPKTRHRVHSGWSNVDWHMLMDSNFGDP 1kqf.1    --------------------------------------------------------------------------------  target    YRLDKRAPCVGEHQLHINPQAARDLNINDGDYVYVDANPADRPYLGAKPDDPFYRVSRCMLRVTYNRAYPYNIVMMKHAP 1kqf.1    --------------------------------------------------------------------------------  target    FIATEKSVKAHETRPDGRALSANTGYQANLRYGSQQSVTRNWHMPMHQTDTLFHKSKVFMGFIFGGEADNHAVNTVPKET 1kqf.1    --------------------------------------------------------------------------------  target    LVRVTKAEDGGMGGKGIWQPATTGFSPDNESDFMKKYLAGELTKVKT 1kqf.1    ----------------------------------------------- ``` | | | | | | | | | | | | | | | | | | | | | | | | | | | | | | | | | | | | | | | | | | | | | | | | | |
|  | 3egw.1.A | Respiratory nitrate reductase 1 alpha chain  *The crystal structure of the NarGHI mutant NarH - C16A* | 0.00 | 0.00 | 12.96 | 0.06 | 343-396 | X-ray | 1.90 | monomer | 2 x MD1, 2 x MGD, 2 x 6MO, 6 x SF4, 4 x F3S, 2 x 3PH, 4 x HEM, 2 x AGA | HHblits | 0.25 |
| ``` target    IMDGKNLVENKLTDSHWFIECMERGAKIVVIAPEYGPPSTKADYWIPIRPQTDAALWLGITRLMIEKKWYDETFVKGFTD 3egw.1    --------------------------------------------------------------------------------  target    FPLLVRTDTLQRLRAHEVFPQYKTSLSADGPSMKIQGLSAEQHAKLGDFVVWDGKTNAPAAITRDDVGATITKKGIDPVL 3egw.1    --------------------------------------------------------------------------------  target    AGSFKVKLVDGKEVEVATLWTLYQDHLKDYDLDTVVEITQAPKEMIEQLAQDIATMKPVAIHQGEGINHWFHATEMNRAA 3egw.1    --------------------------------------------------------------------------------  target    YLPLMLTGNIGRPGAGCQTWAGNYKAALFQGSPWTGPGFKGWVAEDPFDINLNPKAHGKEIHAHAYTKDEEPAYWNHGDL 3egw.1    --------------------------------------------------------------------------------  target    ALIVDTPKFGRKNFTGKTHMPTPTKALIFNNVNLINNAKWAYGMIKNVNPNVEMIVSMDIQMTASIEYADLALPANSWLE 3egw.1    ----------------------NSSYIIAWGSNVPQTRTPDAHFFTEVRYKGTKTVAVTPDYAEIAKLCDLWLAPK----  target    FEGLEITASCSNPFLQIWKGGIPPVFDSRDDLDILAGIANALADVTGEKRFRDYFAFAAADKRGIYIQRLLDTCTTTAGY 3egw.1    --------------------------------------------------------------------------------  target    KLADIMAGKYGPPGGCLLNFRTYPRIPFYEQVHDSEPFHTDTGRMHAYADVPEAIEYGENFIVHREGPEATPYLPNVIVS 3egw.1    --------------------------------------------------------------------------------  target    SNPHIRPEDYGIAADAEHWDDRTIRNIKMPWSKVKETKNFLWEKGFQFYCLTPKTRHRVHSGWSNVDWHMLMDSNFGDPY 3egw.1    --------------------------------------------------------------------------------  target    RLDKRAPCVGEHQLHINPQAARDLNINDGDYVYVDANPADRPYLGAKPDDPFYRVSRCMLRVTYNRAYPYNIVMMKHAPF 3egw.1    --------------------------------------------------------------------------------  target    IATEKSVKAHETRPDGRALSANTGYQANLRYGSQQSVTRNWHMPMHQTDTLFHKSKVFMGFIFGGEADNHAVNTVPKETL 3egw.1    --------------------------------------------------------------------------------  target    VRVTKAEDGGMGGKGIWQPATTGFSPDNESDFMKKYLAGELTKVKT 3egw.1    ---------------------------------------------- ``` | | | | | | | | | | | | | | | | | | | | | | | | | | | | | | | | | | | | | | | | | | | | | | | | | |
|  | 1r27.4.A | Respiratory nitrate reductase 1 alpha chain  *Crystal Structure of NarGH complex* | 0.01 | 0.00 | 12.96 | 0.06 | 343-396 | X-ray | 2.00 | monomer | 4 x MO, 16 x SF4, 8 x MGD, 4 x F3S | HHblits | 0.25 |
| ``` target    IMDGKNLVENKLTDSHWFIECMERGAKIVVIAPEYGPPSTKADYWIPIRPQTDAALWLGITRLMIEKKWYDETFVKGFTD 1r27.4    --------------------------------------------------------------------------------  target    FPLLVRTDTLQRLRAHEVFPQYKTSLSADGPSMKIQGLSAEQHAKLGDFVVWDGKTNAPAAITRDDVGATITKKGIDPVL 1r27.4    --------------------------------------------------------------------------------  target    AGSFKVKLVDGKEVEVATLWTLYQDHLKDYDLDTVVEITQAPKEMIEQLAQDIATMKPVAIHQGEGINHWFHATEMNRAA 1r27.4    --------------------------------------------------------------------------------  target    YLPLMLTGNIGRPGAGCQTWAGNYKAALFQGSPWTGPGFKGWVAEDPFDINLNPKAHGKEIHAHAYTKDEEPAYWNHGDL 1r27.4    --------------------------------------------------------------------------------  target    ALIVDTPKFGRKNFTGKTHMPTPTKALIFNNVNLINNAKWAYGMIKNVNPNVEMIVSMDIQMTASIEYADLALPANSWLE 1r27.4    ----------------------NSSYIIAWGSNVPQTRTPDAHFFTEVRYKGTKTVAVTPDYAEIAKLCDLWLAPK----  target    FEGLEITASCSNPFLQIWKGGIPPVFDSRDDLDILAGIANALADVTGEKRFRDYFAFAAADKRGIYIQRLLDTCTTTAGY 1r27.4    --------------------------------------------------------------------------------  target    KLADIMAGKYGPPGGCLLNFRTYPRIPFYEQVHDSEPFHTDTGRMHAYADVPEAIEYGENFIVHREGPEATPYLPNVIVS 1r27.4    --------------------------------------------------------------------------------  target    SNPHIRPEDYGIAADAEHWDDRTIRNIKMPWSKVKETKNFLWEKGFQFYCLTPKTRHRVHSGWSNVDWHMLMDSNFGDPY 1r27.4    --------------------------------------------------------------------------------  target    RLDKRAPCVGEHQLHINPQAARDLNINDGDYVYVDANPADRPYLGAKPDDPFYRVSRCMLRVTYNRAYPYNIVMMKHAPF 1r27.4    --------------------------------------------------------------------------------  target    IATEKSVKAHETRPDGRALSANTGYQANLRYGSQQSVTRNWHMPMHQTDTLFHKSKVFMGFIFGGEADNHAVNTVPKETL 1r27.4    --------------------------------------------------------------------------------  target    VRVTKAEDGGMGGKGIWQPATTGFSPDNESDFMKKYLAGELTKVKT 1r27.4    ---------------------------------------------- ``` | | | | | | | | | | | | | | | | | | | | | | | | | | | | | | | | | | | | | | | | | | | | | | | | | |
|  | 1q16.1.A | Respiratory nitrate reductase 1 alpha chain  *Crystal structure of Nitrate Reductase A, NarGHI, from Escherichia coli* | 0.00 | 0.00 | 12.96 | 0.06 | 343-396 | X-ray | 1.90 | monomer | 2 x MD1, 1 x 6MO, 2 x HEM, 4 x SF4, 1 x F3S, 1 x AGA, 1 x 3PH | HHblits | 0.25 |
| ``` target    IMDGKNLVENKLTDSHWFIECMERGAKIVVIAPEYGPPSTKADYWIPIRPQTDAALWLGITRLMIEKKWYDETFVKGFTD 1q16.1    --------------------------------------------------------------------------------  target    FPLLVRTDTLQRLRAHEVFPQYKTSLSADGPSMKIQGLSAEQHAKLGDFVVWDGKTNAPAAITRDDVGATITKKGIDPVL 1q16.1    --------------------------------------------------------------------------------  target    AGSFKVKLVDGKEVEVATLWTLYQDHLKDYDLDTVVEITQAPKEMIEQLAQDIATMKPVAIHQGEGINHWFHATEMNRAA 1q16.1    --------------------------------------------------------------------------------  target    YLPLMLTGNIGRPGAGCQTWAGNYKAALFQGSPWTGPGFKGWVAEDPFDINLNPKAHGKEIHAHAYTKDEEPAYWNHGDL 1q16.1    --------------------------------------------------------------------------------  target    ALIVDTPKFGRKNFTGKTHMPTPTKALIFNNVNLINNAKWAYGMIKNVNPNVEMIVSMDIQMTASIEYADLALPANSWLE 1q16.1    ----------------------NSSYIIAWGSNVPQTRTPDAHFFTEVRYKGTKTVAVTPDYAEIAKLCDLWLAPK----  target    FEGLEITASCSNPFLQIWKGGIPPVFDSRDDLDILAGIANALADVTGEKRFRDYFAFAAADKRGIYIQRLLDTCTTTAGY 1q16.1    --------------------------------------------------------------------------------  target    KLADIMAGKYGPPGGCLLNFRTYPRIPFYEQVHDSEPFHTDTGRMHAYADVPEAIEYGENFIVHREGPEATPYLPNVIVS 1q16.1    --------------------------------------------------------------------------------  target    SNPHIRPEDYGIAADAEHWDDRTIRNIKMPWSKVKETKNFLWEKGFQFYCLTPKTRHRVHSGWSNVDWHMLMDSNFGDPY 1q16.1    --------------------------------------------------------------------------------  target    RLDKRAPCVGEHQLHINPQAARDLNINDGDYVYVDANPADRPYLGAKPDDPFYRVSRCMLRVTYNRAYPYNIVMMKHAPF 1q16.1    --------------------------------------------------------------------------------  target    IATEKSVKAHETRPDGRALSANTGYQANLRYGSQQSVTRNWHMPMHQTDTLFHKSKVFMGFIFGGEADNHAVNTVPKETL 1q16.1    --------------------------------------------------------------------------------  target    VRVTKAEDGGMGGKGIWQPATTGFSPDNESDFMKKYLAGELTKVKT 1q16.1    ---------------------------------------------- ``` | | | | | | | | | | | | | | | | | | | | | | | | | | | | | | | | | | | | | | | | | | | | | | | | | |
|  | 3ir5.1.A | Respiratory nitrate reductase 1 alpha chain  *Crystal structure of NarGHI mutant NarG-H49C* | 0.01 | 0.00 | 12.96 | 0.06 | 343-396 | X-ray | 2.30 | monomer | 2 x MD1, 1 x 6MO, 4 x SF4, 1 x AGA, 1 x F3S, 2 x HEM | HHblits | 0.25 |
| ``` target    IMDGKNLVENKLTDSHWFIECMERGAKIVVIAPEYGPPSTKADYWIPIRPQTDAALWLGITRLMIEKKWYDETFVKGFTD 3ir5.1    --------------------------------------------------------------------------------  target    FPLLVRTDTLQRLRAHEVFPQYKTSLSADGPSMKIQGLSAEQHAKLGDFVVWDGKTNAPAAITRDDVGATITKKGIDPVL 3ir5.1    --------------------------------------------------------------------------------  target    AGSFKVKLVDGKEVEVATLWTLYQDHLKDYDLDTVVEITQAPKEMIEQLAQDIATMKPVAIHQGEGINHWFHATEMNRAA 3ir5.1    --------------------------------------------------------------------------------  target    YLPLMLTGNIGRPGAGCQTWAGNYKAALFQGSPWTGPGFKGWVAEDPFDINLNPKAHGKEIHAHAYTKDEEPAYWNHGDL 3ir5.1    --------------------------------------------------------------------------------  target    ALIVDTPKFGRKNFTGKTHMPTPTKALIFNNVNLINNAKWAYGMIKNVNPNVEMIVSMDIQMTASIEYADLALPANSWLE 3ir5.1    ----------------------NSSYIIAWGSNVPQTRTPDAHFFTEVRYKGTKTVAVTPDYAEIAKLCDLWLAPK----  target    FEGLEITASCSNPFLQIWKGGIPPVFDSRDDLDILAGIANALADVTGEKRFRDYFAFAAADKRGIYIQRLLDTCTTTAGY 3ir5.1    --------------------------------------------------------------------------------  target    KLADIMAGKYGPPGGCLLNFRTYPRIPFYEQVHDSEPFHTDTGRMHAYADVPEAIEYGENFIVHREGPEATPYLPNVIVS 3ir5.1    --------------------------------------------------------------------------------  target    SNPHIRPEDYGIAADAEHWDDRTIRNIKMPWSKVKETKNFLWEKGFQFYCLTPKTRHRVHSGWSNVDWHMLMDSNFGDPY 3ir5.1    --------------------------------------------------------------------------------  target    RLDKRAPCVGEHQLHINPQAARDLNINDGDYVYVDANPADRPYLGAKPDDPFYRVSRCMLRVTYNRAYPYNIVMMKHAPF 3ir5.1    --------------------------------------------------------------------------------  target    IATEKSVKAHETRPDGRALSANTGYQANLRYGSQQSVTRNWHMPMHQTDTLFHKSKVFMGFIFGGEADNHAVNTVPKETL 3ir5.1    --------------------------------------------------------------------------------  target    VRVTKAEDGGMGGKGIWQPATTGFSPDNESDFMKKYLAGELTKVKT 3ir5.1    ---------------------------------------------- ``` | | | | | | | | | | | | | | | | | | | | | | | | | | | | | | | | | | | | | | | | | | | | | | | | | |
|  | 3ir6.1.A | Respiratory nitrate reductase 1 alpha chain  *Crystal structure of NarGHI mutant NarG-H49S* | 0.01 | 0.00 | 12.96 | 0.06 | 343-396 | X-ray | 2.80 | monomer | 2 x GDP, 1 x AGA, 3 x SF4, 1 x F3S, 2 x HEM | HHblits | 0.25 |
| ``` target    IMDGKNLVENKLTDSHWFIECMERGAKIVVIAPEYGPPSTKADYWIPIRPQTDAALWLGITRLMIEKKWYDETFVKGFTD 3ir6.1    --------------------------------------------------------------------------------  target    FPLLVRTDTLQRLRAHEVFPQYKTSLSADGPSMKIQGLSAEQHAKLGDFVVWDGKTNAPAAITRDDVGATITKKGIDPVL 3ir6.1    --------------------------------------------------------------------------------  target    AGSFKVKLVDGKEVEVATLWTLYQDHLKDYDLDTVVEITQAPKEMIEQLAQDIATMKPVAIHQGEGINHWFHATEMNRAA 3ir6.1    --------------------------------------------------------------------------------  target    YLPLMLTGNIGRPGAGCQTWAGNYKAALFQGSPWTGPGFKGWVAEDPFDINLNPKAHGKEIHAHAYTKDEEPAYWNHGDL 3ir6.1    --------------------------------------------------------------------------------  target    ALIVDTPKFGRKNFTGKTHMPTPTKALIFNNVNLINNAKWAYGMIKNVNPNVEMIVSMDIQMTASIEYADLALPANSWLE 3ir6.1    ----------------------NSSYIIAWGSNVPQTRTPDAHFFTEVRYKGTKTVAVTPDYAEIAKLCDLWLAPK----  target    FEGLEITASCSNPFLQIWKGGIPPVFDSRDDLDILAGIANALADVTGEKRFRDYFAFAAADKRGIYIQRLLDTCTTTAGY 3ir6.1    --------------------------------------------------------------------------------  target    KLADIMAGKYGPPGGCLLNFRTYPRIPFYEQVHDSEPFHTDTGRMHAYADVPEAIEYGENFIVHREGPEATPYLPNVIVS 3ir6.1    --------------------------------------------------------------------------------  target    SNPHIRPEDYGIAADAEHWDDRTIRNIKMPWSKVKETKNFLWEKGFQFYCLTPKTRHRVHSGWSNVDWHMLMDSNFGDPY 3ir6.1    --------------------------------------------------------------------------------  target    RLDKRAPCVGEHQLHINPQAARDLNINDGDYVYVDANPADRPYLGAKPDDPFYRVSRCMLRVTYNRAYPYNIVMMKHAPF 3ir6.1    --------------------------------------------------------------------------------  target    IATEKSVKAHETRPDGRALSANTGYQANLRYGSQQSVTRNWHMPMHQTDTLFHKSKVFMGFIFGGEADNHAVNTVPKETL 3ir6.1    --------------------------------------------------------------------------------  target    VRVTKAEDGGMGGKGIWQPATTGFSPDNESDFMKKYLAGELTKVKT 3ir6.1    ---------------------------------------------- ``` | | | | | | | | | | | | | | | | | | | | | | | | | | | | | | | | | | | | | | | | | | | | | | | | | |
|  | 7q5y.1.A | NADH dehydrogenase I chain G  *Structure of NADH:ubichinon oxidoreductase (complex I) of the hyperthermophilic eubacterium Aquifex aeolicus* | 0.01 |  | 28.57 | 0.06 | 1-51 | X-ray | 2.70 | hetero-1-1-1-1-1-1-… | 8 x SF4, 2 x FES, 1 x FMN | HHblits | 0.32 |
| ``` target    IMDGKNLVENKLTDSHWFIECMERGAKIVVIAPEYGPPSTKADYWIPIRPQTDAALWLGITRLMIEKKWYDETFVKGFTD 7q5y.1    IIFGEDILEFY-ED-KVFEELKEKLEHLVVVSPYEDGLSEYAHIKIPMSLM-----------------------------  target    FPLLVRTDTLQRLRAHEVFPQYKTSLSADGPSMKIQGLSAEQHAKLGDFVVWDGKTNAPAAITRDDVGATITKKGIDPVL 7q5y.1    --------------------------------------------------------------------------------  target    AGSFKVKLVDGKEVEVATLWTLYQDHLKDYDLDTVVEITQAPKEMIEQLAQDIATMKPVAIHQGEGINHWFHATEMNRAA 7q5y.1    --------------------------------------------------------------------------------  target    YLPLMLTGNIGRPGAGCQTWAGNYKAALFQGSPWTGPGFKGWVAEDPFDINLNPKAHGKEIHAHAYTKDEEPAYWNHGDL 7q5y.1    --------------------------------------------------------------------------------  target    ALIVDTPKFGRKNFTGKTHMPTPTKALIFNNVNLINNAKWAYGMIKNVNPNVEMIVSMDIQMTASIEYADLALPANSWLE 7q5y.1    --------------------------------------------------------------------------------  target    FEGLEITASCSNPFLQIWKGGIPPVFDSRDDLDILAGIANALADVTGEKRFRDYFAFAAADKRGIYIQRLLDTCTTTAGY 7q5y.1    --------------------------------------------------------------------------------  target    KLADIMAGKYGPPGGCLLNFRTYPRIPFYEQVHDSEPFHTDTGRMHAYADVPEAIEYGENFIVHREGPEATPYLPNVIVS 7q5y.1    --------------------------------------------------------------------------------  target    SNPHIRPEDYGIAADAEHWDDRTIRNIKMPWSKVKETKNFLWEKGFQFYCLTPKTRHRVHSGWSNVDWHMLMDSNFGDPY 7q5y.1    --------------------------------------------------------------------------------  target    RLDKRAPCVGEHQLHINPQAARDLNINDGDYVYVDANPADRPYLGAKPDDPFYRVSRCMLRVTYNRAYPYNIVMMKHAPF 7q5y.1    --------------------------------------------------------------------------------  target    IATEKSVKAHETRPDGRALSANTGYQANLRYGSQQSVTRNWHMPMHQTDTLFHKSKVFMGFIFGGEADNHAVNTVPKETL 7q5y.1    --------------------------------------------------------------------------------  target    VRVTKAEDGGMGGKGIWQPATTGFSPDNESDFMKKYLAGELTKVKT 7q5y.1    ---------------------------------------------- ``` | | | | | | | | | | | | | | | | | | | | | | | | | | | | | | | | | | | | | | | | | | | | | | | | | |
|  | 2v3v.1.A | PERIPLASMIC NITRATE REDUCTASE  *A NEW CATALYTIC MECHANISM OF PERIPLASMIC NITRATE REDUCTASE FROM DESULFOVIBRIO DESULFURICANS ATCC 27774 FROM CRYSTALLOGRAPHIC AND EPR DATA AND BASED ON DETAILED ANALYSIS OF THE SIXTH LIGAND* | 0.00 |  | 13.21 | 0.06 | 344-396 | X-ray | 1.99 | monomer | 1 x SF4, 1 x MO, 2 x MGD, 4 x LCP | HHblits | 0.26 |
[truncated: 594,233 more chars]
