# Supplementary material for: Metabolic capacity is maintained despite shifts in microbial diversity in estuary sediments
Source: ISME Commun. 2025 Oct 11;5(1):ycaf182. doi: 10.1093/ismeco/ycaf182 (PMC12687941; doi:10.1093/ismeco/ycaf182)
Supplement: Supplementary_Data_1_ycaf182 [file supplementary_data_1_ycaf182.zip › SWISS-MODEL/13_July_SF_Bin2_scaffold_9994_c1_22545_1/report.html]

13\_July\_SF\_Bin2\_scaffold\_9994\_c1\_2-2545\_1 | Report


|  |  |  |
| --- | --- | --- |
|  |  | SWISS-MODEL Homology Modelling Report |

## Model Building Report

This document lists the results for the homology modelling project "13\_July\_SF\_Bin2\_scaffold\_9994\_c1\_2-2545\_1" submitted to SWISS-MODEL workspace
on March 29, 2023, 7:37 p.m..The submitted primary amino acid sequence is given in Table T1.

If you use any results in your research, please cite the relevant publications:

- Waterhouse, A., Bertoni, M., Bienert, S., Studer, G., Tauriello, G., Gumienny, R.,
  Heer, F.T., de Beer, T.A.P., Rempfer, C., Bordoli, L., Lepore, R., Schwede, T.
  SWISS-MODEL: homology modelling of protein structures and complexes.
  Nucleic Acids Res. 46(W1), W296-W303 (2018).
- Bienert, S., Waterhouse, A., de Beer, T.A.P., Tauriello, G., Studer,
  G., Bordoli, L., Schwede, T. The SWISS-MODEL Repository - new features and
  functionality. Nucleic Acids Res. 45, D313-D319 (2017).
- Studer, G., Tauriello, G., Bienert, S.,
  Biasini, M., Johner, N., Schwede, T. ProMod3 - A versatile homology
  modelling toolbox. PLOS Comp. Biol. 17(1), e1008667 (2021).
- Studer, G., Rempfer, C., Waterhouse, A.M.,
  Gumienny, G., Haas, J., Schwede, T. QMEANDisCo - distance constraints
  applied on model quality estimation. Bioinformatics 36, 1765-1771 (2020).
- Bertoni, M., Kiefer, F., Biasini, M., Bordoli, L.,
  Schwede, T. Modeling protein quaternary structure of homo- and
  hetero-oligomers beyond binary interactions by homology. Scientific
  Reports 7 (2017).

## Results

The SWISS-MODEL template library (SMTL version 2023-03-23, PDB release 2023-03-17) was searched with
for evolutionary related structures matching the target sequence in Table T1. For details on the template search, see Materials and Methods. Overall 428 templates were found (Table T2).

## Models

The following models were built (see Materials and Methods "Model Building"):

| Model #01 | File | Built with | Oligo-State | Ligands | GMQE | QMEANDisCo Global |
| --- | --- | --- | --- | --- | --- | --- |
|  | PDB | ProMod3 3.2.1 | monomer | None | 0.86 | 0.83 ± 0.05 |

|  |  |  |
| --- | --- | --- |
|  |  |  |

| Template | Seq Identity | Oligo-state | QSQE | Found by | Method | Resolution | Seq Similarity | Range | Coverage | Description |
| --- | --- | --- | --- | --- | --- | --- | --- | --- | --- | --- |
| 7b04.1.B | 65.28 | monomer | 0.00 | HHblits | X-ray | 2.97Å | 0.51 | 1 - 845 | 1.00 | Nitrite oxidoreductase subunit A |

  

### Excluded ligands

| Ligand Name.Number | Reason for Exclusion | Description |
| --- | --- | --- |
| CA.10 | Binding site not conserved. | CALCIUM ION |
| CA.11 | Binding site not conserved. | CALCIUM ION |
| F3S.4 | Binding site not conserved. | FE3-S4 CLUSTER |
| HEM.9 | Binding site not conserved. | PROTOPORPHYRIN IX CONTAINING FE |
| MD1.5 | Binding site not conserved. | PHOSPHORIC ACID 4-(2-AMINO-4-OXO-3,4,5,6,-TETRAHYDRO-PTERIDIN-6-YL)-2-HYDROXY-3,4-DIMERCAPTO-BUT-3-EN-YL ESTER GUANYLATE ESTER |
| MD1.6 | Binding site not conserved. | PHOSPHORIC ACID 4-(2-AMINO-4-OXO-3,4,5,6,-TETRAHYDRO-PTERIDIN-6-YL)-2-HYDROXY-3,4-DIMERCAPTO-BUT-3-EN-YL ESTER GUANYLATE ESTER |
| MO.7 | Binding site not conserved. | MOLYBDENUM ATOM |
| SF4.1 | Binding site not conserved. | IRON/SULFUR CLUSTER |
| SF4.2 | Binding site not conserved. | IRON/SULFUR CLUSTER |
| SF4.3 | Binding site not conserved. | IRON/SULFUR CLUSTER |
| SF4.8 | Binding site not conserved. | IRON/SULFUR CLUSTER |

  

```
Target    IMDGKNLVENKLTDSHWFIECMERGAKIVVIAPEYGPPSTKADYWIPIRPQTDAALWLGITRLMIEKKWYDETFVKGFTD  
7b04.1.B  IQTGKNLIENKMPEAHWVTEVMERGGKIVVITPEYSPSAQKADYWIPIRNNTDTALFLGITKILIDNKWYDADYVKKFTD  
  
Target    FPLLVRTDTLQRLRAHEVFPQYKTSLSADGPSMKIQGLSAEQHAKLGDFVVWDGKTNAPAAITRDDVGATITKKGIDPVL  
7b04.1.B  FPLLIRTDTLKRVSPKDIIPNYKLQDISDGPSYHIQGLKDEQREIIGDFVVWDAKSKGPKAITRDDVGETLVKKGIDPVL  
  
Target    AGSFKVKLVDGKEVEVATLWTLYQDHLKDYDLDTVVEITQAPKEMIEQLAQDIATMKPVAIHQGEGINHWFHATEMNRAA  
7b04.1.B  EGSFKLKTIDGKEIEVMTLLEMYKIHLRDYDIDSVVSMTNSPKDLIERLAKDIATIKPVAIHYGEGVNHYFHATLMNRSY  
  
Target    YLPLMLTGNIGRPGAGCQTWAGNYKAALFQGSPWTGPGFKGWVAEDPFDINLNPKAHGKEIHAHAYTKDEEPAYWNHGDL  
7b04.1.B  YLPVMLTGNVGYFGSGSHTWAGNYKAGNFQASKWSGPGFYGWVAEDVFKPNLDPYASAKDLNIKGRALDEEVAYWNHSER  
  
Target    ALIVDTPKFGRKNFTGKTHMPTPTKALIFNNVNLINNAKWAYGMIKNVNPNVEMIVSMDIQMTASIEYADLALPANSWLE  
7b04.1.B  PLIVNTPKYGRKVFTGKTHMPSPTKVLWFTNVNLINNAKHVYQMLKNVNPNIEQIMSTDIEITGSIEYADFAFPANSWVE  
  
Target    FEGLEITASCSNPFLQIWK-GGIPPVFDSRDDLDILAGIANALADVTGEKRFRDYFAFAAADKRGIYIQRLLDTCTTTAG  
7b04.1.B  FQEFEITNSCSNPFIQIWGKTGITPVYESKDDVKILAGMASKLGELLRDKRFEDNWKFAIEGRASVYINRLLDGSTTMKG  
  
Target    YKLADIMAGKYGPPGGCLLNFRTYPRIPFYEQVHDSEPFHTDTGRMHAYADVPEAIEYGENFIVHREGPEATPYLPNVIV  
7b04.1.B  YTCEDILNGKYGEPGVAMLLFRTYPRHPFWEQVHESLPFYTPTGRLQAYNDEPEIIEYGENFIVHREGPEATPYLPNAIV  
  
Target    SSNPHIRPEDYGIAADAEHWDDRTIRNIKMPWSKVKETKNFLWEKGFQFYCLTPKTRHRVHSGWSNVDWHMLMDSNFGDP  
7b04.1.B  STNPYIRPDDYGIPENAEYWEDRTVRNIKKSWEETKKTKNFLWEKGYHFYCVTPKSRHTVHSQWAVTDWNFIWNNNFGDP  
  
Target    YRLDKRAPCVGEHQLHINPQAARDLNINDGDYVYVDANPADRPYLGAKPDDPFYRVSRCMLRVTYNRAYPYNIVMMKHAP  
7b04.1.B  YRMDKRMPGVGEHQIHIHPQAARDLGIEDGDYVYVDANPADRPYEGWKPNDSFYKVSRLMLRAKYNPAYPYNCTMMKHSA  
  
Target    FIATEKSVKAHETRPDGRALSANTGYQANLRYGSQQSVTRNWHMPMHQTDTLFHKSKVFMGFIFGGEADNHAVNTVPKET  
7b04.1.B  WISSDKTVQAHETRPDGRALSP-SGYQSSFRYGSQQSITRDWSMPMHQLDSLFHKAKIGMKFIFGFEADNHCINTVPKET  
  
Target    LVRVTKAEDGGMGGKGIWQPATTGFSPDNESDFMKKYLAGELTKVKT  
7b04.1.B  LVKITKAENGGMGGKGVWDPVKTGYTAGNENDFMKKFLNGELIKVD-
```

  


---

  

| Model #02 | File | Built with | Oligo-State | Ligands | GMQE | QMEANDisCo Global |
| --- | --- | --- | --- | --- | --- | --- |
|  | PDB | ProMod3 3.2.1 | monomer (matching prediction) | None | 0.45 | 0.50 ± 0.05 |

|  |  |  |
| --- | --- | --- |
|  |  |  |

| Template | Seq Identity | Oligo-state | QSQE | Found by | Method | Resolution | Seq Similarity | Range | Coverage | Description |
| --- | --- | --- | --- | --- | --- | --- | --- | --- | --- | --- |
| 1r27.4.A | 22.77 | homo-dimer | - | HHblits | X-ray | 2.00Å | 0.32 | 1 - 812 | 0.82 | Respiratory nitrate reductase 1 alpha chain |

  

### Excluded ligands

| Ligand Name.Number | Reason for Exclusion | Description |
| --- | --- | --- |
| F3S.5 | Binding site not conserved. | FE3-S4 CLUSTER |
| F3S.13 | Binding site not conserved. | FE3-S4 CLUSTER |
| F3S.21 | Binding site not conserved. | FE3-S4 CLUSTER |
| F3S.29 | Binding site not conserved. | FE3-S4 CLUSTER |
| MGD.3 | Binding site not conserved. | 2-AMINO-5,6-DIMERCAPTO-7-METHYL-3,7,8A,9-TETRAHYDRO-8-OXA-1,3,9,10-TETRAAZA-ANTHRACEN-4-ONE GUANOSINE DINUCLEOTIDE |
| MGD.4 | Binding site not conserved. | 2-AMINO-5,6-DIMERCAPTO-7-METHYL-3,7,8A,9-TETRAHYDRO-8-OXA-1,3,9,10-TETRAAZA-ANTHRACEN-4-ONE GUANOSINE DINUCLEOTIDE |
| MGD.11 | Binding site not conserved. | 2-AMINO-5,6-DIMERCAPTO-7-METHYL-3,7,8A,9-TETRAHYDRO-8-OXA-1,3,9,10-TETRAAZA-ANTHRACEN-4-ONE GUANOSINE DINUCLEOTIDE |
| MGD.12 | Binding site not conserved. | 2-AMINO-5,6-DIMERCAPTO-7-METHYL-3,7,8A,9-TETRAHYDRO-8-OXA-1,3,9,10-TETRAAZA-ANTHRACEN-4-ONE GUANOSINE DINUCLEOTIDE |
| MGD.19 | Binding site not conserved. | 2-AMINO-5,6-DIMERCAPTO-7-METHYL-3,7,8A,9-TETRAHYDRO-8-OXA-1,3,9,10-TETRAAZA-ANTHRACEN-4-ONE GUANOSINE DINUCLEOTIDE |
| MGD.20 | Binding site not conserved. | 2-AMINO-5,6-DIMERCAPTO-7-METHYL-3,7,8A,9-TETRAHYDRO-8-OXA-1,3,9,10-TETRAAZA-ANTHRACEN-4-ONE GUANOSINE DINUCLEOTIDE |
| MGD.27 | Binding site not conserved. | 2-AMINO-5,6-DIMERCAPTO-7-METHYL-3,7,8A,9-TETRAHYDRO-8-OXA-1,3,9,10-TETRAAZA-ANTHRACEN-4-ONE GUANOSINE DINUCLEOTIDE |
| MGD.28 | Binding site not conserved. | 2-AMINO-5,6-DIMERCAPTO-7-METHYL-3,7,8A,9-TETRAHYDRO-8-OXA-1,3,9,10-TETRAAZA-ANTHRACEN-4-ONE GUANOSINE DINUCLEOTIDE |
| MO.1 | Binding site not conserved. | MOLYBDENUM ATOM |
| MO.9 | Binding site not conserved. | MOLYBDENUM ATOM |
| MO.17 | Binding site not conserved. | MOLYBDENUM ATOM |
| MO.25 | Binding site not conserved. | MOLYBDENUM ATOM |
| SF4.2 | Binding site not conserved. | IRON/SULFUR CLUSTER |
| SF4.6 | Binding site not conserved. | IRON/SULFUR CLUSTER |
| SF4.7 | Binding site not conserved. | IRON/SULFUR CLUSTER |
| SF4.8 | Binding site not conserved. | IRON/SULFUR CLUSTER |
| SF4.10 | Binding site not conserved. | IRON/SULFUR CLUSTER |
| SF4.14 | Binding site not conserved. | IRON/SULFUR CLUSTER |
| SF4.15 | Binding site not conserved. | IRON/SULFUR CLUSTER |
| SF4.16 | Binding site not conserved. | IRON/SULFUR CLUSTER |
| SF4.18 | Binding site not conserved. | IRON/SULFUR CLUSTER |
| SF4.22 | Binding site not conserved. | IRON/SULFUR CLUSTER |
| SF4.23 | Binding site not conserved. | IRON/SULFUR CLUSTER |
| SF4.24 | Binding site not conserved. | IRON/SULFUR CLUSTER |
| SF4.26 | Binding site not conserved. | IRON/SULFUR CLUSTER |
| SF4.30 | Binding site not conserved. | IRON/SULFUR CLUSTER |
| SF4.31 | Binding site not conserved. | IRON/SULFUR CLUSTER |
| SF4.32 | Binding site not conserved. | IRON/SULFUR CLUSTER |

  

```
Target    IMDGKNLVENKLTDSHWFIECMERGAKIVVIAPEYGPPSTKADYWIPIRPQTDAALWLGITRLMIEKKW------YDETF  
1r27.4.A  IAWGSNVPQTRTPDAHFFTEVRYKGTKTVAVTPDYAEIAKLCDLWLAPKQGTDAAMALAMGHVMLREFHLDNPSQYFTDY  
  
Target    VKGFTDFPLLVRTDT-------LQRLRAHEVFPQYKTSLSADGPSMKIQGLSAEQHAKLGDFVVWDGKTNAPAAITRDDV  
1r27.4.A  VRRYTDMPMLVMLEERDGYYAAGRMLRAADLVDALG-----------------QENNPEWKTVAFNT-NGEMVAPNGS-I  
  
Target    GA------TITK------------------KGID--------------------PVLA-------GSFKVKLVDGKEVEV  
1r27.4.A  GFRWGEKGKWNLEQRDGKTGEETELQLSLLGSQDEIAEVGFPYFGGDGTEHFNKVELENVLLHKLPVKRLQLADGSTALV  
  
Target    ATLWTLYQ------------------DHLKDYDLDTVVEITQAPKEMIEQLAQDIATM-----KPVAIHQGEGINHWFHA  
1r27.4.A  TTVYDLTLANYGLERGLNDVNCATSYDDVKAYTPAWAEQITGVSRSQIIRIAREFADNADKTHGRSMIIVGAGLNHWYHL  
  
Target    TEMNRAAYLPLMLTGNIGRPGAGCQTWAGNYKAALFQG-------SPWTGPGF------------KGWVAEDPFDIN-LN  
1r27.4.A  DMNYRGLINMLIFCGCVGQSGGGWAHYVGQEKLRPQTGWQPLAFALDWQRPARHMNSTSYFYNHSSQWRYETVTAEELLS  
  
Target    PKAHGKEIHA-------HAYTKDEEPAYWNHGDLALIV---------DTPKFG-RKNFTGK--------THMPTPTKALI  
1r27.4.A  PMADKSRYTGHLIDFNVRAERMGWLPSAPQLGTNPLTIAGEAEKAGMNPVDYTVKSLKEGSIRFAAEQPENGKNHPRNLF  
  
Target    FNNVNLINNAKWAYG-M------------------------------IKNVNPNVEMIVSMDIQMTASIEYADLALPANS  
1r27.4.A  IWRSNLLGSSGKGHEFMLKYLLGTEHGIQGKDLGQQGGVKPEEVDWQDNGLEGKLDLVVTLDFRLSSTCLYSDIILPTAT  
  
Target    WLEFEGLEITASCSNPFLQIWKGGIPPVFDSRDDLDILAGIANALADVTGEK------------------------RFRD  
1r27.4.A  WYEKDDMNT--SDMHPFIHPLSAAVDPAWEAKSDWEIYKAIAKKFSEVCVGHLGKETDIVTLPIQHDSAAELAQPLDVKD  
  
Target    YFA---------------------------FAAA------------------DKRGIYIQRL------------------  
1r27.4.A  WKKGECDLIPGKTAPHIMVVERDYPATYERFTSIGPLMEKIGNGGKGIAWNTQSEMDLLRKLNYTKAEGPAKGQPMLNTA  
  
Target    -------LDTCTTTAGY----KLADIMAGKY-G-------PPGGCLLNF---------------------RTYPRIPFYE  
1r27.4.A  IDAAEMILTLAPETNGQVAVKAWAALSEFTGRDHTHLALNKEDE-KIRFRDIQAQPRKIISSPTWSGLEDEHVSYNAGYT  
  
Target    QVHDSEPFHTDTGRMHAYADVPEAIEYGENFIVHREGPEATPYLPNVIVSSNPHIRPEDYGIAADAEHWDDRTIRNIKMP  
1r27.4.A  NVHELIPWRTLSGRQQLYQDHQWMRDFGESLLVYRPPIDTRSVK-------------EVIG-------------------  
  
Target    WSKVKETKNFLWEKGFQFYCLTPKTRHRVHSGWSNVDWHMLMDSNFGDPYRLDKRAPCVGEHQLHINPQAARDLNINDGD  
1r27.4.A  ------QK-SNGNQEKALNFLTPHQKWGIHSTYSDNLLMLTLG---------------RGGPVVWLSEADAKDLGIADND  
  
Target    YVYVDANPADRPYLGAKPDDPFYRVSRCMLRVTYNRAYPYNIVMMKHAPFIATEKSVKAHETRPDGRALSANTGYQANLR  
1r27.4.A  WIEVFNSN-----------------GALTARAVVSQRVPAGMTMMYHAQE----RIVNLPG--------SEITQ----QR  
  
Target    YGSQQSVTRNWHMPMHQTDTLFHKSKVFMGFIFGGEADNHAVNTVPKETLVRVTKAEDGGMGGKGIWQPATTGFSPDNES  
1r27.4.A  GGIHNSVTRITPKPTHMIGGYAHLAYG-----F----NYYGTVGSNRDEFVVVRKMKNIDWL------------------  
  
Target    DFMKKYLAGELTKVKT  
1r27.4.A  ----------------
```

  


---

  

| Model #03 | File | Built with | Oligo-State | Ligands | GMQE | QMEANDisCo Global |
| --- | --- | --- | --- | --- | --- | --- |
|  | PDB | ProMod3 3.2.1 | monomer | None | 0.11 | 0.32 ± 0.05 |

|  |  |  |
| --- | --- | --- |
|  |  |  |

| Template | Seq Identity | Oligo-state | QSQE | Found by | Method | Resolution | Seq Similarity | Range | Coverage | Description |
| --- | --- | --- | --- | --- | --- | --- | --- | --- | --- | --- |
| 2fug.2.C | 17.10 | monomer | 0.00 | HHblits | X-ray | 3.30Å | 0.27 | 367 - 722 | 0.23 | NADH-quinone oxidoreductase chain 3 |

  

### Excluded ligands

| Ligand Name.Number | Reason for Exclusion | Description |
| --- | --- | --- |
| FES.2 | Binding site not conserved. | FE2/S2 (INORGANIC) CLUSTER |
| FES.6 | Binding site not conserved. | FE2/S2 (INORGANIC) CLUSTER |
| FMN.10 | Binding site not conserved. | FLAVIN MONONUCLEOTIDE |
| SF4.1 | Binding site not conserved. | IRON/SULFUR CLUSTER |
| SF4.3 | Binding site not conserved. | IRON/SULFUR CLUSTER |
| SF4.4 | Binding site not conserved. | IRON/SULFUR CLUSTER |
| SF4.5 | Binding site not conserved. | IRON/SULFUR CLUSTER |
| SF4.7 | Binding site not conserved. | IRON/SULFUR CLUSTER |
| SF4.8 | Binding site not conserved. | IRON/SULFUR CLUSTER |
| SF4.9 | Binding site not conserved. | IRON/SULFUR CLUSTER |

  

```
Target    IMDGKNLVENKLTDSHWFIECMERGAKIVVIAPEYGPPSTKADYWIPIRPQTDAALWLGITRLMIEKKWYDETFVKGFTD  
2fug.2.C  --------------------------------------------------------------------------------  
  
Target    FPLLVRTDTLQRLRAHEVFPQYKTSLSADGPSMKIQGLSAEQHAKLGDFVVWDGKTNAPAAITRDDVGATITKKGIDPVL  
2fug.2.C  --------------------------------------------------------------------------------  
  
Target    AGSFKVKLVDGKEVEVATLWTLYQDHLKDYDLDTVVEITQAPKEMIEQLAQDIATMKPVAIHQGEGINHWFHATEMNRAA  
2fug.2.C  --------------------------------------------------------------------------------  
  
Target    YLPLMLTGNIGRPGAGCQTWAGNYKAALFQGSPWTGPGFKGWVAEDPFDINLNPKAHGKEIHAHAYTKDEEPAYWNHGDL  
2fug.2.C  --------------------------------------------------------------------------------  
  
Target    ALIVDTPKFGRKNFTGKTHMPTPTKALIFNNVNLINNAKWAYGMIKNVNPNVEMIVSMDIQMTASI-EYADLALPANSWL  
2fug.2.C  ----------------------------------------------EALKGKRFVVMHLSHLHPLAERYAHVVLPAPTFY  
  
Target    EFEGLEITASCSNPFLQIWKGGIPPVFDSRDDLDILAGIANALADVTGEKRFRDYFAFAAADKRGIYIQRLLDTCTTTAG  
2fug.2.C  EKRGHL---VNLEGRVLPLSPAPIENGEAEGALQVLALLAEALGVRPP------F---R-------LHLEA---------  
  
Target    YKLADIMAGKYGPPGGCLLNFRTYPRIPFYEQVHDSEPFHTDTGRMHAYADVPEAIEYGENFIVHREGPEATPYLPNVIV  
2fug.2.C  --QKALK----------------------------ARKVPEAMGRLSFRLKELR---------------------P----  
  
Target    SSNPHIRPEDYGIAADAEHWDDRTIRNIKMPWSKVKETKNFLWEKGFQFYCLTPKTRHRVHSGWSNVDWHMLMDSNFGDP  
2fug.2.C  ------------------------------------------KERKGAFYLRPTMWKA-----HQAVGKAQE--------  
  
Target    YRLDKRAPCVGEHQLHINPQAARDLNINDGDYVYVDANPADRPYLGAKPDDPFYRVSRCMLRVTYNRAYPYNIVMMKHAP  
2fug.2.C  ---------AARAELWAHPETARAEALPEGAQVAVETPF-----------------GRVEARVVHREDVPKGHLYLSALG  
  
Target    FIATEKSVKAHETRPDGRALSANTGYQANLRYGSQQSVTRNWHMPMHQTDTLFHKSKVFMGFIFGGEADNHAVNTVPKET  
2fug.2.C  PAAG----------------------------------------------------------------------------  
  
Target    LVRVTKAEDGGMGGKGIWQPATTGFSPDNESDFMKKYLAGELTKVKT  
2fug.2.C  -----------------------------------------------
```

  


---

  

## Materials and Methods

## Template Search

Template search with
has been performed against the SWISS-MODEL template library (SMTL, last update: 2023-03-23, last included PDB release: 2023-03-17).

## Template Selection

For each identified template, the template's quality has been predicted from features of the target-template alignment.
The templates with the highest quality have then been selected for model building.

## Model Building

Models are built based on the target-template alignment using ProMod3 (Studer et al.). Coordinates which are conserved between the target and the template are copied from the template to the model. Insertions and deletions are remodelled using a fragment library. Side chains are then rebuilt. Finally, the geometry of the resulting model is regularized by using a force field.

## Model Quality Estimation

The global and per-residue model quality has been assessed using the QMEAN scoring function (Studer et al.).

## Ligand Modelling

Ligands present in the template structure are transferred by homology to the model when the following criteria are met: (a) The ligands are annotated as biologically relevant in the template library, (b) the ligand is in contact with the model, (c) the ligand is not clashing with the protein, (d) the residues in contact with the ligand are conserved between the target and the template. If any of these four criteria is not satisfied, a certain ligand will not be included in the model. The model summary includes information on why and which ligand has not been included.

## Oligomeric State Conservation

The quaternary structure annotation of the template is used to model the target sequence in its oligomeric form. The method (Bertoni et al.) is based on a supervised machine learning algorithm, Support Vector Machines (SVM), which combines interface conservation, structural clustering, and other template features to provide a quaternary structure quality estimate (QSQE). The QSQE score is a number between 0 and 1, reflecting the expected accuracy of the interchain contacts for a model built based a given alignment and template. Higher numbers indicate higher reliability. This complements the GMQE score which estimates the accuracy of the tertiary structure of the resulting model.

## References

- **BLAST**  
  Camacho, C., Coulouris, G., Avagyan, V., Ma, N., Papadopoulos, J.,
  Bealer, K., Madden, T.L. BLAST+: architecture and applications. BMC
  Bioinformatics 10, 421-430 (2009).
- **HHblits**  
  Steinegger, M., Meier, M., Mirdita, M., Vöhringer, H., Haunsberger,
  S. J., Söding, J. HH-suite3 for fast remote homology detection and
  deep protein annotation. BMC Bioinformatics 20, 473 (2019).

## Table T1:

Primary amino acid sequence for which templates were searched and models were built.

IMDGKNLVENKLTDSHWFIECMERGAKIVVIAPEYGPPSTKADYWIPIRPQTDAALWLGITRLMIEKKWYDETFVKGFTDFPLLVRTDTLQRLRAHEVFP  
QYKTSLSADGPSMKIQGLSAEQHAKLGDFVVWDGKTNAPAAITRDDVGATITKKGIDPVLAGSFKVKLVDGKEVEVATLWTLYQDHLKDYDLDTVVEITQ  
APKEMIEQLAQDIATMKPVAIHQGEGINHWFHATEMNRAAYLPLMLTGNIGRPGAGCQTWAGNYKAALFQGSPWTGPGFKGWVAEDPFDINLNPKAHGKE  
IHAHAYTKDEEPAYWNHGDLALIVDTPKFGRKNFTGKTHMPTPTKALIFNNVNLINNAKWAYGMIKNVNPNVEMIVSMDIQMTASIEYADLALPANSWLE  
FEGLEITASCSNPFLQIWKGGIPPVFDSRDDLDILAGIANALADVTGEKRFRDYFAFAAADKRGIYIQRLLDTCTTTAGYKLADIMAGKYGPPGGCLLNF  
RTYPRIPFYEQVHDSEPFHTDTGRMHAYADVPEAIEYGENFIVHREGPEATPYLPNVIVSSNPHIRPEDYGIAADAEHWDDRTIRNIKMPWSKVKETKNF  
LWEKGFQFYCLTPKTRHRVHSGWSNVDWHMLMDSNFGDPYRLDKRAPCVGEHQLHINPQAARDLNINDGDYVYVDANPADRPYLGAKPDDPFYRVSRCML  
RVTYNRAYPYNIVMMKHAPFIATEKSVKAHETRPDGRALSANTGYQANLRYGSQQSVTRNWHMPMHQTDTLFHKSKVFMGFIFGGEADNHAVNTVPKETL  
VRVTKAEDGGMGGKGIWQPATTGFSPDNESDFMKKYLAGELTKVKT

## Table T2:

| Template | Seq Identity | Oligo-state | QSQE | Found by | Method | Resolution | Seq Similarity | Coverage | Description |
| --- | --- | --- | --- | --- | --- | --- | --- | --- | --- |
| 7b04.1.B | 65.28 | monomer | - | HHblits | X-ray | 2.97Å | 0.51 | 1.00 | Nitrite oxidoreductase subunit A |
| 7b04.1.B | 65.48 | monomer | - | BLAST | X-ray | 2.97Å | 0.51 | 1.00 | Nitrite oxidoreductase subunit A |
| 7b04.2.B | 65.48 | monomer | - | BLAST | X-ray | 2.97Å | 0.51 | 1.00 | Nitrite oxidoreductase subunit A |
| 7b04.2.B | 65.28 | monomer | - | HHblits | X-ray | 2.97Å | 0.51 | 1.00 | Nitrite oxidoreductase subunit A |
| 1r27.4.A | 22.77 | homo-dimer | - | HHblits | X-ray | 2.00Å | 0.32 | 0.82 | Respiratory nitrate reductase 1 alpha chain |
| 3egw.1.A | 22.35 | homo-dimer | 0.08 | HHblits | X-ray | 1.90Å | 0.32 | 0.81 | Respiratory nitrate reductase 1 alpha chain |
| 3ir5.1.A | 22.25 | monomer | - | HHblits | X-ray | 2.30Å | 0.32 | 0.82 | Respiratory nitrate reductase 1 alpha chain |
| 1q16.1.A | 22.25 | monomer | - | HHblits | X-ray | 1.90Å | 0.32 | 0.82 | Respiratory nitrate reductase 1 alpha chain |
| 3ir7.1.A | 22.66 | monomer | - | HHblits | X-ray | 2.50Å | 0.32 | 0.82 | Respiratory nitrate reductase 1 alpha chain |
| 3ir6.1.A | 22.53 | monomer | - | HHblits | X-ray | 2.80Å | 0.32 | 0.81 | Respiratory nitrate reductase 1 alpha chain |
| 1e60.1.A | 19.42 | monomer | - | HHblits | X-ray | 2.00Å | 0.29 | 0.61 | Dimethyl sulfoxide/trimethylamine N-oxide reductase |
| 1e18.1.A | 19.61 | monomer | - | HHblits | X-ray | 2.00Å | 0.29 | 0.61 | DMSO REDUCTASE. |
| 1e5v.2.A | 19.42 | monomer | - | HHblits | X-ray | 2.40Å | 0.29 | 0.61 | Dimethyl sulfoxide/trimethylamine N-oxide reductase |
| 4dmr.1.A | 18.87 | monomer | - | HHblits | X-ray | 1.90Å | 0.29 | 0.61 | DMSO REDUCTASE |
| 1dms.1.A | 18.13 | monomer | - | HHblits | X-ray | 1.88Å | 0.29 | 0.61 | DMSO REDUCTASE |
| 3ir6.1.A | 28.63 | monomer | - | BLAST | X-ray | 2.80Å | 0.35 | 0.31 | Respiratory nitrate reductase 1 alpha chain |
| 3ir5.1.A | 28.63 | monomer | - | BLAST | X-ray | 2.30Å | 0.35 | 0.31 | Respiratory nitrate reductase 1 alpha chain |
| 1r27.4.A | 28.63 | monomer | - | BLAST | X-ray | 2.00Å | 0.35 | 0.31 | Respiratory nitrate reductase 1 alpha chain |
| 3ir7.1.A | 28.63 | monomer | - | BLAST | X-ray | 2.50Å | 0.35 | 0.31 | Respiratory nitrate reductase 1 alpha chain |
| 1q16.1.A | 28.63 | monomer | - | BLAST | X-ray | 1.90Å | 0.35 | 0.31 | Respiratory nitrate reductase 1 alpha chain |
| 3egw.1.A | 28.63 | monomer | - | BLAST | X-ray | 1.90Å | 0.35 | 0.31 | Respiratory nitrate reductase 1 alpha chain |
| 6ziy.1.C | 17.10 | monomer | - | HHblits | EM | NA | 0.27 | 0.23 | NADH-quinone oxidoreductase subunit 3 |
| 6q8o.1.C | 17.10 | monomer | - | HHblits | X-ray | 3.61Å | 0.27 | 0.23 | NADH-quinone oxidoreductase subunit 3 |
| 6zjl.1.C | 17.10 | monomer | - | HHblits | EM | NA | 0.27 | 0.23 | NADH-quinone oxidoreductase subunit 3 |
| 3m9s.1.C | 17.10 | monomer | - | HHblits | X-ray | 4.50Å | 0.27 | 0.23 | NADH-quinone oxidoreductase subunit 3 |
| 6zjn.1.C | 17.10 | monomer | - | HHblits | EM | NA | 0.27 | 0.23 | NADH-quinone oxidoreductase subunit 3 |
| 6zjy.1.C | 17.10 | monomer | - | HHblits | EM | NA | 0.27 | 0.23 | NADH-quinone oxidoreductase subunit 3 |
| 2fug.2.C | 17.10 | monomer | - | HHblits | X-ray | 3.30Å | 0.27 | 0.23 | NADH-quinone oxidoreductase chain 3 |
| 6zk9.1.C | 14.04 | monomer | - | HHblits | EM | NA | 0.26 | 0.20 | NADH:ubiquinone oxidoreductase core subunit S1 |
| 7zd6.1.4 | 14.62 | monomer | - | HHblits | EM | NA | 0.26 | 0.20 | NADH-ubiquinone oxidoreductase 75 kDa subunit, mitochondrial |
| 5xtb.1.L | 12.64 | monomer | - | HHblits | EM | NA | 0.26 | 0.21 | NADH-ubiquinone oxidoreductase 75 kDa subunit, mitochondrial |
| 7dgr.10.A | 14.04 | monomer | - | HHblits | EM | NA | 0.26 | 0.20 | NADH-ubiquinone oxidoreductase 75 kDa subunit, mitochondrial |
| 5o31.1.8 | 14.04 | monomer | - | HHblits | EM | 4.13Å | 0.26 | 0.20 | NADH-ubiquinone oxidoreductase 75 kDa subunit, mitochondrial |
| 3ir5.1.A | 12.96 | monomer | - | HHblits | X-ray | 2.30Å | 0.25 | 0.06 | Respiratory nitrate reductase 1 alpha chain |
| 6ljm.1.A | 13.64 | monomer | - | HHblits | X-ray | 1.78Å | 0.28 | 0.05 | NAD-dependent protein deacylase sirtuin-5, mitochondrial |
| 3ir6.1.A | 12.96 | monomer | - | HHblits | X-ray | 2.80Å | 0.25 | 0.06 | Respiratory nitrate reductase 1 alpha chain |
| 1r27.4.A | 12.96 | monomer | - | HHblits | X-ray | 2.00Å | 0.25 | 0.06 | Respiratory nitrate reductase 1 alpha chain |
| 3ir7.1.A | 13.21 | monomer | - | HHblits | X-ray | 2.50Å | 0.25 | 0.06 | Respiratory nitrate reductase 1 alpha chain |
| 1q16.1.A | 12.96 | monomer | - | HHblits | X-ray | 1.90Å | 0.25 | 0.06 | Respiratory nitrate reductase 1 alpha chain |
| 3egw.1.A | 12.96 | monomer | - | HHblits | X-ray | 1.90Å | 0.25 | 0.06 | Respiratory nitrate reductase 1 alpha chain |
| 1dms.1.A | 18.37 | monomer | - | HHblits | X-ray | 1.88Å | 0.29 | 0.06 | DMSO REDUCTASE |
| 1e5v.2.A | 20.83 | monomer | - | HHblits | X-ray | 2.40Å | 0.30 | 0.06 | Dimethyl sulfoxide/trimethylamine N-oxide reductase |
| 4dmr.1.A | 20.83 | monomer | - | HHblits | X-ray | 1.90Å | 0.30 | 0.06 | DMSO REDUCTASE |
| 1e60.1.A | 20.83 | monomer | - | HHblits | X-ray | 2.00Å | 0.30 | 0.06 | Dimethyl sulfoxide/trimethylamine N-oxide reductase |
| 1e18.1.A | 20.83 | monomer | - | HHblits | X-ray | 2.00Å | 0.30 | 0.06 | DMSO REDUCTASE. |
| 1e60.1.A | 12.73 | monomer | - | HHblits | X-ray | 2.00Å | 0.29 | 0.07 | Dimethyl sulfoxide/trimethylamine N-oxide reductase |
| 4dmr.1.A | 13.21 | monomer | - | HHblits | X-ray | 1.90Å | 0.30 | 0.06 | DMSO REDUCTASE |
| 1dms.1.A | 13.21 | monomer | - | HHblits | X-ray | 1.88Å | 0.29 | 0.06 | DMSO REDUCTASE |
| 1e18.1.A | 12.73 | monomer | - | HHblits | X-ray | 2.00Å | 0.29 | 0.07 | DMSO REDUCTASE. |
| 1e5v.2.A | 12.73 | monomer | - | HHblits | X-ray | 2.40Å | 0.29 | 0.07 | Dimethyl sulfoxide/trimethylamine N-oxide reductase |

  
The table above shows the top 50 filtered templates. A further 254 templates were found which were considered to be less suitable for modelling than the filtered list.  
1aa6.1.A, 1cz4.1.A, 1cz5.1.A, 1eu1.1.A, 1fdo.1.A, 1g8j.1.A, 1g8k.1.A, 1h0h.1.A, 1ici.1.A, 1j75.1.A, 1jeo.1.A, 1kqf.1.A, 1m2g.1.A, 1m2h.1.A, 1m2j.1.A, 1m2k.1.A, 1m2n.1.A, 1m2n.1.B, 1ogy.1.A, 1pnq.1.A, 1pnq.2.A, 1q16.1.A, 1r27.4.A, 1tmo.1.A, 1wlf.1.A, 1x94.1.A, 1x94.1.B, 1yc5.1.A, 2b4y.1.A, 2b4y.3.A, 2e7z.1.A, 2h2i.1.A, 2h4h.1.A, 2h59.1.B, 2heo.1.A, 2heo.1.B, 2i2w.1.A, 2i2w.2.B, 2iv2.1.A, 2ivf.1.A, 2ki8.1.A, 2kz3.1.A, 2l1p.1.A, 2lnb.1.A, 2nya.1.A, 2nyr.1.A, 2nyr.1.B, 2o3f.1.A, 2pjh.1.B, 2v3v.1.A, 2v45.1.A, 2vpx.1.D, 2vpz.1.A, 2x3y.1.A, 2xvc.1.A, 3egw.1.A, 3eua.1.A, 3fxa.1.A, 3hu2.1.A, 3ir5.1.A, 3ir6.1.A, 3ir7.1.A, 3iwf.1.A, 3jr3.1.A, 3jwp.1.A, 3nzl.1.A, 3o27.1.A, 3o27.1.B, 3o5a.1.A, 3qc8.1.A, 3qq7.1.A, 3qq8.1.A, 3qwz.1.A, 3riy.2.A, 3sho.1.A, 3sho.1.C, 3tiw.1.A, 3tiw.2.A, 3u31.1.A, 3zg6.1.A, 4aay.1.A, 4bv2.3.A, 4g1c.1.A, 4g1c.2.A, 4ga5.1.A, 4ga6.1.A, 4hda.1.A, 4hda.2.A, 4ivn.1.A, 4kdi.1.A, 4kdi.2.A, 4kdl.1.A, 4rv0.1.A, 4s1w.1.A, 4s1w.1.B, 4twi.1.A, 4utn.1.A, 4utn.2.A, 4v4c.1.A, 4ydd.1.A, 5b6c.1.A, 5cuo.1.A, 5cup.1.A, 5e7o.1.A, 5e7p.1.A, 5epp.1.A, 5g4f.1.A, 5g4f.1.B, 5g4f.1.C, 5g4f.1.D, 5g4f.1.E, 5g4f.1.F, 5g4g.1.A, 5glf.1.A, 5glf.2.A, 5glf.3.A, 5glf.4.A, 5gpn.24.A, 5lu5.1.A, 5lu6.1.A, 5lu7.1.A, 5nqd.1.A, 5o31.1.8, 5t5i.1.B, 5t5i.1.D, 5x4l.1.A, 5x4l.2.A, 5xtb.1.L, 6aco.1.A, 6acp.1.A, 6btm.1.B, 6cz7.1.A, 6enx.1.A, 6eo0.1.A, 6eqs.3.A, 6f0k.1.B, 6g72.1.G, 6gcs.1.A, 6hd3.1.A, 6ljk.1.A, 6lod.1.B, 6qc5.1.C, 6qcf.1.C, 6rfq.1.A, 6rfs.1.A, 6rxm.1.A, 6rxm.2.A, 6rxm.3.A, 6rxm.4.A, 6rxm.5.A, 6rxm.6.A, 6rxo.1.A, 6rxo.2.A, 6rxp.2.A, 6rxq.4.A, 6rxs.1.A, 6s6y.1.B, 6sdr.1.A, 6sdv.1.A, 6tg9.1.A, 6x89.1.H, 6yj4.1.G, 6zk9.1.C, 6zr2.1.G, 7a23.1.O, 7ak5.1.G, 7ak6.1.G, 7aqr.1.F, 7ar7.1.G, 7ar8.1.G, 7arc.1.F, 7b04.1.B, 7b04.2.B, 7bkb.1.F, 7bkb.1.J, 7bkb.1.L, 7dbo.1.A, 7dbo.2.A, 7dg7.1.A, 7dg9.1.A, 7dgr.10.A, 7di0.1.A, 7di0.2.A, 7di0.3.A, 7di1.1.A, 7du6.1.A, 7du7.1.A, 7dvc.1.A, 7dvc.5.A, 7dvf.1.A, 7dvh.1.A, 7dvh.2.A, 7dvh.4.A, 7dww.1.A, 7dww.2.A, 7dxr.1.A, 7dxr.1.B, 7dxr.2.B, 7dxs.1.A, 7dxs.1.B, 7dxs.2.A, 7dxs.2.B, 7dxt.1.A, 7dxu.1.A, 7dxu.1.B, 7dxu.2.B, 7dxv.1.A, 7dxv.1.B, 7dxw.1.A, 7dxx.1.A, 7dxx.1.B, 7dxy.1.A, 7dxz.1.A, 7dxz.2.A, 7dxz.2.B, 7dxz.3.A, 7dyc.1.A, 7dyc.2.A, 7dyc.3.A, 7e5z.1.A, 7en5.1.A, 7en6.1.A, 7en6.1.B, 7en6.1.C, 7en6.1.D, 7l5i.1.A, 7l5s.1.A, 7nz1.1.E, 7p61.1.C, 7p63.1.C, 7q5y.1.A, 7qsd.1.G, 7qv7.1.L, 7qv7.1.O, 7t2r.1.A, 7t30.1.A, 7tgh.58.A, 7v2c.1.L, 7vw6.1.A, 7vxu.1.L, 7wbb.1.A, 7wbb.1.B, 7wbb.1.C, 7wbb.1.D, 7wbb.1.E, 7wbb.1.G, 7z0t.1.G, 7zd6.1.4, 7zm7.1.I, 8b9z.1.G, 8ba0.1.G, 8bqg.1.A, 8e73.55.A, 8e9g.1.G

Swiss Institute of Bioinformatics
Contact Us
