# Supplementary material for: Metabolic capacity is maintained despite shifts in microbial diversity in estuary sediments
Source: ISME Commun. 2025 Oct 11;5(1):ycaf182. doi: 10.1093/ismeco/ycaf182 (PMC12687941; doi:10.1093/ismeco/ycaf182)
Supplement: Supplementary_Data_1_ycaf182 [file supplementary_data_1_ycaf182.zip › SWISS-MODEL/13_July_SF_Bin2_scaffold_20759_c1_11143024_1/templates.html]

13\_July\_SF\_Bin2\_scaffold\_20759\_c1\_1114-3024\_1 | Templates


**Export Alignment**
  
FASTA format
Clustal Format
PNG Image

**Secondary Structure**
  
None
DSSP
PSIPRED
SSpro

**Colour Scheme** 


Fade Mismatches
Enhance Mismatches

Confidencegradient
Confidenceclass
Indels
Chain
Unique Chain
Rainbow
2° Structure
Clustal
Hydrophobic
Size
Charged
Polar
Proline
Ser/Thr
Cysteine
Aliphatic
Aromatic
No Colour

Use QMEANBrane values

|  |  |  |  |
| --- | --- | --- | --- |
| Background |  |  |  |

**3D Viewer**  
NGL
PV

FASTA
Multi FASTA
ClustalW
PNG


SWISS-MODEL

### 13\_July\_SF\_Bin2\_scaffold\_20759\_c1\_1114-3024\_1

### Created: March 29, 2023, 7:36 p.m. at 19:36

- Templates
- Models

Models | Name | Description | GMQE | QSQE | Seq Id | Coverage | Range | Method | Resolution | Oligo-state | Ligands | Found by | Seq Similarity || ✓ | 7b04.1.B | Nitrite oxidoreductase subunit A  *Structure of Nitrite oxidoreductase (Nxr) from the anammox bacterium Kuenenia stuttgartiensis.* | 0.73 | 0.00 | 41.23 | 0.97 | 1-624 | X-ray | 2.97 | monomer | 4 x SF4, 1 x F3S, 2 x MD1, 1 x MO, 1 x HEM, 2 x CA | BLAST | 0.40 |
| ``` target    LARDIAKVPGTTLFAIGMGPNQFFNNDNKDRTQFLLAALTGNIGKIAGNIGSYAGNYRVAMFN-------GVPQYIAENP 7b04.1    LAKDIATIKPVAIH-YGEGVNHYFHATLMNRSYYLPVMLTGNVGYFGSGSHTWAGNYKAGNFQASKWSGPGFYGWVAEDV  target    FDIELD---GAKPARPKLYWRAEPAHYYNHEDHPL-----KMGKTMITGKTHMPTPTKSLWFANANSILGNVKWHFNTVV 7b04.1    FKPNLDPYASAKDLNIKGRALDEEVAYWNHSERPLIVNTPKYGRKVFTGKTHMPSPTKVLWFTNVN-LINNAKHVYQMLK  target    NVLPKMEMIAVQEWWWSTSCEWADIVFAVDAWSELKHPDMCSSVTNPFLTVFPRTPLERPFDTRGDIECLDLVGKQLAKR 7b04.1    NVNPNIEQIMSTDIEITGSIEYADFAFPANSWVEFQEFEITNSCSNPFIQIWGKTGITPVYESKDDVKILAGMASKLGEL  target    TGDRRFADMWKFVEEKKVEVYLQRILDHSSNTKGFKFPELEEKAKKGIP--ALMMTRTNPKTVGYEQVYDSRPWYTKTGR 7b04.1    LRDKRFEDNWKFAIEGRASVYINRLLDGSTTMKGYTCEDILN-GKYGEPGVAMLLFRTYPRHPFWEQVHESLPFYTPTGR  target    LEFYREEDEFIEAGENLPVHREPIDSTFYEPNVIVAPAHPFIKAKGPEAYGVKVDDFDNETRQGRNIVKTWEETKKTVHP 7b04.1    LQAYNDEPEIIEYGENFIVHREGPEATPYLPNAIVS-TNPYIR---PDDYGIPENAEYWEDRTVRNIKKSWEETKKTKNF  target    LAKDGYKFVFHTPKYRHGAHTMPVDTDMVAMLFGPFGDIYRHDKRQPFAAEGYVDIHPDDAKALNIEDGDYVWIDSDPSD 7b04.1    LWEKGYHFYCVTPKSRHTVHSQWAVTDWNFIWNNNFGDPYRMDKRMPGVGEHQIHIHPQAARDLGIEDGDYVYVDANPAD  target    RPFRGWQKNDKDYKFSRLLCRARYYPGTPRGITRMWFNMYGATPGSVEGHESRKDGLAKNPRTGYQAMFRSGSHQSATRG 7b04.1    RPYEGWKPNDSFYKVSRLMLRAKYNPAYPYNCTMMKHSAWISSDKTVQAHETRPDGRALSP-SGYQSSFRYGSQQSITRD  target    WLKPTWMTDSLVRKELFGHAVNKGFLPDVHCPTGAPREAIVKITKAEPGGLNAKGLWRPAALGLRPKYENDKMKDYLAGK 7b04.1    WSMPMHQLDSLFHKAKIGMKFIFGFEADNHCINTVPKETLVKITKAENGGMGGKGVWDPVKTGYTAGNENDFMKKFLNGE  target    FTLAANPKKGGKK 7b04.1    L------------ ``` | | | | | | | | | | | | | | | | | | | | | | | | | | | | | | | | | | | | | | | | | | | | | | | | | |
|  | 7b04.2.B | Nitrite oxidoreductase subunit A  *Structure of Nitrite oxidoreductase (Nxr) from the anammox bacterium Kuenenia stuttgartiensis.* | 0.72 | 0.00 | 41.23 | 0.97 | 1-624 | X-ray | 2.97 | monomer | 4 x SF4, 1 x F3S, 2 x MD1, 1 x MO, 1 x HEM, 2 x CA | BLAST | 0.40 |
| ``` target    LARDIAKVPGTTLFAIGMGPNQFFNNDNKDRTQFLLAALTGNIGKIAGNIGSYAGNYRVAMFN-------GVPQYIAENP 7b04.2    LAKDIATIKPVAIH-YGEGVNHYFHATLMNRSYYLPVMLTGNVGYFGSGSHTWAGNYKAGNFQASKWSGPGFYGWVAEDV  target    FDIELD---GAKPARPKLYWRAEPAHYYNHEDHPL-----KMGKTMITGKTHMPTPTKSLWFANANSILGNVKWHFNTVV 7b04.2    FKPNLDPYASAKDLNIKGRALDEEVAYWNHSERPLIVNTPKYGRKVFTGKTHMPSPTKVLWFTNVN-LINNAKHVYQMLK  target    NVLPKMEMIAVQEWWWSTSCEWADIVFAVDAWSELKHPDMCSSVTNPFLTVFPRTPLERPFDTRGDIECLDLVGKQLAKR 7b04.2    NVNPNIEQIMSTDIEITGSIEYADFAFPANSWVEFQEFEITNSCSNPFIQIWGKTGITPVYESKDDVKILAGMASKLGEL  target    TGDRRFADMWKFVEEKKVEVYLQRILDHSSNTKGFKFPELEEKAKKGIP--ALMMTRTNPKTVGYEQVYDSRPWYTKTGR 7b04.2    LRDKRFEDNWKFAIEGRASVYINRLLDGSTTMKGYTCEDILN-GKYGEPGVAMLLFRTYPRHPFWEQVHESLPFYTPTGR  target    LEFYREEDEFIEAGENLPVHREPIDSTFYEPNVIVAPAHPFIKAKGPEAYGVKVDDFDNETRQGRNIVKTWEETKKTVHP 7b04.2    LQAYNDEPEIIEYGENFIVHREGPEATPYLPNAIVS-TNPYIR---PDDYGIPENAEYWEDRTVRNIKKSWEETKKTKNF  target    LAKDGYKFVFHTPKYRHGAHTMPVDTDMVAMLFGPFGDIYRHDKRQPFAAEGYVDIHPDDAKALNIEDGDYVWIDSDPSD 7b04.2    LWEKGYHFYCVTPKSRHTVHSQWAVTDWNFIWNNNFGDPYRMDKRMPGVGEHQIHIHPQAARDLGIEDGDYVYVDANPAD  target    RPFRGWQKNDKDYKFSRLLCRARYYPGTPRGITRMWFNMYGATPGSVEGHESRKDGLAKNPRTGYQAMFRSGSHQSATRG 7b04.2    RPYEGWKPNDSFYKVSRLMLRAKYNPAYPYNCTMMKHSAWISSDKTVQAHETRPDGRALSP-SGYQSSFRYGSQQSITRD  target    WLKPTWMTDSLVRKELFGHAVNKGFLPDVHCPTGAPREAIVKITKAEPGGLNAKGLWRPAALGLRPKYENDKMKDYLAGK 7b04.2    WSMPMHQLDSLFHKAKIGMKFIFGFEADNHCINTVPKETLVKITKAENGGMGGKGVWDPVKTGYTAGNENDFMKKFLNGE  target    FTLAANPKKGGKK 7b04.2    L------------ ``` | | | | | | | | | | | | | | | | | | | | | | | | | | | | | | | | | | | | | | | | | | | | | | | | | |
|  | 7b04.1.B | Nitrite oxidoreductase subunit A  *Structure of Nitrite oxidoreductase (Nxr) from the anammox bacterium Kuenenia stuttgartiensis.* | 0.72 | 0.00 | 39.51 | 0.97 | 1-627 | X-ray | 2.97 | monomer | 4 x SF4, 1 x F3S, 2 x MD1, 1 x MO, 1 x HEM, 2 x CA | HHblits | 0.39 |
| ``` target    LARDIAKVPGTTLFAIGMGPNQFFNNDNKDRTQFLLAALTGNIGKIAGNIGSYAGNYRVAMFNG-------VPQYIAENP 7b04.1    LAKDIATIKP-VAIHYGEGVNHYFHATLMNRSYYLPVMLTGNVGYFGSGSHTWAGNYKAGNFQASKWSGPGFYGWVAEDV  target    FDIELDGAK------PARPKLYWRAEPAHYYNHEDH-----PLKMGKTMITGKTHMPTPTKSLWFANANSILGNVKWHFN 7b04.1    FKPNLDPYASAKDLNIKGRALDE---EVAYWNHSERPLIVNTPKYGRKVFTGKTHMPSPTKVLWFTNVNLINNA--KHVY  target    TVVNV-LPKMEMIAVQEWWWSTSCEWADIVFAVDAWSELKHPDMCSSVTNPFLTVFPRTPLERPFDTRGDIECLDLVGKQ 7b04.1    QMLKNVNPNIEQIMSTDIEITGSIEYADFAFPANSWVEFQEFEITNSCSNPFIQIWGKTGITPVYESKDDVKILAGMASK  target    LAKRTGDRRFADMWKFVEEKKVEVYLQRILDHSSNTKGFKFPELEEKAK--KGIPALMMTRTNPKTVGYEQVYDSRPWYT 7b04.1    LGELLRDKRFEDNWKFAIEGRASVYINRLLDGSTTMKGYTCEDILNGKYGEPGVAMLLF-RTYPRHPFWEQVHESLPFYT  target    KTGRLEFYREEDEFIEAGENLPVHREPIDSTFYEPNVIVAPAHPFIKAKGPEAYGVKVDDFDNETRQGRNIVKTWEETKK 7b04.1    PTGRLQAYNDEPEIIEYGENFIVHREGPEATPYLPNAIVS-TNPY---IRPDDYGIPENAEYWEDRTVRNIKKSWEETKK  target    TVHPLAKDGYKFVFHTPKYRHGAHTMPVDTDMVAMLFGPFGDIYRHDKRQPFAAEGYVDIHPDDAKALNIEDGDYVWIDS 7b04.1    TKNFLWEKGYHFYCVTPKSRHTVHSQWAVTDWNFIWNNNFGDPYRMDKRMPGVGEHQIHIHPQAARDLGIEDGDYVYVDA  target    DPSDRPFRGWQKNDKDYKFSRLLCRARYYPGTPRGITRMWFNMYGATPGSVEGHESRKDGLAKNPRTGYQAMFRSGSHQS 7b04.1    NPADRPYEGWKPNDSFYKVSRLMLRAKYNPAYPYNCTMMKHSAWISSDKTVQAHETRPDGRALSP-SGYQSSFRYGSQQS  target    ATRGWLKPTWMTDSLVRKELFGHAVNKGFLPDVHCPTGAPREAIVKITKAEPGGLNAKGLWRPAALGLRPKYENDKMKDY 7b04.1    ITRDWSMPMHQLDSLFHKAKIGMKFIFGFEADNHCINTVPKETLVKITKAENGGMGGKGVWDPVKTGYTAGNENDFMKKF  target    LAGKFTLAANPKKGGKK 7b04.1    LNGELIKV--------- ``` | | | | | | | | | | | | | | | | | | | | | | | | | | | | | | | | | | | | | | | | | | | | | | | | | |
|  | 7b04.2.B | Nitrite oxidoreductase subunit A  *Structure of Nitrite oxidoreductase (Nxr) from the anammox bacterium Kuenenia stuttgartiensis.* | 0.71 | 0.00 | 39.51 | 0.97 | 1-627 | X-ray | 2.97 | monomer | 4 x SF4, 1 x F3S, 2 x MD1, 1 x MO, 1 x HEM, 2 x CA | HHblits | 0.39 |
| ``` target    LARDIAKVPGTTLFAIGMGPNQFFNNDNKDRTQFLLAALTGNIGKIAGNIGSYAGNYRVAMFNG-------VPQYIAENP 7b04.2    LAKDIATIKP-VAIHYGEGVNHYFHATLMNRSYYLPVMLTGNVGYFGSGSHTWAGNYKAGNFQASKWSGPGFYGWVAEDV  target    FDIELDGAK------PARPKLYWRAEPAHYYNHEDH-----PLKMGKTMITGKTHMPTPTKSLWFANANSILGNVKWHFN 7b04.2    FKPNLDPYASAKDLNIKGRALDE---EVAYWNHSERPLIVNTPKYGRKVFTGKTHMPSPTKVLWFTNVNLINNA--KHVY  target    TVVNV-LPKMEMIAVQEWWWSTSCEWADIVFAVDAWSELKHPDMCSSVTNPFLTVFPRTPLERPFDTRGDIECLDLVGKQ 7b04.2    QMLKNVNPNIEQIMSTDIEITGSIEYADFAFPANSWVEFQEFEITNSCSNPFIQIWGKTGITPVYESKDDVKILAGMASK  target    LAKRTGDRRFADMWKFVEEKKVEVYLQRILDHSSNTKGFKFPELEEKAK--KGIPALMMTRTNPKTVGYEQVYDSRPWYT 7b04.2    LGELLRDKRFEDNWKFAIEGRASVYINRLLDGSTTMKGYTCEDILNGKYGEPGVAMLLF-RTYPRHPFWEQVHESLPFYT  target    KTGRLEFYREEDEFIEAGENLPVHREPIDSTFYEPNVIVAPAHPFIKAKGPEAYGVKVDDFDNETRQGRNIVKTWEETKK 7b04.2    PTGRLQAYNDEPEIIEYGENFIVHREGPEATPYLPNAIVS-TNPY---IRPDDYGIPENAEYWEDRTVRNIKKSWEETKK  target    TVHPLAKDGYKFVFHTPKYRHGAHTMPVDTDMVAMLFGPFGDIYRHDKRQPFAAEGYVDIHPDDAKALNIEDGDYVWIDS 7b04.2    TKNFLWEKGYHFYCVTPKSRHTVHSQWAVTDWNFIWNNNFGDPYRMDKRMPGVGEHQIHIHPQAARDLGIEDGDYVYVDA  target    DPSDRPFRGWQKNDKDYKFSRLLCRARYYPGTPRGITRMWFNMYGATPGSVEGHESRKDGLAKNPRTGYQAMFRSGSHQS 7b04.2    NPADRPYEGWKPNDSFYKVSRLMLRAKYNPAYPYNCTMMKHSAWISSDKTVQAHETRPDGRALSP-SGYQSSFRYGSQQS  target    ATRGWLKPTWMTDSLVRKELFGHAVNKGFLPDVHCPTGAPREAIVKITKAEPGGLNAKGLWRPAALGLRPKYENDKMKDY 7b04.2    ITRDWSMPMHQLDSLFHKAKIGMKFIFGFEADNHCINTVPKETLVKITKAENGGMGGKGVWDPVKTGYTAGNENDFMKKF  target    LAGKFTLAANPKKGGKK 7b04.2    LNGELIKV--------- ``` | | | | | | | | | | | | | | | | | | | | | | | | | | | | | | | | | | | | | | | | | | | | | | | | | |
|  | 3ir5.1.A | Respiratory nitrate reductase 1 alpha chain  *Crystal structure of NarGHI mutant NarG-H49C* | 0.37 | 0.00 | 22.09 | 0.77 | 1-595 | X-ray | 2.30 | monomer | 2 x MD1, 1 x 6MO, 4 x SF4, 1 x AGA, 1 x F3S, 2 x HEM | HHblits | 0.31 |
| ``` target    LARDIAKVP----GTTLFAIGMGPNQFFNNDNKDRTQFLLAALTGNIGKIAGNIGSYAGNYRVAMFNGVPQY--IAE--N 3ir5.1    IAREFADNADKTHGRSMIIVGAGLNHWYHLDMNYRGLINMLIFCGCVGQSGGGWAHYVGQEKLRPQTGWQPLAFALDWQR  target    PF----------------------DIELDGAKPARPKLYWRA--------EPAHYYNH----EDHPL-----------K- 3ir5.1    PARHMNSTSYFYNHSSQWRYETVTAEELLS-PMADKSRYTGHLIDFNVRAERMGWLPSAPQLGTNPLTIAGEAEKAGMNP  target    ---MGKTMITGKT--------HMPTPTKSLWFANANSILGNVKWHFNT-------------------------------- 3ir5.1    VDYTVKSLKEGSIRFAAEQPENGKNHPRNLFIWRSNLLGSSG--KGHEFMLKYLLGTEHGIQGKDLGQQGGVKPEEVDWQ  target    VVNVLPKMEMIAVQEWWWSTSCEWADIVFAVDAWSELKHPDMCSSVTNPFLTVFPRTPLERPFDTRGDIECLDLVGKQLA 3ir5.1    DNGLEGKLDLVVTLDFRLSSTCLYSDIILPTATWYEKD--DMNTSDMHPFIHPLS-AAVDPAWEAKSDWEIYKAIAKKFS  target    KRTG----DR----RF---------------ADMWK----------------------------FVE------------- 3ir5.1    EVCVGHLGKETDIVTLPIQHDSAAELAQPLDVKDWKKGECDLIPGKTAPHIMVVERDYPATYERFTSIGPLMEKIGNGGK  target    -----EKK-------------------------VEVYLQRILDHSSNTKGF----KFPELEEKAKKGIPALM-------- 3ir5.1    GIAWNTQSEMDLLRKLNYTKAEGPAKGQPMLNTAIDAAEMILTLAPETNGQVAVKAWAALSEFTGRDHTHLALNKEDEKI  target    -MT--------------------RTNPKTVGYEQVYDSRPWYTKTGRLEFYREEDEFIEAGENLPVHREPIDSTFYEPNV 3ir5.1    RFRDIQAQPRKIISSPTWSGLEDEHVSYNAGYTNVHELIPWRTLSGRQQLYQDHQWMRDFGESLLVYRPPIDTRSVKE--  target    IVAPAHPFIKAKGPEAYGVKVDDFDNETRQGRNIVKTWEETKKTVHPLAKDGYKFVFHTPKYRHGAHTMPVDTDMVAMLF 3ir5.1    -------------------------------------VIGQK----SNGNQEKALNFLTPHQKWGIHSTYSDNLLMLTL-  target    GPFGDIYRHDKRQPFAAEGYVDIHPDDAKALNIEDGDYVWIDSDPSDRPFRGWQKNDKDYKFSRLLCRARYYPGTPRGIT 3ir5.1    --------------GRGGPVVWLSEADAKDLGIADNDWIEVFNSN-----------------GALTARAVVSQRVPAGMT  target    RMWFNMYGATPGSVEGHESRKDGLAKNPRTGYQAMFRSGSHQSATRGWLKPTWMTDSLVRKELFGHAVNKGFLPDVHCPT 3ir5.1    MMYHAQER----I--------VNLPGSEIT----QQRGGIHNSVTRITPKPTHMIGGY---------AHLAYGFNYYGTV  target    GAPREAIVKITKAEPGGLNAKGLWRPAALGLRPKYENDKMKDYLAGKFTLAANPKKGGKK 3ir5.1    GSNRDEFVVVRKMKNIDWL----------------------------------------- ``` | | | | | | | | | | | | | | | | | | | | | | | | | | | | | | | | | | | | | | | | | | | | | | | | | |
|  | 3ir7.1.A | Respiratory nitrate reductase 1 alpha chain  *Crystal structure of NarGHI mutant NarG-R94S* | 0.36 | 0.00 | 21.88 | 0.77 | 1-595 | X-ray | 2.50 | monomer | 2 x MD1, 4 x SF4, 1 x 6MO, 1 x AGA, 1 x F3S, 2 x HEM | HHblits | 0.31 |
| ``` target    LARDIAKVP----GTTLFAIGMGPNQFFNNDNKDRTQFLLAALTGNIGKIAGNIGSYAGNYRVAMFNGVPQ--YIAE--N 3ir7.1    IAREFADNADKTHGRSMIIVGAGLNHWYHLDMNYRGLINMLIFCGCVGQSGGGWAHYVGQEKLRPQTGWQPLAFALDWQR  target    PFD----------------------IELDGAKPARPKLYWRA--------EPAHYYN----HEDHPL-----------K- 3ir7.1    PARHMNSTSYFYNHSSQWRYETVTAEELLSP-MADKSRYTGHLIDFNVRAERMGWLPSAPQLGTNPLTIAGEAEKAGMNP  target    ---MGKTMITGKT--------HMPTPTKSLWFANANSILGNVKWHFNT-------------------------------- 3ir7.1    VDYTVKSLKEGSIRFAAEQPENGKNHPRNLFIWRSNLLGSSG--KGHEFMLKYLLGTEHGIQGKDLGQQGGVKPEEVDWQ  target    VVNVLPKMEMIAVQEWWWSTSCEWADIVFAVDAWSELKHPDMCSSVTNPFLTVFPRTPLERPFDTRGDIECLDLVGKQLA 3ir7.1    DNGLEGKLDLVVTLDFRLSSTCLYSDIILPTATWYEKD--DMNTSDMHPFIHPLS-AAVDPAWEAKSDWEIYKAIAKKFS  target    KRTG----DRR----F---------------ADMWK----------------------------FVE------------- 3ir7.1    EVCVGHLGKETDIVTLPIQHDSAAELAQPLDVKDWKKGECDLIPGKTAPHIMVVERDYPATYERFTSIGPLMEKIGNGGK  target    -----EKKVEVYL-------------------------QRILDHSSNTKGF----KFPELEEKAKKGIPALMM------- 3ir7.1    GIAWNTQSEMDLLRKLNYTKAEGPAKGQPMLNTAIDAAEMILTLAPETNGQVAVKAWAALSEFTGRDHTHLALNKEDEKI  target    --T--RT------------------NPKTVGYEQVYDSRPWYTKTGRLEFYREEDEFIEAGENLPVHREPIDSTFYEPNV 3ir7.1    RFRDIQAQPRKIISSPTWSGLEDEHVSYNAGYTNVHELIPWRTLSGRQQLYQDHQWMRDFGESLLVYRPPIDTRSVK-E-  target    IVAPAHPFIKAKGPEAYGVKVDDFDNETRQGRNIVKTWEETKKTVHPLAKDGYKFVFHTPKYRHGAHTMPVDTDMVAMLF 3ir7.1    -------------------------------------VIGQ----KSNGNQEKALNFLTPHQKWGIHSTYSDNLLMLTL-  target    GPFGDIYRHDKRQPFAAEGYVDIHPDDAKALNIEDGDYVWIDSDPSDRPFRGWQKNDKDYKFSRLLCRARYYPGTPRGIT 3ir7.1    --------------GRGGPVVWLSEADAKDLGIADNDWIEVFNSN-----------------GALTARAVVSQRVPAGMT  target    RMWFNMYGATPGSVEGHESRKDGLAKNPRTGYQAMFRSGSHQSATRGWLKPTWMTDSLVRKELFGHAVNKGFLPDVHCPT 3ir7.1    MMYHAQER----I--------VNLPGSEI-T---QQRGGIHNSVTRITPKPTHMIGGY---------AHLAYGFNYYGTV  target    GAPREAIVKITKAEPGGLNAKGLWRPAALGLRPKYENDKMKDYLAGKFTLAANPKKGGKK 3ir7.1    GSNRDEFVVVRKMKNIDWL----------------------------------------- ``` | | | | | | | | | | | | | | | | | | | | | | | | | | | | | | | | | | | | | | | | | | | | | | | | | |
|  | 1q16.1.A | Respiratory nitrate reductase 1 alpha chain  *Crystal structure of Nitrate Reductase A, NarGHI, from Escherichia coli* | 0.37 | 0.00 | 21.88 | 0.77 | 1-595 | X-ray | 1.90 | monomer | 2 x MD1, 1 x 6MO, 2 x HEM, 4 x SF4, 1 x F3S, 1 x AGA, 1 x 3PH | HHblits | 0.31 |
| ``` target    LARDIAKVP----GTTLFAIGMGPNQFFNNDNKDRTQFLLAALTGNIGKIAGNIGSYAGNYRVAMFNGVPQY--IAE--N 1q16.1    IAREFADNADKTHGRSMIIVGAGLNHWYHLDMNYRGLINMLIFCGCVGQSGGGWAHYVGQEKLRPQTGWQPLAFALDWQR  target    PF----------------------DIELDGAKPARPKLYWRA--------EPAHYYNH----EDHPL-----------K- 1q16.1    PARHMNSTSYFYNHSSQWRYETVTAEELLSP-MADKSRYTGHLIDFNVRAERMGWLPSAPQLGTNPLTIAGEAEKAGMNP  target    ---MGKTMITGKT--------HMPTPTKSLWFANANSILGNVKWHFNT-------------------------------- 1q16.1    VDYTVKSLKEGSIRFAAEQPENGKNHPRNLFIWRSNLLGSSG--KGHEFMLKYLLGTEHGIQGKDLGQQGGVKPEEVDWQ  target    VVNVLPKMEMIAVQEWWWSTSCEWADIVFAVDAWSELKHPDMCSSVTNPFLTVFPRTPLERPFDTRGDIECLDLVGKQLA 1q16.1    DNGLEGKLDLVVTLDFRLSSTCLYSDIILPTATWYEKD--DMNTSDMHPFIHPLS-AAVDPAWEAKSDWEIYKAIAKKFS  target    KRTG----DRR----F---------------ADMWK----------------------------FVE------------- 1q16.1    EVCVGHLGKETDIVTLPIQHDSAAELAQPLDVKDWKKGECDLIPGKTAPHIMVVERDYPATYERFTSIGPLMEKIGNGGK  target    -----EKKV-------------------------EVYLQRILDHSSNTKGF----KFPELEEKAKKGIPALMM------- 1q16.1    GIAWNTQSEMDLLRKLNYTKAEGPAKGQPMLNTAIDAAEMILTLAPETNGQVAVKAWAALSEFTGRDHTHLALNKEDEKI  target    --T--RT------------------NPKTVGYEQVYDSRPWYTKTGRLEFYREEDEFIEAGENLPVHREPIDSTFYEPNV 1q16.1    RFRDIQAQPRKIISSPTWSGLEDEHVSYNAGYTNVHELIPWRTLSGRQQLYQDHQWMRDFGESLLVYRPPIDTRSVK---  target    IVAPAHPFIKAKGPEAYGVKVDDFDNETRQGRNIVKTWEETKKTVHPLAKDGYKFVFHTPKYRHGAHTMPVDTDMVAMLF 1q16.1    ------E------------------------------VIGQ----KSNGNQEKALNFLTPHQKWGIHSTYSDNLLMLTL-  target    GPFGDIYRHDKRQPFAAEGYVDIHPDDAKALNIEDGDYVWIDSDPSDRPFRGWQKNDKDYKFSRLLCRARYYPGTPRGIT 1q16.1    --------------GRGGPVVWLSEADAKDLGIADNDWIEVFNSN-----------------GALTARAVVSQRVPAGMT  target    RMWFNMYGATPGSVEGHESRKDGLAKNPRTGYQAMFRSGSHQSATRGWLKPTWMTDSLVRKELFGHAVNKGFLPDVHCPT 1q16.1    MMYHAQERI------------VNLPGSEIT----QQRGGIHNSVTRITPKPTHMIGGY---------AHLAYGFNYYGTV  target    GAPREAIVKITKAEPGGLNAKGLWRPAALGLRPKYENDKMKDYLAGKFTLAANPKKGGKK 1q16.1    GSNRDEFVVVRKMKNIDWL----------------------------------------- ``` | | | | | | | | | | | | | | | | | | | | | | | | | | | | | | | | | | | | | | | | | | | | | | | | | |
| ✓ | 1r27.4.A | Respiratory nitrate reductase 1 alpha chain  *Crystal Structure of NarGH complex* | 0.37 |  | 21.88 | 0.77 | 1-595 | X-ray | 2.00 | homo-dimer | 4 x MO, 16 x SF4, 8 x MGD, 4 x F3S | HHblits | 0.31 |
| ``` target    LARDIAKVP----GTTLFAIGMGPNQFFNNDNKDRTQFLLAALTGNIGKIAGNIGSYAGNYRVAMFNGVPQY--IAE--N 1r27.4    IAREFADNADKTHGRSMIIVGAGLNHWYHLDMNYRGLINMLIFCGCVGQSGGGWAHYVGQEKLRPQTGWQPLAFALDWQR  target    PF----------------------DIELDGAKPARPKLYWRA--------EPAHYYN----HEDHPL-----------K- 1r27.4    PARHMNSTSYFYNHSSQWRYETVTAEELLSP-MADKSRYTGHLIDFNVRAERMGWLPSAPQLGTNPLTIAGEAEKAGMNP  target    ---MGKTMITGKT--------HMPTPTKSLWFANANSILGNVKWHFNT-------------------------------- 1r27.4    VDYTVKSLKEGSIRFAAEQPENGKNHPRNLFIWRSNLLGSSG--KGHEFMLKYLLGTEHGIQGKDLGQQGGVKPEEVDWQ  target    VVNVLPKMEMIAVQEWWWSTSCEWADIVFAVDAWSELKHPDMCSSVTNPFLTVFPRTPLERPFDTRGDIECLDLVGKQLA 1r27.4    DNGLEGKLDLVVTLDFRLSSTCLYSDIILPTATWYEKD--DMNTSDMHPFIHPLS-AAVDPAWEAKSDWEIYKAIAKKFS  target    KRTG----DRR----F---------------ADMWK----------------------------FVE------------- 1r27.4    EVCVGHLGKETDIVTLPIQHDSAAELAQPLDVKDWKKGECDLIPGKTAPHIMVVERDYPATYERFTSIGPLMEKIGNGGK  target    -----EKKVEVY-------------------------LQRILDHSSNTKGF----KFPELEEKAKKGIPALMMT------ 1r27.4    GIAWNTQSEMDLLRKLNYTKAEGPAKGQPMLNTAIDAAEMILTLAPETNGQVAVKAWAALSEFTGRDHTHLALNKEDEKI  target    -----R------------------TNPKTVGYEQVYDSRPWYTKTGRLEFYREEDEFIEAGENLPVHREPIDSTFYEPNV 1r27.4    RFRDIQAQPRKIISSPTWSGLEDEHVSYNAGYTNVHELIPWRTLSGRQQLYQDHQWMRDFGESLLVYRPPIDTRSVKE--  target    IVAPAHPFIKAKGPEAYGVKVDDFDNETRQGRNIVKTWEETKKTVHPLAKDGYKFVFHTPKYRHGAHTMPVDTDMVAMLF 1r27.4    -------------------------------------VIGQ----KSNGNQEKALNFLTPHQKWGIHSTYSDNLLMLTL-  target    GPFGDIYRHDKRQPFAAEGYVDIHPDDAKALNIEDGDYVWIDSDPSDRPFRGWQKNDKDYKFSRLLCRARYYPGTPRGIT 1r27.4    --------------GRGGPVVWLSEADAKDLGIADNDWIEVFNSN-----------------GALTARAVVSQRVPAGMT  target    RMWFNMYGATPGSVEGHESRKDGLAKNPRTGYQAMFRSGSHQSATRGWLKPTWMTDSLVRKELFGHAVNKGFLPDVHCPT 1r27.4    MMYHAQERI------------VNLPGSEIT----QQRGGIHNSVTRITPKPTHMIGGY---------AHLAYGFNYYGTV  target    GAPREAIVKITKAEPGGLNAKGLWRPAALGLRPKYENDKMKDYLAGKFTLAANPKKGGKK 1r27.4    GSNRDEFVVVRKMKNIDWL----------------------------------------- ``` | | | | | | | | | | | | | | | | | | | | | | | | | | | | | | | | | | | | | | | | | | | | | | | | | |
|  | 3egw.1.A | Respiratory nitrate reductase 1 alpha chain  *The crystal structure of the NarGHI mutant NarH - C16A* | 0.37 | 0.10 | 22.22 | 0.76 | 1-592 | X-ray | 1.90 | homo-dimer | 2 x MD1, 2 x MGD, 2 x 6MO, 6 x SF4, 4 x F3S, 2 x 3PH, 4 x HEM, 2 x AGA | HHblits | 0.31 |
| ``` target    LARDIAKVP----GTTLFAIGMGPNQFFNNDNKDRTQFLLAALTGNIGKIAGNIGSYAGNYRVAMFNGVPQY--IAE--N 3egw.1    IAREFADNADKTHGRSMIIVGAGLNHWYHLDMNYRGLINMLIFCGCVGQSGGGWAHYVGQEKLRPQTGWQPLAFALDWQR  target    PFD----------------------IELDGAKPARPKLYWRA--------EPAHYYNH----EDHPL------------- 3egw.1    PARHMNSTSYFYNHSSQWRYETVTAEELLSP-MADKSRYTGHLIDFNVRAERMGWLPSAPQLGTNPLTIAGEAEKAGMNP  target    -K-MGKTMITGKT--------HMPTPTKSLWFANANSILGNVKWHFNT-------------------------------- 3egw.1    VDYTVKSLKEGSIRFAAEQPENGKNHPRNLFIWRSNLLGSSG--KGHEFMLKYLLGTEHGIQGKDLGQQGGVKPEEVDWQ  target    VVNVLPKMEMIAVQEWWWSTSCEWADIVFAVDAWSELKHPDMCSSVTNPFLTVFPRTPLERPFDTRGDIECLDLVGKQLA 3egw.1    DNGLEGKLDLVVTLDFRLSSTCLYSDIILPTATWYEKD--DMNTSDMHPFIHPLS-AAVDPAWEAKSDWEIYKAIAKKFS  target    KRTG----DR----RF---------------ADMWK----------------------------FVE------------- 3egw.1    EVCVGHLGKETDIVTLPIQHDSAAELAQPLDVKDWKKGECDLIPGKTAPHIMVVERDYPATYERFTSIGPLMEKIGNGGK  target    -----EKKVEVYLQR-------------------------ILDHSSNTKGF----KFPELEEKAKKGIPALMMT------ 3egw.1    GIAWNTQSEMDLLRKLNYTKAEGPAKGQPMLNTAIDAAEMILTLAPETNGQVAVKAWAALSEFTGRDHTHLALNKEDEKI  target    -----RT------------------NPKTVGYEQVYDSRPWYTKTGRLEFYREEDEFIEAGENLPVHREPIDSTFYEPNV 3egw.1    RFRDIQAQPRKIISSPTWSGLEDEHVSYNAGYTNVHELIPWRTLSGRQQLYQDHQWMRDFGESLLVYRPPIDTRSVKE--  target    IVAPAHPFIKAKGPEAYGVKVDDFDNETRQGRNIVKTWEETKKTVHPLAKDGYKFVFHTPKYRHGAHTMPVDTDMVAMLF 3egw.1    -------------------------------------VIGQ----KSNGNQEKALNFLTPHQKWGIHSTYSDNLLMLTL-  target    GPFGDIYRHDKRQPFAAEGYVDIHPDDAKALNIEDGDYVWIDSDPSDRPFRGWQKNDKDYKFSRLLCRARYYPGTPRGIT 3egw.1    --------------GRGGPVVWLSEADAKDLGIADNDWIEVFNSN-----------------GALTARAVVSQRVPAGMT  target    RMWFNMYGATPGSVEGHESRKDGLAKNPRTGYQAMFRSGSHQSATRGWLKPTWMTDSLVRKELFGHAVNKGFLPDVHCPT 3egw.1    MMYHAQERI------------VNLPGSEIT----QQRGGIHNSVTRITPKPTHMIGGY---------AHLAYGFNYYGTV  target    GAPREAIVKITKAEPGGLNAKGLWRPAALGLRPKYENDKMKDYLAGKFTLAANPKKGGKK 3egw.1    GSNRDEFVVVRKMKNI-------------------------------------------- ``` | | | | | | | | | | | | | | | | | | | | | | | | | | | | | | | | | | | | | | | | | | | | | | | | | |
|  | 3ir6.1.A | Respiratory nitrate reductase 1 alpha chain  *Crystal structure of NarGHI mutant NarG-H49S* | 0.36 | 0.00 | 22.06 | 0.76 | 1-591 | X-ray | 2.80 | monomer | 2 x GDP, 1 x AGA, 3 x SF4, 1 x F3S, 2 x HEM | HHblits | 0.31 |
| ``` target    LARDIAKVP----GTTLFAIGMGPNQFFNNDNKDRTQFLLAALTGNIGKIAGNIGSYAGNYRVAMFNGVPQY--IAE--N 3ir6.1    IAREFADNADKTHGRSMIIVGAGLNHWYHLDMNYRGLINMLIFCGCVGQSGGGWAHYVGQEKLRPQTGWQPLAFALDWQR  target    PF----------------------DIELDGAKPARPKLYWRA--------EPAHYYNH----EDHPL-----------K- 3ir6.1    PARHMNSTSYFYNHSSQWRYETVTAEELLSP-MADKSRYTGHLIDFNVRAERMGWLPSAPQLGTNPLTIAGEAEKAGMNP  target    ---MGKTMITGKT--------HMPTPTKSLWFANANSILGNVKWHFNT-------------------------------- 3ir6.1    VDYTVKSLKEGSIRFAAEQPENGKNHPRNLFIWRSNLLGSSG--KGHEFMLKYLLGTEHGIQGKDLGQQGGVKPEEVDWQ  target    VVNVLPKMEMIAVQEWWWSTSCEWADIVFAVDAWSELKHPDMCSSVTNPFLTVFPRTPLERPFDTRGDIECLDLVGKQLA 3ir6.1    DNGLEGKLDLVVTLDFRLSSTCLYSDIILPTATWYEKD--DMNTSDMHPFIHPLS-AAVDPAWEAKSDWEIYKAIAKKFS  target    KRTG----DR----RF---------------ADMWK----------------------------FVE------------- 3ir6.1    EVCVGHLGKETDIVTLPIQHDSAAELAQPLDVKDWKKGECDLIPGKTAPHIMVVERDYPATYERFTSIGPLMEKIGNGGK  target    -----EKKVEVYLQ-------------------------RILDHSSNTKGF----KFPELEEKAKKGIPALMM------- 3ir6.1    GIAWNTQSEMDLLRKLNYTKAEGPAKGQPMLNTAIDAAEMILTLAPETNGQVAVKAWAALSEFTGRDHTHLALNKEDEKI  target    --T--------------------RTNPKTVGYEQVYDSRPWYTKTGRLEFYREEDEFIEAGENLPVHREPIDSTFYEPNV 3ir6.1    RFRDIQAQPRKIISSPTWSGLEDEHVSYNAGYTNVHELIPWRTLSGRQQLYQDHQWMRDFGESLLVYRPPIDTRSVKE--  target    IVAPAHPFIKAKGPEAYGVKVDDFDNETRQGRNIVKTWEETKKTVHPLAKDGYKFVFHTPKYRHGAHTMPVDTDMVAMLF 3ir6.1    -------------------------------------VIGQ----KSNGNQEKALNFLTPHQKWGIHSTYSDNLLMLTL-  target    GPFGDIYRHDKRQPFAAEGYVDIHPDDAKALNIEDGDYVWIDSDPSDRPFRGWQKNDKDYKFSRLLCRARYYPGTPRGIT 3ir6.1    --------------GRGGPVVWLSEADAKDLGIADNDWIEVFNSN-----------------GALTARAVVSQRVPAGMT  target    RMWFNMYGATPGSVEGHESRKDGLAKNPRTGYQAMFRSGSHQSATRGWLKPTWMTDSLVRKELFGHAVNKGFLPDVHCPT 3ir6.1    MMYHAQERI------------VNLPGSEI-T---QQRGGIHNSVTRITPKPTHMIGGY---------AHLAYGFNYYGTV  target    GAPREAIVKITKAEPGGLNAKGLWRPAALGLRPKYENDKMKDYLAGKFTLAANPKKGGKK 3ir6.1    GSNRDEFVVVRKMKN--------------------------------------------- ``` | | | | | | | | | | | | | | | | | | | | | | | | | | | | | | | | | | | | | | | | | | | | | | | | | |
|  | 5e7o.1.A | DMSO reductase family type II enzyme, molybdopterin subunit  *Crystal structure of the perchlorate reductase PcrAB mutant W461E of PcrA from Azospira suillum PS* | 0.39 | 0.00 | 24.62 | 0.72 | 1-592 | X-ray | 2.40 | monomer | 4 x SF4, 1 x MO, 1 x MGD, 1 x MD1, 1 x F3S | HHblits | 0.32 |
| ``` target    LARDIAKVPGTTLFAIGMGPNQFFNNDNKDRTQFLLAALTGNIGKIAGNIGSYAGNYRVAMFNGVPQYIAENPFDIELDG 5e7o.1    LAREFATAKP-SMIICGGGTQHWYYSDVLLRAMHLLTALTGTEGTNGGGMNHYIGQEKPAFVAGLVAL--AFPE--GVNK  target    AKPARPKLYWRAEPAHYYNH---E--DHPLKMGKTMITGKT----HMPTPTKSLWFANANSILGNVKWHFNTVV-NVLPK 5e7o.1    QR-FCQTTIWTYIHAEVNDEIISSDIDTEKYLRDSITTGQMPNMPEQGRDPKVFFVYRGNWLNQAK--GQKYVLENLWPK  target    MEMIAVQEWWWSTSCEWADIVFAVDAWSELKHPDMCSSVTNPFLTVFPRTPLERPFDTRGDIECLDLVGKQLAKRTGDRR 5e7o.1    LELIVDINIRMDSTALYSDVVLPSAHWYEKL--DLNVTSEHSYINMTE-PAIKPMWESKTDWQIFLA----LAKRVEMAA  target    F-ADMWKFVE------------------E---KKVEVYLQRILDHSSNTKGFKFPELEEKAKK-GIPALMMTR-TNPKTV 5e7o.1    KRKKYEKFNDEKFKWVRDLSNLWNQMTMDGKLAEDEAAAQYILDNAPQSKGITIQMLREKPQRFKSNWTSPLKEGVPYTP  target    GYEQVYDSRPWYTKTGRLEFYREEDEFIEAGENLPVHREPIDSTFYEPNVIVAPAHPFIKAKGPEAYGVKVDDFDNETRQ 5e7o.1    FQYFVVDKKPWPTLTGRQQFYLDHDTFFDMGVELPTYKAPID--------------------------------------  target    GRNIVKTWEETKKTVHPLAKDGYKFVFHTPKYRHGAHTMPVDTDMVAMLFGPFGDIYRHDKRQPFAAEGYVDIHPDDAKA 5e7o.1    -------------------ADKYPFRFNSPHSRHSVHSTFKDNVLMLR---------------LQRGGPSIEMSPLDAKP  target    LNIEDGDYVWIDSDPSDRPFRGWQKNDKDYKFSRLLCRARYYPGTPRGITRMWFNMYGATPGSVEGHESRKDGLAKNPRT 5e7o.1    LGIKDNDWVEAWNNH-----------------GKVICRVKIRNGEQRGRVSMWHCPELYMD-------------------  target    GYQAMFRSGSHQSATRGWLKPTWMTDSLVRKELFGHAVNKGFLPDVHCPTGAPREAIVKITKAEPGGLNAKGLWRPAALG 5e7o.1    -----LLTGGSQSVCPVRINPTNLVGNY---------GHLFFRPNYYGPAGSQRDVRVNVKRYIGA--------------  target    LRPKYENDKMKDYLAGKFTLAANPKKGGKK 5e7o.1    ------------------------------ ``` | | | | | | | | | | | | | | | | | | | | | | | | | | | | | | | | | | | | | | | | | | | | | | | | | |
|  | 4ydd.1.A | DMSO reductase family type II enzyme, molybdopterin subunit  *Crystal structure of the perchlorate reductase PcrAB from Azospira suillum PS* | 0.39 | 0.00 | 24.40 | 0.72 | 1-592 | X-ray | 1.86 | monomer | 4 x SF4, 1 x MO, 1 x MGD, 1 x MD1, 1 x F3S | HHblits | 0.32 |
| ``` target    LARDIAKVPGTTLFAIGMGPNQFFNNDNKDRTQFLLAALTGNIGKIAGNIGSYAGNYRVAMFNGVPQYIAENPFDIELDG 4ydd.1    LAREFATAKP-SMIICGGGTQHWYYSDVLLRAMHLLTALTGTEGTNGGGMNHYIGQWKPAFVAGLVAL--AFPEGV--NK  target    AKPARPKLYWRAEPAHY----YNHEDHP-LKMGKTMITGKT----HMPTPTKSLWFANANSILGNVKWHFNTVV-NVLPK 4ydd.1    QR-FCQTTIWTYIHAEVNDEIISSDIDTEKYLRDSITTGQMPNMPEQGRDPKVFFVYRGNWLNQA--KGQKYVLENLWPK  target    MEMIAVQEWWWSTSCEWADIVFAVDAWSELKHPDMCSSVTNPFLTVFPRTPLERPFDTRGDIECLDLVGKQLAKRTGDRR 4ydd.1    LELIVDINIRMDSTALYSDVVLPSAHWYEKL--DLNVTSEHSYINMTE-PAIKPMWESKTDWQIFLA----LAKRVEMAA  target    F-ADMWKFVE------------------E---KKVEVYLQRILDHSSNTKGFKFPELEEKAKK-GIPALMMTR-TNPKTV 4ydd.1    KRKKYEKFNDEKFKWVRDLSNLWNQMTMDGKLAEDEAAAQYILDNAPQSKGITIQMLREKPQRFKSNWTSPLKEGVPYTP  target    GYEQVYDSRPWYTKTGRLEFYREEDEFIEAGENLPVHREPIDSTFYEPNVIVAPAHPFIKAKGPEAYGVKVDDFDNETRQ 4ydd.1    FQYFVVDKKPWPTLTGRQQFYLDHDTFFDMGVELPTYKAPID--------------------------------------  target    GRNIVKTWEETKKTVHPLAKDGYKFVFHTPKYRHGAHTMPVDTDMVAMLFGPFGDIYRHDKRQPFAAEGYVDIHPDDAKA 4ydd.1    -------------------ADKYPFRFNSPHSRHSVHSTFKDNVLMLR---------------LQRGGPSIEMSPLDAKP  target    LNIEDGDYVWIDSDPSDRPFRGWQKNDKDYKFSRLLCRARYYPGTPRGITRMWFNMYGATPGSVEGHESRKDGLAKNPRT 4ydd.1    LGIKDNDWVEAWNNH-----------------GKVICRVKIRNGEQRGRVSMWHCPELYMD-------------------  target    GYQAMFRSGSHQSATRGWLKPTWMTDSLVRKELFGHAVNKGFLPDVHCPTGAPREAIVKITKAEPGGLNAKGLWRPAALG 4ydd.1    -----LLTGGSQSVCPVRINPTNLVGNY---------GHLFFRPNYYGPAGSQRDVRVNVKRYIGA--------------  target    LRPKYENDKMKDYLAGKFTLAANPKKGGKK 4ydd.1    ------------------------------ ``` | | | | | | | | | | | | | | | | | | | | | | | | | | | | | | | | | | | | | | | | | | | | | | | | | |
|  | 1kqf.1.A | FORMATE DEHYDROGENASE, NITRATE-INDUCIBLE, MAJOR SUBUNIT  *FORMATE DEHYDROGENASE N FROM E. COLI* | 0.28 |  | 17.06 | 0.67 | 1-502 | X-ray | 1.60 | hetero-oligomer | 3 x 6MO, 15 x SF4, 6 x MGD, 6 x HEM, 3 x CDL | HHblits | 0.28 |
| ``` target    LARDIAKVP---GTTLFAIGMGPNQFFNNDNKDRTQFLLAALTGNIGKIAGNIGSYAGNYRVAMF---N----GVPQYIA 1kqf.1    VCEVLASTSAPDRTTTFLYALGWTQHTVGAQNIRTMAMIQLLLGNMGMAGGGVNALRGHSNIQGLTDLGLLSTSLPGYLT  target    ENPFDIEL----------DGAKPARPKLYWRAEPAHYYNHE----------------------DHPLKMGKTMITGKTHM 1kqf.1    L-PSEKQVDLQSYLEANTPKATLADQVNYWSNYPKFFVSLMKSFYGDAAQKENNWGYDWLPKWDQTYDVIKYFNM---MD  target    PTPTKSLWFANANSILGNVKWHFNTVVNVLPKMEMIAVQEWWWSTSCEWAD-----------------IVFAVDAWSELK 1kqf.1    EGKVTGYFCQGFNPVASF--PDKNKVVSCLSKLKYMVVIDPLVTETSTFWQNHGESNDVDPASIQTEVFRLPSTCFAEED  target    HPDMCSSVTNPFLTVFPRTPLERPFDTRGDIECLDLVGKQLAKRTGDRR-------F----ADMWKFVEEKKV------- 1kqf.1    --GSIAN-SGRWLQWHW-KGQDAPGEARNDGEILAG----IYHHLRELYQSEGGKGVEPLMKMSWNYKQPHEPQSDEVAK  target    ---EVYLQRILDHSSN------TKGFKFPELEEKAKKG--I---------------------------------PALM-- 1kqf.1    ENNGYALEDLYDANGVLIAKKGQLLSSFAHLRDDGTTASSCWIYTGSWTEQGNQMANRDNSDPSGLGNTLGWAWAWPLNR  target    ---MT-R---------TNPKT-VGYE---------QVYDSRPWYTKTGRLEFYREEDEFI----EAG-ENLPVHREPIDS 1kqf.1    RVLYNRASADINGKPWDPKRMLIQWNGSKWTGNDIPDFGNAAPGTPTGPFIMQPEGMGRLFAINKMAEGPFPEHYEPIET  target    TFYEPNVIVAPAHPFIKAKGPEAYGVKVDDFDNETRQGRNIVKTWEETKKTVHPLAKDGYKFVFHTPKYRHGAHTMPVDT 1kqf.1    PL--------GTNPLHPNVV-----------SN------PVVRLYEQDAL--RMGKKEQFPYVGTTYRLTEHFHTWTKHA  target    DMVAMLFGPFGDIYRHDKRQPFAAEGYVDIHPDDAKALNIEDGDYVWIDSDPSDRPFRGWQKNDKDYKFSRLLCRARYYP 1kqf.1    LLNA-------------I---AQPEQFVEISETLAAAKGINNGDRVTVSSKR-----------------GFIRAVAVVTR  target    GTPR--------GITRMWFNMYGATPGSVEGHESRKDGLAKNPRTGYQAMFRSGSHQSATRGWLKPTWMTDSLVRKELFG 1kqf.1    RLKPLNVNGQQVETVGIPIHW-----------------------------------------------------------  target    HAVNKGFLPDVHCPTGAPREAIVKITKAEPGGLNAKGLWRPAALGLRPKYENDKMKDYLAGKFTLAANPKKGGKK 1kqf.1    --------------------------------------------------------------------------- ``` | | | | | | | | | | | | | | | | | | | | | | | | | | | | | | | | | | | | | | | | | | | | | | | | | |
|  | 6cz7.1.A | ArrA  *The arsenate respiratory reductase (Arr) complex from Shewanella sp. ANA-3* | 0.33 |  | 18.38 | 0.66 | 1-502 | X-ray | 1.62 | hetero-1-1-mer | 5 x SF4, 2 x MGD, 1 x MO, 1 x PG5 | HHblits | 0.28 |
| ``` target    LARDIAKVPGTTLFAIGMGPNQFFNNDNKDRTQFLLAALTGNIGKIAGNIGSYAGNYRVAMFNGVPQYIAENPFDIELDG 6cz7.1    IAKDMGAAAPAVQVWTSRGAVMQARGTYTSISCHALNGLFGGIDSKGGLFPGNKTPL-L---KEYPEAKAYM-DEIAAKG  target    AKPARPKLYWRAEPAHYYNH----EDHPLKMGKTMITGKTHMPTPTKSLWFANANSILGNVKWHFNTVVNVLPKMEMIAV 6cz7.1    VKKEKIDQRGRLEFPALAKGKSGGGVITANAANGIRNQ---DPYEIKVMLAYFNNFNFSN--PEGQRWDEALSKVDFMAH  target    QEWWWSTSCEWADIVFAVDA-WSELKHPDMCSSVTNPFLTV-FPRTPLERPFDTRGD-IECLDLVGKQLAKRTGDRRFAD 6cz7.1    ITTNVSEFSWFADVLLPSSHHMFEKW--GVLDSIGNGVAQISIQQPSIKRLWDTRIDESEIPYM----LAKKLADKGFDA  target    MWKFVEE-----------KKVEVYLQRILDHSSN-----------TKGFKFPELEEKAKKGIPALMMTRTNPKTVGYEQV 6cz7.1    PWRYINEQIVDPETGKPAADEAEFAKLMVRYLTAPLWKEDASKYGDKLSSWDEFVQ---KGVWNSS-----P----YKLE  target    YDSRPWYTKTGRLEFYREEDEFIEAGENLPVHREPIDSTFYEPNVIVAPAHPFIKAKGPEAYGVKVDDFDNETRQGRNIV 6cz7.1    ARWGKFKTETTKFEFYSKTLEKA-----LQSHADKH---KVSIDE-VM-KAC---------------DYQ-ARGHL-AFI  target    KTWEETKKTVHPLAKDGYKFVFHTPKYRHGAHTMPVDTDMVAMLFGPFGDIYRHDKRQPFAAEGYVDIHPDDAKALNIED 6cz7.1    PHYEEPYR---FGDESEFPLLLVDQKSRLNKEGRTANSPWYYEFKDVD-----P---GDVANEDVAKFNPIDGKKFGLKD  target    GDYVWIDSDPSDRPFRGWQKNDKDYKFSRLLCRARYYPGTPRGITRMWFNMYGATPGSVEGHESRKDGLAKNPRTGYQAM 6cz7.1    GDEIRITSPV-----------------GMLTCKAKLWEGVRPGTVAKCFGQ-----------------------------  target    FRSGSHQSATRGWLKPTWMTDSLVRKELFGHAVNKGFLPDVHCPTGAPREAIVKITKAEPGGLNAKGLWRPAALGLRPKY 6cz7.1    --------------------------------------------------------------------------------  target    ENDKMKDYLAGKFTLAANPKKGGKK 6cz7.1    ------------------------- ``` | | | | | | | | | | | | | | | | | | | | | | | | | | | | | | | | | | | | | | | | | | | | | | | | | |
|  | 2nya.1.A | Periplasmic nitrate reductase  *Crystal structure of the periplasmic nitrate reductase (NAP) from Escherichia coli* | 0.28 |  | 17.32 | 0.64 | 1-502 | X-ray | 2.50 | monomer | 1 x SF4, 1 x 6MO, 2 x MGD | HHblits | 0.28 |
| ``` target    LARDIAKVPGTTLFAIGMGPNQFFNNDNKDRTQFLLAALTGNIGKIAGNIGSYAGNYRVAMFN-GVPQYIAENPFDIELD 2nya.1    LAQLYADPNKKVISYWTMGFNQHTRGVWANNLVYNLHLLTGKISQPGCGPFSLTGQPSACGTAREVGTFAHRLPADMVVT  target    GAK-PARPKLYWRAEPAHYYNHEDHPLKMGKTMIT-GKTHMPTPTKSLWFANANSILGNVKWHFNTVVNVLPKMEMIAVQ 2nya.1    NEKHRDICEKKWNI------PSGTIPAKIGLHAVAQDRALKDGKLNVYWTMCTNNMQAGPNINEERMPGWRDPRNFIIVS  target    EWWWSTSCEWADIVFAVDAWSELKHPDMCSSVTNPFLTVFPRTPLERPFDTRGDIECLDLVGKQLAKRTGDRRFADMWKF 2nya.1    DPYPTVSALAADLILPTAMWVEKE--GAYGN-AERRTQFWR-QQVQAPGEAKSDLWQLVQ----FSRRFKTEEV---WPE  target    VEEK-----KVEVYLQRILDHSSNTKGFKFPELEEK------AKKGIPALM--------MTR-TNPKTVGYEQV--YDSR 2nya.1    DLLAKKPELRGKTLYEVLYAT-PEVSKFPVSELAEDQLNDESRELGFYLQKGLFEEYAWFGRGHGHDLAPFDDYHKARGL  target    PWYTKTGRLEFYREE---DEFIEAGENLPVHREPIDSTFYEPNVIVAPAHPFIKAKGPEAYGVKVDDFDNETRQGRNIVK 2nya.1    RWPVVNGKETQWRYSEGNDPYVKAGEGYKFYGKP-------------------------------------DGKAVIFAL  target    TWEETKKTVHPLAKDGYKFVFHTPKYRHGAH--TMPVDTDMVAMLFGPFGDIYRHDKRQPFAAEGYVDIHPDDAKALNIE 2nya.1    PFEPAA----EAPDEEYDLWLSTGRVLEHWHTGSMTRRVPELHR----------------AFPEAVLFIHPLDAKARDLR  target    DGDYVWIDSDPSDRPFRGWQKNDKDYKFSRLLCRARYYPG--TPRGITRMWFNMYGATPGSVEGHESRKDGLAKNPRTGY 2nya.1    RGDKVKVVSRR-----------------GEVISIVETRGRNRPPQGLVYMPFFD--------------------------  target    QAMFRSGSHQSATRGWLKPTWMTDSLVRKELFGHAVNKGFLPDVHCPTGAPREAIVKITKAEPGGLNAKGLWRPAALGLR 2nya.1    --------------------------------------------------------------------------------  target    PKYENDKMKDYLAGKFTLAANPKKGGKK 2nya.1    ---------------------------- ``` | | | | | | | | | | | | | | | | | | | | | | | | | | | | | | | | | | | | | | | | | | | | | | | | | |
|  | 7l5i.1.A | Trimethylamine-N-oxide reductase  *Crystal Structure of Haemophilus influenzae MtsZ at pH 7.0* | 0.33 |  | 21.50 | 0.63 | 1-503 | X-ray | 1.73 | monomer | 2 x MGD, 1 x MO, 1 x O | HHblits | 0.30 |
| ``` target    LARDIAKVPGTTLFAIGMGPNQFFNNDNKDRTQFLLAALTGNIGKIAGNIGSYAGNYRVAMFN--G-VPQYIAENPFDIE 7l5i.1    LAADFAS-KR-TMLMGGWGMQRQRHGEQTHWMLVTLASMLGQIGLPGGGFGLSYHYSNGGVPTATGGIIGSITASPS--G  target    LDGAKPA-RPKLYWRAEPAHYYNHEDHPLKMGKTMITGKTHM-PTPTKSLWFANANSILGNVKWHFNTVVNVLPKMEMIA 7l5i.1    KAGAKTWLDDTSKSAF-PLAR--IADVLLHPGKKIQYNGTEITYPDIKAVYWAGGNPFVHH--QDTNTLVKAFQKPDVVI  target    VQEWWWSTSCEWADIVFAVDAWSELKHPDMCSSVTNPFLTVFPRTPLERPFDTRGDIECLDLVGKQLAKRTGDRRFADMW 7l5i.1    VNEVNWTPTARMADIVLPATTSYERNDLTMAGDYSMMSVYPMK-QVVPPQFEAKNDYDIFVE----LAKRAGVEEQ----  target    KFVEEKKVEVYLQRILDHSSN---TKGFKFPELEEKAKK--GIPALMMTRTNPKTVGYEQVY---DSRPWYTKTGRLEFY 7l5i.1    -YTEGKTEMEWLEEFYNAAFSAARANRVAMPRFDKFWAENKPLSFEAGE-AAKKWVRYGEFREDPLLNPLGTPSGKIEIF  target    REEDEFIEAG--ENLPVHREPIDSTFYEPNVIVAPAHPFIKAKGPEAYGVKVDDFDNETRQGRNIVKTWEETKKTVHPLA 7l5i.1    SDVVEKMNYNDCKGHPSWMEPEEF---------------------------------------------------AG-NV  target    KDGYKFVFHTPKYRHGAHTMPVDTDMVAMLFGPFGDIYRHDKRQPFAAEGYVDIHPDDAKALNIEDGDYVWIDSDPSDRP 7l5i.1    TEEYPLALVTPHPYYRLHSQLAHTSLRQK-------------Y-AVNDREPVMIHPEDAAARGIKDGDIVRIHSKR----  target    FRGWQKNDKDYKFSRLLCRARYYPGTPRGITRMWFNMYGATPGSVEGHESRKDGLAKNPRTGYQAMFRSGSHQSATRGWL 7l5i.1    -------------GQVLAGAAVTENIIKGTVALHEGAW------------------------------------------  target    KPTWMTDSLVRKELFGHAVNKGFLPDVHCPTGAPREAIVKITKAEPGGLNAKGLWRPAALGLRPKYENDKMKDYLAGKFT 7l5i.1    --------------------------------------------------------------------------------  target    LAANPKKGGKK 7l5i.1    ----------- ``` | | | | | | | | | | | | | | | | | | | | | | | | | | | | | | | | | | | | | | | | | | | | | | | | | |
|  | 7l5s.1.A | Trimethylamine-N-oxide reductase  *Crystal Structure of Haemophilus influenzae MtsZ at pH 5.5* | 0.33 |  | 21.50 | 0.63 | 1-503 | X-ray | 2.09 | monomer | 1 x O, 2 x MGD, 1 x MO | HHblits | 0.30 |
| ``` target    LARDIAKVPGTTLFAIGMGPNQFFNNDNKDRTQFLLAALTGNIGKIAGNIGSYAGNYRVAMFN--G-VPQYIAENPFDIE 7l5s.1    LAADFAS-KR-TMLMGGWGMQRQRHGEQTHWMLVTLASMLGQIGLPGGGFGLSYHYSNGGVPTATGGIIGSITASPS--G  target    LDGAKPA-RPKLYWRAEPAHYYNHEDHPLKMGKTMITGKTHM-PTPTKSLWFANANSILGNVKWHFNTVVNVLPKMEMIA 7l5s.1    KAGAKTWLDDTSKSAF-PLAR--IADVLLHPGKKIQYNGTEITYPDIKAVYWAGGNPFVHH--QDTNTLVKAFQKPDVVI  target    VQEWWWSTSCEWADIVFAVDAWSELKHPDMCSSVTNPFLTVFPRTPLERPFDTRGDIECLDLVGKQLAKRTGDRRFADMW 7l5s.1    VNEVNWTPTARMADIVLPATTSYERNDLTMAGDYSMMSVYPMK-QVVPPQFEAKNDYDIFVE----LAKRAGVEEQ----  target    KFVEEKKVEVYLQRILDHSSN---TKGFKFPELEEKAKK--GIPALMMTRTNPKTVGYEQVY---DSRPWYTKTGRLEFY 7l5s.1    -YTEGKTEMEWLEEFYNAAFSAARANRVAMPRFDKFWAENKPLSFEAGE-AAKKWVRYGEFREDPLLNPLGTPSGKIEIF  target    REEDEFIEAG--ENLPVHREPIDSTFYEPNVIVAPAHPFIKAKGPEAYGVKVDDFDNETRQGRNIVKTWEETKKTVHPLA 7l5s.1    SDVVEKMNYNDCKGHPSWMEPEEF---------------------------------------------------AG-NV  target    KDGYKFVFHTPKYRHGAHTMPVDTDMVAMLFGPFGDIYRHDKRQPFAAEGYVDIHPDDAKALNIEDGDYVWIDSDPSDRP 7l5s.1    TEEYPLALVTPHPYYRLHSQLAHTSLRQK-------------Y-AVNDREPVMIHPEDAAARGIKDGDIVRIHSKR----  target    FRGWQKNDKDYKFSRLLCRARYYPGTPRGITRMWFNMYGATPGSVEGHESRKDGLAKNPRTGYQAMFRSGSHQSATRGWL 7l5s.1    -------------GQVLAGAAVTENIIKGTVALHEGAW------------------------------------------  target    KPTWMTDSLVRKELFGHAVNKGFLPDVHCPTGAPREAIVKITKAEPGGLNAKGLWRPAALGLRPKYENDKMKDYLAGKFT 7l5s.1    --------------------------------------------------------------------------------  target    LAANPKKGGKK 7l5s.1    ----------- ``` | | | | | | | | | | | | | | | | | | | | | | | | | | | | | | | | | | | | | | | | | | | | | | | | | |
|  | 1e18.1.A | DMSO REDUCTASE.  *TUNGSTEN-SUSBSTITUTED DMSO REDUCTASE FROM RHODOBACTER CAPSULATUS* | 0.33 | 0.00 | 20.45 | 0.62 | 1-502 | X-ray | 2.00 | monomer | 2 x PGD, 1 x 6WO | HHblits | 0.30 |
| ``` target    LARDIAKVPGTTLFAIGMGPNQFFNNDNKDRTQFLLAALTGNIGKIAGNIGSYAGNYRVAMFN-GVPQYIAENPFDIELD 1e18.1    LARLFES-KR-TMLAAGWSMQRMHHGEQAHWMLVTLASMLGQIGLPGGGFGLSYHYSGGGTPSTSGPALAGITD-----G  target    GAKPARPKLYWRAEPAHYY---NHEDHPLKMGKTM-ITGKTHMPTPTKSLWFANANSILGNVKWHFNTVVNVLPKMEMIA 1e18.1    GAATKGPE-WLAASGASVIPVARVVDMLENPGAEFDFNGTRSKFPDVKMAYWVGGNPFVHH--QDRNRMVKAWEKLETFV  target    VQEWWWSTSCEWADIVFAVDAWSELKHPDMC--SSVTNPFLTVFPRTPLERPFDTRGDIECLDLVGKQLAKRTGDRRFAD 1e18.1    VHDFQWTPTARHADIVLPATTSYERN--DIETIGDYSNTGILAMK-KIVEPLYEARSDYDIFAA----VAERLGKGK---  target    MWKFVEEKKVEVYLQRILDHSSN---TKGFKFPELEEKAKKGIPALMMTRTNPKTVGYEQVY---DSRPWYTKTGRLEFY 1e18.1    --EFTEGKDEMGWIKSFYDDAAKQGKAAGVEMPAFDAFWAEGIVEFPVTD-GADFVRYASFREDPLLNPLGTPTGLIEIY  target    REEDEFIEAG--ENLPVHREPIDSTFYEPNVIVAPAHPFIKAKGPEAYGVKVDDFDNETRQGRNIVKTWEETKKTVHPLA 1e18.1    SKNIEKMGYDDCPAHPTWMEPLER---------------------------------------------------LD-GP  target    KDGYKFVFHTPKYRHGAHTMPVDTDMVAMLFGPFGDIYRHDKRQPFAAEGYVDIHPDDAKALNIEDGDYVWIDSDPSDRP 1e18.1    GAKYPLHIAASHPFNRLHSQLN-GTVLREG-------Y------AVQGHEPCLMHPDDAAARGIADGDVVRVHNDR----  target    FRGWQKNDKDYKFSRLLCRARYYPGTPRGITRMWFNMYGATPGSVEGHESRKDGLAKNPRTGYQAMFRSGSHQSATRGWL 1e18.1    -------------GQILTGVKVTDAVMKGVIQIYEGG-------------------------------------------  target    KPTWMTDSLVRKELFGHAVNKGFLPDVHCPTGAPREAIVKITKAEPGGLNAKGLWRPAALGLRPKYENDKMKDYLAGKFT 1e18.1    --------------------------------------------------------------------------------  target    LAANPKKGGKK 1e18.1    ----------- ``` | | | | | | | | | | | | | | | | | | | | | | | | | | | | | | | | | | | | | | | | | | | | | | | | | |
|  | 4dmr.1.A | DMSO REDUCTASE  *REDUCED DMSO REDUCTASE FROM RHODOBACTER CAPSULATUS WITH BOUND DMSO SUBSTRATE* | 0.33 | 0.00 | 20.45 | 0.62 | 1-502 | X-ray | 1.90 | monomer | 2 x PGD, 1 x 4MO, 1 x O | HHblits | 0.30 |
| ``` target    LARDIAKVPGTTLFAIGMGPNQFFNNDNKDRTQFLLAALTGNIGKIAGNIGSYAGNYRVAMF-NGVPQYIAENPFDIELD 4dmr.1    LARLFES-KR-TMLAAGWSMQRMHHGEQAHWMLVTLASMLGQIGLPGGGFGLSYHYSGGGTPSTSGPALAGITD-----G  target    GAKPARPKLYWRAEPAHYY---NHEDHPLKMGKTM-ITGKTHMPTPTKSLWFANANSILGNVKWHFNTVVNVLPKMEMIA 4dmr.1    GAATKGPE-WLAASGASVIPVARVVDMLENPGAEFDFNGTRSKFPDVKMAYWVGGNPFVHH--QDRNRMVKAWEKLETFV  target    VQEWWWSTSCEWADIVFAVDAWSELKHPDMC--SSVTNPFLTVFPRTPLERPFDTRGDIECLDLVGKQLAKRTGDRRFAD 4dmr.1    VHDFQWTPTARHADIVLPATTSYERN--DIETIGDYSNTGILAMK-KIVEPLYEARSDYDIFAA----VAERLGKGA---  target    MWKFVEEKKVEVYLQRILDHSS---NTKGFKFPELEEKAKKGIPALMMTRTNPKTVGYEQVY---DSRPWYTKTGRLEFY 4dmr.1    --EFTEGKDEMGWIKSFYDDAAKQGKAAGVQMPAFDAFWAEGIVEFPVTD-GADFVRYASFREDPLLNPLGTPTGLIEIY  target    REEDEFIEAG--ENLPVHREPIDSTFYEPNVIVAPAHPFIKAKGPEAYGVKVDDFDNETRQGRNIVKTWEETKKTVHPLA 4dmr.1    SKNIEKMGYDDCPAHPTWMEPLER---------------------------------------------------L-DGP  target    KDGYKFVFHTPKYRHGAHTMPVDTDMVAMLFGPFGDIYRHDKRQPFAAEGYVDIHPDDAKALNIEDGDYVWIDSDPSDRP 4dmr.1    GAKYPLHIAASHPFNRLHSQLN-GTVLREG-------Y------AVQGHEPCLMHPDDAAARGIADGDVVRVHNDR----  target    FRGWQKNDKDYKFSRLLCRARYYPGTPRGITRMWFNMYGATPGSVEGHESRKDGLAKNPRTGYQAMFRSGSHQSATRGWL 4dmr.1    -------------GQILTGVKVTDAVMKGVIQIYEGG-------------------------------------------  target    KPTWMTDSLVRKELFGHAVNKGFLPDVHCPTGAPREAIVKITKAEPGGLNAKGLWRPAALGLRPKYENDKMKDYLAGKFT 4dmr.1    --------------------------------------------------------------------------------  target    LAANPKKGGKK 4dmr.1    ----------- ``` | | | | | | | | | | | | | | | | | | | | | | | | | | | | | | | | | | | | | | | | | | | | | | | | | |
|  | 1e60.1.A | Dimethyl sulfoxide/trimethylamine N-oxide reductase  *OXIDIZED DMSO REDUCTASE EXPOSED TO HEPES - Structure II BUFFER* | 0.34 | 0.00 | 20.15 | 0.62 | 1-502 | X-ray | 2.00 | monomer | 2 x PGD, 1 x 2MO | HHblits | 0.30 |
| ``` target    LARDIAKVPGTTLFAIGMGPNQFFNNDNKDRTQFLLAALTGNIGKIAGNIGSYAGNYRVAMF-NGVPQYIAENPFDIELD 1e60.1    LARLFES-KR-TMLAAGWSMQRMHHGEQAHWMLVTLASMLGQIGLPGGGFGLSYHYSGGGTPSTSGPALAGITD-----G  target    GAKPARPKLY--WRAEPAHYYNHEDHPLKMGKTM-ITGKTHMPTPTKSLWFANANSILGNVKWHFNTVVNVLPKMEMIAV 1e60.1    GAATKGPEWLAASGASVIPVARVVDMLENPGAEFDFNGTRSKFPDVKMAYWVGGNPFVHH--QDRNRMVKAWEKLETFVV  target    QEWWWSTSCEWADIVFAVDAWSELKHPDMC--SSVTNPFLTVFPRTPLERPFDTRGDIECLDLVGKQLAKRTGDRRFADM 1e60.1    HDFQWTPTARHADIVLPATTSYERN--DIETIGDYSNTGILAMK-KIVEPLYEARSDYDIFAA----VAERLGKGA----  target    WKFVEEKKVEVYLQRILDHSS---NTKGFKFPELEEKAKKGIPALMMTRTNPKTVGYEQVY---DSRPWYTKTGRLEFYR 1e60.1    -EFTEGKDEMGWIKSFYDDAAKQGKAAGVEMPAFDAFWAEGIVEFPVTD-GADFVRYASFREDPLLNPLGTPTGLIEIYS  target    EEDEFIEAG--ENLPVHREPIDSTFYEPNVIVAPAHPFIKAKGPEAYGVKVDDFDNETRQGRNIVKTWEETKKTVHPLAK 1e60.1    KNIEKMGYDDCPAHPTWMEPLER---------------------------------------------------L-DGPG  target    DGYKFVFHTPKYRHGAHTMPVDTDMVAMLFGPFGDIYRHDKRQPFAAEGYVDIHPDDAKALNIEDGDYVWIDSDPSDRPF 1e60.1    AKYPLHIAASHPFNRLHSQLNG-TVLREG-------Y------AVQGHEPCLMHPDDAAARGIADGDVVRVHNDR-----  target    RGWQKNDKDYKFSRLLCRARYYPGTPRGITRMWFNMYGATPGSVEGHESRKDGLAKNPRTGYQAMFRSGSHQSATRGWLK 1e60.1    ------------GQILTGVKVTDAVMKGVIQIYEGG--------------------------------------------  target    PTWMTDSLVRKELFGHAVNKGFLPDVHCPTGAPREAIVKITKAEPGGLNAKGLWRPAALGLRPKYENDKMKDYLAGKFTL 1e60.1    --------------------------------------------------------------------------------  target    AANPKKGGKK 1e60.1    ---------- ``` | | | | | | | | | | | | | | | | | | | | | | | | | | | | | | | | | | | | | | | | | | | | | | | | | |
|  | 1e5v.2.A | Dimethyl sulfoxide/trimethylamine N-oxide reductase  *OXIDIZED DMSO REDUCTASE EXPOSED TO HEPES BUFFER* | 0.32 | 0.00 | 20.51 | 0.62 | 1-502 | X-ray | 2.40 | monomer | 2 x PGD, 1 x 2MO | HHblits | 0.30 |
| ``` target    LARDIAKVPGTTLFAIGMGPNQFFNNDNKDRTQFLLAALTGNIGKIAGNIGSYAGNYRVAMFN-GVPQYIAENPFDIELD 1e5v.2    LARLFES-KR-TMLAAGWSMQRMHHGEQAHWMLVTLASMLGQIGLPGGGFGLSYHYSGGGTPSTSGPALAGI------TD  target    GA-KPARPKLYWRAEPAHYY---NHEDHPLKMGKTM-ITGKTHMPTPTKSLWFANANSILGNVKWHFNTVVNVLPKMEMI 1e5v.2    GGAATKGPE-WLAASGASVIPVARVVDMLENPGAEFDFNGTRSKFPDVKMAYWVGGNPFVHH--QDRNRMVKAWEKLETF  target    AVQEWWWSTSCEWADIVFAVDAWSELKHPDMC--SSVTNPFLTVFPRTPLERPFDTRGDIECLDLVGKQLAKRTGDRRFA 1e5v.2    VVHDFQWTPTARHADIVLPATTSYERN--DIETIGDYSNTGILAMK-KIVEPLYEARSDYDIFAA----VAERLGKGA--  target    DMWKFVEEKKVEVYLQRILDHSS---NTKGFKFPELEEKAKKGIPALMMTRTNPKTVGYEQVY---DSRPWYTKTGRLEF 1e5v.2    ---EFTEGKDEMGWIKSFYDDAAKQGKAAGVQMPAFDAFWAEGIVEFPVTD-GADFVRYASFREDPLLNPLGTPTGLIEI  target    YREEDEFIEAG--ENLPVHREPIDSTFYEPNVIVAPAHPFIKAKGPEAYGVKVDDFDNETRQGRNIVKTWEETKKTVHPL 1e5v.2    YSKNIEKMGYDDCPAHPTWMEPLER---------------------------------------------------L-DG  target    AKDGYKFVFHTPKYRHGAHTMPVDTDMVAMLFGPFGDIYRHDKRQPFAAEGYVDIHPDDAKALNIEDGDYVWIDSDPSDR 1e5v.2    PGAKYPLHIAASHPFNRLHSQLN-GTVLREG-------Y------AVQGHEPCLMHPDDAAARGIADGDVVRVHNDR---  target    PFRGWQKNDKDYKFSRLLCRARYYPGTPRGITRMWFNMYGATPGSVEGHESRKDGLAKNPRTGYQAMFRSGSHQSATRGW 1e5v.2    --------------GQILTGVKVTDAVMKGVIQIYEGG------------------------------------------  target    LKPTWMTDSLVRKELFGHAVNKGFLPDVHCPTGAPREAIVKITKAEPGGLNAKGLWRPAALGLRPKYENDKMKDYLAGKF 1e5v.2    --------------------------------------------------------------------------------  target    TLAANPKKGGKK 1e5v.2    ------------ ``` | | | | | | | | | | | | | | | | | | | | | | | | | | | | | | | | | | | | | | | | | | | | | | | | | |
|  | 1dms.1.A | DMSO REDUCTASE  *STRUCTURE OF DMSO REDUCTASE* | 0.33 | 0.00 | 20.20 | 0.62 | 1-502 | X-ray | 1.88 | monomer | 2 x PGD, 1 x 2MO | HHblits | 0.30 |
| ``` target    LARDIAKVPGTTLFAIGMGPNQFFNNDNKDRTQFLLAALTGNIGKIAGNIGSYAGNYRVAMF-NGVPQYIAENPFDIELD 1dms.1    LARLFKS-KR-TMLAAGWSMQRMHHGEQAHWMLVTLASMLGQIGLPGGGFGLSYHYSGGGTPSSSGPALSGITDG-----  target    GAKPARPKLYWRAEPAHYYNH---EDHPLKMGKTM-ITGKTHMPTPTKSLWFANANSILGNVKWHFNTVVNVLPKMEMIA 1dms.1    GAATKGPE-WLAASGASVIPVARVVDMLENPGAEFDFNGTRSKFPDVKMAYWVGGNPFVHH--QDRNRMVKAWEKLETFI  target    VQEWWWSTSCEWADIVFAVDAWSELKHPDMC--SSVTNPFLTVFPRTPLERPFDTRGDIECLDLVGKQLAKRTGDRRFAD 1dms.1    VHDFQWTPTARHADIVLPATTSYERN--DIETIGDYSNTGILAMK-KIVEPLYEARSDYDIFAA----VAERLGKGK---  target    MWKFVEEKKVEVYLQRILDHSSN---TKGFKFPELEEKAKKGIPALMMTRTNPKTVGYEQVY---DSRPWYTKTGRLEFY 1dms.1    --EFTEGKDEMGWIKSFYDDAAKQGKAGGVEMPAFDAFWAEGIVEFPVTDG-ADFVRYASFREDPLLNPLGTPTGLIEIY  target    REEDEFIEAG--ENLPVHREPIDSTFYEPNVIVAPAHPFIKAKGPEAYGVKVDDFDNETRQGRNIVKTWEETKKTVHPLA 1dms.1    SKNIEKMGYDDCPAHPTWMEPLER---------------------------------------------------L-DGP  target    KDGYKFVFHTPKYRHGAHTMPVDTDMVAMLFGPFGDIYRHDKRQPFAAEGYVDIHPDDAKALNIEDGDYVWIDSDPSDRP 1dms.1    GAKYPLHIAASHPFNRLHSQLNG-TVLRE-------G------YAVQGHEPCLMHPDDAAARGIADGDVVRVHNDR----  target    FRGWQKNDKDYKFSRLLCRARYYPGTPRGITRMWFNMYGATPGSVEGHESRKDGLAKNPRTGYQAMFRSGSHQSATRGWL 1dms.1    -------------GQILTGVKVTDAVMKGVIQIYEGG-------------------------------------------  target    KPTWMTDSLVRKELFGHAVNKGFLPDVHCPTGAPREAIVKITKAEPGGLNAKGLWRPAALGLRPKYENDKMKDYLAGKFT 1dms.1    --------------------------------------------------------------------------------  target    LAANPKKGGKK 1dms.1    ----------- ``` | | | | | | | | | | | | | | | | | | | | | | | | | | | | | | | | | | | | | | | | | | | | | | | | | |
|  | 7qv7.1.L | Hydrogen dependent carbon dioxide reductase subunit FdhF  *Cryo-EM structure of Hydrogen-dependent CO2 reductase.* | 0.27 |  | 19.44 | 0.61 | 1-504 | EM | 0.00 | hetero-2-6-6-2-mer | 52 x SF4, 6 x 402 | HHblits | 0.30 |
| ``` target    LARDIAKVPGTTLFAIGMGPNQFFNNDNKDRTQFLLAALTGNIGKIAGNIGSYAGNYRVA-MFN-G-VPQYIAENPFDIE 7qv7.1    AARFYGQAQA-AAILYSMGVTQFSHGTGNVVSLANLAVITGNLGRPGAGICPLRGQNNVQGACDVGALPNVL---PGYLD  target    LD-GAKPARPKLYWRAEPAHYYNHEDHPLKMGKTMITGKTHMPTPTKSLWFANANSILGNVKWHFNTVVNVLPKMEMIAV 7qv7.1    VTKEQNRERFEKVWGVKLPS--NIGLRVTEVPDAIL------NKRVRALYIFGENPIMSD--PDSDHLRHALEHLDLLIV  target    QEWWWSTSCEWADIVFAVDAWSELKHPDMCSSVTNPFLTVFPRTPLERPFDTRGDIECLDLVGKQLAKRTGDRRFADMWK 7qv7.1    QDIFLTETARLAHVVLPAACWAEKD--GTFTN-TERRVQRVR-KAVEAPGEAKPDWWIFSQ----IAERMGYTGM----Q  target    FVEEKKVEVYLQRILDHSSN-TKGFKFPELEEKAKKGIPALMMTRTNPKTVGYEQVYDSRPWYTKTGRLEFYREEDEFIE 7qv7.1    YN---NVQEIWDEVRKIVPEKFGGISYARLEKE--KGLAWPCPTEDHT---GTPILYLGGKFATPSGKAQMYPVIFYP--  target    AGENLPVHREPIDSTFYEPNVIVAPAHPFIKAKGPEAYGVKVDDFDNETRQGRNIVKTWEETKKTVHPLAKDGYKFVFHT 7qv7.1    ---NTCICDEGAEKQDF-------NHVI----------------------------VGSI------AELPDEEYPFTLTT  target    PKYRHGAHTMPVD--TDMVAMLFGPFGDIYRHDKRQPFAAEGYVDIHPDDAKALNIEDGDYVWIDSDPSDRPFRGWQKND 7qv7.1    GRRVYHYHTATMTRKSPVI-------------DQ---IAPQELVEINPQDATRLGINDGDFLRVSTRR------------  target    KDYKFSRLLCRARYYPGTPRGITRMWFNMYGATPGSVEGHESRKDGLAKNPRTGYQAMFRSGSHQSATRGWLKPTWMTDS 7qv7.1    -----GYVATRAWVTERVPKGTIFMTFHYWE-------------------------------------------------  target    LVRKELFGHAVNKGFLPDVHCPTGAPREAIVKITKAEPGGLNAKGLWRPAALGLRPKYENDKMKDYLAGKFTLAANPKKG 7qv7.1    --------------------------------------------------------------------------------  target    GKK 7qv7.1    --- ``` | | | | | | | | | | | | | | | | | | | | | | | | | | | | | | | | | | | | | | | | | | | | | | | | | |
|  | 7qv7.1.O | Hydrogen dependent carbon dioxide reductase subunit FdhF  *Cryo-EM structure of Hydrogen-dependent CO2 reductase.* | 0.26 |  | 19.44 | 0.61 | 1-504 | EM | 0.00 | hetero-2-6-6-2-mer | 52 x SF4, 6 x 402 | HHblits | 0.30 |
| ``` target    LARDIAKVPGTTLFAIGMGPNQFFNNDNKDRTQFLLAALTGNIGKIAGNIGSYAGNYRVA-MFN-G-VPQYIAENPFDIE 7qv7.1    AARFYGQAQA-AAILYSMGVTQFSHGTGNVVSLANLAVITGNLGRPGAGICPLRGQNNVQGACDVGALPNVL---PGYLD  target    LD-GAKPARPKLYWRAEPAHYYNHEDHPLKMGKTMITGKTHMPTPTKSLWFANANSILGNVKWHFNTVVNVLPKMEMIAV 7qv7.1    VTKEQNRERFEKVWGVKLPS--NIGLRVTEVPDAIL------NKRVRALYIFGENPIMSD--PDSDHLRHALEHLDLLIV  target    QEWWWSTSCEWADIVFAVDAWSELKHPDMCSSVTNPFLTVFPRTPLERPFDTRGDIECLDLVGKQLAKRTGDRRFADMWK 7qv7.1    QDIFLTETARLAHVVLPAACWAEKD--GTFTN-TERRVQRVR-KAVEAPGEAKPDWWIFSQ----IAERMGYTGM----Q  target    FVEEKKVEVYLQRILDHSSN-TKGFKFPELEEKAKKGIPALMMTRTNPKTVGYEQVYDSRPWYTKTGRLEFYREEDEFIE 7qv7.1    YN---NVQEIWDEVRKIVPEKFGGISYARLEKE--KGLAWPCPTEDHT---GTPILYLGGKFATPSGKAQMYPVIFYP--  target    AGENLPVHREPIDSTFYEPNVIVAPAHPFIKAKGPEAYGVKVDDFDNETRQGRNIVKTWEETKKTVHPLAKDGYKFVFHT 7qv7.1    ---NTCICDEGAEKQDF-------NHVI----------------------------VGSI------AELPDEEYPFTLTT  target    PKYRHGAHTMPVD--TDMVAMLFGPFGDIYRHDKRQPFAAEGYVDIHPDDAKALNIEDGDYVWIDSDPSDRPFRGWQKND 7qv7.1    GRRVYHYHTATMTRKSPVI-------------DQ---IAPQELVEINPQDATRLGINDGDFLRVSTRR------------  target    KDYKFSRLLCRARYYPGTPRGITRMWFNMYGATPGSVEGHESRKDGLAKNPRTGYQAMFRSGSHQSATRGWLKPTWMTDS 7qv7.1    -----GYVATRAWVTERVPKGTIFMTFHYWE-------------------------------------------------  target    LVRKELFGHAVNKGFLPDVHCPTGAPREAIVKITKAEPGGLNAKGLWRPAALGLRPKYENDKMKDYLAGKFTLAANPKKG 7qv7.1    --------------------------------------------------------------------------------  target    GKK 7qv7.1    --- ``` | | | | | | | | | | | | | | | | | | | | | | | | | | | | | | | | | | | | | | | | | | | | | | | | | |
|  | 1tmo.1.A | TRIMETHYLAMINE N-OXIDE REDUCTASE  *TRIMETHYLAMINE N-OXIDE REDUCTASE FROM SHEWANELLA MASSILIA* | 0.34 |  | 18.02 | 0.62 | 1-503 | X-ray | 2.50 | monomer | 2 x 2MD, 1 x 2MO | HHblits | 0.29 |
| ``` target    LARDIAKVPGTTLFAIGMGPNQFFNNDNKDRTQFLLAALTGNIGKIAGNIGSYAGNYRVA-MFNGVPQYIAENPFDIELD 1tmo.1    LAKTLVKGR--TQFMMGWCIQRQQHGEQPYWMAAVLATMIGQIGLPGGGISYGHHYSSIGVPSSGAAAP-GAFPR--NLD  target    GAKPA--RPKLYWRAEPAHYYNHEDHPLKMGKTMIT-G-------KTHMPTPTKSLWFANANSILGNVKWHFNTVVNVLP 1tmo.1    ENQKPLFDSSDFKGA--SSTIP----VARWIDAILEPGKTIDANGSKVVYPDIKMMIFSGNNPWNHH--QDRNRMKQAFH  target    KMEMIAVQEWWWSTSCEWADIVFAVDAWSELKHPDMCSSVTNPFLTVFPRTPLERPFDTRGDIECLDLVGKQLAKRTGDR 1tmo.1    KLECVVTVDVNWTATCRFSDIVLPACTTYERNDIDVYGAYANRGILAMQ-KMVEPLFDSLSDFEIFTR----FAAVLGKE  target    RFADMWKFVEEKKVEVYLQRILDHSSN-----TKGFKFPELEEKAKKGIPALMMTRTNPKTVGYEQV---YDSRPWYTKT 1tmo.1    K-----EYTRNMGEMEWLETLYNECKAANAGKFEMPDFATFWK---QGYVHFGD---GEVWTRHADFRNDPEINPLGTPS  target    GRLEFYREEDEFIEAG--ENLPVHREPIDSTFYEPNVIVAPAHPFIKAKGPEAYGVKVDDFDNETRQGRNIVKTWEETKK 1tmo.1    GLIEIFSRKIDQFGYDDCKGHPTWMEKTERSH------------------------------------------------  target    TVHPLAKDGYKFVFHTPKYRHGAHTMPVDTDMVAMLFGPFGDIYRHDKRQPFAAEGYVDIHPDDAKALNIEDGDYVWIDS 1tmo.1    --GGPGSDKHPIWLQSCHPDKRLHSQMCESREYRET-------------YAVNGREPVYISPVDAKARGIKDGDIVRVFN  target    DPSDRPFRGWQKNDKDYKFSRLLCRARYYPGTPRGITRMWFNMYGATPGSVEGHESRKDGLAKNPRTGYQAMFRSGSHQS 1tmo.1    DR-----------------GQLLAGAVVSDNFPKGIVRIHEGAW------------------------------------  target    ATRGWLKPTWMTDSLVRKELFGHAVNKGFLPDVHCPTGAPREAIVKITKAEPGGLNAKGLWRPAALGLRPKYENDKMKDY 1tmo.1    --------------------------------------------------------------------------------  target    LAGKFTLAANPKKGGKK 1tmo.1    ----------------- ``` | | | | | | | | | | | | | | | | | | | | | | | | | | | | | | | | | | | | | | | | | | | | | | | | | |
|  | 1eu1.1.A | DIMETHYL SULFOXIDE REDUCTASE  *THE CRYSTAL STRUCTURE OF RHODOBACTER SPHAEROIDES DIMETHYLSULFOXIDE REDUCTASE REVEALS TWO DISTINCT MOLYBDENUM COORDINATION ENVIRONMENTS.* | 0.32 |  | 20.10 | 0.61 | 1-502 | X-ray | 1.30 | monomer | 3 x GLC, 1 x CD, 2 x MGD, 1 x 6MO, 2 x O | HHblits | 0.30 |
| ``` target    LARDIAKVPGTTLFAIGMGPNQFFNNDNKDRTQFLLAALTGNIGKIAGNIGSYAGNYRVAMFN-GVPQYIAENPFDIELD 1eu1.1    LARSFVAGR--TMLAAGWSIQRMHHGEQAHWMLVTLASMIGQIGLPGGGFGLSYHYSNGGSPTSDGPALGGIS------D  target    GAKPARPKLYWRAEPAHYYNHEDH-PLKMGKTMIT--------GKTHMPTPTKSLWFANANSILGNVKWHFNTVVNVLPK 1eu1.1    GGKAVE-GAAWLSES----GATSIPCARVVDMLLNPGGEFQFNGATATYPDVKLAYWAGGNPFAH--HQDRNRMLKAWEK  target    MEMIAVQEWWWSTSCEWADIVFAVDAWSELKHPDMC--SSVTNPFLTVFPRTPLERPFDTRGDIECLDLVGKQLAKRTGD 1eu1.1    LETFIVQDFQWTATARHADIVLPATTSYERN--DIESVGDYSNRAILAMK-KVVDPLYEARSDYDIFAA----LAERLGK  target    RRFADMWKFVEEKKVEVYLQRILDHSSN------TKGFKFPELEEKAKKGIPALMMTRTNPKTVGYEQVY---DSRPWYT 1eu1.1    GA-----EFTEGRDEMGWISSFYEAAVKQAEFKNVAMPSFEDFWS---EGIVEFPITEG-ANFVRYADFREDPLFNPLGT  target    KTGRLEFYREEDEFIEAG--ENLPVHREPIDSTFYEPNVIVAPAHPFIKAKGPEAYGVKVDDFDNETRQGRNIVKTWEET 1eu1.1    PSGLIEIYSKNIEKMGYDDCPAHPTWMEPAER------------------------------------------------  target    KKTVHPLAKDGYKFVFHTPKYRHGAHTMPVDTDMVAMLFGPFGDIYRHDKRQPFAAEGYVDIHPDDAKALNIEDGDYVWI 1eu1.1    ---LG-GAGAKYPLHVVASHPKSRLHSQLNGTS-LRD----------LY---AVAGHEPCLINPADAAARGIADGDVLRV  target    DSDPSDRPFRGWQKNDKDYKFSRLLCRARYYPGTPRGITRMWFNMYGATPGSVEGHESRKDGLAKNPRTGYQAMFRSGSH 1eu1.1    FNDR-----------------GQILVGAKVSDAVMPGAIQIYEGG-----------------------------------  target    QSATRGWLKPTWMTDSLVRKELFGHAVNKGFLPDVHCPTGAPREAIVKITKAEPGGLNAKGLWRPAALGLRPKYENDKMK 1eu1.1    --------------------------------------------------------------------------------  target    DYLAGKFTLAANPKKGGKK 1eu1.1    ------------------- ``` | | | | | | | | | | | | | | | | | | | | | | | | | | | | | | | | | | | | | | | | | | | | | | | | | |
|  | 1ogy.1.A | PERIPLASMIC NITRATE REDUCTASE  *Crystal structure of the heterodimeric nitrate reductase from Rhodobacter sphaeroides* | 0.26 |  | 16.20 | 0.62 | 1-502 | X-ray | 3.20 | hetero-1-1-mer | 1 x SF4, 1 x MO, 2 x MGD, 2 x HEC | HHblits | 0.27 |
| ``` target    LARDIAKVPGTTLFAIGMGPNQFFNNDNKDRTQFLLAALTGNIGKIAGNIGSYAGNYRVAMFN-GVPQYIAENPFDIELD 1ogy.1    LAELYADPDRKWMSLWTMGFNQHVRGVWANHMVYNLHLLTGKISEPGNSPFSLTGQPFACGTAREVGTFAHRLPADMVVT  target    -GAKPARPKLYWRAEPAHYYNHEDHPLKMGKTMIT-GKTHMPTPTKSLWFANANSILGNVKWHFNTVVNVLPKMEMIAVQ 1ogy.1    NPEHRAHAEEIWKL------PAGLLPDWVGAHAVEQDRKLHDGEINFYWVQVNNNMQAAPNIDQETYPGYRNPENFIVVS  target    EWWWSTSCEWADIVFAVDAWSELKHPDMCSSVTNPFLTVFPRTPLERPFDTRGDIECLDLVGKQLAKRTGDRRFADMWKF 1ogy.1    DAYPTVTGRAADLVLPAAMWVEKE--GAYGN-AERRTHFWH-QLVEAPGEARSDLWQLME----FSKRFTTDEVW-PEEI  target    VE---EKKVEVYLQRILDHS------------------------SNTKGFKFPELEEKAK---------------KGIPA 1ogy.1    LSAAPAYRGKTLFEVLFANGSVDRFPASDVNPDHANHEAALFGFYPQKG-LFEEYAAFGRGHGHDLAPFDTYHEVRGLHW  target    LMMTRTNPKTVGYEQVYDSRPWYTKTGRLEFYREEDEFIEAGENLPVHREPIDSTFYEPNVIVAPAHPFIKAKGPEAYGV 1ogy.1    PVVEG--EE-TRWRYREGFDPYVKPGEGLRFYGKPDGRAVI-LGVP-YEP------------------------------  target    KVDDFDNETRQGRNIVKTWEETKKTVHPLAKDGYKFVFHTPKYRHGAHTMPVDTDMVAMLFGPFGDIYRHDKRQPFAAEG 1ogy.1    ---------------------PAE----SPDEEFGFWLVTGRVLEHWHSGSMTLRWP-----------ELYK---AFPGA  target    YVDIHPDDAKALNIEDGDYVWIDSDPSDRPFRGWQKNDKDYKFSRLLCRARY--YPGTPRGITRMWFNMYGATPGSVEGH 1ogy.1    VCFMHPEDARSRGLNRGSEVRVISRR-----------------GEIRTRLETRGRNRMPRGVVFVPWFD-----------  target    ESRKDGLAKNPRTGYQAMFRSGSHQSATRGWLKPTWMTDSLVRKELFGHAVNKGFLPDVHCPTGAPREAIVKITKAEPGG 1ogy.1    --------------------------------------------------------------------------------  target    LNAKGLWRPAALGLRPKYENDKMKDYLAGKFTLAANPKKGGKK 1ogy.1    ------------------------------------------- ``` | | | | | | | | | | | | | | | | | | | | | | | | | | | | | | | | | | | | | | | | | | | | | | | | | |
|  | 2e7z.1.A | Acetylene hydratase Ahy  *Acetylene Hydratase from Pelobacter acetylenicus* | 0.29 |  | 16.36 | 0.61 | 1-504 | X-ray | 1.26 | monomer | 1 x SF4, 2 x MGD, 1 x W | HHblits | 0.29 |
| ``` target    LARDIAKVPGTTLFAIGMGPNQFFNNDNKDRTQFLLAALTGNIGKIAGNIGSYAGNY--RVAMF---NGVPQYIAE---- 2e7z.1    AAVMFATESPA-SIPWAVSTDMQKNSCSAIRAQCILRAIVGSFVN-GAEILGAPHSDLVPISKIQMHEALPEEKKKLQLG  target    -NPFDIE-LDG--AKPARPKLYWRAEPAHY-YNHEDHPLKMGKTMITGKTHMPTPTKSLWFANANSILGNVKWHFNTVVN 2e7z.1    TETYPFLTYTGMSALEEPSERVYGVKYFHNMGAFMANPTALFTAMATEK---PYPVKAFFALASNALMGY--ANQQNALK  target    VLPKMEMIAVQEWWWSTSCEWADIVFAVDAWSELKHPDMCSSV-TNPFLTVFPRTPLERPFDTRGDIECLDLVGKQLAKR 2e7z.1    GLMNQDLVVCYDQFMTPTAQLADYVLPGDHWLERP--VVQPNWEGIPFGNTSQ-QVVEPAGEAKDEYYFIRE----LAVR  target    TGDRRFADMWKFVEEKKVEVYLQRILDHSSNTKGFKFPELEEKAKKGIPALMMTRTNPKTVGYEQVYDSRPWYTKTGRLE 2e7z.1    MGLEE---HFPW---KDRLELINYRISP----TGMEWEEYQKQY--TYMSK-----LPDYF----GPEGVGVATPSGKVE  target    FYREEDEFIEAG-ENLPVHREPIDSTFYEPNVIVAPAHPFIKAKGPEAYGVKVDDFDNETRQGRNIVKTWEETKKTVHPL 2e7z.1    LYSSVFE--KLGYDPLPYYHEPLQTEIS-------------------------------------------------DPE  target    AKDGYKFVFHTPKYR-HGAHTMPVDTDMVAMLFGPFGDIYRHDKRQPFAAEGYVDIHPDDAKALNIEDGDYVWIDSDPSD 2e7z.1    LAKEYPLILFAGLREDSNFQSCYHQPGILR-------------D---AEPDPVALLHPKTAQSLGLPSGEWIWVETTH--  target    RPFRGWQKNDKDYKFSRLLCRARYYPGTPRGITRMWFNMYGATPGSVEGHESRKDGLAKNPRTGYQAMFRSGSHQSATRG 2e7z.1    ---------------GRLKLLLKHDGAQPEGTIRIPHGRWC---------------------------------------  target    WLKPTWMTDSLVRKELFGHAVNKGFLPDVHCPTGAPREAIVKITKAEPGGLNAKGLWRPAALGLRPKYENDKMKDYLAGK 2e7z.1    --------------------------------------------------------------------------------  target    FTLAANPKKGGKK 2e7z.1    ------------- ``` | | | | | | | | | | | | | | | | | | | | | | | | | | | | | | | | | | | | | | | | | | | | | | | | | |
|  | 2v45.1.A | PERIPLASMIC NITRATE REDUCTASE  *A NEW CATALYTIC MECHANISM OF PERIPLASMIC NITRATE REDUCTASE FROM DESULFOVIBRIO DESULFURICANS ATCC 27774 FROM CRYSTALLOGRAPHIC AND EPR DATA AND BASED ON DETAILED ANALYSIS OF THE SIXTH LIGAND* | 0.31 |  | 17.78 | 0.61 | 1-502 | X-ray | 2.40 | monomer | 1 x SF4, 1 x MO, 2 x MGD, 1 x LCP | HHblits | 0.28 |
| ``` target    LARDIAKVPGTTLFAIGMGPNQFFNNDNKDRTQFLLAALTGNIGKIAGNIGSYAGNYRVAM---FNG-VPQYIAENPFDI 2v45.1    AARAFAESAA-TMSLWCMGINQRVQGVFANNLIHNLHLITGQICRPGATSFSLTGQPNACGGVRDGGALSHLL---PAGR  target    ELDGAK-PARPKLYWRAEPAHYYNHEDHPLKMGKTMITGKTHMPTPTKSLWFANANSILGNVKWHFNTVVNVLPKME-MI 2v45.1    AIPNAKHRAEMEKLWGLPEGRIAPEP--GYHT-VALFE--ALGRGDVKCMIICETNPAHTL--PNLNKVHKAMSHPESFI  target    AVQEWWWS-TSCEWADIVFAVDAWSELKHPDMCSSVTNPFLTVFPRTPLERPFDTRGDIECLDLVGKQLAKRTGDRRFAD 2v45.1    VCIEAFPDAVTLEYADLVLPPAFWCERD--GVYG-CGERRYSLTE-KAVDPPGQCRPTVNTLVE----FARRAGVDP--Q  target    MWKFVEEKKVEVYLQRILDHSS----NTKGFKFPELEEKAKKGIPALMMTRTNPK-TVGYEQVYDSRPWYTKTGRLEFYR 2v45.1    LVNFR---NAEDVWNEWRMVSKGTTYDFWGMTRERLRKE--SGLIWPCPSEDHPGTSLRYVRGQDPCVPADHPDRFFFYG  target    EEDEFIEAGENLPVHREPIDSTFYEPNVIVAPAHPFIKAKGPEAYGVKVDDFDNETRQGRNIVKTWEETKKTVHPLAKDG 2v45.1    KPDGR------AVIWMRPAK-----------------------------------------------G----AAEEPDAE  target    YKFVFHTPKYRHGAHTMPVDTDMVAMLFGPFGDIYRHDKRQPFAAEGYVDIHPDDAKALNIEDGDYVWIDSDPSDRPFRG 2v45.1    YPLYLTSMRVIDHWHTATMTGK-----------VPELQK---ANPIAFVEINEEDAARTGIKHGDSVIVETRR-------  target    WQKNDKDYKFSRLLCRARYYPGTPRGITRMWFNMYGATPGSVEGHESRKDGLAKNPRTGYQAMFRSGSHQSATRGWLKPT 2v45.1    ----------DAMELPARVSDVCRPGLIAVPFFD----------------------------------------------  target    WMTDSLVRKELFGHAVNKGFLPDVHCPTGAPREAIVKITKAEPGGLNAKGLWRPAALGLRPKYENDKMKDYLAGKFTLAA 2v45.1    --------------------------------------------------------------------------------  target    NPKKGGKK 2v45.1    -------- ``` | | | | | | | | | | | | | | | | | | | | | | | | | | | | | | | | | | | | | | | | | | | | | | | | | |
|  | 2vpz.1.A | THIOSULFATE REDUCTASE  *POLYSULFIDE REDUCTASE NATIVE STRUCTURE* | 0.31 |  | 17.28 | 0.60 | 1-504 | X-ray | 2.40 | hetero-oligomer | 10 x SF4, 4 x MGD, 2 x MO | HHblits | 0.28 |
| ``` target    LARDIAKVPGTTLFAIGMGP-NQFFNNDNKDRTQFLLAALTGNIGKIAGNIGSYAGNYRVAMFNGVPQYIAENPFDIE-L 2vpz.1    VAREMAAHKPRAVL-PPTRHNVWYGDDTYRVMALLYVNVLLGNYGRPGGFYIAQSPYLEKYPLPPLPLEPAAGGCSGPSG  target    DGAKPARPKLYWRAEPAHYYNHEDHPLKMGKTMITGKTHMPTPTKSLWFANANSILGNVKWHFNTVVNVLPKMEMIAVQE 2vpz.1    GDHEPEGFKPR--ADKGKFFARSTAIQELIEPMITGE---PYPIKGLFAYGINLFHSI--PNVPRTKEALKNLDLYVAID  target    WWWSTSCEWADIVFAVDAWSELKHPDMCSSVTNPFLTVFPRTPLERPFDTRGDIECLDLVGKQLAKRTGDRRFADMWKFV 2vpz.1    VLPQEHVMWADVILPEATYLERYDDFVLVAHKTPFIQLRT-PAHEPLFDTKPGWWIARE----LGLRLGLEQ---YFPW-  target    EEKKVEVYLQRILDHSSNTKGFKFPELEEKAKKGIPALMMTRTNPKTVGYEQVYDSRPWYTKTGRLEFYREEDEFIEAG- 2vpz.1    --KTIEEYLETRLQSL----GLDLETMKGMG---TLVQR---GKPWLEDW-EKEGRLPFGTASGKIELYCQRFK--EAGH  target    ENLPVHREPIDSTFYEPNVIVAPAHPFIKAKGPEAYGVKVDDFDNETRQGRNIVKTWEETKKTVHPLAKDGYKFVFHTPK 2vpz.1    QPLPVFTPPEE---------------------------------------------------------PPEGFYRLLYGR  target    YRHGAHTMPVDTDMVAMLFGPFGDIYRHDKRQPFAAEGYVDIHPDDAKALNIEDGDYVWIDSDPSDRPFRGWQKNDKDYK 2vpz.1    SPVHTFARTQNNWVLM-------------E---MDPENEVWIHKEEAKRLGLKEGDYVMLVNQD----------------  target    FSRLLCRA--RYYPGTPRGITRMWFNMYGATPGSVEGHESRKDGLAKNPRTGYQAMFRSGSHQSATRGWLKPTWMTDSLV 2vpz.1    -GVKEGPVRVKPTARIRKDCVYIVHGFGH---------------------------------------------------  target    RKELFGHAVNKGFLPDVHCPTGAPREAIVKITKAEPGGLNAKGLWRPAALGLRPKYENDKMKDYLAGKFTLAANPKKGGK 2vpz.1    --------------------------------------------------------------------------------  target    K 2vpz.1    - ``` | | | | | | | | | | | | | | | | | | | | | | | | | | | | | | | | | | | | | | | | | | | | | | | | | |
|  | 2vpx.1.D | THIOSULFATE REDUCTASE  *POLYSULFIDE REDUCTASE WITH BOUND QUINONE (UQ1)* | 0.30 |  | 17.28 | 0.60 | 1-504 | X-ray | 3.10 | hetero-oligomer | 10 x SF4, 4 x MGD, 2 x MO, 2 x UQ1 | HHblits | 0.28 |
| ``` target    LARDIAKVPGTTLFAIGMGP-NQFFNNDNKDRTQFLLAALTGNIGKIAGNIGSYAGNYRVAMFNGVPQYIAENPFDIE-L 2vpx.1    VAREMAAHKPRAVL-PPTRHNVWYGDDTYRVMALLYVNVLLGNYGRPGGFYIAQSPYLEKYPLPPLPLEPAAGGCSGPSG  target    DGAKPARPKLYWRAEPAHYYNHEDHPLKMGKTMITGKTHMPTPTKSLWFANANSILGNVKWHFNTVVNVLPKMEMIAVQE 2vpx.1    GDHEPEGFKPR--ADKGKFFARSTAIQELIEPMITGE---PYPIKGLFAYGINLFHSI--PNVPRTKEALKNLDLYVAID  target    WWWSTSCEWADIVFAVDAWSELKHPDMCSSVTNPFLTVFPRTPLERPFDTRGDIECLDLVGKQLAKRTGDRRFADMWKFV 2vpx.1    VLPQEHVMWADVILPEATYLERYDDFVLVAHKTPFIQLRT-PAHEPLFDTKPGWWIARE----LGLRLGLEQ---YFPW-  target    EEKKVEVYLQRILDHSSNTKGFKFPELEEKAKKGIPALMMTRTNPKTVGYEQVYDSRPWYTKTGRLEFYREEDEFIEAG- 2vpx.1    --KTIEEYLETRLQSL----GLDLETMKGMG---TLVQR---GKPWLEDW-EKEGRLPFGTASGKIELYCQRFK--EAGH  target    ENLPVHREPIDSTFYEPNVIVAPAHPFIKAKGPEAYGVKVDDFDNETRQGRNIVKTWEETKKTVHPLAKDGYKFVFHTPK 2vpx.1    QPLPVFTPPEE---------------------------------------------------------PPEGFYRLLYGR  target    YRHGAHTMPVDTDMVAMLFGPFGDIYRHDKRQPFAAEGYVDIHPDDAKALNIEDGDYVWIDSDPSDRPFRGWQKNDKDYK 2vpx.1    SPVHTFARTQNNWVLM-------------E---MDPENEVWIHKEEAKRLGLKEGDYVMLVNQD----------------  target    FSRLLCRA--RYYPGTPRGITRMWFNMYGATPGSVEGHESRKDGLAKNPRTGYQAMFRSGSHQSATRGWLKPTWMTDSLV 2vpx.1    -GVKEGPVRVKPTARIRKDCVYIVHGFGH---------------------------------------------------  target    RKELFGHAVNKGFLPDVHCPTGAPREAIVKITKAEPGGLNAKGLWRPAALGLRPKYENDKMKDYLAGKFTLAANPKKGGK 2vpx.1    --------------------------------------------------------------------------------  target    K 2vpx.1    - ``` | | | | | | | | | | | | | | | | | | | | | | | | | | | | | | | | | | | | | | | | | | | | | | | | | |
|  | 1g8k.1.A | ARSENITE OXIDASE  *CRYSTAL STRUCTURE ANALYSIS OF ARSENITE OXIDASE FROM ALCALIGENES FAECALIS* | 0.28 |  | 15.50 | 0.61 | 1-502 | X-ray | 1.64 | hetero-1-1-mer | 3 x HG, 2 x CA, 2 x MGD, 1 x O, 1 x 4MO, 1 x F3S, 1 x FES | HHblits | 0.27 |
| ``` target    LARDIAKVP-----GTTLFAIGMGPNQFFNNDNKDRTQFLLAALTGNIGKIAGNIGSYAGNYRVAMFNGVPQYIAENPFD 1g8k.1    AAEWSYKPKASGQAPRTMHAYEKGIIWGNDNYVIQSALLDLVIATHNVGRRGTGCVRMGGHQEGYTRPPYPGDKKIY-ID  target    IELDGAKPARPKLYWRAEPAHYYNHEDHPLKMGKTMITGKTHMPTPTKSLWFANANSILGNVKWHFNTVVNVLPK-MEMI 1g8k.1    QELIKG-KGRIMTWWGCNNFQ--TS-NNAQALREAILQR---SAIVKQAMQKARGATTE----EMVDVIYEATQNGGLFV  target    AVQEWWWSTSCEWADIVFAVDAWSELKHPDMCSSVTNPFLTVFPRTPLERPFDTRGDIECLDLVGKQLAKRTGDRR---- 1g8k.1    TSINLYPTKLAEAAHLMLPAAHPGEMN--LTS-MNGERRIRLSE-KFMDPPGTAMADCLIAAR----IANALRDMYQKDG  target    ---FADMWKFVEEKKVEVYLQRILDHSSN--------T-----KGFKFPELEEKAKKGIPALMMTRTN-PKTVGYEQVYD 1g8k.1    KAEMAAQFEGFDWKTEEDAFNDGFRRAGQPGAPAIDSQGGSTGHLVTYDRLRKSGNNGVQLPVVSWDESKGLVGTEMLYT  target    SRPWYTKTGRLEFYREEDEFIEAGENLPVHREPIDSTFYEPNVIVAPAHPFIKAKGPEAYGVKVDDFDNETRQGRNIVKT 1g8k.1    EGKFDTDDGKAHFKPAPW------NGLPATVQ------------------------------------------------  target    WEETKKTVHPLAKDGYKFVFHTPKYRHGAHTMPVDTDMVAMLFGPFGDIYRHDKRQPFAAEGYVDIHPDDAKALNIEDGD 1g8k.1    ----------QQKDKYRFWLNNGRNNEVWQTAYHDQYN-----------SLMQE---RYPMAYIEMNPDDCKQLDVTGGD  target    YVWIDSDPSDRPFRGWQKNDKDYKFSRLLCRARYYPGTPRGITRMWFNMYGATPGSVEGHESRKDGLAKNPRTGYQAMFR 1g8k.1    IVEVYNDF-----------------GSTFAMVYPVAEIKRGQTFMLFGY-------------------------------  target    SGSHQSATRGWLKPTWMTDSLVRKELFGHAVNKGFLPDVHCPTGAPREAIVKITKAEPGGLNAKGLWRPAALGLRPKYEN 1g8k.1    --------------------------------------------------------------------------------  target    DKMKDYLAGKFTLAANPKKGGKK 1g8k.1    ----------------------- ``` | | | | | | | | | | | | | | | | | | | | | | | | | | | | | | | | | | | | | | | | | | | | | | | | | |
|  | 1g8j.1.A | ARSENITE OXIDASE  *CRYSTAL STRUCTURE ANALYSIS OF ARSENITE OXIDASE FROM ALCALIGENES FAECALIS* | 0.28 |  | 15.58 | 0.61 | 1-502 | X-ray | 2.03 | hetero-oligomer | 2 x MGD, 1 x O, 1 x 4MO, 1 x F3S, 1 x FES | HHblits | 0.27 |
| ``` target    LARDIAKVP-----GTTLFAIGMGPNQFFNNDNKDRTQFLLAALTGNIGKIAGNIGSYAGNYRVAMFNGVPQYIAENPFD 1g8j.1    AAEWSYKPKASGQAPRTMHAYEKGIIWGNDNYVIQSALLDLVIATHNVGRRGTGCVRMGGHQEGYTRPPYPGDKKIY-ID  target    IELDGAKPARPKLYWRAEPAHYYNHEDHPLKMGKTMITGKTHMPTPTKSLWFANANSILGNVKWHFNTVVNVLPKME-MI 1g8j.1    QELIKG-KGRIMTWWGCNNFQ---TSNNAQALREAILQRS---AIVKQAMQKARGATTEE----MVDVIYEATQNGGLFV  target    AVQEWWWSTSCEWADIVFAVDAWSELKHPDMCSSVTNPFLTVFPRTPLERPFDTRGDIECLDLVGKQLAKRTGDRRF--- 1g8j.1    TSINLYPTKLAEAAHLMLPAAHPGEMN--LTSM-NGERRIRLSE-KFMDPPGTAMADCLIAAR----IANALRDMYQKDG  target    ----ADMWKFVEEKKVEVYLQRILDHSSNT-------------KGFKFPELEEKAKKGIPALMMTRT-NPKTVGYEQVYD 1g8j.1    KAEMAAQFEGFDWKTEEDAFNDGFRRAGQPGAPAIDSQGGSTGHLVTYDRLRKSGNNGVQLPVVSWDESKGLVGTEMLYT  target    SRPWYTKTGRLEFYREEDEFIEAGENLPVHREPIDSTFYEPNVIVAPAHPFIKAKGPEAYGVKVDDFDNETRQGRNIVKT 1g8j.1    EGKFDTDDGKAHFKPAPW------NGLPATVQ------------------------------------------------  target    WEETKKTVHPLAKDGYKFVFHTPKYRHGAHTMPVD--TDMVAMLFGPFGDIYRHDKRQPFAAEGYVDIHPDDAKALNIED 1g8j.1    ----------QQKDKYRFWLNNGRNNEVWQTAYHDQYNSLM-------------QE---RYPMAYIEMNPDDCKQLDVTG  target    GDYVWIDSDPSDRPFRGWQKNDKDYKFSRLLCRARYYPGTPRGITRMWFNMYGATPGSVEGHESRKDGLAKNPRTGYQAM 1g8j.1    GDIVEVYNDF-----------------GSTFAMVYPVAEIKRGQTFMLFGY-----------------------------  target    FRSGSHQSATRGWLKPTWMTDSLVRKELFGHAVNKGFLPDVHCPTGAPREAIVKITKAEPGGLNAKGLWRPAALGLRPKY 1g8j.1    --------------------------------------------------------------------------------  target    ENDKMKDYLAGKFTLAANPKKGGKK 1g8j.1    ------------------------- ``` | | | | | | | | | | | | | | | | | | | | | | | | | | | | | | | | | | | | | | | | | | | | | | | | | |
|  | 1aa6.1.A | FORMATE DEHYDROGENASE H  *REDUCED FORM OF FORMATE DEHYDROGENASE H FROM E. COLI* | 0.31 | 0.00 | 20.65 | 0.58 | 1-502 | X-ray | 2.30 | monomer | 1 x SF4, 2 x MGD, 1 x 4MO | HHblits | 0.31 |
| ``` target    LARDIAKVPGTTLFAIGMGPNQFFNNDNKDRTQFLLAALTGNIGKIAGNIGSYAGNYRVAM-F--NGVPQYIAENPFDIE 1aa6.1    AARMYAQAKS-AAILWGMGVTQFYQGVETVRSLTSLAMLTGNLGKPHAGVNPVRGQNNVQGACDMGALPDTYPG--YQYV  target    LDGAKPARPKLYWRAEPAHYYNHEDHPLKMG---KTMITGKTHMPTPTKSLWFANANSILGNVKWHFNTVVNVLPKMEMI 1aa6.1    KDPANREKFAKAWGV--------ESLPAHTGYRISELPH--RAAHGEVRAAYIMGEDPLQT--DAELSAVRKAFEDLELV  target    AVQEWWWSTSCEWADIVFAVDAWSELKHPDMCSSVTNPFLTVFPRTPLERPFDTRGDIECLDLVGKQLAKRTGDRRFADM 1aa6.1    IVQDIFMTKTASAADVILPSTSWGEHE--GVFTA-ADRGFQRFF-KAVEPKWDLKTDWQIISE----IATRMGYPM----  target    WKFVEEKKVEVYLQRILDHSSNTKGFKFPELEEKAKKGIPALMMTRTNPKTVGYEQVYDSRPWYTKTGRLEFYREEDEFI 1aa6.1    -HYN---NTQEIWDELRHLCPDFYGATYEKMGEL---GFIQWPCRDTSDADQGT-SYLFKEKFDTPNGLAQFFTCDW---  target    EAGENLPVHREPIDSTFYEPNVIVAPAHPFIKAKGPEAYGVKVDDFDNETRQGRNIVKTWEETKKTVHPLAKDGYKFVFH 1aa6.1    ---------VAPID-------------------------------------------------------KLTDEYPMVLS  target    TPKYR--HGAHTMPVDTDMVAMLFGPFGDIYRHDKRQPFAAEGYVDIHPDDAKALNIEDGDYVWIDSDPSDRPFRGWQKN 1aa6.1    TVREVGHYSCRSMTGNCAALAAL-------------AD--EPGYAQINTEDAKRLGIEDEALVWVHSRK-----------  target    DKDYKFSRLLCRARYYPGTPRGITRMWFNMYGATPGSVEGHESRKDGLAKNPRTGYQAMFRSGSHQSATRGWLKPTWMTD 1aa6.1    ------GKIITRAQVSDRPNKGAIYMTYQW--------------------------------------------------  target    SLVRKELFGHAVNKGFLPDVHCPTGAPREAIVKITKAEPGGLNAKGLWRPAALGLRPKYENDKMKDYLAGKFTLAANPKK 1aa6.1    --------------------------------------------------------------------------------  target    GGKK 1aa6.1    ---- ``` | | | | | | | | | | | | | | | | | | | | | | | | | | | | | | | | | | | | | | | | | | | | | | | | | |
|  | 1fdo.1.A | FORMATE DEHYDROGENASE H  *OXIDIZED FORM OF FORMATE DEHYDROGENASE H FROM E. COLI* | 0.31 | 0.00 | 20.65 | 0.58 | 1-502 | X-ray | 2.80 | monomer | 1 x SF4, 2 x MGD, 1 x 6MO | HHblits | 0.31 |
| ``` target    LARDIAKVPGTTLFAIGMGPNQFFNNDNKDRTQFLLAALTGNIGKIAGNIGSYAGNYRVAM-F--NGVPQYIAENPFDIE 1fdo.1    AARMYAQAKS-AAILWGMGVTQFYQGVETVRSLTSLAMLTGNLGKPHAGVNPVRGQNNVQGACDMGALPDTYPG--YQYV  target    LDGAKPARPKLYWRAEPAHYYNHEDHPLKMG---KTMITGKTHMPTPTKSLWFANANSILGNVKWHFNTVVNVLPKMEMI 1fdo.1    KDPANREKFAKAWGV--------ESLPAHTGYRISELPH--RAAHGEVRAAYIMGEDPLQT--DAELSAVRKAFEDLELV  target    AVQEWWWSTSCEWADIVFAVDAWSELKHPDMCSSVTNPFLTVFPRTPLERPFDTRGDIECLDLVGKQLAKRTGDRRFADM 1fdo.1    IVQDIFMTKTASAADVILPSTSWGEHE--GVFTA-ADRGFQRFF-KAVEPKWDLKTDWQIISE----IATRMGYPM----  target    WKFVEEKKVEVYLQRILDHSSNTKGFKFPELEEKAKKGIPALMMTRTNPKTVGYEQVYDSRPWYTKTGRLEFYREEDEFI 1fdo.1    -HYN---NTQEIWDELRHLCPDFYGATYEKMGEL---GFIQWPCRDTSDADQGT-SYLFKEKFDTPNGLAQFFTCDW---  target    EAGENLPVHREPIDSTFYEPNVIVAPAHPFIKAKGPEAYGVKVDDFDNETRQGRNIVKTWEETKKTVHPLAKDGYKFVFH 1fdo.1    ---------VAPID-------------------------------------------------------KLTDEYPMVLS  target    TPKYR--HGAHTMPVDTDMVAMLFGPFGDIYRHDKRQPFAAEGYVDIHPDDAKALNIEDGDYVWIDSDPSDRPFRGWQKN 1fdo.1    TVREVGHYSCRSMTGNCAALAAL-------------AD--EPGYAQINTEDAKRLGIEDEALVWVHSRK-----------  target    DKDYKFSRLLCRARYYPGTPRGITRMWFNMYGATPGSVEGHESRKDGLAKNPRTGYQAMFRSGSHQSATRGWLKPTWMTD 1fdo.1    ------GKIITRAQVSDRPNKGAIYMTYQW--------------------------------------------------  target    SLVRKELFGHAVNKGFLPDVHCPTGAPREAIVKITKAEPGGLNAKGLWRPAALGLRPKYENDKMKDYLAGKFTLAANPKK 1fdo.1    --------------------------------------------------------------------------------  target    GGKK 1fdo.1    ---- ``` | | | | | | | | | | | | | | | | | | | | | | | | | | | | | | | | | | | | | | | | | | | | | | | | | |
|  | 2iv2.1.A | Formate dehydrogenase H  *Reinterpretation of reduced form of formate dehydrogenase H from E. coli* | 0.31 | 0.00 | 20.65 | 0.58 | 1-502 | X-ray | 2.27 | monomer | 1 x SF4, 1 x 2MD, 1 x MGD | HHblits | 0.31 |
| ``` target    LARDIAKVPGTTLFAIGMGPNQFFNNDNKDRTQFLLAALTGNIGKIAGNIGSYAGNYRVAM-F--NGVPQYIAENPFDIE 2iv2.1    AARMYAQAKS-AAILWGMGVTQFYQGVETVRSLTSLAMLTGNLGKPHAGVNPVRGQNNVQGACDMGALPDTYPG--YQYV  target    LDGAKPARPKLYWRAEPAHYYNHEDHPLKMG---KTMITGKTHMPTPTKSLWFANANSILGNVKWHFNTVVNVLPKMEMI 2iv2.1    KDPANREKFAKAWGV--------ESLPAHTGYRISELPH--RAAHGEVRAAYIMGEDPLQT--DAELSAVRKAFEDLELV  target    AVQEWWWSTSCEWADIVFAVDAWSELKHPDMCSSVTNPFLTVFPRTPLERPFDTRGDIECLDLVGKQLAKRTGDRRFADM 2iv2.1    IVQDIFMTKTASAADVILPSTSWGEHE--GVFTA-ADRGFQRFF-KAVEPKWDLKTDWQIISE----IATRMGYPM----  target    WKFVEEKKVEVYLQRILDHSSNTKGFKFPELEEKAKKGIPALMMTRTNPKTVGYEQVYDSRPWYTKTGRLEFYREEDEFI 2iv2.1    -HYN---NTQEIWDELRHLCPDFYGATYEKMGEL---GFIQWPCRDTSDADQGT-SYLFKEKFDTPNGLAQFFTCDW---  target    EAGENLPVHREPIDSTFYEPNVIVAPAHPFIKAKGPEAYGVKVDDFDNETRQGRNIVKTWEETKKTVHPLAKDGYKFVFH 2iv2.1    ---------VAPID-------------------------------------------------------KLTDEYPMVLS  target    TPKYR--HGAHTMPVDTDMVAMLFGPFGDIYRHDKRQPFAAEGYVDIHPDDAKALNIEDGDYVWIDSDPSDRPFRGWQKN 2iv2.1    TVREVGHYSCRSMTGNCAALAAL-------------AD--EPGYAQINTEDAKRLGIEDEALVWVHSRK-----------  target    DKDYKFSRLLCRARYYPGTPRGITRMWFNMYGATPGSVEGHESRKDGLAKNPRTGYQAMFRSGSHQSATRGWLKPTWMTD 2iv2.1    ------GKIITRAQVSDRPNKGAIYMTYQW--------------------------------------------------  target    SLVRKELFGHAVNKGFLPDVHCPTGAPREAIVKITKAEPGGLNAKGLWRPAALGLRPKYENDKMKDYLAGKFTLAANPKK 2iv2.1    --------------------------------------------------------------------------------  target    GGKK 2iv2.1    ---- ``` | | | | | | | | | | | | | | | | | | | | | | | | | | | | | | | | | | | | | | | | | | | | | | | | | |
|  | 7z0t.1.G | Formate dehydrogenase H  *Structure of the Escherichia coli formate hydrogenlyase complex (aerobic preparation, composite structure)* | 0.31 | 0.00 | 20.65 | 0.58 | 1-502 | EM | 0.00 | monomer | 1 x NI, 1 x FCO, 8 x SF4, 1 x FE, 2 x MGD, 1 x 6MO | HHblits | 0.31 |
| ``` target    LARDIAKVPGTTLFAIGMGPNQFFNNDNKDRTQFLLAALTGNIGKIAGNIGSYAGNYRVAM-F--NGVPQYIAENPFDIE 7z0t.1    AARMYAQAKS-AAILWGMGVTQFYQGVETVRSLTSLAMLTGNLGKPHAGVNPVRGQNNVQGACDMGALPDTYPG--YQYV  target    LDGAKPARPKLYWRAEPAHYYNHEDHPLKMG---KTMITGKTHMPTPTKSLWFANANSILGNVKWHFNTVVNVLPKMEMI 7z0t.1    KDPANREKFAKAWGV--------ESLPAHTGYRISELPH--RAAHGEVRAAYIMGEDPLQT--DAELSAVRKAFEDLELV  target    AVQEWWWSTSCEWADIVFAVDAWSELKHPDMCSSVTNPFLTVFPRTPLERPFDTRGDIECLDLVGKQLAKRTGDRRFADM 7z0t.1    IVQDIFMTKTASAADVILPSTSWGEHE--GVFTA-ADRGFQRFF-KAVEPKWDLKTDWQIISE----IATRMGYPM----  target    WKFVEEKKVEVYLQRILDHSSNTKGFKFPELEEKAKKGIPALMMTRTNPKTVGYEQVYDSRPWYTKTGRLEFYREEDEFI 7z0t.1    -HYN---NTQEIWDELRHLCPDFYGATYEKMGEL---GFIQWPCRDTSDADQGT-SYLFKEKFDTPNGLAQFFTCDW---  target    EAGENLPVHREPIDSTFYEPNVIVAPAHPFIKAKGPEAYGVKVDDFDNETRQGRNIVKTWEETKKTVHPLAKDGYKFVFH 7z0t.1    ---------VAPID-------------------------------------------------------KLTDEYPMVLS  target    TPKYR--HGAHTMPVDTDMVAMLFGPFGDIYRHDKRQPFAAEGYVDIHPDDAKALNIEDGDYVWIDSDPSDRPFRGWQKN 7z0t.1    TVREVGHYSCRSMTGNCAALAAL-------------AD--EPGYAQINTEDAKRLGIEDEALVWVHSRK-----------  target    DKDYKFSRLLCRARYYPGTPRGITRMWFNMYGATPGSVEGHESRKDGLAKNPRTGYQAMFRSGSHQSATRGWLKPTWMTD 7z0t.1    ------GKIITRAQVSDRPNKGAIYMTYQW--------------------------------------------------  target    SLVRKELFGHAVNKGFLPDVHCPTGAPREAIVKITKAEPGGLNAKGLWRPAALGLRPKYENDKMKDYLAGKFTLAANPKK 7z0t.1    --------------------------------------------------------------------------------  target    GGKK 7z0t.1    ---- ``` | | | | | | | | | | | | | | | | | | | | | | | | | | | | | | | | | | | | | | | | | | | | | | | | | |
|  | 7bkb.1.F | Formate dehydrogenase  *Formate dehydrogenase - heterodisulfide reductase - formylmethanofuran dehydrogenase complex from Methanospirillum hungatei (hexameric, composite structure)* | 0.28 |  | 18.82 | 0.58 | 1-502 | EM | 0.00 | hetero-2-2-2-2-2-2-… | 48 x SF4, 4 x FAD, 2 x FES, 4 x 9S8, 4 x ZN, 2 x MO, 4 x MGD | HHblits | 0.30 |
| ``` target    LARDIAKVPGTTLFAIGMGPNQFFNNDNKDRTQFLLAALTGNIGKIAGNIGSYAGNYRVAMFNGVPQYIAENPFDIELDG 7bkb.1    IAFRYAKAKNA-VIIYCLGITELTTGTDNVRSMGNLALLTGNVGREGVGVNPLRGQNNVQGACDMGAYPNVYSGYQKCEV  target    A-KPARPKLYWRAEPAHYYNHEDHPLKMGKTMITGKTHMPTPTKSLWFANANSILGNVKWHFNTVVNVLPKMEMIAVQEW 7bkb.1    AENRAKMEKAW--------SVTNLPDWYGATLTEQINQCGDEIKGMYILGLNPVVTY--PSSNHVKAQLEKLDFLVVQDI  target    WWSTSCEWADIVFAVDAWSELKHPDMCSSVTNPFLTVFPRTPLERPFDTRGDIECLDLVGKQLAKRTGDRRFADMWKFVE 7bkb.1    FFTETCQYADVILPGACFAEKD--GTFTS-GERRINRVR-KAVNPPGQAKEDIHIISE----LAAKMGFKG----FEL--  target    EKKVEVYLQRILDHSSNTKGFKFPELEEKAKKGIPALMMTRTNPKTVGYEQVYDSRPWYTKTGRLEFYREEDEFIEAGEN 7bkb.1    -PTAKDVWDDMRAVTPSMFGATYEKLERP--EGICWPCPTEEHPGTP----ILHREKFATADGKGNLFGID---------  target    LPVHREPIDSTFYEPNVIVAPAHPFIKAKGPEAYGVKVDDFDNETRQGRNIVKTWEETKKTVHPLAKDGYKFVFHTPKYR 7bkb.1    ---YRPPAE-------------------------------------------------------VADAEYPFTLMTGRLI  target    HGAHTMPVDTDMVAMLFGPFGDIYRHDKRQPFAAEGYVDIHPDDAKALNIEDGDYVWIDSDPSDRPFRGWQKNDKDYKFS 7bkb.1    FHYHSRTQTDR-----------AADLHR---EVPESYAQINIEDARRLGIKNNEYIKLKSRR-----------------G  target    RLLCRARYYPGTPRGITRMWFNMYGATPGSVEGHESRKDGLAKNPRTGYQAMFRSGSHQSATRGWLKPTWMTDSLVRKEL 7bkb.1    ETTTLARVTDEVAPGVVYMTMHF---------------------------------------------------------  target    FGHAVNKGFLPDVHCPTGAPREAIVKITKAEPGGLNAKGLWRPAALGLRPKYENDKMKDYLAGKFTLAANPKKGGKK 7bkb.1    ----------------------------------------------------------------------------- ``` | | | | | | | | | | | | | | | | | | | | | | | | | | | | | | | | | | | | | | | | | | | | | | | | | |
|  | 4aay.1.A | AROA  *Crystal Structure of the arsenite oxidase protein complex from Rhizobium species strain NT-26* | 0.27 |  | 14.96 | 0.60 | 1-504 | X-ray | 2.70 | hetero-oligomer | 4 x MGD, 2 x O, 2 x 4MO, 2 x F3S, 2 x FES | HHblits | 0.27 |
| ``` target    LARDIAKVPG-----TTLFAIGMGPNQFFNNDNKDRTQFLLAALTGNIGKIAGNIGSYAGNYRVAMFNGVPQYIAENPFD 4aay.1    AAEWIGMPKEGGKRRRVMFGYEKGLIWGNDNYRTNGALVNLALATGNIGRPGGGVVRLGGHQEGYVRPSDAH--VGRPAA  target    I--ELDGAKPARPKLYWRAEPAHYYNHEDHPLKMGKTMITGKTHMPTPTKSLWFANANSILG------N--VKWHFNTVV 4aay.1    YVDQLLIGGQGGVHHIW--------GCD----HYKT------TLNAHEFKRVYKKRTDMVKDAMSAAPYGDREAMVNAIV  target    NVLPKM-EMIAVQEWWWSTSCEWADIVFAVDAWSELKHPDMCSSVTNPFLTVFPRTPLERPFDTRGDIECLDLVGKQLAK 4aay.1    DAINQGGLFAVNVDIIPTKIGEACHVILPAATSGEMN--LTS-MNGERRMRLTE-RYMDPPGQSMPDCLIAARLANTMER  target    RTGD---RRFAD---MWKFVEEKKVEV-YLQRILDHSSNTKGFKFPELEEKAKKGIPALMMTRTNPKTVGYEQVYDSRPW 4aay.1    VLTEMGDVGYAAQFKGFDWQ---TEEDAFMDGYNKNAHGGEFVTYERLSAMGTNGFQEPATGFTDGKIEGTQRLYTDGVF  target    YTKTGRLEFYREEDEFIEAGENLPVHREPIDSTFYEPNVIVAPAHPFIKAKGPEAYGVKVDDFDNETRQGRNIVKTWEET 4aay.1    STDDGKARFMDAPWR---------GLQAPGK-------------------------------------------------  target    KKTVHPLAKDGYKFVFHTPKYRHGAHTMPVD--TDMVAMLFGPFGDIYRHDKRQPFAAEGYVDIHPDDAKALNIEDGDYV 4aay.1    -----QQQKDSHKYLINNGRANVVWQSAYLDQENDFV-------------MD---RFPYPFIEMNPEDMAEAGLKEGDLV  target    WIDSDPSDRPFRGWQKNDKDYKFSRLLCRARYYPGTPRGITRMWFNMYGATPGSVEGHESRKDGLAKNPRTGYQAMFRSG 4aay.1    EIYNDA-----------------GATQAMAYPTPTARRGETFMLFGFPT-------------------------------  target    SHQSATRGWLKPTWMTDSLVRKELFGHAVNKGFLPDVHCPTGAPREAIVKITKAEPGGLNAKGLWRPAALGLRPKYENDK 4aay.1    --------------------------------------------------------------------------------  target    MKDYLAGKFTLAANPKKGGKK 4aay.1    --------------------- ``` | | | | | | | | | | | | | | | | | | | | | | | | | | | | | | | | | | | | | | | | | | | | | | | | | |
|  | 2v3v.1.A | PERIPLASMIC NITRATE REDUCTASE  *A NEW CATALYTIC MECHANISM OF PERIPLASMIC NITRATE REDUCTASE FROM DESULFOVIBRIO DESULFURICANS ATCC 27774 FROM CRYSTALLOGRAPHIC AND EPR DATA AND BASED ON DETAILED ANALYSIS OF THE SIXTH LIGAND* | 0.31 |  | 17.51 | 0.59 | 1-502 | X-ray | 1.99 | monomer | 1 x SF4, 1 x MO, 2 x MGD, 4 x LCP | HHblits | 0.28 |
| ``` target    LARDIAKVPGTTLFAIGMGPNQFFNNDNKDRTQFLLAALTGNIGKIAGNIGSYAGNYRVAM---FNG-VPQYIAENPFDI 2v3v.1    AARAFAESAAT-MSLWCMGINQRVQGVFANNLIHNLHLITGQICRPGATSFSLTGQPNACGGVRDGGALSHLL---PAGR  target    ELDGAK-PARPKLYWRAEPAHYYNHEDHPLKMGKTMITGKTHMPTPTKSLWFANANSILGNVKWHFNTVVNVLPKME-MI 2v3v.1    AIPNAKHRAEMEKLWGLPEGRI--AP-EPGYHTVALFE--ALGRGDVKCMIICETNPAHTL--PNLNKVHKAMSHPESFI  target    AVQEWWWS-TSCEWADIVFAVDAWSELKHPDMCSSVTNPFLTVFPRTPLERPFDTRGDIECLDLVGKQLAKRTGDRRFAD 2v3v.1    VCIEAFPDAVTLEYADLVLPPAFWCERD--GVYG-CGERRYSLTE-KAVDPPGQCRPTVNTLVE----FARRAGVDPQ--  target    MWKFVEEKKVEVYLQRILDHSSN----TKGFKFPELEEKAKKGIPALMMTRTNPKTVGYEQVYD------------SRPW 2v3v.1    LVNF---RNAEDVWNEWRMVSKGTTYDFWGMTRERLRKE--SGLIWPCPSEDHPG-TSLRYVRGQDPCVPADHPDRFFFY  target    YTKTGRLEFYREEDEFIEAGENLPVHREPIDSTFYEPNVIVAPAHPFIKAKGPEAYGVKVDDFDNETRQGRNIVKTWEET 2v3v.1    GKPDGRAVIWMRPA----------------K-----------------------------------------------GA  target    KKTVHPLAKDGYKFVFHTPKYRHGAHTMPVDTDMVAMLFGPFGDIYRHDKRQPFAAEGYVDIHPDDAKALNIEDGDYVWI 2v3v.1    ----AEEPDAEYPLYLTSMRVIDHWHTATMTGK-----------VPELQK---ANPIAFVEINEEDAARTGIKHGDSVIV  target    DSDPSDRPFRGWQKNDKDYKFSRLLCRARYYPGTPRGITRMWFNMYGATPGSVEGHESRKDGLAKNPRTGYQAMFRSGSH 2v3v.1    ETRR-----------------DAMELPARVSDVCRPGLIAVPFFD-----------------------------------  target    QSATRGWLKPTWMTDSLVRKELFGHAVNKGFLPDVHCPTGAPREAIVKITKAEPGGLNAKGLWRPAALGLRPKYENDKMK 2v3v.1    --------------------------------------------------------------------------------  target    DYLAGKFTLAANPKKGGKK 2v3v.1    ------------------- ``` | | | | | | | | | | | | | | | | | | | | | | | | | | | | | | | | | | | | | | | | | | | | | | | | | |
|  | 5nqd.1.A | AroA  *Arsenite oxidase AioAB from Rhizobium sp. str. NT-26 mutant AioBF108A* | 0.27 |  | 15.73 | 0.59 | 1-502 | X-ray | 2.20 | hetero-2-2-mer | 4 x MGD, 2 x O, 2 x 4MO, 2 x F3S, 2 x FES | HHblits | 0.27 |
| ``` target    LARDIAKVPG-----TTLFAIGMGPNQFFNNDNKDRTQFLLAALTGNIGKIAGNIGSYAGNYRVAMFNGVPQYIAENPFD 5nqd.1    AAEWIGMPKEGGKRRRVMFGYEKGLIWGNDNYRTNGALVNLALATGNIGRPGGGVVRLGGHQEGYVRPSDA--HVGRPAA  target    I--ELDGAKPARPKLYWRAEPAHYYNHEDHPLKMGKTMITGKTHMPTPTKSLWFANANSILG------N--VKWHFNTVV 5nqd.1    YVDQLLIGGQGGVHHIWG--------CD----HYKTTL------NAHEFKRVYKKRTDMVKDAMSAAPYGDREAMVNAIV  target    NVLPKM-EMIAVQEWWWSTSCEWADIVFAVDAWSELKHPDMCSSVTNPFLTVFPRTPLERPFDTRGDIECLDLVGKQLAK 5nqd.1    DAINQGGLFAVNVDIIPTKIGEACHVILPAATSGEMN--LTS-MNGERRMRLTE-RYMDPPGQSMPDCLIAAR----LAN  target    RTGDR-------RFA---DMWKFVEEKKVEV-YLQRILDHSSNTKGFKFPELEEKAKKGIPALMMTRTNPKTVGYEQVYD 5nqd.1    TMERVLTEMGDVGYAAQFKGFDWQ---TEEDAFMDGYNKNAHGGEFVTYERLSAMGTNGFQEPATGFTDGKIEGTQRLYT  target    SRPWYTKTGRLEFYREEDEFIEAGENLPVHREPIDSTFYEPNVIVAPAHPFIKAKGPEAYGVKVDDFDNETRQGRNIVKT 5nqd.1    DGVFSTDDGKARFMDAPWR---------GLQAPGK---------------------------------------------  target    WEETKKTVHPLAKDGYKFVFHTPKYRHGAHTMPVD--TDMVAMLFGPFGDIYRHDKRQPFAAEGYVDIHPDDAKALNIED 5nqd.1    ---------QQQKDSHKYLINNGRANVVWQSAYLDQENDFVM-------------D---RFPYPFIEMNPEDMAEAGLKE  target    GDYVWIDSDPSDRPFRGWQKNDKDYKFSRLLCRARYYPGTPRGITRMWFNMYGATPGSVEGHESRKDGLAKNPRTGYQAM 5nqd.1    GDLVEIYNDA-----------------GATQAMAYPTPTARRGETFMLFGF-----------------------------  target    FRSGSHQSATRGWLKPTWMTDSLVRKELFGHAVNKGFLPDVHCPTGAPREAIVKITKAEPGGLNAKGLWRPAALGLRPKY 5nqd.1    --------------------------------------------------------------------------------  target    ENDKMKDYLAGKFTLAANPKKGGKK 5nqd.1    ------------------------- ``` | | | | | | | | | | | | | | | | | | | | | | | | | | | | | | | | | | | | | | | | | | | | | | | | | |
|  | 7e5z.1.A | Formate dehydrogenase  *Dehydrogenase holoenzyme* | 0.25 |  | 17.86 | 0.57 | 1-502 | EM | 0.00 | hetero-1-1-mer | 1 x W, 2 x MGD, 2 x FES, 4 x SF4, 1 x FMN | HHblits | 0.30 |
| ``` target    LARDIAKVPGTTLFAIGMGPNQFFNNDNKDRTQFLLAALTGNIGKIAGNIGSYAGNYRVAMF--NG-VPQYIAENPFDIE 7e5z.1    VARLYARAKS-SLIFWGMGVSQHVHGTDNSRCLIALALITGQIGRPGTGLHPLRGQNNVQGASDAGLIPMVY---PDYQS  target    LDGA-KPARPKLYWRAEPAHYYNHEDHPLKMGKTMITGKTHMPTPTKSLWFANANSILGNVKWHFNTVVNVLPKMEMIAV 7e5z.1    VEKDAVRELFEEFWGQSL-D-PQKGLTVVEIMRAI------HAGEIRGMFVEGENPAMSD--PDLNHARHALAMLDHLVV  target    QEWWWSTSCEWADIVFAVDAWSELKHPDMCSSVTNP--FLTVFPRTPLERPFDTRGDIECLDLVGKQLAKRTGDRRFADM 7e5z.1    QDLFLTETAFHADVVLPASAFAEKA-----GTFTNTDRRVQIAQ-PVVAPPGDARQDWWIIQE----LARRLDLD-----  target    WKFVEEKKVEVYLQRILDHSSNTKGFKFPELEEKAKKGIPALMMTRTNPKTVGYEQVYDSRPWYTKTGRLEFYREEDEFI 7e5z.1    WNYG---GPADIFAEMAQVMPSLNNITWERLERE---GAVTYPVDA--PDQPGN-EIIFYAGFPTESGRAKIVPAAI---  target    EAGENLPVHREPIDSTFYEPNVIVAPAHPFIKAKGPEAYGVKVDDFDNETRQGRNIVKTWEETKKTVHPLAKDGYKFVFH 7e5z.1    ---------VPPDE-------------------------------------------------------VPDDEFPMVLS  target    TPKYRHGAHTMPV--DTDMVAMLFGPFGDIYRHDKRQPFAAEGYVDIHPDDAKALNIEDGDYVWIDSDPSDRPFRGWQKN 7e5z.1    TGRVLEHWHTGSMTRRAGVL-------------DA---LEPEAVAFMAPKELYRLGLRPGGSMRLETRR-----------  target    DKDYKFSRLLCRARYYPGTPRGITRMWFNMYGATPGSVEGHESRKDGLAKNPRTGYQAMFRSGSHQSATRGWLKPTWMTD 7e5z.1    ------GAVVLKVRSDRDVPIGMIFMPFCY--------------------------------------------------  target    SLVRKELFGHAVNKGFLPDVHCPTGAPREAIVKITKAEPGGLNAKGLWRPAALGLRPKYENDKMKDYLAGKFTLAANPKK 7e5z.1    --------------------------------------------------------------------------------  target    GGKK 7e5z.1    ---- ``` | | | | | | | | | | | | | | | | | | | | | | | | | | | | | | | | | | | | | | | | | | | | | | | | | |
|  | 7vw6.1.A | Formate dehydrogenase  *Cryo-EM Structure of Formate Dehydrogenase 1 from Methylorubrum extorquens AM1* | 0.29 |  | 18.56 | 0.57 | 1-502 | EM | 0.00 | hetero-1-1-mer | 4 x SF4, 2 x FES, 2 x MGD, 1 x W, 1 x FMN | HHblits | 0.30 |
| ``` target    LARDIAKVPGTTLFAIGMGPNQFFNNDNKDRTQFLLAALTGNIGKIAGNIGSYAGNYRVAMF--NG-VPQYIAENPFDIE 7vw6.1    VARLYARAKS-SLIFWGMGVSQHVHGTDNSRCLIALALITGQIGRPGTGLHPLRGQNNVQGASDAGLIPMVY---PDYQS  target    LDGA-KPARPKLYWRAEPAHYYNHEDHPLKMGK---TMITGKTHMPTPTKSLWFANANSILGNVKWHFNTVVNVLPKMEM 7vw6.1    VEKDAVRELFEEFWGQ---------SLDPQKGLTVVEIMRA--IHAGEIRGMFVEGENPAMSD--PDLNHARHALAMLDH  target    IAVQEWWWSTSCEWADIVFAVDAWSELKHPDMCSSVTN--PFLTVFPRTPLERPFDTRGDIECLDLVGKQLAKRTGDRRF 7vw6.1    LVVQDLFLTETAFHADVVLPASAFAEKA-----GTFTNTDRRVQIAQ-PVVAPPGDARQDWWIIQE----LARRLDLD--  target    ADMWKFVEEKKVEVYLQRILDHSSNTKGFKFPELEEKAKKGIPALMMTRTNPKTVGYEQVYDSRPWYTKTGRLEFYREED 7vw6.1    ---WNYG---GPADIFAEMAQVMPSLNNITWERLERE---GAVTYPVDA--PDQPGNE-IIFYAGFPTESGRAKIVPAAI  target    EFIEAGENLPVHREPIDSTFYEPNVIVAPAHPFIKAKGPEAYGVKVDDFDNETRQGRNIVKTWEETKKTVHPLAKDGYKF 7vw6.1    --------V----PPDE-------------------------------------------------------VPDDEFPM  target    VFHTPKYRHGAHTMPV--DTDMVAMLFGPFGDIYRHDKRQPFAAEGYVDIHPDDAKALNIEDGDYVWIDSDPSDRPFRGW 7vw6.1    VLSTGRVLEHWHTGSMTRRAGVL-------------DA---LEPEAVAFMAPKELYRLGLRPGGSMRLETRR--------  target    QKNDKDYKFSRLLCRARYYPGTPRGITRMWFNMYGATPGSVEGHESRKDGLAKNPRTGYQAMFRSGSHQSATRGWLKPTW 7vw6.1    ---------GAVVLKVRSDRDVPIGMIFMPFCY-----------------------------------------------  target    MTDSLVRKELFGHAVNKGFLPDVHCPTGAPREAIVKITKAEPGGLNAKGLWRPAALGLRPKYENDKMKDYLAGKFTLAAN 7vw6.1    --------------------------------------------------------------------------------  target    PKKGGKK 7vw6.1    ------- ``` | | | | | | | | | | | | | | | | | | | | | | | | | | | | | | | | | | | | | | | | | | | | | | | | | |
|  | 6tg9.1.A | Formate dehydrogenase subunit alpha  *Cryo-EM Structure of NADH reduced form of NAD+-dependent Formate Dehydrogenase from Rhodobacter capsulatus* | 0.27 |  | 16.99 | 0.56 | 1-502 | EM | 3.24 | hetero-2-2-2-2-mer | 4 x MGD, 2 x 6MO, 4 x FES, 10 x SF4, 2 x H2S, 2 x FMN, 2 x NAI | HHblits | 0.29 |
| ``` target    LARDIAKVPGTTLFAIGMGPNQFFNNDNKDRTQFLLAALTGNIGKIAGNIGSYAGNYRVAM-F--NGVPQYIAENPFDIE 6tg9.1    AARAYAAAPN-AAIYYGLGVTEHSQGSTTVIAIANLAMMTGNIGRPGVGVNPLRGQNNVQGSCDMGSFPHEF---PGYRH  target    LDGA-KPARPKLYWRAEPAHYYNHEDHPLKMGKT---MITGKTHMPTPTKSLWFANANSILGNVKWHFNTVVNVLPKMEM 6tg9.1    VSDDATRGLFERTWGV---------TLSSEPGLRIPNMLDA--AVEGRFKALYVQGEDILQSD--PDTRHVSAGLAAMDL  target    IAVQEWWWSTSCEWADIVFAVDAWSELKHPDMCSSVTN--PFLTVFPRTPLERPFDTRGDIECLDLVGKQLAKRTGDRRF 6tg9.1    VIVHDLFLNETANYAHVFLPGSTFLEKD-----GTFTNAERRINRVR-RVMAPKA-GFADWEVTQM----LANALGAG--  target    ADMWKFVEEKKVEVYLQRILDHSSNTKGFKFPELEEKAKKGIPALMMTRTNPKTVGYEQVYDSRPWYTKTGRLEFYREED 6tg9.1    ---WHYT---HPSEIMAEIAATTPGFAAVTYEMLDARG--SVQWPCNE--------------KAPEGSPIMHVEGFVRGK  target    EFIEAGENLPVHREPIDSTFYEPNVIVAPAHPFIKAKGPEAYGVKVDDFDNETRQGRNIVKTWEETKKTVHPLAKDGYKF 6tg9.1    -----G--------------------------------------------------RFIRTAYLPTD----EKTGPRFPL  target    VFHTPKYRHGAHTMPVDTDMVAMLFGPFGDIYRHDKRQPFAAEGYVDIHPDDAKALNIEDGDYVWIDSDPSDRPFRGWQK 6tg9.1    LLTTGRILSQYNVGAQTRRT-----------EN----TVWHGEDRLEIHPTDAETRGIRDGDWVRLASRA----------  target    NDKDYKFSRLLCRARYYPGTPRGITRMWFNMYGATPGSVEGHESRKDGLAKNPRTGYQAMFRSGSHQSATRGWLKPTWMT 6tg9.1    -------GETTLRATVTDRVSPGVVYTTFHH-------------------------------------------------  target    DSLVRKELFGHAVNKGFLPDVHCPTGAPREAIVKITKAEPGGLNAKGLWRPAALGLRPKYENDKMKDYLAGKFTLAANPK 6tg9.1    --------------------------------------------------------------------------------  target    KGGKK 6tg9.1    ----- ``` | | | | | | | | | | | | | | | | | | | | | | | | | | | | | | | | | | | | | | | | | | | | | | | | | |
|  | 2ivf.1.A | ETHYLBENZENE DEHYDROGENASE ALPHA-SUBUNIT  *ETHYLBENZENE DEHYDROGENASE FROM AROMATOLEUM AROMATICUM* | 0.31 | 0.00 | 21.18 | 0.53 | 119-589 | X-ray | 1.88 | monomer | 1 x MES, 4 x SF4, 1 x MO, 1 x MGD, 1 x MD1, 1 x F3S, 1 x HEM | HHblits | 0.30 |
| ``` target    LARDIAKVPGTTLFAIGMGPNQFFNNDNKDRTQFLLAALTGNIGKIAGNIGSYAGNYRVAMFNGVPQYIAENPFDIELDG 2ivf.1    --------------------------------------------------------------------------------  target    AKPARPKLYWRAEPAHYYNHEDHPLKMGKTMITGKTHMPTPTKSLWFANANSILGNVKWHFNTV--VNVLPKMEMIAVQE 2ivf.1    --------------------------------------DKTPQVYMLLSQNPMRRKR--SGAKMFPDVLFPKLKMIFALE  target    WWWSTSCEWADIVFAVDAWSELKHPDMCSS-VTNPFLTVFPRTPLERPFDTRGDIECLDLVGKQLAKRTGD---RRF--- 2ivf.1    TRMSSSAMYADIVLPCAWYYEKH--EMTTPCSGNPFFTFVD-RSVAPPGECREEWDAIALILKKVGERAAARGLTEFNDH  target    -------ADMW-KFVE---EKKVEVYLQRILDHSSN----TKGFKFPELEEKAKKGIPALMMT--------------RTN 2ivf.1    NGRKRRYDELYKKFTMDGHLLTNEDCLKEMVDINRAVGVFAKDYTYEKFKKE---GQTRFLSMGTGVSRYAHANEVDVTK  target    PKTVGYEQVYDSRPWYTKTGRLEFYREEDEFIEAGENLPVHREPIDSTFYEPNVIVAPAHPFIKAKGPEAYGVKVDDFDN 2ivf.1    PIYPMRWHFDDKKVFPTHTRRAQFYLDHDWYLEAGESLPTHKDTPM----------------------------------  target    ETRQGRNIVKTWEETKKTVHPLAKDGYKFVFHTPKYRHGAHTMPVDTDMVAMLFGPFGDIYRHDKRQPFAAEGYVDIHPD 2ivf.1    ----------------------VGGDHPFKITGGHPRVSIHSTHLTNSHLSRL---------------HRGQPVVHMNSK  target    DAKALNIEDGDYVWIDSDPSDRPFRGWQKNDKDYKFSRLLCRARYYPGTPRGITRMWFNMYGATPGSVEGHESRKDGLAK 2ivf.1    DAAELGIKDGDMAKLFNDF-----------------ADCEIMVRTAPNVQPKQCIVYFWDAHQ-----------------  target    NPRTGYQAMFRS-GSHQSATRGWLKPTWMTDSLVRKELFGHAVNKGFLPDVHCPTG-APREAIVKITKAEPGGLNAKGLW 2ivf.1    ---------YKGWKPYDILLIGMPKPLHLAGGY---------EQFRYYFMNGSPAPVTDRGVRVSIKKA-----------  target    RPAALGLRPKYENDKMKDYLAGKFTLAANPKKGGKK 2ivf.1    ------------------------------------ ``` | | | | | | | | | | | | | | | | | | | | | | | | | | | | | | | | | | | | | | | | | | | | | | | | | |
|  | 4ydd.1.A | DMSO reductase family type II enzyme, molybdopterin subunit  *Crystal structure of the perchlorate reductase PcrAB from Azospira suillum PS* | 0.23 | 0.00 | 25.94 | 0.50 | 1-332 | X-ray | 1.86 | monomer | 4 x SF4, 1 x MO, 1 x MGD, 1 x MD1, 1 x F3S | BLAST | 0.34 |
| ``` target    LARDIAKVPGTTLFAIGMGPNQFFNNDNKDRTQFLLAALTGNIGKIAGNIGSYAGNYRVAMFNGVPQYIAENPFDIELDG 4ydd.1    LAREFATAK-PSMIICGGGTQHWYYSDVLLRAMHLLTALTGTEGTNGGGMNHYIGQWKPAFVAGLVALAFPEGVNKQRFC  target    AKPARPKLYWRAEPAHYYNHEDHPLKMGKTMITGKT-HMPTPTKS--LWFANANSILGNVKWHFNTVVNVLPKMEMIAVQ 4ydd.1    QTTIWTYIHAEVNDEIISSDIDTEKYLRDSITTGQMPNMPEQGRDPKVFFVYRGNWLNQAKGQKYVLENLWPKLELIVDI  target    EWWWSTSCEWADIVFAVDAWSELKHPDMCSSVTNPFLTVFPRTPLERP-FDTRGDIECLDLVGKQL---AKRTGDRRFAD 4ydd.1    NIRMDSTALYSDVVLPSAHWYE----KLDLNVTSEHSYINMTEPAIKPMWESKTDWQIFLALAKRVEMAAKRKKYEKFND  target    -----------MW-------KFVEEKKVEVYLQRILDHSSNTKGFKFPELEEKAKKGIPALMMTRTNPKTVGYEQ----- 4ydd.1    EKFKWVRDLSNLWNQMTMDGKLAED---EAAAQYILDNAPQSKGITIQMLREKPQR----FKSNWTSPLKEGVPYTPFQY  target    -VYDSRPWYTKTGRLEFYREEDEFIEAGENLPVHREPIDSTFYEPNVIVAPAHPFIKAKGPEAYGVKVDDFDNETRQGRN 4ydd.1    FVVDKKPWPTLTGRQQFYLDHDTFFDMGVELPTYKAPIDADKY-------------------------------------  target    IVKTWEETKKTVHPLAKDGYKFVFHTPKYRHGAHTMPVDTDMVAMLFGPFGDIYRHDKRQPFAAEGYVDIHPDDAKALNI 4ydd.1    --------------------------------------------------------------------------------  target    EDGDYVWIDSDPSDRPFRGWQKNDKDYKFSRLLCRARYYPGTPRGITRMWFNMYGATPGSVEGHESRKDGLAKNPRTGYQ 4ydd.1    --------------------------------------------------------------------------------  target    AMFRSGSHQSATRGWLKPTWMTDSLVRKELFGHAVNKGFLPDVHCPTGAPREAIVKITKAEPGGLNAKGLWRPAALGLRP 4ydd.1    --------------------------------------------------------------------------------  target    KYENDKMKDYLAGKFTLAANPKKGGKK 4ydd.1    --------------------------- ``` | | | | | | | | | | | | | | | | | | | | | | | | | | | | | | | | | | | | | | | | | | | | | | | | | |
|  | 5e7o.1.A | DMSO reductase family type II enzyme, molybdopterin subunit  *Crystal structure of the perchlorate reductase PcrAB mutant W461E of PcrA from Azospira suillum PS* | 0.23 | 0.00 | 25.94 | 0.50 | 1-332 | X-ray | 2.40 | monomer | 4 x SF4, 1 x MO, 1 x MGD, 1 x MD1, 1 x F3S | BLAST | 0.33 |
| ``` target    LARDIAKVPGTTLFAIGMGPNQFFNNDNKDRTQFLLAALTGNIGKIAGNIGSYAGNYRVAMFNGVPQYIAENPFDIELDG 5e7o.1    LAREFATAK-PSMIICGGGTQHWYYSDVLLRAMHLLTALTGTEGTNGGGMNHYIGQEKPAFVAGLVALAFPEGVNKQRFC  target    AKPARPKLYWRAEPAHYYNHEDHPLKMGKTMITGKT-HMPTPTKS--LWFANANSILGNVKWHFNTVVNVLPKMEMIAVQ 5e7o.1    QTTIWTYIHAEVNDEIISSDIDTEKYLRDSITTGQMPNMPEQGRDPKVFFVYRGNWLNQAKGQKYVLENLWPKLELIVDI  target    EWWWSTSCEWADIVFAVDAWSELKHPDMCSSVTNPFLTVFPRTPLERP-FDTRGDIECLDLVGKQL---AKRTGDRRFAD 5e7o.1    NIRMDSTALYSDVVLPSAHWYE----KLDLNVTSEHSYINMTEPAIKPMWESKTDWQIFLALAKRVEMAAKRKKYEKFND  target    -----------MW-------KFVEEKKVEVYLQRILDHSSNTKGFKFPELEEKAKKGIPALMMTRTNPKTVGYEQ----- 5e7o.1    EKFKWVRDLSNLWNQMTMDGKLAED---EAAAQYILDNAPQSKGITIQMLREKPQR----FKSNWTSPLKEGVPYTPFQY  target    -VYDSRPWYTKTGRLEFYREEDEFIEAGENLPVHREPIDSTFYEPNVIVAPAHPFIKAKGPEAYGVKVDDFDNETRQGRN 5e7o.1    FVVDKKPWPTLTGRQQFYLDHDTFFDMGVELPTYKAPIDADKY-------------------------------------  target    IVKTWEETKKTVHPLAKDGYKFVFHTPKYRHGAHTMPVDTDMVAMLFGPFGDIYRHDKRQPFAAEGYVDIHPDDAKALNI 5e7o.1    --------------------------------------------------------------------------------  target    EDGDYVWIDSDPSDRPFRGWQKNDKDYKFSRLLCRARYYPGTPRGITRMWFNMYGATPGSVEGHESRKDGLAKNPRTGYQ 5e7o.1    --------------------------------------------------------------------------------  target    AMFRSGSHQSATRGWLKPTWMTDSLVRKELFGHAVNKGFLPDVHCPTGAPREAIVKITKAEPGGLNAKGLWRPAALGLRP 5e7o.1    --------------------------------------------------------------------------------  target    KYENDKMKDYLAGKFTLAANPKKGGKK 5e7o.1    --------------------------- ``` | | | | | | | | | | | | | | | | | | | | | | | | | | | | | | | | | | | | | | | | | | | | | | | | | |
| ✓ | 7p63.1.C | NADH-quinone oxidoreductase  *Complex I from E. coli, DDM/LMNG-purified, under Turnover at pH 6, Closed state* | 0.26 | 0.00 | 12.82 | 0.55 | 1-504 | EM | 0.00 | monomer | 7 x SF4, 1 x FMN, 1 x NAI, 2 x FES, 1 x CA, 1 x DCQ, 4 x LFA, 8 x 3PE | HHblits | 0.26 |
| ``` target    LARDIAKVPGTTLFAIGMGPNQFFNNDNKDRTQFLLAALTGNIGKIAGNIGSYAGNYRVAMFNGVPQYIAENPFDIELDG 7p63.1    IVQALAGAKKP-LIISGTNAG----SLEVIQAAANVAKALKGRGADVGIT-MIARSVNSM---GLGI----------MGG  target    AKPARPKLYWRAEPAHYYNHEDHPLKMGKTMITGKTHMPTPTKSLWFANANSILGNVKWHFNTVVNVLPKMEMIAVQEWW 7p63.1    ---------------------GSLEEALTEL------ETGRADAVVVLE-NDLHRH--ASATRVNAALAKAPLVMVVDHQ  target    WSTSCEWADIVFAVDAWSELKHPDMCSSVTNP--FLTVFPRTPLERPF-----DTRGDIECLDLVGKQLAKRTGDRRFAD 7p63.1    RTAIMENAHLVLSAASFAESD-----GTVINNEGRAQRFF-QVYDPAYYDSKTVMLESWRWLHS----LHSTLLSRE---  target    MWKFVEEKKVEVYLQRILDHSSNTKGFKFPELEEKAK----------------------KGIPALMMTRT---------- 7p63.1    -VDWT---QLDHVIDAVVAKIPELAGIKDAAPDATFRIRGQKLAREPHRYSGRTAMRANISVHEPRQPQDIDTMFTFSME  target    -------NPKT-V-GYEQVY-----DSRPWYTKTGRLEFYREEDEFIEAG-ENLPVHREPIDSTFYEPNVIVAPAHPFIK 7p63.1    GNNQPTAHRSQVPFAWAPGWNSPQAWNKFQDEVGGKLRFGDPGVRLFETSENGLDYFTSVPA------------------  target    AKGPEAYGVKVDDFDNETRQGRNIVKTWEETKKTVHPLAKDGYKFVFHTPKYRHGAHTMPVDTDMVAMLFGPFGDIYRHD 7p63.1    ------------------------------------RFQPQDGKWRIAPYYHLFGSDELSQRAPVFQS------------  target    KRQPFAAEGYVDIHPDDAKALNIEDGDYVWIDSDPSDRPFRGWQKNDKDYKFSRLLCRARYYPGTPRGITRMWFNMYGAT 7p63.1    ----RMPQPYIKLNPADAAKLGVNAGTRVSFSYDG-----------------NTVTLPVEIAEGLTAGQVGLPMGMSG--  target    PGSVEGHESRKDGLAKNPRTGYQAMFRSGSHQSATRGWLKPTWMTDSLVRKELFGHAVNKGFLPDVHCPTGAPREAIVKI 7p63.1    --------------------------------------------------------------------------------  target    TKAEPGGLNAKGLWRPAALGLRPKYENDKMKDYLAGKFTLAANPKKGGKK 7p63.1    -------------------------------------------------- ``` | | | | | | | | | | | | | | | | | | | | | | | | | | | | | | | | | | | | | | | | | | | | | | | | | |
|  | 7p61.1.C | NADH-quinone oxidoreductase  *Complex I from E. coli, DDM-purified, with NADH, Resting state* | 0.25 | 0.00 | 12.82 | 0.55 | 1-504 | EM | 0.00 | monomer | 7 x SF4, 1 x FMN, 1 x NAI, 2 x FES, 1 x CA, 2 x 3PE, 1 x UQ8 | HHblits | 0.26 |
| ``` target    LARDIAKVPGTTLFAIGMGPNQFFNNDNKDRTQFLLAALTGNIGKIAGNIGSYAGNYRVAMFNGVPQYIAENPFDIELDG 7p61.1    IVQALAGAKKP-LIISGTNAG----SLEVIQAAANVAKALKGRGADVGIT-MIARSVNSM---GLGI----------MGG  target    AKPARPKLYWRAEPAHYYNHEDHPLKMGKTMITGKTHMPTPTKSLWFANANSILGNVKWHFNTVVNVLPKMEMIAVQEWW 7p61.1    -G--------------------SLEEALTEL------ETGRADAVVVLE-NDLHRH--ASATRVNAALAKAPLVMVVDHQ  target    WSTSCEWADIVFAVDAWSELKHPDMCSSVTN--PFLTVFPRTPLERPF-----DTRGDIECLDLVGKQLAKRTGDRRFAD 7p61.1    RTAIMENAHLVLSAASFAESD-----GTVINNEGRAQRFF-QVYDPAYYDSKTVMLESWRWLHS----LHSTLLSREV--  target    MWKFVEEKKVEVYLQRILDHSSNTKGFKFPELEEKAK----------------------KGIPALMMTR----------- 7p61.1    --DWT---QLDHVIDAVVAKIPELAGIKDAAPDATFRIRGQKLAREPHRYSGRTAMRANISVHEPRQPQDIDTMFTFSME  target    ------TNPK-T-VGYEQV-----YDSRPWYTKTGRLEFYREEDEFIEAG-ENLPVHREPIDSTFYEPNVIVAPAHPFIK 7p61.1    GNNQPTAHRSQVPFAWAPGWNSPQAWNKFQDEVGGKLRFGDPGVRLFETSENGLDYFTSVPA------------------  target    AKGPEAYGVKVDDFDNETRQGRNIVKTWEETKKTVHPLAKDGYKFVFHTPKYRHGAHTMPVDTDMVAMLFGPFGDIYRHD 7p61.1    ------------------------------------RFQPQDGKWRIAPYYHLFGSDELSQRAPVFQS------------  target    KRQPFAAEGYVDIHPDDAKALNIEDGDYVWIDSDPSDRPFRGWQKNDKDYKFSRLLCRARYYPGTPRGITRMWFNMYGAT 7p61.1    ----RMPQPYIKLNPADAAKLGVNAGTRVSFSYDG-----------------NTVTLPVEIAEGLTAGQVGLPMGMSG--  target    PGSVEGHESRKDGLAKNPRTGYQAMFRSGSHQSATRGWLKPTWMTDSLVRKELFGHAVNKGFLPDVHCPTGAPREAIVKI 7p61.1    --------------------------------------------------------------------------------  target    TKAEPGGLNAKGLWRPAALGLRPKYENDKMKDYLAGKFTLAANPKKGGKK 7p61.1    -------------------------------------------------- ``` | | | | | | | | | | | | | | | | | | | | | | | | | | | | | | | | | | | | | | | | | | | | | | | | | |
|  | 7nz1.1.E | NADH-quinone oxidoreductase subunit G  *Respiratory complex I from Escherichia coli - focused refinement of cytoplasmic arm* | 0.26 | 0.00 | 12.82 | 0.55 | 1-504 | EM | 0.00 | monomer | 7 x SF4, 2 x FES, 1 x FMN, 1 x CA | HHblits | 0.26 |
| ``` target    LARDIAKVPGTTLFAIGMGPNQFFNNDNKDRTQFLLAALTGNIGKIAGNIGSYAGNYRVAMFNGVPQYIAENPFDIELDG 7nz1.1    IVQALAGAKKP-LIISGTNAG----SLEVIQAAANVAKALKGRGADVGIT-MIARSVNS---MGLGI----------MGG  target    AKPARPKLYWRAEPAHYYNHEDHPLKMGKTMITGKTHMPTPTKSLWFANANSILGNVKWHFNTVVNVLPKMEMIAVQEWW 7nz1.1    ---------------------GSLEEALTELE------TGRADAVVVLE-NDLHRH--ASAIRVNAALAKAPLVMVVDHQ  target    WSTSCEWADIVFAVDAWSELKHPDMCSSVTN--PFLTVFPRTPLERPF-----DTRGDIECLDLVGKQLAKRTGDRRFAD 7nz1.1    RTAIMENAHLVLSAASFAESD-----GTVINNEGRAQRFF-QVYDPAYYDSKTVMLESWRWLHS----LHSTLLSRE---  target    MWKFVEEKKVEVYLQRILDHSSNTKGFKFPELEEKAK----------------------KGIPALMMTRT---------- 7nz1.1    -VDWT---QLDHVIDAVVAKIPELAGIKDAAPDATFRIRGQKLAREPHRYSGRTAMRANISVHEPRQPQDIDTMFTFSME  target    -------NPK-TVG-YEQV-----YDSRPWYTKTGRLEFYREEDEFIEAG-ENLPVHREPIDSTFYEPNVIVAPAHPFIK 7nz1.1    GNNQPTAHRSQVPFAWAPGWNSPQAWNKFQDEVGGKLRFGDPGVRLFETSENGLDYFTSVPA------------------  target    AKGPEAYGVKVDDFDNETRQGRNIVKTWEETKKTVHPLAKDGYKFVFHTPKYRHGAHTMPVDTDMVAMLFGPFGDIYRHD 7nz1.1    ------------------------------------RFQPQDGKWRIAPYYHLFGSDELSQRAPVFQS------------  target    KRQPFAAEGYVDIHPDDAKALNIEDGDYVWIDSDPSDRPFRGWQKNDKDYKFSRLLCRARYYPGTPRGITRMWFNMYGAT 7nz1.1    ----RMPQPYIKLNPADAAKLGVNAGTRVSFSYDG-----------------NTVTLPVEIAEGLTAGQVGLPMGMSG--  target    PGSVEGHESRKDGLAKNPRTGYQAMFRSGSHQSATRGWLKPTWMTDSLVRKELFGHAVNKGFLPDVHCPTGAPREAIVKI 7nz1.1    --------------------------------------------------------------------------------  target    TKAEPGGLNAKGLWRPAALGLRPKYENDKMKDYLAGKFTLAANPKKGGKK 7nz1.1    -------------------------------------------------- ``` | | | | | | | | | | | | | | | | | | | | | | | | | | | | | | | | | | | | | | | | | | | | | | | | | |
|  | 4v4c.1.A | Pyrogallol hydroxytransferase large subunit  *Crystal Structure of Pyrogallol-Phloroglucinol Transhydroxylase from Pelobacter acidigallici* | 0.23 |  | 16.33 | 0.46 | 120-502 | X-ray | 2.35 | hetero-oligomer | 2 x CA, 2 x MGD, 1 x 4MO, 3 x SF4 | HHblits | 0.28 |
| ``` target    LARDIAKVPGTTLFAIGMGPNQFFNNDNKDRTQFLLAALTGNIGKIAGNIGSYAGNYRVAMFNGVPQYIAENPFDIELDG 4v4c.1    --------------------------------------------------------------------------------  target    AKPARPKLYWRAEPAHYYNHEDHPLKMGKTMITGKTHMPTPTKSLWFANANSILGNVKWHFNTVVNVL--PKMEMIAVQE 4v4c.1    ---------------------------------------SKIKMFWKYGGPHLGTM--TATNRYAKMYTHDSLEFVVSQS  target    WWWSTSCEWADIVFAVDAWSELKHPDMCSSV-----------TNPFLTVFPRTPLERPFDTRGDIECLDLVGKQLAKRTG 4v4c.1    IWFEGEVPFADIILPACTNFERWDISEFANCSGYIPDNYQLCNHRVISLQA-KCIEPVGESMSDYEIYRL----FAKKLN  target    DRRFADMWKFVEEKKVEVYLQRILDHSSNTKGFKFPELEEKAKKGIPALMMTRTNP---KTVGY-----------E---- 4v4c.1    IEEM-----FSEGKDELAWCEQYFNATDMPKYMTWDEFFKKG---YFVVPDNPNRKKTVALRWFAEGREKDTPDWGPRLN  target    QVYDSRPWYTKTGRLEFYREEDEFI-EAG------ENLPVHREPIDSTFYEPNVIVAPAHPFIKAKGPEAYGVKVDDFDN 4v4c.1    NQVCRKGLQTTTGKVEFIATSLKNFEEQGYIDEHRPSMHTYVP-------------------------------------  target    ETRQGRNIVKTWEETKKTVHPLAKDGYKFVFHTPKYRHGAHTMPVDTD-MVAMLFGPFGDIYRHDKRQPFAAEGYVDIHP 4v4c.1    ----------AWESQKH---SPLAVKYPLGMLSPHPRFSMHTMGDGKNSYMNYIK----DH---RVEVDGYKYWIMRVNS  target    DDAKALNIEDGDYVWIDSDPSDRPFRGWQKNDKDYKFSRLLCRARYYPGTPRGITRMWFNMYGATPGSVEGHESRKDGLA 4v4c.1    IDAEARGIKNGDLIRAYNDR-----------------GSVILAAQVTECLQPGTVHSYESC-------------------  target    KNPRTGYQAMFRSGSHQSATRGWLKPTWMTDSLVRKELFGHAVNKGFLPDVHCPTGAPREAIVKITKAEPGGLNAKGLWR 4v4c.1    --------------------------------------------------------------------------------  target    PAALGLRPKYENDKMKDYLAGKFTLAANPKKGGKK 4v4c.1    ----------------------------------- ``` | | | | | | | | | | | | | | | | | | | | | | | | | | | | | | | | | | | | | | | | | | | | | | | | | |
|  | 6lod.1.B | Fe-S-cluster-containing hydrogenase components 1-like protein  *Cryo-EM structure of the air-oxidized photosynthetic alternative complex III from Roseiflexus castenholzii* | 0.18 |  | 11.29 | 0.39 | 119-505 | EM | 0.00 | hetero-1-1-1-1-1-1-… | 6 x HEC, 2 x EL6, 3 x SF4, 1 x F3S | HHblits | 0.26 |
| ``` target    LARDIAKVPGTTLFAIGMGPNQFFNNDNKDRTQFLLAALTGNIGKIAGNIGSYAGNYRVAMFNGVPQYIAENPFDIELDG 6lod.1    --------------------------------------------------------------------------------  target    AKPARPKLYWRAEPAHYYNHEDHPLKMGKTMITGKTHMPTPTKSLWFANANSILGNVKWHFNTVVNVLPKMEMIAVQEWW 6lod.1    --------------------------------------AGTVEVLLMIESNPVYNA--PADIPFAEALAKVPLSMHVGLY  target    WSTSCEWADIVFAVDAWSELKHPDMCSSVTNPFLTVFPRTPLERPFDTRGDIECLDLVGKQLAKRTGDRRFADMWKFVEE 6lod.1    RDETAQQSVWHINGAHFLEAW-GDVR-A-FDGTTTIVQ-PLIAPLYNGKSAIEVLNV----LLGKPQETG----YQ----  target    KKVEVYLQRILDHSSNTKGFKFPELEEKAKKGIPALMMTRTNPKTVGYEQVYDSRPWYTKTGRLEFYREEDEFIEAGENL 6lod.1    -TLTAYWQTQDASG--NFRVFWNTAL---HDGVITAT------------------Q--ARSRQVTLQ----------QGF  target    PVHREPIDSTFYEPNVIVAPAHPFIKAKGPEAYGVKVDDFDNETRQGRNIVKTWEETKKTVHPLAKDGYKFVFHTPKYRH 6lod.1    ADAAP---------------------------------------------------------PAPTQGLEIVFRPDPSL-  target    GAHTMPVDTDMVAMLFGPFGDIYRHDKRQPFAAEGYVDIHPDDAKALNIEDGDYVWIDSDPSDRPFRGWQKNDKDYKFSR 6lod.1    -WDGAFANNAWLQETPKPYTK---------LTWDNVALMSVRTANALGLKNGDVVRLTYQG-----------------RS  target    LLCRARYYPGTPRGITRMWFNMYGATPGSVEGHESRKDGLAKNPRTGYQAMFRSGSHQSATRGWLKPTWMTDSLVRKELF 6lod.1    VDAPVWVQPGHADDSVTVHFGFGRT-------------------------------------------------------  target    GHAVNKGFLPDVHCPTGAPREAIVKITKAEPGGLNAKGLWRPAALGLRPKYENDKMKDYLAGKFTLAANPKKGGKK 6lod.1    ---------------------------------------------------------------------------- ``` | | | | | | | | | | | | | | | | | | | | | | | | | | | | | | | | | | | | | | | | | | | | | | | | | |
|  | 6f0k.1.B | Fe-S-cluster-containing hydrogenase  *Alternative complex III* | 0.18 |  | 12.85 | 0.39 | 119-506 | EM | 0.00 | hetero-1-1-1-1-1-1-… | 6 x HEC, 1 x F3S, 3 x SF4 | HHblits | 0.25 |
| ``` target    LARDIAKVPGTTLFAIGMGPNQFFNNDNKDRTQFLLAALTGNIGKIAGNIGSYAGNYRVAMFNGVPQYIAENPFDIELDG 6f0k.1    --------------------------------------------------------------------------------  target    AKPARPKLYWRAEPAHYYNHEDHPLKMGKTMITGKTHMPTPTKSLWFANANSILGNVKWHFNTVVNVLPKMEMIAVQEWW 6f0k.1    --------------------------------------AGAVDALLLLNVNPVYDA--PAALGFAEALAQVPEVIHLGLH  target    WSTSCEWADIVFAVDAWSELKHPDMCSSVTNPFLTVFPRTPLERPFDT-RGDIECLDLVGKQLAKRTGDRRFADMWKFVE 6f0k.1    VDETARRSTWHLPSTHYLEAW--GDGRAY-DGTLSVIQ-PLIAPLYEAAHSPLEVLAL----LATGEEQS----AYD---  target    EKKVEVYLQRILDHSSNTKGFKFPELEE-KAKKGIPALMMTRTNPKTVGYEQVYDSRPWYTKTGRLEFYREEDEFIEAGE 6f0k.1    --LVRNTWRRLLAGR----G-AFEQAWQRVLHDGFL------------------PDSGYPTVSLRPNR-----------Q  target    NLPVHREPIDSTFYEPNVIVAPAHPFIKAKGPEAYGVKVDDFDNETRQGRNIVKTWEETKKTVHPLAKDGYKFVFHTPKY 6f0k.1    ALADWPQ------------------------------------------------------------AAEGGLEVVFRLD  target    RHGAHTMPVDTDMVAMLFGPFGDIYRHDKRQPFAAEGYVDIHPDDAKALNIED--------GDYVWIDSDPSDRPFRGWQ 6f0k.1    PTVLDGSFANNAWAQELPDPITK---------IVWDNVAILSPKTAAALGVKAEYHKGVYIADVIELSLDG---------  target    KNDKDYKFSRLLCRARYYPGTPRGITRMWFNMYGATPGSVEGHESRKDGLAKNPRTGYQAMFRSGSHQSATRGWLKPTWM 6f0k.1    --------RAVELPVWVLPGHPDDSITVYLGYGREI--------------------------------------------  target    TDSLVRKELFGHAVNKGFLPDVHCPTGAPREAIVKITKAEPGGLNAKGLWRPAALGLRPKYENDKMKDYLAGKFTLAANP 6f0k.1    --------------------------------------------------------------------------------  target    KKGGKK 6f0k.1    ------ ``` | | | | | | | | | | | | | | | | | | | | | | | | | | | | | | | | | | | | | | | | | | | | | | | | | |
|  | 7tgh.58.A | NADH-ubiquinone oxidoreductase 75 kDa subunit  *Cryo-EM structure of respiratory super-complex CI+III2 from Tetrahymena thermophila* | 0.16 |  | 15.21 | 0.34 | 1-322 | EM | 0.00 | monomer |  | HHblits | 0.27 |
| ``` target    LARDIAKVPGTTLFAIGMGPNQFFNNDNKDRTQFLLAALTGNIGKIAGNIGSYAGNYRVAMFNGVPQYIAENPFDIELDG 7tgh.58   FAERLKKAKLP-MIMVGASALEREDGAELYNTLKVISNKTGVISEEKSWNGFNILHKEM---GRI--------------N  target    AKPARPKLYWRAEPAHYYNHEDHPLKMGKTMITGKTHMPTPTKSLWFANANSILGNVKWHFNTVVNVLPKMEMIAVQEWW 7tgh.58   A----L-------------------ELGIN-PT---SVNKNAKLVFILGADNNLR---------PEDIPADAFVVYFGTH  target    WSTSCEWADIVFAVDAWSELKHPDMCSSVTNPFLTVFPRTPLERPFDTRGDIECLDLVGKQLAKRTGDRRFADMWKFVEE 7tgh.58   GDEGAYYADIILPTAAYTEKN--ATWVN-TEGRVQQGR-LVVMPPGDAREDWQIIRA----LSEEAGVP-----LPYD--  target    KKVEVYLQRILDHSSNTKGFKFPELEEKAKKGIPALMMTRTNPKTVGYEQVYDSRPWYTKTGRLEFYREEDEFIEAG-EN 7tgh.58   -SLEELRYRVAELAP--------HLL-------------------------KYDYIEPTIFGKVALSAQQGVK--TTLSP  target    LPVHREPIDSTFYEPNVIVAPAHPFIKAKGPEAYGVKVDDFDNETRQGRNIVKTWEETKKTVHPLAKDGYKFVFHTPKYR 7tgh.58   TPI-----------------------------------------------------------------------------  target    HGAHTMPVDTDMVAMLFGPFGDIYRHDKRQPFAAEGYVDIHPDDAKALNIEDGDYVWIDSDPSDRPFRGWQKNDKDYKFS 7tgh.58   --------------------------------------------------------------------------------  target    RLLCRARYYPGTPRGITRMWFNMYGATPGSVEGHESRKDGLAKNPRTGYQAMFRSGSHQSATRGWLKPTWMTDSLVRKEL 7tgh.58   --------------------------------------------------------------------------------  target    FGHAVNKGFLPDVHCPTGAPREAIVKITKAEPGGLNAKGLWRPAALGLRPKYENDKMKDYLAGKFTLAANPKKGGKK 7tgh.58   ----------------------------------------------------------------------------- ``` | | | | | | | | | | | | | | | | | | | | | | | | | | | | | | | | | | | | | | | | | | | | | | | | | |
|  | 6sdr.1.A | Formate dehydrogenase, alpha subunit, selenocysteine-containing  *W-formate dehydrogenase from Desulfovibrio vulgaris - Oxidized form* | 0.15 |  | 17.84 | 0.33 | 1-222 | X-ray | 2.10 | hetero-1-1-mer | 2 x MGD, 4 x SF4, 1 x H2S, 1 x W | HHblits | 0.29 |
| ``` target    LARDIAKVP---GTTLFAIGMGPNQFFNNDNKDRTQFLLAALTGNIGKIAGNIGSYAGNYRVAMFN--G-----VPQYIA 6sdr.1    VYKAYAATGKPDKAGTIMYAMGWTQHSVGVQNIRAMAMIQLLLGNIGVAGGGVNALRGESNVQGSTDQGLLAHIWPGYNP  target    ENPFDI----EL-DGAK--PARP--KLYWRAEPAHY----------------YNH---EDHPLKM----GKTMITGKTHM 6sdr.1    V-PNSKAATLELYNAATPQSKDPMSVNWWQNRPKYVASYLKALYPDEEPAAAYDYLPRIDAGRKLTDYFWLNIFEK--MD  target    PTPTKSLWFANANSILGNVKWHFNTVVNVLPKMEMIAVQEWWWSTSCEW--------AD-----IVFAVDAWSELKHPDM 6sdr.1    KGEFKGLFAWGMNPACG--GANANKNRKAMGKLEWLVNVNLFENETSSFWKGPGMNPAEIGTEVFFLPCCVSIEKE--GS  target    CSSVTNPFLTVFPRTPLERPFDTRGDIECLDLVGKQLAKRTGDRRFADMWKFVEEKKVEVYLQRILDHSSNTKGFKFPEL 6sdr.1    VAN-SGRWMQWRY-RGPKPYAETKPDGDIMLDMFKKV-------------------------------------------  target    EEKAKKGIPALMMTRTNPKTVGYEQVYDSRPWYTKTGRLEFYREEDEFIEAGENLPVHREPIDSTFYEPNVIVAPAHPFI 6sdr.1    --------------------------------------------------------------------------------  target    KAKGPEAYGVKVDDFDNETRQGRNIVKTWEETKKTVHPLAKDGYKFVFHTPKYRHGAHTMPVDTDMVAMLFGPFGDIYRH 6sdr.1    --------------------------------------------------------------------------------  target    DKRQPFAAEGYVDIHPDDAKALNIEDGDYVWIDSDPSDRPFRGWQKNDKDYKFSRLLCRARYYPGTPRGITRMWFNMYGA 6sdr.1    --------------------------------------------------------------------------------  target    TPGSVEGHESRKDGLAKNPRTGYQAMFRSGSHQSATRGWLKPTWMTDSLVRKELFGHAVNKGFLPDVHCPTGAPREAIVK 6sdr.1    --------------------------------------------------------------------------------  target    ITKAEPGGLNAKGLWRPAALGLRPKYENDKMKDYLAGKFTLAANPKKGGKK 6sdr.1    --------------------------------------------------- ``` | | | | | | | | | | | | | | | | | | | | | | | | | | | | | | | | | | | | | | | | | | | | | | | | | |
| ✓ | 2ivf.1.A | ETHYLBENZENE DEHYDROGENASE ALPHA-SUBUNIT  *ETHYLBENZENE DEHYDROGENASE FROM AROMATOLEUM AROMATICUM* | 0.12 | 0.00 | 30.93 | 0.31 | 147-342 | X-ray | 1.88 | monomer | 1 x MES, 4 x SF4, 1 x MO, 1 x MGD, 1 x MD1, 1 x F3S, 1 x HEM | BLAST | 0.36 |
| ``` target    LARDIAKVPGTTLFAIGMGPNQFFNNDNKDRTQFLLAALTGNIGKIAGNIGSYAGNYRVAMFNGVPQYIAENPFDIELDG 2ivf.1    --------------------------------------------------------------------------------  target    AKPARPKLYWRAEPAHYYNHEDHPLKMGKTMITGKTHMPTPTKSLWFANANSILGNVKWHFNTVVNVLPKMEMIAVQEWW 2ivf.1    ------------------------------------------------------------------LFPKLKMIFALETR  target    WSTSCEWADIVFAVDAWSELKHPDMCSSVTNPFLTVFPRTPLERPFDTRGDIECLDLVGKQLAKRTG------------- 2ivf.1    MSSSAMYADIVLPC-AWYYEKHEMTTPCSGNPFFTFVDRS-VAPPGECREEWDAIALILKKVGERAAARGLTEFNDHNGR  target    DRRFADMW-KFVEEKKV---EVYLQRILDHSSNTKGFKFPELEEKAKK-GIPALMMTRTNPKTVGYEQVYD-SRPWY--- 2ivf.1    KRRYDELYKKFTMDGHLLTNEDCLKEMVDINRAVGVFAKDYTYEKFKKEGQTRFLSMGTGVSRYAHANEVDVTKPIYPMR  target    ----------TKTGRLEFYREEDEFIEAGENLPVHRE--------PIDSTFYEPNVIVAPAHPFIKAKGPEAYGVKVDDF 2ivf.1    WHFDDKKVFPTHTRRAQFYLDHDWYLEAGESLPTHKDTPMVGGDHPFKITGGHPRVSIHSTH------------------  target    DNETRQGRNIVKTWEETKKTVHPLAKDGYKFVFHTPKYRHGAHTMPVDTDMVAMLFGPFGDIYRHDKRQPFAAEGYVDIH 2ivf.1    --------------------------------------------------------------------------------  target    PDDAKALNIEDGDYVWIDSDPSDRPFRGWQKNDKDYKFSRLLCRARYYPGTPRGITRMWFNMYGATPGSVEGHESRKDGL 2ivf.1    --------------------------------------------------------------------------------  target    AKNPRTGYQAMFRSGSHQSATRGWLKPTWMTDSLVRKELFGHAVNKGFLPDVHCPTGAPREAIVKITKAEPGGLNAKGLW 2ivf.1    --------------------------------------------------------------------------------  target    RPAALGLRPKYENDKMKDYLAGKFTLAANPKKGGKK 2ivf.1    ------------------------------------ ``` | | | | | | | | | | | | | | | | | | | | | | | | | | | | | | | | | | | | | | | | | | | | | | | | | |
|  | 6sdv.1.A | Formate dehydrogenase, alpha subunit, selenocysteine-containing,Formate dehydrogenase, alpha subunit, selenocysteine-containing,W-formate dehydrogenase - alpha subunit  *W-formate dehydrogenase from Desulfovibrio vulgaris - Formate reduced form* | 0.16 |  | 16.90 | 0.33 | 1-222 | X-ray | 1.90 | hetero-1-1-mer | 2 x MGD, 4 x SF4, 1 x W, 1 x H2S | HHblits | 0.28 |
| ``` target    LARDIAKVP---GTTLFAIGMGPNQFFNNDNKDRTQFLLAALTGNIGKIAGNIGSYAGNYRVAM--FNG-----VPQYIA 6sdv.1    VYKAYAATGKPDKAGTIMYAMGWTQHSVGVQNIRAMAMIQLLLGNIGVAGGGVNALRGESNVQGSTDQGLLAHIWPGYNP  target    ENPFDI----EL-DGAK--PARP-KLYWRAEPAHYYN-H------EDHP-----------------LKMGKTMITGKTHM 6sdv.1    V-PNSKAATLELYNAATPQSKDPMSVNWWQNRPKYVASYLKALYPDEEPAAAYDYLPRIDAGRKLTDYFWLNIFE--KMD  target    PTPTKSLWFANANSILGNVKWHFNTVVNVLPKMEMIAVQEWWWSTSCEW--------AD-----IVFAVDAWSELKHPDM 6sdv.1    KGEFKGLFAWGMNPACG--GANANKNRKAMGKLEWLVNVNLFENETSSFWKGPGMNPAEIGTEVFFLPCCVSIEKE--GS  target    CSSVTNPFLTVFPRTPLERPFDTRGDIECLDLVGKQLAKRTGDRRFADMWKFVEEKKVEVYLQRILDHSSNTKGFKFPEL 6sdv.1    VAN-SGRWMQWRY-RGPKPYAETKPDGDIMLDMFKKV-------------------------------------------  target    EEKAKKGIPALMMTRTNPKTVGYEQVYDSRPWYTKTGRLEFYREEDEFIEAGENLPVHREPIDSTFYEPNVIVAPAHPFI 6sdv.1    --------------------------------------------------------------------------------  target    KAKGPEAYGVKVDDFDNETRQGRNIVKTWEETKKTVHPLAKDGYKFVFHTPKYRHGAHTMPVDTDMVAMLFGPFGDIYRH 6sdv.1    --------------------------------------------------------------------------------  target    DKRQPFAAEGYVDIHPDDAKALNIEDGDYVWIDSDPSDRPFRGWQKNDKDYKFSRLLCRARYYPGTPRGITRMWFNMYGA 6sdv.1    --------------------------------------------------------------------------------  target    TPGSVEGHESRKDGLAKNPRTGYQAMFRSGSHQSATRGWLKPTWMTDSLVRKELFGHAVNKGFLPDVHCPTGAPREAIVK 6sdv.1    --------------------------------------------------------------------------------  target    ITKAEPGGLNAKGLWRPAALGLRPKYENDKMKDYLAGKFTLAANPKKGGKK 6sdv.1    --------------------------------------------------- ``` | | | | | | | | | | | | | | | | | | | | | | | | | | | | | | | | | | | | | | | | | | | | | | | | | |
|  | 8bqg.1.A | Formate dehydrogenase, alpha subunit, selenocysteine-containing  *W-formate dehydrogenase from Desulfovibrio vulgaris - Soaking with Formate 1 min* | 0.16 |  | 16.36 | 0.34 | 1-227 | X-ray | 1.95 | hetero-1-1-mer | 2 x MGD, 4 x SF4, 1 x H2S, 1 x W | HHblits | 0.28 |
| ``` target    LARDIAKVP---GTTLFAIGMGPNQFFNNDNKDRTQFLLAALTGNIGKIAGNIGSYAGNYRVAMF--NG-----VPQYIA 8bqg.1    VYKAYAATGKPDKAGTIMYAMGWTQHSVGVQNIRAMAMIQLLLGNIGVAGGGVNALRGESNVQGSTDQGLLAHIWPGYNP  target    ENPFDIE----L-----DGAKPARPKLYWRAEPA-------------------HYYNHEDHP----LKMGKTMITGKTHM 8bqg.1    V-PNSKAATLELYNAATPQSKDPMSVNWWQNRPKYVASYLKALYPDEEPAAAYDYLPRIDAGRKLTDYFWLNIFEK--MD  target    PTPTKSLWFANANSILGNVKWHFNTVVNVLPKMEMIAVQEWWWSTSCEW--------AD-----IVFAVDAWSELKHPDM 8bqg.1    KGEFKGLFAWGMNPACG--GANANKNRKAMGKLEWLVNVNLFENETSSFWKGPGMNPAEIGTEVFFLPCCVSIEKE--GS  target    CSSVTNPFLTVFPRTPLERPFDTRGDIECLDLVGKQLAKRTGDRRFADMWKFVEEKKVEVYLQRILDHSSNTKGFKFPEL 8bqg.1    VAN-SGRWMQWRY-RGPKPYAETKPDGDIMLD----MFKKVR--------------------------------------  target    EEKAKKGIPALMMTRTNPKTVGYEQVYDSRPWYTKTGRLEFYREEDEFIEAGENLPVHREPIDSTFYEPNVIVAPAHPFI 8bqg.1    --------------------------------------------------------------------------------  target    KAKGPEAYGVKVDDFDNETRQGRNIVKTWEETKKTVHPLAKDGYKFVFHTPKYRHGAHTMPVDTDMVAMLFGPFGDIYRH 8bqg.1    --------------------------------------------------------------------------------  target    DKRQPFAAEGYVDIHPDDAKALNIEDGDYVWIDSDPSDRPFRGWQKNDKDYKFSRLLCRARYYPGTPRGITRMWFNMYGA 8bqg.1    --------------------------------------------------------------------------------  target    TPGSVEGHESRKDGLAKNPRTGYQAMFRSGSHQSATRGWLKPTWMTDSLVRKELFGHAVNKGFLPDVHCPTGAPREAIVK 8bqg.1    --------------------------------------------------------------------------------  target    ITKAEPGGLNAKGLWRPAALGLRPKYENDKMKDYLAGKFTLAANPKKGGKK 8bqg.1    --------------------------------------------------- ``` | | | | | | | | | | | | | | | | | | | | | | | | | | | | | | | | | | | | | | | | | | | | | | | | | |
|  | 3o5a.1.A | Periplasmic nitrate reductase  *Crystal Structure of partially reduced Periplasmic Nitrate Reductase from Cupriavidus necator using Ionic Liquids* | 0.14 |  | 18.48 | 0.33 | 1-229 | X-ray | 1.72 | hetero-oligomer | 1 x SF4, 1 x MOS, 2 x MGD, 2 x HEC | HHblits | 0.28 |
| ``` target    LARDIAKVPGTTLFAIGMGPNQFFNNDNKDRTQFLLAALTGNIGKIAGNIGSYAGNYRVAM-FNGVPQYIAENPFDIELD 3o5a.1    LAELYADPNIKVMSLWTMGFNQHTRGTWANNMVYNLHLLTGKIATPGNSPFSLTGQPSACGTAREVGTFSHRLPADMVVT  target    -GAKPARPKLYWRAEPAHYYNHEDHPLKMG-KTMITGKTHMPTPTKSLWFANANSILGNVKWHFNTVVNVL----PKMEM 3o5a.1    NPKHREEAERIWKL------PPGTIPDKPGYDAVLQNRMLKDGKLNAYWVQVNNNMQAAA----NLMEEGLPGYRNPANF  target    IAVQEWWWSTSCEWADIVFAVDAWSELKHPDMCSSVTNPFLTVFPRTPLERPFDTRGDIECLDLVGKQLAKRTGDRRFAD 3o5a.1    IVVSDAYPTVTALAADLVLPSAMWVEKE--GAYGN-AERRTQFWH-QLVDAPGEARSDLWQLVE----FAKRFKVE----  target    MWKFVEEKKVEVYLQRILDHSSNTKGFKFPELEEKAKKGIPALMMTRTNPKTVGYEQVYDSRPWYTKTGRLEFYREEDEF 3o5a.1    --------------------------------------------------------------------------------  target    IEAGENLPVHREPIDSTFYEPNVIVAPAHPFIKAKGPEAYGVKVDDFDNETRQGRNIVKTWEETKKTVHPLAKDGYKFVF 3o5a.1    --------------------------------------------------------------------------------  target    HTPKYRHGAHTMPVDTDMVAMLFGPFGDIYRHDKRQPFAAEGYVDIHPDDAKALNIEDGDYVWIDSDPSDRPFRGWQKND 3o5a.1    --------------------------------------------------------------------------------  target    KDYKFSRLLCRARYYPGTPRGITRMWFNMYGATPGSVEGHESRKDGLAKNPRTGYQAMFRSGSHQSATRGWLKPTWMTDS 3o5a.1    --------------------------------------------------------------------------------  target    LVRKELFGHAVNKGFLPDVHCPTGAPREAIVKITKAEPGGLNAKGLWRPAALGLRPKYENDKMKDYLAGKFTLAANPKKG 3o5a.1    --------------------------------------------------------------------------------  target    GKK 3o5a.1    --- ``` | | | | | | | | | | | | | | | | | | | | | | | | | | | | | | | | | | | | | | | | | | | | | | | | | |
|  | 1h0h.1.A | FORMATE DEHYDROGENASE SUBUNIT ALPHA  *Tungsten containing Formate Dehydrogenase from Desulfovibrio Gigas* | 0.15 |  | 15.42 | 0.34 | 1-227 | X-ray | 1.80 | hetero-1-1-mer | 1 x W, 1 x 2MD, 1 x MGD, 4 x SF4, 1 x CA | HHblits | 0.27 |
| ``` target    LARDIAKVP---GTTLFAIGMGPNQFFNNDNKDRTQFLLAALTGNIGKIAGNIGSYAGNYRVA-M------FNGVPQYIA 1h0h.1    VYDAYCATGKPDKAGTIMYAMGWTQHTVGVQNIRAMSINQLLLGNIGVAGGGVNALRGEANVQGSTDHGLLMHIYPGYLG  target    EN----PF----DIELDG-AKPARPKLYWRAEPAH--------YYNHEDHPLKM--------G-----KTMITGKTHMPT 1h0h.1    TARASIPTYEEYTKKFTPVSKDPQ-SANWWSNFPKYSASYIKSMWPDADLNEAYGYLPKGEDGKDYSWLTLFDD--MFQG  target    PTKSLWFANANSILGNVKWHFNTVVNVLPKMEMIAVQEWWWSTSCEWA-------------DIVFAVDAWSELKHPDMCS 1h0h.1    KIKGFFAWGQNPACS--GANSNKTREALTKLDWMVNVNIFDNETGSFWRGPDMDPKKIKTEVFFLPCAVAIEKE--GSIS  target    SVTNPFLTVFPRTPLERPFDTRGDIECLDLVGKQLAKRTGDRRFADMWKFVEEKKVEVYLQRILDHSSNTKGFKFPELEE 1h0h.1    N-SGRWMQWRY-VGPEPRKNAIPDGDLIVE----LAKRVQ----------------------------------------  target    KAKKGIPALMMTRTNPKTVGYEQVYDSRPWYTKTGRLEFYREEDEFIEAGENLPVHREPIDSTFYEPNVIVAPAHPFIKA 1h0h.1    --------------------------------------------------------------------------------  target    KGPEAYGVKVDDFDNETRQGRNIVKTWEETKKTVHPLAKDGYKFVFHTPKYRHGAHTMPVDTDMVAMLFGPFGDIYRHDK 1h0h.1    --------------------------------------------------------------------------------  target    RQPFAAEGYVDIHPDDAKALNIEDGDYVWIDSDPSDRPFRGWQKNDKDYKFSRLLCRARYYPGTPRGITRMWFNMYGATP 1h0h.1    --------------------------------------------------------------------------------  target    GSVEGHESRKDGLAKNPRTGYQAMFRSGSHQSATRGWLKPTWMTDSLVRKELFGHAVNKGFLPDVHCPTGAPREAIVKIT 1h0h.1    --------------------------------------------------------------------------------  target    KAEPGGLNAKGLWRPAALGLRPKYENDKMKDYLAGKFTLAANPKKGGKK 1h0h.1    ------------------------------------------------- ``` | | | | | | | | | | | | | | | | | | | | | | | | | | | | | | | | | | | | | | | | | | | | | | | | | |
|  | 6zr2.1.G | NADH-ubiquinone oxidoreductase 75 kDa subunit, mitochondrial  *Cryo-EM structure of respiratory complex I in the active state from Mus musculus at 3.1 A* | 0.13 | 0.00 | 16.24 | 0.31 | 1-266 | EM | 3.10 | monomer | 6 x SF4, 4 x PC1, 2 x FES, 1 x FMN, 9 x 3PE, 7 x CDL, 1 x ATP, 1 x NDP, 1 x ZN, 2 x EHZ | HHblits | 0.28 |
| ``` target    LARDIAKVPGTTLFAIGMGPNQFFNNDNKDRTQFLLAALTGNIGKIAGNIGSYAGNYRVAMFNGVPQYIAENPFDIELDG 6zr2.1    FCEVLKDAKKP-MVVLGSSALQRDDGAAILVAVSNMVQKIRVTTGVAAEWKVMNILHRI--ASQV--------------A  target    AKPARPKLYWRAEPAHYYNHEDHPLKMG-KTMITGKTHMPTPTKSLWFANANSILGNVKWHFNTVVNVLPKMEMIAVQEW 6zr2.1    A----L---------------DLGYKPGVEAI------RKNPPKMLFLLGADGG--------CITRQDLPKDCFIVYQGH  target    WWSTSCEWADIVFAVDAWSELKHPDMCSSVTNPFLTV-FPRTPLERPFDTRGDIECLDLVGKQLAKRTGDRRFADMWKFV 6zr2.1    HGDVGAPMADVILPGAAYTEKS-----ATYVNTEGRAQQTKVAVTPPGLAREDWKIIRA----LSEIAGIT-----LPYD  target    EEKKVEVYLQRILDHSSNTKGFKFPELEEKAKKGIPALMMTRTNPKTVGYEQVYDSRPWYTKTGRLEFYREEDEFIEAGE 6zr2.1    ---TLDQVRNRLEEVSPNL--VRYDDIE----------------------------------------------------  target    NLPVHREPIDSTFYEPNVIVAPAHPFIKAKGPEAYGVKVDDFDNETRQGRNIVKTWEETKKTVHPLAKDGYKFVFHTPKY 6zr2.1    --------------------------------------------------------------------------------  target    RHGAHTMPVDTDMVAMLFGPFGDIYRHDKRQPFAAEGYVDIHPDDAKALNIEDGDYVWIDSDPSDRPFRGWQKNDKDYKF 6zr2.1    --------------------------------------------------------------------------------  target    SRLLCRARYYPGTPRGITRMWFNMYGATPGSVEGHESRKDGLAKNPRTGYQAMFRSGSHQSATRGWLKPTWMTDSLVRKE 6zr2.1    --------------------------------------------------------------------------------  target    LFGHAVNKGFLPDVHCPTGAPREAIVKITKAEPGGLNAKGLWRPAALGLRPKYENDKMKDYLAGKFTLAANPKKGGKK 6zr2.1    ------------------------------------------------------------------------------ ``` | | | | | | | | | | | | | | | | | | | | | | | | | | | | | | | | | | | | | | | | | | | | | | | | | |
|  | 6g72.1.G | NADH-ubiquinone oxidoreductase 75 kDa subunit, mitochondrial  *Mouse mitochondrial complex I in the deactive state* | 0.13 | 0.00 | 16.24 | 0.31 | 1-266 | EM | 0.00 | monomer | 6 x SF4, 2 x FES, 1 x FMN, 1 x ADP, 1 x NDP, 1 x ZN, 2 x EHZ | HHblits | 0.28 |
| ``` target    LARDIAKVPGTTLFAIGMGPNQFFNNDNKDRTQFLLAALTGNIGKIAGNIGSYAGNYRVAMFNGVPQYIAENPFDIELDG 6g72.1    FCEVLKDAKKP-MVVLGSSALQRDDGAAILVAVSNMVQKIRVTTGVAAEWKVMNILHRI--ASQV--------------A  target    AKPARPKLYWRAEPAHYYNHEDHPLKMG-KTMITGKTHMPTPTKSLWFANANSILGNVKWHFNTVVNVLPKMEMIAVQEW 6g72.1    A----L---------------DLGYKPGVEAI------RKNPPKMLFLLGADGG--------CITRQDLPKDCFIVYQGH  target    WWSTSCEWADIVFAVDAWSELKHPDMCSSVTNPFLTV-FPRTPLERPFDTRGDIECLDLVGKQLAKRTGDRRFADMWKFV 6g72.1    HGDVGAPMADVILPGAAYTEKS-----ATYVNTEGRAQQTKVAVTPPGLAREDWKIIRA----LSEIAGIT-----LPYD  target    EEKKVEVYLQRILDHSSNTKGFKFPELEEKAKKGIPALMMTRTNPKTVGYEQVYDSRPWYTKTGRLEFYREEDEFIEAGE 6g72.1    ---TLDQVRNRLEEVSPNL--VRYDDIE----------------------------------------------------  target    NLPVHREPIDSTFYEPNVIVAPAHPFIKAKGPEAYGVKVDDFDNETRQGRNIVKTWEETKKTVHPLAKDGYKFVFHTPKY 6g72.1    --------------------------------------------------------------------------------  target    RHGAHTMPVDTDMVAMLFGPFGDIYRHDKRQPFAAEGYVDIHPDDAKALNIEDGDYVWIDSDPSDRPFRGWQKNDKDYKF 6g72.1    --------------------------------------------------------------------------------  target    SRLLCRARYYPGTPRGITRMWFNMYGATPGSVEGHESRKDGLAKNPRTGYQAMFRSGSHQSATRGWLKPTWMTDSLVRKE 6g72.1    --------------------------------------------------------------------------------  target    LFGHAVNKGFLPDVHCPTGAPREAIVKITKAEPGGLNAKGLWRPAALGLRPKYENDKMKDYLAGKFTLAANPKKGGKK 6g72.1    ------------------------------------------------------------------------------ ``` | | | | | | | | | | | | | | | | | | | | | | | | | | | | | | | | | | | | | | | | | | | | | | | | | |
|  | 7ak6.1.G | NADH-ubiquinone oxidoreductase 75 kDa subunit, mitochondrial  *Cryo-EM structure of ND6-P25L mutant respiratory complex I from Mus musculus at 3.8 A* | 0.13 | 0.00 | 16.24 | 0.31 | 1-266 | EM | 0.00 | monomer | 6 x SF4, 1 x PC1, 2 x FES, 1 x FMN, 4 x 3PE, 2 x CDL, 1 x ATP, 1 x NDP, 1 x ZN, 2 x EHZ | HHblits | 0.28 |
| ``` target    LARDIAKVPGTTLFAIGMGPNQFFNNDNKDRTQFLLAALTGNIGKIAGNIGSYAGNYRVAMFNGVPQYIAENPFDIELDG 7ak6.1    FCEVLKDAKKP-MVVLGSSALQRDDGAAILVAVSNMVQKIRVTTGVAAEWKVMNILHRI--ASQV--------------A  target    AKPARPKLYWRAEPAHYYNHEDHPLKMG-KTMITGKTHMPTPTKSLWFANANSILGNVKWHFNTVVNVLPKMEMIAVQEW 7ak6.1    A----L---------------DLGYKPGVEAI------RKNPPKMLFLLGADGG--------CITRQDLPKDCFIVYQGH  target    WWSTSCEWADIVFAVDAWSELKHPDMCSSVTNPFLTV-FPRTPLERPFDTRGDIECLDLVGKQLAKRTGDRRFADMWKFV 7ak6.1    HGDVGAPMADVILPGAAYTEKS-----ATYVNTEGRAQQTKVAVTPPGLAREDWKIIRA----LSEIAGIT-----LPYD  target    EEKKVEVYLQRILDHSSNTKGFKFPELEEKAKKGIPALMMTRTNPKTVGYEQVYDSRPWYTKTGRLEFYREEDEFIEAGE 7ak6.1    ---TLDQVRNRLEEVSPNL--VRYDDIE----------------------------------------------------  target    NLPVHREPIDSTFYEPNVIVAPAHPFIKAKGPEAYGVKVDDFDNETRQGRNIVKTWEETKKTVHPLAKDGYKFVFHTPKY 7ak6.1    --------------------------------------------------------------------------------  target    RHGAHTMPVDTDMVAMLFGPFGDIYRHDKRQPFAAEGYVDIHPDDAKALNIEDGDYVWIDSDPSDRPFRGWQKNDKDYKF 7ak6.1    --------------------------------------------------------------------------------  target    SRLLCRARYYPGTPRGITRMWFNMYGATPGSVEGHESRKDGLAKNPRTGYQAMFRSGSHQSATRGWLKPTWMTDSLVRKE 7ak6.1    --------------------------------------------------------------------------------  target    LFGHAVNKGFLPDVHCPTGAPREAIVKITKAEPGGLNAKGLWRPAALGLRPKYENDKMKDYLAGKFTLAANPKKGGKK 7ak6.1    ------------------------------------------------------------------------------ ``` | | | | | | | | | | | | | | | | | | | | | | | | | | | | | | | | | | | | | | | | | | | | | | | | | |
|  | 7ak5.1.G | NADH-ubiquinone oxidoreductase 75 kDa subunit, mitochondrial  *Cryo-EM structure of respiratory complex I in the deactive state from Mus musculus at 3.2 A* | 0.14 | 0.00 | 15.23 | 0.31 | 1-266 | EM | 0.00 | monomer | 6 x SF4, 2 x PC1, 2 x FES, 1 x FMN, 8 x 3PE, 4 x CDL, 1 x ATP, 1 x NDP, 1 x ZN, 2 x EHZ | HHblits | 0.27 |
| ``` target    LARDIAKVPGTTLFAIGMGPNQFFNNDNKDRTQFLLAALTGNIGKIAGNIGSYAGNYRVAMFNGVPQYIAENPFDIELDG 7ak5.1    FCEVLKDAKKP-MVVLGSSALQRDDGAAILVAVSNMVQKIRVTTGVAAEWKVMNI---------LHRI--ASQV-----A  target    AKPARPKLYWRAEPAHYYNHEDHPLKMG-KTMITGKTHMPTPTKSLWFANANSILGNVKWHFNTVVNVLPKMEMIAVQEW 7ak5.1    A----L---------------DLGYKPGVEAI------RKNPPKMLFLLGADGG--------CITRQDLPKDCFIVYQGH  target    WWSTSCEWADIVFAVDAWSELKHPDMCSSVTNPFLTV-FPRTPLERPFDTRGDIECLDLVGKQLAKRTGDRRFADMWKFV 7ak5.1    HGDVGAPMADVILPGAAYTEKS-----ATYVNTEGRAQQTKVAVTPPGLAREDWKIIRA----LSEIAGIT-----LPYD  target    EEKKVEVYLQRILDHSSNTKGFKFPELEEKAKKGIPALMMTRTNPKTVGYEQVYDSRPWYTKTGRLEFYREEDEFIEAGE 7ak5.1    ---TLDQVRNRLEEVSPNL--VRYDDIE----------------------------------------------------  target    NLPVHREPIDSTFYEPNVIVAPAHPFIKAKGPEAYGVKVDDFDNETRQGRNIVKTWEETKKTVHPLAKDGYKFVFHTPKY 7ak5.1    --------------------------------------------------------------------------------  target    RHGAHTMPVDTDMVAMLFGPFGDIYRHDKRQPFAAEGYVDIHPDDAKALNIEDGDYVWIDSDPSDRPFRGWQKNDKDYKF 7ak5.1    --------------------------------------------------------------------------------  target    SRLLCRARYYPGTPRGITRMWFNMYGATPGSVEGHESRKDGLAKNPRTGYQAMFRSGSHQSATRGWLKPTWMTDSLVRKE 7ak5.1    --------------------------------------------------------------------------------  target    LFGHAVNKGFLPDVHCPTGAPREAIVKITKAEPGGLNAKGLWRPAALGLRPKYENDKMKDYLAGKFTLAANPKKGGKK 7ak5.1    ------------------------------------------------------------------------------ ``` | | | | | | | | | | | | | | | | | | | | | | | | | | | | | | | | | | | | | | | | | | | | | | | | | |
|  | 3m9s.1.C | NADH-quinone oxidoreductase subunit 3  *Crystal structure of respiratory complex I from Thermus thermophilus* | 0.16 | 0.00 | 18.56 | 0.31 | 144-506 | X-ray | 4.50 | monomer | 7 x SF4, 2 x FES, 1 x FMN | HHblits | 0.28 |
| ``` target    LARDIAKVPGTTLFAIGMGPNQFFNNDNKDRTQFLLAALTGNIGKIAGNIGSYAGNYRVAMFNGVPQYIAENPFDIELDG 3m9s.1    --------------------------------------------------------------------------------  target    AKPARPKLYWRAEPAHYYNHEDHPLKMGKTMITGKTHMPTPTKSLWFANANSILGNVKWHFNTVVNVLPKMEMIAVQEWW 3m9s.1    ---------------------------------------------------------------PEEALKGKRFVVMHLSH  target    WSTSC-EWADIVFAVDAWSELKHPDMCSSVTNPFLTV-FPRTPLERPFDTRGDIECLDLVGKQLAKRTGDRRFADMWKFV 3m9s.1    LHPLAERYAHVVLPAPTFYEKR-----GHLVNLEGRVLPLSPAPIENGEAEGALQVLAL----LAEALGVRP-----PFR  target    EEKKVEVYLQRILDHSSNTKGFKFPELEEKAKKGIPALMMTRTNPKTVGYEQVYDSRPWYTKTGRLEFYREEDEFIEAGE 3m9s.1    ---LHLEAQK---------------ALK---------------------------ARKVPEAMGRLSFRLKEL-------  target    NLPVHREPIDSTFYEPNVIVAPAHPFIKAKGPEAYGVKVDDFDNETRQGRNIVKTWEETKKTVHPLAKDGYKFVFHTPKY 3m9s.1    --R----P----------------------------------------------------------KERKGAFYLRPTMW  target    RHGAHTMPVDTDMVAMLFGPFGDIYRHDKRQPFAAEGYVDIHPDDAKALNIEDGDYVWIDSDPSDRPFRGWQKNDKDYKF 3m9s.1    KAHQAVG-----KA-------------Q----EAARAELWAHPETARAEALPEGAQVAVETPF-----------------  target    SRLLCRARYYPGTPRGITRMWFNMYGATPGSVEGHESRKDGLAKNPRTGYQAMFRSGSHQSATRGWLKPTWMTDSLVRKE 3m9s.1    GRVEARVVHREDVPKGHLYLSALGPAAG----------------------------------------------------  target    LFGHAVNKGFLPDVHCPTGAPREAIVKITKAEPGGLNAKGLWRPAALGLRPKYENDKMKDYLAGKFTLAANPKKGGKK 3m9s.1    ------------------------------------------------------------------------------ ``` | | | | | | | | | | | | | | | | | | | | | | | | | | | | | | | | | | | | | | | | | | | | | | | | | |
| ✓ | 2fug.2.C | NADH-quinone oxidoreductase chain 3  *Crystal structure of the hydrophilic domain of respiratory complex I from Thermus thermophilus* | 0.14 | 0.00 | 18.56 | 0.31 | 144-506 | X-ray | 3.30 | monomer | 7 x SF4, 2 x FES, 1 x FMN | HHblits | 0.28 |
| ``` target    LARDIAKVPGTTLFAIGMGPNQFFNNDNKDRTQFLLAALTGNIGKIAGNIGSYAGNYRVAMFNGVPQYIAENPFDIELDG 2fug.2    --------------------------------------------------------------------------------  target    AKPARPKLYWRAEPAHYYNHEDHPLKMGKTMITGKTHMPTPTKSLWFANANSILGNVKWHFNTVVNVLPKMEMIAVQEWW 2fug.2    ---------------------------------------------------------------PEEALKGKRFVVMHLSH  target    WSTSC-EWADIVFAVDAWSELKHPDMCSSVTNPFLTV-FPRTPLERPFDTRGDIECLDLVGKQLAKRTGDRRFADMWKFV 2fug.2    LHPLAERYAHVVLPAPTFYEKR-----GHLVNLEGRVLPLSPAPIENGEAEGALQVLAL----LAEALGVRP-----PFR  target    EEKKVEVYLQRILDHSSNTKGFKFPELEEKAKKGIPALMMTRTNPKTVGYEQVYDSRPWYTKTGRLEFYREEDEFIEAGE 2fug.2    ---LHLEAQK---------------ALK---------------------------ARKVPEAMGRLSFRLKEL-------  target    NLPVHREPIDSTFYEPNVIVAPAHPFIKAKGPEAYGVKVDDFDNETRQGRNIVKTWEETKKTVHPLAKDGYKFVFHTPKY 2fug.2    --R----P----------------------------------------------------------KERKGAFYLRPTMW  target    RHGAHTMPVDTDMVAMLFGPFGDIYRHDKRQPFAAEGYVDIHPDDAKALNIEDGDYVWIDSDPSDRPFRGWQKNDKDYKF 2fug.2    KAHQAVG-----KA-------------Q----EAARAELWAHPETARAEALPEGAQVAVETPF-----------------  target    SRLLCRARYYPGTPRGITRMWFNMYGATPGSVEGHESRKDGLAKNPRTGYQAMFRSGSHQSATRGWLKPTWMTDSLVRKE 2fug.2    GRVEARVVHREDVPKGHLYLSALGPAAG----------------------------------------------------  target    LFGHAVNKGFLPDVHCPTGAPREAIVKITKAEPGGLNAKGLWRPAALGLRPKYENDKMKDYLAGKFTLAANPKKGGKK 2fug.2    ------------------------------------------------------------------------------ ``` | | | | | | | | | | | | | | | | | | | | | | | | | | | | | | | | | | | | | | | | | | | | | | | | | |
|  | 6zjl.1.C | NADH-quinone oxidoreductase subunit 3  *Respiratory complex I from Thermus thermophilus, NAD+ dataset, major state* | 0.15 | 0.00 | 18.56 | 0.31 | 144-506 | EM | 0.00 | monomer | 7 x SF4, 1 x FMN, 2 x FES | HHblits | 0.28 |
| ``` target    LARDIAKVPGTTLFAIGMGPNQFFNNDNKDRTQFLLAALTGNIGKIAGNIGSYAGNYRVAMFNGVPQYIAENPFDIELDG 6zjl.1    --------------------------------------------------------------------------------  target    AKPARPKLYWRAEPAHYYNHEDHPLKMGKTMITGKTHMPTPTKSLWFANANSILGNVKWHFNTVVNVLPKMEMIAVQEWW 6zjl.1    ---------------------------------------------------------------PEEALKGKRFVVMHLSH  target    WSTSC-EWADIVFAVDAWSELKHPDMCSSVTNPFLTV-FPRTPLERPFDTRGDIECLDLVGKQLAKRTGDRRFADMWKFV 6zjl.1    LHPLAERYAHVVLPAPTFYEKR-----GHLVNLEGRVLPLSPAPIENGEAEGALQVLAL----LAEALGVRP-----PFR  target    EEKKVEVYLQRILDHSSNTKGFKFPELEEKAKKGIPALMMTRTNPKTVGYEQVYDSRPWYTKTGRLEFYREEDEFIEAGE 6zjl.1    ---LHLEAQK---------------ALK---------------------------ARKVPEAMGRLSFRLKEL-------  target    NLPVHREPIDSTFYEPNVIVAPAHPFIKAKGPEAYGVKVDDFDNETRQGRNIVKTWEETKKTVHPLAKDGYKFVFHTPKY 6zjl.1    --R----P----------------------------------------------------------KERKGAFYLRPTMW  target    RHGAHTMPVDTDMVAMLFGPFGDIYRHDKRQPFAAEGYVDIHPDDAKALNIEDGDYVWIDSDPSDRPFRGWQKNDKDYKF 6zjl.1    KAHQAVG-----KA-------------Q----EAARAELWAHPETARAEALPEGAQVAVETPF-----------------  target    SRLLCRARYYPGTPRGITRMWFNMYGATPGSVEGHESRKDGLAKNPRTGYQAMFRSGSHQSATRGWLKPTWMTDSLVRKE 6zjl.1    GRVEARVVHREDVPKGHLYLSALGPAAG----------------------------------------------------  target    LFGHAVNKGFLPDVHCPTGAPREAIVKITKAEPGGLNAKGLWRPAALGLRPKYENDKMKDYLAGKFTLAANPKKGGKK 6zjl.1    ------------------------------------------------------------------------------ ``` | | | | | | | | | | | | | | | | | | | | | | | | | | | | | | | | | | | | | | | | | | | | | | | | | |
|  | 6q8o.1.C | NADH-quinone oxidoreductase subunit 3  *Respiratory complex I from Thermus thermophilus with bound Piericidin A* | 0.16 | 0.00 | 18.56 | 0.31 | 144-506 | X-ray | 3.61 | monomer | 7 x SF4, 1 x FMN, 2 x FES, 1 x HQH | HHblits | 0.28 |
| ``` target    LARDIAKVPGTTLFAIGMGPNQFFNNDNKDRTQFLLAALTGNIGKIAGNIGSYAGNYRVAMFNGVPQYIAENPFDIELDG 6q8o.1    --------------------------------------------------------------------------------  target    AKPARPKLYWRAEPAHYYNHEDHPLKMGKTMITGKTHMPTPTKSLWFANANSILGNVKWHFNTVVNVLPKMEMIAVQEWW 6q8o.1    ---------------------------------------------------------------PEEALKGKRFVVMHLSH  target    WSTSC-EWADIVFAVDAWSELKHPDMCSSVTNPFLTV-FPRTPLERPFDTRGDIECLDLVGKQLAKRTGDRRFADMWKFV 6q8o.1    LHPLAERYAHVVLPAPTFYEKR-----GHLVNLEGRVLPLSPAPIENGEAEGALQVLAL----LAEALGVRP-----PFR  target    EEKKVEVYLQRILDHSSNTKGFKFPELEEKAKKGIPALMMTRTNPKTVGYEQVYDSRPWYTKTGRLEFYREEDEFIEAGE 6q8o.1    ---LHLEAQK---------------ALK---------------------------ARKVPEAMGRLSFRLKEL-------  target    NLPVHREPIDSTFYEPNVIVAPAHPFIKAKGPEAYGVKVDDFDNETRQGRNIVKTWEETKKTVHPLAKDGYKFVFHTPKY 6q8o.1    --R----P----------------------------------------------------------KERKGAFYLRPTMW  target    RHGAHTMPVDTDMVAMLFGPFGDIYRHDKRQPFAAEGYVDIHPDDAKALNIEDGDYVWIDSDPSDRPFRGWQKNDKDYKF 6q8o.1    KAHQAVG-----KA-------------Q----EAARAELWAHPETARAEALPEGAQVAVETPF-----------------  target    SRLLCRARYYPGTPRGITRMWFNMYGATPGSVEGHESRKDGLAKNPRTGYQAMFRSGSHQSATRGWLKPTWMTDSLVRKE 6q8o.1    GRVEARVVHREDVPKGHLYLSALGPAAG----------------------------------------------------  target    LFGHAVNKGFLPDVHCPTGAPREAIVKITKAEPGGLNAKGLWRPAALGLRPKYENDKMKDYLAGKFTLAANPKKGGKK 6q8o.1    ------------------------------------------------------------------------------ ``` | | | | | | | | | | | | | | | | | | | | | | | | | | | | | | | | | | | | | | | | | | | | | | | | | |
|  | 6zjy.1.C | NADH-quinone oxidoreductase subunit 3  *Respiratory complex I from Thermus thermophilus, NAD+ dataset, minor state* | 0.15 | 0.00 | 18.56 | 0.31 | 144-506 | EM | 0.00 | monomer | 7 x SF4, 2 x FES | HHblits | 0.28 |
| ``` target    LARDIAKVPGTTLFAIGMGPNQFFNNDNKDRTQFLLAALTGNIGKIAGNIGSYAGNYRVAMFNGVPQYIAENPFDIELDG 6zjy.1    --------------------------------------------------------------------------------  target    AKPARPKLYWRAEPAHYYNHEDHPLKMGKTMITGKTHMPTPTKSLWFANANSILGNVKWHFNTVVNVLPKMEMIAVQEWW 6zjy.1    ---------------------------------------------------------------PEEALKGKRFVVMHLSH  target    WSTSC-EWADIVFAVDAWSELKHPDMCSSVTNPFLTV-FPRTPLERPFDTRGDIECLDLVGKQLAKRTGDRRFADMWKFV 6zjy.1    LHPLAERYAHVVLPAPTFYEKR-----GHLVNLEGRVLPLSPAPIENGEAEGALQVLAL----LAEALGVRP-----PFR  target    EEKKVEVYLQRILDHSSNTKGFKFPELEEKAKKGIPALMMTRTNPKTVGYEQVYDSRPWYTKTGRLEFYREEDEFIEAGE 6zjy.1    ---LHLEAQK---------------ALK---------------------------ARKVPEAMGRLSFRLKEL-------  target    NLPVHREPIDSTFYEPNVIVAPAHPFIKAKGPEAYGVKVDDFDNETRQGRNIVKTWEETKKTVHPLAKDGYKFVFHTPKY 6zjy.1    --R----P----------------------------------------------------------KERKGAFYLRPTMW  target    RHGAHTMPVDTDMVAMLFGPFGDIYRHDKRQPFAAEGYVDIHPDDAKALNIEDGDYVWIDSDPSDRPFRGWQKNDKDYKF 6zjy.1    KAHQAVG-----KA-------------Q----EAARAELWAHPETARAEALPEGAQVAVETPF-----------------  target    SRLLCRARYYPGTPRGITRMWFNMYGATPGSVEGHESRKDGLAKNPRTGYQAMFRSGSHQSATRGWLKPTWMTDSLVRKE 6zjy.1    GRVEARVVHREDVPKGHLYLSALGPAAG----------------------------------------------------  target    LFGHAVNKGFLPDVHCPTGAPREAIVKITKAEPGGLNAKGLWRPAALGLRPKYENDKMKDYLAGKFTLAANPKKGGKK 6zjy.1    ------------------------------------------------------------------------------ ``` | | | | | | | | | | | | | | | | | | | | | | | | | | | | | | | | | | | | | | | | | | | | | | | | | |
|  | 6zjn.1.C | NADH-quinone oxidoreductase subunit 3  *Respiratory complex I from Thermus thermophilus, NADH dataset, minor state* | 0.16 | 0.00 | 18.56 | 0.31 | 144-506 | EM | 0.00 | monomer | 7 x SF4, 2 x FES | HHblits | 0.28 |
| ``` target    LARDIAKVPGTTLFAIGMGPNQFFNNDNKDRTQFLLAALTGNIGKIAGNIGSYAGNYRVAMFNGVPQYIAENPFDIELDG 6zjn.1    --------------------------------------------------------------------------------  target    AKPARPKLYWRAEPAHYYNHEDHPLKMGKTMITGKTHMPTPTKSLWFANANSILGNVKWHFNTVVNVLPKMEMIAVQEWW 6zjn.1    ---------------------------------------------------------------PEEALKGKRFVVMHLSH  target    WSTSC-EWADIVFAVDAWSELKHPDMCSSVTNPFLTV-FPRTPLERPFDTRGDIECLDLVGKQLAKRTGDRRFADMWKFV 6zjn.1    LHPLAERYAHVVLPAPTFYEKR-----GHLVNLEGRVLPLSPAPIENGEAEGALQVLAL----LAEALGVRP-----PFR  target    EEKKVEVYLQRILDHSSNTKGFKFPELEEKAKKGIPALMMTRTNPKTVGYEQVYDSRPWYTKTGRLEFYREEDEFIEAGE 6zjn.1    ---LHLEAQK---------------ALK---------------------------ARKVPEAMGRLSFRLKEL-------  target    NLPVHREPIDSTFYEPNVIVAPAHPFIKAKGPEAYGVKVDDFDNETRQGRNIVKTWEETKKTVHPLAKDGYKFVFHTPKY 6zjn.1    --R----P----------------------------------------------------------KERKGAFYLRPTMW  target    RHGAHTMPVDTDMVAMLFGPFGDIYRHDKRQPFAAEGYVDIHPDDAKALNIEDGDYVWIDSDPSDRPFRGWQKNDKDYKF 6zjn.1    KAHQAVG-----KA-------------Q----EAARAELWAHPETARAEALPEGAQVAVETPF-----------------  target    SRLLCRARYYPGTPRGITRMWFNMYGATPGSVEGHESRKDGLAKNPRTGYQAMFRSGSHQSATRGWLKPTWMTDSLVRKE 6zjn.1    GRVEARVVHREDVPKGHLYLSALGPAAG----------------------------------------------------  target    LFGHAVNKGFLPDVHCPTGAPREAIVKITKAEPGGLNAKGLWRPAALGLRPKYENDKMKDYLAGKFTLAANPKKGGKK 6zjn.1    ------------------------------------------------------------------------------ ``` | | | | | | | | | | | | | | | | | | | | | | | | | | | | | | | | | | | | | | | | | | | | | | | | | |
|  | 6ziy.1.C | NADH-quinone oxidoreductase subunit 3  *Respiratory complex I from Thermus thermophilus, NADH dataset, major state* | 0.15 | 0.00 | 18.56 | 0.31 | 144-506 | EM | 0.00 | monomer | 7 x SF4, 1 x FMN, 1 x NAI, 2 x FES | HHblits | 0.28 |
| ``` target    LARDIAKVPGTTLFAIGMGPNQFFNNDNKDRTQFLLAALTGNIGKIAGNIGSYAGNYRVAMFNGVPQYIAENPFDIELDG 6ziy.1    --------------------------------------------------------------------------------  target    AKPARPKLYWRAEPAHYYNHEDHPLKMGKTMITGKTHMPTPTKSLWFANANSILGNVKWHFNTVVNVLPKMEMIAVQEWW 6ziy.1    ---------------------------------------------------------------PEEALKGKRFVVMHLSH  target    WSTSC-EWADIVFAVDAWSELKHPDMCSSVTNPFLTV-FPRTPLERPFDTRGDIECLDLVGKQLAKRTGDRRFADMWKFV 6ziy.1    LHPLAERYAHVVLPAPTFYEKR-----GHLVNLEGRVLPLSPAPIENGEAEGALQVLAL----LAEALGVRP-----PFR  target    EEKKVEVYLQRILDHSSNTKGFKFPELEEKAKKGIPALMMTRTNPKTVGYEQVYDSRPWYTKTGRLEFYREEDEFIEAGE 6ziy.1    ---LHLEAQK---------------ALK---------------------------ARKVPEAMGRLSFRLKEL-------  target    NLPVHREPIDSTFYEPNVIVAPAHPFIKAKGPEAYGVKVDDFDNETRQGRNIVKTWEETKKTVHPLAKDGYKFVFHTPKY 6ziy.1    --R----P----------------------------------------------------------KERKGAFYLRPTMW  target    RHGAHTMPVDTDMVAMLFGPFGDIYRHDKRQPFAAEGYVDIHPDDAKALNIEDGDYVWIDSDPSDRPFRGWQKNDKDYKF 6ziy.1    KAHQAVG-----KA-------------Q----EAARAELWAHPETARAEALPEGAQVAVETPF-----------------  target    SRLLCRARYYPGTPRGITRMWFNMYGATPGSVEGHESRKDGLAKNPRTGYQAMFRSGSHQSATRGWLKPTWMTDSLVRKE 6ziy.1    GRVEARVVHREDVPKGHLYLSALGPAAG----------------------------------------------------  target    LFGHAVNKGFLPDVHCPTGAPREAIVKITKAEPGGLNAKGLWRPAALGLRPKYENDKMKDYLAGKFTLAANPKKGGKK 6ziy.1    ------------------------------------------------------------------------------ ``` | | | | | | | | | | | | | | | | | | | | | | | | | | | | | | | | | | | | | | | | | | | | | | | | | |
|  | 5t5i.1.B | Tungsten formylmethanofuran dehydrogenase subunit B  *TUNGSTEN-CONTAINING FORMYLMETHANOFURAN DEHYDROGENASE FROM METHANOTHERMOBACTER WOLFEII, ORTHORHOMBIC FORM AT 1.9 A* | 0.13 |  | 13.83 | 0.30 | 1-228 | X-ray | 1.90 | hetero-oligomer | 4 x ZN, 2 x MG, 18 x K, 22 x SF4, 2 x W, 4 x MGD, 2 x H2S, 2 x CA | HHblits | 0.28 |
| ``` target    LARDIAKVPGTTLFAIGMGPNQFFNNDNKDRTQFLLAALTGNIGKIAGNIGSYAGNYRVAMFNGVPQYIAENPFDIELDG 5t5i.1    AVEVLKNAQF-GILFFGMGITHSRGKHRNIDTAIMMVQDLND--YAKWTLIPMRGHYNVTGFNQVCTWESGYPYCV----  target    AKPARPKLYWRAEPAHYYNHEDHPLKMGKTMITGKTHMPTPTKSLWFANANSILGNVKWHFNTVVNVLPKMEMIAVQEWW 5t5i.1    ------D-FSGGEP-R-YNP--GETGANDLL------QNREADAMMVIASDPGAHFP--Q--RALERMAEIP-VIAIEPH  target    WSTSCEWADIVFAVDA-WSELKHPDMCSSVTNPFLTVF-PRTPLERPFDTRGDIECLDLVGKQLAKRTGDRRFADMWKFV 5t5i.1    RTPTTEMADIIIPPAIVGMEAE-----GTAYRMEGVPIRMKKVVDS--DLLSDREILER----LLEKVRE----------  target    EEKKVEVYLQRILDHSSNTKGFKFPELEEKAKKGIPALMMTRTNPKTVGYEQVYDSRPWYTKTGRLEFYREEDEFIEAGE 5t5i.1    --------------------------------------------------------------------------------  target    NLPVHREPIDSTFYEPNVIVAPAHPFIKAKGPEAYGVKVDDFDNETRQGRNIVKTWEETKKTVHPLAKDGYKFVFHTPKY 5t5i.1    --------------------------------------------------------------------------------  target    RHGAHTMPVDTDMVAMLFGPFGDIYRHDKRQPFAAEGYVDIHPDDAKALNIEDGDYVWIDSDPSDRPFRGWQKNDKDYKF 5t5i.1    --------------------------------------------------------------------------------  target    SRLLCRARYYPGTPRGITRMWFNMYGATPGSVEGHESRKDGLAKNPRTGYQAMFRSGSHQSATRGWLKPTWMTDSLVRKE 5t5i.1    --------------------------------------------------------------------------------  target    LFGHAVNKGFLPDVHCPTGAPREAIVKITKAEPGGLNAKGLWRPAALGLRPKYENDKMKDYLAGKFTLAANPKKGGKK 5t5i.1    ------------------------------------------------------------------------------ ``` | | | | | | | | | | | | | | | | | | | | | | | | | | | | | | | | | | | | | | | | | | | | | | | | | |
|  | 6qcf.1.C | NADH:ubiquinone oxidoreductase core subunit S1  *Ovine respiratory complex I FRC open class 6* | 0.12 | 0.00 | 15.43 | 0.30 | 1-255 | EM | 0.00 | monomer | 6 x SF4, 1 x FMN, 2 x FES, 1 x ZN, 1 x NDP, 2 x ZMP | HHblits | 0.27 |
| ``` target    LARDIAKVPGTTLFAIGMGPNQFFNNDNKDRTQFLLAALTGNIGKIAGNIGSYAGNYRVAMFNGVPQYIAENPFDIELDG 6qcf.1    FSQVLQEAKKP-MVVLGSSALQRNDGAAILAAVSNIAQKIRTSSGVTGDWKVMNILHRI------ASQV----------A  target    AKPARPKLYWRAEPAHYYNHEDHPLKM-GKTMITGKTHMPTPTKSLWFANANSILGNVKWHFNTVVNVLPKMEMIAVQEW 6qcf.1    AL-------------------DLGYKPGVEAI------RKNPPKMLFLLGADGGC--------VTRQDLPKDCFIVYQGH  target    WWSTSCEWADIVFAVDAWSELKHPDMCSSVTNPFLTV-FPRTPLERPFDTRGDIECLDLVGKQLAKRTGDRRFADMWKFV 6qcf.1    HGDVGAPIADVILPGAAYTEKS-----ATYVNTEGRAQQTKVAVMPPGLAREDWKIIRA----LSEIAGMT-----LPYD  target    EEKKVEVYLQRILDHSSNTKGFKFPELEEKAKKGIPALMMTRTNPKTVGYEQVYDSRPWYTKTGRLEFYREEDEFIEAGE 6qcf.1    ---TLDQVRNRLEEVSP---------------------------------------------------------------  target    NLPVHREPIDSTFYEPNVIVAPAHPFIKAKGPEAYGVKVDDFDNETRQGRNIVKTWEETKKTVHPLAKDGYKFVFHTPKY 6qcf.1    --------------------------------------------------------------------------------  target    RHGAHTMPVDTDMVAMLFGPFGDIYRHDKRQPFAAEGYVDIHPDDAKALNIEDGDYVWIDSDPSDRPFRGWQKNDKDYKF 6qcf.1    --------------------------------------------------------------------------------  target    SRLLCRARYYPGTPRGITRMWFNMYGATPGSVEGHESRKDGLAKNPRTGYQAMFRSGSHQSATRGWLKPTWMTDSLVRKE 6qcf.1    --------------------------------------------------------------------------------  target    LFGHAVNKGFLPDVHCPTGAPREAIVKITKAEPGGLNAKGLWRPAALGLRPKYENDKMKDYLAGKFTLAANPKKGGKK 6qcf.1    ------------------------------------------------------------------------------ ``` | | | | | | | | | | | | | | | | | | | | | | | | | | | | | | | | | | | | | | | | | | | | | | | | | |
|  | 6qc5.1.C | NADH:ubiquinone oxidoreductase core subunit S1  *Ovine respiratory complex I FRC closed class 1* | 0.12 | 0.00 | 15.43 | 0.30 | 1-255 | EM | 0.00 | monomer | 6 x SF4, 1 x FMN, 2 x FES, 2 x 3PE, 1 x ZN, 1 x NDP, 2 x ZMP, 1 x PC1 | HHblits | 0.27 |
| ``` target    LARDIAKVPGTTLFAIGMGPNQFFNNDNKDRTQFLLAALTGNIGKIAGNIGSYAGNYRVAMFNGVPQYIAENPFDIELDG 6qc5.1    FSQVLQEAKKP-MVVLGSSALQRNDGAAILAAVSNIAQKIRTSSGVTGDWKVMNILHRI------ASQV----------A  target    AKPARPKLYWRAEPAHYYNHEDHPLKM-GKTMITGKTHMPTPTKSLWFANANSILGNVKWHFNTVVNVLPKMEMIAVQEW 6qc5.1    AL-------------------DLGYKPGVEAI------RKNPPKMLFLLGADGGC--------VTRQDLPKDCFIVYQGH  target    WWSTSCEWADIVFAVDAWSELKHPDMCSSVTNPFLTV-FPRTPLERPFDTRGDIECLDLVGKQLAKRTGDRRFADMWKFV 6qc5.1    HGDVGAPIADVILPGAAYTEKS-----ATYVNTEGRAQQTKVAVMPPGLAREDWKIIRA----LSEIAGMT-----LPYD  target    EEKKVEVYLQRILDHSSNTKGFKFPELEEKAKKGIPALMMTRTNPKTVGYEQVYDSRPWYTKTGRLEFYREEDEFIEAGE 6qc5.1    ---TLDQVRNRLEEVSP---------------------------------------------------------------  target    NLPVHREPIDSTFYEPNVIVAPAHPFIKAKGPEAYGVKVDDFDNETRQGRNIVKTWEETKKTVHPLAKDGYKFVFHTPKY 6qc5.1    --------------------------------------------------------------------------------  target    RHGAHTMPVDTDMVAMLFGPFGDIYRHDKRQPFAAEGYVDIHPDDAKALNIEDGDYVWIDSDPSDRPFRGWQKNDKDYKF 6qc5.1    --------------------------------------------------------------------------------  target    SRLLCRARYYPGTPRGITRMWFNMYGATPGSVEGHESRKDGLAKNPRTGYQAMFRSGSHQSATRGWLKPTWMTDSLVRKE 6qc5.1    --------------------------------------------------------------------------------  target    LFGHAVNKGFLPDVHCPTGAPREAIVKITKAEPGGLNAKGLWRPAALGLRPKYENDKMKDYLAGKFTLAANPKKGGKK 6qc5.1    ------------------------------------------------------------------------------ ``` | | | | | | | | | | | | | | | | | | | | | | | | | | | | | | | | | | | | | | | | | | | | | | | | | |
|  | 7dgr.10.A | NADH-ubiquinone oxidoreductase 75 kDa subunit, mitochondrial  *Activity optimized supercomplex state2* | 0.12 | 0.00 | 15.43 | 0.30 | 1-255 | EM | 0.00 | monomer |  | HHblits | 0.27 |
| ``` target    LARDIAKVPGTTLFAIGMGPNQFFNNDNKDRTQFLLAALTGNIGKIAGNIGSYAGNYRVAMFNGVPQYIAENPFDIELDG 7dgr.10   FSQVLQEAKKP-MVILGSSALQRNDGAAILAAVSNIAQKIRTSSGVTGDWKVMNILHRI------ASQV----------A  target    AKPARPKLYWRAEPAHYYNHEDHPLKM-GKTMITGKTHMPTPTKSLWFANANSILGNVKWHFNTVVNVLPKMEMIAVQEW 7dgr.10   A----L---------------DLGYKPGVEAI------QKNPPKMLFLLGADGGC--------ITRQDLPKDCFIVYQGH  target    WWSTSCEWADIVFAVDAWSELKHPDMCSSVTNPFLTV-FPRTPLERPFDTRGDIECLDLVGKQLAKRTGDRRFADMWKFV 7dgr.10   HGDVGAPIADVILPGAAYTEKS-----ATYVNTEGRAQQTKVAVTPPGLAREDWKIIRA----LSEIAGMT-----LPYD  target    EEKKVEVYLQRILDHSSNTKGFKFPELEEKAKKGIPALMMTRTNPKTVGYEQVYDSRPWYTKTGRLEFYREEDEFIEAGE 7dgr.10   ---TLDQVRNRLEEVSP---------------------------------------------------------------  target    NLPVHREPIDSTFYEPNVIVAPAHPFIKAKGPEAYGVKVDDFDNETRQGRNIVKTWEETKKTVHPLAKDGYKFVFHTPKY 7dgr.10   --------------------------------------------------------------------------------  target    RHGAHTMPVDTDMVAMLFGPFGDIYRHDKRQPFAAEGYVDIHPDDAKALNIEDGDYVWIDSDPSDRPFRGWQKNDKDYKF 7dgr.10   --------------------------------------------------------------------------------  target    SRLLCRARYYPGTPRGITRMWFNMYGATPGSVEGHESRKDGLAKNPRTGYQAMFRSGSHQSATRGWLKPTWMTDSLVRKE 7dgr.10   --------------------------------------------------------------------------------  target    LFGHAVNKGFLPDVHCPTGAPREAIVKITKAEPGGLNAKGLWRPAALGLRPKYENDKMKDYLAGKFTLAANPKKGGKK 7dgr.10   ------------------------------------------------------------------------------ ``` | | | | | | | | | | | | | | | | | | | | | | | | | | | | | | | | | | | | | | | | | | | | | | | | | |
|  | 5o31.1.8 | NADH-ubiquinone oxidoreductase 75 kDa subunit, mitochondrial  *Mitochondrial complex I in the deactive state* | 0.12 | 0.00 | 15.43 | 0.30 | 1-255 | EM | 4.13 | monomer | 6 x SF4, 2 x FES, 1 x FMN, 1 x NAP, 1 x ZN | HHblits | 0.27 |
| ``` target    LARDIAKVPGTTLFAIGMGPNQFFNNDNKDRTQFLLAALTGNIGKIAGNIGSYAGNYRVAMFNGVPQYIAENPFDIELDG 5o31.1    FSQVLQEAKKP-MVILGSSALQRNDGAAILAAVSNIAQKIRTSSGVTGDWKVMNILHRI------ASQV----------A  target    AKPARPKLYWRAEPAHYYNHEDHPLKM-GKTMITGKTHMPTPTKSLWFANANSILGNVKWHFNTVVNVLPKMEMIAVQEW 5o31.1    A----L---------------DLGYKPGVEAI------QKNPPKMLFLLGADGGC--------ITRQDLPKDCFIVYQGH  target    WWSTSCEWADIVFAVDAWSELKHPDMCSSVTNPFLTV-FPRTPLERPFDTRGDIECLDLVGKQLAKRTGDRRFADMWKFV 5o31.1    HGDVGAPIADVILPGAAYTEKS-----ATYVNTEGRAQQTKVAVTPPGLAREDWKIIRA----LSEIAGMT-----LPYD  target    EEKKVEVYLQRILDHSSNTKGFKFPELEEKAKKGIPALMMTRTNPKTVGYEQVYDSRPWYTKTGRLEFYREEDEFIEAGE 5o31.1    ---TLDQVRNRLEEVSP---------------------------------------------------------------  target    NLPVHREPIDSTFYEPNVIVAPAHPFIKAKGPEAYGVKVDDFDNETRQGRNIVKTWEETKKTVHPLAKDGYKFVFHTPKY 5o31.1    --------------------------------------------------------------------------------  target    RHGAHTMPVDTDMVAMLFGPFGDIYRHDKRQPFAAEGYVDIHPDDAKALNIEDGDYVWIDSDPSDRPFRGWQKNDKDYKF 5o31.1    --------------------------------------------------------------------------------  target    SRLLCRARYYPGTPRGITRMWFNMYGATPGSVEGHESRKDGLAKNPRTGYQAMFRSGSHQSATRGWLKPTWMTDSLVRKE 5o31.1    --------------------------------------------------------------------------------  target    LFGHAVNKGFLPDVHCPTGAPREAIVKITKAEPGGLNAKGLWRPAALGLRPKYENDKMKDYLAGKFTLAANPKKGGKK 5o31.1    ------------------------------------------------------------------------------ ``` | | | | | | | | | | | | | | | | | | | | | | | | | | | | | | | | | | | | | | | | | | | | | | | | | |
|  | 7zd6.1.4 | NADH-ubiquinone oxidoreductase 75 kDa subunit, mitochondrial  *Complex I from Ovis aries, at pH7.4, Open state* | 0.13 | 0.00 | 15.43 | 0.30 | 1-255 | EM | 0.00 | monomer | 6 x PC1, 14 x 3PE, 1 x DCQ, 2 x ZMP, 1 x AMP, 1 x MYR, 6 x SF4, 1 x FMN, 1 x NAI, 2 x FES, 1 x K, 1 x ZN, 1 x NDP | HHblits | 0.27 |
| ``` target    LARDIAKVPGTTLFAIGMGPNQFFNNDNKDRTQFLLAALTGNIGKIAGNIGSYAGNYRVAMFNGVPQYIAENPFDIELDG 7zd6.1    FSQVLQEAKKP-MVVLGSSALQRNDGAAILAAVSNIAQKIRTSSGVTGDWKVMNI---------LHRIA--SQV-----A  target    AKPARPKLYWRAEPAHYYNHEDHPLKMG-KTMITGKTHMPTPTKSLWFANANSILGNVKWHFNTVVNVLPKMEMIAVQEW 7zd6.1    A----L---------------DLGYKPGVEAI------RKNPPKMLFLLGADGGC--------VTRQDLPKDCFIVYQGH  target    WWSTSCEWADIVFAVDAWSELKHPDMCSSVTNPFLTV-FPRTPLERPFDTRGDIECLDLVGKQLAKRTGDRRFADMWKFV 7zd6.1    HGDVGAPIADVILPGAAYTEKS-----ATYVNTEGRAQQTKVAVMPPGLAREDWKIIRA----LSEIAGMT-----LPYD  target    EEKKVEVYLQRILDHSSNTKGFKFPELEEKAKKGIPALMMTRTNPKTVGYEQVYDSRPWYTKTGRLEFYREEDEFIEAGE 7zd6.1    ---TLDQVRNRLEEVSP---------------------------------------------------------------  target    NLPVHREPIDSTFYEPNVIVAPAHPFIKAKGPEAYGVKVDDFDNETRQGRNIVKTWEETKKTVHPLAKDGYKFVFHTPKY 7zd6.1    --------------------------------------------------------------------------------  target    RHGAHTMPVDTDMVAMLFGPFGDIYRHDKRQPFAAEGYVDIHPDDAKALNIEDGDYVWIDSDPSDRPFRGWQKNDKDYKF 7zd6.1    --------------------------------------------------------------------------------  target    SRLLCRARYYPGTPRGITRMWFNMYGATPGSVEGHESRKDGLAKNPRTGYQAMFRSGSHQSATRGWLKPTWMTDSLVRKE 7zd6.1    --------------------------------------------------------------------------------  target    LFGHAVNKGFLPDVHCPTGAPREAIVKITKAEPGGLNAKGLWRPAALGLRPKYENDKMKDYLAGKFTLAANPKKGGKK 7zd6.1    ------------------------------------------------------------------------------ ``` | | | | | | | | | | | | | | | | | | | | | | | | | | | | | | | | | | | | | | | | | | | | | | | | | |
|  | 5xtb.1.L | NADH-ubiquinone oxidoreductase 75 kDa subunit, mitochondrial  *Cryo-EM structure of human respiratory complex I matrix arm* | 0.11 | 0.00 | 15.43 | 0.30 | 1-255 | EM | 0.00 | monomer | 6 x SF4, 1 x FMN, 1 x 8Q1, 1 x NDP, 2 x FES | HHblits | 0.27 |
| ``` target    LARDIAKVPGTTLFAIGMGPNQFFNNDNKDRTQFLLAALTGNIGKIAGNIGSYAGNYRVAMFNGVPQYIAENPFDIELDG 5xtb.1    FSQVLKEAKKP-MVVLGSSALQRNDGAAILAAVSSIAQKIRMTSGVTGDWKVMNILHRI--ASQV--------------A  target    AKPARPKLYWRAEPAHYYNHEDHPLKM-GKTMITGKTHMPTPTKSLWFANANSILGNVKWHFNTVVNVLPKMEMIAVQEW 5xtb.1    A----LD---------------LGYKPGVEAIR------KNPPKVLFLLGADGGC--------ITRQDLPKDCFIIYQGH  target    WWSTSCEWADIVFAVDAWSELKHPDMCSSVTNPFLTV-FPRTPLERPFDTRGDIECLDLVGKQLAKRTGDRRFADMWKFV 5xtb.1    HGDVGAPIADVILPGAAYTEKS-----ATYVNTEGRAQQTKVAVTPPGLAREDWKIIRA----LSEIAGMT-----LPYD  target    EEKKVEVYLQRILDHSSNTKGFKFPELEEKAKKGIPALMMTRTNPKTVGYEQVYDSRPWYTKTGRLEFYREEDEFIEAGE 5xtb.1    ---TLDQVRNRLEEVSP---------------------------------------------------------------  target    NLPVHREPIDSTFYEPNVIVAPAHPFIKAKGPEAYGVKVDDFDNETRQGRNIVKTWEETKKTVHPLAKDGYKFVFHTPKY 5xtb.1    --------------------------------------------------------------------------------  target    RHGAHTMPVDTDMVAMLFGPFGDIYRHDKRQPFAAEGYVDIHPDDAKALNIEDGDYVWIDSDPSDRPFRGWQKNDKDYKF 5xtb.1    --------------------------------------------------------------------------------  target    SRLLCRARYYPGTPRGITRMWFNMYGATPGSVEGHESRKDGLAKNPRTGYQAMFRSGSHQSATRGWLKPTWMTDSLVRKE 5xtb.1    --------------------------------------------------------------------------------  target    LFGHAVNKGFLPDVHCPTGAPREAIVKITKAEPGGLNAKGLWRPAALGLRPKYENDKMKDYLAGKFTLAANPKKGGKK 5xtb.1    ------------------------------------------------------------------------------ ``` | | | | | | | | | | | | | | | | | | | | | | | | | | | | | | | | | | | | | | | | | | | | | | | | | |
|  | 5gpn.24.A | NADH-ubiquinone oxidoreductase 75 kDa subunit  *Architecture of mammalian respirasome* | 0.11 | 0.00 | 16.04 | 0.29 | 1-254 | EM | 0.00 | monomer |  | HHblits | 0.27 |
| ``` target    LARDIAKVPGTTLFAIGMGPNQFFNNDNKDRTQFLLAALTGNIGKIAGNIGSYAGNYRVAMFNGVPQYIAENPFDIELDG 5gpn.24   FSQILKEAKKP-MVVLGSSALQRSDGTAILAAVSNIAQNIRLSSGVTGDWKVMNILHRI--ASQV--------------A  target    AKPARPKLYWRAEPAHYYNHEDHPLKM-GKTMITGKTHMPTPTKSLWFANANSILGNVKWHFNTVVNVLPKMEMIAVQEW 5gpn.24   A----L---------------DLGYKPGVEAI------RKNPPKVLFLLGADGG--------CITRQDLPKDCFIIYQGH  target    WWSTSCEWADIVFAVDAWSELKHPDMCSSVTNPFLTV-FPRTPLERPFDTRGDIECLDLVGKQLAKRTGDRRFADMWKFV 5gpn.24   HGDVGAPMADVILPGAAYTEKS-----ATYVNTEGRAQQTKVAVTPPGLAREDWKIIRA----LSEIAGMT-----LPYD  target    EEKKVEVYLQRILDHSSNTKGFKFPELEEKAKKGIPALMMTRTNPKTVGYEQVYDSRPWYTKTGRLEFYREEDEFIEAGE 5gpn.24   ---TLDQVRSRLEEVS----------------------------------------------------------------  target    NLPVHREPIDSTFYEPNVIVAPAHPFIKAKGPEAYGVKVDDFDNETRQGRNIVKTWEETKKTVHPLAKDGYKFVFHTPKY 5gpn.24   --------------------------------------------------------------------------------  target    RHGAHTMPVDTDMVAMLFGPFGDIYRHDKRQPFAAEGYVDIHPDDAKALNIEDGDYVWIDSDPSDRPFRGWQKNDKDYKF 5gpn.24   --------------------------------------------------------------------------------  target    SRLLCRARYYPGTPRGITRMWFNMYGATPGSVEGHESRKDGLAKNPRTGYQAMFRSGSHQSATRGWLKPTWMTDSLVRKE 5gpn.24   --------------------------------------------------------------------------------  target    LFGHAVNKGFLPDVHCPTGAPREAIVKITKAEPGGLNAKGLWRPAALGLRPKYENDKMKDYLAGKFTLAANPKKGGKK 5gpn.24   ------------------------------------------------------------------------------ ``` | | | | | | | | | | | | | | | | | | | | | | | | | | | | | | | | | | | | | | | | | | | | | | | | | |
|  | 7qsd.1.G | NADH-ubiquinone oxidoreductase 75 kDa subunit, mitochondrial  *Bovine complex I in the active state at 3.1 A* | 0.13 | 0.00 | 14.89 | 0.30 | 1-255 | EM | 0.00 | monomer | 5 x PC1, 13 x 3PE, 6 x SF4, 2 x FES, 1 x FMN, 4 x CDL, 3 x LMT, 1 x GTP, 1 x MG, 1 x NDP, 1 x ZN, 2 x EHZ | HHblits | 0.27 |
| ``` target    LARDIAKVPGTTLFAIGMGPNQFFNNDNKDRTQFLLAALTGNIGKIAGNIGSYAGNYRVAMFNGVPQYIAENPFDIELDG 7qsd.1    FSQVLQEAKKP-MVILGSSALQRNDGAAILAAVSNIAQKIRTSSGVTGDWKVMNIL---------HRIA--SQV-----A  target    AKPARPKLYWRAEPAHYYNHEDHPLKM-GKTMITGKTHMPTPTKSLWFANANSILGNVKWHFNTVVNVLPKMEMIAVQEW 7qsd.1    A----L---------------DLGYKPGVEAI------QKNPPKMLFLLGADGGC--------ITRQDLPKDCFIVYQGH  target    WWSTSCEWADIVFAVDAWSELKHPDMCSSVTNPFLTV-FPRTPLERPFDTRGDIECLDLVGKQLAKRTGDRRFADMWKFV 7qsd.1    HGDVGAPIADVILPGAAYTEKS-----ATYVNTEGRAQQTKVAVTPPGLAREDWKIIRA----LSEIAGMT-----LPYD  target    EEKKVEVYLQRILDHSSNTKGFKFPELEEKAKKGIPALMMTRTNPKTVGYEQVYDSRPWYTKTGRLEFYREEDEFIEAGE 7qsd.1    ---TLDQVRNRLEEVSP---------------------------------------------------------------  target    NLPVHREPIDSTFYEPNVIVAPAHPFIKAKGPEAYGVKVDDFDNETRQGRNIVKTWEETKKTVHPLAKDGYKFVFHTPKY 7qsd.1    --------------------------------------------------------------------------------  target    RHGAHTMPVDTDMVAMLFGPFGDIYRHDKRQPFAAEGYVDIHPDDAKALNIEDGDYVWIDSDPSDRPFRGWQKNDKDYKF 7qsd.1    --------------------------------------------------------------------------------  target    SRLLCRARYYPGTPRGITRMWFNMYGATPGSVEGHESRKDGLAKNPRTGYQAMFRSGSHQSATRGWLKPTWMTDSLVRKE 7qsd.1    --------------------------------------------------------------------------------  target    LFGHAVNKGFLPDVHCPTGAPREAIVKITKAEPGGLNAKGLWRPAALGLRPKYENDKMKDYLAGKFTLAANPKKGGKK 7qsd.1    ------------------------------------------------------------------------------ ``` | | | | | | | | | | | | | | | | | | | | | | | | | | | | | | | | | | | | | | | | | | | | | | | | | |
|  | 6zk9.1.C | NADH:ubiquinone oxidoreductase core subunit S1  *Peripheral domain of open complex I during turnover* | 0.13 | 0.00 | 15.51 | 0.29 | 1-254 | EM | 0.00 | monomer | 6 x SF4, 1 x FMN, 1 x NAI, 2 x FES, 1 x K, 2 x PC1, 2 x 3PE, 1 x ZN, 1 x NDP, 1 x ZMP, 1 x CDL | HHblits | 0.27 |
| ``` target    LARDIAKVPGTTLFAIGMGPNQFFNNDNKDRTQFLLAALTGNIGKIAGNIGSYAGNYRVAMFNGVPQYIAENPFDIELDG 6zk9.1    FSQVLQEAKKP-MVVLGSSALQRNDGAAILAAVSNIAQKIRTSSGVTGDWKVMNILHRI--ASQ-V-------------A  target    AKPARPKLYWRAEPAHYYNHEDHPLKM-GKTMITGKTHMPTPTKSLWFANANSILGNVKWHFNTVVNVLPKMEMIAVQEW 6zk9.1    A----L---------------DLGYKPGVEAIR------KNPPKMLFLLGADGGC--------VTRQDLPKDCFIVYQGH  target    WWSTSCEWADIVFAVDAWSELKHPDMCSSVTNPFLTV-FPRTPLERPFDTRGDIECLDLVGKQLAKRTGDRRFADMWKFV 6zk9.1    HGDVGAPIADVILPGAAYTEKS-----ATYVNTEGRAQQTKVAVMPPGLAREDWKIIRA----LSEIAGMT-----LPYD  target    EEKKVEVYLQRILDHSSNTKGFKFPELEEKAKKGIPALMMTRTNPKTVGYEQVYDSRPWYTKTGRLEFYREEDEFIEAGE 6zk9.1    ---TLDQVRNRLEEVS----------------------------------------------------------------  target    NLPVHREPIDSTFYEPNVIVAPAHPFIKAKGPEAYGVKVDDFDNETRQGRNIVKTWEETKKTVHPLAKDGYKFVFHTPKY 6zk9.1    --------------------------------------------------------------------------------  target    RHGAHTMPVDTDMVAMLFGPFGDIYRHDKRQPFAAEGYVDIHPDDAKALNIEDGDYVWIDSDPSDRPFRGWQKNDKDYKF 6zk9.1    --------------------------------------------------------------------------------  target    SRLLCRARYYPGTPRGITRMWFNMYGATPGSVEGHESRKDGLAKNPRTGYQAMFRSGSHQSATRGWLKPTWMTDSLVRKE 6zk9.1    --------------------------------------------------------------------------------  target    LFGHAVNKGFLPDVHCPTGAPREAIVKITKAEPGGLNAKGLWRPAALGLRPKYENDKMKDYLAGKFTLAANPKKGGKK 6zk9.1    ------------------------------------------------------------------------------ ``` | | | | | | | | | | | | | | | | | | | | | | | | | | | | | | | | | | | | | | | | | | | | | | | | | |
|  | 7vxu.1.L | NADH-ubiquinone oxidoreductase 75 kDa subunit, mitochondrial  *Matrix arm of deactive state CI from Q10 dataset* | 0.12 | 0.00 | 15.51 | 0.29 | 1-254 | EM | 0.00 | monomer | 6 x SF4, 1 x FMN, 1 x PEE, 1 x PLX, 1 x 8Q1, 1 x NDP, 2 x FES, 1 x MG, 1 x CDL, 1 x ZN | HHblits | 0.27 |
| ``` target    LARDIAKVPGTTLFAIGMGPNQFFNNDNKDRTQFLLAALTGNIGKIAGNIGSYAGNYRVAMFNGVPQYIAENPFDIELDG 7vxu.1    FSQILKEAKKP-MVVLGSSALQRSDGTAILAAVSNIAQNIRLSSGVTGDWKVMNILHRI--ASQV--------------A  target    AKPARPKLYWRAEPAHYYNHEDHPLKM-GKTMITGKTHMPTPTKSLWFANANSILGNVKWHFNTVVNVLPKMEMIAVQEW 7vxu.1    A----LD---------------LGYKPGVEAIR------KNPPKVLFLLGADGG--------CITRQDLPKDCFIIYQGH  target    WWSTSCEWADIVFAVDAWSELKHPDMCSSVTNPFLTV-FPRTPLERPFDTRGDIECLDLVGKQLAKRTGDRRFADMWKFV 7vxu.1    HGDVGAPMADVILPGAAYTEKS-----ATYVNTEGRAQQTKVAVTPPGLAREDWKIIRA----LSEIAGMT-----LPYD  target    EEKKVEVYLQRILDHSSNTKGFKFPELEEKAKKGIPALMMTRTNPKTVGYEQVYDSRPWYTKTGRLEFYREEDEFIEAGE 7vxu.1    ---TLDQVRSRLEEVS----------------------------------------------------------------  target    NLPVHREPIDSTFYEPNVIVAPAHPFIKAKGPEAYGVKVDDFDNETRQGRNIVKTWEETKKTVHPLAKDGYKFVFHTPKY 7vxu.1    --------------------------------------------------------------------------------  target    RHGAHTMPVDTDMVAMLFGPFGDIYRHDKRQPFAAEGYVDIHPDDAKALNIEDGDYVWIDSDPSDRPFRGWQKNDKDYKF 7vxu.1    --------------------------------------------------------------------------------  target    SRLLCRARYYPGTPRGITRMWFNMYGATPGSVEGHESRKDGLAKNPRTGYQAMFRSGSHQSATRGWLKPTWMTDSLVRKE 7vxu.1    --------------------------------------------------------------------------------  target    LFGHAVNKGFLPDVHCPTGAPREAIVKITKAEPGGLNAKGLWRPAALGLRPKYENDKMKDYLAGKFTLAANPKKGGKK 7vxu.1    ------------------------------------------------------------------------------ ``` | | | | | | | | | | | | | | | | | | | | | | | | | | | | | | | | | | | | | | | | | | | | | | | | | |
|  | 7v2c.1.L | NADH-ubiquinone oxidoreductase 75 kDa subunit, mitochondrial  *Active state complex I from Q10 dataset* | 0.12 | 0.00 | 14.97 | 0.29 | 1-254 | EM | 0.00 | monomer | 6 x SF4, 1 x FMN, 10 x PEE, 8 x PLX, 2 x 8Q1, 1 x NDP, 2 x UQ, 11 x CDL, 2 x FES, 1 x MG, 1 x ZN, 1 x ADP | HHblits | 0.27 |
| ``` target    LARDIAKVPGTTLFAIGMGPNQFFNNDNKDRTQFLLAALTGNIGKIAGNIGSYAGNYRVAMFNGVPQYIAENPFDIELDG 7v2c.1    FSQILKEAKKP-MVVLGSSALQRSDGTAILAAVSNIAQNIRLSSGVTGDWKVMNI---------LHRIA--SQV-----A  target    AKPARPKLYWRAEPAHYYNHEDHPLKM-GKTMITGKTHMPTPTKSLWFANANSILGNVKWHFNTVVNVLPKMEMIAVQEW 7v2c.1    A----L---------------DLGYKPGVEAI------RKNPPKVLFLLGADGG--------CITRQDLPKDCFIIYQGH  target    WWSTSCEWADIVFAVDAWSELKHPDMCSSVTNPFLTV-FPRTPLERPFDTRGDIECLDLVGKQLAKRTGDRRFADMWKFV 7v2c.1    HGDVGAPMADVILPGAAYTEKS-----ATYVNTEGRAQQTKVAVTPPGLAREDWKIIRA----LSEIAGMT-----LPYD  target    EEKKVEVYLQRILDHSSNTKGFKFPELEEKAKKGIPALMMTRTNPKTVGYEQVYDSRPWYTKTGRLEFYREEDEFIEAGE 7v2c.1    ---TLDQVRSRLEEVS----------------------------------------------------------------  target    NLPVHREPIDSTFYEPNVIVAPAHPFIKAKGPEAYGVKVDDFDNETRQGRNIVKTWEETKKTVHPLAKDGYKFVFHTPKY 7v2c.1    --------------------------------------------------------------------------------  target    RHGAHTMPVDTDMVAMLFGPFGDIYRHDKRQPFAAEGYVDIHPDDAKALNIEDGDYVWIDSDPSDRPFRGWQKNDKDYKF 7v2c.1    --------------------------------------------------------------------------------  target    SRLLCRARYYPGTPRGITRMWFNMYGATPGSVEGHESRKDGLAKNPRTGYQAMFRSGSHQSATRGWLKPTWMTDSLVRKE 7v2c.1    --------------------------------------------------------------------------------  target    LFGHAVNKGFLPDVHCPTGAPREAIVKITKAEPGGLNAKGLWRPAALGLRPKYENDKMKDYLAGKFTLAANPKKGGKK 7v2c.1    ------------------------------------------------------------------------------ ``` | | | | | | | | | | | | | | | | | | | | | | | | | | | | | | | | | | | | | | | | | | | | | | | | | |
|  | 7t2r.1.A | NiFe hydrogenase subunit A  *Structure of electron bifurcating Ni-Fe hydrogenase complex HydABCSL in FMN-free apo state* | 0.12 |  | 14.44 | 0.29 | 1-254 | EM | 0.00 | hetero-2-2-2-2-2-mer | 6 x FES, 12 x SF4, 2 x 3NI, 2 x FCO | HHblits | 0.26 |
| ``` target    LARDIAKVPGTTLFAIGMGPNQFFNNDNKDRTQFLLAALTGNIGKIAGNIGSYAGNYRVAMFNGVPQYIAENPFDIELDG 7t2r.1    VALMLISARRP-IFIIGGR---ATKSHELVTAACNLAVASKAFFEDGLGVVPLLVSANSL---GAR------------NT  target    AKPARPKLYWRAEPAHYYNHEDHPLKMGKTMITGKTHMPTPTKSLWFANANSILGNVKWHFNTVVNVLPKMEMIAVQEWW 7t2r.1    ----V------V--------SENP-------WLG----RERRDFLYVFSTAMV-----PEEEEILAAISATRFVVVQTPF  target    WS-TSCEWADIVFAVDAWSELKHPDMCSSVTNPFLTV-FPRTPLERPFDTRGDIECLDLVGKQLAKRTGDRRFADMWKFV 7t2r.1    KVRPLVNLADILLPAPAWYERS-----GHFCTIEGERRKLNTIVPPKGEIKSLHYVMDE----FAKKLGVKL-----ERP  target    EEKKVEVYLQRILDHSSNTKGFKFPELEEKAKKGIPALMMTRTNPKTVGYEQVYDSRPWYTKTGRLEFYREEDEFIEAGE 7t2r.1    EVSPCEEIFKSQLRAS----------------------------------------------------------------  target    NLPVHREPIDSTFYEPNVIVAPAHPFIKAKGPEAYGVKVDDFDNETRQGRNIVKTWEETKKTVHPLAKDGYKFVFHTPKY 7t2r.1    --------------------------------------------------------------------------------  target    RHGAHTMPVDTDMVAMLFGPFGDIYRHDKRQPFAAEGYVDIHPDDAKALNIEDGDYVWIDSDPSDRPFRGWQKNDKDYKF 7t2r.1    --------------------------------------------------------------------------------  target    SRLLCRARYYPGTPRGITRMWFNMYGATPGSVEGHESRKDGLAKNPRTGYQAMFRSGSHQSATRGWLKPTWMTDSLVRKE 7t2r.1    --------------------------------------------------------------------------------  target    LFGHAVNKGFLPDVHCPTGAPREAIVKITKAEPGGLNAKGLWRPAALGLRPKYENDKMKDYLAGKFTLAANPKKGGKK 7t2r.1    ------------------------------------------------------------------------------ ``` | | | | | | | | | | | | | | | | | | | | | | | | | | | | | | | | | | | | | | | | | | | | | | | | | |
|  | 7t30.1.A | NiFe hydrogenase subunit A  *Structure of electron bifurcating Ni-Fe hydrogenase complex HydABCSL in FMN/NAD(H) bound state* | 0.12 |  | 14.44 | 0.29 | 1-254 | EM | 0.00 | hetero-2-2-2-2-2-mer | 4 x FES, 12 x SF4, 2 x NAD, 2 x FMN, 2 x 3NI, 2 x FCO | HHblits | 0.26 |
| ``` target    LARDIAKVPGTTLFAIGMGPNQFFNNDNKDRTQFLLAALTGNIGKIAGNIGSYAGNYRVAMFNGVPQYIAENPFDIELDG 7t30.1    VALMLISARRP-IFIIGGR---ATKSHELVTAACNLAVASKAFFEDGLGVVPLLVSANSL---GAR------------NT  target    AKPARPKLYWRAEPAHYYNHEDHPLKMGKTMITGKTHMPTPTKSLWFANANSILGNVKWHFNTVVNVLPKMEMIAVQEWW 7t30.1    ----V------V--------SENP-------WLG----RERRDFLYVFSTAMV-----PEEEEILAAISATRFVVVQTPF  target    WS-TSCEWADIVFAVDAWSELKHPDMCSSVTNPFLTV-FPRTPLERPFDTRGDIECLDLVGKQLAKRTGDRRFADMWKFV 7t30.1    KVRPLVNLADILLPAPAWYERS-----GHFCTIEGERRKLNTIVPPKGEIKSLHYVMDE----FAKKLGVKL-----ERP  target    EEKKVEVYLQRILDHSSNTKGFKFPELEEKAKKGIPALMMTRTNPKTVGYEQVYDSRPWYTKTGRLEFYREEDEFIEAGE 7t30.1    EVSPCEEIFKSQLRAS----------------------------------------------------------------  target    NLPVHREPIDSTFYEPNVIVAPAHPFIKAKGPEAYGVKVDDFDNETRQGRNIVKTWEETKKTVHPLAKDGYKFVFHTPKY 7t30.1    --------------------------------------------------------------------------------  target    RHGAHTMPVDTDMVAMLFGPFGDIYRHDKRQPFAAEGYVDIHPDDAKALNIEDGDYVWIDSDPSDRPFRGWQKNDKDYKF 7t30.1    --------------------------------------------------------------------------------  target    SRLLCRARYYPGTPRGITRMWFNMYGATPGSVEGHESRKDGLAKNPRTGYQAMFRSGSHQSATRGWLKPTWMTDSLVRKE 7t30.1    --------------------------------------------------------------------------------  target    LFGHAVNKGFLPDVHCPTGAPREAIVKITKAEPGGLNAKGLWRPAALGLRPKYENDKMKDYLAGKFTLAANPKKGGKK 7t30.1    ------------------------------------------------------------------------------ ``` | | | | | | | | | | | | | | | | | | | | | | | | | | | | | | | | | | | | | | | | | | | | | | | | | |
|  | 6yj4.1.G | Subunit NUAM of NADH:Ubiquinone Oxidoreductase (Complex I)  *Structure of Yarrowia lipolytica complex I at 2.7 A* | 0.12 |  | 15.93 | 0.29 | 1-254 | EM | 0.00 | hetero-1-1-1-1-1-1-… | 18 x 3PE, 6 x SF4, 5 x LMT, 8 x PLC, 2 x FES, 1 x FMN, 6 x CDL, 1 x NDP, 1 x ZN, 2 x EHZ | HHblits | 0.28 |
| ``` target    LARDIAKVPGTTLFAIGMGPNQFFNNDNKDRTQFLLAALTGN-IGKIAGNIGSYAGNYRVAMFNGVPQYIAENPFDIELD 6yj4.1    FGEVLKNAKNP-LIIVGSGITDREDAGAFFNTIGKFVESTPSVLNENWNGYNVLQRSASRA---GAY------------D  target    GAKPARPKLYWRAEPAHYYNHEDHPLKMGKTMITGKTHMPTPTKSLWFANANSILGNVKWHFNTVVNVLPKMEMIAVQEW 6yj4.1    I----------GF----------TP---SDE------ASKTTPKMVWLLGADEVAA----------SDIPADAFVVYQGH  target    WWSTSCEWADIVFAVDAWSELKHPDMCSSVTNPFLTV-FPRTPLERPFDTRGDIECLDLVGKQLAKRTGDRRFADMWKFV 6yj4.1    NGDVGAQFADVVLPGAAYTEKA-----GTYVNTEGRSQISRAATGPPGGAREDWKILRA----VSEYLGVA-----LPYE  target    EEKKVEVYLQRILDHSSNTKGFKFPELEEKAKKGIPALMMTRTNPKTVGYEQVYDSRPWYTKTGRLEFYREEDEFIEAGE 6yj4.1    ---DAYEVRDRLAEIS----------------------------------------------------------------  target    NLPVHREPIDSTFYEPNVIVAPAHPFIKAKGPEAYGVKVDDFDNETRQGRNIVKTWEETKKTVHPLAKDGYKFVFHTPKY 6yj4.1    --------------------------------------------------------------------------------  target    RHGAHTMPVDTDMVAMLFGPFGDIYRHDKRQPFAAEGYVDIHPDDAKALNIEDGDYVWIDSDPSDRPFRGWQKNDKDYKF 6yj4.1    --------------------------------------------------------------------------------  target    SRLLCRARYYPGTPRGITRMWFNMYGATPGSVEGHESRKDGLAKNPRTGYQAMFRSGSHQSATRGWLKPTWMTDSLVRKE 6yj4.1    --------------------------------------------------------------------------------  target    LFGHAVNKGFLPDVHCPTGAPREAIVKITKAEPGGLNAKGLWRPAALGLRPKYENDKMKDYLAGKFTLAANPKKGGKK 6yj4.1    ------------------------------------------------------------------------------ ``` | | | | | | | | | | | | | | | | | | | | | | | | | | | | | | | | | | | | | | | | | | | | | | | | | |
|  | 6rfs.1.A | Subunit NUAM of NADH:Ubiquinone Oxidoreductase (Complex I)  *Cryo-EM structure of a respiratory complex I mutant lacking NDUFS4* | 0.12 |  | 15.93 | 0.29 | 1-254 | EM | 4.04 | hetero-1-1-1-1-1-1-… | 6 x SF4, 2 x FES, 1 x FMN, 1 x NDP, 1 x ZN, 1 x ZMP | HHblits | 0.28 |
| ``` target    LARDIAKVPGTTLFAIGMGPNQFFNNDNKDRTQFLLAALTGN-IGKIAGNIGSYAGNYRVAMFNGVPQYIAENPFDIELD 6rfs.1    FGEVLKNAKNP-LIIVGSGITDREDAGAFFNTIGKFVESTPSVLNENWNGYNVLQRSASRA---GAY------------D  target    GAKPARPKLYWRAEPAHYYNHEDHPLKMGKTMITGKTHMPTPTKSLWFANANSILGNVKWHFNTVVNVLPKMEMIAVQEW 6rfs.1    I----------GF----------TP---SDE------ASKTTPKMVWLLGADEVAA----------SDIPADAFVVYQGH  target    WWSTSCEWADIVFAVDAWSELKHPDMCSSVTNPFLTV-FPRTPLERPFDTRGDIECLDLVGKQLAKRTGDRRFADMWKFV 6rfs.1    NGDVGAQFADVVLPGAAYTEKA-----GTYVNTEGRSQISRAATGPPGGAREDWKILRA----VSEYLGVA-----LPYE  target    EEKKVEVYLQRILDHSSNTKGFKFPELEEKAKKGIPALMMTRTNPKTVGYEQVYDSRPWYTKTGRLEFYREEDEFIEAGE 6rfs.1    ---DAYEVRDRLAEIS----------------------------------------------------------------  target    NLPVHREPIDSTFYEPNVIVAPAHPFIKAKGPEAYGVKVDDFDNETRQGRNIVKTWEETKKTVHPLAKDGYKFVFHTPKY 6rfs.1    --------------------------------------------------------------------------------  target    RHGAHTMPVDTDMVAMLFGPFGDIYRHDKRQPFAAEGYVDIHPDDAKALNIEDGDYVWIDSDPSDRPFRGWQKNDKDYKF 6rfs.1    --------------------------------------------------------------------------------  target    SRLLCRARYYPGTPRGITRMWFNMYGATPGSVEGHESRKDGLAKNPRTGYQAMFRSGSHQSATRGWLKPTWMTDSLVRKE 6rfs.1    --------------------------------------------------------------------------------  target    LFGHAVNKGFLPDVHCPTGAPREAIVKITKAEPGGLNAKGLWRPAALGLRPKYENDKMKDYLAGKFTLAANPKKGGKK 6rfs.1    ------------------------------------------------------------------------------ ``` | | | | | | | | | | | | | | | | | | | | | | | | | | | | | | | | | | | | | | | | | | | | | | | | | |
|  | 6rfq.1.A | Subunit NUAM of NADH:Ubiquinone Oxidoreductase (Complex I)  *Cryo-EM structure of a respiratory complex I assembly intermediate with NDUFAF2* | 0.12 |  | 15.93 | 0.29 | 1-254 | EM | 3.30 | hetero-1-1-1-1-1-1-… | 6 x SF4, 2 x FES, 1 x FMN, 1 x NDP, 10 x 3PE, 2 x LMN, 4 x CDL, 2 x ZMP, 4 x PLC, 3 x T7X, 1 x CPL | HHblits | 0.28 |
| ``` target    LARDIAKVPGTTLFAIGMGPNQFFNNDNKDRTQFLLAALTGN-IGKIAGNIGSYAGNYRVAMFNGVPQYIAENPFDIELD 6rfq.1    FGEVLKNAKNP-LIIVGSGITDREDAGAFFNTIGKFVESTPSVLNENWNGYNVLQRSASRA---GAY------------D  target    GAKPARPKLYWRAEPAHYYNHEDHPLKMGKTMITGKTHMPTPTKSLWFANANSILGNVKWHFNTVVNVLPKMEMIAVQEW 6rfq.1    I----------GF----------TP---SDE------ASKTTPKMVWLLGADEVAA----------SDIPADAFVVYQGH  target    WWSTSCEWADIVFAVDAWSELKHPDMCSSVTNPFLTV-FPRTPLERPFDTRGDIECLDLVGKQLAKRTGDRRFADMWKFV 6rfq.1    NGDVGAQFADVVLPGAAYTEKA-----GTYVNTEGRSQISRAATGPPGGAREDWKILRA----VSEYLGVA-----LPYE  target    EEKKVEVYLQRILDHSSNTKGFKFPELEEKAKKGIPALMMTRTNPKTVGYEQVYDSRPWYTKTGRLEFYREEDEFIEAGE 6rfq.1    ---DAYEVRDRLAEIS----------------------------------------------------------------  target    NLPVHREPIDSTFYEPNVIVAPAHPFIKAKGPEAYGVKVDDFDNETRQGRNIVKTWEETKKTVHPLAKDGYKFVFHTPKY 6rfq.1    --------------------------------------------------------------------------------  target    RHGAHTMPVDTDMVAMLFGPFGDIYRHDKRQPFAAEGYVDIHPDDAKALNIEDGDYVWIDSDPSDRPFRGWQKNDKDYKF 6rfq.1    --------------------------------------------------------------------------------  target    SRLLCRARYYPGTPRGITRMWFNMYGATPGSVEGHESRKDGLAKNPRTGYQAMFRSGSHQSATRGWLKPTWMTDSLVRKE 6rfq.1    --------------------------------------------------------------------------------  target    LFGHAVNKGFLPDVHCPTGAPREAIVKITKAEPGGLNAKGLWRPAALGLRPKYENDKMKDYLAGKFTLAANPKKGGKK 6rfq.1    ------------------------------------------------------------------------------ ``` | | | | | | | | | | | | | | | | | | | | | | | | | | | | | | | | | | | | | | | | | | | | | | | | | |
|  | 6gcs.1.A | 75-KDA PROTEIN (NUAM)  *Cryo-EM structure of respiratory complex I from Yarrowia lipolytica* | 0.12 |  | 15.93 | 0.29 | 1-254 | EM | 4.32 | hetero-1-1-1-1-1-1-… | 6 x SF4, 2 x FES, 1 x FMN, 1 x NDP, 1 x ZN, 1 x ZMP, 1 x CDL, 3 x 3PE | HHblits | 0.28 |
| ``` target    LARDIAKVPGTTLFAIGMGPNQFFNNDNKDRTQFLLAALTGN-IGKIAGNIGSYAGNYRVAMFNGVPQYIAENPFDIELD 6gcs.1    FGEVLKNAKNP-LIIVGSGITDREDAGAFFNTIGKFVESTPSVLNENWNGYNVLQRSASRA---GAY------------D  target    GAKPARPKLYWRAEPAHYYNHEDHPLKMGKTMITGKTHMPTPTKSLWFANANSILGNVKWHFNTVVNVLPKMEMIAVQEW 6gcs.1    I----------GF----------TP---SDE------ASKTTPKMVWLLGADEVAA----------SDIPADAFVVYQGH  target    WWSTSCEWADIVFAVDAWSELKHPDMCSSVTNPFLTV-FPRTPLERPFDTRGDIECLDLVGKQLAKRTGDRRFADMWKFV 6gcs.1    NGDVGAQFADVVLPGAAYTEKA-----GTYVNTEGRSQISRAATGPPGGAREDWKILRA----VSEYLGVA-----LPYE  target    EEKKVEVYLQRILDHSSNTKGFKFPELEEKAKKGIPALMMTRTNPKTVGYEQVYDSRPWYTKTGRLEFYREEDEFIEAGE 6gcs.1    ---DAYEVRDRLAEIS----------------------------------------------------------------  target    NLPVHREPIDSTFYEPNVIVAPAHPFIKAKGPEAYGVKVDDFDNETRQGRNIVKTWEETKKTVHPLAKDGYKFVFHTPKY 6gcs.1    --------------------------------------------------------------------------------  target    RHGAHTMPVDTDMVAMLFGPFGDIYRHDKRQPFAAEGYVDIHPDDAKALNIEDGDYVWIDSDPSDRPFRGWQKNDKDYKF 6gcs.1    --------------------------------------------------------------------------------  target    SRLLCRARYYPGTPRGITRMWFNMYGATPGSVEGHESRKDGLAKNPRTGYQAMFRSGSHQSATRGWLKPTWMTDSLVRKE 6gcs.1    --------------------------------------------------------------------------------  target    LFGHAVNKGFLPDVHCPTGAPREAIVKITKAEPGGLNAKGLWRPAALGLRPKYENDKMKDYLAGKFTLAANPKKGGKK 6gcs.1    ------------------------------------------------------------------------------ ``` | | | | | | | | | | | | | | | | | | | | | | | | | | | | | | | | | | | | | | | | | | | | | | | | | |
|  | 7zm7.1.I | NADH-ubiquinone oxidoreductase-like protein  *CryoEM structure of mitochondrial complex I from Chaetomium thermophilum (inhibited by DDM)* | 0.13 |  | 16.94 | 0.29 | 2-255 | EM | 0.00 | hetero-1-1-1-1-1-1-… | 4 x PC1, 14 x LMT, 5 x CDL, 8 x 3PE, 2 x FES, 6 x SF4, 1 x FMN, 1 x NDP, 1 x ZN, 2 x ZMP | HHblits | 0.27 |
| ``` target    LARDIAKVPGTTLFAIGMGPNQFFNNDNKDRTQFLLAALTG--NIGKIAGNIGSYAGNYRVAMFNGVPQYIAENPFDIEL 7zm7.1    -GEKLKKAKR-PMIIVGSGVTEHPDAKAFYETVWSFVEKNASNFLTEEWCGYNVLQRAASR-----A-------------  target    DGAKPARPKLYWRAEPAHYYNHEDHPLKMGKTMITGKTHMPTPTKSLWFANANSILGNVKWHFNTVVNVLPKMEMIAVQE 7zm7.1    -GA----FEVGFV------------V--PSPEV------AATKPKFVWLLGADEFDP----------ADVPKDAFIVYQG  target    WWWSTSCEWADIVFAVDAWSELKHPDMCSSVTNPFLTV-FPRTPLERPFDTRGDIECLDLVGKQLAKRTGDRRFADMWKF 7zm7.1    HHGDRGAEIADIVLPGAAYTEKA-----GTYVNTEGRVQMTRAATGLPGAARTDWKIIRA----VSEFLGVP-----LPY  target    VEEKKVEVYLQRILDHSSNTKGFKFPELEEKAKKGIPALMMTRTNPKTVGYEQVYDSRPWYTKTGRLEFYREEDEFIEAG 7zm7.1    D---DVAQLRDRMAEISP--------------------------------------------------------------  target    ENLPVHREPIDSTFYEPNVIVAPAHPFIKAKGPEAYGVKVDDFDNETRQGRNIVKTWEETKKTVHPLAKDGYKFVFHTPK 7zm7.1    --------------------------------------------------------------------------------  target    YRHGAHTMPVDTDMVAMLFGPFGDIYRHDKRQPFAAEGYVDIHPDDAKALNIEDGDYVWIDSDPSDRPFRGWQKNDKDYK 7zm7.1    --------------------------------------------------------------------------------  target    FSRLLCRARYYPGTPRGITRMWFNMYGATPGSVEGHESRKDGLAKNPRTGYQAMFRSGSHQSATRGWLKPTWMTDSLVRK 7zm7.1    --------------------------------------------------------------------------------  target    ELFGHAVNKGFLPDVHCPTGAPREAIVKITKAEPGGLNAKGLWRPAALGLRPKYENDKMKDYLAGKFTLAANPKKGGKK 7zm7.1    ------------------------------------------------------------------------------- ``` | | | | | | | | | | | | | | | | | | | | | | | | | | | | | | | | | | | | | | | | | | | | | | | | | |
|  | 7arc.1.F | 75 kDa  *Cryo-EM structure of Polytomella Complex-I (peripheral arm)* | 0.13 |  | 13.66 | 0.29 | 1-254 | EM | 0.00 | hetero-1-1-1-1-1-1-… | 6 x SF4, 2 x FES, 1 x FMN, 1 x NDP, 1 x ZN, 1 x 8Q1 | HHblits | 0.27 |
| ``` target    LARDIAKVPGTTLFAIGMGPNQFFNNDNKDRTQFLLAALTGNIGKIAGNIGSYAGNYRVAMFNGVPQYIAENPFDIELDG 7arc.1    FFEALKGAKNP-VVIVGSSVLRRDDREAVLKTVNDLVDAAGVVKEGWNGFNVLHDNASRV---AAL------------DI  target    AKPARPKLYWRAEPAHYYNHEDHPLKMGKTMITGKTHMPTPTKSLWFANANSILGNVKWHFNTVVNVLPKMEMIAVQEWW 7arc.1    G-------F-V------------PS----ASA---RTNPVPAKVVYLLGSDDFKD----------EEIPADAFVIYQGHH  target    WSTSCEWADIVFAVDAWSELKHPDMCSSVTNP--FLTVFPRTPLERPFDTRGDIECLDLVGKQLAKRTGDRRFADMWKFV 7arc.1    GDKGAARANVVLPGAAYTEKA-----SLFANTEGRVQTTR-TAVPVLGDAREDWKIIRA----LSEVVGQQ-----LPYD  target    EEKKVEVYLQRILDHSSNTKGFKFPELEEKAKKGIPALMMTRTNPKTVGYEQVYDSRPWYTKTGRLEFYREEDEFIEAGE 7arc.1    ---SQPQVRARLAEVA----------------------------------------------------------------  target    NLPVHREPIDSTFYEPNVIVAPAHPFIKAKGPEAYGVKVDDFDNETRQGRNIVKTWEETKKTVHPLAKDGYKFVFHTPKY 7arc.1    --------------------------------------------------------------------------------  target    RHGAHTMPVDTDMVAMLFGPFGDIYRHDKRQPFAAEGYVDIHPDDAKALNIEDGDYVWIDSDPSDRPFRGWQKNDKDYKF 7arc.1    --------------------------------------------------------------------------------  target    SRLLCRARYYPGTPRGITRMWFNMYGATPGSVEGHESRKDGLAKNPRTGYQAMFRSGSHQSATRGWLKPTWMTDSLVRKE 7arc.1    --------------------------------------------------------------------------------  target    LFGHAVNKGFLPDVHCPTGAPREAIVKITKAEPGGLNAKGLWRPAALGLRPKYENDKMKDYLAGKFTLAANPKKGGKK 7arc.1    ------------------------------------------------------------------------------ ``` | | | | | | | | | | | | | | | | | | | | | | | | | | | | | | | | | | | | | | | | | | | | | | | | | |
|  | 6x89.1.H | NADH dehydrogenase [ubiquinone] iron-sulfur protein 1, mitochondrial  *Vigna radiata mitochondrial complex I\** | 0.12 |  | 14.29 | 0.29 | 1-254 | EM | 0.00 | hetero-1-1-1-1-1-1-… | 1 x NAP, 6 x PC1, 6 x SF4, 2 x FES, 2 x ZN, 1 x FMN | HHblits | 0.27 |
| ``` target    LARDIAKVPGTTLFAIGMGPNQFFNNDNKDRTQFLLAALTGNIGKIAGNIGSYAGNYRVAMFNGVPQYIAENPFDIELDG 6x89.1    FFKTLSDAKNP-VIIVGAGVFERKDQDAIFAAVETIAQKANVVRPDWNGLNVLLLHAAQA---AA------------LDL  target    AKPARPKLYWRAEPAHYYNHEDHPLKMGKTMITGKTHMPTPTKSLWFANANSILGNVKWHFNTVVNVLPKMEMIAVQEWW 6x89.1    G----------L----------VPQS---------EKSLESAKFVYLMGADDVN----------LDKIPDDAFVVYQGHH  target    WSTSCEWADIVFAVDAWSELKHPDMCSSVTNPFLTV-FPRTPLERPFDTRGDIECLDLVGKQLAKRTGDRRFADMWKFVE 6x89.1    GDKSVYRANVILPTAAFSEKE-----GTYQNTEGCTQQTLPAVPTVGDSRDDWKIIRA----LSEVAGVR-----LPYD-  target    EKKVEVYLQRILDHSSNTKGFKFPELEEKAKKGIPALMMTRTNPKTVGYEQVYDSRPWYTKTGRLEFYREEDEFIEAGEN 6x89.1    --TIGAVRARIRNVA-----------------------------------------------------------------  target    LPVHREPIDSTFYEPNVIVAPAHPFIKAKGPEAYGVKVDDFDNETRQGRNIVKTWEETKKTVHPLAKDGYKFVFHTPKYR 6x89.1    --------------------------------------------------------------------------------  target    HGAHTMPVDTDMVAMLFGPFGDIYRHDKRQPFAAEGYVDIHPDDAKALNIEDGDYVWIDSDPSDRPFRGWQKNDKDYKFS 6x89.1    --------------------------------------------------------------------------------  target    RLLCRARYYPGTPRGITRMWFNMYGATPGSVEGHESRKDGLAKNPRTGYQAMFRSGSHQSATRGWLKPTWMTDSLVRKEL 6x89.1    --------------------------------------------------------------------------------  target    FGHAVNKGFLPDVHCPTGAPREAIVKITKAEPGGLNAKGLWRPAALGLRPKYENDKMKDYLAGKFTLAANPKKGGKK 6x89.1    ----------------------------------------------------------------------------- ``` | | | | | | | | | | | | | | | | | | | | | | | | | | | | | | | | | | | | | | | | | | | | | | | | | |
|  | 8e73.55.A | NDUS1  *Vigna radiata supercomplex I+III2 (full bridge)* | 0.13 |  | 14.29 | 0.29 | 1-254 | EM | 0.00 | monomer |  | HHblits | 0.27 |
| ``` target    LARDIAKVPGTTLFAIGMGPNQFFNNDNKDRTQFLLAALTGNIGKIAGNIGSYAGNYRVAMFNGVPQYIAENPFDIELDG 8e73.55   FFKTLSDAKNP-VIIVGAGVFERKDQDAIFAAVETIAQKANVVRPDWNGLNVLLLHAAQA---AA------------LDL  target    AKPARPKLYWRAEPAHYYNHEDHPLKMGKTMITGKTHMPTPTKSLWFANANSILGNVKWHFNTVVNVLPKMEMIAVQEWW 8e73.55   G----------L----------VPQS---------EKSLESAKFVYLMGADDVN----------LDKIPDDAFVVYQGHH  target    WSTSCEWADIVFAVDAWSELKHPDMCSSVTNPFLTV-FPRTPLERPFDTRGDIECLDLVGKQLAKRTGDRRFADMWKFVE 8e73.55   GDKSVYRANVILPTAAFSEKE-----GTYQNTEGCTQQTLPAVPTVGDSRDDWKIIRA----LSEVAGVR-----LPYD-  target    EKKVEVYLQRILDHSSNTKGFKFPELEEKAKKGIPALMMTRTNPKTVGYEQVYDSRPWYTKTGRLEFYREEDEFIEAGEN 8e73.55   --TIGAVRARIRNVA-----------------------------------------------------------------  target    LPVHREPIDSTFYEPNVIVAPAHPFIKAKGPEAYGVKVDDFDNETRQGRNIVKTWEETKKTVHPLAKDGYKFVFHTPKYR 8e73.55   --------------------------------------------------------------------------------  target    HGAHTMPVDTDMVAMLFGPFGDIYRHDKRQPFAAEGYVDIHPDDAKALNIEDGDYVWIDSDPSDRPFRGWQKNDKDYKFS 8e73.55   --------------------------------------------------------------------------------  target    RLLCRARYYPGTPRGITRMWFNMYGATPGSVEGHESRKDGLAKNPRTGYQAMFRSGSHQSATRGWLKPTWMTDSLVRKEL 8e73.55   --------------------------------------------------------------------------------  target    FGHAVNKGFLPDVHCPTGAPREAIVKITKAEPGGLNAKGLWRPAALGLRPKYENDKMKDYLAGKFTLAANPKKGGKK 8e73.55   ----------------------------------------------------------------------------- ``` | | | | | | | | | | | | | | | | | | | | | | | | | | | | | | | | | | | | | | | | | | | | | | | | | |
|  | 8b9z.1.G | NADH-ubiquinone oxidoreductase 75 kDa subunit, mitochondrial  *Drosophila melanogaster complex I in the Active state (Dm1)* | 0.12 |  | 16.94 | 0.29 | 1-255 | EM | 3.28 | hetero-1-1-1-1-1-1-… | 3 x PC1, 16 x 3PE, 6 x SF4, 4 x CDL, 2 x FES, 1 x FMN, 1 x UQ9, 1 x DGT, 1 x NDP, 1 x ZN, 2 x EHZ | HHblits | 0.27 |
| ``` target    LARDIAKVPGTTLFAIGMGPNQFFNNDNKDRTQFLLAALTGNIGKIAG-NIGSYAGNYRVAMFNGVPQYIAENPFDIELD 8b9z.1    FSKVLEGAKKP-AIIIGADLLERADGAAIHATV---AEYCKKLKKPNWNPFNVLQTNAAQ---VGA------------LD  target    GAKPARPKLYWRAEPAHYYNHEDHPLKMGKTMITGKTHMPTPTKSLWFANANSILGNVKWHFNTVVNVLPKMEMIAVQEW 8b9z.1    V----G------Y----------KA-----GAQT---AVKAQPKVLFLLNADAG--------KVTREQLPKDCFVVYIGS  target    WWSTSCEWADIVFAVDAWSELKHPDMCSSVTNPFLTV-FPRTPLERPFDTRGDIECLDLVGKQLAKRTGDRRFADMWKFV 8b9z.1    HGDNGASIADAVLPGAAYTEKQ-----GIYVNTEGRPQQTLPGVSPPGMAREDWKILRA----LSEVVGKP-----LPYD  target    EEKKVEVYLQRILDHSSNTKGFKFPELEEKAKKGIPALMMTRTNPKTVGYEQVYDSRPWYTKTGRLEFYREEDEFIEAGE 8b9z.1    ---NLDELRNRLEDVAP---------------------------------------------------------------  target    NLPVHREPIDSTFYEPNVIVAPAHPFIKAKGPEAYGVKVDDFDNETRQGRNIVKTWEETKKTVHPLAKDGYKFVFHTPKY 8b9z.1    --------------------------------------------------------------------------------  target    RHGAHTMPVDTDMVAMLFGPFGDIYRHDKRQPFAAEGYVDIHPDDAKALNIEDGDYVWIDSDPSDRPFRGWQKNDKDYKF 8b9z.1    --------------------------------------------------------------------------------  target    SRLLCRARYYPGTPRGITRMWFNMYGATPGSVEGHESRKDGLAKNPRTGYQAMFRSGSHQSATRGWLKPTWMTDSLVRKE 8b9z.1    --------------------------------------------------------------------------------  target    LFGHAVNKGFLPDVHCPTGAPREAIVKITKAEPGGLNAKGLWRPAALGLRPKYENDKMKDYLAGKFTLAANPKKGGKK 8b9z.1    ------------------------------------------------------------------------------ ``` | | | | | | | | | | | | | | | | | | | | | | | | | | | | | | | | | | | | | | | | | | | | | | | | | |
|  | 8ba0.1.G | NADH-ubiquinone oxidoreductase 75 kDa subunit, mitochondrial  *Drosophila melanogaster complex I in the Twisted state (Dm2)* | 0.12 |  | 16.94 | 0.29 | 1-255 | EM | 3.68 | hetero-1-1-1-1-1-1-… | 6 x SF4, 6 x 3PE, 2 x FES, 1 x FMN, 2 x CDL, 1 x DGT, 1 x NDP, 1 x ZN, 2 x EHZ | HHblits | 0.27 |
| ``` target    LARDIAKVPGTTLFAIGMGPNQFFNNDNKDRTQFLLAALTGNIGKIAG-NIGSYAGNYRVAMFNGVPQYIAENPFDIELD 8ba0.1    FSKVLEGAKKP-AIIIGADLLERADGAAIHATV---AEYCKKLKKPNWNPFNVLQTNAAQ---VGA------------LD  target    GAKPARPKLYWRAEPAHYYNHEDHPLKMGKTMITGKTHMPTPTKSLWFANANSILGNVKWHFNTVVNVLPKMEMIAVQEW 8ba0.1    V----G------Y----------KA-----GAQT---AVKAQPKVLFLLNADAG--------KVTREQLPKDCFVVYIGS  target    WWSTSCEWADIVFAVDAWSELKHPDMCSSVTNPFLTV-FPRTPLERPFDTRGDIECLDLVGKQLAKRTGDRRFADMWKFV 8ba0.1    HGDNGASIADAVLPGAAYTEKQ-----GIYVNTEGRPQQTLPGVSPPGMAREDWKILRA----LSEVVGKP-----LPYD  target    EEKKVEVYLQRILDHSSNTKGFKFPELEEKAKKGIPALMMTRTNPKTVGYEQVYDSRPWYTKTGRLEFYREEDEFIEAGE 8ba0.1    ---NLDELRNRLEDVAP---------------------------------------------------------------  target    NLPVHREPIDSTFYEPNVIVAPAHPFIKAKGPEAYGVKVDDFDNETRQGRNIVKTWEETKKTVHPLAKDGYKFVFHTPKY 8ba0.1    --------------------------------------------------------------------------------  target    RHGAHTMPVDTDMVAMLFGPFGDIYRHDKRQPFAAEGYVDIHPDDAKALNIEDGDYVWIDSDPSDRPFRGWQKNDKDYKF 8ba0.1    --------------------------------------------------------------------------------  target    SRLLCRARYYPGTPRGITRMWFNMYGATPGSVEGHESRKDGLAKNPRTGYQAMFRSGSHQSATRGWLKPTWMTDSLVRKE 8ba0.1    --------------------------------------------------------------------------------  target    LFGHAVNKGFLPDVHCPTGAPREAIVKITKAEPGGLNAKGLWRPAALGLRPKYENDKMKDYLAGKFTLAANPKKGGKK 8ba0.1    ------------------------------------------------------------------------------ ``` | | | | | | | | | | | | | | | | | | | | | | | | | | | | | | | | | | | | | | | | | | | | | | | | | |
|  | 7ar7.1.G | NADH dehydrogenase [ubiquinone] iron-sulfur protein 1, mitochondrial  *Cryo-EM structure of Arabidopsis thaliana complex-I (open conformation)* | 0.12 |  | 12.64 | 0.29 | 1-254 | EM | 0.00 | hetero-1-1-1-1-1-1-… | 6 x SF4, 2 x FES, 1 x FMN, 1 x UQ9, 3 x PTY, 2 x PC7, 1 x LMN, 1 x NDP, 2 x ZN, 2 x 8Q1, 1 x PGT, 1 x PSF, 1 x T7X | HHblits | 0.26 |
| ``` target    LARDIAKVPGTTLFAIGMGPNQFFNNDNKDRTQFLLAALTGNIGKIAGNIGSYAGNYRVAMFNGVPQYIAENPFDIELDG 7ar7.1    FCTALKNAKNP-AIIVGAGLFNRTDKNAILSSVESIAQANNVVRPDWNGLNFLLQYAAQA---AAL------------DL  target    AKPARPKLYWRAEPAHYYNHEDHPLKMGKTMITGKTHMPTPTKSLWFANANSILGNVKWHFNTVVNVLPKMEMIAVQEWW 7ar7.1    ------G-L-------------IQ----Q-SAK----ALESAKFVYLMGADDVN----------VDKIPKDAFVVYQGHH  target    WSTSCEWADIVFAVDAWSELKHPDMCSSVTNPFLTV-FPRTPLERPFDTRGDIECLDLVGKQLAKRTGDRRFADMWKFVE 7ar7.1    GDKAVYRANVILPASAFTEKE-----GTYENTEGFTQQTVPAVPTVGDARDDWKIVRA----LSEVSGVK-----LPYN-  target    EKKVEVYLQRILDHSSNTKGFKFPELEEKAKKGIPALMMTRTNPKTVGYEQVYDSRPWYTKTGRLEFYREEDEFIEAGEN 7ar7.1    --SIEGVRSRIKSVA-----------------------------------------------------------------  target    LPVHREPIDSTFYEPNVIVAPAHPFIKAKGPEAYGVKVDDFDNETRQGRNIVKTWEETKKTVHPLAKDGYKFVFHTPKYR 7ar7.1    --------------------------------------------------------------------------------  target    HGAHTMPVDTDMVAMLFGPFGDIYRHDKRQPFAAEGYVDIHPDDAKALNIEDGDYVWIDSDPSDRPFRGWQKNDKDYKFS 7ar7.1    --------------------------------------------------------------------------------  target    RLLCRARYYPGTPRGITRMWFNMYGATPGSVEGHESRKDGLAKNPRTGYQAMFRSGSHQSATRGWLKPTWMTDSLVRKEL 7ar7.1    --------------------------------------------------------------------------------  target    FGHAVNKGFLPDVHCPTGAPREAIVKITKAEPGGLNAKGLWRPAALGLRPKYENDKMKDYLAGKFTLAANPKKGGKK 7ar7.1    ----------------------------------------------------------------------------- ``` | | | | | | | | | | | | | | | | | | | | | | | | | | | | | | | | | | | | | | | | | | | | | | | | | |
|  | 7aqr.1.F | NADH dehydrogenase [ubiquinone] iron-sulfur protein 1, mitochondrial  *Cryo-EM structure of Arabidopsis thaliana Complex-I (peripheral arm)* | 0.13 |  | 13.26 | 0.28 | 1-253 | EM | 0.00 | hetero-1-1-1-1-1-1-… | 6 x SF4, 2 x FES, 1 x FMN, 1 x NDP, 1 x ZN, 1 x 8Q1 | HHblits | 0.27 |
| ``` target    LARDIAKVPGTTLFAIGMGPNQFFNNDNKDRTQFLLAALTGNIGKIAGNIGSYAGNYRVAMFNGVPQYIAENPFDIELDG 7aqr.1    FCTALKNAKNP-AIIVGAGLFNRTDKNAILSSVESIAQANNVVRPDWNGLNFLLQYAAQA---AA------------LDL  target    AKPARPKLYWRAEPAHYYNHEDHPLKMGKTMITGKTHMPTPTKSLWFANANSILGNVKWHFNTVVNVLPKMEMIAVQEWW 7aqr.1    ----------GL----------IQ----Q-SAK----ALESAKFVYLMGADDVN----------VDKIPKDAFVVYQGHH  target    WSTSCEWADIVFAVDAWSELKHPDMCSSVTNPFLTV-FPRTPLERPFDTRGDIECLDLVGKQLAKRTGDRRFADMWKFVE 7aqr.1    GDKAVYRANVILPASAFTEKE-----GTYENTEGFTQQTVPAVPTVGDARDDWKIVRA----LSEVSGVK-----LPYN-  target    EKKVEVYLQRILDHSSNTKGFKFPELEEKAKKGIPALMMTRTNPKTVGYEQVYDSRPWYTKTGRLEFYREEDEFIEAGEN 7aqr.1    --SIEGVRSRIKSV------------------------------------------------------------------  target    LPVHREPIDSTFYEPNVIVAPAHPFIKAKGPEAYGVKVDDFDNETRQGRNIVKTWEETKKTVHPLAKDGYKFVFHTPKYR 7aqr.1    --------------------------------------------------------------------------------  target    HGAHTMPVDTDMVAMLFGPFGDIYRHDKRQPFAAEGYVDIHPDDAKALNIEDGDYVWIDSDPSDRPFRGWQKNDKDYKFS 7aqr.1    --------------------------------------------------------------------------------  target    RLLCRARYYPGTPRGITRMWFNMYGATPGSVEGHESRKDGLAKNPRTGYQAMFRSGSHQSATRGWLKPTWMTDSLVRKEL 7aqr.1    --------------------------------------------------------------------------------  target    FGHAVNKGFLPDVHCPTGAPREAIVKITKAEPGGLNAKGLWRPAALGLRPKYENDKMKDYLAGKFTLAANPKKGGKK 7aqr.1    ----------------------------------------------------------------------------- ``` | | | | | | | | | | | | | | | | | | | | | | | | | | | | | | | | | | | | | | | | | | | | | | | | | |
|  | 7a23.1.O | 75kDa  *Plant mitochondrial respiratory complex I* | 0.12 |  | 13.26 | 0.28 | 1-253 | EM | 0.00 | hetero-1-1-1-1-1-1-… | 6 x SF4, 1 x FMN, 2 x T7X, 3 x CDL, 1 x U10, 1 x PEV, 2 x FES, 1 x NDP, 2 x ZN | HHblits | 0.27 |
| ``` target    LARDIAKVPGTTLFAIGMGPNQFFNNDNKDRTQFLLAALTGNIGKIAGNIGSYAGNYRVAMFNGVPQYIAENPFDIELDG 7a23.1    FCTALKNAKNP-AIIVGAGLFNRTDKNAILSSVESIAQANNVVRPDWNGLNFLLQYAAQA---AA------------LDL  target    AKPARPKLYWRAEPAHYYNHEDHPLKMGKTMITGKTHMPTPTKSLWFANANSILGNVKWHFNTVVNVLPKMEMIAVQEWW 7a23.1    ----------GL----------IQ----Q-SAK----ALESAKFVYLMGADDVN----------VDKIPKDAFVVYQGHH  target    WSTSCEWADIVFAVDAWSELKHPDMCSSVTNPFLTV-FPRTPLERPFDTRGDIECLDLVGKQLAKRTGDRRFADMWKFVE 7a23.1    GDKAVYRANVILPASAFTEKE-----GTYENTEGFTQQTVPAVPTVGDARDDWKIVRA----LSEVSGVK-----LPYN-  target    EKKVEVYLQRILDHSSNTKGFKFPELEEKAKKGIPALMMTRTNPKTVGYEQVYDSRPWYTKTGRLEFYREEDEFIEAGEN 7a23.1    --SIEGVRSRIKSV------------------------------------------------------------------  target    LPVHREPIDSTFYEPNVIVAPAHPFIKAKGPEAYGVKVDDFDNETRQGRNIVKTWEETKKTVHPLAKDGYKFVFHTPKYR 7a23.1    --------------------------------------------------------------------------------  target    HGAHTMPVDTDMVAMLFGPFGDIYRHDKRQPFAAEGYVDIHPDDAKALNIEDGDYVWIDSDPSDRPFRGWQKNDKDYKFS 7a23.1    --------------------------------------------------------------------------------  target    RLLCRARYYPGTPRGITRMWFNMYGATPGSVEGHESRKDGLAKNPRTGYQAMFRSGSHQSATRGWLKPTWMTDSLVRKEL 7a23.1    --------------------------------------------------------------------------------  target    FGHAVNKGFLPDVHCPTGAPREAIVKITKAEPGGLNAKGLWRPAALGLRPKYENDKMKDYLAGKFTLAANPKKGGKK 7a23.1    ----------------------------------------------------------------------------- ``` | | | | | | | | | | | | | | | | | | | | | | | | | | | | | | | | | | | | | | | | | | | | | | | | | |
|  | 7ar8.1.G | NADH dehydrogenase [ubiquinone] iron-sulfur protein 1, mitochondrial  *Cryo-EM structure of Arabidopsis thaliana complex-I (closed conformation)* | 0.12 |  | 13.26 | 0.28 | 1-253 | EM | 0.00 | hetero-1-1-1-1-1-1-… | 6 x SF4, 2 x FES, 1 x FMN, 1 x UQ9, 3 x PTY, 2 x PC7, 1 x PGT, 1 x FE, 1 x NDP, 2 x ZN, 2 x 8Q1, 1 x LMN, 1 x PSF, 1 x T7X | HHblits | 0.27 |
| ``` target    LARDIAKVPGTTLFAIGMGPNQFFNNDNKDRTQFLLAALTGNIGKIAGNIGSYAGNYRVAMFNGVPQYIAENPFDIELDG 7ar8.1    FCTALKNAKNP-AIIVGAGLFNRTDKNAILSSVESIAQANNVVRPDWNGLNFLLQYAAQA---AA------------LDL  target    AKPARPKLYWRAEPAHYYNHEDHPLKMGKTMITGKTHMPTPTKSLWFANANSILGNVKWHFNTVVNVLPKMEMIAVQEWW 7ar8.1    ----------GL----------IQ----Q-SAK----ALESAKFVYLMGADDVN----------VDKIPKDAFVVYQGHH  target    WSTSCEWADIVFAVDAWSELKHPDMCSSVTNPFLTV-FPRTPLERPFDTRGDIECLDLVGKQLAKRTGDRRFADMWKFVE 7ar8.1    GDKAVYRANVILPASAFTEKE-----GTYENTEGFTQQTVPAVPTVGDARDDWKIVRA----LSEVSGVK-----LPYN-  target    EKKVEVYLQRILDHSSNTKGFKFPELEEKAKKGIPALMMTRTNPKTVGYEQVYDSRPWYTKTGRLEFYREEDEFIEAGEN 7ar8.1    --SIEGVRSRIKSV------------------------------------------------------------------  target    LPVHREPIDSTFYEPNVIVAPAHPFIKAKGPEAYGVKVDDFDNETRQGRNIVKTWEETKKTVHPLAKDGYKFVFHTPKYR 7ar8.1    --------------------------------------------------------------------------------  target    HGAHTMPVDTDMVAMLFGPFGDIYRHDKRQPFAAEGYVDIHPDDAKALNIEDGDYVWIDSDPSDRPFRGWQKNDKDYKFS 7ar8.1    --------------------------------------------------------------------------------  target    RLLCRARYYPGTPRGITRMWFNMYGATPGSVEGHESRKDGLAKNPRTGYQAMFRSGSHQSATRGWLKPTWMTDSLVRKEL 7ar8.1    --------------------------------------------------------------------------------  target    FGHAVNKGFLPDVHCPTGAPREAIVKITKAEPGGLNAKGLWRPAALGLRPKYENDKMKDYLAGKFTLAANPKKGGKK 7ar8.1    ----------------------------------------------------------------------------- ``` | | | | | | | | | | | | | | | | | | | | | | | | | | | | | | | | | | | | | | | | | | | | | | | | | |
|  | 7bkb.1.L | Formylmethanofuran dehydrogenase, subunit B  *Formate dehydrogenase - heterodisulfide reductase - formylmethanofuran dehydrogenase complex from Methanospirillum hungatei (hexameric, composite structure)* | 0.12 |  | 13.33 | 0.28 | 1-228 | EM | 0.00 | hetero-2-2-2-2-2-2-… | 48 x SF4, 4 x FAD, 2 x FES, 4 x 9S8, 4 x ZN, 2 x MO, 4 x MGD | HHblits | 0.26 |
| ``` target    LARDIAKVPGTTLFAIGMGPNQFFNNDNKDRTQFL------------LAALTGNIGKIAGNIGSYAGNYRVAMFNGVPQY 7bkb.1    VAEIMKNARF-GTTFFGMGLTHTDGRNHNIDIAISLTRDLNKISKWTIMAMRGHYNIAGPGVVWSWTF-------GFPYC  target    IAENPFDIELDGAKPARPKLYWRAEPAHYYNHEDHPLKMGKTMITGKTHMPTPTKSLWFANANSILGNVKWHFNTVVNVL 7bkb.1    LD---L----TKQ--N-----HA-----HMNP--GETSSVDMA------MRDEVDMFINIGTDAAAHFP--IP--AVKQL  target    PKMEMIAVQEWWWSTSCEWADIVFAVDAW-SELKHPDMCSSVTNPFLTVFPRTPLERPFDTRGDIECLDLVGKQLAKRTG 7bkb.1    KKHPW-VTIDPSINMASEISDLHIPVCICGVDVG--GIVYRM-DNVPIQF-RKVIEPPEGVMDDETLLNK----IADRME  target    DRRFADMWKFVEEKKVEVYLQRILDHSSNTKGFKFPELEEKAKKGIPALMMTRTNPKTVGYEQVYDSRPWYTKTGRLEFY 7bkb.1    E-------------------------------------------------------------------------------  target    REEDEFIEAGENLPVHREPIDSTFYEPNVIVAPAHPFIKAKGPEAYGVKVDDFDNETRQGRNIVKTWEETKKTVHPLAKD 7bkb.1    --------------------------------------------------------------------------------  target    GYKFVFHTPKYRHGAHTMPVDTDMVAMLFGPFGDIYRHDKRQPFAAEGYVDIHPDDAKALNIEDGDYVWIDSDPSDRPFR 7bkb.1    --------------------------------------------------------------------------------  target    GWQKNDKDYKFSRLLCRARYYPGTPRGITRMWFNMYGATPGSVEGHESRKDGLAKNPRTGYQAMFRSGSHQSATRGWLKP 7bkb.1    --------------------------------------------------------------------------------  target    TWMTDSLVRKELFGHAVNKGFLPDVHCPTGAPREAIVKITKAEPGGLNAKGLWRPAALGLRPKYENDKMKDYLAGKFTLA 7bkb.1    --------------------------------------------------------------------------------  target    ANPKKGGKK 7bkb.1    --------- ``` | | | | | | | | | | | | | | | | | | | | | | | | | | | | | | | | | | | | | | | | | | | | | | | | | |
|  | 6btm.1.B | Alternative Complex III subunit B  *Structure of Alternative Complex III from Flavobacterium johnsoniae (Wild Type)* | 0.11 |  | 12.66 | 0.25 | 1-219 | EM | 3.40 | hetero-1-1-1-1-1-1-… | 6 x HEC, 1 x F3S, 1 x SF4, 2 x E87 | HHblits | 0.26 |
| ``` target    LARDIAKVPGTTLFAIGMGPNQFFNNDNKDRTQFLLAALTGNIGKIAGNIGSYAGNYRVAMFNGVPQYIAENPFDIELDG 6btm.1    AAQQLKAAGTKGILVSGIED------KNAQLLVLAINQALASEAFSTAGTRQIR--------KGS--------------N  target    AKPARPKLYWRAEPAHYYNHEDHPLKMGKTMITGKTHMPTPTKSLWFANANSILGNVKWHFNTVVNVLPKMEMIAVQEWW 6btm.1    A---V------------------VAQLIKD---M---NAGSVHTLIMSGVNPVYTL--ADSASFVSGLKKVKTSVAFSLK  target    WSTSCEWADIVFAVDAWSELKHPDMCSSVTNPFLTVFPRTPLERPFDTRGDIECLDLVGKQLAKRTGDRRFADMWKFVEE 6btm.1    EDETAAVSTIAAAAPHYLESWG-DVE-ITK-GTYSLTQ-PTIRPIFDTKQFQDVLLSVN---------------------  target    KKVEVYLQRILDHSSNTKGFKFPELEEKAKKGIPALMMTRTNPKTVGYEQVYDSRPWYTKTGRLEFYREEDEFIEAGENL 6btm.1    --------------------------------------------------------------------------------  target    PVHREPIDSTFYEPNVIVAPAHPFIKAKGPEAYGVKVDDFDNETRQGRNIVKTWEETKKTVHPLAKDGYKFVFHTPKYRH 6btm.1    --------------------------------------------------------------------------------  target    GAHTMPVDTDMVAMLFGPFGDIYRHDKRQPFAAEGYVDIHPDDAKALNIEDGDYVWIDSDPSDRPFRGWQKNDKDYKFSR 6btm.1    --------------------------------------------------------------------------------  target    LLCRARYYPGTPRGITRMWFNMYGATPGSVEGHESRKDGLAKNPRTGYQAMFRSGSHQSATRGWLKPTWMTDSLVRKELF 6btm.1    --------------------------------------------------------------------------------  target    GHAVNKGFLPDVHCPTGAPREAIVKITKAEPGGLNAKGLWRPAALGLRPKYENDKMKDYLAGKFTLAANPKKGGKK 6btm.1    ---------------------------------------------------------------------------- ``` | | | | | | | | | | | | | | | | | | | | | | | | | | | | | | | | | | | | | | | | | | | | | | | | | |
|  | 8e9g.1.G | NADH-quinone oxidoreductase subunit G  *Mycobacterial respiratory complex I with both quinone positions modelled* | 0.06 |  | 19.39 | 0.15 | 119-229 | EM | 0.00 | hetero-1-1-1-1-1-1-… |  | HHblits | 0.26 |
| ``` target    LARDIAKVPGTTLFAIGMGPNQFFNNDNKDRTQFLLAALTGNIGKIAGNIGSYAGNYRVAMFNGVPQYIAENPFDIELDG 8e9g.1    --------------------------------------------------------------------------------  target    AKPARPKLYWRAEPAHYYNHEDHPLKMGKTMITGKTHMPTPTKSLWFANANSILGNVKWHFNTVVNVLPKMEMIAVQEWW 8e9g.1    --------------------------------------SGHLAALLVGG-VELGD--LPDPELAVAAVRTTPFVVSLELR  target    WSTSCEWADIVFAVDAWSELKHPDMCSSVTNPFLTVFPRTPLERPFDTRGDIECLDLVGKQLAKRTGDRRFADMWKFVEE 8e9g.1    ESAVTELADVVFPVAPVVEKA-----GSFLNWEGRPRPF-APSLKTNAIPDLRVLHY----LADEIGVD-----------  target    KKVEVYLQRILDHSSNTKGFKFPELEEKAKKGIPALMMTRTNPKTVGYEQVYDSRPWYTKTGRLEFYREEDEFIEAGENL 8e9g.1    --------------------------------------------------------------------------------  target    PVHREPIDSTFYEPNVIVAPAHPFIKAKGPEAYGVKVDDFDNETRQGRNIVKTWEETKKTVHPLAKDGYKFVFHTPKYRH 8e9g.1    --------------------------------------------------------------------------------  target    GAHTMPVDTDMVAMLFGPFGDIYRHDKRQPFAAEGYVDIHPDDAKALNIEDGDYVWIDSDPSDRPFRGWQKNDKDYKFSR 8e9g.1    --------------------------------------------------------------------------------  target    LLCRARYYPGTPRGITRMWFNMYGATPGSVEGHESRKDGLAKNPRTGYQAMFRSGSHQSATRGWLKPTWMTDSLVRKELF 8e9g.1    --------------------------------------------------------------------------------  target    GHAVNKGFLPDVHCPTGAPREAIVKITKAEPGGLNAKGLWRPAALGLRPKYENDKMKDYLAGKFTLAANPKKGGKK 8e9g.1    ---------------------------------------------------------------------------- ``` | | | | | | | | | | | | | | | | | | | | | | | | | | | | | | | | | | | | | | | | | | | | | | | | | |
|  | 7q5y.1.A | NADH dehydrogenase I chain G  *Structure of NADH:ubichinon oxidoreductase (complex I) of the hyperthermophilic eubacterium Aquifex aeolicus* | 0.05 |  | 13.40 | 0.15 | 120-227 | X-ray | 2.70 | hetero-1-1-1-1-1-1-… | 8 x SF4, 2 x FES, 1 x FMN | HHblits | 0.26 |
| ``` target    LARDIAKVPGTTLFAIGMGPNQFFNNDNKDRTQFLLAALTGNIGKIAGNIGSYAGNYRVAMFNGVPQYIAENPFDIELDG 7q5y.1    --------------------------------------------------------------------------------  target    AKPARPKLYWRAEPAHYYNHEDHPLKMGKTMITGKTHMPTPTKSLWFANANSILGNVKWHFNTVVNVLPKMEMIAVQEWW 7q5y.1    ---------------------------------------GDIENLIIFGEDILEFY--ED-KVFEELKEKLEHLVVVSPY  target    WSTSCEWADIVFAVDAWSELKHPDMCSSVTNPFLTVFPRTPLERPFDTRGDIECLDLVGKQLAKRTGDRRFADMWKFVEE 7q5y.1    EDGLSEYAHIKIPMSLMGENE-----GTYKTFFGEVKGKK-FLP--WAFDDLAFWKYLGENFKEEKG-------------  target    KKVEVYLQRILDHSSNTKGFKFPELEEKAKKGIPALMMTRTNPKTVGYEQVYDSRPWYTKTGRLEFYREEDEFIEAGENL 7q5y.1    --------------------------------------------------------------------------------  target    PVHREPIDSTFYEPNVIVAPAHPFIKAKGPEAYGVKVDDFDNETRQGRNIVKTWEETKKTVHPLAKDGYKFVFHTPKYRH 7q5y.1    --------------------------------------------------------------------------------  target    GAHTMPVDTDMVAMLFGPFGDIYRHDKRQPFAAEGYVDIHPDDAKALNIEDGDYVWIDSDPSDRPFRGWQKNDKDYKFSR 7q5y.1    --------------------------------------------------------------------------------  target    LLCRARYYPGTPRGITRMWFNMYGATPGSVEGHESRKDGLAKNPRTGYQAMFRSGSHQSATRGWLKPTWMTDSLVRKELF 7q5y.1    --------------------------------------------------------------------------------  target    GHAVNKGFLPDVHCPTGAPREAIVKITKAEPGGLNAKGLWRPAALGLRPKYENDKMKDYLAGKFTLAANPKKGGKK 7q5y.1    ---------------------------------------------------------------------------- ``` | | | | | | | | | | | | | | | | | | | | | | | | | | | | | | | | | | | | | | | | | | | | | | | | | |
|  | 6s6y.1.B | Tungsten-containing formylmethanofuran dehydrogenase, subunit B  *X-ray crystal structure of the formyltransferase/hydrolase complex (FhcABCD) from Methylorubrum extorquens in complex with methylofuran* | 0.04 |  | 12.09 | 0.14 | 120-228 | X-ray | 3.10 | hetero-2-2-2-2-mer | 1 x MFN, 4 x ZN, 4 x CA, 4 x K, 3 x DGL, 2 x GLU, 1 x IAS | HHblits | 0.25 |
| ``` target    LARDIAKVPGTTLFAIGMGPNQFFNNDNKDRTQFLLAALTGNIGKIAGNIGSYAGNYRVAMFNGVPQYIAENPFDIELDG 6s6y.1    --------------------------------------------------------------------------------  target    AKPARPKLYWRAEPAHYYNHEDHPLKMGKTMITGKTHMPTPTKSLWFANANSILGNVKWHFNTVVNVLPKMEMIAVQE-W 6s6y.1    ---------------------------------------GEADAALWLASLPAP---------RPAWLGSLPTIAIVGEG  target    WWSTSCEWADIVFAVDAW-SELKHPDMCSSVTNPF-LTVFPRTPLERP---FDTRGDIECLDLVGKQLAKRTGDRRFADM 6s6y.1    SQEAAGETAEVVITVGVPGQSVG-----GALWNDRRGVIAYAEASDPAKTPAETETAAGVLTR----IRDRLIE------  target    WKFVEEKKVEVYLQRILDHSSNTKGFKFPELEEKAKKGIPALMMTRTNPKTVGYEQVYDSRPWYTKTGRLEFYREEDEFI 6s6y.1    --------------------------------------------------------------------------------  target    EAGENLPVHREPIDSTFYEPNVIVAPAHPFIKAKGPEAYGVKVDDFDNETRQGRNIVKTWEETKKTVHPLAKDGYKFVFH 6s6y.1    --------------------------------------------------------------------------------  target    TPKYRHGAHTMPVDTDMVAMLFGPFGDIYRHDKRQPFAAEGYVDIHPDDAKALNIEDGDYVWIDSDPSDRPFRGWQKNDK 6s6y.1    --------------------------------------------------------------------------------  target    DYKFSRLLCRARYYPGTPRGITRMWFNMYGATPGSVEGHESRKDGLAKNPRTGYQAMFRSGSHQSATRGWLKPTWMTDSL 6s6y.1    --------------------------------------------------------------------------------  target    VRKELFGHAVNKGFLPDVHCPTGAPREAIVKITKAEPGGLNAKGLWRPAALGLRPKYENDKMKDYLAGKFTLAANPKKGG 6s6y.1    --------------------------------------------------------------------------------  target    KK 6s6y.1    -- ``` | | | | | | | | | | | | | | | | | | | | | | | | | | | | | | | | | | | | | | | | | | | | | | | | | |
|  | 3o5a.1.A | Periplasmic nitrate reductase  *Crystal Structure of partially reduced Periplasmic Nitrate Reductase from Cupriavidus necator using Ionic Liquids* | 0.05 |  | 22.62 | 0.13 | 386-502 | X-ray | 1.72 | hetero-oligomer | 1 x SF4, 1 x MOS, 2 x MGD, 2 x HEC | HHblits | 0.30 |
| ``` target    LARDIAKVPGTTLFAIGMGPNQFFNNDNKDRTQFLLAALTGNIGKIAGNIGSYAGNYRVAMFNGVPQYIAENPFDIELDG 3o5a.1    --------------------------------------------------------------------------------  target    AKPARPKLYWRAEPAHYYNHEDHPLKMGKTMITGKTHMPTPTKSLWFANANSILGNVKWHFNTVVNVLPKMEMIAVQEWW 3o5a.1    --------------------------------------------------------------------------------  target    WSTSCEWADIVFAVDAWSELKHPDMCSSVTNPFLTVFPRTPLERPFDTRGDIECLDLVGKQLAKRTGDRRFADMWKFVEE 3o5a.1    --------------------------------------------------------------------------------  target    KKVEVYLQRILDHSSNTKGFKFPELEEKAKKGIPALMMTRTNPKTVGYEQVYDSRPWYTKTGRLEFYREEDEFIEAGENL 3o5a.1    --------------------------------------------------------------------------------  target    PVHREPIDSTFYEPNVIVAPAHPFIKAKGPEAYGVKVDDFDNETRQGRNIVKTWEETKKTVHPLAKDGYKFVFHTPKYRH 3o5a.1    -----------------------------------------------------------------DKEYPYWLVTGRVLE  target    GAHTMP--VDTDMVAMLFGPFGDIYRHDKRQPFAAEGYVDIHPDDAKALNIEDGDYVWIDSDPSDRPFRGWQKNDKDYKF 3o5a.1    HWHSGSMTRRVPEL-------------YRS---FPNAVVFMHPEDAKALGLRRGVEVEVVSRR-----------------  target    SRLLCRARYY--PGTPRGITRMWFNMYGATPGSVEGHESRKDGLAKNPRTGYQAMFRSGSHQSATRGWLKPTWMTDSLVR 3o5a.1    GRMRSRIETRGRDAPPRGLVFVPWFD------------------------------------------------------  target    KELFGHAVNKGFLPDVHCPTGAPREAIVKITKAEPGGLNAKGLWRPAALGLRPKYENDKMKDYLAGKFTLAANPKKGGKK 3o5a.1    -------------------------------------------------------------------------------- ``` | | | | | | | | | | | | | | | | | | | | | | | | | | | | | | | | | | | | | | | | | | | | | | | | | |
|  | 1h0h.1.A | FORMATE DEHYDROGENASE SUBUNIT ALPHA  *Tungsten containing Formate Dehydrogenase from Desulfovibrio Gigas* | 0.04 |  | 15.48 | 0.13 | 386-502 | X-ray | 1.80 | hetero-1-1-mer | 1 x W, 1 x 2MD, 1 x MGD, 4 x SF4, 1 x CA | HHblits | 0.28 |
| ``` target    LARDIAKVPGTTLFAIGMGPNQFFNNDNKDRTQFLLAALTGNIGKIAGNIGSYAGNYRVAMFNGVPQYIAENPFDIELDG 1h0h.1    --------------------------------------------------------------------------------  target    AKPARPKLYWRAEPAHYYNHEDHPLKMGKTMITGKTHMPTPTKSLWFANANSILGNVKWHFNTVVNVLPKMEMIAVQEWW 1h0h.1    --------------------------------------------------------------------------------  target    WSTSCEWADIVFAVDAWSELKHPDMCSSVTNPFLTVFPRTPLERPFDTRGDIECLDLVGKQLAKRTGDRRFADMWKFVEE 1h0h.1    --------------------------------------------------------------------------------  target    KKVEVYLQRILDHSSNTKGFKFPELEEKAKKGIPALMMTRTNPKTVGYEQVYDSRPWYTKTGRLEFYREEDEFIEAGENL 1h0h.1    --------------------------------------------------------------------------------  target    PVHREPIDSTFYEPNVIVAPAHPFIKAKGPEAYGVKVDDFDNETRQGRNIVKTWEETKKTVHPLAKDGYKFVFHTPKYRH 1h0h.1    -----------------------------------------------------------------DPRYPFICSTYRVTE  target    GA--HTMPVDTDMVAMLFGPFGDIYRHDKRQPFAAEGYVDIHPDDAKALNIEDGDYVWIDSDPSDRPFRGWQKNDKDYKF 1h0h.1    HWQTGLMTRNTPWLLE-------------A---EPQMFCEMSEELATLRGIKNGDKVILESVR-----------------  target    SRLLCRARYYPGTPRG--------ITRMWFNMYGATPGSVEGHESRKDGLAKNPRTGYQAMFRSGSHQSATRGWLKPTWM 1h0h.1    GKLWAKAIITKRIKPFAIQGQQVHMVGIPWHY------------------------------------------------  target    TDSLVRKELFGHAVNKGFLPDVHCPTGAPREAIVKITKAEPGGLNAKGLWRPAALGLRPKYENDKMKDYLAGKFTLAANP 1h0h.1    --------------------------------------------------------------------------------  target    KKGGKK 1h0h.1    ------ ``` | | | | | | | | | | | | | | | | | | | | | | | | | | | | | | | | | | | | | | | | | | | | | | | | | |
|  | 8e9g.1.G | NADH-quinone oxidoreductase subunit G  *Mycobacterial respiratory complex I with both quinone positions modelled* | 0.04 |  | 14.29 | 0.13 | 387-504 | EM | 0.00 | hetero-1-1-1-1-1-1-… |  | HHblits | 0.27 |
| ``` target    LARDIAKVPGTTLFAIGMGPNQFFNNDNKDRTQFLLAALTGNIGKIAGNIGSYAGNYRVAMFNGVPQYIAENPFDIELDG 8e9g.1    --------------------------------------------------------------------------------  target    AKPARPKLYWRAEPAHYYNHEDHPLKMGKTMITGKTHMPTPTKSLWFANANSILGNVKWHFNTVVNVLPKMEMIAVQEWW 8e9g.1    --------------------------------------------------------------------------------  target    WSTSCEWADIVFAVDAWSELKHPDMCSSVTNPFLTVFPRTPLERPFDTRGDIECLDLVGKQLAKRTGDRRFADMWKFVEE 8e9g.1    --------------------------------------------------------------------------------  target    KKVEVYLQRILDHSSNTKGFKFPELEEKAKKGIPALMMTRTNPKTVGYEQVYDSRPWYTKTGRLEFYREEDEFIEAGENL 8e9g.1    --------------------------------------------------------------------------------  target    PVHREPIDSTFYEPNVIVAPAHPFIKAKGPEAYGVKVDDFDNETRQGRNIVKTWEETKKTVHPLAKDGYKFVFHTPKYRH 8e9g.1    ------------------------------------------------------------------GSGQAVLASWRMLL  target    GAHTMPVDTDMVAMLFGPFGDIYRHDKRQPFAAEGYVDIHPDDAKALNIEDGDYVWIDSDPSDRPFRGWQKNDKDYKFSR 8e9g.1    DAGRLQDGEPHLAG----------------TAVRPVARMSAATAAGIGASDGAPVTVSTER-----------------GA  target    LLCRARYYPGTPRGITRMWFNMYGATPGSVEGHESRKDGLAKNPRTGYQAMFRSGSHQSATRGWLKPTWMTDSLVRKELF 8e9g.1    VTLPLAVTD-MPDGVVWLPMNSPG--------------------------------------------------------  target    GHAVNKGFLPDVHCPTGAPREAIVKITKAEPGGLNAKGLWRPAALGLRPKYENDKMKDYLAGKFTLAANPKKGGKK 8e9g.1    ---------------------------------------------------------------------------- ``` | | | | | | | | | | | | | | | | | | | | | | | | | | | | | | | | | | | | | | | | | | | | | | | | | |
|  | 2ki8.1.A | Tungsten formylmethanofuran dehydrogenase, subunit D (FwdD-2)  *Solution NMR structure of tungsten formylmethanofuran dehydrogenase subunit D from Archaeoglobus fulgidus, Northeast Structural Genomics Consortium target AtT7* | 0.05 |  | 17.50 | 0.13 | 388-502 | NMR | 0.00 | monomer |  | HHblits | 0.30 |
| ``` target    LARDIAKVPGTTLFAIGMGPNQFFNNDNKDRTQFLLAALTGNIGKIAGNIGSYAGNYRVAMFNGVPQYIAENPFDIELDG 2ki8.1    --------------------------------------------------------------------------------  target    AKPARPKLYWRAEPAHYYNHEDHPLKMGKTMITGKTHMPTPTKSLWFANANSILGNVKWHFNTVVNVLPKMEMIAVQEWW 2ki8.1    --------------------------------------------------------------------------------  target    WSTSCEWADIVFAVDAWSELKHPDMCSSVTNPFLTVFPRTPLERPFDTRGDIECLDLVGKQLAKRTGDRRFADMWKFVEE 2ki8.1    --------------------------------------------------------------------------------  target    KKVEVYLQRILDHSSNTKGFKFPELEEKAKKGIPALMMTRTNPKTVGYEQVYDSRPWYTKTGRLEFYREEDEFIEAGENL 2ki8.1    --------------------------------------------------------------------------------  target    PVHREPIDSTFYEPNVIVAPAHPFIKAKGPEAYGVKVDDFDNETRQGRNIVKTWEETKKTVHPLAKDGYKFVFHTPKYRH 2ki8.1    -------------------------------------------------------------------MLEVEVISGRTLN  target    GAHTMPVDTDMVAMLFGPFGDIYRHDKRQPFAAEGYVDIHPDDAKALNIEDGDYVWIDSDPSDRPFRGWQKNDKDYKFSR 2ki8.1    QGATVE--EKLT------------EE---YFNAVNYAEINEEDWNALGLQEGDRVKVKTEF-----------------GE  target    LLCRARYYPGTPRGITRMWFNMYGATPGSVEGHESRKDGLAKNPRTGYQAMFRSGSHQSATRGWLKPTWMTDSLVRKELF 2ki8.1    VVVFAKKG-DVPKGMIFIPMGP----------------------------------------------------------  target    GHAVNKGFLPDVHCPTGAPREAIVKITKAEPGGLNAKGLWRPAALGLRPKYENDKMKDYLAGKFTLAANPKKGGKK 2ki8.1    ---------------------------------------------------------------------------- ``` | | | | | | | | | | | | | | | | | | | | | | | | | | | | | | | | | | | | | | | | | | | | | | | | | |
|  | 7bkb.1.J | Formylmethanofuran dehydrogenase, subunit D  *Formate dehydrogenase - heterodisulfide reductase - formylmethanofuran dehydrogenase complex from Methanospirillum hungatei (hexameric, composite structure)* | 0.04 |  | 14.46 | 0.13 | 387-502 | EM | 0.00 | hetero-2-2-2-2-2-2-… | 48 x SF4, 4 x FAD, 2 x FES, 4 x 9S8, 4 x ZN, 2 x MO, 4 x MGD | HHblits | 0.27 |
| ``` target    LARDIAKVPGTTLFAIGMGPNQFFNNDNKDRTQFLLAALTGNIGKIAGNIGSYAGNYRVAMFNGVPQYIAENPFDIELDG 7bkb.1    --------------------------------------------------------------------------------  target    AKPARPKLYWRAEPAHYYNHEDHPLKMGKTMITGKTHMPTPTKSLWFANANSILGNVKWHFNTVVNVLPKMEMIAVQEWW 7bkb.1    --------------------------------------------------------------------------------  target    WSTSCEWADIVFAVDAWSELKHPDMCSSVTNPFLTVFPRTPLERPFDTRGDIECLDLVGKQLAKRTGDRRFADMWKFVEE 7bkb.1    --------------------------------------------------------------------------------  target    KKVEVYLQRILDHSSNTKGFKFPELEEKAKKGIPALMMTRTNPKTVGYEQVYDSRPWYTKTGRLEFYREEDEFIEAGENL 7bkb.1    --------------------------------------------------------------------------------  target    PVHREPIDSTFYEPNVIVAPAHPFIKAKGPEAYGVKVDDFDNETRQGRNIVKTWEETKKTVHPLAKDGYKFVFHTPKYRH 7bkb.1    ------------------------------------------------------------------AKKTLNMITQRAVE  target    GAHTMPVDTDMVAMLFGPFGDIYRHDKRQPFAAEGYVDIHPDDAKALNIEDGDYVWIDSDPSDRPFRGWQKNDKDYKFSR 7bkb.1    EGIAMEI-GKTSR-------------QY--FDACSIIEMNEQDMKELGIMKNTNVRVKSES-----------------GE  target    LLCRARYY-PGTPRGITRMWFNMYGATPGSVEGHESRKDGLAKNPRTGYQAMFRSGSHQSATRGWLKPTWMTDSLVRKEL 7bkb.1    VVVKAVVGRQTCYPGLCHIRQGV---------------------------------------------------------  target    FGHAVNKGFLPDVHCPTGAPREAIVKITKAEPGGLNAKGLWRPAALGLRPKYENDKMKDYLAGKFTLAANPKKGGKK 7bkb.1    ----------------------------------------------------------------------------- ``` | | | | | | | | | | | | | | | | | | | | | | | | | | | | | | | | | | | | | | | | | | | | | | | | | |
|  | 5t5i.1.D | Tungsten formylmethanofuran dehydrogenase subunit fwdD  *TUNGSTEN-CONTAINING FORMYLMETHANOFURAN DEHYDROGENASE FROM METHANOTHERMOBACTER WOLFEII, ORTHORHOMBIC FORM AT 1.9 A* | 0.04 |  | 17.50 | 0.13 | 390-502 | X-ray | 1.90 | hetero-oligomer | 4 x ZN, 2 x MG, 18 x K, 22 x SF4, 2 x W, 4 x MGD, 2 x H2S, 2 x CA | HHblits | 0.30 |
| ``` target    LARDIAKVPGTTLFAIGMGPNQFFNNDNKDRTQFLLAALTGNIGKIAGNIGSYAGNYRVAMFNGVPQYIAENPFDIELDG 5t5i.1    --------------------------------------------------------------------------------  target    AKPARPKLYWRAEPAHYYNHEDHPLKMGKTMITGKTHMPTPTKSLWFANANSILGNVKWHFNTVVNVLPKMEMIAVQEWW 5t5i.1    --------------------------------------------------------------------------------  target    WSTSCEWADIVFAVDAWSELKHPDMCSSVTNPFLTVFPRTPLERPFDTRGDIECLDLVGKQLAKRTGDRRFADMWKFVEE 5t5i.1    --------------------------------------------------------------------------------  target    KKVEVYLQRILDHSSNTKGFKFPELEEKAKKGIPALMMTRTNPKTVGYEQVYDSRPWYTKTGRLEFYREEDEFIEAGENL 5t5i.1    --------------------------------------------------------------------------------  target    PVHREPIDSTFYEPNVIVAPAHPFIKAKGPEAYGVKVDDFDNETRQGRNIVKTWEETKKTVHPLAKDGYKFVFHTPKYRH 5t5i.1    ---------------------------------------------------------------------RVILNTGRTIW  target    GAHTMPVDTDMVAMLFGPFGDIYRHDKRQPFAAEGYVDIHPDDAKALNIEDGDYVWIDSDPSDRPFRGWQKNDKDYKFSR 5t5i.1    QGQAIESGKDLK-------------M---YVDAAAIIQMNPEMMKQLGIAEGDNVKVISEY-----------------GD  target    LLCRAR-YYPGTPRGITRMWFNMYGATPGSVEGHESRKDGLAKNPRTGYQAMFRSGSHQSATRGWLKPTWMTDSLVRKEL 5t5i.1    VVVKAVEAKEPLPEGMVYIPMGP---------------------------------------------------------  target    FGHAVNKGFLPDVHCPTGAPREAIVKITKAEPGGLNAKGLWRPAALGLRPKYENDKMKDYLAGKFTLAANPKKGGKK 5t5i.1    ----------------------------------------------------------------------------- ``` | | | | | | | | | | | | | | | | | | | | | | | | | | | | | | | | | | | | | | | | | | | | | | | | | |
|  | 6sdr.1.A | Formate dehydrogenase, alpha subunit, selenocysteine-containing  *W-formate dehydrogenase from Desulfovibrio vulgaris - Oxidized form* | 0.03 |  | 19.23 | 0.12 | 386-496 | X-ray | 2.10 | hetero-1-1-mer | 2 x MGD, 4 x SF4, 1 x H2S, 1 x W | HHblits | 0.27 |
| ``` target    LARDIAKVPGTTLFAIGMGPNQFFNNDNKDRTQFLLAALTGNIGKIAGNIGSYAGNYRVAMFNGVPQYIAENPFDIELDG 6sdr.1    --------------------------------------------------------------------------------  target    AKPARPKLYWRAEPAHYYNHEDHPLKMGKTMITGKTHMPTPTKSLWFANANSILGNVKWHFNTVVNVLPKMEMIAVQEWW 6sdr.1    --------------------------------------------------------------------------------  target    WSTSCEWADIVFAVDAWSELKHPDMCSSVTNPFLTVFPRTPLERPFDTRGDIECLDLVGKQLAKRTGDRRFADMWKFVEE 6sdr.1    --------------------------------------------------------------------------------  target    KKVEVYLQRILDHSSNTKGFKFPELEEKAKKGIPALMMTRTNPKTVGYEQVYDSRPWYTKTGRLEFYREEDEFIEAGENL 6sdr.1    --------------------------------------------------------------------------------  target    PVHREPIDSTFYEPNVIVAPAHPFIKAKGPEAYGVKVDDFDNETRQGRNIVKTWEETKKTVHPLAKDGYKFVFHTPKYRH 6sdr.1    -----------------------------------------------------------------DPRYPFIGTTYRVTE  target    GAHT--MPVDTDMVAMLFGPFGDIYRHDKRQPFAAEGYVDIHPDDAKALNIEDGDYVWIDSDPSDRPFRGWQKNDKDYKF 6sdr.1    HWQTGLMTRRCAWLV-------------EA---EPQIFCEISKELAKLRGIGNGDTVKVSSLR-----------------  target    SRLLCRARYYPGTPRGITRMWFNMYGATPGSVEGHESRKDGLAKNPRTGYQAMFRSGSHQSATRGWLKPTWMTDSLVRKE 6sdr.1    GALEAVAIVTERIRPFKI--------------------------------------------------------------  target    LFGHAVNKGFLPDVHCPTGAPREAIVKITKAEPGGLNAKGLWRPAALGLRPKYENDKMKDYLAGKFTLAANPKKGGKK 6sdr.1    ------------------------------------------------------------------------------ ``` | | | | | | | | | | | | | | | | | | | | | | | | | | | | | | | | | | | | | | | | | | | | | | | | | |
|  | 6sdv.1.A | Formate dehydrogenase, alpha subunit, selenocysteine-containing,Formate dehydrogenase, alpha subunit, selenocysteine-containing,W-formate dehydrogenase - alpha subunit  *W-formate dehydrogenase from Desulfovibrio vulgaris - Formate reduced form* | 0.04 |  | 19.23 | 0.12 | 386-496 | X-ray | 1.90 | hetero-1-1-mer | 2 x MGD, 4 x SF4, 1 x W, 1 x H2S | HHblits | 0.27 |
| ``` target    LARDIAKVPGTTLFAIGMGPNQFFNNDNKDRTQFLLAALTGNIGKIAGNIGSYAGNYRVAMFNGVPQYIAENPFDIELDG 6sdv.1    --------------------------------------------------------------------------------  target    AKPARPKLYWRAEPAHYYNHEDHPLKMGKTMITGKTHMPTPTKSLWFANANSILGNVKWHFNTVVNVLPKMEMIAVQEWW 6sdv.1    --------------------------------------------------------------------------------  target    WSTSCEWADIVFAVDAWSELKHPDMCSSVTNPFLTVFPRTPLERPFDTRGDIECLDLVGKQLAKRTGDRRFADMWKFVEE 6sdv.1    --------------------------------------------------------------------------------  target    KKVEVYLQRILDHSSNTKGFKFPELEEKAKKGIPALMMTRTNPKTVGYEQVYDSRPWYTKTGRLEFYREEDEFIEAGENL 6sdv.1    --------------------------------------------------------------------------------  target    PVHREPIDSTFYEPNVIVAPAHPFIKAKGPEAYGVKVDDFDNETRQGRNIVKTWEETKKTVHPLAKDGYKFVFHTPKYRH 6sdv.1    -----------------------------------------------------------------DPRYPFIGTTYRVTE  target    GAHT--MPVDTDMVAMLFGPFGDIYRHDKRQPFAAEGYVDIHPDDAKALNIEDGDYVWIDSDPSDRPFRGWQKNDKDYKF 6sdv.1    HWQTGLMTRRCAWLVE-------------A---EPQIFCEISKELAKLRGIGNGDTVKVSSLR-----------------  target    SRLLCRARYYPGTPRGITRMWFNMYGATPGSVEGHESRKDGLAKNPRTGYQAMFRSGSHQSATRGWLKPTWMTDSLVRKE 6sdv.1    GALEAVAIVTERIRPFKI--------------------------------------------------------------  target    LFGHAVNKGFLPDVHCPTGAPREAIVKITKAEPGGLNAKGLWRPAALGLRPKYENDKMKDYLAGKFTLAANPKKGGKK 6sdv.1    ------------------------------------------------------------------------------ ``` | | | | | | | | | | | | | | | | | | | | | | | | | | | | | | | | | | | | | | | | | | | | | | | | | |
|  | 8bqg.1.A | Formate dehydrogenase, alpha subunit, selenocysteine-containing  *W-formate dehydrogenase from Desulfovibrio vulgaris - Soaking with Formate 1 min* | 0.03 |  | 18.18 | 0.12 | 386-495 | X-ray | 1.95 | hetero-1-1-mer | 2 x MGD, 4 x SF4, 1 x H2S, 1 x W | HHblits | 0.26 |
| ``` target    LARDIAKVPGTTLFAIGMGPNQFFNNDNKDRTQFLLAALTGNIGKIAGNIGSYAGNYRVAMFNGVPQYIAENPFDIELDG 8bqg.1    --------------------------------------------------------------------------------  target    AKPARPKLYWRAEPAHYYNHEDHPLKMGKTMITGKTHMPTPTKSLWFANANSILGNVKWHFNTVVNVLPKMEMIAVQEWW 8bqg.1    --------------------------------------------------------------------------------  target    WSTSCEWADIVFAVDAWSELKHPDMCSSVTNPFLTVFPRTPLERPFDTRGDIECLDLVGKQLAKRTGDRRFADMWKFVEE 8bqg.1    --------------------------------------------------------------------------------  target    KKVEVYLQRILDHSSNTKGFKFPELEEKAKKGIPALMMTRTNPKTVGYEQVYDSRPWYTKTGRLEFYREEDEFIEAGENL 8bqg.1    --------------------------------------------------------------------------------  target    PVHREPIDSTFYEPNVIVAPAHPFIKAKGPEAYGVKVDDFDNETRQGRNIVKTWEETKKTVHPLAKDGYKFVFHTPKYRH 8bqg.1    -----------------------------------------------------------------DPRYPFIGTTYRVTE  target    GAHTM--PVDTDMVAMLFGPFGDIYRHDKRQPFAAEGYVDIHPDDAKALNIEDGDYVWIDSDPSDRPFRGWQKNDKDYKF 8bqg.1    HWQTGLMTRRCAWLVE-------------A---EPQIFCEISKELAKLRGIGNGDTVKVSSLR-----------------  target    SRLLCRARYYPGTPRGITRMWFNMYGATPGSVEGHESRKDGLAKNPRTGYQAMFRSGSHQSATRGWLKPTWMTDSLVRKE 8bqg.1    GALEAVAIVTERIRPFK---------------------------------------------------------------  target    LFGHAVNKGFLPDVHCPTGAPREAIVKITKAEPGGLNAKGLWRPAALGLRPKYENDKMKDYLAGKFTLAANPKKGGKK 8bqg.1    ------------------------------------------------------------------------------ ``` | | | | | | | | | | | | | | | | | | | | | | | | | | | | | | | | | | | | | | | | | | | | | | | | | |
|  | 2e7z.1.A | Acetylene hydratase Ahy  *Acetylene Hydratase from Pelobacter acetylenicus* | 0.02 |  | 21.43 | 0.09 | 121-176 | X-ray | 1.26 | monomer | 1 x SF4, 2 x MGD, 1 x W | HHblits | 0.28 |
| ``` target    LARDIAKVPGTTLFAIGMGPNQFFNNDNKDRTQFLLAALTGNIGKIAGNIGSYAGNYRVAMFNGVPQYIAENPFDIELDG 2e7z.1    --------------------------------------------------------------------------------  target    AKPARPKLYWRAEPAHYYNHEDHPLKMGKTMITGKTHMPTPTKSLWFANANSILGNVKWHFNTVVNVLPKMEMIAVQEWW 2e7z.1    ----------------------------------------DSNCLLFIGKNLSNHNWVSQFNDLKAALKRGCKLIVLDPR  target    WSTSCEWADIVFAVDAWSELKHPDMCSSVTNPFLTVFPRTPLERPFDTRGDIECLDLVGKQLAKRTGDRRFADMWKFVEE 2e7z.1    RTKVAEMADIWLPLRY----------------------------------------------------------------  target    KKVEVYLQRILDHSSNTKGFKFPELEEKAKKGIPALMMTRTNPKTVGYEQVYDSRPWYTKTGRLEFYREEDEFIEAGENL 2e7z.1    --------------------------------------------------------------------------------  target    PVHREPIDSTFYEPNVIVAPAHPFIKAKGPEAYGVKVDDFDNETRQGRNIVKTWEETKKTVHPLAKDGYKFVFHTPKYRH 2e7z.1    --------------------------------------------------------------------------------  target    GAHTMPVDTDMVAMLFGPFGDIYRHDKRQPFAAEGYVDIHPDDAKALNIEDGDYVWIDSDPSDRPFRGWQKNDKDYKFSR 2e7z.1    --------------------------------------------------------------------------------  target    LLCRARYYPGTPRGITRMWFNMYGATPGSVEGHESRKDGLAKNPRTGYQAMFRSGSHQSATRGWLKPTWMTDSLVRKELF 2e7z.1    --------------------------------------------------------------------------------  target    GHAVNKGFLPDVHCPTGAPREAIVKITKAEPGGLNAKGLWRPAALGLRPKYENDKMKDYLAGKFTLAANPKKGGKK 2e7z.1    ---------------------------------------------------------------------------- ``` | | | | | | | | | | | | | | | | | | | | | | | | | | | | | | | | | | | | | | | | | | | | | | | | | |
|  | 7b04.1.B | Nitrite oxidoreductase subunit A  *Structure of Nitrite oxidoreductase (Nxr) from the anammox bacterium Kuenenia stuttgartiensis.* | 0.02 |  | 22.22 | 0.08 | 121-175 | X-ray | 2.97 | hetero-1-1-1-mer | 4 x SF4, 1 x F3S, 2 x MD1, 1 x MO, 1 x HEM, 2 x CA | HHblits | 0.28 |
| ``` target    LARDIAKVPGTTLFAIGMGPNQFFNNDNKDRTQFLLAALTGNIGKIAGNIGSYAGNYRVAMFNGVPQYIAENPFDIELDG 7b04.1    --------------------------------------------------------------------------------  target    AKPARPKLYWRAEPAHYYNHEDHPLKMGKTMITGKTHMPTPTKSLWFANANSILGNVKWHFNTVVNVLPKMEMIAVQEWW 7b04.1    ----------------------------------------FSKLLIQTGKNLIENKM-PEAHWVTEVMERGGKIVVITPE  target    WSTSCEWADIVFAVDAWSELKHPDMCSSVTNPFLTVFPRTPLERPFDTRGDIECLDLVGKQLAKRTGDRRFADMWKFVEE 7b04.1    YSPSAQKADYWIPIR-----------------------------------------------------------------  target    KKVEVYLQRILDHSSNTKGFKFPELEEKAKKGIPALMMTRTNPKTVGYEQVYDSRPWYTKTGRLEFYREEDEFIEAGENL 7b04.1    --------------------------------------------------------------------------------  target    PVHREPIDSTFYEPNVIVAPAHPFIKAKGPEAYGVKVDDFDNETRQGRNIVKTWEETKKTVHPLAKDGYKFVFHTPKYRH 7b04.1    --------------------------------------------------------------------------------  target    GAHTMPVDTDMVAMLFGPFGDIYRHDKRQPFAAEGYVDIHPDDAKALNIEDGDYVWIDSDPSDRPFRGWQKNDKDYKFSR 7b04.1    --------------------------------------------------------------------------------  target    LLCRARYYPGTPRGITRMWFNMYGATPGSVEGHESRKDGLAKNPRTGYQAMFRSGSHQSATRGWLKPTWMTDSLVRKELF 7b04.1    --------------------------------------------------------------------------------  target    GHAVNKGFLPDVHCPTGAPREAIVKITKAEPGGLNAKGLWRPAALGLRPKYENDKMKDYLAGKFTLAANPKKGGKK 7b04.1    ---------------------------------------------------------------------------- ``` | | | | | | | | | | | | | | | | | | | | | | | | | | | | | | | | | | | | | | | | | | | | | | | | | |
|  | 7b04.2.B | Nitrite oxidoreductase subunit A  *Structure of Nitrite oxidoreductase (Nxr) from the anammox bacterium Kuenenia stuttgartiensis.* | 0.01 |  | 22.22 | 0.08 | 121-175 | X-ray | 2.97 | hetero-1-1-1-mer | 4 x SF4, 1 x F3S, 2 x MD1, 1 x MO, 1 x HEM, 2 x CA | HHblits | 0.28 |
| ``` target    LARDIAKVPGTTLFAIGMGPNQFFNNDNKDRTQFLLAALTGNIGKIAGNIGSYAGNYRVAMFNGVPQYIAENPFDIELDG 7b04.2    --------------------------------------------------------------------------------  target    AKPARPKLYWRAEPAHYYNHEDHPLKMGKTMITGKTHMPTPTKSLWFANANSILGNVKWHFNTVVNVLPKMEMIAVQEWW 7b04.2    ----------------------------------------FSKLLIQTGKNLIENKM-PEAHWVTEVMERGGKIVVITPE  target    WSTSCEWADIVFAVDAWSELKHPDMCSSVTNPFLTVFPRTPLERPFDTRGDIECLDLVGKQLAKRTGDRRFADMWKFVEE 7b04.2    YSPSAQKADYWIPIR-----------------------------------------------------------------  target    KKVEVYLQRILDHSSNTKGFKFPELEEKAKKGIPALMMTRTNPKTVGYEQVYDSRPWYTKTGRLEFYREEDEFIEAGENL 7b04.2    --------------------------------------------------------------------------------  target    PVHREPIDSTFYEPNVIVAPAHPFIKAKGPEAYGVKVDDFDNETRQGRNIVKTWEETKKTVHPLAKDGYKFVFHTPKYRH 7b04.2    --------------------------------------------------------------------------------  target    GAHTMPVDTDMVAMLFGPFGDIYRHDKRQPFAAEGYVDIHPDDAKALNIEDGDYVWIDSDPSDRPFRGWQKNDKDYKFSR 7b04.2    --------------------------------------------------------------------------------  target    LLCRARYYPGTPRGITRMWFNMYGATPGSVEGHESRKDGLAKNPRTGYQAMFRSGSHQSATRGWLKPTWMTDSLVRKELF 7b04.2    --------------------------------------------------------------------------------  target    GHAVNKGFLPDVHCPTGAPREAIVKITKAEPGGLNAKGLWRPAALGLRPKYENDKMKDYLAGKFTLAANPKKGGKK 7b04.2    ---------------------------------------------------------------------------- ``` | | | | | | | | | | | | | | | | | | | | | | | | | | | | | | | | | | | | | | | | | | | | | | | | | |
|  | 6f0k.1.B | Fe-S-cluster-containing hydrogenase  *Alternative complex III* | 0.01 |  | 10.71 | 0.09 | 121-176 | EM | 0.00 | hetero-1-1-1-1-1-1-… | 6 x HEC, 1 x F3S, 3 x SF4 | HHblits | 0.25 |
| ``` target    LARDIAKVPGTTLFAIGMGPNQFFNNDNKDRTQFLLAALTGNIGKIAGNIGSYAGNYRVAMFNGVPQYIAENPFDIELDG 6f0k.1    --------------------------------------------------------------------------------  target    AKPARPKLYWRAEPAHYYNHEDHPLKMGKTMITGKTHMPTPTKSLWFANANSILG-NVKWHF-NTVVNVL-------PKM 6f0k.1    ----------------------------------------EARVIVSLDADFLGPTDRNFVENTREFAASRRMERPEDEI  target    EMIAVQEWWWSTSCEWADIVFAVDAWSELKHPDMCSSVTNPFLTVFPRTPLERPFDTRGDIECLDLVGKQLAKRTGDRRF 6f0k.1    SRLYVIESTYTVTGGMADHRLRLRA-------------------------------------------------------  target    ADMWKFVEEKKVEVYLQRILDHSSNTKGFKFPELEEKAKKGIPALMMTRTNPKTVGYEQVYDSRPWYTKTGRLEFYREED 6f0k.1    --------------------------------------------------------------------------------  target    EFIEAGENLPVHREPIDSTFYEPNVIVAPAHPFIKAKGPEAYGVKVDDFDNETRQGRNIVKTWEETKKTVHPLAKDGYKF 6f0k.1    --------------------------------------------------------------------------------  target    VFHTPKYRHGAHTMPVDTDMVAMLFGPFGDIYRHDKRQPFAAEGYVDIHPDDAKALNIEDGDYVWIDSDPSDRPFRGWQK 6f0k.1    --------------------------------------------------------------------------------  target    NDKDYKFSRLLCRARYYPGTPRGITRMWFNMYGATPGSVEGHESRKDGLAKNPRTGYQAMFRSGSHQSATRGWLKPTWMT 6f0k.1    --------------------------------------------------------------------------------  target    DSLVRKELFGHAVNKGFLPDVHCPTGAPREAIVKITKAEPGGLNAKGLWRPAALGLRPKYENDKMKDYLAGKFTLAANPK 6f0k.1    --------------------------------------------------------------------------------  target    KGGKK 6f0k.1    ----- ``` | | | | | | | | | | | | | | | | | | | | | | | | | | | | | | | | | | | | | | | | | | | | | | | | | |
|  | 2v45.1.A | PERIPLASMIC NITRATE REDUCTASE  *A NEW CATALYTIC MECHANISM OF PERIPLASMIC NITRATE REDUCTASE FROM DESULFOVIBRIO DESULFURICANS ATCC 27774 FROM CRYSTALLOGRAPHIC AND EPR DATA AND BASED ON DETAILED ANALYSIS OF THE SIXTH LIGAND* | 0.02 |  | 12.73 | 0.09 | 121-176 | X-ray | 2.40 | monomer | 1 x SF4, 1 x MO, 2 x MGD, 1 x LCP | HHblits | 0.26 |
| ``` target    LARDIAKVPGTTLFAIGMGPNQFFNNDNKDRTQFLLAALTGNIGKIAGNIGSYAGNYRVAMFNGVPQYIAENPFDIELDG 2v45.1    --------------------------------------------------------------------------------  target    AKPARPKLYWRAEPAHYYNHEDHPLKMGKTMITGKTHMPTPTKSLWFANANSILGNVKWHFNTVVNVL--PKMEMIAVQE 2v45.1    ----------------------------------------QATCFFIIGSNTSEAHPV-LFRRIARRKQVEPGVKIIVAD  target    WWWSTSCEWADIVFAVDAWSELKHPDMCSSVTNPFLTVFPRTPLERPFDTRGDIECLDLVGKQLAKRTGDRRFADMWKFV 2v45.1    PRRTNTSRIADMHVAFRP--------------------------------------------------------------  target    EEKKVEVYLQRILDHSSNTKGFKFPELEEKAKKGIPALMMTRTNPKTVGYEQVYDSRPWYTKTGRLEFYREEDEFIEAGE 2v45.1    --------------------------------------------------------------------------------  target    NLPVHREPIDSTFYEPNVIVAPAHPFIKAKGPEAYGVKVDDFDNETRQGRNIVKTWEETKKTVHPLAKDGYKFVFHTPKY 2v45.1    --------------------------------------------------------------------------------  target    RHGAHTMPVDTDMVAMLFGPFGDIYRHDKRQPFAAEGYVDIHPDDAKALNIEDGDYVWIDSDPSDRPFRGWQKNDKDYKF 2v45.1    --------------------------------------------------------------------------------  target    SRLLCRARYYPGTPRGITRMWFNMYGATPGSVEGHESRKDGLAKNPRTGYQAMFRSGSHQSATRGWLKPTWMTDSLVRKE 2v45.1    --------------------------------------------------------------------------------  target    LFGHAVNKGFLPDVHCPTGAPREAIVKITKAEPGGLNAKGLWRPAALGLRPKYENDKMKDYLAGKFTLAANPKKGGKK 2v45.1    ------------------------------------------------------------------------------ ``` | | | | | | | | | | | | | | | | | | | | | | | | | | | | | | | | | | | | | | | | | | | | | | | | | |
|  | 1aa6.1.A | FORMATE DEHYDROGENASE H  *REDUCED FORM OF FORMATE DEHYDROGENASE H FROM E. COLI* | 0.02 |  | 18.52 | 0.08 | 121-175 | X-ray | 2.30 | monomer | 1 x SF4, 2 x MGD, 1 x 4MO | HHblits | 0.27 |
| ``` target    LARDIAKVPGTTLFAIGMGPNQFFNNDNKDRTQFLLAALTGNIGKIAGNIGSYAGNYRVAMFNGVPQYIAENPFDIELDG 1aa6.1    --------------------------------------------------------------------------------  target    AKPARPKLYWRAEPAHYYNHEDHPLKMGKTMITGKTHMPTPTKSLWFANANSILGNVKWHFNTVVNVLPKMEMIAVQEWW 1aa6.1    ----------------------------------------NTDLVFVFGYNPADSHPI-VANHVINAKRNGAKIIVCDPR  target    WSTSCEWADIVFAVDAWSELKHPDMCSSVTNPFLTVFPRTPLERPFDTRGDIECLDLVGKQLAKRTGDRRFADMWKFVEE 1aa6.1    KIETARIADMHIALK-----------------------------------------------------------------  target    KKVEVYLQRILDHSSNTKGFKFPELEEKAKKGIPALMMTRTNPKTVGYEQVYDSRPWYTKTGRLEFYREEDEFIEAGENL 1aa6.1    --------------------------------------------------------------------------------  target    PVHREPIDSTFYEPNVIVAPAHPFIKAKGPEAYGVKVDDFDNETRQGRNIVKTWEETKKTVHPLAKDGYKFVFHTPKYRH 1aa6.1    --------------------------------------------------------------------------------  target    GAHTMPVDTDMVAMLFGPFGDIYRHDKRQPFAAEGYVDIHPDDAKALNIEDGDYVWIDSDPSDRPFRGWQKNDKDYKFSR 1aa6.1    --------------------------------------------------------------------------------  target    LLCRARYYPGTPRGITRMWFNMYGATPGSVEGHESRKDGLAKNPRTGYQAMFRSGSHQSATRGWLKPTWMTDSLVRKELF 1aa6.1    --------------------------------------------------------------------------------  target    GHAVNKGFLPDVHCPTGAPREAIVKITKAEPGGLNAKGLWRPAALGLRPKYENDKMKDYLAGKFTLAANPKKGGKK 1aa6.1    ---------------------------------------------------------------------------- ``` | | | | | | | | | | | | | | | | | | | | | | | | | | | | | | | | | | | | | | | | | | | | | | | | | |
|  | 1fdo.1.A | FORMATE DEHYDROGENASE H  *OXIDIZED FORM OF FORMATE DEHYDROGENASE H FROM E. COLI* | 0.02 |  | 18.52 | 0.08 | 121-175 | X-ray | 2.80 | monomer | 1 x SF4, 2 x MGD, 1 x 6MO | HHblits | 0.27 |
| ``` target    LARDIAKVPGTTLFAIGMGPNQFFNNDNKDRTQFLLAALTGNIGKIAGNIGSYAGNYRVAMFNGVPQYIAENPFDIELDG 1fdo.1    --------------------------------------------------------------------------------  target    AKPARPKLYWRAEPAHYYNHEDHPLKMGKTMITGKTHMPTPTKSLWFANANSILGNVKWHFNTVVNVLPKMEMIAVQEWW 1fdo.1    ----------------------------------------NTDLVFVFGYNPADSHPI-VANHVINAKRNGAKIIVCDPR  target    WSTSCEWADIVFAVDAWSELKHPDMCSSVTNPFLTVFPRTPLERPFDTRGDIECLDLVGKQLAKRTGDRRFADMWKFVEE 1fdo.1    KIETARIADMHIALK-----------------------------------------------------------------  target    KKVEVYLQRILDHSSNTKGFKFPELEEKAKKGIPALMMTRTNPKTVGYEQVYDSRPWYTKTGRLEFYREEDEFIEAGENL 1fdo.1    --------------------------------------------------------------------------------  target    PVHREPIDSTFYEPNVIVAPAHPFIKAKGPEAYGVKVDDFDNETRQGRNIVKTWEETKKTVHPLAKDGYKFVFHTPKYRH 1fdo.1    --------------------------------------------------------------------------------  target    GAHTMPVDTDMVAMLFGPFGDIYRHDKRQPFAAEGYVDIHPDDAKALNIEDGDYVWIDSDPSDRPFRGWQKNDKDYKFSR 1fdo.1    --------------------------------------------------------------------------------  target    LLCRARYYPGTPRGITRMWFNMYGATPGSVEGHESRKDGLAKNPRTGYQAMFRSGSHQSATRGWLKPTWMTDSLVRKELF 1fdo.1    --------------------------------------------------------------------------------  target    GHAVNKGFLPDVHCPTGAPREAIVKITKAEPGGLNAKGLWRPAALGLRPKYENDKMKDYLAGKFTLAANPKKGGKK 1fdo.1    ---------------------------------------------------------------------------- ``` | | | | | | | | | | | | | | | | | | | | | | | | | | | | | | | | | | | | | | | | | | | | | | | | | |
|  | 2iv2.1.A | Formate dehydrogenase H  *Reinterpretation of reduced form of formate dehydrogenase H from E. coli* | 0.01 |  | 18.52 | 0.08 | 121-175 | X-ray | 2.27 | monomer | 1 x SF4, 1 x 2MD, 1 x MGD | HHblits | 0.27 |
| ``` target    LARDIAKVPGTTLFAIGMGPNQFFNNDNKDRTQFLLAALTGNIGKIAGNIGSYAGNYRVAMFNGVPQYIAENPFDIELDG 2iv2.1    --------------------------------------------------------------------------------  target    AKPARPKLYWRAEPAHYYNHEDHPLKMGKTMITGKTHMPTPTKSLWFANANSILGNVKWHFNTVVNVLPKMEMIAVQEWW 2iv2.1    ----------------------------------------NTDLVFVFGYNPADSHPI-VANHVINAKRNGAKIIVCDPR  target    WSTSCEWADIVFAVDAWSELKHPDMCSSVTNPFLTVFPRTPLERPFDTRGDIECLDLVGKQLAKRTGDRRFADMWKFVEE 2iv2.1    KIETARIADMHIALK-----------------------------------------------------------------  target    KKVEVYLQRILDHSSNTKGFKFPELEEKAKKGIPALMMTRTNPKTVGYEQVYDSRPWYTKTGRLEFYREEDEFIEAGENL 2iv2.1    --------------------------------------------------------------------------------  target    PVHREPIDSTFYEPNVIVAPAHPFIKAKGPEAYGVKVDDFDNETRQGRNIVKTWEETKKTVHPLAKDGYKFVFHTPKYRH 2iv2.1    --------------------------------------------------------------------------------  target    GAHTMPVDTDMVAMLFGPFGDIYRHDKRQPFAAEGYVDIHPDDAKALNIEDGDYVWIDSDPSDRPFRGWQKNDKDYKFSR 2iv2.1    --------------------------------------------------------------------------------  target    LLCRARYYPGTPRGITRMWFNMYGATPGSVEGHESRKDGLAKNPRTGYQAMFRSGSHQSATRGWLKPTWMTDSLVRKELF 2iv2.1    --------------------------------------------------------------------------------  target    GHAVNKGFLPDVHCPTGAPREAIVKITKAEPGGLNAKGLWRPAALGLRPKYENDKMKDYLAGKFTLAANPKKGGKK 2iv2.1    ---------------------------------------------------------------------------- ``` | | | | | | | | | | | | | | | | | | | | | | | | | | | | | | | | | | | | | | | | | | | | | | | | | |
|  | 7z0t.1.G | Formate dehydrogenase H  *Structure of the Escherichia coli formate hydrogenlyase complex (aerobic preparation, composite structure)* | 0.01 |  | 18.52 | 0.08 | 121-175 | EM | 0.00 | hetero-1-1-1-1-1-1-… | 1 x NI, 1 x FCO, 8 x SF4, 1 x FE, 2 x MGD, 1 x 6MO | HHblits | 0.27 |
| ``` target    LARDIAKVPGTTLFAIGMGPNQFFNNDNKDRTQFLLAALTGNIGKIAGNIGSYAGNYRVAMFNGVPQYIAENPFDIELDG 7z0t.1    --------------------------------------------------------------------------------  target    AKPARPKLYWRAEPAHYYNHEDHPLKMGKTMITGKTHMPTPTKSLWFANANSILGNVKWHFNTVVNVLPKMEMIAVQEWW 7z0t.1    ----------------------------------------NTDLVFVFGYNPADSHPI-VANHVINAKRNGAKIIVCDPR  target    WSTSCEWADIVFAVDAWSELKHPDMCSSVTNPFLTVFPRTPLERPFDTRGDIECLDLVGKQLAKRTGDRRFADMWKFVEE 7z0t.1    KIETARIADMHIALK-----------------------------------------------------------------  target    KKVEVYLQRILDHSSNTKGFKFPELEEKAKKGIPALMMTRTNPKTVGYEQVYDSRPWYTKTGRLEFYREEDEFIEAGENL 7z0t.1    --------------------------------------------------------------------------------  target    PVHREPIDSTFYEPNVIVAPAHPFIKAKGPEAYGVKVDDFDNETRQGRNIVKTWEETKKTVHPLAKDGYKFVFHTPKYRH 7z0t.1    --------------------------------------------------------------------------------  target    GAHTMPVDTDMVAMLFGPFGDIYRHDKRQPFAAEGYVDIHPDDAKALNIEDGDYVWIDSDPSDRPFRGWQKNDKDYKFSR 7z0t.1    --------------------------------------------------------------------------------  target    LLCRARYYPGTPRGITRMWFNMYGATPGSVEGHESRKDGLAKNPRTGYQAMFRSGSHQSATRGWLKPTWMTDSLVRKELF 7z0t.1    --------------------------------------------------------------------------------  target    GHAVNKGFLPDVHCPTGAPREAIVKITKAEPGGLNAKGLWRPAALGLRPKYENDKMKDYLAGKFTLAANPKKGGKK 7z0t.1    ---------------------------------------------------------------------------- ``` | | | | | | | | | | | | | | | | | | | | | | | | | | | | | | | | | | | | | | | | | | | | | | | | | |
|  | 2nya.1.A | Periplasmic nitrate reductase  *Crystal structure of the periplasmic nitrate reductase (NAP) from Escherichia coli* | 0.02 |  | 16.67 | 0.08 | 121-175 | X-ray | 2.50 | monomer | 1 x SF4, 1 x 6MO, 2 x MGD | HHblits | 0.26 |
| ``` target    LARDIAKVPGTTLFAIGMGPNQFFNNDNKDRTQFLLAALTGNIGKIAGNIGSYAGNYRVAMFNGVPQYIAENPFDIELDG 2nya.1    --------------------------------------------------------------------------------  target    AKPARPKLYWRAEPAHYYNHEDHPLKMGKTMITGKTHMPTPTKSLWFANANSILGNVKWHFNTVVNVL--PKMEMIAVQE 2nya.1    ----------------------------------------QADAFVLWGANMAEMHPI-LWSRITNRRLSNQNVTVAVLS  target    WWWSTSCEWADIVFAVDAWSELKHPDMCSSVTNPFLTVFPRTPLERPFDTRGDIECLDLVGKQLAKRTGDRRFADMWKFV 2nya.1    TYQHRSFELADNGIIFT---------------------------------------------------------------  target    EEKKVEVYLQRILDHSSNTKGFKFPELEEKAKKGIPALMMTRTNPKTVGYEQVYDSRPWYTKTGRLEFYREEDEFIEAGE 2nya.1    --------------------------------------------------------------------------------  target    NLPVHREPIDSTFYEPNVIVAPAHPFIKAKGPEAYGVKVDDFDNETRQGRNIVKTWEETKKTVHPLAKDGYKFVFHTPKY 2nya.1    --------------------------------------------------------------------------------  target    RHGAHTMPVDTDMVAMLFGPFGDIYRHDKRQPFAAEGYVDIHPDDAKALNIEDGDYVWIDSDPSDRPFRGWQKNDKDYKF 2nya.1    --------------------------------------------------------------------------------  target    SRLLCRARYYPGTPRGITRMWFNMYGATPGSVEGHESRKDGLAKNPRTGYQAMFRSGSHQSATRGWLKPTWMTDSLVRKE 2nya.1    --------------------------------------------------------------------------------  target    LFGHAVNKGFLPDVHCPTGAPREAIVKITKAEPGGLNAKGLWRPAALGLRPKYENDKMKDYLAGKFTLAANPKKGGKK 2nya.1    ------------------------------------------------------------------------------ ``` | | | | | | | | | | | | | | | | | | | | | | | | | | | | | | | | | | | | | | | | | | | | | | | | | |
|  | 1kqf.1.A | FORMATE DEHYDROGENASE, NITRATE-INDUCIBLE, MAJOR SUBUNIT  *FORMATE DEHYDROGENASE N FROM E. COLI* | 0.02 |  | 11.11 | 0.08 | 121-175 | X-ray | 1.60 | hetero-oligomer | 3 x 6MO, 15 x SF4, 6 x MGD, 6 x HEM, 3 x CDL | HHblits | 0.26 |
| ``` target    LARDIAKVPGTTLFAIGMGPNQFFNNDNKDRTQFLLAALTGNIGKIAGNIGSYAGNYRVAMFNGVPQYIAENPFDIELDG 1kqf.1    --------------------------------------------------------------------------------  target    AKPARPKLYWRAEPAHYYNHEDHPLKMGKTMITGKTHMPTPTKSLWFANANSILGNVKWHFNTVVNVLP-KMEMIAVQEW 1kqf.1    ----------------------------------------NANVVMVMGGNAAEAHPV-GFRWAMEAKNNNDATLIVVDP  target    WWSTSCEWADIVFAVDAWSELKHPDMCSSVTNPFLTVFPRTPLERPFDTRGDIECLDLVGKQLAKRTGDRRFADMWKFVE 1kqf.1    RFTRTASVADIYAPIR----------------------------------------------------------------  target    EKKVEVYLQRILDHSSNTKGFKFPELEEKAKKGIPALMMTRTNPKTVGYEQVYDSRPWYTKTGRLEFYREEDEFIEAGEN 1kqf.1    --------------------------------------------------------------------------------  target    LPVHREPIDSTFYEPNVIVAPAHPFIKAKGPEAYGVKVDDFDNETRQGRNIVKTWEETKKTVHPLAKDGYKFVFHTPKYR 1kqf.1    --------------------------------------------------------------------------------  target    HGAHTMPVDTDMVAMLFGPFGDIYRHDKRQPFAAEGYVDIHPDDAKALNIEDGDYVWIDSDPSDRPFRGWQKNDKDYKFS 1kqf.1    --------------------------------------------------------------------------------  target    RLLCRARYYPGTPRGITRMWFNMYGATPGSVEGHESRKDGLAKNPRTGYQAMFRSGSHQSATRGWLKPTWMTDSLVRKEL 1kqf.1    --------------------------------------------------------------------------------  target    FGHAVNKGFLPDVHCPTGAPREAIVKITKAEPGGLNAKGLWRPAALGLRPKYENDKMKDYLAGKFTLAANPKKGGKK 1kqf.1    ----------------------------------------------------------------------------- ``` | | | | | | | | | | | | | | | | | | | | | | | | | | | | | | | | | | | | | | | | | | | | | | | | | |
|  | 1e18.1.A | DMSO REDUCTASE.  *TUNGSTEN-SUSBSTITUTED DMSO REDUCTASE FROM RHODOBACTER CAPSULATUS* | 0.01 |  | 14.55 | 0.09 | 121-175 | X-ray | 2.00 | monomer | 2 x PGD, 1 x 6WO | HHblits | 0.25 |
| ``` target    LARDIAKVPGTTLFAIGMGPNQFFNNDNKDRTQFLLAALTGNIGKIAGNIGSYAGNYRVAMFNGVPQYIAENPFDIELDG 1e18.1    --------------------------------------------------------------------------------  target    AKPARPKLYWRAEPAHYYNHEDHPLKMGKTMITGKTHMPTPTKSLWFANANSILGNVKWH------FNTVVNVLP-KMEM 1e18.1    ----------------------------------------NTEVMVFWAADPIKTSQIGWVIPEHGAYPGLEALKAKGTK  target    IAVQEWWWSTSCEW-ADIVFAVDAWSELKHPDMCSSVTNPFLTVFPRTPLERPFDTRGDIECLDLVGKQLAKRTGDRRFA 1e18.1    VIVIDPVRTKTVEFFGAEHITPK---------------------------------------------------------  target    DMWKFVEEKKVEVYLQRILDHSSNTKGFKFPELEEKAKKGIPALMMTRTNPKTVGYEQVYDSRPWYTKTGRLEFYREEDE 1e18.1    --------------------------------------------------------------------------------  target    FIEAGENLPVHREPIDSTFYEPNVIVAPAHPFIKAKGPEAYGVKVDDFDNETRQGRNIVKTWEETKKTVHPLAKDGYKFV 1e18.1    --------------------------------------------------------------------------------  target    FHTPKYRHGAHTMPVDTDMVAMLFGPFGDIYRHDKRQPFAAEGYVDIHPDDAKALNIEDGDYVWIDSDPSDRPFRGWQKN 1e18.1    --------------------------------------------------------------------------------  target    DKDYKFSRLLCRARYYPGTPRGITRMWFNMYGATPGSVEGHESRKDGLAKNPRTGYQAMFRSGSHQSATRGWLKPTWMTD 1e18.1    --------------------------------------------------------------------------------  target    SLVRKELFGHAVNKGFLPDVHCPTGAPREAIVKITKAEPGGLNAKGLWRPAALGLRPKYENDKMKDYLAGKFTLAANPKK 1e18.1    --------------------------------------------------------------------------------  target    GGKK 1e18.1    ---- ``` | | | | | | | | | | | | | | | | | | | | | | | | | | | | | | | | | | | | | | | | | | | | | | | | | |
|  | 1e60.1.A | Dimethyl sulfoxide/trimethylamine N-oxide reductase  *OXIDIZED DMSO REDUCTASE EXPOSED TO HEPES - Structure II BUFFER* | 0.01 |  | 14.55 | 0.09 | 121-175 | X-ray | 2.00 | monomer | 2 x PGD, 1 x 2MO | HHblits | 0.25 |
| ``` target    LARDIAKVPGTTLFAIGMGPNQFFNNDNKDRTQFLLAALTGNIGKIAGNIGSYAGNYRVAMFNGVPQYIAENPFDIELDG 1e60.1    --------------------------------------------------------------------------------  target    AKPARPKLYWRAEPAHYYNHEDHPLKMGKTMITGKTHMPTPTKSLWFANANSILGNVKWH------FNTVVNVL-PKMEM 1e60.1    ----------------------------------------NTEVMVFWAADPIKTSQIGWVIPEHGAYPGLEALKAKGTK  target    IAVQEWWWSTSCEW-ADIVFAVDAWSELKHPDMCSSVTNPFLTVFPRTPLERPFDTRGDIECLDLVGKQLAKRTGDRRFA 1e60.1    VIVIDPVRTKTVEFFGAEHITPK---------------------------------------------------------  target    DMWKFVEEKKVEVYLQRILDHSSNTKGFKFPELEEKAKKGIPALMMTRTNPKTVGYEQVYDSRPWYTKTGRLEFYREEDE 1e60.1    --------------------------------------------------------------------------------  target    FIEAGENLPVHREPIDSTFYEPNVIVAPAHPFIKAKGPEAYGVKVDDFDNETRQGRNIVKTWEETKKTVHPLAKDGYKFV 1e60.1    --------------------------------------------------------------------------------  target    FHTPKYRHGAHTMPVDTDMVAMLFGPFGDIYRHDKRQPFAAEGYVDIHPDDAKALNIEDGDYVWIDSDPSDRPFRGWQKN 1e60.1    --------------------------------------------------------------------------------  target    DKDYKFSRLLCRARYYPGTPRGITRMWFNMYGATPGSVEGHESRKDGLAKNPRTGYQAMFRSGSHQSATRGWLKPTWMTD 1e60.1    --------------------------------------------------------------------------------  target    SLVRKELFGHAVNKGFLPDVHCPTGAPREAIVKITKAEPGGLNAKGLWRPAALGLRPKYENDKMKDYLAGKFTLAANPKK 1e60.1    --------------------------------------------------------------------------------  target    GGKK 1e60.1    ---- ``` | | | | | | | | | | | | | | | | | | | | | | | | | | | | | | | | | | | | | | | | | | | | | | | | | |
|  | 4dmr.1.A | DMSO REDUCTASE  *REDUCED DMSO REDUCTASE FROM RHODOBACTER CAPSULATUS WITH BOUND DMSO SUBSTRATE* | 0.01 |  | 14.55 | 0.09 | 121-175 | X-ray | 1.90 | monomer | 2 x PGD, 1 x 4MO, 1 x O | HHblits | 0.25 |
| ``` target    LARDIAKVPGTTLFAIGMGPNQFFNNDNKDRTQFLLAALTGNIGKIAGNIGSYAGNYRVAMFNGVPQYIAENPFDIELDG 4dmr.1    --------------------------------------------------------------------------------  target    AKPARPKLYWRAEPAHYYNHEDHPLKMGKTMITGKTHMPTPTKSLWFANANSILGNVKWH------FNTVVNVL-PKMEM 4dmr.1    ----------------------------------------NTEVMVFWAADPIKTSQIGWVIPEHGAYPGLEALKAKGTK  target    IAVQEWWWSTSCEW-ADIVFAVDAWSELKHPDMCSSVTNPFLTVFPRTPLERPFDTRGDIECLDLVGKQLAKRTGDRRFA 4dmr.1    VIVIDPVRTKTVEFFGAEHITPK---------------------------------------------------------  target    DMWKFVEEKKVEVYLQRILDHSSNTKGFKFPELEEKAKKGIPALMMTRTNPKTVGYEQVYDSRPWYTKTGRLEFYREEDE 4dmr.1    --------------------------------------------------------------------------------  target    FIEAGENLPVHREPIDSTFYEPNVIVAPAHPFIKAKGPEAYGVKVDDFDNETRQGRNIVKTWEETKKTVHPLAKDGYKFV 4dmr.1    --------------------------------------------------------------------------------  target    FHTPKYRHGAHTMPVDTDMVAMLFGPFGDIYRHDKRQPFAAEGYVDIHPDDAKALNIEDGDYVWIDSDPSDRPFRGWQKN 4dmr.1    --------------------------------------------------------------------------------  target    DKDYKFSRLLCRARYYPGTPRGITRMWFNMYGATPGSVEGHESRKDGLAKNPRTGYQAMFRSGSHQSATRGWLKPTWMTD 4dmr.1    --------------------------------------------------------------------------------  target    SLVRKELFGHAVNKGFLPDVHCPTGAPREAIVKITKAEPGGLNAKGLWRPAALGLRPKYENDKMKDYLAGKFTLAANPKK 4dmr.1    --------------------------------------------------------------------------------  target    GGKK 4dmr.1    ---- ``` | | | | | | | | | | | | | | | | | | | | | | | | | | | | | | | | | | | | | | | | | | | | | | | | | |
|  | 1e5v.2.A | Dimethyl sulfoxide/trimethylamine N-oxide reductase  *OXIDIZED DMSO REDUCTASE EXPOSED TO HEPES BUFFER* | 0.01 |  | 14.55 | 0.09 | 121-175 | X-ray | 2.40 | monomer | 2 x PGD, 1 x 2MO | HHblits | 0.25 |
| ``` target    LARDIAKVPGTTLFAIGMGPNQFFNNDNKDRTQFLLAALTGNIGKIAGNIGSYAGNYRVAMFNGVPQYIAENPFDIELDG 1e5v.2    --------------------------------------------------------------------------------  target    AKPARPKLYWRAEPAHYYNHEDHPLKMGKTMITGKTHMPTPTKSLWFANANSILGNVKWH------FNTVVNVL-PKMEM 1e5v.2    ----------------------------------------NTEVMVFWAADPIKTSQIGWVIPEHGAYPGLEALKAKGTK  target    IAVQEWWWSTSCEW-ADIVFAVDAWSELKHPDMCSSVTNPFLTVFPRTPLERPFDTRGDIECLDLVGKQLAKRTGDRRFA 1e5v.2    VIVIDPVRTKTVEFFGAEHITPK---------------------------------------------------------  target    DMWKFVEEKKVEVYLQRILDHSSNTKGFKFPELEEKAKKGIPALMMTRTNPKTVGYEQVYDSRPWYTKTGRLEFYREEDE 1e5v.2    --------------------------------------------------------------------------------  target    FIEAGENLPVHREPIDSTFYEPNVIVAPAHPFIKAKGPEAYGVKVDDFDNETRQGRNIVKTWEETKKTVHPLAKDGYKFV 1e5v.2    --------------------------------------------------------------------------------  target    FHTPKYRHGAHTMPVDTDMVAMLFGPFGDIYRHDKRQPFAAEGYVDIHPDDAKALNIEDGDYVWIDSDPSDRPFRGWQKN 1e5v.2    --------------------------------------------------------------------------------  target    DKDYKFSRLLCRARYYPGTPRGITRMWFNMYGATPGSVEGHESRKDGLAKNPRTGYQAMFRSGSHQSATRGWLKPTWMTD 1e5v.2    --------------------------------------------------------------------------------  target    SLVRKELFGHAVNKGFLPDVHCPTGAPREAIVKITKAEPGGLNAKGLWRPAALGLRPKYENDKMKDYLAGKFTLAANPKK 1e5v.2    --------------------------------------------------------------------------------  target    GGKK 1e5v.2    ---- ``` | | | | | | | | | | | | | | | | | | | | | | | | | | | | | | | | | | | | | | | | | | | | | | | | | |
|  | 1dms.1.A | DMSO REDUCTASE  *STRUCTURE OF DMSO REDUCTASE* | 0.01 |  | 14.55 | 0.09 | 121-175 | X-ray | 1.88 | monomer | 2 x PGD, 1 x 2MO | HHblits | 0.25 |
| ``` target    LARDIAKVPGTTLFAIGMGPNQFFNNDNKDRTQFLLAALTGNIGKIAGNIGSYAGNYRVAMFNGVPQYIAENPFDIELDG 1dms.1    --------------------------------------------------------------------------------  target    AKPARPKLYWRAEPAHYYNHEDHPLKMGKTMITGKTHMPTPTKSLWFANANSILGNVKWH------FNTVVNVLP-KMEM 1dms.1    ----------------------------------------NTEVMVFWAADPIKTSQIGWVIPEHGAYPGLEALKAKGTK  target    IAVQEWWWSTSCEW-ADIVFAVDAWSELKHPDMCSSVTNPFLTVFPRTPLERPFDTRGDIECLDLVGKQLAKRTGDRRFA 1dms.1    VIVIDPVRTKTVEFFGADHVTPK---------------------------------------------------------  target    DMWKFVEEKKVEVYLQRILDHSSNTKGFKFPELEEKAKKGIPALMMTRTNPKTVGYEQVYDSRPWYTKTGRLEFYREEDE 1dms.1    --------------------------------------------------------------------------------  target    FIEAGENLPVHREPIDSTFYEPNVIVAPAHPFIKAKGPEAYGVKVDDFDNETRQGRNIVKTWEETKKTVHPLAKDGYKFV 1dms.1    --------------------------------------------------------------------------------  target    FHTPKYRHGAHTMPVDTDMVAMLFGPFGDIYRHDKRQPFAAEGYVDIHPDDAKALNIEDGDYVWIDSDPSDRPFRGWQKN 1dms.1    --------------------------------------------------------------------------------  target    DKDYKFSRLLCRARYYPGTPRGITRMWFNMYGATPGSVEGHESRKDGLAKNPRTGYQAMFRSGSHQSATRGWLKPTWMTD 1dms.1    --------------------------------------------------------------------------------  target    SLVRKELFGHAVNKGFLPDVHCPTGAPREAIVKITKAEPGGLNAKGLWRPAALGLRPKYENDKMKDYLAGKFTLAANPKK 1dms.1    --------------------------------------------------------------------------------  target    GGKK 1dms.1    ---- ``` | | | | | | | | | | | | | | | | | | | | | | | | | | | | | | | | | | | | | | | | | | | | | | | | | |
|  | 7qv7.1.L | Hydrogen dependent carbon dioxide reductase subunit FdhF  *Cryo-EM structure of Hydrogen-dependent CO2 reductase.* | 0.02 |  | 12.96 | 0.08 | 121-175 | EM | 0.00 | hetero-2-6-6-2-mer | 52 x SF4, 6 x 402 | HHblits | 0.26 |
| ``` target    LARDIAKVPGTTLFAIGMGPNQFFNNDNKDRTQFLLAALTGNIGKIAGNIGSYAGNYRVAMFNGVPQYIAENPFDIELDG 7qv7.1    --------------------------------------------------------------------------------  target    AKPARPKLYWRAEPAHYYNHEDHPLKMGKTMITGKTHMPTPTKSLWFANANSILGNVKWHFNTVVNVLPKMEMIAVQEWW 7qv7.1    ----------------------------------------YSDVIFIIGSNTAECHPL-IAAHVIKAKERGAKLIVADPR  target    WSTSCEWADIVFAVDAWSELKHPDMCSSVTNPFLTVFPRTPLERPFDTRGDIECLDLVGKQLAKRTGDRRFADMWKFVEE 7qv7.1    MNAMVHKADIWLRVP-----------------------------------------------------------------  target    KKVEVYLQRILDHSSNTKGFKFPELEEKAKKGIPALMMTRTNPKTVGYEQVYDSRPWYTKTGRLEFYREEDEFIEAGENL 7qv7.1    --------------------------------------------------------------------------------  target    PVHREPIDSTFYEPNVIVAPAHPFIKAKGPEAYGVKVDDFDNETRQGRNIVKTWEETKKTVHPLAKDGYKFVFHTPKYRH 7qv7.1    --------------------------------------------------------------------------------  target    GAHTMPVDTDMVAMLFGPFGDIYRHDKRQPFAAEGYVDIHPDDAKALNIEDGDYVWIDSDPSDRPFRGWQKNDKDYKFSR 7qv7.1    --------------------------------------------------------------------------------  target    LLCRARYYPGTPRGITRMWFNMYGATPGSVEGHESRKDGLAKNPRTGYQAMFRSGSHQSATRGWLKPTWMTDSLVRKELF 7qv7.1    --------------------------------------------------------------------------------  target    GHAVNKGFLPDVHCPTGAPREAIVKITKAEPGGLNAKGLWRPAALGLRPKYENDKMKDYLAGKFTLAANPKKGGKK 7qv7.1    ---------------------------------------------------------------------------- ``` | | | | | | | | | | | | | | | | | | | | | | | | | | | | | | | | | | | | | | | | | | | | | | | | | |
|  | 7qv7.1.O | Hydrogen dependent carbon dioxide reductase subunit FdhF  *Cryo-EM structure of Hydrogen-dependent CO2 reductase.* | 0.02 |  | 12.96 | 0.08 | 121-175 | EM | 0.00 | hetero-2-6-6-2-mer | 52 x SF4, 6 x 402 | HHblits | 0.26 |
| ``` target    LARDIAKVPGTTLFAIGMGPNQFFNNDNKDRTQFLLAALTGNIGKIAGNIGSYAGNYRVAMFNGVPQYIAENPFDIELDG 7qv7.1    --------------------------------------------------------------------------------  target    AKPARPKLYWRAEPAHYYNHEDHPLKMGKTMITGKTHMPTPTKSLWFANANSILGNVKWHFNTVVNVLPKMEMIAVQEWW 7qv7.1    ----------------------------------------YSDVIFIIGSNTAECHPL-IAAHVIKAKERGAKLIVADPR  target    WSTSCEWADIVFAVDAWSELKHPDMCSSVTNPFLTVFPRTPLERPFDTRGDIECLDLVGKQLAKRTGDRRFADMWKFVEE 7qv7.1    MNAMVHKADIWLRVP-----------------------------------------------------------------  target    KKVEVYLQRILDHSSNTKGFKFPELEEKAKKGIPALMMTRTNPKTVGYEQVYDSRPWYTKTGRLEFYREEDEFIEAGENL 7qv7.1    --------------------------------------------------------------------------------  target    PVHREPIDSTFYEPNVIVAPAHPFIKAKGPEAYGVKVDDFDNETRQGRNIVKTWEETKKTVHPLAKDGYKFVFHTPKYRH 7qv7.1    --------------------------------------------------------------------------------  target    GAHTMPVDTDMVAMLFGPFGDIYRHDKRQPFAAEGYVDIHPDDAKALNIEDGDYVWIDSDPSDRPFRGWQKNDKDYKFSR 7qv7.1    --------------------------------------------------------------------------------  target    LLCRARYYPGTPRGITRMWFNMYGATPGSVEGHESRKDGLAKNPRTGYQAMFRSGSHQSATRGWLKPTWMTDSLVRKELF 7qv7.1    --------------------------------------------------------------------------------  target    GHAVNKGFLPDVHCPTGAPREAIVKITKAEPGGLNAKGLWRPAALGLRPKYENDKMKDYLAGKFTLAANPKKGGKK 7qv7.1    ---------------------------------------------------------------------------- ``` | | | | | | | | | | | | | | | | | | | | | | | | | | | | | | | | | | | | | | | | | | | | | | | | | |
|  | 7bkb.1.F | Formate dehydrogenase  *Formate dehydrogenase - heterodisulfide reductase - formylmethanofuran dehydrogenase complex from Methanospirillum hungatei (hexameric, composite structure)* | 0.02 |  | 9.26 | 0.08 | 121-175 | EM | 0.00 | hetero-2-2-2-2-2-2-… | 48 x SF4, 4 x FAD, 2 x FES, 4 x 9S8, 4 x ZN, 2 x MO, 4 x MGD | HHblits | 0.26 |
| ``` target    LARDIAKVPGTTLFAIGMGPNQFFNNDNKDRTQFLLAALTGNIGKIAGNIGSYAGNYRVAMFNGVPQYIAENPFDIELDG 7bkb.1    --------------------------------------------------------------------------------  target    AKPARPKLYWRAEPAHYYNHEDHPLKMGKTMITGKTHMPTPTKSLWFANANSILGNVKWHFNTVVNVLPKMEMIAVQEWW 7bkb.1    ----------------------------------------NADLILIWGSNAVEAHPL-AGRRIAQAKKKGIQIIAVDPR  target    WSTSCEWADIVFAVDAWSELKHPDMCSSVTNPFLTVFPRTPLERPFDTRGDIECLDLVGKQLAKRTGDRRFADMWKFVEE 7bkb.1    YTMTARLADTYVRFN-----------------------------------------------------------------  target    KKVEVYLQRILDHSSNTKGFKFPELEEKAKKGIPALMMTRTNPKTVGYEQVYDSRPWYTKTGRLEFYREEDEFIEAGENL 7bkb.1    --------------------------------------------------------------------------------  target    PVHREPIDSTFYEPNVIVAPAHPFIKAKGPEAYGVKVDDFDNETRQGRNIVKTWEETKKTVHPLAKDGYKFVFHTPKYRH 7bkb.1    --------------------------------------------------------------------------------  target    GAHTMPVDTDMVAMLFGPFGDIYRHDKRQPFAAEGYVDIHPDDAKALNIEDGDYVWIDSDPSDRPFRGWQKNDKDYKFSR 7bkb.1    --------------------------------------------------------------------------------  target    LLCRARYYPGTPRGITRMWFNMYGATPGSVEGHESRKDGLAKNPRTGYQAMFRSGSHQSATRGWLKPTWMTDSLVRKELF 7bkb.1    --------------------------------------------------------------------------------  target    GHAVNKGFLPDVHCPTGAPREAIVKITKAEPGGLNAKGLWRPAALGLRPKYENDKMKDYLAGKFTLAANPKKGGKK 7bkb.1    ---------------------------------------------------------------------------- ``` | | | | | | | | | | | | | | | | | | | | | | | | | | | | | | | | | | | | | | | | | | | | | | | | | |
|  | 2v3v.1.A | PERIPLASMIC NITRATE REDUCTASE  *A NEW CATALYTIC MECHANISM OF PERIPLASMIC NITRATE REDUCTASE FROM DESULFOVIBRIO DESULFURICANS ATCC 27774 FROM CRYSTALLOGRAPHIC AND EPR DATA AND BASED ON DETAILED ANALYSIS OF THE SIXTH LIGAND* | 0.02 |  | 12.96 | 0.08 | 121-175 | X-ray | 1.99 | monomer | 1 x SF4, 1 x MO, 2 x MGD, 4 x LCP | HHblits | 0.26 |
| ``` target    LARDIAKVPGTTLFAIGMGPNQFFNNDNKDRTQFLLAALTGNIGKIAGNIGSYAGNYRVAMFNGVPQYIAENPFDIELDG 2v3v.1    --------------------------------------------------------------------------------  target    AKPARPKLYWRAEPAHYYNHEDHPLKMGKTMITGKTHMPTPTKSLWFANANSILGNVKWHFNTVVNVL--PKMEMIAVQE 2v3v.1    ----------------------------------------QATCFFIIGSNTSEAHPV-LFRRIARRKQVEPGVKIIVAD  target    WWWSTSCEWADIVFAVDAWSELKHPDMCSSVTNPFLTVFPRTPLERPFDTRGDIECLDLVGKQLAKRTGDRRFADMWKFV 2v3v.1    PRRTNTSRIADMHVAFR---------------------------------------------------------------  target    EEKKVEVYLQRILDHSSNTKGFKFPELEEKAKKGIPALMMTRTNPKTVGYEQVYDSRPWYTKTGRLEFYREEDEFIEAGE 2v3v.1    --------------------------------------------------------------------------------  target    NLPVHREPIDSTFYEPNVIVAPAHPFIKAKGPEAYGVKVDDFDNETRQGRNIVKTWEETKKTVHPLAKDGYKFVFHTPKY 2v3v.1    --------------------------------------------------------------------------------  target    RHGAHTMPVDTDMVAMLFGPFGDIYRHDKRQPFAAEGYVDIHPDDAKALNIEDGDYVWIDSDPSDRPFRGWQKNDKDYKF 2v3v.1    --------------------------------------------------------------------------------  target    SRLLCRARYYPGTPRGITRMWFNMYGATPGSVEGHESRKDGLAKNPRTGYQAMFRSGSHQSATRGWLKPTWMTDSLVRKE 2v3v.1    --------------------------------------------------------------------------------  target    LFGHAVNKGFLPDVHCPTGAPREAIVKITKAEPGGLNAKGLWRPAALGLRPKYENDKMKDYLAGKFTLAANPKKGGKK 2v3v.1    ------------------------------------------------------------------------------ ``` | | | | | | | | | | | | | | | | | | | | | | | | | | | | | | | | | | | | | | | | | | | | | | | | | |
|  | 2vpz.1.A | THIOSULFATE REDUCTASE  *POLYSULFIDE REDUCTASE NATIVE STRUCTURE* | 0.02 |  | 9.09 | 0.09 | 121-175 | X-ray | 2.40 | hetero-oligomer | 10 x SF4, 4 x MGD, 2 x MO | HHblits | 0.24 |
| ``` target    LARDIAKVPGTTLFAIGMGPNQFFNNDNKDRTQFLLAALTGNIGKIAGNIGSYAGNYRVAMFNGVPQYIAENPFDIELDG 2vpz.1    --------------------------------------------------------------------------------  target    AKPARPKLYWRAEPAHYYNHEDHPLKMGKTMITGKTHMPTPTKSLWFANANSILGNVKWHFNTVVNVLPKMEMIAVQEWW 2vpz.1    ----------------------------------------NARYIVLIGHHIGEDTHNTQLQDFALALKNGAKVVVVDPR  target    WSTSCEWADIVFAVDAWSELKHPDMCSSVTNPFLTVFPRTPLERPFDTRGDIECLDLVGKQLAKRTGDRRFADMWKFVEE 2vpz.1    FSTAAAKAHRWLPIK-----------------------------------------------------------------  target    KKVEVYLQRILDHSSNTKGFKFPELEEKAKKGIPALMMTRTNPKTVGYEQVYDSRPWYTKTGRLEFYREEDEFIEAGENL 2vpz.1    --------------------------------------------------------------------------------  target    PVHREPIDSTFYEPNVIVAPAHPFIKAKGPEAYGVKVDDFDNETRQGRNIVKTWEETKKTVHPLAKDGYKFVFHTPKYRH 2vpz.1    --------------------------------------------------------------------------------  target    GAHTMPVDTDMVAMLFGPFGDIYRHDKRQPFAAEGYVDIHPDDAKALNIEDGDYVWIDSDPSDRPFRGWQKNDKDYKFSR 2vpz.1    --------------------------------------------------------------------------------  target    LLCRARYYPGTPRGITRMWFNMYGATPGSVEGHESRKDGLAKNPRTGYQAMFRSGSHQSATRGWLKPTWMTDSLVRKELF 2vpz.1    --------------------------------------------------------------------------------  target    GHAVNKGFLPDVHCPTGAPREAIVKITKAEPGGLNAKGLWRPAALGLRPKYENDKMKDYLAGKFTLAANPKKGGKK 2vpz.1    ---------------------------------------------------------------------------- ``` | | | | | | | | | | | | | | | | | | | | | | | | | | | | | | | | | | | | | | | | | | | | | | | | | |
|  | 2vpx.1.D | THIOSULFATE REDUCTASE  *POLYSULFIDE REDUCTASE WITH BOUND QUINONE (UQ1)* | 0.02 |  | 9.09 | 0.09 | 121-175 | X-ray | 3.10 | hetero-oligomer | 10 x SF4, 4 x MGD, 2 x MO, 2 x UQ1 | HHblits | 0.24 |
| ``` target    LARDIAKVPGTTLFAIGMGPNQFFNNDNKDRTQFLLAALTGNIGKIAGNIGSYAGNYRVAMFNGVPQYIAENPFDIELDG 2vpx.1    --------------------------------------------------------------------------------  target    AKPARPKLYWRAEPAHYYNHEDHPLKMGKTMITGKTHMPTPTKSLWFANANSILGNVKWHFNTVVNVLPKMEMIAVQEWW 2vpx.1    ----------------------------------------NARYIVLIGHHIGEDTHNTQLQDFALALKNGAKVVVVDPR  target    WSTSCEWADIVFAVDAWSELKHPDMCSSVTNPFLTVFPRTPLERPFDTRGDIECLDLVGKQLAKRTGDRRFADMWKFVEE 2vpx.1    FSTAAAKAHRWLPIK-----------------------------------------------------------------  target    KKVEVYLQRILDHSSNTKGFKFPELEEKAKKGIPALMMTRTNPKTVGYEQVYDSRPWYTKTGRLEFYREEDEFIEAGENL 2vpx.1    --------------------------------------------------------------------------------  target    PVHREPIDSTFYEPNVIVAPAHPFIKAKGPEAYGVKVDDFDNETRQGRNIVKTWEETKKTVHPLAKDGYKFVFHTPKYRH 2vpx.1    --------------------------------------------------------------------------------  target    GAHTMPVDTDMVAMLFGPFGDIYRHDKRQPFAAEGYVDIHPDDAKALNIEDGDYVWIDSDPSDRPFRGWQKNDKDYKFSR 2vpx.1    --------------------------------------------------------------------------------  target    LLCRARYYPGTPRGITRMWFNMYGATPGSVEGHESRKDGLAKNPRTGYQAMFRSGSHQSATRGWLKPTWMTDSLVRKELF 2vpx.1    --------------------------------------------------------------------------------  target    GHAVNKGFLPDVHCPTGAPREAIVKITKAEPGGLNAKGLWRPAALGLRPKYENDKMKDYLAGKFTLAANPKKGGKK 2vpx.1    ---------------------------------------------------------------------------- ``` | | | | | | | | | | | | | | | | | | | | | | | | | | | | | | | | | | | | | | | | | | | | | | | | | |
|  | 7l5i.1.A | Trimethylamine-N-oxide reductase  *Crystal Structure of Haemophilus influenzae MtsZ at pH 7.0* | 0.01 |  | 10.91 | 0.09 | 121-175 | X-ray | 1.73 | monomer | 2 x MGD, 1 x MO, 1 x O | HHblits | 0.24 |
| ``` target    LARDIAKVPGTTLFAIGMGPNQFFNNDNKDRTQFLLAALTGNIGKIAGNIGSYAGNYRVAMFNGVPQYIAENPFDIELDG 7l5i.1    --------------------------------------------------------------------------------  target    AKPARPKLYWRAEPAHYYNHEDHPLKMGKTMITGKTHMPTPTKSLWFANANSILGNVKW-------HFNTVVNVLPKMEM 7l5i.1    ----------------------------------------SSDIIVLWSANPLTTMRIAWMSTDQKGIEYFKKFQASGKR  target    IAVQEWWWSTSCEW-ADIVFAVDAWSELKHPDMCSSVTNPFLTVFPRTPLERPFDTRGDIECLDLVGKQLAKRTGDRRFA 7l5i.1    IICIDPQKSETCQMLNAEWIPVN---------------------------------------------------------  target    DMWKFVEEKKVEVYLQRILDHSSNTKGFKFPELEEKAKKGIPALMMTRTNPKTVGYEQVYDSRPWYTKTGRLEFYREEDE 7l5i.1    --------------------------------------------------------------------------------  target    FIEAGENLPVHREPIDSTFYEPNVIVAPAHPFIKAKGPEAYGVKVDDFDNETRQGRNIVKTWEETKKTVHPLAKDGYKFV 7l5i.1    --------------------------------------------------------------------------------  target    FHTPKYRHGAHTMPVDTDMVAMLFGPFGDIYRHDKRQPFAAEGYVDIHPDDAKALNIEDGDYVWIDSDPSDRPFRGWQKN 7l5i.1    --------------------------------------------------------------------------------  target    DKDYKFSRLLCRARYYPGTPRGITRMWFNMYGATPGSVEGHESRKDGLAKNPRTGYQAMFRSGSHQSATRGWLKPTWMTD 7l5i.1    --------------------------------------------------------------------------------  target    SLVRKELFGHAVNKGFLPDVHCPTGAPREAIVKITKAEPGGLNAKGLWRPAALGLRPKYENDKMKDYLAGKFTLAANPKK 7l5i.1    --------------------------------------------------------------------------------  target    GGKK 7l5i.1    ---- ``` | | | | | | | | | | | | | | | | | | | | | | | | | | | | | | | | | | | | | | | | | | | | | | | | | |
|  | 7l5s.1.A | Trimethylamine-N-oxide reductase  *Crystal Structure of Haemophilus influenzae MtsZ at pH 5.5* | 0.01 |  | 10.91 | 0.09 | 121-175 | X-ray | 2.09 | monomer | 1 x O, 2 x MGD, 1 x MO | HHblits | 0.24 |
| ``` target    LARDIAKVPGTTLFAIGMGPNQFFNNDNKDRTQFLLAALTGNIGKIAGNIGSYAGNYRVAMFNGVPQYIAENPFDIELDG 7l5s.1    --------------------------------------------------------------------------------  target    AKPARPKLYWRAEPAHYYNHEDHPLKMGKTMITGKTHMPTPTKSLWFANANSILGNVKW-------HFNTVVNVLPKMEM 7l5s.1    ----------------------------------------SSDIIVLWSANPLTTMRIAWMSTDQKGIEYFKKFQASGKR  target    IAVQEWWWSTSCEW-ADIVFAVDAWSELKHPDMCSSVTNPFLTVFPRTPLERPFDTRGDIECLDLVGKQLAKRTGDRRFA 7l5s.1    IICIDPQKSETCQMLNAEWIPVN---------------------------------------------------------  target    DMWKFVEEKKVEVYLQRILDHSSNTKGFKFPELEEKAKKGIPALMMTRTNPKTVGYEQVYDSRPWYTKTGRLEFYREEDE 7l5s.1    --------------------------------------------------------------------------------  target    FIEAGENLPVHREPIDSTFYEPNVIVAPAHPFIKAKGPEAYGVKVDDFDNETRQGRNIVKTWEETKKTVHPLAKDGYKFV 7l5s.1    --------------------------------------------------------------------------------  target    FHTPKYRHGAHTMPVDTDMVAMLFGPFGDIYRHDKRQPFAAEGYVDIHPDDAKALNIEDGDYVWIDSDPSDRPFRGWQKN 7l5s.1    --------------------------------------------------------------------------------  target    DKDYKFSRLLCRARYYPGTPRGITRMWFNMYGATPGSVEGHESRKDGLAKNPRTGYQAMFRSGSHQSATRGWLKPTWMTD 7l5s.1    --------------------------------------------------------------------------------  target    SLVRKELFGHAVNKGFLPDVHCPTGAPREAIVKITKAEPGGLNAKGLWRPAALGLRPKYENDKMKDYLAGKFTLAANPKK 7l5s.1    --------------------------------------------------------------------------------  target    GGKK 7l5s.1    ---- ``` | | | | | | | | | | | | | | | | | | | | | | | | | | | | | | | | | | | | | | | | | | | | | | | | | |
|  | 4v4c.1.A | Pyrogallol hydroxytransferase large subunit  *Crystal Structure of Pyrogallol-Phloroglucinol Transhydroxylase from Pelobacter acidigallici* | 0.01 |  | 9.09 | 0.09 | 121-175 | X-ray | 2.35 | hetero-oligomer | 2 x CA, 2 x MGD, 1 x 4MO, 3 x SF4 | HHblits | 0.24 |
| ``` target    LARDIAKVPGTTLFAIGMGPNQFFNNDNKDRTQFLLAALTGNIGKIAGNIGSYAGNYRVAMFNGVPQYIAENPFDIELDG 4v4c.1    --------------------------------------------------------------------------------  target    AKPARPKLYWRAEPAHYYNHEDHPLKMGKTMITGKTHMPTPTKSLWFANANSILGNVKWHF--NTVV-NVL-PKMEMIAV 4v4c.1    ----------------------------------------HAEMIVFWSSDPETNSGIYAGFESNIRRQWLKDLGVDFVF  target    QEWWWSTSCE-WADIVFAVDAWSELKHPDMCSSVTNPFLTVFPRTPLERPFDTRGDIECLDLVGKQLAKRTGDRRFADMW 4v4c.1    IDPHMNHTARLVADKWFSPK------------------------------------------------------------  target    KFVEEKKVEVYLQRILDHSSNTKGFKFPELEEKAKKGIPALMMTRTNPKTVGYEQVYDSRPWYTKTGRLEFYREEDEFIE 4v4c.1    --------------------------------------------------------------------------------  target    AGENLPVHREPIDSTFYEPNVIVAPAHPFIKAKGPEAYGVKVDDFDNETRQGRNIVKTWEETKKTVHPLAKDGYKFVFHT 4v4c.1    --------------------------------------------------------------------------------  target    PKYRHGAHTMPVDTDMVAMLFGPFGDIYRHDKRQPFAAEGYVDIHPDDAKALNIEDGDYVWIDSDPSDRPFRGWQKNDKD 4v4c.1    --------------------------------------------------------------------------------  target    YKFSRLLCRARYYPGTPRGITRMWFNMYGATPGSVEGHESRKDGLAKNPRTGYQAMFRSGSHQSATRGWLKPTWMTDSLV 4v4c.1    --------------------------------------------------------------------------------  target    RKELFGHAVNKGFLPDVHCPTGAPREAIVKITKAEPGGLNAKGLWRPAALGLRPKYENDKMKDYLAGKFTLAANPKKGGK 4v4c.1    --------------------------------------------------------------------------------  target    K 4v4c.1    - ``` | | | | | | | | | | | | | | | | | | | | | | | | | | | | | | | | | | | | | | | | | | | | | | | | | |
|  | 2ivf.1.A | ETHYLBENZENE DEHYDROGENASE ALPHA-SUBUNIT  *ETHYLBENZENE DEHYDROGENASE FROM AROMATOLEUM AROMATICUM* | 0.02 |  | 11.11 | 0.08 | 121-175 | X-ray | 1.88 | hetero-oligomer | 1 x MES, 4 x SF4, 1 x MO, 1 x MGD, 1 x MD1, 1 x F3S, 1 x HEM | HHblits | 0.25 |
| ``` target    LARDIAKVPGTTLFAIGMGPNQFFNNDNKDRTQFLLAALTGNIGKIAGNIGSYAGNYRVAMFNGVPQYIAENPFDIELDG 2ivf.1    --------------------------------------------------------------------------------  target    AKPARPKLYWRAEPAHYYNHEDHPLKMGKTMITGKTHMPTPTKSLWFANANSILGNVKWHFNTVVNVLPKMEMIAVQEWW 2ivf.1    ----------------------------------------DAELIFMTCSNWSYTYPS-SYHFLSEARYKGAEVVVIAPD  target    WSTSCEWADIVFAVDAWSELKHPDMCSSVTNPFLTVFPRTPLERPFDTRGDIECLDLVGKQLAKRTGDRRFADMWKFVEE 2ivf.1    FNPTTPAADLHVPVR-----------------------------------------------------------------  target    KKVEVYLQRILDHSSNTKGFKFPELEEKAKKGIPALMMTRTNPKTVGYEQVYDSRPWYTKTGRLEFYREEDEFIEAGENL 2ivf.1    --------------------------------------------------------------------------------  target    PVHREPIDSTFYEPNVIVAPAHPFIKAKGPEAYGVKVDDFDNETRQGRNIVKTWEETKKTVHPLAKDGYKFVFHTPKYRH 2ivf.1    --------------------------------------------------------------------------------  target    GAHTMPVDTDMVAMLFGPFGDIYRHDKRQPFAAEGYVDIHPDDAKALNIEDGDYVWIDSDPSDRPFRGWQKNDKDYKFSR 2ivf.1    --------------------------------------------------------------------------------  target    LLCRARYYPGTPRGITRMWFNMYGATPGSVEGHESRKDGLAKNPRTGYQAMFRSGSHQSATRGWLKPTWMTDSLVRKELF 2ivf.1    --------------------------------------------------------------------------------  target    GHAVNKGFLPDVHCPTGAPREAIVKITKAEPGGLNAKGLWRPAALGLRPKYENDKMKDYLAGKFTLAANPKKGGKK 2ivf.1    ---------------------------------------------------------------------------- ``` | | | | | | | | | | | | | | | | | | | | | | | | | | | | | | | | | | | | | | | | | | | | | | | | | |
|  | 7e5z.1.A | Formate dehydrogenase  *Dehydrogenase holoenzyme* | 0.01 |  | 11.11 | 0.08 | 121-175 | EM | 0.00 | hetero-1-1-mer | 1 x W, 2 x MGD, 2 x FES, 4 x SF4, 1 x FMN | HHblits | 0.25 |
| ``` target    LARDIAKVPGTTLFAIGMGPNQFFNNDNKDRTQFLLAALTGNIGKIAGNIGSYAGNYRVAMFNGVPQYIAENPFDIELDG 7e5z.1    --------------------------------------------------------------------------------  target    AKPARPKLYWRAEPAHYYNHEDHPLKMGKTMITGKTHMPTPTKSLWFANANSILGNVKWHFNTVVNVLPK-MEMIAVQEW 7e5z.1    ----------------------------------------DAEVIVVIGANPTVNHPV-AATFLKNAVKQRGAKLIIMDP  target    WWSTSCEWADIVFAVDAWSELKHPDMCSSVTNPFLTVFPRTPLERPFDTRGDIECLDLVGKQLAKRTGDRRFADMWKFVE 7e5z.1    RRQTLSRHAYRHLAFR----------------------------------------------------------------  target    EKKVEVYLQRILDHSSNTKGFKFPELEEKAKKGIPALMMTRTNPKTVGYEQVYDSRPWYTKTGRLEFYREEDEFIEAGEN 7e5z.1    --------------------------------------------------------------------------------  target    LPVHREPIDSTFYEPNVIVAPAHPFIKAKGPEAYGVKVDDFDNETRQGRNIVKTWEETKKTVHPLAKDGYKFVFHTPKYR 7e5z.1    --------------------------------------------------------------------------------  target    HGAHTMPVDTDMVAMLFGPFGDIYRHDKRQPFAAEGYVDIHPDDAKALNIEDGDYVWIDSDPSDRPFRGWQKNDKDYKFS 7e5z.1    --------------------------------------------------------------------------------  target    RLLCRARYYPGTPRGITRMWFNMYGATPGSVEGHESRKDGLAKNPRTGYQAMFRSGSHQSATRGWLKPTWMTDSLVRKEL 7e5z.1    --------------------------------------------------------------------------------  target    FGHAVNKGFLPDVHCPTGAPREAIVKITKAEPGGLNAKGLWRPAALGLRPKYENDKMKDYLAGKFTLAANPKKGGKK 7e5z.1    ----------------------------------------------------------------------------- ``` | | | | | | | | | | | | | | | | | | | | | | | | | | | | | | | | | | | | | | | | | | | | | | | | | |
|  | 7vw6.1.A | Formate dehydrogenase  *Cryo-EM Structure of Formate Dehydrogenase 1 from Methylorubrum extorquens AM1* | 0.01 |  | 11.11 | 0.08 | 121-175 | EM | 0.00 | hetero-1-1-mer | 4 x SF4, 2 x FES, 2 x MGD, 1 x W, 1 x FMN | HHblits | 0.25 |
| ``` target    LARDIAKVPGTTLFAIGMGPNQFFNNDNKDRTQFLLAALTGNIGKIAGNIGSYAGNYRVAMFNGVPQYIAENPFDIELDG 7vw6.1    --------------------------------------------------------------------------------  target    AKPARPKLYWRAEPAHYYNHEDHPLKMGKTMITGKTHMPTPTKSLWFANANSILGNVKWHFNTVVNVLPK-MEMIAVQEW 7vw6.1    ----------------------------------------DAEVIVVIGANPTVNHPV-AATFLKNAVKQRGAKLIIMDP  target    WWSTSCEWADIVFAVDAWSELKHPDMCSSVTNPFLTVFPRTPLERPFDTRGDIECLDLVGKQLAKRTGDRRFADMWKFVE 7vw6.1    RRQTLSRHAYRHLAFR----------------------------------------------------------------  target    EKKVEVYLQRILDHSSNTKGFKFPELEEKAKKGIPALMMTRTNPKTVGYEQVYDSRPWYTKTGRLEFYREEDEFIEAGEN 7vw6.1    --------------------------------------------------------------------------------  target    LPVHREPIDSTFYEPNVIVAPAHPFIKAKGPEAYGVKVDDFDNETRQGRNIVKTWEETKKTVHPLAKDGYKFVFHTPKYR 7vw6.1    --------------------------------------------------------------------------------  target    HGAHTMPVDTDMVAMLFGPFGDIYRHDKRQPFAAEGYVDIHPDDAKALNIEDGDYVWIDSDPSDRPFRGWQKNDKDYKFS 7vw6.1    --------------------------------------------------------------------------------  target    RLLCRARYYPGTPRGITRMWFNMYGATPGSVEGHESRKDGLAKNPRTGYQAMFRSGSHQSATRGWLKPTWMTDSLVRKEL 7vw6.1    --------------------------------------------------------------------------------  target    FGHAVNKGFLPDVHCPTGAPREAIVKITKAEPGGLNAKGLWRPAALGLRPKYENDKMKDYLAGKFTLAANPKKGGKK 7vw6.1    ----------------------------------------------------------------------------- ``` | | | | | | | | | | | | | | | | | | | | | | | | | | | | | | | | | | | | | | | | | | | | | | | | | |
|  | 1eu1.1.A | DIMETHYL SULFOXIDE REDUCTASE  *THE CRYSTAL STRUCTURE OF RHODOBACTER SPHAEROIDES DIMETHYLSULFOXIDE REDUCTASE REVEALS TWO DISTINCT MOLYBDENUM COORDINATION ENVIRONMENTS.* | 0.01 |  | 10.91 | 0.09 | 121-175 | X-ray | 1.30 | monomer | 3 x GLC, 1 x CD, 2 x MGD, 1 x 6MO, 2 x O | HHblits | 0.24 |
| ``` target    LARDIAKVPGTTLFAIGMGPNQFFNNDNKDRTQFLLAALTGNIGKIAGNIGSYAGNYRVAMFNGVPQYIAENPFDIELDG 1eu1.1    --------------------------------------------------------------------------------  target    AKPARPKLYWRAEPAHYYNHEDHPLKMGKTMITGKTHMPTPTKSLWFANANSILGNVKWH------F-NTVVNVLPKMEM 1eu1.1    ----------------------------------------NTDLMVFWAADPMKTNEIGWVIPDHGAYAGMKALKEKGTR  target    IAVQEWWWSTSCEWAD-IVFAVDAWSELKHPDMCSSVTNPFLTVFPRTPLERPFDTRGDIECLDLVGKQLAKRTGDRRFA 1eu1.1    VICINPVRTETADYFGADVVSPR---------------------------------------------------------  target    DMWKFVEEKKVEVYLQRILDHSSNTKGFKFPELEEKAKKGIPALMMTRTNPKTVGYEQVYDSRPWYTKTGRLEFYREEDE 1eu1.1    --------------------------------------------------------------------------------  target    FIEAGENLPVHREPIDSTFYEPNVIVAPAHPFIKAKGPEAYGVKVDDFDNETRQGRNIVKTWEETKKTVHPLAKDGYKFV 1eu1.1    --------------------------------------------------------------------------------  target    FHTPKYRHGAHTMPVDTDMVAMLFGPFGDIYRHDKRQPFAAEGYVDIHPDDAKALNIEDGDYVWIDSDPSDRPFRGWQKN 1eu1.1    --------------------------------------------------------------------------------  target    DKDYKFSRLLCRARYYPGTPRGITRMWFNMYGATPGSVEGHESRKDGLAKNPRTGYQAMFRSGSHQSATRGWLKPTWMTD 1eu1.1    --------------------------------------------------------------------------------  target    SLVRKELFGHAVNKGFLPDVHCPTGAPREAIVKITKAEPGGLNAKGLWRPAALGLRPKYENDKMKDYLAGKFTLAANPKK 1eu1.1    --------------------------------------------------------------------------------  target    GGKK 1eu1.1    ---- ``` | | | | | | | | | | | | | | | | | | | | | | | | | | | | | | | | | | | | | | | | | | | | | | | | | |
|  | 6cz7.1.A | ArrA  *The arsenate respiratory reductase (Arr) complex from Shewanella sp. ANA-3* | 0.01 |  | 14.81 | 0.08 | 121-175 | X-ray | 1.62 | hetero-1-1-mer | 5 x SF4, 2 x MGD, 1 x MO, 1 x PG5 | HHblits | 0.25 |
| ``` target    LARDIAKVPGTTLFAIGMGPNQFFNNDNKDRTQFLLAALTGNIGKIAGNIGSYAGNYRVAMFNGVPQYIAENPFDIELDG 6cz7.1    --------------------------------------------------------------------------------  target    AKPARPKLYWRAEPAHYYNHEDHPLKMGKTMITGKTHMPTPTKSLWFANANSILGNVKWHFNTVVN--VLPKMEMIAVQE 6cz7.1    ----------------------------------------NAKFILSFGADPIASNRQ-VSFYSQTWGDSLDHAKVVVVD  target    WWWSTSCEWADIVFAVDAWSELKHPDMCSSVTNPFLTVFPRTPLERPFDTRGDIECLDLVGKQLAKRTGDRRFADMWKFV 6cz7.1    PRLSASAAKAHKWIPIE---------------------------------------------------------------  target    EEKKVEVYLQRILDHSSNTKGFKFPELEEKAKKGIPALMMTRTNPKTVGYEQVYDSRPWYTKTGRLEFYREEDEFIEAGE 6cz7.1    --------------------------------------------------------------------------------  target    NLPVHREPIDSTFYEPNVIVAPAHPFIKAKGPEAYGVKVDDFDNETRQGRNIVKTWEETKKTVHPLAKDGYKFVFHTPKY 6cz7.1    --------------------------------------------------------------------------------  target    RHGAHTMPVDTDMVAMLFGPFGDIYRHDKRQPFAAEGYVDIHPDDAKALNIEDGDYVWIDSDPSDRPFRGWQKNDKDYKF 6cz7.1    --------------------------------------------------------------------------------  target    SRLLCRARYYPGTPRGITRMWFNMYGATPGSVEGHESRKDGLAKNPRTGYQAMFRSGSHQSATRGWLKPTWMTDSLVRKE 6cz7.1    --------------------------------------------------------------------------------  target    LFGHAVNKGFLPDVHCPTGAPREAIVKITKAEPGGLNAKGLWRPAALGLRPKYENDKMKDYLAGKFTLAANPKKGGKK 6cz7.1    ------------------------------------------------------------------------------ ``` | | | | | | | | | | | | | | | | | | | | | | | | | | | | | | | | | | | | | | | | | | | | | | | | | |
|  | 1ogy.1.A | PERIPLASMIC NITRATE REDUCTASE  *Crystal structure of the heterodimeric nitrate reductase from Rhodobacter sphaeroides* | 0.01 |  | 9.26 | 0.08 | 121-175 | X-ray | 3.20 | hetero-1-1-mer | 1 x SF4, 1 x MO, 2 x MGD, 2 x HEC | HHblits | 0.25 |
| ``` target    LARDIAKVPGTTLFAIGMGPNQFFNNDNKDRTQFLLAALTGNIGKIAGNIGSYAGNYRVAMFNGVPQYIAENPFDIELDG 1ogy.1    --------------------------------------------------------------------------------  target    AKPARPKLYWRAEPAHYYNHEDHPLKMGKTMITGKTHMPTPTKSLWFANANSILGNVKWHFNTVVNVL--PKMEMIAVQE 1ogy.1    ----------------------------------------AADAFVLWGSNMAEMHPI-LWSRLTDRRLSHEHVRVAVLS  target    WWWSTSCEWADIVFAVDAWSELKHPDMCSSVTNPFLTVFPRTPLERPFDTRGDIECLDLVGKQLAKRTGDRRFADMWKFV 1ogy.1    TFTHRSSDLSDTPIIFR---------------------------------------------------------------  target    EEKKVEVYLQRILDHSSNTKGFKFPELEEKAKKGIPALMMTRTNPKTVGYEQVYDSRPWYTKTGRLEFYREEDEFIEAGE 1ogy.1    --------------------------------------------------------------------------------  target    NLPVHREPIDSTFYEPNVIVAPAHPFIKAKGPEAYGVKVDDFDNETRQGRNIVKTWEETKKTVHPLAKDGYKFVFHTPKY 1ogy.1    --------------------------------------------------------------------------------  target    RHGAHTMPVDTDMVAMLFGPFGDIYRHDKRQPFAAEGYVDIHPDDAKALNIEDGDYVWIDSDPSDRPFRGWQKNDKDYKF 1ogy.1    --------------------------------------------------------------------------------  target    SRLLCRARYYPGTPRGITRMWFNMYGATPGSVEGHESRKDGLAKNPRTGYQAMFRSGSHQSATRGWLKPTWMTDSLVRKE 1ogy.1    --------------------------------------------------------------------------------  target    LFGHAVNKGFLPDVHCPTGAPREAIVKITKAEPGGLNAKGLWRPAALGLRPKYENDKMKDYLAGKFTLAANPKKGGKK 1ogy.1    ------------------------------------------------------------------------------ ``` | | | | | | | | | | | | | | | | | | | | | | | | | | | | | | | | | | | | | | | | | | | | | | | | | |
|  | 2pjh.1.B | Transitional endoplasmic reticulum ATPase  *Strctural Model of the p97 N domain- npl4 UBD complex* | 0.02 |  | 15.09 | 0.08 | 434-502 | NMR | 0.00 | hetero-1-1-mer |  | HHblits | 0.26 |
| ``` target    LARDIAKVPGTTLFAIGMGPNQFFNNDNKDRTQFLLAALTGNIGKIAGNIGSYAGNYRVAMFNGVPQYIAENPFDIELDG 2pjh.1    --------------------------------------------------------------------------------  target    AKPARPKLYWRAEPAHYYNHEDHPLKMGKTMITGKTHMPTPTKSLWFANANSILGNVKWHFNTVVNVLPKMEMIAVQEWW 2pjh.1    --------------------------------------------------------------------------------  target    WSTSCEWADIVFAVDAWSELKHPDMCSSVTNPFLTVFPRTPLERPFDTRGDIECLDLVGKQLAKRTGDRRFADMWKFVEE 2pjh.1    --------------------------------------------------------------------------------  target    KKVEVYLQRILDHSSNTKGFKFPELEEKAKKGIPALMMTRTNPKTVGYEQVYDSRPWYTKTGRLEFYREEDEFIEAGENL 2pjh.1    --------------------------------------------------------------------------------  target    PVHREPIDSTFYEPNVIVAPAHPFIKAKGPEAYGVKVDDFDNETRQGRNIVKTWEETKKTVHPLAKDGYKFVFHTPKYRH 2pjh.1    --------------------------------------------------------------------------------  target    GAHTMPVDTDMVAMLFGPFGDIYRHDKRQPFAAEGYVDIHPDDAKALNIEDGDYVWIDSDPSDRPFRGWQKNDKDYKFSR 2pjh.1    ---------------------------------NSVVSLSQPKMDELQLFRGDTVLLKGKKR----------------RE  target    LLCRARYYPGTPRGITRMWFNMYGATPGSVEGHESRKDGLAKNPRTGYQAMFRSGSHQSATRGWLKPTWMTDSLVRKELF 2pjh.1    AVCIVLSDDTCSDEKIRMNRVV----------------------------------------------------------  target    GHAVNKGFLPDVHCPTGAPREAIVKITKAEPGGLNAKGLWRPAALGLRPKYENDKMKDYLAGKFTLAANPKKGGKK 2pjh.1    ---------------------------------------------------------------------------- ``` | | | | | | | | | | | | | | | | | | | | | | | | | | | | | | | | | | | | | | | | | | | | | | | | | |
|  | 5t5i.1.B | Tungsten formylmethanofuran dehydrogenase subunit B  *TUNGSTEN-CONTAINING FORMYLMETHANOFURAN DEHYDROGENASE FROM METHANOTHERMOBACTER WOLFEII, ORTHORHOMBIC FORM AT 1.9 A* | 0.01 |  | 13.21 | 0.08 | 121-175 | X-ray | 1.90 | hetero-oligomer | 4 x ZN, 2 x MG, 18 x K, 22 x SF4, 2 x W, 4 x MGD, 2 x H2S, 2 x CA | HHblits | 0.26 |
| ``` target    LARDIAKVPGTTLFAIGMGPNQFFNNDNKDRTQFLLAALTGNIGKIAGNIGSYAGNYRVAMFNGVPQYIAENPFDIELDG 5t5i.1    --------------------------------------------------------------------------------  target    AKPARPKLYWRAEPAHYYNHEDHPLKMGKTMITGKTHMPTPTKSLWFANANSILGNVKWHFNT--------VVNVLPKME 5t5i.1    ----------------------------------------RADVVVYWGCNPMHAHP--RHMSRNVFARGFFRERGRSDR  target    MIAVQEWWWSTSCEWADIVFAVDAWSELKHPDMCSSVTNPFLTVFPRTPLERPFDTRGDIECLDLVGKQLAKRTGDRRFA 5t5i.1    TLIVVDPRKTDSAKLADIHLQLD---------------------------------------------------------  target    DMWKFVEEKKVEVYLQRILDHSSNTKGFKFPELEEKAKKGIPALMMTRTNPKTVGYEQVYDSRPWYTKTGRLEFYREEDE 5t5i.1    --------------------------------------------------------------------------------  target    FIEAGENLPVHREPIDSTFYEPNVIVAPAHPFIKAKGPEAYGVKVDDFDNETRQGRNIVKTWEETKKTVHPLAKDGYKFV 5t5i.1    --------------------------------------------------------------------------------  target    FHTPKYRHGAHTMPVDTDMVAMLFGPFGDIYRHDKRQPFAAEGYVDIHPDDAKALNIEDGDYVWIDSDPSDRPFRGWQKN 5t5i.1    --------------------------------------------------------------------------------  target    DKDYKFSRLLCRARYYPGTPRGITRMWFNMYGATPGSVEGHESRKDGLAKNPRTGYQAMFRSGSHQSATRGWLKPTWMTD 5t5i.1    --------------------------------------------------------------------------------  target    SLVRKELFGHAVNKGFLPDVHCPTGAPREAIVKITKAEPGGLNAKGLWRPAALGLRPKYENDKMKDYLAGKFTLAANPKK 5t5i.1    --------------------------------------------------------------------------------  target    GGKK 5t5i.1    ---- ``` | | | | | | | | | | | | | | | | | | | | | | | | | | | | | | | | | | | | | | | | | | | | | | | | | |
|  | 7wbb.1.A | AFG2 isoform 1  *Cryo-EM structure of substrate engaged Drg1 hexamer* | 0.02 |  | 20.00 | 0.08 | 433-498 | EM | 0.00 | hetero-6-1-mer | 11 x ATP | HHblits | 0.30 |
| ``` target    LARDIAKVPGTTLFAIGMGPNQFFNNDNKDRTQFLLAALTGNIGKIAGNIGSYAGNYRVAMFNGVPQYIAENPFDIELDG 7wbb.1    --------------------------------------------------------------------------------  target    AKPARPKLYWRAEPAHYYNHEDHPLKMGKTMITGKTHMPTPTKSLWFANANSILGNVKWHFNTVVNVLPKMEMIAVQEWW 7wbb.1    --------------------------------------------------------------------------------  target    WSTSCEWADIVFAVDAWSELKHPDMCSSVTNPFLTVFPRTPLERPFDTRGDIECLDLVGKQLAKRTGDRRFADMWKFVEE 7wbb.1    --------------------------------------------------------------------------------  target    KKVEVYLQRILDHSSNTKGFKFPELEEKAKKGIPALMMTRTNPKTVGYEQVYDSRPWYTKTGRLEFYREEDEFIEAGENL 7wbb.1    --------------------------------------------------------------------------------  target    PVHREPIDSTFYEPNVIVAPAHPFIKAKGPEAYGVKVDDFDNETRQGRNIVKTWEETKKTVHPLAKDGYKFVFHTPKYRH 7wbb.1    --------------------------------------------------------------------------------  target    GAHTMPVDTDMVAMLFGPFGDIYRHDKRQPFAAEGYVDIHPDDAKALNIEDGDYVWIDSDPSDRPFRGWQKNDKDYKFSR 7wbb.1    --------------------------------ETCTAYIHPNVLSSLEINPGSFCTVGKI-G-----E----------NG  target    LLCRARY--YPGTPRGITRMWFNMYGATPGSVEGHESRKDGLAKNPRTGYQAMFRSGSHQSATRGWLKPTWMTDSLVRKE 7wbb.1    ILVIARAGDEEVHPVNVITL------------------------------------------------------------  target    LFGHAVNKGFLPDVHCPTGAPREAIVKITKAEPGGLNAKGLWRPAALGLRPKYENDKMKDYLAGKFTLAANPKKGGKK 7wbb.1    ------------------------------------------------------------------------------ ``` | | | | | | | | | | | | | | | | | | | | | | | | | | | | | | | | | | | | | | | | | | | | | | | | | |
|  | 7wbb.1.B | AFG2 isoform 1  *Cryo-EM structure of substrate engaged Drg1 hexamer* | 0.02 |  | 20.00 | 0.08 | 433-498 | EM | 0.00 | hetero-6-1-mer | 11 x ATP | HHblits | 0.30 |
| ``` target    LARDIAKVPGTTLFAIGMGPNQFFNNDNKDRTQFLLAALTGNIGKIAGNIGSYAGNYRVAMFNGVPQYIAENPFDIELDG 7wbb.1    --------------------------------------------------------------------------------  target    AKPARPKLYWRAEPAHYYNHEDHPLKMGKTMITGKTHMPTPTKSLWFANANSILGNVKWHFNTVVNVLPKMEMIAVQEWW 7wbb.1    --------------------------------------------------------------------------------  target    WSTSCEWADIVFAVDAWSELKHPDMCSSVTNPFLTVFPRTPLERPFDTRGDIECLDLVGKQLAKRTGDRRFADMWKFVEE 7wbb.1    --------------------------------------------------------------------------------  target    KKVEVYLQRILDHSSNTKGFKFPELEEKAKKGIPALMMTRTNPKTVGYEQVYDSRPWYTKTGRLEFYREEDEFIEAGENL 7wbb.1    --------------------------------------------------------------------------------  target    PVHREPIDSTFYEPNVIVAPAHPFIKAKGPEAYGVKVDDFDNETRQGRNIVKTWEETKKTVHPLAKDGYKFVFHTPKYRH 7wbb.1    --------------------------------------------------------------------------------  target    GAHTMPVDTDMVAMLFGPFGDIYRHDKRQPFAAEGYVDIHPDDAKALNIEDGDYVWIDSDPSDRPFRGWQKNDKDYKFSR 7wbb.1    --------------------------------ETCTAYIHPNVLSSLEINPGSFCTVGKI-G-----E----------NG  target    LLCRARY--YPGTPRGITRMWFNMYGATPGSVEGHESRKDGLAKNPRTGYQAMFRSGSHQSATRGWLKPTWMTDSLVRKE 7wbb.1    ILVIARAGDEEVHPVNVITL------------------------------------------------------------  target    LFGHAVNKGFLPDVHCPTGAPREAIVKITKAEPGGLNAKGLWRPAALGLRPKYENDKMKDYLAGKFTLAANPKKGGKK 7wbb.1    ------------------------------------------------------------------------------ ``` | | | | | | | | | | | | | | | | | | | | | | | | | | | | | | | | | | | | | | | | | | | | | | | | | |
|  | 7wbb.1.C | AFG2 isoform 1  *Cryo-EM structure of substrate engaged Drg1 hexamer* | 0.02 |  | 20.00 | 0.08 | 433-498 | EM | 0.00 | hetero-6-1-mer | 11 x ATP | HHblits | 0.30 |
| ``` target    LARDIAKVPGTTLFAIGMGPNQFFNNDNKDRTQFLLAALTGNIGKIAGNIGSYAGNYRVAMFNGVPQYIAENPFDIELDG 7wbb.1    --------------------------------------------------------------------------------  target    AKPARPKLYWRAEPAHYYNHEDHPLKMGKTMITGKTHMPTPTKSLWFANANSILGNVKWHFNTVVNVLPKMEMIAVQEWW 7wbb.1    --------------------------------------------------------------------------------  target    WSTSCEWADIVFAVDAWSELKHPDMCSSVTNPFLTVFPRTPLERPFDTRGDIECLDLVGKQLAKRTGDRRFADMWKFVEE 7wbb.1    --------------------------------------------------------------------------------  target    KKVEVYLQRILDHSSNTKGFKFPELEEKAKKGIPALMMTRTNPKTVGYEQVYDSRPWYTKTGRLEFYREEDEFIEAGENL 7wbb.1    --------------------------------------------------------------------------------  target    PVHREPIDSTFYEPNVIVAPAHPFIKAKGPEAYGVKVDDFDNETRQGRNIVKTWEETKKTVHPLAKDGYKFVFHTPKYRH 7wbb.1    --------------------------------------------------------------------------------  target    GAHTMPVDTDMVAMLFGPFGDIYRHDKRQPFAAEGYVDIHPDDAKALNIEDGDYVWIDSDPSDRPFRGWQKNDKDYKFSR 7wbb.1    --------------------------------ETCTAYIHPNVLSSLEINPGSFCTVGKI-G-----E----------NG  target    LLCRARY--YPGTPRGITRMWFNMYGATPGSVEGHESRKDGLAKNPRTGYQAMFRSGSHQSATRGWLKPTWMTDSLVRKE 7wbb.1    ILVIARAGDEEVHPVNVITL------------------------------------------------------------  target    LFGHAVNKGFLPDVHCPTGAPREAIVKITKAEPGGLNAKGLWRPAALGLRPKYENDKMKDYLAGKFTLAANPKKGGKK 7wbb.1    ------------------------------------------------------------------------------ ``` | | | | | | | | | | | | | | | | | | | | | | | | | | | | | | | | | | | | | | | | | | | | | | | | | |
|  | 7wbb.1.D | AFG2 isoform 1  *Cryo-EM structure of substrate engaged Drg1 hexamer* | 0.03 |  | 20.00 | 0.08 | 433-498 | EM | 0.00 | hetero-6-1-mer | 11 x ATP | HHblits | 0.30 |
| ``` target    LARDIAKVPGTTLFAIGMGPNQFFNNDNKDRTQFLLAALTGNIGKIAGNIGSYAGNYRVAMFNGVPQYIAENPFDIELDG 7wbb.1    --------------------------------------------------------------------------------  target    AKPARPKLYWRAEPAHYYNHEDHPLKMGKTMITGKTHMPTPTKSLWFANANSILGNVKWHFNTVVNVLPKMEMIAVQEWW 7wbb.1    --------------------------------------------------------------------------------  target    WSTSCEWADIVFAVDAWSELKHPDMCSSVTNPFLTVFPRTPLERPFDTRGDIECLDLVGKQLAKRTGDRRFADMWKFVEE 7wbb.1    --------------------------------------------------------------------------------  target    KKVEVYLQRILDHSSNTKGFKFPELEEKAKKGIPALMMTRTNPKTVGYEQVYDSRPWYTKTGRLEFYREEDEFIEAGENL 7wbb.1    --------------------------------------------------------------------------------  target    PVHREPIDSTFYEPNVIVAPAHPFIKAKGPEAYGVKVDDFDNETRQGRNIVKTWEETKKTVHPLAKDGYKFVFHTPKYRH 7wbb.1    --------------------------------------------------------------------------------  target    GAHTMPVDTDMVAMLFGPFGDIYRHDKRQPFAAEGYVDIHPDDAKALNIEDGDYVWIDSDPSDRPFRGWQKNDKDYKFSR 7wbb.1    --------------------------------ETCTAYIHPNVLSSLEINPGSFCTVGKI-G-----E----------NG  target    LLCRARY--YPGTPRGITRMWFNMYGATPGSVEGHESRKDGLAKNPRTGYQAMFRSGSHQSATRGWLKPTWMTDSLVRKE 7wbb.1    ILVIARAGDEEVHPVNVITL------------------------------------------------------------  target    LFGHAVNKGFLPDVHCPTGAPREAIVKITKAEPGGLNAKGLWRPAALGLRPKYENDKMKDYLAGKFTLAANPKKGGKK 7wbb.1    ------------------------------------------------------------------------------ ``` | | | | | | | | | | | | | | | | | | | | | | | | | | | | | | | | | | | | | | | | | | | | | | | | | |
|  | 7wbb.1.E | AFG2 isoform 1  *Cryo-EM structure of substrate engaged Drg1 hexamer* | 0.02 |  | 20.00 | 0.08 | 433-498 | EM | 0.00 | hetero-6-1-mer | 11 x ATP | HHblits | 0.30 |
| ``` target    LARDIAKVPGTTLFAIGMGPNQFFNNDNKDRTQFLLAALTGNIGKIAGNIGSYAGNYRVAMFNGVPQYIAENPFDIELDG 7wbb.1    --------------------------------------------------------------------------------  target    AKPARPKLYWRAEPAHYYNHEDHPLKMGKTMITGKTHMPTPTKSLWFANANSILGNVKWHFNTVVNVLPKMEMIAVQEWW 7wbb.1    --------------------------------------------------------------------------------  target    WSTSCEWADIVFAVDAWSELKHPDMCSSVTNPFLTVFPRTPLERPFDTRGDIECLDLVGKQLAKRTGDRRFADMWKFVEE 7wbb.1    --------------------------------------------------------------------------------  target    KKVEVYLQRILDHSSNTKGFKFPELEEKAKKGIPALMMTRTNPKTVGYEQVYDSRPWYTKTGRLEFYREEDEFIEAGENL 7wbb.1    --------------------------------------------------------------------------------  target    PVHREPIDSTFYEPNVIVAPAHPFIKAKGPEAYGVKVDDFDNETRQGRNIVKTWEETKKTVHPLAKDGYKFVFHTPKYRH 7wbb.1    --------------------------------------------------------------------------------  target    GAHTMPVDTDMVAMLFGPFGDIYRHDKRQPFAAEGYVDIHPDDAKALNIEDGDYVWIDSDPSDRPFRGWQKNDKDYKFSR 7wbb.1    --------------------------------ETCTAYIHPNVLSSLEINPGSFCTVGKI-G-----E----------NG  target    LLCRARY--YPGTPRGITRMWFNMYGATPGSVEGHESRKDGLAKNPRTGYQAMFRSGSHQSATRGWLKPTWMTDSLVRKE 7wbb.1    ILVIARAGDEEVHPVNVITL------------------------------------------------------------  target    LFGHAVNKGFLPDVHCPTGAPREAIVKITKAEPGGLNAKGLWRPAALGLRPKYENDKMKDYLAGKFTLAANPKKGGKK 7wbb.1    ------------------------------------------------------------------------------ ``` | | | | | | | | | | | | | | | | | | | | | | | | | | | | | | | | | | | | | | | | | | | | | | | | | |
|  | 7wbb.1.G | AFG2 isoform 1  *Cryo-EM structure of substrate engaged Drg1 hexamer* | 0.02 |  | 20.00 | 0.08 | 433-498 | EM | 0.00 | hetero-6-1-mer | 11 x ATP | HHblits | 0.30 |
| ``` target    LARDIAKVPGTTLFAIGMGPNQFFNNDNKDRTQFLLAALTGNIGKIAGNIGSYAGNYRVAMFNGVPQYIAENPFDIELDG 7wbb.1    --------------------------------------------------------------------------------  target    AKPARPKLYWRAEPAHYYNHEDHPLKMGKTMITGKTHMPTPTKSLWFANANSILGNVKWHFNTVVNVLPKMEMIAVQEWW 7wbb.1    --------------------------------------------------------------------------------  target    WSTSCEWADIVFAVDAWSELKHPDMCSSVTNPFLTVFPRTPLERPFDTRGDIECLDLVGKQLAKRTGDRRFADMWKFVEE 7wbb.1    --------------------------------------------------------------------------------  target    KKVEVYLQRILDHSSNTKGFKFPELEEKAKKGIPALMMTRTNPKTVGYEQVYDSRPWYTKTGRLEFYREEDEFIEAGENL 7wbb.1    --------------------------------------------------------------------------------  target    PVHREPIDSTFYEPNVIVAPAHPFIKAKGPEAYGVKVDDFDNETRQGRNIVKTWEETKKTVHPLAKDGYKFVFHTPKYRH 7wbb.1    --------------------------------------------------------------------------------  target    GAHTMPVDTDMVAMLFGPFGDIYRHDKRQPFAAEGYVDIHPDDAKALNIEDGDYVWIDSDPSDRPFRGWQKNDKDYKFSR 7wbb.1    --------------------------------ETCTAYIHPNVLSSLEINPGSFCTVGKI-G-----E----------NG  target    LLCRARY--YPGTPRGITRMWFNMYGATPGSVEGHESRKDGLAKNPRTGYQAMFRSGSHQSATRGWLKPTWMTDSLVRKE 7wbb.1    ILVIARAGDEEVHPVNVITL------------------------------------------------------------  target    LFGHAVNKGFLPDVHCPTGAPREAIVKITKAEPGGLNAKGLWRPAALGLRPKYENDKMKDYLAGKFTLAANPKKGGKK 7wbb.1    ------------------------------------------------------------------------------ ``` | | | | | | | | | | | | | | | | | | | | | | | | | | | | | | | | | | | | | | | | | | | | | | | | | |
|  | 3ir7.1.A | Respiratory nitrate reductase 1 alpha chain  *Crystal structure of NarGHI mutant NarG-R94S* | 0.02 |  | 9.26 | 0.08 | 121-175 | X-ray | 2.50 | hetero-1-1-1-mer | 2 x MD1, 4 x SF4, 1 x 6MO, 1 x AGA, 1 x F3S, 2 x HEM | HHblits | 0.23 |
| ``` target    LARDIAKVPGTTLFAIGMGPNQFFNNDNKDRTQFLLAALTGNIGKIAGNIGSYAGNYRVAMFNGVPQYIAENPFDIELDG 3ir7.1    --------------------------------------------------------------------------------  target    AKPARPKLYWRAEPAHYYNHEDHPLKMGKTMITGKTHMPTPTKSLWFANANSILGNVKWHFNTVVNVLPKMEMIAVQEWW 3ir7.1    ----------------------------------------NSSYIIAWGSNVPQTRT-PDAHFFTEVRYKGTKTVAVTPD  target    WSTSCEWADIVFAVDAWSELKHPDMCSSVTNPFLTVFPRTPLERPFDTRGDIECLDLVGKQLAKRTGDRRFADMWKFVEE 3ir7.1    YAEIAKLCDLWLAPK-----------------------------------------------------------------  target    KKVEVYLQRILDHSSNTKGFKFPELEEKAKKGIPALMMTRTNPKTVGYEQVYDSRPWYTKTGRLEFYREEDEFIEAGENL 3ir7.1    --------------------------------------------------------------------------------  target    PVHREPIDSTFYEPNVIVAPAHPFIKAKGPEAYGVKVDDFDNETRQGRNIVKTWEETKKTVHPLAKDGYKFVFHTPKYRH 3ir7.1    --------------------------------------------------------------------------------  target    GAHTMPVDTDMVAMLFGPFGDIYRHDKRQPFAAEGYVDIHPDDAKALNIEDGDYVWIDSDPSDRPFRGWQKNDKDYKFSR 3ir7.1    --------------------------------------------------------------------------------  target    LLCRARYYPGTPRGITRMWFNMYGATPGSVEGHESRKDGLAKNPRTGYQAMFRSGSHQSATRGWLKPTWMTDSLVRKELF 3ir7.1    --------------------------------------------------------------------------------  target    GHAVNKGFLPDVHCPTGAPREAIVKITKAEPGGLNAKGLWRPAALGLRPKYENDKMKDYLAGKFTLAANPKKGGKK 3ir7.1    ---------------------------------------------------------------------------- ``` | | | | | | | | | | | | | | | | | | | | | | | | | | | | | | | | | | | | | | | | | | | | | | | | | |
|  | 3egw.1.A | Respiratory nitrate reductase 1 alpha chain  *The crystal structure of the NarGHI mutant NarH - C16A* | 0.01 |  | 9.26 | 0.08 | 121-175 | X-ray | 1.90 | hetero-2-2-2-mer | 2 x MD1, 2 x MGD, 2 x 6MO, 6 x SF4, 4 x F3S, 2 x 3PH, 4 x HEM, 2 x AGA | HHblits | 0.23 |
| ``` target    LARDIAKVPGTTLFAIGMGPNQFFNNDNKDRTQFLLAALTGNIGKIAGNIGSYAGNYRVAMFNGVPQYIAENPFDIELDG 3egw.1    --------------------------------------------------------------------------------  target    AKPARPKLYWRAEPAHYYNHEDHPLKMGKTMITGKTHMPTPTKSLWFANANSILGNVKWHFNTVVNVLPKMEMIAVQEWW 3egw.1    ----------------------------------------NSSYIIAWGSNVPQTRT-PDAHFFTEVRYKGTKTVAVTPD  target    WSTSCEWADIVFAVDAWSELKHPDMCSSVTNPFLTVFPRTPLERPFDTRGDIECLDLVGKQLAKRTGDRRFADMWKFVEE 3egw.1    YAEIAKLCDLWLAPK-----------------------------------------------------------------  target    KKVEVYLQRILDHSSNTKGFKFPELEEKAKKGIPALMMTRTNPKTVGYEQVYDSRPWYTKTGRLEFYREEDEFIEAGENL 3egw.1    --------------------------------------------------------------------------------  target    PVHREPIDSTFYEPNVIVAPAHPFIKAKGPEAYGVKVDDFDNETRQGRNIVKTWEETKKTVHPLAKDGYKFVFHTPKYRH 3egw.1    --------------------------------------------------------------------------------  target    GAHTMPVDTDMVAMLFGPFGDIYRHDKRQPFAAEGYVDIHPDDAKALNIEDGDYVWIDSDPSDRPFRGWQKNDKDYKFSR 3egw.1    --------------------------------------------------------------------------------  target    LLCRARYYPGTPRGITRMWFNMYGATPGSVEGHESRKDGLAKNPRTGYQAMFRSGSHQSATRGWLKPTWMTDSLVRKELF 3egw.1    --------------------------------------------------------------------------------  target    GHAVNKGFLPDVHCPTGAPREAIVKITKAEPGGLNAKGLWRPAALGLRPKYENDKMKDYLAGKFTLAANPKKGGKK 3egw.1    ---------------------------------------------------------------------------- ``` | | | | | | | | | | | | | | | | | | | | | | | | | | | | | | | | | | | | | | | | | | | | | | | | | |
|  | 1r27.4.A | Respiratory nitrate reductase 1 alpha chain  *Crystal Structure of NarGH complex* | 0.01 |  | 9.26 | 0.08 | 121-175 | X-ray | 2.00 | hetero-4-4-mer | 4 x MO, 16 x SF4, 8 x MGD, 4 x F3S | HHblits | 0.23 |
| ``` target    LARDIAKVPGTTLFAIGMGPNQFFNNDNKDRTQFLLAALTGNIGKIAGNIGSYAGNYRVAMFNGVPQYIAENPFDIELDG 1r27.4    --------------------------------------------------------------------------------  target    AKPARPKLYWRAEPAHYYNHEDHPLKMGKTMITGKTHMPTPTKSLWFANANSILGNVKWHFNTVVNVLPKMEMIAVQEWW 1r27.4    ----------------------------------------NSSYIIAWGSNVPQTRT-PDAHFFTEVRYKGTKTVAVTPD  target    WSTSCEWADIVFAVDAWSELKHPDMCSSVTNPFLTVFPRTPLERPFDTRGDIECLDLVGKQLAKRTGDRRFADMWKFVEE 1r27.4    YAEIAKLCDLWLAPK-----------------------------------------------------------------  target    KKVEVYLQRILDHSSNTKGFKFPELEEKAKKGIPALMMTRTNPKTVGYEQVYDSRPWYTKTGRLEFYREEDEFIEAGENL 1r27.4    --------------------------------------------------------------------------------  target    PVHREPIDSTFYEPNVIVAPAHPFIKAKGPEAYGVKVDDFDNETRQGRNIVKTWEETKKTVHPLAKDGYKFVFHTPKYRH 1r27.4    --------------------------------------------------------------------------------  target    GAHTMPVDTDMVAMLFGPFGDIYRHDKRQPFAAEGYVDIHPDDAKALNIEDGDYVWIDSDPSDRPFRGWQKNDKDYKFSR 1r27.4    --------------------------------------------------------------------------------  target    LLCRARYYPGTPRGITRMWFNMYGATPGSVEGHESRKDGLAKNPRTGYQAMFRSGSHQSATRGWLKPTWMTDSLVRKELF 1r27.4    --------------------------------------------------------------------------------  target    GHAVNKGFLPDVHCPTGAPREAIVKITKAEPGGLNAKGLWRPAALGLRPKYENDKMKDYLAGKFTLAANPKKGGKK 1r27.4    ---------------------------------------------------------------------------- ``` | | | | | | | | | | | | | | | | | | | | | | | | | | | | | | | | | | | | | | | | | | | | | | | | | |
|  | 3ir5.1.A | Respiratory nitrate reductase 1 alpha chain  *Crystal structure of NarGHI mutant NarG-H49C* | 0.02 |  | 9.26 | 0.08 | 121-175 | X-ray | 2.30 | hetero-1-1-1-mer | 2 x MD1, 1 x 6MO, 4 x SF4, 1 x AGA, 1 x F3S, 2 x HEM | HHblits | 0.23 |
| ``` target    LARDIAKVPGTTLFAIGMGPNQFFNNDNKDRTQFLLAALTGNIGKIAGNIGSYAGNYRVAMFNGVPQYIAENPFDIELDG 3ir5.1    --------------------------------------------------------------------------------  target    AKPARPKLYWRAEPAHYYNHEDHPLKMGKTMITGKTHMPTPTKSLWFANANSILGNVKWHFNTVVNVLPKMEMIAVQEWW 3ir5.1    ----------------------------------------NSSYIIAWGSNVPQTRT-PDAHFFTEVRYKGTKTVAVTPD  target    WSTSCEWADIVFAVDAWSELKHPDMCSSVTNPFLTVFPRTPLERPFDTRGDIECLDLVGKQLAKRTGDRRFADMWKFVEE 3ir5.1    YAEIAKLCDLWLAPK-----------------------------------------------------------------  target    KKVEVYLQRILDHSSNTKGFKFPELEEKAKKGIPALMMTRTNPKTVGYEQVYDSRPWYTKTGRLEFYREEDEFIEAGENL 3ir5.1    --------------------------------------------------------------------------------  target    PVHREPIDSTFYEPNVIVAPAHPFIKAKGPEAYGVKVDDFDNETRQGRNIVKTWEETKKTVHPLAKDGYKFVFHTPKYRH 3ir5.1    --------------------------------------------------------------------------------  target    GAHTMPVDTDMVAMLFGPFGDIYRHDKRQPFAAEGYVDIHPDDAKALNIEDGDYVWIDSDPSDRPFRGWQKNDKDYKFSR 3ir5.1    --------------------------------------------------------------------------------  target    LLCRARYYPGTPRGITRMWFNMYGATPGSVEGHESRKDGLAKNPRTGYQAMFRSGSHQSATRGWLKPTWMTDSLVRKELF 3ir5.1    --------------------------------------------------------------------------------  target    GHAVNKGFLPDVHCPTGAPREAIVKITKAEPGGLNAKGLWRPAALGLRPKYENDKMKDYLAGKFTLAANPKKGGKK 3ir5.1    ---------------------------------------------------------------------------- ``` | | | | | | | | | | | | | | | | | | | | | | | | | | | | | | | | | | | | | | | | | | | | | | | | | |
|  | 1q16.1.A | Respiratory nitrate reductase 1 alpha chain  *Crystal structure of Nitrate Reductase A, NarGHI, from Escherichia coli* | 0.01 |  | 9.26 | 0.08 | 121-175 | X-ray | 1.90 | hetero-oligomer | 2 x MD1, 1 x 6MO, 2 x HEM, 4 x SF4, 1 x F3S, 1 x AGA, 1 x 3PH | HHblits | 0.23 |
| ``` target    LARDIAKVPGTTLFAIGMGPNQFFNNDNKDRTQFLLAALTGNIGKIAGNIGSYAGNYRVAMFNGVPQYIAENPFDIELDG 1q16.1    --------------------------------------------------------------------------------  target    AKPARPKLYWRAEPAHYYNHEDHPLKMGKTMITGKTHMPTPTKSLWFANANSILGNVKWHFNTVVNVLPKMEMIAVQEWW 1q16.1    ----------------------------------------NSSYIIAWGSNVPQTRT-PDAHFFTEVRYKGTKTVAVTPD  target    WSTSCEWADIVFAVDAWSELKHPDMCSSVTNPFLTVFPRTPLERPFDTRGDIECLDLVGKQLAKRTGDRRFADMWKFVEE 1q16.1    YAEIAKLCDLWLAPK-----------------------------------------------------------------  target    KKVEVYLQRILDHSSNTKGFKFPELEEKAKKGIPALMMTRTNPKTVGYEQVYDSRPWYTKTGRLEFYREEDEFIEAGENL 1q16.1    --------------------------------------------------------------------------------  target    PVHREPIDSTFYEPNVIVAPAHPFIKAKGPEAYGVKVDDFDNETRQGRNIVKTWEETKKTVHPLAKDGYKFVFHTPKYRH 1q16.1    --------------------------------------------------------------------------------  target    GAHTMPVDTDMVAMLFGPFGDIYRHDKRQPFAAEGYVDIHPDDAKALNIEDGDYVWIDSDPSDRPFRGWQKNDKDYKFSR 1q16.1    --------------------------------------------------------------------------------  target    LLCRARYYPGTPRGITRMWFNMYGATPGSVEGHESRKDGLAKNPRTGYQAMFRSGSHQSATRGWLKPTWMTDSLVRKELF 1q16.1    --------------------------------------------------------------------------------  target    GHAVNKGFLPDVHCPTGAPREAIVKITKAEPGGLNAKGLWRPAALGLRPKYENDKMKDYLAGKFTLAANPKKGGKK 1q16.1    ---------------------------------------------------------------------------- ``` | | | | | | | | | | | | | | | | | | | | | | | | | | | | | | | | | | | | | | | | | | | | | | | | | |
|  | 3ir6.1.A | Respiratory nitrate reductase 1 alpha chain  *Crystal structure of NarGHI mutant NarG-H49S* | 0.02 |  | 9.26 | 0.08 | 121-175 | X-ray | 2.80 | hetero-1-1-1-mer | 2 x GDP, 1 x AGA, 3 x SF4, 1 x F3S, 2 x HEM | HHblits | 0.23 |
| ``` target    LARDIAKVPGTTLFAIGMGPNQFFNNDNKDRTQFLLAALTGNIGKIAGNIGSYAGNYRVAMFNGVPQYIAENPFDIELDG 3ir6.1    --------------------------------------------------------------------------------  target    AKPARPKLYWRAEPAHYYNHEDHPLKMGKTMITGKTHMPTPTKSLWFANANSILGNVKWHFNTVVNVLPKMEMIAVQEWW 3ir6.1    ----------------------------------------NSSYIIAWGSNVPQTRT-PDAHFFTEVRYKGTKTVAVTPD  target    WSTSCEWADIVFAVDAWSELKHPDMCSSVTNPFLTVFPRTPLERPFDTRGDIECLDLVGKQLAKRTGDRRFADMWKFVEE 3ir6.1    YAEIAKLCDLWLAPK-----------------------------------------------------------------  target    KKVEVYLQRILDHSSNTKGFKFPELEEKAKKGIPALMMTRTNPKTVGYEQVYDSRPWYTKTGRLEFYREEDEFIEAGENL 3ir6.1    --------------------------------------------------------------------------------  target    PVHREPIDSTFYEPNVIVAPAHPFIKAKGPEAYGVKVDDFDNETRQGRNIVKTWEETKKTVHPLAKDGYKFVFHTPKYRH 3ir6.1    --------------------------------------------------------------------------------  target    GAHTMPVDTDMVAMLFGPFGDIYRHDKRQPFAAEGYVDIHPDDAKALNIEDGDYVWIDSDPSDRPFRGWQKNDKDYKFSR 3ir6.1    --------------------------------------------------------------------------------  target    LLCRARYYPGTPRGITRMWFNMYGATPGSVEGHESRKDGLAKNPRTGYQAMFRSGSHQSATRGWLKPTWMTDSLVRKELF 3ir6.1    --------------------------------------------------------------------------------  target    GHAVNKGFLPDVHCPTGAPREAIVKITKAEPGGLNAKGLWRPAALGLRPKYENDKMKDYLAGKFTLAANPKKGGKK 3ir6.1    ---------------------------------------------------------------------------- ``` | | | | | | | | | | | | | | | | | | | | | | | | | | | | | | | | | | | | | | | | | | | | | | | | | |
|  | 4ydd.1.A | DMSO reductase family type II enzyme, molybdopterin subunit  *Crystal structure of the perchlorate reductase PcrAB from Azospira suillum PS* | 0.02 |  | 9.26 | 0.08 | 121-175 | X-ray | 1.86 | hetero-oligomer | 4 x SF4, 1 x MO, 1 x MGD, 1 x MD1, 1 x F3S | HHblits | 0.23 |
| ``` target    LARDIAKVPGTTLFAIGMGPNQFFNNDNKDRTQFLLAALTGNIGKIAGNIGSYAGNYRVAMFNGVPQYIAENPFDIELDG 4ydd.1    --------------------------------------------------------------------------------  target    AKPARPKLYWRAEPAHYYNHEDHPLKMGKTMITGKTHMPTPTKSLWFANANSILGNVKWHFNTVVNVLPKMEMIAVQEWW 4ydd.1    ----------------------------------------NSKYIILWGSNPTQTRI-PDAHFLSEAQLNGAKIVSISPD  target    WSTSCEWADIVFAVDAWSELKHPDMCSSVTNPFLTVFPRTPLERPFDTRGDIECLDLVGKQLAKRTGDRRFADMWKFVEE 4ydd.1    YNSSTIKVDKWIHPQ-----------------------------------------------------------------  target    KKVEVYLQRILDHSSNTKGFKFPELEEKAKKGIPALMMTRTNPKTVGYEQVYDSRPWYTKTGRLEFYREEDEFIEAGENL 4ydd.1    --------------------------------------------------------------------------------  target    PVHREPIDSTFYEPNVIVAPAHPFIKAKGPEAYGVKVDDFDNETRQGRNIVKTWEETKKTVHPLAKDGYKFVFHTPKYRH 4ydd.1    --------------------------------------------------------------------------------  target    GAHTMPVDTDMVAMLFGPFGDIYRHDKRQPFAAEGYVDIHPDDAKALNIEDGDYVWIDSDPSDRPFRGWQKNDKDYKFSR 4ydd.1    --------------------------------------------------------------------------------  target    LLCRARYYPGTPRGITRMWFNMYGATPGSVEGHESRKDGLAKNPRTGYQAMFRSGSHQSATRGWLKPTWMTDSLVRKELF 4ydd.1    --------------------------------------------------------------------------------  target    GHAVNKGFLPDVHCPTGAPREAIVKITKAEPGGLNAKGLWRPAALGLRPKYENDKMKDYLAGKFTLAANPKKGGKK 4ydd.1    ---------------------------------------------------------------------------- ``` | | | | | | | | | | | | | | | | | | | | | | | | | | | | | | | | | | | | | | | | | | | | | | | | | |
|  | 5e7o.1.A | DMSO reductase family type II enzyme, molybdopterin subunit  *Crystal structure of the perchlorate reductase PcrAB mutant W461E of PcrA from Azospira suillum PS* | 0.02 |  | 9.26 | 0.08 | 121-175 | X-ray | 2.40 | hetero-oligomer | 4 x SF4, 1 x MO, 1 x MGD, 1 x MD1, 1 x F3S | HHblits | 0.23 |
| ``` target    LARDIAKVPGTTLFAIGMGPNQFFNNDNKDRTQFLLAALTGNIGKIAGNIGSYAGNYRVAMFNGVPQYIAENPFDIELDG 5e7o.1    --------------------------------------------------------------------------------  target    AKPARPKLYWRAEPAHYYNHEDHPLKMGKTMITGKTHMPTPTKSLWFANANSILGNVKWHFNTVVNVLPKMEMIAVQEWW 5e7o.1    ----------------------------------------NSKYIILWGSNPTQTRI-PDAHFLSEAQLNGAKIVSISPD  target    WSTSCEWADIVFAVDAWSELKHPDMCSSVTNPFLTVFPRTPLERPFDTRGDIECLDLVGKQLAKRTGDRRFADMWKFVEE 5e7o.1    YNSSTIKVDKWIHPQ-----------------------------------------------------------------  target    KKVEVYLQRILDHSSNTKGFKFPELEEKAKKGIPALMMTRTNPKTVGYEQVYDSRPWYTKTGRLEFYREEDEFIEAGENL 5e7o.1    --------------------------------------------------------------------------------  target    PVHREPIDSTFYEPNVIVAPAHPFIKAKGPEAYGVKVDDFDNETRQGRNIVKTWEETKKTVHPLAKDGYKFVFHTPKYRH 5e7o.1    --------------------------------------------------------------------------------  target    GAHTMPVDTDMVAMLFGPFGDIYRHDKRQPFAAEGYVDIHPDDAKALNIEDGDYVWIDSDPSDRPFRGWQKNDKDYKFSR 5e7o.1    --------------------------------------------------------------------------------  target    LLCRARYYPGTPRGITRMWFNMYGATPGSVEGHESRKDGLAKNPRTGYQAMFRSGSHQSATRGWLKPTWMTDSLVRKELF 5e7o.1    --------------------------------------------------------------------------------  target    GHAVNKGFLPDVHCPTGAPREAIVKITKAEPGGLNAKGLWRPAALGLRPKYENDKMKDYLAGKFTLAANPKKGGKK 5e7o.1    ---------------------------------------------------------------------------- ``` | | | | | | | | | | | | | | | | | | | | | | | | | | | | | | | | | | | | | | | | | | | | | | | | | |
|  | 4rv0.1.A | Transitional endoplasmic reticulum ATPase TER94  *Crystal structure of TN complex* | 0.02 |  | 17.65 | 0.08 | 434-500 | X-ray | 2.00 | hetero-oligomer |  | HHblits | 0.27 |
| ``` target    LARDIAKVPGTTLFAIGMGPNQFFNNDNKDRTQFLLAALTGNIGKIAGNIGSYAGNYRVAMFNGVPQYIAENPFDIELDG 4rv0.1    --------------------------------------------------------------------------------  target    AKPARPKLYWRAEPAHYYNHEDHPLKMGKTMITGKTHMPTPTKSLWFANANSILGNVKWHFNTVVNVLPKMEMIAVQEWW 4rv0.1    --------------------------------------------------------------------------------  target    WSTSCEWADIVFAVDAWSELKHPDMCSSVTNPFLTVFPRTPLERPFDTRGDIECLDLVGKQLAKRTGDRRFADMWKFVEE 4rv0.1    --------------------------------------------------------------------------------  target    KKVEVYLQRILDHSSNTKGFKFPELEEKAKKGIPALMMTRTNPKTVGYEQVYDSRPWYTKTGRLEFYREEDEFIEAGENL 4rv0.1    --------------------------------------------------------------------------------  target    PVHREPIDSTFYEPNVIVAPAHPFIKAKGPEAYGVKVDDFDNETRQGRNIVKTWEETKKTVHPLAKDGYKFVFHTPKYRH 4rv0.1    --------------------------------------------------------------------------------  target    GAHTMPVDTDMVAMLFGPFGDIYRHDKRQPFAAEGYVDIHPDDAKALNIEDGDYVWIDSDPSDRPFRGWQKNDKDYKFSR 4rv0.1    ---------------------------------NSVVSLSQAKMDELQLFRGDTVILKGKRR----------------KE  target    LLCRARYYPGTPRGITRMWFNMYGATPGSVEGHESRKDGLAKNPRTGYQAMFRSGSHQSATRGWLKPTWMTDSLVRKELF 4rv0.1    TVCIVLSDDTCPDEKIRMNR------------------------------------------------------------  target    GHAVNKGFLPDVHCPTGAPREAIVKITKAEPGGLNAKGLWRPAALGLRPKYENDKMKDYLAGKFTLAANPKKGGKK 4rv0.1    ---------------------------------------------------------------------------- ``` | | | | | | | | | | | | | | | | | | | | | | | | | | | | | | | | | | | | | | | | | | | | | | | | | |
|  | 1tmo.1.A | TRIMETHYLAMINE N-OXIDE REDUCTASE  *TRIMETHYLAMINE N-OXIDE REDUCTASE FROM SHEWANELLA MASSILIA* | 0.00 |  | 3.64 | 0.09 | 121-175 | X-ray | 2.50 | monomer | 2 x 2MD, 1 x 2MO | HHblits | 0.21 |
| ``` target    LARDIAKVPGTTLFAIGMGPNQFFNNDNKDRTQFLLAALTGNIGKIAGNIGSYAGNYRVAMFNGVPQYIAENPFDIELDG 1tmo.1    --------------------------------------------------------------------------------  target    AKPARPKLYWRAEPAHYYNHEDHPLKMGKTMITGKTHMPTPTKSLWFANANSILGNV-------KWHF---NTVVNVLPK 1tmo.1    ----------------------------------------HSDTIVLWSNDPYKNLQVGWNAETHESFAYLAQLKEKVKQ  target    M-EMIAVQEWWWSTSCE-WADIVFAVDAWSELKHPDMCSSVTNPFLTVFPRTPLERPFDTRGDIECLDLVGKQLAKRTGD 1tmo.1    GKIRVISIDPVVTKTQAYLGCEQLYVN-----------------------------------------------------  target    RRFADMWKFVEEKKVEVYLQRILDHSSNTKGFKFPELEEKAKKGIPALMMTRTNPKTVGYEQVYDSRPWYTKTGRLEFYR 1tmo.1    --------------------------------------------------------------------------------  target    EEDEFIEAGENLPVHREPIDSTFYEPNVIVAPAHPFIKAKGPEAYGVKVDDFDNETRQGRNIVKTWEETKKTVHPLAKDG 1tmo.1    --------------------------------------------------------------------------------  target    YKFVFHTPKYRHGAHTMPVDTDMVAMLFGPFGDIYRHDKRQPFAAEGYVDIHPDDAKALNIEDGDYVWIDSDPSDRPFRG 1tmo.1    --------------------------------------------------------------------------------  target    WQKNDKDYKFSRLLCRARYYPGTPRGITRMWFNMYGATPGSVEGHESRKDGLAKNPRTGYQAMFRSGSHQSATRGWLKPT 1tmo.1    --------------------------------------------------------------------------------  target    WMTDSLVRKELFGHAVNKGFLPDVHCPTGAPREAIVKITKAEPGGLNAKGLWRPAALGLRPKYENDKMKDYLAGKFTLAA 1tmo.1    --------------------------------------------------------------------------------  target    NPKKGGKK 1tmo.1    -------- ``` | | | | | | | | | | | | | | | | | | | | | | | | | | | | | | | | | | | | | | | | | | | | | | | | | |
|  | 3qc8.1.A | Transitional endoplasmic reticulum ATPase  *Crystal Structure of FAF1 UBX Domain In Complex with p97/VCP N Domain Reveals The Conserved FcisP Touch-Turn Motif of UBX Domain Suffering Conformational Change* | 0.02 |  | 15.69 | 0.08 | 434-500 | X-ray | 2.20 | hetero-oligomer |  | HHblits | 0.26 |
| ``` target    LARDIAKVPGTTLFAIGMGPNQFFNNDNKDRTQFLLAALTGNIGKIAGNIGSYAGNYRVAMFNGVPQYIAENPFDIELDG 3qc8.1    --------------------------------------------------------------------------------  target    AKPARPKLYWRAEPAHYYNHEDHPLKMGKTMITGKTHMPTPTKSLWFANANSILGNVKWHFNTVVNVLPKMEMIAVQEWW 3qc8.1    --------------------------------------------------------------------------------  target    WSTSCEWADIVFAVDAWSELKHPDMCSSVTNPFLTVFPRTPLERPFDTRGDIECLDLVGKQLAKRTGDRRFADMWKFVEE 3qc8.1    --------------------------------------------------------------------------------  target    KKVEVYLQRILDHSSNTKGFKFPELEEKAKKGIPALMMTRTNPKTVGYEQVYDSRPWYTKTGRLEFYREEDEFIEAGENL 3qc8.1    --------------------------------------------------------------------------------  target    PVHREPIDSTFYEPNVIVAPAHPFIKAKGPEAYGVKVDDFDNETRQGRNIVKTWEETKKTVHPLAKDGYKFVFHTPKYRH 3qc8.1    --------------------------------------------------------------------------------  target    GAHTMPVDTDMVAMLFGPFGDIYRHDKRQPFAAEGYVDIHPDDAKALNIEDGDYVWIDSDPSDRPFRGWQKNDKDYKFSR 3qc8.1    ---------------------------------NSVVSLSQPKMDELQLFRGDTVLLKGKKR----------------RE  target    LLCRARYYPGTPRGITRMWFNMYGATPGSVEGHESRKDGLAKNPRTGYQAMFRSGSHQSATRGWLKPTWMTDSLVRKELF 3qc8.1    AVCIVLSDDTCSDEKIRMNR------------------------------------------------------------  target    GHAVNKGFLPDVHCPTGAPREAIVKITKAEPGGLNAKGLWRPAALGLRPKYENDKMKDYLAGKFTLAANPKKGGKK 3qc8.1    ---------------------------------------------------------------------------- ``` | | | | | | | | | | | | | | | | | | | | | | | | | | | | | | | | | | | | | | | | | | | | | | | | | |
|  | 5x4l.1.A | Transitional endoplasmic reticulum ATPase  *Crystal structure of the UBX domain of human UBXD7 in complex with p97 N domain* | 0.02 |  | 15.69 | 0.08 | 434-500 | X-ray | 2.40 | hetero-oligomer |  | HHblits | 0.26 |
| ``` target    LARDIAKVPGTTLFAIGMGPNQFFNNDNKDRTQFLLAALTGNIGKIAGNIGSYAGNYRVAMFNGVPQYIAENPFDIELDG 5x4l.1    --------------------------------------------------------------------------------  target    AKPARPKLYWRAEPAHYYNHEDHPLKMGKTMITGKTHMPTPTKSLWFANANSILGNVKWHFNTVVNVLPKMEMIAVQEWW 5x4l.1    --------------------------------------------------------------------------------  target    WSTSCEWADIVFAVDAWSELKHPDMCSSVTNPFLTVFPRTPLERPFDTRGDIECLDLVGKQLAKRTGDRRFADMWKFVEE 5x4l.1    --------------------------------------------------------------------------------  target    KKVEVYLQRILDHSSNTKGFKFPELEEKAKKGIPALMMTRTNPKTVGYEQVYDSRPWYTKTGRLEFYREEDEFIEAGENL 5x4l.1    --------------------------------------------------------------------------------  target    PVHREPIDSTFYEPNVIVAPAHPFIKAKGPEAYGVKVDDFDNETRQGRNIVKTWEETKKTVHPLAKDGYKFVFHTPKYRH 5x4l.1    --------------------------------------------------------------------------------  target    GAHTMPVDTDMVAMLFGPFGDIYRHDKRQPFAAEGYVDIHPDDAKALNIEDGDYVWIDSDPSDRPFRGWQKNDKDYKFSR 5x4l.1    ---------------------------------NSVVSLSQPKMDELQLFRGDTVLLKGKKR----------------RE  target    LLCRARYYPGTPRGITRMWFNMYGATPGSVEGHESRKDGLAKNPRTGYQAMFRSGSHQSATRGWLKPTWMTDSLVRKELF 5x4l.1    AVCIVLSDDTCSDEKIRMNR------------------------------------------------------------  target    GHAVNKGFLPDVHCPTGAPREAIVKITKAEPGGLNAKGLWRPAALGLRPKYENDKMKDYLAGKFTLAANPKKGGKK 5x4l.1    ---------------------------------------------------------------------------- ``` | | | | | | | | | | | | | | | | | | | | | | | | | | | | | | | | | | | | | | | | | | | | | | | | | |
|  | 5x4l.2.A | Transitional endoplasmic reticulum ATPase  *Crystal structure of the UBX domain of human UBXD7 in complex with p97 N domain* | 0.02 |  | 15.69 | 0.08 | 434-500 | X-ray | 2.40 | hetero-oligomer |  | HHblits | 0.26 |
| ``` target    LARDIAKVPGTTLFAIGMGPNQFFNNDNKDRTQFLLAALTGNIGKIAGNIGSYAGNYRVAMFNGVPQYIAENPFDIELDG 5x4l.2    --------------------------------------------------------------------------------  target    AKPARPKLYWRAEPAHYYNHEDHPLKMGKTMITGKTHMPTPTKSLWFANANSILGNVKWHFNTVVNVLPKMEMIAVQEWW 5x4l.2    --------------------------------------------------------------------------------  target    WSTSCEWADIVFAVDAWSELKHPDMCSSVTNPFLTVFPRTPLERPFDTRGDIECLDLVGKQLAKRTGDRRFADMWKFVEE 5x4l.2    --------------------------------------------------------------------------------  target    KKVEVYLQRILDHSSNTKGFKFPELEEKAKKGIPALMMTRTNPKTVGYEQVYDSRPWYTKTGRLEFYREEDEFIEAGENL 5x4l.2    --------------------------------------------------------------------------------  target    PVHREPIDSTFYEPNVIVAPAHPFIKAKGPEAYGVKVDDFDNETRQGRNIVKTWEETKKTVHPLAKDGYKFVFHTPKYRH 5x4l.2    --------------------------------------------------------------------------------  target    GAHTMPVDTDMVAMLFGPFGDIYRHDKRQPFAAEGYVDIHPDDAKALNIEDGDYVWIDSDPSDRPFRGWQKNDKDYKFSR 5x4l.2    ---------------------------------NSVVSLSQPKMDELQLFRGDTVLLKGKKR----------------RE  target    LLCRARYYPGTPRGITRMWFNMYGATPGSVEGHESRKDGLAKNPRTGYQAMFRSGSHQSATRGWLKPTWMTDSLVRKELF 5x4l.2    AVCIVLSDDTCSDEKIRMNR------------------------------------------------------------  target    GHAVNKGFLPDVHCPTGAPREAIVKITKAEPGGLNAKGLWRPAALGLRPKYENDKMKDYLAGKFTLAANPKKGGKK 5x4l.2    ---------------------------------------------------------------------------- ``` | | | | | | | | | | | | | | | | | | | | | | | | | | | | | | | | | | | | | | | | | | | | | | | | | |
|  | 3tiw.1.A | Transitional endoplasmic reticulum ATPase  *Crystal structure of p97N in complex with the C-terminus of gp78* | 0.02 |  | 15.69 | 0.08 | 434-500 | X-ray | 1.80 | hetero-oligomer |  | HHblits | 0.26 |
| ``` target    LARDIAKVPGTTLFAIGMGPNQFFNNDNKDRTQFLLAALTGNIGKIAGNIGSYAGNYRVAMFNGVPQYIAENPFDIELDG 3tiw.1    --------------------------------------------------------------------------------  target    AKPARPKLYWRAEPAHYYNHEDHPLKMGKTMITGKTHMPTPTKSLWFANANSILGNVKWHFNTVVNVLPKMEMIAVQEWW 3tiw.1    --------------------------------------------------------------------------------  target    WSTSCEWADIVFAVDAWSELKHPDMCSSVTNPFLTVFPRTPLERPFDTRGDIECLDLVGKQLAKRTGDRRFADMWKFVEE 3tiw.1    --------------------------------------------------------------------------------  target    KKVEVYLQRILDHSSNTKGFKFPELEEKAKKGIPALMMTRTNPKTVGYEQVYDSRPWYTKTGRLEFYREEDEFIEAGENL 3tiw.1    --------------------------------------------------------------------------------  target    PVHREPIDSTFYEPNVIVAPAHPFIKAKGPEAYGVKVDDFDNETRQGRNIVKTWEETKKTVHPLAKDGYKFVFHTPKYRH 3tiw.1    --------------------------------------------------------------------------------  target    GAHTMPVDTDMVAMLFGPFGDIYRHDKRQPFAAEGYVDIHPDDAKALNIEDGDYVWIDSDPSDRPFRGWQKNDKDYKFSR 3tiw.1    ---------------------------------NSVVSLSQPKMDELQLFRGDTVLLKGKKR----------------RE  target    LLCRARYYPGTPRGITRMWFNMYGATPGSVEGHESRKDGLAKNPRTGYQAMFRSGSHQSATRGWLKPTWMTDSLVRKELF 3tiw.1    AVCIVLSDDTCSDEKIRMNR------------------------------------------------------------  target    GHAVNKGFLPDVHCPTGAPREAIVKITKAEPGGLNAKGLWRPAALGLRPKYENDKMKDYLAGKFTLAANPKKGGKK 3tiw.1    ---------------------------------------------------------------------------- ``` | | | | | | | | | | | | | | | | | | | | | | | | | | | | | | | | | | | | | | | | | | | | | | | | | |
|  | 3tiw.2.A | Transitional endoplasmic reticulum ATPase  *Crystal structure of p97N in complex with the C-terminus of gp78* | 0.02 |  | 15.69 | 0.08 | 434-500 | X-ray | 1.80 | hetero-oligomer |  | HHblits | 0.26 |
| ``` target    LARDIAKVPGTTLFAIGMGPNQFFNNDNKDRTQFLLAALTGNIGKIAGNIGSYAGNYRVAMFNGVPQYIAENPFDIELDG 3tiw.2    --------------------------------------------------------------------------------  target    AKPARPKLYWRAEPAHYYNHEDHPLKMGKTMITGKTHMPTPTKSLWFANANSILGNVKWHFNTVVNVLPKMEMIAVQEWW 3tiw.2    --------------------------------------------------------------------------------  target    WSTSCEWADIVFAVDAWSELKHPDMCSSVTNPFLTVFPRTPLERPFDTRGDIECLDLVGKQLAKRTGDRRFADMWKFVEE 3tiw.2    --------------------------------------------------------------------------------  target    KKVEVYLQRILDHSSNTKGFKFPELEEKAKKGIPALMMTRTNPKTVGYEQVYDSRPWYTKTGRLEFYREEDEFIEAGENL 3tiw.2    --------------------------------------------------------------------------------  target    PVHREPIDSTFYEPNVIVAPAHPFIKAKGPEAYGVKVDDFDNETRQGRNIVKTWEETKKTVHPLAKDGYKFVFHTPKYRH 3tiw.2    --------------------------------------------------------------------------------  target    GAHTMPVDTDMVAMLFGPFGDIYRHDKRQPFAAEGYVDIHPDDAKALNIEDGDYVWIDSDPSDRPFRGWQKNDKDYKFSR 3tiw.2    ---------------------------------NSVVSLSQPKMDELQLFRGDTVLLKGKKR----------------RE  target    LLCRARYYPGTPRGITRMWFNMYGATPGSVEGHESRKDGLAKNPRTGYQAMFRSGSHQSATRGWLKPTWMTDSLVRKELF 3tiw.2    AVCIVLSDDTCSDEKIRMNR------------------------------------------------------------  target    GHAVNKGFLPDVHCPTGAPREAIVKITKAEPGGLNAKGLWRPAALGLRPKYENDKMKDYLAGKFTLAANPKKGGKK 3tiw.2    ---------------------------------------------------------------------------- ``` | | | | | | | | | | | | | | | | | | | | | | | | | | | | | | | | | | | | | | | | | | | | | | | | | |
|  | 4kdl.1.A | Transitional endoplasmic reticulum ATPase  *Crystal structure of p97/VCP N in complex with OTU1 UBXL* | 0.02 |  | 15.69 | 0.08 | 434-500 | X-ray | 1.81 | monomer |  | HHblits | 0.26 |
| ``` target    LARDIAKVPGTTLFAIGMGPNQFFNNDNKDRTQFLLAALTGNIGKIAGNIGSYAGNYRVAMFNGVPQYIAENPFDIELDG 4kdl.1    --------------------------------------------------------------------------------  target    AKPARPKLYWRAEPAHYYNHEDHPLKMGKTMITGKTHMPTPTKSLWFANANSILGNVKWHFNTVVNVLPKMEMIAVQEWW 4kdl.1    --------------------------------------------------------------------------------  target    WSTSCEWADIVFAVDAWSELKHPDMCSSVTNPFLTVFPRTPLERPFDTRGDIECLDLVGKQLAKRTGDRRFADMWKFVEE 4kdl.1    --------------------------------------------------------------------------------  target    KKVEVYLQRILDHSSNTKGFKFPELEEKAKKGIPALMMTRTNPKTVGYEQVYDSRPWYTKTGRLEFYREEDEFIEAGENL 4kdl.1    --------------------------------------------------------------------------------  target    PVHREPIDSTFYEPNVIVAPAHPFIKAKGPEAYGVKVDDFDNETRQGRNIVKTWEETKKTVHPLAKDGYKFVFHTPKYRH 4kdl.1    --------------------------------------------------------------------------------  target    GAHTMPVDTDMVAMLFGPFGDIYRHDKRQPFAAEGYVDIHPDDAKALNIEDGDYVWIDSDPSDRPFRGWQKNDKDYKFSR 4kdl.1    ---------------------------------NSVVSLSQPKMDELQLFRGDTVLLKGKKR----------------RE  target    LLCRARYYPGTPRGITRMWFNMYGATPGSVEGHESRKDGLAKNPRTGYQAMFRSGSHQSATRGWLKPTWMTDSLVRKELF 4kdl.1    AVCIVLSDDTCSDEKIRMNR------------------------------------------------------------  target    GHAVNKGFLPDVHCPTGAPREAIVKITKAEPGGLNAKGLWRPAALGLRPKYENDKMKDYLAGKFTLAANPKKGGKK 4kdl.1    ---------------------------------------------------------------------------- ``` | | | | | | | | | | | | | | | | | | | | | | | | | | | | | | | | | | | | | | | | | | | | | | | | | |
|  | 4kdi.2.A | Transitional endoplasmic reticulum ATPase  *Crystal structure of p97/VCP N in complex with OTU1 UBXL* | 0.02 |  | 15.69 | 0.08 | 434-500 | X-ray | 1.86 | hetero-oligomer |  | HHblits | 0.26 |
| ``` target    LARDIAKVPGTTLFAIGMGPNQFFNNDNKDRTQFLLAALTGNIGKIAGNIGSYAGNYRVAMFNGVPQYIAENPFDIELDG 4kdi.2    --------------------------------------------------------------------------------  target    AKPARPKLYWRAEPAHYYNHEDHPLKMGKTMITGKTHMPTPTKSLWFANANSILGNVKWHFNTVVNVLPKMEMIAVQEWW 4kdi.2    --------------------------------------------------------------------------------  target    WSTSCEWADIVFAVDAWSELKHPDMCSSVTNPFLTVFPRTPLERPFDTRGDIECLDLVGKQLAKRTGDRRFADMWKFVEE 4kdi.2    --------------------------------------------------------------------------------  target    KKVEVYLQRILDHSSNTKGFKFPELEEKAKKGIPALMMTRTNPKTVGYEQVYDSRPWYTKTGRLEFYREEDEFIEAGENL 4kdi.2    --------------------------------------------------------------------------------  target    PVHREPIDSTFYEPNVIVAPAHPFIKAKGPEAYGVKVDDFDNETRQGRNIVKTWEETKKTVHPLAKDGYKFVFHTPKYRH 4kdi.2    --------------------------------------------------------------------------------  target    GAHTMPVDTDMVAMLFGPFGDIYRHDKRQPFAAEGYVDIHPDDAKALNIEDGDYVWIDSDPSDRPFRGWQKNDKDYKFSR 4kdi.2    ---------------------------------NSVVSLSQPKMDELQLFRGDTVLLKGKKR----------------RE  target    LLCRARYYPGTPRGITRMWFNMYGATPGSVEGHESRKDGLAKNPRTGYQAMFRSGSHQSATRGWLKPTWMTDSLVRKELF 4kdi.2    AVCIVLSDDTCSDEKIRMNR------------------------------------------------------------  target    GHAVNKGFLPDVHCPTGAPREAIVKITKAEPGGLNAKGLWRPAALGLRPKYENDKMKDYLAGKFTLAANPKKGGKK 4kdi.2    ---------------------------------------------------------------------------- ``` | | | | | | | | | | | | | | | | | | | | | | | | | | | | | | | | | | | | | | | | | | | | | | | | | |
|  | 4kdi.1.A | Transitional endoplasmic reticulum ATPase  *Crystal structure of p97/VCP N in complex with OTU1 UBXL* | 0.02 |  | 15.69 | 0.08 | 434-500 | X-ray | 1.86 | hetero-oligomer |  | HHblits | 0.26 |
| ``` target    LARDIAKVPGTTLFAIGMGPNQFFNNDNKDRTQFLLAALTGNIGKIAGNIGSYAGNYRVAMFNGVPQYIAENPFDIELDG 4kdi.1    --------------------------------------------------------------------------------  target    AKPARPKLYWRAEPAHYYNHEDHPLKMGKTMITGKTHMPTPTKSLWFANANSILGNVKWHFNTVVNVLPKMEMIAVQEWW 4kdi.1    --------------------------------------------------------------------------------  target    WSTSCEWADIVFAVDAWSELKHPDMCSSVTNPFLTVFPRTPLERPFDTRGDIECLDLVGKQLAKRTGDRRFADMWKFVEE 4kdi.1    --------------------------------------------------------------------------------  target    KKVEVYLQRILDHSSNTKGFKFPELEEKAKKGIPALMMTRTNPKTVGYEQVYDSRPWYTKTGRLEFYREEDEFIEAGENL 4kdi.1    --------------------------------------------------------------------------------  target    PVHREPIDSTFYEPNVIVAPAHPFIKAKGPEAYGVKVDDFDNETRQGRNIVKTWEETKKTVHPLAKDGYKFVFHTPKYRH 4kdi.1    --------------------------------------------------------------------------------  target    GAHTMPVDTDMVAMLFGPFGDIYRHDKRQPFAAEGYVDIHPDDAKALNIEDGDYVWIDSDPSDRPFRGWQKNDKDYKFSR 4kdi.1    ---------------------------------NSVVSLSQPKMDELQLFRGDTVLLKGKKR----------------RE  target    LLCRARYYPGTPRGITRMWFNMYGATPGSVEGHESRKDGLAKNPRTGYQAMFRSGSHQSATRGWLKPTWMTDSLVRKELF 4kdi.1    AVCIVLSDDTCSDEKIRMNR------------------------------------------------------------  target    GHAVNKGFLPDVHCPTGAPREAIVKITKAEPGGLNAKGLWRPAALGLRPKYENDKMKDYLAGKFTLAANPKKGGKK 4kdi.1    ---------------------------------------------------------------------------- ``` | | | | | | | | | | | | | | | | | | | | | | | | | | | | | | | | | | | | | | | | | | | | | | | | | |
|  | 3qwz.1.A | Transitional endoplasmic reticulum ATPase  *Crystal structure of FAF1 UBX-p97N-domain complex* | 0.02 |  | 15.69 | 0.08 | 434-500 | X-ray | 2.00 | hetero-oligomer |  | HHblits | 0.26 |
| ``` target    LARDIAKVPGTTLFAIGMGPNQFFNNDNKDRTQFLLAALTGNIGKIAGNIGSYAGNYRVAMFNGVPQYIAENPFDIELDG 3qwz.1    --------------------------------------------------------------------------------  target    AKPARPKLYWRAEPAHYYNHEDHPLKMGKTMITGKTHMPTPTKSLWFANANSILGNVKWHFNTVVNVLPKMEMIAVQEWW 3qwz.1    --------------------------------------------------------------------------------  target    WSTSCEWADIVFAVDAWSELKHPDMCSSVTNPFLTVFPRTPLERPFDTRGDIECLDLVGKQLAKRTGDRRFADMWKFVEE 3qwz.1    --------------------------------------------------------------------------------  target    KKVEVYLQRILDHSSNTKGFKFPELEEKAKKGIPALMMTRTNPKTVGYEQVYDSRPWYTKTGRLEFYREEDEFIEAGENL 3qwz.1    --------------------------------------------------------------------------------  target    PVHREPIDSTFYEPNVIVAPAHPFIKAKGPEAYGVKVDDFDNETRQGRNIVKTWEETKKTVHPLAKDGYKFVFHTPKYRH 3qwz.1    --------------------------------------------------------------------------------  target    GAHTMPVDTDMVAMLFGPFGDIYRHDKRQPFAAEGYVDIHPDDAKALNIEDGDYVWIDSDPSDRPFRGWQKNDKDYKFSR 3qwz.1    ---------------------------------NSVVSLSQPKMDELQLFRGDTVLLKGKKR----------------RE  target    LLCRARYYPGTPRGITRMWFNMYGATPGSVEGHESRKDGLAKNPRTGYQAMFRSGSHQSATRGWLKPTWMTDSLVRKELF 3qwz.1    AVCIVLSDDTCSDEKIRMNR------------------------------------------------------------  target    GHAVNKGFLPDVHCPTGAPREAIVKITKAEPGGLNAKGLWRPAALGLRPKYENDKMKDYLAGKFTLAANPKKGGKK 3qwz.1    ---------------------------------------------------------------------------- ``` | | | | | | | | | | | | | | | | | | | | | | | | | | | | | | | | | | | | | | | | | | | | | | | | | |
|  | 3qq8.1.A | Transitional endoplasmic reticulum ATPase  *Crystal structure of p97-N in complex with FAF1-UBX* | 0.02 |  | 15.69 | 0.08 | 434-500 | X-ray | 2.00 | hetero-oligomer |  | HHblits | 0.26 |
| ``` target    LARDIAKVPGTTLFAIGMGPNQFFNNDNKDRTQFLLAALTGNIGKIAGNIGSYAGNYRVAMFNGVPQYIAENPFDIELDG 3qq8.1    --------------------------------------------------------------------------------  target    AKPARPKLYWRAEPAHYYNHEDHPLKMGKTMITGKTHMPTPTKSLWFANANSILGNVKWHFNTVVNVLPKMEMIAVQEWW 3qq8.1    --------------------------------------------------------------------------------  target    WSTSCEWADIVFAVDAWSELKHPDMCSSVTNPFLTVFPRTPLERPFDTRGDIECLDLVGKQLAKRTGDRRFADMWKFVEE 3qq8.1    --------------------------------------------------------------------------------  target    KKVEVYLQRILDHSSNTKGFKFPELEEKAKKGIPALMMTRTNPKTVGYEQVYDSRPWYTKTGRLEFYREEDEFIEAGENL 3qq8.1    --------------------------------------------------------------------------------  target    PVHREPIDSTFYEPNVIVAPAHPFIKAKGPEAYGVKVDDFDNETRQGRNIVKTWEETKKTVHPLAKDGYKFVFHTPKYRH 3qq8.1    --------------------------------------------------------------------------------  target    GAHTMPVDTDMVAMLFGPFGDIYRHDKRQPFAAEGYVDIHPDDAKALNIEDGDYVWIDSDPSDRPFRGWQKNDKDYKFSR 3qq8.1    ---------------------------------NSVVSLSQPKMDELQLFRGDTVLLKGKKR----------------RE  target    LLCRARYYPGTPRGITRMWFNMYGATPGSVEGHESRKDGLAKNPRTGYQAMFRSGSHQSATRGWLKPTWMTDSLVRKELF 3qq8.1    AVCIVLSDDTCSDEKIRMNR------------------------------------------------------------  target    GHAVNKGFLPDVHCPTGAPREAIVKITKAEPGGLNAKGLWRPAALGLRPKYENDKMKDYLAGKFTLAANPKKGGKK 3qq8.1    ---------------------------------------------------------------------------- ``` | | | | | | | | | | | | | | | | | | | | | | | | | | | | | | | | | | | | | | | | | | | | | | | | | |
|  | 3qq7.1.A | Transitional endoplasmic reticulum ATPase  *Crystal Structure of the p97 N-terminal domain* | 0.02 |  | 15.69 | 0.08 | 434-500 | X-ray | 2.65 | monomer | 1 x HEZ, 1 x CO | HHblits | 0.26 |
| ``` target    LARDIAKVPGTTLFAIGMGPNQFFNNDNKDRTQFLLAALTGNIGKIAGNIGSYAGNYRVAMFNGVPQYIAENPFDIELDG 3qq7.1    --------------------------------------------------------------------------------  target    AKPARPKLYWRAEPAHYYNHEDHPLKMGKTMITGKTHMPTPTKSLWFANANSILGNVKWHFNTVVNVLPKMEMIAVQEWW 3qq7.1    --------------------------------------------------------------------------------  target    WSTSCEWADIVFAVDAWSELKHPDMCSSVTNPFLTVFPRTPLERPFDTRGDIECLDLVGKQLAKRTGDRRFADMWKFVEE 3qq7.1    --------------------------------------------------------------------------------  target    KKVEVYLQRILDHSSNTKGFKFPELEEKAKKGIPALMMTRTNPKTVGYEQVYDSRPWYTKTGRLEFYREEDEFIEAGENL 3qq7.1    --------------------------------------------------------------------------------  target    PVHREPIDSTFYEPNVIVAPAHPFIKAKGPEAYGVKVDDFDNETRQGRNIVKTWEETKKTVHPLAKDGYKFVFHTPKYRH 3qq7.1    --------------------------------------------------------------------------------  target    GAHTMPVDTDMVAMLFGPFGDIYRHDKRQPFAAEGYVDIHPDDAKALNIEDGDYVWIDSDPSDRPFRGWQKNDKDYKFSR 3qq7.1    ---------------------------------NSVVSLSQPKMDELQLFRGDTVLLKGKKR----------------RE  target    LLCRARYYPGTPRGITRMWFNMYGATPGSVEGHESRKDGLAKNPRTGYQAMFRSGSHQSATRGWLKPTWMTDSLVRKELF 3qq7.1    AVCIVLSDDTCSDEKIRMNR------------------------------------------------------------  target    GHAVNKGFLPDVHCPTGAPREAIVKITKAEPGGLNAKGLWRPAALGLRPKYENDKMKDYLAGKFTLAANPKKGGKK 3qq7.1    ---------------------------------------------------------------------------- ``` | | | | | | | | | | | | | | | | | | | | | | | | | | | | | | | | | | | | | | | | | | | | | | | | | |
|  | 7du7.1.A | mkDPBB\_sym1 protein  *Crystal structure of the rationally designed mkDPBB\_sym1 protein* | 0.02 |  | 25.53 | 0.07 | 434-498 | X-ray | 1.20 | monomer |  | HHblits | 0.32 |
| ``` target    LARDIAKVPGTTLFAIGMGPNQFFNNDNKDRTQFLLAALTGNIGKIAGNIGSYAGNYRVAMFNGVPQYIAENPFDIELDG 7du7.1    --------------------------------------------------------------------------------  target    AKPARPKLYWRAEPAHYYNHEDHPLKMGKTMITGKTHMPTPTKSLWFANANSILGNVKWHFNTVVNVLPKMEMIAVQEWW 7du7.1    --------------------------------------------------------------------------------  target    WSTSCEWADIVFAVDAWSELKHPDMCSSVTNPFLTVFPRTPLERPFDTRGDIECLDLVGKQLAKRTGDRRFADMWKFVEE 7du7.1    --------------------------------------------------------------------------------  target    KKVEVYLQRILDHSSNTKGFKFPELEEKAKKGIPALMMTRTNPKTVGYEQVYDSRPWYTKTGRLEFYREEDEFIEAGENL 7du7.1    --------------------------------------------------------------------------------  target    PVHREPIDSTFYEPNVIVAPAHPFIKAKGPEAYGVKVDDFDNETRQGRNIVKTWEETKKTVHPLAKDGYKFVFHTPKYRH 7du7.1    --------------------------------------------------------------------------------  target    GAHTMPVDTDMVAMLFGPFGDIYRHDKRQPFAAEGYVDIHPDDAKALNIEDGDYVWIDSDPSDRPFRGWQKNDKDYKFSR 7du7.1    ---------------------------------KGIVRMDKASRAKLGVSVGDYVEVKKVL------------------S  target    LLCRARYY--PGTPRGITRMWFNMYGATPGSVEGHESRKDGLAKNPRTGYQAMFRSGSHQSATRGWLKPTWMTDSLVRKE 7du7.1    VKLRVAEAYPEDVGKGIVRM------------------------------------------------------------  target    LFGHAVNKGFLPDVHCPTGAPREAIVKITKAEPGGLNAKGLWRPAALGLRPKYENDKMKDYLAGKFTLAANPKKGGKK 7du7.1    ------------------------------------------------------------------------------ ``` | | | | | | | | | | | | | | | | | | | | | | | | | | | | | | | | | | | | | | | | | | | | | | | | | |
|  | 7di1.1.A | mkDPBB\_sym\_86 protein  *Crystal structure of the rationally designed mkDPBB\_sym\_86 protein* | 0.02 |  | 23.40 | 0.07 | 434-498 | X-ray | 2.10 | monomer |  | HHblits | 0.32 |
| ``` target    LARDIAKVPGTTLFAIGMGPNQFFNNDNKDRTQFLLAALTGNIGKIAGNIGSYAGNYRVAMFNGVPQYIAENPFDIELDG 7di1.1    --------------------------------------------------------------------------------  target    AKPARPKLYWRAEPAHYYNHEDHPLKMGKTMITGKTHMPTPTKSLWFANANSILGNVKWHFNTVVNVLPKMEMIAVQEWW 7di1.1    --------------------------------------------------------------------------------  target    WSTSCEWADIVFAVDAWSELKHPDMCSSVTNPFLTVFPRTPLERPFDTRGDIECLDLVGKQLAKRTGDRRFADMWKFVEE 7di1.1    --------------------------------------------------------------------------------  target    KKVEVYLQRILDHSSNTKGFKFPELEEKAKKGIPALMMTRTNPKTVGYEQVYDSRPWYTKTGRLEFYREEDEFIEAGENL 7di1.1    --------------------------------------------------------------------------------  target    PVHREPIDSTFYEPNVIVAPAHPFIKAKGPEAYGVKVDDFDNETRQGRNIVKTWEETKKTVHPLAKDGYKFVFHTPKYRH 7di1.1    --------------------------------------------------------------------------------  target    GAHTMPVDTDMVAMLFGPFGDIYRHDKRQPFAAEGYVDIHPDDAKALNIEDGDYVWIDSDPSDRPFRGWQKNDKDYKFSR 7di1.1    ---------------------------------KRIVRMDKASRAKLGVSVGDYVEVKKVK------------------S  target    LLCRARYY--PGTPRGITRMWFNMYGATPGSVEGHESRKDGLAKNPRTGYQAMFRSGSHQSATRGWLKPTWMTDSLVRKE 7di1.1    VVARVAEAYPEDVGKGIVRM------------------------------------------------------------  target    LFGHAVNKGFLPDVHCPTGAPREAIVKITKAEPGGLNAKGLWRPAALGLRPKYENDKMKDYLAGKFTLAANPKKGGKK 7di1.1    ------------------------------------------------------------------------------ ``` | | | | | | | | | | | | | | | | | | | | | | | | | | | | | | | | | | | | | | | | | | | | | | | | | |
|  | 5g4g.1.A | VCP-LIKE ATPASE  *Structure of the ATPgS-bound VAT complex* | 0.01 |  | 20.41 | 0.08 | 434-498 | EM | 7.80 | homo-hexamer |  | HHblits | 0.28 |
| ``` target    LARDIAKVPGTTLFAIGMGPNQFFNNDNKDRTQFLLAALTGNIGKIAGNIGSYAGNYRVAMFNGVPQYIAENPFDIELDG 5g4g.1    --------------------------------------------------------------------------------  target    AKPARPKLYWRAEPAHYYNHEDHPLKMGKTMITGKTHMPTPTKSLWFANANSILGNVKWHFNTVVNVLPKMEMIAVQEWW 5g4g.1    --------------------------------------------------------------------------------  target    WSTSCEWADIVFAVDAWSELKHPDMCSSVTNPFLTVFPRTPLERPFDTRGDIECLDLVGKQLAKRTGDRRFADMWKFVEE 5g4g.1    --------------------------------------------------------------------------------  target    KKVEVYLQRILDHSSNTKGFKFPELEEKAKKGIPALMMTRTNPKTVGYEQVYDSRPWYTKTGRLEFYREEDEFIEAGENL 5g4g.1    --------------------------------------------------------------------------------  target    PVHREPIDSTFYEPNVIVAPAHPFIKAKGPEAYGVKVDDFDNETRQGRNIVKTWEETKKTVHPLAKDGYKFVFHTPKYRH 5g4g.1    --------------------------------------------------------------------------------  target    GAHTMPVDTDMVAMLFGPFGDIYRHDKRQPFAAEGYVDIHPDDAKALNIEDGDYVWIDSDPSDRPFRGWQKNDKDYKFSR 5g4g.1    ---------------------------------MSRVRLDESSRRLLDAEIGDVVEIEKVRK----------------TV  target    LLCRARYYPGTPRGITRMWFNMYGATPGSVEGHESRKDGLAKNPRTGYQAMFRSGSHQSATRGWLKPTWMTDSLVRKELF 5g4g.1    GRVYRARPEDENKGIVRI--------------------------------------------------------------  target    GHAVNKGFLPDVHCPTGAPREAIVKITKAEPGGLNAKGLWRPAALGLRPKYENDKMKDYLAGKFTLAANPKKGGKK 5g4g.1    ---------------------------------------------------------------------------- ``` | | | | | | | | | | | | | | | | | | | | | | | | | | | | | | | | | | | | | | | | | | | | | | | | | |
|  | 4ga5.1.A | Putative thymidine phosphorylase  *Crystal structure of AMP phosphorylase C-terminal deletion mutant in the apo-form* | 0.03 |  | 18.37 | 0.08 | 434-499 | X-ray | 3.25 | homo-dimer |  | HHblits | 0.28 |
| ``` target    LARDIAKVPGTTLFAIGMGPNQFFNNDNKDRTQFLLAALTGNIGKIAGNIGSYAGNYRVAMFNGVPQYIAENPFDIELDG 4ga5.1    --------------------------------------------------------------------------------  target    AKPARPKLYWRAEPAHYYNHEDHPLKMGKTMITGKTHMPTPTKSLWFANANSILGNVKWHFNTVVNVLPKMEMIAVQEWW 4ga5.1    --------------------------------------------------------------------------------  target    WSTSCEWADIVFAVDAWSELKHPDMCSSVTNPFLTVFPRTPLERPFDTRGDIECLDLVGKQLAKRTGDRRFADMWKFVEE 4ga5.1    --------------------------------------------------------------------------------  target    KKVEVYLQRILDHSSNTKGFKFPELEEKAKKGIPALMMTRTNPKTVGYEQVYDSRPWYTKTGRLEFYREEDEFIEAGENL 4ga5.1    --------------------------------------------------------------------------------  target    PVHREPIDSTFYEPNVIVAPAHPFIKAKGPEAYGVKVDDFDNETRQGRNIVKTWEETKKTVHPLAKDGYKFVFHTPKYRH 4ga5.1    --------------------------------------------------------------------------------  target    GAHTMPVDTDMVAMLFGPFGDIYRHDKRQPFAAEGYVDIHPDDAKALNIEDGDYVWIDSDPSDRPFRGWQKNDKDYKFSR 4ga5.1    ---------------------------------RYTVLINEEDAKEAKLHPDDLVKIEAGK-----------------KA  target    LLCRARYYPGTPRGITRMWFNMYGATPGSVEGHESRKDGLAKNPRTGYQAMFRSGSHQSATRGWLKPTWMTDSLVRKELF 4ga5.1    VYGSVALSNLVGKGEVGIS-------------------------------------------------------------  target    GHAVNKGFLPDVHCPTGAPREAIVKITKAEPGGLNAKGLWRPAALGLRPKYENDKMKDYLAGKFTLAANPKKGGKK 4ga5.1    ---------------------------------------------------------------------------- ``` | | | | | | | | | | | | | | | | | | | | | | | | | | | | | | | | | | | | | | | | | | | | | | | | | |
|  | 4ga6.1.A | Putative thymidine phosphorylase  *Crystal structure of AMP phosphorylase C-terminal deletion mutant in complex with substrates* | 0.03 |  | 18.37 | 0.08 | 434-499 | X-ray | 2.21 | homo-dimer | 2 x AMP | HHblits | 0.28 |
| ``` target    LARDIAKVPGTTLFAIGMGPNQFFNNDNKDRTQFLLAALTGNIGKIAGNIGSYAGNYRVAMFNGVPQYIAENPFDIELDG 4ga6.1    --------------------------------------------------------------------------------  target    AKPARPKLYWRAEPAHYYNHEDHPLKMGKTMITGKTHMPTPTKSLWFANANSILGNVKWHFNTVVNVLPKMEMIAVQEWW 4ga6.1    --------------------------------------------------------------------------------  target    WSTSCEWADIVFAVDAWSELKHPDMCSSVTNPFLTVFPRTPLERPFDTRGDIECLDLVGKQLAKRTGDRRFADMWKFVEE 4ga6.1    --------------------------------------------------------------------------------  target    KKVEVYLQRILDHSSNTKGFKFPELEEKAKKGIPALMMTRTNPKTVGYEQVYDSRPWYTKTGRLEFYREEDEFIEAGENL 4ga6.1    --------------------------------------------------------------------------------  target    PVHREPIDSTFYEPNVIVAPAHPFIKAKGPEAYGVKVDDFDNETRQGRNIVKTWEETKKTVHPLAKDGYKFVFHTPKYRH 4ga6.1    --------------------------------------------------------------------------------  target    GAHTMPVDTDMVAMLFGPFGDIYRHDKRQPFAAEGYVDIHPDDAKALNIEDGDYVWIDSDPSDRPFRGWQKNDKDYKFSR 4ga6.1    ---------------------------------RYTVLINEEDAKEAKLHPDDLVKIEAGK-----------------KA  target    LLCRARYYPGTPRGITRMWFNMYGATPGSVEGHESRKDGLAKNPRTGYQAMFRSGSHQSATRGWLKPTWMTDSLVRKELF 4ga6.1    VYGSVALSNLVGKGEVGIS-------------------------------------------------------------  target    GHAVNKGFLPDVHCPTGAPREAIVKITKAEPGGLNAKGLWRPAALGLRPKYENDKMKDYLAGKFTLAANPKKGGKK 4ga6.1    ---------------------------------------------------------------------------- ``` | | | | | | | | | | | | | | | | | | | | | | | | | | | | | | | | | | | | | | | | | | | | | | | | | |
|  | 5b6c.1.A | Transitional endoplasmic reticulum ATPase  *Structural Details of Ufd1 binding to p97* | 0.02 |  | 16.00 | 0.08 | 435-500 | X-ray | 1.55 | hetero-oligomer |  | HHblits | 0.26 |
| ``` target    LARDIAKVPGTTLFAIGMGPNQFFNNDNKDRTQFLLAALTGNIGKIAGNIGSYAGNYRVAMFNGVPQYIAENPFDIELDG 5b6c.1    --------------------------------------------------------------------------------  target    AKPARPKLYWRAEPAHYYNHEDHPLKMGKTMITGKTHMPTPTKSLWFANANSILGNVKWHFNTVVNVLPKMEMIAVQEWW 5b6c.1    --------------------------------------------------------------------------------  target    WSTSCEWADIVFAVDAWSELKHPDMCSSVTNPFLTVFPRTPLERPFDTRGDIECLDLVGKQLAKRTGDRRFADMWKFVEE 5b6c.1    --------------------------------------------------------------------------------  target    KKVEVYLQRILDHSSNTKGFKFPELEEKAKKGIPALMMTRTNPKTVGYEQVYDSRPWYTKTGRLEFYREEDEFIEAGENL 5b6c.1    --------------------------------------------------------------------------------  target    PVHREPIDSTFYEPNVIVAPAHPFIKAKGPEAYGVKVDDFDNETRQGRNIVKTWEETKKTVHPLAKDGYKFVFHTPKYRH 5b6c.1    --------------------------------------------------------------------------------  target    GAHTMPVDTDMVAMLFGPFGDIYRHDKRQPFAAEGYVDIHPDDAKALNIEDGDYVWIDSDPSDRPFRGWQKNDKDYKFSR 5b6c.1    ----------------------------------SVVSLSQPKMDELQLFRGDTVLLKGKKR----------------RE  target    LLCRARYYPGTPRGITRMWFNMYGATPGSVEGHESRKDGLAKNPRTGYQAMFRSGSHQSATRGWLKPTWMTDSLVRKELF 5b6c.1    AVCIVLSDDTCSDEKIRMNR------------------------------------------------------------  target    GHAVNKGFLPDVHCPTGAPREAIVKITKAEPGGLNAKGLWRPAALGLRPKYENDKMKDYLAGKFTLAANPKKGGKK 5b6c.1    ---------------------------------------------------------------------------- ``` | | | | | | | | | | | | | | | | | | | | | | | | | | | | | | | | | | | | | | | | | | | | | | | | | |
|  | 5epp.1.A | Transitional endoplasmic reticulum ATPase  *Structural Insights into the Interaction of p97 N-terminus Domain and VBM Motif in Rhomboid Protease, RHBDL4* | 0.02 |  | 16.00 | 0.08 | 435-500 | X-ray | 1.88 | hetero-oligomer |  | HHblits | 0.26 |
| ``` target    LARDIAKVPGTTLFAIGMGPNQFFNNDNKDRTQFLLAALTGNIGKIAGNIGSYAGNYRVAMFNGVPQYIAENPFDIELDG 5epp.1    --------------------------------------------------------------------------------  target    AKPARPKLYWRAEPAHYYNHEDHPLKMGKTMITGKTHMPTPTKSLWFANANSILGNVKWHFNTVVNVLPKMEMIAVQEWW 5epp.1    --------------------------------------------------------------------------------  target    WSTSCEWADIVFAVDAWSELKHPDMCSSVTNPFLTVFPRTPLERPFDTRGDIECLDLVGKQLAKRTGDRRFADMWKFVEE 5epp.1    --------------------------------------------------------------------------------  target    KKVEVYLQRILDHSSNTKGFKFPELEEKAKKGIPALMMTRTNPKTVGYEQVYDSRPWYTKTGRLEFYREEDEFIEAGENL 5epp.1    --------------------------------------------------------------------------------  target    PVHREPIDSTFYEPNVIVAPAHPFIKAKGPEAYGVKVDDFDNETRQGRNIVKTWEETKKTVHPLAKDGYKFVFHTPKYRH 5epp.1    --------------------------------------------------------------------------------  target    GAHTMPVDTDMVAMLFGPFGDIYRHDKRQPFAAEGYVDIHPDDAKALNIEDGDYVWIDSDPSDRPFRGWQKNDKDYKFSR 5epp.1    ----------------------------------SVVSLSQPKMDELQLFRGDTVLLKGKKR----------------RE  target    LLCRARYYPGTPRGITRMWFNMYGATPGSVEGHESRKDGLAKNPRTGYQAMFRSGSHQSATRGWLKPTWMTDSLVRKELF 5epp.1    AVCIVLSDDTCSDEKIRMNR------------------------------------------------------------  target    GHAVNKGFLPDVHCPTGAPREAIVKITKAEPGGLNAKGLWRPAALGLRPKYENDKMKDYLAGKFTLAANPKKGGKK 5epp.1    ---------------------------------------------------------------------------- ``` | | | | | | | | | | | | | | | | | | | | | | | | | | | | | | | | | | | | | | | | | | | | | | | | | |
|  | 5glf.2.A | Transitional endoplasmic reticulum ATPase  *Structural insights into the interaction of p97 N-terminal domain and SHP motif in Derlin-1 rhomboid pseudoprotease* | 0.02 |  | 16.00 | 0.08 | 435-500 | X-ray | 2.25 | hetero-1-1-mer |  | HHblits | 0.26 |
| ``` target    LARDIAKVPGTTLFAIGMGPNQFFNNDNKDRTQFLLAALTGNIGKIAGNIGSYAGNYRVAMFNGVPQYIAENPFDIELDG 5glf.2    --------------------------------------------------------------------------------  target    AKPARPKLYWRAEPAHYYNHEDHPLKMGKTMITGKTHMPTPTKSLWFANANSILGNVKWHFNTVVNVLPKMEMIAVQEWW 5glf.2    --------------------------------------------------------------------------------  target    WSTSCEWADIVFAVDAWSELKHPDMCSSVTNPFLTVFPRTPLERPFDTRGDIECLDLVGKQLAKRTGDRRFADMWKFVEE 5glf.2    --------------------------------------------------------------------------------  target    KKVEVYLQRILDHSSNTKGFKFPELEEKAKKGIPALMMTRTNPKTVGYEQVYDSRPWYTKTGRLEFYREEDEFIEAGENL 5glf.2    --------------------------------------------------------------------------------  target    PVHREPIDSTFYEPNVIVAPAHPFIKAKGPEAYGVKVDDFDNETRQGRNIVKTWEETKKTVHPLAKDGYKFVFHTPKYRH 5glf.2    --------------------------------------------------------------------------------  target    GAHTMPVDTDMVAMLFGPFGDIYRHDKRQPFAAEGYVDIHPDDAKALNIEDGDYVWIDSDPSDRPFRGWQKNDKDYKFSR 5glf.2    ----------------------------------SVVSLSQPKMDELQLFRGDTVLLKGKKR----------------RE  target    LLCRARYYPGTPRGITRMWFNMYGATPGSVEGHESRKDGLAKNPRTGYQAMFRSGSHQSATRGWLKPTWMTDSLVRKELF 5glf.2    AVCIVLSDDTCSDEKIRMNR------------------------------------------------------------  target    GHAVNKGFLPDVHCPTGAPREAIVKITKAEPGGLNAKGLWRPAALGLRPKYENDKMKDYLAGKFTLAANPKKGGKK 5glf.2    ---------------------------------------------------------------------------- ``` | | | | | | | | | | | | | | | | | | | | | | | | | | | | | | | | | | | | | | | | | | | | | | | | | |
|  | 5glf.3.A | Transitional endoplasmic reticulum ATPase  *Structural insights into the interaction of p97 N-terminal domain and SHP motif in Derlin-1 rhomboid pseudoprotease* | 0.02 |  | 16.00 | 0.08 | 435-500 | X-ray | 2.25 | hetero-1-1-mer |  | HHblits | 0.26 |
| ``` target    LARDIAKVPGTTLFAIGMGPNQFFNNDNKDRTQFLLAALTGNIGKIAGNIGSYAGNYRVAMFNGVPQYIAENPFDIELDG 5glf.3    --------------------------------------------------------------------------------  target    AKPARPKLYWRAEPAHYYNHEDHPLKMGKTMITGKTHMPTPTKSLWFANANSILGNVKWHFNTVVNVLPKMEMIAVQEWW 5glf.3    --------------------------------------------------------------------------------  target    WSTSCEWADIVFAVDAWSELKHPDMCSSVTNPFLTVFPRTPLERPFDTRGDIECLDLVGKQLAKRTGDRRFADMWKFVEE 5glf.3    --------------------------------------------------------------------------------  target    KKVEVYLQRILDHSSNTKGFKFPELEEKAKKGIPALMMTRTNPKTVGYEQVYDSRPWYTKTGRLEFYREEDEFIEAGENL 5glf.3    --------------------------------------------------------------------------------  target    PVHREPIDSTFYEPNVIVAPAHPFIKAKGPEAYGVKVDDFDNETRQGRNIVKTWEETKKTVHPLAKDGYKFVFHTPKYRH 5glf.3    --------------------------------------------------------------------------------  target    GAHTMPVDTDMVAMLFGPFGDIYRHDKRQPFAAEGYVDIHPDDAKALNIEDGDYVWIDSDPSDRPFRGWQKNDKDYKFSR 5glf.3    ----------------------------------SVVSLSQPKMDELQLFRGDTVLLKGKKR----------------RE  target    LLCRARYYPGTPRGITRMWFNMYGATPGSVEGHESRKDGLAKNPRTGYQAMFRSGSHQSATRGWLKPTWMTDSLVRKELF 5glf.3    AVCIVLSDDTCSDEKIRMNR------------------------------------------------------------  target    GHAVNKGFLPDVHCPTGAPREAIVKITKAEPGGLNAKGLWRPAALGLRPKYENDKMKDYLAGKFTLAANPKKGGKK 5glf.3    ---------------------------------------------------------------------------- ``` | | | | | | | | | | | | | | | | | | | | | | | | | | | | | | | | | | | | | | | | | | | | | | | | | |
|  | 5glf.1.A | Transitional endoplasmic reticulum ATPase  *Structural insights into the interaction of p97 N-terminal domain and SHP motif in Derlin-1 rhomboid pseudoprotease* | 0.02 |  | 16.00 | 0.08 | 435-500 | X-ray | 2.25 | hetero-1-1-mer |  | HHblits | 0.26 |
| ``` target    LARDIAKVPGTTLFAIGMGPNQFFNNDNKDRTQFLLAALTGNIGKIAGNIGSYAGNYRVAMFNGVPQYIAENPFDIELDG 5glf.1    --------------------------------------------------------------------------------  target    AKPARPKLYWRAEPAHYYNHEDHPLKMGKTMITGKTHMPTPTKSLWFANANSILGNVKWHFNTVVNVLPKMEMIAVQEWW 5glf.1    --------------------------------------------------------------------------------  target    WSTSCEWADIVFAVDAWSELKHPDMCSSVTNPFLTVFPRTPLERPFDTRGDIECLDLVGKQLAKRTGDRRFADMWKFVEE 5glf.1    --------------------------------------------------------------------------------  target    KKVEVYLQRILDHSSNTKGFKFPELEEKAKKGIPALMMTRTNPKTVGYEQVYDSRPWYTKTGRLEFYREEDEFIEAGENL 5glf.1    --------------------------------------------------------------------------------  target    PVHREPIDSTFYEPNVIVAPAHPFIKAKGPEAYGVKVDDFDNETRQGRNIVKTWEETKKTVHPLAKDGYKFVFHTPKYRH 5glf.1    --------------------------------------------------------------------------------  target    GAHTMPVDTDMVAMLFGPFGDIYRHDKRQPFAAEGYVDIHPDDAKALNIEDGDYVWIDSDPSDRPFRGWQKNDKDYKFSR 5glf.1    ----------------------------------SVVSLSQPKMDELQLFRGDTVLLKGKKR----------------RE  target    LLCRARYYPGTPRGITRMWFNMYGATPGSVEGHESRKDGLAKNPRTGYQAMFRSGSHQSATRGWLKPTWMTDSLVRKELF 5glf.1    AVCIVLSDDTCSDEKIRMNR------------------------------------------------------------  target    GHAVNKGFLPDVHCPTGAPREAIVKITKAEPGGLNAKGLWRPAALGLRPKYENDKMKDYLAGKFTLAANPKKGGKK 5glf.1    ---------------------------------------------------------------------------- ``` | | | | | | | | | | | | | | | | | | | | | | | | | | | | | | | | | | | | | | | | | | | | | | | | | |
|  | 5glf.4.A | Transitional endoplasmic reticulum ATPase  *Structural insights into the interaction of p97 N-terminal domain and SHP motif in Derlin-1 rhomboid pseudoprotease* | 0.02 |  | 16.00 | 0.08 | 435-500 | X-ray | 2.25 | hetero-1-1-mer |  | HHblits | 0.26 |
| ``` target    LARDIAKVPGTTLFAIGMGPNQFFNNDNKDRTQFLLAALTGNIGKIAGNIGSYAGNYRVAMFNGVPQYIAENPFDIELDG 5glf.4    --------------------------------------------------------------------------------  target    AKPARPKLYWRAEPAHYYNHEDHPLKMGKTMITGKTHMPTPTKSLWFANANSILGNVKWHFNTVVNVLPKMEMIAVQEWW 5glf.4    --------------------------------------------------------------------------------  target    WSTSCEWADIVFAVDAWSELKHPDMCSSVTNPFLTVFPRTPLERPFDTRGDIECLDLVGKQLAKRTGDRRFADMWKFVEE 5glf.4    --------------------------------------------------------------------------------  target    KKVEVYLQRILDHSSNTKGFKFPELEEKAKKGIPALMMTRTNPKTVGYEQVYDSRPWYTKTGRLEFYREEDEFIEAGENL 5glf.4    --------------------------------------------------------------------------------  target    PVHREPIDSTFYEPNVIVAPAHPFIKAKGPEAYGVKVDDFDNETRQGRNIVKTWEETKKTVHPLAKDGYKFVFHTPKYRH 5glf.4    --------------------------------------------------------------------------------  target    GAHTMPVDTDMVAMLFGPFGDIYRHDKRQPFAAEGYVDIHPDDAKALNIEDGDYVWIDSDPSDRPFRGWQKNDKDYKFSR 5glf.4    ----------------------------------SVVSLSQPKMDELQLFRGDTVLLKGKKR----------------RE  target    LLCRARYYPGTPRGITRMWFNMYGATPGSVEGHESRKDGLAKNPRTGYQAMFRSGSHQSATRGWLKPTWMTDSLVRKELF 5glf.4    AVCIVLSDDTCSDEKIRMNR------------------------------------------------------------  target    GHAVNKGFLPDVHCPTGAPREAIVKITKAEPGGLNAKGLWRPAALGLRPKYENDKMKDYLAGKFTLAANPKKGGKK 5glf.4    ---------------------------------------------------------------------------- ``` | | | | | | | | | | | | | | | | | | | | | | | | | | | | | | | | | | | | | | | | | | | | | | | | | |
|  | 7dg7.1.A | ATPase of the AAA+ class  *DPBB domain of VCP-like ATPase from Methanopyrus kandleri* | 0.02 |  | 21.28 | 0.07 | 434-498 | X-ray | 1.60 | monomer | 2 x IMD, 8 x ZN | HHblits | 0.31 |
| ``` target    LARDIAKVPGTTLFAIGMGPNQFFNNDNKDRTQFLLAALTGNIGKIAGNIGSYAGNYRVAMFNGVPQYIAENPFDIELDG 7dg7.1    --------------------------------------------------------------------------------  target    AKPARPKLYWRAEPAHYYNHEDHPLKMGKTMITGKTHMPTPTKSLWFANANSILGNVKWHFNTVVNVLPKMEMIAVQEWW 7dg7.1    --------------------------------------------------------------------------------  target    WSTSCEWADIVFAVDAWSELKHPDMCSSVTNPFLTVFPRTPLERPFDTRGDIECLDLVGKQLAKRTGDRRFADMWKFVEE 7dg7.1    --------------------------------------------------------------------------------  target    KKVEVYLQRILDHSSNTKGFKFPELEEKAKKGIPALMMTRTNPKTVGYEQVYDSRPWYTKTGRLEFYREEDEFIEAGENL 7dg7.1    --------------------------------------------------------------------------------  target    PVHREPIDSTFYEPNVIVAPAHPFIKAKGPEAYGVKVDDFDNETRQGRNIVKTWEETKKTVHPLAKDGYKFVFHTPKYRH 7dg7.1    --------------------------------------------------------------------------------  target    GAHTMPVDTDMVAMLFGPFGDIYRHDKRQPFAAEGYVDIHPDDAKALNIEDGDYVWIDSDPSDRPFRGWQKNDKDYKFSR 7dg7.1    ---------------------------------KRAVRMDKASRDRIGVSEGDLVKITGSK------------------T  target    LLCRARYY--PGTPRGITRMWFNMYGATPGSVEGHESRKDGLAKNPRTGYQAMFRSGSHQSATRGWLKPTWMTDSLVRKE 7dg7.1    TVARVLPAKKEDVGKGIVRM------------------------------------------------------------  target    LFGHAVNKGFLPDVHCPTGAPREAIVKITKAEPGGLNAKGLWRPAALGLRPKYENDKMKDYLAGKFTLAANPKKGGKK 7dg7.1    ------------------------------------------------------------------------------ ``` | | | | | | | | | | | | | | | | | | | | | | | | | | | | | | | | | | | | | | | | | | | | | | | | | |
|  | 7t2r.1.A | NiFe hydrogenase subunit A  *Structure of electron bifurcating Ni-Fe hydrogenase complex HydABCSL in FMN-free apo state* | 0.01 |  | 5.66 | 0.08 | 121-175 | EM | 0.00 | hetero-2-2-2-2-2-mer | 6 x FES, 12 x SF4, 2 x 3NI, 2 x FCO | HHblits | 0.21 |
| ``` target    LARDIAKVPGTTLFAIGMGPNQFFNNDNKDRTQFLLAALTGNIGKIAGNIGSYAGNYRVAMFNGVPQYIAENPFDIELDG 7t2r.1    --------------------------------------------------------------------------------  target    AKPARPKLYWRAEPAHYYNHEDHPLKMGKTMITGKTHMPTPTKSLWFANANSILGNVKWHF-NTVVNVLPKME-MIAVQE 7t2r.1    ----------------------------------------DSDLIITMFADPQKEAP--VVASYIRVACLHRNAKLMNLS  target    WWWSTSCEWADIVFAVDAWSELKHPDMCSSVTNPFLTVFPRTPLERPFDTRGDIECLDLVGKQLAKRTGDRRFADMWKFV 7t2r.1    YGPSPFPGLVDLDIRLP---------------------------------------------------------------  target    EEKKVEVYLQRILDHSSNTKGFKFPELEEKAKKGIPALMMTRTNPKTVGYEQVYDSRPWYTKTGRLEFYREEDEFIEAGE 7t2r.1    --------------------------------------------------------------------------------  target    NLPVHREPIDSTFYEPNVIVAPAHPFIKAKGPEAYGVKVDDFDNETRQGRNIVKTWEETKKTVHPLAKDGYKFVFHTPKY 7t2r.1    --------------------------------------------------------------------------------  target    RHGAHTMPVDTDMVAMLFGPFGDIYRHDKRQPFAAEGYVDIHPDDAKALNIEDGDYVWIDSDPSDRPFRGWQKNDKDYKF 7t2r.1    --------------------------------------------------------------------------------  target    SRLLCRARYYPGTPRGITRMWFNMYGATPGSVEGHESRKDGLAKNPRTGYQAMFRSGSHQSATRGWLKPTWMTDSLVRKE 7t2r.1    --------------------------------------------------------------------------------  target    LFGHAVNKGFLPDVHCPTGAPREAIVKITKAEPGGLNAKGLWRPAALGLRPKYENDKMKDYLAGKFTLAANPKKGGKK 7t2r.1    ------------------------------------------------------------------------------ ``` | | | | | | | | | | | | | | | | | | | | | | | | | | | | | | | | | | | | | | | | | | | | | | | | | |
|  | 7t30.1.A | NiFe hydrogenase subunit A  *Structure of electron bifurcating Ni-Fe hydrogenase complex HydABCSL in FMN/NAD(H) bound state* | 0.01 |  | 5.66 | 0.08 | 121-175 | EM | 0.00 | hetero-2-2-2-2-2-mer | 4 x FES, 12 x SF4, 2 x NAD, 2 x FMN, 2 x 3NI, 2 x FCO | HHblits | 0.21 |
| ``` target    LARDIAKVPGTTLFAIGMGPNQFFNNDNKDRTQFLLAALTGNIGKIAGNIGSYAGNYRVAMFNGVPQYIAENPFDIELDG 7t30.1    --------------------------------------------------------------------------------  target    AKPARPKLYWRAEPAHYYNHEDHPLKMGKTMITGKTHMPTPTKSLWFANANSILGNVKWHF-NTVVNVLPKME-MIAVQE 7t30.1    ----------------------------------------DSDLIITMFADPQKEAP--VVASYIRVACLHRNAKLMNLS  target    WWWSTSCEWADIVFAVDAWSELKHPDMCSSVTNPFLTVFPRTPLERPFDTRGDIECLDLVGKQLAKRTGDRRFADMWKFV 7t30.1    YGPSPFPGLVDLDIRLP---------------------------------------------------------------  target    EEKKVEVYLQRILDHSSNTKGFKFPELEEKAKKGIPALMMTRTNPKTVGYEQVYDSRPWYTKTGRLEFYREEDEFIEAGE 7t30.1    --------------------------------------------------------------------------------  target    NLPVHREPIDSTFYEPNVIVAPAHPFIKAKGPEAYGVKVDDFDNETRQGRNIVKTWEETKKTVHPLAKDGYKFVFHTPKY 7t30.1    --------------------------------------------------------------------------------  target    RHGAHTMPVDTDMVAMLFGPFGDIYRHDKRQPFAAEGYVDIHPDDAKALNIEDGDYVWIDSDPSDRPFRGWQKNDKDYKF 7t30.1    --------------------------------------------------------------------------------  target    SRLLCRARYYPGTPRGITRMWFNMYGATPGSVEGHESRKDGLAKNPRTGYQAMFRSGSHQSATRGWLKPTWMTDSLVRKE 7t30.1    --------------------------------------------------------------------------------  target    LFGHAVNKGFLPDVHCPTGAPREAIVKITKAEPGGLNAKGLWRPAALGLRPKYENDKMKDYLAGKFTLAANPKKGGKK 7t30.1    ------------------------------------------------------------------------------ ``` | | | | | | | | | | | | | | | | | | | | | | | | | | | | | | | | | | | | | | | | | | | | | | | | | |
|  | 5cup.1.A | Phosphate propanoyltransferase  *Structure of Rhodopseudomonas palustris PduL - phosphate bound form* | 0.00 |  | 29.63 | 0.04 | 433-459 | X-ray | 2.10 | homo-dimer | 4 x ZN | HHblits | 0.39 |
| ``` target    LARDIAKVPGTTLFAIGMGPNQFFNNDNKDRTQFLLAALTGNIGKIAGNIGSYAGNYRVAMFNGVPQYIAENPFDIELDG 5cup.1    --------------------------------------------------------------------------------  target    AKPARPKLYWRAEPAHYYNHEDHPLKMGKTMITGKTHMPTPTKSLWFANANSILGNVKWHFNTVVNVLPKMEMIAVQEWW 5cup.1    --------------------------------------------------------------------------------  target    WSTSCEWADIVFAVDAWSELKHPDMCSSVTNPFLTVFPRTPLERPFDTRGDIECLDLVGKQLAKRTGDRRFADMWKFVEE 5cup.1    --------------------------------------------------------------------------------  target    KKVEVYLQRILDHSSNTKGFKFPELEEKAKKGIPALMMTRTNPKTVGYEQVYDSRPWYTKTGRLEFYREEDEFIEAGENL 5cup.1    --------------------------------------------------------------------------------  target    PVHREPIDSTFYEPNVIVAPAHPFIKAKGPEAYGVKVDDFDNETRQGRNIVKTWEETKKTVHPLAKDGYKFVFHTPKYRH 5cup.1    --------------------------------------------------------------------------------  target    GAHTMPVDTDMVAMLFGPFGDIYRHDKRQPFAAEGYVDIHPDDAKALNIEDGDYVWIDSDPSDRPFRGWQKNDKDYKFSR 5cup.1    --------------------------------AQRHIHMHPSTAAKLGLRNGDEVDVEA---------------------  target    LLCRARYYPGTPRGITRMWFNMYGATPGSVEGHESRKDGLAKNPRTGYQAMFRSGSHQSATRGWLKPTWMTDSLVRKELF 5cup.1    --------------------------------------------------------------------------------  target    GHAVNKGFLPDVHCPTGAPREAIVKITKAEPGGLNAKGLWRPAALGLRPKYENDKMKDYLAGKFTLAANPKKGGKK 5cup.1    ---------------------------------------------------------------------------- ``` | | | | | | | | | | | | | | | | | | | | | | | | | | | | | | | | | | | | | | | | | | | | | | | | | |
|  | 5cuo.1.A | Phosphate propanoyltransferase  *Structure of Rhodopseudomonas palustris PduL - CoA bound form* | 0.00 |  | 29.63 | 0.04 | 433-459 | X-ray | 1.54 | homo-dimer | 2 x COA, 4 x ZN | HHblits | 0.39 |
| ``` target    LARDIAKVPGTTLFAIGMGPNQFFNNDNKDRTQFLLAALTGNIGKIAGNIGSYAGNYRVAMFNGVPQYIAENPFDIELDG 5cuo.1    --------------------------------------------------------------------------------  target    AKPARPKLYWRAEPAHYYNHEDHPLKMGKTMITGKTHMPTPTKSLWFANANSILGNVKWHFNTVVNVLPKMEMIAVQEWW 5cuo.1    --------------------------------------------------------------------------------  target    WSTSCEWADIVFAVDAWSELKHPDMCSSVTNPFLTVFPRTPLERPFDTRGDIECLDLVGKQLAKRTGDRRFADMWKFVEE 5cuo.1    --------------------------------------------------------------------------------  target    KKVEVYLQRILDHSSNTKGFKFPELEEKAKKGIPALMMTRTNPKTVGYEQVYDSRPWYTKTGRLEFYREEDEFIEAGENL 5cuo.1    --------------------------------------------------------------------------------  target    PVHREPIDSTFYEPNVIVAPAHPFIKAKGPEAYGVKVDDFDNETRQGRNIVKTWEETKKTVHPLAKDGYKFVFHTPKYRH 5cuo.1    --------------------------------------------------------------------------------  target    GAHTMPVDTDMVAMLFGPFGDIYRHDKRQPFAAEGYVDIHPDDAKALNIEDGDYVWIDSDPSDRPFRGWQKNDKDYKFSR 5cuo.1    --------------------------------AQRHIHMHPSTAAKLGLRNGDEVDVEA---------------------  target    LLCRARYYPGTPRGITRMWFNMYGATPGSVEGHESRKDGLAKNPRTGYQAMFRSGSHQSATRGWLKPTWMTDSLVRKELF 5cuo.1    --------------------------------------------------------------------------------  target    GHAVNKGFLPDVHCPTGAPREAIVKITKAEPGGLNAKGLWRPAALGLRPKYENDKMKDYLAGKFTLAANPKKGGKK 5cuo.1    ---------------------------------------------------------------------------- ``` | | | | | | | | | | | | | | | | | | | | | | | | | | | | | | | | | | | | | | | | | | | | | | | | | |
|  | 5e7p.1.A | Cell division control protein Cdc48  *Crystal Structure of MSMEG\_0858 (Uniprot A0QQS4), a AAA ATPase.* | 0.00 |  | 32.14 | 0.04 | 434-461 | X-ray | 2.51 | monomer | 2 x ADP | HHblits | 0.35 |
| ``` target    LARDIAKVPGTTLFAIGMGPNQFFNNDNKDRTQFLLAALTGNIGKIAGNIGSYAGNYRVAMFNGVPQYIAENPFDIELDG 5e7p.1    --------------------------------------------------------------------------------  target    AKPARPKLYWRAEPAHYYNHEDHPLKMGKTMITGKTHMPTPTKSLWFANANSILGNVKWHFNTVVNVLPKMEMIAVQEWW 5e7p.1    --------------------------------------------------------------------------------  target    WSTSCEWADIVFAVDAWSELKHPDMCSSVTNPFLTVFPRTPLERPFDTRGDIECLDLVGKQLAKRTGDRRFADMWKFVEE 5e7p.1    --------------------------------------------------------------------------------  target    KKVEVYLQRILDHSSNTKGFKFPELEEKAKKGIPALMMTRTNPKTVGYEQVYDSRPWYTKTGRLEFYREEDEFIEAGENL 5e7p.1    --------------------------------------------------------------------------------  target    PVHREPIDSTFYEPNVIVAPAHPFIKAKGPEAYGVKVDDFDNETRQGRNIVKTWEETKKTVHPLAKDGYKFVFHTPKYRH 5e7p.1    --------------------------------------------------------------------------------  target    GAHTMPVDTDMVAMLFGPFGDIYRHDKRQPFAAEGYVDIHPDDAKALNIEDGDYVWIDSDPSDRPFRGWQKNDKDYKFSR 5e7p.1    ---------------------------------RGVVRLHPEVLAALGIREWDAVALTGTR-------------------  target    LLCRARYYPGTPRGITRMWFNMYGATPGSVEGHESRKDGLAKNPRTGYQAMFRSGSHQSATRGWLKPTWMTDSLVRKELF 5e7p.1    --------------------------------------------------------------------------------  target    GHAVNKGFLPDVHCPTGAPREAIVKITKAEPGGLNAKGLWRPAALGLRPKYENDKMKDYLAGKFTLAANPKKGGKK 5e7p.1    ---------------------------------------------------------------------------- ``` | | | | | | | | | | | | | | | | | | | | | | | | | | | | | | | | | | | | | | | | | | | | | | | | | |
|  | 6hd3.1.A | Cell division control protein 48 homolog A  *Common mode of remodeling AAA ATPases p97/CDC48 by their disassembly cofactors ASPL/PUX1* | 0.00 |  | 25.93 | 0.04 | 435-461 | X-ray | 2.80 | homo-24-mer | 24 x ADP | HHblits | 0.34 |
| ``` target    LARDIAKVPGTTLFAIGMGPNQFFNNDNKDRTQFLLAALTGNIGKIAGNIGSYAGNYRVAMFNGVPQYIAENPFDIELDG 6hd3.1    --------------------------------------------------------------------------------  target    AKPARPKLYWRAEPAHYYNHEDHPLKMGKTMITGKTHMPTPTKSLWFANANSILGNVKWHFNTVVNVLPKMEMIAVQEWW 6hd3.1    --------------------------------------------------------------------------------  target    WSTSCEWADIVFAVDAWSELKHPDMCSSVTNPFLTVFPRTPLERPFDTRGDIECLDLVGKQLAKRTGDRRFADMWKFVEE 6hd3.1    --------------------------------------------------------------------------------  target    KKVEVYLQRILDHSSNTKGFKFPELEEKAKKGIPALMMTRTNPKTVGYEQVYDSRPWYTKTGRLEFYREEDEFIEAGENL 6hd3.1    --------------------------------------------------------------------------------  target    PVHREPIDSTFYEPNVIVAPAHPFIKAKGPEAYGVKVDDFDNETRQGRNIVKTWEETKKTVHPLAKDGYKFVFHTPKYRH 6hd3.1    --------------------------------------------------------------------------------  target    GAHTMPVDTDMVAMLFGPFGDIYRHDKRQPFAAEGYVDIHPDDAKALNIEDGDYVWIDSDPSDRPFRGWQKNDKDYKFSR 6hd3.1    ----------------------------------SVVSLHPATMEKLQLFRGDTILIKGKK-------------------  target    LLCRARYYPGTPRGITRMWFNMYGATPGSVEGHESRKDGLAKNPRTGYQAMFRSGSHQSATRGWLKPTWMTDSLVRKELF 6hd3.1    --------------------------------------------------------------------------------  target    GHAVNKGFLPDVHCPTGAPREAIVKITKAEPGGLNAKGLWRPAALGLRPKYENDKMKDYLAGKFTLAANPKKGGKK 6hd3.1    ---------------------------------------------------------------------------- ``` | | | | | | | | | | | | | | | | | | | | | | | | | | | | | | | | | | | | | | | | | | | | | | | | | |
|  | 5g4f.1.A | VCP-LIKE ATPASE  *Structure of the ADP-bound VAT complex* | 0.00 |  | 25.00 | 0.04 | 434-461 | EM | 7.00 | homo-hexamer |  | HHblits | 0.31 |
| ``` target    LARDIAKVPGTTLFAIGMGPNQFFNNDNKDRTQFLLAALTGNIGKIAGNIGSYAGNYRVAMFNGVPQYIAENPFDIELDG 5g4f.1    --------------------------------------------------------------------------------  target    AKPARPKLYWRAEPAHYYNHEDHPLKMGKTMITGKTHMPTPTKSLWFANANSILGNVKWHFNTVVNVLPKMEMIAVQEWW 5g4f.1    --------------------------------------------------------------------------------  target    WSTSCEWADIVFAVDAWSELKHPDMCSSVTNPFLTVFPRTPLERPFDTRGDIECLDLVGKQLAKRTGDRRFADMWKFVEE 5g4f.1    --------------------------------------------------------------------------------  target    KKVEVYLQRILDHSSNTKGFKFPELEEKAKKGIPALMMTRTNPKTVGYEQVYDSRPWYTKTGRLEFYREEDEFIEAGENL 5g4f.1    --------------------------------------------------------------------------------  target    PVHREPIDSTFYEPNVIVAPAHPFIKAKGPEAYGVKVDDFDNETRQGRNIVKTWEETKKTVHPLAKDGYKFVFHTPKYRH 5g4f.1    --------------------------------------------------------------------------------  target    GAHTMPVDTDMVAMLFGPFGDIYRHDKRQPFAAEGYVDIHPDDAKALNIEDGDYVWIDSDPSDRPFRGWQKNDKDYKFSR 5g4f.1    ---------------------------------MSRVRLDESSRRLLDAEIGDVVEIEKVR-------------------  target    LLCRARYYPGTPRGITRMWFNMYGATPGSVEGHESRKDGLAKNPRTGYQAMFRSGSHQSATRGWLKPTWMTDSLVRKELF 5g4f.1    --------------------------------------------------------------------------------  target    GHAVNKGFLPDVHCPTGAPREAIVKITKAEPGGLNAKGLWRPAALGLRPKYENDKMKDYLAGKFTLAANPKKGGKK 5g4f.1    ---------------------------------------------------------------------------- ``` | | | | | | | | | | | | | | | | | | | | | | | | | | | | | | | | | | | | | | | | | | | | | | | | | |
|  | 5g4f.1.B | VCP-LIKE ATPASE  *Structure of the ADP-bound VAT complex* | 0.00 |  | 25.00 | 0.04 | 434-461 | EM | 7.00 | homo-hexamer |  | HHblits | 0.31 |
| ``` target    LARDIAKVPGTTLFAIGMGPNQFFNNDNKDRTQFLLAALTGNIGKIAGNIGSYAGNYRVAMFNGVPQYIAENPFDIELDG 5g4f.1    --------------------------------------------------------------------------------  target    AKPARPKLYWRAEPAHYYNHEDHPLKMGKTMITGKTHMPTPTKSLWFANANSILGNVKWHFNTVVNVLPKMEMIAVQEWW 5g4f.1    --------------------------------------------------------------------------------  target    WSTSCEWADIVFAVDAWSELKHPDMCSSVTNPFLTVFPRTPLERPFDTRGDIECLDLVGKQLAKRTGDRRFADMWKFVEE 5g4f.1    --------------------------------------------------------------------------------  target    KKVEVYLQRILDHSSNTKGFKFPELEEKAKKGIPALMMTRTNPKTVGYEQVYDSRPWYTKTGRLEFYREEDEFIEAGENL 5g4f.1    --------------------------------------------------------------------------------  target    PVHREPIDSTFYEPNVIVAPAHPFIKAKGPEAYGVKVDDFDNETRQGRNIVKTWEETKKTVHPLAKDGYKFVFHTPKYRH 5g4f.1    --------------------------------------------------------------------------------  target    GAHTMPVDTDMVAMLFGPFGDIYRHDKRQPFAAEGYVDIHPDDAKALNIEDGDYVWIDSDPSDRPFRGWQKNDKDYKFSR 5g4f.1    ---------------------------------MSRVRLDESSRRLLDAEIGDVVEIEKVR-------------------  target    LLCRARYYPGTPRGITRMWFNMYGATPGSVEGHESRKDGLAKNPRTGYQAMFRSGSHQSATRGWLKPTWMTDSLVRKELF 5g4f.1    --------------------------------------------------------------------------------  target    GHAVNKGFLPDVHCPTGAPREAIVKITKAEPGGLNAKGLWRPAALGLRPKYENDKMKDYLAGKFTLAANPKKGGKK 5g4f.1    ---------------------------------------------------------------------------- ``` | | | | | | | | | | | | | | | | | | | | | | | | | | | | | | | | | | | | | | | | | | | | | | | | | |
|  | 5g4f.1.C | VCP-LIKE ATPASE  *Structure of the ADP-bound VAT complex* | 0.00 |  | 25.00 | 0.04 | 434-461 | EM | 7.00 | homo-hexamer |  | HHblits | 0.31 |
| ``` target    LARDIAKVPGTTLFAIGMGPNQFFNNDNKDRTQFLLAALTGNIGKIAGNIGSYAGNYRVAMFNGVPQYIAENPFDIELDG 5g4f.1    --------------------------------------------------------------------------------  target    AKPARPKLYWRAEPAHYYNHEDHPLKMGKTMITGKTHMPTPTKSLWFANANSILGNVKWHFNTVVNVLPKMEMIAVQEWW 5g4f.1    --------------------------------------------------------------------------------  target    WSTSCEWADIVFAVDAWSELKHPDMCSSVTNPFLTVFPRTPLERPFDTRGDIECLDLVGKQLAKRTGDRRFADMWKFVEE 5g4f.1    --------------------------------------------------------------------------------  target    KKVEVYLQRILDHSSNTKGFKFPELEEKAKKGIPALMMTRTNPKTVGYEQVYDSRPWYTKTGRLEFYREEDEFIEAGENL 5g4f.1    --------------------------------------------------------------------------------  target    PVHREPIDSTFYEPNVIVAPAHPFIKAKGPEAYGVKVDDFDNETRQGRNIVKTWEETKKTVHPLAKDGYKFVFHTPKYRH 5g4f.1    --------------------------------------------------------------------------------  target    GAHTMPVDTDMVAMLFGPFGDIYRHDKRQPFAAEGYVDIHPDDAKALNIEDGDYVWIDSDPSDRPFRGWQKNDKDYKFSR 5g4f.1    ---------------------------------MSRVRLDESSRRLLDAEIGDVVEIEKVR-------------------  target    LLCRARYYPGTPRGITRMWFNMYGATPGSVEGHESRKDGLAKNPRTGYQAMFRSGSHQSATRGWLKPTWMTDSLVRKELF 5g4f.1    --------------------------------------------------------------------------------  target    GHAVNKGFLPDVHCPTGAPREAIVKITKAEPGGLNAKGLWRPAALGLRPKYENDKMKDYLAGKFTLAANPKKGGKK 5g4f.1    ---------------------------------------------------------------------------- ``` | | | | | | | | | | | | | | | | | | | | | | | | | | | | | | | | | | | | | | | | | | | | | | | | | |
|  | 5g4f.1.D | VCP-LIKE ATPASE  *Structure of the ADP-bound VAT complex* | 0.00 |  | 25.00 | 0.04 | 434-461 | EM | 7.00 | homo-hexamer |  | HHblits | 0.31 |
| ``` target    LARDIAKVPGTTLFAIGMGPNQFFNNDNKDRTQFLLAALTGNIGKIAGNIGSYAGNYRVAMFNGVPQYIAENPFDIELDG 5g4f.1    --------------------------------------------------------------------------------  target    AKPARPKLYWRAEPAHYYNHEDHPLKMGKTMITGKTHMPTPTKSLWFANANSILGNVKWHFNTVVNVLPKMEMIAVQEWW 5g4f.1    --------------------------------------------------------------------------------  target    WSTSCEWADIVFAVDAWSELKHPDMCSSVTNPFLTVFPRTPLERPFDTRGDIECLDLVGKQLAKRTGDRRFADMWKFVEE 5g4f.1    --------------------------------------------------------------------------------  target    KKVEVYLQRILDHSSNTKGFKFPELEEKAKKGIPALMMTRTNPKTVGYEQVYDSRPWYTKTGRLEFYREEDEFIEAGENL 5g4f.1    --------------------------------------------------------------------------------  target    PVHREPIDSTFYEPNVIVAPAHPFIKAKGPEAYGVKVDDFDNETRQGRNIVKTWEETKKTVHPLAKDGYKFVFHTPKYRH 5g4f.1    --------------------------------------------------------------------------------  target    GAHTMPVDTDMVAMLFGPFGDIYRHDKRQPFAAEGYVDIHPDDAKALNIEDGDYVWIDSDPSDRPFRGWQKNDKDYKFSR 5g4f.1    ---------------------------------MSRVRLDESSRRLLDAEIGDVVEIEKVR-------------------  target    LLCRARYYPGTPRGITRMWFNMYGATPGSVEGHESRKDGLAKNPRTGYQAMFRSGSHQSATRGWLKPTWMTDSLVRKELF 5g4f.1    --------------------------------------------------------------------------------  target    GHAVNKGFLPDVHCPTGAPREAIVKITKAEPGGLNAKGLWRPAALGLRPKYENDKMKDYLAGKFTLAANPKKGGKK 5g4f.1    ---------------------------------------------------------------------------- ``` | | | | | | | | | | | | | | | | | | | | | | | | | | | | | | | | | | | | | | | | | | | | | | | | | |
|  | 5g4f.1.E | VCP-LIKE ATPASE  *Structure of the ADP-bound VAT complex* | 0.00 |  | 25.00 | 0.04 | 434-461 | EM | 7.00 | homo-hexamer |  | HHblits | 0.31 |
| ``` target    LARDIAKVPGTTLFAIGMGPNQFFNNDNKDRTQFLLAALTGNIGKIAGNIGSYAGNYRVAMFNGVPQYIAENPFDIELDG 5g4f.1    --------------------------------------------------------------------------------  target    AKPARPKLYWRAEPAHYYNHEDHPLKMGKTMITGKTHMPTPTKSLWFANANSILGNVKWHFNTVVNVLPKMEMIAVQEWW 5g4f.1    --------------------------------------------------------------------------------  target    WSTSCEWADIVFAVDAWSELKHPDMCSSVTNPFLTVFPRTPLERPFDTRGDIECLDLVGKQLAKRTGDRRFADMWKFVEE 5g4f.1    --------------------------------------------------------------------------------  target    KKVEVYLQRILDHSSNTKGFKFPELEEKAKKGIPALMMTRTNPKTVGYEQVYDSRPWYTKTGRLEFYREEDEFIEAGENL 5g4f.1    --------------------------------------------------------------------------------  target    PVHREPIDSTFYEPNVIVAPAHPFIKAKGPEAYGVKVDDFDNETRQGRNIVKTWEETKKTVHPLAKDGYKFVFHTPKYRH 5g4f.1    --------------------------------------------------------------------------------  target    GAHTMPVDTDMVAMLFGPFGDIYRHDKRQPFAAEGYVDIHPDDAKALNIEDGDYVWIDSDPSDRPFRGWQKNDKDYKFSR 5g4f.1    ---------------------------------MSRVRLDESSRRLLDAEIGDVVEIEKVR-------------------  target    LLCRARYYPGTPRGITRMWFNMYGATPGSVEGHESRKDGLAKNPRTGYQAMFRSGSHQSATRGWLKPTWMTDSLVRKELF 5g4f.1    --------------------------------------------------------------------------------  target    GHAVNKGFLPDVHCPTGAPREAIVKITKAEPGGLNAKGLWRPAALGLRPKYENDKMKDYLAGKFTLAANPKKGGKK 5g4f.1    ---------------------------------------------------------------------------- ``` | | | | | | | | | | | | | | | | | | | | | | | | | | | | | | | | | | | | | | | | | | | | | | | | | |
|  | 5g4f.1.F | VCP-LIKE ATPASE  *Structure of the ADP-bound VAT complex* | 0.00 |  | 25.00 | 0.04 | 434-461 | EM | 7.00 | homo-hexamer |  | HHblits | 0.31 |
| ``` target    LARDIAKVPGTTLFAIGMGPNQFFNNDNKDRTQFLLAALTGNIGKIAGNIGSYAGNYRVAMFNGVPQYIAENPFDIELDG 5g4f.1    --------------------------------------------------------------------------------  target    AKPARPKLYWRAEPAHYYNHEDHPLKMGKTMITGKTHMPTPTKSLWFANANSILGNVKWHFNTVVNVLPKMEMIAVQEWW 5g4f.1    --------------------------------------------------------------------------------  target    WSTSCEWADIVFAVDAWSELKHPDMCSSVTNPFLTVFPRTPLERPFDTRGDIECLDLVGKQLAKRTGDRRFADMWKFVEE 5g4f.1    --------------------------------------------------------------------------------  target    KKVEVYLQRILDHSSNTKGFKFPELEEKAKKGIPALMMTRTNPKTVGYEQVYDSRPWYTKTGRLEFYREEDEFIEAGENL 5g4f.1    --------------------------------------------------------------------------------  target    PVHREPIDSTFYEPNVIVAPAHPFIKAKGPEAYGVKVDDFDNETRQGRNIVKTWEETKKTVHPLAKDGYKFVFHTPKYRH 5g4f.1    --------------------------------------------------------------------------------  target    GAHTMPVDTDMVAMLFGPFGDIYRHDKRQPFAAEGYVDIHPDDAKALNIEDGDYVWIDSDPSDRPFRGWQKNDKDYKFSR 5g4f.1    ---------------------------------MSRVRLDESSRRLLDAEIGDVVEIEKVR-------------------  target    LLCRARYYPGTPRGITRMWFNMYGATPGSVEGHESRKDGLAKNPRTGYQAMFRSGSHQSATRGWLKPTWMTDSLVRKELF 5g4f.1    --------------------------------------------------------------------------------  target    GHAVNKGFLPDVHCPTGAPREAIVKITKAEPGGLNAKGLWRPAALGLRPKYENDKMKDYLAGKFTLAANPKKGGKK 5g4f.1    ---------------------------------------------------------------------------- ``` | | | | | | | | | | | | | | | | | | | | | | | | | | | | | | | | | | | | | | | | | | | | | | | | | |
|  | 2yuj.1.A | Ubiquitin fusion degradation 1-like  *Solution structure of human ubiquitin fusion degradation protein 1 homolog UFD1* | 0.00 |  | 25.00 | 0.04 | 433-460 | NMR | 0.00 | monomer |  | HHblits | 0.30 |
| ``` target    LARDIAKVPGTTLFAIGMGPNQFFNNDNKDRTQFLLAALTGNIGKIAGNIGSYAGNYRVAMFNGVPQYIAENPFDIELDG 2yuj.1    --------------------------------------------------------------------------------  target    AKPARPKLYWRAEPAHYYNHEDHPLKMGKTMITGKTHMPTPTKSLWFANANSILGNVKWHFNTVVNVLPKMEMIAVQEWW 2yuj.1    --------------------------------------------------------------------------------  target    WSTSCEWADIVFAVDAWSELKHPDMCSSVTNPFLTVFPRTPLERPFDTRGDIECLDLVGKQLAKRTGDRRFADMWKFVEE 2yuj.1    --------------------------------------------------------------------------------  target    KKVEVYLQRILDHSSNTKGFKFPELEEKAKKGIPALMMTRTNPKTVGYEQVYDSRPWYTKTGRLEFYREEDEFIEAGENL 2yuj.1    --------------------------------------------------------------------------------  target    PVHREPIDSTFYEPNVIVAPAHPFIKAKGPEAYGVKVDDFDNETRQGRNIVKTWEETKKTVHPLAKDGYKFVFHTPKYRH 2yuj.1    --------------------------------------------------------------------------------  target    GAHTMPVDTDMVAMLFGPFGDIYRHDKRQPFAAEGYVDIHPDDAKALNIEDGDYVWIDSDPSDRPFRGWQKNDKDYKFSR 2yuj.1    --------------------------------DEGICYLPHWMMQNLLLEEGGLVQVESV--------------------  target    LLCRARYYPGTPRGITRMWFNMYGATPGSVEGHESRKDGLAKNPRTGYQAMFRSGSHQSATRGWLKPTWMTDSLVRKELF 2yuj.1    --------------------------------------------------------------------------------  target    GHAVNKGFLPDVHCPTGAPREAIVKITKAEPGGLNAKGLWRPAALGLRPKYENDKMKDYLAGKFTLAANPKKGGKK 2yuj.1    ---------------------------------------------------------------------------- ``` | | | | | | | | | | | | | | | | | | | | | | | | | | | | | | | | | | | | | | | | | | | | | | | | | |
|  | 2ro5.1.A | Stage V sporulation protein T  *RDC-refined solution structure of the N-terminal DNA recognition domain of the Bacillus subtilis transition-state regulator SpoVT* | 0.00 |  | 33.33 | 0.04 | 434-460 | NMR | 0.00 | homo-dimer |  | HHblits | 0.33 |
| ``` target    LARDIAKVPGTTLFAIGMGPNQFFNNDNKDRTQFLLAALTGNIGKIAGNIGSYAGNYRVAMFNGVPQYIAENPFDIELDG 2ro5.1    --------------------------------------------------------------------------------  target    AKPARPKLYWRAEPAHYYNHEDHPLKMGKTMITGKTHMPTPTKSLWFANANSILGNVKWHFNTVVNVLPKMEMIAVQEWW 2ro5.1    --------------------------------------------------------------------------------  target    WSTSCEWADIVFAVDAWSELKHPDMCSSVTNPFLTVFPRTPLERPFDTRGDIECLDLVGKQLAKRTGDRRFADMWKFVEE 2ro5.1    --------------------------------------------------------------------------------  target    KKVEVYLQRILDHSSNTKGFKFPELEEKAKKGIPALMMTRTNPKTVGYEQVYDSRPWYTKTGRLEFYREEDEFIEAGENL 2ro5.1    --------------------------------------------------------------------------------  target    PVHREPIDSTFYEPNVIVAPAHPFIKAKGPEAYGVKVDDFDNETRQGRNIVKTWEETKKTVHPLAKDGYKFVFHTPKYRH 2ro5.1    --------------------------------------------------------------------------------  target    GAHTMPVDTDMVAMLFGPFGDIYRHDKRQPFAAEGYVDIHPDDAKALNIEDGDYVWIDSDPSDRPFRGWQKNDKDYKFSR 2ro5.1    ---------------------------------LGRVVIPKEIRRTLRIREGDPLEIFVD--------------------  target    LLCRARYYPGTPRGITRMWFNMYGATPGSVEGHESRKDGLAKNPRTGYQAMFRSGSHQSATRGWLKPTWMTDSLVRKELF 2ro5.1    --------------------------------------------------------------------------------  target    GHAVNKGFLPDVHCPTGAPREAIVKITKAEPGGLNAKGLWRPAALGLRPKYENDKMKDYLAGKFTLAANPKKGGKK 2ro5.1    ---------------------------------------------------------------------------- ``` | | | | | | | | | | | | | | | | | | | | | | | | | | | | | | | | | | | | | | | | | | | | | | | | | |
|  | 2k1n.1.C | AbrB family transcriptional regulator  *DNA bound structure of the N-terminal domain of AbrB* | 0.00 |  | 28.57 | 0.04 | 434-461 | NMR | 0.00 | homo-tetramer |  | HHblits | 0.30 |
| ``` target    LARDIAKVPGTTLFAIGMGPNQFFNNDNKDRTQFLLAALTGNIGKIAGNIGSYAGNYRVAMFNGVPQYIAENPFDIELDG 2k1n.1    --------------------------------------------------------------------------------  target    AKPARPKLYWRAEPAHYYNHEDHPLKMGKTMITGKTHMPTPTKSLWFANANSILGNVKWHFNTVVNVLPKMEMIAVQEWW 2k1n.1    --------------------------------------------------------------------------------  target    WSTSCEWADIVFAVDAWSELKHPDMCSSVTNPFLTVFPRTPLERPFDTRGDIECLDLVGKQLAKRTGDRRFADMWKFVEE 2k1n.1    --------------------------------------------------------------------------------  target    KKVEVYLQRILDHSSNTKGFKFPELEEKAKKGIPALMMTRTNPKTVGYEQVYDSRPWYTKTGRLEFYREEDEFIEAGENL 2k1n.1    --------------------------------------------------------------------------------  target    PVHREPIDSTFYEPNVIVAPAHPFIKAKGPEAYGVKVDDFDNETRQGRNIVKTWEETKKTVHPLAKDGYKFVFHTPKYRH 2k1n.1    --------------------------------------------------------------------------------  target    GAHTMPVDTDMVAMLFGPFGDIYRHDKRQPFAAEGYVDIHPDDAKALNIEDGDYVWIDSDPSDRPFRGWQKNDKDYKFSR 2k1n.1    ---------------------------------LGRVVIPIELRRTLGIAEKDALEIYVDD-------------------  target    LLCRARYYPGTPRGITRMWFNMYGATPGSVEGHESRKDGLAKNPRTGYQAMFRSGSHQSATRGWLKPTWMTDSLVRKELF 2k1n.1    --------------------------------------------------------------------------------  target    GHAVNKGFLPDVHCPTGAPREAIVKITKAEPGGLNAKGLWRPAALGLRPKYENDKMKDYLAGKFTLAANPKKGGKK 2k1n.1    ---------------------------------------------------------------------------- ``` | | | | | | | | | | | | | | | | | | | | | | | | | | | | | | | | | | | | | | | | | | | | | | | | | |
|  | 2k1n.1.D | AbrB family transcriptional regulator  *DNA bound structure of the N-terminal domain of AbrB* | 0.00 |  | 28.57 | 0.04 | 434-461 | NMR | 0.00 | homo-tetramer |  | HHblits | 0.30 |
| ``` target    LARDIAKVPGTTLFAIGMGPNQFFNNDNKDRTQFLLAALTGNIGKIAGNIGSYAGNYRVAMFNGVPQYIAENPFDIELDG 2k1n.1    --------------------------------------------------------------------------------  target    AKPARPKLYWRAEPAHYYNHEDHPLKMGKTMITGKTHMPTPTKSLWFANANSILGNVKWHFNTVVNVLPKMEMIAVQEWW 2k1n.1    --------------------------------------------------------------------------------  target    WSTSCEWADIVFAVDAWSELKHPDMCSSVTNPFLTVFPRTPLERPFDTRGDIECLDLVGKQLAKRTGDRRFADMWKFVEE 2k1n.1    --------------------------------------------------------------------------------  target    KKVEVYLQRILDHSSNTKGFKFPELEEKAKKGIPALMMTRTNPKTVGYEQVYDSRPWYTKTGRLEFYREEDEFIEAGENL 2k1n.1    --------------------------------------------------------------------------------  target    PVHREPIDSTFYEPNVIVAPAHPFIKAKGPEAYGVKVDDFDNETRQGRNIVKTWEETKKTVHPLAKDGYKFVFHTPKYRH 2k1n.1    --------------------------------------------------------------------------------  target    GAHTMPVDTDMVAMLFGPFGDIYRHDKRQPFAAEGYVDIHPDDAKALNIEDGDYVWIDSDPSDRPFRGWQKNDKDYKFSR 2k1n.1    ---------------------------------LGRVVIPIELRRTLGIAEKDALEIYVDD-------------------  target    LLCRARYYPGTPRGITRMWFNMYGATPGSVEGHESRKDGLAKNPRTGYQAMFRSGSHQSATRGWLKPTWMTDSLVRKELF 2k1n.1    --------------------------------------------------------------------------------  target    GHAVNKGFLPDVHCPTGAPREAIVKITKAEPGGLNAKGLWRPAALGLRPKYENDKMKDYLAGKFTLAANPKKGGKK 2k1n.1    ---------------------------------------------------------------------------- ``` | | | | | | | | | | | | | | | | | | | | | | | | | | | | | | | | | | | | | | | | | | | | | | | | | |
|  | 2k1n.1.E | AbrB family transcriptional regulator  *DNA bound structure of the N-terminal domain of AbrB* | 0.00 |  | 28.57 | 0.04 | 434-461 | NMR | 0.00 | homo-tetramer |  | HHblits | 0.30 |
| ``` target    LARDIAKVPGTTLFAIGMGPNQFFNNDNKDRTQFLLAALTGNIGKIAGNIGSYAGNYRVAMFNGVPQYIAENPFDIELDG 2k1n.1    --------------------------------------------------------------------------------  target    AKPARPKLYWRAEPAHYYNHEDHPLKMGKTMITGKTHMPTPTKSLWFANANSILGNVKWHFNTVVNVLPKMEMIAVQEWW 2k1n.1    --------------------------------------------------------------------------------  target    WSTSCEWADIVFAVDAWSELKHPDMCSSVTNPFLTVFPRTPLERPFDTRGDIECLDLVGKQLAKRTGDRRFADMWKFVEE 2k1n.1    --------------------------------------------------------------------------------  target    KKVEVYLQRILDHSSNTKGFKFPELEEKAKKGIPALMMTRTNPKTVGYEQVYDSRPWYTKTGRLEFYREEDEFIEAGENL 2k1n.1    --------------------------------------------------------------------------------  target    PVHREPIDSTFYEPNVIVAPAHPFIKAKGPEAYGVKVDDFDNETRQGRNIVKTWEETKKTVHPLAKDGYKFVFHTPKYRH 2k1n.1    --------------------------------------------------------------------------------  target    GAHTMPVDTDMVAMLFGPFGDIYRHDKRQPFAAEGYVDIHPDDAKALNIEDGDYVWIDSDPSDRPFRGWQKNDKDYKFSR 2k1n.1    ---------------------------------LGRVVIPIELRRTLGIAEKDALEIYVDD-------------------  target    LLCRARYYPGTPRGITRMWFNMYGATPGSVEGHESRKDGLAKNPRTGYQAMFRSGSHQSATRGWLKPTWMTDSLVRKELF 2k1n.1    --------------------------------------------------------------------------------  target    GHAVNKGFLPDVHCPTGAPREAIVKITKAEPGGLNAKGLWRPAALGLRPKYENDKMKDYLAGKFTLAANPKKGGKK 2k1n.1    ---------------------------------------------------------------------------- ``` | | | | | | | | | | | | | | | | | | | | | | | | | | | | | | | | | | | | | | | | | | | | | | | | | |
|  | 2k1n.1.F | AbrB family transcriptional regulator  *DNA bound structure of the N-terminal domain of AbrB* | 0.00 |  | 28.57 | 0.04 | 434-461 | NMR | 0.00 | homo-tetramer |  | HHblits | 0.30 |
| ``` target    LARDIAKVPGTTLFAIGMGPNQFFNNDNKDRTQFLLAALTGNIGKIAGNIGSYAGNYRVAMFNGVPQYIAENPFDIELDG 2k1n.1    --------------------------------------------------------------------------------  target    AKPARPKLYWRAEPAHYYNHEDHPLKMGKTMITGKTHMPTPTKSLWFANANSILGNVKWHFNTVVNVLPKMEMIAVQEWW 2k1n.1    --------------------------------------------------------------------------------  target    WSTSCEWADIVFAVDAWSELKHPDMCSSVTNPFLTVFPRTPLERPFDTRGDIECLDLVGKQLAKRTGDRRFADMWKFVEE 2k1n.1    --------------------------------------------------------------------------------  target    KKVEVYLQRILDHSSNTKGFKFPELEEKAKKGIPALMMTRTNPKTVGYEQVYDSRPWYTKTGRLEFYREEDEFIEAGENL 2k1n.1    --------------------------------------------------------------------------------  target    PVHREPIDSTFYEPNVIVAPAHPFIKAKGPEAYGVKVDDFDNETRQGRNIVKTWEETKKTVHPLAKDGYKFVFHTPKYRH 2k1n.1    --------------------------------------------------------------------------------  target    GAHTMPVDTDMVAMLFGPFGDIYRHDKRQPFAAEGYVDIHPDDAKALNIEDGDYVWIDSDPSDRPFRGWQKNDKDYKFSR 2k1n.1    ---------------------------------LGRVVIPIELRRTLGIAEKDALEIYVDD-------------------  target    LLCRARYYPGTPRGITRMWFNMYGATPGSVEGHESRKDGLAKNPRTGYQAMFRSGSHQSATRGWLKPTWMTDSLVRKELF 2k1n.1    --------------------------------------------------------------------------------  target    GHAVNKGFLPDVHCPTGAPREAIVKITKAEPGGLNAKGLWRPAALGLRPKYENDKMKDYLAGKFTLAANPKKGGKK 2k1n.1    ---------------------------------------------------------------------------- ``` | | | | | | | | | | | | | | | | | | | | | | | | | | | | | | | | | | | | | | | | | | | | | | | | | |
|  | 7dvf.1.A | reDPBB\_sym2 protein  *Crystal structure of the computationally designed reDPBB\_sym2 protein* | 0.00 |  | 25.93 | 0.04 | 434-460 | X-ray | 1.21 | monomer |  | HHblits | 0.33 |
| ``` target    LARDIAKVPGTTLFAIGMGPNQFFNNDNKDRTQFLLAALTGNIGKIAGNIGSYAGNYRVAMFNGVPQYIAENPFDIELDG 7dvf.1    --------------------------------------------------------------------------------  target    AKPARPKLYWRAEPAHYYNHEDHPLKMGKTMITGKTHMPTPTKSLWFANANSILGNVKWHFNTVVNVLPKMEMIAVQEWW 7dvf.1    --------------------------------------------------------------------------------  target    WSTSCEWADIVFAVDAWSELKHPDMCSSVTNPFLTVFPRTPLERPFDTRGDIECLDLVGKQLAKRTGDRRFADMWKFVEE 7dvf.1    --------------------------------------------------------------------------------  target    KKVEVYLQRILDHSSNTKGFKFPELEEKAKKGIPALMMTRTNPKTVGYEQVYDSRPWYTKTGRLEFYREEDEFIEAGENL 7dvf.1    --------------------------------------------------------------------------------  target    PVHREPIDSTFYEPNVIVAPAHPFIKAKGPEAYGVKVDDFDNETRQGRNIVKTWEETKKTVHPLAKDGYKFVFHTPKYRH 7dvf.1    --------------------------------------------------------------------------------  target    GAHTMPVDTDMVAMLFGPFGDIYRHDKRQPFAAEGYVDIHPDDAKALNIEDGDYVWIDSDPSDRPFRGWQKNDKDYKFSR 7dvf.1    ---------------------------------KGIVRMDKASREKLGVSAGDLVEIKGS--------------------  target    LLCRARYYPGTPRGITRMWFNMYGATPGSVEGHESRKDGLAKNPRTGYQAMFRSGSHQSATRGWLKPTWMTDSLVRKELF 7dvf.1    --------------------------------------------------------------------------------  target    GHAVNKGFLPDVHCPTGAPREAIVKITKAEPGGLNAKGLWRPAALGLRPKYENDKMKDYLAGKFTLAANPKKGGKK 7dvf.1    ---------------------------------------------------------------------------- ``` | | | | | | | | | | | | | | | | | | | | | | | | | | | | | | | | | | | | | | | | | | | | | | | | | |
|  | 1wlf.1.A | Peroxisome biogenesis factor 1  *Structure of the N-terminal domain of PEX1 AAA-ATPase: Characterization of a putative adaptor-binding domain* | 0.00 |  | 22.22 | 0.04 | 434-460 | X-ray | 2.05 | monomer |  | HHblits | 0.33 |
| ``` target    LARDIAKVPGTTLFAIGMGPNQFFNNDNKDRTQFLLAALTGNIGKIAGNIGSYAGNYRVAMFNGVPQYIAENPFDIELDG 1wlf.1    --------------------------------------------------------------------------------  target    AKPARPKLYWRAEPAHYYNHEDHPLKMGKTMITGKTHMPTPTKSLWFANANSILGNVKWHFNTVVNVLPKMEMIAVQEWW 1wlf.1    --------------------------------------------------------------------------------  target    WSTSCEWADIVFAVDAWSELKHPDMCSSVTNPFLTVFPRTPLERPFDTRGDIECLDLVGKQLAKRTGDRRFADMWKFVEE 1wlf.1    --------------------------------------------------------------------------------  target    KKVEVYLQRILDHSSNTKGFKFPELEEKAKKGIPALMMTRTNPKTVGYEQVYDSRPWYTKTGRLEFYREEDEFIEAGENL 1wlf.1    --------------------------------------------------------------------------------  target    PVHREPIDSTFYEPNVIVAPAHPFIKAKGPEAYGVKVDDFDNETRQGRNIVKTWEETKKTVHPLAKDGYKFVFHTPKYRH 1wlf.1    --------------------------------------------------------------------------------  target    GAHTMPVDTDMVAMLFGPFGDIYRHDKRQPFAAEGYVDIHPDDAKALNIEDGDYVWIDSDPSDRPFRGWQKNDKDYKFSR 1wlf.1    ---------------------------------ENVAEINRQVGQKLGLSSGDQVFLRPC--------------------  target    LLCRARYYPGTPRGITRMWFNMYGATPGSVEGHESRKDGLAKNPRTGYQAMFRSGSHQSATRGWLKPTWMTDSLVRKELF 1wlf.1    --------------------------------------------------------------------------------  target    GHAVNKGFLPDVHCPTGAPREAIVKITKAEPGGLNAKGLWRPAALGLRPKYENDKMKDYLAGKFTLAANPKKGGKK 1wlf.1    ---------------------------------------------------------------------------- ``` | | | | | | | | | | | | | | | | | | | | | | | | | | | | | | | | | | | | | | | | | | | | | | | | | |
|  | 7dvc.1.A | reDPBB\_sym1 protein  *Crystal structure of the computationally designed reDPBB\_sym1 protein* | 0.00 |  | 25.93 | 0.04 | 434-460 | X-ray | 1.71 | monomer |  | HHblits | 0.32 |
| ``` target    LARDIAKVPGTTLFAIGMGPNQFFNNDNKDRTQFLLAALTGNIGKIAGNIGSYAGNYRVAMFNGVPQYIAENPFDIELDG 7dvc.1    --------------------------------------------------------------------------------  target    AKPARPKLYWRAEPAHYYNHEDHPLKMGKTMITGKTHMPTPTKSLWFANANSILGNVKWHFNTVVNVLPKMEMIAVQEWW 7dvc.1    --------------------------------------------------------------------------------  target    WSTSCEWADIVFAVDAWSELKHPDMCSSVTNPFLTVFPRTPLERPFDTRGDIECLDLVGKQLAKRTGDRRFADMWKFVEE 7dvc.1    --------------------------------------------------------------------------------  target    KKVEVYLQRILDHSSNTKGFKFPELEEKAKKGIPALMMTRTNPKTVGYEQVYDSRPWYTKTGRLEFYREEDEFIEAGENL 7dvc.1    --------------------------------------------------------------------------------  target    PVHREPIDSTFYEPNVIVAPAHPFIKAKGPEAYGVKVDDFDNETRQGRNIVKTWEETKKTVHPLAKDGYKFVFHTPKYRH 7dvc.1    --------------------------------------------------------------------------------  target    GAHTMPVDTDMVAMLFGPFGDIYRHDKRQPFAAEGYVDIHPDDAKALNIEDGDYVWIDSDPSDRPFRGWQKNDKDYKFSR 7dvc.1    ---------------------------------KGIVRMDKASRDKLGVSAGDLVEIKGS--------------------  target    LLCRARYYPGTPRGITRMWFNMYGATPGSVEGHESRKDGLAKNPRTGYQAMFRSGSHQSATRGWLKPTWMTDSLVRKELF 7dvc.1    --------------------------------------------------------------------------------  target    GHAVNKGFLPDVHCPTGAPREAIVKITKAEPGGLNAKGLWRPAALGLRPKYENDKMKDYLAGKFTLAANPKKGGKK 7dvc.1    ---------------------------------------------------------------------------- ``` | | | | | | | | | | | | | | | | | | | | | | | | | | | | | | | | | | | | | | | | | | | | | | | | | |
|  | 7dvc.5.A | reDPBB\_sym1 protein  *Crystal structure of the computationally designed reDPBB\_sym1 protein* | 0.00 |  | 25.93 | 0.04 | 434-460 | X-ray | 1.71 | monomer |  | HHblits | 0.32 |
| ``` target    LARDIAKVPGTTLFAIGMGPNQFFNNDNKDRTQFLLAALTGNIGKIAGNIGSYAGNYRVAMFNGVPQYIAENPFDIELDG 7dvc.5    --------------------------------------------------------------------------------  target    AKPARPKLYWRAEPAHYYNHEDHPLKMGKTMITGKTHMPTPTKSLWFANANSILGNVKWHFNTVVNVLPKMEMIAVQEWW 7dvc.5    --------------------------------------------------------------------------------  target    WSTSCEWADIVFAVDAWSELKHPDMCSSVTNPFLTVFPRTPLERPFDTRGDIECLDLVGKQLAKRTGDRRFADMWKFVEE 7dvc.5    --------------------------------------------------------------------------------  target    KKVEVYLQRILDHSSNTKGFKFPELEEKAKKGIPALMMTRTNPKTVGYEQVYDSRPWYTKTGRLEFYREEDEFIEAGENL 7dvc.5    --------------------------------------------------------------------------------  target    PVHREPIDSTFYEPNVIVAPAHPFIKAKGPEAYGVKVDDFDNETRQGRNIVKTWEETKKTVHPLAKDGYKFVFHTPKYRH 7dvc.5    --------------------------------------------------------------------------------  target    GAHTMPVDTDMVAMLFGPFGDIYRHDKRQPFAAEGYVDIHPDDAKALNIEDGDYVWIDSDPSDRPFRGWQKNDKDYKFSR 7dvc.5    ---------------------------------KGIVRMDKASRDKLGVSAGDLVEIKGS--------------------  target    LLCRARYYPGTPRGITRMWFNMYGATPGSVEGHESRKDGLAKNPRTGYQAMFRSGSHQSATRGWLKPTWMTDSLVRKELF 7dvc.5    --------------------------------------------------------------------------------  target    GHAVNKGFLPDVHCPTGAPREAIVKITKAEPGGLNAKGLWRPAALGLRPKYENDKMKDYLAGKFTLAANPKKGGKK 7dvc.5    ---------------------------------------------------------------------------- ``` | | | | | | | | | | | | | | | | | | | | | | | | | | | | | | | | | | | | | | | | | | | | | | | | | |
|  | 3hu2.1.A | Transitional endoplasmic reticulum ATPase  *Structure of p97 N-D1 R86A mutant in complex with ATPgS* | 0.00 |  | 17.86 | 0.04 | 434-461 | X-ray | 2.85 | homo-hexamer | 6 x AGS, 6 x MG | HHblits | 0.29 |
| ``` target    LARDIAKVPGTTLFAIGMGPNQFFNNDNKDRTQFLLAALTGNIGKIAGNIGSYAGNYRVAMFNGVPQYIAENPFDIELDG 3hu2.1    --------------------------------------------------------------------------------  target    AKPARPKLYWRAEPAHYYNHEDHPLKMGKTMITGKTHMPTPTKSLWFANANSILGNVKWHFNTVVNVLPKMEMIAVQEWW 3hu2.1    --------------------------------------------------------------------------------  target    WSTSCEWADIVFAVDAWSELKHPDMCSSVTNPFLTVFPRTPLERPFDTRGDIECLDLVGKQLAKRTGDRRFADMWKFVEE 3hu2.1    --------------------------------------------------------------------------------  target    KKVEVYLQRILDHSSNTKGFKFPELEEKAKKGIPALMMTRTNPKTVGYEQVYDSRPWYTKTGRLEFYREEDEFIEAGENL 3hu2.1    --------------------------------------------------------------------------------  target    PVHREPIDSTFYEPNVIVAPAHPFIKAKGPEAYGVKVDDFDNETRQGRNIVKTWEETKKTVHPLAKDGYKFVFHTPKYRH 3hu2.1    --------------------------------------------------------------------------------  target    GAHTMPVDTDMVAMLFGPFGDIYRHDKRQPFAAEGYVDIHPDDAKALNIEDGDYVWIDSDPSDRPFRGWQKNDKDYKFSR 3hu2.1    ---------------------------------NSVVSLSQPKMDELQLFRGDTVLLKGKK-------------------  target    LLCRARYYPGTPRGITRMWFNMYGATPGSVEGHESRKDGLAKNPRTGYQAMFRSGSHQSATRGWLKPTWMTDSLVRKELF 3hu2.1    --------------------------------------------------------------------------------  target    GHAVNKGFLPDVHCPTGAPREAIVKITKAEPGGLNAKGLWRPAALGLRPKYENDKMKDYLAGKFTLAANPKKGGKK 3hu2.1    ---------------------------------------------------------------------------- ``` | | | | | | | | | | | | | | | | | | | | | | | | | | | | | | | | | | | | | | | | | | | | | | | | | |
|  | 3hu1.1.A | Transitional endoplasmic reticulum ATPase  *Structure of p97 N-D1 R95G mutant in complex with ATPgS* | 0.00 |  | 17.86 | 0.04 | 434-461 | X-ray | 2.81 | homo-hexamer | 6 x AGS, 6 x MG | HHblits | 0.29 |
| ``` target    LARDIAKVPGTTLFAIGMGPNQFFNNDNKDRTQFLLAALTGNIGKIAGNIGSYAGNYRVAMFNGVPQYIAENPFDIELDG 3hu1.1    --------------------------------------------------------------------------------  target    AKPARPKLYWRAEPAHYYNHEDHPLKMGKTMITGKTHMPTPTKSLWFANANSILGNVKWHFNTVVNVLPKMEMIAVQEWW 3hu1.1    --------------------------------------------------------------------------------  target    WSTSCEWADIVFAVDAWSELKHPDMCSSVTNPFLTVFPRTPLERPFDTRGDIECLDLVGKQLAKRTGDRRFADMWKFVEE 3hu1.1    --------------------------------------------------------------------------------  target    KKVEVYLQRILDHSSNTKGFKFPELEEKAKKGIPALMMTRTNPKTVGYEQVYDSRPWYTKTGRLEFYREEDEFIEAGENL 3hu1.1    --------------------------------------------------------------------------------  target    PVHREPIDSTFYEPNVIVAPAHPFIKAKGPEAYGVKVDDFDNETRQGRNIVKTWEETKKTVHPLAKDGYKFVFHTPKYRH 3hu1.1    --------------------------------------------------------------------------------  target    GAHTMPVDTDMVAMLFGPFGDIYRHDKRQPFAAEGYVDIHPDDAKALNIEDGDYVWIDSDPSDRPFRGWQKNDKDYKFSR 3hu1.1    ---------------------------------NSVVSLSQPKMDELQLFRGDTVLLKGKK-------------------  target    LLCRARYYPGTPRGITRMWFNMYGATPGSVEGHESRKDGLAKNPRTGYQAMFRSGSHQSATRGWLKPTWMTDSLVRKELF 3hu1.1    --------------------------------------------------------------------------------  target    GHAVNKGFLPDVHCPTGAPREAIVKITKAEPGGLNAKGLWRPAALGLRPKYENDKMKDYLAGKFTLAANPKKGGKK 3hu1.1    ---------------------------------------------------------------------------- ``` | | | | | | | | | | | | | | | | | | | | | | | | | | | | | | | | | | | | | | | | | | | | | | | | | |
|  | 2l66.1.A | Transcriptional regulator, AbrB family  *The DNA-recognition fold of Sso7c4 suggests a new member of SpoVT-AbrB superfamily from archaea.* | 0.00 |  | 25.93 | 0.04 | 434-460 | NMR | 0.00 | homo-dimer |  | HHblits | 0.32 |
| ``` target    LARDIAKVPGTTLFAIGMGPNQFFNNDNKDRTQFLLAALTGNIGKIAGNIGSYAGNYRVAMFNGVPQYIAENPFDIELDG 2l66.1    --------------------------------------------------------------------------------  target    AKPARPKLYWRAEPAHYYNHEDHPLKMGKTMITGKTHMPTPTKSLWFANANSILGNVKWHFNTVVNVLPKMEMIAVQEWW 2l66.1    --------------------------------------------------------------------------------  target    WSTSCEWADIVFAVDAWSELKHPDMCSSVTNPFLTVFPRTPLERPFDTRGDIECLDLVGKQLAKRTGDRRFADMWKFVEE 2l66.1    --------------------------------------------------------------------------------  target    KKVEVYLQRILDHSSNTKGFKFPELEEKAKKGIPALMMTRTNPKTVGYEQVYDSRPWYTKTGRLEFYREEDEFIEAGENL 2l66.1    --------------------------------------------------------------------------------  target    PVHREPIDSTFYEPNVIVAPAHPFIKAKGPEAYGVKVDDFDNETRQGRNIVKTWEETKKTVHPLAKDGYKFVFHTPKYRH 2l66.1    --------------------------------------------------------------------------------  target    GAHTMPVDTDMVAMLFGPFGDIYRHDKRQPFAAEGYVDIHPDDAKALNIEDGDYVWIDSDPSDRPFRGWQKNDKDYKFSR 2l66.1    ---------------------------------NYQVTIPAKVRQKFQIKEGDLVKVTFD--------------------  target    LLCRARYYPGTPRGITRMWFNMYGATPGSVEGHESRKDGLAKNPRTGYQAMFRSGSHQSATRGWLKPTWMTDSLVRKELF 2l66.1    --------------------------------------------------------------------------------  target    GHAVNKGFLPDVHCPTGAPREAIVKITKAEPGGLNAKGLWRPAALGLRPKYENDKMKDYLAGKFTLAANPKKGGKK 2l66.1    ---------------------------------------------------------------------------- ``` | | | | | | | | | | | | | | | | | | | | | | | | | | | | | | | | | | | | | | | | | | | | | | | | | |
|  | 5udf.1.A | Lipoprotein-releasing system transmembrane protein LolE  *Structure of the N-terminal domain of lipoprotein-releasing system transmembrane protein LolE from Acinetobacter baumannii* | 0.00 |  | 21.43 | 0.04 | 434-461 | X-ray | 2.35 | homo-tetramer |  | HHblits | 0.29 |
| ``` target    LARDIAKVPGTTLFAIGMGPNQFFNNDNKDRTQFLLAALTGNIGKIAGNIGSYAGNYRVAMFNGVPQYIAENPFDIELDG 5udf.1    --------------------------------------------------------------------------------  target    AKPARPKLYWRAEPAHYYNHEDHPLKMGKTMITGKTHMPTPTKSLWFANANSILGNVKWHFNTVVNVLPKMEMIAVQEWW 5udf.1    --------------------------------------------------------------------------------  target    WSTSCEWADIVFAVDAWSELKHPDMCSSVTNPFLTVFPRTPLERPFDTRGDIECLDLVGKQLAKRTGDRRFADMWKFVEE 5udf.1    --------------------------------------------------------------------------------  target    KKVEVYLQRILDHSSNTKGFKFPELEEKAKKGIPALMMTRTNPKTVGYEQVYDSRPWYTKTGRLEFYREEDEFIEAGENL 5udf.1    --------------------------------------------------------------------------------  target    PVHREPIDSTFYEPNVIVAPAHPFIKAKGPEAYGVKVDDFDNETRQGRNIVKTWEETKKTVHPLAKDGYKFVFHTPKYRH 5udf.1    --------------------------------------------------------------------------------  target    GAHTMPVDTDMVAMLFGPFGDIYRHDKRQPFAAEGYVDIHPDDAKALNIEDGDYVWIDSDPSDRPFRGWQKNDKDYKFSR 5udf.1    ---------------------------------EFGIVLGKDMADSLGLRLNDSVTLVLPE-------------------  target    LLCRARYYPGTPRGITRMWFNMYGATPGSVEGHESRKDGLAKNPRTGYQAMFRSGSHQSATRGWLKPTWMTDSLVRKELF 5udf.1    --------------------------------------------------------------------------------  target    GHAVNKGFLPDVHCPTGAPREAIVKITKAEPGGLNAKGLWRPAALGLRPKYENDKMKDYLAGKFTLAANPKKGGKK 5udf.1    ---------------------------------------------------------------------------- ``` | | | | | | | | | | | | | | | | | | | | | | | | | | | | | | | | | | | | | | | | | | | | | | | | | |
|  | 7dvh.2.A | reDPBB\_sym4 protein  *Crystal structure of the computationally designed reDPBB\_sym4 protein* | 0.00 |  | 26.92 | 0.04 | 434-459 | X-ray | 1.70 | monomer |  | HHblits | 0.34 |
| ``` target    LARDIAKVPGTTLFAIGMGPNQFFNNDNKDRTQFLLAALTGNIGKIAGNIGSYAGNYRVAMFNGVPQYIAENPFDIELDG 7dvh.2    --------------------------------------------------------------------------------  target    AKPARPKLYWRAEPAHYYNHEDHPLKMGKTMITGKTHMPTPTKSLWFANANSILGNVKWHFNTVVNVLPKMEMIAVQEWW 7dvh.2    --------------------------------------------------------------------------------  target    WSTSCEWADIVFAVDAWSELKHPDMCSSVTNPFLTVFPRTPLERPFDTRGDIECLDLVGKQLAKRTGDRRFADMWKFVEE 7dvh.2    --------------------------------------------------------------------------------  target    KKVEVYLQRILDHSSNTKGFKFPELEEKAKKGIPALMMTRTNPKTVGYEQVYDSRPWYTKTGRLEFYREEDEFIEAGENL 7dvh.2    --------------------------------------------------------------------------------  target    PVHREPIDSTFYEPNVIVAPAHPFIKAKGPEAYGVKVDDFDNETRQGRNIVKTWEETKKTVHPLAKDGYKFVFHTPKYRH 7dvh.2    --------------------------------------------------------------------------------  target    GAHTMPVDTDMVAMLFGPFGDIYRHDKRQPFAAEGYVDIHPDDAKALNIEDGDYVWIDSDPSDRPFRGWQKNDKDYKFSR 7dvh.2    ---------------------------------KGIVRMDKYERQNLGVSVGDYVEVKK---------------------  target    LLCRARYYPGTPRGITRMWFNMYGATPGSVEGHESRKDGLAKNPRTGYQAMFRSGSHQSATRGWLKPTWMTDSLVRKELF 7dvh.2    --------------------------------------------------------------------------------  target    GHAVNKGFLPDVHCPTGAPREAIVKITKAEPGGLNAKGLWRPAALGLRPKYENDKMKDYLAGKFTLAANPKKGGKK 7dvh.2    ---------------------------------------------------------------------------- ``` | | | | | | | | | | | | | | | | | | | | | | | | | | | | | | | | | | | | | | | | | | | | | | | | | |
|  | 7dvh.1.A | reDPBB\_sym4 protein  *Crystal structure of the computationally designed reDPBB\_sym4 protein* | 0.00 |  | 26.92 | 0.04 | 434-459 | X-ray | 1.70 | monomer |  | HHblits | 0.34 |
| ``` target    LARDIAKVPGTTLFAIGMGPNQFFNNDNKDRTQFLLAALTGNIGKIAGNIGSYAGNYRVAMFNGVPQYIAENPFDIELDG 7dvh.1    --------------------------------------------------------------------------------  target    AKPARPKLYWRAEPAHYYNHEDHPLKMGKTMITGKTHMPTPTKSLWFANANSILGNVKWHFNTVVNVLPKMEMIAVQEWW 7dvh.1    --------------------------------------------------------------------------------  target    WSTSCEWADIVFAVDAWSELKHPDMCSSVTNPFLTVFPRTPLERPFDTRGDIECLDLVGKQLAKRTGDRRFADMWKFVEE 7dvh.1    --------------------------------------------------------------------------------  target    KKVEVYLQRILDHSSNTKGFKFPELEEKAKKGIPALMMTRTNPKTVGYEQVYDSRPWYTKTGRLEFYREEDEFIEAGENL 7dvh.1    --------------------------------------------------------------------------------  target    PVHREPIDSTFYEPNVIVAPAHPFIKAKGPEAYGVKVDDFDNETRQGRNIVKTWEETKKTVHPLAKDGYKFVFHTPKYRH 7dvh.1    --------------------------------------------------------------------------------  target    GAHTMPVDTDMVAMLFGPFGDIYRHDKRQPFAAEGYVDIHPDDAKALNIEDGDYVWIDSDPSDRPFRGWQKNDKDYKFSR 7dvh.1    ---------------------------------KGIVRMDKYERQNLGVSVGDYVEVKK---------------------  target    LLCRARYYPGTPRGITRMWFNMYGATPGSVEGHESRKDGLAKNPRTGYQAMFRSGSHQSATRGWLKPTWMTDSLVRKELF 7dvh.1    --------------------------------------------------------------------------------  target    GHAVNKGFLPDVHCPTGAPREAIVKITKAEPGGLNAKGLWRPAALGLRPKYENDKMKDYLAGKFTLAANPKKGGKK 7dvh.1    ---------------------------------------------------------------------------- ``` | | | | | | | | | | | | | | | | | | | | | | | | | | | | | | | | | | | | | | | | | | | | | | | | | |
|  | 7dvh.4.A | reDPBB\_sym4 protein  *Crystal structure of the computationally designed reDPBB\_sym4 protein* | 0.00 |  | 26.92 | 0.04 | 434-459 | X-ray | 1.70 | monomer |  | HHblits | 0.34 |
| ``` target    LARDIAKVPGTTLFAIGMGPNQFFNNDNKDRTQFLLAALTGNIGKIAGNIGSYAGNYRVAMFNGVPQYIAENPFDIELDG 7dvh.4    --------------------------------------------------------------------------------  target    AKPARPKLYWRAEPAHYYNHEDHPLKMGKTMITGKTHMPTPTKSLWFANANSILGNVKWHFNTVVNVLPKMEMIAVQEWW 7dvh.4    --------------------------------------------------------------------------------  target    WSTSCEWADIVFAVDAWSELKHPDMCSSVTNPFLTVFPRTPLERPFDTRGDIECLDLVGKQLAKRTGDRRFADMWKFVEE 7dvh.4    --------------------------------------------------------------------------------  target    KKVEVYLQRILDHSSNTKGFKFPELEEKAKKGIPALMMTRTNPKTVGYEQVYDSRPWYTKTGRLEFYREEDEFIEAGENL 7dvh.4    --------------------------------------------------------------------------------  target    PVHREPIDSTFYEPNVIVAPAHPFIKAKGPEAYGVKVDDFDNETRQGRNIVKTWEETKKTVHPLAKDGYKFVFHTPKYRH 7dvh.4    --------------------------------------------------------------------------------  target    GAHTMPVDTDMVAMLFGPFGDIYRHDKRQPFAAEGYVDIHPDDAKALNIEDGDYVWIDSDPSDRPFRGWQKNDKDYKFSR 7dvh.4    ---------------------------------KGIVRMDKYERQNLGVSVGDYVEVKK---------------------  target    LLCRARYYPGTPRGITRMWFNMYGATPGSVEGHESRKDGLAKNPRTGYQAMFRSGSHQSATRGWLKPTWMTDSLVRKELF 7dvh.4    --------------------------------------------------------------------------------  target    GHAVNKGFLPDVHCPTGAPREAIVKITKAEPGGLNAKGLWRPAALGLRPKYENDKMKDYLAGKFTLAANPKKGGKK 7dvh.4    ---------------------------------------------------------------------------- ``` | | | | | | | | | | | | | | | | | | | | | | | | | | | | | | | | | | | | | | | | | | | | | | | | | |
|  | 7dbo.1.A | VCP-like ATPase  *DPBB domain of VCP-like ATPase from Thermoplasma acidophilum* | 0.00 |  | 25.93 | 0.04 | 434-460 | X-ray | 1.90 | monomer |  | HHblits | 0.31 |
| ``` target    LARDIAKVPGTTLFAIGMGPNQFFNNDNKDRTQFLLAALTGNIGKIAGNIGSYAGNYRVAMFNGVPQYIAENPFDIELDG 7dbo.1    --------------------------------------------------------------------------------  target    AKPARPKLYWRAEPAHYYNHEDHPLKMGKTMITGKTHMPTPTKSLWFANANSILGNVKWHFNTVVNVLPKMEMIAVQEWW 7dbo.1    --------------------------------------------------------------------------------  target    WSTSCEWADIVFAVDAWSELKHPDMCSSVTNPFLTVFPRTPLERPFDTRGDIECLDLVGKQLAKRTGDRRFADMWKFVEE 7dbo.1    --------------------------------------------------------------------------------  target    KKVEVYLQRILDHSSNTKGFKFPELEEKAKKGIPALMMTRTNPKTVGYEQVYDSRPWYTKTGRLEFYREEDEFIEAGENL 7dbo.1    --------------------------------------------------------------------------------  target    PVHREPIDSTFYEPNVIVAPAHPFIKAKGPEAYGVKVDDFDNETRQGRNIVKTWEETKKTVHPLAKDGYKFVFHTPKYRH 7dbo.1    --------------------------------------------------------------------------------  target    GAHTMPVDTDMVAMLFGPFGDIYRHDKRQPFAAEGYVDIHPDDAKALNIEDGDYVWIDSDPSDRPFRGWQKNDKDYKFSR 7dbo.1    ---------------------------------MSRVRLDESSRRLLDAEIGDVVEIEKV--------------------  target    LLCRARYYPGTPRGITRMWFNMYGATPGSVEGHESRKDGLAKNPRTGYQAMFRSGSHQSATRGWLKPTWMTDSLVRKELF 7dbo.1    --------------------------------------------------------------------------------  target    GHAVNKGFLPDVHCPTGAPREAIVKITKAEPGGLNAKGLWRPAALGLRPKYENDKMKDYLAGKFTLAANPKKGGKK 7dbo.1    ---------------------------------------------------------------------------- ``` | | | | | | | | | | | | | | | | | | | | | | | | | | | | | | | | | | | | | | | | | | | | | | | | | |
|  | 7dbo.2.A | VCP-like ATPase  *DPBB domain of VCP-like ATPase from Thermoplasma acidophilum* | 0.00 |  | 25.93 | 0.04 | 434-460 | X-ray | 1.90 | monomer |  | HHblits | 0.31 |
[truncated: 152,508 more chars]
