# Supplementary material for: Metabolic capacity is maintained despite shifts in microbial diversity in estuary sediments
Source: ISME Commun. 2025 Oct 11;5(1):ycaf182. doi: 10.1093/ismeco/ycaf182 (PMC12687941; doi:10.1093/ismeco/ycaf182)
Supplement: Supplementary_Data_1_ycaf182 [file supplementary_data_1_ycaf182.zip › SWISS-MODEL/13_July_SF_Bin2_scaffold_20759_c1_11143024_1/models.html]

13\_July\_SF\_Bin2\_scaffold\_20759\_c1\_1114-3024\_1 | Models


**Export Alignment**
  
FASTA format
Clustal Format
PNG Image

**Secondary Structure**
  
None
DSSP
PSIPRED
SSpro

**Colour Scheme** 


Fade Mismatches
Enhance Mismatches

Confidencegradient
Confidenceclass
Indels
Chain
Unique Chain
Rainbow
2° Structure
Clustal
Hydrophobic
Size
Charged
Polar
Proline
Ser/Thr
Cysteine
Aliphatic
Aromatic
No Colour

Use QMEANBrane values

|  |  |  |  |
| --- | --- | --- | --- |
| Background |  |  |  |

**3D Viewer**  
NGL
PV

FASTA
Multi FASTA
ClustalW
PNG


SWISS-MODEL

### 13\_July\_SF\_Bin2\_scaffold\_20759\_c1\_1114-3024\_1

### Created: March 29, 2023, 7:36 p.m. at 19:36

- Templates
- Models

Order by:
GMQE
QMEANDisCo
Oligo State
Ligands
Seq Identity
Similarity
Coverage

Model 01

- PDB Format *(Display)*
- JSON Format *(Display)*
- Model Report *(Display)*

Oligo-State
:   Monomer

GMQE
:   0.69

QMEANDisCo Global:
:   0.64  ± 0.05

Ligands

QMEANDisCo Local

QMEAN Z-Scores

Template

7b04.1.B

Nitrite oxidoreductase subunit A  
Structure of Nitrite oxidoreductase (Nxr) from the anammox bacterium Kuenenia stuttgartiensis.

Seq Identity
:   41.23%

Coverage

|  |  |
| --- | --- |
| **Biounit Oligo State** | Hetero-trimer |
| **QSQE** | 0.00 |
| **Method** | X-ray, 2.97 Å |
| **Seq Similarity** | 0.40 |
| **Coverage** | 0.97 |
| **Range** | 1-624 |

| Ligand | Added to Model | Description |
| --- | --- | --- |
| CA | ✕ - Binding site not conserved. | CALCIUM ION |
| CA | ✕ - Binding site not conserved. | CALCIUM ION |
| F3S | ✕ - Binding site not conserved. | FE3-S4 CLUSTER |
| HEM | ✕ - Binding site not conserved. | PROTOPORPHYRIN IX CONTAINING FE |
| MD1 | ✕ - Binding site not conserved. | PHOSPHORIC ACID 4-(2-AMINO-4-OXO-3,4,5,6,-TETRAHYDRO-PTERIDIN-6-YL)-2-HYDROXY-3,4-DIMERCAPTO-BUT-3-EN-YL ESTER GUANYLATE ESTER |
| MD1 | ✕ - Binding site not conserved. | PHOSPHORIC ACID 4-(2-AMINO-4-OXO-3,4,5,6,-TETRAHYDRO-PTERIDIN-6-YL)-2-HYDROXY-3,4-DIMERCAPTO-BUT-3-EN-YL ESTER GUANYLATE ESTER |
| MO | ✕ - Binding site not conserved. | MOLYBDENUM ATOM |
| SF4 | ✕ - Binding site not conserved. | IRON/SULFUR CLUSTER |
| SF4 | ✕ - Binding site not conserved. | IRON/SULFUR CLUSTER |
| SF4 | ✕ - Binding site not conserved. | IRON/SULFUR CLUSTER |
| SF4 | ✕ - Binding site not conserved. | IRON/SULFUR CLUSTER |

Model-Template Alignment

|  |  |  |
| --- | --- | --- |
|  |  |  |

Model 02

- PDB Format *(Display)*
- JSON Format *(Display)*
- Model Report *(Display)*

Oligo-State
:   Monomer  
    (matching prediction)

GMQE
:   0.41

QMEANDisCo Global:
:   0.48  ± 0.05

Ligands

QMEANDisCo Local

QMEAN Z-Scores

Template

1r27.4.A

Respiratory nitrate reductase 1 alpha chain  
Crystal Structure of NarGH complex

Seq Identity
:   21.88%

Coverage

|  |  |
| --- | --- |
| **Biounit Oligo State** | Hetero-octamer |
| **QSQE** | - |
| **Method** | X-ray, 2.00 Å |
| **Seq Similarity** | 0.31 |
| **Coverage** | 0.77 |
| **Range** | 1-595 |

| Ligand | Added to Model | Description |
| --- | --- | --- |
| F3S | ✕ - Binding site not conserved. | FE3-S4 CLUSTER |
| F3S | ✕ - Binding site not conserved. | FE3-S4 CLUSTER |
| F3S | ✕ - Binding site not conserved. | FE3-S4 CLUSTER |
| F3S | ✕ - Binding site not conserved. | FE3-S4 CLUSTER |
| MGD | ✕ - Binding site not conserved. | 2-AMINO-5,6-DIMERCAPTO-7-METHYL-3,7,8A,9-TETRAHYDRO-8-OXA-1,3,9,10-TETRAAZA-ANTHRACEN-4-ONE GUANOSINE DINUCLEOTIDE |
| MGD | ✕ - Binding site not conserved. | 2-AMINO-5,6-DIMERCAPTO-7-METHYL-3,7,8A,9-TETRAHYDRO-8-OXA-1,3,9,10-TETRAAZA-ANTHRACEN-4-ONE GUANOSINE DINUCLEOTIDE |
| MGD | ✕ - Binding site not conserved. | 2-AMINO-5,6-DIMERCAPTO-7-METHYL-3,7,8A,9-TETRAHYDRO-8-OXA-1,3,9,10-TETRAAZA-ANTHRACEN-4-ONE GUANOSINE DINUCLEOTIDE |
| MGD | ✕ - Binding site not conserved. | 2-AMINO-5,6-DIMERCAPTO-7-METHYL-3,7,8A,9-TETRAHYDRO-8-OXA-1,3,9,10-TETRAAZA-ANTHRACEN-4-ONE GUANOSINE DINUCLEOTIDE |
| MGD | ✕ - Binding site not conserved. | 2-AMINO-5,6-DIMERCAPTO-7-METHYL-3,7,8A,9-TETRAHYDRO-8-OXA-1,3,9,10-TETRAAZA-ANTHRACEN-4-ONE GUANOSINE DINUCLEOTIDE |
| MGD | ✕ - Binding site not conserved. | 2-AMINO-5,6-DIMERCAPTO-7-METHYL-3,7,8A,9-TETRAHYDRO-8-OXA-1,3,9,10-TETRAAZA-ANTHRACEN-4-ONE GUANOSINE DINUCLEOTIDE |
| MGD | ✕ - Binding site not conserved. | 2-AMINO-5,6-DIMERCAPTO-7-METHYL-3,7,8A,9-TETRAHYDRO-8-OXA-1,3,9,10-TETRAAZA-ANTHRACEN-4-ONE GUANOSINE DINUCLEOTIDE |
| MGD | ✕ - Binding site not conserved. | 2-AMINO-5,6-DIMERCAPTO-7-METHYL-3,7,8A,9-TETRAHYDRO-8-OXA-1,3,9,10-TETRAAZA-ANTHRACEN-4-ONE GUANOSINE DINUCLEOTIDE |
| MO | ✕ - Binding site not conserved. | MOLYBDENUM ATOM |
| MO | ✕ - Binding site not conserved. | MOLYBDENUM ATOM |
| MO | ✕ - Binding site not conserved. | MOLYBDENUM ATOM |
| MO | ✕ - Binding site not conserved. | MOLYBDENUM ATOM |
| SF4 | ✕ - Binding site not conserved. | IRON/SULFUR CLUSTER |
| SF4 | ✕ - Binding site not conserved. | IRON/SULFUR CLUSTER |
| SF4 | ✕ - Binding site not conserved. | IRON/SULFUR CLUSTER |
| SF4 | ✕ - Binding site not conserved. | IRON/SULFUR CLUSTER |
| SF4 | ✕ - Binding site not conserved. | IRON/SULFUR CLUSTER |
| SF4 | ✕ - Binding site not conserved. | IRON/SULFUR CLUSTER |
| SF4 | ✕ - Binding site not conserved. | IRON/SULFUR CLUSTER |
| SF4 | ✕ - Binding site not conserved. | IRON/SULFUR CLUSTER |
| SF4 | ✕ - Binding site not conserved. | IRON/SULFUR CLUSTER |
| SF4 | ✕ - Binding site not conserved. | IRON/SULFUR CLUSTER |
| SF4 | ✕ - Binding site not conserved. | IRON/SULFUR CLUSTER |
| SF4 | ✕ - Binding site not conserved. | IRON/SULFUR CLUSTER |
| SF4 | ✕ - Binding site not conserved. | IRON/SULFUR CLUSTER |
| SF4 | ✕ - Binding site not conserved. | IRON/SULFUR CLUSTER |
| SF4 | ✕ - Binding site not conserved. | IRON/SULFUR CLUSTER |
| SF4 | ✕ - Binding site not conserved. | IRON/SULFUR CLUSTER |

Model-Template Alignment

|  |  |  |
| --- | --- | --- |
|  |  |  |

Model 03

- PDB Format *(Display)*
- JSON Format *(Display)*
- Model Report *(Display)*

Oligo-State
:   Monomer

GMQE
:   0.28

QMEANDisCo Global:
:   0.40  ± 0.05

Ligands

QMEANDisCo Local

QMEAN Z-Scores

Template

7p63.1.C

NADH-quinone oxidoreductase  
Complex I from E. coli, DDM/LMNG-purified, under Turnover at pH 6, Closed state

Seq Identity
:   12.82%

Coverage

|  |  |
| --- | --- |
| **Biounit Oligo State** | Hetero-13-mer |
| **QSQE** | 0.00 |
| **Method** | EM |
| **Seq Similarity** | 0.26 |
| **Coverage** | 0.55 |
| **Range** | 1-504 |

| Ligand | Added to Model | Description |
| --- | --- | --- |
| 3PE | ✕ - Binding site not conserved. | 1,2-Distearoyl-sn-glycerophosphoethanolamine |
| 3PE | ✕ - Binding site not conserved. | 1,2-Distearoyl-sn-glycerophosphoethanolamine |
| 3PE | ✕ - Binding site not conserved. | 1,2-Distearoyl-sn-glycerophosphoethanolamine |
| 3PE | ✕ - Binding site not conserved. | 1,2-Distearoyl-sn-glycerophosphoethanolamine |
| 3PE | ✕ - Binding site not conserved. | 1,2-Distearoyl-sn-glycerophosphoethanolamine |
| 3PE | ✕ - Binding site not conserved. | 1,2-Distearoyl-sn-glycerophosphoethanolamine |
| 3PE | ✕ - Binding site not conserved. | 1,2-Distearoyl-sn-glycerophosphoethanolamine |
| 3PE | ✕ - Binding site not conserved. | 1,2-Distearoyl-sn-glycerophosphoethanolamine |
| CA | ✕ - Binding site not conserved. | CALCIUM ION |
| DCQ | ✕ - Binding site not conserved. | 2-decyl-5,6-dimethoxy-3-methylcyclohexa-2,5-diene-1,4-dione |
| FES | ✕ - Binding site not conserved. | FE2/S2 (INORGANIC) CLUSTER |
| FES | ✕ - Binding site not conserved. | FE2/S2 (INORGANIC) CLUSTER |
| FMN | ✕ - Binding site not conserved. | FLAVIN MONONUCLEOTIDE |
| LFA | ✕ - Binding site not conserved. | EICOSANE |
| LFA | ✕ - Binding site not conserved. | EICOSANE |
| LFA | ✕ - Binding site not conserved. | EICOSANE |
| LFA | ✕ - Binding site not conserved. | EICOSANE |
| NAI | ✕ - Binding site not conserved. | 1,4-DIHYDRONICOTINAMIDE ADENINE DINUCLEOTIDE |
| SF4 | ✕ - Binding site not conserved. | IRON/SULFUR CLUSTER |
| SF4 | ✕ - Binding site not conserved. | IRON/SULFUR CLUSTER |
| SF4 | ✕ - Binding site not conserved. | IRON/SULFUR CLUSTER |
| SF4 | ✕ - Binding site not conserved. | IRON/SULFUR CLUSTER |
| SF4 | ✕ - Binding site not conserved. | IRON/SULFUR CLUSTER |
| SF4 | ✕ - Binding site not conserved. | IRON/SULFUR CLUSTER |
| SF4 | ✕ - Binding site not conserved. | IRON/SULFUR CLUSTER |

Model-Template Alignment

|  |  |  |
| --- | --- | --- |
|  |  |  |

Model 04

- PDB Format *(Display)*
- JSON Format *(Display)*
- Model Report *(Display)*

Oligo-State
:   Monomer

GMQE
:   0.14

QMEANDisCo Global:
:   0.35  ± 0.05

Ligands

QMEANDisCo Local

QMEAN Z-Scores

Template

2fug.2.C

NADH-quinone oxidoreductase chain 3  
Crystal structure of the hydrophilic domain of respiratory complex I from Thermus thermophilus

Seq Identity
:   18.56%

Coverage

|  |  |
| --- | --- |
| **Biounit Oligo State** | Hetero-octamer |
| **QSQE** | 0.00 |
| **Method** | X-ray, 3.30 Å |
| **Seq Similarity** | 0.28 |
| **Coverage** | 0.31 |
| **Range** | 144-505 |

| Ligand | Added to Model | Description |
| --- | --- | --- |
| FES | ✕ - Binding site not conserved. | FE2/S2 (INORGANIC) CLUSTER |
| FES | ✕ - Binding site not conserved. | FE2/S2 (INORGANIC) CLUSTER |
| FMN | ✕ - Binding site not conserved. | FLAVIN MONONUCLEOTIDE |
| SF4 | ✕ - Binding site not conserved. | IRON/SULFUR CLUSTER |
| SF4 | ✕ - Binding site not conserved. | IRON/SULFUR CLUSTER |
| SF4 | ✕ - Binding site not conserved. | IRON/SULFUR CLUSTER |
| SF4 | ✕ - Binding site not conserved. | IRON/SULFUR CLUSTER |
| SF4 | ✕ - Binding site not conserved. | IRON/SULFUR CLUSTER |
| SF4 | ✕ - Binding site not conserved. | IRON/SULFUR CLUSTER |
| SF4 | ✕ - Binding site not conserved. | IRON/SULFUR CLUSTER |

Model-Template Alignment

|  |  |  |
| --- | --- | --- |
|  |  |  |

Model 05

- PDB Format *(Display)*
- JSON Format *(Display)*
- Model Report *(Display)*

Oligo-State
:   Monomer

GMQE
:   0.13

QMEANDisCo Global:
:   0.44  ± 0.06

Ligands

QMEANDisCo Local

QMEAN Z-Scores

Template

2ivf.1.A

ETHYLBENZENE DEHYDROGENASE ALPHA-SUBUNIT  
ETHYLBENZENE DEHYDROGENASE FROM AROMATOLEUM AROMATICUM

Seq Identity
:   30.93%

Coverage

|  |  |
| --- | --- |
| **Biounit Oligo State** | Hetero-trimer |
| **QSQE** | 0.00 |
| **Method** | X-ray, 1.88 Å |
| **Seq Similarity** | 0.36 |
| **Coverage** | 0.31 |
| **Range** | 147-342 |

| Ligand | Added to Model | Description |
| --- | --- | --- |
| ACT | ✕ - Not biologically relevant. | ACETATE ION |
| ACT | ✕ - Not biologically relevant. | ACETATE ION |
| F3S | ✕ - Binding site not conserved. | FE3-S4 CLUSTER |
| GOL | ✕ - Not biologically relevant. | GLYCEROL |
| GOL | ✕ - Not biologically relevant. | GLYCEROL |
| GOL | ✕ - Not biologically relevant. | GLYCEROL |
| GOL | ✕ - Not biologically relevant. | GLYCEROL |
| GOL | ✕ - Not biologically relevant. | GLYCEROL |
| GOL | ✕ - Not biologically relevant. | GLYCEROL |
| GOL | ✕ - Not biologically relevant. | GLYCEROL |
| GOL | ✕ - Not biologically relevant. | GLYCEROL |
| HEM | ✕ - Binding site not conserved. | PROTOPORPHYRIN IX CONTAINING FE |
| MD1 | ✕ - Binding site not conserved. | PHOSPHORIC ACID 4-(2-AMINO-4-OXO-3,4,5,6,-TETRAHYDRO-PTERIDIN-6-YL)-2-HYDROXY-3,4-DIMERCAPTO-BUT-3-EN-YL ESTER GUANYLATE ESTER |
| MES | ✕ - Binding site not conserved. | 2-(N-MORPHOLINO)-ETHANESULFONIC ACID |
| MGD | ✕ - Binding site not conserved. | 2-AMINO-5,6-DIMERCAPTO-7-METHYL-3,7,8A,9-TETRAHYDRO-8-OXA-1,3,9,10-TETRAAZA-ANTHRACEN-4-ONE GUANOSINE DINUCLEOTIDE |
| MO | ✕ - Binding site not conserved. | MOLYBDENUM ATOM |
| PO4 | ✕ - Not biologically relevant. | PHOSPHATE ION |
| SF4 | ✕ - Binding site not conserved. | IRON/SULFUR CLUSTER |
| SF4 | ✕ - Binding site not conserved. | IRON/SULFUR CLUSTER |
| SF4 | ✕ - Binding site not conserved. | IRON/SULFUR CLUSTER |
| SF4 | ✕ - Binding site not conserved. | IRON/SULFUR CLUSTER |

Model-Template Alignment

|  |  |  |
| --- | --- | --- |
|  |  |  |

Apply

Close

Cartoon

- Cartoon
- Tube
- Trace
- Lines
- Ball+Stick
- Licorice
- Hyperball
- Rope
- Surface
- Spacefill
- Outline
- Fog

###### Background

- Transparent

###### Resolution

- Low
- Medium
- High
- Extreme

##### Click model image to view in 3D

##### Click model image to view in 3D

×

### Delete Model - ""

Are you sure you want to delete this model?  
(This really can't be undone!)

Close
Delete Model
