# Supplementary material for: Metabolic capacity is maintained despite shifts in microbial diversity in estuary sediments
Source: ISME Commun. 2025 Oct 11;5(1):ycaf182. doi: 10.1093/ismeco/ycaf182 (PMC12687941; doi:10.1093/ismeco/ycaf182)
Supplement: Supplementary_Data_1_ycaf182 [file supplementary_data_1_ycaf182.zip › SWISS-MODEL/4_1_Oct_SF_Bin32_scaffold_7496_c1_22440_1/templates.html]

4\_1\_Oct\_SF\_Bin32\_scaffold\_7496\_c1\_2-2440\_1 | Templates


**Export Alignment**
  
FASTA format
Clustal Format
PNG Image

**Secondary Structure**
  
None
DSSP
PSIPRED
SSpro

**Colour Scheme** 


Fade Mismatches
Enhance Mismatches

Confidencegradient
Confidenceclass
Indels
Chain
Unique Chain
Rainbow
2° Structure
Clustal
Hydrophobic
Size
Charged
Polar
Proline
Ser/Thr
Cysteine
Aliphatic
Aromatic
No Colour

Use QMEANBrane values

|  |  |  |  |
| --- | --- | --- | --- |
| Background |  |  |  |

**3D Viewer**  
NGL
PV

FASTA
Multi FASTA
ClustalW
PNG


SWISS-MODEL

### 4\_1\_Oct\_SF\_Bin32\_scaffold\_7496\_c1\_2-2440\_1

### Created: March 29, 2023, 8:38 p.m. at 20:38

- Templates
- Models

Models | Name | Description | GMQE | QSQE | Seq Id | Coverage | Range | Method | Resolution | Oligo-state | Ligands | Found by | Seq Similarity || ✓ | 7b04.1.B | Nitrite oxidoreductase subunit A  *Structure of Nitrite oxidoreductase (Nxr) from the anammox bacterium Kuenenia stuttgartiensis.* | 0.72 | 0.00 | 40.77 | 0.95 | 1-811 | X-ray | 2.97 | monomer | 4 x SF4, 1 x F3S, 2 x MD1, 1 x MO, 1 x HEM, 2 x CA | BLAST | 0.41 |
| ``` target    SWDEATTIAAKTMEDVARTFNGDEGARKLLAQGYHPEMVEVMHGAGVQALKLRGGMPLLGIGRIFGFYRFANMLALLDRK 7b04.1    SWDEAFTYASKGIIHITKKYSGPEGAQKLIDQGYPKEMVDRMQGAGTRTFKGRGGMGLLGVIGKYGMYRFNNCLAIVDAH  target    LRPDAPADEILGSRTFDNYAWHTDLPPGHPMVTGSQTVDFDLFSAEHTKLLLIIGMNWICTKMPDGHWIGDARLKGTRVI 7b04.1    NRGVGP-DQALGGRNWSNYTWHGDQAPGHPFSHGLQTSDVDMNDVRFSKLLIQTGKNLIENKMPEAHWVTEVMERGGKIV  target    VISADYMPTANKADEVIILRPGTDAAFFLGVARELIEKGLYDRAAVIERTDLPLLVRLDTGERLDARDVIPGYELAALTN 7b04.1    VITPEYSPSAQKADYWIPIRNNTDTALFLGITKILIDNKWYDADYVKKFTDFPLLIRTDTLKRVSPKDIIPNYKLQDISD  target    YVTLKPDAEIKGNPPPPPFTAGGQVVPTELRDAWGDFVWWDRATGRPRPVSRDEVG---ARFDGDPALLGEFEVELVDGS 7b04.1    ----GPSYHIQG-------------LKDEQREIIGDFVVWDAKSKGPKAITRDDVGETLVKKGIDPVLEGSFKLKTIDGK  target    TVPVRPAFDLLKQYLDESFDLRTASEVCRVPPQAIQSIARQLAANKRETLLAAGMGPNHYFQNDLFGRVQFLVAALTDNI 7b04.1    EIEVMTLLEMYKIHLRD-YDIDSVVSMTNSPKDLIERLAKDIATIK-PVAIHYGEGVNHYFHATLMNRSYYLPVMLTGNV  target    GHLGGNVGSYAGNYRGSVFQA-------MGQWIAEDPFAIEPDL-----TKPATVKRYYKAESAHYWNYGERPLRAVAKD 7b04.1    GYFGSGSHTWAGNYKAGNFQASKWSGPGFYGWVAEDVF--KPNLDPYASAKDLNIKGRALDEEVAYWNHSERPL-IVNTP  target    DEGDLTKGEVLTGKSHMPTPTKLIWFGNSNSLLGNAKWSFDVVKNTLPRQDAVFCNEWHWTSSCEYADLVFPADSWAEFK 7b04.1    KYGR----KVFTGKTHMPSPTKVLWFTNVN-LINNAKHVYQMLKNVNPNIEQIMSTDIEITGSIEYADFAFPANSWVEFQ  target    LPDATASCTNPFLLAFPTTPLKRLYDTRSDYEALALTAKALGELIDEPRMEQYWRGILDGDPTPYLQRIFSGSNATRGIT 7b04.1    EFEITNSCSNPFIQIWGKTGITPVYESKDDVKILAGMASKLGELLRDKRFEDNWKFAIEGRASVYINRLLDGSTTMKGYT  target    YDEL--HESSKRGVPLLMNMRTYPRSGGWEQRQEDKPWYTATGRLEFYRPEPEFQAAGESLPVWREPVDATFYEPNAILS 7b04.1    CEDILNGKYGEPGVAMLL-FRTYPRHPFWEQVHESLPFYTPTGRLQAYNDEPEIIEYGENFIVHREGPEATPYLPNAIVS  target    NAAHPSIAPRAPEDYGVPESQLDVETRQYRNVVRTWAELQQTLHPLQERDPAFRFVFQTPKYRWGAHSTAVDADWISMLF 7b04.1    --TNPYI---RPDDYGIPENAEYWEDRTVRNIKKSWEETKKTKNFLWEK--GYHFYCVTPKSRHTVHSQWAVTDWNFIWN  target    GPFGDPYRRDPRMPWTGEAYLEINPKDAA 7b04.1    NNFGDPYRMDKRMPGVGEHQIHIHPQAA- ``` | | | | | | | | | | | | | | | | | | | | | | | | | | | | | | | | | | | | | | | | | | | | | | | | | |
|  | 7b04.2.B | Nitrite oxidoreductase subunit A  *Structure of Nitrite oxidoreductase (Nxr) from the anammox bacterium Kuenenia stuttgartiensis.* | 0.70 | 0.00 | 40.77 | 0.95 | 1-811 | X-ray | 2.97 | monomer | 4 x SF4, 1 x F3S, 2 x MD1, 1 x MO, 1 x HEM, 2 x CA | BLAST | 0.41 |
| ``` target    SWDEATTIAAKTMEDVARTFNGDEGARKLLAQGYHPEMVEVMHGAGVQALKLRGGMPLLGIGRIFGFYRFANMLALLDRK 7b04.2    SWDEAFTYASKGIIHITKKYSGPEGAQKLIDQGYPKEMVDRMQGAGTRTFKGRGGMGLLGVIGKYGMYRFNNCLAIVDAH  target    LRPDAPADEILGSRTFDNYAWHTDLPPGHPMVTGSQTVDFDLFSAEHTKLLLIIGMNWICTKMPDGHWIGDARLKGTRVI 7b04.2    NRGVGP-DQALGGRNWSNYTWHGDQAPGHPFSHGLQTSDVDMNDVRFSKLLIQTGKNLIENKMPEAHWVTEVMERGGKIV  target    VISADYMPTANKADEVIILRPGTDAAFFLGVARELIEKGLYDRAAVIERTDLPLLVRLDTGERLDARDVIPGYELAALTN 7b04.2    VITPEYSPSAQKADYWIPIRNNTDTALFLGITKILIDNKWYDADYVKKFTDFPLLIRTDTLKRVSPKDIIPNYKLQDISD  target    YVTLKPDAEIKGNPPPPPFTAGGQVVPTELRDAWGDFVWWDRATGRPRPVSRDEVG---ARFDGDPALLGEFEVELVDGS 7b04.2    ----GPSYHIQG-------------LKDEQREIIGDFVVWDAKSKGPKAITRDDVGETLVKKGIDPVLEGSFKLKTIDGK  target    TVPVRPAFDLLKQYLDESFDLRTASEVCRVPPQAIQSIARQLAANKRETLLAAGMGPNHYFQNDLFGRVQFLVAALTDNI 7b04.2    EIEVMTLLEMYKIHLRD-YDIDSVVSMTNSPKDLIERLAKDIATIK-PVAIHYGEGVNHYFHATLMNRSYYLPVMLTGNV  target    GHLGGNVGSYAGNYRGSVFQA-------MGQWIAEDPFAIEPDL-----TKPATVKRYYKAESAHYWNYGERPLRAVAKD 7b04.2    GYFGSGSHTWAGNYKAGNFQASKWSGPGFYGWVAEDVF--KPNLDPYASAKDLNIKGRALDEEVAYWNHSERPL-IVNTP  target    DEGDLTKGEVLTGKSHMPTPTKLIWFGNSNSLLGNAKWSFDVVKNTLPRQDAVFCNEWHWTSSCEYADLVFPADSWAEFK 7b04.2    KYGR----KVFTGKTHMPSPTKVLWFTNVN-LINNAKHVYQMLKNVNPNIEQIMSTDIEITGSIEYADFAFPANSWVEFQ  target    LPDATASCTNPFLLAFPTTPLKRLYDTRSDYEALALTAKALGELIDEPRMEQYWRGILDGDPTPYLQRIFSGSNATRGIT 7b04.2    EFEITNSCSNPFIQIWGKTGITPVYESKDDVKILAGMASKLGELLRDKRFEDNWKFAIEGRASVYINRLLDGSTTMKGYT  target    YDEL--HESSKRGVPLLMNMRTYPRSGGWEQRQEDKPWYTATGRLEFYRPEPEFQAAGESLPVWREPVDATFYEPNAILS 7b04.2    CEDILNGKYGEPGVAMLL-FRTYPRHPFWEQVHESLPFYTPTGRLQAYNDEPEIIEYGENFIVHREGPEATPYLPNAIVS  target    NAAHPSIAPRAPEDYGVPESQLDVETRQYRNVVRTWAELQQTLHPLQERDPAFRFVFQTPKYRWGAHSTAVDADWISMLF 7b04.2    --TNPYI---RPDDYGIPENAEYWEDRTVRNIKKSWEETKKTKNFLWEK--GYHFYCVTPKSRHTVHSQWAVTDWNFIWN  target    GPFGDPYRRDPRMPWTGEAYLEINPKDAA 7b04.2    NNFGDPYRMDKRMPGVGEHQIHIHPQAA- ``` | | | | | | | | | | | | | | | | | | | | | | | | | | | | | | | | | | | | | | | | | | | | | | | | | |
|  | 7b04.1.B | Nitrite oxidoreductase subunit A  *Structure of Nitrite oxidoreductase (Nxr) from the anammox bacterium Kuenenia stuttgartiensis.* | 0.71 | 0.00 | 38.92 | 0.96 | 1-812 | X-ray | 2.97 | monomer | 4 x SF4, 1 x F3S, 2 x MD1, 1 x MO, 1 x HEM, 2 x CA | HHblits | 0.40 |
| ``` target    SWDEATTIAAKTMEDVARTFNGDEGARKLLAQGYHPEMVEVMHGAGVQALKLRGGMPLLGIGRIFGFYRFANMLALLDRK 7b04.1    SWDEAFTYASKGIIHITKKYSGPEGAQKLIDQGYPKEMVDRMQGAGTRTFKGRGGMGLLGVIGKYGMYRFNNCLAIVDAH  target    LRPDAPADEILGSRTFDNYAWHTDLPPGHPMVTGSQTVDFDLFSAEHTKLLLIIGMNWICTKMPDGHWIGDARLKGTRVI 7b04.1    -NRGVGPDQALGGRNWSNYTWHGDQAPGHPFSHGLQTSDVDMNDVRFSKLLIQTGKNLIENKMPEAHWVTEVMERGGKIV  target    VISADYMPTANKADEVIILRPGTDAAFFLGVARELIEKGLYDRAAVIERTDLPLLVRLDTGERLDARDVIPGYELAALTN 7b04.1    VITPEYSPSAQKADYWIPIRNNTDTALFLGITKILIDNKWYDADYVKKFTDFPLLIRTDTLKRVSPKDIIPNYKLQDISD  target    YVTLKPDAEIKGNPPPPPFTAGGQVVPTELRDAWGDFVWWDRATGRPRPVSRDEVG---ARFDGDPALLGEFEVELVDGS 7b04.1    -----------G---PSYHIQG---LKDEQREIIGDFVVWDAKSKGPKAITRDDVGETLVKKGIDPVLEGSFKLKTIDGK  target    TVPVRPAFDLLKQYLDESFDLRTASEVCRVPPQAIQSIARQLAANKRETLLAAGMGPNHYFQNDLFGRVQFLVAALTDNI 7b04.1    EIEVMTLLEMYKIHLR-DYDIDSVVSMTNSPKDLIERLAKDIATIKPVAI-HYGEGVNHYFHATLMNRSYYLPVMLTGNV  target    GHLGGNVGSYAGNYRGSVFQ---AMGQ----WIAEDPFAI-EPDLTKP--ATVKRYYKAESAHYWNYGERPLRAVAKDDE 7b04.1    GYFGSGSHTWAGNYKAGNFQASKWSGPGFYGWVAEDVFKPNLDPYASAKDLNIKGRALDEEVAYWNHSERPLIV-N----  target    GDLTKGEVLTGKSHMPTPTKLIWFGNSNSLLGNAKWSFDVVKNTLPRQDAVFCNEWHWTSSCEYADLVFPADSWAEFKLP 7b04.1    TPKYGRKVFTGKTHMPSPTKVLWFTNVNLINNAKH-VYQMLKNVNPNIEQIMSTDIEITGSIEYADFAFPANSWVEF--Q  target    DATASC--TNPFLLAFPTTPLKRLYDTRSDYEALALTAKALGELIDEPRMEQYWRGILDGDPTPYLQRIFSGSNATRGIT 7b04.1    EFEITNSCSNPFIQIWGKTGITPVYESKDDVKILAGMASKLGELLRDKRFEDNWKFAIEGRASVYINRLLDGSTTMKGYT  target    YDELHESSK--RGVPLLMNMRTYPRSGGWEQRQEDKPWYTATGRLEFYRPEPEFQAAGESLPVWREPVDATFYEPNAILS 7b04.1    CEDILNGKYGEPGVAML-LFRTYPRHPFWEQVHESLPFYTPTGRLQAYNDEPEIIEYGENFIVHREGPEATPYLPNAIVS  target    NAAHPSIAPRAPEDYGVPESQLDVETRQYRNVVRTWAELQQTLHPLQERDPAFRFVFQTPKYRWGAHSTAVDADWISMLF 7b04.1    T--NPYI---RPDDYGIPENAEYWEDRTVRNIKKSWEETKKTKNFLWE--KGYHFYCVTPKSRHTVHSQWAVTDWNFIWN  target    GPFGDPYRRDPRMPWTGEAYLEINPKDAA 7b04.1    NNFGDPYRMDKRMPGVGEHQIHIHPQAAR ``` | | | | | | | | | | | | | | | | | | | | | | | | | | | | | | | | | | | | | | | | | | | | | | | | | |
|  | 7b04.2.B | Nitrite oxidoreductase subunit A  *Structure of Nitrite oxidoreductase (Nxr) from the anammox bacterium Kuenenia stuttgartiensis.* | 0.68 | 0.00 | 38.92 | 0.96 | 1-812 | X-ray | 2.97 | monomer | 4 x SF4, 1 x F3S, 2 x MD1, 1 x MO, 1 x HEM, 2 x CA | HHblits | 0.40 |
| ``` target    SWDEATTIAAKTMEDVARTFNGDEGARKLLAQGYHPEMVEVMHGAGVQALKLRGGMPLLGIGRIFGFYRFANMLALLDRK 7b04.2    SWDEAFTYASKGIIHITKKYSGPEGAQKLIDQGYPKEMVDRMQGAGTRTFKGRGGMGLLGVIGKYGMYRFNNCLAIVDAH  target    LRPDAPADEILGSRTFDNYAWHTDLPPGHPMVTGSQTVDFDLFSAEHTKLLLIIGMNWICTKMPDGHWIGDARLKGTRVI 7b04.2    -NRGVGPDQALGGRNWSNYTWHGDQAPGHPFSHGLQTSDVDMNDVRFSKLLIQTGKNLIENKMPEAHWVTEVMERGGKIV  target    VISADYMPTANKADEVIILRPGTDAAFFLGVARELIEKGLYDRAAVIERTDLPLLVRLDTGERLDARDVIPGYELAALTN 7b04.2    VITPEYSPSAQKADYWIPIRNNTDTALFLGITKILIDNKWYDADYVKKFTDFPLLIRTDTLKRVSPKDIIPNYKLQDISD  target    YVTLKPDAEIKGNPPPPPFTAGGQVVPTELRDAWGDFVWWDRATGRPRPVSRDEVG---ARFDGDPALLGEFEVELVDGS 7b04.2    -----------G---PSYHIQG---LKDEQREIIGDFVVWDAKSKGPKAITRDDVGETLVKKGIDPVLEGSFKLKTIDGK  target    TVPVRPAFDLLKQYLDESFDLRTASEVCRVPPQAIQSIARQLAANKRETLLAAGMGPNHYFQNDLFGRVQFLVAALTDNI 7b04.2    EIEVMTLLEMYKIHLR-DYDIDSVVSMTNSPKDLIERLAKDIATIKPVAI-HYGEGVNHYFHATLMNRSYYLPVMLTGNV  target    GHLGGNVGSYAGNYRGSVFQ---AMGQ----WIAEDPFAI-EPDLTKP--ATVKRYYKAESAHYWNYGERPLRAVAKDDE 7b04.2    GYFGSGSHTWAGNYKAGNFQASKWSGPGFYGWVAEDVFKPNLDPYASAKDLNIKGRALDEEVAYWNHSERPLIV-N----  target    GDLTKGEVLTGKSHMPTPTKLIWFGNSNSLLGNAKWSFDVVKNTLPRQDAVFCNEWHWTSSCEYADLVFPADSWAEFKLP 7b04.2    TPKYGRKVFTGKTHMPSPTKVLWFTNVNLINNAKH-VYQMLKNVNPNIEQIMSTDIEITGSIEYADFAFPANSWVEF--Q  target    DATASC--TNPFLLAFPTTPLKRLYDTRSDYEALALTAKALGELIDEPRMEQYWRGILDGDPTPYLQRIFSGSNATRGIT 7b04.2    EFEITNSCSNPFIQIWGKTGITPVYESKDDVKILAGMASKLGELLRDKRFEDNWKFAIEGRASVYINRLLDGSTTMKGYT  target    YDELHESSK--RGVPLLMNMRTYPRSGGWEQRQEDKPWYTATGRLEFYRPEPEFQAAGESLPVWREPVDATFYEPNAILS 7b04.2    CEDILNGKYGEPGVAML-LFRTYPRHPFWEQVHESLPFYTPTGRLQAYNDEPEIIEYGENFIVHREGPEATPYLPNAIVS  target    NAAHPSIAPRAPEDYGVPESQLDVETRQYRNVVRTWAELQQTLHPLQERDPAFRFVFQTPKYRWGAHSTAVDADWISMLF 7b04.2    T--NPYI---RPDDYGIPENAEYWEDRTVRNIKKSWEETKKTKNFLWE--KGYHFYCVTPKSRHTVHSQWAVTDWNFIWN  target    GPFGDPYRRDPRMPWTGEAYLEINPKDAA 7b04.2    NNFGDPYRMDKRMPGVGEHQIHIHPQAAR ``` | | | | | | | | | | | | | | | | | | | | | | | | | | | | | | | | | | | | | | | | | | | | | | | | | |
|  | 5e7o.1.A | DMSO reductase family type II enzyme, molybdopterin subunit  *Crystal structure of the perchlorate reductase PcrAB mutant W461E of PcrA from Azospira suillum PS* | 0.49 | 0.00 | 26.73 | 0.78 | 1-812 | X-ray | 2.40 | monomer | 4 x SF4, 1 x MO, 1 x MGD, 1 x MD1, 1 x F3S | HHblits | 0.34 |
| ``` target    SWDEATTIAAKTMEDVARTFNGDEGARKLLAQGYHPEMVEVMHGAGVQALKLRGGMPLLGIGRIFGFYRFANMLALLDRK 5e7o.1    TWEEALDMIADKCVDTIKN--------------HAPDCISVYSPVPAVSPV-----------SFSAGHRFA---------  target    LRPDAPADEILGSRTFDNYAWHTDLPPGHPMVTGSQTVDFDLFSAEHTKLLLIIGMNWICTKMPDGHWIGDARLKGTRVI 5e7o.1    --------HYIGAHAHTFYDWYGDHPTGQTQTCGVQGDTCETADWFNSKYIILWGSNPTQTRIPDAHFLSEAQLNGAKIV  target    VISADYMPTANKADEVIILRPGTDAAFFLGVARELIEKGLYDRAAVIERTDLPLLVRLDTGERLDARDVIPGYELAALTN 5e7o.1    SISPDYNSSTIKVDKWIHPQPGTDGALAMAMAHVIIKEKLYDAHSLKEQTDLSYLVRSDTKRFLREADVVAGG-------  target    YVTLKPDAEIKGNPPPPPFTAGGQVVPTELRDAWGDFVWWDRATGRPRPVSRDEV------------------------G 5e7o.1    --------------------------------SKDKFYFWNAKTGKPVIPKGSWGDQPEKKGSPVGFLGRNTFAFPKGYI  target    ARFDGDPALLGEFEVELVDGSTVPVRPAFDLLKQYLDESFDLRTASEVCRVPPQAIQSIARQLAANKRETLLAAGMGPNH 5e7o.1    DLGDLDPALEGKFNMQLLDGKTVEVRPVFEILKSRLMADNTPEKAAKITGVTAKAITELAREFATAKPSMI-ICGGGTQH  target    YFQNDLFGRVQFLVAALTDNIGHLGGNVGSYAGNYRGSVFQAMGQWIAEDPFAIEPDLTKPATVKRYYKAESAHYWNYGE 5e7o.1    WYYSDVLLRAMHLLTALTGTEGTNGGGMNHYIGQEKPAF--VAGLVA--LAFP---EGVNK---QRFCQ---TTIWTYIH  target    RP-----LR---AVAKDDEGDLTKGEVLTGKSHMPTPTKLIWFGNSNSLLGNAKWSFDVVKNTLPRQDAVFCNEWHWTSS 5e7o.1    AEVNDEIISSDIDTEKYLRDSITTGQMPN-MPEQGRDPKVFFVYRGNWLNQAKGQ-KYVLENLWPKLELIVDINIRMDST  target    CEYADLVFPADSWAEFKLPDATASCTNPFLLAFPTTPLKRLYDTRSDYEALALTAKALGELIDEPRM------------- 5e7o.1    ALYSDVVLPSAHWYEKL--DLNVTSEHSYINMT-EPAIKPMWESKTDWQIFLALAKRVEMAAKRKKYEKFNDEKFKWVRD  target    -EQYWRGIL----DGDPTPYLQRIFSGSNATRGITYDELHESSKRGVPLLMN--M-RTYPRSGGWEQRQEDKPWYTATGR 5e7o.1    LSNLWNQMTMDGKLAEDEAAAQYILDNAPQSKGITIQMLREKPQR-FKSNWTSPLKEGVPYTPFQYFVVDKKPWPTLTGR  target    LEFYRPEPEFQAAGESLPVWREPVDATFYEPNAILSNAAHPSIAPRAPEDYGVPESQLDVETRQYRNVVRTWAELQQTLH 5e7o.1    QQFYLDHDTFFDMGVELPTYKAPIDA------------------------------------------------------  target    PLQERDPAFRFVFQTPKYRWGAHSTAVDADWISMLFGPFGDPYRRDPRMPWTGEAYLEINPKDAA 5e7o.1    ------DKYPFRFNSPHSRHSVHSTFKDNVLMLRL---------------QRGGPSIEMSPLDAK ``` | | | | | | | | | | | | | | | | | | | | | | | | | | | | | | | | | | | | | | | | | | | | | | | | | |
|  | 4ydd.1.A | DMSO reductase family type II enzyme, molybdopterin subunit  *Crystal structure of the perchlorate reductase PcrAB from Azospira suillum PS* | 0.48 | 0.00 | 26.30 | 0.78 | 1-812 | X-ray | 1.86 | monomer | 4 x SF4, 1 x MO, 1 x MGD, 1 x MD1, 1 x F3S | HHblits | 0.34 |
| ``` target    SWDEATTIAAKTMEDVARTFNGDEGARKLLAQGYHPEMVEVMHGAGVQALKLRGGMPLLGIGRIFGFYRFANMLALLD-R 4ydd.1    TWEEALDMIADKCVDTIKN--------------HAPDCISVYSPVPAV-----------------------SPVSFSAGH  target    KLRPDAPADEILGSRTFDNYAWHTDLPPGHPMVTGSQTVDFDLFSAEHTKLLLIIGMNWICTKMPDGHWIGDARLKGTRV 4ydd.1    RF------AHYIGAHAHTFYDWYGDHPTGQTQTCGVQGDTCETADWFNSKYIILWGSNPTQTRIPDAHFLSEAQLNGAKI  target    IVISADYMPTANKADEVIILRPGTDAAFFLGVARELIEKGLYDRAAVIERTDLPLLVRLDTGERLDARDVIPGYELAALT 4ydd.1    VSISPDYNSSTIKVDKWIHPQPGTDGALAMAMAHVIIKEKLYDAHSLKEQTDLSYLVRSDTKRFLREADVVAGG------  target    NYVTLKPDAEIKGNPPPPPFTAGGQVVPTELRDAWGDFVWWDRATGRPRPVSRDEVG----------------------- 4ydd.1    ---------------------------------SKDKFYFWNAKTGKPVIPKGSWGDQPEKKGSPVGFLGRNTFAFPKGY  target    -ARFDGDPALLGEFEVELVDGSTVPVRPAFDLLKQYLDESFDLRTASEVCRVPPQAIQSIARQLAANKRETLLAAGMGPN 4ydd.1    IDLGDLDPALEGKFNMQLLDGKTVEVRPVFEILKSRLMADNTPEKAAKITGVTAKAITELAREFATAKPSMI-ICGGGTQ  target    HYFQNDLFGRVQFLVAALTDNIGHLGGNVGSYAGNYRGSVFQAMGQWIAEDPFAIEPDLTKPATVKRYYKAESAHYWNYG 4ydd.1    HWYYSDVLLRAMHLLTALTGTEGTNGGGMNHYIGQWKPAF--VAGLVA--LAFPE---GVNK---QRFCQ---TTIWTYI  target    ERPL-----RA-VA--KDDEGDLTKGEVLTGKSHMPTPTKLIWFGNSNSLLGNAKWSFDVVKNTLPRQDAVFCNEWHWTS 4ydd.1    HAEVNDEIISSDIDTEKYLRDSITTGQMPN-MPEQGRDPKVFFVYRGNWLNQAKGQ-KYVLENLWPKLELIVDINIRMDS  target    SCEYADLVFPADSWAEFKLPDATASCTNPFLLAFPTTPLKRLYDTRSDYEALALTAKALGELIDEPRM------------ 4ydd.1    TALYSDVVLPSAHWYEKL--DLNVTSEHSYINMT-EPAIKPMWESKTDWQIFLALAKRVEMAAKRKKYEKFNDEKFKWVR  target    --EQYWRGIL----DGDPTPYLQRIFSGSNATRGITYDELHESSKRGVPLLMN---MRTYPRSGGWEQRQEDKPWYTATG 4ydd.1    DLSNLWNQMTMDGKLAEDEAAAQYILDNAPQSKGITIQMLREKPQR-FKSNWTSPLKEGVPYTPFQYFVVDKKPWPTLTG  target    RLEFYRPEPEFQAAGESLPVWREPVDATFYEPNAILSNAAHPSIAPRAPEDYGVPESQLDVETRQYRNVVRTWAELQQTL 4ydd.1    RQQFYLDHDTFFDMGVELPTYKAPIDA-----------------------------------------------------  target    HPLQERDPAFRFVFQTPKYRWGAHSTAVDADWISMLFGPFGDPYRRDPRMPWTGEAYLEINPKDAA 4ydd.1    -------DKYPFRFNSPHSRHSVHSTFKDNVLMLRL---------------QRGGPSIEMSPLDAK ``` | | | | | | | | | | | | | | | | | | | | | | | | | | | | | | | | | | | | | | | | | | | | | | | | | |
|  | 2ivf.1.A | ETHYLBENZENE DEHYDROGENASE ALPHA-SUBUNIT  *ETHYLBENZENE DEHYDROGENASE FROM AROMATOLEUM AROMATICUM* | 0.48 | 0.00 | 25.39 | 0.80 | 1-812 | X-ray | 1.88 | monomer | 1 x MES, 4 x SF4, 1 x MO, 1 x MGD, 1 x MD1, 1 x F3S, 1 x HEM | HHblits | 0.32 |
| ``` target    SWDEATTIAAKTMEDVARTFNGDEGARKLLAQGYHPEMVEVMHGAGVQALKLRGGMPLLGIGRIFGFYRFANMLALLDRK 2ivf.1    SWDEAAGDIADSIIDSFEA--------------QGSDGFILDAPHVHAGSI-----------AWGAGFRMTYLMD-----  target    LRPDAPADEILGSRTFDNYAWHTDLPPGHPMVTGSQTVDFDLFSAEHTKLLLIIGMNWICTKMPDGHWIGDARLKGTRVI 2ivf.1    --------GVSPDINVDIG----DTYMGAFHTFGKMHMGYSADNLLDAELIFMTCSNWSYTYPSSYHFLSEARYKGAEVV  target    VISADYMPTANKADEVIILRPGTDAAFFLGVARELIEKGLYDRAAVIERTDLPLLVRLDTGERLDARDVIPGYELAALTN 2ivf.1    VIAPDFNPTTPAADLHVPVRVGSDAAFWLGLSQVMIDEKLFDRQFVCEQTDLPLLVRMDTGKFLSAEDVDGG--------  target    YVTLKPDAEIKGNPPPPPFTAGGQVVPTELRDAWGDFVWWDRATGRPRPVSRDEVGARFDGDPALLGEFEVELVDGSTVP 2ivf.1    --------------------------------EAKQFYFFDEKAGSVRKASRGTLKL--DFMPALEGTFSARLKNGKTIQ  target    VRPAFDLLKQYLDESFDLRTASEVCRVPPQAIQSIARQLAANKRETLLAAGMGPNHYFQNDLFGRVQFLVAALTDNIGHL 2ivf.1    VRTVFEGLREHLK-DYTPEKASAKCGVPVSLIRELGRKVAKKRT--CSYIGFSSAKSYHGDLMERSLFLAMALSGNWGKP  target    GGNVGSYAGNYRGS-------VFQAMGQWIA----EDPFAI-----EPDLTKPATVKRYYK------------------A 2ivf.1    GTGAFAWAYSDDNMVYLGVMSKPTAQGGMDELHQMAEGFNKRTLEADPTSTDEMGNIEFMKVVTSAVGLVPPAMWLYYHV  target    ESAHYWNYGE--RP-LR-AVAKD----DEGDLTKGEVLTGKSHMPTPTKLIWFGNSNSLLGNAKWSFDVVKNTLPRQDAV 2ivf.1    GYDQLWNNKAWTDPALKKSFGAYLDEAKEKGWWTNDHIRP--APDKTPQVYMLLSQNPMRRKRSGAKMFPDVLFPKLKMI  target    FCNEWHWTSSCEYADLVFPADSWAEFKLPDATAS-CTNPFLLAFPTTPLKRLYDTRSDYEALALTAKALGELIDEPR--- 2ivf.1    FALETRMSSSAMYADIVLPCAWYYEKH--EMTTPCSGNPFFTF-VDRSVAPPGECREEWDAIALILKKVGERAAARGLTE  target    ----------MEQYWRGIL----DGDPTPYLQRIFSGSNA----TRGITYDELHESSKRGVPLLMN-MR----------T 2ivf.1    FNDHNGRKRRYDELYKKFTMDGHLLTNEDCLKEMVDINRAVGVFAKDYTYEKFKKEGQTRFLSMGTGVSRYAHANEVDVT  target    YPRSGGWEQRQEDKPWYTATGRLEFYRPEPEFQAAGESLPVWREPVDATFYEPNAILSNAAHPSIAPRAPEDYGVPESQL 2ivf.1    KPIYPMRWHFDDKKVFPTHTRRAQFYLDHDWYLEAGESLPTHKDTPMV--------------------------------  target    DVETRQYRNVVRTWAELQQTLHPLQERDPAFRFVFQTPKYRWGAHSTAVDADWISMLFGPFGDPYRRDPRMPWTGEAYLE 2ivf.1    ---------------------------GGDHPFKITGGHPRVSIHSTHLTNSHLSRL---------------HRGQPVVH  target    INPKDAA 2ivf.1    MNSKDAA ``` | | | | | | | | | | | | | | | | | | | | | | | | | | | | | | | | | | | | | | | | | | | | | | | | | |
|  | 4ydd.1.A | DMSO reductase family type II enzyme, molybdopterin subunit  *Crystal structure of the perchlorate reductase PcrAB from Azospira suillum PS* | 0.39 | 0.00 | 30.81 | 0.68 | 91-696 | X-ray | 1.86 | monomer | 4 x SF4, 1 x MO, 1 x MGD, 1 x MD1, 1 x F3S | BLAST | 0.36 |
| ``` target    SWDEATTIAAKTMEDVARTFNGDEGARKLLAQGYHPEMVEVMHGAGVQALKLRGGMPLLGIGRIFGFYRFANMLALLDRK 4ydd.1    --------------------------------------------------------------------------------  target    LRPDAPADEILGSRTFDNYAWHTDLPPGHPMVTGSQTVDFDLFSAEHTKLLLIIGMNWICTKMPDGHWIGDARLKGTRVI 4ydd.1    ----------IGAHAHTFYDWYGDHPTGQTQTCGVQGDTCETADWFNSKYIILWGSNPTQTRIPDAHFLSEAQLNGAKIV  target    VISADYMPTANKADEVIILRPGTDAAFFLGVARELIEKGLYDRAAVIERTDLPLLVRLDTGERLDARDVIPG--YELAAL 4ydd.1    SISPDYNSSTIKVDKWIHPQPGTDGALAMAMAHVIIKEKLYDAHSLKEQTDLSYLVRSDTKRFLREADVVAGGSKDKFYF  target    TNYVTLKPDAEIKGNPPPPPFTAGGQVVPTELRDAWGDFVWWDRATGRPR----------PVSRDEVGARFDGDPALLGE 4ydd.1    WNAKTGKP------------------VIP---KGSWGD---QPEKKGSPVGFLGRNTFAFPKGYIDLG---DLDPALEGK  target    FEVELVDGSTVPVRPAFDLLKQYLDESFDLRTASEVCRVPPQAIQSIARQLAANKRETLLAAGMGPNHYFQNDLFGRVQF 4ydd.1    FNMQLLDGKTVEVRPVFEILKSRLMADNTPEKAAKITGVTAKAITELAREFATAKPSMIICGG-GTQHWYYSDVLLRAMH  target    LVAALTDNIGHLGGNVGSYAGNYR-----GSVFQAMGQWIAEDPFAIEPDLTKPATVKRYYKAESAHYWNYGERPLRAVA 4ydd.1    LLTALTGTEGTNGGGMNHYIGQWKPAFVAGLVALAFPEGVNKQRFC-------QTTIWTYIHAEVND---------EIIS  target    KD-DEGDLTKGEVLTGKS-HMPTPTK--LIWFGNSNSLLGNAKWSFDVVKNTLPRQDAVFCNEWHWTSSCEYADLVFPAD 4ydd.1    SDIDTEKYLRDSITTGQMPNMPEQGRDPKVFFVYRGNWLNQAKGQKYVLENLWPKLELIVDINIRMDSTALYSDVVLPSA  target    SWAEFKLPDATASCTNPFLLAFPTTPLKRLYDTRSDYEALALTAKALGELIDEPRMEQY--------------WRGI-LD 4ydd.1    HWYE-KLDLNVTSEHSYINMTEPA--IKPMWESKTDWQIFLALAKRVEMAAKRKKYEKFNDEKFKWVRDLSNLWNQMTMD  target    G---DPTPYLQRIFSGSNATRGITYDELHESSKRGVPLLMNMRTYPRSGG--WEQRQ----EDKPWYTATGRLEFYRPEP 4ydd.1    GKLAEDEAAAQYILDNAPQSKGITIQMLREKPQR----FKSNWTSPLKEGVPYTPFQYFVVDKKPWPTLTGRQQFYLDHD  target    EFQAAGESLPVWREPVDATFYEPNAILSNAAHPSIAPRAPEDYGVPESQLDVETRQYRNVVRTWAELQQTLHPLQERDPA 4ydd.1    TFFDMGVELPTYKAPIDADKY-----------------------------------------------------------  target    FRFVFQTPKYRWGAHSTAVDADWISMLFGPFGDPYRRDPRMPWTGEAYLEINPKDAA 4ydd.1    --------------------------------------------------------- ``` | | | | | | | | | | | | | | | | | | | | | | | | | | | | | | | | | | | | | | | | | | | | | | | | | |
|  | 5e7o.1.A | DMSO reductase family type II enzyme, molybdopterin subunit  *Crystal structure of the perchlorate reductase PcrAB mutant W461E of PcrA from Azospira suillum PS* | 0.39 | 0.00 | 30.81 | 0.68 | 91-696 | X-ray | 2.40 | monomer | 4 x SF4, 1 x MO, 1 x MGD, 1 x MD1, 1 x F3S | BLAST | 0.36 |
| ``` target    SWDEATTIAAKTMEDVARTFNGDEGARKLLAQGYHPEMVEVMHGAGVQALKLRGGMPLLGIGRIFGFYRFANMLALLDRK 5e7o.1    --------------------------------------------------------------------------------  target    LRPDAPADEILGSRTFDNYAWHTDLPPGHPMVTGSQTVDFDLFSAEHTKLLLIIGMNWICTKMPDGHWIGDARLKGTRVI 5e7o.1    ----------IGAHAHTFYDWYGDHPTGQTQTCGVQGDTCETADWFNSKYIILWGSNPTQTRIPDAHFLSEAQLNGAKIV  target    VISADYMPTANKADEVIILRPGTDAAFFLGVARELIEKGLYDRAAVIERTDLPLLVRLDTGERLDARDVIPG--YELAAL 5e7o.1    SISPDYNSSTIKVDKWIHPQPGTDGALAMAMAHVIIKEKLYDAHSLKEQTDLSYLVRSDTKRFLREADVVAGGSKDKFYF  target    TNYVTLKPDAEIKGNPPPPPFTAGGQVVPTELRDAWGDFVWWDRATGRPR----------PVSRDEVGARFDGDPALLGE 5e7o.1    WNAKTGKP------------------VIP---KGSWGD---QPEKKGSPVGFLGRNTFAFPKGYIDLG---DLDPALEGK  target    FEVELVDGSTVPVRPAFDLLKQYLDESFDLRTASEVCRVPPQAIQSIARQLAANKRETLLAAGMGPNHYFQNDLFGRVQF 5e7o.1    FNMQLLDGKTVEVRPVFEILKSRLMADNTPEKAAKITGVTAKAITELAREFATAKPSMIICGG-GTQHWYYSDVLLRAMH  target    LVAALTDNIGHLGGNVGSYAGNYR-----GSVFQAMGQWIAEDPFAIEPDLTKPATVKRYYKAESAHYWNYGERPLRAVA 5e7o.1    LLTALTGTEGTNGGGMNHYIGQEKPAFVAGLVALAFPEGVNKQRFC-------QTTIWTYIHAEVND---------EIIS  target    KD-DEGDLTKGEVLTGKS-HMPTPTK--LIWFGNSNSLLGNAKWSFDVVKNTLPRQDAVFCNEWHWTSSCEYADLVFPAD 5e7o.1    SDIDTEKYLRDSITTGQMPNMPEQGRDPKVFFVYRGNWLNQAKGQKYVLENLWPKLELIVDINIRMDSTALYSDVVLPSA  target    SWAEFKLPDATASCTNPFLLAFPTTPLKRLYDTRSDYEALALTAKALGELIDEPRMEQY--------------WRGI-LD 5e7o.1    HWYE-KLDLNVTSEHSYINMTEPA--IKPMWESKTDWQIFLALAKRVEMAAKRKKYEKFNDEKFKWVRDLSNLWNQMTMD  target    G---DPTPYLQRIFSGSNATRGITYDELHESSKRGVPLLMNMRTYPRSGG--WEQRQ----EDKPWYTATGRLEFYRPEP 5e7o.1    GKLAEDEAAAQYILDNAPQSKGITIQMLREKPQR----FKSNWTSPLKEGVPYTPFQYFVVDKKPWPTLTGRQQFYLDHD  target    EFQAAGESLPVWREPVDATFYEPNAILSNAAHPSIAPRAPEDYGVPESQLDVETRQYRNVVRTWAELQQTLHPLQERDPA 5e7o.1    TFFDMGVELPTYKAPIDADKY-----------------------------------------------------------  target    FRFVFQTPKYRWGAHSTAVDADWISMLFGPFGDPYRRDPRMPWTGEAYLEINPKDAA 5e7o.1    --------------------------------------------------------- ``` | | | | | | | | | | | | | | | | | | | | | | | | | | | | | | | | | | | | | | | | | | | | | | | | | |
|  | 6cz7.1.A | ArrA  *The arsenate respiratory reductase (Arr) complex from Shewanella sp. ANA-3* | 0.35 |  | 17.05 | 0.74 | 1-812 | X-ray | 1.62 | hetero-1-1-mer | 5 x SF4, 2 x MGD, 1 x MO, 1 x PG5 | HHblits | 0.28 |
| ``` target    SWDEATTIAAKTMEDVARTFNGDEGARKLLAQGYHPEMVEVMHGAGVQALKLRGGMPLLGIGRIFGFYRFANMLALLDRK 6cz7.1    SWDKALDMLADKIIALRVA--------------NEPHKYALLRGRY------------------------SHINDLLYKK  target    LRPDAPADEILGSRTFDNYAWHTDLP--PGHPMVTGSQTVDFDLFSAEHTKLLLIIGMNWICTKMPDGHWIGD--ARLKG 6cz7.1    M------TNLIGSPNNISHSSVCAEAHKMGPYYLDGN--WGYNQYDVKNAKFILSFGADPIASNRQVSFYSQTWGDSLDH  target    TRVIVISADYMPTANKADEVIILRPGTDAAFFLGVARELIEKGLYDRAAVIERTDLPLLVRLDTGERLDARDVIPGYELA 6cz7.1    AKVVVVDPRLSASAAKAHKWIPIEPGQDSVLALAIAHVALVEGVWHKPFVGDFIEGKNLFKA-------------GKT--  target    ALTNYVTLKPDAEIKGNPPPPPFTAGGQVVPTELRDAWGDFVWWDRATGRPRPVSRDEVGARFDGDPALLGEFEVELVDG 6cz7.1    -----------------------------------VSVESFK--------------------------------E-----  target    STVPVRPAFDLLKQYLDESFDLRTASEVCRVPPQAIQSIARQLAANKRETLLAAGMGPNHYFQNDLFGRVQFLVAALTDN 6cz7.1    --THTYGLVEWWNQALK-DYTPEWASKITGIDPKTIIAIAKDMGAAAPAVQVWTSRGAVMQARGTYTSISCHALNGLFGG  target    IGHLGGNVGSYAGNYRGSVFQAMGQWIAEDPFAIEPDLTKPATVKRYYKAESAHYWNYGERPLRAVAKDDEGDLTKGEVL 6cz7.1    IDSKGGLFPGNKTPLLK-EYPEAKAYMD--EIA-AKGV-KKEKIDQRGRLEFPALAKGK--SGGGV---I-TANAANGIR  target    TGKSHMPTPTKLIWFGNSNSLLGNAKWSFDVVKNTLPRQDAVFCNEWHWTSSCEYADLVFPADS-WAEFKLPDATASCTN 6cz7.1    NQ---DPYEIKVMLAYFNNFNFSNPE--GQRWDEALSKVDFMAHITTNVSEFSWFADVLLPSSHHMFEKW--GVLDSIGN  target    P--FLLAFPTTPLKRLYDTRSD-YEALALTAKALGEL-ID--EPRM-EQYWRG---ILDGDPTPYLQRIFSGS------- 6cz7.1    GVAQIS-IQQPSIKRLWDTRIDESEIPYMLAKKLADKGFDAPWRYINEQIVDPETGKPAADEAEFAKLMVRYLTAPLWKE  target    ----NATRGITYDELHESSKRGVPLLMNMRTYPRSGGWEQRQEDKPWYTATGRLEFYRPEPEFQAAGESLPVWREPVDAT 6cz7.1    DASKYGDKLSSWDEFVQK---GVWNSS---PYK------LEARWGKFKTETTKFEFYSKTLEKA-----LQSHADKHK--  target    FYEPNAILSNAAHPSIAPRAPEDYGVPESQLDVETRQYRNVVRTWAELQQTLHPLQERDPAFRFVFQTPKYRWGAHSTAV 6cz7.1    -VSIDEVMK--A--CDYQARGHLAFIP----------------HYEEP----YRFGD-ESEFPLLLVDQKSRLNKEGRTA  target    DADWISMLFGPFGDPYRRDPRMPWTGEAYLEINPKDAA 6cz7.1    NSPWYYEFKDV----D----PGDVANEDVAKFNPIDGK ``` | | | | | | | | | | | | | | | | | | | | | | | | | | | | | | | | | | | | | | | | | | | | | | | | | |
|  | 5nqd.1.A | AroA  *Arsenite oxidase AioAB from Rhizobium sp. str. NT-26 mutant AioBF108A* | 0.31 |  | 16.37 | 0.70 | 1-812 | X-ray | 2.20 | hetero-2-2-mer | 4 x MGD, 2 x O, 2 x 4MO, 2 x F3S, 2 x FES | HHblits | 0.28 |
| ``` target    SWDEATTIAAKTMEDVARTFNGDEGARKLLAQGYHPEMVEVMHGAGVQALKLRGGMPLLGIGRIFGFYRFANMLALLDRK 5nqd.1    SWDDALDLVARVTAKIVKE--------------KGEDALIVSAFD-------------------HGGAGGGYENTWGTGK  target    LRPDAPADEILGSRTFDNYAWHTDLP--PGHPMVTGSQTVDFDLFSAEHTKLLLIIGMNWICTKMPDG--HWIG------ 5nqd.1    L-----YFEAMKVKNIRIHNRPAYNSEVHGT-RDMGVGELNNCYEDAELADTIVAVGTNALETQTNYFLNHWIPNLRGES  target    ---------DARLKGTRVIVISADYMPTAN------KAD--EVIILRPGTDAAFFLGVARELIEKGLYDRAAVIERTDLP 5nqd.1    LGKKKELMPEEPHEAGRIIIVDPRRTVTVNACEQTAGADNVLHLAINSGTDLALFNALFTYIADKGWVDRDFIDKSTLRE  target    LLVRLDTGERLDARDVIPGYELAALTNYVTLKPDAEIKGNPPPPPFTAGGQVVPTELRDAWGDFVWWDRATGRPRPVSRD 5nqd.1    GTARP---------------------------------------------------------PL--YPA-RG--------  target    EVGARFDGDPALLGEFEVELVDGSTVPVRPAFDLLKQYLD-ESFDLRTASEVCRVPPQAIQSIARQLAANKR-----ETL 5nqd.1    -----------------------VSEANPGHLSSFEDAVEGCRMSIEEAAEITGLDAAQIIKAAEWIGMPKEGGKRRRVM  target    LAAGMGPNHYFQNDLFGRVQFLVAALTDNIGHLGGNVGSYAGNYRGSVFQAMGQWIAEDPFAIEPDLTKPATVKRYYKAE 5nqd.1    FGYEKGLIWGNDNYRTNGALVNLALATGNIGRPGGGVVRLGGHQEGYVR--PSDAHVGRPAA-YVDQLLIGGQG-----G  target    SAHYWNYGERPLRAVAKDDEGDLTKGEVLTGKSHMPTPTKLIWFGNSNSLL------GNAK--WSFDVVKNTLPRQ-DAV 5nqd.1    VHHIWGCD------------------HYKTT--LNAHEFKRVYKKRTDMVKDAMSAAPYGDREAMVNAIVDAINQGGLFA  target    FCNEWHWTSSCEYADLVFPADSWAEFKLPDATASCTNPFLLAFPTTPLKRLYDTRSDYEALALTAKALGELIDEPR---M 5nqd.1    VNVDIIPTKIGEACHVILPAATSGEM---NLTSMNGERRMRLT-ERYMDPPGQSMPDCLIAARLANTMERVLTEMGDVGY  target    EQYWRGILDGDPTPYL-QRIFSGSNATRGITYDELHESSKRGVPLLMNMRTYPRSGGWEQRQEDKPWYTATGRLEFYRPE 5nqd.1    AAQFKGFDWQTEEDAFMDGYNKNAHGGEFVTYERLSAMGTNGFQEPATGFTDGKIEGTQRLYTDGVFSTDDGKARFMDAP  target    PEFQAAGESLPVWREPVDATFYEPNAILSNAAHPSIAPRAPEDYGVPESQLDVETRQYRNVVRTWAELQQTLHPLQERDP 5nqd.1    WR--G-------LQAPGK-Q-------------------------------------------------------Q-QKD  target    AFRFVFQTPKYRWGAHSTAV--DADWISMLFGPFGDPYRRDPRMPWTGEAYLEINPKDAA 5nqd.1    SHKYLINNGRANVVWQSAYLDQENDFVMDR----------------FPYPFIEMNPEDMA ``` | | | | | | | | | | | | | | | | | | | | | | | | | | | | | | | | | | | | | | | | | | | | | | | | | |
|  | 4aay.1.A | AROA  *Crystal Structure of the arsenite oxidase protein complex from Rhizobium species strain NT-26* | 0.31 |  | 16.02 | 0.70 | 1-812 | X-ray | 2.70 | hetero-oligomer | 4 x MGD, 2 x O, 2 x 4MO, 2 x F3S, 2 x FES | HHblits | 0.28 |
| ``` target    SWDEATTIAAKTMEDVARTFNGDEGARKLLAQGYHPEMVEVMHGAGVQALKLRGGMPLLGIGRIFGFYRFANMLALLDRK 4aay.1    SWDDALDLVARVTAKIVKE--------------KGEDALIVSAFDH-------------------GGAGGGYENTWGTGK  target    LRPDAPADEILGSRTFDNYAWHTDLP--PGHPMVTGSQTVDFDLFSAEHTKLLLIIGMNWICTKMPDG--HWIG------ 4aay.1    L-----YFEAMKVKNIRIHNRPAYNSEVHGT-RDMGVGELNNCYEDAELADTIVAVGTNALETQTNYFLNHWIPNLRGES  target    ---------DARLKGTRVIVISADYMPTAN------KAD--EVIILRPGTDAAFFLGVARELIEKGLYDRAAVIERTDLP 4aay.1    LGKKKELMPEEPHEAGRIIIVDPRRTVTVNACEQTAGADNVLHLAINSGTDLALFNALFTYIADKGWVDRDFIDKSTLRE  target    LLVRLDTGERLDARDVIPGYELAALTNYVTLKPDAEIKGNPPPPPFTAGGQVVPTELRDAWGDFVWWDRATGRPRPVSRD 4aay.1    GTARP---------------------------------------------------------PL--YPAR-GV-------  target    EVGARFDGDPALLGEFEVELVDGSTVPVRPAFDLLKQYLD-ESFDLRTASEVCRVPPQAIQSIARQLAANKR-----ETL 4aay.1    ------------------------SEANPGHLSSFEDAVEGCRMSIEEAAEITGLDAAQIIKAAEWIGMPKEGGKRRRVM  target    LAAGMGPNHYFQNDLFGRVQFLVAALTDNIGHLGGNVGSYAGNYRGSVFQAMGQWIAEDPFAIEPDLTKPATVKRYYKAE 4aay.1    FGYEKGLIWGNDNYRTNGALVNLALATGNIGRPGGGVVRLGGHQEGYV-----RPS--DAHVGRPAA-YVDQLLIGGQGG  target    SAHYWNYGERPLRAVAKDDEGDLTKGEVLTGKSHMPTPTKLIWFGNSNSLLG------NAK--WSFDVVKNTLPRQ-DAV 4aay.1    VHHIWGCD------------------HYKTT--LNAHEFKRVYKKRTDMVKDAMSAAPYGDREAMVNAIVDAINQGGLFA  target    FCNEWHWTSSCEYADLVFPADSWAEFKLPDATASCTNPFLLAFPTTPLKRLYDTRSDYEALALTAKALGELIDEPR---M 4aay.1    VNVDIIPTKIGEACHVILPAATSGEM---NLTSMNGERRMRLT-ERYMDPPGQSMPDCLIAARLANTMERVLTEMGDVGY  target    EQYWRGILDGDPTPY-LQRIFSGSNATRGITYDELHESSKRGVPLLMNMRTYPRSGGWEQRQEDKPWYTATGRLEFYRPE 4aay.1    AAQFKGFDWQTEEDAFMDGYNKNAHGGEFVTYERLSAMGTNGFQEPATGFTDGKIEGTQRLYTDGVFSTDDGKARFMDAP  target    PEFQAAGESLPVWREPVDATFYEPNAILSNAAHPSIAPRAPEDYGVPESQLDVETRQYRNVVRTWAELQQTLHPLQERDP 4aay.1    WR--G-------LQAPGKQQ---------------------------------------------------------QKD  target    AFRFVFQTPKYRWGAHSTAV--DADWISMLFGPFGDPYRRDPRMPWTGEAYLEINPKDAA 4aay.1    SHKYLINNGRANVVWQSAYLDQENDFVMDR----------------FPYPFIEMNPEDMA ``` | | | | | | | | | | | | | | | | | | | | | | | | | | | | | | | | | | | | | | | | | | | | | | | | | |
|  | 7qv7.1.L | Hydrogen dependent carbon dioxide reductase subunit FdhF  *Cryo-EM structure of Hydrogen-dependent CO2 reductase.* | 0.36 |  | 18.58 | 0.68 | 1-812 | EM | 0.00 | hetero-2-6-6-2-mer | 52 x SF4, 6 x 402 | HHblits | 0.30 |
| ``` target    SWDEATTIAAKTMEDVARTFNGDEGARKLLAQGYHPEMVEVMHGAGVQALKLRGGMPLLGIGRIFGFYRFANMLALLDRK 7qv7.1    SWDEALNFIANGLKKIKSE--------------YGSDAFAMFCSA-----------------------RATNEDNYAAQK  target    LRPDAPADEILGSRTFDNYAWHTDLP--PGHPMVTGSQTVDFDLFSAE-HTKLLLIIGMNWICTKMPDGHWIGDARLKGT 7qv7.1    F-----ARAVIGINNVDHCARLCHAPTVAGLAMTLGSGAMTNSIPEISTYSDVIFIIGSNTAECHPLIAAHVIKAKERGA  target    RVIVISADYMPTANKADEVIILRPGTDAAFFLGVARELIEKGLYDRAAVIERTDLPLLVRLDTGERLDARDVIPGYELAA 7qv7.1    KLIVADPRMNAMVHKADIWLRVPSGYNIPLINGMIHIIIKEGLVKTDFVKNHAV--------------------G-----  target    LTNYVTLKPDAEIKGNPPPPPFTAGGQVVPTELRDAWGDFVWWDRATGRPRPVSRDEVGARFDGDPALLGEFEVELVDGS 7qv7.1    --------------------------------------------------------------------------------  target    TVPVRPAFDLLKQYLDESFDLRTASEVCRVPPQAIQSIARQLAANKRETLLAAGMGPNHYFQNDLFGRVQFLVAALTDNI 7qv7.1    -------FEEMAKAVE-KYTPEYVEELTGIPKKDLIKAARFYGQAQAAAI-LYSMGVTQFSHGTGNVVSLANLAVITGNL  target    GHLGGNVGSYAGN--YRGSVFQAMGQWIAEDPFAIEPDLTKPATVKRYYKAESAHYWNYGERPLRAVAKDDEGDLTKGEV 7qv7.1    GRPGAGICPLRGQNNVQGAC--DVGALPN--VLPGYLDVTKEQNRERF-----EKVWGVKL-P-------SNIGLRVTEV  target    LTGKSHMPTPTKLIWFGNSNSLLGNAKWSFDVVKNTLPRQDAVFCNEWHWTSSCEYADLVFPADSWAEFKLPDATASCTN 7qv7.1    PDAI--LNKRVRALYIFGENPIMSDPD--SDHLRHALEHLDLLIVQDIFLTETARLAHVVLPAACWAEK---DGTFTNTE  target    PFLLAFPTTPLKRLYDTRSDYEALALTAKALGEL-IDEPRMEQYWRGILDGDPTPYLQRIFSGSN-ATRGITYDELHESS 7qv7.1    RRVQRV-RKAVEAPGEAKPDWWIFSQIAERMGYTGMQYNN------------VQEIWDEVRKIVPEKFGGISYARLEKEK  target    KRGVPLLMNMRTYPRSGGWEQRQEDKPWYTATGRLEFYRPEPEFQAAGESLPVWREPVDATFYEPNAILSNAAHPSIAPR 7qv7.1    --GLAWPCPTEDH---TGTPILYLGGKFATPSGKAQMYPVIFYP--N---TCICDEGAEKQDFNHV--------------  target    APEDYGVPESQLDVETRQYRNVVRTWAELQQTLHPLQERDPAFRFVFQTPKYRWGAHSTA--VDADWISMLFGPFGDPYR 7qv7.1    ----------------------IV--GS----IAEL--PDEEYPFTLTTGRRVYHYHTATMTRKSPVIDQI---------  target    RDPRMPWTGEAYLEINPKDAA 7qv7.1    -------APQELVEINPQDAT ``` | | | | | | | | | | | | | | | | | | | | | | | | | | | | | | | | | | | | | | | | | | | | | | | | | |
|  | 7qv7.1.O | Hydrogen dependent carbon dioxide reductase subunit FdhF  *Cryo-EM structure of Hydrogen-dependent CO2 reductase.* | 0.35 |  | 18.58 | 0.68 | 1-812 | EM | 0.00 | hetero-2-6-6-2-mer | 52 x SF4, 6 x 402 | HHblits | 0.30 |
| ``` target    SWDEATTIAAKTMEDVARTFNGDEGARKLLAQGYHPEMVEVMHGAGVQALKLRGGMPLLGIGRIFGFYRFANMLALLDRK 7qv7.1    SWDEALNFIANGLKKIKSE--------------YGSDAFAMFCSA-----------------------RATNEDNYAAQK  target    LRPDAPADEILGSRTFDNYAWHTDLP--PGHPMVTGSQTVDFDLFSAE-HTKLLLIIGMNWICTKMPDGHWIGDARLKGT 7qv7.1    F-----ARAVIGINNVDHCARLCHAPTVAGLAMTLGSGAMTNSIPEISTYSDVIFIIGSNTAECHPLIAAHVIKAKERGA  target    RVIVISADYMPTANKADEVIILRPGTDAAFFLGVARELIEKGLYDRAAVIERTDLPLLVRLDTGERLDARDVIPGYELAA 7qv7.1    KLIVADPRMNAMVHKADIWLRVPSGYNIPLINGMIHIIIKEGLVKTDFVKNHAV--------------------G-----  target    LTNYVTLKPDAEIKGNPPPPPFTAGGQVVPTELRDAWGDFVWWDRATGRPRPVSRDEVGARFDGDPALLGEFEVELVDGS 7qv7.1    --------------------------------------------------------------------------------  target    TVPVRPAFDLLKQYLDESFDLRTASEVCRVPPQAIQSIARQLAANKRETLLAAGMGPNHYFQNDLFGRVQFLVAALTDNI 7qv7.1    -------FEEMAKAVE-KYTPEYVEELTGIPKKDLIKAARFYGQAQAAAI-LYSMGVTQFSHGTGNVVSLANLAVITGNL  target    GHLGGNVGSYAGN--YRGSVFQAMGQWIAEDPFAIEPDLTKPATVKRYYKAESAHYWNYGERPLRAVAKDDEGDLTKGEV 7qv7.1    GRPGAGICPLRGQNNVQGAC--DVGALPN--VLPGYLDVTKEQNRERF-----EKVWGVKL-P-------SNIGLRVTEV  target    LTGKSHMPTPTKLIWFGNSNSLLGNAKWSFDVVKNTLPRQDAVFCNEWHWTSSCEYADLVFPADSWAEFKLPDATASCTN 7qv7.1    PDAI--LNKRVRALYIFGENPIMSDPD--SDHLRHALEHLDLLIVQDIFLTETARLAHVVLPAACWAEK---DGTFTNTE  target    PFLLAFPTTPLKRLYDTRSDYEALALTAKALGEL-IDEPRMEQYWRGILDGDPTPYLQRIFSGSN-ATRGITYDELHESS 7qv7.1    RRVQRV-RKAVEAPGEAKPDWWIFSQIAERMGYTGMQYNN------------VQEIWDEVRKIVPEKFGGISYARLEKEK  target    KRGVPLLMNMRTYPRSGGWEQRQEDKPWYTATGRLEFYRPEPEFQAAGESLPVWREPVDATFYEPNAILSNAAHPSIAPR 7qv7.1    --GLAWPCPTEDH---TGTPILYLGGKFATPSGKAQMYPVIFYP--N---TCICDEGAEKQDFNHV--------------  target    APEDYGVPESQLDVETRQYRNVVRTWAELQQTLHPLQERDPAFRFVFQTPKYRWGAHSTA--VDADWISMLFGPFGDPYR 7qv7.1    ----------------------IV--GS----IAEL--PDEEYPFTLTTGRRVYHYHTATMTRKSPVIDQI---------  target    RDPRMPWTGEAYLEINPKDAA 7qv7.1    -------APQELVEINPQDAT ``` | | | | | | | | | | | | | | | | | | | | | | | | | | | | | | | | | | | | | | | | | | | | | | | | | |
|  | 2e7z.1.A | Acetylene hydratase Ahy  *Acetylene Hydratase from Pelobacter acetylenicus* | 0.36 |  | 20.48 | 0.67 | 1-812 | X-ray | 1.26 | monomer | 1 x SF4, 2 x MGD, 1 x W | HHblits | 0.29 |
| ``` target    SWDEATTIAAKTMEDVARTFNGDEGARKLLAQGYHPEMVEVMHGAGVQALKLRGGMPLLGIGRIFGFYRFANMLALLDRK 2e7z.1    SWDQALDEIAEKLKKIIAK--------------YGPESLGVSQTEI-----------------------NQQSEYGTLRR  target    LRPDAPADEILGSRTFDNYAWHTDLP--PGHPMVTGSQTVDFDLFSAEHTKLLLIIGMNWICTKMPD-GHWIGDARLKGT 2e7z.1    F------MNLLGSPNWTSAMYMCIGNTAGVHRVTHGS----YSFASFADSNCLLFIGKNLSNHNWVSQFNDLKAALKRGC  target    RVIVISADYMPTANKADEVIILRPGTDAAFFLGVARELIEKGLYDRAAVIERTDLPLLVRLDTGERLDARDVIPGYELAA 2e7z.1    KLIVLDPRRTKVAEMADIWLPLRYGTDAALFLGMINVIINEQLYDKEFVENWCV--------------------G-----  target    LTNYVTLKPDAEIKGNPPPPPFTAGGQVVPTELRDAWGDFVWWDRATGRPRPVSRDEVGARFDGDPALLGEFEVELVDGS 2e7z.1    --------------------------------------------------------------------------------  target    TVPVRPAFDLLKQYLDESFDLRTASEVCRVPPQAIQSIARQLAANKRETLLAAGMGPNHYFQNDLFGRVQFLVAALTDNI 2e7z.1    -------FEELKERVQ-EYPLDKVAEITGCDAGEIRKAAVMFATESPASI-PWAVSTDMQKNSCSAIRAQCILRAIVGSF  target    GHLGGNVGSYAGNYRG-SVFQAMGQWIAE--------DPFAIEPDLTKPATVKRYYKAESAHYWNYGERPLRAVAKDDEG 2e7z.1    VNGAEILGAPHSDLVPISKIQMHEALPEEKKKLQLGTETYPFLTYT----GMSALEE-PSERVYGVKYFH-NMGAFMANP  target    DLTKGEVLTGKSHMPTPTKLIWFGNSNSLLGNAKWSFDVVKNTLPRQDAVFCNEWHWTSSCEYADLVFPADSWAEFKLPD 2e7z.1    TALFTAMATE---KPYPVKAFFALASNALMGYAN--QQNALKGLMNQDLVVCYDQFMTPTAQLADYVLPGDHWLERP---  target    ATASCTN--PFLLAFPTTPLKRLYDTRSDYEALALTAKALGELIDEPRMEQYWRGILDGDPTPYLQRIFSGSNATRGITY 2e7z.1    VVQPNWEGIPFGNT-SQQVVEPAGEAKDEYYFIRELAVRMGLEEHFP-----W-----KDRLELINYRIS----PTGMEW  target    DELHESSKRGVPLLMNMRTYPRSGGWEQRQEDKPWYTATGRLEFYRPEPEFQAAGESLPVWREPVDATFYEPNAILSNAA 2e7z.1    EEYQKQYT--YMS-----KLPD---Y-FGPEGVGVATPSGKVELYSSVFEK-LGYDPLPYYHEPLQTEISDP--------  target    HPSIAPRAPEDYGVPESQLDVETRQYRNVVRTWAELQQTLHPLQERDPAFRFVFQTPKYR-WGAHSTAVDADWISMLFGP 2e7z.1    -----------------------------------------E---LAKEYPLILFAGLREDSNFQSCYHQPGILRDA---  target    FGDPYRRDPRMPWTGEAYLEINPKDAA 2e7z.1    -------------EPDPVALLHPKTAQ ``` | | | | | | | | | | | | | | | | | | | | | | | | | | | | | | | | | | | | | | | | | | | | | | | | | |
|  | 1aa6.1.A | FORMATE DEHYDROGENASE H  *REDUCED FORM OF FORMATE DEHYDROGENASE H FROM E. COLI* | 0.39 | 0.00 | 19.92 | 0.66 | 1-812 | X-ray | 2.30 | monomer | 1 x SF4, 2 x MGD, 1 x 4MO | HHblits | 0.31 |
| ``` target    SWDEATTIAAKTMEDVARTFNGDEGARKLLAQGYHPEMVEVMHGAGVQALKLRGGMPLLGIGRIFGFYRFANMLALLDRK 1aa6.1    SWDEALNYVAERLSAIKEK--------------YGPDAIQTTGSSRGT----------------------GNETNYVMQK  target    LRPDAPADEILGSRTFDNYAWHTDLP--PGHPMVTGSQTVDFDLFSAEHTKLLLIIGMNWICTKMPDGHWIGDARLKGTR 1aa6.1    F-----ARAVIGTNNVDCCARVUHGPSVAGLHQSVGNGAMSNAINEIDNTDLVFVFGYNPADSHPIVANHVINAKRNGAK  target    VIVISADYMPTANKADEVIILRPGTDAAFFLGVARELIEKGLYDRAAVIERTDLPLLVRLDTGERLDARDVIPGYELAAL 1aa6.1    IIVCDPRKIETARIADMHIALKNGSNIALLNAMGHVIIEENLYDKAFVASRTE--------------------G------  target    TNYVTLKPDAEIKGNPPPPPFTAGGQVVPTELRDAWGDFVWWDRATGRPRPVSRDEVGARFDGDPALLGEFEVELVDGST 1aa6.1    --------------------------------------------------------------------------------  target    VPVRPAFDLLKQYLDESFDLRTASEVCRVPPQAIQSIARQLAANKRETLLAAGMGPNHYFQNDLFGRVQFLVAALTDNIG 1aa6.1    ------FEEYRKIVE-GYTPESVEDITGVSASEIRQAARMYAQAKSAAI-LWGMGVTQFYQGVETVRSLTSLAMLTGNLG  target    HLGGNVGSYAGN--YRGSVFQAMGQWIAEDPFAIEPDLTKPATVKRYYKAESAHYWNYGERPLRAVAKDDEGDLTKGEVL 1aa6.1    KPHAGVNPVRGQNNVQGAC--DMGALPD--TYPGYQYVKDPANREKFA-----KAWGVESLP-------AHTGYRISELP  target    TGKSHMPTPTKLIWFGNSNSLLGNAKWSFDVVKNTLPRQDAVFCNEWHWTSSCEYADLVFPADSWAEFKLPDATASCTNP 1aa6.1    HR--AAHGEVRAAYIMGEDPLQTDAE--LSAVRKAFEDLELVIVQDIFMTKTASAADVILPSTSWGEHE--G-VFTAADR  target    FLLAFPTTPLKRLYDTRSDYEALALTAKALGELIDEPRMEQYWRGILDGDPTPYLQRIFSGSNATRGITYDELHESSKRG 1aa6.1    GFQRF-FKAVEPKWDLKTDWQIISEIATRMGYPMHYNNTQ------------EIWDELRHLCPDFYGATYEKMGELG---  target    VPLLMNMRTYPRSGGWEQRQEDKPWYTATGRLEFYRPEPEFQAAGESLPVWREPVDATFYEPNAILSNAAHPSIAPRAPE 1aa6.1    FIQWPCRDTSDAD-QGTSYLFKEKFDTPNGLAQFFTCD------------WVAPIDKL----------------------  target    DYGVPESQLDVETRQYRNVVRTWAELQQTLHPLQERDPAFRFVFQTPKYR--WGAHSTAVDADWISMLFGPFGDPYRRDP 1aa6.1    ------------------------------------TDEYPMVLSTVREVGHYSCRSMTGNCAALAALA-----------  target    RMPWTGEAYLEINPKDAA 1aa6.1    ----DEPGYAQINTEDAK ``` | | | | | | | | | | | | | | | | | | | | | | | | | | | | | | | | | | | | | | | | | | | | | | | | | |
|  | 1fdo.1.A | FORMATE DEHYDROGENASE H  *OXIDIZED FORM OF FORMATE DEHYDROGENASE H FROM E. COLI* | 0.39 | 0.00 | 19.92 | 0.66 | 1-812 | X-ray | 2.80 | monomer | 1 x SF4, 2 x MGD, 1 x 6MO | HHblits | 0.31 |
| ``` target    SWDEATTIAAKTMEDVARTFNGDEGARKLLAQGYHPEMVEVMHGAGVQALKLRGGMPLLGIGRIFGFYRFANMLALLDRK 1fdo.1    SWDEALNYVAERLSAIKEK--------------YGPDAIQTTGSSRGT----------------------GNETNYVMQK  target    LRPDAPADEILGSRTFDNYAWHTDLP--PGHPMVTGSQTVDFDLFSAEHTKLLLIIGMNWICTKMPDGHWIGDARLKGTR 1fdo.1    F-----ARAVIGTNNVDCCARVUHGPSVAGLHQSVGNGAMSNAINEIDNTDLVFVFGYNPADSHPIVANHVINAKRNGAK  target    VIVISADYMPTANKADEVIILRPGTDAAFFLGVARELIEKGLYDRAAVIERTDLPLLVRLDTGERLDARDVIPGYELAAL 1fdo.1    IIVCDPRKIETARIADMHIALKNGSNIALLNAMGHVIIEENLYDKAFVASRTE--------------------G------  target    TNYVTLKPDAEIKGNPPPPPFTAGGQVVPTELRDAWGDFVWWDRATGRPRPVSRDEVGARFDGDPALLGEFEVELVDGST 1fdo.1    --------------------------------------------------------------------------------  target    VPVRPAFDLLKQYLDESFDLRTASEVCRVPPQAIQSIARQLAANKRETLLAAGMGPNHYFQNDLFGRVQFLVAALTDNIG 1fdo.1    ------FEEYRKIVE-GYTPESVEDITGVSASEIRQAARMYAQAKSAAI-LWGMGVTQFYQGVETVRSLTSLAMLTGNLG  target    HLGGNVGSYAGN--YRGSVFQAMGQWIAEDPFAIEPDLTKPATVKRYYKAESAHYWNYGERPLRAVAKDDEGDLTKGEVL 1fdo.1    KPHAGVNPVRGQNNVQGAC--DMGALPD--TYPGYQYVKDPANREKFA-----KAWGVESLP-------AHTGYRISELP  target    TGKSHMPTPTKLIWFGNSNSLLGNAKWSFDVVKNTLPRQDAVFCNEWHWTSSCEYADLVFPADSWAEFKLPDATASCTNP 1fdo.1    HR--AAHGEVRAAYIMGEDPLQTDAE--LSAVRKAFEDLELVIVQDIFMTKTASAADVILPSTSWGEHE--G-VFTAADR  target    FLLAFPTTPLKRLYDTRSDYEALALTAKALGELIDEPRMEQYWRGILDGDPTPYLQRIFSGSNATRGITYDELHESSKRG 1fdo.1    GFQRF-FKAVEPKWDLKTDWQIISEIATRMGYPMHYNNTQ------------EIWDELRHLCPDFYGATYEKMGELG---  target    VPLLMNMRTYPRSGGWEQRQEDKPWYTATGRLEFYRPEPEFQAAGESLPVWREPVDATFYEPNAILSNAAHPSIAPRAPE 1fdo.1    FIQWPCRDTSDAD-QGTSYLFKEKFDTPNGLAQFFTCD------------WVAPIDKL----------------------  target    DYGVPESQLDVETRQYRNVVRTWAELQQTLHPLQERDPAFRFVFQTPKYR--WGAHSTAVDADWISMLFGPFGDPYRRDP 1fdo.1    ------------------------------------TDEYPMVLSTVREVGHYSCRSMTGNCAALAALA-----------  target    RMPWTGEAYLEINPKDAA 1fdo.1    ----DEPGYAQINTEDAK ``` | | | | | | | | | | | | | | | | | | | | | | | | | | | | | | | | | | | | | | | | | | | | | | | | | |
|  | 2iv2.1.A | Formate dehydrogenase H  *Reinterpretation of reduced form of formate dehydrogenase H from E. coli* | 0.39 | 0.00 | 19.92 | 0.66 | 1-812 | X-ray | 2.27 | monomer | 1 x SF4, 1 x 2MD, 1 x MGD | HHblits | 0.31 |
| ``` target    SWDEATTIAAKTMEDVARTFNGDEGARKLLAQGYHPEMVEVMHGAGVQALKLRGGMPLLGIGRIFGFYRFANMLALLDRK 2iv2.1    SWDEALNYVAERLSAIKEK--------------YGPDAIQTTGSSRGT----------------------GNETNYVMQK  target    LRPDAPADEILGSRTFDNYAWHTDLP--PGHPMVTGSQTVDFDLFSAEHTKLLLIIGMNWICTKMPDGHWIGDARLKGTR 2iv2.1    F-----ARAVIGTNNVDCCARVUHGPSVAGLHQSVGNGAMSNAINEIDNTDLVFVFGYNPADSHPIVANHVINAKRNGAK  target    VIVISADYMPTANKADEVIILRPGTDAAFFLGVARELIEKGLYDRAAVIERTDLPLLVRLDTGERLDARDVIPGYELAAL 2iv2.1    IIVCDPRKIETARIADMHIALKNGSNIALLNAMGHVIIEENLYDKAFVASRTE--------------------G------  target    TNYVTLKPDAEIKGNPPPPPFTAGGQVVPTELRDAWGDFVWWDRATGRPRPVSRDEVGARFDGDPALLGEFEVELVDGST 2iv2.1    --------------------------------------------------------------------------------  target    VPVRPAFDLLKQYLDESFDLRTASEVCRVPPQAIQSIARQLAANKRETLLAAGMGPNHYFQNDLFGRVQFLVAALTDNIG 2iv2.1    ------FEEYRKIVE-GYTPESVEDITGVSASEIRQAARMYAQAKSAAI-LWGMGVTQFYQGVETVRSLTSLAMLTGNLG  target    HLGGNVGSYAGN--YRGSVFQAMGQWIAEDPFAIEPDLTKPATVKRYYKAESAHYWNYGERPLRAVAKDDEGDLTKGEVL 2iv2.1    KPHAGVNPVRGQNNVQGAC--DMGALPD--TYPGYQYVKDPANREKFA-----KAWGVESLP-------AHTGYRISELP  target    TGKSHMPTPTKLIWFGNSNSLLGNAKWSFDVVKNTLPRQDAVFCNEWHWTSSCEYADLVFPADSWAEFKLPDATASCTNP 2iv2.1    HR--AAHGEVRAAYIMGEDPLQTDAE--LSAVRKAFEDLELVIVQDIFMTKTASAADVILPSTSWGEHE--G-VFTAADR  target    FLLAFPTTPLKRLYDTRSDYEALALTAKALGELIDEPRMEQYWRGILDGDPTPYLQRIFSGSNATRGITYDELHESSKRG 2iv2.1    GFQRF-FKAVEPKWDLKTDWQIISEIATRMGYPMHYNNTQ------------EIWDELRHLCPDFYGATYEKMGELG---  target    VPLLMNMRTYPRSGGWEQRQEDKPWYTATGRLEFYRPEPEFQAAGESLPVWREPVDATFYEPNAILSNAAHPSIAPRAPE 2iv2.1    FIQWPCRDTSDAD-QGTSYLFKEKFDTPNGLAQFFTCD------------WVAPIDKL----------------------  target    DYGVPESQLDVETRQYRNVVRTWAELQQTLHPLQERDPAFRFVFQTPKYR--WGAHSTAVDADWISMLFGPFGDPYRRDP 2iv2.1    ------------------------------------TDEYPMVLSTVREVGHYSCRSMTGNCAALAALA-----------  target    RMPWTGEAYLEINPKDAA 2iv2.1    ----DEPGYAQINTEDAK ``` | | | | | | | | | | | | | | | | | | | | | | | | | | | | | | | | | | | | | | | | | | | | | | | | | |
|  | 7z0t.1.G | Formate dehydrogenase H  *Structure of the Escherichia coli formate hydrogenlyase complex (aerobic preparation, composite structure)* | 0.39 | 0.00 | 19.92 | 0.66 | 1-812 | EM | 0.00 | monomer | 1 x NI, 1 x FCO, 8 x SF4, 1 x FE, 2 x MGD, 1 x 6MO | HHblits | 0.31 |
| ``` target    SWDEATTIAAKTMEDVARTFNGDEGARKLLAQGYHPEMVEVMHGAGVQALKLRGGMPLLGIGRIFGFYRFANMLALLDRK 7z0t.1    SWDEALNYVAERLSAIKEK--------------YGPDAIQTTGSSRGT----------------------GNETNYVMQK  target    LRPDAPADEILGSRTFDNYAWHTDLP--PGHPMVTGSQTVDFDLFSAEHTKLLLIIGMNWICTKMPDGHWIGDARLKGTR 7z0t.1    F-----ARAVIGTNNVDCCARVUHGPSVAGLHQSVGNGAMSNAINEIDNTDLVFVFGYNPADSHPIVANHVINAKRNGAK  target    VIVISADYMPTANKADEVIILRPGTDAAFFLGVARELIEKGLYDRAAVIERTDLPLLVRLDTGERLDARDVIPGYELAAL 7z0t.1    IIVCDPRKIETARIADMHIALKNGSNIALLNAMGHVIIEENLYDKAFVASRTE--------------------G------  target    TNYVTLKPDAEIKGNPPPPPFTAGGQVVPTELRDAWGDFVWWDRATGRPRPVSRDEVGARFDGDPALLGEFEVELVDGST 7z0t.1    --------------------------------------------------------------------------------  target    VPVRPAFDLLKQYLDESFDLRTASEVCRVPPQAIQSIARQLAANKRETLLAAGMGPNHYFQNDLFGRVQFLVAALTDNIG 7z0t.1    ------FEEYRKIVE-GYTPESVEDITGVSASEIRQAARMYAQAKSAAI-LWGMGVTQFYQGVETVRSLTSLAMLTGNLG  target    HLGGNVGSYAGN--YRGSVFQAMGQWIAEDPFAIEPDLTKPATVKRYYKAESAHYWNYGERPLRAVAKDDEGDLTKGEVL 7z0t.1    KPHAGVNPVRGQNNVQGAC--DMGALPD--TYPGYQYVKDPANREKFA-----KAWGVESLP-------AHTGYRISELP  target    TGKSHMPTPTKLIWFGNSNSLLGNAKWSFDVVKNTLPRQDAVFCNEWHWTSSCEYADLVFPADSWAEFKLPDATASCTNP 7z0t.1    HR--AAHGEVRAAYIMGEDPLQTDAE--LSAVRKAFEDLELVIVQDIFMTKTASAADVILPSTSWGEHE--G-VFTAADR  target    FLLAFPTTPLKRLYDTRSDYEALALTAKALGELIDEPRMEQYWRGILDGDPTPYLQRIFSGSNATRGITYDELHESSKRG 7z0t.1    GFQRF-FKAVEPKWDLKTDWQIISEIATRMGYPMHYNNTQ------------EIWDELRHLCPDFYGATYEKMGELG---  target    VPLLMNMRTYPRSGGWEQRQEDKPWYTATGRLEFYRPEPEFQAAGESLPVWREPVDATFYEPNAILSNAAHPSIAPRAPE 7z0t.1    FIQWPCRDTSDAD-QGTSYLFKEKFDTPNGLAQFFTCD------------WVAPIDKL----------------------  target    DYGVPESQLDVETRQYRNVVRTWAELQQTLHPLQERDPAFRFVFQTPKYR--WGAHSTAVDADWISMLFGPFGDPYRRDP 7z0t.1    ------------------------------------TDEYPMVLSTVREVGHYSCRSMTGNCAALAALA-----------  target    RMPWTGEAYLEINPKDAA 7z0t.1    ----DEPGYAQINTEDAK ``` | | | | | | | | | | | | | | | | | | | | | | | | | | | | | | | | | | | | | | | | | | | | | | | | | |
|  | 1g8k.1.A | ARSENITE OXIDASE  *CRYSTAL STRUCTURE ANALYSIS OF ARSENITE OXIDASE FROM ALCALIGENES FAECALIS* | 0.31 |  | 17.42 | 0.68 | 1-812 | X-ray | 1.64 | hetero-1-1-mer | 3 x HG, 2 x CA, 2 x MGD, 1 x O, 1 x 4MO, 1 x F3S, 1 x FES | HHblits | 0.28 |
| ``` target    SWDEATTIAAKTMEDVARTFNGDEGARKLLAQGYHPEMVEVMHGAGVQALKLRGGMPLLGIGRIFGFYRFANMLALLDRK 1g8k.1    TWDHAMALYAGLIKKTLDK--------------DGPQGVFFSCFDH-------------------GGAGGGFENTWGTGK  target    LRPDAPADEILGSRTFDNYAWHTD-LPPGHPMVTGSQTVDFDLFSAEHTKLLLIIGMNWICTKMPD--GHWI-------- 1g8k.1    L-----MFSAIQTPMVRIHNRPAYNSECHATREMGIGELNNAYEDAQLADVIWSIGNNPYESQTNYFLNHWLPNLQGATT  target    -------GDARLKGTRVIVISADYMPTANKA--------DEVIILRPGTDAAFFLGVARELIEKGLYDRAAVIERTDLPL 1g8k.1    SKKKERFPNENFPQARIIFVDPRETPSVAIARHVAGNDRVLHLAIEPGTDTALFNGLFTYVVEQGWIDKPFIEAHTK---  target    LVRLDTGERLDARDVIPGYELAALTNYVTLKPDAEIKGNPPPPPFTAGGQVVPTELRDAWGDFVWWDRATGRPRPVSRDE 1g8k.1    -----------------GF-------------------------------------------------------------  target    VGARFDGDPALLGEFEVELVDGSTVPVRPAFDLLKQYLDESFDLRTASEVCRVPPQAIQSIARQLAANKR-----ETLLA 1g8k.1    -------------------------------DDAVK-T-NRLSLDECSNITGVPVDMLKRAAEWSYKPKASGQAPRTMHA  target    AGMGPNHYFQNDLFGRVQFLVAALTDNIGHLGGNVGSYAGNYRGSVFQAMGQWIAEDPFAIEPDLTKPATVKRYYKAESA 1g8k.1    YEKGIIWGNDNYVIQSALLDLVIATHNVGRRGTGCVRMGGHQEGYTR---P------PYPGDKKIYIDQELIK-GKGRIM  target    HYWNYGERPLRAVAKDDEGDLTKGEVLTGKSHMPTPTKLIWFGNSNSLLGNAKWSFDVVKNTLPR-QDAVFCNEWHWTSS 1g8k.1    TWWGC--NNFQTS---NNA-QALREAILQR--SAIVKQAMQKARGATTE--E--MVDVIYEATQNGGLFVTSINLYPTKL  target    CEYADLVFPADSWAEFKLPDATASCTNPFLLAFPTTPLKRLYDTRSDYEALALTAKALGELIDEP---RMEQYWRGILDG 1g8k.1    AEAAHLMLPAAHPGEM---NLTSMNGERRIRLS-EKFMDPPGTAMADCLIAARIANALRDMYQKDGKAEMAAQFEGFDWK  target    DPTPYLQRIFSGSN--------AT-----RGITYDELHESSKRGVPLLMNMRTY-PRSGGWEQRQEDKPWYTATGRLEFY 1g8k.1    TEEDAFNDGFRRAGQPGAPAIDSQGGSTGHLVTYDRLRKSGNNGVQLPVVSWDESKGLVGTEMLYTEGKFDTDDGKAHFK  target    RPEPEFQAAGESLPVWREPVDATFYEPNAILSNAAHPSIAPRAPEDYGVPESQLDVETRQYRNVVRTWAELQQTLHPLQE 1g8k.1    PAPW------NGLPATVQQ-------------------------------------------------------------  target    RDPAFRFVFQTPKYRWGAHSTAVD--ADWISMLFGPFGDPYRRDPRMPWTGEAYLEINPKDAA 1g8k.1    QKDKYRFWLNNGRNNEVWQTAYHDQYNSLMQER----------------YPMAYIEMNPDDCK ``` | | | | | | | | | | | | | | | | | | | | | | | | | | | | | | | | | | | | | | | | | | | | | | | | | |
|  | 2vpz.1.A | THIOSULFATE REDUCTASE  *POLYSULFIDE REDUCTASE NATIVE STRUCTURE* | 0.36 |  | 19.74 | 0.66 | 1-812 | X-ray | 2.40 | hetero-oligomer | 10 x SF4, 4 x MGD, 2 x MO | HHblits | 0.29 |
| ``` target    SWDEATTIAAKTMEDVARTFNGDEGARKLLAQGYHPEMVEVMHGAGVQALKLRGGMPLLGIGRIFGFYRFANMLALLDRK 2vpz.1    TWEEALDHIAKKMLEIREK--------------YGPEAIAFFGHG-TGD------------------YWF--------VD  target    LRPDAPADEILGSRTFDNY-AWHTD--LPPGHPMVTGSQTVDFDLFSAEHTKLLLIIGMNWICT-KMPDGHWIGDARLKG 2vpz.1    F-----LPAAWGSPNAAKPSVSLCTAPREVASQWVFGRPIGGHEPIDWENARYIVLIGHHIGEDTHNTQLQDFALALKNG  target    TRVIVISADYMPTANKADEVIILRPGTDAAFFLGVARELIEKGLYDRAAVIERTDLPLLVRLDTGERLDARDVIPGYELA 2vpz.1    AKVVVVDPRFSTAAAKAHRWLPIKPGTDTALLLAWIHVLIYEDLYDKEYVAKYTV--------------------G----  target    ALTNYVTLKPDAEIKGNPPPPPFTAGGQVVPTELRDAWGDFVWWDRATGRPRPVSRDEVGARFDGDPALLGEFEVELVDG 2vpz.1    --------------------------------------------------------------------------------  target    STVPVRPAFDLLKQYLDESFDLRTASEVCRVPPQAIQSIARQLAANKRETLLAAGMGP-NHYFQNDLFGRVQFLVAALTD 2vpz.1    --------FEELKAHVK-DFTPEWAEKHTEIPAQVIREVAREMAAHKPRAVL-PPTRHNVWYGDDTYRVMALLYVNVLLG  target    NIGHLGGNVGSYAGNYRGSVFQAMGQWIAEDPFAIEPDLTKPATVKRYYKAESAHYWNYGERPLRAVAKDDEGDLTKGEV 2vpz.1    NYGRPGGFYIAQSPYLEKYP---LPPLPL-EPAA--GGCSGPSG-GDHEP----EGFKPRADKGKFFARSTAIQELIEPM  target    LTGKSHMPTPTKLIWFGNSNSLLGNAKWSFDVVKNTLPRQDAVFCNEWHWTSSCEYADLVFPADSWAEFKLPDATASCTN 2vpz.1    ITGE---PYPIKGLFAYGINLFHSIPN--VPRTKEALKNLDLYVAIDVLPQEHVMWADVILPEATYLERYDDFVLVAHKT  target    PFLLAFPTTPLKRLYDTRSDYEALALTAKALGELIDEPRMEQYWRGILDGDPTPYLQRIFSGSNATRGITYDELHESSKR 2vpz.1    PFIQL-RTPAHEPLFDTKPGWWIARELGLRLGLEQY-------FP---WKTIEEYLETRLQS----LGLDLETMKGMGT-  target    GVPLLMNMRTYPRSGGWEQRQEDKPWYTATGRLEFYRPEPEFQAAGESLPVWREPVDATFYEPNAILSNAAHPSIAPRAP 2vpz.1    --LVQR---GKPWLEDWE-KEGRLPFGTASGKIELYCQRFKE-AGHQPLPVFTPPEEP----------------------  target    EDYGVPESQLDVETRQYRNVVRTWAELQQTLHPLQERDPAFRFVFQTPKYRWGAHSTAVDADWISMLFGPFGDPYRRDPR 2vpz.1    --------------------------------------PEGFYRLLYGRSPVHTFARTQNNWVLMEM-------------  target    MPWTGEAYLEINPKDAA 2vpz.1    ---DPENEVWIHKEEAK ``` | | | | | | | | | | | | | | | | | | | | | | | | | | | | | | | | | | | | | | | | | | | | | | | | | |
|  | 2vpx.1.D | THIOSULFATE REDUCTASE  *POLYSULFIDE REDUCTASE WITH BOUND QUINONE (UQ1)* | 0.36 |  | 19.74 | 0.66 | 1-812 | X-ray | 3.10 | hetero-oligomer | 10 x SF4, 4 x MGD, 2 x MO, 2 x UQ1 | HHblits | 0.29 |
| ``` target    SWDEATTIAAKTMEDVARTFNGDEGARKLLAQGYHPEMVEVMHGAGVQALKLRGGMPLLGIGRIFGFYRFANMLALLDRK 2vpx.1    TWEEALDHIAKKMLEIREK--------------YGPEAIAFFGHG-TGD------------------YWF--------VD  target    LRPDAPADEILGSRTFDNY-AWHTD--LPPGHPMVTGSQTVDFDLFSAEHTKLLLIIGMNWICT-KMPDGHWIGDARLKG 2vpx.1    F-----LPAAWGSPNAAKPSVSLCTAPREVASQWVFGRPIGGHEPIDWENARYIVLIGHHIGEDTHNTQLQDFALALKNG  target    TRVIVISADYMPTANKADEVIILRPGTDAAFFLGVARELIEKGLYDRAAVIERTDLPLLVRLDTGERLDARDVIPGYELA 2vpx.1    AKVVVVDPRFSTAAAKAHRWLPIKPGTDTALLLAWIHVLIYEDLYDKEYVAKYTV--------------------G----  target    ALTNYVTLKPDAEIKGNPPPPPFTAGGQVVPTELRDAWGDFVWWDRATGRPRPVSRDEVGARFDGDPALLGEFEVELVDG 2vpx.1    --------------------------------------------------------------------------------  target    STVPVRPAFDLLKQYLDESFDLRTASEVCRVPPQAIQSIARQLAANKRETLLAAGMGP-NHYFQNDLFGRVQFLVAALTD 2vpx.1    --------FEELKAHVK-DFTPEWAEKHTEIPAQVIREVAREMAAHKPRAVL-PPTRHNVWYGDDTYRVMALLYVNVLLG  target    NIGHLGGNVGSYAGNYRGSVFQAMGQWIAEDPFAIEPDLTKPATVKRYYKAESAHYWNYGERPLRAVAKDDEGDLTKGEV 2vpx.1    NYGRPGGFYIAQSPYLEKYP---LPPLPL-EPAA--GGCSGPSG-GDHEP----EGFKPRADKGKFFARSTAIQELIEPM  target    LTGKSHMPTPTKLIWFGNSNSLLGNAKWSFDVVKNTLPRQDAVFCNEWHWTSSCEYADLVFPADSWAEFKLPDATASCTN 2vpx.1    ITGE---PYPIKGLFAYGINLFHSIPN--VPRTKEALKNLDLYVAIDVLPQEHVMWADVILPEATYLERYDDFVLVAHKT  target    PFLLAFPTTPLKRLYDTRSDYEALALTAKALGELIDEPRMEQYWRGILDGDPTPYLQRIFSGSNATRGITYDELHESSKR 2vpx.1    PFIQL-RTPAHEPLFDTKPGWWIARELGLRLGLEQY-------FP---WKTIEEYLETRLQS----LGLDLETMKGMGT-  target    GVPLLMNMRTYPRSGGWEQRQEDKPWYTATGRLEFYRPEPEFQAAGESLPVWREPVDATFYEPNAILSNAAHPSIAPRAP 2vpx.1    --LVQR---GKPWLEDWE-KEGRLPFGTASGKIELYCQRFKE-AGHQPLPVFTPPEEP----------------------  target    EDYGVPESQLDVETRQYRNVVRTWAELQQTLHPLQERDPAFRFVFQTPKYRWGAHSTAVDADWISMLFGPFGDPYRRDPR 2vpx.1    --------------------------------------PEGFYRLLYGRSPVHTFARTQNNWVLMEM-------------  target    MPWTGEAYLEINPKDAA 2vpx.1    ---DPENEVWIHKEEAK ``` | | | | | | | | | | | | | | | | | | | | | | | | | | | | | | | | | | | | | | | | | | | | | | | | | |
|  | 7bkb.1.F | Formate dehydrogenase  *Formate dehydrogenase - heterodisulfide reductase - formylmethanofuran dehydrogenase complex from Methanospirillum hungatei (hexameric, composite structure)* | 0.35 | 0.00 | 21.71 | 0.65 | 1-812 | EM | 0.00 | monomer | 48 x SF4, 4 x FAD, 2 x FES, 4 x 9S8, 4 x ZN, 2 x MO, 4 x MGD | HHblits | 0.31 |
| ``` target    SWDEATTIAAKTMEDVARTFNGDEGARKLLAQGYHPEMVEVMHGAGVQALKLRGGMPLLGIGRIFGFYRFANMLALLDRK 7bkb.1    SWDEALDLVAKNLKVIYDK--------------HGPKGLGFQTSC-----------------------RTVNEDCYIFQK  target    LRPDAPADEILGSRTFDNYAWHTDLP--PGHPMVTGSQTVDFDLFSAEHTKLLLIIGMNWICTKMPDGHWIGDARLKGTR 7bkb.1    FA-----RVGFKTNNVDNCARICHGPSVAGLSLSFGSGAATNGFEDALNADLILIWGSNAVEAHPLAGRRIAQAKKKGIQ  target    VIVISADYMPTANKADEVIILRPGTDAAFFLGVARELIEKGLYDRAAVIERTDLPLLVRLDTGERLDARDVIPGYELAAL 7bkb.1    IIAVDPRYTMTARLADTYVRFNPSTHIALANSMMYWIIKEGLEDKKFIQDRVN--------------------G------  target    TNYVTLKPDAEIKGNPPPPPFTAGGQVVPTELRDAWGDFVWWDRATGRPRPVSRDEVGARFDGDPALLGEFEVELVDGST 7bkb.1    --------------------------------------------------------------------------------  target    VPVRPAFDLLKQYLDESFDLRTASEVCRVPPQAIQSIARQLAANKRETLLAAGMGPNHYFQNDLFGRVQFLVAALTDNIG 7bkb.1    ------FEDLKKTVE-NY--ADAEAIHGVPLDVVKDIAFRYAKAKNAVI-IYCLGITELTTGTDNVRSMGNLALLTGNVG  target    HLGGNVGSYAGN--YRGSVFQAMGQWIAEDPFAIEPDLTKPATVKRYYKAESAHYWNYGERPLRAVAKDDEGDLTKGEVL 7bkb.1    REGVGVNPLRGQNNVQGAC--DMGAYPN--VYSGYQKCEVAENRAK-----MEKAWSVTNLP-------DWYGATLTEQI  target    TGKSHMPTPTKLIWFGNSNSLLGNAKWSFDVVKNTLPRQDAVFCNEWHWTSSCEYADLVFPADSWAEFKLPDATASCTNP 7bkb.1    NQ---CGDEIKGMYILGLNPVVTYPS--SNHVKAQLEKLDFLVVQDIFFTETCQYADVILPGACFAEK---DGTFTSGER  target    FLLAFPTTPLKRLYDTRSDYEALALTAKALGEL-IDEPRMEQYWRGILDGDPTPYLQRIFSGSNATRGITYDELHESSKR 7bkb.1    RINRV-RKAVNPPGQAKEDIHIISELAAKMGFKGFELP------------TAKDVWDDMRAVTPSMFGATYEKLERPE--  target    GVPLLMNMRTYPRSGGWEQRQEDKPWYTATGRLEFYRPEPEFQAAGESLPVWREPVDATFYEPNAILSNAAHPSIAPRAP 7bkb.1    GICWPCPTEEHPGTP----ILHREKFATADGKGNLFGID------------YRPPAEVA---------------------  target    EDYGVPESQLDVETRQYRNVVRTWAELQQTLHPLQERDPAFRFVFQTPKYRWGAHSTAVDA--DWISMLFGPFGDPYRRD 7bkb.1    -------------------------------------DAEYPFTLMTGRLIFHYHSRTQTDRAADLHRE-----------  target    PRMPWTGEAYLEINPKDAA 7bkb.1    -----VPESYAQINIEDAR ``` | | | | | | | | | | | | | | | | | | | | | | | | | | | | | | | | | | | | | | | | | | | | | | | | | |
|  | 3ir7.1.A | Respiratory nitrate reductase 1 alpha chain  *Crystal structure of NarGHI mutant NarG-R94S* | 0.36 | 0.00 | 23.52 | 0.62 | 1-588 | X-ray | 2.50 | monomer | 2 x MD1, 4 x SF4, 1 x 6MO, 1 x AGA, 1 x F3S, 2 x HEM | HHblits | 0.32 |
| ``` target    SWDEATTIAAKTMEDVARTFNGDEGARKLLAQGYHPEMVEVMHGAGVQALKLRGGMPLLGIGRIFGFYRFANMLALLDRK 3ir7.1    SWQEVNELIAASNVYTIKN--------------YGPDRVAGFSPIPAMSMV-----------SYASGAR-----------  target    LRPDAPADEILGSRTFDNYAWHTDLPPGHPMVTGSQTVDFDLFSAEHTKLLLIIGMNWICTKMPDGHWIGDARLKGTRVI 3ir7.1    ------YLSLIGGTCLSFYDWYCDLPPASPQTWGEQTDVPESADWYNSSYIIAWGSNVPQTRTPDAHFFTEVRYKGTKTV  target    VISADYMPTANKADEVIILRPGTDAAFFLGVARELIEKGL------YDRAAVIERTDLPLLVRLDT-------GERLDAR 3ir7.1    AVTPDYAEIAKLCDLWLAPKQGTDAAMALAMGHVMLREFHLDNPSQYFTDYVRRYTDMPMLVMLEERDGYYAAGRMLRAA  target    DVIPGYELAALTNYVTLKPDAEIKGNPPPPPFTAGGQVVPTELRDAWGDFVWWDRATGRPRPVSRDEVG-----ARF--- 3ir7.1    DLVDALGQE----------------------------------NNPEWKTVAFNT-NGEMVAPNGSIGFRWGEKGKWNLE  target    ------------------D-------GDPA-----------------LLG---EFEVELVDGSTVPVRPAFDLLK----- 3ir7.1    QRDGKTGEETELQLSLLGSQDEIAEVGFPYFGGDGTEHFNKVELENVLLHKLPVKRLQLADGSTALVTTVYDLTLANYGL  target    -------------QYLDESFDLRTASEVCRVPPQAIQSIARQLAAN-----KRETLLAAGMGPNHYFQNDLFGRVQFLVA 3ir7.1    ERGLNDVNCATSYDDVK-AYTPAWAEQITGVSRSQIIRIAREFADNADKTHGRS-MIIVGAGLNHWYHLDMNYRGLINML  target    ALTDNIGHLGGNVGSYAGNYRGSVFQAMGQWIAEDPFAIEPD------------------------LTKPA-TVKRYYK- 3ir7.1    IFCGCVGQSGGGWAHYVGQEKLRPQTGWQPLAFALDWQRPARHMNSTSYFYNHSSQWRYETVTAEELLSPMADKSRYTGH  target    -------AESAHYWN----YGERPLRAVAKDDEGD-----LTKGEVLTGKS--------HMPTPTKLIWFGNSNSLLGNA 3ir7.1    LIDFNVRAERMGWLPSAPQLGTNPLTIAGEAEKAGMNPVDYTVKSLKEGSIRFAAEQPENGKNHPRNLFIWRSNLLGSSG  target    KWSFDV------------------------------VKNTLPRQDAVFCNEWHWTSSCEYADLVFPADSWAEFKLPDATA 3ir7.1    KGHEFMLKYLLGTEHGIQGKDLGQQGGVKPEEVDWQDNGLEGKLDLVVTLDFRLSSTCLYSDIILPTATWYEK--DDMNT  target    SCTNPFLLAFPTTPLKRLYDTRSDYEALALTAKALGELIDEPRMEQYWRGILDGDPTPYLQRIFSGSNATRGITYDELHE 3ir7.1    SDMHPFIHP-LSAAVDPAWEAKSDWEIYKAIAKKFSE-------------------------------------------  target    SSKRGVPLLMNMRTYPRSGGWEQRQEDKPWYTATGRLEFYRPEPEFQAAGESLPVWREPVDATFYEPNAILSNAAHPSIA 3ir7.1    --------------------------------------------------------------------------------  target    PRAPEDYGVPESQLDVETRQYRNVVRTWAELQQTLHPLQERDPAFRFVFQTPKYRWGAHSTAVDADWISMLFGPFGDPYR 3ir7.1    --------------------------------------------------------------------------------  target    RDPRMPWTGEAYLEINPKDAA 3ir7.1    --------------------- ``` | | | | | | | | | | | | | | | | | | | | | | | | | | | | | | | | | | | | | | | | | | | | | | | | | |
|  | 1q16.1.A | Respiratory nitrate reductase 1 alpha chain  *Crystal structure of Nitrate Reductase A, NarGHI, from Escherichia coli* | 0.35 | 0.00 | 23.52 | 0.62 | 1-588 | X-ray | 1.90 | monomer | 2 x MD1, 1 x 6MO, 2 x HEM, 4 x SF4, 1 x F3S, 1 x AGA, 1 x 3PH | HHblits | 0.32 |
| ``` target    SWDEATTIAAKTMEDVARTFNGDEGARKLLAQGYHPEMVEVMHGAGVQALKLRGGMPLLGIGRIFGFYRFANMLALLDRK 1q16.1    SWQEVNELIAASNVYTIKN--------------YGPDRVAGFSPIPAMSMV-----------SYASGAR-----------  target    LRPDAPADEILGSRTFDNYAWHTDLPPGHPMVTGSQTVDFDLFSAEHTKLLLIIGMNWICTKMPDGHWIGDARLKGTRVI 1q16.1    ------YLSLIGGTCLSFYDWYCDLPPASPQTWGEQTDVPESADWYNSSYIIAWGSNVPQTRTPDAHFFTEVRYKGTKTV  target    VISADYMPTANKADEVIILRPGTDAAFFLGVARELIEKGL------YDRAAVIERTDLPLLVRLDT-------GERLDAR 1q16.1    AVTPDYAEIAKLCDLWLAPKQGTDAAMALAMGHVMLREFHLDNPSQYFTDYVRRYTDMPMLVMLEERDGYYAAGRMLRAA  target    DVIPGYELAALTNYVTLKPDAEIKGNPPPPPFTAGGQVVPTELRDAWGDFVWWDRATGRPRPVSRDEV------------ 1q16.1    DLVDALGQE----------------------------------NNPEWKTVAFNT-NGEMVAPNGSIGFRWGEKGKWNLE  target    -----------------GARF----DGDP-------------ALL----G---EFEVELVDGSTVPVRPAFDLLK----- 1q16.1    QRDGKTGEETELQLSLLGSQDEIAEVGFPYFGGDGTEHFNKVELENVLLHKLPVKRLQLADGSTALVTTVYDLTLANYGL  target    -------------QYLDESFDLRTASEVCRVPPQAIQSIARQLAAN-----KRETLLAAGMGPNHYFQNDLFGRVQFLVA 1q16.1    ERGLNDVNCATSYDDVK-AYTPAWAEQITGVSRSQIIRIAREFADNADKTHGRS-MIIVGAGLNHWYHLDMNYRGLINML  target    ALTDNIGHLGGNVGSYAGNYRGSVFQAMGQWIAEDPFAI-----------E--------PD-----LTKPA-TVKRYYKA 1q16.1    IFCGCVGQSGGGWAHYVGQEKLRPQTGWQPLAFALDWQRPARHMNSTSYFYNHSSQWRYETVTAEELLSPMADKSRYTGH  target    --------ESAHYW----NYGERPLRAVAKDDE-----GDLTKGEVLTGKS--------HMPTPTKLIWFGNSNSLLGNA 1q16.1    LIDFNVRAERMGWLPSAPQLGTNPLTIAGEAEKAGMNPVDYTVKSLKEGSIRFAAEQPENGKNHPRNLFIWRSNLLGSSG  target    KWSFDVV------------------------------KNTLPRQDAVFCNEWHWTSSCEYADLVFPADSWAEFKLPDATA 1q16.1    KGHEFMLKYLLGTEHGIQGKDLGQQGGVKPEEVDWQDNGLEGKLDLVVTLDFRLSSTCLYSDIILPTATWYEK--DDMNT  target    SCTNPFLLAFPTTPLKRLYDTRSDYEALALTAKALGELIDEPRMEQYWRGILDGDPTPYLQRIFSGSNATRGITYDELHE 1q16.1    SDMHPFIHP-LSAAVDPAWEAKSDWEIYKAIAKKFSE-------------------------------------------  target    SSKRGVPLLMNMRTYPRSGGWEQRQEDKPWYTATGRLEFYRPEPEFQAAGESLPVWREPVDATFYEPNAILSNAAHPSIA 1q16.1    --------------------------------------------------------------------------------  target    PRAPEDYGVPESQLDVETRQYRNVVRTWAELQQTLHPLQERDPAFRFVFQTPKYRWGAHSTAVDADWISMLFGPFGDPYR 1q16.1    --------------------------------------------------------------------------------  target    RDPRMPWTGEAYLEINPKDAA 1q16.1    --------------------- ``` | | | | | | | | | | | | | | | | | | | | | | | | | | | | | | | | | | | | | | | | | | | | | | | | | |
|  | 1r27.4.A | Respiratory nitrate reductase 1 alpha chain  *Crystal Structure of NarGH complex* | 0.36 | 0.00 | 23.52 | 0.62 | 1-588 | X-ray | 2.00 | monomer | 4 x MO, 16 x SF4, 8 x MGD, 4 x F3S | HHblits | 0.32 |
| ``` target    SWDEATTIAAKTMEDVARTFNGDEGARKLLAQGYHPEMVEVMHGAGVQALKLRGGMPLLGIGRIFGFYRFANMLALLDRK 1r27.4    SWQEVNELIAASNVYTIKN--------------YGPDRVAGFSPIPAMSMV-----------SYASGAR-----------  target    LRPDAPADEILGSRTFDNYAWHTDLPPGHPMVTGSQTVDFDLFSAEHTKLLLIIGMNWICTKMPDGHWIGDARLKGTRVI 1r27.4    ------YLSLIGGTCLSFYDWYCDLPPASPQTWGEQTDVPESADWYNSSYIIAWGSNVPQTRTPDAHFFTEVRYKGTKTV  target    VISADYMPTANKADEVIILRPGTDAAFFLGVARELIEKGL------YDRAAVIERTDLPLLVRLDT-------GERLDAR 1r27.4    AVTPDYAEIAKLCDLWLAPKQGTDAAMALAMGHVMLREFHLDNPSQYFTDYVRRYTDMPMLVMLEERDGYYAAGRMLRAA  target    DVIPGYELAALTNYVTLKPDAEIKGNPPPPPFTAGGQVVPTELRDAWGDFVWWDRATGRPRPVSRDEV------------ 1r27.4    DLVDALGQE----------------------------------NNPEWKTVAFNT-NGEMVAPNGSIGFRWGEKGKWNLE  target    -----------------GAR----FDGDP-------------ALL-------GEFEVELVDGSTVPVRPAFDLLK----- 1r27.4    QRDGKTGEETELQLSLLGSQDEIAEVGFPYFGGDGTEHFNKVELENVLLHKLPVKRLQLADGSTALVTTVYDLTLANYGL  target    -------------QYLDESFDLRTASEVCRVPPQAIQSIARQLAAN-----KRETLLAAGMGPNHYFQNDLFGRVQFLVA 1r27.4    ERGLNDVNCATSYDDVK-AYTPAWAEQITGVSRSQIIRIAREFADNADKTHGRS-MIIVGAGLNHWYHLDMNYRGLINML  target    ALTDNIGHLGGNVGSYAGNYRGSVFQAMGQWIAEDPFAI-------------------EPD-----LTKPA-TVKRYYKA 1r27.4    IFCGCVGQSGGGWAHYVGQEKLRPQTGWQPLAFALDWQRPARHMNSTSYFYNHSSQWRYETVTAEELLSPMADKSRYTGH  target    --------ESAHYWN----YGERPLRAVAKDDE-----GDLTKGEVLTGKS--------HMPTPTKLIWFGNSNSLLGNA 1r27.4    LIDFNVRAERMGWLPSAPQLGTNPLTIAGEAEKAGMNPVDYTVKSLKEGSIRFAAEQPENGKNHPRNLFIWRSNLLGSSG  target    KWSFDV------------------------------VKNTLPRQDAVFCNEWHWTSSCEYADLVFPADSWAEFKLPDATA 1r27.4    KGHEFMLKYLLGTEHGIQGKDLGQQGGVKPEEVDWQDNGLEGKLDLVVTLDFRLSSTCLYSDIILPTATWYEK--DDMNT  target    SCTNPFLLAFPTTPLKRLYDTRSDYEALALTAKALGELIDEPRMEQYWRGILDGDPTPYLQRIFSGSNATRGITYDELHE 1r27.4    SDMHPFIHP-LSAAVDPAWEAKSDWEIYKAIAKKFSE-------------------------------------------  target    SSKRGVPLLMNMRTYPRSGGWEQRQEDKPWYTATGRLEFYRPEPEFQAAGESLPVWREPVDATFYEPNAILSNAAHPSIA 1r27.4    --------------------------------------------------------------------------------  target    PRAPEDYGVPESQLDVETRQYRNVVRTWAELQQTLHPLQERDPAFRFVFQTPKYRWGAHSTAVDADWISMLFGPFGDPYR 1r27.4    --------------------------------------------------------------------------------  target    RDPRMPWTGEAYLEINPKDAA 1r27.4    --------------------- ``` | | | | | | | | | | | | | | | | | | | | | | | | | | | | | | | | | | | | | | | | | | | | | | | | | |
| ✓ | 3ir5.1.A | Respiratory nitrate reductase 1 alpha chain  *Crystal structure of NarGHI mutant NarG-H49C* | 0.36 | 0.00 | 23.32 | 0.62 | 1-588 | X-ray | 2.30 | monomer | 2 x MD1, 1 x 6MO, 4 x SF4, 1 x AGA, 1 x F3S, 2 x HEM | HHblits | 0.32 |
| ``` target    SWDEATTIAAKTMEDVARTFNGDEGARKLLAQGYHPEMVEVMHGAGVQALKLRGGMPLLGIGRIFGFYRFANMLALLDRK 3ir5.1    SWQEVNELIAASNVYTIKN--------------YGPDRVAGFSPIPAMSMV-----------SYASGAR-----------  target    LRPDAPADEILGSRTFDNYAWHTDLPPGHPMVTGSQTVDFDLFSAEHTKLLLIIGMNWICTKMPDGHWIGDARLKGTRVI 3ir5.1    ------YLSLIGGTCLSFYDWYCDLPPASPQTWGEQTDVPESADWYNSSYIIAWGSNVPQTRTPDAHFFTEVRYKGTKTV  target    VISADYMPTANKADEVIILRPGTDAAFFLGVARELIEKGL------YDRAAVIERTDLPLLVRLD-------TGERLDAR 3ir5.1    AVTPDYAEIAKLCDLWLAPKQGTDAAMALAMGHVMLREFHLDNPSQYFTDYVRRYTDMPMLVMLEERDGYYAAGRMLRAA  target    DVIPGYELAALTNYVTLKPDAEIKGNPPPPPFTAGGQVVPTELRDAWGDFVWWDRATGRPRPVSRDEV------------ 3ir5.1    DLVDALGQE----------------------------------NNPEWKTVAFNT-NGEMVAPNGSIGFRWGEKGKWNLE  target    --------------------------G-ARFD-----------GDPALLG---EFEVELVDGSTVPVRPAFDLLK----- 3ir5.1    QRDGKTGEETELQLSLLGSQDEIAEVGFPYFGGDGTEHFNKVELENVLLHKLPVKRLQLADGSTALVTTVYDLTLANYGL  target    -------------QYLDESFDLRTASEVCRVPPQAIQSIARQLAAN-----KRETLLAAGMGPNHYFQNDLFGRVQFLVA 3ir5.1    ERGLNDVNCATSYDDVK-AYTPAWAEQITGVSRSQIIRIAREFADNADKTHGRS-MIIVGAGLNHWYHLDMNYRGLINML  target    ALTDNIGHLGGNVGSYAGNYRGSVFQAMGQWIAEDPFAI-------------------EPD-----LTKPA-TVKRYYKA 3ir5.1    IFCGCVGQSGGGWAHYVGQEKLRPQTGWQPLAFALDWQRPARHMNSTSYFYNHSSQWRYETVTAEELLSPMADKSRYTGH  target    --------ESAHYWN----YGERPLRAVAKDDEGD-----LTKGEVLTGKS--------HMPTPTKLIWFGNSNSLLGNA 3ir5.1    LIDFNVRAERMGWLPSAPQLGTNPLTIAGEAEKAGMNPVDYTVKSLKEGSIRFAAEQPENGKNHPRNLFIWRSNLLGSSG  target    KWSFDV------------------------------VKNTLPRQDAVFCNEWHWTSSCEYADLVFPADSWAEFKLPDATA 3ir5.1    KGHEFMLKYLLGTEHGIQGKDLGQQGGVKPEEVDWQDNGLEGKLDLVVTLDFRLSSTCLYSDIILPTATWYEK--DDMNT  target    SCTNPFLLAFPTTPLKRLYDTRSDYEALALTAKALGELIDEPRMEQYWRGILDGDPTPYLQRIFSGSNATRGITYDELHE 3ir5.1    SDMHPFIHP-LSAAVDPAWEAKSDWEIYKAIAKKFSE-------------------------------------------  target    SSKRGVPLLMNMRTYPRSGGWEQRQEDKPWYTATGRLEFYRPEPEFQAAGESLPVWREPVDATFYEPNAILSNAAHPSIA 3ir5.1    --------------------------------------------------------------------------------  target    PRAPEDYGVPESQLDVETRQYRNVVRTWAELQQTLHPLQERDPAFRFVFQTPKYRWGAHSTAVDADWISMLFGPFGDPYR 3ir5.1    --------------------------------------------------------------------------------  target    RDPRMPWTGEAYLEINPKDAA 3ir5.1    --------------------- ``` | | | | | | | | | | | | | | | | | | | | | | | | | | | | | | | | | | | | | | | | | | | | | | | | | |
|  | 3egw.1.A | Respiratory nitrate reductase 1 alpha chain  *The crystal structure of the NarGHI mutant NarH - C16A* | 0.35 | 0.00 | 23.32 | 0.62 | 1-588 | X-ray | 1.90 | monomer | 2 x MD1, 2 x MGD, 2 x 6MO, 6 x SF4, 4 x F3S, 2 x 3PH, 4 x HEM, 2 x AGA | HHblits | 0.32 |
| ``` target    SWDEATTIAAKTMEDVARTFNGDEGARKLLAQGYHPEMVEVMHGAGVQALKLRGGMPLLGIGRIFGFYRFANMLALLDRK 3egw.1    SWQEVNELIAASNVYTIKN--------------YGPDRVAGFSPIPAMSMV-----------SYASGAR-----------  target    LRPDAPADEILGSRTFDNYAWHTDLPPGHPMVTGSQTVDFDLFSAEHTKLLLIIGMNWICTKMPDGHWIGDARLKGTRVI 3egw.1    ------YLSLIGGTCLSFYDWYCDLPPASPQTWGEQTDVPESADWYNSSYIIAWGSNVPQTRTPDAHFFTEVRYKGTKTV  target    VISADYMPTANKADEVIILRPGTDAAFFLGVARELIEKGL------YDRAAVIERTDLPLLVRLD-------TGERLDAR 3egw.1    AVTPDYAEIAKLCDLWLAPKQGTDAAMALAMGHVMLREFHLDNPSQYFTDYVRRYTDMPMLVMLEERDGYYAAGRMLRAA  target    DVIPGYELAALTNYVTLKPDAEIKGNPPPPPFTAGGQVVPTELRDAWGDFVWWDRATGRPRPVSRDEV------------ 3egw.1    DLVAALGQEN----------------------------------NPEWKTVAFNT-NGEMVAPNGSIGFRWGEKGKWNLE  target    --------------------------G-ARFD-----------GDPALLG---EFEVELVDGSTVPVRPAFDLLK----- 3egw.1    QRDGKTGEETELQLSLLGSQDEIAEVGFPYFGGDGTEHFNKVELENVLLHKLPVKRLQLADGSTALVTTVYDLTLANYGL  target    -------------QYLDESFDLRTASEVCRVPPQAIQSIARQLAAN-----KRETLLAAGMGPNHYFQNDLFGRVQFLVA 3egw.1    ERGLNDVNCATSYDDVK-AYTPAWAEQITGVSRSQIIRIAREFADNADKTHGRS-MIIVGAGLNHWYHLDMNYRGLINML  target    ALTDNIGHLGGNVGSYAGNYRGSVFQAMGQWIAEDPFA--I---------E--------PD-----LTKPA-TVKRYYKA 3egw.1    IFCGCVGQSGGGWAHYVGQEKLRPQTGWQPLAFALDWQRPARHMNSTSYFYNHSSQWRYETVTAEELLSPMADKSRYTGH  target    --------ESAHYWN----YGERPLRAVAKDDEGD-----LTKGEVLTGKS--------HMPTPTKLIWFGNSNSLLGNA 3egw.1    LIDFNVRAERMGWLPSAPQLGTNPLTIAGEAEKAGMNPVDYTVKSLKEGSIRFAAEQPENGKNHPRNLFIWRSNLLGSSG  target    KWSFDV------------------------------VKNTLPRQDAVFCNEWHWTSSCEYADLVFPADSWAEFKLPDATA 3egw.1    KGHEFMLKYLLGTEHGIQGKDLGQQGGVKPEEVDWQDNGLEGKLDLVVTLDFRLSSTCLYSDIILPTATWYEK--DDMNT  target    SCTNPFLLAFPTTPLKRLYDTRSDYEALALTAKALGELIDEPRMEQYWRGILDGDPTPYLQRIFSGSNATRGITYDELHE 3egw.1    SDMHPFIHP-LSAAVDPAWEAKSDWEIYKAIAKKFSE-------------------------------------------  target    SSKRGVPLLMNMRTYPRSGGWEQRQEDKPWYTATGRLEFYRPEPEFQAAGESLPVWREPVDATFYEPNAILSNAAHPSIA 3egw.1    --------------------------------------------------------------------------------  target    PRAPEDYGVPESQLDVETRQYRNVVRTWAELQQTLHPLQERDPAFRFVFQTPKYRWGAHSTAVDADWISMLFGPFGDPYR 3egw.1    --------------------------------------------------------------------------------  target    RDPRMPWTGEAYLEINPKDAA 3egw.1    --------------------- ``` | | | | | | | | | | | | | | | | | | | | | | | | | | | | | | | | | | | | | | | | | | | | | | | | | |
|  | 3ir6.1.A | Respiratory nitrate reductase 1 alpha chain  *Crystal structure of NarGHI mutant NarG-H49S* | 0.34 | 0.00 | 23.32 | 0.62 | 1-588 | X-ray | 2.80 | monomer | 2 x GDP, 1 x AGA, 3 x SF4, 1 x F3S, 2 x HEM | HHblits | 0.32 |
| ``` target    SWDEATTIAAKTMEDVARTFNGDEGARKLLAQGYHPEMVEVMHGAGVQALKLRGGMPLLGIGRIFGFYRFANMLALLDRK 3ir6.1    SWQEVNELIAASNVYTIKN--------------YGPDRVAGFSPIPAMSMV-----------SYASGAR-----------  target    LRPDAPADEILGSRTFDNYAWHTDLPPGHPMVTGSQTVDFDLFSAEHTKLLLIIGMNWICTKMPDGHWIGDARLKGTRVI 3ir6.1    ------YLSLIGGTCLSFYDWYCDLPPASPQTWGEQTDVPESADWYNSSYIIAWGSNVPQTRTPDAHFFTEVRYKGTKTV  target    VISADYMPTANKADEVIILRPGTDAAFFLGVARELIEKGL------YDRAAVIERTDLPLLVRLD-------TGERLDAR 3ir6.1    AVTPDYAEIAKLCDLWLAPKQGTDAAMALAMGHVMLREFHLDNPSQYFTDYVRRYTDMPMLVMLEERDGYYAAGRMLRAA  target    DVIPGYELAALTNYVTLKPDAEIKGNPPPPPFTAGGQVVPTELRDAWGDFVWWDRATGRPRPVSRDEV------------ 3ir6.1    DLVDALGQEN----------------------------------NPEWKTVAFNT-NGEMVAPNGSIGFRWGEKGKWNLE  target    -----------------GARF----DGDPALL-----------------G---EFEVELVDGSTVPVRPAFDLLK----- 3ir6.1    QRDGKTGEETELQLSLLGSQDEIAEVGFPYFGGDGTEHFNKVELENVLLHKLPVKRLQLADGSTALVTTVYDLTLANYGL  target    -------------QYLDESFDLRTASEVCRVPPQAIQSIARQLAAN-----KRETLLAAGMGPNHYFQNDLFGRVQFLVA 3ir6.1    ERGLNDVNCATSYDDVK-AYTPAWAEQITGVSRSQIIRIAREFADNADKTHGRS-MIIVGAGLNHWYHLDMNYRGLINML  target    ALTDNIGHLGGNVGSYAGNYRGSVFQAMGQWIAEDPFA--I---------E--------PD-----LTKPA-TVKRYYKA 3ir6.1    IFCGCVGQSGGGWAHYVGQEKLRPQTGWQPLAFALDWQRPARHMNSTSYFYNHSSQWRYETVTAEELLSPMADKSRYTGH  target    --------ESAHYWN----YGERPLRAVAKDDE-----GDLTKGEVLTGKS--------HMPTPTKLIWFGNSNSLLGNA 3ir6.1    LIDFNVRAERMGWLPSAPQLGTNPLTIAGEAEKAGMNPVDYTVKSLKEGSIRFAAEQPENGKNHPRNLFIWRSNLLGSSG  target    KWSFDV------------------------------VKNTLPRQDAVFCNEWHWTSSCEYADLVFPADSWAEFKLPDATA 3ir6.1    KGHEFMLKYLLGTEHGIQGKDLGQQGGVKPEEVDWQDNGLEGKLDLVVTLDFRLSSTCLYSDIILPTATWYEK--DDMNT  target    SCTNPFLLAFPTTPLKRLYDTRSDYEALALTAKALGELIDEPRMEQYWRGILDGDPTPYLQRIFSGSNATRGITYDELHE 3ir6.1    SDMHPFIHP-LSAAVDPAWEAKSDWEIYKAIAKKFSE-------------------------------------------  target    SSKRGVPLLMNMRTYPRSGGWEQRQEDKPWYTATGRLEFYRPEPEFQAAGESLPVWREPVDATFYEPNAILSNAAHPSIA 3ir6.1    --------------------------------------------------------------------------------  target    PRAPEDYGVPESQLDVETRQYRNVVRTWAELQQTLHPLQERDPAFRFVFQTPKYRWGAHSTAVDADWISMLFGPFGDPYR 3ir6.1    --------------------------------------------------------------------------------  target    RDPRMPWTGEAYLEINPKDAA 3ir6.1    --------------------- ``` | | | | | | | | | | | | | | | | | | | | | | | | | | | | | | | | | | | | | | | | | | | | | | | | | |
|  | 1eu1.1.A | DIMETHYL SULFOXIDE REDUCTASE  *THE CRYSTAL STRUCTURE OF RHODOBACTER SPHAEROIDES DIMETHYLSULFOXIDE REDUCTASE REVEALS TWO DISTINCT MOLYBDENUM COORDINATION ENVIRONMENTS.* | 0.34 |  | 20.62 | 0.63 | 120-769 | X-ray | 1.30 | monomer | 3 x GLC, 1 x CD, 2 x MGD, 1 x 6MO, 2 x O | HHblits | 0.30 |
| ``` target    SWDEATTIAAKTMEDVARTFNGDEGARKLLAQGYHPEMVEVMHGAGVQALKLRGGMPLLGIGRIFGFYRFANMLALLDRK 1eu1.1    --------------------------------------------------------------------------------  target    LRPDAPADEILGSRTFDNYAWHTDLPPGHPMVTGSQTVDFDLFSAEHTKLLLIIGMNWICTKMPD--------GHWIGDA 1eu1.1    ---------------------------------------AWPVVVENTDLMVFWAADPMKTNEIGWVIPDHGAYAGMKAL  target    RLKGTRVIVISADYMPTANKAD-EVIILRPGTDAAFFLGVARELIEKGLYDRAAVIERTDLPLLVRLDTGERLDARDVIP 1eu1.1    KEKGTRVICINPVRTETADYFGADVVSPRPQTDVALMLGMAHTLYSEDLHDKDFLENCTT--------------------  target    GYELAALTNYVTLKPDAEIKGNPPPPPFTAGGQVVPTELRDAWGDFVWWDRATGRPRPVSRDEVGARFDGDPALLGEFEV 1eu1.1    GFDL--------------------------------------FAAY---------------------------LTGE---  target    ELVDGSTVPVRPAFDLLKQYLDESFDLRTASEVCRVPPQAIQSIARQLAANKRETLLAAGMGPNHYFQNDLFGRVQFLVA 1eu1.1    --SDG-----------------TPKTAEWAAEICGLPAEQIRELARSFVAGR--TMLAAGWSIQRMHHGEQAHWMLVTLA  target    ALTDNIGHLGGNVGSYAGNYRGSV-FQAMGQWIAEDPFAIEPDLTKPATVKRYYKAESAHYWNYGERPLRAVAKDDEGDL 1eu1.1    SMIGQIGLPGGGFGLSYHYSNGGSPTSDGPAL------GGISDGGKAVEGAAWLS-----ESGATSIPCARVV---DMLL  target    TKGE--VLTGKSHMPTPTKLIWFGNSNSLLGNAKWSFDVVKNTLPRQDAVFCNEWHWTSSCEYADLVFPADSWAEFKLPD 1eu1.1    NPGGEFQFNGATATYPDVKLAYWAGGNPFAHHQD--RNRMLKAWEKLETFIVQDFQWTATARHADIVLPATTSYERNDIE  target    ATASCTNPFLLAFPTTPLKRLYDTRSDYEALALTAKALGELIDEPRMEQYWRGILDGDPTPYLQRIFSGS------NATR 1eu1.1    SVGDYSNRAILA-MKKVVDPLYEARSDYDIFAALAERLGKGAEFTEGRDE---------MGWISSFYEAAVKQAEFKNVA  target    GITYDELHESSKRGVPLLMNMRTYPRSGGWEQRQEDKPWYTATGRLEFYRPEPEFQ--AAGESLPVWREPVDA----TFY 1eu1.1    MPSFEDFWSEGIVEFPITE-GANFVRYADFREDPLFNPLGTPSGLIEIYSKNIEKMGYDDCPAHPTWMEPAERLGGAGAK  target    EPNAILSNAAHPSIAPRAPED--------YGVPESQLDVETRQYRN-----VVRTWAELQQTLHPLQERDPAFRFVFQTP 1eu1.1    YPLHVVASHPKSRLHSQLNGTSLRDLYAVAGHEPCLINPADAAARGIADGDVLRVFNDRGQILVGAKVSDAVMPGAIQIY  target    KYRWGAHSTAVDADWISMLFGPFGDPYRRDPRMPWTGEAYLEINPKDAA 1eu1.1    EGGWYD------------------------------------------- ``` | | | | | | | | | | | | | | | | | | | | | | | | | | | | | | | | | | | | | | | | | | | | | | | | | |
|  | 1dms.1.A | DMSO REDUCTASE  *STRUCTURE OF DMSO REDUCTASE* | 0.33 |  | 20.12 | 0.63 | 123-770 | X-ray | 1.88 | monomer | 2 x PGD, 1 x 2MO | HHblits | 0.30 |
| ``` target    SWDEATTIAAKTMEDVARTFNGDEGARKLLAQGYHPEMVEVMHGAGVQALKLRGGMPLLGIGRIFGFYRFANMLALLDRK 1dms.1    --------------------------------------------------------------------------------  target    LRPDAPADEILGSRTFDNYAWHTDLPPGHPMVTGSQTVDFDLFSAEHTKLLLIIGMNWICTKMPDG--------HWIGDA 1dms.1    ------------------------------------------VLAENTEVMVFWAADPIKTSQIGWVIPEHGAYPGLEAL  target    RLKGTRVIVISADYMPTANK-ADEVIILRPGTDAAFFLGVARELIEKGLYDRAAVIERTDLPLLVRLDTGERLDARDVIP 1dms.1    KAKGTKVIVIDPVRTKTVEFFGADHVTPKPQTDVAIMLGMAHTLVAEDLYDKDFIANYTS--------------------  target    GYELAALTNYVTLKPDAEIKGNPPPPPFTAGGQVVPTELRDAWGDFVWWDRATGRPRPVSRDEVGARFDGDPALLGEFEV 1dms.1    GFDK--------------------------------------FLPY---------------------------LMGET--  target    ELVDGSTVPVRPAFDLLKQYLDESFDLRTASEVCRVPPQAIQSIARQLAANKRETLLAAGMGPNHYFQNDLFGRVQFLVA 1dms.1    -------------------DS-TPKTAEWASDISGVPAETIKELARLFKSK-RT-MLAAGWSMQRMHHGEQAHWMLVTLA  target    ALTDNIGHLGGNVGSYAGNYRGSVFQAMGQWIAEDPFAIEPDLTKPATVKRYYKAESAHYWNYGERPLRAV-AKDDEGDL 1dms.1    SMLGQIGLPGGGFGLSYHYSGGGTPSS--SGP---ALSGITDGGAATKGPEWLA-----ASGASVIPVARVVDMLENPGA  target    TKGEVLTGKSHMPTPTKLIWFGNSNSLLGNAKWSFDVVKNTLPRQDAVFCNEWHWTSSCEYADLVFPADSWAEFKLPDAT 1dms.1    EFDFN--GTRSKFPDVKMAYWVGGNPFVHHQD--RNRMVKAWEKLETFIVHDFQWTPTARHADIVLPATTSYERNDIETI  target    ASCTNPFLLAFPTTPLKRLYDTRSDYEALALTAKALGELIDEPRMEQYWRGILDGDPTPYLQRIFSGSN---ATRGI--- 1dms.1    GDYSNTGILA-MKKIVEPLYEARSDYDIFAAVAERLGKGKEFTEGKD---------EMGWIKSFYDDAAKQGKAGGVEMP  target    TYDELHESSKRGVPLLMNM--RTYPRSGGWEQRQEDKPWYTATGRLEFYRPEPEFQ--AAGESLPVWREPVDA----TFY 1dms.1    AFDAFWAE---GIVEFPVTDGADFVRYASFREDPLLNPLGTPTGLIEIYSKNIEKMGYDDCPAHPTWMEPLERLDGPGAK  target    EPNAILSNAAHPSIAPRAPED--------YGVPESQLDVETRQYRN-----VVRTWAELQQTLHPLQERDPAFRFVFQTP 1dms.1    YPLHIAASHPFNRLHSQLNGTVLREGYAVQGHEPCLMHPDDAAARGIADGDVVRVHNDRGQILTGVKVTDAVMKGVIQIY  target    KYRWGAHSTAVDADWISMLFGPFGDPYRRDPRMPWTGEAYLEINPKDAA 1dms.1    EGGWYDP------------------------------------------ ``` | | | | | | | | | | | | | | | | | | | | | | | | | | | | | | | | | | | | | | | | | | | | | | | | | |
|  | 4v4c.1.A | Pyrogallol hydroxytransferase large subunit  *Crystal Structure of Pyrogallol-Phloroglucinol Transhydroxylase from Pelobacter acidigallici* | 0.30 |  | 17.23 | 0.62 | 123-812 | X-ray | 2.35 | hetero-oligomer | 2 x CA, 2 x MGD, 1 x 4MO, 3 x SF4 | HHblits | 0.28 |
| ``` target    SWDEATTIAAKTMEDVARTFNGDEGARKLLAQGYHPEMVEVMHGAGVQALKLRGGMPLLGIGRIFGFYRFANMLALLDRK 4v4c.1    --------------------------------------------------------------------------------  target    LRPDAPADEILGSRTFDNYAWHTDLPPGHPMVTGSQTVDFDLFSAEHTKLLLIIGMNWICTKMPDGHW-----IGDARLK 4v4c.1    ------------------------------------------DGLKHAEMIVFWSSDPETNSGIYAGFESNIRRQWLKDL  target    GTRVIVISADYMPTAN-KADEVIILRPGTDAAFFLGVARELIEKGLYDRAAVIERTDLPLLVRLDTGERLDARDVIPGYE 4v4c.1    GVDFVFIDPHMNHTARLVADKWFSPKIGTDHALSFAIAYTWLKEDSYDKEYVAANAH--------------------GFE  target    LAALTNYVTLKPDAEIKGNPPPPPFTAGGQVVPTELRDAWGDFVWWDRATGRPRPVSRDEVGARFDGDPALLGEFEVELV 4v4c.1    E--------------------------------------WADYVL---------------------------G-----KT  target    DGSTVPVRPAFDLLKQYLDESFDLRTASEVCRVPPQAIQSIARQLAANKRETLLAAGM----GPNHYFQNDLFGRVQFLV 4v4c.1    DG-----------------TPKTCEWAEEESGVPACEIRALARQWAKKNTYLA-AGGLGGWGGACRASHGIEWARGMIAL  target    AALTDNIGHLGGNVGSYAGNYRGSVFQAMGQWIAEDPFAIEPD-----------------LTKPATVKRYYKAESAHYWN 4v4c.1    ATMQG-MGKPGSNMWSTTQGVPLDYEFYFPGYAEGGISGDCENSAAGFKFAWRMFDGKTTFPSPSNLNT-SAGQHIPRLK  target    YGERPLRAVAKDDEGDLT---KGEVLTGKSH---MPTPTKLIWFGNSNSLLGNAKWSFDVVKNTL--PRQDAVFCNEWHW 4v4c.1    IPECIMGGKFQWSGKGFAGGDISHQLHQYEYPAPGYSKIKMFWKYGGPHLGTMTA--TNRYAKMYTHDSLEFVVSQSIWF  target    TSSCEYADLVFPADSWAEFKLPDATASCT------------NPFLLAFPTTPLKRLYDTRSDYEALALTAKALGELIDEP 4v4c.1    EGEVPFADIILPACTNFERWDI-SEFANCSGYIPDNYQLCNHRVISL-QAKCIEPVGESMSDYEIYRLFAKKLNIEEMFS  target    RMEQYWRGILDGDPTPYLQRIFSGSNATRGITYDELHESSKRGVPLLMNMR---TYPRS--------GGWE----QRQED 4v4c.1    E---------GKDELAWCEQYFNATDMPKYMTWDEFFKKGYFVVPDNPNRKKTVALRWFAEGREKDTPDWGPRLNNQVCR  target    KPWYTATGRLEFYRPEPEFQ-------AAGESLPVWREPVDATFYEPNAILSNAAHPSIAPRAPEDYGVPESQLDVETRQ 4v4c.1    KGLQTTTGKVEFIATSLKNFEEQGYIDEHRPSMHTYVPAWESQK------------------------------------  target    YRNVVRTWAELQQTLHPLQERDPAFRFVFQTPKYRWGAHSTAVD-ADWISMLFGPFGDPYRRDPRMP--WTGEAYLEINP 4v4c.1    --------------HSPL---AVKYPLGMLSPHPRFSMHTMGDGKNSYMNYIK---------DHRVEVDGYKYWIMRVNS  target    KDAA 4v4c.1    IDAE ``` | | | | | | | | | | | | | | | | | | | | | | | | | | | | | | | | | | | | | | | | | | | | | | | | | |
|  | 7l5i.1.A | Trimethylamine-N-oxide reductase  *Crystal Structure of Haemophilus influenzae MtsZ at pH 7.0* | 0.33 |  | 19.92 | 0.60 | 124-812 | X-ray | 1.73 | monomer | 2 x MGD, 1 x MO, 1 x O | HHblits | 0.30 |
| ``` target    SWDEATTIAAKTMEDVARTFNGDEGARKLLAQGYHPEMVEVMHGAGVQALKLRGGMPLLGIGRIFGFYRFANMLALLDRK 7l5i.1    --------------------------------------------------------------------------------  target    LRPDAPADEILGSRTFDNYAWHTDLPPGHPMVTGSQTVDFDLFSAEHTKLLLIIGMNWICTKMPD--------GHWIGDA 7l5i.1    -------------------------------------------ILESSDIIVLWSANPLTTMRIAWMSTDQKGIEYFKKF  target    RLKGTRVIVISADYMPTANK-ADEVIILRPGTDAAFFLGVARELIEKGLYDRAAVIERTDLPLLVRLDTGERLDARDVIP 7l5i.1    QASGKRIICIDPQKSETCQMLNAEWIPVNTATDVPLMLGIAHTLVEQGKHDKDFLKKYTS--------------------  target    GYELAALTNYVTLKPDAEIKGNPPPPPFTAGGQVVPTELRDAWGDFVWWDRATGRPRPVSRDEVGARFDGDPALLGEFEV 7l5i.1    GYAK--------------------------------------FEEYL---------------------------LGK---  target    ELVDGSTVPVRPAFDLLKQYLDESFDLRTASEVCRVPPQAIQSIARQLAANKRETLLAAGMGPNHYFQNDLFGRVQFLVA 7l5i.1    --TD----------------G-QPKTAEWAAKICGVPAETIKQLAADFAS-KRT-MLMGGWGMQRQRHGEQTHWMLVTLA  target    ALTDNIGHLGGNVGSYAGNYRGSVFQAMGQWIAEDPFAIEPDLTKPATVKRYYKAESAHYWNYGERPLRAVA-KDDEGDL 7l5i.1    SMLGQIGLPGGGFGLSYHYSNGGVPTATGGIIG-S-ITASPSGK-A-GAKTWLDDTSKSAF-----PLARIADVLLHPGK  target    TKGEVLTGKSHMPTPTKLIWFGNSNSLLGNAKWSFDVVKNTLPRQDAVFCNEWHWTSSCEYADLVFPADSWAEFKLPDAT 7l5i.1    KIQYNGTE--ITYPDIKAVYWAGGNPFVHHQD--TNTLVKAFQKPDVVIVNEVNWTPTARMADIVLPATTSYERNDLTMA  target    ASCTNPFLLAFPTTPLKRLYDTRSDYEALALTAKALGELIDEPRMEQYWRGILDGDPTPYLQRIFSG----SN--ATRGI 7l5i.1    GDYSMMSVYP-MKQVVPPQFEAKNDYDIFVELAKRAGVEEQYTEGK---------TEMEWLEEFYNAAFSAARANRVAMP  target    TYDELHESSKRGVPL--LMNMRTYPRSGGWEQRQEDKPWYTATGRLEFYRPEPEFQ--AAGESLPVWREPVDATFYEPNA 7l5i.1    RFDKFWAENK-PLSFEAGEAAKKWVRYGEFREDPLLNPLGTPSGKIEIFSDVVEKMNYNDCKGHPSWMEPEEFAG-----  target    ILSNAAHPSIAPRAPEDYGVPESQLDVETRQYRNVVRTWAELQQTLHPLQERDPAFRFVFQTPKYRWGAHSTAVDADWIS 7l5i.1    -----------------------------------------------N---VTEEYPLALVTPHPYYRLHSQLAHTSLRQ  target    MLFGPFGDPYRRDPRMPWTGEAYLEINPKDAA 7l5i.1    KYA--------------VNDREPVMIHPEDAA ``` | | | | | | | | | | | | | | | | | | | | | | | | | | | | | | | | | | | | | | | | | | | | | | | | | |
|  | 7l5s.1.A | Trimethylamine-N-oxide reductase  *Crystal Structure of Haemophilus influenzae MtsZ at pH 5.5* | 0.33 |  | 19.92 | 0.60 | 124-812 | X-ray | 2.09 | monomer | 1 x O, 2 x MGD, 1 x MO | HHblits | 0.30 |
| ``` target    SWDEATTIAAKTMEDVARTFNGDEGARKLLAQGYHPEMVEVMHGAGVQALKLRGGMPLLGIGRIFGFYRFANMLALLDRK 7l5s.1    --------------------------------------------------------------------------------  target    LRPDAPADEILGSRTFDNYAWHTDLPPGHPMVTGSQTVDFDLFSAEHTKLLLIIGMNWICTKMPD--------GHWIGDA 7l5s.1    -------------------------------------------ILESSDIIVLWSANPLTTMRIAWMSTDQKGIEYFKKF  target    RLKGTRVIVISADYMPTANK-ADEVIILRPGTDAAFFLGVARELIEKGLYDRAAVIERTDLPLLVRLDTGERLDARDVIP 7l5s.1    QASGKRIICIDPQKSETCQMLNAEWIPVNTATDVPLMLGIAHTLVEQGKHDKDFLKKYTS--------------------  target    GYELAALTNYVTLKPDAEIKGNPPPPPFTAGGQVVPTELRDAWGDFVWWDRATGRPRPVSRDEVGARFDGDPALLGEFEV 7l5s.1    GYAK--------------------------------------FEEYL---------------------------LGK---  target    ELVDGSTVPVRPAFDLLKQYLDESFDLRTASEVCRVPPQAIQSIARQLAANKRETLLAAGMGPNHYFQNDLFGRVQFLVA 7l5s.1    --TD----------------G-QPKTAEWAAKICGVPAETIKQLAADFAS-KRT-MLMGGWGMQRQRHGEQTHWMLVTLA  target    ALTDNIGHLGGNVGSYAGNYRGSVFQAMGQWIAEDPFAIEPDLTKPATVKRYYKAESAHYWNYGERPLRAVA-KDDEGDL 7l5s.1    SMLGQIGLPGGGFGLSYHYSNGGVPTATGGIIG-S-ITASPSGK-A-GAKTWLDDTSKSAF-----PLARIADVLLHPGK  target    TKGEVLTGKSHMPTPTKLIWFGNSNSLLGNAKWSFDVVKNTLPRQDAVFCNEWHWTSSCEYADLVFPADSWAEFKLPDAT 7l5s.1    KIQYNGTE--ITYPDIKAVYWAGGNPFVHHQD--TNTLVKAFQKPDVVIVNEVNWTPTARMADIVLPATTSYERNDLTMA  target    ASCTNPFLLAFPTTPLKRLYDTRSDYEALALTAKALGELIDEPRMEQYWRGILDGDPTPYLQRIFSG----SN--ATRGI 7l5s.1    GDYSMMSVYP-MKQVVPPQFEAKNDYDIFVELAKRAGVEEQYTEGK---------TEMEWLEEFYNAAFSAARANRVAMP  target    TYDELHESSKRGVPL--LMNMRTYPRSGGWEQRQEDKPWYTATGRLEFYRPEPEFQ--AAGESLPVWREPVDATFYEPNA 7l5s.1    RFDKFWAENK-PLSFEAGEAAKKWVRYGEFREDPLLNPLGTPSGKIEIFSDVVEKMNYNDCKGHPSWMEPEEFAG-----  target    ILSNAAHPSIAPRAPEDYGVPESQLDVETRQYRNVVRTWAELQQTLHPLQERDPAFRFVFQTPKYRWGAHSTAVDADWIS 7l5s.1    -----------------------------------------------N---VTEEYPLALVTPHPYYRLHSQLAHTSLRQ  target    MLFGPFGDPYRRDPRMPWTGEAYLEINPKDAA 7l5s.1    KYA--------------VNDREPVMIHPEDAA ``` | | | | | | | | | | | | | | | | | | | | | | | | | | | | | | | | | | | | | | | | | | | | | | | | | |
|  | 1e60.1.A | Dimethyl sulfoxide/trimethylamine N-oxide reductase  *OXIDIZED DMSO REDUCTASE EXPOSED TO HEPES - Structure II BUFFER* | 0.33 | 0.00 | 20.65 | 0.60 | 122-812 | X-ray | 2.00 | monomer | 2 x PGD, 1 x 2MO | HHblits | 0.30 |
| ``` target    SWDEATTIAAKTMEDVARTFNGDEGARKLLAQGYHPEMVEVMHGAGVQALKLRGGMPLLGIGRIFGFYRFANMLALLDRK 1e60.1    --------------------------------------------------------------------------------  target    LRPDAPADEILGSRTFDNYAWHTDLPPGHPMVTGSQTVDFDLFSAEHTKLLLIIGMNWICTKMPDG--------HWIGDA 1e60.1    -----------------------------------------PVLAENTEVMVFWAADPIKTSQIGWVIPEHGAYPGLEAL  target    RLKGTRVIVISADYMPTANK-ADEVIILRPGTDAAFFLGVARELIEKGLYDRAAVIERTDLPLLVRLDTGERLDARDVIP 1e60.1    KAKGTKVIVIDPVRTKTVEFFGAEHITPKPQTDVAIMLGMAHTLVAEDLYDKDFIANYTS--------------------  target    GYELAALTNYVTLKPDAEIKGNPPPPPFTAGGQVVPTELRDAWGDFVWWDRATGRPRPVSRDEVGARFDGDPALLGEFEV 1e60.1    GFDK--------------------------------------FL---------------------------PYLDGET--  target    ELVDGSTVPVRPAFDLLKQYLDESFDLRTASEVCRVPPQAIQSIARQLAANKRETLLAAGMGPNHYFQNDLFGRVQFLVA 1e60.1    -------------------DS-TPKTAEWAEGISGVPAETIKELARLFESK-RT-MLAAGWSMQRMHHGEQAHWMLVTLA  target    ALTDNIGHLGGNVGSYAGNYRGSVFQAMGQWIAEDPFAIEPDLTKPATVKRYYKAESAHYWNYGERPLRAV-AKDDEGDL 1e60.1    SMLGQIGLPGGGFGLSYHYSGGGTPSTSGPA-----LAGITDGGAATKGPEWLA-----ASGASVIPVARVVDMLENPGA  target    TKGEVLTGKSHMPTPTKLIWFGNSNSLLGNAKWSFDVVKNTLPRQDAVFCNEWHWTSSCEYADLVFPADSWAEFKLPDAT 1e60.1    EFDFN--GTRSKFPDVKMAYWVGGNPFVHHQD--RNRMVKAWEKLETFVVHDFQWTPTARHADIVLPATTSYERNDIETI  target    ASCTNPFLLAFPTTPLKRLYDTRSDYEALALTAKALGELIDEPRMEQYWRGILDGDPTPYLQRIFSGSNATRGI---TYD 1e60.1    GDYSNTGILA-MKKIVEPLYEARSDYDIFAAVAERLGKGAEFTEGKDEMGW-----IKSFYDDAAKQ-GKAAGVEMPAFD  target    ELHESSKRGVPLLMNM--RTYPRSGGWEQRQEDKPWYTATGRLEFYRPEPEFQA--AGESLPVWREPVDATFYEPNAILS 1e60.1    AFWAE---GIVEFPVTDGADFVRYASFREDPLLNPLGTPTGLIEIYSKNIEKMGYDDCPAHPTWMEPLERL---------  target    NAAHPSIAPRAPEDYGVPESQLDVETRQYRNVVRTWAELQQTLHPLQERDPAFRFVFQTPKYRWGAHSTAVDADWISMLF 1e60.1    ----------------------------------------------DGPGAKYPLHIAASHPFNRLHSQL-NGTVLREGY  target    GPFGDPYRRDPRMPWTGEAYLEINPKDAA 1e60.1    A-------------VQGHEPCLMHPDDAA ``` | | | | | | | | | | | | | | | | | | | | | | | | | | | | | | | | | | | | | | | | | | | | | | | | | |
|  | 4dmr.1.A | DMSO REDUCTASE  *REDUCED DMSO REDUCTASE FROM RHODOBACTER CAPSULATUS WITH BOUND DMSO SUBSTRATE* | 0.33 | 0.00 | 20.78 | 0.60 | 122-812 | X-ray | 1.90 | monomer | 2 x PGD, 1 x 4MO, 1 x O | HHblits | 0.30 |
| ``` target    SWDEATTIAAKTMEDVARTFNGDEGARKLLAQGYHPEMVEVMHGAGVQALKLRGGMPLLGIGRIFGFYRFANMLALLDRK 4dmr.1    --------------------------------------------------------------------------------  target    LRPDAPADEILGSRTFDNYAWHTDLPPGHPMVTGSQTVDFDLFSAEHTKLLLIIGMNWICTKMPDG--------HWIGDA 4dmr.1    -----------------------------------------PVLAENTEVMVFWAADPIKTSQIGWVIPEHGAYPGLEAL  target    RLKGTRVIVISADYMPTANK-ADEVIILRPGTDAAFFLGVARELIEKGLYDRAAVIERTDLPLLVRLDTGERLDARDVIP 4dmr.1    KAKGTKVIVIDPVRTKTVEFFGAEHITPKPQTDVAIMLGMAHTLVAEDLYDKDFIANYTS--------------------  target    GYELAALTNYVTLKPDAEIKGNPPPPPFTAGGQVVPTELRDAWGDFVWWDRATGRPRPVSRDEVGARFDGDPALLGEFEV 4dmr.1    GFDK--------------------------------------FLP---------------------------YLDGET--  target    ELVDGSTVPVRPAFDLLKQYLDESFDLRTASEVCRVPPQAIQSIARQLAANKRETLLAAGMGPNHYFQNDLFGRVQFLVA 4dmr.1    -------------------DS-TPKTAEWAEGISGVPAETIKELARLFESK-RT-MLAAGWSMQRMHHGEQAHWMLVTLA  target    ALTDNIGHLGGNVGSYAGNYRGSVFQAMGQWIAEDPFAIEPDLTKPATVKRYYKAESAHYWNYGERPL-RAVAKDDEGDL 4dmr.1    SMLGQIGLPGGGFGLSYHYSGGGTPSTSGPA-----LAGITDGGAATKGPEWLA-----ASGASVIPVARVVDMLENPGA  target    TKGEVLTGKSHMPTPTKLIWFGNSNSLLGNAKWSFDVVKNTLPRQDAVFCNEWHWTSSCEYADLVFPADSWAEFKLPDAT 4dmr.1    EFDF--NGTRSKFPDVKMAYWVGGNPFVHHQD--RNRMVKAWEKLETFVVHDFQWTPTARHADIVLPATTSYERNDIETI  target    ASCTNPFLLAFPTTPLKRLYDTRSDYEALALTAKALGELIDEPRMEQYWRGILDGDPTPYLQRIFSGSN---ATRGI--- 4dmr.1    GDYSNTGILA-MKKIVEPLYEARSDYDIFAAVAERLGKGAEFTEGKDE---------MGWIKSFYDDAAKQGKAAGVQMP  target    TYDELHESSKRGVPLLMNM--RTYPRSGGWEQRQEDKPWYTATGRLEFYRPEPEFQ--AAGESLPVWREPVDATFYEPNA 4dmr.1    AFDAFWAE---GIVEFPVTDGADFVRYASFREDPLLNPLGTPTGLIEIYSKNIEKMGYDDCPAHPTWMEPLERLD-----  target    ILSNAAHPSIAPRAPEDYGVPESQLDVETRQYRNVVRTWAELQQTLHPLQERDPAFRFVFQTPKYRWGAHSTAVDADWIS 4dmr.1    --------------------------------------------------GPGAKYPLHIAASHPFNRLHSQL-NGTVLR  target    MLFGPFGDPYRRDPRMPWTGEAYLEINPKDAA 4dmr.1    EGYA-------------VQGHEPCLMHPDDAA ``` | | | | | | | | | | | | | | | | | | | | | | | | | | | | | | | | | | | | | | | | | | | | | | | | | |
|  | 1e18.1.A | DMSO REDUCTASE.  *TUNGSTEN-SUSBSTITUTED DMSO REDUCTASE FROM RHODOBACTER CAPSULATUS* | 0.33 | 0.00 | 20.78 | 0.60 | 122-812 | X-ray | 2.00 | monomer | 2 x PGD, 1 x 6WO | HHblits | 0.30 |
| ``` target    SWDEATTIAAKTMEDVARTFNGDEGARKLLAQGYHPEMVEVMHGAGVQALKLRGGMPLLGIGRIFGFYRFANMLALLDRK 1e18.1    --------------------------------------------------------------------------------  target    LRPDAPADEILGSRTFDNYAWHTDLPPGHPMVTGSQTVDFDLFSAEHTKLLLIIGMNWICTKMPDG--------HWIGDA 1e18.1    -----------------------------------------PVLAENTEVMVFWAADPIKTSQIGWVIPEHGAYPGLEAL  target    RLKGTRVIVISADYMPTANK-ADEVIILRPGTDAAFFLGVARELIEKGLYDRAAVIERTDLPLLVRLDTGERLDARDVIP 1e18.1    KAKGTKVIVIDPVRTKTVEFFGAEHITPKPQTDVAIMLGMAHTLVAEDLYDKDFIANYTS--------------------  target    GYELAALTNYVTLKPDAEIKGNPPPPPFTAGGQVVPTELRDAWGDFVWWDRATGRPRPVSRDEVGARFDGDPALLGEFEV 1e18.1    GFDK--------------------------------------FL---------------------------PYLDGET--  target    ELVDGSTVPVRPAFDLLKQYLDESFDLRTASEVCRVPPQAIQSIARQLAANKRETLLAAGMGPNHYFQNDLFGRVQFLVA 1e18.1    -------------------DS-TPKTAEWAEGISGVPAETIKELARLFESK-RT-MLAAGWSMQRMHHGEQAHWMLVTLA  target    ALTDNIGHLGGNVGSYAGNYRGSVFQAMGQWIAEDPFAIEPDLTKPATVKRYYKAESAHYWNYGERPLRAV-AKDDEGDL 1e18.1    SMLGQIGLPGGGFGLSYHYSGGGTPSTSGPA-----LAGITDGGAATKGPEWLA-----ASGASVIPVARVVDMLENPGA  target    TKGEVLTGKSHMPTPTKLIWFGNSNSLLGNAKWSFDVVKNTLPRQDAVFCNEWHWTSSCEYADLVFPADSWAEFKLPDAT 1e18.1    EFDFN--GTRSKFPDVKMAYWVGGNPFVHHQD--RNRMVKAWEKLETFVVHDFQWTPTARHADIVLPATTSYERNDIETI  target    ASCTNPFLLAFPTTPLKRLYDTRSDYEALALTAKALGELIDEPRMEQYWRGILDGDPTPYLQRIFSGSN---ATRGI--- 1e18.1    GDYSNTGILA-MKKIVEPLYEARSDYDIFAAVAERLGKGKEFTEGKD---------EMGWIKSFYDDAAKQGKAAGVEMP  target    TYDELHESSKRGVPLLMNM--RTYPRSGGWEQRQEDKPWYTATGRLEFYRPEPEFQ--AAGESLPVWREPVDATFYEPNA 1e18.1    AFDAFWAE---GIVEFPVTDGADFVRYASFREDPLLNPLGTPTGLIEIYSKNIEKMGYDDCPAHPTWMEPLERL------  target    ILSNAAHPSIAPRAPEDYGVPESQLDVETRQYRNVVRTWAELQQTLHPLQERDPAFRFVFQTPKYRWGAHSTAVDADWIS 1e18.1    -------------------------------------------------DGPGAKYPLHIAASHPFNRLHSQL-NGTVLR  target    MLFGPFGDPYRRDPRMPWTGEAYLEINPKDAA 1e18.1    EGYA-------------VQGHEPCLMHPDDAA ``` | | | | | | | | | | | | | | | | | | | | | | | | | | | | | | | | | | | | | | | | | | | | | | | | | |
|  | 1e5v.2.A | Dimethyl sulfoxide/trimethylamine N-oxide reductase  *OXIDIZED DMSO REDUCTASE EXPOSED TO HEPES BUFFER* | 0.33 | 0.00 | 20.82 | 0.60 | 123-812 | X-ray | 2.40 | monomer | 2 x PGD, 1 x 2MO | HHblits | 0.30 |
| ``` target    SWDEATTIAAKTMEDVARTFNGDEGARKLLAQGYHPEMVEVMHGAGVQALKLRGGMPLLGIGRIFGFYRFANMLALLDRK 1e5v.2    --------------------------------------------------------------------------------  target    LRPDAPADEILGSRTFDNYAWHTDLPPGHPMVTGSQTVDFDLFSAEHTKLLLIIGMNWICTKMPDG--------HWIGDA 1e5v.2    ------------------------------------------VLAENTEVMVFWAADPIKTSQIGWVIPEHGAYPGLEAL  target    RLKGTRVIVISADYMPTANK-ADEVIILRPGTDAAFFLGVARELIEKGLYDRAAVIERTDLPLLVRLDTGERLDARDVIP 1e5v.2    KAKGTKVIVIDPVRTKTVEFFGAEHITPKPQTDVAIMLGMAHTLVAEDLYDKDFIANYTS--------------------  target    GYELAALTNYVTLKPDAEIKGNPPPPPFTAGGQVVPTELRDAWGDFVWWDRATGRPRPVSRDEVGARFDGDPALLGEFEV 1e5v.2    GFDK--------------------------------------FLP---------------------------YLDGET--  target    ELVDGSTVPVRPAFDLLKQYLDESFDLRTASEVCRVPPQAIQSIARQLAANKRETLLAAGMGPNHYFQNDLFGRVQFLVA 1e5v.2    -------------------DS-TPKTAEWAEGISGVPAETIKELARLFESK-R-TMLAAGWSMQRMHHGEQAHWMLVTLA  target    ALTDNIGHLGGNVGSYAGNYRGSVFQAMGQWIAEDPFAIEPDLTKPATVKRYYKAESAHYWNYGERPLRA-VAKDDEGDL 1e5v.2    SMLGQIGLPGGGFGLSYHYSGGGTPSTSGPA-----LAGITDGGAATKGPEWLA-----ASGASVIPVARVVDMLENPGA  target    TKGEVLTGKSHMPTPTKLIWFGNSNSLLGNAKWSFDVVKNTLPRQDAVFCNEWHWTSSCEYADLVFPADSWAEFKLPDAT 1e5v.2    EFDF--NGTRSKFPDVKMAYWVGGNPFVHHQD--RNRMVKAWEKLETFVVHDFQWTPTARHADIVLPATTSYERNDIETI  target    ASCTNPFLLAFPTTPLKRLYDTRSDYEALALTAKALGELIDEPRMEQYWRGILDGDPTPYLQRIFSGSN---ATRGI--- 1e5v.2    GDYSNTGILA-MKKIVEPLYEARSDYDIFAAVAERLGKGAEFTEGKDE---------MGWIKSFYDDAAKQGKAAGVQMP  target    TYDELHESSKRGVPLLMNM--RTYPRSGGWEQRQEDKPWYTATGRLEFYRPEPEFQ--AAGESLPVWREPVDATFYEPNA 1e5v.2    AFDAFWAE---GIVEFPVTDGADFVRYASFREDPLLNPLGTPTGLIEIYSKNIEKMGYDDCPAHPTWMEPLERLD-----  target    ILSNAAHPSIAPRAPEDYGVPESQLDVETRQYRNVVRTWAELQQTLHPLQERDPAFRFVFQTPKYRWGAHSTAVDADWIS 1e5v.2    --------------------------------------------------GPGAKYPLHIAASHPFNRLHSQL-NGTVLR  target    MLFGPFGDPYRRDPRMPWTGEAYLEINPKDAA 1e5v.2    EGYA-------------VQGHEPCLMHPDDAA ``` | | | | | | | | | | | | | | | | | | | | | | | | | | | | | | | | | | | | | | | | | | | | | | | | | |
|  | 1kqf.1.A | FORMATE DEHYDROGENASE, NITRATE-INDUCIBLE, MAJOR SUBUNIT  *FORMATE DEHYDROGENASE N FROM E. COLI* | 0.33 |  | 19.34 | 0.60 | 1-588 | X-ray | 1.60 | hetero-oligomer | 3 x 6MO, 15 x SF4, 6 x MGD, 6 x HEM, 3 x CDL | HHblits | 0.29 |
| ``` target    SWDEATTIAAKTMEDVARTFNGDEGARKLLAQGYHPEMVEVMHGAGVQALKLRGGMPLLGIGRIFGFYRFANMLALLDRK 1kqf.1    SWEEAFSRIAKLMKADRDANFIEK----------NEQGVTVNRWL-S--------------TGMLCASGASNETGMLTQK  target    LRPDAPADEILGSRTFDNYAWHTDLP--PGHPMVTGSQTVDFDLFSAEHTKLLLIIGMNWICTKMPDGHWIGDAR-LKGT 1kqf.1    FA------RSLGMLAVDNQARVUHGPTVASLAPTFGRGAMTNHWVDIKNANVVMVMGGNAAEAHPVGFRWAMEAKNNNDA  target    RVIVISADYMPTANKADEVIILRPGTDAAFFLGVARELIEKGLYDRAAVIERTDLPLLVRLDTGERLDARDVIPGYELAA 1kqf.1    TLIVVDPRFTRTASVADIYAPIRSGTDITFLSGVLRYLIENNKINAEYVKHYTNASLLVRDDF-AFEDGLF--SGYDAEK  target    LTNYVTLKPDAEIKGNPPPPPFTAGGQVVPTELRDAWGDFVWWDRATGRPRPVSRDEVGARFDGDPALLGEFEVELVDGS 1kqf.1    R----------------------------------QY-DKSSWNYQL----------------------DENGYAKRDET  target    TVPVRPAFDLLKQYLDESFDLRTASEVCRVPPQAIQSIARQLAANK---RETLLAAGMGPNHYFQNDLFGRVQFLVAALT 1kqf.1    LTHPRCVWNLLKEHVS-RYTPDVVENICGTPKADFLKVCEVLASTSAPDRTTTFLYALGWTQHTVGAQNIRTMAMIQLLL  target    DNIGHLGGNVGSYAGNYRGSVFQAMGQWIAEDPFAIEPDLTKPAT--VKRYYKA--------ESAHYWNYG----ERPLR 1kqf.1    GNMGMAGGGVNALRGHSNIQGLTDLGLLSTSL--PGYLTLPSEKQVDLQSYLEANTPKATLADQVNYWSNYPKFFVSLMK  target    A-----------VAK----DDEGDLTKGEVLTGKSHMPTPTKLIWFGNSNSLLGNAKWSFDVVKNTLPRQDAVFCNEWHW 1kqf.1    SFYGDAAQKENNWGYDWLPKWDQTYDVIKYFNM--MDEGKVTGYFCQGFNPVASFPD--KNKVVSCLSKLKYMVVIDPLV  target    TSSCEYAD-----------------LVFPADSWAEFKLPDATASCTNPFLLAFPTTPLKRLYDTRSDYEALALTAKALGE 1kqf.1    TETSTFWQNHGESNDVDPASIQTEVFRLPSTCFAEED---GSIANSGRWLQW-HWKGQDAPGEARNDGEILAGIYHHLRE  target    LIDEPRMEQYWRGILDGDPTPYLQRIFSGSNATRGITYDELHESSKRGVPLLMNMRTYPRSGGWEQRQEDKPWYTATGRL 1kqf.1    --------------------------------------------------------------------------------  target    EFYRPEPEFQAAGESLPVWREPVDATFYEPNAILSNAAHPSIAPRAPEDYGVPESQLDVETRQYRNVVRTWAELQQTLHP 1kqf.1    --------------------------------------------------------------------------------  target    LQERDPAFRFVFQTPKYRWGAHSTAVDADWISMLFGPFGDPYRRDPRMPWTGEAYLEINPKDAA 1kqf.1    ---------------------------------------------------------------- ``` | | | | | | | | | | | | | | | | | | | | | | | | | | | | | | | | | | | | | | | | | | | | | | | | | |
|  | 1tmo.1.A | TRIMETHYLAMINE N-OXIDE REDUCTASE  *TRIMETHYLAMINE N-OXIDE REDUCTASE FROM SHEWANELLA MASSILIA* | 0.31 |  | 17.90 | 0.60 | 122-812 | X-ray | 2.50 | monomer | 2 x 2MD, 1 x 2MO | HHblits | 0.29 |
| ``` target    SWDEATTIAAKTMEDVARTFNGDEGARKLLAQGYHPEMVEVMHGAGVQALKLRGGMPLLGIGRIFGFYRFANMLALLDRK 1tmo.1    --------------------------------------------------------------------------------  target    LRPDAPADEILGSRTFDNYAWHTDLPPGHPMVTGSQTVDFDLFSAEHTKLLLIIGMNWICTKMP--------DG---HWI 1tmo.1    -----------------------------------------PLILEHSDTIVLWSNDPYKNLQVGWNAETHESFAYLAQL  target    GDARL-KGTRVIVISADYMPTANK-ADEVIILRPGTDAAFFLGVARELIEKGLYDRAAVIERTDLPLLVRLDTGERLDAR 1tmo.1    KEKVKQGKIRVISIDPVVTKTQAYLGCEQLYVNPQTDVTLMLAIAHEMISKKLYDDKFIQGYSL----------------  target    DVIPGYELAALTNYVTLKPDAEIKGNPPPPPFTAGGQVVPTELRDAWGDFVWWDRATGRPRPVSRDEVGARFDGDPALLG 1tmo.1    ----GFEE--------------------------------------FVP---------------------------YVMG  target    EFEVELVDGSTVPVRPAFDLLKQYLDESFDLRTASEVCRVPPQAIQSIARQLAANKRETLLAAGMGPNHYFQNDLFGRVQ 1tmo.1    T-----KDG-----------------VAKTPEWAAPICGVEAHVIRDLAKTLVKGRT--QFMMGWCIQRQQHGEQPYWMA  target    FLVAALTDNIGHLGGNVGSYAGNY------RGSVFQAMGQWIAEDPFAIEPDLTKPATVKRYYKAESAHYWNYGERPLRA 1tmo.1    AVLATMIGQIGLPGGGISYGHHYSSIGVPSSGAA--APGAFPR--NLDENQKPLF--DSSDFKG-----ASS--TIPVAR  target    VAK-DDEGDLTKGEVLTGKSHMPTPTKLIWFGNSNSLLGNAKWSFDVVKNTLPRQDAVFCNEWHWTSSCEYADLVFPADS 1tmo.1    WIDAILEPGKTIDANGSK--VVYPDIKMMIFSGNNPWNHHQD--RNRMKQAFHKLECVVTVDVNWTATCRFSDIVLPACT  target    WAEFKLPDATASCTNPFLLAFPTTPLKRLYDTRSDYEALALTAKALGELIDEPRMEQYWRGILDGDPTPYLQRIFSGSN- 1tmo.1    TYERNDIDVYGAYANRGILA-MQKMVEPLFDSLSDFEIFTRFAAVLGKEKEYTRN---------MGEMEWLETLYNECKA  target    ----ATRGITYDELHESSKRGVPLLMNMRTYPRSGGWEQRQEDKPWYTATGRLEFYRPEPEFQ--AAGESLPVWREPVDA 1tmo.1    ANAGKFEMPDFATFWKQG---YVHFGDGEVWTRHADFRNDPEINPLGTPSGLIEIFSRKIDQFGYDDCKGHPTWMEKTER  target    TFYEPNAILSNAAHPSIAPRAPEDYGVPESQLDVETRQYRNVVRTWAELQQTLHPLQERDPAFRFVFQTPKYRWGAHSTA 1tmo.1    SHG--------------------------------------------------GPG---SDKHPIWLQSCHPDKRLHSQM  target    VDADWISMLFGPFGDPYRRDPRMPWTGEAYLEINPKDAA 1tmo.1    CESREYRETYA-------------VNGREPVYISPVDAK ``` | | | | | | | | | | | | | | | | | | | | | | | | | | | | | | | | | | | | | | | | | | | | | | | | | |
|  | 8bqg.1.A | Formate dehydrogenase, alpha subunit, selenocysteine-containing  *W-formate dehydrogenase from Desulfovibrio vulgaris - Soaking with Formate 1 min* | 0.32 |  | 18.43 | 0.59 | 1-588 | X-ray | 1.95 | hetero-1-1-mer | 2 x MGD, 4 x SF4, 1 x H2S, 1 x W | HHblits | 0.29 |
| ``` target    SWDEATTIAAKTMEDVARTFNGDEGARKLLAQGYHPEMVEVMHGAGVQALKLRGGMPLLGIGRIFGFYRFANMLALLDRK 8bqg.1    TWDFALTEIAKRIKKTRDASFTEKNAAGD--LVNRTEAIASF-----------------------GSAAMDNEECWAYGN  target    LRPDAPADEILGSRTFDNYAWHTDLP--PGHPMVTGSQTVDFDLFSAEHTKLLLIIGMNWICTKMPDGHWIGDARLKGTR 8bqg.1    IL------RSLGLVYIEHQARIUHSPTVPALAESFGRGAMTNHWNDLANSDCILIMGSNAAENHPIAFKWVLRAKDKGAT  target    VIVISADYMPTANKADEVIILRPGTDAAFFLGVARELIEKGLYDRAAVIERTDLPLLVRLDTGERLDARDVIPGYELAAL 8bqg.1    LIHVDPRFTRTSARCDVYAPIRSGADIPFLGGLIKYILDNKLYFTDYVREYTNASLIVGEKFS---FKDGLFSGYDAA--  target    TNYVTLKPDAEIKGNPPPPPFTAGGQVVPTELRDAWGDFV-WWDRATGRPRPVSRDEVGARFDGDPALLGEFEVELVDGS 8bqg.1    --------------------------------NKKYDKSMWAFELDAN---------------------G---VPKRDPA  target    TVPVRPAFDLLKQYLDESFDLRTASEVCRVPPQAIQSIARQLAANK---RETLLAAGMGPNHYFQNDLFGRVQFLVAALT 8bqg.1    LKHPRCVINLLKKHYE-RYNLDKVAAITGTSKEQLQQVYKAYAATGKPDKAGTIMYAMGWTQHSVGVQNIRAMAMIQLLL  target    DNIGHLGGNVGSYAGNY--RGSVFQAMGQWIAEDPFAIEPDLTKPA--TVKRYY-------KAESAHYWNYG-------- 8bqg.1    GNIGVAGGGVNALRGESNVQGST--DQGLLAHIWP--GYNPVPNSKAATLELYNAATPQSKDPMSVNWWQNRPKYVASYL  target    ------ERPL---RAVAKDDEG----DLTKGEVLTGKSHMPTPTKLIWFGNSNSLLGNAKWSFDVVKNTLPRQDAVFCNE 8bqg.1    KALYPDEEPAAAYDYLPRIDAGRKLTDYFWLNIFEK--MDKGEFKGLFAWGMNPACGGAN--ANKNRKAMGKLEWLVNVN  target    WHWTSSCEY--------AD-----LVFPADSWAEFKLPDATASCTNPFLLAFPTTPLKRLYDTRSDYEALALTAKALGEL 8bqg.1    LFENETSSFWKGPGMNPAEIGTEVFFLPCCVSIEKE--GS-VANSGRWMQW-RYRGPKPYAETKPDGDIMLDMFKKVRE-  target    IDEPRMEQYWRGILDGDPTPYLQRIFSGSNATRGITYDELHESSKRGVPLLMNMRTYPRSGGWEQRQEDKPWYTATGRLE 8bqg.1    --------------------------------------------------------------------------------  target    FYRPEPEFQAAGESLPVWREPVDATFYEPNAILSNAAHPSIAPRAPEDYGVPESQLDVETRQYRNVVRTWAELQQTLHPL 8bqg.1    --------------------------------------------------------------------------------  target    QERDPAFRFVFQTPKYRWGAHSTAVDADWISMLFGPFGDPYRRDPRMPWTGEAYLEINPKDAA 8bqg.1    --------------------------------------------------------------- ``` | | | | | | | | | | | | | | | | | | | | | | | | | | | | | | | | | | | | | | | | | | | | | | | | | |
|  | 6sdr.1.A | Formate dehydrogenase, alpha subunit, selenocysteine-containing  *W-formate dehydrogenase from Desulfovibrio vulgaris - Oxidized form* | 0.31 |  | 18.63 | 0.59 | 1-588 | X-ray | 2.10 | hetero-1-1-mer | 2 x MGD, 4 x SF4, 1 x H2S, 1 x W | HHblits | 0.29 |
| ``` target    SWDEATTIAAKTMEDVARTFNGDEGARKLLAQGYHPEMVEVMHGAGV-QALKLRGGMPLLGIGRIFGFYRFANMLALLDR 6sdr.1    TWDFALTEIAKRIKKTRDAS--------------FTEKNAAGDLVNRTEAI------------ASFGSAAMDNEECWAYG  target    KLRPDAPADEILGSRTFDNYAWHTDLP--PGHPMVTGSQTVDFDLFSAEHTKLLLIIGMNWICTKMPDGHWIGDARLKGT 6sdr.1    NIL------RSLGLVYIEHQARIUHSPTVPALAESFGRGAMTNHWNDLANSDCILIMGSNAAENHPIAFKWVLRAKDKGA  target    RVIVISADYMPTANKADEVIILRPGTDAAFFLGVARELIEKGLYDRAAVIERTDLPLLVRLDTGERLDARDVIPGYELAA 6sdr.1    TLIHVDPRFTRTSARCDVYAPIRSGADIPFLGGLIKYILDNKLYFTDYVREYTNASLIVGEKFS---FKDGLFSGYDAA-  target    LTNYVTLKPDAEIKGNPPPPPFTAGGQVVPTELRDAWGDFVWWDRATGRPRPVSRDEVGARFDGDPALLGEFEVELVDGS 6sdr.1    N---------------------------------KKYDKS-MWAFE-------------------LDANG---VPKRDPA  target    TVPVRPAFDLLKQYLDESFDLRTASEVCRVPPQAIQSIARQLAANK---RETLLAAGMGPNHYFQNDLFGRVQFLVAALT 6sdr.1    LKHPRCVINLLKKHYE-RYNLDKVAAITGTSKEQLQQVYKAYAATGKPDKAGTIMYAMGWTQHSVGVQNIRAMAMIQLLL  target    DNIGHLGGNVGSYAGNY--RGSVFQAMGQWIAEDPFAIEPDLTKPA--TVKRYY-------KAESAHYWNYGE------- 6sdr.1    GNIGVAGGGVNALRGESNVQGST--DQGLLAHIWP--GYNPVPNSKAATLELYNAATPQSKDPMSVNWWQNRPKYVASYL  target    -------RPL---RAVAKDDEG-DL---TKGEVLTGKSHMPTPTKLIWFGNSNSLLGNAKWSFDVVKNTLPRQDAVFCNE 6sdr.1    KALYPDEEPAAAYDYLPRIDAGRKLTDYFWLNIFEK--MDKGEFKGLFAWGMNPACGGAN--ANKNRKAMGKLEWLVNVN  target    WHWTSSCEY--------AD-----LVFPADSWAEFKLPDATASCTNPFLLAFPTTPLKRLYDTRSDYEALALTAKALGEL 6sdr.1    LFENETSSFWKGPGMNPAEIGTEVFFLPCCVSIEKE--GS-VANSGRWMQW-RYRGPKPYAETKPDGDIMLDMFKKVRE-  target    IDEPRMEQYWRGILDGDPTPYLQRIFSGSNATRGITYDELHESSKRGVPLLMNMRTYPRSGGWEQRQEDKPWYTATGRLE 6sdr.1    --------------------------------------------------------------------------------  target    FYRPEPEFQAAGESLPVWREPVDATFYEPNAILSNAAHPSIAPRAPEDYGVPESQLDVETRQYRNVVRTWAELQQTLHPL 6sdr.1    --------------------------------------------------------------------------------  target    QERDPAFRFVFQTPKYRWGAHSTAVDADWISMLFGPFGDPYRRDPRMPWTGEAYLEINPKDAA 6sdr.1    --------------------------------------------------------------- ``` | | | | | | | | | | | | | | | | | | | | | | | | | | | | | | | | | | | | | | | | | | | | | | | | | |
|  | 6sdv.1.A | Formate dehydrogenase, alpha subunit, selenocysteine-containing,Formate dehydrogenase, alpha subunit, selenocysteine-containing,W-formate dehydrogenase - alpha subunit  *W-formate dehydrogenase from Desulfovibrio vulgaris - Formate reduced form* | 0.31 |  | 18.26 | 0.59 | 1-588 | X-ray | 1.90 | hetero-1-1-mer | 2 x MGD, 4 x SF4, 1 x W, 1 x H2S | HHblits | 0.29 |
| ``` target    SWDEATTIAAKTMEDVARTFNGDEGARKLLAQGYHPEMVEVMHGAG-VQALKLRGGMPLLGIGRIFGFYRFANMLALLDR 6sdv.1    TWDFALTEIAKRIKKTRDASF--------------TEKNAAGDLVNRTEAI------------ASFGSAAMDNEECWAYG  target    KLRPDAPADEILGSRTFDNYAWHTDLP--PGHPMVTGSQTVDFDLFSAEHTKLLLIIGMNWICTKMPDGHWIGDARLKGT 6sdv.1    NIL------RSLGLVYIEHQARIUHSPTVPALAESFGRGAMTNHWNDLANSDCILIMGSNAAENHPIAFKWVLRAKDKGA  target    RVIVISADYMPTANKADEVIILRPGTDAAFFLGVARELIEKGLYDRAAVIERTDLPLLVRLDTGERLDARDVIPGYELAA 6sdv.1    TLIHVDPRFTRTSARCDVYAPIRSGADIPFLGGLIKYILDNKLYFTDYVREYTNASLIVGEKFS---FKDGLFSGYDAA-  target    LTNYVTLKPDAEIKGNPPPPPFTAGGQVVPTELRDAWGDFVWWDRATGRPRPVSRDEVGARFDGDPALLGEFE-VELVDG 6sdv.1    ---------------------------------------NKKYDKSM------------------WAFELDANGVPKRDP  target    STVPVRPAFDLLKQYLDESFDLRTASEVCRVPPQAIQSIARQLAANK---RETLLAAGMGPNHYFQNDLFGRVQFLVAAL 6sdv.1    ALKHPRCVINLLKKHYE-RYNLDKVAAITGTSKEQLQQVYKAYAATGKPDKAGTIMYAMGWTQHSVGVQNIRAMAMIQLL  target    TDNIGHLGGNVGSYAGNY--RGSVFQAMGQWIAEDPFAIEPDLTKPA--TVKRY-------YKAESAHYWNYGER----- 6sdv.1    LGNIGVAGGGVNALRGESNVQGST--DQGLLAHIW--PGYNPVPNSKAATLELYNAATPQSKDPMSVNWWQNRPKYVASY  target    ---------PL---RAVAKDD-EG---DLTKGEVLTGKSHMPTPTKLIWFGNSNSLLGNAKWSFDVVKNTLPRQDAVFCN 6sdv.1    LKALYPDEEPAAAYDYLPRIDAGRKLTDYFWLNIFEK--MDKGEFKGLFAWGMNPACGGAN--ANKNRKAMGKLEWLVNV  target    EWHWTSSCEY--------AD-----LVFPADSWAEFKLPDATASCTNPFLLAFPTTPLKRLYDTRSDYEALALTAKALGE 6sdv.1    NLFENETSSFWKGPGMNPAEIGTEVFFLPCCVSIEKE--GS-VANSGRWMQW-RYRGPKPYAETKPDGDIMLDMFKKVRE  target    LIDEPRMEQYWRGILDGDPTPYLQRIFSGSNATRGITYDELHESSKRGVPLLMNMRTYPRSGGWEQRQEDKPWYTATGRL 6sdv.1    --------------------------------------------------------------------------------  target    EFYRPEPEFQAAGESLPVWREPVDATFYEPNAILSNAAHPSIAPRAPEDYGVPESQLDVETRQYRNVVRTWAELQQTLHP 6sdv.1    --------------------------------------------------------------------------------  target    LQERDPAFRFVFQTPKYRWGAHSTAVDADWISMLFGPFGDPYRRDPRMPWTGEAYLEINPKDAA 6sdv.1    ---------------------------------------------------------------- ``` | | | | | | | | | | | | | | | | | | | | | | | | | | | | | | | | | | | | | | | | | | | | | | | | | |
|  | 7e5z.1.A | Formate dehydrogenase  *Dehydrogenase holoenzyme* | 0.30 |  | 19.20 | 0.58 | 1-673 | EM | 0.00 | hetero-1-1-mer | 1 x W, 2 x MGD, 2 x FES, 4 x SF4, 1 x FMN | HHblits | 0.30 |
| ``` target    SWDEATTIAAKTMEDVARTFNGDEGARKLLAQGYHPEMVEVMHGAGVQALKLRGGMPLLGIGRIFGFYRFANMLALLDRK 7e5z.1    TWEEALDRAAGGLKAIRDT--------------NGRKALAGF-----------------------GSAKGSNEEAYLFQK  target    LRPDAPADEILGSRTFDNYAWHTDLP--PGHPMVTGSQTVDFDLFSAEHTKLLLIIGMNWICTKMPDGHWIGDA-RLKGT 7e5z.1    L-----VRLGFGTNNVDHCTRLCHASSVAALMEGLNSGAVTAPFSAALDAEVIVVIGANPTVNHPVAATFLKNAVKQRGA  target    RVIVISADYMPTANKADEVIILRPGTDAAFFLGVARELIEKGLYDRAAVIERTDLPLLVRLDTGERLDARDVIPGYELAA 7e5z.1    KLIIMDPRRQTLSRHAYRHLAFRPGSDVAMLNAMLNVIVTEGLYDEQYIAGYTE--------------------N-----  target    LTNYVTLKPDAEIKGNPPPPPFTAGGQVVPTELRDAWGDFVWWDRATGRPRPVSRDEVGARFDGDPALLGEFEVELVDGS 7e5z.1    --------------------------------------------------------------------------------  target    TVPVRPAFDLLKQYLDESFDLRTASEVCRVPPQAIQSIARQLAANKRETLLAAGMGPNHYFQNDLFGRVQFLVAALTDNI 7e5z.1    -------FEALREKIV-DFTPEKMASVCGIDAETLREVARLYARAKSSLI-FWGMGVSQHVHGTDNSRCLIALALITGQI  target    GHLGGNVGSYAG--NYRGSVFQAMGQWIAEDPFAIEPDLTKPATVKRYYKAESAHYWNYGERPLRAVAKDDEGDLTKGEV 7e5z.1    GRPGTGLHPLRGQNNVQGAS--DAGLIP--MVYPDYQSVEKDAVREL-----FEEFWGQS---LD-----PQKGLTVVEI  target    LTGKSHMPTPTKLIWFGNSNSLLGNAKWSFDVVKNTLPRQDAVFCNEWHWTSSCEYADLVFPADSWAEFKLPDATASCTN 7e5z.1    MRAI--HAGEIRGMFVEGENPAMSDPD--LNHARHALAMLDHLVVQDLFLTETAFHADVVLPASAFAEK---AGTFTNTD  target    PFLLAFPTTPLKRLYDTRSDYEALALTAKALGELIDEPRMEQYWRGILDGDPTPYLQRIFSGSNATRGITYDELHESSKR 7e5z.1    RRVQIA-QPVVAPPGDARQDWWIIQELARRLDLDWNYG------------GPADIFAEMAQVMPSLNNITWERLEREG--  target    GVPLLMNMRTYPRSGGWEQRQEDKPWYTATGRLEFYRPEPEFQAAGESLPVWREPVDATFYEPNAILSNAAHPSIAPRAP 7e5z.1    -AVTYPV-DA-PDQPGNE-IIFYAGFPTESGRAKIVPA------------------------------------------  target    EDYGVPESQLDVETRQYRNVVRTWAELQQTLHPLQERDPAFRFVFQTPKYRWGAHSTAVDADWISMLFGPFGDPYRRDPR 7e5z.1    --------------------------------------------------------------------------------  target    MPWTGEAYLEINPKDAA 7e5z.1    ----------------- ``` | | | | | | | | | | | | | | | | | | | | | | | | | | | | | | | | | | | | | | | | | | | | | | | | | |
|  | 7vw6.1.A | Formate dehydrogenase  *Cryo-EM Structure of Formate Dehydrogenase 1 from Methylorubrum extorquens AM1* | 0.34 |  | 19.20 | 0.58 | 1-673 | EM | 0.00 | hetero-1-1-mer | 4 x SF4, 2 x FES, 2 x MGD, 1 x W, 1 x FMN | HHblits | 0.30 |
| ``` target    SWDEATTIAAKTMEDVARTFNGDEGARKLLAQGYHPEMVEVMHGAGVQALKLRGGMPLLGIGRIFGFYRFANMLALLDRK 7vw6.1    TWEEALDRAAGGLKAIRDT--------------NGRKALAGF-----------------------GSAKGSNEEAYLFQK  target    LRPDAPADEILGSRTFDNYAWHTDLP--PGHPMVTGSQTVDFDLFSAEHTKLLLIIGMNWICTKMPDGHWIGDA-RLKGT 7vw6.1    L-----VRLGFGTNNVDHCTRLCHASSVAALMEGLNSGAVTAPFSAALDAEVIVVIGANPTVNHPVAATFLKNAVKQRGA  target    RVIVISADYMPTANKADEVIILRPGTDAAFFLGVARELIEKGLYDRAAVIERTDLPLLVRLDTGERLDARDVIPGYELAA 7vw6.1    KLIIMDPRRQTLSRHAYRHLAFRPGSDVAMLNAMLNVIVTEGLYDEQYIAGYTE--------------------N-----  target    LTNYVTLKPDAEIKGNPPPPPFTAGGQVVPTELRDAWGDFVWWDRATGRPRPVSRDEVGARFDGDPALLGEFEVELVDGS 7vw6.1    --------------------------------------------------------------------------------  target    TVPVRPAFDLLKQYLDESFDLRTASEVCRVPPQAIQSIARQLAANKRETLLAAGMGPNHYFQNDLFGRVQFLVAALTDNI 7vw6.1    -------FEALREKIV-DFTPEKMASVCGIDAETLREVARLYARAKSSLI-FWGMGVSQHVHGTDNSRCLIALALITGQI  target    GHLGGNVGSYAG--NYRGSVFQAMGQWIAEDPFAIEPDLTKPATVKRYYKAESAHYWNYGERPLRAVAKDDEGDLTKGEV 7vw6.1    GRPGTGLHPLRGQNNVQGAS--DAGLIP--MVYPDYQSVEKDAVREL-----FEEFWGQS---LD-----PQKGLTVVEI  target    LTGKSHMPTPTKLIWFGNSNSLLGNAKWSFDVVKNTLPRQDAVFCNEWHWTSSCEYADLVFPADSWAEFKLPDATASCTN 7vw6.1    MRAI--HAGEIRGMFVEGENPAMSDPD--LNHARHALAMLDHLVVQDLFLTETAFHADVVLPASAFAEK---AGTFTNTD  target    PFLLAFPTTPLKRLYDTRSDYEALALTAKALGELIDEPRMEQYWRGILDGDPTPYLQRIFSGSNATRGITYDELHESSKR 7vw6.1    RRVQIA-QPVVAPPGDARQDWWIIQELARRLDLDWNYG------------GPADIFAEMAQVMPSLNNITWERLEREG--  target    GVPLLMNMRTYPRSGGWEQRQEDKPWYTATGRLEFYRPEPEFQAAGESLPVWREPVDATFYEPNAILSNAAHPSIAPRAP 7vw6.1    -AVTYPV-DA-PDQPGNE-IIFYAGFPTESGRAKIVPA------------------------------------------  target    EDYGVPESQLDVETRQYRNVVRTWAELQQTLHPLQERDPAFRFVFQTPKYRWGAHSTAVDADWISMLFGPFGDPYRRDPR 7vw6.1    --------------------------------------------------------------------------------  target    MPWTGEAYLEINPKDAA 7vw6.1    ----------------- ``` | | | | | | | | | | | | | | | | | | | | | | | | | | | | | | | | | | | | | | | | | | | | | | | | | |
|  | 1h0h.1.A | FORMATE DEHYDROGENASE SUBUNIT ALPHA  *Tungsten containing Formate Dehydrogenase from Desulfovibrio Gigas* | 0.32 |  | 17.01 | 0.59 | 1-588 | X-ray | 1.80 | hetero-1-1-mer | 1 x W, 1 x 2MD, 1 x MGD, 4 x SF4, 1 x CA | HHblits | 0.28 |
| ``` target    SWDEATTIAAKTMEDVARTFNGDEGARKLLAQGYHPEMVEVMHGAGVQALKLRGGMPLLGIGRIFGFYRFANMLALLDRK 1h0h.1    SWDWMLDTIAERVAKTREATFVTK---------NAKG-QVVNRCD---GI------------ASVGSAAMDNEECWIYQA  target    LRPDAPADEILGSRTFDNYAWHTDLP--PGHPMVTGSQTVDFDLFSAEHTKLLLIIGMNWICTKMPDGHWIGDARLKGTR 1h0h.1    WL------RSLGLFYIEHQARIUHSATVAALAESYGRGAMTNHWIDLKNSDVILMMGSNPAENHPISFKWVMRAKDKGAT  target    VIVISADYMPTANKADEVIILRPGTDAAFFLGVARELIEKGLYDRAAVIERTDLPLLVRLDTGERLDARDVIPGYELAAL 1h0h.1    LIHVDPRYTRTSTKCDLYAPLRSGSDIAFLNGMTKYILEKELYFKDYVVNYTNASFIVGEGF---AFEEGL---------  target    TNYVTLKPDAEIKGNPPPPPFTAGGQVVPTELRDAWGDFVWWDRATGRPRPVSRDEVGARFDGDPALLGEFEVELVDGST 1h0h.1    --------------------------------------FAGYNKETRKYDKSKW-------GFERDENGNP---KRDETL  target    VPVRPAFDLLKQYLDESFDLRTASEVCRVPPQAIQSIARQLAAN----KRETLLAAGMGPNHYFQNDLFGRVQFLVAALT 1h0h.1    KHPRCVFQIMKKHYE-RYDLDKISAICGTPKELILKVYDAYCATGKPDKAG-TIMYAMGWTQHTVGVQNIRAMSINQLLL  target    DNIGHLGGNVGSYAGNY--RGSVFQAMGQWIAEDPFAIEPDL-------------------TKPATVKRYYKA------E 1h0h.1    GNIGVAGGGVNALRGEANVQGST--DHGLLM--HIYPGYLGTARASIPTYEEYTKKFTPVSKDPQSANWWSNFPKYSASY  target    SAHYWNYGERPLRAVAKD----DEGDLTKGEVLTGKSHMPTPTKLIWFGNSNSLLGNAKWSFDVVKNTLPRQDAVFCNEW 1h0h.1    IKSMWPDADLNE-AYGYLPKGEDGKDYSWLTLFDDM--FQGKIKGFFAWGQNPACSGAN--SNKTREALTKLDWMVNVNI  target    HWTSSCEYA-------------DLVFPADSWAEFKLPDATASCTNPFLLAFPTTPLKRLYDTRSDYEALALTAKALGELI 1h0h.1    FDNETGSFWRGPDMDPKKIKTEVFFLPCAVAIEKE--G-SISNSGRWMQW-RYVGPEPRKNAIPDGDLIVELAKRVQK--  target    DEPRMEQYWRGILDGDPTPYLQRIFSGSNATRGITYDELHESSKRGVPLLMNMRTYPRSGGWEQRQEDKPWYTATGRLEF 1h0h.1    --------------------------------------------------------------------------------  target    YRPEPEFQAAGESLPVWREPVDATFYEPNAILSNAAHPSIAPRAPEDYGVPESQLDVETRQYRNVVRTWAELQQTLHPLQ 1h0h.1    --------------------------------------------------------------------------------  target    ERDPAFRFVFQTPKYRWGAHSTAVDADWISMLFGPFGDPYRRDPRMPWTGEAYLEINPKDAA 1h0h.1    -------------------------------------------------------------- ``` | | | | | | | | | | | | | | | | | | | | | | | | | | | | | | | | | | | | | | | | | | | | | | | | | |
|  | 6lod.1.B | Fe-S-cluster-containing hydrogenase components 1-like protein  *Cryo-EM structure of the air-oxidized photosynthetic alternative complex III from Roseiflexus castenholzii* | 0.28 |  | 12.19 | 0.60 | 1-770 | EM | 0.00 | hetero-1-1-1-1-1-1-… | 6 x HEC, 2 x EL6, 3 x SF4, 1 x F3S | HHblits | 0.26 |
| ``` target    SWDEATTIAAKTMEDVARTFNGDEGARKLLAQGYHPEMVEVMHGAGVQALKLRGGMPLLGIGRIFGFYRFANMLALLDRK 6lod.1    TWDAFVAAATAAMQAQTAK--------------QGAGL-RVLSGSL-------------------TSPTLIAQKQQLLTQ  target    LRPDAPADEILGSRTFDNYAWHT--DLPPGHPMVTGSQTVDFDLFSAEHTKLLLIIGMNWICTKMPD---GHWIGDAR-- 6lod.1    F----------PQAKWYEYEPVGRDNANAGARLAFGAD--VHTIYRLDTAKVIVGFDADFTAPSPTGVRMARQLADGRRI  target    ----LKGTRVIVISADYMPTANKADEVIILRPGTDAAFFLGVARELIEKGLYDRAAVIERTDLPLLVRLDTGERLDARDV 6lod.1    RKGTKEVNRLYLAESTPSITGLLADHRLPVRSSQIEHLVRALATLVGVPNV-----------------------------  target    IPGYELAALTNYVTLKPDAEIKGNPPPPPFTAGGQVVPTELRDAWGDFVWWDRATGRPRPVSRDEVGARFDGDPALLGEF 6lod.1    --------------------------------------------------------------------------------  target    EVELVDGSTVPVRPAFDLLKQYLDESFDLRTASEVCRVPPQAIQSIARQLAANKRETLLAAGMGPNHYFQNDLFGRVQFL 6lod.1    -----------------------------AAGAPLSDTEKKWVEAAAKDLQANRGACVVLVGES--QPP---VVHALGHA  target    VAALTDNIGHLGGNVGSYAGNYRGSVFQAMGQWIAEDPFAIEPDLTKPATVKRYYKAESAHYWNYGERPLRAVAKDDEGD 6lod.1    INAQLGNVGST---VVYTEP-V---------E----D----DPS---G-GI----------------AA-----------  target    LTKGEVLTGKSHMPTPTKLIWFGNSNSLLGNAKWSFDVVKNTLPRQDAVFCNEWHWTSSCEYADLVFPADSWAEFKLPDA 6lod.1    --LSALTQEM--NAGTVEVLLMIESNPVYNAPA--DIPFAEALAKVPLSMHVGLYRDETAQQSVWHINGAHFLEAW--G-  target    TASCTNPFLLAFPTTPLKRLYDTRSDYEALALTAKALGELIDEPRMEQYWRGILDGDPTPYLQRIFSGSNATRGITYDEL 6lod.1    DVRAFDGTTT-IVQPLIAPLYNGKSAIEVLNVLLGKPQET-GYQTLTAYWQTQ------------DA--SGNFRVFWNTA  target    HESSKRGVPLLMNMRTYPRSGGWEQRQEDKPWYTATGRLEFYRPEPE-FQAAG-ESLPVWREPVDATFYEPNAILSNAAH 6lod.1    LHD---GVITATQARSRQ-VTL-QQGFADAAPPAPTQGLEIVFRPDPSLWDGAFANNAWLQET-----PKPYTKLTWDNV  target    PSIAPRAPEDYGVPESQLDVETRQYRNVVRTWAELQQTLHPLQERDPAFRFVFQTPKYRWGAHSTAVDADWISMLFGPFG 6lod.1    ALMSVRTANALGLKNG----------DVVRLTYQGRSVDAPVWVQPGHADDSVTVHFGFGRTA-----------------  target    DPYRRDPRMPWTGEAYLEINPKDAA 6lod.1    ------------------------- ``` | | | | | | | | | | | | | | | | | | | | | | | | | | | | | | | | | | | | | | | | | | | | | | | | | |
|  | 1ogy.1.A | PERIPLASMIC NITRATE REDUCTASE  *Crystal structure of the heterodimeric nitrate reductase from Rhodobacter sphaeroides* | 0.30 |  | 19.56 | 0.55 | 1-589 | X-ray | 3.20 | hetero-1-1-mer | 1 x SF4, 1 x MO, 2 x MGD, 2 x HEC | HHblits | 0.28 |
| ``` target    SWDEATTIAAKTMEDVARTFNGDEGARKLLAQGYHPEMVEVMHGAGVQALKLRGGMPLLGIGRIFGFYRFANMLALLDRK 1ogy.1    SWDEAFDVMAAQAKLVLKE--------------KAPEAVGMFGSG-----------------------QWTIWEGYAASK  target    LRPDAPADEILGSRTFDNYAWHTD--LPPGHPMVTGSQTVDFDLFSAEHTKLLLIIGMNWICTKMPDGHWIGDAR--LKG 1ogy.1    L-----MRAGFRSNNLDPNARHCMASAATAFMRTFGMDEPMGCYDDFEAADAFVLWGSNMAEMHPILWSRLTDRRLSHEH  target    TRVIVISADYMPTANKADEVIILRPGTDAAFFLGVARELIEKGLYDRAAVIERTDLPLLVRLDTGERLDARDVIPGYELA 1ogy.1    VRVAVLSTFTHRSSDLSDTPIIFRPGTDRAILNYIAHHIISTGRVNRDFVDRHTNFALGAT-DIGYGLRPEHQ-------  target    ALTNYVTLKPDAEIKGNPPPPPFTAGGQVVPTELRDAWGDFVWWDRATGRPRPVSRDEVGARFDGDPALLGEFEVELVDG 1ogy.1    -----------------------------------------LQLAA-----------------------KG------AAD  target    STVPVRPAFDLLKQYLDESFDLRTASEVCRVPPQAIQSIARQLAANKRETLLAAGMGPNHYFQNDLFGRVQFLVAALTDN 1ogy.1    AGAMTPTDFETFAALVS-EYTLEKAAEISGVEPALLEELAELYADPDRKWMSLWTMGFNQHVRGVWANHMVYNLHLLTGK  target    IGHLGGNVGSYAGNYRGSV-FQAMGQWIAEDPFAIEPDLTKPATVKRYYKAESAHYWNYGERPLRAVAKDDEGDLTKGEV 1ogy.1    ISEPGNSPFSLTGQPFACGTAREVGTFAHRLP--ADMVVTNPEHRAHA-----EEIWKLPA-GLLP----DWVGAHAVEQ  target    LTGKSHMPTPTKLIWFGNSNSLLGNAKWSFDVVKNTLPRQDAVFCNEWHWTSSCEYADLVFPADSWAEFKLPDATASCTN 1ogy.1    DRKL--HDGEINFYWVQVNNNMQAAPNIDQETYPGYRNPENFIVVSDAYPTVTGRAADLVLPAAMWVEKE--G-AYGNAE  target    PFLLAFPTTPLKRLYDTRSDYEALALTAKALGELIDEPRMEQYWRGILDGDPTPYLQRIFSGSNATRGITYDELHESSKR 1ogy.1    RRTHF-WHQLVEAPGEARSDLWQLMEFSKRFTTD----------------------------------------------  target    GVPLLMNMRTYPRSGGWEQRQEDKPWYTATGRLEFYRPEPEFQAAGESLPVWREPVDATFYEPNAILSNAAHPSIAPRAP 1ogy.1    --------------------------------------------------------------------------------  target    EDYGVPESQLDVETRQYRNVVRTWAELQQTLHPLQERDPAFRFVFQTPKYRWGAHSTAVDADWISMLFGPFGDPYRRDPR 1ogy.1    --------------------------------------------------------------------------------  target    MPWTGEAYLEINPKDAA 1ogy.1    ----------------- ``` | | | | | | | | | | | | | | | | | | | | | | | | | | | | | | | | | | | | | | | | | | | | | | | | | |
|  | 6tg9.1.A | Formate dehydrogenase subunit alpha  *Cryo-EM Structure of NADH reduced form of NAD+-dependent Formate Dehydrogenase from Rhodobacter capsulatus* | 0.32 |  | 20.41 | 0.54 | 1-632 | EM | 3.24 | hetero-2-2-2-2-mer | 4 x MGD, 2 x 6MO, 4 x FES, 10 x SF4, 2 x H2S, 2 x FMN, 2 x NAI | HHblits | 0.30 |
| ``` target    SWDEATTIAAKTMEDVARTFNGDEGARKLLAQGYHPEMVEVMHGAGVQALKLRGGMPLLGIGRIFGFYRFANMLALLDRK 6tg9.1    NWTEALDFTATRLRALRDS--------------HGADALGVIT-----------------------SSRCTNEETYLVQK  target    LRPDAPADEILGSRTFDNYAWHTDLP--PGHPMVTGSQTVDFDLFSAEHTKLLLIIGMNWICTKMPDGHWIGDARLKGTR 6tg9.1    L-----ARAVFGTNNTDTCARVCHSPTGYGLKQTFGTSAGTQDFDSVEETDLALVIGANPTDGHPVFASRLRKRLRAGAK  target    VIVISADYMPTAN----KADEVIILRPGTDAAFFLGVARELIEKGLYDRAAVIERTDLPLLVRLDTGERLDARDVIPGYE 6tg9.1    LIVVDPRRIDLLNTPHRGEAWHLQLKPGTNVAVMTAMAHVIVTEQIFDKRFIGDRCD---------------------WD  target    LAALTNYVTLKPDAEIKGNPPPPPFTAGGQVVPTELRDAWGDFVWWDRATGRPRPVSRDEVGARFDGDPALLGEFEVELV 6tg9.1    --------------------------------------------------------------------------------  target    DGSTVPVRPAFDLLKQYLD-ESFDLRTASEVCRVPPQAIQSIARQLAANKRETLLAAGMGPNHYFQNDLFGRVQFLVAAL 6tg9.1    ---------EWADYAEFVANPEYAPEAVESLTGVPAGLLRQAARAYAAAPNA-AIYYGLGVTEHSQGSTTVIAIANLAMM  target    TDNIGHLGGNVGSYAGN--YRGSVFQAMGQWIAEDPFAIEPDLTKPATVKRYYKAESAHYWNYGERPLRAVAKDDEGDLT 6tg9.1    TGNIGRPGVGVNPLRGQNNVQGSC--DMGSFPH--EFPGYRHVSDDATRGL-----FERTWGVTL-S-------SEPGLR  target    KGEVLTGKSHMPTPTKLIWFGNSNSLLGNAKWSFDVVKNTLPRQDAVFCNEWHWTSSCEYADLVFPADSWAEFKLPDATA 6tg9.1    IPNMLDAA--VEGRFKALYVQGEDILQSDPD--TRHVSAGLAAMDLVIVHDLFLNETANYAHVFLPGSTFLEK---DGTF  target    SCTNPFLLAFPTTPLKRLYDTRSDYEALALTAKALGELIDEPRMEQYWRGILDGDPTPYLQRIFSGSNATRGITYDELHE 6tg9.1    TNAERRINRV-RRVMAPKA-GFADWEVTQMLANALGAGWHY------------THPSEIMAEIAATTPGFAAVTYEMLDA  target    SSKRGVPLLMNMRTYPRSGGWEQRQEDKPWYTATGRLEFYRPEPEFQAAGESLPVWREPVDATFYEPNAILSNAAHPSIA 6tg9.1    R-------------------------------------------------------------------------------  target    PRAPEDYGVPESQLDVETRQYRNVVRTWAELQQTLHPLQERDPAFRFVFQTPKYRWGAHSTAVDADWISMLFGPFGDPYR 6tg9.1    --------------------------------------------------------------------------------  target    RDPRMPWTGEAYLEINPKDAA 6tg9.1    --------------------- ``` | | | | | | | | | | | | | | | | | | | | | | | | | | | | | | | | | | | | | | | | | | | | | | | | | |
|  | 8e9g.1.G | NADH-quinone oxidoreductase subunit G  *Mycobacterial respiratory complex I with both quinone positions modelled* | 0.24 |  | 15.95 | 0.57 | 1-769 | EM | 0.00 | hetero-1-1-1-1-1-1-… |  | HHblits | 0.26 |
| ``` target    SWDEATTIAAKTMEDVARTFNGDEGARKLLAQGYHPEMVEVMHGAGVQALKLRGGMPLLGIGRIFGFYRFANMLALLDRK 8e9g.1    SWSEALTVAAAGLLTAAG-------------------STGV-----------------------LVGGRCTVEDAYAYAK  target    LRPDAPADEILGSRTFDNYAWHTDLP--PGHPMVTGSQTVDFDLFSAEHTKLLLIIGMNWICTKMPDGHWIGDA-RLKGT 8e9g.1    F-----ARMVLNTNDVDFRARPHSAEEAEFLAAHVAGQTMGLRYAELENAPTVLLAGFEPEEESPIVFLRLRKGVRKNGV  target    RVIVISADYMP-TANKADEVIILRPGTDAAFFLGVARELIEKGLYDRAAVIERTDLPLLVRLDTGERLDARDVIPGYELA 8e9g.1    QVVAVAPWASRGLTKLAGTVVPTVPGDEPAALDGMHD---------DDRLRR----------------------------  target    ALTNYVTLKPDAEIKGNPPPPPFTAGGQVVPTELRDAWGDFVWWDRATGRPRPVSRDEVGARFDGDPALLGEFEVELVDG 8e9g.1    --------------------------------------------------------------------------------  target    STVPVRPAFDLLKQYLDESFDLRTASEVCRVPPQAIQSIARQLAANKRETLLAAGMGPNHYFQNDLFGRVQFLVAALTDN 8e9g.1    ------------------PGAVILVGERLATSPGALSAAVRLAAATGAR-LAWIPRR----AGERGAIEAGALPNLLPGG  target    IGHLGGNVGSYAGNYRGSVFQAMGQWIAEDPFAIEPDLTKPATVKRYYKAESAHYWNYGERPLRAVAKDDEGDLTKGEVL 8e9g.1    RPVDDADARA--------------EV------------------A--------RAWYISALPE-------APGRDTAAIL  target    TGKSHMPTPTKLIWFGNSNSLLGNAKWSFDVVKNTLPRQDAVFCNEWHWTSSCEYADLVFPADSWAEFKLPDATASCTNP 8e9g.1    STA--ASGHLAALLVGG-VELGDLPD--PELAVAAVRTTPFVVSLELRESAVTELADVVFPVAPVVEK---AGSFLNWEG  target    FLLAFPTTPLKRLYDTRSDYEALALTAKALGELIDEPRMEQYWRGILDGDPTPYLQRIFSGSNATRGITYDELHESSKRG 8e9g.1    RPRPF-APSL--KTNAIPDLRVLHYLADEIGVDLALPT------------AEAADAELAQLG------TWGGAR------  target    VPLLMNMRTYPRSGGWEQRQEDKPWYTATGRLEFYRPEPEFQAAG--ESLPVWREPVDATFYEPNAILSNAAHPSIAPRA 8e9g.1    ---------PPA-P-T--APPTARPEAGSGQAVLASWRMLLDAGRLQDGEPHLAG------------TAVRPVARMSAAT  target    PEDYGVPESQLDVETRQYRNVVRTWAELQQTLHPLQERDPAFRFVFQTPKYRWGAHSTAVDADWISMLFGPFGDPYRRDP 8e9g.1    AAGIGASDG----------APVTVSTERGAVTLPLAVTD-MPDGVVWLPMNSPGS-------------------------  target    RMPWTGEAYLEINPKDAA 8e9g.1    ------------------ ``` | | | | | | | | | | | | | | | | | | | | | | | | | | | | | | | | | | | | | | | | | | | | | | | | | |
|  | 3o5a.1.A | Periplasmic nitrate reductase  *Crystal Structure of partially reduced Periplasmic Nitrate Reductase from Cupriavidus necator using Ionic Liquids* | 0.30 |  | 17.78 | 0.55 | 1-589 | X-ray | 1.72 | hetero-oligomer | 1 x SF4, 1 x MOS, 2 x MGD, 2 x HEC | HHblits | 0.28 |
| ``` target    SWDEATTIAAKTMEDVARTFNGDEGARKLLAQGYHPEMVEVMHGAGVQALKLRGGMPLLGIGRIFGFYRFANMLALLDRK 3o5a.1    TWDQAFDEMERQFKRVLKE--------------KGPTAVGMFG-SGQ----------------------WTVWEGYAAAK  target    LRPDAPADEILGSRTFDNYAWHTDLP--PGHPMVTGSQTVDFDLFSAEHTKLLLIIGMNWICTKMPDGHWIGDAR--LKG 3o5a.1    L-----YKAGFRSNNIDPNARHCMASAAAGFMRTFGMDEPMGCYDDFEAADAFVLWGSNMAEMHPILWTRVTDRRLSHPK  target    TRVIVISADYMPTANKADEVIILRPGTDAAFFLGVARELIEKGLYDRAAVIERTDLPLLVRLDTGERLDARDVIPGYELA 3o5a.1    TRVVVLSTFTHRCFDLADIGIIFKPQTDLAMLNYIANYIIRNNKVNKDFVNKHTVFKEGVTDIG-YGLRPDHPLQKA---  target    ALTNYVTLKPDAEIKGNPPPPPFTAGGQVVPTELRDAWGDFVWWDRATGRPRPVSRDEVGARFDGDPALLGEFEVELVDG 3o5a.1    ------------------------------------------------------------------------AK-N-ASD  target    STVPVRPAFDLLKQYLDESFDLRTASEVCRVPPQAIQSIARQLAANKRETLLAAGMGPNHYFQNDLFGRVQFLVAALTDN 3o5a.1    PGAAKVITFDEFAKFVS-KYDADYVSKLSAVPKAKLDQLAELYADPNIKVMSLWTMGFNQHTRGTWANNMVYNLHLLTGK  target    IGHLGGNVGSYAGNYRGSV-FQAMGQWIAEDPFAIEPDLTKPATVKRYYKAESAHYWNYGERPLRAVAKDDEGDLTKGEV 3o5a.1    IATPGNSPFSLTGQPSACGTAREVGTFSH--RLPADMVVTNPKHREE-----AERIWKLPPGTI-P----DKPGY--DAV  target    LTGKSHMPTPTKLIWFGNSNSLLGNAKWSFDVVKNTLPRQDAVFCNEWHWTSSCEYADLVFPADSWAEFKLPDATASCTN 3o5a.1    LQNRMLKDGKLNAYWVQVNNNMQAAANLMEEGLPGYRNPANFIVVSDAYPTVTALAADLVLPSAMWVEKE---GAYGNAE  target    PFLLAFPTTPLKRLYDTRSDYEALALTAKALGELIDEPRMEQYWRGILDGDPTPYLQRIFSGSNATRGITYDELHESSKR 3o5a.1    RRTQFW-HQLVDAPGEARSDLWQLVEFAKRFKVE----------------------------------------------  target    GVPLLMNMRTYPRSGGWEQRQEDKPWYTATGRLEFYRPEPEFQAAGESLPVWREPVDATFYEPNAILSNAAHPSIAPRAP 3o5a.1    --------------------------------------------------------------------------------  target    EDYGVPESQLDVETRQYRNVVRTWAELQQTLHPLQERDPAFRFVFQTPKYRWGAHSTAVDADWISMLFGPFGDPYRRDPR 3o5a.1    --------------------------------------------------------------------------------  target    MPWTGEAYLEINPKDAA 3o5a.1    ----------------- ``` | | | | | | | | | | | | | | | | | | | | | | | | | | | | | | | | | | | | | | | | | | | | | | | | | |
|  | 2nya.1.A | Periplasmic nitrate reductase  *Crystal structure of the periplasmic nitrate reductase (NAP) from Escherichia coli* | 0.30 |  | 15.85 | 0.55 | 1-589 | X-ray | 2.50 | monomer | 1 x SF4, 1 x 6MO, 2 x MGD | HHblits | 0.28 |
| ``` target    SWDEATTIAAKTMEDVARTFNGDEGARKLLAQGYHPEMVEVMHGAGVQALKLRGGMPLLGIGRIFGFYRFANMLALLDRK 2nya.1    TWDQAFDVMEEKFKTALKE--------------KGPESIGMFGS-G----------------------QWTIWEGYAASK  target    LRPDAPADEILGSRTFDNYAWHTD--LPPGHPMVTGSQTVDFDLFSAEHTKLLLIIGMNWICTKMPDGHWIGDAR--LKG 2nya.1    L-----FKAGFRSNNIDPNARHCMASAVVGFMRTFGMDEPMGCYDDIEQADAFVLWGANMAEMHPILWSRITNRRLSNQN  target    TRVIVISADYMPTANKADEVIILRPGTDAAFFLGVARELIEKGLYDRAAVIERTDLPLLVRLDTGERLDARDVIPGYELA 2nya.1    VTVAVLSTYQHRSFELADNGIIFTPQSDLVILNYIANYIIQNNAINQDFFSKHVNLRKGATD-IGYGLRPTHPLEKA---  target    ALTNYVTLKPDAEIKGNPPPPPFTAGGQVVPTELRDAWGDFVWWDRATGRPRPVSRDEVGARFDGDPALLGEFEVELVDG 2nya.1    ------------------------------------------------------------------------A---KNPG  target    STVPVRPAFDLLKQYLDESFDLRTASEVCRVPPQAIQSIARQLAANKRETLLAAGMGPNHYFQNDLFGRVQFLVAALTDN 2nya.1    SDASEPMSFEDYKAFVA-EYTLEKTAEMTGVPKDQLEQLAQLYADPNKKVISYWTMGFNQHTRGVWANNLVYNLHLLTGK  target    IGHLGGNVGSYAGNY--RGSVFQAMGQWIAEDPFAIEPDLTKPATVKRYYKAESAHYWNYGERPLRAVAKDDEGDLTKGE 2nya.1    ISQPGCGPFSLTGQPSACGTA-REVGTFAHR--LPADMVVTNEKHRDIC-----EKKWNIPSGTI-P----AKIGLHAVA  target    VLTGKSHMPTPTKLIWFGNSNSLLGNAKWSFDVVKNTLPRQDAVFCNEWHWTSSCEYADLVFPADSWAEFKLPDATASCT 2nya.1    QDR--ALKDGKLNVYWTMCTNNMQAGPNINEERMPGWRDPRNFIIVSDPYPTVSALAADLILPTAMWVEKE--G-AYGNA  target    NPFLLAFPTTPLKRLYDTRSDYEALALTAKALGELIDEPRMEQYWRGILDGDPTPYLQRIFSGSNATRGITYDELHESSK 2nya.1    ERRTQFW-RQQVQAPGEAKSDLWQLVQFSRRFKTE---------------------------------------------  target    RGVPLLMNMRTYPRSGGWEQRQEDKPWYTATGRLEFYRPEPEFQAAGESLPVWREPVDATFYEPNAILSNAAHPSIAPRA 2nya.1    --------------------------------------------------------------------------------  target    PEDYGVPESQLDVETRQYRNVVRTWAELQQTLHPLQERDPAFRFVFQTPKYRWGAHSTAVDADWISMLFGPFGDPYRRDP 2nya.1    --------------------------------------------------------------------------------  target    RMPWTGEAYLEINPKDAA 2nya.1    ------------------ ``` | | | | | | | | | | | | | | | | | | | | | | | | | | | | | | | | | | | | | | | | | | | | | | | | | |
|  | 2v45.1.A | PERIPLASMIC NITRATE REDUCTASE  *A NEW CATALYTIC MECHANISM OF PERIPLASMIC NITRATE REDUCTASE FROM DESULFOVIBRIO DESULFURICANS ATCC 27774 FROM CRYSTALLOGRAPHIC AND EPR DATA AND BASED ON DETAILED ANALYSIS OF THE SIXTH LIGAND* | 0.29 |  | 19.71 | 0.52 | 1-589 | X-ray | 2.40 | monomer | 1 x SF4, 1 x MO, 2 x MGD, 1 x LCP | HHblits | 0.29 |
| ``` target    SWDEATTIAAKTMEDVARTFNGDEGARKLLAQGYHPEMVEVMHGAGVQALKLRGGMPLLGIGRIFGFYRFANMLALLDRK 2v45.1    SWDEALDLMASRFRSSIDM--------------YGPNSVAWYGSG-----------------------QCLTEESYVANK  target    LRPDAPADEILGSRTFDNYAWHTDL--PPGHPMVTGSQTVDFDLFSAEHTKLLLIIGMNWICTKMPDGHWIGDAR--LKG 2v45.1    I-----FKGGFGTNNVDGNPRLCMASAVGGYVTSFGKDEPMGTYADIDQATCFFIIGSNTSEAHPVLFRRIARRKQVEPG  target    TRVIVISADYMPTANKADEVIILRPGTDAAFFLGVARELIEKGLYDRAAVIERTDLPLLVRLDTGERLDARDVIPGYELA 2v45.1    VKIIVADPRRTNTSRIADMHVAFRPGTDLAFMHSMAWVIINEELDNPRFWQRYVNF--------------------MDA-  target    ALTNYVTLKPDAEIKGNPPPPPFTAGGQVVPTELRDAWGDFVWWDRATGRPRPVSRDEVGARFDGDPALLGEFEVELVDG 2v45.1    --------------------------------------------------------------------------------  target    STVPVRPAFDLLKQYLDESFDLRTASEVCRVPPQAIQSIARQLAANKRETLLAAGMGPNHYFQNDLFGRVQFLVAALTDN 2v45.1    --EGKPSDFEGYKAFLE-NYRPEKVAEICRVPVEQIYGAARAFAESAATMS-LWCMGINQRVQGVFANNLIHNLHLITGQ  target    IGHLGGNVGSYAGN--YRGSVFQAMGQWIAEDPFAIEPDLTKPATVKRYYKAESAHYWNYGERPLRAVAKDDEGDLTKGE 2v45.1    ICRPGATSFSLTGQPNACGGVR-DGGALSH--LLPAGRAIPNAKHRA-----EMEKLWGLPEGRIA-----PEPGYHTVA  target    VLTGKSHMPTPTKLIWFGNSNSLLGNAKWSFDVVKNTLPRQD-AVFCNEWHWT-SSCEYADLVFPADSWAEFKLPDATAS 2v45.1    LFEA--LGRGDVKCMIICETNPAHTLPN--LNKVHKAMSHPESFIVCIEAFPDAVTLEYADLVLPPAFWCER---DGVYG  target    CTNPFLLAFPTTPLKRLYDTRSDYEALALTAKALGELIDEPRMEQYWRGILDGDPTPYLQRIFSGSNATRGITYDELHES 2v45.1    CGERRYSL-TEKAVDPPGQCRPTVNTLVEFARRAGVD-------------------------------------------  target    SKRGVPLLMNMRTYPRSGGWEQRQEDKPWYTATGRLEFYRPEPEFQAAGESLPVWREPVDATFYEPNAILSNAAHPSIAP 2v45.1    --------------------------------------------------------------------------------  target    RAPEDYGVPESQLDVETRQYRNVVRTWAELQQTLHPLQERDPAFRFVFQTPKYRWGAHSTAVDADWISMLFGPFGDPYRR 2v45.1    --------------------------------------------------------------------------------  target    DPRMPWTGEAYLEINPKDAA 2v45.1    -------------------- ``` | | | | | | | | | | | | | | | | | | | | | | | | | | | | | | | | | | | | | | | | | | | | | | | | | |
|  | 2v3v.1.A | PERIPLASMIC NITRATE REDUCTASE  *A NEW CATALYTIC MECHANISM OF PERIPLASMIC NITRATE REDUCTASE FROM DESULFOVIBRIO DESULFURICANS ATCC 27774 FROM CRYSTALLOGRAPHIC AND EPR DATA AND BASED ON DETAILED ANALYSIS OF THE SIXTH LIGAND* | 0.30 |  | 19.24 | 0.52 | 1-589 | X-ray | 1.99 | monomer | 1 x SF4, 1 x MO, 2 x MGD, 4 x LCP | HHblits | 0.28 |
| ``` target    SWDEATTIAAKTMEDVARTFNGDEGARKLLAQGYHPEMVEVMHGAGVQALKLRGGMPLLGIGRIFGFYRFANMLALLDRK 2v3v.1    SWDEALDLMASRFRSSIDM--------------YGPNSVAWYGSG-----------------------QCLTEESYVANK  target    LRPDAPADEILGSRTFDNYAWHTDL--PPGHPMVTGSQTVDFDLFSAEHTKLLLIIGMNWICTKMPDGHWIGDAR--LKG 2v3v.1    I-----FKGGFGTNNVDGNPRLCMASAVGGYVTSFGKDEPMGTYADIDQATCFFIIGSNTSEAHPVLFRRIARRKQVEPG  target    TRVIVISADYMPTANKADEVIILRPGTDAAFFLGVARELIEKGLYDRAAVIERTDLPLLVRLDTGERLDARDVIPGYELA 2v3v.1    VKIIVADPRRTNTSRIADMHVAFRPGTDLAFMHSMAWVIINEELDNPRFWQRYVNF--------------------MDA-  target    ALTNYVTLKPDAEIKGNPPPPPFTAGGQVVPTELRDAWGDFVWWDRATGRPRPVSRDEVGARFDGDPALLGEFEVELVDG 2v3v.1    --------------------------------------------------------------------------------  target    STVPVRPAFDLLKQYLDESFDLRTASEVCRVPPQAIQSIARQLAANKRETLLAAGMGPNHYFQNDLFGRVQFLVAALTDN 2v3v.1    --EGKPSDFEGYKAFLE-NYRPEKVAEICRVPVEQIYGAARAFAESAATMS-LWCMGINQRVQGVFANNLIHNLHLITGQ  target    IGHLGGNVGSYAGN--YRGSVFQAMGQWIAEDPFAIEPDLTKPATVKRYYKAESAHYWNYGERPLRAVAKDDEGDLTKGE 2v3v.1    ICRPGATSFSLTGQPNACGGV-RDGGALSH--LLPAGRAIPNAKHRAEM-----EKLWGLPEGRIA-----PEPGYHTVA  target    VLTGKSHMPTPTKLIWFGNSNSLLGNAKWSFDVVKNTLPRQD-AVFCNEWHWT-SSCEYADLVFPADSWAEFKLPDATAS 2v3v.1    LFEA--LGRGDVKCMIICETNPAHTLPN--LNKVHKAMSHPESFIVCIEAFPDAVTLEYADLVLPPAFWCERD---GVYG  target    CTNPFLLAFPTTPLKRLYDTRSDYEALALTAKALGELIDEPRMEQYWRGILDGDPTPYLQRIFSGSNATRGITYDELHES 2v3v.1    CGERRYSL-TEKAVDPPGQCRPTVNTLVEFARRAGVD-------------------------------------------  target    SKRGVPLLMNMRTYPRSGGWEQRQEDKPWYTATGRLEFYRPEPEFQAAGESLPVWREPVDATFYEPNAILSNAAHPSIAP 2v3v.1    --------------------------------------------------------------------------------  target    RAPEDYGVPESQLDVETRQYRNVVRTWAELQQTLHPLQERDPAFRFVFQTPKYRWGAHSTAVDADWISMLFGPFGDPYRR 2v3v.1    --------------------------------------------------------------------------------  target    DPRMPWTGEAYLEINPKDAA 2v3v.1    -------------------- ``` | | | | | | | | | | | | | | | | | | | | | | | | | | | | | | | | | | | | | | | | | | | | | | | | | |
|  | 1g8j.1.A | ARSENITE OXIDASE  *CRYSTAL STRUCTURE ANALYSIS OF ARSENITE OXIDASE FROM ALCALIGENES FAECALIS* | 0.26 |  | 16.75 | 0.50 | 1-588 | X-ray | 2.03 | hetero-oligomer | 2 x MGD, 1 x O, 1 x 4MO, 1 x F3S, 1 x FES | HHblits | 0.28 |
| ``` target    SWDEATTIAAKTMEDVARTFNGDEGARKLLAQGYHPEMVEVMHGAGVQALKLRGGMPLLGIGRIFGFYRFANMLALLDRK 1g8j.1    TWDHAMALYAGLIKKTLDS--------------DGPQGVFFSCFDHG-------------------GAGGGFENTWGTGK  target    LRPDAPADEILGSRTFDNYA-----WHTDLPPGHPMVTGSQTVDFDLFSAEHTKLLLIIGMNWICTKMPDG--HWI---- 1g8j.1    L-----MFSAIQTPMVRIHNRPAYNSECH----ATREMGIGELNNAYEDAQLADVIWSIGNNPYESQTNYFLNHWLPNLQ  target    -----------GDARLKGTRVIVISADYMPTANKA--------DEVIILRPGTDAAFFLGVARELIEKGLYDRAAVIERT 1g8j.1    GATTSKKKERFPNENFPQARIIFVDPRETPSVAIARHVAGNDRVLHLAIEPGTDTALFNGLFTYVVEQGWIDKPFIEAHT  target    DLPLLVRLDTGERLDARDVIPGYELAALTNYVTLKPDAEIKGNPPPPPFTAGGQVVPTELRDAWGDFVWWDRATGRPRPV 1g8j.1    K--------------------GF---------------------------------------------------------  target    SRDEVGARFDGDPALLGEFEVELVDGSTVPVRPAFDLLKQYLDESFDLRTASEVCRVPPQAIQSIARQLAANK-----RE 1g8j.1    -----------------------------------DDAVK-T-NRLSLDECSNITGVPVDMLKRAAEWSYKPKASGQAPR  target    TLLAAGMGPNHYFQNDLFGRVQFLVAALTDNIGHLGGNVGSYAGNYRGSVFQAMGQWIAEDPFAIEPDLTKPATVKRYYK 1g8j.1    TMHAYEKGIIWGNDNYVIQSALLDLVIATHNVGRRGTGCVRMGGHQEGYT-----R----PPYPGDKKIYIDQELIK-GK  target    AESAHYWNYGERPLRAVAKDDEGDLTKGEVLTGKSHMPTPTKLIWFGNSNSLLGNAKWSFDVVKNTLPRQD-AVFCNEWH 1g8j.1    GRIMTWWGC--NNFQTS---NNAQALREAILQR---SAIVKQAMQKARGATTEEM----VDVIYEATQNGGLFVTSINLY  target    WTSSCEYADLVFPADSWAEFKLPDATASCTNPFLLAFPTTPLKRLYDTRSDYEALALTAKALGELIDEPRMEQYWRGILD 1g8j.1    PTKLAEAAHLMLPAAHPGEM---NLTSMNGERRIRLS-EKFMDPPGTAMADCLIAARIANALRD----------------  target    GDPTPYLQRIFSGSNATRGITYDELHESSKRGVPLLMNMRTYPRSGGWEQRQEDKPWYTATGRLEFYRPEPEFQAAGESL 1g8j.1    --------------------------------------------------------------------------------  target    PVWREPVDATFYEPNAILSNAAHPSIAPRAPEDYGVPESQLDVETRQYRNVVRTWAELQQTLHPLQERDPAFRFVFQTPK 1g8j.1    --------------------------------------------------------------------------------  target    YRWGAHSTAVDADWISMLFGPFGDPYRRDPRMPWTGEAYLEINPKDAA 1g8j.1    ------------------------------------------------ ``` | | | | | | | | | | | | | | | | | | | | | | | | | | | | | | | | | | | | | | | | | | | | | | | | | |
|  | 3ir5.1.A | Respiratory nitrate reductase 1 alpha chain  *Crystal structure of NarGHI mutant NarG-H49C* | 0.19 | 0.00 | 31.10 | 0.42 | 90-451 | X-ray | 2.30 | monomer | 2 x MD1, 1 x 6MO, 4 x SF4, 1 x AGA, 1 x F3S, 2 x HEM | BLAST | 0.36 |
| ``` target    SWDEATTIAAKTMEDVARTFNGDEGARKLLAQGYHPEMVEVMHGAGVQALKLRGGMPLLGIGRIFGFYRFANMLALLDRK 3ir5.1    --------------------------------------------------------------------------------  target    LRPDAPADEILGSRTFDNYAWHTDLPPGHPMVTGSQTVDFDLFSAEHTKLLLIIGMNWICTKMPDGHWIGDARLKGTRVI 3ir5.1    ---------LIGGTCLSFYDWYCDLPPASPQTWGEQTDVPESADWYNSSYIIAWGSNVPQTRTPDAHFFTEVRYKGTKTV  target    VISADYMPTANKADEVIILRPGTDAAFFLGVARELIEKGLYDRAA------VIERTDLPLLVRLDTGERLDARDVIPGYE 3ir5.1    AVTPDYAEIAKLCDLWLAPKQGTDAAMALAMGHVMLREFHLDNPSQYFTDYVRRYTDMPMLVMLE------ERD---GYY  target    LAALTNYVTLKPDAEIKGNPP---PPPFTAGGQVVP--TELRDAWGDFVWW-----DRATGRPRPV------SRDEVGA- 3ir5.1    AAGRMLRAADLVDALGQENNPEWKTVAFNTNGEMVAPNGSIGFRWGEKGKWNLEQRDGKTGEETELQLSLLGSQDEIAEV  target    ---RFDGD-----------PALLGEFEV---ELVDGSTVPVRPAFDL------LKQYLDE-----SFDLRTA------SE 3ir5.1    GFPYFGGDGTEHFNKVELENVLLHKLPVKRLQLADGSTALVTTVYDLTLANYGLERGLNDVNCATSYDDVKAYTPAWAEQ  target    VCRVPPQAIQSIARQLAANKRET----LLAAGMGPNHYFQNDLFGRVQFLVAALTDNIGHLGGNVGSYAGNYRGSVFQAM 3ir5.1    ITGVSRSQIIRIAREFADNADKTHGRSMIIVGAGLNHWYHLDMNYRGLINMLIFCGCVGQSGGGWAHYVGQEK---LRPQ  target    GQWIAEDPFAIEPDLTKPATVKRYYKAESAHYWNYGERPLRAVAKDDEGDLTKGEVLTGKSHMPTPTKLIWFGNSNSLLG 3ir5.1    TGW---QPLAFALDWQRPA---RHMNSTSYFY------------------------------------------------  target    NAKWSFDVVKNTLPRQDAVFCNEWHWTSSCEYADLVFPADSWAEFKLPDATASCTNPFLLAFPTTPLKRLYDTRSDYEAL 3ir5.1    --------------------------------------------------------------------------------  target    ALTAKALGELIDEPRMEQYWRGILDGDPTPYLQRIFSGSNATRGITYDELHESSKRGVPLLMNMRTYPRSGGWEQRQEDK 3ir5.1    --------------------------------------------------------------------------------  target    PWYTATGRLEFYRPEPEFQAAGESLPVWREPVDATFYEPNAILSNAAHPSIAPRAPEDYGVPESQLDVETRQYRNVVRTW 3ir5.1    --------------------------------------------------------------------------------  target    AELQQTLHPLQERDPAFRFVFQTPKYRWGAHSTAVDADWISMLFGPFGDPYRRDPRMPWTGEAYLEINPKDAA 3ir5.1    ------------------------------------------------------------------------- ``` | | | | | | | | | | | | | | | | | | | | | | | | | | | | | | | | | | | | | | | | | | | | | | | | | |
|  | 1r27.4.A | Respiratory nitrate reductase 1 alpha chain  *Crystal Structure of NarGH complex* | 0.19 | 0.00 | 31.10 | 0.42 | 90-451 | X-ray | 2.00 | monomer | 4 x MO, 16 x SF4, 8 x MGD, 4 x F3S | BLAST | 0.36 |
| ``` target    SWDEATTIAAKTMEDVARTFNGDEGARKLLAQGYHPEMVEVMHGAGVQALKLRGGMPLLGIGRIFGFYRFANMLALLDRK 1r27.4    --------------------------------------------------------------------------------  target    LRPDAPADEILGSRTFDNYAWHTDLPPGHPMVTGSQTVDFDLFSAEHTKLLLIIGMNWICTKMPDGHWIGDARLKGTRVI 1r27.4    ---------LIGGTCLSFYDWYCDLPPASPQTWGEQTDVPESADWYNSSYIIAWGSNVPQTRTPDAHFFTEVRYKGTKTV  target    VISADYMPTANKADEVIILRPGTDAAFFLGVARELIEKGLYDRAA------VIERTDLPLLVRLDTGERLDARDVIPGYE 1r27.4    AVTPDYAEIAKLCDLWLAPKQGTDAAMALAMGHVMLREFHLDNPSQYFTDYVRRYTDMPMLVMLE------ERD---GYY  target    LAALTNYVTLKPDAEIKGNPP---PPPFTAGGQVVP--TELRDAWGDFVWW-----DRATGRPRPV------SRDEVGA- 1r27.4    AAGRMLRAADLVDALGQENNPEWKTVAFNTNGEMVAPNGSIGFRWGEKGKWNLEQRDGKTGEETELQLSLLGSQDEIAEV  target    ---RFDGD-----------PALLGEFEV---ELVDGSTVPVRPAFDL------LKQYLDE-----SFDLRTA------SE 1r27.4    GFPYFGGDGTEHFNKVELENVLLHKLPVKRLQLADGSTALVTTVYDLTLANYGLERGLNDVNCATSYDDVKAYTPAWAEQ  target    VCRVPPQAIQSIARQLAANKRET----LLAAGMGPNHYFQNDLFGRVQFLVAALTDNIGHLGGNVGSYAGNYRGSVFQAM 1r27.4    ITGVSRSQIIRIAREFADNADKTHGRSMIIVGAGLNHWYHLDMNYRGLINMLIFCGCVGQSGGGWAHYVGQEK---LRPQ  target    GQWIAEDPFAIEPDLTKPATVKRYYKAESAHYWNYGERPLRAVAKDDEGDLTKGEVLTGKSHMPTPTKLIWFGNSNSLLG 1r27.4    TGW---QPLAFALDWQRPA---RHMNSTSYFY------------------------------------------------  target    NAKWSFDVVKNTLPRQDAVFCNEWHWTSSCEYADLVFPADSWAEFKLPDATASCTNPFLLAFPTTPLKRLYDTRSDYEAL 1r27.4    --------------------------------------------------------------------------------  target    ALTAKALGELIDEPRMEQYWRGILDGDPTPYLQRIFSGSNATRGITYDELHESSKRGVPLLMNMRTYPRSGGWEQRQEDK 1r27.4    --------------------------------------------------------------------------------  target    PWYTATGRLEFYRPEPEFQAAGESLPVWREPVDATFYEPNAILSNAAHPSIAPRAPEDYGVPESQLDVETRQYRNVVRTW 1r27.4    --------------------------------------------------------------------------------  target    AELQQTLHPLQERDPAFRFVFQTPKYRWGAHSTAVDADWISMLFGPFGDPYRRDPRMPWTGEAYLEINPKDAA 1r27.4    ------------------------------------------------------------------------- ``` | | | | | | | | | | | | | | | | | | | | | | | | | | | | | | | | | | | | | | | | | | | | | | | | | |
| ✓ | 3ir7.1.A | Respiratory nitrate reductase 1 alpha chain  *Crystal structure of NarGHI mutant NarG-R94S* | 0.19 | 0.00 | 31.10 | 0.42 | 90-451 | X-ray | 2.50 | monomer | 2 x MD1, 4 x SF4, 1 x 6MO, 1 x AGA, 1 x F3S, 2 x HEM | BLAST | 0.36 |
| ``` target    SWDEATTIAAKTMEDVARTFNGDEGARKLLAQGYHPEMVEVMHGAGVQALKLRGGMPLLGIGRIFGFYRFANMLALLDRK 3ir7.1    --------------------------------------------------------------------------------  target    LRPDAPADEILGSRTFDNYAWHTDLPPGHPMVTGSQTVDFDLFSAEHTKLLLIIGMNWICTKMPDGHWIGDARLKGTRVI 3ir7.1    ---------LIGGTCLSFYDWYCDLPPASPQTWGEQTDVPESADWYNSSYIIAWGSNVPQTRTPDAHFFTEVRYKGTKTV  target    VISADYMPTANKADEVIILRPGTDAAFFLGVARELIEKGLYDRAA------VIERTDLPLLVRLDTGERLDARDVIPGYE 3ir7.1    AVTPDYAEIAKLCDLWLAPKQGTDAAMALAMGHVMLREFHLDNPSQYFTDYVRRYTDMPMLVMLE------ERD---GYY  target    LAALTNYVTLKPDAEIKGNPP---PPPFTAGGQVVP--TELRDAWGDFVWW-----DRATGRPRPV------SRDEVGA- 3ir7.1    AAGRMLRAADLVDALGQENNPEWKTVAFNTNGEMVAPNGSIGFRWGEKGKWNLEQRDGKTGEETELQLSLLGSQDEIAEV  target    ---RFDGD-----------PALLGEFEV---ELVDGSTVPVRPAFDL------LKQYLDE-----SFDLRTA------SE 3ir7.1    GFPYFGGDGTEHFNKVELENVLLHKLPVKRLQLADGSTALVTTVYDLTLANYGLERGLNDVNCATSYDDVKAYTPAWAEQ  target    VCRVPPQAIQSIARQLAANKRET----LLAAGMGPNHYFQNDLFGRVQFLVAALTDNIGHLGGNVGSYAGNYRGSVFQAM 3ir7.1    ITGVSRSQIIRIAREFADNADKTHGRSMIIVGAGLNHWYHLDMNYRGLINMLIFCGCVGQSGGGWAHYVGQEK---LRPQ  target    GQWIAEDPFAIEPDLTKPATVKRYYKAESAHYWNYGERPLRAVAKDDEGDLTKGEVLTGKSHMPTPTKLIWFGNSNSLLG 3ir7.1    TGW---QPLAFALDWQRPA---RHMNSTSYFY------------------------------------------------  target    NAKWSFDVVKNTLPRQDAVFCNEWHWTSSCEYADLVFPADSWAEFKLPDATASCTNPFLLAFPTTPLKRLYDTRSDYEAL 3ir7.1    --------------------------------------------------------------------------------  target    ALTAKALGELIDEPRMEQYWRGILDGDPTPYLQRIFSGSNATRGITYDELHESSKRGVPLLMNMRTYPRSGGWEQRQEDK 3ir7.1    --------------------------------------------------------------------------------  target    PWYTATGRLEFYRPEPEFQAAGESLPVWREPVDATFYEPNAILSNAAHPSIAPRAPEDYGVPESQLDVETRQYRNVVRTW 3ir7.1    --------------------------------------------------------------------------------  target    AELQQTLHPLQERDPAFRFVFQTPKYRWGAHSTAVDADWISMLFGPFGDPYRRDPRMPWTGEAYLEINPKDAA 3ir7.1    ------------------------------------------------------------------------- ``` | | | | | | | | | | | | | | | | | | | | | | | | | | | | | | | | | | | | | | | | | | | | | | | | | |
|  | 1q16.1.A | Respiratory nitrate reductase 1 alpha chain  *Crystal structure of Nitrate Reductase A, NarGHI, from Escherichia coli* | 0.19 | 0.00 | 31.10 | 0.42 | 90-451 | X-ray | 1.90 | monomer | 2 x MD1, 1 x 6MO, 2 x HEM, 4 x SF4, 1 x F3S, 1 x AGA, 1 x 3PH | BLAST | 0.36 |
| ``` target    SWDEATTIAAKTMEDVARTFNGDEGARKLLAQGYHPEMVEVMHGAGVQALKLRGGMPLLGIGRIFGFYRFANMLALLDRK 1q16.1    --------------------------------------------------------------------------------  target    LRPDAPADEILGSRTFDNYAWHTDLPPGHPMVTGSQTVDFDLFSAEHTKLLLIIGMNWICTKMPDGHWIGDARLKGTRVI 1q16.1    ---------LIGGTCLSFYDWYCDLPPASPQTWGEQTDVPESADWYNSSYIIAWGSNVPQTRTPDAHFFTEVRYKGTKTV  target    VISADYMPTANKADEVIILRPGTDAAFFLGVARELIEKGLYDRAA------VIERTDLPLLVRLDTGERLDARDVIPGYE 1q16.1    AVTPDYAEIAKLCDLWLAPKQGTDAAMALAMGHVMLREFHLDNPSQYFTDYVRRYTDMPMLVMLE------ERD---GYY  target    LAALTNYVTLKPDAEIKGNPP---PPPFTAGGQVVP--TELRDAWGDFVWW-----DRATGRPRPV------SRDEVGA- 1q16.1    AAGRMLRAADLVDALGQENNPEWKTVAFNTNGEMVAPNGSIGFRWGEKGKWNLEQRDGKTGEETELQLSLLGSQDEIAEV  target    ---RFDGD-----------PALLGEFEV---ELVDGSTVPVRPAFDL------LKQYLDE-----SFDLRTA------SE 1q16.1    GFPYFGGDGTEHFNKVELENVLLHKLPVKRLQLADGSTALVTTVYDLTLANYGLERGLNDVNCATSYDDVKAYTPAWAEQ  target    VCRVPPQAIQSIARQLAANKRET----LLAAGMGPNHYFQNDLFGRVQFLVAALTDNIGHLGGNVGSYAGNYRGSVFQAM 1q16.1    ITGVSRSQIIRIAREFADNADKTHGRSMIIVGAGLNHWYHLDMNYRGLINMLIFCGCVGQSGGGWAHYVGQEK---LRPQ  target    GQWIAEDPFAIEPDLTKPATVKRYYKAESAHYWNYGERPLRAVAKDDEGDLTKGEVLTGKSHMPTPTKLIWFGNSNSLLG 1q16.1    TGW---QPLAFALDWQRPA---RHMNSTSYFY------------------------------------------------  target    NAKWSFDVVKNTLPRQDAVFCNEWHWTSSCEYADLVFPADSWAEFKLPDATASCTNPFLLAFPTTPLKRLYDTRSDYEAL 1q16.1    --------------------------------------------------------------------------------  target    ALTAKALGELIDEPRMEQYWRGILDGDPTPYLQRIFSGSNATRGITYDELHESSKRGVPLLMNMRTYPRSGGWEQRQEDK 1q16.1    --------------------------------------------------------------------------------  target    PWYTATGRLEFYRPEPEFQAAGESLPVWREPVDATFYEPNAILSNAAHPSIAPRAPEDYGVPESQLDVETRQYRNVVRTW 1q16.1    --------------------------------------------------------------------------------  target    AELQQTLHPLQERDPAFRFVFQTPKYRWGAHSTAVDADWISMLFGPFGDPYRRDPRMPWTGEAYLEINPKDAA 1q16.1    ------------------------------------------------------------------------- ``` | | | | | | | | | | | | | | | | | | | | | | | | | | | | | | | | | | | | | | | | | | | | | | | | | |
|  | 3ir6.1.A | Respiratory nitrate reductase 1 alpha chain  *Crystal structure of NarGHI mutant NarG-H49S* | 0.18 | 0.00 | 31.10 | 0.42 | 90-451 | X-ray | 2.80 | monomer | 2 x GDP, 1 x AGA, 3 x SF4, 1 x F3S, 2 x HEM | BLAST | 0.36 |
| ``` target    SWDEATTIAAKTMEDVARTFNGDEGARKLLAQGYHPEMVEVMHGAGVQALKLRGGMPLLGIGRIFGFYRFANMLALLDRK 3ir6.1    --------------------------------------------------------------------------------  target    LRPDAPADEILGSRTFDNYAWHTDLPPGHPMVTGSQTVDFDLFSAEHTKLLLIIGMNWICTKMPDGHWIGDARLKGTRVI 3ir6.1    ---------LIGGTCLSFYDWYCDLPPASPQTWGEQTDVPESADWYNSSYIIAWGSNVPQTRTPDAHFFTEVRYKGTKTV  target    VISADYMPTANKADEVIILRPGTDAAFFLGVARELIEKGLYDRAA------VIERTDLPLLVRLDTGERLDARDVIPGYE 3ir6.1    AVTPDYAEIAKLCDLWLAPKQGTDAAMALAMGHVMLREFHLDNPSQYFTDYVRRYTDMPMLVMLE------ERD---GYY  target    LAALTNYVTLKPDAEIKGNPP---PPPFTAGGQVVP--TELRDAWGDFVWW-----DRATGRPRPV------SRDEVGA- 3ir6.1    AAGRMLRAADLVDALGQENNPEWKTVAFNTNGEMVAPNGSIGFRWGEKGKWNLEQRDGKTGEETELQLSLLGSQDEIAEV  target    ---RFDGD-----------PALLGEFEV---ELVDGSTVPVRPAFDL------LKQYLDE-----SFDLRTA------SE 3ir6.1    GFPYFGGDGTEHFNKVELENVLLHKLPVKRLQLADGSTALVTTVYDLTLANYGLERGLNDVNCATSYDDVKAYTPAWAEQ  target    VCRVPPQAIQSIARQLAANKRET----LLAAGMGPNHYFQNDLFGRVQFLVAALTDNIGHLGGNVGSYAGNYRGSVFQAM 3ir6.1    ITGVSRSQIIRIAREFADNADKTHGRSMIIVGAGLNHWYHLDMNYRGLINMLIFCGCVGQSGGGWAHYVGQEK---LRPQ  target    GQWIAEDPFAIEPDLTKPATVKRYYKAESAHYWNYGERPLRAVAKDDEGDLTKGEVLTGKSHMPTPTKLIWFGNSNSLLG 3ir6.1    TGW---QPLAFALDWQRPA---RHMNSTSYFY------------------------------------------------  target    NAKWSFDVVKNTLPRQDAVFCNEWHWTSSCEYADLVFPADSWAEFKLPDATASCTNPFLLAFPTTPLKRLYDTRSDYEAL 3ir6.1    --------------------------------------------------------------------------------  target    ALTAKALGELIDEPRMEQYWRGILDGDPTPYLQRIFSGSNATRGITYDELHESSKRGVPLLMNMRTYPRSGGWEQRQEDK 3ir6.1    --------------------------------------------------------------------------------  target    PWYTATGRLEFYRPEPEFQAAGESLPVWREPVDATFYEPNAILSNAAHPSIAPRAPEDYGVPESQLDVETRQYRNVVRTW 3ir6.1    --------------------------------------------------------------------------------  target    AELQQTLHPLQERDPAFRFVFQTPKYRWGAHSTAVDADWISMLFGPFGDPYRRDPRMPWTGEAYLEINPKDAA 3ir6.1    ------------------------------------------------------------------------- ``` | | | | | | | | | | | | | | | | | | | | | | | | | | | | | | | | | | | | | | | | | | | | | | | | | |
|  | 3egw.1.A | Respiratory nitrate reductase 1 alpha chain  *The crystal structure of the NarGHI mutant NarH - C16A* | 0.19 | 0.00 | 31.66 | 0.42 | 90-451 | X-ray | 1.90 | monomer | 2 x MD1, 2 x MGD, 2 x 6MO, 6 x SF4, 4 x F3S, 2 x 3PH, 4 x HEM, 2 x AGA | BLAST | 0.36 |
| ``` target    SWDEATTIAAKTMEDVARTFNGDEGARKLLAQGYHPEMVEVMHGAGVQALKLRGGMPLLGIGRIFGFYRFANMLALLDRK 3egw.1    --------------------------------------------------------------------------------  target    LRPDAPADEILGSRTFDNYAWHTDLPPGHPMVTGSQTVDFDLFSAEHTKLLLIIGMNWICTKMPDGHWIGDARLKGTRVI 3egw.1    ---------LIGGTCLSFYDWYCDLPPASPQTWGEQTDVPESADWYNSSYIIAWGSNVPQTRTPDAHFFTEVRYKGTKTV  target    VISADYMPTANKADEVIILRPGTDAAFFLGVARELIEKGLYDRAA------VIERTDLPLLVRLD-------TGERLDAR 3egw.1    AVTPDYAEIAKLCDLWLAPKQGTDAAMALAMGHVMLREFHLDNPSQYFTDYVRRYTDMPMLVMLEERDGYYAAGRMLRAA  target    DVIPGYELAALTNYVTLKPDAEIKGNP--PPPPFTAGGQVVP--TELRDAWGDFVWW-----DRATGRPRPV------SR 3egw.1    DLV-----AALGQ----------ENNPEWKTVAFNTNGEMVAPNGSIGFRWGEKGKWNLEQRDGKTGEETELQLSLLGSQ  target    DEVGA----RFDGD-----------PALLGEFEV---ELVDGSTVPVRPAFDL------LKQYLDE-----SFDLRTA-- 3egw.1    DEIAEVGFPYFGGDGTEHFNKVELENVLLHKLPVKRLQLADGSTALVTTVYDLTLANYGLERGLNDVNCATSYDDVKAYT  target    ----SEVCRVPPQAIQSIARQLAANKRET----LLAAGMGPNHYFQNDLFGRVQFLVAALTDNIGHLGGNVGSYAGNYRG 3egw.1    PAWAEQITGVSRSQIIRIAREFADNADKTHGRSMIIVGAGLNHWYHLDMNYRGLINMLIFCGCVGQSGGGWAHYVGQEK-  target    SVFQAMGQWIAEDPFAIEPDLTKPATVKRYYKAESAHYWNYGERPLRAVAKDDEGDLTKGEVLTGKSHMPTPTKLIWFGN 3egw.1    --LRPQTGW---QPLAFALDWQRPA---RHMNSTSYFY------------------------------------------  target    SNSLLGNAKWSFDVVKNTLPRQDAVFCNEWHWTSSCEYADLVFPADSWAEFKLPDATASCTNPFLLAFPTTPLKRLYDTR 3egw.1    --------------------------------------------------------------------------------  target    SDYEALALTAKALGELIDEPRMEQYWRGILDGDPTPYLQRIFSGSNATRGITYDELHESSKRGVPLLMNMRTYPRSGGWE 3egw.1    --------------------------------------------------------------------------------  target    QRQEDKPWYTATGRLEFYRPEPEFQAAGESLPVWREPVDATFYEPNAILSNAAHPSIAPRAPEDYGVPESQLDVETRQYR 3egw.1    --------------------------------------------------------------------------------  target    NVVRTWAELQQTLHPLQERDPAFRFVFQTPKYRWGAHSTAVDADWISMLFGPFGDPYRRDPRMPWTGEAYLEINPKDAA 3egw.1    ------------------------------------------------------------------------------- ``` | | | | | | | | | | | | | | | | | | | | | | | | | | | | | | | | | | | | | | | | | | | | | | | | | |
|  | 7t2r.1.A | NiFe hydrogenase subunit A  *Structure of electron bifurcating Ni-Fe hydrogenase complex HydABCSL in FMN-free apo state* | 0.22 |  | 13.15 | 0.45 | 1-589 | EM | 0.00 | hetero-2-2-2-2-2-mer | 6 x FES, 12 x SF4, 2 x 3NI, 2 x FCO | HHblits | 0.26 |
| ``` target    SWDEATTIAAKTMEDVARTFNGDEGARKLLAQGYHPEMVEVMHGAGVQALKLRGGMPLLGIGRIFGFYRFANMLALLDRK 7t2r.1    TWEEALALASAEFKKAYDQE--------------KAG--------------------------AILSSLCTDEELTLFSA  target    LRPDAPADEILGSRTFDNYAWHTDLP--PGHPMV--TGSQTVDFDLFSAEHTKLLLIIGMNWICTKMPDGHWIGDAR-LK 7t2r.1    L-----FRNALKMKHIDTFDGDIIRGFFKGFMPFREQGV-RPFTAAHHILDSDLIITMFADPQKEAPVVASYIRVACLHR  target    GTRVIVISADYMPTANKADEVIILRPGTDAAFFLGVARELIEKGLYDRAAVIERTDLPLLVRLDTGERLDARDVIPGYEL 7t2r.1    NAKLMNLSYGPSPFPGLVDLDIRLPEGQAVPKALSNLAEIIGKISLGPSDMASFGE-----------------FEAG---  target    AALTNYVTLKPDAEIKGNPPPPPFTAGGQVVPTELRDAWGDFVWWDRATGRPRPVSRDEVGARFDGDPALLGEFEVELVD 7t2r.1    --------------------------------------------------------------------------------  target    GSTVPVRPAFDLLKQYLDESFDLRTASEVCRVPPQAIQSIARQLAANKRETLLAAGMGPNHYFQNDLFGRVQFLVAALTD 7t2r.1    ---------AGKALSSY--RESIEESARAMGLDPKIAEEVALMLISARRPIFI-IGGRA---TKSHELVTAACNLAVASK  target    NIGHLGGNVGSYAGNYRGSVFQAMGQWIAEDPFAIEPDLTKPATVKRYYKAESAHYWNYGERPLRAVAKDDEGDLTKGEV 7t2r.1    AFFEDGLGVVPLLVSAN-----SLGAR---------NTVVSE-------------------NP---------------W-  target    LTGKSHMPTPTKLIWFGNSNSLLGNAKWSFDVVKNTLPRQDAVFCNEWHWT-SSCEYADLVFPADSWAEFKLPDATASCT 7t2r.1    -LG----RERRDFLYVFSTAMV---PE--EEEILAAISATRFVVVQTPFKVRPLVNLADILLPAPAWYER---SGHFCTI  target    NPFLLAFPTTPLKRLYDTRSDYEALALTAKALGELIDEPRMEQYWRGILDGDPTPYLQRIFSGSNATRGITYDELHESSK 7t2r.1    EGERRKL-NTIVPPKGEIKSLHYVMDEFAKKLGVK---------------------------------------------  target    RGVPLLMNMRTYPRSGGWEQRQEDKPWYTATGRLEFYRPEPEFQAAGESLPVWREPVDATFYEPNAILSNAAHPSIAPRA 7t2r.1    --------------------------------------------------------------------------------  target    PEDYGVPESQLDVETRQYRNVVRTWAELQQTLHPLQERDPAFRFVFQTPKYRWGAHSTAVDADWISMLFGPFGDPYRRDP 7t2r.1    --------------------------------------------------------------------------------  target    RMPWTGEAYLEINPKDAA 7t2r.1    ------------------ ``` | | | | | | | | | | | | | | | | | | | | | | | | | | | | | | | | | | | | | | | | | | | | | | | | | |
|  | 7t30.1.A | NiFe hydrogenase subunit A  *Structure of electron bifurcating Ni-Fe hydrogenase complex HydABCSL in FMN/NAD(H) bound state* | 0.21 |  | 13.15 | 0.45 | 1-589 | EM | 0.00 | hetero-2-2-2-2-2-mer | 4 x FES, 12 x SF4, 2 x NAD, 2 x FMN, 2 x 3NI, 2 x FCO | HHblits | 0.26 |
| ``` target    SWDEATTIAAKTMEDVARTFNGDEGARKLLAQGYHPEMVEVMHGAGVQALKLRGGMPLLGIGRIFGFYRFANMLALLDRK 7t30.1    TWEEALALASAEFKKAYDQE--------------KAG--------------------------AILSSLCTDEELTLFSA  target    LRPDAPADEILGSRTFDNYAWHTDLP--PGHPMV--TGSQTVDFDLFSAEHTKLLLIIGMNWICTKMPDGHWIGDAR-LK 7t30.1    L-----FRNALKMKHIDTFDGDIIRGFFKGFMPFREQGV-RPFTAAHHILDSDLIITMFADPQKEAPVVASYIRVACLHR  target    GTRVIVISADYMPTANKADEVIILRPGTDAAFFLGVARELIEKGLYDRAAVIERTDLPLLVRLDTGERLDARDVIPGYEL 7t30.1    NAKLMNLSYGPSPFPGLVDLDIRLPEGQAVPKALSNLAEIIGKISLGPSDMASFGE-----------------FEAG---  target    AALTNYVTLKPDAEIKGNPPPPPFTAGGQVVPTELRDAWGDFVWWDRATGRPRPVSRDEVGARFDGDPALLGEFEVELVD 7t30.1    --------------------------------------------------------------------------------  target    GSTVPVRPAFDLLKQYLDESFDLRTASEVCRVPPQAIQSIARQLAANKRETLLAAGMGPNHYFQNDLFGRVQFLVAALTD 7t30.1    ---------AGKALSSY--RESIEESARAMGLDPKIAEEVALMLISARRPIFI-IGGRA---TKSHELVTAACNLAVASK  target    NIGHLGGNVGSYAGNYRGSVFQAMGQWIAEDPFAIEPDLTKPATVKRYYKAESAHYWNYGERPLRAVAKDDEGDLTKGEV 7t30.1    AFFEDGLGVVPLLVSAN-----SLGAR---------NTVVSE-------------------NP---------------W-  target    LTGKSHMPTPTKLIWFGNSNSLLGNAKWSFDVVKNTLPRQDAVFCNEWHWT-SSCEYADLVFPADSWAEFKLPDATASCT 7t30.1    -LG----RERRDFLYVFSTAMV---PE--EEEILAAISATRFVVVQTPFKVRPLVNLADILLPAPAWYER---SGHFCTI  target    NPFLLAFPTTPLKRLYDTRSDYEALALTAKALGELIDEPRMEQYWRGILDGDPTPYLQRIFSGSNATRGITYDELHESSK 7t30.1    EGERRKL-NTIVPPKGEIKSLHYVMDEFAKKLGVK---------------------------------------------  target    RGVPLLMNMRTYPRSGGWEQRQEDKPWYTATGRLEFYRPEPEFQAAGESLPVWREPVDATFYEPNAILSNAAHPSIAPRA 7t30.1    --------------------------------------------------------------------------------  target    PEDYGVPESQLDVETRQYRNVVRTWAELQQTLHPLQERDPAFRFVFQTPKYRWGAHSTAVDADWISMLFGPFGDPYRRDP 7t30.1    --------------------------------------------------------------------------------  target    RMPWTGEAYLEINPKDAA 7t30.1    ------------------ ``` | | | | | | | | | | | | | | | | | | | | | | | | | | | | | | | | | | | | | | | | | | | | | | | | | |
|  | 7p63.1.C | NADH-quinone oxidoreductase  *Complex I from E. coli, DDM/LMNG-purified, under Turnover at pH 6, Closed state* | 0.21 |  | 17.72 | 0.41 | 1-588 | EM | 0.00 | hetero-1-1-1-1-1-1-… | 7 x SF4, 1 x FMN, 1 x NAI, 2 x FES, 1 x CA, 1 x DCQ, 4 x LFA, 8 x 3PE | HHblits | 0.27 |
| ``` target    SWDEATTIAAKTMEDVARTFNGDEGARKLLAQGYHPEMVEVMHGAGVQALKLRGGMPLLGIGRIFGFYRFANMLALLDRK 7p63.1    NAEQAMQGAADILRQSKKVIG-------------------------------------------IGSPRASVESNFALRE  target    LRPDAPADEILGSRTFDNYAWHTDLP---PGHPMVTGSQTVDFDLFSAEHTKLLLIIGMNWICTKMPDGHWIGDARLKGT 7p63.1    L---------VGEENFYTGIAHGEQERLQLALKVLREGGIYTPALREIESYDAVLVLGEDVTQTGARVALAVRQAVKGKA  target    R--------------------------VIVISADYMPTANKADEVIILRPGTDAAFFLGVARELIEKGLYDRAAVIERTD 7p63.1    REMAAAQKVADWQIAAILNIGQRAKHPLFVTNVDDTRLDDIAAWTYRAPVEDQARLGFAIAHALDNSAP-----------  target    LPLLVRLDTGERLDARDVIPGYELAALTNYVTLKPDAEIKGNPPPPPFTAGGQVVPTELRDAWGDFVWWDRATGRPRPVS 7p63.1    --------------------------------------------------------------------------------  target    RDEVGARFDGDPALLGEFEVELVDGSTVPVRPAFDLLKQYLDESFDLRTASEVCRVPPQAIQSIARQLAANKRETLLAAG 7p63.1    ---------------------------------------AV-DGIEPEL--------QSKIDVIVQALAGAKKPLI-ISG  target    MGPNHYFQNDLFGRVQFLVAALTDNIGHLGGNVGSYAG-NYRGSVFQAMGQWIAEDPFAIEPDLTKPATVKRYYKAESAH 7p63.1    TNAG----SLEVIQAAANVAKALKGRGADVGITMIARSVNSMG-------LG----IMG----G----------------  target    YWNYGERPLRAVAKDDEGDLTKGEVLTGKSHMPTPTKLIWFGNSNSLLGNAKWSFDVVKNTLPRQDAVFCNEWHWTSSCE 7p63.1    -------------------GSLEEALTEL--ETGRADAVVVLE-NDLHRHAS--ATRVNAALAKAPLVMVVDHQRTAIME  target    YADLVFPADSWAEFKLPDATASCTNPFLLAFPTTPLKRLY-----DTRSDYEALALTAKALGELIDEPRMEQYWRGILDG 7p63.1    NAHLVLSAASFAES---DGTVINNEGRAQRF-FQVYDPAYYDSKTVMLESWRWLHSLHSTLLS-----------------  target    DPTPYLQRIFSGSNATRGITYDELHESSKRGVPLLMNMRTYPRSGGWEQRQEDKPWYTATGRLEFYRPEPEFQAAGESLP 7p63.1    --------------------------------------------------------------------------------  target    VWREPVDATFYEPNAILSNAAHPSIAPRAPEDYGVPESQLDVETRQYRNVVRTWAELQQTLHPLQERDPAFRFVFQTPKY 7p63.1    --------------------------------------------------------------------------------  target    RWGAHSTAVDADWISMLFGPFGDPYRRDPRMPWTGEAYLEINPKDAA 7p63.1    ----------------------------------------------- ``` | | | | | | | | | | | | | | | | | | | | | | | | | | | | | | | | | | | | | | | | | | | | | | | | | |
|  | 7nz1.1.E | NADH-quinone oxidoreductase subunit G  *Respiratory complex I from Escherichia coli - focused refinement of cytoplasmic arm* | 0.22 |  | 17.72 | 0.41 | 1-588 | EM | 0.00 | hetero-1-1-1-1-1-1-… | 7 x SF4, 2 x FES, 1 x FMN, 1 x CA | HHblits | 0.27 |
| ``` target    SWDEATTIAAKTMEDVARTFNGDEGARKLLAQGYHPEMVEVMHGAGVQALKLRGGMPLLGIGRIFGFYRFANMLALLDRK 7nz1.1    NAEQAMQGAADILRQSKKVIG-------------------------------------------IGSPRASVESNFALRE  target    LRPDAPADEILGSRTFDNYAWHTDLP---PGHPMVTGSQTVDFDLFSAEHTKLLLIIGMNWICTKMPDGHWIGDARLKGT 7nz1.1    L---------VGEENFYTGIAHGEQERLQLALKVLREGGIYTPALREIESYDAVLVLGEDVTQTGARVALAVRQAVKGKA  target    R--------------------------VIVISADYMPTANKADEVIILRPGTDAAFFLGVARELIEKGLYDRAAVIERTD 7nz1.1    REMAAAQKVADWQIAAILNIGQRAKHPLFVTNVDDTRLDDIAAWTYRAPVEDQARLGFAIAHALDNSAP-----------  target    LPLLVRLDTGERLDARDVIPGYELAALTNYVTLKPDAEIKGNPPPPPFTAGGQVVPTELRDAWGDFVWWDRATGRPRPVS 7nz1.1    --------------------------------------------------------------------------------  target    RDEVGARFDGDPALLGEFEVELVDGSTVPVRPAFDLLKQYLDESFDLRTASEVCRVPPQAIQSIARQLAANKRETLLAAG 7nz1.1    ---------------------------------------AV-DGIEPEL--------QSKIDVIVQALAGAKKPLI-ISG  target    MGPNHYFQNDLFGRVQFLVAALTDNIGHLGGNVGSYAG-NYRGSVFQAMGQWIAEDPFAIEPDLTKPATVKRYYKAESAH 7nz1.1    TNAG----SLEVIQAAANVAKALKGRGADVGITMIARSVNSM-------GLG----IMG----G----------------  target    YWNYGERPLRAVAKDDEGDLTKGEVLTGKSHMPTPTKLIWFGNSNSLLGNAKWSFDVVKNTLPRQDAVFCNEWHWTSSCE 7nz1.1    -------------------GSLEEALTEL--ETGRADAVVVLE-NDLHRHAS--AIRVNAALAKAPLVMVVDHQRTAIME  target    YADLVFPADSWAEFKLPDATASCTNPFLLAFPTTPLKRLY-----DTRSDYEALALTAKALGELIDEPRMEQYWRGILDG 7nz1.1    NAHLVLSAASFAES---DGTVINNEGRAQRF-FQVYDPAYYDSKTVMLESWRWLHSLHSTLLS-----------------  target    DPTPYLQRIFSGSNATRGITYDELHESSKRGVPLLMNMRTYPRSGGWEQRQEDKPWYTATGRLEFYRPEPEFQAAGESLP 7nz1.1    --------------------------------------------------------------------------------  target    VWREPVDATFYEPNAILSNAAHPSIAPRAPEDYGVPESQLDVETRQYRNVVRTWAELQQTLHPLQERDPAFRFVFQTPKY 7nz1.1    --------------------------------------------------------------------------------  target    RWGAHSTAVDADWISMLFGPFGDPYRRDPRMPWTGEAYLEINPKDAA 7nz1.1    ----------------------------------------------- ``` | | | | | | | | | | | | | | | | | | | | | | | | | | | | | | | | | | | | | | | | | | | | | | | | | |
|  | 7p61.1.C | NADH-quinone oxidoreductase  *Complex I from E. coli, DDM-purified, with NADH, Resting state* | 0.21 |  | 17.72 | 0.41 | 1-588 | EM | 0.00 | hetero-1-1-1-1-1-1-… | 7 x SF4, 1 x FMN, 1 x NAI, 2 x FES, 1 x CA, 2 x 3PE, 1 x UQ8 | HHblits | 0.27 |
| ``` target    SWDEATTIAAKTMEDVARTFNGDEGARKLLAQGYHPEMVEVMHGAGVQALKLRGGMPLLGIGRIFGFYRFANMLALLDRK 7p61.1    NAEQAMQGAADILRQSKKVI--------------------G-----------------------IGSPRASVESNFALRE  target    LRPDAPADEILGSRTFDNYAWHTDLP---PGHPMVTGSQTVDFDLFSAEHTKLLLIIGMNWICTKMPDGHWIGDARLKGT 7p61.1    L---------VGEENFYTGIAHGEQERLQLALKVLREGGIYTPALREIESYDAVLVLGEDVTQTGARVALAVRQAVKGKA  target    R--------------------------VIVISADYMPTANKADEVIILRPGTDAAFFLGVARELIEKGLYDRAAVIERTD 7p61.1    REMAAAQKVADWQIAAILNIGQRAKHPLFVTNVDDTRLDDIAAWTYRAPVEDQARLGFAIAHALDNSAP-----------  target    LPLLVRLDTGERLDARDVIPGYELAALTNYVTLKPDAEIKGNPPPPPFTAGGQVVPTELRDAWGDFVWWDRATGRPRPVS 7p61.1    --------------------------------------------------------------------------------  target    RDEVGARFDGDPALLGEFEVELVDGSTVPVRPAFDLLKQYLDESFDLRTASEVCRVPPQAIQSIARQLAANKRETLLAAG 7p61.1    ---------------------------------------AV-DGIEPEL--------QSKIDVIVQALAGAKKPLI-ISG  target    MGPNHYFQNDLFGRVQFLVAALTDNIGHLGGNVGSYAG-NYRGSVFQAMGQWIAEDPFAIEPDLTKPATVKRYYKAESAH 7p61.1    TNAG----SLEVIQAAANVAKALKGRGADVGITMIARSVNSMG-------LG----IMG----G----------------  target    YWNYGERPLRAVAKDDEGDLTKGEVLTGKSHMPTPTKLIWFGNSNSLLGNAKWSFDVVKNTLPRQDAVFCNEWHWTSSCE 7p61.1    -------------------GSLEEALTEL--ETGRADAVVVLE-NDLHRHAS--ATRVNAALAKAPLVMVVDHQRTAIME  target    YADLVFPADSWAEFKLPDATASCTNPFLLAFPTTPLKRLY-----DTRSDYEALALTAKALGELIDEPRMEQYWRGILDG 7p61.1    NAHLVLSAASFAES---DGTVINNEGRAQRF-FQVYDPAYYDSKTVMLESWRWLHSLHSTLLS-----------------  target    DPTPYLQRIFSGSNATRGITYDELHESSKRGVPLLMNMRTYPRSGGWEQRQEDKPWYTATGRLEFYRPEPEFQAAGESLP 7p61.1    --------------------------------------------------------------------------------  target    VWREPVDATFYEPNAILSNAAHPSIAPRAPEDYGVPESQLDVETRQYRNVVRTWAELQQTLHPLQERDPAFRFVFQTPKY 7p61.1    --------------------------------------------------------------------------------  target    RWGAHSTAVDADWISMLFGPFGDPYRRDPRMPWTGEAYLEINPKDAA 7p61.1    ----------------------------------------------- ``` | | | | | | | | | | | | | | | | | | | | | | | | | | | | | | | | | | | | | | | | | | | | | | | | | |
|  | 7zm7.1.I | NADH-ubiquinone oxidoreductase-like protein  *CryoEM structure of mitochondrial complex I from Chaetomium thermophilum (inhibited by DDM)* | 0.19 |  | 16.08 | 0.38 | 1-589 | EM | 0.00 | hetero-1-1-1-1-1-1-… | 4 x PC1, 14 x LMT, 5 x CDL, 8 x 3PE, 2 x FES, 6 x SF4, 1 x FMN, 1 x NDP, 1 x ZN, 2 x ZMP | HHblits | 0.28 |
| ``` target    SWDEATTIAAKTMEDVARTFNGDEGARKLLAQGYHPEMVEVMHGAGVQALKLRGGMPLLGIGRIFGFYRFANMLALLDRK 7zm7.1    TWEQALTEIAHAYQTLAPKE--------------NE--FKVI-----------------------AGQLVEVESLVAMKD  target    LRPDAPADEILGSRTFDNYAWHTDLPPGHPMVTGSQ-TVDFDLFSAEHTKLLLIIGMNWICTKMPDGHWIGDAR-LKGTR 7zm7.1    LA------NRLGSENLALDFPGGSQPLAHGVDIRSNYLFNSKIWGIEEADAILLVGTNPRHEAAVLNARIRKQWLRSDLE  target    VIVISADYMPTANKADEVIILRPGTDAAFFLGVARELIEKGLYDRAAVIERTDLPLLVRLDTGERLDARDVIPGYELAAL 7zm7.1    IAAVGQPWESTFDYEH------LGTDLAALKNALSGPF------------------------------------------  target    TNYVTLKPDAEIKGNPPPPPFTAGGQVVPTELRDAWGDFVWWDRATGRPRPVSRDEVGARFDGDPALLGEFEVELVDGST 7zm7.1    --------------------------------------------------------------------------------  target    VPVRPAFDLLKQYLDESFDLRTASEVCRVPPQAIQSIARQLAANKRETLLAAGMGPNHYFQNDLFGRVQFLVAALTD--N 7zm7.1    -------------------------------------GEKLKKAKRP-MIIVGSGVTEHPDAKAFYETVWSFVEKNASNF  target    IGHLGGNVGSYAGNYRGSVFQAMGQWIAEDPFAIEPDLTKPATVKRYYKAESAHYWNYGERPLRAVAKDDEGDLTKGEVL 7zm7.1    LTEEWCGYNVLQRAAS-----RAGAF----------------EV----------GFV----V---P---------SPEV-  target    TGKSHMPTPTKLIWFGNSNSLLGNAKWSFDVVKNTLPRQDAVFCNEWHWTSSCEYADLVFPADSWAEFKLPDATASCTNP 7zm7.1    -----AATKPKFVWLLGADEFDP----------ADVPKDAFIVYQGHHGDRGAEIADIVLPGAAYTEK---AGTYVNTEG  target    FLLAFPTTPLKRLYDTRSDYEALALTAKALGELIDEPRMEQYWRGILDGDPTPYLQRIFSGSNATRGITYDELHESSKRG 7zm7.1    RVQMT-RAATGLPGAARTDWKIIRAVSEFLGVP-----------------------------------------------  target    VPLLMNMRTYPRSGGWEQRQEDKPWYTATGRLEFYRPEPEFQAAGESLPVWREPVDATFYEPNAILSNAAHPSIAPRAPE 7zm7.1    --------------------------------------------------------------------------------  target    DYGVPESQLDVETRQYRNVVRTWAELQQTLHPLQERDPAFRFVFQTPKYRWGAHSTAVDADWISMLFGPFGDPYRRDPRM 7zm7.1    --------------------------------------------------------------------------------  target    PWTGEAYLEINPKDAA 7zm7.1    ---------------- ``` | | | | | | | | | | | | | | | | | | | | | | | | | | | | | | | | | | | | | | | | | | | | | | | | | |
|  | 6zr2.1.G | NADH-ubiquinone oxidoreductase 75 kDa subunit, mitochondrial  *Cryo-EM structure of respiratory complex I in the active state from Mus musculus at 3.1 A* | 0.19 | 0.00 | 11.46 | 0.39 | 1-589 | EM | 3.10 | monomer | 6 x SF4, 4 x PC1, 2 x FES, 1 x FMN, 9 x 3PE, 7 x CDL, 1 x ATP, 1 x NDP, 1 x ZN, 2 x EHZ | HHblits | 0.25 |
| ``` target    SWDEATTIAAKTMEDVARTFNGDEGARKLLAQGYHPEMVEVMHGAGVQALKLRGGMPLLGIGRIFGFYRFANMLALLDRK 6zr2.1    SWEDALSRVAGMLQNF------------------EGNAVAAI-----------------------AGGLVDAEALVALKD  target    LRPDAPADEILGSRTFDNYAWHTDLPPGHPMVTGSQ-TVDFDLFSAEHTKLLLIIGMNWICTKMPDGHWIGDAR-LKGTR 6zr2.1    LLN------KVDSDNLCTEEIFPTEGA--GTDLRSNYLLNTTIAGVEEADVVLLVGTNPRFEAPLFNARIRKSWLHNDLK  target    VIVISADYMPTANKADEVIILRPGTDAAFFLGVARELIEKGLYDRAAVIERTDLPLLVRLDTGERLDARDVIPGYELAAL 6zr2.1    VALIGSPVDLTYRYDHLGDSP------------------------KILQDIAS--------------------G------  target    TNYVTLKPDAEIKGNPPPPPFTAGGQVVPTELRDAWGDFVWWDRATGRPRPVSRDEVGARFDGDPALLGEFEVELVDGST 6zr2.1    --------------------------------------------------------------------------------  target    VPVRPAFDLLKQYLDESFDLRTASEVCRVPPQAIQSIARQLAANKRETLLAAGMGPNHYFQNDLFGRVQFLVAALTDNIG 6zr2.1    ---------------------------------RHSFCEVLKDAKKPMV-VLGSSALQRDDGAAILVAVSNMVQKIRVTT  target    HLGGNVGSYAGNYRGSVFQAMGQWIAEDPFAIEPDLTKPATVKRYYKAESAHYWNYGERPLRAVAKDDEGDLTKGEVLTG 6zr2.1    GVAAEWKVMN------------IL------H---RIASQVAA-----------LDL---GYK-------PGV--EAI---  target    KSHMPTPTKLIWFGNSNSLLGNAKWSFDVVKNTLPRQDAVFCNEWHWTSSCEYADLVFPADSWAEFKLPDATASCTNPFL 6zr2.1    ---RKNPPKMLFLLGADGG--------CITRQDLPKDCFIVYQGHHGDVGAPMADVILPGAAYTEK---SATYVNTEGRA  target    LAFPTTPLKRLYDTRSDYEALALTAKALGELIDEPRMEQYWRGILDGDPTPYLQRIFSGSNATRGITYDELHESSKRGVP 6zr2.1    QQT-KVAVTPPGLAREDWKIIRALSEIAGIT-------------------------------------------------  target    LLMNMRTYPRSGGWEQRQEDKPWYTATGRLEFYRPEPEFQAAGESLPVWREPVDATFYEPNAILSNAAHPSIAPRAPEDY 6zr2.1    --------------------------------------------------------------------------------  target    GVPESQLDVETRQYRNVVRTWAELQQTLHPLQERDPAFRFVFQTPKYRWGAHSTAVDADWISMLFGPFGDPYRRDPRMPW 6zr2.1    --------------------------------------------------------------------------------  target    TGEAYLEINPKDAA 6zr2.1    -------------- ``` | | | | | | | | | | | | | | | | | | | | | | | | | | | | | | | | | | | | | | | | | | | | | | | | | |
|  | 6g72.1.G | NADH-ubiquinone oxidoreductase 75 kDa subunit, mitochondrial  *Mouse mitochondrial complex I in the deactive state* | 0.18 | 0.00 | 11.46 | 0.39 | 1-589 | EM | 0.00 | monomer | 6 x SF4, 2 x FES, 1 x FMN, 1 x ADP, 1 x NDP, 1 x ZN, 2 x EHZ | HHblits | 0.25 |
| ``` target    SWDEATTIAAKTMEDVARTFNGDEGARKLLAQGYHPEMVEVMHGAGVQALKLRGGMPLLGIGRIFGFYRFANMLALLDRK 6g72.1    SWEDALSRVAGMLQNF------------------EGNAVAAI-----------------------AGGLVDAEALVALKD  target    LRPDAPADEILGSRTFDNYAWHTDLPPGHPMVTGSQ-TVDFDLFSAEHTKLLLIIGMNWICTKMPDGHWIGDAR-LKGTR 6g72.1    LLN------KVDSDNLCTEEIFPTEGA--GTDLRSNYLLNTTIAGVEEADVVLLVGTNPRFEAPLFNARIRKSWLHNDLK  target    VIVISADYMPTANKADEVIILRPGTDAAFFLGVARELIEKGLYDRAAVIERTDLPLLVRLDTGERLDARDVIPGYELAAL 6g72.1    VALIGSPVDLTYRYDHLGDSP------------------------KILQDIAS--------------------G------  target    TNYVTLKPDAEIKGNPPPPPFTAGGQVVPTELRDAWGDFVWWDRATGRPRPVSRDEVGARFDGDPALLGEFEVELVDGST 6g72.1    --------------------------------------------------------------------------------  target    VPVRPAFDLLKQYLDESFDLRTASEVCRVPPQAIQSIARQLAANKRETLLAAGMGPNHYFQNDLFGRVQFLVAALTDNIG 6g72.1    ---------------------------------RHSFCEVLKDAKKPMV-VLGSSALQRDDGAAILVAVSNMVQKIRVTT  target    HLGGNVGSYAGNYRGSVFQAMGQWIAEDPFAIEPDLTKPATVKRYYKAESAHYWNYGERPLRAVAKDDEGDLTKGEVLTG 6g72.1    GVAAEWKVMN------------IL------H---RIASQVAA-----------LDL---GYK-------PGV--EAI---  target    KSHMPTPTKLIWFGNSNSLLGNAKWSFDVVKNTLPRQDAVFCNEWHWTSSCEYADLVFPADSWAEFKLPDATASCTNPFL 6g72.1    ---RKNPPKMLFLLGADGG--------CITRQDLPKDCFIVYQGHHGDVGAPMADVILPGAAYTEK---SATYVNTEGRA  target    LAFPTTPLKRLYDTRSDYEALALTAKALGELIDEPRMEQYWRGILDGDPTPYLQRIFSGSNATRGITYDELHESSKRGVP 6g72.1    QQT-KVAVTPPGLAREDWKIIRALSEIAGIT-------------------------------------------------  target    LLMNMRTYPRSGGWEQRQEDKPWYTATGRLEFYRPEPEFQAAGESLPVWREPVDATFYEPNAILSNAAHPSIAPRAPEDY 6g72.1    --------------------------------------------------------------------------------  target    GVPESQLDVETRQYRNVVRTWAELQQTLHPLQERDPAFRFVFQTPKYRWGAHSTAVDADWISMLFGPFGDPYRRDPRMPW 6g72.1    --------------------------------------------------------------------------------  target    TGEAYLEINPKDAA 6g72.1    -------------- ``` | | | | | | | | | | | | | | | | | | | | | | | | | | | | | | | | | | | | | | | | | | | | | | | | | |
|  | 7ak6.1.G | NADH-ubiquinone oxidoreductase 75 kDa subunit, mitochondrial  *Cryo-EM structure of ND6-P25L mutant respiratory complex I from Mus musculus at 3.8 A* | 0.18 | 0.00 | 11.46 | 0.39 | 1-589 | EM | 0.00 | monomer | 6 x SF4, 1 x PC1, 2 x FES, 1 x FMN, 4 x 3PE, 2 x CDL, 1 x ATP, 1 x NDP, 1 x ZN, 2 x EHZ | HHblits | 0.25 |
| ``` target    SWDEATTIAAKTMEDVARTFNGDEGARKLLAQGYHPEMVEVMHGAGVQALKLRGGMPLLGIGRIFGFYRFANMLALLDRK 7ak6.1    SWEDALSRVAGMLQNF------------------EGNAVAAI-----------------------AGGLVDAEALVALKD  target    LRPDAPADEILGSRTFDNYAWHTDLPPGHPMVTGSQ-TVDFDLFSAEHTKLLLIIGMNWICTKMPDGHWIGDAR-LKGTR 7ak6.1    LLN------KVDSDNLCTEEIFPTEGA--GTDLRSNYLLNTTIAGVEEADVVLLVGTNPRFEAPLFNARIRKSWLHNDLK  target    VIVISADYMPTANKADEVIILRPGTDAAFFLGVARELIEKGLYDRAAVIERTDLPLLVRLDTGERLDARDVIPGYELAAL 7ak6.1    VALIGSPVDLTYRYDHLGDSP------------------------KILQDIAS--------------------G------  target    TNYVTLKPDAEIKGNPPPPPFTAGGQVVPTELRDAWGDFVWWDRATGRPRPVSRDEVGARFDGDPALLGEFEVELVDGST 7ak6.1    --------------------------------------------------------------------------------  target    VPVRPAFDLLKQYLDESFDLRTASEVCRVPPQAIQSIARQLAANKRETLLAAGMGPNHYFQNDLFGRVQFLVAALTDNIG 7ak6.1    ---------------------------------RHSFCEVLKDAKKPMV-VLGSSALQRDDGAAILVAVSNMVQKIRVTT  target    HLGGNVGSYAGNYRGSVFQAMGQWIAEDPFAIEPDLTKPATVKRYYKAESAHYWNYGERPLRAVAKDDEGDLTKGEVLTG 7ak6.1    GVAAEWKVMN------------IL------H---RIASQVAA-----------LDL---GYK-------PGV--EAI---  target    KSHMPTPTKLIWFGNSNSLLGNAKWSFDVVKNTLPRQDAVFCNEWHWTSSCEYADLVFPADSWAEFKLPDATASCTNPFL 7ak6.1    ---RKNPPKMLFLLGADGG--------CITRQDLPKDCFIVYQGHHGDVGAPMADVILPGAAYTEK---SATYVNTEGRA  target    LAFPTTPLKRLYDTRSDYEALALTAKALGELIDEPRMEQYWRGILDGDPTPYLQRIFSGSNATRGITYDELHESSKRGVP 7ak6.1    QQT-KVAVTPPGLAREDWKIIRALSEIAGIT-------------------------------------------------  target    LLMNMRTYPRSGGWEQRQEDKPWYTATGRLEFYRPEPEFQAAGESLPVWREPVDATFYEPNAILSNAAHPSIAPRAPEDY 7ak6.1    --------------------------------------------------------------------------------  target    GVPESQLDVETRQYRNVVRTWAELQQTLHPLQERDPAFRFVFQTPKYRWGAHSTAVDADWISMLFGPFGDPYRRDPRMPW 7ak6.1    --------------------------------------------------------------------------------  target    TGEAYLEINPKDAA 7ak6.1    -------------- ``` | | | | | | | | | | | | | | | | | | | | | | | | | | | | | | | | | | | | | | | | | | | | | | | | | |
|  | 7ak5.1.G | NADH-ubiquinone oxidoreductase 75 kDa subunit, mitochondrial  *Cryo-EM structure of respiratory complex I in the deactive state from Mus musculus at 3.2 A* | 0.18 | 0.00 | 11.46 | 0.39 | 1-589 | EM | 0.00 | monomer | 6 x SF4, 2 x PC1, 2 x FES, 1 x FMN, 8 x 3PE, 4 x CDL, 1 x ATP, 1 x NDP, 1 x ZN, 2 x EHZ | HHblits | 0.25 |
| ``` target    SWDEATTIAAKTMEDVARTFNGDEGARKLLAQGYHPEMVEVMHGAGVQALKLRGGMPLLGIGRIFGFYRFANMLALLDRK 7ak5.1    SWEDALSRVAGMLQNF------------------EGNAVAAI-----------------------AGGLVDAEALVALKD  target    LRPDAPADEILGSRTFDNYAWHTDLPPGHPMVTGSQ-TVDFDLFSAEHTKLLLIIGMNWICTKMPDGHWIGDAR-LKGTR 7ak5.1    LLN------KVDSDNLCTEEIFPTEG--AGTDLRSNYLLNTTIAGVEEADVVLLVGTNPRFEAPLFNARIRKSWLHNDLK  target    VIVISADYMPTANKADEVIILRPGTDAAFFLGVARELIEKGLYDRAAVIERTDLPLLVRLDTGERLDARDVIPGYELAAL 7ak5.1    VALIGSPVDLTYRYDHLGDSP------------------------KILQDIAS--------------------G------  target    TNYVTLKPDAEIKGNPPPPPFTAGGQVVPTELRDAWGDFVWWDRATGRPRPVSRDEVGARFDGDPALLGEFEVELVDGST 7ak5.1    --------------------------------------------------------------------------------  target    VPVRPAFDLLKQYLDESFDLRTASEVCRVPPQAIQSIARQLAANKRETLLAAGMGPNHYFQNDLFGRVQFLVAALTDNIG 7ak5.1    ---------------------------------RHSFCEVLKDAKKPMV-VLGSSALQRDDGAAILVAVSNMVQKIRVTT  target    HLGGNVGSYAGNYRGSVFQAMGQWIAEDPFAIEPDLTKPATVKRYYKAESAHYWNYGERPLRAVAKDDEGDLTKGEVLTG 7ak5.1    GVAAEWKVMN------------IL------H---RIASQVAA-----------LDL---GYK-------PGV--EAI---  target    KSHMPTPTKLIWFGNSNSLLGNAKWSFDVVKNTLPRQDAVFCNEWHWTSSCEYADLVFPADSWAEFKLPDATASCTNPFL 7ak5.1    ---RKNPPKMLFLLGADGG--------CITRQDLPKDCFIVYQGHHGDVGAPMADVILPGAAYTEK---SATYVNTEGRA  target    LAFPTTPLKRLYDTRSDYEALALTAKALGELIDEPRMEQYWRGILDGDPTPYLQRIFSGSNATRGITYDELHESSKRGVP 7ak5.1    QQT-KVAVTPPGLAREDWKIIRALSEIAGIT-------------------------------------------------  target    LLMNMRTYPRSGGWEQRQEDKPWYTATGRLEFYRPEPEFQAAGESLPVWREPVDATFYEPNAILSNAAHPSIAPRAPEDY 7ak5.1    --------------------------------------------------------------------------------  target    GVPESQLDVETRQYRNVVRTWAELQQTLHPLQERDPAFRFVFQTPKYRWGAHSTAVDADWISMLFGPFGDPYRRDPRMPW 7ak5.1    --------------------------------------------------------------------------------  target    TGEAYLEINPKDAA 7ak5.1    -------------- ``` | | | | | | | | | | | | | | | | | | | | | | | | | | | | | | | | | | | | | | | | | | | | | | | | | |
|  | 7bkb.1.L | Formylmethanofuran dehydrogenase, subunit B  *Formate dehydrogenase - heterodisulfide reductase - formylmethanofuran dehydrogenase complex from Methanospirillum hungatei (hexameric, composite structure)* | 0.16 |  | 16.49 | 0.35 | 113-589 | EM | 0.00 | hetero-2-2-2-2-2-2-… | 48 x SF4, 4 x FAD, 2 x FES, 4 x 9S8, 4 x ZN, 2 x MO, 4 x MGD | HHblits | 0.27 |
| ``` target    SWDEATTIAAKTMEDVARTFNGDEGARKLLAQGYHPEMVEVMHGAGVQALKLRGGMPLLGIGRIFGFYRFANMLALLDRK 7bkb.1    --------------------------------------------------------------------------------  target    LRPDAPADEILGSRTFDNYAWHTDLPPGHPMVTGSQTVDFDLFSA-EHTKLLLIIGMNWICTKMPDGHWI--------GD 7bkb.1    --------------------------------FDNGYPSCTLGEVKNRADVIVYWGSNPAHAHPRHMSRYSIFPRGFFTG  target    ARLKGTRVIVISADYMPTANKADEVIILRPGTDAAFFLGVARELIEKGLYDRAAVIERTDLPLLVRLDTGERLDARDVIP 7bkb.1    KGQKKRTVIVIDPRFTDTANVADYHLQVKQGHDYELFNAFRMVIHGHG--------------------------------  target    GYELAALTNYVTLKPDAEIKGNPPPPPFTAGGQVVPTELRDAWGDFVWWDRATGRPRPVSRDEVGARFDGDPALLGEFEV 7bkb.1    --------------------------------------------------------------------------------  target    ELVDGSTVPVRPAFDLLKQYLDESFDLRTASEVCRVPPQAIQSIARQLAANKRETLLAAGMGPNHYFQNDLFGRVQF--- 7bkb.1    --------------------------KDLPDEVAGIKKETILEVAEIMKNARFGTT-FFGMGLTHTDGRNHNIDIAISLT  target    ---------LVAALTDNIGHLGGNVGSYAGNYRGSVFQAMGQWIAEDPFAIEPDLTKPATVKRYYKAESAHYWNYGERPL 7bkb.1    RDLNKISKWTIMAMRGHYNIAGPGVVWSWTF----------GF------PYCLDLTKQ-N---H------AHMN----P-  target    RAVAKDDEGDLTKGEVLTGKSHMPTPTKLIWFGNSNSLLGNAKWSFDVVKNTLPRQDAVFCNEWHWTSSCEYADLVFPAD 7bkb.1    ---------GE--TSSVDM--AMRDEVDMFINIGTDAAAHFPI---P-AVKQLKKHPW-VTIDPSINMASEISDLHIPVC  target    SW-AEFKLPDATASCTNPFLLAFPTTPLKRLYDTRSDYEALALTAKALGELIDEPRMEQYWRGILDGDPTPYLQRIFSGS 7bkb.1    ICGVDV---GGIVYRMDNVPIQF-RKVIEPPEGVMDDETLLNKIADRMEEL-----------------------------  target    NATRGITYDELHESSKRGVPLLMNMRTYPRSGGWEQRQEDKPWYTATGRLEFYRPEPEFQAAGESLPVWREPVDATFYEP 7bkb.1    --------------------------------------------------------------------------------  target    NAILSNAAHPSIAPRAPEDYGVPESQLDVETRQYRNVVRTWAELQQTLHPLQERDPAFRFVFQTPKYRWGAHSTAVDADW 7bkb.1    --------------------------------------------------------------------------------  target    ISMLFGPFGDPYRRDPRMPWTGEAYLEINPKDAA 7bkb.1    ---------------------------------- ``` | | | | | | | | | | | | | | | | | | | | | | | | | | | | | | | | | | | | | | | | | | | | | | | | | |
|  | 5t5i.1.B | Tungsten formylmethanofuran dehydrogenase subunit B  *TUNGSTEN-CONTAINING FORMYLMETHANOFURAN DEHYDROGENASE FROM METHANOTHERMOBACTER WOLFEII, ORTHORHOMBIC FORM AT 1.9 A* | 0.17 |  | 16.25 | 0.35 | 120-588 | X-ray | 1.90 | hetero-oligomer | 4 x ZN, 2 x MG, 18 x K, 22 x SF4, 2 x W, 4 x MGD, 2 x H2S, 2 x CA | HHblits | 0.27 |
| ``` target    SWDEATTIAAKTMEDVARTFNGDEGARKLLAQGYHPEMVEVMHGAGVQALKLRGGMPLLGIGRIFGFYRFANMLALLDRK 5t5i.1    --------------------------------------------------------------------------------  target    LRPDAPADEILGSRTFDNYAWHTDLPPGHPMVTGSQTVDFDLFSA-EHTKLLLIIGMNWICTKMPDGHW-------IGDA 5t5i.1    ---------------------------------------CTFGEVKNRADVVVYWGCNPMHAHPRHMSRNVFARGFFRER  target    RLKGTRVIVISADYMPTANKADEVIILRPGTDAAFFLGVARELIEKGLYDRAAVIERTDLPLLVRLDTGERLDARDVIPG 5t5i.1    GRSDRTLIVVDPRKTDSAKLADIHLQLDFDRDYELLDAMRACLLGHE---------------------------------  target    YELAALTNYVTLKPDAEIKGNPPPPPFTAGGQVVPTELRDAWGDFVWWDRATGRPRPVSRDEVGARFDGDPALLGEFEVE 5t5i.1    --------------------------------------------------------------------------------  target    LVDGSTVPVRPAFDLLKQYLDESFDLRTASEVCRVPPQAIQSIARQLAANKRETLLAAGMGPNHYFQNDLFGRVQFLVAA 5t5i.1    --------------------------ILYDEVAGVPREQIEEAVEVLKNAQFGIL-FFGMGITHSRGKHRNIDTAIMMVQ  target    LTDNIGHLGGNVGSYAGNY--RGSVFQAMGQWIAEDPFAIEPDLTKPATVKRYYKAESAHYWNYGERPLRAVAKDDEGDL 5t5i.1    DLNDY--AKWTLIPMRGHYNVTGFN--QVCTWES--GYPYCVD-----------------FSGGE-PRYNP------GET  target    TKGEVLTGKSHMPTPTKLIWFGNSNSLLGNAKWSFDVVKNTLPRQDAVFCNEWHWTSSCEYADLVFPADS-WAEFKLPDA 5t5i.1    GANDLL-----QNREADAMMVIASDPGAHFPQ----RALERMAEIP-VIAIEPHRTPTTEMADIIIPPAIVGMEA---EG  target    TASCTNPFLLAFPTTPLKRLYDTRSDYEALALTAKALGELIDEPRMEQYWRGILDGDPTPYLQRIFSGSNATRGITYDEL 5t5i.1    TAYRMEGVPIRM-KKVVDS--DLLSDREILERLLEKVRE-----------------------------------------  target    HESSKRGVPLLMNMRTYPRSGGWEQRQEDKPWYTATGRLEFYRPEPEFQAAGESLPVWREPVDATFYEPNAILSNAAHPS 5t5i.1    --------------------------------------------------------------------------------  target    IAPRAPEDYGVPESQLDVETRQYRNVVRTWAELQQTLHPLQERDPAFRFVFQTPKYRWGAHSTAVDADWISMLFGPFGDP 5t5i.1    --------------------------------------------------------------------------------  target    YRRDPRMPWTGEAYLEINPKDAA 5t5i.1    ----------------------- ``` | | | | | | | | | | | | | | | | | | | | | | | | | | | | | | | | | | | | | | | | | | | | | | | | | |
|  | 2ivf.1.A | ETHYLBENZENE DEHYDROGENASE ALPHA-SUBUNIT  *ETHYLBENZENE DEHYDROGENASE FROM AROMATOLEUM AROMATICUM* | 0.21 | 0.00 | 39.42 | 0.30 | 121-411 | X-ray | 1.88 | monomer | 1 x MES, 4 x SF4, 1 x MO, 1 x MGD, 1 x MD1, 1 x F3S, 1 x HEM | BLAST | 0.39 |
| ``` target    SWDEATTIAAKTMEDVARTFNGDEGARKLLAQGYHPEMVEVMHGAGVQALKLRGGMPLLGIGRIFGFYRFANMLALLDRK 2ivf.1    --------------------------------------------------------------------------------  target    LRPDAPADEILGSRTFDNYAWHTDLPPGHPMVTGSQTVDFDLFSAEHTKLLLIIGMNWICTKMPDGHWIGDARLKGTRVI 2ivf.1    ----------------------------------------NLLDAE---LIFMTCSNWSYTYPSSYHFLSEARYKGAEVV  target    VISADYMPTANKADEVIILRPGTDAAFFLGVARELIEKGLYDRAAVIERTDLPLLVRLDTGERLDARDVIPGYELAALTN 2ivf.1    VIAPDFNPTTPAADLHVPVRVGSDAAFWLGLSQVMIDEKLFDRQFVCEQTDLPLLVRMDTGKFLSAEDV-----------  target    YVTLKPDAEIKGNPPPPPFTAGGQVVPTELRDAWGDFVWWDRATGRPRPVSRDEVGARFDGDPALLGEFEVELVDGSTVP 2ivf.1    --------------------DGGEA---------KQFYFFDEKAGSVRKASRGTL--KLDFMPALEGTFSARLKNGKTIQ  target    VRPAFDLLKQYLDESFDLRTASEVCRVPPQAIQSIARQLAANKRETLLAAGMGPNHYFQNDLFGRVQFLVAALTDNIGHL 2ivf.1    VRTVFEGLREHLKD-YTPEKASAKCGVPVSLIRELGRKVA--KKRTCSYIGFSSAKSYHGDLMERSLFLAMALSGNWGKP  target    GGNVGSYAGNYRGSVFQAMGQWIAEDPFAIEPDLTKPATVKRYYKAESAHYWNYGERPLRAVAKDDEGDLTKGEVLTGKS 2ivf.1    G--TGAFAWAY---------------------------------------------------------------------  target    HMPTPTKLIWFGNSNSLLGNAKWSFDVVKNTLPRQDAVFCNEWHWTSSCEYADLVFPADSWAEFKLPDATASCTNPFLLA 2ivf.1    --------------------------------------------------------------------------------  target    FPTTPLKRLYDTRSDYEALALTAKALGELIDEPRMEQYWRGILDGDPTPYLQRIFSGSNATRGITYDELHESSKRGVPLL 2ivf.1    --------------------------------------------------------------------------------  target    MNMRTYPRSGGWEQRQEDKPWYTATGRLEFYRPEPEFQAAGESLPVWREPVDATFYEPNAILSNAAHPSIAPRAPEDYGV 2ivf.1    --------------------------------------------------------------------------------  target    PESQLDVETRQYRNVVRTWAELQQTLHPLQERDPAFRFVFQTPKYRWGAHSTAVDADWISMLFGPFGDPYRRDPRMPWTG 2ivf.1    --------------------------------------------------------------------------------  target    EAYLEINPKDAA 2ivf.1    ------------ ``` | | | | | | | | | | | | | | | | | | | | | | | | | | | | | | | | | | | | | | | | | | | | | | | | | |
|  | 6f0k.1.B | Fe-S-cluster-containing hydrogenase  *Alternative complex III* | 0.15 |  | 17.58 | 0.34 | 107-588 | EM | 0.00 | hetero-1-1-1-1-1-1-… | 6 x HEC, 1 x F3S, 3 x SF4 | HHblits | 0.27 |
| ``` target    SWDEATTIAAKTMEDVARTFNGDEGARKLLAQGYHPEMVEVMHGAGVQALKLRGGMPLLGIGRIFGFYRFANMLALLDRK 6f0k.1    --------------------------------------------------------------------------------  target    LRPDAPADEILGSRTFDNYAWHTDLPPGHPMVTGSQTVDFDLFSAEHTKLLLIIGMNWICTK-MPD---G------HWIG 6f0k.1    --------------------------LGLQQAFGRPV--RARYRFSEARVIVSLDADFLGPTDRNFVENTREFAASRRME  target    DARLKGTRVIVISADYMPTANKADEVIILRPGTDAAFFLGVARELIEKGLYDRAAVIERTDLPLLVRLDTGERLDARDVI 6f0k.1    RPEDEISRLYVIESTYTVTGGMADHRLRLRAGDIPAFAAALAAELGVGELRE----------------------------  target    PGYELAALTNYVTLKPDAEIKGNPPPPPFTAGGQVVPTELRDAWGDFVWWDRATGRPRPVSRDEVGARFDGDPALLGEFE 6f0k.1    --------------------------------------------------------------------------------  target    VELVDGSTVPVRPAFDLLKQYLDESFDLRTASEVCRVPPQAIQSIARQLAANKRETLLAAGMGPNHYFQNDLFGRVQFLV 6f0k.1    -----------------------------AGARFAGH--PYVVEIARDLRAAGARGVVLAGETQ---PPA--VHALCAVI  target    AALTDNIGHLGGNVGSYAGNYRGSVFQAMGQWIAEDPFAIEPDLTKPATVKRYYKAESAHYWNYGERPLRAVAKDDEGDL 6f0k.1    NDLLGSLGRTVILH---A-------------LD--E-----PATAQHA-------------------ALA--------EL  target    TKGEVLTGKSHMPTPTKLIWFGNSNSLLGNAKWSFDVVKNTLPRQDAVFCNEWHWTSSCEYADLVFPADSWAEFKLPDAT 6f0k.1    -VQAMQ------AGAVDALLLLNVNPVYDAPA--ALGFAEALAQVPEVIHLGLHVDETARRSTWHLPSTHYLEAW--GDG  target    ASCTNPFLLAFPTTPLKRLYDT-RSDYEALALTAKALGELIDEPRMEQYWRGILDGDPTPYLQRIFSGSNATRGITYDEL 6f0k.1    RA-YDGTL-SVIQPLIAPLYEAAHSPLEVLALLATGEEQ-----------------------------------------  target    HESSKRGVPLLMNMRTYPRSGGWEQRQEDKPWYTATGRLEFYRPEPEFQAAGESLPVWREPVDATFYEPNAILSNAAHPS 6f0k.1    --------------------------------------------------------------------------------  target    IAPRAPEDYGVPESQLDVETRQYRNVVRTWAELQQTLHPLQERDPAFRFVFQTPKYRWGAHSTAVDADWISMLFGPFGDP 6f0k.1    --------------------------------------------------------------------------------  target    YRRDPRMPWTGEAYLEINPKDAA 6f0k.1    ----------------------- ``` | | | | | | | | | | | | | | | | | | | | | | | | | | | | | | | | | | | | | | | | | | | | | | | | | |
|  | 6btm.1.B | Alternative Complex III subunit B  *Structure of Alternative Complex III from Flavobacterium johnsoniae (Wild Type)* | 0.16 |  | 9.70 | 0.33 | 107-587 | EM | 3.40 | hetero-1-1-1-1-1-1-… | 6 x HEC, 1 x F3S, 1 x SF4, 2 x E87 | HHblits | 0.26 |
| ``` target    SWDEATTIAAKTMEDVARTFNGDEGARKLLAQGYHPEMVEVMHGAGVQALKLRGGMPLLGIGRIFGFYRFANMLALLDRK 6btm.1    --------------------------------------------------------------------------------  target    LRPDAPADEILGSRTFDNYAWHTDLPPGHPMVTGSQTVDFDLFSAEHTKLLLIIGMNWICTKMPD--GHWIGDARL---- 6btm.1    --------------------------DAFETVYGER--ALVDYDFSKASLIVSVGADFLGDWQGGGYDAGYAKGRIPQNG  target    KGTRVIVISADYMPTANKADEVIILRPGTDAAFFLGVARELIEKGLYDRAAVIERTDLPLLVRLDTGERLDARDVIPGYE 6btm.1    KMSRHFQFESNMTLSGAAADKRVPMTTADQKQALVQIYNIVVGASVP---------------------------------  target    LAALTNYVTLKPDAEIKGNPPPPPFTAGGQVVPTELRDAWGDFVWWDRATGRPRPVSRDEVGARFDGDPALLGEFEVELV 6btm.1    --------------------------------------------------------------------------------  target    DGSTVPVRPAFDLLKQYLDESFDLRTASEVCRVPPQAIQSIARQLAANKRETLLAAGMGPNHYFQNDLFGRVQFLVAALT 6btm.1    ---------------------VS------LDAKFKAEVVKAAQQLKAAGTKGILVSGIED------KNAQLLVLAINQAL  target    DNIGHLGGNVGSYAGNYRGSVFQAMGQWIAEDPFAIEPDLTKPATVKRYYKAESAHYWNYGERPLRAVAKDDEGDLTKGE 6btm.1    ASEAFSTAGTRQI-----------------------RK--GSNAV-------------------V-------------AQ  target    VLTGKSHMPTPTKLIWFGNSNSLLGNAKWSFDVVKNTLPRQDAVFCNEWHWTSSCEYADLVFPADSWAEFKLPDATASCT 6btm.1    LIKD--MNAGSVHTLIMSGVNPVYTLAD--SASFVSGLKKVKTSVAFSLKEDETAAVSTIAAAAPHYLESWG-D-VEIT-  target    NPFLLAFPTTPLKRLYDTRSDYEALALTAKALGELIDEPRMEQYWRGILDGDPTPYLQRIFSGSNATRGITYDELHESSK 6btm.1    KGT-YSLTQPTIRPIFDTKQFQDVLLSVNGTPG-----------------------------------------------  target    RGVPLLMNMRTYPRSGGWEQRQEDKPWYTATGRLEFYRPEPEFQAAGESLPVWREPVDATFYEPNAILSNAAHPSIAPRA 6btm.1    --------------------------------------------------------------------------------  target    PEDYGVPESQLDVETRQYRNVVRTWAELQQTLHPLQERDPAFRFVFQTPKYRWGAHSTAVDADWISMLFGPFGDPYRRDP 6btm.1    --------------------------------------------------------------------------------  target    RMPWTGEAYLEINPKDAA 6btm.1    ------------------ ``` | | | | | | | | | | | | | | | | | | | | | | | | | | | | | | | | | | | | | | | | | | | | | | | | | |
|  | 7arc.1.F | 75 kDa  *Cryo-EM structure of Polytomella Complex-I (peripheral arm)* | 0.15 |  | 15.35 | 0.30 | 119-589 | EM | 0.00 | hetero-1-1-1-1-1-1-… | 6 x SF4, 2 x FES, 1 x FMN, 1 x NDP, 1 x ZN, 1 x 8Q1 | HHblits | 0.28 |
| ``` target    SWDEATTIAAKTMEDVARTFNGDEGARKLLAQGYHPEMVEVMHGAGVQALKLRGGMPLLGIGRIFGFYRFANMLALLDRK 7arc.1    --------------------------------------------------------------------------------  target    LRPDAPADEILGSRTFDNYAWHTDLPPGHPMVTGSQTVDFDLFSAEHTKLLLIIGMNWICTKMPDGHWIGDARLKGTRVI 7arc.1    --------------------------------------NTTIASIEKADVILLVGTNPRFESPVFNARLRKVFLDGAKVG  target    VISADYMPTANKADEVIILRPGTDAAFFLGVARELIEKGLYDRAAVIERTDLPLLVRLDTGERLDARDVIPGYELAALTN 7arc.1    LVGEKVDLT------YAYQHLGADVAALESLASGK---------------------------------------------  target    YVTLKPDAEIKGNPPPPPFTAGGQVVPTELRDAWGDFVWWDRATGRPRPVSRDEVGARFDGDPALLGEFEVELVDGSTVP 7arc.1    --------------------------------------------------------------------------------  target    VRPAFDLLKQYLDESFDLRTASEVCRVPPQAIQSIARQLAANKRETLLAAGMGPNHYFQNDLFGRVQFLVAALTDNIGHL 7arc.1    --------------------------------GAFFEALKGAKNPVV-IVGSSVLRRDDREAVLKTVNDLVDAAGVVKEG  target    GGNVGSYAGNYRGSVFQAMGQWIAEDPFAIEPDLTKPATVKRYYKAESAHYWNYGERPLRAVAKDDEGDLTKGEVLTGKS 7arc.1    WNGFNVLHDNAS-----RVAAL----------DIG---------------FV-----P--------------SAS-AR--  target    HMPTPTKLIWFGNSNSLLGNAKWSFDVVKNTLPRQDAVFCNEWHWTSSCEYADLVFPADSWAEFKLPDATASCTNPFLLA 7arc.1    TNPVPAKVVYLLGSDDFKD----------EEIPADAFVIYQGHHGDKGAARANVVLPGAAYTEK---ASLFANTEGRVQT  target    FPTTPLKRLYDTRSDYEALALTAKALGELIDEPRMEQYWRGILDGDPTPYLQRIFSGSNATRGITYDELHESSKRGVPLL 7arc.1    T-RTAVPVLGDAREDWKIIRALSEVVGQQ---------------------------------------------------  target    MNMRTYPRSGGWEQRQEDKPWYTATGRLEFYRPEPEFQAAGESLPVWREPVDATFYEPNAILSNAAHPSIAPRAPEDYGV 7arc.1    --------------------------------------------------------------------------------  target    PESQLDVETRQYRNVVRTWAELQQTLHPLQERDPAFRFVFQTPKYRWGAHSTAVDADWISMLFGPFGDPYRRDPRMPWTG 7arc.1    --------------------------------------------------------------------------------  target    EAYLEINPKDAA 7arc.1    ------------ ``` | | | | | | | | | | | | | | | | | | | | | | | | | | | | | | | | | | | | | | | | | | | | | | | | | |
|  | 7dgr.10.A | NADH-ubiquinone oxidoreductase 75 kDa subunit, mitochondrial  *Activity optimized supercomplex state2* | 0.14 |  | 12.65 | 0.30 | 119-589 | EM | 0.00 | monomer |  | HHblits | 0.26 |
| ``` target    SWDEATTIAAKTMEDVARTFNGDEGARKLLAQGYHPEMVEVMHGAGVQALKLRGGMPLLGIGRIFGFYRFANMLALLDRK 7dgr.10   --------------------------------------------------------------------------------  target    LRPDAPADEILGSRTFDNYAWHTDLPPGHPMVTGSQTVDFDLFSAEHTKLLLIIGMNWICTKMPDGHWIGDARL-KGTRV 7dgr.10   --------------------------------------NTTIAGVEEADVVLLVGTNPRFEAPLFNARIRKSWLHNDLKV  target    IVISADYMPTANKADEVIILRPGTDAAFFLGVARELIEKGLYDRAAVIERTDLPLLVRLDTGERLDARDVIPGYELAALT 7dgr.10   ALIGSPVDLTYRYDHLGDSPKILQDI------------------------AS--------------------G-------  target    NYVTLKPDAEIKGNPPPPPFTAGGQVVPTELRDAWGDFVWWDRATGRPRPVSRDEVGARFDGDPALLGEFEVELVDGSTV 7dgr.10   --------------------------------------------------------------------------------  target    PVRPAFDLLKQYLDESFDLRTASEVCRVPPQAIQSIARQLAANKRETLLAAGMGPNHYFQNDLFGRVQFLVAALTDNIGH 7dgr.10   --------------------------------SHPFSQVLQEAKKPMV-ILGSSALQRNDGAAILAAVSNIAQKIRTSSG  target    LGGNVGSYAGNYRGSVFQAMGQWIAEDPFAIEPDLTKPATVKRYYKAESAHYWNYGERPLRAVAKDDEGDLTKGEVLTGK 7dgr.10   VTGDWKVMN--IL-------HR---------I---ASQVAA-----------LDL---GYKP-------G--VEAI----  target    SHMPTPTKLIWFGNSNSLLGNAKWSFDVVKNTLPRQDAVFCNEWHWTSSCEYADLVFPADSWAEFKLPDATASCTNPFLL 7dgr.10   --QKNPPKMLFLLGADGG--------CITRQDLPKDCFIVYQGHHGDVGAPIADVILPGAAYTEK---SATYVNTEGRAQ  target    AFPTTPLKRLYDTRSDYEALALTAKALGELIDEPRMEQYWRGILDGDPTPYLQRIFSGSNATRGITYDELHESSKRGVPL 7dgr.10   QT-KVAVTPPGLAREDWKIIRALSEIAGMT--------------------------------------------------  target    LMNMRTYPRSGGWEQRQEDKPWYTATGRLEFYRPEPEFQAAGESLPVWREPVDATFYEPNAILSNAAHPSIAPRAPEDYG 7dgr.10   --------------------------------------------------------------------------------  target    VPESQLDVETRQYRNVVRTWAELQQTLHPLQERDPAFRFVFQTPKYRWGAHSTAVDADWISMLFGPFGDPYRRDPRMPWT 7dgr.10   --------------------------------------------------------------------------------  target    GEAYLEINPKDAA 7dgr.10   ------------- ``` | | | | | | | | | | | | | | | | | | | | | | | | | | | | | | | | | | | | | | | | | | | | | | | | | |
|  | 5o31.1.8 | NADH-ubiquinone oxidoreductase 75 kDa subunit, mitochondrial  *Mitochondrial complex I in the deactive state* | 0.14 |  | 12.65 | 0.30 | 119-589 | EM | 4.13 | hetero-1-1-1-1-1-1-… | 6 x SF4, 2 x FES, 1 x FMN, 1 x NAP, 1 x ZN | HHblits | 0.26 |
| ``` target    SWDEATTIAAKTMEDVARTFNGDEGARKLLAQGYHPEMVEVMHGAGVQALKLRGGMPLLGIGRIFGFYRFANMLALLDRK 5o31.1    --------------------------------------------------------------------------------  target    LRPDAPADEILGSRTFDNYAWHTDLPPGHPMVTGSQTVDFDLFSAEHTKLLLIIGMNWICTKMPDGHWIGDARL-KGTRV 5o31.1    --------------------------------------NTTIAGVEEADVVLLVGTNPRFEAPLFNARIRKSWLHNDLKV  target    IVISADYMPTANKADEVIILRPGTDAAFFLGVARELIEKGLYDRAAVIERTDLPLLVRLDTGERLDARDVIPGYELAALT 5o31.1    ALIGSPVDLTYRYDHLGDSPKILQDI------------------------AS--------------------G-------  target    NYVTLKPDAEIKGNPPPPPFTAGGQVVPTELRDAWGDFVWWDRATGRPRPVSRDEVGARFDGDPALLGEFEVELVDGSTV 5o31.1    --------------------------------------------------------------------------------  target    PVRPAFDLLKQYLDESFDLRTASEVCRVPPQAIQSIARQLAANKRETLLAAGMGPNHYFQNDLFGRVQFLVAALTDNIGH 5o31.1    --------------------------------SHPFSQVLQEAKKPMV-ILGSSALQRNDGAAILAAVSNIAQKIRTSSG  target    LGGNVGSYAGNYRGSVFQAMGQWIAEDPFAIEPDLTKPATVKRYYKAESAHYWNYGERPLRAVAKDDEGDLTKGEVLTGK 5o31.1    VTGDWKVMN--IL-------HR---------I---ASQVAA-----------LDL---GYKP-------G--VEAI----  target    SHMPTPTKLIWFGNSNSLLGNAKWSFDVVKNTLPRQDAVFCNEWHWTSSCEYADLVFPADSWAEFKLPDATASCTNPFLL 5o31.1    --QKNPPKMLFLLGADGG--------CITRQDLPKDCFIVYQGHHGDVGAPIADVILPGAAYTEK---SATYVNTEGRAQ  target    AFPTTPLKRLYDTRSDYEALALTAKALGELIDEPRMEQYWRGILDGDPTPYLQRIFSGSNATRGITYDELHESSKRGVPL 5o31.1    QT-KVAVTPPGLAREDWKIIRALSEIAGMT--------------------------------------------------  target    LMNMRTYPRSGGWEQRQEDKPWYTATGRLEFYRPEPEFQAAGESLPVWREPVDATFYEPNAILSNAAHPSIAPRAPEDYG 5o31.1    --------------------------------------------------------------------------------  target    VPESQLDVETRQYRNVVRTWAELQQTLHPLQERDPAFRFVFQTPKYRWGAHSTAVDADWISMLFGPFGDPYRRDPRMPWT 5o31.1    --------------------------------------------------------------------------------  target    GEAYLEINPKDAA 5o31.1    ------------- ``` | | | | | | | | | | | | | | | | | | | | | | | | | | | | | | | | | | | | | | | | | | | | | | | | | |
|  | 7aqr.1.F | NADH dehydrogenase [ubiquinone] iron-sulfur protein 1, mitochondrial  *Cryo-EM structure of Arabidopsis thaliana Complex-I (peripheral arm)* | 0.14 |  | 13.39 | 0.29 | 119-589 | EM | 0.00 | hetero-1-1-1-1-1-1-… | 6 x SF4, 2 x FES, 1 x FMN, 1 x NDP, 1 x ZN, 1 x 8Q1 | HHblits | 0.27 |
| ``` target    SWDEATTIAAKTMEDVARTFNGDEGARKLLAQGYHPEMVEVMHGAGVQALKLRGGMPLLGIGRIFGFYRFANMLALLDRK 7aqr.1    --------------------------------------------------------------------------------  target    LRPDAPADEILGSRTFDNYAWHTDLPPGHPMVTGSQTVDFDLFSAEHTKLLLIIGMNWICTKMPDGHWIGDAR-LKGTRV 7aqr.1    --------------------------------------NTSISGLENADLFLLIGTQPRVEAAMVNARICKTVRASNAKV  target    IVISADYMPTANKADEVIILRPGTDAAFFLGVARELIEKGLYDRAAVIERTDLPLLVRLDTGERLDARDVIPGYELAALT 7aqr.1    GYVGPPAEFN--YDCKHLGTGPDTLKEI----------------------------------------------------  target    NYVTLKPDAEIKGNPPPPPFTAGGQVVPTELRDAWGDFVWWDRATGRPRPVSRDEVGARFDGDPALLGEFEVELVDGSTV 7aqr.1    --------------------------------------------------------------------------------  target    PVRPAFDLLKQYLDESFDLRTASEVCRVPPQAIQSIARQLAANKRETLLAAGMGPNHYFQNDLFGRVQFLVAALTDNIGH 7aqr.1    -----------------------------AEGRHPFCTALKNAKNPAI-IVGAGLFNRTDKNAILSSVESIAQANNVVRP  target    LGGNVGSYAGNYRGSVFQAMGQWIAEDPFAIEPDLTKPATVKRYYKAESAHYWNYGERPLRAVAKDDEGDLTKGEVLTGK 7aqr.1    DWNGLNFLLQYAA-----QAAAL----------DL------------------G----------------LIQ-QSAK--  target    SHMPTPTKLIWFGNSNSLLGNAKWSFDVVKNTLPRQDAVFCNEWHWTSSCEYADLVFPADSWAEFKLPDATASCTNPFLL 7aqr.1    --ALESAKFVYLMGADDVN----------VDKIPKDAFVVYQGHHGDKAVYRANVILPASAFTEK---EGTYENTEGFTQ  target    AFPTTPLKRLYDTRSDYEALALTAKALGELIDEPRMEQYWRGILDGDPTPYLQRIFSGSNATRGITYDELHESSKRGVPL 7aqr.1    QT-VPAVPTVGDARDDWKIVRALSEVSGVK--------------------------------------------------  target    LMNMRTYPRSGGWEQRQEDKPWYTATGRLEFYRPEPEFQAAGESLPVWREPVDATFYEPNAILSNAAHPSIAPRAPEDYG 7aqr.1    --------------------------------------------------------------------------------  target    VPESQLDVETRQYRNVVRTWAELQQTLHPLQERDPAFRFVFQTPKYRWGAHSTAVDADWISMLFGPFGDPYRRDPRMPWT 7aqr.1    --------------------------------------------------------------------------------  target    GEAYLEINPKDAA 7aqr.1    ------------- ``` | | | | | | | | | | | | | | | | | | | | | | | | | | | | | | | | | | | | | | | | | | | | | | | | | |
|  | 7a23.1.O | 75kDa  *Plant mitochondrial respiratory complex I* | 0.14 |  | 13.39 | 0.29 | 119-589 | EM | 0.00 | hetero-1-1-1-1-1-1-… | 6 x SF4, 1 x FMN, 2 x T7X, 3 x CDL, 1 x U10, 1 x PEV, 2 x FES, 1 x NDP, 2 x ZN | HHblits | 0.27 |
| ``` target    SWDEATTIAAKTMEDVARTFNGDEGARKLLAQGYHPEMVEVMHGAGVQALKLRGGMPLLGIGRIFGFYRFANMLALLDRK 7a23.1    --------------------------------------------------------------------------------  target    LRPDAPADEILGSRTFDNYAWHTDLPPGHPMVTGSQTVDFDLFSAEHTKLLLIIGMNWICTKMPDGHWIGDAR-LKGTRV 7a23.1    --------------------------------------NTSISGLENADLFLLIGTQPRVEAAMVNARICKTVRASNAKV  target    IVISADYMPTANKADEVIILRPGTDAAFFLGVARELIEKGLYDRAAVIERTDLPLLVRLDTGERLDARDVIPGYELAALT 7a23.1    GYVGPPAEFN--YDCKHLGTGPDTLKEI----------------------------------------------------  target    NYVTLKPDAEIKGNPPPPPFTAGGQVVPTELRDAWGDFVWWDRATGRPRPVSRDEVGARFDGDPALLGEFEVELVDGSTV 7a23.1    --------------------------------------------------------------------------------  target    PVRPAFDLLKQYLDESFDLRTASEVCRVPPQAIQSIARQLAANKRETLLAAGMGPNHYFQNDLFGRVQFLVAALTDNIGH 7a23.1    -----------------------------AEGRHPFCTALKNAKNPAI-IVGAGLFNRTDKNAILSSVESIAQANNVVRP  target    LGGNVGSYAGNYRGSVFQAMGQWIAEDPFAIEPDLTKPATVKRYYKAESAHYWNYGERPLRAVAKDDEGDLTKGEVLTGK 7a23.1    DWNGLNFLLQYAA-----QAAAL----------DL------------------G----------------LIQ-QSAK--  target    SHMPTPTKLIWFGNSNSLLGNAKWSFDVVKNTLPRQDAVFCNEWHWTSSCEYADLVFPADSWAEFKLPDATASCTNPFLL 7a23.1    --ALESAKFVYLMGADDVN----------VDKIPKDAFVVYQGHHGDKAVYRANVILPASAFTEK---EGTYENTEGFTQ  target    AFPTTPLKRLYDTRSDYEALALTAKALGELIDEPRMEQYWRGILDGDPTPYLQRIFSGSNATRGITYDELHESSKRGVPL 7a23.1    QT-VPAVPTVGDARDDWKIVRALSEVSGVK--------------------------------------------------  target    LMNMRTYPRSGGWEQRQEDKPWYTATGRLEFYRPEPEFQAAGESLPVWREPVDATFYEPNAILSNAAHPSIAPRAPEDYG 7a23.1    --------------------------------------------------------------------------------  target    VPESQLDVETRQYRNVVRTWAELQQTLHPLQERDPAFRFVFQTPKYRWGAHSTAVDADWISMLFGPFGDPYRRDPRMPWT 7a23.1    --------------------------------------------------------------------------------  target    GEAYLEINPKDAA 7a23.1    ------------- ``` | | | | | | | | | | | | | | | | | | | | | | | | | | | | | | | | | | | | | | | | | | | | | | | | | |
|  | 7ar8.1.G | NADH dehydrogenase [ubiquinone] iron-sulfur protein 1, mitochondrial  *Cryo-EM structure of Arabidopsis thaliana complex-I (closed conformation)* | 0.14 |  | 13.39 | 0.29 | 119-589 | EM | 0.00 | hetero-1-1-1-1-1-1-… | 6 x SF4, 2 x FES, 1 x FMN, 1 x UQ9, 3 x PTY, 2 x PC7, 1 x PGT, 1 x FE, 1 x NDP, 2 x ZN, 2 x 8Q1, 1 x LMN, 1 x PSF, 1 x T7X | HHblits | 0.27 |
| ``` target    SWDEATTIAAKTMEDVARTFNGDEGARKLLAQGYHPEMVEVMHGAGVQALKLRGGMPLLGIGRIFGFYRFANMLALLDRK 7ar8.1    --------------------------------------------------------------------------------  target    LRPDAPADEILGSRTFDNYAWHTDLPPGHPMVTGSQTVDFDLFSAEHTKLLLIIGMNWICTKMPDGHWIGDAR-LKGTRV 7ar8.1    --------------------------------------NTSISGLENADLFLLIGTQPRVEAAMVNARICKTVRASNAKV  target    IVISADYMPTANKADEVIILRPGTDAAFFLGVARELIEKGLYDRAAVIERTDLPLLVRLDTGERLDARDVIPGYELAALT 7ar8.1    GYVGPPAEFN--YDCKHLGTGPDTLKEI----------------------------------------------------  target    NYVTLKPDAEIKGNPPPPPFTAGGQVVPTELRDAWGDFVWWDRATGRPRPVSRDEVGARFDGDPALLGEFEVELVDGSTV 7ar8.1    --------------------------------------------------------------------------------  target    PVRPAFDLLKQYLDESFDLRTASEVCRVPPQAIQSIARQLAANKRETLLAAGMGPNHYFQNDLFGRVQFLVAALTDNIGH 7ar8.1    -----------------------------AEGRHPFCTALKNAKNPAI-IVGAGLFNRTDKNAILSSVESIAQANNVVRP  target    LGGNVGSYAGNYRGSVFQAMGQWIAEDPFAIEPDLTKPATVKRYYKAESAHYWNYGERPLRAVAKDDEGDLTKGEVLTGK 7ar8.1    DWNGLNFLLQYAA-----QAAAL----------DL------------------G----------------LIQ-QSAK--  target    SHMPTPTKLIWFGNSNSLLGNAKWSFDVVKNTLPRQDAVFCNEWHWTSSCEYADLVFPADSWAEFKLPDATASCTNPFLL 7ar8.1    --ALESAKFVYLMGADDVN----------VDKIPKDAFVVYQGHHGDKAVYRANVILPASAFTEK---EGTYENTEGFTQ  target    AFPTTPLKRLYDTRSDYEALALTAKALGELIDEPRMEQYWRGILDGDPTPYLQRIFSGSNATRGITYDELHESSKRGVPL 7ar8.1    QT-VPAVPTVGDARDDWKIVRALSEVSGVK--------------------------------------------------  target    LMNMRTYPRSGGWEQRQEDKPWYTATGRLEFYRPEPEFQAAGESLPVWREPVDATFYEPNAILSNAAHPSIAPRAPEDYG 7ar8.1    --------------------------------------------------------------------------------  target    VPESQLDVETRQYRNVVRTWAELQQTLHPLQERDPAFRFVFQTPKYRWGAHSTAVDADWISMLFGPFGDPYRRDPRMPWT 7ar8.1    --------------------------------------------------------------------------------  target    GEAYLEINPKDAA 7ar8.1    ------------- ``` | | | | | | | | | | | | | | | | | | | | | | | | | | | | | | | | | | | | | | | | | | | | | | | | | |
|  | 7ar7.1.G | NADH dehydrogenase [ubiquinone] iron-sulfur protein 1, mitochondrial  *Cryo-EM structure of Arabidopsis thaliana complex-I (open conformation)* | 0.14 |  | 12.97 | 0.29 | 119-589 | EM | 0.00 | hetero-1-1-1-1-1-1-… | 6 x SF4, 2 x FES, 1 x FMN, 1 x UQ9, 3 x PTY, 2 x PC7, 1 x LMN, 1 x NDP, 2 x ZN, 2 x 8Q1, 1 x PGT, 1 x PSF, 1 x T7X | HHblits | 0.27 |
| ``` target    SWDEATTIAAKTMEDVARTFNGDEGARKLLAQGYHPEMVEVMHGAGVQALKLRGGMPLLGIGRIFGFYRFANMLALLDRK 7ar7.1    --------------------------------------------------------------------------------  target    LRPDAPADEILGSRTFDNYAWHTDLPPGHPMVTGSQTVDFDLFSAEHTKLLLIIGMNWICTKMPDGHWIGDAR-LKGTRV 7ar7.1    --------------------------------------NTSISGLENADLFLLIGTQPRVEAAMVNARICKTVRASNAKV  target    IVISADYMPTANKADEVIILRPGTDAAFFLGVARELIEKGLYDRAAVIERTDLPLLVRLDTGERLDARDVIPGYELAALT 7ar7.1    GYVGPPAEFN--YDCKHLGTGPDTLKEI----------------------------------------------------  target    NYVTLKPDAEIKGNPPPPPFTAGGQVVPTELRDAWGDFVWWDRATGRPRPVSRDEVGARFDGDPALLGEFEVELVDGSTV 7ar7.1    --------------------------------------------------------------------------------  target    PVRPAFDLLKQYLDESFDLRTASEVCRVPPQAIQSIARQLAANKRETLLAAGMGPNHYFQNDLFGRVQFLVAALTDNIGH 7ar7.1    -----------------------------AEGRHPFCTALKNAKNPAI-IVGAGLFNRTDKNAILSSVESIAQANNVVRP  target    LGGNVGSYAGNYRGSVFQAMGQWIAEDPFAIEPDLTKPATVKRYYKAESAHYWNYGERPLRAVAKDDEGDLTKGEVLTGK 7ar7.1    DWNGLNFLLQYAA-----QAAAL----------DL----G---------------------LI---------Q-QSAK--  target    SHMPTPTKLIWFGNSNSLLGNAKWSFDVVKNTLPRQDAVFCNEWHWTSSCEYADLVFPADSWAEFKLPDATASCTNPFLL 7ar7.1    --ALESAKFVYLMGADDVN----------VDKIPKDAFVVYQGHHGDKAVYRANVILPASAFTEK---EGTYENTEGFTQ  target    AFPTTPLKRLYDTRSDYEALALTAKALGELIDEPRMEQYWRGILDGDPTPYLQRIFSGSNATRGITYDELHESSKRGVPL 7ar7.1    QT-VPAVPTVGDARDDWKIVRALSEVSGVK--------------------------------------------------  target    LMNMRTYPRSGGWEQRQEDKPWYTATGRLEFYRPEPEFQAAGESLPVWREPVDATFYEPNAILSNAAHPSIAPRAPEDYG 7ar7.1    --------------------------------------------------------------------------------  target    VPESQLDVETRQYRNVVRTWAELQQTLHPLQERDPAFRFVFQTPKYRWGAHSTAVDADWISMLFGPFGDPYRRDPRMPWT 7ar7.1    --------------------------------------------------------------------------------  target    GEAYLEINPKDAA 7ar7.1    ------------- ``` | | | | | | | | | | | | | | | | | | | | | | | | | | | | | | | | | | | | | | | | | | | | | | | | | |
|  | 6x89.1.H | NADH dehydrogenase [ubiquinone] iron-sulfur protein 1, mitochondrial  *Vigna radiata mitochondrial complex I\** | 0.14 |  | 13.39 | 0.29 | 119-589 | EM | 0.00 | hetero-1-1-1-1-1-1-… | 1 x NAP, 6 x PC1, 6 x SF4, 2 x FES, 2 x ZN, 1 x FMN | HHblits | 0.27 |
| ``` target    SWDEATTIAAKTMEDVARTFNGDEGARKLLAQGYHPEMVEVMHGAGVQALKLRGGMPLLGIGRIFGFYRFANMLALLDRK 6x89.1    --------------------------------------------------------------------------------  target    LRPDAPADEILGSRTFDNYAWHTDLPPGHPMVTGSQTVDFDLFSAEHTKLLLIIGMNWICTKMPDGHWIGDARL-KGTRV 6x89.1    --------------------------------------NTSIAGLEKADVFLLVGTQPRVEAAMVNARIRKTVRSNQAKV  target    IVISADYMPTANKADEVIILRPGTDAAFFLGVARELIEKGLYDRAAVIERTDLPLLVRLDTGERLDARDVIPGYELAALT 6x89.1    GYIGPATDFN--YDHKHLGTDPQTLVEIAE------------------------------------------GR------  target    NYVTLKPDAEIKGNPPPPPFTAGGQVVPTELRDAWGDFVWWDRATGRPRPVSRDEVGARFDGDPALLGEFEVELVDGSTV 6x89.1    --------------------------------------------------------------------------------  target    PVRPAFDLLKQYLDESFDLRTASEVCRVPPQAIQSIARQLAANKRETLLAAGMGPNHYFQNDLFGRVQFLVAALTDNIGH 6x89.1    ---------------------------------HPFFKTLSDAKNPVI-IVGAGVFERKDQDAIFAAVETIAQKANVVRP  target    LGGNVGSYAGNYRGSVFQAMGQWIAEDPFAIEPDLTKPATVKRYYKAESAHYWNYGERPLRAVAKDDEGDLTKGEVLTGK 6x89.1    DWNGLNVLLLHAA-----QAAAL----------DL----GL----------------VP--------------QSE----  target    SHMPTPTKLIWFGNSNSLLGNAKWSFDVVKNTLPRQDAVFCNEWHWTSSCEYADLVFPADSWAEFKLPDATASCTNPFLL 6x89.1    -KSLESAKFVYLMGADDVN----------LDKIPDDAFVVYQGHHGDKSVYRANVILPTAAFSEK---EGTYQNTEGCTQ  target    AFPTTPLKRLYDTRSDYEALALTAKALGELIDEPRMEQYWRGILDGDPTPYLQRIFSGSNATRGITYDELHESSKRGVPL 6x89.1    QT-LPAVPTVGDSRDDWKIIRALSEVAGVR--------------------------------------------------  target    LMNMRTYPRSGGWEQRQEDKPWYTATGRLEFYRPEPEFQAAGESLPVWREPVDATFYEPNAILSNAAHPSIAPRAPEDYG 6x89.1    --------------------------------------------------------------------------------  target    VPESQLDVETRQYRNVVRTWAELQQTLHPLQERDPAFRFVFQTPKYRWGAHSTAVDADWISMLFGPFGDPYRRDPRMPWT 6x89.1    --------------------------------------------------------------------------------  target    GEAYLEINPKDAA 6x89.1    ------------- ``` | | | | | | | | | | | | | | | | | | | | | | | | | | | | | | | | | | | | | | | | | | | | | | | | | |
|  | 8e73.55.A | NDUS1  *Vigna radiata supercomplex I+III2 (full bridge)* | 0.15 |  | 13.39 | 0.29 | 119-589 | EM | 0.00 | monomer |  | HHblits | 0.27 |
| ``` target    SWDEATTIAAKTMEDVARTFNGDEGARKLLAQGYHPEMVEVMHGAGVQALKLRGGMPLLGIGRIFGFYRFANMLALLDRK 8e73.55   --------------------------------------------------------------------------------  target    LRPDAPADEILGSRTFDNYAWHTDLPPGHPMVTGSQTVDFDLFSAEHTKLLLIIGMNWICTKMPDGHWIGDARL-KGTRV 8e73.55   --------------------------------------NTSIAGLEKADVFLLVGTQPRVEAAMVNARIRKTVRSNQAKV  target    IVISADYMPTANKADEVIILRPGTDAAFFLGVARELIEKGLYDRAAVIERTDLPLLVRLDTGERLDARDVIPGYELAALT 8e73.55   GYIGPATDFN--YDHKHLGTDPQTLVEIAE------------------------------------------GR------  target    NYVTLKPDAEIKGNPPPPPFTAGGQVVPTELRDAWGDFVWWDRATGRPRPVSRDEVGARFDGDPALLGEFEVELVDGSTV 8e73.55   --------------------------------------------------------------------------------  target    PVRPAFDLLKQYLDESFDLRTASEVCRVPPQAIQSIARQLAANKRETLLAAGMGPNHYFQNDLFGRVQFLVAALTDNIGH 8e73.55   ---------------------------------HPFFKTLSDAKNPVI-IVGAGVFERKDQDAIFAAVETIAQKANVVRP  target    LGGNVGSYAGNYRGSVFQAMGQWIAEDPFAIEPDLTKPATVKRYYKAESAHYWNYGERPLRAVAKDDEGDLTKGEVLTGK 8e73.55   DWNGLNVLLLHAA-----QAAAL----------DL----GL----------------VP--------------QSE----  target    SHMPTPTKLIWFGNSNSLLGNAKWSFDVVKNTLPRQDAVFCNEWHWTSSCEYADLVFPADSWAEFKLPDATASCTNPFLL 8e73.55   -KSLESAKFVYLMGADDVN----------LDKIPDDAFVVYQGHHGDKSVYRANVILPTAAFSEK---EGTYQNTEGCTQ  target    AFPTTPLKRLYDTRSDYEALALTAKALGELIDEPRMEQYWRGILDGDPTPYLQRIFSGSNATRGITYDELHESSKRGVPL 8e73.55   QT-LPAVPTVGDSRDDWKIIRALSEVAGVR--------------------------------------------------  target    LMNMRTYPRSGGWEQRQEDKPWYTATGRLEFYRPEPEFQAAGESLPVWREPVDATFYEPNAILSNAAHPSIAPRAPEDYG 8e73.55   --------------------------------------------------------------------------------  target    VPESQLDVETRQYRNVVRTWAELQQTLHPLQERDPAFRFVFQTPKYRWGAHSTAVDADWISMLFGPFGDPYRRDPRMPWT 8e73.55   --------------------------------------------------------------------------------  target    GEAYLEINPKDAA 8e73.55   ------------- ``` | | | | | | | | | | | | | | | | | | | | | | | | | | | | | | | | | | | | | | | | | | | | | | | | | |
|  | 5xtb.1.L | NADH-ubiquinone oxidoreductase 75 kDa subunit, mitochondrial  *Cryo-EM structure of human respiratory complex I matrix arm* | 0.09 |  | 13.14 | 0.22 | 352-589 | EM | 0.00 | hetero-1-1-1-1-1-1-… | 6 x SF4, 1 x FMN, 1 x 8Q1, 1 x NDP, 2 x FES | HHblits | 0.27 |
| ``` target    SWDEATTIAAKTMEDVARTFNGDEGARKLLAQGYHPEMVEVMHGAGVQALKLRGGMPLLGIGRIFGFYRFANMLALLDRK 5xtb.1    --------------------------------------------------------------------------------  target    LRPDAPADEILGSRTFDNYAWHTDLPPGHPMVTGSQTVDFDLFSAEHTKLLLIIGMNWICTKMPDGHWIGDARLKGTRVI 5xtb.1    --------------------------------------------------------------------------------  target    VISADYMPTANKADEVIILRPGTDAAFFLGVARELIEKGLYDRAAVIERTDLPLLVRLDTGERLDARDVIPGYELAALTN 5xtb.1    --------------------------------------------------------------------------------  target    YVTLKPDAEIKGNPPPPPFTAGGQVVPTELRDAWGDFVWWDRATGRPRPVSRDEVGARFDGDPALLGEFEVELVDGSTVP 5xtb.1    --------------------------------------------------------------------------------  target    VRPAFDLLKQYLDESFDLRTASEVCRVPPQAIQSIARQLAANKRETLLAAGMGPNHYFQNDLFGRVQFLVAALTDNIGHL 5xtb.1    -------------------------------SHPFSQVLKEAKKPMV-VLGSSALQRNDGAAILAAVSSIAQKIRMTSGV  target    GGNVGSYAGNYRGSVFQAMGQWIAEDPFAIEPDLTKPATVKRYYKAESAHYWNYGERPLRAVAKDDEGDLTKGEVLTGKS 5xtb.1    TGDWKVMNILHRIA--SQVAA------------------------------LDLGYKP----------G--VEAIR----  target    HMPTPTKLIWFGNSNSLLGNAKWSFDVVKNTLPRQDAVFCNEWHWTSSCEYADLVFPADSWAEFKLPDATASCTNPFLLA 5xtb.1    --KNPPKVLFLLGADGG--------CITRQDLPKDCFIIYQGHHGDVGAPIADVILPGAAYTEK---SATYVNTEGRAQQ  target    FPTTPLKRLYDTRSDYEALALTAKALGELIDEPRMEQYWRGILDGDPTPYLQRIFSGSNATRGITYDELHESSKRGVPLL 5xtb.1    T-KVAVTPPGLAREDWKIIRALSEIAGMT---------------------------------------------------  target    MNMRTYPRSGGWEQRQEDKPWYTATGRLEFYRPEPEFQAAGESLPVWREPVDATFYEPNAILSNAAHPSIAPRAPEDYGV 5xtb.1    --------------------------------------------------------------------------------  target    PESQLDVETRQYRNVVRTWAELQQTLHPLQERDPAFRFVFQTPKYRWGAHSTAVDADWISMLFGPFGDPYRRDPRMPWTG 5xtb.1    --------------------------------------------------------------------------------  target    EAYLEINPKDAA 5xtb.1    ------------ ``` | | | | | | | | | | | | | | | | | | | | | | | | | | | | | | | | | | | | | | | | | | | | | | | | | |
|  | 7tgh.58.A | NADH-ubiquinone oxidoreductase 75 kDa subunit  *Cryo-EM structure of respiratory super-complex CI+III2 from Tetrahymena thermophila* | 0.10 |  | 16.28 | 0.21 | 350-589 | EM | 0.00 | monomer |  | HHblits | 0.28 |
| ``` target    SWDEATTIAAKTMEDVARTFNGDEGARKLLAQGYHPEMVEVMHGAGVQALKLRGGMPLLGIGRIFGFYRFANMLALLDRK 7tgh.58   --------------------------------------------------------------------------------  target    LRPDAPADEILGSRTFDNYAWHTDLPPGHPMVTGSQTVDFDLFSAEHTKLLLIIGMNWICTKMPDGHWIGDARLKGTRVI 7tgh.58   --------------------------------------------------------------------------------  target    VISADYMPTANKADEVIILRPGTDAAFFLGVARELIEKGLYDRAAVIERTDLPLLVRLDTGERLDARDVIPGYELAALTN 7tgh.58   --------------------------------------------------------------------------------  target    YVTLKPDAEIKGNPPPPPFTAGGQVVPTELRDAWGDFVWWDRATGRPRPVSRDEVGARFDGDPALLGEFEVELVDGSTVP 7tgh.58   --------------------------------------------------------------------------------  target    VRPAFDLLKQYLDESFDLRTASEVCRVPPQAIQSIARQLAANKRETLLAAGMGPNHYFQNDLFGRVQFLVAALTDNIGHL 7tgh.58   -----------------------------DGTHPFAERLKKAKLPMI-MVGASALEREDGAELYNTLKVISNKTGVISEE  target    GGNVGSYAGNYRGSVFQAMGQWIAEDPFAIEPDLTKPATVKRYYKAESAHYWNYGERPLRAVAKDDEGDLTKGEVLTGKS 7tgh.58   KSWNGFNILHKE------MGR------------IN---A------------L-----EL---------GINPT-------  target    HMPTPTKLIWFGNSNSLLGNAKWSFDVVKNTLPRQDAVFCNEWHWTSSCEYADLVFPADSWAEFKLPDATASCTNPFLLA 7tgh.58   SVNKNAKLVFILGADNNLRP---------EDIPADAFVVYFGTHGDEGAYYADIILPTAAYTEK---NATWVNTEGRVQQ  target    FPTTPLKRLYDTRSDYEALALTAKALGELIDEPRMEQYWRGILDGDPTPYLQRIFSGSNATRGITYDELHESSKRGVPLL 7tgh.58   G-RLVVMPPGDAREDWQIIRALSEEAGVP---------------------------------------------------  target    MNMRTYPRSGGWEQRQEDKPWYTATGRLEFYRPEPEFQAAGESLPVWREPVDATFYEPNAILSNAAHPSIAPRAPEDYGV 7tgh.58   --------------------------------------------------------------------------------  target    PESQLDVETRQYRNVVRTWAELQQTLHPLQERDPAFRFVFQTPKYRWGAHSTAVDADWISMLFGPFGDPYRRDPRMPWTG 7tgh.58   --------------------------------------------------------------------------------  target    EAYLEINPKDAA 7tgh.58   ------------ ``` | | | | | | | | | | | | | | | | | | | | | | | | | | | | | | | | | | | | | | | | | | | | | | | | | |
|  | 6zk9.1.C | NADH:ubiquinone oxidoreductase core subunit S1  *Peripheral domain of open complex I during turnover* | 0.10 |  | 12.21 | 0.21 | 355-589 | EM | 0.00 | hetero-1-1-1-1-1-1-… | 6 x SF4, 1 x FMN, 1 x NAI, 2 x FES, 1 x K, 2 x PC1, 2 x 3PE, 1 x ZN, 1 x NDP, 1 x ZMP, 1 x CDL | HHblits | 0.27 |
| ``` target    SWDEATTIAAKTMEDVARTFNGDEGARKLLAQGYHPEMVEVMHGAGVQALKLRGGMPLLGIGRIFGFYRFANMLALLDRK 6zk9.1    --------------------------------------------------------------------------------  target    LRPDAPADEILGSRTFDNYAWHTDLPPGHPMVTGSQTVDFDLFSAEHTKLLLIIGMNWICTKMPDGHWIGDARLKGTRVI 6zk9.1    --------------------------------------------------------------------------------  target    VISADYMPTANKADEVIILRPGTDAAFFLGVARELIEKGLYDRAAVIERTDLPLLVRLDTGERLDARDVIPGYELAALTN 6zk9.1    --------------------------------------------------------------------------------  target    YVTLKPDAEIKGNPPPPPFTAGGQVVPTELRDAWGDFVWWDRATGRPRPVSRDEVGARFDGDPALLGEFEVELVDGSTVP 6zk9.1    --------------------------------------------------------------------------------  target    VRPAFDLLKQYLDESFDLRTASEVCRVPPQAIQSIARQLAANKRETLLAAGMGPNHYFQNDLFGRVQFLVAALTDNIGHL 6zk9.1    ----------------------------------FSQVLQEAKKPMV-VLGSSALQRNDGAAILAAVSNIAQKIRTSSGV  target    GGNVGSYAGNYRGSVFQAMGQWIAEDPFAIEPDLTKPATVKRYYKAESAHYWNYGERPLRAVAKDDEGDLTKGEVLTGKS 6zk9.1    TGDWKVMN--IL-------HR------------IASQVAA-----------LDL---GYKP-------G--VEAIR----  target    HMPTPTKLIWFGNSNSLLGNAKWSFDVVKNTLPRQDAVFCNEWHWTSSCEYADLVFPADSWAEFKLPDATASCTNPFLLA 6zk9.1    --KNPPKMLFLLGADGGC--------VTRQDLPKDCFIVYQGHHGDVGAPIADVILPGAAYTEK---SATYVNTEGRAQQ  target    FPTTPLKRLYDTRSDYEALALTAKALGELIDEPRMEQYWRGILDGDPTPYLQRIFSGSNATRGITYDELHESSKRGVPLL 6zk9.1    T-KVAVMPPGLAREDWKIIRALSEIAGMT---------------------------------------------------  target    MNMRTYPRSGGWEQRQEDKPWYTATGRLEFYRPEPEFQAAGESLPVWREPVDATFYEPNAILSNAAHPSIAPRAPEDYGV 6zk9.1    --------------------------------------------------------------------------------  target    PESQLDVETRQYRNVVRTWAELQQTLHPLQERDPAFRFVFQTPKYRWGAHSTAVDADWISMLFGPFGDPYRRDPRMPWTG 6zk9.1    --------------------------------------------------------------------------------  target    EAYLEINPKDAA 6zk9.1    ------------ ``` | | | | | | | | | | | | | | | | | | | | | | | | | | | | | | | | | | | | | | | | | | | | | | | | | |
|  | 6yj4.1.G | Subunit NUAM of NADH:Ubiquinone Oxidoreductase (Complex I)  *Structure of Yarrowia lipolytica complex I at 2.7 A* | 0.09 |  | 16.17 | 0.21 | 354-589 | EM | 0.00 | hetero-1-1-1-1-1-1-… | 18 x 3PE, 6 x SF4, 5 x LMT, 8 x PLC, 2 x FES, 1 x FMN, 6 x CDL, 1 x NDP, 1 x ZN, 2 x EHZ | HHblits | 0.29 |
| ``` target    SWDEATTIAAKTMEDVARTFNGDEGARKLLAQGYHPEMVEVMHGAGVQALKLRGGMPLLGIGRIFGFYRFANMLALLDRK 6yj4.1    --------------------------------------------------------------------------------  target    LRPDAPADEILGSRTFDNYAWHTDLPPGHPMVTGSQTVDFDLFSAEHTKLLLIIGMNWICTKMPDGHWIGDARLKGTRVI 6yj4.1    --------------------------------------------------------------------------------  target    VISADYMPTANKADEVIILRPGTDAAFFLGVARELIEKGLYDRAAVIERTDLPLLVRLDTGERLDARDVIPGYELAALTN 6yj4.1    --------------------------------------------------------------------------------  target    YVTLKPDAEIKGNPPPPPFTAGGQVVPTELRDAWGDFVWWDRATGRPRPVSRDEVGARFDGDPALLGEFEVELVDGSTVP 6yj4.1    --------------------------------------------------------------------------------  target    VRPAFDLLKQYLDESFDLRTASEVCRVPPQAIQSIARQLAANKRETLLAAGMGPNHYFQNDLFGRVQFLVAALTDN-IGH 6yj4.1    ---------------------------------EFGEVLKNAKNPLI-IVGSGITDREDAGAFFNTIGKFVESTPSVLNE  target    LGGNVGSYAGNYRGSVFQAMGQWIAEDPFAIEPDLTKPATVKRYYKAESAHYWNYGERPLRAVAKDDEGDLTKGEVLTGK 6yj4.1    NWNGYNVLQRSAS-----RAGAY----------DI------------------GF--TPS-------------DEA----  target    SHMPTPTKLIWFGNSNSLLGNAKWSFDVVKNTLPRQDAVFCNEWHWTSSCEYADLVFPADSWAEFKLPDATASCTNPFLL 6yj4.1    --SKTTPKMVWLLGADEVAAS----------DIPADAFVVYQGHNGDVGAQFADVVLPGAAYTEK---AGTYVNTEGRSQ  target    AFPTTPLKRLYDTRSDYEALALTAKALGELIDEPRMEQYWRGILDGDPTPYLQRIFSGSNATRGITYDELHESSKRGVPL 6yj4.1    IS-RAATGPPGGAREDWKILRAVSEYLGVA--------------------------------------------------  target    LMNMRTYPRSGGWEQRQEDKPWYTATGRLEFYRPEPEFQAAGESLPVWREPVDATFYEPNAILSNAAHPSIAPRAPEDYG 6yj4.1    --------------------------------------------------------------------------------  target    VPESQLDVETRQYRNVVRTWAELQQTLHPLQERDPAFRFVFQTPKYRWGAHSTAVDADWISMLFGPFGDPYRRDPRMPWT 6yj4.1    --------------------------------------------------------------------------------  target    GEAYLEINPKDAA 6yj4.1    ------------- ``` | | | | | | | | | | | | | | | | | | | | | | | | | | | | | | | | | | | | | | | | | | | | | | | | | |
|  | 6rfs.1.A | Subunit NUAM of NADH:Ubiquinone Oxidoreductase (Complex I)  *Cryo-EM structure of a respiratory complex I mutant lacking NDUFS4* | 0.09 |  | 16.17 | 0.21 | 354-589 | EM | 4.04 | hetero-1-1-1-1-1-1-… | 6 x SF4, 2 x FES, 1 x FMN, 1 x NDP, 1 x ZN, 1 x ZMP | HHblits | 0.29 |
| ``` target    SWDEATTIAAKTMEDVARTFNGDEGARKLLAQGYHPEMVEVMHGAGVQALKLRGGMPLLGIGRIFGFYRFANMLALLDRK 6rfs.1    --------------------------------------------------------------------------------  target    LRPDAPADEILGSRTFDNYAWHTDLPPGHPMVTGSQTVDFDLFSAEHTKLLLIIGMNWICTKMPDGHWIGDARLKGTRVI 6rfs.1    --------------------------------------------------------------------------------  target    VISADYMPTANKADEVIILRPGTDAAFFLGVARELIEKGLYDRAAVIERTDLPLLVRLDTGERLDARDVIPGYELAALTN 6rfs.1    --------------------------------------------------------------------------------  target    YVTLKPDAEIKGNPPPPPFTAGGQVVPTELRDAWGDFVWWDRATGRPRPVSRDEVGARFDGDPALLGEFEVELVDGSTVP 6rfs.1    --------------------------------------------------------------------------------  target    VRPAFDLLKQYLDESFDLRTASEVCRVPPQAIQSIARQLAANKRETLLAAGMGPNHYFQNDLFGRVQFLVAALTDN-IGH 6rfs.1    ---------------------------------EFGEVLKNAKNPLI-IVGSGITDREDAGAFFNTIGKFVESTPSVLNE  target    LGGNVGSYAGNYRGSVFQAMGQWIAEDPFAIEPDLTKPATVKRYYKAESAHYWNYGERPLRAVAKDDEGDLTKGEVLTGK 6rfs.1    NWNGYNVLQRSAS-----RAGAY----------DI------------------GF--TPS-------------DEA----  target    SHMPTPTKLIWFGNSNSLLGNAKWSFDVVKNTLPRQDAVFCNEWHWTSSCEYADLVFPADSWAEFKLPDATASCTNPFLL 6rfs.1    --SKTTPKMVWLLGADEVAAS----------DIPADAFVVYQGHNGDVGAQFADVVLPGAAYTEK---AGTYVNTEGRSQ  target    AFPTTPLKRLYDTRSDYEALALTAKALGELIDEPRMEQYWRGILDGDPTPYLQRIFSGSNATRGITYDELHESSKRGVPL 6rfs.1    IS-RAATGPPGGAREDWKILRAVSEYLGVA--------------------------------------------------  target    LMNMRTYPRSGGWEQRQEDKPWYTATGRLEFYRPEPEFQAAGESLPVWREPVDATFYEPNAILSNAAHPSIAPRAPEDYG 6rfs.1    --------------------------------------------------------------------------------  target    VPESQLDVETRQYRNVVRTWAELQQTLHPLQERDPAFRFVFQTPKYRWGAHSTAVDADWISMLFGPFGDPYRRDPRMPWT 6rfs.1    --------------------------------------------------------------------------------  target    GEAYLEINPKDAA 6rfs.1    ------------- ``` | | | | | | | | | | | | | | | | | | | | | | | | | | | | | | | | | | | | | | | | | | | | | | | | | |
|  | 6rfq.1.A | Subunit NUAM of NADH:Ubiquinone Oxidoreductase (Complex I)  *Cryo-EM structure of a respiratory complex I assembly intermediate with NDUFAF2* | 0.09 |  | 16.17 | 0.21 | 354-589 | EM | 3.30 | hetero-1-1-1-1-1-1-… | 6 x SF4, 2 x FES, 1 x FMN, 1 x NDP, 10 x 3PE, 2 x LMN, 4 x CDL, 2 x ZMP, 4 x PLC, 3 x T7X, 1 x CPL | HHblits | 0.29 |
| ``` target    SWDEATTIAAKTMEDVARTFNGDEGARKLLAQGYHPEMVEVMHGAGVQALKLRGGMPLLGIGRIFGFYRFANMLALLDRK 6rfq.1    --------------------------------------------------------------------------------  target    LRPDAPADEILGSRTFDNYAWHTDLPPGHPMVTGSQTVDFDLFSAEHTKLLLIIGMNWICTKMPDGHWIGDARLKGTRVI 6rfq.1    --------------------------------------------------------------------------------  target    VISADYMPTANKADEVIILRPGTDAAFFLGVARELIEKGLYDRAAVIERTDLPLLVRLDTGERLDARDVIPGYELAALTN 6rfq.1    --------------------------------------------------------------------------------  target    YVTLKPDAEIKGNPPPPPFTAGGQVVPTELRDAWGDFVWWDRATGRPRPVSRDEVGARFDGDPALLGEFEVELVDGSTVP 6rfq.1    --------------------------------------------------------------------------------  target    VRPAFDLLKQYLDESFDLRTASEVCRVPPQAIQSIARQLAANKRETLLAAGMGPNHYFQNDLFGRVQFLVAALTDN-IGH 6rfq.1    ---------------------------------EFGEVLKNAKNPLI-IVGSGITDREDAGAFFNTIGKFVESTPSVLNE  target    LGGNVGSYAGNYRGSVFQAMGQWIAEDPFAIEPDLTKPATVKRYYKAESAHYWNYGERPLRAVAKDDEGDLTKGEVLTGK 6rfq.1    NWNGYNVLQRSAS-----RAGAY----------DI------------------GF--TPS-------------DEA----  target    SHMPTPTKLIWFGNSNSLLGNAKWSFDVVKNTLPRQDAVFCNEWHWTSSCEYADLVFPADSWAEFKLPDATASCTNPFLL 6rfq.1    --SKTTPKMVWLLGADEVAAS----------DIPADAFVVYQGHNGDVGAQFADVVLPGAAYTEK---AGTYVNTEGRSQ  target    AFPTTPLKRLYDTRSDYEALALTAKALGELIDEPRMEQYWRGILDGDPTPYLQRIFSGSNATRGITYDELHESSKRGVPL 6rfq.1    IS-RAATGPPGGAREDWKILRAVSEYLGVA--------------------------------------------------  target    LMNMRTYPRSGGWEQRQEDKPWYTATGRLEFYRPEPEFQAAGESLPVWREPVDATFYEPNAILSNAAHPSIAPRAPEDYG 6rfq.1    --------------------------------------------------------------------------------  target    VPESQLDVETRQYRNVVRTWAELQQTLHPLQERDPAFRFVFQTPKYRWGAHSTAVDADWISMLFGPFGDPYRRDPRMPWT 6rfq.1    --------------------------------------------------------------------------------  target    GEAYLEINPKDAA 6rfq.1    ------------- ``` | | | | | | | | | | | | | | | | | | | | | | | | | | | | | | | | | | | | | | | | | | | | | | | | | |
|  | 6gcs.1.A | 75-KDA PROTEIN (NUAM)  *Cryo-EM structure of respiratory complex I from Yarrowia lipolytica* | 0.09 |  | 16.17 | 0.21 | 354-589 | EM | 4.32 | hetero-1-1-1-1-1-1-… | 6 x SF4, 2 x FES, 1 x FMN, 1 x NDP, 1 x ZN, 1 x ZMP, 1 x CDL, 3 x 3PE | HHblits | 0.29 |
| ``` target    SWDEATTIAAKTMEDVARTFNGDEGARKLLAQGYHPEMVEVMHGAGVQALKLRGGMPLLGIGRIFGFYRFANMLALLDRK 6gcs.1    --------------------------------------------------------------------------------  target    LRPDAPADEILGSRTFDNYAWHTDLPPGHPMVTGSQTVDFDLFSAEHTKLLLIIGMNWICTKMPDGHWIGDARLKGTRVI 6gcs.1    --------------------------------------------------------------------------------  target    VISADYMPTANKADEVIILRPGTDAAFFLGVARELIEKGLYDRAAVIERTDLPLLVRLDTGERLDARDVIPGYELAALTN 6gcs.1    --------------------------------------------------------------------------------  target    YVTLKPDAEIKGNPPPPPFTAGGQVVPTELRDAWGDFVWWDRATGRPRPVSRDEVGARFDGDPALLGEFEVELVDGSTVP 6gcs.1    --------------------------------------------------------------------------------  target    VRPAFDLLKQYLDESFDLRTASEVCRVPPQAIQSIARQLAANKRETLLAAGMGPNHYFQNDLFGRVQFLVAALTDN-IGH 6gcs.1    ---------------------------------EFGEVLKNAKNPLI-IVGSGITDREDAGAFFNTIGKFVESTPSVLNE  target    LGGNVGSYAGNYRGSVFQAMGQWIAEDPFAIEPDLTKPATVKRYYKAESAHYWNYGERPLRAVAKDDEGDLTKGEVLTGK 6gcs.1    NWNGYNVLQRSAS-----RAGAY----------DI------------------GF--TPS-------------DEA----  target    SHMPTPTKLIWFGNSNSLLGNAKWSFDVVKNTLPRQDAVFCNEWHWTSSCEYADLVFPADSWAEFKLPDATASCTNPFLL 6gcs.1    --SKTTPKMVWLLGADEVAAS----------DIPADAFVVYQGHNGDVGAQFADVVLPGAAYTEK---AGTYVNTEGRSQ  target    AFPTTPLKRLYDTRSDYEALALTAKALGELIDEPRMEQYWRGILDGDPTPYLQRIFSGSNATRGITYDELHESSKRGVPL 6gcs.1    IS-RAATGPPGGAREDWKILRAVSEYLGVA--------------------------------------------------  target    LMNMRTYPRSGGWEQRQEDKPWYTATGRLEFYRPEPEFQAAGESLPVWREPVDATFYEPNAILSNAAHPSIAPRAPEDYG 6gcs.1    --------------------------------------------------------------------------------  target    VPESQLDVETRQYRNVVRTWAELQQTLHPLQERDPAFRFVFQTPKYRWGAHSTAVDADWISMLFGPFGDPYRRDPRMPWT 6gcs.1    --------------------------------------------------------------------------------  target    GEAYLEINPKDAA 6gcs.1    ------------- ``` | | | | | | | | | | | | | | | | | | | | | | | | | | | | | | | | | | | | | | | | | | | | | | | | | |
|  | 3ir5.1.A | Respiratory nitrate reductase 1 alpha chain  *Crystal structure of NarGHI mutant NarG-H49C* | 0.03 | 0.00 | 17.24 | 0.14 | 654-769 | X-ray | 2.30 | monomer | 2 x MD1, 1 x 6MO, 4 x SF4, 1 x AGA, 1 x F3S, 2 x HEM | HHblits | 0.27 |
| ``` target    SWDEATTIAAKTMEDVARTFNGDEGARKLLAQGYHPEMVEVMHGAGVQALKLRGGMPLLGIGRIFGFYRFANMLALLDRK 3ir5.1    --------------------------------------------------------------------------------  target    LRPDAPADEILGSRTFDNYAWHTDLPPGHPMVTGSQTVDFDLFSAEHTKLLLIIGMNWICTKMPDGHWIGDARLKGTRVI 3ir5.1    --------------------------------------------------------------------------------  target    VISADYMPTANKADEVIILRPGTDAAFFLGVARELIEKGLYDRAAVIERTDLPLLVRLDTGERLDARDVIPGYELAALTN 3ir5.1    --------------------------------------------------------------------------------  target    YVTLKPDAEIKGNPPPPPFTAGGQVVPTELRDAWGDFVWWDRATGRPRPVSRDEVGARFDGDPALLGEFEVELVDGSTVP 3ir5.1    --------------------------------------------------------------------------------  target    VRPAFDLLKQYLDESFDLRTASEVCRVPPQAIQSIARQLAANKRETLLAAGMGPNHYFQNDLFGRVQFLVAALTDNIGHL 3ir5.1    --------------------------------------------------------------------------------  target    GGNVGSYAGNYRGSVFQAMGQWIAEDPFAIEPDLTKPATVKRYYKAESAHYWNYGERPLRAVAKDDEGDLTKGEVLTGKS 3ir5.1    --------------------------------------------------------------------------------  target    HMPTPTKLIWFGNSNSLLGNAKWSFDVVKNTLPRQDAVFCNEWHWTSSCEYADLVFPADSWAEFKLPDATASCTNPFLLA 3ir5.1    --------------------------------------------------------------------------------  target    FPTTPLKRLYDTRSDYEALALTAKALGELIDEPRMEQYWRGILDGDPTPYLQRIFSGSNATRGITYDELHESSKRGVPLL 3ir5.1    --------------------------------------------------------------------------------  target    MNMRTYPRSGGWEQRQEDKPWYTATGRLEFYRPEPEFQAAGESLPVWREPVDAT-------------FYEPNAILSNAAH 3ir5.1    -------------NVHELIPWRTLSGRQQLYQDHQWMRDFGESLLVYRPPIDTRSVKEVIGQKSNGNQEKALNFLTPHQK  target    PSIAPRAPED-------YGVPESQLDVETRQYRN-----VVRTWAELQQTLHPLQERDPAFRFVFQTPKYRWGAHSTAVD 3ir5.1    WGIHSTYSDNLLMLTLGRGGPVVWLSEADAKDLGIADNDWIEVFNSNGALTARAVVSQRVPAGMTMMYHAQERI------  target    ADWISMLFGPFGDPYRRDPRMPWTGEAYLEINPKDAA 3ir5.1    ------------------------------------- ``` | | | | | | | | | | | | | | | | | | | | | | | | | | | | | | | | | | | | | | | | | | | | | | | | | |
|  | 3egw.1.A | Respiratory nitrate reductase 1 alpha chain  *The crystal structure of the NarGHI mutant NarH - C16A* | 0.03 | 0.00 | 17.24 | 0.14 | 654-769 | X-ray | 1.90 | monomer | 2 x MD1, 2 x MGD, 2 x 6MO, 6 x SF4, 4 x F3S, 2 x 3PH, 4 x HEM, 2 x AGA | HHblits | 0.27 |
| ``` target    SWDEATTIAAKTMEDVARTFNGDEGARKLLAQGYHPEMVEVMHGAGVQALKLRGGMPLLGIGRIFGFYRFANMLALLDRK 3egw.1    --------------------------------------------------------------------------------  target    LRPDAPADEILGSRTFDNYAWHTDLPPGHPMVTGSQTVDFDLFSAEHTKLLLIIGMNWICTKMPDGHWIGDARLKGTRVI 3egw.1    --------------------------------------------------------------------------------  target    VISADYMPTANKADEVIILRPGTDAAFFLGVARELIEKGLYDRAAVIERTDLPLLVRLDTGERLDARDVIPGYELAALTN 3egw.1    --------------------------------------------------------------------------------  target    YVTLKPDAEIKGNPPPPPFTAGGQVVPTELRDAWGDFVWWDRATGRPRPVSRDEVGARFDGDPALLGEFEVELVDGSTVP 3egw.1    --------------------------------------------------------------------------------  target    VRPAFDLLKQYLDESFDLRTASEVCRVPPQAIQSIARQLAANKRETLLAAGMGPNHYFQNDLFGRVQFLVAALTDNIGHL 3egw.1    --------------------------------------------------------------------------------  target    GGNVGSYAGNYRGSVFQAMGQWIAEDPFAIEPDLTKPATVKRYYKAESAHYWNYGERPLRAVAKDDEGDLTKGEVLTGKS 3egw.1    --------------------------------------------------------------------------------  target    HMPTPTKLIWFGNSNSLLGNAKWSFDVVKNTLPRQDAVFCNEWHWTSSCEYADLVFPADSWAEFKLPDATASCTNPFLLA 3egw.1    --------------------------------------------------------------------------------  target    FPTTPLKRLYDTRSDYEALALTAKALGELIDEPRMEQYWRGILDGDPTPYLQRIFSGSNATRGITYDELHESSKRGVPLL 3egw.1    --------------------------------------------------------------------------------  target    MNMRTYPRSGGWEQRQEDKPWYTATGRLEFYRPEPEFQAAGESLPVWREPVDAT-------------FYEPNAILSNAAH 3egw.1    -------------NVHELIPWRTLSGRQQLYQDHQWMRDFGESLLVYRPPIDTRSVKEVIGQKSNGNQEKALNFLTPHQK  target    PSIAPRAPED-------YGVPESQLDVETRQYRN-----VVRTWAELQQTLHPLQERDPAFRFVFQTPKYRWGAHSTAVD 3egw.1    WGIHSTYSDNLLMLTLGRGGPVVWLSEADAKDLGIADNDWIEVFNSNGALTARAVVSQRVPAGMTMMYHAQERI------  target    ADWISMLFGPFGDPYRRDPRMPWTGEAYLEINPKDAA 3egw.1    ------------------------------------- ``` | | | | | | | | | | | | | | | | | | | | | | | | | | | | | | | | | | | | | | | | | | | | | | | | | |
| ✓ | 1r27.4.A | Respiratory nitrate reductase 1 alpha chain  *Crystal Structure of NarGH complex* | 0.03 | 0.00 | 17.24 | 0.14 | 654-769 | X-ray | 2.00 | monomer | 4 x MO, 16 x SF4, 8 x MGD, 4 x F3S | HHblits | 0.27 |
| ``` target    SWDEATTIAAKTMEDVARTFNGDEGARKLLAQGYHPEMVEVMHGAGVQALKLRGGMPLLGIGRIFGFYRFANMLALLDRK 1r27.4    --------------------------------------------------------------------------------  target    LRPDAPADEILGSRTFDNYAWHTDLPPGHPMVTGSQTVDFDLFSAEHTKLLLIIGMNWICTKMPDGHWIGDARLKGTRVI 1r27.4    --------------------------------------------------------------------------------  target    VISADYMPTANKADEVIILRPGTDAAFFLGVARELIEKGLYDRAAVIERTDLPLLVRLDTGERLDARDVIPGYELAALTN 1r27.4    --------------------------------------------------------------------------------  target    YVTLKPDAEIKGNPPPPPFTAGGQVVPTELRDAWGDFVWWDRATGRPRPVSRDEVGARFDGDPALLGEFEVELVDGSTVP 1r27.4    --------------------------------------------------------------------------------  target    VRPAFDLLKQYLDESFDLRTASEVCRVPPQAIQSIARQLAANKRETLLAAGMGPNHYFQNDLFGRVQFLVAALTDNIGHL 1r27.4    --------------------------------------------------------------------------------  target    GGNVGSYAGNYRGSVFQAMGQWIAEDPFAIEPDLTKPATVKRYYKAESAHYWNYGERPLRAVAKDDEGDLTKGEVLTGKS 1r27.4    --------------------------------------------------------------------------------  target    HMPTPTKLIWFGNSNSLLGNAKWSFDVVKNTLPRQDAVFCNEWHWTSSCEYADLVFPADSWAEFKLPDATASCTNPFLLA 1r27.4    --------------------------------------------------------------------------------  target    FPTTPLKRLYDTRSDYEALALTAKALGELIDEPRMEQYWRGILDGDPTPYLQRIFSGSNATRGITYDELHESSKRGVPLL 1r27.4    --------------------------------------------------------------------------------  target    MNMRTYPRSGGWEQRQEDKPWYTATGRLEFYRPEPEFQAAGESLPVWREPVDAT-------------FYEPNAILSNAAH 1r27.4    -------------NVHELIPWRTLSGRQQLYQDHQWMRDFGESLLVYRPPIDTRSVKEVIGQKSNGNQEKALNFLTPHQK  target    PSIAPRAPED-------YGVPESQLDVETRQYRN-----VVRTWAELQQTLHPLQERDPAFRFVFQTPKYRWGAHSTAVD 1r27.4    WGIHSTYSDNLLMLTLGRGGPVVWLSEADAKDLGIADNDWIEVFNSNGALTARAVVSQRVPAGMTMMYHAQERI------  target    ADWISMLFGPFGDPYRRDPRMPWTGEAYLEINPKDAA 1r27.4    ------------------------------------- ``` | | | | | | | | | | | | | | | | | | | | | | | | | | | | | | | | | | | | | | | | | | | | | | | | | |
|  | 1q16.1.A | Respiratory nitrate reductase 1 alpha chain  *Crystal structure of Nitrate Reductase A, NarGHI, from Escherichia coli* | 0.03 | 0.00 | 17.39 | 0.14 | 654-768 | X-ray | 1.90 | monomer | 2 x MD1, 1 x 6MO, 2 x HEM, 4 x SF4, 1 x F3S, 1 x AGA, 1 x 3PH | HHblits | 0.27 |
| ``` target    SWDEATTIAAKTMEDVARTFNGDEGARKLLAQGYHPEMVEVMHGAGVQALKLRGGMPLLGIGRIFGFYRFANMLALLDRK 1q16.1    --------------------------------------------------------------------------------  target    LRPDAPADEILGSRTFDNYAWHTDLPPGHPMVTGSQTVDFDLFSAEHTKLLLIIGMNWICTKMPDGHWIGDARLKGTRVI 1q16.1    --------------------------------------------------------------------------------  target    VISADYMPTANKADEVIILRPGTDAAFFLGVARELIEKGLYDRAAVIERTDLPLLVRLDTGERLDARDVIPGYELAALTN 1q16.1    --------------------------------------------------------------------------------  target    YVTLKPDAEIKGNPPPPPFTAGGQVVPTELRDAWGDFVWWDRATGRPRPVSRDEVGARFDGDPALLGEFEVELVDGSTVP 1q16.1    --------------------------------------------------------------------------------  target    VRPAFDLLKQYLDESFDLRTASEVCRVPPQAIQSIARQLAANKRETLLAAGMGPNHYFQNDLFGRVQFLVAALTDNIGHL 1q16.1    --------------------------------------------------------------------------------  target    GGNVGSYAGNYRGSVFQAMGQWIAEDPFAIEPDLTKPATVKRYYKAESAHYWNYGERPLRAVAKDDEGDLTKGEVLTGKS 1q16.1    --------------------------------------------------------------------------------  target    HMPTPTKLIWFGNSNSLLGNAKWSFDVVKNTLPRQDAVFCNEWHWTSSCEYADLVFPADSWAEFKLPDATASCTNPFLLA 1q16.1    --------------------------------------------------------------------------------  target    FPTTPLKRLYDTRSDYEALALTAKALGELIDEPRMEQYWRGILDGDPTPYLQRIFSGSNATRGITYDELHESSKRGVPLL 1q16.1    --------------------------------------------------------------------------------  target    MNMRTYPRSGGWEQRQEDKPWYTATGRLEFYRPEPEFQAAGESLPVWREPVDA-------------TFYEPNAILSNAAH 1q16.1    -------------NVHELIPWRTLSGRQQLYQDHQWMRDFGESLLVYRPPIDTRSVKEVIGQKSNGNQEKALNFLTPHQK  target    PSIAPRAPED-------YGVPESQLDVETRQYRN-----VVRTWAELQQTLHPLQERDPAFRFVFQTPKYRWGAHSTAVD 1q16.1    WGIHSTYSDNLLMLTLGRGGPVVWLSEADAKDLGIADNDWIEVFNSNGALTARAVVSQRVPAGMTMMYHAQER-------  target    ADWISMLFGPFGDPYRRDPRMPWTGEAYLEINPKDAA 1q16.1    ------------------------------------- ``` | | | | | | | | | | | | | | | | | | | | | | | | | | | | | | | | | | | | | | | | | | | | | | | | | |
|  | 3ir7.1.A | Respiratory nitrate reductase 1 alpha chain  *Crystal structure of NarGHI mutant NarG-R94S* | 0.03 | 0.00 | 17.39 | 0.14 | 654-768 | X-ray | 2.50 | monomer | 2 x MD1, 4 x SF4, 1 x 6MO, 1 x AGA, 1 x F3S, 2 x HEM | HHblits | 0.27 |
| ``` target    SWDEATTIAAKTMEDVARTFNGDEGARKLLAQGYHPEMVEVMHGAGVQALKLRGGMPLLGIGRIFGFYRFANMLALLDRK 3ir7.1    --------------------------------------------------------------------------------  target    LRPDAPADEILGSRTFDNYAWHTDLPPGHPMVTGSQTVDFDLFSAEHTKLLLIIGMNWICTKMPDGHWIGDARLKGTRVI 3ir7.1    --------------------------------------------------------------------------------  target    VISADYMPTANKADEVIILRPGTDAAFFLGVARELIEKGLYDRAAVIERTDLPLLVRLDTGERLDARDVIPGYELAALTN 3ir7.1    --------------------------------------------------------------------------------  target    YVTLKPDAEIKGNPPPPPFTAGGQVVPTELRDAWGDFVWWDRATGRPRPVSRDEVGARFDGDPALLGEFEVELVDGSTVP 3ir7.1    --------------------------------------------------------------------------------  target    VRPAFDLLKQYLDESFDLRTASEVCRVPPQAIQSIARQLAANKRETLLAAGMGPNHYFQNDLFGRVQFLVAALTDNIGHL 3ir7.1    --------------------------------------------------------------------------------  target    GGNVGSYAGNYRGSVFQAMGQWIAEDPFAIEPDLTKPATVKRYYKAESAHYWNYGERPLRAVAKDDEGDLTKGEVLTGKS 3ir7.1    --------------------------------------------------------------------------------  target    HMPTPTKLIWFGNSNSLLGNAKWSFDVVKNTLPRQDAVFCNEWHWTSSCEYADLVFPADSWAEFKLPDATASCTNPFLLA 3ir7.1    --------------------------------------------------------------------------------  target    FPTTPLKRLYDTRSDYEALALTAKALGELIDEPRMEQYWRGILDGDPTPYLQRIFSGSNATRGITYDELHESSKRGVPLL 3ir7.1    --------------------------------------------------------------------------------  target    MNMRTYPRSGGWEQRQEDKPWYTATGRLEFYRPEPEFQAAGESLPVWREPVDAT-------------FYEPNAILSNAAH 3ir7.1    -------------NVHELIPWRTLSGRQQLYQDHQWMRDFGESLLVYRPPIDTRSVKEVIGQKSNGNQEKALNFLTPHQK  target    PSIAPRAPED-------YGVPESQLDVETRQYRN-----VVRTWAELQQTLHPLQERDPAFRFVFQTPKYRWGAHSTAVD 3ir7.1    WGIHSTYSDNLLMLTLGRGGPVVWLSEADAKDLGIADNDWIEVFNSNGALTARAVVSQRVPAGMTMMYHAQER-------  target    ADWISMLFGPFGDPYRRDPRMPWTGEAYLEINPKDAA 3ir7.1    ------------------------------------- ``` | | | | | | | | | | | | | | | | | | | | | | | | | | | | | | | | | | | | | | | | | | | | | | | | | |
|  | 3ir6.1.A | Respiratory nitrate reductase 1 alpha chain  *Crystal structure of NarGHI mutant NarG-H49S* | 0.03 | 0.00 | 17.54 | 0.14 | 655-768 | X-ray | 2.80 | monomer | 2 x GDP, 1 x AGA, 3 x SF4, 1 x F3S, 2 x HEM | HHblits | 0.27 |
| ``` target    SWDEATTIAAKTMEDVARTFNGDEGARKLLAQGYHPEMVEVMHGAGVQALKLRGGMPLLGIGRIFGFYRFANMLALLDRK 3ir6.1    --------------------------------------------------------------------------------  target    LRPDAPADEILGSRTFDNYAWHTDLPPGHPMVTGSQTVDFDLFSAEHTKLLLIIGMNWICTKMPDGHWIGDARLKGTRVI 3ir6.1    --------------------------------------------------------------------------------  target    VISADYMPTANKADEVIILRPGTDAAFFLGVARELIEKGLYDRAAVIERTDLPLLVRLDTGERLDARDVIPGYELAALTN 3ir6.1    --------------------------------------------------------------------------------  target    YVTLKPDAEIKGNPPPPPFTAGGQVVPTELRDAWGDFVWWDRATGRPRPVSRDEVGARFDGDPALLGEFEVELVDGSTVP 3ir6.1    --------------------------------------------------------------------------------  target    VRPAFDLLKQYLDESFDLRTASEVCRVPPQAIQSIARQLAANKRETLLAAGMGPNHYFQNDLFGRVQFLVAALTDNIGHL 3ir6.1    --------------------------------------------------------------------------------  target    GGNVGSYAGNYRGSVFQAMGQWIAEDPFAIEPDLTKPATVKRYYKAESAHYWNYGERPLRAVAKDDEGDLTKGEVLTGKS 3ir6.1    --------------------------------------------------------------------------------  target    HMPTPTKLIWFGNSNSLLGNAKWSFDVVKNTLPRQDAVFCNEWHWTSSCEYADLVFPADSWAEFKLPDATASCTNPFLLA 3ir6.1    --------------------------------------------------------------------------------  target    FPTTPLKRLYDTRSDYEALALTAKALGELIDEPRMEQYWRGILDGDPTPYLQRIFSGSNATRGITYDELHESSKRGVPLL 3ir6.1    --------------------------------------------------------------------------------  target    MNMRTYPRSGGWEQRQEDKPWYTATGRLEFYRPEPEFQAAGESLPVWREPVDAT-------------FYEPNAILSNAAH 3ir6.1    --------------VHELIPWRTLSGRQQLYQDHQWMRDFGESLLVYRPPIDTRSVKEVIGQKSNGNQEKALNFLTPHQK  target    PSIAPRAPED-------YGVPESQLDVETRQYRN-----VVRTWAELQQTLHPLQERDPAFRFVFQTPKYRWGAHSTAVD 3ir6.1    WGIHSTYSDNLLMLTLGRGGPVVWLSEADAKDLGIADNDWIEVFNSNGALTARAVVSQRVPAGMTMMYHAQER-------  target    ADWISMLFGPFGDPYRRDPRMPWTGEAYLEINPKDAA 3ir6.1    ------------------------------------- ``` | | | | | | | | | | | | | | | | | | | | | | | | | | | | | | | | | | | | | | | | | | | | | | | | | |
|  | 7zd6.1.4 | NADH-ubiquinone oxidoreductase 75 kDa subunit, mitochondrial  *Complex I from Ovis aries, at pH7.4, Open state* | 0.05 | 0.00 | 17.02 | 0.12 | 484-589 | EM | 0.00 | monomer | 6 x PC1, 14 x 3PE, 1 x DCQ, 2 x ZMP, 1 x AMP, 1 x MYR, 6 x SF4, 1 x FMN, 1 x NAI, 2 x FES, 1 x K, 1 x ZN, 1 x NDP | HHblits | 0.30 |
| ``` target    SWDEATTIAAKTMEDVARTFNGDEGARKLLAQGYHPEMVEVMHGAGVQALKLRGGMPLLGIGRIFGFYRFANMLALLDRK 7zd6.1    --------------------------------------------------------------------------------  target    LRPDAPADEILGSRTFDNYAWHTDLPPGHPMVTGSQTVDFDLFSAEHTKLLLIIGMNWICTKMPDGHWIGDARLKGTRVI 7zd6.1    --------------------------------------------------------------------------------  target    VISADYMPTANKADEVIILRPGTDAAFFLGVARELIEKGLYDRAAVIERTDLPLLVRLDTGERLDARDVIPGYELAALTN 7zd6.1    --------------------------------------------------------------------------------  target    YVTLKPDAEIKGNPPPPPFTAGGQVVPTELRDAWGDFVWWDRATGRPRPVSRDEVGARFDGDPALLGEFEVELVDGSTVP 7zd6.1    --------------------------------------------------------------------------------  target    VRPAFDLLKQYLDESFDLRTASEVCRVPPQAIQSIARQLAANKRETLLAAGMGPNHYFQNDLFGRVQFLVAALTDNIGHL 7zd6.1    --------------------------------------------------------------------------------  target    GGNVGSYAGNYRGSVFQAMGQWIAEDPFAIEPDLTKPATVKRYYKAESAHYWNYGERPLRAVAKDDEGDLTKGEVLTGKS 7zd6.1    --------------------------------------------------------------------------------  target    HMPTPTKLIWFGNSNSLLGNAKWSFDVVKNTLPRQDAVFCNEWHWTSSCEYADLVFPADSWAEFKLPDATASCTNPFLLA 7zd6.1    ---NPPKMLFLLGADGGC--------VTRQDLPKDCFIVYQGHHGDVGAPIADVILPGAAYTEK---SATYVNTEGRAQQ  target    FPTTPLKRLYDTRSDYEALALTAKALGELIDEPRMEQYWRGILDGDPTPYLQRIFSGSNATRGITYDELHESSKRGVPLL 7zd6.1    T-KVAVMPPGLAREDWKIIRALSEIAGMT---------------------------------------------------  target    MNMRTYPRSGGWEQRQEDKPWYTATGRLEFYRPEPEFQAAGESLPVWREPVDATFYEPNAILSNAAHPSIAPRAPEDYGV 7zd6.1    --------------------------------------------------------------------------------  target    PESQLDVETRQYRNVVRTWAELQQTLHPLQERDPAFRFVFQTPKYRWGAHSTAVDADWISMLFGPFGDPYRRDPRMPWTG 7zd6.1    --------------------------------------------------------------------------------  target    EAYLEINPKDAA 7zd6.1    ------------ ``` | | | | | | | | | | | | | | | | | | | | | | | | | | | | | | | | | | | | | | | | | | | | | | | | | |
|  | 6qcf.1.C | NADH:ubiquinone oxidoreductase core subunit S1  *Ovine respiratory complex I FRC open class 6* | 0.05 | 0.00 | 17.02 | 0.12 | 484-589 | EM | 0.00 | monomer | 6 x SF4, 1 x FMN, 2 x FES, 1 x ZN, 1 x NDP, 2 x ZMP | HHblits | 0.30 |
| ``` target    SWDEATTIAAKTMEDVARTFNGDEGARKLLAQGYHPEMVEVMHGAGVQALKLRGGMPLLGIGRIFGFYRFANMLALLDRK 6qcf.1    --------------------------------------------------------------------------------  target    LRPDAPADEILGSRTFDNYAWHTDLPPGHPMVTGSQTVDFDLFSAEHTKLLLIIGMNWICTKMPDGHWIGDARLKGTRVI 6qcf.1    --------------------------------------------------------------------------------  target    VISADYMPTANKADEVIILRPGTDAAFFLGVARELIEKGLYDRAAVIERTDLPLLVRLDTGERLDARDVIPGYELAALTN 6qcf.1    --------------------------------------------------------------------------------  target    YVTLKPDAEIKGNPPPPPFTAGGQVVPTELRDAWGDFVWWDRATGRPRPVSRDEVGARFDGDPALLGEFEVELVDGSTVP 6qcf.1    --------------------------------------------------------------------------------  target    VRPAFDLLKQYLDESFDLRTASEVCRVPPQAIQSIARQLAANKRETLLAAGMGPNHYFQNDLFGRVQFLVAALTDNIGHL 6qcf.1    --------------------------------------------------------------------------------  target    GGNVGSYAGNYRGSVFQAMGQWIAEDPFAIEPDLTKPATVKRYYKAESAHYWNYGERPLRAVAKDDEGDLTKGEVLTGKS 6qcf.1    --------------------------------------------------------------------------------  target    HMPTPTKLIWFGNSNSLLGNAKWSFDVVKNTLPRQDAVFCNEWHWTSSCEYADLVFPADSWAEFKLPDATASCTNPFLLA 6qcf.1    ---NPPKMLFLLGADGGC--------VTRQDLPKDCFIVYQGHHGDVGAPIADVILPGAAYTEK---SATYVNTEGRAQQ  target    FPTTPLKRLYDTRSDYEALALTAKALGELIDEPRMEQYWRGILDGDPTPYLQRIFSGSNATRGITYDELHESSKRGVPLL 6qcf.1    T-KVAVMPPGLAREDWKIIRALSEIAGMT---------------------------------------------------  target    MNMRTYPRSGGWEQRQEDKPWYTATGRLEFYRPEPEFQAAGESLPVWREPVDATFYEPNAILSNAAHPSIAPRAPEDYGV 6qcf.1    --------------------------------------------------------------------------------  target    PESQLDVETRQYRNVVRTWAELQQTLHPLQERDPAFRFVFQTPKYRWGAHSTAVDADWISMLFGPFGDPYRRDPRMPWTG 6qcf.1    --------------------------------------------------------------------------------  target    EAYLEINPKDAA 6qcf.1    ------------ ``` | | | | | | | | | | | | | | | | | | | | | | | | | | | | | | | | | | | | | | | | | | | | | | | | | |
|  | 6qc5.1.C | NADH:ubiquinone oxidoreductase core subunit S1  *Ovine respiratory complex I FRC closed class 1* | 0.05 | 0.00 | 17.02 | 0.12 | 484-589 | EM | 0.00 | monomer | 6 x SF4, 1 x FMN, 2 x FES, 2 x 3PE, 1 x ZN, 1 x NDP, 2 x ZMP, 1 x PC1 | HHblits | 0.30 |
| ``` target    SWDEATTIAAKTMEDVARTFNGDEGARKLLAQGYHPEMVEVMHGAGVQALKLRGGMPLLGIGRIFGFYRFANMLALLDRK 6qc5.1    --------------------------------------------------------------------------------  target    LRPDAPADEILGSRTFDNYAWHTDLPPGHPMVTGSQTVDFDLFSAEHTKLLLIIGMNWICTKMPDGHWIGDARLKGTRVI 6qc5.1    --------------------------------------------------------------------------------  target    VISADYMPTANKADEVIILRPGTDAAFFLGVARELIEKGLYDRAAVIERTDLPLLVRLDTGERLDARDVIPGYELAALTN 6qc5.1    --------------------------------------------------------------------------------  target    YVTLKPDAEIKGNPPPPPFTAGGQVVPTELRDAWGDFVWWDRATGRPRPVSRDEVGARFDGDPALLGEFEVELVDGSTVP 6qc5.1    --------------------------------------------------------------------------------  target    VRPAFDLLKQYLDESFDLRTASEVCRVPPQAIQSIARQLAANKRETLLAAGMGPNHYFQNDLFGRVQFLVAALTDNIGHL 6qc5.1    --------------------------------------------------------------------------------  target    GGNVGSYAGNYRGSVFQAMGQWIAEDPFAIEPDLTKPATVKRYYKAESAHYWNYGERPLRAVAKDDEGDLTKGEVLTGKS 6qc5.1    --------------------------------------------------------------------------------  target    HMPTPTKLIWFGNSNSLLGNAKWSFDVVKNTLPRQDAVFCNEWHWTSSCEYADLVFPADSWAEFKLPDATASCTNPFLLA 6qc5.1    ---NPPKMLFLLGADGGC--------VTRQDLPKDCFIVYQGHHGDVGAPIADVILPGAAYTEK---SATYVNTEGRAQQ  target    FPTTPLKRLYDTRSDYEALALTAKALGELIDEPRMEQYWRGILDGDPTPYLQRIFSGSNATRGITYDELHESSKRGVPLL 6qc5.1    T-KVAVMPPGLAREDWKIIRALSEIAGMT---------------------------------------------------  target    MNMRTYPRSGGWEQRQEDKPWYTATGRLEFYRPEPEFQAAGESLPVWREPVDATFYEPNAILSNAAHPSIAPRAPEDYGV 6qc5.1    --------------------------------------------------------------------------------  target    PESQLDVETRQYRNVVRTWAELQQTLHPLQERDPAFRFVFQTPKYRWGAHSTAVDADWISMLFGPFGDPYRRDPRMPWTG 6qc5.1    --------------------------------------------------------------------------------  target    EAYLEINPKDAA 6qc5.1    ------------ ``` | | | | | | | | | | | | | | | | | | | | | | | | | | | | | | | | | | | | | | | | | | | | | | | | | |
|  | 5gpn.24.A | NADH-ubiquinone oxidoreductase 75 kDa subunit  *Architecture of mammalian respirasome* | 0.05 | 0.00 | 15.96 | 0.12 | 484-589 | EM | 0.00 | monomer |  | HHblits | 0.29 |
| ``` target    SWDEATTIAAKTMEDVARTFNGDEGARKLLAQGYHPEMVEVMHGAGVQALKLRGGMPLLGIGRIFGFYRFANMLALLDRK 5gpn.24   --------------------------------------------------------------------------------  target    LRPDAPADEILGSRTFDNYAWHTDLPPGHPMVTGSQTVDFDLFSAEHTKLLLIIGMNWICTKMPDGHWIGDARLKGTRVI 5gpn.24   --------------------------------------------------------------------------------  target    VISADYMPTANKADEVIILRPGTDAAFFLGVARELIEKGLYDRAAVIERTDLPLLVRLDTGERLDARDVIPGYELAALTN 5gpn.24   --------------------------------------------------------------------------------  target    YVTLKPDAEIKGNPPPPPFTAGGQVVPTELRDAWGDFVWWDRATGRPRPVSRDEVGARFDGDPALLGEFEVELVDGSTVP 5gpn.24   --------------------------------------------------------------------------------  target    VRPAFDLLKQYLDESFDLRTASEVCRVPPQAIQSIARQLAANKRETLLAAGMGPNHYFQNDLFGRVQFLVAALTDNIGHL 5gpn.24   --------------------------------------------------------------------------------  target    GGNVGSYAGNYRGSVFQAMGQWIAEDPFAIEPDLTKPATVKRYYKAESAHYWNYGERPLRAVAKDDEGDLTKGEVLTGKS 5gpn.24   --------------------------------------------------------------------------------  target    HMPTPTKLIWFGNSNSLLGNAKWSFDVVKNTLPRQDAVFCNEWHWTSSCEYADLVFPADSWAEFKLPDATASCTNPFLLA 5gpn.24   ---NPPKVLFLLGADGG--------CITRQDLPKDCFIIYQGHHGDVGAPMADVILPGAAYTEK---SATYVNTEGRAQQ  target    FPTTPLKRLYDTRSDYEALALTAKALGELIDEPRMEQYWRGILDGDPTPYLQRIFSGSNATRGITYDELHESSKRGVPLL 5gpn.24   T-KVAVTPPGLAREDWKIIRALSEIAGMT---------------------------------------------------  target    MNMRTYPRSGGWEQRQEDKPWYTATGRLEFYRPEPEFQAAGESLPVWREPVDATFYEPNAILSNAAHPSIAPRAPEDYGV 5gpn.24   --------------------------------------------------------------------------------  target    PESQLDVETRQYRNVVRTWAELQQTLHPLQERDPAFRFVFQTPKYRWGAHSTAVDADWISMLFGPFGDPYRRDPRMPWTG 5gpn.24   --------------------------------------------------------------------------------  target    EAYLEINPKDAA 5gpn.24   ------------ ``` | | | | | | | | | | | | | | | | | | | | | | | | | | | | | | | | | | | | | | | | | | | | | | | | | |
|  | 7qsd.1.G | NADH-ubiquinone oxidoreductase 75 kDa subunit, mitochondrial  *Bovine complex I in the active state at 3.1 A* | 0.05 |  | 15.96 | 0.12 | 484-589 | EM | 0.00 | hetero-1-1-1-1-1-1-… | 5 x PC1, 13 x 3PE, 6 x SF4, 2 x FES, 1 x FMN, 4 x CDL, 3 x LMT, 1 x GTP, 1 x MG, 1 x NDP, 1 x ZN, 2 x EHZ | HHblits | 0.29 |
| ``` target    SWDEATTIAAKTMEDVARTFNGDEGARKLLAQGYHPEMVEVMHGAGVQALKLRGGMPLLGIGRIFGFYRFANMLALLDRK 7qsd.1    --------------------------------------------------------------------------------  target    LRPDAPADEILGSRTFDNYAWHTDLPPGHPMVTGSQTVDFDLFSAEHTKLLLIIGMNWICTKMPDGHWIGDARLKGTRVI 7qsd.1    --------------------------------------------------------------------------------  target    VISADYMPTANKADEVIILRPGTDAAFFLGVARELIEKGLYDRAAVIERTDLPLLVRLDTGERLDARDVIPGYELAALTN 7qsd.1    --------------------------------------------------------------------------------  target    YVTLKPDAEIKGNPPPPPFTAGGQVVPTELRDAWGDFVWWDRATGRPRPVSRDEVGARFDGDPALLGEFEVELVDGSTVP 7qsd.1    --------------------------------------------------------------------------------  target    VRPAFDLLKQYLDESFDLRTASEVCRVPPQAIQSIARQLAANKRETLLAAGMGPNHYFQNDLFGRVQFLVAALTDNIGHL 7qsd.1    --------------------------------------------------------------------------------  target    GGNVGSYAGNYRGSVFQAMGQWIAEDPFAIEPDLTKPATVKRYYKAESAHYWNYGERPLRAVAKDDEGDLTKGEVLTGKS 7qsd.1    --------------------------------------------------------------------------------  target    HMPTPTKLIWFGNSNSLLGNAKWSFDVVKNTLPRQDAVFCNEWHWTSSCEYADLVFPADSWAEFKLPDATASCTNPFLLA 7qsd.1    ---NPPKMLFLLGADGG--------CITRQDLPKDCFIVYQGHHGDVGAPIADVILPGAAYTEK---SATYVNTEGRAQQ  target    FPTTPLKRLYDTRSDYEALALTAKALGELIDEPRMEQYWRGILDGDPTPYLQRIFSGSNATRGITYDELHESSKRGVPLL 7qsd.1    T-KVAVTPPGLAREDWKIIRALSEIAGMT---------------------------------------------------  target    MNMRTYPRSGGWEQRQEDKPWYTATGRLEFYRPEPEFQAAGESLPVWREPVDATFYEPNAILSNAAHPSIAPRAPEDYGV 7qsd.1    --------------------------------------------------------------------------------  target    PESQLDVETRQYRNVVRTWAELQQTLHPLQERDPAFRFVFQTPKYRWGAHSTAVDADWISMLFGPFGDPYRRDPRMPWTG 7qsd.1    --------------------------------------------------------------------------------  target    EAYLEINPKDAA 7qsd.1    ------------ ``` | | | | | | | | | | | | | | | | | | | | | | | | | | | | | | | | | | | | | | | | | | | | | | | | | |
|  | 7vxu.1.L | NADH-ubiquinone oxidoreductase 75 kDa subunit, mitochondrial  *Matrix arm of deactive state CI from Q10 dataset* | 0.05 |  | 15.96 | 0.12 | 484-589 | EM | 0.00 | hetero-1-1-1-1-1-1-… | 6 x SF4, 1 x FMN, 1 x PEE, 1 x PLX, 1 x 8Q1, 1 x NDP, 2 x FES, 1 x MG, 1 x CDL, 1 x ZN | HHblits | 0.29 |
| ``` target    SWDEATTIAAKTMEDVARTFNGDEGARKLLAQGYHPEMVEVMHGAGVQALKLRGGMPLLGIGRIFGFYRFANMLALLDRK 7vxu.1    --------------------------------------------------------------------------------  target    LRPDAPADEILGSRTFDNYAWHTDLPPGHPMVTGSQTVDFDLFSAEHTKLLLIIGMNWICTKMPDGHWIGDARLKGTRVI 7vxu.1    --------------------------------------------------------------------------------  target    VISADYMPTANKADEVIILRPGTDAAFFLGVARELIEKGLYDRAAVIERTDLPLLVRLDTGERLDARDVIPGYELAALTN 7vxu.1    --------------------------------------------------------------------------------  target    YVTLKPDAEIKGNPPPPPFTAGGQVVPTELRDAWGDFVWWDRATGRPRPVSRDEVGARFDGDPALLGEFEVELVDGSTVP 7vxu.1    --------------------------------------------------------------------------------  target    VRPAFDLLKQYLDESFDLRTASEVCRVPPQAIQSIARQLAANKRETLLAAGMGPNHYFQNDLFGRVQFLVAALTDNIGHL 7vxu.1    --------------------------------------------------------------------------------  target    GGNVGSYAGNYRGSVFQAMGQWIAEDPFAIEPDLTKPATVKRYYKAESAHYWNYGERPLRAVAKDDEGDLTKGEVLTGKS 7vxu.1    --------------------------------------------------------------------------------  target    HMPTPTKLIWFGNSNSLLGNAKWSFDVVKNTLPRQDAVFCNEWHWTSSCEYADLVFPADSWAEFKLPDATASCTNPFLLA 7vxu.1    ---NPPKVLFLLGADGG--------CITRQDLPKDCFIIYQGHHGDVGAPMADVILPGAAYTEK---SATYVNTEGRAQQ  target    FPTTPLKRLYDTRSDYEALALTAKALGELIDEPRMEQYWRGILDGDPTPYLQRIFSGSNATRGITYDELHESSKRGVPLL 7vxu.1    T-KVAVTPPGLAREDWKIIRALSEIAGMT---------------------------------------------------  target    MNMRTYPRSGGWEQRQEDKPWYTATGRLEFYRPEPEFQAAGESLPVWREPVDATFYEPNAILSNAAHPSIAPRAPEDYGV 7vxu.1    --------------------------------------------------------------------------------  target    PESQLDVETRQYRNVVRTWAELQQTLHPLQERDPAFRFVFQTPKYRWGAHSTAVDADWISMLFGPFGDPYRRDPRMPWTG 7vxu.1    --------------------------------------------------------------------------------  target    EAYLEINPKDAA 7vxu.1    ------------ ``` | | | | | | | | | | | | | | | | | | | | | | | | | | | | | | | | | | | | | | | | | | | | | | | | | |
|  | 7v2c.1.L | NADH-ubiquinone oxidoreductase 75 kDa subunit, mitochondrial  *Active state complex I from Q10 dataset* | 0.05 |  | 15.96 | 0.12 | 484-589 | EM | 0.00 | hetero-1-1-1-1-1-2-… | 6 x SF4, 1 x FMN, 10 x PEE, 8 x PLX, 2 x 8Q1, 1 x NDP, 2 x UQ, 11 x CDL, 2 x FES, 1 x MG, 1 x ZN, 1 x ADP | HHblits | 0.29 |
| ``` target    SWDEATTIAAKTMEDVARTFNGDEGARKLLAQGYHPEMVEVMHGAGVQALKLRGGMPLLGIGRIFGFYRFANMLALLDRK 7v2c.1    --------------------------------------------------------------------------------  target    LRPDAPADEILGSRTFDNYAWHTDLPPGHPMVTGSQTVDFDLFSAEHTKLLLIIGMNWICTKMPDGHWIGDARLKGTRVI 7v2c.1    --------------------------------------------------------------------------------  target    VISADYMPTANKADEVIILRPGTDAAFFLGVARELIEKGLYDRAAVIERTDLPLLVRLDTGERLDARDVIPGYELAALTN 7v2c.1    --------------------------------------------------------------------------------  target    YVTLKPDAEIKGNPPPPPFTAGGQVVPTELRDAWGDFVWWDRATGRPRPVSRDEVGARFDGDPALLGEFEVELVDGSTVP 7v2c.1    --------------------------------------------------------------------------------  target    VRPAFDLLKQYLDESFDLRTASEVCRVPPQAIQSIARQLAANKRETLLAAGMGPNHYFQNDLFGRVQFLVAALTDNIGHL 7v2c.1    --------------------------------------------------------------------------------  target    GGNVGSYAGNYRGSVFQAMGQWIAEDPFAIEPDLTKPATVKRYYKAESAHYWNYGERPLRAVAKDDEGDLTKGEVLTGKS 7v2c.1    --------------------------------------------------------------------------------  target    HMPTPTKLIWFGNSNSLLGNAKWSFDVVKNTLPRQDAVFCNEWHWTSSCEYADLVFPADSWAEFKLPDATASCTNPFLLA 7v2c.1    ---NPPKVLFLLGADGG--------CITRQDLPKDCFIIYQGHHGDVGAPMADVILPGAAYTEK---SATYVNTEGRAQQ  target    FPTTPLKRLYDTRSDYEALALTAKALGELIDEPRMEQYWRGILDGDPTPYLQRIFSGSNATRGITYDELHESSKRGVPLL 7v2c.1    T-KVAVTPPGLAREDWKIIRALSEIAGMT---------------------------------------------------  target    MNMRTYPRSGGWEQRQEDKPWYTATGRLEFYRPEPEFQAAGESLPVWREPVDATFYEPNAILSNAAHPSIAPRAPEDYGV 7v2c.1    --------------------------------------------------------------------------------  target    PESQLDVETRQYRNVVRTWAELQQTLHPLQERDPAFRFVFQTPKYRWGAHSTAVDADWISMLFGPFGDPYRRDPRMPWTG 7v2c.1    --------------------------------------------------------------------------------  target    EAYLEINPKDAA 7v2c.1    ------------ ``` | | | | | | | | | | | | | | | | | | | | | | | | | | | | | | | | | | | | | | | | | | | | | | | | | |
|  | 8b9z.1.G | NADH-ubiquinone oxidoreductase 75 kDa subunit, mitochondrial  *Drosophila melanogaster complex I in the Active state (Dm1)* | 0.05 |  | 18.09 | 0.12 | 484-589 | EM | 3.28 | hetero-1-1-1-1-1-1-… | 3 x PC1, 16 x 3PE, 6 x SF4, 4 x CDL, 2 x FES, 1 x FMN, 1 x UQ9, 1 x DGT, 1 x NDP, 1 x ZN, 2 x EHZ | HHblits | 0.29 |
| ``` target    SWDEATTIAAKTMEDVARTFNGDEGARKLLAQGYHPEMVEVMHGAGVQALKLRGGMPLLGIGRIFGFYRFANMLALLDRK 8b9z.1    --------------------------------------------------------------------------------  target    LRPDAPADEILGSRTFDNYAWHTDLPPGHPMVTGSQTVDFDLFSAEHTKLLLIIGMNWICTKMPDGHWIGDARLKGTRVI 8b9z.1    --------------------------------------------------------------------------------  target    VISADYMPTANKADEVIILRPGTDAAFFLGVARELIEKGLYDRAAVIERTDLPLLVRLDTGERLDARDVIPGYELAALTN 8b9z.1    --------------------------------------------------------------------------------  target    YVTLKPDAEIKGNPPPPPFTAGGQVVPTELRDAWGDFVWWDRATGRPRPVSRDEVGARFDGDPALLGEFEVELVDGSTVP 8b9z.1    --------------------------------------------------------------------------------  target    VRPAFDLLKQYLDESFDLRTASEVCRVPPQAIQSIARQLAANKRETLLAAGMGPNHYFQNDLFGRVQFLVAALTDNIGHL 8b9z.1    --------------------------------------------------------------------------------  target    GGNVGSYAGNYRGSVFQAMGQWIAEDPFAIEPDLTKPATVKRYYKAESAHYWNYGERPLRAVAKDDEGDLTKGEVLTGKS 8b9z.1    --------------------------------------------------------------------------------  target    HMPTPTKLIWFGNSNSLLGNAKWSFDVVKNTLPRQDAVFCNEWHWTSSCEYADLVFPADSWAEFKLPDATASCTNPFLLA 8b9z.1    ---AQPKVLFLLNADAG--------KVTREQLPKDCFVVYIGSHGDNGASIADAVLPGAAYTEK---QGIYVNTEGRPQQ  target    FPTTPLKRLYDTRSDYEALALTAKALGELIDEPRMEQYWRGILDGDPTPYLQRIFSGSNATRGITYDELHESSKRGVPLL 8b9z.1    T-LPGVSPPGMAREDWKILRALSEVVGKP---------------------------------------------------  target    MNMRTYPRSGGWEQRQEDKPWYTATGRLEFYRPEPEFQAAGESLPVWREPVDATFYEPNAILSNAAHPSIAPRAPEDYGV 8b9z.1    --------------------------------------------------------------------------------  target    PESQLDVETRQYRNVVRTWAELQQTLHPLQERDPAFRFVFQTPKYRWGAHSTAVDADWISMLFGPFGDPYRRDPRMPWTG 8b9z.1    --------------------------------------------------------------------------------  target    EAYLEINPKDAA 8b9z.1    ------------ ``` | | | | | | | | | | | | | | | | | | | | | | | | | | | | | | | | | | | | | | | | | | | | | | | | | |
|  | 8ba0.1.G | NADH-ubiquinone oxidoreductase 75 kDa subunit, mitochondrial  *Drosophila melanogaster complex I in the Twisted state (Dm2)* | 0.05 |  | 18.09 | 0.12 | 484-589 | EM | 3.68 | hetero-1-1-1-1-1-1-… | 6 x SF4, 6 x 3PE, 2 x FES, 1 x FMN, 2 x CDL, 1 x DGT, 1 x NDP, 1 x ZN, 2 x EHZ | HHblits | 0.29 |
| ``` target    SWDEATTIAAKTMEDVARTFNGDEGARKLLAQGYHPEMVEVMHGAGVQALKLRGGMPLLGIGRIFGFYRFANMLALLDRK 8ba0.1    --------------------------------------------------------------------------------  target    LRPDAPADEILGSRTFDNYAWHTDLPPGHPMVTGSQTVDFDLFSAEHTKLLLIIGMNWICTKMPDGHWIGDARLKGTRVI 8ba0.1    --------------------------------------------------------------------------------  target    VISADYMPTANKADEVIILRPGTDAAFFLGVARELIEKGLYDRAAVIERTDLPLLVRLDTGERLDARDVIPGYELAALTN 8ba0.1    --------------------------------------------------------------------------------  target    YVTLKPDAEIKGNPPPPPFTAGGQVVPTELRDAWGDFVWWDRATGRPRPVSRDEVGARFDGDPALLGEFEVELVDGSTVP 8ba0.1    --------------------------------------------------------------------------------  target    VRPAFDLLKQYLDESFDLRTASEVCRVPPQAIQSIARQLAANKRETLLAAGMGPNHYFQNDLFGRVQFLVAALTDNIGHL 8ba0.1    --------------------------------------------------------------------------------  target    GGNVGSYAGNYRGSVFQAMGQWIAEDPFAIEPDLTKPATVKRYYKAESAHYWNYGERPLRAVAKDDEGDLTKGEVLTGKS 8ba0.1    --------------------------------------------------------------------------------  target    HMPTPTKLIWFGNSNSLLGNAKWSFDVVKNTLPRQDAVFCNEWHWTSSCEYADLVFPADSWAEFKLPDATASCTNPFLLA 8ba0.1    ---AQPKVLFLLNADAG--------KVTREQLPKDCFVVYIGSHGDNGASIADAVLPGAAYTEK---QGIYVNTEGRPQQ  target    FPTTPLKRLYDTRSDYEALALTAKALGELIDEPRMEQYWRGILDGDPTPYLQRIFSGSNATRGITYDELHESSKRGVPLL 8ba0.1    T-LPGVSPPGMAREDWKILRALSEVVGKP---------------------------------------------------  target    MNMRTYPRSGGWEQRQEDKPWYTATGRLEFYRPEPEFQAAGESLPVWREPVDATFYEPNAILSNAAHPSIAPRAPEDYGV 8ba0.1    --------------------------------------------------------------------------------  target    PESQLDVETRQYRNVVRTWAELQQTLHPLQERDPAFRFVFQTPKYRWGAHSTAVDADWISMLFGPFGDPYRRDPRMPWTG 8ba0.1    --------------------------------------------------------------------------------  target    EAYLEINPKDAA 8ba0.1    ------------ ``` | | | | | | | | | | | | | | | | | | | | | | | | | | | | | | | | | | | | | | | | | | | | | | | | | |
|  | 7q5y.1.A | NADH dehydrogenase I chain G  *Structure of NADH:ubichinon oxidoreductase (complex I) of the hyperthermophilic eubacterium Aquifex aeolicus* | 0.04 |  | 10.53 | 0.12 | 484-589 | X-ray | 2.70 | hetero-1-1-1-1-1-1-… | 8 x SF4, 2 x FES, 1 x FMN | HHblits | 0.24 |
| ``` target    SWDEATTIAAKTMEDVARTFNGDEGARKLLAQGYHPEMVEVMHGAGVQALKLRGGMPLLGIGRIFGFYRFANMLALLDRK 7q5y.1    --------------------------------------------------------------------------------  target    LRPDAPADEILGSRTFDNYAWHTDLPPGHPMVTGSQTVDFDLFSAEHTKLLLIIGMNWICTKMPDGHWIGDARLKGTRVI 7q5y.1    --------------------------------------------------------------------------------  target    VISADYMPTANKADEVIILRPGTDAAFFLGVARELIEKGLYDRAAVIERTDLPLLVRLDTGERLDARDVIPGYELAALTN 7q5y.1    --------------------------------------------------------------------------------  target    YVTLKPDAEIKGNPPPPPFTAGGQVVPTELRDAWGDFVWWDRATGRPRPVSRDEVGARFDGDPALLGEFEVELVDGSTVP 7q5y.1    --------------------------------------------------------------------------------  target    VRPAFDLLKQYLDESFDLRTASEVCRVPPQAIQSIARQLAANKRETLLAAGMGPNHYFQNDLFGRVQFLVAALTDNIGHL 7q5y.1    --------------------------------------------------------------------------------  target    GGNVGSYAGNYRGSVFQAMGQWIAEDPFAIEPDLTKPATVKRYYKAESAHYWNYGERPLRAVAKDDEGDLTKGEVLTGKS 7q5y.1    --------------------------------------------------------------------------------  target    HMPTPTKLIWFGNSNSLLGNAKWSFDVVKNTLPRQDAVFCNEWHWTSSCEYADLVFPADSWAEFKLPDATASCTNPFLLA 7q5y.1    ---GDIENLIIFGEDILEFYED---KVFEELKEKLEHLVVVSPYEDGLSEYAHIKIPMSLMGEN---EGTYKTFFGEVKG  target    FPTTPLKRLYDTRSDYEALALTAKALGELIDEPRMEQYWRGILDGDPTPYLQRIFSGSNATRGITYDELHESSKRGVPLL 7q5y.1    K--K-FLP--WAFDDLAFWKYLGENFKEE---------------------------------------------------  target    MNMRTYPRSGGWEQRQEDKPWYTATGRLEFYRPEPEFQAAGESLPVWREPVDATFYEPNAILSNAAHPSIAPRAPEDYGV 7q5y.1    --------------------------------------------------------------------------------  target    PESQLDVETRQYRNVVRTWAELQQTLHPLQERDPAFRFVFQTPKYRWGAHSTAVDADWISMLFGPFGDPYRRDPRMPWTG 7q5y.1    --------------------------------------------------------------------------------  target    EAYLEINPKDAA 7q5y.1    ------------ ``` | | | | | | | | | | | | | | | | | | | | | | | | | | | | | | | | | | | | | | | | | | | | | | | | | |
|  | 6s6y.1.B | Tungsten-containing formylmethanofuran dehydrogenase, subunit B  *X-ray crystal structure of the formyltransferase/hydrolase complex (FhcABCD) from Methylorubrum extorquens in complex with methylofuran* | 0.04 |  | 10.87 | 0.11 | 484-588 | X-ray | 3.10 | hetero-2-2-2-2-mer | 1 x MFN, 4 x ZN, 4 x CA, 4 x K, 3 x DGL, 2 x GLU, 1 x IAS | HHblits | 0.23 |
| ``` target    SWDEATTIAAKTMEDVARTFNGDEGARKLLAQGYHPEMVEVMHGAGVQALKLRGGMPLLGIGRIFGFYRFANMLALLDRK 6s6y.1    --------------------------------------------------------------------------------  target    LRPDAPADEILGSRTFDNYAWHTDLPPGHPMVTGSQTVDFDLFSAEHTKLLLIIGMNWICTKMPDGHWIGDARLKGTRVI 6s6y.1    --------------------------------------------------------------------------------  target    VISADYMPTANKADEVIILRPGTDAAFFLGVARELIEKGLYDRAAVIERTDLPLLVRLDTGERLDARDVIPGYELAALTN 6s6y.1    --------------------------------------------------------------------------------  target    YVTLKPDAEIKGNPPPPPFTAGGQVVPTELRDAWGDFVWWDRATGRPRPVSRDEVGARFDGDPALLGEFEVELVDGSTVP 6s6y.1    --------------------------------------------------------------------------------  target    VRPAFDLLKQYLDESFDLRTASEVCRVPPQAIQSIARQLAANKRETLLAAGMGPNHYFQNDLFGRVQFLVAALTDNIGHL 6s6y.1    --------------------------------------------------------------------------------  target    GGNVGSYAGNYRGSVFQAMGQWIAEDPFAIEPDLTKPATVKRYYKAESAHYWNYGERPLRAVAKDDEGDLTKGEVLTGKS 6s6y.1    --------------------------------------------------------------------------------  target    HMPTPTKLIWFGNSNSLLGNAKWSFDVVKNTLPRQDAVFCNE-WHWTSSCEYADLVFPADSW-AEFKLPDATASCTNPFL 6s6y.1    ---GEADAALWLASLPAP---------RPAWLGSLPTIAIVGEGSQEAAGETAEVVITVGVPGQSV---GGALWNDRRGV  target    LAFPTTPLKRL---YDTRSDYEALALTAKALGELIDEPRMEQYWRGILDGDPTPYLQRIFSGSNATRGITYDELHESSKR 6s6y.1    IAY-AEASDPAKTPAETETAAGVLTRIRDRLIE-----------------------------------------------  target    GVPLLMNMRTYPRSGGWEQRQEDKPWYTATGRLEFYRPEPEFQAAGESLPVWREPVDATFYEPNAILSNAAHPSIAPRAP 6s6y.1    --------------------------------------------------------------------------------  target    EDYGVPESQLDVETRQYRNVVRTWAELQQTLHPLQERDPAFRFVFQTPKYRWGAHSTAVDADWISMLFGPFGDPYRRDPR 6s6y.1    --------------------------------------------------------------------------------  target    MPWTGEAYLEINPKDAA 6s6y.1    ----------------- ``` | | | | | | | | | | | | | | | | | | | | | | | | | | | | | | | | | | | | | | | | | | | | | | | | | |
|  | 3m9s.1.C | NADH-quinone oxidoreductase subunit 3  *Crystal structure of respiratory complex I from Thermus thermophilus* | 0.04 | 0.00 | 21.79 | 0.10 | 508-589 | X-ray | 4.50 | monomer | 7 x SF4, 2 x FES, 1 x FMN | HHblits | 0.29 |
| ``` target    SWDEATTIAAKTMEDVARTFNGDEGARKLLAQGYHPEMVEVMHGAGVQALKLRGGMPLLGIGRIFGFYRFANMLALLDRK 3m9s.1    --------------------------------------------------------------------------------  target    LRPDAPADEILGSRTFDNYAWHTDLPPGHPMVTGSQTVDFDLFSAEHTKLLLIIGMNWICTKMPDGHWIGDARLKGTRVI 3m9s.1    --------------------------------------------------------------------------------  target    VISADYMPTANKADEVIILRPGTDAAFFLGVARELIEKGLYDRAAVIERTDLPLLVRLDTGERLDARDVIPGYELAALTN 3m9s.1    --------------------------------------------------------------------------------  target    YVTLKPDAEIKGNPPPPPFTAGGQVVPTELRDAWGDFVWWDRATGRPRPVSRDEVGARFDGDPALLGEFEVELVDGSTVP 3m9s.1    --------------------------------------------------------------------------------  target    VRPAFDLLKQYLDESFDLRTASEVCRVPPQAIQSIARQLAANKRETLLAAGMGPNHYFQNDLFGRVQFLVAALTDNIGHL 3m9s.1    --------------------------------------------------------------------------------  target    GGNVGSYAGNYRGSVFQAMGQWIAEDPFAIEPDLTKPATVKRYYKAESAHYWNYGERPLRAVAKDDEGDLTKGEVLTGKS 3m9s.1    --------------------------------------------------------------------------------  target    HMPTPTKLIWFGNSNSLLGNAKWSFDVVKNTLPRQDAVFCNEWHWTSSC-EYADLVFPADSWAEFKLPDATASCTNPFLL 3m9s.1    ---------------------------PEEALKGKRFVVMHLSHLHPLAERYAHVVLPAPTFYEK---RGHLVNLEGRVL  target    AFPTTPLKRLYDTRSDYEALALTAKALGELIDEPRMEQYWRGILDGDPTPYLQRIFSGSNATRGITYDELHESSKRGVPL 3m9s.1    PL-SPAPIENGEAEGALQVLALLAEALGVR--------------------------------------------------  target    LMNMRTYPRSGGWEQRQEDKPWYTATGRLEFYRPEPEFQAAGESLPVWREPVDATFYEPNAILSNAAHPSIAPRAPEDYG 3m9s.1    --------------------------------------------------------------------------------  target    VPESQLDVETRQYRNVVRTWAELQQTLHPLQERDPAFRFVFQTPKYRWGAHSTAVDADWISMLFGPFGDPYRRDPRMPWT 3m9s.1    --------------------------------------------------------------------------------  target    GEAYLEINPKDAA 3m9s.1    ------------- ``` | | | | | | | | | | | | | | | | | | | | | | | | | | | | | | | | | | | | | | | | | | | | | | | | | |
|  | 2fug.2.C | NADH-quinone oxidoreductase chain 3  *Crystal structure of the hydrophilic domain of respiratory complex I from Thermus thermophilus* | 0.04 | 0.00 | 21.79 | 0.10 | 508-589 | X-ray | 3.30 | monomer | 7 x SF4, 2 x FES, 1 x FMN | HHblits | 0.29 |
| ``` target    SWDEATTIAAKTMEDVARTFNGDEGARKLLAQGYHPEMVEVMHGAGVQALKLRGGMPLLGIGRIFGFYRFANMLALLDRK 2fug.2    --------------------------------------------------------------------------------  target    LRPDAPADEILGSRTFDNYAWHTDLPPGHPMVTGSQTVDFDLFSAEHTKLLLIIGMNWICTKMPDGHWIGDARLKGTRVI 2fug.2    --------------------------------------------------------------------------------  target    VISADYMPTANKADEVIILRPGTDAAFFLGVARELIEKGLYDRAAVIERTDLPLLVRLDTGERLDARDVIPGYELAALTN 2fug.2    --------------------------------------------------------------------------------  target    YVTLKPDAEIKGNPPPPPFTAGGQVVPTELRDAWGDFVWWDRATGRPRPVSRDEVGARFDGDPALLGEFEVELVDGSTVP 2fug.2    --------------------------------------------------------------------------------  target    VRPAFDLLKQYLDESFDLRTASEVCRVPPQAIQSIARQLAANKRETLLAAGMGPNHYFQNDLFGRVQFLVAALTDNIGHL 2fug.2    --------------------------------------------------------------------------------  target    GGNVGSYAGNYRGSVFQAMGQWIAEDPFAIEPDLTKPATVKRYYKAESAHYWNYGERPLRAVAKDDEGDLTKGEVLTGKS 2fug.2    --------------------------------------------------------------------------------  target    HMPTPTKLIWFGNSNSLLGNAKWSFDVVKNTLPRQDAVFCNEWHWTSSC-EYADLVFPADSWAEFKLPDATASCTNPFLL 2fug.2    ---------------------------PEEALKGKRFVVMHLSHLHPLAERYAHVVLPAPTFYEK---RGHLVNLEGRVL  target    AFPTTPLKRLYDTRSDYEALALTAKALGELIDEPRMEQYWRGILDGDPTPYLQRIFSGSNATRGITYDELHESSKRGVPL 2fug.2    PL-SPAPIENGEAEGALQVLALLAEALGVR--------------------------------------------------  target    LMNMRTYPRSGGWEQRQEDKPWYTATGRLEFYRPEPEFQAAGESLPVWREPVDATFYEPNAILSNAAHPSIAPRAPEDYG 2fug.2    --------------------------------------------------------------------------------  target    VPESQLDVETRQYRNVVRTWAELQQTLHPLQERDPAFRFVFQTPKYRWGAHSTAVDADWISMLFGPFGDPYRRDPRMPWT 2fug.2    --------------------------------------------------------------------------------  target    GEAYLEINPKDAA 2fug.2    ------------- ``` | | | | | | | | | | | | | | | | | | | | | | | | | | | | | | | | | | | | | | | | | | | | | | | | | |
|  | 6zjl.1.C | NADH-quinone oxidoreductase subunit 3  *Respiratory complex I from Thermus thermophilus, NAD+ dataset, major state* | 0.04 | 0.00 | 21.79 | 0.10 | 508-589 | EM | 0.00 | monomer | 7 x SF4, 1 x FMN, 2 x FES | HHblits | 0.29 |
| ``` target    SWDEATTIAAKTMEDVARTFNGDEGARKLLAQGYHPEMVEVMHGAGVQALKLRGGMPLLGIGRIFGFYRFANMLALLDRK 6zjl.1    --------------------------------------------------------------------------------  target    LRPDAPADEILGSRTFDNYAWHTDLPPGHPMVTGSQTVDFDLFSAEHTKLLLIIGMNWICTKMPDGHWIGDARLKGTRVI 6zjl.1    --------------------------------------------------------------------------------  target    VISADYMPTANKADEVIILRPGTDAAFFLGVARELIEKGLYDRAAVIERTDLPLLVRLDTGERLDARDVIPGYELAALTN 6zjl.1    --------------------------------------------------------------------------------  target    YVTLKPDAEIKGNPPPPPFTAGGQVVPTELRDAWGDFVWWDRATGRPRPVSRDEVGARFDGDPALLGEFEVELVDGSTVP 6zjl.1    --------------------------------------------------------------------------------  target    VRPAFDLLKQYLDESFDLRTASEVCRVPPQAIQSIARQLAANKRETLLAAGMGPNHYFQNDLFGRVQFLVAALTDNIGHL 6zjl.1    --------------------------------------------------------------------------------  target    GGNVGSYAGNYRGSVFQAMGQWIAEDPFAIEPDLTKPATVKRYYKAESAHYWNYGERPLRAVAKDDEGDLTKGEVLTGKS 6zjl.1    --------------------------------------------------------------------------------  target    HMPTPTKLIWFGNSNSLLGNAKWSFDVVKNTLPRQDAVFCNEWHWTSSC-EYADLVFPADSWAEFKLPDATASCTNPFLL 6zjl.1    ---------------------------PEEALKGKRFVVMHLSHLHPLAERYAHVVLPAPTFYEK---RGHLVNLEGRVL  target    AFPTTPLKRLYDTRSDYEALALTAKALGELIDEPRMEQYWRGILDGDPTPYLQRIFSGSNATRGITYDELHESSKRGVPL 6zjl.1    PL-SPAPIENGEAEGALQVLALLAEALGVR--------------------------------------------------  target    LMNMRTYPRSGGWEQRQEDKPWYTATGRLEFYRPEPEFQAAGESLPVWREPVDATFYEPNAILSNAAHPSIAPRAPEDYG 6zjl.1    --------------------------------------------------------------------------------  target    VPESQLDVETRQYRNVVRTWAELQQTLHPLQERDPAFRFVFQTPKYRWGAHSTAVDADWISMLFGPFGDPYRRDPRMPWT 6zjl.1    --------------------------------------------------------------------------------  target    GEAYLEINPKDAA 6zjl.1    ------------- ``` | | | | | | | | | | | | | | | | | | | | | | | | | | | | | | | | | | | | | | | | | | | | | | | | | |
|  | 6q8o.1.C | NADH-quinone oxidoreductase subunit 3  *Respiratory complex I from Thermus thermophilus with bound Piericidin A* | 0.04 | 0.00 | 21.79 | 0.10 | 508-589 | X-ray | 3.61 | monomer | 7 x SF4, 1 x FMN, 2 x FES, 1 x HQH | HHblits | 0.29 |
| ``` target    SWDEATTIAAKTMEDVARTFNGDEGARKLLAQGYHPEMVEVMHGAGVQALKLRGGMPLLGIGRIFGFYRFANMLALLDRK 6q8o.1    --------------------------------------------------------------------------------  target    LRPDAPADEILGSRTFDNYAWHTDLPPGHPMVTGSQTVDFDLFSAEHTKLLLIIGMNWICTKMPDGHWIGDARLKGTRVI 6q8o.1    --------------------------------------------------------------------------------  target    VISADYMPTANKADEVIILRPGTDAAFFLGVARELIEKGLYDRAAVIERTDLPLLVRLDTGERLDARDVIPGYELAALTN 6q8o.1    --------------------------------------------------------------------------------  target    YVTLKPDAEIKGNPPPPPFTAGGQVVPTELRDAWGDFVWWDRATGRPRPVSRDEVGARFDGDPALLGEFEVELVDGSTVP 6q8o.1    --------------------------------------------------------------------------------  target    VRPAFDLLKQYLDESFDLRTASEVCRVPPQAIQSIARQLAANKRETLLAAGMGPNHYFQNDLFGRVQFLVAALTDNIGHL 6q8o.1    --------------------------------------------------------------------------------  target    GGNVGSYAGNYRGSVFQAMGQWIAEDPFAIEPDLTKPATVKRYYKAESAHYWNYGERPLRAVAKDDEGDLTKGEVLTGKS 6q8o.1    --------------------------------------------------------------------------------  target    HMPTPTKLIWFGNSNSLLGNAKWSFDVVKNTLPRQDAVFCNEWHWTSSC-EYADLVFPADSWAEFKLPDATASCTNPFLL 6q8o.1    ---------------------------PEEALKGKRFVVMHLSHLHPLAERYAHVVLPAPTFYEK---RGHLVNLEGRVL  target    AFPTTPLKRLYDTRSDYEALALTAKALGELIDEPRMEQYWRGILDGDPTPYLQRIFSGSNATRGITYDELHESSKRGVPL 6q8o.1    PL-SPAPIENGEAEGALQVLALLAEALGVR--------------------------------------------------  target    LMNMRTYPRSGGWEQRQEDKPWYTATGRLEFYRPEPEFQAAGESLPVWREPVDATFYEPNAILSNAAHPSIAPRAPEDYG 6q8o.1    --------------------------------------------------------------------------------  target    VPESQLDVETRQYRNVVRTWAELQQTLHPLQERDPAFRFVFQTPKYRWGAHSTAVDADWISMLFGPFGDPYRRDPRMPWT 6q8o.1    --------------------------------------------------------------------------------  target    GEAYLEINPKDAA 6q8o.1    ------------- ``` | | | | | | | | | | | | | | | | | | | | | | | | | | | | | | | | | | | | | | | | | | | | | | | | | |
|  | 6zjy.1.C | NADH-quinone oxidoreductase subunit 3  *Respiratory complex I from Thermus thermophilus, NAD+ dataset, minor state* | 0.04 | 0.00 | 21.79 | 0.10 | 508-589 | EM | 0.00 | monomer | 7 x SF4, 2 x FES | HHblits | 0.29 |
| ``` target    SWDEATTIAAKTMEDVARTFNGDEGARKLLAQGYHPEMVEVMHGAGVQALKLRGGMPLLGIGRIFGFYRFANMLALLDRK 6zjy.1    --------------------------------------------------------------------------------  target    LRPDAPADEILGSRTFDNYAWHTDLPPGHPMVTGSQTVDFDLFSAEHTKLLLIIGMNWICTKMPDGHWIGDARLKGTRVI 6zjy.1    --------------------------------------------------------------------------------  target    VISADYMPTANKADEVIILRPGTDAAFFLGVARELIEKGLYDRAAVIERTDLPLLVRLDTGERLDARDVIPGYELAALTN 6zjy.1    --------------------------------------------------------------------------------  target    YVTLKPDAEIKGNPPPPPFTAGGQVVPTELRDAWGDFVWWDRATGRPRPVSRDEVGARFDGDPALLGEFEVELVDGSTVP 6zjy.1    --------------------------------------------------------------------------------  target    VRPAFDLLKQYLDESFDLRTASEVCRVPPQAIQSIARQLAANKRETLLAAGMGPNHYFQNDLFGRVQFLVAALTDNIGHL 6zjy.1    --------------------------------------------------------------------------------  target    GGNVGSYAGNYRGSVFQAMGQWIAEDPFAIEPDLTKPATVKRYYKAESAHYWNYGERPLRAVAKDDEGDLTKGEVLTGKS 6zjy.1    --------------------------------------------------------------------------------  target    HMPTPTKLIWFGNSNSLLGNAKWSFDVVKNTLPRQDAVFCNEWHWTSSC-EYADLVFPADSWAEFKLPDATASCTNPFLL 6zjy.1    ---------------------------PEEALKGKRFVVMHLSHLHPLAERYAHVVLPAPTFYEK---RGHLVNLEGRVL  target    AFPTTPLKRLYDTRSDYEALALTAKALGELIDEPRMEQYWRGILDGDPTPYLQRIFSGSNATRGITYDELHESSKRGVPL 6zjy.1    PL-SPAPIENGEAEGALQVLALLAEALGVR--------------------------------------------------  target    LMNMRTYPRSGGWEQRQEDKPWYTATGRLEFYRPEPEFQAAGESLPVWREPVDATFYEPNAILSNAAHPSIAPRAPEDYG 6zjy.1    --------------------------------------------------------------------------------  target    VPESQLDVETRQYRNVVRTWAELQQTLHPLQERDPAFRFVFQTPKYRWGAHSTAVDADWISMLFGPFGDPYRRDPRMPWT 6zjy.1    --------------------------------------------------------------------------------  target    GEAYLEINPKDAA 6zjy.1    ------------- ``` | | | | | | | | | | | | | | | | | | | | | | | | | | | | | | | | | | | | | | | | | | | | | | | | | |
|  | 6zjn.1.C | NADH-quinone oxidoreductase subunit 3  *Respiratory complex I from Thermus thermophilus, NADH dataset, minor state* | 0.04 |  | 21.79 | 0.10 | 508-589 | EM | 0.00 | hetero-1-1-1-1-1-1-… | 7 x SF4, 2 x FES | HHblits | 0.29 |
| ``` target    SWDEATTIAAKTMEDVARTFNGDEGARKLLAQGYHPEMVEVMHGAGVQALKLRGGMPLLGIGRIFGFYRFANMLALLDRK 6zjn.1    --------------------------------------------------------------------------------  target    LRPDAPADEILGSRTFDNYAWHTDLPPGHPMVTGSQTVDFDLFSAEHTKLLLIIGMNWICTKMPDGHWIGDARLKGTRVI 6zjn.1    --------------------------------------------------------------------------------  target    VISADYMPTANKADEVIILRPGTDAAFFLGVARELIEKGLYDRAAVIERTDLPLLVRLDTGERLDARDVIPGYELAALTN 6zjn.1    --------------------------------------------------------------------------------  target    YVTLKPDAEIKGNPPPPPFTAGGQVVPTELRDAWGDFVWWDRATGRPRPVSRDEVGARFDGDPALLGEFEVELVDGSTVP 6zjn.1    --------------------------------------------------------------------------------  target    VRPAFDLLKQYLDESFDLRTASEVCRVPPQAIQSIARQLAANKRETLLAAGMGPNHYFQNDLFGRVQFLVAALTDNIGHL 6zjn.1    --------------------------------------------------------------------------------  target    GGNVGSYAGNYRGSVFQAMGQWIAEDPFAIEPDLTKPATVKRYYKAESAHYWNYGERPLRAVAKDDEGDLTKGEVLTGKS 6zjn.1    --------------------------------------------------------------------------------  target    HMPTPTKLIWFGNSNSLLGNAKWSFDVVKNTLPRQDAVFCNEWHWTSSC-EYADLVFPADSWAEFKLPDATASCTNPFLL 6zjn.1    ---------------------------PEEALKGKRFVVMHLSHLHPLAERYAHVVLPAPTFYEK---RGHLVNLEGRVL  target    AFPTTPLKRLYDTRSDYEALALTAKALGELIDEPRMEQYWRGILDGDPTPYLQRIFSGSNATRGITYDELHESSKRGVPL 6zjn.1    PL-SPAPIENGEAEGALQVLALLAEALGVR--------------------------------------------------  target    LMNMRTYPRSGGWEQRQEDKPWYTATGRLEFYRPEPEFQAAGESLPVWREPVDATFYEPNAILSNAAHPSIAPRAPEDYG 6zjn.1    --------------------------------------------------------------------------------  target    VPESQLDVETRQYRNVVRTWAELQQTLHPLQERDPAFRFVFQTPKYRWGAHSTAVDADWISMLFGPFGDPYRRDPRMPWT 6zjn.1    --------------------------------------------------------------------------------  target    GEAYLEINPKDAA 6zjn.1    ------------- ``` | | | | | | | | | | | | | | | | | | | | | | | | | | | | | | | | | | | | | | | | | | | | | | | | | |
|  | 6ziy.1.C | NADH-quinone oxidoreductase subunit 3  *Respiratory complex I from Thermus thermophilus, NADH dataset, major state* | 0.04 |  | 21.79 | 0.10 | 508-589 | EM | 0.00 | hetero-1-1-1-1-1-1-… | 7 x SF4, 1 x FMN, 1 x NAI, 2 x FES | HHblits | 0.29 |
| ``` target    SWDEATTIAAKTMEDVARTFNGDEGARKLLAQGYHPEMVEVMHGAGVQALKLRGGMPLLGIGRIFGFYRFANMLALLDRK 6ziy.1    --------------------------------------------------------------------------------  target    LRPDAPADEILGSRTFDNYAWHTDLPPGHPMVTGSQTVDFDLFSAEHTKLLLIIGMNWICTKMPDGHWIGDARLKGTRVI 6ziy.1    --------------------------------------------------------------------------------  target    VISADYMPTANKADEVIILRPGTDAAFFLGVARELIEKGLYDRAAVIERTDLPLLVRLDTGERLDARDVIPGYELAALTN 6ziy.1    --------------------------------------------------------------------------------  target    YVTLKPDAEIKGNPPPPPFTAGGQVVPTELRDAWGDFVWWDRATGRPRPVSRDEVGARFDGDPALLGEFEVELVDGSTVP 6ziy.1    --------------------------------------------------------------------------------  target    VRPAFDLLKQYLDESFDLRTASEVCRVPPQAIQSIARQLAANKRETLLAAGMGPNHYFQNDLFGRVQFLVAALTDNIGHL 6ziy.1    --------------------------------------------------------------------------------  target    GGNVGSYAGNYRGSVFQAMGQWIAEDPFAIEPDLTKPATVKRYYKAESAHYWNYGERPLRAVAKDDEGDLTKGEVLTGKS 6ziy.1    --------------------------------------------------------------------------------  target    HMPTPTKLIWFGNSNSLLGNAKWSFDVVKNTLPRQDAVFCNEWHWTSSC-EYADLVFPADSWAEFKLPDATASCTNPFLL 6ziy.1    ---------------------------PEEALKGKRFVVMHLSHLHPLAERYAHVVLPAPTFYEK---RGHLVNLEGRVL  target    AFPTTPLKRLYDTRSDYEALALTAKALGELIDEPRMEQYWRGILDGDPTPYLQRIFSGSNATRGITYDELHESSKRGVPL 6ziy.1    PL-SPAPIENGEAEGALQVLALLAEALGVR--------------------------------------------------  target    LMNMRTYPRSGGWEQRQEDKPWYTATGRLEFYRPEPEFQAAGESLPVWREPVDATFYEPNAILSNAAHPSIAPRAPEDYG 6ziy.1    --------------------------------------------------------------------------------  target    VPESQLDVETRQYRNVVRTWAELQQTLHPLQERDPAFRFVFQTPKYRWGAHSTAVDADWISMLFGPFGDPYRRDPRMPWT 6ziy.1    --------------------------------------------------------------------------------  target    GEAYLEINPKDAA 6ziy.1    ------------- ``` | | | | | | | | | | | | | | | | | | | | | | | | | | | | | | | | | | | | | | | | | | | | | | | | | |
|  | 7b04.1.B | Nitrite oxidoreductase subunit A  *Structure of Nitrite oxidoreductase (Nxr) from the anammox bacterium Kuenenia stuttgartiensis.* | 0.01 |  | 5.41 | 0.09 | 695-768 | X-ray | 2.97 | hetero-1-1-1-mer | 4 x SF4, 1 x F3S, 2 x MD1, 1 x MO, 1 x HEM, 2 x CA | HHblits | 0.23 |
| ``` target    SWDEATTIAAKTMEDVARTFNGDEGARKLLAQGYHPEMVEVMHGAGVQALKLRGGMPLLGIGRIFGFYRFANMLALLDRK 7b04.1    --------------------------------------------------------------------------------  target    LRPDAPADEILGSRTFDNYAWHTDLPPGHPMVTGSQTVDFDLFSAEHTKLLLIIGMNWICTKMPDGHWIGDARLKGTRVI 7b04.1    --------------------------------------------------------------------------------  target    VISADYMPTANKADEVIILRPGTDAAFFLGVARELIEKGLYDRAAVIERTDLPLLVRLDTGERLDARDVIPGYELAALTN 7b04.1    --------------------------------------------------------------------------------  target    YVTLKPDAEIKGNPPPPPFTAGGQVVPTELRDAWGDFVWWDRATGRPRPVSRDEVGARFDGDPALLGEFEVELVDGSTVP 7b04.1    --------------------------------------------------------------------------------  target    VRPAFDLLKQYLDESFDLRTASEVCRVPPQAIQSIARQLAANKRETLLAAGMGPNHYFQNDLFGRVQFLVAALTDNIGHL 7b04.1    --------------------------------------------------------------------------------  target    GGNVGSYAGNYRGSVFQAMGQWIAEDPFAIEPDLTKPATVKRYYKAESAHYWNYGERPLRAVAKDDEGDLTKGEVLTGKS 7b04.1    --------------------------------------------------------------------------------  target    HMPTPTKLIWFGNSNSLLGNAKWSFDVVKNTLPRQDAVFCNEWHWTSSCEYADLVFPADSWAEFKLPDATASCTNPFLLA 7b04.1    --------------------------------------------------------------------------------  target    FPTTPLKRLYDTRSDYEALALTAKALGELIDEPRMEQYWRGILDGDPTPYLQRIFSGSNATRGITYDELHESSKRGVPLL 7b04.1    --------------------------------------------------------------------------------  target    MNMRTYPRSGGWEQRQEDKPWYTATGRLEFYRPEPEFQAAGESLPVWREPVDATFYEPNAILSNAAHPSIAPRAPED--- 7b04.1    ------------------------------------------------------KGYHFYCVTPKSRHTVHSQWAVTDWN  target    -------------------YGVPESQLDVETRQYRN-----VVRTWAE-----------------LQQTLHPLQERDPAF 7b04.1    FIWNNNFGDPYRMDKRMPGVGEHQIHIHPQAARDLGIEDGDYVYVDANPADRPYEGWKPNDSFYKVSRLMLRAKYNPAYP  target    RFVFQTPKYRWGAHSTAVDADWISMLFGPFGDPYRRDPRMPWTGEAYLEINPKDAA 7b04.1    YNCTMMKHSAWI-------------------------------------------- ``` | | | | | | | | | | | | | | | | | | | | | | | | | | | | | | | | | | | | | | | | | | | | | | | | | |
|  | 7b04.2.B | Nitrite oxidoreductase subunit A  *Structure of Nitrite oxidoreductase (Nxr) from the anammox bacterium Kuenenia stuttgartiensis.* | 0.01 |  | 5.41 | 0.09 | 695-768 | X-ray | 2.97 | hetero-1-1-1-mer | 4 x SF4, 1 x F3S, 2 x MD1, 1 x MO, 1 x HEM, 2 x CA | HHblits | 0.23 |
| ``` target    SWDEATTIAAKTMEDVARTFNGDEGARKLLAQGYHPEMVEVMHGAGVQALKLRGGMPLLGIGRIFGFYRFANMLALLDRK 7b04.2    --------------------------------------------------------------------------------  target    LRPDAPADEILGSRTFDNYAWHTDLPPGHPMVTGSQTVDFDLFSAEHTKLLLIIGMNWICTKMPDGHWIGDARLKGTRVI 7b04.2    --------------------------------------------------------------------------------  target    VISADYMPTANKADEVIILRPGTDAAFFLGVARELIEKGLYDRAAVIERTDLPLLVRLDTGERLDARDVIPGYELAALTN 7b04.2    --------------------------------------------------------------------------------  target    YVTLKPDAEIKGNPPPPPFTAGGQVVPTELRDAWGDFVWWDRATGRPRPVSRDEVGARFDGDPALLGEFEVELVDGSTVP 7b04.2    --------------------------------------------------------------------------------  target    VRPAFDLLKQYLDESFDLRTASEVCRVPPQAIQSIARQLAANKRETLLAAGMGPNHYFQNDLFGRVQFLVAALTDNIGHL 7b04.2    --------------------------------------------------------------------------------  target    GGNVGSYAGNYRGSVFQAMGQWIAEDPFAIEPDLTKPATVKRYYKAESAHYWNYGERPLRAVAKDDEGDLTKGEVLTGKS 7b04.2    --------------------------------------------------------------------------------  target    HMPTPTKLIWFGNSNSLLGNAKWSFDVVKNTLPRQDAVFCNEWHWTSSCEYADLVFPADSWAEFKLPDATASCTNPFLLA 7b04.2    --------------------------------------------------------------------------------  target    FPTTPLKRLYDTRSDYEALALTAKALGELIDEPRMEQYWRGILDGDPTPYLQRIFSGSNATRGITYDELHESSKRGVPLL 7b04.2    --------------------------------------------------------------------------------  target    MNMRTYPRSGGWEQRQEDKPWYTATGRLEFYRPEPEFQAAGESLPVWREPVDATFYEPNAILSNAAHPSIAPRAPED--- 7b04.2    ------------------------------------------------------KGYHFYCVTPKSRHTVHSQWAVTDWN  target    -------------------YGVPESQLDVETRQYRN-----VVRTWAE-----------------LQQTLHPLQERDPAF 7b04.2    FIWNNNFGDPYRMDKRMPGVGEHQIHIHPQAARDLGIEDGDYVYVDANPADRPYEGWKPNDSFYKVSRLMLRAKYNPAYP  target    RFVFQTPKYRWGAHSTAVDADWISMLFGPFGDPYRRDPRMPWTGEAYLEINPKDAA 7b04.2    YNCTMMKHSAWI-------------------------------------------- ``` | | | | | | | | | | | | | | | | | | | | | | | | | | | | | | | | | | | | | | | | | | | | | | | | | |
|  | 3etn.1.A | putative phosphosugar isomerase involved in capsule formation  *Crystal structure of putative phosphosugar isomerase involved in capsule formation (YP\_209877.1) from Bacteroides fragilis NCTC 9343 at 1.70 A resolution* | 0.02 |  | 22.81 | 0.07 | 126-184 | X-ray | 1.70 | homo-tetramer | 4 x CMK | HHblits | 0.30 |
| ``` target    SWDEATTIAAKTMEDVARTFNGDEGARKLLAQGYHPEMVEVMHGAGVQALKLRGGMPLLGIGRIFGFYRFANMLALLDRK 3etn.1    --------------------------------------------------------------------------------  target    LRPDAPADEILGSRTFDNYAWHTDLPPGHPMVTGSQTVDFDLFSAEHTKLLLIIGMNWICTKMPDGHWIGDARL--KGTR 3etn.1    ---------------------------------------------QENDLLLLISNSG-KTR-EIVELTQLAHNLNPGLK  target    VIVISAD-YMPTANKADEVIILRPGTDAAFFLGVARELIEKGLYDRAAVIERTDLPLLVRLDTGERLDARDVIPGYELAA 3etn.1    FIVITGNPDSPLASESDVCLSTGHPAE-----------------------------------------------------  target    LTNYVTLKPDAEIKGNPPPPPFTAGGQVVPTELRDAWGDFVWWDRATGRPRPVSRDEVGARFDGDPALLGEFEVELVDGS 3etn.1    --------------------------------------------------------------------------------  target    TVPVRPAFDLLKQYLDESFDLRTASEVCRVPPQAIQSIARQLAANKRETLLAAGMGPNHYFQNDLFGRVQFLVAALTDNI 3etn.1    --------------------------------------------------------------------------------  target    GHLGGNVGSYAGNYRGSVFQAMGQWIAEDPFAIEPDLTKPATVKRYYKAESAHYWNYGERPLRAVAKDDEGDLTKGEVLT 3etn.1    --------------------------------------------------------------------------------  target    GKSHMPTPTKLIWFGNSNSLLGNAKWSFDVVKNTLPRQDAVFCNEWHWTSSCEYADLVFPADSWAEFKLPDATASCTNPF 3etn.1    --------------------------------------------------------------------------------  target    LLAFPTTPLKRLYDTRSDYEALALTAKALGELIDEPRMEQYWRGILDGDPTPYLQRIFSGSNATRGITYDELHESSKRGV 3etn.1    --------------------------------------------------------------------------------  target    PLLMNMRTYPRSGGWEQRQEDKPWYTATGRLEFYRPEPEFQAAGESLPVWREPVDATFYEPNAILSNAAHPSIAPRAPED 3etn.1    --------------------------------------------------------------------------------  target    YGVPESQLDVETRQYRNVVRTWAELQQTLHPLQERDPAFRFVFQTPKYRWGAHSTAVDADWISMLFGPFGDPYRRDPRMP 3etn.1    --------------------------------------------------------------------------------  target    WTGEAYLEINPKDAA 3etn.1    --------------- ``` | | | | | | | | | | | | | | | | | | | | | | | | | | | | | | | | | | | | | | | | | | | | | | | | | |
|  | 2e7z.1.A | Acetylene hydratase Ahy  *Acetylene Hydratase from Pelobacter acetylenicus* | 0.01 |  | 20.69 | 0.07 | 486-543 | X-ray | 1.26 | monomer | 1 x SF4, 2 x MGD, 1 x W | HHblits | 0.29 |
| ``` target    SWDEATTIAAKTMEDVARTFNGDEGARKLLAQGYHPEMVEVMHGAGVQALKLRGGMPLLGIGRIFGFYRFANMLALLDRK 2e7z.1    --------------------------------------------------------------------------------  target    LRPDAPADEILGSRTFDNYAWHTDLPPGHPMVTGSQTVDFDLFSAEHTKLLLIIGMNWICTKMPDGHWIGDARLKGTRVI 2e7z.1    --------------------------------------------------------------------------------  target    VISADYMPTANKADEVIILRPGTDAAFFLGVARELIEKGLYDRAAVIERTDLPLLVRLDTGERLDARDVIPGYELAALTN 2e7z.1    --------------------------------------------------------------------------------  target    YVTLKPDAEIKGNPPPPPFTAGGQVVPTELRDAWGDFVWWDRATGRPRPVSRDEVGARFDGDPALLGEFEVELVDGSTVP 2e7z.1    --------------------------------------------------------------------------------  target    VRPAFDLLKQYLDESFDLRTASEVCRVPPQAIQSIARQLAANKRETLLAAGMGPNHYFQNDLFGRVQFLVAALTDNIGHL 2e7z.1    --------------------------------------------------------------------------------  target    GGNVGSYAGNYRGSVFQAMGQWIAEDPFAIEPDLTKPATVKRYYKAESAHYWNYGERPLRAVAKDDEGDLTKGEVLTGKS 2e7z.1    --------------------------------------------------------------------------------  target    HMPTPTKLIWFGNSNSLLGNAKWSFDVVKNTLPRQDAVFCNEWHWTSSCEYADLVFPADSWAEFKLPDATASCTNPFLLA 2e7z.1    -----SNCLLFIGKNLSNHNWVSQFNDLKAALKRGCKLIVLDPRRTKVAEMADIWLPLRYGTD-----------------  target    FPTTPLKRLYDTRSDYEALALTAKALGELIDEPRMEQYWRGILDGDPTPYLQRIFSGSNATRGITYDELHESSKRGVPLL 2e7z.1    --------------------------------------------------------------------------------  target    MNMRTYPRSGGWEQRQEDKPWYTATGRLEFYRPEPEFQAAGESLPVWREPVDATFYEPNAILSNAAHPSIAPRAPEDYGV 2e7z.1    --------------------------------------------------------------------------------  target    PESQLDVETRQYRNVVRTWAELQQTLHPLQERDPAFRFVFQTPKYRWGAHSTAVDADWISMLFGPFGDPYRRDPRMPWTG 2e7z.1    --------------------------------------------------------------------------------  target    EAYLEINPKDAA 2e7z.1    ------------ ``` | | | | | | | | | | | | | | | | | | | | | | | | | | | | | | | | | | | | | | | | | | | | | | | | | |
|  | 1jeo.1.A | HYPOTHETICAL PROTEIN MJ1247  *Crystal Structure of the Hypothetical Protein MJ1247 from Methanococcus jannaschii at 2.0 A Resolution Infers a Molecular Function of 3-Hexulose-6-Phosphate isomerase.* | 0.01 |  | 18.64 | 0.07 | 125-185 | X-ray | 2.00 | monomer |  | HHblits | 0.26 |
| ``` target    SWDEATTIAAKTMEDVARTFNGDEGARKLLAQGYHPEMVEVMHGAGVQALKLRGGMPLLGIGRIFGFYRFANMLALLDRK 1jeo.1    --------------------------------------------------------------------------------  target    LRPDAPADEILGSRTFDNYAWHTDLPPGHPMVTGSQTVDFDLFSAEHTKLLLIIGMNWICTKMPDGHWIGDARLKGTRVI 1jeo.1    --------------------------------------------YEKDDLLILISGSGRTE--SVLTVAKKAKNINNNII  target    VISADYMPTANKADEVIILRPGTDAAFFLGVARELIEKGLYDRAAVIERTDLPLLVRLDTGERLDARDVIPGYELAALTN 1jeo.1    AIVCECGNVVEFADLTIPLEVKKSK-------------------------------------------------------  target    YVTLKPDAEIKGNPPPPPFTAGGQVVPTELRDAWGDFVWWDRATGRPRPVSRDEVGARFDGDPALLGEFEVELVDGSTVP 1jeo.1    --------------------------------------------------------------------------------  target    VRPAFDLLKQYLDESFDLRTASEVCRVPPQAIQSIARQLAANKRETLLAAGMGPNHYFQNDLFGRVQFLVAALTDNIGHL 1jeo.1    --------------------------------------------------------------------------------  target    GGNVGSYAGNYRGSVFQAMGQWIAEDPFAIEPDLTKPATVKRYYKAESAHYWNYGERPLRAVAKDDEGDLTKGEVLTGKS 1jeo.1    --------------------------------------------------------------------------------  target    HMPTPTKLIWFGNSNSLLGNAKWSFDVVKNTLPRQDAVFCNEWHWTSSCEYADLVFPADSWAEFKLPDATASCTNPFLLA 1jeo.1    --------------------------------------------------------------------------------  target    FPTTPLKRLYDTRSDYEALALTAKALGELIDEPRMEQYWRGILDGDPTPYLQRIFSGSNATRGITYDELHESSKRGVPLL 1jeo.1    --------------------------------------------------------------------------------  target    MNMRTYPRSGGWEQRQEDKPWYTATGRLEFYRPEPEFQAAGESLPVWREPVDATFYEPNAILSNAAHPSIAPRAPEDYGV 1jeo.1    --------------------------------------------------------------------------------  target    PESQLDVETRQYRNVVRTWAELQQTLHPLQERDPAFRFVFQTPKYRWGAHSTAVDADWISMLFGPFGDPYRRDPRMPWTG 1jeo.1    --------------------------------------------------------------------------------  target    EAYLEINPKDAA 1jeo.1    ------------ ``` | | | | | | | | | | | | | | | | | | | | | | | | | | | | | | | | | | | | | | | | | | | | | | | | | |
|  | 7bkb.1.F | Formate dehydrogenase  *Formate dehydrogenase - heterodisulfide reductase - formylmethanofuran dehydrogenase complex from Methanospirillum hungatei (hexameric, composite structure)* | 0.01 |  | 17.54 | 0.07 | 126-183 | EM | 0.00 | hetero-2-2-2-2-2-2-… | 48 x SF4, 4 x FAD, 2 x FES, 4 x 9S8, 4 x ZN, 2 x MO, 4 x MGD | HHblits | 0.28 |
| ``` target    SWDEATTIAAKTMEDVARTFNGDEGARKLLAQGYHPEMVEVMHGAGVQALKLRGGMPLLGIGRIFGFYRFANMLALLDRK 7bkb.1    --------------------------------------------------------------------------------  target    LRPDAPADEILGSRTFDNYAWHTDLPPGHPMVTGSQTVDFDLFSAEHTKLLLIIGMNWICTKMPDGHWIGDARLKGTRVI 7bkb.1    ---------------------------------------------DEIKGMYILGLNPVVTYP-SSNHVKAQLEKLDFLV  target    VISADYMPTANKADEVIILRPGTDAAFFLGVARELIEKGLYDRAAVIERTDLPLLVRLDTGERLDARDVIPGYELAALTN 7bkb.1    VQDIFFTETCQYADVILPGACFA---------------------------------------------------------  target    YVTLKPDAEIKGNPPPPPFTAGGQVVPTELRDAWGDFVWWDRATGRPRPVSRDEVGARFDGDPALLGEFEVELVDGSTVP 7bkb.1    --------------------------------------------------------------------------------  target    VRPAFDLLKQYLDESFDLRTASEVCRVPPQAIQSIARQLAANKRETLLAAGMGPNHYFQNDLFGRVQFLVAALTDNIGHL 7bkb.1    --------------------------------------------------------------------------------  target    GGNVGSYAGNYRGSVFQAMGQWIAEDPFAIEPDLTKPATVKRYYKAESAHYWNYGERPLRAVAKDDEGDLTKGEVLTGKS 7bkb.1    --------------------------------------------------------------------------------  target    HMPTPTKLIWFGNSNSLLGNAKWSFDVVKNTLPRQDAVFCNEWHWTSSCEYADLVFPADSWAEFKLPDATASCTNPFLLA 7bkb.1    --------------------------------------------------------------------------------  target    FPTTPLKRLYDTRSDYEALALTAKALGELIDEPRMEQYWRGILDGDPTPYLQRIFSGSNATRGITYDELHESSKRGVPLL 7bkb.1    --------------------------------------------------------------------------------  target    MNMRTYPRSGGWEQRQEDKPWYTATGRLEFYRPEPEFQAAGESLPVWREPVDATFYEPNAILSNAAHPSIAPRAPEDYGV 7bkb.1    --------------------------------------------------------------------------------  target    PESQLDVETRQYRNVVRTWAELQQTLHPLQERDPAFRFVFQTPKYRWGAHSTAVDADWISMLFGPFGDPYRRDPRMPWTG 7bkb.1    --------------------------------------------------------------------------------  target    EAYLEINPKDAA 7bkb.1    ------------ ``` | | | | | | | | | | | | | | | | | | | | | | | | | | | | | | | | | | | | | | | | | | | | | | | | | |
|  | 3sho.1.A | Transcriptional regulator, RpiR family  *Crystal structure of RpiR transcription factor from Sphaerobacter thermophilus (sugar isomerase domain)* | 0.01 |  | 16.07 | 0.07 | 127-184 | X-ray | 1.80 | homo-tetramer |  | HHblits | 0.28 |
| ``` target    SWDEATTIAAKTMEDVARTFNGDEGARKLLAQGYHPEMVEVMHGAGVQALKLRGGMPLLGIGRIFGFYRFANMLALLDRK 3sho.1    --------------------------------------------------------------------------------  target    LRPDAPADEILGSRTFDNYAWHTDLPPGHPMVTGSQTVDFDLFSAEHTKLLLIIGMNWICTKMPDGHWIGDARLKGTRVI 3sho.1    ----------------------------------------------PTDLMIGVSVWRYLR--DTVAALAGAAERGVPTM  target    VISAD-YMPTANKADEVIILRPGTDAAFFLGVARELIEKGLYDRAAVIERTDLPLLVRLDTGERLDARDVIPGYELAALT 3sho.1    ALTDSSVSPPARIADHVLVAATRGV-------------------------------------------------------  target    NYVTLKPDAEIKGNPPPPPFTAGGQVVPTELRDAWGDFVWWDRATGRPRPVSRDEVGARFDGDPALLGEFEVELVDGSTV 3sho.1    --------------------------------------------------------------------------------  target    PVRPAFDLLKQYLDESFDLRTASEVCRVPPQAIQSIARQLAANKRETLLAAGMGPNHYFQNDLFGRVQFLVAALTDNIGH 3sho.1    --------------------------------------------------------------------------------  target    LGGNVGSYAGNYRGSVFQAMGQWIAEDPFAIEPDLTKPATVKRYYKAESAHYWNYGERPLRAVAKDDEGDLTKGEVLTGK 3sho.1    --------------------------------------------------------------------------------  target    SHMPTPTKLIWFGNSNSLLGNAKWSFDVVKNTLPRQDAVFCNEWHWTSSCEYADLVFPADSWAEFKLPDATASCTNPFLL 3sho.1    --------------------------------------------------------------------------------  target    AFPTTPLKRLYDTRSDYEALALTAKALGELIDEPRMEQYWRGILDGDPTPYLQRIFSGSNATRGITYDELHESSKRGVPL 3sho.1    --------------------------------------------------------------------------------  target    LMNMRTYPRSGGWEQRQEDKPWYTATGRLEFYRPEPEFQAAGESLPVWREPVDATFYEPNAILSNAAHPSIAPRAPEDYG 3sho.1    --------------------------------------------------------------------------------  target    VPESQLDVETRQYRNVVRTWAELQQTLHPLQERDPAFRFVFQTPKYRWGAHSTAVDADWISMLFGPFGDPYRRDPRMPWT 3sho.1    --------------------------------------------------------------------------------  target    GEAYLEINPKDAA 3sho.1    ------------- ``` | | | | | | | | | | | | | | | | | | | | | | | | | | | | | | | | | | | | | | | | | | | | | | | | | |
|  | 3sho.1.C | Transcriptional regulator, RpiR family  *Crystal structure of RpiR transcription factor from Sphaerobacter thermophilus (sugar isomerase domain)* | 0.01 |  | 16.07 | 0.07 | 127-184 | X-ray | 1.80 | homo-tetramer |  | HHblits | 0.28 |
| ``` target    SWDEATTIAAKTMEDVARTFNGDEGARKLLAQGYHPEMVEVMHGAGVQALKLRGGMPLLGIGRIFGFYRFANMLALLDRK 3sho.1    --------------------------------------------------------------------------------  target    LRPDAPADEILGSRTFDNYAWHTDLPPGHPMVTGSQTVDFDLFSAEHTKLLLIIGMNWICTKMPDGHWIGDARLKGTRVI 3sho.1    ----------------------------------------------PTDLMIGVSVWRYLR--DTVAALAGAAERGVPTM  target    VISAD-YMPTANKADEVIILRPGTDAAFFLGVARELIEKGLYDRAAVIERTDLPLLVRLDTGERLDARDVIPGYELAALT 3sho.1    ALTDSSVSPPARIADHVLVAATRGV-------------------------------------------------------  target    NYVTLKPDAEIKGNPPPPPFTAGGQVVPTELRDAWGDFVWWDRATGRPRPVSRDEVGARFDGDPALLGEFEVELVDGSTV 3sho.1    --------------------------------------------------------------------------------  target    PVRPAFDLLKQYLDESFDLRTASEVCRVPPQAIQSIARQLAANKRETLLAAGMGPNHYFQNDLFGRVQFLVAALTDNIGH 3sho.1    --------------------------------------------------------------------------------  target    LGGNVGSYAGNYRGSVFQAMGQWIAEDPFAIEPDLTKPATVKRYYKAESAHYWNYGERPLRAVAKDDEGDLTKGEVLTGK 3sho.1    --------------------------------------------------------------------------------  target    SHMPTPTKLIWFGNSNSLLGNAKWSFDVVKNTLPRQDAVFCNEWHWTSSCEYADLVFPADSWAEFKLPDATASCTNPFLL 3sho.1    --------------------------------------------------------------------------------  target    AFPTTPLKRLYDTRSDYEALALTAKALGELIDEPRMEQYWRGILDGDPTPYLQRIFSGSNATRGITYDELHESSKRGVPL 3sho.1    --------------------------------------------------------------------------------  target    LMNMRTYPRSGGWEQRQEDKPWYTATGRLEFYRPEPEFQAAGESLPVWREPVDATFYEPNAILSNAAHPSIAPRAPEDYG 3sho.1    --------------------------------------------------------------------------------  target    VPESQLDVETRQYRNVVRTWAELQQTLHPLQERDPAFRFVFQTPKYRWGAHSTAVDADWISMLFGPFGDPYRRDPRMPWT 3sho.1    --------------------------------------------------------------------------------  target    GEAYLEINPKDAA 3sho.1    ------------- ``` | | | | | | | | | | | | | | | | | | | | | | | | | | | | | | | | | | | | | | | | | | | | | | | | | |
|  | 1x94.1.A | putative Phosphoheptose isomerase  *Crystal Structure of a Hypothetical protein* | 0.01 |  | 19.30 | 0.07 | 126-184 | X-ray | 2.50 | homo-dimer |  | HHblits | 0.27 |
| ``` target    SWDEATTIAAKTMEDVARTFNGDEGARKLLAQGYHPEMVEVMHGAGVQALKLRGGMPLLGIGRIFGFYRFANMLALLDRK 1x94.1    --------------------------------------------------------------------------------  target    LRPDAPADEILGSRTFDNYAWHTDLPPGHPMVTGSQTVDFDLFSAEHTKLLLIIGMNWICTKMPDGHWIGDARLKGTRVI 1x94.1    ---------------------------------------------AKGDVLFGLSTSGN-SG-NILKAIEAAKAKGMKTI  target    VISA-DYMPTANKADEVIILRPGTDAAFFLGVARELIEKGLYDRAAVIERTDLPLLVRLDTGERLDARDVIPGYELAALT 1x94.1    ALTGKDGGKMAGLADVEIRVPHFGY-------------------------------------------------------  target    NYVTLKPDAEIKGNPPPPPFTAGGQVVPTELRDAWGDFVWWDRATGRPRPVSRDEVGARFDGDPALLGEFEVELVDGSTV 1x94.1    --------------------------------------------------------------------------------  target    PVRPAFDLLKQYLDESFDLRTASEVCRVPPQAIQSIARQLAANKRETLLAAGMGPNHYFQNDLFGRVQFLVAALTDNIGH 1x94.1    --------------------------------------------------------------------------------  target    LGGNVGSYAGNYRGSVFQAMGQWIAEDPFAIEPDLTKPATVKRYYKAESAHYWNYGERPLRAVAKDDEGDLTKGEVLTGK 1x94.1    --------------------------------------------------------------------------------  target    SHMPTPTKLIWFGNSNSLLGNAKWSFDVVKNTLPRQDAVFCNEWHWTSSCEYADLVFPADSWAEFKLPDATASCTNPFLL 1x94.1    --------------------------------------------------------------------------------  target    AFPTTPLKRLYDTRSDYEALALTAKALGELIDEPRMEQYWRGILDGDPTPYLQRIFSGSNATRGITYDELHESSKRGVPL 1x94.1    --------------------------------------------------------------------------------  target    LMNMRTYPRSGGWEQRQEDKPWYTATGRLEFYRPEPEFQAAGESLPVWREPVDATFYEPNAILSNAAHPSIAPRAPEDYG 1x94.1    --------------------------------------------------------------------------------  target    VPESQLDVETRQYRNVVRTWAELQQTLHPLQERDPAFRFVFQTPKYRWGAHSTAVDADWISMLFGPFGDPYRRDPRMPWT 1x94.1    --------------------------------------------------------------------------------  target    GEAYLEINPKDAA 1x94.1    ------------- ``` | | | | | | | | | | | | | | | | | | | | | | | | | | | | | | | | | | | | | | | | | | | | | | | | | |
|  | 1x94.1.B | putative Phosphoheptose isomerase  *Crystal Structure of a Hypothetical protein* | 0.01 |  | 19.30 | 0.07 | 126-184 | X-ray | 2.50 | homo-dimer |  | HHblits | 0.27 |
| ``` target    SWDEATTIAAKTMEDVARTFNGDEGARKLLAQGYHPEMVEVMHGAGVQALKLRGGMPLLGIGRIFGFYRFANMLALLDRK 1x94.1    --------------------------------------------------------------------------------  target    LRPDAPADEILGSRTFDNYAWHTDLPPGHPMVTGSQTVDFDLFSAEHTKLLLIIGMNWICTKMPDGHWIGDARLKGTRVI 1x94.1    ---------------------------------------------AKGDVLFGLSTSGN-SG-NILKAIEAAKAKGMKTI  target    VISA-DYMPTANKADEVIILRPGTDAAFFLGVARELIEKGLYDRAAVIERTDLPLLVRLDTGERLDARDVIPGYELAALT 1x94.1    ALTGKDGGKMAGLADVEIRVPHFGY-------------------------------------------------------  target    NYVTLKPDAEIKGNPPPPPFTAGGQVVPTELRDAWGDFVWWDRATGRPRPVSRDEVGARFDGDPALLGEFEVELVDGSTV 1x94.1    --------------------------------------------------------------------------------  target    PVRPAFDLLKQYLDESFDLRTASEVCRVPPQAIQSIARQLAANKRETLLAAGMGPNHYFQNDLFGRVQFLVAALTDNIGH 1x94.1    --------------------------------------------------------------------------------  target    LGGNVGSYAGNYRGSVFQAMGQWIAEDPFAIEPDLTKPATVKRYYKAESAHYWNYGERPLRAVAKDDEGDLTKGEVLTGK 1x94.1    --------------------------------------------------------------------------------  target    SHMPTPTKLIWFGNSNSLLGNAKWSFDVVKNTLPRQDAVFCNEWHWTSSCEYADLVFPADSWAEFKLPDATASCTNPFLL 1x94.1    --------------------------------------------------------------------------------  target    AFPTTPLKRLYDTRSDYEALALTAKALGELIDEPRMEQYWRGILDGDPTPYLQRIFSGSNATRGITYDELHESSKRGVPL 1x94.1    --------------------------------------------------------------------------------  target    LMNMRTYPRSGGWEQRQEDKPWYTATGRLEFYRPEPEFQAAGESLPVWREPVDATFYEPNAILSNAAHPSIAPRAPEDYG 1x94.1    --------------------------------------------------------------------------------  target    VPESQLDVETRQYRNVVRTWAELQQTLHPLQERDPAFRFVFQTPKYRWGAHSTAVDADWISMLFGPFGDPYRRDPRMPWT 1x94.1    --------------------------------------------------------------------------------  target    GEAYLEINPKDAA 1x94.1    ------------- ``` | | | | | | | | | | | | | | | | | | | | | | | | | | | | | | | | | | | | | | | | | | | | | | | | | |
|  | 3hba.1.A | Putative phosphosugar isomerase  *Crystal structure of a putative phosphosugar isomerase (sden\_2705) from shewanella denitrificans os217 at 2.00 A resolution* | 0.01 |  | 21.82 | 0.07 | 128-184 | X-ray | 2.00 | homo-dimer |  | HHblits | 0.29 |
| ``` target    SWDEATTIAAKTMEDVARTFNGDEGARKLLAQGYHPEMVEVMHGAGVQALKLRGGMPLLGIGRIFGFYRFANMLALLDRK 3hba.1    --------------------------------------------------------------------------------  target    LRPDAPADEILGSRTFDNYAWHTDLPPGHPMVTGSQTVDFDLFSAEHTKLLLIIGMNWICTKMPDGHWIGDARLKGTRVI 3hba.1    -----------------------------------------------GGLVIVISQSGRS--PDILAQARMAKNAGAFCV  target    VISAD-YMPTANKADEVIILRPGTDAAFFLGVARELIEKGLYDRAAVIERTDLPLLVRLDTGERLDARDVIPGYELAALT 3hba.1    ALVNDETAPIKDIVDVVIPLRAGEE-------------------------------------------------------  target    NYVTLKPDAEIKGNPPPPPFTAGGQVVPTELRDAWGDFVWWDRATGRPRPVSRDEVGARFDGDPALLGEFEVELVDGSTV 3hba.1    --------------------------------------------------------------------------------  target    PVRPAFDLLKQYLDESFDLRTASEVCRVPPQAIQSIARQLAANKRETLLAAGMGPNHYFQNDLFGRVQFLVAALTDNIGH 3hba.1    --------------------------------------------------------------------------------  target    LGGNVGSYAGNYRGSVFQAMGQWIAEDPFAIEPDLTKPATVKRYYKAESAHYWNYGERPLRAVAKDDEGDLTKGEVLTGK 3hba.1    --------------------------------------------------------------------------------  target    SHMPTPTKLIWFGNSNSLLGNAKWSFDVVKNTLPRQDAVFCNEWHWTSSCEYADLVFPADSWAEFKLPDATASCTNPFLL 3hba.1    --------------------------------------------------------------------------------  target    AFPTTPLKRLYDTRSDYEALALTAKALGELIDEPRMEQYWRGILDGDPTPYLQRIFSGSNATRGITYDELHESSKRGVPL 3hba.1    --------------------------------------------------------------------------------  target    LMNMRTYPRSGGWEQRQEDKPWYTATGRLEFYRPEPEFQAAGESLPVWREPVDATFYEPNAILSNAAHPSIAPRAPEDYG 3hba.1    --------------------------------------------------------------------------------  target    VPESQLDVETRQYRNVVRTWAELQQTLHPLQERDPAFRFVFQTPKYRWGAHSTAVDADWISMLFGPFGDPYRRDPRMPWT 3hba.1    --------------------------------------------------------------------------------  target    GEAYLEINPKDAA 3hba.1    ------------- ``` | | | | | | | | | | | | | | | | | | | | | | | | | | | | | | | | | | | | | | | | | | | | | | | | | |
|  | 3hba.1.B | Putative phosphosugar isomerase  *Crystal structure of a putative phosphosugar isomerase (sden\_2705) from shewanella denitrificans os217 at 2.00 A resolution* | 0.01 |  | 21.82 | 0.07 | 128-184 | X-ray | 2.00 | homo-dimer |  | HHblits | 0.29 |
| ``` target    SWDEATTIAAKTMEDVARTFNGDEGARKLLAQGYHPEMVEVMHGAGVQALKLRGGMPLLGIGRIFGFYRFANMLALLDRK 3hba.1    --------------------------------------------------------------------------------  target    LRPDAPADEILGSRTFDNYAWHTDLPPGHPMVTGSQTVDFDLFSAEHTKLLLIIGMNWICTKMPDGHWIGDARLKGTRVI 3hba.1    -----------------------------------------------GGLVIVISQSGRS--PDILAQARMAKNAGAFCV  target    VISAD-YMPTANKADEVIILRPGTDAAFFLGVARELIEKGLYDRAAVIERTDLPLLVRLDTGERLDARDVIPGYELAALT 3hba.1    ALVNDETAPIKDIVDVVIPLRAGEE-------------------------------------------------------  target    NYVTLKPDAEIKGNPPPPPFTAGGQVVPTELRDAWGDFVWWDRATGRPRPVSRDEVGARFDGDPALLGEFEVELVDGSTV 3hba.1    --------------------------------------------------------------------------------  target    PVRPAFDLLKQYLDESFDLRTASEVCRVPPQAIQSIARQLAANKRETLLAAGMGPNHYFQNDLFGRVQFLVAALTDNIGH 3hba.1    --------------------------------------------------------------------------------  target    LGGNVGSYAGNYRGSVFQAMGQWIAEDPFAIEPDLTKPATVKRYYKAESAHYWNYGERPLRAVAKDDEGDLTKGEVLTGK 3hba.1    --------------------------------------------------------------------------------  target    SHMPTPTKLIWFGNSNSLLGNAKWSFDVVKNTLPRQDAVFCNEWHWTSSCEYADLVFPADSWAEFKLPDATASCTNPFLL 3hba.1    --------------------------------------------------------------------------------  target    AFPTTPLKRLYDTRSDYEALALTAKALGELIDEPRMEQYWRGILDGDPTPYLQRIFSGSNATRGITYDELHESSKRGVPL 3hba.1    --------------------------------------------------------------------------------  target    LMNMRTYPRSGGWEQRQEDKPWYTATGRLEFYRPEPEFQAAGESLPVWREPVDATFYEPNAILSNAAHPSIAPRAPEDYG 3hba.1    --------------------------------------------------------------------------------  target    VPESQLDVETRQYRNVVRTWAELQQTLHPLQERDPAFRFVFQTPKYRWGAHSTAVDADWISMLFGPFGDPYRRDPRMPWT 3hba.1    --------------------------------------------------------------------------------  target    GEAYLEINPKDAA 3hba.1    ------------- ``` | | | | | | | | | | | | | | | | | | | | | | | | | | | | | | | | | | | | | | | | | | | | | | | | | |
|  | 7en6.1.A | HTH-type transcriptional regulator MurR  *The crystal structure of Escherichia coli MurR in apo form* | 0.01 |  | 17.54 | 0.07 | 126-184 | X-ray | 2.28 | homo-tetramer |  | HHblits | 0.26 |
| ``` target    SWDEATTIAAKTMEDVARTFNGDEGARKLLAQGYHPEMVEVMHGAGVQALKLRGGMPLLGIGRIFGFYRFANMLALLDRK 7en6.1    --------------------------------------------------------------------------------  target    LRPDAPADEILGSRTFDNYAWHTDLPPGHPMVTGSQTVDFDLFSAEHTKLLLIIGMNWICTKMPDGHWIGDARLKGTRVI 7en6.1    ---------------------------------------------KKGDVQIAISYSGSKK--EIVLCAEAARKQGATVI  target    VISAD-YMPTANKADEVIILRPGTDAAFFLGVARELIEKGLYDRAAVIERTDLPLLVRLDTGERLDARDVIPGYELAALT 7en6.1    AITSLTDSPLRRLAHFTLDTVSGET-------------------------------------------------------  target    NYVTLKPDAEIKGNPPPPPFTAGGQVVPTELRDAWGDFVWWDRATGRPRPVSRDEVGARFDGDPALLGEFEVELVDGSTV 7en6.1    --------------------------------------------------------------------------------  target    PVRPAFDLLKQYLDESFDLRTASEVCRVPPQAIQSIARQLAANKRETLLAAGMGPNHYFQNDLFGRVQFLVAALTDNIGH 7en6.1    --------------------------------------------------------------------------------  target    LGGNVGSYAGNYRGSVFQAMGQWIAEDPFAIEPDLTKPATVKRYYKAESAHYWNYGERPLRAVAKDDEGDLTKGEVLTGK 7en6.1    --------------------------------------------------------------------------------  target    SHMPTPTKLIWFGNSNSLLGNAKWSFDVVKNTLPRQDAVFCNEWHWTSSCEYADLVFPADSWAEFKLPDATASCTNPFLL 7en6.1    --------------------------------------------------------------------------------  target    AFPTTPLKRLYDTRSDYEALALTAKALGELIDEPRMEQYWRGILDGDPTPYLQRIFSGSNATRGITYDELHESSKRGVPL 7en6.1    --------------------------------------------------------------------------------  target    LMNMRTYPRSGGWEQRQEDKPWYTATGRLEFYRPEPEFQAAGESLPVWREPVDATFYEPNAILSNAAHPSIAPRAPEDYG 7en6.1    --------------------------------------------------------------------------------  target    VPESQLDVETRQYRNVVRTWAELQQTLHPLQERDPAFRFVFQTPKYRWGAHSTAVDADWISMLFGPFGDPYRRDPRMPWT 7en6.1    --------------------------------------------------------------------------------  target    GEAYLEINPKDAA 7en6.1    ------------- ``` | | | | | | | | | | | | | | | | | | | | | | | | | | | | | | | | | | | | | | | | | | | | | | | | | |
|  | 7en6.1.B | HTH-type transcriptional regulator MurR  *The crystal structure of Escherichia coli MurR in apo form* | 0.01 |  | 17.54 | 0.07 | 126-184 | X-ray | 2.28 | homo-tetramer |  | HHblits | 0.26 |
| ``` target    SWDEATTIAAKTMEDVARTFNGDEGARKLLAQGYHPEMVEVMHGAGVQALKLRGGMPLLGIGRIFGFYRFANMLALLDRK 7en6.1    --------------------------------------------------------------------------------  target    LRPDAPADEILGSRTFDNYAWHTDLPPGHPMVTGSQTVDFDLFSAEHTKLLLIIGMNWICTKMPDGHWIGDARLKGTRVI 7en6.1    ---------------------------------------------KKGDVQIAISYSGSKK--EIVLCAEAARKQGATVI  target    VISAD-YMPTANKADEVIILRPGTDAAFFLGVARELIEKGLYDRAAVIERTDLPLLVRLDTGERLDARDVIPGYELAALT 7en6.1    AITSLTDSPLRRLAHFTLDTVSGET-------------------------------------------------------  target    NYVTLKPDAEIKGNPPPPPFTAGGQVVPTELRDAWGDFVWWDRATGRPRPVSRDEVGARFDGDPALLGEFEVELVDGSTV 7en6.1    --------------------------------------------------------------------------------  target    PVRPAFDLLKQYLDESFDLRTASEVCRVPPQAIQSIARQLAANKRETLLAAGMGPNHYFQNDLFGRVQFLVAALTDNIGH 7en6.1    --------------------------------------------------------------------------------  target    LGGNVGSYAGNYRGSVFQAMGQWIAEDPFAIEPDLTKPATVKRYYKAESAHYWNYGERPLRAVAKDDEGDLTKGEVLTGK 7en6.1    --------------------------------------------------------------------------------  target    SHMPTPTKLIWFGNSNSLLGNAKWSFDVVKNTLPRQDAVFCNEWHWTSSCEYADLVFPADSWAEFKLPDATASCTNPFLL 7en6.1    --------------------------------------------------------------------------------  target    AFPTTPLKRLYDTRSDYEALALTAKALGELIDEPRMEQYWRGILDGDPTPYLQRIFSGSNATRGITYDELHESSKRGVPL 7en6.1    --------------------------------------------------------------------------------  target    LMNMRTYPRSGGWEQRQEDKPWYTATGRLEFYRPEPEFQAAGESLPVWREPVDATFYEPNAILSNAAHPSIAPRAPEDYG 7en6.1    --------------------------------------------------------------------------------  target    VPESQLDVETRQYRNVVRTWAELQQTLHPLQERDPAFRFVFQTPKYRWGAHSTAVDADWISMLFGPFGDPYRRDPRMPWT 7en6.1    --------------------------------------------------------------------------------  target    GEAYLEINPKDAA 7en6.1    ------------- ``` | | | | | | | | | | | | | | | | | | | | | | | | | | | | | | | | | | | | | | | | | | | | | | | | | |
|  | 7en6.1.C | HTH-type transcriptional regulator MurR  *The crystal structure of Escherichia coli MurR in apo form* | 0.01 |  | 17.54 | 0.07 | 126-184 | X-ray | 2.28 | homo-tetramer |  | HHblits | 0.26 |
| ``` target    SWDEATTIAAKTMEDVARTFNGDEGARKLLAQGYHPEMVEVMHGAGVQALKLRGGMPLLGIGRIFGFYRFANMLALLDRK 7en6.1    --------------------------------------------------------------------------------  target    LRPDAPADEILGSRTFDNYAWHTDLPPGHPMVTGSQTVDFDLFSAEHTKLLLIIGMNWICTKMPDGHWIGDARLKGTRVI 7en6.1    ---------------------------------------------KKGDVQIAISYSGSKK--EIVLCAEAARKQGATVI  target    VISAD-YMPTANKADEVIILRPGTDAAFFLGVARELIEKGLYDRAAVIERTDLPLLVRLDTGERLDARDVIPGYELAALT 7en6.1    AITSLTDSPLRRLAHFTLDTVSGET-------------------------------------------------------  target    NYVTLKPDAEIKGNPPPPPFTAGGQVVPTELRDAWGDFVWWDRATGRPRPVSRDEVGARFDGDPALLGEFEVELVDGSTV 7en6.1    --------------------------------------------------------------------------------  target    PVRPAFDLLKQYLDESFDLRTASEVCRVPPQAIQSIARQLAANKRETLLAAGMGPNHYFQNDLFGRVQFLVAALTDNIGH 7en6.1    --------------------------------------------------------------------------------  target    LGGNVGSYAGNYRGSVFQAMGQWIAEDPFAIEPDLTKPATVKRYYKAESAHYWNYGERPLRAVAKDDEGDLTKGEVLTGK 7en6.1    --------------------------------------------------------------------------------  target    SHMPTPTKLIWFGNSNSLLGNAKWSFDVVKNTLPRQDAVFCNEWHWTSSCEYADLVFPADSWAEFKLPDATASCTNPFLL 7en6.1    --------------------------------------------------------------------------------  target    AFPTTPLKRLYDTRSDYEALALTAKALGELIDEPRMEQYWRGILDGDPTPYLQRIFSGSNATRGITYDELHESSKRGVPL 7en6.1    --------------------------------------------------------------------------------  target    LMNMRTYPRSGGWEQRQEDKPWYTATGRLEFYRPEPEFQAAGESLPVWREPVDATFYEPNAILSNAAHPSIAPRAPEDYG 7en6.1    --------------------------------------------------------------------------------  target    VPESQLDVETRQYRNVVRTWAELQQTLHPLQERDPAFRFVFQTPKYRWGAHSTAVDADWISMLFGPFGDPYRRDPRMPWT 7en6.1    --------------------------------------------------------------------------------  target    GEAYLEINPKDAA 7en6.1    ------------- ``` | | | | | | | | | | | | | | | | | | | | | | | | | | | | | | | | | | | | | | | | | | | | | | | | | |
|  | 7en6.1.D | HTH-type transcriptional regulator MurR  *The crystal structure of Escherichia coli MurR in apo form* | 0.01 |  | 17.54 | 0.07 | 126-184 | X-ray | 2.28 | homo-tetramer |  | HHblits | 0.26 |
| ``` target    SWDEATTIAAKTMEDVARTFNGDEGARKLLAQGYHPEMVEVMHGAGVQALKLRGGMPLLGIGRIFGFYRFANMLALLDRK 7en6.1    --------------------------------------------------------------------------------  target    LRPDAPADEILGSRTFDNYAWHTDLPPGHPMVTGSQTVDFDLFSAEHTKLLLIIGMNWICTKMPDGHWIGDARLKGTRVI 7en6.1    ---------------------------------------------KKGDVQIAISYSGSKK--EIVLCAEAARKQGATVI  target    VISAD-YMPTANKADEVIILRPGTDAAFFLGVARELIEKGLYDRAAVIERTDLPLLVRLDTGERLDARDVIPGYELAALT 7en6.1    AITSLTDSPLRRLAHFTLDTVSGET-------------------------------------------------------  target    NYVTLKPDAEIKGNPPPPPFTAGGQVVPTELRDAWGDFVWWDRATGRPRPVSRDEVGARFDGDPALLGEFEVELVDGSTV 7en6.1    --------------------------------------------------------------------------------  target    PVRPAFDLLKQYLDESFDLRTASEVCRVPPQAIQSIARQLAANKRETLLAAGMGPNHYFQNDLFGRVQFLVAALTDNIGH 7en6.1    --------------------------------------------------------------------------------  target    LGGNVGSYAGNYRGSVFQAMGQWIAEDPFAIEPDLTKPATVKRYYKAESAHYWNYGERPLRAVAKDDEGDLTKGEVLTGK 7en6.1    --------------------------------------------------------------------------------  target    SHMPTPTKLIWFGNSNSLLGNAKWSFDVVKNTLPRQDAVFCNEWHWTSSCEYADLVFPADSWAEFKLPDATASCTNPFLL 7en6.1    --------------------------------------------------------------------------------  target    AFPTTPLKRLYDTRSDYEALALTAKALGELIDEPRMEQYWRGILDGDPTPYLQRIFSGSNATRGITYDELHESSKRGVPL 7en6.1    --------------------------------------------------------------------------------  target    LMNMRTYPRSGGWEQRQEDKPWYTATGRLEFYRPEPEFQAAGESLPVWREPVDATFYEPNAILSNAAHPSIAPRAPEDYG 7en6.1    --------------------------------------------------------------------------------  target    VPESQLDVETRQYRNVVRTWAELQQTLHPLQERDPAFRFVFQTPKYRWGAHSTAVDADWISMLFGPFGDPYRRDPRMPWT 7en6.1    --------------------------------------------------------------------------------  target    GEAYLEINPKDAA 7en6.1    ------------- ``` | | | | | | | | | | | | | | | | | | | | | | | | | | | | | | | | | | | | | | | | | | | | | | | | | |
|  | 7en5.1.A | HTH-type transcriptional regulator MurR  *The crystal structure of Escherichia coli MurR in complex with N-acetylglucosamine-6-phosphate* | 0.01 |  | 17.54 | 0.07 | 126-184 | X-ray | 1.25 | homo-tetramer | 4 x 4QY, 4 x MXE | HHblits | 0.26 |
| ``` target    SWDEATTIAAKTMEDVARTFNGDEGARKLLAQGYHPEMVEVMHGAGVQALKLRGGMPLLGIGRIFGFYRFANMLALLDRK 7en5.1    --------------------------------------------------------------------------------  target    LRPDAPADEILGSRTFDNYAWHTDLPPGHPMVTGSQTVDFDLFSAEHTKLLLIIGMNWICTKMPDGHWIGDARLKGTRVI 7en5.1    ---------------------------------------------KKGDVQIAISYSGSKK--EIVLCAEAARKQGATVI  target    VISAD-YMPTANKADEVIILRPGTDAAFFLGVARELIEKGLYDRAAVIERTDLPLLVRLDTGERLDARDVIPGYELAALT 7en5.1    AITSLTDSPLRRLAHFTLDTVSGET-------------------------------------------------------  target    NYVTLKPDAEIKGNPPPPPFTAGGQVVPTELRDAWGDFVWWDRATGRPRPVSRDEVGARFDGDPALLGEFEVELVDGSTV 7en5.1    --------------------------------------------------------------------------------  target    PVRPAFDLLKQYLDESFDLRTASEVCRVPPQAIQSIARQLAANKRETLLAAGMGPNHYFQNDLFGRVQFLVAALTDNIGH 7en5.1    --------------------------------------------------------------------------------  target    LGGNVGSYAGNYRGSVFQAMGQWIAEDPFAIEPDLTKPATVKRYYKAESAHYWNYGERPLRAVAKDDEGDLTKGEVLTGK 7en5.1    --------------------------------------------------------------------------------  target    SHMPTPTKLIWFGNSNSLLGNAKWSFDVVKNTLPRQDAVFCNEWHWTSSCEYADLVFPADSWAEFKLPDATASCTNPFLL 7en5.1    --------------------------------------------------------------------------------  target    AFPTTPLKRLYDTRSDYEALALTAKALGELIDEPRMEQYWRGILDGDPTPYLQRIFSGSNATRGITYDELHESSKRGVPL 7en5.1    --------------------------------------------------------------------------------  target    LMNMRTYPRSGGWEQRQEDKPWYTATGRLEFYRPEPEFQAAGESLPVWREPVDATFYEPNAILSNAAHPSIAPRAPEDYG 7en5.1    --------------------------------------------------------------------------------  target    VPESQLDVETRQYRNVVRTWAELQQTLHPLQERDPAFRFVFQTPKYRWGAHSTAVDADWISMLFGPFGDPYRRDPRMPWT 7en5.1    --------------------------------------------------------------------------------  target    GEAYLEINPKDAA 7en5.1    ------------- ``` | | | | | | | | | | | | | | | | | | | | | | | | | | | | | | | | | | | | | | | | | | | | | | | | | |
|  | 7l5i.1.A | Trimethylamine-N-oxide reductase  *Crystal Structure of Haemophilus influenzae MtsZ at pH 7.0* | 0.01 |  | 27.78 | 0.07 | 127-181 | X-ray | 1.73 | monomer | 2 x MGD, 1 x MO, 1 x O | HHblits | 0.30 |
| ``` target    SWDEATTIAAKTMEDVARTFNGDEGARKLLAQGYHPEMVEVMHGAGVQALKLRGGMPLLGIGRIFGFYRFANMLALLDRK 7l5i.1    --------------------------------------------------------------------------------  target    LRPDAPADEILGSRTFDNYAWHTDLPPGHPMVTGSQTVDFDLFSAEHTKLLLIIGMNWICTKMPDGHWIGDARLKGTRVI 7l5i.1    ----------------------------------------------DIKAVYWAGGNPFVHH-QDTNTLVKAFQKPDVVI  target    VISADYMPTANKADEVIILRPGTDAAFFLGVARELIEKGLYDRAAVIERTDLPLLVRLDTGERLDARDVIPGYELAALTN 7l5i.1    VNEVNWTPTARMADIVLPATT-----------------------------------------------------------  target    YVTLKPDAEIKGNPPPPPFTAGGQVVPTELRDAWGDFVWWDRATGRPRPVSRDEVGARFDGDPALLGEFEVELVDGSTVP 7l5i.1    --------------------------------------------------------------------------------  target    VRPAFDLLKQYLDESFDLRTASEVCRVPPQAIQSIARQLAANKRETLLAAGMGPNHYFQNDLFGRVQFLVAALTDNIGHL 7l5i.1    --------------------------------------------------------------------------------  target    GGNVGSYAGNYRGSVFQAMGQWIAEDPFAIEPDLTKPATVKRYYKAESAHYWNYGERPLRAVAKDDEGDLTKGEVLTGKS 7l5i.1    --------------------------------------------------------------------------------  target    HMPTPTKLIWFGNSNSLLGNAKWSFDVVKNTLPRQDAVFCNEWHWTSSCEYADLVFPADSWAEFKLPDATASCTNPFLLA 7l5i.1    --------------------------------------------------------------------------------  target    FPTTPLKRLYDTRSDYEALALTAKALGELIDEPRMEQYWRGILDGDPTPYLQRIFSGSNATRGITYDELHESSKRGVPLL 7l5i.1    --------------------------------------------------------------------------------  target    MNMRTYPRSGGWEQRQEDKPWYTATGRLEFYRPEPEFQAAGESLPVWREPVDATFYEPNAILSNAAHPSIAPRAPEDYGV 7l5i.1    --------------------------------------------------------------------------------  target    PESQLDVETRQYRNVVRTWAELQQTLHPLQERDPAFRFVFQTPKYRWGAHSTAVDADWISMLFGPFGDPYRRDPRMPWTG 7l5i.1    --------------------------------------------------------------------------------  target    EAYLEINPKDAA 7l5i.1    ------------ ``` | | | | | | | | | | | | | | | | | | | | | | | | | | | | | | | | | | | | | | | | | | | | | | | | | |
|  | 7l5s.1.A | Trimethylamine-N-oxide reductase  *Crystal Structure of Haemophilus influenzae MtsZ at pH 5.5* | 0.01 |  | 27.78 | 0.07 | 127-181 | X-ray | 2.09 | monomer | 1 x O, 2 x MGD, 1 x MO | HHblits | 0.30 |
| ``` target    SWDEATTIAAKTMEDVARTFNGDEGARKLLAQGYHPEMVEVMHGAGVQALKLRGGMPLLGIGRIFGFYRFANMLALLDRK 7l5s.1    --------------------------------------------------------------------------------  target    LRPDAPADEILGSRTFDNYAWHTDLPPGHPMVTGSQTVDFDLFSAEHTKLLLIIGMNWICTKMPDGHWIGDARLKGTRVI 7l5s.1    ----------------------------------------------DIKAVYWAGGNPFVHH-QDTNTLVKAFQKPDVVI  target    VISADYMPTANKADEVIILRPGTDAAFFLGVARELIEKGLYDRAAVIERTDLPLLVRLDTGERLDARDVIPGYELAALTN 7l5s.1    VNEVNWTPTARMADIVLPATT-----------------------------------------------------------  target    YVTLKPDAEIKGNPPPPPFTAGGQVVPTELRDAWGDFVWWDRATGRPRPVSRDEVGARFDGDPALLGEFEVELVDGSTVP 7l5s.1    --------------------------------------------------------------------------------  target    VRPAFDLLKQYLDESFDLRTASEVCRVPPQAIQSIARQLAANKRETLLAAGMGPNHYFQNDLFGRVQFLVAALTDNIGHL 7l5s.1    --------------------------------------------------------------------------------  target    GGNVGSYAGNYRGSVFQAMGQWIAEDPFAIEPDLTKPATVKRYYKAESAHYWNYGERPLRAVAKDDEGDLTKGEVLTGKS 7l5s.1    --------------------------------------------------------------------------------  target    HMPTPTKLIWFGNSNSLLGNAKWSFDVVKNTLPRQDAVFCNEWHWTSSCEYADLVFPADSWAEFKLPDATASCTNPFLLA 7l5s.1    --------------------------------------------------------------------------------  target    FPTTPLKRLYDTRSDYEALALTAKALGELIDEPRMEQYWRGILDGDPTPYLQRIFSGSNATRGITYDELHESSKRGVPLL 7l5s.1    --------------------------------------------------------------------------------  target    MNMRTYPRSGGWEQRQEDKPWYTATGRLEFYRPEPEFQAAGESLPVWREPVDATFYEPNAILSNAAHPSIAPRAPEDYGV 7l5s.1    --------------------------------------------------------------------------------  target    PESQLDVETRQYRNVVRTWAELQQTLHPLQERDPAFRFVFQTPKYRWGAHSTAVDADWISMLFGPFGDPYRRDPRMPWTG 7l5s.1    --------------------------------------------------------------------------------  target    EAYLEINPKDAA 7l5s.1    ------------ ``` | | | | | | | | | | | | | | | | | | | | | | | | | | | | | | | | | | | | | | | | | | | | | | | | | |
|  | 1s7g.1.D | NAD-dependent deacetylase 2  *Structural Basis for the Mechanism and Regulation of Sir2 Enzymes* | 0.01 |  | 20.75 | 0.07 | 124-178 | X-ray | 2.30 | homo-pentamer | 9 x ZN, 3 x NAD, 1 x APR, 1 x 2PE | HHblits | 0.32 |
| ``` target    SWDEATTIAAKTMEDVARTFNGDEGARKLLAQGYHPEMVEVMHGAGVQALKLRGGMPLLGIGRIFGFYRFANMLALLDRK 1s7g.1    --------------------------------------------------------------------------------  target    LRPDAPADEILGSRTFDNYAWHTDLPPGHPMVTGSQTVDFDLFSAEHTKLLLIIGMNWICTKMPDGHWIGDARLKGTRVI 1s7g.1    -------------------------------------------EAKHCDAFMVVGSSLVV--YPAAELPYIAKKAGAKMI  target    VISADYMPTANKADEVIILRPGTDAAFFLGVARELIEKGLYDRAAVIERTDLPLLVRLDTGERLDARDVIPGYELAALTN 1s7g.1    IVNAEPTMADPIFDVKII--------------------------------------------------------------  target    YVTLKPDAEIKGNPPPPPFTAGGQVVPTELRDAWGDFVWWDRATGRPRPVSRDEVGARFDGDPALLGEFEVELVDGSTVP 1s7g.1    --------------------------------------------------------------------------------  target    VRPAFDLLKQYLDESFDLRTASEVCRVPPQAIQSIARQLAANKRETLLAAGMGPNHYFQNDLFGRVQFLVAALTDNIGHL 1s7g.1    --------------------------------------------------------------------------------  target    GGNVGSYAGNYRGSVFQAMGQWIAEDPFAIEPDLTKPATVKRYYKAESAHYWNYGERPLRAVAKDDEGDLTKGEVLTGKS 1s7g.1    --------------------------------------------------------------------------------  target    HMPTPTKLIWFGNSNSLLGNAKWSFDVVKNTLPRQDAVFCNEWHWTSSCEYADLVFPADSWAEFKLPDATASCTNPFLLA 1s7g.1    --------------------------------------------------------------------------------  target    FPTTPLKRLYDTRSDYEALALTAKALGELIDEPRMEQYWRGILDGDPTPYLQRIFSGSNATRGITYDELHESSKRGVPLL 1s7g.1    --------------------------------------------------------------------------------  target    MNMRTYPRSGGWEQRQEDKPWYTATGRLEFYRPEPEFQAAGESLPVWREPVDATFYEPNAILSNAAHPSIAPRAPEDYGV 1s7g.1    --------------------------------------------------------------------------------  target    PESQLDVETRQYRNVVRTWAELQQTLHPLQERDPAFRFVFQTPKYRWGAHSTAVDADWISMLFGPFGDPYRRDPRMPWTG 1s7g.1    --------------------------------------------------------------------------------  target    EAYLEINPKDAA 1s7g.1    ------------ ``` | | | | | | | | | | | | | | | | | | | | | | | | | | | | | | | | | | | | | | | | | | | | | | | | | |
|  | 1ma3.1.A | Transcriptional regulatory protein, Sir2 family  *Structure of a Sir2 enzyme bound to an acetylated p53 peptide* | 0.02 |  | 20.75 | 0.07 | 124-178 | X-ray | 2.00 | hetero-oligomer | 1 x ZN, 1 x MES | HHblits | 0.32 |
| ``` target    SWDEATTIAAKTMEDVARTFNGDEGARKLLAQGYHPEMVEVMHGAGVQALKLRGGMPLLGIGRIFGFYRFANMLALLDRK 1ma3.1    --------------------------------------------------------------------------------  target    LRPDAPADEILGSRTFDNYAWHTDLPPGHPMVTGSQTVDFDLFSAEHTKLLLIIGMNWICTKMPDGHWIGDARLKGTRVI 1ma3.1    -------------------------------------------EAKHCDAFMVVGSSLVV--YPAAELPYIAKKAGAKMI  target    VISADYMPTANKADEVIILRPGTDAAFFLGVARELIEKGLYDRAAVIERTDLPLLVRLDTGERLDARDVIPGYELAALTN 1ma3.1    IVNAEPTMADPIFDVKII--------------------------------------------------------------  target    YVTLKPDAEIKGNPPPPPFTAGGQVVPTELRDAWGDFVWWDRATGRPRPVSRDEVGARFDGDPALLGEFEVELVDGSTVP 1ma3.1    --------------------------------------------------------------------------------  target    VRPAFDLLKQYLDESFDLRTASEVCRVPPQAIQSIARQLAANKRETLLAAGMGPNHYFQNDLFGRVQFLVAALTDNIGHL 1ma3.1    --------------------------------------------------------------------------------  target    GGNVGSYAGNYRGSVFQAMGQWIAEDPFAIEPDLTKPATVKRYYKAESAHYWNYGERPLRAVAKDDEGDLTKGEVLTGKS 1ma3.1    --------------------------------------------------------------------------------  target    HMPTPTKLIWFGNSNSLLGNAKWSFDVVKNTLPRQDAVFCNEWHWTSSCEYADLVFPADSWAEFKLPDATASCTNPFLLA 1ma3.1    --------------------------------------------------------------------------------  target    FPTTPLKRLYDTRSDYEALALTAKALGELIDEPRMEQYWRGILDGDPTPYLQRIFSGSNATRGITYDELHESSKRGVPLL 1ma3.1    --------------------------------------------------------------------------------  target    MNMRTYPRSGGWEQRQEDKPWYTATGRLEFYRPEPEFQAAGESLPVWREPVDATFYEPNAILSNAAHPSIAPRAPEDYGV 1ma3.1    --------------------------------------------------------------------------------  target    PESQLDVETRQYRNVVRTWAELQQTLHPLQERDPAFRFVFQTPKYRWGAHSTAVDADWISMLFGPFGDPYRRDPRMPWTG 1ma3.1    --------------------------------------------------------------------------------  target    EAYLEINPKDAA 1ma3.1    ------------ ``` | | | | | | | | | | | | | | | | | | | | | | | | | | | | | | | | | | | | | | | | | | | | | | | | | |
|  | 1s7g.1.B | NAD-dependent deacetylase 2  *Structural Basis for the Mechanism and Regulation of Sir2 Enzymes* | 0.01 |  | 20.75 | 0.07 | 124-178 | X-ray | 2.30 | homo-pentamer | 9 x ZN, 3 x NAD, 1 x APR, 1 x 2PE | HHblits | 0.32 |
| ``` target    SWDEATTIAAKTMEDVARTFNGDEGARKLLAQGYHPEMVEVMHGAGVQALKLRGGMPLLGIGRIFGFYRFANMLALLDRK 1s7g.1    --------------------------------------------------------------------------------  target    LRPDAPADEILGSRTFDNYAWHTDLPPGHPMVTGSQTVDFDLFSAEHTKLLLIIGMNWICTKMPDGHWIGDARLKGTRVI 1s7g.1    -------------------------------------------EAKHCDAFMVVGSSLVV--YPAAELPYIAKKAGAKMI  target    VISADYMPTANKADEVIILRPGTDAAFFLGVARELIEKGLYDRAAVIERTDLPLLVRLDTGERLDARDVIPGYELAALTN 1s7g.1    IVNAEPTMADPIFDVKII--------------------------------------------------------------  target    YVTLKPDAEIKGNPPPPPFTAGGQVVPTELRDAWGDFVWWDRATGRPRPVSRDEVGARFDGDPALLGEFEVELVDGSTVP 1s7g.1    --------------------------------------------------------------------------------  target    VRPAFDLLKQYLDESFDLRTASEVCRVPPQAIQSIARQLAANKRETLLAAGMGPNHYFQNDLFGRVQFLVAALTDNIGHL 1s7g.1    --------------------------------------------------------------------------------  target    GGNVGSYAGNYRGSVFQAMGQWIAEDPFAIEPDLTKPATVKRYYKAESAHYWNYGERPLRAVAKDDEGDLTKGEVLTGKS 1s7g.1    --------------------------------------------------------------------------------  target    HMPTPTKLIWFGNSNSLLGNAKWSFDVVKNTLPRQDAVFCNEWHWTSSCEYADLVFPADSWAEFKLPDATASCTNPFLLA 1s7g.1    --------------------------------------------------------------------------------  target    FPTTPLKRLYDTRSDYEALALTAKALGELIDEPRMEQYWRGILDGDPTPYLQRIFSGSNATRGITYDELHESSKRGVPLL 1s7g.1    --------------------------------------------------------------------------------  target    MNMRTYPRSGGWEQRQEDKPWYTATGRLEFYRPEPEFQAAGESLPVWREPVDATFYEPNAILSNAAHPSIAPRAPEDYGV 1s7g.1    --------------------------------------------------------------------------------  target    PESQLDVETRQYRNVVRTWAELQQTLHPLQERDPAFRFVFQTPKYRWGAHSTAVDADWISMLFGPFGDPYRRDPRMPWTG 1s7g.1    --------------------------------------------------------------------------------  target    EAYLEINPKDAA 1s7g.1    ------------ ``` | | | | | | | | | | | | | | | | | | | | | | | | | | | | | | | | | | | | | | | | | | | | | | | | | |
|  | 1s7g.1.A | NAD-dependent deacetylase 2  *Structural Basis for the Mechanism and Regulation of Sir2 Enzymes* | 0.01 |  | 20.75 | 0.07 | 124-178 | X-ray | 2.30 | homo-pentamer | 9 x ZN, 3 x NAD, 1 x APR, 1 x 2PE | HHblits | 0.32 |
| ``` target    SWDEATTIAAKTMEDVARTFNGDEGARKLLAQGYHPEMVEVMHGAGVQALKLRGGMPLLGIGRIFGFYRFANMLALLDRK 1s7g.1    --------------------------------------------------------------------------------  target    LRPDAPADEILGSRTFDNYAWHTDLPPGHPMVTGSQTVDFDLFSAEHTKLLLIIGMNWICTKMPDGHWIGDARLKGTRVI 1s7g.1    -------------------------------------------EAKHCDAFMVVGSSLVV--YPAAELPYIAKKAGAKMI  target    VISADYMPTANKADEVIILRPGTDAAFFLGVARELIEKGLYDRAAVIERTDLPLLVRLDTGERLDARDVIPGYELAALTN 1s7g.1    IVNAEPTMADPIFDVKII--------------------------------------------------------------  target    YVTLKPDAEIKGNPPPPPFTAGGQVVPTELRDAWGDFVWWDRATGRPRPVSRDEVGARFDGDPALLGEFEVELVDGSTVP 1s7g.1    --------------------------------------------------------------------------------  target    VRPAFDLLKQYLDESFDLRTASEVCRVPPQAIQSIARQLAANKRETLLAAGMGPNHYFQNDLFGRVQFLVAALTDNIGHL 1s7g.1    --------------------------------------------------------------------------------  target    GGNVGSYAGNYRGSVFQAMGQWIAEDPFAIEPDLTKPATVKRYYKAESAHYWNYGERPLRAVAKDDEGDLTKGEVLTGKS 1s7g.1    --------------------------------------------------------------------------------  target    HMPTPTKLIWFGNSNSLLGNAKWSFDVVKNTLPRQDAVFCNEWHWTSSCEYADLVFPADSWAEFKLPDATASCTNPFLLA 1s7g.1    --------------------------------------------------------------------------------  target    FPTTPLKRLYDTRSDYEALALTAKALGELIDEPRMEQYWRGILDGDPTPYLQRIFSGSNATRGITYDELHESSKRGVPLL 1s7g.1    --------------------------------------------------------------------------------  target    MNMRTYPRSGGWEQRQEDKPWYTATGRLEFYRPEPEFQAAGESLPVWREPVDATFYEPNAILSNAAHPSIAPRAPEDYGV 1s7g.1    --------------------------------------------------------------------------------  target    PESQLDVETRQYRNVVRTWAELQQTLHPLQERDPAFRFVFQTPKYRWGAHSTAVDADWISMLFGPFGDPYRRDPRMPWTG 1s7g.1    --------------------------------------------------------------------------------  target    EAYLEINPKDAA 1s7g.1    ------------ ``` | | | | | | | | | | | | | | | | | | | | | | | | | | | | | | | | | | | | | | | | | | | | | | | | | |
|  | 1s7g.1.C | NAD-dependent deacetylase 2  *Structural Basis for the Mechanism and Regulation of Sir2 Enzymes* | 0.01 |  | 20.75 | 0.07 | 124-178 | X-ray | 2.30 | homo-pentamer | 9 x ZN, 3 x NAD, 1 x APR, 1 x 2PE | HHblits | 0.32 |
| ``` target    SWDEATTIAAKTMEDVARTFNGDEGARKLLAQGYHPEMVEVMHGAGVQALKLRGGMPLLGIGRIFGFYRFANMLALLDRK 1s7g.1    --------------------------------------------------------------------------------  target    LRPDAPADEILGSRTFDNYAWHTDLPPGHPMVTGSQTVDFDLFSAEHTKLLLIIGMNWICTKMPDGHWIGDARLKGTRVI 1s7g.1    -------------------------------------------EAKHCDAFMVVGSSLVV--YPAAELPYIAKKAGAKMI  target    VISADYMPTANKADEVIILRPGTDAAFFLGVARELIEKGLYDRAAVIERTDLPLLVRLDTGERLDARDVIPGYELAALTN 1s7g.1    IVNAEPTMADPIFDVKII--------------------------------------------------------------  target    YVTLKPDAEIKGNPPPPPFTAGGQVVPTELRDAWGDFVWWDRATGRPRPVSRDEVGARFDGDPALLGEFEVELVDGSTVP 1s7g.1    --------------------------------------------------------------------------------  target    VRPAFDLLKQYLDESFDLRTASEVCRVPPQAIQSIARQLAANKRETLLAAGMGPNHYFQNDLFGRVQFLVAALTDNIGHL 1s7g.1    --------------------------------------------------------------------------------  target    GGNVGSYAGNYRGSVFQAMGQWIAEDPFAIEPDLTKPATVKRYYKAESAHYWNYGERPLRAVAKDDEGDLTKGEVLTGKS 1s7g.1    --------------------------------------------------------------------------------  target    HMPTPTKLIWFGNSNSLLGNAKWSFDVVKNTLPRQDAVFCNEWHWTSSCEYADLVFPADSWAEFKLPDATASCTNPFLLA 1s7g.1    --------------------------------------------------------------------------------  target    FPTTPLKRLYDTRSDYEALALTAKALGELIDEPRMEQYWRGILDGDPTPYLQRIFSGSNATRGITYDELHESSKRGVPLL 1s7g.1    --------------------------------------------------------------------------------  target    MNMRTYPRSGGWEQRQEDKPWYTATGRLEFYRPEPEFQAAGESLPVWREPVDATFYEPNAILSNAAHPSIAPRAPEDYGV 1s7g.1    --------------------------------------------------------------------------------  target    PESQLDVETRQYRNVVRTWAELQQTLHPLQERDPAFRFVFQTPKYRWGAHSTAVDADWISMLFGPFGDPYRRDPRMPWTG 1s7g.1    --------------------------------------------------------------------------------  target    EAYLEINPKDAA 1s7g.1    ------------ ``` | | | | | | | | | | | | | | | | | | | | | | | | | | | | | | | | | | | | | | | | | | | | | | | | | |
|  | 1s7g.1.E | NAD-dependent deacetylase 2  *Structural Basis for the Mechanism and Regulation of Sir2 Enzymes* | 0.01 |  | 20.75 | 0.07 | 124-178 | X-ray | 2.30 | homo-pentamer | 9 x ZN, 3 x NAD, 1 x APR, 1 x 2PE | HHblits | 0.32 |
| ``` target    SWDEATTIAAKTMEDVARTFNGDEGARKLLAQGYHPEMVEVMHGAGVQALKLRGGMPLLGIGRIFGFYRFANMLALLDRK 1s7g.1    --------------------------------------------------------------------------------  target    LRPDAPADEILGSRTFDNYAWHTDLPPGHPMVTGSQTVDFDLFSAEHTKLLLIIGMNWICTKMPDGHWIGDARLKGTRVI 1s7g.1    -------------------------------------------EAKHCDAFMVVGSSLVV--YPAAELPYIAKKAGAKMI  target    VISADYMPTANKADEVIILRPGTDAAFFLGVARELIEKGLYDRAAVIERTDLPLLVRLDTGERLDARDVIPGYELAALTN 1s7g.1    IVNAEPTMADPIFDVKII--------------------------------------------------------------  target    YVTLKPDAEIKGNPPPPPFTAGGQVVPTELRDAWGDFVWWDRATGRPRPVSRDEVGARFDGDPALLGEFEVELVDGSTVP 1s7g.1    --------------------------------------------------------------------------------  target    VRPAFDLLKQYLDESFDLRTASEVCRVPPQAIQSIARQLAANKRETLLAAGMGPNHYFQNDLFGRVQFLVAALTDNIGHL 1s7g.1    --------------------------------------------------------------------------------  target    GGNVGSYAGNYRGSVFQAMGQWIAEDPFAIEPDLTKPATVKRYYKAESAHYWNYGERPLRAVAKDDEGDLTKGEVLTGKS 1s7g.1    --------------------------------------------------------------------------------  target    HMPTPTKLIWFGNSNSLLGNAKWSFDVVKNTLPRQDAVFCNEWHWTSSCEYADLVFPADSWAEFKLPDATASCTNPFLLA 1s7g.1    --------------------------------------------------------------------------------  target    FPTTPLKRLYDTRSDYEALALTAKALGELIDEPRMEQYWRGILDGDPTPYLQRIFSGSNATRGITYDELHESSKRGVPLL 1s7g.1    --------------------------------------------------------------------------------  target    MNMRTYPRSGGWEQRQEDKPWYTATGRLEFYRPEPEFQAAGESLPVWREPVDATFYEPNAILSNAAHPSIAPRAPEDYGV 1s7g.1    --------------------------------------------------------------------------------  target    PESQLDVETRQYRNVVRTWAELQQTLHPLQERDPAFRFVFQTPKYRWGAHSTAVDADWISMLFGPFGDPYRRDPRMPWTG 1s7g.1    --------------------------------------------------------------------------------  target    EAYLEINPKDAA 1s7g.1    ------------ ``` | | | | | | | | | | | | | | | | | | | | | | | | | | | | | | | | | | | | | | | | | | | | | | | | | |
|  | 4twj.1.A | NAD-dependent protein deacylase 2  *The structure of Sir2Af2 bound to a myristoylated histone peptide* | 0.01 |  | 20.75 | 0.07 | 124-178 | X-ray | 1.65 | hetero-1-1-mer | 1 x ZN | HHblits | 0.32 |
| ``` target    SWDEATTIAAKTMEDVARTFNGDEGARKLLAQGYHPEMVEVMHGAGVQALKLRGGMPLLGIGRIFGFYRFANMLALLDRK 4twj.1    --------------------------------------------------------------------------------  target    LRPDAPADEILGSRTFDNYAWHTDLPPGHPMVTGSQTVDFDLFSAEHTKLLLIIGMNWICTKMPDGHWIGDARLKGTRVI 4twj.1    -------------------------------------------EAKHCDAFMVVGSSLVV--YPAAELPYIAKKAGAKMI  target    VISADYMPTANKADEVIILRPGTDAAFFLGVARELIEKGLYDRAAVIERTDLPLLVRLDTGERLDARDVIPGYELAALTN 4twj.1    IVNAEPTMADPIFDVKII--------------------------------------------------------------  target    YVTLKPDAEIKGNPPPPPFTAGGQVVPTELRDAWGDFVWWDRATGRPRPVSRDEVGARFDGDPALLGEFEVELVDGSTVP 4twj.1    --------------------------------------------------------------------------------  target    VRPAFDLLKQYLDESFDLRTASEVCRVPPQAIQSIARQLAANKRETLLAAGMGPNHYFQNDLFGRVQFLVAALTDNIGHL 4twj.1    --------------------------------------------------------------------------------  target    GGNVGSYAGNYRGSVFQAMGQWIAEDPFAIEPDLTKPATVKRYYKAESAHYWNYGERPLRAVAKDDEGDLTKGEVLTGKS 4twj.1    --------------------------------------------------------------------------------  target    HMPTPTKLIWFGNSNSLLGNAKWSFDVVKNTLPRQDAVFCNEWHWTSSCEYADLVFPADSWAEFKLPDATASCTNPFLLA 4twj.1    --------------------------------------------------------------------------------  target    FPTTPLKRLYDTRSDYEALALTAKALGELIDEPRMEQYWRGILDGDPTPYLQRIFSGSNATRGITYDELHESSKRGVPLL 4twj.1    --------------------------------------------------------------------------------  target    MNMRTYPRSGGWEQRQEDKPWYTATGRLEFYRPEPEFQAAGESLPVWREPVDATFYEPNAILSNAAHPSIAPRAPEDYGV 4twj.1    --------------------------------------------------------------------------------  target    PESQLDVETRQYRNVVRTWAELQQTLHPLQERDPAFRFVFQTPKYRWGAHSTAVDADWISMLFGPFGDPYRRDPRMPWTG 4twj.1    --------------------------------------------------------------------------------  target    EAYLEINPKDAA 4twj.1    ------------ ``` | | | | | | | | | | | | | | | | | | | | | | | | | | | | | | | | | | | | | | | | | | | | | | | | | |
|  | 4s12.1.A | N-acetylmuramic acid 6-phosphate etherase  *1.55 Angstrom Crystal Structure of N-acetylmuramic acid 6-phosphate Etherase from Yersinia enterocolitica.* | 0.01 |  | 21.43 | 0.07 | 127-184 | X-ray | 1.55 | homo-dimer |  | HHblits | 0.27 |
| ``` target    SWDEATTIAAKTMEDVARTFNGDEGARKLLAQGYHPEMVEVMHGAGVQALKLRGGMPLLGIGRIFGFYRFANMLALLDRK 4s12.1    --------------------------------------------------------------------------------  target    LRPDAPADEILGSRTFDNYAWHTDLPPGHPMVTGSQTVDFDLFSAEHTKLLLIIGMNWICTKMPDGHWIGDARLKGTRVI 4s12.1    ----------------------------------------------ATDMVVGLAASG-RT-PYVIGALRFARQLGCPTA  target    VISAD-YMPTANKADEVIILRPGTDAAFFLGVARELIEKGLYDRAAVIERTDLPLLVRLDTGERLDARDVIPGYELAALT 4s12.1    AISCNPDSPIAQEALVAISPVVGPE-------------------------------------------------------  target    NYVTLKPDAEIKGNPPPPPFTAGGQVVPTELRDAWGDFVWWDRATGRPRPVSRDEVGARFDGDPALLGEFEVELVDGSTV 4s12.1    --------------------------------------------------------------------------------  target    PVRPAFDLLKQYLDESFDLRTASEVCRVPPQAIQSIARQLAANKRETLLAAGMGPNHYFQNDLFGRVQFLVAALTDNIGH 4s12.1    --------------------------------------------------------------------------------  target    LGGNVGSYAGNYRGSVFQAMGQWIAEDPFAIEPDLTKPATVKRYYKAESAHYWNYGERPLRAVAKDDEGDLTKGEVLTGK 4s12.1    --------------------------------------------------------------------------------  target    SHMPTPTKLIWFGNSNSLLGNAKWSFDVVKNTLPRQDAVFCNEWHWTSSCEYADLVFPADSWAEFKLPDATASCTNPFLL 4s12.1    --------------------------------------------------------------------------------  target    AFPTTPLKRLYDTRSDYEALALTAKALGELIDEPRMEQYWRGILDGDPTPYLQRIFSGSNATRGITYDELHESSKRGVPL 4s12.1    --------------------------------------------------------------------------------  target    LMNMRTYPRSGGWEQRQEDKPWYTATGRLEFYRPEPEFQAAGESLPVWREPVDATFYEPNAILSNAAHPSIAPRAPEDYG 4s12.1    --------------------------------------------------------------------------------  target    VPESQLDVETRQYRNVVRTWAELQQTLHPLQERDPAFRFVFQTPKYRWGAHSTAVDADWISMLFGPFGDPYRRDPRMPWT 4s12.1    --------------------------------------------------------------------------------  target    GEAYLEINPKDAA 4s12.1    ------------- ``` | | | | | | | | | | | | | | | | | | | | | | | | | | | | | | | | | | | | | | | | | | | | | | | | | |
|  | 4s12.2.A | N-acetylmuramic acid 6-phosphate etherase  *1.55 Angstrom Crystal Structure of N-acetylmuramic acid 6-phosphate Etherase from Yersinia enterocolitica.* | 0.01 |  | 21.43 | 0.07 | 127-184 | X-ray | 1.55 | homo-dimer |  | HHblits | 0.27 |
| ``` target    SWDEATTIAAKTMEDVARTFNGDEGARKLLAQGYHPEMVEVMHGAGVQALKLRGGMPLLGIGRIFGFYRFANMLALLDRK 4s12.2    --------------------------------------------------------------------------------  target    LRPDAPADEILGSRTFDNYAWHTDLPPGHPMVTGSQTVDFDLFSAEHTKLLLIIGMNWICTKMPDGHWIGDARLKGTRVI 4s12.2    ----------------------------------------------ATDMVVGLAASG-RT-PYVIGALRFARQLGCPTA  target    VISAD-YMPTANKADEVIILRPGTDAAFFLGVARELIEKGLYDRAAVIERTDLPLLVRLDTGERLDARDVIPGYELAALT 4s12.2    AISCNPDSPIAQEALVAISPVVGPE-------------------------------------------------------  target    NYVTLKPDAEIKGNPPPPPFTAGGQVVPTELRDAWGDFVWWDRATGRPRPVSRDEVGARFDGDPALLGEFEVELVDGSTV 4s12.2    --------------------------------------------------------------------------------  target    PVRPAFDLLKQYLDESFDLRTASEVCRVPPQAIQSIARQLAANKRETLLAAGMGPNHYFQNDLFGRVQFLVAALTDNIGH 4s12.2    --------------------------------------------------------------------------------  target    LGGNVGSYAGNYRGSVFQAMGQWIAEDPFAIEPDLTKPATVKRYYKAESAHYWNYGERPLRAVAKDDEGDLTKGEVLTGK 4s12.2    --------------------------------------------------------------------------------  target    SHMPTPTKLIWFGNSNSLLGNAKWSFDVVKNTLPRQDAVFCNEWHWTSSCEYADLVFPADSWAEFKLPDATASCTNPFLL 4s12.2    --------------------------------------------------------------------------------  target    AFPTTPLKRLYDTRSDYEALALTAKALGELIDEPRMEQYWRGILDGDPTPYLQRIFSGSNATRGITYDELHESSKRGVPL 4s12.2    --------------------------------------------------------------------------------  target    LMNMRTYPRSGGWEQRQEDKPWYTATGRLEFYRPEPEFQAAGESLPVWREPVDATFYEPNAILSNAAHPSIAPRAPEDYG 4s12.2    --------------------------------------------------------------------------------  target    VPESQLDVETRQYRNVVRTWAELQQTLHPLQERDPAFRFVFQTPKYRWGAHSTAVDADWISMLFGPFGDPYRRDPRMPWT 4s12.2    --------------------------------------------------------------------------------  target    GEAYLEINPKDAA 4s12.2    ------------- ``` | | | | | | | | | | | | | | | | | | | | | | | | | | | | | | | | | | | | | | | | | | | | | | | | | |
|  | 4s12.2.B | N-acetylmuramic acid 6-phosphate etherase  *1.55 Angstrom Crystal Structure of N-acetylmuramic acid 6-phosphate Etherase from Yersinia enterocolitica.* | 0.01 |  | 21.43 | 0.07 | 127-184 | X-ray | 1.55 | homo-dimer |  | HHblits | 0.27 |
| ``` target    SWDEATTIAAKTMEDVARTFNGDEGARKLLAQGYHPEMVEVMHGAGVQALKLRGGMPLLGIGRIFGFYRFANMLALLDRK 4s12.2    --------------------------------------------------------------------------------  target    LRPDAPADEILGSRTFDNYAWHTDLPPGHPMVTGSQTVDFDLFSAEHTKLLLIIGMNWICTKMPDGHWIGDARLKGTRVI 4s12.2    ----------------------------------------------ATDMVVGLAASG-RT-PYVIGALRFARQLGCPTA  target    VISAD-YMPTANKADEVIILRPGTDAAFFLGVARELIEKGLYDRAAVIERTDLPLLVRLDTGERLDARDVIPGYELAALT 4s12.2    AISCNPDSPIAQEALVAISPVVGPE-------------------------------------------------------  target    NYVTLKPDAEIKGNPPPPPFTAGGQVVPTELRDAWGDFVWWDRATGRPRPVSRDEVGARFDGDPALLGEFEVELVDGSTV 4s12.2    --------------------------------------------------------------------------------  target    PVRPAFDLLKQYLDESFDLRTASEVCRVPPQAIQSIARQLAANKRETLLAAGMGPNHYFQNDLFGRVQFLVAALTDNIGH 4s12.2    --------------------------------------------------------------------------------  target    LGGNVGSYAGNYRGSVFQAMGQWIAEDPFAIEPDLTKPATVKRYYKAESAHYWNYGERPLRAVAKDDEGDLTKGEVLTGK 4s12.2    --------------------------------------------------------------------------------  target    SHMPTPTKLIWFGNSNSLLGNAKWSFDVVKNTLPRQDAVFCNEWHWTSSCEYADLVFPADSWAEFKLPDATASCTNPFLL 4s12.2    --------------------------------------------------------------------------------  target    AFPTTPLKRLYDTRSDYEALALTAKALGELIDEPRMEQYWRGILDGDPTPYLQRIFSGSNATRGITYDELHESSKRGVPL 4s12.2    --------------------------------------------------------------------------------  target    LMNMRTYPRSGGWEQRQEDKPWYTATGRLEFYRPEPEFQAAGESLPVWREPVDATFYEPNAILSNAAHPSIAPRAPEDYG 4s12.2    --------------------------------------------------------------------------------  target    VPESQLDVETRQYRNVVRTWAELQQTLHPLQERDPAFRFVFQTPKYRWGAHSTAVDADWISMLFGPFGDPYRRDPRMPWT 4s12.2    --------------------------------------------------------------------------------  target    GEAYLEINPKDAA 4s12.2    ------------- ``` | | | | | | | | | | | | | | | | | | | | | | | | | | | | | | | | | | | | | | | | | | | | | | | | | |
|  | 1m2j.1.A | Silent Information Regulator 2  *Sir2 homologue H80N mutant-ADP ribose complex* | 0.02 |  | 18.52 | 0.07 | 124-179 | X-ray | 1.70 | monomer | 1 x ZN, 1 x APR | HHblits | 0.30 |
| ``` target    SWDEATTIAAKTMEDVARTFNGDEGARKLLAQGYHPEMVEVMHGAGVQALKLRGGMPLLGIGRIFGFYRFANMLALLDRK 1m2j.1    --------------------------------------------------------------------------------  target    LRPDAPADEILGSRTFDNYAWHTDLPPGHPMVTGSQTVDFDLFSAEHTKLLLIIGMNWICTKMPDGHWIGDARLKGTRVI 1m2j.1    -------------------------------------------EVERADVIIVAGTSAVV--QPAASLPLIVKQRGGAII  target    VISADYMPTANKADEVIILRPGTDAAFFLGVARELIEKGLYDRAAVIERTDLPLLVRLDTGERLDARDVIPGYELAALTN 1m2j.1    EINPDETPLTPIADYSLRG-------------------------------------------------------------  target    YVTLKPDAEIKGNPPPPPFTAGGQVVPTELRDAWGDFVWWDRATGRPRPVSRDEVGARFDGDPALLGEFEVELVDGSTVP 1m2j.1    --------------------------------------------------------------------------------  target    VRPAFDLLKQYLDESFDLRTASEVCRVPPQAIQSIARQLAANKRETLLAAGMGPNHYFQNDLFGRVQFLVAALTDNIGHL 1m2j.1    --------------------------------------------------------------------------------  target    GGNVGSYAGNYRGSVFQAMGQWIAEDPFAIEPDLTKPATVKRYYKAESAHYWNYGERPLRAVAKDDEGDLTKGEVLTGKS 1m2j.1    --------------------------------------------------------------------------------  target    HMPTPTKLIWFGNSNSLLGNAKWSFDVVKNTLPRQDAVFCNEWHWTSSCEYADLVFPADSWAEFKLPDATASCTNPFLLA 1m2j.1    --------------------------------------------------------------------------------  target    FPTTPLKRLYDTRSDYEALALTAKALGELIDEPRMEQYWRGILDGDPTPYLQRIFSGSNATRGITYDELHESSKRGVPLL 1m2j.1    --------------------------------------------------------------------------------  target    MNMRTYPRSGGWEQRQEDKPWYTATGRLEFYRPEPEFQAAGESLPVWREPVDATFYEPNAILSNAAHPSIAPRAPEDYGV 1m2j.1    --------------------------------------------------------------------------------  target    PESQLDVETRQYRNVVRTWAELQQTLHPLQERDPAFRFVFQTPKYRWGAHSTAVDADWISMLFGPFGDPYRRDPRMPWTG 1m2j.1    --------------------------------------------------------------------------------  target    EAYLEINPKDAA 1m2j.1    ------------ ``` | | | | | | | | | | | | | | | | | | | | | | | | | | | | | | | | | | | | | | | | | | | | | | | | | |
|  | 1ici.1.A | TRANSCRIPTIONAL REGULATORY PROTEIN, SIR2 FAMILY  *CRYSTAL STRUCTURE OF A SIR2 HOMOLOG-NAD COMPLEX* | 0.02 |  | 18.52 | 0.07 | 124-179 | X-ray | 2.10 | homo-dimer | 2 x ZN, 2 x NAD | HHblits | 0.30 |
| ``` target    SWDEATTIAAKTMEDVARTFNGDEGARKLLAQGYHPEMVEVMHGAGVQALKLRGGMPLLGIGRIFGFYRFANMLALLDRK 1ici.1    --------------------------------------------------------------------------------  target    LRPDAPADEILGSRTFDNYAWHTDLPPGHPMVTGSQTVDFDLFSAEHTKLLLIIGMNWICTKMPDGHWIGDARLKGTRVI 1ici.1    -------------------------------------------EVERADVIIVAGTSAVV--QPAASLPLIVKQRGGAII  target    VISADYMPTANKADEVIILRPGTDAAFFLGVARELIEKGLYDRAAVIERTDLPLLVRLDTGERLDARDVIPGYELAALTN 1ici.1    EINPDETPLTPIADYSLRG-------------------------------------------------------------  target    YVTLKPDAEIKGNPPPPPFTAGGQVVPTELRDAWGDFVWWDRATGRPRPVSRDEVGARFDGDPALLGEFEVELVDGSTVP 1ici.1    --------------------------------------------------------------------------------  target    VRPAFDLLKQYLDESFDLRTASEVCRVPPQAIQSIARQLAANKRETLLAAGMGPNHYFQNDLFGRVQFLVAALTDNIGHL 1ici.1    --------------------------------------------------------------------------------  target    GGNVGSYAGNYRGSVFQAMGQWIAEDPFAIEPDLTKPATVKRYYKAESAHYWNYGERPLRAVAKDDEGDLTKGEVLTGKS 1ici.1    --------------------------------------------------------------------------------  target    HMPTPTKLIWFGNSNSLLGNAKWSFDVVKNTLPRQDAVFCNEWHWTSSCEYADLVFPADSWAEFKLPDATASCTNPFLLA 1ici.1    --------------------------------------------------------------------------------  target    FPTTPLKRLYDTRSDYEALALTAKALGELIDEPRMEQYWRGILDGDPTPYLQRIFSGSNATRGITYDELHESSKRGVPLL 1ici.1    --------------------------------------------------------------------------------  target    MNMRTYPRSGGWEQRQEDKPWYTATGRLEFYRPEPEFQAAGESLPVWREPVDATFYEPNAILSNAAHPSIAPRAPEDYGV 1ici.1    --------------------------------------------------------------------------------  target    PESQLDVETRQYRNVVRTWAELQQTLHPLQERDPAFRFVFQTPKYRWGAHSTAVDADWISMLFGPFGDPYRRDPRMPWTG 1ici.1    --------------------------------------------------------------------------------  target    EAYLEINPKDAA 1ici.1    ------------ ``` | | | | | | | | | | | | | | | | | | | | | | | | | | | | | | | | | | | | | | | | | | | | | | | | | |
|  | 1dms.1.A | DMSO REDUCTASE  *STRUCTURE OF DMSO REDUCTASE* | 0.01 |  | 25.93 | 0.07 | 127-181 | X-ray | 1.88 | monomer | 2 x PGD, 1 x 2MO | HHblits | 0.30 |
| ``` target    SWDEATTIAAKTMEDVARTFNGDEGARKLLAQGYHPEMVEVMHGAGVQALKLRGGMPLLGIGRIFGFYRFANMLALLDRK 1dms.1    --------------------------------------------------------------------------------  target    LRPDAPADEILGSRTFDNYAWHTDLPPGHPMVTGSQTVDFDLFSAEHTKLLLIIGMNWICTKMPDGHWIGDARLKGTRVI 1dms.1    ----------------------------------------------DVKMAYWVGGNPFVHHQ-DRNRMVKAWEKLETFI  target    VISADYMPTANKADEVIILRPGTDAAFFLGVARELIEKGLYDRAAVIERTDLPLLVRLDTGERLDARDVIPGYELAALTN 1dms.1    VHDFQWTPTARHADIVLPATT-----------------------------------------------------------  target    YVTLKPDAEIKGNPPPPPFTAGGQVVPTELRDAWGDFVWWDRATGRPRPVSRDEVGARFDGDPALLGEFEVELVDGSTVP 1dms.1    --------------------------------------------------------------------------------  target    VRPAFDLLKQYLDESFDLRTASEVCRVPPQAIQSIARQLAANKRETLLAAGMGPNHYFQNDLFGRVQFLVAALTDNIGHL 1dms.1    --------------------------------------------------------------------------------  target    GGNVGSYAGNYRGSVFQAMGQWIAEDPFAIEPDLTKPATVKRYYKAESAHYWNYGERPLRAVAKDDEGDLTKGEVLTGKS 1dms.1    --------------------------------------------------------------------------------  target    HMPTPTKLIWFGNSNSLLGNAKWSFDVVKNTLPRQDAVFCNEWHWTSSCEYADLVFPADSWAEFKLPDATASCTNPFLLA 1dms.1    --------------------------------------------------------------------------------  target    FPTTPLKRLYDTRSDYEALALTAKALGELIDEPRMEQYWRGILDGDPTPYLQRIFSGSNATRGITYDELHESSKRGVPLL 1dms.1    --------------------------------------------------------------------------------  target    MNMRTYPRSGGWEQRQEDKPWYTATGRLEFYRPEPEFQAAGESLPVWREPVDATFYEPNAILSNAAHPSIAPRAPEDYGV 1dms.1    --------------------------------------------------------------------------------  target    PESQLDVETRQYRNVVRTWAELQQTLHPLQERDPAFRFVFQTPKYRWGAHSTAVDADWISMLFGPFGDPYRRDPRMPWTG 1dms.1    --------------------------------------------------------------------------------  target    EAYLEINPKDAA 1dms.1    ------------ ``` | | | | | | | | | | | | | | | | | | | | | | | | | | | | | | | | | | | | | | | | | | | | | | | | | |
|  | 3riy.2.A | NAD-dependent deacetylase sirtuin-5  *Sirt5 is an NAD-dependent protein lysine demalonylase and desuccinylase* | 0.01 |  | 16.67 | 0.07 | 123-178 | X-ray | 1.55 | hetero-oligomer | 1 x ZN, 1 x NAD | HHblits | 0.30 |
| ``` target    SWDEATTIAAKTMEDVARTFNGDEGARKLLAQGYHPEMVEVMHGAGVQALKLRGGMPLLGIGRIFGFYRFANMLALLDRK 3riy.2    --------------------------------------------------------------------------------  target    LRPDAPADEILGSRTFDNYAWHTDLPPGHPMVTGSQTVDFDLFSAEHTKLLLIIGMNWICTKMPDGHWIGDARLKGTRVI 3riy.2    ------------------------------------------RELAHCDLCLVVGTSSVV--YPAAMFAPQVAARGVPVA  target    VISADYMPTANKADEVIILRPGTDAAFFLGVARELIEKGLYDRAAVIERTDLPLLVRLDTGERLDARDVIPGYELAALTN 3riy.2    EFNTETTPATNRFRFHFQ--------------------------------------------------------------  target    YVTLKPDAEIKGNPPPPPFTAGGQVVPTELRDAWGDFVWWDRATGRPRPVSRDEVGARFDGDPALLGEFEVELVDGSTVP 3riy.2    --------------------------------------------------------------------------------  target    VRPAFDLLKQYLDESFDLRTASEVCRVPPQAIQSIARQLAANKRETLLAAGMGPNHYFQNDLFGRVQFLVAALTDNIGHL 3riy.2    --------------------------------------------------------------------------------  target    GGNVGSYAGNYRGSVFQAMGQWIAEDPFAIEPDLTKPATVKRYYKAESAHYWNYGERPLRAVAKDDEGDLTKGEVLTGKS 3riy.2    --------------------------------------------------------------------------------  target    HMPTPTKLIWFGNSNSLLGNAKWSFDVVKNTLPRQDAVFCNEWHWTSSCEYADLVFPADSWAEFKLPDATASCTNPFLLA 3riy.2    --------------------------------------------------------------------------------  target    FPTTPLKRLYDTRSDYEALALTAKALGELIDEPRMEQYWRGILDGDPTPYLQRIFSGSNATRGITYDELHESSKRGVPLL 3riy.2    --------------------------------------------------------------------------------  target    MNMRTYPRSGGWEQRQEDKPWYTATGRLEFYRPEPEFQAAGESLPVWREPVDATFYEPNAILSNAAHPSIAPRAPEDYGV 3riy.2    --------------------------------------------------------------------------------  target    PESQLDVETRQYRNVVRTWAELQQTLHPLQERDPAFRFVFQTPKYRWGAHSTAVDADWISMLFGPFGDPYRRDPRMPWTG 3riy.2    --------------------------------------------------------------------------------  target    EAYLEINPKDAA 3riy.2    ------------ ``` | | | | | | | | | | | | | | | | | | | | | | | | | | | | | | | | | | | | | | | | | | | | | | | | | |
|  | 2i2w.1.A | Phosphoheptose isomerase  *Crystal Structure of Escherichia Coli Phosphoheptose Isomerase* | 0.01 |  | 25.45 | 0.07 | 126-182 | X-ray | 1.95 | homo-dimer |  | HHblits | 0.28 |
| ``` target    SWDEATTIAAKTMEDVARTFNGDEGARKLLAQGYHPEMVEVMHGAGVQALKLRGGMPLLGIGRIFGFYRFANMLALLDRK 2i2w.1    --------------------------------------------------------------------------------  target    LRPDAPADEILGSRTFDNYAWHTDLPPGHPMVTGSQTVDFDLFSAEHTKLLLIIGMNWICTKMPDGHWIGDARLKGTRVI 2i2w.1    ---------------------------------------------REGDVLLGISTSGNSA--NVIKAIAAAREKGMKVI  target    VISADY-MPTANKADEVIILRPGTDAAFFLGVARELIEKGLYDRAAVIERTDLPLLVRLDTGERLDARDVIPGYELAALT 2i2w.1    TLTGKDGGKMAGTADIEIRVPHF---------------------------------------------------------  target    NYVTLKPDAEIKGNPPPPPFTAGGQVVPTELRDAWGDFVWWDRATGRPRPVSRDEVGARFDGDPALLGEFEVELVDGSTV 2i2w.1    --------------------------------------------------------------------------------  target    PVRPAFDLLKQYLDESFDLRTASEVCRVPPQAIQSIARQLAANKRETLLAAGMGPNHYFQNDLFGRVQFLVAALTDNIGH 2i2w.1    --------------------------------------------------------------------------------  target    LGGNVGSYAGNYRGSVFQAMGQWIAEDPFAIEPDLTKPATVKRYYKAESAHYWNYGERPLRAVAKDDEGDLTKGEVLTGK 2i2w.1    --------------------------------------------------------------------------------  target    SHMPTPTKLIWFGNSNSLLGNAKWSFDVVKNTLPRQDAVFCNEWHWTSSCEYADLVFPADSWAEFKLPDATASCTNPFLL 2i2w.1    --------------------------------------------------------------------------------  target    AFPTTPLKRLYDTRSDYEALALTAKALGELIDEPRMEQYWRGILDGDPTPYLQRIFSGSNATRGITYDELHESSKRGVPL 2i2w.1    --------------------------------------------------------------------------------  target    LMNMRTYPRSGGWEQRQEDKPWYTATGRLEFYRPEPEFQAAGESLPVWREPVDATFYEPNAILSNAAHPSIAPRAPEDYG 2i2w.1    --------------------------------------------------------------------------------  target    VPESQLDVETRQYRNVVRTWAELQQTLHPLQERDPAFRFVFQTPKYRWGAHSTAVDADWISMLFGPFGDPYRRDPRMPWT 2i2w.1    --------------------------------------------------------------------------------  target    GEAYLEINPKDAA 2i2w.1    ------------- ``` | | | | | | | | | | | | | | | | | | | | | | | | | | | | | | | | | | | | | | | | | | | | | | | | | |
|  | 2i2w.2.B | Phosphoheptose isomerase  *Crystal Structure of Escherichia Coli Phosphoheptose Isomerase* | 0.01 |  | 25.45 | 0.07 | 126-182 | X-ray | 1.95 | homo-dimer |  | HHblits | 0.28 |
| ``` target    SWDEATTIAAKTMEDVARTFNGDEGARKLLAQGYHPEMVEVMHGAGVQALKLRGGMPLLGIGRIFGFYRFANMLALLDRK 2i2w.2    --------------------------------------------------------------------------------  target    LRPDAPADEILGSRTFDNYAWHTDLPPGHPMVTGSQTVDFDLFSAEHTKLLLIIGMNWICTKMPDGHWIGDARLKGTRVI 2i2w.2    ---------------------------------------------REGDVLLGISTSGNSA--NVIKAIAAAREKGMKVI  target    VISADY-MPTANKADEVIILRPGTDAAFFLGVARELIEKGLYDRAAVIERTDLPLLVRLDTGERLDARDVIPGYELAALT 2i2w.2    TLTGKDGGKMAGTADIEIRVPHF---------------------------------------------------------  target    NYVTLKPDAEIKGNPPPPPFTAGGQVVPTELRDAWGDFVWWDRATGRPRPVSRDEVGARFDGDPALLGEFEVELVDGSTV 2i2w.2    --------------------------------------------------------------------------------  target    PVRPAFDLLKQYLDESFDLRTASEVCRVPPQAIQSIARQLAANKRETLLAAGMGPNHYFQNDLFGRVQFLVAALTDNIGH 2i2w.2    --------------------------------------------------------------------------------  target    LGGNVGSYAGNYRGSVFQAMGQWIAEDPFAIEPDLTKPATVKRYYKAESAHYWNYGERPLRAVAKDDEGDLTKGEVLTGK 2i2w.2    --------------------------------------------------------------------------------  target    SHMPTPTKLIWFGNSNSLLGNAKWSFDVVKNTLPRQDAVFCNEWHWTSSCEYADLVFPADSWAEFKLPDATASCTNPFLL 2i2w.2    --------------------------------------------------------------------------------  target    AFPTTPLKRLYDTRSDYEALALTAKALGELIDEPRMEQYWRGILDGDPTPYLQRIFSGSNATRGITYDELHESSKRGVPL 2i2w.2    --------------------------------------------------------------------------------  target    LMNMRTYPRSGGWEQRQEDKPWYTATGRLEFYRPEPEFQAAGESLPVWREPVDATFYEPNAILSNAAHPSIAPRAPEDYG 2i2w.2    --------------------------------------------------------------------------------  target    VPESQLDVETRQYRNVVRTWAELQQTLHPLQERDPAFRFVFQTPKYRWGAHSTAVDADWISMLFGPFGDPYRRDPRMPWT 2i2w.2    --------------------------------------------------------------------------------  target    GEAYLEINPKDAA 2i2w.2    ------------- ``` | | | | | | | | | | | | | | | | | | | | | | | | | | | | | | | | | | | | | | | | | | | | | | | | | |
|  | 3k35.1.A | NAD-dependent deacetylase sirtuin-6  *Crystal Structure of Human SIRT6* | 0.01 |  | 16.67 | 0.07 | 123-178 | X-ray | 2.00 | monomer | 1 x ZN, 1 x APR | HHblits | 0.29 |
| ``` target    SWDEATTIAAKTMEDVARTFNGDEGARKLLAQGYHPEMVEVMHGAGVQALKLRGGMPLLGIGRIFGFYRFANMLALLDRK 3k35.1    --------------------------------------------------------------------------------  target    LRPDAPADEILGSRTFDNYAWHTDLPPGHPMVTGSQTVDFDLFSAEHTKLLLIIGMNWICTKMPDGHWIGDARLKGTRVI 3k35.1    ------------------------------------------EASRNADLSITLGTSLQI--RPSGNLPLATKRRGGRLV  target    VISADYMPTANKADEVIILRPGTDAAFFLGVARELIEKGLYDRAAVIERTDLPLLVRLDTGERLDARDVIPGYELAALTN 3k35.1    IVNLQPTKHDRHADLRIH--------------------------------------------------------------  target    YVTLKPDAEIKGNPPPPPFTAGGQVVPTELRDAWGDFVWWDRATGRPRPVSRDEVGARFDGDPALLGEFEVELVDGSTVP 3k35.1    --------------------------------------------------------------------------------  target    VRPAFDLLKQYLDESFDLRTASEVCRVPPQAIQSIARQLAANKRETLLAAGMGPNHYFQNDLFGRVQFLVAALTDNIGHL 3k35.1    --------------------------------------------------------------------------------  target    GGNVGSYAGNYRGSVFQAMGQWIAEDPFAIEPDLTKPATVKRYYKAESAHYWNYGERPLRAVAKDDEGDLTKGEVLTGKS 3k35.1    --------------------------------------------------------------------------------  target    HMPTPTKLIWFGNSNSLLGNAKWSFDVVKNTLPRQDAVFCNEWHWTSSCEYADLVFPADSWAEFKLPDATASCTNPFLLA 3k35.1    --------------------------------------------------------------------------------  target    FPTTPLKRLYDTRSDYEALALTAKALGELIDEPRMEQYWRGILDGDPTPYLQRIFSGSNATRGITYDELHESSKRGVPLL 3k35.1    --------------------------------------------------------------------------------  target    MNMRTYPRSGGWEQRQEDKPWYTATGRLEFYRPEPEFQAAGESLPVWREPVDATFYEPNAILSNAAHPSIAPRAPEDYGV 3k35.1    --------------------------------------------------------------------------------  target    PESQLDVETRQYRNVVRTWAELQQTLHPLQERDPAFRFVFQTPKYRWGAHSTAVDADWISMLFGPFGDPYRRDPRMPWTG 3k35.1    --------------------------------------------------------------------------------  target    EAYLEINPKDAA 3k35.1    ------------ ``` | | | | | | | | | | | | | | | | | | | | | | | | | | | | | | | | | | | | | | | | | | | | | | | | | |
|  | 2v3v.1.A | PERIPLASMIC NITRATE REDUCTASE  *A NEW CATALYTIC MECHANISM OF PERIPLASMIC NITRATE REDUCTASE FROM DESULFOVIBRIO DESULFURICANS ATCC 27774 FROM CRYSTALLOGRAPHIC AND EPR DATA AND BASED ON DETAILED ANALYSIS OF THE SIXTH LIGAND* | 0.01 |  | 21.82 | 0.07 | 127-182 | X-ray | 1.99 | monomer | 1 x SF4, 1 x MO, 2 x MGD, 4 x LCP | HHblits | 0.28 |
| ``` target    SWDEATTIAAKTMEDVARTFNGDEGARKLLAQGYHPEMVEVMHGAGVQALKLRGGMPLLGIGRIFGFYRFANMLALLDRK 2v3v.1    --------------------------------------------------------------------------------  target    LRPDAPADEILGSRTFDNYAWHTDLPPGHPMVTGSQTVDFDLFSAEHTKLLLIIGMNWICTKMPDGHWIGDARLKG-TRV 2v3v.1    ----------------------------------------------DVKCMIICETNPAHTL-PNLNKVHKAMSHPESFI  target    IVISADYM-PTANKADEVIILRPGTDAAFFLGVARELIEKGLYDRAAVIERTDLPLLVRLDTGERLDARDVIPGYELAAL 2v3v.1    VCIEAFPDAVTLEYADLVLPPAFW--------------------------------------------------------  target    TNYVTLKPDAEIKGNPPPPPFTAGGQVVPTELRDAWGDFVWWDRATGRPRPVSRDEVGARFDGDPALLGEFEVELVDGST 2v3v.1    --------------------------------------------------------------------------------  target    VPVRPAFDLLKQYLDESFDLRTASEVCRVPPQAIQSIARQLAANKRETLLAAGMGPNHYFQNDLFGRVQFLVAALTDNIG 2v3v.1    --------------------------------------------------------------------------------  target    HLGGNVGSYAGNYRGSVFQAMGQWIAEDPFAIEPDLTKPATVKRYYKAESAHYWNYGERPLRAVAKDDEGDLTKGEVLTG 2v3v.1    --------------------------------------------------------------------------------  target    KSHMPTPTKLIWFGNSNSLLGNAKWSFDVVKNTLPRQDAVFCNEWHWTSSCEYADLVFPADSWAEFKLPDATASCTNPFL 2v3v.1    --------------------------------------------------------------------------------  target    LAFPTTPLKRLYDTRSDYEALALTAKALGELIDEPRMEQYWRGILDGDPTPYLQRIFSGSNATRGITYDELHESSKRGVP 2v3v.1    --------------------------------------------------------------------------------  target    LLMNMRTYPRSGGWEQRQEDKPWYTATGRLEFYRPEPEFQAAGESLPVWREPVDATFYEPNAILSNAAHPSIAPRAPEDY 2v3v.1    --------------------------------------------------------------------------------  target    GVPESQLDVETRQYRNVVRTWAELQQTLHPLQERDPAFRFVFQTPKYRWGAHSTAVDADWISMLFGPFGDPYRRDPRMPW 2v3v.1    --------------------------------------------------------------------------------  target    TGEAYLEINPKDAA 2v3v.1    -------------- ``` | | | | | | | | | | | | | | | | | | | | | | | | | | | | | | | | | | | | | | | | | | | | | | | | | |
|  | 6cz7.1.A | ArrA  *The arsenate respiratory reductase (Arr) complex from Shewanella sp. ANA-3* | 0.01 |  | 18.18 | 0.07 | 126-181 | X-ray | 1.62 | hetero-1-1-mer | 5 x SF4, 2 x MGD, 1 x MO, 1 x PG5 | HHblits | 0.28 |
| ``` target    SWDEATTIAAKTMEDVARTFNGDEGARKLLAQGYHPEMVEVMHGAGVQALKLRGGMPLLGIGRIFGFYRFANMLALLDRK 6cz7.1    --------------------------------------------------------------------------------  target    LRPDAPADEILGSRTFDNYAWHTDLPPGHPMVTGSQTVDFDLFSAEHTKLLLIIGMNWICTKMPDGHWIGDARLKGTRVI 6cz7.1    ---------------------------------------------YEIKVMLAYFNNFNFSN-PEGQRWDEALSKVDFMA  target    VISADYMPTANKADEVIILRPGTDAAFFLGVARELIEKGLYDRAAVIERTDLPLLVRLDTGERLDARDVIPGYELAALTN 6cz7.1    HITTNVSEFSWFADVLLPSSH-----------------------------------------------------------  target    YVTLKPDAEIKGNPPPPPFTAGGQVVPTELRDAWGDFVWWDRATGRPRPVSRDEVGARFDGDPALLGEFEVELVDGSTVP 6cz7.1    --------------------------------------------------------------------------------  target    VRPAFDLLKQYLDESFDLRTASEVCRVPPQAIQSIARQLAANKRETLLAAGMGPNHYFQNDLFGRVQFLVAALTDNIGHL 6cz7.1    --------------------------------------------------------------------------------  target    GGNVGSYAGNYRGSVFQAMGQWIAEDPFAIEPDLTKPATVKRYYKAESAHYWNYGERPLRAVAKDDEGDLTKGEVLTGKS 6cz7.1    --------------------------------------------------------------------------------  target    HMPTPTKLIWFGNSNSLLGNAKWSFDVVKNTLPRQDAVFCNEWHWTSSCEYADLVFPADSWAEFKLPDATASCTNPFLLA 6cz7.1    --------------------------------------------------------------------------------  target    FPTTPLKRLYDTRSDYEALALTAKALGELIDEPRMEQYWRGILDGDPTPYLQRIFSGSNATRGITYDELHESSKRGVPLL 6cz7.1    --------------------------------------------------------------------------------  target    MNMRTYPRSGGWEQRQEDKPWYTATGRLEFYRPEPEFQAAGESLPVWREPVDATFYEPNAILSNAAHPSIAPRAPEDYGV 6cz7.1    --------------------------------------------------------------------------------  target    PESQLDVETRQYRNVVRTWAELQQTLHPLQERDPAFRFVFQTPKYRWGAHSTAVDADWISMLFGPFGDPYRRDPRMPWTG 6cz7.1    --------------------------------------------------------------------------------  target    EAYLEINPKDAA 6cz7.1    ------------ ``` | | | | | | | | | | | | | | | | | | | | | | | | | | | | | | | | | | | | | | | | | | | | | | | | | |
|  | 4ydd.1.A | DMSO reductase family type II enzyme, molybdopterin subunit  *Crystal structure of the perchlorate reductase PcrAB from Azospira suillum PS* | 0.01 |  | 16.36 | 0.07 | 127-181 | X-ray | 1.86 | hetero-oligomer | 4 x SF4, 1 x MO, 1 x MGD, 1 x MD1, 1 x F3S | HHblits | 0.28 |
| ``` target    SWDEATTIAAKTMEDVARTFNGDEGARKLLAQGYHPEMVEVMHGAGVQALKLRGGMPLLGIGRIFGFYRFANMLALLDRK 4ydd.1    --------------------------------------------------------------------------------  target    LRPDAPADEILGSRTFDNYAWHTDLPPGHPMVTGSQTVDFDLFSAEHTKLLLIIGMNWICTKMPDGHWIGDARLKGTRVI 4ydd.1    ----------------------------------------------DPKVFFVYRGNWLNQAKGQKYVLENLWPKLELIV  target    VISADYMPTANKADEVIILRPGTDAAFFLGVARELIEKGLYDRAAVIERTDLPLLVRLDTGERLDARDVIPGYELAALTN 4ydd.1    DINIRMDSTALYSDVVLPSAH-----------------------------------------------------------  target    YVTLKPDAEIKGNPPPPPFTAGGQVVPTELRDAWGDFVWWDRATGRPRPVSRDEVGARFDGDPALLGEFEVELVDGSTVP 4ydd.1    --------------------------------------------------------------------------------  target    VRPAFDLLKQYLDESFDLRTASEVCRVPPQAIQSIARQLAANKRETLLAAGMGPNHYFQNDLFGRVQFLVAALTDNIGHL 4ydd.1    --------------------------------------------------------------------------------  target    GGNVGSYAGNYRGSVFQAMGQWIAEDPFAIEPDLTKPATVKRYYKAESAHYWNYGERPLRAVAKDDEGDLTKGEVLTGKS 4ydd.1    --------------------------------------------------------------------------------  target    HMPTPTKLIWFGNSNSLLGNAKWSFDVVKNTLPRQDAVFCNEWHWTSSCEYADLVFPADSWAEFKLPDATASCTNPFLLA 4ydd.1    --------------------------------------------------------------------------------  target    FPTTPLKRLYDTRSDYEALALTAKALGELIDEPRMEQYWRGILDGDPTPYLQRIFSGSNATRGITYDELHESSKRGVPLL 4ydd.1    --------------------------------------------------------------------------------  target    MNMRTYPRSGGWEQRQEDKPWYTATGRLEFYRPEPEFQAAGESLPVWREPVDATFYEPNAILSNAAHPSIAPRAPEDYGV 4ydd.1    --------------------------------------------------------------------------------  target    PESQLDVETRQYRNVVRTWAELQQTLHPLQERDPAFRFVFQTPKYRWGAHSTAVDADWISMLFGPFGDPYRRDPRMPWTG 4ydd.1    --------------------------------------------------------------------------------  target    EAYLEINPKDAA 4ydd.1    ------------ ``` | | | | | | | | | | | | | | | | | | | | | | | | | | | | | | | | | | | | | | | | | | | | | | | | | |
|  | 1m2g.1.A | Silent Information Regulator 2  *Sir2 homologue-ADP ribose complex* | 0.02 |  | 18.87 | 0.07 | 124-178 | X-ray | 1.70 | monomer | 1 x ZN, 1 x APR | HHblits | 0.30 |
| ``` target    SWDEATTIAAKTMEDVARTFNGDEGARKLLAQGYHPEMVEVMHGAGVQALKLRGGMPLLGIGRIFGFYRFANMLALLDRK 1m2g.1    --------------------------------------------------------------------------------  target    LRPDAPADEILGSRTFDNYAWHTDLPPGHPMVTGSQTVDFDLFSAEHTKLLLIIGMNWICTKMPDGHWIGDARLKGTRVI 1m2g.1    -------------------------------------------EVERADVIIVAGTSAVVQ--PAASLPLIVKQRGGAII  target    VISADYMPTANKADEVIILRPGTDAAFFLGVARELIEKGLYDRAAVIERTDLPLLVRLDTGERLDARDVIPGYELAALTN 1m2g.1    EINPDETPLTPIADYSLR--------------------------------------------------------------  target    YVTLKPDAEIKGNPPPPPFTAGGQVVPTELRDAWGDFVWWDRATGRPRPVSRDEVGARFDGDPALLGEFEVELVDGSTVP 1m2g.1    --------------------------------------------------------------------------------  target    VRPAFDLLKQYLDESFDLRTASEVCRVPPQAIQSIARQLAANKRETLLAAGMGPNHYFQNDLFGRVQFLVAALTDNIGHL 1m2g.1    --------------------------------------------------------------------------------  target    GGNVGSYAGNYRGSVFQAMGQWIAEDPFAIEPDLTKPATVKRYYKAESAHYWNYGERPLRAVAKDDEGDLTKGEVLTGKS 1m2g.1    --------------------------------------------------------------------------------  target    HMPTPTKLIWFGNSNSLLGNAKWSFDVVKNTLPRQDAVFCNEWHWTSSCEYADLVFPADSWAEFKLPDATASCTNPFLLA 1m2g.1    --------------------------------------------------------------------------------  target    FPTTPLKRLYDTRSDYEALALTAKALGELIDEPRMEQYWRGILDGDPTPYLQRIFSGSNATRGITYDELHESSKRGVPLL 1m2g.1    --------------------------------------------------------------------------------  target    MNMRTYPRSGGWEQRQEDKPWYTATGRLEFYRPEPEFQAAGESLPVWREPVDATFYEPNAILSNAAHPSIAPRAPEDYGV 1m2g.1    --------------------------------------------------------------------------------  target    PESQLDVETRQYRNVVRTWAELQQTLHPLQERDPAFRFVFQTPKYRWGAHSTAVDADWISMLFGPFGDPYRRDPRMPWTG 1m2g.1    --------------------------------------------------------------------------------  target    EAYLEINPKDAA 1m2g.1    ------------ ``` | | | | | | | | | | | | | | | | | | | | | | | | | | | | | | | | | | | | | | | | | | | | | | | | | |
|  | 1m2n.1.A | Silent Information Regulator 2  *Sir2 homologues (D102G/F159A/R170A) mutant-2'-O-acetyl ADP ribose complex* | 0.02 |  | 18.87 | 0.07 | 124-178 | X-ray | 2.60 | homo-dimer | 2 x ZN, 2 x OAD | HHblits | 0.30 |
| ``` target    SWDEATTIAAKTMEDVARTFNGDEGARKLLAQGYHPEMVEVMHGAGVQALKLRGGMPLLGIGRIFGFYRFANMLALLDRK 1m2n.1    --------------------------------------------------------------------------------  target    LRPDAPADEILGSRTFDNYAWHTDLPPGHPMVTGSQTVDFDLFSAEHTKLLLIIGMNWICTKMPDGHWIGDARLKGTRVI 1m2n.1    -------------------------------------------EVERADVIIVAGTSAVVQ--PAASLPLIVKQRGGAII  target    VISADYMPTANKADEVIILRPGTDAAFFLGVARELIEKGLYDRAAVIERTDLPLLVRLDTGERLDARDVIPGYELAALTN 1m2n.1    EINPDETPLTPIADYSLR--------------------------------------------------------------  target    YVTLKPDAEIKGNPPPPPFTAGGQVVPTELRDAWGDFVWWDRATGRPRPVSRDEVGARFDGDPALLGEFEVELVDGSTVP 1m2n.1    --------------------------------------------------------------------------------  target    VRPAFDLLKQYLDESFDLRTASEVCRVPPQAIQSIARQLAANKRETLLAAGMGPNHYFQNDLFGRVQFLVAALTDNIGHL 1m2n.1    --------------------------------------------------------------------------------  target    GGNVGSYAGNYRGSVFQAMGQWIAEDPFAIEPDLTKPATVKRYYKAESAHYWNYGERPLRAVAKDDEGDLTKGEVLTGKS 1m2n.1    --------------------------------------------------------------------------------  target    HMPTPTKLIWFGNSNSLLGNAKWSFDVVKNTLPRQDAVFCNEWHWTSSCEYADLVFPADSWAEFKLPDATASCTNPFLLA 1m2n.1    --------------------------------------------------------------------------------  target    FPTTPLKRLYDTRSDYEALALTAKALGELIDEPRMEQYWRGILDGDPTPYLQRIFSGSNATRGITYDELHESSKRGVPLL 1m2n.1    --------------------------------------------------------------------------------  target    MNMRTYPRSGGWEQRQEDKPWYTATGRLEFYRPEPEFQAAGESLPVWREPVDATFYEPNAILSNAAHPSIAPRAPEDYGV 1m2n.1    --------------------------------------------------------------------------------  target    PESQLDVETRQYRNVVRTWAELQQTLHPLQERDPAFRFVFQTPKYRWGAHSTAVDADWISMLFGPFGDPYRRDPRMPWTG 1m2n.1    --------------------------------------------------------------------------------  target    EAYLEINPKDAA 1m2n.1    ------------ ``` | | | | | | | | | | | | | | | | | | | | | | | | | | | | | | | | | | | | | | | | | | | | | | | | | |
|  | 1m2n.1.B | Silent Information Regulator 2  *Sir2 homologues (D102G/F159A/R170A) mutant-2'-O-acetyl ADP ribose complex* | 0.01 |  | 18.87 | 0.07 | 124-178 | X-ray | 2.60 | homo-dimer | 2 x ZN, 2 x OAD | HHblits | 0.30 |
| ``` target    SWDEATTIAAKTMEDVARTFNGDEGARKLLAQGYHPEMVEVMHGAGVQALKLRGGMPLLGIGRIFGFYRFANMLALLDRK 1m2n.1    --------------------------------------------------------------------------------  target    LRPDAPADEILGSRTFDNYAWHTDLPPGHPMVTGSQTVDFDLFSAEHTKLLLIIGMNWICTKMPDGHWIGDARLKGTRVI 1m2n.1    -------------------------------------------EVERADVIIVAGTSAVVQ--PAASLPLIVKQRGGAII  target    VISADYMPTANKADEVIILRPGTDAAFFLGVARELIEKGLYDRAAVIERTDLPLLVRLDTGERLDARDVIPGYELAALTN 1m2n.1    EINPDETPLTPIADYSLR--------------------------------------------------------------  target    YVTLKPDAEIKGNPPPPPFTAGGQVVPTELRDAWGDFVWWDRATGRPRPVSRDEVGARFDGDPALLGEFEVELVDGSTVP 1m2n.1    --------------------------------------------------------------------------------  target    VRPAFDLLKQYLDESFDLRTASEVCRVPPQAIQSIARQLAANKRETLLAAGMGPNHYFQNDLFGRVQFLVAALTDNIGHL 1m2n.1    --------------------------------------------------------------------------------  target    GGNVGSYAGNYRGSVFQAMGQWIAEDPFAIEPDLTKPATVKRYYKAESAHYWNYGERPLRAVAKDDEGDLTKGEVLTGKS 1m2n.1    --------------------------------------------------------------------------------  target    HMPTPTKLIWFGNSNSLLGNAKWSFDVVKNTLPRQDAVFCNEWHWTSSCEYADLVFPADSWAEFKLPDATASCTNPFLLA 1m2n.1    --------------------------------------------------------------------------------  target    FPTTPLKRLYDTRSDYEALALTAKALGELIDEPRMEQYWRGILDGDPTPYLQRIFSGSNATRGITYDELHESSKRGVPLL 1m2n.1    --------------------------------------------------------------------------------  target    MNMRTYPRSGGWEQRQEDKPWYTATGRLEFYRPEPEFQAAGESLPVWREPVDATFYEPNAILSNAAHPSIAPRAPEDYGV 1m2n.1    --------------------------------------------------------------------------------  target    PESQLDVETRQYRNVVRTWAELQQTLHPLQERDPAFRFVFQTPKYRWGAHSTAVDADWISMLFGPFGDPYRRDPRMPWTG 1m2n.1    --------------------------------------------------------------------------------  target    EAYLEINPKDAA 1m2n.1    ------------ ``` | | | | | | | | | | | | | | | | | | | | | | | | | | | | | | | | | | | | | | | | | | | | | | | | | |
|  | 6enx.1.A | NAD-dependent protein deacylase sirtuin-5, mitochondrial  *Zebrafish Sirt5 in complex with stalled bicyclic intermediate of inhibitory compound 10* | 0.01 |  | 16.67 | 0.07 | 123-178 | X-ray | 1.95 | monomer | 1 x ZN, 1 x BJW | HHblits | 0.29 |
| ``` target    SWDEATTIAAKTMEDVARTFNGDEGARKLLAQGYHPEMVEVMHGAGVQALKLRGGMPLLGIGRIFGFYRFANMLALLDRK 6enx.1    --------------------------------------------------------------------------------  target    LRPDAPADEILGSRTFDNYAWHTDLPPGHPMVTGSQTVDFDLFSAEHTKLLLIIGMNWICTKMPDGHWIGDARLKGTRVI 6enx.1    ------------------------------------------RELEKCDLCLVVGTSSIV--YPAAMFAPQVASRGVPVA  target    VISADYMPTANKADEVIILRPGTDAAFFLGVARELIEKGLYDRAAVIERTDLPLLVRLDTGERLDARDVIPGYELAALTN 6enx.1    EFNMECTPATQRFKYHFE--------------------------------------------------------------  target    YVTLKPDAEIKGNPPPPPFTAGGQVVPTELRDAWGDFVWWDRATGRPRPVSRDEVGARFDGDPALLGEFEVELVDGSTVP 6enx.1    --------------------------------------------------------------------------------  target    VRPAFDLLKQYLDESFDLRTASEVCRVPPQAIQSIARQLAANKRETLLAAGMGPNHYFQNDLFGRVQFLVAALTDNIGHL 6enx.1    --------------------------------------------------------------------------------  target    GGNVGSYAGNYRGSVFQAMGQWIAEDPFAIEPDLTKPATVKRYYKAESAHYWNYGERPLRAVAKDDEGDLTKGEVLTGKS 6enx.1    --------------------------------------------------------------------------------  target    HMPTPTKLIWFGNSNSLLGNAKWSFDVVKNTLPRQDAVFCNEWHWTSSCEYADLVFPADSWAEFKLPDATASCTNPFLLA 6enx.1    --------------------------------------------------------------------------------  target    FPTTPLKRLYDTRSDYEALALTAKALGELIDEPRMEQYWRGILDGDPTPYLQRIFSGSNATRGITYDELHESSKRGVPLL 6enx.1    --------------------------------------------------------------------------------  target    MNMRTYPRSGGWEQRQEDKPWYTATGRLEFYRPEPEFQAAGESLPVWREPVDATFYEPNAILSNAAHPSIAPRAPEDYGV 6enx.1    --------------------------------------------------------------------------------  target    PESQLDVETRQYRNVVRTWAELQQTLHPLQERDPAFRFVFQTPKYRWGAHSTAVDADWISMLFGPFGDPYRRDPRMPWTG 6enx.1    --------------------------------------------------------------------------------  target    EAYLEINPKDAA 6enx.1    ------------ ``` | | | | | | | | | | | | | | | | | | | | | | | | | | | | | | | | | | | | | | | | | | | | | | | | | |
|  | 1s5p.1.A | NAD-dependent deacetylase  *Structure and substrate binding properties of cobB, a Sir2 homolog protein deacetylase from Eschericia coli.* | 0.01 |  | 15.09 | 0.07 | 124-178 | X-ray | 1.96 | monomer | 1 x ZN, 1 x LYS-GLY-GLY-ALA-ALY-ARG-HIS-ARG | HHblits | 0.30 |
| ``` target    SWDEATTIAAKTMEDVARTFNGDEGARKLLAQGYHPEMVEVMHGAGVQALKLRGGMPLLGIGRIFGFYRFANMLALLDRK 1s5p.1    --------------------------------------------------------------------------------  target    LRPDAPADEILGSRTFDNYAWHTDLPPGHPMVTGSQTVDFDLFSAEHTKLLLIIGMNWICTKMPDGHWIGDARLKGTRVI 1s5p.1    -------------------------------------------ALSMADIFIAIGTSGHV--YPAAGFVHEAKLHGAHTV  target    VISADYMPTANKADEVIILRPGTDAAFFLGVARELIEKGLYDRAAVIERTDLPLLVRLDTGERLDARDVIPGYELAALTN 1s5p.1    ELNLEPSQVGNEFAEKYY--------------------------------------------------------------  target    YVTLKPDAEIKGNPPPPPFTAGGQVVPTELRDAWGDFVWWDRATGRPRPVSRDEVGARFDGDPALLGEFEVELVDGSTVP 1s5p.1    --------------------------------------------------------------------------------  target    VRPAFDLLKQYLDESFDLRTASEVCRVPPQAIQSIARQLAANKRETLLAAGMGPNHYFQNDLFGRVQFLVAALTDNIGHL 1s5p.1    --------------------------------------------------------------------------------  target    GGNVGSYAGNYRGSVFQAMGQWIAEDPFAIEPDLTKPATVKRYYKAESAHYWNYGERPLRAVAKDDEGDLTKGEVLTGKS 1s5p.1    --------------------------------------------------------------------------------  target    HMPTPTKLIWFGNSNSLLGNAKWSFDVVKNTLPRQDAVFCNEWHWTSSCEYADLVFPADSWAEFKLPDATASCTNPFLLA 1s5p.1    --------------------------------------------------------------------------------  target    FPTTPLKRLYDTRSDYEALALTAKALGELIDEPRMEQYWRGILDGDPTPYLQRIFSGSNATRGITYDELHESSKRGVPLL 1s5p.1    --------------------------------------------------------------------------------  target    MNMRTYPRSGGWEQRQEDKPWYTATGRLEFYRPEPEFQAAGESLPVWREPVDATFYEPNAILSNAAHPSIAPRAPEDYGV 1s5p.1    --------------------------------------------------------------------------------  target    PESQLDVETRQYRNVVRTWAELQQTLHPLQERDPAFRFVFQTPKYRWGAHSTAVDADWISMLFGPFGDPYRRDPRMPWTG 1s5p.1    --------------------------------------------------------------------------------  target    EAYLEINPKDAA 1s5p.1    ------------ ``` | | | | | | | | | | | | | | | | | | | | | | | | | | | | | | | | | | | | | | | | | | | | | | | | | |
|  | 5xhs.1.A | NAD-dependent protein deacylase sirtuin-5, mitochondrial  *Crystal structure of SIRT5 complexed with a fluorogenic small-molecule substrate SuBKA* | 0.01 |  | 16.98 | 0.07 | 124-178 | X-ray | 2.19 | monomer | 1 x PHQ, 1 x SLL, 1 x MCM, 1 x ZN | HHblits | 0.30 |
| ``` target    SWDEATTIAAKTMEDVARTFNGDEGARKLLAQGYHPEMVEVMHGAGVQALKLRGGMPLLGIGRIFGFYRFANMLALLDRK 5xhs.1    --------------------------------------------------------------------------------  target    LRPDAPADEILGSRTFDNYAWHTDLPPGHPMVTGSQTVDFDLFSAEHTKLLLIIGMNWICTKMPDGHWIGDARLKGTRVI 5xhs.1    -------------------------------------------ELAHCDLCLVVGTSSVV--YPAAMFAPQVAARGVPVA  target    VISADYMPTANKADEVIILRPGTDAAFFLGVARELIEKGLYDRAAVIERTDLPLLVRLDTGERLDARDVIPGYELAALTN 5xhs.1    EFNTETTPATNRFRFHFQ--------------------------------------------------------------  target    YVTLKPDAEIKGNPPPPPFTAGGQVVPTELRDAWGDFVWWDRATGRPRPVSRDEVGARFDGDPALLGEFEVELVDGSTVP 5xhs.1    --------------------------------------------------------------------------------  target    VRPAFDLLKQYLDESFDLRTASEVCRVPPQAIQSIARQLAANKRETLLAAGMGPNHYFQNDLFGRVQFLVAALTDNIGHL 5xhs.1    --------------------------------------------------------------------------------  target    GGNVGSYAGNYRGSVFQAMGQWIAEDPFAIEPDLTKPATVKRYYKAESAHYWNYGERPLRAVAKDDEGDLTKGEVLTGKS 5xhs.1    --------------------------------------------------------------------------------  target    HMPTPTKLIWFGNSNSLLGNAKWSFDVVKNTLPRQDAVFCNEWHWTSSCEYADLVFPADSWAEFKLPDATASCTNPFLLA 5xhs.1    --------------------------------------------------------------------------------  target    FPTTPLKRLYDTRSDYEALALTAKALGELIDEPRMEQYWRGILDGDPTPYLQRIFSGSNATRGITYDELHESSKRGVPLL 5xhs.1    --------------------------------------------------------------------------------  target    MNMRTYPRSGGWEQRQEDKPWYTATGRLEFYRPEPEFQAAGESLPVWREPVDATFYEPNAILSNAAHPSIAPRAPEDYGV 5xhs.1    --------------------------------------------------------------------------------  target    PESQLDVETRQYRNVVRTWAELQQTLHPLQERDPAFRFVFQTPKYRWGAHSTAVDADWISMLFGPFGDPYRRDPRMPWTG 5xhs.1    --------------------------------------------------------------------------------  target    EAYLEINPKDAA 5xhs.1    ------------ ``` | | | | | | | | | | | | | | | | | | | | | | | | | | | | | | | | | | | | | | | | | | | | | | | | | |
|  | 6ljm.1.A | NAD-dependent protein deacylase sirtuin-5, mitochondrial  *Crystal structure of human Sirt5 in complex with the fluorogenic tetrapeptide substrate P13* | 0.01 |  | 16.98 | 0.07 | 124-178 | X-ray | 1.78 | monomer | 1 x ZN, 1 x SIN, 1 x MCM, 1 x SER-LEU-GLY-LYS | HHblits | 0.30 |
| ``` target    SWDEATTIAAKTMEDVARTFNGDEGARKLLAQGYHPEMVEVMHGAGVQALKLRGGMPLLGIGRIFGFYRFANMLALLDRK 6ljm.1    --------------------------------------------------------------------------------  target    LRPDAPADEILGSRTFDNYAWHTDLPPGHPMVTGSQTVDFDLFSAEHTKLLLIIGMNWICTKMPDGHWIGDARLKGTRVI 6ljm.1    -------------------------------------------ELAHCDLCLVVGTSSVV--YPAAMFAPQVAARGVPVA  target    VISADYMPTANKADEVIILRPGTDAAFFLGVARELIEKGLYDRAAVIERTDLPLLVRLDTGERLDARDVIPGYELAALTN 6ljm.1    EFNTETTPATNRFRFHFQ--------------------------------------------------------------  target    YVTLKPDAEIKGNPPPPPFTAGGQVVPTELRDAWGDFVWWDRATGRPRPVSRDEVGARFDGDPALLGEFEVELVDGSTVP 6ljm.1    --------------------------------------------------------------------------------  target    VRPAFDLLKQYLDESFDLRTASEVCRVPPQAIQSIARQLAANKRETLLAAGMGPNHYFQNDLFGRVQFLVAALTDNIGHL 6ljm.1    --------------------------------------------------------------------------------  target    GGNVGSYAGNYRGSVFQAMGQWIAEDPFAIEPDLTKPATVKRYYKAESAHYWNYGERPLRAVAKDDEGDLTKGEVLTGKS 6ljm.1    --------------------------------------------------------------------------------  target    HMPTPTKLIWFGNSNSLLGNAKWSFDVVKNTLPRQDAVFCNEWHWTSSCEYADLVFPADSWAEFKLPDATASCTNPFLLA 6ljm.1    --------------------------------------------------------------------------------  target    FPTTPLKRLYDTRSDYEALALTAKALGELIDEPRMEQYWRGILDGDPTPYLQRIFSGSNATRGITYDELHESSKRGVPLL 6ljm.1    --------------------------------------------------------------------------------  target    MNMRTYPRSGGWEQRQEDKPWYTATGRLEFYRPEPEFQAAGESLPVWREPVDATFYEPNAILSNAAHPSIAPRAPEDYGV 6ljm.1    --------------------------------------------------------------------------------  target    PESQLDVETRQYRNVVRTWAELQQTLHPLQERDPAFRFVFQTPKYRWGAHSTAVDADWISMLFGPFGDPYRRDPRMPWTG 6ljm.1    --------------------------------------------------------------------------------  target    EAYLEINPKDAA 6ljm.1    ------------ ``` | | | | | | | | | | | | | | | | | | | | | | | | | | | | | | | | | | | | | | | | | | | | | | | | | |
|  | 6ljk.1.A | NAD-dependent protein deacylase sirtuin-5, mitochondrial  *Crystal structure of human Sirt5 in complex with an internally quenched fluorescent substrate GluIQF* | 0.01 |  | 16.98 | 0.07 | 124-178 | X-ray | 1.39 | monomer | 1 x ZN, 1 x GUA, 1 x BE2-SER-ALA-ILE-LYS-SER-NIY-GLY-SET | HHblits | 0.30 |
| ``` target    SWDEATTIAAKTMEDVARTFNGDEGARKLLAQGYHPEMVEVMHGAGVQALKLRGGMPLLGIGRIFGFYRFANMLALLDRK 6ljk.1    --------------------------------------------------------------------------------  target    LRPDAPADEILGSRTFDNYAWHTDLPPGHPMVTGSQTVDFDLFSAEHTKLLLIIGMNWICTKMPDGHWIGDARLKGTRVI 6ljk.1    -------------------------------------------ELAHCDLCLVVGTSSVV--YPAAMFAPQVAARGVPVA  target    VISADYMPTANKADEVIILRPGTDAAFFLGVARELIEKGLYDRAAVIERTDLPLLVRLDTGERLDARDVIPGYELAALTN 6ljk.1    EFNTETTPATNRFRFHFQ--------------------------------------------------------------  target    YVTLKPDAEIKGNPPPPPFTAGGQVVPTELRDAWGDFVWWDRATGRPRPVSRDEVGARFDGDPALLGEFEVELVDGSTVP 6ljk.1    --------------------------------------------------------------------------------  target    VRPAFDLLKQYLDESFDLRTASEVCRVPPQAIQSIARQLAANKRETLLAAGMGPNHYFQNDLFGRVQFLVAALTDNIGHL 6ljk.1    --------------------------------------------------------------------------------  target    GGNVGSYAGNYRGSVFQAMGQWIAEDPFAIEPDLTKPATVKRYYKAESAHYWNYGERPLRAVAKDDEGDLTKGEVLTGKS 6ljk.1    --------------------------------------------------------------------------------  target    HMPTPTKLIWFGNSNSLLGNAKWSFDVVKNTLPRQDAVFCNEWHWTSSCEYADLVFPADSWAEFKLPDATASCTNPFLLA 6ljk.1    --------------------------------------------------------------------------------  target    FPTTPLKRLYDTRSDYEALALTAKALGELIDEPRMEQYWRGILDGDPTPYLQRIFSGSNATRGITYDELHESSKRGVPLL 6ljk.1    --------------------------------------------------------------------------------  target    MNMRTYPRSGGWEQRQEDKPWYTATGRLEFYRPEPEFQAAGESLPVWREPVDATFYEPNAILSNAAHPSIAPRAPEDYGV 6ljk.1    --------------------------------------------------------------------------------  target    PESQLDVETRQYRNVVRTWAELQQTLHPLQERDPAFRFVFQTPKYRWGAHSTAVDADWISMLFGPFGDPYRRDPRMPWTG 6ljk.1    --------------------------------------------------------------------------------  target    EAYLEINPKDAA 6ljk.1    ------------ ``` | | | | | | | | | | | | | | | | | | | | | | | | | | | | | | | | | | | | | | | | | | | | | | | | | |
|  | 6acp.1.A | NAD-dependent protein deacylase sirtuin-5, mitochondrial  *histone lysine desuccinylase Sirt5 in complex with succinyl peptide H4K91* | 0.01 |  | 16.98 | 0.07 | 124-178 | X-ray | 2.30 | monomer | 1 x ZN, 1 x TYR-ALA-LEU-SLL-ARG-GLN-GLY | HHblits | 0.30 |
| ``` target    SWDEATTIAAKTMEDVARTFNGDEGARKLLAQGYHPEMVEVMHGAGVQALKLRGGMPLLGIGRIFGFYRFANMLALLDRK 6acp.1    --------------------------------------------------------------------------------  target    LRPDAPADEILGSRTFDNYAWHTDLPPGHPMVTGSQTVDFDLFSAEHTKLLLIIGMNWICTKMPDGHWIGDARLKGTRVI 6acp.1    -------------------------------------------ELAHCDLCLVVGTSSVV--YPAAMFAPQVAARGVPVA  target    VISADYMPTANKADEVIILRPGTDAAFFLGVARELIEKGLYDRAAVIERTDLPLLVRLDTGERLDARDVIPGYELAALTN 6acp.1    EFNTETTPATNRFRFHFQ--------------------------------------------------------------  target    YVTLKPDAEIKGNPPPPPFTAGGQVVPTELRDAWGDFVWWDRATGRPRPVSRDEVGARFDGDPALLGEFEVELVDGSTVP 6acp.1    --------------------------------------------------------------------------------  target    VRPAFDLLKQYLDESFDLRTASEVCRVPPQAIQSIARQLAANKRETLLAAGMGPNHYFQNDLFGRVQFLVAALTDNIGHL 6acp.1    --------------------------------------------------------------------------------  target    GGNVGSYAGNYRGSVFQAMGQWIAEDPFAIEPDLTKPATVKRYYKAESAHYWNYGERPLRAVAKDDEGDLTKGEVLTGKS 6acp.1    --------------------------------------------------------------------------------  target    HMPTPTKLIWFGNSNSLLGNAKWSFDVVKNTLPRQDAVFCNEWHWTSSCEYADLVFPADSWAEFKLPDATASCTNPFLLA 6acp.1    --------------------------------------------------------------------------------  target    FPTTPLKRLYDTRSDYEALALTAKALGELIDEPRMEQYWRGILDGDPTPYLQRIFSGSNATRGITYDELHESSKRGVPLL 6acp.1    --------------------------------------------------------------------------------  target    MNMRTYPRSGGWEQRQEDKPWYTATGRLEFYRPEPEFQAAGESLPVWREPVDATFYEPNAILSNAAHPSIAPRAPEDYGV 6acp.1    --------------------------------------------------------------------------------  target    PESQLDVETRQYRNVVRTWAELQQTLHPLQERDPAFRFVFQTPKYRWGAHSTAVDADWISMLFGPFGDPYRRDPRMPWTG 6acp.1    --------------------------------------------------------------------------------  target    EAYLEINPKDAA 6acp.1    ------------ ``` | | | | | | | | | | | | | | | | | | | | | | | | | | | | | | | | | | | | | | | | | | | | | | | | | |
|  | 4g1c.2.A | NAD-dependent protein deacylase sirtuin-5, mitochondrial  *Human SIRT5 bound to Succ-IDH2 and Carba-NAD* | 0.01 |  | 16.98 | 0.07 | 124-178 | X-ray | 1.94 | monomer | 1 x ZN, 1 x ACE-ALA-VAL-SLL-CYS-ALA-NH2 | HHblits | 0.30 |
| ``` target    SWDEATTIAAKTMEDVARTFNGDEGARKLLAQGYHPEMVEVMHGAGVQALKLRGGMPLLGIGRIFGFYRFANMLALLDRK 4g1c.2    --------------------------------------------------------------------------------  target    LRPDAPADEILGSRTFDNYAWHTDLPPGHPMVTGSQTVDFDLFSAEHTKLLLIIGMNWICTKMPDGHWIGDARLKGTRVI 4g1c.2    -------------------------------------------ELAHCDLCLVVGTSSVV--YPAAMFAPQVAARGVPVA  target    VISADYMPTANKADEVIILRPGTDAAFFLGVARELIEKGLYDRAAVIERTDLPLLVRLDTGERLDARDVIPGYELAALTN 4g1c.2    EFNTETTPATNRFRFHFQ--------------------------------------------------------------  target    YVTLKPDAEIKGNPPPPPFTAGGQVVPTELRDAWGDFVWWDRATGRPRPVSRDEVGARFDGDPALLGEFEVELVDGSTVP 4g1c.2    --------------------------------------------------------------------------------  target    VRPAFDLLKQYLDESFDLRTASEVCRVPPQAIQSIARQLAANKRETLLAAGMGPNHYFQNDLFGRVQFLVAALTDNIGHL 4g1c.2    --------------------------------------------------------------------------------  target    GGNVGSYAGNYRGSVFQAMGQWIAEDPFAIEPDLTKPATVKRYYKAESAHYWNYGERPLRAVAKDDEGDLTKGEVLTGKS 4g1c.2    --------------------------------------------------------------------------------  target    HMPTPTKLIWFGNSNSLLGNAKWSFDVVKNTLPRQDAVFCNEWHWTSSCEYADLVFPADSWAEFKLPDATASCTNPFLLA 4g1c.2    --------------------------------------------------------------------------------  target    FPTTPLKRLYDTRSDYEALALTAKALGELIDEPRMEQYWRGILDGDPTPYLQRIFSGSNATRGITYDELHESSKRGVPLL 4g1c.2    --------------------------------------------------------------------------------  target    MNMRTYPRSGGWEQRQEDKPWYTATGRLEFYRPEPEFQAAGESLPVWREPVDATFYEPNAILSNAAHPSIAPRAPEDYGV 4g1c.2    --------------------------------------------------------------------------------  target    PESQLDVETRQYRNVVRTWAELQQTLHPLQERDPAFRFVFQTPKYRWGAHSTAVDADWISMLFGPFGDPYRRDPRMPWTG 4g1c.2    --------------------------------------------------------------------------------  target    EAYLEINPKDAA 4g1c.2    ------------ ``` | | | | | | | | | | | | | | | | | | | | | | | | | | | | | | | | | | | | | | | | | | | | | | | | | |
|  | 4g1c.1.A | NAD-dependent protein deacylase sirtuin-5, mitochondrial  *Human SIRT5 bound to Succ-IDH2 and Carba-NAD* | 0.01 |  | 16.98 | 0.07 | 124-178 | X-ray | 1.94 | monomer | 1 x ZN, 1 x CNA, 1 x ACE-ALA-VAL-SLL-CYS-ALA-NH2 | HHblits | 0.30 |
| ``` target    SWDEATTIAAKTMEDVARTFNGDEGARKLLAQGYHPEMVEVMHGAGVQALKLRGGMPLLGIGRIFGFYRFANMLALLDRK 4g1c.1    --------------------------------------------------------------------------------  target    LRPDAPADEILGSRTFDNYAWHTDLPPGHPMVTGSQTVDFDLFSAEHTKLLLIIGMNWICTKMPDGHWIGDARLKGTRVI 4g1c.1    -------------------------------------------ELAHCDLCLVVGTSSVV--YPAAMFAPQVAARGVPVA  target    VISADYMPTANKADEVIILRPGTDAAFFLGVARELIEKGLYDRAAVIERTDLPLLVRLDTGERLDARDVIPGYELAALTN 4g1c.1    EFNTETTPATNRFRFHFQ--------------------------------------------------------------  target    YVTLKPDAEIKGNPPPPPFTAGGQVVPTELRDAWGDFVWWDRATGRPRPVSRDEVGARFDGDPALLGEFEVELVDGSTVP 4g1c.1    --------------------------------------------------------------------------------  target    VRPAFDLLKQYLDESFDLRTASEVCRVPPQAIQSIARQLAANKRETLLAAGMGPNHYFQNDLFGRVQFLVAALTDNIGHL 4g1c.1    --------------------------------------------------------------------------------  target    GGNVGSYAGNYRGSVFQAMGQWIAEDPFAIEPDLTKPATVKRYYKAESAHYWNYGERPLRAVAKDDEGDLTKGEVLTGKS 4g1c.1    --------------------------------------------------------------------------------  target    HMPTPTKLIWFGNSNSLLGNAKWSFDVVKNTLPRQDAVFCNEWHWTSSCEYADLVFPADSWAEFKLPDATASCTNPFLLA 4g1c.1    --------------------------------------------------------------------------------  target    FPTTPLKRLYDTRSDYEALALTAKALGELIDEPRMEQYWRGILDGDPTPYLQRIFSGSNATRGITYDELHESSKRGVPLL 4g1c.1    --------------------------------------------------------------------------------  target    MNMRTYPRSGGWEQRQEDKPWYTATGRLEFYRPEPEFQAAGESLPVWREPVDATFYEPNAILSNAAHPSIAPRAPEDYGV 4g1c.1    --------------------------------------------------------------------------------  target    PESQLDVETRQYRNVVRTWAELQQTLHPLQERDPAFRFVFQTPKYRWGAHSTAVDADWISMLFGPFGDPYRRDPRMPWTG 4g1c.1    --------------------------------------------------------------------------------  target    EAYLEINPKDAA 4g1c.1    ------------ ``` | | | | | | | | | | | | | | | | | | | | | | | | | | | | | | | | | | | | | | | | | | | | | | | | | |
|  | 1aa6.1.A | FORMATE DEHYDROGENASE H  *REDUCED FORM OF FORMATE DEHYDROGENASE H FROM E. COLI* | 0.01 |  | 20.00 | 0.07 | 126-181 | X-ray | 2.30 | monomer | 1 x SF4, 2 x MGD, 1 x 4MO | HHblits | 0.27 |
| ``` target    SWDEATTIAAKTMEDVARTFNGDEGARKLLAQGYHPEMVEVMHGAGVQALKLRGGMPLLGIGRIFGFYRFANMLALLDRK 1aa6.1    --------------------------------------------------------------------------------  target    LRPDAPADEILGSRTFDNYAWHTDLPPGHPMVTGSQTVDFDLFSAEHTKLLLIIGMNWICTKMPDGHWIGDARLKGTRVI 1aa6.1    ---------------------------------------------GEVRAAYIMGEDPLQTDA-ELSAVRKAFEDLELVI  target    VISADYMPTANKADEVIILRPGTDAAFFLGVARELIEKGLYDRAAVIERTDLPLLVRLDTGERLDARDVIPGYELAALTN 1aa6.1    VQDIFMTKTASAADVILPSTS-----------------------------------------------------------  target    YVTLKPDAEIKGNPPPPPFTAGGQVVPTELRDAWGDFVWWDRATGRPRPVSRDEVGARFDGDPALLGEFEVELVDGSTVP 1aa6.1    --------------------------------------------------------------------------------  target    VRPAFDLLKQYLDESFDLRTASEVCRVPPQAIQSIARQLAANKRETLLAAGMGPNHYFQNDLFGRVQFLVAALTDNIGHL 1aa6.1    --------------------------------------------------------------------------------  target    GGNVGSYAGNYRGSVFQAMGQWIAEDPFAIEPDLTKPATVKRYYKAESAHYWNYGERPLRAVAKDDEGDLTKGEVLTGKS 1aa6.1    --------------------------------------------------------------------------------  target    HMPTPTKLIWFGNSNSLLGNAKWSFDVVKNTLPRQDAVFCNEWHWTSSCEYADLVFPADSWAEFKLPDATASCTNPFLLA 1aa6.1    --------------------------------------------------------------------------------  target    FPTTPLKRLYDTRSDYEALALTAKALGELIDEPRMEQYWRGILDGDPTPYLQRIFSGSNATRGITYDELHESSKRGVPLL 1aa6.1    --------------------------------------------------------------------------------  target    MNMRTYPRSGGWEQRQEDKPWYTATGRLEFYRPEPEFQAAGESLPVWREPVDATFYEPNAILSNAAHPSIAPRAPEDYGV 1aa6.1    --------------------------------------------------------------------------------  target    PESQLDVETRQYRNVVRTWAELQQTLHPLQERDPAFRFVFQTPKYRWGAHSTAVDADWISMLFGPFGDPYRRDPRMPWTG 1aa6.1    --------------------------------------------------------------------------------  target    EAYLEINPKDAA 1aa6.1    ------------ ``` | | | | | | | | | | | | | | | | | | | | | | | | | | | | | | | | | | | | | | | | | | | | | | | | | |
|  | 1fdo.1.A | FORMATE DEHYDROGENASE H  *OXIDIZED FORM OF FORMATE DEHYDROGENASE H FROM E. COLI* | 0.01 |  | 20.00 | 0.07 | 126-181 | X-ray | 2.80 | monomer | 1 x SF4, 2 x MGD, 1 x 6MO | HHblits | 0.27 |
| ``` target    SWDEATTIAAKTMEDVARTFNGDEGARKLLAQGYHPEMVEVMHGAGVQALKLRGGMPLLGIGRIFGFYRFANMLALLDRK 1fdo.1    --------------------------------------------------------------------------------  target    LRPDAPADEILGSRTFDNYAWHTDLPPGHPMVTGSQTVDFDLFSAEHTKLLLIIGMNWICTKMPDGHWIGDARLKGTRVI 1fdo.1    ---------------------------------------------GEVRAAYIMGEDPLQTDA-ELSAVRKAFEDLELVI  target    VISADYMPTANKADEVIILRPGTDAAFFLGVARELIEKGLYDRAAVIERTDLPLLVRLDTGERLDARDVIPGYELAALTN 1fdo.1    VQDIFMTKTASAADVILPSTS-----------------------------------------------------------  target    YVTLKPDAEIKGNPPPPPFTAGGQVVPTELRDAWGDFVWWDRATGRPRPVSRDEVGARFDGDPALLGEFEVELVDGSTVP 1fdo.1    --------------------------------------------------------------------------------  target    VRPAFDLLKQYLDESFDLRTASEVCRVPPQAIQSIARQLAANKRETLLAAGMGPNHYFQNDLFGRVQFLVAALTDNIGHL 1fdo.1    --------------------------------------------------------------------------------  target    GGNVGSYAGNYRGSVFQAMGQWIAEDPFAIEPDLTKPATVKRYYKAESAHYWNYGERPLRAVAKDDEGDLTKGEVLTGKS 1fdo.1    --------------------------------------------------------------------------------  target    HMPTPTKLIWFGNSNSLLGNAKWSFDVVKNTLPRQDAVFCNEWHWTSSCEYADLVFPADSWAEFKLPDATASCTNPFLLA 1fdo.1    --------------------------------------------------------------------------------  target    FPTTPLKRLYDTRSDYEALALTAKALGELIDEPRMEQYWRGILDGDPTPYLQRIFSGSNATRGITYDELHESSKRGVPLL 1fdo.1    --------------------------------------------------------------------------------  target    MNMRTYPRSGGWEQRQEDKPWYTATGRLEFYRPEPEFQAAGESLPVWREPVDATFYEPNAILSNAAHPSIAPRAPEDYGV 1fdo.1    --------------------------------------------------------------------------------  target    PESQLDVETRQYRNVVRTWAELQQTLHPLQERDPAFRFVFQTPKYRWGAHSTAVDADWISMLFGPFGDPYRRDPRMPWTG 1fdo.1    --------------------------------------------------------------------------------  target    EAYLEINPKDAA 1fdo.1    ------------ ``` | | | | | | | | | | | | | | | | | | | | | | | | | | | | | | | | | | | | | | | | | | | | | | | | | |
|  | 2iv2.1.A | Formate dehydrogenase H  *Reinterpretation of reduced form of formate dehydrogenase H from E. coli* | 0.01 |  | 20.00 | 0.07 | 126-181 | X-ray | 2.27 | monomer | 1 x SF4, 1 x 2MD, 1 x MGD | HHblits | 0.27 |
| ``` target    SWDEATTIAAKTMEDVARTFNGDEGARKLLAQGYHPEMVEVMHGAGVQALKLRGGMPLLGIGRIFGFYRFANMLALLDRK 2iv2.1    --------------------------------------------------------------------------------  target    LRPDAPADEILGSRTFDNYAWHTDLPPGHPMVTGSQTVDFDLFSAEHTKLLLIIGMNWICTKMPDGHWIGDARLKGTRVI 2iv2.1    ---------------------------------------------GEVRAAYIMGEDPLQTDA-ELSAVRKAFEDLELVI  target    VISADYMPTANKADEVIILRPGTDAAFFLGVARELIEKGLYDRAAVIERTDLPLLVRLDTGERLDARDVIPGYELAALTN 2iv2.1    VQDIFMTKTASAADVILPSTS-----------------------------------------------------------  target    YVTLKPDAEIKGNPPPPPFTAGGQVVPTELRDAWGDFVWWDRATGRPRPVSRDEVGARFDGDPALLGEFEVELVDGSTVP 2iv2.1    --------------------------------------------------------------------------------  target    VRPAFDLLKQYLDESFDLRTASEVCRVPPQAIQSIARQLAANKRETLLAAGMGPNHYFQNDLFGRVQFLVAALTDNIGHL 2iv2.1    --------------------------------------------------------------------------------  target    GGNVGSYAGNYRGSVFQAMGQWIAEDPFAIEPDLTKPATVKRYYKAESAHYWNYGERPLRAVAKDDEGDLTKGEVLTGKS 2iv2.1    --------------------------------------------------------------------------------  target    HMPTPTKLIWFGNSNSLLGNAKWSFDVVKNTLPRQDAVFCNEWHWTSSCEYADLVFPADSWAEFKLPDATASCTNPFLLA 2iv2.1    --------------------------------------------------------------------------------  target    FPTTPLKRLYDTRSDYEALALTAKALGELIDEPRMEQYWRGILDGDPTPYLQRIFSGSNATRGITYDELHESSKRGVPLL 2iv2.1    --------------------------------------------------------------------------------  target    MNMRTYPRSGGWEQRQEDKPWYTATGRLEFYRPEPEFQAAGESLPVWREPVDATFYEPNAILSNAAHPSIAPRAPEDYGV 2iv2.1    --------------------------------------------------------------------------------  target    PESQLDVETRQYRNVVRTWAELQQTLHPLQERDPAFRFVFQTPKYRWGAHSTAVDADWISMLFGPFGDPYRRDPRMPWTG 2iv2.1    --------------------------------------------------------------------------------  target    EAYLEINPKDAA 2iv2.1    ------------ ``` | | | | | | | | | | | | | | | | | | | | | | | | | | | | | | | | | | | | | | | | | | | | | | | | | |
|  | 7z0t.1.G | Formate dehydrogenase H  *Structure of the Escherichia coli formate hydrogenlyase complex (aerobic preparation, composite structure)* | 0.01 |  | 20.00 | 0.07 | 126-181 | EM | 0.00 | hetero-1-1-1-1-1-1-… | 1 x NI, 1 x FCO, 8 x SF4, 1 x FE, 2 x MGD, 1 x 6MO | HHblits | 0.27 |
| ``` target    SWDEATTIAAKTMEDVARTFNGDEGARKLLAQGYHPEMVEVMHGAGVQALKLRGGMPLLGIGRIFGFYRFANMLALLDRK 7z0t.1    --------------------------------------------------------------------------------  target    LRPDAPADEILGSRTFDNYAWHTDLPPGHPMVTGSQTVDFDLFSAEHTKLLLIIGMNWICTKMPDGHWIGDARLKGTRVI 7z0t.1    ---------------------------------------------GEVRAAYIMGEDPLQTDA-ELSAVRKAFEDLELVI  target    VISADYMPTANKADEVIILRPGTDAAFFLGVARELIEKGLYDRAAVIERTDLPLLVRLDTGERLDARDVIPGYELAALTN 7z0t.1    VQDIFMTKTASAADVILPSTS-----------------------------------------------------------  target    YVTLKPDAEIKGNPPPPPFTAGGQVVPTELRDAWGDFVWWDRATGRPRPVSRDEVGARFDGDPALLGEFEVELVDGSTVP 7z0t.1    --------------------------------------------------------------------------------  target    VRPAFDLLKQYLDESFDLRTASEVCRVPPQAIQSIARQLAANKRETLLAAGMGPNHYFQNDLFGRVQFLVAALTDNIGHL 7z0t.1    --------------------------------------------------------------------------------  target    GGNVGSYAGNYRGSVFQAMGQWIAEDPFAIEPDLTKPATVKRYYKAESAHYWNYGERPLRAVAKDDEGDLTKGEVLTGKS 7z0t.1    --------------------------------------------------------------------------------  target    HMPTPTKLIWFGNSNSLLGNAKWSFDVVKNTLPRQDAVFCNEWHWTSSCEYADLVFPADSWAEFKLPDATASCTNPFLLA 7z0t.1    --------------------------------------------------------------------------------  target    FPTTPLKRLYDTRSDYEALALTAKALGELIDEPRMEQYWRGILDGDPTPYLQRIFSGSNATRGITYDELHESSKRGVPLL 7z0t.1    --------------------------------------------------------------------------------  target    MNMRTYPRSGGWEQRQEDKPWYTATGRLEFYRPEPEFQAAGESLPVWREPVDATFYEPNAILSNAAHPSIAPRAPEDYGV 7z0t.1    --------------------------------------------------------------------------------  target    PESQLDVETRQYRNVVRTWAELQQTLHPLQERDPAFRFVFQTPKYRWGAHSTAVDADWISMLFGPFGDPYRRDPRMPWTG 7z0t.1    --------------------------------------------------------------------------------  target    EAYLEINPKDAA 7z0t.1    ------------ ``` | | | | | | | | | | | | | | | | | | | | | | | | | | | | | | | | | | | | | | | | | | | | | | | | | |
|  | 4ivn.1.A | Transcriptional regulator  *The Vibrio vulnificus NanR protein complexed with ManNAc-6P* | 0.01 |  | 12.73 | 0.07 | 126-182 | X-ray | 1.90 | homo-dimer | 2 x BMX | HHblits | 0.27 |
| ``` target    SWDEATTIAAKTMEDVARTFNGDEGARKLLAQGYHPEMVEVMHGAGVQALKLRGGMPLLGIGRIFGFYRFANMLALLDRK 4ivn.1    --------------------------------------------------------------------------------  target    LRPDAPADEILGSRTFDNYAWHTDLPPGHPMVTGSQTVDFDLFSAEHTKLLLIIGMNWICTKMPDGHWIGDARLKGTRVI 4ivn.1    ---------------------------------------------SQGDLWFAVSSSGSTK--EVIHAAGLAYKRDIPVV  target    VISAD-YMPTANKADEVIILRPGTDAAFFLGVARELIEKGLYDRAAVIERTDLPLLVRLDTGERLDARDVIPGYELAALT 4ivn.1    SLTNINHSPLSSLSTEMLVAARP---------------------------------------------------------  target    NYVTLKPDAEIKGNPPPPPFTAGGQVVPTELRDAWGDFVWWDRATGRPRPVSRDEVGARFDGDPALLGEFEVELVDGSTV 4ivn.1    --------------------------------------------------------------------------------  target    PVRPAFDLLKQYLDESFDLRTASEVCRVPPQAIQSIARQLAANKRETLLAAGMGPNHYFQNDLFGRVQFLVAALTDNIGH 4ivn.1    --------------------------------------------------------------------------------  target    LGGNVGSYAGNYRGSVFQAMGQWIAEDPFAIEPDLTKPATVKRYYKAESAHYWNYGERPLRAVAKDDEGDLTKGEVLTGK 4ivn.1    --------------------------------------------------------------------------------  target    SHMPTPTKLIWFGNSNSLLGNAKWSFDVVKNTLPRQDAVFCNEWHWTSSCEYADLVFPADSWAEFKLPDATASCTNPFLL 4ivn.1    --------------------------------------------------------------------------------  target    AFPTTPLKRLYDTRSDYEALALTAKALGELIDEPRMEQYWRGILDGDPTPYLQRIFSGSNATRGITYDELHESSKRGVPL 4ivn.1    --------------------------------------------------------------------------------  target    LMNMRTYPRSGGWEQRQEDKPWYTATGRLEFYRPEPEFQAAGESLPVWREPVDATFYEPNAILSNAAHPSIAPRAPEDYG 4ivn.1    --------------------------------------------------------------------------------  target    VPESQLDVETRQYRNVVRTWAELQQTLHPLQERDPAFRFVFQTPKYRWGAHSTAVDADWISMLFGPFGDPYRRDPRMPWT 4ivn.1    --------------------------------------------------------------------------------  target    GEAYLEINPKDAA 4ivn.1    ------------- ``` | | | | | | | | | | | | | | | | | | | | | | | | | | | | | | | | | | | | | | | | | | | | | | | | | |
|  | 1e5v.2.A | Dimethyl sulfoxide/trimethylamine N-oxide reductase  *OXIDIZED DMSO REDUCTASE EXPOSED TO HEPES BUFFER* | 0.01 |  | 24.53 | 0.07 | 127-180 | X-ray | 2.40 | monomer | 2 x PGD, 1 x 2MO | HHblits | 0.30 |
| ``` target    SWDEATTIAAKTMEDVARTFNGDEGARKLLAQGYHPEMVEVMHGAGVQALKLRGGMPLLGIGRIFGFYRFANMLALLDRK 1e5v.2    --------------------------------------------------------------------------------  target    LRPDAPADEILGSRTFDNYAWHTDLPPGHPMVTGSQTVDFDLFSAEHTKLLLIIGMNWICTKMPDGHWIGDARLKGTRVI 1e5v.2    ----------------------------------------------DVKMAYWVGGNPFVHHQ-DRNRMVKAWEKLETFV  target    VISADYMPTANKADEVIILRPGTDAAFFLGVARELIEKGLYDRAAVIERTDLPLLVRLDTGERLDARDVIPGYELAALTN 1e5v.2    VHDFQWTPTARHADIVLPAT------------------------------------------------------------  target    YVTLKPDAEIKGNPPPPPFTAGGQVVPTELRDAWGDFVWWDRATGRPRPVSRDEVGARFDGDPALLGEFEVELVDGSTVP 1e5v.2    --------------------------------------------------------------------------------  target    VRPAFDLLKQYLDESFDLRTASEVCRVPPQAIQSIARQLAANKRETLLAAGMGPNHYFQNDLFGRVQFLVAALTDNIGHL 1e5v.2    --------------------------------------------------------------------------------  target    GGNVGSYAGNYRGSVFQAMGQWIAEDPFAIEPDLTKPATVKRYYKAESAHYWNYGERPLRAVAKDDEGDLTKGEVLTGKS 1e5v.2    --------------------------------------------------------------------------------  target    HMPTPTKLIWFGNSNSLLGNAKWSFDVVKNTLPRQDAVFCNEWHWTSSCEYADLVFPADSWAEFKLPDATASCTNPFLLA 1e5v.2    --------------------------------------------------------------------------------  target    FPTTPLKRLYDTRSDYEALALTAKALGELIDEPRMEQYWRGILDGDPTPYLQRIFSGSNATRGITYDELHESSKRGVPLL 1e5v.2    --------------------------------------------------------------------------------  target    MNMRTYPRSGGWEQRQEDKPWYTATGRLEFYRPEPEFQAAGESLPVWREPVDATFYEPNAILSNAAHPSIAPRAPEDYGV 1e5v.2    --------------------------------------------------------------------------------  target    PESQLDVETRQYRNVVRTWAELQQTLHPLQERDPAFRFVFQTPKYRWGAHSTAVDADWISMLFGPFGDPYRRDPRMPWTG 1e5v.2    --------------------------------------------------------------------------------  target    EAYLEINPKDAA 1e5v.2    ------------ ``` | | | | | | | | | | | | | | | | | | | | | | | | | | | | | | | | | | | | | | | | | | | | | | | | | |
|  | 4dmr.1.A | DMSO REDUCTASE  *REDUCED DMSO REDUCTASE FROM RHODOBACTER CAPSULATUS WITH BOUND DMSO SUBSTRATE* | 0.01 |  | 24.53 | 0.07 | 127-180 | X-ray | 1.90 | monomer | 2 x PGD, 1 x 4MO, 1 x O | HHblits | 0.30 |
| ``` target    SWDEATTIAAKTMEDVARTFNGDEGARKLLAQGYHPEMVEVMHGAGVQALKLRGGMPLLGIGRIFGFYRFANMLALLDRK 4dmr.1    --------------------------------------------------------------------------------  target    LRPDAPADEILGSRTFDNYAWHTDLPPGHPMVTGSQTVDFDLFSAEHTKLLLIIGMNWICTKMPDGHWIGDARLKGTRVI 4dmr.1    ----------------------------------------------DVKMAYWVGGNPFVHHQ-DRNRMVKAWEKLETFV  target    VISADYMPTANKADEVIILRPGTDAAFFLGVARELIEKGLYDRAAVIERTDLPLLVRLDTGERLDARDVIPGYELAALTN 4dmr.1    VHDFQWTPTARHADIVLPAT------------------------------------------------------------  target    YVTLKPDAEIKGNPPPPPFTAGGQVVPTELRDAWGDFVWWDRATGRPRPVSRDEVGARFDGDPALLGEFEVELVDGSTVP 4dmr.1    --------------------------------------------------------------------------------  target    VRPAFDLLKQYLDESFDLRTASEVCRVPPQAIQSIARQLAANKRETLLAAGMGPNHYFQNDLFGRVQFLVAALTDNIGHL 4dmr.1    --------------------------------------------------------------------------------  target    GGNVGSYAGNYRGSVFQAMGQWIAEDPFAIEPDLTKPATVKRYYKAESAHYWNYGERPLRAVAKDDEGDLTKGEVLTGKS 4dmr.1    --------------------------------------------------------------------------------  target    HMPTPTKLIWFGNSNSLLGNAKWSFDVVKNTLPRQDAVFCNEWHWTSSCEYADLVFPADSWAEFKLPDATASCTNPFLLA 4dmr.1    --------------------------------------------------------------------------------  target    FPTTPLKRLYDTRSDYEALALTAKALGELIDEPRMEQYWRGILDGDPTPYLQRIFSGSNATRGITYDELHESSKRGVPLL 4dmr.1    --------------------------------------------------------------------------------  target    MNMRTYPRSGGWEQRQEDKPWYTATGRLEFYRPEPEFQAAGESLPVWREPVDATFYEPNAILSNAAHPSIAPRAPEDYGV 4dmr.1    --------------------------------------------------------------------------------  target    PESQLDVETRQYRNVVRTWAELQQTLHPLQERDPAFRFVFQTPKYRWGAHSTAVDADWISMLFGPFGDPYRRDPRMPWTG 4dmr.1    --------------------------------------------------------------------------------  target    EAYLEINPKDAA 4dmr.1    ------------ ``` | | | | | | | | | | | | | | | | | | | | | | | | | | | | | | | | | | | | | | | | | | | | | | | | | |
|  | 1e60.1.A | Dimethyl sulfoxide/trimethylamine N-oxide reductase  *OXIDIZED DMSO REDUCTASE EXPOSED TO HEPES - Structure II BUFFER* | 0.01 |  | 24.53 | 0.07 | 127-180 | X-ray | 2.00 | monomer | 2 x PGD, 1 x 2MO | HHblits | 0.30 |
| ``` target    SWDEATTIAAKTMEDVARTFNGDEGARKLLAQGYHPEMVEVMHGAGVQALKLRGGMPLLGIGRIFGFYRFANMLALLDRK 1e60.1    --------------------------------------------------------------------------------  target    LRPDAPADEILGSRTFDNYAWHTDLPPGHPMVTGSQTVDFDLFSAEHTKLLLIIGMNWICTKMPDGHWIGDARLKGTRVI 1e60.1    ----------------------------------------------DVKMAYWVGGNPFVHHQ-DRNRMVKAWEKLETFV  target    VISADYMPTANKADEVIILRPGTDAAFFLGVARELIEKGLYDRAAVIERTDLPLLVRLDTGERLDARDVIPGYELAALTN 1e60.1    VHDFQWTPTARHADIVLPAT------------------------------------------------------------  target    YVTLKPDAEIKGNPPPPPFTAGGQVVPTELRDAWGDFVWWDRATGRPRPVSRDEVGARFDGDPALLGEFEVELVDGSTVP 1e60.1    --------------------------------------------------------------------------------  target    VRPAFDLLKQYLDESFDLRTASEVCRVPPQAIQSIARQLAANKRETLLAAGMGPNHYFQNDLFGRVQFLVAALTDNIGHL 1e60.1    --------------------------------------------------------------------------------  target    GGNVGSYAGNYRGSVFQAMGQWIAEDPFAIEPDLTKPATVKRYYKAESAHYWNYGERPLRAVAKDDEGDLTKGEVLTGKS 1e60.1    --------------------------------------------------------------------------------  target    HMPTPTKLIWFGNSNSLLGNAKWSFDVVKNTLPRQDAVFCNEWHWTSSCEYADLVFPADSWAEFKLPDATASCTNPFLLA 1e60.1    --------------------------------------------------------------------------------  target    FPTTPLKRLYDTRSDYEALALTAKALGELIDEPRMEQYWRGILDGDPTPYLQRIFSGSNATRGITYDELHESSKRGVPLL 1e60.1    --------------------------------------------------------------------------------  target    MNMRTYPRSGGWEQRQEDKPWYTATGRLEFYRPEPEFQAAGESLPVWREPVDATFYEPNAILSNAAHPSIAPRAPEDYGV 1e60.1    --------------------------------------------------------------------------------  target    PESQLDVETRQYRNVVRTWAELQQTLHPLQERDPAFRFVFQTPKYRWGAHSTAVDADWISMLFGPFGDPYRRDPRMPWTG 1e60.1    --------------------------------------------------------------------------------  target    EAYLEINPKDAA 1e60.1    ------------ ``` | | | | | | | | | | | | | | | | | | | | | | | | | | | | | | | | | | | | | | | | | | | | | | | | | |
|  | 1e18.1.A | DMSO REDUCTASE.  *TUNGSTEN-SUSBSTITUTED DMSO REDUCTASE FROM RHODOBACTER CAPSULATUS* | 0.01 |  | 24.53 | 0.07 | 127-180 | X-ray | 2.00 | monomer | 2 x PGD, 1 x 6WO | HHblits | 0.30 |
| ``` target    SWDEATTIAAKTMEDVARTFNGDEGARKLLAQGYHPEMVEVMHGAGVQALKLRGGMPLLGIGRIFGFYRFANMLALLDRK 1e18.1    --------------------------------------------------------------------------------  target    LRPDAPADEILGSRTFDNYAWHTDLPPGHPMVTGSQTVDFDLFSAEHTKLLLIIGMNWICTKMPDGHWIGDARLKGTRVI 1e18.1    ----------------------------------------------DVKMAYWVGGNPFVHHQ-DRNRMVKAWEKLETFV  target    VISADYMPTANKADEVIILRPGTDAAFFLGVARELIEKGLYDRAAVIERTDLPLLVRLDTGERLDARDVIPGYELAALTN 1e18.1    VHDFQWTPTARHADIVLPAT------------------------------------------------------------  target    YVTLKPDAEIKGNPPPPPFTAGGQVVPTELRDAWGDFVWWDRATGRPRPVSRDEVGARFDGDPALLGEFEVELVDGSTVP 1e18.1    --------------------------------------------------------------------------------  target    VRPAFDLLKQYLDESFDLRTASEVCRVPPQAIQSIARQLAANKRETLLAAGMGPNHYFQNDLFGRVQFLVAALTDNIGHL 1e18.1    --------------------------------------------------------------------------------  target    GGNVGSYAGNYRGSVFQAMGQWIAEDPFAIEPDLTKPATVKRYYKAESAHYWNYGERPLRAVAKDDEGDLTKGEVLTGKS 1e18.1    --------------------------------------------------------------------------------  target    HMPTPTKLIWFGNSNSLLGNAKWSFDVVKNTLPRQDAVFCNEWHWTSSCEYADLVFPADSWAEFKLPDATASCTNPFLLA 1e18.1    --------------------------------------------------------------------------------  target    FPTTPLKRLYDTRSDYEALALTAKALGELIDEPRMEQYWRGILDGDPTPYLQRIFSGSNATRGITYDELHESSKRGVPLL 1e18.1    --------------------------------------------------------------------------------  target    MNMRTYPRSGGWEQRQEDKPWYTATGRLEFYRPEPEFQAAGESLPVWREPVDATFYEPNAILSNAAHPSIAPRAPEDYGV 1e18.1    --------------------------------------------------------------------------------  target    PESQLDVETRQYRNVVRTWAELQQTLHPLQERDPAFRFVFQTPKYRWGAHSTAVDADWISMLFGPFGDPYRRDPRMPWTG 1e18.1    --------------------------------------------------------------------------------  target    EAYLEINPKDAA 1e18.1    ------------ ``` | | | | | | | | | | | | | | | | | | | | | | | | | | | | | | | | | | | | | | | | | | | | | | | | | |
|  | 1tmo.1.A | TRIMETHYLAMINE N-OXIDE REDUCTASE  *TRIMETHYLAMINE N-OXIDE REDUCTASE FROM SHEWANELLA MASSILIA* | 0.01 |  | 18.52 | 0.07 | 127-181 | X-ray | 2.50 | monomer | 2 x 2MD, 1 x 2MO | HHblits | 0.28 |
[truncated: 356,632 more chars]
