# Supplementary material for: Metabolic capacity is maintained despite shifts in microbial diversity in estuary sediments
Source: ISME Commun. 2025 Oct 11;5(1):ycaf182. doi: 10.1093/ismeco/ycaf182 (PMC12687941; doi:10.1093/ismeco/ycaf182)
Supplement: Supplementary_Data_1_ycaf182 [file supplementary_data_1_ycaf182.zip › SWISS-MODEL/13_May_SF_Bin61_scaffold_51921_c131277_1/templates.html]

13\_May\_SF\_Bin61\_scaffold\_51921\_c1:3-1277\_1 | Templates


**Export Alignment**
  
FASTA format
Clustal Format
PNG Image

**Secondary Structure**
  
None
DSSP
PSIPRED
SSpro

**Colour Scheme** 


Fade Mismatches
Enhance Mismatches

Confidencegradient
Confidenceclass
Indels
Chain
Unique Chain
Rainbow
2° Structure
Clustal
Hydrophobic
Size
Charged
Polar
Proline
Ser/Thr
Cysteine
Aliphatic
Aromatic
No Colour

Use QMEANBrane values

|  |  |  |  |
| --- | --- | --- | --- |
| Background |  |  |  |

**3D Viewer**  
NGL
PV

FASTA
Multi FASTA
ClustalW
PNG


SWISS-MODEL

### 13\_May\_SF\_Bin61\_scaffold\_51921\_c1:3-1277\_1

### Created: March 29, 2023, 5:44 p.m. at 17:44

- Templates
- Models

Models | Name | Description | GMQE | QSQE | Seq Id | Coverage | Range | Method | Resolution | Oligo-state | Ligands | Found by | Seq Similarity ||  | 7b04.1.B | Nitrite oxidoreductase subunit A  *Structure of Nitrite oxidoreductase (Nxr) from the anammox bacterium Kuenenia stuttgartiensis.* | 0.73 | 0.00 | 43.24 | 0.96 | 1-413 | X-ray | 2.97 | monomer | 4 x SF4, 1 x F3S, 2 x MD1, 1 x MO, 1 x HEM, 2 x CA | BLAST | 0.41 |
| ``` target    EVLARVGHKLAEQTGDARFADVWKLVDEKRTDAHLQRILDHSSNTKGYDALDLEAKAKKGIP--TLMMNRTYPKAVGYEQ 7b04.1    KILAGMASKLGELLRDKRFEDNWKFAIEGRASVYINRLLDGSTTMKGYTCEDI-LNGKYGEPGVAMLLFRTYPRHPFWEQ  target    VADSRPWYTKSGRLEFYRDEDEFIEAGENLPVHREPIDSTFYEPNVIVSAPHEALRPAGPEDYGVELSDMSGEIRQGRNV 7b04.1    VHESLPFYTPTGRLQAYNDEPEIIEYGENFIVHREGPEATPYLPNAIVST-NPYIRP---DDYGIPENAEYWEDRTVRNI  target    VKAWAELKKTPHPLAKDGYRFVFHTPKYRHGAHTMPIDTDMVAMLFGPFGDVYRHDRRTPYVAEGYVDIHPSDAREIGVE 7b04.1    KKSWEETKKTKNFLWEKGYHFYCVTPKSRHTVHSQWAVTDWNFIWNNNFGDPYRMDKRMPGVGEHQIHIHPQAARDLGIE  target    DGDYVFIDSDPEDRPFRGWQKNKRDYEFSRLLCRARYYPGTPRGVTRMWFNMYGATPGSVEGQKSREDGLAKNPRTNYQA 7b04.1    DGDYVYVDANPADRPYEGWKPNDSFYKVSRLMLRAKYNPAYPYNCTMMKHSAWISSDKTVQAHETRPDGRALSP-SGYQS  target    MFRSGSHQSATRGWLKPTWMTDSLVRKGLFGQSIGKGFLPDVHCPTGAPRESIVKITKAEPGGLGAEGLWRPAALGLRPG 7b04.1    SFRYGSQQSITRDWSMPMHQLDSLFHKAKIGMKFIFGFEADNHCINTVPKETLVKITKAENGGMGGKGVWDPVKTGYTAG  target    YESKSMKTYLDGGYVDDADRQGGQG 7b04.1    NENDFMKKFLNGELI---------- ``` | | | | | | | | | | | | | | | | | | | | | | | | | | | | | | | | | | | | | | | | | | | | | | | | | |
|  | 7b04.2.B | Nitrite oxidoreductase subunit A  *Structure of Nitrite oxidoreductase (Nxr) from the anammox bacterium Kuenenia stuttgartiensis.* | 0.71 | 0.00 | 43.24 | 0.96 | 1-413 | X-ray | 2.97 | monomer | 4 x SF4, 1 x F3S, 2 x MD1, 1 x MO, 1 x HEM, 2 x CA | BLAST | 0.41 |
| ``` target    EVLARVGHKLAEQTGDARFADVWKLVDEKRTDAHLQRILDHSSNTKGYDALDLEAKAKKGIP--TLMMNRTYPKAVGYEQ 7b04.2    KILAGMASKLGELLRDKRFEDNWKFAIEGRASVYINRLLDGSTTMKGYTCEDI-LNGKYGEPGVAMLLFRTYPRHPFWEQ  target    VADSRPWYTKSGRLEFYRDEDEFIEAGENLPVHREPIDSTFYEPNVIVSAPHEALRPAGPEDYGVELSDMSGEIRQGRNV 7b04.2    VHESLPFYTPTGRLQAYNDEPEIIEYGENFIVHREGPEATPYLPNAIVST-NPYIRP---DDYGIPENAEYWEDRTVRNI  target    VKAWAELKKTPHPLAKDGYRFVFHTPKYRHGAHTMPIDTDMVAMLFGPFGDVYRHDRRTPYVAEGYVDIHPSDAREIGVE 7b04.2    KKSWEETKKTKNFLWEKGYHFYCVTPKSRHTVHSQWAVTDWNFIWNNNFGDPYRMDKRMPGVGEHQIHIHPQAARDLGIE  target    DGDYVFIDSDPEDRPFRGWQKNKRDYEFSRLLCRARYYPGTPRGVTRMWFNMYGATPGSVEGQKSREDGLAKNPRTNYQA 7b04.2    DGDYVYVDANPADRPYEGWKPNDSFYKVSRLMLRAKYNPAYPYNCTMMKHSAWISSDKTVQAHETRPDGRALSP-SGYQS  target    MFRSGSHQSATRGWLKPTWMTDSLVRKGLFGQSIGKGFLPDVHCPTGAPRESIVKITKAEPGGLGAEGLWRPAALGLRPG 7b04.2    SFRYGSQQSITRDWSMPMHQLDSLFHKAKIGMKFIFGFEADNHCINTVPKETLVKITKAENGGMGGKGVWDPVKTGYTAG  target    YESKSMKTYLDGGYVDDADRQGGQG 7b04.2    NENDFMKKFLNGELI---------- ``` | | | | | | | | | | | | | | | | | | | | | | | | | | | | | | | | | | | | | | | | | | | | | | | | | |
| ✓ | 7b04.1.B | Nitrite oxidoreductase subunit A  *Structure of Nitrite oxidoreductase (Nxr) from the anammox bacterium Kuenenia stuttgartiensis.* | 0.73 | 0.00 | 42.51 | 0.96 | 1-415 | X-ray | 2.97 | monomer | 4 x SF4, 1 x F3S, 2 x MD1, 1 x MO, 1 x HEM, 2 x CA | HHblits | 0.41 |
| ``` target    EVLARVGHKLAEQTGDARFADVWKLVDEKRTDAHLQRILDHSSNTKGYDALDLEAKAKKGIP----TLMMNRTYPKAVGY 7b04.1    KILAGMASKLGELLRDKRFEDNWKFAIEGRASVYINRLLDGSTTMKGYTCEDIL---NGKYGEPGVAMLLFRTYPRHPFW  target    EQVADSRPWYTKSGRLEFYRDEDEFIEAGENLPVHREPIDSTFYEPNVIVSAPHEALRPAGPEDYGVELSDMSGEIRQGR 7b04.1    EQVHESLPFYTPTGRLQAYNDEPEIIEYGENFIVHREGPEATPYLPNAIVS-TNPYIR---PDDYGIPENAEYWEDRTVR  target    NVVKAWAELKKTPHPLAKDGYRFVFHTPKYRHGAHTMPIDTDMVAMLFGPFGDVYRHDRRTPYVAEGYVDIHPSDAREIG 7b04.1    NIKKSWEETKKTKNFLWEKGYHFYCVTPKSRHTVHSQWAVTDWNFIWNNNFGDPYRMDKRMPGVGEHQIHIHPQAARDLG  target    VEDGDYVFIDSDPEDRPFRGWQKNKRDYEFSRLLCRARYYPGTPRGVTRMWFNMYGATPGSVEGQKSREDGLAKNPRTNY 7b04.1    IEDGDYVYVDANPADRPYEGWKPNDSFYKVSRLMLRAKYNPAYPYNCTMMKHSAWISSDKTVQAHETRPDGRALSP-SGY  target    QAMFRSGSHQSATRGWLKPTWMTDSLVRKGLFGQSIGKGFLPDVHCPTGAPRESIVKITKAEPGGLGAEGLWRPAALGLR 7b04.1    QSSFRYGSQQSITRDWSMPMHQLDSLFHKAKIGMKFIFGFEADNHCINTVPKETLVKITKAENGGMGGKGVWDPVKTGYT  target    PGYESKSMKTYLDGGYVDDADRQGGQG 7b04.1    AGNENDFMKKFLNGELIKV-------- ``` | | | | | | | | | | | | | | | | | | | | | | | | | | | | | | | | | | | | | | | | | | | | | | | | | |
|  | 7b04.2.B | Nitrite oxidoreductase subunit A  *Structure of Nitrite oxidoreductase (Nxr) from the anammox bacterium Kuenenia stuttgartiensis.* | 0.71 | 0.00 | 42.51 | 0.96 | 1-415 | X-ray | 2.97 | monomer | 4 x SF4, 1 x F3S, 2 x MD1, 1 x MO, 1 x HEM, 2 x CA | HHblits | 0.41 |
| ``` target    EVLARVGHKLAEQTGDARFADVWKLVDEKRTDAHLQRILDHSSNTKGYDALDLEAKAKKGIP----TLMMNRTYPKAVGY 7b04.2    KILAGMASKLGELLRDKRFEDNWKFAIEGRASVYINRLLDGSTTMKGYTCEDIL---NGKYGEPGVAMLLFRTYPRHPFW  target    EQVADSRPWYTKSGRLEFYRDEDEFIEAGENLPVHREPIDSTFYEPNVIVSAPHEALRPAGPEDYGVELSDMSGEIRQGR 7b04.2    EQVHESLPFYTPTGRLQAYNDEPEIIEYGENFIVHREGPEATPYLPNAIVS-TNPYIR---PDDYGIPENAEYWEDRTVR  target    NVVKAWAELKKTPHPLAKDGYRFVFHTPKYRHGAHTMPIDTDMVAMLFGPFGDVYRHDRRTPYVAEGYVDIHPSDAREIG 7b04.2    NIKKSWEETKKTKNFLWEKGYHFYCVTPKSRHTVHSQWAVTDWNFIWNNNFGDPYRMDKRMPGVGEHQIHIHPQAARDLG  target    VEDGDYVFIDSDPEDRPFRGWQKNKRDYEFSRLLCRARYYPGTPRGVTRMWFNMYGATPGSVEGQKSREDGLAKNPRTNY 7b04.2    IEDGDYVYVDANPADRPYEGWKPNDSFYKVSRLMLRAKYNPAYPYNCTMMKHSAWISSDKTVQAHETRPDGRALSP-SGY  target    QAMFRSGSHQSATRGWLKPTWMTDSLVRKGLFGQSIGKGFLPDVHCPTGAPRESIVKITKAEPGGLGAEGLWRPAALGLR 7b04.2    QSSFRYGSQQSITRDWSMPMHQLDSLFHKAKIGMKFIFGFEADNHCINTVPKETLVKITKAENGGMGGKGVWDPVKTGYT  target    PGYESKSMKTYLDGGYVDDADRQGGQG 7b04.2    AGNENDFMKKFLNGELIKV-------- ``` | | | | | | | | | | | | | | | | | | | | | | | | | | | | | | | | | | | | | | | | | | | | | | | | | |
|  | 2ivf.1.A | ETHYLBENZENE DEHYDROGENASE ALPHA-SUBUNIT  *ETHYLBENZENE DEHYDROGENASE FROM AROMATOLEUM AROMATICUM* | 0.31 |  | 21.43 | 0.60 | 1-377 | X-ray | 1.88 | hetero-oligomer | 1 x MES, 4 x SF4, 1 x MO, 1 x MGD, 1 x MD1, 1 x F3S, 1 x HEM | HHblits | 0.30 |
| ``` target    EVLARVGHKLAEQTGDA-------------RFADVWK-LVDEK---RTDAHLQRILDHSSNTK----GYDALDLEAKAKK 2ivf.1    DAIALILKKVGERAAARGLTEFNDHNGRKRRYDELYKKFTMDGHLLTNEDCLKEMVDINRAVGVFAKDYT---YEKFKKE  target    GIPTLMMNR--------------TYPKAVGYEQVADSRPWYTKSGRLEFYRDEDEFIEAGENLPVHREPIDSTFYEPNVI 2ivf.1    GQTRFLSMGTGVSRYAHANEVDVTKPIYPMRWHFDDKKVFPTHTRRAQFYLDHDWYLEAGESLPTHKDTPMV--------  target    VSAPHEALRPAGPEDYGVELSDMSGEIRQGRNVVKAWAELKKTPHPLAKDGYRFVFHTPKYRHGAHTMPIDTDMVAMLFG 2ivf.1    ------------------------------------------------GGDHPFKITGGHPRVSIHSTHLTNSHLSRLH-  target    PFGDVYRHDRRTPYVAEGYVDIHPSDAREIGVEDGDYVFIDSDPEDRPFRGWQKNKRDYEFSRLLCRARYYPGTPRGVTR 2ivf.1    --------------RGQPVVHMNSKDAAELGIKDGDMAKLFNDF-----------------ADCEIMVRTAPNVQPKQCI  target    MWFNMYGATPGSVEGQKSREDGLAKNPRTNYQAMFRSGSHQSATRGWLKPTWMTDSLVRKGLFGQSIGKGFLPDVHCPTG 2ivf.1    VYFWDAHQ-----------Y--------KG----W--KPYDILLIGMPKPLHLA------GGYEQF---RYYFMNGSPAP  target    -APRESIVKITKAEPGGLGAEGLWRPAALGLRPGYESKSMKTYLDGGYVDDADRQGGQG 2ivf.1    VTDRGVRVSIKKA---------------------------------------------- ``` | | | | | | | | | | | | | | | | | | | | | | | | | | | | | | | | | | | | | | | | | | | | | | | | | |
|  | 5e7o.1.A | DMSO reductase family type II enzyme, molybdopterin subunit  *Crystal structure of the perchlorate reductase PcrAB mutant W461E of PcrA from Azospira suillum PS* | 0.34 | 0.00 | 29.46 | 0.53 | 32-380 | X-ray | 2.40 | monomer | 4 x SF4, 1 x MO, 1 x MGD, 1 x MD1, 1 x F3S | HHblits | 0.35 |
| ``` target    EVLARVGHKLAEQTGDARFADVWKLVDEKRTDAHLQRILDHSSNTKGYDALDLEAKAKKGIPTLMM-----NRTYPKAVG 5e7o.1    -------------------------------EAAAQYILDNAPQSKGIT---IQMLREKPQRFKSNWTSPLKEGVPYTPF  target    YEQVADSRPWYTKSGRLEFYRDEDEFIEAGENLPVHREPIDSTFYEPNVIVSAPHEALRPAGPEDYGVELSDMSGEIRQG 5e7o.1    QYFVVDKKPWPTLTGRQQFYLDHDTFFDMGVELPTYKAPID---------------------------------------  target    RNVVKAWAELKKTPHPLAKDGYRFVFHTPKYRHGAHTMPIDTDMVAMLFGPFGDVYRHDRRTPYVAEGYVDIHPSDAREI 5e7o.1    ------------------ADKYPFRFNSPHSRHSVHSTFKDNVLMLRL----------QR-----GGPSIEMSPLDAKPL  target    GVEDGDYVFIDSDPEDRPFRGWQKNKRDYEFSRLLCRARYYPGTPRGVTRMWFNMYGATPGSVEGQKSREDGLAKNPRTN 5e7o.1    GIKDNDWVEAWNNH-----------------GKVICRVKIRNGEQRGRVSMWHCPEL-----------------------  target    YQAMFRSGSHQSATRGWLKPTWMTDSLVRKGLFGQSIGKGFLPDVHCPTGAPRESIVKITKAEPGGLGAEGLWRPAALGL 5e7o.1    YMD-LLTGGSQSVCPVRINPTNLV------GNYGHL---FFRPNYYGPAGSQRDVRVNVKRYIGA---------------  target    RPGYESKSMKTYLDGGYVDDADRQGGQG 5e7o.1    ---------------------------- ``` | | | | | | | | | | | | | | | | | | | | | | | | | | | | | | | | | | | | | | | | | | | | | | | | | |
|  | 4ydd.1.A | DMSO reductase family type II enzyme, molybdopterin subunit  *Crystal structure of the perchlorate reductase PcrAB from Azospira suillum PS* | 0.34 | 0.00 | 29.60 | 0.53 | 33-380 | X-ray | 1.86 | monomer | 4 x SF4, 1 x MO, 1 x MGD, 1 x MD1, 1 x F3S | HHblits | 0.35 |
| ``` target    EVLARVGHKLAEQTGDARFADVWKLVDEKRTDAHLQRILDHSSNTKGYDALDLEAKAKKGIPTLM-----MNRTYPKAVG 4ydd.1    --------------------------------AAAQYILDNAPQSKGIT---IQMLREKPQRFKSNWTSPLKEGVPYTPF  target    YEQVADSRPWYTKSGRLEFYRDEDEFIEAGENLPVHREPIDSTFYEPNVIVSAPHEALRPAGPEDYGVELSDMSGEIRQG 4ydd.1    QYFVVDKKPWPTLTGRQQFYLDHDTFFDMGVELPTYKAPID---------------------------------------  target    RNVVKAWAELKKTPHPLAKDGYRFVFHTPKYRHGAHTMPIDTDMVAMLFGPFGDVYRHDRRTPYVAEGYVDIHPSDAREI 4ydd.1    ------------------ADKYPFRFNSPHSRHSVHSTFKDNVLMLRLQ----------R-----GGPSIEMSPLDAKPL  target    GVEDGDYVFIDSDPEDRPFRGWQKNKRDYEFSRLLCRARYYPGTPRGVTRMWFNMYGATPGSVEGQKSREDGLAKNPRTN 4ydd.1    GIKDNDWVEAWNNH-----------------GKVICRVKIRNGEQRGRVSMWHCPEL-----------------------  target    YQAMFRSGSHQSATRGWLKPTWMTDSLVRKGLFGQSIGKGFLPDVHCPTGAPRESIVKITKAEPGGLGAEGLWRPAALGL 4ydd.1    YMD-LLTGGSQSVCPVRINPTNLV------GNYGHL---FFRPNYYGPAGSQRDVRVNVKRYIGA---------------  target    RPGYESKSMKTYLDGGYVDDADRQGGQG 4ydd.1    ---------------------------- ``` | | | | | | | | | | | | | | | | | | | | | | | | | | | | | | | | | | | | | | | | | | | | | | | | | |
|  | 1r27.4.A | Respiratory nitrate reductase 1 alpha chain  *Crystal Structure of NarGH complex* | 0.33 | 0.00 | 27.49 | 0.50 | 73-383 | X-ray | 2.00 | monomer | 4 x MO, 16 x SF4, 8 x MGD, 4 x F3S | HHblits | 0.33 |
| ``` target    EVLARVGHKLAEQTGDARFADVWKLVDEKRTDAHLQRILDHSSNTKGYDALDLEAKAKKGIPTLMMNRTYPKAVGYEQVA 1r27.4    ------------------------------------------------------------------------NAGYTNVH  target    DSRPWYTKSGRLEFYRDEDEFIEAGENLPVHREPIDSTFYEPNVIVSAPHEALRPAGPEDYGVELSDMSGEIRQGRNVVK 1r27.4    ELIPWRTLSGRQQLYQDHQWMRDFGESLLVYRPPIDTRSVKE-----------------VIG------------------  target    AWAELKKTPHPLAKDGYRFVFHTPKYRHGAHTMPIDTDMVAMLFGPFGDVYRHDRRTPYVAEGYVDIHPSDAREIGVEDG 1r27.4    -------QKS-NGNQEKALNFLTPHQKWGIHSTYSDNLLMLTLG----------R-----GGPVVWLSEADAKDLGIADN  target    DYVFIDSDPEDRPFRGWQKNKRDYEFSRLLCRARYYPGTPRGVTRMWFNMYGATPGSVEGQKSREDGLAKNPRTNYQAMF 1r27.4    DWIEVFNSN-----------------GALTARAVVSQRVPAGMTMMYHAQ----ERIVNLPGSEI------------TQQ  target    RSGSHQSATRGWLKPTWMTDSLVRKGLFGQSIGKGFLPDVHCPTGAPRESIVKITKAEPGGLGAEGLWRPAALGLRPGYE 1r27.4    RGGIHNSVTRITPKPTHMI------GGYAHL---AYGFNYYGTVGSNRDEFVVVRKMKNIDWL-----------------  target    SKSMKTYLDGGYVDDADRQGGQG 1r27.4    ----------------------- ``` | | | | | | | | | | | | | | | | | | | | | | | | | | | | | | | | | | | | | | | | | | | | | | | | | |
|  | 1q16.1.A | Respiratory nitrate reductase 1 alpha chain  *Crystal structure of Nitrate Reductase A, NarGHI, from Escherichia coli* | 0.33 | 0.00 | 27.01 | 0.50 | 73-383 | X-ray | 1.90 | monomer | 2 x MD1, 1 x 6MO, 2 x HEM, 4 x SF4, 1 x F3S, 1 x AGA, 1 x 3PH | HHblits | 0.33 |
| ``` target    EVLARVGHKLAEQTGDARFADVWKLVDEKRTDAHLQRILDHSSNTKGYDALDLEAKAKKGIPTLMMNRTYPKAVGYEQVA 1q16.1    ------------------------------------------------------------------------NAGYTNVH  target    DSRPWYTKSGRLEFYRDEDEFIEAGENLPVHREPIDSTFYEPNVIVSAPHEALRPAGPEDYGVELSDMSGEIRQGRNVVK 1q16.1    ELIPWRTLSGRQQLYQDHQWMRDFGESLLVYRPPIDTRSVKE-----------------VIG------------------  target    AWAELKKTPHPLAKDGYRFVFHTPKYRHGAHTMPIDTDMVAMLFGPFGDVYRHDRRTPYVAEGYVDIHPSDAREIGVEDG 1q16.1    -------QKSN-GNQEKALNFLTPHQKWGIHSTYSDNLLMLTLG---------------RGGPVVWLSEADAKDLGIADN  target    DYVFIDSDPEDRPFRGWQKNKRDYEFSRLLCRARYYPGTPRGVTRMWFNMYGATPGSVEGQKSREDGLAKNPRTNYQAMF 1q16.1    DWIEVFNSN-----------------GALTARAVVSQRVPAGMTMMYHAQ----ERIVNLPGSEI------------TQQ  target    RSGSHQSATRGWLKPTWMTDSLVRKGLFGQSIGKGFLPDVHCPTGAPRESIVKITKAEPGGLGAEGLWRPAALGLRPGYE 1q16.1    RGGIHNSVTRITPKPTHMI------GGYAHL---AYGFNYYGTVGSNRDEFVVVRKMKNIDWL-----------------  target    SKSMKTYLDGGYVDDADRQGGQG 1q16.1    ----------------------- ``` | | | | | | | | | | | | | | | | | | | | | | | | | | | | | | | | | | | | | | | | | | | | | | | | | |
|  | 3ir5.1.A | Respiratory nitrate reductase 1 alpha chain  *Crystal structure of NarGHI mutant NarG-H49C* | 0.33 | 0.00 | 27.62 | 0.50 | 74-383 | X-ray | 2.30 | monomer | 2 x MD1, 1 x 6MO, 4 x SF4, 1 x AGA, 1 x F3S, 2 x HEM | HHblits | 0.33 |
| ``` target    EVLARVGHKLAEQTGDARFADVWKLVDEKRTDAHLQRILDHSSNTKGYDALDLEAKAKKGIPTLMMNRTYPKAVGYEQVA 3ir5.1    -------------------------------------------------------------------------AGYTNVH  target    DSRPWYTKSGRLEFYRDEDEFIEAGENLPVHREPIDSTFYEPNVIVSAPHEALRPAGPEDYGVELSDMSGEIRQGRNVVK 3ir5.1    ELIPWRTLSGRQQLYQDHQWMRDFGESLLVYRPPIDTRSVKE-----------------VIG------------------  target    AWAELKKTPHPLAKDGYRFVFHTPKYRHGAHTMPIDTDMVAMLFGPFGDVYRHDRRTPYVAEGYVDIHPSDAREIGVEDG 3ir5.1    -------QKSN-GNQEKALNFLTPHQKWGIHSTYSDNLLMLTLG----------R-----GGPVVWLSEADAKDLGIADN  target    DYVFIDSDPEDRPFRGWQKNKRDYEFSRLLCRARYYPGTPRGVTRMWFNMYGATPGSVEGQKSREDGLAKNPRTNYQAMF 3ir5.1    DWIEVFNSN-----------------GALTARAVVSQRVPAGMTMMYHAQ----ERIVNLPGSEI------------TQQ  target    RSGSHQSATRGWLKPTWMTDSLVRKGLFGQSIGKGFLPDVHCPTGAPRESIVKITKAEPGGLGAEGLWRPAALGLRPGYE 3ir5.1    RGGIHNSVTRITPKPTHMI------GGYAHL---AYGFNYYGTVGSNRDEFVVVRKMKNIDWL-----------------  target    SKSMKTYLDGGYVDDADRQGGQG 3ir5.1    ----------------------- ``` | | | | | | | | | | | | | | | | | | | | | | | | | | | | | | | | | | | | | | | | | | | | | | | | | |
|  | 3ir7.1.A | Respiratory nitrate reductase 1 alpha chain  *Crystal structure of NarGHI mutant NarG-R94S* | 0.32 | 0.00 | 26.67 | 0.50 | 74-383 | X-ray | 2.50 | monomer | 2 x MD1, 4 x SF4, 1 x 6MO, 1 x AGA, 1 x F3S, 2 x HEM | HHblits | 0.32 |
| ``` target    EVLARVGHKLAEQTGDARFADVWKLVDEKRTDAHLQRILDHSSNTKGYDALDLEAKAKKGIPTLMMNRTYPKAVGYEQVA 3ir7.1    -------------------------------------------------------------------------AGYTNVH  target    DSRPWYTKSGRLEFYRDEDEFIEAGENLPVHREPIDSTFYEPNVIVSAPHEALRPAGPEDYGVELSDMSGEIRQGRNVVK 3ir7.1    ELIPWRTLSGRQQLYQDHQWMRDFGESLLVYRPPIDTRSVKE--------------------------------------  target    AWAELKKTPHPLAKDGYRFVFHTPKYRHGAHTMPIDTDMVAMLFGPFGDVYRHDRRTPYVAEGYVDIHPSDAREIGVEDG 3ir7.1    ----VIGQKSNG-NQEKALNFLTPHQKWGIHSTYSDNLLMLTLG---------------RGGPVVWLSEADAKDLGIADN  target    DYVFIDSDPEDRPFRGWQKNKRDYEFSRLLCRARYYPGTPRGVTRMWFNMYGATPGSVEGQKSREDGLAKNPRTNYQAMF 3ir7.1    DWIEVFNSN-----------------GALTARAVVSQRVPAGMTMMYHAQ----ERIVNLPGSEI------------TQQ  target    RSGSHQSATRGWLKPTWMTDSLVRKGLFGQSIGKGFLPDVHCPTGAPRESIVKITKAEPGGLGAEGLWRPAALGLRPGYE 3ir7.1    RGGIHNSVTRITPKPTHMI------GGYAHL---AYGFNYYGTVGSNRDEFVVVRKMKNIDWL-----------------  target    SKSMKTYLDGGYVDDADRQGGQG 3ir7.1    ----------------------- ``` | | | | | | | | | | | | | | | | | | | | | | | | | | | | | | | | | | | | | | | | | | | | | | | | | |
|  | 3egw.1.A | Respiratory nitrate reductase 1 alpha chain  *The crystal structure of the NarGHI mutant NarH - C16A* | 0.33 | 0.00 | 27.54 | 0.49 | 74-380 | X-ray | 1.90 | monomer | 2 x MD1, 2 x MGD, 2 x 6MO, 6 x SF4, 4 x F3S, 2 x 3PH, 4 x HEM, 2 x AGA | HHblits | 0.33 |
| ``` target    EVLARVGHKLAEQTGDARFADVWKLVDEKRTDAHLQRILDHSSNTKGYDALDLEAKAKKGIPTLMMNRTYPKAVGYEQVA 3egw.1    -------------------------------------------------------------------------AGYTNVH  target    DSRPWYTKSGRLEFYRDEDEFIEAGENLPVHREPIDSTFYEPNVIVSAPHEALRPAGPEDYGVELSDMSGEIRQGRNVVK 3egw.1    ELIPWRTLSGRQQLYQDHQWMRDFGESLLVYRPPIDTRSVKE-----------------VIG------------------  target    AWAELKKTPHPLAKDGYRFVFHTPKYRHGAHTMPIDTDMVAMLFGPFGDVYRHDRRTPYVAEGYVDIHPSDAREIGVEDG 3egw.1    -------QKSN-GNQEKALNFLTPHQKWGIHSTYSDNLLMLTLG---------------RGGPVVWLSEADAKDLGIADN  target    DYVFIDSDPEDRPFRGWQKNKRDYEFSRLLCRARYYPGTPRGVTRMWFNMYGATPGSVEGQKSREDGLAKNPRTNYQAMF 3egw.1    DWIEVFNSN-----------------GALTARAVVSQRVPAGMTMMYHAQ----ERIVNLPGSEI------------TQQ  target    RSGSHQSATRGWLKPTWMTDSLVRKGLFGQSIGKGFLPDVHCPTGAPRESIVKITKAEPGGLGAEGLWRPAALGLRPGYE 3egw.1    RGGIHNSVTRITPKPTHMI------GGYAHL---AYGFNYYGTVGSNRDEFVVVRKMKNI--------------------  target    SKSMKTYLDGGYVDDADRQGGQG 3egw.1    ----------------------- ``` | | | | | | | | | | | | | | | | | | | | | | | | | | | | | | | | | | | | | | | | | | | | | | | | | |
|  | 3ir6.1.A | Respiratory nitrate reductase 1 alpha chain  *Crystal structure of NarGHI mutant NarG-H49S* | 0.32 | 0.00 | 27.05 | 0.49 | 74-380 | X-ray | 2.80 | monomer | 2 x GDP, 1 x AGA, 3 x SF4, 1 x F3S, 2 x HEM | HHblits | 0.33 |
| ``` target    EVLARVGHKLAEQTGDARFADVWKLVDEKRTDAHLQRILDHSSNTKGYDALDLEAKAKKGIPTLMMNRTYPKAVGYEQVA 3ir6.1    -------------------------------------------------------------------------AGYTNVH  target    DSRPWYTKSGRLEFYRDEDEFIEAGENLPVHREPIDSTFYEPNVIVSAPHEALRPAGPEDYGVELSDMSGEIRQGRNVVK 3ir6.1    ELIPWRTLSGRQQLYQDHQWMRDFGESLLVYRPPIDTRSVKE--------------------------------------  target    AWAELKKTPHPLAKDGYRFVFHTPKYRHGAHTMPIDTDMVAMLFGPFGDVYRHDRRTPYVAEGYVDIHPSDAREIGVEDG 3ir6.1    ----VIGQKSNG-NQEKALNFLTPHQKWGIHSTYSDNLLMLTLG---------------RGGPVVWLSEADAKDLGIADN  target    DYVFIDSDPEDRPFRGWQKNKRDYEFSRLLCRARYYPGTPRGVTRMWFNMYGATPGSVEGQKSREDGLAKNPRTNYQAMF 3ir6.1    DWIEVFNSN-----------------GALTARAVVSQRVPAGMTMMYHAQ----ERIVNLPGSE---------I---TQQ  target    RSGSHQSATRGWLKPTWMTDSLVRKGLFGQSIGKGFLPDVHCPTGAPRESIVKITKAEPGGLGAEGLWRPAALGLRPGYE 3ir6.1    RGGIHNSVTRITPKPTHMI------GGYAHL---AYGFNYYGTVGSNRDEFVVVRKMKNI--------------------  target    SKSMKTYLDGGYVDDADRQGGQG 3ir6.1    ----------------------- ``` | | | | | | | | | | | | | | | | | | | | | | | | | | | | | | | | | | | | | | | | | | | | | | | | | |
|  | 2e7z.1.A | Acetylene hydratase Ahy  *Acetylene Hydratase from Pelobacter acetylenicus* | 0.12 |  | 17.97 | 0.30 | 81-292 | X-ray | 1.26 | monomer | 1 x SF4, 2 x MGD, 1 x W | HHblits | 0.31 |
| ``` target    EVLARVGHKLAEQTGDARFADVWKLVDEKRTDAHLQRILDHSSNTKGYDALDLEAKAKKGIPTLMMNRTYPKAVGYEQVA 2e7z.1    --------------------------------------------------------------------------------  target    DSRPWYTKSGRLEFYRDEDEFIEAGE-NLPVHREPIDSTFYEPNVIVSAPHEALRPAGPEDYGVELSDMSGEIRQGRNVV 2e7z.1    EGVGVATPSGKVELYSS--VFEKLGYDPLPYYHEPLQTEIS---------------------------------------  target    KAWAELKKTPHPLAKDGYRFVFHTPKYR-HGAHTMPIDTDMVAMLFGPFGDVYRHDRRTPYVAEGYVDIHPSDAREIGVE 2e7z.1    ----------DPELAKEYPLILFAGLREDSNFQSCYHQPGILRDA----------------EPDPVALLHPKTAQSLGLP  target    DGDYVFIDSDPEDRPFRGWQKNKRDYEFSRLLCRARYYPGTPRGVTRMWFNMYGATPGSVEGQKSREDGLAKNPRTNYQA 2e7z.1    SGEWIWVETTH-----------------GRLKLLLKHDGAQPEGTIRIPHGRWC--------------------------  target    MFRSGSHQSATRGWLKPTWMTDSLVRKGLFGQSIGKGFLPDVHCPTGAPRESIVKITKAEPGGLGAEGLWRPAALGLRPG 2e7z.1    --------------------------------------------------------------------------------  target    YESKSMKTYLDGGYVDDADRQGGQG 2e7z.1    ------------------------- ``` | | | | | | | | | | | | | | | | | | | | | | | | | | | | | | | | | | | | | | | | | | | | | | | | | |
|  | 4ydd.1.A | DMSO reductase family type II enzyme, molybdopterin subunit  *Crystal structure of the perchlorate reductase PcrAB from Azospira suillum PS* | 0.07 | 0.00 | 37.65 | 0.20 | 32-120 | X-ray | 1.86 | monomer | 4 x SF4, 1 x MO, 1 x MGD, 1 x MD1, 1 x F3S | BLAST | 0.39 |
| ``` target    EVLARVGHKLAEQTGDARFADVWKLVDEKRTDAHLQRILDHSSNTKGYDALDLEAKAKKGIPTLMMNRTYPKAVGYEQ-- 4ydd.1    -------------------------------EAAAQYILDNAPQSKGITIQMLREKPQR----FKSNWTSPLKEGVPYTP  target    ----VADSRPWYTKSGRLEFYRDEDEFIEAGENLPVHREPIDSTFYEPNVIVSAPHEALRPAGPEDYGVELSDMSGEIRQ 4ydd.1    FQYFVVDKKPWPTLTGRQQFYLDHDTFFDMGVELPTYKAPIDADKY----------------------------------  target    GRNVVKAWAELKKTPHPLAKDGYRFVFHTPKYRHGAHTMPIDTDMVAMLFGPFGDVYRHDRRTPYVAEGYVDIHPSDARE 4ydd.1    --------------------------------------------------------------------------------  target    IGVEDGDYVFIDSDPEDRPFRGWQKNKRDYEFSRLLCRARYYPGTPRGVTRMWFNMYGATPGSVEGQKSREDGLAKNPRT 4ydd.1    --------------------------------------------------------------------------------  target    NYQAMFRSGSHQSATRGWLKPTWMTDSLVRKGLFGQSIGKGFLPDVHCPTGAPRESIVKITKAEPGGLGAEGLWRPAALG 4ydd.1    --------------------------------------------------------------------------------  target    LRPGYESKSMKTYLDGGYVDDADRQGGQG 4ydd.1    ----------------------------- ``` | | | | | | | | | | | | | | | | | | | | | | | | | | | | | | | | | | | | | | | | | | | | | | | | | |
|  | 5e7o.1.A | DMSO reductase family type II enzyme, molybdopterin subunit  *Crystal structure of the perchlorate reductase PcrAB mutant W461E of PcrA from Azospira suillum PS* | 0.07 | 0.00 | 37.65 | 0.20 | 32-120 | X-ray | 2.40 | monomer | 4 x SF4, 1 x MO, 1 x MGD, 1 x MD1, 1 x F3S | BLAST | 0.39 |
| ``` target    EVLARVGHKLAEQTGDARFADVWKLVDEKRTDAHLQRILDHSSNTKGYDALDLEAKAKKGIPTLMMNRTYPKAVGYEQ-- 5e7o.1    -------------------------------EAAAQYILDNAPQSKGITIQMLREKPQR----FKSNWTSPLKEGVPYTP  target    ----VADSRPWYTKSGRLEFYRDEDEFIEAGENLPVHREPIDSTFYEPNVIVSAPHEALRPAGPEDYGVELSDMSGEIRQ 5e7o.1    FQYFVVDKKPWPTLTGRQQFYLDHDTFFDMGVELPTYKAPIDADKY----------------------------------  target    GRNVVKAWAELKKTPHPLAKDGYRFVFHTPKYRHGAHTMPIDTDMVAMLFGPFGDVYRHDRRTPYVAEGYVDIHPSDARE 5e7o.1    --------------------------------------------------------------------------------  target    IGVEDGDYVFIDSDPEDRPFRGWQKNKRDYEFSRLLCRARYYPGTPRGVTRMWFNMYGATPGSVEGQKSREDGLAKNPRT 5e7o.1    --------------------------------------------------------------------------------  target    NYQAMFRSGSHQSATRGWLKPTWMTDSLVRKGLFGQSIGKGFLPDVHCPTGAPRESIVKITKAEPGGLGAEGLWRPAALG 5e7o.1    --------------------------------------------------------------------------------  target    LRPGYESKSMKTYLDGGYVDDADRQGGQG 5e7o.1    ----------------------------- ``` | | | | | | | | | | | | | | | | | | | | | | | | | | | | | | | | | | | | | | | | | | | | | | | | | |
|  | 6cz7.1.A | ArrA  *The arsenate respiratory reductase (Arr) complex from Shewanella sp. ANA-3* | 0.09 |  | 18.68 | 0.22 | 175-290 | X-ray | 1.62 | hetero-1-1-mer | 5 x SF4, 2 x MGD, 1 x MO, 1 x PG5 | HHblits | 0.30 |
| ``` target    EVLARVGHKLAEQTGDARFADVWKLVDEKRTDAHLQRILDHSSNTKGYDALDLEAKAKKGIPTLMMNRTYPKAVGYEQVA 6cz7.1    --------------------------------------------------------------------------------  target    DSRPWYTKSGRLEFYRDEDEFIEAGENLPVHREPIDSTFYEPNVIVSAPHEALRPAGPEDYGVELSDMSGEIRQGRNVVK 6cz7.1    --------------------------------------------------------------------------------  target    AWAELKKTPHPLAKDGYRFVFHTPKYRHGAHTMPIDTDMVAMLFGPFGDVYRHDRRTPYVAEGYVDIHPSDAREIGVEDG 6cz7.1    --------------SEFPLLLVDQKSRLNKEGRTANSPWYYEFKDV--------DPGDVANEDVAKFNPIDGKKFGLKDG  target    DYVFIDSDPEDRPFRGWQKNKRDYEFSRLLCRARYYPGTPRGVTRMWFNMYGATPGSVEGQKSREDGLAKNPRTNYQAMF 6cz7.1    DEIRITSPV-----------------GMLTCKAKLWEGVRPGTVAKCFGQ------------------------------  target    RSGSHQSATRGWLKPTWMTDSLVRKGLFGQSIGKGFLPDVHCPTGAPRESIVKITKAEPGGLGAEGLWRPAALGLRPGYE 6cz7.1    --------------------------------------------------------------------------------  target    SKSMKTYLDGGYVDDADRQGGQG 6cz7.1    ----------------------- ``` | | | | | | | | | | | | | | | | | | | | | | | | | | | | | | | | | | | | | | | | | | | | | | | | | |
|  | 4v4c.1.A | Pyrogallol hydroxytransferase large subunit  *Crystal Structure of Pyrogallol-Phloroglucinol Transhydroxylase from Pelobacter acidigallici* | 0.09 |  | 17.39 | 0.22 | 175-290 | X-ray | 2.35 | hetero-oligomer | 2 x CA, 2 x MGD, 1 x 4MO, 3 x SF4 | HHblits | 0.29 |
| ``` target    EVLARVGHKLAEQTGDARFADVWKLVDEKRTDAHLQRILDHSSNTKGYDALDLEAKAKKGIPTLMMNRTYPKAVGYEQVA 4v4c.1    --------------------------------------------------------------------------------  target    DSRPWYTKSGRLEFYRDEDEFIEAGENLPVHREPIDSTFYEPNVIVSAPHEALRPAGPEDYGVELSDMSGEIRQGRNVVK 4v4c.1    --------------------------------------------------------------------------------  target    AWAELKKTPHPLAKDGYRFVFHTPKYRHGAHTMPID-TDMVAMLFGPFGDVYRHDRRTPYVAEGYVDIHPSDAREIGVED 4v4c.1    --------------VKYPLGMLSPHPRFSMHTMGDGKNSYMNYIK----DH-RV--EVDGYKYWIMRVNSIDAEARGIKN  target    GDYVFIDSDPEDRPFRGWQKNKRDYEFSRLLCRARYYPGTPRGVTRMWFNMYGATPGSVEGQKSREDGLAKNPRTNYQAM 4v4c.1    GDLIRAYNDR-----------------GSVILAAQVTECLQPGTVHSYESC-----------------------------  target    FRSGSHQSATRGWLKPTWMTDSLVRKGLFGQSIGKGFLPDVHCPTGAPRESIVKITKAEPGGLGAEGLWRPAALGLRPGY 4v4c.1    --------------------------------------------------------------------------------  target    ESKSMKTYLDGGYVDDADRQGGQG 4v4c.1    ------------------------ ``` | | | | | | | | | | | | | | | | | | | | | | | | | | | | | | | | | | | | | | | | | | | | | | | | | |
|  | 6lod.1.B | Fe-S-cluster-containing hydrogenase components 1-like protein  *Cryo-EM structure of the air-oxidized photosynthetic alternative complex III from Roseiflexus castenholzii* | 0.07 |  | 14.13 | 0.22 | 175-294 | EM | 0.00 | hetero-1-1-1-1-1-1-… | 6 x HEC, 2 x EL6, 3 x SF4, 1 x F3S | HHblits | 0.27 |
| ``` target    EVLARVGHKLAEQTGDARFADVWKLVDEKRTDAHLQRILDHSSNTKGYDALDLEAKAKKGIPTLMMNRTYPKAVGYEQVA 6lod.1    --------------------------------------------------------------------------------  target    DSRPWYTKSGRLEFYRDEDEFIEAGENLPVHREPIDSTFYEPNVIVSAPHEALRPAGPEDYGVELSDMSGEIRQGRNVVK 6lod.1    --------------------------------------------------------------------------------  target    AWAELKKTPHPLAKDGYRFVFHTPKYRHGAHTMPIDTDMVAMLFGPFGDVYRHDRRTPYVAEGYVDIHPSDAREIGVEDG 6lod.1    --------------QGLEIVFRPDP--SLWDGAFANNAWLQETPKP----YTK-----LTWDNVALMSVRTANALGLKNG  target    DYVFIDSDPEDRPFRGWQKNKRDYEFSRLLCRARYYPGTPRGVTRMWFNMYGATPGSVEGQKSREDGLAKNPRTNYQAMF 6lod.1    DVVRLTYQG-----------------RSVDAPVWVQPGHADDSVTVHFGFGRTA--------------------------  target    RSGSHQSATRGWLKPTWMTDSLVRKGLFGQSIGKGFLPDVHCPTGAPRESIVKITKAEPGGLGAEGLWRPAALGLRPGYE 6lod.1    --------------------------------------------------------------------------------  target    SKSMKTYLDGGYVDDADRQGGQG 6lod.1    ----------------------- ``` | | | | | | | | | | | | | | | | | | | | | | | | | | | | | | | | | | | | | | | | | | | | | | | | | |
|  | 1aa6.1.A | FORMATE DEHYDROGENASE H  *REDUCED FORM OF FORMATE DEHYDROGENASE H FROM E. COLI* | 0.08 | 0.00 | 25.00 | 0.20 | 175-290 | X-ray | 2.30 | monomer | 1 x SF4, 2 x MGD, 1 x 4MO | HHblits | 0.33 |
| ``` target    EVLARVGHKLAEQTGDARFADVWKLVDEKRTDAHLQRILDHSSNTKGYDALDLEAKAKKGIPTLMMNRTYPKAVGYEQVA 1aa6.1    --------------------------------------------------------------------------------  target    DSRPWYTKSGRLEFYRDEDEFIEAGENLPVHREPIDSTFYEPNVIVSAPHEALRPAGPEDYGVELSDMSGEIRQGRNVVK 1aa6.1    --------------------------------------------------------------------------------  target    AWAELKKTPHPLAKDGYRFVFHTPKYR--HGAHTMPIDTDMVAMLFGPFGDVYRHDRRTPYVAEGYVDIHPSDAREIGVE 1aa6.1    --------------DEYPMVLSTVREVGHYSCRSMTGNCAALAALA----------DE-----PGYAQINTEDAKRLGIE  target    DGDYVFIDSDPEDRPFRGWQKNKRDYEFSRLLCRARYYPGTPRGVTRMWFNMYGATPGSVEGQKSREDGLAKNPRTNYQA 1aa6.1    DEALVWVHSRK-----------------GKIITRAQVSDRPNKGAIYMTYQW----------------------------  target    MFRSGSHQSATRGWLKPTWMTDSLVRKGLFGQSIGKGFLPDVHCPTGAPRESIVKITKAEPGGLGAEGLWRPAALGLRPG 1aa6.1    --------------------------------------------------------------------------------  target    YESKSMKTYLDGGYVDDADRQGGQG 1aa6.1    ------------------------- ``` | | | | | | | | | | | | | | | | | | | | | | | | | | | | | | | | | | | | | | | | | | | | | | | | | |
|  | 1fdo.1.A | FORMATE DEHYDROGENASE H  *OXIDIZED FORM OF FORMATE DEHYDROGENASE H FROM E. COLI* | 0.08 | 0.00 | 25.00 | 0.20 | 175-290 | X-ray | 2.80 | monomer | 1 x SF4, 2 x MGD, 1 x 6MO | HHblits | 0.33 |
| ``` target    EVLARVGHKLAEQTGDARFADVWKLVDEKRTDAHLQRILDHSSNTKGYDALDLEAKAKKGIPTLMMNRTYPKAVGYEQVA 1fdo.1    --------------------------------------------------------------------------------  target    DSRPWYTKSGRLEFYRDEDEFIEAGENLPVHREPIDSTFYEPNVIVSAPHEALRPAGPEDYGVELSDMSGEIRQGRNVVK 1fdo.1    --------------------------------------------------------------------------------  target    AWAELKKTPHPLAKDGYRFVFHTPKYR--HGAHTMPIDTDMVAMLFGPFGDVYRHDRRTPYVAEGYVDIHPSDAREIGVE 1fdo.1    --------------DEYPMVLSTVREVGHYSCRSMTGNCAALAALA----------DE-----PGYAQINTEDAKRLGIE  target    DGDYVFIDSDPEDRPFRGWQKNKRDYEFSRLLCRARYYPGTPRGVTRMWFNMYGATPGSVEGQKSREDGLAKNPRTNYQA 1fdo.1    DEALVWVHSRK-----------------GKIITRAQVSDRPNKGAIYMTYQW----------------------------  target    MFRSGSHQSATRGWLKPTWMTDSLVRKGLFGQSIGKGFLPDVHCPTGAPRESIVKITKAEPGGLGAEGLWRPAALGLRPG 1fdo.1    --------------------------------------------------------------------------------  target    YESKSMKTYLDGGYVDDADRQGGQG 1fdo.1    ------------------------- ``` | | | | | | | | | | | | | | | | | | | | | | | | | | | | | | | | | | | | | | | | | | | | | | | | | |
|  | 2iv2.1.A | Formate dehydrogenase H  *Reinterpretation of reduced form of formate dehydrogenase H from E. coli* | 0.08 | 0.00 | 25.00 | 0.20 | 175-290 | X-ray | 2.27 | monomer | 1 x SF4, 1 x 2MD, 1 x MGD | HHblits | 0.33 |
| ``` target    EVLARVGHKLAEQTGDARFADVWKLVDEKRTDAHLQRILDHSSNTKGYDALDLEAKAKKGIPTLMMNRTYPKAVGYEQVA 2iv2.1    --------------------------------------------------------------------------------  target    DSRPWYTKSGRLEFYRDEDEFIEAGENLPVHREPIDSTFYEPNVIVSAPHEALRPAGPEDYGVELSDMSGEIRQGRNVVK 2iv2.1    --------------------------------------------------------------------------------  target    AWAELKKTPHPLAKDGYRFVFHTPKYR--HGAHTMPIDTDMVAMLFGPFGDVYRHDRRTPYVAEGYVDIHPSDAREIGVE 2iv2.1    --------------DEYPMVLSTVREVGHYSCRSMTGNCAALAALA----------DE-----PGYAQINTEDAKRLGIE  target    DGDYVFIDSDPEDRPFRGWQKNKRDYEFSRLLCRARYYPGTPRGVTRMWFNMYGATPGSVEGQKSREDGLAKNPRTNYQA 2iv2.1    DEALVWVHSRK-----------------GKIITRAQVSDRPNKGAIYMTYQW----------------------------  target    MFRSGSHQSATRGWLKPTWMTDSLVRKGLFGQSIGKGFLPDVHCPTGAPRESIVKITKAEPGGLGAEGLWRPAALGLRPG 2iv2.1    --------------------------------------------------------------------------------  target    YESKSMKTYLDGGYVDDADRQGGQG 2iv2.1    ------------------------- ``` | | | | | | | | | | | | | | | | | | | | | | | | | | | | | | | | | | | | | | | | | | | | | | | | | |
|  | 7z0t.1.G | Formate dehydrogenase H  *Structure of the Escherichia coli formate hydrogenlyase complex (aerobic preparation, composite structure)* | 0.08 | 0.00 | 25.00 | 0.20 | 175-290 | EM | 0.00 | monomer | 1 x NI, 1 x FCO, 8 x SF4, 1 x FE, 2 x MGD, 1 x 6MO | HHblits | 0.33 |
| ``` target    EVLARVGHKLAEQTGDARFADVWKLVDEKRTDAHLQRILDHSSNTKGYDALDLEAKAKKGIPTLMMNRTYPKAVGYEQVA 7z0t.1    --------------------------------------------------------------------------------  target    DSRPWYTKSGRLEFYRDEDEFIEAGENLPVHREPIDSTFYEPNVIVSAPHEALRPAGPEDYGVELSDMSGEIRQGRNVVK 7z0t.1    --------------------------------------------------------------------------------  target    AWAELKKTPHPLAKDGYRFVFHTPKYR--HGAHTMPIDTDMVAMLFGPFGDVYRHDRRTPYVAEGYVDIHPSDAREIGVE 7z0t.1    --------------DEYPMVLSTVREVGHYSCRSMTGNCAALAALA----------DE-----PGYAQINTEDAKRLGIE  target    DGDYVFIDSDPEDRPFRGWQKNKRDYEFSRLLCRARYYPGTPRGVTRMWFNMYGATPGSVEGQKSREDGLAKNPRTNYQA 7z0t.1    DEALVWVHSRK-----------------GKIITRAQVSDRPNKGAIYMTYQW----------------------------  target    MFRSGSHQSATRGWLKPTWMTDSLVRKGLFGQSIGKGFLPDVHCPTGAPRESIVKITKAEPGGLGAEGLWRPAALGLRPG 7z0t.1    --------------------------------------------------------------------------------  target    YESKSMKTYLDGGYVDDADRQGGQG 7z0t.1    ------------------------- ``` | | | | | | | | | | | | | | | | | | | | | | | | | | | | | | | | | | | | | | | | | | | | | | | | | |
|  | 6f0k.1.B | Fe-S-cluster-containing hydrogenase  *Alternative complex III* | 0.06 |  | 13.04 | 0.22 | 177-294 | EM | 0.00 | hetero-1-1-1-1-1-1-… | 6 x HEC, 1 x F3S, 3 x SF4 | HHblits | 0.25 |
| ``` target    EVLARVGHKLAEQTGDARFADVWKLVDEKRTDAHLQRILDHSSNTKGYDALDLEAKAKKGIPTLMMNRTYPKAVGYEQVA 6f0k.1    --------------------------------------------------------------------------------  target    DSRPWYTKSGRLEFYRDEDEFIEAGENLPVHREPIDSTFYEPNVIVSAPHEALRPAGPEDYGVELSDMSGEIRQGRNVVK 6f0k.1    --------------------------------------------------------------------------------  target    AWAELKKTPHPLAKDGYRFVFHTPKYRHGAHTMPIDTDMVAMLFGPFGDVYRHDRRTPYVAEGYVDIHPSDAREIGVED- 6f0k.1    ----------------GGLEVVFRLDPTVLDGSFANNAWAQELPDP----ITK-----IVWDNVAILSPKTAAALGVKAE  target    -------GDYVFIDSDPEDRPFRGWQKNKRDYEFSRLLCRARYYPGTPRGVTRMWFNMYGATPGSVEGQKSREDGLAKNP 6f0k.1    YHKGVYIADVIELSLDG-----------------RAVELPVWVLPGHPDDSITVYLGYGREI------------------  target    RTNYQAMFRSGSHQSATRGWLKPTWMTDSLVRKGLFGQSIGKGFLPDVHCPTGAPRESIVKITKAEPGGLGAEGLWRPAA 6f0k.1    --------------------------------------------------------------------------------  target    LGLRPGYESKSMKTYLDGGYVDDADRQGGQG 6f0k.1    ------------------------------- ``` | | | | | | | | | | | | | | | | | | | | | | | | | | | | | | | | | | | | | | | | | | | | | | | | | |
|  | 1tmo.1.A | TRIMETHYLAMINE N-OXIDE REDUCTASE  *TRIMETHYLAMINE N-OXIDE REDUCTASE FROM SHEWANELLA MASSILIA* | 0.08 |  | 22.09 | 0.20 | 175-290 | X-ray | 2.50 | monomer | 2 x 2MD, 1 x 2MO | HHblits | 0.31 |
| ``` target    EVLARVGHKLAEQTGDARFADVWKLVDEKRTDAHLQRILDHSSNTKGYDALDLEAKAKKGIPTLMMNRTYPKAVGYEQVA 1tmo.1    --------------------------------------------------------------------------------  target    DSRPWYTKSGRLEFYRDEDEFIEAGENLPVHREPIDSTFYEPNVIVSAPHEALRPAGPEDYGVELSDMSGEIRQGRNVVK 1tmo.1    --------------------------------------------------------------------------------  target    AWAELKKTPHPLAKDGYRFVFHTPKYRHGAHTMPIDTDMVAMLFGPFGDVYRHDRRTPYVAEGYVDIHPSDAREIGVEDG 1tmo.1    --------------DKHPIWLQSCHPDKRLHSQMCESREYRETY-------AV------NGREPVYISPVDAKARGIKDG  target    DYVFIDSDPEDRPFRGWQKNKRDYEFSRLLCRARYYPGTPRGVTRMWFNMYGATPGSVEGQKSREDGLAKNPRTNYQAMF 1tmo.1    DIVRVFNDR-----------------GQLLAGAVVSDNFPKGIVRIHEGA------------------------------  target    RSGSHQSATRGWLKPTWMTDSLVRKGLFGQSIGKGFLPDVHCPTGAPRESIVKITKAEPGGLGAEGLWRPAALGLRPGYE 1tmo.1    --------------------------------------------------------------------------------  target    SKSMKTYLDGGYVDDADRQGGQG 1tmo.1    ----------------------- ``` | | | | | | | | | | | | | | | | | | | | | | | | | | | | | | | | | | | | | | | | | | | | | | | | | |
|  | 7l5i.1.A | Trimethylamine-N-oxide reductase  *Crystal Structure of Haemophilus influenzae MtsZ at pH 7.0* | 0.10 | 0.00 | 24.71 | 0.20 | 175-290 | X-ray | 1.73 | monomer | 2 x MGD, 1 x MO, 1 x O | HHblits | 0.31 |
| ``` target    EVLARVGHKLAEQTGDARFADVWKLVDEKRTDAHLQRILDHSSNTKGYDALDLEAKAKKGIPTLMMNRTYPKAVGYEQVA 7l5i.1    --------------------------------------------------------------------------------  target    DSRPWYTKSGRLEFYRDEDEFIEAGENLPVHREPIDSTFYEPNVIVSAPHEALRPAGPEDYGVELSDMSGEIRQGRNVVK 7l5i.1    --------------------------------------------------------------------------------  target    AWAELKKTPHPLAKDGYRFVFHTPKYRHGAHTMPIDTDMVAMLFGPFGDVYRHDRRTPYVAEGYVDIHPSDAREIGVEDG 7l5i.1    --------------EEYPLALVTPHPYYRLHSQLAHTSLRQKYA--------V------NDREPVMIHPEDAAARGIKDG  target    DYVFIDSDPEDRPFRGWQKNKRDYEFSRLLCRARYYPGTPRGVTRMWFNMYGATPGSVEGQKSREDGLAKNPRTNYQAMF 7l5i.1    DIVRIHSKR-----------------GQVLAGAAVTENIIKGTVALHEGA------------------------------  target    RSGSHQSATRGWLKPTWMTDSLVRKGLFGQSIGKGFLPDVHCPTGAPRESIVKITKAEPGGLGAEGLWRPAALGLRPGYE 7l5i.1    --------------------------------------------------------------------------------  target    SKSMKTYLDGGYVDDADRQGGQG 7l5i.1    ----------------------- ``` | | | | | | | | | | | | | | | | | | | | | | | | | | | | | | | | | | | | | | | | | | | | | | | | | |
|  | 7l5s.1.A | Trimethylamine-N-oxide reductase  *Crystal Structure of Haemophilus influenzae MtsZ at pH 5.5* | 0.10 | 0.00 | 24.71 | 0.20 | 175-290 | X-ray | 2.09 | monomer | 1 x O, 2 x MGD, 1 x MO | HHblits | 0.31 |
| ``` target    EVLARVGHKLAEQTGDARFADVWKLVDEKRTDAHLQRILDHSSNTKGYDALDLEAKAKKGIPTLMMNRTYPKAVGYEQVA 7l5s.1    --------------------------------------------------------------------------------  target    DSRPWYTKSGRLEFYRDEDEFIEAGENLPVHREPIDSTFYEPNVIVSAPHEALRPAGPEDYGVELSDMSGEIRQGRNVVK 7l5s.1    --------------------------------------------------------------------------------  target    AWAELKKTPHPLAKDGYRFVFHTPKYRHGAHTMPIDTDMVAMLFGPFGDVYRHDRRTPYVAEGYVDIHPSDAREIGVEDG 7l5s.1    --------------EEYPLALVTPHPYYRLHSQLAHTSLRQKYA--------V------NDREPVMIHPEDAAARGIKDG  target    DYVFIDSDPEDRPFRGWQKNKRDYEFSRLLCRARYYPGTPRGVTRMWFNMYGATPGSVEGQKSREDGLAKNPRTNYQAMF 7l5s.1    DIVRIHSKR-----------------GQVLAGAAVTENIIKGTVALHEGA------------------------------  target    RSGSHQSATRGWLKPTWMTDSLVRKGLFGQSIGKGFLPDVHCPTGAPRESIVKITKAEPGGLGAEGLWRPAALGLRPGYE 7l5s.1    --------------------------------------------------------------------------------  target    SKSMKTYLDGGYVDDADRQGGQG 7l5s.1    ----------------------- ``` | | | | | | | | | | | | | | | | | | | | | | | | | | | | | | | | | | | | | | | | | | | | | | | | | |
|  | 4aay.1.A | AROA  *Crystal Structure of the arsenite oxidase protein complex from Rhizobium species strain NT-26* | 0.08 | 0.00 | 24.10 | 0.20 | 175-290 | X-ray | 2.70 | monomer | 4 x MGD, 2 x O, 2 x 4MO, 2 x F3S, 2 x FES | HHblits | 0.33 |
| ``` target    EVLARVGHKLAEQTGDARFADVWKLVDEKRTDAHLQRILDHSSNTKGYDALDLEAKAKKGIPTLMMNRTYPKAVGYEQVA 4aay.1    --------------------------------------------------------------------------------  target    DSRPWYTKSGRLEFYRDEDEFIEAGENLPVHREPIDSTFYEPNVIVSAPHEALRPAGPEDYGVELSDMSGEIRQGRNVVK 4aay.1    --------------------------------------------------------------------------------  target    AWAELKKTPHPLAKDGYRFVFHTPKYRHGAHTMPID--TDMVAMLFGPFGDVYRHDRRTPYVAEGYVDIHPSDAREIGVE 4aay.1    --------------DSHKYLINNGRANVVWQSAYLDQENDFVMDR----------------FPYPFIEMNPEDMAEAGLK  target    DGDYVFIDSDPEDRPFRGWQKNKRDYEFSRLLCRARYYPGTPRGVTRMWFNMYGATPGSVEGQKSREDGLAKNPRTNYQA 4aay.1    EGDLVEIYNDA-----------------GATQAMAYPTPTARRGETFMLFGF----------------------------  target    MFRSGSHQSATRGWLKPTWMTDSLVRKGLFGQSIGKGFLPDVHCPTGAPRESIVKITKAEPGGLGAEGLWRPAALGLRPG 4aay.1    --------------------------------------------------------------------------------  target    YESKSMKTYLDGGYVDDADRQGGQG 4aay.1    ------------------------- ``` | | | | | | | | | | | | | | | | | | | | | | | | | | | | | | | | | | | | | | | | | | | | | | | | | |
|  | 5nqd.1.A | AroA  *Arsenite oxidase AioAB from Rhizobium sp. str. NT-26 mutant AioBF108A* | 0.08 | 0.00 | 24.10 | 0.20 | 175-290 | X-ray | 2.20 | monomer | 4 x MGD, 2 x O, 2 x 4MO, 2 x F3S, 2 x FES | HHblits | 0.33 |
| ``` target    EVLARVGHKLAEQTGDARFADVWKLVDEKRTDAHLQRILDHSSNTKGYDALDLEAKAKKGIPTLMMNRTYPKAVGYEQVA 5nqd.1    --------------------------------------------------------------------------------  target    DSRPWYTKSGRLEFYRDEDEFIEAGENLPVHREPIDSTFYEPNVIVSAPHEALRPAGPEDYGVELSDMSGEIRQGRNVVK 5nqd.1    --------------------------------------------------------------------------------  target    AWAELKKTPHPLAKDGYRFVFHTPKYRHGAHTMPID--TDMVAMLFGPFGDVYRHDRRTPYVAEGYVDIHPSDAREIGVE 5nqd.1    --------------DSHKYLINNGRANVVWQSAYLDQENDFVMDR----------------FPYPFIEMNPEDMAEAGLK  target    DGDYVFIDSDPEDRPFRGWQKNKRDYEFSRLLCRARYYPGTPRGVTRMWFNMYGATPGSVEGQKSREDGLAKNPRTNYQA 5nqd.1    EGDLVEIYNDA-----------------GATQAMAYPTPTARRGETFMLFGF----------------------------  target    MFRSGSHQSATRGWLKPTWMTDSLVRKGLFGQSIGKGFLPDVHCPTGAPRESIVKITKAEPGGLGAEGLWRPAALGLRPG 5nqd.1    --------------------------------------------------------------------------------  target    YESKSMKTYLDGGYVDDADRQGGQG 5nqd.1    ------------------------- ``` | | | | | | | | | | | | | | | | | | | | | | | | | | | | | | | | | | | | | | | | | | | | | | | | | |
|  | 1e5v.2.A | Dimethyl sulfoxide/trimethylamine N-oxide reductase  *OXIDIZED DMSO REDUCTASE EXPOSED TO HEPES BUFFER* | 0.08 | 0.00 | 17.44 | 0.20 | 175-291 | X-ray | 2.40 | monomer | 2 x PGD, 1 x 2MO | HHblits | 0.30 |
| ``` target    EVLARVGHKLAEQTGDARFADVWKLVDEKRTDAHLQRILDHSSNTKGYDALDLEAKAKKGIPTLMMNRTYPKAVGYEQVA 1e5v.2    --------------------------------------------------------------------------------  target    DSRPWYTKSGRLEFYRDEDEFIEAGENLPVHREPIDSTFYEPNVIVSAPHEALRPAGPEDYGVELSDMSGEIRQGRNVVK 1e5v.2    --------------------------------------------------------------------------------  target    AWAELKKTPHPLAKDGYRFVFHTPKYRHGAHTMPIDTDMVAMLFGPFGDVYRHDRRTPYVAEGYVDIHPSDAREIGVEDG 1e5v.2    --------------AKYPLHIAASHPFNRLHSQL-NGTVLREGY-------AV------QGHEPCLMHPDDAAARGIADG  target    DYVFIDSDPEDRPFRGWQKNKRDYEFSRLLCRARYYPGTPRGVTRMWFNMYGATPGSVEGQKSREDGLAKNPRTNYQAMF 1e5v.2    DVVRVHNDR-----------------GQILTGVKVTDAVMKGVIQIYEGGW-----------------------------  target    RSGSHQSATRGWLKPTWMTDSLVRKGLFGQSIGKGFLPDVHCPTGAPRESIVKITKAEPGGLGAEGLWRPAALGLRPGYE 1e5v.2    --------------------------------------------------------------------------------  target    SKSMKTYLDGGYVDDADRQGGQG 1e5v.2    ----------------------- ``` | | | | | | | | | | | | | | | | | | | | | | | | | | | | | | | | | | | | | | | | | | | | | | | | | |
|  | 1e18.1.A | DMSO REDUCTASE.  *TUNGSTEN-SUSBSTITUTED DMSO REDUCTASE FROM RHODOBACTER CAPSULATUS* | 0.08 | 0.00 | 17.44 | 0.20 | 175-291 | X-ray | 2.00 | monomer | 2 x PGD, 1 x 6WO | HHblits | 0.29 |
| ``` target    EVLARVGHKLAEQTGDARFADVWKLVDEKRTDAHLQRILDHSSNTKGYDALDLEAKAKKGIPTLMMNRTYPKAVGYEQVA 1e18.1    --------------------------------------------------------------------------------  target    DSRPWYTKSGRLEFYRDEDEFIEAGENLPVHREPIDSTFYEPNVIVSAPHEALRPAGPEDYGVELSDMSGEIRQGRNVVK 1e18.1    --------------------------------------------------------------------------------  target    AWAELKKTPHPLAKDGYRFVFHTPKYRHGAHTMPIDTDMVAMLFGPFGDVYRHDRRTPYVAEGYVDIHPSDAREIGVEDG 1e18.1    --------------AKYPLHIAASHPFNRLHSQLN-GTVLREGY-------AV------QGHEPCLMHPDDAAARGIADG  target    DYVFIDSDPEDRPFRGWQKNKRDYEFSRLLCRARYYPGTPRGVTRMWFNMYGATPGSVEGQKSREDGLAKNPRTNYQAMF 1e18.1    DVVRVHNDR-----------------GQILTGVKVTDAVMKGVIQIYEGGW-----------------------------  target    RSGSHQSATRGWLKPTWMTDSLVRKGLFGQSIGKGFLPDVHCPTGAPRESIVKITKAEPGGLGAEGLWRPAALGLRPGYE 1e18.1    --------------------------------------------------------------------------------  target    SKSMKTYLDGGYVDDADRQGGQG 1e18.1    ----------------------- ``` | | | | | | | | | | | | | | | | | | | | | | | | | | | | | | | | | | | | | | | | | | | | | | | | | |
|  | 1g8k.1.A | ARSENITE OXIDASE  *CRYSTAL STRUCTURE ANALYSIS OF ARSENITE OXIDASE FROM ALCALIGENES FAECALIS* | 0.08 | 0.00 | 22.89 | 0.20 | 175-290 | X-ray | 1.64 | monomer | 3 x HG, 2 x CA, 2 x MGD, 1 x O, 1 x 4MO, 1 x F3S, 1 x FES | HHblits | 0.32 |
| ``` target    EVLARVGHKLAEQTGDARFADVWKLVDEKRTDAHLQRILDHSSNTKGYDALDLEAKAKKGIPTLMMNRTYPKAVGYEQVA 1g8k.1    --------------------------------------------------------------------------------  target    DSRPWYTKSGRLEFYRDEDEFIEAGENLPVHREPIDSTFYEPNVIVSAPHEALRPAGPEDYGVELSDMSGEIRQGRNVVK 1g8k.1    --------------------------------------------------------------------------------  target    AWAELKKTPHPLAKDGYRFVFHTPKYRHGAHTMPID--TDMVAMLFGPFGDVYRHDRRTPYVAEGYVDIHPSDAREIGVE 1g8k.1    --------------DKYRFWLNNGRNNEVWQTAYHDQYNSLMQER----------------YPMAYIEMNPDDCKQLDVT  target    DGDYVFIDSDPEDRPFRGWQKNKRDYEFSRLLCRARYYPGTPRGVTRMWFNMYGATPGSVEGQKSREDGLAKNPRTNYQA 1g8k.1    GGDIVEVYNDF-----------------GSTFAMVYPVAEIKRGQTFMLFGY----------------------------  target    MFRSGSHQSATRGWLKPTWMTDSLVRKGLFGQSIGKGFLPDVHCPTGAPRESIVKITKAEPGGLGAEGLWRPAALGLRPG 1g8k.1    --------------------------------------------------------------------------------  target    YESKSMKTYLDGGYVDDADRQGGQG 1g8k.1    ------------------------- ``` | | | | | | | | | | | | | | | | | | | | | | | | | | | | | | | | | | | | | | | | | | | | | | | | | |
|  | 1g8j.1.A | ARSENITE OXIDASE  *CRYSTAL STRUCTURE ANALYSIS OF ARSENITE OXIDASE FROM ALCALIGENES FAECALIS* | 0.08 | 0.00 | 22.89 | 0.20 | 175-290 | X-ray | 2.03 | monomer | 2 x MGD, 1 x O, 1 x 4MO, 1 x F3S, 1 x FES | HHblits | 0.32 |
| ``` target    EVLARVGHKLAEQTGDARFADVWKLVDEKRTDAHLQRILDHSSNTKGYDALDLEAKAKKGIPTLMMNRTYPKAVGYEQVA 1g8j.1    --------------------------------------------------------------------------------  target    DSRPWYTKSGRLEFYRDEDEFIEAGENLPVHREPIDSTFYEPNVIVSAPHEALRPAGPEDYGVELSDMSGEIRQGRNVVK 1g8j.1    --------------------------------------------------------------------------------  target    AWAELKKTPHPLAKDGYRFVFHTPKYRHGAHTMPID--TDMVAMLFGPFGDVYRHDRRTPYVAEGYVDIHPSDAREIGVE 1g8j.1    --------------DKYRFWLNNGRNNEVWQTAYHDQYNSLMQER----------------YPMAYIEMNPDDCKQLDVT  target    DGDYVFIDSDPEDRPFRGWQKNKRDYEFSRLLCRARYYPGTPRGVTRMWFNMYGATPGSVEGQKSREDGLAKNPRTNYQA 1g8j.1    GGDIVEVYNDF-----------------GSTFAMVYPVAEIKRGQTFMLFGY----------------------------  target    MFRSGSHQSATRGWLKPTWMTDSLVRKGLFGQSIGKGFLPDVHCPTGAPRESIVKITKAEPGGLGAEGLWRPAALGLRPG 1g8j.1    --------------------------------------------------------------------------------  target    YESKSMKTYLDGGYVDDADRQGGQG 1g8j.1    ------------------------- ``` | | | | | | | | | | | | | | | | | | | | | | | | | | | | | | | | | | | | | | | | | | | | | | | | | |
|  | 6tg9.1.A | Formate dehydrogenase subunit alpha  *Cryo-EM Structure of NADH reduced form of NAD+-dependent Formate Dehydrogenase from Rhodobacter capsulatus* | 0.07 |  | 22.62 | 0.20 | 175-290 | EM | 3.24 | hetero-2-2-2-2-mer | 4 x MGD, 2 x 6MO, 4 x FES, 10 x SF4, 2 x H2S, 2 x FMN, 2 x NAI | HHblits | 0.31 |
| ``` target    EVLARVGHKLAEQTGDARFADVWKLVDEKRTDAHLQRILDHSSNTKGYDALDLEAKAKKGIPTLMMNRTYPKAVGYEQVA 6tg9.1    --------------------------------------------------------------------------------  target    DSRPWYTKSGRLEFYRDEDEFIEAGENLPVHREPIDSTFYEPNVIVSAPHEALRPAGPEDYGVELSDMSGEIRQGRNVVK 6tg9.1    --------------------------------------------------------------------------------  target    AWAELKKTPHPLAKDGYRFVFHTPKYRHGAHTMPIDTDMVAMLFGPFGDVYRHDRRTPYVAEGYVDIHPSDAREIGVEDG 6tg9.1    --------------PRFPLLLTTGRILSQYNVGAQTRRTE---------------NTVWHGEDRLEIHPTDAETRGIRDG  target    DYVFIDSDPEDRPFRGWQKNKRDYEFSRLLCRARYYPGTPRGVTRMWFNMYGATPGSVEGQKSREDGLAKNPRTNYQAMF 6tg9.1    DWVRLASRA-----------------GETTLRATVTDRVSPGVVYTTFHH------------------------------  target    RSGSHQSATRGWLKPTWMTDSLVRKGLFGQSIGKGFLPDVHCPTGAPRESIVKITKAEPGGLGAEGLWRPAALGLRPGYE 6tg9.1    --------------------------------------------------------------------------------  target    SKSMKTYLDGGYVDDADRQGGQG 6tg9.1    ----------------------- ``` | | | | | | | | | | | | | | | | | | | | | | | | | | | | | | | | | | | | | | | | | | | | | | | | | |
|  | 1eu1.1.A | DIMETHYL SULFOXIDE REDUCTASE  *THE CRYSTAL STRUCTURE OF RHODOBACTER SPHAEROIDES DIMETHYLSULFOXIDE REDUCTASE REVEALS TWO DISTINCT MOLYBDENUM COORDINATION ENVIRONMENTS.* | 0.08 |  | 18.82 | 0.20 | 175-290 | X-ray | 1.30 | monomer | 3 x GLC, 1 x CD, 2 x MGD, 1 x 6MO, 2 x O | HHblits | 0.29 |
| ``` target    EVLARVGHKLAEQTGDARFADVWKLVDEKRTDAHLQRILDHSSNTKGYDALDLEAKAKKGIPTLMMNRTYPKAVGYEQVA 1eu1.1    --------------------------------------------------------------------------------  target    DSRPWYTKSGRLEFYRDEDEFIEAGENLPVHREPIDSTFYEPNVIVSAPHEALRPAGPEDYGVELSDMSGEIRQGRNVVK 1eu1.1    --------------------------------------------------------------------------------  target    AWAELKKTPHPLAKDGYRFVFHTPKYRHGAHTMPIDTDMVAMLFGPFGDVYRHDRRTPYVAEGYVDIHPSDAREIGVEDG 1eu1.1    --------------AKYPLHVVASHPKSRLHSQLNGT-SLRDLY----------A---VAGHEPCLINPADAAARGIADG  target    DYVFIDSDPEDRPFRGWQKNKRDYEFSRLLCRARYYPGTPRGVTRMWFNMYGATPGSVEGQKSREDGLAKNPRTNYQAMF 1eu1.1    DVLRVFNDR-----------------GQILVGAKVSDAVMPGAIQIYEGG------------------------------  target    RSGSHQSATRGWLKPTWMTDSLVRKGLFGQSIGKGFLPDVHCPTGAPRESIVKITKAEPGGLGAEGLWRPAALGLRPGYE 1eu1.1    --------------------------------------------------------------------------------  target    SKSMKTYLDGGYVDDADRQGGQG 1eu1.1    ----------------------- ``` | | | | | | | | | | | | | | | | | | | | | | | | | | | | | | | | | | | | | | | | | | | | | | | | | |
|  | 4dmr.1.A | DMSO REDUCTASE  *REDUCED DMSO REDUCTASE FROM RHODOBACTER CAPSULATUS WITH BOUND DMSO SUBSTRATE* | 0.08 | 0.00 | 17.65 | 0.20 | 175-290 | X-ray | 1.90 | monomer | 2 x PGD, 1 x 4MO, 1 x O | HHblits | 0.29 |
| ``` target    EVLARVGHKLAEQTGDARFADVWKLVDEKRTDAHLQRILDHSSNTKGYDALDLEAKAKKGIPTLMMNRTYPKAVGYEQVA 4dmr.1    --------------------------------------------------------------------------------  target    DSRPWYTKSGRLEFYRDEDEFIEAGENLPVHREPIDSTFYEPNVIVSAPHEALRPAGPEDYGVELSDMSGEIRQGRNVVK 4dmr.1    --------------------------------------------------------------------------------  target    AWAELKKTPHPLAKDGYRFVFHTPKYRHGAHTMPIDTDMVAMLFGPFGDVYRHDRRTPYVAEGYVDIHPSDAREIGVEDG 4dmr.1    --------------AKYPLHIAASHPFNRLHSQL-NGTVLREGY-------AV------QGHEPCLMHPDDAAARGIADG  target    DYVFIDSDPEDRPFRGWQKNKRDYEFSRLLCRARYYPGTPRGVTRMWFNMYGATPGSVEGQKSREDGLAKNPRTNYQAMF 4dmr.1    DVVRVHNDR-----------------GQILTGVKVTDAVMKGVIQIYEGG------------------------------  target    RSGSHQSATRGWLKPTWMTDSLVRKGLFGQSIGKGFLPDVHCPTGAPRESIVKITKAEPGGLGAEGLWRPAALGLRPGYE 4dmr.1    --------------------------------------------------------------------------------  target    SKSMKTYLDGGYVDDADRQGGQG 4dmr.1    ----------------------- ``` | | | | | | | | | | | | | | | | | | | | | | | | | | | | | | | | | | | | | | | | | | | | | | | | | |
|  | 1dms.1.A | DMSO REDUCTASE  *STRUCTURE OF DMSO REDUCTASE* | 0.08 | 0.00 | 17.65 | 0.20 | 175-290 | X-ray | 1.88 | monomer | 2 x PGD, 1 x 2MO | HHblits | 0.29 |
| ``` target    EVLARVGHKLAEQTGDARFADVWKLVDEKRTDAHLQRILDHSSNTKGYDALDLEAKAKKGIPTLMMNRTYPKAVGYEQVA 1dms.1    --------------------------------------------------------------------------------  target    DSRPWYTKSGRLEFYRDEDEFIEAGENLPVHREPIDSTFYEPNVIVSAPHEALRPAGPEDYGVELSDMSGEIRQGRNVVK 1dms.1    --------------------------------------------------------------------------------  target    AWAELKKTPHPLAKDGYRFVFHTPKYRHGAHTMPIDTDMVAMLFGPFGDVYRHDRRTPYVAEGYVDIHPSDAREIGVEDG 1dms.1    --------------AKYPLHIAASHPFNRLHSQLN-GTVLREGY-------------AVQGHEPCLMHPDDAAARGIADG  target    DYVFIDSDPEDRPFRGWQKNKRDYEFSRLLCRARYYPGTPRGVTRMWFNMYGATPGSVEGQKSREDGLAKNPRTNYQAMF 1dms.1    DVVRVHNDR-----------------GQILTGVKVTDAVMKGVIQIYEGG------------------------------  target    RSGSHQSATRGWLKPTWMTDSLVRKGLFGQSIGKGFLPDVHCPTGAPRESIVKITKAEPGGLGAEGLWRPAALGLRPGYE 1dms.1    --------------------------------------------------------------------------------  target    SKSMKTYLDGGYVDDADRQGGQG 1dms.1    ----------------------- ``` | | | | | | | | | | | | | | | | | | | | | | | | | | | | | | | | | | | | | | | | | | | | | | | | | |
|  | 1e60.1.A | Dimethyl sulfoxide/trimethylamine N-oxide reductase  *OXIDIZED DMSO REDUCTASE EXPOSED TO HEPES - Structure II BUFFER* | 0.08 | 0.00 | 17.65 | 0.20 | 175-290 | X-ray | 2.00 | monomer | 2 x PGD, 1 x 2MO | HHblits | 0.29 |
| ``` target    EVLARVGHKLAEQTGDARFADVWKLVDEKRTDAHLQRILDHSSNTKGYDALDLEAKAKKGIPTLMMNRTYPKAVGYEQVA 1e60.1    --------------------------------------------------------------------------------  target    DSRPWYTKSGRLEFYRDEDEFIEAGENLPVHREPIDSTFYEPNVIVSAPHEALRPAGPEDYGVELSDMSGEIRQGRNVVK 1e60.1    --------------------------------------------------------------------------------  target    AWAELKKTPHPLAKDGYRFVFHTPKYRHGAHTMPIDTDMVAMLFGPFGDVYRHDRRTPYVAEGYVDIHPSDAREIGVEDG 1e60.1    --------------AKYPLHIAASHPFNRLHSQLN-GTVLREGY-------AV------QGHEPCLMHPDDAAARGIADG  target    DYVFIDSDPEDRPFRGWQKNKRDYEFSRLLCRARYYPGTPRGVTRMWFNMYGATPGSVEGQKSREDGLAKNPRTNYQAMF 1e60.1    DVVRVHNDR-----------------GQILTGVKVTDAVMKGVIQIYEGG------------------------------  target    RSGSHQSATRGWLKPTWMTDSLVRKGLFGQSIGKGFLPDVHCPTGAPRESIVKITKAEPGGLGAEGLWRPAALGLRPGYE 1e60.1    --------------------------------------------------------------------------------  target    SKSMKTYLDGGYVDDADRQGGQG 1e60.1    ----------------------- ``` | | | | | | | | | | | | | | | | | | | | | | | | | | | | | | | | | | | | | | | | | | | | | | | | | |
|  | 2nya.1.A | Periplasmic nitrate reductase  *Crystal structure of the periplasmic nitrate reductase (NAP) from Escherichia coli* | 0.08 |  | 21.69 | 0.20 | 175-290 | X-ray | 2.50 | monomer | 1 x SF4, 1 x 6MO, 2 x MGD | HHblits | 0.30 |
| ``` target    EVLARVGHKLAEQTGDARFADVWKLVDEKRTDAHLQRILDHSSNTKGYDALDLEAKAKKGIPTLMMNRTYPKAVGYEQVA 2nya.1    --------------------------------------------------------------------------------  target    DSRPWYTKSGRLEFYRDEDEFIEAGENLPVHREPIDSTFYEPNVIVSAPHEALRPAGPEDYGVELSDMSGEIRQGRNVVK 2nya.1    --------------------------------------------------------------------------------  target    AWAELKKTPHPLAKDGYRFVFHTPKYRHGAH--TMPIDTDMVAMLFGPFGDVYRHDRRTPYVAEGYVDIHPSDAREIGVE 2nya.1    --------------EEYDLWLSTGRVLEHWHTGSMTRRVPELHRA----------------FPEAVLFIHPLDAKARDLR  target    DGDYVFIDSDPEDRPFRGWQKNKRDYEFSRLLCRARYYPG--TPRGVTRMWFNMYGATPGSVEGQKSREDGLAKNPRTNY 2nya.1    RGDKVKVVSRR-----------------GEVISIVETRGRNRPPQGLVYMPFFD--------------------------  target    QAMFRSGSHQSATRGWLKPTWMTDSLVRKGLFGQSIGKGFLPDVHCPTGAPRESIVKITKAEPGGLGAEGLWRPAALGLR 2nya.1    --------------------------------------------------------------------------------  target    PGYESKSMKTYLDGGYVDDADRQGGQG 2nya.1    --------------------------- ``` | | | | | | | | | | | | | | | | | | | | | | | | | | | | | | | | | | | | | | | | | | | | | | | | | |
|  | 7vw6.1.A | Formate dehydrogenase  *Cryo-EM Structure of Formate Dehydrogenase 1 from Methylorubrum extorquens AM1* | 0.08 |  | 18.07 | 0.20 | 175-290 | EM | 0.00 | hetero-1-1-mer | 4 x SF4, 2 x FES, 2 x MGD, 1 x W, 1 x FMN | HHblits | 0.29 |
| ``` target    EVLARVGHKLAEQTGDARFADVWKLVDEKRTDAHLQRILDHSSNTKGYDALDLEAKAKKGIPTLMMNRTYPKAVGYEQVA 7vw6.1    --------------------------------------------------------------------------------  target    DSRPWYTKSGRLEFYRDEDEFIEAGENLPVHREPIDSTFYEPNVIVSAPHEALRPAGPEDYGVELSDMSGEIRQGRNVVK 7vw6.1    --------------------------------------------------------------------------------  target    AWAELKKTPHPLAKDGYRFVFHTPKYRHGAHTMP--IDTDMVAMLFGPFGDVYRHDRRTPYVAEGYVDIHPSDAREIGVE 7vw6.1    --------------DEFPMVLSTGRVLEHWHTGSMTRRAGVLDAL----------------EPEAVAFMAPKELYRLGLR  target    DGDYVFIDSDPEDRPFRGWQKNKRDYEFSRLLCRARYYPGTPRGVTRMWFNMYGATPGSVEGQKSREDGLAKNPRTNYQA 7vw6.1    PGGSMRLETRR-----------------GAVVLKVRSDRDVPIGMIFMPFCY----------------------------  target    MFRSGSHQSATRGWLKPTWMTDSLVRKGLFGQSIGKGFLPDVHCPTGAPRESIVKITKAEPGGLGAEGLWRPAALGLRPG 7vw6.1    --------------------------------------------------------------------------------  target    YESKSMKTYLDGGYVDDADRQGGQG 7vw6.1    ------------------------- ``` | | | | | | | | | | | | | | | | | | | | | | | | | | | | | | | | | | | | | | | | | | | | | | | | | |
|  | 7p63.1.C | NADH-quinone oxidoreductase  *Complex I from E. coli, DDM/LMNG-purified, under Turnover at pH 6, Closed state* | 0.07 |  | 15.66 | 0.20 | 175-290 | EM | 0.00 | hetero-1-1-1-1-1-1-… | 7 x SF4, 1 x FMN, 1 x NAI, 2 x FES, 1 x CA, 1 x DCQ, 4 x LFA, 8 x 3PE | HHblits | 0.29 |
| ``` target    EVLARVGHKLAEQTGDARFADVWKLVDEKRTDAHLQRILDHSSNTKGYDALDLEAKAKKGIPTLMMNRTYPKAVGYEQVA 7p63.1    --------------------------------------------------------------------------------  target    DSRPWYTKSGRLEFYRDEDEFIEAGENLPVHREPIDSTFYEPNVIVSAPHEALRPAGPEDYGVELSDMSGEIRQGRNVVK 7p63.1    --------------------------------------------------------------------------------  target    AWAELKKTPHPLAKDGYRFVFHTPKYRHGAHTMPIDTDMVAMLFGPFGDVYRHDRRTPYVAEGYVDIHPSDAREIGVEDG 7p63.1    --------------QDGKWRIAPYYHLFGSDELSQRAPVFQSR----------------MPQPYIKLNPADAAKLGVNAG  target    DYVFIDSDPEDRPFRGWQKNKRDYEFSRLLCRARYYPGTPRGVTRMWFNMYGATPGSVEGQKSREDGLAKNPRTNYQAMF 7p63.1    TRVSFSYDG-----------------NTVTLPVEIAEGLTAGQVGLPMGM------------------------------  target    RSGSHQSATRGWLKPTWMTDSLVRKGLFGQSIGKGFLPDVHCPTGAPRESIVKITKAEPGGLGAEGLWRPAALGLRPGYE 7p63.1    --------------------------------------------------------------------------------  target    SKSMKTYLDGGYVDDADRQGGQG 7p63.1    ----------------------- ``` | | | | | | | | | | | | | | | | | | | | | | | | | | | | | | | | | | | | | | | | | | | | | | | | | |
|  | 7p61.1.C | NADH-quinone oxidoreductase  *Complex I from E. coli, DDM-purified, with NADH, Resting state* | 0.07 |  | 15.66 | 0.20 | 175-290 | EM | 0.00 | hetero-1-1-1-1-1-1-… | 7 x SF4, 1 x FMN, 1 x NAI, 2 x FES, 1 x CA, 2 x 3PE, 1 x UQ8 | HHblits | 0.29 |
| ``` target    EVLARVGHKLAEQTGDARFADVWKLVDEKRTDAHLQRILDHSSNTKGYDALDLEAKAKKGIPTLMMNRTYPKAVGYEQVA 7p61.1    --------------------------------------------------------------------------------  target    DSRPWYTKSGRLEFYRDEDEFIEAGENLPVHREPIDSTFYEPNVIVSAPHEALRPAGPEDYGVELSDMSGEIRQGRNVVK 7p61.1    --------------------------------------------------------------------------------  target    AWAELKKTPHPLAKDGYRFVFHTPKYRHGAHTMPIDTDMVAMLFGPFGDVYRHDRRTPYVAEGYVDIHPSDAREIGVEDG 7p61.1    --------------QDGKWRIAPYYHLFGSDELSQRAPVFQSR----------------MPQPYIKLNPADAAKLGVNAG  target    DYVFIDSDPEDRPFRGWQKNKRDYEFSRLLCRARYYPGTPRGVTRMWFNMYGATPGSVEGQKSREDGLAKNPRTNYQAMF 7p61.1    TRVSFSYDG-----------------NTVTLPVEIAEGLTAGQVGLPMGM------------------------------  target    RSGSHQSATRGWLKPTWMTDSLVRKGLFGQSIGKGFLPDVHCPTGAPRESIVKITKAEPGGLGAEGLWRPAALGLRPGYE 7p61.1    --------------------------------------------------------------------------------  target    SKSMKTYLDGGYVDDADRQGGQG 7p61.1    ----------------------- ``` | | | | | | | | | | | | | | | | | | | | | | | | | | | | | | | | | | | | | | | | | | | | | | | | | |
|  | 7nz1.1.E | NADH-quinone oxidoreductase subunit G  *Respiratory complex I from Escherichia coli - focused refinement of cytoplasmic arm* | 0.07 |  | 15.66 | 0.20 | 175-290 | EM | 0.00 | hetero-1-1-1-1-1-1-… | 7 x SF4, 2 x FES, 1 x FMN, 1 x CA | HHblits | 0.29 |
| ``` target    EVLARVGHKLAEQTGDARFADVWKLVDEKRTDAHLQRILDHSSNTKGYDALDLEAKAKKGIPTLMMNRTYPKAVGYEQVA 7nz1.1    --------------------------------------------------------------------------------  target    DSRPWYTKSGRLEFYRDEDEFIEAGENLPVHREPIDSTFYEPNVIVSAPHEALRPAGPEDYGVELSDMSGEIRQGRNVVK 7nz1.1    --------------------------------------------------------------------------------  target    AWAELKKTPHPLAKDGYRFVFHTPKYRHGAHTMPIDTDMVAMLFGPFGDVYRHDRRTPYVAEGYVDIHPSDAREIGVEDG 7nz1.1    --------------QDGKWRIAPYYHLFGSDELSQRAPVFQSR----------------MPQPYIKLNPADAAKLGVNAG  target    DYVFIDSDPEDRPFRGWQKNKRDYEFSRLLCRARYYPGTPRGVTRMWFNMYGATPGSVEGQKSREDGLAKNPRTNYQAMF 7nz1.1    TRVSFSYDG-----------------NTVTLPVEIAEGLTAGQVGLPMGM------------------------------  target    RSGSHQSATRGWLKPTWMTDSLVRKGLFGQSIGKGFLPDVHCPTGAPRESIVKITKAEPGGLGAEGLWRPAALGLRPGYE 7nz1.1    --------------------------------------------------------------------------------  target    SKSMKTYLDGGYVDDADRQGGQG 7nz1.1    ----------------------- ``` | | | | | | | | | | | | | | | | | | | | | | | | | | | | | | | | | | | | | | | | | | | | | | | | | |
|  | 2v45.1.A | PERIPLASMIC NITRATE REDUCTASE  *A NEW CATALYTIC MECHANISM OF PERIPLASMIC NITRATE REDUCTASE FROM DESULFOVIBRIO DESULFURICANS ATCC 27774 FROM CRYSTALLOGRAPHIC AND EPR DATA AND BASED ON DETAILED ANALYSIS OF THE SIXTH LIGAND* | 0.07 |  | 18.07 | 0.20 | 175-290 | X-ray | 2.40 | monomer | 1 x SF4, 1 x MO, 2 x MGD, 1 x LCP | HHblits | 0.29 |
| ``` target    EVLARVGHKLAEQTGDARFADVWKLVDEKRTDAHLQRILDHSSNTKGYDALDLEAKAKKGIPTLMMNRTYPKAVGYEQVA 2v45.1    --------------------------------------------------------------------------------  target    DSRPWYTKSGRLEFYRDEDEFIEAGENLPVHREPIDSTFYEPNVIVSAPHEALRPAGPEDYGVELSDMSGEIRQGRNVVK 2v45.1    --------------------------------------------------------------------------------  target    AWAELKKTPHPLAKDGYRFVFHTPKYRHGAHTMPID--TDMVAMLFGPFGDVYRHDRRTPYVAEGYVDIHPSDAREIGVE 2v45.1    --------------AEYPLYLTSMRVIDHWHTATMTGKVPELQKA----------------NPIAFVEINEEDAARTGIK  target    DGDYVFIDSDPEDRPFRGWQKNKRDYEFSRLLCRARYYPGTPRGVTRMWFNMYGATPGSVEGQKSREDGLAKNPRTNYQA 2v45.1    HGDSVIVETRR-----------------DAMELPARVSDVCRPGLIAVPFFD----------------------------  target    MFRSGSHQSATRGWLKPTWMTDSLVRKGLFGQSIGKGFLPDVHCPTGAPRESIVKITKAEPGGLGAEGLWRPAALGLRPG 2v45.1    --------------------------------------------------------------------------------  target    YESKSMKTYLDGGYVDDADRQGGQG 2v45.1    ------------------------- ``` | | | | | | | | | | | | | | | | | | | | | | | | | | | | | | | | | | | | | | | | | | | | | | | | | |
|  | 2v3v.1.A | PERIPLASMIC NITRATE REDUCTASE  *A NEW CATALYTIC MECHANISM OF PERIPLASMIC NITRATE REDUCTASE FROM DESULFOVIBRIO DESULFURICANS ATCC 27774 FROM CRYSTALLOGRAPHIC AND EPR DATA AND BASED ON DETAILED ANALYSIS OF THE SIXTH LIGAND* | 0.07 |  | 18.07 | 0.20 | 175-290 | X-ray | 1.99 | monomer | 1 x SF4, 1 x MO, 2 x MGD, 4 x LCP | HHblits | 0.29 |
| ``` target    EVLARVGHKLAEQTGDARFADVWKLVDEKRTDAHLQRILDHSSNTKGYDALDLEAKAKKGIPTLMMNRTYPKAVGYEQVA 2v3v.1    --------------------------------------------------------------------------------  target    DSRPWYTKSGRLEFYRDEDEFIEAGENLPVHREPIDSTFYEPNVIVSAPHEALRPAGPEDYGVELSDMSGEIRQGRNVVK 2v3v.1    --------------------------------------------------------------------------------  target    AWAELKKTPHPLAKDGYRFVFHTPKYRHGAHTMPIDT--DMVAMLFGPFGDVYRHDRRTPYVAEGYVDIHPSDAREIGVE 2v3v.1    --------------AEYPLYLTSMRVIDHWHTATMTGKVPELQKA----------------NPIAFVEINEEDAARTGIK  target    DGDYVFIDSDPEDRPFRGWQKNKRDYEFSRLLCRARYYPGTPRGVTRMWFNMYGATPGSVEGQKSREDGLAKNPRTNYQA 2v3v.1    HGDSVIVETRR-----------------DAMELPARVSDVCRPGLIAVPFFD----------------------------  target    MFRSGSHQSATRGWLKPTWMTDSLVRKGLFGQSIGKGFLPDVHCPTGAPRESIVKITKAEPGGLGAEGLWRPAALGLRPG 2v3v.1    --------------------------------------------------------------------------------  target    YESKSMKTYLDGGYVDDADRQGGQG 2v3v.1    ------------------------- ``` | | | | | | | | | | | | | | | | | | | | | | | | | | | | | | | | | | | | | | | | | | | | | | | | | |
|  | 1kqf.1.A | FORMATE DEHYDROGENASE, NITRATE-INDUCIBLE, MAJOR SUBUNIT  *FORMATE DEHYDROGENASE N FROM E. COLI* | 0.08 |  | 18.07 | 0.20 | 175-290 | X-ray | 1.60 | hetero-oligomer | 3 x 6MO, 15 x SF4, 6 x MGD, 6 x HEM, 3 x CDL | HHblits | 0.28 |
| ``` target    EVLARVGHKLAEQTGDARFADVWKLVDEKRTDAHLQRILDHSSNTKGYDALDLEAKAKKGIPTLMMNRTYPKAVGYEQVA 1kqf.1    --------------------------------------------------------------------------------  target    DSRPWYTKSGRLEFYRDEDEFIEAGENLPVHREPIDSTFYEPNVIVSAPHEALRPAGPEDYGVELSDMSGEIRQGRNVVK 1kqf.1    --------------------------------------------------------------------------------  target    AWAELKKTPHPLAKDGYRFVFHTPKYRHGAHTMPIDTDMVAMLFGPFGDVYRHDRRTPYVAEGYVDIHPSDAREIGVEDG 1kqf.1    --------------EQFPYVGTTYRLTEHFHTWTKHALLNA-IA---------------QPEQFVEISETLAAAKGINNG  target    DYVFIDSDPEDRPFRGWQKNKRDYEFSRLLCRARYYPGTPR--------GVTRMWFNMYGATPGSVEGQKSREDGLAKNP 1kqf.1    DRVTVSSKR-----------------GFIRAVAVVTRRLKPLNVNGQQVETVGIPIHW----------------------  target    RTNYQAMFRSGSHQSATRGWLKPTWMTDSLVRKGLFGQSIGKGFLPDVHCPTGAPRESIVKITKAEPGGLGAEGLWRPAA 1kqf.1    --------------------------------------------------------------------------------  target    LGLRPGYESKSMKTYLDGGYVDDADRQGGQG 1kqf.1    ------------------------------- ``` | | | | | | | | | | | | | | | | | | | | | | | | | | | | | | | | | | | | | | | | | | | | | | | | | |
|  | 1ogy.1.A | PERIPLASMIC NITRATE REDUCTASE  *Crystal structure of the heterodimeric nitrate reductase from Rhodobacter sphaeroides* | 0.07 |  | 20.99 | 0.19 | 175-288 | X-ray | 3.20 | hetero-1-1-mer | 1 x SF4, 1 x MO, 2 x MGD, 2 x HEC | HHblits | 0.30 |
| ``` target    EVLARVGHKLAEQTGDARFADVWKLVDEKRTDAHLQRILDHSSNTKGYDALDLEAKAKKGIPTLMMNRTYPKAVGYEQVA 1ogy.1    --------------------------------------------------------------------------------  target    DSRPWYTKSGRLEFYRDEDEFIEAGENLPVHREPIDSTFYEPNVIVSAPHEALRPAGPEDYGVELSDMSGEIRQGRNVVK 1ogy.1    --------------------------------------------------------------------------------  target    AWAELKKTPHPLAKDGYRFVFHTPKYRHGAHTMPIDT--DMVAMLFGPFGDVYRHDRRTPYVAEGYVDIHPSDAREIGVE 1ogy.1    --------------EEFGFWLVTGRVLEHWHSGSMTLRWPELYKA----------------FPGAVCFMHPEDARSRGLN  target    DGDYVFIDSDPEDRPFRGWQKNKRDYEFSRLLCRARY--YPGTPRGVTRMWFNMYGATPGSVEGQKSREDGLAKNPRTNY 1ogy.1    RGSEVRVISRR-----------------GEIRTRLETRGRNRMPRGVVFVPW----------------------------  target    QAMFRSGSHQSATRGWLKPTWMTDSLVRKGLFGQSIGKGFLPDVHCPTGAPRESIVKITKAEPGGLGAEGLWRPAALGLR 1ogy.1    --------------------------------------------------------------------------------  target    PGYESKSMKTYLDGGYVDDADRQGGQG 1ogy.1    --------------------------- ``` | | | | | | | | | | | | | | | | | | | | | | | | | | | | | | | | | | | | | | | | | | | | | | | | | |
|  | 5t5i.1.D | Tungsten formylmethanofuran dehydrogenase subunit fwdD  *TUNGSTEN-CONTAINING FORMYLMETHANOFURAN DEHYDROGENASE FROM METHANOTHERMOBACTER WOLFEII, ORTHORHOMBIC FORM AT 1.9 A* | 0.06 |  | 16.25 | 0.19 | 178-290 | X-ray | 1.90 | hetero-oligomer | 4 x ZN, 2 x MG, 18 x K, 22 x SF4, 2 x W, 4 x MGD, 2 x H2S, 2 x CA | HHblits | 0.30 |
| ``` target    EVLARVGHKLAEQTGDARFADVWKLVDEKRTDAHLQRILDHSSNTKGYDALDLEAKAKKGIPTLMMNRTYPKAVGYEQVA 5t5i.1    --------------------------------------------------------------------------------  target    DSRPWYTKSGRLEFYRDEDEFIEAGENLPVHREPIDSTFYEPNVIVSAPHEALRPAGPEDYGVELSDMSGEIRQGRNVVK 5t5i.1    --------------------------------------------------------------------------------  target    AWAELKKTPHPLAKDGYRFVFHTPKYRHGAHTMPIDTDMVAMLFGPFGDVYRHDRRTPYVAEGYVDIHPSDAREIGVEDG 5t5i.1    -----------------RVILNTGRTIWQGQAIESGKDLKMYV----------------DAAAIIQMNPEMMKQLGIAEG  target    DYVFIDSDPEDRPFRGWQKNKRDYEFSRLLCRAR-YYPGTPRGVTRMWFNMYGATPGSVEGQKSREDGLAKNPRTNYQAM 5t5i.1    DNVKVISEY-----------------GDVVVKAVEAKEPLPEGMVYIPMGP-----------------------------  target    FRSGSHQSATRGWLKPTWMTDSLVRKGLFGQSIGKGFLPDVHCPTGAPRESIVKITKAEPGGLGAEGLWRPAALGLRPGY 5t5i.1    --------------------------------------------------------------------------------  target    ESKSMKTYLDGGYVDDADRQGGQG 5t5i.1    ------------------------ ``` | | | | | | | | | | | | | | | | | | | | | | | | | | | | | | | | | | | | | | | | | | | | | | | | | |
|  | 3o5a.1.A | Periplasmic nitrate reductase  *Crystal Structure of partially reduced Periplasmic Nitrate Reductase from Cupriavidus necator using Ionic Liquids* | 0.07 |  | 21.25 | 0.19 | 175-287 | X-ray | 1.72 | hetero-oligomer | 1 x SF4, 1 x MOS, 2 x MGD, 2 x HEC | HHblits | 0.30 |
| ``` target    EVLARVGHKLAEQTGDARFADVWKLVDEKRTDAHLQRILDHSSNTKGYDALDLEAKAKKGIPTLMMNRTYPKAVGYEQVA 3o5a.1    --------------------------------------------------------------------------------  target    DSRPWYTKSGRLEFYRDEDEFIEAGENLPVHREPIDSTFYEPNVIVSAPHEALRPAGPEDYGVELSDMSGEIRQGRNVVK 3o5a.1    --------------------------------------------------------------------------------  target    AWAELKKTPHPLAKDGYRFVFHTPKYRHGAHTM--PIDTDMVAMLFGPFGDVYRHDRRTPYVAEGYVDIHPSDAREIGVE 3o5a.1    --------------KEYPYWLVTGRVLEHWHSGSMTRRVPELYRS----------------FPNAVVFMHPEDAKALGLR  target    DGDYVFIDSDPEDRPFRGWQKNKRDYEFSRLLCRARYY--PGTPRGVTRMWFNMYGATPGSVEGQKSREDGLAKNPRTNY 3o5a.1    RGVEVEVVSRR-----------------GRMRSRIETRGRDAPPRGLVFVP-----------------------------  target    QAMFRSGSHQSATRGWLKPTWMTDSLVRKGLFGQSIGKGFLPDVHCPTGAPRESIVKITKAEPGGLGAEGLWRPAALGLR 3o5a.1    --------------------------------------------------------------------------------  target    PGYESKSMKTYLDGGYVDDADRQGGQG 3o5a.1    --------------------------- ``` | | | | | | | | | | | | | | | | | | | | | | | | | | | | | | | | | | | | | | | | | | | | | | | | | |
|  | 7bkb.1.J | Formylmethanofuran dehydrogenase, subunit D  *Formate dehydrogenase - heterodisulfide reductase - formylmethanofuran dehydrogenase complex from Methanospirillum hungatei (hexameric, composite structure)* | 0.06 |  | 13.25 | 0.20 | 175-290 | EM | 0.00 | hetero-2-2-2-2-2-2-… | 48 x SF4, 4 x FAD, 2 x FES, 4 x 9S8, 4 x ZN, 2 x MO, 4 x MGD | HHblits | 0.27 |
| ``` target    EVLARVGHKLAEQTGDARFADVWKLVDEKRTDAHLQRILDHSSNTKGYDALDLEAKAKKGIPTLMMNRTYPKAVGYEQVA 7bkb.1    --------------------------------------------------------------------------------  target    DSRPWYTKSGRLEFYRDEDEFIEAGENLPVHREPIDSTFYEPNVIVSAPHEALRPAGPEDYGVELSDMSGEIRQGRNVVK 7bkb.1    --------------------------------------------------------------------------------  target    AWAELKKTPHPLAKDGYRFVFHTPKYRHGAHTMPIDTDMVAMLFGPFGDVYRHDRRTPYVAEGYVDIHPSDAREIGVEDG 7bkb.1    --------------AKKTLNMITQRAVEEGIAMEI-GKTSRQY------------F---DACSIIEMNEQDMKELGIMKN  target    DYVFIDSDPEDRPFRGWQKNKRDYEFSRLLCRARYY-PGTPRGVTRMWFNMYGATPGSVEGQKSREDGLAKNPRTNYQAM 7bkb.1    TNVRVKSES-----------------GEVVVKAVVGRQTCYPGLCHIRQGV-----------------------------  target    FRSGSHQSATRGWLKPTWMTDSLVRKGLFGQSIGKGFLPDVHCPTGAPRESIVKITKAEPGGLGAEGLWRPAALGLRPGY 7bkb.1    --------------------------------------------------------------------------------  target    ESKSMKTYLDGGYVDDADRQGGQG 7bkb.1    ------------------------ ``` | | | | | | | | | | | | | | | | | | | | | | | | | | | | | | | | | | | | | | | | | | | | | | | | | |
|  | 2ki8.1.A | Tungsten formylmethanofuran dehydrogenase, subunit D (FwdD-2)  *Solution NMR structure of tungsten formylmethanofuran dehydrogenase subunit D from Archaeoglobus fulgidus, Northeast Structural Genomics Consortium target AtT7* | 0.07 |  | 15.00 | 0.19 | 176-290 | NMR | 0.00 | monomer |  | HHblits | 0.29 |
| ``` target    EVLARVGHKLAEQTGDARFADVWKLVDEKRTDAHLQRILDHSSNTKGYDALDLEAKAKKGIPTLMMNRTYPKAVGYEQVA 2ki8.1    --------------------------------------------------------------------------------  target    DSRPWYTKSGRLEFYRDEDEFIEAGENLPVHREPIDSTFYEPNVIVSAPHEALRPAGPEDYGVELSDMSGEIRQGRNVVK 2ki8.1    --------------------------------------------------------------------------------  target    AWAELKKTPHPLAKDGYRFVFHTPKYRHGAHTMPIDTDMVAMLFGPFGDVYRHDRRTPYVAEGYVDIHPSDAREIGVEDG 2ki8.1    ---------------MLEVEVISGRTLNQGATVE--EKLTE----------EYF-----NAVNYAEINEEDWNALGLQEG  target    DYVFIDSDPEDRPFRGWQKNKRDYEFSRLLCRARYYPGTPRGVTRMWFNMYGATPGSVEGQKSREDGLAKNPRTNYQAMF 2ki8.1    DRVKVKTEF-----------------GEVVVFAKKG-DVPKGMIFIPMGP------------------------------  target    RSGSHQSATRGWLKPTWMTDSLVRKGLFGQSIGKGFLPDVHCPTGAPRESIVKITKAEPGGLGAEGLWRPAALGLRPGYE 2ki8.1    --------------------------------------------------------------------------------  target    SKSMKTYLDGGYVDDADRQGGQG 2ki8.1    ----------------------- ``` | | | | | | | | | | | | | | | | | | | | | | | | | | | | | | | | | | | | | | | | | | | | | | | | | |
|  | 8e9g.1.G | NADH-quinone oxidoreductase subunit G  *Mycobacterial respiratory complex I with both quinone positions modelled* | 0.07 |  | 15.85 | 0.19 | 175-290 | EM | 0.00 | hetero-1-1-1-1-1-1-… |  | HHblits | 0.27 |
| ``` target    EVLARVGHKLAEQTGDARFADVWKLVDEKRTDAHLQRILDHSSNTKGYDALDLEAKAKKGIPTLMMNRTYPKAVGYEQVA 8e9g.1    --------------------------------------------------------------------------------  target    DSRPWYTKSGRLEFYRDEDEFIEAGENLPVHREPIDSTFYEPNVIVSAPHEALRPAGPEDYGVELSDMSGEIRQGRNVVK 8e9g.1    --------------------------------------------------------------------------------  target    AWAELKKTPHPLAKDGYRFVFHTPKYRHGAHTMPIDTDMVAMLFGPFGDVYRHDRRTPYVAEGYVDIHPSDAREIGVEDG 8e9g.1    --------------GSGQAVLASWRMLLDAGRLQDGEPHLAGT----------------AVRPVARMSAATAAGIGASDG  target    DYVFIDSDPEDRPFRGWQKNKRDYEFSRLLCRARYYPGTPRGVTRMWFNMYGATPGSVEGQKSREDGLAKNPRTNYQAMF 8e9g.1    APVTVSTER-----------------GAVTLPLAVTD-MPDGVVWLPMNS------------------------------  target    RSGSHQSATRGWLKPTWMTDSLVRKGLFGQSIGKGFLPDVHCPTGAPRESIVKITKAEPGGLGAEGLWRPAALGLRPGYE 8e9g.1    --------------------------------------------------------------------------------  target    SKSMKTYLDGGYVDDADRQGGQG 8e9g.1    ----------------------- ``` | | | | | | | | | | | | | | | | | | | | | | | | | | | | | | | | | | | | | | | | | | | | | | | | | |
|  | 2vpz.1.A | THIOSULFATE REDUCTASE  *POLYSULFIDE REDUCTASE NATIVE STRUCTURE* | 0.06 |  | 13.92 | 0.19 | 179-290 | X-ray | 2.40 | hetero-oligomer | 10 x SF4, 4 x MGD, 2 x MO | HHblits | 0.28 |
| ``` target    EVLARVGHKLAEQTGDARFADVWKLVDEKRTDAHLQRILDHSSNTKGYDALDLEAKAKKGIPTLMMNRTYPKAVGYEQVA 2vpz.1    --------------------------------------------------------------------------------  target    DSRPWYTKSGRLEFYRDEDEFIEAGENLPVHREPIDSTFYEPNVIVSAPHEALRPAGPEDYGVELSDMSGEIRQGRNVVK 2vpz.1    --------------------------------------------------------------------------------  target    AWAELKKTPHPLAKDGYRFVFHTPKYRHGAHTMPIDTDMVAMLFGPFGDVYRHDRRTPYVAEGYVDIHPSDAREIGVEDG 2vpz.1    ------------------YRLLYGRSPVHTFARTQNNWVLMEM----------------DPENEVWIHKEEAKRLGLKEG  target    DYVFIDSDPEDRPFRGWQKNKRDYEFSRL--LCRARYYPGTPRGVTRMWFNMYGATPGSVEGQKSREDGLAKNPRTNYQA 2vpz.1    DYVMLVNQD-----------------GVKEGPVRVKPTARIRKDCVYIVHGF----------------------------  target    MFRSGSHQSATRGWLKPTWMTDSLVRKGLFGQSIGKGFLPDVHCPTGAPRESIVKITKAEPGGLGAEGLWRPAALGLRPG 2vpz.1    --------------------------------------------------------------------------------  target    YESKSMKTYLDGGYVDDADRQGGQG 2vpz.1    ------------------------- ``` | | | | | | | | | | | | | | | | | | | | | | | | | | | | | | | | | | | | | | | | | | | | | | | | | |
|  | 2vpx.1.D | THIOSULFATE REDUCTASE  *POLYSULFIDE REDUCTASE WITH BOUND QUINONE (UQ1)* | 0.06 |  | 13.92 | 0.19 | 179-290 | X-ray | 3.10 | hetero-oligomer | 10 x SF4, 4 x MGD, 2 x MO, 2 x UQ1 | HHblits | 0.28 |
| ``` target    EVLARVGHKLAEQTGDARFADVWKLVDEKRTDAHLQRILDHSSNTKGYDALDLEAKAKKGIPTLMMNRTYPKAVGYEQVA 2vpx.1    --------------------------------------------------------------------------------  target    DSRPWYTKSGRLEFYRDEDEFIEAGENLPVHREPIDSTFYEPNVIVSAPHEALRPAGPEDYGVELSDMSGEIRQGRNVVK 2vpx.1    --------------------------------------------------------------------------------  target    AWAELKKTPHPLAKDGYRFVFHTPKYRHGAHTMPIDTDMVAMLFGPFGDVYRHDRRTPYVAEGYVDIHPSDAREIGVEDG 2vpx.1    ------------------YRLLYGRSPVHTFARTQNNWVLMEM----------------DPENEVWIHKEEAKRLGLKEG  target    DYVFIDSDPEDRPFRGWQKNKRDYEFSRL--LCRARYYPGTPRGVTRMWFNMYGATPGSVEGQKSREDGLAKNPRTNYQA 2vpx.1    DYVMLVNQD-----------------GVKEGPVRVKPTARIRKDCVYIVHGF----------------------------  target    MFRSGSHQSATRGWLKPTWMTDSLVRKGLFGQSIGKGFLPDVHCPTGAPRESIVKITKAEPGGLGAEGLWRPAALGLRPG 2vpx.1    --------------------------------------------------------------------------------  target    YESKSMKTYLDGGYVDDADRQGGQG 2vpx.1    ------------------------- ``` | | | | | | | | | | | | | | | | | | | | | | | | | | | | | | | | | | | | | | | | | | | | | | | | | |
|  | 1h0h.1.A | FORMATE DEHYDROGENASE SUBUNIT ALPHA  *Tungsten containing Formate Dehydrogenase from Desulfovibrio Gigas* | 0.06 |  | 16.88 | 0.18 | 175-284 | X-ray | 1.80 | hetero-1-1-mer | 1 x W, 1 x 2MD, 1 x MGD, 4 x SF4, 1 x CA | HHblits | 0.28 |
| ``` target    EVLARVGHKLAEQTGDARFADVWKLVDEKRTDAHLQRILDHSSNTKGYDALDLEAKAKKGIPTLMMNRTYPKAVGYEQVA 1h0h.1    --------------------------------------------------------------------------------  target    DSRPWYTKSGRLEFYRDEDEFIEAGENLPVHREPIDSTFYEPNVIVSAPHEALRPAGPEDYGVELSDMSGEIRQGRNVVK 1h0h.1    --------------------------------------------------------------------------------  target    AWAELKKTPHPLAKDGYRFVFHTPKYR--HGAHTMPIDTDMVAMLFGPFGDVYRHDRRTPYVAEGYVDIHPSDAREIGVE 1h0h.1    --------------PRYPFICSTYRVTEHWQTGLMTRNTPWLLEA----------------EPQMFCEMSEELATLRGIK  target    DGDYVFIDSDPEDRPFRGWQKNKRDYEFSRLLCRARYYPGTPRGVTRMWFNMYGATPGSVEGQKSREDGLAKNPRTNYQA 1h0h.1    NGDKVILESVR-----------------GKLWAKAIITKRIKPFAI----------------------------------  target    MFRSGSHQSATRGWLKPTWMTDSLVRKGLFGQSIGKGFLPDVHCPTGAPRESIVKITKAEPGGLGAEGLWRPAALGLRPG 1h0h.1    --------------------------------------------------------------------------------  target    YESKSMKTYLDGGYVDDADRQGGQG 1h0h.1    ------------------------- ``` | | | | | | | | | | | | | | | | | | | | | | | | | | | | | | | | | | | | | | | | | | | | | | | | | |
|  | 8bqg.1.A | Formate dehydrogenase, alpha subunit, selenocysteine-containing  *W-formate dehydrogenase from Desulfovibrio vulgaris - Soaking with Formate 1 min* | 0.06 |  | 18.18 | 0.18 | 175-284 | X-ray | 1.95 | hetero-1-1-mer | 2 x MGD, 4 x SF4, 1 x H2S, 1 x W | HHblits | 0.27 |
| ``` target    EVLARVGHKLAEQTGDARFADVWKLVDEKRTDAHLQRILDHSSNTKGYDALDLEAKAKKGIPTLMMNRTYPKAVGYEQVA 8bqg.1    --------------------------------------------------------------------------------  target    DSRPWYTKSGRLEFYRDEDEFIEAGENLPVHREPIDSTFYEPNVIVSAPHEALRPAGPEDYGVELSDMSGEIRQGRNVVK 8bqg.1    --------------------------------------------------------------------------------  target    AWAELKKTPHPLAKDGYRFVFHTPKYRHGAHT--MPIDTDMVAMLFGPFGDVYRHDRRTPYVAEGYVDIHPSDAREIGVE 8bqg.1    --------------PRYPFIGTTYRVTEHWQTGLMTRRCAWLVEA----------------EPQIFCEISKELAKLRGIG  target    DGDYVFIDSDPEDRPFRGWQKNKRDYEFSRLLCRARYYPGTPRGVTRMWFNMYGATPGSVEGQKSREDGLAKNPRTNYQA 8bqg.1    NGDTVKVSSLR-----------------GALEAVAIVTERIRPFKI----------------------------------  target    MFRSGSHQSATRGWLKPTWMTDSLVRKGLFGQSIGKGFLPDVHCPTGAPRESIVKITKAEPGGLGAEGLWRPAALGLRPG 8bqg.1    --------------------------------------------------------------------------------  target    YESKSMKTYLDGGYVDDADRQGGQG 8bqg.1    ------------------------- ``` | | | | | | | | | | | | | | | | | | | | | | | | | | | | | | | | | | | | | | | | | | | | | | | | | |
|  | 6sdv.1.A | Formate dehydrogenase, alpha subunit, selenocysteine-containing,Formate dehydrogenase, alpha subunit, selenocysteine-containing,W-formate dehydrogenase - alpha subunit  *W-formate dehydrogenase from Desulfovibrio vulgaris - Formate reduced form* | 0.06 |  | 16.88 | 0.18 | 175-284 | X-ray | 1.90 | hetero-1-1-mer | 2 x MGD, 4 x SF4, 1 x W, 1 x H2S | HHblits | 0.26 |
| ``` target    EVLARVGHKLAEQTGDARFADVWKLVDEKRTDAHLQRILDHSSNTKGYDALDLEAKAKKGIPTLMMNRTYPKAVGYEQVA 6sdv.1    --------------------------------------------------------------------------------  target    DSRPWYTKSGRLEFYRDEDEFIEAGENLPVHREPIDSTFYEPNVIVSAPHEALRPAGPEDYGVELSDMSGEIRQGRNVVK 6sdv.1    --------------------------------------------------------------------------------  target    AWAELKKTPHPLAKDGYRFVFHTPKYRHGAH--TMPIDTDMVAMLFGPFGDVYRHDRRTPYVAEGYVDIHPSDAREIGVE 6sdv.1    --------------PRYPFIGTTYRVTEHWQTGLMTRRCAWLVEA----------------EPQIFCEISKELAKLRGIG  target    DGDYVFIDSDPEDRPFRGWQKNKRDYEFSRLLCRARYYPGTPRGVTRMWFNMYGATPGSVEGQKSREDGLAKNPRTNYQA 6sdv.1    NGDTVKVSSLR-----------------GALEAVAIVTERIRPFKI----------------------------------  target    MFRSGSHQSATRGWLKPTWMTDSLVRKGLFGQSIGKGFLPDVHCPTGAPRESIVKITKAEPGGLGAEGLWRPAALGLRPG 6sdv.1    --------------------------------------------------------------------------------  target    YESKSMKTYLDGGYVDDADRQGGQG 6sdv.1    ------------------------- ``` | | | | | | | | | | | | | | | | | | | | | | | | | | | | | | | | | | | | | | | | | | | | | | | | | |
|  | 6sdr.1.A | Formate dehydrogenase, alpha subunit, selenocysteine-containing  *W-formate dehydrogenase from Desulfovibrio vulgaris - Oxidized form* | 0.06 |  | 16.88 | 0.18 | 175-284 | X-ray | 2.10 | hetero-1-1-mer | 2 x MGD, 4 x SF4, 1 x H2S, 1 x W | HHblits | 0.26 |
| ``` target    EVLARVGHKLAEQTGDARFADVWKLVDEKRTDAHLQRILDHSSNTKGYDALDLEAKAKKGIPTLMMNRTYPKAVGYEQVA 6sdr.1    --------------------------------------------------------------------------------  target    DSRPWYTKSGRLEFYRDEDEFIEAGENLPVHREPIDSTFYEPNVIVSAPHEALRPAGPEDYGVELSDMSGEIRQGRNVVK 6sdr.1    --------------------------------------------------------------------------------  target    AWAELKKTPHPLAKDGYRFVFHTPKYRHGAH--TMPIDTDMVAMLFGPFGDVYRHDRRTPYVAEGYVDIHPSDAREIGVE 6sdr.1    --------------PRYPFIGTTYRVTEHWQTGLMTRRCAWLVEA----------------EPQIFCEISKELAKLRGIG  target    DGDYVFIDSDPEDRPFRGWQKNKRDYEFSRLLCRARYYPGTPRGVTRMWFNMYGATPGSVEGQKSREDGLAKNPRTNYQA 6sdr.1    NGDTVKVSSLR-----------------GALEAVAIVTERIRPFKI----------------------------------  target    MFRSGSHQSATRGWLKPTWMTDSLVRKGLFGQSIGKGFLPDVHCPTGAPRESIVKITKAEPGGLGAEGLWRPAALGLRPG 6sdr.1    --------------------------------------------------------------------------------  target    YESKSMKTYLDGGYVDDADRQGGQG 6sdr.1    ------------------------- ``` | | | | | | | | | | | | | | | | | | | | | | | | | | | | | | | | | | | | | | | | | | | | | | | | | |
|  | 7wbb.1.A | AFG2 isoform 1  *Cryo-EM structure of substrate engaged Drg1 hexamer* | 0.04 | 0.00 | 19.30 | 0.13 | 212-287 | EM | 0.00 | homo-tetramer | 11 x ATP | HHblits | 0.30 |
| ``` target    EVLARVGHKLAEQTGDARFADVWKLVDEKRTDAHLQRILDHSSNTKGYDALDLEAKAKKGIPTLMMNRTYPKAVGYEQVA 7wbb.1    --------------------------------------------------------------------------------  target    DSRPWYTKSGRLEFYRDEDEFIEAGENLPVHREPIDSTFYEPNVIVSAPHEALRPAGPEDYGVELSDMSGEIRQGRNVVK 7wbb.1    --------------------------------------------------------------------------------  target    AWAELKKTPHPLAKDGYRFVFHTPKYRHGAHTMPIDTDMVAMLFGPFGDVYRHDRRTPYVAEGYVDIHPSDAREIGVEDG 7wbb.1    ---------------------------------------------------DHGKETC-----TAYIHPNVLSSLEINPG  target    DYVFIDSDPEDRPFRGWQKNKRDYEFSRLLCRARYYPGTPRGVTRMWFNMYGATPGSVEGQKSREDGLAKNPRTNYQAMF 7wbb.1    SFCTVGKI-------GEN-------GILVIARAGDEEVHPVNVITLS---------------------------------  target    RSGSHQSATRGWLKPTWMTDSLVRKGLFGQSIGKGFLPDVHCPTGAPRESIVKITKAEPGGLGAEGLWRPAALGLRPGYE 7wbb.1    --------------------------------------------------------------------------------  target    SKSMKTYLDGGYVDDADRQGGQG 7wbb.1    ----------------------- ``` | | | | | | | | | | | | | | | | | | | | | | | | | | | | | | | | | | | | | | | | | | | | | | | | | |
|  | 7wbb.1.B | AFG2 isoform 1  *Cryo-EM structure of substrate engaged Drg1 hexamer* | 0.04 | 0.00 | 19.30 | 0.13 | 212-287 | EM | 0.00 | homo-tetramer | 11 x ATP | HHblits | 0.30 |
| ``` target    EVLARVGHKLAEQTGDARFADVWKLVDEKRTDAHLQRILDHSSNTKGYDALDLEAKAKKGIPTLMMNRTYPKAVGYEQVA 7wbb.1    --------------------------------------------------------------------------------  target    DSRPWYTKSGRLEFYRDEDEFIEAGENLPVHREPIDSTFYEPNVIVSAPHEALRPAGPEDYGVELSDMSGEIRQGRNVVK 7wbb.1    --------------------------------------------------------------------------------  target    AWAELKKTPHPLAKDGYRFVFHTPKYRHGAHTMPIDTDMVAMLFGPFGDVYRHDRRTPYVAEGYVDIHPSDAREIGVEDG 7wbb.1    ---------------------------------------------------DHGKETC-----TAYIHPNVLSSLEINPG  target    DYVFIDSDPEDRPFRGWQKNKRDYEFSRLLCRARYYPGTPRGVTRMWFNMYGATPGSVEGQKSREDGLAKNPRTNYQAMF 7wbb.1    SFCTVGKI-------GEN-------GILVIARAGDEEVHPVNVITLS---------------------------------  target    RSGSHQSATRGWLKPTWMTDSLVRKGLFGQSIGKGFLPDVHCPTGAPRESIVKITKAEPGGLGAEGLWRPAALGLRPGYE 7wbb.1    --------------------------------------------------------------------------------  target    SKSMKTYLDGGYVDDADRQGGQG 7wbb.1    ----------------------- ``` | | | | | | | | | | | | | | | | | | | | | | | | | | | | | | | | | | | | | | | | | | | | | | | | | |
|  | 7wbb.1.C | AFG2 isoform 1  *Cryo-EM structure of substrate engaged Drg1 hexamer* | 0.04 | 0.00 | 19.30 | 0.13 | 212-287 | EM | 0.00 | homo-tetramer | 11 x ATP | HHblits | 0.30 |
| ``` target    EVLARVGHKLAEQTGDARFADVWKLVDEKRTDAHLQRILDHSSNTKGYDALDLEAKAKKGIPTLMMNRTYPKAVGYEQVA 7wbb.1    --------------------------------------------------------------------------------  target    DSRPWYTKSGRLEFYRDEDEFIEAGENLPVHREPIDSTFYEPNVIVSAPHEALRPAGPEDYGVELSDMSGEIRQGRNVVK 7wbb.1    --------------------------------------------------------------------------------  target    AWAELKKTPHPLAKDGYRFVFHTPKYRHGAHTMPIDTDMVAMLFGPFGDVYRHDRRTPYVAEGYVDIHPSDAREIGVEDG 7wbb.1    ---------------------------------------------------DHGKETC-----TAYIHPNVLSSLEINPG  target    DYVFIDSDPEDRPFRGWQKNKRDYEFSRLLCRARYYPGTPRGVTRMWFNMYGATPGSVEGQKSREDGLAKNPRTNYQAMF 7wbb.1    SFCTVGKI-------GEN-------GILVIARAGDEEVHPVNVITLS---------------------------------  target    RSGSHQSATRGWLKPTWMTDSLVRKGLFGQSIGKGFLPDVHCPTGAPRESIVKITKAEPGGLGAEGLWRPAALGLRPGYE 7wbb.1    --------------------------------------------------------------------------------  target    SKSMKTYLDGGYVDDADRQGGQG 7wbb.1    ----------------------- ``` | | | | | | | | | | | | | | | | | | | | | | | | | | | | | | | | | | | | | | | | | | | | | | | | | |
|  | 7wbb.1.D | AFG2 isoform 1  *Cryo-EM structure of substrate engaged Drg1 hexamer* | 0.04 | 0.00 | 19.30 | 0.13 | 212-287 | EM | 0.00 | homo-tetramer | 11 x ATP | HHblits | 0.30 |
| ``` target    EVLARVGHKLAEQTGDARFADVWKLVDEKRTDAHLQRILDHSSNTKGYDALDLEAKAKKGIPTLMMNRTYPKAVGYEQVA 7wbb.1    --------------------------------------------------------------------------------  target    DSRPWYTKSGRLEFYRDEDEFIEAGENLPVHREPIDSTFYEPNVIVSAPHEALRPAGPEDYGVELSDMSGEIRQGRNVVK 7wbb.1    --------------------------------------------------------------------------------  target    AWAELKKTPHPLAKDGYRFVFHTPKYRHGAHTMPIDTDMVAMLFGPFGDVYRHDRRTPYVAEGYVDIHPSDAREIGVEDG 7wbb.1    ---------------------------------------------------DHGKETC-----TAYIHPNVLSSLEINPG  target    DYVFIDSDPEDRPFRGWQKNKRDYEFSRLLCRARYYPGTPRGVTRMWFNMYGATPGSVEGQKSREDGLAKNPRTNYQAMF 7wbb.1    SFCTVGKI-------GEN-------GILVIARAGDEEVHPVNVITLS---------------------------------  target    RSGSHQSATRGWLKPTWMTDSLVRKGLFGQSIGKGFLPDVHCPTGAPRESIVKITKAEPGGLGAEGLWRPAALGLRPGYE 7wbb.1    --------------------------------------------------------------------------------  target    SKSMKTYLDGGYVDDADRQGGQG 7wbb.1    ----------------------- ``` | | | | | | | | | | | | | | | | | | | | | | | | | | | | | | | | | | | | | | | | | | | | | | | | | |
|  | 7wbb.1.E | AFG2 isoform 1  *Cryo-EM structure of substrate engaged Drg1 hexamer* | 0.04 | 0.00 | 19.30 | 0.13 | 212-287 | EM | 0.00 | homo-tetramer | 11 x ATP | HHblits | 0.30 |
| ``` target    EVLARVGHKLAEQTGDARFADVWKLVDEKRTDAHLQRILDHSSNTKGYDALDLEAKAKKGIPTLMMNRTYPKAVGYEQVA 7wbb.1    --------------------------------------------------------------------------------  target    DSRPWYTKSGRLEFYRDEDEFIEAGENLPVHREPIDSTFYEPNVIVSAPHEALRPAGPEDYGVELSDMSGEIRQGRNVVK 7wbb.1    --------------------------------------------------------------------------------  target    AWAELKKTPHPLAKDGYRFVFHTPKYRHGAHTMPIDTDMVAMLFGPFGDVYRHDRRTPYVAEGYVDIHPSDAREIGVEDG 7wbb.1    ---------------------------------------------------DHGKETC-----TAYIHPNVLSSLEINPG  target    DYVFIDSDPEDRPFRGWQKNKRDYEFSRLLCRARYYPGTPRGVTRMWFNMYGATPGSVEGQKSREDGLAKNPRTNYQAMF 7wbb.1    SFCTVGKI-------GEN-------GILVIARAGDEEVHPVNVITLS---------------------------------  target    RSGSHQSATRGWLKPTWMTDSLVRKGLFGQSIGKGFLPDVHCPTGAPRESIVKITKAEPGGLGAEGLWRPAALGLRPGYE 7wbb.1    --------------------------------------------------------------------------------  target    SKSMKTYLDGGYVDDADRQGGQG 7wbb.1    ----------------------- ``` | | | | | | | | | | | | | | | | | | | | | | | | | | | | | | | | | | | | | | | | | | | | | | | | | |
|  | 7wbb.1.G | AFG2 isoform 1  *Cryo-EM structure of substrate engaged Drg1 hexamer* | 0.04 | 0.00 | 19.30 | 0.13 | 212-287 | EM | 0.00 | homo-tetramer | 11 x ATP | HHblits | 0.30 |
| ``` target    EVLARVGHKLAEQTGDARFADVWKLVDEKRTDAHLQRILDHSSNTKGYDALDLEAKAKKGIPTLMMNRTYPKAVGYEQVA 7wbb.1    --------------------------------------------------------------------------------  target    DSRPWYTKSGRLEFYRDEDEFIEAGENLPVHREPIDSTFYEPNVIVSAPHEALRPAGPEDYGVELSDMSGEIRQGRNVVK 7wbb.1    --------------------------------------------------------------------------------  target    AWAELKKTPHPLAKDGYRFVFHTPKYRHGAHTMPIDTDMVAMLFGPFGDVYRHDRRTPYVAEGYVDIHPSDAREIGVEDG 7wbb.1    ---------------------------------------------------DHGKETC-----TAYIHPNVLSSLEINPG  target    DYVFIDSDPEDRPFRGWQKNKRDYEFSRLLCRARYYPGTPRGVTRMWFNMYGATPGSVEGQKSREDGLAKNPRTNYQAMF 7wbb.1    SFCTVGKI-------GEN-------GILVIARAGDEEVHPVNVITLS---------------------------------  target    RSGSHQSATRGWLKPTWMTDSLVRKGLFGQSIGKGFLPDVHCPTGAPRESIVKITKAEPGGLGAEGLWRPAALGLRPGYE 7wbb.1    --------------------------------------------------------------------------------  target    SKSMKTYLDGGYVDDADRQGGQG 7wbb.1    ----------------------- ``` | | | | | | | | | | | | | | | | | | | | | | | | | | | | | | | | | | | | | | | | | | | | | | | | | |
|  | 3m9s.1.C | NADH-quinone oxidoreductase subunit 3  *Crystal structure of respiratory complex I from Thermus thermophilus* | 0.05 | 0.00 | 19.23 | 0.12 | 222-290 | X-ray | 4.50 | monomer | 7 x SF4, 2 x FES, 1 x FMN | HHblits | 0.30 |
| ``` target    EVLARVGHKLAEQTGDARFADVWKLVDEKRTDAHLQRILDHSSNTKGYDALDLEAKAKKGIPTLMMNRTYPKAVGYEQVA 3m9s.1    --------------------------------------------------------------------------------  target    DSRPWYTKSGRLEFYRDEDEFIEAGENLPVHREPIDSTFYEPNVIVSAPHEALRPAGPEDYGVELSDMSGEIRQGRNVVK 3m9s.1    --------------------------------------------------------------------------------  target    AWAELKKTPHPLAKDGYRFVFHTPKYRHGAHTMPIDTDMVAMLFGPFGDVYRHDRRTPYVAEGYVDIHPSDAREIGVEDG 3m9s.1    -------------------------------------------------------------RAELWAHPETARAEALPEG  target    DYVFIDSDPEDRPFRGWQKNKRDYEFSRLLCRARYYPGTPRGVTRMWFNMYGATPGSVEGQKSREDGLAKNPRTNYQAMF 3m9s.1    AQVAVETPF-----------------GRVEARVVHREDVPKGHLYLSALG------------------------------  target    RSGSHQSATRGWLKPTWMTDSLVRKGLFGQSIGKGFLPDVHCPTGAPRESIVKITKAEPGGLGAEGLWRPAALGLRPGYE 3m9s.1    --------------------------------------------------------------------------------  target    SKSMKTYLDGGYVDDADRQGGQG 3m9s.1    ----------------------- ``` | | | | | | | | | | | | | | | | | | | | | | | | | | | | | | | | | | | | | | | | | | | | | | | | | |
|  | 2fug.2.C | NADH-quinone oxidoreductase chain 3  *Crystal structure of the hydrophilic domain of respiratory complex I from Thermus thermophilus* | 0.03 | 0.00 | 19.23 | 0.12 | 222-290 | X-ray | 3.30 | monomer | 7 x SF4, 2 x FES, 1 x FMN | HHblits | 0.30 |
| ``` target    EVLARVGHKLAEQTGDARFADVWKLVDEKRTDAHLQRILDHSSNTKGYDALDLEAKAKKGIPTLMMNRTYPKAVGYEQVA 2fug.2    --------------------------------------------------------------------------------  target    DSRPWYTKSGRLEFYRDEDEFIEAGENLPVHREPIDSTFYEPNVIVSAPHEALRPAGPEDYGVELSDMSGEIRQGRNVVK 2fug.2    --------------------------------------------------------------------------------  target    AWAELKKTPHPLAKDGYRFVFHTPKYRHGAHTMPIDTDMVAMLFGPFGDVYRHDRRTPYVAEGYVDIHPSDAREIGVEDG 2fug.2    -------------------------------------------------------------RAELWAHPETARAEALPEG  target    DYVFIDSDPEDRPFRGWQKNKRDYEFSRLLCRARYYPGTPRGVTRMWFNMYGATPGSVEGQKSREDGLAKNPRTNYQAMF 2fug.2    AQVAVETPF-----------------GRVEARVVHREDVPKGHLYLSALG------------------------------  target    RSGSHQSATRGWLKPTWMTDSLVRKGLFGQSIGKGFLPDVHCPTGAPRESIVKITKAEPGGLGAEGLWRPAALGLRPGYE 2fug.2    --------------------------------------------------------------------------------  target    SKSMKTYLDGGYVDDADRQGGQG 2fug.2    ----------------------- ``` | | | | | | | | | | | | | | | | | | | | | | | | | | | | | | | | | | | | | | | | | | | | | | | | | |
|  | 6zjl.1.C | NADH-quinone oxidoreductase subunit 3  *Respiratory complex I from Thermus thermophilus, NAD+ dataset, major state* | 0.05 | 0.00 | 19.23 | 0.12 | 222-290 | EM | 0.00 | monomer | 7 x SF4, 1 x FMN, 2 x FES | HHblits | 0.30 |
| ``` target    EVLARVGHKLAEQTGDARFADVWKLVDEKRTDAHLQRILDHSSNTKGYDALDLEAKAKKGIPTLMMNRTYPKAVGYEQVA 6zjl.1    --------------------------------------------------------------------------------  target    DSRPWYTKSGRLEFYRDEDEFIEAGENLPVHREPIDSTFYEPNVIVSAPHEALRPAGPEDYGVELSDMSGEIRQGRNVVK 6zjl.1    --------------------------------------------------------------------------------  target    AWAELKKTPHPLAKDGYRFVFHTPKYRHGAHTMPIDTDMVAMLFGPFGDVYRHDRRTPYVAEGYVDIHPSDAREIGVEDG 6zjl.1    -------------------------------------------------------------RAELWAHPETARAEALPEG  target    DYVFIDSDPEDRPFRGWQKNKRDYEFSRLLCRARYYPGTPRGVTRMWFNMYGATPGSVEGQKSREDGLAKNPRTNYQAMF 6zjl.1    AQVAVETPF-----------------GRVEARVVHREDVPKGHLYLSALG------------------------------  target    RSGSHQSATRGWLKPTWMTDSLVRKGLFGQSIGKGFLPDVHCPTGAPRESIVKITKAEPGGLGAEGLWRPAALGLRPGYE 6zjl.1    --------------------------------------------------------------------------------  target    SKSMKTYLDGGYVDDADRQGGQG 6zjl.1    ----------------------- ``` | | | | | | | | | | | | | | | | | | | | | | | | | | | | | | | | | | | | | | | | | | | | | | | | | |
|  | 6q8o.1.C | NADH-quinone oxidoreductase subunit 3  *Respiratory complex I from Thermus thermophilus with bound Piericidin A* | 0.05 | 0.00 | 19.23 | 0.12 | 222-290 | X-ray | 3.61 | monomer | 7 x SF4, 1 x FMN, 2 x FES, 1 x HQH | HHblits | 0.30 |
| ``` target    EVLARVGHKLAEQTGDARFADVWKLVDEKRTDAHLQRILDHSSNTKGYDALDLEAKAKKGIPTLMMNRTYPKAVGYEQVA 6q8o.1    --------------------------------------------------------------------------------  target    DSRPWYTKSGRLEFYRDEDEFIEAGENLPVHREPIDSTFYEPNVIVSAPHEALRPAGPEDYGVELSDMSGEIRQGRNVVK 6q8o.1    --------------------------------------------------------------------------------  target    AWAELKKTPHPLAKDGYRFVFHTPKYRHGAHTMPIDTDMVAMLFGPFGDVYRHDRRTPYVAEGYVDIHPSDAREIGVEDG 6q8o.1    -------------------------------------------------------------RAELWAHPETARAEALPEG  target    DYVFIDSDPEDRPFRGWQKNKRDYEFSRLLCRARYYPGTPRGVTRMWFNMYGATPGSVEGQKSREDGLAKNPRTNYQAMF 6q8o.1    AQVAVETPF-----------------GRVEARVVHREDVPKGHLYLSALG------------------------------  target    RSGSHQSATRGWLKPTWMTDSLVRKGLFGQSIGKGFLPDVHCPTGAPRESIVKITKAEPGGLGAEGLWRPAALGLRPGYE 6q8o.1    --------------------------------------------------------------------------------  target    SKSMKTYLDGGYVDDADRQGGQG 6q8o.1    ----------------------- ``` | | | | | | | | | | | | | | | | | | | | | | | | | | | | | | | | | | | | | | | | | | | | | | | | | |
|  | 6zjy.1.C | NADH-quinone oxidoreductase subunit 3  *Respiratory complex I from Thermus thermophilus, NAD+ dataset, minor state* | 0.05 | 0.00 | 19.23 | 0.12 | 222-290 | EM | 0.00 | monomer | 7 x SF4, 2 x FES | HHblits | 0.30 |
| ``` target    EVLARVGHKLAEQTGDARFADVWKLVDEKRTDAHLQRILDHSSNTKGYDALDLEAKAKKGIPTLMMNRTYPKAVGYEQVA 6zjy.1    --------------------------------------------------------------------------------  target    DSRPWYTKSGRLEFYRDEDEFIEAGENLPVHREPIDSTFYEPNVIVSAPHEALRPAGPEDYGVELSDMSGEIRQGRNVVK 6zjy.1    --------------------------------------------------------------------------------  target    AWAELKKTPHPLAKDGYRFVFHTPKYRHGAHTMPIDTDMVAMLFGPFGDVYRHDRRTPYVAEGYVDIHPSDAREIGVEDG 6zjy.1    -------------------------------------------------------------RAELWAHPETARAEALPEG  target    DYVFIDSDPEDRPFRGWQKNKRDYEFSRLLCRARYYPGTPRGVTRMWFNMYGATPGSVEGQKSREDGLAKNPRTNYQAMF 6zjy.1    AQVAVETPF-----------------GRVEARVVHREDVPKGHLYLSALG------------------------------  target    RSGSHQSATRGWLKPTWMTDSLVRKGLFGQSIGKGFLPDVHCPTGAPRESIVKITKAEPGGLGAEGLWRPAALGLRPGYE 6zjy.1    --------------------------------------------------------------------------------  target    SKSMKTYLDGGYVDDADRQGGQG 6zjy.1    ----------------------- ``` | | | | | | | | | | | | | | | | | | | | | | | | | | | | | | | | | | | | | | | | | | | | | | | | | |
|  | 6zjn.1.C | NADH-quinone oxidoreductase subunit 3  *Respiratory complex I from Thermus thermophilus, NADH dataset, minor state* | 0.06 | 0.00 | 19.23 | 0.12 | 222-290 | EM | 0.00 | monomer | 7 x SF4, 2 x FES | HHblits | 0.30 |
| ``` target    EVLARVGHKLAEQTGDARFADVWKLVDEKRTDAHLQRILDHSSNTKGYDALDLEAKAKKGIPTLMMNRTYPKAVGYEQVA 6zjn.1    --------------------------------------------------------------------------------  target    DSRPWYTKSGRLEFYRDEDEFIEAGENLPVHREPIDSTFYEPNVIVSAPHEALRPAGPEDYGVELSDMSGEIRQGRNVVK 6zjn.1    --------------------------------------------------------------------------------  target    AWAELKKTPHPLAKDGYRFVFHTPKYRHGAHTMPIDTDMVAMLFGPFGDVYRHDRRTPYVAEGYVDIHPSDAREIGVEDG 6zjn.1    -------------------------------------------------------------RAELWAHPETARAEALPEG  target    DYVFIDSDPEDRPFRGWQKNKRDYEFSRLLCRARYYPGTPRGVTRMWFNMYGATPGSVEGQKSREDGLAKNPRTNYQAMF 6zjn.1    AQVAVETPF-----------------GRVEARVVHREDVPKGHLYLSALG------------------------------  target    RSGSHQSATRGWLKPTWMTDSLVRKGLFGQSIGKGFLPDVHCPTGAPRESIVKITKAEPGGLGAEGLWRPAALGLRPGYE 6zjn.1    --------------------------------------------------------------------------------  target    SKSMKTYLDGGYVDDADRQGGQG 6zjn.1    ----------------------- ``` | | | | | | | | | | | | | | | | | | | | | | | | | | | | | | | | | | | | | | | | | | | | | | | | | |
|  | 6ziy.1.C | NADH-quinone oxidoreductase subunit 3  *Respiratory complex I from Thermus thermophilus, NADH dataset, major state* | 0.04 | 0.00 | 19.23 | 0.12 | 222-290 | EM | 0.00 | monomer | 7 x SF4, 1 x FMN, 1 x NAI, 2 x FES | HHblits | 0.30 |
| ``` target    EVLARVGHKLAEQTGDARFADVWKLVDEKRTDAHLQRILDHSSNTKGYDALDLEAKAKKGIPTLMMNRTYPKAVGYEQVA 6ziy.1    --------------------------------------------------------------------------------  target    DSRPWYTKSGRLEFYRDEDEFIEAGENLPVHREPIDSTFYEPNVIVSAPHEALRPAGPEDYGVELSDMSGEIRQGRNVVK 6ziy.1    --------------------------------------------------------------------------------  target    AWAELKKTPHPLAKDGYRFVFHTPKYRHGAHTMPIDTDMVAMLFGPFGDVYRHDRRTPYVAEGYVDIHPSDAREIGVEDG 6ziy.1    -------------------------------------------------------------RAELWAHPETARAEALPEG  target    DYVFIDSDPEDRPFRGWQKNKRDYEFSRLLCRARYYPGTPRGVTRMWFNMYGATPGSVEGQKSREDGLAKNPRTNYQAMF 6ziy.1    AQVAVETPF-----------------GRVEARVVHREDVPKGHLYLSALG------------------------------  target    RSGSHQSATRGWLKPTWMTDSLVRKGLFGQSIGKGFLPDVHCPTGAPRESIVKITKAEPGGLGAEGLWRPAALGLRPGYE 6ziy.1    --------------------------------------------------------------------------------  target    SKSMKTYLDGGYVDDADRQGGQG 6ziy.1    ----------------------- ``` | | | | | | | | | | | | | | | | | | | | | | | | | | | | | | | | | | | | | | | | | | | | | | | | | |
|  | 2d9r.1.A | conserved hypothetical protein  *Structure of Conserved Protein of Unknown Function PG0164 from Porphyromonas gingivalis [W83]* | 0.03 |  | 16.00 | 0.12 | 185-246 | X-ray | 2.01 | monomer |  | HHblits | 0.27 |
| ``` target    EVLARVGHKLAEQTGDARFADVWKLVDEKRTDAHLQRILDHSSNTKGYDALDLEAKAKKGIPTLMMNRTYPKAVGYEQVA 2d9r.1    --------------------------------------------------------------------------------  target    DSRPWYTKSGRLEFYRDEDEFIEAGENLPVHREPIDSTFYEPNVIVSAPHEALRPAGPEDYGVELSDMSGEIRQGRNVVK 2d9r.1    --------------------------------------------------------------------------------  target    AWAELKKTPHPLAKDGYRFVFHTPKYRHGAHTMPIDTDMVAMLFGPFGDVYRHDRRTPYVAEGYVDIHPSDAREIGVEDG 2d9r.1    ------------------------KGRVRVNATFDGYPYTGYIV-R------MGLP-----CHILGLRQDIRRAIGKQPG  target    DYVFIDSDPEDRPFRGWQKNKRDYEFSRLLCRARYYPGTPRGVTRMWFNMYGATPGSVEGQKSREDGLAKNPRTNYQAMF 2d9r.1    DSVYVT--------------------------------------------------------------------------  target    RSGSHQSATRGWLKPTWMTDSLVRKGLFGQSIGKGFLPDVHCPTGAPRESIVKITKAEPGGLGAEGLWRPAALGLRPGYE 2d9r.1    --------------------------------------------------------------------------------  target    SKSMKTYLDGGYVDDADRQGGQG 2d9r.1    ----------------------- ``` | | | | | | | | | | | | | | | | | | | | | | | | | | | | | | | | | | | | | | | | | | | | | | | | | |
|  | 4kdl.1.A | Transitional endoplasmic reticulum ATPase  *Crystal structure of p97/VCP N in complex with OTU1 UBXL* | 0.03 | 0.00 | 16.00 | 0.12 | 224-289 | X-ray | 1.81 | monomer |  | HHblits | 0.26 |
| ``` target    EVLARVGHKLAEQTGDARFADVWKLVDEKRTDAHLQRILDHSSNTKGYDALDLEAKAKKGIPTLMMNRTYPKAVGYEQVA 4kdl.1    --------------------------------------------------------------------------------  target    DSRPWYTKSGRLEFYRDEDEFIEAGENLPVHREPIDSTFYEPNVIVSAPHEALRPAGPEDYGVELSDMSGEIRQGRNVVK 4kdl.1    --------------------------------------------------------------------------------  target    AWAELKKTPHPLAKDGYRFVFHTPKYRHGAHTMPIDTDMVAMLFGPFGDVYRHDRRTPYVAEGYVDIHPSDAREIGVEDG 4kdl.1    ---------------------------------------------------------------VVSLSQPKMDELQLFRG  target    DYVFIDSDPEDRPFRGWQKNKRDYEFSRLLCRARYYPGTPRGVTRMWFNMYGATPGSVEGQKSREDGLAKNPRTNYQAMF 4kdl.1    DTVLLKGKKR----------------REAVCIVLSDDTCSDEKIRMNRV-------------------------------  target    RSGSHQSATRGWLKPTWMTDSLVRKGLFGQSIGKGFLPDVHCPTGAPRESIVKITKAEPGGLGAEGLWRPAALGLRPGYE 4kdl.1    --------------------------------------------------------------------------------  target    SKSMKTYLDGGYVDDADRQGGQG 4kdl.1    ----------------------- ``` | | | | | | | | | | | | | | | | | | | | | | | | | | | | | | | | | | | | | | | | | | | | | | | | | |
|  | 4kdi.2.A | Transitional endoplasmic reticulum ATPase  *Crystal structure of p97/VCP N in complex with OTU1 UBXL* | 0.03 | 0.00 | 16.00 | 0.12 | 224-289 | X-ray | 1.86 | monomer |  | HHblits | 0.26 |
| ``` target    EVLARVGHKLAEQTGDARFADVWKLVDEKRTDAHLQRILDHSSNTKGYDALDLEAKAKKGIPTLMMNRTYPKAVGYEQVA 4kdi.2    --------------------------------------------------------------------------------  target    DSRPWYTKSGRLEFYRDEDEFIEAGENLPVHREPIDSTFYEPNVIVSAPHEALRPAGPEDYGVELSDMSGEIRQGRNVVK 4kdi.2    --------------------------------------------------------------------------------  target    AWAELKKTPHPLAKDGYRFVFHTPKYRHGAHTMPIDTDMVAMLFGPFGDVYRHDRRTPYVAEGYVDIHPSDAREIGVEDG 4kdi.2    ---------------------------------------------------------------VVSLSQPKMDELQLFRG  target    DYVFIDSDPEDRPFRGWQKNKRDYEFSRLLCRARYYPGTPRGVTRMWFNMYGATPGSVEGQKSREDGLAKNPRTNYQAMF 4kdi.2    DTVLLKGKKR----------------REAVCIVLSDDTCSDEKIRMNRV-------------------------------  target    RSGSHQSATRGWLKPTWMTDSLVRKGLFGQSIGKGFLPDVHCPTGAPRESIVKITKAEPGGLGAEGLWRPAALGLRPGYE 4kdi.2    --------------------------------------------------------------------------------  target    SKSMKTYLDGGYVDDADRQGGQG 4kdi.2    ----------------------- ``` | | | | | | | | | | | | | | | | | | | | | | | | | | | | | | | | | | | | | | | | | | | | | | | | | |
|  | 4kdi.1.A | Transitional endoplasmic reticulum ATPase  *Crystal structure of p97/VCP N in complex with OTU1 UBXL* | 0.03 | 0.00 | 16.00 | 0.12 | 224-289 | X-ray | 1.86 | monomer |  | HHblits | 0.26 |
| ``` target    EVLARVGHKLAEQTGDARFADVWKLVDEKRTDAHLQRILDHSSNTKGYDALDLEAKAKKGIPTLMMNRTYPKAVGYEQVA 4kdi.1    --------------------------------------------------------------------------------  target    DSRPWYTKSGRLEFYRDEDEFIEAGENLPVHREPIDSTFYEPNVIVSAPHEALRPAGPEDYGVELSDMSGEIRQGRNVVK 4kdi.1    --------------------------------------------------------------------------------  target    AWAELKKTPHPLAKDGYRFVFHTPKYRHGAHTMPIDTDMVAMLFGPFGDVYRHDRRTPYVAEGYVDIHPSDAREIGVEDG 4kdi.1    ---------------------------------------------------------------VVSLSQPKMDELQLFRG  target    DYVFIDSDPEDRPFRGWQKNKRDYEFSRLLCRARYYPGTPRGVTRMWFNMYGATPGSVEGQKSREDGLAKNPRTNYQAMF 4kdi.1    DTVLLKGKKR----------------REAVCIVLSDDTCSDEKIRMNRV-------------------------------  target    RSGSHQSATRGWLKPTWMTDSLVRKGLFGQSIGKGFLPDVHCPTGAPRESIVKITKAEPGGLGAEGLWRPAALGLRPGYE 4kdi.1    --------------------------------------------------------------------------------  target    SKSMKTYLDGGYVDDADRQGGQG 4kdi.1    ----------------------- ``` | | | | | | | | | | | | | | | | | | | | | | | | | | | | | | | | | | | | | | | | | | | | | | | | | |
|  | 3qwz.1.A | Transitional endoplasmic reticulum ATPase  *Crystal structure of FAF1 UBX-p97N-domain complex* | 0.03 | 0.00 | 16.33 | 0.12 | 224-288 | X-ray | 2.00 | monomer |  | HHblits | 0.27 |
| ``` target    EVLARVGHKLAEQTGDARFADVWKLVDEKRTDAHLQRILDHSSNTKGYDALDLEAKAKKGIPTLMMNRTYPKAVGYEQVA 3qwz.1    --------------------------------------------------------------------------------  target    DSRPWYTKSGRLEFYRDEDEFIEAGENLPVHREPIDSTFYEPNVIVSAPHEALRPAGPEDYGVELSDMSGEIRQGRNVVK 3qwz.1    --------------------------------------------------------------------------------  target    AWAELKKTPHPLAKDGYRFVFHTPKYRHGAHTMPIDTDMVAMLFGPFGDVYRHDRRTPYVAEGYVDIHPSDAREIGVEDG 3qwz.1    ---------------------------------------------------------------VVSLSQPKMDELQLFRG  target    DYVFIDSDPEDRPFRGWQKNKRDYEFSRLLCRARYYPGTPRGVTRMWFNMYGATPGSVEGQKSREDGLAKNPRTNYQAMF 3qwz.1    DTVLLKGKKR----------------REAVCIVLSDDTCSDEKIRMNR--------------------------------  target    RSGSHQSATRGWLKPTWMTDSLVRKGLFGQSIGKGFLPDVHCPTGAPRESIVKITKAEPGGLGAEGLWRPAALGLRPGYE 3qwz.1    --------------------------------------------------------------------------------  target    SKSMKTYLDGGYVDDADRQGGQG 3qwz.1    ----------------------- ``` | | | | | | | | | | | | | | | | | | | | | | | | | | | | | | | | | | | | | | | | | | | | | | | | | |
|  | 5g4g.1.A | VCP-LIKE ATPASE  *Structure of the ATPgS-bound VAT complex* | 0.02 | 0.00 | 21.28 | 0.11 | 224-286 | EM | 7.80 | monomer |  | HHblits | 0.29 |
| ``` target    EVLARVGHKLAEQTGDARFADVWKLVDEKRTDAHLQRILDHSSNTKGYDALDLEAKAKKGIPTLMMNRTYPKAVGYEQVA 5g4g.1    --------------------------------------------------------------------------------  target    DSRPWYTKSGRLEFYRDEDEFIEAGENLPVHREPIDSTFYEPNVIVSAPHEALRPAGPEDYGVELSDMSGEIRQGRNVVK 5g4g.1    --------------------------------------------------------------------------------  target    AWAELKKTPHPLAKDGYRFVFHTPKYRHGAHTMPIDTDMVAMLFGPFGDVYRHDRRTPYVAEGYVDIHPSDAREIGVEDG 5g4g.1    ---------------------------------------------------------------RVRLDESSRRLLDAEIG  target    DYVFIDSDPEDRPFRGWQKNKRDYEFSRLLCRARYYPGTPRGVTRMWFNMYGATPGSVEGQKSREDGLAKNPRTNYQAMF 5g4g.1    DVVEIEKVRK----------------TVGRVYRARPEDENKGIVRI----------------------------------  target    RSGSHQSATRGWLKPTWMTDSLVRKGLFGQSIGKGFLPDVHCPTGAPRESIVKITKAEPGGLGAEGLWRPAALGLRPGYE 5g4g.1    --------------------------------------------------------------------------------  target    SKSMKTYLDGGYVDDADRQGGQG 5g4g.1    ----------------------- ``` | | | | | | | | | | | | | | | | | | | | | | | | | | | | | | | | | | | | | | | | | | | | | | | | | |
|  | 3qc8.1.A | Transitional endoplasmic reticulum ATPase  *Crystal Structure of FAF1 UBX Domain In Complex with p97/VCP N Domain Reveals The Conserved FcisP Touch-Turn Motif of UBX Domain Suffering Conformational Change* | 0.03 | 0.00 | 16.67 | 0.11 | 224-287 | X-ray | 2.20 | monomer |  | HHblits | 0.27 |
| ``` target    EVLARVGHKLAEQTGDARFADVWKLVDEKRTDAHLQRILDHSSNTKGYDALDLEAKAKKGIPTLMMNRTYPKAVGYEQVA 3qc8.1    --------------------------------------------------------------------------------  target    DSRPWYTKSGRLEFYRDEDEFIEAGENLPVHREPIDSTFYEPNVIVSAPHEALRPAGPEDYGVELSDMSGEIRQGRNVVK 3qc8.1    --------------------------------------------------------------------------------  target    AWAELKKTPHPLAKDGYRFVFHTPKYRHGAHTMPIDTDMVAMLFGPFGDVYRHDRRTPYVAEGYVDIHPSDAREIGVEDG 3qc8.1    ---------------------------------------------------------------VVSLSQPKMDELQLFRG  target    DYVFIDSDPEDRPFRGWQKNKRDYEFSRLLCRARYYPGTPRGVTRMWFNMYGATPGSVEGQKSREDGLAKNPRTNYQAMF 3qc8.1    DTVLLKGKKR----------------REAVCIVLSDDTCSDEKIRMN---------------------------------  target    RSGSHQSATRGWLKPTWMTDSLVRKGLFGQSIGKGFLPDVHCPTGAPRESIVKITKAEPGGLGAEGLWRPAALGLRPGYE 3qc8.1    --------------------------------------------------------------------------------  target    SKSMKTYLDGGYVDDADRQGGQG 3qc8.1    ----------------------- ``` | | | | | | | | | | | | | | | | | | | | | | | | | | | | | | | | | | | | | | | | | | | | | | | | | |
|  | 3tiw.1.A | Transitional endoplasmic reticulum ATPase  *Crystal structure of p97N in complex with the C-terminus of gp78* | 0.04 | 0.00 | 17.02 | 0.11 | 224-286 | X-ray | 1.80 | monomer |  | HHblits | 0.28 |
| ``` target    EVLARVGHKLAEQTGDARFADVWKLVDEKRTDAHLQRILDHSSNTKGYDALDLEAKAKKGIPTLMMNRTYPKAVGYEQVA 3tiw.1    --------------------------------------------------------------------------------  target    DSRPWYTKSGRLEFYRDEDEFIEAGENLPVHREPIDSTFYEPNVIVSAPHEALRPAGPEDYGVELSDMSGEIRQGRNVVK 3tiw.1    --------------------------------------------------------------------------------  target    AWAELKKTPHPLAKDGYRFVFHTPKYRHGAHTMPIDTDMVAMLFGPFGDVYRHDRRTPYVAEGYVDIHPSDAREIGVEDG 3tiw.1    ---------------------------------------------------------------VVSLSQPKMDELQLFRG  target    DYVFIDSDPEDRPFRGWQKNKRDYEFSRLLCRARYYPGTPRGVTRMWFNMYGATPGSVEGQKSREDGLAKNPRTNYQAMF 3tiw.1    DTVLLKGKKR----------------REAVCIVLSDDTCSDEKIRM----------------------------------  target    RSGSHQSATRGWLKPTWMTDSLVRKGLFGQSIGKGFLPDVHCPTGAPRESIVKITKAEPGGLGAEGLWRPAALGLRPGYE 3tiw.1    --------------------------------------------------------------------------------  target    SKSMKTYLDGGYVDDADRQGGQG 3tiw.1    ----------------------- ``` | | | | | | | | | | | | | | | | | | | | | | | | | | | | | | | | | | | | | | | | | | | | | | | | | |
|  | 3tiw.2.A | Transitional endoplasmic reticulum ATPase  *Crystal structure of p97N in complex with the C-terminus of gp78* | 0.04 | 0.00 | 17.02 | 0.11 | 224-286 | X-ray | 1.80 | monomer |  | HHblits | 0.28 |
| ``` target    EVLARVGHKLAEQTGDARFADVWKLVDEKRTDAHLQRILDHSSNTKGYDALDLEAKAKKGIPTLMMNRTYPKAVGYEQVA 3tiw.2    --------------------------------------------------------------------------------  target    DSRPWYTKSGRLEFYRDEDEFIEAGENLPVHREPIDSTFYEPNVIVSAPHEALRPAGPEDYGVELSDMSGEIRQGRNVVK 3tiw.2    --------------------------------------------------------------------------------  target    AWAELKKTPHPLAKDGYRFVFHTPKYRHGAHTMPIDTDMVAMLFGPFGDVYRHDRRTPYVAEGYVDIHPSDAREIGVEDG 3tiw.2    ---------------------------------------------------------------VVSLSQPKMDELQLFRG  target    DYVFIDSDPEDRPFRGWQKNKRDYEFSRLLCRARYYPGTPRGVTRMWFNMYGATPGSVEGQKSREDGLAKNPRTNYQAMF 3tiw.2    DTVLLKGKKR----------------REAVCIVLSDDTCSDEKIRM----------------------------------  target    RSGSHQSATRGWLKPTWMTDSLVRKGLFGQSIGKGFLPDVHCPTGAPRESIVKITKAEPGGLGAEGLWRPAALGLRPGYE 3tiw.2    --------------------------------------------------------------------------------  target    SKSMKTYLDGGYVDDADRQGGQG 3tiw.2    ----------------------- ``` | | | | | | | | | | | | | | | | | | | | | | | | | | | | | | | | | | | | | | | | | | | | | | | | | |
|  | 4ga5.1.A | Putative thymidine phosphorylase  *Crystal structure of AMP phosphorylase C-terminal deletion mutant in the apo-form* | 0.04 |  | 19.57 | 0.11 | 224-286 | X-ray | 3.25 | homo-dimer |  | HHblits | 0.29 |
| ``` target    EVLARVGHKLAEQTGDARFADVWKLVDEKRTDAHLQRILDHSSNTKGYDALDLEAKAKKGIPTLMMNRTYPKAVGYEQVA 4ga5.1    --------------------------------------------------------------------------------  target    DSRPWYTKSGRLEFYRDEDEFIEAGENLPVHREPIDSTFYEPNVIVSAPHEALRPAGPEDYGVELSDMSGEIRQGRNVVK 4ga5.1    --------------------------------------------------------------------------------  target    AWAELKKTPHPLAKDGYRFVFHTPKYRHGAHTMPIDTDMVAMLFGPFGDVYRHDRRTPYVAEGYVDIHPSDAREIGVEDG 4ga5.1    ---------------------------------------------------------------TVLINEEDAKEAKLHPD  target    DYVFIDSDPEDRPFRGWQKNKRDYEFSRLLCRARYYPGTPRGVTRMWFNMYGATPGSVEGQKSREDGLAKNPRTNYQAMF 4ga5.1    DLVKIEAGK-----------------KAVYGSVALSNLVGKGEVGI----------------------------------  target    RSGSHQSATRGWLKPTWMTDSLVRKGLFGQSIGKGFLPDVHCPTGAPRESIVKITKAEPGGLGAEGLWRPAALGLRPGYE 4ga5.1    --------------------------------------------------------------------------------  target    SKSMKTYLDGGYVDDADRQGGQG 4ga5.1    ----------------------- ``` | | | | | | | | | | | | | | | | | | | | | | | | | | | | | | | | | | | | | | | | | | | | | | | | | |
|  | 4ga6.1.A | Putative thymidine phosphorylase  *Crystal structure of AMP phosphorylase C-terminal deletion mutant in complex with substrates* | 0.04 |  | 19.57 | 0.11 | 224-286 | X-ray | 2.21 | homo-dimer | 2 x AMP | HHblits | 0.29 |
| ``` target    EVLARVGHKLAEQTGDARFADVWKLVDEKRTDAHLQRILDHSSNTKGYDALDLEAKAKKGIPTLMMNRTYPKAVGYEQVA 4ga6.1    --------------------------------------------------------------------------------  target    DSRPWYTKSGRLEFYRDEDEFIEAGENLPVHREPIDSTFYEPNVIVSAPHEALRPAGPEDYGVELSDMSGEIRQGRNVVK 4ga6.1    --------------------------------------------------------------------------------  target    AWAELKKTPHPLAKDGYRFVFHTPKYRHGAHTMPIDTDMVAMLFGPFGDVYRHDRRTPYVAEGYVDIHPSDAREIGVEDG 4ga6.1    ---------------------------------------------------------------TVLINEEDAKEAKLHPD  target    DYVFIDSDPEDRPFRGWQKNKRDYEFSRLLCRARYYPGTPRGVTRMWFNMYGATPGSVEGQKSREDGLAKNPRTNYQAMF 4ga6.1    DLVKIEAGK-----------------KAVYGSVALSNLVGKGEVGI----------------------------------  target    RSGSHQSATRGWLKPTWMTDSLVRKGLFGQSIGKGFLPDVHCPTGAPRESIVKITKAEPGGLGAEGLWRPAALGLRPGYE 4ga6.1    --------------------------------------------------------------------------------  target    SKSMKTYLDGGYVDDADRQGGQG 4ga6.1    ----------------------- ``` | | | | | | | | | | | | | | | | | | | | | | | | | | | | | | | | | | | | | | | | | | | | | | | | | |
|  | 3plx.1.B | Aspartate 1-decarboxylase  *The crystal structure of aspartate alpha-decarboxylase from Campylobacter jejuni subsp. jejuni NCTC 11168* | 0.03 |  | 15.56 | 0.11 | 225-286 | X-ray | 1.75 | hetero-oligomer |  | HHblits | 0.28 |
| ``` target    EVLARVGHKLAEQTGDARFADVWKLVDEKRTDAHLQRILDHSSNTKGYDALDLEAKAKKGIPTLMMNRTYPKAVGYEQVA 3plx.1    --------------------------------------------------------------------------------  target    DSRPWYTKSGRLEFYRDEDEFIEAGENLPVHREPIDSTFYEPNVIVSAPHEALRPAGPEDYGVELSDMSGEIRQGRNVVK 3plx.1    --------------------------------------------------------------------------------  target    AWAELKKTPHPLAKDGYRFVFHTPKYRHGAHTMPIDTDMVAMLFGPFGDVYRHDRRTPYVAEGYVDIHPSDAREIGVEDG 3plx.1    ----------------------------------------------------------------ISIDEKLLQASGILEY  target    DYVFIDSDPEDRPFRGWQKNKRDYEFSRLLCRARYYPGTPRGVTRMWFNMYGATPGSVEGQKSREDGLAKNPRTNYQAMF 3plx.1    EKVQVVNVNN-----G----------ARFETYTIATQ--EEGVVCL----------------------------------  target    RSGSHQSATRGWLKPTWMTDSLVRKGLFGQSIGKGFLPDVHCPTGAPRESIVKITKAEPGGLGAEGLWRPAALGLRPGYE 3plx.1    --------------------------------------------------------------------------------  target    SKSMKTYLDGGYVDDADRQGGQG 3plx.1    ----------------------- ``` | | | | | | | | | | | | | | | | | | | | | | | | | | | | | | | | | | | | | | | | | | | | | | | | | |
|  | 1uhd.1.B | Aspartate 1-decarboxylase alpha chain  *Crystal structure of aspartate decarboxylase, pyruvoly group bound form* | 0.03 |  | 20.00 | 0.11 | 225-286 | X-ray | 2.00 | hetero-oligomer |  | HHblits | 0.27 |
| ``` target    EVLARVGHKLAEQTGDARFADVWKLVDEKRTDAHLQRILDHSSNTKGYDALDLEAKAKKGIPTLMMNRTYPKAVGYEQVA 1uhd.1    --------------------------------------------------------------------------------  target    DSRPWYTKSGRLEFYRDEDEFIEAGENLPVHREPIDSTFYEPNVIVSAPHEALRPAGPEDYGVELSDMSGEIRQGRNVVK 1uhd.1    --------------------------------------------------------------------------------  target    AWAELKKTPHPLAKDGYRFVFHTPKYRHGAHTMPIDTDMVAMLFGPFGDVYRHDRRTPYVAEGYVDIHPSDAREIGVEDG 1uhd.1    ----------------------------------------------------------------ITIDEDLAKLAKLREG  target    DYVFIDSDPEDRPFRGWQKNKRDYEFSRLLCRARYYPGTPRGVTRMWFNMYGATPGSVEGQKSREDGLAKNPRTNYQAMF 1uhd.1    MKVEIVDVNN-----G----------ERFSTYVILGKK--RGEICV----------------------------------  target    RSGSHQSATRGWLKPTWMTDSLVRKGLFGQSIGKGFLPDVHCPTGAPRESIVKITKAEPGGLGAEGLWRPAALGLRPGYE 1uhd.1    --------------------------------------------------------------------------------  target    SKSMKTYLDGGYVDDADRQGGQG 1uhd.1    ----------------------- ``` | | | | | | | | | | | | | | | | | | | | | | | | | | | | | | | | | | | | | | | | | | | | | | | | | |
|  | 1uhe.1.B | Aspartate 1-decarboxylase alpha chain  *Crystal structure of aspartate decarboxylase, isoaspargine complex* | 0.03 |  | 20.45 | 0.10 | 226-286 | X-ray | 1.55 | hetero-oligomer | 4 x NSN | HHblits | 0.27 |
| ``` target    EVLARVGHKLAEQTGDARFADVWKLVDEKRTDAHLQRILDHSSNTKGYDALDLEAKAKKGIPTLMMNRTYPKAVGYEQVA 1uhe.1    --------------------------------------------------------------------------------  target    DSRPWYTKSGRLEFYRDEDEFIEAGENLPVHREPIDSTFYEPNVIVSAPHEALRPAGPEDYGVELSDMSGEIRQGRNVVK 1uhe.1    --------------------------------------------------------------------------------  target    AWAELKKTPHPLAKDGYRFVFHTPKYRHGAHTMPIDTDMVAMLFGPFGDVYRHDRRTPYVAEGYVDIHPSDAREIGVEDG 1uhe.1    -----------------------------------------------------------------TIDEDLAKLAKLREG  target    DYVFIDSDPEDRPFRGWQKNKRDYEFSRLLCRARYYPGTPRGVTRMWFNMYGATPGSVEGQKSREDGLAKNPRTNYQAMF 1uhe.1    MKVEIVDVNN-----G----------ERFSTYVILGKK--RGEICV----------------------------------  target    RSGSHQSATRGWLKPTWMTDSLVRKGLFGQSIGKGFLPDVHCPTGAPRESIVKITKAEPGGLGAEGLWRPAALGLRPGYE 1uhe.1    --------------------------------------------------------------------------------  target    SKSMKTYLDGGYVDDADRQGGQG 1uhe.1    ----------------------- ``` | | | | | | | | | | | | | | | | | | | | | | | | | | | | | | | | | | | | | | | | | | | | | | | | | |
|  | 5cup.1.A | Phosphate propanoyltransferase  *Structure of Rhodopseudomonas palustris PduL - phosphate bound form* | 0.00 |  | 35.71 | 0.07 | 220-247 | X-ray | 2.10 | homo-dimer | 4 x ZN | HHblits | 0.41 |
| ``` target    EVLARVGHKLAEQTGDARFADVWKLVDEKRTDAHLQRILDHSSNTKGYDALDLEAKAKKGIPTLMMNRTYPKAVGYEQVA 5cup.1    --------------------------------------------------------------------------------  target    DSRPWYTKSGRLEFYRDEDEFIEAGENLPVHREPIDSTFYEPNVIVSAPHEALRPAGPEDYGVELSDMSGEIRQGRNVVK 5cup.1    --------------------------------------------------------------------------------  target    AWAELKKTPHPLAKDGYRFVFHTPKYRHGAHTMPIDTDMVAMLFGPFGDVYRHDRRTPYVAEGYVDIHPSDAREIGVEDG 5cup.1    -----------------------------------------------------------VAQRHIHMHPSTAAKLGLRNG  target    DYVFIDSDPEDRPFRGWQKNKRDYEFSRLLCRARYYPGTPRGVTRMWFNMYGATPGSVEGQKSREDGLAKNPRTNYQAMF 5cup.1    DEVDVEA-------------------------------------------------------------------------  target    RSGSHQSATRGWLKPTWMTDSLVRKGLFGQSIGKGFLPDVHCPTGAPRESIVKITKAEPGGLGAEGLWRPAALGLRPGYE 5cup.1    --------------------------------------------------------------------------------  target    SKSMKTYLDGGYVDDADRQGGQG 5cup.1    ----------------------- ``` | | | | | | | | | | | | | | | | | | | | | | | | | | | | | | | | | | | | | | | | | | | | | | | | | |
|  | 5cuo.1.A | Phosphate propanoyltransferase  *Structure of Rhodopseudomonas palustris PduL - CoA bound form* | 0.00 |  | 35.71 | 0.07 | 220-247 | X-ray | 1.54 | homo-dimer | 2 x COA, 4 x ZN | HHblits | 0.41 |
| ``` target    EVLARVGHKLAEQTGDARFADVWKLVDEKRTDAHLQRILDHSSNTKGYDALDLEAKAKKGIPTLMMNRTYPKAVGYEQVA 5cuo.1    --------------------------------------------------------------------------------  target    DSRPWYTKSGRLEFYRDEDEFIEAGENLPVHREPIDSTFYEPNVIVSAPHEALRPAGPEDYGVELSDMSGEIRQGRNVVK 5cuo.1    --------------------------------------------------------------------------------  target    AWAELKKTPHPLAKDGYRFVFHTPKYRHGAHTMPIDTDMVAMLFGPFGDVYRHDRRTPYVAEGYVDIHPSDAREIGVEDG 5cuo.1    -----------------------------------------------------------VAQRHIHMHPSTAAKLGLRNG  target    DYVFIDSDPEDRPFRGWQKNKRDYEFSRLLCRARYYPGTPRGVTRMWFNMYGATPGSVEGQKSREDGLAKNPRTNYQAMF 5cuo.1    DEVDVEA-------------------------------------------------------------------------  target    RSGSHQSATRGWLKPTWMTDSLVRKGLFGQSIGKGFLPDVHCPTGAPRESIVKITKAEPGGLGAEGLWRPAALGLRPGYE 5cuo.1    --------------------------------------------------------------------------------  target    SKSMKTYLDGGYVDDADRQGGQG 5cuo.1    ----------------------- ``` | | | | | | | | | | | | | | | | | | | | | | | | | | | | | | | | | | | | | | | | | | | | | | | | | |
|  | 1wlf.1.A | Peroxisome biogenesis factor 1  *Structure of the N-terminal domain of PEX1 AAA-ATPase: Characterization of a putative adaptor-binding domain* | 0.00 |  | 25.00 | 0.07 | 221-248 | X-ray | 2.05 | monomer |  | HHblits | 0.35 |
| ``` target    EVLARVGHKLAEQTGDARFADVWKLVDEKRTDAHLQRILDHSSNTKGYDALDLEAKAKKGIPTLMMNRTYPKAVGYEQVA 1wlf.1    --------------------------------------------------------------------------------  target    DSRPWYTKSGRLEFYRDEDEFIEAGENLPVHREPIDSTFYEPNVIVSAPHEALRPAGPEDYGVELSDMSGEIRQGRNVVK 1wlf.1    --------------------------------------------------------------------------------  target    AWAELKKTPHPLAKDGYRFVFHTPKYRHGAHTMPIDTDMVAMLFGPFGDVYRHDRRTPYVAEGYVDIHPSDAREIGVEDG 1wlf.1    ------------------------------------------------------------SENVAEINRQVGQKLGLSSG  target    DYVFIDSDPEDRPFRGWQKNKRDYEFSRLLCRARYYPGTPRGVTRMWFNMYGATPGSVEGQKSREDGLAKNPRTNYQAMF 1wlf.1    DQVFLRPC------------------------------------------------------------------------  target    RSGSHQSATRGWLKPTWMTDSLVRKGLFGQSIGKGFLPDVHCPTGAPRESIVKITKAEPGGLGAEGLWRPAALGLRPGYE 1wlf.1    --------------------------------------------------------------------------------  target    SKSMKTYLDGGYVDDADRQGGQG 1wlf.1    ----------------------- ``` | | | | | | | | | | | | | | | | | | | | | | | | | | | | | | | | | | | | | | | | | | | | | | | | | |
|  | 7dvf.1.A | reDPBB\_sym2 protein  *Crystal structure of the computationally designed reDPBB\_sym2 protein* | 0.00 |  | 29.63 | 0.06 | 222-248 | X-ray | 1.21 | monomer |  | HHblits | 0.35 |
| ``` target    EVLARVGHKLAEQTGDARFADVWKLVDEKRTDAHLQRILDHSSNTKGYDALDLEAKAKKGIPTLMMNRTYPKAVGYEQVA 7dvf.1    --------------------------------------------------------------------------------  target    DSRPWYTKSGRLEFYRDEDEFIEAGENLPVHREPIDSTFYEPNVIVSAPHEALRPAGPEDYGVELSDMSGEIRQGRNVVK 7dvf.1    --------------------------------------------------------------------------------  target    AWAELKKTPHPLAKDGYRFVFHTPKYRHGAHTMPIDTDMVAMLFGPFGDVYRHDRRTPYVAEGYVDIHPSDAREIGVEDG 7dvf.1    -------------------------------------------------------------KGIVRMDKASREKLGVSAG  target    DYVFIDSDPEDRPFRGWQKNKRDYEFSRLLCRARYYPGTPRGVTRMWFNMYGATPGSVEGQKSREDGLAKNPRTNYQAMF 7dvf.1    DLVEIKGS------------------------------------------------------------------------  target    RSGSHQSATRGWLKPTWMTDSLVRKGLFGQSIGKGFLPDVHCPTGAPRESIVKITKAEPGGLGAEGLWRPAALGLRPGYE 7dvf.1    --------------------------------------------------------------------------------  target    SKSMKTYLDGGYVDDADRQGGQG 7dvf.1    ----------------------- ``` | | | | | | | | | | | | | | | | | | | | | | | | | | | | | | | | | | | | | | | | | | | | | | | | | |
|  | 7mdx.1.B | Lipoprotein-releasing system transmembrane protein  *LolCDE nucleotide-free* | 0.01 |  | 28.57 | 0.07 | 221-248 | EM | 0.00 | hetero-1-1-2-mer | 1 x YPC-DSN-DAL-DAL-ALA-ALA | HHblits | 0.31 |
| ``` target    EVLARVGHKLAEQTGDARFADVWKLVDEKRTDAHLQRILDHSSNTKGYDALDLEAKAKKGIPTLMMNRTYPKAVGYEQVA 7mdx.1    --------------------------------------------------------------------------------  target    DSRPWYTKSGRLEFYRDEDEFIEAGENLPVHREPIDSTFYEPNVIVSAPHEALRPAGPEDYGVELSDMSGEIRQGRNVVK 7mdx.1    --------------------------------------------------------------------------------  target    AWAELKKTPHPLAKDGYRFVFHTPKYRHGAHTMPIDTDMVAMLFGPFGDVYRHDRRTPYVAEGYVDIHPSDAREIGVEDG 7mdx.1    ------------------------------------------------------------GEQQIIIGKGVADALKVKQG  target    DYVFIDSDPEDRPFRGWQKNKRDYEFSRLLCRARYYPGTPRGVTRMWFNMYGATPGSVEGQKSREDGLAKNPRTNYQAMF 7mdx.1    DWVSIMIP------------------------------------------------------------------------  target    RSGSHQSATRGWLKPTWMTDSLVRKGLFGQSIGKGFLPDVHCPTGAPRESIVKITKAEPGGLGAEGLWRPAALGLRPGYE 7mdx.1    --------------------------------------------------------------------------------  target    SKSMKTYLDGGYVDDADRQGGQG 7mdx.1    ----------------------- ``` | | | | | | | | | | | | | | | | | | | | | | | | | | | | | | | | | | | | | | | | | | | | | | | | | |
|  | 4cs0.1.A | ASPARTATE 1-DECARBOXYLASE  *Direct visualisation of strain-induced protein post-translational modification* | 0.00 |  | 17.24 | 0.07 | 221-249 | X-ray | 2.10 | hetero-oligomer | 4 x ACO, 4 x MG | HHblits | 0.27 |
| ``` target    EVLARVGHKLAEQTGDARFADVWKLVDEKRTDAHLQRILDHSSNTKGYDALDLEAKAKKGIPTLMMNRTYPKAVGYEQVA 4cs0.1    --------------------------------------------------------------------------------  target    DSRPWYTKSGRLEFYRDEDEFIEAGENLPVHREPIDSTFYEPNVIVSAPHEALRPAGPEDYGVELSDMSGEIRQGRNVVK 4cs0.1    --------------------------------------------------------------------------------  target    AWAELKKTPHPLAKDGYRFVFHTPKYRHGAHTMPIDTDMVAMLFGPFGDVYRHDRRTPYVAEGYVDIHPSDAREIGVEDG 4cs0.1    ------------------------------------------------------------YEGACAIDQDFLDAAGILEN  target    DYVFIDSDPEDRPFRGWQKNKRDYEFSRLLCRARYYPGTPRGVTRMWFNMYGATPGSVEGQKSREDGLAKNPRTNYQAMF 4cs0.1    EAIDIWNVT-----------------------------------------------------------------------  target    RSGSHQSATRGWLKPTWMTDSLVRKGLFGQSIGKGFLPDVHCPTGAPRESIVKITKAEPGGLGAEGLWRPAALGLRPGYE 4cs0.1    --------------------------------------------------------------------------------  target    SKSMKTYLDGGYVDDADRQGGQG 4cs0.1    ----------------------- ``` | | | | | | | | | | | | | | | | | | | | | | | | | | | | | | | | | | | | | | | | | | | | | | | | | |
|  | 1pt1.1.A | Aspartate 1-decarboxylase  *Unprocessed Pyruvoyl Dependent Aspartate Decarboxylase with Histidine 11 Mutated to Alanine* | 0.00 |  | 17.24 | 0.07 | 221-249 | X-ray | 1.90 | homo-tetramer |  | HHblits | 0.27 |
| ``` target    EVLARVGHKLAEQTGDARFADVWKLVDEKRTDAHLQRILDHSSNTKGYDALDLEAKAKKGIPTLMMNRTYPKAVGYEQVA 1pt1.1    --------------------------------------------------------------------------------  target    DSRPWYTKSGRLEFYRDEDEFIEAGENLPVHREPIDSTFYEPNVIVSAPHEALRPAGPEDYGVELSDMSGEIRQGRNVVK 1pt1.1    --------------------------------------------------------------------------------  target    AWAELKKTPHPLAKDGYRFVFHTPKYRHGAHTMPIDTDMVAMLFGPFGDVYRHDRRTPYVAEGYVDIHPSDAREIGVEDG 1pt1.1    ------------------------------------------------------------YEGSCAIDQDFLDAAGILEN  target    DYVFIDSDPEDRPFRGWQKNKRDYEFSRLLCRARYYPGTPRGVTRMWFNMYGATPGSVEGQKSREDGLAKNPRTNYQAMF 1pt1.1    EAIDIWNVT-----------------------------------------------------------------------  target    RSGSHQSATRGWLKPTWMTDSLVRKGLFGQSIGKGFLPDVHCPTGAPRESIVKITKAEPGGLGAEGLWRPAALGLRPGYE 1pt1.1    --------------------------------------------------------------------------------  target    SKSMKTYLDGGYVDDADRQGGQG 1pt1.1    ----------------------- ``` | | | | | | | | | | | | | | | | | | | | | | | | | | | | | | | | | | | | | | | | | | | | | | | | | |
|  | 7du7.1.A | mkDPBB\_sym1 protein  *Crystal structure of the rationally designed mkDPBB\_sym1 protein* | 0.00 |  | 30.77 | 0.06 | 223-248 | X-ray | 1.20 | monomer |  | HHblits | 0.35 |
| ``` target    EVLARVGHKLAEQTGDARFADVWKLVDEKRTDAHLQRILDHSSNTKGYDALDLEAKAKKGIPTLMMNRTYPKAVGYEQVA 7du7.1    --------------------------------------------------------------------------------  target    DSRPWYTKSGRLEFYRDEDEFIEAGENLPVHREPIDSTFYEPNVIVSAPHEALRPAGPEDYGVELSDMSGEIRQGRNVVK 7du7.1    --------------------------------------------------------------------------------  target    AWAELKKTPHPLAKDGYRFVFHTPKYRHGAHTMPIDTDMVAMLFGPFGDVYRHDRRTPYVAEGYVDIHPSDAREIGVEDG 7du7.1    --------------------------------------------------------------GIVRMDKASRAKLGVSVG  target    DYVFIDSDPEDRPFRGWQKNKRDYEFSRLLCRARYYPGTPRGVTRMWFNMYGATPGSVEGQKSREDGLAKNPRTNYQAMF 7du7.1    DYVEVKKV------------------------------------------------------------------------  target    RSGSHQSATRGWLKPTWMTDSLVRKGLFGQSIGKGFLPDVHCPTGAPRESIVKITKAEPGGLGAEGLWRPAALGLRPGYE 7du7.1    --------------------------------------------------------------------------------  target    SKSMKTYLDGGYVDDADRQGGQG 7du7.1    ----------------------- ``` | | | | | | | | | | | | | | | | | | | | | | | | | | | | | | | | | | | | | | | | | | | | | | | | | |
|  | 7dvc.1.A | reDPBB\_sym1 protein  *Crystal structure of the computationally designed reDPBB\_sym1 protein* | 0.00 |  | 30.77 | 0.06 | 223-248 | X-ray | 1.71 | monomer |  | HHblits | 0.34 |
| ``` target    EVLARVGHKLAEQTGDARFADVWKLVDEKRTDAHLQRILDHSSNTKGYDALDLEAKAKKGIPTLMMNRTYPKAVGYEQVA 7dvc.1    --------------------------------------------------------------------------------  target    DSRPWYTKSGRLEFYRDEDEFIEAGENLPVHREPIDSTFYEPNVIVSAPHEALRPAGPEDYGVELSDMSGEIRQGRNVVK 7dvc.1    --------------------------------------------------------------------------------  target    AWAELKKTPHPLAKDGYRFVFHTPKYRHGAHTMPIDTDMVAMLFGPFGDVYRHDRRTPYVAEGYVDIHPSDAREIGVEDG 7dvc.1    --------------------------------------------------------------GIVRMDKASRDKLGVSAG  target    DYVFIDSDPEDRPFRGWQKNKRDYEFSRLLCRARYYPGTPRGVTRMWFNMYGATPGSVEGQKSREDGLAKNPRTNYQAMF 7dvc.1    DLVEIKGS------------------------------------------------------------------------  target    RSGSHQSATRGWLKPTWMTDSLVRKGLFGQSIGKGFLPDVHCPTGAPRESIVKITKAEPGGLGAEGLWRPAALGLRPGYE 7dvc.1    --------------------------------------------------------------------------------  target    SKSMKTYLDGGYVDDADRQGGQG 7dvc.1    ----------------------- ``` | | | | | | | | | | | | | | | | | | | | | | | | | | | | | | | | | | | | | | | | | | | | | | | | | |
|  | 7dvc.5.A | reDPBB\_sym1 protein  *Crystal structure of the computationally designed reDPBB\_sym1 protein* | 0.00 |  | 30.77 | 0.06 | 223-248 | X-ray | 1.71 | monomer |  | HHblits | 0.34 |
| ``` target    EVLARVGHKLAEQTGDARFADVWKLVDEKRTDAHLQRILDHSSNTKGYDALDLEAKAKKGIPTLMMNRTYPKAVGYEQVA 7dvc.5    --------------------------------------------------------------------------------  target    DSRPWYTKSGRLEFYRDEDEFIEAGENLPVHREPIDSTFYEPNVIVSAPHEALRPAGPEDYGVELSDMSGEIRQGRNVVK 7dvc.5    --------------------------------------------------------------------------------  target    AWAELKKTPHPLAKDGYRFVFHTPKYRHGAHTMPIDTDMVAMLFGPFGDVYRHDRRTPYVAEGYVDIHPSDAREIGVEDG 7dvc.5    --------------------------------------------------------------GIVRMDKASRDKLGVSAG  target    DYVFIDSDPEDRPFRGWQKNKRDYEFSRLLCRARYYPGTPRGVTRMWFNMYGATPGSVEGQKSREDGLAKNPRTNYQAMF 7dvc.5    DLVEIKGS------------------------------------------------------------------------  target    RSGSHQSATRGWLKPTWMTDSLVRKGLFGQSIGKGFLPDVHCPTGAPRESIVKITKAEPGGLGAEGLWRPAALGLRPGYE 7dvc.5    --------------------------------------------------------------------------------  target    SKSMKTYLDGGYVDDADRQGGQG 7dvc.5    ----------------------- ``` | | | | | | | | | | | | | | | | | | | | | | | | | | | | | | | | | | | | | | | | | | | | | | | | | |
|  | 6hd3.1.A | Cell division control protein 48 homolog A  *Common mode of remodeling AAA ATPases p97/CDC48 by their disassembly cofactors ASPL/PUX1* | 0.00 |  | 23.08 | 0.06 | 224-249 | X-ray | 2.80 | homo-24-mer | 24 x ADP | HHblits | 0.34 |
| ``` target    EVLARVGHKLAEQTGDARFADVWKLVDEKRTDAHLQRILDHSSNTKGYDALDLEAKAKKGIPTLMMNRTYPKAVGYEQVA 6hd3.1    --------------------------------------------------------------------------------  target    DSRPWYTKSGRLEFYRDEDEFIEAGENLPVHREPIDSTFYEPNVIVSAPHEALRPAGPEDYGVELSDMSGEIRQGRNVVK 6hd3.1    --------------------------------------------------------------------------------  target    AWAELKKTPHPLAKDGYRFVFHTPKYRHGAHTMPIDTDMVAMLFGPFGDVYRHDRRTPYVAEGYVDIHPSDAREIGVEDG 6hd3.1    ---------------------------------------------------------------VVSLHPATMEKLQLFRG  target    DYVFIDSDPEDRPFRGWQKNKRDYEFSRLLCRARYYPGTPRGVTRMWFNMYGATPGSVEGQKSREDGLAKNPRTNYQAMF 6hd3.1    DTILIKGKK-----------------------------------------------------------------------  target    RSGSHQSATRGWLKPTWMTDSLVRKGLFGQSIGKGFLPDVHCPTGAPRESIVKITKAEPGGLGAEGLWRPAALGLRPGYE 6hd3.1    --------------------------------------------------------------------------------  target    SKSMKTYLDGGYVDDADRQGGQG 6hd3.1    ----------------------- ``` | | | | | | | | | | | | | | | | | | | | | | | | | | | | | | | | | | | | | | | | | | | | | | | | | |
|  | 3pjy.1.A | Hypothetical signal peptide protein  *Crystal structure of a putative transcription regulator (R01717) from Sinorhizobium meliloti 1021 at 1.55 A resolution* | 0.00 |  | 18.52 | 0.06 | 223-249 | X-ray | 1.55 | homo-dimer |  | HHblits | 0.31 |
| ``` target    EVLARVGHKLAEQTGDARFADVWKLVDEKRTDAHLQRILDHSSNTKGYDALDLEAKAKKGIPTLMMNRTYPKAVGYEQVA 3pjy.1    --------------------------------------------------------------------------------  target    DSRPWYTKSGRLEFYRDEDEFIEAGENLPVHREPIDSTFYEPNVIVSAPHEALRPAGPEDYGVELSDMSGEIRQGRNVVK 3pjy.1    --------------------------------------------------------------------------------  target    AWAELKKTPHPLAKDGYRFVFHTPKYRHGAHTMPIDTDMVAMLFGPFGDVYRHDRRTPYVAEGYVDIHPSDAREIGVEDG 3pjy.1    --------------------------------------------------------------YVLELNAGTVKRLGVSPG  target    DYVFIDSDPEDRPFRGWQKNKRDYEFSRLLCRARYYPGTPRGVTRMWFNMYGATPGSVEGQKSREDGLAKNPRTNYQAMF 3pjy.1    DRLEGAGLP-----------------------------------------------------------------------  target    RSGSHQSATRGWLKPTWMTDSLVRKGLFGQSIGKGFLPDVHCPTGAPRESIVKITKAEPGGLGAEGLWRPAALGLRPGYE 3pjy.1    --------------------------------------------------------------------------------  target    SKSMKTYLDGGYVDDADRQGGQG 3pjy.1    ----------------------- ``` | | | | | | | | | | | | | | | | | | | | | | | | | | | | | | | | | | | | | | | | | | | | | | | | | |
|  | 3pjy.1.B | Hypothetical signal peptide protein  *Crystal structure of a putative transcription regulator (R01717) from Sinorhizobium meliloti 1021 at 1.55 A resolution* | 0.00 |  | 18.52 | 0.06 | 223-249 | X-ray | 1.55 | homo-dimer |  | HHblits | 0.31 |
| ``` target    EVLARVGHKLAEQTGDARFADVWKLVDEKRTDAHLQRILDHSSNTKGYDALDLEAKAKKGIPTLMMNRTYPKAVGYEQVA 3pjy.1    --------------------------------------------------------------------------------  target    DSRPWYTKSGRLEFYRDEDEFIEAGENLPVHREPIDSTFYEPNVIVSAPHEALRPAGPEDYGVELSDMSGEIRQGRNVVK 3pjy.1    --------------------------------------------------------------------------------  target    AWAELKKTPHPLAKDGYRFVFHTPKYRHGAHTMPIDTDMVAMLFGPFGDVYRHDRRTPYVAEGYVDIHPSDAREIGVEDG 3pjy.1    --------------------------------------------------------------YVLELNAGTVKRLGVSPG  target    DYVFIDSDPEDRPFRGWQKNKRDYEFSRLLCRARYYPGTPRGVTRMWFNMYGATPGSVEGQKSREDGLAKNPRTNYQAMF 3pjy.1    DRLEGAGLP-----------------------------------------------------------------------  target    RSGSHQSATRGWLKPTWMTDSLVRKGLFGQSIGKGFLPDVHCPTGAPRESIVKITKAEPGGLGAEGLWRPAALGLRPGYE 3pjy.1    --------------------------------------------------------------------------------  target    SKSMKTYLDGGYVDDADRQGGQG 3pjy.1    ----------------------- ``` | | | | | | | | | | | | | | | | | | | | | | | | | | | | | | | | | | | | | | | | | | | | | | | | | |
|  | 7du7.1.A | mkDPBB\_sym1 protein  *Crystal structure of the rationally designed mkDPBB\_sym1 protein* | 0.00 |  | 32.00 | 0.06 | 222-246 | X-ray | 1.20 | monomer |  | HHblits | 0.37 |
| ``` target    EVLARVGHKLAEQTGDARFADVWKLVDEKRTDAHLQRILDHSSNTKGYDALDLEAKAKKGIPTLMMNRTYPKAVGYEQVA 7du7.1    --------------------------------------------------------------------------------  target    DSRPWYTKSGRLEFYRDEDEFIEAGENLPVHREPIDSTFYEPNVIVSAPHEALRPAGPEDYGVELSDMSGEIRQGRNVVK 7du7.1    --------------------------------------------------------------------------------  target    AWAELKKTPHPLAKDGYRFVFHTPKYRHGAHTMPIDTDMVAMLFGPFGDVYRHDRRTPYVAEGYVDIHPSDAREIGVEDG 7du7.1    -------------------------------------------------------------KGIVRMDKASRAKLGVSVG  target    DYVFIDSDPEDRPFRGWQKNKRDYEFSRLLCRARYYPGTPRGVTRMWFNMYGATPGSVEGQKSREDGLAKNPRTNYQAMF 7du7.1    DYVEVK--------------------------------------------------------------------------  target    RSGSHQSATRGWLKPTWMTDSLVRKGLFGQSIGKGFLPDVHCPTGAPRESIVKITKAEPGGLGAEGLWRPAALGLRPGYE 7du7.1    --------------------------------------------------------------------------------  target    SKSMKTYLDGGYVDDADRQGGQG 7du7.1    ----------------------- ``` | | | | | | | | | | | | | | | | | | | | | | | | | | | | | | | | | | | | | | | | | | | | | | | | | |
|  | 1pqh.1.A | Aspartate 1-decarboxylase  *Serine 25 to Threonine mutation of aspartate decarboxylase* | 0.00 |  | 17.86 | 0.07 | 222-249 | X-ray | 1.29 | homo-tetramer | 6 x MLA | HHblits | 0.28 |
| ``` target    EVLARVGHKLAEQTGDARFADVWKLVDEKRTDAHLQRILDHSSNTKGYDALDLEAKAKKGIPTLMMNRTYPKAVGYEQVA 1pqh.1    --------------------------------------------------------------------------------  target    DSRPWYTKSGRLEFYRDEDEFIEAGENLPVHREPIDSTFYEPNVIVSAPHEALRPAGPEDYGVELSDMSGEIRQGRNVVK 1pqh.1    --------------------------------------------------------------------------------  target    AWAELKKTPHPLAKDGYRFVFHTPKYRHGAHTMPIDTDMVAMLFGPFGDVYRHDRRTPYVAEGYVDIHPSDAREIGVEDG 1pqh.1    -------------------------------------------------------------EGTCAIDQDFLDAAGILEN  target    DYVFIDSDPEDRPFRGWQKNKRDYEFSRLLCRARYYPGTPRGVTRMWFNMYGATPGSVEGQKSREDGLAKNPRTNYQAMF 1pqh.1    EAIDIWNVT-----------------------------------------------------------------------  target    RSGSHQSATRGWLKPTWMTDSLVRKGLFGQSIGKGFLPDVHCPTGAPRESIVKITKAEPGGLGAEGLWRPAALGLRPGYE 1pqh.1    --------------------------------------------------------------------------------  target    SKSMKTYLDGGYVDDADRQGGQG 1pqh.1    ----------------------- ``` | | | | | | | | | | | | | | | | | | | | | | | | | | | | | | | | | | | | | | | | | | | | | | | | | |
|  | 7dvh.2.A | reDPBB\_sym4 protein  *Crystal structure of the computationally designed reDPBB\_sym4 protein* | 0.00 |  | 32.00 | 0.06 | 223-247 | X-ray | 1.70 | monomer |  | HHblits | 0.37 |
| ``` target    EVLARVGHKLAEQTGDARFADVWKLVDEKRTDAHLQRILDHSSNTKGYDALDLEAKAKKGIPTLMMNRTYPKAVGYEQVA 7dvh.2    --------------------------------------------------------------------------------  target    DSRPWYTKSGRLEFYRDEDEFIEAGENLPVHREPIDSTFYEPNVIVSAPHEALRPAGPEDYGVELSDMSGEIRQGRNVVK 7dvh.2    --------------------------------------------------------------------------------  target    AWAELKKTPHPLAKDGYRFVFHTPKYRHGAHTMPIDTDMVAMLFGPFGDVYRHDRRTPYVAEGYVDIHPSDAREIGVEDG 7dvh.2    --------------------------------------------------------------GIVRMDKYERQNLGVSVG  target    DYVFIDSDPEDRPFRGWQKNKRDYEFSRLLCRARYYPGTPRGVTRMWFNMYGATPGSVEGQKSREDGLAKNPRTNYQAMF 7dvh.2    DYVEVKK-------------------------------------------------------------------------  target    RSGSHQSATRGWLKPTWMTDSLVRKGLFGQSIGKGFLPDVHCPTGAPRESIVKITKAEPGGLGAEGLWRPAALGLRPGYE 7dvh.2    --------------------------------------------------------------------------------  target    SKSMKTYLDGGYVDDADRQGGQG 7dvh.2    ----------------------- ``` | | | | | | | | | | | | | | | | | | | | | | | | | | | | | | | | | | | | | | | | | | | | | | | | | |
|  | 7dvh.1.A | reDPBB\_sym4 protein  *Crystal structure of the computationally designed reDPBB\_sym4 protein* | 0.00 |  | 32.00 | 0.06 | 223-247 | X-ray | 1.70 | monomer |  | HHblits | 0.37 |
| ``` target    EVLARVGHKLAEQTGDARFADVWKLVDEKRTDAHLQRILDHSSNTKGYDALDLEAKAKKGIPTLMMNRTYPKAVGYEQVA 7dvh.1    --------------------------------------------------------------------------------  target    DSRPWYTKSGRLEFYRDEDEFIEAGENLPVHREPIDSTFYEPNVIVSAPHEALRPAGPEDYGVELSDMSGEIRQGRNVVK 7dvh.1    --------------------------------------------------------------------------------  target    AWAELKKTPHPLAKDGYRFVFHTPKYRHGAHTMPIDTDMVAMLFGPFGDVYRHDRRTPYVAEGYVDIHPSDAREIGVEDG 7dvh.1    --------------------------------------------------------------GIVRMDKYERQNLGVSVG  target    DYVFIDSDPEDRPFRGWQKNKRDYEFSRLLCRARYYPGTPRGVTRMWFNMYGATPGSVEGQKSREDGLAKNPRTNYQAMF 7dvh.1    DYVEVKK-------------------------------------------------------------------------  target    RSGSHQSATRGWLKPTWMTDSLVRKGLFGQSIGKGFLPDVHCPTGAPRESIVKITKAEPGGLGAEGLWRPAALGLRPGYE 7dvh.1    --------------------------------------------------------------------------------  target    SKSMKTYLDGGYVDDADRQGGQG 7dvh.1    ----------------------- ``` | | | | | | | | | | | | | | | | | | | | | | | | | | | | | | | | | | | | | | | | | | | | | | | | | |
|  | 7dvh.4.A | reDPBB\_sym4 protein  *Crystal structure of the computationally designed reDPBB\_sym4 protein* | 0.00 |  | 32.00 | 0.06 | 223-247 | X-ray | 1.70 | monomer |  | HHblits | 0.37 |
| ``` target    EVLARVGHKLAEQTGDARFADVWKLVDEKRTDAHLQRILDHSSNTKGYDALDLEAKAKKGIPTLMMNRTYPKAVGYEQVA 7dvh.4    --------------------------------------------------------------------------------  target    DSRPWYTKSGRLEFYRDEDEFIEAGENLPVHREPIDSTFYEPNVIVSAPHEALRPAGPEDYGVELSDMSGEIRQGRNVVK 7dvh.4    --------------------------------------------------------------------------------  target    AWAELKKTPHPLAKDGYRFVFHTPKYRHGAHTMPIDTDMVAMLFGPFGDVYRHDRRTPYVAEGYVDIHPSDAREIGVEDG 7dvh.4    --------------------------------------------------------------GIVRMDKYERQNLGVSVG  target    DYVFIDSDPEDRPFRGWQKNKRDYEFSRLLCRARYYPGTPRGVTRMWFNMYGATPGSVEGQKSREDGLAKNPRTNYQAMF 7dvh.4    DYVEVKK-------------------------------------------------------------------------  target    RSGSHQSATRGWLKPTWMTDSLVRKGLFGQSIGKGFLPDVHCPTGAPRESIVKITKAEPGGLGAEGLWRPAALGLRPGYE 7dvh.4    --------------------------------------------------------------------------------  target    SKSMKTYLDGGYVDDADRQGGQG 7dvh.4    ----------------------- ``` | | | | | | | | | | | | | | | | | | | | | | | | | | | | | | | | | | | | | | | | | | | | | | | | | |
|  | 7di1.1.A | mkDPBB\_sym\_86 protein  *Crystal structure of the rationally designed mkDPBB\_sym\_86 protein* | 0.00 |  | 32.00 | 0.06 | 222-246 | X-ray | 2.10 | monomer |  | HHblits | 0.37 |
| ``` target    EVLARVGHKLAEQTGDARFADVWKLVDEKRTDAHLQRILDHSSNTKGYDALDLEAKAKKGIPTLMMNRTYPKAVGYEQVA 7di1.1    --------------------------------------------------------------------------------  target    DSRPWYTKSGRLEFYRDEDEFIEAGENLPVHREPIDSTFYEPNVIVSAPHEALRPAGPEDYGVELSDMSGEIRQGRNVVK 7di1.1    --------------------------------------------------------------------------------  target    AWAELKKTPHPLAKDGYRFVFHTPKYRHGAHTMPIDTDMVAMLFGPFGDVYRHDRRTPYVAEGYVDIHPSDAREIGVEDG 7di1.1    -------------------------------------------------------------KGIVRMDKYERAKLGVSVG  target    DYVFIDSDPEDRPFRGWQKNKRDYEFSRLLCRARYYPGTPRGVTRMWFNMYGATPGSVEGQKSREDGLAKNPRTNYQAMF 7di1.1    DYVEVK--------------------------------------------------------------------------  target    RSGSHQSATRGWLKPTWMTDSLVRKGLFGQSIGKGFLPDVHCPTGAPRESIVKITKAEPGGLGAEGLWRPAALGLRPGYE 7di1.1    --------------------------------------------------------------------------------  target    SKSMKTYLDGGYVDDADRQGGQG 7di1.1    ----------------------- ``` | | | | | | | | | | | | | | | | | | | | | | | | | | | | | | | | | | | | | | | | | | | | | | | | | |
|  | 6f49.1.A | Lipoprotein-releasing system transmembrane protein LolC,Lipoprotein-releasing system transmembrane protein LolC  *Periplasmic domain of LolC lacking the Hook.* | 0.00 |  | 22.22 | 0.06 | 222-248 | X-ray | 2.02 | monomer |  | HHblits | 0.30 |
| ``` target    EVLARVGHKLAEQTGDARFADVWKLVDEKRTDAHLQRILDHSSNTKGYDALDLEAKAKKGIPTLMMNRTYPKAVGYEQVA 6f49.1    --------------------------------------------------------------------------------  target    DSRPWYTKSGRLEFYRDEDEFIEAGENLPVHREPIDSTFYEPNVIVSAPHEALRPAGPEDYGVELSDMSGEIRQGRNVVK 6f49.1    --------------------------------------------------------------------------------  target    AWAELKKTPHPLAKDGYRFVFHTPKYRHGAHTMPIDTDMVAMLFGPFGDVYRHDRRTPYVAEGYVDIHPSDAREIGVEDG 6f49.1    -------------------------------------------------------------KYNVILGEQLASQLGVNRG  target    DYVFIDSDPEDRPFRGWQKNKRDYEFSRLLCRARYYPGTPRGVTRMWFNMYGATPGSVEGQKSREDGLAKNPRTNYQAMF 6f49.1    DQIRVMVG------------------------------------------------------------------------  target    RSGSHQSATRGWLKPTWMTDSLVRKGLFGQSIGKGFLPDVHCPTGAPRESIVKITKAEPGGLGAEGLWRPAALGLRPGYE 6f49.1    --------------------------------------------------------------------------------  target    SKSMKTYLDGGYVDDADRQGGQG 6f49.1    ----------------------- ``` | | | | | | | | | | | | | | | | | | | | | | | | | | | | | | | | | | | | | | | | | | | | | | | | | |
|  | 3oug.1.A | Aspartate 1-decarboxylase  *Crystal structure of cleaved L-aspartate-alpha-decarboxylase from Francisella tularensis* | 0.00 |  | 10.71 | 0.07 | 222-249 | X-ray | 1.55 | homo-tetramer |  | HHblits | 0.27 |
| ``` target    EVLARVGHKLAEQTGDARFADVWKLVDEKRTDAHLQRILDHSSNTKGYDALDLEAKAKKGIPTLMMNRTYPKAVGYEQVA 3oug.1    --------------------------------------------------------------------------------  target    DSRPWYTKSGRLEFYRDEDEFIEAGENLPVHREPIDSTFYEPNVIVSAPHEALRPAGPEDYGVELSDMSGEIRQGRNVVK 3oug.1    --------------------------------------------------------------------------------  target    AWAELKKTPHPLAKDGYRFVFHTPKYRHGAHTMPIDTDMVAMLFGPFGDVYRHDRRTPYVAEGYVDIHPSDAREIGVEDG 3oug.1    -------------------------------------------------------------VGSITIDSEIMKQANIIEN  target    DYVFIDSDPEDRPFRGWQKNKRDYEFSRLLCRARYYPGTPRGVTRMWFNMYGATPGSVEGQKSREDGLAKNPRTNYQAMF 3oug.1    EKVQVVNLN-----------------------------------------------------------------------  target    RSGSHQSATRGWLKPTWMTDSLVRKGLFGQSIGKGFLPDVHCPTGAPRESIVKITKAEPGGLGAEGLWRPAALGLRPGYE 3oug.1    --------------------------------------------------------------------------------  target    SKSMKTYLDGGYVDDADRQGGQG 3oug.1    ----------------------- ``` | | | | | | | | | | | | | | | | | | | | | | | | | | | | | | | | | | | | | | | | | | | | | | | | | |
|  | 2c45.1.A | Aspartate 1-decarboxylase  *NATIVE PRECURSOR OF PYRUVOYL DEPENDENT ASPARTATE DECARBOXYLASE* | 0.01 |  | 21.43 | 0.07 | 222-249 | X-ray | 2.99 | homo-tetramer |  | HHblits | 0.27 |
| ``` target    EVLARVGHKLAEQTGDARFADVWKLVDEKRTDAHLQRILDHSSNTKGYDALDLEAKAKKGIPTLMMNRTYPKAVGYEQVA 2c45.1    --------------------------------------------------------------------------------  target    DSRPWYTKSGRLEFYRDEDEFIEAGENLPVHREPIDSTFYEPNVIVSAPHEALRPAGPEDYGVELSDMSGEIRQGRNVVK 2c45.1    --------------------------------------------------------------------------------  target    AWAELKKTPHPLAKDGYRFVFHTPKYRHGAHTMPIDTDMVAMLFGPFGDVYRHDRRTPYVAEGYVDIHPSDAREIGVEDG 2c45.1    -------------------------------------------------------------VGSVTIDADLMDAADLLEG  target    DYVFIDSDPEDRPFRGWQKNKRDYEFSRLLCRARYYPGTPRGVTRMWFNMYGATPGSVEGQKSREDGLAKNPRTNYQAMF 2c45.1    EQVTIVDID-----------------------------------------------------------------------  target    RSGSHQSATRGWLKPTWMTDSLVRKGLFGQSIGKGFLPDVHCPTGAPRESIVKITKAEPGGLGAEGLWRPAALGLRPGYE 2c45.1    --------------------------------------------------------------------------------  target    SKSMKTYLDGGYVDDADRQGGQG 2c45.1    ----------------------- ``` | | | | | | | | | | | | | | | | | | | | | | | | | | | | | | | | | | | | | | | | | | | | | | | | | |
|  | 7dxy.1.A | mk2h\_deltaMILPS  *Crystal structure of the chemically synthesized mk2h\_deltaMILPS peptide homodimer* | 0.00 |  | 28.00 | 0.06 | 222-246 | X-ray | 1.40 | homo-dimer |  | HHblits | 0.35 |
| ``` target    EVLARVGHKLAEQTGDARFADVWKLVDEKRTDAHLQRILDHSSNTKGYDALDLEAKAKKGIPTLMMNRTYPKAVGYEQVA 7dxy.1    --------------------------------------------------------------------------------  target    DSRPWYTKSGRLEFYRDEDEFIEAGENLPVHREPIDSTFYEPNVIVSAPHEALRPAGPEDYGVELSDMSGEIRQGRNVVK 7dxy.1    --------------------------------------------------------------------------------  target    AWAELKKTPHPLAKDGYRFVFHTPKYRHGAHTMPIDTDMVAMLFGPFGDVYRHDRRTPYVAEGYVDIHPSDAREIGVEDG 7dxy.1    -------------------------------------------------------------KRVVRVDKYERAKVGVKVG  target    DYVFIDSDPEDRPFRGWQKNKRDYEFSRLLCRARYYPGTPRGVTRMWFNMYGATPGSVEGQKSREDGLAKNPRTNYQAMF 7dxy.1    DYVEVK--------------------------------------------------------------------------  target    RSGSHQSATRGWLKPTWMTDSLVRKGLFGQSIGKGFLPDVHCPTGAPRESIVKITKAEPGGLGAEGLWRPAALGLRPGYE 7dxy.1    --------------------------------------------------------------------------------  target    SKSMKTYLDGGYVDDADRQGGQG 7dxy.1    ----------------------- ``` | | | | | | | | | | | | | | | | | | | | | | | | | | | | | | | | | | | | | | | | | | | | | | | | | |
|  | 7dxx.1.A | mk2h\_deltaMILPS protein  *Crystal structure of the mk2h\_deltaMILPS peptide homodimer* | 0.00 |  | 28.00 | 0.06 | 222-246 | X-ray | 1.40 | homo-dimer | 1 x MLA | HHblits | 0.35 |
| ``` target    EVLARVGHKLAEQTGDARFADVWKLVDEKRTDAHLQRILDHSSNTKGYDALDLEAKAKKGIPTLMMNRTYPKAVGYEQVA 7dxx.1    --------------------------------------------------------------------------------  target    DSRPWYTKSGRLEFYRDEDEFIEAGENLPVHREPIDSTFYEPNVIVSAPHEALRPAGPEDYGVELSDMSGEIRQGRNVVK 7dxx.1    --------------------------------------------------------------------------------  target    AWAELKKTPHPLAKDGYRFVFHTPKYRHGAHTMPIDTDMVAMLFGPFGDVYRHDRRTPYVAEGYVDIHPSDAREIGVEDG 7dxx.1    -------------------------------------------------------------KRVVRVDKYERAKVGVKVG  target    DYVFIDSDPEDRPFRGWQKNKRDYEFSRLLCRARYYPGTPRGVTRMWFNMYGATPGSVEGQKSREDGLAKNPRTNYQAMF 7dxx.1    DYVEVK--------------------------------------------------------------------------  target    RSGSHQSATRGWLKPTWMTDSLVRKGLFGQSIGKGFLPDVHCPTGAPRESIVKITKAEPGGLGAEGLWRPAALGLRPGYE 7dxx.1    --------------------------------------------------------------------------------  target    SKSMKTYLDGGYVDDADRQGGQG 7dxx.1    ----------------------- ``` | | | | | | | | | | | | | | | | | | | | | | | | | | | | | | | | | | | | | | | | | | | | | | | | | |
|  | 7dxx.1.B | mk2h\_deltaMILPS protein  *Crystal structure of the mk2h\_deltaMILPS peptide homodimer* | 0.00 |  | 28.00 | 0.06 | 222-246 | X-ray | 1.40 | homo-dimer | 1 x MLA | HHblits | 0.35 |
| ``` target    EVLARVGHKLAEQTGDARFADVWKLVDEKRTDAHLQRILDHSSNTKGYDALDLEAKAKKGIPTLMMNRTYPKAVGYEQVA 7dxx.1    --------------------------------------------------------------------------------  target    DSRPWYTKSGRLEFYRDEDEFIEAGENLPVHREPIDSTFYEPNVIVSAPHEALRPAGPEDYGVELSDMSGEIRQGRNVVK 7dxx.1    --------------------------------------------------------------------------------  target    AWAELKKTPHPLAKDGYRFVFHTPKYRHGAHTMPIDTDMVAMLFGPFGDVYRHDRRTPYVAEGYVDIHPSDAREIGVEDG 7dxx.1    -------------------------------------------------------------KRVVRVDKYERAKVGVKVG  target    DYVFIDSDPEDRPFRGWQKNKRDYEFSRLLCRARYYPGTPRGVTRMWFNMYGATPGSVEGQKSREDGLAKNPRTNYQAMF 7dxx.1    DYVEVK--------------------------------------------------------------------------  target    RSGSHQSATRGWLKPTWMTDSLVRKGLFGQSIGKGFLPDVHCPTGAPRESIVKITKAEPGGLGAEGLWRPAALGLRPGYE 7dxx.1    --------------------------------------------------------------------------------  target    SKSMKTYLDGGYVDDADRQGGQG 7dxx.1    ----------------------- ``` | | | | | | | | | | | | | | | | | | | | | | | | | | | | | | | | | | | | | | | | | | | | | | | | | |
|  | 7dvf.1.A | reDPBB\_sym2 protein  *Crystal structure of the computationally designed reDPBB\_sym2 protein* | 0.00 |  | 32.00 | 0.06 | 222-246 | X-ray | 1.21 | monomer |  | HHblits | 0.35 |
| ``` target    EVLARVGHKLAEQTGDARFADVWKLVDEKRTDAHLQRILDHSSNTKGYDALDLEAKAKKGIPTLMMNRTYPKAVGYEQVA 7dvf.1    --------------------------------------------------------------------------------  target    DSRPWYTKSGRLEFYRDEDEFIEAGENLPVHREPIDSTFYEPNVIVSAPHEALRPAGPEDYGVELSDMSGEIRQGRNVVK 7dvf.1    --------------------------------------------------------------------------------  target    AWAELKKTPHPLAKDGYRFVFHTPKYRHGAHTMPIDTDMVAMLFGPFGDVYRHDRRTPYVAEGYVDIHPSDAREIGVEDG 7dvf.1    -------------------------------------------------------------KGIVRMDKASREKLGVSAG  target    DYVFIDSDPEDRPFRGWQKNKRDYEFSRLLCRARYYPGTPRGVTRMWFNMYGATPGSVEGQKSREDGLAKNPRTNYQAMF 7dvf.1    DLVEIK--------------------------------------------------------------------------  target    RSGSHQSATRGWLKPTWMTDSLVRKGLFGQSIGKGFLPDVHCPTGAPRESIVKITKAEPGGLGAEGLWRPAALGLRPGYE 7dvf.1    --------------------------------------------------------------------------------  target    SKSMKTYLDGGYVDDADRQGGQG 7dvf.1    ----------------------- ``` | | | | | | | | | | | | | | | | | | | | | | | | | | | | | | | | | | | | | | | | | | | | | | | | | |
|  | 1pqf.1.A | Aspartate 1-decarboxylase  *Glycine 24 to Serine mutation of aspartate decarboxylase* | 0.00 |  | 14.29 | 0.07 | 222-249 | X-ray | 2.00 | homo-tetramer |  | HHblits | 0.26 |
| ``` target    EVLARVGHKLAEQTGDARFADVWKLVDEKRTDAHLQRILDHSSNTKGYDALDLEAKAKKGIPTLMMNRTYPKAVGYEQVA 1pqf.1    --------------------------------------------------------------------------------  target    DSRPWYTKSGRLEFYRDEDEFIEAGENLPVHREPIDSTFYEPNVIVSAPHEALRPAGPEDYGVELSDMSGEIRQGRNVVK 1pqf.1    --------------------------------------------------------------------------------  target    AWAELKKTPHPLAKDGYRFVFHTPKYRHGAHTMPIDTDMVAMLFGPFGDVYRHDRRTPYVAEGYVDIHPSDAREIGVEDG 1pqf.1    -------------------------------------------------------------ESSCAIDQDFLDAAGILEN  target    DYVFIDSDPEDRPFRGWQKNKRDYEFSRLLCRARYYPGTPRGVTRMWFNMYGATPGSVEGQKSREDGLAKNPRTNYQAMF 1pqf.1    EAIDIWNVT-----------------------------------------------------------------------  target    RSGSHQSATRGWLKPTWMTDSLVRKGLFGQSIGKGFLPDVHCPTGAPRESIVKITKAEPGGLGAEGLWRPAALGLRPGYE 1pqf.1    --------------------------------------------------------------------------------  target    SKSMKTYLDGGYVDDADRQGGQG 1pqf.1    ----------------------- ``` | | | | | | | | | | | | | | | | | | | | | | | | | | | | | | | | | | | | | | | | | | | | | | | | | |
|  | 7dxw.1.A | mk2h\_deltaMIL protein  *Crystal structure of the mk2h\_deltaMIL peptide homodimer* | 0.00 |  | 28.00 | 0.06 | 222-246 | X-ray | 1.51 | homo-dimer |  | HHblits | 0.35 |
| ``` target    EVLARVGHKLAEQTGDARFADVWKLVDEKRTDAHLQRILDHSSNTKGYDALDLEAKAKKGIPTLMMNRTYPKAVGYEQVA 7dxw.1    --------------------------------------------------------------------------------  target    DSRPWYTKSGRLEFYRDEDEFIEAGENLPVHREPIDSTFYEPNVIVSAPHEALRPAGPEDYGVELSDMSGEIRQGRNVVK 7dxw.1    --------------------------------------------------------------------------------  target    AWAELKKTPHPLAKDGYRFVFHTPKYRHGAHTMPIDTDMVAMLFGPFGDVYRHDRRTPYVAEGYVDIHPSDAREIGVEDG 7dxw.1    -------------------------------------------------------------KRVVRVDKYERAKVGVSVG  target    DYVFIDSDPEDRPFRGWQKNKRDYEFSRLLCRARYYPGTPRGVTRMWFNMYGATPGSVEGQKSREDGLAKNPRTNYQAMF 7dxw.1    DYVEVK--------------------------------------------------------------------------  target    RSGSHQSATRGWLKPTWMTDSLVRKGLFGQSIGKGFLPDVHCPTGAPRESIVKITKAEPGGLGAEGLWRPAALGLRPGYE 7dxw.1    --------------------------------------------------------------------------------  target    SKSMKTYLDGGYVDDADRQGGQG 7dxw.1    ----------------------- ``` | | | | | | | | | | | | | | | | | | | | | | | | | | | | | | | | | | | | | | | | | | | | | | | | | |
|  | 5g4f.1.A | VCP-LIKE ATPASE  *Structure of the ADP-bound VAT complex* | 0.00 |  | 30.77 | 0.06 | 224-249 | EM | 7.00 | homo-hexamer |  | HHblits | 0.32 |
| ``` target    EVLARVGHKLAEQTGDARFADVWKLVDEKRTDAHLQRILDHSSNTKGYDALDLEAKAKKGIPTLMMNRTYPKAVGYEQVA 5g4f.1    --------------------------------------------------------------------------------  target    DSRPWYTKSGRLEFYRDEDEFIEAGENLPVHREPIDSTFYEPNVIVSAPHEALRPAGPEDYGVELSDMSGEIRQGRNVVK 5g4f.1    --------------------------------------------------------------------------------  target    AWAELKKTPHPLAKDGYRFVFHTPKYRHGAHTMPIDTDMVAMLFGPFGDVYRHDRRTPYVAEGYVDIHPSDAREIGVEDG 5g4f.1    ---------------------------------------------------------------RVRLDESSRRLLDAEIG  target    DYVFIDSDPEDRPFRGWQKNKRDYEFSRLLCRARYYPGTPRGVTRMWFNMYGATPGSVEGQKSREDGLAKNPRTNYQAMF 5g4f.1    DVVEIEKVR-----------------------------------------------------------------------  target    RSGSHQSATRGWLKPTWMTDSLVRKGLFGQSIGKGFLPDVHCPTGAPRESIVKITKAEPGGLGAEGLWRPAALGLRPGYE 5g4f.1    --------------------------------------------------------------------------------  target    SKSMKTYLDGGYVDDADRQGGQG 5g4f.1    ----------------------- ``` | | | | | | | | | | | | | | | | | | | | | | | | | | | | | | | | | | | | | | | | | | | | | | | | | |
|  | 5g4f.1.B | VCP-LIKE ATPASE  *Structure of the ADP-bound VAT complex* | 0.00 |  | 30.77 | 0.06 | 224-249 | EM | 7.00 | homo-hexamer |  | HHblits | 0.32 |
| ``` target    EVLARVGHKLAEQTGDARFADVWKLVDEKRTDAHLQRILDHSSNTKGYDALDLEAKAKKGIPTLMMNRTYPKAVGYEQVA 5g4f.1    --------------------------------------------------------------------------------  target    DSRPWYTKSGRLEFYRDEDEFIEAGENLPVHREPIDSTFYEPNVIVSAPHEALRPAGPEDYGVELSDMSGEIRQGRNVVK 5g4f.1    --------------------------------------------------------------------------------  target    AWAELKKTPHPLAKDGYRFVFHTPKYRHGAHTMPIDTDMVAMLFGPFGDVYRHDRRTPYVAEGYVDIHPSDAREIGVEDG 5g4f.1    ---------------------------------------------------------------RVRLDESSRRLLDAEIG  target    DYVFIDSDPEDRPFRGWQKNKRDYEFSRLLCRARYYPGTPRGVTRMWFNMYGATPGSVEGQKSREDGLAKNPRTNYQAMF 5g4f.1    DVVEIEKVR-----------------------------------------------------------------------  target    RSGSHQSATRGWLKPTWMTDSLVRKGLFGQSIGKGFLPDVHCPTGAPRESIVKITKAEPGGLGAEGLWRPAALGLRPGYE 5g4f.1    --------------------------------------------------------------------------------  target    SKSMKTYLDGGYVDDADRQGGQG 5g4f.1    ----------------------- ``` | | | | | | | | | | | | | | | | | | | | | | | | | | | | | | | | | | | | | | | | | | | | | | | | | |
|  | 5g4f.1.C | VCP-LIKE ATPASE  *Structure of the ADP-bound VAT complex* | 0.00 |  | 30.77 | 0.06 | 224-249 | EM | 7.00 | homo-hexamer |  | HHblits | 0.32 |
| ``` target    EVLARVGHKLAEQTGDARFADVWKLVDEKRTDAHLQRILDHSSNTKGYDALDLEAKAKKGIPTLMMNRTYPKAVGYEQVA 5g4f.1    --------------------------------------------------------------------------------  target    DSRPWYTKSGRLEFYRDEDEFIEAGENLPVHREPIDSTFYEPNVIVSAPHEALRPAGPEDYGVELSDMSGEIRQGRNVVK 5g4f.1    --------------------------------------------------------------------------------  target    AWAELKKTPHPLAKDGYRFVFHTPKYRHGAHTMPIDTDMVAMLFGPFGDVYRHDRRTPYVAEGYVDIHPSDAREIGVEDG 5g4f.1    ---------------------------------------------------------------RVRLDESSRRLLDAEIG  target    DYVFIDSDPEDRPFRGWQKNKRDYEFSRLLCRARYYPGTPRGVTRMWFNMYGATPGSVEGQKSREDGLAKNPRTNYQAMF 5g4f.1    DVVEIEKVR-----------------------------------------------------------------------  target    RSGSHQSATRGWLKPTWMTDSLVRKGLFGQSIGKGFLPDVHCPTGAPRESIVKITKAEPGGLGAEGLWRPAALGLRPGYE 5g4f.1    --------------------------------------------------------------------------------  target    SKSMKTYLDGGYVDDADRQGGQG 5g4f.1    ----------------------- ``` | | | | | | | | | | | | | | | | | | | | | | | | | | | | | | | | | | | | | | | | | | | | | | | | | |
|  | 5g4f.1.D | VCP-LIKE ATPASE  *Structure of the ADP-bound VAT complex* | 0.00 |  | 30.77 | 0.06 | 224-249 | EM | 7.00 | homo-hexamer |  | HHblits | 0.32 |
| ``` target    EVLARVGHKLAEQTGDARFADVWKLVDEKRTDAHLQRILDHSSNTKGYDALDLEAKAKKGIPTLMMNRTYPKAVGYEQVA 5g4f.1    --------------------------------------------------------------------------------  target    DSRPWYTKSGRLEFYRDEDEFIEAGENLPVHREPIDSTFYEPNVIVSAPHEALRPAGPEDYGVELSDMSGEIRQGRNVVK 5g4f.1    --------------------------------------------------------------------------------  target    AWAELKKTPHPLAKDGYRFVFHTPKYRHGAHTMPIDTDMVAMLFGPFGDVYRHDRRTPYVAEGYVDIHPSDAREIGVEDG 5g4f.1    ---------------------------------------------------------------RVRLDESSRRLLDAEIG  target    DYVFIDSDPEDRPFRGWQKNKRDYEFSRLLCRARYYPGTPRGVTRMWFNMYGATPGSVEGQKSREDGLAKNPRTNYQAMF 5g4f.1    DVVEIEKVR-----------------------------------------------------------------------  target    RSGSHQSATRGWLKPTWMTDSLVRKGLFGQSIGKGFLPDVHCPTGAPRESIVKITKAEPGGLGAEGLWRPAALGLRPGYE 5g4f.1    --------------------------------------------------------------------------------  target    SKSMKTYLDGGYVDDADRQGGQG 5g4f.1    ----------------------- ``` | | | | | | | | | | | | | | | | | | | | | | | | | | | | | | | | | | | | | | | | | | | | | | | | | |
|  | 5g4f.1.E | VCP-LIKE ATPASE  *Structure of the ADP-bound VAT complex* | 0.00 |  | 30.77 | 0.06 | 224-249 | EM | 7.00 | homo-hexamer |  | HHblits | 0.32 |
| ``` target    EVLARVGHKLAEQTGDARFADVWKLVDEKRTDAHLQRILDHSSNTKGYDALDLEAKAKKGIPTLMMNRTYPKAVGYEQVA 5g4f.1    --------------------------------------------------------------------------------  target    DSRPWYTKSGRLEFYRDEDEFIEAGENLPVHREPIDSTFYEPNVIVSAPHEALRPAGPEDYGVELSDMSGEIRQGRNVVK 5g4f.1    --------------------------------------------------------------------------------  target    AWAELKKTPHPLAKDGYRFVFHTPKYRHGAHTMPIDTDMVAMLFGPFGDVYRHDRRTPYVAEGYVDIHPSDAREIGVEDG 5g4f.1    ---------------------------------------------------------------RVRLDESSRRLLDAEIG  target    DYVFIDSDPEDRPFRGWQKNKRDYEFSRLLCRARYYPGTPRGVTRMWFNMYGATPGSVEGQKSREDGLAKNPRTNYQAMF 5g4f.1    DVVEIEKVR-----------------------------------------------------------------------  target    RSGSHQSATRGWLKPTWMTDSLVRKGLFGQSIGKGFLPDVHCPTGAPRESIVKITKAEPGGLGAEGLWRPAALGLRPGYE 5g4f.1    --------------------------------------------------------------------------------  target    SKSMKTYLDGGYVDDADRQGGQG 5g4f.1    ----------------------- ``` | | | | | | | | | | | | | | | | | | | | | | | | | | | | | | | | | | | | | | | | | | | | | | | | | |
|  | 5g4f.1.F | VCP-LIKE ATPASE  *Structure of the ADP-bound VAT complex* | 0.00 |  | 30.77 | 0.06 | 224-249 | EM | 7.00 | homo-hexamer |  | HHblits | 0.32 |
| ``` target    EVLARVGHKLAEQTGDARFADVWKLVDEKRTDAHLQRILDHSSNTKGYDALDLEAKAKKGIPTLMMNRTYPKAVGYEQVA 5g4f.1    --------------------------------------------------------------------------------  target    DSRPWYTKSGRLEFYRDEDEFIEAGENLPVHREPIDSTFYEPNVIVSAPHEALRPAGPEDYGVELSDMSGEIRQGRNVVK 5g4f.1    --------------------------------------------------------------------------------  target    AWAELKKTPHPLAKDGYRFVFHTPKYRHGAHTMPIDTDMVAMLFGPFGDVYRHDRRTPYVAEGYVDIHPSDAREIGVEDG 5g4f.1    ---------------------------------------------------------------RVRLDESSRRLLDAEIG  target    DYVFIDSDPEDRPFRGWQKNKRDYEFSRLLCRARYYPGTPRGVTRMWFNMYGATPGSVEGQKSREDGLAKNPRTNYQAMF 5g4f.1    DVVEIEKVR-----------------------------------------------------------------------  target    RSGSHQSATRGWLKPTWMTDSLVRKGLFGQSIGKGFLPDVHCPTGAPRESIVKITKAEPGGLGAEGLWRPAALGLRPGYE 5g4f.1    --------------------------------------------------------------------------------  target    SKSMKTYLDGGYVDDADRQGGQG 5g4f.1    ----------------------- ``` | | | | | | | | | | | | | | | | | | | | | | | | | | | | | | | | | | | | | | | | | | | | | | | | | |
|  | 7dvc.1.A | reDPBB\_sym1 protein  *Crystal structure of the computationally designed reDPBB\_sym1 protein* | 0.00 |  | 32.00 | 0.06 | 222-246 | X-ray | 1.71 | monomer |  | HHblits | 0.35 |
| ``` target    EVLARVGHKLAEQTGDARFADVWKLVDEKRTDAHLQRILDHSSNTKGYDALDLEAKAKKGIPTLMMNRTYPKAVGYEQVA 7dvc.1    --------------------------------------------------------------------------------  target    DSRPWYTKSGRLEFYRDEDEFIEAGENLPVHREPIDSTFYEPNVIVSAPHEALRPAGPEDYGVELSDMSGEIRQGRNVVK 7dvc.1    --------------------------------------------------------------------------------  target    AWAELKKTPHPLAKDGYRFVFHTPKYRHGAHTMPIDTDMVAMLFGPFGDVYRHDRRTPYVAEGYVDIHPSDAREIGVEDG 7dvc.1    -------------------------------------------------------------KGIVRMDKASRDKLGVSAG  target    DYVFIDSDPEDRPFRGWQKNKRDYEFSRLLCRARYYPGTPRGVTRMWFNMYGATPGSVEGQKSREDGLAKNPRTNYQAMF 7dvc.1    DLVEIK--------------------------------------------------------------------------  target    RSGSHQSATRGWLKPTWMTDSLVRKGLFGQSIGKGFLPDVHCPTGAPRESIVKITKAEPGGLGAEGLWRPAALGLRPGYE 7dvc.1    --------------------------------------------------------------------------------  target    SKSMKTYLDGGYVDDADRQGGQG 7dvc.1    ----------------------- ``` | | | | | | | | | | | | | | | | | | | | | | | | | | | | | | | | | | | | | | | | | | | | | | | | | |
|  | 7dvc.5.A | reDPBB\_sym1 protein  *Crystal structure of the computationally designed reDPBB\_sym1 protein* | 0.00 |  | 32.00 | 0.06 | 222-246 | X-ray | 1.71 | monomer |  | HHblits | 0.35 |
| ``` target    EVLARVGHKLAEQTGDARFADVWKLVDEKRTDAHLQRILDHSSNTKGYDALDLEAKAKKGIPTLMMNRTYPKAVGYEQVA 7dvc.5    --------------------------------------------------------------------------------  target    DSRPWYTKSGRLEFYRDEDEFIEAGENLPVHREPIDSTFYEPNVIVSAPHEALRPAGPEDYGVELSDMSGEIRQGRNVVK 7dvc.5    --------------------------------------------------------------------------------  target    AWAELKKTPHPLAKDGYRFVFHTPKYRHGAHTMPIDTDMVAMLFGPFGDVYRHDRRTPYVAEGYVDIHPSDAREIGVEDG 7dvc.5    -------------------------------------------------------------KGIVRMDKASRDKLGVSAG  target    DYVFIDSDPEDRPFRGWQKNKRDYEFSRLLCRARYYPGTPRGVTRMWFNMYGATPGSVEGQKSREDGLAKNPRTNYQAMF 7dvc.5    DLVEIK--------------------------------------------------------------------------  target    RSGSHQSATRGWLKPTWMTDSLVRKGLFGQSIGKGFLPDVHCPTGAPRESIVKITKAEPGGLGAEGLWRPAALGLRPGYE 7dvc.5    --------------------------------------------------------------------------------  target    SKSMKTYLDGGYVDDADRQGGQG 7dvc.5    ----------------------- ``` | | | | | | | | | | | | | | | | | | | | | | | | | | | | | | | | | | | | | | | | | | | | | | | | | |
|  | 7dxu.1.B | mk2h\_dP protein  *Crystal structure of the mk2h\_deltaP peptide homodimer* | 0.00 |  | 28.00 | 0.06 | 222-246 | X-ray | 2.31 | homo-dimer |  | HHblits | 0.34 |
| ``` target    EVLARVGHKLAEQTGDARFADVWKLVDEKRTDAHLQRILDHSSNTKGYDALDLEAKAKKGIPTLMMNRTYPKAVGYEQVA 7dxu.1    --------------------------------------------------------------------------------  target    DSRPWYTKSGRLEFYRDEDEFIEAGENLPVHREPIDSTFYEPNVIVSAPHEALRPAGPEDYGVELSDMSGEIRQGRNVVK 7dxu.1    --------------------------------------------------------------------------------  target    AWAELKKTPHPLAKDGYRFVFHTPKYRHGAHTMPIDTDMVAMLFGPFGDVYRHDRRTPYVAEGYVDIHPSDAREIGVEDG 7dxu.1    -------------------------------------------------------------KRIVRMDKYERAKLGVSVG  target    DYVFIDSDPEDRPFRGWQKNKRDYEFSRLLCRARYYPGTPRGVTRMWFNMYGATPGSVEGQKSREDGLAKNPRTNYQAMF 7dxu.1    DYVEVK--------------------------------------------------------------------------  target    RSGSHQSATRGWLKPTWMTDSLVRKGLFGQSIGKGFLPDVHCPTGAPRESIVKITKAEPGGLGAEGLWRPAALGLRPGYE 7dxu.1    --------------------------------------------------------------------------------  target    SKSMKTYLDGGYVDDADRQGGQG 7dxu.1    ----------------------- ``` | | | | | | | | | | | | | | | | | | | | | | | | | | | | | | | | | | | | | | | | | | | | | | | | | |
|  | 7dxu.1.A | mk2h\_dP protein  *Crystal structure of the mk2h\_deltaP peptide homodimer* | 0.00 |  | 28.00 | 0.06 | 222-246 | X-ray | 2.31 | homo-dimer |  | HHblits | 0.34 |
| ``` target    EVLARVGHKLAEQTGDARFADVWKLVDEKRTDAHLQRILDHSSNTKGYDALDLEAKAKKGIPTLMMNRTYPKAVGYEQVA 7dxu.1    --------------------------------------------------------------------------------  target    DSRPWYTKSGRLEFYRDEDEFIEAGENLPVHREPIDSTFYEPNVIVSAPHEALRPAGPEDYGVELSDMSGEIRQGRNVVK 7dxu.1    --------------------------------------------------------------------------------  target    AWAELKKTPHPLAKDGYRFVFHTPKYRHGAHTMPIDTDMVAMLFGPFGDVYRHDRRTPYVAEGYVDIHPSDAREIGVEDG 7dxu.1    -------------------------------------------------------------KRIVRMDKYERAKLGVSVG  target    DYVFIDSDPEDRPFRGWQKNKRDYEFSRLLCRARYYPGTPRGVTRMWFNMYGATPGSVEGQKSREDGLAKNPRTNYQAMF 7dxu.1    DYVEVK--------------------------------------------------------------------------  target    RSGSHQSATRGWLKPTWMTDSLVRKGLFGQSIGKGFLPDVHCPTGAPRESIVKITKAEPGGLGAEGLWRPAALGLRPGYE 7dxu.1    --------------------------------------------------------------------------------  target    SKSMKTYLDGGYVDDADRQGGQG 7dxu.1    ----------------------- ``` | | | | | | | | | | | | | | | | | | | | | | | | | | | | | | | | | | | | | | | | | | | | | | | | | |
|  | 7dxu.2.B | mk2h\_dP protein  *Crystal structure of the mk2h\_deltaP peptide homodimer* | 0.00 |  | 28.00 | 0.06 | 222-246 | X-ray | 2.31 | homo-dimer |  | HHblits | 0.34 |
| ``` target    EVLARVGHKLAEQTGDARFADVWKLVDEKRTDAHLQRILDHSSNTKGYDALDLEAKAKKGIPTLMMNRTYPKAVGYEQVA 7dxu.2    --------------------------------------------------------------------------------  target    DSRPWYTKSGRLEFYRDEDEFIEAGENLPVHREPIDSTFYEPNVIVSAPHEALRPAGPEDYGVELSDMSGEIRQGRNVVK 7dxu.2    --------------------------------------------------------------------------------  target    AWAELKKTPHPLAKDGYRFVFHTPKYRHGAHTMPIDTDMVAMLFGPFGDVYRHDRRTPYVAEGYVDIHPSDAREIGVEDG 7dxu.2    -------------------------------------------------------------KRIVRMDKYERAKLGVSVG  target    DYVFIDSDPEDRPFRGWQKNKRDYEFSRLLCRARYYPGTPRGVTRMWFNMYGATPGSVEGQKSREDGLAKNPRTNYQAMF 7dxu.2    DYVEVK--------------------------------------------------------------------------  target    RSGSHQSATRGWLKPTWMTDSLVRKGLFGQSIGKGFLPDVHCPTGAPRESIVKITKAEPGGLGAEGLWRPAALGLRPGYE 7dxu.2    --------------------------------------------------------------------------------  target    SKSMKTYLDGGYVDDADRQGGQG 7dxu.2    ----------------------- ``` | | | | | | | | | | | | | | | | | | | | | | | | | | | | | | | | | | | | | | | | | | | | | | | | | |
|  | 7dxr.1.B | mk2h protein  *Crystal structure of the mk2h peptide homodimer.* | 0.00 |  | 28.00 | 0.06 | 222-246 | X-ray | 1.60 | homo-dimer |  | HHblits | 0.34 |
| ``` target    EVLARVGHKLAEQTGDARFADVWKLVDEKRTDAHLQRILDHSSNTKGYDALDLEAKAKKGIPTLMMNRTYPKAVGYEQVA 7dxr.1    --------------------------------------------------------------------------------  target    DSRPWYTKSGRLEFYRDEDEFIEAGENLPVHREPIDSTFYEPNVIVSAPHEALRPAGPEDYGVELSDMSGEIRQGRNVVK 7dxr.1    --------------------------------------------------------------------------------  target    AWAELKKTPHPLAKDGYRFVFHTPKYRHGAHTMPIDTDMVAMLFGPFGDVYRHDRRTPYVAEGYVDIHPSDAREIGVEDG 7dxr.1    -------------------------------------------------------------KRIVRMDKYERAKLGVSVG  target    DYVFIDSDPEDRPFRGWQKNKRDYEFSRLLCRARYYPGTPRGVTRMWFNMYGATPGSVEGQKSREDGLAKNPRTNYQAMF 7dxr.1    DYVEVK--------------------------------------------------------------------------  target    RSGSHQSATRGWLKPTWMTDSLVRKGLFGQSIGKGFLPDVHCPTGAPRESIVKITKAEPGGLGAEGLWRPAALGLRPGYE 7dxr.1    --------------------------------------------------------------------------------  target    SKSMKTYLDGGYVDDADRQGGQG 7dxr.1    ----------------------- ``` | | | | | | | | | | | | | | | | | | | | | | | | | | | | | | | | | | | | | | | | | | | | | | | | | |
|  | 7dxr.1.A | mk2h protein  *Crystal structure of the mk2h peptide homodimer.* | 0.00 |  | 28.00 | 0.06 | 222-246 | X-ray | 1.60 | homo-dimer |  | HHblits | 0.34 |
| ``` target    EVLARVGHKLAEQTGDARFADVWKLVDEKRTDAHLQRILDHSSNTKGYDALDLEAKAKKGIPTLMMNRTYPKAVGYEQVA 7dxr.1    --------------------------------------------------------------------------------  target    DSRPWYTKSGRLEFYRDEDEFIEAGENLPVHREPIDSTFYEPNVIVSAPHEALRPAGPEDYGVELSDMSGEIRQGRNVVK 7dxr.1    --------------------------------------------------------------------------------  target    AWAELKKTPHPLAKDGYRFVFHTPKYRHGAHTMPIDTDMVAMLFGPFGDVYRHDRRTPYVAEGYVDIHPSDAREIGVEDG 7dxr.1    -------------------------------------------------------------KRIVRMDKYERAKLGVSVG  target    DYVFIDSDPEDRPFRGWQKNKRDYEFSRLLCRARYYPGTPRGVTRMWFNMYGATPGSVEGQKSREDGLAKNPRTNYQAMF 7dxr.1    DYVEVK--------------------------------------------------------------------------  target    RSGSHQSATRGWLKPTWMTDSLVRKGLFGQSIGKGFLPDVHCPTGAPRESIVKITKAEPGGLGAEGLWRPAALGLRPGYE 7dxr.1    --------------------------------------------------------------------------------  target    SKSMKTYLDGGYVDDADRQGGQG 7dxr.1    ----------------------- ``` | | | | | | | | | | | | | | | | | | | | | | | | | | | | | | | | | | | | | | | | | | | | | | | | | |
|  | 7dxr.2.B | mk2h protein  *Crystal structure of the mk2h peptide homodimer.* | 0.00 |  | 28.00 | 0.06 | 222-246 | X-ray | 1.60 | homo-dimer | 1 x CXS | HHblits | 0.34 |
| ``` target    EVLARVGHKLAEQTGDARFADVWKLVDEKRTDAHLQRILDHSSNTKGYDALDLEAKAKKGIPTLMMNRTYPKAVGYEQVA 7dxr.2    --------------------------------------------------------------------------------  target    DSRPWYTKSGRLEFYRDEDEFIEAGENLPVHREPIDSTFYEPNVIVSAPHEALRPAGPEDYGVELSDMSGEIRQGRNVVK 7dxr.2    --------------------------------------------------------------------------------  target    AWAELKKTPHPLAKDGYRFVFHTPKYRHGAHTMPIDTDMVAMLFGPFGDVYRHDRRTPYVAEGYVDIHPSDAREIGVEDG 7dxr.2    -------------------------------------------------------------KRIVRMDKYERAKLGVSVG  target    DYVFIDSDPEDRPFRGWQKNKRDYEFSRLLCRARYYPGTPRGVTRMWFNMYGATPGSVEGQKSREDGLAKNPRTNYQAMF 7dxr.2    DYVEVK--------------------------------------------------------------------------  target    RSGSHQSATRGWLKPTWMTDSLVRKGLFGQSIGKGFLPDVHCPTGAPRESIVKITKAEPGGLGAEGLWRPAALGLRPGYE 7dxr.2    --------------------------------------------------------------------------------  target    SKSMKTYLDGGYVDDADRQGGQG 7dxr.2    ----------------------- ``` | | | | | | | | | | | | | | | | | | | | | | | | | | | | | | | | | | | | | | | | | | | | | | | | | |
|  | 7dxt.1.A | mk2h protein  *Crystal structure of the chemically synthesized mk2h peptide homodimer* | 0.00 |  | 28.00 | 0.06 | 222-246 | X-ray | 1.80 | homo-dimer |  | HHblits | 0.34 |
| ``` target    EVLARVGHKLAEQTGDARFADVWKLVDEKRTDAHLQRILDHSSNTKGYDALDLEAKAKKGIPTLMMNRTYPKAVGYEQVA 7dxt.1    --------------------------------------------------------------------------------  target    DSRPWYTKSGRLEFYRDEDEFIEAGENLPVHREPIDSTFYEPNVIVSAPHEALRPAGPEDYGVELSDMSGEIRQGRNVVK 7dxt.1    --------------------------------------------------------------------------------  target    AWAELKKTPHPLAKDGYRFVFHTPKYRHGAHTMPIDTDMVAMLFGPFGDVYRHDRRTPYVAEGYVDIHPSDAREIGVEDG 7dxt.1    -------------------------------------------------------------KRIVRMDKYERAKLGVSVG  target    DYVFIDSDPEDRPFRGWQKNKRDYEFSRLLCRARYYPGTPRGVTRMWFNMYGATPGSVEGQKSREDGLAKNPRTNYQAMF 7dxt.1    DYVEVK--------------------------------------------------------------------------  target    RSGSHQSATRGWLKPTWMTDSLVRKGLFGQSIGKGFLPDVHCPTGAPRESIVKITKAEPGGLGAEGLWRPAALGLRPGYE 7dxt.1    --------------------------------------------------------------------------------  target    SKSMKTYLDGGYVDDADRQGGQG 7dxt.1    ----------------------- ``` | | | | | | | | | | | | | | | | | | | | | | | | | | | | | | | | | | | | | | | | | | | | | | | | | |
|  | 7du6.1.A | mkDPBB\_sym2 protein  *Crystal structure of the rationally designed mkDPBB\_sym2 protein* | 0.00 |  | 28.00 | 0.06 | 222-246 | X-ray | 1.60 | monomer |  | HHblits | 0.34 |
| ``` target    EVLARVGHKLAEQTGDARFADVWKLVDEKRTDAHLQRILDHSSNTKGYDALDLEAKAKKGIPTLMMNRTYPKAVGYEQVA 7du6.1    --------------------------------------------------------------------------------  target    DSRPWYTKSGRLEFYRDEDEFIEAGENLPVHREPIDSTFYEPNVIVSAPHEALRPAGPEDYGVELSDMSGEIRQGRNVVK 7du6.1    --------------------------------------------------------------------------------  target    AWAELKKTPHPLAKDGYRFVFHTPKYRHGAHTMPIDTDMVAMLFGPFGDVYRHDRRTPYVAEGYVDIHPSDAREIGVEDG 7du6.1    -------------------------------------------------------------KRIVRMDKYERAKLGVSVG  target    DYVFIDSDPEDRPFRGWQKNKRDYEFSRLLCRARYYPGTPRGVTRMWFNMYGATPGSVEGQKSREDGLAKNPRTNYQAMF 7du6.1    DYVEVK--------------------------------------------------------------------------  target    RSGSHQSATRGWLKPTWMTDSLVRKGLFGQSIGKGFLPDVHCPTGAPRESIVKITKAEPGGLGAEGLWRPAALGLRPGYE 7du6.1    --------------------------------------------------------------------------------  target    SKSMKTYLDGGYVDDADRQGGQG 7du6.1    ----------------------- ``` | | | | | | | | | | | | | | | | | | | | | | | | | | | | | | | | | | | | | | | | | | | | | | | | | |
|  | 5e7p.1.A | Cell division control protein Cdc48  *Crystal Structure of MSMEG\_0858 (Uniprot A0QQS4), a AAA ATPase.* | 0.00 |  | 24.00 | 0.06 | 224-248 | X-ray | 2.51 | monomer | 2 x ADP | HHblits | 0.34 |
| ``` target    EVLARVGHKLAEQTGDARFADVWKLVDEKRTDAHLQRILDHSSNTKGYDALDLEAKAKKGIPTLMMNRTYPKAVGYEQVA 5e7p.1    --------------------------------------------------------------------------------  target    DSRPWYTKSGRLEFYRDEDEFIEAGENLPVHREPIDSTFYEPNVIVSAPHEALRPAGPEDYGVELSDMSGEIRQGRNVVK 5e7p.1    --------------------------------------------------------------------------------  target    AWAELKKTPHPLAKDGYRFVFHTPKYRHGAHTMPIDTDMVAMLFGPFGDVYRHDRRTPYVAEGYVDIHPSDAREIGVEDG 5e7p.1    ---------------------------------------------------------------VVRLHPEVLAALGIREW  target    DYVFIDSDPEDRPFRGWQKNKRDYEFSRLLCRARYYPGTPRGVTRMWFNMYGATPGSVEGQKSREDGLAKNPRTNYQAMF 5e7p.1    DAVALTGT------------------------------------------------------------------------  target    RSGSHQSATRGWLKPTWMTDSLVRKGLFGQSIGKGFLPDVHCPTGAPRESIVKITKAEPGGLGAEGLWRPAALGLRPGYE 5e7p.1    --------------------------------------------------------------------------------  target    SKSMKTYLDGGYVDDADRQGGQG 5e7p.1    ----------------------- ``` | | | | | | | | | | | | | | | | | | | | | | | | | | | | | | | | | | | | | | | | | | | | | | | | | |
|  | 7di0.1.A | apDPBB\_sym\_79 protein  *Crystal structure of the rationally designed apDPBB\_sym\_79 protein* | 0.00 |  | 32.00 | 0.06 | 222-246 | X-ray | 1.60 | monomer |  | HHblits | 0.34 |
| ``` target    EVLARVGHKLAEQTGDARFADVWKLVDEKRTDAHLQRILDHSSNTKGYDALDLEAKAKKGIPTLMMNRTYPKAVGYEQVA 7di0.1    --------------------------------------------------------------------------------  target    DSRPWYTKSGRLEFYRDEDEFIEAGENLPVHREPIDSTFYEPNVIVSAPHEALRPAGPEDYGVELSDMSGEIRQGRNVVK 7di0.1    --------------------------------------------------------------------------------  target    AWAELKKTPHPLAKDGYRFVFHTPKYRHGAHTMPIDTDMVAMLFGPFGDVYRHDRRTPYVAEGYVDIHPSDAREIGVEDG 7di0.1    -------------------------------------------------------------RGIVRMDKYLRAALGVSVG  target    DYVFIDSDPEDRPFRGWQKNKRDYEFSRLLCRARYYPGTPRGVTRMWFNMYGATPGSVEGQKSREDGLAKNPRTNYQAMF 7di0.1    DYVEVK--------------------------------------------------------------------------  target    RSGSHQSATRGWLKPTWMTDSLVRKGLFGQSIGKGFLPDVHCPTGAPRESIVKITKAEPGGLGAEGLWRPAALGLRPGYE 7di0.1    --------------------------------------------------------------------------------  target    SKSMKTYLDGGYVDDADRQGGQG 7di0.1    ----------------------- ``` | | | | | | | | | | | | | | | | | | | | | | | | | | | | | | | | | | | | | | | | | | | | | | | | | |
|  | 7di0.2.A | apDPBB\_sym\_79 protein  *Crystal structure of the rationally designed apDPBB\_sym\_79 protein* | 0.00 |  | 32.00 | 0.06 | 222-246 | X-ray | 1.60 | monomer |  | HHblits | 0.34 |
| ``` target    EVLARVGHKLAEQTGDARFADVWKLVDEKRTDAHLQRILDHSSNTKGYDALDLEAKAKKGIPTLMMNRTYPKAVGYEQVA 7di0.2    --------------------------------------------------------------------------------  target    DSRPWYTKSGRLEFYRDEDEFIEAGENLPVHREPIDSTFYEPNVIVSAPHEALRPAGPEDYGVELSDMSGEIRQGRNVVK 7di0.2    --------------------------------------------------------------------------------  target    AWAELKKTPHPLAKDGYRFVFHTPKYRHGAHTMPIDTDMVAMLFGPFGDVYRHDRRTPYVAEGYVDIHPSDAREIGVEDG 7di0.2    -------------------------------------------------------------RGIVRMDKYLRAALGVSVG  target    DYVFIDSDPEDRPFRGWQKNKRDYEFSRLLCRARYYPGTPRGVTRMWFNMYGATPGSVEGQKSREDGLAKNPRTNYQAMF 7di0.2    DYVEVK--------------------------------------------------------------------------  target    RSGSHQSATRGWLKPTWMTDSLVRKGLFGQSIGKGFLPDVHCPTGAPRESIVKITKAEPGGLGAEGLWRPAALGLRPGYE 7di0.2    --------------------------------------------------------------------------------  target    SKSMKTYLDGGYVDDADRQGGQG 7di0.2    ----------------------- ``` | | | | | | | | | | | | | | | | | | | | | | | | | | | | | | | | | | | | | | | | | | | | | | | | | |
|  | 7di0.3.A | apDPBB\_sym\_79 protein  *Crystal structure of the rationally designed apDPBB\_sym\_79 protein* | 0.00 |  | 32.00 | 0.06 | 222-246 | X-ray | 1.60 | monomer |  | HHblits | 0.34 |
| ``` target    EVLARVGHKLAEQTGDARFADVWKLVDEKRTDAHLQRILDHSSNTKGYDALDLEAKAKKGIPTLMMNRTYPKAVGYEQVA 7di0.3    --------------------------------------------------------------------------------  target    DSRPWYTKSGRLEFYRDEDEFIEAGENLPVHREPIDSTFYEPNVIVSAPHEALRPAGPEDYGVELSDMSGEIRQGRNVVK 7di0.3    --------------------------------------------------------------------------------  target    AWAELKKTPHPLAKDGYRFVFHTPKYRHGAHTMPIDTDMVAMLFGPFGDVYRHDRRTPYVAEGYVDIHPSDAREIGVEDG 7di0.3    -------------------------------------------------------------RGIVRMDKYLRAALGVSVG  target    DYVFIDSDPEDRPFRGWQKNKRDYEFSRLLCRARYYPGTPRGVTRMWFNMYGATPGSVEGQKSREDGLAKNPRTNYQAMF 7di0.3    DYVEVK--------------------------------------------------------------------------  target    RSGSHQSATRGWLKPTWMTDSLVRKGLFGQSIGKGFLPDVHCPTGAPRESIVKITKAEPGGLGAEGLWRPAALGLRPGYE 7di0.3    --------------------------------------------------------------------------------  target    SKSMKTYLDGGYVDDADRQGGQG 7di0.3    ----------------------- ``` | | | | | | | | | | | | | | | | | | | | | | | | | | | | | | | | | | | | | | | | | | | | | | | | | |
|  | 7di1.1.A | mkDPBB\_sym\_86 protein  *Crystal structure of the rationally designed mkDPBB\_sym\_86 protein* | 0.00 |  | 28.00 | 0.06 | 224-248 | X-ray | 2.10 | monomer |  | HHblits | 0.34 |
| ``` target    EVLARVGHKLAEQTGDARFADVWKLVDEKRTDAHLQRILDHSSNTKGYDALDLEAKAKKGIPTLMMNRTYPKAVGYEQVA 7di1.1    --------------------------------------------------------------------------------  target    DSRPWYTKSGRLEFYRDEDEFIEAGENLPVHREPIDSTFYEPNVIVSAPHEALRPAGPEDYGVELSDMSGEIRQGRNVVK 7di1.1    --------------------------------------------------------------------------------  target    AWAELKKTPHPLAKDGYRFVFHTPKYRHGAHTMPIDTDMVAMLFGPFGDVYRHDRRTPYVAEGYVDIHPSDAREIGVEDG 7di1.1    ---------------------------------------------------------------IVRMDKASRAKLGVSVG  target    DYVFIDSDPEDRPFRGWQKNKRDYEFSRLLCRARYYPGTPRGVTRMWFNMYGATPGSVEGQKSREDGLAKNPRTNYQAMF 7di1.1    DYVEVKKV------------------------------------------------------------------------  target    RSGSHQSATRGWLKPTWMTDSLVRKGLFGQSIGKGFLPDVHCPTGAPRESIVKITKAEPGGLGAEGLWRPAALGLRPGYE 7di1.1    --------------------------------------------------------------------------------  target    SKSMKTYLDGGYVDDADRQGGQG 7di1.1    ----------------------- ``` | | | | | | | | | | | | | | | | | | | | | | | | | | | | | | | | | | | | | | | | | | | | | | | | | |
|  | 7dg7.1.A | ATPase of the AAA+ class  *DPBB domain of VCP-like ATPase from Methanopyrus kandleri* | 0.00 |  | 32.00 | 0.06 | 224-248 | X-ray | 1.60 | monomer | 2 x IMD, 8 x ZN | HHblits | 0.34 |
| ``` target    EVLARVGHKLAEQTGDARFADVWKLVDEKRTDAHLQRILDHSSNTKGYDALDLEAKAKKGIPTLMMNRTYPKAVGYEQVA 7dg7.1    --------------------------------------------------------------------------------  target    DSRPWYTKSGRLEFYRDEDEFIEAGENLPVHREPIDSTFYEPNVIVSAPHEALRPAGPEDYGVELSDMSGEIRQGRNVVK 7dg7.1    --------------------------------------------------------------------------------  target    AWAELKKTPHPLAKDGYRFVFHTPKYRHGAHTMPIDTDMVAMLFGPFGDVYRHDRRTPYVAEGYVDIHPSDAREIGVEDG 7dg7.1    ---------------------------------------------------------------AVRMDKASRDRIGVSEG  target    DYVFIDSDPEDRPFRGWQKNKRDYEFSRLLCRARYYPGTPRGVTRMWFNMYGATPGSVEGQKSREDGLAKNPRTNYQAMF 7dg7.1    DLVKITGS------------------------------------------------------------------------  target    RSGSHQSATRGWLKPTWMTDSLVRKGLFGQSIGKGFLPDVHCPTGAPRESIVKITKAEPGGLGAEGLWRPAALGLRPGYE 7dg7.1    --------------------------------------------------------------------------------  target    SKSMKTYLDGGYVDDADRQGGQG 7dg7.1    ----------------------- ``` | | | | | | | | | | | | | | | | | | | | | | | | | | | | | | | | | | | | | | | | | | | | | | | | | |
|  | 7du6.1.A | mkDPBB\_sym2 protein  *Crystal structure of the rationally designed mkDPBB\_sym2 protein* | 0.00 |  | 28.00 | 0.06 | 224-248 | X-ray | 1.60 | monomer |  | HHblits | 0.34 |
| ``` target    EVLARVGHKLAEQTGDARFADVWKLVDEKRTDAHLQRILDHSSNTKGYDALDLEAKAKKGIPTLMMNRTYPKAVGYEQVA 7du6.1    --------------------------------------------------------------------------------  target    DSRPWYTKSGRLEFYRDEDEFIEAGENLPVHREPIDSTFYEPNVIVSAPHEALRPAGPEDYGVELSDMSGEIRQGRNVVK 7du6.1    --------------------------------------------------------------------------------  target    AWAELKKTPHPLAKDGYRFVFHTPKYRHGAHTMPIDTDMVAMLFGPFGDVYRHDRRTPYVAEGYVDIHPSDAREIGVEDG 7du6.1    ---------------------------------------------------------------IVRMDKYERAKLGVSVG  target    DYVFIDSDPEDRPFRGWQKNKRDYEFSRLLCRARYYPGTPRGVTRMWFNMYGATPGSVEGQKSREDGLAKNPRTNYQAMF 7du6.1    DYVEVKKV------------------------------------------------------------------------  target    RSGSHQSATRGWLKPTWMTDSLVRKGLFGQSIGKGFLPDVHCPTGAPRESIVKITKAEPGGLGAEGLWRPAALGLRPGYE 7du6.1    --------------------------------------------------------------------------------  target    SKSMKTYLDGGYVDDADRQGGQG 7du6.1    ----------------------- ``` | | | | | | | | | | | | | | | | | | | | | | | | | | | | | | | | | | | | | | | | | | | | | | | | | |
|  | 7dg9.1.A | Cell division control protein 48, AAA family  *DPBB domain of VCP-like ATPase from Aeropyrum pernix* | 0.00 |  | 37.50 | 0.06 | 224-247 | X-ray | 1.60 | monomer | 4 x ZN | HHblits | 0.37 |
| ``` target    EVLARVGHKLAEQTGDARFADVWKLVDEKRTDAHLQRILDHSSNTKGYDALDLEAKAKKGIPTLMMNRTYPKAVGYEQVA 7dg9.1    --------------------------------------------------------------------------------  target    DSRPWYTKSGRLEFYRDEDEFIEAGENLPVHREPIDSTFYEPNVIVSAPHEALRPAGPEDYGVELSDMSGEIRQGRNVVK 7dg9.1    --------------------------------------------------------------------------------  target    AWAELKKTPHPLAKDGYRFVFHTPKYRHGAHTMPIDTDMVAMLFGPFGDVYRHDRRTPYVAEGYVDIHPSDAREIGVEDG 7dg9.1    ---------------------------------------------------------------IVRIDRQTAARLGVEVG  target    DYVFIDSDPEDRPFRGWQKNKRDYEFSRLLCRARYYPGTPRGVTRMWFNMYGATPGSVEGQKSREDGLAKNPRTNYQAMF 7dg9.1    DFVKVSK-------------------------------------------------------------------------  target    RSGSHQSATRGWLKPTWMTDSLVRKGLFGQSIGKGFLPDVHCPTGAPRESIVKITKAEPGGLGAEGLWRPAALGLRPGYE 7dg9.1    --------------------------------------------------------------------------------  target    SKSMKTYLDGGYVDDADRQGGQG 7dg9.1    ----------------------- ``` | | | | | | | | | | | | | | | | | | | | | | | | | | | | | | | | | | | | | | | | | | | | | | | | | |
|  | 7di0.1.A | apDPBB\_sym\_79 protein  *Crystal structure of the rationally designed apDPBB\_sym\_79 protein* | 0.00 |  | 28.00 | 0.06 | 224-248 | X-ray | 1.60 | monomer |  | HHblits | 0.33 |
| ``` target    EVLARVGHKLAEQTGDARFADVWKLVDEKRTDAHLQRILDHSSNTKGYDALDLEAKAKKGIPTLMMNRTYPKAVGYEQVA 7di0.1    --------------------------------------------------------------------------------  target    DSRPWYTKSGRLEFYRDEDEFIEAGENLPVHREPIDSTFYEPNVIVSAPHEALRPAGPEDYGVELSDMSGEIRQGRNVVK 7di0.1    --------------------------------------------------------------------------------  target    AWAELKKTPHPLAKDGYRFVFHTPKYRHGAHTMPIDTDMVAMLFGPFGDVYRHDRRTPYVAEGYVDIHPSDAREIGVEDG 7di0.1    ---------------------------------------------------------------IVRMDKQTRARLGVSVG  target    DYVFIDSDPEDRPFRGWQKNKRDYEFSRLLCRARYYPGTPRGVTRMWFNMYGATPGSVEGQKSREDGLAKNPRTNYQAMF 7di0.1    DYVEVKKV------------------------------------------------------------------------  target    RSGSHQSATRGWLKPTWMTDSLVRKGLFGQSIGKGFLPDVHCPTGAPRESIVKITKAEPGGLGAEGLWRPAALGLRPGYE 7di0.1    --------------------------------------------------------------------------------  target    SKSMKTYLDGGYVDDADRQGGQG 7di0.1    ----------------------- ``` | | | | | | | | | | | | | | | | | | | | | | | | | | | | | | | | | | | | | | | | | | | | | | | | | |
|  | 7di0.2.A | apDPBB\_sym\_79 protein  *Crystal structure of the rationally designed apDPBB\_sym\_79 protein* | 0.00 |  | 28.00 | 0.06 | 224-248 | X-ray | 1.60 | monomer |  | HHblits | 0.33 |
| ``` target    EVLARVGHKLAEQTGDARFADVWKLVDEKRTDAHLQRILDHSSNTKGYDALDLEAKAKKGIPTLMMNRTYPKAVGYEQVA 7di0.2    --------------------------------------------------------------------------------  target    DSRPWYTKSGRLEFYRDEDEFIEAGENLPVHREPIDSTFYEPNVIVSAPHEALRPAGPEDYGVELSDMSGEIRQGRNVVK 7di0.2    --------------------------------------------------------------------------------  target    AWAELKKTPHPLAKDGYRFVFHTPKYRHGAHTMPIDTDMVAMLFGPFGDVYRHDRRTPYVAEGYVDIHPSDAREIGVEDG 7di0.2    ---------------------------------------------------------------IVRMDKQTRARLGVSVG  target    DYVFIDSDPEDRPFRGWQKNKRDYEFSRLLCRARYYPGTPRGVTRMWFNMYGATPGSVEGQKSREDGLAKNPRTNYQAMF 7di0.2    DYVEVKKV------------------------------------------------------------------------  target    RSGSHQSATRGWLKPTWMTDSLVRKGLFGQSIGKGFLPDVHCPTGAPRESIVKITKAEPGGLGAEGLWRPAALGLRPGYE 7di0.2    --------------------------------------------------------------------------------  target    SKSMKTYLDGGYVDDADRQGGQG 7di0.2    ----------------------- ``` | | | | | | | | | | | | | | | | | | | | | | | | | | | | | | | | | | | | | | | | | | | | | | | | | |
|  | 7di0.3.A | apDPBB\_sym\_79 protein  *Crystal structure of the rationally designed apDPBB\_sym\_79 protein* | 0.00 |  | 28.00 | 0.06 | 224-248 | X-ray | 1.60 | monomer |  | HHblits | 0.33 |
| ``` target    EVLARVGHKLAEQTGDARFADVWKLVDEKRTDAHLQRILDHSSNTKGYDALDLEAKAKKGIPTLMMNRTYPKAVGYEQVA 7di0.3    --------------------------------------------------------------------------------  target    DSRPWYTKSGRLEFYRDEDEFIEAGENLPVHREPIDSTFYEPNVIVSAPHEALRPAGPEDYGVELSDMSGEIRQGRNVVK 7di0.3    --------------------------------------------------------------------------------  target    AWAELKKTPHPLAKDGYRFVFHTPKYRHGAHTMPIDTDMVAMLFGPFGDVYRHDRRTPYVAEGYVDIHPSDAREIGVEDG 7di0.3    ---------------------------------------------------------------IVRMDKQTRARLGVSVG  target    DYVFIDSDPEDRPFRGWQKNKRDYEFSRLLCRARYYPGTPRGVTRMWFNMYGATPGSVEGQKSREDGLAKNPRTNYQAMF 7di0.3    DYVEVKKV------------------------------------------------------------------------  target    RSGSHQSATRGWLKPTWMTDSLVRKGLFGQSIGKGFLPDVHCPTGAPRESIVKITKAEPGGLGAEGLWRPAALGLRPGYE 7di0.3    --------------------------------------------------------------------------------  target    SKSMKTYLDGGYVDDADRQGGQG 7di0.3    ----------------------- ``` | | | | | | | | | | | | | | | | | | | | | | | | | | | | | | | | | | | | | | | | | | | | | | | | | |
|  | 7dxs.1.A | ap1h protein  *Crystal structure of the ap1h peptide homodimer.* | 0.00 |  | 33.33 | 0.06 | 223-246 | X-ray | 2.10 | homo-dimer |  | HHblits | 0.36 |
| ``` target    EVLARVGHKLAEQTGDARFADVWKLVDEKRTDAHLQRILDHSSNTKGYDALDLEAKAKKGIPTLMMNRTYPKAVGYEQVA 7dxs.1    --------------------------------------------------------------------------------  target    DSRPWYTKSGRLEFYRDEDEFIEAGENLPVHREPIDSTFYEPNVIVSAPHEALRPAGPEDYGVELSDMSGEIRQGRNVVK 7dxs.1    --------------------------------------------------------------------------------  target    AWAELKKTPHPLAKDGYRFVFHTPKYRHGAHTMPIDTDMVAMLFGPFGDVYRHDRRTPYVAEGYVDIHPSDAREIGVEDG 7dxs.1    --------------------------------------------------------------GIVRMDKQTRAKLGVSVG  target    DYVFIDSDPEDRPFRGWQKNKRDYEFSRLLCRARYYPGTPRGVTRMWFNMYGATPGSVEGQKSREDGLAKNPRTNYQAMF 7dxs.1    DYVEVK--------------------------------------------------------------------------  target    RSGSHQSATRGWLKPTWMTDSLVRKGLFGQSIGKGFLPDVHCPTGAPRESIVKITKAEPGGLGAEGLWRPAALGLRPGYE 7dxs.1    --------------------------------------------------------------------------------  target    SKSMKTYLDGGYVDDADRQGGQG 7dxs.1    ----------------------- ``` | | | | | | | | | | | | | | | | | | | | | | | | | | | | | | | | | | | | | | | | | | | | | | | | | |
|  | 7dxs.1.B | ap1h protein  *Crystal structure of the ap1h peptide homodimer.* | 0.00 |  | 33.33 | 0.06 | 223-246 | X-ray | 2.10 | homo-dimer |  | HHblits | 0.36 |
| ``` target    EVLARVGHKLAEQTGDARFADVWKLVDEKRTDAHLQRILDHSSNTKGYDALDLEAKAKKGIPTLMMNRTYPKAVGYEQVA 7dxs.1    --------------------------------------------------------------------------------  target    DSRPWYTKSGRLEFYRDEDEFIEAGENLPVHREPIDSTFYEPNVIVSAPHEALRPAGPEDYGVELSDMSGEIRQGRNVVK 7dxs.1    --------------------------------------------------------------------------------  target    AWAELKKTPHPLAKDGYRFVFHTPKYRHGAHTMPIDTDMVAMLFGPFGDVYRHDRRTPYVAEGYVDIHPSDAREIGVEDG 7dxs.1    --------------------------------------------------------------GIVRMDKQTRAKLGVSVG  target    DYVFIDSDPEDRPFRGWQKNKRDYEFSRLLCRARYYPGTPRGVTRMWFNMYGATPGSVEGQKSREDGLAKNPRTNYQAMF 7dxs.1    DYVEVK--------------------------------------------------------------------------  target    RSGSHQSATRGWLKPTWMTDSLVRKGLFGQSIGKGFLPDVHCPTGAPRESIVKITKAEPGGLGAEGLWRPAALGLRPGYE 7dxs.1    --------------------------------------------------------------------------------  target    SKSMKTYLDGGYVDDADRQGGQG 7dxs.1    ----------------------- ``` | | | | | | | | | | | | | | | | | | | | | | | | | | | | | | | | | | | | | | | | | | | | | | | | | |
|  | 7dxs.2.A | ap1h protein  *Crystal structure of the ap1h peptide homodimer.* | 0.00 |  | 33.33 | 0.06 | 223-246 | X-ray | 2.10 | homo-dimer |  | HHblits | 0.36 |
| ``` target    EVLARVGHKLAEQTGDARFADVWKLVDEKRTDAHLQRILDHSSNTKGYDALDLEAKAKKGIPTLMMNRTYPKAVGYEQVA 7dxs.2    --------------------------------------------------------------------------------  target    DSRPWYTKSGRLEFYRDEDEFIEAGENLPVHREPIDSTFYEPNVIVSAPHEALRPAGPEDYGVELSDMSGEIRQGRNVVK 7dxs.2    --------------------------------------------------------------------------------  target    AWAELKKTPHPLAKDGYRFVFHTPKYRHGAHTMPIDTDMVAMLFGPFGDVYRHDRRTPYVAEGYVDIHPSDAREIGVEDG 7dxs.2    --------------------------------------------------------------GIVRMDKQTRAKLGVSVG  target    DYVFIDSDPEDRPFRGWQKNKRDYEFSRLLCRARYYPGTPRGVTRMWFNMYGATPGSVEGQKSREDGLAKNPRTNYQAMF 7dxs.2    DYVEVK--------------------------------------------------------------------------  target    RSGSHQSATRGWLKPTWMTDSLVRKGLFGQSIGKGFLPDVHCPTGAPRESIVKITKAEPGGLGAEGLWRPAALGLRPGYE 7dxs.2    --------------------------------------------------------------------------------  target    SKSMKTYLDGGYVDDADRQGGQG 7dxs.2    ----------------------- ``` | | | | | | | | | | | | | | | | | | | | | | | | | | | | | | | | | | | | | | | | | | | | | | | | | |
|  | 7dxs.2.B | ap1h protein  *Crystal structure of the ap1h peptide homodimer.* | 0.00 |  | 33.33 | 0.06 | 223-246 | X-ray | 2.10 | homo-dimer |  | HHblits | 0.36 |
| ``` target    EVLARVGHKLAEQTGDARFADVWKLVDEKRTDAHLQRILDHSSNTKGYDALDLEAKAKKGIPTLMMNRTYPKAVGYEQVA 7dxs.2    --------------------------------------------------------------------------------  target    DSRPWYTKSGRLEFYRDEDEFIEAGENLPVHREPIDSTFYEPNVIVSAPHEALRPAGPEDYGVELSDMSGEIRQGRNVVK 7dxs.2    --------------------------------------------------------------------------------  target    AWAELKKTPHPLAKDGYRFVFHTPKYRHGAHTMPIDTDMVAMLFGPFGDVYRHDRRTPYVAEGYVDIHPSDAREIGVEDG 7dxs.2    --------------------------------------------------------------GIVRMDKQTRAKLGVSVG  target    DYVFIDSDPEDRPFRGWQKNKRDYEFSRLLCRARYYPGTPRGVTRMWFNMYGATPGSVEGQKSREDGLAKNPRTNYQAMF 7dxs.2    DYVEVK--------------------------------------------------------------------------  target    RSGSHQSATRGWLKPTWMTDSLVRKGLFGQSIGKGFLPDVHCPTGAPRESIVKITKAEPGGLGAEGLWRPAALGLRPGYE 7dxs.2    --------------------------------------------------------------------------------  target    SKSMKTYLDGGYVDDADRQGGQG 7dxs.2    ----------------------- ``` | | | | | | | | | | | | | | | | | | | | | | | | | | | | | | | | | | | | | | | | | | | | | | | | | |
|  | 7dxv.1.A | mk2h\_dY protein  *Crystal structure of the mk2h\_deltaY peptide homodimer* | 0.00 |  | 24.00 | 0.06 | 222-246 | X-ray | 2.30 | homo-dimer |  | HHblits | 0.33 |
| ``` target    EVLARVGHKLAEQTGDARFADVWKLVDEKRTDAHLQRILDHSSNTKGYDALDLEAKAKKGIPTLMMNRTYPKAVGYEQVA 7dxv.1    --------------------------------------------------------------------------------  target    DSRPWYTKSGRLEFYRDEDEFIEAGENLPVHREPIDSTFYEPNVIVSAPHEALRPAGPEDYGVELSDMSGEIRQGRNVVK 7dxv.1    --------------------------------------------------------------------------------  target    AWAELKKTPHPLAKDGYRFVFHTPKYRHGAHTMPIDTDMVAMLFGPFGDVYRHDRRTPYVAEGYVDIHPSDAREIGVEDG 7dxv.1    -------------------------------------------------------------KRIVRMDKAERAKLGVSVG  target    DYVFIDSDPEDRPFRGWQKNKRDYEFSRLLCRARYYPGTPRGVTRMWFNMYGATPGSVEGQKSREDGLAKNPRTNYQAMF 7dxv.1    DVVEVK--------------------------------------------------------------------------  target    RSGSHQSATRGWLKPTWMTDSLVRKGLFGQSIGKGFLPDVHCPTGAPRESIVKITKAEPGGLGAEGLWRPAALGLRPGYE 7dxv.1    --------------------------------------------------------------------------------  target    SKSMKTYLDGGYVDDADRQGGQG 7dxv.1    ----------------------- ``` | | | | | | | | | | | | | | | | | | | | | | | | | | | | | | | | | | | | | | | | | | | | | | | | | |
|  | 7dxv.1.B | mk2h\_dY protein  *Crystal structure of the mk2h\_deltaY peptide homodimer* | 0.00 |  | 24.00 | 0.06 | 222-246 | X-ray | 2.30 | homo-dimer |  | HHblits | 0.33 |
| ``` target    EVLARVGHKLAEQTGDARFADVWKLVDEKRTDAHLQRILDHSSNTKGYDALDLEAKAKKGIPTLMMNRTYPKAVGYEQVA 7dxv.1    --------------------------------------------------------------------------------  target    DSRPWYTKSGRLEFYRDEDEFIEAGENLPVHREPIDSTFYEPNVIVSAPHEALRPAGPEDYGVELSDMSGEIRQGRNVVK 7dxv.1    --------------------------------------------------------------------------------  target    AWAELKKTPHPLAKDGYRFVFHTPKYRHGAHTMPIDTDMVAMLFGPFGDVYRHDRRTPYVAEGYVDIHPSDAREIGVEDG 7dxv.1    -------------------------------------------------------------KRIVRMDKAERAKLGVSVG  target    DYVFIDSDPEDRPFRGWQKNKRDYEFSRLLCRARYYPGTPRGVTRMWFNMYGATPGSVEGQKSREDGLAKNPRTNYQAMF 7dxv.1    DVVEVK--------------------------------------------------------------------------  target    RSGSHQSATRGWLKPTWMTDSLVRKGLFGQSIGKGFLPDVHCPTGAPRESIVKITKAEPGGLGAEGLWRPAALGLRPGYE 7dxv.1    --------------------------------------------------------------------------------  target    SKSMKTYLDGGYVDDADRQGGQG 7dxv.1    ----------------------- ``` | | | | | | | | | | | | | | | | | | | | | | | | | | | | | | | | | | | | | | | | | | | | | | | | | |
|  | 7dg7.1.A | ATPase of the AAA+ class  *DPBB domain of VCP-like ATPase from Methanopyrus kandleri* | 0.00 |  | 24.00 | 0.06 | 222-246 | X-ray | 1.60 | monomer | 2 x IMD, 8 x ZN | HHblits | 0.33 |
| ``` target    EVLARVGHKLAEQTGDARFADVWKLVDEKRTDAHLQRILDHSSNTKGYDALDLEAKAKKGIPTLMMNRTYPKAVGYEQVA 7dg7.1    --------------------------------------------------------------------------------  target    DSRPWYTKSGRLEFYRDEDEFIEAGENLPVHREPIDSTFYEPNVIVSAPHEALRPAGPEDYGVELSDMSGEIRQGRNVVK 7dg7.1    --------------------------------------------------------------------------------  target    AWAELKKTPHPLAKDGYRFVFHTPKYRHGAHTMPIDTDMVAMLFGPFGDVYRHDRRTPYVAEGYVDIHPSDAREIGVEDG 7dg7.1    -------------------------------------------------------------KGIVRMDKYERQNAGASVG  target    DYVFIDSDPEDRPFRGWQKNKRDYEFSRLLCRARYYPGTPRGVTRMWFNMYGATPGSVEGQKSREDGLAKNPRTNYQAMF 7dg7.1    EPVEVD--------------------------------------------------------------------------  target    RSGSHQSATRGWLKPTWMTDSLVRKGLFGQSIGKGFLPDVHCPTGAPRESIVKITKAEPGGLGAEGLWRPAALGLRPGYE 7dg7.1    --------------------------------------------------------------------------------  target    SKSMKTYLDGGYVDDADRQGGQG 7dg7.1    ----------------------- ``` | | | | | | | | | | | | | | | | | | | | | | | | | | | | | | | | | | | | | | | | | | | | | | | | | |
|  | 2pjh.1.B | Transitional endoplasmic reticulum ATPase  *Strctural Model of the p97 N domain- npl4 UBD complex* | 0.00 |  | 19.23 | 0.06 | 223-248 | NMR | 0.00 | hetero-1-1-mer |  | HHblits | 0.30 |
| ``` target    EVLARVGHKLAEQTGDARFADVWKLVDEKRTDAHLQRILDHSSNTKGYDALDLEAKAKKGIPTLMMNRTYPKAVGYEQVA 2pjh.1    --------------------------------------------------------------------------------  target    DSRPWYTKSGRLEFYRDEDEFIEAGENLPVHREPIDSTFYEPNVIVSAPHEALRPAGPEDYGVELSDMSGEIRQGRNVVK 2pjh.1    --------------------------------------------------------------------------------  target    AWAELKKTPHPLAKDGYRFVFHTPKYRHGAHTMPIDTDMVAMLFGPFGDVYRHDRRTPYVAEGYVDIHPSDAREIGVEDG 2pjh.1    --------------------------------------------------------------SVVSLSQPKMDELQLFRG  target    DYVFIDSDPEDRPFRGWQKNKRDYEFSRLLCRARYYPGTPRGVTRMWFNMYGATPGSVEGQKSREDGLAKNPRTNYQAMF 2pjh.1    DTVLLKGK------------------------------------------------------------------------  target    RSGSHQSATRGWLKPTWMTDSLVRKGLFGQSIGKGFLPDVHCPTGAPRESIVKITKAEPGGLGAEGLWRPAALGLRPGYE 2pjh.1    --------------------------------------------------------------------------------  target    SKSMKTYLDGGYVDDADRQGGQG 2pjh.1    ----------------------- ``` | | | | | | | | | | | | | | | | | | | | | | | | | | | | | | | | | | | | | | | | | | | | | | | | | |
|  | 3hu1.1.A | Transitional endoplasmic reticulum ATPase  *Structure of p97 N-D1 R95G mutant in complex with ATPgS* | 0.00 |  | 19.23 | 0.06 | 224-249 | X-ray | 2.81 | homo-hexamer | 6 x AGS, 6 x MG | HHblits | 0.30 |
| ``` target    EVLARVGHKLAEQTGDARFADVWKLVDEKRTDAHLQRILDHSSNTKGYDALDLEAKAKKGIPTLMMNRTYPKAVGYEQVA 3hu1.1    --------------------------------------------------------------------------------  target    DSRPWYTKSGRLEFYRDEDEFIEAGENLPVHREPIDSTFYEPNVIVSAPHEALRPAGPEDYGVELSDMSGEIRQGRNVVK 3hu1.1    --------------------------------------------------------------------------------  target    AWAELKKTPHPLAKDGYRFVFHTPKYRHGAHTMPIDTDMVAMLFGPFGDVYRHDRRTPYVAEGYVDIHPSDAREIGVEDG 3hu1.1    ---------------------------------------------------------------VVSLSQPKMDELQLFRG  target    DYVFIDSDPEDRPFRGWQKNKRDYEFSRLLCRARYYPGTPRGVTRMWFNMYGATPGSVEGQKSREDGLAKNPRTNYQAMF 3hu1.1    DTVLLKGKK-----------------------------------------------------------------------  target    RSGSHQSATRGWLKPTWMTDSLVRKGLFGQSIGKGFLPDVHCPTGAPRESIVKITKAEPGGLGAEGLWRPAALGLRPGYE 3hu1.1    --------------------------------------------------------------------------------  target    SKSMKTYLDGGYVDDADRQGGQG 3hu1.1    ----------------------- ``` | | | | | | | | | | | | | | | | | | | | | | | | | | | | | | | | | | | | | | | | | | | | | | | | | |
|  | 7dbo.1.A | VCP-like ATPase  *DPBB domain of VCP-like ATPase from Thermoplasma acidophilum* | 0.00 |  | 32.00 | 0.06 | 224-248 | X-ray | 1.90 | monomer |  | HHblits | 0.33 |
| ``` target    EVLARVGHKLAEQTGDARFADVWKLVDEKRTDAHLQRILDHSSNTKGYDALDLEAKAKKGIPTLMMNRTYPKAVGYEQVA 7dbo.1    --------------------------------------------------------------------------------  target    DSRPWYTKSGRLEFYRDEDEFIEAGENLPVHREPIDSTFYEPNVIVSAPHEALRPAGPEDYGVELSDMSGEIRQGRNVVK 7dbo.1    --------------------------------------------------------------------------------  target    AWAELKKTPHPLAKDGYRFVFHTPKYRHGAHTMPIDTDMVAMLFGPFGDVYRHDRRTPYVAEGYVDIHPSDAREIGVEDG 7dbo.1    ---------------------------------------------------------------RVRLDESSRRLLDAEIG  target    DYVFIDSDPEDRPFRGWQKNKRDYEFSRLLCRARYYPGTPRGVTRMWFNMYGATPGSVEGQKSREDGLAKNPRTNYQAMF 7dbo.1    DVVEIEKV------------------------------------------------------------------------  target    RSGSHQSATRGWLKPTWMTDSLVRKGLFGQSIGKGFLPDVHCPTGAPRESIVKITKAEPGGLGAEGLWRPAALGLRPGYE 7dbo.1    --------------------------------------------------------------------------------  target    SKSMKTYLDGGYVDDADRQGGQG 7dbo.1    ----------------------- ``` | | | | | | | | | | | | | | | | | | | | | | | | | | | | | | | | | | | | | | | | | | | | | | | | | |
|  | 7dbo.2.A | VCP-like ATPase  *DPBB domain of VCP-like ATPase from Thermoplasma acidophilum* | 0.00 |  | 32.00 | 0.06 | 224-248 | X-ray | 1.90 | monomer |  | HHblits | 0.33 |
| ``` target    EVLARVGHKLAEQTGDARFADVWKLVDEKRTDAHLQRILDHSSNTKGYDALDLEAKAKKGIPTLMMNRTYPKAVGYEQVA 7dbo.2    --------------------------------------------------------------------------------  target    DSRPWYTKSGRLEFYRDEDEFIEAGENLPVHREPIDSTFYEPNVIVSAPHEALRPAGPEDYGVELSDMSGEIRQGRNVVK 7dbo.2    --------------------------------------------------------------------------------  target    AWAELKKTPHPLAKDGYRFVFHTPKYRHGAHTMPIDTDMVAMLFGPFGDVYRHDRRTPYVAEGYVDIHPSDAREIGVEDG 7dbo.2    ---------------------------------------------------------------RVRLDESSRRLLDAEIG  target    DYVFIDSDPEDRPFRGWQKNKRDYEFSRLLCRARYYPGTPRGVTRMWFNMYGATPGSVEGQKSREDGLAKNPRTNYQAMF 7dbo.2    DVVEIEKV------------------------------------------------------------------------  target    RSGSHQSATRGWLKPTWMTDSLVRKGLFGQSIGKGFLPDVHCPTGAPRESIVKITKAEPGGLGAEGLWRPAALGLRPGYE 7dbo.2    --------------------------------------------------------------------------------  target    SKSMKTYLDGGYVDDADRQGGQG 7dbo.2    ----------------------- ``` | | | | | | | | | | | | | | | | | | | | | | | | | | | | | | | | | | | | | | | | | | | | | | | | | |
|  | 1cz4.1.A | VCP-LIKE ATPASE  *NMR STRUCTURE OF VAT-N: THE N-TERMINAL DOMAIN OF VAT (VCP-LIKE ATPASE OF THERMOPLASMA)* | 0.00 |  | 32.00 | 0.06 | 224-248 | NMR | 0.00 | monomer |  | HHblits | 0.33 |
| ``` target    EVLARVGHKLAEQTGDARFADVWKLVDEKRTDAHLQRILDHSSNTKGYDALDLEAKAKKGIPTLMMNRTYPKAVGYEQVA 1cz4.1    --------------------------------------------------------------------------------  target    DSRPWYTKSGRLEFYRDEDEFIEAGENLPVHREPIDSTFYEPNVIVSAPHEALRPAGPEDYGVELSDMSGEIRQGRNVVK 1cz4.1    --------------------------------------------------------------------------------  target    AWAELKKTPHPLAKDGYRFVFHTPKYRHGAHTMPIDTDMVAMLFGPFGDVYRHDRRTPYVAEGYVDIHPSDAREIGVEDG 1cz4.1    ---------------------------------------------------------------RVRLDESSRRLLDAEIG  target    DYVFIDSDPEDRPFRGWQKNKRDYEFSRLLCRARYYPGTPRGVTRMWFNMYGATPGSVEGQKSREDGLAKNPRTNYQAMF 1cz4.1    DVVEIEKV------------------------------------------------------------------------  target    RSGSHQSATRGWLKPTWMTDSLVRKGLFGQSIGKGFLPDVHCPTGAPRESIVKITKAEPGGLGAEGLWRPAALGLRPGYE 1cz4.1    --------------------------------------------------------------------------------  target    SKSMKTYLDGGYVDDADRQGGQG 1cz4.1    ----------------------- ``` | | | | | | | | | | | | | | | | | | | | | | | | | | | | | | | | | | | | | | | | | | | | | | | | | |
|  | 1cz5.1.A | VCP-LIKE ATPASE  *NMR STRUCTURE OF VAT-N: THE N-TERMINAL DOMAIN OF VAT (VCP-LIKE ATPASE OF THERMOPLASMA)* | 0.00 |  | 32.00 | 0.06 | 224-248 | NMR | 0.00 | monomer |  | HHblits | 0.33 |
| ``` target    EVLARVGHKLAEQTGDARFADVWKLVDEKRTDAHLQRILDHSSNTKGYDALDLEAKAKKGIPTLMMNRTYPKAVGYEQVA 1cz5.1    --------------------------------------------------------------------------------  target    DSRPWYTKSGRLEFYRDEDEFIEAGENLPVHREPIDSTFYEPNVIVSAPHEALRPAGPEDYGVELSDMSGEIRQGRNVVK 1cz5.1    --------------------------------------------------------------------------------  target    AWAELKKTPHPLAKDGYRFVFHTPKYRHGAHTMPIDTDMVAMLFGPFGDVYRHDRRTPYVAEGYVDIHPSDAREIGVEDG 1cz5.1    ---------------------------------------------------------------RVRLDESSRRLLDAEIG  target    DYVFIDSDPEDRPFRGWQKNKRDYEFSRLLCRARYYPGTPRGVTRMWFNMYGATPGSVEGQKSREDGLAKNPRTNYQAMF 1cz5.1    DVVEIEKV------------------------------------------------------------------------  target    RSGSHQSATRGWLKPTWMTDSLVRKGLFGQSIGKGFLPDVHCPTGAPRESIVKITKAEPGGLGAEGLWRPAALGLRPGYE 1cz5.1    --------------------------------------------------------------------------------  target    SKSMKTYLDGGYVDDADRQGGQG 1cz5.1    ----------------------- ``` | | | | | | | | | | | | | | | | | | | | | | | | | | | | | | | | | | | | | | | | | | | | | | | | | |
|  | 7dww.1.A | msDPBB\_sym2 protein  *Crystal structure of the computationally designed msDPBB\_sym2 protein* | 0.00 |  | 32.00 | 0.06 | 222-246 | X-ray | 1.80 | monomer |  | HHblits | 0.32 |
| ``` target    EVLARVGHKLAEQTGDARFADVWKLVDEKRTDAHLQRILDHSSNTKGYDALDLEAKAKKGIPTLMMNRTYPKAVGYEQVA 7dww.1    --------------------------------------------------------------------------------  target    DSRPWYTKSGRLEFYRDEDEFIEAGENLPVHREPIDSTFYEPNVIVSAPHEALRPAGPEDYGVELSDMSGEIRQGRNVVK 7dww.1    --------------------------------------------------------------------------------  target    AWAELKKTPHPLAKDGYRFVFHTPKYRHGAHTMPIDTDMVAMLFGPFGDVYRHDRRTPYVAEGYVDIHPSDAREIGVEDG 7dww.1    -------------------------------------------------------------KNIVRMDEELMRLLGVKVG  target    DYVFIDSDPEDRPFRGWQKNKRDYEFSRLLCRARYYPGTPRGVTRMWFNMYGATPGSVEGQKSREDGLAKNPRTNYQAMF 7dww.1    DLVEIM--------------------------------------------------------------------------  target    RSGSHQSATRGWLKPTWMTDSLVRKGLFGQSIGKGFLPDVHCPTGAPRESIVKITKAEPGGLGAEGLWRPAALGLRPGYE 7dww.1    --------------------------------------------------------------------------------  target    SKSMKTYLDGGYVDDADRQGGQG 7dww.1    ----------------------- ``` | | | | | | | | | | | | | | | | | | | | | | | | | | | | | | | | | | | | | | | | | | | | | | | | | |
|  | 7dww.2.A | msDPBB\_sym2 protein  *Crystal structure of the computationally designed msDPBB\_sym2 protein* | 0.00 |  | 32.00 | 0.06 | 222-246 | X-ray | 1.80 | monomer |  | HHblits | 0.32 |
| ``` target    EVLARVGHKLAEQTGDARFADVWKLVDEKRTDAHLQRILDHSSNTKGYDALDLEAKAKKGIPTLMMNRTYPKAVGYEQVA 7dww.2    --------------------------------------------------------------------------------  target    DSRPWYTKSGRLEFYRDEDEFIEAGENLPVHREPIDSTFYEPNVIVSAPHEALRPAGPEDYGVELSDMSGEIRQGRNVVK 7dww.2    --------------------------------------------------------------------------------  target    AWAELKKTPHPLAKDGYRFVFHTPKYRHGAHTMPIDTDMVAMLFGPFGDVYRHDRRTPYVAEGYVDIHPSDAREIGVEDG 7dww.2    -------------------------------------------------------------KNIVRMDEELMRLLGVKVG  target    DYVFIDSDPEDRPFRGWQKNKRDYEFSRLLCRARYYPGTPRGVTRMWFNMYGATPGSVEGQKSREDGLAKNPRTNYQAMF 7dww.2    DLVEIM--------------------------------------------------------------------------  target    RSGSHQSATRGWLKPTWMTDSLVRKGLFGQSIGKGFLPDVHCPTGAPRESIVKITKAEPGGLGAEGLWRPAALGLRPGYE 7dww.2    --------------------------------------------------------------------------------  target    SKSMKTYLDGGYVDDADRQGGQG 7dww.2    ----------------------- ``` | | | | | | | | | | | | | | | | | | | | | | | | | | | | | | | | | | | | | | | | | | | | | | | | | |
|  | 7dg9.1.A | Cell division control protein 48, AAA family  *DPBB domain of VCP-like ATPase from Aeropyrum pernix* | 0.00 |  | 24.00 | 0.06 | 222-246 | X-ray | 1.60 | monomer | 4 x ZN | HHblits | 0.32 |
| ``` target    EVLARVGHKLAEQTGDARFADVWKLVDEKRTDAHLQRILDHSSNTKGYDALDLEAKAKKGIPTLMMNRTYPKAVGYEQVA 7dg9.1    --------------------------------------------------------------------------------  target    DSRPWYTKSGRLEFYRDEDEFIEAGENLPVHREPIDSTFYEPNVIVSAPHEALRPAGPEDYGVELSDMSGEIRQGRNVVK 7dg9.1    --------------------------------------------------------------------------------  target    AWAELKKTPHPLAKDGYRFVFHTPKYRHGAHTMPIDTDMVAMLFGPFGDVYRHDRRTPYVAEGYVDIHPSDAREIGVEDG 7dg9.1    -------------------------------------------------------------RGIIRMDGYLRAALGVTVG  target    DYVFIDSDPEDRPFRGWQKNKRDYEFSRLLCRARYYPGTPRGVTRMWFNMYGATPGSVEGQKSREDGLAKNPRTNYQAMF 7dg9.1    DTVTVE--------------------------------------------------------------------------  target    RSGSHQSATRGWLKPTWMTDSLVRKGLFGQSIGKGFLPDVHCPTGAPRESIVKITKAEPGGLGAEGLWRPAALGLRPGYE 7dg9.1    --------------------------------------------------------------------------------  target    SKSMKTYLDGGYVDDADRQGGQG 7dg9.1    ----------------------- ``` | | | | | | | | | | | | | | | | | | | | | | | | | | | | | | | | | | | | | | | | | | | | | | | | | |
|  | 7dbo.1.A | VCP-like ATPase  *DPBB domain of VCP-like ATPase from Thermoplasma acidophilum* | 0.00 |  | 28.00 | 0.06 | 222-246 | X-ray | 1.90 | monomer |  | HHblits | 0.31 |
| ``` target    EVLARVGHKLAEQTGDARFADVWKLVDEKRTDAHLQRILDHSSNTKGYDALDLEAKAKKGIPTLMMNRTYPKAVGYEQVA 7dbo.1    --------------------------------------------------------------------------------  target    DSRPWYTKSGRLEFYRDEDEFIEAGENLPVHREPIDSTFYEPNVIVSAPHEALRPAGPEDYGVELSDMSGEIRQGRNVVK 7dbo.1    --------------------------------------------------------------------------------  target    AWAELKKTPHPLAKDGYRFVFHTPKYRHGAHTMPIDTDMVAMLFGPFGDVYRHDRRTPYVAEGYVDIHPSDAREIGVEDG 7dbo.1    -------------------------------------------------------------KGIVRIDSVMRNNCGASIG  target    DYVFIDSDPEDRPFRGWQKNKRDYEFSRLLCRARYYPGTPRGVTRMWFNMYGATPGSVEGQKSREDGLAKNPRTNYQAMF 7dbo.1    DKVKVR--------------------------------------------------------------------------  target    RSGSHQSATRGWLKPTWMTDSLVRKGLFGQSIGKGFLPDVHCPTGAPRESIVKITKAEPGGLGAEGLWRPAALGLRPGYE 7dbo.1    --------------------------------------------------------------------------------  target    SKSMKTYLDGGYVDDADRQGGQG 7dbo.1    ----------------------- ``` | | | | | | | | | | | | | | | | | | | | | | | | | | | | | | | | | | | | | | | | | | | | | | | | | |
|  | 7dbo.2.A | VCP-like ATPase  *DPBB domain of VCP-like ATPase from Thermoplasma acidophilum* | 0.00 |  | 28.00 | 0.06 | 222-246 | X-ray | 1.90 | monomer |  | HHblits | 0.31 |
| ``` target    EVLARVGHKLAEQTGDARFADVWKLVDEKRTDAHLQRILDHSSNTKGYDALDLEAKAKKGIPTLMMNRTYPKAVGYEQVA 7dbo.2    --------------------------------------------------------------------------------  target    DSRPWYTKSGRLEFYRDEDEFIEAGENLPVHREPIDSTFYEPNVIVSAPHEALRPAGPEDYGVELSDMSGEIRQGRNVVK 7dbo.2    --------------------------------------------------------------------------------  target    AWAELKKTPHPLAKDGYRFVFHTPKYRHGAHTMPIDTDMVAMLFGPFGDVYRHDRRTPYVAEGYVDIHPSDAREIGVEDG 7dbo.2    -------------------------------------------------------------KGIVRIDSVMRNNCGASIG  target    DYVFIDSDPEDRPFRGWQKNKRDYEFSRLLCRARYYPGTPRGVTRMWFNMYGATPGSVEGQKSREDGLAKNPRTNYQAMF 7dbo.2    DKVKVR--------------------------------------------------------------------------  target    RSGSHQSATRGWLKPTWMTDSLVRKGLFGQSIGKGFLPDVHCPTGAPRESIVKITKAEPGGLGAEGLWRPAALGLRPGYE 7dbo.2    --------------------------------------------------------------------------------  target    SKSMKTYLDGGYVDDADRQGGQG 7dbo.2    ----------------------- ``` | | | | | | | | | | | | | | | | | | | | | | | | | | | | | | | | | | | | | | | | | | | | | | | | | |
|  | 7dww.1.A | msDPBB\_sym2 protein  *Crystal structure of the computationally designed msDPBB\_sym2 protein* | 0.00 |  | 32.00 | 0.06 | 224-248 | X-ray | 1.80 | monomer |  | HHblits | 0.31 |
| ``` target    EVLARVGHKLAEQTGDARFADVWKLVDEKRTDAHLQRILDHSSNTKGYDALDLEAKAKKGIPTLMMNRTYPKAVGYEQVA 7dww.1    --------------------------------------------------------------------------------  target    DSRPWYTKSGRLEFYRDEDEFIEAGENLPVHREPIDSTFYEPNVIVSAPHEALRPAGPEDYGVELSDMSGEIRQGRNVVK 7dww.1    --------------------------------------------------------------------------------  target    AWAELKKTPHPLAKDGYRFVFHTPKYRHGAHTMPIDTDMVAMLFGPFGDVYRHDRRTPYVAEGYVDIHPSDAREIGVEDG 7dww.1    ---------------------------------------------------------------IVRMDEELMRLLGVKVG  target    DYVFIDSDPEDRPFRGWQKNKRDYEFSRLLCRARYYPGTPRGVTRMWFNMYGATPGSVEGQKSREDGLAKNPRTNYQAMF 7dww.1    DLVEIMKV------------------------------------------------------------------------  target    RSGSHQSATRGWLKPTWMTDSLVRKGLFGQSIGKGFLPDVHCPTGAPRESIVKITKAEPGGLGAEGLWRPAALGLRPGYE 7dww.1    --------------------------------------------------------------------------------  target    SKSMKTYLDGGYVDDADRQGGQG 7dww.1    ----------------------- ``` | | | | | | | | | | | | | | | | | | | | | | | | | | | | | | | | | | | | | | | | | | | | | | | | | |
|  | 7dww.2.A | msDPBB\_sym2 protein  *Crystal structure of the computationally designed msDPBB\_sym2 protein* | 0.00 |  | 32.00 | 0.06 | 224-248 | X-ray | 1.80 | monomer |  | HHblits | 0.31 |
| ``` target    EVLARVGHKLAEQTGDARFADVWKLVDEKRTDAHLQRILDHSSNTKGYDALDLEAKAKKGIPTLMMNRTYPKAVGYEQVA 7dww.2    --------------------------------------------------------------------------------  target    DSRPWYTKSGRLEFYRDEDEFIEAGENLPVHREPIDSTFYEPNVIVSAPHEALRPAGPEDYGVELSDMSGEIRQGRNVVK 7dww.2    --------------------------------------------------------------------------------  target    AWAELKKTPHPLAKDGYRFVFHTPKYRHGAHTMPIDTDMVAMLFGPFGDVYRHDRRTPYVAEGYVDIHPSDAREIGVEDG 7dww.2    ---------------------------------------------------------------IVRMDEELMRLLGVKVG  target    DYVFIDSDPEDRPFRGWQKNKRDYEFSRLLCRARYYPGTPRGVTRMWFNMYGATPGSVEGQKSREDGLAKNPRTNYQAMF 7dww.2    DLVEIMKV------------------------------------------------------------------------  target    RSGSHQSATRGWLKPTWMTDSLVRKGLFGQSIGKGFLPDVHCPTGAPRESIVKITKAEPGGLGAEGLWRPAALGLRPGYE 7dww.2    --------------------------------------------------------------------------------  target    SKSMKTYLDGGYVDDADRQGGQG 7dww.2    ----------------------- ``` | | | | | | | | | | | | | | | | | | | | | | | | | | | | | | | | | | | | | | | | | | | | | | | | | |
|  | 7dxz.1.A | mk2h\_deltaMILPYS protein  *Crystal structure of the chemically synthesized mk2h\_deltaMILPYS peptide homodimer in complex with malonate* | 0.00 |  | 25.00 | 0.06 | 223-246 | X-ray | 1.90 | homo-dimer | 4 x MLA | HHblits | 0.34 |
| ``` target    EVLARVGHKLAEQTGDARFADVWKLVDEKRTDAHLQRILDHSSNTKGYDALDLEAKAKKGIPTLMMNRTYPKAVGYEQVA 7dxz.1    --------------------------------------------------------------------------------  target    DSRPWYTKSGRLEFYRDEDEFIEAGENLPVHREPIDSTFYEPNVIVSAPHEALRPAGPEDYGVELSDMSGEIRQGRNVVK 7dxz.1    --------------------------------------------------------------------------------  target    AWAELKKTPHPLAKDGYRFVFHTPKYRHGAHTMPIDTDMVAMLFGPFGDVYRHDRRTPYVAEGYVDIHPSDAREIGVEDG 7dxz.1    --------------------------------------------------------------RVVRVDKAERAKVGVKVG  target    DYVFIDSDPEDRPFRGWQKNKRDYEFSRLLCRARYYPGTPRGVTRMWFNMYGATPGSVEGQKSREDGLAKNPRTNYQAMF 7dxz.1    DVVEVK--------------------------------------------------------------------------  target    RSGSHQSATRGWLKPTWMTDSLVRKGLFGQSIGKGFLPDVHCPTGAPRESIVKITKAEPGGLGAEGLWRPAALGLRPGYE 7dxz.1    --------------------------------------------------------------------------------  target    SKSMKTYLDGGYVDDADRQGGQG 7dxz.1    ----------------------- ``` | | | | | | | | | | | | | | | | | | | | | | | | | | | | | | | | | | | | | | | | | | | | | | | | | |
|  | 7dxz.2.A | mk2h\_deltaMILPYS protein  *Crystal structure of the chemically synthesized mk2h\_deltaMILPYS peptide homodimer in complex with malonate* | 0.00 |  | 25.00 | 0.06 | 223-246 | X-ray | 1.90 | homo-dimer | 3 x MLA | HHblits | 0.34 |
| ``` target    EVLARVGHKLAEQTGDARFADVWKLVDEKRTDAHLQRILDHSSNTKGYDALDLEAKAKKGIPTLMMNRTYPKAVGYEQVA 7dxz.2    --------------------------------------------------------------------------------  target    DSRPWYTKSGRLEFYRDEDEFIEAGENLPVHREPIDSTFYEPNVIVSAPHEALRPAGPEDYGVELSDMSGEIRQGRNVVK 7dxz.2    --------------------------------------------------------------------------------  target    AWAELKKTPHPLAKDGYRFVFHTPKYRHGAHTMPIDTDMVAMLFGPFGDVYRHDRRTPYVAEGYVDIHPSDAREIGVEDG 7dxz.2    --------------------------------------------------------------RVVRVDKAERAKVGVKVG  target    DYVFIDSDPEDRPFRGWQKNKRDYEFSRLLCRARYYPGTPRGVTRMWFNMYGATPGSVEGQKSREDGLAKNPRTNYQAMF 7dxz.2    DVVEVK--------------------------------------------------------------------------  target    RSGSHQSATRGWLKPTWMTDSLVRKGLFGQSIGKGFLPDVHCPTGAPRESIVKITKAEPGGLGAEGLWRPAALGLRPGYE 7dxz.2    --------------------------------------------------------------------------------  target    SKSMKTYLDGGYVDDADRQGGQG 7dxz.2    ----------------------- ``` | | | | | | | | | | | | | | | | | | | | | | | | | | | | | | | | | | | | | | | | | | | | | | | | | |
|  | 7dxz.2.B | mk2h\_deltaMILPYS protein  *Crystal structure of the chemically synthesized mk2h\_deltaMILPYS peptide homodimer in complex with malonate* | 0.00 |  | 25.00 | 0.06 | 223-246 | X-ray | 1.90 | homo-dimer | 3 x MLA | HHblits | 0.34 |
| ``` target    EVLARVGHKLAEQTGDARFADVWKLVDEKRTDAHLQRILDHSSNTKGYDALDLEAKAKKGIPTLMMNRTYPKAVGYEQVA 7dxz.2    --------------------------------------------------------------------------------  target    DSRPWYTKSGRLEFYRDEDEFIEAGENLPVHREPIDSTFYEPNVIVSAPHEALRPAGPEDYGVELSDMSGEIRQGRNVVK 7dxz.2    --------------------------------------------------------------------------------  target    AWAELKKTPHPLAKDGYRFVFHTPKYRHGAHTMPIDTDMVAMLFGPFGDVYRHDRRTPYVAEGYVDIHPSDAREIGVEDG 7dxz.2    --------------------------------------------------------------RVVRVDKAERAKVGVKVG  target    DYVFIDSDPEDRPFRGWQKNKRDYEFSRLLCRARYYPGTPRGVTRMWFNMYGATPGSVEGQKSREDGLAKNPRTNYQAMF 7dxz.2    DVVEVK--------------------------------------------------------------------------  target    RSGSHQSATRGWLKPTWMTDSLVRKGLFGQSIGKGFLPDVHCPTGAPRESIVKITKAEPGGLGAEGLWRPAALGLRPGYE 7dxz.2    --------------------------------------------------------------------------------  target    SKSMKTYLDGGYVDDADRQGGQG 7dxz.2    ----------------------- ``` | | | | | | | | | | | | | | | | | | | | | | | | | | | | | | | | | | | | | | | | | | | | | | | | | |
|  | 7dxz.3.A | mk2h\_deltaMILPYS protein  *Crystal structure of the chemically synthesized mk2h\_deltaMILPYS peptide homodimer in complex with malonate* | 0.00 |  | 25.00 | 0.06 | 223-246 | X-ray | 1.90 | homo-dimer | 4 x MLA | HHblits | 0.34 |
| ``` target    EVLARVGHKLAEQTGDARFADVWKLVDEKRTDAHLQRILDHSSNTKGYDALDLEAKAKKGIPTLMMNRTYPKAVGYEQVA 7dxz.3    --------------------------------------------------------------------------------  target    DSRPWYTKSGRLEFYRDEDEFIEAGENLPVHREPIDSTFYEPNVIVSAPHEALRPAGPEDYGVELSDMSGEIRQGRNVVK 7dxz.3    --------------------------------------------------------------------------------  target    AWAELKKTPHPLAKDGYRFVFHTPKYRHGAHTMPIDTDMVAMLFGPFGDVYRHDRRTPYVAEGYVDIHPSDAREIGVEDG 7dxz.3    --------------------------------------------------------------RVVRVDKAERAKVGVKVG  target    DYVFIDSDPEDRPFRGWQKNKRDYEFSRLLCRARYYPGTPRGVTRMWFNMYGATPGSVEGQKSREDGLAKNPRTNYQAMF 7dxz.3    DVVEVK--------------------------------------------------------------------------  target    RSGSHQSATRGWLKPTWMTDSLVRKGLFGQSIGKGFLPDVHCPTGAPRESIVKITKAEPGGLGAEGLWRPAALGLRPGYE 7dxz.3    --------------------------------------------------------------------------------  target    SKSMKTYLDGGYVDDADRQGGQG 7dxz.3    ----------------------- ``` | | | | | | | | | | | | | | | | | | | | | | | | | | | | | | | | | | | | | | | | | | | | | | | | | |
|  | 7dyc.1.A | mk2h\_deltaMILPYS protein  *Crystal structure of the chemically synthesized mk2h\_deltaMILPYS peptide homodimer in complex with malate* | 0.00 |  | 25.00 | 0.06 | 223-246 | X-ray | 2.30 | homo-dimer | 2 x MLT | HHblits | 0.34 |
| ``` target    EVLARVGHKLAEQTGDARFADVWKLVDEKRTDAHLQRILDHSSNTKGYDALDLEAKAKKGIPTLMMNRTYPKAVGYEQVA 7dyc.1    --------------------------------------------------------------------------------  target    DSRPWYTKSGRLEFYRDEDEFIEAGENLPVHREPIDSTFYEPNVIVSAPHEALRPAGPEDYGVELSDMSGEIRQGRNVVK 7dyc.1    --------------------------------------------------------------------------------  target    AWAELKKTPHPLAKDGYRFVFHTPKYRHGAHTMPIDTDMVAMLFGPFGDVYRHDRRTPYVAEGYVDIHPSDAREIGVEDG 7dyc.1    --------------------------------------------------------------RVVRVDKAERAKVGVKVG  target    DYVFIDSDPEDRPFRGWQKNKRDYEFSRLLCRARYYPGTPRGVTRMWFNMYGATPGSVEGQKSREDGLAKNPRTNYQAMF 7dyc.1    DVVEVK--------------------------------------------------------------------------  target    RSGSHQSATRGWLKPTWMTDSLVRKGLFGQSIGKGFLPDVHCPTGAPRESIVKITKAEPGGLGAEGLWRPAALGLRPGYE 7dyc.1    --------------------------------------------------------------------------------  target    SKSMKTYLDGGYVDDADRQGGQG 7dyc.1    ----------------------- ``` | | | | | | | | | | | | | | | | | | | | | | | | | | | | | | | | | | | | | | | | | | | | | | | | | |
|  | 7dyc.2.A | mk2h\_deltaMILPYS protein  *Crystal structure of the chemically synthesized mk2h\_deltaMILPYS peptide homodimer in complex with malate* | 0.00 |  | 25.00 | 0.06 | 223-246 | X-ray | 2.30 | homo-dimer |  | HHblits | 0.34 |
| ``` target    EVLARVGHKLAEQTGDARFADVWKLVDEKRTDAHLQRILDHSSNTKGYDALDLEAKAKKGIPTLMMNRTYPKAVGYEQVA 7dyc.2    --------------------------------------------------------------------------------  target    DSRPWYTKSGRLEFYRDEDEFIEAGENLPVHREPIDSTFYEPNVIVSAPHEALRPAGPEDYGVELSDMSGEIRQGRNVVK 7dyc.2    --------------------------------------------------------------------------------  target    AWAELKKTPHPLAKDGYRFVFHTPKYRHGAHTMPIDTDMVAMLFGPFGDVYRHDRRTPYVAEGYVDIHPSDAREIGVEDG 7dyc.2    --------------------------------------------------------------RVVRVDKAERAKVGVKVG  target    DYVFIDSDPEDRPFRGWQKNKRDYEFSRLLCRARYYPGTPRGVTRMWFNMYGATPGSVEGQKSREDGLAKNPRTNYQAMF 7dyc.2    DVVEVK--------------------------------------------------------------------------  target    RSGSHQSATRGWLKPTWMTDSLVRKGLFGQSIGKGFLPDVHCPTGAPRESIVKITKAEPGGLGAEGLWRPAALGLRPGYE 7dyc.2    --------------------------------------------------------------------------------  target    SKSMKTYLDGGYVDDADRQGGQG 7dyc.2    ----------------------- ``` | | | | | | | | | | | | | | | | | | | | | | | | | | | | | | | | | | | | | | | | | | | | | | | | | |
|  | 7dyc.3.A | mk2h\_deltaMILPYS protein  *Crystal structure of the chemically synthesized mk2h\_deltaMILPYS peptide homodimer in complex with malate* | 0.00 |  | 25.00 | 0.06 | 223-246 | X-ray | 2.30 | homo-dimer | 2 x LMR | HHblits | 0.34 |
| ``` target    EVLARVGHKLAEQTGDARFADVWKLVDEKRTDAHLQRILDHSSNTKGYDALDLEAKAKKGIPTLMMNRTYPKAVGYEQVA 7dyc.3    --------------------------------------------------------------------------------  target    DSRPWYTKSGRLEFYRDEDEFIEAGENLPVHREPIDSTFYEPNVIVSAPHEALRPAGPEDYGVELSDMSGEIRQGRNVVK 7dyc.3    --------------------------------------------------------------------------------  target    AWAELKKTPHPLAKDGYRFVFHTPKYRHGAHTMPIDTDMVAMLFGPFGDVYRHDRRTPYVAEGYVDIHPSDAREIGVEDG 7dyc.3    --------------------------------------------------------------RVVRVDKAERAKVGVKVG  target    DYVFIDSDPEDRPFRGWQKNKRDYEFSRLLCRARYYPGTPRGVTRMWFNMYGATPGSVEGQKSREDGLAKNPRTNYQAMF 7dyc.3    DVVEVK--------------------------------------------------------------------------  target    RSGSHQSATRGWLKPTWMTDSLVRKGLFGQSIGKGFLPDVHCPTGAPRESIVKITKAEPGGLGAEGLWRPAALGLRPGYE 7dyc.3    --------------------------------------------------------------------------------  target    SKSMKTYLDGGYVDDADRQGGQG 7dyc.3    ----------------------- ``` | | | | | | | | | | | | | | | | | | | | | | | | | | | | | | | | | | | | | | | | | | | | | | | | | |
|  | 4rv0.1.A | Transitional endoplasmic reticulum ATPase TER94  *Crystal structure of TN complex* | 0.00 |  | 20.00 | 0.06 | 224-248 | X-ray | 2.00 | hetero-oligomer |  | HHblits | 0.31 |
| ``` target    EVLARVGHKLAEQTGDARFADVWKLVDEKRTDAHLQRILDHSSNTKGYDALDLEAKAKKGIPTLMMNRTYPKAVGYEQVA 4rv0.1    --------------------------------------------------------------------------------  target    DSRPWYTKSGRLEFYRDEDEFIEAGENLPVHREPIDSTFYEPNVIVSAPHEALRPAGPEDYGVELSDMSGEIRQGRNVVK 4rv0.1    --------------------------------------------------------------------------------  target    AWAELKKTPHPLAKDGYRFVFHTPKYRHGAHTMPIDTDMVAMLFGPFGDVYRHDRRTPYVAEGYVDIHPSDAREIGVEDG 4rv0.1    ---------------------------------------------------------------VVSLSQAKMDELQLFRG  target    DYVFIDSDPEDRPFRGWQKNKRDYEFSRLLCRARYYPGTPRGVTRMWFNMYGATPGSVEGQKSREDGLAKNPRTNYQAMF 4rv0.1    DTVILKGK------------------------------------------------------------------------  target    RSGSHQSATRGWLKPTWMTDSLVRKGLFGQSIGKGFLPDVHCPTGAPRESIVKITKAEPGGLGAEGLWRPAALGLRPGYE 4rv0.1    --------------------------------------------------------------------------------  target    SKSMKTYLDGGYVDDADRQGGQG 4rv0.1    ----------------------- ``` | | | | | | | | | | | | | | | | | | | | | | | | | | | | | | | | | | | | | | | | | | | | | | | | | |
|  | 3o27.1.A | Putative uncharacterized protein  *The crystal structure of C68 from the hybrid virus-plasmid pSSVx* | 0.00 |  | 16.00 | 0.06 | 224-248 | X-ray | 2.80 | homo-dimer |  | HHblits | 0.31 |
| ``` target    EVLARVGHKLAEQTGDARFADVWKLVDEKRTDAHLQRILDHSSNTKGYDALDLEAKAKKGIPTLMMNRTYPKAVGYEQVA 3o27.1    --------------------------------------------------------------------------------  target    DSRPWYTKSGRLEFYRDEDEFIEAGENLPVHREPIDSTFYEPNVIVSAPHEALRPAGPEDYGVELSDMSGEIRQGRNVVK 3o27.1    --------------------------------------------------------------------------------  target    AWAELKKTPHPLAKDGYRFVFHTPKYRHGAHTMPIDTDMVAMLFGPFGDVYRHDRRTPYVAEGYVDIHPSDAREIGVEDG 3o27.1    ---------------------------------------------------------------YLLIPKDIAEALDIKPD  target    DYVFIDSDPEDRPFRGWQKNKRDYEFSRLLCRARYYPGTPRGVTRMWFNMYGATPGSVEGQKSREDGLAKNPRTNYQAMF 3o27.1    DTFILNME------------------------------------------------------------------------  target    RSGSHQSATRGWLKPTWMTDSLVRKGLFGQSIGKGFLPDVHCPTGAPRESIVKITKAEPGGLGAEGLWRPAALGLRPGYE 3o27.1    --------------------------------------------------------------------------------  target    SKSMKTYLDGGYVDDADRQGGQG 3o27.1    ----------------------- ``` | | | | | | | | | | | | | | | | | | | | | | | | | | | | | | | | | | | | | | | | | | | | | | | | | |
|  | 3o27.1.B | Putative uncharacterized protein  *The crystal structure of C68 from the hybrid virus-plasmid pSSVx* | 0.00 |  | 16.00 | 0.06 | 224-248 | X-ray | 2.80 | homo-dimer |  | HHblits | 0.31 |
| ``` target    EVLARVGHKLAEQTGDARFADVWKLVDEKRTDAHLQRILDHSSNTKGYDALDLEAKAKKGIPTLMMNRTYPKAVGYEQVA 3o27.1    --------------------------------------------------------------------------------  target    DSRPWYTKSGRLEFYRDEDEFIEAGENLPVHREPIDSTFYEPNVIVSAPHEALRPAGPEDYGVELSDMSGEIRQGRNVVK 3o27.1    --------------------------------------------------------------------------------  target    AWAELKKTPHPLAKDGYRFVFHTPKYRHGAHTMPIDTDMVAMLFGPFGDVYRHDRRTPYVAEGYVDIHPSDAREIGVEDG 3o27.1    ---------------------------------------------------------------YLLIPKDIAEALDIKPD  target    DYVFIDSDPEDRPFRGWQKNKRDYEFSRLLCRARYYPGTPRGVTRMWFNMYGATPGSVEGQKSREDGLAKNPRTNYQAMF 3o27.1    DTFILNME------------------------------------------------------------------------  target    RSGSHQSATRGWLKPTWMTDSLVRKGLFGQSIGKGFLPDVHCPTGAPRESIVKITKAEPGGLGAEGLWRPAALGLRPGYE 3o27.1    --------------------------------------------------------------------------------  target    SKSMKTYLDGGYVDDADRQGGQG 3o27.1    ----------------------- ``` | | | | | | | | | | | | | | | | | | | | | | | | | | | | | | | | | | | | | | | | | | | | | | | | | |
|  | 5x4l.1.A | Transitional endoplasmic reticulum ATPase  *Crystal structure of the UBX domain of human UBXD7 in complex with p97 N domain* | 0.00 |  | 20.00 | 0.06 | 224-248 | X-ray | 2.40 | hetero-oligomer |  | HHblits | 0.30 |
| ``` target    EVLARVGHKLAEQTGDARFADVWKLVDEKRTDAHLQRILDHSSNTKGYDALDLEAKAKKGIPTLMMNRTYPKAVGYEQVA 5x4l.1    --------------------------------------------------------------------------------  target    DSRPWYTKSGRLEFYRDEDEFIEAGENLPVHREPIDSTFYEPNVIVSAPHEALRPAGPEDYGVELSDMSGEIRQGRNVVK 5x4l.1    --------------------------------------------------------------------------------  target    AWAELKKTPHPLAKDGYRFVFHTPKYRHGAHTMPIDTDMVAMLFGPFGDVYRHDRRTPYVAEGYVDIHPSDAREIGVEDG 5x4l.1    ---------------------------------------------------------------VVSLSQPKMDELQLFRG  target    DYVFIDSDPEDRPFRGWQKNKRDYEFSRLLCRARYYPGTPRGVTRMWFNMYGATPGSVEGQKSREDGLAKNPRTNYQAMF 5x4l.1    DTVLLKGK------------------------------------------------------------------------  target    RSGSHQSATRGWLKPTWMTDSLVRKGLFGQSIGKGFLPDVHCPTGAPRESIVKITKAEPGGLGAEGLWRPAALGLRPGYE 5x4l.1    --------------------------------------------------------------------------------  target    SKSMKTYLDGGYVDDADRQGGQG 5x4l.1    ----------------------- ``` | | | | | | | | | | | | | | | | | | | | | | | | | | | | | | | | | | | | | | | | | | | | | | | | | |
|  | 5x4l.2.A | Transitional endoplasmic reticulum ATPase  *Crystal structure of the UBX domain of human UBXD7 in complex with p97 N domain* | 0.00 |  | 20.00 | 0.06 | 224-248 | X-ray | 2.40 | hetero-oligomer |  | HHblits | 0.30 |
| ``` target    EVLARVGHKLAEQTGDARFADVWKLVDEKRTDAHLQRILDHSSNTKGYDALDLEAKAKKGIPTLMMNRTYPKAVGYEQVA 5x4l.2    --------------------------------------------------------------------------------  target    DSRPWYTKSGRLEFYRDEDEFIEAGENLPVHREPIDSTFYEPNVIVSAPHEALRPAGPEDYGVELSDMSGEIRQGRNVVK 5x4l.2    --------------------------------------------------------------------------------  target    AWAELKKTPHPLAKDGYRFVFHTPKYRHGAHTMPIDTDMVAMLFGPFGDVYRHDRRTPYVAEGYVDIHPSDAREIGVEDG 5x4l.2    ---------------------------------------------------------------VVSLSQPKMDELQLFRG  target    DYVFIDSDPEDRPFRGWQKNKRDYEFSRLLCRARYYPGTPRGVTRMWFNMYGATPGSVEGQKSREDGLAKNPRTNYQAMF 5x4l.2    DTVLLKGK------------------------------------------------------------------------  target    RSGSHQSATRGWLKPTWMTDSLVRKGLFGQSIGKGFLPDVHCPTGAPRESIVKITKAEPGGLGAEGLWRPAALGLRPGYE 5x4l.2    --------------------------------------------------------------------------------  target    SKSMKTYLDGGYVDDADRQGGQG 5x4l.2    ----------------------- ``` | | | | | | | | | | | | | | | | | | | | | | | | | | | | | | | | | | | | | | | | | | | | | | | | | |
|  | 5b6c.1.A | Transitional endoplasmic reticulum ATPase  *Structural Details of Ufd1 binding to p97* | 0.00 |  | 20.00 | 0.06 | 224-248 | X-ray | 1.55 | hetero-oligomer |  | HHblits | 0.30 |
| ``` target    EVLARVGHKLAEQTGDARFADVWKLVDEKRTDAHLQRILDHSSNTKGYDALDLEAKAKKGIPTLMMNRTYPKAVGYEQVA 5b6c.1    --------------------------------------------------------------------------------  target    DSRPWYTKSGRLEFYRDEDEFIEAGENLPVHREPIDSTFYEPNVIVSAPHEALRPAGPEDYGVELSDMSGEIRQGRNVVK 5b6c.1    --------------------------------------------------------------------------------  target    AWAELKKTPHPLAKDGYRFVFHTPKYRHGAHTMPIDTDMVAMLFGPFGDVYRHDRRTPYVAEGYVDIHPSDAREIGVEDG 5b6c.1    ---------------------------------------------------------------VVSLSQPKMDELQLFRG  target    DYVFIDSDPEDRPFRGWQKNKRDYEFSRLLCRARYYPGTPRGVTRMWFNMYGATPGSVEGQKSREDGLAKNPRTNYQAMF 5b6c.1    DTVLLKGK------------------------------------------------------------------------  target    RSGSHQSATRGWLKPTWMTDSLVRKGLFGQSIGKGFLPDVHCPTGAPRESIVKITKAEPGGLGAEGLWRPAALGLRPGYE 5b6c.1    --------------------------------------------------------------------------------  target    SKSMKTYLDGGYVDDADRQGGQG 5b6c.1    ----------------------- ``` | | | | | | | | | | | | | | | | | | | | | | | | | | | | | | | | | | | | | | | | | | | | | | | | | |
|  | 5epp.1.A | Transitional endoplasmic reticulum ATPase  *Structural Insights into the Interaction of p97 N-terminus Domain and VBM Motif in Rhomboid Protease, RHBDL4* | 0.00 |  | 20.00 | 0.06 | 224-248 | X-ray | 1.88 | hetero-oligomer |  | HHblits | 0.30 |
| ``` target    EVLARVGHKLAEQTGDARFADVWKLVDEKRTDAHLQRILDHSSNTKGYDALDLEAKAKKGIPTLMMNRTYPKAVGYEQVA 5epp.1    --------------------------------------------------------------------------------  target    DSRPWYTKSGRLEFYRDEDEFIEAGENLPVHREPIDSTFYEPNVIVSAPHEALRPAGPEDYGVELSDMSGEIRQGRNVVK 5epp.1    --------------------------------------------------------------------------------  target    AWAELKKTPHPLAKDGYRFVFHTPKYRHGAHTMPIDTDMVAMLFGPFGDVYRHDRRTPYVAEGYVDIHPSDAREIGVEDG 5epp.1    ---------------------------------------------------------------VVSLSQPKMDELQLFRG  target    DYVFIDSDPEDRPFRGWQKNKRDYEFSRLLCRARYYPGTPRGVTRMWFNMYGATPGSVEGQKSREDGLAKNPRTNYQAMF 5epp.1    DTVLLKGK------------------------------------------------------------------------  target    RSGSHQSATRGWLKPTWMTDSLVRKGLFGQSIGKGFLPDVHCPTGAPRESIVKITKAEPGGLGAEGLWRPAALGLRPGYE 5epp.1    --------------------------------------------------------------------------------  target    SKSMKTYLDGGYVDDADRQGGQG 5epp.1    ----------------------- ``` | | | | | | | | | | | | | | | | | | | | | | | | | | | | | | | | | | | | | | | | | | | | | | | | | |
|  | 5glf.2.A | Transitional endoplasmic reticulum ATPase  *Structural insights into the interaction of p97 N-terminal domain and SHP motif in Derlin-1 rhomboid pseudoprotease* | 0.00 |  | 20.00 | 0.06 | 224-248 | X-ray | 2.25 | hetero-1-1-mer |  | HHblits | 0.30 |
| ``` target    EVLARVGHKLAEQTGDARFADVWKLVDEKRTDAHLQRILDHSSNTKGYDALDLEAKAKKGIPTLMMNRTYPKAVGYEQVA 5glf.2    --------------------------------------------------------------------------------  target    DSRPWYTKSGRLEFYRDEDEFIEAGENLPVHREPIDSTFYEPNVIVSAPHEALRPAGPEDYGVELSDMSGEIRQGRNVVK 5glf.2    --------------------------------------------------------------------------------  target    AWAELKKTPHPLAKDGYRFVFHTPKYRHGAHTMPIDTDMVAMLFGPFGDVYRHDRRTPYVAEGYVDIHPSDAREIGVEDG 5glf.2    ---------------------------------------------------------------VVSLSQPKMDELQLFRG  target    DYVFIDSDPEDRPFRGWQKNKRDYEFSRLLCRARYYPGTPRGVTRMWFNMYGATPGSVEGQKSREDGLAKNPRTNYQAMF 5glf.2    DTVLLKGK------------------------------------------------------------------------  target    RSGSHQSATRGWLKPTWMTDSLVRKGLFGQSIGKGFLPDVHCPTGAPRESIVKITKAEPGGLGAEGLWRPAALGLRPGYE 5glf.2    --------------------------------------------------------------------------------  target    SKSMKTYLDGGYVDDADRQGGQG 5glf.2    ----------------------- ``` | | | | | | | | | | | | | | | | | | | | | | | | | | | | | | | | | | | | | | | | | | | | | | | | | |
|  | 5glf.3.A | Transitional endoplasmic reticulum ATPase  *Structural insights into the interaction of p97 N-terminal domain and SHP motif in Derlin-1 rhomboid pseudoprotease* | 0.00 |  | 20.00 | 0.06 | 224-248 | X-ray | 2.25 | hetero-1-1-mer |  | HHblits | 0.30 |
| ``` target    EVLARVGHKLAEQTGDARFADVWKLVDEKRTDAHLQRILDHSSNTKGYDALDLEAKAKKGIPTLMMNRTYPKAVGYEQVA 5glf.3    --------------------------------------------------------------------------------  target    DSRPWYTKSGRLEFYRDEDEFIEAGENLPVHREPIDSTFYEPNVIVSAPHEALRPAGPEDYGVELSDMSGEIRQGRNVVK 5glf.3    --------------------------------------------------------------------------------  target    AWAELKKTPHPLAKDGYRFVFHTPKYRHGAHTMPIDTDMVAMLFGPFGDVYRHDRRTPYVAEGYVDIHPSDAREIGVEDG 5glf.3    ---------------------------------------------------------------VVSLSQPKMDELQLFRG  target    DYVFIDSDPEDRPFRGWQKNKRDYEFSRLLCRARYYPGTPRGVTRMWFNMYGATPGSVEGQKSREDGLAKNPRTNYQAMF 5glf.3    DTVLLKGK------------------------------------------------------------------------  target    RSGSHQSATRGWLKPTWMTDSLVRKGLFGQSIGKGFLPDVHCPTGAPRESIVKITKAEPGGLGAEGLWRPAALGLRPGYE 5glf.3    --------------------------------------------------------------------------------  target    SKSMKTYLDGGYVDDADRQGGQG 5glf.3    ----------------------- ``` | | | | | | | | | | | | | | | | | | | | | | | | | | | | | | | | | | | | | | | | | | | | | | | | | |
|  | 5glf.1.A | Transitional endoplasmic reticulum ATPase  *Structural insights into the interaction of p97 N-terminal domain and SHP motif in Derlin-1 rhomboid pseudoprotease* | 0.00 |  | 20.00 | 0.06 | 224-248 | X-ray | 2.25 | hetero-1-1-mer |  | HHblits | 0.30 |
| ``` target    EVLARVGHKLAEQTGDARFADVWKLVDEKRTDAHLQRILDHSSNTKGYDALDLEAKAKKGIPTLMMNRTYPKAVGYEQVA 5glf.1    --------------------------------------------------------------------------------  target    DSRPWYTKSGRLEFYRDEDEFIEAGENLPVHREPIDSTFYEPNVIVSAPHEALRPAGPEDYGVELSDMSGEIRQGRNVVK 5glf.1    --------------------------------------------------------------------------------  target    AWAELKKTPHPLAKDGYRFVFHTPKYRHGAHTMPIDTDMVAMLFGPFGDVYRHDRRTPYVAEGYVDIHPSDAREIGVEDG 5glf.1    ---------------------------------------------------------------VVSLSQPKMDELQLFRG  target    DYVFIDSDPEDRPFRGWQKNKRDYEFSRLLCRARYYPGTPRGVTRMWFNMYGATPGSVEGQKSREDGLAKNPRTNYQAMF 5glf.1    DTVLLKGK------------------------------------------------------------------------  target    RSGSHQSATRGWLKPTWMTDSLVRKGLFGQSIGKGFLPDVHCPTGAPRESIVKITKAEPGGLGAEGLWRPAALGLRPGYE 5glf.1    --------------------------------------------------------------------------------  target    SKSMKTYLDGGYVDDADRQGGQG 5glf.1    ----------------------- ``` | | | | | | | | | | | | | | | | | | | | | | | | | | | | | | | | | | | | | | | | | | | | | | | | | |
|  | 5glf.4.A | Transitional endoplasmic reticulum ATPase  *Structural insights into the interaction of p97 N-terminal domain and SHP motif in Derlin-1 rhomboid pseudoprotease* | 0.00 |  | 20.00 | 0.06 | 224-248 | X-ray | 2.25 | hetero-1-1-mer |  | HHblits | 0.30 |
| ``` target    EVLARVGHKLAEQTGDARFADVWKLVDEKRTDAHLQRILDHSSNTKGYDALDLEAKAKKGIPTLMMNRTYPKAVGYEQVA 5glf.4    --------------------------------------------------------------------------------  target    DSRPWYTKSGRLEFYRDEDEFIEAGENLPVHREPIDSTFYEPNVIVSAPHEALRPAGPEDYGVELSDMSGEIRQGRNVVK 5glf.4    --------------------------------------------------------------------------------  target    AWAELKKTPHPLAKDGYRFVFHTPKYRHGAHTMPIDTDMVAMLFGPFGDVYRHDRRTPYVAEGYVDIHPSDAREIGVEDG 5glf.4    ---------------------------------------------------------------VVSLSQPKMDELQLFRG  target    DYVFIDSDPEDRPFRGWQKNKRDYEFSRLLCRARYYPGTPRGVTRMWFNMYGATPGSVEGQKSREDGLAKNPRTNYQAMF 5glf.4    DTVLLKGK------------------------------------------------------------------------  target    RSGSHQSATRGWLKPTWMTDSLVRKGLFGQSIGKGFLPDVHCPTGAPRESIVKITKAEPGGLGAEGLWRPAALGLRPGYE 5glf.4    --------------------------------------------------------------------------------  target    SKSMKTYLDGGYVDDADRQGGQG 5glf.4    ----------------------- ``` | | | | | | | | | | | | | | | | | | | | | | | | | | | | | | | | | | | | | | | | | | | | | | | | | |
|  | 3qq8.1.A | Transitional endoplasmic reticulum ATPase  *Crystal structure of p97-N in complex with FAF1-UBX* | 0.00 |  | 20.00 | 0.06 | 224-248 | X-ray | 2.00 | hetero-oligomer |  | HHblits | 0.30 |
| ``` target    EVLARVGHKLAEQTGDARFADVWKLVDEKRTDAHLQRILDHSSNTKGYDALDLEAKAKKGIPTLMMNRTYPKAVGYEQVA 3qq8.1    --------------------------------------------------------------------------------  target    DSRPWYTKSGRLEFYRDEDEFIEAGENLPVHREPIDSTFYEPNVIVSAPHEALRPAGPEDYGVELSDMSGEIRQGRNVVK 3qq8.1    --------------------------------------------------------------------------------  target    AWAELKKTPHPLAKDGYRFVFHTPKYRHGAHTMPIDTDMVAMLFGPFGDVYRHDRRTPYVAEGYVDIHPSDAREIGVEDG 3qq8.1    ---------------------------------------------------------------VVSLSQPKMDELQLFRG  target    DYVFIDSDPEDRPFRGWQKNKRDYEFSRLLCRARYYPGTPRGVTRMWFNMYGATPGSVEGQKSREDGLAKNPRTNYQAMF 3qq8.1    DTVLLKGK------------------------------------------------------------------------  target    RSGSHQSATRGWLKPTWMTDSLVRKGLFGQSIGKGFLPDVHCPTGAPRESIVKITKAEPGGLGAEGLWRPAALGLRPGYE 3qq8.1    --------------------------------------------------------------------------------  target    SKSMKTYLDGGYVDDADRQGGQG 3qq8.1    ----------------------- ``` | | | | | | | | | | | | | | | | | | | | | | | | | | | | | | | | | | | | | | | | | | | | | | | | | |
|  | 3qq7.1.A | Transitional endoplasmic reticulum ATPase  *Crystal Structure of the p97 N-terminal domain* | 0.00 |  | 20.00 | 0.06 | 224-248 | X-ray | 2.65 | monomer | 1 x HEZ, 1 x CO | HHblits | 0.30 |
| ``` target    EVLARVGHKLAEQTGDARFADVWKLVDEKRTDAHLQRILDHSSNTKGYDALDLEAKAKKGIPTLMMNRTYPKAVGYEQVA 3qq7.1    --------------------------------------------------------------------------------  target    DSRPWYTKSGRLEFYRDEDEFIEAGENLPVHREPIDSTFYEPNVIVSAPHEALRPAGPEDYGVELSDMSGEIRQGRNVVK 3qq7.1    --------------------------------------------------------------------------------  target    AWAELKKTPHPLAKDGYRFVFHTPKYRHGAHTMPIDTDMVAMLFGPFGDVYRHDRRTPYVAEGYVDIHPSDAREIGVEDG 3qq7.1    ---------------------------------------------------------------VVSLSQPKMDELQLFRG  target    DYVFIDSDPEDRPFRGWQKNKRDYEFSRLLCRARYYPGTPRGVTRMWFNMYGATPGSVEGQKSREDGLAKNPRTNYQAMF 3qq7.1    DTVLLKGK------------------------------------------------------------------------  target    RSGSHQSATRGWLKPTWMTDSLVRKGLFGQSIGKGFLPDVHCPTGAPRESIVKITKAEPGGLGAEGLWRPAALGLRPGYE 3qq7.1    --------------------------------------------------------------------------------  target    SKSMKTYLDGGYVDDADRQGGQG 3qq7.1    ----------------------- ``` | | | | | | | | | | | | | | | | | | | | | | | | | | | | | | | | | | | | | | | | | | | | | | | | | |
|  | 5udf.1.A | Lipoprotein-releasing system transmembrane protein LolE  *Structure of the N-terminal domain of lipoprotein-releasing system transmembrane protein LolE from Acinetobacter baumannii* | 0.00 |  | 15.38 | 0.06 | 223-248 | X-ray | 2.35 | homo-tetramer |  | HHblits | 0.26 |
| ``` target    EVLARVGHKLAEQTGDARFADVWKLVDEKRTDAHLQRILDHSSNTKGYDALDLEAKAKKGIPTLMMNRTYPKAVGYEQVA 5udf.1    --------------------------------------------------------------------------------  target    DSRPWYTKSGRLEFYRDEDEFIEAGENLPVHREPIDSTFYEPNVIVSAPHEALRPAGPEDYGVELSDMSGEIRQGRNVVK 5udf.1    --------------------------------------------------------------------------------  target    AWAELKKTPHPLAKDGYRFVFHTPKYRHGAHTMPIDTDMVAMLFGPFGDVYRHDRRTPYVAEGYVDIHPSDAREIGVEDG 5udf.1    --------------------------------------------------------------FGIVLGKDMADSLGLRLN  target    DYVFIDSDPEDRPFRGWQKNKRDYEFSRLLCRARYYPGTPRGVTRMWFNMYGATPGSVEGQKSREDGLAKNPRTNYQAMF 5udf.1    DSVTLVLP------------------------------------------------------------------------  target    RSGSHQSATRGWLKPTWMTDSLVRKGLFGQSIGKGFLPDVHCPTGAPRESIVKITKAEPGGLGAEGLWRPAALGLRPGYE 5udf.1    --------------------------------------------------------------------------------  target    SKSMKTYLDGGYVDDADRQGGQG 5udf.1    ----------------------- ``` | | | | | | | | | | | | | | | | | | | | | | | | | | | | | | | | | | | | | | | | | | | | | | | | | |
|  | 1yle.1.A | Arginine N-succinyltransferase, alpha chain  *The structure of arginine/ornithine succinyltransferase subunit AI from Pseudomonas aeruginosa.* | 0.00 |  | 25.00 | 0.06 | 224-247 | X-ray | 1.70 | monomer | 1 x CA | HHblits | 0.31 |
| ``` target    EVLARVGHKLAEQTGDARFADVWKLVDEKRTDAHLQRILDHSSNTKGYDALDLEAKAKKGIPTLMMNRTYPKAVGYEQVA 1yle.1    --------------------------------------------------------------------------------  target    DSRPWYTKSGRLEFYRDEDEFIEAGENLPVHREPIDSTFYEPNVIVSAPHEALRPAGPEDYGVELSDMSGEIRQGRNVVK 1yle.1    --------------------------------------------------------------------------------  target    AWAELKKTPHPLAKDGYRFVFHTPKYRHGAHTMPIDTDMVAMLFGPFGDVYRHDRRTPYVAEGYVDIHPSDAREIGVEDG 1yle.1    ---------------------------------------------------------------PVALSVEAAEALGVGEG  target    DYVFIDSDPEDRPFRGWQKNKRDYEFSRLLCRARYYPGTPRGVTRMWFNMYGATPGSVEGQKSREDGLAKNPRTNYQAMF 1yle.1    ASVRLVA-------------------------------------------------------------------------  target    RSGSHQSATRGWLKPTWMTDSLVRKGLFGQSIGKGFLPDVHCPTGAPRESIVKITKAEPGGLGAEGLWRPAALGLRPGYE 1yle.1    --------------------------------------------------------------------------------  target    SKSMKTYLDGGYVDDADRQGGQG 1yle.1    ----------------------- ``` | | | | | | | | | | | | | | | | | | | | | | | | | | | | | | | | | | | | | | | | | | | | | | | | | |
|  | 5cup.1.A | Phosphate propanoyltransferase  *Structure of Rhodopseudomonas palustris PduL - phosphate bound form* | 0.00 |  | 20.83 | 0.06 | 224-247 | X-ray | 2.10 | homo-dimer | 4 x ZN | HHblits | 0.30 |
| ``` target    EVLARVGHKLAEQTGDARFADVWKLVDEKRTDAHLQRILDHSSNTKGYDALDLEAKAKKGIPTLMMNRTYPKAVGYEQVA 5cup.1    --------------------------------------------------------------------------------  target    DSRPWYTKSGRLEFYRDEDEFIEAGENLPVHREPIDSTFYEPNVIVSAPHEALRPAGPEDYGVELSDMSGEIRQGRNVVK 5cup.1    --------------------------------------------------------------------------------  target    AWAELKKTPHPLAKDGYRFVFHTPKYRHGAHTMPIDTDMVAMLFGPFGDVYRHDRRTPYVAEGYVDIHPSDAREIGVEDG 5cup.1    ---------------------------------------------------------------EMHIDVEEANALCLKND  target    DYVFIDSDPEDRPFRGWQKNKRDYEFSRLLCRARYYPGTPRGVTRMWFNMYGATPGSVEGQKSREDGLAKNPRTNYQAMF 5cup.1    DVVRICK-------------------------------------------------------------------------  target    RSGSHQSATRGWLKPTWMTDSLVRKGLFGQSIGKGFLPDVHCPTGAPRESIVKITKAEPGGLGAEGLWRPAALGLRPGYE 5cup.1    --------------------------------------------------------------------------------  target    SKSMKTYLDGGYVDDADRQGGQG 5cup.1    ----------------------- ``` | | | | | | | | | | | | | | | | | | | | | | | | | | | | | | | | | | | | | | | | | | | | | | | | | |
|  | 5cuo.1.A | Phosphate propanoyltransferase  *Structure of Rhodopseudomonas palustris PduL - CoA bound form* | 0.00 |  | 20.83 | 0.06 | 224-247 | X-ray | 1.54 | homo-dimer | 2 x COA, 4 x ZN | HHblits | 0.30 |
| ``` target    EVLARVGHKLAEQTGDARFADVWKLVDEKRTDAHLQRILDHSSNTKGYDALDLEAKAKKGIPTLMMNRTYPKAVGYEQVA 5cuo.1    --------------------------------------------------------------------------------  target    DSRPWYTKSGRLEFYRDEDEFIEAGENLPVHREPIDSTFYEPNVIVSAPHEALRPAGPEDYGVELSDMSGEIRQGRNVVK 5cuo.1    --------------------------------------------------------------------------------  target    AWAELKKTPHPLAKDGYRFVFHTPKYRHGAHTMPIDTDMVAMLFGPFGDVYRHDRRTPYVAEGYVDIHPSDAREIGVEDG 5cuo.1    ---------------------------------------------------------------EMHIDVEEANALCLKND  target    DYVFIDSDPEDRPFRGWQKNKRDYEFSRLLCRARYYPGTPRGVTRMWFNMYGATPGSVEGQKSREDGLAKNPRTNYQAMF 5cuo.1    DVVRICK-------------------------------------------------------------------------  target    RSGSHQSATRGWLKPTWMTDSLVRKGLFGQSIGKGFLPDVHCPTGAPRESIVKITKAEPGGLGAEGLWRPAALGLRPGYE 5cuo.1    --------------------------------------------------------------------------------  target    SKSMKTYLDGGYVDDADRQGGQG 5cuo.1    ----------------------- ``` | | | | | | | | | | | | | | | | | | | | | | | | | | | | | | | | | | | | | | | | | | | | | | | | | |
