# Supplementary material for: Metabolic capacity is maintained despite shifts in microbial diversity in estuary sediments
Source: ISME Commun. 2025 Oct 11;5(1):ycaf182. doi: 10.1093/ismeco/ycaf182 (PMC12687941; doi:10.1093/ismeco/ycaf182)
Supplement: Supplementary_Data_1_ycaf182 [file supplementary_data_1_ycaf182.zip › SWISS-MODEL/4_1_May_SF_Bin52_scaffold_9609_c1_40994944_1/templates.html]

4\_1\_May\_SF\_Bin52\_scaffold\_9609\_c1\_4099-4944\_1 | Templates


**Export Alignment**
  
FASTA format
Clustal Format
PNG Image

**Secondary Structure**
  
None
DSSP
PSIPRED
SSpro

**Colour Scheme** 


Fade Mismatches
Enhance Mismatches

Confidencegradient
Confidenceclass
Indels
Chain
Unique Chain
Rainbow
2° Structure
Clustal
Hydrophobic
Size
Charged
Polar
Proline
Ser/Thr
Cysteine
Aliphatic
Aromatic
No Colour

Use QMEANBrane values

|  |  |  |  |
| --- | --- | --- | --- |
| Background |  |  |  |

**3D Viewer**  
NGL
PV

FASTA
Multi FASTA
ClustalW
PNG


SWISS-MODEL

### 4\_1\_May\_SF\_Bin52\_scaffold\_9609\_c1\_4099-4944\_1

### Created: March 29, 2023, 8:38 p.m. at 20:38

- Templates
- Models

Models | Name | Description | GMQE | QSQE | Seq Id | Coverage | Range | Method | Resolution | Oligo-state | Ligands | Found by | Seq Similarity || ✓ | 7b04.1.B | Nitrite oxidoreductase subunit A  *Structure of Nitrite oxidoreductase (Nxr) from the anammox bacterium Kuenenia stuttgartiensis.* | 0.69 | 0.00 | 38.29 | 0.96 | 3-274 | X-ray | 2.97 | monomer | 4 x SF4, 1 x F3S, 2 x MD1, 1 x MO, 1 x HEM, 2 x CA | BLAST | 0.39 |
| ``` target    GLGVPESQLDVETRQYRNVVRTWAELQQTLHPLQERDPAFRFVFQTPKYRWGAHSTAVDADWISMLFGPFGDPYRRDPRM 7b04.1    --GIPENAEYWEDRTVRNIKKSWEETKKTKNFLWEK--GYHFYCVTPKSRHTVHSQWAVTDWNFIWNNNFGDPYRMDKRM  target    PWTGEAYLEINPKDAAELGLADGDYAWVDADPEDRPYRGWNEDDPYYEVARAMMRVRIYTGMSRGVIRTWFNMYAATPAT 7b04.1    PGVGEHQIHIHPQAARDLGIEDGDYVYVDANPADRPYEGWKPNDSFYKVSRLMLRAKYNPAYPYNCTMMKHSAWISSDKT  target    VANQKATPGNPARNEQTRYVALFRYGSHQSGTRAWLRPTQQTDSLVRKGYFGQVIGTGFEADVHSVSGAPKEAFVKIEKA 7b04.1    VQAHETRPDGRALSP-SGYQSSFRYGSQQSITRDWSMPMHQLDSLFHKAKIGMKFIFGFEADNHCINTVPKETLVKITKA  target    EDGGIGAERLWRPLTLGLRPEAPSAALTAYLAGDYSGTKGS 7b04.1    ENGGMGGKGVWDPVKTGYTAGNENDFMKKFLNGE------- ``` | | | | | | | | | | | | | | | | | | | | | | | | | | | | | | | | | | | | | | | | | | | | | | | | | |
|  | 7b04.2.B | Nitrite oxidoreductase subunit A  *Structure of Nitrite oxidoreductase (Nxr) from the anammox bacterium Kuenenia stuttgartiensis.* | 0.67 | 0.00 | 38.29 | 0.96 | 3-274 | X-ray | 2.97 | monomer | 4 x SF4, 1 x F3S, 2 x MD1, 1 x MO, 1 x HEM, 2 x CA | BLAST | 0.39 |
| ``` target    GLGVPESQLDVETRQYRNVVRTWAELQQTLHPLQERDPAFRFVFQTPKYRWGAHSTAVDADWISMLFGPFGDPYRRDPRM 7b04.2    --GIPENAEYWEDRTVRNIKKSWEETKKTKNFLWEK--GYHFYCVTPKSRHTVHSQWAVTDWNFIWNNNFGDPYRMDKRM  target    PWTGEAYLEINPKDAAELGLADGDYAWVDADPEDRPYRGWNEDDPYYEVARAMMRVRIYTGMSRGVIRTWFNMYAATPAT 7b04.2    PGVGEHQIHIHPQAARDLGIEDGDYVYVDANPADRPYEGWKPNDSFYKVSRLMLRAKYNPAYPYNCTMMKHSAWISSDKT  target    VANQKATPGNPARNEQTRYVALFRYGSHQSGTRAWLRPTQQTDSLVRKGYFGQVIGTGFEADVHSVSGAPKEAFVKIEKA 7b04.2    VQAHETRPDGRALSP-SGYQSSFRYGSQQSITRDWSMPMHQLDSLFHKAKIGMKFIFGFEADNHCINTVPKETLVKITKA  target    EDGGIGAERLWRPLTLGLRPEAPSAALTAYLAGDYSGTKGS 7b04.2    ENGGMGGKGVWDPVKTGYTAGNENDFMKKFLNGE------- ``` | | | | | | | | | | | | | | | | | | | | | | | | | | | | | | | | | | | | | | | | | | | | | | | | | |
|  | 7b04.1.B | Nitrite oxidoreductase subunit A  *Structure of Nitrite oxidoreductase (Nxr) from the anammox bacterium Kuenenia stuttgartiensis.* | 0.68 | 0.00 | 37.45 | 0.95 | 10-279 | X-ray | 2.97 | monomer | 4 x SF4, 1 x F3S, 2 x MD1, 1 x MO, 1 x HEM, 2 x CA | HHblits | 0.39 |
| ``` target    GLGVPESQLDVETRQYRNVVRTWAELQQTLHPLQERDPAFRFVFQTPKYRWGAHSTAVDADWISMLFGPFGDPYRRDPRM 7b04.1    ---------YWEDRTVRNIKKSWEETKKTKNFLWE--KGYHFYCVTPKSRHTVHSQWAVTDWNFIWNNNFGDPYRMDKRM  target    PWTGEAYLEINPKDAAELGLADGDYAWVDADPEDRPYRGWNEDDPYYEVARAMMRVRIYTGMSRGVIRTWFNMYAATPAT 7b04.1    PGVGEHQIHIHPQAARDLGIEDGDYVYVDANPADRPYEGWKPNDSFYKVSRLMLRAKYNPAYPYNCTMMKHSAWISSDKT  target    VANQKATPGNPARNEQTRYVALFRYGSHQSGTRAWLRPTQQTDSLVRKGYFGQVIGTGFEADVHSVSGAPKEAFVKIEKA 7b04.1    VQAHETRPDGRALSP-SGYQSSFRYGSQQSITRDWSMPMHQLDSLFHKAKIGMKFIFGFEADNHCINTVPKETLVKITKA  target    EDGGIGAERLWRPLTLGLRPEAPSAALTAYLAGDYSGTKGS 7b04.1    ENGGMGGKGVWDPVKTGYTAGNENDFMKKFLNGELIKVD-- ``` | | | | | | | | | | | | | | | | | | | | | | | | | | | | | | | | | | | | | | | | | | | | | | | | | |
|  | 7b04.2.B | Nitrite oxidoreductase subunit A  *Structure of Nitrite oxidoreductase (Nxr) from the anammox bacterium Kuenenia stuttgartiensis.* | 0.66 | 0.00 | 37.45 | 0.95 | 10-279 | X-ray | 2.97 | monomer | 4 x SF4, 1 x F3S, 2 x MD1, 1 x MO, 1 x HEM, 2 x CA | HHblits | 0.39 |
| ``` target    GLGVPESQLDVETRQYRNVVRTWAELQQTLHPLQERDPAFRFVFQTPKYRWGAHSTAVDADWISMLFGPFGDPYRRDPRM 7b04.2    ---------YWEDRTVRNIKKSWEETKKTKNFLWE--KGYHFYCVTPKSRHTVHSQWAVTDWNFIWNNNFGDPYRMDKRM  target    PWTGEAYLEINPKDAAELGLADGDYAWVDADPEDRPYRGWNEDDPYYEVARAMMRVRIYTGMSRGVIRTWFNMYAATPAT 7b04.2    PGVGEHQIHIHPQAARDLGIEDGDYVYVDANPADRPYEGWKPNDSFYKVSRLMLRAKYNPAYPYNCTMMKHSAWISSDKT  target    VANQKATPGNPARNEQTRYVALFRYGSHQSGTRAWLRPTQQTDSLVRKGYFGQVIGTGFEADVHSVSGAPKEAFVKIEKA 7b04.2    VQAHETRPDGRALSP-SGYQSSFRYGSQQSITRDWSMPMHQLDSLFHKAKIGMKFIFGFEADNHCINTVPKETLVKITKA  target    EDGGIGAERLWRPLTLGLRPEAPSAALTAYLAGDYSGTKGS 7b04.2    ENGGMGGKGVWDPVKTGYTAGNENDFMKKFLNGELIKVD-- ``` | | | | | | | | | | | | | | | | | | | | | | | | | | | | | | | | | | | | | | | | | | | | | | | | | |
|  | 3ir5.1.A | Respiratory nitrate reductase 1 alpha chain  *Crystal structure of NarGHI mutant NarG-H49C* | 0.33 | 0.00 | 23.18 | 0.54 | 38-245 | X-ray | 2.30 | monomer | 2 x MD1, 1 x 6MO, 4 x SF4, 1 x AGA, 1 x F3S, 2 x HEM | HHblits | 0.32 |
| ``` target    GLGVPESQLDVETRQYRNVVRTWAELQQTLHPLQERDPAFRFVFQTPKYRWGAHSTAVDADWISMLFGPFGDPYRRDPRM 3ir5.1    -------------------------------------QEKALNFLTPHQKWGIHSTYSDNLLMLTL--------------  target    PWTGEAYLEINPKDAAELGLADGDYAWVDADPEDRPYRGWNEDDPYYEVARAMMRVRIYTGMSRGVIRTWFNMYAATPAT 3ir5.1    -GRGGPVVWLSEADAKDLGIADNDWIEVFNS-----------------NGALTARAVVSQRVPAGMTMMYHAQ---ERIV  target    VANQKATPGNPARNEQTRYVALFRYGSHQSGTRAWLRPTQQTDSLVRKGYFGQVIGTGFEADVHSVSGAPKEAFVKIEKA 3ir5.1    -----NLP-------GSE-ITQQRGGIHNSVTRITPKPTH----MI--GGYAHL---AYGFNYYGTVGSNRDEFVVVRKM  target    EDGGIGAERLWRPLTLGLRPEAPSAALTAYLAGDYSGTKGS 3ir5.1    KNIDW------------------------------------ ``` | | | | | | | | | | | | | | | | | | | | | | | | | | | | | | | | | | | | | | | | | | | | | | | | | |
|  | 1q16.1.A | Respiratory nitrate reductase 1 alpha chain  *Crystal structure of Nitrate Reductase A, NarGHI, from Escherichia coli* | 0.33 | 0.00 | 23.18 | 0.54 | 38-245 | X-ray | 1.90 | monomer | 2 x MD1, 1 x 6MO, 2 x HEM, 4 x SF4, 1 x F3S, 1 x AGA, 1 x 3PH | HHblits | 0.32 |
| ``` target    GLGVPESQLDVETRQYRNVVRTWAELQQTLHPLQERDPAFRFVFQTPKYRWGAHSTAVDADWISMLFGPFGDPYRRDPRM 1q16.1    -------------------------------------QEKALNFLTPHQKWGIHSTYSDNLLMLTL--------------  target    PWTGEAYLEINPKDAAELGLADGDYAWVDADPEDRPYRGWNEDDPYYEVARAMMRVRIYTGMSRGVIRTWFNMYAATPAT 1q16.1    -GRGGPVVWLSEADAKDLGIADNDWIEVFNS-----------------NGALTARAVVSQRVPAGMTMMYHAQ---ERI-  target    VANQKATPGNPARNEQTRYVALFRYGSHQSGTRAWLRPTQQTDSLVRKGYFGQVIGTGFEADVHSVSGAPKEAFVKIEKA 1q16.1    ----VNLP-------GS-EITQQRGGIHNSVTRITPKPTH----MI--GGYAHL---AYGFNYYGTVGSNRDEFVVVRKM  target    EDGGIGAERLWRPLTLGLRPEAPSAALTAYLAGDYSGTKGS 1q16.1    KNIDW------------------------------------ ``` | | | | | | | | | | | | | | | | | | | | | | | | | | | | | | | | | | | | | | | | | | | | | | | | | |
|  | 1r27.4.A | Respiratory nitrate reductase 1 alpha chain  *Crystal Structure of NarGH complex* | 0.32 | 0.00 | 23.18 | 0.54 | 38-245 | X-ray | 2.00 | monomer | 4 x MO, 16 x SF4, 8 x MGD, 4 x F3S | HHblits | 0.32 |
| ``` target    GLGVPESQLDVETRQYRNVVRTWAELQQTLHPLQERDPAFRFVFQTPKYRWGAHSTAVDADWISMLFGPFGDPYRRDPRM 1r27.4    -------------------------------------QEKALNFLTPHQKWGIHSTYSDNLLMLTL--------------  target    PWTGEAYLEINPKDAAELGLADGDYAWVDADPEDRPYRGWNEDDPYYEVARAMMRVRIYTGMSRGVIRTWFNMYAATPAT 1r27.4    -GRGGPVVWLSEADAKDLGIADNDWIEVFNS-----------------NGALTARAVVSQRVPAGMTMMYHAQ---ERI-  target    VANQKATPGNPARNEQTRYVALFRYGSHQSGTRAWLRPTQQTDSLVRKGYFGQVIGTGFEADVHSVSGAPKEAFVKIEKA 1r27.4    ----VNLP-------G-SEITQQRGGIHNSVTRITPKPTH----MI--GGYAHL---AYGFNYYGTVGSNRDEFVVVRKM  target    EDGGIGAERLWRPLTLGLRPEAPSAALTAYLAGDYSGTKGS 1r27.4    KNIDW------------------------------------ ``` | | | | | | | | | | | | | | | | | | | | | | | | | | | | | | | | | | | | | | | | | | | | | | | | | |
|  | 3ir7.1.A | Respiratory nitrate reductase 1 alpha chain  *Crystal structure of NarGHI mutant NarG-R94S* | 0.33 | 0.00 | 22.52 | 0.54 | 38-245 | X-ray | 2.50 | monomer | 2 x MD1, 4 x SF4, 1 x 6MO, 1 x AGA, 1 x F3S, 2 x HEM | HHblits | 0.32 |
| ``` target    GLGVPESQLDVETRQYRNVVRTWAELQQTLHPLQERDPAFRFVFQTPKYRWGAHSTAVDADWISMLFGPFGDPYRRDPRM 3ir7.1    -------------------------------------QEKALNFLTPHQKWGIHSTYSDNLLMLTL--------------  target    PWTGEAYLEINPKDAAELGLADGDYAWVDADPEDRPYRGWNEDDPYYEVARAMMRVRIYTGMSRGVIRTWFNMYAATPAT 3ir7.1    -GRGGPVVWLSEADAKDLGIADNDWIEVFNS-----------------NGALTARAVVSQRVPAGMTMMYHAQ---ERI-  target    VANQKATPGNPARNEQTRYVALFRYGSHQSGTRAWLRPTQQTDSLVRKGYFGQVIGTGFEADVHSVSGAPKEAFVKIEKA 3ir7.1    -----------VNLPGS-EITQQRGGIHNSVTRITPKPTH----MI--GGYAHL---AYGFNYYGTVGSNRDEFVVVRKM  target    EDGGIGAERLWRPLTLGLRPEAPSAALTAYLAGDYSGTKGS 3ir7.1    KNIDW------------------------------------ ``` | | | | | | | | | | | | | | | | | | | | | | | | | | | | | | | | | | | | | | | | | | | | | | | | | |
|  | 3ir6.1.A | Respiratory nitrate reductase 1 alpha chain  *Crystal structure of NarGHI mutant NarG-H49S* | 0.32 | 0.00 | 23.65 | 0.53 | 38-242 | X-ray | 2.80 | monomer | 2 x GDP, 1 x AGA, 3 x SF4, 1 x F3S, 2 x HEM | HHblits | 0.32 |
| ``` target    GLGVPESQLDVETRQYRNVVRTWAELQQTLHPLQERDPAFRFVFQTPKYRWGAHSTAVDADWISMLFGPFGDPYRRDPRM 3ir6.1    -------------------------------------QEKALNFLTPHQKWGIHSTYSDNLLMLTL--------------  target    PWTGEAYLEINPKDAAELGLADGDYAWVDADPEDRPYRGWNEDDPYYEVARAMMRVRIYTGMSRGVIRTWFNMYAATPAT 3ir6.1    -GRGGPVVWLSEADAKDLGIADNDWIEVFNS-----------------NGALTARAVVSQRVPAGMTMMYHAQ---ERIV  target    VANQKATPGNPARNEQTRYVALFRYGSHQSGTRAWLRPTQQTDSLVRKGYFGQVIGTGFEADVHSVSGAPKEAFVKIEKA 3ir6.1    -----NLP-------GS-EITQQRGGIHNSVTRITPKPTH----MI--GGYAHL---AYGFNYYGTVGSNRDEFVVVRKM  target    EDGGIGAERLWRPLTLGLRPEAPSAALTAYLAGDYSGTKGS 3ir6.1    KN--------------------------------------- ``` | | | | | | | | | | | | | | | | | | | | | | | | | | | | | | | | | | | | | | | | | | | | | | | | | |
|  | 3egw.1.A | Respiratory nitrate reductase 1 alpha chain  *The crystal structure of the NarGHI mutant NarH - C16A* | 0.32 | 0.00 | 23.65 | 0.53 | 38-242 | X-ray | 1.90 | monomer | 2 x MD1, 2 x MGD, 2 x 6MO, 6 x SF4, 4 x F3S, 2 x 3PH, 4 x HEM, 2 x AGA | HHblits | 0.32 |
| ``` target    GLGVPESQLDVETRQYRNVVRTWAELQQTLHPLQERDPAFRFVFQTPKYRWGAHSTAVDADWISMLFGPFGDPYRRDPRM 3egw.1    -------------------------------------QEKALNFLTPHQKWGIHSTYSDNLLMLTL--------------  target    PWTGEAYLEINPKDAAELGLADGDYAWVDADPEDRPYRGWNEDDPYYEVARAMMRVRIYTGMSRGVIRTWFNMYAATPAT 3egw.1    -GRGGPVVWLSEADAKDLGIADNDWIEVFNS-----------------NGALTARAVVSQRVPAGMTMMYHAQ---ERIV  target    VANQKATPGNPARNEQTRYVALFRYGSHQSGTRAWLRPTQQTDSLVRKGYFGQVIGTGFEADVHSVSGAPKEAFVKIEKA 3egw.1    -----NLP-GS-------EITQQRGGIHNSVTRITPKPTH----MI--GGYAHL---AYGFNYYGTVGSNRDEFVVVRKM  target    EDGGIGAERLWRPLTLGLRPEAPSAALTAYLAGDYSGTKGS 3egw.1    KN--------------------------------------- ``` | | | | | | | | | | | | | | | | | | | | | | | | | | | | | | | | | | | | | | | | | | | | | | | | | |
|  | 5e7o.1.A | DMSO reductase family type II enzyme, molybdopterin subunit  *Crystal structure of the perchlorate reductase PcrAB mutant W461E of PcrA from Azospira suillum PS* | 0.31 | 0.00 | 27.14 | 0.50 | 38-243 | X-ray | 2.40 | monomer | 4 x SF4, 1 x MO, 1 x MGD, 1 x MD1, 1 x F3S | HHblits | 0.34 |
| ``` target    GLGVPESQLDVETRQYRNVVRTWAELQQTLHPLQERDPAFRFVFQTPKYRWGAHSTAVDADWISMLFGPFGDPYRRDPRM 5e7o.1    -------------------------------------DKYPFRFNSPHSRHSVHSTFKDNVLMLRL--------------  target    PWTGEAYLEINPKDAAELGLADGDYAWVDADPEDRPYRGWNEDDPYYEVARAMMRVRIYTGMSRGVIRTWFNMYAATPAT 5e7o.1    -QRGGPSIEMSPLDAKPLGIKDNDWVEAWNN-----------------HGKVICRVKIRNGEQRGRVSMWHCP---EL--  target    VANQKATPGNPARNEQTRYVALFRY-GSHQSGTRAWLRPTQQTDSLVRKGYFGQVIGTGFEADVHSVSGAPKEAFVKIEK 5e7o.1    ------------------Y--MDLLTGGSQSVCPVRINPTN----LV--GNYGHL---FFRPNYYGPAGSQRDVRVNVKR  target    AEDGGIGAERLWRPLTLGLRPEAPSAALTAYLAGDYSGTKGS 5e7o.1    YIGA-------------------------------------- ``` | | | | | | | | | | | | | | | | | | | | | | | | | | | | | | | | | | | | | | | | | | | | | | | | | |
|  | 4ydd.1.A | DMSO reductase family type II enzyme, molybdopterin subunit  *Crystal structure of the perchlorate reductase PcrAB from Azospira suillum PS* | 0.31 | 0.00 | 27.14 | 0.50 | 38-243 | X-ray | 1.86 | monomer | 4 x SF4, 1 x MO, 1 x MGD, 1 x MD1, 1 x F3S | HHblits | 0.34 |
| ``` target    GLGVPESQLDVETRQYRNVVRTWAELQQTLHPLQERDPAFRFVFQTPKYRWGAHSTAVDADWISMLFGPFGDPYRRDPRM 4ydd.1    -------------------------------------DKYPFRFNSPHSRHSVHSTFKDNVLMLRL--------------  target    PWTGEAYLEINPKDAAELGLADGDYAWVDADPEDRPYRGWNEDDPYYEVARAMMRVRIYTGMSRGVIRTWFNMYAATPAT 4ydd.1    -QRGGPSIEMSPLDAKPLGIKDNDWVEAWNN-----------------HGKVICRVKIRNGEQRGRVSMWHCP---EL--  target    VANQKATPGNPARNEQTRYVALFRY-GSHQSGTRAWLRPTQQTDSLVRKGYFGQVIGTGFEADVHSVSGAPKEAFVKIEK 4ydd.1    ------------------Y--MDLLTGGSQSVCPVRINPTN----LV--GNYGHL---FFRPNYYGPAGSQRDVRVNVKR  target    AEDGGIGAERLWRPLTLGLRPEAPSAALTAYLAGDYSGTKGS 4ydd.1    YIGA-------------------------------------- ``` | | | | | | | | | | | | | | | | | | | | | | | | | | | | | | | | | | | | | | | | | | | | | | | | | |
|  | 2ivf.1.A | ETHYLBENZENE DEHYDROGENASE ALPHA-SUBUNIT  *ETHYLBENZENE DEHYDROGENASE FROM AROMATOLEUM AROMATICUM* | 0.28 | 0.00 | 25.00 | 0.48 | 38-240 | X-ray | 1.88 | monomer | 1 x MES, 4 x SF4, 1 x MO, 1 x MGD, 1 x MD1, 1 x F3S, 1 x HEM | HHblits | 0.30 |
| ``` target    GLGVPESQLDVETRQYRNVVRTWAELQQTLHPLQERDPAFRFVFQTPKYRWGAHSTAVDADWISMLFGPFGDPYRRDPRM 2ivf.1    -------------------------------------GDHPFKITGGHPRVSIHSTHLTNSHLSRL--------------  target    PWTGEAYLEINPKDAAELGLADGDYAWVDADPEDRPYRGWNEDDPYYEVARAMMRVRIYTGMSRGVIRTWFNMYAATPAT 2ivf.1    -HRGQPVVHMNSKDAAELGIKDGDMAKLFND-----------------FADCEIMVRTAPNVQPKQCIVYFWD---AH--  target    VANQKATPGNPARNEQTRYVALFRY-GSHQSGTRAWLRPTQQTDSLVRKGYFGQVIGTGFEADVHSVSG-APKEAFVKIE 2ivf.1    ---------------------QYKGWKPYDILLIGMPKPLH----LA--GGYEQF---RYYFMNGSPAPVTDRGVRVSIK  target    KAEDGGIGAERLWRPLTLGLRPEAPSAALTAYLAGDYSGTKGS 2ivf.1    KA----------------------------------------- ``` | | | | | | | | | | | | | | | | | | | | | | | | | | | | | | | | | | | | | | | | | | | | | | | | | |
|  | 6cz7.1.A | ArrA  *The arsenate respiratory reductase (Arr) complex from Shewanella sp. ANA-3* | 0.14 | 0.00 | 18.68 | 0.32 | 38-153 | X-ray | 1.62 | monomer | 5 x SF4, 2 x MGD, 1 x MO, 1 x PG5 | HHblits | 0.31 |
| ``` target    GLGVPESQLDVETRQYRNVVRTWAELQQTLHPLQERDPAFRFVFQTPKYRWGAHSTAVDADWISMLFGPFGDPYRRDPRM 6cz7.1    -------------------------------------SEFPLLLVDQKSRLNKEGRTANSPWYYEFKD----VD----PG  target    PWTGEAYLEINPKDAAELGLADGDYAWVDADPEDRPYRGWNEDDPYYEVARAMMRVRIYTGMSRGVIRTWFNMYAATPAT 6cz7.1    DVANEDVAKFNPIDGKKFGLKDGDEIRITSP-----------------VGMLTCKAKLWEGVRPGTVAKCFGQ-------  target    VANQKATPGNPARNEQTRYVALFRYGSHQSGTRAWLRPTQQTDSLVRKGYFGQVIGTGFEADVHSVSGAPKEAFVKIEKA 6cz7.1    --------------------------------------------------------------------------------  target    EDGGIGAERLWRPLTLGLRPEAPSAALTAYLAGDYSGTKGS 6cz7.1    ----------------------------------------- ``` | | | | | | | | | | | | | | | | | | | | | | | | | | | | | | | | | | | | | | | | | | | | | | | | | |
|  | 4v4c.1.A | Pyrogallol hydroxytransferase large subunit  *Crystal Structure of Pyrogallol-Phloroglucinol Transhydroxylase from Pelobacter acidigallici* | 0.12 | 0.00 | 14.44 | 0.32 | 38-153 | X-ray | 2.35 | monomer | 2 x CA, 2 x MGD, 1 x 4MO, 3 x SF4 | HHblits | 0.30 |
| ``` target    GLGVPESQLDVETRQYRNVVRTWAELQQTLHPLQERDPAFRFVFQTPKYRWGAHSTAVDAD-WISMLFGPFGDPYRRDPR 4v4c.1    -------------------------------------VKYPLGMLSPHPRFSMHTMGDGKNSYMNYI---------KDHR  target    MP--WTGEAYLEINPKDAAELGLADGDYAWVDADPEDRPYRGWNEDDPYYEVARAMMRVRIYTGMSRGVIRTWFNMYAAT 4v4c.1    VEVDGYKYWIMRVNSIDAEARGIKNGDLIRAYND-----------------RGSVILAAQVTECLQPGTVHSYESC----  target    PATVANQKATPGNPARNEQTRYVALFRYGSHQSGTRAWLRPTQQTDSLVRKGYFGQVIGTGFEADVHSVSGAPKEAFVKI 4v4c.1    --------------------------------------------------------------------------------  target    EKAEDGGIGAERLWRPLTLGLRPEAPSAALTAYLAGDYSGTKGS 4v4c.1    -------------------------------------------- ``` | | | | | | | | | | | | | | | | | | | | | | | | | | | | | | | | | | | | | | | | | | | | | | | | | |
|  | 6tg9.1.A | Formate dehydrogenase subunit alpha  *Cryo-EM Structure of NADH reduced form of NAD+-dependent Formate Dehydrogenase from Rhodobacter capsulatus* | 0.12 | 0.00 | 27.38 | 0.30 | 38-153 | EM | 3.24 | monomer | 4 x MGD, 2 x 6MO, 4 x FES, 10 x SF4, 2 x H2S, 2 x FMN, 2 x NAI | HHblits | 0.33 |
| ``` target    GLGVPESQLDVETRQYRNVVRTWAELQQTLHPLQERDPAFRFVFQTPKYRWGAHSTAVDADWISMLFGPFGDPYRRDPRM 6tg9.1    -------------------------------------PRFPLLLTTGRILSQYNVGAQTRRT---------------ENT  target    PWTGEAYLEINPKDAAELGLADGDYAWVDADPEDRPYRGWNEDDPYYEVARAMMRVRIYTGMSRGVIRTWFNMYAATPAT 6tg9.1    VWHGEDRLEIHPTDAETRGIRDGDWVRLASR-----------------AGETTLRATVTDRVSPGVVYTTFHH-------  target    VANQKATPGNPARNEQTRYVALFRYGSHQSGTRAWLRPTQQTDSLVRKGYFGQVIGTGFEADVHSVSGAPKEAFVKIEKA 6tg9.1    --------------------------------------------------------------------------------  target    EDGGIGAERLWRPLTLGLRPEAPSAALTAYLAGDYSGTKGS 6tg9.1    ----------------------------------------- ``` | | | | | | | | | | | | | | | | | | | | | | | | | | | | | | | | | | | | | | | | | | | | | | | | | |
|  | 1aa6.1.A | FORMATE DEHYDROGENASE H  *REDUCED FORM OF FORMATE DEHYDROGENASE H FROM E. COLI* | 0.11 | 0.00 | 20.24 | 0.30 | 38-153 | X-ray | 2.30 | monomer | 1 x SF4, 2 x MGD, 1 x 4MO | HHblits | 0.32 |
| ``` target    GLGVPESQLDVETRQYRNVVRTWAELQQTLHPLQERDPAFRFVFQTPKYR--WGAHSTAVDADWISMLFGPFGDPYRRDP 1aa6.1    -------------------------------------DEYPMVLSTVREVGHYSCRSMTGNCAALAAL------------  target    RMPWTGEAYLEINPKDAAELGLADGDYAWVDADPEDRPYRGWNEDDPYYEVARAMMRVRIYTGMSRGVIRTWFNMYAATP 1aa6.1    ---ADEPGYAQINTEDAKRLGIEDEALVWVHSR-----------------KGKIITRAQVSDRPNKGAIYMTYQW-----  target    ATVANQKATPGNPARNEQTRYVALFRYGSHQSGTRAWLRPTQQTDSLVRKGYFGQVIGTGFEADVHSVSGAPKEAFVKIE 1aa6.1    --------------------------------------------------------------------------------  target    KAEDGGIGAERLWRPLTLGLRPEAPSAALTAYLAGDYSGTKGS 1aa6.1    ------------------------------------------- ``` | | | | | | | | | | | | | | | | | | | | | | | | | | | | | | | | | | | | | | | | | | | | | | | | | |
|  | 1fdo.1.A | FORMATE DEHYDROGENASE H  *OXIDIZED FORM OF FORMATE DEHYDROGENASE H FROM E. COLI* | 0.11 | 0.00 | 20.24 | 0.30 | 38-153 | X-ray | 2.80 | monomer | 1 x SF4, 2 x MGD, 1 x 6MO | HHblits | 0.32 |
| ``` target    GLGVPESQLDVETRQYRNVVRTWAELQQTLHPLQERDPAFRFVFQTPKYR--WGAHSTAVDADWISMLFGPFGDPYRRDP 1fdo.1    -------------------------------------DEYPMVLSTVREVGHYSCRSMTGNCAALAAL------------  target    RMPWTGEAYLEINPKDAAELGLADGDYAWVDADPEDRPYRGWNEDDPYYEVARAMMRVRIYTGMSRGVIRTWFNMYAATP 1fdo.1    ---ADEPGYAQINTEDAKRLGIEDEALVWVHSR-----------------KGKIITRAQVSDRPNKGAIYMTYQW-----  target    ATVANQKATPGNPARNEQTRYVALFRYGSHQSGTRAWLRPTQQTDSLVRKGYFGQVIGTGFEADVHSVSGAPKEAFVKIE 1fdo.1    --------------------------------------------------------------------------------  target    KAEDGGIGAERLWRPLTLGLRPEAPSAALTAYLAGDYSGTKGS 1fdo.1    ------------------------------------------- ``` | | | | | | | | | | | | | | | | | | | | | | | | | | | | | | | | | | | | | | | | | | | | | | | | | |
|  | 2iv2.1.A | Formate dehydrogenase H  *Reinterpretation of reduced form of formate dehydrogenase H from E. coli* | 0.12 | 0.00 | 20.24 | 0.30 | 38-153 | X-ray | 2.27 | monomer | 1 x SF4, 1 x 2MD, 1 x MGD | HHblits | 0.32 |
| ``` target    GLGVPESQLDVETRQYRNVVRTWAELQQTLHPLQERDPAFRFVFQTPKYR--WGAHSTAVDADWISMLFGPFGDPYRRDP 2iv2.1    -------------------------------------DEYPMVLSTVREVGHYSCRSMTGNCAALAAL------------  target    RMPWTGEAYLEINPKDAAELGLADGDYAWVDADPEDRPYRGWNEDDPYYEVARAMMRVRIYTGMSRGVIRTWFNMYAATP 2iv2.1    ---ADEPGYAQINTEDAKRLGIEDEALVWVHSR-----------------KGKIITRAQVSDRPNKGAIYMTYQW-----  target    ATVANQKATPGNPARNEQTRYVALFRYGSHQSGTRAWLRPTQQTDSLVRKGYFGQVIGTGFEADVHSVSGAPKEAFVKIE 2iv2.1    --------------------------------------------------------------------------------  target    KAEDGGIGAERLWRPLTLGLRPEAPSAALTAYLAGDYSGTKGS 2iv2.1    ------------------------------------------- ``` | | | | | | | | | | | | | | | | | | | | | | | | | | | | | | | | | | | | | | | | | | | | | | | | | |
|  | 7z0t.1.G | Formate dehydrogenase H  *Structure of the Escherichia coli formate hydrogenlyase complex (aerobic preparation, composite structure)* | 0.11 | 0.00 | 20.24 | 0.30 | 38-153 | EM | 0.00 | monomer | 1 x NI, 1 x FCO, 8 x SF4, 1 x FE, 2 x MGD, 1 x 6MO | HHblits | 0.32 |
| ``` target    GLGVPESQLDVETRQYRNVVRTWAELQQTLHPLQERDPAFRFVFQTPKYR--WGAHSTAVDADWISMLFGPFGDPYRRDP 7z0t.1    -------------------------------------DEYPMVLSTVREVGHYSCRSMTGNCAALAAL------------  target    RMPWTGEAYLEINPKDAAELGLADGDYAWVDADPEDRPYRGWNEDDPYYEVARAMMRVRIYTGMSRGVIRTWFNMYAATP 7z0t.1    ---ADEPGYAQINTEDAKRLGIEDEALVWVHSR-----------------KGKIITRAQVSDRPNKGAIYMTYQW-----  target    ATVANQKATPGNPARNEQTRYVALFRYGSHQSGTRAWLRPTQQTDSLVRKGYFGQVIGTGFEADVHSVSGAPKEAFVKIE 7z0t.1    --------------------------------------------------------------------------------  target    KAEDGGIGAERLWRPLTLGLRPEAPSAALTAYLAGDYSGTKGS 7z0t.1    ------------------------------------------- ``` | | | | | | | | | | | | | | | | | | | | | | | | | | | | | | | | | | | | | | | | | | | | | | | | | |
|  | 1eu1.1.A | DIMETHYL SULFOXIDE REDUCTASE  *THE CRYSTAL STRUCTURE OF RHODOBACTER SPHAEROIDES DIMETHYLSULFOXIDE REDUCTASE REVEALS TWO DISTINCT MOLYBDENUM COORDINATION ENVIRONMENTS.* | 0.12 | 0.00 | 22.35 | 0.30 | 38-153 | X-ray | 1.30 | monomer | 3 x GLC, 1 x CD, 2 x MGD, 1 x 6MO, 2 x O | HHblits | 0.31 |
| ``` target    GLGVPESQLDVETRQYRNVVRTWAELQQTLHPLQERDPAFRFVFQTPKYRWGAHSTAVDADWISMLFGPFGDPYRRDPRM 1eu1.1    -------------------------------------AKYPLHVVASHPKSRLHSQLNGTS-LRDLY-------------  target    PWTGEAYLEINPKDAAELGLADGDYAWVDADPEDRPYRGWNEDDPYYEVARAMMRVRIYTGMSRGVIRTWFNMYAATPAT 1eu1.1    AVAGHEPCLINPADAAARGIADGDVLRVFND-----------------RGQILVGAKVSDAVMPGAIQIYEGG-------  target    VANQKATPGNPARNEQTRYVALFRYGSHQSGTRAWLRPTQQTDSLVRKGYFGQVIGTGFEADVHSVSGAPKEAFVKIEKA 1eu1.1    --------------------------------------------------------------------------------  target    EDGGIGAERLWRPLTLGLRPEAPSAALTAYLAGDYSGTKGS 1eu1.1    ----------------------------------------- ``` | | | | | | | | | | | | | | | | | | | | | | | | | | | | | | | | | | | | | | | | | | | | | | | | | |
|  | 4dmr.1.A | DMSO REDUCTASE  *REDUCED DMSO REDUCTASE FROM RHODOBACTER CAPSULATUS WITH BOUND DMSO SUBSTRATE* | 0.13 | 0.00 | 22.35 | 0.30 | 38-153 | X-ray | 1.90 | monomer | 2 x PGD, 1 x 4MO, 1 x O | HHblits | 0.30 |
| ``` target    GLGVPESQLDVETRQYRNVVRTWAELQQTLHPLQERDPAFRFVFQTPKYRWGAHSTAVDADWISMLFGPFGDPYRRDPRM 4dmr.1    -------------------------------------AKYPLHIAASHPFNRLHSQLN-GTVLREG-------Y------  target    PWTGEAYLEINPKDAAELGLADGDYAWVDADPEDRPYRGWNEDDPYYEVARAMMRVRIYTGMSRGVIRTWFNMYAATPAT 4dmr.1    AVQGHEPCLMHPDDAAARGIADGDVVRVHND-----------------RGQILTGVKVTDAVMKGVIQIYEGG-------  target    VANQKATPGNPARNEQTRYVALFRYGSHQSGTRAWLRPTQQTDSLVRKGYFGQVIGTGFEADVHSVSGAPKEAFVKIEKA 4dmr.1    --------------------------------------------------------------------------------  target    EDGGIGAERLWRPLTLGLRPEAPSAALTAYLAGDYSGTKGS 4dmr.1    ----------------------------------------- ``` | | | | | | | | | | | | | | | | | | | | | | | | | | | | | | | | | | | | | | | | | | | | | | | | | |
|  | 1tmo.1.A | TRIMETHYLAMINE N-OXIDE REDUCTASE  *TRIMETHYLAMINE N-OXIDE REDUCTASE FROM SHEWANELLA MASSILIA* | 0.12 |  | 18.60 | 0.31 | 38-153 | X-ray | 2.50 | monomer | 2 x 2MD, 1 x 2MO | HHblits | 0.29 |
| ``` target    GLGVPESQLDVETRQYRNVVRTWAELQQTLHPLQERDPAFRFVFQTPKYRWGAHSTAVDADWISMLFGPFGDPYRRDPRM 1tmo.1    -------------------------------------DKHPIWLQSCHPDKRLHSQMCESREYRETY-------------  target    PWTGEAYLEINPKDAAELGLADGDYAWVDADPEDRPYRGWNEDDPYYEVARAMMRVRIYTGMSRGVIRTWFNMYAATPAT 1tmo.1    AVNGREPVYISPVDAKARGIKDGDIVRVFND-----------------RGQLLAGAVVSDNFPKGIVRIHEGA-------  target    VANQKATPGNPARNEQTRYVALFRYGSHQSGTRAWLRPTQQTDSLVRKGYFGQVIGTGFEADVHSVSGAPKEAFVKIEKA 1tmo.1    --------------------------------------------------------------------------------  target    EDGGIGAERLWRPLTLGLRPEAPSAALTAYLAGDYSGTKGS 1tmo.1    ----------------------------------------- ``` | | | | | | | | | | | | | | | | | | | | | | | | | | | | | | | | | | | | | | | | | | | | | | | | | |
|  | 1e5v.2.A | Dimethyl sulfoxide/trimethylamine N-oxide reductase  *OXIDIZED DMSO REDUCTASE EXPOSED TO HEPES BUFFER* | 0.12 | 0.00 | 21.18 | 0.30 | 38-153 | X-ray | 2.40 | monomer | 2 x PGD, 1 x 2MO | HHblits | 0.30 |
| ``` target    GLGVPESQLDVETRQYRNVVRTWAELQQTLHPLQERDPAFRFVFQTPKYRWGAHSTAVDADWISMLFGPFGDPYRRDPRM 1e5v.2    -------------------------------------AKYPLHIAASHPFNRLHSQLN-GTVLREGY-------------  target    PWTGEAYLEINPKDAAELGLADGDYAWVDADPEDRPYRGWNEDDPYYEVARAMMRVRIYTGMSRGVIRTWFNMYAATPAT 1e5v.2    AVQGHEPCLMHPDDAAARGIADGDVVRVHND-----------------RGQILTGVKVTDAVMKGVIQIYEGG-------  target    VANQKATPGNPARNEQTRYVALFRYGSHQSGTRAWLRPTQQTDSLVRKGYFGQVIGTGFEADVHSVSGAPKEAFVKIEKA 1e5v.2    --------------------------------------------------------------------------------  target    EDGGIGAERLWRPLTLGLRPEAPSAALTAYLAGDYSGTKGS 1e5v.2    ----------------------------------------- ``` | | | | | | | | | | | | | | | | | | | | | | | | | | | | | | | | | | | | | | | | | | | | | | | | | |
|  | 4aay.1.A | AROA  *Crystal Structure of the arsenite oxidase protein complex from Rhizobium species strain NT-26* | 0.11 | 0.00 | 18.82 | 0.30 | 38-153 | X-ray | 2.70 | homo-dimer | 4 x MGD, 2 x O, 2 x 4MO, 2 x F3S, 2 x FES | HHblits | 0.30 |
| ``` target    GLGVPESQLDVETRQYRNVVRTWAELQQTLHPLQERDPAFRFVFQTPKYRWGAHSTAVDADWISMLFGPFGDPYRRDPRM 4aay.1    -------------------------------------DSHKYLINNGRANVVWQSAYLDQENDFVMD-------------  target    PWTGEAYLEINPKDAAELGLADGDYAWVDADPEDRPYRGWNEDDPYYEVARAMMRVRIYTGMSRGVIRTWFNMYAATPAT 4aay.1    -RFPYPFIEMNPEDMAEAGLKEGDLVEIYND-----------------AGATQAMAYPTPTARRGETFMLFGF-------  target    VANQKATPGNPARNEQTRYVALFRYGSHQSGTRAWLRPTQQTDSLVRKGYFGQVIGTGFEADVHSVSGAPKEAFVKIEKA 4aay.1    --------------------------------------------------------------------------------  target    EDGGIGAERLWRPLTLGLRPEAPSAALTAYLAGDYSGTKGS 4aay.1    ----------------------------------------- ``` | | | | | | | | | | | | | | | | | | | | | | | | | | | | | | | | | | | | | | | | | | | | | | | | | |
|  | 5nqd.1.A | AroA  *Arsenite oxidase AioAB from Rhizobium sp. str. NT-26 mutant AioBF108A* | 0.11 | 0.00 | 18.82 | 0.30 | 38-153 | X-ray | 2.20 | homo-dimer | 4 x MGD, 2 x O, 2 x 4MO, 2 x F3S, 2 x FES | HHblits | 0.30 |
| ``` target    GLGVPESQLDVETRQYRNVVRTWAELQQTLHPLQERDPAFRFVFQTPKYRWGAHSTAVDADWISMLFGPFGDPYRRDPRM 5nqd.1    -------------------------------------DSHKYLINNGRANVVWQSAYLDQENDFVMD-------------  target    PWTGEAYLEINPKDAAELGLADGDYAWVDADPEDRPYRGWNEDDPYYEVARAMMRVRIYTGMSRGVIRTWFNMYAATPAT 5nqd.1    -RFPYPFIEMNPEDMAEAGLKEGDLVEIYND-----------------AGATQAMAYPTPTARRGETFMLFGF-------  target    VANQKATPGNPARNEQTRYVALFRYGSHQSGTRAWLRPTQQTDSLVRKGYFGQVIGTGFEADVHSVSGAPKEAFVKIEKA 5nqd.1    --------------------------------------------------------------------------------  target    EDGGIGAERLWRPLTLGLRPEAPSAALTAYLAGDYSGTKGS 5nqd.1    ----------------------------------------- ``` | | | | | | | | | | | | | | | | | | | | | | | | | | | | | | | | | | | | | | | | | | | | | | | | | |
|  | 7l5i.1.A | Trimethylamine-N-oxide reductase  *Crystal Structure of Haemophilus influenzae MtsZ at pH 7.0* | 0.13 | 0.00 | 16.47 | 0.30 | 38-153 | X-ray | 1.73 | monomer | 2 x MGD, 1 x MO, 1 x O | HHblits | 0.30 |
| ``` target    GLGVPESQLDVETRQYRNVVRTWAELQQTLHPLQERDPAFRFVFQTPKYRWGAHSTAVDADWISMLFGPFGDPYRRDPRM 7l5i.1    -------------------------------------EEYPLALVTPHPYYRLHSQLAHTSLRQKYA-------------  target    PWTGEAYLEINPKDAAELGLADGDYAWVDADPEDRPYRGWNEDDPYYEVARAMMRVRIYTGMSRGVIRTWFNMYAATPAT 7l5i.1    -VNDREPVMIHPEDAAARGIKDGDIVRIHSK-----------------RGQVLAGAAVTENIIKGTVALHEGA-------  target    VANQKATPGNPARNEQTRYVALFRYGSHQSGTRAWLRPTQQTDSLVRKGYFGQVIGTGFEADVHSVSGAPKEAFVKIEKA 7l5i.1    --------------------------------------------------------------------------------  target    EDGGIGAERLWRPLTLGLRPEAPSAALTAYLAGDYSGTKGS 7l5i.1    ----------------------------------------- ``` | | | | | | | | | | | | | | | | | | | | | | | | | | | | | | | | | | | | | | | | | | | | | | | | | |
|  | 7l5s.1.A | Trimethylamine-N-oxide reductase  *Crystal Structure of Haemophilus influenzae MtsZ at pH 5.5* | 0.14 | 0.00 | 16.47 | 0.30 | 38-153 | X-ray | 2.09 | monomer | 1 x O, 2 x MGD, 1 x MO | HHblits | 0.30 |
| ``` target    GLGVPESQLDVETRQYRNVVRTWAELQQTLHPLQERDPAFRFVFQTPKYRWGAHSTAVDADWISMLFGPFGDPYRRDPRM 7l5s.1    -------------------------------------EEYPLALVTPHPYYRLHSQLAHTSLRQKYA-------------  target    PWTGEAYLEINPKDAAELGLADGDYAWVDADPEDRPYRGWNEDDPYYEVARAMMRVRIYTGMSRGVIRTWFNMYAATPAT 7l5s.1    -VNDREPVMIHPEDAAARGIKDGDIVRIHSK-----------------RGQVLAGAAVTENIIKGTVALHEGA-------  target    VANQKATPGNPARNEQTRYVALFRYGSHQSGTRAWLRPTQQTDSLVRKGYFGQVIGTGFEADVHSVSGAPKEAFVKIEKA 7l5s.1    --------------------------------------------------------------------------------  target    EDGGIGAERLWRPLTLGLRPEAPSAALTAYLAGDYSGTKGS 7l5s.1    ----------------------------------------- ``` | | | | | | | | | | | | | | | | | | | | | | | | | | | | | | | | | | | | | | | | | | | | | | | | | |
|  | 1e60.1.A | Dimethyl sulfoxide/trimethylamine N-oxide reductase  *OXIDIZED DMSO REDUCTASE EXPOSED TO HEPES - Structure II BUFFER* | 0.12 | 0.00 | 21.18 | 0.30 | 38-153 | X-ray | 2.00 | monomer | 2 x PGD, 1 x 2MO | HHblits | 0.29 |
| ``` target    GLGVPESQLDVETRQYRNVVRTWAELQQTLHPLQERDPAFRFVFQTPKYRWGAHSTAVDADWISMLFGPFGDPYRRDPRM 1e60.1    -------------------------------------AKYPLHIAASHPFNRLHSQLN-GTVLREG-------------Y  target    PWTGEAYLEINPKDAAELGLADGDYAWVDADPEDRPYRGWNEDDPYYEVARAMMRVRIYTGMSRGVIRTWFNMYAATPAT 1e60.1    AVQGHEPCLMHPDDAAARGIADGDVVRVHND-----------------RGQILTGVKVTDAVMKGVIQIYEGG-------  target    VANQKATPGNPARNEQTRYVALFRYGSHQSGTRAWLRPTQQTDSLVRKGYFGQVIGTGFEADVHSVSGAPKEAFVKIEKA 1e60.1    --------------------------------------------------------------------------------  target    EDGGIGAERLWRPLTLGLRPEAPSAALTAYLAGDYSGTKGS 1e60.1    ----------------------------------------- ``` | | | | | | | | | | | | | | | | | | | | | | | | | | | | | | | | | | | | | | | | | | | | | | | | | |
|  | 1e18.1.A | DMSO REDUCTASE.  *TUNGSTEN-SUSBSTITUTED DMSO REDUCTASE FROM RHODOBACTER CAPSULATUS* | 0.12 | 0.00 | 21.18 | 0.30 | 38-153 | X-ray | 2.00 | monomer | 2 x PGD, 1 x 6WO | HHblits | 0.29 |
| ``` target    GLGVPESQLDVETRQYRNVVRTWAELQQTLHPLQERDPAFRFVFQTPKYRWGAHSTAVDADWISMLFGPFGDPYRRDPRM 1e18.1    -------------------------------------AKYPLHIAASHPFNRLHSQLN-GTVLREG-------------Y  target    PWTGEAYLEINPKDAAELGLADGDYAWVDADPEDRPYRGWNEDDPYYEVARAMMRVRIYTGMSRGVIRTWFNMYAATPAT 1e18.1    AVQGHEPCLMHPDDAAARGIADGDVVRVHND-----------------RGQILTGVKVTDAVMKGVIQIYEGG-------  target    VANQKATPGNPARNEQTRYVALFRYGSHQSGTRAWLRPTQQTDSLVRKGYFGQVIGTGFEADVHSVSGAPKEAFVKIEKA 1e18.1    --------------------------------------------------------------------------------  target    EDGGIGAERLWRPLTLGLRPEAPSAALTAYLAGDYSGTKGS 1e18.1    ----------------------------------------- ``` | | | | | | | | | | | | | | | | | | | | | | | | | | | | | | | | | | | | | | | | | | | | | | | | | |
|  | 1dms.1.A | DMSO REDUCTASE  *STRUCTURE OF DMSO REDUCTASE* | 0.12 | 0.00 | 21.18 | 0.30 | 38-153 | X-ray | 1.88 | monomer | 2 x PGD, 1 x 2MO | HHblits | 0.29 |
| ``` target    GLGVPESQLDVETRQYRNVVRTWAELQQTLHPLQERDPAFRFVFQTPKYRWGAHSTAVDADWISMLFGPFGDPYRRDPRM 1dms.1    -------------------------------------AKYPLHIAASHPFNRLHSQLNG-TVLREG-------------Y  target    PWTGEAYLEINPKDAAELGLADGDYAWVDADPEDRPYRGWNEDDPYYEVARAMMRVRIYTGMSRGVIRTWFNMYAATPAT 1dms.1    AVQGHEPCLMHPDDAAARGIADGDVVRVHND-----------------RGQILTGVKVTDAVMKGVIQIYEGG-------  target    VANQKATPGNPARNEQTRYVALFRYGSHQSGTRAWLRPTQQTDSLVRKGYFGQVIGTGFEADVHSVSGAPKEAFVKIEKA 1dms.1    --------------------------------------------------------------------------------  target    EDGGIGAERLWRPLTLGLRPEAPSAALTAYLAGDYSGTKGS 1dms.1    ----------------------------------------- ``` | | | | | | | | | | | | | | | | | | | | | | | | | | | | | | | | | | | | | | | | | | | | | | | | | |
|  | 6f0k.1.B | Fe-S-cluster-containing hydrogenase  *Alternative complex III* | 0.12 |  | 17.24 | 0.31 | 41-153 | EM | 0.00 | hetero-1-1-1-1-1-1-… | 6 x HEC, 1 x F3S, 3 x SF4 | HHblits | 0.27 |
| ``` target    GLGVPESQLDVETRQYRNVVRTWAELQQTLHPLQERDPAFRFVFQTPKYRWGAHSTAVDADWISMLFGPFGDPYRRDPRM 6f0k.1    ----------------------------------------GLEVVFRLDPTVLDGSFANNAWAQELP---------DPIT  target    PWTGEAYLEINPKDAAELGLAD--------GDYAWVDADPEDRPYRGWNEDDPYYEVARAMMRVRIYTGMSRGVIRTWFN 6f0k.1    KIVWDNVAILSPKTAAALGVKAEYHKGVYIADVIELSLD-----------------GRAVELPVWVLPGHPDDSITVYLG  target    MYAATPATVANQKATPGNPARNEQTRYVALFRYGSHQSGTRAWLRPTQQTDSLVRKGYFGQVIGTGFEADVHSVSGAPKE 6f0k.1    Y-------------------------------------------------------------------------------  target    AFVKIEKAEDGGIGAERLWRPLTLGLRPEAPSAALTAYLAGDYSGTKGS 6f0k.1    ------------------------------------------------- ``` | | | | | | | | | | | | | | | | | | | | | | | | | | | | | | | | | | | | | | | | | | | | | | | | | |
|  | 1ogy.1.A | PERIPLASMIC NITRATE REDUCTASE  *Crystal structure of the heterodimeric nitrate reductase from Rhodobacter sphaeroides* | 0.11 |  | 22.35 | 0.30 | 38-153 | X-ray | 3.20 | hetero-1-1-mer | 1 x SF4, 1 x MO, 2 x MGD, 2 x HEC | HHblits | 0.29 |
| ``` target    GLGVPESQLDVETRQYRNVVRTWAELQQTLHPLQERDPAFRFVFQTPKYRWGAHSTAVDADWISMLFGPFGDPYRRDPRM 1ogy.1    -------------------------------------EEFGFWLVTGRVLEHWHSGSMTLRWPELYK-------------  target    PWTGEAYLEINPKDAAELGLADGDYAWVDADPEDRPYRGWNEDDPYYEVARAMMRVRI--YTGMSRGVIRTWFNMYAATP 1ogy.1    -AFPGAVCFMHPEDARSRGLNRGSEVRVISR-----------------RGEIRTRLETRGRNRMPRGVVFVPWFD-----  target    ATVANQKATPGNPARNEQTRYVALFRYGSHQSGTRAWLRPTQQTDSLVRKGYFGQVIGTGFEADVHSVSGAPKEAFVKIE 1ogy.1    --------------------------------------------------------------------------------  target    KAEDGGIGAERLWRPLTLGLRPEAPSAALTAYLAGDYSGTKGS 1ogy.1    ------------------------------------------- ``` | | | | | | | | | | | | | | | | | | | | | | | | | | | | | | | | | | | | | | | | | | | | | | | | | |
|  | 2v45.1.A | PERIPLASMIC NITRATE REDUCTASE  *A NEW CATALYTIC MECHANISM OF PERIPLASMIC NITRATE REDUCTASE FROM DESULFOVIBRIO DESULFURICANS ATCC 27774 FROM CRYSTALLOGRAPHIC AND EPR DATA AND BASED ON DETAILED ANALYSIS OF THE SIXTH LIGAND* | 0.11 |  | 18.82 | 0.30 | 38-153 | X-ray | 2.40 | monomer | 1 x SF4, 1 x MO, 2 x MGD, 1 x LCP | HHblits | 0.29 |
| ``` target    GLGVPESQLDVETRQYRNVVRTWAELQQTLHPLQERDPAFRFVFQTPKYRWGAHSTAVDADWISMLFGPFGDPYRRDPRM 2v45.1    -------------------------------------AEYPLYLTSMRVIDHWHTATMTGKVPELQK-------------  target    PWTGEAYLEINPKDAAELGLADGDYAWVDADPEDRPYRGWNEDDPYYEVARAMMRVRIYTGMSRGVIRTWFNMYAATPAT 2v45.1    -ANPIAFVEINEEDAARTGIKHGDSVIVETR-----------------RDAMELPARVSDVCRPGLIAVPFFD-------  target    VANQKATPGNPARNEQTRYVALFRYGSHQSGTRAWLRPTQQTDSLVRKGYFGQVIGTGFEADVHSVSGAPKEAFVKIEKA 2v45.1    --------------------------------------------------------------------------------  target    EDGGIGAERLWRPLTLGLRPEAPSAALTAYLAGDYSGTKGS 2v45.1    ----------------------------------------- ``` | | | | | | | | | | | | | | | | | | | | | | | | | | | | | | | | | | | | | | | | | | | | | | | | | |
|  | 2v3v.1.A | PERIPLASMIC NITRATE REDUCTASE  *A NEW CATALYTIC MECHANISM OF PERIPLASMIC NITRATE REDUCTASE FROM DESULFOVIBRIO DESULFURICANS ATCC 27774 FROM CRYSTALLOGRAPHIC AND EPR DATA AND BASED ON DETAILED ANALYSIS OF THE SIXTH LIGAND* | 0.11 |  | 18.82 | 0.30 | 38-153 | X-ray | 1.99 | monomer | 1 x SF4, 1 x MO, 2 x MGD, 4 x LCP | HHblits | 0.29 |
| ``` target    GLGVPESQLDVETRQYRNVVRTWAELQQTLHPLQERDPAFRFVFQTPKYRWGAHSTAVDADWISMLFGPFGDPYRRDPRM 2v3v.1    -------------------------------------AEYPLYLTSMRVIDHWHTATMTGKVPELQK-------------  target    PWTGEAYLEINPKDAAELGLADGDYAWVDADPEDRPYRGWNEDDPYYEVARAMMRVRIYTGMSRGVIRTWFNMYAATPAT 2v3v.1    -ANPIAFVEINEEDAARTGIKHGDSVIVETR-----------------RDAMELPARVSDVCRPGLIAVPFFD-------  target    VANQKATPGNPARNEQTRYVALFRYGSHQSGTRAWLRPTQQTDSLVRKGYFGQVIGTGFEADVHSVSGAPKEAFVKIEKA 2v3v.1    --------------------------------------------------------------------------------  target    EDGGIGAERLWRPLTLGLRPEAPSAALTAYLAGDYSGTKGS 2v3v.1    ----------------------------------------- ``` | | | | | | | | | | | | | | | | | | | | | | | | | | | | | | | | | | | | | | | | | | | | | | | | | |
|  | 7p63.1.C | NADH-quinone oxidoreductase  *Complex I from E. coli, DDM/LMNG-purified, under Turnover at pH 6, Closed state* | 0.13 | 0.00 | 20.48 | 0.30 | 38-153 | EM | 0.00 | monomer | 7 x SF4, 1 x FMN, 1 x NAI, 2 x FES, 1 x CA, 1 x DCQ, 4 x LFA, 8 x 3PE | HHblits | 0.30 |
| ``` target    GLGVPESQLDVETRQYRNVVRTWAELQQTLHPLQERDPAFRFVFQTPKYRWGAHSTAVDADWISMLFGPFGDPYRRDPRM 7p63.1    -------------------------------------QDGKWRIAPYYHLFGSDELSQRAPVFQSR--------------  target    PWTGEAYLEINPKDAAELGLADGDYAWVDADPEDRPYRGWNEDDPYYEVARAMMRVRIYTGMSRGVIRTWFNMYAATPAT 7p63.1    --MPQPYIKLNPADAAKLGVNAGTRVSFSYD-----------------GNTVTLPVEIAEGLTAGQVGLPMGM-------  target    VANQKATPGNPARNEQTRYVALFRYGSHQSGTRAWLRPTQQTDSLVRKGYFGQVIGTGFEADVHSVSGAPKEAFVKIEKA 7p63.1    --------------------------------------------------------------------------------  target    EDGGIGAERLWRPLTLGLRPEAPSAALTAYLAGDYSGTKGS 7p63.1    ----------------------------------------- ``` | | | | | | | | | | | | | | | | | | | | | | | | | | | | | | | | | | | | | | | | | | | | | | | | | |
|  | 7nz1.1.E | NADH-quinone oxidoreductase subunit G  *Respiratory complex I from Escherichia coli - focused refinement of cytoplasmic arm* | 0.13 | 0.00 | 20.48 | 0.30 | 38-153 | EM | 0.00 | monomer | 7 x SF4, 2 x FES, 1 x FMN, 1 x CA | HHblits | 0.30 |
| ``` target    GLGVPESQLDVETRQYRNVVRTWAELQQTLHPLQERDPAFRFVFQTPKYRWGAHSTAVDADWISMLFGPFGDPYRRDPRM 7nz1.1    -------------------------------------QDGKWRIAPYYHLFGSDELSQRAPVFQSR--------------  target    PWTGEAYLEINPKDAAELGLADGDYAWVDADPEDRPYRGWNEDDPYYEVARAMMRVRIYTGMSRGVIRTWFNMYAATPAT 7nz1.1    --MPQPYIKLNPADAAKLGVNAGTRVSFSYD-----------------GNTVTLPVEIAEGLTAGQVGLPMGM-------  target    VANQKATPGNPARNEQTRYVALFRYGSHQSGTRAWLRPTQQTDSLVRKGYFGQVIGTGFEADVHSVSGAPKEAFVKIEKA 7nz1.1    --------------------------------------------------------------------------------  target    EDGGIGAERLWRPLTLGLRPEAPSAALTAYLAGDYSGTKGS 7nz1.1    ----------------------------------------- ``` | | | | | | | | | | | | | | | | | | | | | | | | | | | | | | | | | | | | | | | | | | | | | | | | | |
|  | 7p61.1.C | NADH-quinone oxidoreductase  *Complex I from E. coli, DDM-purified, with NADH, Resting state* | 0.12 | 0.00 | 20.48 | 0.30 | 38-153 | EM | 0.00 | monomer | 7 x SF4, 1 x FMN, 1 x NAI, 2 x FES, 1 x CA, 2 x 3PE, 1 x UQ8 | HHblits | 0.30 |
| ``` target    GLGVPESQLDVETRQYRNVVRTWAELQQTLHPLQERDPAFRFVFQTPKYRWGAHSTAVDADWISMLFGPFGDPYRRDPRM 7p61.1    -------------------------------------QDGKWRIAPYYHLFGSDELSQRAPVFQSR--------------  target    PWTGEAYLEINPKDAAELGLADGDYAWVDADPEDRPYRGWNEDDPYYEVARAMMRVRIYTGMSRGVIRTWFNMYAATPAT 7p61.1    --MPQPYIKLNPADAAKLGVNAGTRVSFSYD-----------------GNTVTLPVEIAEGLTAGQVGLPMGM-------  target    VANQKATPGNPARNEQTRYVALFRYGSHQSGTRAWLRPTQQTDSLVRKGYFGQVIGTGFEADVHSVSGAPKEAFVKIEKA 7p61.1    --------------------------------------------------------------------------------  target    EDGGIGAERLWRPLTLGLRPEAPSAALTAYLAGDYSGTKGS 7p61.1    ----------------------------------------- ``` | | | | | | | | | | | | | | | | | | | | | | | | | | | | | | | | | | | | | | | | | | | | | | | | | |
|  | 1g8j.1.A | ARSENITE OXIDASE  *CRYSTAL STRUCTURE ANALYSIS OF ARSENITE OXIDASE FROM ALCALIGENES FAECALIS* | 0.11 | 0.00 | 21.69 | 0.30 | 38-153 | X-ray | 2.03 | monomer | 2 x MGD, 1 x O, 1 x 4MO, 1 x F3S, 1 x FES | HHblits | 0.30 |
| ``` target    GLGVPESQLDVETRQYRNVVRTWAELQQTLHPLQERDPAFRFVFQTPKYRWGAHSTAVDA--DWISMLFGPFGDPYRRDP 1g8j.1    -------------------------------------DKYRFWLNNGRNNEVWQTAYHDQYNSLMQER------------  target    RMPWTGEAYLEINPKDAAELGLADGDYAWVDADPEDRPYRGWNEDDPYYEVARAMMRVRIYTGMSRGVIRTWFNMYAATP 1g8j.1    ----YPMAYIEMNPDDCKQLDVTGGDIVEVYND-----------------FGSTFAMVYPVAEIKRGQTFMLFGY-----  target    ATVANQKATPGNPARNEQTRYVALFRYGSHQSGTRAWLRPTQQTDSLVRKGYFGQVIGTGFEADVHSVSGAPKEAFVKIE 1g8j.1    --------------------------------------------------------------------------------  target    KAEDGGIGAERLWRPLTLGLRPEAPSAALTAYLAGDYSGTKGS 1g8j.1    ------------------------------------------- ``` | | | | | | | | | | | | | | | | | | | | | | | | | | | | | | | | | | | | | | | | | | | | | | | | | |
|  | 7vw6.1.A | Formate dehydrogenase  *Cryo-EM Structure of Formate Dehydrogenase 1 from Methylorubrum extorquens AM1* | 0.12 | 0.00 | 22.89 | 0.30 | 38-153 | EM | 0.00 | monomer | 4 x SF4, 2 x FES, 2 x MGD, 1 x W, 1 x FMN | HHblits | 0.30 |
| ``` target    GLGVPESQLDVETRQYRNVVRTWAELQQTLHPLQERDPAFRFVFQTPKYRWGAHSTAVD--ADWISMLFGPFGDPYRRDP 7vw6.1    -------------------------------------DEFPMVLSTGRVLEHWHTGSMTRRAGVLDAL------------  target    RMPWTGEAYLEINPKDAAELGLADGDYAWVDADPEDRPYRGWNEDDPYYEVARAMMRVRIYTGMSRGVIRTWFNMYAATP 7vw6.1    ----EPEAVAFMAPKELYRLGLRPGGSMRLETR-----------------RGAVVLKVRSDRDVPIGMIFMPFCY-----  target    ATVANQKATPGNPARNEQTRYVALFRYGSHQSGTRAWLRPTQQTDSLVRKGYFGQVIGTGFEADVHSVSGAPKEAFVKIE 7vw6.1    --------------------------------------------------------------------------------  target    KAEDGGIGAERLWRPLTLGLRPEAPSAALTAYLAGDYSGTKGS 7vw6.1    ------------------------------------------- ``` | | | | | | | | | | | | | | | | | | | | | | | | | | | | | | | | | | | | | | | | | | | | | | | | | |
|  | 1g8k.1.A | ARSENITE OXIDASE  *CRYSTAL STRUCTURE ANALYSIS OF ARSENITE OXIDASE FROM ALCALIGENES FAECALIS* | 0.11 | 0.00 | 21.69 | 0.30 | 38-153 | X-ray | 1.64 | monomer | 3 x HG, 2 x CA, 2 x MGD, 1 x O, 1 x 4MO, 1 x F3S, 1 x FES | HHblits | 0.30 |
| ``` target    GLGVPESQLDVETRQYRNVVRTWAELQQTLHPLQERDPAFRFVFQTPKYRWGAHSTAVDA--DWISMLFGPFGDPYRRDP 1g8k.1    -------------------------------------DKYRFWLNNGRNNEVWQTAYHDQYNSLMQE-------------  target    RMPWTGEAYLEINPKDAAELGLADGDYAWVDADPEDRPYRGWNEDDPYYEVARAMMRVRIYTGMSRGVIRTWFNMYAATP 1g8k.1    ---RYPMAYIEMNPDDCKQLDVTGGDIVEVYND-----------------FGSTFAMVYPVAEIKRGQTFMLFGY-----  target    ATVANQKATPGNPARNEQTRYVALFRYGSHQSGTRAWLRPTQQTDSLVRKGYFGQVIGTGFEADVHSVSGAPKEAFVKIE 1g8k.1    --------------------------------------------------------------------------------  target    KAEDGGIGAERLWRPLTLGLRPEAPSAALTAYLAGDYSGTKGS 1g8k.1    ------------------------------------------- ``` | | | | | | | | | | | | | | | | | | | | | | | | | | | | | | | | | | | | | | | | | | | | | | | | | |
|  | 2e7z.1.A | Acetylene hydratase Ahy  *Acetylene Hydratase from Pelobacter acetylenicus* | 0.11 |  | 16.87 | 0.30 | 38-153 | X-ray | 1.26 | monomer | 1 x SF4, 2 x MGD, 1 x W | HHblits | 0.29 |
| ``` target    GLGVPESQLDVETRQYRNVVRTWAELQQTLHPLQERDPAFRFVFQTPKYR-WGAHSTAVDADWISMLFGPFGDPYRRDPR 2e7z.1    -------------------------------------KEYPLILFAGLREDSNFQSCYHQPGILRDA-------------  target    MPWTGEAYLEINPKDAAELGLADGDYAWVDADPEDRPYRGWNEDDPYYEVARAMMRVRIYTGMSRGVIRTWFNMYAATPA 2e7z.1    ---EPDPVALLHPKTAQSLGLPSGEWIWVETT-----------------HGRLKLLLKHDGAQPEGTIRIPHGR------  target    TVANQKATPGNPARNEQTRYVALFRYGSHQSGTRAWLRPTQQTDSLVRKGYFGQVIGTGFEADVHSVSGAPKEAFVKIEK 2e7z.1    --------------------------------------------------------------------------------  target    AEDGGIGAERLWRPLTLGLRPEAPSAALTAYLAGDYSGTKGS 2e7z.1    ------------------------------------------ ``` | | | | | | | | | | | | | | | | | | | | | | | | | | | | | | | | | | | | | | | | | | | | | | | | | |
|  | 5t5i.1.D | Tungsten formylmethanofuran dehydrogenase subunit fwdD  *TUNGSTEN-CONTAINING FORMYLMETHANOFURAN DEHYDROGENASE FROM METHANOTHERMOBACTER WOLFEII, ORTHORHOMBIC FORM AT 1.9 A* | 0.10 | 0.00 | 17.50 | 0.28 | 41-153 | X-ray | 1.90 | homo-dimer | 4 x ZN, 2 x MG, 18 x K, 22 x SF4, 2 x W, 4 x MGD, 2 x H2S, 2 x CA | HHblits | 0.31 |
| ``` target    GLGVPESQLDVETRQYRNVVRTWAELQQTLHPLQERDPAFRFVFQTPKYRWGAHSTAVDADWISMLFGPFGDPYRRDPRM 5t5i.1    ----------------------------------------RVILNTGRTIWQGQAIESGKDLKMY---------------  target    PWTGEAYLEINPKDAAELGLADGDYAWVDADPEDRPYRGWNEDDPYYEVARAMMRVR-IYTGMSRGVIRTWFNMYAATPA 5t5i.1    -VDAAAIIQMNPEMMKQLGIAEGDNVKVISE-----------------YGDVVVKAVEAKEPLPEGMVYIPMGP------  target    TVANQKATPGNPARNEQTRYVALFRYGSHQSGTRAWLRPTQQTDSLVRKGYFGQVIGTGFEADVHSVSGAPKEAFVKIEK 5t5i.1    --------------------------------------------------------------------------------  target    AEDGGIGAERLWRPLTLGLRPEAPSAALTAYLAGDYSGTKGS 5t5i.1    ------------------------------------------ ``` | | | | | | | | | | | | | | | | | | | | | | | | | | | | | | | | | | | | | | | | | | | | | | | | | |
|  | 2nya.1.A | Periplasmic nitrate reductase  *Crystal structure of the periplasmic nitrate reductase (NAP) from Escherichia coli* | 0.12 |  | 20.48 | 0.30 | 38-153 | X-ray | 2.50 | monomer | 1 x SF4, 1 x 6MO, 2 x MGD | HHblits | 0.28 |
| ``` target    GLGVPESQLDVETRQYRNVVRTWAELQQTLHPLQERDPAFRFVFQTPKYRWGAH--STAVDADWISMLFGPFGDPYRRDP 2nya.1    -------------------------------------EEYDLWLSTGRVLEHWHTGSMTRRVPELHRA------------  target    RMPWTGEAYLEINPKDAAELGLADGDYAWVDADPEDRPYRGWNEDDPYYEVARAMMRVRIYTG--MSRGVIRTWFNMYAA 2nya.1    ----FPEAVLFIHPLDAKARDLRRGDKVKVVSR-----------------RGEVISIVETRGRNRPPQGLVYMPFFD---  target    TPATVANQKATPGNPARNEQTRYVALFRYGSHQSGTRAWLRPTQQTDSLVRKGYFGQVIGTGFEADVHSVSGAPKEAFVK 2nya.1    --------------------------------------------------------------------------------  target    IEKAEDGGIGAERLWRPLTLGLRPEAPSAALTAYLAGDYSGTKGS 2nya.1    --------------------------------------------- ``` | | | | | | | | | | | | | | | | | | | | | | | | | | | | | | | | | | | | | | | | | | | | | | | | | |
|  | 1kqf.1.A | FORMATE DEHYDROGENASE, NITRATE-INDUCIBLE, MAJOR SUBUNIT  *FORMATE DEHYDROGENASE N FROM E. COLI* | 0.12 |  | 15.66 | 0.30 | 38-153 | X-ray | 1.60 | hetero-oligomer | 3 x 6MO, 15 x SF4, 6 x MGD, 6 x HEM, 3 x CDL | HHblits | 0.27 |
| ``` target    GLGVPESQLDVETRQYRNVVRTWAELQQTLHPLQERDPAFRFVFQTPKYRWGAHSTAVDADWISMLFGPFGDPYRRDPRM 1kqf.1    -------------------------------------EQFPYVGTTYRLTEHFHTWTK-HALLNAI--------------  target    PWTGEAYLEINPKDAAELGLADGDYAWVDADPEDRPYRGWNEDDPYYEVARAMMRVRIYTGMSR--------GVIRTWFN 1kqf.1    -AQPEQFVEISETLAAAKGINNGDRVTVSSK-----------------RGFIRAVAVVTRRLKPLNVNGQQVETVGIPIH  target    MYAATPATVANQKATPGNPARNEQTRYVALFRYGSHQSGTRAWLRPTQQTDSLVRKGYFGQVIGTGFEADVHSVSGAPKE 1kqf.1    W-------------------------------------------------------------------------------  target    AFVKIEKAEDGGIGAERLWRPLTLGLRPEAPSAALTAYLAGDYSGTKGS 1kqf.1    ------------------------------------------------- ``` | | | | | | | | | | | | | | | | | | | | | | | | | | | | | | | | | | | | | | | | | | | | | | | | | |
|  | 3o5a.1.A | Periplasmic nitrate reductase  *Crystal Structure of partially reduced Periplasmic Nitrate Reductase from Cupriavidus necator using Ionic Liquids* | 0.11 |  | 19.75 | 0.29 | 38-151 | X-ray | 1.72 | hetero-oligomer | 1 x SF4, 1 x MOS, 2 x MGD, 2 x HEC | HHblits | 0.29 |
| ``` target    GLGVPESQLDVETRQYRNVVRTWAELQQTLHPLQERDPAFRFVFQTPKYRWGAHSTA--VDADWISMLFGPFGDPYRRDP 3o5a.1    -------------------------------------KEYPYWLVTGRVLEHWHSGSMTRRVPELYRS------------  target    RMPWTGEAYLEINPKDAAELGLADGDYAWVDADPEDRPYRGWNEDDPYYEVARAMMRVRIY--TGMSRGVIRTWFNMYAA 3o5a.1    ----FPNAVVFMHPEDAKALGLRRGVEVEVVSR-----------------RGRMRSRIETRGRDAPPRGLVFVPW-----  target    TPATVANQKATPGNPARNEQTRYVALFRYGSHQSGTRAWLRPTQQTDSLVRKGYFGQVIGTGFEADVHSVSGAPKEAFVK 3o5a.1    --------------------------------------------------------------------------------  target    IEKAEDGGIGAERLWRPLTLGLRPEAPSAALTAYLAGDYSGTKGS 3o5a.1    --------------------------------------------- ``` | | | | | | | | | | | | | | | | | | | | | | | | | | | | | | | | | | | | | | | | | | | | | | | | | |
|  | 7bkb.1.J | Formylmethanofuran dehydrogenase, subunit D  *Formate dehydrogenase - heterodisulfide reductase - formylmethanofuran dehydrogenase complex from Methanospirillum hungatei (hexameric, composite structure)* | 0.09 |  | 10.84 | 0.30 | 38-153 | EM | 0.00 | hetero-2-2-2-2-2-2-… | 48 x SF4, 4 x FAD, 2 x FES, 4 x 9S8, 4 x ZN, 2 x MO, 4 x MGD | HHblits | 0.26 |
| ``` target    GLGVPESQLDVETRQYRNVVRTWAELQQTLHPLQERDPAFRFVFQTPKYRWGAHSTAVDADWISMLFGPFGDPYRRDPRM 7bkb.1    -------------------------------------AKKTLNMITQRAVEEGIAMEI-GKTSRQY--------------  target    PWTGEAYLEINPKDAAELGLADGDYAWVDADPEDRPYRGWNEDDPYYEVARAMMRVRIY-TGMSRGVIRTWFNMYAATPA 7bkb.1    -FDACSIIEMNEQDMKELGIMKNTNVRVKSE-----------------SGEVVVKAVVGRQTCYPGLCHIRQGV------  target    TVANQKATPGNPARNEQTRYVALFRYGSHQSGTRAWLRPTQQTDSLVRKGYFGQVIGTGFEADVHSVSGAPKEAFVKIEK 7bkb.1    --------------------------------------------------------------------------------  target    AEDGGIGAERLWRPLTLGLRPEAPSAALTAYLAGDYSGTKGS 7bkb.1    ------------------------------------------ ``` | | | | | | | | | | | | | | | | | | | | | | | | | | | | | | | | | | | | | | | | | | | | | | | | | |
|  | 8e9g.1.G | NADH-quinone oxidoreductase subunit G  *Mycobacterial respiratory complex I with both quinone positions modelled* | 0.11 |  | 14.81 | 0.29 | 39-153 | EM | 0.00 | hetero-1-1-1-1-1-1-… |  | HHblits | 0.27 |
| ``` target    GLGVPESQLDVETRQYRNVVRTWAELQQTLHPLQERDPAFRFVFQTPKYRWGAHSTAVDADWISMLFGPFGDPYRRDPRM 8e9g.1    --------------------------------------SGQAVLASWRMLLDAGRLQDGEPHLAGT--------------  target    PWTGEAYLEINPKDAAELGLADGDYAWVDADPEDRPYRGWNEDDPYYEVARAMMRVRIYTGMSRGVIRTWFNMYAATPAT 8e9g.1    --AVRPVARMSAATAAGIGASDGAPVTVSTE-----------------RGAVTLPLAVTD-MPDGVVWLPMNS-------  target    VANQKATPGNPARNEQTRYVALFRYGSHQSGTRAWLRPTQQTDSLVRKGYFGQVIGTGFEADVHSVSGAPKEAFVKIEKA 8e9g.1    --------------------------------------------------------------------------------  target    EDGGIGAERLWRPLTLGLRPEAPSAALTAYLAGDYSGTKGS 8e9g.1    ----------------------------------------- ``` | | | | | | | | | | | | | | | | | | | | | | | | | | | | | | | | | | | | | | | | | | | | | | | | | |
|  | 2ki8.1.A | Tungsten formylmethanofuran dehydrogenase, subunit D (FwdD-2)  *Solution NMR structure of tungsten formylmethanofuran dehydrogenase subunit D from Archaeoglobus fulgidus, Northeast Structural Genomics Consortium target AtT7* | 0.10 |  | 16.25 | 0.28 | 39-153 | NMR | 0.00 | monomer |  | HHblits | 0.28 |
| ``` target    GLGVPESQLDVETRQYRNVVRTWAELQQTLHPLQERDPAFRFVFQTPKYRWGAHSTAVDADWISMLFGPFGDPYRRDPRM 2ki8.1    --------------------------------------MLEVEVISGRTLNQGATVE--EKLTEEY--------------  target    PWTGEAYLEINPKDAAELGLADGDYAWVDADPEDRPYRGWNEDDPYYEVARAMMRVRIYTGMSRGVIRTWFNMYAATPAT 2ki8.1    -FNAVNYAEINEEDWNALGLQEGDRVKVKTE-----------------FGEVVVFAKKG-DVPKGMIFIPMGP-------  target    VANQKATPGNPARNEQTRYVALFRYGSHQSGTRAWLRPTQQTDSLVRKGYFGQVIGTGFEADVHSVSGAPKEAFVKIEKA 2ki8.1    --------------------------------------------------------------------------------  target    EDGGIGAERLWRPLTLGLRPEAPSAALTAYLAGDYSGTKGS 2ki8.1    ----------------------------------------- ``` | | | | | | | | | | | | | | | | | | | | | | | | | | | | | | | | | | | | | | | | | | | | | | | | | |
|  | 1h0h.1.A | FORMATE DEHYDROGENASE SUBUNIT ALPHA  *Tungsten containing Formate Dehydrogenase from Desulfovibrio Gigas* | 0.09 | 0.00 | 15.58 | 0.27 | 38-147 | X-ray | 1.80 | monomer | 1 x W, 1 x 2MD, 1 x MGD, 4 x SF4, 1 x CA | HHblits | 0.30 |
| ``` target    GLGVPESQLDVETRQYRNVVRTWAELQQTLHPLQERDPAFRFVFQTPKYR--WGAHSTAVDADWISMLFGPFGDPYRRDP 1h0h.1    -------------------------------------PRYPFICSTYRVTEHWQTGLMTRNTPWLLEA------------  target    RMPWTGEAYLEINPKDAAELGLADGDYAWVDADPEDRPYRGWNEDDPYYEVARAMMRVRIYTGMSRGVIRTWFNMYAATP 1h0h.1    ----EPQMFCEMSEELATLRGIKNGDKVILESV-----------------RGKLWAKAIITKRIKPFAI-----------  target    ATVANQKATPGNPARNEQTRYVALFRYGSHQSGTRAWLRPTQQTDSLVRKGYFGQVIGTGFEADVHSVSGAPKEAFVKIE 1h0h.1    --------------------------------------------------------------------------------  target    KAEDGGIGAERLWRPLTLGLRPEAPSAALTAYLAGDYSGTKGS 1h0h.1    ------------------------------------------- ``` | | | | | | | | | | | | | | | | | | | | | | | | | | | | | | | | | | | | | | | | | | | | | | | | | |
|  | 6sdv.1.A | Formate dehydrogenase, alpha subunit, selenocysteine-containing,Formate dehydrogenase, alpha subunit, selenocysteine-containing,W-formate dehydrogenase - alpha subunit  *W-formate dehydrogenase from Desulfovibrio vulgaris - Formate reduced form* | 0.09 |  | 15.58 | 0.27 | 38-147 | X-ray | 1.90 | hetero-1-1-mer | 2 x MGD, 4 x SF4, 1 x W, 1 x H2S | HHblits | 0.27 |
| ``` target    GLGVPESQLDVETRQYRNVVRTWAELQQTLHPLQERDPAFRFVFQTPKYRWGAHS--TAVDADWISMLFGPFGDPYRRDP 6sdv.1    -------------------------------------PRYPFIGTTYRVTEHWQTGLMTRRCAWLVEA------------  target    RMPWTGEAYLEINPKDAAELGLADGDYAWVDADPEDRPYRGWNEDDPYYEVARAMMRVRIYTGMSRGVIRTWFNMYAATP 6sdv.1    ----EPQIFCEISKELAKLRGIGNGDTVKVSSL-----------------RGALEAVAIVTERIRPFKI-----------  target    ATVANQKATPGNPARNEQTRYVALFRYGSHQSGTRAWLRPTQQTDSLVRKGYFGQVIGTGFEADVHSVSGAPKEAFVKIE 6sdv.1    --------------------------------------------------------------------------------  target    KAEDGGIGAERLWRPLTLGLRPEAPSAALTAYLAGDYSGTKGS 6sdv.1    ------------------------------------------- ``` | | | | | | | | | | | | | | | | | | | | | | | | | | | | | | | | | | | | | | | | | | | | | | | | | |
|  | 6sdr.1.A | Formate dehydrogenase, alpha subunit, selenocysteine-containing  *W-formate dehydrogenase from Desulfovibrio vulgaris - Oxidized form* | 0.09 |  | 15.58 | 0.27 | 38-147 | X-ray | 2.10 | hetero-1-1-mer | 2 x MGD, 4 x SF4, 1 x H2S, 1 x W | HHblits | 0.27 |
| ``` target    GLGVPESQLDVETRQYRNVVRTWAELQQTLHPLQERDPAFRFVFQTPKYRWGAHS--TAVDADWISMLFGPFGDPYRRDP 6sdr.1    -------------------------------------PRYPFIGTTYRVTEHWQTGLMTRRCAWLVEA------------  target    RMPWTGEAYLEINPKDAAELGLADGDYAWVDADPEDRPYRGWNEDDPYYEVARAMMRVRIYTGMSRGVIRTWFNMYAATP 6sdr.1    ----EPQIFCEISKELAKLRGIGNGDTVKVSSL-----------------RGALEAVAIVTERIRPFKI-----------  target    ATVANQKATPGNPARNEQTRYVALFRYGSHQSGTRAWLRPTQQTDSLVRKGYFGQVIGTGFEADVHSVSGAPKEAFVKIE 6sdr.1    --------------------------------------------------------------------------------  target    KAEDGGIGAERLWRPLTLGLRPEAPSAALTAYLAGDYSGTKGS 6sdr.1    ------------------------------------------- ``` | | | | | | | | | | | | | | | | | | | | | | | | | | | | | | | | | | | | | | | | | | | | | | | | | |
|  | 8bqg.1.A | Formate dehydrogenase, alpha subunit, selenocysteine-containing  *W-formate dehydrogenase from Desulfovibrio vulgaris - Soaking with Formate 1 min* | 0.09 |  | 15.58 | 0.27 | 38-147 | X-ray | 1.95 | hetero-1-1-mer | 2 x MGD, 4 x SF4, 1 x H2S, 1 x W | HHblits | 0.27 |
| ``` target    GLGVPESQLDVETRQYRNVVRTWAELQQTLHPLQERDPAFRFVFQTPKYRWGAHST--AVDADWISMLFGPFGDPYRRDP 8bqg.1    -------------------------------------PRYPFIGTTYRVTEHWQTGLMTRRCAWLVEA------------  target    RMPWTGEAYLEINPKDAAELGLADGDYAWVDADPEDRPYRGWNEDDPYYEVARAMMRVRIYTGMSRGVIRTWFNMYAATP 8bqg.1    ----EPQIFCEISKELAKLRGIGNGDTVKVSSL-----------------RGALEAVAIVTERIRPFKI-----------  target    ATVANQKATPGNPARNEQTRYVALFRYGSHQSGTRAWLRPTQQTDSLVRKGYFGQVIGTGFEADVHSVSGAPKEAFVKIE 8bqg.1    --------------------------------------------------------------------------------  target    KAEDGGIGAERLWRPLTLGLRPEAPSAALTAYLAGDYSGTKGS 8bqg.1    ------------------------------------------- ``` | | | | | | | | | | | | | | | | | | | | | | | | | | | | | | | | | | | | | | | | | | | | | | | | | |
| ✓ | 3hu1.1.A | Transitional endoplasmic reticulum ATPase  *Structure of p97 N-D1 R95G mutant in complex with ATPgS* | 0.07 | 0.00 | 17.91 | 0.24 | 86-153 | X-ray | 2.81 | monomer | 6 x AGS, 6 x MG | HHblits | 0.26 |
| ``` target    GLGVPESQLDVETRQYRNVVRTWAELQQTLHPLQERDPAFRFVFQTPKYRWGAHSTAVDADWISMLFGPFGDPYRRDPRM 3hu1.1    --------------------------------------------------------------------------------  target    PWTGEAYLEINPKDAAELGLADGDYAWVDADPEDRPYRGWN--EDDPYYEVARAMMRVRIYTGMSRGVIRTWFNMYAATP 3hu1.1    -----SVVSLSQPKMDELQLFRGDTVLLKGKKR-REAVCIVLSDDTCSDEKIRMNRVVRNNLRVGLGDVISIQPC-----  target    ATVANQKATPGNPARNEQTRYVALFRYGSHQSGTRAWLRPTQQTDSLVRKGYFGQVIGTGFEADVHSVSGAPKEAFVKIE 3hu1.1    --------------------------------------------------------------------------------  target    KAEDGGIGAERLWRPLTLGLRPEAPSAALTAYLAGDYSGTKGS 3hu1.1    ------------------------------------------- ``` | | | | | | | | | | | | | | | | | | | | | | | | | | | | | | | | | | | | | | | | | | | | | | | | | |
|  | 3m9s.1.C | NADH-quinone oxidoreductase subunit 3  *Crystal structure of respiratory complex I from Thermus thermophilus* | 0.08 | 0.00 | 21.43 | 0.20 | 83-155 | X-ray | 4.50 | monomer | 7 x SF4, 2 x FES, 1 x FMN | HHblits | 0.28 |
| ``` target    GLGVPESQLDVETRQYRNVVRTWAELQQTLHPLQERDPAFRFVFQTPKYRWGAHSTAVDADWISMLFGPFGDPYRRDPRM 3m9s.1    --------------------------------------------------------------------------------  target    PWTGEAYLEINPKDAAELGLADGDYAWVDADPEDRPYRGWNEDDPYYEVARAMMRVRIYTGMSRGVIRTWFNMYAATPAT 3m9s.1    --AARAELWAHPETARAEALPEGAQVAVETP-----------------FGRVEARVVHREDVPKGHLYLSALGPA-----  target    VANQKATPGNPARNEQTRYVALFRYGSHQSGTRAWLRPTQQTDSLVRKGYFGQVIGTGFEADVHSVSGAPKEAFVKIEKA 3m9s.1    --------------------------------------------------------------------------------  target    EDGGIGAERLWRPLTLGLRPEAPSAALTAYLAGDYSGTKGS 3m9s.1    ----------------------------------------- ``` | | | | | | | | | | | | | | | | | | | | | | | | | | | | | | | | | | | | | | | | | | | | | | | | | |
|  | 2fug.2.C | NADH-quinone oxidoreductase chain 3  *Crystal structure of the hydrophilic domain of respiratory complex I from Thermus thermophilus* | 0.05 | 0.00 | 21.43 | 0.20 | 83-155 | X-ray | 3.30 | monomer | 7 x SF4, 2 x FES, 1 x FMN | HHblits | 0.28 |
| ``` target    GLGVPESQLDVETRQYRNVVRTWAELQQTLHPLQERDPAFRFVFQTPKYRWGAHSTAVDADWISMLFGPFGDPYRRDPRM 2fug.2    --------------------------------------------------------------------------------  target    PWTGEAYLEINPKDAAELGLADGDYAWVDADPEDRPYRGWNEDDPYYEVARAMMRVRIYTGMSRGVIRTWFNMYAATPAT 2fug.2    --AARAELWAHPETARAEALPEGAQVAVETP-----------------FGRVEARVVHREDVPKGHLYLSALGPA-----  target    VANQKATPGNPARNEQTRYVALFRYGSHQSGTRAWLRPTQQTDSLVRKGYFGQVIGTGFEADVHSVSGAPKEAFVKIEKA 2fug.2    --------------------------------------------------------------------------------  target    EDGGIGAERLWRPLTLGLRPEAPSAALTAYLAGDYSGTKGS 2fug.2    ----------------------------------------- ``` | | | | | | | | | | | | | | | | | | | | | | | | | | | | | | | | | | | | | | | | | | | | | | | | | |
|  | 6zjl.1.C | NADH-quinone oxidoreductase subunit 3  *Respiratory complex I from Thermus thermophilus, NAD+ dataset, major state* | 0.07 | 0.00 | 21.43 | 0.20 | 83-155 | EM | 0.00 | monomer | 7 x SF4, 1 x FMN, 2 x FES | HHblits | 0.28 |
| ``` target    GLGVPESQLDVETRQYRNVVRTWAELQQTLHPLQERDPAFRFVFQTPKYRWGAHSTAVDADWISMLFGPFGDPYRRDPRM 6zjl.1    --------------------------------------------------------------------------------  target    PWTGEAYLEINPKDAAELGLADGDYAWVDADPEDRPYRGWNEDDPYYEVARAMMRVRIYTGMSRGVIRTWFNMYAATPAT 6zjl.1    --AARAELWAHPETARAEALPEGAQVAVETP-----------------FGRVEARVVHREDVPKGHLYLSALGPA-----  target    VANQKATPGNPARNEQTRYVALFRYGSHQSGTRAWLRPTQQTDSLVRKGYFGQVIGTGFEADVHSVSGAPKEAFVKIEKA 6zjl.1    --------------------------------------------------------------------------------  target    EDGGIGAERLWRPLTLGLRPEAPSAALTAYLAGDYSGTKGS 6zjl.1    ----------------------------------------- ``` | | | | | | | | | | | | | | | | | | | | | | | | | | | | | | | | | | | | | | | | | | | | | | | | | |
|  | 6q8o.1.C | NADH-quinone oxidoreductase subunit 3  *Respiratory complex I from Thermus thermophilus with bound Piericidin A* | 0.08 | 0.00 | 21.43 | 0.20 | 83-155 | X-ray | 3.61 | monomer | 7 x SF4, 1 x FMN, 2 x FES, 1 x HQH | HHblits | 0.28 |
| ``` target    GLGVPESQLDVETRQYRNVVRTWAELQQTLHPLQERDPAFRFVFQTPKYRWGAHSTAVDADWISMLFGPFGDPYRRDPRM 6q8o.1    --------------------------------------------------------------------------------  target    PWTGEAYLEINPKDAAELGLADGDYAWVDADPEDRPYRGWNEDDPYYEVARAMMRVRIYTGMSRGVIRTWFNMYAATPAT 6q8o.1    --AARAELWAHPETARAEALPEGAQVAVETP-----------------FGRVEARVVHREDVPKGHLYLSALGPA-----  target    VANQKATPGNPARNEQTRYVALFRYGSHQSGTRAWLRPTQQTDSLVRKGYFGQVIGTGFEADVHSVSGAPKEAFVKIEKA 6q8o.1    --------------------------------------------------------------------------------  target    EDGGIGAERLWRPLTLGLRPEAPSAALTAYLAGDYSGTKGS 6q8o.1    ----------------------------------------- ``` | | | | | | | | | | | | | | | | | | | | | | | | | | | | | | | | | | | | | | | | | | | | | | | | | |
|  | 6zjy.1.C | NADH-quinone oxidoreductase subunit 3  *Respiratory complex I from Thermus thermophilus, NAD+ dataset, minor state* | 0.08 | 0.00 | 21.43 | 0.20 | 83-155 | EM | 0.00 | monomer | 7 x SF4, 2 x FES | HHblits | 0.28 |
| ``` target    GLGVPESQLDVETRQYRNVVRTWAELQQTLHPLQERDPAFRFVFQTPKYRWGAHSTAVDADWISMLFGPFGDPYRRDPRM 6zjy.1    --------------------------------------------------------------------------------  target    PWTGEAYLEINPKDAAELGLADGDYAWVDADPEDRPYRGWNEDDPYYEVARAMMRVRIYTGMSRGVIRTWFNMYAATPAT 6zjy.1    --AARAELWAHPETARAEALPEGAQVAVETP-----------------FGRVEARVVHREDVPKGHLYLSALGPA-----  target    VANQKATPGNPARNEQTRYVALFRYGSHQSGTRAWLRPTQQTDSLVRKGYFGQVIGTGFEADVHSVSGAPKEAFVKIEKA 6zjy.1    --------------------------------------------------------------------------------  target    EDGGIGAERLWRPLTLGLRPEAPSAALTAYLAGDYSGTKGS 6zjy.1    ----------------------------------------- ``` | | | | | | | | | | | | | | | | | | | | | | | | | | | | | | | | | | | | | | | | | | | | | | | | | |
|  | 6zjn.1.C | NADH-quinone oxidoreductase subunit 3  *Respiratory complex I from Thermus thermophilus, NADH dataset, minor state* | 0.08 | 0.00 | 21.43 | 0.20 | 83-155 | EM | 0.00 | monomer | 7 x SF4, 2 x FES | HHblits | 0.28 |
| ``` target    GLGVPESQLDVETRQYRNVVRTWAELQQTLHPLQERDPAFRFVFQTPKYRWGAHSTAVDADWISMLFGPFGDPYRRDPRM 6zjn.1    --------------------------------------------------------------------------------  target    PWTGEAYLEINPKDAAELGLADGDYAWVDADPEDRPYRGWNEDDPYYEVARAMMRVRIYTGMSRGVIRTWFNMYAATPAT 6zjn.1    --AARAELWAHPETARAEALPEGAQVAVETP-----------------FGRVEARVVHREDVPKGHLYLSALGPA-----  target    VANQKATPGNPARNEQTRYVALFRYGSHQSGTRAWLRPTQQTDSLVRKGYFGQVIGTGFEADVHSVSGAPKEAFVKIEKA 6zjn.1    --------------------------------------------------------------------------------  target    EDGGIGAERLWRPLTLGLRPEAPSAALTAYLAGDYSGTKGS 6zjn.1    ----------------------------------------- ``` | | | | | | | | | | | | | | | | | | | | | | | | | | | | | | | | | | | | | | | | | | | | | | | | | |
|  | 6ziy.1.C | NADH-quinone oxidoreductase subunit 3  *Respiratory complex I from Thermus thermophilus, NADH dataset, major state* | 0.07 | 0.00 | 21.43 | 0.20 | 83-155 | EM | 0.00 | monomer | 7 x SF4, 1 x FMN, 1 x NAI, 2 x FES | HHblits | 0.28 |
| ``` target    GLGVPESQLDVETRQYRNVVRTWAELQQTLHPLQERDPAFRFVFQTPKYRWGAHSTAVDADWISMLFGPFGDPYRRDPRM 6ziy.1    --------------------------------------------------------------------------------  target    PWTGEAYLEINPKDAAELGLADGDYAWVDADPEDRPYRGWNEDDPYYEVARAMMRVRIYTGMSRGVIRTWFNMYAATPAT 6ziy.1    --AARAELWAHPETARAEALPEGAQVAVETP-----------------FGRVEARVVHREDVPKGHLYLSALGPA-----  target    VANQKATPGNPARNEQTRYVALFRYGSHQSGTRAWLRPTQQTDSLVRKGYFGQVIGTGFEADVHSVSGAPKEAFVKIEKA 6ziy.1    --------------------------------------------------------------------------------  target    EDGGIGAERLWRPLTLGLRPEAPSAALTAYLAGDYSGTKGS 6ziy.1    ----------------------------------------- ``` | | | | | | | | | | | | | | | | | | | | | | | | | | | | | | | | | | | | | | | | | | | | | | | | | |
|  | 6lod.1.B | Fe-S-cluster-containing hydrogenase components 1-like protein  *Cryo-EM structure of the air-oxidized photosynthetic alternative complex III from Roseiflexus castenholzii* | 0.06 |  | 18.52 | 0.19 | 83-153 | EM | 0.00 | hetero-1-1-1-1-1-1-… | 6 x HEC, 2 x EL6, 3 x SF4, 1 x F3S | HHblits | 0.29 |
| ``` target    GLGVPESQLDVETRQYRNVVRTWAELQQTLHPLQERDPAFRFVFQTPKYRWGAHSTAVDADWISMLFGPFGDPYRRDPRM 6lod.1    --------------------------------------------------------------------------------  target    PWTGEAYLEINPKDAAELGLADGDYAWVDADPEDRPYRGWNEDDPYYEVARAMMRVRIYTGMSRGVIRTWFNMYAATPAT 6lod.1    --TWDNVALMSVRTANALGLKNGDVVRLTYQ-----------------GRSVDAPVWVQPGHADDSVTVHFGF-------  target    VANQKATPGNPARNEQTRYVALFRYGSHQSGTRAWLRPTQQTDSLVRKGYFGQVIGTGFEADVHSVSGAPKEAFVKIEKA 6lod.1    --------------------------------------------------------------------------------  target    EDGGIGAERLWRPLTLGLRPEAPSAALTAYLAGDYSGTKGS 6lod.1    ----------------------------------------- ``` | | | | | | | | | | | | | | | | | | | | | | | | | | | | | | | | | | | | | | | | | | | | | | | | | |
|  | 2vpz.1.A | THIOSULFATE REDUCTASE  *POLYSULFIDE REDUCTASE NATIVE STRUCTURE* | 0.06 | 0.00 | 20.75 | 0.19 | 84-153 | X-ray | 2.40 | monomer | 10 x SF4, 4 x MGD, 2 x MO | HHblits | 0.30 |
| ``` target    GLGVPESQLDVETRQYRNVVRTWAELQQTLHPLQERDPAFRFVFQTPKYRWGAHSTAVDADWISMLFGPFGDPYRRDPRM 2vpz.1    --------------------------------------------------------------------------------  target    PWTGEAYLEINPKDAAELGLADGDYAWVDADPEDRPYRGWNEDDPYYEVARAM--MRVRIYTGMSRGVIRTWFNMYAATP 2vpz.1    ---PENEVWIHKEEAKRLGLKEGDYVMLVNQ-----------------DGVKEGPVRVKPTARIRKDCVYIVHGF-----  target    ATVANQKATPGNPARNEQTRYVALFRYGSHQSGTRAWLRPTQQTDSLVRKGYFGQVIGTGFEADVHSVSGAPKEAFVKIE 2vpz.1    --------------------------------------------------------------------------------  target    KAEDGGIGAERLWRPLTLGLRPEAPSAALTAYLAGDYSGTKGS 2vpz.1    ------------------------------------------- ``` | | | | | | | | | | | | | | | | | | | | | | | | | | | | | | | | | | | | | | | | | | | | | | | | | |
|  | 2vpx.1.D | THIOSULFATE REDUCTASE  *POLYSULFIDE REDUCTASE WITH BOUND QUINONE (UQ1)* | 0.06 | 0.00 | 20.75 | 0.19 | 84-153 | X-ray | 3.10 | monomer | 10 x SF4, 4 x MGD, 2 x MO, 2 x UQ1 | HHblits | 0.30 |
| ``` target    GLGVPESQLDVETRQYRNVVRTWAELQQTLHPLQERDPAFRFVFQTPKYRWGAHSTAVDADWISMLFGPFGDPYRRDPRM 2vpx.1    --------------------------------------------------------------------------------  target    PWTGEAYLEINPKDAAELGLADGDYAWVDADPEDRPYRGWNEDDPYYEVARAM--MRVRIYTGMSRGVIRTWFNMYAATP 2vpx.1    ---PENEVWIHKEEAKRLGLKEGDYVMLVNQ-----------------DGVKEGPVRVKPTARIRKDCVYIVHGF-----  target    ATVANQKATPGNPARNEQTRYVALFRYGSHQSGTRAWLRPTQQTDSLVRKGYFGQVIGTGFEADVHSVSGAPKEAFVKIE 2vpx.1    --------------------------------------------------------------------------------  target    KAEDGGIGAERLWRPLTLGLRPEAPSAALTAYLAGDYSGTKGS 2vpx.1    ------------------------------------------- ``` | | | | | | | | | | | | | | | | | | | | | | | | | | | | | | | | | | | | | | | | | | | | | | | | | |
|  | 5cup.1.A | Phosphate propanoyltransferase  *Structure of Rhodopseudomonas palustris PduL - phosphate bound form* | 0.03 | 0.00 | 28.89 | 0.16 | 64-110 | X-ray | 2.10 | monomer | 4 x ZN | HHblits | 0.35 |
| ``` target    GLGVPESQLDVETRQYRNVVRTWAELQQTLHPLQERDPAFRFVFQTPKYRWGAHSTAVDADWISMLFGPFGDPYRRDPRM 5cup.1    ---------------------------------------------------------------LTIIGPQGSVTKD--HG  target    PWTGEAYLEINPKDAAELGLADGDYAWVDADPEDRPYRGWNEDDPYYEVARAMMRVRIYTGMSRGVIRTWFNMYAATPAT 5cup.1    VIVAQRHIHMHPSTAAKLGLRNGDEVDVEA--------------------------------------------------  target    VANQKATPGNPARNEQTRYVALFRYGSHQSGTRAWLRPTQQTDSLVRKGYFGQVIGTGFEADVHSVSGAPKEAFVKIEKA 5cup.1    --------------------------------------------------------------------------------  target    EDGGIGAERLWRPLTLGLRPEAPSAALTAYLAGDYSGTKGS 5cup.1    ----------------------------------------- ``` | | | | | | | | | | | | | | | | | | | | | | | | | | | | | | | | | | | | | | | | | | | | | | | | | |
|  | 5cuo.1.A | Phosphate propanoyltransferase  *Structure of Rhodopseudomonas palustris PduL - CoA bound form* | 0.04 | 0.00 | 28.89 | 0.16 | 64-110 | X-ray | 1.54 | monomer | 2 x COA, 4 x ZN | HHblits | 0.35 |
| ``` target    GLGVPESQLDVETRQYRNVVRTWAELQQTLHPLQERDPAFRFVFQTPKYRWGAHSTAVDADWISMLFGPFGDPYRRDPRM 5cuo.1    ---------------------------------------------------------------LTIIGPQGSVTKD--HG  target    PWTGEAYLEINPKDAAELGLADGDYAWVDADPEDRPYRGWNEDDPYYEVARAMMRVRIYTGMSRGVIRTWFNMYAATPAT 5cuo.1    VIVAQRHIHMHPSTAAKLGLRNGDEVDVEA--------------------------------------------------  target    VANQKATPGNPARNEQTRYVALFRYGSHQSGTRAWLRPTQQTDSLVRKGYFGQVIGTGFEADVHSVSGAPKEAFVKIEKA 5cuo.1    --------------------------------------------------------------------------------  target    EDGGIGAERLWRPLTLGLRPEAPSAALTAYLAGDYSGTKGS 5cuo.1    ----------------------------------------- ``` | | | | | | | | | | | | | | | | | | | | | | | | | | | | | | | | | | | | | | | | | | | | | | | | | |
|  | 2d9r.1.A | conserved hypothetical protein  *Structure of Conserved Protein of Unknown Function PG0164 from Porphyromonas gingivalis [W83]* | 0.04 |  | 14.00 | 0.18 | 49-111 | X-ray | 2.01 | monomer |  | HHblits | 0.26 |
| ``` target    GLGVPESQLDVETRQYRNVVRTWAELQQTLHPLQERDPAFRFVFQTPKYRWGAHSTAVDADWISMLFGPFGDPYRRDPRM 2d9r.1    ------------------------------------------------GRVRVNATFDGYPYTGYI-------------V  target    PWT-GEAYLEINPKDAAELGLADGDYAWVDADPEDRPYRGWNEDDPYYEVARAMMRVRIYTGMSRGVIRTWFNMYAATPA 2d9r.1    RMGLPCHILGLRQDIRRAIGKQPGDSVYVTLL------------------------------------------------  target    TVANQKATPGNPARNEQTRYVALFRYGSHQSGTRAWLRPTQQTDSLVRKGYFGQVIGTGFEADVHSVSGAPKEAFVKIEK 2d9r.1    --------------------------------------------------------------------------------  target    AEDGGIGAERLWRPLTLGLRPEAPSAALTAYLAGDYSGTKGS 2d9r.1    ------------------------------------------ ``` | | | | | | | | | | | | | | | | | | | | | | | | | | | | | | | | | | | | | | | | | | | | | | | | | |
|  | 6wkk.1.G | Gp26 capsid decoration protein  *Phage G gp27 major capsid proteins and gp26 decoration proteins* | 0.03 |  | 32.43 | 0.13 | 83-119 | EM | 0.00 | hetero-6-18-mer |  | HHblits | 0.35 |
| ``` target    GLGVPESQLDVETRQYRNVVRTWAELQQTLHPLQERDPAFRFVFQTPKYRWGAHSTAVDADWISMLFGPFGDPYRRDPRM 6wkk.1    --------------------------------------------------------------------------------  target    PWTGEAYLEINPKDAAELGLADGDYAWVDADPEDRPYRGWNEDDPYYEVARAMMRVRIYTGMSRGVIRTWFNMYAATPAT 6wkk.1    --GGEYYVNISRTSLTAAGIAAGDEITCDADGKMIKFTG-----------------------------------------  target    VANQKATPGNPARNEQTRYVALFRYGSHQSGTRAWLRPTQQTDSLVRKGYFGQVIGTGFEADVHSVSGAPKEAFVKIEKA 6wkk.1    --------------------------------------------------------------------------------  target    EDGGIGAERLWRPLTLGLRPEAPSAALTAYLAGDYSGTKGS 6wkk.1    ----------------------------------------- ``` | | | | | | | | | | | | | | | | | | | | | | | | | | | | | | | | | | | | | | | | | | | | | | | | | |
|  | 6wkk.1.H | Gp26 capsid decoration protein  *Phage G gp27 major capsid proteins and gp26 decoration proteins* | 0.01 |  | 32.43 | 0.13 | 83-119 | EM | 0.00 | hetero-6-18-mer |  | HHblits | 0.35 |
| ``` target    GLGVPESQLDVETRQYRNVVRTWAELQQTLHPLQERDPAFRFVFQTPKYRWGAHSTAVDADWISMLFGPFGDPYRRDPRM 6wkk.1    --------------------------------------------------------------------------------  target    PWTGEAYLEINPKDAAELGLADGDYAWVDADPEDRPYRGWNEDDPYYEVARAMMRVRIYTGMSRGVIRTWFNMYAATPAT 6wkk.1    --GGEYYVNISRTSLTAAGIAAGDEITCDADGKMIKFTG-----------------------------------------  target    VANQKATPGNPARNEQTRYVALFRYGSHQSGTRAWLRPTQQTDSLVRKGYFGQVIGTGFEADVHSVSGAPKEAFVKIEKA 6wkk.1    --------------------------------------------------------------------------------  target    EDGGIGAERLWRPLTLGLRPEAPSAALTAYLAGDYSGTKGS 6wkk.1    ----------------------------------------- ``` | | | | | | | | | | | | | | | | | | | | | | | | | | | | | | | | | | | | | | | | | | | | | | | | | |
|  | 6wkk.1.I | Gp26 capsid decoration protein  *Phage G gp27 major capsid proteins and gp26 decoration proteins* | 0.01 |  | 32.43 | 0.13 | 83-119 | EM | 0.00 | hetero-6-18-mer |  | HHblits | 0.35 |
| ``` target    GLGVPESQLDVETRQYRNVVRTWAELQQTLHPLQERDPAFRFVFQTPKYRWGAHSTAVDADWISMLFGPFGDPYRRDPRM 6wkk.1    --------------------------------------------------------------------------------  target    PWTGEAYLEINPKDAAELGLADGDYAWVDADPEDRPYRGWNEDDPYYEVARAMMRVRIYTGMSRGVIRTWFNMYAATPAT 6wkk.1    --GGEYYVNISRTSLTAAGIAAGDEITCDADGKMIKFTG-----------------------------------------  target    VANQKATPGNPARNEQTRYVALFRYGSHQSGTRAWLRPTQQTDSLVRKGYFGQVIGTGFEADVHSVSGAPKEAFVKIEKA 6wkk.1    --------------------------------------------------------------------------------  target    EDGGIGAERLWRPLTLGLRPEAPSAALTAYLAGDYSGTKGS 6wkk.1    ----------------------------------------- ``` | | | | | | | | | | | | | | | | | | | | | | | | | | | | | | | | | | | | | | | | | | | | | | | | | |
|  | 6wkk.1.J | Gp26 capsid decoration protein  *Phage G gp27 major capsid proteins and gp26 decoration proteins* | 0.01 |  | 32.43 | 0.13 | 83-119 | EM | 0.00 | hetero-6-18-mer |  | HHblits | 0.35 |
| ``` target    GLGVPESQLDVETRQYRNVVRTWAELQQTLHPLQERDPAFRFVFQTPKYRWGAHSTAVDADWISMLFGPFGDPYRRDPRM 6wkk.1    --------------------------------------------------------------------------------  target    PWTGEAYLEINPKDAAELGLADGDYAWVDADPEDRPYRGWNEDDPYYEVARAMMRVRIYTGMSRGVIRTWFNMYAATPAT 6wkk.1    --GGEYYVNISRTSLTAAGIAAGDEITCDADGKMIKFTG-----------------------------------------  target    VANQKATPGNPARNEQTRYVALFRYGSHQSGTRAWLRPTQQTDSLVRKGYFGQVIGTGFEADVHSVSGAPKEAFVKIEKA 6wkk.1    --------------------------------------------------------------------------------  target    EDGGIGAERLWRPLTLGLRPEAPSAALTAYLAGDYSGTKGS 6wkk.1    ----------------------------------------- ``` | | | | | | | | | | | | | | | | | | | | | | | | | | | | | | | | | | | | | | | | | | | | | | | | | |
|  | 6wkk.1.K | Gp26 capsid decoration protein  *Phage G gp27 major capsid proteins and gp26 decoration proteins* | 0.01 |  | 32.43 | 0.13 | 83-119 | EM | 0.00 | hetero-6-18-mer |  | HHblits | 0.35 |
| ``` target    GLGVPESQLDVETRQYRNVVRTWAELQQTLHPLQERDPAFRFVFQTPKYRWGAHSTAVDADWISMLFGPFGDPYRRDPRM 6wkk.1    --------------------------------------------------------------------------------  target    PWTGEAYLEINPKDAAELGLADGDYAWVDADPEDRPYRGWNEDDPYYEVARAMMRVRIYTGMSRGVIRTWFNMYAATPAT 6wkk.1    --GGEYYVNISRTSLTAAGIAAGDEITCDADGKMIKFTG-----------------------------------------  target    VANQKATPGNPARNEQTRYVALFRYGSHQSGTRAWLRPTQQTDSLVRKGYFGQVIGTGFEADVHSVSGAPKEAFVKIEKA 6wkk.1    --------------------------------------------------------------------------------  target    EDGGIGAERLWRPLTLGLRPEAPSAALTAYLAGDYSGTKGS 6wkk.1    ----------------------------------------- ``` | | | | | | | | | | | | | | | | | | | | | | | | | | | | | | | | | | | | | | | | | | | | | | | | | |
|  | 6wkk.1.L | Gp26 capsid decoration protein  *Phage G gp27 major capsid proteins and gp26 decoration proteins* | 0.02 |  | 32.43 | 0.13 | 83-119 | EM | 0.00 | hetero-6-18-mer |  | HHblits | 0.35 |
| ``` target    GLGVPESQLDVETRQYRNVVRTWAELQQTLHPLQERDPAFRFVFQTPKYRWGAHSTAVDADWISMLFGPFGDPYRRDPRM 6wkk.1    --------------------------------------------------------------------------------  target    PWTGEAYLEINPKDAAELGLADGDYAWVDADPEDRPYRGWNEDDPYYEVARAMMRVRIYTGMSRGVIRTWFNMYAATPAT 6wkk.1    --GGEYYVNISRTSLTAAGIAAGDEITCDADGKMIKFTG-----------------------------------------  target    VANQKATPGNPARNEQTRYVALFRYGSHQSGTRAWLRPTQQTDSLVRKGYFGQVIGTGFEADVHSVSGAPKEAFVKIEKA 6wkk.1    --------------------------------------------------------------------------------  target    EDGGIGAERLWRPLTLGLRPEAPSAALTAYLAGDYSGTKGS 6wkk.1    ----------------------------------------- ``` | | | | | | | | | | | | | | | | | | | | | | | | | | | | | | | | | | | | | | | | | | | | | | | | | |
|  | 6wkk.1.M | Gp26 capsid decoration protein  *Phage G gp27 major capsid proteins and gp26 decoration proteins* | 0.01 |  | 32.43 | 0.13 | 83-119 | EM | 0.00 | hetero-6-18-mer |  | HHblits | 0.35 |
| ``` target    GLGVPESQLDVETRQYRNVVRTWAELQQTLHPLQERDPAFRFVFQTPKYRWGAHSTAVDADWISMLFGPFGDPYRRDPRM 6wkk.1    --------------------------------------------------------------------------------  target    PWTGEAYLEINPKDAAELGLADGDYAWVDADPEDRPYRGWNEDDPYYEVARAMMRVRIYTGMSRGVIRTWFNMYAATPAT 6wkk.1    --GGEYYVNISRTSLTAAGIAAGDEITCDADGKMIKFTG-----------------------------------------  target    VANQKATPGNPARNEQTRYVALFRYGSHQSGTRAWLRPTQQTDSLVRKGYFGQVIGTGFEADVHSVSGAPKEAFVKIEKA 6wkk.1    --------------------------------------------------------------------------------  target    EDGGIGAERLWRPLTLGLRPEAPSAALTAYLAGDYSGTKGS 6wkk.1    ----------------------------------------- ``` | | | | | | | | | | | | | | | | | | | | | | | | | | | | | | | | | | | | | | | | | | | | | | | | | |
|  | 6wkk.1.N | Gp26 capsid decoration protein  *Phage G gp27 major capsid proteins and gp26 decoration proteins* | 0.01 |  | 32.43 | 0.13 | 83-119 | EM | 0.00 | hetero-6-18-mer |  | HHblits | 0.35 |
| ``` target    GLGVPESQLDVETRQYRNVVRTWAELQQTLHPLQERDPAFRFVFQTPKYRWGAHSTAVDADWISMLFGPFGDPYRRDPRM 6wkk.1    --------------------------------------------------------------------------------  target    PWTGEAYLEINPKDAAELGLADGDYAWVDADPEDRPYRGWNEDDPYYEVARAMMRVRIYTGMSRGVIRTWFNMYAATPAT 6wkk.1    --GGEYYVNISRTSLTAAGIAAGDEITCDADGKMIKFTG-----------------------------------------  target    VANQKATPGNPARNEQTRYVALFRYGSHQSGTRAWLRPTQQTDSLVRKGYFGQVIGTGFEADVHSVSGAPKEAFVKIEKA 6wkk.1    --------------------------------------------------------------------------------  target    EDGGIGAERLWRPLTLGLRPEAPSAALTAYLAGDYSGTKGS 6wkk.1    ----------------------------------------- ``` | | | | | | | | | | | | | | | | | | | | | | | | | | | | | | | | | | | | | | | | | | | | | | | | | |
|  | 6wkk.1.O | Gp26 capsid decoration protein  *Phage G gp27 major capsid proteins and gp26 decoration proteins* | 0.02 |  | 32.43 | 0.13 | 83-119 | EM | 0.00 | hetero-6-18-mer |  | HHblits | 0.35 |
| ``` target    GLGVPESQLDVETRQYRNVVRTWAELQQTLHPLQERDPAFRFVFQTPKYRWGAHSTAVDADWISMLFGPFGDPYRRDPRM 6wkk.1    --------------------------------------------------------------------------------  target    PWTGEAYLEINPKDAAELGLADGDYAWVDADPEDRPYRGWNEDDPYYEVARAMMRVRIYTGMSRGVIRTWFNMYAATPAT 6wkk.1    --GGEYYVNISRTSLTAAGIAAGDEITCDADGKMIKFTG-----------------------------------------  target    VANQKATPGNPARNEQTRYVALFRYGSHQSGTRAWLRPTQQTDSLVRKGYFGQVIGTGFEADVHSVSGAPKEAFVKIEKA 6wkk.1    --------------------------------------------------------------------------------  target    EDGGIGAERLWRPLTLGLRPEAPSAALTAYLAGDYSGTKGS 6wkk.1    ----------------------------------------- ``` | | | | | | | | | | | | | | | | | | | | | | | | | | | | | | | | | | | | | | | | | | | | | | | | | |
|  | 6wkk.1.P | Gp26 capsid decoration protein  *Phage G gp27 major capsid proteins and gp26 decoration proteins* | 0.03 |  | 32.43 | 0.13 | 83-119 | EM | 0.00 | hetero-6-18-mer |  | HHblits | 0.35 |
| ``` target    GLGVPESQLDVETRQYRNVVRTWAELQQTLHPLQERDPAFRFVFQTPKYRWGAHSTAVDADWISMLFGPFGDPYRRDPRM 6wkk.1    --------------------------------------------------------------------------------  target    PWTGEAYLEINPKDAAELGLADGDYAWVDADPEDRPYRGWNEDDPYYEVARAMMRVRIYTGMSRGVIRTWFNMYAATPAT 6wkk.1    --GGEYYVNISRTSLTAAGIAAGDEITCDADGKMIKFTG-----------------------------------------  target    VANQKATPGNPARNEQTRYVALFRYGSHQSGTRAWLRPTQQTDSLVRKGYFGQVIGTGFEADVHSVSGAPKEAFVKIEKA 6wkk.1    --------------------------------------------------------------------------------  target    EDGGIGAERLWRPLTLGLRPEAPSAALTAYLAGDYSGTKGS 6wkk.1    ----------------------------------------- ``` | | | | | | | | | | | | | | | | | | | | | | | | | | | | | | | | | | | | | | | | | | | | | | | | | |
|  | 6wkk.1.Q | Gp26 capsid decoration protein  *Phage G gp27 major capsid proteins and gp26 decoration proteins* | 0.00 |  | 32.43 | 0.13 | 83-119 | EM | 0.00 | hetero-6-18-mer |  | HHblits | 0.35 |
| ``` target    GLGVPESQLDVETRQYRNVVRTWAELQQTLHPLQERDPAFRFVFQTPKYRWGAHSTAVDADWISMLFGPFGDPYRRDPRM 6wkk.1    --------------------------------------------------------------------------------  target    PWTGEAYLEINPKDAAELGLADGDYAWVDADPEDRPYRGWNEDDPYYEVARAMMRVRIYTGMSRGVIRTWFNMYAATPAT 6wkk.1    --GGEYYVNISRTSLTAAGIAAGDEITCDADGKMIKFTG-----------------------------------------  target    VANQKATPGNPARNEQTRYVALFRYGSHQSGTRAWLRPTQQTDSLVRKGYFGQVIGTGFEADVHSVSGAPKEAFVKIEKA 6wkk.1    --------------------------------------------------------------------------------  target    EDGGIGAERLWRPLTLGLRPEAPSAALTAYLAGDYSGTKGS 6wkk.1    ----------------------------------------- ``` | | | | | | | | | | | | | | | | | | | | | | | | | | | | | | | | | | | | | | | | | | | | | | | | | |
|  | 6wkk.1.R | Gp26 capsid decoration protein  *Phage G gp27 major capsid proteins and gp26 decoration proteins* | 0.03 |  | 32.43 | 0.13 | 83-119 | EM | 0.00 | hetero-6-18-mer |  | HHblits | 0.35 |
| ``` target    GLGVPESQLDVETRQYRNVVRTWAELQQTLHPLQERDPAFRFVFQTPKYRWGAHSTAVDADWISMLFGPFGDPYRRDPRM 6wkk.1    --------------------------------------------------------------------------------  target    PWTGEAYLEINPKDAAELGLADGDYAWVDADPEDRPYRGWNEDDPYYEVARAMMRVRIYTGMSRGVIRTWFNMYAATPAT 6wkk.1    --GGEYYVNISRTSLTAAGIAAGDEITCDADGKMIKFTG-----------------------------------------  target    VANQKATPGNPARNEQTRYVALFRYGSHQSGTRAWLRPTQQTDSLVRKGYFGQVIGTGFEADVHSVSGAPKEAFVKIEKA 6wkk.1    --------------------------------------------------------------------------------  target    EDGGIGAERLWRPLTLGLRPEAPSAALTAYLAGDYSGTKGS 6wkk.1    ----------------------------------------- ``` | | | | | | | | | | | | | | | | | | | | | | | | | | | | | | | | | | | | | | | | | | | | | | | | | |
|  | 6wkk.1.S | Gp26 capsid decoration protein  *Phage G gp27 major capsid proteins and gp26 decoration proteins* | 0.04 |  | 32.43 | 0.13 | 83-119 | EM | 0.00 | hetero-6-18-mer |  | HHblits | 0.35 |
| ``` target    GLGVPESQLDVETRQYRNVVRTWAELQQTLHPLQERDPAFRFVFQTPKYRWGAHSTAVDADWISMLFGPFGDPYRRDPRM 6wkk.1    --------------------------------------------------------------------------------  target    PWTGEAYLEINPKDAAELGLADGDYAWVDADPEDRPYRGWNEDDPYYEVARAMMRVRIYTGMSRGVIRTWFNMYAATPAT 6wkk.1    --GGEYYVNISRTSLTAAGIAAGDEITCDADGKMIKFTG-----------------------------------------  target    VANQKATPGNPARNEQTRYVALFRYGSHQSGTRAWLRPTQQTDSLVRKGYFGQVIGTGFEADVHSVSGAPKEAFVKIEKA 6wkk.1    --------------------------------------------------------------------------------  target    EDGGIGAERLWRPLTLGLRPEAPSAALTAYLAGDYSGTKGS 6wkk.1    ----------------------------------------- ``` | | | | | | | | | | | | | | | | | | | | | | | | | | | | | | | | | | | | | | | | | | | | | | | | | |
|  | 6wkk.1.T | Gp26 capsid decoration protein  *Phage G gp27 major capsid proteins and gp26 decoration proteins* | 0.02 |  | 32.43 | 0.13 | 83-119 | EM | 0.00 | hetero-6-18-mer |  | HHblits | 0.35 |
| ``` target    GLGVPESQLDVETRQYRNVVRTWAELQQTLHPLQERDPAFRFVFQTPKYRWGAHSTAVDADWISMLFGPFGDPYRRDPRM 6wkk.1    --------------------------------------------------------------------------------  target    PWTGEAYLEINPKDAAELGLADGDYAWVDADPEDRPYRGWNEDDPYYEVARAMMRVRIYTGMSRGVIRTWFNMYAATPAT 6wkk.1    --GGEYYVNISRTSLTAAGIAAGDEITCDADGKMIKFTG-----------------------------------------  target    VANQKATPGNPARNEQTRYVALFRYGSHQSGTRAWLRPTQQTDSLVRKGYFGQVIGTGFEADVHSVSGAPKEAFVKIEKA 6wkk.1    --------------------------------------------------------------------------------  target    EDGGIGAERLWRPLTLGLRPEAPSAALTAYLAGDYSGTKGS 6wkk.1    ----------------------------------------- ``` | | | | | | | | | | | | | | | | | | | | | | | | | | | | | | | | | | | | | | | | | | | | | | | | | |
|  | 6wkk.1.U | Gp26 capsid decoration protein  *Phage G gp27 major capsid proteins and gp26 decoration proteins* | 0.02 |  | 32.43 | 0.13 | 83-119 | EM | 0.00 | hetero-6-18-mer |  | HHblits | 0.35 |
| ``` target    GLGVPESQLDVETRQYRNVVRTWAELQQTLHPLQERDPAFRFVFQTPKYRWGAHSTAVDADWISMLFGPFGDPYRRDPRM 6wkk.1    --------------------------------------------------------------------------------  target    PWTGEAYLEINPKDAAELGLADGDYAWVDADPEDRPYRGWNEDDPYYEVARAMMRVRIYTGMSRGVIRTWFNMYAATPAT 6wkk.1    --GGEYYVNISRTSLTAAGIAAGDEITCDADGKMIKFTG-----------------------------------------  target    VANQKATPGNPARNEQTRYVALFRYGSHQSGTRAWLRPTQQTDSLVRKGYFGQVIGTGFEADVHSVSGAPKEAFVKIEKA 6wkk.1    --------------------------------------------------------------------------------  target    EDGGIGAERLWRPLTLGLRPEAPSAALTAYLAGDYSGTKGS 6wkk.1    ----------------------------------------- ``` | | | | | | | | | | | | | | | | | | | | | | | | | | | | | | | | | | | | | | | | | | | | | | | | | |
|  | 6wkk.1.V | Gp26 capsid decoration protein  *Phage G gp27 major capsid proteins and gp26 decoration proteins* | 0.03 |  | 32.43 | 0.13 | 83-119 | EM | 0.00 | hetero-6-18-mer |  | HHblits | 0.35 |
| ``` target    GLGVPESQLDVETRQYRNVVRTWAELQQTLHPLQERDPAFRFVFQTPKYRWGAHSTAVDADWISMLFGPFGDPYRRDPRM 6wkk.1    --------------------------------------------------------------------------------  target    PWTGEAYLEINPKDAAELGLADGDYAWVDADPEDRPYRGWNEDDPYYEVARAMMRVRIYTGMSRGVIRTWFNMYAATPAT 6wkk.1    --GGEYYVNISRTSLTAAGIAAGDEITCDADGKMIKFTG-----------------------------------------  target    VANQKATPGNPARNEQTRYVALFRYGSHQSGTRAWLRPTQQTDSLVRKGYFGQVIGTGFEADVHSVSGAPKEAFVKIEKA 6wkk.1    --------------------------------------------------------------------------------  target    EDGGIGAERLWRPLTLGLRPEAPSAALTAYLAGDYSGTKGS 6wkk.1    ----------------------------------------- ``` | | | | | | | | | | | | | | | | | | | | | | | | | | | | | | | | | | | | | | | | | | | | | | | | | |
|  | 6wkk.1.W | Gp26 capsid decoration protein  *Phage G gp27 major capsid proteins and gp26 decoration proteins* | 0.01 |  | 32.43 | 0.13 | 83-119 | EM | 0.00 | hetero-6-18-mer |  | HHblits | 0.35 |
| ``` target    GLGVPESQLDVETRQYRNVVRTWAELQQTLHPLQERDPAFRFVFQTPKYRWGAHSTAVDADWISMLFGPFGDPYRRDPRM 6wkk.1    --------------------------------------------------------------------------------  target    PWTGEAYLEINPKDAAELGLADGDYAWVDADPEDRPYRGWNEDDPYYEVARAMMRVRIYTGMSRGVIRTWFNMYAATPAT 6wkk.1    --GGEYYVNISRTSLTAAGIAAGDEITCDADGKMIKFTG-----------------------------------------  target    VANQKATPGNPARNEQTRYVALFRYGSHQSGTRAWLRPTQQTDSLVRKGYFGQVIGTGFEADVHSVSGAPKEAFVKIEKA 6wkk.1    --------------------------------------------------------------------------------  target    EDGGIGAERLWRPLTLGLRPEAPSAALTAYLAGDYSGTKGS 6wkk.1    ----------------------------------------- ``` | | | | | | | | | | | | | | | | | | | | | | | | | | | | | | | | | | | | | | | | | | | | | | | | | |
|  | 6wkk.1.X | Gp26 capsid decoration protein  *Phage G gp27 major capsid proteins and gp26 decoration proteins* | 0.01 |  | 32.43 | 0.13 | 83-119 | EM | 0.00 | hetero-6-18-mer |  | HHblits | 0.35 |
| ``` target    GLGVPESQLDVETRQYRNVVRTWAELQQTLHPLQERDPAFRFVFQTPKYRWGAHSTAVDADWISMLFGPFGDPYRRDPRM 6wkk.1    --------------------------------------------------------------------------------  target    PWTGEAYLEINPKDAAELGLADGDYAWVDADPEDRPYRGWNEDDPYYEVARAMMRVRIYTGMSRGVIRTWFNMYAATPAT 6wkk.1    --GGEYYVNISRTSLTAAGIAAGDEITCDADGKMIKFTG-----------------------------------------  target    VANQKATPGNPARNEQTRYVALFRYGSHQSGTRAWLRPTQQTDSLVRKGYFGQVIGTGFEADVHSVSGAPKEAFVKIEKA 6wkk.1    --------------------------------------------------------------------------------  target    EDGGIGAERLWRPLTLGLRPEAPSAALTAYLAGDYSGTKGS 6wkk.1    ----------------------------------------- ``` | | | | | | | | | | | | | | | | | | | | | | | | | | | | | | | | | | | | | | | | | | | | | | | | | |
|  | 3pjy.1.A | Hypothetical signal peptide protein  *Crystal structure of a putative transcription regulator (R01717) from Sinorhizobium meliloti 1021 at 1.55 A resolution* | 0.01 |  | 25.00 | 0.11 | 82-113 | X-ray | 1.55 | homo-dimer |  | HHblits | 0.30 |
| ``` target    GLGVPESQLDVETRQYRNVVRTWAELQQTLHPLQERDPAFRFVFQTPKYRWGAHSTAVDADWISMLFGPFGDPYRRDPRM 3pjy.1    --------------------------------------------------------------------------------  target    PWTGEAYLEINPKDAAELGLADGDYAWVDADPEDRPYRGWNEDDPYYEVARAMMRVRIYTGMSRGVIRTWFNMYAATPAT 3pjy.1    -EPVAYVLELNAGTVKRLGVSPGDRLEGAGLPA-----------------------------------------------  target    VANQKATPGNPARNEQTRYVALFRYGSHQSGTRAWLRPTQQTDSLVRKGYFGQVIGTGFEADVHSVSGAPKEAFVKIEKA 3pjy.1    --------------------------------------------------------------------------------  target    EDGGIGAERLWRPLTLGLRPEAPSAALTAYLAGDYSGTKGS 3pjy.1    ----------------------------------------- ``` | | | | | | | | | | | | | | | | | | | | | | | | | | | | | | | | | | | | | | | | | | | | | | | | | |
|  | 3pjy.1.B | Hypothetical signal peptide protein  *Crystal structure of a putative transcription regulator (R01717) from Sinorhizobium meliloti 1021 at 1.55 A resolution* | 0.01 |  | 25.00 | 0.11 | 82-113 | X-ray | 1.55 | homo-dimer |  | HHblits | 0.30 |
| ``` target    GLGVPESQLDVETRQYRNVVRTWAELQQTLHPLQERDPAFRFVFQTPKYRWGAHSTAVDADWISMLFGPFGDPYRRDPRM 3pjy.1    --------------------------------------------------------------------------------  target    PWTGEAYLEINPKDAAELGLADGDYAWVDADPEDRPYRGWNEDDPYYEVARAMMRVRIYTGMSRGVIRTWFNMYAATPAT 3pjy.1    -EPVAYVLELNAGTVKRLGVSPGDRLEGAGLPA-----------------------------------------------  target    VANQKATPGNPARNEQTRYVALFRYGSHQSGTRAWLRPTQQTDSLVRKGYFGQVIGTGFEADVHSVSGAPKEAFVKIEKA 3pjy.1    --------------------------------------------------------------------------------  target    EDGGIGAERLWRPLTLGLRPEAPSAALTAYLAGDYSGTKGS 3pjy.1    ----------------------------------------- ``` | | | | | | | | | | | | | | | | | | | | | | | | | | | | | | | | | | | | | | | | | | | | | | | | | |
|  | 7du6.1.A | mkDPBB\_sym2 protein  *Crystal structure of the rationally designed mkDPBB\_sym2 protein* | 0.02 |  | 26.67 | 0.11 | 82-111 | X-ray | 1.60 | monomer |  | HHblits | 0.34 |
| ``` target    GLGVPESQLDVETRQYRNVVRTWAELQQTLHPLQERDPAFRFVFQTPKYRWGAHSTAVDADWISMLFGPFGDPYRRDPRM 7du6.1    --------------------------------------------------------------------------------  target    PWTGEAYLEINPKDAAELGLADGDYAWVDADPEDRPYRGWNEDDPYYEVARAMMRVRIYTGMSRGVIRTWFNMYAATPAT 7du6.1    -DVGKRIVRMDKYERAKLGVSVGDYVEVKKV-------------------------------------------------  target    VANQKATPGNPARNEQTRYVALFRYGSHQSGTRAWLRPTQQTDSLVRKGYFGQVIGTGFEADVHSVSGAPKEAFVKIEKA 7du6.1    --------------------------------------------------------------------------------  target    EDGGIGAERLWRPLTLGLRPEAPSAALTAYLAGDYSGTKGS 7du6.1    ----------------------------------------- ``` | | | | | | | | | | | | | | | | | | | | | | | | | | | | | | | | | | | | | | | | | | | | | | | | | |
|  | 7di0.1.A | apDPBB\_sym\_79 protein  *Crystal structure of the rationally designed apDPBB\_sym\_79 protein* | 0.01 |  | 27.59 | 0.10 | 83-111 | X-ray | 1.60 | monomer |  | HHblits | 0.34 |
| ``` target    GLGVPESQLDVETRQYRNVVRTWAELQQTLHPLQERDPAFRFVFQTPKYRWGAHSTAVDADWISMLFGPFGDPYRRDPRM 7di0.1    --------------------------------------------------------------------------------  target    PWTGEAYLEINPKDAAELGLADGDYAWVDADPEDRPYRGWNEDDPYYEVARAMMRVRIYTGMSRGVIRTWFNMYAATPAT 7di0.1    --VGRKIVRMDKQTRARLGVSVGDYVEVKKV-------------------------------------------------  target    VANQKATPGNPARNEQTRYVALFRYGSHQSGTRAWLRPTQQTDSLVRKGYFGQVIGTGFEADVHSVSGAPKEAFVKIEKA 7di0.1    --------------------------------------------------------------------------------  target    EDGGIGAERLWRPLTLGLRPEAPSAALTAYLAGDYSGTKGS 7di0.1    ----------------------------------------- ``` | | | | | | | | | | | | | | | | | | | | | | | | | | | | | | | | | | | | | | | | | | | | | | | | | |
|  | 7di0.2.A | apDPBB\_sym\_79 protein  *Crystal structure of the rationally designed apDPBB\_sym\_79 protein* | 0.01 |  | 27.59 | 0.10 | 83-111 | X-ray | 1.60 | monomer |  | HHblits | 0.34 |
| ``` target    GLGVPESQLDVETRQYRNVVRTWAELQQTLHPLQERDPAFRFVFQTPKYRWGAHSTAVDADWISMLFGPFGDPYRRDPRM 7di0.2    --------------------------------------------------------------------------------  target    PWTGEAYLEINPKDAAELGLADGDYAWVDADPEDRPYRGWNEDDPYYEVARAMMRVRIYTGMSRGVIRTWFNMYAATPAT 7di0.2    --VGRKIVRMDKQTRARLGVSVGDYVEVKKV-------------------------------------------------  target    VANQKATPGNPARNEQTRYVALFRYGSHQSGTRAWLRPTQQTDSLVRKGYFGQVIGTGFEADVHSVSGAPKEAFVKIEKA 7di0.2    --------------------------------------------------------------------------------  target    EDGGIGAERLWRPLTLGLRPEAPSAALTAYLAGDYSGTKGS 7di0.2    ----------------------------------------- ``` | | | | | | | | | | | | | | | | | | | | | | | | | | | | | | | | | | | | | | | | | | | | | | | | | |
|  | 7di0.3.A | apDPBB\_sym\_79 protein  *Crystal structure of the rationally designed apDPBB\_sym\_79 protein* | 0.01 |  | 27.59 | 0.10 | 83-111 | X-ray | 1.60 | monomer |  | HHblits | 0.34 |
| ``` target    GLGVPESQLDVETRQYRNVVRTWAELQQTLHPLQERDPAFRFVFQTPKYRWGAHSTAVDADWISMLFGPFGDPYRRDPRM 7di0.3    --------------------------------------------------------------------------------  target    PWTGEAYLEINPKDAAELGLADGDYAWVDADPEDRPYRGWNEDDPYYEVARAMMRVRIYTGMSRGVIRTWFNMYAATPAT 7di0.3    --VGRKIVRMDKQTRARLGVSVGDYVEVKKV-------------------------------------------------  target    VANQKATPGNPARNEQTRYVALFRYGSHQSGTRAWLRPTQQTDSLVRKGYFGQVIGTGFEADVHSVSGAPKEAFVKIEKA 7di0.3    --------------------------------------------------------------------------------  target    EDGGIGAERLWRPLTLGLRPEAPSAALTAYLAGDYSGTKGS 7di0.3    ----------------------------------------- ``` | | | | | | | | | | | | | | | | | | | | | | | | | | | | | | | | | | | | | | | | | | | | | | | | | |
|  | 1wlf.1.A | Peroxisome biogenesis factor 1  *Structure of the N-terminal domain of PEX1 AAA-ATPase: Characterization of a putative adaptor-binding domain* | 0.00 |  | 31.03 | 0.10 | 83-111 | X-ray | 2.05 | monomer |  | HHblits | 0.34 |
| ``` target    GLGVPESQLDVETRQYRNVVRTWAELQQTLHPLQERDPAFRFVFQTPKYRWGAHSTAVDADWISMLFGPFGDPYRRDPRM 1wlf.1    --------------------------------------------------------------------------------  target    PWTGEAYLEINPKDAAELGLADGDYAWVDADPEDRPYRGWNEDDPYYEVARAMMRVRIYTGMSRGVIRTWFNMYAATPAT 1wlf.1    --QSENVAEINRQVGQKLGLSSGDQVFLRPC-------------------------------------------------  target    VANQKATPGNPARNEQTRYVALFRYGSHQSGTRAWLRPTQQTDSLVRKGYFGQVIGTGFEADVHSVSGAPKEAFVKIEKA 1wlf.1    --------------------------------------------------------------------------------  target    EDGGIGAERLWRPLTLGLRPEAPSAALTAYLAGDYSGTKGS 1wlf.1    ----------------------------------------- ``` | | | | | | | | | | | | | | | | | | | | | | | | | | | | | | | | | | | | | | | | | | | | | | | | | |
|  | 7dvf.1.A | reDPBB\_sym2 protein  *Crystal structure of the computationally designed reDPBB\_sym2 protein* | 0.02 |  | 16.67 | 0.11 | 82-111 | X-ray | 1.21 | monomer |  | HHblits | 0.32 |
| ``` target    GLGVPESQLDVETRQYRNVVRTWAELQQTLHPLQERDPAFRFVFQTPKYRWGAHSTAVDADWISMLFGPFGDPYRRDPRM 7dvf.1    --------------------------------------------------------------------------------  target    PWTGEAYLEINPKDAAELGLADGDYAWVDADPEDRPYRGWNEDDPYYEVARAMMRVRIYTGMSRGVIRTWFNMYAATPAT 7dvf.1    -DVGKGIVRMDKASREKLGVSAGDLVEIKGS-------------------------------------------------  target    VANQKATPGNPARNEQTRYVALFRYGSHQSGTRAWLRPTQQTDSLVRKGYFGQVIGTGFEADVHSVSGAPKEAFVKIEKA 7dvf.1    --------------------------------------------------------------------------------  target    EDGGIGAERLWRPLTLGLRPEAPSAALTAYLAGDYSGTKGS 7dvf.1    ----------------------------------------- ``` | | | | | | | | | | | | | | | | | | | | | | | | | | | | | | | | | | | | | | | | | | | | | | | | | |
|  | 7dvc.1.A | reDPBB\_sym1 protein  *Crystal structure of the computationally designed reDPBB\_sym1 protein* | 0.02 |  | 16.67 | 0.11 | 82-111 | X-ray | 1.71 | monomer |  | HHblits | 0.31 |
| ``` target    GLGVPESQLDVETRQYRNVVRTWAELQQTLHPLQERDPAFRFVFQTPKYRWGAHSTAVDADWISMLFGPFGDPYRRDPRM 7dvc.1    --------------------------------------------------------------------------------  target    PWTGEAYLEINPKDAAELGLADGDYAWVDADPEDRPYRGWNEDDPYYEVARAMMRVRIYTGMSRGVIRTWFNMYAATPAT 7dvc.1    -DVGKGIVRMDKASRDKLGVSAGDLVEIKGS-------------------------------------------------  target    VANQKATPGNPARNEQTRYVALFRYGSHQSGTRAWLRPTQQTDSLVRKGYFGQVIGTGFEADVHSVSGAPKEAFVKIEKA 7dvc.1    --------------------------------------------------------------------------------  target    EDGGIGAERLWRPLTLGLRPEAPSAALTAYLAGDYSGTKGS 7dvc.1    ----------------------------------------- ``` | | | | | | | | | | | | | | | | | | | | | | | | | | | | | | | | | | | | | | | | | | | | | | | | | |
|  | 7dvc.5.A | reDPBB\_sym1 protein  *Crystal structure of the computationally designed reDPBB\_sym1 protein* | 0.02 |  | 16.67 | 0.11 | 82-111 | X-ray | 1.71 | monomer |  | HHblits | 0.31 |
| ``` target    GLGVPESQLDVETRQYRNVVRTWAELQQTLHPLQERDPAFRFVFQTPKYRWGAHSTAVDADWISMLFGPFGDPYRRDPRM 7dvc.5    --------------------------------------------------------------------------------  target    PWTGEAYLEINPKDAAELGLADGDYAWVDADPEDRPYRGWNEDDPYYEVARAMMRVRIYTGMSRGVIRTWFNMYAATPAT 7dvc.5    -DVGKGIVRMDKASRDKLGVSAGDLVEIKGS-------------------------------------------------  target    VANQKATPGNPARNEQTRYVALFRYGSHQSGTRAWLRPTQQTDSLVRKGYFGQVIGTGFEADVHSVSGAPKEAFVKIEKA 7dvc.5    --------------------------------------------------------------------------------  target    EDGGIGAERLWRPLTLGLRPEAPSAALTAYLAGDYSGTKGS 7dvc.5    ----------------------------------------- ``` | | | | | | | | | | | | | | | | | | | | | | | | | | | | | | | | | | | | | | | | | | | | | | | | | |
|  | 7dg9.1.A | Cell division control protein 48, AAA family  *DPBB domain of VCP-like ATPase from Aeropyrum pernix* | 0.00 |  | 32.14 | 0.10 | 83-110 | X-ray | 1.60 | monomer | 4 x ZN | HHblits | 0.36 |
| ``` target    GLGVPESQLDVETRQYRNVVRTWAELQQTLHPLQERDPAFRFVFQTPKYRWGAHSTAVDADWISMLFGPFGDPYRRDPRM 7dg9.1    --------------------------------------------------------------------------------  target    PWTGEAYLEINPKDAAELGLADGDYAWVDADPEDRPYRGWNEDDPYYEVARAMMRVRIYTGMSRGVIRTWFNMYAATPAT 7dg9.1    --VGRKIVRIDRQTAARLGVEVGDFVKVSK--------------------------------------------------  target    VANQKATPGNPARNEQTRYVALFRYGSHQSGTRAWLRPTQQTDSLVRKGYFGQVIGTGFEADVHSVSGAPKEAFVKIEKA 7dg9.1    --------------------------------------------------------------------------------  target    EDGGIGAERLWRPLTLGLRPEAPSAALTAYLAGDYSGTKGS 7dg9.1    ----------------------------------------- ``` | | | | | | | | | | | | | | | | | | | | | | | | | | | | | | | | | | | | | | | | | | | | | | | | | |
|  | 7dxv.1.A | mk2h\_dY protein  *Crystal structure of the mk2h\_deltaY peptide homodimer* | 0.00 |  | 24.14 | 0.10 | 81-109 | X-ray | 2.30 | homo-dimer |  | HHblits | 0.33 |
| ``` target    GLGVPESQLDVETRQYRNVVRTWAELQQTLHPLQERDPAFRFVFQTPKYRWGAHSTAVDADWISMLFGPFGDPYRRDPRM 7dxv.1    --------------------------------------------------------------------------------  target    PWTGEAYLEINPKDAAELGLADGDYAWVDADPEDRPYRGWNEDDPYYEVARAMMRVRIYTGMSRGVIRTWFNMYAATPAT 7dxv.1    EDVGKRIVRMDKAERAKLGVSVGDVVEVK---------------------------------------------------  target    VANQKATPGNPARNEQTRYVALFRYGSHQSGTRAWLRPTQQTDSLVRKGYFGQVIGTGFEADVHSVSGAPKEAFVKIEKA 7dxv.1    --------------------------------------------------------------------------------  target    EDGGIGAERLWRPLTLGLRPEAPSAALTAYLAGDYSGTKGS 7dxv.1    ----------------------------------------- ``` | | | | | | | | | | | | | | | | | | | | | | | | | | | | | | | | | | | | | | | | | | | | | | | | | |
|  | 7dxv.1.B | mk2h\_dY protein  *Crystal structure of the mk2h\_deltaY peptide homodimer* | 0.00 |  | 24.14 | 0.10 | 81-109 | X-ray | 2.30 | homo-dimer |  | HHblits | 0.33 |
| ``` target    GLGVPESQLDVETRQYRNVVRTWAELQQTLHPLQERDPAFRFVFQTPKYRWGAHSTAVDADWISMLFGPFGDPYRRDPRM 7dxv.1    --------------------------------------------------------------------------------  target    PWTGEAYLEINPKDAAELGLADGDYAWVDADPEDRPYRGWNEDDPYYEVARAMMRVRIYTGMSRGVIRTWFNMYAATPAT 7dxv.1    EDVGKRIVRMDKAERAKLGVSVGDVVEVK---------------------------------------------------  target    VANQKATPGNPARNEQTRYVALFRYGSHQSGTRAWLRPTQQTDSLVRKGYFGQVIGTGFEADVHSVSGAPKEAFVKIEKA 7dxv.1    --------------------------------------------------------------------------------  target    EDGGIGAERLWRPLTLGLRPEAPSAALTAYLAGDYSGTKGS 7dxv.1    ----------------------------------------- ``` | | | | | | | | | | | | | | | | | | | | | | | | | | | | | | | | | | | | | | | | | | | | | | | | | |
|  | 7arh.1.A | Lipoprotein-releasing ABC transporter permease subunit LolC  *LolCDE in complex with lipoprotein* | 0.02 |  | 23.33 | 0.11 | 82-111 | EM | 0.00 | hetero-1-1-2-1-mer | 1 x Z41, 1 x PLM | HHblits | 0.30 |
| ``` target    GLGVPESQLDVETRQYRNVVRTWAELQQTLHPLQERDPAFRFVFQTPKYRWGAHSTAVDADWISMLFGPFGDPYRRDPRM 7arh.1    --------------------------------------------------------------------------------  target    PWTGEAYLEINPKDAAELGLADGDYAWVDADPEDRPYRGWNEDDPYYEVARAMMRVRIYTGMSRGVIRTWFNMYAATPAT 7arh.1    -EPGKYNVILGEQLASQLGVNRGDQIRVMVP-------------------------------------------------  target    VANQKATPGNPARNEQTRYVALFRYGSHQSGTRAWLRPTQQTDSLVRKGYFGQVIGTGFEADVHSVSGAPKEAFVKIEKA 7arh.1    --------------------------------------------------------------------------------  target    EDGGIGAERLWRPLTLGLRPEAPSAALTAYLAGDYSGTKGS 7arh.1    ----------------------------------------- ``` | | | | | | | | | | | | | | | | | | | | | | | | | | | | | | | | | | | | | | | | | | | | | | | | | |
|  | 7ari.1.A | Lipoprotein-releasing ABC transporter permease subunit LolC  *LolCDE apo structure* | 0.02 |  | 23.33 | 0.11 | 82-111 | EM | 0.00 | hetero-1-1-2-mer |  | HHblits | 0.30 |
| ``` target    GLGVPESQLDVETRQYRNVVRTWAELQQTLHPLQERDPAFRFVFQTPKYRWGAHSTAVDADWISMLFGPFGDPYRRDPRM 7ari.1    --------------------------------------------------------------------------------  target    PWTGEAYLEINPKDAAELGLADGDYAWVDADPEDRPYRGWNEDDPYYEVARAMMRVRIYTGMSRGVIRTWFNMYAATPAT 7ari.1    -EPGKYNVILGEQLASQLGVNRGDQIRVMVP-------------------------------------------------  target    VANQKATPGNPARNEQTRYVALFRYGSHQSGTRAWLRPTQQTDSLVRKGYFGQVIGTGFEADVHSVSGAPKEAFVKIEKA 7ari.1    --------------------------------------------------------------------------------  target    EDGGIGAERLWRPLTLGLRPEAPSAALTAYLAGDYSGTKGS 7ari.1    ----------------------------------------- ``` | | | | | | | | | | | | | | | | | | | | | | | | | | | | | | | | | | | | | | | | | | | | | | | | | |
|  | 7arj.1.A | Lipoprotein-releasing ABC transporter permease subunit LolC  *LolCDE in complex with lipoprotein and AMPPNP complex undimerized form* | 0.02 |  | 23.33 | 0.11 | 82-111 | EM | 0.00 | hetero-1-1-2-1-mer | 2 x ANP, 2 x MG, 1 x Z41, 1 x PLM | HHblits | 0.30 |
| ``` target    GLGVPESQLDVETRQYRNVVRTWAELQQTLHPLQERDPAFRFVFQTPKYRWGAHSTAVDADWISMLFGPFGDPYRRDPRM 7arj.1    --------------------------------------------------------------------------------  target    PWTGEAYLEINPKDAAELGLADGDYAWVDADPEDRPYRGWNEDDPYYEVARAMMRVRIYTGMSRGVIRTWFNMYAATPAT 7arj.1    -EPGKYNVILGEQLASQLGVNRGDQIRVMVP-------------------------------------------------  target    VANQKATPGNPARNEQTRYVALFRYGSHQSGTRAWLRPTQQTDSLVRKGYFGQVIGTGFEADVHSVSGAPKEAFVKIEKA 7arj.1    --------------------------------------------------------------------------------  target    EDGGIGAERLWRPLTLGLRPEAPSAALTAYLAGDYSGTKGS 7arj.1    ----------------------------------------- ``` | | | | | | | | | | | | | | | | | | | | | | | | | | | | | | | | | | | | | | | | | | | | | | | | | |
|  | 7ark.1.A | Lipoprotein-releasing ABC transporter permease subunit LolC  *LolCDE in complex with AMP-PNP in the closed NBD state* | 0.01 |  | 23.33 | 0.11 | 82-111 | EM | 0.00 | hetero-1-1-2-mer | 2 x ANP, 2 x MG | HHblits | 0.30 |
| ``` target    GLGVPESQLDVETRQYRNVVRTWAELQQTLHPLQERDPAFRFVFQTPKYRWGAHSTAVDADWISMLFGPFGDPYRRDPRM 7ark.1    --------------------------------------------------------------------------------  target    PWTGEAYLEINPKDAAELGLADGDYAWVDADPEDRPYRGWNEDDPYYEVARAMMRVRIYTGMSRGVIRTWFNMYAATPAT 7ark.1    -EPGKYNVILGEQLASQLGVNRGDQIRVMVP-------------------------------------------------  target    VANQKATPGNPARNEQTRYVALFRYGSHQSGTRAWLRPTQQTDSLVRKGYFGQVIGTGFEADVHSVSGAPKEAFVKIEKA 7ark.1    --------------------------------------------------------------------------------  target    EDGGIGAERLWRPLTLGLRPEAPSAALTAYLAGDYSGTKGS 7ark.1    ----------------------------------------- ``` | | | | | | | | | | | | | | | | | | | | | | | | | | | | | | | | | | | | | | | | | | | | | | | | | |
|  | 7arl.1.A | Lipoprotein-releasing ABC transporter permease subunit LolC  *LolCDE in complex with lipoprotein and ADP* | 0.02 |  | 23.33 | 0.11 | 82-111 | EM | 0.00 | hetero-1-1-2-1-mer | 1 x Z41, 2 x ADP, 2 x MG, 1 x PLM | HHblits | 0.30 |
| ``` target    GLGVPESQLDVETRQYRNVVRTWAELQQTLHPLQERDPAFRFVFQTPKYRWGAHSTAVDADWISMLFGPFGDPYRRDPRM 7arl.1    --------------------------------------------------------------------------------  target    PWTGEAYLEINPKDAAELGLADGDYAWVDADPEDRPYRGWNEDDPYYEVARAMMRVRIYTGMSRGVIRTWFNMYAATPAT 7arl.1    -EPGKYNVILGEQLASQLGVNRGDQIRVMVP-------------------------------------------------  target    VANQKATPGNPARNEQTRYVALFRYGSHQSGTRAWLRPTQQTDSLVRKGYFGQVIGTGFEADVHSVSGAPKEAFVKIEKA 7arl.1    --------------------------------------------------------------------------------  target    EDGGIGAERLWRPLTLGLRPEAPSAALTAYLAGDYSGTKGS 7arl.1    ----------------------------------------- ``` | | | | | | | | | | | | | | | | | | | | | | | | | | | | | | | | | | | | | | | | | | | | | | | | | |
|  | 7arm.1.A | Lipoprotein-releasing ABC transporter permease subunit LolC  *LolCDE in complex with lipoprotein and LolA* | 0.02 |  | 23.33 | 0.11 | 82-111 | EM | 0.00 | hetero-1-1-2-1-1-mer | 1 x Z41, 1 x PLM | HHblits | 0.30 |
| ``` target    GLGVPESQLDVETRQYRNVVRTWAELQQTLHPLQERDPAFRFVFQTPKYRWGAHSTAVDADWISMLFGPFGDPYRRDPRM 7arm.1    --------------------------------------------------------------------------------  target    PWTGEAYLEINPKDAAELGLADGDYAWVDADPEDRPYRGWNEDDPYYEVARAMMRVRIYTGMSRGVIRTWFNMYAATPAT 7arm.1    -EPGKYNVILGEQLASQLGVNRGDQIRVMVP-------------------------------------------------  target    VANQKATPGNPARNEQTRYVALFRYGSHQSGTRAWLRPTQQTDSLVRKGYFGQVIGTGFEADVHSVSGAPKEAFVKIEKA 7arm.1    --------------------------------------------------------------------------------  target    EDGGIGAERLWRPLTLGLRPEAPSAALTAYLAGDYSGTKGS 7arm.1    ----------------------------------------- ``` | | | | | | | | | | | | | | | | | | | | | | | | | | | | | | | | | | | | | | | | | | | | | | | | | |
|  | 7mdy.1.B | Lipo-releasing system transmembrane protein lolC  *LolCDE nucleotide-bound* | 0.01 |  | 23.33 | 0.11 | 82-111 | EM | 0.00 | hetero-1-1-2-mer | 2 x MG | HHblits | 0.30 |
| ``` target    GLGVPESQLDVETRQYRNVVRTWAELQQTLHPLQERDPAFRFVFQTPKYRWGAHSTAVDADWISMLFGPFGDPYRRDPRM 7mdy.1    --------------------------------------------------------------------------------  target    PWTGEAYLEINPKDAAELGLADGDYAWVDADPEDRPYRGWNEDDPYYEVARAMMRVRIYTGMSRGVIRTWFNMYAATPAT 7mdy.1    -EPGKYNVILGEQLASQLGVNRGDQIRVMVP-------------------------------------------------  target    VANQKATPGNPARNEQTRYVALFRYGSHQSGTRAWLRPTQQTDSLVRKGYFGQVIGTGFEADVHSVSGAPKEAFVKIEKA 7mdy.1    --------------------------------------------------------------------------------  target    EDGGIGAERLWRPLTLGLRPEAPSAALTAYLAGDYSGTKGS 7mdy.1    ----------------------------------------- ``` | | | | | | | | | | | | | | | | | | | | | | | | | | | | | | | | | | | | | | | | | | | | | | | | | |
|  | 7v8i.1.A | Lipoprotein-releasing system transmembrane protein LolC  *LolCD(E171Q)E with bound AMPPNP in nanodiscs* | 0.01 |  | 23.33 | 0.11 | 82-111 | EM | 0.00 | hetero-1-2-1-mer | 2 x MG, 2 x ANP | HHblits | 0.30 |
| ``` target    GLGVPESQLDVETRQYRNVVRTWAELQQTLHPLQERDPAFRFVFQTPKYRWGAHSTAVDADWISMLFGPFGDPYRRDPRM 7v8i.1    --------------------------------------------------------------------------------  target    PWTGEAYLEINPKDAAELGLADGDYAWVDADPEDRPYRGWNEDDPYYEVARAMMRVRIYTGMSRGVIRTWFNMYAATPAT 7v8i.1    -EPGKYNVILGEQLASQLGVNRGDQIRVMVP-------------------------------------------------  target    VANQKATPGNPARNEQTRYVALFRYGSHQSGTRAWLRPTQQTDSLVRKGYFGQVIGTGFEADVHSVSGAPKEAFVKIEKA 7v8i.1    --------------------------------------------------------------------------------  target    EDGGIGAERLWRPLTLGLRPEAPSAALTAYLAGDYSGTKGS 7v8i.1    ----------------------------------------- ``` | | | | | | | | | | | | | | | | | | | | | | | | | | | | | | | | | | | | | | | | | | | | | | | | | |
|  | 7v8m.1.A | Lipoprotein-releasing system transmembrane protein LolC  *LolCDE-apo in nanodiscs* | 0.02 |  | 23.33 | 0.11 | 82-111 | EM | 0.00 | hetero-1-2-1-mer |  | HHblits | 0.30 |
| ``` target    GLGVPESQLDVETRQYRNVVRTWAELQQTLHPLQERDPAFRFVFQTPKYRWGAHSTAVDADWISMLFGPFGDPYRRDPRM 7v8m.1    --------------------------------------------------------------------------------  target    PWTGEAYLEINPKDAAELGLADGDYAWVDADPEDRPYRGWNEDDPYYEVARAMMRVRIYTGMSRGVIRTWFNMYAATPAT 7v8m.1    -EPGKYNVILGEQLASQLGVNRGDQIRVMVP-------------------------------------------------  target    VANQKATPGNPARNEQTRYVALFRYGSHQSGTRAWLRPTQQTDSLVRKGYFGQVIGTGFEADVHSVSGAPKEAFVKIEKA 7v8m.1    --------------------------------------------------------------------------------  target    EDGGIGAERLWRPLTLGLRPEAPSAALTAYLAGDYSGTKGS 7v8m.1    ----------------------------------------- ``` | | | | | | | | | | | | | | | | | | | | | | | | | | | | | | | | | | | | | | | | | | | | | | | | | |
|  | 7v8l.1.C | Lipoprotein-releasing system transmembrane protein LolC  *LolCDE with bound RcsF in nanodiscs* | 0.02 |  | 23.33 | 0.11 | 82-111 | EM | 0.00 | hetero-1-1-1-2-mer | 1 x PCJ | HHblits | 0.30 |
| ``` target    GLGVPESQLDVETRQYRNVVRTWAELQQTLHPLQERDPAFRFVFQTPKYRWGAHSTAVDADWISMLFGPFGDPYRRDPRM 7v8l.1    --------------------------------------------------------------------------------  target    PWTGEAYLEINPKDAAELGLADGDYAWVDADPEDRPYRGWNEDDPYYEVARAMMRVRIYTGMSRGVIRTWFNMYAATPAT 7v8l.1    -EPGKYNVILGEQLASQLGVNRGDQIRVMVP-------------------------------------------------  target    VANQKATPGNPARNEQTRYVALFRYGSHQSGTRAWLRPTQQTDSLVRKGYFGQVIGTGFEADVHSVSGAPKEAFVKIEKA 7v8l.1    --------------------------------------------------------------------------------  target    EDGGIGAERLWRPLTLGLRPEAPSAALTAYLAGDYSGTKGS 7v8l.1    ----------------------------------------- ``` | | | | | | | | | | | | | | | | | | | | | | | | | | | | | | | | | | | | | | | | | | | | | | | | | |
|  | 7dxu.1.B | mk2h\_dP protein  *Crystal structure of the mk2h\_deltaP peptide homodimer* | 0.00 |  | 28.57 | 0.10 | 82-109 | X-ray | 2.31 | homo-dimer |  | HHblits | 0.35 |
| ``` target    GLGVPESQLDVETRQYRNVVRTWAELQQTLHPLQERDPAFRFVFQTPKYRWGAHSTAVDADWISMLFGPFGDPYRRDPRM 7dxu.1    --------------------------------------------------------------------------------  target    PWTGEAYLEINPKDAAELGLADGDYAWVDADPEDRPYRGWNEDDPYYEVARAMMRVRIYTGMSRGVIRTWFNMYAATPAT 7dxu.1    -DVGKRIVRMDKYERAKLGVSVGDYVEVK---------------------------------------------------  target    VANQKATPGNPARNEQTRYVALFRYGSHQSGTRAWLRPTQQTDSLVRKGYFGQVIGTGFEADVHSVSGAPKEAFVKIEKA 7dxu.1    --------------------------------------------------------------------------------  target    EDGGIGAERLWRPLTLGLRPEAPSAALTAYLAGDYSGTKGS 7dxu.1    ----------------------------------------- ``` | | | | | | | | | | | | | | | | | | | | | | | | | | | | | | | | | | | | | | | | | | | | | | | | | |
|  | 7dxu.1.A | mk2h\_dP protein  *Crystal structure of the mk2h\_deltaP peptide homodimer* | 0.00 |  | 28.57 | 0.10 | 82-109 | X-ray | 2.31 | homo-dimer |  | HHblits | 0.35 |
| ``` target    GLGVPESQLDVETRQYRNVVRTWAELQQTLHPLQERDPAFRFVFQTPKYRWGAHSTAVDADWISMLFGPFGDPYRRDPRM 7dxu.1    --------------------------------------------------------------------------------  target    PWTGEAYLEINPKDAAELGLADGDYAWVDADPEDRPYRGWNEDDPYYEVARAMMRVRIYTGMSRGVIRTWFNMYAATPAT 7dxu.1    -DVGKRIVRMDKYERAKLGVSVGDYVEVK---------------------------------------------------  target    VANQKATPGNPARNEQTRYVALFRYGSHQSGTRAWLRPTQQTDSLVRKGYFGQVIGTGFEADVHSVSGAPKEAFVKIEKA 7dxu.1    --------------------------------------------------------------------------------  target    EDGGIGAERLWRPLTLGLRPEAPSAALTAYLAGDYSGTKGS 7dxu.1    ----------------------------------------- ``` | | | | | | | | | | | | | | | | | | | | | | | | | | | | | | | | | | | | | | | | | | | | | | | | | |
|  | 7dxu.2.B | mk2h\_dP protein  *Crystal structure of the mk2h\_deltaP peptide homodimer* | 0.00 |  | 28.57 | 0.10 | 82-109 | X-ray | 2.31 | homo-dimer |  | HHblits | 0.35 |
| ``` target    GLGVPESQLDVETRQYRNVVRTWAELQQTLHPLQERDPAFRFVFQTPKYRWGAHSTAVDADWISMLFGPFGDPYRRDPRM 7dxu.2    --------------------------------------------------------------------------------  target    PWTGEAYLEINPKDAAELGLADGDYAWVDADPEDRPYRGWNEDDPYYEVARAMMRVRIYTGMSRGVIRTWFNMYAATPAT 7dxu.2    -DVGKRIVRMDKYERAKLGVSVGDYVEVK---------------------------------------------------  target    VANQKATPGNPARNEQTRYVALFRYGSHQSGTRAWLRPTQQTDSLVRKGYFGQVIGTGFEADVHSVSGAPKEAFVKIEKA 7dxu.2    --------------------------------------------------------------------------------  target    EDGGIGAERLWRPLTLGLRPEAPSAALTAYLAGDYSGTKGS 7dxu.2    ----------------------------------------- ``` | | | | | | | | | | | | | | | | | | | | | | | | | | | | | | | | | | | | | | | | | | | | | | | | | |
|  | 7du7.1.A | mkDPBB\_sym1 protein  *Crystal structure of the rationally designed mkDPBB\_sym1 protein* | 0.01 |  | 28.57 | 0.10 | 84-111 | X-ray | 1.20 | monomer |  | HHblits | 0.35 |
| ``` target    GLGVPESQLDVETRQYRNVVRTWAELQQTLHPLQERDPAFRFVFQTPKYRWGAHSTAVDADWISMLFGPFGDPYRRDPRM 7du7.1    --------------------------------------------------------------------------------  target    PWTGEAYLEINPKDAAELGLADGDYAWVDADPEDRPYRGWNEDDPYYEVARAMMRVRIYTGMSRGVIRTWFNMYAATPAT 7du7.1    ---GKGIVRMDKASRAKLGVSVGDYVEVKKV-------------------------------------------------  target    VANQKATPGNPARNEQTRYVALFRYGSHQSGTRAWLRPTQQTDSLVRKGYFGQVIGTGFEADVHSVSGAPKEAFVKIEKA 7du7.1    --------------------------------------------------------------------------------  target    EDGGIGAERLWRPLTLGLRPEAPSAALTAYLAGDYSGTKGS 7du7.1    ----------------------------------------- ``` | | | | | | | | | | | | | | | | | | | | | | | | | | | | | | | | | | | | | | | | | | | | | | | | | |
|  | 7dxs.1.A | ap1h protein  *Crystal structure of the ap1h peptide homodimer.* | 0.00 |  | 28.57 | 0.10 | 82-109 | X-ray | 2.10 | homo-dimer |  | HHblits | 0.35 |
| ``` target    GLGVPESQLDVETRQYRNVVRTWAELQQTLHPLQERDPAFRFVFQTPKYRWGAHSTAVDADWISMLFGPFGDPYRRDPRM 7dxs.1    --------------------------------------------------------------------------------  target    PWTGEAYLEINPKDAAELGLADGDYAWVDADPEDRPYRGWNEDDPYYEVARAMMRVRIYTGMSRGVIRTWFNMYAATPAT 7dxs.1    -DVGRGIVRMDKQTRAKLGVSVGDYVEVK---------------------------------------------------  target    VANQKATPGNPARNEQTRYVALFRYGSHQSGTRAWLRPTQQTDSLVRKGYFGQVIGTGFEADVHSVSGAPKEAFVKIEKA 7dxs.1    --------------------------------------------------------------------------------  target    EDGGIGAERLWRPLTLGLRPEAPSAALTAYLAGDYSGTKGS 7dxs.1    ----------------------------------------- ``` | | | | | | | | | | | | | | | | | | | | | | | | | | | | | | | | | | | | | | | | | | | | | | | | | |
|  | 7dxs.1.B | ap1h protein  *Crystal structure of the ap1h peptide homodimer.* | 0.00 |  | 28.57 | 0.10 | 82-109 | X-ray | 2.10 | homo-dimer |  | HHblits | 0.35 |
| ``` target    GLGVPESQLDVETRQYRNVVRTWAELQQTLHPLQERDPAFRFVFQTPKYRWGAHSTAVDADWISMLFGPFGDPYRRDPRM 7dxs.1    --------------------------------------------------------------------------------  target    PWTGEAYLEINPKDAAELGLADGDYAWVDADPEDRPYRGWNEDDPYYEVARAMMRVRIYTGMSRGVIRTWFNMYAATPAT 7dxs.1    -DVGRGIVRMDKQTRAKLGVSVGDYVEVK---------------------------------------------------  target    VANQKATPGNPARNEQTRYVALFRYGSHQSGTRAWLRPTQQTDSLVRKGYFGQVIGTGFEADVHSVSGAPKEAFVKIEKA 7dxs.1    --------------------------------------------------------------------------------  target    EDGGIGAERLWRPLTLGLRPEAPSAALTAYLAGDYSGTKGS 7dxs.1    ----------------------------------------- ``` | | | | | | | | | | | | | | | | | | | | | | | | | | | | | | | | | | | | | | | | | | | | | | | | | |
|  | 7dxs.2.A | ap1h protein  *Crystal structure of the ap1h peptide homodimer.* | 0.00 |  | 28.57 | 0.10 | 82-109 | X-ray | 2.10 | homo-dimer |  | HHblits | 0.35 |
| ``` target    GLGVPESQLDVETRQYRNVVRTWAELQQTLHPLQERDPAFRFVFQTPKYRWGAHSTAVDADWISMLFGPFGDPYRRDPRM 7dxs.2    --------------------------------------------------------------------------------  target    PWTGEAYLEINPKDAAELGLADGDYAWVDADPEDRPYRGWNEDDPYYEVARAMMRVRIYTGMSRGVIRTWFNMYAATPAT 7dxs.2    -DVGRGIVRMDKQTRAKLGVSVGDYVEVK---------------------------------------------------  target    VANQKATPGNPARNEQTRYVALFRYGSHQSGTRAWLRPTQQTDSLVRKGYFGQVIGTGFEADVHSVSGAPKEAFVKIEKA 7dxs.2    --------------------------------------------------------------------------------  target    EDGGIGAERLWRPLTLGLRPEAPSAALTAYLAGDYSGTKGS 7dxs.2    ----------------------------------------- ``` | | | | | | | | | | | | | | | | | | | | | | | | | | | | | | | | | | | | | | | | | | | | | | | | | |
|  | 7dxs.2.B | ap1h protein  *Crystal structure of the ap1h peptide homodimer.* | 0.00 |  | 28.57 | 0.10 | 82-109 | X-ray | 2.10 | homo-dimer |  | HHblits | 0.35 |
| ``` target    GLGVPESQLDVETRQYRNVVRTWAELQQTLHPLQERDPAFRFVFQTPKYRWGAHSTAVDADWISMLFGPFGDPYRRDPRM 7dxs.2    --------------------------------------------------------------------------------  target    PWTGEAYLEINPKDAAELGLADGDYAWVDADPEDRPYRGWNEDDPYYEVARAMMRVRIYTGMSRGVIRTWFNMYAATPAT 7dxs.2    -DVGRGIVRMDKQTRAKLGVSVGDYVEVK---------------------------------------------------  target    VANQKATPGNPARNEQTRYVALFRYGSHQSGTRAWLRPTQQTDSLVRKGYFGQVIGTGFEADVHSVSGAPKEAFVKIEKA 7dxs.2    --------------------------------------------------------------------------------  target    EDGGIGAERLWRPLTLGLRPEAPSAALTAYLAGDYSGTKGS 7dxs.2    ----------------------------------------- ``` | | | | | | | | | | | | | | | | | | | | | | | | | | | | | | | | | | | | | | | | | | | | | | | | | |
|  | 7di1.1.A | mkDPBB\_sym\_86 protein  *Crystal structure of the rationally designed mkDPBB\_sym\_86 protein* | 0.02 |  | 28.57 | 0.10 | 84-111 | X-ray | 2.10 | monomer |  | HHblits | 0.35 |
| ``` target    GLGVPESQLDVETRQYRNVVRTWAELQQTLHPLQERDPAFRFVFQTPKYRWGAHSTAVDADWISMLFGPFGDPYRRDPRM 7di1.1    --------------------------------------------------------------------------------  target    PWTGEAYLEINPKDAAELGLADGDYAWVDADPEDRPYRGWNEDDPYYEVARAMMRVRIYTGMSRGVIRTWFNMYAATPAT 7di1.1    ---GKRIVRMDKASRAKLGVSVGDYVEVKKV-------------------------------------------------  target    VANQKATPGNPARNEQTRYVALFRYGSHQSGTRAWLRPTQQTDSLVRKGYFGQVIGTGFEADVHSVSGAPKEAFVKIEKA 7di1.1    --------------------------------------------------------------------------------  target    EDGGIGAERLWRPLTLGLRPEAPSAALTAYLAGDYSGTKGS 7di1.1    ----------------------------------------- ``` | | | | | | | | | | | | | | | | | | | | | | | | | | | | | | | | | | | | | | | | | | | | | | | | | |
|  | 7dxy.1.A | mk2h\_deltaMILPS  *Crystal structure of the chemically synthesized mk2h\_deltaMILPS peptide homodimer* | 0.00 |  | 25.00 | 0.10 | 82-109 | X-ray | 1.40 | homo-dimer |  | HHblits | 0.34 |
| ``` target    GLGVPESQLDVETRQYRNVVRTWAELQQTLHPLQERDPAFRFVFQTPKYRWGAHSTAVDADWISMLFGPFGDPYRRDPRM 7dxy.1    --------------------------------------------------------------------------------  target    PWTGEAYLEINPKDAAELGLADGDYAWVDADPEDRPYRGWNEDDPYYEVARAMMRVRIYTGMSRGVIRTWFNMYAATPAT 7dxy.1    -DVGKRVVRVDKYERAKVGVKVGDYVEVK---------------------------------------------------  target    VANQKATPGNPARNEQTRYVALFRYGSHQSGTRAWLRPTQQTDSLVRKGYFGQVIGTGFEADVHSVSGAPKEAFVKIEKA 7dxy.1    --------------------------------------------------------------------------------  target    EDGGIGAERLWRPLTLGLRPEAPSAALTAYLAGDYSGTKGS 7dxy.1    ----------------------------------------- ``` | | | | | | | | | | | | | | | | | | | | | | | | | | | | | | | | | | | | | | | | | | | | | | | | | |
|  | 7dvh.2.A | reDPBB\_sym4 protein  *Crystal structure of the computationally designed reDPBB\_sym4 protein* | 0.02 |  | 25.00 | 0.10 | 84-111 | X-ray | 1.70 | monomer |  | HHblits | 0.34 |
| ``` target    GLGVPESQLDVETRQYRNVVRTWAELQQTLHPLQERDPAFRFVFQTPKYRWGAHSTAVDADWISMLFGPFGDPYRRDPRM 7dvh.2    --------------------------------------------------------------------------------  target    PWTGEAYLEINPKDAAELGLADGDYAWVDADPEDRPYRGWNEDDPYYEVARAMMRVRIYTGMSRGVIRTWFNMYAATPAT 7dvh.2    ---GKGIVRMDKYERQNLGVSVGDYVEVKKA-------------------------------------------------  target    VANQKATPGNPARNEQTRYVALFRYGSHQSGTRAWLRPTQQTDSLVRKGYFGQVIGTGFEADVHSVSGAPKEAFVKIEKA 7dvh.2    --------------------------------------------------------------------------------  target    EDGGIGAERLWRPLTLGLRPEAPSAALTAYLAGDYSGTKGS 7dvh.2    ----------------------------------------- ``` | | | | | | | | | | | | | | | | | | | | | | | | | | | | | | | | | | | | | | | | | | | | | | | | | |
|  | 7dvh.1.A | reDPBB\_sym4 protein  *Crystal structure of the computationally designed reDPBB\_sym4 protein* | 0.02 |  | 25.00 | 0.10 | 84-111 | X-ray | 1.70 | monomer |  | HHblits | 0.34 |
| ``` target    GLGVPESQLDVETRQYRNVVRTWAELQQTLHPLQERDPAFRFVFQTPKYRWGAHSTAVDADWISMLFGPFGDPYRRDPRM 7dvh.1    --------------------------------------------------------------------------------  target    PWTGEAYLEINPKDAAELGLADGDYAWVDADPEDRPYRGWNEDDPYYEVARAMMRVRIYTGMSRGVIRTWFNMYAATPAT 7dvh.1    ---GKGIVRMDKYERQNLGVSVGDYVEVKKA-------------------------------------------------  target    VANQKATPGNPARNEQTRYVALFRYGSHQSGTRAWLRPTQQTDSLVRKGYFGQVIGTGFEADVHSVSGAPKEAFVKIEKA 7dvh.1    --------------------------------------------------------------------------------  target    EDGGIGAERLWRPLTLGLRPEAPSAALTAYLAGDYSGTKGS 7dvh.1    ----------------------------------------- ``` | | | | | | | | | | | | | | | | | | | | | | | | | | | | | | | | | | | | | | | | | | | | | | | | | |
|  | 7dvh.4.A | reDPBB\_sym4 protein  *Crystal structure of the computationally designed reDPBB\_sym4 protein* | 0.02 |  | 25.00 | 0.10 | 84-111 | X-ray | 1.70 | monomer |  | HHblits | 0.34 |
| ``` target    GLGVPESQLDVETRQYRNVVRTWAELQQTLHPLQERDPAFRFVFQTPKYRWGAHSTAVDADWISMLFGPFGDPYRRDPRM 7dvh.4    --------------------------------------------------------------------------------  target    PWTGEAYLEINPKDAAELGLADGDYAWVDADPEDRPYRGWNEDDPYYEVARAMMRVRIYTGMSRGVIRTWFNMYAATPAT 7dvh.4    ---GKGIVRMDKYERQNLGVSVGDYVEVKKA-------------------------------------------------  target    VANQKATPGNPARNEQTRYVALFRYGSHQSGTRAWLRPTQQTDSLVRKGYFGQVIGTGFEADVHSVSGAPKEAFVKIEKA 7dvh.4    --------------------------------------------------------------------------------  target    EDGGIGAERLWRPLTLGLRPEAPSAALTAYLAGDYSGTKGS 7dvh.4    ----------------------------------------- ``` | | | | | | | | | | | | | | | | | | | | | | | | | | | | | | | | | | | | | | | | | | | | | | | | | |
|  | 4ga5.1.A | Putative thymidine phosphorylase  *Crystal structure of AMP phosphorylase C-terminal deletion mutant in the apo-form* | 0.02 |  | 32.14 | 0.10 | 84-111 | X-ray | 3.25 | homo-dimer |  | HHblits | 0.34 |
| ``` target    GLGVPESQLDVETRQYRNVVRTWAELQQTLHPLQERDPAFRFVFQTPKYRWGAHSTAVDADWISMLFGPFGDPYRRDPRM 4ga5.1    --------------------------------------------------------------------------------  target    PWTGEAYLEINPKDAAELGLADGDYAWVDADPEDRPYRGWNEDDPYYEVARAMMRVRIYTGMSRGVIRTWFNMYAATPAT 4ga5.1    ---GRYTVLINEEDAKEAKLHPDDLVKIEAG-------------------------------------------------  target    VANQKATPGNPARNEQTRYVALFRYGSHQSGTRAWLRPTQQTDSLVRKGYFGQVIGTGFEADVHSVSGAPKEAFVKIEKA 4ga5.1    --------------------------------------------------------------------------------  target    EDGGIGAERLWRPLTLGLRPEAPSAALTAYLAGDYSGTKGS 4ga5.1    ----------------------------------------- ``` | | | | | | | | | | | | | | | | | | | | | | | | | | | | | | | | | | | | | | | | | | | | | | | | | |
|  | 4ga6.1.A | Putative thymidine phosphorylase  *Crystal structure of AMP phosphorylase C-terminal deletion mutant in complex with substrates* | 0.02 |  | 32.14 | 0.10 | 84-111 | X-ray | 2.21 | homo-dimer | 2 x AMP | HHblits | 0.34 |
| ``` target    GLGVPESQLDVETRQYRNVVRTWAELQQTLHPLQERDPAFRFVFQTPKYRWGAHSTAVDADWISMLFGPFGDPYRRDPRM 4ga6.1    --------------------------------------------------------------------------------  target    PWTGEAYLEINPKDAAELGLADGDYAWVDADPEDRPYRGWNEDDPYYEVARAMMRVRIYTGMSRGVIRTWFNMYAATPAT 4ga6.1    ---GRYTVLINEEDAKEAKLHPDDLVKIEAG-------------------------------------------------  target    VANQKATPGNPARNEQTRYVALFRYGSHQSGTRAWLRPTQQTDSLVRKGYFGQVIGTGFEADVHSVSGAPKEAFVKIEKA 4ga6.1    --------------------------------------------------------------------------------  target    EDGGIGAERLWRPLTLGLRPEAPSAALTAYLAGDYSGTKGS 4ga6.1    ----------------------------------------- ``` | | | | | | | | | | | | | | | | | | | | | | | | | | | | | | | | | | | | | | | | | | | | | | | | | |
|  | 7mdx.1.A | Lipo-releasing system transmembrane protein lolC  *LolCDE nucleotide-free* | 0.02 |  | 24.14 | 0.10 | 83-111 | EM | 0.00 | hetero-1-1-2-mer | 1 x YPC-DSN-DAL-DAL-ALA-ALA | HHblits | 0.31 |
| ``` target    GLGVPESQLDVETRQYRNVVRTWAELQQTLHPLQERDPAFRFVFQTPKYRWGAHSTAVDADWISMLFGPFGDPYRRDPRM 7mdx.1    --------------------------------------------------------------------------------  target    PWTGEAYLEINPKDAAELGLADGDYAWVDADPEDRPYRGWNEDDPYYEVARAMMRVRIYTGMSRGVIRTWFNMYAATPAT 7mdx.1    --PGKYNVILGEQLASQLGVNRGDQIRVMVP-------------------------------------------------  target    VANQKATPGNPARNEQTRYVALFRYGSHQSGTRAWLRPTQQTDSLVRKGYFGQVIGTGFEADVHSVSGAPKEAFVKIEKA 7mdx.1    --------------------------------------------------------------------------------  target    EDGGIGAERLWRPLTLGLRPEAPSAALTAYLAGDYSGTKGS 7mdx.1    ----------------------------------------- ``` | | | | | | | | | | | | | | | | | | | | | | | | | | | | | | | | | | | | | | | | | | | | | | | | | |
|  | 7mdx.1.B | Lipoprotein-releasing system transmembrane protein  *LolCDE nucleotide-free* | 0.02 |  | 24.14 | 0.10 | 83-111 | EM | 0.00 | hetero-1-1-2-mer | 1 x YPC-DSN-DAL-DAL-ALA-ALA | HHblits | 0.31 |
| ``` target    GLGVPESQLDVETRQYRNVVRTWAELQQTLHPLQERDPAFRFVFQTPKYRWGAHSTAVDADWISMLFGPFGDPYRRDPRM 7mdx.1    --------------------------------------------------------------------------------  target    PWTGEAYLEINPKDAAELGLADGDYAWVDADPEDRPYRGWNEDDPYYEVARAMMRVRIYTGMSRGVIRTWFNMYAATPAT 7mdx.1    --AGEQQIIIGKGVADALKVKQGDWVSIMIP-------------------------------------------------  target    VANQKATPGNPARNEQTRYVALFRYGSHQSGTRAWLRPTQQTDSLVRKGYFGQVIGTGFEADVHSVSGAPKEAFVKIEKA 7mdx.1    --------------------------------------------------------------------------------  target    EDGGIGAERLWRPLTLGLRPEAPSAALTAYLAGDYSGTKGS 7mdx.1    ----------------------------------------- ``` | | | | | | | | | | | | | | | | | | | | | | | | | | | | | | | | | | | | | | | | | | | | | | | | | |
|  | 7arh.1.B | Lipoprotein-releasing system transmembrane protein LolE  *LolCDE in complex with lipoprotein* | 0.02 |  | 24.14 | 0.10 | 83-111 | EM | 0.00 | hetero-1-1-2-1-mer | 1 x Z41, 1 x PLM | HHblits | 0.31 |
| ``` target    GLGVPESQLDVETRQYRNVVRTWAELQQTLHPLQERDPAFRFVFQTPKYRWGAHSTAVDADWISMLFGPFGDPYRRDPRM 7arh.1    --------------------------------------------------------------------------------  target    PWTGEAYLEINPKDAAELGLADGDYAWVDADPEDRPYRGWNEDDPYYEVARAMMRVRIYTGMSRGVIRTWFNMYAATPAT 7arh.1    --AGEQQIIIGKGVADALKVKQGDWVSIMIP-------------------------------------------------  target    VANQKATPGNPARNEQTRYVALFRYGSHQSGTRAWLRPTQQTDSLVRKGYFGQVIGTGFEADVHSVSGAPKEAFVKIEKA 7arh.1    --------------------------------------------------------------------------------  target    EDGGIGAERLWRPLTLGLRPEAPSAALTAYLAGDYSGTKGS 7arh.1    ----------------------------------------- ``` | | | | | | | | | | | | | | | | | | | | | | | | | | | | | | | | | | | | | | | | | | | | | | | | | |
|  | 7ari.1.B | Lipoprotein-releasing system transmembrane protein LolE  *LolCDE apo structure* | 0.01 |  | 24.14 | 0.10 | 83-111 | EM | 0.00 | hetero-1-1-2-mer |  | HHblits | 0.31 |
| ``` target    GLGVPESQLDVETRQYRNVVRTWAELQQTLHPLQERDPAFRFVFQTPKYRWGAHSTAVDADWISMLFGPFGDPYRRDPRM 7ari.1    --------------------------------------------------------------------------------  target    PWTGEAYLEINPKDAAELGLADGDYAWVDADPEDRPYRGWNEDDPYYEVARAMMRVRIYTGMSRGVIRTWFNMYAATPAT 7ari.1    --AGEQQIIIGKGVADALKVKQGDWVSIMIP-------------------------------------------------  target    VANQKATPGNPARNEQTRYVALFRYGSHQSGTRAWLRPTQQTDSLVRKGYFGQVIGTGFEADVHSVSGAPKEAFVKIEKA 7ari.1    --------------------------------------------------------------------------------  target    EDGGIGAERLWRPLTLGLRPEAPSAALTAYLAGDYSGTKGS 7ari.1    ----------------------------------------- ``` | | | | | | | | | | | | | | | | | | | | | | | | | | | | | | | | | | | | | | | | | | | | | | | | | |
|  | 7arj.1.B | Lipoprotein-releasing system transmembrane protein LolE  *LolCDE in complex with lipoprotein and AMPPNP complex undimerized form* | 0.02 |  | 24.14 | 0.10 | 83-111 | EM | 0.00 | hetero-1-1-2-1-mer | 2 x ANP, 2 x MG, 1 x Z41, 1 x PLM | HHblits | 0.31 |
| ``` target    GLGVPESQLDVETRQYRNVVRTWAELQQTLHPLQERDPAFRFVFQTPKYRWGAHSTAVDADWISMLFGPFGDPYRRDPRM 7arj.1    --------------------------------------------------------------------------------  target    PWTGEAYLEINPKDAAELGLADGDYAWVDADPEDRPYRGWNEDDPYYEVARAMMRVRIYTGMSRGVIRTWFNMYAATPAT 7arj.1    --AGEQQIIIGKGVADALKVKQGDWVSIMIP-------------------------------------------------  target    VANQKATPGNPARNEQTRYVALFRYGSHQSGTRAWLRPTQQTDSLVRKGYFGQVIGTGFEADVHSVSGAPKEAFVKIEKA 7arj.1    --------------------------------------------------------------------------------  target    EDGGIGAERLWRPLTLGLRPEAPSAALTAYLAGDYSGTKGS 7arj.1    ----------------------------------------- ``` | | | | | | | | | | | | | | | | | | | | | | | | | | | | | | | | | | | | | | | | | | | | | | | | | |
|  | 7ark.1.B | Lipoprotein-releasing system transmembrane protein LolE  *LolCDE in complex with AMP-PNP in the closed NBD state* | 0.01 |  | 24.14 | 0.10 | 83-111 | EM | 0.00 | hetero-1-1-2-mer | 2 x ANP, 2 x MG | HHblits | 0.31 |
| ``` target    GLGVPESQLDVETRQYRNVVRTWAELQQTLHPLQERDPAFRFVFQTPKYRWGAHSTAVDADWISMLFGPFGDPYRRDPRM 7ark.1    --------------------------------------------------------------------------------  target    PWTGEAYLEINPKDAAELGLADGDYAWVDADPEDRPYRGWNEDDPYYEVARAMMRVRIYTGMSRGVIRTWFNMYAATPAT 7ark.1    --AGEQQIIIGKGVADALKVKQGDWVSIMIP-------------------------------------------------  target    VANQKATPGNPARNEQTRYVALFRYGSHQSGTRAWLRPTQQTDSLVRKGYFGQVIGTGFEADVHSVSGAPKEAFVKIEKA 7ark.1    --------------------------------------------------------------------------------  target    EDGGIGAERLWRPLTLGLRPEAPSAALTAYLAGDYSGTKGS 7ark.1    ----------------------------------------- ``` | | | | | | | | | | | | | | | | | | | | | | | | | | | | | | | | | | | | | | | | | | | | | | | | | |
|  | 7arl.1.B | Lipoprotein-releasing system transmembrane protein LolE  *LolCDE in complex with lipoprotein and ADP* | 0.02 |  | 24.14 | 0.10 | 83-111 | EM | 0.00 | hetero-1-1-2-1-mer | 1 x Z41, 2 x ADP, 2 x MG, 1 x PLM | HHblits | 0.31 |
| ``` target    GLGVPESQLDVETRQYRNVVRTWAELQQTLHPLQERDPAFRFVFQTPKYRWGAHSTAVDADWISMLFGPFGDPYRRDPRM 7arl.1    --------------------------------------------------------------------------------  target    PWTGEAYLEINPKDAAELGLADGDYAWVDADPEDRPYRGWNEDDPYYEVARAMMRVRIYTGMSRGVIRTWFNMYAATPAT 7arl.1    --AGEQQIIIGKGVADALKVKQGDWVSIMIP-------------------------------------------------  target    VANQKATPGNPARNEQTRYVALFRYGSHQSGTRAWLRPTQQTDSLVRKGYFGQVIGTGFEADVHSVSGAPKEAFVKIEKA 7arl.1    --------------------------------------------------------------------------------  target    EDGGIGAERLWRPLTLGLRPEAPSAALTAYLAGDYSGTKGS 7arl.1    ----------------------------------------- ``` | | | | | | | | | | | | | | | | | | | | | | | | | | | | | | | | | | | | | | | | | | | | | | | | | |
|  | 7arm.1.B | Lipoprotein-releasing system transmembrane protein LolE  *LolCDE in complex with lipoprotein and LolA* | 0.02 |  | 24.14 | 0.10 | 83-111 | EM | 0.00 | hetero-1-1-2-1-1-mer | 1 x Z41, 1 x PLM | HHblits | 0.31 |
| ``` target    GLGVPESQLDVETRQYRNVVRTWAELQQTLHPLQERDPAFRFVFQTPKYRWGAHSTAVDADWISMLFGPFGDPYRRDPRM 7arm.1    --------------------------------------------------------------------------------  target    PWTGEAYLEINPKDAAELGLADGDYAWVDADPEDRPYRGWNEDDPYYEVARAMMRVRIYTGMSRGVIRTWFNMYAATPAT 7arm.1    --AGEQQIIIGKGVADALKVKQGDWVSIMIP-------------------------------------------------  target    VANQKATPGNPARNEQTRYVALFRYGSHQSGTRAWLRPTQQTDSLVRKGYFGQVIGTGFEADVHSVSGAPKEAFVKIEKA 7arm.1    --------------------------------------------------------------------------------  target    EDGGIGAERLWRPLTLGLRPEAPSAALTAYLAGDYSGTKGS 7arm.1    ----------------------------------------- ``` | | | | | | | | | | | | | | | | | | | | | | | | | | | | | | | | | | | | | | | | | | | | | | | | | |
|  | 7mdy.1.A | Lipoprotein transporter subunit LolE  *LolCDE nucleotide-bound* | 0.01 |  | 24.14 | 0.10 | 83-111 | EM | 0.00 | hetero-1-1-2-mer | 2 x MG | HHblits | 0.31 |
| ``` target    GLGVPESQLDVETRQYRNVVRTWAELQQTLHPLQERDPAFRFVFQTPKYRWGAHSTAVDADWISMLFGPFGDPYRRDPRM 7mdy.1    --------------------------------------------------------------------------------  target    PWTGEAYLEINPKDAAELGLADGDYAWVDADPEDRPYRGWNEDDPYYEVARAMMRVRIYTGMSRGVIRTWFNMYAATPAT 7mdy.1    --AGEQQIIIGKGVADALKVKQGDWVSIMIP-------------------------------------------------  target    VANQKATPGNPARNEQTRYVALFRYGSHQSGTRAWLRPTQQTDSLVRKGYFGQVIGTGFEADVHSVSGAPKEAFVKIEKA 7mdy.1    --------------------------------------------------------------------------------  target    EDGGIGAERLWRPLTLGLRPEAPSAALTAYLAGDYSGTKGS 7mdy.1    ----------------------------------------- ``` | | | | | | | | | | | | | | | | | | | | | | | | | | | | | | | | | | | | | | | | | | | | | | | | | |
|  | 7v8m.1.C | Lipoprotein-releasing system transmembrane protein LolE  *LolCDE-apo in nanodiscs* | 0.01 |  | 24.14 | 0.10 | 83-111 | EM | 0.00 | hetero-1-2-1-mer |  | HHblits | 0.31 |
| ``` target    GLGVPESQLDVETRQYRNVVRTWAELQQTLHPLQERDPAFRFVFQTPKYRWGAHSTAVDADWISMLFGPFGDPYRRDPRM 7v8m.1    --------------------------------------------------------------------------------  target    PWTGEAYLEINPKDAAELGLADGDYAWVDADPEDRPYRGWNEDDPYYEVARAMMRVRIYTGMSRGVIRTWFNMYAATPAT 7v8m.1    --AGEQQIIIGKGVADALKVKQGDWVSIMIP-------------------------------------------------  target    VANQKATPGNPARNEQTRYVALFRYGSHQSGTRAWLRPTQQTDSLVRKGYFGQVIGTGFEADVHSVSGAPKEAFVKIEKA 7v8m.1    --------------------------------------------------------------------------------  target    EDGGIGAERLWRPLTLGLRPEAPSAALTAYLAGDYSGTKGS 7v8m.1    ----------------------------------------- ``` | | | | | | | | | | | | | | | | | | | | | | | | | | | | | | | | | | | | | | | | | | | | | | | | | |
|  | 7v8i.1.C | Lipoprotein-releasing system transmembrane protein LolE  *LolCD(E171Q)E with bound AMPPNP in nanodiscs* | 0.02 |  | 24.14 | 0.10 | 83-111 | EM | 0.00 | hetero-1-2-1-mer | 2 x MG, 2 x ANP | HHblits | 0.31 |
| ``` target    GLGVPESQLDVETRQYRNVVRTWAELQQTLHPLQERDPAFRFVFQTPKYRWGAHSTAVDADWISMLFGPFGDPYRRDPRM 7v8i.1    --------------------------------------------------------------------------------  target    PWTGEAYLEINPKDAAELGLADGDYAWVDADPEDRPYRGWNEDDPYYEVARAMMRVRIYTGMSRGVIRTWFNMYAATPAT 7v8i.1    --AGEQQIIIGKGVADALKVKQGDWVSIMIP-------------------------------------------------  target    VANQKATPGNPARNEQTRYVALFRYGSHQSGTRAWLRPTQQTDSLVRKGYFGQVIGTGFEADVHSVSGAPKEAFVKIEKA 7v8i.1    --------------------------------------------------------------------------------  target    EDGGIGAERLWRPLTLGLRPEAPSAALTAYLAGDYSGTKGS 7v8i.1    ----------------------------------------- ``` | | | | | | | | | | | | | | | | | | | | | | | | | | | | | | | | | | | | | | | | | | | | | | | | | |
|  | 7v8l.1.A | Lipoprotein-releasing system transmembrane protein LolE  *LolCDE with bound RcsF in nanodiscs* | 0.02 |  | 24.14 | 0.10 | 83-111 | EM | 0.00 | hetero-1-1-1-2-mer | 1 x PCJ | HHblits | 0.31 |
| ``` target    GLGVPESQLDVETRQYRNVVRTWAELQQTLHPLQERDPAFRFVFQTPKYRWGAHSTAVDADWISMLFGPFGDPYRRDPRM 7v8l.1    --------------------------------------------------------------------------------  target    PWTGEAYLEINPKDAAELGLADGDYAWVDADPEDRPYRGWNEDDPYYEVARAMMRVRIYTGMSRGVIRTWFNMYAATPAT 7v8l.1    --AGEQQIIIGKGVADALKVKQGDWVSIMIP-------------------------------------------------  target    VANQKATPGNPARNEQTRYVALFRYGSHQSGTRAWLRPTQQTDSLVRKGYFGQVIGTGFEADVHSVSGAPKEAFVKIEKA 7v8l.1    --------------------------------------------------------------------------------  target    EDGGIGAERLWRPLTLGLRPEAPSAALTAYLAGDYSGTKGS 7v8l.1    ----------------------------------------- ``` | | | | | | | | | | | | | | | | | | | | | | | | | | | | | | | | | | | | | | | | | | | | | | | | | |
|  | 7dxr.1.B | mk2h protein  *Crystal structure of the mk2h peptide homodimer.* | 0.00 |  | 29.63 | 0.10 | 83-109 | X-ray | 1.60 | homo-dimer |  | HHblits | 0.36 |
| ``` target    GLGVPESQLDVETRQYRNVVRTWAELQQTLHPLQERDPAFRFVFQTPKYRWGAHSTAVDADWISMLFGPFGDPYRRDPRM 7dxr.1    --------------------------------------------------------------------------------  target    PWTGEAYLEINPKDAAELGLADGDYAWVDADPEDRPYRGWNEDDPYYEVARAMMRVRIYTGMSRGVIRTWFNMYAATPAT 7dxr.1    --VGKRIVRMDKYERAKLGVSVGDYVEVK---------------------------------------------------  target    VANQKATPGNPARNEQTRYVALFRYGSHQSGTRAWLRPTQQTDSLVRKGYFGQVIGTGFEADVHSVSGAPKEAFVKIEKA 7dxr.1    --------------------------------------------------------------------------------  target    EDGGIGAERLWRPLTLGLRPEAPSAALTAYLAGDYSGTKGS 7dxr.1    ----------------------------------------- ``` | | | | | | | | | | | | | | | | | | | | | | | | | | | | | | | | | | | | | | | | | | | | | | | | | |
|  | 7dxr.1.A | mk2h protein  *Crystal structure of the mk2h peptide homodimer.* | 0.00 |  | 29.63 | 0.10 | 83-109 | X-ray | 1.60 | homo-dimer |  | HHblits | 0.36 |
| ``` target    GLGVPESQLDVETRQYRNVVRTWAELQQTLHPLQERDPAFRFVFQTPKYRWGAHSTAVDADWISMLFGPFGDPYRRDPRM 7dxr.1    --------------------------------------------------------------------------------  target    PWTGEAYLEINPKDAAELGLADGDYAWVDADPEDRPYRGWNEDDPYYEVARAMMRVRIYTGMSRGVIRTWFNMYAATPAT 7dxr.1    --VGKRIVRMDKYERAKLGVSVGDYVEVK---------------------------------------------------  target    VANQKATPGNPARNEQTRYVALFRYGSHQSGTRAWLRPTQQTDSLVRKGYFGQVIGTGFEADVHSVSGAPKEAFVKIEKA 7dxr.1    --------------------------------------------------------------------------------  target    EDGGIGAERLWRPLTLGLRPEAPSAALTAYLAGDYSGTKGS 7dxr.1    ----------------------------------------- ``` | | | | | | | | | | | | | | | | | | | | | | | | | | | | | | | | | | | | | | | | | | | | | | | | | |
|  | 7dxr.2.B | mk2h protein  *Crystal structure of the mk2h peptide homodimer.* | 0.00 |  | 29.63 | 0.10 | 83-109 | X-ray | 1.60 | homo-dimer | 1 x CXS | HHblits | 0.36 |
| ``` target    GLGVPESQLDVETRQYRNVVRTWAELQQTLHPLQERDPAFRFVFQTPKYRWGAHSTAVDADWISMLFGPFGDPYRRDPRM 7dxr.2    --------------------------------------------------------------------------------  target    PWTGEAYLEINPKDAAELGLADGDYAWVDADPEDRPYRGWNEDDPYYEVARAMMRVRIYTGMSRGVIRTWFNMYAATPAT 7dxr.2    --VGKRIVRMDKYERAKLGVSVGDYVEVK---------------------------------------------------  target    VANQKATPGNPARNEQTRYVALFRYGSHQSGTRAWLRPTQQTDSLVRKGYFGQVIGTGFEADVHSVSGAPKEAFVKIEKA 7dxr.2    --------------------------------------------------------------------------------  target    EDGGIGAERLWRPLTLGLRPEAPSAALTAYLAGDYSGTKGS 7dxr.2    ----------------------------------------- ``` | | | | | | | | | | | | | | | | | | | | | | | | | | | | | | | | | | | | | | | | | | | | | | | | | |
|  | 7dxt.1.A | mk2h protein  *Crystal structure of the chemically synthesized mk2h peptide homodimer* | 0.00 |  | 29.63 | 0.10 | 83-109 | X-ray | 1.80 | homo-dimer |  | HHblits | 0.36 |
| ``` target    GLGVPESQLDVETRQYRNVVRTWAELQQTLHPLQERDPAFRFVFQTPKYRWGAHSTAVDADWISMLFGPFGDPYRRDPRM 7dxt.1    --------------------------------------------------------------------------------  target    PWTGEAYLEINPKDAAELGLADGDYAWVDADPEDRPYRGWNEDDPYYEVARAMMRVRIYTGMSRGVIRTWFNMYAATPAT 7dxt.1    --VGKRIVRMDKYERAKLGVSVGDYVEVK---------------------------------------------------  target    VANQKATPGNPARNEQTRYVALFRYGSHQSGTRAWLRPTQQTDSLVRKGYFGQVIGTGFEADVHSVSGAPKEAFVKIEKA 7dxt.1    --------------------------------------------------------------------------------  target    EDGGIGAERLWRPLTLGLRPEAPSAALTAYLAGDYSGTKGS 7dxt.1    ----------------------------------------- ``` | | | | | | | | | | | | | | | | | | | | | | | | | | | | | | | | | | | | | | | | | | | | | | | | | |
|  | 5e7p.1.A | Cell division control protein Cdc48  *Crystal Structure of MSMEG\_0858 (Uniprot A0QQS4), a AAA ATPase.* | 0.02 |  | 17.24 | 0.10 | 82-110 | X-ray | 2.51 | monomer | 2 x ADP | HHblits | 0.31 |
| ``` target    GLGVPESQLDVETRQYRNVVRTWAELQQTLHPLQERDPAFRFVFQTPKYRWGAHSTAVDADWISMLFGPFGDPYRRDPRM 5e7p.1    --------------------------------------------------------------------------------  target    PWTGEAYLEINPKDAAELGLADGDYAWVDADPEDRPYRGWNEDDPYYEVARAMMRVRIYTGMSRGVIRTWFNMYAATPAT 5e7p.1    -DSRRGVVRLHPEVLAALGIREWDAVALTG--------------------------------------------------  target    VANQKATPGNPARNEQTRYVALFRYGSHQSGTRAWLRPTQQTDSLVRKGYFGQVIGTGFEADVHSVSGAPKEAFVKIEKA 5e7p.1    --------------------------------------------------------------------------------  target    EDGGIGAERLWRPLTLGLRPEAPSAALTAYLAGDYSGTKGS 5e7p.1    ----------------------------------------- ``` | | | | | | | | | | | | | | | | | | | | | | | | | | | | | | | | | | | | | | | | | | | | | | | | | |
|  | 7dxw.1.A | mk2h\_deltaMIL protein  *Crystal structure of the mk2h\_deltaMIL peptide homodimer* | 0.00 |  | 25.93 | 0.10 | 83-109 | X-ray | 1.51 | homo-dimer |  | HHblits | 0.36 |
| ``` target    GLGVPESQLDVETRQYRNVVRTWAELQQTLHPLQERDPAFRFVFQTPKYRWGAHSTAVDADWISMLFGPFGDPYRRDPRM 7dxw.1    --------------------------------------------------------------------------------  target    PWTGEAYLEINPKDAAELGLADGDYAWVDADPEDRPYRGWNEDDPYYEVARAMMRVRIYTGMSRGVIRTWFNMYAATPAT 7dxw.1    --VGKRVVRVDKYERAKVGVSVGDYVEVK---------------------------------------------------  target    VANQKATPGNPARNEQTRYVALFRYGSHQSGTRAWLRPTQQTDSLVRKGYFGQVIGTGFEADVHSVSGAPKEAFVKIEKA 7dxw.1    --------------------------------------------------------------------------------  target    EDGGIGAERLWRPLTLGLRPEAPSAALTAYLAGDYSGTKGS 7dxw.1    ----------------------------------------- ``` | | | | | | | | | | | | | | | | | | | | | | | | | | | | | | | | | | | | | | | | | | | | | | | | | |
|  | 7dxx.1.A | mk2h\_deltaMILPS protein  *Crystal structure of the mk2h\_deltaMILPS peptide homodimer* | 0.00 |  | 25.93 | 0.10 | 83-109 | X-ray | 1.40 | homo-dimer | 1 x MLA | HHblits | 0.36 |
| ``` target    GLGVPESQLDVETRQYRNVVRTWAELQQTLHPLQERDPAFRFVFQTPKYRWGAHSTAVDADWISMLFGPFGDPYRRDPRM 7dxx.1    --------------------------------------------------------------------------------  target    PWTGEAYLEINPKDAAELGLADGDYAWVDADPEDRPYRGWNEDDPYYEVARAMMRVRIYTGMSRGVIRTWFNMYAATPAT 7dxx.1    --VGKRVVRVDKYERAKVGVKVGDYVEVK---------------------------------------------------  target    VANQKATPGNPARNEQTRYVALFRYGSHQSGTRAWLRPTQQTDSLVRKGYFGQVIGTGFEADVHSVSGAPKEAFVKIEKA 7dxx.1    --------------------------------------------------------------------------------  target    EDGGIGAERLWRPLTLGLRPEAPSAALTAYLAGDYSGTKGS 7dxx.1    ----------------------------------------- ``` | | | | | | | | | | | | | | | | | | | | | | | | | | | | | | | | | | | | | | | | | | | | | | | | | |
|  | 7dxx.1.B | mk2h\_deltaMILPS protein  *Crystal structure of the mk2h\_deltaMILPS peptide homodimer* | 0.00 |  | 25.93 | 0.10 | 83-109 | X-ray | 1.40 | homo-dimer | 1 x MLA | HHblits | 0.36 |
| ``` target    GLGVPESQLDVETRQYRNVVRTWAELQQTLHPLQERDPAFRFVFQTPKYRWGAHSTAVDADWISMLFGPFGDPYRRDPRM 7dxx.1    --------------------------------------------------------------------------------  target    PWTGEAYLEINPKDAAELGLADGDYAWVDADPEDRPYRGWNEDDPYYEVARAMMRVRIYTGMSRGVIRTWFNMYAATPAT 7dxx.1    --VGKRVVRVDKYERAKVGVKVGDYVEVK---------------------------------------------------  target    VANQKATPGNPARNEQTRYVALFRYGSHQSGTRAWLRPTQQTDSLVRKGYFGQVIGTGFEADVHSVSGAPKEAFVKIEKA 7dxx.1    --------------------------------------------------------------------------------  target    EDGGIGAERLWRPLTLGLRPEAPSAALTAYLAGDYSGTKGS 7dxx.1    ----------------------------------------- ``` | | | | | | | | | | | | | | | | | | | | | | | | | | | | | | | | | | | | | | | | | | | | | | | | | |
|  | 7dxz.1.A | mk2h\_deltaMILPYS protein  *Crystal structure of the chemically synthesized mk2h\_deltaMILPYS peptide homodimer in complex with malonate* | 0.00 |  | 21.43 | 0.10 | 82-109 | X-ray | 1.90 | homo-dimer | 4 x MLA | HHblits | 0.33 |
| ``` target    GLGVPESQLDVETRQYRNVVRTWAELQQTLHPLQERDPAFRFVFQTPKYRWGAHSTAVDADWISMLFGPFGDPYRRDPRM 7dxz.1    --------------------------------------------------------------------------------  target    PWTGEAYLEINPKDAAELGLADGDYAWVDADPEDRPYRGWNEDDPYYEVARAMMRVRIYTGMSRGVIRTWFNMYAATPAT 7dxz.1    -DVGKRVVRVDKAERAKVGVKVGDVVEVK---------------------------------------------------  target    VANQKATPGNPARNEQTRYVALFRYGSHQSGTRAWLRPTQQTDSLVRKGYFGQVIGTGFEADVHSVSGAPKEAFVKIEKA 7dxz.1    --------------------------------------------------------------------------------  target    EDGGIGAERLWRPLTLGLRPEAPSAALTAYLAGDYSGTKGS 7dxz.1    ----------------------------------------- ``` | | | | | | | | | | | | | | | | | | | | | | | | | | | | | | | | | | | | | | | | | | | | | | | | | |
|  | 7dxz.2.A | mk2h\_deltaMILPYS protein  *Crystal structure of the chemically synthesized mk2h\_deltaMILPYS peptide homodimer in complex with malonate* | 0.00 |  | 21.43 | 0.10 | 82-109 | X-ray | 1.90 | homo-dimer | 3 x MLA | HHblits | 0.33 |
| ``` target    GLGVPESQLDVETRQYRNVVRTWAELQQTLHPLQERDPAFRFVFQTPKYRWGAHSTAVDADWISMLFGPFGDPYRRDPRM 7dxz.2    --------------------------------------------------------------------------------  target    PWTGEAYLEINPKDAAELGLADGDYAWVDADPEDRPYRGWNEDDPYYEVARAMMRVRIYTGMSRGVIRTWFNMYAATPAT 7dxz.2    -DVGKRVVRVDKAERAKVGVKVGDVVEVK---------------------------------------------------  target    VANQKATPGNPARNEQTRYVALFRYGSHQSGTRAWLRPTQQTDSLVRKGYFGQVIGTGFEADVHSVSGAPKEAFVKIEKA 7dxz.2    --------------------------------------------------------------------------------  target    EDGGIGAERLWRPLTLGLRPEAPSAALTAYLAGDYSGTKGS 7dxz.2    ----------------------------------------- ``` | | | | | | | | | | | | | | | | | | | | | | | | | | | | | | | | | | | | | | | | | | | | | | | | | |
|  | 7dxz.2.B | mk2h\_deltaMILPYS protein  *Crystal structure of the chemically synthesized mk2h\_deltaMILPYS peptide homodimer in complex with malonate* | 0.00 |  | 21.43 | 0.10 | 82-109 | X-ray | 1.90 | homo-dimer | 3 x MLA | HHblits | 0.33 |
| ``` target    GLGVPESQLDVETRQYRNVVRTWAELQQTLHPLQERDPAFRFVFQTPKYRWGAHSTAVDADWISMLFGPFGDPYRRDPRM 7dxz.2    --------------------------------------------------------------------------------  target    PWTGEAYLEINPKDAAELGLADGDYAWVDADPEDRPYRGWNEDDPYYEVARAMMRVRIYTGMSRGVIRTWFNMYAATPAT 7dxz.2    -DVGKRVVRVDKAERAKVGVKVGDVVEVK---------------------------------------------------  target    VANQKATPGNPARNEQTRYVALFRYGSHQSGTRAWLRPTQQTDSLVRKGYFGQVIGTGFEADVHSVSGAPKEAFVKIEKA 7dxz.2    --------------------------------------------------------------------------------  target    EDGGIGAERLWRPLTLGLRPEAPSAALTAYLAGDYSGTKGS 7dxz.2    ----------------------------------------- ``` | | | | | | | | | | | | | | | | | | | | | | | | | | | | | | | | | | | | | | | | | | | | | | | | | |
|  | 7dxz.3.A | mk2h\_deltaMILPYS protein  *Crystal structure of the chemically synthesized mk2h\_deltaMILPYS peptide homodimer in complex with malonate* | 0.00 |  | 21.43 | 0.10 | 82-109 | X-ray | 1.90 | homo-dimer | 4 x MLA | HHblits | 0.33 |
| ``` target    GLGVPESQLDVETRQYRNVVRTWAELQQTLHPLQERDPAFRFVFQTPKYRWGAHSTAVDADWISMLFGPFGDPYRRDPRM 7dxz.3    --------------------------------------------------------------------------------  target    PWTGEAYLEINPKDAAELGLADGDYAWVDADPEDRPYRGWNEDDPYYEVARAMMRVRIYTGMSRGVIRTWFNMYAATPAT 7dxz.3    -DVGKRVVRVDKAERAKVGVKVGDVVEVK---------------------------------------------------  target    VANQKATPGNPARNEQTRYVALFRYGSHQSGTRAWLRPTQQTDSLVRKGYFGQVIGTGFEADVHSVSGAPKEAFVKIEKA 7dxz.3    --------------------------------------------------------------------------------  target    EDGGIGAERLWRPLTLGLRPEAPSAALTAYLAGDYSGTKGS 7dxz.3    ----------------------------------------- ``` | | | | | | | | | | | | | | | | | | | | | | | | | | | | | | | | | | | | | | | | | | | | | | | | | |
|  | 7dyc.1.A | mk2h\_deltaMILPYS protein  *Crystal structure of the chemically synthesized mk2h\_deltaMILPYS peptide homodimer in complex with malate* | 0.00 |  | 21.43 | 0.10 | 82-109 | X-ray | 2.30 | homo-dimer | 2 x MLT | HHblits | 0.33 |
| ``` target    GLGVPESQLDVETRQYRNVVRTWAELQQTLHPLQERDPAFRFVFQTPKYRWGAHSTAVDADWISMLFGPFGDPYRRDPRM 7dyc.1    --------------------------------------------------------------------------------  target    PWTGEAYLEINPKDAAELGLADGDYAWVDADPEDRPYRGWNEDDPYYEVARAMMRVRIYTGMSRGVIRTWFNMYAATPAT 7dyc.1    -DVGKRVVRVDKAERAKVGVKVGDVVEVK---------------------------------------------------  target    VANQKATPGNPARNEQTRYVALFRYGSHQSGTRAWLRPTQQTDSLVRKGYFGQVIGTGFEADVHSVSGAPKEAFVKIEKA 7dyc.1    --------------------------------------------------------------------------------  target    EDGGIGAERLWRPLTLGLRPEAPSAALTAYLAGDYSGTKGS 7dyc.1    ----------------------------------------- ``` | | | | | | | | | | | | | | | | | | | | | | | | | | | | | | | | | | | | | | | | | | | | | | | | | |
|  | 7dyc.2.A | mk2h\_deltaMILPYS protein  *Crystal structure of the chemically synthesized mk2h\_deltaMILPYS peptide homodimer in complex with malate* | 0.00 |  | 21.43 | 0.10 | 82-109 | X-ray | 2.30 | homo-dimer |  | HHblits | 0.33 |
| ``` target    GLGVPESQLDVETRQYRNVVRTWAELQQTLHPLQERDPAFRFVFQTPKYRWGAHSTAVDADWISMLFGPFGDPYRRDPRM 7dyc.2    --------------------------------------------------------------------------------  target    PWTGEAYLEINPKDAAELGLADGDYAWVDADPEDRPYRGWNEDDPYYEVARAMMRVRIYTGMSRGVIRTWFNMYAATPAT 7dyc.2    -DVGKRVVRVDKAERAKVGVKVGDVVEVK---------------------------------------------------  target    VANQKATPGNPARNEQTRYVALFRYGSHQSGTRAWLRPTQQTDSLVRKGYFGQVIGTGFEADVHSVSGAPKEAFVKIEKA 7dyc.2    --------------------------------------------------------------------------------  target    EDGGIGAERLWRPLTLGLRPEAPSAALTAYLAGDYSGTKGS 7dyc.2    ----------------------------------------- ``` | | | | | | | | | | | | | | | | | | | | | | | | | | | | | | | | | | | | | | | | | | | | | | | | | |
|  | 7dyc.3.A | mk2h\_deltaMILPYS protein  *Crystal structure of the chemically synthesized mk2h\_deltaMILPYS peptide homodimer in complex with malate* | 0.00 |  | 21.43 | 0.10 | 82-109 | X-ray | 2.30 | homo-dimer | 2 x LMR | HHblits | 0.33 |
| ``` target    GLGVPESQLDVETRQYRNVVRTWAELQQTLHPLQERDPAFRFVFQTPKYRWGAHSTAVDADWISMLFGPFGDPYRRDPRM 7dyc.3    --------------------------------------------------------------------------------  target    PWTGEAYLEINPKDAAELGLADGDYAWVDADPEDRPYRGWNEDDPYYEVARAMMRVRIYTGMSRGVIRTWFNMYAATPAT 7dyc.3    -DVGKRVVRVDKAERAKVGVKVGDVVEVK---------------------------------------------------  target    VANQKATPGNPARNEQTRYVALFRYGSHQSGTRAWLRPTQQTDSLVRKGYFGQVIGTGFEADVHSVSGAPKEAFVKIEKA 7dyc.3    --------------------------------------------------------------------------------  target    EDGGIGAERLWRPLTLGLRPEAPSAALTAYLAGDYSGTKGS 7dyc.3    ----------------------------------------- ``` | | | | | | | | | | | | | | | | | | | | | | | | | | | | | | | | | | | | | | | | | | | | | | | | | |
|  | 7dg7.1.A | ATPase of the AAA+ class  *DPBB domain of VCP-like ATPase from Methanopyrus kandleri* | 0.02 |  | 14.29 | 0.10 | 84-111 | X-ray | 1.60 | monomer | 2 x IMD, 8 x ZN | HHblits | 0.32 |
| ``` target    GLGVPESQLDVETRQYRNVVRTWAELQQTLHPLQERDPAFRFVFQTPKYRWGAHSTAVDADWISMLFGPFGDPYRRDPRM 7dg7.1    --------------------------------------------------------------------------------  target    PWTGEAYLEINPKDAAELGLADGDYAWVDADPEDRPYRGWNEDDPYYEVARAMMRVRIYTGMSRGVIRTWFNMYAATPAT 7dg7.1    ---GKRAVRMDKASRDRIGVSEGDLVKITGS-------------------------------------------------  target    VANQKATPGNPARNEQTRYVALFRYGSHQSGTRAWLRPTQQTDSLVRKGYFGQVIGTGFEADVHSVSGAPKEAFVKIEKA 7dg7.1    --------------------------------------------------------------------------------  target    EDGGIGAERLWRPLTLGLRPEAPSAALTAYLAGDYSGTKGS 7dg7.1    ----------------------------------------- ``` | | | | | | | | | | | | | | | | | | | | | | | | | | | | | | | | | | | | | | | | | | | | | | | | | |
|  | 3idw.1.A | Actin cytoskeleton-regulatory complex protein SLA1  *Crystal structure of Sla1 homology domain 2* | 0.00 |  | 28.57 | 0.10 | 84-111 | X-ray | 1.85 | monomer |  | HHblits | 0.32 |
| ``` target    GLGVPESQLDVETRQYRNVVRTWAELQQTLHPLQERDPAFRFVFQTPKYRWGAHSTAVDADWISMLFGPFGDPYRRDPRM 3idw.1    --------------------------------------------------------------------------------  target    PWTGEAYLEINPKDAAELGLADGDYAWVDADPEDRPYRGWNEDDPYYEVARAMMRVRIYTGMSRGVIRTWFNMYAATPAT 3idw.1    ---EDMMPDINNSMLRTLGLREGDIVRVMKH-------------------------------------------------  target    VANQKATPGNPARNEQTRYVALFRYGSHQSGTRAWLRPTQQTDSLVRKGYFGQVIGTGFEADVHSVSGAPKEAFVKIEKA 3idw.1    --------------------------------------------------------------------------------  target    EDGGIGAERLWRPLTLGLRPEAPSAALTAYLAGDYSGTKGS 3idw.1    ----------------------------------------- ``` | | | | | | | | | | | | | | | | | | | | | | | | | | | | | | | | | | | | | | | | | | | | | | | | | |
|  | 7dww.1.A | msDPBB\_sym2 protein  *Crystal structure of the computationally designed msDPBB\_sym2 protein* | 0.02 |  | 17.24 | 0.10 | 83-111 | X-ray | 1.80 | monomer |  | HHblits | 0.29 |
| ``` target    GLGVPESQLDVETRQYRNVVRTWAELQQTLHPLQERDPAFRFVFQTPKYRWGAHSTAVDADWISMLFGPFGDPYRRDPRM 7dww.1    --------------------------------------------------------------------------------  target    PWTGEAYLEINPKDAAELGLADGDYAWVDADPEDRPYRGWNEDDPYYEVARAMMRVRIYTGMSRGVIRTWFNMYAATPAT 7dww.1    --VGKNIVRMDEELMRLLGVKVGDLVEIMKV-------------------------------------------------  target    VANQKATPGNPARNEQTRYVALFRYGSHQSGTRAWLRPTQQTDSLVRKGYFGQVIGTGFEADVHSVSGAPKEAFVKIEKA 7dww.1    --------------------------------------------------------------------------------  target    EDGGIGAERLWRPLTLGLRPEAPSAALTAYLAGDYSGTKGS 7dww.1    ----------------------------------------- ``` | | | | | | | | | | | | | | | | | | | | | | | | | | | | | | | | | | | | | | | | | | | | | | | | | |
|  | 7dww.2.A | msDPBB\_sym2 protein  *Crystal structure of the computationally designed msDPBB\_sym2 protein* | 0.01 |  | 17.24 | 0.10 | 83-111 | X-ray | 1.80 | monomer |  | HHblits | 0.29 |
| ``` target    GLGVPESQLDVETRQYRNVVRTWAELQQTLHPLQERDPAFRFVFQTPKYRWGAHSTAVDADWISMLFGPFGDPYRRDPRM 7dww.2    --------------------------------------------------------------------------------  target    PWTGEAYLEINPKDAAELGLADGDYAWVDADPEDRPYRGWNEDDPYYEVARAMMRVRIYTGMSRGVIRTWFNMYAATPAT 7dww.2    --VGKNIVRMDEELMRLLGVKVGDLVEIMKV-------------------------------------------------  target    VANQKATPGNPARNEQTRYVALFRYGSHQSGTRAWLRPTQQTDSLVRKGYFGQVIGTGFEADVHSVSGAPKEAFVKIEKA 7dww.2    --------------------------------------------------------------------------------  target    EDGGIGAERLWRPLTLGLRPEAPSAALTAYLAGDYSGTKGS 7dww.2    ----------------------------------------- ``` | | | | | | | | | | | | | | | | | | | | | | | | | | | | | | | | | | | | | | | | | | | | | | | | | |
|  | 6f49.1.A | Lipoprotein-releasing system transmembrane protein LolC,Lipoprotein-releasing system transmembrane protein LolC  *Periplasmic domain of LolC lacking the Hook.* | 0.02 |  | 25.00 | 0.10 | 84-111 | X-ray | 2.02 | monomer |  | HHblits | 0.31 |
| ``` target    GLGVPESQLDVETRQYRNVVRTWAELQQTLHPLQERDPAFRFVFQTPKYRWGAHSTAVDADWISMLFGPFGDPYRRDPRM 6f49.1    --------------------------------------------------------------------------------  target    PWTGEAYLEINPKDAAELGLADGDYAWVDADPEDRPYRGWNEDDPYYEVARAMMRVRIYTGMSRGVIRTWFNMYAATPAT 6f49.1    ---GKYNVILGEQLASQLGVNRGDQIRVMVG-------------------------------------------------  target    VANQKATPGNPARNEQTRYVALFRYGSHQSGTRAWLRPTQQTDSLVRKGYFGQVIGTGFEADVHSVSGAPKEAFVKIEKA 6f49.1    --------------------------------------------------------------------------------  target    EDGGIGAERLWRPLTLGLRPEAPSAALTAYLAGDYSGTKGS 6f49.1    ----------------------------------------- ``` | | | | | | | | | | | | | | | | | | | | | | | | | | | | | | | | | | | | | | | | | | | | | | | | | |
|  | 5naa.1.A | Lipoprotein-releasing system transmembrane protein LolC  *Lipoprotein-releasing system transmembrane protein LolC* | 0.01 |  | 25.00 | 0.10 | 84-111 | X-ray | 1.88 | monomer |  | HHblits | 0.31 |
| ``` target    GLGVPESQLDVETRQYRNVVRTWAELQQTLHPLQERDPAFRFVFQTPKYRWGAHSTAVDADWISMLFGPFGDPYRRDPRM 5naa.1    --------------------------------------------------------------------------------  target    PWTGEAYLEINPKDAAELGLADGDYAWVDADPEDRPYRGWNEDDPYYEVARAMMRVRIYTGMSRGVIRTWFNMYAATPAT 5naa.1    ---GKYNVILGEQLASQLGVNRGDQIRVMVP-------------------------------------------------  target    VANQKATPGNPARNEQTRYVALFRYGSHQSGTRAWLRPTQQTDSLVRKGYFGQVIGTGFEADVHSVSGAPKEAFVKIEKA 5naa.1    --------------------------------------------------------------------------------  target    EDGGIGAERLWRPLTLGLRPEAPSAALTAYLAGDYSGTKGS 5naa.1    ----------------------------------------- ``` | | | | | | | | | | | | | | | | | | | | | | | | | | | | | | | | | | | | | | | | | | | | | | | | | |
|  | 5naa.2.A | Lipoprotein-releasing system transmembrane protein LolC  *Lipoprotein-releasing system transmembrane protein LolC* | 0.02 |  | 25.00 | 0.10 | 84-111 | X-ray | 1.88 | monomer |  | HHblits | 0.31 |
| ``` target    GLGVPESQLDVETRQYRNVVRTWAELQQTLHPLQERDPAFRFVFQTPKYRWGAHSTAVDADWISMLFGPFGDPYRRDPRM 5naa.2    --------------------------------------------------------------------------------  target    PWTGEAYLEINPKDAAELGLADGDYAWVDADPEDRPYRGWNEDDPYYEVARAMMRVRIYTGMSRGVIRTWFNMYAATPAT 5naa.2    ---GKYNVILGEQLASQLGVNRGDQIRVMVP-------------------------------------------------  target    VANQKATPGNPARNEQTRYVALFRYGSHQSGTRAWLRPTQQTDSLVRKGYFGQVIGTGFEADVHSVSGAPKEAFVKIEKA 5naa.2    --------------------------------------------------------------------------------  target    EDGGIGAERLWRPLTLGLRPEAPSAALTAYLAGDYSGTKGS 5naa.2    ----------------------------------------- ``` | | | | | | | | | | | | | | | | | | | | | | | | | | | | | | | | | | | | | | | | | | | | | | | | | |
|  | 6f3z.1.A | Lipoprotein-releasing system transmembrane protein LolC  *Complex of E. coli LolA and periplasmic domain of LolC* | 0.02 |  | 25.00 | 0.10 | 84-111 | X-ray | 2.00 | hetero-1-1-mer |  | HHblits | 0.31 |
| ``` target    GLGVPESQLDVETRQYRNVVRTWAELQQTLHPLQERDPAFRFVFQTPKYRWGAHSTAVDADWISMLFGPFGDPYRRDPRM 6f3z.1    --------------------------------------------------------------------------------  target    PWTGEAYLEINPKDAAELGLADGDYAWVDADPEDRPYRGWNEDDPYYEVARAMMRVRIYTGMSRGVIRTWFNMYAATPAT 6f3z.1    ---GKYNVILGEQLASQLGVNRGDQIRVMVP-------------------------------------------------  target    VANQKATPGNPARNEQTRYVALFRYGSHQSGTRAWLRPTQQTDSLVRKGYFGQVIGTGFEADVHSVSGAPKEAFVKIEKA 6f3z.1    --------------------------------------------------------------------------------  target    EDGGIGAERLWRPLTLGLRPEAPSAALTAYLAGDYSGTKGS 6f3z.1    ----------------------------------------- ``` | | | | | | | | | | | | | | | | | | | | | | | | | | | | | | | | | | | | | | | | | | | | | | | | | |
|  | 6f3z.2.A | Lipoprotein-releasing system transmembrane protein LolC  *Complex of E. coli LolA and periplasmic domain of LolC* | 0.02 |  | 25.00 | 0.10 | 84-111 | X-ray | 2.00 | hetero-1-1-mer |  | HHblits | 0.31 |
| ``` target    GLGVPESQLDVETRQYRNVVRTWAELQQTLHPLQERDPAFRFVFQTPKYRWGAHSTAVDADWISMLFGPFGDPYRRDPRM 6f3z.2    --------------------------------------------------------------------------------  target    PWTGEAYLEINPKDAAELGLADGDYAWVDADPEDRPYRGWNEDDPYYEVARAMMRVRIYTGMSRGVIRTWFNMYAATPAT 6f3z.2    ---GKYNVILGEQLASQLGVNRGDQIRVMVP-------------------------------------------------  target    VANQKATPGNPARNEQTRYVALFRYGSHQSGTRAWLRPTQQTDSLVRKGYFGQVIGTGFEADVHSVSGAPKEAFVKIEKA 6f3z.2    --------------------------------------------------------------------------------  target    EDGGIGAERLWRPLTLGLRPEAPSAALTAYLAGDYSGTKGS 6f3z.2    ----------------------------------------- ``` | | | | | | | | | | | | | | | | | | | | | | | | | | | | | | | | | | | | | | | | | | | | | | | | | |
|  | 7di1.1.A | mkDPBB\_sym\_86 protein  *Crystal structure of the rationally designed mkDPBB\_sym\_86 protein* | 0.00 |  | 30.77 | 0.09 | 84-109 | X-ray | 2.10 | monomer |  | HHblits | 0.37 |
| ``` target    GLGVPESQLDVETRQYRNVVRTWAELQQTLHPLQERDPAFRFVFQTPKYRWGAHSTAVDADWISMLFGPFGDPYRRDPRM 7di1.1    --------------------------------------------------------------------------------  target    PWTGEAYLEINPKDAAELGLADGDYAWVDADPEDRPYRGWNEDDPYYEVARAMMRVRIYTGMSRGVIRTWFNMYAATPAT 7di1.1    ---GKGIVRMDKYERAKLGVSVGDYVEVK---------------------------------------------------  target    VANQKATPGNPARNEQTRYVALFRYGSHQSGTRAWLRPTQQTDSLVRKGYFGQVIGTGFEADVHSVSGAPKEAFVKIEKA 7di1.1    --------------------------------------------------------------------------------  target    EDGGIGAERLWRPLTLGLRPEAPSAALTAYLAGDYSGTKGS 7di1.1    ----------------------------------------- ``` | | | | | | | | | | | | | | | | | | | | | | | | | | | | | | | | | | | | | | | | | | | | | | | | | |
|  | 7dbo.1.A | VCP-like ATPase  *DPBB domain of VCP-like ATPase from Thermoplasma acidophilum* | 0.02 |  | 13.79 | 0.10 | 83-111 | X-ray | 1.90 | monomer |  | HHblits | 0.28 |
| ``` target    GLGVPESQLDVETRQYRNVVRTWAELQQTLHPLQERDPAFRFVFQTPKYRWGAHSTAVDADWISMLFGPFGDPYRRDPRM 7dbo.1    --------------------------------------------------------------------------------  target    PWTGEAYLEINPKDAAELGLADGDYAWVDADPEDRPYRGWNEDDPYYEVARAMMRVRIYTGMSRGVIRTWFNMYAATPAT 7dbo.1    --PGMSRVRLDESSRRLLDAEIGDVVEIEKV-------------------------------------------------  target    VANQKATPGNPARNEQTRYVALFRYGSHQSGTRAWLRPTQQTDSLVRKGYFGQVIGTGFEADVHSVSGAPKEAFVKIEKA 7dbo.1    --------------------------------------------------------------------------------  target    EDGGIGAERLWRPLTLGLRPEAPSAALTAYLAGDYSGTKGS 7dbo.1    ----------------------------------------- ``` | | | | | | | | | | | | | | | | | | | | | | | | | | | | | | | | | | | | | | | | | | | | | | | | | |
|  | 7dbo.2.A | VCP-like ATPase  *DPBB domain of VCP-like ATPase from Thermoplasma acidophilum* | 0.02 |  | 13.79 | 0.10 | 83-111 | X-ray | 1.90 | monomer |  | HHblits | 0.28 |
| ``` target    GLGVPESQLDVETRQYRNVVRTWAELQQTLHPLQERDPAFRFVFQTPKYRWGAHSTAVDADWISMLFGPFGDPYRRDPRM 7dbo.2    --------------------------------------------------------------------------------  target    PWTGEAYLEINPKDAAELGLADGDYAWVDADPEDRPYRGWNEDDPYYEVARAMMRVRIYTGMSRGVIRTWFNMYAATPAT 7dbo.2    --PGMSRVRLDESSRRLLDAEIGDVVEIEKV-------------------------------------------------  target    VANQKATPGNPARNEQTRYVALFRYGSHQSGTRAWLRPTQQTDSLVRKGYFGQVIGTGFEADVHSVSGAPKEAFVKIEKA 7dbo.2    --------------------------------------------------------------------------------  target    EDGGIGAERLWRPLTLGLRPEAPSAALTAYLAGDYSGTKGS 7dbo.2    ----------------------------------------- ``` | | | | | | | | | | | | | | | | | | | | | | | | | | | | | | | | | | | | | | | | | | | | | | | | | |
|  | 1cz4.1.A | VCP-LIKE ATPASE  *NMR STRUCTURE OF VAT-N: THE N-TERMINAL DOMAIN OF VAT (VCP-LIKE ATPASE OF THERMOPLASMA)* | 0.02 |  | 13.79 | 0.10 | 83-111 | NMR | 0.00 | monomer |  | HHblits | 0.28 |
| ``` target    GLGVPESQLDVETRQYRNVVRTWAELQQTLHPLQERDPAFRFVFQTPKYRWGAHSTAVDADWISMLFGPFGDPYRRDPRM 1cz4.1    --------------------------------------------------------------------------------  target    PWTGEAYLEINPKDAAELGLADGDYAWVDADPEDRPYRGWNEDDPYYEVARAMMRVRIYTGMSRGVIRTWFNMYAATPAT 1cz4.1    --PGMSRVRLDESSRRLLDAEIGDVVEIEKV-------------------------------------------------  target    VANQKATPGNPARNEQTRYVALFRYGSHQSGTRAWLRPTQQTDSLVRKGYFGQVIGTGFEADVHSVSGAPKEAFVKIEKA 1cz4.1    --------------------------------------------------------------------------------  target    EDGGIGAERLWRPLTLGLRPEAPSAALTAYLAGDYSGTKGS 1cz4.1    ----------------------------------------- ``` | | | | | | | | | | | | | | | | | | | | | | | | | | | | | | | | | | | | | | | | | | | | | | | | | |
|  | 1cz5.1.A | VCP-LIKE ATPASE  *NMR STRUCTURE OF VAT-N: THE N-TERMINAL DOMAIN OF VAT (VCP-LIKE ATPASE OF THERMOPLASMA)* | 0.02 |  | 13.79 | 0.10 | 83-111 | NMR | 0.00 | monomer |  | HHblits | 0.28 |
| ``` target    GLGVPESQLDVETRQYRNVVRTWAELQQTLHPLQERDPAFRFVFQTPKYRWGAHSTAVDADWISMLFGPFGDPYRRDPRM 1cz5.1    --------------------------------------------------------------------------------  target    PWTGEAYLEINPKDAAELGLADGDYAWVDADPEDRPYRGWNEDDPYYEVARAMMRVRIYTGMSRGVIRTWFNMYAATPAT 1cz5.1    --PGMSRVRLDESSRRLLDAEIGDVVEIEKV-------------------------------------------------  target    VANQKATPGNPARNEQTRYVALFRYGSHQSGTRAWLRPTQQTDSLVRKGYFGQVIGTGFEADVHSVSGAPKEAFVKIEKA 1cz5.1    --------------------------------------------------------------------------------  target    EDGGIGAERLWRPLTLGLRPEAPSAALTAYLAGDYSGTKGS 1cz5.1    ----------------------------------------- ``` | | | | | | | | | | | | | | | | | | | | | | | | | | | | | | | | | | | | | | | | | | | | | | | | | |
|  | 5g4g.1.A | VCP-LIKE ATPASE  *Structure of the ATPgS-bound VAT complex* | 0.01 |  | 13.79 | 0.10 | 83-111 | EM | 7.80 | homo-hexamer |  | HHblits | 0.28 |
| ``` target    GLGVPESQLDVETRQYRNVVRTWAELQQTLHPLQERDPAFRFVFQTPKYRWGAHSTAVDADWISMLFGPFGDPYRRDPRM 5g4g.1    --------------------------------------------------------------------------------  target    PWTGEAYLEINPKDAAELGLADGDYAWVDADPEDRPYRGWNEDDPYYEVARAMMRVRIYTGMSRGVIRTWFNMYAATPAT 5g4g.1    --PGMSRVRLDESSRRLLDAEIGDVVEIEKV-------------------------------------------------  target    VANQKATPGNPARNEQTRYVALFRYGSHQSGTRAWLRPTQQTDSLVRKGYFGQVIGTGFEADVHSVSGAPKEAFVKIEKA 5g4g.1    --------------------------------------------------------------------------------  target    EDGGIGAERLWRPLTLGLRPEAPSAALTAYLAGDYSGTKGS 5g4g.1    ----------------------------------------- ``` | | | | | | | | | | | | | | | | | | | | | | | | | | | | | | | | | | | | | | | | | | | | | | | | | |
|  | 5g4f.1.A | VCP-LIKE ATPASE  *Structure of the ADP-bound VAT complex* | 0.02 |  | 13.79 | 0.10 | 83-111 | EM | 7.00 | homo-hexamer |  | HHblits | 0.28 |
| ``` target    GLGVPESQLDVETRQYRNVVRTWAELQQTLHPLQERDPAFRFVFQTPKYRWGAHSTAVDADWISMLFGPFGDPYRRDPRM 5g4f.1    --------------------------------------------------------------------------------  target    PWTGEAYLEINPKDAAELGLADGDYAWVDADPEDRPYRGWNEDDPYYEVARAMMRVRIYTGMSRGVIRTWFNMYAATPAT 5g4f.1    --PGMSRVRLDESSRRLLDAEIGDVVEIEKV-------------------------------------------------  target    VANQKATPGNPARNEQTRYVALFRYGSHQSGTRAWLRPTQQTDSLVRKGYFGQVIGTGFEADVHSVSGAPKEAFVKIEKA 5g4f.1    --------------------------------------------------------------------------------  target    EDGGIGAERLWRPLTLGLRPEAPSAALTAYLAGDYSGTKGS 5g4f.1    ----------------------------------------- ``` | | | | | | | | | | | | | | | | | | | | | | | | | | | | | | | | | | | | | | | | | | | | | | | | | |
|  | 5g4f.1.B | VCP-LIKE ATPASE  *Structure of the ADP-bound VAT complex* | 0.02 |  | 13.79 | 0.10 | 83-111 | EM | 7.00 | homo-hexamer |  | HHblits | 0.28 |
| ``` target    GLGVPESQLDVETRQYRNVVRTWAELQQTLHPLQERDPAFRFVFQTPKYRWGAHSTAVDADWISMLFGPFGDPYRRDPRM 5g4f.1    --------------------------------------------------------------------------------  target    PWTGEAYLEINPKDAAELGLADGDYAWVDADPEDRPYRGWNEDDPYYEVARAMMRVRIYTGMSRGVIRTWFNMYAATPAT 5g4f.1    --PGMSRVRLDESSRRLLDAEIGDVVEIEKV-------------------------------------------------  target    VANQKATPGNPARNEQTRYVALFRYGSHQSGTRAWLRPTQQTDSLVRKGYFGQVIGTGFEADVHSVSGAPKEAFVKIEKA 5g4f.1    --------------------------------------------------------------------------------  target    EDGGIGAERLWRPLTLGLRPEAPSAALTAYLAGDYSGTKGS 5g4f.1    ----------------------------------------- ``` | | | | | | | | | | | | | | | | | | | | | | | | | | | | | | | | | | | | | | | | | | | | | | | | | |
|  | 5g4f.1.C | VCP-LIKE ATPASE  *Structure of the ADP-bound VAT complex* | 0.02 |  | 13.79 | 0.10 | 83-111 | EM | 7.00 | homo-hexamer |  | HHblits | 0.28 |
| ``` target    GLGVPESQLDVETRQYRNVVRTWAELQQTLHPLQERDPAFRFVFQTPKYRWGAHSTAVDADWISMLFGPFGDPYRRDPRM 5g4f.1    --------------------------------------------------------------------------------  target    PWTGEAYLEINPKDAAELGLADGDYAWVDADPEDRPYRGWNEDDPYYEVARAMMRVRIYTGMSRGVIRTWFNMYAATPAT 5g4f.1    --PGMSRVRLDESSRRLLDAEIGDVVEIEKV-------------------------------------------------  target    VANQKATPGNPARNEQTRYVALFRYGSHQSGTRAWLRPTQQTDSLVRKGYFGQVIGTGFEADVHSVSGAPKEAFVKIEKA 5g4f.1    --------------------------------------------------------------------------------  target    EDGGIGAERLWRPLTLGLRPEAPSAALTAYLAGDYSGTKGS 5g4f.1    ----------------------------------------- ``` | | | | | | | | | | | | | | | | | | | | | | | | | | | | | | | | | | | | | | | | | | | | | | | | | |
|  | 5g4f.1.D | VCP-LIKE ATPASE  *Structure of the ADP-bound VAT complex* | 0.02 |  | 13.79 | 0.10 | 83-111 | EM | 7.00 | homo-hexamer |  | HHblits | 0.28 |
| ``` target    GLGVPESQLDVETRQYRNVVRTWAELQQTLHPLQERDPAFRFVFQTPKYRWGAHSTAVDADWISMLFGPFGDPYRRDPRM 5g4f.1    --------------------------------------------------------------------------------  target    PWTGEAYLEINPKDAAELGLADGDYAWVDADPEDRPYRGWNEDDPYYEVARAMMRVRIYTGMSRGVIRTWFNMYAATPAT 5g4f.1    --PGMSRVRLDESSRRLLDAEIGDVVEIEKV-------------------------------------------------  target    VANQKATPGNPARNEQTRYVALFRYGSHQSGTRAWLRPTQQTDSLVRKGYFGQVIGTGFEADVHSVSGAPKEAFVKIEKA 5g4f.1    --------------------------------------------------------------------------------  target    EDGGIGAERLWRPLTLGLRPEAPSAALTAYLAGDYSGTKGS 5g4f.1    ----------------------------------------- ``` | | | | | | | | | | | | | | | | | | | | | | | | | | | | | | | | | | | | | | | | | | | | | | | | | |
|  | 5g4f.1.E | VCP-LIKE ATPASE  *Structure of the ADP-bound VAT complex* | 0.02 |  | 13.79 | 0.10 | 83-111 | EM | 7.00 | homo-hexamer |  | HHblits | 0.28 |
| ``` target    GLGVPESQLDVETRQYRNVVRTWAELQQTLHPLQERDPAFRFVFQTPKYRWGAHSTAVDADWISMLFGPFGDPYRRDPRM 5g4f.1    --------------------------------------------------------------------------------  target    PWTGEAYLEINPKDAAELGLADGDYAWVDADPEDRPYRGWNEDDPYYEVARAMMRVRIYTGMSRGVIRTWFNMYAATPAT 5g4f.1    --PGMSRVRLDESSRRLLDAEIGDVVEIEKV-------------------------------------------------  target    VANQKATPGNPARNEQTRYVALFRYGSHQSGTRAWLRPTQQTDSLVRKGYFGQVIGTGFEADVHSVSGAPKEAFVKIEKA 5g4f.1    --------------------------------------------------------------------------------  target    EDGGIGAERLWRPLTLGLRPEAPSAALTAYLAGDYSGTKGS 5g4f.1    ----------------------------------------- ``` | | | | | | | | | | | | | | | | | | | | | | | | | | | | | | | | | | | | | | | | | | | | | | | | | |
|  | 5g4f.1.F | VCP-LIKE ATPASE  *Structure of the ADP-bound VAT complex* | 0.02 |  | 13.79 | 0.10 | 83-111 | EM | 7.00 | homo-hexamer |  | HHblits | 0.28 |
| ``` target    GLGVPESQLDVETRQYRNVVRTWAELQQTLHPLQERDPAFRFVFQTPKYRWGAHSTAVDADWISMLFGPFGDPYRRDPRM 5g4f.1    --------------------------------------------------------------------------------  target    PWTGEAYLEINPKDAAELGLADGDYAWVDADPEDRPYRGWNEDDPYYEVARAMMRVRIYTGMSRGVIRTWFNMYAATPAT 5g4f.1    --PGMSRVRLDESSRRLLDAEIGDVVEIEKV-------------------------------------------------  target    VANQKATPGNPARNEQTRYVALFRYGSHQSGTRAWLRPTQQTDSLVRKGYFGQVIGTGFEADVHSVSGAPKEAFVKIEKA 5g4f.1    --------------------------------------------------------------------------------  target    EDGGIGAERLWRPLTLGLRPEAPSAALTAYLAGDYSGTKGS 5g4f.1    ----------------------------------------- ``` | | | | | | | | | | | | | | | | | | | | | | | | | | | | | | | | | | | | | | | | | | | | | | | | | |
|  | 7du7.1.A | mkDPBB\_sym1 protein  *Crystal structure of the rationally designed mkDPBB\_sym1 protein* | 0.00 |  | 30.77 | 0.09 | 84-109 | X-ray | 1.20 | monomer |  | HHblits | 0.37 |
| ``` target    GLGVPESQLDVETRQYRNVVRTWAELQQTLHPLQERDPAFRFVFQTPKYRWGAHSTAVDADWISMLFGPFGDPYRRDPRM 7du7.1    --------------------------------------------------------------------------------  target    PWTGEAYLEINPKDAAELGLADGDYAWVDADPEDRPYRGWNEDDPYYEVARAMMRVRIYTGMSRGVIRTWFNMYAATPAT 7du7.1    ---GKGIVRMDKASRAKLGVSVGDYVEVK---------------------------------------------------  target    VANQKATPGNPARNEQTRYVALFRYGSHQSGTRAWLRPTQQTDSLVRKGYFGQVIGTGFEADVHSVSGAPKEAFVKIEKA 7du7.1    --------------------------------------------------------------------------------  target    EDGGIGAERLWRPLTLGLRPEAPSAALTAYLAGDYSGTKGS 7du7.1    ----------------------------------------- ``` | | | | | | | | | | | | | | | | | | | | | | | | | | | | | | | | | | | | | | | | | | | | | | | | | |
|  | 7du6.1.A | mkDPBB\_sym2 protein  *Crystal structure of the rationally designed mkDPBB\_sym2 protein* | 0.00 |  | 30.77 | 0.09 | 84-109 | X-ray | 1.60 | monomer |  | HHblits | 0.37 |
| ``` target    GLGVPESQLDVETRQYRNVVRTWAELQQTLHPLQERDPAFRFVFQTPKYRWGAHSTAVDADWISMLFGPFGDPYRRDPRM 7du6.1    --------------------------------------------------------------------------------  target    PWTGEAYLEINPKDAAELGLADGDYAWVDADPEDRPYRGWNEDDPYYEVARAMMRVRIYTGMSRGVIRTWFNMYAATPAT 7du6.1    ---GKRIVRMDKYERAKLGVSVGDYVEVK---------------------------------------------------  target    VANQKATPGNPARNEQTRYVALFRYGSHQSGTRAWLRPTQQTDSLVRKGYFGQVIGTGFEADVHSVSGAPKEAFVKIEKA 7du6.1    --------------------------------------------------------------------------------  target    EDGGIGAERLWRPLTLGLRPEAPSAALTAYLAGDYSGTKGS 7du6.1    ----------------------------------------- ``` | | | | | | | | | | | | | | | | | | | | | | | | | | | | | | | | | | | | | | | | | | | | | | | | | |
|  | 2l66.1.A | Transcriptional regulator, AbrB family  *The DNA-recognition fold of Sso7c4 suggests a new member of SpoVT-AbrB superfamily from archaea.* | 0.01 |  | 21.43 | 0.10 | 85-112 | NMR | 0.00 | homo-dimer |  | HHblits | 0.30 |
| ``` target    GLGVPESQLDVETRQYRNVVRTWAELQQTLHPLQERDPAFRFVFQTPKYRWGAHSTAVDADWISMLFGPFGDPYRRDPRM 2l66.1    --------------------------------------------------------------------------------  target    PWTGEAYLEINPKDAAELGLADGDYAWVDADPEDRPYRGWNEDDPYYEVARAMMRVRIYTGMSRGVIRTWFNMYAATPAT 2l66.1    ----NYQVTIPAKVRQKFQIKEGDLVKVTFDE------------------------------------------------  target    VANQKATPGNPARNEQTRYVALFRYGSHQSGTRAWLRPTQQTDSLVRKGYFGQVIGTGFEADVHSVSGAPKEAFVKIEKA 2l66.1    --------------------------------------------------------------------------------  target    EDGGIGAERLWRPLTLGLRPEAPSAALTAYLAGDYSGTKGS 2l66.1    ----------------------------------------- ``` | | | | | | | | | | | | | | | | | | | | | | | | | | | | | | | | | | | | | | | | | | | | | | | | | |
|  | 5udf.1.A | Lipoprotein-releasing system transmembrane protein LolE  *Structure of the N-terminal domain of lipoprotein-releasing system transmembrane protein LolE from Acinetobacter baumannii* | 0.02 |  | 25.00 | 0.10 | 84-111 | X-ray | 2.35 | homo-tetramer |  | HHblits | 0.30 |
| ``` target    GLGVPESQLDVETRQYRNVVRTWAELQQTLHPLQERDPAFRFVFQTPKYRWGAHSTAVDADWISMLFGPFGDPYRRDPRM 5udf.1    --------------------------------------------------------------------------------  target    PWTGEAYLEINPKDAAELGLADGDYAWVDADPEDRPYRGWNEDDPYYEVARAMMRVRIYTGMSRGVIRTWFNMYAATPAT 5udf.1    ---GEFGIVLGKDMADSLGLRLNDSVTLVLP-------------------------------------------------  target    VANQKATPGNPARNEQTRYVALFRYGSHQSGTRAWLRPTQQTDSLVRKGYFGQVIGTGFEADVHSVSGAPKEAFVKIEKA 5udf.1    --------------------------------------------------------------------------------  target    EDGGIGAERLWRPLTLGLRPEAPSAALTAYLAGDYSGTKGS 5udf.1    ----------------------------------------- ``` | | | | | | | | | | | | | | | | | | | | | | | | | | | | | | | | | | | | | | | | | | | | | | | | | |
|  | 4rv0.1.A | Transitional endoplasmic reticulum ATPase TER94  *Crystal structure of TN complex* | 0.02 |  | 17.86 | 0.10 | 84-111 | X-ray | 2.00 | hetero-oligomer |  | HHblits | 0.29 |
| ``` target    GLGVPESQLDVETRQYRNVVRTWAELQQTLHPLQERDPAFRFVFQTPKYRWGAHSTAVDADWISMLFGPFGDPYRRDPRM 4rv0.1    --------------------------------------------------------------------------------  target    PWTGEAYLEINPKDAAELGLADGDYAWVDADPEDRPYRGWNEDDPYYEVARAMMRVRIYTGMSRGVIRTWFNMYAATPAT 4rv0.1    ---DNSVVSLSQAKMDELQLFRGDTVILKGK-------------------------------------------------  target    VANQKATPGNPARNEQTRYVALFRYGSHQSGTRAWLRPTQQTDSLVRKGYFGQVIGTGFEADVHSVSGAPKEAFVKIEKA 4rv0.1    --------------------------------------------------------------------------------  target    EDGGIGAERLWRPLTLGLRPEAPSAALTAYLAGDYSGTKGS 4rv0.1    ----------------------------------------- ``` | | | | | | | | | | | | | | | | | | | | | | | | | | | | | | | | | | | | | | | | | | | | | | | | | |
|  | 7wbb.1.A | AFG2 isoform 1  *Cryo-EM structure of substrate engaged Drg1 hexamer* | 0.01 |  | 17.86 | 0.10 | 83-110 | EM | 0.00 | hetero-6-1-mer | 11 x ATP | HHblits | 0.29 |
| ``` target    GLGVPESQLDVETRQYRNVVRTWAELQQTLHPLQERDPAFRFVFQTPKYRWGAHSTAVDADWISMLFGPFGDPYRRDPRM 7wbb.1    --------------------------------------------------------------------------------  target    PWTGEAYLEINPKDAAELGLADGDYAWVDADPEDRPYRGWNEDDPYYEVARAMMRVRIYTGMSRGVIRTWFNMYAATPAT 7wbb.1    --KETCTAYIHPNVLSSLEINPGSFCTVGK--------------------------------------------------  target    VANQKATPGNPARNEQTRYVALFRYGSHQSGTRAWLRPTQQTDSLVRKGYFGQVIGTGFEADVHSVSGAPKEAFVKIEKA 7wbb.1    --------------------------------------------------------------------------------  target    EDGGIGAERLWRPLTLGLRPEAPSAALTAYLAGDYSGTKGS 7wbb.1    ----------------------------------------- ``` | | | | | | | | | | | | | | | | | | | | | | | | | | | | | | | | | | | | | | | | | | | | | | | | | |
|  | 7wbb.1.B | AFG2 isoform 1  *Cryo-EM structure of substrate engaged Drg1 hexamer* | 0.01 |  | 17.86 | 0.10 | 83-110 | EM | 0.00 | hetero-6-1-mer | 11 x ATP | HHblits | 0.29 |
| ``` target    GLGVPESQLDVETRQYRNVVRTWAELQQTLHPLQERDPAFRFVFQTPKYRWGAHSTAVDADWISMLFGPFGDPYRRDPRM 7wbb.1    --------------------------------------------------------------------------------  target    PWTGEAYLEINPKDAAELGLADGDYAWVDADPEDRPYRGWNEDDPYYEVARAMMRVRIYTGMSRGVIRTWFNMYAATPAT 7wbb.1    --KETCTAYIHPNVLSSLEINPGSFCTVGK--------------------------------------------------  target    VANQKATPGNPARNEQTRYVALFRYGSHQSGTRAWLRPTQQTDSLVRKGYFGQVIGTGFEADVHSVSGAPKEAFVKIEKA 7wbb.1    --------------------------------------------------------------------------------  target    EDGGIGAERLWRPLTLGLRPEAPSAALTAYLAGDYSGTKGS 7wbb.1    ----------------------------------------- ``` | | | | | | | | | | | | | | | | | | | | | | | | | | | | | | | | | | | | | | | | | | | | | | | | | |
|  | 7wbb.1.C | AFG2 isoform 1  *Cryo-EM structure of substrate engaged Drg1 hexamer* | 0.02 |  | 17.86 | 0.10 | 83-110 | EM | 0.00 | hetero-6-1-mer | 11 x ATP | HHblits | 0.29 |
| ``` target    GLGVPESQLDVETRQYRNVVRTWAELQQTLHPLQERDPAFRFVFQTPKYRWGAHSTAVDADWISMLFGPFGDPYRRDPRM 7wbb.1    --------------------------------------------------------------------------------  target    PWTGEAYLEINPKDAAELGLADGDYAWVDADPEDRPYRGWNEDDPYYEVARAMMRVRIYTGMSRGVIRTWFNMYAATPAT 7wbb.1    --KETCTAYIHPNVLSSLEINPGSFCTVGK--------------------------------------------------  target    VANQKATPGNPARNEQTRYVALFRYGSHQSGTRAWLRPTQQTDSLVRKGYFGQVIGTGFEADVHSVSGAPKEAFVKIEKA 7wbb.1    --------------------------------------------------------------------------------  target    EDGGIGAERLWRPLTLGLRPEAPSAALTAYLAGDYSGTKGS 7wbb.1    ----------------------------------------- ``` | | | | | | | | | | | | | | | | | | | | | | | | | | | | | | | | | | | | | | | | | | | | | | | | | |
|  | 7wbb.1.D | AFG2 isoform 1  *Cryo-EM structure of substrate engaged Drg1 hexamer* | 0.02 |  | 17.86 | 0.10 | 83-110 | EM | 0.00 | hetero-6-1-mer | 11 x ATP | HHblits | 0.29 |
| ``` target    GLGVPESQLDVETRQYRNVVRTWAELQQTLHPLQERDPAFRFVFQTPKYRWGAHSTAVDADWISMLFGPFGDPYRRDPRM 7wbb.1    --------------------------------------------------------------------------------  target    PWTGEAYLEINPKDAAELGLADGDYAWVDADPEDRPYRGWNEDDPYYEVARAMMRVRIYTGMSRGVIRTWFNMYAATPAT 7wbb.1    --KETCTAYIHPNVLSSLEINPGSFCTVGK--------------------------------------------------  target    VANQKATPGNPARNEQTRYVALFRYGSHQSGTRAWLRPTQQTDSLVRKGYFGQVIGTGFEADVHSVSGAPKEAFVKIEKA 7wbb.1    --------------------------------------------------------------------------------  target    EDGGIGAERLWRPLTLGLRPEAPSAALTAYLAGDYSGTKGS 7wbb.1    ----------------------------------------- ``` | | | | | | | | | | | | | | | | | | | | | | | | | | | | | | | | | | | | | | | | | | | | | | | | | |
|  | 7wbb.1.E | AFG2 isoform 1  *Cryo-EM structure of substrate engaged Drg1 hexamer* | 0.01 |  | 17.86 | 0.10 | 83-110 | EM | 0.00 | hetero-6-1-mer | 11 x ATP | HHblits | 0.29 |
| ``` target    GLGVPESQLDVETRQYRNVVRTWAELQQTLHPLQERDPAFRFVFQTPKYRWGAHSTAVDADWISMLFGPFGDPYRRDPRM 7wbb.1    --------------------------------------------------------------------------------  target    PWTGEAYLEINPKDAAELGLADGDYAWVDADPEDRPYRGWNEDDPYYEVARAMMRVRIYTGMSRGVIRTWFNMYAATPAT 7wbb.1    --KETCTAYIHPNVLSSLEINPGSFCTVGK--------------------------------------------------  target    VANQKATPGNPARNEQTRYVALFRYGSHQSGTRAWLRPTQQTDSLVRKGYFGQVIGTGFEADVHSVSGAPKEAFVKIEKA 7wbb.1    --------------------------------------------------------------------------------  target    EDGGIGAERLWRPLTLGLRPEAPSAALTAYLAGDYSGTKGS 7wbb.1    ----------------------------------------- ``` | | | | | | | | | | | | | | | | | | | | | | | | | | | | | | | | | | | | | | | | | | | | | | | | | |
|  | 7wbb.1.G | AFG2 isoform 1  *Cryo-EM structure of substrate engaged Drg1 hexamer* | 0.01 |  | 17.86 | 0.10 | 83-110 | EM | 0.00 | hetero-6-1-mer | 11 x ATP | HHblits | 0.29 |
| ``` target    GLGVPESQLDVETRQYRNVVRTWAELQQTLHPLQERDPAFRFVFQTPKYRWGAHSTAVDADWISMLFGPFGDPYRRDPRM 7wbb.1    --------------------------------------------------------------------------------  target    PWTGEAYLEINPKDAAELGLADGDYAWVDADPEDRPYRGWNEDDPYYEVARAMMRVRIYTGMSRGVIRTWFNMYAATPAT 7wbb.1    --KETCTAYIHPNVLSSLEINPGSFCTVGK--------------------------------------------------  target    VANQKATPGNPARNEQTRYVALFRYGSHQSGTRAWLRPTQQTDSLVRKGYFGQVIGTGFEADVHSVSGAPKEAFVKIEKA 7wbb.1    --------------------------------------------------------------------------------  target    EDGGIGAERLWRPLTLGLRPEAPSAALTAYLAGDYSGTKGS 7wbb.1    ----------------------------------------- ``` | | | | | | | | | | | | | | | | | | | | | | | | | | | | | | | | | | | | | | | | | | | | | | | | | |
|  | 7di0.1.A | apDPBB\_sym\_79 protein  *Crystal structure of the rationally designed apDPBB\_sym\_79 protein* | 0.00 |  | 30.77 | 0.09 | 84-109 | X-ray | 1.60 | monomer |  | HHblits | 0.35 |
| ``` target    GLGVPESQLDVETRQYRNVVRTWAELQQTLHPLQERDPAFRFVFQTPKYRWGAHSTAVDADWISMLFGPFGDPYRRDPRM 7di0.1    --------------------------------------------------------------------------------  target    PWTGEAYLEINPKDAAELGLADGDYAWVDADPEDRPYRGWNEDDPYYEVARAMMRVRIYTGMSRGVIRTWFNMYAATPAT 7di0.1    ---GRGIVRMDKYLRAALGVSVGDYVEVK---------------------------------------------------  target    VANQKATPGNPARNEQTRYVALFRYGSHQSGTRAWLRPTQQTDSLVRKGYFGQVIGTGFEADVHSVSGAPKEAFVKIEKA 7di0.1    --------------------------------------------------------------------------------  target    EDGGIGAERLWRPLTLGLRPEAPSAALTAYLAGDYSGTKGS 7di0.1    ----------------------------------------- ``` | | | | | | | | | | | | | | | | | | | | | | | | | | | | | | | | | | | | | | | | | | | | | | | | | |
|  | 7di0.2.A | apDPBB\_sym\_79 protein  *Crystal structure of the rationally designed apDPBB\_sym\_79 protein* | 0.00 |  | 30.77 | 0.09 | 84-109 | X-ray | 1.60 | monomer |  | HHblits | 0.35 |
| ``` target    GLGVPESQLDVETRQYRNVVRTWAELQQTLHPLQERDPAFRFVFQTPKYRWGAHSTAVDADWISMLFGPFGDPYRRDPRM 7di0.2    --------------------------------------------------------------------------------  target    PWTGEAYLEINPKDAAELGLADGDYAWVDADPEDRPYRGWNEDDPYYEVARAMMRVRIYTGMSRGVIRTWFNMYAATPAT 7di0.2    ---GRGIVRMDKYLRAALGVSVGDYVEVK---------------------------------------------------  target    VANQKATPGNPARNEQTRYVALFRYGSHQSGTRAWLRPTQQTDSLVRKGYFGQVIGTGFEADVHSVSGAPKEAFVKIEKA 7di0.2    --------------------------------------------------------------------------------  target    EDGGIGAERLWRPLTLGLRPEAPSAALTAYLAGDYSGTKGS 7di0.2    ----------------------------------------- ``` | | | | | | | | | | | | | | | | | | | | | | | | | | | | | | | | | | | | | | | | | | | | | | | | | |
|  | 7di0.3.A | apDPBB\_sym\_79 protein  *Crystal structure of the rationally designed apDPBB\_sym\_79 protein* | 0.00 |  | 30.77 | 0.09 | 84-109 | X-ray | 1.60 | monomer |  | HHblits | 0.35 |
| ``` target    GLGVPESQLDVETRQYRNVVRTWAELQQTLHPLQERDPAFRFVFQTPKYRWGAHSTAVDADWISMLFGPFGDPYRRDPRM 7di0.3    --------------------------------------------------------------------------------  target    PWTGEAYLEINPKDAAELGLADGDYAWVDADPEDRPYRGWNEDDPYYEVARAMMRVRIYTGMSRGVIRTWFNMYAATPAT 7di0.3    ---GRGIVRMDKYLRAALGVSVGDYVEVK---------------------------------------------------  target    VANQKATPGNPARNEQTRYVALFRYGSHQSGTRAWLRPTQQTDSLVRKGYFGQVIGTGFEADVHSVSGAPKEAFVKIEKA 7di0.3    --------------------------------------------------------------------------------  target    EDGGIGAERLWRPLTLGLRPEAPSAALTAYLAGDYSGTKGS 7di0.3    ----------------------------------------- ``` | | | | | | | | | | | | | | | | | | | | | | | | | | | | | | | | | | | | | | | | | | | | | | | | | |
|  | 7dvf.1.A | reDPBB\_sym2 protein  *Crystal structure of the computationally designed reDPBB\_sym2 protein* | 0.01 |  | 19.23 | 0.09 | 84-109 | X-ray | 1.21 | monomer |  | HHblits | 0.33 |
| ``` target    GLGVPESQLDVETRQYRNVVRTWAELQQTLHPLQERDPAFRFVFQTPKYRWGAHSTAVDADWISMLFGPFGDPYRRDPRM 7dvf.1    --------------------------------------------------------------------------------  target    PWTGEAYLEINPKDAAELGLADGDYAWVDADPEDRPYRGWNEDDPYYEVARAMMRVRIYTGMSRGVIRTWFNMYAATPAT 7dvf.1    ---GKGIVRMDKASREKLGVSAGDLVEIK---------------------------------------------------  target    VANQKATPGNPARNEQTRYVALFRYGSHQSGTRAWLRPTQQTDSLVRKGYFGQVIGTGFEADVHSVSGAPKEAFVKIEKA 7dvf.1    --------------------------------------------------------------------------------  target    EDGGIGAERLWRPLTLGLRPEAPSAALTAYLAGDYSGTKGS 7dvf.1    ----------------------------------------- ``` | | | | | | | | | | | | | | | | | | | | | | | | | | | | | | | | | | | | | | | | | | | | | | | | | |
|  | 5itm.1.A | AbrB family transcriptional regulator  *The structure of truncated histone-like protein* | 0.00 |  | 22.22 | 0.10 | 85-111 | X-ray | 1.40 | homo-hexamer |  | HHblits | 0.30 |
| ``` target    GLGVPESQLDVETRQYRNVVRTWAELQQTLHPLQERDPAFRFVFQTPKYRWGAHSTAVDADWISMLFGPFGDPYRRDPRM 5itm.1    --------------------------------------------------------------------------------  target    PWTGEAYLEINPKDAAELGLADGDYAWVDADPEDRPYRGWNEDDPYYEVARAMMRVRIYTGMSRGVIRTWFNMYAATPAT 5itm.1    ----NYQVTIPAKVRQKFQIKEGDLVKVTFD-------------------------------------------------  target    VANQKATPGNPARNEQTRYVALFRYGSHQSGTRAWLRPTQQTDSLVRKGYFGQVIGTGFEADVHSVSGAPKEAFVKIEKA 5itm.1    --------------------------------------------------------------------------------  target    EDGGIGAERLWRPLTLGLRPEAPSAALTAYLAGDYSGTKGS 5itm.1    ----------------------------------------- ``` | | | | | | | | | | | | | | | | | | | | | | | | | | | | | | | | | | | | | | | | | | | | | | | | | |
|  | 5itm.1.B | AbrB family transcriptional regulator  *The structure of truncated histone-like protein* | 0.00 |  | 22.22 | 0.10 | 85-111 | X-ray | 1.40 | homo-hexamer |  | HHblits | 0.30 |
| ``` target    GLGVPESQLDVETRQYRNVVRTWAELQQTLHPLQERDPAFRFVFQTPKYRWGAHSTAVDADWISMLFGPFGDPYRRDPRM 5itm.1    --------------------------------------------------------------------------------  target    PWTGEAYLEINPKDAAELGLADGDYAWVDADPEDRPYRGWNEDDPYYEVARAMMRVRIYTGMSRGVIRTWFNMYAATPAT 5itm.1    ----NYQVTIPAKVRQKFQIKEGDLVKVTFD-------------------------------------------------  target    VANQKATPGNPARNEQTRYVALFRYGSHQSGTRAWLRPTQQTDSLVRKGYFGQVIGTGFEADVHSVSGAPKEAFVKIEKA 5itm.1    --------------------------------------------------------------------------------  target    EDGGIGAERLWRPLTLGLRPEAPSAALTAYLAGDYSGTKGS 5itm.1    ----------------------------------------- ``` | | | | | | | | | | | | | | | | | | | | | | | | | | | | | | | | | | | | | | | | | | | | | | | | | |
|  | 5itm.1.E | AbrB family transcriptional regulator  *The structure of truncated histone-like protein* | 0.00 |  | 22.22 | 0.10 | 85-111 | X-ray | 1.40 | homo-hexamer |  | HHblits | 0.30 |
| ``` target    GLGVPESQLDVETRQYRNVVRTWAELQQTLHPLQERDPAFRFVFQTPKYRWGAHSTAVDADWISMLFGPFGDPYRRDPRM 5itm.1    --------------------------------------------------------------------------------  target    PWTGEAYLEINPKDAAELGLADGDYAWVDADPEDRPYRGWNEDDPYYEVARAMMRVRIYTGMSRGVIRTWFNMYAATPAT 5itm.1    ----NYQVTIPAKVRQKFQIKEGDLVKVTFD-------------------------------------------------  target    VANQKATPGNPARNEQTRYVALFRYGSHQSGTRAWLRPTQQTDSLVRKGYFGQVIGTGFEADVHSVSGAPKEAFVKIEKA 5itm.1    --------------------------------------------------------------------------------  target    EDGGIGAERLWRPLTLGLRPEAPSAALTAYLAGDYSGTKGS 5itm.1    ----------------------------------------- ``` | | | | | | | | | | | | | | | | | | | | | | | | | | | | | | | | | | | | | | | | | | | | | | | | | |
|  | 7dvc.1.A | reDPBB\_sym1 protein  *Crystal structure of the computationally designed reDPBB\_sym1 protein* | 0.01 |  | 19.23 | 0.09 | 84-109 | X-ray | 1.71 | monomer |  | HHblits | 0.33 |
| ``` target    GLGVPESQLDVETRQYRNVVRTWAELQQTLHPLQERDPAFRFVFQTPKYRWGAHSTAVDADWISMLFGPFGDPYRRDPRM 7dvc.1    --------------------------------------------------------------------------------  target    PWTGEAYLEINPKDAAELGLADGDYAWVDADPEDRPYRGWNEDDPYYEVARAMMRVRIYTGMSRGVIRTWFNMYAATPAT 7dvc.1    ---GKGIVRMDKASRDKLGVSAGDLVEIK---------------------------------------------------  target    VANQKATPGNPARNEQTRYVALFRYGSHQSGTRAWLRPTQQTDSLVRKGYFGQVIGTGFEADVHSVSGAPKEAFVKIEKA 7dvc.1    --------------------------------------------------------------------------------  target    EDGGIGAERLWRPLTLGLRPEAPSAALTAYLAGDYSGTKGS 7dvc.1    ----------------------------------------- ``` | | | | | | | | | | | | | | | | | | | | | | | | | | | | | | | | | | | | | | | | | | | | | | | | | |
|  | 7dvc.5.A | reDPBB\_sym1 protein  *Crystal structure of the computationally designed reDPBB\_sym1 protein* | 0.01 |  | 19.23 | 0.09 | 84-109 | X-ray | 1.71 | monomer |  | HHblits | 0.33 |
| ``` target    GLGVPESQLDVETRQYRNVVRTWAELQQTLHPLQERDPAFRFVFQTPKYRWGAHSTAVDADWISMLFGPFGDPYRRDPRM 7dvc.5    --------------------------------------------------------------------------------  target    PWTGEAYLEINPKDAAELGLADGDYAWVDADPEDRPYRGWNEDDPYYEVARAMMRVRIYTGMSRGVIRTWFNMYAATPAT 7dvc.5    ---GKGIVRMDKASRDKLGVSAGDLVEIK---------------------------------------------------  target    VANQKATPGNPARNEQTRYVALFRYGSHQSGTRAWLRPTQQTDSLVRKGYFGQVIGTGFEADVHSVSGAPKEAFVKIEKA 7dvc.5    --------------------------------------------------------------------------------  target    EDGGIGAERLWRPLTLGLRPEAPSAALTAYLAGDYSGTKGS 7dvc.5    ----------------------------------------- ``` | | | | | | | | | | | | | | | | | | | | | | | | | | | | | | | | | | | | | | | | | | | | | | | | | |
|  | 7dg9.1.A | Cell division control protein 48, AAA family  *DPBB domain of VCP-like ATPase from Aeropyrum pernix* | 0.01 |  | 26.92 | 0.09 | 84-109 | X-ray | 1.60 | monomer | 4 x ZN | HHblits | 0.33 |
| ``` target    GLGVPESQLDVETRQYRNVVRTWAELQQTLHPLQERDPAFRFVFQTPKYRWGAHSTAVDADWISMLFGPFGDPYRRDPRM 7dg9.1    --------------------------------------------------------------------------------  target    PWTGEAYLEINPKDAAELGLADGDYAWVDADPEDRPYRGWNEDDPYYEVARAMMRVRIYTGMSRGVIRTWFNMYAATPAT 7dg9.1    ---GRGIIRMDGYLRAALGVTVGDTVTVE---------------------------------------------------  target    VANQKATPGNPARNEQTRYVALFRYGSHQSGTRAWLRPTQQTDSLVRKGYFGQVIGTGFEADVHSVSGAPKEAFVKIEKA 7dg9.1    --------------------------------------------------------------------------------  target    EDGGIGAERLWRPLTLGLRPEAPSAALTAYLAGDYSGTKGS 7dg9.1    ----------------------------------------- ``` | | | | | | | | | | | | | | | | | | | | | | | | | | | | | | | | | | | | | | | | | | | | | | | | | |
|  | 3qc8.1.A | Transitional endoplasmic reticulum ATPase  *Crystal Structure of FAF1 UBX Domain In Complex with p97/VCP N Domain Reveals The Conserved FcisP Touch-Turn Motif of UBX Domain Suffering Conformational Change* | 0.01 |  | 18.52 | 0.10 | 85-111 | X-ray | 2.20 | hetero-oligomer |  | HHblits | 0.30 |
| ``` target    GLGVPESQLDVETRQYRNVVRTWAELQQTLHPLQERDPAFRFVFQTPKYRWGAHSTAVDADWISMLFGPFGDPYRRDPRM 3qc8.1    --------------------------------------------------------------------------------  target    PWTGEAYLEINPKDAAELGLADGDYAWVDADPEDRPYRGWNEDDPYYEVARAMMRVRIYTGMSRGVIRTWFNMYAATPAT 3qc8.1    ----NSVVSLSQPKMDELQLFRGDTVLLKGK-------------------------------------------------  target    VANQKATPGNPARNEQTRYVALFRYGSHQSGTRAWLRPTQQTDSLVRKGYFGQVIGTGFEADVHSVSGAPKEAFVKIEKA 3qc8.1    --------------------------------------------------------------------------------  target    EDGGIGAERLWRPLTLGLRPEAPSAALTAYLAGDYSGTKGS 3qc8.1    ----------------------------------------- ``` | | | | | | | | | | | | | | | | | | | | | | | | | | | | | | | | | | | | | | | | | | | | | | | | | |
|  | 5b6c.1.A | Transitional endoplasmic reticulum ATPase  *Structural Details of Ufd1 binding to p97* | 0.01 |  | 18.52 | 0.10 | 85-111 | X-ray | 1.55 | hetero-oligomer |  | HHblits | 0.30 |
| ``` target    GLGVPESQLDVETRQYRNVVRTWAELQQTLHPLQERDPAFRFVFQTPKYRWGAHSTAVDADWISMLFGPFGDPYRRDPRM 5b6c.1    --------------------------------------------------------------------------------  target    PWTGEAYLEINPKDAAELGLADGDYAWVDADPEDRPYRGWNEDDPYYEVARAMMRVRIYTGMSRGVIRTWFNMYAATPAT 5b6c.1    ----NSVVSLSQPKMDELQLFRGDTVLLKGK-------------------------------------------------  target    VANQKATPGNPARNEQTRYVALFRYGSHQSGTRAWLRPTQQTDSLVRKGYFGQVIGTGFEADVHSVSGAPKEAFVKIEKA 5b6c.1    --------------------------------------------------------------------------------  target    EDGGIGAERLWRPLTLGLRPEAPSAALTAYLAGDYSGTKGS 5b6c.1    ----------------------------------------- ``` | | | | | | | | | | | | | | | | | | | | | | | | | | | | | | | | | | | | | | | | | | | | | | | | | |
|  | 5x4l.1.A | Transitional endoplasmic reticulum ATPase  *Crystal structure of the UBX domain of human UBXD7 in complex with p97 N domain* | 0.01 |  | 18.52 | 0.10 | 85-111 | X-ray | 2.40 | hetero-oligomer |  | HHblits | 0.30 |
| ``` target    GLGVPESQLDVETRQYRNVVRTWAELQQTLHPLQERDPAFRFVFQTPKYRWGAHSTAVDADWISMLFGPFGDPYRRDPRM 5x4l.1    --------------------------------------------------------------------------------  target    PWTGEAYLEINPKDAAELGLADGDYAWVDADPEDRPYRGWNEDDPYYEVARAMMRVRIYTGMSRGVIRTWFNMYAATPAT 5x4l.1    ----NSVVSLSQPKMDELQLFRGDTVLLKGK-------------------------------------------------  target    VANQKATPGNPARNEQTRYVALFRYGSHQSGTRAWLRPTQQTDSLVRKGYFGQVIGTGFEADVHSVSGAPKEAFVKIEKA 5x4l.1    --------------------------------------------------------------------------------  target    EDGGIGAERLWRPLTLGLRPEAPSAALTAYLAGDYSGTKGS 5x4l.1    ----------------------------------------- ``` | | | | | | | | | | | | | | | | | | | | | | | | | | | | | | | | | | | | | | | | | | | | | | | | | |
|  | 5x4l.2.A | Transitional endoplasmic reticulum ATPase  *Crystal structure of the UBX domain of human UBXD7 in complex with p97 N domain* | 0.01 |  | 18.52 | 0.10 | 85-111 | X-ray | 2.40 | hetero-oligomer |  | HHblits | 0.30 |
| ``` target    GLGVPESQLDVETRQYRNVVRTWAELQQTLHPLQERDPAFRFVFQTPKYRWGAHSTAVDADWISMLFGPFGDPYRRDPRM 5x4l.2    --------------------------------------------------------------------------------  target    PWTGEAYLEINPKDAAELGLADGDYAWVDADPEDRPYRGWNEDDPYYEVARAMMRVRIYTGMSRGVIRTWFNMYAATPAT 5x4l.2    ----NSVVSLSQPKMDELQLFRGDTVLLKGK-------------------------------------------------  target    VANQKATPGNPARNEQTRYVALFRYGSHQSGTRAWLRPTQQTDSLVRKGYFGQVIGTGFEADVHSVSGAPKEAFVKIEKA 5x4l.2    --------------------------------------------------------------------------------  target    EDGGIGAERLWRPLTLGLRPEAPSAALTAYLAGDYSGTKGS 5x4l.2    ----------------------------------------- ``` | | | | | | | | | | | | | | | | | | | | | | | | | | | | | | | | | | | | | | | | | | | | | | | | | |
|  | 5epp.1.A | Transitional endoplasmic reticulum ATPase  *Structural Insights into the Interaction of p97 N-terminus Domain and VBM Motif in Rhomboid Protease, RHBDL4* | 0.01 |  | 18.52 | 0.10 | 85-111 | X-ray | 1.88 | hetero-oligomer |  | HHblits | 0.30 |
| ``` target    GLGVPESQLDVETRQYRNVVRTWAELQQTLHPLQERDPAFRFVFQTPKYRWGAHSTAVDADWISMLFGPFGDPYRRDPRM 5epp.1    --------------------------------------------------------------------------------  target    PWTGEAYLEINPKDAAELGLADGDYAWVDADPEDRPYRGWNEDDPYYEVARAMMRVRIYTGMSRGVIRTWFNMYAATPAT 5epp.1    ----NSVVSLSQPKMDELQLFRGDTVLLKGK-------------------------------------------------  target    VANQKATPGNPARNEQTRYVALFRYGSHQSGTRAWLRPTQQTDSLVRKGYFGQVIGTGFEADVHSVSGAPKEAFVKIEKA 5epp.1    --------------------------------------------------------------------------------  target    EDGGIGAERLWRPLTLGLRPEAPSAALTAYLAGDYSGTKGS 5epp.1    ----------------------------------------- ``` | | | | | | | | | | | | | | | | | | | | | | | | | | | | | | | | | | | | | | | | | | | | | | | | | |
|  | 5glf.2.A | Transitional endoplasmic reticulum ATPase  *Structural insights into the interaction of p97 N-terminal domain and SHP motif in Derlin-1 rhomboid pseudoprotease* | 0.01 |  | 18.52 | 0.10 | 85-111 | X-ray | 2.25 | hetero-1-1-mer |  | HHblits | 0.30 |
| ``` target    GLGVPESQLDVETRQYRNVVRTWAELQQTLHPLQERDPAFRFVFQTPKYRWGAHSTAVDADWISMLFGPFGDPYRRDPRM 5glf.2    --------------------------------------------------------------------------------  target    PWTGEAYLEINPKDAAELGLADGDYAWVDADPEDRPYRGWNEDDPYYEVARAMMRVRIYTGMSRGVIRTWFNMYAATPAT 5glf.2    ----NSVVSLSQPKMDELQLFRGDTVLLKGK-------------------------------------------------  target    VANQKATPGNPARNEQTRYVALFRYGSHQSGTRAWLRPTQQTDSLVRKGYFGQVIGTGFEADVHSVSGAPKEAFVKIEKA 5glf.2    --------------------------------------------------------------------------------  target    EDGGIGAERLWRPLTLGLRPEAPSAALTAYLAGDYSGTKGS 5glf.2    ----------------------------------------- ``` | | | | | | | | | | | | | | | | | | | | | | | | | | | | | | | | | | | | | | | | | | | | | | | | | |
|  | 5glf.3.A | Transitional endoplasmic reticulum ATPase  *Structural insights into the interaction of p97 N-terminal domain and SHP motif in Derlin-1 rhomboid pseudoprotease* | 0.01 |  | 18.52 | 0.10 | 85-111 | X-ray | 2.25 | hetero-1-1-mer |  | HHblits | 0.30 |
| ``` target    GLGVPESQLDVETRQYRNVVRTWAELQQTLHPLQERDPAFRFVFQTPKYRWGAHSTAVDADWISMLFGPFGDPYRRDPRM 5glf.3    --------------------------------------------------------------------------------  target    PWTGEAYLEINPKDAAELGLADGDYAWVDADPEDRPYRGWNEDDPYYEVARAMMRVRIYTGMSRGVIRTWFNMYAATPAT 5glf.3    ----NSVVSLSQPKMDELQLFRGDTVLLKGK-------------------------------------------------  target    VANQKATPGNPARNEQTRYVALFRYGSHQSGTRAWLRPTQQTDSLVRKGYFGQVIGTGFEADVHSVSGAPKEAFVKIEKA 5glf.3    --------------------------------------------------------------------------------  target    EDGGIGAERLWRPLTLGLRPEAPSAALTAYLAGDYSGTKGS 5glf.3    ----------------------------------------- ``` | | | | | | | | | | | | | | | | | | | | | | | | | | | | | | | | | | | | | | | | | | | | | | | | | |
|  | 5glf.1.A | Transitional endoplasmic reticulum ATPase  *Structural insights into the interaction of p97 N-terminal domain and SHP motif in Derlin-1 rhomboid pseudoprotease* | 0.01 |  | 18.52 | 0.10 | 85-111 | X-ray | 2.25 | hetero-1-1-mer |  | HHblits | 0.30 |
| ``` target    GLGVPESQLDVETRQYRNVVRTWAELQQTLHPLQERDPAFRFVFQTPKYRWGAHSTAVDADWISMLFGPFGDPYRRDPRM 5glf.1    --------------------------------------------------------------------------------  target    PWTGEAYLEINPKDAAELGLADGDYAWVDADPEDRPYRGWNEDDPYYEVARAMMRVRIYTGMSRGVIRTWFNMYAATPAT 5glf.1    ----NSVVSLSQPKMDELQLFRGDTVLLKGK-------------------------------------------------  target    VANQKATPGNPARNEQTRYVALFRYGSHQSGTRAWLRPTQQTDSLVRKGYFGQVIGTGFEADVHSVSGAPKEAFVKIEKA 5glf.1    --------------------------------------------------------------------------------  target    EDGGIGAERLWRPLTLGLRPEAPSAALTAYLAGDYSGTKGS 5glf.1    ----------------------------------------- ``` | | | | | | | | | | | | | | | | | | | | | | | | | | | | | | | | | | | | | | | | | | | | | | | | | |
|  | 5glf.4.A | Transitional endoplasmic reticulum ATPase  *Structural insights into the interaction of p97 N-terminal domain and SHP motif in Derlin-1 rhomboid pseudoprotease* | 0.01 |  | 18.52 | 0.10 | 85-111 | X-ray | 2.25 | hetero-1-1-mer |  | HHblits | 0.30 |
| ``` target    GLGVPESQLDVETRQYRNVVRTWAELQQTLHPLQERDPAFRFVFQTPKYRWGAHSTAVDADWISMLFGPFGDPYRRDPRM 5glf.4    --------------------------------------------------------------------------------  target    PWTGEAYLEINPKDAAELGLADGDYAWVDADPEDRPYRGWNEDDPYYEVARAMMRVRIYTGMSRGVIRTWFNMYAATPAT 5glf.4    ----NSVVSLSQPKMDELQLFRGDTVLLKGK-------------------------------------------------  target    VANQKATPGNPARNEQTRYVALFRYGSHQSGTRAWLRPTQQTDSLVRKGYFGQVIGTGFEADVHSVSGAPKEAFVKIEKA 5glf.4    --------------------------------------------------------------------------------  target    EDGGIGAERLWRPLTLGLRPEAPSAALTAYLAGDYSGTKGS 5glf.4    ----------------------------------------- ``` | | | | | | | | | | | | | | | | | | | | | | | | | | | | | | | | | | | | | | | | | | | | | | | | | |
|  | 4kdl.1.A | Transitional endoplasmic reticulum ATPase  *Crystal structure of p97/VCP N in complex with OTU1 UBXL* | 0.01 |  | 18.52 | 0.10 | 85-111 | X-ray | 1.81 | monomer |  | HHblits | 0.30 |
| ``` target    GLGVPESQLDVETRQYRNVVRTWAELQQTLHPLQERDPAFRFVFQTPKYRWGAHSTAVDADWISMLFGPFGDPYRRDPRM 4kdl.1    --------------------------------------------------------------------------------  target    PWTGEAYLEINPKDAAELGLADGDYAWVDADPEDRPYRGWNEDDPYYEVARAMMRVRIYTGMSRGVIRTWFNMYAATPAT 4kdl.1    ----NSVVSLSQPKMDELQLFRGDTVLLKGK-------------------------------------------------  target    VANQKATPGNPARNEQTRYVALFRYGSHQSGTRAWLRPTQQTDSLVRKGYFGQVIGTGFEADVHSVSGAPKEAFVKIEKA 4kdl.1    --------------------------------------------------------------------------------  target    EDGGIGAERLWRPLTLGLRPEAPSAALTAYLAGDYSGTKGS 4kdl.1    ----------------------------------------- ``` | | | | | | | | | | | | | | | | | | | | | | | | | | | | | | | | | | | | | | | | | | | | | | | | | |
|  | 4kdi.2.A | Transitional endoplasmic reticulum ATPase  *Crystal structure of p97/VCP N in complex with OTU1 UBXL* | 0.01 |  | 18.52 | 0.10 | 85-111 | X-ray | 1.86 | hetero-oligomer |  | HHblits | 0.30 |
| ``` target    GLGVPESQLDVETRQYRNVVRTWAELQQTLHPLQERDPAFRFVFQTPKYRWGAHSTAVDADWISMLFGPFGDPYRRDPRM 4kdi.2    --------------------------------------------------------------------------------  target    PWTGEAYLEINPKDAAELGLADGDYAWVDADPEDRPYRGWNEDDPYYEVARAMMRVRIYTGMSRGVIRTWFNMYAATPAT 4kdi.2    ----NSVVSLSQPKMDELQLFRGDTVLLKGK-------------------------------------------------  target    VANQKATPGNPARNEQTRYVALFRYGSHQSGTRAWLRPTQQTDSLVRKGYFGQVIGTGFEADVHSVSGAPKEAFVKIEKA 4kdi.2    --------------------------------------------------------------------------------  target    EDGGIGAERLWRPLTLGLRPEAPSAALTAYLAGDYSGTKGS 4kdi.2    ----------------------------------------- ``` | | | | | | | | | | | | | | | | | | | | | | | | | | | | | | | | | | | | | | | | | | | | | | | | | |
|  | 4kdi.1.A | Transitional endoplasmic reticulum ATPase  *Crystal structure of p97/VCP N in complex with OTU1 UBXL* | 0.01 |  | 18.52 | 0.10 | 85-111 | X-ray | 1.86 | hetero-oligomer |  | HHblits | 0.30 |
| ``` target    GLGVPESQLDVETRQYRNVVRTWAELQQTLHPLQERDPAFRFVFQTPKYRWGAHSTAVDADWISMLFGPFGDPYRRDPRM 4kdi.1    --------------------------------------------------------------------------------  target    PWTGEAYLEINPKDAAELGLADGDYAWVDADPEDRPYRGWNEDDPYYEVARAMMRVRIYTGMSRGVIRTWFNMYAATPAT 4kdi.1    ----NSVVSLSQPKMDELQLFRGDTVLLKGK-------------------------------------------------  target    VANQKATPGNPARNEQTRYVALFRYGSHQSGTRAWLRPTQQTDSLVRKGYFGQVIGTGFEADVHSVSGAPKEAFVKIEKA 4kdi.1    --------------------------------------------------------------------------------  target    EDGGIGAERLWRPLTLGLRPEAPSAALTAYLAGDYSGTKGS 4kdi.1    ----------------------------------------- ``` | | | | | | | | | | | | | | | | | | | | | | | | | | | | | | | | | | | | | | | | | | | | | | | | | |
|  | 3tiw.1.A | Transitional endoplasmic reticulum ATPase  *Crystal structure of p97N in complex with the C-terminus of gp78* | 0.01 |  | 18.52 | 0.10 | 85-111 | X-ray | 1.80 | hetero-oligomer |  | HHblits | 0.30 |
| ``` target    GLGVPESQLDVETRQYRNVVRTWAELQQTLHPLQERDPAFRFVFQTPKYRWGAHSTAVDADWISMLFGPFGDPYRRDPRM 3tiw.1    --------------------------------------------------------------------------------  target    PWTGEAYLEINPKDAAELGLADGDYAWVDADPEDRPYRGWNEDDPYYEVARAMMRVRIYTGMSRGVIRTWFNMYAATPAT 3tiw.1    ----NSVVSLSQPKMDELQLFRGDTVLLKGK-------------------------------------------------  target    VANQKATPGNPARNEQTRYVALFRYGSHQSGTRAWLRPTQQTDSLVRKGYFGQVIGTGFEADVHSVSGAPKEAFVKIEKA 3tiw.1    --------------------------------------------------------------------------------  target    EDGGIGAERLWRPLTLGLRPEAPSAALTAYLAGDYSGTKGS 3tiw.1    ----------------------------------------- ``` | | | | | | | | | | | | | | | | | | | | | | | | | | | | | | | | | | | | | | | | | | | | | | | | | |
|  | 3tiw.2.A | Transitional endoplasmic reticulum ATPase  *Crystal structure of p97N in complex with the C-terminus of gp78* | 0.01 |  | 18.52 | 0.10 | 85-111 | X-ray | 1.80 | hetero-oligomer |  | HHblits | 0.30 |
| ``` target    GLGVPESQLDVETRQYRNVVRTWAELQQTLHPLQERDPAFRFVFQTPKYRWGAHSTAVDADWISMLFGPFGDPYRRDPRM 3tiw.2    --------------------------------------------------------------------------------  target    PWTGEAYLEINPKDAAELGLADGDYAWVDADPEDRPYRGWNEDDPYYEVARAMMRVRIYTGMSRGVIRTWFNMYAATPAT 3tiw.2    ----NSVVSLSQPKMDELQLFRGDTVLLKGK-------------------------------------------------  target    VANQKATPGNPARNEQTRYVALFRYGSHQSGTRAWLRPTQQTDSLVRKGYFGQVIGTGFEADVHSVSGAPKEAFVKIEKA 3tiw.2    --------------------------------------------------------------------------------  target    EDGGIGAERLWRPLTLGLRPEAPSAALTAYLAGDYSGTKGS 3tiw.2    ----------------------------------------- ``` | | | | | | | | | | | | | | | | | | | | | | | | | | | | | | | | | | | | | | | | | | | | | | | | | |
|  | 3qwz.1.A | Transitional endoplasmic reticulum ATPase  *Crystal structure of FAF1 UBX-p97N-domain complex* | 0.01 |  | 18.52 | 0.10 | 85-111 | X-ray | 2.00 | hetero-oligomer |  | HHblits | 0.30 |
| ``` target    GLGVPESQLDVETRQYRNVVRTWAELQQTLHPLQERDPAFRFVFQTPKYRWGAHSTAVDADWISMLFGPFGDPYRRDPRM 3qwz.1    --------------------------------------------------------------------------------  target    PWTGEAYLEINPKDAAELGLADGDYAWVDADPEDRPYRGWNEDDPYYEVARAMMRVRIYTGMSRGVIRTWFNMYAATPAT 3qwz.1    ----NSVVSLSQPKMDELQLFRGDTVLLKGK-------------------------------------------------  target    VANQKATPGNPARNEQTRYVALFRYGSHQSGTRAWLRPTQQTDSLVRKGYFGQVIGTGFEADVHSVSGAPKEAFVKIEKA 3qwz.1    --------------------------------------------------------------------------------  target    EDGGIGAERLWRPLTLGLRPEAPSAALTAYLAGDYSGTKGS 3qwz.1    ----------------------------------------- ``` | | | | | | | | | | | | | | | | | | | | | | | | | | | | | | | | | | | | | | | | | | | | | | | | | |
|  | 3qq8.1.A | Transitional endoplasmic reticulum ATPase  *Crystal structure of p97-N in complex with FAF1-UBX* | 0.01 |  | 18.52 | 0.10 | 85-111 | X-ray | 2.00 | hetero-oligomer |  | HHblits | 0.30 |
| ``` target    GLGVPESQLDVETRQYRNVVRTWAELQQTLHPLQERDPAFRFVFQTPKYRWGAHSTAVDADWISMLFGPFGDPYRRDPRM 3qq8.1    --------------------------------------------------------------------------------  target    PWTGEAYLEINPKDAAELGLADGDYAWVDADPEDRPYRGWNEDDPYYEVARAMMRVRIYTGMSRGVIRTWFNMYAATPAT 3qq8.1    ----NSVVSLSQPKMDELQLFRGDTVLLKGK-------------------------------------------------  target    VANQKATPGNPARNEQTRYVALFRYGSHQSGTRAWLRPTQQTDSLVRKGYFGQVIGTGFEADVHSVSGAPKEAFVKIEKA 3qq8.1    --------------------------------------------------------------------------------  target    EDGGIGAERLWRPLTLGLRPEAPSAALTAYLAGDYSGTKGS 3qq8.1    ----------------------------------------- ``` | | | | | | | | | | | | | | | | | | | | | | | | | | | | | | | | | | | | | | | | | | | | | | | | | |
|  | 3qq7.1.A | Transitional endoplasmic reticulum ATPase  *Crystal Structure of the p97 N-terminal domain* | 0.01 |  | 18.52 | 0.10 | 85-111 | X-ray | 2.65 | monomer | 1 x HEZ, 1 x CO | HHblits | 0.30 |
| ``` target    GLGVPESQLDVETRQYRNVVRTWAELQQTLHPLQERDPAFRFVFQTPKYRWGAHSTAVDADWISMLFGPFGDPYRRDPRM 3qq7.1    --------------------------------------------------------------------------------  target    PWTGEAYLEINPKDAAELGLADGDYAWVDADPEDRPYRGWNEDDPYYEVARAMMRVRIYTGMSRGVIRTWFNMYAATPAT 3qq7.1    ----NSVVSLSQPKMDELQLFRGDTVLLKGK-------------------------------------------------  target    VANQKATPGNPARNEQTRYVALFRYGSHQSGTRAWLRPTQQTDSLVRKGYFGQVIGTGFEADVHSVSGAPKEAFVKIEKA 3qq7.1    --------------------------------------------------------------------------------  target    EDGGIGAERLWRPLTLGLRPEAPSAALTAYLAGDYSGTKGS 3qq7.1    ----------------------------------------- ``` | | | | | | | | | | | | | | | | | | | | | | | | | | | | | | | | | | | | | | | | | | | | | | | | | |
|  | 2pjh.1.B | Transitional endoplasmic reticulum ATPase  *Strctural Model of the p97 N domain- npl4 UBD complex* | 0.01 |  | 18.52 | 0.10 | 85-111 | NMR | 0.00 | hetero-1-1-mer |  | HHblits | 0.30 |
| ``` target    GLGVPESQLDVETRQYRNVVRTWAELQQTLHPLQERDPAFRFVFQTPKYRWGAHSTAVDADWISMLFGPFGDPYRRDPRM 2pjh.1    --------------------------------------------------------------------------------  target    PWTGEAYLEINPKDAAELGLADGDYAWVDADPEDRPYRGWNEDDPYYEVARAMMRVRIYTGMSRGVIRTWFNMYAATPAT 2pjh.1    ----NSVVSLSQPKMDELQLFRGDTVLLKGK-------------------------------------------------  target    VANQKATPGNPARNEQTRYVALFRYGSHQSGTRAWLRPTQQTDSLVRKGYFGQVIGTGFEADVHSVSGAPKEAFVKIEKA 2pjh.1    --------------------------------------------------------------------------------  target    EDGGIGAERLWRPLTLGLRPEAPSAALTAYLAGDYSGTKGS 2pjh.1    ----------------------------------------- ``` | | | | | | | | | | | | | | | | | | | | | | | | | | | | | | | | | | | | | | | | | | | | | | | | | |
|  | 7dg7.1.A | ATPase of the AAA+ class  *DPBB domain of VCP-like ATPase from Methanopyrus kandleri* | 0.01 |  | 19.23 | 0.09 | 84-109 | X-ray | 1.60 | monomer | 2 x IMD, 8 x ZN | HHblits | 0.32 |
| ``` target    GLGVPESQLDVETRQYRNVVRTWAELQQTLHPLQERDPAFRFVFQTPKYRWGAHSTAVDADWISMLFGPFGDPYRRDPRM 7dg7.1    --------------------------------------------------------------------------------  target    PWTGEAYLEINPKDAAELGLADGDYAWVDADPEDRPYRGWNEDDPYYEVARAMMRVRIYTGMSRGVIRTWFNMYAATPAT 7dg7.1    ---GKGIVRMDKYERQNAGASVGEPVEVD---------------------------------------------------  target    VANQKATPGNPARNEQTRYVALFRYGSHQSGTRAWLRPTQQTDSLVRKGYFGQVIGTGFEADVHSVSGAPKEAFVKIEKA 7dg7.1    --------------------------------------------------------------------------------  target    EDGGIGAERLWRPLTLGLRPEAPSAALTAYLAGDYSGTKGS 7dg7.1    ----------------------------------------- ``` | | | | | | | | | | | | | | | | | | | | | | | | | | | | | | | | | | | | | | | | | | | | | | | | | |
|  | 2w1t.1.A | STAGE V SPORULATION PROTEIN T  *Crystal Structure of B. subtilis SpoVT* | 0.01 |  | 18.52 | 0.10 | 86-112 | X-ray | 2.60 | hetero-1-1-mer |  | HHblits | 0.28 |
| ``` target    GLGVPESQLDVETRQYRNVVRTWAELQQTLHPLQERDPAFRFVFQTPKYRWGAHSTAVDADWISMLFGPFGDPYRRDPRM 2w1t.1    --------------------------------------------------------------------------------  target    PWTGEAYLEINPKDAAELGLADGDYAWVDADPEDRPYRGWNEDDPYYEVARAMMRVRIYTGMSRGVIRTWFNMYAATPAT 2w1t.1    -----GRVVIPKEIRRTLRIREGDPLEIFVDR------------------------------------------------  target    VANQKATPGNPARNEQTRYVALFRYGSHQSGTRAWLRPTQQTDSLVRKGYFGQVIGTGFEADVHSVSGAPKEAFVKIEKA 2w1t.1    --------------------------------------------------------------------------------  target    EDGGIGAERLWRPLTLGLRPEAPSAALTAYLAGDYSGTKGS 2w1t.1    ----------------------------------------- ``` | | | | | | | | | | | | | | | | | | | | | | | | | | | | | | | | | | | | | | | | | | | | | | | | | |
|  | 6hd3.1.A | Cell division control protein 48 homolog A  *Common mode of remodeling AAA ATPases p97/CDC48 by their disassembly cofactors ASPL/PUX1* | 0.01 |  | 19.23 | 0.09 | 86-111 | X-ray | 2.80 | homo-24-mer | 24 x ADP | HHblits | 0.31 |
| ``` target    GLGVPESQLDVETRQYRNVVRTWAELQQTLHPLQERDPAFRFVFQTPKYRWGAHSTAVDADWISMLFGPFGDPYRRDPRM 6hd3.1    --------------------------------------------------------------------------------  target    PWTGEAYLEINPKDAAELGLADGDYAWVDADPEDRPYRGWNEDDPYYEVARAMMRVRIYTGMSRGVIRTWFNMYAATPAT 6hd3.1    -----SVVSLHPATMEKLQLFRGDTILIKGK-------------------------------------------------  target    VANQKATPGNPARNEQTRYVALFRYGSHQSGTRAWLRPTQQTDSLVRKGYFGQVIGTGFEADVHSVSGAPKEAFVKIEKA 6hd3.1    --------------------------------------------------------------------------------  target    EDGGIGAERLWRPLTLGLRPEAPSAALTAYLAGDYSGTKGS 6hd3.1    ----------------------------------------- ``` | | | | | | | | | | | | | | | | | | | | | | | | | | | | | | | | | | | | | | | | | | | | | | | | | |
|  | 7dww.1.A | msDPBB\_sym2 protein  *Crystal structure of the computationally designed msDPBB\_sym2 protein* | 0.01 |  | 19.23 | 0.09 | 84-109 | X-ray | 1.80 | monomer |  | HHblits | 0.30 |
| ``` target    GLGVPESQLDVETRQYRNVVRTWAELQQTLHPLQERDPAFRFVFQTPKYRWGAHSTAVDADWISMLFGPFGDPYRRDPRM 7dww.1    --------------------------------------------------------------------------------  target    PWTGEAYLEINPKDAAELGLADGDYAWVDADPEDRPYRGWNEDDPYYEVARAMMRVRIYTGMSRGVIRTWFNMYAATPAT 7dww.1    ---GKNIVRMDEELMRLLGVKVGDLVEIM---------------------------------------------------  target    VANQKATPGNPARNEQTRYVALFRYGSHQSGTRAWLRPTQQTDSLVRKGYFGQVIGTGFEADVHSVSGAPKEAFVKIEKA 7dww.1    --------------------------------------------------------------------------------  target    EDGGIGAERLWRPLTLGLRPEAPSAALTAYLAGDYSGTKGS 7dww.1    ----------------------------------------- ``` | | | | | | | | | | | | | | | | | | | | | | | | | | | | | | | | | | | | | | | | | | | | | | | | | |
|  | 7dww.2.A | msDPBB\_sym2 protein  *Crystal structure of the computationally designed msDPBB\_sym2 protein* | 0.01 |  | 19.23 | 0.09 | 84-109 | X-ray | 1.80 | monomer |  | HHblits | 0.30 |
| ``` target    GLGVPESQLDVETRQYRNVVRTWAELQQTLHPLQERDPAFRFVFQTPKYRWGAHSTAVDADWISMLFGPFGDPYRRDPRM 7dww.2    --------------------------------------------------------------------------------  target    PWTGEAYLEINPKDAAELGLADGDYAWVDADPEDRPYRGWNEDDPYYEVARAMMRVRIYTGMSRGVIRTWFNMYAATPAT 7dww.2    ---GKNIVRMDEELMRLLGVKVGDLVEIM---------------------------------------------------  target    VANQKATPGNPARNEQTRYVALFRYGSHQSGTRAWLRPTQQTDSLVRKGYFGQVIGTGFEADVHSVSGAPKEAFVKIEKA 7dww.2    --------------------------------------------------------------------------------  target    EDGGIGAERLWRPLTLGLRPEAPSAALTAYLAGDYSGTKGS 7dww.2    ----------------------------------------- ``` | | | | | | | | | | | | | | | | | | | | | | | | | | | | | | | | | | | | | | | | | | | | | | | | | |
|  | 3o27.1.A | Putative uncharacterized protein  *The crystal structure of C68 from the hybrid virus-plasmid pSSVx* | 0.01 |  | 23.08 | 0.09 | 86-111 | X-ray | 2.80 | homo-dimer |  | HHblits | 0.29 |
| ``` target    GLGVPESQLDVETRQYRNVVRTWAELQQTLHPLQERDPAFRFVFQTPKYRWGAHSTAVDADWISMLFGPFGDPYRRDPRM 3o27.1    --------------------------------------------------------------------------------  target    PWTGEAYLEINPKDAAELGLADGDYAWVDADPEDRPYRGWNEDDPYYEVARAMMRVRIYTGMSRGVIRTWFNMYAATPAT 3o27.1    -----FYLLIPKDIAEALDIKPDDTFILNME-------------------------------------------------  target    VANQKATPGNPARNEQTRYVALFRYGSHQSGTRAWLRPTQQTDSLVRKGYFGQVIGTGFEADVHSVSGAPKEAFVKIEKA 3o27.1    --------------------------------------------------------------------------------  target    EDGGIGAERLWRPLTLGLRPEAPSAALTAYLAGDYSGTKGS 3o27.1    ----------------------------------------- ``` | | | | | | | | | | | | | | | | | | | | | | | | | | | | | | | | | | | | | | | | | | | | | | | | | |
|  | 3o27.1.B | Putative uncharacterized protein  *The crystal structure of C68 from the hybrid virus-plasmid pSSVx* | 0.00 |  | 23.08 | 0.09 | 86-111 | X-ray | 2.80 | homo-dimer |  | HHblits | 0.29 |
| ``` target    GLGVPESQLDVETRQYRNVVRTWAELQQTLHPLQERDPAFRFVFQTPKYRWGAHSTAVDADWISMLFGPFGDPYRRDPRM 3o27.1    --------------------------------------------------------------------------------  target    PWTGEAYLEINPKDAAELGLADGDYAWVDADPEDRPYRGWNEDDPYYEVARAMMRVRIYTGMSRGVIRTWFNMYAATPAT 3o27.1    -----FYLLIPKDIAEALDIKPDDTFILNME-------------------------------------------------  target    VANQKATPGNPARNEQTRYVALFRYGSHQSGTRAWLRPTQQTDSLVRKGYFGQVIGTGFEADVHSVSGAPKEAFVKIEKA 3o27.1    --------------------------------------------------------------------------------  target    EDGGIGAERLWRPLTLGLRPEAPSAALTAYLAGDYSGTKGS 3o27.1    ----------------------------------------- ``` | | | | | | | | | | | | | | | | | | | | | | | | | | | | | | | | | | | | | | | | | | | | | | | | | |
|  | 7dbo.1.A | VCP-like ATPase  *DPBB domain of VCP-like ATPase from Thermoplasma acidophilum* | 0.01 |  | 19.23 | 0.09 | 84-109 | X-ray | 1.90 | monomer |  | HHblits | 0.29 |
| ``` target    GLGVPESQLDVETRQYRNVVRTWAELQQTLHPLQERDPAFRFVFQTPKYRWGAHSTAVDADWISMLFGPFGDPYRRDPRM 7dbo.1    --------------------------------------------------------------------------------  target    PWTGEAYLEINPKDAAELGLADGDYAWVDADPEDRPYRGWNEDDPYYEVARAMMRVRIYTGMSRGVIRTWFNMYAATPAT 7dbo.1    ---NKGIVRIDSVMRNNCGASIGDKVKVR---------------------------------------------------  target    VANQKATPGNPARNEQTRYVALFRYGSHQSGTRAWLRPTQQTDSLVRKGYFGQVIGTGFEADVHSVSGAPKEAFVKIEKA 7dbo.1    --------------------------------------------------------------------------------  target    EDGGIGAERLWRPLTLGLRPEAPSAALTAYLAGDYSGTKGS 7dbo.1    ----------------------------------------- ``` | | | | | | | | | | | | | | | | | | | | | | | | | | | | | | | | | | | | | | | | | | | | | | | | | |
|  | 7dbo.2.A | VCP-like ATPase  *DPBB domain of VCP-like ATPase from Thermoplasma acidophilum* | 0.01 |  | 19.23 | 0.09 | 84-109 | X-ray | 1.90 | monomer |  | HHblits | 0.29 |
| ``` target    GLGVPESQLDVETRQYRNVVRTWAELQQTLHPLQERDPAFRFVFQTPKYRWGAHSTAVDADWISMLFGPFGDPYRRDPRM 7dbo.2    --------------------------------------------------------------------------------  target    PWTGEAYLEINPKDAAELGLADGDYAWVDADPEDRPYRGWNEDDPYYEVARAMMRVRIYTGMSRGVIRTWFNMYAATPAT 7dbo.2    ---NKGIVRIDSVMRNNCGASIGDKVKVR---------------------------------------------------  target    VANQKATPGNPARNEQTRYVALFRYGSHQSGTRAWLRPTQQTDSLVRKGYFGQVIGTGFEADVHSVSGAPKEAFVKIEKA 7dbo.2    --------------------------------------------------------------------------------  target    EDGGIGAERLWRPLTLGLRPEAPSAALTAYLAGDYSGTKGS 7dbo.2    ----------------------------------------- ``` | | | | | | | | | | | | | | | | | | | | | | | | | | | | | | | | | | | | | | | | | | | | | | | | | |
|  | 5cup.1.A | Phosphate propanoyltransferase  *Structure of Rhodopseudomonas palustris PduL - phosphate bound form* | 0.00 |  | 20.00 | 0.09 | 86-110 | X-ray | 2.10 | homo-dimer | 4 x ZN | HHblits | 0.29 |
| ``` target    GLGVPESQLDVETRQYRNVVRTWAELQQTLHPLQERDPAFRFVFQTPKYRWGAHSTAVDADWISMLFGPFGDPYRRDPRM 5cup.1    --------------------------------------------------------------------------------  target    PWTGEAYLEINPKDAAELGLADGDYAWVDADPEDRPYRGWNEDDPYYEVARAMMRVRIYTGMSRGVIRTWFNMYAATPAT 5cup.1    -----DEMHIDVEEANALCLKNDDVVRICK--------------------------------------------------  target    VANQKATPGNPARNEQTRYVALFRYGSHQSGTRAWLRPTQQTDSLVRKGYFGQVIGTGFEADVHSVSGAPKEAFVKIEKA 5cup.1    --------------------------------------------------------------------------------  target    EDGGIGAERLWRPLTLGLRPEAPSAALTAYLAGDYSGTKGS 5cup.1    ----------------------------------------- ``` | | | | | | | | | | | | | | | | | | | | | | | | | | | | | | | | | | | | | | | | | | | | | | | | | |
|  | 5cuo.1.A | Phosphate propanoyltransferase  *Structure of Rhodopseudomonas palustris PduL - CoA bound form* | 0.00 |  | 20.00 | 0.09 | 86-110 | X-ray | 1.54 | homo-dimer | 2 x COA, 4 x ZN | HHblits | 0.29 |
| ``` target    GLGVPESQLDVETRQYRNVVRTWAELQQTLHPLQERDPAFRFVFQTPKYRWGAHSTAVDADWISMLFGPFGDPYRRDPRM 5cuo.1    --------------------------------------------------------------------------------  target    PWTGEAYLEINPKDAAELGLADGDYAWVDADPEDRPYRGWNEDDPYYEVARAMMRVRIYTGMSRGVIRTWFNMYAATPAT 5cuo.1    -----DEMHIDVEEANALCLKNDDVVRICK--------------------------------------------------  target    VANQKATPGNPARNEQTRYVALFRYGSHQSGTRAWLRPTQQTDSLVRKGYFGQVIGTGFEADVHSVSGAPKEAFVKIEKA 5cuo.1    --------------------------------------------------------------------------------  target    EDGGIGAERLWRPLTLGLRPEAPSAALTAYLAGDYSGTKGS 5cuo.1    ----------------------------------------- ``` | | | | | | | | | | | | | | | | | | | | | | | | | | | | | | | | | | | | | | | | | | | | | | | | | |
|  | 1yle.1.A | Arginine N-succinyltransferase, alpha chain  *The structure of arginine/ornithine succinyltransferase subunit AI from Pseudomonas aeruginosa.* | 0.00 |  | 16.67 | 0.09 | 86-109 | X-ray | 1.70 | monomer | 1 x CA | HHblits | 0.29 |
| ``` target    GLGVPESQLDVETRQYRNVVRTWAELQQTLHPLQERDPAFRFVFQTPKYRWGAHSTAVDADWISMLFGPFGDPYRRDPRM 1yle.1    --------------------------------------------------------------------------------  target    PWTGEAYLEINPKDAAELGLADGDYAWVDADPEDRPYRGWNEDDPYYEVARAMMRVRIYTGMSRGVIRTWFNMYAATPAT 1yle.1    -----KPVALSVEAAEALGVGEGASVRLV---------------------------------------------------  target    VANQKATPGNPARNEQTRYVALFRYGSHQSGTRAWLRPTQQTDSLVRKGYFGQVIGTGFEADVHSVSGAPKEAFVKIEKA 1yle.1    --------------------------------------------------------------------------------  target    EDGGIGAERLWRPLTLGLRPEAPSAALTAYLAGDYSGTKGS 1yle.1    ----------------------------------------- ``` | | | | | | | | | | | | | | | | | | | | | | | | | | | | | | | | | | | | | | | | | | | | | | | | | |
