# Supplementary material for: Metabolic capacity is maintained despite shifts in microbial diversity in estuary sediments
Source: ISME Commun. 2025 Oct 11;5(1):ycaf182. doi: 10.1093/ismeco/ycaf182 (PMC12687941; doi:10.1093/ismeco/ycaf182)
Supplement: Supplementary_Data_1_ycaf182 [file supplementary_data_1_ycaf182.zip › SWISS-MODEL/4_1_May_SF_Bin52_scaffold_5716_c1_37766595_1/templates.html]

4\_1\_May\_SF\_Bin52\_scaffold\_5716\_c1\_3776-6595\_1 | Templates


**Export Alignment**
  
FASTA format
Clustal Format
PNG Image

**Secondary Structure**
  
None
DSSP
PSIPRED
SSpro

**Colour Scheme** 


Fade Mismatches
Enhance Mismatches

Confidencegradient
Confidenceclass
Indels
Chain
Unique Chain
Rainbow
2° Structure
Clustal
Hydrophobic
Size
Charged
Polar
Proline
Ser/Thr
Cysteine
Aliphatic
Aromatic
No Colour

Use QMEANBrane values

|  |  |  |  |
| --- | --- | --- | --- |
| Background |  |  |  |

**3D Viewer**  
NGL
PV

FASTA
Multi FASTA
ClustalW
PNG


SWISS-MODEL

### 4\_1\_May\_SF\_Bin52\_scaffold\_5716\_c1\_3776-6595\_1

### Created: March 29, 2023, 8:37 p.m. at 20:37

- Templates
- Models

Models | Name | Description | GMQE | QSQE | Seq Id | Coverage | Range | Method | Resolution | Oligo-state | Ligands | Found by | Seq Similarity || ✓ | 7b04.1.B | Nitrite oxidoreductase subunit A  *Structure of Nitrite oxidoreductase (Nxr) from the anammox bacterium Kuenenia stuttgartiensis.* | 0.68 | 0.00 | 39.66 | 0.95 | 2-938 | X-ray | 2.97 | monomer | 4 x SF4, 1 x F3S, 2 x MD1, 1 x MO, 1 x HEM, 2 x CA | HHblits | 0.40 |
| ``` target    AQGVSRRQLLGRALALGSGAALADLLGPARFLSPAGAATAGAVVPGNPLRVMPDRTWEQIYRNQFEDDSTFVFTCAPNDT 7b04.1    -MKLTRRAFLQVAGATGATLTLAKNAMAFRLLKP-------AVVVDNPLDTYPDRRWESVYRDQYQYDRTFTYCCSPNDT  target    HNCLLRAHVKNGVVVRISPTYGYGEATDLYGNRASHRWDPRTCQKGLILSRRFYSERRVKAPMIRKGFKDWVEAGYPRND 7b04.1    HACRIRAFVRNNVMMRVEQNYDHQNYSDLYGNKATRNWNPRMCLKGYTFHRRVYGPYRLRYPLIRKGWKRWADDGFPELT  target    DGTP-QMDVTLRGSDDWIRISWDEATTIAAKTMEDVARTF-NGDEGARKLLAQGYHPEMVEVMHGAGVQALKLRGGMPLL 7b04.1    PENKTKYMFDNRGNDELLRASWDEAFTYASKGIIHITKKYSGPEG-AQKLIDQGYPKEMVDRMQGAGTRTFKGRGGMGLL  target    GIGRIFGFYRFANMLALLDRKLRPDAPADEILGSRTFDNYAWHTDLPPGHPMVTGSQTVDFDLFSAEHTKLLLIIGMNWI 7b04.1    GVIGKYGMYRFNNCLAIVDAHNRG-VGPDQALGGRNWSNYTWHGDQAPGHPFSHGLQTSDVDMNDVRFSKLLIQTGKNLI  target    CTKMPDGHWIGDARLKGTRVIVISADYMPTANKADEVIILRPGTDAAFFLGVARELIEKGLYDRAAVIERTDLPLLVRLD 7b04.1    ENKMPEAHWVTEVMERGGKIVVITPEYSPSAQKADYWIPIRNNTDTALFLGITKILIDNKWYDADYVKKFTDFPLLIRTD  target    TGERLDARDVIPGYELAALTNYVTLKPDAEIKGNPPPPPFTAGGQVVPTELRDAWGDFVWWDRATGRPRPVSRDEVG--- 7b04.1    TLKRVSPKDIIPNYKLQDISD-----------G---PSYHIQG---LKDEQREIIGDFVVWDAKSKGPKAITRDDVGETL  target    ARFDGDPALLGEFEVELVDGSTVPVRPAFDLLKQYLDESFDLRTASEVCRVPPQAIQSIARQLAANKRETLLAAGMGPNH 7b04.1    VKKGIDPVLEGSFKLKTIDGKEIEVMTLLEMYKIHLR-DYDIDSVVSMTNSPKDLIERLAKDIATIKPVA-IHYGEGVNH  target    YFQNDLFGRVQFLVAALTDNIGHLGGNVGSYAGNYRGSVFQA---MG-Q---WIAEDPFAIEPDLTKPA------TVKRY 7b04.1    YFHATLMNRSYYLPVMLTGNVGYFGSGSHTWAGNYKAGNFQASKWSGPGFYGWVAEDVF--KPNL-DPYASAKDLNIKGR  target    YKAESAHYWNYGERPLRAVAKDDEGDLTKGEVLTGKSHMPTPTKLIWFGNSNSLLGNAKWSFDVVKNTLPRQDAVFCNEW 7b04.1    ALDEEVAYWNHSERPLIV-NT---P-KYGRKVFTGKTHMPSPTKVLWFTNVNLINNAKH-VYQMLKNVNPNIEQIMSTDI  target    HWTSSCEYADLVFPADSWAEFKLPDATAS--CTNPFLLAFPTTPLKRLYDTRSDYEALALTAKALGELIDEPRMEQYWRG 7b04.1    EITGSIEYADFAFPANSWVEFQ--EFEITNSCSNPFIQIWGKTGITPVYESKDDVKILAGMASKLGELLRDKRFEDNWKF  target    ILDGDPTPYLQRIFSGSNATRGITYDELHESSK--RGVPLLMNMRTYPRSGGWEQRQEDKPWYTATGRLEFYRPEPEFQA 7b04.1    AIEGRASVYINRLLDGSTTMKGYTCEDILNGKYGEPGVAML-LFRTYPRHPFWEQVHESLPFYTPTGRLQAYNDEPEIIE  target    AGESLPVWREPVDATFYEPNAILSNAAHPSIAPRAPEDYGVPESQLDVETRQYRNVVRTWAELQQTLHPLQERDPAFRFV 7b04.1    YGENFIVHREGPEATPYLPNAIVS--TNPYIR---PDDYGIPENAEYWEDRTVRNIKKSWEETKKTKNFLWE--KGYHFY  target    F 7b04.1    - ``` | | | | | | | | | | | | | | | | | | | | | | | | | | | | | | | | | | | | | | | | | | | | | | | | | |
|  | 7b04.2.B | Nitrite oxidoreductase subunit A  *Structure of Nitrite oxidoreductase (Nxr) from the anammox bacterium Kuenenia stuttgartiensis.* | 0.65 | 0.00 | 39.66 | 0.95 | 2-938 | X-ray | 2.97 | monomer | 4 x SF4, 1 x F3S, 2 x MD1, 1 x MO, 1 x HEM, 2 x CA | HHblits | 0.40 |
| ``` target    AQGVSRRQLLGRALALGSGAALADLLGPARFLSPAGAATAGAVVPGNPLRVMPDRTWEQIYRNQFEDDSTFVFTCAPNDT 7b04.2    -MKLTRRAFLQVAGATGATLTLAKNAMAFRLLKP-------AVVVDNPLDTYPDRRWESVYRDQYQYDRTFTYCCSPNDT  target    HNCLLRAHVKNGVVVRISPTYGYGEATDLYGNRASHRWDPRTCQKGLILSRRFYSERRVKAPMIRKGFKDWVEAGYPRND 7b04.2    HACRIRAFVRNNVMMRVEQNYDHQNYSDLYGNKATRNWNPRMCLKGYTFHRRVYGPYRLRYPLIRKGWKRWADDGFPELT  target    DGTP-QMDVTLRGSDDWIRISWDEATTIAAKTMEDVARTF-NGDEGARKLLAQGYHPEMVEVMHGAGVQALKLRGGMPLL 7b04.2    PENKTKYMFDNRGNDELLRASWDEAFTYASKGIIHITKKYSGPEG-AQKLIDQGYPKEMVDRMQGAGTRTFKGRGGMGLL  target    GIGRIFGFYRFANMLALLDRKLRPDAPADEILGSRTFDNYAWHTDLPPGHPMVTGSQTVDFDLFSAEHTKLLLIIGMNWI 7b04.2    GVIGKYGMYRFNNCLAIVDAHNRG-VGPDQALGGRNWSNYTWHGDQAPGHPFSHGLQTSDVDMNDVRFSKLLIQTGKNLI  target    CTKMPDGHWIGDARLKGTRVIVISADYMPTANKADEVIILRPGTDAAFFLGVARELIEKGLYDRAAVIERTDLPLLVRLD 7b04.2    ENKMPEAHWVTEVMERGGKIVVITPEYSPSAQKADYWIPIRNNTDTALFLGITKILIDNKWYDADYVKKFTDFPLLIRTD  target    TGERLDARDVIPGYELAALTNYVTLKPDAEIKGNPPPPPFTAGGQVVPTELRDAWGDFVWWDRATGRPRPVSRDEVG--- 7b04.2    TLKRVSPKDIIPNYKLQDISD-----------G---PSYHIQG---LKDEQREIIGDFVVWDAKSKGPKAITRDDVGETL  target    ARFDGDPALLGEFEVELVDGSTVPVRPAFDLLKQYLDESFDLRTASEVCRVPPQAIQSIARQLAANKRETLLAAGMGPNH 7b04.2    VKKGIDPVLEGSFKLKTIDGKEIEVMTLLEMYKIHLR-DYDIDSVVSMTNSPKDLIERLAKDIATIKPVA-IHYGEGVNH  target    YFQNDLFGRVQFLVAALTDNIGHLGGNVGSYAGNYRGSVFQA---MG-Q---WIAEDPFAIEPDLTKPA------TVKRY 7b04.2    YFHATLMNRSYYLPVMLTGNVGYFGSGSHTWAGNYKAGNFQASKWSGPGFYGWVAEDVF--KPNL-DPYASAKDLNIKGR  target    YKAESAHYWNYGERPLRAVAKDDEGDLTKGEVLTGKSHMPTPTKLIWFGNSNSLLGNAKWSFDVVKNTLPRQDAVFCNEW 7b04.2    ALDEEVAYWNHSERPLIV-NT---P-KYGRKVFTGKTHMPSPTKVLWFTNVNLINNAKH-VYQMLKNVNPNIEQIMSTDI  target    HWTSSCEYADLVFPADSWAEFKLPDATAS--CTNPFLLAFPTTPLKRLYDTRSDYEALALTAKALGELIDEPRMEQYWRG 7b04.2    EITGSIEYADFAFPANSWVEFQ--EFEITNSCSNPFIQIWGKTGITPVYESKDDVKILAGMASKLGELLRDKRFEDNWKF  target    ILDGDPTPYLQRIFSGSNATRGITYDELHESSK--RGVPLLMNMRTYPRSGGWEQRQEDKPWYTATGRLEFYRPEPEFQA 7b04.2    AIEGRASVYINRLLDGSTTMKGYTCEDILNGKYGEPGVAML-LFRTYPRHPFWEQVHESLPFYTPTGRLQAYNDEPEIIE  target    AGESLPVWREPVDATFYEPNAILSNAAHPSIAPRAPEDYGVPESQLDVETRQYRNVVRTWAELQQTLHPLQERDPAFRFV 7b04.2    YGENFIVHREGPEATPYLPNAIVS--TNPYIR---PDDYGIPENAEYWEDRTVRNIKKSWEETKKTKNFLWE--KGYHFY  target    F 7b04.2    - ``` | | | | | | | | | | | | | | | | | | | | | | | | | | | | | | | | | | | | | | | | | | | | | | | | | |
| ✓ | 7b04.1.B | Nitrite oxidoreductase subunit A  *Structure of Nitrite oxidoreductase (Nxr) from the anammox bacterium Kuenenia stuttgartiensis.* | 0.70 | 0.00 | 41.29 | 0.91 | 47-938 | X-ray | 2.97 | monomer | 4 x SF4, 1 x F3S, 2 x MD1, 1 x MO, 1 x HEM, 2 x CA | BLAST | 0.41 |
| ``` target    AQGVSRRQLLGRALALGSGAALADLLGPARFLSPAGAATAGAVVPGNPLRVMPDRTWEQIYRNQFEDDSTFVFTCAPNDT 7b04.1    ----------------------------------------------NPLDTYPDRRWESVYRDQYQYDRTFTYCCSPNDT  target    HNCLLRAHVKNGVVVRISPTYGYGEATDLYGNRASHRWDPRTCQKGLILSRRFYSERRVKAPMIRKGFKDWVEAGYPRND 7b04.1    HACRIRAFVRNNVMMRVEQNYDHQNYSDLYGNKATRNWNPRMCLKGYTFHRRVYGPYRLRYPLIRKGWKRWADDGFP---  target    DGTPQMDVTL----RGSDDWIRISWDEATTIAAKTMEDVARTFNGDEGARKLLAQGYHPEMVEVMHGAGVQALKLRGGMP 7b04.1    ELTPENKTKYMFDNRGNDELLRASWDEAFTYASKGIIHITKKYSGPEGAQKLIDQGYPKEMVDRMQGAGTRTFKGRGGMG  target    LLGIGRIFGFYRFANMLALLDRKLRPDAPADEILGSRTFDNYAWHTDLPPGHPMVTGSQTVDFDLFSAEHTKLLLIIGMN 7b04.1    LLGVIGKYGMYRFNNCLAIVDAHNRGVGP-DQALGGRNWSNYTWHGDQAPGHPFSHGLQTSDVDMNDVRFSKLLIQTGKN  target    WICTKMPDGHWIGDARLKGTRVIVISADYMPTANKADEVIILRPGTDAAFFLGVARELIEKGLYDRAAVIERTDLPLLVR 7b04.1    LIENKMPEAHWVTEVMERGGKIVVITPEYSPSAQKADYWIPIRNNTDTALFLGITKILIDNKWYDADYVKKFTDFPLLIR  target    LDTGERLDARDVIPGYELAALTNYVTLKPDAEIKGNPPPPPFTAGGQVVPTELRDAWGDFVWWDRATGRPRPVSRDEVG- 7b04.1    TDTLKRVSPKDIIPNYKLQDISD----GPSYHIQG-------------LKDEQREIIGDFVVWDAKSKGPKAITRDDVGE  target    --ARFDGDPALLGEFEVELVDGSTVPVRPAFDLLKQYLDESFDLRTASEVCRVPPQAIQSIARQLAANKRETLLAAGMGP 7b04.1    TLVKKGIDPVLEGSFKLKTIDGKEIEVMTLLEMYKIHLRD-YDIDSVVSMTNSPKDLIERLAKDIATIK-PVAIHYGEGV  target    NHYFQNDLFGRVQFLVAALTDNIGHLGGNVGSYAGNYRGSVFQA-------MGQWIAEDPFAIEPDL-----TKPATVKR 7b04.1    NHYFHATLMNRSYYLPVMLTGNVGYFGSGSHTWAGNYKAGNFQASKWSGPGFYGWVAEDVF--KPNLDPYASAKDLNIKG  target    YYKAESAHYWNYGERPLRAVAKDDEGDLTKGEVLTGKSHMPTPTKLIWFGNSNSLLGNAKWSFDVVKNTLPRQDAVFCNE 7b04.1    RALDEEVAYWNHSERPL-IVNTPKYGR----KVFTGKTHMPSPTKVLWFTNVN-LINNAKHVYQMLKNVNPNIEQIMSTD  target    WHWTSSCEYADLVFPADSWAEFKLPDATASCTNPFLLAFPTTPLKRLYDTRSDYEALALTAKALGELIDEPRMEQYWRGI 7b04.1    IEITGSIEYADFAFPANSWVEFQEFEITNSCSNPFIQIWGKTGITPVYESKDDVKILAGMASKLGELLRDKRFEDNWKFA  target    LDGDPTPYLQRIFSGSNATRGITYDEL--HESSKRGVPLLMNMRTYPRSGGWEQRQEDKPWYTATGRLEFYRPEPEFQAA 7b04.1    IEGRASVYINRLLDGSTTMKGYTCEDILNGKYGEPGVAMLL-FRTYPRHPFWEQVHESLPFYTPTGRLQAYNDEPEIIEY  target    GESLPVWREPVDATFYEPNAILSNAAHPSIAPRAPEDYGVPESQLDVETRQYRNVVRTWAELQQTLHPLQERDPAFRFVF 7b04.1    GENFIVHREGPEATPYLPNAIVS--TNPYI---RPDDYGIPENAEYWEDRTVRNIKKSWEETKKTKNFLWEKGYHFYCV- ``` | | | | | | | | | | | | | | | | | | | | | | | | | | | | | | | | | | | | | | | | | | | | | | | | | |
|  | 7b04.2.B | Nitrite oxidoreductase subunit A  *Structure of Nitrite oxidoreductase (Nxr) from the anammox bacterium Kuenenia stuttgartiensis.* | 0.68 | 0.00 | 41.29 | 0.91 | 47-938 | X-ray | 2.97 | monomer | 4 x SF4, 1 x F3S, 2 x MD1, 1 x MO, 1 x HEM, 2 x CA | BLAST | 0.41 |
| ``` target    AQGVSRRQLLGRALALGSGAALADLLGPARFLSPAGAATAGAVVPGNPLRVMPDRTWEQIYRNQFEDDSTFVFTCAPNDT 7b04.2    ----------------------------------------------NPLDTYPDRRWESVYRDQYQYDRTFTYCCSPNDT  target    HNCLLRAHVKNGVVVRISPTYGYGEATDLYGNRASHRWDPRTCQKGLILSRRFYSERRVKAPMIRKGFKDWVEAGYPRND 7b04.2    HACRIRAFVRNNVMMRVEQNYDHQNYSDLYGNKATRNWNPRMCLKGYTFHRRVYGPYRLRYPLIRKGWKRWADDGFP---  target    DGTPQMDVTL----RGSDDWIRISWDEATTIAAKTMEDVARTFNGDEGARKLLAQGYHPEMVEVMHGAGVQALKLRGGMP 7b04.2    ELTPENKTKYMFDNRGNDELLRASWDEAFTYASKGIIHITKKYSGPEGAQKLIDQGYPKEMVDRMQGAGTRTFKGRGGMG  target    LLGIGRIFGFYRFANMLALLDRKLRPDAPADEILGSRTFDNYAWHTDLPPGHPMVTGSQTVDFDLFSAEHTKLLLIIGMN 7b04.2    LLGVIGKYGMYRFNNCLAIVDAHNRGVGP-DQALGGRNWSNYTWHGDQAPGHPFSHGLQTSDVDMNDVRFSKLLIQTGKN  target    WICTKMPDGHWIGDARLKGTRVIVISADYMPTANKADEVIILRPGTDAAFFLGVARELIEKGLYDRAAVIERTDLPLLVR 7b04.2    LIENKMPEAHWVTEVMERGGKIVVITPEYSPSAQKADYWIPIRNNTDTALFLGITKILIDNKWYDADYVKKFTDFPLLIR  target    LDTGERLDARDVIPGYELAALTNYVTLKPDAEIKGNPPPPPFTAGGQVVPTELRDAWGDFVWWDRATGRPRPVSRDEVG- 7b04.2    TDTLKRVSPKDIIPNYKLQDISD----GPSYHIQG-------------LKDEQREIIGDFVVWDAKSKGPKAITRDDVGE  target    --ARFDGDPALLGEFEVELVDGSTVPVRPAFDLLKQYLDESFDLRTASEVCRVPPQAIQSIARQLAANKRETLLAAGMGP 7b04.2    TLVKKGIDPVLEGSFKLKTIDGKEIEVMTLLEMYKIHLRD-YDIDSVVSMTNSPKDLIERLAKDIATIK-PVAIHYGEGV  target    NHYFQNDLFGRVQFLVAALTDNIGHLGGNVGSYAGNYRGSVFQA-------MGQWIAEDPFAIEPDL-----TKPATVKR 7b04.2    NHYFHATLMNRSYYLPVMLTGNVGYFGSGSHTWAGNYKAGNFQASKWSGPGFYGWVAEDVF--KPNLDPYASAKDLNIKG  target    YYKAESAHYWNYGERPLRAVAKDDEGDLTKGEVLTGKSHMPTPTKLIWFGNSNSLLGNAKWSFDVVKNTLPRQDAVFCNE 7b04.2    RALDEEVAYWNHSERPL-IVNTPKYGR----KVFTGKTHMPSPTKVLWFTNVN-LINNAKHVYQMLKNVNPNIEQIMSTD  target    WHWTSSCEYADLVFPADSWAEFKLPDATASCTNPFLLAFPTTPLKRLYDTRSDYEALALTAKALGELIDEPRMEQYWRGI 7b04.2    IEITGSIEYADFAFPANSWVEFQEFEITNSCSNPFIQIWGKTGITPVYESKDDVKILAGMASKLGELLRDKRFEDNWKFA  target    LDGDPTPYLQRIFSGSNATRGITYDEL--HESSKRGVPLLMNMRTYPRSGGWEQRQEDKPWYTATGRLEFYRPEPEFQAA 7b04.2    IEGRASVYINRLLDGSTTMKGYTCEDILNGKYGEPGVAMLL-FRTYPRHPFWEQVHESLPFYTPTGRLQAYNDEPEIIEY  target    GESLPVWREPVDATFYEPNAILSNAAHPSIAPRAPEDYGVPESQLDVETRQYRNVVRTWAELQQTLHPLQERDPAFRFVF 7b04.2    GENFIVHREGPEATPYLPNAIVS--TNPYI---RPDDYGIPENAEYWEDRTVRNIKKSWEETKKTKNFLWEKGYHFYCV- ``` | | | | | | | | | | | | | | | | | | | | | | | | | | | | | | | | | | | | | | | | | | | | | | | | | |
|  | 2ivf.1.A | ETHYLBENZENE DEHYDROGENASE ALPHA-SUBUNIT  *ETHYLBENZENE DEHYDROGENASE FROM AROMATOLEUM AROMATICUM* | 0.46 | 0.00 | 25.73 | 0.80 | 2-883 | X-ray | 1.88 | monomer | 1 x MES, 4 x SF4, 1 x MO, 1 x MGD, 1 x MD1, 1 x F3S, 1 x HEM | HHblits | 0.32 |
| ``` target    AQGVSRRQLLGRALALGSGAALADLLGPARFLSPAGAATAGAVVPGNPLRVM-PDRTWEQIYRNQFEDDSTFVFTCAPND 2ivf.1    -QDQHRRDFLKRSGAAVLSLSLSSLAT--------GV-VPGFLKDAQAGTKAPGYASWEDIYRKEWKWDKVNWGSHLNIC  target    --THNCLLRAHVKNGVVVRISPTYGYGEATDLYGNRASHRWDPRTCQKGLILSRRFYSERRVKAPMIRKGFKDWVEAGYP 2ivf.1    WPQGSCKFYVYVRNGIVWREEQAAQTPA-----CNVDYVDYNPLGCQKGSAFNNNLYGDERVKYPLKRVG----------  target    RNDDGTPQMDVTLRGSDDWIRISWDEATTIAAKTMEDVARTFNGDEGARKLLAQGYHPEMVEVMHGAGVQALKLRGGMPL 2ivf.1    ------------KRGEGKWKRVSWDEAAGDIADSIIDSFEAQGS--------------DGFILDAPHVHAG-----SIA-  target    LGIGRIFGFYRFANMLALLDRKLRPDAPADEILGSRTFDNYAWHTDLPPGHPMVTGSQTVDFDLFSAEHTKLLLIIGMNW 2ivf.1    -----WGAGFRMTYLMD-------------GVSPDINVD----IGDTYMGAFHTFGKMHMGYSADNLLDAELIFMTCSNW  target    ICTKMPDGHWIGDARLKGTRVIVISADYMPTANKADEVIILRPGTDAAFFLGVARELIEKGLYDRAAVIERTDLPLLVRL 2ivf.1    SYTYPSSYHFLSEARYKGAEVVVIAPDFNPTTPAADLHVPVRVGSDAAFWLGLSQVMIDEKLFDRQFVCEQTDLPLLVRM  target    DTGERLDARDVIPGYELAALTNYVTLKPDAEIKGNPPPPPFTAGGQVVPTELRDAWGDFVWWDRATGRPRPVSRDEVGAR 2ivf.1    DTGKFLSAEDVDGG----------------------------------------EAKQFYFFDEKAGSVRKASRGTLKL-  target    FDGDPALLGEFEVELVDGSTVPVRPAFDLLKQYLDESFDLRTASEVCRVPPQAIQSIARQLAANKRETLLAAGMGPNHYF 2ivf.1    -DFMPALEGTFSARLKNGKTIQVRTVFEGLREHLK-DYTPEKASAKCGVPVSLIRELGRKVAKKR--TCSYIGFSSAKSY  target    QNDLFGRVQFLVAALTDNIGHLGGNVGSYAGNYRGSVF-------QAMGQWI---A-EDPFAI-----EPDLTKPATVKR 2ivf.1    HGDLMERSLFLAMALSGNWGKPGTGAFAWAYSDDNMVYLGVMSKPTAQGGMDELHQMAEGFNKRTLEADPTSTDEMGNIE  target    YYKA------------------ESAHYWNYG--ERP-LR----AVA-KDDEGDLTKGEVLTGKSHMPTPTKLIWFGNSNS 2ivf.1    FMKVVTSAVGLVPPAMWLYYHVGYDQLWNNKAWTDPALKKSFGAYLDEAKEKGWWTNDHIR--PAPDKTPQVYMLLSQNP  target    LLGNAKWSFDVVKNTLPRQDAVFCNEWHWTSSCEYADLVFPADSWAEFKLPDATAS-CTNPFLLAFPTTPLKRLYDTRSD 2ivf.1    MRRKRSGAKMFPDVLFPKLKMIFALETRMSSSAMYADIVLPCAWYYEKH--EMTTPCSGNPFFTF-VDRSVAPPGECREE  target    YEALALTAKALGELIDEPR-------------MEQYWRGIL----DGDPTPYLQRIFSGSN----ATRGITYDELHESSK 2ivf.1    WDAIALILKKVGERAAARGLTEFNDHNGRKRRYDELYKKFTMDGHLLTNEDCLKEMVDINRAVGVFAKDYTYEKFKKEGQ  target    RGVPLLMN-MR----------TYPRSGGWEQRQEDKPWYTATGRLEFYRPEPEFQAAGESLPVWREPVDATFYEPNAILS 2ivf.1    TRFLSMGTGVSRYAHANEVDVTKPIYPMRWHFDDKKVFPTHTRRAQFYLDHDWYLEAGESLPTHKDTPMVGGDHPFKITG  target    NAAHPSIAPRAPEDYGVPESQLDVETRQYRNVVRTWAELQQTLHPLQERDPAFRFVF 2ivf.1    G-------------------------------------------------------- ``` | | | | | | | | | | | | | | | | | | | | | | | | | | | | | | | | | | | | | | | | | | | | | | | | | |
|  | 4ydd.1.A | DMSO reductase family type II enzyme, molybdopterin subunit  *Crystal structure of the perchlorate reductase PcrAB from Azospira suillum PS* | 0.46 | 0.00 | 30.14 | 0.75 | 57-875 | X-ray | 1.86 | monomer | 4 x SF4, 1 x MO, 1 x MGD, 1 x MD1, 1 x F3S | BLAST | 0.36 |
| ``` target    AQGVSRRQLLGRALALGSGAALADLLGPARFLSPAGAATAGAVVPGNPLRVMPDRTWEQIYRNQFEDDSTFVFTCAPNDT 4ydd.1    --------------------------------------------------------WENFHRTQWSWDKKTRGAHLVNCT  target    HNCLLRAHVKNGVVVRISPTYGYGEATDLYGNRASHRWDPRTCQKGLILSRRFYSERRVKAPMIRKGFKDWVEAGYPRND 4ydd.1    GACPHFVYSKDGVVMREE------QSKDIAPMPNIPEYNPRGCNKGECGHDYMYGPHRIKYPLIRVG-------------  target    DGTPQMDVTLRGSDDWIRISWDEATTIAAKTMEDVARTFNGDEGARKLLAQGYHPEMVEVMHGA-GVQALKLRGGMPLLG 4ydd.1    ---------ERGEGKWRRATWEEALDMIADKCVDTIKN--------------HAPDCISVYSPVPAVSPVSFSAG-----  target    IGRIFGFYRFANMLALLDRKLRPDAPADEILGSRTFDNYAWHTDLPPGHPMVTGSQTVDFDLFSAEHTKLLLIIGMNWIC 4ydd.1    -------HRFAHYI-----------------GAHAHTFYDWYGDHPTGQTQTCGVQGDTCETADWFNSKYIILWGSNPTQ  target    TKMPDGHWIGDARLKGTRVIVISADYMPTANKADEVIILRPGTDAAFFLGVARELIEKGLYDRAAVIERTDLPLLVRLDT 4ydd.1    TRIPDAHFLSEAQLNGAKIVSISPDYNSSTIKVDKWIHPQPGTDGALAMAMAHVIIKEKLYDAHSLKEQTDLSYLVRSDT  target    GERLDARDVIPG--YELAALTNYVTLKPDAEIKGNPPPPPFTAGGQVVPTELRDAWGDFVWWDRATGRPR---------- 4ydd.1    KRFLREADVVAGGSKDKFYFWNAKTGKP------------------VIP---KGSWGD---QPEKKGSPVGFLGRNTFAF  target    PVSRDEVGARFDGDPALLGEFEVELVDGSTVPVRPAFDLLKQYLDESFDLRTASEVCRVPPQAIQSIARQLAANKRETLL 4ydd.1    PKGYIDLG---DLDPALEGKFNMQLLDGKTVEVRPVFEILKSRLMADNTPEKAAKITGVTAKAITELAREFATAKPSMII  target    AAGMGPNHYFQNDLFGRVQFLVAALTDNIGHLGGNVGSYAGNYRGSVFQAMGQWIAEDPFAIEPDLTKPATVKRYYKAES 4ydd.1    CGG-GTQHWYYSDVLLRAMHLLTALTGTEGTNGGGMNHYIGQWKPAFVAGLVA------------LAFPEGVNKQRFCQT  target    AHYWNY--GERPLRAVAKD-DEGDLTKGEVLTGKS-HMPTPTK--LIWFGNSNSLLGNAKWSFDVVKNTLPRQDAVFCNE 4ydd.1    T-IWTYIHAEVNDEIISSDIDTEKYLRDSITTGQMPNMPEQGRDPKVFFVYRGNWLNQAKGQKYVLENLWPKLELIVDIN  target    WHWTSSCEYADLVFPADSWAEFKLPDATASCTNPFLLAFPTTPLKRLYDTRSDYEALALTAKALGELIDEPRMEQY---- 4ydd.1    IRMDSTALYSDVVLPSAHWYE-KLDLNVTSEHSYINMTEPA--IKPMWESKTDWQIFLALAKRVEMAAKRKKYEKFNDEK  target    ----------WRGI-LDG---DPTPYLQRIFSGSNATRGITYDELHESSKRGVPLLMNMRTYPRSGG--WEQRQ----ED 4ydd.1    FKWVRDLSNLWNQMTMDGKLAEDEAAAQYILDNAPQSKGITIQMLREKPQR----FKSNWTSPLKEGVPYTPFQYFVVDK  target    KPWYTATGRLEFYRPEPEFQAAGESLPVWREPVDATFYEPNAILSNAAHPSIAPRAPEDYGVPESQLDVETRQYRNVVRT 4ydd.1    KPWPTLTGRQQFYLDHDTFFDMGVELPTYKAPIDADKY------------------------------------------  target    WAELQQTLHPLQERDPAFRFVF 4ydd.1    ---------------------- ``` | | | | | | | | | | | | | | | | | | | | | | | | | | | | | | | | | | | | | | | | | | | | | | | | | |
|  | 5e7o.1.A | DMSO reductase family type II enzyme, molybdopterin subunit  *Crystal structure of the perchlorate reductase PcrAB mutant W461E of PcrA from Azospira suillum PS* | 0.45 | 0.00 | 30.14 | 0.75 | 57-875 | X-ray | 2.40 | monomer | 4 x SF4, 1 x MO, 1 x MGD, 1 x MD1, 1 x F3S | BLAST | 0.36 |
| ``` target    AQGVSRRQLLGRALALGSGAALADLLGPARFLSPAGAATAGAVVPGNPLRVMPDRTWEQIYRNQFEDDSTFVFTCAPNDT 5e7o.1    --------------------------------------------------------WENFHRTQWSWDKKTRGAHLVNCT  target    HNCLLRAHVKNGVVVRISPTYGYGEATDLYGNRASHRWDPRTCQKGLILSRRFYSERRVKAPMIRKGFKDWVEAGYPRND 5e7o.1    GACPHFVYSKDGVVMREE------QSKDIAPMPNIPEYNPRGCNKGECGHDYMYGPHRIKYPLIRVG-------------  target    DGTPQMDVTLRGSDDWIRISWDEATTIAAKTMEDVARTFNGDEGARKLLAQGYHPEMVEVMHGA-GVQALKLRGGMPLLG 5e7o.1    ---------ERGEGKWRRATWEEALDMIADKCVDTIKN--------------HAPDCISVYSPVPAVSPVSFSAG-----  target    IGRIFGFYRFANMLALLDRKLRPDAPADEILGSRTFDNYAWHTDLPPGHPMVTGSQTVDFDLFSAEHTKLLLIIGMNWIC 5e7o.1    -------HRFAHYI-----------------GAHAHTFYDWYGDHPTGQTQTCGVQGDTCETADWFNSKYIILWGSNPTQ  target    TKMPDGHWIGDARLKGTRVIVISADYMPTANKADEVIILRPGTDAAFFLGVARELIEKGLYDRAAVIERTDLPLLVRLDT 5e7o.1    TRIPDAHFLSEAQLNGAKIVSISPDYNSSTIKVDKWIHPQPGTDGALAMAMAHVIIKEKLYDAHSLKEQTDLSYLVRSDT  target    GERLDARDVIPG--YELAALTNYVTLKPDAEIKGNPPPPPFTAGGQVVPTELRDAWGDFVWWDRATGRPR---------- 5e7o.1    KRFLREADVVAGGSKDKFYFWNAKTGKP------------------VIP---KGSWGD---QPEKKGSPVGFLGRNTFAF  target    PVSRDEVGARFDGDPALLGEFEVELVDGSTVPVRPAFDLLKQYLDESFDLRTASEVCRVPPQAIQSIARQLAANKRETLL 5e7o.1    PKGYIDLG---DLDPALEGKFNMQLLDGKTVEVRPVFEILKSRLMADNTPEKAAKITGVTAKAITELAREFATAKPSMII  target    AAGMGPNHYFQNDLFGRVQFLVAALTDNIGHLGGNVGSYAGNYRGSVFQAMGQWIAEDPFAIEPDLTKPATVKRYYKAES 5e7o.1    CGG-GTQHWYYSDVLLRAMHLLTALTGTEGTNGGGMNHYIGQEKPAFVAGLVA------------LAFPEGVNKQRFCQT  target    AHYWNY--GERPLRAVAKD-DEGDLTKGEVLTGKS-HMPTPTK--LIWFGNSNSLLGNAKWSFDVVKNTLPRQDAVFCNE 5e7o.1    T-IWTYIHAEVNDEIISSDIDTEKYLRDSITTGQMPNMPEQGRDPKVFFVYRGNWLNQAKGQKYVLENLWPKLELIVDIN  target    WHWTSSCEYADLVFPADSWAEFKLPDATASCTNPFLLAFPTTPLKRLYDTRSDYEALALTAKALGELIDEPRMEQY---- 5e7o.1    IRMDSTALYSDVVLPSAHWYE-KLDLNVTSEHSYINMTEPA--IKPMWESKTDWQIFLALAKRVEMAAKRKKYEKFNDEK  target    ----------WRGI-LDG---DPTPYLQRIFSGSNATRGITYDELHESSKRGVPLLMNMRTYPRSGG--WEQRQ----ED 5e7o.1    FKWVRDLSNLWNQMTMDGKLAEDEAAAQYILDNAPQSKGITIQMLREKPQR----FKSNWTSPLKEGVPYTPFQYFVVDK  target    KPWYTATGRLEFYRPEPEFQAAGESLPVWREPVDATFYEPNAILSNAAHPSIAPRAPEDYGVPESQLDVETRQYRNVVRT 5e7o.1    KPWPTLTGRQQFYLDHDTFFDMGVELPTYKAPIDADKY------------------------------------------  target    WAELQQTLHPLQERDPAFRFVF 5e7o.1    ---------------------- ``` | | | | | | | | | | | | | | | | | | | | | | | | | | | | | | | | | | | | | | | | | | | | | | | | | |
|  | 4ydd.1.A | DMSO reductase family type II enzyme, molybdopterin subunit  *Crystal structure of the perchlorate reductase PcrAB from Azospira suillum PS* | 0.47 | 0.00 | 26.60 | 0.75 | 53-884 | X-ray | 1.86 | monomer | 4 x SF4, 1 x MO, 1 x MGD, 1 x MD1, 1 x F3S | HHblits | 0.34 |
| ``` target    AQGVSRRQLLGRALALGSGAALADLLGPARFLSPAGAATAGAVVPGNPLRVMPDRTWEQIYRNQFEDDSTFVFTCAPNDT 4ydd.1    ----------------------------------------------------EYSGWENFHRTQWSWDKKTRGAHLVNCT  target    HNCLLRAHVKNGVVVRISPTYGYGEATDLYGNRASHRWDPRTCQKGLILSRRFYSERRVKAPMIRKGFKDWVEAGYPRND 4ydd.1    GACPHFVYSKDGVVMREEQSKD------IAPMPNIPEYNPRGCNKGECGHDYMYGPHRIKYPLIRVG-------------  target    DGTPQMDVTLRGSDDWIRISWDEATTIAAKTMEDVARTFNGDEGARKLLAQGYHPEMVEVMHGAGVQALKLRGGMPLLGI 4ydd.1    ---------ERGEGKWRRATWEEALDMIADKCVDTIKNHA--------------PDCISVYSPVPAVSP--------V--  target    GRIFGFYRFANMLALLDRKLRPDAPADEILGSRTFDNYAWHTDLPPGHPMVTGSQTVDFDLFSAEHTKLLLIIGMNWICT 4ydd.1    -SFSAGHRFAH-----------------YIGAHAHTFYDWYGDHPTGQTQTCGVQGDTCETADWFNSKYIILWGSNPTQT  target    KMPDGHWIGDARLKGTRVIVISADYMPTANKADEVIILRPGTDAAFFLGVARELIEKGLYDRAAVIERTDLPLLVRLDTG 4ydd.1    RIPDAHFLSEAQLNGAKIVSISPDYNSSTIKVDKWIHPQPGTDGALAMAMAHVIIKEKLYDAHSLKEQTDLSYLVRSDTK  target    ERLDARDVIPGYELAALTNYVTLKPDAEIKGNPPPPPFTAGGQVVPTELRDAWGDFVWWDRATGRPRPVSRDEVG----- 4ydd.1    RFLREADVVAGG---------------------------------------SKDKFYFWNAKTGKPVIPKGSWGDQPEKK  target    -------------------ARFDGDPALLGEFEVELVDGSTVPVRPAFDLLKQYLDESFDLRTASEVCRVPPQAIQSIAR 4ydd.1    GSPVGFLGRNTFAFPKGYIDLGDLDPALEGKFNMQLLDGKTVEVRPVFEILKSRLMADNTPEKAAKITGVTAKAITELAR  target    QLAANKRETLLAAGMGPNHYFQNDLFGRVQFLVAALTDNIGHLGGNVGSYAGNYRGSVFQAMGQWIAEDPFAIEPDLTKP 4ydd.1    EFATAKPS-MIICGGGTQHWYYSDVLLRAMHLLTALTGTEGTNGGGMNHYIGQWKPAFV--AGLVA--LAFP---EGVNK  target    ATVKRYYKAESAHYWNYGERPLR-AV-AK-DDEGDLTKGEVLTGK----SHMPTPTKLIWFGNSNSLLGNAKWSFDVVKN 4ydd.1    ---QRFCQ---TTIWTYIHAEVNDEIISSDIDTEKYLRDSITTGQMPNMPEQGRDPKVFFVYRGNWLNQAKGQ-KYVLEN  target    TLPRQDAVFCNEWHWTSSCEYADLVFPADSWAEFKLPDATASCTNPFLLAFPTTPLKRLYDTRSDYEALALTAKALGELI 4ydd.1    LWPKLELIVDINIRMDSTALYSDVVLPSAHWYEKL--DLNVTSEHSYINMT-EPAIKPMWESKTDWQIFLALAKRVEMAA  target    DEPRM--------------EQYWRGILD----GDPTPYLQRIFSGSNATRGITYDELHESSKRGVPLLMN--M-RTYPRS 4ydd.1    KRKKYEKFNDEKFKWVRDLSNLWNQMTMDGKLAEDEAAAQYILDNAPQSKGITIQMLREKPQR-FKSNWTSPLKEGVPYT  target    GGWEQRQEDKPWYTATGRLEFYRPEPEFQAAGESLPVWREPVDATFYEPNAILSNAAHPSIAPRAPEDYGVPESQLDVET 4ydd.1    PFQYFVVDKKPWPTLTGRQQFYLDHDTFFDMGVELPTYKAPIDA-DKYPFRFNSPH------------------------  target    RQYRNVVRTWAELQQTLHPLQERDPAFRFVF 4ydd.1    ------------------------------- ``` | | | | | | | | | | | | | | | | | | | | | | | | | | | | | | | | | | | | | | | | | | | | | | | | | |
|  | 5e7o.1.A | DMSO reductase family type II enzyme, molybdopterin subunit  *Crystal structure of the perchlorate reductase PcrAB mutant W461E of PcrA from Azospira suillum PS* | 0.48 | 0.00 | 26.60 | 0.75 | 53-884 | X-ray | 2.40 | monomer | 4 x SF4, 1 x MO, 1 x MGD, 1 x MD1, 1 x F3S | HHblits | 0.33 |
| ``` target    AQGVSRRQLLGRALALGSGAALADLLGPARFLSPAGAATAGAVVPGNPLRVMPDRTWEQIYRNQFEDDSTFVFTCAPNDT 5e7o.1    ----------------------------------------------------EYSGWENFHRTQWSWDKKTRGAHLVNCT  target    HNCLLRAHVKNGVVVRISPTYGYGEATDLYGNRASHRWDPRTCQKGLILSRRFYSERRVKAPMIRKGFKDWVEAGYPRND 5e7o.1    GACPHFVYSKDGVVMREEQSKD------IAPMPNIPEYNPRGCNKGECGHDYMYGPHRIKYPLIRVG-------------  target    DGTPQMDVTLRGSDDWIRISWDEATTIAAKTMEDVARTFNGDEGARKLLAQGYHPEMVEVMHGAGVQALKLRGGMPLLGI 5e7o.1    ---------ERGEGKWRRATWEEALDMIADKCVDTIKNHA--------------PDCISVYSPVPAVSP-----------  target    GRIFGFYRFANMLALLDRKLRPDAPADEILGSRTFDNYAWHTDLPPGHPMVTGSQTVDFDLFSAEHTKLLLIIGMNWICT 5e7o.1    VSFSAGHRFAH-----------------YIGAHAHTFYDWYGDHPTGQTQTCGVQGDTCETADWFNSKYIILWGSNPTQT  target    KMPDGHWIGDARLKGTRVIVISADYMPTANKADEVIILRPGTDAAFFLGVARELIEKGLYDRAAVIERTDLPLLVRLDTG 5e7o.1    RIPDAHFLSEAQLNGAKIVSISPDYNSSTIKVDKWIHPQPGTDGALAMAMAHVIIKEKLYDAHSLKEQTDLSYLVRSDTK  target    ERLDARDVIPGYELAALTNYVTLKPDAEIKGNPPPPPFTAGGQVVPTELRDAWGDFVWWDRATGRPRPVSRDEVG----- 5e7o.1    RFLREADVVAGG---------------------------------------SKDKFYFWNAKTGKPVIPKGSWGDQPEKK  target    -------------------ARFDGDPALLGEFEVELVDGSTVPVRPAFDLLKQYLDESFDLRTASEVCRVPPQAIQSIAR 5e7o.1    GSPVGFLGRNTFAFPKGYIDLGDLDPALEGKFNMQLLDGKTVEVRPVFEILKSRLMADNTPEKAAKITGVTAKAITELAR  target    QLAANKRETLLAAGMGPNHYFQNDLFGRVQFLVAALTDNIGHLGGNVGSYAGNYRGSVFQAMGQWIAEDPFAIEPDLTKP 5e7o.1    EFATAKPS-MIICGGGTQHWYYSDVLLRAMHLLTALTGTEGTNGGGMNHYIGQEKPAFV--AGLVA--LAFP---EGVNK  target    ATVKRYYKAESAHYWNYGERPL--RAVAK-DDEGDLTKGEVLTGK----SHMPTPTKLIWFGNSNSLLGNAKWSFDVVKN 5e7o.1    ---QRFCQ---TTIWTYIHAEVNDEIISSDIDTEKYLRDSITTGQMPNMPEQGRDPKVFFVYRGNWLNQAKGQ-KYVLEN  target    TLPRQDAVFCNEWHWTSSCEYADLVFPADSWAEFKLPDATASCTNPFLLAFPTTPLKRLYDTRSDYEALALTAKALGELI 5e7o.1    LWPKLELIVDINIRMDSTALYSDVVLPSAHWYEKL--DLNVTSEHSYINMT-EPAIKPMWESKTDWQIFLALAKRVEMAA  target    DEPRM--------------EQYWRGIL----DGDPTPYLQRIFSGSNATRGITYDELHESSKRG---VPLLMNMRTYPRS 5e7o.1    KRKKYEKFNDEKFKWVRDLSNLWNQMTMDGKLAEDEAAAQYILDNAPQSKGITIQMLREKPQRFKSNWTSPLK-EGVPYT  target    GGWEQRQEDKPWYTATGRLEFYRPEPEFQAAGESLPVWREPVDATFYEPNAILSNAAHPSIAPRAPEDYGVPESQLDVET 5e7o.1    PFQYFVVDKKPWPTLTGRQQFYLDHDTFFDMGVELPTYKAPIDA-DKYPFRFNSPH------------------------  target    RQYRNVVRTWAELQQTLHPLQERDPAFRFVF 5e7o.1    ------------------------------- ``` | | | | | | | | | | | | | | | | | | | | | | | | | | | | | | | | | | | | | | | | | | | | | | | | | |
|  | 1r27.4.A | Respiratory nitrate reductase 1 alpha chain  *Crystal Structure of NarGH complex* | 0.40 | 0.19 | 24.16 | 0.70 | 1-767 | X-ray | 2.00 | homo-dimer | 4 x MO, 16 x SF4, 8 x MGD, 4 x F3S | HHblits | 0.32 |
| ``` target    AQGVSRRQLLGRALALGSGAALADLLGPARFLSPAGAATAGAVVPGNPLRVMPDRTWEQIYRNQFEDDSTFVFTCAPNDT 1r27.4    SKFLDRFRYFKQKGETFADGHG----------------------QLL----NTNRDWEDGYRQRWQHDKIVRSTHGVNCT  target    HNCLLRAHVKNGVVVRISPTYGYGEATDLYGNRASHRWDPRTCQKGLILSRRFYSERRVKAPMIRKGF-KDWVEAGY--- 1r27.4    GSCSWKIYVKNGLVTWETQQTDYPR-----TRPDLPNHEPRGCPRGASYSWYLYSANRLKYPMMRKRLMKMWREAKALHS  target    -PR-------NDDGTPQMDVTLRGSDDWIRISWDEATTIAAKTMEDVARTFNGDEGARKLLAQGYHPEMVEVMHGAGVQA 1r27.4    DPVEAWASIIEDADKAKSFKQARGRGGFVRSSWQEVNELIAASNVYTIKNYG--------------PDRVAGFSPIPAMS  target    LKLRGGMPLLGIGRIFGFYRFANMLALLDRKLRPDAPADEILGSRTFDNYAWHTDLPPGHPMVTGSQTVDFDLFSAEHTK 1r27.4    MV-----------SYASGARYL-----------------SLIGGTCLSFYDWYCDLPPASPQTWGEQTDVPESADWYNSS  target    LLLIIGMNWICTKMPDGHWIGDARLKGTRVIVISADYMPTANKADEVIILRPGTDAAFFLGVARELIEKGL------YDR 1r27.4    YIIAWGSNVPQTRTPDAHFFTEVRYKGTKTVAVTPDYAEIAKLCDLWLAPKQGTDAAMALAMGHVMLREFHLDNPSQYFT  target    AAVIERTDLPLLVRLDT-------GERLDARDVIPGYELAALTNYVTLKPDAEIKGNPPPPPFTAGGQVVPTELRDAWGD 1r27.4    DYVRRYTDMPMLVMLEERDGYYAAGRMLRAADLVDALGQEN----------------------------------NPEWK  target    FVWWDRATGRPRPVSRDE-----------------------------------V---G-ARFDG-------DPALL---- 1r27.4    TVAFNT-NGEMVAPNGSIGFRWGEKGKWNLEQRDGKTGEETELQLSLLGSQDEIAEVGFPYFGGDGTEHFNKVELENVLL  target    ---GEFEVELVDGSTVPVRPAFDLLK------------------QYLDESFDLRTASEVCRVPPQAIQSIARQLAAN--- 1r27.4    HKLPVKRLQLADGSTALVTTVYDLTLANYGLERGLNDVNCATSYDDV-KAYTPAWAEQITGVSRSQIIRIAREFADNADK  target    --KRETLLAAGMGPNHYFQNDLFGRVQFLVAALTDNIGHLGGNVGSYAGNYRGSVFQAMGQWIAEDPFAI---------- 1r27.4    THGR-SMIIVGAGLNHWYHLDMNYRGLINMLIFCGCVGQSGGGWAHYVGQEKLRPQTGWQPLAFALDWQRPARHMNSTSY  target    -E-------------PDLTKPA-TVKRYYK--------AESAHYW----NYGERPLRAVAKDD-----EGDLTKGEVLTG 1r27.4    FYNHSSQWRYETVTAEELLSPMADKSRYTGHLIDFNVRAERMGWLPSAPQLGTNPLTIAGEAEKAGMNPVDYTVKSLKEG  target    KS--------HMPTPTKLIWFGNSNSLLGNAKWSFDVV------------------------------KNTLPRQDAVFC 1r27.4    SIRFAAEQPENGKNHPRNLFIWRSNLLGSSGKGHEFMLKYLLGTEHGIQGKDLGQQGGVKPEEVDWQDNGLEGKLDLVVT  target    NEWHWTSSCEYADLVFPADSWAEFKLPDATASCTNPFLLAFPTTPLKRLYDTRSDYEALALTAKALGELIDEPRMEQYWR 1r27.4    LDFRLSSTCLYSDIILPTATWYEK--DDMNTSDMHPFIHPL-SAAVDPAWEAKSDWEIYKAIAKKFSE------------  target    GILDGDPTPYLQRIFSGSNATRGITYDELHESSKRGVPLLMNMRTYPRSGGWEQRQEDKPWYTATGRLEFYRPEPEFQAA 1r27.4    --------------------------------------------------------------------------------  target    GESLPVWREPVDATFYEPNAILSNAAHPSIAPRAPEDYGVPESQLDVETRQYRNVVRTWAELQQTLHPLQERDPAFRFVF 1r27.4    -------------------------------------------------------------------------------- ``` | | | | | | | | | | | | | | | | | | | | | | | | | | | | | | | | | | | | | | | | | | | | | | | | | |
|  | 3ir7.1.A | Respiratory nitrate reductase 1 alpha chain  *Crystal structure of NarGHI mutant NarG-R94S* | 0.39 | 0.00 | 24.01 | 0.70 | 1-767 | X-ray | 2.50 | monomer | 2 x MD1, 4 x SF4, 1 x 6MO, 1 x AGA, 1 x F3S, 2 x HEM | HHblits | 0.32 |
| ``` target    AQGVSRRQLLGRALALGSGAALADLLGPARFLSPAGAATAGAVVPGNPLRVMPDRTWEQIYRNQFEDDSTFVFTCAPNDT 3ir7.1    SKFLDRFRYFKQKGETFADGHG----------------------QL----LNTNRDWEDGYRQRWQHDKIVRSTHGVNCT  target    HNCLLRAHVKNGVVVRISPTYGYGEATDLYGNRASHRWDPRTCQKGLILSRRFYSERRVKAPMIRKGF-KDWVEAGYP-- 3ir7.1    GSCSWKIYVKNGLVTWETQQTDYPR-----TRPDLPNHEPRGCPSGASYSWYLYSANRLKYPMMRKRLMKMWREAKALHS  target    --R-------NDDGTPQMDVTLRGSDDWIRISWDEATTIAAKTMEDVARTFNGDEGARKLLAQGYHPEMVEVMHGAGVQA 3ir7.1    DPVEAWASIIEDADKAKSFKQARGRGGFVRSSWQEVNELIAASNVYTIKNYG--------------PDRVAGFSPIPAMS  target    LKLRGGMPLLGIGRIFGFYRFANMLALLDRKLRPDAPADEILGSRTFDNYAWHTDLPPGHPMVTGSQTVDFDLFSAEHTK 3ir7.1    MV-----------SYASGARYL-----------------SLIGGTCLSFYDWYCDLPPASPQTWGEQTDVPESADWYNSS  target    LLLIIGMNWICTKMPDGHWIGDARLKGTRVIVISADYMPTANKADEVIILRPGTDAAFFLGVARELIEKGL------YDR 3ir7.1    YIIAWGSNVPQTRTPDAHFFTEVRYKGTKTVAVTPDYAEIAKLCDLWLAPKQGTDAAMALAMGHVMLREFHLDNPSQYFT  target    AAVIERTDLPLLVRLDT-------GERLDARDVIPGYELAALTNYVTLKPDAEIKGNPPPPPFTAGGQVVPTELRDAWGD 3ir7.1    DYVRRYTDMPMLVMLEERDGYYAAGRMLRAADLVDALGQEN----------------------------------NPEWK  target    FVWWDRATGRPRPVSRDE-----------------------------------V---G-ARFD-----------GDPALL 3ir7.1    TVAFNT-NGEMVAPNGSIGFRWGEKGKWNLEQRDGKTGEETELQLSLLGSQDEIAEVGFPYFGGDGTEHFNKVELENVLL  target    G---EFEVELVDGSTVPVRPAFDLLK------------------QYLDESFDLRTASEVCRVPPQAIQSIARQLAAN--- 3ir7.1    HKLPVKRLQLADGSTALVTTVYDLTLANYGLERGLNDVNCATSYDDV-KAYTPAWAEQITGVSRSQIIRIAREFADNADK  target    --KRETLLAAGMGPNHYFQNDLFGRVQFLVAALTDNIGHLGGNVGSYAGNYRGSVFQAMGQWIAEDPFAIEP-------- 3ir7.1    THGR-SMIIVGAGLNHWYHLDMNYRGLINMLIFCGCVGQSGGGWAHYVGQEKLRPQTGWQPLAFALDWQRPARHMNSTSY  target    ----------------DLTKPA-TVKRYYK--------AESAHYW----NYGERPLRAVAKDD-----EGDLTKGEVLTG 3ir7.1    FYNHSSQWRYETVTAEELLSPMADKSRYTGHLIDFNVRAERMGWLPSAPQLGTNPLTIAGEAEKAGMNPVDYTVKSLKEG  target    KS--------HMPTPTKLIWFGNSNSLLGNAKWSFDV------------------------------VKNTLPRQDAVFC 3ir7.1    SIRFAAEQPENGKNHPRNLFIWRSNLLGSSGKGHEFMLKYLLGTEHGIQGKDLGQQGGVKPEEVDWQDNGLEGKLDLVVT  target    NEWHWTSSCEYADLVFPADSWAEFKLPDATASCTNPFLLAFPTTPLKRLYDTRSDYEALALTAKALGELIDEPRMEQYWR 3ir7.1    LDFRLSSTCLYSDIILPTATWYEK--DDMNTSDMHPFIHPL-SAAVDPAWEAKSDWEIYKAIAKKFSE------------  target    GILDGDPTPYLQRIFSGSNATRGITYDELHESSKRGVPLLMNMRTYPRSGGWEQRQEDKPWYTATGRLEFYRPEPEFQAA 3ir7.1    --------------------------------------------------------------------------------  target    GESLPVWREPVDATFYEPNAILSNAAHPSIAPRAPEDYGVPESQLDVETRQYRNVVRTWAELQQTLHPLQERDPAFRFVF 3ir7.1    -------------------------------------------------------------------------------- ``` | | | | | | | | | | | | | | | | | | | | | | | | | | | | | | | | | | | | | | | | | | | | | | | | | |
|  | 3ir6.1.A | Respiratory nitrate reductase 1 alpha chain  *Crystal structure of NarGHI mutant NarG-H49S* | 0.38 | 0.00 | 24.20 | 0.70 | 2-767 | X-ray | 2.80 | monomer | 2 x GDP, 1 x AGA, 3 x SF4, 1 x F3S, 2 x HEM | HHblits | 0.32 |
| ``` target    AQGVSRRQLLGRALALGSGAALADLLGPARFLSPAGAATAGAVVPGNPLRVMPDRTWEQIYRNQFEDDSTFVFTCAPNDT 3ir6.1    -KFLDRFRYFKQKGETFADGH-GQ---------------------LL----NTNRDWEDGYRQRWQHDKIVRSTSGVNCT  target    HNCLLRAHVKNGVVVRISPTYGYGEATDLYGNRASHRWDPRTCQKGLILSRRFYSERRVKAPMIRKGF-KDWVEAGY--- 3ir6.1    GSCSWKIYVKNGLVTWETQQTDYPR-----TRPDLPNHEPRGCPRGASYSWYLYSANRLKYPMMRKRLMKMWREAKALHS  target    -PR-------NDDGTPQMDVTLRGSDDWIRISWDEATTIAAKTMEDVARTFNGDEGARKLLAQGYHPEMVEVMHGAGVQA 3ir6.1    DPVEAWASIIEDADKAKSFKQARGRGGFVRSSWQEVNELIAASNVYTIKNYG--------------PDRVAGFSPIPAMS  target    LKLRGGMPLLGIGRIFGFYRFANMLALLDRKLRPDAPADEILGSRTFDNYAWHTDLPPGHPMVTGSQTVDFDLFSAEHTK 3ir6.1    MV-----------SYASGARYL-----------------SLIGGTCLSFYDWYCDLPPASPQTWGEQTDVPESADWYNSS  target    LLLIIGMNWICTKMPDGHWIGDARLKGTRVIVISADYMPTANKADEVIILRPGTDAAFFLGVARELIEKGL------YDR 3ir6.1    YIIAWGSNVPQTRTPDAHFFTEVRYKGTKTVAVTPDYAEIAKLCDLWLAPKQGTDAAMALAMGHVMLREFHLDNPSQYFT  target    AAVIERTDLPLLVRLD-------TGERLDARDVIPGYELAALTNYVTLKPDAEIKGNPPPPPFTAGGQVVPTELRDAWGD 3ir6.1    DYVRRYTDMPMLVMLEERDGYYAAGRMLRAADLVDALGQEN----------------------------------NPEWK  target    FVWWDRATGRPRPVSRDE-----------------------------VGARF----DGDPA-------------LL---- 3ir6.1    TVAFNT-NGEMVAPNGSIGFRWGEKGKWNLEQRDGKTGEETELQLSLLGSQDEIAEVGFPYFGGDGTEHFNKVELENVLL  target    G---EFEVELVDGSTVPVRPAFDLLK------------------QYLDESFDLRTASEVCRVPPQAIQSIARQLAAN--- 3ir6.1    HKLPVKRLQLADGSTALVTTVYDLTLANYGLERGLNDVNCATSYDDV-KAYTPAWAEQITGVSRSQIIRIAREFADNADK  target    --KRETLLAAGMGPNHYFQNDLFGRVQFLVAALTDNIGHLGGNVGSYAGNYRGSVFQAMGQWIAEDPFAI---------- 3ir6.1    THGR-SMIIVGAGLNHWYHLDMNYRGLINMLIFCGCVGQSGGGWAHYVGQEKLRPQTGWQPLAFALDWQRPARHMNSTSY  target    -EP-------------DLTKPA-TVKRYYK--------AESAHYW----NYGERPLRAVAKDD-----EGDLTKGEVLTG 3ir6.1    FYNHSSQWRYETVTAEELLSPMADKSRYTGHLIDFNVRAERMGWLPSAPQLGTNPLTIAGEAEKAGMNPVDYTVKSLKEG  target    KS--------HMPTPTKLIWFGNSNSLLGNAKWSFDV------------------------------VKNTLPRQDAVFC 3ir6.1    SIRFAAEQPENGKNHPRNLFIWRSNLLGSSGKGHEFMLKYLLGTEHGIQGKDLGQQGGVKPEEVDWQDNGLEGKLDLVVT  target    NEWHWTSSCEYADLVFPADSWAEFKLPDATASCTNPFLLAFPTTPLKRLYDTRSDYEALALTAKALGELIDEPRMEQYWR 3ir6.1    LDFRLSSTCLYSDIILPTATWYEK--DDMNTSDMHPFIHPL-SAAVDPAWEAKSDWEIYKAIAKKFSE------------  target    GILDGDPTPYLQRIFSGSNATRGITYDELHESSKRGVPLLMNMRTYPRSGGWEQRQEDKPWYTATGRLEFYRPEPEFQAA 3ir6.1    --------------------------------------------------------------------------------  target    GESLPVWREPVDATFYEPNAILSNAAHPSIAPRAPEDYGVPESQLDVETRQYRNVVRTWAELQQTLHPLQERDPAFRFVF 3ir6.1    -------------------------------------------------------------------------------- ``` | | | | | | | | | | | | | | | | | | | | | | | | | | | | | | | | | | | | | | | | | | | | | | | | | |
| ✓ | 3ir5.1.A | Respiratory nitrate reductase 1 alpha chain  *Crystal structure of NarGHI mutant NarG-H49C* | 0.40 | 0.00 | 25.36 | 0.67 | 54-767 | X-ray | 2.30 | monomer | 2 x MD1, 1 x 6MO, 4 x SF4, 1 x AGA, 1 x F3S, 2 x HEM | HHblits | 0.33 |
| ``` target    AQGVSRRQLLGRALALGSGAALADLLGPARFLSPAGAATAGAVVPGNPLRVMPDRTWEQIYRNQFEDDSTFVFTCAPNDT 3ir5.1    -----------------------------------------------------NRDWEDGYRQRWQHDKIVRSTCGVNCT  target    HNCLLRAHVKNGVVVRISPTYGYGEATDLYGNRASHRWDPRTCQKGLILSRRFYSERRVKAPMIRKGF-KDWVEAGY--- 3ir5.1    GSCSWKIYVKNGLVTWETQQTDYPR-----TRPDLPNHEPRGCPRGASYSWYLYSANRLKYPMMRKRLMKMWREAKALHS  target    -PR-------NDDGTPQMDVTLRGSDDWIRISWDEATTIAAKTMEDVARTFNGDEGARKLLAQGYHPEMVEVMHGAGVQA 3ir5.1    DPVEAWASIIEDADKAKSFKQARGRGGFVRSSWQEVNELIAASNVYTIKNYG--------------PDRVAGFSPIPAMS  target    LKLRGGMPLLGIGRIFGFYRFANMLALLDRKLRPDAPADEILGSRTFDNYAWHTDLPPGHPMVTGSQTVDFDLFSAEHTK 3ir5.1    MV-----------SYASGARYLS-----------------LIGGTCLSFYDWYCDLPPASPQTWGEQTDVPESADWYNSS  target    LLLIIGMNWICTKMPDGHWIGDARLKGTRVIVISADYMPTANKADEVIILRPGTDAAFFLGVARELIEKGL------YDR 3ir5.1    YIIAWGSNVPQTRTPDAHFFTEVRYKGTKTVAVTPDYAEIAKLCDLWLAPKQGTDAAMALAMGHVMLREFHLDNPSQYFT  target    AAVIERTDLPLLVRLD-------TGERLDARDVIPGYELAALTNYVTLKPDAEIKGNPPPPPFTAGGQVVPTELRDAWGD 3ir5.1    DYVRRYTDMPMLVMLEERDGYYAAGRMLRAADLVDALGQEN----------------------------------NPEWK  target    FVWWDRATGRPRPVSRDE--------------------------------------VG-ARFD-----------GDPALL 3ir5.1    TVAFNT-NGEMVAPNGSIGFRWGEKGKWNLEQRDGKTGEETELQLSLLGSQDEIAEVGFPYFGGDGTEHFNKVELENVLL  target    G---EFEVELVDGSTVPVRPAFDLLK------------------QYLDESFDLRTASEVCRVPPQAIQSIARQLAAN--- 3ir5.1    HKLPVKRLQLADGSTALVTTVYDLTLANYGLERGLNDVNCATSYDDV-KAYTPAWAEQITGVSRSQIIRIAREFADNADK  target    --KRETLLAAGMGPNHYFQNDLFGRVQFLVAALTDNIGHLGGNVGSYAGNYRGSVFQAMGQWIAEDPFAIEP-------- 3ir5.1    THGR-SMIIVGAGLNHWYHLDMNYRGLINMLIFCGCVGQSGGGWAHYVGQEKLRPQTGWQPLAFALDWQRPARHMNSTSY  target    ----------------DLTKPA-TVKRYYK--------AESAHYW----NYGERPLRAVAKDD-----EGDLTKGEVLTG 3ir5.1    FYNHSSQWRYETVTAEELLSPMADKSRYTGHLIDFNVRAERMGWLPSAPQLGTNPLTIAGEAEKAGMNPVDYTVKSLKEG  target    KS--------HMPTPTKLIWFGNSNSLLGNAKWSFDVV------------------------------KNTLPRQDAVFC 3ir5.1    SIRFAAEQPENGKNHPRNLFIWRSNLLGSSGKGHEFMLKYLLGTEHGIQGKDLGQQGGVKPEEVDWQDNGLEGKLDLVVT  target    NEWHWTSSCEYADLVFPADSWAEFKLPDATASCTNPFLLAFPTTPLKRLYDTRSDYEALALTAKALGELIDEPRMEQYWR 3ir5.1    LDFRLSSTCLYSDIILPTATWYEK--DDMNTSDMHPFIHPL-SAAVDPAWEAKSDWEIYKAIAKKFSE------------  target    GILDGDPTPYLQRIFSGSNATRGITYDELHESSKRGVPLLMNMRTYPRSGGWEQRQEDKPWYTATGRLEFYRPEPEFQAA 3ir5.1    --------------------------------------------------------------------------------  target    GESLPVWREPVDATFYEPNAILSNAAHPSIAPRAPEDYGVPESQLDVETRQYRNVVRTWAELQQTLHPLQERDPAFRFVF 3ir5.1    -------------------------------------------------------------------------------- ``` | | | | | | | | | | | | | | | | | | | | | | | | | | | | | | | | | | | | | | | | | | | | | | | | | |
|  | 1q16.1.A | Respiratory nitrate reductase 1 alpha chain  *Crystal structure of Nitrate Reductase A, NarGHI, from Escherichia coli* | 0.40 | 0.00 | 25.56 | 0.67 | 53-767 | X-ray | 1.90 | monomer | 2 x MD1, 1 x 6MO, 2 x HEM, 4 x SF4, 1 x F3S, 1 x AGA, 1 x 3PH | HHblits | 0.33 |
| ``` target    AQGVSRRQLLGRALALGSGAALADLLGPARFLSPAGAATAGAVVPGNPLRVMPDRTWEQIYRNQFEDDSTFVFTCAPNDT 1q16.1    ----------------------------------------------------TNRDWEDGYRQRWQHDKIVRSTHGVNCT  target    HNCLLRAHVKNGVVVRISPTYGYGEATDLYGNRASHRWDPRTCQKGLILSRRFYSERRVKAPMIRKGF-KDWVEAGY--- 1q16.1    GSCSWKIYVKNGLVTWETQQTDYPR-----TRPDLPNHEPRGCPRGASYSWYLYSANRLKYPMMRKRLMKMWREAKALHS  target    -PR-------NDDGTPQMDVTLRGSDDWIRISWDEATTIAAKTMEDVARTFNGDEGARKLLAQGYHPEMVEVMHGAGVQA 1q16.1    DPVEAWASIIEDADKAKSFKQARGRGGFVRSSWQEVNELIAASNVYTIKNYG--------------PDRVAGFSPIPAMS  target    LKLRGGMPLLGIGRIFGFYRFANMLALLDRKLRPDAPADEILGSRTFDNYAWHTDLPPGHPMVTGSQTVDFDLFSAEHTK 1q16.1    MV-----------SYASGARYL-----------------SLIGGTCLSFYDWYCDLPPASPQTWGEQTDVPESADWYNSS  target    LLLIIGMNWICTKMPDGHWIGDARLKGTRVIVISADYMPTANKADEVIILRPGTDAAFFLGVARELIEKGL------YDR 1q16.1    YIIAWGSNVPQTRTPDAHFFTEVRYKGTKTVAVTPDYAEIAKLCDLWLAPKQGTDAAMALAMGHVMLREFHLDNPSQYFT  target    AAVIERTDLPLLVRLDT-------GERLDARDVIPGYELAALTNYVTLKPDAEIKGNPPPPPFTAGGQVVPTELRDAWGD 1q16.1    DYVRRYTDMPMLVMLEERDGYYAAGRMLRAADLVDALGQEN----------------------------------NPEWK  target    FVWWDRATGRPRPVSRDE-----------------------------------V---G-ARFDG-------DPALL---- 1q16.1    TVAFNT-NGEMVAPNGSIGFRWGEKGKWNLEQRDGKTGEETELQLSLLGSQDEIAEVGFPYFGGDGTEHFNKVELENVLL  target    ---GEFEVELVDGSTVPVRPAFDLLK------------------QYLDESFDLRTASEVCRVPPQAIQSIARQLAAN--- 1q16.1    HKLPVKRLQLADGSTALVTTVYDLTLANYGLERGLNDVNCATSYDDV-KAYTPAWAEQITGVSRSQIIRIAREFADNADK  target    --KRETLLAAGMGPNHYFQNDLFGRVQFLVAALTDNIGHLGGNVGSYAGNYRGSVFQAMGQ------------------- 1q16.1    THGR-SMIIVGAGLNHWYHLDMNYRGLINMLIFCGCVGQSGGGWAHYVGQEKLRPQTGWQPLAFALDWQRPARHMNSTSY  target    -------WIAEDPFAIEPDLTKPA-TVKRYYK--------AESAHY----WNYGERPLRAVAKDD-----EGDLTKGEVL 1q16.1    FYNHSSQWRYETVTA--EELLSPMADKSRYTGHLIDFNVRAERMGWLPSAPQLGTNPLTIAGEAEKAGMNPVDYTVKSLK  target    TGKS--------HMPTPTKLIWFGNSNSLLGNAKWSFDVV------------------------------KNTLPRQDAV 1q16.1    EGSIRFAAEQPENGKNHPRNLFIWRSNLLGSSGKGHEFMLKYLLGTEHGIQGKDLGQQGGVKPEEVDWQDNGLEGKLDLV  target    FCNEWHWTSSCEYADLVFPADSWAEFKLPDATASCTNPFLLAFPTTPLKRLYDTRSDYEALALTAKALGELIDEPRMEQY 1q16.1    VTLDFRLSSTCLYSDIILPTATWYEK--DDMNTSDMHPFIHPL-SAAVDPAWEAKSDWEIYKAIAKKFSE----------  target    WRGILDGDPTPYLQRIFSGSNATRGITYDELHESSKRGVPLLMNMRTYPRSGGWEQRQEDKPWYTATGRLEFYRPEPEFQ 1q16.1    --------------------------------------------------------------------------------  target    AAGESLPVWREPVDATFYEPNAILSNAAHPSIAPRAPEDYGVPESQLDVETRQYRNVVRTWAELQQTLHPLQERDPAFRF 1q16.1    --------------------------------------------------------------------------------  target    VF 1q16.1    -- ``` | | | | | | | | | | | | | | | | | | | | | | | | | | | | | | | | | | | | | | | | | | | | | | | | | |
|  | 3egw.1.A | Respiratory nitrate reductase 1 alpha chain  *The crystal structure of the NarGHI mutant NarH - C16A* | 0.39 | 0.17 | 24.84 | 0.67 | 53-767 | X-ray | 1.90 | homo-dimer | 2 x MD1, 2 x MGD, 2 x 6MO, 6 x SF4, 4 x F3S, 2 x 3PH, 4 x HEM, 2 x AGA | HHblits | 0.32 |
| ``` target    AQGVSRRQLLGRALALGSGAALADLLGPARFLSPAGAATAGAVVPGNPLRVMPDRTWEQIYRNQFEDDSTFVFTCAPNDT 3egw.1    ----------------------------------------------------TNRDWEDGYRQRWQHDKIVRSTHGVNCT  target    HNCLLRAHVKNGVVVRISPTYGYGEATDLYGNRASHRWDPRTCQKGLILSRRFYSERRVKAPMIRKGF-KDWVEAGY--- 3egw.1    GSCSWKIYVKNGLVTWETQQTDYPR-----TRPDLPNHEPRGCPRGASYSWYLYSANRLKYPMMRKRLMKMWREAKALHS  target    -PR-------NDDGTPQMDVTLRGSDDWIRISWDEATTIAAKTMEDVARTFNGDEGARKLLAQGYHPEMVEVMHGAGVQA 3egw.1    DPVEAWASIIEDADKAKSFKQARGRGGFVRSSWQEVNELIAASNVYTIKNYG--------------PDRVAGFSPIPAMS  target    LKLRGGMPLLGIGRIFGFYRFANMLALLDRKLRPDAPADEILGSRTFDNYAWHTDLPPGHPMVTGSQTVDFDLFSAEHTK 3egw.1    MV-----------SYASGARYL-----------------SLIGGTCLSFYDWYCDLPPASPQTWGEQTDVPESADWYNSS  target    LLLIIGMNWICTKMPDGHWIGDARLKGTRVIVISADYMPTANKADEVIILRPGTDAAFFLGVARELIEKGL------YDR 3egw.1    YIIAWGSNVPQTRTPDAHFFTEVRYKGTKTVAVTPDYAEIAKLCDLWLAPKQGTDAAMALAMGHVMLREFHLDNPSQYFT  target    AAVIERTDLPLLVRLD-------TGERLDARDVIPGYELAALTNYVTLKPDAEIKGNPPPPPFTAGGQVVPTELRDAWGD 3egw.1    DYVRRYTDMPMLVMLEERDGYYAAGRMLRAADLVAALGQENN----------------------------------PEWK  target    FVWWDRATGRPRPVSRDE-----------------------------------V----GARFD-----------GDPALL 3egw.1    TVAFNT-NGEMVAPNGSIGFRWGEKGKWNLEQRDGKTGEETELQLSLLGSQDEIAEVGFPYFGGDGTEHFNKVELENVLL  target    G---EFEVELVDGSTVPVRPAFDLLK------------------QYLDESFDLRTASEVCRVPPQAIQSIARQLAAN--- 3egw.1    HKLPVKRLQLADGSTALVTTVYDLTLANYGLERGLNDVNCATSYDDV-KAYTPAWAEQITGVSRSQIIRIAREFADNADK  target    --KRETLLAAGMGPNHYFQNDLFGRVQFLVAALTDNIGHLGGNVGSYAGNYRGSVFQAMGQWIAEDPFAI---------- 3egw.1    THGR-SMIIVGAGLNHWYHLDMNYRGLINMLIFCGCVGQSGGGWAHYVGQEKLRPQTGWQPLAFALDWQRPARHMNSTSY  target    -E-------------PDLTKPA-TVKRYYK--------AESAHYW----NYGERPLRAVAKDD-----EGDLTKGEVLTG 3egw.1    FYNHSSQWRYETVTAEELLSPMADKSRYTGHLIDFNVRAERMGWLPSAPQLGTNPLTIAGEAEKAGMNPVDYTVKSLKEG  target    KS--------HMPTPTKLIWFGNSNSLLGNAKWSFDV------------------------------VKNTLPRQDAVFC 3egw.1    SIRFAAEQPENGKNHPRNLFIWRSNLLGSSGKGHEFMLKYLLGTEHGIQGKDLGQQGGVKPEEVDWQDNGLEGKLDLVVT  target    NEWHWTSSCEYADLVFPADSWAEFKLPDATASCTNPFLLAFPTTPLKRLYDTRSDYEALALTAKALGELIDEPRMEQYWR 3egw.1    LDFRLSSTCLYSDIILPTATWYEK--DDMNTSDMHPFIHPL-SAAVDPAWEAKSDWEIYKAIAKKFSE------------  target    GILDGDPTPYLQRIFSGSNATRGITYDELHESSKRGVPLLMNMRTYPRSGGWEQRQEDKPWYTATGRLEFYRPEPEFQAA 3egw.1    --------------------------------------------------------------------------------  target    GESLPVWREPVDATFYEPNAILSNAAHPSIAPRAPEDYGVPESQLDVETRQYRNVVRTWAELQQTLHPLQERDPAFRFVF 3egw.1    -------------------------------------------------------------------------------- ``` | | | | | | | | | | | | | | | | | | | | | | | | | | | | | | | | | | | | | | | | | | | | | | | | | |
|  | 1tmo.1.A | TRIMETHYLAMINE N-OXIDE REDUCTASE  *TRIMETHYLAMINE N-OXIDE REDUCTASE FROM SHEWANELLA MASSILIA* | 0.34 |  | 18.29 | 0.69 | 4-885 | X-ray | 2.50 | monomer | 2 x 2MD, 1 x 2MO | HHblits | 0.29 |
| ``` target    AQGVSRRQLLGRALALGSGAALADLLGPARFLSPAGAATAGAVVPGNPLRVMPDRTWEQIYRNQFEDDSTFVFTCAPNDT 1tmo.1    ---MNRRDFLKGIASSSFVV-------------------LGGSSVLTPLNALAKAG---------INEDEWLTTGSH-F-  target    HNCLLRAHVKNGVVVRISPTYGYGEATDLYGNRASHRWDPRTCQKGLILSRRFYSERRVKAPMIRKGFKDWVEAGYPRND 1tmo.1    --GAFKMKRKNGVIAEVKPFDL------------DKYPT--DMING--IRGMVYNPSRVRYPMVRLDFL---LKGH----  target    DGTPQMDVTLRGSDDWIRISWDEATTIAAKTMEDVARTFNGDEGARKLLAQGYHPEMVEVMHGAGVQALKLRGGMPLLGI 1tmo.1    ----KSNTHQRGDFRFVRVTWDKALTLFKHSLDEVQTQYGP--------------SGLHAGQTGWRATGQL-----H---  target    GRIFGFYRFANMLALLDRKLRPDAPADEILGSRTFDNYAWHTDLPPGHPMVTGSQ----TVDFDLFSAEHTKLLLIIGMN 1tmo.1    ---SSTSHMQRAVGMHGNYV-------KKIGDYS--T----GAGQTILPYVLGSTEVYAQGTSWPLILEHSDTIVLWSND  target    WICTKMP--------DG---HWIGDARL-KGTRVIVISADYMPTANK-ADEVIILRPGTDAAFFLGVARELIEKGLYDRA 1tmo.1    PYKNLQVGWNAETHESFAYLAQLKEKVKQGKIRVISIDPVVTKTQAYLGCEQLYVNPQTDVTLMLAIAHEMISKKLYDDK  target    AVIERTDLPLLVRLDTGERLDARDVIPGYELAALTNYVTLKPDAEIKGNPPPPPFTAGGQVVPTELRDAWGDFVWWDRAT 1tmo.1    FIQGYSL--------------------GFEE--F------------------------------------VP--------  target    GRPRPVSRDEVGARFDGDPALLGEFEVELVDGSTVPVRPAFDLLKQYLDESFDLRTASEVCRVPPQAIQSIARQLAANKR 1tmo.1    -------------------YVMGT-----KDG-----------------VAKTPEWAAPICGVEAHVIRDLAKTLVKGRT  target    ETLLAAGMGPNHYFQNDLFGRVQFLVAALTDNIGHLGGNVGSYAGNY------RGSVFQAMGQWIAEDPFAIEPDLTKPA 1tmo.1    --QFMMGWCIQRQQHGEQPYWMAAVLATMIGQIGLPGGGISYGHHYSSIGVPSSGAA--APGAFPRN--LD---ENQKP-  target    TVKRYYKAESAHYWN-YGERPLRAVAKD-DEGDLTKGEVLTGKSHMPTPTKLIWFGNSNSLLGNAKWSFDVVKNTLPRQD 1tmo.1    ----LFD--SSDFKGASSTIPVARWIDAILEPGKTIDANGSK--VVYPDIKMMIFSGNNPWNHHQD--RNRMKQAFHKLE  target    AVFCNEWHWTSSCEYADLVFPADSWAEFKLPDATASCTNPFLLAFPTTPLKRLYDTRSDYEALALTAKALGELIDEPRME 1tmo.1    CVVTVDVNWTATCRFSDIVLPACTTYERNDIDVYGAYANRGILA-MQKMVEPLFDSLSDFEIFTRFAAVLGKEKEYTRN-  target    QYWRGILDGDPTPYLQRIFSGS----N-ATRGITYDELHESSKRGVPLLMNMRTYPRSGGWEQRQEDKPWYTATGRLEFY 1tmo.1    --------MGEMEWLETLYNECKAANAGKFEMPDFATFWKQG---YVHFGDGEVWTRHADFRNDPEINPLGTPSGLIEIF  target    RPEPEFQ--AAGESLPVWREPVDAT------FYEPNAILSNAAHPSIAPRAPEDYGVPESQLDVETRQYRNVVRTWAELQ 1tmo.1    SRKIDQFGYDDCKGHPTWMEKTERSHGGPGSDKHPIWLQSCHP-------------------------------------  target    QTLHPLQERDPAFRFVF 1tmo.1    ----------------- ``` | | | | | | | | | | | | | | | | | | | | | | | | | | | | | | | | | | | | | | | | | | | | | | | | | |
|  | 1eu1.1.A | DIMETHYL SULFOXIDE REDUCTASE  *THE CRYSTAL STRUCTURE OF RHODOBACTER SPHAEROIDES DIMETHYLSULFOXIDE REDUCTASE REVEALS TWO DISTINCT MOLYBDENUM COORDINATION ENVIRONMENTS.* | 0.34 |  | 21.53 | 0.65 | 72-883 | X-ray | 1.30 | monomer | 3 x GLC, 1 x CD, 2 x MGD, 1 x 6MO, 2 x O | HHblits | 0.31 |
| ``` target    AQGVSRRQLLGRALALGSGAALADLLGPARFLSPAGAATAGAVVPGNPLRVMPDRTWEQIYRNQFEDDSTFVFTCAPNDT 1eu1.1    -----------------------------------------------------------------------NGEVMSGCH  target    HNCLLRAHVKNGVVVRISPTYGYGEATDLYGNRASHRWDPRTCQKGLILSRRFYSERRVKAPMIRKGF-KDWVEAGYPRN 1eu1.1    WGV-FKARVENGRAVAFEPW------------DKDPAPSHQLPG----VLDSIYSPTRIKYPMVRREFLEKGVNA-----  target    DDGTPQMDVTLRGSDDWIRISWDEATTIAAKTMEDVARTFNGDEGARKLLAQGYHPEMVEVMHGAGVQALKLRGGMPLLG 1eu1.1    -------DRSTRGNGDFVRVTWDEALDLVARELKRVQESYGPTG--------------TFGGSYGWKSP----GR--LHN  target    IGRIFGFYRFANMLALLDRKLRPDAPADEILGSRTFDNYAWHTDLPPGHPMVTGSQ----TVDFDLFSAEHTKLLLIIGM 1eu1.1    C-QVL-MRRALNLAGGFV----------NSSGD--YS----TAAAQIIMPHVMGTLEVYEQQTAWPVVVENTDLMVFWAA  target    NWICTKMPD--------GHWIGDARLKGTRVIVISADYMPTANKAD-EVIILRPGTDAAFFLGVARELIEKGLYDRAAVI 1eu1.1    DPMKTNEIGWVIPDHGAYAGMKALKEKGTRVICINPVRTETADYFGADVVSPRPQTDVALMLGMAHTLYSEDLHDKDFLE  target    ERTDLPLLVRLDTGERLDARDVIPGYELAALTNYVTLKPDAEIKGNPPPPPFTAGGQVVPTELRDAWGDFVWWDRATGRP 1eu1.1    NCTT--------------------GFDL--------------------------------------FAAY----------  target    RPVSRDEVGARFDGDPALLGEFEVELVDGSTVPVRPAFDLLKQYLDESFDLRTASEVCRVPPQAIQSIARQLAANKRETL 1eu1.1    -----------------LTGE-----SDG-----------------TPKTAEWAAEICGLPAEQIRELARSFVAGR--TM  target    LAAGMGPNHYFQNDLFGRVQFLVAALTDNIGHLGGNVGSYAGNYRGSV-FQAMGQWIAEDPFAIEPDLTKPATVKRYYKA 1eu1.1    LAAGWSIQRMHHGEQAHWMLVTLASMIGQIGLPGGGFGLSYHYSNGGSPTSDGPAL----G-GISDGGKAV-EGAAWLS-  target    ESAHYWNYGERPLRAVAKDDEGDLTKGE--VLTGKSHMPTPTKLIWFGNSNSLLGNAKWSFDVVKNTLPRQDAVFCNEWH 1eu1.1    ----ESGATSIPCARVVD---MLLNPGGEFQFNGATATYPDVKLAYWAGGNPFAHHQD--RNRMLKAWEKLETFIVQDFQ  target    WTSSCEYADLVFPADSWAEFKLPDATASCTNPFLLAFPTTPLKRLYDTRSDYEALALTAKALGELIDEPRMEQYWRGILD 1eu1.1    WTATARHADIVLPATTSYERNDIESVGDYSNRAILA-MKKVVDPLYEARSDYDIFAALAERLGKGAEFTEGRDEMGWI--  target    GDPTPYLQRIFSG--SNATRGITYDELHESSKRGVPLLMNMRTYPRSGGWEQRQEDKPWYTATGRLEFYRPEPEFQ--AA 1eu1.1    ---SSFYEAAVKQAEFKNVAMPSFEDFWSEGIVEFPITE-GANFVRYADFREDPLFNPLGTPSGLIEIYSKNIEKMGYDD  target    GESLPVWREPVDA----TFYEPNAILSNAAHPSIAPRAPEDYGVPESQLDVETRQYRNVVRTWAELQQTLHPLQERDPAF 1eu1.1    CPAHPTWMEPAERLGGAGAKYPLHVVAS----------------------------------------------------  target    RFVF 1eu1.1    ---- ``` | | | | | | | | | | | | | | | | | | | | | | | | | | | | | | | | | | | | | | | | | | | | | | | | | |
|  | 1dms.1.A | DMSO REDUCTASE  *STRUCTURE OF DMSO REDUCTASE* | 0.34 |  | 21.02 | 0.65 | 72-883 | X-ray | 1.88 | monomer | 2 x PGD, 1 x 2MO | HHblits | 0.30 |
| ``` target    AQGVSRRQLLGRALALGSGAALADLLGPARFLSPAGAATAGAVVPGNPLRVMPDRTWEQIYRNQFEDDSTFVFTCAPNDT 1dms.1    -----------------------------------------------------------------------ANGTVMSGS  target    HNCLLRAHVKNGVVVRISPTYGYGEATDLYGNRASHRWDPRTCQKGLILSRRFYSERRVKAPMIRKGF-KDWVEAGYPRN 1dms.1    HWGVFTATVENGRATAFTPWE------------KDP----HPTPMLEGVLDSIYSPTRIKYPMVRREFLEKGVN------  target    DDGTPQMDVTLRGSDDWIRISWDEATTIAAKTMEDVARTFNGDEGARKLLAQGYHPEMVEVMHGAGVQALKLRGGMPLLG 1dms.1    ------ADRSTRGNGDFVRVSWDQALDLVAAEVKRVEETYGPQ--------------GVFGGSYGWKSPGRL-------H  target    IGRIFGFYRFANMLALLDRKLRPDAPADEILGSRTFD-NYAWHTDLPPGHPMVTGSQ----TVDFDLFSAEHTKLLLIIG 1dms.1    NCT-TLLRRMLTL----------------AGGYVNGAGDYSTGAA-QVIMPHVVGTLEVYEQQTAWPVLAENTEVMVFWA  target    MNWICTKMPDG--------HWIGDARLKGTRVIVISADYMPTANK-ADEVIILRPGTDAAFFLGVARELIEKGLYDRAAV 1dms.1    ADPIKTSQIGWVIPEHGAYPGLEALKAKGTKVIVIDPVRTKTVEFFGADHVTPKPQTDVAIMLGMAHTLVAEDLYDKDFI  target    IERTDLPLLVRLDTGERLDARDVIPGYELAALTNYVTLKPDAEIKGNPPPPPFTAGGQVVPTELRDAWGDFVWWDRATGR 1dms.1    ANYTS--------------------GFDK--F------------------------------------LPY---------  target    PRPVSRDEVGARFDGDPALLGEFEVELVDGSTVPVRPAFDLLKQYLDESFDLRTASEVCRVPPQAIQSIARQLAANKRET 1dms.1    ------------------LMGET-----D----------------S-TPKTAEWASDISGVPAETIKELARLFKSK-RT-  target    LLAAGMGPNHYFQNDLFGRVQFLVAALTDNIGHLGGNVGSYAGNYRGSVFQAMGQWIAEDPFAIEPDLTKPATVKRYYKA 1dms.1    MLAAGWSMQRMHHGEQAHWMLVTLASMLGQIGLPGGGFGLSYHYSGGGTPSSSGP-----ALSGITDGGAATKGPEWLA-  target    ESAHYWNYGERPLRAVAK-DDEGDLTKGEVLTGKSHMPTPTKLIWFGNSNSLLGNAKWSFDVVKNTLPRQDAVFCNEWHW 1dms.1    ----ASGASVIPVARVVDMLENPGAEFDFN--GTRSKFPDVKMAYWVGGNPFVHHQD--RNRMVKAWEKLETFIVHDFQW  target    TSSCEYADLVFPADSWAEFKLPDATASCTNPFLLAFPTTPLKRLYDTRSDYEALALTAKALGELIDEPRMEQYWRGILDG 1dms.1    TPTARHADIVLPATTSYERNDIETIGDYSNTGILA-MKKIVEPLYEARSDYDIFAAVAERLGKGKEFTEGKD--------  target    DPTPYLQRIFSGSN---ATRGI---TYDELHESSKRGVPLLMNM--RTYPRSGGWEQRQEDKPWYTATGRLEFYRPEPEF 1dms.1    -EMGWIKSFYDDAAKQGKAGGVEMPAFDAFWAEG---IVEFPVTDGADFVRYASFREDPLLNPLGTPTGLIEIYSKNIEK  target    Q--AAGESLPVWREPVDA----TFYEPNAILSNAAHPSIAPRAPEDYGVPESQLDVETRQYRNVVRTWAELQQTLHPLQE 1dms.1    MGYDDCPAHPTWMEPLERLDGPGAKYPLHIAAS-----------------------------------------------  target    RDPAFRFVF 1dms.1    --------- ``` | | | | | | | | | | | | | | | | | | | | | | | | | | | | | | | | | | | | | | | | | | | | | | | | | |
|  | 1e18.1.A | DMSO REDUCTASE.  *TUNGSTEN-SUSBSTITUTED DMSO REDUCTASE FROM RHODOBACTER CAPSULATUS* | 0.33 | 0.00 | 21.88 | 0.65 | 74-883 | X-ray | 2.00 | monomer | 2 x PGD, 1 x 6WO | HHblits | 0.30 |
| ``` target    AQGVSRRQLLGRALALGSGAALADLLGPARFLSPAGAATAGAVVPGNPLRVMPDRTWEQIYRNQFEDDSTFVFTCAPNDT 1e18.1    -------------------------------------------------------------------------NGTVMSG  target    HNCL-LRAHVKNGVVVRISPTYGYGEATDLYGNRASHRWDPRTCQKGLILSRRFYSERRVKAPMIRKGF-KDWVEAGYPR 1e18.1    SHWGVFTATVENGRATAFTPWE------------KDPH----PSPMLAGVLDSIYSPTRIKYPMVRREFLEKGVN-----  target    NDDGTPQMDVTLRGSDDWIRISWDEATTIAAKTMEDVARTFNGDEGARKLLAQGYHPEMVEVMHGAGVQALKLRGGMPLL 1e18.1    -------ADRSTRGNGDFVRVSWDQALDLVAAEVKRVEETYGPQ--------------GVFGGSYGWKS-----------  target    GIGRIFGFYRFANMLALLDRKLRPDAPADEILGSRTFD-NYAWHTD-----LPPGHPMVTGSQTVDFDLFSAEHTKLLLI 1e18.1    -------PGRLHNCT-TLLRRML-----TLAGGYVNGAGDYSTGAAQVIMPHVVGTLEVYEQQ--TAWPVLAENTEVMVF  target    IGMNWICTKMPDG--------HWIGDARLKGTRVIVISADYMPTANK-ADEVIILRPGTDAAFFLGVARELIEKGLYDRA 1e18.1    WAADPIKTSQIGWVIPEHGAYPGLEALKAKGTKVIVIDPVRTKTVEFFGAEHITPKPQTDVAIMLGMAHTLVAEDLYDKD  target    AVIERTDLPLLVRLDTGERLDARDVIPGYELAALTNYVTLKPDAEIKGNPPPPPFTAGGQVVPTELRDAWGDFVWWDRAT 1e18.1    FIANYTS--------------------GFDK--F------------------------------------L---------  target    GRPRPVSRDEVGARFDGDPALLGEFEVELVDGSTVPVRPAFDLLKQYLDESFDLRTASEVCRVPPQAIQSIARQLAANKR 1e18.1    ------------------PYLDGET---------------------DS-TPKTAEWAEGISGVPAETIKELARLFESK-R  target    ETLLAAGMGPNHYFQNDLFGRVQFLVAALTDNIGHLGGNVGSYAGNYRGSVFQAMGQWIAEDPFAIEPDLTKPATVKRYY 1e18.1    -TMLAAGWSMQRMHHGEQAHWMLVTLASMLGQIGLPGGGFGLSYHYSGGGTPSTSGPA-----LAGITDGGAATKGPEWL  target    KAESAHYWNYGERPLRAVAK-DDEGDLTKGEVLTGKSHMPTPTKLIWFGNSNSLLGNAKWSFDVVKNTLPRQDAVFCNEW 1e18.1    A-----ASGASVIPVARVVDMLENPGAEFDFN--GTRSKFPDVKMAYWVGGNPFVHHQD--RNRMVKAWEKLETFVVHDF  target    HWTSSCEYADLVFPADSWAEFKLPDATASCTNPFLLAFPTTPLKRLYDTRSDYEALALTAKALGELIDEPRMEQYWRGIL 1e18.1    QWTPTARHADIVLPATTSYERNDIETIGDYSNTGILA-MKKIVEPLYEARSDYDIFAAVAERLGKGKEFTEGKDE-----  target    DGDPTPYLQRIFSGSN---ATRG---ITYDELHESSKRGVPLLMNMRTYPRSGGWEQRQEDKPWYTATGRLEFYRPEPEF 1e18.1    ----MGWIKSFYDDAAKQGKAAGVEMPAFDAFWAEGIVEFPVT-DGADFVRYASFREDPLLNPLGTPTGLIEIYSKNIEK  target    Q--AAGESLPVWREPVDA----TFYEPNAILSNAAHPSIAPRAPEDYGVPESQLDVETRQYRNVVRTWAELQQTLHPLQE 1e18.1    MGYDDCPAHPTWMEPLERLDGPGAKYPLHIAAS-----------------------------------------------  target    RDPAFRFVF 1e18.1    --------- ``` | | | | | | | | | | | | | | | | | | | | | | | | | | | | | | | | | | | | | | | | | | | | | | | | | |
|  | 1e60.1.A | Dimethyl sulfoxide/trimethylamine N-oxide reductase  *OXIDIZED DMSO REDUCTASE EXPOSED TO HEPES - Structure II BUFFER* | 0.35 | 0.00 | 21.31 | 0.65 | 73-883 | X-ray | 2.00 | monomer | 2 x PGD, 1 x 2MO | HHblits | 0.30 |
| ``` target    AQGVSRRQLLGRALALGSGAALADLLGPARFLSPAGAATAGAVVPGNPLRVMPDRTWEQIYRNQFEDDSTFVFTCAPNDT 1e60.1    ------------------------------------------------------------------------ANGTVMSG  target    HNCL-LRAHVKNGVVVRISPTYGYGEATDLYGNRASHRWDPRTCQKGLILSRRFYSERRVKAPMIRKGF-KDWVEAGYPR 1e60.1    SHWGVFTATVENGRATAFTPWE------------KDPH----PSPMLAGVLDSIYSPTRIKYPMVRREFLEKGV------  target    NDDGTPQMDVTLRGSDDWIRISWDEATTIAAKTMEDVARTFNGDEGARKLLAQGYHPEMVEVMHGAGVQALKLRGGMPLL 1e60.1    ------NADRSTRGNGDFVRVSWDQALDLVAAEVKRVEETYGPE--------------GVFGGSYGWKSPGRL-------  target    GIGRIFGFYRFANMLALLDRKLRPDAPADEILGSRTFD-NYAWHTD-----LPPGHPMVTGSQTVDFDLFSAEHTKLLLI 1e60.1    HNCT-TLLRRMLTL----------------AGGYVNGAGDYSTGAAQVIMPHVVGTLEVYEQQ--TAWPVLAENTEVMVF  target    IGMNWICTKMPDG--------HWIGDARLKGTRVIVISADYMPTANK-ADEVIILRPGTDAAFFLGVARELIEKGLYDRA 1e60.1    WAADPIKTSQIGWVIPEHGAYPGLEALKAKGTKVIVIDPVRTKTVEFFGAEHITPKPQTDVAIMLGMAHTLVAEDLYDKD  target    AVIERTDLPLLVRLDTGERLDARDVIPGYELAALTNYVTLKPDAEIKGNPPPPPFTAGGQVVPTELRDAWGDFVWWDRAT 1e60.1    FIANYTS--------------------GFDK--F------------------------------------L---------  target    GRPRPVSRDEVGARFDGDPALLGEFEVELVDGSTVPVRPAFDLLKQYLDESFDLRTASEVCRVPPQAIQSIARQLAANKR 1e60.1    ------------------PYLDGET---------------------DS-TPKTAEWAEGISGVPAETIKELARLFESK-R  target    ETLLAAGMGPNHYFQNDLFGRVQFLVAALTDNIGHLGGNVGSYAGNYRGSVFQAMGQWIAEDPFAIEPDLTKPATVKRYY 1e60.1    -TMLAAGWSMQRMHHGEQAHWMLVTLASMLGQIGLPGGGFGLSYHYSGGGTPSTSGPA-----LAGITDGGAATKGPEWL  target    KAESAHYWNYGERPLRAVAK-DDEGDLTKGEVLTGKSHMPTPTKLIWFGNSNSLLGNAKWSFDVVKNTLPRQDAVFCNEW 1e60.1    A-----ASGASVIPVARVVDMLENPGAEFDFN--GTRSKFPDVKMAYWVGGNPFVHHQD--RNRMVKAWEKLETFVVHDF  target    HWTSSCEYADLVFPADSWAEFKLPDATASCTNPFLLAFPTTPLKRLYDTRSDYEALALTAKALGELIDEPRMEQYWRGIL 1e60.1    QWTPTARHADIVLPATTSYERNDIETIGDYSNTGILA-MKKIVEPLYEARSDYDIFAAVAERLGKGAEFTEGKDEMGW--  target    DGDPTPYLQRIFSGSNATRGI---TYDELHESSKRGVPLLMNM--RTYPRSGGWEQRQEDKPWYTATGRLEFYRPEPEFQ 1e60.1    ---IKSFYDDAAKQG-KAAGVEMPAFDAFWAEG---IVEFPVTDGADFVRYASFREDPLLNPLGTPTGLIEIYSKNIEKM  target    --AAGESLPVWREPVDA----TFYEPNAILSNAAHPSIAPRAPEDYGVPESQLDVETRQYRNVVRTWAELQQTLHPLQER 1e60.1    GYDDCPAHPTWMEPLERLDGPGAKYPLHIAAS------------------------------------------------  target    DPAFRFVF 1e60.1    -------- ``` | | | | | | | | | | | | | | | | | | | | | | | | | | | | | | | | | | | | | | | | | | | | | | | | | |
|  | 1e5v.2.A | Dimethyl sulfoxide/trimethylamine N-oxide reductase  *OXIDIZED DMSO REDUCTASE EXPOSED TO HEPES BUFFER* | 0.34 | 0.00 | 21.55 | 0.65 | 74-882 | X-ray | 2.40 | monomer | 2 x PGD, 1 x 2MO | HHblits | 0.30 |
| ``` target    AQGVSRRQLLGRALALGSGAALADLLGPARFLSPAGAATAGAVVPGNPLRVMPDRTWEQIYRNQFEDDSTFVFTCAPNDT 1e5v.2    -------------------------------------------------------------------------NGTVMSG  target    HNCL-LRAHVKNGVVVRISPTYGYGEATDLYGNRASHRWDPRTCQKGLILSRRFYSERRVKAPMIRKGF-KDWVEAGYPR 1e5v.2    SHWGVFTATVENGRATAFTPWE------------KDPH----PSPMLAGVLDSIYSPTRIKYPMVRREFLEKGV------  target    NDDGTPQMDVTLRGSDDWIRISWDEATTIAAKTMEDVARTFNGDEGARKLLAQGYHPEMVEVMHGAGVQALKLRGGMPLL 1e5v.2    ------NADRSTRGNGDFVRVSWDQALDLVAAEVKRVEETYGP--------------EGVFGGSYGWKSPGRL-------  target    GIGRIFGFYRFANMLALLDRKLRPDAPADEILGSRTFD-NYAW---H--TDLPPGHPMVTGSQTVDFDLFSAEHTKLLLI 1e5v.2    HNCT-TLLRRMLTLA----------------GGYVNGAGDYSTGAAQVIMPHVVGTLEVYEQQT--AWPVLAENTEVMVF  target    IGMNWICTKMPDG--------HWIGDARLKGTRVIVISADYMPTANK-ADEVIILRPGTDAAFFLGVARELIEKGLYDRA 1e5v.2    WAADPIKTSQIGWVIPEHGAYPGLEALKAKGTKVIVIDPVRTKTVEFFGAEHITPKPQTDVAIMLGMAHTLVAEDLYDKD  target    AVIERTDLPLLVRLDTGERLDARDVIPGYELAALTNYVTLKPDAEIKGNPPPPPFTAGGQVVPTELRDAWGDFVWWDRAT 1e5v.2    FIANYTS--------------------GFDK--F------------------------------------LP--------  target    GRPRPVSRDEVGARFDGDPALLGEFEVELVDGSTVPVRPAFDLLKQYLDESFDLRTASEVCRVPPQAIQSIARQLAANKR 1e5v.2    -------------------YLDGET---------------------DS-TPKTAEWAEGISGVPAETIKELARLFESK-R  target    ETLLAAGMGPNHYFQNDLFGRVQFLVAALTDNIGHLGGNVGSYAGNYRGSVFQAMGQWIAEDPFAIEPDLTKPATVKRYY 1e5v.2    -TMLAAGWSMQRMHHGEQAHWMLVTLASMLGQIGLPGGGFGLSYHYSGGGTPSTSGPA-----LAGITDGGAATKGPEWL  target    KAESAHYWNYGERPLRAVA-KDDEGDLTKGEVLTGKSHMPTPTKLIWFGNSNSLLGNAKWSFDVVKNTLPRQDAVFCNEW 1e5v.2    A-----ASGASVIPVARVVDMLENPGAEFDF--NGTRSKFPDVKMAYWVGGNPFVHHQD--RNRMVKAWEKLETFVVHDF  target    HWTSSCEYADLVFPADSWAEFKLPDATASCTNPFLLAFPTTPLKRLYDTRSDYEALALTAKALGELIDEPRMEQYWRGIL 1e5v.2    QWTPTARHADIVLPATTSYERNDIETIGDYSNTGILA-MKKIVEPLYEARSDYDIFAAVAERLGKGAEFTEGKDEMGW--  target    DGDPTPYLQRIFSGSNATRGI---TYDELHESSKRGVPLLMNM--RTYPRSGGWEQRQEDKPWYTATGRLEFYRPEPEFQ 1e5v.2    ---IKSFYDDAAKQ-GKAAGVQMPAFDAFWAEG---IVEFPVTDGADFVRYASFREDPLLNPLGTPTGLIEIYSKNIEKM  target    --AAGESLPVWREPVDA----TFYEPNAILSNAAHPSIAPRAPEDYGVPESQLDVETRQYRNVVRTWAELQQTLHPLQER 1e5v.2    GYDDCPAHPTWMEPLERLDGPGAKYPLHIAA-------------------------------------------------  target    DPAFRFVF 1e5v.2    -------- ``` | | | | | | | | | | | | | | | | | | | | | | | | | | | | | | | | | | | | | | | | | | | | | | | | | |
|  | 4dmr.1.A | DMSO REDUCTASE  *REDUCED DMSO REDUCTASE FROM RHODOBACTER CAPSULATUS WITH BOUND DMSO SUBSTRATE* | 0.34 | 0.00 | 21.02 | 0.65 | 74-883 | X-ray | 1.90 | monomer | 2 x PGD, 1 x 4MO, 1 x O | HHblits | 0.30 |
| ``` target    AQGVSRRQLLGRALALGSGAALADLLGPARFLSPAGAATAGAVVPGNPLRVMPDRTWEQIYRNQFEDDSTFVFTCAPNDT 4dmr.1    -------------------------------------------------------------------------NGTVMSG  target    HNCL-LRAHVKNGVVVRISPTYGYGEATDLYGNRASHRWDPRTCQKGLILSRRFYSERRVKAPMIRKGF-KDWVEAGYPR 4dmr.1    SHWGVFTATVENGRATAFTPWE------------KDPH----PSPMLAGVLDSIYSPTRIKYPMVRREFLEKGV------  target    NDDGTPQMDVTLRGSDDWIRISWDEATTIAAKTMEDVARTFNGDEGARKLLAQGYHPEMVEVMHGAGVQALKLRGGMPLL 4dmr.1    ------NADRSTRGNGDFVRVSWDQALDLVAAEVKRVEETYGPS--------------GVFGGSYGWKSPGRL-------  target    GIGRIFGFYRFANMLALLDRKLRPDAPADEILGSRTFD-NYAWHT-----DLPPGHPMVTGSQTVDFDLFSAEHTKLLLI 4dmr.1    HNCT-TLLRRMLTLA----------------GGYVNGAGDYSTGAAQVIMPHVVGTLEVYEQQ--TAWPVLAENTEVMVF  target    IGMNWICTKMPDG--------HWIGDARLKGTRVIVISADYMPTANK-ADEVIILRPGTDAAFFLGVARELIEKGLYDRA 4dmr.1    WAADPIKTSQIGWVIPEHGAYPGLEALKAKGTKVIVIDPVRTKTVEFFGAEHITPKPQTDVAIMLGMAHTLVAEDLYDKD  target    AVIERTDLPLLVRLDTGERLDARDVIPGYELAALTNYVTLKPDAEIKGNPPPPPFTAGGQVVPTELRDAWGDFVWWDRAT 4dmr.1    FIANYTS--------------------GFDK--F------------------------------------LP--------  target    GRPRPVSRDEVGARFDGDPALLGEFEVELVDGSTVPVRPAFDLLKQYLDESFDLRTASEVCRVPPQAIQSIARQLAANKR 4dmr.1    -------------------YLDGET---------------------DS-TPKTAEWAEGISGVPAETIKELARLFESK-R  target    ETLLAAGMGPNHYFQNDLFGRVQFLVAALTDNIGHLGGNVGSYAGNYRGSVFQAMGQWIAEDPFAIEPDLTKPATVKRYY 4dmr.1    -TMLAAGWSMQRMHHGEQAHWMLVTLASMLGQIGLPGGGFGLSYHYSGGGTPSTSGPAL--AGI--TDGGAAT-KGPEWL  target    KAESAHYWNYGERPLRAV-AKDDEGDLTKGEVLTGKSHMPTPTKLIWFGNSNSLLGNAKWSFDVVKNTLPRQDAVFCNEW 4dmr.1    A-----ASGASVIPVARVVDMLENPGAEFDFN--GTRSKFPDVKMAYWVGGNPFVHHQD--RNRMVKAWEKLETFVVHDF  target    HWTSSCEYADLVFPADSWAEFKLPDATASCTNPFLLAFPTTPLKRLYDTRSDYEALALTAKALGELIDEPRMEQYWRGIL 4dmr.1    QWTPTARHADIVLPATTSYERNDIETIGDYSNTGILA-MKKIVEPLYEARSDYDIFAAVAERLGKGAEFTEGKDEMGW--  target    DGDPTPYLQRIFSGSNATRGI---TYDELHESSKRGVPLLMNM--RTYPRSGGWEQRQEDKPWYTATGRLEFYRPEPEFQ 4dmr.1    ---IKSFYDDAAKQ-GKAAGVQMPAFDAFWAE---GIVEFPVTDGADFVRYASFREDPLLNPLGTPTGLIEIYSKNIEKM  target    --AAGESLPVWREPVDA----TFYEPNAILSNAAHPSIAPRAPEDYGVPESQLDVETRQYRNVVRTWAELQQTLHPLQER 4dmr.1    GYDDCPAHPTWMEPLERLDGPGAKYPLHIAAS------------------------------------------------  target    DPAFRFVF 4dmr.1    -------- ``` | | | | | | | | | | | | | | | | | | | | | | | | | | | | | | | | | | | | | | | | | | | | | | | | | |
|  | 6sdr.1.A | Formate dehydrogenase, alpha subunit, selenocysteine-containing  *W-formate dehydrogenase from Desulfovibrio vulgaris - Oxidized form* | 0.33 | 0.00 | 18.18 | 0.64 | 2-767 | X-ray | 2.10 | monomer | 2 x MGD, 4 x SF4, 1 x H2S, 1 x W | HHblits | 0.29 |
| ``` target    AQGVSRRQLLGRALALGSGAALADLLGPARFLSPAGAATAGAVVPGNPLRVMPDRTWEQIYRNQFEDDSTFVFTCAPNDT 6sdr.1    -MTVTRRHFLKLSAGAAVAGAFT-----------------GLGLSLAPTVARAELQ-----KLQW--A-KQTTSICCYCA  target    HNCLLRAHVK---NGVVVRISPTYGYGEATDLYGNRASHRWDPRTCQKGLILSRRFYSERRVKAPMIRKGFKDWVEAGYP 6sdr.1    VGCGLIVHTAKDGQGRAVNVEGD------------PDHPINEGSLCPKGASIFQLGENDQRGTQPLYRAP----------  target    RNDDGTPQMDVTLRGSDDWIRISWDEATTIAAKTMEDVARTFNGDEGARKLLAQGYHPEMVEVMHGAGVQALKLRGGMPL 6sdr.1    --------------FSDTWKPVTWDFALTEIAKRIKKTRDASFTEKNAAGDL--VNRTEAIASFGS--------------  target    LGIGRIFGFYRFANMLALLDRKLRPDAPADEILGSRTFDNYAWHTD--LPPGHPMVTGSQTVDFDLFSAEHTKLLLIIGM 6sdr.1    ---------AAMDNEECWAYGNIL------RSLGLVYIEHQARIUHSPTVPALAESFGRGAMTNHWNDLANSDCILIMGS  target    NWICTKMPDGHWIGDARLKGTRVIVISADYMPTANKADEVIILRPGTDAAFFLGVARELIEKGLYDRAAVIERTDLPLLV 6sdr.1    NAAENHPIAFKWVLRAKDKGATLIHVDPRFTRTSARCDVYAPIRSGADIPFLGGLIKYILDNKLYFTDYVREYTNASLIV  target    RLDTGERLDARDVIPGYELAALTNYVTLKPDAEIKGNPPPPPFTAGGQVVPTELRDAWGDFVWWDRATGRPRPVSRDEVG 6sdr.1    GEKFS--F-KDGLFSGYDAA-N---------------------------------KKYDKS-MWAFE-------------  target    ARFDGDPALLGEFEVELVDGSTVPVRPAFDLLKQYLDESFDLRTASEVCRVPPQAIQSIARQLAANK---RETLLAAGMG 6sdr.1    ------LDANG---VPKRDPALKHPRCVINLLKKHYE-RYNLDKVAAITGTSKEQLQQVYKAYAATGKPDKAGTIMYAMG  target    PNHYFQNDLFGRVQFLVAALTDNIGHLGGNVGSYAGNYRGSVFQAMGQWIAEDPFAIEPDLTKPATVKRYY-------KA 6sdr.1    WTQHSVGVQNIRAMAMIQLLLGNIGVAGGGVNALRGESNVQGSTDQGLLAHIWPGYNPVPNSKAATLELYNAATPQSKDP  target    ESAHYWNYGE--------------RPL---RAVAKDDEG----DLTKGEVLTGKSHMPTPTKLIWFGNSNSLLGNAKWSF 6sdr.1    MSVNWWQNRPKYVASYLKALYPDEEPAAAYDYLPRIDAGRKLTDYFWLNIFEK--MDKGEFKGLFAWGMNPACGGAN--A  target    DVVKNTLPRQDAVFCNEWHWTSSCEY--------AD-----LVFPADSWAEFKLPDATASCTNPFLLAFPTTPLKRLYDT 6sdr.1    NKNRKAMGKLEWLVNVNLFENETSSFWKGPGMNPAEIGTEVFFLPCCVSIEKE--GSV-ANSGRWMQW-RYRGPKPYAET  target    RSDYEALALTAKALGELIDEPRMEQYWRGILDGDPTPYLQRIFSGSNATRGITYDELHESSKRGVPLLMNMRTYPRSGGW 6sdr.1    KPDGDIMLDMFKKVRE----------------------------------------------------------------  target    EQRQEDKPWYTATGRLEFYRPEPEFQAAGESLPVWREPVDATFYEPNAILSNAAHPSIAPRAPEDYGVPESQLDVETRQY 6sdr.1    --------------------------------------------------------------------------------  target    RNVVRTWAELQQTLHPLQERDPAFRFVF 6sdr.1    ---------------------------- ``` | | | | | | | | | | | | | | | | | | | | | | | | | | | | | | | | | | | | | | | | | | | | | | | | | |
| ✓ | 6sdv.1.A | Formate dehydrogenase, alpha subunit, selenocysteine-containing,Formate dehydrogenase, alpha subunit, selenocysteine-containing,W-formate dehydrogenase - alpha subunit  *W-formate dehydrogenase from Desulfovibrio vulgaris - Formate reduced form* | 0.34 | 0.00 | 18.11 | 0.64 | 2-767 | X-ray | 1.90 | monomer | 2 x MGD, 4 x SF4, 1 x W, 1 x H2S | HHblits | 0.29 |
| ``` target    AQGVSRRQLLGRALALGSGAALADLLGPARFLSPAGAATAGAVVPGNPLRVMPDRTWEQIYRNQFEDDSTFVFTCAPNDT 6sdv.1    -MTVTRRHFLKLSAGAAVAGAFT-----------------GLGLSLAPTVARAELQ-----KL--QWA-KQTTSICCYCA  target    HNCLLRAHVK---NGVVVRISPTYGYGEATDLYGNRASHRWDPRTCQKGLILSRRFYSERRVKAPMIRKGFKDWVEAGYP 6sdv.1    VGCGLIVHTAKDGQGRAVNVEGD------------PDHPINEGSLCPKGASIFQLGENDQRGTQPLYRAP----------  target    RNDDGTPQMDVTLRGSDDWIRISWDEATTIAAKTMEDVARTFNGDEGARKLLAQGYHPEMVEVMHGAGVQALKLRGGMPL 6sdv.1    --------------FSDTWKPVTWDFALTEIAKRIKKTRDASFTEKNAAGDL--VNRTEAIASFG---------------  target    LGIGRIFGFYRFANMLALLDRKLRPDAPADEILGSRTFDNYAWHTD--LPPGHPMVTGSQTVDFDLFSAEHTKLLLIIGM 6sdv.1    --------SAAMDNEECWAYGNIL------RSLGLVYIEHQARIUHSPTVPALAESFGRGAMTNHWNDLANSDCILIMGS  target    NWICTKMPDGHWIGDARLKGTRVIVISADYMPTANKADEVIILRPGTDAAFFLGVARELIEKGLYDRAAVIERTDLPLLV 6sdv.1    NAAENHPIAFKWVLRAKDKGATLIHVDPRFTRTSARCDVYAPIRSGADIPFLGGLIKYILDNKLYFTDYVREYTNASLIV  target    RLDTGERLDARDVIPGYELAALTNYVTLKPDAEIKGNPPPPPFTAGGQVVPTELRDAWGDFVWWDRATGRPRPVSRDEVG 6sdv.1    GEKFS---FKDGLFSGYDAA-N---------------------------------KKYDKS-MWAFE------LD--A--  target    ARFDGDPALLGEFEVELVDGSTVPVRPAFDLLKQYLDESFDLRTASEVCRVPPQAIQSIARQLAANK---RETLLAAGMG 6sdv.1    ---------NG---VPKRDPALKHPRCVINLLKKHYE-RYNLDKVAAITGTSKEQLQQVYKAYAATGKPDKAGTIMYAMG  target    PNHYFQNDLFGRVQFLVAALTDNIGHLGGNVGSYAGNY--RGSVFQAMGQWIAEDPFAI-EPDLT-------KPATVKRY 6sdv.1    WTQHSVGVQNIRAMAMIQLLLGNIGVAGGGVNALRGESNVQGST--DQGLLAHIWPGYNPVPNSKAATLELYNAATPQSK  target    YKAESAHYWNYGER--------------PL---RAVAKDDE-G---DLTKGEVLTGKSHMPTPTKLIWFGNSNSLLGNAK 6sdv.1    DP-MSVNWWQNRPKYVASYLKALYPDEEPAAAYDYLPRIDAGRKLTDYFWLNIFEK--MDKGEFKGLFAWGMNPACGGAN  target    WSFDVVKNTLPRQDAVFCNEWHWTSSCEY--------AD-----LVFPADSWAEFKLPDATASCTNPFLLAFPTTPLKRL 6sdv.1    --ANKNRKAMGKLEWLVNVNLFENETSSFWKGPGMNPAEIGTEVFFLPCCVSIEKE--GSV-ANSGRWMQW-RYRGPKPY  target    YDTRSDYEALALTAKALGELIDEPRMEQYWRGILDGDPTPYLQRIFSGSNATRGITYDELHESSKRGVPLLMNMRTYPRS 6sdv.1    AETKPDGDIMLDMFKKVRE-------------------------------------------------------------  target    GGWEQRQEDKPWYTATGRLEFYRPEPEFQAAGESLPVWREPVDATFYEPNAILSNAAHPSIAPRAPEDYGVPESQLDVET 6sdv.1    --------------------------------------------------------------------------------  target    RQYRNVVRTWAELQQTLHPLQERDPAFRFVF 6sdv.1    ------------------------------- ``` | | | | | | | | | | | | | | | | | | | | | | | | | | | | | | | | | | | | | | | | | | | | | | | | | |
|  | 1kqf.1.A | FORMATE DEHYDROGENASE, NITRATE-INDUCIBLE, MAJOR SUBUNIT  *FORMATE DEHYDROGENASE N FROM E. COLI* | 0.33 |  | 20.30 | 0.63 | 2-767 | X-ray | 1.60 | hetero-oligomer | 3 x 6MO, 15 x SF4, 6 x MGD, 6 x HEM, 3 x CDL | HHblits | 0.29 |
| ``` target    AQGVSRRQLLGRALALGSGAALADLLGPARFLSPAGAATAGAVVPGNPLRVMP-DRTWEQIYRNQFEDDSTFVFTCAPND 1kqf.1    -MDVSRRQFFKICAGGMAGTTVAAL-------------------GFAPKQALAQARNYK------LLRA-KEIRNTCTYC  target    THNCLLRAHVKNG-------VVVRISPTYGYGEATDLYGNRASHRWDPRTCQKGLILSRRFYSERRVKAPMIRKGFKDWV 1kqf.1    SVGCGLLMYSLGDGAKNAREAIYHIEG------------DPDHPVSRGALCPKGAGLLDYVNSENRLRYPEYRAP-----  target    EAGYPRNDDGTPQMDVTLRGSDDWIRISWDEATTIAAKTMEDVARTFNGDEGARKLLAQGYHPE-----MVEVMHGAGVQ 1kqf.1    -------------------GSDKWQRISWEEAFSRIAKLMKADRDANFIEKNE-------QGVTVNRWLSTGMLCA----  target    ALKLRGGMPLLGIGRIFGFYRFANMLALLDRKLRPDAPADEILGSRTFDNYAWHTD--LPPGHPMVTGSQTVDFDLFSAE 1kqf.1    -------------------SGASNETGMLTQKFA------RSLGMLAVDNQARVUHGPTVASLAPTFGRGAMTNHWVDIK  target    HTKLLLIIGMNWICTKMPDGHWIGDARL-KGTRVIVISADYMPTANKADEVIILRPGTDAAFFLGVARELIEKGLYDRAA 1kqf.1    NANVVMVMGGNAAEAHPVGFRWAMEAKNNNDATLIVVDPRFTRTASVADIYAPIRSGTDITFLSGVLRYLIENNKINAEY  target    VIERTDLPLLVRLDTGERLDARDVIPGYELAALTNYVTLKPDAEIKGNPPPPPFTAGGQVVPTELRDAWGDFVWWDRATG 1kqf.1    VKHYTNASLLVRDDF-AFEDGLF--SGYDAEK----------------------------------RQY-DKSSWNYQLD  target    RPRPVSRDEVGARFDGDPALLGEFEVELVDGSTVPVRPAFDLLKQYLDESFDLRTASEVCRVPPQAIQSIARQLAANK-- 1kqf.1    ----------------------ENGYAKRDETLTHPRCVWNLLKEHVS-RYTPDVVENICGTPKADFLKVCEVLASTSAP  target    -RETLLAAGMGPNHYFQNDLFGRVQFLVAALTDNIGHLGGNVGSYAGNYRGSVFQAMGQWIAEDPFAIEPDLTKPAT--V 1kqf.1    DRTTTFLYALGWTQHTVGAQNIRTMAMIQLLLGNMGMAGGGVNALRGHSNIQGLTDLGLLSTSLPGY--LTLPSEKQVDL  target    KRYYKA--------ESAHYWN----YGERPLRA----------------VAKDDEGDLTKGEVLTGKSHMPTPTKLIWFG 1kqf.1    QSYLEANTPKATLADQVNYWSNYPKFFVSLMKSFYGDAAQKENNWGYDWLPKW-DQTYDVIKYFNM--MDEGKVTGYFCQ  target    NSNSLLGNAKWSFDVVKNTLPRQDAVFCNEWHWTSSCEYAD-----------------LVFPADSWAEFKLPDATASCTN 1kqf.1    GFNPVASFPD--KNKVVSCLSKLKYMVVIDPLVTETSTFWQNHGESNDVDPASIQTEVFRLPSTCFAEED--GS-IANSG  target    PFLLAFPTTPLKRLYDTRSDYEALALTAKALGELIDEPRMEQYWRGILDGDPTPYLQRIFSGSNATRGITYDELHESSKR 1kqf.1    RWLQW-HWKGQDAPGEARNDGEILAGIYHHLRE-----------------------------------------------  target    GVPLLMNMRTYPRSGGWEQRQEDKPWYTATGRLEFYRPEPEFQAAGESLPVWREPVDATFYEPNAILSNAAHPSIAPRAP 1kqf.1    --------------------------------------------------------------------------------  target    EDYGVPESQLDVETRQYRNVVRTWAELQQTLHPLQERDPAFRFVF 1kqf.1    --------------------------------------------- ``` | | | | | | | | | | | | | | | | | | | | | | | | | | | | | | | | | | | | | | | | | | | | | | | | | |
|  | 7vw6.1.A | Formate dehydrogenase  *Cryo-EM Structure of Formate Dehydrogenase 1 from Methylorubrum extorquens AM1* | 0.36 |  | 18.32 | 0.63 | 36-852 | EM | 0.00 | hetero-1-1-mer | 4 x SF4, 2 x FES, 2 x MGD, 1 x W, 1 x FMN | HHblits | 0.29 |
| ``` target    AQGVSRRQLLGRALALGSGAALADLLGPARFLSPAGAATAGAVVPGNPLRVMPDRTWEQIYRNQFEDDSTFVFTCAPNDT 7vw6.1    -----------------------------------TCVACGECVQACPTGALMPAAYLDANQTRTVYPDREVKSLCPYCG  target    HNCLLRAHVKNGVVVRISPTYGYGEATDLYGNRASHRWDPRTCQKGLILSRRFYSERRVKAPMIRKGFKDWVEAGYPRND 7vw6.1    VGCQVSYKVKDERIVYAEGV-------------NGPANQNRLCVKGRFGFDYVHHPHRLTVPLIRLENVP-----KDAN-  target    DGTPQMDVTLRGSDDWIRISWDEATTIAAKTMEDVARTFNGDEGARKLLAQGYHPEMVEVMHGAGVQALKLRGGMPLLGI 7vw6.1    ----DQVDPANPWTHFREATWEEALDRAAGGLKAIRDTNGR--------------KALAGF-------------------  target    GRIFGFYRFANMLALLDRKLRPDAPADEILGSRTFDNYAWHTDLP--PGHPMVTGSQTVDFDLFSAEHTKLLLIIGMNWI 7vw6.1    ----GSAKGSNEEAYLFQKLV-----RLGFGTNNVDHCTRLCHASSVAALMEGLNSGAVTAPFSAALDAEVIVVIGANPT  target    CTKMPDGHWIGDAR-LKGTRVIVISADYMPTANKADEVIILRPGTDAAFFLGVARELIEKGLYDRAAVIERTDLPLLVRL 7vw6.1    VNHPVAATFLKNAVKQRGAKLIIMDPRRQTLSRHAYRHLAFRPGSDVAMLNAMLNVIVTEGLYDEQYIAGYTE-------  target    DTGERLDARDVIPGYELAALTNYVTLKPDAEIKGNPPPPPFTAGGQVVPTELRDAWGDFVWWDRATGRPRPVSRDEVGAR 7vw6.1    -------------N------------------------------------------------------------------  target    FDGDPALLGEFEVELVDGSTVPVRPAFDLLKQYLDESFDLRTASEVCRVPPQAIQSIARQLAANKRETLLAAGMGPNHYF 7vw6.1    --------------------------FEALREKIV-DFTPEKMASVCGIDAETLREVARLYARAKSS-LIFWGMGVSQHV  target    QNDLFGRVQFLVAALTDNIGHLGGNVGSYAGN--YRGSVFQAMGQWIAEDPFAIEPDLTKPATVKRYYKAESAHYWNYGE 7vw6.1    HGTDNSRCLIALALITGQIGRPGTGLHPLRGQNNVQGAS--DAGLIPM--VYPDYQSVEKDAVRE-----LFEEFWGQSL  target    RPLRAVAKDDEGDLTKGEVLTGKSHMPTPTKLIWFGNSNSLLGNAKWSFDVVKNTLPRQDAVFCNEWHWTSSCEYADLVF 7vw6.1    DP--------QKGLTVVEIMRAI--HAGEIRGMFVEGENPAMSDPD--LNHARHALAMLDHLVVQDLFLTETAFHADVVL  target    PADSWAEFKLPDATASCTNPFLLAFPTTPLKRLYDTRSDYEALALTAKALGELIDEPRMEQYWRGILDGDPTPYLQRIFS 7vw6.1    PASAFAEKA---GTFTNTDRRVQIA-QPVVAPPGDARQDWWIIQELARRLDLDWNYGGPADI------------FAEMAQ  target    GSNATRGITYDELHESSKRGVPLLMNMRTYPRSGGWEQRQEDKPWYTATGRLEFYRPEPEFQAAGESLPVWREPVDATFY 7vw6.1    VMPSLNNITWERLEREGA--VTYPVDAPDQP---GNE-IIFYAGFPTESGRAKIVPA-----------------------  target    EPNAILSNAAHPSIAPRAPEDYGVPESQLDVETRQYRNVVRTWAELQQTLHPLQERDPAFRFVF 7vw6.1    ---------------------------------------------------------------- ``` | | | | | | | | | | | | | | | | | | | | | | | | | | | | | | | | | | | | | | | | | | | | | | | | | |
|  | 7e5z.1.A | Formate dehydrogenase  *Dehydrogenase holoenzyme* | 0.31 |  | 18.15 | 0.63 | 36-852 | EM | 0.00 | hetero-1-1-mer | 1 x W, 2 x MGD, 2 x FES, 4 x SF4, 1 x FMN | HHblits | 0.29 |
| ``` target    AQGVSRRQLLGRALALGSGAALADLLGPARFLSPAGAATAGAVVPGNPLRVMPDRTWEQIYRNQFEDDSTFVFTCAPNDT 7e5z.1    -----------------------------------TCVACGECVQACPTGALMPAAYLDANQTRTVYPDREVKSLCPYCG  target    HNCLLRAHVKNGVVVRISPTYGYGEATDLYGNRASHRWDPRTCQKGLILSRRFYSERRVKAPMIRKGFKDWVEAGYPRND 7e5z.1    VGCQVSYKVKDERIVYAEGV-------------NGPANQNRLCVKGRFGFDYVHHPHRLTVPLIRLENVP---KD-----  target    DGTPQMDVTLRGSDDWIRISWDEATTIAAKTMEDVARTFNGDEGARKLLAQGYHPEMVEVMHGAGVQALKLRGGMPLLGI 7e5z.1    --ANDQVDPANPWTHFREATWEEALDRAAGGLKAIRDTNGR--------------KALAGF-------------------  target    GRIFGFYRFANMLALLDRKLRPDAPADEILGSRTFDNYAWHTDLP--PGHPMVTGSQTVDFDLFSAEHTKLLLIIGMNWI 7e5z.1    ----GSAKGSNEEAYLFQKLV-----RLGFGTNNVDHCTRLCHASSVAALMEGLNSGAVTAPFSAALDAEVIVVIGANPT  target    CTKMPDGHWIGDA-RLKGTRVIVISADYMPTANKADEVIILRPGTDAAFFLGVARELIEKGLYDRAAVIERTDLPLLVRL 7e5z.1    VNHPVAATFLKNAVKQRGAKLIIMDPRRQTLSRHAYRHLAFRPGSDVAMLNAMLNVIVTEGLYDEQYIAGYTE-------  target    DTGERLDARDVIPGYELAALTNYVTLKPDAEIKGNPPPPPFTAGGQVVPTELRDAWGDFVWWDRATGRPRPVSRDEVGAR 7e5z.1    -------------N------------------------------------------------------------------  target    FDGDPALLGEFEVELVDGSTVPVRPAFDLLKQYLDESFDLRTASEVCRVPPQAIQSIARQLAANKRETLLAAGMGPNHYF 7e5z.1    --------------------------FEALREKIV-DFTPEKMASVCGIDAETLREVARLYARAKSS-LIFWGMGVSQHV  target    QNDLFGRVQFLVAALTDNIGHLGGNVGSYAGN--YRGSVFQAMGQWIAEDPFAIEPDLTKPATVKRYYKAESAHYWNYGE 7e5z.1    HGTDNSRCLIALALITGQIGRPGTGLHPLRGQNNVQGAS--DAGLIPM--VYPDYQSVEKDAVRE-----LFEEFWGQSL  target    RPLRAVAKDDEGDLTKGEVLTGKSHMPTPTKLIWFGNSNSLLGNAKWSFDVVKNTLPRQDAVFCNEWHWTSSCEYADLVF 7e5z.1    DP--------QKGLTVVEIMRAI--HAGEIRGMFVEGENPAMSDPD--LNHARHALAMLDHLVVQDLFLTETAFHADVVL  target    PADSWAEFKLPDATASCTNPFLLAFPTTPLKRLYDTRSDYEALALTAKALGELIDEPRMEQYWRGILDGDPTPYLQRIFS 7e5z.1    PASAFAEKA---GTFTNTDRRVQIA-QPVVAPPGDARQDWWIIQELARRLDLDWNYGGPAD------------IFAEMAQ  target    GSNATRGITYDELHESSKRGVPLLMNMRTYPRSGGWEQRQEDKPWYTATGRLEFYRPEPEFQAAGESLPVWREPVDATFY 7e5z.1    VMPSLNNITWERLEREGA--VTYPVDAPDQP---GNE-IIFYAGFPTESGRAKIVPA-----------------------  target    EPNAILSNAAHPSIAPRAPEDYGVPESQLDVETRQYRNVVRTWAELQQTLHPLQERDPAFRFVF 7e5z.1    ---------------------------------------------------------------- ``` | | | | | | | | | | | | | | | | | | | | | | | | | | | | | | | | | | | | | | | | | | | | | | | | | |
|  | 2e7z.1.A | Acetylene hydratase Ahy  *Acetylene Hydratase from Pelobacter acetylenicus* | 0.35 |  | 22.15 | 0.62 | 72-883 | X-ray | 1.26 | monomer | 1 x SF4, 2 x MGD, 1 x W | HHblits | 0.30 |
| ``` target    AQGVSRRQLLGRALALGSGAALADLLGPARFLSPAGAATAGAVVPGNPLRVMPDRTWEQIYRNQFEDDSTFVFTCAPNDT 2e7z.1    -----------------------------------------------------------------------KHVVCQSCD  target    HNCLLRAHVK-NGVVVRISPTYGYGEATDLYGNRASHRW-DPRTCQKGLILSRRFYSERRVKAPMIRKGFKDWVEAGYPR 2e7z.1    INCVVEAEVKADGKIQTKSISE------------PHPTTPPNSICMKSVNADTIRTHKDRVLYPLKNVGS----------  target    NDDGTPQMDVTLRGSDDWIRISWDEATTIAAKTMEDVARTFNGDEGARKLLAQGYHPEMVEVMHGAGVQALKLRGGMPLL 2e7z.1    -----------KRGEQRWERISWDQALDEIAEKLKKIIAKYG--------------PESLGVSQTEINQQS---------  target    GIGRIFGFYRFANMLALLDRKLRPDAPADEILGSRTFDNYAWHTDLP--PGHPMVTGSQTVDFDLFSAEHTKLLLIIGMN 2e7z.1    ---EYGTLRRFMN-----------------LLGSPNWTSAMYMCIGNTAGVHRVTHGS----YSFASFADSNCLLFIGKN  target    WICTKMPD-GHWIGDARLKGTRVIVISADYMPTANKADEVIILRPGTDAAFFLGVARELIEKGLYDRAAVIERTDLPLLV 2e7z.1    LSNHNWVSQFNDLKAALKRGCKLIVLDPRRTKVAEMADIWLPLRYGTDAALFLGMINVIINEQLYDKEFVENWCV-----  target    RLDTGERLDARDVIPGYELAALTNYVTLKPDAEIKGNPPPPPFTAGGQVVPTELRDAWGDFVWWDRATGRPRPVSRDEVG 2e7z.1    ---------------G----------------------------------------------------------------  target    ARFDGDPALLGEFEVELVDGSTVPVRPAFDLLKQYLDESFDLRTASEVCRVPPQAIQSIARQLAANKRETLLAAGMGPNH 2e7z.1    ----------------------------FEELKERVQ-EYPLDKVAEITGCDAGEIRKAAVMFATESPASI-PWAVSTDM  target    YFQNDLFGRVQFLVAALTDNIGHLGGNVGSYAGNYRG-SVFQAMGQWIAE--------DPFAIEPDLTKPATVKRYYKAE 2e7z.1    QKNSCSAIRAQCILRAIVGSFVNGAEILGAPHSDLVPISKIQMHEALPEEKKKLQLGTETYPF---LT-YTGMSALEE-P  target    SAHYWNYGERPLRAVAKDDEGDLTKGEVLTGKSHMPTPTKLIWFGNSNSLLGNAKWSFDVVKNTLPRQDAVFCNEWHWTS 2e7z.1    SERVYGVKYFH-NMGAFMANPTALFTAMATE---KPYPVKAFFALASNALMGYAN--QQNALKGLMNQDLVVCYDQFMTP  target    SCEYADLVFPADSWAEFKLPDATASC-TNPFLLAFPTTPLKRLYDTRSDYEALALTAKALGELIDEPRMEQYWRGILDGD 2e7z.1    TAQLADYVLPGDHWLERP--VVQPNWEGIPFGNT-SQQVVEPAGEAKDEYYFIRELAVRMGLEEHFP----------WKD  target    PTPYLQRIFSGSNATRGITYDELHESSKRGVPLLMNMRTYPRSGGWEQRQEDKPWYTATGRLEFYRPEPEFQAAGESLPV 2e7z.1    RLELINYRIS----PTGMEWEEYQKQYT--YMSK--L---PD---Y-FGPEGVGVATPSGKVELYSSVFE-KLGYDPLPY  target    WREPVDA-------TFYEPNAILSNAAHPSIAPRAPEDYGVPESQLDVETRQYRNVVRTWAELQQTLHPLQERDPAFRFV 2e7z.1    YHEPLQTEISDPELAKEYPLILFAG-------------------------------------------------------  target    F 2e7z.1    - ``` | | | | | | | | | | | | | | | | | | | | | | | | | | | | | | | | | | | | | | | | | | | | | | | | | |
|  | 2vpz.1.A | THIOSULFATE REDUCTASE  *POLYSULFIDE REDUCTASE NATIVE STRUCTURE* | 0.36 |  | 21.05 | 0.61 | 71-871 | X-ray | 2.40 | hetero-oligomer | 10 x SF4, 4 x MGD, 2 x MO | HHblits | 0.30 |
| ``` target    AQGVSRRQLLGRALALGSGAALADLLGPARFLSPAGAATAGAVVPGNPLRVMPDRTWEQIYRNQFEDDSTFVFTCAPNDT 2vpz.1    ----------------------------------------------------------------------SVYQICEGCF  target    HNCLLRAHVKNGVVVRISPTYGYGEATDLYGNRASHRWDPRTCQKGLILSRRFYSERRVKAPMIRKGFKDWVEAGYPRND 2vpz.1    WRCGIVAHAVGNRVYKVEG------------YEANPKSRGRLCPRGQGAPQTTYDPDRLKRPLIRVEGS-----------  target    DGTPQMDVTLRGSDDWIRISWDEATTIAAKTMEDVARTFNGDEGARKLLAQGYHPEMVEVMHGAGVQALKLRGGMPLLGI 2vpz.1    ---------QRGEGKYRVATWEEALDHIAKKMLEIREKYGP--------------EAIAFFGH-GTGDYW----------  target    GRIFGFYRFANMLALLDRKLRPDAPADEILGSRTFDNY-AWHT--DLPPGHPMVTGSQTVDFDLFSAEHTKLLLIIGMNW 2vpz.1    -----FVDFL----------------PAAWGSPNAAKPSVSLCTAPREVASQWVFGRPIGGHEPIDWENARYIVLIGHHI  target    ICT-KMPDGHWIGDARLKGTRVIVISADYMPTANKADEVIILRPGTDAAFFLGVARELIEKGLYDRAAVIERTDLPLLVR 2vpz.1    GEDTHNTQLQDFALALKNGAKVVVVDPRFSTAAAKAHRWLPIKPGTDTALLLAWIHVLIYEDLYDKEYVAKYTV------  target    LDTGERLDARDVIPGYELAALTNYVTLKPDAEIKGNPPPPPFTAGGQVVPTELRDAWGDFVWWDRATGRPRPVSRDEVGA 2vpz.1    --------------G-----------------------------------------------------------------  target    RFDGDPALLGEFEVELVDGSTVPVRPAFDLLKQYLDESFDLRTASEVCRVPPQAIQSIARQLAANKRETLLAAGMGPNH- 2vpz.1    ---------------------------FEELKAHVK-DFTPEWAEKHTEIPAQVIREVAREMAAHKPRAVL-PPTRHNVW  target    YFQNDLFGRVQFLVAALTDNIGHLGGNVGSYAGNYRGSVFQAMGQWIAEDPFAIEPDLTKPATVKRYYKAESAHYWNYGE 2vpz.1    YGDDTYRVMALLYVNVLLGNYGRPGGFYIAQSPYLEKYPL---PPLP-LEPAAG--GCSGPSG-GDHEP---EGFKPRAD  target    RPLRAVAKDDEGDLTKGEVLTGKSHMPTPTKLIWFGNSNSLLGNAKWSFDVVKNTLPRQDAVFCNEWHWTSSCEYADLVF 2vpz.1    -KGKFFARSTAIQELIEPMITGE---PYPIKGLFAYGINLFHSIPN--VPRTKEALKNLDLYVAIDVLPQEHVMWADVIL  target    PADSWAEFKLPDATASCTNPFLLAFPTTPLKRLYDTRSDYEALALTAKALGELIDEPRMEQYWRGILDGDPTPYLQRIFS 2vpz.1    PEATYLERYDDFVLVAHKTPFIQLR-TPAHEPLFDTKPGWWIARELGLRLGLEQYFP----------WKTIEEYLETRLQ  target    GSNATRGITYDELHESSKRGVPLLMNMRTYPRSGGWEQRQEDKPWYTATGRLEFYRPEPEFQAAGESLPVWREPVDATFY 2vpz.1    S----LGLDLETMKGMGT---LVQR---GKPWLEDWE-KEGRLPFGTASGKIELYCQRFK-EAGHQPLPVFTPPEE----  target    EPNAILSNAAHPSIAPRAPEDYGVPESQLDVETRQYRNVVRTWAELQQTLHPLQERDPAFRFVF 2vpz.1    ---------------------------------------------------------------- ``` | | | | | | | | | | | | | | | | | | | | | | | | | | | | | | | | | | | | | | | | | | | | | | | | | |
|  | 2vpx.1.D | THIOSULFATE REDUCTASE  *POLYSULFIDE REDUCTASE WITH BOUND QUINONE (UQ1)* | 0.35 |  | 21.05 | 0.61 | 71-871 | X-ray | 3.10 | hetero-oligomer | 10 x SF4, 4 x MGD, 2 x MO, 2 x UQ1 | HHblits | 0.30 |
| ``` target    AQGVSRRQLLGRALALGSGAALADLLGPARFLSPAGAATAGAVVPGNPLRVMPDRTWEQIYRNQFEDDSTFVFTCAPNDT 2vpx.1    ----------------------------------------------------------------------SVYQICEGCF  target    HNCLLRAHVKNGVVVRISPTYGYGEATDLYGNRASHRWDPRTCQKGLILSRRFYSERRVKAPMIRKGFKDWVEAGYPRND 2vpx.1    WRCGIVAHAVGNRVYKVEG------------YEANPKSRGRLCPRGQGAPQTTYDPDRLKRPLIRVEGS-----------  target    DGTPQMDVTLRGSDDWIRISWDEATTIAAKTMEDVARTFNGDEGARKLLAQGYHPEMVEVMHGAGVQALKLRGGMPLLGI 2vpx.1    ---------QRGEGKYRVATWEEALDHIAKKMLEIREKYGP--------------EAIAFFGH-GTGDYW----------  target    GRIFGFYRFANMLALLDRKLRPDAPADEILGSRTFDNY-AWHT--DLPPGHPMVTGSQTVDFDLFSAEHTKLLLIIGMNW 2vpx.1    -----FVDFL----------------PAAWGSPNAAKPSVSLCTAPREVASQWVFGRPIGGHEPIDWENARYIVLIGHHI  target    ICT-KMPDGHWIGDARLKGTRVIVISADYMPTANKADEVIILRPGTDAAFFLGVARELIEKGLYDRAAVIERTDLPLLVR 2vpx.1    GEDTHNTQLQDFALALKNGAKVVVVDPRFSTAAAKAHRWLPIKPGTDTALLLAWIHVLIYEDLYDKEYVAKYTV------  target    LDTGERLDARDVIPGYELAALTNYVTLKPDAEIKGNPPPPPFTAGGQVVPTELRDAWGDFVWWDRATGRPRPVSRDEVGA 2vpx.1    --------------G-----------------------------------------------------------------  target    RFDGDPALLGEFEVELVDGSTVPVRPAFDLLKQYLDESFDLRTASEVCRVPPQAIQSIARQLAANKRETLLAAGMGPNH- 2vpx.1    ---------------------------FEELKAHVK-DFTPEWAEKHTEIPAQVIREVAREMAAHKPRAVL-PPTRHNVW  target    YFQNDLFGRVQFLVAALTDNIGHLGGNVGSYAGNYRGSVFQAMGQWIAEDPFAIEPDLTKPATVKRYYKAESAHYWNYGE 2vpx.1    YGDDTYRVMALLYVNVLLGNYGRPGGFYIAQSPYLEKYPL---PPLP-LEPAAG--GCSGPSG-GDHEP---EGFKPRAD  target    RPLRAVAKDDEGDLTKGEVLTGKSHMPTPTKLIWFGNSNSLLGNAKWSFDVVKNTLPRQDAVFCNEWHWTSSCEYADLVF 2vpx.1    -KGKFFARSTAIQELIEPMITGE---PYPIKGLFAYGINLFHSIPN--VPRTKEALKNLDLYVAIDVLPQEHVMWADVIL  target    PADSWAEFKLPDATASCTNPFLLAFPTTPLKRLYDTRSDYEALALTAKALGELIDEPRMEQYWRGILDGDPTPYLQRIFS 2vpx.1    PEATYLERYDDFVLVAHKTPFIQLR-TPAHEPLFDTKPGWWIARELGLRLGLEQYFP----------WKTIEEYLETRLQ  target    GSNATRGITYDELHESSKRGVPLLMNMRTYPRSGGWEQRQEDKPWYTATGRLEFYRPEPEFQAAGESLPVWREPVDATFY 2vpx.1    S----LGLDLETMKGMGT---LVQR---GKPWLEDWE-KEGRLPFGTASGKIELYCQRFK-EAGHQPLPVFTPPEE----  target    EPNAILSNAAHPSIAPRAPEDYGVPESQLDVETRQYRNVVRTWAELQQTLHPLQERDPAFRFVF 2vpx.1    ---------------------------------------------------------------- ``` | | | | | | | | | | | | | | | | | | | | | | | | | | | | | | | | | | | | | | | | | | | | | | | | | |
|  | 4aay.1.A | AROA  *Crystal Structure of the arsenite oxidase protein complex from Rhizobium species strain NT-26* | 0.29 |  | 15.98 | 0.62 | 68-853 | X-ray | 2.70 | hetero-oligomer | 4 x MGD, 2 x O, 2 x 4MO, 2 x F3S, 2 x FES | HHblits | 0.28 |
| ``` target    AQGVSRRQLLGRALALGSGAALADLLGPARFLSPAGAATAGAVVPGNPLRVMPDRTWEQIYRNQFEDDSTFVFTCAPNDT 4aay.1    -------------------------------------------------------------------DAKKHNVTCHFCI  target    HNCLLRAHV-----------------------------------------KNGVVVRISPTYGYGEATDLYGNRASHRWD 4aay.1    VGCGYHAYTWPINKQGGTDPQNNIFGVDLSEQQQAESDAWYSPSMYNVVKQDGRDVHVVIKPD----------HECVVNS  target    PRTCQKGLILSRRFY------SERRVKAPMIRKGFKDWVEAGYPRNDDGTPQMDVTLRGSDDWIRISWDEATTIAAKTME 4aay.1    GLGSVRGARMAETSFSEARNTQQQRLTDPLVWRY--------------------------GQMQPTSWDDALDLVARVTA  target    DVARTFNGDEGARKLLAQGYHPEMVEVMHG--AGVQALKLRGGMPLLGIGRIFGFYRFANMLALLDRKLRPDAPADEILG 4aay.1    KIVKEKGE--------------DALIVSAFDHGGA---------------------GGGYENTWGTGKL-----YFEAMK  target    SRTFDNYAWHTDLP--PGHPMVTGSQTVDFDLFSAEHTKLLLIIGMNWICTKMPDG--HWIG---------------DAR 4aay.1    VKNIRIHNRPAYNSEVHGT-RDMGVGELNNCYEDAELADTIVAVGTNALETQTNYFLNHWIPNLRGESLGKKKELMPEEP  target    LKGTRVIVISADYMPTAN------KAD--EVIILRPGTDAAFFLGVARELIEKGLYDRAAVIERTDLPLLVRLDTGERLD 4aay.1    HEAGRIIIVDPRRTVTVNACEQTAGADNVLHLAINSGTDLALFNALFTYIADKGWVDRDFIDKSTLREGTARP-------  target    ARDVIPGYELAALTNYVTLKPDAEIKGNPPPPPFTAGGQVVPTELRDAWGDFVWWDRATGRPRPVSRDEVGARFDGDPAL 4aay.1    --------------------------------------------------P--LYPA-RGV-------------------  target    LGEFEVELVDGSTVPVRPAFDLLKQYLD-ESFDLRTASEVCRVPPQAIQSIARQLAANKR-----ETLLAAGMGPNHYFQ 4aay.1    --------SE----ANPGHLSSFEDAVEGCRMSIEEAAEITGLDAAQIIKAAEWIGMPKEGGKRRRVMFGYEKGLIWGND  target    NDLFGRVQFLVAALTDNIGHLGGNVGSYAGNYRGSVFQAMGQWIAEDPFAIEPDLTKPAT-VKRYY---KAESAHYWNYG 4aay.1    NYRTNGALVNLALATGNIGRPGGGVVRLGGHQEGYV-----RPS--DAH-----VGRPAAYVDQLLIGGQGGVHHIWGCD  target    ERPLRAVAKDDEGDLTKGEVLTGKSHMPTPTKLIWFGNSNSLL------GNAK--WSFDVVKNTLPRQ-DAVFCNEWHWT 4aay.1    --HY-------------KTTL-----NAHEFKRVYKKRTDMVKDAMSAAPYGDREAMVNAIVDAINQGGLFAVNVDIIPT  target    SSCEYADLVFPADSWAEFKLPDATASCTNPFLLAFPTTPLKRLYDTRSDYEALALTAKALGELIDEPR---MEQYWRGIL 4aay.1    KIGEACHVILPAATSGEMN---LTSMNGERRMRLT-ERYMDPPGQSMPDCLIAARLANTMERVLTEMGDVGYAAQFKGFD  target    DGDPTPYL-QRIFSGSNATRGITYDELHESSKRGVPLLMNMRTYPRSGGWEQRQEDKPWYTATGRLEFYRPEPEFQAAGE 4aay.1    WQTEEDAFMDGYNKNAHGGEFVTYERLSAMGTNGFQEPATGFTDGKIEGTQRLYTDGVFSTDDGKARFMDAP--------  target    SLPVWREPVDATFYEPNAILSNAAHPSIAPRAPEDYGVPESQLDVETRQYRNVVRTWAELQQTLHPLQERDPAFRFVF 4aay.1    ------------------------------------------------------------------------------ ``` | | | | | | | | | | | | | | | | | | | | | | | | | | | | | | | | | | | | | | | | | | | | | | | | | |
|  | 5nqd.1.A | AroA  *Arsenite oxidase AioAB from Rhizobium sp. str. NT-26 mutant AioBF108A* | 0.30 |  | 15.81 | 0.62 | 68-853 | X-ray | 2.20 | hetero-2-2-mer | 4 x MGD, 2 x O, 2 x 4MO, 2 x F3S, 2 x FES | HHblits | 0.28 |
| ``` target    AQGVSRRQLLGRALALGSGAALADLLGPARFLSPAGAATAGAVVPGNPLRVMPDRTWEQIYRNQFEDDSTFVFTCAPNDT 5nqd.1    -------------------------------------------------------------------DAKKHNVTCHFCI  target    HNCLLRAHV-----------------------------------------KNGVVVRISPTYGYGEATDLYGNRASHRWD 5nqd.1    VGCGYHAYTWPINKQGGTDPQNNIFGVDLSEQQQAESDAWYSPSMYNVVKQDGRDVHVVIKPD----------HECVVNS  target    PRTCQKGLILSRRFY------SERRVKAPMIRKGFKDWVEAGYPRNDDGTPQMDVTLRGSDDWIRISWDEATTIAAKTME 5nqd.1    GLGSVRGARMAETSFSEARNTQQQRLTDPLVWRY--------------------------GQMQPTSWDDALDLVARVTA  target    DVARTFNGDEGARKLLAQGYHPEMVEVMHG--AGVQALKLRGGMPLLGIGRIFGFYRFANMLALLDRKLRPDAPADEILG 5nqd.1    KIVKEKGE--------------DALIVSAFDHGGA---------------------GGGYENTWGTGKL-----YFEAMK  target    SRTFDNYAWHTDLP--PGHPMVTGSQTVDFDLFSAEHTKLLLIIGMNWICTKMPDG--HWIG---------------DAR 5nqd.1    VKNIRIHNRPAYNSEVHGT-RDMGVGELNNCYEDAELADTIVAVGTNALETQTNYFLNHWIPNLRGESLGKKKELMPEEP  target    LKGTRVIVISADYMPTAN------KAD--EVIILRPGTDAAFFLGVARELIEKGLYDRAAVIERTDLPLLVRLDTGERLD 5nqd.1    HEAGRIIIVDPRRTVTVNACEQTAGADNVLHLAINSGTDLALFNALFTYIADKGWVDRDFIDKSTLREGTARP-------  target    ARDVIPGYELAALTNYVTLKPDAEIKGNPPPPPFTAGGQVVPTELRDAWGDFVWWDRATGRPRPVSRDEVGARFDGDPAL 5nqd.1    --------------------------------------------------PL--YPA-RG--------------------  target    LGEFEVELVDGSTVPVRPAFDLLKQYLD-ESFDLRTASEVCRVPPQAIQSIARQLAANKR-----ETLLAAGMGPNHYFQ 5nqd.1    -----------VSEANPGHLSSFEDAVEGCRMSIEEAAEITGLDAAQIIKAAEWIGMPKEGGKRRRVMFGYEKGLIWGND  target    NDLFGRVQFLVAALTDNIGHLGGNVGSYAGNYRGSVFQAMGQWIAEDPFAIEPDLTKPAT-VKRYY---KAESAHYWNYG 5nqd.1    NYRTNGALVNLALATGNIGRPGGGVVRLGGHQEGYVRP--SD-----AHV-----GRPAAYVDQLLIGGQGGVHHIWGCD  target    ERPLRAVAKDDEGDLTKGEVLTGKSHMPTPTKLIWFGNSNSLL------GNAK--WSFDVVKNTLPRQ-DAVFCNEWHWT 5nqd.1    --HY------------KTTL------NAHEFKRVYKKRTDMVKDAMSAAPYGDREAMVNAIVDAINQGGLFAVNVDIIPT  target    SSCEYADLVFPADSWAEFKLPDATASCTNPFLLAFPTTPLKRLYDTRSDYEALALTAKALGELIDEPR---MEQYWRGIL 5nqd.1    KIGEACHVILPAATSGEMN---LTSMNGERRMRLT-ERYMDPPGQSMPDCLIAARLANTMERVLTEMGDVGYAAQFKGFD  target    DGDPTPYL-QRIFSGSNATRGITYDELHESSKRGVPLLMNMRTYPRSGGWEQRQEDKPWYTATGRLEFYRPEPEFQAAGE 5nqd.1    WQTEEDAFMDGYNKNAHGGEFVTYERLSAMGTNGFQEPATGFTDGKIEGTQRLYTDGVFSTDDGKARFMDAP--------  target    SLPVWREPVDATFYEPNAILSNAAHPSIAPRAPEDYGVPESQLDVETRQYRNVVRTWAELQQTLHPLQERDPAFRFVF 5nqd.1    ------------------------------------------------------------------------------ ``` | | | | | | | | | | | | | | | | | | | | | | | | | | | | | | | | | | | | | | | | | | | | | | | | | |
|  | 1g8j.1.A | ARSENITE OXIDASE  *CRYSTAL STRUCTURE ANALYSIS OF ARSENITE OXIDASE FROM ALCALIGENES FAECALIS* | 0.31 |  | 16.81 | 0.61 | 47-852 | X-ray | 2.03 | hetero-oligomer | 2 x MGD, 1 x O, 1 x 4MO, 1 x F3S, 1 x FES | HHblits | 0.28 |
| ``` target    AQGVSRRQLLGRALALGSGAALADLLGPARFLSPAGAATAGAVVPGNPLRVMPDRTWEQIYRNQFEDDSTFVFTCAPNDT 1g8j.1    ----------------------------------------------CPNDRITLPPA----------NAQRTNMTCHFCI  target    HNCLLRAHVKN-----GV--------------------------------------VVRISPTYGYGEATDLYGNRASHR 1g8j.1    VGCGYHVYKWPELEEGGRAPEQNALGLDFRKQLPPLASTLTPAMTNVVTEHDGARYDIMVVP------------DKACVV  target    WDPRTCQKGLILSRRFYSE-----RRVKAPMIRKGFKDWVEAGYPRNDDGTPQMDVTLRGSDDWIRISWDEATTIAAKTM 1g8j.1    NSGLSSTRGGKMASYMYTPTGDGKERLSAPRLYAA--------------------------DEWVDTTWDHAMALYAGLI  target    EDVARTFNGDEGARKLLAQGYHPEMVEVMHGA--GVQALKLRGGMPLLGIGRIFGFYRFANMLALLDRKLRPDAPADEIL 1g8j.1    KKTLDSDG--------------PQGVFFSCFDHGGAG---------------------GGFENTWGTGKL-----MFSAI  target    GSRTFDNYAW--HTDLPPGHPMVTGSQTVDFDLFSAEHTKLLLIIGMNWICTKMPD--GHWIG---------------DA 1g8j.1    QTPMVRIHNRPAYNSECH-ATREMGIGELNNAYEDAQLADVIWSIGNNPYESQTNYFLNHWLPNLQGATTSKKKERFPNE  target    RLKGTRVIVISADYMPTANKA--------DEVIILRPGTDAAFFLGVARELIEKGLYDRAAVIERTDLPLLVRLDTGERL 1g8j.1    NFPQARIIFVDPRETPSVAIARHVAGNDRVLHLAIEPGTDTALFNGLFTYVVEQGWIDKPFIEAHTK-------------  target    DARDVIPGYELAALTNYVTLKPDAEIKGNPPPPPFTAGGQVVPTELRDAWGDFVWWDRATGRPRPVSRDEVGARFDGDPA 1g8j.1    -------GF-----------------------------------------------------------------------  target    LLGEFEVELVDGSTVPVRPAFDLLKQYLDESFDLRTASEVCRVPPQAIQSIARQLAANK-----RETLLAAGMGPNHYFQ 1g8j.1    ---------------------DDAVK-T-NRLSLDECSNITGVPVDMLKRAAEWSYKPKASGQAPRTMHAYEKGIIWGND  target    NDLFGRVQFLVAALTDNIGHLGGNVGSYAGNYRGSVFQAMGQWIAEDPFAIEPDLTKPATVKRYYKAESAHYWNYGERPL 1g8j.1    NYVIQSALLDLVIATHNVGRRGTGCVRMGGHQEGYTR---PP----YPGDKKIYIDQE-----LIKGKGRIMTWWGCNNF  target    RAVAKDDEGDLTKGEVLTGKSHMPTPTKLIWFGNSNSLLGNAKWSFDVVKNTLPRQD-AVFCNEWHWTSSCEYADLVFPA 1g8j.1    QTSNN---AQALREAILQR---SAIVKQAMQKARGATTEEM----VDVIYEATQNGGLFVTSINLYPTKLAEAAHLMLPA  target    DSWAEFKLPDATASCTNPFLLAFPTTPLKRLYDTRSDYEALALTAKALGELIDEPR---MEQYWRGILDGDPTPYLQRIF 1g8j.1    AHPGEMN---LTSMNGERRIRLS-EKFMDPPGTAMADCLIAARIANALRDMYQKDGKAEMAAQFEGFDWKTEEDAFNDGF  target    SGSN-------------ATRGITYDELHESSKRGVPLLMNM-RTYPRSGGWEQRQEDKPWYTATGRLEFYRPEPEFQAAG 1g8j.1    RRAGQPGAPAIDSQGGSTGHLVTYDRLRKSGNNGVQLPVVSWDESKGLVGTEMLYTEGKFDTDDGKAHFKPA--------  target    ESLPVWREPVDATFYEPNAILSNAAHPSIAPRAPEDYGVPESQLDVETRQYRNVVRTWAELQQTLHPLQERDPAFRFVF 1g8j.1    ------------------------------------------------------------------------------- ``` | | | | | | | | | | | | | | | | | | | | | | | | | | | | | | | | | | | | | | | | | | | | | | | | | |
|  | 8bqg.1.A | Formate dehydrogenase, alpha subunit, selenocysteine-containing  *W-formate dehydrogenase from Desulfovibrio vulgaris - Soaking with Formate 1 min* | 0.33 | 0.00 | 18.52 | 0.60 | 54-767 | X-ray | 1.95 | monomer | 2 x MGD, 4 x SF4, 1 x H2S, 1 x W | HHblits | 0.29 |
| ``` target    AQGVSRRQLLGRALALGSGAALADLLGPARFLSPAGAATAGAVVPGNPLRVMPDRTWEQIYRNQFEDDSTFVFTCAPNDT 8bqg.1    -----------------------------------------------------ELQ-----KLQW--A-KQTTSICCYCA  target    HNCLLRAHVK---NGVVVRISPTYGYGEATDLYGNRASHRWDPRTCQKGLILSRRFYSERRVKAPMIRKGFKDWVEAGYP 8bqg.1    VGCGLIVHTAKDGQGRAVNVEGD------------PDHPINEGSLCPKGASIFQLGENDQRGTQPLYRAP----------  target    RNDDGTPQMDVTLRGSDDWIRISWDEATTIAAKTMEDVARTFNGDEGARKLLAQGYHPEMVEVMHGAGVQALKLRGGMPL 8bqg.1    --------------FSDTWKPVTWDFALTEIAKRIKKTRDASFTEKNAAGDL--VNRTEAIASFGSA-------------  target    LGIGRIFGFYRFANMLALLDRKLRPDAPADEILGSRTFDNYAWHTDL--PPGHPMVTGSQTVDFDLFSAEHTKLLLIIGM 8bqg.1    ----------AMDNEECWAYGNIL------RSLGLVYIEHQARIUHSPTVPALAESFGRGAMTNHWNDLANSDCILIMGS  target    NWICTKMPDGHWIGDARLKGTRVIVISADYMPTANKADEVIILRPGTDAAFFLGVARELIEKGLYDRAAVIERTDLPLLV 8bqg.1    NAAENHPIAFKWVLRAKDKGATLIHVDPRFTRTSARCDVYAPIRSGADIPFLGGLIKYILDNKLYFTDYVREYTNASLIV  target    RLDTGERLDARDVIPGYELAALTNYVTLKPDAEIKGNPPPPPFTAGGQVVPTELRDAWGDF-VWWDRATGRPRPVSRDEV 8bqg.1    GEKFS---FKDGLFSGYDAA----------------------------------NKKYDKSMWAFELDAN----------  target    GARFDGDPALLGEFEVELVDGSTVPVRPAFDLLKQYLDESFDLRTASEVCRVPPQAIQSIARQLAANK---RETLLAAGM 8bqg.1    -----------G---VPKRDPALKHPRCVINLLKKHYE-RYNLDKVAAITGTSKEQLQQVYKAYAATGKPDKAGTIMYAM  target    GPNHYFQNDLFGRVQFLVAALTDNIGHLGGNVGSYAGNYR--GSVFQAMGQWIAEDPFAIEPDLTKPATVKRYY------ 8bqg.1    GWTQHSVGVQNIRAMAMIQLLLGNIGVAGGGVNALRGESNVQGST--DQGLLAHIWPGYNPVPNSKAATLELYNAATPQS  target    -KAESAHYWNY-------------G-ERPL---RAVAKDDE----GDLTKGEVLTGKSHMPTPTKLIWFGNSNSLLGNAK 8bqg.1    KDPMSVNWWQNRPKYVASYLKALYPDEEPAAAYDYLPRIDAGRKLTDYFWLNIFEKM--DKGEFKGLFAWGMNPACGGAN  target    WSFDVVKNTLPRQDAVFCNEWHWTSSCEY--------AD-----LVFPADSWAEFKLPDATASCTNPFLLAFPTTPLKRL 8bqg.1    --ANKNRKAMGKLEWLVNVNLFENETSSFWKGPGMNPAEIGTEVFFLPCCVSIEKE--GSV-ANSGRWMQW-RYRGPKPY  target    YDTRSDYEALALTAKALGELIDEPRMEQYWRGILDGDPTPYLQRIFSGSNATRGITYDELHESSKRGVPLLMNMRTYPRS 8bqg.1    AETKPDGDIMLDMFKKVRE-------------------------------------------------------------  target    GGWEQRQEDKPWYTATGRLEFYRPEPEFQAAGESLPVWREPVDATFYEPNAILSNAAHPSIAPRAPEDYGVPESQLDVET 8bqg.1    --------------------------------------------------------------------------------  target    RQYRNVVRTWAELQQTLHPLQERDPAFRFVF 8bqg.1    ------------------------------- ``` | | | | | | | | | | | | | | | | | | | | | | | | | | | | | | | | | | | | | | | | | | | | | | | | | |
|  | 1aa6.1.A | FORMATE DEHYDROGENASE H  *REDUCED FORM OF FORMATE DEHYDROGENASE H FROM E. COLI* | 0.35 | 0.00 | 20.18 | 0.59 | 72-853 | X-ray | 2.30 | monomer | 1 x SF4, 2 x MGD, 1 x 4MO | HHblits | 0.31 |
| ``` target    AQGVSRRQLLGRALALGSGAALADLLGPARFLSPAGAATAGAVVPGNPLRVMPDRTWEQIYRNQFEDDSTFVFTCAPNDT 1aa6.1    -----------------------------------------------------------------------VVTVCPYCA  target    HNCLLRAHVKNGVVVRISPTYGYGEATDLYGNRASHRWDPRTCQKGLILSRRFYSE----RRVKAPMIRKGFKDWVEAGY 1aa6.1    SGCKINLVVDNGKIVRAEAAQ-------------GKTNQGTLCLKGYYGWDFINDTQILTPRLKTPMIRRQ---------  target    PRNDDGTPQMDVTLRGSDDWIRISWDEATTIAAKTMEDVARTFNGDEGARKLLAQGYHPEMVEVMHGAGVQALKLRGGMP 1aa6.1    ---------------RGGKLEPVSWDEALNYVAERLSAIKEKYGP--------------DAIQTTGSSRGTGN-------  target    LLGIGRIFGFYRFANMLALLDRKLRPDAPADEILGSRTFDNYAWHTDL--PPGHPMVTGSQTVDFDLFSAEHTKLLLIIG 1aa6.1    ----ETNYVMQKFAR----------------AVIGTNNVDCCARVUHGPSVAGLHQSVGNGAMSNAINEIDNTDLVFVFG  target    MNWICTKMPDGHWIGDARLKGTRVIVISADYMPTANKADEVIILRPGTDAAFFLGVARELIEKGLYDRAAVIERTDLPLL 1aa6.1    YNPADSHPIVANHVINAKRNGAKIIVCDPRKIETARIADMHIALKNGSNIALLNAMGHVIIEENLYDKAFVASRTE----  target    VRLDTGERLDARDVIPGYELAALTNYVTLKPDAEIKGNPPPPPFTAGGQVVPTELRDAWGDFVWWDRATGRPRPVSRDEV 1aa6.1    ----------------G---------------------------------------------------------------  target    GARFDGDPALLGEFEVELVDGSTVPVRPAFDLLKQYLDESFDLRTASEVCRVPPQAIQSIARQLAANKRETLLAAGMGPN 1aa6.1    -----------------------------FEEYRKIVE-GYTPESVEDITGVSASEIRQAARMYAQAKSAAI-LWGMGVT  target    HYFQNDLFGRVQFLVAALTDNIGHLGGNVGSYAGN--YRGSVFQAMGQWIAEDPFAIEPDLTKPATVKRYYKAESAHYWN 1aa6.1    QFYQGVETVRSLTSLAMLTGNLGKPHAGVNPVRGQNNVQGAC--DMGALPD--TYPGYQYVKDPANREKF-----AKAWG  target    YGERPLRAVAKDDEGDLTKGEVLTGKSHMPTPTKLIWFGNSNSLLGNAKWSFDVVKNTLPRQDAVFCNEWHWTSSCEYAD 1aa6.1    VESLPA-------HTGYRISELPHRA--AHGEVRAAYIMGEDPLQTDAE--LSAVRKAFEDLELVIVQDIFMTKTASAAD  target    LVFPADSWAEFKLPDATASCTNPFLLAFPTTPLKRLYDTRSDYEALALTAKALGELIDEPRMEQYWRGILDGDPTPYLQR 1aa6.1    VILPSTSWGEHE--GV-FTAADRGFQRF-FKAVEPKWDLKTDWQIISEIATRMGYPMHYNNTQEIW------------DE  target    IFSGSNATRGITYDELHESSKRGVPLLMNMRTYPRSGGWEQRQEDKPWYTATGRLEFYRPEPEFQAAGESLPVWREPVDA 1aa6.1    LRHLCPDFYGATYEKMGELG---FIQWPCRDTSDADQG-TSYLFKEKFDTPNGLAQFFTCD-------------------  target    TFYEPNAILSNAAHPSIAPRAPEDYGVPESQLDVETRQYRNVVRTWAELQQTLHPLQERDPAFRFVF 1aa6.1    ------------------------------------------------------------------- ``` | | | | | | | | | | | | | | | | | | | | | | | | | | | | | | | | | | | | | | | | | | | | | | | | | |
|  | 1fdo.1.A | FORMATE DEHYDROGENASE H  *OXIDIZED FORM OF FORMATE DEHYDROGENASE H FROM E. COLI* | 0.35 | 0.00 | 20.18 | 0.59 | 72-853 | X-ray | 2.80 | monomer | 1 x SF4, 2 x MGD, 1 x 6MO | HHblits | 0.31 |
| ``` target    AQGVSRRQLLGRALALGSGAALADLLGPARFLSPAGAATAGAVVPGNPLRVMPDRTWEQIYRNQFEDDSTFVFTCAPNDT 1fdo.1    -----------------------------------------------------------------------VVTVCPYCA  target    HNCLLRAHVKNGVVVRISPTYGYGEATDLYGNRASHRWDPRTCQKGLILSRRFYSE----RRVKAPMIRKGFKDWVEAGY 1fdo.1    SGCKINLVVDNGKIVRAEAAQ-------------GKTNQGTLCLKGYYGWDFINDTQILTPRLKTPMIRRQ---------  target    PRNDDGTPQMDVTLRGSDDWIRISWDEATTIAAKTMEDVARTFNGDEGARKLLAQGYHPEMVEVMHGAGVQALKLRGGMP 1fdo.1    ---------------RGGKLEPVSWDEALNYVAERLSAIKEKYGP--------------DAIQTTGSSRGTGN-------  target    LLGIGRIFGFYRFANMLALLDRKLRPDAPADEILGSRTFDNYAWHTDL--PPGHPMVTGSQTVDFDLFSAEHTKLLLIIG 1fdo.1    ----ETNYVMQKFAR----------------AVIGTNNVDCCARVUHGPSVAGLHQSVGNGAMSNAINEIDNTDLVFVFG  target    MNWICTKMPDGHWIGDARLKGTRVIVISADYMPTANKADEVIILRPGTDAAFFLGVARELIEKGLYDRAAVIERTDLPLL 1fdo.1    YNPADSHPIVANHVINAKRNGAKIIVCDPRKIETARIADMHIALKNGSNIALLNAMGHVIIEENLYDKAFVASRTE----  target    VRLDTGERLDARDVIPGYELAALTNYVTLKPDAEIKGNPPPPPFTAGGQVVPTELRDAWGDFVWWDRATGRPRPVSRDEV 1fdo.1    ----------------G---------------------------------------------------------------  target    GARFDGDPALLGEFEVELVDGSTVPVRPAFDLLKQYLDESFDLRTASEVCRVPPQAIQSIARQLAANKRETLLAAGMGPN 1fdo.1    -----------------------------FEEYRKIVE-GYTPESVEDITGVSASEIRQAARMYAQAKSAAI-LWGMGVT  target    HYFQNDLFGRVQFLVAALTDNIGHLGGNVGSYAGN--YRGSVFQAMGQWIAEDPFAIEPDLTKPATVKRYYKAESAHYWN 1fdo.1    QFYQGVETVRSLTSLAMLTGNLGKPHAGVNPVRGQNNVQGAC--DMGALPD--TYPGYQYVKDPANREKF-----AKAWG  target    YGERPLRAVAKDDEGDLTKGEVLTGKSHMPTPTKLIWFGNSNSLLGNAKWSFDVVKNTLPRQDAVFCNEWHWTSSCEYAD 1fdo.1    VESLPA-------HTGYRISELPHRA--AHGEVRAAYIMGEDPLQTDAE--LSAVRKAFEDLELVIVQDIFMTKTASAAD  target    LVFPADSWAEFKLPDATASCTNPFLLAFPTTPLKRLYDTRSDYEALALTAKALGELIDEPRMEQYWRGILDGDPTPYLQR 1fdo.1    VILPSTSWGEHE--GV-FTAADRGFQRF-FKAVEPKWDLKTDWQIISEIATRMGYPMHYNNTQEIW------------DE  target    IFSGSNATRGITYDELHESSKRGVPLLMNMRTYPRSGGWEQRQEDKPWYTATGRLEFYRPEPEFQAAGESLPVWREPVDA 1fdo.1    LRHLCPDFYGATYEKMGELG---FIQWPCRDTSDADQG-TSYLFKEKFDTPNGLAQFFTCD-------------------  target    TFYEPNAILSNAAHPSIAPRAPEDYGVPESQLDVETRQYRNVVRTWAELQQTLHPLQERDPAFRFVF 1fdo.1    ------------------------------------------------------------------- ``` | | | | | | | | | | | | | | | | | | | | | | | | | | | | | | | | | | | | | | | | | | | | | | | | | |
|  | 2iv2.1.A | Formate dehydrogenase H  *Reinterpretation of reduced form of formate dehydrogenase H from E. coli* | 0.35 | 0.00 | 20.18 | 0.59 | 72-853 | X-ray | 2.27 | monomer | 1 x SF4, 1 x 2MD, 1 x MGD | HHblits | 0.31 |
| ``` target    AQGVSRRQLLGRALALGSGAALADLLGPARFLSPAGAATAGAVVPGNPLRVMPDRTWEQIYRNQFEDDSTFVFTCAPNDT 2iv2.1    -----------------------------------------------------------------------VVTVCPYCA  target    HNCLLRAHVKNGVVVRISPTYGYGEATDLYGNRASHRWDPRTCQKGLILSRRFYSE----RRVKAPMIRKGFKDWVEAGY 2iv2.1    SGCKINLVVDNGKIVRAEAAQ-------------GKTNQGTLCLKGYYGWDFINDTQILTPRLKTPMIRRQ---------  target    PRNDDGTPQMDVTLRGSDDWIRISWDEATTIAAKTMEDVARTFNGDEGARKLLAQGYHPEMVEVMHGAGVQALKLRGGMP 2iv2.1    ---------------RGGKLEPVSWDEALNYVAERLSAIKEKYGP--------------DAIQTTGSSRGTGN-------  target    LLGIGRIFGFYRFANMLALLDRKLRPDAPADEILGSRTFDNYAWHTDL--PPGHPMVTGSQTVDFDLFSAEHTKLLLIIG 2iv2.1    ----ETNYVMQKFAR----------------AVIGTNNVDCCARVUHGPSVAGLHQSVGNGAMSNAINEIDNTDLVFVFG  target    MNWICTKMPDGHWIGDARLKGTRVIVISADYMPTANKADEVIILRPGTDAAFFLGVARELIEKGLYDRAAVIERTDLPLL 2iv2.1    YNPADSHPIVANHVINAKRNGAKIIVCDPRKIETARIADMHIALKNGSNIALLNAMGHVIIEENLYDKAFVASRTE----  target    VRLDTGERLDARDVIPGYELAALTNYVTLKPDAEIKGNPPPPPFTAGGQVVPTELRDAWGDFVWWDRATGRPRPVSRDEV 2iv2.1    ----------------G---------------------------------------------------------------  target    GARFDGDPALLGEFEVELVDGSTVPVRPAFDLLKQYLDESFDLRTASEVCRVPPQAIQSIARQLAANKRETLLAAGMGPN 2iv2.1    -----------------------------FEEYRKIVE-GYTPESVEDITGVSASEIRQAARMYAQAKSAAI-LWGMGVT  target    HYFQNDLFGRVQFLVAALTDNIGHLGGNVGSYAGN--YRGSVFQAMGQWIAEDPFAIEPDLTKPATVKRYYKAESAHYWN 2iv2.1    QFYQGVETVRSLTSLAMLTGNLGKPHAGVNPVRGQNNVQGAC--DMGALPD--TYPGYQYVKDPANREKF-----AKAWG  target    YGERPLRAVAKDDEGDLTKGEVLTGKSHMPTPTKLIWFGNSNSLLGNAKWSFDVVKNTLPRQDAVFCNEWHWTSSCEYAD 2iv2.1    VESLPA-------HTGYRISELPHRA--AHGEVRAAYIMGEDPLQTDAE--LSAVRKAFEDLELVIVQDIFMTKTASAAD  target    LVFPADSWAEFKLPDATASCTNPFLLAFPTTPLKRLYDTRSDYEALALTAKALGELIDEPRMEQYWRGILDGDPTPYLQR 2iv2.1    VILPSTSWGEHE--GV-FTAADRGFQRF-FKAVEPKWDLKTDWQIISEIATRMGYPMHYNNTQEIW------------DE  target    IFSGSNATRGITYDELHESSKRGVPLLMNMRTYPRSGGWEQRQEDKPWYTATGRLEFYRPEPEFQAAGESLPVWREPVDA 2iv2.1    LRHLCPDFYGATYEKMGELG---FIQWPCRDTSDADQG-TSYLFKEKFDTPNGLAQFFTCD-------------------  target    TFYEPNAILSNAAHPSIAPRAPEDYGVPESQLDVETRQYRNVVRTWAELQQTLHPLQERDPAFRFVF 2iv2.1    ------------------------------------------------------------------- ``` | | | | | | | | | | | | | | | | | | | | | | | | | | | | | | | | | | | | | | | | | | | | | | | | | |
|  | 7z0t.1.G | Formate dehydrogenase H  *Structure of the Escherichia coli formate hydrogenlyase complex (aerobic preparation, composite structure)* | 0.35 | 0.00 | 20.18 | 0.59 | 72-853 | EM | 0.00 | monomer | 1 x NI, 1 x FCO, 8 x SF4, 1 x FE, 2 x MGD, 1 x 6MO | HHblits | 0.31 |
| ``` target    AQGVSRRQLLGRALALGSGAALADLLGPARFLSPAGAATAGAVVPGNPLRVMPDRTWEQIYRNQFEDDSTFVFTCAPNDT 7z0t.1    -----------------------------------------------------------------------VVTVCPYCA  target    HNCLLRAHVKNGVVVRISPTYGYGEATDLYGNRASHRWDPRTCQKGLILSRRFYSE----RRVKAPMIRKGFKDWVEAGY 7z0t.1    SGCKINLVVDNGKIVRAEAAQ-------------GKTNQGTLCLKGYYGWDFINDTQILTPRLKTPMIRRQ---------  target    PRNDDGTPQMDVTLRGSDDWIRISWDEATTIAAKTMEDVARTFNGDEGARKLLAQGYHPEMVEVMHGAGVQALKLRGGMP 7z0t.1    ---------------RGGKLEPVSWDEALNYVAERLSAIKEKYGP--------------DAIQTTGSSRGTGN-------  target    LLGIGRIFGFYRFANMLALLDRKLRPDAPADEILGSRTFDNYAWHTDL--PPGHPMVTGSQTVDFDLFSAEHTKLLLIIG 7z0t.1    ----ETNYVMQKFAR----------------AVIGTNNVDCCARVUHGPSVAGLHQSVGNGAMSNAINEIDNTDLVFVFG  target    MNWICTKMPDGHWIGDARLKGTRVIVISADYMPTANKADEVIILRPGTDAAFFLGVARELIEKGLYDRAAVIERTDLPLL 7z0t.1    YNPADSHPIVANHVINAKRNGAKIIVCDPRKIETARIADMHIALKNGSNIALLNAMGHVIIEENLYDKAFVASRTE----  target    VRLDTGERLDARDVIPGYELAALTNYVTLKPDAEIKGNPPPPPFTAGGQVVPTELRDAWGDFVWWDRATGRPRPVSRDEV 7z0t.1    ----------------G---------------------------------------------------------------  target    GARFDGDPALLGEFEVELVDGSTVPVRPAFDLLKQYLDESFDLRTASEVCRVPPQAIQSIARQLAANKRETLLAAGMGPN 7z0t.1    -----------------------------FEEYRKIVE-GYTPESVEDITGVSASEIRQAARMYAQAKSAAI-LWGMGVT  target    HYFQNDLFGRVQFLVAALTDNIGHLGGNVGSYAGN--YRGSVFQAMGQWIAEDPFAIEPDLTKPATVKRYYKAESAHYWN 7z0t.1    QFYQGVETVRSLTSLAMLTGNLGKPHAGVNPVRGQNNVQGAC--DMGALPD--TYPGYQYVKDPANREKF-----AKAWG  target    YGERPLRAVAKDDEGDLTKGEVLTGKSHMPTPTKLIWFGNSNSLLGNAKWSFDVVKNTLPRQDAVFCNEWHWTSSCEYAD 7z0t.1    VESLPA-------HTGYRISELPHRA--AHGEVRAAYIMGEDPLQTDAE--LSAVRKAFEDLELVIVQDIFMTKTASAAD  target    LVFPADSWAEFKLPDATASCTNPFLLAFPTTPLKRLYDTRSDYEALALTAKALGELIDEPRMEQYWRGILDGDPTPYLQR 7z0t.1    VILPSTSWGEHE--GV-FTAADRGFQRF-FKAVEPKWDLKTDWQIISEIATRMGYPMHYNNTQEIW------------DE  target    IFSGSNATRGITYDELHESSKRGVPLLMNMRTYPRSGGWEQRQEDKPWYTATGRLEFYRPEPEFQAAGESLPVWREPVDA 7z0t.1    LRHLCPDFYGATYEKMGELG---FIQWPCRDTSDADQG-TSYLFKEKFDTPNGLAQFFTCD-------------------  target    TFYEPNAILSNAAHPSIAPRAPEDYGVPESQLDVETRQYRNVVRTWAELQQTLHPLQERDPAFRFVF 7z0t.1    ------------------------------------------------------------------- ``` | | | | | | | | | | | | | | | | | | | | | | | | | | | | | | | | | | | | | | | | | | | | | | | | | |
|  | 1h0h.1.A | FORMATE DEHYDROGENASE SUBUNIT ALPHA  *Tungsten containing Formate Dehydrogenase from Desulfovibrio Gigas* | 0.32 |  | 18.04 | 0.60 | 70-767 | X-ray | 1.80 | hetero-1-1-mer | 1 x W, 1 x 2MD, 1 x MGD, 4 x SF4, 1 x CA | HHblits | 0.29 |
| ``` target    AQGVSRRQLLGRALALGSGAALADLLGPARFLSPAGAATAGAVVPGNPLRVMPDRTWEQIYRNQFEDDSTFVFTCAPNDT 1h0h.1    ---------------------------------------------------------------------KQTTSVCCYCS  target    HNCLLRAHV--KNGVVVRISPTYGYGEATDLYGNRASHRWDPRTCQKGLILSRRFYSERRVKAPMIRKGFKDWVEAGYPR 1h0h.1    VGCGLIVHTDKKTNRAINVEGD------------PDHPINEGSLCAKGASTWQLAENERRPANPLYRA------------  target    NDDGTPQMDVTLRGSDDWIRISWDEATTIAAKTMEDVARTFNGDEGARKLLAQGYHPEMVEVMHGAGVQALKLRGGMPLL 1h0h.1    ------------PGSDQWEEKSWDWMLDTIAERVAKTREATFVTKNAKGQV--VNRCDGIASV-----------------  target    GIGRIFGFYRFANMLALLDRKLRPDAPADEILGSRTFDNYAWHTDL--PPGHPMVTGSQTVDFDLFSAEHTKLLLIIGMN 1h0h.1    ------GSAAMDNEECWIYQAWL------RSLGLFYIEHQARIUHSATVAALAESYGRGAMTNHWIDLKNSDVILMMGSN  target    WICTKMPDGHWIGDARLKGTRVIVISADYMPTANKADEVIILRPGTDAAFFLGVARELIEKGLYDRAAVIERTDLPLLVR 1h0h.1    PAENHPISFKWVMRAKDKGATLIHVDPRYTRTSTKCDLYAPLRSGSDIAFLNGMTKYILEKELYFKDYVVNYTNASFIVG  target    LDTGERLDARDVIPGYELAALTNYVTLKPDAEIKGNPPPPPFTAGGQVVPTELRDAWGDFVWWDRATGRPRPVSRDEVGA 1h0h.1    EGF---AFEEGL-----------------------------------------------FAGYNKETRKYDKSKW-----  target    RFDGDPALLGEFEVELVDGSTVPVRPAFDLLKQYLDESFDLRTASEVCRVPPQAIQSIARQLAANK---RETLLAAGMGP 1h0h.1    --GFERDENGNP---KRDETLKHPRCVFQIMKKHYE-RYDLDKISAICGTPKELILKVYDAYCATGKPDKAGTIMYAMGW  target    NHYFQNDLFGRVQFLVAALTDNIGHLGGNVGSYAGNY--RGSVFQAMGQWIAEDPFAIEPDLTK-PA---TVKRYY---K 1h0h.1    TQHTVGVQNIRAMSINQLLLGNIGVAGGGVNALRGEANVQGST--DHGLLMHIYPGYLGTARASIPTYEEYTKKFTPVSK  target    A-ESAHYWN-YGER--PLR--AVAKDD-------------EGDLTKGEVLTGKSHMPTPTKLIWFGNSNSLLGNAKWSFD 1h0h.1    DPQSANWWSNFPKYSASYIKSMWPDADLNEAYGYLPKGEDGKDYSWLTLFDDM--FQGKIKGFFAWGQNPACSGAN--SN  target    VVKNTLPRQDAVFCNEWHWTSSCEYA-------------DLVFPADSWAEFKLPDATASCTNPFLLAFPTTPLKRLYDTR 1h0h.1    KTREALTKLDWMVNVNIFDNETGSFWRGPDMDPKKIKTEVFFLPCAVAIEKE--GS-ISNSGRWMQW-RYVGPEPRKNAI  target    SDYEALALTAKALGELIDEPRMEQYWRGILDGDPTPYLQRIFSGSNATRGITYDELHESSKRGVPLLMNMRTYPRSGGWE 1h0h.1    PDGDLIVELAKRVQK-----------------------------------------------------------------  target    QRQEDKPWYTATGRLEFYRPEPEFQAAGESLPVWREPVDATFYEPNAILSNAAHPSIAPRAPEDYGVPESQLDVETRQYR 1h0h.1    --------------------------------------------------------------------------------  target    NVVRTWAELQQTLHPLQERDPAFRFVF 1h0h.1    --------------------------- ``` | | | | | | | | | | | | | | | | | | | | | | | | | | | | | | | | | | | | | | | | | | | | | | | | | |
|  | 7bkb.1.F | Formate dehydrogenase  *Formate dehydrogenase - heterodisulfide reductase - formylmethanofuran dehydrogenase complex from Methanospirillum hungatei (hexameric, composite structure)* | 0.34 |  | 21.83 | 0.58 | 69-852 | EM | 0.00 | hetero-2-2-2-2-2-2-… | 48 x SF4, 4 x FAD, 2 x FES, 4 x 9S8, 4 x ZN, 2 x MO, 4 x MGD | HHblits | 0.31 |
| ``` target    AQGVSRRQLLGRALALGSGAALADLLGPARFLSPAGAATAGAVVPGNPLRVMPDRTWEQIYRNQFEDDSTFVFTCAPNDT 7bkb.1    --------------------------------------------------------------------MKYVATTCPYCG  target    HNCLLRAHVKNGVVVRISPTYGYGEATDLYGNRASHRWDPRTCQKGLILSRRFYSERRVKAPMIRKGFKDWVEAGYPRND 7bkb.1    VGCTLNLVVSNGKVVGVEPN------------QRSPINEGKLCPKGVTCWEHIHSPDRLTTPLIKKD-------------  target    DGTPQMDVTLRGSDDWIRISWDEATTIAAKTMEDVARTFNGDEGARKLLAQGYHPEMVEVMHGAGVQALKLRGGMPLLGI 7bkb.1    -------------GKFIEASWDEALDLVAKNLKVIYDKHGP--------------KGLGFQTSC----------------  target    GRIFGFYRFANMLALLDRKLRPDAPADEILGSRTFDNYAWHTDLP--PGHPMVTGSQTVDFDLFSAEHTKLLLIIGMNWI 7bkb.1    -------RTVNEDCYIFQKFA-----RVGFKTNNVDNCARICHGPSVAGLSLSFGSGAATNGFEDALNADLILIWGSNAV  target    CTKMPDGHWIGDARLKGTRVIVISADYMPTANKADEVIILRPGTDAAFFLGVARELIEKGLYDRAAVIERTDLPLLVRLD 7bkb.1    EAHPLAGRRIAQAKKKGIQIIAVDPRYTMTARLADTYVRFNPSTHIALANSMMYWIIKEGLEDKKFIQDRVN--------  target    TGERLDARDVIPGYELAALTNYVTLKPDAEIKGNPPPPPFTAGGQVVPTELRDAWGDFVWWDRATGRPRPVSRDEVGARF 7bkb.1    ------------G-------------------------------------------------------------------  target    DGDPALLGEFEVELVDGSTVPVRPAFDLLKQYLDESFDLRTASEVCRVPPQAIQSIARQLAANKRETLLAAGMGPNHYFQ 7bkb.1    -------------------------FEDLKKTVE-NY--ADAEAIHGVPLDVVKDIAFRYAKAKNA-VIIYCLGITELTT  target    NDLFGRVQFLVAALTDNIGHLGGNVGSYAGN--YRGSVFQAMGQWIAEDPFAIEPDLTKPATVKRYYKAESAHYWNYGER 7bkb.1    GTDNVRSMGNLALLTGNVGREGVGVNPLRGQNNVQGAC--DMGAYPN--VYSGYQKCEVAENRA-----KMEKAWSVTNL  target    PLRAVAKDDEGDLTKGEVLTGKSHMPTPTKLIWFGNSNSLLGNAKWSFDVVKNTLPRQDAVFCNEWHWTSSCEYADLVFP 7bkb.1    P--DW-----YGATLTEQINQ---CGDEIKGMYILGLNPVVTYPS--SNHVKAQLEKLDFLVVQDIFFTETCQYADVILP  target    ADSWAEFKLPDATASCTNPFLLAFPTTPLKRLYDTRSDYEALALTAKALGELI-DEPRMEQYWRGILDGDPTPYLQRIFS 7bkb.1    GACFAEKD---GTFTSGERRINRV-RKAVNPPGQAKEDIHIISELAAKMGFKGFELPTAKD------------VWDDMRA  target    GSNATRGITYDELHESSKRGVPLLMNMRTYPRSGGWEQRQEDKPWYTATGRLEFYRPEPEFQAAGESLPVWREPVDATFY 7bkb.1    VTPSMFGATYEKLERPE--GICWPCPTEEHPGTP----ILHREKFATADGKGNLFGI-----------------------  target    EPNAILSNAAHPSIAPRAPEDYGVPESQLDVETRQYRNVVRTWAELQQTLHPLQERDPAFRFVF 7bkb.1    ---------------------------------------------------------------- ``` | | | | | | | | | | | | | | | | | | | | | | | | | | | | | | | | | | | | | | | | | | | | | | | | | |
|  | 1g8k.1.A | ARSENITE OXIDASE  *CRYSTAL STRUCTURE ANALYSIS OF ARSENITE OXIDASE FROM ALCALIGENES FAECALIS* | 0.30 |  | 16.78 | 0.60 | 70-852 | X-ray | 1.64 | hetero-1-1-mer | 3 x HG, 2 x CA, 2 x MGD, 1 x O, 1 x 4MO, 1 x F3S, 1 x FES | HHblits | 0.28 |
| ``` target    AQGVSRRQLLGRALALGSGAALADLLGPARFLSPAGAATAGAVVPGNPLRVMPDRTWEQIYRNQFEDDSTFVFTCAPNDT 1g8k.1    ---------------------------------------------------------------------QRTNMTCHFCI  target    HNCLLRAHVKN-----GV--------------------------------------VVRISPTYGYGEATDLYGNRASHR 1g8k.1    VGCGYHVYKWPELEEGGRAPEQNALGLDFRKQLPPLAVTLTPAMTNVVTEHDGARYDIMVVP------------DKACVV  target    WDPRTCQKGLILSRRFYSE-----RRVKAPMIRKGFKDWVEAGYPRNDDGTPQMDVTLRGSDDWIRISWDEATTIAAKTM 1g8k.1    NSGLSSTRGGKMASYMYTPTGDGKERLSAPRLYAA--------------------------DEWVDTTWDHAMALYAGLI  target    EDVARTFNGDEGARKLLAQGYHPEMVEVMHGAGVQALKLRGGMPLLGIGRIFGFYRFANMLALLDRKLRPDAPADEILGS 1g8k.1    KKTLDKDG--------------PQGVFFSCFDHG-------------------GAGGGFENTWGTGKL-----MFSAIQT  target    RTFDNYAW--HTDLPPGHPMVTGSQTVDFDLFSAEHTKLLLIIGMNWICTKMPD--GHWI---------------GDARL 1g8k.1    PMVRIHNRPAYNSECH-ATREMGIGELNNAYEDAQLADVIWSIGNNPYESQTNYFLNHWLPNLQGATTSKKKERFPNENF  target    KGTRVIVISADYMPTANKA--------DEVIILRPGTDAAFFLGVARELIEKGLYDRAAVIERTDLPLLVRLDTGERLDA 1g8k.1    PQARIIFVDPRETPSVAIARHVAGNDRVLHLAIEPGTDTALFNGLFTYVVEQGWIDKPFIEAHTK---------------  target    RDVIPGYELAALTNYVTLKPDAEIKGNPPPPPFTAGGQVVPTELRDAWGDFVWWDRATGRPRPVSRDEVGARFDGDPALL 1g8k.1    -----GF-------------------------------------------------------------------------  target    GEFEVELVDGSTVPVRPAFDLLKQYLDESFDLRTASEVCRVPPQAIQSIARQLAANK-----RETLLAAGMGPNHYFQND 1g8k.1    -------------------DDAVK-T-NRLSLDECSNITGVPVDMLKRAAEWSYKPKASGQAPRTMHAYEKGIIWGNDNY  target    LFGRVQFLVAALTDNIGHLGGNVGSYAGNYRGSVFQAMGQWIAEDPFAIEPDLTKPATVKRYYKAESAHYWNYGERPLRA 1g8k.1    VIQSALLDLVIATHNVGRRGTGCVRMGGHQEGYTR---PPY----P--GDKKIYIDQEL---IKGKGRIMTWWGCNNFQT  target    VAKDDEGDLTKGEVLTGKSHMPTPTKLIWFGNSNSLLGNAKWSFDVVKNTLPR-QDAVFCNEWHWTSSCEYADLVFPADS 1g8k.1    SNN---A-QALREAILQR--SAIVKQAMQKARGATTEE----MVDVIYEATQNGGLFVTSINLYPTKLAEAAHLMLPAAH  target    WAEFKLPDATASCTNPFLLAFPTTPLKRLYDTRSDYEALALTAKALGELIDEP---RMEQYWRGILDGDPTPYLQRIFSG 1g8k.1    PGEMN---LTSMNGERRIRLS-EKFMDPPGTAMADCLIAARIANALRDMYQKDGKAEMAAQFEGFDWKTEEDAFNDGFRR  target    SN-------------ATRGITYDELHESSKRGVPLLMNMRT-YPRSGGWEQRQEDKPWYTATGRLEFYRPEPEFQAAGES 1g8k.1    AGQPGAPAIDSQGGSTGHLVTYDRLRKSGNNGVQLPVVSWDESKGLVGTEMLYTEGKFDTDDGKAHFKPA----------  target    LPVWREPVDATFYEPNAILSNAAHPSIAPRAPEDYGVPESQLDVETRQYRNVVRTWAELQQTLHPLQERDPAFRFVF 1g8k.1    ----------------------------------------------------------------------------- ``` | | | | | | | | | | | | | | | | | | | | | | | | | | | | | | | | | | | | | | | | | | | | | | | | | |
|  | 3ir5.1.A | Respiratory nitrate reductase 1 alpha chain  *Crystal structure of NarGHI mutant NarG-H49C* | 0.26 | 0.00 | 31.76 | 0.54 | 54-630 | X-ray | 2.30 | monomer | 2 x MD1, 1 x 6MO, 4 x SF4, 1 x AGA, 1 x F3S, 2 x HEM | BLAST | 0.36 |
| ``` target    AQGVSRRQLLGRALALGSGAALADLLGPARFLSPAGAATAGAVVPGNPLRVMPDRTWEQIYRNQFEDDSTFVFTCAPNDT 3ir5.1    -----------------------------------------------------NRDWEDGYRQRWQHDKIVRSTCGVNCT  target    HNCLLRAHVKNGVVVRISPTYGYGEATDLYGNRASHRWDPRTCQKGLILSRRFYSERRVKAPMIRKGF-KDWVEAGYPRN 3ir5.1    GSCSWKIYVKNGLVTWETQQTDYPRTRPDLPNH-----EPRGCPRGASYSWYLYSANRLKYPMMRKRLMKMWREAKALHS  target    DDGTPQMDVT-----------LRGSDDWIRISWDEATTIAAKTMEDVARTFNGDEGARKLLAQGYHP--EMVEVMHGAGV 3ir5.1    DPVEAWASIIEDADKAKSFKQARGRGGFVRSSWQEVNELIAASNVYTIKNYGPDRVA------GFSPIPAMSMVSYASGA  target    QALKLRGGMPLLGIGRIFGFYRFANMLALLDRKLRPDAPADEILGSRTFDNYAWHTDLPPGHPMVTGSQTVDFDLFSAEH 3ir5.1    RYLSLIGGTCL------------------------------------SF--YDWYCDLPPASPQTWGEQTDVPESADWYN  target    TKLLLIIGMNWICTKMPDGHWIGDARLKGTRVIVISADYMPTANKADEVIILRPGTDAAFFLGVARELIEKGLYDRAA-- 3ir5.1    SSYIIAWGSNVPQTRTPDAHFFTEVRYKGTKTVAVTPDYAEIAKLCDLWLAPKQGTDAAMALAMGHVMLREFHLDNPSQY  target    ----VIERTDLPLLVRLDTGERLDARDVIPGYELAALTNYVTLKPDAEIKGNPP---PPPFTAGGQVVP--TELRDAWGD 3ir5.1    FTDYVRRYTDMPMLVMLE------ERD---GYYAAGRMLRAADLVDALGQENNPEWKTVAFNTNGEMVAPNGSIGFRWGE  target    FVWW-----DRATGRPRPV------SRDEVGA----RFDGD-----------PALLGEFEV---ELVDGSTVPVRPAFDL 3ir5.1    KGKWNLEQRDGKTGEETELQLSLLGSQDEIAEVGFPYFGGDGTEHFNKVELENVLLHKLPVKRLQLADGSTALVTTVYDL  target    ------LKQYLDE-----SFDLRTA------SEVCRVPPQAIQSIARQLAANKRET----LLAAGMGPNHYFQNDLFGRV 3ir5.1    TLANYGLERGLNDVNCATSYDDVKAYTPAWAEQITGVSRSQIIRIAREFADNADKTHGRSMIIVGAGLNHWYHLDMNYRG  target    QFLVAALTDNIGHLGGNVGSYAGNYRGSVFQAMGQWIAEDPFAIEPDLTKPATVKRYYKAESAHYWNYGERPLRAVAKDD 3ir5.1    LINMLIFCGCVGQSGGGWAHYVGQEK---LRPQTGW---QPLAFALDWQRPA---RHMNSTSYFY---------------  target    EGDLTKGEVLTGKSHMPTPTKLIWFGNSNSLLGNAKWSFDVVKNTLPRQDAVFCNEWHWTSSCEYADLVFPADSWAEFKL 3ir5.1    --------------------------------------------------------------------------------  target    PDATASCTNPFLLAFPTTPLKRLYDTRSDYEALALTAKALGELIDEPRMEQYWRGILDGDPTPYLQRIFSGSNATRGITY 3ir5.1    --------------------------------------------------------------------------------  target    DELHESSKRGVPLLMNMRTYPRSGGWEQRQEDKPWYTATGRLEFYRPEPEFQAAGESLPVWREPVDATFYEPNAILSNAA 3ir5.1    --------------------------------------------------------------------------------  target    HPSIAPRAPEDYGVPESQLDVETRQYRNVVRTWAELQQTLHPLQERDPAFRFVF 3ir5.1    ------------------------------------------------------ ``` | | | | | | | | | | | | | | | | | | | | | | | | | | | | | | | | | | | | | | | | | | | | | | | | | |
|  | 3ir6.1.A | Respiratory nitrate reductase 1 alpha chain  *Crystal structure of NarGHI mutant NarG-H49S* | 0.26 | 0.00 | 31.57 | 0.54 | 54-630 | X-ray | 2.80 | monomer | 2 x GDP, 1 x AGA, 3 x SF4, 1 x F3S, 2 x HEM | BLAST | 0.36 |
| ``` target    AQGVSRRQLLGRALALGSGAALADLLGPARFLSPAGAATAGAVVPGNPLRVMPDRTWEQIYRNQFEDDSTFVFTCAPNDT 3ir6.1    -----------------------------------------------------NRDWEDGYRQRWQHDKIVRSTSGVNCT  target    HNCLLRAHVKNGVVVRISPTYGYGEATDLYGNRASHRWDPRTCQKGLILSRRFYSERRVKAPMIRKGF-KDWVEAGYPRN 3ir6.1    GSCSWKIYVKNGLVTWETQQTDYPRTRPDLPNH-----EPRGCPRGASYSWYLYSANRLKYPMMRKRLMKMWREAKALHS  target    DDGTPQMDVT-----------LRGSDDWIRISWDEATTIAAKTMEDVARTFNGDEGARKLLAQGYHP--EMVEVMHGAGV 3ir6.1    DPVEAWASIIEDADKAKSFKQARGRGGFVRSSWQEVNELIAASNVYTIKNYGPDRVA------GFSPIPAMSMVSYASGA  target    QALKLRGGMPLLGIGRIFGFYRFANMLALLDRKLRPDAPADEILGSRTFDNYAWHTDLPPGHPMVTGSQTVDFDLFSAEH 3ir6.1    RYLSLIGGTCL------------------------------------SF--YDWYCDLPPASPQTWGEQTDVPESADWYN  target    TKLLLIIGMNWICTKMPDGHWIGDARLKGTRVIVISADYMPTANKADEVIILRPGTDAAFFLGVARELIEKGLYDRAA-- 3ir6.1    SSYIIAWGSNVPQTRTPDAHFFTEVRYKGTKTVAVTPDYAEIAKLCDLWLAPKQGTDAAMALAMGHVMLREFHLDNPSQY  target    ----VIERTDLPLLVRLDTGERLDARDVIPGYELAALTNYVTLKPDAEIKGNPP---PPPFTAGGQVVP--TELRDAWGD 3ir6.1    FTDYVRRYTDMPMLVMLE------ERD---GYYAAGRMLRAADLVDALGQENNPEWKTVAFNTNGEMVAPNGSIGFRWGE  target    FVWW-----DRATGRPRPV------SRDEVGA----RFDGD-----------PALLGEFEV---ELVDGSTVPVRPAFDL 3ir6.1    KGKWNLEQRDGKTGEETELQLSLLGSQDEIAEVGFPYFGGDGTEHFNKVELENVLLHKLPVKRLQLADGSTALVTTVYDL  target    ------LKQYLDE-----SFDLRTA------SEVCRVPPQAIQSIARQLAANKRET----LLAAGMGPNHYFQNDLFGRV 3ir6.1    TLANYGLERGLNDVNCATSYDDVKAYTPAWAEQITGVSRSQIIRIAREFADNADKTHGRSMIIVGAGLNHWYHLDMNYRG  target    QFLVAALTDNIGHLGGNVGSYAGNYRGSVFQAMGQWIAEDPFAIEPDLTKPATVKRYYKAESAHYWNYGERPLRAVAKDD 3ir6.1    LINMLIFCGCVGQSGGGWAHYVGQEK---LRPQTGW---QPLAFALDWQRPA---RHMNSTSYFY---------------  target    EGDLTKGEVLTGKSHMPTPTKLIWFGNSNSLLGNAKWSFDVVKNTLPRQDAVFCNEWHWTSSCEYADLVFPADSWAEFKL 3ir6.1    --------------------------------------------------------------------------------  target    PDATASCTNPFLLAFPTTPLKRLYDTRSDYEALALTAKALGELIDEPRMEQYWRGILDGDPTPYLQRIFSGSNATRGITY 3ir6.1    --------------------------------------------------------------------------------  target    DELHESSKRGVPLLMNMRTYPRSGGWEQRQEDKPWYTATGRLEFYRPEPEFQAAGESLPVWREPVDATFYEPNAILSNAA 3ir6.1    --------------------------------------------------------------------------------  target    HPSIAPRAPEDYGVPESQLDVETRQYRNVVRTWAELQQTLHPLQERDPAFRFVF 3ir6.1    ------------------------------------------------------ ``` | | | | | | | | | | | | | | | | | | | | | | | | | | | | | | | | | | | | | | | | | | | | | | | | | |
|  | 1r27.4.A | Respiratory nitrate reductase 1 alpha chain  *Crystal Structure of NarGH complex* | 0.26 | 0.09 | 31.57 | 0.54 | 54-630 | X-ray | 2.00 | homo-dimer | 4 x MO, 16 x SF4, 8 x MGD, 4 x F3S | BLAST | 0.36 |
| ``` target    AQGVSRRQLLGRALALGSGAALADLLGPARFLSPAGAATAGAVVPGNPLRVMPDRTWEQIYRNQFEDDSTFVFTCAPNDT 1r27.4    -----------------------------------------------------NRDWEDGYRQRWQHDKIVRSTHGVNCT  target    HNCLLRAHVKNGVVVRISPTYGYGEATDLYGNRASHRWDPRTCQKGLILSRRFYSERRVKAPMIRKGF-KDWVEAGYPRN 1r27.4    GSCSWKIYVKNGLVTWETQQTDYPRTRPDLPNH-----EPRGCPRGASYSWYLYSANRLKYPMMRKRLMKMWREAKALHS  target    DDGTPQMDVT-----------LRGSDDWIRISWDEATTIAAKTMEDVARTFNGDEGARKLLAQGYHP--EMVEVMHGAGV 1r27.4    DPVEAWASIIEDADKAKSFKQARGRGGFVRSSWQEVNELIAASNVYTIKNYGPDRVA------GFSPIPAMSMVSYASGA  target    QALKLRGGMPLLGIGRIFGFYRFANMLALLDRKLRPDAPADEILGSRTFDNYAWHTDLPPGHPMVTGSQTVDFDLFSAEH 1r27.4    RYLSLIGGTCL------------------------------------SF--YDWYCDLPPASPQTWGEQTDVPESADWYN  target    TKLLLIIGMNWICTKMPDGHWIGDARLKGTRVIVISADYMPTANKADEVIILRPGTDAAFFLGVARELIEKGLYDRAA-- 1r27.4    SSYIIAWGSNVPQTRTPDAHFFTEVRYKGTKTVAVTPDYAEIAKLCDLWLAPKQGTDAAMALAMGHVMLREFHLDNPSQY  target    ----VIERTDLPLLVRLDTGERLDARDVIPGYELAALTNYVTLKPDAEIKGNPP---PPPFTAGGQVVP--TELRDAWGD 1r27.4    FTDYVRRYTDMPMLVMLE------ERD---GYYAAGRMLRAADLVDALGQENNPEWKTVAFNTNGEMVAPNGSIGFRWGE  target    FVWW-----DRATGRPRPV------SRDEVGA----RFDGD-----------PALLGEFEV---ELVDGSTVPVRPAFDL 1r27.4    KGKWNLEQRDGKTGEETELQLSLLGSQDEIAEVGFPYFGGDGTEHFNKVELENVLLHKLPVKRLQLADGSTALVTTVYDL  target    ------LKQYLDE-----SFDLRTA------SEVCRVPPQAIQSIARQLAANKRET----LLAAGMGPNHYFQNDLFGRV 1r27.4    TLANYGLERGLNDVNCATSYDDVKAYTPAWAEQITGVSRSQIIRIAREFADNADKTHGRSMIIVGAGLNHWYHLDMNYRG  target    QFLVAALTDNIGHLGGNVGSYAGNYRGSVFQAMGQWIAEDPFAIEPDLTKPATVKRYYKAESAHYWNYGERPLRAVAKDD 1r27.4    LINMLIFCGCVGQSGGGWAHYVGQEK---LRPQTGW---QPLAFALDWQRPA---RHMNSTSYFY---------------  target    EGDLTKGEVLTGKSHMPTPTKLIWFGNSNSLLGNAKWSFDVVKNTLPRQDAVFCNEWHWTSSCEYADLVFPADSWAEFKL 1r27.4    --------------------------------------------------------------------------------  target    PDATASCTNPFLLAFPTTPLKRLYDTRSDYEALALTAKALGELIDEPRMEQYWRGILDGDPTPYLQRIFSGSNATRGITY 1r27.4    --------------------------------------------------------------------------------  target    DELHESSKRGVPLLMNMRTYPRSGGWEQRQEDKPWYTATGRLEFYRPEPEFQAAGESLPVWREPVDATFYEPNAILSNAA 1r27.4    --------------------------------------------------------------------------------  target    HPSIAPRAPEDYGVPESQLDVETRQYRNVVRTWAELQQTLHPLQERDPAFRFVF 1r27.4    ------------------------------------------------------ ``` | | | | | | | | | | | | | | | | | | | | | | | | | | | | | | | | | | | | | | | | | | | | | | | | | |
|  | 1q16.1.A | Respiratory nitrate reductase 1 alpha chain  *Crystal structure of Nitrate Reductase A, NarGHI, from Escherichia coli* | 0.26 | 0.00 | 31.57 | 0.54 | 54-630 | X-ray | 1.90 | monomer | 2 x MD1, 1 x 6MO, 2 x HEM, 4 x SF4, 1 x F3S, 1 x AGA, 1 x 3PH | BLAST | 0.36 |
| ``` target    AQGVSRRQLLGRALALGSGAALADLLGPARFLSPAGAATAGAVVPGNPLRVMPDRTWEQIYRNQFEDDSTFVFTCAPNDT 1q16.1    -----------------------------------------------------NRDWEDGYRQRWQHDKIVRSTHGVNCT  target    HNCLLRAHVKNGVVVRISPTYGYGEATDLYGNRASHRWDPRTCQKGLILSRRFYSERRVKAPMIRKGF-KDWVEAGYPRN 1q16.1    GSCSWKIYVKNGLVTWETQQTDYPRTRPDLPNH-----EPRGCPRGASYSWYLYSANRLKYPMMRKRLMKMWREAKALHS  target    DDGTPQMDVT-----------LRGSDDWIRISWDEATTIAAKTMEDVARTFNGDEGARKLLAQGYHP--EMVEVMHGAGV 1q16.1    DPVEAWASIIEDADKAKSFKQARGRGGFVRSSWQEVNELIAASNVYTIKNYGPDRVA------GFSPIPAMSMVSYASGA  target    QALKLRGGMPLLGIGRIFGFYRFANMLALLDRKLRPDAPADEILGSRTFDNYAWHTDLPPGHPMVTGSQTVDFDLFSAEH 1q16.1    RYLSLIGGTCL------------------------------------SF--YDWYCDLPPASPQTWGEQTDVPESADWYN  target    TKLLLIIGMNWICTKMPDGHWIGDARLKGTRVIVISADYMPTANKADEVIILRPGTDAAFFLGVARELIEKGLYDRAA-- 1q16.1    SSYIIAWGSNVPQTRTPDAHFFTEVRYKGTKTVAVTPDYAEIAKLCDLWLAPKQGTDAAMALAMGHVMLREFHLDNPSQY  target    ----VIERTDLPLLVRLDTGERLDARDVIPGYELAALTNYVTLKPDAEIKGNPP---PPPFTAGGQVVP--TELRDAWGD 1q16.1    FTDYVRRYTDMPMLVMLE------ERD---GYYAAGRMLRAADLVDALGQENNPEWKTVAFNTNGEMVAPNGSIGFRWGE  target    FVWW-----DRATGRPRPV------SRDEVGA----RFDGD-----------PALLGEFEV---ELVDGSTVPVRPAFDL 1q16.1    KGKWNLEQRDGKTGEETELQLSLLGSQDEIAEVGFPYFGGDGTEHFNKVELENVLLHKLPVKRLQLADGSTALVTTVYDL  target    ------LKQYLDE-----SFDLRTA------SEVCRVPPQAIQSIARQLAANKRET----LLAAGMGPNHYFQNDLFGRV 1q16.1    TLANYGLERGLNDVNCATSYDDVKAYTPAWAEQITGVSRSQIIRIAREFADNADKTHGRSMIIVGAGLNHWYHLDMNYRG  target    QFLVAALTDNIGHLGGNVGSYAGNYRGSVFQAMGQWIAEDPFAIEPDLTKPATVKRYYKAESAHYWNYGERPLRAVAKDD 1q16.1    LINMLIFCGCVGQSGGGWAHYVGQEK---LRPQTGW---QPLAFALDWQRPA---RHMNSTSYFY---------------  target    EGDLTKGEVLTGKSHMPTPTKLIWFGNSNSLLGNAKWSFDVVKNTLPRQDAVFCNEWHWTSSCEYADLVFPADSWAEFKL 1q16.1    --------------------------------------------------------------------------------  target    PDATASCTNPFLLAFPTTPLKRLYDTRSDYEALALTAKALGELIDEPRMEQYWRGILDGDPTPYLQRIFSGSNATRGITY 1q16.1    --------------------------------------------------------------------------------  target    DELHESSKRGVPLLMNMRTYPRSGGWEQRQEDKPWYTATGRLEFYRPEPEFQAAGESLPVWREPVDATFYEPNAILSNAA 1q16.1    --------------------------------------------------------------------------------  target    HPSIAPRAPEDYGVPESQLDVETRQYRNVVRTWAELQQTLHPLQERDPAFRFVF 1q16.1    ------------------------------------------------------ ``` | | | | | | | | | | | | | | | | | | | | | | | | | | | | | | | | | | | | | | | | | | | | | | | | | |
| ✓ | 3ir7.1.A | Respiratory nitrate reductase 1 alpha chain  *Crystal structure of NarGHI mutant NarG-R94S* | 0.27 | 0.00 | 31.57 | 0.54 | 54-630 | X-ray | 2.50 | monomer | 2 x MD1, 4 x SF4, 1 x 6MO, 1 x AGA, 1 x F3S, 2 x HEM | BLAST | 0.36 |
| ``` target    AQGVSRRQLLGRALALGSGAALADLLGPARFLSPAGAATAGAVVPGNPLRVMPDRTWEQIYRNQFEDDSTFVFTCAPNDT 3ir7.1    -----------------------------------------------------NRDWEDGYRQRWQHDKIVRSTHGVNCT  target    HNCLLRAHVKNGVVVRISPTYGYGEATDLYGNRASHRWDPRTCQKGLILSRRFYSERRVKAPMIRKGF-KDWVEAGYPRN 3ir7.1    GSCSWKIYVKNGLVTWETQQTDYPRTRPDLPNH-----EPRGCPSGASYSWYLYSANRLKYPMMRKRLMKMWREAKALHS  target    DDGTPQMDVT-----------LRGSDDWIRISWDEATTIAAKTMEDVARTFNGDEGARKLLAQGYHP--EMVEVMHGAGV 3ir7.1    DPVEAWASIIEDADKAKSFKQARGRGGFVRSSWQEVNELIAASNVYTIKNYGPDRVA------GFSPIPAMSMVSYASGA  target    QALKLRGGMPLLGIGRIFGFYRFANMLALLDRKLRPDAPADEILGSRTFDNYAWHTDLPPGHPMVTGSQTVDFDLFSAEH 3ir7.1    RYLSLIGGTCL------------------------------------SF--YDWYCDLPPASPQTWGEQTDVPESADWYN  target    TKLLLIIGMNWICTKMPDGHWIGDARLKGTRVIVISADYMPTANKADEVIILRPGTDAAFFLGVARELIEKGLYDRAA-- 3ir7.1    SSYIIAWGSNVPQTRTPDAHFFTEVRYKGTKTVAVTPDYAEIAKLCDLWLAPKQGTDAAMALAMGHVMLREFHLDNPSQY  target    ----VIERTDLPLLVRLDTGERLDARDVIPGYELAALTNYVTLKPDAEIKGNPP---PPPFTAGGQVVP--TELRDAWGD 3ir7.1    FTDYVRRYTDMPMLVMLE------ERD---GYYAAGRMLRAADLVDALGQENNPEWKTVAFNTNGEMVAPNGSIGFRWGE  target    FVWW-----DRATGRPRPV------SRDEVGA----RFDGD-----------PALLGEFEV---ELVDGSTVPVRPAFDL 3ir7.1    KGKWNLEQRDGKTGEETELQLSLLGSQDEIAEVGFPYFGGDGTEHFNKVELENVLLHKLPVKRLQLADGSTALVTTVYDL  target    ------LKQYLDE-----SFDLRTA------SEVCRVPPQAIQSIARQLAANKRET----LLAAGMGPNHYFQNDLFGRV 3ir7.1    TLANYGLERGLNDVNCATSYDDVKAYTPAWAEQITGVSRSQIIRIAREFADNADKTHGRSMIIVGAGLNHWYHLDMNYRG  target    QFLVAALTDNIGHLGGNVGSYAGNYRGSVFQAMGQWIAEDPFAIEPDLTKPATVKRYYKAESAHYWNYGERPLRAVAKDD 3ir7.1    LINMLIFCGCVGQSGGGWAHYVGQEK---LRPQTGW---QPLAFALDWQRPA---RHMNSTSYFY---------------  target    EGDLTKGEVLTGKSHMPTPTKLIWFGNSNSLLGNAKWSFDVVKNTLPRQDAVFCNEWHWTSSCEYADLVFPADSWAEFKL 3ir7.1    --------------------------------------------------------------------------------  target    PDATASCTNPFLLAFPTTPLKRLYDTRSDYEALALTAKALGELIDEPRMEQYWRGILDGDPTPYLQRIFSGSNATRGITY 3ir7.1    --------------------------------------------------------------------------------  target    DELHESSKRGVPLLMNMRTYPRSGGWEQRQEDKPWYTATGRLEFYRPEPEFQAAGESLPVWREPVDATFYEPNAILSNAA 3ir7.1    --------------------------------------------------------------------------------  target    HPSIAPRAPEDYGVPESQLDVETRQYRNVVRTWAELQQTLHPLQERDPAFRFVF 3ir7.1    ------------------------------------------------------ ``` | | | | | | | | | | | | | | | | | | | | | | | | | | | | | | | | | | | | | | | | | | | | | | | | | |
|  | 7qv7.1.L | Hydrogen dependent carbon dioxide reductase subunit FdhF  *Cryo-EM structure of Hydrogen-dependent CO2 reductase.* | 0.33 |  | 18.98 | 0.58 | 71-854 | EM | 0.00 | hetero-2-6-6-2-mer | 52 x SF4, 6 x 402 | HHblits | 0.30 |
| ``` target    AQGVSRRQLLGRALALGSGAALADLLGPARFLSPAGAATAGAVVPGNPLRVMPDRTWEQIYRNQFEDDSTFVFTCAPNDT 7qv7.1    ----------------------------------------------------------------------KVLTTCPYCG  target    HNCLLRAHVKNGVVVRISPTYGYGEATDLYGNRASHRWDPRTCQKGLILSRRFYSERRVKAPMIRKGFKDWVEAGYPRND 7qv7.1    TGCGLYLKVENEKIVGVEPD------------KLHPVNQGELCIKGYYGYKYVHDPRRLTSPLIKKN-------------  target    DGTPQMDVTLRGSDDWIRISWDEATTIAAKTMEDVARTFNGDEGARKLLAQGYHPEMVEVMHGAGVQALKLRGGMPLLGI 7qv7.1    -------------GKFVPVSWDEALNFIANGLKKIKSEYG--------------SDAFAMFCSAR---------------  target    GRIFGFYRFANMLALLDRKLRPDAPADEILGSRTFDNYAWHTDLP--PGHPMVTGSQTVDFDLFSAE-HTKLLLIIGMNW 7qv7.1    --------ATNEDNYAAQKFA-----RAVIGINNVDHCARLCHAPTVAGLAMTLGSGAMTNSIPEISTYSDVIFIIGSNT  target    ICTKMPDGHWIGDARLKGTRVIVISADYMPTANKADEVIILRPGTDAAFFLGVARELIEKGLYDRAAVIERTDLPLLVRL 7qv7.1    AECHPLIAAHVIKAKERGAKLIVADPRMNAMVHKADIWLRVPSGYNIPLINGMIHIIIKEGLVKTDFVKNHAV-------  target    DTGERLDARDVIPGYELAALTNYVTLKPDAEIKGNPPPPPFTAGGQVVPTELRDAWGDFVWWDRATGRPRPVSRDEVGAR 7qv7.1    -------------G------------------------------------------------------------------  target    FDGDPALLGEFEVELVDGSTVPVRPAFDLLKQYLDESFDLRTASEVCRVPPQAIQSIARQLAANKRETLLAAGMGPNHYF 7qv7.1    --------------------------FEEMAKAVE-KYTPEYVEELTGIPKKDLIKAARFYGQAQAAA-ILYSMGVTQFS  target    QNDLFGRVQFLVAALTDNIGHLGGNVGSYAGN--YRGSVFQAMGQWIAEDPFAIEPDLTKPATVKRYYKAESAHYWNYGE 7qv7.1    HGTGNVVSLANLAVITGNLGRPGAGICPLRGQNNVQGAC--DVGALPNVLPG--YLDVTKEQNRERF-----EKVWGVK-  target    RPLRAVAKDDEGDLTKGEVLTGKSHMPTPTKLIWFGNSNSLLGNAKWSFDVVKNTLPRQDAVFCNEWHWTSSCEYADLVF 7qv7.1    LP-------SNIGLRVTEVPDAI--LNKRVRALYIFGENPIMSDPD--SDHLRHALEHLDLLIVQDIFLTETARLAHVVL  target    PADSWAEFKLPDATASCTNPFLLAFPTTPLKRLYDTRSDYEALALTAKALGELI-DEPRMEQYWRGILDGDPTPYLQRIF 7qv7.1    PAACWAEKD---GTFTNTERRVQRV-RKAVEAPGEAKPDWWIFSQIAERMGYTGMQYNNVQEIW------------DEVR  target    SGSN-ATRGITYDELHESSKRGVPLLMNMRTYPRSGGWEQRQEDKPWYTATGRLEFYRPEPEFQAAGESLPVWREPVDAT 7qv7.1    KIVPEKFGGISYARLEKEK--GLAWPCPTED---HTGTPILYLGGKFATPSGKAQMYPVIF-------------------  target    FYEPNAILSNAAHPSIAPRAPEDYGVPESQLDVETRQYRNVVRTWAELQQTLHPLQERDPAFRFVF 7qv7.1    ------------------------------------------------------------------ ``` | | | | | | | | | | | | | | | | | | | | | | | | | | | | | | | | | | | | | | | | | | | | | | | | | |
|  | 7qv7.1.O | Hydrogen dependent carbon dioxide reductase subunit FdhF  *Cryo-EM structure of Hydrogen-dependent CO2 reductase.* | 0.33 |  | 18.98 | 0.58 | 71-854 | EM | 0.00 | hetero-2-6-6-2-mer | 52 x SF4, 6 x 402 | HHblits | 0.30 |
| ``` target    AQGVSRRQLLGRALALGSGAALADLLGPARFLSPAGAATAGAVVPGNPLRVMPDRTWEQIYRNQFEDDSTFVFTCAPNDT 7qv7.1    ----------------------------------------------------------------------KVLTTCPYCG  target    HNCLLRAHVKNGVVVRISPTYGYGEATDLYGNRASHRWDPRTCQKGLILSRRFYSERRVKAPMIRKGFKDWVEAGYPRND 7qv7.1    TGCGLYLKVENEKIVGVEPD------------KLHPVNQGELCIKGYYGYKYVHDPRRLTSPLIKKN-------------  target    DGTPQMDVTLRGSDDWIRISWDEATTIAAKTMEDVARTFNGDEGARKLLAQGYHPEMVEVMHGAGVQALKLRGGMPLLGI 7qv7.1    -------------GKFVPVSWDEALNFIANGLKKIKSEYG--------------SDAFAMFCSAR---------------  target    GRIFGFYRFANMLALLDRKLRPDAPADEILGSRTFDNYAWHTDLP--PGHPMVTGSQTVDFDLFSAE-HTKLLLIIGMNW 7qv7.1    --------ATNEDNYAAQKFA-----RAVIGINNVDHCARLCHAPTVAGLAMTLGSGAMTNSIPEISTYSDVIFIIGSNT  target    ICTKMPDGHWIGDARLKGTRVIVISADYMPTANKADEVIILRPGTDAAFFLGVARELIEKGLYDRAAVIERTDLPLLVRL 7qv7.1    AECHPLIAAHVIKAKERGAKLIVADPRMNAMVHKADIWLRVPSGYNIPLINGMIHIIIKEGLVKTDFVKNHAV-------  target    DTGERLDARDVIPGYELAALTNYVTLKPDAEIKGNPPPPPFTAGGQVVPTELRDAWGDFVWWDRATGRPRPVSRDEVGAR 7qv7.1    -------------G------------------------------------------------------------------  target    FDGDPALLGEFEVELVDGSTVPVRPAFDLLKQYLDESFDLRTASEVCRVPPQAIQSIARQLAANKRETLLAAGMGPNHYF 7qv7.1    --------------------------FEEMAKAVE-KYTPEYVEELTGIPKKDLIKAARFYGQAQAAA-ILYSMGVTQFS  target    QNDLFGRVQFLVAALTDNIGHLGGNVGSYAGN--YRGSVFQAMGQWIAEDPFAIEPDLTKPATVKRYYKAESAHYWNYGE 7qv7.1    HGTGNVVSLANLAVITGNLGRPGAGICPLRGQNNVQGAC--DVGALPNVLPG--YLDVTKEQNRERF-----EKVWGVK-  target    RPLRAVAKDDEGDLTKGEVLTGKSHMPTPTKLIWFGNSNSLLGNAKWSFDVVKNTLPRQDAVFCNEWHWTSSCEYADLVF 7qv7.1    LP-------SNIGLRVTEVPDAI--LNKRVRALYIFGENPIMSDPD--SDHLRHALEHLDLLIVQDIFLTETARLAHVVL  target    PADSWAEFKLPDATASCTNPFLLAFPTTPLKRLYDTRSDYEALALTAKALGELI-DEPRMEQYWRGILDGDPTPYLQRIF 7qv7.1    PAACWAEKD---GTFTNTERRVQRV-RKAVEAPGEAKPDWWIFSQIAERMGYTGMQYNNVQEIW------------DEVR  target    SGSN-ATRGITYDELHESSKRGVPLLMNMRTYPRSGGWEQRQEDKPWYTATGRLEFYRPEPEFQAAGESLPVWREPVDAT 7qv7.1    KIVPEKFGGISYARLEKEK--GLAWPCPTED---HTGTPILYLGGKFATPSGKAQMYPVIF-------------------  target    FYEPNAILSNAAHPSIAPRAPEDYGVPESQLDVETRQYRNVVRTWAELQQTLHPLQERDPAFRFVF 7qv7.1    ------------------------------------------------------------------ ``` | | | | | | | | | | | | | | | | | | | | | | | | | | | | | | | | | | | | | | | | | | | | | | | | | |
|  | 3egw.1.A | Respiratory nitrate reductase 1 alpha chain  *The crystal structure of the NarGHI mutant NarH - C16A* | 0.27 | 0.09 | 31.94 | 0.54 | 54-630 | X-ray | 1.90 | homo-dimer | 2 x MD1, 2 x MGD, 2 x 6MO, 6 x SF4, 4 x F3S, 2 x 3PH, 4 x HEM, 2 x AGA | BLAST | 0.36 |
| ``` target    AQGVSRRQLLGRALALGSGAALADLLGPARFLSPAGAATAGAVVPGNPLRVMPDRTWEQIYRNQFEDDSTFVFTCAPNDT 3egw.1    -----------------------------------------------------NRDWEDGYRQRWQHDKIVRSTHGVNCT  target    HNCLLRAHVKNGVVVRISPTYGYGEATDLYGNRASHRWDPRTCQKGLILSRRFYSERRVKAPMIRKGF-KDWVEAGYPRN 3egw.1    GSCSWKIYVKNGLVTWETQQTDYPRTRPDLPNH-----EPRGCPRGASYSWYLYSANRLKYPMMRKRLMKMWREAKALHS  target    DDGTPQMDVT-----------LRGSDDWIRISWDEATTIAAKTMEDVARTFNGDEGARKLLAQGYHP--EMVEVMHGAGV 3egw.1    DPVEAWASIIEDADKAKSFKQARGRGGFVRSSWQEVNELIAASNVYTIKNYGPDRVA------GFSPIPAMSMVSYASGA  target    QALKLRGGMPLLGIGRIFGFYRFANMLALLDRKLRPDAPADEILGSRTFDNYAWHTDLPPGHPMVTGSQTVDFDLFSAEH 3egw.1    RYLSLIGGTCL------------------------------------SF--YDWYCDLPPASPQTWGEQTDVPESADWYN  target    TKLLLIIGMNWICTKMPDGHWIGDARLKGTRVIVISADYMPTANKADEVIILRPGTDAAFFLGVARELIEKGLYDRAA-- 3egw.1    SSYIIAWGSNVPQTRTPDAHFFTEVRYKGTKTVAVTPDYAEIAKLCDLWLAPKQGTDAAMALAMGHVMLREFHLDNPSQY  target    ----VIERTDLPLLVRLD-------TGERLDARDVIPGYELAALTNYVTLKPDAEIKGNP--PPPPFTAGGQVVP--TEL 3egw.1    FTDYVRRYTDMPMLVMLEERDGYYAAGRMLRAADLV-----AALGQ----------ENNPEWKTVAFNTNGEMVAPNGSI  target    RDAWGDFVWW-----DRATGRPRPV------SRDEVGA----RFDGD-----------PALLGEFEV---ELVDGSTVPV 3egw.1    GFRWGEKGKWNLEQRDGKTGEETELQLSLLGSQDEIAEVGFPYFGGDGTEHFNKVELENVLLHKLPVKRLQLADGSTALV  target    RPAFDL------LKQYLDE-----SFDLRTA------SEVCRVPPQAIQSIARQLAANKRET----LLAAGMGPNHYFQN 3egw.1    TTVYDLTLANYGLERGLNDVNCATSYDDVKAYTPAWAEQITGVSRSQIIRIAREFADNADKTHGRSMIIVGAGLNHWYHL  target    DLFGRVQFLVAALTDNIGHLGGNVGSYAGNYRGSVFQAMGQWIAEDPFAIEPDLTKPATVKRYYKAESAHYWNYGERPLR 3egw.1    DMNYRGLINMLIFCGCVGQSGGGWAHYVGQEK---LRPQTGW---QPLAFALDWQRPA---RHMNSTSYFY---------  target    AVAKDDEGDLTKGEVLTGKSHMPTPTKLIWFGNSNSLLGNAKWSFDVVKNTLPRQDAVFCNEWHWTSSCEYADLVFPADS 3egw.1    --------------------------------------------------------------------------------  target    WAEFKLPDATASCTNPFLLAFPTTPLKRLYDTRSDYEALALTAKALGELIDEPRMEQYWRGILDGDPTPYLQRIFSGSNA 3egw.1    --------------------------------------------------------------------------------  target    TRGITYDELHESSKRGVPLLMNMRTYPRSGGWEQRQEDKPWYTATGRLEFYRPEPEFQAAGESLPVWREPVDATFYEPNA 3egw.1    --------------------------------------------------------------------------------  target    ILSNAAHPSIAPRAPEDYGVPESQLDVETRQYRNVVRTWAELQQTLHPLQERDPAFRFVF 3egw.1    ------------------------------------------------------------ ``` | | | | | | | | | | | | | | | | | | | | | | | | | | | | | | | | | | | | | | | | | | | | | | | | | |
|  | 6tg9.1.A | Formate dehydrogenase subunit alpha  *Cryo-EM Structure of NADH reduced form of NAD+-dependent Formate Dehydrogenase from Rhodobacter capsulatus* | 0.32 |  | 20.22 | 0.58 | 36-811 | EM | 3.24 | hetero-2-2-2-2-mer | 4 x MGD, 2 x 6MO, 4 x FES, 10 x SF4, 2 x H2S, 2 x FMN, 2 x NAI | HHblits | 0.30 |
| ``` target    AQGVSRRQLLGRALALGSGAALADLLGPARFLSPAGAATAGAVVPGNPLRVMPDRTWEQIYRNQFEDDSTFVFTCAPNDT 6tg9.1    -----------------------------------DCVSCGACVQACPTATLVEKSV----ERIGTPE-RKVVTTCAYCG  target    HNCLLRAHVKNGVVVRISPTYGYGEATDLYGNRASHRWDPRTCQKGLILSRRFYSERRVKAPMIRKGFKDWVEAGYPRND 6tg9.1    VGCSFEAHMLGDQLVRMVPW------------KGGAANRGHSCVKGRFAYGYATHQDRILKPMIRD--------------  target    DGTPQMDVTLRGSDDWIRISWDEATTIAAKTMEDVARTFNGDEGARKLLAQGYHPEMVEVMHGAGVQALKLRGGMPLLGI 6tg9.1    ----------KITDPWREVNWTEALDFTATRLRALRDSHG--------------ADALGVITS-----------------  target    GRIFGFYRFANMLALLDRKLRPDAPADEILGSRTFDNYAWHTDLP--PGHPMVTGSQTVDFDLFSAEHTKLLLIIGMNWI 6tg9.1    ------SRCTNEETYLVQKLA-----RAVFGTNNTDTCARVCHSPTGYGLKQTFGTSAGTQDFDSVEETDLALVIGANPT  target    CTKMPDGHWIGDARLKGTRVIVISADYMPTAN----KADEVIILRPGTDAAFFLGVARELIEKGLYDRAAVIERTDLPLL 6tg9.1    DGHPVFASRLRKRLRAGAKLIVVDPRRIDLLNTPHRGEAWHLQLKPGTNVAVMTAMAHVIVTEQIFDKRFIGDRCDWD--  target    VRLDTGERLDARDVIPGYELAALTNYVTLKPDAEIKGNPPPPPFTAGGQVVPTELRDAWGDFVWWDRATGRPRPVSRDEV 6tg9.1    ----------------EW--------------------------------------------------------------  target    GARFDGDPALLGEFEVELVDGSTVPVRPAFDLLKQYLD-ESFDLRTASEVCRVPPQAIQSIARQLAANKRETLLAAGMGP 6tg9.1    ------------------------------ADYAEFVANPEYAPEAVESLTGVPAGLLRQAARAYAAAPNA-AIYYGLGV  target    NHYFQNDLFGRVQFLVAALTDNIGHLGGNVGSYAGN--YRGSVFQAMGQWIAEDPFAIEPDLTKPATVKRYYKAESAHYW 6tg9.1    TEHSQGSTTVIAIANLAMMTGNIGRPGVGVNPLRGQNNVQGSC--DMGSFPHEFPG--YRHVSDDATRG-----LFERTW  target    NYGERPLRAVAKDDEGDLTKGEVLTGKSHMPTPTKLIWFGNSNSLLGNAKWSFDVVKNTLPRQDAVFCNEWHWTSSCEYA 6tg9.1    GVTL-SS-------EPGLRIPNMLDAA--VEGRFKALYVQGEDILQSDPD--TRHVSAGLAAMDLVIVHDLFLNETANYA  target    DLVFPADSWAEFKLPDATASCTNPFLLAFPTTPLKRLYDTRSDYEALALTAKALGELIDEPRMEQYWRGILDGDPTPYLQ 6tg9.1    HVFLPGSTFLEKD---GTFTNAERRINRV-RRVMAPKA-GFADWEVTQMLANALGAGWHYTHP------------SEIMA  target    RIFSGSNATRGITYDELHESSKRGVPLLMNMRTYPRSGGWEQRQEDKPWYTATGRLEFYRPEPEFQAAGESLPVWREPVD 6tg9.1    EIAATTPGFAAVTYEMLDAR------------------------------------------------------------  target    ATFYEPNAILSNAAHPSIAPRAPEDYGVPESQLDVETRQYRNVVRTWAELQQTLHPLQERDPAFRFVF 6tg9.1    -------------------------------------------------------------------- ``` | | | | | | | | | | | | | | | | | | | | | | | | | | | | | | | | | | | | | | | | | | | | | | | | | |
|  | 4v4c.1.A | Pyrogallol hydroxytransferase large subunit  *Crystal Structure of Pyrogallol-Phloroglucinol Transhydroxylase from Pelobacter acidigallici* | 0.30 |  | 19.77 | 0.57 | 71-768 | X-ray | 2.35 | hetero-oligomer | 2 x CA, 2 x MGD, 1 x 4MO, 3 x SF4 | HHblits | 0.29 |
| ``` target    AQGVSRRQLLGRALALGSGAALADLLGPARFLSPAGAATAGAVVPGNPLRVMPDRTWEQIYRNQFEDDSTFVFTCAPNDT 4v4c.1    ----------------------------------------------------------------------EVVRLTN-SS  target    HNCLLRAHVKNGVVVRISPTYGYGE------ATDLYGNRASHRWDPRTCQKGLILSRRFYSERRVKAPMIRKGFKDWVEA 4v4c.1    TGGPVFVYVKDGKIIRMTPMDFDDAVDAPSWKIEARGKTFTPPRKTSIAPYTAGFKSMIYSDLRIPYPMKRKSFDP---N  target    GYPRNDDGTPQMDVTLRGSD--------DWIRISWDEATTIAAKTMEDVARTFNGDEGARKLLAQGYHPEMVEVMHGAGV 4v4c.1    G---------ERNPQLRGAGLSKQDPWSDYERISWDEATDIVVAEINRIKHAYGP--------------SAILSTPSSHH  target    QALKLRGGMPLLGIGRIFGFYRFANMLALLDRKLRPDAPADEILGSRTFDNYA-----WH--TDLPPGHPMVTGSQTVDF 4v4c.1    MWG--NVGYR------HSTYFRFMNM-----------------MGFTYADHNPDSWEGWHWGGMHMWGFSWRLGNPEQYD  target    D-LFSAEHTKLLLIIGMNWICTKMPDGHW-----IGDARLKGTRVIVISADYMPTAN-KADEVIILRPGTDAAFFLGVAR 4v4c.1    LLEDGLKHAEMIVFWSSDPETNSGIYAGFESNIRRQWLKDLGVDFVFIDPHMNHTARLVADKWFSPKIGTDHALSFAIAY  target    ELIEKGLYDRAAVIERTDLPLLVRLDTGERLDARDVIPGYELAALTNYVTLKPDAEIKGNPPPPPFTAGGQVVPTELRDA 4v4c.1    TWLKEDSYDKEYVAANAH--------------------GFEE--------------------------------------  target    WGDFVWWDRATGRPRPVSRDEVGARFDGDPALLGEFEVELVDGSTVPVRPAFDLLKQYLDESFDLRTASEVCRVPPQAIQ 4v4c.1    WADYVL---------------------------G-----KTDG-----------------TPKTCEWAEEESGVPACEIR  target    SIARQLAANKRETLLAAGM----GPNHYFQNDLFGRVQFLVAALTDNIGHLGGNVGSYAGNYRGSVFQAMGQWIAEDPFA 4v4c.1    ALARQWAKKNTY-LAAGGLGGWGGACRASHGIEWARGMIALATMQG-MGKPGSNMWSTTQGVPLDYEFYFPGYAEGGISG  target    I-EP----------------DLTKPATVKRYYKAESAHYWNYGER----PLRAVAKDDEGDLTKGEVLTGKSH---MPTP 4v4c.1    DCENSAAGFKFAWRMFDGKTTFPSPSNLNTS-AGQHIPRLKIPECIMGGKFQWSGKGFAGGD-ISHQLHQYEYPAPGYSK  target    TKLIWFGNSNSLLGNAKWSFDVVKNTL--PRQDAVFCNEWHWTSSCEYADLVFPADSWAEFKLPDATASC---------- 4v4c.1    IKMFWKYGGPHLGTMTA--TNRYAKMYTHDSLEFVVSQSIWFEGEVPFADIILPACTNFERWDI-SEFANCSGYIPDNYQ  target    --TNPFLLAFPTTPLKRLYDTRSDYEALALTAKALGELIDEPRMEQYWRGILDGDPTPYLQRIFSGSNATRGITYDELHE 4v4c.1    LCNHRVISL-QAKCIEPVGESMSDYEIYRLFAKKLNIE------------------------------------------  target    SSKRGVPLLMNMRTYPRSGGWEQRQEDKPWYTATGRLEFYRPEPEFQAAGESLPVWREPVDATFYEPNAILSNAAHPSIA 4v4c.1    --------------------------------------------------------------------------------  target    PRAPEDYGVPESQLDVETRQYRNVVRTWAELQQTLHPLQERDPAFRFVF 4v4c.1    ------------------------------------------------- ``` | | | | | | | | | | | | | | | | | | | | | | | | | | | | | | | | | | | | | | | | | | | | | | | | | |
|  | 7l5i.1.A | Trimethylamine-N-oxide reductase  *Crystal Structure of Haemophilus influenzae MtsZ at pH 7.0* | 0.30 | 0.00 | 22.85 | 0.55 | 55-768 | X-ray | 1.73 | monomer | 2 x MGD, 1 x MO, 1 x O | HHblits | 0.31 |
| ``` target    AQGVSRRQLLGRALALGSGAALADLLGPARFLSPAGAATAGAVVPGNPLRVMPDRTWEQIYRNQFEDDSTFVFTCAPNDT 7l5i.1    ------------------------------------------------------KEA-------------EMKTVVTAAH  target    HNCLLRAHVKNGVVVRISPTYGYGEATDLYGNRASHRWDPRTCQKGLILSRRFYSERRVKAPMIRKGFKDWVEAGYPRND 7l5i.1    -WGSIGVVVQDGKVVKSGPAIE-------------PAVPNELQT---VVADQLYSEARVKCPMVRKGFLA---------N  target    DGTPQMDVTLRGSDDWIRISWDEATTIAAKTMEDVARTFNGDEGARKLLAQGYHPEMVEVMHGAGVQALKLRGGMPLLGI 7l5i.1    PG--KSDTTMRGRDEWVRVSWDEALDLVHNQLKRVRDEHGST--------------GIFAGSYGWFSC-----GS-L-H-  target    GRIFGFYRFANMLALLDRKLRPDAPADEILGSRTFDNYAWHTDLPPGHPMVTGSQT---VDFDL-FSAEHTKLLLIIGMN 7l5i.1    ASRTLLQRYMNATGGFV-------------GHK--GDYS-TGAAQVIMPHVLGTIEVYEQQTSWESILESSDIIVLWSAN  target    WICTKMPD--------GHWIGDARLKGTRVIVISADYMPTANK-ADEVIILRPGTDAAFFLGVARELIEKGLYDRAAVIE 7l5i.1    PLTTMRIAWMSTDQKGIEYFKKFQASGKRIICIDPQKSETCQMLNAEWIPVNTATDVPLMLGIAHTLVEQGKHDKDFLKK  target    RTDLPLLVRLDTGERLDARDVIPGYELAALTNYVTLKPDAEIKGNPPPPPFTAGGQVVPTELRDAWGDFVWWDRATGRPR 7l5i.1    YTS--------------------GYAK--F------------------------------------EEYL----------  target    PVSRDEVGARFDGDPALLGEFEVELVDGSTVPVRPAFDLLKQYLDESFDLRTASEVCRVPPQAIQSIARQLAANKRETLL 7l5i.1    -----------------LGK-----TDG-----------------QPKTAEWAAKICGVPAETIKQLAADFAS-KRT-ML  target    AAGMGPNHYFQNDLFGRVQFLVAALTDNIGHLGGNVGSYAGNYRGSVFQAMGQWIAEDPFAIEPDLTKPATVKRYYKAES 7l5i.1    MGGWGMQRQRHGEQTHWMLVTLASMLGQIGLPGGGFGLSYHYSNGGVPTATGGIIG-SI-TASPSG-KA-GAKTWLDDTS  target    AHYWNYGERPLRAVAK-DDEGDLTKGEVLTGKSHMPTPTKLIWFGNSNSLLGNAKWSFDVVKNTLPRQDAVFCNEWHWTS 7l5i.1    KSA-----FPLARIADVLLHPGKKIQYNGTEI--TYPDIKAVYWAGGNPFVHHQD--TNTLVKAFQKPDVVIVNEVNWTP  target    SCEYADLVFPADSWAEFKLPDATASCTNPFLLAFPTTPLKRLYDTRSDYEALALTAKALGELIDEPRMEQYWRGILDGDP 7l5i.1    TARMADIVLPATTSYERNDLTMAGDYSMMSVYPM-KQVVPPQFEAKNDYDIFVELAKRAGVE------------------  target    TPYLQRIFSGSNATRGITYDELHESSKRGVPLLMNMRTYPRSGGWEQRQEDKPWYTATGRLEFYRPEPEFQAAGESLPVW 7l5i.1    --------------------------------------------------------------------------------  target    REPVDATFYEPNAILSNAAHPSIAPRAPEDYGVPESQLDVETRQYRNVVRTWAELQQTLHPLQERDPAFRFVF 7l5i.1    ------------------------------------------------------------------------- ``` | | | | | | | | | | | | | | | | | | | | | | | | | | | | | | | | | | | | | | | | | | | | | | | | | |
|  | 7l5s.1.A | Trimethylamine-N-oxide reductase  *Crystal Structure of Haemophilus influenzae MtsZ at pH 5.5* | 0.31 | 0.00 | 22.85 | 0.55 | 55-768 | X-ray | 2.09 | monomer | 1 x O, 2 x MGD, 1 x MO | HHblits | 0.31 |
| ``` target    AQGVSRRQLLGRALALGSGAALADLLGPARFLSPAGAATAGAVVPGNPLRVMPDRTWEQIYRNQFEDDSTFVFTCAPNDT 7l5s.1    ------------------------------------------------------KEA-------------EMKTVVTAAH  target    HNCLLRAHVKNGVVVRISPTYGYGEATDLYGNRASHRWDPRTCQKGLILSRRFYSERRVKAPMIRKGFKDWVEAGYPRND 7l5s.1    -WGSIGVVVQDGKVVKSGPAIE-------------PAVPNELQT---VVADQLYSEARVKCPMVRKGFLA---------N  target    DGTPQMDVTLRGSDDWIRISWDEATTIAAKTMEDVARTFNGDEGARKLLAQGYHPEMVEVMHGAGVQALKLRGGMPLLGI 7l5s.1    PG--KSDTTMRGRDEWVRVSWDEALDLVHNQLKRVRDEHGST--------------GIFAGSYGWFSC-----GS-L-H-  target    GRIFGFYRFANMLALLDRKLRPDAPADEILGSRTFDNYAWHTDLPPGHPMVTGSQT---VDFDL-FSAEHTKLLLIIGMN 7l5s.1    ASRTLLQRYMNATGGFV-------------GHK--GDYS-TGAAQVIMPHVLGTIEVYEQQTSWESILESSDIIVLWSAN  target    WICTKMPD--------GHWIGDARLKGTRVIVISADYMPTANK-ADEVIILRPGTDAAFFLGVARELIEKGLYDRAAVIE 7l5s.1    PLTTMRIAWMSTDQKGIEYFKKFQASGKRIICIDPQKSETCQMLNAEWIPVNTATDVPLMLGIAHTLVEQGKHDKDFLKK  target    RTDLPLLVRLDTGERLDARDVIPGYELAALTNYVTLKPDAEIKGNPPPPPFTAGGQVVPTELRDAWGDFVWWDRATGRPR 7l5s.1    YTS--------------------GYAK--F------------------------------------EEYL----------  target    PVSRDEVGARFDGDPALLGEFEVELVDGSTVPVRPAFDLLKQYLDESFDLRTASEVCRVPPQAIQSIARQLAANKRETLL 7l5s.1    -----------------LGK-----TDG-----------------QPKTAEWAAKICGVPAETIKQLAADFAS-KRT-ML  target    AAGMGPNHYFQNDLFGRVQFLVAALTDNIGHLGGNVGSYAGNYRGSVFQAMGQWIAEDPFAIEPDLTKPATVKRYYKAES 7l5s.1    MGGWGMQRQRHGEQTHWMLVTLASMLGQIGLPGGGFGLSYHYSNGGVPTATGGIIG-SI-TASPSG-KA-GAKTWLDDTS  target    AHYWNYGERPLRAVAK-DDEGDLTKGEVLTGKSHMPTPTKLIWFGNSNSLLGNAKWSFDVVKNTLPRQDAVFCNEWHWTS 7l5s.1    KSA-----FPLARIADVLLHPGKKIQYNGTEI--TYPDIKAVYWAGGNPFVHHQD--TNTLVKAFQKPDVVIVNEVNWTP  target    SCEYADLVFPADSWAEFKLPDATASCTNPFLLAFPTTPLKRLYDTRSDYEALALTAKALGELIDEPRMEQYWRGILDGDP 7l5s.1    TARMADIVLPATTSYERNDLTMAGDYSMMSVYPM-KQVVPPQFEAKNDYDIFVELAKRAGVE------------------  target    TPYLQRIFSGSNATRGITYDELHESSKRGVPLLMNMRTYPRSGGWEQRQEDKPWYTATGRLEFYRPEPEFQAAGESLPVW 7l5s.1    --------------------------------------------------------------------------------  target    REPVDATFYEPNAILSNAAHPSIAPRAPEDYGVPESQLDVETRQYRNVVRTWAELQQTLHPLQERDPAFRFVF 7l5s.1    ------------------------------------------------------------------------- ``` | | | | | | | | | | | | | | | | | | | | | | | | | | | | | | | | | | | | | | | | | | | | | | | | | |
|  | 1ogy.1.A | PERIPLASMIC NITRATE REDUCTASE  *Crystal structure of the heterodimeric nitrate reductase from Rhodobacter sphaeroides* | 0.30 |  | 18.68 | 0.56 | 69-768 | X-ray | 3.20 | hetero-1-1-mer | 1 x SF4, 1 x MO, 2 x MGD, 2 x HEC | HHblits | 0.28 |
| ``` target    AQGVSRRQLLGRALALGSGAALADLLGPARFLSPAGAATAGAVVPGNPLRVMPDRTWEQIYRNQFEDDSTFVFTCAPNDT 1ogy.1    --------------------------------------------------------------------IRWSKAPCRFCG  target    HNCLLRAHVKNGVVVRISPTYGYGEATDLYGNRASHRWDPRTCQKGLILSRRFYSERRVKAPMIRKGFKDWVEAGYPRND 1ogy.1    TGCGVMVGTRDGQVVATHGD------------TQAEVNRGLNCVKGYFLSKIMYGEDRLTTPLLRMKDG-----------  target    DGTPQMDVTLRGSDDWIRISWDEATTIAAKTMEDVARTFNGDEGARKLLAQGYHPEMVEVMHGAGVQALKLRGGMPLLGI 1ogy.1    --------VYHKEGEFAPVSWDEAFDVMAAQAKLVLKEKA--------------PEAVGMFGSG-QWTIW----------  target    GRIFGFYRFANMLALLDRKLRPDAPADEILGSRTFDNYAWHT--DLPPGHPMVTGSQTVDFDLFSAEHTKLLLIIGMNWI 1ogy.1    -EGYAASKLMR----------------AGFRSNNLDPNARHCMASAATAFMRTFGMDEPMGCYDDFEAADAFVLWGSNMA  target    CTKMPDGHWIGDAR--LKGTRVIVISADYMPTANKADEVIILRPGTDAAFFLGVARELIEKGLYDRAAVIERTDLPLLVR 1ogy.1    EMHPILWSRLTDRRLSHEHVRVAVLSTFTHRSSDLSDTPIIFRPGTDRAILNYIAHHIISTGRVNRDFVDRHTNFALGAT  target    LDTGERLDARDVIPGYELAALTNYVTLKPDAEIKGNPPPPPFTAGGQVVPTELRDAWGDFVWWDRATGRPRPVSRDEVGA 1ogy.1    -DIGYGLRPEH------------------------------------------------QLQLAA---------------  target    RFDGDPALLGEFEVELVDGSTVPVRPAFDLLKQYLDESFDLRTASEVCRVPPQAIQSIARQLAANKRETLLAAGMGPNHY 1ogy.1    --------------KGAADAGAMTPTDFETFAALVS-EYTLEKAAEISGVEPALLEELAELYADPDRKWMSLWTMGFNQH  target    FQNDLFGRVQFLVAALTDNIGHLGGNVGSYAGNYRGSV-FQAMGQWIAEDPFAIEPDLTKPATVKRYYKAESAHYWNYGE 1ogy.1    VRGVWANHMVYNLHLLTGKISEPGNSPFSLTGQPFACGTAREVGTFAHRLPADM--VVTNPEHRAHA-----EEIWKLPA  target    RPLRAVAKDDEGDLTKGEVLTGKSHMPTPTKLIWFGNSNSLLGNAKWSFDVVKNTLPRQDAVFCNEWHWTSSCEYADLVF 1ogy.1    -GL--LPD--WVGAHAVEQDRKL--HDGEINFYWVQVNNNMQAAPNIDQETYPGYRNPENFIVVSDAYPTVTGRAADLVL  target    PADSWAEFKLPDATASCTNPFLLAFPTTPLKRLYDTRSDYEALALTAKALGELIDEPRMEQYWRGILDGDPTPYLQRIFS 1ogy.1    PAAMWVEKE--GA-YGNAERRTHFW-HQLVEAPGEARSDLWQLMEFSKRFTTD---------------------------  target    GSNATRGITYDELHESSKRGVPLLMNMRTYPRSGGWEQRQEDKPWYTATGRLEFYRPEPEFQAAGESLPVWREPVDATFY 1ogy.1    --------------------------------------------------------------------------------  target    EPNAILSNAAHPSIAPRAPEDYGVPESQLDVETRQYRNVVRTWAELQQTLHPLQERDPAFRFVF 1ogy.1    ---------------------------------------------------------------- ``` | | | | | | | | | | | | | | | | | | | | | | | | | | | | | | | | | | | | | | | | | | | | | | | | | |
|  | 3o5a.1.A | Periplasmic nitrate reductase  *Crystal Structure of partially reduced Periplasmic Nitrate Reductase from Cupriavidus necator using Ionic Liquids* | 0.30 |  | 17.80 | 0.56 | 71-768 | X-ray | 1.72 | hetero-oligomer | 1 x SF4, 1 x MOS, 2 x MGD, 2 x HEC | HHblits | 0.28 |
| ``` target    AQGVSRRQLLGRALALGSGAALADLLGPARFLSPAGAATAGAVVPGNPLRVMPDRTWEQIYRNQFEDDSTFVFTCAPNDT 3o5a.1    ----------------------------------------------------------------------WSKAPCRFCG  target    HNCLLRAHVKNGVVVRISPTYGYGEATDLYGNRASHRWDPRTCQKGLILSRRFYSERRVKAPMIRKGFKDWVEAGYPRND 3o5a.1    TGCGVTVAVKDNKVVATQGD------------PQAEVNKGLNCVKGYFLSKIMYGQDRLTRPLMRMKNG-----------  target    DGTPQMDVTLRGSDDWIRISWDEATTIAAKTMEDVARTFNGDEGARKLLAQGYHPEMVEVMHGAGVQALKLRGGMPLLGI 3o5a.1    --------KYDKNGDFAPVTWDQAFDEMERQFKRVLKEKG--------------PTAVGMFGS-GQWTVW----------  target    GRIFGFYRFANMLALLDRKLRPDAPADEILGSRTFDNYAWHTD--LPPGHPMVTGSQTVDFDLFSAEHTKLLLIIGMNWI 3o5a.1    -EGYAAAKLYK----------------AGFRSNNIDPNARHCMASAAAGFMRTFGMDEPMGCYDDFEAADAFVLWGSNMA  target    CTKMPDGHWIGDAR--LKGTRVIVISADYMPTANKADEVIILRPGTDAAFFLGVARELIEKGLYDRAAVIERTDLPLLVR 3o5a.1    EMHPILWTRVTDRRLSHPKTRVVVLSTFTHRCFDLADIGIIFKPQTDLAMLNYIANYIIRNNKVNKDFVNKHTVFKEGVT  target    LDTGERLDARDVIPGYELAALTNYVTLKPDAEIKGNPPPPPFTAGGQVVPTELRDAWGDFVWWDRATGRPRPVSRDEVGA 3o5a.1    DIG-YGLRPDHPLQKA-----------------------------------------------------AK---------  target    RFDGDPALLGEFEVELVDGSTVPVRPAFDLLKQYLDESFDLRTASEVCRVPPQAIQSIARQLAANKRETLLAAGMGPNHY 3o5a.1    ---------------NASDPGAAKVITFDEFAKFVS-KYDADYVSKLSAVPKAKLDQLAELYADPNIKVMSLWTMGFNQH  target    FQNDLFGRVQFLVAALTDNIGHLGGNVGSYAGNYRGSV-FQAMGQWIAEDPFAIEPDLTKPATVKRYYKAESAHYWNYGE 3o5a.1    TRGTWANNMVYNLHLLTGKIATPGNSPFSLTGQPSACGTAREVGTFSHRLPA--DMVVTNPKHREE-----AERIWKLPP  target    RPLRAVAKDDEGDLTKGEVLTGKSHMPTPTKLIWFGNSNSLLGNAKWSFDVVKNTLPRQDAVFCNEWHWTSSCEYADLVF 3o5a.1    GT---IPD--KPGY--DAVLQNRMLKDGKLNAYWVQVNNNMQAAANLMEEGLPGYRNPANFIVVSDAYPTVTALAADLVL  target    PADSWAEFKLPDATASCTNPFLLAFPTTPLKRLYDTRSDYEALALTAKALGELIDEPRMEQYWRGILDGDPTPYLQRIFS 3o5a.1    PSAMWVEKE--GA-YGNAERRTQFW-HQLVDAPGEARSDLWQLVEFAKRFKVE---------------------------  target    GSNATRGITYDELHESSKRGVPLLMNMRTYPRSGGWEQRQEDKPWYTATGRLEFYRPEPEFQAAGESLPVWREPVDATFY 3o5a.1    --------------------------------------------------------------------------------  target    EPNAILSNAAHPSIAPRAPEDYGVPESQLDVETRQYRNVVRTWAELQQTLHPLQERDPAFRFVF 3o5a.1    ---------------------------------------------------------------- ``` | | | | | | | | | | | | | | | | | | | | | | | | | | | | | | | | | | | | | | | | | | | | | | | | | |
|  | 2nya.1.A | Periplasmic nitrate reductase  *Crystal structure of the periplasmic nitrate reductase (NAP) from Escherichia coli* | 0.30 |  | 16.00 | 0.56 | 72-768 | X-ray | 2.50 | monomer | 1 x SF4, 1 x 6MO, 2 x MGD | HHblits | 0.28 |
| ``` target    AQGVSRRQLLGRALALGSGAALADLLGPARFLSPAGAATAGAVVPGNPLRVMPDRTWEQIYRNQFEDDSTFVFTCAPNDT 2nya.1    -----------------------------------------------------------------------DKAPCRFCG  target    HNCLLRAHVKNGVVVRISPTYGYGEATDLYGNRASHRWDPRTCQKGLILSRRFYSERRVKAPMIRKGFKDWVEAGYPRND 2nya.1    TGCGVLVGTQQGRVVACQG------------DPDAPVNRGLNCIKGYFLPKIMYGKDRLTQPLLRMKNG-----------  target    DGTPQMDVTLRGSDDWIRISWDEATTIAAKTMEDVARTFNGDEGARKLLAQGYHPEMVEVMHGAGVQALKLRGGMPLLGI 2nya.1    --------KYDKEGEFTPITWDQAFDVMEEKFKTALKEKG--------------PESIGMFGS-GQWTIW----------  target    GRIFGFYRFANMLALLDRKLRPDAPADEILGSRTFDNYAWHT--DLPPGHPMVTGSQTVDFDLFSAEHTKLLLIIGMNWI 2nya.1    -EGYAASKLFK----------------AGFRSNNIDPNARHCMASAVVGFMRTFGMDEPMGCYDDIEQADAFVLWGANMA  target    CTKMPDGHWIGDAR--LKGTRVIVISADYMPTANKADEVIILRPGTDAAFFLGVARELIEKGLYDRAAVIERTDLPLLVR 2nya.1    EMHPILWSRITNRRLSNQNVTVAVLSTYQHRSFELADNGIIFTPQSDLVILNYIANYIIQNNAINQDFFSKHVNLRKGAT  target    LDTGERLDARDVIPGYELAALTNYVTLKPDAEIKGNPPPPPFTAGGQVVPTELRDAWGDFVWWDRATGRPRPVSRDEVGA 2nya.1    D-IGYGLRPTHPLEKA----------------------------------------------------------------  target    RFDGDPALLGEFEVELVDGSTVPVRPAFDLLKQYLDESFDLRTASEVCRVPPQAIQSIARQLAANKRETLLAAGMGPNHY 2nya.1    --------------AKNPGSDASEPMSFEDYKAFVA-EYTLEKTAEMTGVPKDQLEQLAQLYADPNKKVISYWTMGFNQH  target    FQNDLFGRVQFLVAALTDNIGHLGGNVGSYAGNY--RGSVFQAMGQWIAEDPFAIEPDLTKPATVKRYYKAESAHYWNYG 2nya.1    TRGVWANNLVYNLHLLTGKISQPGCGPFSLTGQPSACGTAR-EVGTFAHRLPAD--MVVTNEKHRD-----ICEKKWNIP  target    ERPLRAVAKDDEGDLTKGEVLTGKSHMPTPTKLIWFGNSNSLLGNAKWSFDVVKNTLPRQDAVFCNEWHWTSSCEYADLV 2nya.1    SGTI---PA--KIGLHAVAQDR--ALKDGKLNVYWTMCTNNMQAGPNINEERMPGWRDPRNFIIVSDPYPTVSALAADLI  target    FPADSWAEFKLPDATASCTNPFLLAFPTTPLKRLYDTRSDYEALALTAKALGELIDEPRMEQYWRGILDGDPTPYLQRIF 2nya.1    LPTAMWVEKE--GA-YGNAERRTQFW-RQQVQAPGEAKSDLWQLVQFSRRFKTE--------------------------  target    SGSNATRGITYDELHESSKRGVPLLMNMRTYPRSGGWEQRQEDKPWYTATGRLEFYRPEPEFQAAGESLPVWREPVDATF 2nya.1    --------------------------------------------------------------------------------  target    YEPNAILSNAAHPSIAPRAPEDYGVPESQLDVETRQYRNVVRTWAELQQTLHPLQERDPAFRFVF 2nya.1    ----------------------------------------------------------------- ``` | | | | | | | | | | | | | | | | | | | | | | | | | | | | | | | | | | | | | | | | | | | | | | | | | |
|  | 6cz7.1.A | ArrA  *The arsenate respiratory reductase (Arr) complex from Shewanella sp. ANA-3* | 0.31 |  | 17.15 | 0.55 | 71-767 | X-ray | 1.62 | hetero-1-1-mer | 5 x SF4, 2 x MGD, 1 x MO, 1 x PG5 | HHblits | 0.29 |
| ``` target    AQGVSRRQLLGRALALGSGAALADLLGPARFLSPAGAATAGAVVPGNPLRVMPDRTWEQIYRNQFEDDSTFVFTCAPNDT 6cz7.1    ----------------------------------------------------------------------WLATTCQGCT  target    HNCLLRAHVKNGVVVRISPTYGYGEATDLYGNRASHRWDPRTCQKGLILSRRFYSERRVKAPMIRKGFKDWVEAGYPRND 6cz7.1    SWCAKQIYVMDGRALKVRGN------------PNSGVHGMSSCPRQHLSLQQVYDPDRLRTPMMRTNPKK----------  target    DGTPQMDVTLRGSDDWIRISWDEATTIAAKTMEDVARTFNGDEGARKLLAQGYHPEMVEVMHGAGVQALKLRGGMPLLGI 6cz7.1    --------GRDQDPKFVPISWDKALDMLADKIIALRVANE--------------PHKYALLRGRYSHI----N-------  target    GRIFGFYRFANMLALLDRKLRPDAPADEILGSRTFDNYAWHTDLP--PGHPMVTGSQTVDFDLFSAEHTKLLLIIGMNWI 6cz7.1    --DLLYKKMTN-----------------LIGSPNNISHSSVCAEAHKMGPYYLDGN--WGYNQYDVKNAKFILSFGADPI  target    CTKMPDGHWIG--DARLKGTRVIVISADYMPTANKADEVIILRPGTDAAFFLGVARELIEKGLYDRAAVIERTDLPLLVR 6cz7.1    ASNRQVSFYSQTWGDSLDHAKVVVVDPRLSASAAKAHKWIPIEPGQDSVLALAIAHVALVEGVWHKPFVGDFIEGKNLFK  target    LDTGERLDARDVIPGYELAALTNYVTLKPDAEIKGNPPPPPFTAGGQVVPTELRDAWGDFVWWDRATGRPRPVSRDEVGA 6cz7.1    A--GKTV------------------------------------------------SVESFK-------------------  target    RFDGDPALLGEFEVELVDGSTVPVRPAFDLLKQYLDESFDLRTASEVCRVPPQAIQSIARQLAANKRETLLAAGMGPNHY 6cz7.1    -------------E-------THTYGLVEWWNQALK-DYTPEWASKITGIDPKTIIAIAKDMGAAAPAVQVWTSRGAVMQ  target    FQNDLFGRVQFLVAALTDNIGHLGGNVGSYAGNYRGSVFQAMGQWIAEDPFAIEPDLTKPATVKRYYKAESAHYWNYGER 6cz7.1    ARGTYTSISCHALNGLFGGIDSKGGLFPGNKTPLLK-EYPEAKAYM-DEI-A-AKGV-KKEKIDQRGRLEFPALAKG--K  target    PLRAVAKDDEGDLTKGEVLTGKSHMPTPTKLIWFGNSNSLLGNAKWSFDVVKNTLPRQDAVFCNEWHWTSSCEYADLVFP 6cz7.1    SGGGVI----TANAANGIRNQ---DPYEIKVMLAYFNNFNFSNPE--GQRWDEALSKVDFMAHITTNVSEFSWFADVLLP  target    ADS-WAEFKLPDATASCTNPF-LLAFPTTPLKRLYDTRSD-YEALALTAKALGELIDEPRMEQYWRGILDGDPTPYLQRI 6cz7.1    SSHHMFEKW--GVLDSIGNGVAQISIQQPSIKRLWDTRIDESEIPYMLAKKLAD--------------------------  target    FSGSNATRGITYDELHESSKRGVPLLMNMRTYPRSGGWEQRQEDKPWYTATGRLEFYRPEPEFQAAGESLPVWREPVDAT 6cz7.1    --------------------------------------------------------------------------------  target    FYEPNAILSNAAHPSIAPRAPEDYGVPESQLDVETRQYRNVVRTWAELQQTLHPLQERDPAFRFVF 6cz7.1    ------------------------------------------------------------------ ``` | | | | | | | | | | | | | | | | | | | | | | | | | | | | | | | | | | | | | | | | | | | | | | | | | |
|  | 2v45.1.A | PERIPLASMIC NITRATE REDUCTASE  *A NEW CATALYTIC MECHANISM OF PERIPLASMIC NITRATE REDUCTASE FROM DESULFOVIBRIO DESULFURICANS ATCC 27774 FROM CRYSTALLOGRAPHIC AND EPR DATA AND BASED ON DETAILED ANALYSIS OF THE SIXTH LIGAND* | 0.29 |  | 19.84 | 0.53 | 70-768 | X-ray | 2.40 | monomer | 1 x SF4, 1 x MO, 2 x MGD, 1 x LCP | HHblits | 0.29 |
| ``` target    AQGVSRRQLLGRALALGSGAALADLLGPARFLSPAGAATAGAVVPGNPLRVMPDRTWEQIYRNQFEDDSTFVFTCAPNDT 2v45.1    ---------------------------------------------------------------------KWVKGVCRYCG  target    HNCLLRAHVKNGVVVRISPTYGYGEATDLYGNRASHRWDPRTCQKGLILSRRFYSERRVKAPMIRKGFKDWVEAGYPRND 2v45.1    TGCGVLVGVKDGKAVAIQGNP------------NNH-NAGLLCLKGSLLIPVLNSKERVTQPLVRRH-------------  target    DGTPQMDVTLRGSDDWIRISWDEATTIAAKTMEDVARTFNGDEGARKLLAQGYHPEMVEVMHGAGVQALKLRGGMPLLGI 2v45.1    -----------KGGKLEPVSWDEALDLMASRFRSSIDMYGP--------------NSVAWYGSGQ---------------  target    GRIFGFYRFANMLALLDRKLRPDAPADEILGSRTFDNYAWHTD--LPPGHPMVTGSQTVDFDLFSAEHTKLLLIIGMNWI 2v45.1    --------CLTEESYVANKI-----FKGGFGTNNVDGNPRLCMASAVGGYVTSFGKDEPMGTYADIDQATCFFIIGSNTS  target    CTKMPDGHWIGDAR--LKGTRVIVISADYMPTANKADEVIILRPGTDAAFFLGVARELIEKGLYDRAAVIERTDLPLLVR 2v45.1    EAHPVLFRRIARRKQVEPGVKIIVADPRRTNTSRIADMHVAFRPGTDLAFMHSMAWVIINEELDNPRFWQRYVNF-----  target    LDTGERLDARDVIPGYELAALTNYVTLKPDAEIKGNPPPPPFTAGGQVVPTELRDAWGDFVWWDRATGRPRPVSRDEVGA 2v45.1    ---------------MDA--------------------------------------------------------------  target    RFDGDPALLGEFEVELVDGSTVPVRPAFDLLKQYLDESFDLRTASEVCRVPPQAIQSIARQLAANKRETLLAAGMGPNHY 2v45.1    ---------------------EGKPSDFEGYKAFLE-NYRPEKVAEICRVPVEQIYGAARAFAESAAT-MSLWCMGINQR  target    FQNDLFGRVQFLVAALTDNIGHLGGNVGSYAGNY--RGSVFQAMGQWIAEDPFAIEPDLTKPATVKRYYKAESAHYWNYG 2v45.1    VQGVFANNLIHNLHLITGQICRPGATSFSLTGQPNACGGVR-DGGALSHLLPAG--RAIPNAKHRA-----EMEKLWGLP  target    ERPLRAVAKDDEGDLTKGEVLTGKSHMPTPTKLIWFGNSNSLLGNAKWSFDVVKNTLPRQD-AVFCNEWHWT-SSCEYAD 2v45.1    EGRIAP-----EPGYHTVALFEAL--GRGDVKCMIICETNPAHTLPN--LNKVHKAMSHPESFIVCIEAFPDAVTLEYAD  target    LVFPADSWAEFKLPDATASCTNPFLLAFPTTPLKRLYDTRSDYEALALTAKALGELIDEPRMEQYWRGILDGDPTPYLQR 2v45.1    LVLPPAFWCERD--G-VYGCGERRYSL-TEKAVDPPGQCRPTVNTLVEFARRAGVD------------------------  target    IFSGSNATRGITYDELHESSKRGVPLLMNMRTYPRSGGWEQRQEDKPWYTATGRLEFYRPEPEFQAAGESLPVWREPVDA 2v45.1    --------------------------------------------------------------------------------  target    TFYEPNAILSNAAHPSIAPRAPEDYGVPESQLDVETRQYRNVVRTWAELQQTLHPLQERDPAFRFVF 2v45.1    ------------------------------------------------------------------- ``` | | | | | | | | | | | | | | | | | | | | | | | | | | | | | | | | | | | | | | | | | | | | | | | | | |
|  | 2v3v.1.A | PERIPLASMIC NITRATE REDUCTASE  *A NEW CATALYTIC MECHANISM OF PERIPLASMIC NITRATE REDUCTASE FROM DESULFOVIBRIO DESULFURICANS ATCC 27774 FROM CRYSTALLOGRAPHIC AND EPR DATA AND BASED ON DETAILED ANALYSIS OF THE SIXTH LIGAND* | 0.30 |  | 19.68 | 0.53 | 71-768 | X-ray | 1.99 | monomer | 1 x SF4, 1 x MO, 2 x MGD, 4 x LCP | HHblits | 0.29 |
| ``` target    AQGVSRRQLLGRALALGSGAALADLLGPARFLSPAGAATAGAVVPGNPLRVMPDRTWEQIYRNQFEDDSTFVFTCAPNDT 2v3v.1    ----------------------------------------------------------------------WVKGVCRYCG  target    HNCLLRAHVKNGVVVRISPTYGYGEATDLYGNRASHRWDPRTCQKGLILSRRFYSERRVKAPMIRKGFKDWVEAGYPRND 2v3v.1    TGCGVLVGVKDGKAVAIQGD------------PNNH-NAGLLCLKGSLLIPVLNSKERVTQPLVRRH-------------  target    DGTPQMDVTLRGSDDWIRISWDEATTIAAKTMEDVARTFNGDEGARKLLAQGYHPEMVEVMHGAGVQALKLRGGMPLLGI 2v3v.1    -----------KGGKLEPVSWDEALDLMASRFRSSIDMYGP--------------NSVAWYGSGQ---------------  target    GRIFGFYRFANMLALLDRKLRPDAPADEILGSRTFDNYAWHTD--LPPGHPMVTGSQTVDFDLFSAEHTKLLLIIGMNWI 2v3v.1    --------CLTEESYVANKI-----FKGGFGTNNVDGNPRLCMASAVGGYVTSFGKDEPMGTYADIDQATCFFIIGSNTS  target    CTKMPDGHWIGDAR--LKGTRVIVISADYMPTANKADEVIILRPGTDAAFFLGVARELIEKGLYDRAAVIERTDLPLLVR 2v3v.1    EAHPVLFRRIARRKQVEPGVKIIVADPRRTNTSRIADMHVAFRPGTDLAFMHSMAWVIINEELDNPRFWQRYVNF-----  target    LDTGERLDARDVIPGYELAALTNYVTLKPDAEIKGNPPPPPFTAGGQVVPTELRDAWGDFVWWDRATGRPRPVSRDEVGA 2v3v.1    ---------------MDA--------------------------------------------------------------  target    RFDGDPALLGEFEVELVDGSTVPVRPAFDLLKQYLDESFDLRTASEVCRVPPQAIQSIARQLAANKRETLLAAGMGPNHY 2v3v.1    ---------------------EGKPSDFEGYKAFLE-NYRPEKVAEICRVPVEQIYGAARAFAESAAT-MSLWCMGINQR  target    FQNDLFGRVQFLVAALTDNIGHLGGNVGSYAGNY--RGSVFQAMGQWIAEDPFAIEPDLTKPATVKRYYKAESAHYWNYG 2v3v.1    VQGVFANNLIHNLHLITGQICRPGATSFSLTGQPNACGGVR-DGGALSHLLPAG--RAIPNAKHRAE-----MEKLWGLP  target    ERPLRAVAKDDEGDLTKGEVLTGKSHMPTPTKLIWFGNSNSLLGNAKWSFDVVKNTLPRQD-AVFCNEWHWT-SSCEYAD 2v3v.1    EGRIAP-----EPGYHTVALFEAL--GRGDVKCMIICETNPAHTLPN--LNKVHKAMSHPESFIVCIEAFPDAVTLEYAD  target    LVFPADSWAEFKLPDATASCTNPFLLAFPTTPLKRLYDTRSDYEALALTAKALGELIDEPRMEQYWRGILDGDPTPYLQR 2v3v.1    LVLPPAFWCERD--G-VYGCGERRYSL-TEKAVDPPGQCRPTVNTLVEFARRAGVD------------------------  target    IFSGSNATRGITYDELHESSKRGVPLLMNMRTYPRSGGWEQRQEDKPWYTATGRLEFYRPEPEFQAAGESLPVWREPVDA 2v3v.1    --------------------------------------------------------------------------------  target    TFYEPNAILSNAAHPSIAPRAPEDYGVPESQLDVETRQYRNVVRTWAELQQTLHPLQERDPAFRFVF 2v3v.1    ------------------------------------------------------------------- ``` | | | | | | | | | | | | | | | | | | | | | | | | | | | | | | | | | | | | | | | | | | | | | | | | | |
|  | 2ivf.1.A | ETHYLBENZENE DEHYDROGENASE ALPHA-SUBUNIT  *ETHYLBENZENE DEHYDROGENASE FROM AROMATOLEUM AROMATICUM* | 0.28 | 0.00 | 35.66 | 0.44 | 56-590 | X-ray | 1.88 | monomer | 1 x MES, 4 x SF4, 1 x MO, 1 x MGD, 1 x MD1, 1 x F3S, 1 x HEM | BLAST | 0.38 |
| ``` target    AQGVSRRQLLGRALALGSGAALADLLGPARFLSPAGAATAGAVVPGNPLRVMPDRTWEQIYRNQFEDDS----TFVFTCA 2ivf.1    -------------------------------------------------------SWEDIYRKEWKWDKVNWGSHLNICW  target    PNDTHNCLLRAHVKNGVVVRISPTYGYGEATDLYGNRASHRWDPRTCQKGLILSRRFYSERRVKAPMIRKGFKDWVEAGY 2ivf.1    PQGS--CKFYVYVRNGIVWREEQA-----AQTPACNVDYVDYNPLGCQKGSAFNNNLYGDERVKYPLKRVG---------  target    PRNDDGTPQMDVTLRGSDDWIRISWDEATTIAAKTMEDVARTFNGDEGARKLLAQGYHPEMVEVMHGAGVQALKLRGGMP 2ivf.1    -------------KRGEGKWKRVSWDEA---AGDIADSIIDSFEA-QGSDGFILDAPHVHAGSIAWGAGFRMTYLMDG--  target    LLGIGRIFGFYRFANMLALLDRKLRPDAPADEILGSRTFDNY--AWHTDLPPGHPMVTGSQTVDFDLFSAEHTKLLLIIG 2ivf.1    -----------------------VSPDINVD--IG----DTYMGAFHT---------FGKMHMGYSADNLLDAELIFMTC  target    MNWICTKMPDGHWIGDARLKGTRVIVISADYMPTANKADEVIILRPGTDAAFFLGVARELIEKGLYDRAAVIERTDLPLL 2ivf.1    SNWSYTYPSSYHFLSEARYKGAEVVVIAPDFNPTTPAADLHVPVRVGSDAAFWLGLSQVMIDEKLFDRQFVCEQTDLPLL  target    VRLDTGERLDARDVIPGYELAALTNYVTLKPDAEIKGNPPPPPFTAGGQVVPTELRDAWGDFVWWDRATGRPRPVSRDEV 2ivf.1    VRMDTGKFLSAEDV-------------------------------DGGEA---------KQFYFFDEKAGSVRKASRGTL  target    GARFDGDPALLGEFEVELVDGSTVPVRPAFDLLKQYLDESFDLRTASEVCRVPPQAIQSIARQLAANKRETLLAAGMGPN 2ivf.1    --KLDFMPALEGTFSARLKNGKTIQVRTVFEGLREHLKD-YTPEKASAKCGVPVSLIRELGRKVA--KKRTCSYIGFSSA  target    HYFQNDLFGRVQFLVAALTDNIGHLGGNVGSYAGNYRGSVFQAMGQWIAEDPFAIEPDLTKPATVKRYYKAESAHYWNYG 2ivf.1    KSYHGDLMERSLFLAMALSGNWGKPG--TGAFAWAY--------------------------------------------  target    ERPLRAVAKDDEGDLTKGEVLTGKSHMPTPTKLIWFGNSNSLLGNAKWSFDVVKNTLPRQDAVFCNEWHWTSSCEYADLV 2ivf.1    --------------------------------------------------------------------------------  target    FPADSWAEFKLPDATASCTNPFLLAFPTTPLKRLYDTRSDYEALALTAKALGELIDEPRMEQYWRGILDGDPTPYLQRIF 2ivf.1    --------------------------------------------------------------------------------  target    SGSNATRGITYDELHESSKRGVPLLMNMRTYPRSGGWEQRQEDKPWYTATGRLEFYRPEPEFQAAGESLPVWREPVDATF 2ivf.1    --------------------------------------------------------------------------------  target    YEPNAILSNAAHPSIAPRAPEDYGVPESQLDVETRQYRNVVRTWAELQQTLHPLQERDPAFRFVF 2ivf.1    ----------------------------------------------------------------- ``` | | | | | | | | | | | | | | | | | | | | | | | | | | | | | | | | | | | | | | | | | | | | | | | | | |
|  | 7t2r.1.A | NiFe hydrogenase subunit A  *Structure of electron bifurcating Ni-Fe hydrogenase complex HydABCSL in FMN-free apo state* | 0.25 |  | 15.17 | 0.50 | 37-768 | EM | 0.00 | hetero-2-2-2-2-2-mer | 6 x FES, 12 x SF4, 2 x 3NI, 2 x FCO | HHblits | 0.27 |
| ``` target    AQGVSRRQLLGRALALGSGAALADLLGPARFLSPAGAATAGAVVPGNPLRVMPDRTWEQIYRNQFEDDSTFVFTCAPNDT 7t2r.1    ------------------------------------CVNCGACAQSCPTGTITIREFAYRGR-RSE-CDAVVESVCPLCA  target    HNCLLRAHVKNGVVVRISPTYGYGEATDLYGNRASHRWDPRTCQKGLILSRRFYSERRVKAPMIRKGFKDWVEAGYPRND 7t2r.1    VGCKIKTYVRTGSIVRVEGT------------GVEEPDGGQLCHMGRWWLPESTERERVTVPLIREG-------------  target    DGTPQMDVTLRGSDDWIRISWDEATTIAAKTMEDVARTFNGDEGARKLLAQGYHPEMVEVMHGAGVQALKLRGGMPLLGI 7t2r.1    -------------ASYREATWEEALALASAEFKKAYDQE--------------KAGAI----------------------  target    GRIFGFYRFANMLALLDRKLRPDAPADEILGSRTFDNYAWHTDL--PPGHP-M-VTGSQTVDFDLFSAEHTKLLLIIGMN 7t2r.1    ----LSSLCTDEELTLFSALF-----RNALKMKHIDTFDGDIIRGFFKGFMPFREQGV-RPFTAAHHILDSDLIITMFAD  target    WICTKMPDGHWIGDAR-LKGTRVIVISADYMPTANKADEVIILRPGTDAAFFLGVARELIEKGLYDRAAVIERTDLPLLV 7t2r.1    PQKEAPVVASYIRVACLHRNAKLMNLSYGPSPFPGLVDLDIRLPEGQAVPKALSNLAEIIGKISLGPSDMASFGE-----  target    RLDTGERLDARDVIPGYELAALTNYVTLKPDAEIKGNPPPPPFTAGGQVVPTELRDAWGDFVWWDRATGRPRPVSRDEVG 7t2r.1    ------------FEAG----------------------------------------------------------------  target    ARFDGDPALLGEFEVELVDGSTVPVRPAFDLLKQYLDESFDLRTASEVCRVPPQAIQSIARQLAANKRETLLAAGMGPNH 7t2r.1    ----------------------------AGKALSSY--RESIEESARAMGLDPKIAEEVALMLISARRPIFI-IGGRA--  target    YFQNDLFGRVQFLVAALTDNIGHLGGNVGSYAGNYRGSVFQAMGQWIAEDPFAIEPDLTKPATVKRYYKAESAHYWNYGE 7t2r.1    -TKSHELVTAACNLAVASKAFFEDGLGVVPLLVSAN-----SLGAR----------NT----V--------------VSE  target    RPLRAVAKDDEGDLTKGEVLTGKSHMPTPTKLIWFGNSNSLLGNAKWSFDVVKNTLPRQDAVFCNEWHWT-SSCEYADLV 7t2r.1    NP---------------W--LG----RERRDFLYVFSTAMV---P--EEEEILAAISATRFVVVQTPFKVRPLVNLADIL  target    FPADSWAEFKLPDATASCTNPFLLAFPTTPLKRLYDTRSDYEALALTAKALGELIDEPRMEQYWRGILDGDPTPYLQRIF 7t2r.1    LPAPAWYERS---GHFCTIEGERRKL-NTIVPPKGEIKSLHYVMDEFAKKLGVK--------------------------  target    SGSNATRGITYDELHESSKRGVPLLMNMRTYPRSGGWEQRQEDKPWYTATGRLEFYRPEPEFQAAGESLPVWREPVDATF 7t2r.1    --------------------------------------------------------------------------------  target    YEPNAILSNAAHPSIAPRAPEDYGVPESQLDVETRQYRNVVRTWAELQQTLHPLQERDPAFRFVF 7t2r.1    ----------------------------------------------------------------- ``` | | | | | | | | | | | | | | | | | | | | | | | | | | | | | | | | | | | | | | | | | | | | | | | | | |
|  | 7t30.1.A | NiFe hydrogenase subunit A  *Structure of electron bifurcating Ni-Fe hydrogenase complex HydABCSL in FMN/NAD(H) bound state* | 0.25 |  | 15.17 | 0.50 | 37-768 | EM | 0.00 | hetero-2-2-2-2-2-mer | 4 x FES, 12 x SF4, 2 x NAD, 2 x FMN, 2 x 3NI, 2 x FCO | HHblits | 0.27 |
| ``` target    AQGVSRRQLLGRALALGSGAALADLLGPARFLSPAGAATAGAVVPGNPLRVMPDRTWEQIYRNQFEDDSTFVFTCAPNDT 7t30.1    ------------------------------------CVNCGACAQSCPTGTITIREFAYRGR-RSE-CDAVVESVCPLCA  target    HNCLLRAHVKNGVVVRISPTYGYGEATDLYGNRASHRWDPRTCQKGLILSRRFYSERRVKAPMIRKGFKDWVEAGYPRND 7t30.1    VGCKIKTYVRTGSIVRVEGT------------GVEEPDGGQLCHMGRWWLPESTERERVTVPLIREG-------------  target    DGTPQMDVTLRGSDDWIRISWDEATTIAAKTMEDVARTFNGDEGARKLLAQGYHPEMVEVMHGAGVQALKLRGGMPLLGI 7t30.1    -------------ASYREATWEEALALASAEFKKAYDQE--------------KAGAI----------------------  target    GRIFGFYRFANMLALLDRKLRPDAPADEILGSRTFDNYAWHTDL--PPGHP-M-VTGSQTVDFDLFSAEHTKLLLIIGMN 7t30.1    ----LSSLCTDEELTLFSALF-----RNALKMKHIDTFDGDIIRGFFKGFMPFREQGV-RPFTAAHHILDSDLIITMFAD  target    WICTKMPDGHWIGDAR-LKGTRVIVISADYMPTANKADEVIILRPGTDAAFFLGVARELIEKGLYDRAAVIERTDLPLLV 7t30.1    PQKEAPVVASYIRVACLHRNAKLMNLSYGPSPFPGLVDLDIRLPEGQAVPKALSNLAEIIGKISLGPSDMASFGE-----  target    RLDTGERLDARDVIPGYELAALTNYVTLKPDAEIKGNPPPPPFTAGGQVVPTELRDAWGDFVWWDRATGRPRPVSRDEVG 7t30.1    ------------FEAG----------------------------------------------------------------  target    ARFDGDPALLGEFEVELVDGSTVPVRPAFDLLKQYLDESFDLRTASEVCRVPPQAIQSIARQLAANKRETLLAAGMGPNH 7t30.1    ----------------------------AGKALSSY--RESIEESARAMGLDPKIAEEVALMLISARRPIFI-IGGRA--  target    YFQNDLFGRVQFLVAALTDNIGHLGGNVGSYAGNYRGSVFQAMGQWIAEDPFAIEPDLTKPATVKRYYKAESAHYWNYGE 7t30.1    -TKSHELVTAACNLAVASKAFFEDGLGVVPLLVSAN-----SLGAR----------NT----V--------------VSE  target    RPLRAVAKDDEGDLTKGEVLTGKSHMPTPTKLIWFGNSNSLLGNAKWSFDVVKNTLPRQDAVFCNEWHWT-SSCEYADLV 7t30.1    NP---------------W--LG----RERRDFLYVFSTAMV---P--EEEEILAAISATRFVVVQTPFKVRPLVNLADIL  target    FPADSWAEFKLPDATASCTNPFLLAFPTTPLKRLYDTRSDYEALALTAKALGELIDEPRMEQYWRGILDGDPTPYLQRIF 7t30.1    LPAPAWYERS---GHFCTIEGERRKL-NTIVPPKGEIKSLHYVMDEFAKKLGVK--------------------------  target    SGSNATRGITYDELHESSKRGVPLLMNMRTYPRSGGWEQRQEDKPWYTATGRLEFYRPEPEFQAAGESLPVWREPVDATF 7t30.1    --------------------------------------------------------------------------------  target    YEPNAILSNAAHPSIAPRAPEDYGVPESQLDVETRQYRNVVRTWAELQQTLHPLQERDPAFRFVF 7t30.1    ----------------------------------------------------------------- ``` | | | | | | | | | | | | | | | | | | | | | | | | | | | | | | | | | | | | | | | | | | | | | | | | | |
|  | 7p63.1.C | NADH-quinone oxidoreductase  *Complex I from E. coli, DDM/LMNG-purified, under Turnover at pH 6, Closed state* | 0.25 | 0.00 | 18.76 | 0.47 | 36-767 | EM | 0.00 | monomer | 7 x SF4, 1 x FMN, 1 x NAI, 2 x FES, 1 x CA, 1 x DCQ, 4 x LFA, 8 x 3PE | HHblits | 0.28 |
| ``` target    AQGVSRRQLLGRALALGSGAALADLLGPARFLSPAGAATAGAVVPGNPLRVMPDRTWEQIYRNQFEDDSTFVFTCAPNDT 7p63.1    -----------------------------------ESEFSGNLVEICPTGVFTDKTHSERYNR--KWDMQFAPSICQQCS  target    HNCLLRAHVKNGVVVRISPTYGYGEATDLYGNRASHRWDPRTCQKGLILSRRFYSERRVKAPMIRKGFKDWVEAGYPRND 7p63.1    IGCNISPGERYGELRRIENR------------YNGTVNHYFLCDRGRFGYGYVNLKDRPRQPVQRRG-------------  target    DGTPQMDVTLRGSDDWIRISWDEATTIAAKTMEDVARTFNGDEGARKLLAQGYHPEMVEVMHGAGVQALKLRGGMPLLGI 7p63.1    -------------DDFITLNAEQAMQGAADILRQSKKVIG--------I-------------------------------  target    GRIFGFYRFANMLALLDRKLRPDAPADEILGSRTFDNYAWHTDLP---PGHPMVTGSQTVDFDLFSAEHTKLLLIIGMNW 7p63.1    ----GSPRASVESNFALR---------ELVGEENFYTGIAHGEQERLQLALKVLREGGIYTPALREIESYDAVLVLGEDV  target    ICTKMPDGHWIGDARLKGTR--------------------------VIVISADYMPTANKADEVIILRPGTDAAFFLGVA 7p63.1    TQTGARVALAVRQAVKGKAREMAAAQKVADWQIAAILNIGQRAKHPLFVTNVDDTRLDDIAAWTYRAPVEDQARLGFAIA  target    RELIEKGLYDRAAVIERTDLPLLVRLDTGERLDARDVIPGYELAALTNYVTLKPDAEIKGNPPPPPFTAGGQVVPTELRD 7p63.1    HALDNSAP----A-------------------------------------------------------------------  target    AWGDFVWWDRATGRPRPVSRDEVGARFDGDPALLGEFEVELVDGSTVPVRPAFDLLKQYLDESFDLRTASEVCRVPPQAI 7p63.1    -----------------------------------------------------------V-DGIEPEL--------QSKI  target    QSIARQLAANKRETLLAAGMGPNHYFQNDLFGRVQFLVAALTDNIGHLGGNVGSYAG-NYRGSVFQAMGQWIAEDPFAIE 7p63.1    DVIVQALAGAKKPLI-ISGTNAG----SLEVIQAAANVAKALKGRGADVGITMIARSVNSMG-------LG---------  target    PDLTKPATVKRYYKAESAHYWNYGERPLRAVAKDDEGDLTKGEVLTGKSHMPTPTKLIWFGNSNSLLGNAKWSFDVVKNT 7p63.1    -IM--------------------GGGS-------------LEEALTEL--ETGRADAVVVLE-NDLHRHAS--ATRVNAA  target    LPRQDAVFCNEWHWTSSCEYADLVFPADSWAEFKLPDATASCTNPFLLAFPTTPLKRLY-----DTRSDYEALALTAKAL 7p63.1    LAKAPLVMVVDHQRTAIMENAHLVLSAASFAESD---GTVINNEGRAQRF-FQVYDPAYYDSKTVMLESWRWLHSLHSTL  target    GELIDEPRMEQYWRGILDGDPTPYLQRIFSGSNATRGITYDELHESSKRGVPLLMNMRTYPRSGGWEQRQEDKPWYTATG 7p63.1    LS------------------------------------------------------------------------------  target    RLEFYRPEPEFQAAGESLPVWREPVDATFYEPNAILSNAAHPSIAPRAPEDYGVPESQLDVETRQYRNVVRTWAELQQTL 7p63.1    --------------------------------------------------------------------------------  target    HPLQERDPAFRFVF 7p63.1    -------------- ``` | | | | | | | | | | | | | | | | | | | | | | | | | | | | | | | | | | | | | | | | | | | | | | | | | |
|  | 7p61.1.C | NADH-quinone oxidoreductase  *Complex I from E. coli, DDM-purified, with NADH, Resting state* | 0.25 | 0.00 | 18.54 | 0.47 | 36-767 | EM | 0.00 | monomer | 7 x SF4, 1 x FMN, 1 x NAI, 2 x FES, 1 x CA, 2 x 3PE, 1 x UQ8 | HHblits | 0.27 |
| ``` target    AQGVSRRQLLGRALALGSGAALADLLGPARFLSPAGAATAGAVVPGNPLRVMPDRTWEQIYRNQFEDDSTFVFTCAPNDT 7p61.1    -----------------------------------ESEFSGNLVEICPTGVFTDKTHSERYNR--KWDMQFAPSICQQCS  target    HNCLLRAHVKNGVVVRISPTYGYGEATDLYGNRASHRWDPRTCQKGLILSRRFYSERRVKAPMIRKGFKDWVEAGYPRND 7p61.1    IGCNISPGERYGELRRIENR------------YNGTVNHYFLCDRGRFGYGYVNLKDRPRQPVQRRG-------------  target    DGTPQMDVTLRGSDDWIRISWDEATTIAAKTMEDVARTFNGDEGARKLLAQGYHPEMVEVMHGAGVQALKLRGGMPLLGI 7p61.1    -------------DDFITLNAEQAMQGAADILRQSKKVIG--------I-------------------------------  target    GRIFGFYRFANMLALLDRKLRPDAPADEILGSRTFDNYAWHTDL---PPGHPMVTGSQTVDFDLFSAEHTKLLLIIGMNW 7p61.1    ----GSPRASVESNFALRE---------LVGEENFYTGIAHGEQERLQLALKVLREGGIYTPALREIESYDAVLVLGEDV  target    ICTKMPDGHWIGDARLKGTR--------------------------VIVISADYMPTANKADEVIILRPGTDAAFFLGVA 7p61.1    TQTGARVALAVRQAVKGKAREMAAAQKVADWQIAAILNIGQRAKHPLFVTNVDDTRLDDIAAWTYRAPVEDQARLGFAIA  target    RELIEKGLYDRAAVIERTDLPLLVRLDTGERLDARDVIPGYELAALTNYVTLKPDAEIKGNPPPPPFTAGGQVVPTELRD 7p61.1    HALDNSAP-------A----------------------------------------------------------------  target    AWGDFVWWDRATGRPRPVSRDEVGARFDGDPALLGEFEVELVDGSTVPVRPAFDLLKQYLDESFDLRTASEVCRVPPQAI 7p61.1    -----------------------------------------------------------V-DGIEPEL--------QSKI  target    QSIARQLAANKRETLLAAGMGPNHYFQNDLFGRVQFLVAALTDNIGHLGGNVGSYAG-NYRGSVFQAMGQWIAEDPFAIE 7p61.1    DVIVQALAGAKKP-LIISGTNAG----SLEVIQAAANVAKALKGRGADVGITMIARSVNSMG-------LG---------  target    PDLTKPATVKRYYKAESAHYWNYGERPLRAVAKDDEGDLTKGEVLTGKSHMPTPTKLIWFGNSNSLLGNAKWSFDVVKNT 7p61.1    -IM--------------------GGGS-------------LEEALTEL--ETGRADAVVVLE-NDLHRHAS--ATRVNAA  target    LPRQDAVFCNEWHWTSSCEYADLVFPADSWAEFKLPDATASCTNPFLLAFPTTPLKRLY-----DTRSDYEALALTAKAL 7p61.1    LAKAPLVMVVDHQRTAIMENAHLVLSAASFAESD---GTVINNEGRAQRF-FQVYDPAYYDSKTVMLESWRWLHSLHSTL  target    GELIDEPRMEQYWRGILDGDPTPYLQRIFSGSNATRGITYDELHESSKRGVPLLMNMRTYPRSGGWEQRQEDKPWYTATG 7p61.1    LS------------------------------------------------------------------------------  target    RLEFYRPEPEFQAAGESLPVWREPVDATFYEPNAILSNAAHPSIAPRAPEDYGVPESQLDVETRQYRNVVRTWAELQQTL 7p61.1    --------------------------------------------------------------------------------  target    HPLQERDPAFRFVF 7p61.1    -------------- ``` | | | | | | | | | | | | | | | | | | | | | | | | | | | | | | | | | | | | | | | | | | | | | | | | | |
|  | 7nz1.1.E | NADH-quinone oxidoreductase subunit G  *Respiratory complex I from Escherichia coli - focused refinement of cytoplasmic arm* | 0.26 | 0.00 | 18.31 | 0.47 | 36-767 | EM | 0.00 | monomer | 7 x SF4, 2 x FES, 1 x FMN, 1 x CA | HHblits | 0.27 |
| ``` target    AQGVSRRQLLGRALALGSGAALADLLGPARFLSPAGAATAGAVVPGNPLRVMPDRTWEQIYRNQFEDDSTFVFTCAPNDT 7nz1.1    -----------------------------------ESEFSGNLVEICPTGVFTDKTHSERYNR--KWDMQFAPSICQQCS  target    HNCLLRAHVKNGVVVRISPTYGYGEATDLYGNRASHRWDPRTCQKGLILSRRFYSERRVKAPMIRKGFKDWVEAGYPRND 7nz1.1    IGCNISPGERYGELRRIENR------------YNGTVNHYFLCDRGRFGYGYVNLKDRPRQPVQRRG-------------  target    DGTPQMDVTLRGSDDWIRISWDEATTIAAKTMEDVARTFNGDEGARKLLAQGYHPEMVEVMHGAGVQALKLRGGMPLLGI 7nz1.1    -------------DDFITLNAEQAMQGAADILRQSKKVIG--------I-------------------------------  target    GRIFGFYRFANMLALLDRKLRPDAPADEILGSRTFDNYAWHTDL---PPGHPMVTGSQTVDFDLFSAEHTKLLLIIGMNW 7nz1.1    ----GSPRASVESNFALRE---------LVGEENFYTGIAHGEQERLQLALKVLREGGIYTPALREIESYDAVLVLGEDV  target    ICTKMPDGHWIGDARLKGTR--------------------------VIVISADYMPTANKADEVIILRPGTDAAFFLGVA 7nz1.1    TQTGARVALAVRQAVKGKAREMAAAQKVADWQIAAILNIGQRAKHPLFVTNVDDTRLDDIAAWTYRAPVEDQARLGFAIA  target    RELIEKGLYDRAAVIERTDLPLLVRLDTGERLDARDVIPGYELAALTNYVTLKPDAEIKGNPPPPPFTAGGQVVPTELRD 7nz1.1    HALDNSAP-------A----------------------------------------------------------------  target    AWGDFVWWDRATGRPRPVSRDEVGARFDGDPALLGEFEVELVDGSTVPVRPAFDLLKQYLDESFDLRTASEVCRVPPQAI 7nz1.1    -----------------------------------------------------------V-DGIEPEL--------QSKI  target    QSIARQLAANKRETLLAAGMGPNHYFQNDLFGRVQFLVAALTDNIGHLGGNVGSYAG-NYRGSVFQAMGQWIAEDPFAIE 7nz1.1    DVIVQALAGAKKPL-IISGTNAG----SLEVIQAAANVAKALKGRGADVGITMIARSVNSM-------GLG---------  target    PDLTKPATVKRYYKAESAHYWNYGERPLRAVAKDDEGDLTKGEVLTGKSHMPTPTKLIWFGNSNSLLGNAKWSFDVVKNT 7nz1.1    -IM--------------------GGGS-------------LEEALTEL--ETGRADAVVVLE-NDLHRHAS--AIRVNAA  target    LPRQDAVFCNEWHWTSSCEYADLVFPADSWAEFKLPDATASCTNPFLLAFPTTPLKRLY-----DTRSDYEALALTAKAL 7nz1.1    LAKAPLVMVVDHQRTAIMENAHLVLSAASFAESD---GTVINNEGRAQRF-FQVYDPAYYDSKTVMLESWRWLHSLHSTL  target    GELIDEPRMEQYWRGILDGDPTPYLQRIFSGSNATRGITYDELHESSKRGVPLLMNMRTYPRSGGWEQRQEDKPWYTATG 7nz1.1    LS------------------------------------------------------------------------------  target    RLEFYRPEPEFQAAGESLPVWREPVDATFYEPNAILSNAAHPSIAPRAPEDYGVPESQLDVETRQYRNVVRTWAELQQTL 7nz1.1    --------------------------------------------------------------------------------  target    HPLQERDPAFRFVF 7nz1.1    -------------- ``` | | | | | | | | | | | | | | | | | | | | | | | | | | | | | | | | | | | | | | | | | | | | | | | | | |
|  | 6yj4.1.G | Subunit NUAM of NADH:Ubiquinone Oxidoreductase (Complex I)  *Structure of Yarrowia lipolytica complex I at 2.7 A* | 0.24 | 0.00 | 17.23 | 0.44 | 37-769 | EM | 0.00 | monomer | 18 x 3PE, 6 x SF4, 5 x LMT, 8 x PLC, 2 x FES, 1 x FMN, 6 x CDL, 1 x NDP, 1 x ZN, 2 x EHZ | HHblits | 0.29 |
| ``` target    AQGVSRRQLLGRALALGSGAALADLLGPARFLSPAGAATAGAVVPGNPLRVMPDRTWEQIYRNQFEDDSTFVFTCAPNDT 6yj4.1    ------------------------------------TELSGNVIDLCPVGALTNKPYAFRAR---PWELKKTESIDVMDA  target    HNCLLRAHVKNGVVVRISPTYGYGEATDLYGNRASHRWDPRTCQKGLILSRRFYSERRVKAPMIRKGFKDWVEAGYPRND 6yj4.1    VGSNIRIDSKGVEVMRVIPRV------------HEDVNEEWINDKSRFACDGLK-TQRLTTPLIRVG-------------  target    DGTPQMDVTLRGSDDWIRISWDEATTIAAKTMEDVARTFNGDEGARKLLAQGYHPEMVEVMHGAGVQALKLRGGMPLLGI 6yj4.1    -------------DKFVNATWDDALSTIAKAYQQKAP--K--------------GDEFKAVAGALVEV------------  target    GRIFGFYRFANMLALLDRKLRPDAPADEILGSRTFDNYAWHTDLPPGHPMVTGSQ-TVDFDLFSAEHTKLLLIIGMNWIC 6yj4.1    ESMVALKDMTN-----------------ALGSENTTTDTPNGNSAPAHGITFRSNYLFNSSIAGIEDADAILLVGTNPRR  target    TKMPDGHWIGDAR-LKGTRVIVISADYMPTANKADEVIILRPGTDAAFFLGVARELIEKGLYDRAAVIERTDLPLLVRLD 6yj4.1    EAAVMNARIRKAWLRQELEIASVGPTLDATFDVAEL----------------------------------GN--------  target    TGERLDARDVIPGYELAALTNYVTLKPDAEIKGNPPPPPFTAGGQVVPTELRDAWGDFVWWDRATGRPRPVSRDEVGARF 6yj4.1    ------------TH------------------------------------------------------------------  target    DGDPALLGEFEVELVDGSTVPVRPAFDLLKQYLDESFDLRTASEVCRVPPQAIQSIARQLAANKRETLLAAGMGPNHYFQ 6yj4.1    --------------------------ADLEKALS-------------------GEFGEVLKNAKNP-LIIVGSGITDRED  target    NDLFGRVQFLVAALTDNI-GHLGGNVGSYAGNYRGSVFQAMGQWIAEDPFAIEPDLTKPATVKRYYKAESAHYWNYGERP 6yj4.1    AGAFFNTIGKFVESTPSVLNENWNGYNVLQRSAS-----RAGAY----------------D--------------IGFTP  target    LRAVAKDDEGDLTKGEVLTGKSHMPTPTKLIWFGNSNSLLGNAKWSFDVVKNTLPRQDAVFCNEWHWTSSCEYADLVFPA 6yj4.1    S-------------DEA------SKTTPKMVWLLGADEVAAS----------DIPADAFVVYQGHNGDVGAQFADVVLPG  target    DSWAEFKLPDATASCTNPFLLAFPTTPLKRLYDTRSDYEALALTAKALGELIDEPRMEQYWRGILDGDPTPYLQRIFSGS 6yj4.1    AAYTEKA---GTYVNTEGRSQIS-RAATGPPGGAREDWKILRAVSEYLGVAL----------------------------  target    NATRGITYDELHESSKRGVPLLMNMRTYPRSGGWEQRQEDKPWYTATGRLEFYRPEPEFQAAGESLPVWREPVDATFYEP 6yj4.1    --------------------------------------------------------------------------------  target    NAILSNAAHPSIAPRAPEDYGVPESQLDVETRQYRNVVRTWAELQQTLHPLQERDPAFRFVF 6yj4.1    -------------------------------------------------------------- ``` | | | | | | | | | | | | | | | | | | | | | | | | | | | | | | | | | | | | | | | | | | | | | | | | | |
|  | 6rfs.1.A | Subunit NUAM of NADH:Ubiquinone Oxidoreductase (Complex I)  *Cryo-EM structure of a respiratory complex I mutant lacking NDUFS4* | 0.24 | 0.00 | 17.23 | 0.44 | 37-769 | EM | 4.04 | monomer | 6 x SF4, 2 x FES, 1 x FMN, 1 x NDP, 1 x ZN, 1 x ZMP | HHblits | 0.29 |
| ``` target    AQGVSRRQLLGRALALGSGAALADLLGPARFLSPAGAATAGAVVPGNPLRVMPDRTWEQIYRNQFEDDSTFVFTCAPNDT 6rfs.1    ------------------------------------TELSGNVIDLCPVGALTNKPYAFRAR---PWELKKTESIDVMDA  target    HNCLLRAHVKNGVVVRISPTYGYGEATDLYGNRASHRWDPRTCQKGLILSRRFYSERRVKAPMIRKGFKDWVEAGYPRND 6rfs.1    VGSNIRIDSKGVEVMRVIPRV------------HEDVNEEWINDKSRFACDGLK-TQRLTTPLIRVG-------------  target    DGTPQMDVTLRGSDDWIRISWDEATTIAAKTMEDVARTFNGDEGARKLLAQGYHPEMVEVMHGAGVQALKLRGGMPLLGI 6rfs.1    -------------DKFVNATWDDALSTIAKAYQQKAP--K--------------GDEFKAVAGALVEV------------  target    GRIFGFYRFANMLALLDRKLRPDAPADEILGSRTFDNYAWHTDLPPGHPMVTGSQ-TVDFDLFSAEHTKLLLIIGMNWIC 6rfs.1    ESMVALKDMTN-----------------ALGSENTTTDTPNGNSAPAHGITFRSNYLFNSSIAGIEDADAILLVGTNPRR  target    TKMPDGHWIGDAR-LKGTRVIVISADYMPTANKADEVIILRPGTDAAFFLGVARELIEKGLYDRAAVIERTDLPLLVRLD 6rfs.1    EAAVMNARIRKAWLRQELEIASVGPTLDATFDVAEL----------------------------------GN--------  target    TGERLDARDVIPGYELAALTNYVTLKPDAEIKGNPPPPPFTAGGQVVPTELRDAWGDFVWWDRATGRPRPVSRDEVGARF 6rfs.1    ------------TH------------------------------------------------------------------  target    DGDPALLGEFEVELVDGSTVPVRPAFDLLKQYLDESFDLRTASEVCRVPPQAIQSIARQLAANKRETLLAAGMGPNHYFQ 6rfs.1    --------------------------ADLEKALS-------------------GEFGEVLKNAKNP-LIIVGSGITDRED  target    NDLFGRVQFLVAALTDNI-GHLGGNVGSYAGNYRGSVFQAMGQWIAEDPFAIEPDLTKPATVKRYYKAESAHYWNYGERP 6rfs.1    AGAFFNTIGKFVESTPSVLNENWNGYNVLQRSAS-----RAGAY----------------D--------------IGFTP  target    LRAVAKDDEGDLTKGEVLTGKSHMPTPTKLIWFGNSNSLLGNAKWSFDVVKNTLPRQDAVFCNEWHWTSSCEYADLVFPA 6rfs.1    S-------------DEA------SKTTPKMVWLLGADEVAAS----------DIPADAFVVYQGHNGDVGAQFADVVLPG  target    DSWAEFKLPDATASCTNPFLLAFPTTPLKRLYDTRSDYEALALTAKALGELIDEPRMEQYWRGILDGDPTPYLQRIFSGS 6rfs.1    AAYTEKA---GTYVNTEGRSQIS-RAATGPPGGAREDWKILRAVSEYLGVAL----------------------------  target    NATRGITYDELHESSKRGVPLLMNMRTYPRSGGWEQRQEDKPWYTATGRLEFYRPEPEFQAAGESLPVWREPVDATFYEP 6rfs.1    --------------------------------------------------------------------------------  target    NAILSNAAHPSIAPRAPEDYGVPESQLDVETRQYRNVVRTWAELQQTLHPLQERDPAFRFVF 6rfs.1    -------------------------------------------------------------- ``` | | | | | | | | | | | | | | | | | | | | | | | | | | | | | | | | | | | | | | | | | | | | | | | | | |
|  | 6rfq.1.A | Subunit NUAM of NADH:Ubiquinone Oxidoreductase (Complex I)  *Cryo-EM structure of a respiratory complex I assembly intermediate with NDUFAF2* | 0.24 | 0.00 | 17.23 | 0.44 | 37-769 | EM | 3.30 | monomer | 6 x SF4, 2 x FES, 1 x FMN, 1 x NDP, 10 x 3PE, 2 x LMN, 4 x CDL, 2 x ZMP, 4 x PLC, 3 x T7X, 1 x CPL | HHblits | 0.29 |
| ``` target    AQGVSRRQLLGRALALGSGAALADLLGPARFLSPAGAATAGAVVPGNPLRVMPDRTWEQIYRNQFEDDSTFVFTCAPNDT 6rfq.1    ------------------------------------TELSGNVIDLCPVGALTNKPYAFRAR---PWELKKTESIDVMDA  target    HNCLLRAHVKNGVVVRISPTYGYGEATDLYGNRASHRWDPRTCQKGLILSRRFYSERRVKAPMIRKGFKDWVEAGYPRND 6rfq.1    VGSNIRIDSKGVEVMRVIPRV------------HEDVNEEWINDKSRFACDGLK-TQRLTTPLIRVG-------------  target    DGTPQMDVTLRGSDDWIRISWDEATTIAAKTMEDVARTFNGDEGARKLLAQGYHPEMVEVMHGAGVQALKLRGGMPLLGI 6rfq.1    -------------DKFVNATWDDALSTIAKAYQQKAP--K--------------GDEFKAVAGALVEV------------  target    GRIFGFYRFANMLALLDRKLRPDAPADEILGSRTFDNYAWHTDLPPGHPMVTGSQ-TVDFDLFSAEHTKLLLIIGMNWIC 6rfq.1    ESMVALKDMTN-----------------ALGSENTTTDTPNGNSAPAHGITFRSNYLFNSSIAGIEDADAILLVGTNPRR  target    TKMPDGHWIGDAR-LKGTRVIVISADYMPTANKADEVIILRPGTDAAFFLGVARELIEKGLYDRAAVIERTDLPLLVRLD 6rfq.1    EAAVMNARIRKAWLRQELEIASVGPTLDATFDVAEL----------------------------------GN--------  target    TGERLDARDVIPGYELAALTNYVTLKPDAEIKGNPPPPPFTAGGQVVPTELRDAWGDFVWWDRATGRPRPVSRDEVGARF 6rfq.1    ------------TH------------------------------------------------------------------  target    DGDPALLGEFEVELVDGSTVPVRPAFDLLKQYLDESFDLRTASEVCRVPPQAIQSIARQLAANKRETLLAAGMGPNHYFQ 6rfq.1    --------------------------ADLEKALS-------------------GEFGEVLKNAKNP-LIIVGSGITDRED  target    NDLFGRVQFLVAALTDNI-GHLGGNVGSYAGNYRGSVFQAMGQWIAEDPFAIEPDLTKPATVKRYYKAESAHYWNYGERP 6rfq.1    AGAFFNTIGKFVESTPSVLNENWNGYNVLQRSAS-----RAGAY----------------D--------------IGFTP  target    LRAVAKDDEGDLTKGEVLTGKSHMPTPTKLIWFGNSNSLLGNAKWSFDVVKNTLPRQDAVFCNEWHWTSSCEYADLVFPA 6rfq.1    S-------------DEA------SKTTPKMVWLLGADEVAAS----------DIPADAFVVYQGHNGDVGAQFADVVLPG  target    DSWAEFKLPDATASCTNPFLLAFPTTPLKRLYDTRSDYEALALTAKALGELIDEPRMEQYWRGILDGDPTPYLQRIFSGS 6rfq.1    AAYTEKA---GTYVNTEGRSQIS-RAATGPPGGAREDWKILRAVSEYLGVAL----------------------------  target    NATRGITYDELHESSKRGVPLLMNMRTYPRSGGWEQRQEDKPWYTATGRLEFYRPEPEFQAAGESLPVWREPVDATFYEP 6rfq.1    --------------------------------------------------------------------------------  target    NAILSNAAHPSIAPRAPEDYGVPESQLDVETRQYRNVVRTWAELQQTLHPLQERDPAFRFVF 6rfq.1    -------------------------------------------------------------- ``` | | | | | | | | | | | | | | | | | | | | | | | | | | | | | | | | | | | | | | | | | | | | | | | | | |
|  | 6gcs.1.A | 75-KDA PROTEIN (NUAM)  *Cryo-EM structure of respiratory complex I from Yarrowia lipolytica* | 0.24 | 0.00 | 17.23 | 0.44 | 37-769 | EM | 4.32 | monomer | 6 x SF4, 2 x FES, 1 x FMN, 1 x NDP, 1 x ZN, 1 x ZMP, 1 x CDL, 3 x 3PE | HHblits | 0.29 |
| ``` target    AQGVSRRQLLGRALALGSGAALADLLGPARFLSPAGAATAGAVVPGNPLRVMPDRTWEQIYRNQFEDDSTFVFTCAPNDT 6gcs.1    ------------------------------------TELSGNVIDLCPVGALTNKPYAFRAR---PWELKKTESIDVMDA  target    HNCLLRAHVKNGVVVRISPTYGYGEATDLYGNRASHRWDPRTCQKGLILSRRFYSERRVKAPMIRKGFKDWVEAGYPRND 6gcs.1    VGSNIRIDSKGVEVMRVIPRV------------HEDVNEEWINDKSRFACDGLK-TQRLTTPLIRVG-------------  target    DGTPQMDVTLRGSDDWIRISWDEATTIAAKTMEDVARTFNGDEGARKLLAQGYHPEMVEVMHGAGVQALKLRGGMPLLGI 6gcs.1    -------------DKFVNATWDDALSTIAKAYQQKAP--K--------------GDEFKAVAGALVEV------------  target    GRIFGFYRFANMLALLDRKLRPDAPADEILGSRTFDNYAWHTDLPPGHPMVTGSQ-TVDFDLFSAEHTKLLLIIGMNWIC 6gcs.1    ESMVALKDMTN-----------------ALGSENTTTDTPNGNSAPAHGITFRSNYLFNSSIAGIEDADAILLVGTNPRR  target    TKMPDGHWIGDAR-LKGTRVIVISADYMPTANKADEVIILRPGTDAAFFLGVARELIEKGLYDRAAVIERTDLPLLVRLD 6gcs.1    EAAVMNARIRKAWLRQELEIASVGPTLDATFDVAEL----------------------------------GN--------  target    TGERLDARDVIPGYELAALTNYVTLKPDAEIKGNPPPPPFTAGGQVVPTELRDAWGDFVWWDRATGRPRPVSRDEVGARF 6gcs.1    ------------TH------------------------------------------------------------------  target    DGDPALLGEFEVELVDGSTVPVRPAFDLLKQYLDESFDLRTASEVCRVPPQAIQSIARQLAANKRETLLAAGMGPNHYFQ 6gcs.1    --------------------------ADLEKALS-------------------GEFGEVLKNAKNP-LIIVGSGITDRED  target    NDLFGRVQFLVAALTDNI-GHLGGNVGSYAGNYRGSVFQAMGQWIAEDPFAIEPDLTKPATVKRYYKAESAHYWNYGERP 6gcs.1    AGAFFNTIGKFVESTPSVLNENWNGYNVLQRSAS-----RAGAY----------------D--------------IGFTP  target    LRAVAKDDEGDLTKGEVLTGKSHMPTPTKLIWFGNSNSLLGNAKWSFDVVKNTLPRQDAVFCNEWHWTSSCEYADLVFPA 6gcs.1    S-------------DEA------SKTTPKMVWLLGADEVAAS----------DIPADAFVVYQGHNGDVGAQFADVVLPG  target    DSWAEFKLPDATASCTNPFLLAFPTTPLKRLYDTRSDYEALALTAKALGELIDEPRMEQYWRGILDGDPTPYLQRIFSGS 6gcs.1    AAYTEKA---GTYVNTEGRSQIS-RAATGPPGGAREDWKILRAVSEYLGVAL----------------------------  target    NATRGITYDELHESSKRGVPLLMNMRTYPRSGGWEQRQEDKPWYTATGRLEFYRPEPEFQAAGESLPVWREPVDATFYEP 6gcs.1    --------------------------------------------------------------------------------  target    NAILSNAAHPSIAPRAPEDYGVPESQLDVETRQYRNVVRTWAELQQTLHPLQERDPAFRFVF 6gcs.1    -------------------------------------------------------------- ``` | | | | | | | | | | | | | | | | | | | | | | | | | | | | | | | | | | | | | | | | | | | | | | | | | |
|  | 7dgr.10.A | NADH-ubiquinone oxidoreductase 75 kDa subunit, mitochondrial  *Activity optimized supercomplex state2* | 0.20 | 0.00 | 13.60 | 0.45 | 37-770 | EM | 0.00 | monomer |  | HHblits | 0.27 |
| ``` target    AQGVSRRQLLGRALALGSGAALADLLGPARFLSPAGAATAGAVVPGNPLRVMPDRTWEQIYRNQFEDDSTFVFTCAPNDT 7dgr.10   ------------------------------------SELSGNIIDICPVGALTSKPYAFTAR---PWETRKTESIDVMDA  target    HNCLLRAHVKNGVVVRISPTYGYGEATDLYGNRASHRWDPRTCQKGLILSRRFYSERRVKAPMIRKGFKDWVEAGYPRND 7dgr.10   VGSNIVVSTRTGEVMRILPR------------MHEDINEEWISDKTRFAYDGLK-RQRLTEPMVRNE-------------  target    DGTPQMDVTLRGSDDWIRISWDEATTIAAKTMEDVARTFNGDEGARKLLAQGYHPEMVEVMHGAGVQALKLRGGMPLLGI 7dgr.10   ------------KGLLTHTTWEDALSRVAGMLQSF----Q--------------GNDVAAIAGG----------------  target    GRIFGFYRFANMLALLDRKLRPDAPADEILGSRTFDNYAWHTDLPPGHPMVTGSQTVDFDLFSAEHTKLLLIIGMNWICT 7dgr.10   -------LVDAEALIALKDLL------NRVDSDTLCTEEVFPTAGAGTDLR-SNYLLNTTIAGVEEADVVLLVGTNPRFE  target    KMPDGHWIGDARL-KGTRVIVISADYMPTANKADEVIILRPGTDAAFFLGVARELIEKGLYDRAAVIERTDLPLLVRLDT 7dgr.10   APLFNARIRKSWLHNDLKVALIGSPVDLTYRYDHLGDSPKILQDIA------------------------S---------  target    GERLDARDVIPGYELAALTNYVTLKPDAEIKGNPPPPPFTAGGQVVPTELRDAWGDFVWWDRATGRPRPVSRDEVGARFD 7dgr.10   --------------------------------------------------------------------------------  target    GDPALLGEFEVELVDGSTVPVRPAFDLLKQYLDESFDLRTASEVCRVPPQAIQSIARQLAANKRETLLAAGMGPNHYFQN 7dgr.10   ----------------------------------G----------------SHPFSQVLQEAKKP-MVILGSSALQRNDG  target    DLFGRVQFLVAALTDNIGHLGGNVGSYAGNYRGSVFQAMGQWIAEDPFAIEPDLTKPATVKRYYKAESAHYWNYGERPLR 7dgr.10   AAILAAVSNIAQKIRTSSGVTGDWKVMNI---------LHR------------IAS--QVA---------ALDLGYKP--  target    AVAKDDEGDLTKGEVLTGKSHMPTPTKLIWFGNSNSLLGNAKWSFDVVKNTLPRQDAVFCNEWHWTSSCEYADLVFPADS 7dgr.10   --------G--VEAIQ------KNPPKMLFLLGADGG--------CITRQDLPKDCFIVYQGHHGDVGAPIADVILPGAA  target    WAEFKLPDATASCTNPFLLAFPTTPLKRLYDTRSDYEALALTAKALGELIDEPRMEQYWRGILDGDPTPYLQRIFSGSNA 7dgr.10   YTEK---SATYVNTEGRAQQT-KVAVTPPGLAREDWKIIRALSEIAGMTLP-----------------------------  target    TRGITYDELHESSKRGVPLLMNMRTYPRSGGWEQRQEDKPWYTATGRLEFYRPEPEFQAAGESLPVWREPVDATFYEPNA 7dgr.10   --------------------------------------------------------------------------------  target    ILSNAAHPSIAPRAPEDYGVPESQLDVETRQYRNVVRTWAELQQTLHPLQERDPAFRFVF 7dgr.10   ------------------------------------------------------------ ``` | | | | | | | | | | | | | | | | | | | | | | | | | | | | | | | | | | | | | | | | | | | | | | | | | |
|  | 5o31.1.8 | NADH-ubiquinone oxidoreductase 75 kDa subunit, mitochondrial  *Mitochondrial complex I in the deactive state* | 0.21 | 0.00 | 13.60 | 0.45 | 37-770 | EM | 4.13 | monomer | 6 x SF4, 2 x FES, 1 x FMN, 1 x NAP, 1 x ZN | HHblits | 0.27 |
| ``` target    AQGVSRRQLLGRALALGSGAALADLLGPARFLSPAGAATAGAVVPGNPLRVMPDRTWEQIYRNQFEDDSTFVFTCAPNDT 5o31.1    ------------------------------------SELSGNIIDICPVGALTSKPYAFTAR---PWETRKTESIDVMDA  target    HNCLLRAHVKNGVVVRISPTYGYGEATDLYGNRASHRWDPRTCQKGLILSRRFYSERRVKAPMIRKGFKDWVEAGYPRND 5o31.1    VGSNIVVSTRTGEVMRILPR------------MHEDINEEWISDKTRFAYDGLK-RQRLTEPMVRNE-------------  target    DGTPQMDVTLRGSDDWIRISWDEATTIAAKTMEDVARTFNGDEGARKLLAQGYHPEMVEVMHGAGVQALKLRGGMPLLGI 5o31.1    ------------KGLLTHTTWEDALSRVAGMLQSF----Q--------------GNDVAAIAGG----------------  target    GRIFGFYRFANMLALLDRKLRPDAPADEILGSRTFDNYAWHTDLPPGHPMVTGSQTVDFDLFSAEHTKLLLIIGMNWICT 5o31.1    -------LVDAEALIALKDLL------NRVDSDTLCTEEVFPTAGAGTDLR-SNYLLNTTIAGVEEADVVLLVGTNPRFE  target    KMPDGHWIGDARL-KGTRVIVISADYMPTANKADEVIILRPGTDAAFFLGVARELIEKGLYDRAAVIERTDLPLLVRLDT 5o31.1    APLFNARIRKSWLHNDLKVALIGSPVDLTYRYDHLGDSPKILQDIA------------------------S---------  target    GERLDARDVIPGYELAALTNYVTLKPDAEIKGNPPPPPFTAGGQVVPTELRDAWGDFVWWDRATGRPRPVSRDEVGARFD 5o31.1    --------------------------------------------------------------------------------  target    GDPALLGEFEVELVDGSTVPVRPAFDLLKQYLDESFDLRTASEVCRVPPQAIQSIARQLAANKRETLLAAGMGPNHYFQN 5o31.1    ----------------------------------G----------------SHPFSQVLQEAKKP-MVILGSSALQRNDG  target    DLFGRVQFLVAALTDNIGHLGGNVGSYAGNYRGSVFQAMGQWIAEDPFAIEPDLTKPATVKRYYKAESAHYWNYGERPLR 5o31.1    AAILAAVSNIAQKIRTSSGVTGDWKVMNI---------LHR------------IAS--QVA---------ALDLGYKP--  target    AVAKDDEGDLTKGEVLTGKSHMPTPTKLIWFGNSNSLLGNAKWSFDVVKNTLPRQDAVFCNEWHWTSSCEYADLVFPADS 5o31.1    --------G--VEAIQ------KNPPKMLFLLGADGG--------CITRQDLPKDCFIVYQGHHGDVGAPIADVILPGAA  target    WAEFKLPDATASCTNPFLLAFPTTPLKRLYDTRSDYEALALTAKALGELIDEPRMEQYWRGILDGDPTPYLQRIFSGSNA 5o31.1    YTEK---SATYVNTEGRAQQT-KVAVTPPGLAREDWKIIRALSEIAGMTLP-----------------------------  target    TRGITYDELHESSKRGVPLLMNMRTYPRSGGWEQRQEDKPWYTATGRLEFYRPEPEFQAAGESLPVWREPVDATFYEPNA 5o31.1    --------------------------------------------------------------------------------  target    ILSNAAHPSIAPRAPEDYGVPESQLDVETRQYRNVVRTWAELQQTLHPLQERDPAFRFVF 5o31.1    ------------------------------------------------------------ ``` | | | | | | | | | | | | | | | | | | | | | | | | | | | | | | | | | | | | | | | | | | | | | | | | | |
|  | 6zk9.1.C | NADH:ubiquinone oxidoreductase core subunit S1  *Peripheral domain of open complex I during turnover* | 0.23 | 0.00 | 13.37 | 0.45 | 37-770 | EM | 0.00 | monomer | 6 x SF4, 1 x FMN, 1 x NAI, 2 x FES, 1 x K, 2 x PC1, 2 x 3PE, 1 x ZN, 1 x NDP, 1 x ZMP, 1 x CDL | HHblits | 0.27 |
| ``` target    AQGVSRRQLLGRALALGSGAALADLLGPARFLSPAGAATAGAVVPGNPLRVMPDRTWEQIYRNQFEDDSTFVFTCAPNDT 6zk9.1    ------------------------------------SELSGNIIDICPVGALTSKPYAFTAR---PWETRKTESIDVMDA  target    HNCLLRAHVKNGVVVRISPTYGYGEATDLYGNRASHRWDPRTCQKGLILSRRFYSERRVKAPMIRKGFKDWVEAGYPRND 6zk9.1    VGSNIVVSTRTGEVMRILPR------------MHEDINEEWISDKTRFAYDGLK-RQRLTEPMVRNE-------------  target    DGTPQMDVTLRGSDDWIRISWDEATTIAAKTMEDVARTFNGDEGARKLLAQGYHPEMVEVMHGAGVQALKLRGGMPLLGI 6zk9.1    ------------KGLLTHTTWEDALSRVAGMLQSCQ------------------GNDVAAIAGG----------------  target    GRIFGFYRFANMLALLDRKLRPDAPADEILGSRTFDNYAWHTDLPPGHPMVTGSQTVDFDLFSAEHTKLLLIIGMNWICT 6zk9.1    -------LVDAEALIALKDLL------NRVDSDTLCTEEVFPTAGAGTDLR-SNYLLNTTIAGVEEADVVLLVGTNPRFE  target    KMPDGHWIGDARL-KGTRVIVISADYMPTANKADEVIILRPGTDAAFFLGVARELIEKGLYDRAAVIERTDLPLLVRLDT 6zk9.1    APLFNARIRKSWLHNDLKVALIGSPVDLTYRYDHLGD------SP------------------KILQDIAS---------  target    GERLDARDVIPGYELAALTNYVTLKPDAEIKGNPPPPPFTAGGQVVPTELRDAWGDFVWWDRATGRPRPVSRDEVGARFD 6zk9.1    --------------------------------------------------------------------------------  target    GDPALLGEFEVELVDGSTVPVRPAFDLLKQYLDESFDLRTASEVCRVPPQAIQSIARQLAANKRETLLAAGMGPNHYFQN 6zk9.1    --------------------------------------------------GSHPFSQVLQEAKKP-MVVLGSSALQRNDG  target    DLFGRVQFLVAALTDNIGHLGGNVGSYAGNYRGSVFQAMGQWIAEDPFAIEPDLTKPATVKRYYKAESAHYWNYGERPLR 6zk9.1    AAILAAVSNIAQKIRTSSGVTGDWKVMN--I----------L------H---RIAS--QVA---------ALDLGYKP--  target    AVAKDDEGDLTKGEVLTGKSHMPTPTKLIWFGNSNSLLGNAKWSFDVVKNTLPRQDAVFCNEWHWTSSCEYADLVFPADS 6zk9.1    --------G--VEAIR------KNPPKMLFLLGADGGC--------VTRQDLPKDCFIVYQGHHGDVGAPIADVILPGAA  target    WAEFKLPDATASCTNPFLLAFPTTPLKRLYDTRSDYEALALTAKALGELIDEPRMEQYWRGILDGDPTPYLQRIFSGSNA 6zk9.1    YTEK---SATYVNTEGRAQQT-KVAVMPPGLAREDWKIIRALSEIAGMTLP-----------------------------  target    TRGITYDELHESSKRGVPLLMNMRTYPRSGGWEQRQEDKPWYTATGRLEFYRPEPEFQAAGESLPVWREPVDATFYEPNA 6zk9.1    --------------------------------------------------------------------------------  target    ILSNAAHPSIAPRAPEDYGVPESQLDVETRQYRNVVRTWAELQQTLHPLQERDPAFRFVF 6zk9.1    ------------------------------------------------------------ ``` | | | | | | | | | | | | | | | | | | | | | | | | | | | | | | | | | | | | | | | | | | | | | | | | | |
|  | 5xtb.1.L | NADH-ubiquinone oxidoreductase 75 kDa subunit, mitochondrial  *Cryo-EM structure of human respiratory complex I matrix arm* | 0.22 |  | 13.64 | 0.45 | 37-769 | EM | 0.00 | hetero-1-1-1-1-1-1-… | 6 x SF4, 1 x FMN, 1 x 8Q1, 1 x NDP, 2 x FES | HHblits | 0.27 |
| ``` target    AQGVSRRQLLGRALALGSGAALADLLGPARFLSPAGAATAGAVVPGNPLRVMPDRTWEQIYRNQFEDDSTFVFTCAPNDT 5xtb.1    ------------------------------------SELSGNIIDICPVGALTSKPYAFTAR---PWETRKTESIDVMDA  target    HNCLLRAHVKNGVVVRISPTYGYGEATDLYGNRASHRWDPRTCQKGLILSRRFYSERRVKAPMIRKGFKDWVEAGYPRND 5xtb.1    VGSNIVVSTRTGEVMRILPR------------MHEDINEEWISDKTRFAYDGLK-RQRLTEPMVRNE-------------  target    DGTPQMDVTLRGSDDWIRISWDEATTIAAKTMEDVARTFNGDEGARKLLAQGYHPEMVEVMHGAGVQALKLRGGMPLLGI 5xtb.1    ------------KGLLTYTSWEDALSRVAGMLQSFQ------------------GKDVAAIAGG----------------  target    GRIFGFYRFANMLALLDRKLRPDAPADEILGSRTFDNYAWHTDLPPGHPMVTGSQTVDFDLFSAEHTKLLLIIGMNWICT 5xtb.1    -------LVDAEALVALKDLL------NRVDSDTLCTEEVFPTAGAGTDLRSN-YLLNTTIAGVEEADVVLLVGTNPRFE  target    KMPDGHWIGDARL-KGTRVIVISADYMPTANKADEVIILRPGTDAAFFLGVARELIEKGLYDRAAVIERTDLPLLVRLDT 5xtb.1    APLFNARIRKSWLHNDLKVALIGSPVDLTYTYD--H----LGDSPKIL--------------------------------  target    GERLDARDVIPGYELAALTNYVTLKPDAEIKGNPPPPPFTAGGQVVPTELRDAWGDFVWWDRATGRPRPVSRDEVGARFD 5xtb.1    --------------------------------------------------------------------------------  target    GDPALLGEFEVELVDGSTVPVRPAFDLLKQYLDESFDLRTASEVCRVPPQAIQSIARQLAANKRETLLAAGMGPNHYFQN 5xtb.1    ----------------------------QD-IA----------------SGSHPFSQVLKEAKKP-MVVLGSSALQRNDG  target    DLFGRVQFLVAALTDNIGHLGGNVGSYAGNYRGSVFQAMGQWIAEDPFAIEPDLTKPATVKRYYKAESAHYWNYGERPLR 5xtb.1    AAILAAVSSIAQKIRMTSGVTGDWKVMNILH---------------R------IAS--QVA---------ALDLGYKPG-  target    AVAKDDEGDLTKGEVLTGKSHMPTPTKLIWFGNSNSLLGNAKWSFDVVKNTLPRQDAVFCNEWHWTSSCEYADLVFPADS 5xtb.1    -----------VEAIR------KNPPKVLFLLGADGG--------CITRQDLPKDCFIIYQGHHGDVGAPIADVILPGAA  target    WAEFKLPDATASCTNPFLLAFPTTPLKRLYDTRSDYEALALTAKALGELIDEPRMEQYWRGILDGDPTPYLQRIFSGSNA 5xtb.1    YTEKS---ATYVNTEGRAQQT-KVAVTPPGLAREDWKIIRALSEIAGMTL------------------------------  target    TRGITYDELHESSKRGVPLLMNMRTYPRSGGWEQRQEDKPWYTATGRLEFYRPEPEFQAAGESLPVWREPVDATFYEPNA 5xtb.1    --------------------------------------------------------------------------------  target    ILSNAAHPSIAPRAPEDYGVPESQLDVETRQYRNVVRTWAELQQTLHPLQERDPAFRFVF 5xtb.1    ------------------------------------------------------------ ``` | | | | | | | | | | | | | | | | | | | | | | | | | | | | | | | | | | | | | | | | | | | | | | | | | |
|  | 7zd6.1.4 | NADH-ubiquinone oxidoreductase 75 kDa subunit, mitochondrial  *Complex I from Ovis aries, at pH7.4, Open state* | 0.22 | 0.00 | 13.37 | 0.45 | 37-770 | EM | 0.00 | monomer | 6 x PC1, 14 x 3PE, 1 x DCQ, 2 x ZMP, 1 x AMP, 1 x MYR, 6 x SF4, 1 x FMN, 1 x NAI, 2 x FES, 1 x K, 1 x ZN, 1 x NDP | HHblits | 0.27 |
| ``` target    AQGVSRRQLLGRALALGSGAALADLLGPARFLSPAGAATAGAVVPGNPLRVMPDRTWEQIYRNQFEDDSTFVFTCAPNDT 7zd6.1    ------------------------------------SELSGNIIDICPVGALTSKPYAFTAR---PWETRKTESIDVMDA  target    HNCLLRAHVKNGVVVRISPTYGYGEATDLYGNRASHRWDPRTCQKGLILSRRFYSERRVKAPMIRKGFKDWVEAGYPRND 7zd6.1    VGSNIVVSTRTGEVMRILPR------------MHEDINEEWISDKTRFAYDGLK-RQRLTEPMVRNE-------------  target    DGTPQMDVTLRGSDDWIRISWDEATTIAAKTMEDVARTFNGDEGARKLLAQGYHPEMVEVMHGAGVQALKLRGGMPLLGI 7zd6.1    ------------KGLLTHTTWEDALSRVAGMLQSCQ------------------GNDVAAIAGG----------------  target    GRIFGFYRFANMLALLDRKLRPDAPADEILGSRTFDNYAWHTDLPPGHPMVTGSQTVDFDLFSAEHTKLLLIIGMNWICT 7zd6.1    -------LVDAEALIALKDLL------NRVDSDTLCTEEVFPTAGAGTDLR-SNYLLNTTIAGVEEADVVLLVGTNPRFE  target    KMPDGHWIGDARL-KGTRVIVISADYMPTANKADEVIILRPGTDAAFFLGVARELIEKGLYDRAAVIERTDLPLLVRLDT 7zd6.1    APLFNARIRKSWLHNDLKVALIGSPVDLTYRYDHLGD------SP------------------KILQDIAS---------  target    GERLDARDVIPGYELAALTNYVTLKPDAEIKGNPPPPPFTAGGQVVPTELRDAWGDFVWWDRATGRPRPVSRDEVGARFD 7zd6.1    --------------------------------------------------------------------------------  target    GDPALLGEFEVELVDGSTVPVRPAFDLLKQYLDESFDLRTASEVCRVPPQAIQSIARQLAANKRETLLAAGMGPNHYFQN 7zd6.1    --------------------------------------------------GSHPFSQVLQEAKKP-MVVLGSSALQRNDG  target    DLFGRVQFLVAALTDNIGHLGGNVGSYAGNYRGSVFQAMGQWIAEDPFAIEPDLTKPATVKRYYKAESAHYWNYGERPLR 7zd6.1    AAILAAVSNIAQKIRTSSGVTGDWKVMN------------IL------H---RIAS--QVA---------ALDLGYKP--  target    AVAKDDEGDLTKGEVLTGKSHMPTPTKLIWFGNSNSLLGNAKWSFDVVKNTLPRQDAVFCNEWHWTSSCEYADLVFPADS 7zd6.1    --------G--VEAIR------KNPPKMLFLLGADGG--------CVTRQDLPKDCFIVYQGHHGDVGAPIADVILPGAA  target    WAEFKLPDATASCTNPFLLAFPTTPLKRLYDTRSDYEALALTAKALGELIDEPRMEQYWRGILDGDPTPYLQRIFSGSNA 7zd6.1    YTEK---SATYVNTEGRAQQT-KVAVMPPGLAREDWKIIRALSEIAGMTLP-----------------------------  target    TRGITYDELHESSKRGVPLLMNMRTYPRSGGWEQRQEDKPWYTATGRLEFYRPEPEFQAAGESLPVWREPVDATFYEPNA 7zd6.1    --------------------------------------------------------------------------------  target    ILSNAAHPSIAPRAPEDYGVPESQLDVETRQYRNVVRTWAELQQTLHPLQERDPAFRFVF 7zd6.1    ------------------------------------------------------------ ``` | | | | | | | | | | | | | | | | | | | | | | | | | | | | | | | | | | | | | | | | | | | | | | | | | |
|  | 6qcf.1.C | NADH:ubiquinone oxidoreductase core subunit S1  *Ovine respiratory complex I FRC open class 6* | 0.22 | 0.00 | 13.40 | 0.45 | 37-769 | EM | 0.00 | monomer | 6 x SF4, 1 x FMN, 2 x FES, 1 x ZN, 1 x NDP, 2 x ZMP | HHblits | 0.27 |
| ``` target    AQGVSRRQLLGRALALGSGAALADLLGPARFLSPAGAATAGAVVPGNPLRVMPDRTWEQIYRNQFEDDSTFVFTCAPNDT 6qcf.1    ------------------------------------SELSGNIIDICPVGALTSKPYAFTAR---PWETRKTESIDVMDA  target    HNCLLRAHVKNGVVVRISPTYGYGEATDLYGNRASHRWDPRTCQKGLILSRRFYSERRVKAPMIRKGFKDWVEAGYPRND 6qcf.1    VGSNIVVSTRTGEVMRILPR------------MHEDINEEWISDKTRFAYDGLK-RQRLTEPMVRNE-------------  target    DGTPQMDVTLRGSDDWIRISWDEATTIAAKTMEDVARTFNGDEGARKLLAQGYHPEMVEVMHGAGVQALKLRGGMPLLGI 6qcf.1    ------------KGLLTHTTWEDALSRVAGMLQSCQ------------------GNDVAAIAGG----------------  target    GRIFGFYRFANMLALLDRKLRPDAPADEILGSRTFDNYAWHTDLPPGHPMVTGSQTVDFDLFSAEHTKLLLIIGMNWICT 6qcf.1    -------LVDAEALIALKDLL------NRVDSDTLCTEEVFPTAGAGTDLR-SNYLLNTTIAGVEEADVVLLVGTNPRFE  target    KMPDGHWIGDARL-KGTRVIVISADYMPTANKADEVIILRPGTDAAFFLGVARELIEKGLYDRAAVIERTDLPLLVRLDT 6qcf.1    APLFNARIRKSWLHNDLKVALIGSPVDLTYRYDHLGDSP------------------------KILQDIAS---------  target    GERLDARDVIPGYELAALTNYVTLKPDAEIKGNPPPPPFTAGGQVVPTELRDAWGDFVWWDRATGRPRPVSRDEVGARFD 6qcf.1    --------------------------------------------------------------------------------  target    GDPALLGEFEVELVDGSTVPVRPAFDLLKQYLDESFDLRTASEVCRVPPQAIQSIARQLAANKRETLLAAGMGPNHYFQN 6qcf.1    ----------------------------------G----------------SHPFSQVLQEAKKP-MVVLGSSALQRNDG  target    DLFGRVQFLVAALTDNIGHLGGNVGSYAGNYRGSVFQAMGQWIAEDPFAIEPDLTKPATVKRYYKAESAHYWNYGERPLR 6qcf.1    AAILAAVSNIAQKIRTSSGVTGDWKVMN--I-------LHR------------IAS--QVA---------ALDLGYKPG-  target    AVAKDDEGDLTKGEVLTGKSHMPTPTKLIWFGNSNSLLGNAKWSFDVVKNTLPRQDAVFCNEWHWTSSCEYADLVFPADS 6qcf.1    -----------VEAIR------KNPPKMLFLLGADGGC--------VTRQDLPKDCFIVYQGHHGDVGAPIADVILPGAA  target    WAEFKLPDATASCTNPFLLAFPTTPLKRLYDTRSDYEALALTAKALGELIDEPRMEQYWRGILDGDPTPYLQRIFSGSNA 6qcf.1    YTEKS---ATYVNTEGRAQQT-KVAVMPPGLAREDWKIIRALSEIAGMTL------------------------------  target    TRGITYDELHESSKRGVPLLMNMRTYPRSGGWEQRQEDKPWYTATGRLEFYRPEPEFQAAGESLPVWREPVDATFYEPNA 6qcf.1    --------------------------------------------------------------------------------  target    ILSNAAHPSIAPRAPEDYGVPESQLDVETRQYRNVVRTWAELQQTLHPLQERDPAFRFVF 6qcf.1    ------------------------------------------------------------ ``` | | | | | | | | | | | | | | | | | | | | | | | | | | | | | | | | | | | | | | | | | | | | | | | | | |
|  | 6qc5.1.C | NADH:ubiquinone oxidoreductase core subunit S1  *Ovine respiratory complex I FRC closed class 1* | 0.22 | 0.00 | 13.40 | 0.45 | 37-769 | EM | 0.00 | monomer | 6 x SF4, 1 x FMN, 2 x FES, 2 x 3PE, 1 x ZN, 1 x NDP, 2 x ZMP, 1 x PC1 | HHblits | 0.27 |
| ``` target    AQGVSRRQLLGRALALGSGAALADLLGPARFLSPAGAATAGAVVPGNPLRVMPDRTWEQIYRNQFEDDSTFVFTCAPNDT 6qc5.1    ------------------------------------SELSGNIIDICPVGALTSKPYAFTAR---PWETRKTESIDVMDA  target    HNCLLRAHVKNGVVVRISPTYGYGEATDLYGNRASHRWDPRTCQKGLILSRRFYSERRVKAPMIRKGFKDWVEAGYPRND 6qc5.1    VGSNIVVSTRTGEVMRILPR------------MHEDINEEWISDKTRFAYDGLK-RQRLTEPMVRNE-------------  target    DGTPQMDVTLRGSDDWIRISWDEATTIAAKTMEDVARTFNGDEGARKLLAQGYHPEMVEVMHGAGVQALKLRGGMPLLGI 6qc5.1    ------------KGLLTHTTWEDALSRVAGMLQSCQ------------------GNDVAAIAGG----------------  target    GRIFGFYRFANMLALLDRKLRPDAPADEILGSRTFDNYAWHTDLPPGHPMVTGSQTVDFDLFSAEHTKLLLIIGMNWICT 6qc5.1    -------LVDAEALIALKDLL------NRVDSDTLCTEEVFPTAGAGTDLR-SNYLLNTTIAGVEEADVVLLVGTNPRFE  target    KMPDGHWIGDARL-KGTRVIVISADYMPTANKADEVIILRPGTDAAFFLGVARELIEKGLYDRAAVIERTDLPLLVRLDT 6qc5.1    APLFNARIRKSWLHNDLKVALIGSPVDLTYRYDHLGDSP------------------------KILQDIAS---------  target    GERLDARDVIPGYELAALTNYVTLKPDAEIKGNPPPPPFTAGGQVVPTELRDAWGDFVWWDRATGRPRPVSRDEVGARFD 6qc5.1    --------------------------------------------------------------------------------  target    GDPALLGEFEVELVDGSTVPVRPAFDLLKQYLDESFDLRTASEVCRVPPQAIQSIARQLAANKRETLLAAGMGPNHYFQN 6qc5.1    ----------------------------------G----------------SHPFSQVLQEAKKP-MVVLGSSALQRNDG  target    DLFGRVQFLVAALTDNIGHLGGNVGSYAGNYRGSVFQAMGQWIAEDPFAIEPDLTKPATVKRYYKAESAHYWNYGERPLR 6qc5.1    AAILAAVSNIAQKIRTSSGVTGDWKVMN--I-------LHR------------IAS--QVA---------ALDLGYKPG-  target    AVAKDDEGDLTKGEVLTGKSHMPTPTKLIWFGNSNSLLGNAKWSFDVVKNTLPRQDAVFCNEWHWTSSCEYADLVFPADS 6qc5.1    -----------VEAIR------KNPPKMLFLLGADGGC--------VTRQDLPKDCFIVYQGHHGDVGAPIADVILPGAA  target    WAEFKLPDATASCTNPFLLAFPTTPLKRLYDTRSDYEALALTAKALGELIDEPRMEQYWRGILDGDPTPYLQRIFSGSNA 6qc5.1    YTEKS---ATYVNTEGRAQQT-KVAVMPPGLAREDWKIIRALSEIAGMTL------------------------------  target    TRGITYDELHESSKRGVPLLMNMRTYPRSGGWEQRQEDKPWYTATGRLEFYRPEPEFQAAGESLPVWREPVDATFYEPNA 6qc5.1    --------------------------------------------------------------------------------  target    ILSNAAHPSIAPRAPEDYGVPESQLDVETRQYRNVVRTWAELQQTLHPLQERDPAFRFVF 6qc5.1    ------------------------------------------------------------ ``` | | | | | | | | | | | | | | | | | | | | | | | | | | | | | | | | | | | | | | | | | | | | | | | | | |
|  | 7qsd.1.G | NADH-ubiquinone oxidoreductase 75 kDa subunit, mitochondrial  *Bovine complex I in the active state at 3.1 A* | 0.22 | 0.00 | 13.40 | 0.45 | 37-769 | EM | 0.00 | monomer | 5 x PC1, 13 x 3PE, 6 x SF4, 2 x FES, 1 x FMN, 4 x CDL, 3 x LMT, 1 x GTP, 1 x MG, 1 x NDP, 1 x ZN, 2 x EHZ | HHblits | 0.27 |
| ``` target    AQGVSRRQLLGRALALGSGAALADLLGPARFLSPAGAATAGAVVPGNPLRVMPDRTWEQIYRNQFEDDSTFVFTCAPNDT 7qsd.1    ------------------------------------SELSGNIIDICPVGALTSKPYAFTAR---PWETRKTESIDVMDA  target    HNCLLRAHVKNGVVVRISPTYGYGEATDLYGNRASHRWDPRTCQKGLILSRRFYSERRVKAPMIRKGFKDWVEAGYPRND 7qsd.1    VGSNIVVSTRTGEVMRILPRM------------HEDINEEWISDKTRFAYDGLK-RQRLTEPMVRNE-------------  target    DGTPQMDVTLRGSDDWIRISWDEATTIAAKTMEDVARTFNGDEGARKLLAQGYHPEMVEVMHGAGVQALKLRGGMPLLGI 7qsd.1    ------------KGLLTHTTWEDALSRVAGMLQSF----Q--------------GNDVAAIAGG----------------  target    GRIFGFYRFANMLALLDRKLRPDAPADEILGSRTFDNYAWHTDLPPGHPMVTGSQTVDFDLFSAEHTKLLLIIGMNWICT 7qsd.1    -------LVDAEALIALKDLL------NRVDSDTLCTEEVFPTAGAGTDLR-SNYLLNTTIAGVEEADVVLLVGTNPRFE  target    KMPDGHWIGDARL-KGTRVIVISADYMPTANKADEVIILRPGTDAAFFLGVARELIEKGLYDRAAVIERTDLPLLVRLDT 7qsd.1    APLFNARIRKSWLHNDLKVALIGSPVDLTYRYDHLGDSPKILQD------------------------IAS---------  target    GERLDARDVIPGYELAALTNYVTLKPDAEIKGNPPPPPFTAGGQVVPTELRDAWGDFVWWDRATGRPRPVSRDEVGARFD 7qsd.1    --------------------------------------------------------------------------------  target    GDPALLGEFEVELVDGSTVPVRPAFDLLKQYLDESFDLRTASEVCRVPPQAIQSIARQLAANKRETLLAAGMGPNHYFQN 7qsd.1    ----------------------------------G----------------SHPFSQVLQEAKKP-MVILGSSALQRNDG  target    DLFGRVQFLVAALTDNIGHLGGNVGSYAGNYRGSVFQAMGQWIAEDPFAIEPDLTKPATVKRYYKAESAHYWNYGERPLR 7qsd.1    AAILAAVSNIAQKIRTSSGVTGDWKVMN------------IL------H---RIAS--QVA---------ALDLGYKP--  target    AVAKDDEGDLTKGEVLTGKSHMPTPTKLIWFGNSNSLLGNAKWSFDVVKNTLPRQDAVFCNEWHWTSSCEYADLVFPADS 7qsd.1    --------G--VEAIQ------KNPPKMLFLLGADGG--------CITRQDLPKDCFIVYQGHHGDVGAPIADVILPGAA  target    WAEFKLPDATASCTNPFLLAFPTTPLKRLYDTRSDYEALALTAKALGELIDEPRMEQYWRGILDGDPTPYLQRIFSGSNA 7qsd.1    YTEK---SATYVNTEGRAQQT-KVAVTPPGLAREDWKIIRALSEIAGMTL------------------------------  target    TRGITYDELHESSKRGVPLLMNMRTYPRSGGWEQRQEDKPWYTATGRLEFYRPEPEFQAAGESLPVWREPVDATFYEPNA 7qsd.1    --------------------------------------------------------------------------------  target    ILSNAAHPSIAPRAPEDYGVPESQLDVETRQYRNVVRTWAELQQTLHPLQERDPAFRFVF 7qsd.1    ------------------------------------------------------------ ``` | | | | | | | | | | | | | | | | | | | | | | | | | | | | | | | | | | | | | | | | | | | | | | | | | |
|  | 7vxu.1.L | NADH-ubiquinone oxidoreductase 75 kDa subunit, mitochondrial  *Matrix arm of deactive state CI from Q10 dataset* | 0.21 | 0.00 | 13.16 | 0.45 | 37-769 | EM | 0.00 | monomer | 6 x SF4, 1 x FMN, 1 x PEE, 1 x PLX, 1 x 8Q1, 1 x NDP, 2 x FES, 1 x MG, 1 x CDL, 1 x ZN | HHblits | 0.27 |
| ``` target    AQGVSRRQLLGRALALGSGAALADLLGPARFLSPAGAATAGAVVPGNPLRVMPDRTWEQIYRNQFEDDSTFVFTCAPNDT 7vxu.1    ------------------------------------SELSGNIIDICPVGALTSKPYAFTAR---PWETRKTESIDVMDA  target    HNCLLRAHVKNGVVVRISPTYGYGEATDLYGNRASHRWDPRTCQKGLILSRRFYSERRVKAPMIRKGFKDWVEAGYPRND 7vxu.1    VGSNIVVSTRTGEVMRILPRM------------HEDINEEWISDKTRFAYDGLK-RQRLTQPMIRNE-------------  target    DGTPQMDVTLRGSDDWIRISWDEATTIAAKTMEDVARTFNGDEGARKLLAQGYHPEMVEVMHGAGVQALKLRGGMPLLGI 7vxu.1    ------------KGLLTYTTWEDALSRVAGMLQSFQ------------------GNDVAAIAGG----------------  target    GRIFGFYRFANMLALLDRKLRPDAPADEILGSRTFDNYAWHTDLPPGHPMVTGSQTVDFDLFSAEHTKLLLIIGMNWICT 7vxu.1    -------LVDAEALVALKDLL------NRVDSDSLCTEEVFPTAGAGTDLRSN-YLLNTTIAGVEEADVILLVGTNPRFE  target    KMPDGHWIGDARL-KGTRVIVISADYMPTANKADEVIILRPGTDAAFFLGVARELIEKGLYDRAAVIERTDLPLLVRLDT 7vxu.1    APLFNARIRKSWLHNDLKVALIGSPVDLTYRYDHLGDSPK------------------------ILQDIAS---------  target    GERLDARDVIPGYELAALTNYVTLKPDAEIKGNPPPPPFTAGGQVVPTELRDAWGDFVWWDRATGRPRPVSRDEVGARFD 7vxu.1    --------------------------------------------------------------------------------  target    GDPALLGEFEVELVDGSTVPVRPAFDLLKQYLDESFDLRTASEVCRVPPQAIQSIARQLAANKRETLLAAGMGPNHYFQN 7vxu.1    ----------------------------------G----------------NHPFSQILKEAKKP-MVVLGSSALQRSDG  target    DLFGRVQFLVAALTDNIGHLGGNVGSYAGNYRGSVFQAMGQWIAEDPFAIEPDLTKPATVKRYYKAESAHYWNYGERPLR 7vxu.1    TAILAAVSNIAQNIRLSSGVTGDWKVMNI---------LHR------------IAS--QVA---------ALDLGYKP--  target    AVAKDDEGDLTKGEVLTGKSHMPTPTKLIWFGNSNSLLGNAKWSFDVVKNTLPRQDAVFCNEWHWTSSCEYADLVFPADS 7vxu.1    --------G--VEAIR------KNPPKVLFLLGADGG--------CITRQDLPKDCFIIYQGHHGDVGAPMADVILPGAA  target    WAEFKLPDATASCTNPFLLAFPTTPLKRLYDTRSDYEALALTAKALGELIDEPRMEQYWRGILDGDPTPYLQRIFSGSNA 7vxu.1    YTEKS---ATYVNTEGRAQQT-KVAVTPPGLAREDWKIIRALSEIAGMTL------------------------------  target    TRGITYDELHESSKRGVPLLMNMRTYPRSGGWEQRQEDKPWYTATGRLEFYRPEPEFQAAGESLPVWREPVDATFYEPNA 7vxu.1    --------------------------------------------------------------------------------  target    ILSNAAHPSIAPRAPEDYGVPESQLDVETRQYRNVVRTWAELQQTLHPLQERDPAFRFVF 7vxu.1    ------------------------------------------------------------ ``` | | | | | | | | | | | | | | | | | | | | | | | | | | | | | | | | | | | | | | | | | | | | | | | | | |
|  | 5gpn.24.A | NADH-ubiquinone oxidoreductase 75 kDa subunit  *Architecture of mammalian respirasome* | 0.21 |  | 13.16 | 0.45 | 37-769 | EM | 0.00 | monomer |  | HHblits | 0.27 |
| ``` target    AQGVSRRQLLGRALALGSGAALADLLGPARFLSPAGAATAGAVVPGNPLRVMPDRTWEQIYRNQFEDDSTFVFTCAPNDT 5gpn.24   ------------------------------------SELSGNIIDICPVGALTSKPYAFTAR---PWETRKTESIDVMDA  target    HNCLLRAHVKNGVVVRISPTYGYGEATDLYGNRASHRWDPRTCQKGLILSRRFYSERRVKAPMIRKGFKDWVEAGYPRND 5gpn.24   VGSNIVVSTRTGEVMRILPRM------------HEDINEEWISDKTRFAYDGLK-RQRLTQPMIRNE-------------  target    DGTPQMDVTLRGSDDWIRISWDEATTIAAKTMEDVARTFNGDEGARKLLAQGYHPEMVEVMHGAGVQALKLRGGMPLLGI 5gpn.24   ------------KGLLTYTTWEDALSRVAGMLQSF----Q--------------GNDVAAIAGG----------------  target    GRIFGFYRFANMLALLDRKLRPDAPADEILGSRTFDNYAWHTDLPPGHPMVTGSQTVDFDLFSAEHTKLLLIIGMNWICT 5gpn.24   -------LVDAEALVALKDLL------NRVDSDSLCTEEVFPTAGAGTDLRSN-YLLNTTIAGVEEADVILLVGTNPRFE  target    KMPDGHWIGDARL-KGTRVIVISADYMPTANKADEVIILRPGTDAAFFLGVARELIEKGLYDRAAVIERTDLPLLVRLDT 5gpn.24   APLFNARIRKSWLHNDLKVALIGSPVDLTYRYDHLGDSPK------------------------ILQDIAS---------  target    GERLDARDVIPGYELAALTNYVTLKPDAEIKGNPPPPPFTAGGQVVPTELRDAWGDFVWWDRATGRPRPVSRDEVGARFD 5gpn.24   --------------------------------------------------------------------------------  target    GDPALLGEFEVELVDGSTVPVRPAFDLLKQYLDESFDLRTASEVCRVPPQAIQSIARQLAANKRETLLAAGMGPNHYFQN 5gpn.24   ----------------------------------G----------------NHPFSQILKEAKKP-MVVLGSSALQRSDG  target    DLFGRVQFLVAALTDNIGHLGGNVGSYAGNYRGSVFQAMGQWIAEDPFAIEPDLTKPATVKRYYKAESAHYWNYGERPLR 5gpn.24   TAILAAVSNIAQNIRLSSGVTGDWKVMN------------------ILH---RIAS--QVA---------ALDLGYKP--  target    AVAKDDEGDLTKGEVLTGKSHMPTPTKLIWFGNSNSLLGNAKWSFDVVKNTLPRQDAVFCNEWHWTSSCEYADLVFPADS 5gpn.24   --------G--VEAIR------KNPPKVLFLLGADGG--------CITRQDLPKDCFIIYQGHHGDVGAPMADVILPGAA  target    WAEFKLPDATASCTNPFLLAFPTTPLKRLYDTRSDYEALALTAKALGELIDEPRMEQYWRGILDGDPTPYLQRIFSGSNA 5gpn.24   YTEK---SATYVNTEGRAQQT-KVAVTPPGLAREDWKIIRALSEIAGMTL------------------------------  target    TRGITYDELHESSKRGVPLLMNMRTYPRSGGWEQRQEDKPWYTATGRLEFYRPEPEFQAAGESLPVWREPVDATFYEPNA 5gpn.24   --------------------------------------------------------------------------------  target    ILSNAAHPSIAPRAPEDYGVPESQLDVETRQYRNVVRTWAELQQTLHPLQERDPAFRFVF 5gpn.24   ------------------------------------------------------------ ``` | | | | | | | | | | | | | | | | | | | | | | | | | | | | | | | | | | | | | | | | | | | | | | | | | |
|  | 7zm7.1.I | NADH-ubiquinone oxidoreductase-like protein  *CryoEM structure of mitochondrial complex I from Chaetomium thermophilum (inhibited by DDM)* | 0.23 |  | 15.46 | 0.44 | 37-770 | EM | 0.00 | hetero-1-1-1-1-1-1-… | 4 x PC1, 14 x LMT, 5 x CDL, 8 x 3PE, 2 x FES, 6 x SF4, 1 x FMN, 1 x NDP, 1 x ZN, 2 x ZMP | HHblits | 0.27 |
| ``` target    AQGVSRRQLLGRALALGSGAALADLLGPARFLSPAGAATAGAVVPGNPLRVMPDRTWEQIYRNQFEDDSTFVFTCAPNDT 7zm7.1    ------------------------------------SELSGNVIDLCPVGALTSKPYAFRAR---PWELKRTESIDVLDG  target    HNCLLRAHVKNGVVVRISPTYGYGEATDLYGNRASHRWDPRTCQKGLILSRRFYSERRVKAPMIRKGFKDWVEAGYPRND 7zm7.1    LGSNIRVDSRGLEVMRILPRL------------NDDVNEEWINDKTRFACDGLK-TQRLTMPLVRRD-------------  target    DGTPQMDVTLRGSDDWIRISWDEATTIAAKTMEDVARTFNGDEGARKLLAQGYHPEMVEVMHGAGVQALKLRGGMPLLGI 7zm7.1    -------------GKFEPATWEQALTEIAHAYQTLAPKENE----------------FKVIAGQ----------------  target    GRIFGFYRFANMLALLDRKLRPDAPADEILGSRTFDNYAWHTDLPPGHPMVTGSQ-TVDFDLFSAEHTKLLLIIGMNWIC 7zm7.1    -------LVEVESLVAMKDLA------NRLGSENLALDFPGGSQPLAHGVDIRSNYLFNSKIWGIEEADAILLVGTNPRH  target    TKMPDGHWIGDAR-LKGTRVIVISADYMPTANKADEVIILRPGTDAAFFLGVARELIEKGLYDRAAVIERTDLPLLVRLD 7zm7.1    EAAVLNARIRKQWLRSDLEIAAVGQPWESTFDYEH------LGTDLAALKNALSGPF-----------------------  target    TGERLDARDVIPGYELAALTNYVTLKPDAEIKGNPPPPPFTAGGQVVPTELRDAWGDFVWWDRATGRPRPVSRDEVGARF 7zm7.1    --------------------------------------------------------------------------------  target    DGDPALLGEFEVELVDGSTVPVRPAFDLLKQYLDESFDLRTASEVCRVPPQAIQSIARQLAANKRETLLAAGMGPNHYFQ 7zm7.1    --------------------------------------------------------GEKLKKAKRP-MIIVGSGVTEHPD  target    NDLFGRVQFLVAALTDN--IGHLGGNVGSYAGNYRGSVFQAMGQWIAEDPFAIEPDLTKPATVKRYYKAESAHYWNYGER 7zm7.1    AKAFYETVWSFVEKNASNFLTEEWCGYNVLQRAA------------------------S--RAGAF-------EVGF--V  target    PLRAVAKDDEGDLTKGEVLTGKSHMPTPTKLIWFGNSNSLLGNAKWSFDVVKNTLPRQDAVFCNEWHWTSSCEYADLVFP 7zm7.1    V---P---------SPEV------AATKPKFVWLLGADEFDP----------ADVPKDAFIVYQGHHGDRGAEIADIVLP  target    ADSWAEFKLPDATASCTNPFLLAFPTTPLKRLYDTRSDYEALALTAKALGELIDEPRMEQYWRGILDGDPTPYLQRIFSG 7zm7.1    GAAYTEK---AGTYVNTEGRVQMT-RAATGLPGAARTDWKIIRAVSEFLGVPLP--------------------------  target    SNATRGITYDELHESSKRGVPLLMNMRTYPRSGGWEQRQEDKPWYTATGRLEFYRPEPEFQAAGESLPVWREPVDATFYE 7zm7.1    --------------------------------------------------------------------------------  target    PNAILSNAAHPSIAPRAPEDYGVPESQLDVETRQYRNVVRTWAELQQTLHPLQERDPAFRFVF 7zm7.1    --------------------------------------------------------------- ``` | | | | | | | | | | | | | | | | | | | | | | | | | | | | | | | | | | | | | | | | | | | | | | | | | |
|  | 7tgh.58.A | NADH-ubiquinone oxidoreductase 75 kDa subunit  *Cryo-EM structure of respiratory super-complex CI+III2 from Tetrahymena thermophila* | 0.23 |  | 16.02 | 0.44 | 36-768 | EM | 0.00 | monomer |  | HHblits | 0.28 |
| ``` target    AQGVSRRQLLGRALALGSGAALADLLGPARFLSPAGAATAGAVVPGNPLRVMPDRTWEQIYRNQFEDDSTFVFTCAPNDT 7tgh.58   -----------------------------------NTELSGNVVDVCPVGALTNAPYAFTSR---PWELKSFYTSDVFDT  target    HNCLLRAHVKNGVVVRISPTYGYGEATDLYGNRASHRWDPRTCQKGLILSRRFYSERRVKAPMIRKGFKDWVEAGYPRND 7tgh.58   LGSAIQVDTRGPEIMRVLPR------------IHEEINEEWISDKTRHAFDGLK-RQRINSPMKRSK-------------  target    DGTPQMDVTLRGSDDWIRISWDEATTIAAKTMEDVARTFNGDEGARKLLAQGYHPEMVEVMHGAGVQALKLRGGMPLLGI 7tgh.58   ------------DGNYEDIFWEEAIQTISKKCLNTPS------------------DQIGAIIGEFADI------------  target    GRIFGFYRFANMLALLDRKLRPDAPADEILGSRTFDNYAWHTDLPPGHPMVTGSQTVDFDLFSAEHTKLLLIIGMNWICT 7tgh.58   ESITALKDFLN-----------------RLDVDNFEV-RQHGNLKVSPDFR-ANYLMNSKITGVEDADVLLLVGCNPRYE  target    KMPDGHWIGDARLKGTRVIVISADYMPTANKADEVIILRPGTDAAFFLGVARELIEKGLYDRAAVIERTDLPLLVRLDTG 7tgh.58   APVLNARILKSTRKNLKVFNIGTNQDLN--YKNVHL----GNSTKV----------------------------------  target    ERLDARDVIPGYELAALTNYVTLKPDAEIKGNPPPPPFTAGGQVVPTELRDAWGDFVWWDRATGRPRPVSRDEVGARFDG 7tgh.58   --------------------------------------------------------------------------------  target    DPALLGEFEVELVDGSTVPVRPAFDLLKQYLDESFDLRTASEVCRVPPQAIQSIARQLAANKRETLLAAGMGPNHYFQND 7tgh.58   -------------------------------------------LKEIADGTHPFAERLKKAKLPMI-MVGASALEREDGA  target    LFGRVQFLVAALTDNIGHLGGNVGSYAGNYRGSVFQAMGQWIAEDPFAIEPDLTKPATVKRYYKAESAHYWNYGERPLRA 7tgh.58   ELYNTLKVISNKTGVISEEKSWNGFNILHK------EMG------------RI-N--A------------LELG------  target    VAKDDEGDLTKGEVLTGKSHMPTPTKLIWFGNSNSLLGNAKWSFDVVKNTLPRQDAVFCNEWHWTSSCEYADLVFPADSW 7tgh.58   --------INPT-------SVNKNAKLVFILGADNNLR---------PEDIPADAFVVYFGTHGDEGAYYADIILPTAAY  target    AEFKLPDATASCTNPFLLAFPTTPLKRLYDTRSDYEALALTAKALGELIDEPRMEQYWRGILDGDPTPYLQRIFSGSNAT 7tgh.58   TEKN---ATWVNTEGRVQQG-RLVVMPPGDAREDWQIIRALSEEAGVP--------------------------------  target    RGITYDELHESSKRGVPLLMNMRTYPRSGGWEQRQEDKPWYTATGRLEFYRPEPEFQAAGESLPVWREPVDATFYEPNAI 7tgh.58   --------------------------------------------------------------------------------  target    LSNAAHPSIAPRAPEDYGVPESQLDVETRQYRNVVRTWAELQQTLHPLQERDPAFRFVF 7tgh.58   ----------------------------------------------------------- ``` | | | | | | | | | | | | | | | | | | | | | | | | | | | | | | | | | | | | | | | | | | | | | | | | | |
|  | 7v2c.1.L | NADH-ubiquinone oxidoreductase 75 kDa subunit, mitochondrial  *Active state complex I from Q10 dataset* | 0.22 |  | 13.19 | 0.44 | 37-768 | EM | 0.00 | hetero-1-1-1-1-1-2-… | 6 x SF4, 1 x FMN, 10 x PEE, 8 x PLX, 2 x 8Q1, 1 x NDP, 2 x UQ, 11 x CDL, 2 x FES, 1 x MG, 1 x ZN, 1 x ADP | HHblits | 0.27 |
| ``` target    AQGVSRRQLLGRALALGSGAALADLLGPARFLSPAGAATAGAVVPGNPLRVMPDRTWEQIYRNQFEDDSTFVFTCAPNDT 7v2c.1    ------------------------------------SELSGNIIDICPVGALTSKPYAFTAR---PWETRKTESIDVMDA  target    HNCLLRAHVKNGVVVRISPTYGYGEATDLYGNRASHRWDPRTCQKGLILSRRFYSERRVKAPMIRKGFKDWVEAGYPRND 7v2c.1    VGSNIVVSTRTGEVMRILPRM------------HEDINEEWISDKTRFAYDGLK-RQRLTQPMIRNE-------------  target    DGTPQMDVTLRGSDDWIRISWDEATTIAAKTMEDVARTFNGDEGARKLLAQGYHPEMVEVMHGAGVQALKLRGGMPLLGI 7v2c.1    ------------KGLLTYTTWEDALSRVAGMLQSFQ------------------GNDVAAIAGG----------------  target    GRIFGFYRFANMLALLDRKLRPDAPADEILGSRTFDNYAWHTDLPPGHPMVTGSQTVDFDLFSAEHTKLLLIIGMNWICT 7v2c.1    -------LVDAEALVALKDLL------NRVDSDSLCTEEVFPTAGAGTDLR-SNYLLNTTIAGVEEADVILLVGTNPRFE  target    KMPDGHWIGDARL-KGTRVIVISADYMPTANKADEVIILRPGTDAAFFLGVARELIEKGLYDRAAVIERTDLPLLVRLDT 7v2c.1    APLFNARIRKSWLHNDLKVALIGSPVDLTYRYDHLGDSPK------------------------ILQDIAS---------  target    GERLDARDVIPGYELAALTNYVTLKPDAEIKGNPPPPPFTAGGQVVPTELRDAWGDFVWWDRATGRPRPVSRDEVGARFD 7v2c.1    --------------------------------------------------------------------------------  target    GDPALLGEFEVELVDGSTVPVRPAFDLLKQYLDESFDLRTASEVCRVPPQAIQSIARQLAANKRETLLAAGMGPNHYFQN 7v2c.1    ----------------------------------G----------------NHPFSQILKEAKKP-MVVLGSSALQRSDG  target    DLFGRVQFLVAALTDNIGHLGGNVGSYAGNYRGSVFQAMGQWIAEDPFAIEPDLTKPATVKRYYKAESAHYWNYGERPLR 7v2c.1    TAILAAVSNIAQNIRLSSGVTGDWKVMN------------------ILH---RIAS--QVA---------ALDLGYKP--  target    AVAKDDEGDLTKGEVLTGKSHMPTPTKLIWFGNSNSLLGNAKWSFDVVKNTLPRQDAVFCNEWHWTSSCEYADLVFPADS 7v2c.1    --------G--VEAIR------KNPPKVLFLLGADGG--------CITRQDLPKDCFIIYQGHHGDVGAPMADVILPGAA  target    WAEFKLPDATASCTNPFLLAFPTTPLKRLYDTRSDYEALALTAKALGELIDEPRMEQYWRGILDGDPTPYLQRIFSGSNA 7v2c.1    YTEK---SATYVNTEGRAQQT-KVAVTPPGLAREDWKIIRALSEIAGMT-------------------------------  target    TRGITYDELHESSKRGVPLLMNMRTYPRSGGWEQRQEDKPWYTATGRLEFYRPEPEFQAAGESLPVWREPVDATFYEPNA 7v2c.1    --------------------------------------------------------------------------------  target    ILSNAAHPSIAPRAPEDYGVPESQLDVETRQYRNVVRTWAELQQTLHPLQERDPAFRFVF 7v2c.1    ------------------------------------------------------------ ``` | | | | | | | | | | | | | | | | | | | | | | | | | | | | | | | | | | | | | | | | | | | | | | | | | |
|  | 6zr2.1.G | NADH-ubiquinone oxidoreductase 75 kDa subunit, mitochondrial  *Cryo-EM structure of respiratory complex I in the active state from Mus musculus at 3.1 A* | 0.23 |  | 13.16 | 0.45 | 37-769 | EM | 3.10 | hetero-1-1-1-1-1-1-… | 6 x SF4, 4 x PC1, 2 x FES, 1 x FMN, 9 x 3PE, 7 x CDL, 1 x ATP, 1 x NDP, 1 x ZN, 2 x EHZ | HHblits | 0.26 |
| ``` target    AQGVSRRQLLGRALALGSGAALADLLGPARFLSPAGAATAGAVVPGNPLRVMPDRTWEQIYRNQFEDDSTFVFTCAPNDT 6zr2.1    ------------------------------------SELSGNVIDICPVGALTSKPYAFTAR---PWETRKTESIDVMDA  target    HNCLLRAHVKNGVVVRISPTYGYGEATDLYGNRASHRWDPRTCQKGLILSRRFYSERRVKAPMIRKGFKDWVEAGYPRND 6zr2.1    VGSNIVVSTRTGEVMRILPR------------MHEDINEEWISDKTRFAYDGLK-RQRLTEPMVRNE-------------  target    DGTPQMDVTLRGSDDWIRISWDEATTIAAKTMEDVARTFNGDEGARKLLAQGYHPEMVEVMHGAGVQALKLRGGMPLLGI 6zr2.1    ------------KGLLTYTSWEDALSRVAGMLQNF------------------EGNAVAAIAG-----------------  target    GRIFGFYRFANMLALLDRKLRPDAPADEILGSRTFDNYAWHTDLPPGHPMVTGSQTVDFDLFSAEHTKLLLIIGMNWICT 6zr2.1    ------GLVDAEALVALKDLL------NKVDSDNLCTEEIFPTEGAGTDLRSNY-LLNTTIAGVEEADVVLLVGTNPRFE  target    KMPDGHWIGDAR-LKGTRVIVISADYMPTANKADEVIILRPGTDAAFFLGVARELIEKGLYDRAAVIERTDLPLLVRLDT 6zr2.1    APLFNARIRKSWLHNDLKVALIGSPVDLTYRYDHLGDSP------------------------KILQDIAS---------  target    GERLDARDVIPGYELAALTNYVTLKPDAEIKGNPPPPPFTAGGQVVPTELRDAWGDFVWWDRATGRPRPVSRDEVGARFD 6zr2.1    -----------G--------------------------------------------------------------------  target    GDPALLGEFEVELVDGSTVPVRPAFDLLKQYLDESFDLRTASEVCRVPPQAIQSIARQLAANKRETLLAAGMGPNHYFQN 6zr2.1    ---------------------------------------------------RHSFCEVLKDAKKP-MVVLGSSALQRDDG  target    DLFGRVQFLVAALTDNIGHLGGNVGSYAGNYRGSVFQAMGQWIAEDPFAIEPDLTKPATVKRYYKAESAHYWNYGERPLR 6zr2.1    AAILVAVSNMVQKIRVTTGVAAEWKVMN------------I------LH---RIAS--QVA---------ALDLGYKP--  target    AVAKDDEGDLTKGEVLTGKSHMPTPTKLIWFGNSNSLLGNAKWSFDVVKNTLPRQDAVFCNEWHWTSSCEYADLVFPADS 6zr2.1    --------G--VEAIR------KNPPKMLFLLGADGG--------CITRQDLPKDCFIVYQGHHGDVGAPMADVILPGAA  target    WAEFKLPDATASCTNPFLLAFPTTPLKRLYDTRSDYEALALTAKALGELIDEPRMEQYWRGILDGDPTPYLQRIFSGSNA 6zr2.1    YTEK---SATYVNTEGRAQQT-KVAVTPPGLAREDWKIIRALSEIAGITL------------------------------  target    TRGITYDELHESSKRGVPLLMNMRTYPRSGGWEQRQEDKPWYTATGRLEFYRPEPEFQAAGESLPVWREPVDATFYEPNA 6zr2.1    --------------------------------------------------------------------------------  target    ILSNAAHPSIAPRAPEDYGVPESQLDVETRQYRNVVRTWAELQQTLHPLQERDPAFRFVF 6zr2.1    ------------------------------------------------------------ ``` | | | | | | | | | | | | | | | | | | | | | | | | | | | | | | | | | | | | | | | | | | | | | | | | | |
|  | 6g72.1.G | NADH-ubiquinone oxidoreductase 75 kDa subunit, mitochondrial  *Mouse mitochondrial complex I in the deactive state* | 0.22 |  | 13.16 | 0.45 | 37-769 | EM | 0.00 | hetero-1-1-1-1-1-1-… | 6 x SF4, 2 x FES, 1 x FMN, 1 x ADP, 1 x NDP, 1 x ZN, 2 x EHZ | HHblits | 0.26 |
| ``` target    AQGVSRRQLLGRALALGSGAALADLLGPARFLSPAGAATAGAVVPGNPLRVMPDRTWEQIYRNQFEDDSTFVFTCAPNDT 6g72.1    ------------------------------------SELSGNVIDICPVGALTSKPYAFTAR---PWETRKTESIDVMDA  target    HNCLLRAHVKNGVVVRISPTYGYGEATDLYGNRASHRWDPRTCQKGLILSRRFYSERRVKAPMIRKGFKDWVEAGYPRND 6g72.1    VGSNIVVSTRTGEVMRILPR------------MHEDINEEWISDKTRFAYDGLK-RQRLTEPMVRNE-------------  target    DGTPQMDVTLRGSDDWIRISWDEATTIAAKTMEDVARTFNGDEGARKLLAQGYHPEMVEVMHGAGVQALKLRGGMPLLGI 6g72.1    ------------KGLLTYTSWEDALSRVAGMLQNF------------------EGNAVAAIAG-----------------  target    GRIFGFYRFANMLALLDRKLRPDAPADEILGSRTFDNYAWHTDLPPGHPMVTGSQTVDFDLFSAEHTKLLLIIGMNWICT 6g72.1    ------GLVDAEALVALKDLL------NKVDSDNLCTEEIFPTEGAGTDLRSNY-LLNTTIAGVEEADVVLLVGTNPRFE  target    KMPDGHWIGDAR-LKGTRVIVISADYMPTANKADEVIILRPGTDAAFFLGVARELIEKGLYDRAAVIERTDLPLLVRLDT 6g72.1    APLFNARIRKSWLHNDLKVALIGSPVDLTYRYDHLGDSP------------------------KILQDIAS---------  target    GERLDARDVIPGYELAALTNYVTLKPDAEIKGNPPPPPFTAGGQVVPTELRDAWGDFVWWDRATGRPRPVSRDEVGARFD 6g72.1    -----------G--------------------------------------------------------------------  target    GDPALLGEFEVELVDGSTVPVRPAFDLLKQYLDESFDLRTASEVCRVPPQAIQSIARQLAANKRETLLAAGMGPNHYFQN 6g72.1    ---------------------------------------------------RHSFCEVLKDAKKP-MVVLGSSALQRDDG  target    DLFGRVQFLVAALTDNIGHLGGNVGSYAGNYRGSVFQAMGQWIAEDPFAIEPDLTKPATVKRYYKAESAHYWNYGERPLR 6g72.1    AAILVAVSNMVQKIRVTTGVAAEWKVMN------------I------LH---RIAS--QVA---------ALDLGYKP--  target    AVAKDDEGDLTKGEVLTGKSHMPTPTKLIWFGNSNSLLGNAKWSFDVVKNTLPRQDAVFCNEWHWTSSCEYADLVFPADS 6g72.1    --------G--VEAIR------KNPPKMLFLLGADGG--------CITRQDLPKDCFIVYQGHHGDVGAPMADVILPGAA  target    WAEFKLPDATASCTNPFLLAFPTTPLKRLYDTRSDYEALALTAKALGELIDEPRMEQYWRGILDGDPTPYLQRIFSGSNA 6g72.1    YTEK---SATYVNTEGRAQQT-KVAVTPPGLAREDWKIIRALSEIAGITL------------------------------  target    TRGITYDELHESSKRGVPLLMNMRTYPRSGGWEQRQEDKPWYTATGRLEFYRPEPEFQAAGESLPVWREPVDATFYEPNA 6g72.1    --------------------------------------------------------------------------------  target    ILSNAAHPSIAPRAPEDYGVPESQLDVETRQYRNVVRTWAELQQTLHPLQERDPAFRFVF 6g72.1    ------------------------------------------------------------ ``` | | | | | | | | | | | | | | | | | | | | | | | | | | | | | | | | | | | | | | | | | | | | | | | | | |
|  | 7ak6.1.G | NADH-ubiquinone oxidoreductase 75 kDa subunit, mitochondrial  *Cryo-EM structure of ND6-P25L mutant respiratory complex I from Mus musculus at 3.8 A* | 0.22 |  | 13.16 | 0.45 | 37-769 | EM | 0.00 | hetero-1-1-1-1-1-1-… | 6 x SF4, 1 x PC1, 2 x FES, 1 x FMN, 4 x 3PE, 2 x CDL, 1 x ATP, 1 x NDP, 1 x ZN, 2 x EHZ | HHblits | 0.26 |
| ``` target    AQGVSRRQLLGRALALGSGAALADLLGPARFLSPAGAATAGAVVPGNPLRVMPDRTWEQIYRNQFEDDSTFVFTCAPNDT 7ak6.1    ------------------------------------SELSGNVIDICPVGALTSKPYAFTAR---PWETRKTESIDVMDA  target    HNCLLRAHVKNGVVVRISPTYGYGEATDLYGNRASHRWDPRTCQKGLILSRRFYSERRVKAPMIRKGFKDWVEAGYPRND 7ak6.1    VGSNIVVSTRTGEVMRILPR------------MHEDINEEWISDKTRFAYDGLK-RQRLTEPMVRNE-------------  target    DGTPQMDVTLRGSDDWIRISWDEATTIAAKTMEDVARTFNGDEGARKLLAQGYHPEMVEVMHGAGVQALKLRGGMPLLGI 7ak6.1    ------------KGLLTYTSWEDALSRVAGMLQNF------------------EGNAVAAIAG-----------------  target    GRIFGFYRFANMLALLDRKLRPDAPADEILGSRTFDNYAWHTDLPPGHPMVTGSQTVDFDLFSAEHTKLLLIIGMNWICT 7ak6.1    ------GLVDAEALVALKDLL------NKVDSDNLCTEEIFPTEGAGTDLRSNY-LLNTTIAGVEEADVVLLVGTNPRFE  target    KMPDGHWIGDAR-LKGTRVIVISADYMPTANKADEVIILRPGTDAAFFLGVARELIEKGLYDRAAVIERTDLPLLVRLDT 7ak6.1    APLFNARIRKSWLHNDLKVALIGSPVDLTYRYDHLGDSP------------------------KILQDIAS---------  target    GERLDARDVIPGYELAALTNYVTLKPDAEIKGNPPPPPFTAGGQVVPTELRDAWGDFVWWDRATGRPRPVSRDEVGARFD 7ak6.1    -----------G--------------------------------------------------------------------  target    GDPALLGEFEVELVDGSTVPVRPAFDLLKQYLDESFDLRTASEVCRVPPQAIQSIARQLAANKRETLLAAGMGPNHYFQN 7ak6.1    ---------------------------------------------------RHSFCEVLKDAKKP-MVVLGSSALQRDDG  target    DLFGRVQFLVAALTDNIGHLGGNVGSYAGNYRGSVFQAMGQWIAEDPFAIEPDLTKPATVKRYYKAESAHYWNYGERPLR 7ak6.1    AAILVAVSNMVQKIRVTTGVAAEWKVMN------------I------LH---RIAS--QVA---------ALDLGYKP--  target    AVAKDDEGDLTKGEVLTGKSHMPTPTKLIWFGNSNSLLGNAKWSFDVVKNTLPRQDAVFCNEWHWTSSCEYADLVFPADS 7ak6.1    --------G--VEAIR------KNPPKMLFLLGADGG--------CITRQDLPKDCFIVYQGHHGDVGAPMADVILPGAA  target    WAEFKLPDATASCTNPFLLAFPTTPLKRLYDTRSDYEALALTAKALGELIDEPRMEQYWRGILDGDPTPYLQRIFSGSNA 7ak6.1    YTEK---SATYVNTEGRAQQT-KVAVTPPGLAREDWKIIRALSEIAGITL------------------------------  target    TRGITYDELHESSKRGVPLLMNMRTYPRSGGWEQRQEDKPWYTATGRLEFYRPEPEFQAAGESLPVWREPVDATFYEPNA 7ak6.1    --------------------------------------------------------------------------------  target    ILSNAAHPSIAPRAPEDYGVPESQLDVETRQYRNVVRTWAELQQTLHPLQERDPAFRFVF 7ak6.1    ------------------------------------------------------------ ``` | | | | | | | | | | | | | | | | | | | | | | | | | | | | | | | | | | | | | | | | | | | | | | | | | |
|  | 7ak5.1.G | NADH-ubiquinone oxidoreductase 75 kDa subunit, mitochondrial  *Cryo-EM structure of respiratory complex I in the deactive state from Mus musculus at 3.2 A* | 0.22 |  | 13.16 | 0.45 | 37-769 | EM | 0.00 | hetero-1-1-1-1-1-1-… | 6 x SF4, 2 x PC1, 2 x FES, 1 x FMN, 8 x 3PE, 4 x CDL, 1 x ATP, 1 x NDP, 1 x ZN, 2 x EHZ | HHblits | 0.26 |
| ``` target    AQGVSRRQLLGRALALGSGAALADLLGPARFLSPAGAATAGAVVPGNPLRVMPDRTWEQIYRNQFEDDSTFVFTCAPNDT 7ak5.1    ------------------------------------SELSGNVIDICPVGALTSKPYAFTAR---PWETRKTESIDVMDA  target    HNCLLRAHVKNGVVVRISPTYGYGEATDLYGNRASHRWDPRTCQKGLILSRRFYSERRVKAPMIRKGFKDWVEAGYPRND 7ak5.1    VGSNIVVSTRTGEVMRILPR------------MHEDINEEWISDKTRFAYDGLK-RQRLTEPMVRNE-------------  target    DGTPQMDVTLRGSDDWIRISWDEATTIAAKTMEDVARTFNGDEGARKLLAQGYHPEMVEVMHGAGVQALKLRGGMPLLGI 7ak5.1    ------------KGLLTYTSWEDALSRVAGMLQNF------------------EGNAVAAIAG-----------------  target    GRIFGFYRFANMLALLDRKLRPDAPADEILGSRTFDNYAWHTDLPPGHPMVTGSQTVDFDLFSAEHTKLLLIIGMNWICT 7ak5.1    ------GLVDAEALVALKDLL------NKVDSDNLCTEEIFPTEGAGTDLRSNY-LLNTTIAGVEEADVVLLVGTNPRFE  target    KMPDGHWIGDAR-LKGTRVIVISADYMPTANKADEVIILRPGTDAAFFLGVARELIEKGLYDRAAVIERTDLPLLVRLDT 7ak5.1    APLFNARIRKSWLHNDLKVALIGSPVDLTYRYDHLGDSP------------------------KILQDIAS---------  target    GERLDARDVIPGYELAALTNYVTLKPDAEIKGNPPPPPFTAGGQVVPTELRDAWGDFVWWDRATGRPRPVSRDEVGARFD 7ak5.1    -----------G--------------------------------------------------------------------  target    GDPALLGEFEVELVDGSTVPVRPAFDLLKQYLDESFDLRTASEVCRVPPQAIQSIARQLAANKRETLLAAGMGPNHYFQN 7ak5.1    ---------------------------------------------------RHSFCEVLKDAKKP-MVVLGSSALQRDDG  target    DLFGRVQFLVAALTDNIGHLGGNVGSYAGNYRGSVFQAMGQWIAEDPFAIEPDLTKPATVKRYYKAESAHYWNYGERPLR 7ak5.1    AAILVAVSNMVQKIRVTTGVAAEWKVMN------------IL------H---RIAS--QVA---------ALDLGYKP--  target    AVAKDDEGDLTKGEVLTGKSHMPTPTKLIWFGNSNSLLGNAKWSFDVVKNTLPRQDAVFCNEWHWTSSCEYADLVFPADS 7ak5.1    --------G--VEAIR------KNPPKMLFLLGADGG--------CITRQDLPKDCFIVYQGHHGDVGAPMADVILPGAA  target    WAEFKLPDATASCTNPFLLAFPTTPLKRLYDTRSDYEALALTAKALGELIDEPRMEQYWRGILDGDPTPYLQRIFSGSNA 7ak5.1    YTEK---SATYVNTEGRAQQT-KVAVTPPGLAREDWKIIRALSEIAGITL------------------------------  target    TRGITYDELHESSKRGVPLLMNMRTYPRSGGWEQRQEDKPWYTATGRLEFYRPEPEFQAAGESLPVWREPVDATFYEPNA 7ak5.1    --------------------------------------------------------------------------------  target    ILSNAAHPSIAPRAPEDYGVPESQLDVETRQYRNVVRTWAELQQTLHPLQERDPAFRFVF 7ak5.1    ------------------------------------------------------------ ``` | | | | | | | | | | | | | | | | | | | | | | | | | | | | | | | | | | | | | | | | | | | | | | | | | |
|  | 7arc.1.F | 75 kDa  *Cryo-EM structure of Polytomella Complex-I (peripheral arm)* | 0.23 |  | 14.63 | 0.44 | 37-768 | EM | 0.00 | hetero-1-1-1-1-1-1-… | 6 x SF4, 2 x FES, 1 x FMN, 1 x NDP, 1 x ZN, 1 x 8Q1 | HHblits | 0.28 |
| ``` target    AQGVSRRQLLGRALALGSGAALADLLGPARFLSPAGAATAGAVVPGNPLRVMPDRTWEQIYRNQFEDDSTFVFTCAPNDT 7arc.1    ------------------------------------SELSGNVIDLCPVGALLSKPYAFTAR---SWELKGTETIDVSDA  target    HNCLLRAHVKNGVVVRISPTYGYGEATDLYGNRASHRWDPRTCQKGLILSRRFYSERRVKAPMIRKGFKDWVEAGYPRND 7arc.1    LGSNIKVDCRGTEVMRITPR------------LNDAINEEWLSDKGRFQYDGLK-RQRLNTPLVKGA-------------  target    DGTPQMDVTLRGSDDWIRISWDEATTIAAKTMEDVARTFNGDEGARKLLAQGYHPEMVEVMHGAGVQALKLRGGMPLLGI 7arc.1    -------------KGLENATWSAAFDAIRTAIAGAKG------------------NELKAIAGKLADA------------  target    GRIFGFYRFANMLALLDRKLRPDAPADEILGSRTFDNYAWHTDLPPGHPMVTGSQTVDFDLFSAEHTKLLLIIGMNWICT 7arc.1    ESMIALKDLFN-----------------KLGSGNLIHEDGSATLSADVRSS---YIANTTIASIEKADVILLVGTNPRFE  target    KMPDGHWIGDARLKGTRVIVISADYMPTANKADEVIILRPGTDAAFFLGVARELIEKGLYDRAAVIERTDLPLLVRLDTG 7arc.1    SPVFNARLRKVFLDGAKVGLVGEKVDLT------YAYQHLGADVAALESLASG---------------------------  target    ERLDARDVIPGYELAALTNYVTLKPDAEIKGNPPPPPFTAGGQVVPTELRDAWGDFVWWDRATGRPRPVSRDEVGARFDG 7arc.1    --------------------------------------------------------------------------------  target    DPALLGEFEVELVDGSTVPVRPAFDLLKQYLDESFDLRTASEVCRVPPQAIQSIARQLAANKRETLLAAGMGPNHYFQND 7arc.1    --------------------------------------------------KGAFFEALKGAKNP-VVIVGSSVLRRDDRE  target    LFGRVQFLVAALTDNIGHLGGNVGSYAGNYRGSVFQAMGQWIAEDPFAIEPDLTKPATVKRYYKAESAHYWNYGERPLRA 7arc.1    AVLKTVNDLVDAAGVVKEGWNGFNVLHDNASR-----VAAL----------DIG---------------FV-----P---  target    VAKDDEGDLTKGEVLTGKSHMPTPTKLIWFGNSNSLLGNAKWSFDVVKNTLPRQDAVFCNEWHWTSSCEYADLVFPADSW 7arc.1    -----------SAS-AR--TNPVPAKVVYLLGSDDFKD----------EEIPADAFVIYQGHHGDKGAARANVVLPGAAY  target    AEFKLPDATASCTNPFLLAFPTTPLKRLYDTRSDYEALALTAKALGELIDEPRMEQYWRGILDGDPTPYLQRIFSGSNAT 7arc.1    TEKA---SLFANTEGRVQTT-RTAVPVLGDAREDWKIIRALSEVVGQQ--------------------------------  target    RGITYDELHESSKRGVPLLMNMRTYPRSGGWEQRQEDKPWYTATGRLEFYRPEPEFQAAGESLPVWREPVDATFYEPNAI 7arc.1    --------------------------------------------------------------------------------  target    LSNAAHPSIAPRAPEDYGVPESQLDVETRQYRNVVRTWAELQQTLHPLQERDPAFRFVF 7arc.1    ----------------------------------------------------------- ``` | | | | | | | | | | | | | | | | | | | | | | | | | | | | | | | | | | | | | | | | | | | | | | | | | |
|  | 5t5i.1.B | Tungsten formylmethanofuran dehydrogenase subunit B  *TUNGSTEN-CONTAINING FORMYLMETHANOFURAN DEHYDROGENASE FROM METHANOTHERMOBACTER WOLFEII, ORTHORHOMBIC FORM AT 1.9 A* | 0.22 |  | 17.52 | 0.44 | 71-768 | X-ray | 1.90 | hetero-oligomer | 4 x ZN, 2 x MG, 18 x K, 22 x SF4, 2 x W, 4 x MGD, 2 x H2S, 2 x CA | HHblits | 0.27 |
| ``` target    AQGVSRRQLLGRALALGSGAALADLLGPARFLSPAGAATAGAVVPGNPLRVMPDRTWEQIYRNQFEDDSTFVFTCAPNDT 5t5i.1    ----------------------------------------------------------------------VKNVVCPFCG  target    HNCL-LRAHVKNGVVVRISPTYGYGEATDLYGNRASHRWDPRTCQKGLILSRRFYSERRVKAPMIRKGFKDWVEAGYPRN 5t5i.1    TLCDDIICKVEGNEIVGT----------------------INACRIGHSKFVHAEGAMRYKKPLIRKN------------  target    DDGTPQMDVTLRGSDDWIRISWDEATTIAAKTMEDVARTFNGDEGARKLLAQGYHPEMVEVMHGAGVQALKLRGGMPLLG 5t5i.1    --------------GEFVEVSYDEAIDKAAKILAESKRP--------LM----YG---------WSCTEC----------  target    IGRIFGFYRFANMLALLDRKLRPDAPADEILGSRTFDNYAWHTDL--PPGHPMVTGSQTVDFDLFSA-EHTKLLLIIGMN 5t5i.1    -EAQAVGVELAEE-----------------AGA-VIDNTASVCHGPSVLALQ-DVGYP--ICTFGEVKNRADVVVYWGCN  target    WICTKMPDGHW-------IGDARLKGTRVIVISADYMPTANKADEVIILRPGTDAAFFLGVARELIEKGLYDRAAVIERT 5t5i.1    PMHAHPRHMSRNVFARGFFRERGRSDRTLIVVDPRKTDSAKLADIHLQLDFDRDYELLDAMRACLLGHE-----------  target    DLPLLVRLDTGERLDARDVIPGYELAALTNYVTLKPDAEIKGNPPPPPFTAGGQVVPTELRDAWGDFVWWDRATGRPRPV 5t5i.1    --------------------------------------------------------------------------------  target    SRDEVGARFDGDPALLGEFEVELVDGSTVPVRPAFDLLKQYLDESFDLRTASEVCRVPPQAIQSIARQLAANKRETLLAA 5t5i.1    ------------------------------------------------ILYDEVAGVPREQIEEAVEVLKNAQFG-ILFF  target    GMGPNHYFQNDLFGRVQFLVAALTDNIGHLGGNVGSYAGN--YRGSVFQAMGQWIAEDPFAIEPDLTKPATVKRYYKAES 5t5i.1    GMGITHSRGKHRNIDTAIMMVQDLNDY--AKWTLIPMRGHYNVTGFN--QVCTWES--GYPY--------CV--------  target    AHYWNYGERPLRAVAKDDEGDLTKGEVLTGKSHMPTPTKLIWFGNSNSLLGNAKWSFDVVKNTLPRQDAVFCNEWHWTSS 5t5i.1    -DFSGGE-PRYNP------GETGANDLL-----QNREADAMMVIASDPGAHFPQ----RALERMAEIP-VIAIEPHRTPT  target    CEYADLVFPADS-WAEFKLPDATASCTNPFLLAFPTTPLKRLYDTRSDYEALALTAKALGELIDEPRMEQYWRGILDGDP 5t5i.1    TEMADIIIPPAIVGMEAE---GTAYRMEGVPIRM-KKVVDS--DLLSDREILERLLEKVREY------------------  target    TPYLQRIFSGSNATRGITYDELHESSKRGVPLLMNMRTYPRSGGWEQRQEDKPWYTATGRLEFYRPEPEFQAAGESLPVW 5t5i.1    --------------------------------------------------------------------------------  target    REPVDATFYEPNAILSNAAHPSIAPRAPEDYGVPESQLDVETRQYRNVVRTWAELQQTLHPLQERDPAFRFVF 5t5i.1    ------------------------------------------------------------------------- ``` | | | | | | | | | | | | | | | | | | | | | | | | | | | | | | | | | | | | | | | | | | | | | | | | | |
|  | 8b9z.1.G | NADH-ubiquinone oxidoreductase 75 kDa subunit, mitochondrial  *Drosophila melanogaster complex I in the Active state (Dm1)* | 0.22 |  | 15.37 | 0.44 | 37-770 | EM | 3.28 | hetero-1-1-1-1-1-1-… | 3 x PC1, 16 x 3PE, 6 x SF4, 4 x CDL, 2 x FES, 1 x FMN, 1 x UQ9, 1 x DGT, 1 x NDP, 1 x ZN, 2 x EHZ | HHblits | 0.27 |
| ``` target    AQGVSRRQLLGRALALGSGAALADLLGPARFLSPAGAATAGAVVPGNPLRVMPDRTWEQIYRNQFEDDSTFVFTCAPNDT 8b9z.1    ------------------------------------TELSGNVIDLCPVGALTNKPYSFVAR---PWEIRKVSSIDVLDA  target    HNCLLRAHVKNGVVVRISPTYGYGEATDLYGNRASHRWDPRTCQKGLILSRRFYSERRVKAPMIRKGFKDWVEAGYPRND 8b9z.1    VGSNIVVSTRTNEVLRILPRE------------NEDVNEEWLADKSRFACDGLK-RQRLVAPMVRMP-------------  target    DGTPQMDVTLRGSDDWIRISWDEATTIAAKTMEDVARTFNGDEGARKLLAQGYHPEMVEVMHGAGVQALKLRGGMPLLGI 8b9z.1    ------------NGELQAVEWEGALIAVAKAIKAAGG-------------------QIAGISGQ----------------  target    GRIFGFYRFANMLALLDRKLRPDAPADEILGSRTFDNYAWHTDLPPGHPMVTGSQTVDFDLFSAEHTKLLLIIGMNWICT 8b9z.1    -------LADLEAQVALKDLL------NRLGSEVVATEQGFIAGGTDN---RANYLLNSTIAGLEEADAVLLVGTNPRYE  target    KMPDGHWIGDAR-LKGTRVIVISADYMPTANKADEVIILRPGTDAAFFLGVARELIEKGLYDRAAVIERTDLPLLVRLDT 8b9z.1    APLVNTRLRKAYVHNELQIASIGPKIDLS------YDHENLGADAALVKDVCSG--------------------------  target    GERLDARDVIPGYELAALTNYVTLKPDAEIKGNPPPPPFTAGGQVVPTELRDAWGDFVWWDRATGRPRPVSRDEVGARFD 8b9z.1    --------------------------------------------------------------------------------  target    GDPALLGEFEVELVDGSTVPVRPAFDLLKQYLDESFDLRTASEVCRVPPQAIQSIARQLAANKRETLLAAGMGPNHYFQN 8b9z.1    ---------------------------------------------------AHAFSKVLEGAKKP-AIIIGADLLERADG  target    DLFGRVQFLVAALTDNIGHLGGNV-GSYAGNYRGSVFQAMGQWIAEDPFAIEPDLTKPATVKRYYKAESAHYWNYGERPL 8b9z.1    AAI---HATVAEYCKKLKKPNWNPFNVLQTNAA-----QVGAL----------D--------------------VGYKAG  target    RAVAKDDEGDLTKGEVLTGKSHMPTPTKLIWFGNSNSLLGNAKWSFDVVKNTLPRQDAVFCNEWHWTSSCEYADLVFPAD 8b9z.1    ------------AQTA------VKAQPKVLFLLNADAG--------KVTREQLPKDCFVVYIGSHGDNGASIADAVLPGA  target    SWAEFKLPDATASCTNPFLLAFPTTPLKRLYDTRSDYEALALTAKALGELIDEPRMEQYWRGILDGDPTPYLQRIFSGSN 8b9z.1    AYTEK---QGIYVNTEGRPQQT-LPGVSPPGMAREDWKILRALSEVVGKPLP----------------------------  target    ATRGITYDELHESSKRGVPLLMNMRTYPRSGGWEQRQEDKPWYTATGRLEFYRPEPEFQAAGESLPVWREPVDATFYEPN 8b9z.1    --------------------------------------------------------------------------------  target    AILSNAAHPSIAPRAPEDYGVPESQLDVETRQYRNVVRTWAELQQTLHPLQERDPAFRFVF 8b9z.1    ------------------------------------------------------------- ``` | | | | | | | | | | | | | | | | | | | | | | | | | | | | | | | | | | | | | | | | | | | | | | | | | |
|  | 8ba0.1.G | NADH-ubiquinone oxidoreductase 75 kDa subunit, mitochondrial  *Drosophila melanogaster complex I in the Twisted state (Dm2)* | 0.22 |  | 15.37 | 0.44 | 37-770 | EM | 3.68 | hetero-1-1-1-1-1-1-… | 6 x SF4, 6 x 3PE, 2 x FES, 1 x FMN, 2 x CDL, 1 x DGT, 1 x NDP, 1 x ZN, 2 x EHZ | HHblits | 0.27 |
| ``` target    AQGVSRRQLLGRALALGSGAALADLLGPARFLSPAGAATAGAVVPGNPLRVMPDRTWEQIYRNQFEDDSTFVFTCAPNDT 8ba0.1    ------------------------------------TELSGNVIDLCPVGALTNKPYSFVAR---PWEIRKVSSIDVLDA  target    HNCLLRAHVKNGVVVRISPTYGYGEATDLYGNRASHRWDPRTCQKGLILSRRFYSERRVKAPMIRKGFKDWVEAGYPRND 8ba0.1    VGSNIVVSTRTNEVLRILPRE------------NEDVNEEWLADKSRFACDGLK-RQRLVAPMVRMP-------------  target    DGTPQMDVTLRGSDDWIRISWDEATTIAAKTMEDVARTFNGDEGARKLLAQGYHPEMVEVMHGAGVQALKLRGGMPLLGI 8ba0.1    ------------NGELQAVEWEGALIAVAKAIKAAGG-------------------QIAGISGQ----------------  target    GRIFGFYRFANMLALLDRKLRPDAPADEILGSRTFDNYAWHTDLPPGHPMVTGSQTVDFDLFSAEHTKLLLIIGMNWICT 8ba0.1    -------LADLEAQVALKDLL------NRLGSEVVATEQGFIAGGTDN---RANYLLNSTIAGLEEADAVLLVGTNPRYE  target    KMPDGHWIGDAR-LKGTRVIVISADYMPTANKADEVIILRPGTDAAFFLGVARELIEKGLYDRAAVIERTDLPLLVRLDT 8ba0.1    APLVNTRLRKAYVHNELQIASIGPKIDLS------YDHENLGADAALVKDVCSG--------------------------  target    GERLDARDVIPGYELAALTNYVTLKPDAEIKGNPPPPPFTAGGQVVPTELRDAWGDFVWWDRATGRPRPVSRDEVGARFD 8ba0.1    --------------------------------------------------------------------------------  target    GDPALLGEFEVELVDGSTVPVRPAFDLLKQYLDESFDLRTASEVCRVPPQAIQSIARQLAANKRETLLAAGMGPNHYFQN 8ba0.1    ---------------------------------------------------AHAFSKVLEGAKKP-AIIIGADLLERADG  target    DLFGRVQFLVAALTDNIGHLGGNV-GSYAGNYRGSVFQAMGQWIAEDPFAIEPDLTKPATVKRYYKAESAHYWNYGERPL 8ba0.1    AAI---HATVAEYCKKLKKPNWNPFNVLQTNAA-----QVGAL----------D--------------------VGYKAG  target    RAVAKDDEGDLTKGEVLTGKSHMPTPTKLIWFGNSNSLLGNAKWSFDVVKNTLPRQDAVFCNEWHWTSSCEYADLVFPAD 8ba0.1    ------------AQTA------VKAQPKVLFLLNADAG--------KVTREQLPKDCFVVYIGSHGDNGASIADAVLPGA  target    SWAEFKLPDATASCTNPFLLAFPTTPLKRLYDTRSDYEALALTAKALGELIDEPRMEQYWRGILDGDPTPYLQRIFSGSN 8ba0.1    AYTEK---QGIYVNTEGRPQQT-LPGVSPPGMAREDWKILRALSEVVGKPLP----------------------------  target    ATRGITYDELHESSKRGVPLLMNMRTYPRSGGWEQRQEDKPWYTATGRLEFYRPEPEFQAAGESLPVWREPVDATFYEPN 8ba0.1    --------------------------------------------------------------------------------  target    AILSNAAHPSIAPRAPEDYGVPESQLDVETRQYRNVVRTWAELQQTLHPLQERDPAFRFVF 8ba0.1    ------------------------------------------------------------- ``` | | | | | | | | | | | | | | | | | | | | | | | | | | | | | | | | | | | | | | | | | | | | | | | | | |
|  | 6x89.1.H | NADH dehydrogenase [ubiquinone] iron-sulfur protein 1, mitochondrial  *Vigna radiata mitochondrial complex I\** | 0.22 |  | 15.37 | 0.44 | 37-769 | EM | 0.00 | hetero-1-1-1-1-1-1-… | 1 x NAP, 6 x PC1, 6 x SF4, 2 x FES, 2 x ZN, 1 x FMN | HHblits | 0.27 |
| ``` target    AQGVSRRQLLGRALALGSGAALADLLGPARFLSPAGAATAGAVVPGNPLRVMPDRTWEQIYRNQFEDDSTFVFTCAPNDT 6x89.1    ------------------------------------SELSGNVIDICPVGALTSKPFAFKAR---NWELKGTETIDVTDA  target    HNCLLRAHVKNGVVVRISPTYGYGEATDLYGNRASHRWDPRTCQKGLILSRRFYSERRVKAPMIRKGFKDWVEAGYPRND 6x89.1    VGSNIRIDSRGPEVMRIVPRL------------NEDINEEWISDKTRFCYDGLK-RQRLNDPMIRGP-------------  target    DGTPQMDVTLRGSDDWIRISWDEATTIAAKTMEDVARTFNGDEGARKLLAQGYHPEMVEVMHGAGVQALKLRGGMPLLGI 6x89.1    ------------DGRFKAVNWRDALSVIADIAHQV----K--------------PEEIVGVAGKLSDA------------  target    GRIFGFYRFANMLALLDRKLRPDAPADEILGSRTFDNYAWHTDLPPGHPMVTGSQTVDFDLFSAEHTKLLLIIGMNWICT 6x89.1    ESMIALKDFLN-----------------RMGSNDVWGEGIGVNTNADF--RSGY-IMNTSIAGLEKADVFLLVGTQPRVE  target    KMPDGHWIGDARL-KGTRVIVISADYMPTANKADEVIILRPGTDAAFFLGVARELIEKGLYDRAAVIERTDLPLLVRLDT 6x89.1    AAMVNARIRKTVRSNQAKVGYIGPATDFN--YDHKHLGTDPQTLVEIAEG------------------------------  target    GERLDARDVIPGYELAALTNYVTLKPDAEIKGNPPPPPFTAGGQVVPTELRDAWGDFVWWDRATGRPRPVSRDEVGARFD 6x89.1    ------------R-------------------------------------------------------------------  target    GDPALLGEFEVELVDGSTVPVRPAFDLLKQYLDESFDLRTASEVCRVPPQAIQSIARQLAANKRETLLAAGMGPNHYFQN 6x89.1    ----------------------------------------------------HPFFKTLSDAKNP-VIIVGAGVFERKDQ  target    DLFGRVQFLVAALTDNIGHLGGNVGSYAGNYRGSVFQAMGQWIAEDPFAIEPDLTKPATVKRYYKAESAHYWNYGERPLR 6x89.1    DAIFAAVETIAQKANVVRPDWNGLNVLLLHAA-----QAAAL----------DL--------------------GLVP--  target    AVAKDDEGDLTKGEVLTGKSHMPTPTKLIWFGNSNSLLGNAKWSFDVVKNTLPRQDAVFCNEWHWTSSCEYADLVFPADS 6x89.1    ------------QSE-K----SLESAKFVYLMGADDVN----------LDKIPDDAFVVYQGHHGDKSVYRANVILPTAA  target    WAEFKLPDATASCTNPFLLAFPTTPLKRLYDTRSDYEALALTAKALGELIDEPRMEQYWRGILDGDPTPYLQRIFSGSNA 6x89.1    FSEKE---GTYQNTEGCTQQT-LPAVPTVGDSRDDWKIIRALSEVAGVRL------------------------------  target    TRGITYDELHESSKRGVPLLMNMRTYPRSGGWEQRQEDKPWYTATGRLEFYRPEPEFQAAGESLPVWREPVDATFYEPNA 6x89.1    --------------------------------------------------------------------------------  target    ILSNAAHPSIAPRAPEDYGVPESQLDVETRQYRNVVRTWAELQQTLHPLQERDPAFRFVF 6x89.1    ------------------------------------------------------------ ``` | | | | | | | | | | | | | | | | | | | | | | | | | | | | | | | | | | | | | | | | | | | | | | | | | |
|  | 8e73.55.A | NDUS1  *Vigna radiata supercomplex I+III2 (full bridge)* | 0.24 |  | 15.37 | 0.44 | 37-769 | EM | 0.00 | monomer |  | HHblits | 0.27 |
| ``` target    AQGVSRRQLLGRALALGSGAALADLLGPARFLSPAGAATAGAVVPGNPLRVMPDRTWEQIYRNQFEDDSTFVFTCAPNDT 8e73.55   ------------------------------------SELSGNVIDICPVGALTSKPFAFKAR---NWELKGTETIDVTDA  target    HNCLLRAHVKNGVVVRISPTYGYGEATDLYGNRASHRWDPRTCQKGLILSRRFYSERRVKAPMIRKGFKDWVEAGYPRND 8e73.55   VGSNIRIDSRGPEVMRIVPRL------------NEDINEEWISDKTRFCYDGLK-RQRLNDPMIRGP-------------  target    DGTPQMDVTLRGSDDWIRISWDEATTIAAKTMEDVARTFNGDEGARKLLAQGYHPEMVEVMHGAGVQALKLRGGMPLLGI 8e73.55   ------------DGRFKAVNWRDALSVIADIAHQV----K--------------PEEIVGVAGKLSDA------------  target    GRIFGFYRFANMLALLDRKLRPDAPADEILGSRTFDNYAWHTDLPPGHPMVTGSQTVDFDLFSAEHTKLLLIIGMNWICT 8e73.55   ESMIALKDFLN-----------------RMGSNDVWGEGIGVNTNADF--RSGY-IMNTSIAGLEKADVFLLVGTQPRVE  target    KMPDGHWIGDARL-KGTRVIVISADYMPTANKADEVIILRPGTDAAFFLGVARELIEKGLYDRAAVIERTDLPLLVRLDT 8e73.55   AAMVNARIRKTVRSNQAKVGYIGPATDFN--YDHKHLGTDPQTLVEIAEG------------------------------  target    GERLDARDVIPGYELAALTNYVTLKPDAEIKGNPPPPPFTAGGQVVPTELRDAWGDFVWWDRATGRPRPVSRDEVGARFD 8e73.55   ------------R-------------------------------------------------------------------  target    GDPALLGEFEVELVDGSTVPVRPAFDLLKQYLDESFDLRTASEVCRVPPQAIQSIARQLAANKRETLLAAGMGPNHYFQN 8e73.55   ----------------------------------------------------HPFFKTLSDAKNP-VIIVGAGVFERKDQ  target    DLFGRVQFLVAALTDNIGHLGGNVGSYAGNYRGSVFQAMGQWIAEDPFAIEPDLTKPATVKRYYKAESAHYWNYGERPLR 8e73.55   DAIFAAVETIAQKANVVRPDWNGLNVLLLHAA-----QAAAL----------DL--------------------GLVP--  target    AVAKDDEGDLTKGEVLTGKSHMPTPTKLIWFGNSNSLLGNAKWSFDVVKNTLPRQDAVFCNEWHWTSSCEYADLVFPADS 8e73.55   ------------QSE-K----SLESAKFVYLMGADDVN----------LDKIPDDAFVVYQGHHGDKSVYRANVILPTAA  target    WAEFKLPDATASCTNPFLLAFPTTPLKRLYDTRSDYEALALTAKALGELIDEPRMEQYWRGILDGDPTPYLQRIFSGSNA 8e73.55   FSEKE---GTYQNTEGCTQQT-LPAVPTVGDSRDDWKIIRALSEVAGVRL------------------------------  target    TRGITYDELHESSKRGVPLLMNMRTYPRSGGWEQRQEDKPWYTATGRLEFYRPEPEFQAAGESLPVWREPVDATFYEPNA 8e73.55   --------------------------------------------------------------------------------  target    ILSNAAHPSIAPRAPEDYGVPESQLDVETRQYRNVVRTWAELQQTLHPLQERDPAFRFVF 8e73.55   ------------------------------------------------------------ ``` | | | | | | | | | | | | | | | | | | | | | | | | | | | | | | | | | | | | | | | | | | | | | | | | | |
|  | 7bkb.1.L | Formylmethanofuran dehydrogenase, subunit B  *Formate dehydrogenase - heterodisulfide reductase - formylmethanofuran dehydrogenase complex from Methanospirillum hungatei (hexameric, composite structure)* | 0.22 |  | 16.79 | 0.44 | 70-768 | EM | 0.00 | hetero-2-2-2-2-2-2-… | 48 x SF4, 4 x FAD, 2 x FES, 4 x 9S8, 4 x ZN, 2 x MO, 4 x MGD | HHblits | 0.27 |
| ``` target    AQGVSRRQLLGRALALGSGAALADLLGPARFLSPAGAATAGAVVPGNPLRVMPDRTWEQIYRNQFEDDSTFVFTCAPNDT 7bkb.1    ---------------------------------------------------------------------VIENVGCPYCG  target    HNCL-LRAHVKNGVVVRISPTYGYGEATDLYGNRASHRWDPRTCQKGLILSRRFYSERRVKAPMIRKGFKDWVEAGYPRN 7bkb.1    CSCDDVRITVSDDG--------------------KDILEVENVCAIGTEIFKHGCSKDRIRLPRMRQP------------  target    DDGTPQMDVTLRGSDDWIRISWDEATTIAAKTMEDVARTFNGDEGARKLLAQGYHPEMVEVMHGAGVQALKLRGGMPLLG 7bkb.1    -------------DGSMKDISYEEAIDWTARHLLKAKKPL--------MY----G---------FGSTNC--E-------  target    IGRIFGFYRFANMLALLDRKLRPDAPADEILGSRTFDNYAWHTDLPPGHPMVTGSQTVDFDLFSA-EHTKLLLIIGMNWI 7bkb.1    --GQAAAARVMEI-----------------AGG-MLDNCATICHGP-SFLAIFDNGYPSCTLGEVKNRADVIVYWGSNPA  target    CTKMPDGHWI--------GDARLKGTRVIVISADYMPTANKADEVIILRPGTDAAFFLGVARELIEKGLYDRAAVIERTD 7bkb.1    HAHPRHMSRYSIFPRGFFTGKGQKKRTVIVIDPRFTDTANVADYHLQVKQGHDYELFNAFRMVIHGHG------------  target    LPLLVRLDTGERLDARDVIPGYELAALTNYVTLKPDAEIKGNPPPPPFTAGGQVVPTELRDAWGDFVWWDRATGRPRPVS 7bkb.1    --------------------------------------------------------------------------------  target    RDEVGARFDGDPALLGEFEVELVDGSTVPVRPAFDLLKQYLDESFDLRTASEVCRVPPQAIQSIARQLAANKRETLLAAG 7bkb.1    ----------------------------------------------KDLPDEVAGIKKETILEVAEIMKNARFG-TTFFG  target    MGPNHYFQNDLFGRVQF------------LVAALTDNIGHLGGNVGSYAGNYRGSVFQAMGQWIAEDPFAIEPDLTKPAT 7bkb.1    MGLTHTDGRNHNIDIAISLTRDLNKISKWTIMAMRGHYNIAGPGVVWSWTF----------GF----PYC--LDLTKQ-N  target    VKRYYKAESAHYWNYGERPLRAVAKDDEGDLTKGEVLTGKSHMPTPTKLIWFGNSNSLLGNAKWSFDVVKNTLPRQDAVF 7bkb.1    ---H------AHMN----P----------GE--TSSVDM--AMRDEVDMFINIGTDAAAHFPIP----AVKQLKKHPW-V  target    CNEWHWTSSCEYADLVFPADSW-AEFKLPDATASCTNPFLLAFPTTPLKRLYDTRSDYEALALTAKALGELIDEPRMEQY 7bkb.1    TIDPSINMASEISDLHIPVCICGVDVG---GIVYRMDNVPIQF-RKVIEPPEGVMDDETLLNKIADRMEEL---------  target    WRGILDGDPTPYLQRIFSGSNATRGITYDELHESSKRGVPLLMNMRTYPRSGGWEQRQEDKPWYTATGRLEFYRPEPEFQ 7bkb.1    --------------------------------------------------------------------------------  target    AAGESLPVWREPVDATFYEPNAILSNAAHPSIAPRAPEDYGVPESQLDVETRQYRNVVRTWAELQQTLHPLQERDPAFRF 7bkb.1    --------------------------------------------------------------------------------  target    VF 7bkb.1    -- ``` | | | | | | | | | | | | | | | | | | | | | | | | | | | | | | | | | | | | | | | | | | | | | | | | | |
|  | 7aqr.1.F | NADH dehydrogenase [ubiquinone] iron-sulfur protein 1, mitochondrial  *Cryo-EM structure of Arabidopsis thaliana Complex-I (peripheral arm)* | 0.23 |  | 14.39 | 0.44 | 37-769 | EM | 0.00 | hetero-1-1-1-1-1-1-… | 6 x SF4, 2 x FES, 1 x FMN, 1 x NDP, 1 x ZN, 1 x 8Q1 | HHblits | 0.27 |
| ``` target    AQGVSRRQLLGRALALGSGAALADLLGPARFLSPAGAATAGAVVPGNPLRVMPDRTWEQIYRNQFEDDSTFVFTCAPNDT 7aqr.1    ------------------------------------SELSGNVIDICPVGALTSKPFAFKAR-NW--ELKATETIDVSDA  target    HNCLLRAHVKNGVVVRISPTYGYGEATDLYGNRASHRWDPRTCQKGLILSRRFYSERRVKAPMIRKGFKDWVEAGYPRND 7aqr.1    VGSNIRVDSRGPEVMRIIPRL------------NEDINEEWISDKTRFCYDGLK-RQRLSDPMIRDS-------------  target    DGTPQMDVTLRGSDDWIRISWDEATTIAAKTMEDVARTFNGDEGARKLLAQGYHPEMVEVMHGAGVQALKLRGGMPLLGI 7aqr.1    ------------DGRFKAVSWRDALAVVGDIIHQVK------------------PDEIVGVAGQLSDA------------  target    GRIFGFYRFANMLALLDRKLRPDAPADEILGSRTFDNYAWHTDLPPGHPMVTGSQTVDFDLFSAEHTKLLLIIGMNWICT 7aqr.1    ESMMVLKDFVN-----------------RMGSDNVWCEGTAAGVDADLRY---SYLMNTSISGLENADLFLLIGTQPRVE  target    KMPDGHWIGDAR-LKGTRVIVISADYMPTANKADEVIILRPGTDAAFFLGVARELIEKGLYDRAAVIERTDLPLLVRLDT 7aqr.1    AAMVNARICKTVRASNAKVGYVGPPAEFN--YDCKHLGTGPDTLKEI---------------------------------  target    GERLDARDVIPGYELAALTNYVTLKPDAEIKGNPPPPPFTAGGQVVPTELRDAWGDFVWWDRATGRPRPVSRDEVGARFD 7aqr.1    --------------------------------------------------------------------------------  target    GDPALLGEFEVELVDGSTVPVRPAFDLLKQYLDESFDLRTASEVCRVPPQAIQSIARQLAANKRETLLAAGMGPNHYFQN 7aqr.1    ------------------------------------------------AEGRHPFCTALKNAKNP-AIIVGAGLFNRTDK  target    DLFGRVQFLVAALTDNIGHLGGNVGSYAGNYRGSVFQAMGQWIAEDPFAIEPDLTKPATVKRYYKAESAHYWNYGERPLR 7aqr.1    NAILSSVESIAQANNVVRPDWNGLNFLLQYAA-----QAAAL----------------D--------------LG-----  target    AVAKDDEGDLTKGEVLTGKSHMPTPTKLIWFGNSNSLLGNAKWSFDVVKNTLPRQDAVFCNEWHWTSSCEYADLVFPADS 7aqr.1    LIQ----------QSAKA----LESAKFVYLMGADDVN----------VDKIPKDAFVVYQGHHGDKAVYRANVILPASA  target    WAEFKLPDATASCTNPFLLAFPTTPLKRLYDTRSDYEALALTAKALGELIDEPRMEQYWRGILDGDPTPYLQRIFSGSNA 7aqr.1    FTEKE---GTYENTEGFTQQT-VPAVPTVGDARDDWKIVRALSEVSGVKL------------------------------  target    TRGITYDELHESSKRGVPLLMNMRTYPRSGGWEQRQEDKPWYTATGRLEFYRPEPEFQAAGESLPVWREPVDATFYEPNA 7aqr.1    --------------------------------------------------------------------------------  target    ILSNAAHPSIAPRAPEDYGVPESQLDVETRQYRNVVRTWAELQQTLHPLQERDPAFRFVF 7aqr.1    ------------------------------------------------------------ ``` | | | | | | | | | | | | | | | | | | | | | | | | | | | | | | | | | | | | | | | | | | | | | | | | | |
|  | 7a23.1.O | 75kDa  *Plant mitochondrial respiratory complex I* | 0.22 |  | 14.39 | 0.44 | 37-769 | EM | 0.00 | hetero-1-1-1-1-1-1-… | 6 x SF4, 1 x FMN, 2 x T7X, 3 x CDL, 1 x U10, 1 x PEV, 2 x FES, 1 x NDP, 2 x ZN | HHblits | 0.27 |
| ``` target    AQGVSRRQLLGRALALGSGAALADLLGPARFLSPAGAATAGAVVPGNPLRVMPDRTWEQIYRNQFEDDSTFVFTCAPNDT 7a23.1    ------------------------------------SELSGNVIDICPVGALTSKPFAFKAR-NW--ELKATETIDVSDA  target    HNCLLRAHVKNGVVVRISPTYGYGEATDLYGNRASHRWDPRTCQKGLILSRRFYSERRVKAPMIRKGFKDWVEAGYPRND 7a23.1    VGSNIRVDSRGPEVMRIIPRL------------NEDINEEWISDKTRFCYDGLK-RQRLSDPMIRDS-------------  target    DGTPQMDVTLRGSDDWIRISWDEATTIAAKTMEDVARTFNGDEGARKLLAQGYHPEMVEVMHGAGVQALKLRGGMPLLGI 7a23.1    ------------DGRFKAVSWRDALAVVGDIIHQVK------------------PDEIVGVAGQLSDA------------  target    GRIFGFYRFANMLALLDRKLRPDAPADEILGSRTFDNYAWHTDLPPGHPMVTGSQTVDFDLFSAEHTKLLLIIGMNWICT 7a23.1    ESMMVLKDFVN-----------------RMGSDNVWCEGTAAGVDADLRY---SYLMNTSISGLENADLFLLIGTQPRVE  target    KMPDGHWIGDAR-LKGTRVIVISADYMPTANKADEVIILRPGTDAAFFLGVARELIEKGLYDRAAVIERTDLPLLVRLDT 7a23.1    AAMVNARICKTVRASNAKVGYVGPPAEFN--YDCKHLGTGPDTLKEI---------------------------------  target    GERLDARDVIPGYELAALTNYVTLKPDAEIKGNPPPPPFTAGGQVVPTELRDAWGDFVWWDRATGRPRPVSRDEVGARFD 7a23.1    --------------------------------------------------------------------------------  target    GDPALLGEFEVELVDGSTVPVRPAFDLLKQYLDESFDLRTASEVCRVPPQAIQSIARQLAANKRETLLAAGMGPNHYFQN 7a23.1    ------------------------------------------------AEGRHPFCTALKNAKNP-AIIVGAGLFNRTDK  target    DLFGRVQFLVAALTDNIGHLGGNVGSYAGNYRGSVFQAMGQWIAEDPFAIEPDLTKPATVKRYYKAESAHYWNYGERPLR 7a23.1    NAILSSVESIAQANNVVRPDWNGLNFLLQYAA-----QAAAL----------------D--------------LG-----  target    AVAKDDEGDLTKGEVLTGKSHMPTPTKLIWFGNSNSLLGNAKWSFDVVKNTLPRQDAVFCNEWHWTSSCEYADLVFPADS 7a23.1    LIQ----------QSAKA----LESAKFVYLMGADDVN----------VDKIPKDAFVVYQGHHGDKAVYRANVILPASA  target    WAEFKLPDATASCTNPFLLAFPTTPLKRLYDTRSDYEALALTAKALGELIDEPRMEQYWRGILDGDPTPYLQRIFSGSNA 7a23.1    FTEKE---GTYENTEGFTQQT-VPAVPTVGDARDDWKIVRALSEVSGVKL------------------------------  target    TRGITYDELHESSKRGVPLLMNMRTYPRSGGWEQRQEDKPWYTATGRLEFYRPEPEFQAAGESLPVWREPVDATFYEPNA 7a23.1    --------------------------------------------------------------------------------  target    ILSNAAHPSIAPRAPEDYGVPESQLDVETRQYRNVVRTWAELQQTLHPLQERDPAFRFVF 7a23.1    ------------------------------------------------------------ ``` | | | | | | | | | | | | | | | | | | | | | | | | | | | | | | | | | | | | | | | | | | | | | | | | | |
|  | 7ar8.1.G | NADH dehydrogenase [ubiquinone] iron-sulfur protein 1, mitochondrial  *Cryo-EM structure of Arabidopsis thaliana complex-I (closed conformation)* | 0.22 |  | 14.39 | 0.44 | 37-769 | EM | 0.00 | hetero-1-1-1-1-1-1-… | 6 x SF4, 2 x FES, 1 x FMN, 1 x UQ9, 3 x PTY, 2 x PC7, 1 x PGT, 1 x FE, 1 x NDP, 2 x ZN, 2 x 8Q1, 1 x LMN, 1 x PSF, 1 x T7X | HHblits | 0.27 |
| ``` target    AQGVSRRQLLGRALALGSGAALADLLGPARFLSPAGAATAGAVVPGNPLRVMPDRTWEQIYRNQFEDDSTFVFTCAPNDT 7ar8.1    ------------------------------------SELSGNVIDICPVGALTSKPFAFKAR-NW--ELKATETIDVSDA  target    HNCLLRAHVKNGVVVRISPTYGYGEATDLYGNRASHRWDPRTCQKGLILSRRFYSERRVKAPMIRKGFKDWVEAGYPRND 7ar8.1    VGSNIRVDSRGPEVMRIIPRL------------NEDINEEWISDKTRFCYDGLK-RQRLSDPMIRDS-------------  target    DGTPQMDVTLRGSDDWIRISWDEATTIAAKTMEDVARTFNGDEGARKLLAQGYHPEMVEVMHGAGVQALKLRGGMPLLGI 7ar8.1    ------------DGRFKAVSWRDALAVVGDIIHQVK------------------PDEIVGVAGQLSDA------------  target    GRIFGFYRFANMLALLDRKLRPDAPADEILGSRTFDNYAWHTDLPPGHPMVTGSQTVDFDLFSAEHTKLLLIIGMNWICT 7ar8.1    ESMMVLKDFVN-----------------RMGSDNVWCEGTAAGVDADLRY---SYLMNTSISGLENADLFLLIGTQPRVE  target    KMPDGHWIGDAR-LKGTRVIVISADYMPTANKADEVIILRPGTDAAFFLGVARELIEKGLYDRAAVIERTDLPLLVRLDT 7ar8.1    AAMVNARICKTVRASNAKVGYVGPPAEFN--YDCKHLGTGPDTLKEI---------------------------------  target    GERLDARDVIPGYELAALTNYVTLKPDAEIKGNPPPPPFTAGGQVVPTELRDAWGDFVWWDRATGRPRPVSRDEVGARFD 7ar8.1    --------------------------------------------------------------------------------  target    GDPALLGEFEVELVDGSTVPVRPAFDLLKQYLDESFDLRTASEVCRVPPQAIQSIARQLAANKRETLLAAGMGPNHYFQN 7ar8.1    ------------------------------------------------AEGRHPFCTALKNAKNP-AIIVGAGLFNRTDK  target    DLFGRVQFLVAALTDNIGHLGGNVGSYAGNYRGSVFQAMGQWIAEDPFAIEPDLTKPATVKRYYKAESAHYWNYGERPLR 7ar8.1    NAILSSVESIAQANNVVRPDWNGLNFLLQYAA-----QAAAL----------------D--------------LG-----  target    AVAKDDEGDLTKGEVLTGKSHMPTPTKLIWFGNSNSLLGNAKWSFDVVKNTLPRQDAVFCNEWHWTSSCEYADLVFPADS 7ar8.1    LIQ----------QSAKA----LESAKFVYLMGADDVN----------VDKIPKDAFVVYQGHHGDKAVYRANVILPASA  target    WAEFKLPDATASCTNPFLLAFPTTPLKRLYDTRSDYEALALTAKALGELIDEPRMEQYWRGILDGDPTPYLQRIFSGSNA 7ar8.1    FTEKE---GTYENTEGFTQQT-VPAVPTVGDARDDWKIVRALSEVSGVKL------------------------------  target    TRGITYDELHESSKRGVPLLMNMRTYPRSGGWEQRQEDKPWYTATGRLEFYRPEPEFQAAGESLPVWREPVDATFYEPNA 7ar8.1    --------------------------------------------------------------------------------  target    ILSNAAHPSIAPRAPEDYGVPESQLDVETRQYRNVVRTWAELQQTLHPLQERDPAFRFVF 7ar8.1    ------------------------------------------------------------ ``` | | | | | | | | | | | | | | | | | | | | | | | | | | | | | | | | | | | | | | | | | | | | | | | | | |
|  | 7ar7.1.G | NADH dehydrogenase [ubiquinone] iron-sulfur protein 1, mitochondrial  *Cryo-EM structure of Arabidopsis thaliana complex-I (open conformation)* | 0.23 |  | 14.39 | 0.44 | 37-769 | EM | 0.00 | hetero-1-1-1-1-1-1-… | 6 x SF4, 2 x FES, 1 x FMN, 1 x UQ9, 3 x PTY, 2 x PC7, 1 x LMN, 1 x NDP, 2 x ZN, 2 x 8Q1, 1 x PGT, 1 x PSF, 1 x T7X | HHblits | 0.27 |
| ``` target    AQGVSRRQLLGRALALGSGAALADLLGPARFLSPAGAATAGAVVPGNPLRVMPDRTWEQIYRNQFEDDSTFVFTCAPNDT 7ar7.1    ------------------------------------SELSGNVIDICPVGALTSKPFAFKAR---NWELKATETIDVSDA  target    HNCLLRAHVKNGVVVRISPTYGYGEATDLYGNRASHRWDPRTCQKGLILSRRFYSERRVKAPMIRKGFKDWVEAGYPRND 7ar7.1    VGSNIRVDSRGPEVMRIIPR------------LNEDINEEWISDKTRFCYDGLK-RQRLSDPMIRDS-------------  target    DGTPQMDVTLRGSDDWIRISWDEATTIAAKTMEDVARTFNGDEGARKLLAQGYHPEMVEVMHGAGVQALKLRGGMPLLGI 7ar7.1    ------------DGRFKAVSWRDALAVVGDIIHQVK------------------PDEIVGVAGQLSDA------------  target    GRIFGFYRFANMLALLDRKLRPDAPADEILGSRTFDNYAWHTDLPPGHPMVTGSQTVDFDLFSAEHTKLLLIIGMNWICT 7ar7.1    ESMMVLKDFVN-----------------RMGSDNVWCEGTAAGVDADLRY---SYLMNTSISGLENADLFLLIGTQPRVE  target    KMPDGHWIGDAR-LKGTRVIVISADYMPTANKADEVIILRPGTDAAFFLGVARELIEKGLYDRAAVIERTDLPLLVRLDT 7ar7.1    AAMVNARICKTVRASNAKVGYVGPPAEFN--YDCKHLGTGPDTLKEI---------------------------------  target    GERLDARDVIPGYELAALTNYVTLKPDAEIKGNPPPPPFTAGGQVVPTELRDAWGDFVWWDRATGRPRPVSRDEVGARFD 7ar7.1    --------------------------------------------------------------------------------  target    GDPALLGEFEVELVDGSTVPVRPAFDLLKQYLDESFDLRTASEVCRVPPQAIQSIARQLAANKRETLLAAGMGPNHYFQN 7ar7.1    ------------------------------------------------AEGRHPFCTALKNAKNP-AIIVGAGLFNRTDK  target    DLFGRVQFLVAALTDNIGHLGGNVGSYAGNYRGSVFQAMGQWIAEDPFAIEPDLTKPATVKRYYKAESAHYWNYGERPLR 7ar7.1    NAILSSVESIAQANNVVRPDWNGLNFLLQYAA-----QAAAL----------------D--------------LG-----  target    AVAKDDEGDLTKGEVLTGKSHMPTPTKLIWFGNSNSLLGNAKWSFDVVKNTLPRQDAVFCNEWHWTSSCEYADLVFPADS 7ar7.1    LI----------QQSAKA----LESAKFVYLMGADDVN----------VDKIPKDAFVVYQGHHGDKAVYRANVILPASA  target    WAEFKLPDATASCTNPFLLAFPTTPLKRLYDTRSDYEALALTAKALGELIDEPRMEQYWRGILDGDPTPYLQRIFSGSNA 7ar7.1    FTEKE---GTYENTEGFTQQT-VPAVPTVGDARDDWKIVRALSEVSGVKL------------------------------  target    TRGITYDELHESSKRGVPLLMNMRTYPRSGGWEQRQEDKPWYTATGRLEFYRPEPEFQAAGESLPVWREPVDATFYEPNA 7ar7.1    --------------------------------------------------------------------------------  target    ILSNAAHPSIAPRAPEDYGVPESQLDVETRQYRNVVRTWAELQQTLHPLQERDPAFRFVF 7ar7.1    ------------------------------------------------------------ ``` | | | | | | | | | | | | | | | | | | | | | | | | | | | | | | | | | | | | | | | | | | | | | | | | | |
|  | 6lod.1.B | Fe-S-cluster-containing hydrogenase components 1-like protein  *Cryo-EM structure of the air-oxidized photosynthetic alternative complex III from Roseiflexus castenholzii* | 0.21 |  | 13.86 | 0.43 | 71-767 | EM | 0.00 | hetero-1-1-1-1-1-1-… | 6 x HEC, 2 x EL6, 3 x SF4, 1 x F3S | HHblits | 0.26 |
| ``` target    AQGVSRRQLLGRALALGSGAALADLLGPARFLSPAGAATAGAVVPGNPLRVMPDRTWEQIYRNQFEDDSTFVFTCAPNDT 6lod.1    ----------------------------------------------------------------------FFATAVTFAG  target    HNCLLRAHVKNGVVVRISPTYGYGEATDLYGNRASHRWDPRTCQKGLILSRRFYSERRVKAPMIRKGFKDWVEAGYPRND 6lod.1    FGVGLLVESHEGRPTKIEG------------NPDHPASLGSTDLITQAMILTMYDPDRSQAPTNA---------------  target    DGTPQMDVTLRGSDDWIRISWDEATTIAAKTMEDVARTFNGDEGARKLLAQGYHPEMVEVMHGAGVQALKLRGGMPLLGI 6lod.1    ---------------GQETTWDAFVAAATAAMQAQTAKQGA--------------GL-RVLSGSLT--------------  target    GRIFGFYRFANMLALLDRKLRPDAPADEILGSRTFDNYAW--HTDLPPGHPMVTGSQTVDFDLFSAEHTKLLLIIGMNWI 6lod.1    -----SPTLIAQKQQLLTQ----------FPQAKWYEYEPVGRDNANAGARLAFGAD--VHTIYRLDTAKVIVGFDADFT  target    CTKMPD---GHWIGDAR------LKGTRVIVISADYMPTANKADEVIILRPGTDAAFFLGVARELIEKGLYDRAAVIERT 6lod.1    APSPTGVRMARQLADGRRIRKGTKEVNRLYLAESTPSITGLLADHRLPVRSSQIEHLVRALATLVGVPNV----------  target    DLPLLVRLDTGERLDARDVIPGYELAALTNYVTLKPDAEIKGNPPPPPFTAGGQVVPTELRDAWGDFVWWDRATGRPRPV 6lod.1    --------------------------------------------------------------------------------  target    SRDEVGARFDGDPALLGEFEVELVDGSTVPVRPAFDLLKQYLDESFDLRTASEVCRVPPQAIQSIARQLAANKRETLLAA 6lod.1    ------------------------------------------------AAGAPLSDTEKKWVEAAAKDLQANRGACVVLV  target    GMGPNHYFQNDLFGRVQFLVAALTDNIGHLGGNVGSYAGNYRGSVFQAMGQWIAEDPFAIEPDLTKPATVKRYYKAESAH 6lod.1    GESQ--PPV---VHALGHAINAQLGNVGST---VVYTE------------------PVE---DDPSG-GI----------  target    YWNYGERPLRAVAKDDEGDLTKGEVLTGKSHMPTPTKLIWFGNSNSLLGNAKWSFDVVKNTLPRQDAVFCNEWHWTSSCE 6lod.1    ------AALSA--------L-TQEMN------AGTVEVLLMIESNPVYNAPA--DIPFAEALAKVPLSMHVGLYRDETAQ  target    YADLVFPADSWAEFKLPDATASCTNPFLLAFPTTPLKRLYDTRSDYEALALTAKALGELIDEPRMEQYWRGILDGDPTPY 6lod.1    QSVWHINGAHFLEAWG-DV--RAFDGTT-TIVQPLIAPLYNGKSAIEVLNVLLGKPQE----------------------  target    LQRIFSGSNATRGITYDELHESSKRGVPLLMNMRTYPRSGGWEQRQEDKPWYTATGRLEFYRPEPEFQAAGESLPVWREP 6lod.1    --------------------------------------------------------------------------------  target    VDATFYEPNAILSNAAHPSIAPRAPEDYGVPESQLDVETRQYRNVVRTWAELQQTLHPLQERDPAFRFVF 6lod.1    ---------------------------------------------------------------------- ``` | | | | | | | | | | | | | | | | | | | | | | | | | | | | | | | | | | | | | | | | | | | | | | | | | |
|  | 6f0k.1.B | Fe-S-cluster-containing hydrogenase  *Alternative complex III* | 0.20 |  | 15.71 | 0.43 | 69-767 | EM | 0.00 | hetero-1-1-1-1-1-1-… | 6 x HEC, 1 x F3S, 3 x SF4 | HHblits | 0.27 |
| ``` target    AQGVSRRQLLGRALALGSGAALADLLGPARFLSPAGAATAGAVVPGNPLRVMPDRTWEQIYRNQFEDDSTFVFTCAPNDT 6f0k.1    --------------------------------------------------------------------PLYYATAMPFRG  target    HNCLLRAHVKNGVVVRISPTYGYGEATDLYGNRASHRWDPRTCQKGLILSRRFYSERRVKAPMIRKGFKDWVEAGYPRND 6f0k.1    SVRPLLVESHEGRPTKIEG------------NPDHPLSRGATGVFEQASLLNLYDPDRSQQVLRK-G-------------  target    DGTPQMDVTLRGSDDWIRISWDEATTIAAKTMEDVARTFNGDEGARKLLAQGYHPEMVEVMHGAGVQALKLRGGMPLLGI 6f0k.1    ----------------EPASWGDFVQFARSLAAE----A--------------GTKRLAVLCEPSSSPT--------LA-  target    GRIFGFYRFANMLALLDRKLRPDAPADEILGSRTFDNYAWHT--DLPPGHPMVTGSQTVDFDLFSAEHTKLLLIIGMNWI 6f0k.1    -AL--RRELER-----------------RYAQVRWVTYRPEGDDHEALGLQQAFGRPVR--ARYRFSEARVIVSLDADFL  target    CTK-MPD---G------HWIGDARLKGTRVIVISADYMPTANKADEVIILRPGTDAAFFLGVARELIEKGLYDRAAVIER 6f0k.1    GPTDRNFVENTREFAASRRMERPEDEISRLYVIESTYTVTGGMADHRLRLRAGDIPAFAAALAAELGVGELRE-------  target    TDLPLLVRLDTGERLDARDVIPGYELAALTNYVTLKPDAEIKGNPPPPPFTAGGQVVPTELRDAWGDFVWWDRATGRPRP 6f0k.1    --------------------------------------------------------------------------------  target    VSRDEVGARFDGDPALLGEFEVELVDGSTVPVRPAFDLLKQYLDESFDLRTASEVCRVPPQAIQSIARQLAANKRETLLA 6f0k.1    --------------------------------------------------AGARFAGH--PYVVEIARDLRAAGARGVVL  target    AGMGPNHYFQNDLFGRVQFLVAALTDNIGHLGGNVGSYAGNYRGSVFQAMGQWIAEDPFAIEPDLTKPATVKRYYKAESA 6f0k.1    AGETQP---P--AVHALCAVINDLLGSLGRTVILH----------------AL--DEP-----ATAQHA-----------  target    HYWNYGERPLRAVAKDDEGDLTKGEVLTGKSHMPTPTKLIWFGNSNSLLGNAKWSFDVVKNTLPRQDAVFCNEWHWTSSC 6f0k.1    --------ALAE--------L-VQAMQ------AGAVDALLLLNVNPVYDAPA--ALGFAEALAQVPEVIHLGLHVDETA  target    EYADLVFPADSWAEFKLPDATASCTNPFLLAFPTTPLKRLYDT-RSDYEALALTAKALGELIDEPRMEQYWRGILDGDPT 6f0k.1    RRSTWHLPSTHYLEAWG--DGRAY-DGT-LSVIQPLIAPLYEAAHSPLEVLALLATGEEQ--------------------  target    PYLQRIFSGSNATRGITYDELHESSKRGVPLLMNMRTYPRSGGWEQRQEDKPWYTATGRLEFYRPEPEFQAAGESLPVWR 6f0k.1    --------------------------------------------------------------------------------  target    EPVDATFYEPNAILSNAAHPSIAPRAPEDYGVPESQLDVETRQYRNVVRTWAELQQTLHPLQERDPAFRFVF 6f0k.1    ------------------------------------------------------------------------ ``` | | | | | | | | | | | | | | | | | | | | | | | | | | | | | | | | | | | | | | | | | | | | | | | | | |
|  | 6btm.1.B | Alternative Complex III subunit B  *Structure of Alternative Complex III from Flavobacterium johnsoniae (Wild Type)* | 0.21 |  | 11.39 | 0.42 | 71-766 | EM | 3.40 | hetero-1-1-1-1-1-1-… | 6 x HEC, 1 x F3S, 1 x SF4, 2 x E87 | HHblits | 0.26 |
| ``` target    AQGVSRRQLLGRALALGSGAALADLLGPARFLSPAGAATAGAVVPGNPLRVMPDRTWEQIYRNQFEDDSTFVFTCAPNDT 6btm.1    ----------------------------------------------------------------------YYATTVFDGF  target    HNCLLRAHVKNGVVVRISPTYGYGEATDLYGNRASHRWDPRTCQKGLILSRRFYSERRVKAPMIRKGFKDWVEAGYPRND 6btm.1    DFANLLVKTREGRPIKIEN------------NTIAGAK-FSANARIHASILGLYDSMRLKEPKLDG--------------  target    DGTPQMDVTLRGSDDWIRISWDEATTIAAKTMEDVARTFNGDEGARKLLAQGYHPEMVEVMHGAGVQALKLRGGMPLLGI 6btm.1    ----------------KNSSWSAVDLKIKSSLADAKAK-GGQ---------------VVLLTNTLAS--------PTT--  target    GRIFGFYRFANMLALLDRKLRPDAPADEILGSRTFDNYAWHT--DLPPGHPMVTGSQTVDFDLFSAEHTKLLLIIGMNWI 6btm.1    EKL--IGEFIA-----------------KNPNAKHVVYDAVSSSDALDAFETVYGER--ALVDYDFSKASLIVSVGADFL  target    CTKMPD--GHWIGDARL----KGTRVIVISADYMPTANKADEVIILRPGTDAAFFLGVARELIEKGLYDRAAVIERTDLP 6btm.1    GDWQGGGYDAGYAKGRIPQNGKMSRHFQFESNMTLSGAAADKRVPMTTADQKQALVQIYNIVVGASVP------------  target    LLVRLDTGERLDARDVIPGYELAALTNYVTLKPDAEIKGNPPPPPFTAGGQVVPTELRDAWGDFVWWDRATGRPRPVSRD 6btm.1    --------------------------------------------------------------------------------  target    EVGARFDGDPALLGEFEVELVDGSTVPVRPAFDLLKQYLDESFDLRTASEVCRVPPQAIQSIARQLAANKRETLLAAGMG 6btm.1    ------------------------------------------VS------LDAKFKAEVVKAAQQLKAAGTKGILVSGIE  target    PNHYFQNDLFGRVQFLVAALTDNIGHLGGNVGSYAGNYRGSVFQAMGQWIAEDPFAIEPDLTKPATVKRYYKAESAHYWN 6btm.1    DK------NAQLLVLAINQALASEAFSTAGTRQ-------------------IR------KGSNAV--------------  target    YGERPLRAVAKDDEGDLTKGEVLTGKSHMPTPTKLIWFGNSNSLLGNAKWSFDVVKNTLPRQDAVFCNEWHWTSSCEYAD 6btm.1    -----VAQ--------L-IKDM------NAGSVHTLIMSGVNPVYTLAD--SASFVSGLKKVKTSVAFSLKEDETAAVST  target    LVFPADSWAEFKLPDATASCTNPFLLAFPTTPLKRLYDTRSDYEALALTAKALGELIDEPRMEQYWRGILDGDPTPYLQR 6btm.1    IAAAAPHYLESWG-DVE-ITK-G-TYSLTQPTIRPIFDTKQFQDVLLSVNGTPG--------------------------  target    IFSGSNATRGITYDELHESSKRGVPLLMNMRTYPRSGGWEQRQEDKPWYTATGRLEFYRPEPEFQAAGESLPVWREPVDA 6btm.1    --------------------------------------------------------------------------------  target    TFYEPNAILSNAAHPSIAPRAPEDYGVPESQLDVETRQYRNVVRTWAELQQTLHPLQERDPAFRFVF 6btm.1    ------------------------------------------------------------------- ``` | | | | | | | | | | | | | | | | | | | | | | | | | | | | | | | | | | | | | | | | | | | | | | | | | |
|  | 1eu1.1.A | DIMETHYL SULFOXIDE REDUCTASE  *THE CRYSTAL STRUCTURE OF RHODOBACTER SPHAEROIDES DIMETHYLSULFOXIDE REDUCTASE REVEALS TWO DISTINCT MOLYBDENUM COORDINATION ENVIRONMENTS.* | 0.11 |  | 26.56 | 0.27 | 503-778 | X-ray | 1.30 | monomer | 3 x GLC, 1 x CD, 2 x MGD, 1 x 6MO, 2 x O | BLAST | 0.33 |
| ``` target    AQGVSRRQLLGRALALGSGAALADLLGPARFLSPAGAATAGAVVPGNPLRVMPDRTWEQIYRNQFEDDSTFVFTCAPNDT 1eu1.1    --------------------------------------------------------------------------------  target    HNCLLRAHVKNGVVVRISPTYGYGEATDLYGNRASHRWDPRTCQKGLILSRRFYSERRVKAPMIRKGFKDWVEAGYPRND 1eu1.1    --------------------------------------------------------------------------------  target    DGTPQMDVTLRGSDDWIRISWDEATTIAAKTMEDVARTFNGDEGARKLLAQGYHPEMVEVMHGAGVQALKLRGGMPLLGI 1eu1.1    --------------------------------------------------------------------------------  target    GRIFGFYRFANMLALLDRKLRPDAPADEILGSRTFDNYAWHTDLPPGHPMVTGSQTVDFDLFSAEHTKLLLIIGMNWICT 1eu1.1    --------------------------------------------------------------------------------  target    KMPDGHWIGDARLKGTRVIVISADYMPTANKADEVIILRPGTDAAFFLGVARELIEKGLYDRAAVIERTDLPLLVRLDTG 1eu1.1    --------------------------------------------------------------------------------  target    ERLDARDVIPGYELAALTNYVTLKPDAEIKGNPPPPPFTAGGQVVPTELRDAWGDFVWWDRATGRPRPVSRDEVGARFDG 1eu1.1    --------------------------------------------------------------------------------  target    DPALLGEFEVELVDGSTVPVRPAFDLLKQYLDESFD-----LRTASEVCRVPPQAIQSIARQLAANKRETLLAAGMGPNH 1eu1.1    ----------------------GFDLFAAYLTGESDGTPKTAEWAAEICGLPAEQIRELARSFVAGR--TMLAAGWSIQR  target    YFQNDLFGRVQFLVAALTDNIGHLGGNVG-SYAGNYRGSVFQAMGQWIAEDPFAIEPDLTKPATVKRYYKAESAHYW--N 1eu1.1    MHHGEQAHWMLVTLASMIGQIGLPGGGFGLSYHYSNGGS------------PTSDGPAL---GGISDGGKAVEGAAWLSE  target    YGERPLRAVAKDDEGDLTKGEV-LTGKSHMPTPTKLIWFGNSNSLLGNAKWSFDVVKNTLPRQDAVFCNEWHWTSSCEYA 1eu1.1    SGATSIPCARVVDMLLNPGGEFQFNGATATYPDVKLAYWAGGNPFAHHQ--DRNRMLKAWEKLETFIVQDFQWTATARHA  target    DLVFPADSWAEFKLPDATASCTNPFLLAFPTTPLKRLYDTRSDYEALALTAKALGELID--EPRMEQYWRGILDGDPTPY 1eu1.1    DIVLPATTSYERNDIESVGDYSNRAILAMKKV-VDPLYEARSDYDIFAALAERLGKGAEFTEGRDEMGW-----------  target    LQRIFSGSNATRGITYDELHESSKRGVPLLMNMRTYPRSGGWEQRQEDKPWYTATGRLEFYRPEPEFQAAGESLPVWREP 1eu1.1    --------------------------------------------------------------------------------  target    VDATFYEPNAILSNAAHPSIAPRAPEDYGVPESQLDVETRQYRNVVRTWAELQQTLHPLQERDPAFRFVF 1eu1.1    ---------------------------------------------------------------------- ``` | | | | | | | | | | | | | | | | | | | | | | | | | | | | | | | | | | | | | | | | | | | | | | | | | |
|  | 1eu1.1.A | DIMETHYL SULFOXIDE REDUCTASE  *THE CRYSTAL STRUCTURE OF RHODOBACTER SPHAEROIDES DIMETHYLSULFOXIDE REDUCTASE REVEALS TWO DISTINCT MOLYBDENUM COORDINATION ENVIRONMENTS.* | 0.09 |  | 34.17 | 0.26 | 80-382 | X-ray | 1.30 | monomer | 3 x GLC, 1 x CD, 2 x MGD, 1 x 6MO, 2 x O | BLAST | 0.37 |
| ``` target    AQGVSRRQLLGRALALGSGAALADLLGPARFLSPAGAATAGAVVPGNPLRVMPDRTWEQIYRNQFEDDSTFVFTCAPNDT 1eu1.1    -------------------------------------------------------------------------------C  target    HNCLLRAHVKNGVVVRISPTYGYGEATDLYGNRASHRWD----PRTCQKGLILSRRFYSERRVKAPMIRKGFKDWVEAGY 1eu1.1    HWGVFKARVENGRAVAFEP------------------WDKDPAPSHQLPGVLDS--IYSPTRIKYPMVRREF---LEKGV  target    PRNDDGTPQMDVTLRGSDDWIRISWDEATTIAAKTMEDVARTFNGDEGARKLLAQGYHPEMVEVMHGAGV---QALKLRG 1eu1.1    --------NADRSTRGNGDFVRVTWDEALDLVARELKRVQESY----GPTGTFGGSYGWKSPGRLHNCQVLMRRALNLAG  target    GMPLLGIGRIFGFYRFANMLALLDRKLRPDAPADEILGSRTFDNYAWHTDLPPGHPMVTGSQTVDFDLFSAEHTKLLLII 1eu1.1    GFV-----NSSGDYSTAAAQIIMPH----------VMG--TLEVYEQQTAWP------VVVENTDLMVFWAADPMKTNEI  target    GMNWICTKMPD-GHWIGDARL--KGTRVIVISADYMPTANK-ADEVIILRPGTDAAFFLGVARELIEKGLYDRAAVIERT 1eu1.1    G--WV---IPDHGAYAGMKALKEKGTRVICINPVRTETADYFGADVVSPRPQTDVALMLGMAHTLYSEDLHDK-------  target    DLPLLVRLDTGERLDARDVIPGYELAALTNYVTLKPDAEIKGNPPPPPFTAGGQVVPTELRDAWGDFVWWDRATGRPRPV 1eu1.1    --------------------------------------------------------------------------------  target    SRDEVGARFDGDPALLGEFEVELVDGSTVPVRPAFDLLKQYLDESFDLRTASEVCRVPPQAIQSIARQLAANKRETLLAA 1eu1.1    --------------------------------------------------------------------------------  target    GMGPNHYFQNDLFGRVQFLVAALTDNIGHLGGNVGSYAGNYRGSVFQAMGQWIAEDPFAIEPDLTKPATVKRYYKAESAH 1eu1.1    --------------------------------------------------------------------------------  target    YWNYGERPLRAVAKDDEGDLTKGEVLTGKSHMPTPTKLIWFGNSNSLLGNAKWSFDVVKNTLPRQDAVFCNEWHWTSSCE 1eu1.1    --------------------------------------------------------------------------------  target    YADLVFPADSWAEFKLPDATASCTNPFLLAFPTTPLKRLYDTRSDYEALALTAKALGELIDEPRMEQYWRGILDGDPTPY 1eu1.1    --------------------------------------------------------------------------------  target    LQRIFSGSNATRGITYDELHESSKRGVPLLMNMRTYPRSGGWEQRQEDKPWYTATGRLEFYRPEPEFQAAGESLPVWREP 1eu1.1    --------------------------------------------------------------------------------  target    VDATFYEPNAILSNAAHPSIAPRAPEDYGVPESQLDVETRQYRNVVRTWAELQQTLHPLQERDPAFRFVF 1eu1.1    ---------------------------------------------------------------------- ``` | | | | | | | | | | | | | | | | | | | | | | | | | | | | | | | | | | | | | | | | | | | | | | | | | |
|  | 8e9g.1.G | NADH-quinone oxidoreductase subunit G  *Mycobacterial respiratory complex I with both quinone positions modelled* | 0.13 |  | 19.43 | 0.26 | 37-371 | EM | 0.00 | hetero-1-1-1-1-1-1-… |  | HHblits | 0.28 |
| ``` target    AQGVSRRQLLGRALALGSGAALADLLGPARFLSPAGAATAGAVVPGNPLRVMPDRTWEQIYRNQFEDDSTFVFTCAPNDT 8e9g.1    ------------------------------------SYFSGNTVQICPVGALTGTAYRFRAR---PFDLVSSPSVCEHCA  target    HNCLLRAHVKNGVVVRISPTYGYGEATDLYGNRASHRWDPRTCQKGLILSRRFYSERRVKAPMIRKGFKDWVEAGYPRND 8e9g.1    SGCAQRTDHRRGKVLRRLAG------------DEPEVNEEWNCDKGRWAFTYATVGDRITTPMLRDG-------------  target    DGTPQMDVTLRGSDDWIRISWDEATTIAAKTMEDVARTFNGDEGARKLLAQGYHPEMVEVMHGAGVQALKLRGGMPLLGI 8e9g.1    -------------GVLRPASWSEALTVAAAGLLTAAGS-------------------TGVLVG-----------------  target    GRIFGFYRFANMLALLDRKLRPDAPADEILGSRTFDNYAWHTDLP--PGHPMVTGSQTVDFDLFSAEHTKLLLIIGMNWI 8e9g.1    ------GRCTVEDAYAYAKFA-----RMVLNTNDVDFRARPHSAEEAEFLAAHVAGQTMGLRYAELENAPTVLLAGFEPE  target    CTKMPDGHWIGDA-RLKGTRVIVISADYMP-TANKADEVIILRPGTDAAFFLGVARELIEKGLYDRAAVIERTDLPLLVR 8e9g.1    EESPIVFLRLRKGVRKNGVQVVAVAPWASRGLTKLAGTVVPTVPGDEPAALDGMH-------------------------  target    LDTGERLDARDVIPGYELAALTNYVTLKPDAEIKGNPPPPPFTAGGQVVPTELRDAWGDFVWWDRATGRPRPVSRDEVGA 8e9g.1    --------------------------------------------------------------------------------  target    RFDGDPALLGEFEVELVDGSTVPVRPAFDLLKQYLDESFDLRTASEVCRVPPQAIQSIARQLAANKRETLLAAGMGPNHY 8e9g.1    --------------------------------------------------------------------------------  target    FQNDLFGRVQFLVAALTDNIGHLGGNVGSYAGNYRGSVFQAMGQWIAEDPFAIEPDLTKPATVKRYYKAESAHYWNYGER 8e9g.1    --------------------------------------------------------------------------------  target    PLRAVAKDDEGDLTKGEVLTGKSHMPTPTKLIWFGNSNSLLGNAKWSFDVVKNTLPRQDAVFCNEWHWTSSCEYADLVFP 8e9g.1    --------------------------------------------------------------------------------  target    ADSWAEFKLPDATASCTNPFLLAFPTTPLKRLYDTRSDYEALALTAKALGELIDEPRMEQYWRGILDGDPTPYLQRIFSG 8e9g.1    --------------------------------------------------------------------------------  target    SNATRGITYDELHESSKRGVPLLMNMRTYPRSGGWEQRQEDKPWYTATGRLEFYRPEPEFQAAGESLPVWREPVDATFYE 8e9g.1    --------------------------------------------------------------------------------  target    PNAILSNAAHPSIAPRAPEDYGVPESQLDVETRQYRNVVRTWAELQQTLHPLQERDPAFRFVF 8e9g.1    --------------------------------------------------------------- ``` | | | | | | | | | | | | | | | | | | | | | | | | | | | | | | | | | | | | | | | | | | | | | | | | | |
|  | 7l5i.1.A | Trimethylamine-N-oxide reductase  *Crystal Structure of Haemophilus influenzae MtsZ at pH 7.0* | 0.08 |  | 29.03 | 0.23 | 128-382 | X-ray | 1.73 | monomer | 2 x MGD, 1 x MO, 1 x O | BLAST | 0.35 |
| ``` target    AQGVSRRQLLGRALALGSGAALADLLGPARFLSPAGAATAGAVVPGNPLRVMPDRTWEQIYRNQFEDDSTFVFTCAPNDT 7l5i.1    --------------------------------------------------------------------------------  target    HNCLLRAHVKNGVVVRISPTYGYGEATDLYGNRASHRWDPRTCQKGLILSRRFYSERRVKAPMIRKGFKDWVEAGYPRND 7l5i.1    -----------------------------------------------VVADQLYSEARVKCPMVRKGFL-----------  target    DGTP-QMDVTLRGSDDWIRISWDEATTIAAKTMEDVARTFNGDEGARKLLAQGYHPEMVEVMHGAGVQALKLRGGMPLLG 7l5i.1    -ANPGKSDTTMRGRDEWVRVSWDEALDLVHNQLKRV----RDEHGSTGIFAGSYGWFSCGSLHAS--RTLLQRYMNATGG  target    IGRIFGFYRFANMLALLDRKLRPDAPADEILGSRTFDNYAWHTDLPPGHPMVTGSQTVDFDLFSAEHTKLLLIIGMNWIC 7l5i.1    FVGHKGDYSTGAAQVIMPHVLG----TIEVYEQQT----SWESILESSDIIV---------LWSANP---LTTMRIAWMS  target    TKMPDGHWIGDARLKGTRVIVISADYMPTANKAD-EVIILRPGTDAAFFLGVARELIEKGLYDRAAVIERTDLPLLVRLD 7l5i.1    TDQKGIEYFKKFQASGKRIICIDPQKSETCQMLNAEWIPVNTATDVPLMLGIAHTLVEQGKHDK----------------  target    TGERLDARDVIPGYELAALTNYVTLKPDAEIKGNPPPPPFTAGGQVVPTELRDAWGDFVWWDRATGRPRPVSRDEVGARF 7l5i.1    --------------------------------------------------------------------------------  target    DGDPALLGEFEVELVDGSTVPVRPAFDLLKQYLDESFDLRTASEVCRVPPQAIQSIARQLAANKRETLLAAGMGPNHYFQ 7l5i.1    --------------------------------------------------------------------------------  target    NDLFGRVQFLVAALTDNIGHLGGNVGSYAGNYRGSVFQAMGQWIAEDPFAIEPDLTKPATVKRYYKAESAHYWNYGERPL 7l5i.1    --------------------------------------------------------------------------------  target    RAVAKDDEGDLTKGEVLTGKSHMPTPTKLIWFGNSNSLLGNAKWSFDVVKNTLPRQDAVFCNEWHWTSSCEYADLVFPAD 7l5i.1    --------------------------------------------------------------------------------  target    SWAEFKLPDATASCTNPFLLAFPTTPLKRLYDTRSDYEALALTAKALGELIDEPRMEQYWRGILDGDPTPYLQRIFSGSN 7l5i.1    --------------------------------------------------------------------------------  target    ATRGITYDELHESSKRGVPLLMNMRTYPRSGGWEQRQEDKPWYTATGRLEFYRPEPEFQAAGESLPVWREPVDATFYEPN 7l5i.1    --------------------------------------------------------------------------------  target    AILSNAAHPSIAPRAPEDYGVPESQLDVETRQYRNVVRTWAELQQTLHPLQERDPAFRFVF 7l5i.1    ------------------------------------------------------------- ``` | | | | | | | | | | | | | | | | | | | | | | | | | | | | | | | | | | | | | | | | | | | | | | | | | |
|  | 7l5s.1.A | Trimethylamine-N-oxide reductase  *Crystal Structure of Haemophilus influenzae MtsZ at pH 5.5* | 0.08 |  | 29.03 | 0.23 | 128-382 | X-ray | 2.09 | monomer | 1 x O, 2 x MGD, 1 x MO | BLAST | 0.35 |
| ``` target    AQGVSRRQLLGRALALGSGAALADLLGPARFLSPAGAATAGAVVPGNPLRVMPDRTWEQIYRNQFEDDSTFVFTCAPNDT 7l5s.1    --------------------------------------------------------------------------------  target    HNCLLRAHVKNGVVVRISPTYGYGEATDLYGNRASHRWDPRTCQKGLILSRRFYSERRVKAPMIRKGFKDWVEAGYPRND 7l5s.1    -----------------------------------------------VVADQLYSEARVKCPMVRKGFL-----------  target    DGTP-QMDVTLRGSDDWIRISWDEATTIAAKTMEDVARTFNGDEGARKLLAQGYHPEMVEVMHGAGVQALKLRGGMPLLG 7l5s.1    -ANPGKSDTTMRGRDEWVRVSWDEALDLVHNQLKRV----RDEHGSTGIFAGSYGWFSCGSLHAS--RTLLQRYMNATGG  target    IGRIFGFYRFANMLALLDRKLRPDAPADEILGSRTFDNYAWHTDLPPGHPMVTGSQTVDFDLFSAEHTKLLLIIGMNWIC 7l5s.1    FVGHKGDYSTGAAQVIMPHVLG----TIEVYEQQT----SWESILESSDIIV---------LWSANP---LTTMRIAWMS  target    TKMPDGHWIGDARLKGTRVIVISADYMPTANKAD-EVIILRPGTDAAFFLGVARELIEKGLYDRAAVIERTDLPLLVRLD 7l5s.1    TDQKGIEYFKKFQASGKRIICIDPQKSETCQMLNAEWIPVNTATDVPLMLGIAHTLVEQGKHDK----------------  target    TGERLDARDVIPGYELAALTNYVTLKPDAEIKGNPPPPPFTAGGQVVPTELRDAWGDFVWWDRATGRPRPVSRDEVGARF 7l5s.1    --------------------------------------------------------------------------------  target    DGDPALLGEFEVELVDGSTVPVRPAFDLLKQYLDESFDLRTASEVCRVPPQAIQSIARQLAANKRETLLAAGMGPNHYFQ 7l5s.1    --------------------------------------------------------------------------------  target    NDLFGRVQFLVAALTDNIGHLGGNVGSYAGNYRGSVFQAMGQWIAEDPFAIEPDLTKPATVKRYYKAESAHYWNYGERPL 7l5s.1    --------------------------------------------------------------------------------  target    RAVAKDDEGDLTKGEVLTGKSHMPTPTKLIWFGNSNSLLGNAKWSFDVVKNTLPRQDAVFCNEWHWTSSCEYADLVFPAD 7l5s.1    --------------------------------------------------------------------------------  target    SWAEFKLPDATASCTNPFLLAFPTTPLKRLYDTRSDYEALALTAKALGELIDEPRMEQYWRGILDGDPTPYLQRIFSGSN 7l5s.1    --------------------------------------------------------------------------------  target    ATRGITYDELHESSKRGVPLLMNMRTYPRSGGWEQRQEDKPWYTATGRLEFYRPEPEFQAAGESLPVWREPVDATFYEPN 7l5s.1    --------------------------------------------------------------------------------  target    AILSNAAHPSIAPRAPEDYGVPESQLDVETRQYRNVVRTWAELQQTLHPLQERDPAFRFVF 7l5s.1    ------------------------------------------------------------- ``` | | | | | | | | | | | | | | | | | | | | | | | | | | | | | | | | | | | | | | | | | | | | | | | | | |
|  | 3m9s.1.C | NADH-quinone oxidoreductase subunit 3  *Crystal structure of respiratory complex I from Thermus thermophilus* | 0.12 |  | 15.97 | 0.25 | 37-372 | X-ray | 4.50 | hetero-oligomer | 7 x SF4, 2 x FES, 1 x FMN | HHblits | 0.28 |
| ``` target    AQGVSRRQLLGRALALGSGAALADLLGPARFLSPAGAATAGAVVPGNPLRVMPDRTWEQIYRNQFEDDSTFVFTCAPNDT 3m9s.1    ------------------------------------SGFSGNITDICPVGALLDLTARF--RAR-NWEMEETPTTCALCP  target    HNCLLRAHVKNGVVVRISPTYGYGEATDLYGNRASHRWDPRTCQKGLILSRRFYSERRVKAPMIRKGFKDWVEAGYPRND 3m9s.1    VGCGITADTRSGELLRIRAR------------EVPEVNEIWICDAGRFGHEW-ADQNRLKTPLVRKE-------------  target    DGTPQMDVTLRGSDDWIRISWDEATTIAAKTMEDVARTFNGDEGARKLLAQGYHPEMVEVMHGAGVQALKLRGGMPLLGI 3m9s.1    -------------GRLVEATWEEAFLALKEGLKEARG------------------EEVGL--------------------  target    GRIFGFYRFANMLALLDRKLRPDAPADEILGSRTFDNYAWHTDLPPGHPMVTGSQTVDFDLFSAEHTKLLLIIGMNWICT 3m9s.1    ---YLAHDATLEEGLLASELA------KALKTPHLDFQGRTAAPA--------SLFPPASLEDLLQADFALVLG-DPTEE  target    KMPDGHWIGDA-------------------------RLKGTRVIVISADYMPTANKADEVIILRPGTDAAFFLGVARELI 3m9s.1    APILHLRLSEFVRDLKPPHRYNHGTPFADLQIKERMPRRTDKMALFAPYRAPLMKWAAIHEVHRPGEEREILLALLG---  target    EKGLYDRAAVIERTDLPLLVRLDTGERLDARDVIPGYELAALTNYVTLKPDAEIKGNPPPPPFTAGGQVVPTELRDAWGD 3m9s.1    --------------------------------------------------------------------------------  target    FVWWDRATGRPRPVSRDEVGARFDGDPALLGEFEVELVDGSTVPVRPAFDLLKQYLDESFDLRTASEVCRVPPQAIQSIA 3m9s.1    --------------------------------------------------------------------------------  target    RQLAANKRETLLAAGMGPNHYFQNDLFGRVQFLVAALTDNIGHLGGNVGSYAGNYRGSVFQAMGQWIAEDPFAIEPDLTK 3m9s.1    --------------------------------------------------------------------------------  target    PATVKRYYKAESAHYWNYGERPLRAVAKDDEGDLTKGEVLTGKSHMPTPTKLIWFGNSNSLLGNAKWSFDVVKNTLPRQD 3m9s.1    --------------------------------------------------------------------------------  target    AVFCNEWHWTSSCEYADLVFPADSWAEFKLPDATASCTNPFLLAFPTTPLKRLYDTRSDYEALALTAKALGELIDEPRME 3m9s.1    --------------------------------------------------------------------------------  target    QYWRGILDGDPTPYLQRIFSGSNATRGITYDELHESSKRGVPLLMNMRTYPRSGGWEQRQEDKPWYTATGRLEFYRPEPE 3m9s.1    --------------------------------------------------------------------------------  target    FQAAGESLPVWREPVDATFYEPNAILSNAAHPSIAPRAPEDYGVPESQLDVETRQYRNVVRTWAELQQTLHPLQERDPAF 3m9s.1    --------------------------------------------------------------------------------  target    RFVF 3m9s.1    ---- ``` | | | | | | | | | | | | | | | | | | | | | | | | | | | | | | | | | | | | | | | | | | | | | | | | | |
|  | 2fug.2.C | NADH-quinone oxidoreductase chain 3  *Crystal structure of the hydrophilic domain of respiratory complex I from Thermus thermophilus* | 0.12 |  | 15.97 | 0.25 | 37-372 | X-ray | 3.30 | hetero-1-1-1-1-1-1-… | 7 x SF4, 2 x FES, 1 x FMN | HHblits | 0.28 |
| ``` target    AQGVSRRQLLGRALALGSGAALADLLGPARFLSPAGAATAGAVVPGNPLRVMPDRTWEQIYRNQFEDDSTFVFTCAPNDT 2fug.2    ------------------------------------SGFSGNITDICPVGALLDLTARF--RAR-NWEMEETPTTCALCP  target    HNCLLRAHVKNGVVVRISPTYGYGEATDLYGNRASHRWDPRTCQKGLILSRRFYSERRVKAPMIRKGFKDWVEAGYPRND 2fug.2    VGCGITADTRSGELLRIRAR------------EVPEVNEIWICDAGRFGHEW-ADQNRLKTPLVRKE-------------  target    DGTPQMDVTLRGSDDWIRISWDEATTIAAKTMEDVARTFNGDEGARKLLAQGYHPEMVEVMHGAGVQALKLRGGMPLLGI 2fug.2    -------------GRLVEATWEEAFLALKEGLKEARG------------------EEVGL--------------------  target    GRIFGFYRFANMLALLDRKLRPDAPADEILGSRTFDNYAWHTDLPPGHPMVTGSQTVDFDLFSAEHTKLLLIIGMNWICT 2fug.2    ---YLAHDATLEEGLLASELA------KALKTPHLDFQGRTAAPA--------SLFPPASLEDLLQADFALVLG-DPTEE  target    KMPDGHWIGDA-------------------------RLKGTRVIVISADYMPTANKADEVIILRPGTDAAFFLGVARELI 2fug.2    APILHLRLSEFVRDLKPPHRYNHGTPFADLQIKERMPRRTDKMALFAPYRAPLMKWAAIHEVHRPGEEREILLALLG---  target    EKGLYDRAAVIERTDLPLLVRLDTGERLDARDVIPGYELAALTNYVTLKPDAEIKGNPPPPPFTAGGQVVPTELRDAWGD 2fug.2    --------------------------------------------------------------------------------  target    FVWWDRATGRPRPVSRDEVGARFDGDPALLGEFEVELVDGSTVPVRPAFDLLKQYLDESFDLRTASEVCRVPPQAIQSIA 2fug.2    --------------------------------------------------------------------------------  target    RQLAANKRETLLAAGMGPNHYFQNDLFGRVQFLVAALTDNIGHLGGNVGSYAGNYRGSVFQAMGQWIAEDPFAIEPDLTK 2fug.2    --------------------------------------------------------------------------------  target    PATVKRYYKAESAHYWNYGERPLRAVAKDDEGDLTKGEVLTGKSHMPTPTKLIWFGNSNSLLGNAKWSFDVVKNTLPRQD 2fug.2    --------------------------------------------------------------------------------  target    AVFCNEWHWTSSCEYADLVFPADSWAEFKLPDATASCTNPFLLAFPTTPLKRLYDTRSDYEALALTAKALGELIDEPRME 2fug.2    --------------------------------------------------------------------------------  target    QYWRGILDGDPTPYLQRIFSGSNATRGITYDELHESSKRGVPLLMNMRTYPRSGGWEQRQEDKPWYTATGRLEFYRPEPE 2fug.2    --------------------------------------------------------------------------------  target    FQAAGESLPVWREPVDATFYEPNAILSNAAHPSIAPRAPEDYGVPESQLDVETRQYRNVVRTWAELQQTLHPLQERDPAF 2fug.2    --------------------------------------------------------------------------------  target    RFVF 2fug.2    ---- ``` | | | | | | | | | | | | | | | | | | | | | | | | | | | | | | | | | | | | | | | | | | | | | | | | | |
|  | 6zjl.1.C | NADH-quinone oxidoreductase subunit 3  *Respiratory complex I from Thermus thermophilus, NAD+ dataset, major state* | 0.12 |  | 15.97 | 0.25 | 37-372 | EM | 0.00 | hetero-1-1-1-1-1-1-… | 7 x SF4, 1 x FMN, 2 x FES | HHblits | 0.28 |
| ``` target    AQGVSRRQLLGRALALGSGAALADLLGPARFLSPAGAATAGAVVPGNPLRVMPDRTWEQIYRNQFEDDSTFVFTCAPNDT 6zjl.1    ------------------------------------SGFSGNITDICPVGALLDLTARF--RAR-NWEMEETPTTCALCP  target    HNCLLRAHVKNGVVVRISPTYGYGEATDLYGNRASHRWDPRTCQKGLILSRRFYSERRVKAPMIRKGFKDWVEAGYPRND 6zjl.1    VGCGITADTRSGELLRIRAR------------EVPEVNEIWICDAGRFGHEW-ADQNRLKTPLVRKE-------------  target    DGTPQMDVTLRGSDDWIRISWDEATTIAAKTMEDVARTFNGDEGARKLLAQGYHPEMVEVMHGAGVQALKLRGGMPLLGI 6zjl.1    -------------GRLVEATWEEAFLALKEGLKEARG------------------EEVGL--------------------  target    GRIFGFYRFANMLALLDRKLRPDAPADEILGSRTFDNYAWHTDLPPGHPMVTGSQTVDFDLFSAEHTKLLLIIGMNWICT 6zjl.1    ---YLAHDATLEEGLLASELA------KALKTPHLDFQGRTAAPA--------SLFPPASLEDLLQADFALVLG-DPTEE  target    KMPDGHWIGDA-------------------------RLKGTRVIVISADYMPTANKADEVIILRPGTDAAFFLGVARELI 6zjl.1    APILHLRLSEFVRDLKPPHRYNHGTPFADLQIKERMPRRTDKMALFAPYRAPLMKWAAIHEVHRPGEEREILLALLG---  target    EKGLYDRAAVIERTDLPLLVRLDTGERLDARDVIPGYELAALTNYVTLKPDAEIKGNPPPPPFTAGGQVVPTELRDAWGD 6zjl.1    --------------------------------------------------------------------------------  target    FVWWDRATGRPRPVSRDEVGARFDGDPALLGEFEVELVDGSTVPVRPAFDLLKQYLDESFDLRTASEVCRVPPQAIQSIA 6zjl.1    --------------------------------------------------------------------------------  target    RQLAANKRETLLAAGMGPNHYFQNDLFGRVQFLVAALTDNIGHLGGNVGSYAGNYRGSVFQAMGQWIAEDPFAIEPDLTK 6zjl.1    --------------------------------------------------------------------------------  target    PATVKRYYKAESAHYWNYGERPLRAVAKDDEGDLTKGEVLTGKSHMPTPTKLIWFGNSNSLLGNAKWSFDVVKNTLPRQD 6zjl.1    --------------------------------------------------------------------------------  target    AVFCNEWHWTSSCEYADLVFPADSWAEFKLPDATASCTNPFLLAFPTTPLKRLYDTRSDYEALALTAKALGELIDEPRME 6zjl.1    --------------------------------------------------------------------------------  target    QYWRGILDGDPTPYLQRIFSGSNATRGITYDELHESSKRGVPLLMNMRTYPRSGGWEQRQEDKPWYTATGRLEFYRPEPE 6zjl.1    --------------------------------------------------------------------------------  target    FQAAGESLPVWREPVDATFYEPNAILSNAAHPSIAPRAPEDYGVPESQLDVETRQYRNVVRTWAELQQTLHPLQERDPAF 6zjl.1    --------------------------------------------------------------------------------  target    RFVF 6zjl.1    ---- ``` | | | | | | | | | | | | | | | | | | | | | | | | | | | | | | | | | | | | | | | | | | | | | | | | | |
|  | 6q8o.1.C | NADH-quinone oxidoreductase subunit 3  *Respiratory complex I from Thermus thermophilus with bound Piericidin A* | 0.12 |  | 15.97 | 0.25 | 37-372 | X-ray | 3.61 | hetero-1-1-1-1-1-1-… | 7 x SF4, 1 x FMN, 2 x FES, 1 x HQH | HHblits | 0.28 |
| ``` target    AQGVSRRQLLGRALALGSGAALADLLGPARFLSPAGAATAGAVVPGNPLRVMPDRTWEQIYRNQFEDDSTFVFTCAPNDT 6q8o.1    ------------------------------------SGFSGNITDICPVGALLDLTARF--RAR-NWEMEETPTTCALCP  target    HNCLLRAHVKNGVVVRISPTYGYGEATDLYGNRASHRWDPRTCQKGLILSRRFYSERRVKAPMIRKGFKDWVEAGYPRND 6q8o.1    VGCGITADTRSGELLRIRAR------------EVPEVNEIWICDAGRFGHEW-ADQNRLKTPLVRKE-------------  target    DGTPQMDVTLRGSDDWIRISWDEATTIAAKTMEDVARTFNGDEGARKLLAQGYHPEMVEVMHGAGVQALKLRGGMPLLGI 6q8o.1    -------------GRLVEATWEEAFLALKEGLKEARG------------------EEVGL--------------------  target    GRIFGFYRFANMLALLDRKLRPDAPADEILGSRTFDNYAWHTDLPPGHPMVTGSQTVDFDLFSAEHTKLLLIIGMNWICT 6q8o.1    ---YLAHDATLEEGLLASELA------KALKTPHLDFQGRTAAPA--------SLFPPASLEDLLQADFALVLG-DPTEE  target    KMPDGHWIGDA-------------------------RLKGTRVIVISADYMPTANKADEVIILRPGTDAAFFLGVARELI 6q8o.1    APILHLRLSEFVRDLKPPHRYNHGTPFADLQIKERMPRRTDKMALFAPYRAPLMKWAAIHEVHRPGEEREILLALLG---  target    EKGLYDRAAVIERTDLPLLVRLDTGERLDARDVIPGYELAALTNYVTLKPDAEIKGNPPPPPFTAGGQVVPTELRDAWGD 6q8o.1    --------------------------------------------------------------------------------  target    FVWWDRATGRPRPVSRDEVGARFDGDPALLGEFEVELVDGSTVPVRPAFDLLKQYLDESFDLRTASEVCRVPPQAIQSIA 6q8o.1    --------------------------------------------------------------------------------  target    RQLAANKRETLLAAGMGPNHYFQNDLFGRVQFLVAALTDNIGHLGGNVGSYAGNYRGSVFQAMGQWIAEDPFAIEPDLTK 6q8o.1    --------------------------------------------------------------------------------  target    PATVKRYYKAESAHYWNYGERPLRAVAKDDEGDLTKGEVLTGKSHMPTPTKLIWFGNSNSLLGNAKWSFDVVKNTLPRQD 6q8o.1    --------------------------------------------------------------------------------  target    AVFCNEWHWTSSCEYADLVFPADSWAEFKLPDATASCTNPFLLAFPTTPLKRLYDTRSDYEALALTAKALGELIDEPRME 6q8o.1    --------------------------------------------------------------------------------  target    QYWRGILDGDPTPYLQRIFSGSNATRGITYDELHESSKRGVPLLMNMRTYPRSGGWEQRQEDKPWYTATGRLEFYRPEPE 6q8o.1    --------------------------------------------------------------------------------  target    FQAAGESLPVWREPVDATFYEPNAILSNAAHPSIAPRAPEDYGVPESQLDVETRQYRNVVRTWAELQQTLHPLQERDPAF 6q8o.1    --------------------------------------------------------------------------------  target    RFVF 6q8o.1    ---- ``` | | | | | | | | | | | | | | | | | | | | | | | | | | | | | | | | | | | | | | | | | | | | | | | | | |
|  | 6zjy.1.C | NADH-quinone oxidoreductase subunit 3  *Respiratory complex I from Thermus thermophilus, NAD+ dataset, minor state* | 0.12 |  | 15.97 | 0.25 | 37-372 | EM | 0.00 | hetero-1-1-1-1-1-1-… | 7 x SF4, 2 x FES | HHblits | 0.28 |
| ``` target    AQGVSRRQLLGRALALGSGAALADLLGPARFLSPAGAATAGAVVPGNPLRVMPDRTWEQIYRNQFEDDSTFVFTCAPNDT 6zjy.1    ------------------------------------SGFSGNITDICPVGALLDLTARF--RAR-NWEMEETPTTCALCP  target    HNCLLRAHVKNGVVVRISPTYGYGEATDLYGNRASHRWDPRTCQKGLILSRRFYSERRVKAPMIRKGFKDWVEAGYPRND 6zjy.1    VGCGITADTRSGELLRIRAR------------EVPEVNEIWICDAGRFGHEW-ADQNRLKTPLVRKE-------------  target    DGTPQMDVTLRGSDDWIRISWDEATTIAAKTMEDVARTFNGDEGARKLLAQGYHPEMVEVMHGAGVQALKLRGGMPLLGI 6zjy.1    -------------GRLVEATWEEAFLALKEGLKEARG------------------EEVGL--------------------  target    GRIFGFYRFANMLALLDRKLRPDAPADEILGSRTFDNYAWHTDLPPGHPMVTGSQTVDFDLFSAEHTKLLLIIGMNWICT 6zjy.1    ---YLAHDATLEEGLLASELA------KALKTPHLDFQGRTAAPA--------SLFPPASLEDLLQADFALVLG-DPTEE  target    KMPDGHWIGDA-------------------------RLKGTRVIVISADYMPTANKADEVIILRPGTDAAFFLGVARELI 6zjy.1    APILHLRLSEFVRDLKPPHRYNHGTPFADLQIKERMPRRTDKMALFAPYRAPLMKWAAIHEVHRPGEEREILLALLG---  target    EKGLYDRAAVIERTDLPLLVRLDTGERLDARDVIPGYELAALTNYVTLKPDAEIKGNPPPPPFTAGGQVVPTELRDAWGD 6zjy.1    --------------------------------------------------------------------------------  target    FVWWDRATGRPRPVSRDEVGARFDGDPALLGEFEVELVDGSTVPVRPAFDLLKQYLDESFDLRTASEVCRVPPQAIQSIA 6zjy.1    --------------------------------------------------------------------------------  target    RQLAANKRETLLAAGMGPNHYFQNDLFGRVQFLVAALTDNIGHLGGNVGSYAGNYRGSVFQAMGQWIAEDPFAIEPDLTK 6zjy.1    --------------------------------------------------------------------------------  target    PATVKRYYKAESAHYWNYGERPLRAVAKDDEGDLTKGEVLTGKSHMPTPTKLIWFGNSNSLLGNAKWSFDVVKNTLPRQD 6zjy.1    --------------------------------------------------------------------------------  target    AVFCNEWHWTSSCEYADLVFPADSWAEFKLPDATASCTNPFLLAFPTTPLKRLYDTRSDYEALALTAKALGELIDEPRME 6zjy.1    --------------------------------------------------------------------------------  target    QYWRGILDGDPTPYLQRIFSGSNATRGITYDELHESSKRGVPLLMNMRTYPRSGGWEQRQEDKPWYTATGRLEFYRPEPE 6zjy.1    --------------------------------------------------------------------------------  target    FQAAGESLPVWREPVDATFYEPNAILSNAAHPSIAPRAPEDYGVPESQLDVETRQYRNVVRTWAELQQTLHPLQERDPAF 6zjy.1    --------------------------------------------------------------------------------  target    RFVF 6zjy.1    ---- ``` | | | | | | | | | | | | | | | | | | | | | | | | | | | | | | | | | | | | | | | | | | | | | | | | | |
|  | 6zjn.1.C | NADH-quinone oxidoreductase subunit 3  *Respiratory complex I from Thermus thermophilus, NADH dataset, minor state* | 0.12 |  | 15.97 | 0.25 | 37-372 | EM | 0.00 | hetero-1-1-1-1-1-1-… | 7 x SF4, 2 x FES | HHblits | 0.28 |
| ``` target    AQGVSRRQLLGRALALGSGAALADLLGPARFLSPAGAATAGAVVPGNPLRVMPDRTWEQIYRNQFEDDSTFVFTCAPNDT 6zjn.1    ------------------------------------SGFSGNITDICPVGALLDLTARF--RAR-NWEMEETPTTCALCP  target    HNCLLRAHVKNGVVVRISPTYGYGEATDLYGNRASHRWDPRTCQKGLILSRRFYSERRVKAPMIRKGFKDWVEAGYPRND 6zjn.1    VGCGITADTRSGELLRIRAR------------EVPEVNEIWICDAGRFGHEW-ADQNRLKTPLVRKE-------------  target    DGTPQMDVTLRGSDDWIRISWDEATTIAAKTMEDVARTFNGDEGARKLLAQGYHPEMVEVMHGAGVQALKLRGGMPLLGI 6zjn.1    -------------GRLVEATWEEAFLALKEGLKEARG------------------EEVGL--------------------  target    GRIFGFYRFANMLALLDRKLRPDAPADEILGSRTFDNYAWHTDLPPGHPMVTGSQTVDFDLFSAEHTKLLLIIGMNWICT 6zjn.1    ---YLAHDATLEEGLLASELA------KALKTPHLDFQGRTAAPA--------SLFPPASLEDLLQADFALVLG-DPTEE  target    KMPDGHWIGDA-------------------------RLKGTRVIVISADYMPTANKADEVIILRPGTDAAFFLGVARELI 6zjn.1    APILHLRLSEFVRDLKPPHRYNHGTPFADLQIKERMPRRTDKMALFAPYRAPLMKWAAIHEVHRPGEEREILLALLG---  target    EKGLYDRAAVIERTDLPLLVRLDTGERLDARDVIPGYELAALTNYVTLKPDAEIKGNPPPPPFTAGGQVVPTELRDAWGD 6zjn.1    --------------------------------------------------------------------------------  target    FVWWDRATGRPRPVSRDEVGARFDGDPALLGEFEVELVDGSTVPVRPAFDLLKQYLDESFDLRTASEVCRVPPQAIQSIA 6zjn.1    --------------------------------------------------------------------------------  target    RQLAANKRETLLAAGMGPNHYFQNDLFGRVQFLVAALTDNIGHLGGNVGSYAGNYRGSVFQAMGQWIAEDPFAIEPDLTK 6zjn.1    --------------------------------------------------------------------------------  target    PATVKRYYKAESAHYWNYGERPLRAVAKDDEGDLTKGEVLTGKSHMPTPTKLIWFGNSNSLLGNAKWSFDVVKNTLPRQD 6zjn.1    --------------------------------------------------------------------------------  target    AVFCNEWHWTSSCEYADLVFPADSWAEFKLPDATASCTNPFLLAFPTTPLKRLYDTRSDYEALALTAKALGELIDEPRME 6zjn.1    --------------------------------------------------------------------------------  target    QYWRGILDGDPTPYLQRIFSGSNATRGITYDELHESSKRGVPLLMNMRTYPRSGGWEQRQEDKPWYTATGRLEFYRPEPE 6zjn.1    --------------------------------------------------------------------------------  target    FQAAGESLPVWREPVDATFYEPNAILSNAAHPSIAPRAPEDYGVPESQLDVETRQYRNVVRTWAELQQTLHPLQERDPAF 6zjn.1    --------------------------------------------------------------------------------  target    RFVF 6zjn.1    ---- ``` | | | | | | | | | | | | | | | | | | | | | | | | | | | | | | | | | | | | | | | | | | | | | | | | | |
|  | 6ziy.1.C | NADH-quinone oxidoreductase subunit 3  *Respiratory complex I from Thermus thermophilus, NADH dataset, major state* | 0.12 |  | 15.97 | 0.25 | 37-372 | EM | 0.00 | hetero-1-1-1-1-1-1-… | 7 x SF4, 1 x FMN, 1 x NAI, 2 x FES | HHblits | 0.28 |
| ``` target    AQGVSRRQLLGRALALGSGAALADLLGPARFLSPAGAATAGAVVPGNPLRVMPDRTWEQIYRNQFEDDSTFVFTCAPNDT 6ziy.1    ------------------------------------SGFSGNITDICPVGALLDLTARF--RAR-NWEMEETPTTCALCP  target    HNCLLRAHVKNGVVVRISPTYGYGEATDLYGNRASHRWDPRTCQKGLILSRRFYSERRVKAPMIRKGFKDWVEAGYPRND 6ziy.1    VGCGITADTRSGELLRIRAR------------EVPEVNEIWICDAGRFGHEW-ADQNRLKTPLVRKE-------------  target    DGTPQMDVTLRGSDDWIRISWDEATTIAAKTMEDVARTFNGDEGARKLLAQGYHPEMVEVMHGAGVQALKLRGGMPLLGI 6ziy.1    -------------GRLVEATWEEAFLALKEGLKEARG------------------EEVGL--------------------  target    GRIFGFYRFANMLALLDRKLRPDAPADEILGSRTFDNYAWHTDLPPGHPMVTGSQTVDFDLFSAEHTKLLLIIGMNWICT 6ziy.1    ---YLAHDATLEEGLLASELA------KALKTPHLDFQGRTAAPA--------SLFPPASLEDLLQADFALVLG-DPTEE  target    KMPDGHWIGDA-------------------------RLKGTRVIVISADYMPTANKADEVIILRPGTDAAFFLGVARELI 6ziy.1    APILHLRLSEFVRDLKPPHRYNHGTPFADLQIKERMPRRTDKMALFAPYRAPLMKWAAIHEVHRPGEEREILLALLG---  target    EKGLYDRAAVIERTDLPLLVRLDTGERLDARDVIPGYELAALTNYVTLKPDAEIKGNPPPPPFTAGGQVVPTELRDAWGD 6ziy.1    --------------------------------------------------------------------------------  target    FVWWDRATGRPRPVSRDEVGARFDGDPALLGEFEVELVDGSTVPVRPAFDLLKQYLDESFDLRTASEVCRVPPQAIQSIA 6ziy.1    --------------------------------------------------------------------------------  target    RQLAANKRETLLAAGMGPNHYFQNDLFGRVQFLVAALTDNIGHLGGNVGSYAGNYRGSVFQAMGQWIAEDPFAIEPDLTK 6ziy.1    --------------------------------------------------------------------------------  target    PATVKRYYKAESAHYWNYGERPLRAVAKDDEGDLTKGEVLTGKSHMPTPTKLIWFGNSNSLLGNAKWSFDVVKNTLPRQD 6ziy.1    --------------------------------------------------------------------------------  target    AVFCNEWHWTSSCEYADLVFPADSWAEFKLPDATASCTNPFLLAFPTTPLKRLYDTRSDYEALALTAKALGELIDEPRME 6ziy.1    --------------------------------------------------------------------------------  target    QYWRGILDGDPTPYLQRIFSGSNATRGITYDELHESSKRGVPLLMNMRTYPRSGGWEQRQEDKPWYTATGRLEFYRPEPE 6ziy.1    --------------------------------------------------------------------------------  target    FQAAGESLPVWREPVDATFYEPNAILSNAAHPSIAPRAPEDYGVPESQLDVETRQYRNVVRTWAELQQTLHPLQERDPAF 6ziy.1    --------------------------------------------------------------------------------  target    RFVF 6ziy.1    ---- ``` | | | | | | | | | | | | | | | | | | | | | | | | | | | | | | | | | | | | | | | | | | | | | | | | | |
|  | 1dms.1.A | DMSO REDUCTASE  *STRUCTURE OF DMSO REDUCTASE* | 0.08 |  | 31.13 | 0.23 | 133-382 | X-ray | 1.88 | monomer | 2 x PGD, 1 x 2MO | BLAST | 0.36 |
| ``` target    AQGVSRRQLLGRALALGSGAALADLLGPARFLSPAGAATAGAVVPGNPLRVMPDRTWEQIYRNQFEDDSTFVFTCAPNDT 1dms.1    --------------------------------------------------------------------------------  target    HNCLLRAHVKNGVVVRISPTYGYGEATDLYGNRASHRWDPRTCQKGLILSRRFYSERRVKAPMIRKGFKDWVEAGYPRND 1dms.1    ----------------------------------------------------IYSPTRIKYPMVRREF---LEKGV----  target    DGTPQMDVTLRGSDDWIRISWDEATTIAAKTMEDVARTFNGDEGARKLLAQGYHPEMVEVMHGAGVQALKLRGGMPLLGI 1dms.1    ----NADRSTRGNGDFVRVSWDQALDLVAAEVKRVEETY----GPQGVFGGSYGWKSPGRLHNC---TTLLRRMLTLAG-  target    GRIFGFYRFANMLALLDRKLRPDAPADEILGSRTFDNYAWHTDLPPGHPMVTGSQTVDFDLFSAEHTKLLLIIGMNWICT 1dms.1    GYVNGAGDYSTGAA---QVIMP-----HVVG--TLEVYEQQTAWP-----VLAENTEVMVFWAADPIKTSQI---GWVIP  target    KMPDGHWIGDARLKGTRVIVISADYMPTAN--KADEVIILRPGTDAAFFLGVARELIEKGLYDRAAVIERTDLPLLVRLD 1dms.1    EHGAYPGLEALKAKGTKVIVIDPVRTKTVEFFGADHVTP-KPQTDVAIMLGMAHTLVAEDLYDK----------------  target    TGERLDARDVIPGYELAALTNYVTLKPDAEIKGNPPPPPFTAGGQVVPTELRDAWGDFVWWDRATGRPRPVSRDEVGARF 1dms.1    --------------------------------------------------------------------------------  target    DGDPALLGEFEVELVDGSTVPVRPAFDLLKQYLDESFDLRTASEVCRVPPQAIQSIARQLAANKRETLLAAGMGPNHYFQ 1dms.1    --------------------------------------------------------------------------------  target    NDLFGRVQFLVAALTDNIGHLGGNVGSYAGNYRGSVFQAMGQWIAEDPFAIEPDLTKPATVKRYYKAESAHYWNYGERPL 1dms.1    --------------------------------------------------------------------------------  target    RAVAKDDEGDLTKGEVLTGKSHMPTPTKLIWFGNSNSLLGNAKWSFDVVKNTLPRQDAVFCNEWHWTSSCEYADLVFPAD 1dms.1    --------------------------------------------------------------------------------  target    SWAEFKLPDATASCTNPFLLAFPTTPLKRLYDTRSDYEALALTAKALGELIDEPRMEQYWRGILDGDPTPYLQRIFSGSN 1dms.1    --------------------------------------------------------------------------------  target    ATRGITYDELHESSKRGVPLLMNMRTYPRSGGWEQRQEDKPWYTATGRLEFYRPEPEFQAAGESLPVWREPVDATFYEPN 1dms.1    --------------------------------------------------------------------------------  target    AILSNAAHPSIAPRAPEDYGVPESQLDVETRQYRNVVRTWAELQQTLHPLQERDPAFRFVF 1dms.1    ------------------------------------------------------------- ``` | | | | | | | | | | | | | | | | | | | | | | | | | | | | | | | | | | | | | | | | | | | | | | | | | |
|  | 1e18.1.A | DMSO REDUCTASE.  *TUNGSTEN-SUSBSTITUTED DMSO REDUCTASE FROM RHODOBACTER CAPSULATUS* | 0.09 |  | 30.52 | 0.23 | 133-382 | X-ray | 2.00 | monomer | 2 x PGD, 1 x 6WO | BLAST | 0.35 |
| ``` target    AQGVSRRQLLGRALALGSGAALADLLGPARFLSPAGAATAGAVVPGNPLRVMPDRTWEQIYRNQFEDDSTFVFTCAPNDT 1e18.1    --------------------------------------------------------------------------------  target    HNCLLRAHVKNGVVVRISPTYGYGEATDLYGNRASHRWDPRTCQKGLILSRRFYSERRVKAPMIRKGFKDWVEAGYPRND 1e18.1    ----------------------------------------------------IYSPTRIKYPMVRREF---LEKGV----  target    DGTPQMDVTLRGSDDWIRISWDEATTIAAKTMEDVARTFNGDEGARKLLAQGYHPEMVEVMHGAGVQALKLRGGMPLLGI 1e18.1    ----NADRSTRGNGDFVRVSWDQALDLVAAEVKRVEETY----GPQGVFGGSYGWKSPGRLHNC---TTLLRRMLTLAG-  target    GRIFGFYRFANMLALLDRKLRPDAPADEILGSRTFDNYAWHTDLPPGHPMVTGSQTVDFDLFSAEHTKLLLIIGMNWICT 1e18.1    GYVNGAGDYSTGAAQV---IMP-----HVVG--TLEVYEQQTAWP-----VLAENTEVMVFWAADPIKTSQI---GWVIP  target    KMPDGHWIGDARLKGTRVIVISADYMPTAN-KADEVIILRPGTDAAFFLGVARELIEKGLYDRAAVIERTDLPLLVRLDT 1e18.1    EHGAYPGLEALKAKGTKVIVIDPVRTKTVEFFGAEHITPKPQTDVAIMLGMAHTLVAEDLYDK-----------------  target    GERLDARDVIPGYELAALTNYVTLKPDAEIKGNPPPPPFTAGGQVVPTELRDAWGDFVWWDRATGRPRPVSRDEVGARFD 1e18.1    --------------------------------------------------------------------------------  target    GDPALLGEFEVELVDGSTVPVRPAFDLLKQYLDESFDLRTASEVCRVPPQAIQSIARQLAANKRETLLAAGMGPNHYFQN 1e18.1    --------------------------------------------------------------------------------  target    DLFGRVQFLVAALTDNIGHLGGNVGSYAGNYRGSVFQAMGQWIAEDPFAIEPDLTKPATVKRYYKAESAHYWNYGERPLR 1e18.1    --------------------------------------------------------------------------------  target    AVAKDDEGDLTKGEVLTGKSHMPTPTKLIWFGNSNSLLGNAKWSFDVVKNTLPRQDAVFCNEWHWTSSCEYADLVFPADS 1e18.1    --------------------------------------------------------------------------------  target    WAEFKLPDATASCTNPFLLAFPTTPLKRLYDTRSDYEALALTAKALGELIDEPRMEQYWRGILDGDPTPYLQRIFSGSNA 1e18.1    --------------------------------------------------------------------------------  target    TRGITYDELHESSKRGVPLLMNMRTYPRSGGWEQRQEDKPWYTATGRLEFYRPEPEFQAAGESLPVWREPVDATFYEPNA 1e18.1    --------------------------------------------------------------------------------  target    ILSNAAHPSIAPRAPEDYGVPESQLDVETRQYRNVVRTWAELQQTLHPLQERDPAFRFVF 1e18.1    ------------------------------------------------------------ ``` | | | | | | | | | | | | | | | | | | | | | | | | | | | | | | | | | | | | | | | | | | | | | | | | | |
|  | 1e5v.2.A | Dimethyl sulfoxide/trimethylamine N-oxide reductase  *OXIDIZED DMSO REDUCTASE EXPOSED TO HEPES BUFFER* | 0.08 |  | 30.52 | 0.23 | 133-382 | X-ray | 2.40 | monomer | 2 x PGD, 1 x 2MO | BLAST | 0.35 |
| ``` target    AQGVSRRQLLGRALALGSGAALADLLGPARFLSPAGAATAGAVVPGNPLRVMPDRTWEQIYRNQFEDDSTFVFTCAPNDT 1e5v.2    --------------------------------------------------------------------------------  target    HNCLLRAHVKNGVVVRISPTYGYGEATDLYGNRASHRWDPRTCQKGLILSRRFYSERRVKAPMIRKGFKDWVEAGYPRND 1e5v.2    ----------------------------------------------------IYSPTRIKYPMVRREF---LEKGV----  target    DGTPQMDVTLRGSDDWIRISWDEATTIAAKTMEDVARTFNGDEGARKLLAQGYHPEMVEVMHGAGVQALKLRGGMPLLGI 1e5v.2    ----NADRSTRGNGDFVRVSWDQALDLVAAEVKRVEETY----GPEGVFGGSYGWKSPGRLHNC---TTLLRRMLTLAG-  target    GRIFGFYRFANMLALLDRKLRPDAPADEILGSRTFDNYAWHTDLPPGHPMVTGSQTVDFDLFSAEHTKLLLIIGMNWICT 1e5v.2    GYVNGAGDYSTGAAQV---IMP-----HVVG--TLEVYEQQTAWP-----VLAENTEVMVFWAADPIKTSQI---GWVIP  target    KMPDGHWIGDARLKGTRVIVISADYMPTAN-KADEVIILRPGTDAAFFLGVARELIEKGLYDRAAVIERTDLPLLVRLDT 1e5v.2    EHGAYPGLEALKAKGTKVIVIDPVRTKTVEFFGAEHITPKPQTDVAIMLGMAHTLVAEDLYDK-----------------  target    GERLDARDVIPGYELAALTNYVTLKPDAEIKGNPPPPPFTAGGQVVPTELRDAWGDFVWWDRATGRPRPVSRDEVGARFD 1e5v.2    --------------------------------------------------------------------------------  target    GDPALLGEFEVELVDGSTVPVRPAFDLLKQYLDESFDLRTASEVCRVPPQAIQSIARQLAANKRETLLAAGMGPNHYFQN 1e5v.2    --------------------------------------------------------------------------------  target    DLFGRVQFLVAALTDNIGHLGGNVGSYAGNYRGSVFQAMGQWIAEDPFAIEPDLTKPATVKRYYKAESAHYWNYGERPLR 1e5v.2    --------------------------------------------------------------------------------  target    AVAKDDEGDLTKGEVLTGKSHMPTPTKLIWFGNSNSLLGNAKWSFDVVKNTLPRQDAVFCNEWHWTSSCEYADLVFPADS 1e5v.2    --------------------------------------------------------------------------------  target    WAEFKLPDATASCTNPFLLAFPTTPLKRLYDTRSDYEALALTAKALGELIDEPRMEQYWRGILDGDPTPYLQRIFSGSNA 1e5v.2    --------------------------------------------------------------------------------  target    TRGITYDELHESSKRGVPLLMNMRTYPRSGGWEQRQEDKPWYTATGRLEFYRPEPEFQAAGESLPVWREPVDATFYEPNA 1e5v.2    --------------------------------------------------------------------------------  target    ILSNAAHPSIAPRAPEDYGVPESQLDVETRQYRNVVRTWAELQQTLHPLQERDPAFRFVF 1e5v.2    ------------------------------------------------------------ ``` | | | | | | | | | | | | | | | | | | | | | | | | | | | | | | | | | | | | | | | | | | | | | | | | | |
|  | 1e60.1.A | Dimethyl sulfoxide/trimethylamine N-oxide reductase  *OXIDIZED DMSO REDUCTASE EXPOSED TO HEPES - Structure II BUFFER* | 0.09 |  | 30.52 | 0.23 | 133-382 | X-ray | 2.00 | monomer | 2 x PGD, 1 x 2MO | BLAST | 0.35 |
| ``` target    AQGVSRRQLLGRALALGSGAALADLLGPARFLSPAGAATAGAVVPGNPLRVMPDRTWEQIYRNQFEDDSTFVFTCAPNDT 1e60.1    --------------------------------------------------------------------------------  target    HNCLLRAHVKNGVVVRISPTYGYGEATDLYGNRASHRWDPRTCQKGLILSRRFYSERRVKAPMIRKGFKDWVEAGYPRND 1e60.1    ----------------------------------------------------IYSPTRIKYPMVRREF---LEKGV----  target    DGTPQMDVTLRGSDDWIRISWDEATTIAAKTMEDVARTFNGDEGARKLLAQGYHPEMVEVMHGAGVQALKLRGGMPLLGI 1e60.1    ----NADRSTRGNGDFVRVSWDQALDLVAAEVKRVEETY----GPEGVFGGSYGWKSPGRLHNC---TTLLRRMLTLAG-  target    GRIFGFYRFANMLALLDRKLRPDAPADEILGSRTFDNYAWHTDLPPGHPMVTGSQTVDFDLFSAEHTKLLLIIGMNWICT 1e60.1    GYVNGAGDYSTGAAQV---IMP-----HVVG--TLEVYEQQTAWP-----VLAENTEVMVFWAADPIKTSQI---GWVIP  target    KMPDGHWIGDARLKGTRVIVISADYMPTAN-KADEVIILRPGTDAAFFLGVARELIEKGLYDRAAVIERTDLPLLVRLDT 1e60.1    EHGAYPGLEALKAKGTKVIVIDPVRTKTVEFFGAEHITPKPQTDVAIMLGMAHTLVAEDLYDK-----------------  target    GERLDARDVIPGYELAALTNYVTLKPDAEIKGNPPPPPFTAGGQVVPTELRDAWGDFVWWDRATGRPRPVSRDEVGARFD 1e60.1    --------------------------------------------------------------------------------  target    GDPALLGEFEVELVDGSTVPVRPAFDLLKQYLDESFDLRTASEVCRVPPQAIQSIARQLAANKRETLLAAGMGPNHYFQN 1e60.1    --------------------------------------------------------------------------------  target    DLFGRVQFLVAALTDNIGHLGGNVGSYAGNYRGSVFQAMGQWIAEDPFAIEPDLTKPATVKRYYKAESAHYWNYGERPLR 1e60.1    --------------------------------------------------------------------------------  target    AVAKDDEGDLTKGEVLTGKSHMPTPTKLIWFGNSNSLLGNAKWSFDVVKNTLPRQDAVFCNEWHWTSSCEYADLVFPADS 1e60.1    --------------------------------------------------------------------------------  target    WAEFKLPDATASCTNPFLLAFPTTPLKRLYDTRSDYEALALTAKALGELIDEPRMEQYWRGILDGDPTPYLQRIFSGSNA 1e60.1    --------------------------------------------------------------------------------  target    TRGITYDELHESSKRGVPLLMNMRTYPRSGGWEQRQEDKPWYTATGRLEFYRPEPEFQAAGESLPVWREPVDATFYEPNA 1e60.1    --------------------------------------------------------------------------------  target    ILSNAAHPSIAPRAPEDYGVPESQLDVETRQYRNVVRTWAELQQTLHPLQERDPAFRFVF 1e60.1    ------------------------------------------------------------ ``` | | | | | | | | | | | | | | | | | | | | | | | | | | | | | | | | | | | | | | | | | | | | | | | | | |
|  | 4dmr.1.A | DMSO REDUCTASE  *REDUCED DMSO REDUCTASE FROM RHODOBACTER CAPSULATUS WITH BOUND DMSO SUBSTRATE* | 0.09 |  | 30.52 | 0.23 | 133-382 | X-ray | 1.90 | monomer | 2 x PGD, 1 x 4MO, 1 x O | BLAST | 0.35 |
| ``` target    AQGVSRRQLLGRALALGSGAALADLLGPARFLSPAGAATAGAVVPGNPLRVMPDRTWEQIYRNQFEDDSTFVFTCAPNDT 4dmr.1    --------------------------------------------------------------------------------  target    HNCLLRAHVKNGVVVRISPTYGYGEATDLYGNRASHRWDPRTCQKGLILSRRFYSERRVKAPMIRKGFKDWVEAGYPRND 4dmr.1    ----------------------------------------------------IYSPTRIKYPMVRREF---LEKGV----  target    DGTPQMDVTLRGSDDWIRISWDEATTIAAKTMEDVARTFNGDEGARKLLAQGYHPEMVEVMHGAGVQALKLRGGMPLLGI 4dmr.1    ----NADRSTRGNGDFVRVSWDQALDLVAAEVKRVEETY----GPSGVFGGSYGWKSPGRLHNC---TTLLRRMLTLAG-  target    GRIFGFYRFANMLALLDRKLRPDAPADEILGSRTFDNYAWHTDLPPGHPMVTGSQTVDFDLFSAEHTKLLLIIGMNWICT 4dmr.1    GYVNGAGDYSTGAAQV---IMP-----HVVG--TLEVYEQQTAWP-----VLAENTEVMVFWAADPIKTSQI---GWVIP  target    KMPDGHWIGDARLKGTRVIVISADYMPTAN-KADEVIILRPGTDAAFFLGVARELIEKGLYDRAAVIERTDLPLLVRLDT 4dmr.1    EHGAYPGLEALKAKGTKVIVIDPVRTKTVEFFGAEHITPKPQTDVAIMLGMAHTLVAEDLYDK-----------------  target    GERLDARDVIPGYELAALTNYVTLKPDAEIKGNPPPPPFTAGGQVVPTELRDAWGDFVWWDRATGRPRPVSRDEVGARFD 4dmr.1    --------------------------------------------------------------------------------  target    GDPALLGEFEVELVDGSTVPVRPAFDLLKQYLDESFDLRTASEVCRVPPQAIQSIARQLAANKRETLLAAGMGPNHYFQN 4dmr.1    --------------------------------------------------------------------------------  target    DLFGRVQFLVAALTDNIGHLGGNVGSYAGNYRGSVFQAMGQWIAEDPFAIEPDLTKPATVKRYYKAESAHYWNYGERPLR 4dmr.1    --------------------------------------------------------------------------------  target    AVAKDDEGDLTKGEVLTGKSHMPTPTKLIWFGNSNSLLGNAKWSFDVVKNTLPRQDAVFCNEWHWTSSCEYADLVFPADS 4dmr.1    --------------------------------------------------------------------------------  target    WAEFKLPDATASCTNPFLLAFPTTPLKRLYDTRSDYEALALTAKALGELIDEPRMEQYWRGILDGDPTPYLQRIFSGSNA 4dmr.1    --------------------------------------------------------------------------------  target    TRGITYDELHESSKRGVPLLMNMRTYPRSGGWEQRQEDKPWYTATGRLEFYRPEPEFQAAGESLPVWREPVDATFYEPNA 4dmr.1    --------------------------------------------------------------------------------  target    ILSNAAHPSIAPRAPEDYGVPESQLDVETRQYRNVVRTWAELQQTLHPLQERDPAFRFVF 4dmr.1    ------------------------------------------------------------ ``` | | | | | | | | | | | | | | | | | | | | | | | | | | | | | | | | | | | | | | | | | | | | | | | | | |
|  | 7q5y.1.A | NADH dehydrogenase I chain G  *Structure of NADH:ubichinon oxidoreductase (complex I) of the hyperthermophilic eubacterium Aquifex aeolicus* | 0.03 |  | 12.04 | 0.12 | 36-194 | X-ray | 2.70 | hetero-1-1-1-1-1-1-… | 8 x SF4, 2 x FES, 1 x FMN | HHblits | 0.26 |
| ``` target    AQGVSRRQLLGRALALGSGAALADLLGPARFLSPAGAATAGAVVPGNPLRVMPDRTWEQIYRNQFEDDSTFVFTCAPNDT 7q5y.1    -----------------------------------TCEMCGICVHVCPVGAIISKPFKYW-SRSW--LLEKGRTVCNLCP  target    HNCLLRAHVKNG------VVVRISPTYGYGEATDLYGNRASHRWDPRTCQKGLILSRRFYSERRVKAPMIRKGFKDWVEA 7q5y.1    VGCEIQIEYGVGDWRSKRKVYRTK-----------------PTDELNICAKGFFGYDSINHKRLLKTKVGKR--------  target    GYPRNDDGTPQMDVTLRGSDDWIRISWDEATTIAAKTMEDVARTFNGDEGARKLLAQGYHPEMVEVMHGAGVQALKLRGG 7q5y.1    -----------------------EETPGNVVNLLTTILTE----------------------------------------  target    MPLLGIGRIFGFYRFANMLALLDRKLRPDAPADEILGSRTFDNYAWHTDLPPGHPMVTGSQTVDFDLFSAEHTKLLLIIG 7q5y.1    --------------------------------------------------------------------------------  target    MNWICTKMPDGHWIGDARLKGTRVIVISADYMPTANKADEVIILRPGTDAAFFLGVARELIEKGLYDRAAVIERTDLPLL 7q5y.1    --------------------------------------------------------------------------------  target    VRLDTGERLDARDVIPGYELAALTNYVTLKPDAEIKGNPPPPPFTAGGQVVPTELRDAWGDFVWWDRATGRPRPVSRDEV 7q5y.1    --------------------------------------------------------------------------------  target    GARFDGDPALLGEFEVELVDGSTVPVRPAFDLLKQYLDESFDLRTASEVCRVPPQAIQSIARQLAANKRETLLAAGMGPN 7q5y.1    --------------------------------------------------------------------------------  target    HYFQNDLFGRVQFLVAALTDNIGHLGGNVGSYAGNYRGSVFQAMGQWIAEDPFAIEPDLTKPATVKRYYKAESAHYWNYG 7q5y.1    --------------------------------------------------------------------------------  target    ERPLRAVAKDDEGDLTKGEVLTGKSHMPTPTKLIWFGNSNSLLGNAKWSFDVVKNTLPRQDAVFCNEWHWTSSCEYADLV 7q5y.1    --------------------------------------------------------------------------------  target    FPADSWAEFKLPDATASCTNPFLLAFPTTPLKRLYDTRSDYEALALTAKALGELIDEPRMEQYWRGILDGDPTPYLQRIF 7q5y.1    --------------------------------------------------------------------------------  target    SGSNATRGITYDELHESSKRGVPLLMNMRTYPRSGGWEQRQEDKPWYTATGRLEFYRPEPEFQAAGESLPVWREPVDATF 7q5y.1    --------------------------------------------------------------------------------  target    YEPNAILSNAAHPSIAPRAPEDYGVPESQLDVETRQYRNVVRTWAELQQTLHPLQERDPAFRFVF 7q5y.1    ----------------------------------------------------------------- ``` | | | | | | | | | | | | | | | | | | | | | | | | | | | | | | | | | | | | | | | | | | | | | | | | | |
|  | 7q5y.1.A | NADH dehydrogenase I chain G  *Structure of NADH:ubichinon oxidoreductase (complex I) of the hyperthermophilic eubacterium Aquifex aeolicus* | 0.04 |  | 10.42 | 0.10 | 663-769 | X-ray | 2.70 | hetero-1-1-1-1-1-1-… | 8 x SF4, 2 x FES, 1 x FMN | HHblits | 0.24 |
| ``` target    AQGVSRRQLLGRALALGSGAALADLLGPARFLSPAGAATAGAVVPGNPLRVMPDRTWEQIYRNQFEDDSTFVFTCAPNDT 7q5y.1    --------------------------------------------------------------------------------  target    HNCLLRAHVKNGVVVRISPTYGYGEATDLYGNRASHRWDPRTCQKGLILSRRFYSERRVKAPMIRKGFKDWVEAGYPRND 7q5y.1    --------------------------------------------------------------------------------  target    DGTPQMDVTLRGSDDWIRISWDEATTIAAKTMEDVARTFNGDEGARKLLAQGYHPEMVEVMHGAGVQALKLRGGMPLLGI 7q5y.1    --------------------------------------------------------------------------------  target    GRIFGFYRFANMLALLDRKLRPDAPADEILGSRTFDNYAWHTDLPPGHPMVTGSQTVDFDLFSAEHTKLLLIIGMNWICT 7q5y.1    --------------------------------------------------------------------------------  target    KMPDGHWIGDARLKGTRVIVISADYMPTANKADEVIILRPGTDAAFFLGVARELIEKGLYDRAAVIERTDLPLLVRLDTG 7q5y.1    --------------------------------------------------------------------------------  target    ERLDARDVIPGYELAALTNYVTLKPDAEIKGNPPPPPFTAGGQVVPTELRDAWGDFVWWDRATGRPRPVSRDEVGARFDG 7q5y.1    --------------------------------------------------------------------------------  target    DPALLGEFEVELVDGSTVPVRPAFDLLKQYLDESFDLRTASEVCRVPPQAIQSIARQLAANKRETLLAAGMGPNHYFQND 7q5y.1    --------------------------------------------------------------------------------  target    LFGRVQFLVAALTDNIGHLGGNVGSYAGNYRGSVFQAMGQWIAEDPFAIEPDLTKPATVKRYYKAESAHYWNYGERPLRA 7q5y.1    --------------------------------------------------------------------------------  target    VAKDDEGDLTKGEVLTGKSHMPTPTKLIWFGNSNSLLGNAKWSFDVVKNTLPRQDAVFCNEWHWTSSCEYADLVFPADSW 7q5y.1    ----------------------GDIENLIIFGEDILEFYED---KVFEELKEKLEHLVVVSPYEDGLSEYAHIKIPMSLM  target    AEFKLPDATASCTNPFLLAFPTTPLKRLYDTRSDYEALALTAKALGELIDEPRMEQYWRGILDGDPTPYLQRIFSGSNAT 7q5y.1    GEN---EGTYKTFFGEVKGK---KFLP--WAFDDLAFWKYLGENFKEEK-------------------------------  target    RGITYDELHESSKRGVPLLMNMRTYPRSGGWEQRQEDKPWYTATGRLEFYRPEPEFQAAGESLPVWREPVDATFYEPNAI 7q5y.1    --------------------------------------------------------------------------------  target    LSNAAHPSIAPRAPEDYGVPESQLDVETRQYRNVVRTWAELQQTLHPLQERDPAFRFVF 7q5y.1    ----------------------------------------------------------- ``` | | | | | | | | | | | | | | | | | | | | | | | | | | | | | | | | | | | | | | | | | | | | | | | | | |
|  | 6s6y.1.B | Tungsten-containing formylmethanofuran dehydrogenase, subunit B  *X-ray crystal structure of the formyltransferase/hydrolase complex (FhcABCD) from Methylorubrum extorquens in complex with methylofuran* | 0.04 |  | 10.75 | 0.10 | 663-768 | X-ray | 3.10 | hetero-2-2-2-2-mer | 1 x MFN, 4 x ZN, 4 x CA, 4 x K, 3 x DGL, 2 x GLU, 1 x IAS | HHblits | 0.23 |
| ``` target    AQGVSRRQLLGRALALGSGAALADLLGPARFLSPAGAATAGAVVPGNPLRVMPDRTWEQIYRNQFEDDSTFVFTCAPNDT 6s6y.1    --------------------------------------------------------------------------------  target    HNCLLRAHVKNGVVVRISPTYGYGEATDLYGNRASHRWDPRTCQKGLILSRRFYSERRVKAPMIRKGFKDWVEAGYPRND 6s6y.1    --------------------------------------------------------------------------------  target    DGTPQMDVTLRGSDDWIRISWDEATTIAAKTMEDVARTFNGDEGARKLLAQGYHPEMVEVMHGAGVQALKLRGGMPLLGI 6s6y.1    --------------------------------------------------------------------------------  target    GRIFGFYRFANMLALLDRKLRPDAPADEILGSRTFDNYAWHTDLPPGHPMVTGSQTVDFDLFSAEHTKLLLIIGMNWICT 6s6y.1    --------------------------------------------------------------------------------  target    KMPDGHWIGDARLKGTRVIVISADYMPTANKADEVIILRPGTDAAFFLGVARELIEKGLYDRAAVIERTDLPLLVRLDTG 6s6y.1    --------------------------------------------------------------------------------  target    ERLDARDVIPGYELAALTNYVTLKPDAEIKGNPPPPPFTAGGQVVPTELRDAWGDFVWWDRATGRPRPVSRDEVGARFDG 6s6y.1    --------------------------------------------------------------------------------  target    DPALLGEFEVELVDGSTVPVRPAFDLLKQYLDESFDLRTASEVCRVPPQAIQSIARQLAANKRETLLAAGMGPNHYFQND 6s6y.1    --------------------------------------------------------------------------------  target    LFGRVQFLVAALTDNIGHLGGNVGSYAGNYRGSVFQAMGQWIAEDPFAIEPDLTKPATVKRYYKAESAHYWNYGERPLRA 6s6y.1    --------------------------------------------------------------------------------  target    VAKDDEGDLTKGEVLTGKSHMPTPTKLIWFGNSNSLLGNAKWSFDVVKNTLPRQDAVFCNE-WHWTSSCEYADLVFPADS 6s6y.1    ----------------------GEADAALWLASLPAP---------RPAWLGSLPTIAIVGEGSQEAAGETAEVVITVGV  target    W-AEFKLPDATASCTNPFLLAFPTTPLKRL---YDTRSDYEALALTAKALGELIDEPRMEQYWRGILDGDPTPYLQRIFS 6s6y.1    PGQSV---GGALWNDRRGVIAY-AEASDPAKTPAETETAAGVLTRIRDRLIEK---------------------------  target    GSNATRGITYDELHESSKRGVPLLMNMRTYPRSGGWEQRQEDKPWYTATGRLEFYRPEPEFQAAGESLPVWREPVDATFY 6s6y.1    --------------------------------------------------------------------------------  target    EPNAILSNAAHPSIAPRAPEDYGVPESQLDVETRQYRNVVRTWAELQQTLHPLQERDPAFRFVF 6s6y.1    ---------------------------------------------------------------- ``` | | | | | | | | | | | | | | | | | | | | | | | | | | | | | | | | | | | | | | | | | | | | | | | | | |
|  | 3ir5.1.A | Respiratory nitrate reductase 1 alpha chain  *Crystal structure of NarGHI mutant NarG-H49C* | 0.01 |  | 23.81 | 0.09 | 832-915 | X-ray | 2.30 | hetero-1-1-1-mer | 2 x MD1, 1 x 6MO, 4 x SF4, 1 x AGA, 1 x F3S, 2 x HEM | HHblits | 0.30 |
| ``` target    AQGVSRRQLLGRALALGSGAALADLLGPARFLSPAGAATAGAVVPGNPLRVMPDRTWEQIYRNQFEDDSTFVFTCAPNDT 3ir5.1    --------------------------------------------------------------------------------  target    HNCLLRAHVKNGVVVRISPTYGYGEATDLYGNRASHRWDPRTCQKGLILSRRFYSERRVKAPMIRKGFKDWVEAGYPRND 3ir5.1    --------------------------------------------------------------------------------  target    DGTPQMDVTLRGSDDWIRISWDEATTIAAKTMEDVARTFNGDEGARKLLAQGYHPEMVEVMHGAGVQALKLRGGMPLLGI 3ir5.1    --------------------------------------------------------------------------------  target    GRIFGFYRFANMLALLDRKLRPDAPADEILGSRTFDNYAWHTDLPPGHPMVTGSQTVDFDLFSAEHTKLLLIIGMNWICT 3ir5.1    --------------------------------------------------------------------------------  target    KMPDGHWIGDARLKGTRVIVISADYMPTANKADEVIILRPGTDAAFFLGVARELIEKGLYDRAAVIERTDLPLLVRLDTG 3ir5.1    --------------------------------------------------------------------------------  target    ERLDARDVIPGYELAALTNYVTLKPDAEIKGNPPPPPFTAGGQVVPTELRDAWGDFVWWDRATGRPRPVSRDEVGARFDG 3ir5.1    --------------------------------------------------------------------------------  target    DPALLGEFEVELVDGSTVPVRPAFDLLKQYLDESFDLRTASEVCRVPPQAIQSIARQLAANKRETLLAAGMGPNHYFQND 3ir5.1    --------------------------------------------------------------------------------  target    LFGRVQFLVAALTDNIGHLGGNVGSYAGNYRGSVFQAMGQWIAEDPFAIEPDLTKPATVKRYYKAESAHYWNYGERPLRA 3ir5.1    --------------------------------------------------------------------------------  target    VAKDDEGDLTKGEVLTGKSHMPTPTKLIWFGNSNSLLGNAKWSFDVVKNTLPRQDAVFCNEWHWTSSCEYADLVFPADSW 3ir5.1    --------------------------------------------------------------------------------  target    AEFKLPDATASCTNPFLLAFPTTPLKRLYDTRSDYEALALTAKALGELIDEPRMEQYWRGILDGDPTPYLQRIFSGSNAT 3ir5.1    --------------------------------------------------------------------------------  target    RGITYDELHESSKRGVPLLMNMRTYPRSGGWEQRQEDKPWYTATGRLEFYRPEPEFQAAGESLPVWREPVDAT------- 3ir5.1    -------------------------------TNVHELIPWRTLSGRQQLYQDHQWMRDFGESLLVYRPPIDTRSVKEVIG  target    ------FYEPNAILSNAAHPSIAPRAP-------EDYGVPESQLDVETRQYRNVVRTWAELQQTLHPLQERDPAFRFVF 3ir5.1    QKSNGNQEKALNFLTPHQKWGIHSTYSDNLLMLTLGRGGPVVWLSEADAKDLGIA------------------------ ``` | | | | | | | | | | | | | | | | | | | | | | | | | | | | | | | | | | | | | | | | | | | | | | | | | |
|  | 1q16.1.A | Respiratory nitrate reductase 1 alpha chain  *Crystal structure of Nitrate Reductase A, NarGHI, from Escherichia coli* | 0.02 |  | 24.10 | 0.09 | 833-915 | X-ray | 1.90 | hetero-oligomer | 2 x MD1, 1 x 6MO, 2 x HEM, 4 x SF4, 1 x F3S, 1 x AGA, 1 x 3PH | HHblits | 0.30 |
| ``` target    AQGVSRRQLLGRALALGSGAALADLLGPARFLSPAGAATAGAVVPGNPLRVMPDRTWEQIYRNQFEDDSTFVFTCAPNDT 1q16.1    --------------------------------------------------------------------------------  target    HNCLLRAHVKNGVVVRISPTYGYGEATDLYGNRASHRWDPRTCQKGLILSRRFYSERRVKAPMIRKGFKDWVEAGYPRND 1q16.1    --------------------------------------------------------------------------------  target    DGTPQMDVTLRGSDDWIRISWDEATTIAAKTMEDVARTFNGDEGARKLLAQGYHPEMVEVMHGAGVQALKLRGGMPLLGI 1q16.1    --------------------------------------------------------------------------------  target    GRIFGFYRFANMLALLDRKLRPDAPADEILGSRTFDNYAWHTDLPPGHPMVTGSQTVDFDLFSAEHTKLLLIIGMNWICT 1q16.1    --------------------------------------------------------------------------------  target    KMPDGHWIGDARLKGTRVIVISADYMPTANKADEVIILRPGTDAAFFLGVARELIEKGLYDRAAVIERTDLPLLVRLDTG 1q16.1    --------------------------------------------------------------------------------  target    ERLDARDVIPGYELAALTNYVTLKPDAEIKGNPPPPPFTAGGQVVPTELRDAWGDFVWWDRATGRPRPVSRDEVGARFDG 1q16.1    --------------------------------------------------------------------------------  target    DPALLGEFEVELVDGSTVPVRPAFDLLKQYLDESFDLRTASEVCRVPPQAIQSIARQLAANKRETLLAAGMGPNHYFQND 1q16.1    --------------------------------------------------------------------------------  target    LFGRVQFLVAALTDNIGHLGGNVGSYAGNYRGSVFQAMGQWIAEDPFAIEPDLTKPATVKRYYKAESAHYWNYGERPLRA 1q16.1    --------------------------------------------------------------------------------  target    VAKDDEGDLTKGEVLTGKSHMPTPTKLIWFGNSNSLLGNAKWSFDVVKNTLPRQDAVFCNEWHWTSSCEYADLVFPADSW 1q16.1    --------------------------------------------------------------------------------  target    AEFKLPDATASCTNPFLLAFPTTPLKRLYDTRSDYEALALTAKALGELIDEPRMEQYWRGILDGDPTPYLQRIFSGSNAT 1q16.1    --------------------------------------------------------------------------------  target    RGITYDELHESSKRGVPLLMNMRTYPRSGGWEQRQEDKPWYTATGRLEFYRPEPEFQAAGESLPVWREPVDA-------- 1q16.1    --------------------------------NVHELIPWRTLSGRQQLYQDHQWMRDFGESLLVYRPPIDTRSVKEVIG  target    -----TFYEPNAILSNAAHPSIAPRAP-------EDYGVPESQLDVETRQYRNVVRTWAELQQTLHPLQERDPAFRFVF 1q16.1    QKSNGNQEKALNFLTPHQKWGIHSTYSDNLLMLTLGRGGPVVWLSEADAKDLGIA------------------------ ``` | | | | | | | | | | | | | | | | | | | | | | | | | | | | | | | | | | | | | | | | | | | | | | | | | |
|  | 1r27.4.A | Respiratory nitrate reductase 1 alpha chain  *Crystal Structure of NarGH complex* | 0.01 |  | 24.10 | 0.09 | 833-915 | X-ray | 2.00 | hetero-4-4-mer | 4 x MO, 16 x SF4, 8 x MGD, 4 x F3S | HHblits | 0.30 |
| ``` target    AQGVSRRQLLGRALALGSGAALADLLGPARFLSPAGAATAGAVVPGNPLRVMPDRTWEQIYRNQFEDDSTFVFTCAPNDT 1r27.4    --------------------------------------------------------------------------------  target    HNCLLRAHVKNGVVVRISPTYGYGEATDLYGNRASHRWDPRTCQKGLILSRRFYSERRVKAPMIRKGFKDWVEAGYPRND 1r27.4    --------------------------------------------------------------------------------  target    DGTPQMDVTLRGSDDWIRISWDEATTIAAKTMEDVARTFNGDEGARKLLAQGYHPEMVEVMHGAGVQALKLRGGMPLLGI 1r27.4    --------------------------------------------------------------------------------  target    GRIFGFYRFANMLALLDRKLRPDAPADEILGSRTFDNYAWHTDLPPGHPMVTGSQTVDFDLFSAEHTKLLLIIGMNWICT 1r27.4    --------------------------------------------------------------------------------  target    KMPDGHWIGDARLKGTRVIVISADYMPTANKADEVIILRPGTDAAFFLGVARELIEKGLYDRAAVIERTDLPLLVRLDTG 1r27.4    --------------------------------------------------------------------------------  target    ERLDARDVIPGYELAALTNYVTLKPDAEIKGNPPPPPFTAGGQVVPTELRDAWGDFVWWDRATGRPRPVSRDEVGARFDG 1r27.4    --------------------------------------------------------------------------------  target    DPALLGEFEVELVDGSTVPVRPAFDLLKQYLDESFDLRTASEVCRVPPQAIQSIARQLAANKRETLLAAGMGPNHYFQND 1r27.4    --------------------------------------------------------------------------------  target    LFGRVQFLVAALTDNIGHLGGNVGSYAGNYRGSVFQAMGQWIAEDPFAIEPDLTKPATVKRYYKAESAHYWNYGERPLRA 1r27.4    --------------------------------------------------------------------------------  target    VAKDDEGDLTKGEVLTGKSHMPTPTKLIWFGNSNSLLGNAKWSFDVVKNTLPRQDAVFCNEWHWTSSCEYADLVFPADSW 1r27.4    --------------------------------------------------------------------------------  target    AEFKLPDATASCTNPFLLAFPTTPLKRLYDTRSDYEALALTAKALGELIDEPRMEQYWRGILDGDPTPYLQRIFSGSNAT 1r27.4    --------------------------------------------------------------------------------  target    RGITYDELHESSKRGVPLLMNMRTYPRSGGWEQRQEDKPWYTATGRLEFYRPEPEFQAAGESLPVWREPVDA-------- 1r27.4    --------------------------------NVHELIPWRTLSGRQQLYQDHQWMRDFGESLLVYRPPIDTRSVKEVIG  target    -----TFYEPNAILSNAAHPSIAPRA------PE-DYGVPESQLDVETRQYRNVVRTWAELQQTLHPLQERDPAFRFVF 1r27.4    QKSNGNQEKALNFLTPHQKWGIHSTYSDNLLMLTLGRGGPVVWLSEADAKDLGIA------------------------ ``` | | | | | | | | | | | | | | | | | | | | | | | | | | | | | | | | | | | | | | | | | | | | | | | | | |
|  | 3egw.1.A | Respiratory nitrate reductase 1 alpha chain  *The crystal structure of the NarGHI mutant NarH - C16A* | 0.01 |  | 24.10 | 0.09 | 833-915 | X-ray | 1.90 | hetero-2-2-2-mer | 2 x MD1, 2 x MGD, 2 x 6MO, 6 x SF4, 4 x F3S, 2 x 3PH, 4 x HEM, 2 x AGA | HHblits | 0.30 |
| ``` target    AQGVSRRQLLGRALALGSGAALADLLGPARFLSPAGAATAGAVVPGNPLRVMPDRTWEQIYRNQFEDDSTFVFTCAPNDT 3egw.1    --------------------------------------------------------------------------------  target    HNCLLRAHVKNGVVVRISPTYGYGEATDLYGNRASHRWDPRTCQKGLILSRRFYSERRVKAPMIRKGFKDWVEAGYPRND 3egw.1    --------------------------------------------------------------------------------  target    DGTPQMDVTLRGSDDWIRISWDEATTIAAKTMEDVARTFNGDEGARKLLAQGYHPEMVEVMHGAGVQALKLRGGMPLLGI 3egw.1    --------------------------------------------------------------------------------  target    GRIFGFYRFANMLALLDRKLRPDAPADEILGSRTFDNYAWHTDLPPGHPMVTGSQTVDFDLFSAEHTKLLLIIGMNWICT 3egw.1    --------------------------------------------------------------------------------  target    KMPDGHWIGDARLKGTRVIVISADYMPTANKADEVIILRPGTDAAFFLGVARELIEKGLYDRAAVIERTDLPLLVRLDTG 3egw.1    --------------------------------------------------------------------------------  target    ERLDARDVIPGYELAALTNYVTLKPDAEIKGNPPPPPFTAGGQVVPTELRDAWGDFVWWDRATGRPRPVSRDEVGARFDG 3egw.1    --------------------------------------------------------------------------------  target    DPALLGEFEVELVDGSTVPVRPAFDLLKQYLDESFDLRTASEVCRVPPQAIQSIARQLAANKRETLLAAGMGPNHYFQND 3egw.1    --------------------------------------------------------------------------------  target    LFGRVQFLVAALTDNIGHLGGNVGSYAGNYRGSVFQAMGQWIAEDPFAIEPDLTKPATVKRYYKAESAHYWNYGERPLRA 3egw.1    --------------------------------------------------------------------------------  target    VAKDDEGDLTKGEVLTGKSHMPTPTKLIWFGNSNSLLGNAKWSFDVVKNTLPRQDAVFCNEWHWTSSCEYADLVFPADSW 3egw.1    --------------------------------------------------------------------------------  target    AEFKLPDATASCTNPFLLAFPTTPLKRLYDTRSDYEALALTAKALGELIDEPRMEQYWRGILDGDPTPYLQRIFSGSNAT 3egw.1    --------------------------------------------------------------------------------  target    RGITYDELHESSKRGVPLLMNMRTYPRSGGWEQRQEDKPWYTATGRLEFYRPEPEFQAAGESLPVWREPVDAT------- 3egw.1    --------------------------------NVHELIPWRTLSGRQQLYQDHQWMRDFGESLLVYRPPIDTRSVKEVIG  target    ------FYEPNAILSNAAHPSIAPR------APED-YGVPESQLDVETRQYRNVVRTWAELQQTLHPLQERDPAFRFVF 3egw.1    QKSNGNQEKALNFLTPHQKWGIHSTYSDNLLMLTLGRGGPVVWLSEADAKDLGIA------------------------ ``` | | | | | | | | | | | | | | | | | | | | | | | | | | | | | | | | | | | | | | | | | | | | | | | | | |
|  | 3ir6.1.A | Respiratory nitrate reductase 1 alpha chain  *Crystal structure of NarGHI mutant NarG-H49S* | 0.01 |  | 24.10 | 0.09 | 833-915 | X-ray | 2.80 | hetero-1-1-1-mer | 2 x GDP, 1 x AGA, 3 x SF4, 1 x F3S, 2 x HEM | HHblits | 0.30 |
| ``` target    AQGVSRRQLLGRALALGSGAALADLLGPARFLSPAGAATAGAVVPGNPLRVMPDRTWEQIYRNQFEDDSTFVFTCAPNDT 3ir6.1    --------------------------------------------------------------------------------  target    HNCLLRAHVKNGVVVRISPTYGYGEATDLYGNRASHRWDPRTCQKGLILSRRFYSERRVKAPMIRKGFKDWVEAGYPRND 3ir6.1    --------------------------------------------------------------------------------  target    DGTPQMDVTLRGSDDWIRISWDEATTIAAKTMEDVARTFNGDEGARKLLAQGYHPEMVEVMHGAGVQALKLRGGMPLLGI 3ir6.1    --------------------------------------------------------------------------------  target    GRIFGFYRFANMLALLDRKLRPDAPADEILGSRTFDNYAWHTDLPPGHPMVTGSQTVDFDLFSAEHTKLLLIIGMNWICT 3ir6.1    --------------------------------------------------------------------------------  target    KMPDGHWIGDARLKGTRVIVISADYMPTANKADEVIILRPGTDAAFFLGVARELIEKGLYDRAAVIERTDLPLLVRLDTG 3ir6.1    --------------------------------------------------------------------------------  target    ERLDARDVIPGYELAALTNYVTLKPDAEIKGNPPPPPFTAGGQVVPTELRDAWGDFVWWDRATGRPRPVSRDEVGARFDG 3ir6.1    --------------------------------------------------------------------------------  target    DPALLGEFEVELVDGSTVPVRPAFDLLKQYLDESFDLRTASEVCRVPPQAIQSIARQLAANKRETLLAAGMGPNHYFQND 3ir6.1    --------------------------------------------------------------------------------  target    LFGRVQFLVAALTDNIGHLGGNVGSYAGNYRGSVFQAMGQWIAEDPFAIEPDLTKPATVKRYYKAESAHYWNYGERPLRA 3ir6.1    --------------------------------------------------------------------------------  target    VAKDDEGDLTKGEVLTGKSHMPTPTKLIWFGNSNSLLGNAKWSFDVVKNTLPRQDAVFCNEWHWTSSCEYADLVFPADSW 3ir6.1    --------------------------------------------------------------------------------  target    AEFKLPDATASCTNPFLLAFPTTPLKRLYDTRSDYEALALTAKALGELIDEPRMEQYWRGILDGDPTPYLQRIFSGSNAT 3ir6.1    --------------------------------------------------------------------------------  target    RGITYDELHESSKRGVPLLMNMRTYPRSGGWEQRQEDKPWYTATGRLEFYRPEPEFQAAGESLPVWREPVDAT------- 3ir6.1    --------------------------------NVHELIPWRTLSGRQQLYQDHQWMRDFGESLLVYRPPIDTRSVKEVIG  target    ------FYEPNAILSNAAHPSIAPRA------PED-YGVPESQLDVETRQYRNVVRTWAELQQTLHPLQERDPAFRFVF 3ir6.1    QKSNGNQEKALNFLTPHQKWGIHSTYSDNLLMLTLGRGGPVVWLSEADAKDLGIA------------------------ ``` | | | | | | | | | | | | | | | | | | | | | | | | | | | | | | | | | | | | | | | | | | | | | | | | | |
|  | 3ir7.1.A | Respiratory nitrate reductase 1 alpha chain  *Crystal structure of NarGHI mutant NarG-R94S* | 0.01 |  | 25.00 | 0.09 | 836-915 | X-ray | 2.50 | hetero-1-1-1-mer | 2 x MD1, 4 x SF4, 1 x 6MO, 1 x AGA, 1 x F3S, 2 x HEM | HHblits | 0.31 |
| ``` target    AQGVSRRQLLGRALALGSGAALADLLGPARFLSPAGAATAGAVVPGNPLRVMPDRTWEQIYRNQFEDDSTFVFTCAPNDT 3ir7.1    --------------------------------------------------------------------------------  target    HNCLLRAHVKNGVVVRISPTYGYGEATDLYGNRASHRWDPRTCQKGLILSRRFYSERRVKAPMIRKGFKDWVEAGYPRND 3ir7.1    --------------------------------------------------------------------------------  target    DGTPQMDVTLRGSDDWIRISWDEATTIAAKTMEDVARTFNGDEGARKLLAQGYHPEMVEVMHGAGVQALKLRGGMPLLGI 3ir7.1    --------------------------------------------------------------------------------  target    GRIFGFYRFANMLALLDRKLRPDAPADEILGSRTFDNYAWHTDLPPGHPMVTGSQTVDFDLFSAEHTKLLLIIGMNWICT 3ir7.1    --------------------------------------------------------------------------------  target    KMPDGHWIGDARLKGTRVIVISADYMPTANKADEVIILRPGTDAAFFLGVARELIEKGLYDRAAVIERTDLPLLVRLDTG 3ir7.1    --------------------------------------------------------------------------------  target    ERLDARDVIPGYELAALTNYVTLKPDAEIKGNPPPPPFTAGGQVVPTELRDAWGDFVWWDRATGRPRPVSRDEVGARFDG 3ir7.1    --------------------------------------------------------------------------------  target    DPALLGEFEVELVDGSTVPVRPAFDLLKQYLDESFDLRTASEVCRVPPQAIQSIARQLAANKRETLLAAGMGPNHYFQND 3ir7.1    --------------------------------------------------------------------------------  target    LFGRVQFLVAALTDNIGHLGGNVGSYAGNYRGSVFQAMGQWIAEDPFAIEPDLTKPATVKRYYKAESAHYWNYGERPLRA 3ir7.1    --------------------------------------------------------------------------------  target    VAKDDEGDLTKGEVLTGKSHMPTPTKLIWFGNSNSLLGNAKWSFDVVKNTLPRQDAVFCNEWHWTSSCEYADLVFPADSW 3ir7.1    --------------------------------------------------------------------------------  target    AEFKLPDATASCTNPFLLAFPTTPLKRLYDTRSDYEALALTAKALGELIDEPRMEQYWRGILDGDPTPYLQRIFSGSNAT 3ir7.1    --------------------------------------------------------------------------------  target    RGITYDELHESSKRGVPLLMNMRTYPRSGGWEQRQEDKPWYTATGRLEFYRPEPEFQAAGESLPVWREPVDA-------- 3ir7.1    -----------------------------------ELIPWRTLSGRQQLYQDHQWMRDFGESLLVYRPPIDTRSVKEVIG  target    -----TFYEPNAILSNAAHPSIAP------RAPED-YGVPESQLDVETRQYRNVVRTWAELQQTLHPLQERDPAFRFVF 3ir7.1    QKSNGNQEKALNFLTPHQKWGIHSTYSDNLLMLTLGRGGPVVWLSEADAKDLGIA------------------------ ``` | | | | | | | | | | | | | | | | | | | | | | | | | | | | | | | | | | | | | | | | | | | | | | | | | |
|  | 4wd3.1.A | L-amino acid ligase  *Crystal structure of an L-amino acid ligase RizA* | 0.01 |  | 19.12 | 0.07 | 310-381 | X-ray | 2.80 | homo-dimer |  | HHblits | 0.30 |
| ``` target    AQGVSRRQLLGRALALGSGAALADLLGPARFLSPAGAATAGAVVPGNPLRVMPDRTWEQIYRNQFEDDSTFVFTCAPNDT 4wd3.1    --------------------------------------------------------------------------------  target    HNCLLRAHVKNGVVVRISPTYGYGEATDLYGNRASHRWDPRTCQKGLILSRRFYSERRVKAPMIRKGFKDWVEAGYPRND 4wd3.1    --------------------------------------------------------------------------------  target    DGTPQMDVTLRGSDDWIRISWDEATTIAAKTMEDVARTFNGDEGARKLLAQGYHPEMVEVMHGAGVQALKLRGGMPLLGI 4wd3.1    --------------------------------------------------------------------------------  target    GRIFGFYRFANMLALLDRKLRPDAPADEILGSRTFDNYAWHTDLPPGHPMVTGSQTVDFDLFSAEHTKLLLIIGMNWICT 4wd3.1    ---------------------------------------------------------------------ILLINSDKP--  target    KMPDGHWIGDARLKG--TRVIVISADYMPT--ANKADEVIILRPGTDAAFFLGVARELIEKGLYDRAAVIERTDLPLLVR 4wd3.1    --EPIQFFQKDKETNDSINISVITRSCYAPLYSHWADHVYIVDDVTDLTVMKSLMLEILKVGPID---------------  target    LDTGERLDARDVIPGYELAALTNYVTLKPDAEIKGNPPPPPFTAGGQVVPTELRDAWGDFVWWDRATGRPRPVSRDEVGA 4wd3.1    --------------------------------------------------------------------------------  target    RFDGDPALLGEFEVELVDGSTVPVRPAFDLLKQYLDESFDLRTASEVCRVPPQAIQSIARQLAANKRETLLAAGMGPNHY 4wd3.1    --------------------------------------------------------------------------------  target    FQNDLFGRVQFLVAALTDNIGHLGGNVGSYAGNYRGSVFQAMGQWIAEDPFAIEPDLTKPATVKRYYKAESAHYWNYGER 4wd3.1    --------------------------------------------------------------------------------  target    PLRAVAKDDEGDLTKGEVLTGKSHMPTPTKLIWFGNSNSLLGNAKWSFDVVKNTLPRQDAVFCNEWHWTSSCEYADLVFP 4wd3.1    --------------------------------------------------------------------------------  target    ADSWAEFKLPDATASCTNPFLLAFPTTPLKRLYDTRSDYEALALTAKALGELIDEPRMEQYWRGILDGDPTPYLQRIFSG 4wd3.1    --------------------------------------------------------------------------------  target    SNATRGITYDELHESSKRGVPLLMNMRTYPRSGGWEQRQEDKPWYTATGRLEFYRPEPEFQAAGESLPVWREPVDATFYE 4wd3.1    --------------------------------------------------------------------------------  target    PNAILSNAAHPSIAPRAPEDYGVPESQLDVETRQYRNVVRTWAELQQTLHPLQERDPAFRFVF 4wd3.1    --------------------------------------------------------------- ``` | | | | | | | | | | | | | | | | | | | | | | | | | | | | | | | | | | | | | | | | | | | | | | | | | |
|  | 3etn.1.A | putative phosphosugar isomerase involved in capsule formation  *Crystal structure of putative phosphosugar isomerase involved in capsule formation (YP\_209877.1) from Bacteroides fragilis NCTC 9343 at 1.70 A resolution* | 0.01 |  | 23.21 | 0.06 | 305-362 | X-ray | 1.70 | homo-tetramer | 4 x CMK | HHblits | 0.30 |
| ``` target    AQGVSRRQLLGRALALGSGAALADLLGPARFLSPAGAATAGAVVPGNPLRVMPDRTWEQIYRNQFEDDSTFVFTCAPNDT 3etn.1    --------------------------------------------------------------------------------  target    HNCLLRAHVKNGVVVRISPTYGYGEATDLYGNRASHRWDPRTCQKGLILSRRFYSERRVKAPMIRKGFKDWVEAGYPRND 3etn.1    --------------------------------------------------------------------------------  target    DGTPQMDVTLRGSDDWIRISWDEATTIAAKTMEDVARTFNGDEGARKLLAQGYHPEMVEVMHGAGVQALKLRGGMPLLGI 3etn.1    --------------------------------------------------------------------------------  target    GRIFGFYRFANMLALLDRKLRPDAPADEILGSRTFDNYAWHTDLPPGHPMVTGSQTVDFDLFSAEHTKLLLIIGMNWICT 3etn.1    ----------------------------------------------------------------QENDLLLLISNSG-KT  target    KMPDGHWIGDARL--KGTRVIVISADY-MPTANKADEVIILRPGTDAAFFLGVARELIEKGLYDRAAVIERTDLPLLVRL 3etn.1    R-EIVELTQLAHNLNPGLKFIVITGNPDSPLASESDVCLSTGHPA-----------------------------------  target    DTGERLDARDVIPGYELAALTNYVTLKPDAEIKGNPPPPPFTAGGQVVPTELRDAWGDFVWWDRATGRPRPVSRDEVGAR 3etn.1    --------------------------------------------------------------------------------  target    FDGDPALLGEFEVELVDGSTVPVRPAFDLLKQYLDESFDLRTASEVCRVPPQAIQSIARQLAANKRETLLAAGMGPNHYF 3etn.1    --------------------------------------------------------------------------------  target    QNDLFGRVQFLVAALTDNIGHLGGNVGSYAGNYRGSVFQAMGQWIAEDPFAIEPDLTKPATVKRYYKAESAHYWNYGERP 3etn.1    --------------------------------------------------------------------------------  target    LRAVAKDDEGDLTKGEVLTGKSHMPTPTKLIWFGNSNSLLGNAKWSFDVVKNTLPRQDAVFCNEWHWTSSCEYADLVFPA 3etn.1    --------------------------------------------------------------------------------  target    DSWAEFKLPDATASCTNPFLLAFPTTPLKRLYDTRSDYEALALTAKALGELIDEPRMEQYWRGILDGDPTPYLQRIFSGS 3etn.1    --------------------------------------------------------------------------------  target    NATRGITYDELHESSKRGVPLLMNMRTYPRSGGWEQRQEDKPWYTATGRLEFYRPEPEFQAAGESLPVWREPVDATFYEP 3etn.1    --------------------------------------------------------------------------------  target    NAILSNAAHPSIAPRAPEDYGVPESQLDVETRQYRNVVRTWAELQQTLHPLQERDPAFRFVF 3etn.1    -------------------------------------------------------------- ``` | | | | | | | | | | | | | | | | | | | | | | | | | | | | | | | | | | | | | | | | | | | | | | | | | |
|  | 3fxa.1.A | SIS domain protein  *Crystal structure of a putative sugar-phosphate isomerase (lmof2365\_0531) from listeria monocytogenes str. 4b f2365 at 1.60 A resolution* | 0.00 |  | 15.79 | 0.06 | 305-363 | X-ray | 1.60 | homo-tetramer |  | HHblits | 0.28 |
| ``` target    AQGVSRRQLLGRALALGSGAALADLLGPARFLSPAGAATAGAVVPGNPLRVMPDRTWEQIYRNQFEDDSTFVFTCAPNDT 3fxa.1    --------------------------------------------------------------------------------  target    HNCLLRAHVKNGVVVRISPTYGYGEATDLYGNRASHRWDPRTCQKGLILSRRFYSERRVKAPMIRKGFKDWVEAGYPRND 3fxa.1    --------------------------------------------------------------------------------  target    DGTPQMDVTLRGSDDWIRISWDEATTIAAKTMEDVARTFNGDEGARKLLAQGYHPEMVEVMHGAGVQALKLRGGMPLLGI 3fxa.1    --------------------------------------------------------------------------------  target    GRIFGFYRFANMLALLDRKLRPDAPADEILGSRTFDNYAWHTDLPPGHPMVTGSQTVDFDLFSAEHTKLLLIIGMNWICT 3fxa.1    ----------------------------------------------------------------QKEDILILISKGGNT-  target    KMPDGHWIGDARLKGTRVIVISAD-YMPTANKADEVIILRPGTDAAFFLGVARELIEKGLYDRAAVIERTDLPLLVRLDT 3fxa.1    -GELLNLIPACKTKGSTLIGVTENPDSVIAKEADIFFPVSVSKE------------------------------------  target    GERLDARDVIPGYELAALTNYVTLKPDAEIKGNPPPPPFTAGGQVVPTELRDAWGDFVWWDRATGRPRPVSRDEVGARFD 3fxa.1    --------------------------------------------------------------------------------  target    GDPALLGEFEVELVDGSTVPVRPAFDLLKQYLDESFDLRTASEVCRVPPQAIQSIARQLAANKRETLLAAGMGPNHYFQN 3fxa.1    --------------------------------------------------------------------------------  target    DLFGRVQFLVAALTDNIGHLGGNVGSYAGNYRGSVFQAMGQWIAEDPFAIEPDLTKPATVKRYYKAESAHYWNYGERPLR 3fxa.1    --------------------------------------------------------------------------------  target    AVAKDDEGDLTKGEVLTGKSHMPTPTKLIWFGNSNSLLGNAKWSFDVVKNTLPRQDAVFCNEWHWTSSCEYADLVFPADS 3fxa.1    --------------------------------------------------------------------------------  target    WAEFKLPDATASCTNPFLLAFPTTPLKRLYDTRSDYEALALTAKALGELIDEPRMEQYWRGILDGDPTPYLQRIFSGSNA 3fxa.1    --------------------------------------------------------------------------------  target    TRGITYDELHESSKRGVPLLMNMRTYPRSGGWEQRQEDKPWYTATGRLEFYRPEPEFQAAGESLPVWREPVDATFYEPNA 3fxa.1    --------------------------------------------------------------------------------  target    ILSNAAHPSIAPRAPEDYGVPESQLDVETRQYRNVVRTWAELQQTLHPLQERDPAFRFVF 3fxa.1    ------------------------------------------------------------ ``` | | | | | | | | | | | | | | | | | | | | | | | | | | | | | | | | | | | | | | | | | | | | | | | | | |
|  | 1jeo.1.A | HYPOTHETICAL PROTEIN MJ1247  *Crystal Structure of the Hypothetical Protein MJ1247 from Methanococcus jannaschii at 2.0 A Resolution Infers a Molecular Function of 3-Hexulose-6-Phosphate isomerase.* | 0.00 |  | 18.97 | 0.06 | 305-364 | X-ray | 2.00 | monomer |  | HHblits | 0.27 |
| ``` target    AQGVSRRQLLGRALALGSGAALADLLGPARFLSPAGAATAGAVVPGNPLRVMPDRTWEQIYRNQFEDDSTFVFTCAPNDT 1jeo.1    --------------------------------------------------------------------------------  target    HNCLLRAHVKNGVVVRISPTYGYGEATDLYGNRASHRWDPRTCQKGLILSRRFYSERRVKAPMIRKGFKDWVEAGYPRND 1jeo.1    --------------------------------------------------------------------------------  target    DGTPQMDVTLRGSDDWIRISWDEATTIAAKTMEDVARTFNGDEGARKLLAQGYHPEMVEVMHGAGVQALKLRGGMPLLGI 1jeo.1    --------------------------------------------------------------------------------  target    GRIFGFYRFANMLALLDRKLRPDAPADEILGSRTFDNYAWHTDLPPGHPMVTGSQTVDFDLFSAEHTKLLLIIGMNWICT 1jeo.1    ----------------------------------------------------------------EKDDLLILISGSGRTE  target    KMPDGHWIGDARLKGTRVIVISADYMPTANKADEVIILRPGTDAAFFLGVARELIEKGLYDRAAVIERTDLPLLVRLDTG 1jeo.1    --SVLTVAKKAKNINNNIIAIVCECGNVVEFADLTIPLEVKKSK------------------------------------  target    ERLDARDVIPGYELAALTNYVTLKPDAEIKGNPPPPPFTAGGQVVPTELRDAWGDFVWWDRATGRPRPVSRDEVGARFDG 1jeo.1    --------------------------------------------------------------------------------  target    DPALLGEFEVELVDGSTVPVRPAFDLLKQYLDESFDLRTASEVCRVPPQAIQSIARQLAANKRETLLAAGMGPNHYFQND 1jeo.1    --------------------------------------------------------------------------------  target    LFGRVQFLVAALTDNIGHLGGNVGSYAGNYRGSVFQAMGQWIAEDPFAIEPDLTKPATVKRYYKAESAHYWNYGERPLRA 1jeo.1    --------------------------------------------------------------------------------  target    VAKDDEGDLTKGEVLTGKSHMPTPTKLIWFGNSNSLLGNAKWSFDVVKNTLPRQDAVFCNEWHWTSSCEYADLVFPADSW 1jeo.1    --------------------------------------------------------------------------------  target    AEFKLPDATASCTNPFLLAFPTTPLKRLYDTRSDYEALALTAKALGELIDEPRMEQYWRGILDGDPTPYLQRIFSGSNAT 1jeo.1    --------------------------------------------------------------------------------  target    RGITYDELHESSKRGVPLLMNMRTYPRSGGWEQRQEDKPWYTATGRLEFYRPEPEFQAAGESLPVWREPVDATFYEPNAI 1jeo.1    --------------------------------------------------------------------------------  target    LSNAAHPSIAPRAPEDYGVPESQLDVETRQYRNVVRTWAELQQTLHPLQERDPAFRFVF 1jeo.1    ----------------------------------------------------------- ``` | | | | | | | | | | | | | | | | | | | | | | | | | | | | | | | | | | | | | | | | | | | | | | | | | |
|  | 7bkb.1.F | Formate dehydrogenase  *Formate dehydrogenase - heterodisulfide reductase - formylmethanofuran dehydrogenase complex from Methanospirillum hungatei (hexameric, composite structure)* | 0.01 |  | 17.86 | 0.06 | 305-361 | EM | 0.00 | hetero-2-2-2-2-2-2-… | 48 x SF4, 4 x FAD, 2 x FES, 4 x 9S8, 4 x ZN, 2 x MO, 4 x MGD | HHblits | 0.28 |
| ``` target    AQGVSRRQLLGRALALGSGAALADLLGPARFLSPAGAATAGAVVPGNPLRVMPDRTWEQIYRNQFEDDSTFVFTCAPNDT 7bkb.1    --------------------------------------------------------------------------------  target    HNCLLRAHVKNGVVVRISPTYGYGEATDLYGNRASHRWDPRTCQKGLILSRRFYSERRVKAPMIRKGFKDWVEAGYPRND 7bkb.1    --------------------------------------------------------------------------------  target    DGTPQMDVTLRGSDDWIRISWDEATTIAAKTMEDVARTFNGDEGARKLLAQGYHPEMVEVMHGAGVQALKLRGGMPLLGI 7bkb.1    --------------------------------------------------------------------------------  target    GRIFGFYRFANMLALLDRKLRPDAPADEILGSRTFDNYAWHTDLPPGHPMVTGSQTVDFDLFSAEHTKLLLIIGMNWICT 7bkb.1    ----------------------------------------------------------------DEIKGMYILGLNPVVT  target    KMPDGHWIGDARLKGTRVIVISADYMPTANKADEVIILRPGTDAAFFLGVARELIEKGLYDRAAVIERTDLPLLVRLDTG 7bkb.1    YP-SSNHVKAQLEKLDFLVVQDIFFTETCQYADVILPGACF---------------------------------------  target    ERLDARDVIPGYELAALTNYVTLKPDAEIKGNPPPPPFTAGGQVVPTELRDAWGDFVWWDRATGRPRPVSRDEVGARFDG 7bkb.1    --------------------------------------------------------------------------------  target    DPALLGEFEVELVDGSTVPVRPAFDLLKQYLDESFDLRTASEVCRVPPQAIQSIARQLAANKRETLLAAGMGPNHYFQND 7bkb.1    --------------------------------------------------------------------------------  target    LFGRVQFLVAALTDNIGHLGGNVGSYAGNYRGSVFQAMGQWIAEDPFAIEPDLTKPATVKRYYKAESAHYWNYGERPLRA 7bkb.1    --------------------------------------------------------------------------------  target    VAKDDEGDLTKGEVLTGKSHMPTPTKLIWFGNSNSLLGNAKWSFDVVKNTLPRQDAVFCNEWHWTSSCEYADLVFPADSW 7bkb.1    --------------------------------------------------------------------------------  target    AEFKLPDATASCTNPFLLAFPTTPLKRLYDTRSDYEALALTAKALGELIDEPRMEQYWRGILDGDPTPYLQRIFSGSNAT 7bkb.1    --------------------------------------------------------------------------------  target    RGITYDELHESSKRGVPLLMNMRTYPRSGGWEQRQEDKPWYTATGRLEFYRPEPEFQAAGESLPVWREPVDATFYEPNAI 7bkb.1    --------------------------------------------------------------------------------  target    LSNAAHPSIAPRAPEDYGVPESQLDVETRQYRNVVRTWAELQQTLHPLQERDPAFRFVF 7bkb.1    ----------------------------------------------------------- ``` | | | | | | | | | | | | | | | | | | | | | | | | | | | | | | | | | | | | | | | | | | | | | | | | | |
|  | 2e7z.1.A | Acetylene hydratase Ahy  *Acetylene Hydratase from Pelobacter acetylenicus* | 0.00 |  | 21.43 | 0.06 | 665-720 | X-ray | 1.26 | monomer | 1 x SF4, 2 x MGD, 1 x W | HHblits | 0.28 |
| ``` target    AQGVSRRQLLGRALALGSGAALADLLGPARFLSPAGAATAGAVVPGNPLRVMPDRTWEQIYRNQFEDDSTFVFTCAPNDT 2e7z.1    --------------------------------------------------------------------------------  target    HNCLLRAHVKNGVVVRISPTYGYGEATDLYGNRASHRWDPRTCQKGLILSRRFYSERRVKAPMIRKGFKDWVEAGYPRND 2e7z.1    --------------------------------------------------------------------------------  target    DGTPQMDVTLRGSDDWIRISWDEATTIAAKTMEDVARTFNGDEGARKLLAQGYHPEMVEVMHGAGVQALKLRGGMPLLGI 2e7z.1    --------------------------------------------------------------------------------  target    GRIFGFYRFANMLALLDRKLRPDAPADEILGSRTFDNYAWHTDLPPGHPMVTGSQTVDFDLFSAEHTKLLLIIGMNWICT 2e7z.1    --------------------------------------------------------------------------------  target    KMPDGHWIGDARLKGTRVIVISADYMPTANKADEVIILRPGTDAAFFLGVARELIEKGLYDRAAVIERTDLPLLVRLDTG 2e7z.1    --------------------------------------------------------------------------------  target    ERLDARDVIPGYELAALTNYVTLKPDAEIKGNPPPPPFTAGGQVVPTELRDAWGDFVWWDRATGRPRPVSRDEVGARFDG 2e7z.1    --------------------------------------------------------------------------------  target    DPALLGEFEVELVDGSTVPVRPAFDLLKQYLDESFDLRTASEVCRVPPQAIQSIARQLAANKRETLLAAGMGPNHYFQND 2e7z.1    --------------------------------------------------------------------------------  target    LFGRVQFLVAALTDNIGHLGGNVGSYAGNYRGSVFQAMGQWIAEDPFAIEPDLTKPATVKRYYKAESAHYWNYGERPLRA 2e7z.1    --------------------------------------------------------------------------------  target    VAKDDEGDLTKGEVLTGKSHMPTPTKLIWFGNSNSLLGNAKWSFDVVKNTLPRQDAVFCNEWHWTSSCEYADLVFPADSW 2e7z.1    ------------------------SNCLLFIGKNLSNHNWVSQFNDLKAALKRGCKLIVLDPRRTKVAEMADIWLPLRYG  target    AEFKLPDATASCTNPFLLAFPTTPLKRLYDTRSDYEALALTAKALGELIDEPRMEQYWRGILDGDPTPYLQRIFSGSNAT 2e7z.1    --------------------------------------------------------------------------------  target    RGITYDELHESSKRGVPLLMNMRTYPRSGGWEQRQEDKPWYTATGRLEFYRPEPEFQAAGESLPVWREPVDATFYEPNAI 2e7z.1    --------------------------------------------------------------------------------  target    LSNAAHPSIAPRAPEDYGVPESQLDVETRQYRNVVRTWAELQQTLHPLQERDPAFRFVF 2e7z.1    ----------------------------------------------------------- ``` | | | | | | | | | | | | | | | | | | | | | | | | | | | | | | | | | | | | | | | | | | | | | | | | | |
|  | 3sho.1.A | Transcriptional regulator, RpiR family  *Crystal structure of RpiR transcription factor from Sphaerobacter thermophilus (sugar isomerase domain)* | 0.00 |  | 16.07 | 0.06 | 306-363 | X-ray | 1.80 | homo-tetramer |  | HHblits | 0.28 |
| ``` target    AQGVSRRQLLGRALALGSGAALADLLGPARFLSPAGAATAGAVVPGNPLRVMPDRTWEQIYRNQFEDDSTFVFTCAPNDT 3sho.1    --------------------------------------------------------------------------------  target    HNCLLRAHVKNGVVVRISPTYGYGEATDLYGNRASHRWDPRTCQKGLILSRRFYSERRVKAPMIRKGFKDWVEAGYPRND 3sho.1    --------------------------------------------------------------------------------  target    DGTPQMDVTLRGSDDWIRISWDEATTIAAKTMEDVARTFNGDEGARKLLAQGYHPEMVEVMHGAGVQALKLRGGMPLLGI 3sho.1    --------------------------------------------------------------------------------  target    GRIFGFYRFANMLALLDRKLRPDAPADEILGSRTFDNYAWHTDLPPGHPMVTGSQTVDFDLFSAEHTKLLLIIGMNWICT 3sho.1    -----------------------------------------------------------------PTDLMIGVSVWRYLR  target    KMPDGHWIGDARLKGTRVIVISADY-MPTANKADEVIILRPGTDAAFFLGVARELIEKGLYDRAAVIERTDLPLLVRLDT 3sho.1    --DTVAALAGAAERGVPTMALTDSSVSPPARIADHVLVAATRGV------------------------------------  target    GERLDARDVIPGYELAALTNYVTLKPDAEIKGNPPPPPFTAGGQVVPTELRDAWGDFVWWDRATGRPRPVSRDEVGARFD 3sho.1    --------------------------------------------------------------------------------  target    GDPALLGEFEVELVDGSTVPVRPAFDLLKQYLDESFDLRTASEVCRVPPQAIQSIARQLAANKRETLLAAGMGPNHYFQN 3sho.1    --------------------------------------------------------------------------------  target    DLFGRVQFLVAALTDNIGHLGGNVGSYAGNYRGSVFQAMGQWIAEDPFAIEPDLTKPATVKRYYKAESAHYWNYGERPLR 3sho.1    --------------------------------------------------------------------------------  target    AVAKDDEGDLTKGEVLTGKSHMPTPTKLIWFGNSNSLLGNAKWSFDVVKNTLPRQDAVFCNEWHWTSSCEYADLVFPADS 3sho.1    --------------------------------------------------------------------------------  target    WAEFKLPDATASCTNPFLLAFPTTPLKRLYDTRSDYEALALTAKALGELIDEPRMEQYWRGILDGDPTPYLQRIFSGSNA 3sho.1    --------------------------------------------------------------------------------  target    TRGITYDELHESSKRGVPLLMNMRTYPRSGGWEQRQEDKPWYTATGRLEFYRPEPEFQAAGESLPVWREPVDATFYEPNA 3sho.1    --------------------------------------------------------------------------------  target    ILSNAAHPSIAPRAPEDYGVPESQLDVETRQYRNVVRTWAELQQTLHPLQERDPAFRFVF 3sho.1    ------------------------------------------------------------ ``` | | | | | | | | | | | | | | | | | | | | | | | | | | | | | | | | | | | | | | | | | | | | | | | | | |
|  | 3sho.1.C | Transcriptional regulator, RpiR family  *Crystal structure of RpiR transcription factor from Sphaerobacter thermophilus (sugar isomerase domain)* | 0.00 |  | 16.07 | 0.06 | 306-363 | X-ray | 1.80 | homo-tetramer |  | HHblits | 0.28 |
| ``` target    AQGVSRRQLLGRALALGSGAALADLLGPARFLSPAGAATAGAVVPGNPLRVMPDRTWEQIYRNQFEDDSTFVFTCAPNDT 3sho.1    --------------------------------------------------------------------------------  target    HNCLLRAHVKNGVVVRISPTYGYGEATDLYGNRASHRWDPRTCQKGLILSRRFYSERRVKAPMIRKGFKDWVEAGYPRND 3sho.1    --------------------------------------------------------------------------------  target    DGTPQMDVTLRGSDDWIRISWDEATTIAAKTMEDVARTFNGDEGARKLLAQGYHPEMVEVMHGAGVQALKLRGGMPLLGI 3sho.1    --------------------------------------------------------------------------------  target    GRIFGFYRFANMLALLDRKLRPDAPADEILGSRTFDNYAWHTDLPPGHPMVTGSQTVDFDLFSAEHTKLLLIIGMNWICT 3sho.1    -----------------------------------------------------------------PTDLMIGVSVWRYLR  target    KMPDGHWIGDARLKGTRVIVISADY-MPTANKADEVIILRPGTDAAFFLGVARELIEKGLYDRAAVIERTDLPLLVRLDT 3sho.1    --DTVAALAGAAERGVPTMALTDSSVSPPARIADHVLVAATRGV------------------------------------  target    GERLDARDVIPGYELAALTNYVTLKPDAEIKGNPPPPPFTAGGQVVPTELRDAWGDFVWWDRATGRPRPVSRDEVGARFD 3sho.1    --------------------------------------------------------------------------------  target    GDPALLGEFEVELVDGSTVPVRPAFDLLKQYLDESFDLRTASEVCRVPPQAIQSIARQLAANKRETLLAAGMGPNHYFQN 3sho.1    --------------------------------------------------------------------------------  target    DLFGRVQFLVAALTDNIGHLGGNVGSYAGNYRGSVFQAMGQWIAEDPFAIEPDLTKPATVKRYYKAESAHYWNYGERPLR 3sho.1    --------------------------------------------------------------------------------  target    AVAKDDEGDLTKGEVLTGKSHMPTPTKLIWFGNSNSLLGNAKWSFDVVKNTLPRQDAVFCNEWHWTSSCEYADLVFPADS 3sho.1    --------------------------------------------------------------------------------  target    WAEFKLPDATASCTNPFLLAFPTTPLKRLYDTRSDYEALALTAKALGELIDEPRMEQYWRGILDGDPTPYLQRIFSGSNA 3sho.1    --------------------------------------------------------------------------------  target    TRGITYDELHESSKRGVPLLMNMRTYPRSGGWEQRQEDKPWYTATGRLEFYRPEPEFQAAGESLPVWREPVDATFYEPNA 3sho.1    --------------------------------------------------------------------------------  target    ILSNAAHPSIAPRAPEDYGVPESQLDVETRQYRNVVRTWAELQQTLHPLQERDPAFRFVF 3sho.1    ------------------------------------------------------------ ``` | | | | | | | | | | | | | | | | | | | | | | | | | | | | | | | | | | | | | | | | | | | | | | | | | |
|  | 7en6.1.A | HTH-type transcriptional regulator MurR  *The crystal structure of Escherichia coli MurR in apo form* | 0.01 |  | 17.54 | 0.06 | 305-363 | X-ray | 2.28 | homo-tetramer |  | HHblits | 0.26 |
| ``` target    AQGVSRRQLLGRALALGSGAALADLLGPARFLSPAGAATAGAVVPGNPLRVMPDRTWEQIYRNQFEDDSTFVFTCAPNDT 7en6.1    --------------------------------------------------------------------------------  target    HNCLLRAHVKNGVVVRISPTYGYGEATDLYGNRASHRWDPRTCQKGLILSRRFYSERRVKAPMIRKGFKDWVEAGYPRND 7en6.1    --------------------------------------------------------------------------------  target    DGTPQMDVTLRGSDDWIRISWDEATTIAAKTMEDVARTFNGDEGARKLLAQGYHPEMVEVMHGAGVQALKLRGGMPLLGI 7en6.1    --------------------------------------------------------------------------------  target    GRIFGFYRFANMLALLDRKLRPDAPADEILGSRTFDNYAWHTDLPPGHPMVTGSQTVDFDLFSAEHTKLLLIIGMNWICT 7en6.1    ----------------------------------------------------------------KKGDVQIAISYSGSKK  target    KMPDGHWIGDARLKGTRVIVISAD-YMPTANKADEVIILRPGTDAAFFLGVARELIEKGLYDRAAVIERTDLPLLVRLDT 7en6.1    --EIVLCAEAARKQGATVIAITSLTDSPLRRLAHFTLDTVSGET------------------------------------  target    GERLDARDVIPGYELAALTNYVTLKPDAEIKGNPPPPPFTAGGQVVPTELRDAWGDFVWWDRATGRPRPVSRDEVGARFD 7en6.1    --------------------------------------------------------------------------------  target    GDPALLGEFEVELVDGSTVPVRPAFDLLKQYLDESFDLRTASEVCRVPPQAIQSIARQLAANKRETLLAAGMGPNHYFQN 7en6.1    --------------------------------------------------------------------------------  target    DLFGRVQFLVAALTDNIGHLGGNVGSYAGNYRGSVFQAMGQWIAEDPFAIEPDLTKPATVKRYYKAESAHYWNYGERPLR 7en6.1    --------------------------------------------------------------------------------  target    AVAKDDEGDLTKGEVLTGKSHMPTPTKLIWFGNSNSLLGNAKWSFDVVKNTLPRQDAVFCNEWHWTSSCEYADLVFPADS 7en6.1    --------------------------------------------------------------------------------  target    WAEFKLPDATASCTNPFLLAFPTTPLKRLYDTRSDYEALALTAKALGELIDEPRMEQYWRGILDGDPTPYLQRIFSGSNA 7en6.1    --------------------------------------------------------------------------------  target    TRGITYDELHESSKRGVPLLMNMRTYPRSGGWEQRQEDKPWYTATGRLEFYRPEPEFQAAGESLPVWREPVDATFYEPNA 7en6.1    --------------------------------------------------------------------------------  target    ILSNAAHPSIAPRAPEDYGVPESQLDVETRQYRNVVRTWAELQQTLHPLQERDPAFRFVF 7en6.1    ------------------------------------------------------------ ``` | | | | | | | | | | | | | | | | | | | | | | | | | | | | | | | | | | | | | | | | | | | | | | | | | |
|  | 7en6.1.B | HTH-type transcriptional regulator MurR  *The crystal structure of Escherichia coli MurR in apo form* | 0.00 |  | 17.54 | 0.06 | 305-363 | X-ray | 2.28 | homo-tetramer |  | HHblits | 0.26 |
| ``` target    AQGVSRRQLLGRALALGSGAALADLLGPARFLSPAGAATAGAVVPGNPLRVMPDRTWEQIYRNQFEDDSTFVFTCAPNDT 7en6.1    --------------------------------------------------------------------------------  target    HNCLLRAHVKNGVVVRISPTYGYGEATDLYGNRASHRWDPRTCQKGLILSRRFYSERRVKAPMIRKGFKDWVEAGYPRND 7en6.1    --------------------------------------------------------------------------------  target    DGTPQMDVTLRGSDDWIRISWDEATTIAAKTMEDVARTFNGDEGARKLLAQGYHPEMVEVMHGAGVQALKLRGGMPLLGI 7en6.1    --------------------------------------------------------------------------------  target    GRIFGFYRFANMLALLDRKLRPDAPADEILGSRTFDNYAWHTDLPPGHPMVTGSQTVDFDLFSAEHTKLLLIIGMNWICT 7en6.1    ----------------------------------------------------------------KKGDVQIAISYSGSKK  target    KMPDGHWIGDARLKGTRVIVISAD-YMPTANKADEVIILRPGTDAAFFLGVARELIEKGLYDRAAVIERTDLPLLVRLDT 7en6.1    --EIVLCAEAARKQGATVIAITSLTDSPLRRLAHFTLDTVSGET------------------------------------  target    GERLDARDVIPGYELAALTNYVTLKPDAEIKGNPPPPPFTAGGQVVPTELRDAWGDFVWWDRATGRPRPVSRDEVGARFD 7en6.1    --------------------------------------------------------------------------------  target    GDPALLGEFEVELVDGSTVPVRPAFDLLKQYLDESFDLRTASEVCRVPPQAIQSIARQLAANKRETLLAAGMGPNHYFQN 7en6.1    --------------------------------------------------------------------------------  target    DLFGRVQFLVAALTDNIGHLGGNVGSYAGNYRGSVFQAMGQWIAEDPFAIEPDLTKPATVKRYYKAESAHYWNYGERPLR 7en6.1    --------------------------------------------------------------------------------  target    AVAKDDEGDLTKGEVLTGKSHMPTPTKLIWFGNSNSLLGNAKWSFDVVKNTLPRQDAVFCNEWHWTSSCEYADLVFPADS 7en6.1    --------------------------------------------------------------------------------  target    WAEFKLPDATASCTNPFLLAFPTTPLKRLYDTRSDYEALALTAKALGELIDEPRMEQYWRGILDGDPTPYLQRIFSGSNA 7en6.1    --------------------------------------------------------------------------------  target    TRGITYDELHESSKRGVPLLMNMRTYPRSGGWEQRQEDKPWYTATGRLEFYRPEPEFQAAGESLPVWREPVDATFYEPNA 7en6.1    --------------------------------------------------------------------------------  target    ILSNAAHPSIAPRAPEDYGVPESQLDVETRQYRNVVRTWAELQQTLHPLQERDPAFRFVF 7en6.1    ------------------------------------------------------------ ``` | | | | | | | | | | | | | | | | | | | | | | | | | | | | | | | | | | | | | | | | | | | | | | | | | |
|  | 7en6.1.C | HTH-type transcriptional regulator MurR  *The crystal structure of Escherichia coli MurR in apo form* | 0.01 |  | 17.54 | 0.06 | 305-363 | X-ray | 2.28 | homo-tetramer |  | HHblits | 0.26 |
| ``` target    AQGVSRRQLLGRALALGSGAALADLLGPARFLSPAGAATAGAVVPGNPLRVMPDRTWEQIYRNQFEDDSTFVFTCAPNDT 7en6.1    --------------------------------------------------------------------------------  target    HNCLLRAHVKNGVVVRISPTYGYGEATDLYGNRASHRWDPRTCQKGLILSRRFYSERRVKAPMIRKGFKDWVEAGYPRND 7en6.1    --------------------------------------------------------------------------------  target    DGTPQMDVTLRGSDDWIRISWDEATTIAAKTMEDVARTFNGDEGARKLLAQGYHPEMVEVMHGAGVQALKLRGGMPLLGI 7en6.1    --------------------------------------------------------------------------------  target    GRIFGFYRFANMLALLDRKLRPDAPADEILGSRTFDNYAWHTDLPPGHPMVTGSQTVDFDLFSAEHTKLLLIIGMNWICT 7en6.1    ----------------------------------------------------------------KKGDVQIAISYSGSKK  target    KMPDGHWIGDARLKGTRVIVISAD-YMPTANKADEVIILRPGTDAAFFLGVARELIEKGLYDRAAVIERTDLPLLVRLDT 7en6.1    --EIVLCAEAARKQGATVIAITSLTDSPLRRLAHFTLDTVSGET------------------------------------  target    GERLDARDVIPGYELAALTNYVTLKPDAEIKGNPPPPPFTAGGQVVPTELRDAWGDFVWWDRATGRPRPVSRDEVGARFD 7en6.1    --------------------------------------------------------------------------------  target    GDPALLGEFEVELVDGSTVPVRPAFDLLKQYLDESFDLRTASEVCRVPPQAIQSIARQLAANKRETLLAAGMGPNHYFQN 7en6.1    --------------------------------------------------------------------------------  target    DLFGRVQFLVAALTDNIGHLGGNVGSYAGNYRGSVFQAMGQWIAEDPFAIEPDLTKPATVKRYYKAESAHYWNYGERPLR 7en6.1    --------------------------------------------------------------------------------  target    AVAKDDEGDLTKGEVLTGKSHMPTPTKLIWFGNSNSLLGNAKWSFDVVKNTLPRQDAVFCNEWHWTSSCEYADLVFPADS 7en6.1    --------------------------------------------------------------------------------  target    WAEFKLPDATASCTNPFLLAFPTTPLKRLYDTRSDYEALALTAKALGELIDEPRMEQYWRGILDGDPTPYLQRIFSGSNA 7en6.1    --------------------------------------------------------------------------------  target    TRGITYDELHESSKRGVPLLMNMRTYPRSGGWEQRQEDKPWYTATGRLEFYRPEPEFQAAGESLPVWREPVDATFYEPNA 7en6.1    --------------------------------------------------------------------------------  target    ILSNAAHPSIAPRAPEDYGVPESQLDVETRQYRNVVRTWAELQQTLHPLQERDPAFRFVF 7en6.1    ------------------------------------------------------------ ``` | | | | | | | | | | | | | | | | | | | | | | | | | | | | | | | | | | | | | | | | | | | | | | | | | |
|  | 7en6.1.D | HTH-type transcriptional regulator MurR  *The crystal structure of Escherichia coli MurR in apo form* | 0.01 |  | 17.54 | 0.06 | 305-363 | X-ray | 2.28 | homo-tetramer |  | HHblits | 0.26 |
| ``` target    AQGVSRRQLLGRALALGSGAALADLLGPARFLSPAGAATAGAVVPGNPLRVMPDRTWEQIYRNQFEDDSTFVFTCAPNDT 7en6.1    --------------------------------------------------------------------------------  target    HNCLLRAHVKNGVVVRISPTYGYGEATDLYGNRASHRWDPRTCQKGLILSRRFYSERRVKAPMIRKGFKDWVEAGYPRND 7en6.1    --------------------------------------------------------------------------------  target    DGTPQMDVTLRGSDDWIRISWDEATTIAAKTMEDVARTFNGDEGARKLLAQGYHPEMVEVMHGAGVQALKLRGGMPLLGI 7en6.1    --------------------------------------------------------------------------------  target    GRIFGFYRFANMLALLDRKLRPDAPADEILGSRTFDNYAWHTDLPPGHPMVTGSQTVDFDLFSAEHTKLLLIIGMNWICT 7en6.1    ----------------------------------------------------------------KKGDVQIAISYSGSKK  target    KMPDGHWIGDARLKGTRVIVISAD-YMPTANKADEVIILRPGTDAAFFLGVARELIEKGLYDRAAVIERTDLPLLVRLDT 7en6.1    --EIVLCAEAARKQGATVIAITSLTDSPLRRLAHFTLDTVSGET------------------------------------  target    GERLDARDVIPGYELAALTNYVTLKPDAEIKGNPPPPPFTAGGQVVPTELRDAWGDFVWWDRATGRPRPVSRDEVGARFD 7en6.1    --------------------------------------------------------------------------------  target    GDPALLGEFEVELVDGSTVPVRPAFDLLKQYLDESFDLRTASEVCRVPPQAIQSIARQLAANKRETLLAAGMGPNHYFQN 7en6.1    --------------------------------------------------------------------------------  target    DLFGRVQFLVAALTDNIGHLGGNVGSYAGNYRGSVFQAMGQWIAEDPFAIEPDLTKPATVKRYYKAESAHYWNYGERPLR 7en6.1    --------------------------------------------------------------------------------  target    AVAKDDEGDLTKGEVLTGKSHMPTPTKLIWFGNSNSLLGNAKWSFDVVKNTLPRQDAVFCNEWHWTSSCEYADLVFPADS 7en6.1    --------------------------------------------------------------------------------  target    WAEFKLPDATASCTNPFLLAFPTTPLKRLYDTRSDYEALALTAKALGELIDEPRMEQYWRGILDGDPTPYLQRIFSGSNA 7en6.1    --------------------------------------------------------------------------------  target    TRGITYDELHESSKRGVPLLMNMRTYPRSGGWEQRQEDKPWYTATGRLEFYRPEPEFQAAGESLPVWREPVDATFYEPNA 7en6.1    --------------------------------------------------------------------------------  target    ILSNAAHPSIAPRAPEDYGVPESQLDVETRQYRNVVRTWAELQQTLHPLQERDPAFRFVF 7en6.1    ------------------------------------------------------------ ``` | | | | | | | | | | | | | | | | | | | | | | | | | | | | | | | | | | | | | | | | | | | | | | | | | |
|  | 1s7g.1.D | NAD-dependent deacetylase 2  *Structural Basis for the Mechanism and Regulation of Sir2 Enzymes* | 0.01 |  | 20.75 | 0.06 | 303-357 | X-ray | 2.30 | homo-pentamer | 9 x ZN, 3 x NAD, 1 x APR, 1 x 2PE | HHblits | 0.32 |
| ``` target    AQGVSRRQLLGRALALGSGAALADLLGPARFLSPAGAATAGAVVPGNPLRVMPDRTWEQIYRNQFEDDSTFVFTCAPNDT 1s7g.1    --------------------------------------------------------------------------------  target    HNCLLRAHVKNGVVVRISPTYGYGEATDLYGNRASHRWDPRTCQKGLILSRRFYSERRVKAPMIRKGFKDWVEAGYPRND 1s7g.1    --------------------------------------------------------------------------------  target    DGTPQMDVTLRGSDDWIRISWDEATTIAAKTMEDVARTFNGDEGARKLLAQGYHPEMVEVMHGAGVQALKLRGGMPLLGI 1s7g.1    --------------------------------------------------------------------------------  target    GRIFGFYRFANMLALLDRKLRPDAPADEILGSRTFDNYAWHTDLPPGHPMVTGSQTVDFDLFSAEHTKLLLIIGMNWICT 1s7g.1    --------------------------------------------------------------EAKHCDAFMVVGSSLVV-  target    KMPDGHWIGDARLKGTRVIVISADYMPTANKADEVIILRPGTDAAFFLGVARELIEKGLYDRAAVIERTDLPLLVRLDTG 1s7g.1    -YPAAELPYIAKKAGAKMIIVNAEPTMADPIFDVKII-------------------------------------------  target    ERLDARDVIPGYELAALTNYVTLKPDAEIKGNPPPPPFTAGGQVVPTELRDAWGDFVWWDRATGRPRPVSRDEVGARFDG 1s7g.1    --------------------------------------------------------------------------------  target    DPALLGEFEVELVDGSTVPVRPAFDLLKQYLDESFDLRTASEVCRVPPQAIQSIARQLAANKRETLLAAGMGPNHYFQND 1s7g.1    --------------------------------------------------------------------------------  target    LFGRVQFLVAALTDNIGHLGGNVGSYAGNYRGSVFQAMGQWIAEDPFAIEPDLTKPATVKRYYKAESAHYWNYGERPLRA 1s7g.1    --------------------------------------------------------------------------------  target    VAKDDEGDLTKGEVLTGKSHMPTPTKLIWFGNSNSLLGNAKWSFDVVKNTLPRQDAVFCNEWHWTSSCEYADLVFPADSW 1s7g.1    --------------------------------------------------------------------------------  target    AEFKLPDATASCTNPFLLAFPTTPLKRLYDTRSDYEALALTAKALGELIDEPRMEQYWRGILDGDPTPYLQRIFSGSNAT 1s7g.1    --------------------------------------------------------------------------------  target    RGITYDELHESSKRGVPLLMNMRTYPRSGGWEQRQEDKPWYTATGRLEFYRPEPEFQAAGESLPVWREPVDATFYEPNAI 1s7g.1    --------------------------------------------------------------------------------  target    LSNAAHPSIAPRAPEDYGVPESQLDVETRQYRNVVRTWAELQQTLHPLQERDPAFRFVF 1s7g.1    ----------------------------------------------------------- ``` | | | | | | | | | | | | | | | | | | | | | | | | | | | | | | | | | | | | | | | | | | | | | | | | | |
|  | 1ma3.1.A | Transcriptional regulatory protein, Sir2 family  *Structure of a Sir2 enzyme bound to an acetylated p53 peptide* | 0.01 |  | 20.75 | 0.06 | 303-357 | X-ray | 2.00 | hetero-oligomer | 1 x ZN, 1 x MES | HHblits | 0.32 |
| ``` target    AQGVSRRQLLGRALALGSGAALADLLGPARFLSPAGAATAGAVVPGNPLRVMPDRTWEQIYRNQFEDDSTFVFTCAPNDT 1ma3.1    --------------------------------------------------------------------------------  target    HNCLLRAHVKNGVVVRISPTYGYGEATDLYGNRASHRWDPRTCQKGLILSRRFYSERRVKAPMIRKGFKDWVEAGYPRND 1ma3.1    --------------------------------------------------------------------------------  target    DGTPQMDVTLRGSDDWIRISWDEATTIAAKTMEDVARTFNGDEGARKLLAQGYHPEMVEVMHGAGVQALKLRGGMPLLGI 1ma3.1    --------------------------------------------------------------------------------  target    GRIFGFYRFANMLALLDRKLRPDAPADEILGSRTFDNYAWHTDLPPGHPMVTGSQTVDFDLFSAEHTKLLLIIGMNWICT 1ma3.1    --------------------------------------------------------------EAKHCDAFMVVGSSLVV-  target    KMPDGHWIGDARLKGTRVIVISADYMPTANKADEVIILRPGTDAAFFLGVARELIEKGLYDRAAVIERTDLPLLVRLDTG 1ma3.1    -YPAAELPYIAKKAGAKMIIVNAEPTMADPIFDVKII-------------------------------------------  target    ERLDARDVIPGYELAALTNYVTLKPDAEIKGNPPPPPFTAGGQVVPTELRDAWGDFVWWDRATGRPRPVSRDEVGARFDG 1ma3.1    --------------------------------------------------------------------------------  target    DPALLGEFEVELVDGSTVPVRPAFDLLKQYLDESFDLRTASEVCRVPPQAIQSIARQLAANKRETLLAAGMGPNHYFQND 1ma3.1    --------------------------------------------------------------------------------  target    LFGRVQFLVAALTDNIGHLGGNVGSYAGNYRGSVFQAMGQWIAEDPFAIEPDLTKPATVKRYYKAESAHYWNYGERPLRA 1ma3.1    --------------------------------------------------------------------------------  target    VAKDDEGDLTKGEVLTGKSHMPTPTKLIWFGNSNSLLGNAKWSFDVVKNTLPRQDAVFCNEWHWTSSCEYADLVFPADSW 1ma3.1    --------------------------------------------------------------------------------  target    AEFKLPDATASCTNPFLLAFPTTPLKRLYDTRSDYEALALTAKALGELIDEPRMEQYWRGILDGDPTPYLQRIFSGSNAT 1ma3.1    --------------------------------------------------------------------------------  target    RGITYDELHESSKRGVPLLMNMRTYPRSGGWEQRQEDKPWYTATGRLEFYRPEPEFQAAGESLPVWREPVDATFYEPNAI 1ma3.1    --------------------------------------------------------------------------------  target    LSNAAHPSIAPRAPEDYGVPESQLDVETRQYRNVVRTWAELQQTLHPLQERDPAFRFVF 1ma3.1    ----------------------------------------------------------- ``` | | | | | | | | | | | | | | | | | | | | | | | | | | | | | | | | | | | | | | | | | | | | | | | | | |
|  | 1s7g.1.B | NAD-dependent deacetylase 2  *Structural Basis for the Mechanism and Regulation of Sir2 Enzymes* | 0.01 |  | 20.75 | 0.06 | 303-357 | X-ray | 2.30 | homo-pentamer | 9 x ZN, 3 x NAD, 1 x APR, 1 x 2PE | HHblits | 0.32 |
| ``` target    AQGVSRRQLLGRALALGSGAALADLLGPARFLSPAGAATAGAVVPGNPLRVMPDRTWEQIYRNQFEDDSTFVFTCAPNDT 1s7g.1    --------------------------------------------------------------------------------  target    HNCLLRAHVKNGVVVRISPTYGYGEATDLYGNRASHRWDPRTCQKGLILSRRFYSERRVKAPMIRKGFKDWVEAGYPRND 1s7g.1    --------------------------------------------------------------------------------  target    DGTPQMDVTLRGSDDWIRISWDEATTIAAKTMEDVARTFNGDEGARKLLAQGYHPEMVEVMHGAGVQALKLRGGMPLLGI 1s7g.1    --------------------------------------------------------------------------------  target    GRIFGFYRFANMLALLDRKLRPDAPADEILGSRTFDNYAWHTDLPPGHPMVTGSQTVDFDLFSAEHTKLLLIIGMNWICT 1s7g.1    --------------------------------------------------------------EAKHCDAFMVVGSSLVV-  target    KMPDGHWIGDARLKGTRVIVISADYMPTANKADEVIILRPGTDAAFFLGVARELIEKGLYDRAAVIERTDLPLLVRLDTG 1s7g.1    -YPAAELPYIAKKAGAKMIIVNAEPTMADPIFDVKII-------------------------------------------  target    ERLDARDVIPGYELAALTNYVTLKPDAEIKGNPPPPPFTAGGQVVPTELRDAWGDFVWWDRATGRPRPVSRDEVGARFDG 1s7g.1    --------------------------------------------------------------------------------  target    DPALLGEFEVELVDGSTVPVRPAFDLLKQYLDESFDLRTASEVCRVPPQAIQSIARQLAANKRETLLAAGMGPNHYFQND 1s7g.1    --------------------------------------------------------------------------------  target    LFGRVQFLVAALTDNIGHLGGNVGSYAGNYRGSVFQAMGQWIAEDPFAIEPDLTKPATVKRYYKAESAHYWNYGERPLRA 1s7g.1    --------------------------------------------------------------------------------  target    VAKDDEGDLTKGEVLTGKSHMPTPTKLIWFGNSNSLLGNAKWSFDVVKNTLPRQDAVFCNEWHWTSSCEYADLVFPADSW 1s7g.1    --------------------------------------------------------------------------------  target    AEFKLPDATASCTNPFLLAFPTTPLKRLYDTRSDYEALALTAKALGELIDEPRMEQYWRGILDGDPTPYLQRIFSGSNAT 1s7g.1    --------------------------------------------------------------------------------  target    RGITYDELHESSKRGVPLLMNMRTYPRSGGWEQRQEDKPWYTATGRLEFYRPEPEFQAAGESLPVWREPVDATFYEPNAI 1s7g.1    --------------------------------------------------------------------------------  target    LSNAAHPSIAPRAPEDYGVPESQLDVETRQYRNVVRTWAELQQTLHPLQERDPAFRFVF 1s7g.1    ----------------------------------------------------------- ``` | | | | | | | | | | | | | | | | | | | | | | | | | | | | | | | | | | | | | | | | | | | | | | | | | |
|  | 1s7g.1.A | NAD-dependent deacetylase 2  *Structural Basis for the Mechanism and Regulation of Sir2 Enzymes* | 0.01 |  | 20.75 | 0.06 | 303-357 | X-ray | 2.30 | homo-pentamer | 9 x ZN, 3 x NAD, 1 x APR, 1 x 2PE | HHblits | 0.32 |
| ``` target    AQGVSRRQLLGRALALGSGAALADLLGPARFLSPAGAATAGAVVPGNPLRVMPDRTWEQIYRNQFEDDSTFVFTCAPNDT 1s7g.1    --------------------------------------------------------------------------------  target    HNCLLRAHVKNGVVVRISPTYGYGEATDLYGNRASHRWDPRTCQKGLILSRRFYSERRVKAPMIRKGFKDWVEAGYPRND 1s7g.1    --------------------------------------------------------------------------------  target    DGTPQMDVTLRGSDDWIRISWDEATTIAAKTMEDVARTFNGDEGARKLLAQGYHPEMVEVMHGAGVQALKLRGGMPLLGI 1s7g.1    --------------------------------------------------------------------------------  target    GRIFGFYRFANMLALLDRKLRPDAPADEILGSRTFDNYAWHTDLPPGHPMVTGSQTVDFDLFSAEHTKLLLIIGMNWICT 1s7g.1    --------------------------------------------------------------EAKHCDAFMVVGSSLVV-  target    KMPDGHWIGDARLKGTRVIVISADYMPTANKADEVIILRPGTDAAFFLGVARELIEKGLYDRAAVIERTDLPLLVRLDTG 1s7g.1    -YPAAELPYIAKKAGAKMIIVNAEPTMADPIFDVKII-------------------------------------------  target    ERLDARDVIPGYELAALTNYVTLKPDAEIKGNPPPPPFTAGGQVVPTELRDAWGDFVWWDRATGRPRPVSRDEVGARFDG 1s7g.1    --------------------------------------------------------------------------------  target    DPALLGEFEVELVDGSTVPVRPAFDLLKQYLDESFDLRTASEVCRVPPQAIQSIARQLAANKRETLLAAGMGPNHYFQND 1s7g.1    --------------------------------------------------------------------------------  target    LFGRVQFLVAALTDNIGHLGGNVGSYAGNYRGSVFQAMGQWIAEDPFAIEPDLTKPATVKRYYKAESAHYWNYGERPLRA 1s7g.1    --------------------------------------------------------------------------------  target    VAKDDEGDLTKGEVLTGKSHMPTPTKLIWFGNSNSLLGNAKWSFDVVKNTLPRQDAVFCNEWHWTSSCEYADLVFPADSW 1s7g.1    --------------------------------------------------------------------------------  target    AEFKLPDATASCTNPFLLAFPTTPLKRLYDTRSDYEALALTAKALGELIDEPRMEQYWRGILDGDPTPYLQRIFSGSNAT 1s7g.1    --------------------------------------------------------------------------------  target    RGITYDELHESSKRGVPLLMNMRTYPRSGGWEQRQEDKPWYTATGRLEFYRPEPEFQAAGESLPVWREPVDATFYEPNAI 1s7g.1    --------------------------------------------------------------------------------  target    LSNAAHPSIAPRAPEDYGVPESQLDVETRQYRNVVRTWAELQQTLHPLQERDPAFRFVF 1s7g.1    ----------------------------------------------------------- ``` | | | | | | | | | | | | | | | | | | | | | | | | | | | | | | | | | | | | | | | | | | | | | | | | | |
|  | 1s7g.1.C | NAD-dependent deacetylase 2  *Structural Basis for the Mechanism and Regulation of Sir2 Enzymes* | 0.01 |  | 20.75 | 0.06 | 303-357 | X-ray | 2.30 | homo-pentamer | 9 x ZN, 3 x NAD, 1 x APR, 1 x 2PE | HHblits | 0.32 |
| ``` target    AQGVSRRQLLGRALALGSGAALADLLGPARFLSPAGAATAGAVVPGNPLRVMPDRTWEQIYRNQFEDDSTFVFTCAPNDT 1s7g.1    --------------------------------------------------------------------------------  target    HNCLLRAHVKNGVVVRISPTYGYGEATDLYGNRASHRWDPRTCQKGLILSRRFYSERRVKAPMIRKGFKDWVEAGYPRND 1s7g.1    --------------------------------------------------------------------------------  target    DGTPQMDVTLRGSDDWIRISWDEATTIAAKTMEDVARTFNGDEGARKLLAQGYHPEMVEVMHGAGVQALKLRGGMPLLGI 1s7g.1    --------------------------------------------------------------------------------  target    GRIFGFYRFANMLALLDRKLRPDAPADEILGSRTFDNYAWHTDLPPGHPMVTGSQTVDFDLFSAEHTKLLLIIGMNWICT 1s7g.1    --------------------------------------------------------------EAKHCDAFMVVGSSLVV-  target    KMPDGHWIGDARLKGTRVIVISADYMPTANKADEVIILRPGTDAAFFLGVARELIEKGLYDRAAVIERTDLPLLVRLDTG 1s7g.1    -YPAAELPYIAKKAGAKMIIVNAEPTMADPIFDVKII-------------------------------------------  target    ERLDARDVIPGYELAALTNYVTLKPDAEIKGNPPPPPFTAGGQVVPTELRDAWGDFVWWDRATGRPRPVSRDEVGARFDG 1s7g.1    --------------------------------------------------------------------------------  target    DPALLGEFEVELVDGSTVPVRPAFDLLKQYLDESFDLRTASEVCRVPPQAIQSIARQLAANKRETLLAAGMGPNHYFQND 1s7g.1    --------------------------------------------------------------------------------  target    LFGRVQFLVAALTDNIGHLGGNVGSYAGNYRGSVFQAMGQWIAEDPFAIEPDLTKPATVKRYYKAESAHYWNYGERPLRA 1s7g.1    --------------------------------------------------------------------------------  target    VAKDDEGDLTKGEVLTGKSHMPTPTKLIWFGNSNSLLGNAKWSFDVVKNTLPRQDAVFCNEWHWTSSCEYADLVFPADSW 1s7g.1    --------------------------------------------------------------------------------  target    AEFKLPDATASCTNPFLLAFPTTPLKRLYDTRSDYEALALTAKALGELIDEPRMEQYWRGILDGDPTPYLQRIFSGSNAT 1s7g.1    --------------------------------------------------------------------------------  target    RGITYDELHESSKRGVPLLMNMRTYPRSGGWEQRQEDKPWYTATGRLEFYRPEPEFQAAGESLPVWREPVDATFYEPNAI 1s7g.1    --------------------------------------------------------------------------------  target    LSNAAHPSIAPRAPEDYGVPESQLDVETRQYRNVVRTWAELQQTLHPLQERDPAFRFVF 1s7g.1    ----------------------------------------------------------- ``` | | | | | | | | | | | | | | | | | | | | | | | | | | | | | | | | | | | | | | | | | | | | | | | | | |
|  | 1s7g.1.E | NAD-dependent deacetylase 2  *Structural Basis for the Mechanism and Regulation of Sir2 Enzymes* | 0.01 |  | 20.75 | 0.06 | 303-357 | X-ray | 2.30 | homo-pentamer | 9 x ZN, 3 x NAD, 1 x APR, 1 x 2PE | HHblits | 0.32 |
| ``` target    AQGVSRRQLLGRALALGSGAALADLLGPARFLSPAGAATAGAVVPGNPLRVMPDRTWEQIYRNQFEDDSTFVFTCAPNDT 1s7g.1    --------------------------------------------------------------------------------  target    HNCLLRAHVKNGVVVRISPTYGYGEATDLYGNRASHRWDPRTCQKGLILSRRFYSERRVKAPMIRKGFKDWVEAGYPRND 1s7g.1    --------------------------------------------------------------------------------  target    DGTPQMDVTLRGSDDWIRISWDEATTIAAKTMEDVARTFNGDEGARKLLAQGYHPEMVEVMHGAGVQALKLRGGMPLLGI 1s7g.1    --------------------------------------------------------------------------------  target    GRIFGFYRFANMLALLDRKLRPDAPADEILGSRTFDNYAWHTDLPPGHPMVTGSQTVDFDLFSAEHTKLLLIIGMNWICT 1s7g.1    --------------------------------------------------------------EAKHCDAFMVVGSSLVV-  target    KMPDGHWIGDARLKGTRVIVISADYMPTANKADEVIILRPGTDAAFFLGVARELIEKGLYDRAAVIERTDLPLLVRLDTG 1s7g.1    -YPAAELPYIAKKAGAKMIIVNAEPTMADPIFDVKII-------------------------------------------  target    ERLDARDVIPGYELAALTNYVTLKPDAEIKGNPPPPPFTAGGQVVPTELRDAWGDFVWWDRATGRPRPVSRDEVGARFDG 1s7g.1    --------------------------------------------------------------------------------  target    DPALLGEFEVELVDGSTVPVRPAFDLLKQYLDESFDLRTASEVCRVPPQAIQSIARQLAANKRETLLAAGMGPNHYFQND 1s7g.1    --------------------------------------------------------------------------------  target    LFGRVQFLVAALTDNIGHLGGNVGSYAGNYRGSVFQAMGQWIAEDPFAIEPDLTKPATVKRYYKAESAHYWNYGERPLRA 1s7g.1    --------------------------------------------------------------------------------  target    VAKDDEGDLTKGEVLTGKSHMPTPTKLIWFGNSNSLLGNAKWSFDVVKNTLPRQDAVFCNEWHWTSSCEYADLVFPADSW 1s7g.1    --------------------------------------------------------------------------------  target    AEFKLPDATASCTNPFLLAFPTTPLKRLYDTRSDYEALALTAKALGELIDEPRMEQYWRGILDGDPTPYLQRIFSGSNAT 1s7g.1    --------------------------------------------------------------------------------  target    RGITYDELHESSKRGVPLLMNMRTYPRSGGWEQRQEDKPWYTATGRLEFYRPEPEFQAAGESLPVWREPVDATFYEPNAI 1s7g.1    --------------------------------------------------------------------------------  target    LSNAAHPSIAPRAPEDYGVPESQLDVETRQYRNVVRTWAELQQTLHPLQERDPAFRFVF 1s7g.1    ----------------------------------------------------------- ``` | | | | | | | | | | | | | | | | | | | | | | | | | | | | | | | | | | | | | | | | | | | | | | | | | |
|  | 4twj.1.A | NAD-dependent protein deacylase 2  *The structure of Sir2Af2 bound to a myristoylated histone peptide* | 0.01 |  | 20.75 | 0.06 | 303-357 | X-ray | 1.65 | hetero-1-1-mer | 1 x ZN | HHblits | 0.32 |
| ``` target    AQGVSRRQLLGRALALGSGAALADLLGPARFLSPAGAATAGAVVPGNPLRVMPDRTWEQIYRNQFEDDSTFVFTCAPNDT 4twj.1    --------------------------------------------------------------------------------  target    HNCLLRAHVKNGVVVRISPTYGYGEATDLYGNRASHRWDPRTCQKGLILSRRFYSERRVKAPMIRKGFKDWVEAGYPRND 4twj.1    --------------------------------------------------------------------------------  target    DGTPQMDVTLRGSDDWIRISWDEATTIAAKTMEDVARTFNGDEGARKLLAQGYHPEMVEVMHGAGVQALKLRGGMPLLGI 4twj.1    --------------------------------------------------------------------------------  target    GRIFGFYRFANMLALLDRKLRPDAPADEILGSRTFDNYAWHTDLPPGHPMVTGSQTVDFDLFSAEHTKLLLIIGMNWICT 4twj.1    --------------------------------------------------------------EAKHCDAFMVVGSSLVV-  target    KMPDGHWIGDARLKGTRVIVISADYMPTANKADEVIILRPGTDAAFFLGVARELIEKGLYDRAAVIERTDLPLLVRLDTG 4twj.1    -YPAAELPYIAKKAGAKMIIVNAEPTMADPIFDVKII-------------------------------------------  target    ERLDARDVIPGYELAALTNYVTLKPDAEIKGNPPPPPFTAGGQVVPTELRDAWGDFVWWDRATGRPRPVSRDEVGARFDG 4twj.1    --------------------------------------------------------------------------------  target    DPALLGEFEVELVDGSTVPVRPAFDLLKQYLDESFDLRTASEVCRVPPQAIQSIARQLAANKRETLLAAGMGPNHYFQND 4twj.1    --------------------------------------------------------------------------------  target    LFGRVQFLVAALTDNIGHLGGNVGSYAGNYRGSVFQAMGQWIAEDPFAIEPDLTKPATVKRYYKAESAHYWNYGERPLRA 4twj.1    --------------------------------------------------------------------------------  target    VAKDDEGDLTKGEVLTGKSHMPTPTKLIWFGNSNSLLGNAKWSFDVVKNTLPRQDAVFCNEWHWTSSCEYADLVFPADSW 4twj.1    --------------------------------------------------------------------------------  target    AEFKLPDATASCTNPFLLAFPTTPLKRLYDTRSDYEALALTAKALGELIDEPRMEQYWRGILDGDPTPYLQRIFSGSNAT 4twj.1    --------------------------------------------------------------------------------  target    RGITYDELHESSKRGVPLLMNMRTYPRSGGWEQRQEDKPWYTATGRLEFYRPEPEFQAAGESLPVWREPVDATFYEPNAI 4twj.1    --------------------------------------------------------------------------------  target    LSNAAHPSIAPRAPEDYGVPESQLDVETRQYRNVVRTWAELQQTLHPLQERDPAFRFVF 4twj.1    ----------------------------------------------------------- ``` | | | | | | | | | | | | | | | | | | | | | | | | | | | | | | | | | | | | | | | | | | | | | | | | | |
|  | 1ici.1.A | TRANSCRIPTIONAL REGULATORY PROTEIN, SIR2 FAMILY  *CRYSTAL STRUCTURE OF A SIR2 HOMOLOG-NAD COMPLEX* | 0.01 |  | 18.52 | 0.06 | 303-358 | X-ray | 2.10 | homo-dimer | 2 x ZN, 2 x NAD | HHblits | 0.30 |
| ``` target    AQGVSRRQLLGRALALGSGAALADLLGPARFLSPAGAATAGAVVPGNPLRVMPDRTWEQIYRNQFEDDSTFVFTCAPNDT 1ici.1    --------------------------------------------------------------------------------  target    HNCLLRAHVKNGVVVRISPTYGYGEATDLYGNRASHRWDPRTCQKGLILSRRFYSERRVKAPMIRKGFKDWVEAGYPRND 1ici.1    --------------------------------------------------------------------------------  target    DGTPQMDVTLRGSDDWIRISWDEATTIAAKTMEDVARTFNGDEGARKLLAQGYHPEMVEVMHGAGVQALKLRGGMPLLGI 1ici.1    --------------------------------------------------------------------------------  target    GRIFGFYRFANMLALLDRKLRPDAPADEILGSRTFDNYAWHTDLPPGHPMVTGSQTVDFDLFSAEHTKLLLIIGMNWICT 1ici.1    --------------------------------------------------------------EVERADVIIVAGTSAVVQ  target    KMPDGHWIGDARLKGTRVIVISADYMPTANKADEVIILRPGTDAAFFLGVARELIEKGLYDRAAVIERTDLPLLVRLDTG 1ici.1    --PAASLPLIVKQRGGAIIEINPDETPLTPIADYSLRG------------------------------------------  target    ERLDARDVIPGYELAALTNYVTLKPDAEIKGNPPPPPFTAGGQVVPTELRDAWGDFVWWDRATGRPRPVSRDEVGARFDG 1ici.1    --------------------------------------------------------------------------------  target    DPALLGEFEVELVDGSTVPVRPAFDLLKQYLDESFDLRTASEVCRVPPQAIQSIARQLAANKRETLLAAGMGPNHYFQND 1ici.1    --------------------------------------------------------------------------------  target    LFGRVQFLVAALTDNIGHLGGNVGSYAGNYRGSVFQAMGQWIAEDPFAIEPDLTKPATVKRYYKAESAHYWNYGERPLRA 1ici.1    --------------------------------------------------------------------------------  target    VAKDDEGDLTKGEVLTGKSHMPTPTKLIWFGNSNSLLGNAKWSFDVVKNTLPRQDAVFCNEWHWTSSCEYADLVFPADSW 1ici.1    --------------------------------------------------------------------------------  target    AEFKLPDATASCTNPFLLAFPTTPLKRLYDTRSDYEALALTAKALGELIDEPRMEQYWRGILDGDPTPYLQRIFSGSNAT 1ici.1    --------------------------------------------------------------------------------  target    RGITYDELHESSKRGVPLLMNMRTYPRSGGWEQRQEDKPWYTATGRLEFYRPEPEFQAAGESLPVWREPVDATFYEPNAI 1ici.1    --------------------------------------------------------------------------------  target    LSNAAHPSIAPRAPEDYGVPESQLDVETRQYRNVVRTWAELQQTLHPLQERDPAFRFVF 1ici.1    ----------------------------------------------------------- ``` | | | | | | | | | | | | | | | | | | | | | | | | | | | | | | | | | | | | | | | | | | | | | | | | | |
|  | 2i2w.1.A | Phosphoheptose isomerase  *Crystal Structure of Escherichia Coli Phosphoheptose Isomerase* | 0.01 |  | 25.45 | 0.06 | 305-361 | X-ray | 1.95 | homo-dimer |  | HHblits | 0.28 |
| ``` target    AQGVSRRQLLGRALALGSGAALADLLGPARFLSPAGAATAGAVVPGNPLRVMPDRTWEQIYRNQFEDDSTFVFTCAPNDT 2i2w.1    --------------------------------------------------------------------------------  target    HNCLLRAHVKNGVVVRISPTYGYGEATDLYGNRASHRWDPRTCQKGLILSRRFYSERRVKAPMIRKGFKDWVEAGYPRND 2i2w.1    --------------------------------------------------------------------------------  target    DGTPQMDVTLRGSDDWIRISWDEATTIAAKTMEDVARTFNGDEGARKLLAQGYHPEMVEVMHGAGVQALKLRGGMPLLGI 2i2w.1    --------------------------------------------------------------------------------  target    GRIFGFYRFANMLALLDRKLRPDAPADEILGSRTFDNYAWHTDLPPGHPMVTGSQTVDFDLFSAEHTKLLLIIGMNWICT 2i2w.1    ----------------------------------------------------------------REGDVLLGISTSGNSA  target    KMPDGHWIGDARLKGTRVIVISADYM-PTANKADEVIILRPGTDAAFFLGVARELIEKGLYDRAAVIERTDLPLLVRLDT 2i2w.1    --NVIKAIAAAREKGMKVITLTGKDGGKMAGTADIEIRVPHF--------------------------------------  target    GERLDARDVIPGYELAALTNYVTLKPDAEIKGNPPPPPFTAGGQVVPTELRDAWGDFVWWDRATGRPRPVSRDEVGARFD 2i2w.1    --------------------------------------------------------------------------------  target    GDPALLGEFEVELVDGSTVPVRPAFDLLKQYLDESFDLRTASEVCRVPPQAIQSIARQLAANKRETLLAAGMGPNHYFQN 2i2w.1    --------------------------------------------------------------------------------  target    DLFGRVQFLVAALTDNIGHLGGNVGSYAGNYRGSVFQAMGQWIAEDPFAIEPDLTKPATVKRYYKAESAHYWNYGERPLR 2i2w.1    --------------------------------------------------------------------------------  target    AVAKDDEGDLTKGEVLTGKSHMPTPTKLIWFGNSNSLLGNAKWSFDVVKNTLPRQDAVFCNEWHWTSSCEYADLVFPADS 2i2w.1    --------------------------------------------------------------------------------  target    WAEFKLPDATASCTNPFLLAFPTTPLKRLYDTRSDYEALALTAKALGELIDEPRMEQYWRGILDGDPTPYLQRIFSGSNA 2i2w.1    --------------------------------------------------------------------------------  target    TRGITYDELHESSKRGVPLLMNMRTYPRSGGWEQRQEDKPWYTATGRLEFYRPEPEFQAAGESLPVWREPVDATFYEPNA 2i2w.1    --------------------------------------------------------------------------------  target    ILSNAAHPSIAPRAPEDYGVPESQLDVETRQYRNVVRTWAELQQTLHPLQERDPAFRFVF 2i2w.1    ------------------------------------------------------------ ``` | | | | | | | | | | | | | | | | | | | | | | | | | | | | | | | | | | | | | | | | | | | | | | | | | |
|  | 2i2w.2.B | Phosphoheptose isomerase  *Crystal Structure of Escherichia Coli Phosphoheptose Isomerase* | 0.01 |  | 25.45 | 0.06 | 305-361 | X-ray | 1.95 | homo-dimer |  | HHblits | 0.28 |
| ``` target    AQGVSRRQLLGRALALGSGAALADLLGPARFLSPAGAATAGAVVPGNPLRVMPDRTWEQIYRNQFEDDSTFVFTCAPNDT 2i2w.2    --------------------------------------------------------------------------------  target    HNCLLRAHVKNGVVVRISPTYGYGEATDLYGNRASHRWDPRTCQKGLILSRRFYSERRVKAPMIRKGFKDWVEAGYPRND 2i2w.2    --------------------------------------------------------------------------------  target    DGTPQMDVTLRGSDDWIRISWDEATTIAAKTMEDVARTFNGDEGARKLLAQGYHPEMVEVMHGAGVQALKLRGGMPLLGI 2i2w.2    --------------------------------------------------------------------------------  target    GRIFGFYRFANMLALLDRKLRPDAPADEILGSRTFDNYAWHTDLPPGHPMVTGSQTVDFDLFSAEHTKLLLIIGMNWICT 2i2w.2    ----------------------------------------------------------------REGDVLLGISTSGNSA  target    KMPDGHWIGDARLKGTRVIVISADYM-PTANKADEVIILRPGTDAAFFLGVARELIEKGLYDRAAVIERTDLPLLVRLDT 2i2w.2    --NVIKAIAAAREKGMKVITLTGKDGGKMAGTADIEIRVPHF--------------------------------------  target    GERLDARDVIPGYELAALTNYVTLKPDAEIKGNPPPPPFTAGGQVVPTELRDAWGDFVWWDRATGRPRPVSRDEVGARFD 2i2w.2    --------------------------------------------------------------------------------  target    GDPALLGEFEVELVDGSTVPVRPAFDLLKQYLDESFDLRTASEVCRVPPQAIQSIARQLAANKRETLLAAGMGPNHYFQN 2i2w.2    --------------------------------------------------------------------------------  target    DLFGRVQFLVAALTDNIGHLGGNVGSYAGNYRGSVFQAMGQWIAEDPFAIEPDLTKPATVKRYYKAESAHYWNYGERPLR 2i2w.2    --------------------------------------------------------------------------------  target    AVAKDDEGDLTKGEVLTGKSHMPTPTKLIWFGNSNSLLGNAKWSFDVVKNTLPRQDAVFCNEWHWTSSCEYADLVFPADS 2i2w.2    --------------------------------------------------------------------------------  target    WAEFKLPDATASCTNPFLLAFPTTPLKRLYDTRSDYEALALTAKALGELIDEPRMEQYWRGILDGDPTPYLQRIFSGSNA 2i2w.2    --------------------------------------------------------------------------------  target    TRGITYDELHESSKRGVPLLMNMRTYPRSGGWEQRQEDKPWYTATGRLEFYRPEPEFQAAGESLPVWREPVDATFYEPNA 2i2w.2    --------------------------------------------------------------------------------  target    ILSNAAHPSIAPRAPEDYGVPESQLDVETRQYRNVVRTWAELQQTLHPLQERDPAFRFVF 2i2w.2    ------------------------------------------------------------ ``` | | | | | | | | | | | | | | | | | | | | | | | | | | | | | | | | | | | | | | | | | | | | | | | | | |
|  | 2v3v.1.A | PERIPLASMIC NITRATE REDUCTASE  *A NEW CATALYTIC MECHANISM OF PERIPLASMIC NITRATE REDUCTASE FROM DESULFOVIBRIO DESULFURICANS ATCC 27774 FROM CRYSTALLOGRAPHIC AND EPR DATA AND BASED ON DETAILED ANALYSIS OF THE SIXTH LIGAND* | 0.01 |  | 19.64 | 0.06 | 305-361 | X-ray | 1.99 | monomer | 1 x SF4, 1 x MO, 2 x MGD, 4 x LCP | HHblits | 0.27 |
| ``` target    AQGVSRRQLLGRALALGSGAALADLLGPARFLSPAGAATAGAVVPGNPLRVMPDRTWEQIYRNQFEDDSTFVFTCAPNDT 2v3v.1    --------------------------------------------------------------------------------  target    HNCLLRAHVKNGVVVRISPTYGYGEATDLYGNRASHRWDPRTCQKGLILSRRFYSERRVKAPMIRKGFKDWVEAGYPRND 2v3v.1    --------------------------------------------------------------------------------  target    DGTPQMDVTLRGSDDWIRISWDEATTIAAKTMEDVARTFNGDEGARKLLAQGYHPEMVEVMHGAGVQALKLRGGMPLLGI 2v3v.1    --------------------------------------------------------------------------------  target    GRIFGFYRFANMLALLDRKLRPDAPADEILGSRTFDNYAWHTDLPPGHPMVTGSQTVDFDLFSAEHTKLLLIIGMNWICT 2v3v.1    ----------------------------------------------------------------GDVKCMIICETNPAHT  target    KMPDGHWIGDARLKG-TRVIVISADYM-PTANKADEVIILRPGTDAAFFLGVARELIEKGLYDRAAVIERTDLPLLVRLD 2v3v.1    LP-NLNKVHKAMSHPESFIVCIEAFPDAVTLEYADLVLPPAFW-------------------------------------  target    TGERLDARDVIPGYELAALTNYVTLKPDAEIKGNPPPPPFTAGGQVVPTELRDAWGDFVWWDRATGRPRPVSRDEVGARF 2v3v.1    --------------------------------------------------------------------------------  target    DGDPALLGEFEVELVDGSTVPVRPAFDLLKQYLDESFDLRTASEVCRVPPQAIQSIARQLAANKRETLLAAGMGPNHYFQ 2v3v.1    --------------------------------------------------------------------------------  target    NDLFGRVQFLVAALTDNIGHLGGNVGSYAGNYRGSVFQAMGQWIAEDPFAIEPDLTKPATVKRYYKAESAHYWNYGERPL 2v3v.1    --------------------------------------------------------------------------------  target    RAVAKDDEGDLTKGEVLTGKSHMPTPTKLIWFGNSNSLLGNAKWSFDVVKNTLPRQDAVFCNEWHWTSSCEYADLVFPAD 2v3v.1    --------------------------------------------------------------------------------  target    SWAEFKLPDATASCTNPFLLAFPTTPLKRLYDTRSDYEALALTAKALGELIDEPRMEQYWRGILDGDPTPYLQRIFSGSN 2v3v.1    --------------------------------------------------------------------------------  target    ATRGITYDELHESSKRGVPLLMNMRTYPRSGGWEQRQEDKPWYTATGRLEFYRPEPEFQAAGESLPVWREPVDATFYEPN 2v3v.1    --------------------------------------------------------------------------------  target    AILSNAAHPSIAPRAPEDYGVPESQLDVETRQYRNVVRTWAELQQTLHPLQERDPAFRFVF 2v3v.1    ------------------------------------------------------------- ``` | | | | | | | | | | | | | | | | | | | | | | | | | | | | | | | | | | | | | | | | | | | | | | | | | |
|  | 7en5.1.A | HTH-type transcriptional regulator MurR  *The crystal structure of Escherichia coli MurR in complex with N-acetylglucosamine-6-phosphate* | 0.01 |  | 17.86 | 0.06 | 305-362 | X-ray | 1.25 | homo-tetramer | 4 x 4QY, 4 x MXE | HHblits | 0.26 |
| ``` target    AQGVSRRQLLGRALALGSGAALADLLGPARFLSPAGAATAGAVVPGNPLRVMPDRTWEQIYRNQFEDDSTFVFTCAPNDT 7en5.1    --------------------------------------------------------------------------------  target    HNCLLRAHVKNGVVVRISPTYGYGEATDLYGNRASHRWDPRTCQKGLILSRRFYSERRVKAPMIRKGFKDWVEAGYPRND 7en5.1    --------------------------------------------------------------------------------  target    DGTPQMDVTLRGSDDWIRISWDEATTIAAKTMEDVARTFNGDEGARKLLAQGYHPEMVEVMHGAGVQALKLRGGMPLLGI 7en5.1    --------------------------------------------------------------------------------  target    GRIFGFYRFANMLALLDRKLRPDAPADEILGSRTFDNYAWHTDLPPGHPMVTGSQTVDFDLFSAEHTKLLLIIGMNWICT 7en5.1    ----------------------------------------------------------------KKGDVQIAISYSGSKK  target    KMPDGHWIGDARLKGTRVIVISADY-MPTANKADEVIILRPGTDAAFFLGVARELIEKGLYDRAAVIERTDLPLLVRLDT 7en5.1    --EIVLCAEAARKQGATVIAITSLTDSPLRRLAHFTLDTVSGE-------------------------------------  target    GERLDARDVIPGYELAALTNYVTLKPDAEIKGNPPPPPFTAGGQVVPTELRDAWGDFVWWDRATGRPRPVSRDEVGARFD 7en5.1    --------------------------------------------------------------------------------  target    GDPALLGEFEVELVDGSTVPVRPAFDLLKQYLDESFDLRTASEVCRVPPQAIQSIARQLAANKRETLLAAGMGPNHYFQN 7en5.1    --------------------------------------------------------------------------------  target    DLFGRVQFLVAALTDNIGHLGGNVGSYAGNYRGSVFQAMGQWIAEDPFAIEPDLTKPATVKRYYKAESAHYWNYGERPLR 7en5.1    --------------------------------------------------------------------------------  target    AVAKDDEGDLTKGEVLTGKSHMPTPTKLIWFGNSNSLLGNAKWSFDVVKNTLPRQDAVFCNEWHWTSSCEYADLVFPADS 7en5.1    --------------------------------------------------------------------------------  target    WAEFKLPDATASCTNPFLLAFPTTPLKRLYDTRSDYEALALTAKALGELIDEPRMEQYWRGILDGDPTPYLQRIFSGSNA 7en5.1    --------------------------------------------------------------------------------  target    TRGITYDELHESSKRGVPLLMNMRTYPRSGGWEQRQEDKPWYTATGRLEFYRPEPEFQAAGESLPVWREPVDATFYEPNA 7en5.1    --------------------------------------------------------------------------------  target    ILSNAAHPSIAPRAPEDYGVPESQLDVETRQYRNVVRTWAELQQTLHPLQERDPAFRFVF 7en5.1    ------------------------------------------------------------ ``` | | | | | | | | | | | | | | | | | | | | | | | | | | | | | | | | | | | | | | | | | | | | | | | | | |
|  | 7l5i.1.A | Trimethylamine-N-oxide reductase  *Crystal Structure of Haemophilus influenzae MtsZ at pH 7.0* | 0.01 |  | 28.30 | 0.06 | 306-359 | X-ray | 1.73 | monomer | 2 x MGD, 1 x MO, 1 x O | HHblits | 0.31 |
| ``` target    AQGVSRRQLLGRALALGSGAALADLLGPARFLSPAGAATAGAVVPGNPLRVMPDRTWEQIYRNQFEDDSTFVFTCAPNDT 7l5i.1    --------------------------------------------------------------------------------  target    HNCLLRAHVKNGVVVRISPTYGYGEATDLYGNRASHRWDPRTCQKGLILSRRFYSERRVKAPMIRKGFKDWVEAGYPRND 7l5i.1    --------------------------------------------------------------------------------  target    DGTPQMDVTLRGSDDWIRISWDEATTIAAKTMEDVARTFNGDEGARKLLAQGYHPEMVEVMHGAGVQALKLRGGMPLLGI 7l5i.1    --------------------------------------------------------------------------------  target    GRIFGFYRFANMLALLDRKLRPDAPADEILGSRTFDNYAWHTDLPPGHPMVTGSQTVDFDLFSAEHTKLLLIIGMNWICT 7l5i.1    -----------------------------------------------------------------DIKAVYWAGGNPFVH  target    KMPDGHWIGDARLKGTRVIVISADYMPTANKADEVIILRPGTDAAFFLGVARELIEKGLYDRAAVIERTDLPLLVRLDTG 7l5i.1    H-QDTNTLVKAFQKPDVVIVNEVNWTPTARMADIVLPAT-----------------------------------------  target    ERLDARDVIPGYELAALTNYVTLKPDAEIKGNPPPPPFTAGGQVVPTELRDAWGDFVWWDRATGRPRPVSRDEVGARFDG 7l5i.1    --------------------------------------------------------------------------------  target    DPALLGEFEVELVDGSTVPVRPAFDLLKQYLDESFDLRTASEVCRVPPQAIQSIARQLAANKRETLLAAGMGPNHYFQND 7l5i.1    --------------------------------------------------------------------------------  target    LFGRVQFLVAALTDNIGHLGGNVGSYAGNYRGSVFQAMGQWIAEDPFAIEPDLTKPATVKRYYKAESAHYWNYGERPLRA 7l5i.1    --------------------------------------------------------------------------------  target    VAKDDEGDLTKGEVLTGKSHMPTPTKLIWFGNSNSLLGNAKWSFDVVKNTLPRQDAVFCNEWHWTSSCEYADLVFPADSW 7l5i.1    --------------------------------------------------------------------------------  target    AEFKLPDATASCTNPFLLAFPTTPLKRLYDTRSDYEALALTAKALGELIDEPRMEQYWRGILDGDPTPYLQRIFSGSNAT 7l5i.1    --------------------------------------------------------------------------------  target    RGITYDELHESSKRGVPLLMNMRTYPRSGGWEQRQEDKPWYTATGRLEFYRPEPEFQAAGESLPVWREPVDATFYEPNAI 7l5i.1    --------------------------------------------------------------------------------  target    LSNAAHPSIAPRAPEDYGVPESQLDVETRQYRNVVRTWAELQQTLHPLQERDPAFRFVF 7l5i.1    ----------------------------------------------------------- ``` | | | | | | | | | | | | | | | | | | | | | | | | | | | | | | | | | | | | | | | | | | | | | | | | | |
|  | 7l5s.1.A | Trimethylamine-N-oxide reductase  *Crystal Structure of Haemophilus influenzae MtsZ at pH 5.5* | 0.01 |  | 28.30 | 0.06 | 306-359 | X-ray | 2.09 | monomer | 1 x O, 2 x MGD, 1 x MO | HHblits | 0.31 |
| ``` target    AQGVSRRQLLGRALALGSGAALADLLGPARFLSPAGAATAGAVVPGNPLRVMPDRTWEQIYRNQFEDDSTFVFTCAPNDT 7l5s.1    --------------------------------------------------------------------------------  target    HNCLLRAHVKNGVVVRISPTYGYGEATDLYGNRASHRWDPRTCQKGLILSRRFYSERRVKAPMIRKGFKDWVEAGYPRND 7l5s.1    --------------------------------------------------------------------------------  target    DGTPQMDVTLRGSDDWIRISWDEATTIAAKTMEDVARTFNGDEGARKLLAQGYHPEMVEVMHGAGVQALKLRGGMPLLGI 7l5s.1    --------------------------------------------------------------------------------  target    GRIFGFYRFANMLALLDRKLRPDAPADEILGSRTFDNYAWHTDLPPGHPMVTGSQTVDFDLFSAEHTKLLLIIGMNWICT 7l5s.1    -----------------------------------------------------------------DIKAVYWAGGNPFVH  target    KMPDGHWIGDARLKGTRVIVISADYMPTANKADEVIILRPGTDAAFFLGVARELIEKGLYDRAAVIERTDLPLLVRLDTG 7l5s.1    H-QDTNTLVKAFQKPDVVIVNEVNWTPTARMADIVLPAT-----------------------------------------  target    ERLDARDVIPGYELAALTNYVTLKPDAEIKGNPPPPPFTAGGQVVPTELRDAWGDFVWWDRATGRPRPVSRDEVGARFDG 7l5s.1    --------------------------------------------------------------------------------  target    DPALLGEFEVELVDGSTVPVRPAFDLLKQYLDESFDLRTASEVCRVPPQAIQSIARQLAANKRETLLAAGMGPNHYFQND 7l5s.1    --------------------------------------------------------------------------------  target    LFGRVQFLVAALTDNIGHLGGNVGSYAGNYRGSVFQAMGQWIAEDPFAIEPDLTKPATVKRYYKAESAHYWNYGERPLRA 7l5s.1    --------------------------------------------------------------------------------  target    VAKDDEGDLTKGEVLTGKSHMPTPTKLIWFGNSNSLLGNAKWSFDVVKNTLPRQDAVFCNEWHWTSSCEYADLVFPADSW 7l5s.1    --------------------------------------------------------------------------------  target    AEFKLPDATASCTNPFLLAFPTTPLKRLYDTRSDYEALALTAKALGELIDEPRMEQYWRGILDGDPTPYLQRIFSGSNAT 7l5s.1    --------------------------------------------------------------------------------  target    RGITYDELHESSKRGVPLLMNMRTYPRSGGWEQRQEDKPWYTATGRLEFYRPEPEFQAAGESLPVWREPVDATFYEPNAI 7l5s.1    --------------------------------------------------------------------------------  target    LSNAAHPSIAPRAPEDYGVPESQLDVETRQYRNVVRTWAELQQTLHPLQERDPAFRFVF 7l5s.1    ----------------------------------------------------------- ``` | | | | | | | | | | | | | | | | | | | | | | | | | | | | | | | | | | | | | | | | | | | | | | | | | |
|  | 4ydd.1.A | DMSO reductase family type II enzyme, molybdopterin subunit  *Crystal structure of the perchlorate reductase PcrAB from Azospira suillum PS* | 0.01 |  | 16.36 | 0.06 | 306-360 | X-ray | 1.86 | hetero-oligomer | 4 x SF4, 1 x MO, 1 x MGD, 1 x MD1, 1 x F3S | HHblits | 0.28 |
| ``` target    AQGVSRRQLLGRALALGSGAALADLLGPARFLSPAGAATAGAVVPGNPLRVMPDRTWEQIYRNQFEDDSTFVFTCAPNDT 4ydd.1    --------------------------------------------------------------------------------  target    HNCLLRAHVKNGVVVRISPTYGYGEATDLYGNRASHRWDPRTCQKGLILSRRFYSERRVKAPMIRKGFKDWVEAGYPRND 4ydd.1    --------------------------------------------------------------------------------  target    DGTPQMDVTLRGSDDWIRISWDEATTIAAKTMEDVARTFNGDEGARKLLAQGYHPEMVEVMHGAGVQALKLRGGMPLLGI 4ydd.1    --------------------------------------------------------------------------------  target    GRIFGFYRFANMLALLDRKLRPDAPADEILGSRTFDNYAWHTDLPPGHPMVTGSQTVDFDLFSAEHTKLLLIIGMNWICT 4ydd.1    -----------------------------------------------------------------DPKVFFVYRGNWLNQ  target    KMPDGHWIGDARLKGTRVIVISADYMPTANKADEVIILRPGTDAAFFLGVARELIEKGLYDRAAVIERTDLPLLVRLDTG 4ydd.1    AKGQKYVLENLWPKLELIVDINIRMDSTALYSDVVLPSAH----------------------------------------  target    ERLDARDVIPGYELAALTNYVTLKPDAEIKGNPPPPPFTAGGQVVPTELRDAWGDFVWWDRATGRPRPVSRDEVGARFDG 4ydd.1    --------------------------------------------------------------------------------  target    DPALLGEFEVELVDGSTVPVRPAFDLLKQYLDESFDLRTASEVCRVPPQAIQSIARQLAANKRETLLAAGMGPNHYFQND 4ydd.1    --------------------------------------------------------------------------------  target    LFGRVQFLVAALTDNIGHLGGNVGSYAGNYRGSVFQAMGQWIAEDPFAIEPDLTKPATVKRYYKAESAHYWNYGERPLRA 4ydd.1    --------------------------------------------------------------------------------  target    VAKDDEGDLTKGEVLTGKSHMPTPTKLIWFGNSNSLLGNAKWSFDVVKNTLPRQDAVFCNEWHWTSSCEYADLVFPADSW 4ydd.1    --------------------------------------------------------------------------------  target    AEFKLPDATASCTNPFLLAFPTTPLKRLYDTRSDYEALALTAKALGELIDEPRMEQYWRGILDGDPTPYLQRIFSGSNAT 4ydd.1    --------------------------------------------------------------------------------  target    RGITYDELHESSKRGVPLLMNMRTYPRSGGWEQRQEDKPWYTATGRLEFYRPEPEFQAAGESLPVWREPVDATFYEPNAI 4ydd.1    --------------------------------------------------------------------------------  target    LSNAAHPSIAPRAPEDYGVPESQLDVETRQYRNVVRTWAELQQTLHPLQERDPAFRFVF 4ydd.1    ----------------------------------------------------------- ``` | | | | | | | | | | | | | | | | | | | | | | | | | | | | | | | | | | | | | | | | | | | | | | | | | |
|  | 5e7o.1.A | DMSO reductase family type II enzyme, molybdopterin subunit  *Crystal structure of the perchlorate reductase PcrAB mutant W461E of PcrA from Azospira suillum PS* | 0.01 |  | 16.36 | 0.06 | 306-360 | X-ray | 2.40 | hetero-oligomer | 4 x SF4, 1 x MO, 1 x MGD, 1 x MD1, 1 x F3S | HHblits | 0.28 |
| ``` target    AQGVSRRQLLGRALALGSGAALADLLGPARFLSPAGAATAGAVVPGNPLRVMPDRTWEQIYRNQFEDDSTFVFTCAPNDT 5e7o.1    --------------------------------------------------------------------------------  target    HNCLLRAHVKNGVVVRISPTYGYGEATDLYGNRASHRWDPRTCQKGLILSRRFYSERRVKAPMIRKGFKDWVEAGYPRND 5e7o.1    --------------------------------------------------------------------------------  target    DGTPQMDVTLRGSDDWIRISWDEATTIAAKTMEDVARTFNGDEGARKLLAQGYHPEMVEVMHGAGVQALKLRGGMPLLGI 5e7o.1    --------------------------------------------------------------------------------  target    GRIFGFYRFANMLALLDRKLRPDAPADEILGSRTFDNYAWHTDLPPGHPMVTGSQTVDFDLFSAEHTKLLLIIGMNWICT 5e7o.1    -----------------------------------------------------------------DPKVFFVYRGNWLNQ  target    KMPDGHWIGDARLKGTRVIVISADYMPTANKADEVIILRPGTDAAFFLGVARELIEKGLYDRAAVIERTDLPLLVRLDTG 5e7o.1    AKGQKYVLENLWPKLELIVDINIRMDSTALYSDVVLPSAH----------------------------------------  target    ERLDARDVIPGYELAALTNYVTLKPDAEIKGNPPPPPFTAGGQVVPTELRDAWGDFVWWDRATGRPRPVSRDEVGARFDG 5e7o.1    --------------------------------------------------------------------------------  target    DPALLGEFEVELVDGSTVPVRPAFDLLKQYLDESFDLRTASEVCRVPPQAIQSIARQLAANKRETLLAAGMGPNHYFQND 5e7o.1    --------------------------------------------------------------------------------  target    LFGRVQFLVAALTDNIGHLGGNVGSYAGNYRGSVFQAMGQWIAEDPFAIEPDLTKPATVKRYYKAESAHYWNYGERPLRA 5e7o.1    --------------------------------------------------------------------------------  target    VAKDDEGDLTKGEVLTGKSHMPTPTKLIWFGNSNSLLGNAKWSFDVVKNTLPRQDAVFCNEWHWTSSCEYADLVFPADSW 5e7o.1    --------------------------------------------------------------------------------  target    AEFKLPDATASCTNPFLLAFPTTPLKRLYDTRSDYEALALTAKALGELIDEPRMEQYWRGILDGDPTPYLQRIFSGSNAT 5e7o.1    --------------------------------------------------------------------------------  target    RGITYDELHESSKRGVPLLMNMRTYPRSGGWEQRQEDKPWYTATGRLEFYRPEPEFQAAGESLPVWREPVDATFYEPNAI 5e7o.1    --------------------------------------------------------------------------------  target    LSNAAHPSIAPRAPEDYGVPESQLDVETRQYRNVVRTWAELQQTLHPLQERDPAFRFVF 5e7o.1    ----------------------------------------------------------- ``` | | | | | | | | | | | | | | | | | | | | | | | | | | | | | | | | | | | | | | | | | | | | | | | | | |
|  | 1m2j.1.A | Silent Information Regulator 2  *Sir2 homologue H80N mutant-ADP ribose complex* | 0.01 |  | 18.87 | 0.06 | 303-357 | X-ray | 1.70 | monomer | 1 x ZN, 1 x APR | HHblits | 0.30 |
[truncated: 650,201 more chars]
