# Supplementary material for: Metabolic capacity is maintained despite shifts in microbial diversity in estuary sediments
Source: ISME Commun. 2025 Oct 11;5(1):ycaf182. doi: 10.1093/ismeco/ycaf182 (PMC12687941; doi:10.1093/ismeco/ycaf182)
Supplement: Supplementary_Data_1_ycaf182 [file supplementary_data_1_ycaf182.zip › SWISS-MODEL/4_1_May_SF_Bin52_scaffold_5716_c1_37766595_1/report.html]

4\_1\_May\_SF\_Bin52\_scaffold\_5716\_c1\_3776-6595\_1 | Report


|  |  |  |
| --- | --- | --- |
|  |  | SWISS-MODEL Homology Modelling Report |

## Model Building Report

This document lists the results for the homology modelling project "4\_1\_May\_SF\_Bin52\_scaffold\_5716\_c1\_3776-6595\_1" submitted to SWISS-MODEL workspace
on March 29, 2023, 8:37 p.m..The submitted primary amino acid sequence is given in Table T1.

If you use any results in your research, please cite the relevant publications:

- Waterhouse, A., Bertoni, M., Bienert, S., Studer, G., Tauriello, G., Gumienny, R.,
  Heer, F.T., de Beer, T.A.P., Rempfer, C., Bordoli, L., Lepore, R., Schwede, T.
  SWISS-MODEL: homology modelling of protein structures and complexes.
  Nucleic Acids Res. 46(W1), W296-W303 (2018).
- Bienert, S., Waterhouse, A., de Beer, T.A.P., Tauriello, G., Studer,
  G., Bordoli, L., Schwede, T. The SWISS-MODEL Repository - new features and
  functionality. Nucleic Acids Res. 45, D313-D319 (2017).
- Studer, G., Tauriello, G., Bienert, S.,
  Biasini, M., Johner, N., Schwede, T. ProMod3 - A versatile homology
  modelling toolbox. PLOS Comp. Biol. 17(1), e1008667 (2021).
- Studer, G., Rempfer, C., Waterhouse, A.M.,
  Gumienny, G., Haas, J., Schwede, T. QMEANDisCo - distance constraints
  applied on model quality estimation. Bioinformatics 36, 1765-1771 (2020).
- Bertoni, M., Kiefer, F., Biasini, M., Bordoli, L.,
  Schwede, T. Modeling protein quaternary structure of homo- and
  hetero-oligomers beyond binary interactions by homology. Scientific
  Reports 7 (2017).

## Results

The SWISS-MODEL template library (SMTL version 2023-03-23, PDB release 2023-03-17) was searched with
for evolutionary related structures matching the target sequence in Table T1. For details on the template search, see Materials and Methods. Overall 411 templates were found (Table T2).

## Models

The following models were built (see Materials and Methods "Model Building"):

| Model #02 | File | Built with | Oligo-State | Ligands | GMQE | QMEANDisCo Global |
| --- | --- | --- | --- | --- | --- | --- |
|  | PDB | ProMod3 3.2.1 | monomer | 1 x MO: MOLYBDENUM ATOM; | 0.68 | 0.69 ± 0.05 |

|  |  |  |
| --- | --- | --- |
|  |  |  |

| Template | Seq Identity | Oligo-state | QSQE | Found by | Method | Resolution | Seq Similarity | Range | Coverage | Description |
| --- | --- | --- | --- | --- | --- | --- | --- | --- | --- | --- |
| 7b04.1.B | 39.66 | monomer | 0.00 | HHblits | X-ray | 2.97Å | 0.40 | 29 - 938 | 0.95 | Nitrite oxidoreductase subunit A |

  

### Included Ligands

| Ligand | Description |
| --- | --- |
| 1 x MO | MOLYBDENUM ATOM |

  

### Excluded ligands

| Ligand Name.Number | Reason for Exclusion | Description |
| --- | --- | --- |
| CA.10 | Binding site not conserved. | CALCIUM ION |
| CA.11 | Binding site not conserved. | CALCIUM ION |
| F3S.4 | Binding site not conserved. | FE3-S4 CLUSTER |
| HEM.9 | Binding site not conserved. | PROTOPORPHYRIN IX CONTAINING FE |
| MD1.5 | Binding site not conserved. | PHOSPHORIC ACID 4-(2-AMINO-4-OXO-3,4,5,6,-TETRAHYDRO-PTERIDIN-6-YL)-2-HYDROXY-3,4-DIMERCAPTO-BUT-3-EN-YL ESTER GUANYLATE ESTER |
| MD1.6 | Binding site not conserved. | PHOSPHORIC ACID 4-(2-AMINO-4-OXO-3,4,5,6,-TETRAHYDRO-PTERIDIN-6-YL)-2-HYDROXY-3,4-DIMERCAPTO-BUT-3-EN-YL ESTER GUANYLATE ESTER |
| SF4.1 | Binding site not conserved. | IRON/SULFUR CLUSTER |
| SF4.2 | Binding site not conserved. | IRON/SULFUR CLUSTER |
| SF4.3 | Binding site not conserved. | IRON/SULFUR CLUSTER |
| SF4.8 | Binding site not conserved. | IRON/SULFUR CLUSTER |

  

```
Target    AQGVSRRQLLGRALALGSGAALADLLGPARFLSPAGAATAGAVVPGNPLRVMPDRTWEQIYRNQFEDDSTFVFTCAPNDT  
7b04.1.B  -MKLTRRAFLQVAGATGATLTLAKNAMAFRLLKP-------AVVVDNPLDTYPDRRWESVYRDQYQYDRTFTYCCSPNDT  
  
Target    HNCLLRAHVKNGVVVRISPTYGYGEATDLYGNRASHRWDPRTCQKGLILSRRFYSERRVKAPMIRKGFKDWVEAGYPRND  
7b04.1.B  HACRIRAFVRNNVMMRVEQNYDHQNYSDLYGNKATRNWNPRMCLKGYTFHRRVYGPYRLRYPLIRKGWKRWADDGFPELT  
  
Target    DGTP-QMDVTLRGSDDWIRISWDEATTIAAKTMEDVARTF-NGDEGARKLLAQGYHPEMVEVMHGAGVQALKLRGGMPLL  
7b04.1.B  PENKTKYMFDNRGNDELLRASWDEAFTYASKGIIHITKKYSGPEG-AQKLIDQGYPKEMVDRMQGAGTRTFKGRGGMGLL  
  
Target    GIGRIFGFYRFANMLALLDRKLRPDAPADEILGSRTFDNYAWHTDLPPGHPMVTGSQTVDFDLFSAEHTKLLLIIGMNWI  
7b04.1.B  GVIGKYGMYRFNNCLAIVDAHNRG-VGPDQALGGRNWSNYTWHGDQAPGHPFSHGLQTSDVDMNDVRFSKLLIQTGKNLI  
  
Target    CTKMPDGHWIGDARLKGTRVIVISADYMPTANKADEVIILRPGTDAAFFLGVARELIEKGLYDRAAVIERTDLPLLVRLD  
7b04.1.B  ENKMPEAHWVTEVMERGGKIVVITPEYSPSAQKADYWIPIRNNTDTALFLGITKILIDNKWYDADYVKKFTDFPLLIRTD  
  
Target    TGERLDARDVIPGYELAALTNYVTLKPDAEIKGNPPPPPFTAGGQVVPTELRDAWGDFVWWDRATGRPRPVSRDEVG---  
7b04.1.B  TLKRVSPKDIIPNYKLQDISD-----------G---PSYHIQG---LKDEQREIIGDFVVWDAKSKGPKAITRDDVGETL  
  
Target    ARFDGDPALLGEFEVELVDGSTVPVRPAFDLLKQYLDESFDLRTASEVCRVPPQAIQSIARQLAANKRETLLAAGMGPNH  
7b04.1.B  VKKGIDPVLEGSFKLKTIDGKEIEVMTLLEMYKIHLR-DYDIDSVVSMTNSPKDLIERLAKDIATIKPVA-IHYGEGVNH  
  
Target    YFQNDLFGRVQFLVAALTDNIGHLGGNVGSYAGNYRGSVFQA---MG-Q---WIAEDPFAIEPDLTKPA------TVKRY  
7b04.1.B  YFHATLMNRSYYLPVMLTGNVGYFGSGSHTWAGNYKAGNFQASKWSGPGFYGWVAEDVF--KPNL-DPYASAKDLNIKGR  
  
Target    YKAESAHYWNYGERPLRAVAKDDEGDLTKGEVLTGKSHMPTPTKLIWFGNSNSLLGNAKWSFDVVKNTLPRQDAVFCNEW  
7b04.1.B  ALDEEVAYWNHSERPLIV-NT---P-KYGRKVFTGKTHMPSPTKVLWFTNVNLINNAKH-VYQMLKNVNPNIEQIMSTDI  
  
Target    HWTSSCEYADLVFPADSWAEFKLPDATAS--CTNPFLLAFPTTPLKRLYDTRSDYEALALTAKALGELIDEPRMEQYWRG  
7b04.1.B  EITGSIEYADFAFPANSWVEFQ--EFEITNSCSNPFIQIWGKTGITPVYESKDDVKILAGMASKLGELLRDKRFEDNWKF  
  
Target    ILDGDPTPYLQRIFSGSNATRGITYDELHESSK--RGVPLLMNMRTYPRSGGWEQRQEDKPWYTATGRLEFYRPEPEFQA  
7b04.1.B  AIEGRASVYINRLLDGSTTMKGYTCEDILNGKYGEPGVAML-LFRTYPRHPFWEQVHESLPFYTPTGRLQAYNDEPEIIE  
  
Target    AGESLPVWREPVDATFYEPNAILSNAAHPSIAPRAPEDYGVPESQLDVETRQYRNVVRTWAELQQTLHPLQERDPAFRFV  
7b04.1.B  YGENFIVHREGPEATPYLPNAIVS--TNPYIR---PDDYGIPENAEYWEDRTVRNIKKSWEETKKTKNFLWE--KGYHFY  
  
Target    F  
7b04.1.B  -
```

  


---

  

| Model #01 | File | Built with | Oligo-State | Ligands | GMQE | QMEANDisCo Global |
| --- | --- | --- | --- | --- | --- | --- |
|  | PDB | ProMod3 3.2.1 | monomer | 1 x MO: MOLYBDENUM ATOM; | 0.67 | 0.68 ± 0.05 |

|  |  |  |
| --- | --- | --- |
|  |  |  |

| Template | Seq Identity | Oligo-state | QSQE | Found by | Method | Resolution | Seq Similarity | Range | Coverage | Description |
| --- | --- | --- | --- | --- | --- | --- | --- | --- | --- | --- |
| 7b04.1.B | 41.29 | monomer | 0.00 | BLAST | X-ray | 2.97Å | 0.41 | 47 - 938 | 0.91 | Nitrite oxidoreductase subunit A |

  

### Included Ligands

| Ligand | Description |
| --- | --- |
| 1 x MO | MOLYBDENUM ATOM |

  

### Excluded ligands

| Ligand Name.Number | Reason for Exclusion | Description |
| --- | --- | --- |
| CA.10 | Binding site not conserved. | CALCIUM ION |
| CA.11 | Binding site not conserved. | CALCIUM ION |
| F3S.4 | Binding site not conserved. | FE3-S4 CLUSTER |
| HEM.9 | Binding site not conserved. | PROTOPORPHYRIN IX CONTAINING FE |
| MD1.5 | Binding site not conserved. | PHOSPHORIC ACID 4-(2-AMINO-4-OXO-3,4,5,6,-TETRAHYDRO-PTERIDIN-6-YL)-2-HYDROXY-3,4-DIMERCAPTO-BUT-3-EN-YL ESTER GUANYLATE ESTER |
| MD1.6 | Binding site not conserved. | PHOSPHORIC ACID 4-(2-AMINO-4-OXO-3,4,5,6,-TETRAHYDRO-PTERIDIN-6-YL)-2-HYDROXY-3,4-DIMERCAPTO-BUT-3-EN-YL ESTER GUANYLATE ESTER |
| SF4.1 | Binding site not conserved. | IRON/SULFUR CLUSTER |
| SF4.2 | Binding site not conserved. | IRON/SULFUR CLUSTER |
| SF4.3 | Binding site not conserved. | IRON/SULFUR CLUSTER |
| SF4.8 | Binding site not conserved. | IRON/SULFUR CLUSTER |

  

```
Target    AQGVSRRQLLGRALALGSGAALADLLGPARFLSPAGAATAGAVVPGNPLRVMPDRTWEQIYRNQFEDDSTFVFTCAPNDT  
7b04.1.B  ----------------------------------------------NPLDTYPDRRWESVYRDQYQYDRTFTYCCSPNDT  
  
Target    HNCLLRAHVKNGVVVRISPTYGYGEATDLYGNRASHRWDPRTCQKGLILSRRFYSERRVKAPMIRKGFKDWVEAGYPRND  
7b04.1.B  HACRIRAFVRNNVMMRVEQNYDHQNYSDLYGNKATRNWNPRMCLKGYTFHRRVYGPYRLRYPLIRKGWKRWADDGFP---  
  
Target    DGTPQMDVTL----RGSDDWIRISWDEATTIAAKTMEDVARTFNGDEGARKLLAQGYHPEMVEVMHGAGVQALKLRGGMP  
7b04.1.B  ELTPENKTKYMFDNRGNDELLRASWDEAFTYASKGIIHITKKYSGPEGAQKLIDQGYPKEMVDRMQGAGTRTFKGRGGMG  
  
Target    LLGIGRIFGFYRFANMLALLDRKLRPDAPADEILGSRTFDNYAWHTDLPPGHPMVTGSQTVDFDLFSAEHTKLLLIIGMN  
7b04.1.B  LLGVIGKYGMYRFNNCLAIVDAHNRGVGP-DQALGGRNWSNYTWHGDQAPGHPFSHGLQTSDVDMNDVRFSKLLIQTGKN  
  
Target    WICTKMPDGHWIGDARLKGTRVIVISADYMPTANKADEVIILRPGTDAAFFLGVARELIEKGLYDRAAVIERTDLPLLVR  
7b04.1.B  LIENKMPEAHWVTEVMERGGKIVVITPEYSPSAQKADYWIPIRNNTDTALFLGITKILIDNKWYDADYVKKFTDFPLLIR  
  
Target    LDTGERLDARDVIPGYELAALTNYVTLKPDAEIKGNPPPPPFTAGGQVVPTELRDAWGDFVWWDRATGRPRPVSRDEVG-  
7b04.1.B  TDTLKRVSPKDIIPNYKLQDISD----GPSYHIQG-------------LKDEQREIIGDFVVWDAKSKGPKAITRDDVGE  
  
Target    --ARFDGDPALLGEFEVELVDGSTVPVRPAFDLLKQYLDESFDLRTASEVCRVPPQAIQSIARQLAANKRETLLAAGMGP  
7b04.1.B  TLVKKGIDPVLEGSFKLKTIDGKEIEVMTLLEMYKIHLRD-YDIDSVVSMTNSPKDLIERLAKDIATIK-PVAIHYGEGV  
  
Target    NHYFQNDLFGRVQFLVAALTDNIGHLGGNVGSYAGNYRGSVFQA-------MGQWIAEDPFAIEPDL-----TKPATVKR  
7b04.1.B  NHYFHATLMNRSYYLPVMLTGNVGYFGSGSHTWAGNYKAGNFQASKWSGPGFYGWVAEDVF--KPNLDPYASAKDLNIKG  
  
Target    YYKAESAHYWNYGERPLRAVAKDDEGDLTKGEVLTGKSHMPTPTKLIWFGNSNSLLGNAKWSFDVVKNTLPRQDAVFCNE  
7b04.1.B  RALDEEVAYWNHSERPL-IVNTPKYGR----KVFTGKTHMPSPTKVLWFTNVN-LINNAKHVYQMLKNVNPNIEQIMSTD  
  
Target    WHWTSSCEYADLVFPADSWAEFKLPDATASCTNPFLLAFPTTPLKRLYDTRSDYEALALTAKALGELIDEPRMEQYWRGI  
7b04.1.B  IEITGSIEYADFAFPANSWVEFQEFEITNSCSNPFIQIWGKTGITPVYESKDDVKILAGMASKLGELLRDKRFEDNWKFA  
  
Target    LDGDPTPYLQRIFSGSNATRGITYDEL--HESSKRGVPLLMNMRTYPRSGGWEQRQEDKPWYTATGRLEFYRPEPEFQAA  
7b04.1.B  IEGRASVYINRLLDGSTTMKGYTCEDILNGKYGEPGVAMLL-FRTYPRHPFWEQVHESLPFYTPTGRLQAYNDEPEIIEY  
  
Target    GESLPVWREPVDATFYEPNAILSNAAHPSIAPRAPEDYGVPESQLDVETRQYRNVVRTWAELQQTLHPLQERDPAFRFVF  
7b04.1.B  GENFIVHREGPEATPYLPNAIVS--TNPYI---RPDDYGIPENAEYWEDRTVRNIKKSWEETKKTKNFLWEKGYHFYCV-
```

  


---

  

| Model #03 | File | Built with | Oligo-State | Ligands | GMQE | QMEANDisCo Global |
| --- | --- | --- | --- | --- | --- | --- |
|  | PDB | ProMod3 3.2.1 | monomer | 1 x 6MO: MOLYBDENUM(VI) ION; | 0.40 | 0.54 ± 0.05 |

|  |  |  |
| --- | --- | --- |
|  |  |  |

| Template | Seq Identity | Oligo-state | QSQE | Found by | Method | Resolution | Seq Similarity | Range | Coverage | Description |
| --- | --- | --- | --- | --- | --- | --- | --- | --- | --- | --- |
| 3ir5.1.A | 25.36 | monomer | 0.00 | HHblits | X-ray | 2.30Å | 0.33 | 54 - 767 | 0.67 | Respiratory nitrate reductase 1 alpha chain |

  

### Included Ligands

| Ligand | Description |
| --- | --- |
| 1 x 6MO | MOLYBDENUM(VI) ION |

  

### Excluded ligands

| Ligand Name.Number | Reason for Exclusion | Description |
| --- | --- | --- |
| AGA.5 | Binding site not conserved. | (1S)-2-{[{[(2S)-2,3-DIHYDROXYPROPYL]OXY}(HYDROXY)PHOSPHORYL]OXY}-1-[(PENTANOYLOXY)METHYL]ETHYL OCTANOATE |
| F3S.9 | Binding site not conserved. | FE3-S4 CLUSTER |
| HEM.10 | Binding site not conserved. | PROTOPORPHYRIN IX CONTAINING FE |
| HEM.11 | Binding site not conserved. | PROTOPORPHYRIN IX CONTAINING FE |
| MD1.1 | Binding site not conserved. | PHOSPHORIC ACID 4-(2-AMINO-4-OXO-3,4,5,6,-TETRAHYDRO-PTERIDIN-6-YL)-2-HYDROXY-3,4-DIMERCAPTO-BUT-3-EN-YL ESTER GUANYLATE ESTER |
| MD1.2 | Binding site not conserved. | PHOSPHORIC ACID 4-(2-AMINO-4-OXO-3,4,5,6,-TETRAHYDRO-PTERIDIN-6-YL)-2-HYDROXY-3,4-DIMERCAPTO-BUT-3-EN-YL ESTER GUANYLATE ESTER |
| SF4.4 | Binding site not conserved. | IRON/SULFUR CLUSTER |
| SF4.6 | Binding site not conserved. | IRON/SULFUR CLUSTER |
| SF4.7 | Binding site not conserved. | IRON/SULFUR CLUSTER |
| SF4.8 | Binding site not conserved. | IRON/SULFUR CLUSTER |

  

```
Target    AQGVSRRQLLGRALALGSGAALADLLGPARFLSPAGAATAGAVVPGNPLRVMPDRTWEQIYRNQFEDDSTFVFTCAPNDT  
3ir5.1.A  -----------------------------------------------------NRDWEDGYRQRWQHDKIVRSTCGVNCT  
  
Target    HNCLLRAHVKNGVVVRISPTYGYGEATDLYGNRASHRWDPRTCQKGLILSRRFYSERRVKAPMIRKGF-KDWVEAGY---  
3ir5.1.A  GSCSWKIYVKNGLVTWETQQTDYPR-----TRPDLPNHEPRGCPRGASYSWYLYSANRLKYPMMRKRLMKMWREAKALHS  
  
Target    -PR-------NDDGTPQMDVTLRGSDDWIRISWDEATTIAAKTMEDVARTFNGDEGARKLLAQGYHPEMVEVMHGAGVQA  
3ir5.1.A  DPVEAWASIIEDADKAKSFKQARGRGGFVRSSWQEVNELIAASNVYTIKNYG--------------PDRVAGFSPIPAMS  
  
Target    LKLRGGMPLLGIGRIFGFYRFANMLALLDRKLRPDAPADEILGSRTFDNYAWHTDLPPGHPMVTGSQTVDFDLFSAEHTK  
3ir5.1.A  MV-----------SYASGARYLS-----------------LIGGTCLSFYDWYCDLPPASPQTWGEQTDVPESADWYNSS  
  
Target    LLLIIGMNWICTKMPDGHWIGDARLKGTRVIVISADYMPTANKADEVIILRPGTDAAFFLGVARELIEKGL------YDR  
3ir5.1.A  YIIAWGSNVPQTRTPDAHFFTEVRYKGTKTVAVTPDYAEIAKLCDLWLAPKQGTDAAMALAMGHVMLREFHLDNPSQYFT  
  
Target    AAVIERTDLPLLVRLD-------TGERLDARDVIPGYELAALTNYVTLKPDAEIKGNPPPPPFTAGGQVVPTELRDAWGD  
3ir5.1.A  DYVRRYTDMPMLVMLEERDGYYAAGRMLRAADLVDALGQEN----------------------------------NPEWK  
  
Target    FVWWDRATGRPRPVSRDE--------------------------------------VG-ARFD-----------GDPALL  
3ir5.1.A  TVAFNT-NGEMVAPNGSIGFRWGEKGKWNLEQRDGKTGEETELQLSLLGSQDEIAEVGFPYFGGDGTEHFNKVELENVLL  
  
Target    G---EFEVELVDGSTVPVRPAFDLLK------------------QYLDESFDLRTASEVCRVPPQAIQSIARQLAAN---  
3ir5.1.A  HKLPVKRLQLADGSTALVTTVYDLTLANYGLERGLNDVNCATSYDDV-KAYTPAWAEQITGVSRSQIIRIAREFADNADK  
  
Target    --KRETLLAAGMGPNHYFQNDLFGRVQFLVAALTDNIGHLGGNVGSYAGNYRGSVFQAMGQWIAEDPFAIEP--------  
3ir5.1.A  THGR-SMIIVGAGLNHWYHLDMNYRGLINMLIFCGCVGQSGGGWAHYVGQEKLRPQTGWQPLAFALDWQRPARHMNSTSY  
  
Target    ----------------DLTKPA-TVKRYYK--------AESAHYW----NYGERPLRAVAKDD-----EGDLTKGEVLTG  
3ir5.1.A  FYNHSSQWRYETVTAEELLSPMADKSRYTGHLIDFNVRAERMGWLPSAPQLGTNPLTIAGEAEKAGMNPVDYTVKSLKEG  
  
Target    KS--------HMPTPTKLIWFGNSNSLLGNAKWSFDVV------------------------------KNTLPRQDAVFC  
3ir5.1.A  SIRFAAEQPENGKNHPRNLFIWRSNLLGSSGKGHEFMLKYLLGTEHGIQGKDLGQQGGVKPEEVDWQDNGLEGKLDLVVT  
  
Target    NEWHWTSSCEYADLVFPADSWAEFKLPDATASCTNPFLLAFPTTPLKRLYDTRSDYEALALTAKALGELIDEPRMEQYWR  
3ir5.1.A  LDFRLSSTCLYSDIILPTATWYEK--DDMNTSDMHPFIHPL-SAAVDPAWEAKSDWEIYKAIAKKFSE------------  
  
Target    GILDGDPTPYLQRIFSGSNATRGITYDELHESSKRGVPLLMNMRTYPRSGGWEQRQEDKPWYTATGRLEFYRPEPEFQAA  
3ir5.1.A  --------------------------------------------------------------------------------  
  
Target    GESLPVWREPVDATFYEPNAILSNAAHPSIAPRAPEDYGVPESQLDVETRQYRNVVRTWAELQQTLHPLQERDPAFRFVF  
3ir5.1.A  --------------------------------------------------------------------------------
```

  


---

  

| Model #04 | File | Built with | Oligo-State | Ligands | GMQE | QMEANDisCo Global |
| --- | --- | --- | --- | --- | --- | --- |
|  | PDB | ProMod3 3.2.1 | monomer | None | 0.37 | 0.48 ± 0.05 |

|  |  |  |
| --- | --- | --- |
|  |  |  |

| Template | Seq Identity | Oligo-state | QSQE | Found by | Method | Resolution | Seq Similarity | Range | Coverage | Description |
| --- | --- | --- | --- | --- | --- | --- | --- | --- | --- | --- |
| 6sdv.1.A | 18.11 | monomer | 0.00 | HHblits | X-ray | 1.90Å | 0.29 | 54 - 767 | 0.64 | Formate dehydrogenase, alpha subunit, selenocysteine-containing,Formate dehydrogenase, alpha subunit, selenocysteine-containing,W-formate dehydrogenase - alpha subunit |

  

### Excluded ligands

| Ligand Name.Number | Reason for Exclusion | Description |
| --- | --- | --- |
| GOL.6 | Not biologically relevant. | GLYCEROL |
| GOL.7 | Not biologically relevant. | GLYCEROL |
| GOL.8 | Not biologically relevant. | GLYCEROL |
| GOL.9 | Not biologically relevant. | GLYCEROL |
| GOL.10 | Not biologically relevant. | GLYCEROL |
| GOL.11 | Not biologically relevant. | GLYCEROL |
| GOL.12 | Not biologically relevant. | GLYCEROL |
| GOL.13 | Not biologically relevant. | GLYCEROL |
| GOL.14 | Not biologically relevant. | GLYCEROL |
| GOL.15 | Not biologically relevant. | GLYCEROL |
| H2S.5 | Binding site not conserved. | HYDROSULFURIC ACID |
| MGD.1 | Binding site not conserved. | 2-AMINO-5,6-DIMERCAPTO-7-METHYL-3,7,8A,9-TETRAHYDRO-8-OXA-1,3,9,10-TETRAAZA-ANTHRACEN-4-ONE GUANOSINE DINUCLEOTIDE |
| MGD.2 | Binding site not conserved. | 2-AMINO-5,6-DIMERCAPTO-7-METHYL-3,7,8A,9-TETRAHYDRO-8-OXA-1,3,9,10-TETRAAZA-ANTHRACEN-4-ONE GUANOSINE DINUCLEOTIDE |
| NO3.16 | Not biologically relevant. | NITRATE ION |
| NO3.17 | Not biologically relevant. | NITRATE ION |
| PEG.18 | Not biologically relevant. | DI(HYDROXYETHYL)ETHER |
| SF4.3 | Binding site not conserved. | IRON/SULFUR CLUSTER |
| SF4.19 | Binding site not conserved. | IRON/SULFUR CLUSTER |
| SF4.20 | Binding site not conserved. | IRON/SULFUR CLUSTER |
| SF4.21 | Binding site not conserved. | IRON/SULFUR CLUSTER |
| W.4 | Not in contact with model. | TUNGSTEN ION |

  

```
Target    AQGVSRRQLLGRALALGSGAALADLLGPARFLSPAGAATAGAVVPGNPLRVMPDRTWEQIYRNQFEDDSTFVFTCAPNDT  
6sdv.1.A  -MTVTRRHFLKLSAGAAVAGAFT-----------------GLGLSLAPTVARAELQ-----KL--QWA-KQTTSICCYCA  
  
Target    HNCLLRAHVK---NGVVVRISPTYGYGEATDLYGNRASHRWDPRTCQKGLILSRRFYSERRVKAPMIRKGFKDWVEAGYP  
6sdv.1.A  VGCGLIVHTAKDGQGRAVNVEGD------------PDHPINEGSLCPKGASIFQLGENDQRGTQPLYRAP----------  
  
Target    RNDDGTPQMDVTLRGSDDWIRISWDEATTIAAKTMEDVARTFNGDEGARKLLAQGYHPEMVEVMHGAGVQALKLRGGMPL  
6sdv.1.A  --------------FSDTWKPVTWDFALTEIAKRIKKTRDASFTEKNAAGDL--VNRTEAIASFG---------------  
  
Target    LGIGRIFGFYRFANMLALLDRKLRPDAPADEILGSRTFDNYAWHTD--LPPGHPMVTGSQTVDFDLFSAEHTKLLLIIGM  
6sdv.1.A  --------SAAMDNEECWAYGNIL------RSLGLVYIEHQARIUHSPTVPALAESFGRGAMTNHWNDLANSDCILIMGS  
  
Target    NWICTKMPDGHWIGDARLKGTRVIVISADYMPTANKADEVIILRPGTDAAFFLGVARELIEKGLYDRAAVIERTDLPLLV  
6sdv.1.A  NAAENHPIAFKWVLRAKDKGATLIHVDPRFTRTSARCDVYAPIRSGADIPFLGGLIKYILDNKLYFTDYVREYTNASLIV  
  
Target    RLDTGERLDARDVIPGYELAALTNYVTLKPDAEIKGNPPPPPFTAGGQVVPTELRDAWGDFVWWDRATGRPRPVSRDEVG  
6sdv.1.A  GEKFS---FKDGLFSGYDAA-N---------------------------------KKYDKS-MWAFE------LD--A--  
  
Target    ARFDGDPALLGEFEVELVDGSTVPVRPAFDLLKQYLDESFDLRTASEVCRVPPQAIQSIARQLAANK---RETLLAAGMG  
6sdv.1.A  ---------NG---VPKRDPALKHPRCVINLLKKHYE-RYNLDKVAAITGTSKEQLQQVYKAYAATGKPDKAGTIMYAMG  
  
Target    PNHYFQNDLFGRVQFLVAALTDNIGHLGGNVGSYAGNY--RGSVFQAMGQWIAEDPFAI-EPDLT-------KPATVKRY  
6sdv.1.A  WTQHSVGVQNIRAMAMIQLLLGNIGVAGGGVNALRGESNVQGST--DQGLLAHIWPGYNPVPNSKAATLELYNAATPQSK  
  
Target    YKAESAHYWNYGER--------------PL---RAVAKDDE-G---DLTKGEVLTGKSHMPTPTKLIWFGNSNSLLGNAK  
6sdv.1.A  DP-MSVNWWQNRPKYVASYLKALYPDEEPAAAYDYLPRIDAGRKLTDYFWLNIFEK--MDKGEFKGLFAWGMNPACGGAN  
  
Target    WSFDVVKNTLPRQDAVFCNEWHWTSSCEY--------AD-----LVFPADSWAEFKLPDATASCTNPFLLAFPTTPLKRL  
6sdv.1.A  --ANKNRKAMGKLEWLVNVNLFENETSSFWKGPGMNPAEIGTEVFFLPCCVSIEKE--GSV-ANSGRWMQW-RYRGPKPY  
  
Target    YDTRSDYEALALTAKALGELIDEPRMEQYWRGILDGDPTPYLQRIFSGSNATRGITYDELHESSKRGVPLLMNMRTYPRS  
6sdv.1.A  AETKPDGDIMLDMFKKVRE-------------------------------------------------------------  
  
Target    GGWEQRQEDKPWYTATGRLEFYRPEPEFQAAGESLPVWREPVDATFYEPNAILSNAAHPSIAPRAPEDYGVPESQLDVET  
6sdv.1.A  --------------------------------------------------------------------------------  
  
Target    RQYRNVVRTWAELQQTLHPLQERDPAFRFVF  
6sdv.1.A  -------------------------------
```

  


---

  

| Model #05 | File | Built with | Oligo-State | Ligands | GMQE | QMEANDisCo Global |
| --- | --- | --- | --- | --- | --- | --- |
|  | PDB | ProMod3 3.2.1 | monomer | 1 x 6MO: MOLYBDENUM(VI) ION; | 0.26 | 0.45 ± 0.05 |

|  |  |  |
| --- | --- | --- |
|  |  |  |

| Template | Seq Identity | Oligo-state | QSQE | Found by | Method | Resolution | Seq Similarity | Range | Coverage | Description |
| --- | --- | --- | --- | --- | --- | --- | --- | --- | --- | --- |
| 3ir7.1.A | 31.57 | monomer | 0.00 | BLAST | X-ray | 2.50Å | 0.36 | 54 - 630 | 0.54 | Respiratory nitrate reductase 1 alpha chain |

  

### Included Ligands

| Ligand | Description |
| --- | --- |
| 1 x 6MO | MOLYBDENUM(VI) ION |

  

### Excluded ligands

| Ligand Name.Number | Reason for Exclusion | Description |
| --- | --- | --- |
| AGA.5 | Binding site not conserved. | (1S)-2-{[{[(2S)-2,3-DIHYDROXYPROPYL]OXY}(HYDROXY)PHOSPHORYL]OXY}-1-[(PENTANOYLOXY)METHYL]ETHYL OCTANOATE |
| F3S.9 | Binding site not conserved. | FE3-S4 CLUSTER |
| HEM.10 | Binding site not conserved. | PROTOPORPHYRIN IX CONTAINING FE |
| HEM.11 | Binding site not conserved. | PROTOPORPHYRIN IX CONTAINING FE |
| MD1.1 | Binding site not conserved. | PHOSPHORIC ACID 4-(2-AMINO-4-OXO-3,4,5,6,-TETRAHYDRO-PTERIDIN-6-YL)-2-HYDROXY-3,4-DIMERCAPTO-BUT-3-EN-YL ESTER GUANYLATE ESTER |
| MD1.2 | Binding site not conserved. | PHOSPHORIC ACID 4-(2-AMINO-4-OXO-3,4,5,6,-TETRAHYDRO-PTERIDIN-6-YL)-2-HYDROXY-3,4-DIMERCAPTO-BUT-3-EN-YL ESTER GUANYLATE ESTER |
| SF4.3 | Binding site not conserved. | IRON/SULFUR CLUSTER |
| SF4.6 | Binding site not conserved. | IRON/SULFUR CLUSTER |
| SF4.7 | Binding site not conserved. | IRON/SULFUR CLUSTER |
| SF4.8 | Binding site not conserved. | IRON/SULFUR CLUSTER |

  

```
Target    AQGVSRRQLLGRALALGSGAALADLLGPARFLSPAGAATAGAVVPGNPLRVMPDRTWEQIYRNQFEDDSTFVFTCAPNDT  
3ir7.1.A  -----------------------------------------------------NRDWEDGYRQRWQHDKIVRSTHGVNCT  
  
Target    HNCLLRAHVKNGVVVRISPTYGYGEATDLYGNRASHRWDPRTCQKGLILSRRFYSERRVKAPMIRKGF-KDWVEAGYPRN  
3ir7.1.A  GSCSWKIYVKNGLVTWETQQTDYPRTRPDLPNH-----EPRGCPSGASYSWYLYSANRLKYPMMRKRLMKMWREAKALHS  
  
Target    DDGTPQMDVT-----------LRGSDDWIRISWDEATTIAAKTMEDVARTFNGDEGARKLLAQGYHP--EMVEVMHGAGV  
3ir7.1.A  DPVEAWASIIEDADKAKSFKQARGRGGFVRSSWQEVNELIAASNVYTIKNYGPDRVA------GFSPIPAMSMVSYASGA  
  
Target    QALKLRGGMPLLGIGRIFGFYRFANMLALLDRKLRPDAPADEILGSRTFDNYAWHTDLPPGHPMVTGSQTVDFDLFSAEH  
3ir7.1.A  RYLSLIGGTCL------------------------------------SF--YDWYCDLPPASPQTWGEQTDVPESADWYN  
  
Target    TKLLLIIGMNWICTKMPDGHWIGDARLKGTRVIVISADYMPTANKADEVIILRPGTDAAFFLGVARELIEKGLYDRAA--  
3ir7.1.A  SSYIIAWGSNVPQTRTPDAHFFTEVRYKGTKTVAVTPDYAEIAKLCDLWLAPKQGTDAAMALAMGHVMLREFHLDNPSQY  
  
Target    ----VIERTDLPLLVRLDTGERLDARDVIPGYELAALTNYVTLKPDAEIKGNPP---PPPFTAGGQVVP--TELRDAWGD  
3ir7.1.A  FTDYVRRYTDMPMLVMLE------ERD---GYYAAGRMLRAADLVDALGQENNPEWKTVAFNTNGEMVAPNGSIGFRWGE  
  
Target    FVWW-----DRATGRPRPV------SRDEVGA----RFDGD-----------PALLGEFEV---ELVDGSTVPVRPAFDL  
3ir7.1.A  KGKWNLEQRDGKTGEETELQLSLLGSQDEIAEVGFPYFGGDGTEHFNKVELENVLLHKLPVKRLQLADGSTALVTTVYDL  
  
Target    ------LKQYLDE-----SFDLRTA------SEVCRVPPQAIQSIARQLAANKRET----LLAAGMGPNHYFQNDLFGRV  
3ir7.1.A  TLANYGLERGLNDVNCATSYDDVKAYTPAWAEQITGVSRSQIIRIAREFADNADKTHGRSMIIVGAGLNHWYHLDMNYRG  
  
Target    QFLVAALTDNIGHLGGNVGSYAGNYRGSVFQAMGQWIAEDPFAIEPDLTKPATVKRYYKAESAHYWNYGERPLRAVAKDD  
3ir7.1.A  LINMLIFCGCVGQSGGGWAHYVGQEK---LRPQTGW---QPLAFALDWQRPA---RHMNSTSYFY---------------  
  
Target    EGDLTKGEVLTGKSHMPTPTKLIWFGNSNSLLGNAKWSFDVVKNTLPRQDAVFCNEWHWTSSCEYADLVFPADSWAEFKL  
3ir7.1.A  --------------------------------------------------------------------------------  
  
Target    PDATASCTNPFLLAFPTTPLKRLYDTRSDYEALALTAKALGELIDEPRMEQYWRGILDGDPTPYLQRIFSGSNATRGITY  
3ir7.1.A  --------------------------------------------------------------------------------  
  
Target    DELHESSKRGVPLLMNMRTYPRSGGWEQRQEDKPWYTATGRLEFYRPEPEFQAAGESLPVWREPVDATFYEPNAILSNAA  
3ir7.1.A  --------------------------------------------------------------------------------  
  
Target    HPSIAPRAPEDYGVPESQLDVETRQYRNVVRTWAELQQTLHPLQERDPAFRFVF  
3ir7.1.A  ------------------------------------------------------
```

  


---

  

## Materials and Methods

## Template Search

Template search with
has been performed against the SWISS-MODEL template library (SMTL, last update: 2023-03-23, last included PDB release: 2023-03-17).

## Template Selection

For each identified template, the template's quality has been predicted from features of the target-template alignment.
The templates with the highest quality have then been selected for model building.

## Model Building

Models are built based on the target-template alignment using ProMod3 (Studer et al.). Coordinates which are conserved between the target and the template are copied from the template to the model. Insertions and deletions are remodelled using a fragment library. Side chains are then rebuilt. Finally, the geometry of the resulting model is regularized by using a force field.

## Model Quality Estimation

The global and per-residue model quality has been assessed using the QMEAN scoring function (Studer et al.).

## Ligand Modelling

Ligands present in the template structure are transferred by homology to the model when the following criteria are met: (a) The ligands are annotated as biologically relevant in the template library, (b) the ligand is in contact with the model, (c) the ligand is not clashing with the protein, (d) the residues in contact with the ligand are conserved between the target and the template. If any of these four criteria is not satisfied, a certain ligand will not be included in the model. The model summary includes information on why and which ligand has not been included.

## Oligomeric State Conservation

The quaternary structure annotation of the template is used to model the target sequence in its oligomeric form. The method (Bertoni et al.) is based on a supervised machine learning algorithm, Support Vector Machines (SVM), which combines interface conservation, structural clustering, and other template features to provide a quaternary structure quality estimate (QSQE). The QSQE score is a number between 0 and 1, reflecting the expected accuracy of the interchain contacts for a model built based a given alignment and template. Higher numbers indicate higher reliability. This complements the GMQE score which estimates the accuracy of the tertiary structure of the resulting model.

## References

- **BLAST**  
  Camacho, C., Coulouris, G., Avagyan, V., Ma, N., Papadopoulos, J.,
  Bealer, K., Madden, T.L. BLAST+: architecture and applications. BMC
  Bioinformatics 10, 421-430 (2009).
- **HHblits**  
  Steinegger, M., Meier, M., Mirdita, M., Vöhringer, H., Haunsberger,
  S. J., Söding, J. HH-suite3 for fast remote homology detection and
  deep protein annotation. BMC Bioinformatics 20, 473 (2019).

## Table T1:

Primary amino acid sequence for which templates were searched and models were built.

AQGVSRRQLLGRALALGSGAALADLLGPARFLSPAGAATAGAVVPGNPLRVMPDRTWEQIYRNQFEDDSTFVFTCAPNDTHNCLLRAHVKNGVVVRISPT  
YGYGEATDLYGNRASHRWDPRTCQKGLILSRRFYSERRVKAPMIRKGFKDWVEAGYPRNDDGTPQMDVTLRGSDDWIRISWDEATTIAAKTMEDVARTFN  
GDEGARKLLAQGYHPEMVEVMHGAGVQALKLRGGMPLLGIGRIFGFYRFANMLALLDRKLRPDAPADEILGSRTFDNYAWHTDLPPGHPMVTGSQTVDFD  
LFSAEHTKLLLIIGMNWICTKMPDGHWIGDARLKGTRVIVISADYMPTANKADEVIILRPGTDAAFFLGVARELIEKGLYDRAAVIERTDLPLLVRLDTG  
ERLDARDVIPGYELAALTNYVTLKPDAEIKGNPPPPPFTAGGQVVPTELRDAWGDFVWWDRATGRPRPVSRDEVGARFDGDPALLGEFEVELVDGSTVPV  
RPAFDLLKQYLDESFDLRTASEVCRVPPQAIQSIARQLAANKRETLLAAGMGPNHYFQNDLFGRVQFLVAALTDNIGHLGGNVGSYAGNYRGSVFQAMGQ  
WIAEDPFAIEPDLTKPATVKRYYKAESAHYWNYGERPLRAVAKDDEGDLTKGEVLTGKSHMPTPTKLIWFGNSNSLLGNAKWSFDVVKNTLPRQDAVFCN  
EWHWTSSCEYADLVFPADSWAEFKLPDATASCTNPFLLAFPTTPLKRLYDTRSDYEALALTAKALGELIDEPRMEQYWRGILDGDPTPYLQRIFSGSNAT  
RGITYDELHESSKRGVPLLMNMRTYPRSGGWEQRQEDKPWYTATGRLEFYRPEPEFQAAGESLPVWREPVDATFYEPNAILSNAAHPSIAPRAPEDYGVP  
ESQLDVETRQYRNVVRTWAELQQTLHPLQERDPAFRFVF

## Table T2:

| Template | Seq Identity | Oligo-state | QSQE | Found by | Method | Resolution | Seq Similarity | Coverage | Description |
| --- | --- | --- | --- | --- | --- | --- | --- | --- | --- |
| 7b04.1.B | 41.29 | monomer | - | BLAST | X-ray | 2.97Å | 0.41 | 0.91 | Nitrite oxidoreductase subunit A |
| 7b04.2.B | 41.29 | monomer | - | BLAST | X-ray | 2.97Å | 0.41 | 0.91 | Nitrite oxidoreductase subunit A |
| 7b04.1.B | 39.66 | monomer | - | HHblits | X-ray | 2.97Å | 0.40 | 0.95 | Nitrite oxidoreductase subunit A |
| 7b04.2.B | 39.66 | monomer | - | HHblits | X-ray | 2.97Å | 0.40 | 0.95 | Nitrite oxidoreductase subunit A |
| 5e7o.1.A | 26.60 | monomer | - | HHblits | X-ray | 2.40Å | 0.33 | 0.75 | DMSO reductase family type II enzyme, molybdopterin subunit |
| 4ydd.1.A | 26.60 | monomer | - | HHblits | X-ray | 1.86Å | 0.34 | 0.75 | DMSO reductase family type II enzyme, molybdopterin subunit |
| 2ivf.1.A | 25.73 | monomer | - | HHblits | X-ray | 1.88Å | 0.32 | 0.80 | ETHYLBENZENE DEHYDROGENASE ALPHA-SUBUNIT |
| 4ydd.1.A | 30.14 | monomer | - | BLAST | X-ray | 1.86Å | 0.36 | 0.75 | DMSO reductase family type II enzyme, molybdopterin subunit |
| 5e7o.1.A | 30.14 | monomer | - | BLAST | X-ray | 2.40Å | 0.36 | 0.75 | DMSO reductase family type II enzyme, molybdopterin subunit |
| 3ir5.1.A | 25.36 | monomer | - | HHblits | X-ray | 2.30Å | 0.33 | 0.67 | Respiratory nitrate reductase 1 alpha chain |
| 1q16.1.A | 25.56 | monomer | - | HHblits | X-ray | 1.90Å | 0.33 | 0.67 | Respiratory nitrate reductase 1 alpha chain |
| 1r27.4.A | 24.16 | homo-dimer | 0.19 | HHblits | X-ray | 2.00Å | 0.32 | 0.70 | Respiratory nitrate reductase 1 alpha chain |
| 3ir7.1.A | 24.01 | monomer | - | HHblits | X-ray | 2.50Å | 0.32 | 0.70 | Respiratory nitrate reductase 1 alpha chain |
| 3egw.1.A | 24.84 | homo-dimer | 0.17 | HHblits | X-ray | 1.90Å | 0.32 | 0.67 | Respiratory nitrate reductase 1 alpha chain |
| 3ir6.1.A | 24.20 | monomer | - | HHblits | X-ray | 2.80Å | 0.32 | 0.70 | Respiratory nitrate reductase 1 alpha chain |
| 2iv2.1.A | 20.18 | monomer | - | HHblits | X-ray | 2.27Å | 0.31 | 0.59 | Formate dehydrogenase H |
| 1aa6.1.A | 20.18 | monomer | - | HHblits | X-ray | 2.30Å | 0.31 | 0.59 | FORMATE DEHYDROGENASE H |
| 1e60.1.A | 21.31 | monomer | - | HHblits | X-ray | 2.00Å | 0.30 | 0.65 | Dimethyl sulfoxide/trimethylamine N-oxide reductase |
| 1fdo.1.A | 20.18 | monomer | - | HHblits | X-ray | 2.80Å | 0.31 | 0.59 | FORMATE DEHYDROGENASE H |
| 7z0t.1.G | 20.18 | monomer | - | HHblits | EM | NA | 0.31 | 0.59 | Formate dehydrogenase H |
| 4dmr.1.A | 21.02 | monomer | - | HHblits | X-ray | 1.90Å | 0.30 | 0.65 | DMSO REDUCTASE |
| 1e5v.2.A | 21.55 | monomer | - | HHblits | X-ray | 2.40Å | 0.30 | 0.65 | Dimethyl sulfoxide/trimethylamine N-oxide reductase |
| 6sdv.1.A | 18.11 | monomer | - | HHblits | X-ray | 1.90Å | 0.29 | 0.64 | Formate dehydrogenase, alpha subunit, selenocysteine-containing,Formate dehydrogenase, alpha subunit, selenocysteine-containing,W-formate dehydrogenase - alpha subunit |
| 6sdr.1.A | 18.18 | monomer | - | HHblits | X-ray | 2.10Å | 0.29 | 0.64 | Formate dehydrogenase, alpha subunit, selenocysteine-containing |
| 1e18.1.A | 21.88 | monomer | - | HHblits | X-ray | 2.00Å | 0.30 | 0.65 | DMSO REDUCTASE. |
| 8bqg.1.A | 18.52 | monomer | - | HHblits | X-ray | 1.95Å | 0.29 | 0.60 | Formate dehydrogenase, alpha subunit, selenocysteine-containing |
| 7l5s.1.A | 22.85 | monomer | - | HHblits | X-ray | 2.09Å | 0.31 | 0.55 | Trimethylamine-N-oxide reductase |
| 7l5i.1.A | 22.85 | monomer | - | HHblits | X-ray | 1.73Å | 0.31 | 0.55 | Trimethylamine-N-oxide reductase |
| 2ivf.1.A | 35.66 | monomer | - | BLAST | X-ray | 1.88Å | 0.38 | 0.44 | ETHYLBENZENE DEHYDROGENASE ALPHA-SUBUNIT |
| 3egw.1.A | 31.94 | homo-dimer | 0.09 | BLAST | X-ray | 1.90Å | 0.36 | 0.54 | Respiratory nitrate reductase 1 alpha chain |
| 3ir7.1.A | 31.57 | monomer | - | BLAST | X-ray | 2.50Å | 0.36 | 0.54 | Respiratory nitrate reductase 1 alpha chain |
| 3ir5.1.A | 31.76 | monomer | - | BLAST | X-ray | 2.30Å | 0.36 | 0.54 | Respiratory nitrate reductase 1 alpha chain |
| 1q16.1.A | 31.57 | monomer | - | BLAST | X-ray | 1.90Å | 0.36 | 0.54 | Respiratory nitrate reductase 1 alpha chain |
| 1r27.4.A | 31.57 | homo-dimer | 0.09 | BLAST | X-ray | 2.00Å | 0.36 | 0.54 | Respiratory nitrate reductase 1 alpha chain |
| 3ir6.1.A | 31.57 | monomer | - | BLAST | X-ray | 2.80Å | 0.36 | 0.54 | Respiratory nitrate reductase 1 alpha chain |
| 7nz1.1.E | 18.31 | monomer | - | HHblits | EM | NA | 0.27 | 0.47 | NADH-quinone oxidoreductase subunit G |
| 7p63.1.C | 18.76 | monomer | - | HHblits | EM | NA | 0.28 | 0.47 | NADH-quinone oxidoreductase |
| 7p61.1.C | 18.54 | monomer | - | HHblits | EM | NA | 0.27 | 0.47 | NADH-quinone oxidoreductase |
| 6yj4.1.G | 17.23 | monomer | - | HHblits | EM | NA | 0.29 | 0.44 | Subunit NUAM of NADH:Ubiquinone Oxidoreductase (Complex I) |
| 6rfs.1.A | 17.23 | monomer | - | HHblits | EM | 4.04Å | 0.29 | 0.44 | Subunit NUAM of NADH:Ubiquinone Oxidoreductase (Complex I) |
| 6rfq.1.A | 17.23 | monomer | - | HHblits | EM | 3.30Å | 0.29 | 0.44 | Subunit NUAM of NADH:Ubiquinone Oxidoreductase (Complex I) |
| 6gcs.1.A | 17.23 | monomer | - | HHblits | EM | 4.32Å | 0.29 | 0.44 | 75-KDA PROTEIN (NUAM) |
| 6zk9.1.C | 13.37 | monomer | - | HHblits | EM | NA | 0.27 | 0.45 | NADH:ubiquinone oxidoreductase core subunit S1 |
| 7zd6.1.4 | 13.37 | monomer | - | HHblits | EM | NA | 0.27 | 0.45 | NADH-ubiquinone oxidoreductase 75 kDa subunit, mitochondrial |
| 6qc5.1.C | 13.40 | monomer | - | HHblits | EM | NA | 0.27 | 0.45 | NADH:ubiquinone oxidoreductase core subunit S1 |
| 6qcf.1.C | 13.40 | monomer | - | HHblits | EM | NA | 0.27 | 0.45 | NADH:ubiquinone oxidoreductase core subunit S1 |
| 7qsd.1.G | 13.40 | monomer | - | HHblits | EM | NA | 0.27 | 0.45 | NADH-ubiquinone oxidoreductase 75 kDa subunit, mitochondrial |
| 7vxu.1.L | 13.16 | monomer | - | HHblits | EM | NA | 0.27 | 0.45 | NADH-ubiquinone oxidoreductase 75 kDa subunit, mitochondrial |
| 5o31.1.8 | 13.60 | monomer | - | HHblits | EM | 4.13Å | 0.27 | 0.45 | NADH-ubiquinone oxidoreductase 75 kDa subunit, mitochondrial |
| 7dgr.10.A | 13.60 | monomer | - | HHblits | EM | NA | 0.27 | 0.45 | NADH-ubiquinone oxidoreductase 75 kDa subunit, mitochondrial |

  
The table above shows the top 50 filtered templates. A further 281 templates were found which were considered to be less suitable for modelling than the filtered list.  
1aa6.1.A, 1be3.1.E, 1bgy.1.P, 1dms.1.A, 1e18.1.A, 1e5v.2.A, 1e60.1.A, 1eu1.1.A, 1fdo.1.A, 1g8j.1.A, 1g8k.1.A, 1h0h.1.A, 1ici.1.A, 1jeo.1.A, 1kb9.1.E, 1kqf.1.A, 1kqf.1.B, 1l0l.1.E, 1l0n.1.E, 1m2g.1.A, 1m2h.1.A, 1m2j.1.A, 1m2k.1.A, 1m2n.1.A, 1m2n.1.B, 1ma3.1.A, 1ntk.1.E, 1ntm.1.E, 1ogy.1.A, 1q16.1.A, 1q90.1.E, 1r27.4.A, 1s5p.1.A, 1s7g.1.A, 1s7g.1.B, 1s7g.1.C, 1s7g.1.D, 1s7g.1.E, 1sqb.1.E, 1sqp.1.P, 1sqq.1.P, 1sqv.1.E, 1tmo.1.A, 1vf5.1.D, 1vf5.1.L, 1x94.1.A, 1x94.1.B, 1yc5.1.A, 2a3n.1.A, 2b4y.1.A, 2b4y.3.A, 2d2c.1.D, 2d2c.1.L, 2e75.1.D, 2e76.1.D, 2e7z.1.A, 2fug.2.C, 2fyn.1.C, 2fyn.2.C, 2fyu.1.E, 2h2i.1.A, 2h4h.1.A, 2h59.1.B, 2i2w.1.A, 2i2w.2.B, 2iv2.1.A, 2ivf.1.A, 2nya.1.A, 2nyr.1.A, 2nyr.1.B, 2pq4.1.B, 2qjk.1.C, 2qjp.1.C, 2qjy.3.F, 2v3v.1.A, 2v45.1.A, 2vpx.1.D, 2vpz.1.A, 2x3y.1.A, 2ybb.1.b, 3cf4.1.B, 3cwb.1.E, 3egw.1.A, 3etn.1.A, 3eua.1.A, 3fj1.1.A, 3fxa.1.A, 3h1h.1.E, 3h1h.1.O, 3h1i.1.E, 3ir5.1.A, 3ir6.1.A, 3ir7.1.A, 3jr3.1.A, 3jwp.1.A, 3k35.1.A, 3l75.1.E, 3l75.1.O, 3m9s.1.C, 3o5a.1.A, 3pki.1.A, 3riy.2.A, 3sho.1.A, 3sho.1.C, 3u31.1.A, 3zg6.1.A, 4aay.1.A, 4bv2.3.A, 4dmr.1.A, 4g1c.1.A, 4g1c.2.A, 4h44.1.D, 4hda.1.A, 4hda.2.A, 4ivn.1.A, 4nvs.1.A, 4ogq.1.L, 4pv1.1.L, 4twi.1.A, 4twj.1.A, 4u3f.1.E, 4utn.1.A, 4utn.2.A, 4v4c.1.A, 4wd3.1.A, 4ydd.1.A, 5bwl.1.A, 5e7o.1.A, 5gpn.24.A, 5j8k.55.A, 5kkz.1.C, 5kli.1.C, 5klv.1.P, 5lu5.1.A, 5lu6.1.A, 5lu7.1.A, 5mf6.1.A, 5nqd.1.A, 5o31.1.8, 5oj7.1.A, 5ojn.1.A, 5okd.1.E, 5t5i.1.B, 5x16.1.A, 5xhs.1.A, 5xtb.1.L, 5xte.1.C, 5y2f.1.A, 6aco.1.A, 6acp.1.A, 6adq.1.L, 6btm.1.B, 6cz7.1.A, 6enx.1.A, 6eo0.1.A, 6eqs.3.A, 6f0k.1.B, 6fky.1.A, 6fky.2.A, 6flg.1.A, 6g72.1.G, 6gcs.1.A, 6giq.1.E, 6hu9.1.E, 6ljk.1.A, 6ljm.1.A, 6lod.1.B, 6nhg.1.E, 6nin.1.C, 6q8o.1.C, 6q9e.1.E, 6q9e.1.O, 6qbx.15.A, 6qbx.5.A, 6qc2.33.A, 6qc2.43.A, 6qc3.15.A, 6qc3.5.A, 6qc4.15.A, 6qc4.5.A, 6qc5.1.C, 6qcf.1.C, 6rfq.1.A, 6rfs.1.A, 6rxj.1.A, 6rxm.1.A, 6rxm.2.A, 6rxm.3.A, 6rxm.4.A, 6rxm.5.A, 6rxm.6.A, 6rxo.1.A, 6rxo.2.A, 6rxp.2.A, 6rxq.4.A, 6rxs.1.A, 6s6y.1.B, 6sdr.1.A, 6sdv.1.A, 6t0b.1.E, 6t0b.1.O, 6t15.1.E, 6t15.1.O, 6tg9.1.A, 6x89.1.H, 6xvg.3.A, 6yj4.1.G, 6ymx.1.Q, 6ymx.1.Z, 6ziy.1.C, 6zjl.1.C, 6zjn.1.C, 6zjy.1.C, 6zk9.1.C, 6zr2.1.G, 7a23.1.O, 7ak5.1.G, 7ak6.1.G, 7aqr.1.F, 7ar7.1.G, 7ar8.1.G, 7arc.1.F, 7b04.1.B, 7b04.2.B, 7bkb.1.F, 7bkb.1.L, 7cl0.1.A, 7dgr.10.A, 7dgr.60.A, 7dgs.50.A, 7dgs.60.A, 7e1v.1.P, 7e5z.1.A, 7en5.1.A, 7en6.1.A, 7en6.1.B, 7en6.1.C, 7en6.1.D, 7l5i.1.A, 7l5s.1.A, 7nz1.1.E, 7o37.1.E, 7o37.1.O, 7o3c.1.E, 7o3c.1.O, 7o3h.1.E, 7o3h.1.O, 7p61.1.C, 7p63.1.C, 7q5y.1.A, 7qsd.1.G, 7qv7.1.L, 7qv7.1.O, 7r0w.1.L, 7r0w.1.Q, 7rh5.1.V, 7rja.1.H, 7rjb.1.I, 7t2r.1.A, 7t30.1.A, 7tce.2.F, 7tgh.58.A, 7tlj.1.C, 7tz6.1.E, 7tz6.1.P, 7v2c.1.L, 7vw6.1.A, 7vxu.1.L, 7z0t.1.G, 7zd6.1.4, 7zm7.1.I, 7zxy.1.D, 7zxy.1.L, 8asi.1.A, 8asi.1.E, 8asj.1.E, 8b9z.1.G, 8ba0.1.G, 8bqg.1.A, 8e73.55.A, 8e9g.1.G

Swiss Institute of Bioinformatics
Contact Us
