# Supplementary material for: Metabolic capacity is maintained despite shifts in microbial diversity in estuary sediments
Source: ISME Commun. 2025 Oct 11;5(1):ycaf182. doi: 10.1093/ismeco/ycaf182 (PMC12687941; doi:10.1093/ismeco/ycaf182)
Supplement: Supplementary_Data_1_ycaf182 [file supplementary_data_1_ycaf182.zip › SWISS-MODEL/13_May_SF_Bin61_scaffold_30185_c122613481_1/templates.html]

13\_May\_SF\_Bin61\_scaffold\_30185\_c1:2261-3481\_1 | Templates


**Export Alignment**
  
FASTA format
Clustal Format
PNG Image

**Secondary Structure**
  
None
DSSP
PSIPRED
SSpro

**Colour Scheme** 


Fade Mismatches
Enhance Mismatches

Confidencegradient
Confidenceclass
Indels
Chain
Unique Chain
Rainbow
2° Structure
Clustal
Hydrophobic
Size
Charged
Polar
Proline
Ser/Thr
Cysteine
Aliphatic
Aromatic
No Colour

Use QMEANBrane values

|  |  |  |  |
| --- | --- | --- | --- |
| Background |  |  |  |

**3D Viewer**  
NGL
PV

FASTA
Multi FASTA
ClustalW
PNG


SWISS-MODEL

### 13\_May\_SF\_Bin61\_scaffold\_30185\_c1:2261-3481\_1

### Created: March 29, 2023, 5:42 p.m. at 17:42

- Templates
- Models

Models | Name | Description | GMQE | QSQE | Seq Id | Coverage | Range | Method | Resolution | Oligo-state | Ligands | Found by | Seq Similarity ||  | 7b04.1.B | Nitrite oxidoreductase subunit A  *Structure of Nitrite oxidoreductase (Nxr) from the anammox bacterium Kuenenia stuttgartiensis.* | 0.75 | 0.00 | 44.08 | 0.98 | 4-401 | X-ray | 2.97 | monomer | 4 x SF4, 1 x F3S, 2 x MD1, 1 x MO, 1 x HEM, 2 x CA | HHblits | 0.42 |
| ``` target    TARREITRRGFLGTAAGAGFAAFVVSATRA----WGLEAIENPLARYPDREWERVYRDLWRYDSKFTFLCAPNDTHNCLL 7b04.1    ---MKLTRRAFLQVAGATGATLTLAKNAMAFRLLKPAVVVDNPLDTYPDRRWESVYRDQYQYDRTFTYCCSPNDTHACRI  target    DAYVRSGVMTRIGPTMRYGEARDLDGNRASARWDPRVCQKGLALTRRFYGDRRLRHCMVRAGFKRWVDEGFPRGEDGKPP 7b04.1    RAFVRNNVMMRVEQNYDHQNYSDLYGNKATRNWNPRMCLKGYTFHRRVYGPYRLRYPLIRKGWKRWADDGFPELTPENKT  target    K-EYFQRARDEWVRASHDEAAAVVAATLANIAATY-SGEEGAQRLRDQGYEEETIEAMGGAGVQAMKFRGGMPLLGMTRV 7b04.1    KYMFDNRGNDELLRASWDEAFTYASKGIIHITKKYSGPEGAQKL-IDQGYPKEMVDRMQGAGTRTFKGRGGMGLLGVIGK  target    FGLYRMANSMALLDAKVRGVGPDEARGARGFDNYSWHTDLPPGHPMVTGQQTVDFDLNSVELAKNVVVWGMNWITTKMPD 7b04.1    YGMYRFNNCLAIVDAHNRGVGPDQALGGRNWSNYTWHGDQAPGHPFSHGLQTSDVDMNDVRFSKLLIQTGKNLIENKMPE  target    AHWLTEARLKGTRIIVIACEYSSTSSKADDAIVVRPGTTPALALGLSHVIMRDKLYDADYVRRWTDLPMLVRTDTLKYLS 7b04.1    AHWVTEVMERGGKIVVITPEYSPSAQKADYWIPIRNNTDTALFLGITKILIDNKWYDADYVKKFTDFPLLIRTDTLKRVS  target    AEDVFGGGPAPL 7b04.1    PKDIIPN----- ``` | | | | | | | | | | | | | | | | | | | | | | | | | | | | | | | | | | | | | | | | | | | | | | | | | |
|  | 7b04.2.B | Nitrite oxidoreductase subunit A  *Structure of Nitrite oxidoreductase (Nxr) from the anammox bacterium Kuenenia stuttgartiensis.* | 0.75 | 0.00 | 44.08 | 0.98 | 4-401 | X-ray | 2.97 | monomer | 4 x SF4, 1 x F3S, 2 x MD1, 1 x MO, 1 x HEM, 2 x CA | HHblits | 0.42 |
| ``` target    TARREITRRGFLGTAAGAGFAAFVVSATRA----WGLEAIENPLARYPDREWERVYRDLWRYDSKFTFLCAPNDTHNCLL 7b04.2    ---MKLTRRAFLQVAGATGATLTLAKNAMAFRLLKPAVVVDNPLDTYPDRRWESVYRDQYQYDRTFTYCCSPNDTHACRI  target    DAYVRSGVMTRIGPTMRYGEARDLDGNRASARWDPRVCQKGLALTRRFYGDRRLRHCMVRAGFKRWVDEGFPRGEDGKPP 7b04.2    RAFVRNNVMMRVEQNYDHQNYSDLYGNKATRNWNPRMCLKGYTFHRRVYGPYRLRYPLIRKGWKRWADDGFPELTPENKT  target    K-EYFQRARDEWVRASHDEAAAVVAATLANIAATY-SGEEGAQRLRDQGYEEETIEAMGGAGVQAMKFRGGMPLLGMTRV 7b04.2    KYMFDNRGNDELLRASWDEAFTYASKGIIHITKKYSGPEGAQKL-IDQGYPKEMVDRMQGAGTRTFKGRGGMGLLGVIGK  target    FGLYRMANSMALLDAKVRGVGPDEARGARGFDNYSWHTDLPPGHPMVTGQQTVDFDLNSVELAKNVVVWGMNWITTKMPD 7b04.2    YGMYRFNNCLAIVDAHNRGVGPDQALGGRNWSNYTWHGDQAPGHPFSHGLQTSDVDMNDVRFSKLLIQTGKNLIENKMPE  target    AHWLTEARLKGTRIIVIACEYSSTSSKADDAIVVRPGTTPALALGLSHVIMRDKLYDADYVRRWTDLPMLVRTDTLKYLS 7b04.2    AHWVTEVMERGGKIVVITPEYSPSAQKADYWIPIRNNTDTALFLGITKILIDNKWYDADYVKKFTDFPLLIRTDTLKRVS  target    AEDVFGGGPAPL 7b04.2    PKDIIPN----- ``` | | | | | | | | | | | | | | | | | | | | | | | | | | | | | | | | | | | | | | | | | | | | | | | | | |
| ✓ | 7b04.1.B | Nitrite oxidoreductase subunit A  *Structure of Nitrite oxidoreductase (Nxr) from the anammox bacterium Kuenenia stuttgartiensis.* | 0.76 | 0.00 | 47.67 | 0.90 | 35-399 | X-ray | 2.97 | monomer | 4 x SF4, 1 x F3S, 2 x MD1, 1 x MO, 1 x HEM, 2 x CA | BLAST | 0.44 |
| ``` target    TARREITRRGFLGTAAGAGFAAFVVSATRAWGLEAIENPLARYPDREWERVYRDLWRYDSKFTFLCAPNDTHNCLLDAYV 7b04.1    ----------------------------------VVDNPLDTYPDRRWESVYRDQYQYDRTFTYCCSPNDTHACRIRAFV  target    RSGVMTRIGPTMRYGEARDLDGNRASARWDPRVCQKGLALTRRFYGDRRLRHCMVRAGFKRWVDEGFPRGEDGKPPKEYF 7b04.1    RNNVMMRVEQNYDHQNYSDLYGNKATRNWNPRMCLKGYTFHRRVYGPYRLRYPLIRKGWKRWADDGFPELTPENKTKYMF  target    -QRARDEWVRASHDEAAAVVAATLANIAATYSGEEGAQRLRDQGYEEETIEAMGGAGVQAMKFRGGMPLLGMTRVFGLYR 7b04.1    DNRGNDELLRASWDEAFTYASKGIIHITKKYSGPEGAQKLIDQGYPKEMVDRMQGAGTRTFKGRGGMGLLGVIGKYGMYR  target    MANSMALLDAKVRGVGPDEARGARGFDNYSWHTDLPPGHPMVTGQQTVDFDLNSVELAKNVVVWGMNWITTKMPDAHWLT 7b04.1    FNNCLAIVDAHNRGVGPDQALGGRNWSNYTWHGDQAPGHPFSHGLQTSDVDMNDVRFSKLLIQTGKNLIENKMPEAHWVT  target    EARLKGTRIIVIACEYSSTSSKADDAIVVRPGTTPALALGLSHVIMRDKLYDADYVRRWTDLPMLVRTDTLKYLSAEDVF 7b04.1    EVMERGGKIVVITPEYSPSAQKADYWIPIRNNTDTALFLGITKILIDNKWYDADYVKKFTDFPLLIRTDTLKRVSPKDII  target    GGGPAPL 7b04.1    ------- ``` | | | | | | | | | | | | | | | | | | | | | | | | | | | | | | | | | | | | | | | | | | | | | | | | | |
|  | 7b04.2.B | Nitrite oxidoreductase subunit A  *Structure of Nitrite oxidoreductase (Nxr) from the anammox bacterium Kuenenia stuttgartiensis.* | 0.75 | 0.00 | 47.67 | 0.90 | 35-399 | X-ray | 2.97 | monomer | 4 x SF4, 1 x F3S, 2 x MD1, 1 x MO, 1 x HEM, 2 x CA | BLAST | 0.44 |
| ``` target    TARREITRRGFLGTAAGAGFAAFVVSATRAWGLEAIENPLARYPDREWERVYRDLWRYDSKFTFLCAPNDTHNCLLDAYV 7b04.2    ----------------------------------VVDNPLDTYPDRRWESVYRDQYQYDRTFTYCCSPNDTHACRIRAFV  target    RSGVMTRIGPTMRYGEARDLDGNRASARWDPRVCQKGLALTRRFYGDRRLRHCMVRAGFKRWVDEGFPRGEDGKPPKEYF 7b04.2    RNNVMMRVEQNYDHQNYSDLYGNKATRNWNPRMCLKGYTFHRRVYGPYRLRYPLIRKGWKRWADDGFPELTPENKTKYMF  target    -QRARDEWVRASHDEAAAVVAATLANIAATYSGEEGAQRLRDQGYEEETIEAMGGAGVQAMKFRGGMPLLGMTRVFGLYR 7b04.2    DNRGNDELLRASWDEAFTYASKGIIHITKKYSGPEGAQKLIDQGYPKEMVDRMQGAGTRTFKGRGGMGLLGVIGKYGMYR  target    MANSMALLDAKVRGVGPDEARGARGFDNYSWHTDLPPGHPMVTGQQTVDFDLNSVELAKNVVVWGMNWITTKMPDAHWLT 7b04.2    FNNCLAIVDAHNRGVGPDQALGGRNWSNYTWHGDQAPGHPFSHGLQTSDVDMNDVRFSKLLIQTGKNLIENKMPEAHWVT  target    EARLKGTRIIVIACEYSSTSSKADDAIVVRPGTTPALALGLSHVIMRDKLYDADYVRRWTDLPMLVRTDTLKYLSAEDVF 7b04.2    EVMERGGKIVVITPEYSPSAQKADYWIPIRNNTDTALFLGITKILIDNKWYDADYVKKFTDFPLLIRTDTLKRVSPKDII  target    GGGPAPL 7b04.2    ------- ``` | | | | | | | | | | | | | | | | | | | | | | | | | | | | | | | | | | | | | | | | | | | | | | | | | |
|  | 3ir5.1.A | Respiratory nitrate reductase 1 alpha chain  *Crystal structure of NarGHI mutant NarG-H49C* | 0.53 | 0.00 | 27.43 | 0.83 | 2-401 | X-ray | 2.30 | monomer | 2 x MD1, 1 x 6MO, 4 x SF4, 1 x AGA, 1 x F3S, 2 x HEM | HHblits | 0.34 |
| ``` target    TARREITRRGFLGTAAGAGFAAFVVSATRAWGLEAIENPLARYPDREWERVYRDLWRYDSKFTFLCAPNDTHNCLLDAYV 3ir5.1    -MSKFLDRFRYFKQKGETFA-----D-GH-GQ-------L-LNTNRDWEDGYRQRWQHDKIVRSTCGVNCTGSCSWKIYV  target    RSGVMTRIGPTMRYGEARDLDGNRASARWDPRVCQKGLALTRRFYGDRRLRHCMVRAGFK-RWVDEGF---------PRG 3ir5.1    KNGLVTWETQQTDYPR-----TRPDLPNHEPRGCPRGASYSWYLYSANRLKYPMMRKRLMKMWREAKALHSDPVEAWASI  target    -EDGKPPKEY-FQRARDEWVRASHDEAAAVVAATLANIAATYSGEEGAQRLRDQGYEEETIEAMGGAGVQAMKFRGGMPL 3ir5.1    IEDADKAKSFKQARGRGGFVRSSWQEVNELIAASNVYTIKNYGPDRVAGF--------------SPIPAMSMV-------  target    LGMTRVFGLYRMANSMALLDAKVRGVGPDEARGARGFDNYSWHTDLPPGHPMVTGQQTVDFDLNSVELAKNVVVWGMNWI 3ir5.1    ----SYASGARYLSLI----------------GGTCLSFYDWYCDLPPASPQTWGEQTDVPESADWYNSSYIIAWGSNVP  target    TTKMPDAHWLTEARLKGTRIIVIACEYSSTSSKADDAIVVRPGTTPALALGLSHVIMRDKL------YDADYVRRWTDLP 3ir5.1    QTRTPDAHFFTEVRYKGTKTVAVTPDYAEIAKLCDLWLAPKQGTDAAMALAMGHVMLREFHLDNPSQYFTDYVRRYTDMP  target    MLVRTD-------TLKYLSAEDVFGGGPAPL 3ir5.1    MLVMLEERDGYYAAGRMLRAADLVDA----- ``` | | | | | | | | | | | | | | | | | | | | | | | | | | | | | | | | | | | | | | | | | | | | | | | | | |
|  | 3ir6.1.A | Respiratory nitrate reductase 1 alpha chain  *Crystal structure of NarGHI mutant NarG-H49S* | 0.49 | 0.00 | 27.43 | 0.83 | 2-401 | X-ray | 2.80 | monomer | 2 x GDP, 1 x AGA, 3 x SF4, 1 x F3S, 2 x HEM | HHblits | 0.34 |
| ``` target    TARREITRRGFLGTAAGAGFAAFVVSATRAWGLEAIENPLARYPDREWERVYRDLWRYDSKFTFLCAPNDTHNCLLDAYV 3ir6.1    -MSKFLDRFRYFKQKGETFADGHG----Q---L--------LNTNRDWEDGYRQRWQHDKIVRSTSGVNCTGSCSWKIYV  target    RSGVMTRIGPTMRYGEARDLDGNRASARWDPRVCQKGLALTRRFYGDRRLRHCMVRAGFK-RWVDEGF----P---RG-- 3ir6.1    KNGLVTWETQQTDYPR-----TRPDLPNHEPRGCPRGASYSWYLYSANRLKYPMMRKRLMKMWREAKALHSDPVEAWASI  target    -EDGKPPK-EYFQRARDEWVRASHDEAAAVVAATLANIAATYSGEEGAQRLRDQGYEEETIEAMGGAGVQAMKFRGGMPL 3ir6.1    IEDADKAKSFKQARGRGGFVRSSWQEVNELIAASNVYTIKNYGPDRVAGF--------------SPIPAMSMV-------  target    LGMTRVFGLYRMANSMALLDAKVRGVGPDEARGARGFDNYSWHTDLPPGHPMVTGQQTVDFDLNSVELAKNVVVWGMNWI 3ir6.1    ----SYASGARYLSL----------------IGGTCLSFYDWYCDLPPASPQTWGEQTDVPESADWYNSSYIIAWGSNVP  target    TTKMPDAHWLTEARLKGTRIIVIACEYSSTSSKADDAIVVRPGTTPALALGLSHVIMRDKL------YDADYVRRWTDLP 3ir6.1    QTRTPDAHFFTEVRYKGTKTVAVTPDYAEIAKLCDLWLAPKQGTDAAMALAMGHVMLREFHLDNPSQYFTDYVRRYTDMP  target    MLVRTD-------TLKYLSAEDVFGGGPAPL 3ir6.1    MLVMLEERDGYYAAGRMLRAADLVDA----- ``` | | | | | | | | | | | | | | | | | | | | | | | | | | | | | | | | | | | | | | | | | | | | | | | | | |
|  | 3ir7.1.A | Respiratory nitrate reductase 1 alpha chain  *Crystal structure of NarGHI mutant NarG-R94S* | 0.52 | 0.00 | 27.22 | 0.83 | 3-401 | X-ray | 2.50 | monomer | 2 x MD1, 4 x SF4, 1 x 6MO, 1 x AGA, 1 x F3S, 2 x HEM | HHblits | 0.34 |
| ``` target    TARREITRRGFLGTAAGAGFAAFVVSATRAWGLEAIENPLARYPDREWERVYRDLWRYDSKFTFLCAPNDTHNCLLDAYV 3ir7.1    --SKFLDRFRYFKQKGETFADGH----GQ---L--------LNTNRDWEDGYRQRWQHDKIVRSTHGVNCTGSCSWKIYV  target    RSGVMTRIGPTMRYGEARDLDGNRASARWDPRVCQKGLALTRRFYGDRRLRHCMVRAGFK-RWVDEGFP---------R- 3ir7.1    KNGLVTWETQQTDYPR-----TRPDLPNHEPRGCPSGASYSWYLYSANRLKYPMMRKRLMKMWREAKALHSDPVEAWASI  target    GEDGKPPKEY-FQRARDEWVRASHDEAAAVVAATLANIAATYSGEEGAQRLRDQGYEEETIEAMGGAGVQAMKFRGGMPL 3ir7.1    IEDADKAKSFKQARGRGGFVRSSWQEVNELIAASNVYTIKNYGPDRVAG--------------FSPIPAMSMV-------  target    LGMTRVFGLYRMANSMALLDAKVRGVGPDEARGARGFDNYSWHTDLPPGHPMVTGQQTVDFDLNSVELAKNVVVWGMNWI 3ir7.1    ----SYASGARYLSL----------------IGGTCLSFYDWYCDLPPASPQTWGEQTDVPESADWYNSSYIIAWGSNVP  target    TTKMPDAHWLTEARLKGTRIIVIACEYSSTSSKADDAIVVRPGTTPALALGLSHVIMRDKL------YDADYVRRWTDLP 3ir7.1    QTRTPDAHFFTEVRYKGTKTVAVTPDYAEIAKLCDLWLAPKQGTDAAMALAMGHVMLREFHLDNPSQYFTDYVRRYTDMP  target    MLVRTDT-------LKYLSAEDVFGGGPAPL 3ir7.1    MLVMLEERDGYYAAGRMLRAADLVDA----- ``` | | | | | | | | | | | | | | | | | | | | | | | | | | | | | | | | | | | | | | | | | | | | | | | | | |
|  | 1r27.4.A | Respiratory nitrate reductase 1 alpha chain  *Crystal Structure of NarGH complex* | 0.53 | 0.20 | 27.30 | 0.83 | 4-401 | X-ray | 2.00 | homo-dimer | 4 x MO, 16 x SF4, 8 x MGD, 4 x F3S | HHblits | 0.34 |
| ``` target    TARREITRRGFLGTAAGAGFAAFVVSATRAWGLEAIENPLARYPDREWERVYRDLWRYDSKFTFLCAPNDTHNCLLDAYV 1r27.4    ---KFLDRFRYFKQKGETFADGH----GQ---L--------LNTNRDWEDGYRQRWQHDKIVRSTHGVNCTGSCSWKIYV  target    RSGVMTRIGPTMRYGEARDLDGNRASARWDPRVCQKGLALTRRFYGDRRLRHCMVRAGFK-RWVDEGF-------PRG-- 1r27.4    KNGLVTWETQQTDYPR-----TRPDLPNHEPRGCPRGASYSWYLYSANRLKYPMMRKRLMKMWREAKALHSDPVEAWASI  target    -EDGKPPKE-YFQRARDEWVRASHDEAAAVVAATLANIAATYSGEEGAQRLRDQGYEEETIEAMGGAGVQAMKFRGGMPL 1r27.4    IEDADKAKSFKQARGRGGFVRSSWQEVNELIAASNVYTIKNYGPDRVAGF--------------SPIPAMSMV-------  target    LGMTRVFGLYRMANSMALLDAKVRGVGPDEARGARGFDNYSWHTDLPPGHPMVTGQQTVDFDLNSVELAKNVVVWGMNWI 1r27.4    ----SYASGARYLSLI----------------GGTCLSFYDWYCDLPPASPQTWGEQTDVPESADWYNSSYIIAWGSNVP  target    TTKMPDAHWLTEARLKGTRIIVIACEYSSTSSKADDAIVVRPGTTPALALGLSHVIMRDKL------YDADYVRRWTDLP 1r27.4    QTRTPDAHFFTEVRYKGTKTVAVTPDYAEIAKLCDLWLAPKQGTDAAMALAMGHVMLREFHLDNPSQYFTDYVRRYTDMP  target    MLVRTDT-------LKYLSAEDVFGGGPAPL 1r27.4    MLVMLEERDGYYAAGRMLRAADLVDA----- ``` | | | | | | | | | | | | | | | | | | | | | | | | | | | | | | | | | | | | | | | | | | | | | | | | | |
|  | 1q16.1.A | Respiratory nitrate reductase 1 alpha chain  *Crystal structure of Nitrate Reductase A, NarGHI, from Escherichia coli* | 0.53 | 0.00 | 27.22 | 0.83 | 3-401 | X-ray | 1.90 | monomer | 2 x MD1, 1 x 6MO, 2 x HEM, 4 x SF4, 1 x F3S, 1 x AGA, 1 x 3PH | HHblits | 0.34 |
| ``` target    TARREITRRGFLGTAAGAGFAAFVVSATRAWGLEAIENPLARYPDREWERVYRDLWRYDSKFTFLCAPNDTHNCLLDAYV 1q16.1    --SKFLDRFRYFKQKGETFAD----GHG---QL--------LNTNRDWEDGYRQRWQHDKIVRSTHGVNCTGSCSWKIYV  target    RSGVMTRIGPTMRYGEARDLDGNRASARWDPRVCQKGLALTRRFYGDRRLRHCMVRAGFK-RWVDEGF---------PR- 1q16.1    KNGLVTWETQQTDYPR-----TRPDLPNHEPRGCPRGASYSWYLYSANRLKYPMMRKRLMKMWREAKALHSDPVEAWASI  target    GEDGKPPK-EYFQRARDEWVRASHDEAAAVVAATLANIAATYSGEEGAQRLRDQGYEEETIEAMGGAGVQAMKFRGGMPL 1q16.1    IEDADKAKSFKQARGRGGFVRSSWQEVNELIAASNVYTIKNYGPDRVAGF--------------SPIPAMSMV-------  target    LGMTRVFGLYRMANSMALLDAKVRGVGPDEARGARGFDNYSWHTDLPPGHPMVTGQQTVDFDLNSVELAKNVVVWGMNWI 1q16.1    ----SYASGARYLSL----------------IGGTCLSFYDWYCDLPPASPQTWGEQTDVPESADWYNSSYIIAWGSNVP  target    TTKMPDAHWLTEARLKGTRIIVIACEYSSTSSKADDAIVVRPGTTPALALGLSHVIMRDKL------YDADYVRRWTDLP 1q16.1    QTRTPDAHFFTEVRYKGTKTVAVTPDYAEIAKLCDLWLAPKQGTDAAMALAMGHVMLREFHLDNPSQYFTDYVRRYTDMP  target    MLVRTD-------TLKYLSAEDVFGGGPAPL 1q16.1    MLVMLEERDGYYAAGRMLRAADLVDA----- ``` | | | | | | | | | | | | | | | | | | | | | | | | | | | | | | | | | | | | | | | | | | | | | | | | | |
|  | 2ivf.1.A | ETHYLBENZENE DEHYDROGENASE ALPHA-SUBUNIT  *ETHYLBENZENE DEHYDROGENASE FROM AROMATOLEUM AROMATICUM* | 0.50 | 0.00 | 29.48 | 0.81 | 4-400 | X-ray | 1.88 | monomer | 1 x MES, 4 x SF4, 1 x MO, 1 x MGD, 1 x MD1, 1 x F3S, 1 x HEM | HHblits | 0.33 |
| ``` target    TARREITRRGFLGTAAGAGFAAFVVSATR--A-WGLEAIENPLARYPDREWERVYRDLWRYDSKFTFLCAPND--THNCL 2ivf.1    ---QDQHRRDFLKRSGAAVLSLSLSSLATGVVPGFLKDAQAGTKAPGYASWEDIYRKEWKWDKVNWGSHLNICWPQGSCK  target    LDAYVRSGVMTRIGPTMRYGEARDLDGNRASARWDPRVCQKGLALTRRFYGDRRLRHCMVRAGFKRWVDEGFPRGEDGKP 2ivf.1    FYVYVRNGIVWREEQAAQTPA-----CNVDYVDYNPLGCQKGSAFNNNLYGDERVKYPLKRVG---------KRG-----  target    PKEYFQRARDEWVRASHDEAAAVVAATLANIAATYSGEEGAQRLRDQGYEEETIEAMGGAGVQAMKFRGGMPLLGMTRVF 2ivf.1    --------EGKWKRVSWDEAAGDIADSIIDSFEAQGSDGFI--------------LDAPHVHA-----GSIAW------G  target    GLYRMANSMALLDAKVRGVGPDEARGARGFDNYSWHTDLPPGHPMVTGQQTVDFDLNSVELAKNVVVWGMNWITTKMPDA 2ivf.1    AGFRMTYLMD---GVS------------PDINV-DIGDTYMGAFHTFGKMHMGYSADNLLDAELIFMTCSNWSYTYPSSY  target    HWLTEARLKGTRIIVIACEYSSTSSKADDAIVVRPGTTPALALGLSHVIMRDKLYDADYVRRWTDLPMLVRTDTLKYLSA 2ivf.1    HFLSEARYKGAEVVVIAPDFNPTTPAADLHVPVRVGSDAAFWLGLSQVMIDEKLFDRQFVCEQTDLPLLVRMDTGKFLSA  target    EDVFGGGPAPL 2ivf.1    EDVDG------ ``` | | | | | | | | | | | | | | | | | | | | | | | | | | | | | | | | | | | | | | | | | | | | | | | | | |
|  | 3egw.1.A | Respiratory nitrate reductase 1 alpha chain  *The crystal structure of the NarGHI mutant NarH - C16A* | 0.52 | 0.18 | 28.85 | 0.77 | 44-401 | X-ray | 1.90 | homo-dimer | 2 x MD1, 2 x MGD, 2 x 6MO, 6 x SF4, 4 x F3S, 2 x 3PH, 4 x HEM, 2 x AGA | HHblits | 0.35 |
| ``` target    TARREITRRGFLGTAAGAGFAAFVVSATRAWGLEAIENPLARYPDREWERVYRDLWRYDSKFTFLCAPNDTHNCLLDAYV 3egw.1    -------------------------------------------TNRDWEDGYRQRWQHDKIVRSTHGVNCTGSCSWKIYV  target    RSGVMTRIGPTMRYGEARDLDGNRASARWDPRVCQKGLALTRRFYGDRRLRHCMVRAGFK-RWVDEGF-------PRG-- 3egw.1    KNGLVTWETQQTDYPR-----TRPDLPNHEPRGCPRGASYSWYLYSANRLKYPMMRKRLMKMWREAKALHSDPVEAWASI  target    -EDGKPPKE-YFQRARDEWVRASHDEAAAVVAATLANIAATYSGEEGAQRLRDQGYEEETIEAMGGAGVQAMKFRGGMPL 3egw.1    IEDADKAKSFKQARGRGGFVRSSWQEVNELIAASNVYTIKNYGPDRVAGF--------------SPIPAMSMV-------  target    LGMTRVFGLYRMANSMALLDAKVRGVGPDEARGARGFDNYSWHTDLPPGHPMVTGQQTVDFDLNSVELAKNVVVWGMNWI 3egw.1    ----SYASGARYLSLI----------------GGTCLSFYDWYCDLPPASPQTWGEQTDVPESADWYNSSYIIAWGSNVP  target    TTKMPDAHWLTEARLKGTRIIVIACEYSSTSSKADDAIVVRPGTTPALALGLSHVIMRDKL------YDADYVRRWTDLP 3egw.1    QTRTPDAHFFTEVRYKGTKTVAVTPDYAEIAKLCDLWLAPKQGTDAAMALAMGHVMLREFHLDNPSQYFTDYVRRYTDMP  target    MLVRTD-------TLKYLSAEDVFGGGPAPL 3egw.1    MLVMLEERDGYYAAGRMLRAADLVAA----- ``` | | | | | | | | | | | | | | | | | | | | | | | | | | | | | | | | | | | | | | | | | | | | | | | | | |
|  | 3ir5.1.A | Respiratory nitrate reductase 1 alpha chain  *Crystal structure of NarGHI mutant NarG-H49C* | 0.46 | 0.00 | 32.99 | 0.72 | 45-385 | X-ray | 2.30 | monomer | 2 x MD1, 1 x 6MO, 4 x SF4, 1 x AGA, 1 x F3S, 2 x HEM | BLAST | 0.37 |
| ``` target    TARREITRRGFLGTAAGAGFAAFVVSATRAWGLEAIENPLARYPDREWERVYRDLWRYDSKFTFLCAPNDTHNCLLDAYV 3ir5.1    --------------------------------------------NRDWEDGYRQRWQHDKIVRSTCGVNCTGSCSWKIYV  target    RSGVMTRIGPTMRYGEAR-DLDGNRASARWDPRVCQKGLALTRRFYGDRRLRHCMVRAGF-KRW----------VDEGFP 3ir5.1    KNGLVTWETQQTDYPRTRPDLPNH------EPRGCPRGASYSWYLYSANRLKYPMMRKRLMKMWREAKALHSDPVEAWAS  target    RGEDGKPPKEYFQ-RARDEWVRASHDEAAAVVAATLANIAATYSGEEGAQRLRDQGYEEETIEAMGGAGVQAMKFRGGMP 3ir5.1    IIEDADKAKSFKQARGRGGFVRSSW---------------------------------QEVNELIAASNVYTIKNYGPDR  target    LLGMTRVFGLYRMANSMALLDAKVRGVGPDEARGARGFDNYSWHTDLPPGHPMVTGQQTVDFDLNSVELAKNVVVWGMNW 3ir5.1    VAGFSPI-----PAMSMVSYASGARYL---SLIGGTCLSFYDWYCDLPPASPQTWGEQTDVPESADWYNSSYIIAWGSNV  target    ITTKMPDAHWLTEARLKGTRIIVIACEYSSTSSKADDAIVVRPGTTPALALGLSHVIMRD------KLYDADYVRRWTDL 3ir5.1    PQTRTPDAHFFTEVRYKGTKTVAVTPDYAEIAKLCDLWLAPKQGTDAAMALAMGHVMLREFHLDNPSQYFTDYVRRYTDM  target    PMLVRTDTLKYLSAEDVFGGGPAPL 3ir5.1    PMLV--------------------- ``` | | | | | | | | | | | | | | | | | | | | | | | | | | | | | | | | | | | | | | | | | | | | | | | | | |
|  | 3ir6.1.A | Respiratory nitrate reductase 1 alpha chain  *Crystal structure of NarGHI mutant NarG-H49S* | 0.44 | 0.00 | 32.65 | 0.72 | 45-385 | X-ray | 2.80 | monomer | 2 x GDP, 1 x AGA, 3 x SF4, 1 x F3S, 2 x HEM | BLAST | 0.37 |
| ``` target    TARREITRRGFLGTAAGAGFAAFVVSATRAWGLEAIENPLARYPDREWERVYRDLWRYDSKFTFLCAPNDTHNCLLDAYV 3ir6.1    --------------------------------------------NRDWEDGYRQRWQHDKIVRSTSGVNCTGSCSWKIYV  target    RSGVMTRIGPTMRYGEAR-DLDGNRASARWDPRVCQKGLALTRRFYGDRRLRHCMVRAGF-KRW----------VDEGFP 3ir6.1    KNGLVTWETQQTDYPRTRPDLPNH------EPRGCPRGASYSWYLYSANRLKYPMMRKRLMKMWREAKALHSDPVEAWAS  target    RGEDGKPPKEYFQ-RARDEWVRASHDEAAAVVAATLANIAATYSGEEGAQRLRDQGYEEETIEAMGGAGVQAMKFRGGMP 3ir6.1    IIEDADKAKSFKQARGRGGFVRSSW---------------------------------QEVNELIAASNVYTIKNYGPDR  target    LLGMTRVFGLYRMANSMALLDAKVRGVGPDEARGARGFDNYSWHTDLPPGHPMVTGQQTVDFDLNSVELAKNVVVWGMNW 3ir6.1    VAGFSPI-----PAMSMVSYASGARYL---SLIGGTCLSFYDWYCDLPPASPQTWGEQTDVPESADWYNSSYIIAWGSNV  target    ITTKMPDAHWLTEARLKGTRIIVIACEYSSTSSKADDAIVVRPGTTPALALGLSHVIMRD------KLYDADYVRRWTDL 3ir6.1    PQTRTPDAHFFTEVRYKGTKTVAVTPDYAEIAKLCDLWLAPKQGTDAAMALAMGHVMLREFHLDNPSQYFTDYVRRYTDM  target    PMLVRTDTLKYLSAEDVFGGGPAPL 3ir6.1    PMLV--------------------- ``` | | | | | | | | | | | | | | | | | | | | | | | | | | | | | | | | | | | | | | | | | | | | | | | | | |
|  | 1q16.1.A | Respiratory nitrate reductase 1 alpha chain  *Crystal structure of Nitrate Reductase A, NarGHI, from Escherichia coli* | 0.45 | 0.00 | 32.65 | 0.72 | 45-385 | X-ray | 1.90 | monomer | 2 x MD1, 1 x 6MO, 2 x HEM, 4 x SF4, 1 x F3S, 1 x AGA, 1 x 3PH | BLAST | 0.37 |
| ``` target    TARREITRRGFLGTAAGAGFAAFVVSATRAWGLEAIENPLARYPDREWERVYRDLWRYDSKFTFLCAPNDTHNCLLDAYV 1q16.1    --------------------------------------------NRDWEDGYRQRWQHDKIVRSTHGVNCTGSCSWKIYV  target    RSGVMTRIGPTMRYGEAR-DLDGNRASARWDPRVCQKGLALTRRFYGDRRLRHCMVRAGF-KRW----------VDEGFP 1q16.1    KNGLVTWETQQTDYPRTRPDLPNH------EPRGCPRGASYSWYLYSANRLKYPMMRKRLMKMWREAKALHSDPVEAWAS  target    RGEDGKPPKEYFQ-RARDEWVRASHDEAAAVVAATLANIAATYSGEEGAQRLRDQGYEEETIEAMGGAGVQAMKFRGGMP 1q16.1    IIEDADKAKSFKQARGRGGFVRSSW---------------------------------QEVNELIAASNVYTIKNYGPDR  target    LLGMTRVFGLYRMANSMALLDAKVRGVGPDEARGARGFDNYSWHTDLPPGHPMVTGQQTVDFDLNSVELAKNVVVWGMNW 1q16.1    VAGFSPI-----PAMSMVSYASGARYL---SLIGGTCLSFYDWYCDLPPASPQTWGEQTDVPESADWYNSSYIIAWGSNV  target    ITTKMPDAHWLTEARLKGTRIIVIACEYSSTSSKADDAIVVRPGTTPALALGLSHVIMRD------KLYDADYVRRWTDL 1q16.1    PQTRTPDAHFFTEVRYKGTKTVAVTPDYAEIAKLCDLWLAPKQGTDAAMALAMGHVMLREFHLDNPSQYFTDYVRRYTDM  target    PMLVRTDTLKYLSAEDVFGGGPAPL 1q16.1    PMLV--------------------- ``` | | | | | | | | | | | | | | | | | | | | | | | | | | | | | | | | | | | | | | | | | | | | | | | | | |
|  | 3egw.1.A | Respiratory nitrate reductase 1 alpha chain  *The crystal structure of the NarGHI mutant NarH - C16A* | 0.45 | 0.13 | 32.65 | 0.72 | 45-385 | X-ray | 1.90 | homo-dimer | 2 x MD1, 2 x MGD, 2 x 6MO, 6 x SF4, 4 x F3S, 2 x 3PH, 4 x HEM, 2 x AGA | BLAST | 0.37 |
| ``` target    TARREITRRGFLGTAAGAGFAAFVVSATRAWGLEAIENPLARYPDREWERVYRDLWRYDSKFTFLCAPNDTHNCLLDAYV 3egw.1    --------------------------------------------NRDWEDGYRQRWQHDKIVRSTHGVNCTGSCSWKIYV  target    RSGVMTRIGPTMRYGEAR-DLDGNRASARWDPRVCQKGLALTRRFYGDRRLRHCMVRAGF-KRW----------VDEGFP 3egw.1    KNGLVTWETQQTDYPRTRPDLPNH------EPRGCPRGASYSWYLYSANRLKYPMMRKRLMKMWREAKALHSDPVEAWAS  target    RGEDGKPPKEYFQ-RARDEWVRASHDEAAAVVAATLANIAATYSGEEGAQRLRDQGYEEETIEAMGGAGVQAMKFRGGMP 3egw.1    IIEDADKAKSFKQARGRGGFVRSSW---------------------------------QEVNELIAASNVYTIKNYGPDR  target    LLGMTRVFGLYRMANSMALLDAKVRGVGPDEARGARGFDNYSWHTDLPPGHPMVTGQQTVDFDLNSVELAKNVVVWGMNW 3egw.1    VAGFSPI-----PAMSMVSYASGARYL---SLIGGTCLSFYDWYCDLPPASPQTWGEQTDVPESADWYNSSYIIAWGSNV  target    ITTKMPDAHWLTEARLKGTRIIVIACEYSSTSSKADDAIVVRPGTTPALALGLSHVIMRD------KLYDADYVRRWTDL 3egw.1    PQTRTPDAHFFTEVRYKGTKTVAVTPDYAEIAKLCDLWLAPKQGTDAAMALAMGHVMLREFHLDNPSQYFTDYVRRYTDM  target    PMLVRTDTLKYLSAEDVFGGGPAPL 3egw.1    PMLV--------------------- ``` | | | | | | | | | | | | | | | | | | | | | | | | | | | | | | | | | | | | | | | | | | | | | | | | | |
|  | 1r27.4.A | Respiratory nitrate reductase 1 alpha chain  *Crystal Structure of NarGH complex* | 0.45 | 0.15 | 32.65 | 0.72 | 45-385 | X-ray | 2.00 | homo-dimer | 4 x MO, 16 x SF4, 8 x MGD, 4 x F3S | BLAST | 0.37 |
| ``` target    TARREITRRGFLGTAAGAGFAAFVVSATRAWGLEAIENPLARYPDREWERVYRDLWRYDSKFTFLCAPNDTHNCLLDAYV 1r27.4    --------------------------------------------NRDWEDGYRQRWQHDKIVRSTHGVNCTGSCSWKIYV  target    RSGVMTRIGPTMRYGEAR-DLDGNRASARWDPRVCQKGLALTRRFYGDRRLRHCMVRAGF-KRW----------VDEGFP 1r27.4    KNGLVTWETQQTDYPRTRPDLPNH------EPRGCPRGASYSWYLYSANRLKYPMMRKRLMKMWREAKALHSDPVEAWAS  target    RGEDGKPPKEYFQ-RARDEWVRASHDEAAAVVAATLANIAATYSGEEGAQRLRDQGYEEETIEAMGGAGVQAMKFRGGMP 1r27.4    IIEDADKAKSFKQARGRGGFVRSSW---------------------------------QEVNELIAASNVYTIKNYGPDR  target    LLGMTRVFGLYRMANSMALLDAKVRGVGPDEARGARGFDNYSWHTDLPPGHPMVTGQQTVDFDLNSVELAKNVVVWGMNW 1r27.4    VAGFSPI-----PAMSMVSYASGARYL---SLIGGTCLSFYDWYCDLPPASPQTWGEQTDVPESADWYNSSYIIAWGSNV  target    ITTKMPDAHWLTEARLKGTRIIVIACEYSSTSSKADDAIVVRPGTTPALALGLSHVIMRD------KLYDADYVRRWTDL 1r27.4    PQTRTPDAHFFTEVRYKGTKTVAVTPDYAEIAKLCDLWLAPKQGTDAAMALAMGHVMLREFHLDNPSQYFTDYVRRYTDM  target    PMLVRTDTLKYLSAEDVFGGGPAPL 1r27.4    PMLV--------------------- ``` | | | | | | | | | | | | | | | | | | | | | | | | | | | | | | | | | | | | | | | | | | | | | | | | | |
|  | 3ir7.1.A | Respiratory nitrate reductase 1 alpha chain  *Crystal structure of NarGHI mutant NarG-R94S* | 0.45 | 0.00 | 32.65 | 0.72 | 45-385 | X-ray | 2.50 | monomer | 2 x MD1, 4 x SF4, 1 x 6MO, 1 x AGA, 1 x F3S, 2 x HEM | BLAST | 0.37 |
| ``` target    TARREITRRGFLGTAAGAGFAAFVVSATRAWGLEAIENPLARYPDREWERVYRDLWRYDSKFTFLCAPNDTHNCLLDAYV 3ir7.1    --------------------------------------------NRDWEDGYRQRWQHDKIVRSTHGVNCTGSCSWKIYV  target    RSGVMTRIGPTMRYGEAR-DLDGNRASARWDPRVCQKGLALTRRFYGDRRLRHCMVRAGF-KRW----------VDEGFP 3ir7.1    KNGLVTWETQQTDYPRTRPDLPNH------EPRGCPSGASYSWYLYSANRLKYPMMRKRLMKMWREAKALHSDPVEAWAS  target    RGEDGKPPKEYFQ-RARDEWVRASHDEAAAVVAATLANIAATYSGEEGAQRLRDQGYEEETIEAMGGAGVQAMKFRGGMP 3ir7.1    IIEDADKAKSFKQARGRGGFVRSSW---------------------------------QEVNELIAASNVYTIKNYGPDR  target    LLGMTRVFGLYRMANSMALLDAKVRGVGPDEARGARGFDNYSWHTDLPPGHPMVTGQQTVDFDLNSVELAKNVVVWGMNW 3ir7.1    VAGFSPI-----PAMSMVSYASGARYL---SLIGGTCLSFYDWYCDLPPASPQTWGEQTDVPESADWYNSSYIIAWGSNV  target    ITTKMPDAHWLTEARLKGTRIIVIACEYSSTSSKADDAIVVRPGTTPALALGLSHVIMRD------KLYDADYVRRWTDL 3ir7.1    PQTRTPDAHFFTEVRYKGTKTVAVTPDYAEIAKLCDLWLAPKQGTDAAMALAMGHVMLREFHLDNPSQYFTDYVRRYTDM  target    PMLVRTDTLKYLSAEDVFGGGPAPL 3ir7.1    PMLV--------------------- ``` | | | | | | | | | | | | | | | | | | | | | | | | | | | | | | | | | | | | | | | | | | | | | | | | | |
|  | 4ydd.1.A | DMSO reductase family type II enzyme, molybdopterin subunit  *Crystal structure of the perchlorate reductase PcrAB from Azospira suillum PS* | 0.53 | 0.00 | 30.48 | 0.72 | 42-402 | X-ray | 1.86 | monomer | 4 x SF4, 1 x MO, 1 x MGD, 1 x MD1, 1 x F3S | HHblits | 0.36 |
| ``` target    TARREITRRGFLGTAAGAGFAAFVVSATRAWGLEAIENPLARYPDREWERVYRDLWRYDSKFTFLCAPNDTHNCLLDAYV 4ydd.1    -----------------------------------------AFEYSGWENFHRTQWSWDKKTRGAHLVNCTGACPHFVYS  target    RSGVMTRIGPTMRYGEARDLDGNRASARWDPRVCQKGLALTRRFYGDRRLRHCMVRAGFKRWVDEGFPRGEDGKPPKEYF 4ydd.1    KDGVVMREEQSKD------IAPMPNIPEYNPRGCNKGECGHDYMYGPHRIKYPLIRVG---------ERG----------  target    QRARDEWVRASHDEAAAVVAATLANIAATYSGEEGAQRLRDQGYEEETIEAMGGAGVQAMKFRGGMPLLGMTRVFGLYRM 4ydd.1    ---EGKWRRATWEEALDMIADKCVDTIKNHAPDCISVYSPVPAVSPVSFSA-------GHRF---------AHYIGAHA-  target    ANSMALLDAKVRGVGPDEARGARGFDNYSWHTDLPPGHPMVTGQQTVDFDLNSVELAKNVVVWGMNWITTKMPDAHWLTE 4ydd.1    ------------------------HTFYDWYGDHPTGQTQTCGVQGDTCETADWFNSKYIILWGSNPTQTRIPDAHFLSE  target    ARLKGTRIIVIACEYSSTSSKADDAIVVRPGTTPALALGLSHVIMRDKLYDADYVRRWTDLPMLVRTDTLKYLSAEDVFG 4ydd.1    AQLNGAKIVSISPDYNSSTIKVDKWIHPQPGTDGALAMAMAHVIIKEKLYDAHSLKEQTDLSYLVRSDTKRFLREADVVA  target    GGPAPL 4ydd.1    GG---- ``` | | | | | | | | | | | | | | | | | | | | | | | | | | | | | | | | | | | | | | | | | | | | | | | | | |
|  | 1kqf.1.A | FORMATE DEHYDROGENASE, NITRATE-INDUCIBLE, MAJOR SUBUNIT  *FORMATE DEHYDROGENASE N FROM E. COLI* | 0.44 |  | 22.58 | 0.76 | 4-394 | X-ray | 1.60 | hetero-oligomer | 3 x 6MO, 15 x SF4, 6 x MGD, 6 x HEM, 3 x CDL | HHblits | 0.31 |
| ``` target    TARREITRRGFLGTAAGAGFAAFVVSATRAWGLEAIENPLARYPDREWERVYRDLWRYDSKFTFLCAPNDTHNCLLDAYV 1kqf.1    ---MDVSRRQFFKICAGGMAGTTVAA-LGFAPKQA----LA--QARNYK------LLRAKEIRNTCT-YCSVGCGLLMYS  target    RS-G------VMTRIGPTMRYGEARDLDGNRASARWDPRVCQKGLALTRRFYGDRRLRHCMVRAGFKRWVDEGFPRGEDG 1kqf.1    LGDGAKNAREAIYHI------------EGDPDHPVSRGALCPKGAGLLDYVNSENRLRYPEYRAP-----------G---  target    KPPKEYFQRARDEWVRASHDEAAAVVAATLANIAATYSGEEGAQRLRDQGYEEETIEAMGGAGVQAMKFRGGMPLLGMTR 1kqf.1    ----------SDKWQRISWEEAFSRIAKLMKADRDANFIEKNEQGVTVN--RWLSTGMLCASGASNET------------  target    VFGLYRMANSMALLDAKVRGVGPDEARGARGFDNYS--WHTDLPPGHPMVTGQQTVDFDLNSVELAKNVVVWGMNWITTK 1kqf.1    GMLTQKFARSL----------------GMLAVDNQARVUHGPTVASLAPTFGRGAMTNHWVDIKNANVVMVMGGNAAEAH  target    MPDAHWLTEAR-LKGTRIIVIACEYSSTSSKADDAIVVRPGTTPALALGLSHVIMRDKLYDADYVRRWTDLPMLVRTDTL 1kqf.1    PVGFRWAMEAKNNNDATLIVVDPRFTRTASVADIYAPIRSGTDITFLSGVLRYLIENNKINAEYVKHYTNASLLVRDDF-  target    KYLSAEDVFGGGPAPL 1kqf.1    AFED------------ ``` | | | | | | | | | | | | | | | | | | | | | | | | | | | | | | | | | | | | | | | | | | | | | | | | | |
|  | 5e7o.1.A | DMSO reductase family type II enzyme, molybdopterin subunit  *Crystal structure of the perchlorate reductase PcrAB mutant W461E of PcrA from Azospira suillum PS* | 0.53 | 0.00 | 31.49 | 0.71 | 43-401 | X-ray | 2.40 | monomer | 4 x SF4, 1 x MO, 1 x MGD, 1 x MD1, 1 x F3S | HHblits | 0.36 |
| ``` target    TARREITRRGFLGTAAGAGFAAFVVSATRAWGLEAIENPLARYPDREWERVYRDLWRYDSKFTFLCAPNDTHNCLLDAYV 5e7o.1    ------------------------------------------FEYSGWENFHRTQWSWDKKTRGAHLVNCTGACPHFVYS  target    RSGVMTRIGPTMRYGEARDLDGNRASARWDPRVCQKGLALTRRFYGDRRLRHCMVRAGFKRWVDEGFPRGEDGKPPKEYF 5e7o.1    KDGVVMREEQSKD------IAPMPNIPEYNPRGCNKGECGHDYMYGPHRIKYPLIRVG---------ERG----------  target    QRARDEWVRASHDEAAAVVAATLANIAATYSGEEGAQRLRDQGYEEETIEAMGGAG-VQAMKFRGGMPLLGMTRVFGLYR 5e7o.1    ---EGKWRRATWEEALDMIADKCVDTIKNHAPDCISV--------------YSPVPAVSPVSFS------------AGHR  target    MANSMALLDAKVRGVGPDEARGARGFDNYSWHTDLPPGHPMVTGQQTVDFDLNSVELAKNVVVWGMNWITTKMPDAHWLT 5e7o.1    FAHYI----------------GAHAHTFYDWYGDHPTGQTQTCGVQGDTCETADWFNSKYIILWGSNPTQTRIPDAHFLS  target    EARLKGTRIIVIACEYSSTSSKADDAIVVRPGTTPALALGLSHVIMRDKLYDADYVRRWTDLPMLVRTDTLKYLSAEDVF 5e7o.1    EAQLNGAKIVSISPDYNSSTIKVDKWIHPQPGTDGALAMAMAHVIIKEKLYDAHSLKEQTDLSYLVRSDTKRFLREADVV  target    GGGPAPL 5e7o.1    AG----- ``` | | | | | | | | | | | | | | | | | | | | | | | | | | | | | | | | | | | | | | | | | | | | | | | | | |
|  | 5e7o.1.A | DMSO reductase family type II enzyme, molybdopterin subunit  *Crystal structure of the perchlorate reductase PcrAB mutant W461E of PcrA from Azospira suillum PS* | 0.51 | 0.00 | 31.82 | 0.70 | 48-402 | X-ray | 2.40 | monomer | 4 x SF4, 1 x MO, 1 x MGD, 1 x MD1, 1 x F3S | BLAST | 0.37 |
| ``` target    TARREITRRGFLGTAAGAGFAAFVVSATRAWGLEAIENPLARYPDREWERVYRDLWRYDSKFTFLCAPNDTHNCLLDAYV 5e7o.1    -----------------------------------------------WENFHRTQWSWDKKTRGAHLVNCTGACPHFVYS  target    RSGVMTRIGPTMRYGEARDLDGNRASARWDPRVCQKGLALTRRFYGDRRLRHCMVRAGFKRWVDEGFPRGEDGKPPKEYF 5e7o.1    KDGVV------MREEQSKDIAPMPNIPEYNPRGCNKGECGHDYMYGPHRIKYPLIRVG----------------------  target    QRARDEWVRASHDEAAAVVAATLANIAATYSGEEGAQRLRDQGYEEETIEAMGGAGVQAMKFRGGMPLLGMTRVFGLYRM 5e7o.1    ERGEGKWRRA--------------------TWEEALDMIADK-----CVDTIKNHAPDCISVYSPVPAVSPVSFSAGHRF  target    ANSMALLDAKVRGVGPDEARGARGFDNYSWHTDLPPGHPMVTGQQTVDFDLNSVELAKNVVVWGMNWITTKMPDAHWLTE 5e7o.1    AHYIG-------------AHAHTFYDWYGDH---PTGQTQTCGVQGDTCETADWFNSKYIILWGSNPTQTRIPDAHFLSE  target    ARLKGTRIIVIACEYSSTSSKADDAIVVRPGTTPALALGLSHVIMRDKLYDADYVRRWTDLPMLVRTDTLKYLSAEDVFG 5e7o.1    AQLNGAKIVSISPDYNSSTIKVDKWIHPQPGTDGALAMAMAHVIIKEKLYDAHSLKEQTDLSYLVRSDTKRFLREADVVA  target    GGPAPL 5e7o.1    GG---- ``` | | | | | | | | | | | | | | | | | | | | | | | | | | | | | | | | | | | | | | | | | | | | | | | | | |
|  | 4ydd.1.A | DMSO reductase family type II enzyme, molybdopterin subunit  *Crystal structure of the perchlorate reductase PcrAB from Azospira suillum PS* | 0.51 | 0.00 | 31.82 | 0.70 | 48-402 | X-ray | 1.86 | monomer | 4 x SF4, 1 x MO, 1 x MGD, 1 x MD1, 1 x F3S | BLAST | 0.37 |
| ``` target    TARREITRRGFLGTAAGAGFAAFVVSATRAWGLEAIENPLARYPDREWERVYRDLWRYDSKFTFLCAPNDTHNCLLDAYV 4ydd.1    -----------------------------------------------WENFHRTQWSWDKKTRGAHLVNCTGACPHFVYS  target    RSGVMTRIGPTMRYGEARDLDGNRASARWDPRVCQKGLALTRRFYGDRRLRHCMVRAGFKRWVDEGFPRGEDGKPPKEYF 4ydd.1    KDGVV------MREEQSKDIAPMPNIPEYNPRGCNKGECGHDYMYGPHRIKYPLIRVG----------------------  target    QRARDEWVRASHDEAAAVVAATLANIAATYSGEEGAQRLRDQGYEEETIEAMGGAGVQAMKFRGGMPLLGMTRVFGLYRM 4ydd.1    ERGEGKWRRA--------------------TWEEALDMIADK-----CVDTIKNHAPDCISVYSPVPAVSPVSFSAGHRF  target    ANSMALLDAKVRGVGPDEARGARGFDNYSWHTDLPPGHPMVTGQQTVDFDLNSVELAKNVVVWGMNWITTKMPDAHWLTE 4ydd.1    AHYIG-------------AHAHTFYDWYGDH---PTGQTQTCGVQGDTCETADWFNSKYIILWGSNPTQTRIPDAHFLSE  target    ARLKGTRIIVIACEYSSTSSKADDAIVVRPGTTPALALGLSHVIMRDKLYDADYVRRWTDLPMLVRTDTLKYLSAEDVFG 4ydd.1    AQLNGAKIVSISPDYNSSTIKVDKWIHPQPGTDGALAMAMAHVIIKEKLYDAHSLKEQTDLSYLVRSDTKRFLREADVVA  target    GGPAPL 4ydd.1    GG---- ``` | | | | | | | | | | | | | | | | | | | | | | | | | | | | | | | | | | | | | | | | | | | | | | | | | |
|  | 2ivf.1.A | ETHYLBENZENE DEHYDROGENASE ALPHA-SUBUNIT  *ETHYLBENZENE DEHYDROGENASE FROM AROMATOLEUM AROMATICUM* | 0.42 | 0.00 | 34.51 | 0.70 | 48-401 | X-ray | 1.88 | monomer | 1 x MES, 4 x SF4, 1 x MO, 1 x MGD, 1 x MD1, 1 x F3S, 1 x HEM | BLAST | 0.37 |
| ``` target    TARREITRRGFLGTAAGAGFAAFVVSATRAWGLEAIENPLARYPDREWERVYRDLWRYDS----KFTFLCAPNDTHNCLL 2ivf.1    -----------------------------------------------WEDIYRKEWKWDKVNWGSHLNICWPQGS--CKF  target    DAYVRSGVMTRIGPTMRYGEARDLDGNRASARWDPRVCQKGLALTRRFYGDRRLRHCMVRAGFKRWVDEGFPRGEDGKPP 2ivf.1    YVYVRNGIVWR-----EEQAAQTPACNVDYVDYNPLGCQKGSAFNNNLYGDERVKY----------------------PL  target    KEYFQRARDEWVRASHDEAAAVVAATLANIAATYSGEEGAQRLRDQGYEEETIEAMGGAGVQAMKFRGGMPLLGMTRVFG 2ivf.1    KRVGKRGEGKWKRV--------------------SWDEAAGDIADSIIDSFEAQGSDGFILDAPHVHAGSIAWGAG----  target    LYRMANSMALLDAKVRGVGPDEARGARGFDNYSWHTDLPPGHPMVTGQQTVDFDLNSVELAKNVVVWGMNWITTKMPDAH 2ivf.1    -FRMT---YLMD----GVSPDINVDIGDTYMGAFHT---------FGKMHMGYSADNLLDAELIFMTCSNWSYTYPSSYH  target    WLTEARLKGTRIIVIACEYSSTSSKADDAIVVRPGTTPALALGLSHVIMRDKLYDADYVRRWTDLPMLVRTDTLKYLSAE 2ivf.1    FLSEARYKGAEVVVIAPDFNPTTPAADLHVPVRVGSDAAFWLGLSQVMIDEKLFDRQFVCEQTDLPLLVRMDTGKFLSAE  target    DVFGGGPAPL 2ivf.1    DVDGG----- ``` | | | | | | | | | | | | | | | | | | | | | | | | | | | | | | | | | | | | | | | | | | | | | | | | | |
|  | 6sdv.1.A | Formate dehydrogenase, alpha subunit, selenocysteine-containing,Formate dehydrogenase, alpha subunit, selenocysteine-containing,W-formate dehydrogenase - alpha subunit  *W-formate dehydrogenase from Desulfovibrio vulgaris - Formate reduced form* | 0.44 |  | 21.17 | 0.76 | 4-388 | X-ray | 1.90 | hetero-1-1-mer | 2 x MGD, 4 x SF4, 1 x W, 1 x H2S | HHblits | 0.30 |
| ``` target    TARREITRRGFLGTAAGAGFAAFVVSATRAWGLEAIENPLARYPDREWERVYRDLWRYDSKFTFLCAPNDTHNCLLDAYV 6sdv.1    ---MTVTRRHFLKLSAGAAVAGAFTGLGLSLAPTV---ARAE-LQ---------KLQWAKQTTSICC-YCAVGCGLIVHT  target    R---SGVMTRIGPTMRYGEARDLDGNRASARWDPRVCQKGLALTRRFYGDRRLRHCMVRAGFKRWVDEGFPRGEDGKPPK 6sdv.1    AKDGQGRAVNVE------------GDPDHPINEGSLCPKGASIFQLGENDQRGTQPLYRAPF------------------  target    EYFQRARDEWVRASHDEAAAVVAATLANIAATYSGEEGAQRLRDQGYEEETIEAMGGAGVQAMKFRGGMPLLGMTRVFGL 6sdv.1    ------SDTWKPVTWDFALTEIAKRIKKTRDASFTEKNAAGDLVNR--TEAIASFGSAAMDNEEC---WAYGNILRSLGL  target    YRMANSMALLDAKVRGVGPDEARGARGFDNYSWHTDLPPGHPMVTGQQTVDFDLNSVELAKNVVVWGMNWITTKMPDAHW 6sdv.1    VYIEHQ----------------------ARIUH-SPTVPALAESFGRGAMTNHWNDLANSDCILIMGSNAAENHPIAFKW  target    LTEARLKGTRIIVIACEYSSTSSKADDAIVVRPGTTPALALGLSHVIMRDKLYDADYVRRWTDLPMLVRTDTLKYLSAED 6sdv.1    VLRAKDKGATLIHVDPRFTRTSARCDVYAPIRSGADIPFLGGLIKYILDNKLYFTDYVREYTNASLIVGEK---------  target    VFGGGPAPL 6sdv.1    --------- ``` | | | | | | | | | | | | | | | | | | | | | | | | | | | | | | | | | | | | | | | | | | | | | | | | | |
|  | 6sdr.1.A | Formate dehydrogenase, alpha subunit, selenocysteine-containing  *W-formate dehydrogenase from Desulfovibrio vulgaris - Oxidized form* | 0.45 |  | 21.31 | 0.75 | 4-388 | X-ray | 2.10 | hetero-1-1-mer | 2 x MGD, 4 x SF4, 1 x H2S, 1 x W | HHblits | 0.31 |
| ``` target    TARREITRRGFLGTAAGAGFAAFVVSATRAWGLEAIENPLARYPDREWERVYRDLWRYDSKFTFLCAPNDTHNCLLDAYV 6sdr.1    ---MTVTRRHFLKLSAGAAVAGAFTGLGLSLAPTV---ARAE-LQK---------LQWAKQTTSICC-YCAVGCGLIVHT  target    R---SGVMTRIGPTMRYGEARDLDGNRASARWDPRVCQKGLALTRRFYGDRRLRHCMVRAGFKRWVDEGFPRGEDGKPPK 6sdr.1    AKDGQGRAVNVE------------GDPDHPINEGSLCPKGASIFQLGENDQRGTQPLYRAPF------------------  target    EYFQRARDEWVRASHDEAAAVVAATLANIAATYSGEEGAQRLRDQGYEEETIEAMGGAGVQAMKFRGGMPLLGMTRVFGL 6sdr.1    ------SDTWKPVTWDFALTEIAKRIKKTRDASFTEKNAAGDLV--NRTEAIASFGSAAMDNE------------ECWAY  target    YRMANSMALLDAKVRGVGPDEARGARGFDNY--SWHTDLPPGHPMVTGQQTVDFDLNSVELAKNVVVWGMNWITTKMPDA 6sdr.1    GNILRSL----------------GLVYIEHQARIUHSPTVPALAESFGRGAMTNHWNDLANSDCILIMGSNAAENHPIAF  target    HWLTEARLKGTRIIVIACEYSSTSSKADDAIVVRPGTTPALALGLSHVIMRDKLYDADYVRRWTDLPMLVRTDTLKYLSA 6sdr.1    KWVLRAKDKGATLIHVDPRFTRTSARCDVYAPIRSGADIPFLGGLIKYILDNKLYFTDYVREYTNASLIVGEK-------  target    EDVFGGGPAPL 6sdr.1    ----------- ``` | | | | | | | | | | | | | | | | | | | | | | | | | | | | | | | | | | | | | | | | | | | | | | | | | |
|  | 1e5v.2.A | Dimethyl sulfoxide/trimethylamine N-oxide reductase  *OXIDIZED DMSO REDUCTASE EXPOSED TO HEPES BUFFER* | 0.40 |  | 20.88 | 0.73 | 2-383 | X-ray | 2.40 | monomer | 2 x PGD, 1 x 2MO | HHblits | 0.29 |
| ``` target    TARREITRRGFLGTAAGAGFAAFVVSATRAWGLEAIENPLARYPDREWERVYRDLWRYDSKFTFLCAPNDTHNCL-LDAY 1e5v.2    -LRAELYRRAFLSYSVAPGALGMFGRSLL--AK-------GARAE---------------ALANGT-VMSGSHWGVFTAT  target    VRSGVMTRIGPTMRYGEARDLDGNRASARWDPRVCQKGLALTRRFYGDRRLRHCMVRAGFKRWVDEGFPRGEDGKPPKEY 1e5v.2    VENGRATAFT------------PWEKDPHP----SPMLAGVLDSIYSPTRIKYPMVRREFLE-------KGVN----ADR  target    FQRARDEWVRASHDEAAAVVAATLANIAATYSGEEGAQRLRDQGYEEETIEAMGGAGVQAMKFRGGMPLLGMTRVFGLYR 1e5v.2    STRGNGDFVRVSWDQALDLVAAEVKRVEETYGPEGVFGGSY--GW--KSPGRLHNCTTLLRRMLT--------LAGGYVN  target    MANSMALLDAKVRGVGPDEARGARGFDNYSWHTDLPPGHPMVTGQQTVDFDLNSVELAKNVVVWGMNWITTKMPD----- 1e5v.2    GAGD-------Y------------STGAAQVIMPHVVGTLEVYEQQ--TAWPVLAENTEVMVFWAADPIKTSQIGWVIPE  target    ---AHWLTEARLKGTRIIVIACEYSSTSSK-ADDAIVVRPGTTPALALGLSHVIMRDKLYDADYVRRWTDLPMLVRTDTL 1e5v.2    HGAYPGLEALKAKGTKVIVIDPVRTKTVEFFGAEHITPKPQTDVAIMLGMAHTLVAEDLYDKDFIANYTSGFD-------  target    KYLSAEDVFGGGPAPL 1e5v.2    ---------------- ``` | | | | | | | | | | | | | | | | | | | | | | | | | | | | | | | | | | | | | | | | | | | | | | | | | |
|  | 4dmr.1.A | DMSO REDUCTASE  *REDUCED DMSO REDUCTASE FROM RHODOBACTER CAPSULATUS WITH BOUND DMSO SUBSTRATE* | 0.39 |  | 22.95 | 0.72 | 2-383 | X-ray | 1.90 | monomer | 2 x PGD, 1 x 4MO, 1 x O | HHblits | 0.30 |
| ``` target    TARREITRRGFLGTAAGAGFAAFVVSATRAWGLEAIENPLARYPDREWERVYRDLWRYDSKFTFLCAPNDTHNCL-LDAY 4dmr.1    -LRAELYRRAFLSYSVAPGALGMFGRSLL--A-------KGARA--E-------------ALANGT-VMSGSHWGVFTAT  target    VRSGVMTRIGPTMRYGEARDLDGNRASARWDPRVCQKGLALTRRFYGDRRLRHCMVRAGFKRWVDEGFPRGEDGKPPKEY 4dmr.1    VENGRATAFT------------PWEKDPHP----SPMLAGVLDSIYSPTRIKYPMVRREFL-------EKGVN----ADR  target    FQRARDEWVRASHDEAAAVVAATLANIAATYSGEEGAQRLRDQGYEEETIEAMGGAGVQAMKFRGGMPLLGMTRVFGLYR 4dmr.1    STRGNGDFVRVSWDQALDLVAAEVKRVEETYGPSGVFGGSY--GW--KSPGRLHNC----TTL--------------LRR  target    MANSMALLDAKVRGVGPDEARGARGFDNYSWH-----TDLPPGHPMVTGQQTVDFDLNSVELAKNVVVWGMNWITTKMPD 4dmr.1    MLTLAGGY---V-N----------GAGDYSTGAAQVIMPHVVGTLEVYEQQ--TAWPVLAENTEVMVFWAADPIKTSQIG  target    A--------HWLTEARLKGTRIIVIACEYSSTSSK-ADDAIVVRPGTTPALALGLSHVIMRDKLYDADYVRRWTDLPMLV 4dmr.1    WVIPEHGAYPGLEALKAKGTKVIVIDPVRTKTVEFFGAEHITPKPQTDVAIMLGMAHTLVAEDLYDKDFIANYTSGFD--  target    RTDTLKYLSAEDVFGGGPAPL 4dmr.1    --------------------- ``` | | | | | | | | | | | | | | | | | | | | | | | | | | | | | | | | | | | | | | | | | | | | | | | | | |
|  | 1e18.1.A | DMSO REDUCTASE.  *TUNGSTEN-SUSBSTITUTED DMSO REDUCTASE FROM RHODOBACTER CAPSULATUS* | 0.40 |  | 22.26 | 0.72 | 2-383 | X-ray | 2.00 | monomer | 2 x PGD, 1 x 6WO | HHblits | 0.30 |
| ``` target    TARREITRRGFLGTAAGAGFAAFVVSATRAWGLEAIENPLARYPDREWERVYRDLWRYDSKFTFLCAPNDTHNCL-LDAY 1e18.1    -LRAELYRRAFLSYSVAPGALGMFGRSLLA---------KGARA--E-------------ALANGT-VMSGSHWGVFTAT  target    VRSGVMTRIGPTMRYGEARDLDGNRASARWDPRVCQKGLALTRRFYGDRRLRHCMVRAGFKRWVDEGFPRGEDGKPPKEY 1e18.1    VENGRATAFT------------PWEKDPHP----SPMLAGVLDSIYSPTRIKYPMVRREFLE-------KGVN----ADR  target    FQRARDEWVRASHDEAAAVVAATLANIAATYSGEEGAQRLRDQGYEEETIEAMGGAGVQAMKFRGGMPLLGMTRVFGLYR 1e18.1    STRGNGDFVRVSWDQALDLVAAEVKRVEETYGPQGVFGGS----YGWKSPGRLHNCTTLLRRMLT--------LAGGYV-  target    MANSMALLDAKVRGVGPDEARGARGFDNYSWHT-----DLPPGHPMVTGQQTVDFDLNSVELAKNVVVWGMNWITTKMPD 1e18.1    --NG---------------------AGDYSTGAAQVIMPHVVGTLEVYEQQ--TAWPVLAENTEVMVFWAADPIKTSQIG  target    A--------HWLTEARLKGTRIIVIACEYSSTSSK-ADDAIVVRPGTTPALALGLSHVIMRDKLYDADYVRRWTDLPMLV 1e18.1    WVIPEHGAYPGLEALKAKGTKVIVIDPVRTKTVEFFGAEHITPKPQTDVAIMLGMAHTLVAEDLYDKDFIANYTSGFD--  target    RTDTLKYLSAEDVFGGGPAPL 1e18.1    --------------------- ``` | | | | | | | | | | | | | | | | | | | | | | | | | | | | | | | | | | | | | | | | | | | | | | | | | |
|  | 1e60.1.A | Dimethyl sulfoxide/trimethylamine N-oxide reductase  *OXIDIZED DMSO REDUCTASE EXPOSED TO HEPES - Structure II BUFFER* | 0.41 |  | 22.26 | 0.72 | 2-383 | X-ray | 2.00 | monomer | 2 x PGD, 1 x 2MO | HHblits | 0.30 |
| ``` target    TARREITRRGFLGTAAGAGFAAFVVSATRAWGLEAIENPLARYPDREWERVYRDLWRYDSKFTFLCAPNDTHNCL-LDAY 1e60.1    -LRAELYRRAFLSYSVAPGALGMFGRSLLA----K-----GARA--E-------------ALANGT-VMSGSHWGVFTAT  target    VRSGVMTRIGPTMRYGEARDLDGNRASARWDPRVCQKGLALTRRFYGDRRLRHCMVRAGFKRWVDEGFPRGEDGKPPKEY 1e60.1    VENGRATAFT------------PWEKDPHP----SPMLAGVLDSIYSPTRIKYPMVRREFL-------EKGVN----ADR  target    FQRARDEWVRASHDEAAAVVAATLANIAATYSGEEGAQRLRDQGYEEETIEAMGGAGVQAMKFRGGMPLLGMTRVFGLYR 1e60.1    STRGNGDFVRVSWDQALDLVAAEVKRVEETYGPEGVFGGS----YGWKSPGRLHNCTTLLRRML---T-----LAGGYVN  target    MANSMALLDAKVRGVGPDEARGARGFDNYSWHT-----DLPPGHPMVTGQQTVDFDLNSVELAKNVVVWGMNWITTKMPD 1e60.1    G------------------------AGDYSTGAAQVIMPHVVGTLEVYEQQ--TAWPVLAENTEVMVFWAADPIKTSQIG  target    --------AHWLTEARLKGTRIIVIACEYSSTSSK-ADDAIVVRPGTTPALALGLSHVIMRDKLYDADYVRRWTDLPMLV 1e60.1    WVIPEHGAYPGLEALKAKGTKVIVIDPVRTKTVEFFGAEHITPKPQTDVAIMLGMAHTLVAEDLYDKDFIANYTSGFD--  target    RTDTLKYLSAEDVFGGGPAPL 1e60.1    --------------------- ``` | | | | | | | | | | | | | | | | | | | | | | | | | | | | | | | | | | | | | | | | | | | | | | | | | |
|  | 2vpz.1.A | THIOSULFATE REDUCTASE  *POLYSULFIDE REDUCTASE NATIVE STRUCTURE* | 0.42 | 0.00 | 20.21 | 0.71 | 6-384 | X-ray | 2.40 | monomer | 10 x SF4, 4 x MGD, 2 x MO | HHblits | 0.30 |
| ``` target    TARREITRRGFLGTAAGAGFAAFVVSATRAWGLEAIENPLARYPDREWERVYRDLWRYDSKFTFLCAPNDTHNCLLDAYV 2vpz.1    -----MQRREFLKLSALGVGAMALRG-SGP--A--------KALKAPW------YAQEVKSVYQICE-GCFWRCGIVAHA  target    RSGVMTRIGPTMRYGEARDLDGNRASARWDPRVCQKGLALTRRFYGDRRLRHCMVRAGFKRWVDEGFPRGEDGKPPKEYF 2vpz.1    VGNRVYKVE------------GYEANPKSRGRLCPRGQGAPQTTYDPDRLKRPLIRVEGS-------Q------------  target    QRARDEWVRASHDEAAAVVAATLANIAATYSGEEGAQRLRDQGYEEETIEAMGGAGVQAMKFRGGMPLLGMTRVFGLYRM 2vpz.1    -RGEGKYRVATWEEALDHIAKKMLEIREKYGPEAIAF--------------FGHG-TGDYWFVD-----FLPAAWGSPNA  target    ANSMALLDAKVRGVGPDEARGARGFDNYSWHTDLPPGHPMVTGQQTVDFDLNSVELAKNVVVWGMNWITTK-MPDAHWLT 2vpz.1    AKPS---------------------VSL-CTAPREVASQWVFGRPIGGHEPIDWENARYIVLIGHHIGEDTHNTQLQDFA  target    EARLKGTRIIVIACEYSSTSSKADDAIVVRPGTTPALALGLSHVIMRDKLYDADYVRRWTDLPMLVRTDTLKYLSAEDVF 2vpz.1    LALKNGAKVVVVDPRFSTAAAKAHRWLPIKPGTDTALLLAWIHVLIYEDLYDKEYVAKYTVGFEE---------------  target    GGGPAPL 2vpz.1    ------- ``` | | | | | | | | | | | | | | | | | | | | | | | | | | | | | | | | | | | | | | | | | | | | | | | | | |
|  | 2vpx.1.D | THIOSULFATE REDUCTASE  *POLYSULFIDE REDUCTASE WITH BOUND QUINONE (UQ1)* | 0.42 | 0.00 | 20.21 | 0.71 | 6-384 | X-ray | 3.10 | monomer | 10 x SF4, 4 x MGD, 2 x MO, 2 x UQ1 | HHblits | 0.30 |
| ``` target    TARREITRRGFLGTAAGAGFAAFVVSATRAWGLEAIENPLARYPDREWERVYRDLWRYDSKFTFLCAPNDTHNCLLDAYV 2vpx.1    -----MQRREFLKLSALGVGAMALRG-SGP--A--------KALKAPW------YAQEVKSVYQICE-GCFWRCGIVAHA  target    RSGVMTRIGPTMRYGEARDLDGNRASARWDPRVCQKGLALTRRFYGDRRLRHCMVRAGFKRWVDEGFPRGEDGKPPKEYF 2vpx.1    VGNRVYKVE------------GYEANPKSRGRLCPRGQGAPQTTYDPDRLKRPLIRVEGS-------Q------------  target    QRARDEWVRASHDEAAAVVAATLANIAATYSGEEGAQRLRDQGYEEETIEAMGGAGVQAMKFRGGMPLLGMTRVFGLYRM 2vpx.1    -RGEGKYRVATWEEALDHIAKKMLEIREKYGPEAIAF--------------FGHG-TGDYWFVD-----FLPAAWGSPNA  target    ANSMALLDAKVRGVGPDEARGARGFDNYSWHTDLPPGHPMVTGQQTVDFDLNSVELAKNVVVWGMNWITTK-MPDAHWLT 2vpx.1    AKPS---------------------VSL-CTAPREVASQWVFGRPIGGHEPIDWENARYIVLIGHHIGEDTHNTQLQDFA  target    EARLKGTRIIVIACEYSSTSSKADDAIVVRPGTTPALALGLSHVIMRDKLYDADYVRRWTDLPMLVRTDTLKYLSAEDVF 2vpx.1    LALKNGAKVVVVDPRFSTAAAKAHRWLPIKPGTDTALLLAWIHVLIYEDLYDKEYVAKYTVGFEE---------------  target    GGGPAPL 2vpx.1    ------- ``` | | | | | | | | | | | | | | | | | | | | | | | | | | | | | | | | | | | | | | | | | | | | | | | | | |
|  | 8bqg.1.A | Formate dehydrogenase, alpha subunit, selenocysteine-containing  *W-formate dehydrogenase from Desulfovibrio vulgaris - Soaking with Formate 1 min* | 0.43 |  | 20.15 | 0.67 | 42-389 | X-ray | 1.95 | hetero-1-1-mer | 2 x MGD, 4 x SF4, 1 x H2S, 1 x W | HHblits | 0.30 |
| ``` target    TARREITRRGFLGTAAGAGFAAFVVSATRAWGLEAIENPLARYPDREWERVYRDLWRYDSKFTFLCAPNDTHNCLLDAYV 8bqg.1    -----------------------------------------E-LQ---------KLQWAKQTTSICC-YCAVGCGLIVHT  target    R---SGVMTRIGPTMRYGEARDLDGNRASARWDPRVCQKGLALTRRFYGDRRLRHCMVRAGFKRWVDEGFPRGEDGKPPK 8bqg.1    AKDGQGRAVNVE------------GDPDHPINEGSLCPKGASIFQLGENDQRGTQPLYRAPF------------------  target    EYFQRARDEWVRASHDEAAAVVAATLANIAATYSGEEGAQRLRDQGYEEETIEAMGGAGVQAMKFRGGMPLLGMTRVFGL 8bqg.1    ------SDTWKPVTWDFALTEIAKRIKKTRDASFTEKNAAGDLV--NRTEAIASFGSAAMDNEEC---WAYGNILRSLGL  target    YRMANSMALLDAKVRGVGPDEARGARGFDNYSWHTDLPPGHPMVTGQQTVDFDLNSVELAKNVVVWGMNWITTKMPDAHW 8bqg.1    VYIEH----------------------QARIUH-SPTVPALAESFGRGAMTNHWNDLANSDCILIMGSNAAENHPIAFKW  target    LTEARLKGTRIIVIACEYSSTSSKADDAIVVRPGTTPALALGLSHVIMRDKLYDADYVRRWTDLPMLVRTDTLKYLSAED 8bqg.1    VLRAKDKGATLIHVDPRFTRTSARCDVYAPIRSGADIPFLGGLIKYILDNKLYFTDYVREYTNASLIVGEKF--------  target    VFGGGPAPL 8bqg.1    --------- ``` | | | | | | | | | | | | | | | | | | | | | | | | | | | | | | | | | | | | | | | | | | | | | | | | | |
|  | 6f0k.1.B | Fe-S-cluster-containing hydrogenase  *Alternative complex III* | 0.31 |  | 16.79 | 0.69 | 4-386 | EM | 0.00 | hetero-1-1-1-1-1-1-… | 6 x HEC, 1 x F3S, 3 x SF4 | HHblits | 0.27 |
| ``` target    TARREITRRGFLGTAAGAGFAAFVVSATRAWGLEAIENPLARYPDREWERVYRDLWRYDSKFTFLCAPNDTHNCLLDAYV 6f0k.1    ---SGTSRRQFLQIMGASMALAGLTACRR--PV-EKILPYV----RQPEE----IIPGIPLYYATAMPFRGSVRPLLVES  target    RSGVMTRIGPTMRYGEARDLDGNRASARWDPRVCQKGLALTRRFYGDRRLRHCMVRAGFKRWVDEGFPRGEDGKPPKEYF 6f0k.1    HEGRPTKI------------EGNPDHPLSRGATGVFEQASLLNLYDPDRSQQVLRKG-----------------------  target    QRARDEWVRASHDEAAAVVAATLANIAATYSGEEGAQRLRDQGYEEETIEAMGGAGVQAMKFRGGMPLLGMTRVFGLYRM 6f0k.1    -------EPASWGDFVQFARSLA----AEAGTKR--------------LAVLCEPSSSPTL-AA--LRRELERRYAQVR-  target    ANSMALLDAKVRGVGPDEARGARGFDNYSWH--TDLPPGHPMVTGQQTVDFDLNSVELAKNVVVWGMNWITTK-MPD--- 6f0k.1    ------------------------WVTYRPEGDDHEALGLQQAFGRPV--RARYRFSEARVIVSLDADFLGPTDRNFVEN  target    A------HWLTEARLKGTRIIVIACEYSSTSSKADDAIVVRPGTTPALALGLSHVIMRDKLYDADYVRRWTDLPMLVRTD 6f0k.1    TREFAASRRMERPEDEISRLYVIESTYTVTGGMADHRLRLRAGDIPAFAAALAAELGVGELREA--GARFAGHPYVVE--  target    TLKYLSAEDVFGGGPAPL 6f0k.1    ------------------ ``` | | | | | | | | | | | | | | | | | | | | | | | | | | | | | | | | | | | | | | | | | | | | | | | | | |
|  | 4v4c.1.A | Pyrogallol hydroxytransferase large subunit  *Crystal Structure of Pyrogallol-Phloroglucinol Transhydroxylase from Pelobacter acidigallici* | 0.37 |  | 17.22 | 0.67 | 64-382 | X-ray | 2.35 | hetero-oligomer | 2 x CA, 2 x MGD, 1 x 4MO, 3 x SF4 | HHblits | 0.28 |
| ``` target    TARREITRRGFLGTAAGAGFAAFVVSATRAWGLEAIENPLARYPDREWERVYRDLWRYDSKFTFLCAPNDTHNCLLDAYV 4v4c.1    ---------------------------------------------------------------VRLTN-SSTGGPVFVYV  target    RSGVMTRIGPTMR-------YGEARDLDGNRASARWDPRVCQKGLALTRRFYGDRRLRHCMVRAGFKRWVDEGFPRGEDG 4v4c.1    KDGKIIRMTPMDFDDAVDAPSWKI-EARGKTFTPPRKTSIAPYTAGFKSMIYSDLRIPYPMKRKSFD-------PNGE--  target    KPPKEYFQRAR--------DEWVRASHDEAAAVVAATLANIAATYSGEEGAQRLRDQGY-EEETIEAMGGAGVQAMKFRG 4v4c.1    ---RNPQLRGAGLSKQDPWSDYERISWDEATDIVVAEINRIKHAYGPSAILSTPSSHHMWGNVGY--------RHSTY--  target    GMPLLGMTRVFGLYRMANSMALLDAKVRGVGPDEARGARGFDNYSWHTDLPPGHPMVTGQQTVDFD-LNSVELAKNVVVW 4v4c.1    ----FRFMNMMGFTYADHNP------------------DSWEGWHWGGMHMWGFSWRLGNPEQYDLLEDGLKHAEMIVFW  target    GMNWITTKMPDAHW-----LTEARLKGTRIIVIACEYSSTSS-KADDAIVVRPGTTPALALGLSHVIMRDKLYDADYVRR 4v4c.1    SSDPETNSGIYAGFESNIRRQWLKDLGVDFVFIDPHMNHTARLVADKWFSPKIGTDHALSFAIAYTWLKEDSYDKEYVAA  target    WTDLPMLVRTDTLKYLSAEDVFGGGPAPL 4v4c.1    NAHGF------------------------ ``` | | | | | | | | | | | | | | | | | | | | | | | | | | | | | | | | | | | | | | | | | | | | | | | | | |
|  | 1h0h.1.A | FORMATE DEHYDROGENASE SUBUNIT ALPHA  *Tungsten containing Formate Dehydrogenase from Desulfovibrio Gigas* | 0.41 |  | 19.47 | 0.65 | 60-388 | X-ray | 1.80 | hetero-1-1-mer | 1 x W, 1 x 2MD, 1 x MGD, 4 x SF4, 1 x CA | HHblits | 0.30 |
| ``` target    TARREITRRGFLGTAAGAGFAAFVVSATRAWGLEAIENPLARYPDREWERVYRDLWRYDSKFTFLCAPNDTHNCLLDAYV 1h0h.1    -----------------------------------------------------------KQTTSVCC-YCSVGCGLIVHT  target    --RSGVMTRIGPTMRYGEARDLDGNRASARWDPRVCQKGLALTRRFYGDRRLRHCMVRAGFKRWVDEGFPRGEDGKPPKE 1h0h.1    DKKTNRAINVE------------GDPDHPINEGSLCAKGASTWQLAENERRPANPLYRAP-----------G--------  target    YFQRARDEWVRASHDEAAAVVAATLANIAATYSGEEGAQRLRDQGYEEETIEAMGGAGVQAMKFRGGMPLLGMTRVFGLY 1h0h.1    -----SDQWEEKSWDWMLDTIAERVAKTREATFVTKNAKGQVVN--RCDGIASVGSAAMDNE------------ECWIYQ  target    RMANSMALLDAKVRGVGPDEARGARGFDNYS--WHTDLPPGHPMVTGQQTVDFDLNSVELAKNVVVWGMNWITTKMPDAH 1h0h.1    AWLRSL----------------GLFYIEHQARIUHSATVAALAESYGRGAMTNHWIDLKNSDVILMMGSNPAENHPISFK  target    WLTEARLKGTRIIVIACEYSSTSSKADDAIVVRPGTTPALALGLSHVIMRDKLYDADYVRRWTDLPMLVRTDTLKYLSAE 1h0h.1    WVMRAKDKGATLIHVDPRYTRTSTKCDLYAPLRSGSDIAFLNGMTKYILEKELYFKDYVVNYTNASFIVGEG--------  target    DVFGGGPAPL 1h0h.1    ---------- ``` | | | | | | | | | | | | | | | | | | | | | | | | | | | | | | | | | | | | | | | | | | | | | | | | | |
|  | 7e5z.1.A | Formate dehydrogenase  *Dehydrogenase holoenzyme* | 0.36 |  | 19.77 | 0.65 | 58-383 | EM | 0.00 | hetero-1-1-mer | 1 x W, 2 x MGD, 2 x FES, 4 x SF4, 1 x FMN | HHblits | 0.28 |
| ``` target    TARREITRRGFLGTAAGAGFAAFVVSATRAWGLEAIENPLARYPDREWERVYRDLWRYDSKFTFLCAPNDTHNCLLDAYV 7e5z.1    ---------------------------------------------------------PDREVKSLCP-YCGVGCQVSYKV  target    RSGVMTRIGPTMRYGEARDLDGNRASARWDPRVCQKGLALTRRFYGDRRLRHCMVRAGFKRWVDEGFPRGEDGKPPKEYF 7e5z.1    KDERIVYAEG------------V-NGPANQNRLCVKGRFGFDYVHHPHRLTVPLIRLENV-------PKDAND---QVDP  target    QRARDEWVRASHDEAAAVVAATLANIAATYSGEEGAQRLRDQGYEEETIEAMGGAGVQAMKFRGGMPLLGMTRVFGLYRM 7e5z.1    ANPWTHFREATWEEALDRAAGGLKAIRDTNGRKALAGFGSAKGSNEEAYL--------FQKLVR--------LGFGTNNV  target    ANSMALLDAKVRGVGPDEARGARGFDNYSWHTDLPPGHPMVTGQQTVDFDLNSVELAKNVVVWGMNWITTKMPDAHWLTE 7e5z.1    DHCTR-----------------------LCHASSVAALMEGLNSGAVTAPFSAALDAEVIVVIGANPTVNHPVAATFLKN  target    A-RLKGTRIIVIACEYSSTSSKADDAIVVRPGTTPALALGLSHVIMRDKLYDADYVRRWTDLPMLVRTDTLKYLSAEDVF 7e5z.1    AVKQRGAKLIIMDPRRQTLSRHAYRHLAFRPGSDVAMLNAMLNVIVTEGLYDEQYIAGYTENFE----------------  target    GGGPAPL 7e5z.1    ------- ``` | | | | | | | | | | | | | | | | | | | | | | | | | | | | | | | | | | | | | | | | | | | | | | | | | |
|  | 7vw6.1.A | Formate dehydrogenase  *Cryo-EM Structure of Formate Dehydrogenase 1 from Methylorubrum extorquens AM1* | 0.40 |  | 19.77 | 0.65 | 58-383 | EM | 0.00 | hetero-1-1-mer | 4 x SF4, 2 x FES, 2 x MGD, 1 x W, 1 x FMN | HHblits | 0.28 |
| ``` target    TARREITRRGFLGTAAGAGFAAFVVSATRAWGLEAIENPLARYPDREWERVYRDLWRYDSKFTFLCAPNDTHNCLLDAYV 7vw6.1    ---------------------------------------------------------PDREVKSLCP-YCGVGCQVSYKV  target    RSGVMTRIGPTMRYGEARDLDGNRASARWDPRVCQKGLALTRRFYGDRRLRHCMVRAGFKRWVDEGFPRGEDGKPPKEYF 7vw6.1    KDERIVYAEG------------V-NGPANQNRLCVKGRFGFDYVHHPHRLTVPLIRLENV-------PKDAND---QVDP  target    QRARDEWVRASHDEAAAVVAATLANIAATYSGEEGAQRLRDQGYEEETIEAMGGAGVQAMKFRGGMPLLGMTRVFGLYRM 7vw6.1    ANPWTHFREATWEEALDRAAGGLKAIRDTNGRKALAGFGSAKGSNEEAYL--------FQKLVR--------LGFGTNNV  target    ANSMALLDAKVRGVGPDEARGARGFDNYSWHTDLPPGHPMVTGQQTVDFDLNSVELAKNVVVWGMNWITTKMPDAHWLTE 7vw6.1    DHCTR-----------------------LCHASSVAALMEGLNSGAVTAPFSAALDAEVIVVIGANPTVNHPVAATFLKN  target    A-RLKGTRIIVIACEYSSTSSKADDAIVVRPGTTPALALGLSHVIMRDKLYDADYVRRWTDLPMLVRTDTLKYLSAEDVF 7vw6.1    AVKQRGAKLIIMDPRRQTLSRHAYRHLAFRPGSDVAMLNAMLNVIVTEGLYDEQYIAGYTENFE----------------  target    GGGPAPL 7vw6.1    ------- ``` | | | | | | | | | | | | | | | | | | | | | | | | | | | | | | | | | | | | | | | | | | | | | | | | | |
|  | 1ogy.1.A | PERIPLASMIC NITRATE REDUCTASE  *Crystal structure of the heterodimeric nitrate reductase from Rhodobacter sphaeroides* | 0.40 |  | 20.31 | 0.64 | 55-387 | X-ray | 3.20 | hetero-1-1-mer | 1 x SF4, 1 x MO, 2 x MGD, 2 x HEC | HHblits | 0.29 |
| ``` target    TARREITRRGFLGTAAGAGFAAFVVSATRAWGLEAIENPLARYPDREWERVYRDLWRYDSKFTFLCAPNDTHNCLLDAYV 1ogy.1    ------------------------------------------------------EALRIRWSKAPCR-FCGTGCGVMVGT  target    RSGVMTRIGPTMRYGEARDLDGNRASARWDPRVCQKGLALTRRFYGDRRLRHCMVRAGFKRWVDEGFPRGEDGKPPKEYF 1ogy.1    RDGQVVATH------------GDTQAEVNRGLNCVKGYFLSKIMYGEDRLTTPLLRMKDG---------V----------  target    QRARDEWVRASHDEAAAVVAATLANIAATYSGEEGAQRLRDQGYEEETIEAMGGAGVQAMKFRGGMPLLGMTR-VFGLYR 1ogy.1    YHKEGEFAPVSWDEAFDVMAAQAKLVLKEKAPEAVG--------------MFGSGQWTIWE---GYAASKLMRAGFRSNN  target    MANSMALLDAKVRGVGPDEARGARGFDNYSWHTDLPPGHPMVTGQQTVDFDLNSVELAKNVVVWGMNWITTKMPDAHWLT 1ogy.1    LDP----------------------NARH-CMASAATAFMRTFGMDEPMGCYDDFEAADAFVLWGSNMAEMHPILWSRLT  target    EAR--LKGTRIIVIACEYSSTSSKADDAIVVRPGTTPALALGLSHVIMRDKLYDADYVRRWTDLPMLVRTDTLKYLSAED 1ogy.1    DRRLSHEHVRVAVLSTFTHRSSDLSDTPIIFRPGTDRAILNYIAHHIISTGRVNRDFVDRHTNFALGATD----------  target    VFGGGPAPL 1ogy.1    --------- ``` | | | | | | | | | | | | | | | | | | | | | | | | | | | | | | | | | | | | | | | | | | | | | | | | | |
|  | 1dms.1.A | DMSO REDUCTASE  *STRUCTURE OF DMSO REDUCTASE* | 0.36 |  | 22.44 | 0.63 | 64-382 | X-ray | 1.88 | monomer | 2 x PGD, 1 x 2MO | HHblits | 0.30 |
| ``` target    TARREITRRGFLGTAAGAGFAAFVVSATRAWGLEAIENPLARYPDREWERVYRDLWRYDSKFTFLCAPNDTHNCLLDAYV 1dms.1    ---------------------------------------------------------------NGTVMSGSHWGVFTATV  target    RSGVMTRIGPTMRYGEARDLDGNRASARWDPRVCQKGLALTRRFYGDRRLRHCMVRAGFKRWVDEGFPRGEDGKPPKEYF 1dms.1    ENGRATAFT------------PWEKDPHP----TPMLEGVLDSIYSPTRIKYPMVRREFLE-------KGVN----ADRS  target    QRARDEWVRASHDEAAAVVAATLANIAATYSGEEGAQRLRDQGYEEETIEAMGGAGVQAMKFRGGMPLLGMTRVFGLYRM 1dms.1    TRGNGDFVRVSWDQALDLVAAEVKRVEETYGPQGVFGGS----YGWKSPGRLHNCTTLLRRML---T-----LAGGYVNG  target    ANSMALLDAKVRGVGPDEARGARGFDNYSWHT-----DLPPGHPMVTGQQTVDFDLNSVELAKNVVVWGMNWITTKMPDA 1dms.1    A------------------------GDYSTGAAQVIMPHVVGTLEVYEQQ--TAWPVLAENTEVMVFWAADPIKTSQIGW  target    --------HWLTEARLKGTRIIVIACEYSSTSSK-ADDAIVVRPGTTPALALGLSHVIMRDKLYDADYVRRWTDLPMLVR 1dms.1    VIPEHGAYPGLEALKAKGTKVIVIDPVRTKTVEFFGADHVTPKPQTDVAIMLGMAHTLVAEDLYDKDFIANYTSGF----  target    TDTLKYLSAEDVFGGGPAPL 1dms.1    -------------------- ``` | | | | | | | | | | | | | | | | | | | | | | | | | | | | | | | | | | | | | | | | | | | | | | | | | |
|  | 3o5a.1.A | Periplasmic nitrate reductase  *Crystal Structure of partially reduced Periplasmic Nitrate Reductase from Cupriavidus necator using Ionic Liquids* | 0.40 |  | 16.35 | 0.65 | 55-389 | X-ray | 1.72 | hetero-oligomer | 1 x SF4, 1 x MOS, 2 x MGD, 2 x HEC | HHblits | 0.27 |
| ``` target    TARREITRRGFLGTAAGAGFAAFVVSATRAWGLEAIENPLARYPDREWERVYRDLWRYDSKFTFLCAPNDTHNCLLDAYV 3o5a.1    ------------------------------------------------------EVTKLKWSKAPCR-FCGTGCGVTVAV  target    RSGVMTRIGPTMRYGEARDLDGNRASARWDPRVCQKGLALTRRFYGDRRLRHCMVRAGFKRWVDEGFPRGEDGKPPKEYF 3o5a.1    KDNKVVATQ------------GDPQAEVNKGLNCVKGYFLSKIMYGQDRLTRPLMRMKNG-------K------------  target    QRARDEWVRASHDEAAAVVAATLANIAATYSGEEGAQRLRDQGYEEETIEAMGGAGVQA-MKFRGGMPLLGMTRVFGLYR 3o5a.1    YDKNGDFAPVTWDQAFDEMERQFKRVLKEKGPTAVGM--------------FGSGQWTVWEGYAA-AKL--YKAGFRSNN  target    MANSMALLDAKVRGVGPDEARGARGFDNYSWHTDLPPGHPMVTGQQTVDFDLNSVELAKNVVVWGMNWITTKMPDAHWLT 3o5a.1    IDPN----------------------ARHCMA-SAAAGFMRTFGMDEPMGCYDDFEAADAFVLWGSNMAEMHPILWTRVT  target    EAR--LKGTRIIVIACEYSSTSSKADDAIVVRPGTTPALALGLSHVIMRDKLYDADYVRRWTDLPMLVRTDTLKYLSAED 3o5a.1    DRRLSHPKTRVVVLSTFTHRCFDLADIGIIFKPQTDLAMLNYIANYIIRNNKVNKDFVNKHTVFKEGVTDIG--------  target    VFGGGPAPL 3o5a.1    --------- ``` | | | | | | | | | | | | | | | | | | | | | | | | | | | | | | | | | | | | | | | | | | | | | | | | | |
|  | 1eu1.1.A | DIMETHYL SULFOXIDE REDUCTASE  *THE CRYSTAL STRUCTURE OF RHODOBACTER SPHAEROIDES DIMETHYLSULFOXIDE REDUCTASE REVEALS TWO DISTINCT MOLYBDENUM COORDINATION ENVIRONMENTS.* | 0.32 |  | 21.48 | 0.63 | 65-386 | X-ray | 1.30 | monomer | 3 x GLC, 1 x CD, 2 x MGD, 1 x 6MO, 2 x O | HHblits | 0.29 |
| ``` target    TARREITRRGFLGTAAGAGFAAFVVSATRAWGLEAIENPLARYPDREWERVYRDLWRYDSKFTFLCAPNDTHNCLLDAYV 1eu1.1    ----------------------------------------------------------------EVMSGCHWGV-FKARV  target    RSGVMTRIGPTMRYGEARDLDGNRASARWDPRVCQKGLALTRRFYGDRRLRHCMVRAGFKRWVDEGFPRGEDGKPPKEYF 1eu1.1    ENGRAVAFE------------PWDKDPAPSHQLPG----VLDSIYSPTRIKYPMVRREFL-------EKGVNA----DRS  target    QRARDEWVRASHDEAAAVVAATLANIAATYSGEEGAQRLRDQGYEEETIEAMGGAGVQAMKFRGGMPLLGMTRVFGLYR- 1eu1.1    TRGNGDFVRVTWDEALDLVARELKRVQESYGPTGTFGGSYGWKS-PGRLH-------NCQVLM--------RRALNLAGG  target    MANSMALLDAKVRGVGPDEARGARGFDNYSWHTDLPPGHPMVTGQQ----TVDFDLNSVELAKNVVVWGMNWITTKMPD- 1eu1.1    FVNS---------------------SGDYSTAA-AQIIMPHVMGTLEVYEQQTAWPVVVENTDLMVFWAADPMKTNEIGW  target    -------AHWLTEARLKGTRIIVIACEYSSTSSKAD-DAIVVRPGTTPALALGLSHVIMRDKLYDADYVRRWTDLPMLVR 1eu1.1    VIPDHGAYAGMKALKEKGTRVICINPVRTETADYFGADVVSPRPQTDVALMLGMAHTLYSEDLHDKDFLENCTTGFDLFA  target    TDTLKYLSAEDVFGGGPAPL 1eu1.1    -------------------- ``` | | | | | | | | | | | | | | | | | | | | | | | | | | | | | | | | | | | | | | | | | | | | | | | | | |
|  | 7l5i.1.A | Trimethylamine-N-oxide reductase  *Crystal Structure of Haemophilus influenzae MtsZ at pH 7.0* | 0.35 |  | 23.11 | 0.62 | 73-387 | X-ray | 1.73 | monomer | 2 x MGD, 1 x MO, 1 x O | HHblits | 0.31 |
| ``` target    TARREITRRGFLGTAAGAGFAAFVVSATRAWGLEAIENPLARYPDREWERVYRDLWRYDSKFTFLCAPNDTHNCLLDAYV 7l5i.1    ------------------------------------------------------------------------WGSIGVVV  target    RSGVMTRIGPTMRYGEARDLDGNRASARWDPRVCQKGLALTRRFYGDRRLRHCMVRAGFKRWVDEGFPRGEDGKPPKEYF 7l5i.1    QDGKVVKSGPA------------IE-PAVPNELQT---VVADQLYSEARVKCPMVRKGFLA--------NP--G-KSDTT  target    QRARDEWVRASHDEAAAVVAATLANIAATYSGEEGAQRLRDQGYEEETIEAMGGAGVQAMKFRGGMPLLGMTRVFGLYRM 7l5i.1    MRGRDEWVRVSWDEALDLVHNQLKRVRDEHGSTGIFAGSYG-WFSCGSL--------HAS-------------RTLLQRY  target    ANSMALLDAKVRGVGPDEARGARGFDNYSWHTDLPPGHPMVTGQQ---TVDFDL-NSVELAKNVVVWGMNWITTKMPD-- 7l5i.1    MNATGGF---V-G----------HKGDYSTGA-AQVIMPHVLGTIEVYEQQTSWESILESSDIIVLWSANPLTTMRIAWM  target    ------AHWLTEARLKGTRIIVIACEYSSTSSK-ADDAIVVRPGTTPALALGLSHVIMRDKLYDADYVRRWTDLPMLVRT 7l5i.1    STDQKGIEYFKKFQASGKRIICIDPQKSETCQMLNAEWIPVNTATDVPLMLGIAHTLVEQGKHDKDFLKKYTSGYAKFEE  target    DTLKYLSAEDVFGGGPAPL 7l5i.1    ------------------- ``` | | | | | | | | | | | | | | | | | | | | | | | | | | | | | | | | | | | | | | | | | | | | | | | | | |
|  | 7l5s.1.A | Trimethylamine-N-oxide reductase  *Crystal Structure of Haemophilus influenzae MtsZ at pH 5.5* | 0.34 |  | 23.11 | 0.62 | 73-387 | X-ray | 2.09 | monomer | 1 x O, 2 x MGD, 1 x MO | HHblits | 0.31 |
| ``` target    TARREITRRGFLGTAAGAGFAAFVVSATRAWGLEAIENPLARYPDREWERVYRDLWRYDSKFTFLCAPNDTHNCLLDAYV 7l5s.1    ------------------------------------------------------------------------WGSIGVVV  target    RSGVMTRIGPTMRYGEARDLDGNRASARWDPRVCQKGLALTRRFYGDRRLRHCMVRAGFKRWVDEGFPRGEDGKPPKEYF 7l5s.1    QDGKVVKSGPA------------IE-PAVPNELQT---VVADQLYSEARVKCPMVRKGFLA--------NP--G-KSDTT  target    QRARDEWVRASHDEAAAVVAATLANIAATYSGEEGAQRLRDQGYEEETIEAMGGAGVQAMKFRGGMPLLGMTRVFGLYRM 7l5s.1    MRGRDEWVRVSWDEALDLVHNQLKRVRDEHGSTGIFAGSYG-WFSCGSL--------HAS-------------RTLLQRY  target    ANSMALLDAKVRGVGPDEARGARGFDNYSWHTDLPPGHPMVTGQQ---TVDFDL-NSVELAKNVVVWGMNWITTKMPD-- 7l5s.1    MNATGGF---V-G----------HKGDYSTGA-AQVIMPHVLGTIEVYEQQTSWESILESSDIIVLWSANPLTTMRIAWM  target    ------AHWLTEARLKGTRIIVIACEYSSTSSK-ADDAIVVRPGTTPALALGLSHVIMRDKLYDADYVRRWTDLPMLVRT 7l5s.1    STDQKGIEYFKKFQASGKRIICIDPQKSETCQMLNAEWIPVNTATDVPLMLGIAHTLVEQGKHDKDFLKKYTSGYAKFEE  target    DTLKYLSAEDVFGGGPAPL 7l5s.1    ------------------- ``` | | | | | | | | | | | | | | | | | | | | | | | | | | | | | | | | | | | | | | | | | | | | | | | | | |
|  | 1g8j.1.A | ARSENITE OXIDASE  *CRYSTAL STRUCTURE ANALYSIS OF ARSENITE OXIDASE FROM ALCALIGENES FAECALIS* | 0.30 |  | 17.56 | 0.65 | 60-385 | X-ray | 2.03 | hetero-oligomer | 2 x MGD, 1 x O, 1 x 4MO, 1 x F3S, 1 x FES | HHblits | 0.27 |
| ``` target    TARREITRRGFLGTAAGAGFAAFVVSATRAWGLEAIENPLARYPDREWERVYRDLWRYDSKFTFLCAPNDTHNCLLDAYV 1g8j.1    -----------------------------------------------------------QRTNMTC-HFCIVGCGYHVYK  target    RS-----GVMTRIG-PTMRYG-----------------------E--ARDLDGNRASARWDPRVCQKGLALTRRFYGD-- 1g8j.1    WPELEEGGRAPEQNALGLDFRKQLPPLASTLTPAMTNVVTEHDGARYDIMVVPDKACVVNSGLSSTRGGKMASYMYTPTG  target    ---RRLRHCMVRAGFKRWVDEGFPRGEDGKPPKEYFQRARDEWVRASHDEAAAVVAATLANIAATYSGEEGAQRLRDQGY 1g8j.1    DGKERLSAPRLYAA--------------------------DEWVDTTWDHAMALYAGLIKKTLDSDGPQGVFFSCFDH--  target    EEETIEAMGGAGVQA-MKFRGGMPLLGMTRVFGLYRMANSMALLDAKVRGVGPDEARGARGFDNYSWHTDLPPGHPMVTG 1g8j.1    --------GGAGGGFENTWG--TGK-LMFSAIQTPMVRIH--------------------NRPAYNSEC---H-ATREMG  target    QQTVDFDLNSVELAKNVVVWGMNWITTKMPDA--HWL---------------TEARLKGTRIIVIACEYSSTSSKA---- 1g8j.1    IGELNNAYEDAQLADVIWSIGNNPYESQTNYFLNHWLPNLQGATTSKKKERFPNENFPQARIIFVDPRETPSVAIARHVA  target    ----DDAIVVRPGTTPALALGLSHVIMRDKLYDADYVRRWTDLPMLVRTDTLKYLSAEDVFGGGPAPL 1g8j.1    GNDRVLHLAIEPGTDTALFNGLFTYVVEQGWIDKPFIEAHTKGFDDA--------------------- ``` | | | | | | | | | | | | | | | | | | | | | | | | | | | | | | | | | | | | | | | | | | | | | | | | | |
|  | 6cz7.1.A | ArrA  *The arsenate respiratory reductase (Arr) complex from Shewanella sp. ANA-3* | 0.38 |  | 19.69 | 0.63 | 58-385 | X-ray | 1.62 | hetero-1-1-mer | 5 x SF4, 2 x MGD, 1 x MO, 1 x PG5 | HHblits | 0.30 |
| ``` target    TARREITRRGFLGTAAGAGFAAFVVSATRAWGLEAIENPLARYPDREWERVYRDLWRYDSKFTFLCAPNDTHNCLLDAYV 6cz7.1    ---------------------------------------------------------VGEWLATTCQ-GCTSWCAKQIYV  target    RSGVMTRIGPTMRYGEARDLDGNRASARWDPRVCQKGLALTRRFYGDRRLRHCMVRAGFKRWVDEGFPRGEDGKPPKEYF 6cz7.1    MDGRALKVR------------GNPNSGVHGMSSCPRQHLSLQQVYDPDRLRTPMMRTNPK--------KG----------  target    QRARDEWVRASHDEAAAVVAATLANIAATYSGEEGAQRLRDQGYEEETIEAMGGAGVQAMKFRGGMPLLGMTRVFGLYRM 6cz7.1    RDQDPKFVPISWDKALDMLADKIIALRVANEPHKYA--------------LLRGRYSHINDL----LYKKMTNLIGSPNN  target    ANSMALLDAKVRGVGPDEARGARGFDNYSWHTDLPPGHPMVTGQQTVDFDLNSVELAKNVVVWGMNWITTKMPDAHWLT- 6cz7.1    ISHS----------------------SVCAEA-HKMGPYYLDGN--WGYNQYDVKNAKFILSFGADPIASNRQVSFYSQT  target    -EARLKGTRIIVIACEYSSTSSKADDAIVVRPGTTPALALGLSHVIMRDKLYDADYVRRWTDLPMLVRTDTLKYLSAEDV 6cz7.1    WGDSLDHAKVVVVDPRLSASAAKAHKWIPIEPGQDSVLALAIAHVALVEGVWHKPFVGDFIEGKNLF-------------  target    FGGGPAPL 6cz7.1    -------- ``` | | | | | | | | | | | | | | | | | | | | | | | | | | | | | | | | | | | | | | | | | | | | | | | | | |
|  | 5nqd.1.A | AroA  *Arsenite oxidase AioAB from Rhizobium sp. str. NT-26 mutant AioBF108A* | 0.30 |  | 19.77 | 0.64 | 61-382 | X-ray | 2.20 | hetero-2-2-mer | 4 x MGD, 2 x O, 2 x 4MO, 2 x F3S, 2 x FES | HHblits | 0.28 |
| ``` target    TARREITRRGFLGTAAGAGFAAFVVSATRAWGLEAIENPLARYPDREWERVYRDLWRYDSKFTFLCAPNDTHNCLLDAYV 5nqd.1    ------------------------------------------------------------KHNVTC-HFCIVGCGYHAYT  target    R--------------SGVMTRIGPTMR---------------YGEARDLDGNRAS--ARWDPRVCQKGLALTRRFY---- 5nqd.1    WPINKQGGTDPQNNIFGVDLSEQQQAESDAWYSPSMYNVVKQDGRDVHVVIKPDHECVVNSGLGSVRGARMAETSFSEAR  target    --GDRRLRHCMVRAGFKRWVDEGFPRGEDGKPPKEYFQRARDEWVRASHDEAAAVVAATLANIAATYSGEEGAQRLRDQG 5nqd.1    NTQQQRLTDPLVWRY--------------------------GQMQPTSWDDALDLVARVTAKIVKEKGEDALIVSAFD--  target    YEEETIEAMGGAGVQAMKFRGGMPLLG-MTRVFGLYRMANSMALLDAKVRGVGPDEARGARGFDNYSWHTDLPPGHPMVT 5nqd.1    --------HGGAGGGYE---NTWGTGKLYFEAMKVKNIRIHN----------------------RPAYNSE-VHGT-RDM  target    GQQTVDFDLNSVELAKNVVVWGMNWITTKMPDA--HWLT---------------EARLKGTRIIVIACEYSSTSS----- 5nqd.1    GVGELNNCYEDAELADTIVAVGTNALETQTNYFLNHWIPNLRGESLGKKKELMPEEPHEAGRIIIVDPRRTVTVNACEQT  target    -KAD--DAIVVRPGTTPALALGLSHVIMRDKLYDADYVRRWTDLPMLVRTDTLKYLSAEDVFGGGPAPL 5nqd.1    AGADNVLHLAINSGTDLALFNALFTYIADKGWVDRDFIDKSTLRE------------------------ ``` | | | | | | | | | | | | | | | | | | | | | | | | | | | | | | | | | | | | | | | | | | | | | | | | | |
|  | 1g8k.1.A | ARSENITE OXIDASE  *CRYSTAL STRUCTURE ANALYSIS OF ARSENITE OXIDASE FROM ALCALIGENES FAECALIS* | 0.31 |  | 17.69 | 0.64 | 60-383 | X-ray | 1.64 | hetero-1-1-mer | 3 x HG, 2 x CA, 2 x MGD, 1 x O, 1 x 4MO, 1 x F3S, 1 x FES | HHblits | 0.27 |
| ``` target    TARREITRRGFLGTAAGAGFAAFVVSATRAWGLEAIENPLARYPDREWERVYRDLWRYDSKFTFLCAPNDTHNCLLDAYV 1g8k.1    -----------------------------------------------------------QRTNMTC-HFCIVGCGYHVYK  target    RS-----GVMT-RIGPTMRYGE-------------------------ARDLDGNRASARWDPRVCQKGLALTRRFYGD-- 1g8k.1    WPELEEGGRAPEQNALGLDFRKQLPPLAVTLTPAMTNVVTEHDGARYDIMVVPDKACVVNSGLSSTRGGKMASYMYTPTG  target    ---RRLRHCMVRAGFKRWVDEGFPRGEDGKPPKEYFQRARDEWVRASHDEAAAVVAATLANIAATYSGEEGAQRLRDQGY 1g8k.1    DGKERLSAPRLYAA--------------------------DEWVDTTWDHAMALYAGLIKKTLDKDGPQGVFFSCFDH--  target    EEETIEAMGGAGVQAMKFRGGMPLLG-MTRVFGLYRMANSMALLDAKVRGVGPDEARGARGFDNYSWHTDLPPGHPMVTG 1g8k.1    --------GGAGGGFE---NTWGTGKLMFSAIQTPMVRIH-----------------------NRPAYNSECH-ATREMG  target    QQTVDFDLNSVELAKNVVVWGMNWITTKMPD--AHWL---------------TEARLKGTRIIVIACEYSSTSSKA---- 1g8k.1    IGELNNAYEDAQLADVIWSIGNNPYESQTNYFLNHWLPNLQGATTSKKKERFPNENFPQARIIFVDPRETPSVAIARHVA  target    ----DDAIVVRPGTTPALALGLSHVIMRDKLYDADYVRRWTDLPMLVRTDTLKYLSAEDVFGGGPAPL 1g8k.1    GNDRVLHLAIEPGTDTALFNGLFTYVVEQGWIDKPFIEAHTKGFD----------------------- ``` | | | | | | | | | | | | | | | | | | | | | | | | | | | | | | | | | | | | | | | | | | | | | | | | | |
|  | 2nya.1.A | Periplasmic nitrate reductase  *Crystal structure of the periplasmic nitrate reductase (NAP) from Escherichia coli* | 0.39 |  | 14.89 | 0.65 | 62-396 | X-ray | 2.50 | monomer | 1 x SF4, 1 x 6MO, 2 x MGD | HHblits | 0.27 |
| ``` target    TARREITRRGFLGTAAGAGFAAFVVSATRAWGLEAIENPLARYPDREWERVYRDLWRYDSKFTFLCAPNDTHNCLLDAYV 2nya.1    -------------------------------------------------------------DKAPCR-FCGTGCGVLVGT  target    RSGVMTRIGPTMRYGEARDLDGNRASARWDPRVCQKGLALTRRFYGDRRLRHCMVRAGFKRWVDEGFPRGEDGKPPKEYF 2nya.1    QQGRVVACQ------------GDPDAPVNRGLNCIKGYFLPKIMYGKDRLTQPLLRMKNG-------------------K  target    QRARDEWVRASHDEAAAVVAATLANIAATYSGEEGAQRLRDQGYEEETIEAMGGAGVQAMKFRGGMPLLGMT-RVFGLYR 2nya.1    YDKEGEFTPITWDQAFDVMEEKFKTALKEKGPESIGM--------------FGSGQWTIWEG---YAASKLFKAGFRSNN  target    MANSMALLDAKVRGVGPDEARGARGFDNYSWHTDLPPGHPMVTGQQTVDFDLNSVELAKNVVVWGMNWITTKMPDAHWLT 2nya.1    IDPN----------------------ARH-CMASAVVGFMRTFGMDEPMGCYDDIEQADAFVLWGANMAEMHPILWSRIT  target    EAR--LKGTRIIVIACEYSSTSSKADDAIVVRPGTTPALALGLSHVIMRDKLYDADYVRRWTDLPMLVRTDTLKYLSAED 2nya.1    NRRLSNQNVTVAVLSTYQHRSFELADNGIIFTPQSDLVILNYIANYIIQNNAINQDFFSKHVNLRKGATDI-GYGLRPT-  target    VFGGGPAPL 2nya.1    --------- ``` | | | | | | | | | | | | | | | | | | | | | | | | | | | | | | | | | | | | | | | | | | | | | | | | | |
|  | 4aay.1.A | AROA  *Crystal Structure of the arsenite oxidase protein complex from Rhizobium species strain NT-26* | 0.30 |  | 19.38 | 0.64 | 61-382 | X-ray | 2.70 | hetero-oligomer | 4 x MGD, 2 x O, 2 x 4MO, 2 x F3S, 2 x FES | HHblits | 0.28 |
| ``` target    TARREITRRGFLGTAAGAGFAAFVVSATRAWGLEAIENPLARYPDREWERVYRDLWRYDSKFTFLCAPNDTHNCLLDAYV 4aay.1    ------------------------------------------------------------KHNVTC-HFCIVGCGYHAYT  target    R--------------SGVMTRIGPTMRYG---------------EARDLDGNRAS--ARWDPRVCQKGLALTRRFY---- 4aay.1    WPINKQGGTDPQNNIFGVDLSEQQQAESDAWYSPSMYNVVKQDGRDVHVVIKPDHECVVNSGLGSVRGARMAETSFSEAR  target    --GDRRLRHCMVRAGFKRWVDEGFPRGEDGKPPKEYFQRARDEWVRASHDEAAAVVAATLANIAATYSGEEGAQRLRDQG 4aay.1    NTQQQRLTDPLVWRY--------------------------GQMQPTSWDDALDLVARVTAKIVKEKGEDALIVSAFD--  target    YEEETIEAMGGAGVQAMKFRGGMPLLG-MTRVFGLYRMANSMALLDAKVRGVGPDEARGARGFDNYSWHTDLPPGHPMVT 4aay.1    --------HGGAGGGYE---NTWGTGKLYFEAMKVKNIRIHN----------------------RPAYNS-EVHGT-RDM  target    GQQTVDFDLNSVELAKNVVVWGMNWITTKMPDA--HWLT---------------EARLKGTRIIVIACEYSSTSS----- 4aay.1    GVGELNNCYEDAELADTIVAVGTNALETQTNYFLNHWIPNLRGESLGKKKELMPEEPHEAGRIIIVDPRRTVTVNACEQT  target    -KAD--DAIVVRPGTTPALALGLSHVIMRDKLYDADYVRRWTDLPMLVRTDTLKYLSAEDVFGGGPAPL 4aay.1    AGADNVLHLAINSGTDLALFNALFTYIADKGWVDRDFIDKSTLRE------------------------ ``` | | | | | | | | | | | | | | | | | | | | | | | | | | | | | | | | | | | | | | | | | | | | | | | | | |
|  | 1tmo.1.A | TRIMETHYLAMINE N-OXIDE REDUCTASE  *TRIMETHYLAMINE N-OXIDE REDUCTASE FROM SHEWANELLA MASSILIA* | 0.30 |  | 18.18 | 0.62 | 63-385 | X-ray | 2.50 | monomer | 2 x 2MD, 1 x 2MO | HHblits | 0.29 |
| ``` target    TARREITRRGFLGTAAGAGFAAFVVSATRAWGLEAIENPLARYPDREWERVYRDLWRYDSKFTFLCAPNDTHNCLLDAYV 1tmo.1    --------------------------------------------------------------LTTGSH-F---GAFKMKR  target    RSGVMTRIGPTMRYGEARDLDGNRASARWDPRVCQKGLALTRRFYGDRRLRHCMVRAGFKRWVDEGFPRGEDGKPPKEYF 1tmo.1    KNGVIAEVKP------------FDLDKYPTD--MING--IRGMVYNPSRVRYPMVRLDFL-------LKGHK----SNTH  target    QRARDEWVRASHDEAAAVVAATLANIAATYSGEEGAQRLRD-QGYEEETIEAMGGAGVQAMKFRGGMPLLGMTRVFGLY- 1tmo.1    QRGDFRFVRVTWDKALTLFKHSLDEVQTQYGPSGLHAGQTGWRATGQLHS--------STSHM---------QRAVGMHG  target    RMANSMALLDAKVRGVGPDEARGARGFDNYSWHTDLPPGHPMVTGQQ----TVDFDLNSVELAKNVVVWGMNWITTKMP- 1tmo.1    NYVKK---------------------IGDYSTGAG-QTILPYVLGSTEVYAQGTSWPLILEHSDTIVLWSNDPYKNLQVG  target    -------DAH---WLTEARL-KGTRIIVIACEYSSTSSK-ADDAIVVRPGTTPALALGLSHVIMRDKLYDADYVRRWTDL 1tmo.1    WNAETHESFAYLAQLKEKVKQGKIRVISIDPVVTKTQAYLGCEQLYVNPQTDVTLMLAIAHEMISKKLYDDKFIQGYSLG  target    PMLVRTDTLKYLSAEDVFGGGPAPL 1tmo.1    FEEF--------------------- ``` | | | | | | | | | | | | | | | | | | | | | | | | | | | | | | | | | | | | | | | | | | | | | | | | | |
|  | 1aa6.1.A | FORMATE DEHYDROGENASE H  *REDUCED FORM OF FORMATE DEHYDROGENASE H FROM E. COLI* | 0.39 |  | 23.29 | 0.61 | 61-385 | X-ray | 2.30 | monomer | 1 x SF4, 2 x MGD, 1 x 4MO | HHblits | 0.30 |
| ``` target    TARREITRRGFLGTAAGAGFAAFVVSATRAWGLEAIENPLARYPDREWERVYRDLWRYDSKFTFLCAPNDTHNCLLDAYV 1aa6.1    ------------------------------------------------------------KVVTVCP-YCASGCKINLVV  target    RSGVMTRIGPTMRYGEARDLDGNRASARWDPRVCQKGLALTRRFYGD----RRLRHCMVRAGFKRWVDEGFPRGEDGKPP 1aa6.1    DNGKIVRAEA------------AQ-GKTNQGTLCLKGYYGWDFINDTQILTPRLKTPMIRRQR-----------------  target    KEYFQRARDEWVRASHDEAAAVVAATLANIAATYSGEEGAQRLRDQGYEEETIEAMGGAGVQAMKFRGGMPLLGMTRVFG 1aa6.1    -------GGKLEPVSWDEALNYVAERLSAIKEKYGPDAIQTTGSSRGTGNETNY-------VMQKFAR--------AVIG  target    LYRMANSMALLDAKVRGVGPDEARGARGFDNYSWHTDLPPGHPMVTGQQTVDFDLNSVELAKNVVVWGMNWITTKMPDAH 1aa6.1    TNNVDCC----------------------ARV-UHGPSVAGLHQSVGNGAMSNAINEIDNTDLVFVFGYNPADSHPIVAN  target    WLTEARLKGTRIIVIACEYSSTSSKADDAIVVRPGTTPALALGLSHVIMRDKLYDADYVRRWTDLPMLVRTDTLKYLSAE 1aa6.1    HVINAKRNGAKIIVCDPRKIETARIADMHIALKNGSNIALLNAMGHVIIEENLYDKAFVASRTEGFEEY-----------  target    DVFGGGPAPL 1aa6.1    ---------- ``` | | | | | | | | | | | | | | | | | | | | | | | | | | | | | | | | | | | | | | | | | | | | | | | | | |
|  | 1fdo.1.A | FORMATE DEHYDROGENASE H  *OXIDIZED FORM OF FORMATE DEHYDROGENASE H FROM E. COLI* | 0.40 |  | 23.29 | 0.61 | 61-385 | X-ray | 2.80 | monomer | 1 x SF4, 2 x MGD, 1 x 6MO | HHblits | 0.30 |
| ``` target    TARREITRRGFLGTAAGAGFAAFVVSATRAWGLEAIENPLARYPDREWERVYRDLWRYDSKFTFLCAPNDTHNCLLDAYV 1fdo.1    ------------------------------------------------------------KVVTVCP-YCASGCKINLVV  target    RSGVMTRIGPTMRYGEARDLDGNRASARWDPRVCQKGLALTRRFYGD----RRLRHCMVRAGFKRWVDEGFPRGEDGKPP 1fdo.1    DNGKIVRAEA------------AQ-GKTNQGTLCLKGYYGWDFINDTQILTPRLKTPMIRRQR-----------------  target    KEYFQRARDEWVRASHDEAAAVVAATLANIAATYSGEEGAQRLRDQGYEEETIEAMGGAGVQAMKFRGGMPLLGMTRVFG 1fdo.1    -------GGKLEPVSWDEALNYVAERLSAIKEKYGPDAIQTTGSSRGTGNETNY-------VMQKFAR--------AVIG  target    LYRMANSMALLDAKVRGVGPDEARGARGFDNYSWHTDLPPGHPMVTGQQTVDFDLNSVELAKNVVVWGMNWITTKMPDAH 1fdo.1    TNNVDCC----------------------ARV-UHGPSVAGLHQSVGNGAMSNAINEIDNTDLVFVFGYNPADSHPIVAN  target    WLTEARLKGTRIIVIACEYSSTSSKADDAIVVRPGTTPALALGLSHVIMRDKLYDADYVRRWTDLPMLVRTDTLKYLSAE 1fdo.1    HVINAKRNGAKIIVCDPRKIETARIADMHIALKNGSNIALLNAMGHVIIEENLYDKAFVASRTEGFEEY-----------  target    DVFGGGPAPL 1fdo.1    ---------- ``` | | | | | | | | | | | | | | | | | | | | | | | | | | | | | | | | | | | | | | | | | | | | | | | | | |
|  | 2iv2.1.A | Formate dehydrogenase H  *Reinterpretation of reduced form of formate dehydrogenase H from E. coli* | 0.40 |  | 23.29 | 0.61 | 61-385 | X-ray | 2.27 | monomer | 1 x SF4, 1 x 2MD, 1 x MGD | HHblits | 0.30 |
| ``` target    TARREITRRGFLGTAAGAGFAAFVVSATRAWGLEAIENPLARYPDREWERVYRDLWRYDSKFTFLCAPNDTHNCLLDAYV 2iv2.1    ------------------------------------------------------------KVVTVCP-YCASGCKINLVV  target    RSGVMTRIGPTMRYGEARDLDGNRASARWDPRVCQKGLALTRRFYGD----RRLRHCMVRAGFKRWVDEGFPRGEDGKPP 2iv2.1    DNGKIVRAEA------------AQ-GKTNQGTLCLKGYYGWDFINDTQILTPRLKTPMIRRQR-----------------  target    KEYFQRARDEWVRASHDEAAAVVAATLANIAATYSGEEGAQRLRDQGYEEETIEAMGGAGVQAMKFRGGMPLLGMTRVFG 2iv2.1    -------GGKLEPVSWDEALNYVAERLSAIKEKYGPDAIQTTGSSRGTGNETNY-------VMQKFAR--------AVIG  target    LYRMANSMALLDAKVRGVGPDEARGARGFDNYSWHTDLPPGHPMVTGQQTVDFDLNSVELAKNVVVWGMNWITTKMPDAH 2iv2.1    TNNVDCC----------------------ARV-UHGPSVAGLHQSVGNGAMSNAINEIDNTDLVFVFGYNPADSHPIVAN  target    WLTEARLKGTRIIVIACEYSSTSSKADDAIVVRPGTTPALALGLSHVIMRDKLYDADYVRRWTDLPMLVRTDTLKYLSAE 2iv2.1    HVINAKRNGAKIIVCDPRKIETARIADMHIALKNGSNIALLNAMGHVIIEENLYDKAFVASRTEGFEEY-----------  target    DVFGGGPAPL 2iv2.1    ---------- ``` | | | | | | | | | | | | | | | | | | | | | | | | | | | | | | | | | | | | | | | | | | | | | | | | | |
|  | 7z0t.1.G | Formate dehydrogenase H  *Structure of the Escherichia coli formate hydrogenlyase complex (aerobic preparation, composite structure)* | 0.39 |  | 23.29 | 0.61 | 61-385 | EM | 0.00 | hetero-1-1-1-1-1-1-… | 1 x NI, 1 x FCO, 8 x SF4, 1 x FE, 2 x MGD, 1 x 6MO | HHblits | 0.30 |
| ``` target    TARREITRRGFLGTAAGAGFAAFVVSATRAWGLEAIENPLARYPDREWERVYRDLWRYDSKFTFLCAPNDTHNCLLDAYV 7z0t.1    ------------------------------------------------------------KVVTVCP-YCASGCKINLVV  target    RSGVMTRIGPTMRYGEARDLDGNRASARWDPRVCQKGLALTRRFYGD----RRLRHCMVRAGFKRWVDEGFPRGEDGKPP 7z0t.1    DNGKIVRAEA------------AQ-GKTNQGTLCLKGYYGWDFINDTQILTPRLKTPMIRRQR-----------------  target    KEYFQRARDEWVRASHDEAAAVVAATLANIAATYSGEEGAQRLRDQGYEEETIEAMGGAGVQAMKFRGGMPLLGMTRVFG 7z0t.1    -------GGKLEPVSWDEALNYVAERLSAIKEKYGPDAIQTTGSSRGTGNETNY-------VMQKFAR--------AVIG  target    LYRMANSMALLDAKVRGVGPDEARGARGFDNYSWHTDLPPGHPMVTGQQTVDFDLNSVELAKNVVVWGMNWITTKMPDAH 7z0t.1    TNNVDCC----------------------ARV-UHGPSVAGLHQSVGNGAMSNAINEIDNTDLVFVFGYNPADSHPIVAN  target    WLTEARLKGTRIIVIACEYSSTSSKADDAIVVRPGTTPALALGLSHVIMRDKLYDADYVRRWTDLPMLVRTDTLKYLSAE 7z0t.1    HVINAKRNGAKIIVCDPRKIETARIADMHIALKNGSNIALLNAMGHVIIEENLYDKAFVASRTEGFEEY-----------  target    DVFGGGPAPL 7z0t.1    ---------- ``` | | | | | | | | | | | | | | | | | | | | | | | | | | | | | | | | | | | | | | | | | | | | | | | | | |
|  | 6tg9.1.A | Formate dehydrogenase subunit alpha  *Cryo-EM Structure of NADH reduced form of NAD+-dependent Formate Dehydrogenase from Rhodobacter capsulatus* | 0.41 |  | 20.08 | 0.61 | 56-380 | EM | 3.24 | hetero-2-2-2-2-mer | 4 x MGD, 2 x 6MO, 4 x FES, 10 x SF4, 2 x H2S, 2 x FMN, 2 x NAI | HHblits | 0.29 |
| ``` target    TARREITRRGFLGTAAGAGFAAFVVSATRAWGLEAIENPLARYPDREWERVYRDLWRYDSKFTFLCAPNDTHNCLLDAYV 6tg9.1    -------------------------------------------------------GTPERKVVTTCA-YCGVGCSFEAHM  target    RSGVMTRIGPTMRYGEARDLDGNRASARWDPRVCQKGLALTRRFYGDRRLRHCMVRAGFKRWVDEGFPRGEDGKPPKEYF 6tg9.1    LGDQLVRMV------------PWKGGAANRGHSCVKGRFAYGYATHQDRILKPMIRDK-----------I----------  target    QRARDEWVRASHDEAAAVVAATLANIAATYSGEEGAQRLRDQGYEEETIEAMGGAGVQAMKFRGGMPLLGMTRVFGLYRM 6tg9.1    ---TDPWREVNWTEALDFTATRLRALRDSHGADALGVITSSRCTNEETYL--------VQKLA--------RAVFGTNNT  target    ANSMALLDAKVRGVGPDEARGARGFDNYSWHTDLPPGHPMVTGQQTVDFDLNSVELAKNVVVWGMNWITTKMPDAHWLTE 6tg9.1    DTCAR----------------------V-CHSPTGYGLKQTFGTSAGTQDFDSVEETDLALVIGANPTDGHPVFASRLRK  target    ARLKGTRIIVIACEYSSTS----SKADDAIVVRPGTTPALALGLSHVIMRDKLYDADYVRRWTDLPMLVRTDTLKYLSAE 6tg9.1    RLRAGAKLIVVDPRRIDLLNTPHRGEAWHLQLKPGTNVAVMTAMAHVIVTEQIFDKRFIGDRCD----------------  target    DVFGGGPAPL 6tg9.1    ---------- ``` | | | | | | | | | | | | | | | | | | | | | | | | | | | | | | | | | | | | | | | | | | | | | | | | | |
|  | 7qv7.1.L | Hydrogen dependent carbon dioxide reductase subunit FdhF  *Cryo-EM structure of Hydrogen-dependent CO2 reductase.* | 0.38 |  | 20.33 | 0.61 | 60-383 | EM | 0.00 | hetero-2-6-6-2-mer | 52 x SF4, 6 x 402 | HHblits | 0.30 |
| ``` target    TARREITRRGFLGTAAGAGFAAFVVSATRAWGLEAIENPLARYPDREWERVYRDLWRYDSKFTFLCAPNDTHNCLLDAYV 7qv7.1    -----------------------------------------------------------EKVLTTCP-YCGTGCGLYLKV  target    RSGVMTRIGPTMRYGEARDLDGNRASARWDPRVCQKGLALTRRFYGDRRLRHCMVRAGFKRWVDEGFPRGEDGKPPKEYF 7qv7.1    ENEKIVGVE------------PDKLHPVNQGELCIKGYYGYKYVHDPRRLTSPLIKKN----------------------  target    QRARDEWVRASHDEAAAVVAATLANIAATYSGEEGAQRLRDQGYEEETIEAMGGAGVQAMKFRGGMPLLGMTRVFGLYRM 7qv7.1    ----GKFVPVSWDEALNFIANGLKKIKSEYGSDAFAMFCSARATNEDNYA--------AQKFAR--------AVIGINNV  target    ANSMALLDAKVRGVGPDEARGARGFDNYSWHTDLPPGHPMVTGQQTVDFDLNSVE-LAKNVVVWGMNWITTKMPDAHWLT 7qv7.1    DHCA----------------------R-LCHAPTVAGLAMTLGSGAMTNSIPEISTYSDVIFIIGSNTAECHPLIAAHVI  target    EARLKGTRIIVIACEYSSTSSKADDAIVVRPGTTPALALGLSHVIMRDKLYDADYVRRWTDLPMLVRTDTLKYLSAEDVF 7qv7.1    KAKERGAKLIVADPRMNAMVHKADIWLRVPSGYNIPLINGMIHIIIKEGLVKTDFVKNHAVGFE----------------  target    GGGPAPL 7qv7.1    ------- ``` | | | | | | | | | | | | | | | | | | | | | | | | | | | | | | | | | | | | | | | | | | | | | | | | | |
|  | 7qv7.1.O | Hydrogen dependent carbon dioxide reductase subunit FdhF  *Cryo-EM structure of Hydrogen-dependent CO2 reductase.* | 0.38 |  | 20.33 | 0.61 | 60-383 | EM | 0.00 | hetero-2-6-6-2-mer | 52 x SF4, 6 x 402 | HHblits | 0.30 |
| ``` target    TARREITRRGFLGTAAGAGFAAFVVSATRAWGLEAIENPLARYPDREWERVYRDLWRYDSKFTFLCAPNDTHNCLLDAYV 7qv7.1    -----------------------------------------------------------EKVLTTCP-YCGTGCGLYLKV  target    RSGVMTRIGPTMRYGEARDLDGNRASARWDPRVCQKGLALTRRFYGDRRLRHCMVRAGFKRWVDEGFPRGEDGKPPKEYF 7qv7.1    ENEKIVGVE------------PDKLHPVNQGELCIKGYYGYKYVHDPRRLTSPLIKKN----------------------  target    QRARDEWVRASHDEAAAVVAATLANIAATYSGEEGAQRLRDQGYEEETIEAMGGAGVQAMKFRGGMPLLGMTRVFGLYRM 7qv7.1    ----GKFVPVSWDEALNFIANGLKKIKSEYGSDAFAMFCSARATNEDNYA--------AQKFAR--------AVIGINNV  target    ANSMALLDAKVRGVGPDEARGARGFDNYSWHTDLPPGHPMVTGQQTVDFDLNSVE-LAKNVVVWGMNWITTKMPDAHWLT 7qv7.1    DHCA----------------------R-LCHAPTVAGLAMTLGSGAMTNSIPEISTYSDVIFIIGSNTAECHPLIAAHVI  target    EARLKGTRIIVIACEYSSTSSKADDAIVVRPGTTPALALGLSHVIMRDKLYDADYVRRWTDLPMLVRTDTLKYLSAEDVF 7qv7.1    KAKERGAKLIVADPRMNAMVHKADIWLRVPSGYNIPLINGMIHIIIKEGLVKTDFVKNHAVGFE----------------  target    GGGPAPL 7qv7.1    ------- ``` | | | | | | | | | | | | | | | | | | | | | | | | | | | | | | | | | | | | | | | | | | | | | | | | | |
|  | 7bkb.1.F | Formate dehydrogenase  *Formate dehydrogenase - heterodisulfide reductase - formylmethanofuran dehydrogenase complex from Methanospirillum hungatei (hexameric, composite structure)* | 0.40 |  | 20.41 | 0.60 | 60-382 | EM | 0.00 | hetero-2-2-2-2-2-2-… | 48 x SF4, 4 x FAD, 2 x FES, 4 x 9S8, 4 x ZN, 2 x MO, 4 x MGD | HHblits | 0.30 |
| ``` target    TARREITRRGFLGTAAGAGFAAFVVSATRAWGLEAIENPLARYPDREWERVYRDLWRYDSKFTFLCAPNDTHNCLLDAYV 7bkb.1    -----------------------------------------------------------KYVATTCP-YCGVGCTLNLVV  target    RSGVMTRIGPTMRYGEARDLDGNRASARWDPRVCQKGLALTRRFYGDRRLRHCMVRAGFKRWVDEGFPRGEDGKPPKEYF 7bkb.1    SNGKVVGVE------------PNQRSPINEGKLCPKGVTCWEHIHSPDRLTTPLIKKD----------------------  target    QRARDEWVRASHDEAAAVVAATLANIAATYSGEEGAQRLRDQGYEEETIEAMGGAGVQAMKFRGGMPLLGMTRVFGLYRM 7bkb.1    ----GKFIEASWDEALDLVAKNLKVIYDKHGPKGLGFQTSCRTVNEDCYI--------FQKFAR--------VGFKTNNV  target    ANSMALLDAKVRGVGPDEARGARGFDNYSWHTDLPPGHPMVTGQQTVDFDLNSVELAKNVVVWGMNWITTKMPDAHWLTE 7bkb.1    DNCA----------------------R-ICHGPSVAGLSLSFGSGAATNGFEDALNADLILIWGSNAVEAHPLAGRRIAQ  target    ARLKGTRIIVIACEYSSTSSKADDAIVVRPGTTPALALGLSHVIMRDKLYDADYVRRWTDLPMLVRTDTLKYLSAEDVFG 7bkb.1    AKKKGIQIIAVDPRYTMTARLADTYVRFNPSTHIALANSMMYWIIKEGLEDKKFIQDRVNGF------------------  target    GGPAPL 7bkb.1    ------ ``` | | | | | | | | | | | | | | | | | | | | | | | | | | | | | | | | | | | | | | | | | | | | | | | | | |
|  | 2e7z.1.A | Acetylene hydratase Ahy  *Acetylene Hydratase from Pelobacter acetylenicus* | 0.36 |  | 22.22 | 0.60 | 63-383 | X-ray | 1.26 | monomer | 1 x SF4, 2 x MGD, 1 x W | HHblits | 0.31 |
| ``` target    TARREITRRGFLGTAAGAGFAAFVVSATRAWGLEAIENPLARYPDREWERVYRDLWRYDSKFTFLCAPNDTHNCLLDAYV 2e7z.1    --------------------------------------------------------------HVVCQ-SCDINCVVEAEV  target    R-SGVMTRIGPTMRYGEARDLDGNRASARW-DPRVCQKGLALTRRFYGDRRLRHCMVRAGFKRWVDEGFPRGEDGKPPKE 2e7z.1    KADGKIQTKS------------ISEPHPTTPPNSICMKSVNADTIRTHKDRVLYPLKNVGS--------KRG--------  target    YFQRARDEWVRASHDEAAAVVAATLANIAATYSGEEGAQRLRDQGYEEETIEAMGGAGVQAMKFRGGMPLLGMTRVFGLY 2e7z.1    -----EQRWERISWDQALDEIAEKLKKIIAKYGPESLGVS--------------QTEINQQSEYG---TLRRFMNLLGSP  target    RMANSMALLDAKVRGVGPDEARGARGFDNYSWHTDLPPGHPMVTGQQTVDFDLNSVELAKNVVVWGMNWITTKMPD-AHW 2e7z.1    NWTSAM-----------------------YMCIGNTAGVHRVTHGS----YSFASFADSNCLLFIGKNLSNHNWVSQFND  target    LTEARLKGTRIIVIACEYSSTSSKADDAIVVRPGTTPALALGLSHVIMRDKLYDADYVRRWTDLPMLVRTDTLKYLSAED 2e7z.1    LKAALKRGCKLIVLDPRRTKVAEMADIWLPLRYGTDAALFLGMINVIINEQLYDKEFVENWCVGFE--------------  target    VFGGGPAPL 2e7z.1    --------- ``` | | | | | | | | | | | | | | | | | | | | | | | | | | | | | | | | | | | | | | | | | | | | | | | | | |
|  | 2v3v.1.A | PERIPLASMIC NITRATE REDUCTASE  *A NEW CATALYTIC MECHANISM OF PERIPLASMIC NITRATE REDUCTASE FROM DESULFOVIBRIO DESULFURICANS ATCC 27774 FROM CRYSTALLOGRAPHIC AND EPR DATA AND BASED ON DETAILED ANALYSIS OF THE SIXTH LIGAND* | 0.38 |  | 19.84 | 0.61 | 58-381 | X-ray | 1.99 | monomer | 1 x SF4, 1 x MO, 2 x MGD, 4 x LCP | HHblits | 0.29 |
| ``` target    TARREITRRGFLGTAAGAGFAAFVVSATRAWGLEAIENPLARYPDREWERVYRDLWRYDSKFTFLCAPNDTHNCLLDAYV 2v3v.1    ---------------------------------------------------------PEKWVKGVC-RYCGTGCGVLVGV  target    RSGVMTRIGPTMRYGEARDLDGNRASARWDPRVCQKGLALTRRFYGDRRLRHCMVRAGFKRWVDEGFPRGEDGKPPKEYF 2v3v.1    KDGKAVAIQ------------GDPNNH-NAGLLCLKGSLLIPVLNSKERVTQPLVRRHK---------------------  target    QRARDEWVRASHDEAAAVVAATLANIAATYSGEEGAQRLRDQGYEEETIEAMGGAGVQAMKFRGGMPLLGMTRVFGLYRM 2v3v.1    ---GGKLEPVSWDEALDLMASRFRSSIDMYGPNSVAWYGSGQCLTEESYV--------ANKI--------FKGGFGTNNV  target    ANSMALLDAKVRGVGPDEARGARGFDNYSWHTDLPPGHPMVTGQQTVDFDLNSVELAKNVVVWGMNWITTKMPDAHWLTE 2v3v.1    DGN----------------------PRLCMA-SAVGGYVTSFGKDEPMGTYADIDQATCFFIIGSNTSEAHPVLFRRIAR  target    AR--LKGTRIIVIACEYSSTSSKADDAIVVRPGTTPALALGLSHVIMRDKLYDADYVRRWTDLPMLVRTDTLKYLSAEDV 2v3v.1    RKQVEPGVKIIVADPRRTNTSRIADMHVAFRPGTDLAFMHSMAWVIINEELDNPRFWQRYVNF-----------------  target    FGGGPAPL 2v3v.1    -------- ``` | | | | | | | | | | | | | | | | | | | | | | | | | | | | | | | | | | | | | | | | | | | | | | | | | |
|  | 2v45.1.A | PERIPLASMIC NITRATE REDUCTASE  *A NEW CATALYTIC MECHANISM OF PERIPLASMIC NITRATE REDUCTASE FROM DESULFOVIBRIO DESULFURICANS ATCC 27774 FROM CRYSTALLOGRAPHIC AND EPR DATA AND BASED ON DETAILED ANALYSIS OF THE SIXTH LIGAND* | 0.38 |  | 19.92 | 0.61 | 59-381 | X-ray | 2.40 | monomer | 1 x SF4, 1 x MO, 2 x MGD, 1 x LCP | HHblits | 0.29 |
| ``` target    TARREITRRGFLGTAAGAGFAAFVVSATRAWGLEAIENPLARYPDREWERVYRDLWRYDSKFTFLCAPNDTHNCLLDAYV 2v45.1    ----------------------------------------------------------EKWVKGVC-RYCGTGCGVLVGV  target    RSGVMTRIGPTMRYGEARDLDGNRASARWDPRVCQKGLALTRRFYGDRRLRHCMVRAGFKRWVDEGFPRGEDGKPPKEYF 2v45.1    KDGKAVAIQG------------NPNNH-NAGLLCLKGSLLIPVLNSKERVTQPLVRRHK---------------------  target    QRARDEWVRASHDEAAAVVAATLANIAATYSGEEGAQRLRDQGYEEETIEAMGGAGVQAMKFRGGMPLLGMTRVFGLYRM 2v45.1    ---GGKLEPVSWDEALDLMASRFRSSIDMYGPNSVAWYGSGQCLTEESYV--------ANKI--------FKGGFGTNNV  target    ANSMALLDAKVRGVGPDEARGARGFDNYSWHTDLPPGHPMVTGQQTVDFDLNSVELAKNVVVWGMNWITTKMPDAHWLTE 2v45.1    DGNP----------------------RL-CMASAVGGYVTSFGKDEPMGTYADIDQATCFFIIGSNTSEAHPVLFRRIAR  target    AR--LKGTRIIVIACEYSSTSSKADDAIVVRPGTTPALALGLSHVIMRDKLYDADYVRRWTDLPMLVRTDTLKYLSAEDV 2v45.1    RKQVEPGVKIIVADPRRTNTSRIADMHVAFRPGTDLAFMHSMAWVIINEELDNPRFWQRYVNF-----------------  target    FGGGPAPL 2v45.1    -------- ``` | | | | | | | | | | | | | | | | | | | | | | | | | | | | | | | | | | | | | | | | | | | | | | | | | |
|  | 7t2r.1.A | NiFe hydrogenase subunit A  *Structure of electron bifurcating Ni-Fe hydrogenase complex HydABCSL in FMN-free apo state* | 0.31 |  | 14.10 | 0.58 | 56-371 | EM | 0.00 | hetero-2-2-2-2-2-mer | 6 x FES, 12 x SF4, 2 x 3NI, 2 x FCO | HHblits | 0.26 |
| ``` target    TARREITRRGFLGTAAGAGFAAFVVSATRAWGLEAIENPLARYPDREWERVYRDLWRYDSKFTFLCAPNDTHNCLLDAYV 7t2r.1    -------------------------------------------------------SECDAVVESVCP-LCAVGCKIKTYV  target    RSGVMTRIGPTMRYGEARDLDGNRASARWDPRVCQKGLALTRRFYGDRRLRHCMVRAGFKRWVDEGFPRGEDGKPPKEYF 7t2r.1    RTGSIVRVE------------GTGVEEPDGGQLCHMGRWWLPESTERERVTVPLIREG----------------------  target    QRARDEWVRASHDEAAAVVAATLANIAATYSGEEGAQRLRDQGYEEETIEAMGGAGVQAMKFRGGMPLLGMTRVFGLYRM 7t2r.1    ----ASYREATWEEALALASAEFKKAYDQEKAGAI---LSSLCTDEELTL--------FSALF--------RNALKMKHI  target    ANSMALLDAKVRGVGPDEARGARGFDNYSWHTDLPPGHPM--VTGQQTVDFDLNSVELAKNVVVWGMNWITTKMPDAHWL 7t2r.1    DTFDGD----------------------IIRG-FFKGFMPFREQGV-RPFTAAHHILDSDLIITMFADPQKEAPVVASYI  target    TEA-RLKGTRIIVIACEYSSTSSKADDAIVVRPGTTPALALGLSHVIMRDKLYDADYVRRWTDLPMLVRTDTLKYLSAED 7t2r.1    RVACLHRNAKLMNLSYGPSPFPGLVDLDIRLPEGQAVPKALSNLAEIIGKISLG--------------------------  target    VFGGGPAPL 7t2r.1    --------- ``` | | | | | | | | | | | | | | | | | | | | | | | | | | | | | | | | | | | | | | | | | | | | | | | | | |
|  | 7t30.1.A | NiFe hydrogenase subunit A  *Structure of electron bifurcating Ni-Fe hydrogenase complex HydABCSL in FMN/NAD(H) bound state* | 0.31 |  | 14.10 | 0.58 | 56-371 | EM | 0.00 | hetero-2-2-2-2-2-mer | 4 x FES, 12 x SF4, 2 x NAD, 2 x FMN, 2 x 3NI, 2 x FCO | HHblits | 0.26 |
| ``` target    TARREITRRGFLGTAAGAGFAAFVVSATRAWGLEAIENPLARYPDREWERVYRDLWRYDSKFTFLCAPNDTHNCLLDAYV 7t30.1    -------------------------------------------------------SECDAVVESVCP-LCAVGCKIKTYV  target    RSGVMTRIGPTMRYGEARDLDGNRASARWDPRVCQKGLALTRRFYGDRRLRHCMVRAGFKRWVDEGFPRGEDGKPPKEYF 7t30.1    RTGSIVRVE------------GTGVEEPDGGQLCHMGRWWLPESTERERVTVPLIREG----------------------  target    QRARDEWVRASHDEAAAVVAATLANIAATYSGEEGAQRLRDQGYEEETIEAMGGAGVQAMKFRGGMPLLGMTRVFGLYRM 7t30.1    ----ASYREATWEEALALASAEFKKAYDQEKAGAI---LSSLCTDEELTL--------FSALF--------RNALKMKHI  target    ANSMALLDAKVRGVGPDEARGARGFDNYSWHTDLPPGHPM--VTGQQTVDFDLNSVELAKNVVVWGMNWITTKMPDAHWL 7t30.1    DTFDGD----------------------IIRG-FFKGFMPFREQGV-RPFTAAHHILDSDLIITMFADPQKEAPVVASYI  target    TEA-RLKGTRIIVIACEYSSTSSKADDAIVVRPGTTPALALGLSHVIMRDKLYDADYVRRWTDLPMLVRTDTLKYLSAED 7t30.1    RVACLHRNAKLMNLSYGPSPFPGLVDLDIRLPEGQAVPKALSNLAEIIGKISLG--------------------------  target    VFGGGPAPL 7t30.1    --------- ``` | | | | | | | | | | | | | | | | | | | | | | | | | | | | | | | | | | | | | | | | | | | | | | | | | |
|  | 7p61.1.C | NADH-quinone oxidoreductase  *Complex I from E. coli, DDM-purified, with NADH, Resting state* | 0.27 |  | 15.32 | 0.55 | 60-368 | EM | 0.00 | hetero-1-1-1-1-1-1-… | 7 x SF4, 1 x FMN, 1 x NAI, 2 x FES, 1 x CA, 2 x 3PE, 1 x UQ8 | HHblits | 0.27 |
| ``` target    TARREITRRGFLGTAAGAGFAAFVVSATRAWGLEAIENPLARYPDREWERVYRDLWRYDSKFTFLCAPNDTHNCLLDAYV 7p61.1    -----------------------------------------------------------QFAPSICQ-QCSIGCNISPGE  target    RSGVMTRIGPTMRYGEARDLDGNRASARWDPRVCQKGLALTRRFYGDRRLRHCMVRAGFKRWVDEGFPRGEDGKPPKEYF 7p61.1    RYGELRRIE------------NRYNGTVNHYFLCDRGRFGYGYVNLKDRPRQPVQRRG----------------------  target    QRARDEWVRASHDEAAAVVAATLANIAATYSGEEGAQRLRDQGYEEETIEAMGGAGVQAMKFRGGMPLLGMTRVFGLYRM 7p61.1    ----DDFITLNAEQAMQGAADILRQSKKVIGIGS------PRASVESNF-A-------------------LRELVGEENF  target    ANSMALLDAKVRGVGPDEARGARGFDNYSWHTDLPPGHPMVTGQQTVDFDLNSVELAKNVVVWGMNWITTKMPDAHWLTE 7p61.1    YTGIA----------------------HGEQERLQLALKVLREGGIYTPALREIESYDAVLVLGEDVTQTGARVALAVRQ  target    ARLKGTR--------------------------IIVIACEYSSTSSKADDAIVVRPGTTPALALGLSHVIMRDKLYDADY 7p61.1    AVKGKAREMAAAQKVADWQIAAILNIGQRAKHPLFVTNVDDTRLDDIAAWTYRAPVEDQARLGFAIAHALDNSA------  target    VRRWTDLPMLVRTDTLKYLSAEDVFGGGPAPL 7p61.1    -------------------------------- ``` | | | | | | | | | | | | | | | | | | | | | | | | | | | | | | | | | | | | | | | | | | | | | | | | | |
|  | 8e9g.1.G | NADH-quinone oxidoreductase subunit G  *Mycobacterial respiratory complex I with both quinone positions modelled* | 0.29 |  | 18.72 | 0.54 | 60-363 | EM | 0.00 | hetero-1-1-1-1-1-1-… |  | HHblits | 0.28 |
| ``` target    TARREITRRGFLGTAAGAGFAAFVVSATRAWGLEAIENPLARYPDREWERVYRDLWRYDSKFTFLCAPNDTHNCLLDAYV 8e9g.1    -----------------------------------------------------------VSSPSVCEH-CASGCAQRTDH  target    RSGVMTRIGPTMRYGEARDLDGNRASARWDPRVCQKGLALTRRFYGDRRLRHCMVRAGFKRWVDEGFPRGEDGKPPKEYF 8e9g.1    RRGKVLRRL------------AGDEPEVNEEWNCDKGRWAFTYATVGDRITTPMLRDG----------------------  target    QRARDEWVRASHDEAAAVVAATLANIAATYSGEEGAQRLRDQGYEEETIEAMGGAGVQAMKFRGGMPLLGMTRVFGLYRM 8e9g.1    ----GVLRPASWSEALTVAAAGLLTAAGST-------------------GVLVGGRCTVE------------DAYAYAKF  target    ANSMALLDAKVRGVGPDEARGARGFDNY--SWHTDLPPGHPMVTGQQTVDFDLNSVELAKNVVVWGMNWITTKMPDAHWL 8e9g.1    ARMVL---------------NTNDVDFRARPHSAEEAEFLAAHVAGQTMGLRYAELENAPTVLLAGFEPEEESPIVFLRL  target    TEA-RLKGTRIIVIACEYSS-TSSKADDAIVVRPGTTPALALGLSHVIMRDKLYDADYVRRWTDLPMLVRTDTLKYLSAE 8e9g.1    RKGVRKNGVQVVAVAPWASRGLTKLAGTVVPTVPGDEPAALDGMHDD---------------------------------  target    DVFGGGPAPL 8e9g.1    ---------- ``` | | | | | | | | | | | | | | | | | | | | | | | | | | | | | | | | | | | | | | | | | | | | | | | | | |
|  | 6lod.1.B | Fe-S-cluster-containing hydrogenase components 1-like protein  *Cryo-EM structure of the air-oxidized photosynthetic alternative complex III from Roseiflexus castenholzii* | 0.24 |  | 14.41 | 0.55 | 63-370 | EM | 0.00 | hetero-1-1-1-1-1-1-… | 6 x HEC, 2 x EL6, 3 x SF4, 1 x F3S | HHblits | 0.26 |
| ``` target    TARREITRRGFLGTAAGAGFAAFVVSATRAWGLEAIENPLARYPDREWERVYRDLWRYDSKFTFLCAPNDTHNCLLDAYV 6lod.1    --------------------------------------------------------------ATAV-TFAGFGVGLLVES  target    RSGVMTRIGPTMRYGEARDLDGNRASARWDPRVCQKGLALTRRFYGDRRLRHCMVRAGFKRWVDEGFPRGEDGKPPKEYF 6lod.1    HEGRPTKI------------EGNPDHPASLGSTDLITQAMILTMYDPDRSQAPTNA------------------------  target    QRARDEWVRASHDEAAAVVAATLANIAATYSGEEGAQRLRDQGYEEETIEAMGGAGVQAMKFRGGMPLLGMTRVFGLYRM 6lod.1    ------GQETTWDAFVAAATAAMQAQTAKQGAGL---------------RVLSGSLTSP-TLIA-Q-KQQLLTQFPQAKW  target    ANSMALLDAKVRGVGPDEARGARGFDNYSWHTDLPPGHPMVTGQQTVDFDLNSVELAKNVVVWGMNWITTKMPD---AHW 6lod.1    YE----------------------YEPVGR-DNANAGARLAFGAD--VHTIYRLDTAKVIVGFDADFTAPSPTGVRMARQ  target    LTEAR------LKGTRIIVIACEYSSTSSKADDAIVVRPGTTPALALGLSHVIMRDKLYDADYVRRWTDLPMLVRTDTLK 6lod.1    LADGRRIRKGTKEVNRLYLAESTPSITGLLADHRLPVRSSQIEHLVRALATLVGVPNVA---------------------  target    YLSAEDVFGGGPAPL 6lod.1    --------------- ``` | | | | | | | | | | | | | | | | | | | | | | | | | | | | | | | | | | | | | | | | | | | | | | | | | |
|  | 7nz1.1.E | NADH-quinone oxidoreductase subunit G  *Respiratory complex I from Escherichia coli - focused refinement of cytoplasmic arm* | 0.27 |  | 15.38 | 0.54 | 60-367 | EM | 0.00 | hetero-1-1-1-1-1-1-… | 7 x SF4, 2 x FES, 1 x FMN, 1 x CA | HHblits | 0.27 |
| ``` target    TARREITRRGFLGTAAGAGFAAFVVSATRAWGLEAIENPLARYPDREWERVYRDLWRYDSKFTFLCAPNDTHNCLLDAYV 7nz1.1    -----------------------------------------------------------QFAPSICQ-QCSIGCNISPGE  target    RSGVMTRIGPTMRYGEARDLDGNRASARWDPRVCQKGLALTRRFYGDRRLRHCMVRAGFKRWVDEGFPRGEDGKPPKEYF 7nz1.1    RYGELRRIE------------NRYNGTVNHYFLCDRGRFGYGYVNLKDRPRQPVQRRG----------------------  target    QRARDEWVRASHDEAAAVVAATLANIAATYSGEEGAQRLRDQGYEEETIEAMGGAGVQAMKFRGGMPLLGMTRVFGLYRM 7nz1.1    ----DDFITLNAEQAMQGAADILRQSKKVIGIGS------PRASVESNF-A-------------------LRELVGEENF  target    ANSMALLDAKVRGVGPDEARGARGFDNYSWHTDLPPGHPMVTGQQTVDFDLNSVELAKNVVVWGMNWITTKMPDAHWLTE 7nz1.1    YTGIAH----------------------GEQERLQLALKVLREGGIYTPALREIESYDAVLVLGEDVTQTGARVALAVRQ  target    ARLKGTR--------------------------IIVIACEYSSTSSKADDAIVVRPGTTPALALGLSHVIMRDKLYDADY 7nz1.1    AVKGKAREMAAAQKVADWQIAAILNIGQRAKHPLFVTNVDDTRLDDIAAWTYRAPVEDQARLGFAIAHALDNS-------  target    VRRWTDLPMLVRTDTLKYLSAEDVFGGGPAPL 7nz1.1    -------------------------------- ``` | | | | | | | | | | | | | | | | | | | | | | | | | | | | | | | | | | | | | | | | | | | | | | | | | |
|  | 7p63.1.C | NADH-quinone oxidoreductase  *Complex I from E. coli, DDM/LMNG-purified, under Turnover at pH 6, Closed state* | 0.27 |  | 15.38 | 0.54 | 60-367 | EM | 0.00 | hetero-1-1-1-1-1-1-… | 7 x SF4, 1 x FMN, 1 x NAI, 2 x FES, 1 x CA, 1 x DCQ, 4 x LFA, 8 x 3PE | HHblits | 0.27 |
| ``` target    TARREITRRGFLGTAAGAGFAAFVVSATRAWGLEAIENPLARYPDREWERVYRDLWRYDSKFTFLCAPNDTHNCLLDAYV 7p63.1    -----------------------------------------------------------QFAPSICQ-QCSIGCNISPGE  target    RSGVMTRIGPTMRYGEARDLDGNRASARWDPRVCQKGLALTRRFYGDRRLRHCMVRAGFKRWVDEGFPRGEDGKPPKEYF 7p63.1    RYGELRRIE------------NRYNGTVNHYFLCDRGRFGYGYVNLKDRPRQPVQRRG----------------------  target    QRARDEWVRASHDEAAAVVAATLANIAATYSGEEGAQRLRDQGYEEETIEAMGGAGVQAMKFRGGMPLLGMTRVFGLYRM 7p63.1    ----DDFITLNAEQAMQGAADILRQSKKVIGIGS------PRASVESNF-A-------------------LRELVGEENF  target    ANSMALLDAKVRGVGPDEARGARGFDNYSWHTDLPPGHPMVTGQQTVDFDLNSVELAKNVVVWGMNWITTKMPDAHWLTE 7p63.1    YTGIAH----------------------GEQERLQLALKVLREGGIYTPALREIESYDAVLVLGEDVTQTGARVALAVRQ  target    ARLKGTR--------------------------IIVIACEYSSTSSKADDAIVVRPGTTPALALGLSHVIMRDKLYDADY 7p63.1    AVKGKAREMAAAQKVADWQIAAILNIGQRAKHPLFVTNVDDTRLDDIAAWTYRAPVEDQARLGFAIAHALDNS-------  target    VRRWTDLPMLVRTDTLKYLSAEDVFGGGPAPL 7p63.1    -------------------------------- ``` | | | | | | | | | | | | | | | | | | | | | | | | | | | | | | | | | | | | | | | | | | | | | | | | | |
|  | 7zm7.1.I | NADH-ubiquinone oxidoreductase-like protein  *CryoEM structure of mitochondrial complex I from Chaetomium thermophilum (inhibited by DDM)* | 0.26 |  | 14.61 | 0.54 | 61-369 | EM | 0.00 | hetero-1-1-1-1-1-1-… | 4 x PC1, 14 x LMT, 5 x CDL, 8 x 3PE, 2 x FES, 6 x SF4, 1 x FMN, 1 x NDP, 1 x ZN, 2 x ZMP | HHblits | 0.26 |
| ``` target    TARREITRRGFLGTAAGAGFAAFVVSATRAWGLEAIENPLARYPDREWERVYRDLWRYDSKFTFLCAPNDTHNCLLDAYV 7zm7.1    ------------------------------------------------------------RTESID-VLDGLGSNIRVDS  target    RSGVMTRIGPTMRYGEARDLDGNRASARWDPRVCQKGLALTRRFYGDRRLRHCMVRAGFKRWVDEGFPRGEDGKPPKEYF 7zm7.1    RGLEVMRILP------------RLNDDVNEEWINDKTRFACDGLK-TQRLTMPLVRRD----------------------  target    QRARDEWVRASHDEAAAVVAATLANIAATYSGEEGAQRLRDQGYEEETIEAMGGAGVQAMKFRGGMPLLGMTRVFGLYRM 7zm7.1    ----GKFEPATWEQALTEIAHAYQTLAPKENEFK----------------VIAGQLVEVESL---VAMKDLANRLGSENL  target    ANSMALLDAKVRGVGPDEARGARGFDNYSWHTDLPPGHPMVTGQQ-TVDFDLNSVELAKNVVVWGMNWITTKMPDAHWLT 7zm7.1    ALDF-------------------------PGGSQPLAHGVDIRSNYLFNSKIWGIEEADAILLVGTNPRHEAAVLNARIR  target    EAR-LKGTRIIVIACEYSSTSSKADDAIVVRPGTTPALALGLSHVIMRDKLYDADYVRRWTDLPMLVRTDTLKYLSAEDV 7zm7.1    KQWLRSDLEIAAVGQPWESTFDYEH------LGTDLAALKNALSGPFGEKL-----------------------------  target    FGGGPAPL 7zm7.1    -------- ``` | | | | | | | | | | | | | | | | | | | | | | | | | | | | | | | | | | | | | | | | | | | | | | | | | |
|  | 6btm.1.B | Alternative Complex III subunit B  *Structure of Alternative Complex III from Flavobacterium johnsoniae (Wild Type)* | 0.24 |  | 11.98 | 0.53 | 62-368 | EM | 3.40 | hetero-1-1-1-1-1-1-… | 6 x HEC, 1 x F3S, 1 x SF4, 2 x E87 | HHblits | 0.26 |
| ``` target    TARREITRRGFLGTAAGAGFAAFVVSATRAWGLEAIENPLARYPDREWERVYRDLWRYDSKFTFLCAPNDTHNCLLDAYV 6btm.1    -------------------------------------------------------------YATTV-FDGFDFANLLVKT  target    RSGVMTRIGPTMRYGEARDLDGNRASARWDPRVCQKGLALTRRFYGDRRLRHCMVRAGFKRWVDEGFPRGEDGKPPKEYF 6btm.1    REGRPIKI------------ENNTIAGAK-FSANARIHASILGLYDSMRLKEPKLDG-----------------------  target    QRARDEWVRASHDEAAAVVAATLANIAATYSGEEGAQRLRDQGYEEETIEAMGGAGVQAMKFRGGMPLLGMTRVFGLYRM 6btm.1    -------KNSSWSAVDLKIKSSLADAKAK-GGQ---------------VVLLTNTLASP-TTEK--LIGEFIAKNPN---  target    ANSMALLDAKVRGVGPDEARGARGFDNYSWH--TDLPPGHPMVTGQQTVDFDLNSVELAKNVVVWGMNWITTKMPD--AH 6btm.1    ----------------------AKHVVYDAVSSSDALDAFETVYGER--ALVDYDFSKASLIVSVGADFLGDWQGGGYDA  target    WLTEARL----KGTRIIVIACEYSSTSSKADDAIVVRPGTTPALALGLSHVIMRDKLYDADYVRRWTDLPMLVRTDTLKY 6btm.1    GYAKGRIPQNGKMSRHFQFESNMTLSGAAADKRVPMTTADQKQALVQIYNIVVGAS------------------------  target    LSAEDVFGGGPAPL 6btm.1    -------------- ``` | | | | | | | | | | | | | | | | | | | | | | | | | | | | | | | | | | | | | | | | | | | | | | | | | |
|  | 7bkb.1.L | Formylmethanofuran dehydrogenase, subunit B  *Formate dehydrogenase - heterodisulfide reductase - formylmethanofuran dehydrogenase complex from Methanospirillum hungatei (hexameric, composite structure)* | 0.24 |  | 17.45 | 0.52 | 61-368 | EM | 0.00 | hetero-2-2-2-2-2-2-… | 48 x SF4, 4 x FAD, 2 x FES, 4 x 9S8, 4 x ZN, 2 x MO, 4 x MGD | HHblits | 0.27 |
| ``` target    TARREITRRGFLGTAAGAGFAAFVVSATRAWGLEAIENPLARYPDREWERVYRDLWRYDSKFTFLCAPNDTHNCL-LDAY 7bkb.1    ------------------------------------------------------------IENVGCPY-CGCSCDDVRIT  target    VRSGVMTRIGPTMRYGEARDLDGNRASARWDPRVCQKGLALTRRFYGDRRLRHCMVRAGFKRWVDEGFPRGEDGKPPKEY 7bkb.1    VSDDG--------------------KDILEVENVCAIGTEIFKHGCSKDRIRLPRMRQP---------------------  target    FQRARDEWVRASHDEAAAVVAATLANIAATYSGEEGAQRLRDQGYEEETIEAMGGAGVQAMKFRGGMPLLGMTRVFGLYR 7bkb.1    ----DGSMKDISYEEAIDWTARHLLKAKKPL------------------MYGFGSTNCEGQ-A----AAARVMEIAGG-M  target    MANSMALLDAKVRGVGPDEARGARGFDNYSWHTDLPPGHPMVTGQQTVDFDLNSV-ELAKNVVVWGMNWITTKMPDAHWL 7bkb.1    LDN----------------------CATICHGP----SFLAIFDNGYPSCTLGEVKNRADVIVYWGSNPAHAHPRHMSRY  target    --------TEARLKGTRIIVIACEYSSTSSKADDAIVVRPGTTPALALGLSHVIMRDKLYDADYVRRWTDLPMLVRTDTL 7bkb.1    SIFPRGFFTGKGQKKRTVIVIDPRFTDTANVADYHLQVKQGHDYELFNAFRMVIHGHG----------------------  target    KYLSAEDVFGGGPAPL 7bkb.1    ---------------- ``` | | | | | | | | | | | | | | | | | | | | | | | | | | | | | | | | | | | | | | | | | | | | | | | | | |
|  | 3m9s.1.C | NADH-quinone oxidoreductase subunit 3  *Crystal structure of respiratory complex I from Thermus thermophilus* | 0.27 | 0.00 | 17.92 | 0.52 | 60-366 | X-ray | 4.50 | monomer | 7 x SF4, 2 x FES, 1 x FMN | HHblits | 0.27 |
| ``` target    TARREITRRGFLGTAAGAGFAAFVVSATRAWGLEAIENPLARYPDREWERVYRDLWRYDSKFTFLCAPNDTHNCLLDAYV 3m9s.1    -----------------------------------------------------------EETPTTCAL-CPVGCGITADT  target    RSGVMTRIGPTMRYGEARDLDGNRASARWDPRVCQKGLALTRRFYGDRRLRHCMVRAGFKRWVDEGFPRGEDGKPPKEYF 3m9s.1    RSGELLRIR------------AREVPEVNEIWICDAGRFGHEW-ADQNRLKTPLVRKE----------------------  target    QRARDEWVRASHDEAAAVVAATLANIAATYSGEEGAQRLRDQGYEEETIEAMGGAGVQAMKFRGGMPLLGMTRVFGLYRM 3m9s.1    ----GRLVEATWEEAFLALKEGLKEAR----GEE--------------VGLYLAHDATLE------------EGLLASEL  target    ANSMALLDAKVRGVGPDEARGARGFDNYSWHTDLPPGHPMVTGQQTVDFDLNSVELAKNVVVWGMNWITTKMPDAHWLTE 3m9s.1    AKAL----------------KTP---HLDFQGRTAA-PA----SLFPPASLEDLLQADFALVLG-DPTEEAPILHLRLSE  target    -------------------------ARLKGTRIIVIACEYSSTSSKADDAIVVRPGTTPALALGLSHVIMRDKLYDADYV 3m9s.1    FVRDLKPPHRYNHGTPFADLQIKERMPRRTDKMALFAPYRAPLMKWAAIHEVHRPGEEREILLALLGDKEG---------  target    RRWTDLPMLVRTDTLKYLSAEDVFGGGPAPL 3m9s.1    ------------------------------- ``` | | | | | | | | | | | | | | | | | | | | | | | | | | | | | | | | | | | | | | | | | | | | | | | | | |
|  | 2fug.2.C | NADH-quinone oxidoreductase chain 3  *Crystal structure of the hydrophilic domain of respiratory complex I from Thermus thermophilus* | 0.26 | 0.00 | 17.92 | 0.52 | 60-366 | X-ray | 3.30 | monomer | 7 x SF4, 2 x FES, 1 x FMN | HHblits | 0.27 |
| ``` target    TARREITRRGFLGTAAGAGFAAFVVSATRAWGLEAIENPLARYPDREWERVYRDLWRYDSKFTFLCAPNDTHNCLLDAYV 2fug.2    -----------------------------------------------------------EETPTTCAL-CPVGCGITADT  target    RSGVMTRIGPTMRYGEARDLDGNRASARWDPRVCQKGLALTRRFYGDRRLRHCMVRAGFKRWVDEGFPRGEDGKPPKEYF 2fug.2    RSGELLRIR------------AREVPEVNEIWICDAGRFGHEW-ADQNRLKTPLVRKE----------------------  target    QRARDEWVRASHDEAAAVVAATLANIAATYSGEEGAQRLRDQGYEEETIEAMGGAGVQAMKFRGGMPLLGMTRVFGLYRM 2fug.2    ----GRLVEATWEEAFLALKEGLKEAR----GEE--------------VGLYLAHDATLE------------EGLLASEL  target    ANSMALLDAKVRGVGPDEARGARGFDNYSWHTDLPPGHPMVTGQQTVDFDLNSVELAKNVVVWGMNWITTKMPDAHWLTE 2fug.2    AKAL----------------KTP---HLDFQGRTAA-PA----SLFPPASLEDLLQADFALVLG-DPTEEAPILHLRLSE  target    -------------------------ARLKGTRIIVIACEYSSTSSKADDAIVVRPGTTPALALGLSHVIMRDKLYDADYV 2fug.2    FVRDLKPPHRYNHGTPFADLQIKERMPRRTDKMALFAPYRAPLMKWAAIHEVHRPGEEREILLALLGDKEG---------  target    RRWTDLPMLVRTDTLKYLSAEDVFGGGPAPL 2fug.2    ------------------------------- ``` | | | | | | | | | | | | | | | | | | | | | | | | | | | | | | | | | | | | | | | | | | | | | | | | | |
|  | 6zjl.1.C | NADH-quinone oxidoreductase subunit 3  *Respiratory complex I from Thermus thermophilus, NAD+ dataset, major state* | 0.25 | 0.00 | 17.92 | 0.52 | 60-366 | EM | 0.00 | monomer | 7 x SF4, 1 x FMN, 2 x FES | HHblits | 0.27 |
| ``` target    TARREITRRGFLGTAAGAGFAAFVVSATRAWGLEAIENPLARYPDREWERVYRDLWRYDSKFTFLCAPNDTHNCLLDAYV 6zjl.1    -----------------------------------------------------------EETPTTCAL-CPVGCGITADT  target    RSGVMTRIGPTMRYGEARDLDGNRASARWDPRVCQKGLALTRRFYGDRRLRHCMVRAGFKRWVDEGFPRGEDGKPPKEYF 6zjl.1    RSGELLRIR------------AREVPEVNEIWICDAGRFGHEW-ADQNRLKTPLVRKE----------------------  target    QRARDEWVRASHDEAAAVVAATLANIAATYSGEEGAQRLRDQGYEEETIEAMGGAGVQAMKFRGGMPLLGMTRVFGLYRM 6zjl.1    ----GRLVEATWEEAFLALKEGLKEAR----GEE--------------VGLYLAHDATLE------------EGLLASEL  target    ANSMALLDAKVRGVGPDEARGARGFDNYSWHTDLPPGHPMVTGQQTVDFDLNSVELAKNVVVWGMNWITTKMPDAHWLTE 6zjl.1    AKAL----------------KTP---HLDFQGRTAA-PA----SLFPPASLEDLLQADFALVLG-DPTEEAPILHLRLSE  target    -------------------------ARLKGTRIIVIACEYSSTSSKADDAIVVRPGTTPALALGLSHVIMRDKLYDADYV 6zjl.1    FVRDLKPPHRYNHGTPFADLQIKERMPRRTDKMALFAPYRAPLMKWAAIHEVHRPGEEREILLALLGDKEG---------  target    RRWTDLPMLVRTDTLKYLSAEDVFGGGPAPL 6zjl.1    ------------------------------- ``` | | | | | | | | | | | | | | | | | | | | | | | | | | | | | | | | | | | | | | | | | | | | | | | | | |
|  | 6q8o.1.C | NADH-quinone oxidoreductase subunit 3  *Respiratory complex I from Thermus thermophilus with bound Piericidin A* | 0.27 | 0.00 | 17.92 | 0.52 | 60-366 | X-ray | 3.61 | monomer | 7 x SF4, 1 x FMN, 2 x FES, 1 x HQH | HHblits | 0.27 |
| ``` target    TARREITRRGFLGTAAGAGFAAFVVSATRAWGLEAIENPLARYPDREWERVYRDLWRYDSKFTFLCAPNDTHNCLLDAYV 6q8o.1    -----------------------------------------------------------EETPTTCAL-CPVGCGITADT  target    RSGVMTRIGPTMRYGEARDLDGNRASARWDPRVCQKGLALTRRFYGDRRLRHCMVRAGFKRWVDEGFPRGEDGKPPKEYF 6q8o.1    RSGELLRIR------------AREVPEVNEIWICDAGRFGHEW-ADQNRLKTPLVRKE----------------------  target    QRARDEWVRASHDEAAAVVAATLANIAATYSGEEGAQRLRDQGYEEETIEAMGGAGVQAMKFRGGMPLLGMTRVFGLYRM 6q8o.1    ----GRLVEATWEEAFLALKEGLKEAR----GEE--------------VGLYLAHDATLE------------EGLLASEL  target    ANSMALLDAKVRGVGPDEARGARGFDNYSWHTDLPPGHPMVTGQQTVDFDLNSVELAKNVVVWGMNWITTKMPDAHWLTE 6q8o.1    AKAL----------------KTP---HLDFQGRTAA-PA----SLFPPASLEDLLQADFALVLG-DPTEEAPILHLRLSE  target    -------------------------ARLKGTRIIVIACEYSSTSSKADDAIVVRPGTTPALALGLSHVIMRDKLYDADYV 6q8o.1    FVRDLKPPHRYNHGTPFADLQIKERMPRRTDKMALFAPYRAPLMKWAAIHEVHRPGEEREILLALLGDKEG---------  target    RRWTDLPMLVRTDTLKYLSAEDVFGGGPAPL 6q8o.1    ------------------------------- ``` | | | | | | | | | | | | | | | | | | | | | | | | | | | | | | | | | | | | | | | | | | | | | | | | | |
|  | 6zjy.1.C | NADH-quinone oxidoreductase subunit 3  *Respiratory complex I from Thermus thermophilus, NAD+ dataset, minor state* | 0.25 | 0.00 | 17.92 | 0.52 | 60-366 | EM | 0.00 | monomer | 7 x SF4, 2 x FES | HHblits | 0.27 |
| ``` target    TARREITRRGFLGTAAGAGFAAFVVSATRAWGLEAIENPLARYPDREWERVYRDLWRYDSKFTFLCAPNDTHNCLLDAYV 6zjy.1    -----------------------------------------------------------EETPTTCAL-CPVGCGITADT  target    RSGVMTRIGPTMRYGEARDLDGNRASARWDPRVCQKGLALTRRFYGDRRLRHCMVRAGFKRWVDEGFPRGEDGKPPKEYF 6zjy.1    RSGELLRIR------------AREVPEVNEIWICDAGRFGHEW-ADQNRLKTPLVRKE----------------------  target    QRARDEWVRASHDEAAAVVAATLANIAATYSGEEGAQRLRDQGYEEETIEAMGGAGVQAMKFRGGMPLLGMTRVFGLYRM 6zjy.1    ----GRLVEATWEEAFLALKEGLKEAR----GEE--------------VGLYLAHDATLE------------EGLLASEL  target    ANSMALLDAKVRGVGPDEARGARGFDNYSWHTDLPPGHPMVTGQQTVDFDLNSVELAKNVVVWGMNWITTKMPDAHWLTE 6zjy.1    AKAL----------------KTP---HLDFQGRTAA-PA----SLFPPASLEDLLQADFALVLG-DPTEEAPILHLRLSE  target    -------------------------ARLKGTRIIVIACEYSSTSSKADDAIVVRPGTTPALALGLSHVIMRDKLYDADYV 6zjy.1    FVRDLKPPHRYNHGTPFADLQIKERMPRRTDKMALFAPYRAPLMKWAAIHEVHRPGEEREILLALLGDKEG---------  target    RRWTDLPMLVRTDTLKYLSAEDVFGGGPAPL 6zjy.1    ------------------------------- ``` | | | | | | | | | | | | | | | | | | | | | | | | | | | | | | | | | | | | | | | | | | | | | | | | | |
|  | 6zjn.1.C | NADH-quinone oxidoreductase subunit 3  *Respiratory complex I from Thermus thermophilus, NADH dataset, minor state* | 0.26 | 0.00 | 17.92 | 0.52 | 60-366 | EM | 0.00 | monomer | 7 x SF4, 2 x FES | HHblits | 0.27 |
| ``` target    TARREITRRGFLGTAAGAGFAAFVVSATRAWGLEAIENPLARYPDREWERVYRDLWRYDSKFTFLCAPNDTHNCLLDAYV 6zjn.1    -----------------------------------------------------------EETPTTCAL-CPVGCGITADT  target    RSGVMTRIGPTMRYGEARDLDGNRASARWDPRVCQKGLALTRRFYGDRRLRHCMVRAGFKRWVDEGFPRGEDGKPPKEYF 6zjn.1    RSGELLRIR------------AREVPEVNEIWICDAGRFGHEW-ADQNRLKTPLVRKE----------------------  target    QRARDEWVRASHDEAAAVVAATLANIAATYSGEEGAQRLRDQGYEEETIEAMGGAGVQAMKFRGGMPLLGMTRVFGLYRM 6zjn.1    ----GRLVEATWEEAFLALKEGLKEAR----GEE--------------VGLYLAHDATLE------------EGLLASEL  target    ANSMALLDAKVRGVGPDEARGARGFDNYSWHTDLPPGHPMVTGQQTVDFDLNSVELAKNVVVWGMNWITTKMPDAHWLTE 6zjn.1    AKAL----------------KTP---HLDFQGRTAA-PA----SLFPPASLEDLLQADFALVLG-DPTEEAPILHLRLSE  target    -------------------------ARLKGTRIIVIACEYSSTSSKADDAIVVRPGTTPALALGLSHVIMRDKLYDADYV 6zjn.1    FVRDLKPPHRYNHGTPFADLQIKERMPRRTDKMALFAPYRAPLMKWAAIHEVHRPGEEREILLALLGDKEG---------  target    RRWTDLPMLVRTDTLKYLSAEDVFGGGPAPL 6zjn.1    ------------------------------- ``` | | | | | | | | | | | | | | | | | | | | | | | | | | | | | | | | | | | | | | | | | | | | | | | | | |
|  | 6ziy.1.C | NADH-quinone oxidoreductase subunit 3  *Respiratory complex I from Thermus thermophilus, NADH dataset, major state* | 0.25 | 0.00 | 17.92 | 0.52 | 60-366 | EM | 0.00 | monomer | 7 x SF4, 1 x FMN, 1 x NAI, 2 x FES | HHblits | 0.27 |
| ``` target    TARREITRRGFLGTAAGAGFAAFVVSATRAWGLEAIENPLARYPDREWERVYRDLWRYDSKFTFLCAPNDTHNCLLDAYV 6ziy.1    -----------------------------------------------------------EETPTTCAL-CPVGCGITADT  target    RSGVMTRIGPTMRYGEARDLDGNRASARWDPRVCQKGLALTRRFYGDRRLRHCMVRAGFKRWVDEGFPRGEDGKPPKEYF 6ziy.1    RSGELLRIR------------AREVPEVNEIWICDAGRFGHEW-ADQNRLKTPLVRKE----------------------  target    QRARDEWVRASHDEAAAVVAATLANIAATYSGEEGAQRLRDQGYEEETIEAMGGAGVQAMKFRGGMPLLGMTRVFGLYRM 6ziy.1    ----GRLVEATWEEAFLALKEGLKEAR----GEE--------------VGLYLAHDATLE------------EGLLASEL  target    ANSMALLDAKVRGVGPDEARGARGFDNYSWHTDLPPGHPMVTGQQTVDFDLNSVELAKNVVVWGMNWITTKMPDAHWLTE 6ziy.1    AKAL----------------KTP---HLDFQGRTAA-PA----SLFPPASLEDLLQADFALVLG-DPTEEAPILHLRLSE  target    -------------------------ARLKGTRIIVIACEYSSTSSKADDAIVVRPGTTPALALGLSHVIMRDKLYDADYV 6ziy.1    FVRDLKPPHRYNHGTPFADLQIKERMPRRTDKMALFAPYRAPLMKWAAIHEVHRPGEEREILLALLGDKEG---------  target    RRWTDLPMLVRTDTLKYLSAEDVFGGGPAPL 6ziy.1    ------------------------------- ``` | | | | | | | | | | | | | | | | | | | | | | | | | | | | | | | | | | | | | | | | | | | | | | | | | |
|  | 5xtb.1.L | NADH-ubiquinone oxidoreductase 75 kDa subunit, mitochondrial  *Cryo-EM structure of human respiratory complex I matrix arm* | 0.25 |  | 16.19 | 0.52 | 61-362 | EM | 0.00 | hetero-1-1-1-1-1-1-… | 6 x SF4, 1 x FMN, 1 x 8Q1, 1 x NDP, 2 x FES | HHblits | 0.27 |
| ``` target    TARREITRRGFLGTAAGAGFAAFVVSATRAWGLEAIENPLARYPDREWERVYRDLWRYDSKFTFLCAPNDTHNCLLDAYV 5xtb.1    ------------------------------------------------------------KTESID-VMDAVGSNIVVST  target    RSGVMTRIGPTMRYGEARDLDGNRASARWDPRVCQKGLALTRRFYGDRRLRHCMVRAGFKRWVDEGFPRGEDGKPPKEYF 5xtb.1    RTGEVMRIL------------PRMHEDINEEWISDKTRFAYDGLK-RQRLTEPMVRNE----------------------  target    QRARDEWVRASHDEAAAVVAATLANIAATYSGEEGAQRLRDQGYEEETIEAMGGAGVQAMKFRGGMPLLGMTRVFGLYRM 5xtb.1    ---KGLLTYTSWEDALSRVAGMLQSF----QGKDV--------------AAIAGGLVDAEAL---VALKDLLNRVDSDTL  target    ANSMALLDAKVRGVGPDEARGARGFDNYSWHTDLPPGHPMVTGQQTVDFDLNSVELAKNVVVWGMNWITTKMPDAHWLTE 5xtb.1    CTEEV----------------------FP-TAGAGTDLRSNY---LLNTTIAGVEEADVVLLVGTNPRFEAPLFNARIRK  target    ARL-KGTRIIVIACEYSSTSSKADDAIVVRPGTTPALALGLSHVIMRDKLYDADYVRRWTDLPMLVRTDTLKYLSAEDVF 5xtb.1    SWLHNDLKVALIGSPVDLTYTYD------HLGDSPKILQDIAS-------------------------------------  target    GGGPAPL 5xtb.1    ------- ``` | | | | | | | | | | | | | | | | | | | | | | | | | | | | | | | | | | | | | | | | | | | | | | | | | |
|  | 5t5i.1.B | Tungsten formylmethanofuran dehydrogenase subunit B  *TUNGSTEN-CONTAINING FORMYLMETHANOFURAN DEHYDROGENASE FROM METHANOTHERMOBACTER WOLFEII, ORTHORHOMBIC FORM AT 1.9 A* | 0.24 |  | 17.39 | 0.51 | 61-368 | X-ray | 1.90 | hetero-oligomer | 4 x ZN, 2 x MG, 18 x K, 22 x SF4, 2 x W, 4 x MGD, 2 x H2S, 2 x CA | HHblits | 0.27 |
| ``` target    TARREITRRGFLGTAAGAGFAAFVVSATRAWGLEAIENPLARYPDREWERVYRDLWRYDSKFTFLCAPNDTHNCL-LDAY 5t5i.1    ------------------------------------------------------------VKNVVCPF-CGTLCDDIICK  target    VRSGVMTRIGPTMRYGEARDLDGNRASARWDPRVCQKGLALTRRFYGDRRLRHCMVRAGFKRWVDEGFPRGEDGKPPKEY 5t5i.1    VEGNEIVGT----------------------INACRIGHSKFVHAEGAMRYKKPLIRKN---------------------  target    FQRARDEWVRASHDEAAAVVAATLANIAAT--YSGEEGAQRLRDQGYEEETIEAMGGAGVQAMKFRGGMPLLGMTRVFGL 5t5i.1    -----GEFVEVSYDEAIDKAAKILAESKRPLMYGWS---------CTECEAQA-------VGVELA---------EEAGA  target    YRMANSMALLDAKVRGVGPDEARGARGFDNYSWHTDLPPGHPMVTGQQTVDFDLNSV-ELAKNVVVWGMNWITTKMPDAH 5t5i.1    -VIDN----------------------TASVCHGPS-VLALQ-DVGYP--ICTFGEVKNRADVVVYWGCNPMHAHPRHMS  target    W-------LTEARLKGTRIIVIACEYSSTSSKADDAIVVRPGTTPALALGLSHVIMRDKLYDADYVRRWTDLPMLVRTDT 5t5i.1    RNVFARGFFRERGRSDRTLIVVDPRKTDSAKLADIHLQLDFDRDYELLDAMRACLLGHE---------------------  target    LKYLSAEDVFGGGPAPL 5t5i.1    ----------------- ``` | | | | | | | | | | | | | | | | | | | | | | | | | | | | | | | | | | | | | | | | | | | | | | | | | |
|  | 7arc.1.F | 75 kDa  *Cryo-EM structure of Polytomella Complex-I (peripheral arm)* | 0.26 |  | 15.87 | 0.51 | 61-363 | EM | 0.00 | hetero-1-1-1-1-1-1-… | 6 x SF4, 2 x FES, 1 x FMN, 1 x NDP, 1 x ZN, 1 x 8Q1 | HHblits | 0.27 |
| ``` target    TARREITRRGFLGTAAGAGFAAFVVSATRAWGLEAIENPLARYPDREWERVYRDLWRYDSKFTFLCAPNDTHNCLLDAYV 7arc.1    ------------------------------------------------------------GTETID-VSDALGSNIKVDC  target    RSGVMTRIGPTMRYGEARDLDGNRASARWDPRVCQKGLALTRRFYGDRRLRHCMVRAGFKRWVDEGFPRGEDGKPPKEYF 7arc.1    RGTEVMRIT------------PRLNDAINEEWLSDKGRFQYDGLK-RQRLNTPLVKGA----------------------  target    QRARDEWVRASHDEAAAVVAATLANIAATYSGEEGAQRLRDQGYEEETIEAMGGAGVQAMKFRGGMPLLGMTRVFGLYRM 7arc.1    ----KGLENATWSAAFDAIRTAIAGAK----GNE--------------LKAIAGKLADAESM---IALKDLFNKLGSGNL  target    ANSMALLDAKVRGVGPDEARGARGFDNYSWHTDLPPGHPMVTGQQTVDFDLNSVELAKNVVVWGMNWITTKMPDAHWLTE 7arc.1    IHEDG-------------------------SATLSADVRSSY---IANTTIASIEKADVILLVGTNPRFESPVFNARLRK  target    ARLKGTRIIVIACEYSSTSSKADDAIVVRPGTTPALALGLSHVIMRDKLYDADYVRRWTDLPMLVRTDTLKYLSAEDVFG 7arc.1    VFLDGAKVGLVGEKVDLT------YAYQHLGADVAALESLASG-------------------------------------  target    GGPAPL 7arc.1    ------ ``` | | | | | | | | | | | | | | | | | | | | | | | | | | | | | | | | | | | | | | | | | | | | | | | | | |
|  | 7dgr.10.A | NADH-ubiquinone oxidoreductase 75 kDa subunit, mitochondrial  *Activity optimized supercomplex state2* | 0.24 | 0.00 | 15.17 | 0.52 | 60-356 | EM | 0.00 | monomer |  | HHblits | 0.26 |
| ``` target    TARREITRRGFLGTAAGAGFAAFVVSATRAWGLEAIENPLARYPDREWERVYRDLWRYDSKFTFLCAPNDTHNCLLDAYV 7dgr.10   -----------------------------------------------------------RKTESID-VMDAVGSNIVVST  target    RSGVMTRIGPTMRYGEARDLDGNRASARWDPRVCQKGLALTRRFYGDRRLRHCMVRAGFKRWVDEGFPRGEDGKPPKEYF 7dgr.10   RTGEVMRIL------------PRMHEDINEEWISDKTRFAYDGLK-RQRLTEPMVRNE----------------------  target    QRARDEWVRASHDEAAAVVAATLANIAATYSGEEGAQRLRDQGYEEETIEAMGGAGVQAMKFRGGMPLLGMTRVFGLYRM 7dgr.10   ---KGLLTHTTWEDALSRVAGMLQSF----QGNDV--------------AAIAGGLVDAEAL---IALKDLLNRVDSDTL  target    ANSMALLDAKVRGVGPDEARGARGFDNYSWHTDLPPGHPMVTGQQTVDFDLNSVELAKNVVVWGMNWITTKMPDAHWLTE 7dgr.10   CTEE----------------------VFP-TAGAGTDLRSNY---LLNTTIAGVEEADVVLLVGTNPRFEAPLFNARIRK  target    ARL-KGTRIIVIACEYSSTSSKADDAIVVRPGTTPALALGLSHVIMRDKLYDADYVRRWTDLPMLVRTDTLKYLSAEDVF 7dgr.10   SWLHNDLKVALIGSPVDLTYRYDHLGDSPKILQDIAS-------------------------------------------  target    GGGPAPL 7dgr.10   ------- ``` | | | | | | | | | | | | | | | | | | | | | | | | | | | | | | | | | | | | | | | | | | | | | | | | | |
|  | 5o31.1.8 | NADH-ubiquinone oxidoreductase 75 kDa subunit, mitochondrial  *Mitochondrial complex I in the deactive state* | 0.24 | 0.00 | 15.17 | 0.52 | 60-356 | EM | 4.13 | monomer | 6 x SF4, 2 x FES, 1 x FMN, 1 x NAP, 1 x ZN | HHblits | 0.26 |
| ``` target    TARREITRRGFLGTAAGAGFAAFVVSATRAWGLEAIENPLARYPDREWERVYRDLWRYDSKFTFLCAPNDTHNCLLDAYV 5o31.1    -----------------------------------------------------------RKTESID-VMDAVGSNIVVST  target    RSGVMTRIGPTMRYGEARDLDGNRASARWDPRVCQKGLALTRRFYGDRRLRHCMVRAGFKRWVDEGFPRGEDGKPPKEYF 5o31.1    RTGEVMRIL------------PRMHEDINEEWISDKTRFAYDGLK-RQRLTEPMVRNE----------------------  target    QRARDEWVRASHDEAAAVVAATLANIAATYSGEEGAQRLRDQGYEEETIEAMGGAGVQAMKFRGGMPLLGMTRVFGLYRM 5o31.1    ---KGLLTHTTWEDALSRVAGMLQSF----QGNDV--------------AAIAGGLVDAEAL---IALKDLLNRVDSDTL  target    ANSMALLDAKVRGVGPDEARGARGFDNYSWHTDLPPGHPMVTGQQTVDFDLNSVELAKNVVVWGMNWITTKMPDAHWLTE 5o31.1    CTEE----------------------VFP-TAGAGTDLRSNY---LLNTTIAGVEEADVVLLVGTNPRFEAPLFNARIRK  target    ARL-KGTRIIVIACEYSSTSSKADDAIVVRPGTTPALALGLSHVIMRDKLYDADYVRRWTDLPMLVRTDTLKYLSAEDVF 5o31.1    SWLHNDLKVALIGSPVDLTYRYDHLGDSPKILQDIAS-------------------------------------------  target    GGGPAPL 5o31.1    ------- ``` | | | | | | | | | | | | | | | | | | | | | | | | | | | | | | | | | | | | | | | | | | | | | | | | | |
|  | 7qsd.1.G | NADH-ubiquinone oxidoreductase 75 kDa subunit, mitochondrial  *Bovine complex I in the active state at 3.1 A* | 0.25 | 0.00 | 15.31 | 0.51 | 61-355 | EM | 0.00 | monomer | 5 x PC1, 13 x 3PE, 6 x SF4, 2 x FES, 1 x FMN, 4 x CDL, 3 x LMT, 1 x GTP, 1 x MG, 1 x NDP, 1 x ZN, 2 x EHZ | HHblits | 0.26 |
| ``` target    TARREITRRGFLGTAAGAGFAAFVVSATRAWGLEAIENPLARYPDREWERVYRDLWRYDSKFTFLCAPNDTHNCLLDAYV 7qsd.1    ------------------------------------------------------------KTESIDV-MDAVGSNIVVST  target    RSGVMTRIGPTMRYGEARDLDGNRASARWDPRVCQKGLALTRRFYGDRRLRHCMVRAGFKRWVDEGFPRGEDGKPPKEYF 7qsd.1    RTGEVMRIL------------PRMHEDINEEWISDKTRFAYDGLK-RQRLTEPMVRNE----------------------  target    QRARDEWVRASHDEAAAVVAATLANIAATYSGEEGAQRLRDQGYEEETIEAMGGAGVQAMKFRGGMPLLGMTRVFGLYRM 7qsd.1    ---KGLLTHTTWEDALSRVAGMLQSF----QGNDV--------------AAIAGGLVDAEAL---IALKDLLNRVDSDTL  target    ANSMALLDAKVRGVGPDEARGARGFDNYSWHTDLPPGHPMVTGQQTVDFDLNSVELAKNVVVWGMNWITTKMPDAHWLTE 7qsd.1    CTEE----------------------VFP-TAGAGTDLRSN---YLLNTTIAGVEEADVVLLVGTNPRFEAPLFNARIRK  target    ARL-KGTRIIVIACEYSSTSSKADDAIVVRPGTTPALALGLSHVIMRDKLYDADYVRRWTDLPMLVRTDTLKYLSAEDVF 7qsd.1    SWLHNDLKVALIGSPVDLTYRYDHLGDSPKILQDIA--------------------------------------------  target    GGGPAPL 7qsd.1    ------- ``` | | | | | | | | | | | | | | | | | | | | | | | | | | | | | | | | | | | | | | | | | | | | | | | | | |
|  | 6zr2.1.G | NADH-ubiquinone oxidoreductase 75 kDa subunit, mitochondrial  *Cryo-EM structure of respiratory complex I in the active state from Mus musculus at 3.1 A* | 0.25 | 0.00 | 15.79 | 0.51 | 60-354 | EM | 3.10 | monomer | 6 x SF4, 4 x PC1, 2 x FES, 1 x FMN, 9 x 3PE, 7 x CDL, 1 x ATP, 1 x NDP, 1 x ZN, 2 x EHZ | HHblits | 0.26 |
| ``` target    TARREITRRGFLGTAAGAGFAAFVVSATRAWGLEAIENPLARYPDREWERVYRDLWRYDSKFTFLCAPNDTHNCLLDAYV 6zr2.1    -----------------------------------------------------------RKTESIDV-MDAVGSNIVVST  target    RSGVMTRIGPTMRYGEARDLDGNRASARWDPRVCQKGLALTRRFYGDRRLRHCMVRAGFKRWVDEGFPRGEDGKPPKEYF 6zr2.1    RTGEVMRIL------------PRMHEDINEEWISDKTRFAYDGLK-RQRLTEPMVRNE----------------------  target    QRARDEWVRASHDEAAAVVAATLANIAATYSGEEGAQRLRDQGYEEETIEAMGGAGVQAMKFRGGMPLLGMTRVFGLYRM 6zr2.1    ---KGLLTYTSWEDALSRVAGMLQN----FEGNAVA--------------AIAGGLVDAEAL---VALKDLLNKVDSDNL  target    ANSMALLDAKVRGVGPDEARGARGFDNYSWHTDLPPGHPMVTGQQTVDFDLNSVELAKNVVVWGMNWITTKMPDAHWLTE 6zr2.1    CTEEI----------------------F-PTEGAGTDLRSNY---LLNTTIAGVEEADVVLLVGTNPRFEAPLFNARIRK  target    AR-LKGTRIIVIACEYSSTSSKADDAIVVRPGTTPALALGLSHVIMRDKLYDADYVRRWTDLPMLVRTDTLKYLSAEDVF 6zr2.1    SWLHNDLKVALIGSPVDLTYRYDHLGDSPKILQDI---------------------------------------------  target    GGGPAPL 6zr2.1    ------- ``` | | | | | | | | | | | | | | | | | | | | | | | | | | | | | | | | | | | | | | | | | | | | | | | | | |
|  | 6g72.1.G | NADH-ubiquinone oxidoreductase 75 kDa subunit, mitochondrial  *Mouse mitochondrial complex I in the deactive state* | 0.25 | 0.00 | 15.79 | 0.51 | 60-354 | EM | 0.00 | monomer | 6 x SF4, 2 x FES, 1 x FMN, 1 x ADP, 1 x NDP, 1 x ZN, 2 x EHZ | HHblits | 0.26 |
| ``` target    TARREITRRGFLGTAAGAGFAAFVVSATRAWGLEAIENPLARYPDREWERVYRDLWRYDSKFTFLCAPNDTHNCLLDAYV 6g72.1    -----------------------------------------------------------RKTESIDV-MDAVGSNIVVST  target    RSGVMTRIGPTMRYGEARDLDGNRASARWDPRVCQKGLALTRRFYGDRRLRHCMVRAGFKRWVDEGFPRGEDGKPPKEYF 6g72.1    RTGEVMRIL------------PRMHEDINEEWISDKTRFAYDGLK-RQRLTEPMVRNE----------------------  target    QRARDEWVRASHDEAAAVVAATLANIAATYSGEEGAQRLRDQGYEEETIEAMGGAGVQAMKFRGGMPLLGMTRVFGLYRM 6g72.1    ---KGLLTYTSWEDALSRVAGMLQN----FEGNAVA--------------AIAGGLVDAEAL---VALKDLLNKVDSDNL  target    ANSMALLDAKVRGVGPDEARGARGFDNYSWHTDLPPGHPMVTGQQTVDFDLNSVELAKNVVVWGMNWITTKMPDAHWLTE 6g72.1    CTEEI----------------------F-PTEGAGTDLRSNY---LLNTTIAGVEEADVVLLVGTNPRFEAPLFNARIRK  target    AR-LKGTRIIVIACEYSSTSSKADDAIVVRPGTTPALALGLSHVIMRDKLYDADYVRRWTDLPMLVRTDTLKYLSAEDVF 6g72.1    SWLHNDLKVALIGSPVDLTYRYDHLGDSPKILQDI---------------------------------------------  target    GGGPAPL 6g72.1    ------- ``` | | | | | | | | | | | | | | | | | | | | | | | | | | | | | | | | | | | | | | | | | | | | | | | | | |
|  | 7ak6.1.G | NADH-ubiquinone oxidoreductase 75 kDa subunit, mitochondrial  *Cryo-EM structure of ND6-P25L mutant respiratory complex I from Mus musculus at 3.8 A* | 0.25 | 0.00 | 15.79 | 0.51 | 60-354 | EM | 0.00 | monomer | 6 x SF4, 1 x PC1, 2 x FES, 1 x FMN, 4 x 3PE, 2 x CDL, 1 x ATP, 1 x NDP, 1 x ZN, 2 x EHZ | HHblits | 0.26 |
| ``` target    TARREITRRGFLGTAAGAGFAAFVVSATRAWGLEAIENPLARYPDREWERVYRDLWRYDSKFTFLCAPNDTHNCLLDAYV 7ak6.1    -----------------------------------------------------------RKTESIDV-MDAVGSNIVVST  target    RSGVMTRIGPTMRYGEARDLDGNRASARWDPRVCQKGLALTRRFYGDRRLRHCMVRAGFKRWVDEGFPRGEDGKPPKEYF 7ak6.1    RTGEVMRIL------------PRMHEDINEEWISDKTRFAYDGLK-RQRLTEPMVRNE----------------------  target    QRARDEWVRASHDEAAAVVAATLANIAATYSGEEGAQRLRDQGYEEETIEAMGGAGVQAMKFRGGMPLLGMTRVFGLYRM 7ak6.1    ---KGLLTYTSWEDALSRVAGMLQN----FEGNAVA--------------AIAGGLVDAEAL---VALKDLLNKVDSDNL  target    ANSMALLDAKVRGVGPDEARGARGFDNYSWHTDLPPGHPMVTGQQTVDFDLNSVELAKNVVVWGMNWITTKMPDAHWLTE 7ak6.1    CTEEI----------------------F-PTEGAGTDLRSNY---LLNTTIAGVEEADVVLLVGTNPRFEAPLFNARIRK  target    AR-LKGTRIIVIACEYSSTSSKADDAIVVRPGTTPALALGLSHVIMRDKLYDADYVRRWTDLPMLVRTDTLKYLSAEDVF 7ak6.1    SWLHNDLKVALIGSPVDLTYRYDHLGDSPKILQDI---------------------------------------------  target    GGGPAPL 7ak6.1    ------- ``` | | | | | | | | | | | | | | | | | | | | | | | | | | | | | | | | | | | | | | | | | | | | | | | | | |
|  | 8b9z.1.G | NADH-ubiquinone oxidoreductase 75 kDa subunit, mitochondrial  *Drosophila melanogaster complex I in the Active state (Dm1)* | 0.24 |  | 15.31 | 0.51 | 60-363 | EM | 3.28 | hetero-1-1-1-1-1-1-… | 3 x PC1, 16 x 3PE, 6 x SF4, 4 x CDL, 2 x FES, 1 x FMN, 1 x UQ9, 1 x DGT, 1 x NDP, 1 x ZN, 2 x EHZ | HHblits | 0.26 |
| ``` target    TARREITRRGFLGTAAGAGFAAFVVSATRAWGLEAIENPLARYPDREWERVYRDLWRYDSKFTFLCAPNDTHNCLLDAYV 8b9z.1    -----------------------------------------------------------RKVSSID-VLDAVGSNIVVST  target    RSGVMTRIGPTMRYGEARDLDGNRASARWDPRVCQKGLALTRRFYGDRRLRHCMVRAGFKRWVDEGFPRGEDGKPPKEYF 8b9z.1    RTNEVLRIL------------PRENEDVNEEWLADKSRFACDGLK-RQRLVAPMVRMP----------------------  target    QRARDEWVRASHDEAAAVVAATLANIAATYSGEEGAQRLRDQGYEEETIEAMGGAGVQAMKFRGGMPLLGMTRVFGLYRM 8b9z.1    ---NGELQAVEWEGALIAVAKAIKAAGGQ-------------------IAGISGQLADLEAQ---VALKDLLNRLGSEVV  target    ANSMALLDAKVRGVGPDEARGARGFDNYSWHTDLPPGHPMVTGQQTVDFDLNSVELAKNVVVWGMNWITTKMPDAHWLTE 8b9z.1    ATEQG----------------------F---IAGGTDN---RANYLLNSTIAGLEEADAVLLVGTNPRYEAPLVNTRLRK  target    AR-LKGTRIIVIACEYSSTSSKADDAIVVRPGTTPALALGLSHVIMRDKLYDADYVRRWTDLPMLVRTDTLKYLSAEDVF 8b9z.1    AYVHNELQIASIGPKIDLS------YDHENLGADAALVKDVCSG------------------------------------  target    GGGPAPL 8b9z.1    ------- ``` | | | | | | | | | | | | | | | | | | | | | | | | | | | | | | | | | | | | | | | | | | | | | | | | | |
|  | 8ba0.1.G | NADH-ubiquinone oxidoreductase 75 kDa subunit, mitochondrial  *Drosophila melanogaster complex I in the Twisted state (Dm2)* | 0.25 |  | 15.31 | 0.51 | 60-363 | EM | 3.68 | hetero-1-1-1-1-1-1-… | 6 x SF4, 6 x 3PE, 2 x FES, 1 x FMN, 2 x CDL, 1 x DGT, 1 x NDP, 1 x ZN, 2 x EHZ | HHblits | 0.26 |
| ``` target    TARREITRRGFLGTAAGAGFAAFVVSATRAWGLEAIENPLARYPDREWERVYRDLWRYDSKFTFLCAPNDTHNCLLDAYV 8ba0.1    -----------------------------------------------------------RKVSSID-VLDAVGSNIVVST  target    RSGVMTRIGPTMRYGEARDLDGNRASARWDPRVCQKGLALTRRFYGDRRLRHCMVRAGFKRWVDEGFPRGEDGKPPKEYF 8ba0.1    RTNEVLRIL------------PRENEDVNEEWLADKSRFACDGLK-RQRLVAPMVRMP----------------------  target    QRARDEWVRASHDEAAAVVAATLANIAATYSGEEGAQRLRDQGYEEETIEAMGGAGVQAMKFRGGMPLLGMTRVFGLYRM 8ba0.1    ---NGELQAVEWEGALIAVAKAIKAAGGQ-------------------IAGISGQLADLEAQ---VALKDLLNRLGSEVV  target    ANSMALLDAKVRGVGPDEARGARGFDNYSWHTDLPPGHPMVTGQQTVDFDLNSVELAKNVVVWGMNWITTKMPDAHWLTE 8ba0.1    ATEQG----------------------F---IAGGTDN---RANYLLNSTIAGLEEADAVLLVGTNPRYEAPLVNTRLRK  target    AR-LKGTRIIVIACEYSSTSSKADDAIVVRPGTTPALALGLSHVIMRDKLYDADYVRRWTDLPMLVRTDTLKYLSAEDVF 8ba0.1    AYVHNELQIASIGPKIDLS------YDHENLGADAALVKDVCSG------------------------------------  target    GGGPAPL 8ba0.1    ------- ``` | | | | | | | | | | | | | | | | | | | | | | | | | | | | | | | | | | | | | | | | | | | | | | | | | |
|  | 7tgh.58.A | NADH-ubiquinone oxidoreductase 75 kDa subunit  *Cryo-EM structure of respiratory super-complex CI+III2 from Tetrahymena thermophila* | 0.25 |  | 11.43 | 0.52 | 61-363 | EM | 0.00 | monomer |  | HHblits | 0.25 |
| ``` target    TARREITRRGFLGTAAGAGFAAFVVSATRAWGLEAIENPLARYPDREWERVYRDLWRYDSKFTFLCAPNDTHNCLLDAYV 7tgh.58   ------------------------------------------------------------SFYTSD-VFDTLGSAIQVDT  target    RSGVMTRIGPTMRYGEARDLDGNRASARWDPRVCQKGLALTRRFYGDRRLRHCMVRAGFKRWVDEGFPRGEDGKPPKEYF 7tgh.58   RGPEIMRVL------------PRIHEEINEEWISDKTRHAFDGLK-RQRINSPMKRSK----------------------  target    QRARDEWVRASHDEAAAVVAATLANIAATYSGEEGAQRLRDQGYEEETIEAMGGAGVQAMKFRGGMPLLGMTRVFGLYRM 7tgh.58   ---DGNYEDIFWEEAIQTISKKCLNTPSD----QIG--------------AIIGEFADIESI---TALKDFLNRLDVDNF  target    ANSMALLDAKVRGVGPDEARGARGFDNYSWHTDLPPGHPMVTGQQTVDFDLNSVELAKNVVVWGMNWITTKMPDAHWLTE 7tgh.58   EVR--------------------QHGNLKVSP----DFRANY---LMNSKITGVEDADVLLLVGCNPRYEAPVLNARILK  target    ARLKGTRIIVIACEYSSTSSKADDAIVVRPGTTPALALGLSHVIMRDKLYDADYVRRWTDLPMLVRTDTLKYLSAEDVFG 7tgh.58   STRKNLKVFNIGTNQDL--NYKNVHL----GNSTKVLKEIADG-------------------------------------  target    GGPAPL 7tgh.58   ------ ``` | | | | | | | | | | | | | | | | | | | | | | | | | | | | | | | | | | | | | | | | | | | | | | | | | |
|  | 7zd6.1.4 | NADH-ubiquinone oxidoreductase 75 kDa subunit, mitochondrial  *Complex I from Ovis aries, at pH7.4, Open state* | 0.25 | 0.00 | 14.98 | 0.51 | 60-352 | EM | 0.00 | monomer | 6 x PC1, 14 x 3PE, 1 x DCQ, 2 x ZMP, 1 x AMP, 1 x MYR, 6 x SF4, 1 x FMN, 1 x NAI, 2 x FES, 1 x K, 1 x ZN, 1 x NDP | HHblits | 0.26 |
| ``` target    TARREITRRGFLGTAAGAGFAAFVVSATRAWGLEAIENPLARYPDREWERVYRDLWRYDSKFTFLCAPNDTHNCLLDAYV 7zd6.1    -----------------------------------------------------------RKTESIDV-MDAVGSNIVVST  target    RSGVMTRIGPTMRYGEARDLDGNRASARWDPRVCQKGLALTRRFYGDRRLRHCMVRAGFKRWVDEGFPRGEDGKPPKEYF 7zd6.1    RTGEVMRIL------------PRMHEDINEEWISDKTRFAYDGLK-RQRLTEPMVRNE----------------------  target    QRARDEWVRASHDEAAAVVAATLANIAATYSGEEGAQRLRDQGYEEETIEAMGGAGVQAMKFRGGMPLLGMTRVFGLYRM 7zd6.1    ---KGLLTHTTWEDALSRVAGMLQSC----QGNDV--------------AAIAGGLVDAEAL---IALKDLLNRVDSDTL  target    ANSMALLDAKVRGVGPDEARGARGFDNYSWHTDLPPGHPMVTGQQTVDFDLNSVELAKNVVVWGMNWITTKMPDAHWLTE 7zd6.1    CTEEV----------------------FP-TAGAGTDL---RSNYLLNTTIAGVEEADVVLLVGTNPRFEAPLFNARIRK  target    ARL-KGTRIIVIACEYSSTSSKADDAIVVRPGTTPALALGLSHVIMRDKLYDADYVRRWTDLPMLVRTDTLKYLSAEDVF 7zd6.1    SWLHNDLKVALIGSPVDLTYRYDHLGDSPKILQ-----------------------------------------------  target    GGGPAPL 7zd6.1    ------- ``` | | | | | | | | | | | | | | | | | | | | | | | | | | | | | | | | | | | | | | | | | | | | | | | | | |
|  | 6qcf.1.C | NADH:ubiquinone oxidoreductase core subunit S1  *Ovine respiratory complex I FRC open class 6* | 0.25 | 0.00 | 14.98 | 0.51 | 61-353 | EM | 0.00 | monomer | 6 x SF4, 1 x FMN, 2 x FES, 1 x ZN, 1 x NDP, 2 x ZMP | HHblits | 0.26 |
| ``` target    TARREITRRGFLGTAAGAGFAAFVVSATRAWGLEAIENPLARYPDREWERVYRDLWRYDSKFTFLCAPNDTHNCLLDAYV 6qcf.1    ------------------------------------------------------------KTESID-VMDAVGSNIVVST  target    RSGVMTRIGPTMRYGEARDLDGNRASARWDPRVCQKGLALTRRFYGDRRLRHCMVRAGFKRWVDEGFPRGEDGKPPKEYF 6qcf.1    RTGEVMRIL------------PRMHEDINEEWISDKTRFAYDGLK-RQRLTEPMVRNE----------------------  target    QRARDEWVRASHDEAAAVVAATLANIAATYSGEEGAQRLRDQGYEEETIEAMGGAGVQAMKFRGGMPLLGMTRVFGLYRM 6qcf.1    ---KGLLTHTTWEDALSRVAGMLQSC----QGNDV--------------AAIAGGLVDAEAL---IALKDLLNRVDSDTL  target    ANSMALLDAKVRGVGPDEARGARGFDNYSWHTDLPPGHPMVTGQQTVDFDLNSVELAKNVVVWGMNWITTKMPDAHWLTE 6qcf.1    CTEEV----------------------FP-TAGAGTDL---RSNYLLNTTIAGVEEADVVLLVGTNPRFEAPLFNARIRK  target    ARL-KGTRIIVIACEYSSTSSKADDAIVVRPGTTPALALGLSHVIMRDKLYDADYVRRWTDLPMLVRTDTLKYLSAEDVF 6qcf.1    SWLHNDLKVALIGSPVDLTYRYDHLGDSPKILQD----------------------------------------------  target    GGGPAPL 6qcf.1    ------- ``` | | | | | | | | | | | | | | | | | | | | | | | | | | | | | | | | | | | | | | | | | | | | | | | | | |
|  | 6qc5.1.C | NADH:ubiquinone oxidoreductase core subunit S1  *Ovine respiratory complex I FRC closed class 1* | 0.25 |  | 14.98 | 0.51 | 61-353 | EM | 0.00 | hetero-1-1-1-1-1-1-… | 6 x SF4, 1 x FMN, 2 x FES, 2 x 3PE, 1 x ZN, 1 x NDP, 2 x ZMP, 1 x PC1 | HHblits | 0.26 |
| ``` target    TARREITRRGFLGTAAGAGFAAFVVSATRAWGLEAIENPLARYPDREWERVYRDLWRYDSKFTFLCAPNDTHNCLLDAYV 6qc5.1    ------------------------------------------------------------KTESID-VMDAVGSNIVVST  target    RSGVMTRIGPTMRYGEARDLDGNRASARWDPRVCQKGLALTRRFYGDRRLRHCMVRAGFKRWVDEGFPRGEDGKPPKEYF 6qc5.1    RTGEVMRIL------------PRMHEDINEEWISDKTRFAYDGLK-RQRLTEPMVRNE----------------------  target    QRARDEWVRASHDEAAAVVAATLANIAATYSGEEGAQRLRDQGYEEETIEAMGGAGVQAMKFRGGMPLLGMTRVFGLYRM 6qc5.1    ---KGLLTHTTWEDALSRVAGMLQSC----QGNDV--------------AAIAGGLVDAEAL---IALKDLLNRVDSDTL  target    ANSMALLDAKVRGVGPDEARGARGFDNYSWHTDLPPGHPMVTGQQTVDFDLNSVELAKNVVVWGMNWITTKMPDAHWLTE 6qc5.1    CTEEV----------------------FP-TAGAGTDL---RSNYLLNTTIAGVEEADVVLLVGTNPRFEAPLFNARIRK  target    ARL-KGTRIIVIACEYSSTSSKADDAIVVRPGTTPALALGLSHVIMRDKLYDADYVRRWTDLPMLVRTDTLKYLSAEDVF 6qc5.1    SWLHNDLKVALIGSPVDLTYRYDHLGDSPKILQD----------------------------------------------  target    GGGPAPL 6qc5.1    ------- ``` | | | | | | | | | | | | | | | | | | | | | | | | | | | | | | | | | | | | | | | | | | | | | | | | | |
|  | 7ar7.1.G | NADH dehydrogenase [ubiquinone] iron-sulfur protein 1, mitochondrial  *Cryo-EM structure of Arabidopsis thaliana complex-I (open conformation)* | 0.25 |  | 13.46 | 0.51 | 61-358 | EM | 0.00 | hetero-1-1-1-1-1-1-… | 6 x SF4, 2 x FES, 1 x FMN, 1 x UQ9, 3 x PTY, 2 x PC7, 1 x LMN, 1 x NDP, 2 x ZN, 2 x 8Q1, 1 x PGT, 1 x PSF, 1 x T7X | HHblits | 0.26 |
| ``` target    TARREITRRGFLGTAAGAGFAAFVVSATRAWGLEAIENPLARYPDREWERVYRDLWRYDSKFTFLCAPNDTHNCLLDAYV 7ar7.1    ------------------------------------------------------------ATETID-VSDAVGSNIRVDS  target    RSGVMTRIGPTMRYGEARDLDGNRASARWDPRVCQKGLALTRRFYGDRRLRHCMVRAGFKRWVDEGFPRGEDGKPPKEYF 7ar7.1    RGPEVMRII------------PRLNEDINEEWISDKTRFCYDGLK-RQRLSDPMIRDS----------------------  target    QRARDEWVRASHDEAAAVVAATLANIAATYSGEEGAQRLRDQGYEEETIEAMGGAGVQAMKFRGGMPLLGMTRVFGLYRM 7ar7.1    ---DGRFKAVSWRDALAVVGDIIHQV----KPDE--------------IVGVAGQLSDAESM---MVLKDFVNRMGSDNV  target    ANSMALLDAKVRGVGPDEARGARGFDNYSWHTDLPPGHPMVTGQQTVDFDLNSVELAKNVVVWGMNWITTKMPDAHWLTE 7ar7.1    WCEG-------------------------TAAGVDADLRYSY---LMNTSISGLENADLFLLIGTQPRVEAAMVNARICK  target    A-RLKGTRIIVIACEYSSTSSKADDAIVVRPGTTPALALGLSHVIMRDKLYDADYVRRWTDLPMLVRTDTLKYLSAEDVF 7ar7.1    TVRASNAKVGYVGPPAEFN--YDCKHLGTGPDTLKEIAE-----------------------------------------  target    GGGPAPL 7ar7.1    ------- ``` | | | | | | | | | | | | | | | | | | | | | | | | | | | | | | | | | | | | | | | | | | | | | | | | | |
|  | 5gpn.24.A | NADH-ubiquinone oxidoreductase 75 kDa subunit  *Architecture of mammalian respirasome* | 0.23 |  | 14.49 | 0.51 | 61-353 | EM | 0.00 | monomer |  | HHblits | 0.26 |
| ``` target    TARREITRRGFLGTAAGAGFAAFVVSATRAWGLEAIENPLARYPDREWERVYRDLWRYDSKFTFLCAPNDTHNCLLDAYV 5gpn.24   ------------------------------------------------------------KTESIDV-MDAVGSNIVVST  target    RSGVMTRIGPTMRYGEARDLDGNRASARWDPRVCQKGLALTRRFYGDRRLRHCMVRAGFKRWVDEGFPRGEDGKPPKEYF 5gpn.24   RTGEVMRILP------------RMHEDINEEWISDKTRFAYDGLK-RQRLTQPMIRNE----------------------  target    QRARDEWVRASHDEAAAVVAATLANIAATYSGEEGAQRLRDQGYEEETIEAMGGAGVQAMKFRGGMPLLGMTRVFGLYRM 5gpn.24   ---KGLLTYTTWEDALSRVAGMLQSF----QGNDV--------------AAIAGGLVDAEAL---VALKDLLNRVDSDSL  target    ANSMALLDAKVRGVGPDEARGARGFDNYSWHTDLPPGHPMVTGQQTVDFDLNSVELAKNVVVWGMNWITTKMPDAHWLTE 5gpn.24   CTEE----------------------VFPT-AGAGTDLRSNYL---LNTTIAGVEEADVILLVGTNPRFEAPLFNARIRK  target    ARL-KGTRIIVIACEYSSTSSKADDAIVVRPGTTPALALGLSHVIMRDKLYDADYVRRWTDLPMLVRTDTLKYLSAEDVF 5gpn.24   SWLHNDLKVALIGSPVDLTYRYDHLGDSPKILQD----------------------------------------------  target    GGGPAPL 5gpn.24   ------- ``` | | | | | | | | | | | | | | | | | | | | | | | | | | | | | | | | | | | | | | | | | | | | | | | | | |
|  | 6x89.1.H | NADH dehydrogenase [ubiquinone] iron-sulfur protein 1, mitochondrial  *Vigna radiata mitochondrial complex I\** | 0.24 |  | 12.92 | 0.51 | 61-359 | EM | 0.00 | hetero-1-1-1-1-1-1-… | 1 x NAP, 6 x PC1, 6 x SF4, 2 x FES, 2 x ZN, 1 x FMN | HHblits | 0.25 |
| ``` target    TARREITRRGFLGTAAGAGFAAFVVSATRAWGLEAIENPLARYPDREWERVYRDLWRYDSKFTFLCAPNDTHNCLLDAYV 6x89.1    ------------------------------------------------------------GTETIDV-TDAVGSNIRIDS  target    RSGVMTRIGPTMRYGEARDLDGNRASARWDPRVCQKGLALTRRFYGDRRLRHCMVRAGFKRWVDEGFPRGEDGKPPKEYF 6x89.1    RGPEVMRIVP------------RLNEDINEEWISDKTRFCYDGLK-RQRLNDPMIRGP----------------------  target    QRARDEWVRASHDEAAAVVAATLANIAATYSGEEGAQRLRDQGYEEETIEAMGGAGVQAMKFRGGMPLLGMTRVFGLYRM 6x89.1    ---DGRFKAVNWRDALSVIADIAHQV----KPEEI--------------VGVAGKLSDAESMI---ALKDFLNRMGSNDV  target    ANSMALLDAKVRGVGPDEARGARGFDNYSWHTDLPPGHPMVTGQQTVDFDLNSVELAKNVVVWGMNWITTKMPDAHWLTE 6x89.1    WGEG-------------------------IGVNTNADFRSGY---IMNTSIAGLEKADVFLLVGTQPRVEAAMVNARIRK  target    ARL-KGTRIIVIACEYSSTSSKADDAIVVRPGTTPALALGLSHVIMRDKLYDADYVRRWTDLPMLVRTDTLKYLSAEDVF 6x89.1    TVRSNQAKVGYIGPATDFN--YDHKHLGTDPQTLVEIAEG----------------------------------------  target    GGGPAPL 6x89.1    ------- ``` | | | | | | | | | | | | | | | | | | | | | | | | | | | | | | | | | | | | | | | | | | | | | | | | | |
|  | 8e73.55.A | NDUS1  *Vigna radiata supercomplex I+III2 (full bridge)* | 0.25 |  | 12.92 | 0.51 | 61-359 | EM | 0.00 | monomer |  | HHblits | 0.25 |
| ``` target    TARREITRRGFLGTAAGAGFAAFVVSATRAWGLEAIENPLARYPDREWERVYRDLWRYDSKFTFLCAPNDTHNCLLDAYV 8e73.55   ------------------------------------------------------------GTETIDV-TDAVGSNIRIDS  target    RSGVMTRIGPTMRYGEARDLDGNRASARWDPRVCQKGLALTRRFYGDRRLRHCMVRAGFKRWVDEGFPRGEDGKPPKEYF 8e73.55   RGPEVMRIVP------------RLNEDINEEWISDKTRFCYDGLK-RQRLNDPMIRGP----------------------  target    QRARDEWVRASHDEAAAVVAATLANIAATYSGEEGAQRLRDQGYEEETIEAMGGAGVQAMKFRGGMPLLGMTRVFGLYRM 8e73.55   ---DGRFKAVNWRDALSVIADIAHQV----KPEEI--------------VGVAGKLSDAESMI---ALKDFLNRMGSNDV  target    ANSMALLDAKVRGVGPDEARGARGFDNYSWHTDLPPGHPMVTGQQTVDFDLNSVELAKNVVVWGMNWITTKMPDAHWLTE 8e73.55   WGEG-------------------------IGVNTNADFRSGY---IMNTSIAGLEKADVFLLVGTQPRVEAAMVNARIRK  target    ARL-KGTRIIVIACEYSSTSSKADDAIVVRPGTTPALALGLSHVIMRDKLYDADYVRRWTDLPMLVRTDTLKYLSAEDVF 8e73.55   TVRSNQAKVGYIGPATDFN--YDHKHLGTDPQTLVEIAEG----------------------------------------  target    GGGPAPL 8e73.55   ------- ``` | | | | | | | | | | | | | | | | | | | | | | | | | | | | | | | | | | | | | | | | | | | | | | | | | |
|  | 7aqr.1.F | NADH dehydrogenase [ubiquinone] iron-sulfur protein 1, mitochondrial  *Cryo-EM structure of Arabidopsis thaliana Complex-I (peripheral arm)* | 0.25 |  | 12.98 | 0.51 | 61-358 | EM | 0.00 | hetero-1-1-1-1-1-1-… | 6 x SF4, 2 x FES, 1 x FMN, 1 x NDP, 1 x ZN, 1 x 8Q1 | HHblits | 0.25 |
| ``` target    TARREITRRGFLGTAAGAGFAAFVVSATRAWGLEAIENPLARYPDREWERVYRDLWRYDSKFTFLCAPNDTHNCLLDAYV 7aqr.1    ------------------------------------------------------------ATETIDV-SDAVGSNIRVDS  target    RSGVMTRIGPTMRYGEARDLDGNRASARWDPRVCQKGLALTRRFYGDRRLRHCMVRAGFKRWVDEGFPRGEDGKPPKEYF 7aqr.1    RGPEVMRII------------PRLNEDINEEWISDKTRFCYDGLK-RQRLSDPMIRDS----------------------  target    QRARDEWVRASHDEAAAVVAATLANIAATYSGEEGAQRLRDQGYEEETIEAMGGAGVQAMKFRGGMPLLGMTRVFGLYRM 7aqr.1    ---DGRFKAVSWRDALAVVGDIIHQV----KPDEI--------------VGVAGQLSDAESM---MVLKDFVNRMGSDNV  target    ANSMALLDAKVRGVGPDEARGARGFDNYSWHTDLPPGHPMVTGQQTVDFDLNSVELAKNVVVWGMNWITTKMPDAHWLTE 7aqr.1    WCEG-------------------------TAAGVDADLRYSY---LMNTSISGLENADLFLLIGTQPRVEAAMVNARICK  target    A-RLKGTRIIVIACEYSSTSSKADDAIVVRPGTTPALALGLSHVIMRDKLYDADYVRRWTDLPMLVRTDTLKYLSAEDVF 7aqr.1    TVRASNAKVGYVGPPAEFN--YDCKHLGTGPDTLKEIAE-----------------------------------------  target    GGGPAPL 7aqr.1    ------- ``` | | | | | | | | | | | | | | | | | | | | | | | | | | | | | | | | | | | | | | | | | | | | | | | | | |
|  | 7a23.1.O | 75kDa  *Plant mitochondrial respiratory complex I* | 0.25 |  | 12.98 | 0.51 | 61-358 | EM | 0.00 | hetero-1-1-1-1-1-1-… | 6 x SF4, 1 x FMN, 2 x T7X, 3 x CDL, 1 x U10, 1 x PEV, 2 x FES, 1 x NDP, 2 x ZN | HHblits | 0.25 |
| ``` target    TARREITRRGFLGTAAGAGFAAFVVSATRAWGLEAIENPLARYPDREWERVYRDLWRYDSKFTFLCAPNDTHNCLLDAYV 7a23.1    ------------------------------------------------------------ATETIDV-SDAVGSNIRVDS  target    RSGVMTRIGPTMRYGEARDLDGNRASARWDPRVCQKGLALTRRFYGDRRLRHCMVRAGFKRWVDEGFPRGEDGKPPKEYF 7a23.1    RGPEVMRII------------PRLNEDINEEWISDKTRFCYDGLK-RQRLSDPMIRDS----------------------  target    QRARDEWVRASHDEAAAVVAATLANIAATYSGEEGAQRLRDQGYEEETIEAMGGAGVQAMKFRGGMPLLGMTRVFGLYRM 7a23.1    ---DGRFKAVSWRDALAVVGDIIHQV----KPDEI--------------VGVAGQLSDAESM---MVLKDFVNRMGSDNV  target    ANSMALLDAKVRGVGPDEARGARGFDNYSWHTDLPPGHPMVTGQQTVDFDLNSVELAKNVVVWGMNWITTKMPDAHWLTE 7a23.1    WCEG-------------------------TAAGVDADLRYSY---LMNTSISGLENADLFLLIGTQPRVEAAMVNARICK  target    A-RLKGTRIIVIACEYSSTSSKADDAIVVRPGTTPALALGLSHVIMRDKLYDADYVRRWTDLPMLVRTDTLKYLSAEDVF 7a23.1    TVRASNAKVGYVGPPAEFN--YDCKHLGTGPDTLKEIAE-----------------------------------------  target    GGGPAPL 7a23.1    ------- ``` | | | | | | | | | | | | | | | | | | | | | | | | | | | | | | | | | | | | | | | | | | | | | | | | | |
|  | 7ar8.1.G | NADH dehydrogenase [ubiquinone] iron-sulfur protein 1, mitochondrial  *Cryo-EM structure of Arabidopsis thaliana complex-I (closed conformation)* | 0.24 |  | 12.98 | 0.51 | 61-358 | EM | 0.00 | hetero-1-1-1-1-1-1-… | 6 x SF4, 2 x FES, 1 x FMN, 1 x UQ9, 3 x PTY, 2 x PC7, 1 x PGT, 1 x FE, 1 x NDP, 2 x ZN, 2 x 8Q1, 1 x LMN, 1 x PSF, 1 x T7X | HHblits | 0.25 |
| ``` target    TARREITRRGFLGTAAGAGFAAFVVSATRAWGLEAIENPLARYPDREWERVYRDLWRYDSKFTFLCAPNDTHNCLLDAYV 7ar8.1    ------------------------------------------------------------ATETIDV-SDAVGSNIRVDS  target    RSGVMTRIGPTMRYGEARDLDGNRASARWDPRVCQKGLALTRRFYGDRRLRHCMVRAGFKRWVDEGFPRGEDGKPPKEYF 7ar8.1    RGPEVMRII------------PRLNEDINEEWISDKTRFCYDGLK-RQRLSDPMIRDS----------------------  target    QRARDEWVRASHDEAAAVVAATLANIAATYSGEEGAQRLRDQGYEEETIEAMGGAGVQAMKFRGGMPLLGMTRVFGLYRM 7ar8.1    ---DGRFKAVSWRDALAVVGDIIHQV----KPDEI--------------VGVAGQLSDAESM---MVLKDFVNRMGSDNV  target    ANSMALLDAKVRGVGPDEARGARGFDNYSWHTDLPPGHPMVTGQQTVDFDLNSVELAKNVVVWGMNWITTKMPDAHWLTE 7ar8.1    WCEG-------------------------TAAGVDADLRYSY---LMNTSISGLENADLFLLIGTQPRVEAAMVNARICK  target    A-RLKGTRIIVIACEYSSTSSKADDAIVVRPGTTPALALGLSHVIMRDKLYDADYVRRWTDLPMLVRTDTLKYLSAEDVF 7ar8.1    TVRASNAKVGYVGPPAEFN--YDCKHLGTGPDTLKEIAE-----------------------------------------  target    GGGPAPL 7ar8.1    ------- ``` | | | | | | | | | | | | | | | | | | | | | | | | | | | | | | | | | | | | | | | | | | | | | | | | | |
|  | 7vxu.1.L | NADH-ubiquinone oxidoreductase 75 kDa subunit, mitochondrial  *Matrix arm of deactive state CI from Q10 dataset* | 0.25 |  | 14.56 | 0.51 | 61-352 | EM | 0.00 | hetero-1-1-1-1-1-1-… | 6 x SF4, 1 x FMN, 1 x PEE, 1 x PLX, 1 x 8Q1, 1 x NDP, 2 x FES, 1 x MG, 1 x CDL, 1 x ZN | HHblits | 0.26 |
| ``` target    TARREITRRGFLGTAAGAGFAAFVVSATRAWGLEAIENPLARYPDREWERVYRDLWRYDSKFTFLCAPNDTHNCLLDAYV 7vxu.1    ------------------------------------------------------------KTESID-VMDAVGSNIVVST  target    RSGVMTRIGPTMRYGEARDLDGNRASARWDPRVCQKGLALTRRFYGDRRLRHCMVRAGFKRWVDEGFPRGEDGKPPKEYF 7vxu.1    RTGEVMRILP------------RMHEDINEEWISDKTRFAYDGLK-RQRLTQPMIRNE----------------------  target    QRARDEWVRASHDEAAAVVAATLANIAATYSGEEGAQRLRDQGYEEETIEAMGGAGVQAMKFRGGMPLLGMTRVFGLYRM 7vxu.1    ---KGLLTYTTWEDALSRVAGMLQSF----QGND--------------VAAIAGGLVDAEAL---VALKDLLNRVDSDSL  target    ANSMALLDAKVRGVGPDEARGARGFDNYSWHTDLPPGHPMVTGQQTVDFDLNSVELAKNVVVWGMNWITTKMPDAHWLTE 7vxu.1    CTEE----------------------VFP-TAGAGTDLRSNYL---LNTTIAGVEEADVILLVGTNPRFEAPLFNARIRK  target    ARL-KGTRIIVIACEYSSTSSKADDAIVVRPGTTPALALGLSHVIMRDKLYDADYVRRWTDLPMLVRTDTLKYLSAEDVF 7vxu.1    SWLHNDLKVALIGSPVDLTYRYDHLGDSPKILQ-----------------------------------------------  target    GGGPAPL 7vxu.1    ------- ``` | | | | | | | | | | | | | | | | | | | | | | | | | | | | | | | | | | | | | | | | | | | | | | | | | |
|  | 6yj4.1.G | Subunit NUAM of NADH:Ubiquinone Oxidoreductase (Complex I)  *Structure of Yarrowia lipolytica complex I at 2.7 A* | 0.26 |  | 17.50 | 0.49 | 61-344 | EM | 0.00 | hetero-1-1-1-1-1-1-… | 18 x 3PE, 6 x SF4, 5 x LMT, 8 x PLC, 2 x FES, 1 x FMN, 6 x CDL, 1 x NDP, 1 x ZN, 2 x EHZ | HHblits | 0.28 |
| ``` target    TARREITRRGFLGTAAGAGFAAFVVSATRAWGLEAIENPLARYPDREWERVYRDLWRYDSKFTFLCAPNDTHNCLLDAYV 6yj4.1    ------------------------------------------------------------KTESID-VMDAVGSNIRIDS  target    RSGVMTRIGPTMRYGEARDLDGNRASARWDPRVCQKGLALTRRFYGDRRLRHCMVRAGFKRWVDEGFPRGEDGKPPKEYF 6yj4.1    KGVEVMRVI------------PRVHEDVNEEWINDKSRFACDGLK-TQRLTTPLIRVG----------------------  target    QRARDEWVRASHDEAAAVVAATLANIAATYSGEEGAQRLRDQGYEEETIEAMGGAGVQAMKFRGGMPLLGMTRVFGLYRM 6yj4.1    ----DKFVNATWDDALSTIAKAYQQKA--PKGDE--------------FKAVAGALVEVESMV---ALKDMTNALGSENT  target    ANSMALLDAKVRGVGPDEARGARGFDNYSWHTDLPPGHPMVTGQQ-TVDFDLNSVELAKNVVVWGMNWITTKMPDAHWLT 6yj4.1    TTD-------------------------TPNGNSAPAHGITFRSNYLFNSSIAGIEDADAILLVGTNPRREAAVMNARIR  target    EA-RLKGTRIIVIACEYSSTSSKADDAIVVRPGTTPALALGLSHVIMRDKLYDADYVRRWTDLPMLVRTDTLKYLSAEDV 6yj4.1    KAWLRQELEIASVGPTLDATFDVAEL------------------------------------------------------  target    FGGGPAPL 6yj4.1    -------- ``` | | | | | | | | | | | | | | | | | | | | | | | | | | | | | | | | | | | | | | | | | | | | | | | | | |
|  | 6rfs.1.A | Subunit NUAM of NADH:Ubiquinone Oxidoreductase (Complex I)  *Cryo-EM structure of a respiratory complex I mutant lacking NDUFS4* | 0.25 |  | 17.50 | 0.49 | 61-344 | EM | 4.04 | hetero-1-1-1-1-1-1-… | 6 x SF4, 2 x FES, 1 x FMN, 1 x NDP, 1 x ZN, 1 x ZMP | HHblits | 0.28 |
| ``` target    TARREITRRGFLGTAAGAGFAAFVVSATRAWGLEAIENPLARYPDREWERVYRDLWRYDSKFTFLCAPNDTHNCLLDAYV 6rfs.1    ------------------------------------------------------------KTESID-VMDAVGSNIRIDS  target    RSGVMTRIGPTMRYGEARDLDGNRASARWDPRVCQKGLALTRRFYGDRRLRHCMVRAGFKRWVDEGFPRGEDGKPPKEYF 6rfs.1    KGVEVMRVI------------PRVHEDVNEEWINDKSRFACDGLK-TQRLTTPLIRVG----------------------  target    QRARDEWVRASHDEAAAVVAATLANIAATYSGEEGAQRLRDQGYEEETIEAMGGAGVQAMKFRGGMPLLGMTRVFGLYRM 6rfs.1    ----DKFVNATWDDALSTIAKAYQQKA--PKGDE--------------FKAVAGALVEVESMV---ALKDMTNALGSENT  target    ANSMALLDAKVRGVGPDEARGARGFDNYSWHTDLPPGHPMVTGQQ-TVDFDLNSVELAKNVVVWGMNWITTKMPDAHWLT 6rfs.1    TTD-------------------------TPNGNSAPAHGITFRSNYLFNSSIAGIEDADAILLVGTNPRREAAVMNARIR  target    EA-RLKGTRIIVIACEYSSTSSKADDAIVVRPGTTPALALGLSHVIMRDKLYDADYVRRWTDLPMLVRTDTLKYLSAEDV 6rfs.1    KAWLRQELEIASVGPTLDATFDVAEL------------------------------------------------------  target    FGGGPAPL 6rfs.1    -------- ``` | | | | | | | | | | | | | | | | | | | | | | | | | | | | | | | | | | | | | | | | | | | | | | | | | |
|  | 6rfq.1.A | Subunit NUAM of NADH:Ubiquinone Oxidoreductase (Complex I)  *Cryo-EM structure of a respiratory complex I assembly intermediate with NDUFAF2* | 0.25 |  | 17.50 | 0.49 | 61-344 | EM | 3.30 | hetero-1-1-1-1-1-1-… | 6 x SF4, 2 x FES, 1 x FMN, 1 x NDP, 10 x 3PE, 2 x LMN, 4 x CDL, 2 x ZMP, 4 x PLC, 3 x T7X, 1 x CPL | HHblits | 0.28 |
| ``` target    TARREITRRGFLGTAAGAGFAAFVVSATRAWGLEAIENPLARYPDREWERVYRDLWRYDSKFTFLCAPNDTHNCLLDAYV 6rfq.1    ------------------------------------------------------------KTESID-VMDAVGSNIRIDS  target    RSGVMTRIGPTMRYGEARDLDGNRASARWDPRVCQKGLALTRRFYGDRRLRHCMVRAGFKRWVDEGFPRGEDGKPPKEYF 6rfq.1    KGVEVMRVI------------PRVHEDVNEEWINDKSRFACDGLK-TQRLTTPLIRVG----------------------  target    QRARDEWVRASHDEAAAVVAATLANIAATYSGEEGAQRLRDQGYEEETIEAMGGAGVQAMKFRGGMPLLGMTRVFGLYRM 6rfq.1    ----DKFVNATWDDALSTIAKAYQQKA--PKGDE--------------FKAVAGALVEVESMV---ALKDMTNALGSENT  target    ANSMALLDAKVRGVGPDEARGARGFDNYSWHTDLPPGHPMVTGQQ-TVDFDLNSVELAKNVVVWGMNWITTKMPDAHWLT 6rfq.1    TTD-------------------------TPNGNSAPAHGITFRSNYLFNSSIAGIEDADAILLVGTNPRREAAVMNARIR  target    EA-RLKGTRIIVIACEYSSTSSKADDAIVVRPGTTPALALGLSHVIMRDKLYDADYVRRWTDLPMLVRTDTLKYLSAEDV 6rfq.1    KAWLRQELEIASVGPTLDATFDVAEL------------------------------------------------------  target    FGGGPAPL 6rfq.1    -------- ``` | | | | | | | | | | | | | | | | | | | | | | | | | | | | | | | | | | | | | | | | | | | | | | | | | |
|  | 6gcs.1.A | 75-KDA PROTEIN (NUAM)  *Cryo-EM structure of respiratory complex I from Yarrowia lipolytica* | 0.25 |  | 17.50 | 0.49 | 61-344 | EM | 4.32 | hetero-1-1-1-1-1-1-… | 6 x SF4, 2 x FES, 1 x FMN, 1 x NDP, 1 x ZN, 1 x ZMP, 1 x CDL, 3 x 3PE | HHblits | 0.28 |
| ``` target    TARREITRRGFLGTAAGAGFAAFVVSATRAWGLEAIENPLARYPDREWERVYRDLWRYDSKFTFLCAPNDTHNCLLDAYV 6gcs.1    ------------------------------------------------------------KTESID-VMDAVGSNIRIDS  target    RSGVMTRIGPTMRYGEARDLDGNRASARWDPRVCQKGLALTRRFYGDRRLRHCMVRAGFKRWVDEGFPRGEDGKPPKEYF 6gcs.1    KGVEVMRVI------------PRVHEDVNEEWINDKSRFACDGLK-TQRLTTPLIRVG----------------------  target    QRARDEWVRASHDEAAAVVAATLANIAATYSGEEGAQRLRDQGYEEETIEAMGGAGVQAMKFRGGMPLLGMTRVFGLYRM 6gcs.1    ----DKFVNATWDDALSTIAKAYQQKA--PKGDE--------------FKAVAGALVEVESMV---ALKDMTNALGSENT  target    ANSMALLDAKVRGVGPDEARGARGFDNYSWHTDLPPGHPMVTGQQ-TVDFDLNSVELAKNVVVWGMNWITTKMPDAHWLT 6gcs.1    TTD-------------------------TPNGNSAPAHGITFRSNYLFNSSIAGIEDADAILLVGTNPRREAAVMNARIR  target    EA-RLKGTRIIVIACEYSSTSSKADDAIVVRPGTTPALALGLSHVIMRDKLYDADYVRRWTDLPMLVRTDTLKYLSAEDV 6gcs.1    KAWLRQELEIASVGPTLDATFDVAEL------------------------------------------------------  target    FGGGPAPL 6gcs.1    -------- ``` | | | | | | | | | | | | | | | | | | | | | | | | | | | | | | | | | | | | | | | | | | | | | | | | | |
|  | 7v2c.1.L | NADH-ubiquinone oxidoreductase 75 kDa subunit, mitochondrial  *Active state complex I from Q10 dataset* | 0.25 |  | 14.08 | 0.51 | 61-352 | EM | 0.00 | hetero-1-1-1-1-1-2-… | 6 x SF4, 1 x FMN, 10 x PEE, 8 x PLX, 2 x 8Q1, 1 x NDP, 2 x UQ, 11 x CDL, 2 x FES, 1 x MG, 1 x ZN, 1 x ADP | HHblits | 0.26 |
| ``` target    TARREITRRGFLGTAAGAGFAAFVVSATRAWGLEAIENPLARYPDREWERVYRDLWRYDSKFTFLCAPNDTHNCLLDAYV 7v2c.1    ------------------------------------------------------------KTESID-VMDAVGSNIVVST  target    RSGVMTRIGPTMRYGEARDLDGNRASARWDPRVCQKGLALTRRFYGDRRLRHCMVRAGFKRWVDEGFPRGEDGKPPKEYF 7v2c.1    RTGEVMRIL------------PRMHEDINEEWISDKTRFAYDGLK-RQRLTQPMIRNE----------------------  target    QRARDEWVRASHDEAAAVVAATLANIAATYSGEEGAQRLRDQGYEEETIEAMGGAGVQAMKFRGGMPLLGMTRVFGLYRM 7v2c.1    ---KGLLTYTTWEDALSRVAGMLQSF----QGND--------------VAAIAGGLVDAEAL---VALKDLLNRVDSDSL  target    ANSMALLDAKVRGVGPDEARGARGFDNYSWHTDLPPGHPMVTGQQTVDFDLNSVELAKNVVVWGMNWITTKMPDAHWLTE 7v2c.1    CTEE----------------------VFPT-AGAGTDLRSNYL---LNTTIAGVEEADVILLVGTNPRFEAPLFNARIRK  target    ARL-KGTRIIVIACEYSSTSSKADDAIVVRPGTTPALALGLSHVIMRDKLYDADYVRRWTDLPMLVRTDTLKYLSAEDVF 7v2c.1    SWLHNDLKVALIGSPVDLTYRYDHLGDSPKILQ-----------------------------------------------  target    GGGPAPL 7v2c.1    ------- ``` | | | | | | | | | | | | | | | | | | | | | | | | | | | | | | | | | | | | | | | | | | | | | | | | | |
|  | 6zk9.1.C | NADH:ubiquinone oxidoreductase core subunit S1  *Peripheral domain of open complex I during turnover* | 0.25 |  | 15.12 | 0.50 | 60-350 | EM | 0.00 | hetero-1-1-1-1-1-1-… | 6 x SF4, 1 x FMN, 1 x NAI, 2 x FES, 1 x K, 2 x PC1, 2 x 3PE, 1 x ZN, 1 x NDP, 1 x ZMP, 1 x CDL | HHblits | 0.26 |
| ``` target    TARREITRRGFLGTAAGAGFAAFVVSATRAWGLEAIENPLARYPDREWERVYRDLWRYDSKFTFLCAPNDTHNCLLDAYV 6zk9.1    -----------------------------------------------------------RKTESIDV-MDAVGSNIVVST  target    RSGVMTRIGPTMRYGEARDLDGNRASARWDPRVCQKGLALTRRFYGDRRLRHCMVRAGFKRWVDEGFPRGEDGKPPKEYF 6zk9.1    RTGEVMRIL------------PRMHEDINEEWISDKTRFAYDGLK-RQRLTEPMVRNE----------------------  target    QRARDEWVRASHDEAAAVVAATLANIAATYSGEEGAQRLRDQGYEEETIEAMGGAGVQAMKFRGGMPLLGMTRVFGLYRM 6zk9.1    ---KGLLTHTTWEDALSRVAGMLQSC----QGNDV--------------AAIAGGLVDAEAL---IALKDLLNRVDSDTL  target    ANSMALLDAKVRGVGPDEARGARGFDNYSWHTDLPPGHPMVTGQQTVDFDLNSVELAKNVVVWGMNWITTKMPDAHWLTE 6zk9.1    CTEEV-----------------------FPTAGAGTDLR---SNYLLNTTIAGVEEADVVLLVGTNPRFEAPLFNARIRK  target    ARL-KGTRIIVIACEYSSTSSKADDAIVVRPGTTPALALGLSHVIMRDKLYDADYVRRWTDLPMLVRTDTLKYLSAEDVF 6zk9.1    SWLHNDLKVALIGSPVDLTYRYDHLGDSPKI-------------------------------------------------  target    GGGPAPL 6zk9.1    ------- ``` | | | | | | | | | | | | | | | | | | | | | | | | | | | | | | | | | | | | | | | | | | | | | | | | | |
|  | 7ak5.1.G | NADH-ubiquinone oxidoreductase 75 kDa subunit, mitochondrial  *Cryo-EM structure of respiratory complex I in the deactive state from Mus musculus at 3.2 A* | 0.25 |  | 14.08 | 0.51 | 60-351 | EM | 0.00 | hetero-1-1-1-1-1-1-… | 6 x SF4, 2 x PC1, 2 x FES, 1 x FMN, 8 x 3PE, 4 x CDL, 1 x ATP, 1 x NDP, 1 x ZN, 2 x EHZ | HHblits | 0.25 |
| ``` target    TARREITRRGFLGTAAGAGFAAFVVSATRAWGLEAIENPLARYPDREWERVYRDLWRYDSKFTFLCAPNDTHNCLLDAYV 7ak5.1    -----------------------------------------------------------RKTESIDV-MDAVGSNIVVST  target    RSGVMTRIGPTMRYGEARDLDGNRASARWDPRVCQKGLALTRRFYGDRRLRHCMVRAGFKRWVDEGFPRGEDGKPPKEYF 7ak5.1    RTGEVMRIL------------PRMHEDINEEWISDKTRFAYDGLK-RQRLTEPMVRNE----------------------  target    QRARDEWVRASHDEAAAVVAATLANIAATYSGEEGAQRLRDQGYEEETIEAMGGAGVQAMKFRGGMPLLGMTRVFGLYRM 7ak5.1    ---KGLLTYTSWEDALSRVAGMLQN----FEGNAVAAIAGGLVDAEALVA--------LKDL---------LNKVDSDNL  target    ANSMALLDAKVRGVGPDEARGARGFDNYSWHTDLPPGHPMVTGQQTVDFDLNSVELAKNVVVWGMNWITTKMPDAHWLTE 7ak5.1    CTEEI----------------------F-PTEGAGTDLRSNY---LLNTTIAGVEEADVVLLVGTNPRFEAPLFNARIRK  target    AR-LKGTRIIVIACEYSSTSSKADDAIVVRPGTTPALALGLSHVIMRDKLYDADYVRRWTDLPMLVRTDTLKYLSAEDVF 7ak5.1    SWLHNDLKVALIGSPVDLTYRYDHLGDSPKIL------------------------------------------------  target    GGGPAPL 7ak5.1    ------- ``` | | | | | | | | | | | | | | | | | | | | | | | | | | | | | | | | | | | | | | | | | | | | | | | | | |
|  | 7q5y.1.A | NADH dehydrogenase I chain G  *Structure of NADH:ubichinon oxidoreductase (complex I) of the hyperthermophilic eubacterium Aquifex aeolicus* | 0.07 |  | 14.29 | 0.19 | 60-185 | X-ray | 2.70 | hetero-1-1-1-1-1-1-… | 8 x SF4, 2 x FES, 1 x FMN | HHblits | 0.27 |
| ``` target    TARREITRRGFLGTAAGAGFAAFVVSATRAWGLEAIENPLARYPDREWERVYRDLWRYDSKFTFLCAPNDTHNCLLDAYV 7q5y.1    -----------------------------------------------------------EKGRTVC-NLCPVGCEIQIEY  target    RSGV------MTRIGPTMRYGEARDLDGNRASARWDPRVCQKGLALTRRFYGDRRLRHCMVRAGFKRWVDEGFPRGEDGK 7q5y.1    GVGDWRSKRKVYRT-----------------KPTDELNICAKGFFGYDSINHKRLLKTKVGKR-----------------  target    PPKEYFQRARDEWVRASHDEAAAVVAATLANIAATYSGEEGAQRLRDQGYEEETIEAMGGAGVQAMKFRGGMPLLGMTRV 7q5y.1    --------------EETPGNVVNLLTTILTE-------------------------------------------------  target    FGLYRMANSMALLDAKVRGVGPDEARGARGFDNYSWHTDLPPGHPMVTGQQTVDFDLNSVELAKNVVVWGMNWITTKMPD 7q5y.1    --------------------------------------------------------------------------------  target    AHWLTEARLKGTRIIVIACEYSSTSSKADDAIVVRPGTTPALALGLSHVIMRDKLYDADYVRRWTDLPMLVRTDTLKYLS 7q5y.1    --------------------------------------------------------------------------------  target    AEDVFGGGPAPL 7q5y.1    ------------ ``` | | | | | | | | | | | | | | | | | | | | | | | | | | | | | | | | | | | | | | | | | | | | | | | | | |
|  | 7cl0.1.A | NAD-dependent protein deacetylase sirtuin-6  *Crystal structure of human SIRT6* | 0.05 | 0.00 | 12.68 | 0.17 | 292-366 | X-ray | 2.53 | monomer | 1 x AR6, 1 x ZN, 1 x G4U, 1 x THR-ALA-ARG-LYS-SER-THR-GLY | HHblits | 0.28 |
| ``` target    TARREITRRGFLGTAAGAGFAAFVVSATRAWGLEAIENPLARYPDREWERVYRDLWRYDSKFTFLCAPNDTHNCLLDAYV 7cl0.1    --------------------------------------------------------------------------------  target    RSGVMTRIGPTMRYGEARDLDGNRASARWDPRVCQKGLALTRRFYGDRRLRHCMVRAGFKRWVDEGFPRGEDGKPPKEYF 7cl0.1    --------------------------------------------------------------------------------  target    QRARDEWVRASHDEAAAVVAATLANIAATYSGEEGAQRLRDQGYEEETIEAMGGAGVQAMKFRGGMPLLGMTRVFGLYRM 7cl0.1    --------------------------------------------------------------------------------  target    ANSMALLDAKVRGVGPDEARGARGFDNYSWHTDLPPGHPMVTGQQTVDFDLNSVELAKNVVVWGMNWITTKMPDAHWLTE 7cl0.1    ---------------------------------------------------EASRNADLSITLGTSLQIR--PSGNLPLA  target    ARLKGTRIIVIACEYSSTSSKADDAIVVRPGTTPALALGLSHVIMRDKLYDADYVRRWTDLPMLVRTDTLKYLSAEDVFG 7cl0.1    TKRRGGRLVIVNLQPTKHDRHADLRIH--GYVDEVMTRLMKHLGLE----------------------------------  target    GGPAPL 7cl0.1    ------ ``` | | | | | | | | | | | | | | | | | | | | | | | | | | | | | | | | | | | | | | | | | | | | | | | | | |
|  | 1jeo.1.A | HYPOTHETICAL PROTEIN MJ1247  *Crystal Structure of the Hypothetical Protein MJ1247 from Methanococcus jannaschii at 2.0 A Resolution Infers a Molecular Function of 3-Hexulose-6-Phosphate isomerase.* | 0.04 |  | 11.11 | 0.18 | 292-366 | X-ray | 2.00 | monomer |  | HHblits | 0.26 |
| ``` target    TARREITRRGFLGTAAGAGFAAFVVSATRAWGLEAIENPLARYPDREWERVYRDLWRYDSKFTFLCAPNDTHNCLLDAYV 1jeo.1    --------------------------------------------------------------------------------  target    RSGVMTRIGPTMRYGEARDLDGNRASARWDPRVCQKGLALTRRFYGDRRLRHCMVRAGFKRWVDEGFPRGEDGKPPKEYF 1jeo.1    --------------------------------------------------------------------------------  target    QRARDEWVRASHDEAAAVVAATLANIAATYSGEEGAQRLRDQGYEEETIEAMGGAGVQAMKFRGGMPLLGMTRVFGLYRM 1jeo.1    --------------------------------------------------------------------------------  target    ANSMALLDAKVRGVGPDEARGARGFDNYSWHTDLPPGHPMVTGQQTVDFDLNSVELAKNVVVWGMNWITTKMPDAHWLT- 1jeo.1    ---------------------------------------------------DRIIKAKKIFIFGVGRSGY---IGRCFAM  target    EARLKGTRIIVIACEYSSTSSKADDAIVVRPGTTPALALGLSHVIMRDKLYDADYVRRWTDLPMLVRTDTLKYLSAEDVF 1jeo.1    RLMHLGFKSYFVGETTTPSYEKDDLLILISGSGRTESVLTVAKKAKN---------------------------------  target    GGGPAPL 1jeo.1    ------- ``` | | | | | | | | | | | | | | | | | | | | | | | | | | | | | | | | | | | | | | | | | | | | | | | | | |
|  | 6s6y.1.B | Tungsten-containing formylmethanofuran dehydrogenase, subunit B  *X-ray crystal structure of the formyltransferase/hydrolase complex (FhcABCD) from Methylorubrum extorquens in complex with methylofuran* | 0.05 |  | 9.46 | 0.18 | 291-367 | X-ray | 3.10 | hetero-2-2-2-2-mer | 1 x MFN, 4 x ZN, 4 x CA, 4 x K, 3 x DGL, 2 x GLU, 1 x IAS | HHblits | 0.23 |
| ``` target    TARREITRRGFLGTAAGAGFAAFVVSATRAWGLEAIENPLARYPDREWERVYRDLWRYDSKFTFLCAPNDTHNCLLDAYV 6s6y.1    --------------------------------------------------------------------------------  target    RSGVMTRIGPTMRYGEARDLDGNRASARWDPRVCQKGLALTRRFYGDRRLRHCMVRAGFKRWVDEGFPRGEDGKPPKEYF 6s6y.1    --------------------------------------------------------------------------------  target    QRARDEWVRASHDEAAAVVAATLANIAATYSGEEGAQRLRDQGYEEETIEAMGGAGVQAMKFRGGMPLLGMTRVFGLYRM 6s6y.1    --------------------------------------------------------------------------------  target    ANSMALLDAKVRGVGPDEARGARGFDNYSWHTDLPPGHPMVTGQQTVDFDLNSV-ELAKNVVVWGMNWITTKMPDAHWLT 6s6y.1    --------------------------------------------------RAETIGRADVILIVGNRPWDGEL-IAEIAA  target    EAR------LKGTRIIVIACEYSSTSSKADDAIVVRPGTTPALALGLSHVIMRDKLYDADYVRRWTDLPMLVRTDTLKYL 6s6y.1    AAPSRGRAAGAERALLSLGGPQNGAIR--HVAYAADAGGLTISLGHLRAFAKGH--------------------------  target    SAEDVFGGGPAPL 6s6y.1    ------------- ``` | | | | | | | | | | | | | | | | | | | | | | | | | | | | | | | | | | | | | | | | | | | | | | | | | |
|  | 3pki.1.A | NAD-dependent deacetylase sirtuin-6  *Human SIRT6 crystal structure in complex with ADP ribose* | 0.05 | 0.00 | 13.24 | 0.17 | 292-363 | X-ray | 2.04 | monomer | 1 x ZN, 1 x AR6 | HHblits | 0.28 |
| ``` target    TARREITRRGFLGTAAGAGFAAFVVSATRAWGLEAIENPLARYPDREWERVYRDLWRYDSKFTFLCAPNDTHNCLLDAYV 3pki.1    --------------------------------------------------------------------------------  target    RSGVMTRIGPTMRYGEARDLDGNRASARWDPRVCQKGLALTRRFYGDRRLRHCMVRAGFKRWVDEGFPRGEDGKPPKEYF 3pki.1    --------------------------------------------------------------------------------  target    QRARDEWVRASHDEAAAVVAATLANIAATYSGEEGAQRLRDQGYEEETIEAMGGAGVQAMKFRGGMPLLGMTRVFGLYRM 3pki.1    --------------------------------------------------------------------------------  target    ANSMALLDAKVRGVGPDEARGARGFDNYSWHTDLPPGHPMVTGQQTVDFDLNSVELAKNVVVWGMNWITTKMPDAHWLTE 3pki.1    ---------------------------------------------------EASRNADLSITLGTSLQI--RPSGNLPLA  target    ARLKGTRIIVIACEYSSTSSKADDAIVVRPGTTPALALGLSHVIMRDKLYDADYVRRWTDLPMLVRTDTLKYLSAEDVFG 3pki.1    TKRRGGRLVIVNLQPTKHDRHADLRIHG--YVDEVMTRLMEHL-------------------------------------  target    GGPAPL 3pki.1    ------ ``` | | | | | | | | | | | | | | | | | | | | | | | | | | | | | | | | | | | | | | | | | | | | | | | | | |
|  | 2vpz.1.A | THIOSULFATE REDUCTASE  *POLYSULFIDE REDUCTASE NATIVE STRUCTURE* | 0.05 | 0.00 | 34.55 | 0.14 | 325-379 | X-ray | 2.40 | monomer | 10 x SF4, 4 x MGD, 2 x MO | BLAST | 0.40 |
| ``` target    TARREITRRGFLGTAAGAGFAAFVVSATRAWGLEAIENPLARYPDREWERVYRDLWRYDSKFTFLCAPNDTHNCLLDAYV 2vpz.1    --------------------------------------------------------------------------------  target    RSGVMTRIGPTMRYGEARDLDGNRASARWDPRVCQKGLALTRRFYGDRRLRHCMVRAGFKRWVDEGFPRGEDGKPPKEYF 2vpz.1    --------------------------------------------------------------------------------  target    QRARDEWVRASHDEAAAVVAATLANIAATYSGEEGAQRLRDQGYEEETIEAMGGAGVQAMKFRGGMPLLGMTRVFGLYRM 2vpz.1    --------------------------------------------------------------------------------  target    ANSMALLDAKVRGVGPDEARGARGFDNYSWHTDLPPGHPMVTGQQTVDFDLNSVELAKNVVVWGMNWITTKMPDAHWLTE 2vpz.1    --------------------------------------------------------------------------------  target    ARLKGTRIIVIACEYSSTSSKADDAIVVRPGTTPALALGLSHVIMRDKLYDADYVRRWTDLPMLVRTDTLKYLSAEDVFG 2vpz.1    ----GAKVVVVDPRFSTAAAKAHRWLPIKPGTDTALLLAWIHVLIYEDLYDKEYVAKYT---------------------  target    GGPAPL 2vpz.1    ------ ``` | | | | | | | | | | | | | | | | | | | | | | | | | | | | | | | | | | | | | | | | | | | | | | | | | |
|  | 2vpx.1.D | THIOSULFATE REDUCTASE  *POLYSULFIDE REDUCTASE WITH BOUND QUINONE (UQ1)* | 0.05 | 0.00 | 34.55 | 0.14 | 325-379 | X-ray | 3.10 | monomer | 10 x SF4, 4 x MGD, 2 x MO, 2 x UQ1 | BLAST | 0.40 |
| ``` target    TARREITRRGFLGTAAGAGFAAFVVSATRAWGLEAIENPLARYPDREWERVYRDLWRYDSKFTFLCAPNDTHNCLLDAYV 2vpx.1    --------------------------------------------------------------------------------  target    RSGVMTRIGPTMRYGEARDLDGNRASARWDPRVCQKGLALTRRFYGDRRLRHCMVRAGFKRWVDEGFPRGEDGKPPKEYF 2vpx.1    --------------------------------------------------------------------------------  target    QRARDEWVRASHDEAAAVVAATLANIAATYSGEEGAQRLRDQGYEEETIEAMGGAGVQAMKFRGGMPLLGMTRVFGLYRM 2vpx.1    --------------------------------------------------------------------------------  target    ANSMALLDAKVRGVGPDEARGARGFDNYSWHTDLPPGHPMVTGQQTVDFDLNSVELAKNVVVWGMNWITTKMPDAHWLTE 2vpx.1    --------------------------------------------------------------------------------  target    ARLKGTRIIVIACEYSSTSSKADDAIVVRPGTTPALALGLSHVIMRDKLYDADYVRRWTDLPMLVRTDTLKYLSAEDVFG 2vpx.1    ----GAKVVVVDPRFSTAAAKAHRWLPIKPGTDTALLLAWIHVLIYEDLYDKEYVAKYT---------------------  target    GGPAPL 2vpx.1    ------ ``` | | | | | | | | | | | | | | | | | | | | | | | | | | | | | | | | | | | | | | | | | | | | | | | | | |
|  | 1ici.1.A | TRANSCRIPTIONAL REGULATORY PROTEIN, SIR2 FAMILY  *CRYSTAL STRUCTURE OF A SIR2 HOMOLOG-NAD COMPLEX* | 0.04 | 0.00 | 23.64 | 0.14 | 292-348 | X-ray | 2.10 | monomer | 2 x ZN, 2 x NAD | HHblits | 0.30 |
| ``` target    TARREITRRGFLGTAAGAGFAAFVVSATRAWGLEAIENPLARYPDREWERVYRDLWRYDSKFTFLCAPNDTHNCLLDAYV 1ici.1    --------------------------------------------------------------------------------  target    RSGVMTRIGPTMRYGEARDLDGNRASARWDPRVCQKGLALTRRFYGDRRLRHCMVRAGFKRWVDEGFPRGEDGKPPKEYF 1ici.1    --------------------------------------------------------------------------------  target    QRARDEWVRASHDEAAAVVAATLANIAATYSGEEGAQRLRDQGYEEETIEAMGGAGVQAMKFRGGMPLLGMTRVFGLYRM 1ici.1    --------------------------------------------------------------------------------  target    ANSMALLDAKVRGVGPDEARGARGFDNYSWHTDLPPGHPMVTGQQTVDFDLNSVELAKNVVVWGMNWITTKMPDAHWLTE 1ici.1    ---------------------------------------------------REVERADVIIVAGTSAVV--QPAASLPLI  target    ARLKGTRIIVIACEYSSTSSKADDAIVVRPGTTPALALGLSHVIMRDKLYDADYVRRWTDLPMLVRTDTLKYLSAEDVFG 1ici.1    VKQRGGAIIEINPDETPLTPIADYSLRG----------------------------------------------------  target    GGPAPL 1ici.1    ------ ``` | | | | | | | | | | | | | | | | | | | | | | | | | | | | | | | | | | | | | | | | | | | | | | | | | |
|  | 1jeo.1.A | HYPOTHETICAL PROTEIN MJ1247  *Crystal Structure of the Hypothetical Protein MJ1247 from Methanococcus jannaschii at 2.0 A Resolution Infers a Molecular Function of 3-Hexulose-6-Phosphate isomerase.* | 0.03 |  | 17.54 | 0.14 | 296-354 | X-ray | 2.00 | monomer |  | HHblits | 0.27 |
| ``` target    TARREITRRGFLGTAAGAGFAAFVVSATRAWGLEAIENPLARYPDREWERVYRDLWRYDSKFTFLCAPNDTHNCLLDAYV 1jeo.1    --------------------------------------------------------------------------------  target    RSGVMTRIGPTMRYGEARDLDGNRASARWDPRVCQKGLALTRRFYGDRRLRHCMVRAGFKRWVDEGFPRGEDGKPPKEYF 1jeo.1    --------------------------------------------------------------------------------  target    QRARDEWVRASHDEAAAVVAATLANIAATYSGEEGAQRLRDQGYEEETIEAMGGAGVQAMKFRGGMPLLGMTRVFGLYRM 1jeo.1    --------------------------------------------------------------------------------  target    ANSMALLDAKVRGVGPDEARGARGFDNYSWHTDLPPGHPMVTGQQTVDFDLNSVELAKNVVVWGMNWITTKMPDAHWLTE 1jeo.1    -------------------------------------------------------KDDLLILISGSGRTE--SVLTVAKK  target    ARLKGTRIIVIACEYSSTSSKADDAIVVRPGTTPALALGLSHVIMRDKLYDADYVRRWTDLPMLVRTDTLKYLSAEDVFG 1jeo.1    AKNINNNIIAIVCECGNVVEFADLTIPLEVKKSK----------------------------------------------  target    GGPAPL 1jeo.1    ------ ``` | | | | | | | | | | | | | | | | | | | | | | | | | | | | | | | | | | | | | | | | | | | | | | | | | |
|  | 1s7g.1.D | NAD-dependent deacetylase 2  *Structural Basis for the Mechanism and Regulation of Sir2 Enzymes* | 0.03 | 0.00 | 18.18 | 0.14 | 292-348 | X-ray | 2.30 | homo-trimer | 9 x ZN, 3 x NAD, 1 x APR, 1 x 2PE | HHblits | 0.29 |
| ``` target    TARREITRRGFLGTAAGAGFAAFVVSATRAWGLEAIENPLARYPDREWERVYRDLWRYDSKFTFLCAPNDTHNCLLDAYV 1s7g.1    --------------------------------------------------------------------------------  target    RSGVMTRIGPTMRYGEARDLDGNRASARWDPRVCQKGLALTRRFYGDRRLRHCMVRAGFKRWVDEGFPRGEDGKPPKEYF 1s7g.1    --------------------------------------------------------------------------------  target    QRARDEWVRASHDEAAAVVAATLANIAATYSGEEGAQRLRDQGYEEETIEAMGGAGVQAMKFRGGMPLLGMTRVFGLYRM 1s7g.1    --------------------------------------------------------------------------------  target    ANSMALLDAKVRGVGPDEARGARGFDNYSWHTDLPPGHPMVTGQQTVDFDLNSVELAKNVVVWGMNWITTKMPDAHWLTE 1s7g.1    ---------------------------------------------------EEAKHCDAFMVVGSSLVV--YPAAELPYI  target    ARLKGTRIIVIACEYSSTSSKADDAIVVRPGTTPALALGLSHVIMRDKLYDADYVRRWTDLPMLVRTDTLKYLSAEDVFG 1s7g.1    AKKAGAKMIIVNAEPTMADPIFDVKIIG----------------------------------------------------  target    GGPAPL 1s7g.1    ------ ``` | | | | | | | | | | | | | | | | | | | | | | | | | | | | | | | | | | | | | | | | | | | | | | | | | |
|  | 1ma3.1.A | Transcriptional regulatory protein, Sir2 family  *Structure of a Sir2 enzyme bound to an acetylated p53 peptide* | 0.04 | 0.00 | 18.18 | 0.14 | 292-348 | X-ray | 2.00 | monomer | 1 x ZN, 1 x MES | HHblits | 0.29 |
| ``` target    TARREITRRGFLGTAAGAGFAAFVVSATRAWGLEAIENPLARYPDREWERVYRDLWRYDSKFTFLCAPNDTHNCLLDAYV 1ma3.1    --------------------------------------------------------------------------------  target    RSGVMTRIGPTMRYGEARDLDGNRASARWDPRVCQKGLALTRRFYGDRRLRHCMVRAGFKRWVDEGFPRGEDGKPPKEYF 1ma3.1    --------------------------------------------------------------------------------  target    QRARDEWVRASHDEAAAVVAATLANIAATYSGEEGAQRLRDQGYEEETIEAMGGAGVQAMKFRGGMPLLGMTRVFGLYRM 1ma3.1    --------------------------------------------------------------------------------  target    ANSMALLDAKVRGVGPDEARGARGFDNYSWHTDLPPGHPMVTGQQTVDFDLNSVELAKNVVVWGMNWITTKMPDAHWLTE 1ma3.1    ---------------------------------------------------EEAKHCDAFMVVGSSLVV--YPAAELPYI  target    ARLKGTRIIVIACEYSSTSSKADDAIVVRPGTTPALALGLSHVIMRDKLYDADYVRRWTDLPMLVRTDTLKYLSAEDVFG 1ma3.1    AKKAGAKMIIVNAEPTMADPIFDVKIIG----------------------------------------------------  target    GGPAPL 1ma3.1    ------ ``` | | | | | | | | | | | | | | | | | | | | | | | | | | | | | | | | | | | | | | | | | | | | | | | | | |
|  | 1s7g.1.B | NAD-dependent deacetylase 2  *Structural Basis for the Mechanism and Regulation of Sir2 Enzymes* | 0.03 | 0.00 | 18.18 | 0.14 | 292-348 | X-ray | 2.30 | homo-trimer | 9 x ZN, 3 x NAD, 1 x APR, 1 x 2PE | HHblits | 0.29 |
| ``` target    TARREITRRGFLGTAAGAGFAAFVVSATRAWGLEAIENPLARYPDREWERVYRDLWRYDSKFTFLCAPNDTHNCLLDAYV 1s7g.1    --------------------------------------------------------------------------------  target    RSGVMTRIGPTMRYGEARDLDGNRASARWDPRVCQKGLALTRRFYGDRRLRHCMVRAGFKRWVDEGFPRGEDGKPPKEYF 1s7g.1    --------------------------------------------------------------------------------  target    QRARDEWVRASHDEAAAVVAATLANIAATYSGEEGAQRLRDQGYEEETIEAMGGAGVQAMKFRGGMPLLGMTRVFGLYRM 1s7g.1    --------------------------------------------------------------------------------  target    ANSMALLDAKVRGVGPDEARGARGFDNYSWHTDLPPGHPMVTGQQTVDFDLNSVELAKNVVVWGMNWITTKMPDAHWLTE 1s7g.1    ---------------------------------------------------EEAKHCDAFMVVGSSLVV--YPAAELPYI  target    ARLKGTRIIVIACEYSSTSSKADDAIVVRPGTTPALALGLSHVIMRDKLYDADYVRRWTDLPMLVRTDTLKYLSAEDVFG 1s7g.1    AKKAGAKMIIVNAEPTMADPIFDVKIIG----------------------------------------------------  target    GGPAPL 1s7g.1    ------ ``` | | | | | | | | | | | | | | | | | | | | | | | | | | | | | | | | | | | | | | | | | | | | | | | | | |
|  | 1s7g.1.A | NAD-dependent deacetylase 2  *Structural Basis for the Mechanism and Regulation of Sir2 Enzymes* | 0.03 | 0.00 | 18.18 | 0.14 | 292-348 | X-ray | 2.30 | homo-trimer | 9 x ZN, 3 x NAD, 1 x APR, 1 x 2PE | HHblits | 0.29 |
| ``` target    TARREITRRGFLGTAAGAGFAAFVVSATRAWGLEAIENPLARYPDREWERVYRDLWRYDSKFTFLCAPNDTHNCLLDAYV 1s7g.1    --------------------------------------------------------------------------------  target    RSGVMTRIGPTMRYGEARDLDGNRASARWDPRVCQKGLALTRRFYGDRRLRHCMVRAGFKRWVDEGFPRGEDGKPPKEYF 1s7g.1    --------------------------------------------------------------------------------  target    QRARDEWVRASHDEAAAVVAATLANIAATYSGEEGAQRLRDQGYEEETIEAMGGAGVQAMKFRGGMPLLGMTRVFGLYRM 1s7g.1    --------------------------------------------------------------------------------  target    ANSMALLDAKVRGVGPDEARGARGFDNYSWHTDLPPGHPMVTGQQTVDFDLNSVELAKNVVVWGMNWITTKMPDAHWLTE 1s7g.1    ---------------------------------------------------EEAKHCDAFMVVGSSLVV--YPAAELPYI  target    ARLKGTRIIVIACEYSSTSSKADDAIVVRPGTTPALALGLSHVIMRDKLYDADYVRRWTDLPMLVRTDTLKYLSAEDVFG 1s7g.1    AKKAGAKMIIVNAEPTMADPIFDVKIIG----------------------------------------------------  target    GGPAPL 1s7g.1    ------ ``` | | | | | | | | | | | | | | | | | | | | | | | | | | | | | | | | | | | | | | | | | | | | | | | | | |
|  | 1s7g.1.C | NAD-dependent deacetylase 2  *Structural Basis for the Mechanism and Regulation of Sir2 Enzymes* | 0.03 | 0.00 | 18.18 | 0.14 | 292-348 | X-ray | 2.30 | homo-trimer | 9 x ZN, 3 x NAD, 1 x APR, 1 x 2PE | HHblits | 0.29 |
| ``` target    TARREITRRGFLGTAAGAGFAAFVVSATRAWGLEAIENPLARYPDREWERVYRDLWRYDSKFTFLCAPNDTHNCLLDAYV 1s7g.1    --------------------------------------------------------------------------------  target    RSGVMTRIGPTMRYGEARDLDGNRASARWDPRVCQKGLALTRRFYGDRRLRHCMVRAGFKRWVDEGFPRGEDGKPPKEYF 1s7g.1    --------------------------------------------------------------------------------  target    QRARDEWVRASHDEAAAVVAATLANIAATYSGEEGAQRLRDQGYEEETIEAMGGAGVQAMKFRGGMPLLGMTRVFGLYRM 1s7g.1    --------------------------------------------------------------------------------  target    ANSMALLDAKVRGVGPDEARGARGFDNYSWHTDLPPGHPMVTGQQTVDFDLNSVELAKNVVVWGMNWITTKMPDAHWLTE 1s7g.1    ---------------------------------------------------EEAKHCDAFMVVGSSLVV--YPAAELPYI  target    ARLKGTRIIVIACEYSSTSSKADDAIVVRPGTTPALALGLSHVIMRDKLYDADYVRRWTDLPMLVRTDTLKYLSAEDVFG 1s7g.1    AKKAGAKMIIVNAEPTMADPIFDVKIIG----------------------------------------------------  target    GGPAPL 1s7g.1    ------ ``` | | | | | | | | | | | | | | | | | | | | | | | | | | | | | | | | | | | | | | | | | | | | | | | | | |
|  | 1s7g.1.E | NAD-dependent deacetylase 2  *Structural Basis for the Mechanism and Regulation of Sir2 Enzymes* | 0.04 | 0.00 | 18.18 | 0.14 | 292-348 | X-ray | 2.30 | homo-trimer | 9 x ZN, 3 x NAD, 1 x APR, 1 x 2PE | HHblits | 0.29 |
| ``` target    TARREITRRGFLGTAAGAGFAAFVVSATRAWGLEAIENPLARYPDREWERVYRDLWRYDSKFTFLCAPNDTHNCLLDAYV 1s7g.1    --------------------------------------------------------------------------------  target    RSGVMTRIGPTMRYGEARDLDGNRASARWDPRVCQKGLALTRRFYGDRRLRHCMVRAGFKRWVDEGFPRGEDGKPPKEYF 1s7g.1    --------------------------------------------------------------------------------  target    QRARDEWVRASHDEAAAVVAATLANIAATYSGEEGAQRLRDQGYEEETIEAMGGAGVQAMKFRGGMPLLGMTRVFGLYRM 1s7g.1    --------------------------------------------------------------------------------  target    ANSMALLDAKVRGVGPDEARGARGFDNYSWHTDLPPGHPMVTGQQTVDFDLNSVELAKNVVVWGMNWITTKMPDAHWLTE 1s7g.1    ---------------------------------------------------EEAKHCDAFMVVGSSLVV--YPAAELPYI  target    ARLKGTRIIVIACEYSSTSSKADDAIVVRPGTTPALALGLSHVIMRDKLYDADYVRRWTDLPMLVRTDTLKYLSAEDVFG 1s7g.1    AKKAGAKMIIVNAEPTMADPIFDVKIIG----------------------------------------------------  target    GGPAPL 1s7g.1    ------ ``` | | | | | | | | | | | | | | | | | | | | | | | | | | | | | | | | | | | | | | | | | | | | | | | | | |
|  | 4twj.1.A | NAD-dependent protein deacylase 2  *The structure of Sir2Af2 bound to a myristoylated histone peptide* | 0.04 |  | 18.18 | 0.14 | 292-348 | X-ray | 1.65 | hetero-1-1-mer | 1 x ZN | HHblits | 0.29 |
| ``` target    TARREITRRGFLGTAAGAGFAAFVVSATRAWGLEAIENPLARYPDREWERVYRDLWRYDSKFTFLCAPNDTHNCLLDAYV 4twj.1    --------------------------------------------------------------------------------  target    RSGVMTRIGPTMRYGEARDLDGNRASARWDPRVCQKGLALTRRFYGDRRLRHCMVRAGFKRWVDEGFPRGEDGKPPKEYF 4twj.1    --------------------------------------------------------------------------------  target    QRARDEWVRASHDEAAAVVAATLANIAATYSGEEGAQRLRDQGYEEETIEAMGGAGVQAMKFRGGMPLLGMTRVFGLYRM 4twj.1    --------------------------------------------------------------------------------  target    ANSMALLDAKVRGVGPDEARGARGFDNYSWHTDLPPGHPMVTGQQTVDFDLNSVELAKNVVVWGMNWITTKMPDAHWLTE 4twj.1    ---------------------------------------------------EEAKHCDAFMVVGSSLVV--YPAAELPYI  target    ARLKGTRIIVIACEYSSTSSKADDAIVVRPGTTPALALGLSHVIMRDKLYDADYVRRWTDLPMLVRTDTLKYLSAEDVFG 4twj.1    AKKAGAKMIIVNAEPTMADPIFDVKIIG----------------------------------------------------  target    GGPAPL 4twj.1    ------ ``` | | | | | | | | | | | | | | | | | | | | | | | | | | | | | | | | | | | | | | | | | | | | | | | | | |
|  | 1m2g.1.A | Silent Information Regulator 2  *Sir2 homologue-ADP ribose complex* | 0.03 |  | 24.07 | 0.13 | 292-347 | X-ray | 1.70 | monomer | 1 x ZN, 1 x APR | HHblits | 0.30 |
| ``` target    TARREITRRGFLGTAAGAGFAAFVVSATRAWGLEAIENPLARYPDREWERVYRDLWRYDSKFTFLCAPNDTHNCLLDAYV 1m2g.1    --------------------------------------------------------------------------------  target    RSGVMTRIGPTMRYGEARDLDGNRASARWDPRVCQKGLALTRRFYGDRRLRHCMVRAGFKRWVDEGFPRGEDGKPPKEYF 1m2g.1    --------------------------------------------------------------------------------  target    QRARDEWVRASHDEAAAVVAATLANIAATYSGEEGAQRLRDQGYEEETIEAMGGAGVQAMKFRGGMPLLGMTRVFGLYRM 1m2g.1    --------------------------------------------------------------------------------  target    ANSMALLDAKVRGVGPDEARGARGFDNYSWHTDLPPGHPMVTGQQTVDFDLNSVELAKNVVVWGMNWITTKMPDAHWLTE 1m2g.1    ---------------------------------------------------REVERADVIIVAGTSAVVQ--PAASLPLI  target    ARLKGTRIIVIACEYSSTSSKADDAIVVRPGTTPALALGLSHVIMRDKLYDADYVRRWTDLPMLVRTDTLKYLSAEDVFG 1m2g.1    VKQRGGAIIEINPDETPLTPIADYSLR-----------------------------------------------------  target    GGPAPL 1m2g.1    ------ ``` | | | | | | | | | | | | | | | | | | | | | | | | | | | | | | | | | | | | | | | | | | | | | | | | | |
|  | 4twi.1.A | NAD-dependent protein deacylase 1  *The structure of Sir2Af1 bound to a succinylated histone peptide* | 0.03 |  | 24.07 | 0.13 | 292-347 | X-ray | 1.79 | hetero-1-1-mer | 1 x ZN | HHblits | 0.30 |
| ``` target    TARREITRRGFLGTAAGAGFAAFVVSATRAWGLEAIENPLARYPDREWERVYRDLWRYDSKFTFLCAPNDTHNCLLDAYV 4twi.1    --------------------------------------------------------------------------------  target    RSGVMTRIGPTMRYGEARDLDGNRASARWDPRVCQKGLALTRRFYGDRRLRHCMVRAGFKRWVDEGFPRGEDGKPPKEYF 4twi.1    --------------------------------------------------------------------------------  target    QRARDEWVRASHDEAAAVVAATLANIAATYSGEEGAQRLRDQGYEEETIEAMGGAGVQAMKFRGGMPLLGMTRVFGLYRM 4twi.1    --------------------------------------------------------------------------------  target    ANSMALLDAKVRGVGPDEARGARGFDNYSWHTDLPPGHPMVTGQQTVDFDLNSVELAKNVVVWGMNWITTKMPDAHWLTE 4twi.1    ---------------------------------------------------REVERADVIIVAGTSAVVQ--PAASLPLI  target    ARLKGTRIIVIACEYSSTSSKADDAIVVRPGTTPALALGLSHVIMRDKLYDADYVRRWTDLPMLVRTDTLKYLSAEDVFG 4twi.1    VKQRGGAIIEINPDETPLTPIADYSLR-----------------------------------------------------  target    GGPAPL 4twi.1    ------ ``` | | | | | | | | | | | | | | | | | | | | | | | | | | | | | | | | | | | | | | | | | | | | | | | | | |
|  | 6rxo.1.A | NAD-dependent protein deacylase  *Crystal structure of CobB Ac2 (A76G, I131C, V162A) in complex with H4K16-Buturyl peptide* | 0.03 |  | 18.52 | 0.13 | 292-347 | X-ray | 1.95 | hetero-1-1-mer | 1 x ZN | HHblits | 0.30 |
| ``` target    TARREITRRGFLGTAAGAGFAAFVVSATRAWGLEAIENPLARYPDREWERVYRDLWRYDSKFTFLCAPNDTHNCLLDAYV 6rxo.1    --------------------------------------------------------------------------------  target    RSGVMTRIGPTMRYGEARDLDGNRASARWDPRVCQKGLALTRRFYGDRRLRHCMVRAGFKRWVDEGFPRGEDGKPPKEYF 6rxo.1    --------------------------------------------------------------------------------  target    QRARDEWVRASHDEAAAVVAATLANIAATYSGEEGAQRLRDQGYEEETIEAMGGAGVQAMKFRGGMPLLGMTRVFGLYRM 6rxo.1    --------------------------------------------------------------------------------  target    ANSMALLDAKVRGVGPDEARGARGFDNYSWHTDLPPGHPMVTGQQTVDFDLNSVELAKNVVVWGMNWITTKMPDAHWLTE 6rxo.1    ---------------------------------------------------MALSMADIFIAIGTSGHV--YPAAGFVHE  target    ARLKGTRIIVIACEYSSTSSKADDAIVVRPGTTPALALGLSHVIMRDKLYDADYVRRWTDLPMLVRTDTLKYLSAEDVFG 6rxo.1    AKLHGAHTVELNLEPSQVGNEFAEKYY-----------------------------------------------------  target    GGPAPL 6rxo.1    ------ ``` | | | | | | | | | | | | | | | | | | | | | | | | | | | | | | | | | | | | | | | | | | | | | | | | | |
|  | 6rxm.1.A | NAD-dependent protein deacylase  *Crystal structure of CobB Ac2 (A76G, I131C, V162G) in complex with H4K16-Acetyl peptide* | 0.03 |  | 18.52 | 0.13 | 292-347 | X-ray | 1.92 | hetero-1-1-mer | 1 x ZN | HHblits | 0.30 |
| ``` target    TARREITRRGFLGTAAGAGFAAFVVSATRAWGLEAIENPLARYPDREWERVYRDLWRYDSKFTFLCAPNDTHNCLLDAYV 6rxm.1    --------------------------------------------------------------------------------  target    RSGVMTRIGPTMRYGEARDLDGNRASARWDPRVCQKGLALTRRFYGDRRLRHCMVRAGFKRWVDEGFPRGEDGKPPKEYF 6rxm.1    --------------------------------------------------------------------------------  target    QRARDEWVRASHDEAAAVVAATLANIAATYSGEEGAQRLRDQGYEEETIEAMGGAGVQAMKFRGGMPLLGMTRVFGLYRM 6rxm.1    --------------------------------------------------------------------------------  target    ANSMALLDAKVRGVGPDEARGARGFDNYSWHTDLPPGHPMVTGQQTVDFDLNSVELAKNVVVWGMNWITTKMPDAHWLTE 6rxm.1    ---------------------------------------------------MALSMADIFIAIGTSGHV--YPAAGFVHE  target    ARLKGTRIIVIACEYSSTSSKADDAIVVRPGTTPALALGLSHVIMRDKLYDADYVRRWTDLPMLVRTDTLKYLSAEDVFG 6rxm.1    AKLHGAHTVELNLEPSQVGNEFAEKYY-----------------------------------------------------  target    GGPAPL 6rxm.1    ------ ``` | | | | | | | | | | | | | | | | | | | | | | | | | | | | | | | | | | | | | | | | | | | | | | | | | |
|  | 6rxm.3.A | NAD-dependent protein deacylase  *Crystal structure of CobB Ac2 (A76G, I131C, V162G) in complex with H4K16-Acetyl peptide* | 0.03 |  | 18.52 | 0.13 | 292-347 | X-ray | 1.92 | hetero-1-1-mer | 1 x ZN | HHblits | 0.30 |
| ``` target    TARREITRRGFLGTAAGAGFAAFVVSATRAWGLEAIENPLARYPDREWERVYRDLWRYDSKFTFLCAPNDTHNCLLDAYV 6rxm.3    --------------------------------------------------------------------------------  target    RSGVMTRIGPTMRYGEARDLDGNRASARWDPRVCQKGLALTRRFYGDRRLRHCMVRAGFKRWVDEGFPRGEDGKPPKEYF 6rxm.3    --------------------------------------------------------------------------------  target    QRARDEWVRASHDEAAAVVAATLANIAATYSGEEGAQRLRDQGYEEETIEAMGGAGVQAMKFRGGMPLLGMTRVFGLYRM 6rxm.3    --------------------------------------------------------------------------------  target    ANSMALLDAKVRGVGPDEARGARGFDNYSWHTDLPPGHPMVTGQQTVDFDLNSVELAKNVVVWGMNWITTKMPDAHWLTE 6rxm.3    ---------------------------------------------------MALSMADIFIAIGTSGHV--YPAAGFVHE  target    ARLKGTRIIVIACEYSSTSSKADDAIVVRPGTTPALALGLSHVIMRDKLYDADYVRRWTDLPMLVRTDTLKYLSAEDVFG 6rxm.3    AKLHGAHTVELNLEPSQVGNEFAEKYY-----------------------------------------------------  target    GGPAPL 6rxm.3    ------ ``` | | | | | | | | | | | | | | | | | | | | | | | | | | | | | | | | | | | | | | | | | | | | | | | | | |
|  | 6rxm.4.A | NAD-dependent protein deacylase  *Crystal structure of CobB Ac2 (A76G, I131C, V162G) in complex with H4K16-Acetyl peptide* | 0.03 |  | 18.52 | 0.13 | 292-347 | X-ray | 1.92 | hetero-1-1-mer | 1 x ZN | HHblits | 0.30 |
| ``` target    TARREITRRGFLGTAAGAGFAAFVVSATRAWGLEAIENPLARYPDREWERVYRDLWRYDSKFTFLCAPNDTHNCLLDAYV 6rxm.4    --------------------------------------------------------------------------------  target    RSGVMTRIGPTMRYGEARDLDGNRASARWDPRVCQKGLALTRRFYGDRRLRHCMVRAGFKRWVDEGFPRGEDGKPPKEYF 6rxm.4    --------------------------------------------------------------------------------  target    QRARDEWVRASHDEAAAVVAATLANIAATYSGEEGAQRLRDQGYEEETIEAMGGAGVQAMKFRGGMPLLGMTRVFGLYRM 6rxm.4    --------------------------------------------------------------------------------  target    ANSMALLDAKVRGVGPDEARGARGFDNYSWHTDLPPGHPMVTGQQTVDFDLNSVELAKNVVVWGMNWITTKMPDAHWLTE 6rxm.4    ---------------------------------------------------MALSMADIFIAIGTSGHV--YPAAGFVHE  target    ARLKGTRIIVIACEYSSTSSKADDAIVVRPGTTPALALGLSHVIMRDKLYDADYVRRWTDLPMLVRTDTLKYLSAEDVFG 6rxm.4    AKLHGAHTVELNLEPSQVGNEFAEKYY-----------------------------------------------------  target    GGPAPL 6rxm.4    ------ ``` | | | | | | | | | | | | | | | | | | | | | | | | | | | | | | | | | | | | | | | | | | | | | | | | | |
|  | 6rxm.5.A | NAD-dependent protein deacylase  *Crystal structure of CobB Ac2 (A76G, I131C, V162G) in complex with H4K16-Acetyl peptide* | 0.03 |  | 18.52 | 0.13 | 292-347 | X-ray | 1.92 | hetero-1-1-mer | 1 x ZN | HHblits | 0.30 |
| ``` target    TARREITRRGFLGTAAGAGFAAFVVSATRAWGLEAIENPLARYPDREWERVYRDLWRYDSKFTFLCAPNDTHNCLLDAYV 6rxm.5    --------------------------------------------------------------------------------  target    RSGVMTRIGPTMRYGEARDLDGNRASARWDPRVCQKGLALTRRFYGDRRLRHCMVRAGFKRWVDEGFPRGEDGKPPKEYF 6rxm.5    --------------------------------------------------------------------------------  target    QRARDEWVRASHDEAAAVVAATLANIAATYSGEEGAQRLRDQGYEEETIEAMGGAGVQAMKFRGGMPLLGMTRVFGLYRM 6rxm.5    --------------------------------------------------------------------------------  target    ANSMALLDAKVRGVGPDEARGARGFDNYSWHTDLPPGHPMVTGQQTVDFDLNSVELAKNVVVWGMNWITTKMPDAHWLTE 6rxm.5    ---------------------------------------------------MALSMADIFIAIGTSGHV--YPAAGFVHE  target    ARLKGTRIIVIACEYSSTSSKADDAIVVRPGTTPALALGLSHVIMRDKLYDADYVRRWTDLPMLVRTDTLKYLSAEDVFG 6rxm.5    AKLHGAHTVELNLEPSQVGNEFAEKYY-----------------------------------------------------  target    GGPAPL 6rxm.5    ------ ``` | | | | | | | | | | | | | | | | | | | | | | | | | | | | | | | | | | | | | | | | | | | | | | | | | |
|  | 6rxm.6.A | NAD-dependent protein deacylase  *Crystal structure of CobB Ac2 (A76G, I131C, V162G) in complex with H4K16-Acetyl peptide* | 0.03 |  | 18.52 | 0.13 | 292-347 | X-ray | 1.92 | hetero-1-1-mer | 1 x ZN | HHblits | 0.30 |
| ``` target    TARREITRRGFLGTAAGAGFAAFVVSATRAWGLEAIENPLARYPDREWERVYRDLWRYDSKFTFLCAPNDTHNCLLDAYV 6rxm.6    --------------------------------------------------------------------------------  target    RSGVMTRIGPTMRYGEARDLDGNRASARWDPRVCQKGLALTRRFYGDRRLRHCMVRAGFKRWVDEGFPRGEDGKPPKEYF 6rxm.6    --------------------------------------------------------------------------------  target    QRARDEWVRASHDEAAAVVAATLANIAATYSGEEGAQRLRDQGYEEETIEAMGGAGVQAMKFRGGMPLLGMTRVFGLYRM 6rxm.6    --------------------------------------------------------------------------------  target    ANSMALLDAKVRGVGPDEARGARGFDNYSWHTDLPPGHPMVTGQQTVDFDLNSVELAKNVVVWGMNWITTKMPDAHWLTE 6rxm.6    ---------------------------------------------------MALSMADIFIAIGTSGHV--YPAAGFVHE  target    ARLKGTRIIVIACEYSSTSSKADDAIVVRPGTTPALALGLSHVIMRDKLYDADYVRRWTDLPMLVRTDTLKYLSAEDVFG 6rxm.6    AKLHGAHTVELNLEPSQVGNEFAEKYY-----------------------------------------------------  target    GGPAPL 6rxm.6    ------ ``` | | | | | | | | | | | | | | | | | | | | | | | | | | | | | | | | | | | | | | | | | | | | | | | | | |
|  | 6rxm.2.A | NAD-dependent protein deacylase  *Crystal structure of CobB Ac2 (A76G, I131C, V162G) in complex with H4K16-Acetyl peptide* | 0.03 |  | 18.52 | 0.13 | 292-347 | X-ray | 1.92 | hetero-1-1-mer | 1 x ZN | HHblits | 0.30 |
| ``` target    TARREITRRGFLGTAAGAGFAAFVVSATRAWGLEAIENPLARYPDREWERVYRDLWRYDSKFTFLCAPNDTHNCLLDAYV 6rxm.2    --------------------------------------------------------------------------------  target    RSGVMTRIGPTMRYGEARDLDGNRASARWDPRVCQKGLALTRRFYGDRRLRHCMVRAGFKRWVDEGFPRGEDGKPPKEYF 6rxm.2    --------------------------------------------------------------------------------  target    QRARDEWVRASHDEAAAVVAATLANIAATYSGEEGAQRLRDQGYEEETIEAMGGAGVQAMKFRGGMPLLGMTRVFGLYRM 6rxm.2    --------------------------------------------------------------------------------  target    ANSMALLDAKVRGVGPDEARGARGFDNYSWHTDLPPGHPMVTGQQTVDFDLNSVELAKNVVVWGMNWITTKMPDAHWLTE 6rxm.2    ---------------------------------------------------MALSMADIFIAIGTSGHV--YPAAGFVHE  target    ARLKGTRIIVIACEYSSTSSKADDAIVVRPGTTPALALGLSHVIMRDKLYDADYVRRWTDLPMLVRTDTLKYLSAEDVFG 6rxm.2    AKLHGAHTVELNLEPSQVGNEFAEKYY-----------------------------------------------------  target    GGPAPL 6rxm.2    ------ ``` | | | | | | | | | | | | | | | | | | | | | | | | | | | | | | | | | | | | | | | | | | | | | | | | | |
|  | 6rxo.2.A | NAD-dependent protein deacylase  *Crystal structure of CobB Ac2 (A76G, I131C, V162A) in complex with H4K16-Buturyl peptide* | 0.03 |  | 18.52 | 0.13 | 292-347 | X-ray | 1.95 | hetero-1-1-mer | 1 x ZN | HHblits | 0.30 |
| ``` target    TARREITRRGFLGTAAGAGFAAFVVSATRAWGLEAIENPLARYPDREWERVYRDLWRYDSKFTFLCAPNDTHNCLLDAYV 6rxo.2    --------------------------------------------------------------------------------  target    RSGVMTRIGPTMRYGEARDLDGNRASARWDPRVCQKGLALTRRFYGDRRLRHCMVRAGFKRWVDEGFPRGEDGKPPKEYF 6rxo.2    --------------------------------------------------------------------------------  target    QRARDEWVRASHDEAAAVVAATLANIAATYSGEEGAQRLRDQGYEEETIEAMGGAGVQAMKFRGGMPLLGMTRVFGLYRM 6rxo.2    --------------------------------------------------------------------------------  target    ANSMALLDAKVRGVGPDEARGARGFDNYSWHTDLPPGHPMVTGQQTVDFDLNSVELAKNVVVWGMNWITTKMPDAHWLTE 6rxo.2    ---------------------------------------------------MALSMADIFIAIGTSGHV--YPAAGFVHE  target    ARLKGTRIIVIACEYSSTSSKADDAIVVRPGTTPALALGLSHVIMRDKLYDADYVRRWTDLPMLVRTDTLKYLSAEDVFG 6rxo.2    AKLHGAHTVELNLEPSQVGNEFAEKYY-----------------------------------------------------  target    GGPAPL 6rxo.2    ------ ``` | | | | | | | | | | | | | | | | | | | | | | | | | | | | | | | | | | | | | | | | | | | | | | | | | |
|  | 6rxp.2.A | NAD-dependent protein deacylase  *Crystal structure of CobB Ac2 (A76G,I131C,V162A) in complex with H4K16-Crotonyl peptide* | 0.03 |  | 18.52 | 0.13 | 292-347 | X-ray | 1.80 | hetero-1-1-mer | 1 x ZN | HHblits | 0.30 |
| ``` target    TARREITRRGFLGTAAGAGFAAFVVSATRAWGLEAIENPLARYPDREWERVYRDLWRYDSKFTFLCAPNDTHNCLLDAYV 6rxp.2    --------------------------------------------------------------------------------  target    RSGVMTRIGPTMRYGEARDLDGNRASARWDPRVCQKGLALTRRFYGDRRLRHCMVRAGFKRWVDEGFPRGEDGKPPKEYF 6rxp.2    --------------------------------------------------------------------------------  target    QRARDEWVRASHDEAAAVVAATLANIAATYSGEEGAQRLRDQGYEEETIEAMGGAGVQAMKFRGGMPLLGMTRVFGLYRM 6rxp.2    --------------------------------------------------------------------------------  target    ANSMALLDAKVRGVGPDEARGARGFDNYSWHTDLPPGHPMVTGQQTVDFDLNSVELAKNVVVWGMNWITTKMPDAHWLTE 6rxp.2    ---------------------------------------------------MALSMADIFIAIGTSGHV--YPAAGFVHE  target    ARLKGTRIIVIACEYSSTSSKADDAIVVRPGTTPALALGLSHVIMRDKLYDADYVRRWTDLPMLVRTDTLKYLSAEDVFG 6rxp.2    AKLHGAHTVELNLEPSQVGNEFAEKYY-----------------------------------------------------  target    GGPAPL 6rxp.2    ------ ``` | | | | | | | | | | | | | | | | | | | | | | | | | | | | | | | | | | | | | | | | | | | | | | | | | |
|  | 6rxq.4.A | NAD-dependent protein deacylase  *Crystal structure of CobB Ac2 (A76G,I131C,V162A) in complex with H4K16Cr-2'OH-ADPr peptide intermediate after soaking* | 0.03 |  | 18.52 | 0.13 | 292-347 | X-ray | 1.70 | hetero-1-1-mer | 1 x KMQ | HHblits | 0.30 |
| ``` target    TARREITRRGFLGTAAGAGFAAFVVSATRAWGLEAIENPLARYPDREWERVYRDLWRYDSKFTFLCAPNDTHNCLLDAYV 6rxq.4    --------------------------------------------------------------------------------  target    RSGVMTRIGPTMRYGEARDLDGNRASARWDPRVCQKGLALTRRFYGDRRLRHCMVRAGFKRWVDEGFPRGEDGKPPKEYF 6rxq.4    --------------------------------------------------------------------------------  target    QRARDEWVRASHDEAAAVVAATLANIAATYSGEEGAQRLRDQGYEEETIEAMGGAGVQAMKFRGGMPLLGMTRVFGLYRM 6rxq.4    --------------------------------------------------------------------------------  target    ANSMALLDAKVRGVGPDEARGARGFDNYSWHTDLPPGHPMVTGQQTVDFDLNSVELAKNVVVWGMNWITTKMPDAHWLTE 6rxq.4    ---------------------------------------------------MALSMADIFIAIGTSGHV--YPAAGFVHE  target    ARLKGTRIIVIACEYSSTSSKADDAIVVRPGTTPALALGLSHVIMRDKLYDADYVRRWTDLPMLVRTDTLKYLSAEDVFG 6rxq.4    AKLHGAHTVELNLEPSQVGNEFAEKYY-----------------------------------------------------  target    GGPAPL 6rxq.4    ------ ``` | | | | | | | | | | | | | | | | | | | | | | | | | | | | | | | | | | | | | | | | | | | | | | | | | |
|  | 7t2r.1.A | NiFe hydrogenase subunit A  *Structure of electron bifurcating Ni-Fe hydrogenase complex HydABCSL in FMN-free apo state* | 0.02 |  | 10.34 | 0.14 | 296-357 | EM | 0.00 | hetero-2-2-2-2-2-mer | 6 x FES, 12 x SF4, 2 x 3NI, 2 x FCO | HHblits | 0.25 |
| ``` target    TARREITRRGFLGTAAGAGFAAFVVSATRAWGLEAIENPLARYPDREWERVYRDLWRYDSKFTFLCAPNDTHNCLLDAYV 7t2r.1    --------------------------------------------------------------------------------  target    RSGVMTRIGPTMRYGEARDLDGNRASARWDPRVCQKGLALTRRFYGDRRLRHCMVRAGFKRWVDEGFPRGEDGKPPKEYF 7t2r.1    --------------------------------------------------------------------------------  target    QRARDEWVRASHDEAAAVVAATLANIAATYSGEEGAQRLRDQGYEEETIEAMGGAGVQAMKFRGGMPLLGMTRVFGLYRM 7t2r.1    --------------------------------------------------------------------------------  target    ANSMALLDAKVRGVGPDEARGARGFDNYSWHTDLPPGHPMVTGQQTVDFDLNSVELAKNVVVWGMNWITTKMPDAHWLTE 7t2r.1    -------------------------------------------------------RRDFLYVFSTAMV----PEEEEILA  target    ARLKGTRIIVIACEYS-STSSKADDAIVVRPG--TTPALALGLSHVIMRDKLYDADYVRRWTDLPMLVRTDTLKYLSAED 7t2r.1    AISATRFVVVQTPFKVRPLVNLADILLPAPAWYERSGHFC----------------------------------------  target    VFGGGPAPL 7t2r.1    --------- ``` | | | | | | | | | | | | | | | | | | | | | | | | | | | | | | | | | | | | | | | | | | | | | | | | | |
|  | 7t30.1.A | NiFe hydrogenase subunit A  *Structure of electron bifurcating Ni-Fe hydrogenase complex HydABCSL in FMN/NAD(H) bound state* | 0.02 |  | 10.34 | 0.14 | 296-357 | EM | 0.00 | hetero-2-2-2-2-2-mer | 4 x FES, 12 x SF4, 2 x NAD, 2 x FMN, 2 x 3NI, 2 x FCO | HHblits | 0.25 |
| ``` target    TARREITRRGFLGTAAGAGFAAFVVSATRAWGLEAIENPLARYPDREWERVYRDLWRYDSKFTFLCAPNDTHNCLLDAYV 7t30.1    --------------------------------------------------------------------------------  target    RSGVMTRIGPTMRYGEARDLDGNRASARWDPRVCQKGLALTRRFYGDRRLRHCMVRAGFKRWVDEGFPRGEDGKPPKEYF 7t30.1    --------------------------------------------------------------------------------  target    QRARDEWVRASHDEAAAVVAATLANIAATYSGEEGAQRLRDQGYEEETIEAMGGAGVQAMKFRGGMPLLGMTRVFGLYRM 7t30.1    --------------------------------------------------------------------------------  target    ANSMALLDAKVRGVGPDEARGARGFDNYSWHTDLPPGHPMVTGQQTVDFDLNSVELAKNVVVWGMNWITTKMPDAHWLTE 7t30.1    -------------------------------------------------------RRDFLYVFSTAMV----PEEEEILA  target    ARLKGTRIIVIACEYS-STSSKADDAIVVRPG--TTPALALGLSHVIMRDKLYDADYVRRWTDLPMLVRTDTLKYLSAED 7t30.1    AISATRFVVVQTPFKVRPLVNLADILLPAPAWYERSGHFC----------------------------------------  target    VFGGGPAPL 7t30.1    --------- ``` | | | | | | | | | | | | | | | | | | | | | | | | | | | | | | | | | | | | | | | | | | | | | | | | | |
|  | 4lzj.1.A | N-acetylmuramic acid 6-phosphate etherase  *Crystal Structure of MurQ from H.influenzae with bound inhibitor* | 0.03 |  | 25.00 | 0.14 | 296-353 | X-ray | 2.41 | homo-dimer |  | HHblits | 0.27 |
| ``` target    TARREITRRGFLGTAAGAGFAAFVVSATRAWGLEAIENPLARYPDREWERVYRDLWRYDSKFTFLCAPNDTHNCLLDAYV 4lzj.1    --------------------------------------------------------------------------------  target    RSGVMTRIGPTMRYGEARDLDGNRASARWDPRVCQKGLALTRRFYGDRRLRHCMVRAGFKRWVDEGFPRGEDGKPPKEYF 4lzj.1    --------------------------------------------------------------------------------  target    QRARDEWVRASHDEAAAVVAATLANIAATYSGEEGAQRLRDQGYEEETIEAMGGAGVQAMKFRGGMPLLGMTRVFGLYRM 4lzj.1    --------------------------------------------------------------------------------  target    ANSMALLDAKVRGVGPDEARGARGFDNYSWHTDLPPGHPMVTGQQTVDFDLNSVELAKNVVVWGMNWITTKMPDAHWLTE 4lzj.1    -------------------------------------------------------KNDVLVGIAASG-RT-PYVIAGLQY  target    ARLKGTRIIVIACE-YSSTSSKADDAIVVRPGTTPALALGLSHVIMRDKLYDADYVRRWTDLPMLVRTDTLKYLSAEDVF 4lzj.1    AKSLGALTISIASNPKSEMAEIADIAIETIVGPE----------------------------------------------  target    GGGPAPL 4lzj.1    ------- ``` | | | | | | | | | | | | | | | | | | | | | | | | | | | | | | | | | | | | | | | | | | | | | | | | | |
|  | 4lzj.1.B | N-acetylmuramic acid 6-phosphate etherase  *Crystal Structure of MurQ from H.influenzae with bound inhibitor* | 0.03 |  | 25.00 | 0.14 | 296-353 | X-ray | 2.41 | homo-dimer |  | HHblits | 0.27 |
| ``` target    TARREITRRGFLGTAAGAGFAAFVVSATRAWGLEAIENPLARYPDREWERVYRDLWRYDSKFTFLCAPNDTHNCLLDAYV 4lzj.1    --------------------------------------------------------------------------------  target    RSGVMTRIGPTMRYGEARDLDGNRASARWDPRVCQKGLALTRRFYGDRRLRHCMVRAGFKRWVDEGFPRGEDGKPPKEYF 4lzj.1    --------------------------------------------------------------------------------  target    QRARDEWVRASHDEAAAVVAATLANIAATYSGEEGAQRLRDQGYEEETIEAMGGAGVQAMKFRGGMPLLGMTRVFGLYRM 4lzj.1    --------------------------------------------------------------------------------  target    ANSMALLDAKVRGVGPDEARGARGFDNYSWHTDLPPGHPMVTGQQTVDFDLNSVELAKNVVVWGMNWITTKMPDAHWLTE 4lzj.1    -------------------------------------------------------KNDVLVGIAASG-RT-PYVIAGLQY  target    ARLKGTRIIVIACE-YSSTSSKADDAIVVRPGTTPALALGLSHVIMRDKLYDADYVRRWTDLPMLVRTDTLKYLSAEDVF 4lzj.1    AKSLGALTISIASNPKSEMAEIADIAIETIVGPE----------------------------------------------  target    GGGPAPL 4lzj.1    ------- ``` | | | | | | | | | | | | | | | | | | | | | | | | | | | | | | | | | | | | | | | | | | | | | | | | | |
|  | 4lzj.2.B | N-acetylmuramic acid 6-phosphate etherase  *Crystal Structure of MurQ from H.influenzae with bound inhibitor* | 0.03 |  | 25.00 | 0.14 | 296-353 | X-ray | 2.41 | homo-dimer |  | HHblits | 0.27 |
| ``` target    TARREITRRGFLGTAAGAGFAAFVVSATRAWGLEAIENPLARYPDREWERVYRDLWRYDSKFTFLCAPNDTHNCLLDAYV 4lzj.2    --------------------------------------------------------------------------------  target    RSGVMTRIGPTMRYGEARDLDGNRASARWDPRVCQKGLALTRRFYGDRRLRHCMVRAGFKRWVDEGFPRGEDGKPPKEYF 4lzj.2    --------------------------------------------------------------------------------  target    QRARDEWVRASHDEAAAVVAATLANIAATYSGEEGAQRLRDQGYEEETIEAMGGAGVQAMKFRGGMPLLGMTRVFGLYRM 4lzj.2    --------------------------------------------------------------------------------  target    ANSMALLDAKVRGVGPDEARGARGFDNYSWHTDLPPGHPMVTGQQTVDFDLNSVELAKNVVVWGMNWITTKMPDAHWLTE 4lzj.2    -------------------------------------------------------KNDVLVGIAASG-RT-PYVIAGLQY  target    ARLKGTRIIVIACE-YSSTSSKADDAIVVRPGTTPALALGLSHVIMRDKLYDADYVRRWTDLPMLVRTDTLKYLSAEDVF 4lzj.2    AKSLGALTISIASNPKSEMAEIADIAIETIVGPE----------------------------------------------  target    GGGPAPL 4lzj.2    ------- ``` | | | | | | | | | | | | | | | | | | | | | | | | | | | | | | | | | | | | | | | | | | | | | | | | | |
|  | 4m0d.1.A | N-acetylmuramic acid 6-phosphate etherase  *Crystal structure of MurQ from H.influenzae in apo form* | 0.03 |  | 25.00 | 0.14 | 296-353 | X-ray | 2.58 | homo-dimer |  | HHblits | 0.27 |
| ``` target    TARREITRRGFLGTAAGAGFAAFVVSATRAWGLEAIENPLARYPDREWERVYRDLWRYDSKFTFLCAPNDTHNCLLDAYV 4m0d.1    --------------------------------------------------------------------------------  target    RSGVMTRIGPTMRYGEARDLDGNRASARWDPRVCQKGLALTRRFYGDRRLRHCMVRAGFKRWVDEGFPRGEDGKPPKEYF 4m0d.1    --------------------------------------------------------------------------------  target    QRARDEWVRASHDEAAAVVAATLANIAATYSGEEGAQRLRDQGYEEETIEAMGGAGVQAMKFRGGMPLLGMTRVFGLYRM 4m0d.1    --------------------------------------------------------------------------------  target    ANSMALLDAKVRGVGPDEARGARGFDNYSWHTDLPPGHPMVTGQQTVDFDLNSVELAKNVVVWGMNWITTKMPDAHWLTE 4m0d.1    -------------------------------------------------------KNDVLVGIAASG-RT-PYVIAGLQY  target    ARLKGTRIIVIACE-YSSTSSKADDAIVVRPGTTPALALGLSHVIMRDKLYDADYVRRWTDLPMLVRTDTLKYLSAEDVF 4m0d.1    AKSLGALTISIASNPKSEMAEIADIAIETIVGPE----------------------------------------------  target    GGGPAPL 4m0d.1    ------- ``` | | | | | | | | | | | | | | | | | | | | | | | | | | | | | | | | | | | | | | | | | | | | | | | | | |
|  | 4m0d.2.A | N-acetylmuramic acid 6-phosphate etherase  *Crystal structure of MurQ from H.influenzae in apo form* | 0.03 |  | 25.00 | 0.14 | 296-353 | X-ray | 2.58 | homo-dimer |  | HHblits | 0.27 |
| ``` target    TARREITRRGFLGTAAGAGFAAFVVSATRAWGLEAIENPLARYPDREWERVYRDLWRYDSKFTFLCAPNDTHNCLLDAYV 4m0d.2    --------------------------------------------------------------------------------  target    RSGVMTRIGPTMRYGEARDLDGNRASARWDPRVCQKGLALTRRFYGDRRLRHCMVRAGFKRWVDEGFPRGEDGKPPKEYF 4m0d.2    --------------------------------------------------------------------------------  target    QRARDEWVRASHDEAAAVVAATLANIAATYSGEEGAQRLRDQGYEEETIEAMGGAGVQAMKFRGGMPLLGMTRVFGLYRM 4m0d.2    --------------------------------------------------------------------------------  target    ANSMALLDAKVRGVGPDEARGARGFDNYSWHTDLPPGHPMVTGQQTVDFDLNSVELAKNVVVWGMNWITTKMPDAHWLTE 4m0d.2    -------------------------------------------------------KNDVLVGIAASG-RT-PYVIAGLQY  target    ARLKGTRIIVIACE-YSSTSSKADDAIVVRPGTTPALALGLSHVIMRDKLYDADYVRRWTDLPMLVRTDTLKYLSAEDVF 4m0d.2    AKSLGALTISIASNPKSEMAEIADIAIETIVGPE----------------------------------------------  target    GGGPAPL 4m0d.2    ------- ``` | | | | | | | | | | | | | | | | | | | | | | | | | | | | | | | | | | | | | | | | | | | | | | | | | |
|  | 4m0d.2.B | N-acetylmuramic acid 6-phosphate etherase  *Crystal structure of MurQ from H.influenzae in apo form* | 0.03 |  | 25.00 | 0.14 | 296-353 | X-ray | 2.58 | homo-dimer |  | HHblits | 0.27 |
| ``` target    TARREITRRGFLGTAAGAGFAAFVVSATRAWGLEAIENPLARYPDREWERVYRDLWRYDSKFTFLCAPNDTHNCLLDAYV 4m0d.2    --------------------------------------------------------------------------------  target    RSGVMTRIGPTMRYGEARDLDGNRASARWDPRVCQKGLALTRRFYGDRRLRHCMVRAGFKRWVDEGFPRGEDGKPPKEYF 4m0d.2    --------------------------------------------------------------------------------  target    QRARDEWVRASHDEAAAVVAATLANIAATYSGEEGAQRLRDQGYEEETIEAMGGAGVQAMKFRGGMPLLGMTRVFGLYRM 4m0d.2    --------------------------------------------------------------------------------  target    ANSMALLDAKVRGVGPDEARGARGFDNYSWHTDLPPGHPMVTGQQTVDFDLNSVELAKNVVVWGMNWITTKMPDAHWLTE 4m0d.2    -------------------------------------------------------KNDVLVGIAASG-RT-PYVIAGLQY  target    ARLKGTRIIVIACE-YSSTSSKADDAIVVRPGTTPALALGLSHVIMRDKLYDADYVRRWTDLPMLVRTDTLKYLSAEDVF 4m0d.2    AKSLGALTISIASNPKSEMAEIADIAIETIVGPE----------------------------------------------  target    GGGPAPL 4m0d.2    ------- ``` | | | | | | | | | | | | | | | | | | | | | | | | | | | | | | | | | | | | | | | | | | | | | | | | | |
|  | 1q16.1.A | Respiratory nitrate reductase 1 alpha chain  *Crystal structure of Nitrate Reductase A, NarGHI, from Escherichia coli* | 0.02 |  | 16.67 | 0.13 | 297-350 | X-ray | 1.90 | hetero-oligomer | 2 x MD1, 1 x 6MO, 2 x HEM, 4 x SF4, 1 x F3S, 1 x AGA, 1 x 3PH | HHblits | 0.30 |
| ``` target    TARREITRRGFLGTAAGAGFAAFVVSATRAWGLEAIENPLARYPDREWERVYRDLWRYDSKFTFLCAPNDTHNCLLDAYV 1q16.1    --------------------------------------------------------------------------------  target    RSGVMTRIGPTMRYGEARDLDGNRASARWDPRVCQKGLALTRRFYGDRRLRHCMVRAGFKRWVDEGFPRGEDGKPPKEYF 1q16.1    --------------------------------------------------------------------------------  target    QRARDEWVRASHDEAAAVVAATLANIAATYSGEEGAQRLRDQGYEEETIEAMGGAGVQAMKFRGGMPLLGMTRVFGLYRM 1q16.1    --------------------------------------------------------------------------------  target    ANSMALLDAKVRGVGPDEARGARGFDNYSWHTDLPPGHPMVTGQQTVDFDLNSVELAKNVVVWGMNWITTKMPDAH---- 1q16.1    --------------------------------------------------------PRNLFIWRSNLLGSSGKGHEFMLK  target    --------------------------WLT-EARLKGTRIIVIACEYSSTSSKADDAIVVRPGTTPALALGLSHVIMRDKL 1q16.1    YLLGTEHGIQGKDLGQQGGVKPEEVDWQDNGLEGKLDLVVTLDFRLSSTCLYSDIILPTAT-------------------  target    YDADYVRRWTDLPMLVRTDTLKYLSAEDVFGGGPAPL 1q16.1    ------------------------------------- ``` | | | | | | | | | | | | | | | | | | | | | | | | | | | | | | | | | | | | | | | | | | | | | | | | | |
|  | 3egw.1.A | Respiratory nitrate reductase 1 alpha chain  *The crystal structure of the NarGHI mutant NarH - C16A* | 0.02 |  | 16.67 | 0.13 | 297-350 | X-ray | 1.90 | hetero-2-2-2-mer | 2 x MD1, 2 x MGD, 2 x 6MO, 6 x SF4, 4 x F3S, 2 x 3PH, 4 x HEM, 2 x AGA | HHblits | 0.30 |
| ``` target    TARREITRRGFLGTAAGAGFAAFVVSATRAWGLEAIENPLARYPDREWERVYRDLWRYDSKFTFLCAPNDTHNCLLDAYV 3egw.1    --------------------------------------------------------------------------------  target    RSGVMTRIGPTMRYGEARDLDGNRASARWDPRVCQKGLALTRRFYGDRRLRHCMVRAGFKRWVDEGFPRGEDGKPPKEYF 3egw.1    --------------------------------------------------------------------------------  target    QRARDEWVRASHDEAAAVVAATLANIAATYSGEEGAQRLRDQGYEEETIEAMGGAGVQAMKFRGGMPLLGMTRVFGLYRM 3egw.1    --------------------------------------------------------------------------------  target    ANSMALLDAKVRGVGPDEARGARGFDNYSWHTDLPPGHPMVTGQQTVDFDLNSVELAKNVVVWGMNWITTKMPDAH---- 3egw.1    --------------------------------------------------------PRNLFIWRSNLLGSSGKGHEFMLK  target    --------------------------WLT-EARLKGTRIIVIACEYSSTSSKADDAIVVRPGTTPALALGLSHVIMRDKL 3egw.1    YLLGTEHGIQGKDLGQQGGVKPEEVDWQDNGLEGKLDLVVTLDFRLSSTCLYSDIILPTAT-------------------  target    YDADYVRRWTDLPMLVRTDTLKYLSAEDVFGGGPAPL 3egw.1    ------------------------------------- ``` | | | | | | | | | | | | | | | | | | | | | | | | | | | | | | | | | | | | | | | | | | | | | | | | | |
|  | 5ltz.1.A | Phosphoheptose isomerase  *GmhA\_mutant Q175E* | 0.03 |  | 14.29 | 0.14 | 297-354 | X-ray | 1.67 | homo-tetramer | 4 x ZN, 4 x I22 | HHblits | 0.27 |
| ``` target    TARREITRRGFLGTAAGAGFAAFVVSATRAWGLEAIENPLARYPDREWERVYRDLWRYDSKFTFLCAPNDTHNCLLDAYV 5ltz.1    --------------------------------------------------------------------------------  target    RSGVMTRIGPTMRYGEARDLDGNRASARWDPRVCQKGLALTRRFYGDRRLRHCMVRAGFKRWVDEGFPRGEDGKPPKEYF 5ltz.1    --------------------------------------------------------------------------------  target    QRARDEWVRASHDEAAAVVAATLANIAATYSGEEGAQRLRDQGYEEETIEAMGGAGVQAMKFRGGMPLLGMTRVFGLYRM 5ltz.1    --------------------------------------------------------------------------------  target    ANSMALLDAKVRGVGPDEARGARGFDNYSWHTDLPPGHPMVTGQQTVDFDLNSVELAKNVVVWGMNWITTKMPDAHWLTE 5ltz.1    --------------------------------------------------------GDVLIGYSTSG-KS-PNILAAFRE  target    ARLKGTRIIVIAC-EYSSTSSKADDAIVVRPGTTPALALGLSHVIMRDKLYDADYVRRWTDLPMLVRTDTLKYLSAEDVF 5ltz.1    AKAKGMTCVGFTGNRGGEMRELCDLLLEVPSADTP---------------------------------------------  target    GGGPAPL 5ltz.1    ------- ``` | | | | | | | | | | | | | | | | | | | | | | | | | | | | | | | | | | | | | | | | | | | | | | | | | |
|  | 1viv.1.A | Hypothetical protein yckF  *Crystal structure of a hypothetical protein* | 0.03 |  | 16.07 | 0.14 | 296-353 | X-ray | 2.60 | homo-dimer |  | HHblits | 0.27 |
| ``` target    TARREITRRGFLGTAAGAGFAAFVVSATRAWGLEAIENPLARYPDREWERVYRDLWRYDSKFTFLCAPNDTHNCLLDAYV 1viv.1    --------------------------------------------------------------------------------  target    RSGVMTRIGPTMRYGEARDLDGNRASARWDPRVCQKGLALTRRFYGDRRLRHCMVRAGFKRWVDEGFPRGEDGKPPKEYF 1viv.1    --------------------------------------------------------------------------------  target    QRARDEWVRASHDEAAAVVAATLANIAATYSGEEGAQRLRDQGYEEETIEAMGGAGVQAMKFRGGMPLLGMTRVFGLYRM 1viv.1    --------------------------------------------------------------------------------  target    ANSMALLDAKVRGVGPDEARGARGFDNYSWHTDLPPGHPMVTGQQTVDFDLNSVELAKNVVVWGMNWITTKMPDAHWLTE 1viv.1    -------------------------------------------------------EGDLVIIGSGSGE-T-KSLIHTAAK  target    ARLKGTRIIVIACE-YSSTSSKADDAIVVRPGTTPALALGLSHVIMRDKLYDADYVRRWTDLPMLVRTDTLKYLSAEDVF 1viv.1    AKSLHGIVAALTINPESSIGKQADLIIRMPGSPK----------------------------------------------  target    GGGPAPL 1viv.1    ------- ``` | | | | | | | | | | | | | | | | | | | | | | | | | | | | | | | | | | | | | | | | | | | | | | | | | |
|  | 1m2h.1.A | Silent Information Regulator 2  *Sir2 homologue S24A mutant-ADP ribose complex* | 0.03 |  | 24.53 | 0.13 | 292-346 | X-ray | 1.80 | monomer | 1 x ZN, 1 x APR | HHblits | 0.31 |
| ``` target    TARREITRRGFLGTAAGAGFAAFVVSATRAWGLEAIENPLARYPDREWERVYRDLWRYDSKFTFLCAPNDTHNCLLDAYV 1m2h.1    --------------------------------------------------------------------------------  target    RSGVMTRIGPTMRYGEARDLDGNRASARWDPRVCQKGLALTRRFYGDRRLRHCMVRAGFKRWVDEGFPRGEDGKPPKEYF 1m2h.1    --------------------------------------------------------------------------------  target    QRARDEWVRASHDEAAAVVAATLANIAATYSGEEGAQRLRDQGYEEETIEAMGGAGVQAMKFRGGMPLLGMTRVFGLYRM 1m2h.1    --------------------------------------------------------------------------------  target    ANSMALLDAKVRGVGPDEARGARGFDNYSWHTDLPPGHPMVTGQQTVDFDLNSVELAKNVVVWGMNWITTKMPDAHWLTE 1m2h.1    ---------------------------------------------------REVERADVIIVAGTSAVV--QPAASLPLI  target    ARLKGTRIIVIACEYSSTSSKADDAIVVRPGTTPALALGLSHVIMRDKLYDADYVRRWTDLPMLVRTDTLKYLSAEDVFG 1m2h.1    VKQRGGAIIEINPDETPLTPIADYSL------------------------------------------------------  target    GGPAPL 1m2h.1    ------ ``` | | | | | | | | | | | | | | | | | | | | | | | | | | | | | | | | | | | | | | | | | | | | | | | | | |
|  | 1m2k.1.A | Silent Information Regulator 2  *Sir2 homologue F159A mutant-ADP ribose complex* | 0.03 |  | 24.53 | 0.13 | 292-346 | X-ray | 1.47 | monomer | 1 x ZN, 1 x APR | HHblits | 0.31 |
| ``` target    TARREITRRGFLGTAAGAGFAAFVVSATRAWGLEAIENPLARYPDREWERVYRDLWRYDSKFTFLCAPNDTHNCLLDAYV 1m2k.1    --------------------------------------------------------------------------------  target    RSGVMTRIGPTMRYGEARDLDGNRASARWDPRVCQKGLALTRRFYGDRRLRHCMVRAGFKRWVDEGFPRGEDGKPPKEYF 1m2k.1    --------------------------------------------------------------------------------  target    QRARDEWVRASHDEAAAVVAATLANIAATYSGEEGAQRLRDQGYEEETIEAMGGAGVQAMKFRGGMPLLGMTRVFGLYRM 1m2k.1    --------------------------------------------------------------------------------  target    ANSMALLDAKVRGVGPDEARGARGFDNYSWHTDLPPGHPMVTGQQTVDFDLNSVELAKNVVVWGMNWITTKMPDAHWLTE 1m2k.1    ---------------------------------------------------REVERADVIIVAGTSAVV--QPAASLPLI  target    ARLKGTRIIVIACEYSSTSSKADDAIVVRPGTTPALALGLSHVIMRDKLYDADYVRRWTDLPMLVRTDTLKYLSAEDVFG 1m2k.1    VKQRGGAIIEINPDETPLTPIADYSL------------------------------------------------------  target    GGPAPL 1m2k.1    ------ ``` | | | | | | | | | | | | | | | | | | | | | | | | | | | | | | | | | | | | | | | | | | | | | | | | | |
|  | 1dms.1.A | DMSO REDUCTASE  *STRUCTURE OF DMSO REDUCTASE* | 0.04 |  | 20.00 | 0.14 | 296-351 | X-ray | 1.88 | monomer | 2 x PGD, 1 x 2MO | HHblits | 0.28 |
| ``` target    TARREITRRGFLGTAAGAGFAAFVVSATRAWGLEAIENPLARYPDREWERVYRDLWRYDSKFTFLCAPNDTHNCLLDAYV 1dms.1    --------------------------------------------------------------------------------  target    RSGVMTRIGPTMRYGEARDLDGNRASARWDPRVCQKGLALTRRFYGDRRLRHCMVRAGFKRWVDEGFPRGEDGKPPKEYF 1dms.1    --------------------------------------------------------------------------------  target    QRARDEWVRASHDEAAAVVAATLANIAATYSGEEGAQRLRDQGYEEETIEAMGGAGVQAMKFRGGMPLLGMTRVFGLYRM 1dms.1    --------------------------------------------------------------------------------  target    ANSMALLDAKVRGVGPDEARGARGFDNYSWHTDLPPGHPMVTGQQTVDFDLNSVELAKNVVVWGMNWITTKMPDAHWLTE 1dms.1    -------------------------------------------------------DVKMAYWVGGNPFVHHQ-DRNRMVK  target    ARLKGTRIIVIACEYSSTSSKADDAIVVRPGTTPALALGLSHVIMRDKLYDADYVRRWTDLPMLVRTDTLKYLSAEDVFG 1dms.1    AWEKLETFIVHDFQWTPTARHADIVLPATTS-------------------------------------------------  target    GGPAPL 1dms.1    ------ ``` | | | | | | | | | | | | | | | | | | | | | | | | | | | | | | | | | | | | | | | | | | | | | | | | | |
|  | 4ydd.1.A | DMSO reductase family type II enzyme, molybdopterin subunit  *Crystal structure of the perchlorate reductase PcrAB from Azospira suillum PS* | 0.03 |  | 20.00 | 0.14 | 296-350 | X-ray | 1.86 | hetero-oligomer | 4 x SF4, 1 x MO, 1 x MGD, 1 x MD1, 1 x F3S | HHblits | 0.28 |
| ``` target    TARREITRRGFLGTAAGAGFAAFVVSATRAWGLEAIENPLARYPDREWERVYRDLWRYDSKFTFLCAPNDTHNCLLDAYV 4ydd.1    --------------------------------------------------------------------------------  target    RSGVMTRIGPTMRYGEARDLDGNRASARWDPRVCQKGLALTRRFYGDRRLRHCMVRAGFKRWVDEGFPRGEDGKPPKEYF 4ydd.1    --------------------------------------------------------------------------------  target    QRARDEWVRASHDEAAAVVAATLANIAATYSGEEGAQRLRDQGYEEETIEAMGGAGVQAMKFRGGMPLLGMTRVFGLYRM 4ydd.1    --------------------------------------------------------------------------------  target    ANSMALLDAKVRGVGPDEARGARGFDNYSWHTDLPPGHPMVTGQQTVDFDLNSVELAKNVVVWGMNWITTKMPDAHWLTE 4ydd.1    -------------------------------------------------------DPKVFFVYRGNWLNQAKGQKYVLEN  target    ARLKGTRIIVIACEYSSTSSKADDAIVVRPGTTPALALGLSHVIMRDKLYDADYVRRWTDLPMLVRTDTLKYLSAEDVFG 4ydd.1    LWPKLELIVDINIRMDSTALYSDVVLPSAH--------------------------------------------------  target    GGPAPL 4ydd.1    ------ ``` | | | | | | | | | | | | | | | | | | | | | | | | | | | | | | | | | | | | | | | | | | | | | | | | | |
|  | 7l5i.1.A | Trimethylamine-N-oxide reductase  *Crystal Structure of Haemophilus influenzae MtsZ at pH 7.0* | 0.04 |  | 24.07 | 0.13 | 296-350 | X-ray | 1.73 | monomer | 2 x MGD, 1 x MO, 1 x O | HHblits | 0.29 |
| ``` target    TARREITRRGFLGTAAGAGFAAFVVSATRAWGLEAIENPLARYPDREWERVYRDLWRYDSKFTFLCAPNDTHNCLLDAYV 7l5i.1    --------------------------------------------------------------------------------  target    RSGVMTRIGPTMRYGEARDLDGNRASARWDPRVCQKGLALTRRFYGDRRLRHCMVRAGFKRWVDEGFPRGEDGKPPKEYF 7l5i.1    --------------------------------------------------------------------------------  target    QRARDEWVRASHDEAAAVVAATLANIAATYSGEEGAQRLRDQGYEEETIEAMGGAGVQAMKFRGGMPLLGMTRVFGLYRM 7l5i.1    --------------------------------------------------------------------------------  target    ANSMALLDAKVRGVGPDEARGARGFDNYSWHTDLPPGHPMVTGQQTVDFDLNSVELAKNVVVWGMNWITTKMPDAHWLTE 7l5i.1    -------------------------------------------------------DIKAVYWAGGNPFVHHQ-DTNTLVK  target    ARLKGTRIIVIACEYSSTSSKADDAIVVRPGTTPALALGLSHVIMRDKLYDADYVRRWTDLPMLVRTDTLKYLSAEDVFG 7l5i.1    AFQKPDVVIVNEVNWTPTARMADIVLPATT--------------------------------------------------  target    GGPAPL 7l5i.1    ------ ``` | | | | | | | | | | | | | | | | | | | | | | | | | | | | | | | | | | | | | | | | | | | | | | | | | |
|  | 7l5s.1.A | Trimethylamine-N-oxide reductase  *Crystal Structure of Haemophilus influenzae MtsZ at pH 5.5* | 0.04 |  | 24.07 | 0.13 | 296-350 | X-ray | 2.09 | monomer | 1 x O, 2 x MGD, 1 x MO | HHblits | 0.29 |
| ``` target    TARREITRRGFLGTAAGAGFAAFVVSATRAWGLEAIENPLARYPDREWERVYRDLWRYDSKFTFLCAPNDTHNCLLDAYV 7l5s.1    --------------------------------------------------------------------------------  target    RSGVMTRIGPTMRYGEARDLDGNRASARWDPRVCQKGLALTRRFYGDRRLRHCMVRAGFKRWVDEGFPRGEDGKPPKEYF 7l5s.1    --------------------------------------------------------------------------------  target    QRARDEWVRASHDEAAAVVAATLANIAATYSGEEGAQRLRDQGYEEETIEAMGGAGVQAMKFRGGMPLLGMTRVFGLYRM 7l5s.1    --------------------------------------------------------------------------------  target    ANSMALLDAKVRGVGPDEARGARGFDNYSWHTDLPPGHPMVTGQQTVDFDLNSVELAKNVVVWGMNWITTKMPDAHWLTE 7l5s.1    -------------------------------------------------------DIKAVYWAGGNPFVHHQ-DTNTLVK  target    ARLKGTRIIVIACEYSSTSSKADDAIVVRPGTTPALALGLSHVIMRDKLYDADYVRRWTDLPMLVRTDTLKYLSAEDVFG 7l5s.1    AFQKPDVVIVNEVNWTPTARMADIVLPATT--------------------------------------------------  target    GGPAPL 7l5s.1    ------ ``` | | | | | | | | | | | | | | | | | | | | | | | | | | | | | | | | | | | | | | | | | | | | | | | | | |
|  | 1eu1.1.A | DIMETHYL SULFOXIDE REDUCTASE  *THE CRYSTAL STRUCTURE OF RHODOBACTER SPHAEROIDES DIMETHYLSULFOXIDE REDUCTASE REVEALS TWO DISTINCT MOLYBDENUM COORDINATION ENVIRONMENTS.* | 0.04 |  | 20.00 | 0.14 | 296-351 | X-ray | 1.30 | monomer | 3 x GLC, 1 x CD, 2 x MGD, 1 x 6MO, 2 x O | HHblits | 0.28 |
| ``` target    TARREITRRGFLGTAAGAGFAAFVVSATRAWGLEAIENPLARYPDREWERVYRDLWRYDSKFTFLCAPNDTHNCLLDAYV 1eu1.1    --------------------------------------------------------------------------------  target    RSGVMTRIGPTMRYGEARDLDGNRASARWDPRVCQKGLALTRRFYGDRRLRHCMVRAGFKRWVDEGFPRGEDGKPPKEYF 1eu1.1    --------------------------------------------------------------------------------  target    QRARDEWVRASHDEAAAVVAATLANIAATYSGEEGAQRLRDQGYEEETIEAMGGAGVQAMKFRGGMPLLGMTRVFGLYRM 1eu1.1    --------------------------------------------------------------------------------  target    ANSMALLDAKVRGVGPDEARGARGFDNYSWHTDLPPGHPMVTGQQTVDFDLNSVELAKNVVVWGMNWITTKMPDAHWLTE 1eu1.1    -------------------------------------------------------DVKLAYWAGGNPFAHH-QDRNRMLK  target    ARLKGTRIIVIACEYSSTSSKADDAIVVRPGTTPALALGLSHVIMRDKLYDADYVRRWTDLPMLVRTDTLKYLSAEDVFG 1eu1.1    AWEKLETFIVQDFQWTATARHADIVLPATTS-------------------------------------------------  target    GGPAPL 1eu1.1    ------ ``` | | | | | | | | | | | | | | | | | | | | | | | | | | | | | | | | | | | | | | | | | | | | | | | | | |
|  | 1e18.1.A | DMSO REDUCTASE.  *TUNGSTEN-SUSBSTITUTED DMSO REDUCTASE FROM RHODOBACTER CAPSULATUS* | 0.04 |  | 18.18 | 0.14 | 296-351 | X-ray | 2.00 | monomer | 2 x PGD, 1 x 6WO | HHblits | 0.28 |
| ``` target    TARREITRRGFLGTAAGAGFAAFVVSATRAWGLEAIENPLARYPDREWERVYRDLWRYDSKFTFLCAPNDTHNCLLDAYV 1e18.1    --------------------------------------------------------------------------------  target    RSGVMTRIGPTMRYGEARDLDGNRASARWDPRVCQKGLALTRRFYGDRRLRHCMVRAGFKRWVDEGFPRGEDGKPPKEYF 1e18.1    --------------------------------------------------------------------------------  target    QRARDEWVRASHDEAAAVVAATLANIAATYSGEEGAQRLRDQGYEEETIEAMGGAGVQAMKFRGGMPLLGMTRVFGLYRM 1e18.1    --------------------------------------------------------------------------------  target    ANSMALLDAKVRGVGPDEARGARGFDNYSWHTDLPPGHPMVTGQQTVDFDLNSVELAKNVVVWGMNWITTKMPDAHWLTE 1e18.1    -------------------------------------------------------DVKMAYWVGGNPFVHHQ-DRNRMVK  target    ARLKGTRIIVIACEYSSTSSKADDAIVVRPGTTPALALGLSHVIMRDKLYDADYVRRWTDLPMLVRTDTLKYLSAEDVFG 1e18.1    AWEKLETFVVHDFQWTPTARHADIVLPATTS-------------------------------------------------  target    GGPAPL 1e18.1    ------ ``` | | | | | | | | | | | | | | | | | | | | | | | | | | | | | | | | | | | | | | | | | | | | | | | | | |
|  | 1m2j.1.A | Silent Information Regulator 2  *Sir2 homologue H80N mutant-ADP ribose complex* | 0.04 |  | 24.53 | 0.13 | 293-347 | X-ray | 1.70 | monomer | 1 x ZN, 1 x APR | HHblits | 0.31 |
| ``` target    TARREITRRGFLGTAAGAGFAAFVVSATRAWGLEAIENPLARYPDREWERVYRDLWRYDSKFTFLCAPNDTHNCLLDAYV 1m2j.1    --------------------------------------------------------------------------------  target    RSGVMTRIGPTMRYGEARDLDGNRASARWDPRVCQKGLALTRRFYGDRRLRHCMVRAGFKRWVDEGFPRGEDGKPPKEYF 1m2j.1    --------------------------------------------------------------------------------  target    QRARDEWVRASHDEAAAVVAATLANIAATYSGEEGAQRLRDQGYEEETIEAMGGAGVQAMKFRGGMPLLGMTRVFGLYRM 1m2j.1    --------------------------------------------------------------------------------  target    ANSMALLDAKVRGVGPDEARGARGFDNYSWHTDLPPGHPMVTGQQTVDFDLNSVELAKNVVVWGMNWITTKMPDAHWLTE 1m2j.1    ----------------------------------------------------EVERADVIIVAGTSAVV--QPAASLPLI  target    ARLKGTRIIVIACEYSSTSSKADDAIVVRPGTTPALALGLSHVIMRDKLYDADYVRRWTDLPMLVRTDTLKYLSAEDVFG 1m2j.1    VKQRGGAIIEINPDETPLTPIADYSLR-----------------------------------------------------  target    GGPAPL 1m2j.1    ------ ``` | | | | | | | | | | | | | | | | | | | | | | | | | | | | | | | | | | | | | | | | | | | | | | | | | |
|  | 1s5p.1.A | NAD-dependent deacetylase  *Structure and substrate binding properties of cobB, a Sir2 homolog protein deacetylase from Eschericia coli.* | 0.03 |  | 18.87 | 0.13 | 293-347 | X-ray | 1.96 | monomer | 1 x ZN, 1 x LYS-GLY-GLY-ALA-ALY-ARG-HIS-ARG | HHblits | 0.31 |
| ``` target    TARREITRRGFLGTAAGAGFAAFVVSATRAWGLEAIENPLARYPDREWERVYRDLWRYDSKFTFLCAPNDTHNCLLDAYV 1s5p.1    --------------------------------------------------------------------------------  target    RSGVMTRIGPTMRYGEARDLDGNRASARWDPRVCQKGLALTRRFYGDRRLRHCMVRAGFKRWVDEGFPRGEDGKPPKEYF 1s5p.1    --------------------------------------------------------------------------------  target    QRARDEWVRASHDEAAAVVAATLANIAATYSGEEGAQRLRDQGYEEETIEAMGGAGVQAMKFRGGMPLLGMTRVFGLYRM 1s5p.1    --------------------------------------------------------------------------------  target    ANSMALLDAKVRGVGPDEARGARGFDNYSWHTDLPPGHPMVTGQQTVDFDLNSVELAKNVVVWGMNWITTKMPDAHWLTE 1s5p.1    ----------------------------------------------------ALSMADIFIAIGTSGHV--YPAAGFVHE  target    ARLKGTRIIVIACEYSSTSSKADDAIVVRPGTTPALALGLSHVIMRDKLYDADYVRRWTDLPMLVRTDTLKYLSAEDVFG 1s5p.1    AKLHGAHTVELNLEPSQVGNEFAEKYY-----------------------------------------------------  target    GGPAPL 1s5p.1    ------ ``` | | | | | | | | | | | | | | | | | | | | | | | | | | | | | | | | | | | | | | | | | | | | | | | | | |
|  | 6rxs.1.A | NAD-dependent protein deacylase  *Crystal structure of CobB Ac3(A76G,Y92A, I131L, V187Y) in complex with H4K16-Acetyl peptide* | 0.03 |  | 18.87 | 0.13 | 292-346 | X-ray | 1.60 | hetero-1-1-mer | 1 x ZN | HHblits | 0.31 |
| ``` target    TARREITRRGFLGTAAGAGFAAFVVSATRAWGLEAIENPLARYPDREWERVYRDLWRYDSKFTFLCAPNDTHNCLLDAYV 6rxs.1    --------------------------------------------------------------------------------  target    RSGVMTRIGPTMRYGEARDLDGNRASARWDPRVCQKGLALTRRFYGDRRLRHCMVRAGFKRWVDEGFPRGEDGKPPKEYF 6rxs.1    --------------------------------------------------------------------------------  target    QRARDEWVRASHDEAAAVVAATLANIAATYSGEEGAQRLRDQGYEEETIEAMGGAGVQAMKFRGGMPLLGMTRVFGLYRM 6rxs.1    --------------------------------------------------------------------------------  target    ANSMALLDAKVRGVGPDEARGARGFDNYSWHTDLPPGHPMVTGQQTVDFDLNSVELAKNVVVWGMNWITTKMPDAHWLTE 6rxs.1    ---------------------------------------------------MALSMADIFIAIGTSGHV--YPAAGFVHE  target    ARLKGTRIIVIACEYSSTSSKADDAIVVRPGTTPALALGLSHVIMRDKLYDADYVRRWTDLPMLVRTDTLKYLSAEDVFG 6rxs.1    AKLHGAHTVELNLEPSQVGNEFAEKY------------------------------------------------------  target    GGPAPL 6rxs.1    ------ ``` | | | | | | | | | | | | | | | | | | | | | | | | | | | | | | | | | | | | | | | | | | | | | | | | | |
|  | 1m2n.1.A | Silent Information Regulator 2  *Sir2 homologues (D102G/F159A/R170A) mutant-2'-O-acetyl ADP ribose complex* | 0.04 |  | 24.53 | 0.13 | 293-347 | X-ray | 2.60 | homo-dimer | 2 x ZN, 2 x OAD | HHblits | 0.31 |
| ``` target    TARREITRRGFLGTAAGAGFAAFVVSATRAWGLEAIENPLARYPDREWERVYRDLWRYDSKFTFLCAPNDTHNCLLDAYV 1m2n.1    --------------------------------------------------------------------------------  target    RSGVMTRIGPTMRYGEARDLDGNRASARWDPRVCQKGLALTRRFYGDRRLRHCMVRAGFKRWVDEGFPRGEDGKPPKEYF 1m2n.1    --------------------------------------------------------------------------------  target    QRARDEWVRASHDEAAAVVAATLANIAATYSGEEGAQRLRDQGYEEETIEAMGGAGVQAMKFRGGMPLLGMTRVFGLYRM 1m2n.1    --------------------------------------------------------------------------------  target    ANSMALLDAKVRGVGPDEARGARGFDNYSWHTDLPPGHPMVTGQQTVDFDLNSVELAKNVVVWGMNWITTKMPDAHWLTE 1m2n.1    ----------------------------------------------------EVERADVIIVAGTSAVVQ--PAASLPLI  target    ARLKGTRIIVIACEYSSTSSKADDAIVVRPGTTPALALGLSHVIMRDKLYDADYVRRWTDLPMLVRTDTLKYLSAEDVFG 1m2n.1    VKQRGGAIIEINPDETPLTPIADYSLR-----------------------------------------------------  target    GGPAPL 1m2n.1    ------ ``` | | | | | | | | | | | | | | | | | | | | | | | | | | | | | | | | | | | | | | | | | | | | | | | | | |
|  | 1m2n.1.B | Silent Information Regulator 2  *Sir2 homologues (D102G/F159A/R170A) mutant-2'-O-acetyl ADP ribose complex* | 0.03 |  | 24.53 | 0.13 | 293-347 | X-ray | 2.60 | homo-dimer | 2 x ZN, 2 x OAD | HHblits | 0.31 |
| ``` target    TARREITRRGFLGTAAGAGFAAFVVSATRAWGLEAIENPLARYPDREWERVYRDLWRYDSKFTFLCAPNDTHNCLLDAYV 1m2n.1    --------------------------------------------------------------------------------  target    RSGVMTRIGPTMRYGEARDLDGNRASARWDPRVCQKGLALTRRFYGDRRLRHCMVRAGFKRWVDEGFPRGEDGKPPKEYF 1m2n.1    --------------------------------------------------------------------------------  target    QRARDEWVRASHDEAAAVVAATLANIAATYSGEEGAQRLRDQGYEEETIEAMGGAGVQAMKFRGGMPLLGMTRVFGLYRM 1m2n.1    --------------------------------------------------------------------------------  target    ANSMALLDAKVRGVGPDEARGARGFDNYSWHTDLPPGHPMVTGQQTVDFDLNSVELAKNVVVWGMNWITTKMPDAHWLTE 1m2n.1    ----------------------------------------------------EVERADVIIVAGTSAVVQ--PAASLPLI  target    ARLKGTRIIVIACEYSSTSSKADDAIVVRPGTTPALALGLSHVIMRDKLYDADYVRRWTDLPMLVRTDTLKYLSAEDVFG 1m2n.1    VKQRGGAIIEINPDETPLTPIADYSLR-----------------------------------------------------  target    GGPAPL 1m2n.1    ------ ``` | | | | | | | | | | | | | | | | | | | | | | | | | | | | | | | | | | | | | | | | | | | | | | | | | |
|  | 7bkb.1.F | Formate dehydrogenase  *Formate dehydrogenase - heterodisulfide reductase - formylmethanofuran dehydrogenase complex from Methanospirillum hungatei (hexameric, composite structure)* | 0.04 |  | 16.36 | 0.14 | 296-351 | EM | 0.00 | hetero-2-2-2-2-2-2-… | 48 x SF4, 4 x FAD, 2 x FES, 4 x 9S8, 4 x ZN, 2 x MO, 4 x MGD | HHblits | 0.28 |
| ``` target    TARREITRRGFLGTAAGAGFAAFVVSATRAWGLEAIENPLARYPDREWERVYRDLWRYDSKFTFLCAPNDTHNCLLDAYV 7bkb.1    --------------------------------------------------------------------------------  target    RSGVMTRIGPTMRYGEARDLDGNRASARWDPRVCQKGLALTRRFYGDRRLRHCMVRAGFKRWVDEGFPRGEDGKPPKEYF 7bkb.1    --------------------------------------------------------------------------------  target    QRARDEWVRASHDEAAAVVAATLANIAATYSGEEGAQRLRDQGYEEETIEAMGGAGVQAMKFRGGMPLLGMTRVFGLYRM 7bkb.1    --------------------------------------------------------------------------------  target    ANSMALLDAKVRGVGPDEARGARGFDNYSWHTDLPPGHPMVTGQQTVDFDLNSVELAKNVVVWGMNWITTKMPDAHWLTE 7bkb.1    -------------------------------------------------------EIKGMYILGLNPVVTYP-SSNHVKA  target    ARLKGTRIIVIACEYSSTSSKADDAIVVRPGTTPALALGLSHVIMRDKLYDADYVRRWTDLPMLVRTDTLKYLSAEDVFG 7bkb.1    QLEKLDFLVVQDIFFTETCQYADVILPGACF-------------------------------------------------  target    GGPAPL 7bkb.1    ------ ``` | | | | | | | | | | | | | | | | | | | | | | | | | | | | | | | | | | | | | | | | | | | | | | | | | |
|  | 7en6.1.A | HTH-type transcriptional regulator MurR  *The crystal structure of Escherichia coli MurR in apo form* | 0.03 |  | 16.07 | 0.14 | 296-353 | X-ray | 2.28 | homo-tetramer |  | HHblits | 0.26 |
| ``` target    TARREITRRGFLGTAAGAGFAAFVVSATRAWGLEAIENPLARYPDREWERVYRDLWRYDSKFTFLCAPNDTHNCLLDAYV 7en6.1    --------------------------------------------------------------------------------  target    RSGVMTRIGPTMRYGEARDLDGNRASARWDPRVCQKGLALTRRFYGDRRLRHCMVRAGFKRWVDEGFPRGEDGKPPKEYF 7en6.1    --------------------------------------------------------------------------------  target    QRARDEWVRASHDEAAAVVAATLANIAATYSGEEGAQRLRDQGYEEETIEAMGGAGVQAMKFRGGMPLLGMTRVFGLYRM 7en6.1    --------------------------------------------------------------------------------  target    ANSMALLDAKVRGVGPDEARGARGFDNYSWHTDLPPGHPMVTGQQTVDFDLNSVELAKNVVVWGMNWITTKMPDAHWLTE 7en6.1    -------------------------------------------------------KGDVQIAISYSGSK--KEIVLCAEA  target    ARLKGTRIIVIAC-EYSSTSSKADDAIVVRPGTTPALALGLSHVIMRDKLYDADYVRRWTDLPMLVRTDTLKYLSAEDVF 7en6.1    ARKQGATVIAITSLTDSPLRRLAHFTLDTVSGET----------------------------------------------  target    GGGPAPL 7en6.1    ------- ``` | | | | | | | | | | | | | | | | | | | | | | | | | | | | | | | | | | | | | | | | | | | | | | | | | |
|  | 7en6.1.B | HTH-type transcriptional regulator MurR  *The crystal structure of Escherichia coli MurR in apo form* | 0.03 |  | 16.07 | 0.14 | 296-353 | X-ray | 2.28 | homo-tetramer |  | HHblits | 0.26 |
| ``` target    TARREITRRGFLGTAAGAGFAAFVVSATRAWGLEAIENPLARYPDREWERVYRDLWRYDSKFTFLCAPNDTHNCLLDAYV 7en6.1    --------------------------------------------------------------------------------  target    RSGVMTRIGPTMRYGEARDLDGNRASARWDPRVCQKGLALTRRFYGDRRLRHCMVRAGFKRWVDEGFPRGEDGKPPKEYF 7en6.1    --------------------------------------------------------------------------------  target    QRARDEWVRASHDEAAAVVAATLANIAATYSGEEGAQRLRDQGYEEETIEAMGGAGVQAMKFRGGMPLLGMTRVFGLYRM 7en6.1    --------------------------------------------------------------------------------  target    ANSMALLDAKVRGVGPDEARGARGFDNYSWHTDLPPGHPMVTGQQTVDFDLNSVELAKNVVVWGMNWITTKMPDAHWLTE 7en6.1    -------------------------------------------------------KGDVQIAISYSGSK--KEIVLCAEA  target    ARLKGTRIIVIAC-EYSSTSSKADDAIVVRPGTTPALALGLSHVIMRDKLYDADYVRRWTDLPMLVRTDTLKYLSAEDVF 7en6.1    ARKQGATVIAITSLTDSPLRRLAHFTLDTVSGET----------------------------------------------  target    GGGPAPL 7en6.1    ------- ``` | | | | | | | | | | | | | | | | | | | | | | | | | | | | | | | | | | | | | | | | | | | | | | | | | |
|  | 7en6.1.C | HTH-type transcriptional regulator MurR  *The crystal structure of Escherichia coli MurR in apo form* | 0.03 |  | 16.07 | 0.14 | 296-353 | X-ray | 2.28 | homo-tetramer |  | HHblits | 0.26 |
| ``` target    TARREITRRGFLGTAAGAGFAAFVVSATRAWGLEAIENPLARYPDREWERVYRDLWRYDSKFTFLCAPNDTHNCLLDAYV 7en6.1    --------------------------------------------------------------------------------  target    RSGVMTRIGPTMRYGEARDLDGNRASARWDPRVCQKGLALTRRFYGDRRLRHCMVRAGFKRWVDEGFPRGEDGKPPKEYF 7en6.1    --------------------------------------------------------------------------------  target    QRARDEWVRASHDEAAAVVAATLANIAATYSGEEGAQRLRDQGYEEETIEAMGGAGVQAMKFRGGMPLLGMTRVFGLYRM 7en6.1    --------------------------------------------------------------------------------  target    ANSMALLDAKVRGVGPDEARGARGFDNYSWHTDLPPGHPMVTGQQTVDFDLNSVELAKNVVVWGMNWITTKMPDAHWLTE 7en6.1    -------------------------------------------------------KGDVQIAISYSGSK--KEIVLCAEA  target    ARLKGTRIIVIAC-EYSSTSSKADDAIVVRPGTTPALALGLSHVIMRDKLYDADYVRRWTDLPMLVRTDTLKYLSAEDVF 7en6.1    ARKQGATVIAITSLTDSPLRRLAHFTLDTVSGET----------------------------------------------  target    GGGPAPL 7en6.1    ------- ``` | | | | | | | | | | | | | | | | | | | | | | | | | | | | | | | | | | | | | | | | | | | | | | | | | |
|  | 7en6.1.D | HTH-type transcriptional regulator MurR  *The crystal structure of Escherichia coli MurR in apo form* | 0.03 |  | 16.07 | 0.14 | 296-353 | X-ray | 2.28 | homo-tetramer |  | HHblits | 0.26 |
| ``` target    TARREITRRGFLGTAAGAGFAAFVVSATRAWGLEAIENPLARYPDREWERVYRDLWRYDSKFTFLCAPNDTHNCLLDAYV 7en6.1    --------------------------------------------------------------------------------  target    RSGVMTRIGPTMRYGEARDLDGNRASARWDPRVCQKGLALTRRFYGDRRLRHCMVRAGFKRWVDEGFPRGEDGKPPKEYF 7en6.1    --------------------------------------------------------------------------------  target    QRARDEWVRASHDEAAAVVAATLANIAATYSGEEGAQRLRDQGYEEETIEAMGGAGVQAMKFRGGMPLLGMTRVFGLYRM 7en6.1    --------------------------------------------------------------------------------  target    ANSMALLDAKVRGVGPDEARGARGFDNYSWHTDLPPGHPMVTGQQTVDFDLNSVELAKNVVVWGMNWITTKMPDAHWLTE 7en6.1    -------------------------------------------------------KGDVQIAISYSGSK--KEIVLCAEA  target    ARLKGTRIIVIAC-EYSSTSSKADDAIVVRPGTTPALALGLSHVIMRDKLYDADYVRRWTDLPMLVRTDTLKYLSAEDVF 7en6.1    ARKQGATVIAITSLTDSPLRRLAHFTLDTVSGET----------------------------------------------  target    GGGPAPL 7en6.1    ------- ``` | | | | | | | | | | | | | | | | | | | | | | | | | | | | | | | | | | | | | | | | | | | | | | | | | |
|  | 7en5.1.A | HTH-type transcriptional regulator MurR  *The crystal structure of Escherichia coli MurR in complex with N-acetylglucosamine-6-phosphate* | 0.03 |  | 16.07 | 0.14 | 296-353 | X-ray | 1.25 | homo-tetramer | 4 x 4QY, 4 x MXE | HHblits | 0.26 |
| ``` target    TARREITRRGFLGTAAGAGFAAFVVSATRAWGLEAIENPLARYPDREWERVYRDLWRYDSKFTFLCAPNDTHNCLLDAYV 7en5.1    --------------------------------------------------------------------------------  target    RSGVMTRIGPTMRYGEARDLDGNRASARWDPRVCQKGLALTRRFYGDRRLRHCMVRAGFKRWVDEGFPRGEDGKPPKEYF 7en5.1    --------------------------------------------------------------------------------  target    QRARDEWVRASHDEAAAVVAATLANIAATYSGEEGAQRLRDQGYEEETIEAMGGAGVQAMKFRGGMPLLGMTRVFGLYRM 7en5.1    --------------------------------------------------------------------------------  target    ANSMALLDAKVRGVGPDEARGARGFDNYSWHTDLPPGHPMVTGQQTVDFDLNSVELAKNVVVWGMNWITTKMPDAHWLTE 7en5.1    -------------------------------------------------------KGDVQIAISYSGSK--KEIVLCAEA  target    ARLKGTRIIVIAC-EYSSTSSKADDAIVVRPGTTPALALGLSHVIMRDKLYDADYVRRWTDLPMLVRTDTLKYLSAEDVF 7en5.1    ARKQGATVIAITSLTDSPLRRLAHFTLDTVSGET----------------------------------------------  target    GGGPAPL 7en5.1    ------- ``` | | | | | | | | | | | | | | | | | | | | | | | | | | | | | | | | | | | | | | | | | | | | | | | | | |
|  | 1tk9.1.A | Phosphoheptose isomerase 1  *Crystal Structure of Phosphoheptose isomerase 1* | 0.03 |  | 10.53 | 0.14 | 296-354 | X-ray | 2.10 | homo-tetramer |  | HHblits | 0.25 |
| ``` target    TARREITRRGFLGTAAGAGFAAFVVSATRAWGLEAIENPLARYPDREWERVYRDLWRYDSKFTFLCAPNDTHNCLLDAYV 1tk9.1    --------------------------------------------------------------------------------  target    RSGVMTRIGPTMRYGEARDLDGNRASARWDPRVCQKGLALTRRFYGDRRLRHCMVRAGFKRWVDEGFPRGEDGKPPKEYF 1tk9.1    --------------------------------------------------------------------------------  target    QRARDEWVRASHDEAAAVVAATLANIAATYSGEEGAQRLRDQGYEEETIEAMGGAGVQAMKFRGGMPLLGMTRVFGLYRM 1tk9.1    --------------------------------------------------------------------------------  target    ANSMALLDAKVRGVGPDEARGARGFDNYSWHTDLPPGHPMVTGQQTVDFDLNSVELAKNVVVWGMNWITTKMPDAHWLTE 1tk9.1    -------------------------------------------------------EKDVLIGISTSG-KS-PNVLEALKK  target    ARLKGTRIIVIACEY-SSTSSKADDAIVVRPGTTPALALGLSHVIMRDKLYDADYVRRWTDLPMLVRTDTLKYLSAEDVF 1tk9.1    AKELNMLCLGLSGKGGGMMNKLCDHNLVVPSDDTA---------------------------------------------  target    GGGPAPL 1tk9.1    ------- ``` | | | | | | | | | | | | | | | | | | | | | | | | | | | | | | | | | | | | | | | | | | | | | | | | | |
|  | 1nri.1.A | Hypothetical protein HI0754  *Crystal Structure of Putative Phosphosugar Isomerase HI0754 from Haemophilus influenzae* | 0.03 |  | 25.45 | 0.14 | 296-352 | X-ray | 1.90 | homo-dimer |  | HHblits | 0.27 |
| ``` target    TARREITRRGFLGTAAGAGFAAFVVSATRAWGLEAIENPLARYPDREWERVYRDLWRYDSKFTFLCAPNDTHNCLLDAYV 1nri.1    --------------------------------------------------------------------------------  target    RSGVMTRIGPTMRYGEARDLDGNRASARWDPRVCQKGLALTRRFYGDRRLRHCMVRAGFKRWVDEGFPRGEDGKPPKEYF 1nri.1    --------------------------------------------------------------------------------  target    QRARDEWVRASHDEAAAVVAATLANIAATYSGEEGAQRLRDQGYEEETIEAMGGAGVQAMKFRGGMPLLGMTRVFGLYRM 1nri.1    --------------------------------------------------------------------------------  target    ANSMALLDAKVRGVGPDEARGARGFDNYSWHTDLPPGHPMVTGQQTVDFDLNSVELAKNVVVWGMNWITTKMPDAHWLTE 1nri.1    -------------------------------------------------------KNDVLVGIAASG-RT-PYVIAGLQY  target    ARLKGTRIIVIAC-EYSSTSSKADDAIVVRPGTTPALALGLSHVIMRDKLYDADYVRRWTDLPMLVRTDTLKYLSAEDVF 1nri.1    AKSLGALTISIASNPKSEMAEIADIAIETIVGP-----------------------------------------------  target    GGGPAPL 1nri.1    ------- ``` | | | | | | | | | | | | | | | | | | | | | | | | | | | | | | | | | | | | | | | | | | | | | | | | | |
|  | 5mf6.1.A | NAD-dependent protein deacetylase sirtuin-6  *Human Sirt6 in complex with activator UBCS039* | 0.03 |  | 14.81 | 0.13 | 292-347 | X-ray | 1.87 | monomer | 1 x AR6, 1 x ZN, 1 x 7M2 | HHblits | 0.29 |
| ``` target    TARREITRRGFLGTAAGAGFAAFVVSATRAWGLEAIENPLARYPDREWERVYRDLWRYDSKFTFLCAPNDTHNCLLDAYV 5mf6.1    --------------------------------------------------------------------------------  target    RSGVMTRIGPTMRYGEARDLDGNRASARWDPRVCQKGLALTRRFYGDRRLRHCMVRAGFKRWVDEGFPRGEDGKPPKEYF 5mf6.1    --------------------------------------------------------------------------------  target    QRARDEWVRASHDEAAAVVAATLANIAATYSGEEGAQRLRDQGYEEETIEAMGGAGVQAMKFRGGMPLLGMTRVFGLYRM 5mf6.1    --------------------------------------------------------------------------------  target    ANSMALLDAKVRGVGPDEARGARGFDNYSWHTDLPPGHPMVTGQQTVDFDLNSVELAKNVVVWGMNWITTKMPDAHWLTE 5mf6.1    ---------------------------------------------------EASRNADLSITLGTSLQI--RPSGNLPLA  target    ARLKGTRIIVIACEYSSTSSKADDAIVVRPGTTPALALGLSHVIMRDKLYDADYVRRWTDLPMLVRTDTLKYLSAEDVFG 5mf6.1    TKRRGGRLVIVNLQPTKHDRHADLRIH-----------------------------------------------------  target    GGPAPL 5mf6.1    ------ ``` | | | | | | | | | | | | | | | | | | | | | | | | | | | | | | | | | | | | | | | | | | | | | | | | | |
|  | 3zg6.1.A | NAD-DEPENDENT PROTEIN DEACETYLASE SIRTUIN-6  *The novel de-long chain fatty acid function of human sirt6* | 0.03 |  | 14.81 | 0.13 | 292-347 | X-ray | 2.20 | hetero-oligomer | 1 x ZN, 1 x APR | HHblits | 0.29 |
| ``` target    TARREITRRGFLGTAAGAGFAAFVVSATRAWGLEAIENPLARYPDREWERVYRDLWRYDSKFTFLCAPNDTHNCLLDAYV 3zg6.1    --------------------------------------------------------------------------------  target    RSGVMTRIGPTMRYGEARDLDGNRASARWDPRVCQKGLALTRRFYGDRRLRHCMVRAGFKRWVDEGFPRGEDGKPPKEYF 3zg6.1    --------------------------------------------------------------------------------  target    QRARDEWVRASHDEAAAVVAATLANIAATYSGEEGAQRLRDQGYEEETIEAMGGAGVQAMKFRGGMPLLGMTRVFGLYRM 3zg6.1    --------------------------------------------------------------------------------  target    ANSMALLDAKVRGVGPDEARGARGFDNYSWHTDLPPGHPMVTGQQTVDFDLNSVELAKNVVVWGMNWITTKMPDAHWLTE 3zg6.1    ---------------------------------------------------EASRNADLSITLGTSLQI--RPSGNLPLA  target    ARLKGTRIIVIACEYSSTSSKADDAIVVRPGTTPALALGLSHVIMRDKLYDADYVRRWTDLPMLVRTDTLKYLSAEDVFG 3zg6.1    TKRRGGRLVIVNLQPTKHDRHADLRIH-----------------------------------------------------  target    GGPAPL 3zg6.1    ------ ``` | | | | | | | | | | | | | | | | | | | | | | | | | | | | | | | | | | | | | | | | | | | | | | | | | |
|  | 6xvg.3.A | NAD-dependent protein deacetylase sirtuin-6  *Human Sirt6 3-318 in complex with ADP-ribose and the activator MDL-801* | 0.03 |  | 14.81 | 0.13 | 292-347 | X-ray | 2.10 | monomer | 1 x AR6, 1 x ZN, 1 x 8L9 | HHblits | 0.29 |
| ``` target    TARREITRRGFLGTAAGAGFAAFVVSATRAWGLEAIENPLARYPDREWERVYRDLWRYDSKFTFLCAPNDTHNCLLDAYV 6xvg.3    --------------------------------------------------------------------------------  target    RSGVMTRIGPTMRYGEARDLDGNRASARWDPRVCQKGLALTRRFYGDRRLRHCMVRAGFKRWVDEGFPRGEDGKPPKEYF 6xvg.3    --------------------------------------------------------------------------------  target    QRARDEWVRASHDEAAAVVAATLANIAATYSGEEGAQRLRDQGYEEETIEAMGGAGVQAMKFRGGMPLLGMTRVFGLYRM 6xvg.3    --------------------------------------------------------------------------------  target    ANSMALLDAKVRGVGPDEARGARGFDNYSWHTDLPPGHPMVTGQQTVDFDLNSVELAKNVVVWGMNWITTKMPDAHWLTE 6xvg.3    ---------------------------------------------------EASRNADLSITLGTSLQI--RPSGNLPLA  target    ARLKGTRIIVIACEYSSTSSKADDAIVVRPGTTPALALGLSHVIMRDKLYDADYVRRWTDLPMLVRTDTLKYLSAEDVFG 6xvg.3    TKRRGGRLVIVNLQPTKHDRHADLRIH-----------------------------------------------------  target    GGPAPL 6xvg.3    ------ ``` | | | | | | | | | | | | | | | | | | | | | | | | | | | | | | | | | | | | | | | | | | | | | | | | | |
|  | 5y2f.1.A | NAD-dependent protein deacetylase sirtuin-6  *Human SIRT6 in complex with allosteric activator MDL-801* | 0.03 |  | 14.81 | 0.13 | 292-347 | X-ray | 2.53 | monomer | 1 x AR6, 1 x 8L9, 1 x ZN, 1 x HDR, 1 x THR-ALA-ARG-LYS-SER-THR-GLY-GLY | HHblits | 0.29 |
| ``` target    TARREITRRGFLGTAAGAGFAAFVVSATRAWGLEAIENPLARYPDREWERVYRDLWRYDSKFTFLCAPNDTHNCLLDAYV 5y2f.1    --------------------------------------------------------------------------------  target    RSGVMTRIGPTMRYGEARDLDGNRASARWDPRVCQKGLALTRRFYGDRRLRHCMVRAGFKRWVDEGFPRGEDGKPPKEYF 5y2f.1    --------------------------------------------------------------------------------  target    QRARDEWVRASHDEAAAVVAATLANIAATYSGEEGAQRLRDQGYEEETIEAMGGAGVQAMKFRGGMPLLGMTRVFGLYRM 5y2f.1    --------------------------------------------------------------------------------  target    ANSMALLDAKVRGVGPDEARGARGFDNYSWHTDLPPGHPMVTGQQTVDFDLNSVELAKNVVVWGMNWITTKMPDAHWLTE 5y2f.1    ---------------------------------------------------EASRNADLSITLGTSLQI--RPSGNLPLA  target    ARLKGTRIIVIACEYSSTSSKADDAIVVRPGTTPALALGLSHVIMRDKLYDADYVRRWTDLPMLVRTDTLKYLSAEDVFG 5y2f.1    TKRRGGRLVIVNLQPTKHDRHADLRIH-----------------------------------------------------  target    GGPAPL 5y2f.1    ------ ``` | | | | | | | | | | | | | | | | | | | | | | | | | | | | | | | | | | | | | | | | | | | | | | | | | |
|  | 5x16.1.A | NAD-dependent protein deacetylase sirtuin-6  *Sirt6 apo structure* | 0.03 |  | 14.81 | 0.13 | 292-347 | X-ray | 1.97 | monomer | 1 x AR6, 1 x ZN | HHblits | 0.29 |
| ``` target    TARREITRRGFLGTAAGAGFAAFVVSATRAWGLEAIENPLARYPDREWERVYRDLWRYDSKFTFLCAPNDTHNCLLDAYV 5x16.1    --------------------------------------------------------------------------------  target    RSGVMTRIGPTMRYGEARDLDGNRASARWDPRVCQKGLALTRRFYGDRRLRHCMVRAGFKRWVDEGFPRGEDGKPPKEYF 5x16.1    --------------------------------------------------------------------------------  target    QRARDEWVRASHDEAAAVVAATLANIAATYSGEEGAQRLRDQGYEEETIEAMGGAGVQAMKFRGGMPLLGMTRVFGLYRM 5x16.1    --------------------------------------------------------------------------------  target    ANSMALLDAKVRGVGPDEARGARGFDNYSWHTDLPPGHPMVTGQQTVDFDLNSVELAKNVVVWGMNWITTKMPDAHWLTE 5x16.1    ---------------------------------------------------EASRNADLSITLGTSLQI--RPSGNLPLA  target    ARLKGTRIIVIACEYSSTSSKADDAIVVRPGTTPALALGLSHVIMRDKLYDADYVRRWTDLPMLVRTDTLKYLSAEDVFG 5x16.1    TKRRGGRLVIVNLQPTKHDRHADLRIH-----------------------------------------------------  target    GGPAPL 5x16.1    ------ ``` | | | | | | | | | | | | | | | | | | | | | | | | | | | | | | | | | | | | | | | | | | | | | | | | | |
|  | 3k35.1.A | NAD-dependent deacetylase sirtuin-6  *Crystal Structure of Human SIRT6* | 0.03 |  | 14.81 | 0.13 | 292-347 | X-ray | 2.00 | monomer | 1 x ZN, 1 x APR | HHblits | 0.29 |
| ``` target    TARREITRRGFLGTAAGAGFAAFVVSATRAWGLEAIENPLARYPDREWERVYRDLWRYDSKFTFLCAPNDTHNCLLDAYV 3k35.1    --------------------------------------------------------------------------------  target    RSGVMTRIGPTMRYGEARDLDGNRASARWDPRVCQKGLALTRRFYGDRRLRHCMVRAGFKRWVDEGFPRGEDGKPPKEYF 3k35.1    --------------------------------------------------------------------------------  target    QRARDEWVRASHDEAAAVVAATLANIAATYSGEEGAQRLRDQGYEEETIEAMGGAGVQAMKFRGGMPLLGMTRVFGLYRM 3k35.1    --------------------------------------------------------------------------------  target    ANSMALLDAKVRGVGPDEARGARGFDNYSWHTDLPPGHPMVTGQQTVDFDLNSVELAKNVVVWGMNWITTKMPDAHWLTE 3k35.1    ---------------------------------------------------EASRNADLSITLGTSLQI--RPSGNLPLA  target    ARLKGTRIIVIACEYSSTSSKADDAIVVRPGTTPALALGLSHVIMRDKLYDADYVRRWTDLPMLVRTDTLKYLSAEDVFG 3k35.1    TKRRGGRLVIVNLQPTKHDRHADLRIH-----------------------------------------------------  target    GGPAPL 3k35.1    ------ ``` | | | | | | | | | | | | | | | | | | | | | | | | | | | | | | | | | | | | | | | | | | | | | | | | | |
|  | 8bqg.1.A | Formate dehydrogenase, alpha subunit, selenocysteine-containing  *W-formate dehydrogenase from Desulfovibrio vulgaris - Soaking with Formate 1 min* | 0.03 |  | 22.22 | 0.13 | 295-349 | X-ray | 1.95 | hetero-1-1-mer | 2 x MGD, 4 x SF4, 1 x H2S, 1 x W | HHblits | 0.29 |
| ``` target    TARREITRRGFLGTAAGAGFAAFVVSATRAWGLEAIENPLARYPDREWERVYRDLWRYDSKFTFLCAPNDTHNCLLDAYV 8bqg.1    --------------------------------------------------------------------------------  target    RSGVMTRIGPTMRYGEARDLDGNRASARWDPRVCQKGLALTRRFYGDRRLRHCMVRAGFKRWVDEGFPRGEDGKPPKEYF 8bqg.1    --------------------------------------------------------------------------------  target    QRARDEWVRASHDEAAAVVAATLANIAATYSGEEGAQRLRDQGYEEETIEAMGGAGVQAMKFRGGMPLLGMTRVFGLYRM 8bqg.1    --------------------------------------------------------------------------------  target    ANSMALLDAKVRGVGPDEARGARGFDNYSWHTDLPPGHPMVTGQQTVDFDLNSVELAKNVVVWGMNWITTKMPDAHWLTE 8bqg.1    ------------------------------------------------------GEFKGLFAWGMNPACGGA-NANKNRK  target    ARLKGTRIIVIACEYSSTSSK--------AD-----DAIVVRPGTTPALALGLSHVIMRDKLYDADYVRRWTDLPMLVRT 8bqg.1    AMGKLEWLVNVNLFENETSSFWKGPGMNPAEIGTEVFFLPCC--------------------------------------  target    DTLKYLSAEDVFGGGPAPL 8bqg.1    ------------------- ``` | | | | | | | | | | | | | | | | | | | | | | | | | | | | | | | | | | | | | | | | | | | | | | | | | |
|  | 5lu6.1.A | Phosphoheptose isomerase  *Heptose isomerase mutant - H64Q* | 0.03 |  | 12.50 | 0.14 | 296-353 | X-ray | 1.67 | homo-tetramer | 4 x I22 | HHblits | 0.26 |
| ``` target    TARREITRRGFLGTAAGAGFAAFVVSATRAWGLEAIENPLARYPDREWERVYRDLWRYDSKFTFLCAPNDTHNCLLDAYV 5lu6.1    --------------------------------------------------------------------------------  target    RSGVMTRIGPTMRYGEARDLDGNRASARWDPRVCQKGLALTRRFYGDRRLRHCMVRAGFKRWVDEGFPRGEDGKPPKEYF 5lu6.1    --------------------------------------------------------------------------------  target    QRARDEWVRASHDEAAAVVAATLANIAATYSGEEGAQRLRDQGYEEETIEAMGGAGVQAMKFRGGMPLLGMTRVFGLYRM 5lu6.1    --------------------------------------------------------------------------------  target    ANSMALLDAKVRGVGPDEARGARGFDNYSWHTDLPPGHPMVTGQQTVDFDLNSVELAKNVVVWGMNWITTKMPDAHWLTE 5lu6.1    -------------------------------------------------------EGDVLIGYSTSGK-S-PNILAAFRE  target    ARLKGTRIIVIAC-EYSSTSSKADDAIVVRPGTTPALALGLSHVIMRDKLYDADYVRRWTDLPMLVRTDTLKYLSAEDVF 5lu6.1    AKAKGMTCVGFTGNRGGEMRELCDLLLEVPSADT----------------------------------------------  target    GGGPAPL 5lu6.1    ------- ``` | | | | | | | | | | | | | | | | | | | | | | | | | | | | | | | | | | | | | | | | | | | | | | | | | |
|  | 5e7o.1.A | DMSO reductase family type II enzyme, molybdopterin subunit  *Crystal structure of the perchlorate reductase PcrAB mutant W461E of PcrA from Azospira suillum PS* | 0.03 |  | 20.37 | 0.13 | 297-350 | X-ray | 2.40 | hetero-oligomer | 4 x SF4, 1 x MO, 1 x MGD, 1 x MD1, 1 x F3S | HHblits | 0.29 |
| ``` target    TARREITRRGFLGTAAGAGFAAFVVSATRAWGLEAIENPLARYPDREWERVYRDLWRYDSKFTFLCAPNDTHNCLLDAYV 5e7o.1    --------------------------------------------------------------------------------  target    RSGVMTRIGPTMRYGEARDLDGNRASARWDPRVCQKGLALTRRFYGDRRLRHCMVRAGFKRWVDEGFPRGEDGKPPKEYF 5e7o.1    --------------------------------------------------------------------------------  target    QRARDEWVRASHDEAAAVVAATLANIAATYSGEEGAQRLRDQGYEEETIEAMGGAGVQAMKFRGGMPLLGMTRVFGLYRM 5e7o.1    --------------------------------------------------------------------------------  target    ANSMALLDAKVRGVGPDEARGARGFDNYSWHTDLPPGHPMVTGQQTVDFDLNSVELAKNVVVWGMNWITTKMPDAHWLTE 5e7o.1    --------------------------------------------------------PKVFFVYRGNWLNQAKGQKYVLEN  target    ARLKGTRIIVIACEYSSTSSKADDAIVVRPGTTPALALGLSHVIMRDKLYDADYVRRWTDLPMLVRTDTLKYLSAEDVFG 5e7o.1    LWPKLELIVDINIRMDSTALYSDVVLPSAH--------------------------------------------------  target    GGPAPL 5e7o.1    ------ ``` | | | | | | | | | | | | | | | | | | | | | | | | | | | | | | | | | | | | | | | | | | | | | | | | | |
|  | 6enx.1.A | NAD-dependent protein deacylase sirtuin-5, mitochondrial  *Zebrafish Sirt5 in complex with stalled bicyclic intermediate of inhibitory compound 10* | 0.03 |  | 14.81 | 0.13 | 292-347 | X-ray | 1.95 | monomer | 1 x ZN, 1 x BJW | HHblits | 0.28 |
| ``` target    TARREITRRGFLGTAAGAGFAAFVVSATRAWGLEAIENPLARYPDREWERVYRDLWRYDSKFTFLCAPNDTHNCLLDAYV 6enx.1    --------------------------------------------------------------------------------  target    RSGVMTRIGPTMRYGEARDLDGNRASARWDPRVCQKGLALTRRFYGDRRLRHCMVRAGFKRWVDEGFPRGEDGKPPKEYF 6enx.1    --------------------------------------------------------------------------------  target    QRARDEWVRASHDEAAAVVAATLANIAATYSGEEGAQRLRDQGYEEETIEAMGGAGVQAMKFRGGMPLLGMTRVFGLYRM 6enx.1    --------------------------------------------------------------------------------  target    ANSMALLDAKVRGVGPDEARGARGFDNYSWHTDLPPGHPMVTGQQTVDFDLNSVELAKNVVVWGMNWITTKMPDAHWLTE 6enx.1    ---------------------------------------------------RELEKCDLCLVVGTSSIV--YPAAMFAPQ  target    ARLKGTRIIVIACEYSSTSSKADDAIVVRPGTTPALALGLSHVIMRDKLYDADYVRRWTDLPMLVRTDTLKYLSAEDVFG 6enx.1    VASRGVPVAEFNMECTPATQRFKYHFE-----------------------------------------------------  target    GGPAPL 6enx.1    ------ ``` | | | | | | | | | | | | | | | | | | | | | | | | | | | | | | | | | | | | | | | | | | | | | | | | | |
|  | 4utn.1.A | NAD-DEPENDENT PROTEIN DEACYLASE SIRTUIN-5, MITOCHONDRIAL  *Crystal structure of zebrafish Sirtuin 5 in complex with succinylated CPS1-peptide* | 0.03 |  | 14.81 | 0.13 | 292-347 | X-ray | 3.00 | monomer | 1 x ZN, 1 x BEZ-GLY-VAL-LEU-SLL-GLU-TYR-GLY-VAL | HHblits | 0.28 |
| ``` target    TARREITRRGFLGTAAGAGFAAFVVSATRAWGLEAIENPLARYPDREWERVYRDLWRYDSKFTFLCAPNDTHNCLLDAYV 4utn.1    --------------------------------------------------------------------------------  target    RSGVMTRIGPTMRYGEARDLDGNRASARWDPRVCQKGLALTRRFYGDRRLRHCMVRAGFKRWVDEGFPRGEDGKPPKEYF 4utn.1    --------------------------------------------------------------------------------  target    QRARDEWVRASHDEAAAVVAATLANIAATYSGEEGAQRLRDQGYEEETIEAMGGAGVQAMKFRGGMPLLGMTRVFGLYRM 4utn.1    --------------------------------------------------------------------------------  target    ANSMALLDAKVRGVGPDEARGARGFDNYSWHTDLPPGHPMVTGQQTVDFDLNSVELAKNVVVWGMNWITTKMPDAHWLTE 4utn.1    ---------------------------------------------------RELEKCDLCLVVGTSSIV--YPAAMFAPQ  target    ARLKGTRIIVIACEYSSTSSKADDAIVVRPGTTPALALGLSHVIMRDKLYDADYVRRWTDLPMLVRTDTLKYLSAEDVFG 4utn.1    VASRGVPVAEFNMECTPATQRFKYHFE-----------------------------------------------------  target    GGPAPL 4utn.1    ------ ``` | | | | | | | | | | | | | | | | | | | | | | | | | | | | | | | | | | | | | | | | | | | | | | | | | |
|  | 4utn.2.A | NAD-DEPENDENT PROTEIN DEACYLASE SIRTUIN-5, MITOCHONDRIAL  *Crystal structure of zebrafish Sirtuin 5 in complex with succinylated CPS1-peptide* | 0.03 |  | 14.81 | 0.13 | 292-347 | X-ray | 3.00 | monomer | 1 x ZN | HHblits | 0.28 |
| ``` target    TARREITRRGFLGTAAGAGFAAFVVSATRAWGLEAIENPLARYPDREWERVYRDLWRYDSKFTFLCAPNDTHNCLLDAYV 4utn.2    --------------------------------------------------------------------------------  target    RSGVMTRIGPTMRYGEARDLDGNRASARWDPRVCQKGLALTRRFYGDRRLRHCMVRAGFKRWVDEGFPRGEDGKPPKEYF 4utn.2    --------------------------------------------------------------------------------  target    QRARDEWVRASHDEAAAVVAATLANIAATYSGEEGAQRLRDQGYEEETIEAMGGAGVQAMKFRGGMPLLGMTRVFGLYRM 4utn.2    --------------------------------------------------------------------------------  target    ANSMALLDAKVRGVGPDEARGARGFDNYSWHTDLPPGHPMVTGQQTVDFDLNSVELAKNVVVWGMNWITTKMPDAHWLTE 4utn.2    ---------------------------------------------------RELEKCDLCLVVGTSSIV--YPAAMFAPQ  target    ARLKGTRIIVIACEYSSTSSKADDAIVVRPGTTPALALGLSHVIMRDKLYDADYVRRWTDLPMLVRTDTLKYLSAEDVFG 4utn.2    VASRGVPVAEFNMECTPATQRFKYHFE-----------------------------------------------------  target    GGPAPL 4utn.2    ------ ``` | | | | | | | | | | | | | | | | | | | | | | | | | | | | | | | | | | | | | | | | | | | | | | | | | |
|  | 6rxj.1.A | NAD-dependent protein deacylase  *Crystal structure of CobB wt in complex with H4K16-Acetyl peptide* | 0.03 |  | 19.23 | 0.13 | 293-346 | X-ray | 1.60 | hetero-1-1-mer | 1 x ZN | HHblits | 0.31 |
| ``` target    TARREITRRGFLGTAAGAGFAAFVVSATRAWGLEAIENPLARYPDREWERVYRDLWRYDSKFTFLCAPNDTHNCLLDAYV 6rxj.1    --------------------------------------------------------------------------------  target    RSGVMTRIGPTMRYGEARDLDGNRASARWDPRVCQKGLALTRRFYGDRRLRHCMVRAGFKRWVDEGFPRGEDGKPPKEYF 6rxj.1    --------------------------------------------------------------------------------  target    QRARDEWVRASHDEAAAVVAATLANIAATYSGEEGAQRLRDQGYEEETIEAMGGAGVQAMKFRGGMPLLGMTRVFGLYRM 6rxj.1    --------------------------------------------------------------------------------  target    ANSMALLDAKVRGVGPDEARGARGFDNYSWHTDLPPGHPMVTGQQTVDFDLNSVELAKNVVVWGMNWITTKMPDAHWLTE 6rxj.1    ----------------------------------------------------ALSMADIFIAIGTSGHV--YPAAGFVHE  target    ARLKGTRIIVIACEYSSTSSKADDAIVVRPGTTPALALGLSHVIMRDKLYDADYVRRWTDLPMLVRTDTLKYLSAEDVFG 6rxj.1    AKLHGAHTVELNLEPSQVGNEFAEKY------------------------------------------------------  target    GGPAPL 6rxj.1    ------ ``` | | | | | | | | | | | | | | | | | | | | | | | | | | | | | | | | | | | | | | | | | | | | | | | | | |
|  | 1e5v.2.A | Dimethyl sulfoxide/trimethylamine N-oxide reductase  *OXIDIZED DMSO REDUCTASE EXPOSED TO HEPES BUFFER* | 0.04 |  | 18.52 | 0.13 | 296-350 | X-ray | 2.40 | monomer | 2 x PGD, 1 x 2MO | HHblits | 0.28 |
| ``` target    TARREITRRGFLGTAAGAGFAAFVVSATRAWGLEAIENPLARYPDREWERVYRDLWRYDSKFTFLCAPNDTHNCLLDAYV 1e5v.2    --------------------------------------------------------------------------------  target    RSGVMTRIGPTMRYGEARDLDGNRASARWDPRVCQKGLALTRRFYGDRRLRHCMVRAGFKRWVDEGFPRGEDGKPPKEYF 1e5v.2    --------------------------------------------------------------------------------  target    QRARDEWVRASHDEAAAVVAATLANIAATYSGEEGAQRLRDQGYEEETIEAMGGAGVQAMKFRGGMPLLGMTRVFGLYRM 1e5v.2    --------------------------------------------------------------------------------  target    ANSMALLDAKVRGVGPDEARGARGFDNYSWHTDLPPGHPMVTGQQTVDFDLNSVELAKNVVVWGMNWITTKMPDAHWLTE 1e5v.2    -------------------------------------------------------DVKMAYWVGGNPFVHHQ-DRNRMVK  target    ARLKGTRIIVIACEYSSTSSKADDAIVVRPGTTPALALGLSHVIMRDKLYDADYVRRWTDLPMLVRTDTLKYLSAEDVFG 1e5v.2    AWEKLETFVVHDFQWTPTARHADIVLPATT--------------------------------------------------  target    GGPAPL 1e5v.2    ------ ``` | | | | | | | | | | | | | | | | | | | | | | | | | | | | | | | | | | | | | | | | | | | | | | | | | |
|  | 4dmr.1.A | DMSO REDUCTASE  *REDUCED DMSO REDUCTASE FROM RHODOBACTER CAPSULATUS WITH BOUND DMSO SUBSTRATE* | 0.04 |  | 18.52 | 0.13 | 296-350 | X-ray | 1.90 | monomer | 2 x PGD, 1 x 4MO, 1 x O | HHblits | 0.28 |
| ``` target    TARREITRRGFLGTAAGAGFAAFVVSATRAWGLEAIENPLARYPDREWERVYRDLWRYDSKFTFLCAPNDTHNCLLDAYV 4dmr.1    --------------------------------------------------------------------------------  target    RSGVMTRIGPTMRYGEARDLDGNRASARWDPRVCQKGLALTRRFYGDRRLRHCMVRAGFKRWVDEGFPRGEDGKPPKEYF 4dmr.1    --------------------------------------------------------------------------------  target    QRARDEWVRASHDEAAAVVAATLANIAATYSGEEGAQRLRDQGYEEETIEAMGGAGVQAMKFRGGMPLLGMTRVFGLYRM 4dmr.1    --------------------------------------------------------------------------------  target    ANSMALLDAKVRGVGPDEARGARGFDNYSWHTDLPPGHPMVTGQQTVDFDLNSVELAKNVVVWGMNWITTKMPDAHWLTE 4dmr.1    -------------------------------------------------------DVKMAYWVGGNPFVHHQ-DRNRMVK  target    ARLKGTRIIVIACEYSSTSSKADDAIVVRPGTTPALALGLSHVIMRDKLYDADYVRRWTDLPMLVRTDTLKYLSAEDVFG 4dmr.1    AWEKLETFVVHDFQWTPTARHADIVLPATT--------------------------------------------------  target    GGPAPL 4dmr.1    ------ ``` | | | | | | | | | | | | | | | | | | | | | | | | | | | | | | | | | | | | | | | | | | | | | | | | | |
|  | 1e60.1.A | Dimethyl sulfoxide/trimethylamine N-oxide reductase  *OXIDIZED DMSO REDUCTASE EXPOSED TO HEPES - Structure II BUFFER* | 0.03 |  | 18.52 | 0.13 | 296-350 | X-ray | 2.00 | monomer | 2 x PGD, 1 x 2MO | HHblits | 0.28 |
| ``` target    TARREITRRGFLGTAAGAGFAAFVVSATRAWGLEAIENPLARYPDREWERVYRDLWRYDSKFTFLCAPNDTHNCLLDAYV 1e60.1    --------------------------------------------------------------------------------  target    RSGVMTRIGPTMRYGEARDLDGNRASARWDPRVCQKGLALTRRFYGDRRLRHCMVRAGFKRWVDEGFPRGEDGKPPKEYF 1e60.1    --------------------------------------------------------------------------------  target    QRARDEWVRASHDEAAAVVAATLANIAATYSGEEGAQRLRDQGYEEETIEAMGGAGVQAMKFRGGMPLLGMTRVFGLYRM 1e60.1    --------------------------------------------------------------------------------  target    ANSMALLDAKVRGVGPDEARGARGFDNYSWHTDLPPGHPMVTGQQTVDFDLNSVELAKNVVVWGMNWITTKMPDAHWLTE 1e60.1    -------------------------------------------------------DVKMAYWVGGNPFVHHQ-DRNRMVK  target    ARLKGTRIIVIACEYSSTSSKADDAIVVRPGTTPALALGLSHVIMRDKLYDADYVRRWTDLPMLVRTDTLKYLSAEDVFG 1e60.1    AWEKLETFVVHDFQWTPTARHADIVLPATT--------------------------------------------------  target    GGPAPL 1e60.1    ------ ``` | | | | | | | | | | | | | | | | | | | | | | | | | | | | | | | | | | | | | | | | | | | | | | | | | |
|  | 7b04.1.B | Nitrite oxidoreductase subunit A  *Structure of Nitrite oxidoreductase (Nxr) from the anammox bacterium Kuenenia stuttgartiensis.* | 0.03 |  | 16.36 | 0.14 | 296-350 | X-ray | 2.97 | hetero-1-1-1-mer | 4 x SF4, 1 x F3S, 2 x MD1, 1 x MO, 1 x HEM, 2 x CA | HHblits | 0.26 |
| ``` target    TARREITRRGFLGTAAGAGFAAFVVSATRAWGLEAIENPLARYPDREWERVYRDLWRYDSKFTFLCAPNDTHNCLLDAYV 7b04.1    --------------------------------------------------------------------------------  target    RSGVMTRIGPTMRYGEARDLDGNRASARWDPRVCQKGLALTRRFYGDRRLRHCMVRAGFKRWVDEGFPRGEDGKPPKEYF 7b04.1    --------------------------------------------------------------------------------  target    QRARDEWVRASHDEAAAVVAATLANIAATYSGEEGAQRLRDQGYEEETIEAMGGAGVQAMKFRGGMPLLGMTRVFGLYRM 7b04.1    --------------------------------------------------------------------------------  target    ANSMALLDAKVRGVGPDEARGARGFDNYSWHTDLPPGHPMVTGQQTVDFDLNSVELAKNVVVWGMNWITTKMPDAHWLTE 7b04.1    -------------------------------------------------------PTKVLWFTNVNLINNAKHVYQMLKN  target    ARLKGTRIIVIACEYSSTSSKADDAIVVRPGTTPALALGLSHVIMRDKLYDADYVRRWTDLPMLVRTDTLKYLSAEDVFG 7b04.1    VNPNIEQIMSTDIEITGSIEYADFAFPANS--------------------------------------------------  target    GGPAPL 7b04.1    ------ ``` | | | | | | | | | | | | | | | | | | | | | | | | | | | | | | | | | | | | | | | | | | | | | | | | | |
|  | 7b04.2.B | Nitrite oxidoreductase subunit A  *Structure of Nitrite oxidoreductase (Nxr) from the anammox bacterium Kuenenia stuttgartiensis.* | 0.03 |  | 16.36 | 0.14 | 296-350 | X-ray | 2.97 | hetero-1-1-1-mer | 4 x SF4, 1 x F3S, 2 x MD1, 1 x MO, 1 x HEM, 2 x CA | HHblits | 0.26 |
| ``` target    TARREITRRGFLGTAAGAGFAAFVVSATRAWGLEAIENPLARYPDREWERVYRDLWRYDSKFTFLCAPNDTHNCLLDAYV 7b04.2    --------------------------------------------------------------------------------  target    RSGVMTRIGPTMRYGEARDLDGNRASARWDPRVCQKGLALTRRFYGDRRLRHCMVRAGFKRWVDEGFPRGEDGKPPKEYF 7b04.2    --------------------------------------------------------------------------------  target    QRARDEWVRASHDEAAAVVAATLANIAATYSGEEGAQRLRDQGYEEETIEAMGGAGVQAMKFRGGMPLLGMTRVFGLYRM 7b04.2    --------------------------------------------------------------------------------  target    ANSMALLDAKVRGVGPDEARGARGFDNYSWHTDLPPGHPMVTGQQTVDFDLNSVELAKNVVVWGMNWITTKMPDAHWLTE 7b04.2    -------------------------------------------------------PTKVLWFTNVNLINNAKHVYQMLKN  target    ARLKGTRIIVIACEYSSTSSKADDAIVVRPGTTPALALGLSHVIMRDKLYDADYVRRWTDLPMLVRTDTLKYLSAEDVFG 7b04.2    VNPNIEQIMSTDIEITGSIEYADFAFPANS--------------------------------------------------  target    GGPAPL 7b04.2    ------ ``` | | | | | | | | | | | | | | | | | | | | | | | | | | | | | | | | | | | | | | | | | | | | | | | | | |
|  | 4aay.1.A | AROA  *Crystal Structure of the arsenite oxidase protein complex from Rhizobium species strain NT-26* | 0.02 |  | 8.93 | 0.14 | 296-351 | X-ray | 2.70 | hetero-oligomer | 4 x MGD, 2 x O, 2 x 4MO, 2 x F3S, 2 x FES | HHblits | 0.25 |
| ``` target    TARREITRRGFLGTAAGAGFAAFVVSATRAWGLEAIENPLARYPDREWERVYRDLWRYDSKFTFLCAPNDTHNCLLDAYV 4aay.1    --------------------------------------------------------------------------------  target    RSGVMTRIGPTMRYGEARDLDGNRASARWDPRVCQKGLALTRRFYGDRRLRHCMVRAGFKRWVDEGFPRGEDGKPPKEYF 4aay.1    --------------------------------------------------------------------------------  target    QRARDEWVRASHDEAAAVVAATLANIAATYSGEEGAQRLRDQGYEEETIEAMGGAGVQAMKFRGGMPLLGMTRVFGLYRM 4aay.1    --------------------------------------------------------------------------------  target    ANSMALLDAKVRGVGPDEARGARGFDNYSWHTDLPPGHPMVTGQQTVDFDLNSVELAKNVVVWGMNWITTK-----MPD- 4aay.1    -------------------------------------------------------EFKRVYKKRTDMVKDAMSAAPYGDR  target    ---AHWLTEARLKG-TRIIVIACEYSSTSSKADDAIVVRPGTTPALALGLSHVIMRDKLYDADYVRRWTDLPMLVRTDTL 4aay.1    EAMVNAIVDAINQGGLFAVNVDIIPTKIGEACHVILPAATS---------------------------------------  target    KYLSAEDVFGGGPAPL 4aay.1    ---------------- ``` | | | | | | | | | | | | | | | | | | | | | | | | | | | | | | | | | | | | | | | | | | | | | | | | | |
|  | 1tmo.1.A | TRIMETHYLAMINE N-OXIDE REDUCTASE  *TRIMETHYLAMINE N-OXIDE REDUCTASE FROM SHEWANELLA MASSILIA* | 0.03 |  | 14.81 | 0.13 | 296-350 | X-ray | 2.50 | monomer | 2 x 2MD, 1 x 2MO | HHblits | 0.28 |
| ``` target    TARREITRRGFLGTAAGAGFAAFVVSATRAWGLEAIENPLARYPDREWERVYRDLWRYDSKFTFLCAPNDTHNCLLDAYV 1tmo.1    --------------------------------------------------------------------------------  target    RSGVMTRIGPTMRYGEARDLDGNRASARWDPRVCQKGLALTRRFYGDRRLRHCMVRAGFKRWVDEGFPRGEDGKPPKEYF 1tmo.1    --------------------------------------------------------------------------------  target    QRARDEWVRASHDEAAAVVAATLANIAATYSGEEGAQRLRDQGYEEETIEAMGGAGVQAMKFRGGMPLLGMTRVFGLYRM 1tmo.1    --------------------------------------------------------------------------------  target    ANSMALLDAKVRGVGPDEARGARGFDNYSWHTDLPPGHPMVTGQQTVDFDLNSVELAKNVVVWGMNWITTKMPDAHWLTE 1tmo.1    -------------------------------------------------------DIKMMIFSGNNPWNHH-QDRNRMKQ  target    ARLKGTRIIVIACEYSSTSSKADDAIVVRPGTTPALALGLSHVIMRDKLYDADYVRRWTDLPMLVRTDTLKYLSAEDVFG 1tmo.1    AFHKLECVVTVDVNWTATCRFSDIVLPACT--------------------------------------------------  target    GGPAPL 1tmo.1    ------ ``` | | | | | | | | | | | | | | | | | | | | | | | | | | | | | | | | | | | | | | | | | | | | | | | | | |
|  | 2zj4.1.A | Glucosamine--fructose-6-phosphate aminotransferase [isomerizing] 1  *Isomerase domain of human glucose:fructose-6-phosphate amidotransferase* | 0.04 |  | 16.67 | 0.13 | 298-353 | X-ray | 2.20 | homo-dimer | 2 x AGP | HHblits | 0.28 |
| ``` target    TARREITRRGFLGTAAGAGFAAFVVSATRAWGLEAIENPLARYPDREWERVYRDLWRYDSKFTFLCAPNDTHNCLLDAYV 2zj4.1    --------------------------------------------------------------------------------  target    RSGVMTRIGPTMRYGEARDLDGNRASARWDPRVCQKGLALTRRFYGDRRLRHCMVRAGFKRWVDEGFPRGEDGKPPKEYF 2zj4.1    --------------------------------------------------------------------------------  target    QRARDEWVRASHDEAAAVVAATLANIAATYSGEEGAQRLRDQGYEEETIEAMGGAGVQAMKFRGGMPLLGMTRVFGLYRM 2zj4.1    --------------------------------------------------------------------------------  target    ANSMALLDAKVRGVGPDEARGARGFDNYSWHTDLPPGHPMVTGQQTVDFDLNSVELAKNVVVWGMNWITTKMPDAHWLTE 2zj4.1    ---------------------------------------------------------DVCFFLSQS-GET-ADTLMGLRY  target    ARLKGTRIIVIA-CEYSSTSSKADDAIVVRPGTTPALALGLSHVIMRDKLYDADYVRRWTDLPMLVRTDTLKYLSAEDVF 2zj4.1    CKERGALTVGITNTVGSSISRETDCGVHINAGPE----------------------------------------------  target    GGGPAPL 2zj4.1    ------- ``` | | | | | | | | | | | | | | | | | | | | | | | | | | | | | | | | | | | | | | | | | | | | | | | | | |
|  | 5lu7.1.A | Phosphoheptose isomerase  *Heptose isomerase GmhA mutant - D61A* | 0.03 |  | 12.73 | 0.14 | 297-353 | X-ray | 1.92 | homo-tetramer | 4 x ZN, 4 x M7P | HHblits | 0.26 |
| ``` target    TARREITRRGFLGTAAGAGFAAFVVSATRAWGLEAIENPLARYPDREWERVYRDLWRYDSKFTFLCAPNDTHNCLLDAYV 5lu7.1    --------------------------------------------------------------------------------  target    RSGVMTRIGPTMRYGEARDLDGNRASARWDPRVCQKGLALTRRFYGDRRLRHCMVRAGFKRWVDEGFPRGEDGKPPKEYF 5lu7.1    --------------------------------------------------------------------------------  target    QRARDEWVRASHDEAAAVVAATLANIAATYSGEEGAQRLRDQGYEEETIEAMGGAGVQAMKFRGGMPLLGMTRVFGLYRM 5lu7.1    --------------------------------------------------------------------------------  target    ANSMALLDAKVRGVGPDEARGARGFDNYSWHTDLPPGHPMVTGQQTVDFDLNSVELAKNVVVWGMNWITTKMPDAHWLTE 5lu7.1    --------------------------------------------------------GDVLIGYSTS-GKS-PNILAAFRE  target    ARLKGTRIIVIAC-EYSSTSSKADDAIVVRPGTTPALALGLSHVIMRDKLYDADYVRRWTDLPMLVRTDTLKYLSAEDVF 5lu7.1    AKAKGMTCVGFTGNRGGEMRELCDLLLEVPSADT----------------------------------------------  target    GGGPAPL 5lu7.1    ------- ``` | | | | | | | | | | | | | | | | | | | | | | | | | | | | | | | | | | | | | | | | | | | | | | | | | |
|  | 2xbl.1.A | PHOSPHOHEPTOSE ISOMERASE  *Crystal structure of GmhA from Burkholderia pseudomallei in complex with product* | 0.04 |  | 12.73 | 0.14 | 297-353 | X-ray | 1.62 | homo-tetramer | 4 x ZN, 4 x M7P | HHblits | 0.26 |
| ``` target    TARREITRRGFLGTAAGAGFAAFVVSATRAWGLEAIENPLARYPDREWERVYRDLWRYDSKFTFLCAPNDTHNCLLDAYV 2xbl.1    --------------------------------------------------------------------------------  target    RSGVMTRIGPTMRYGEARDLDGNRASARWDPRVCQKGLALTRRFYGDRRLRHCMVRAGFKRWVDEGFPRGEDGKPPKEYF 2xbl.1    --------------------------------------------------------------------------------  target    QRARDEWVRASHDEAAAVVAATLANIAATYSGEEGAQRLRDQGYEEETIEAMGGAGVQAMKFRGGMPLLGMTRVFGLYRM 2xbl.1    --------------------------------------------------------------------------------  target    ANSMALLDAKVRGVGPDEARGARGFDNYSWHTDLPPGHPMVTGQQTVDFDLNSVELAKNVVVWGMNWITTKMPDAHWLTE 2xbl.1    --------------------------------------------------------GDVLIGYSTS-GKS-PNILAAFRE  target    ARLKGTRIIVIAC-EYSSTSSKADDAIVVRPGTTPALALGLSHVIMRDKLYDADYVRRWTDLPMLVRTDTLKYLSAEDVF 2xbl.1    AKAKGMTCVGFTGNRGGEMRELCDLLLEVPSADT----------------------------------------------  target    GGGPAPL 2xbl.1    ------- ``` | | | | | | | | | | | | | | | | | | | | | | | | | | | | | | | | | | | | | | | | | | | | | | | | | |
|  | 6sdr.1.A | Formate dehydrogenase, alpha subunit, selenocysteine-containing  *W-formate dehydrogenase from Desulfovibrio vulgaris - Oxidized form* | 0.03 |  | 22.64 | 0.13 | 296-349 | X-ray | 2.10 | hetero-1-1-mer | 2 x MGD, 4 x SF4, 1 x H2S, 1 x W | HHblits | 0.29 |
| ``` target    TARREITRRGFLGTAAGAGFAAFVVSATRAWGLEAIENPLARYPDREWERVYRDLWRYDSKFTFLCAPNDTHNCLLDAYV 6sdr.1    --------------------------------------------------------------------------------  target    RSGVMTRIGPTMRYGEARDLDGNRASARWDPRVCQKGLALTRRFYGDRRLRHCMVRAGFKRWVDEGFPRGEDGKPPKEYF 6sdr.1    --------------------------------------------------------------------------------  target    QRARDEWVRASHDEAAAVVAATLANIAATYSGEEGAQRLRDQGYEEETIEAMGGAGVQAMKFRGGMPLLGMTRVFGLYRM 6sdr.1    --------------------------------------------------------------------------------  target    ANSMALLDAKVRGVGPDEARGARGFDNYSWHTDLPPGHPMVTGQQTVDFDLNSVELAKNVVVWGMNWITTKMPDAHWLTE 6sdr.1    -------------------------------------------------------EFKGLFAWGMNPACGGA-NANKNRK  target    ARLKGTRIIVIACEYSSTSSK--------AD-----DAIVVRPGTTPALALGLSHVIMRDKLYDADYVRRWTDLPMLVRT 6sdr.1    AMGKLEWLVNVNLFENETSSFWKGPGMNPAEIGTEVFFLPCC--------------------------------------  target    DTLKYLSAEDVFGGGPAPL 6sdr.1    ------------------- ``` | | | | | | | | | | | | | | | | | | | | | | | | | | | | | | | | | | | | | | | | | | | | | | | | | |
|  | 3etn.1.A | putative phosphosugar isomerase involved in capsule formation  *Crystal structure of putative phosphosugar isomerase involved in capsule formation (YP\_209877.1) from Bacteroides fragilis NCTC 9343 at 1.70 A resolution* | 0.03 |  | 16.36 | 0.14 | 297-353 | X-ray | 1.70 | homo-tetramer | 4 x CMK | HHblits | 0.26 |
| ``` target    TARREITRRGFLGTAAGAGFAAFVVSATRAWGLEAIENPLARYPDREWERVYRDLWRYDSKFTFLCAPNDTHNCLLDAYV 3etn.1    --------------------------------------------------------------------------------  target    RSGVMTRIGPTMRYGEARDLDGNRASARWDPRVCQKGLALTRRFYGDRRLRHCMVRAGFKRWVDEGFPRGEDGKPPKEYF 3etn.1    --------------------------------------------------------------------------------  target    QRARDEWVRASHDEAAAVVAATLANIAATYSGEEGAQRLRDQGYEEETIEAMGGAGVQAMKFRGGMPLLGMTRVFGLYRM 3etn.1    --------------------------------------------------------------------------------  target    ANSMALLDAKVRGVGPDEARGARGFDNYSWHTDLPPGHPMVTGQQTVDFDLNSVELAKNVVVWGMNWITTKMPDAHWLTE 3etn.1    --------------------------------------------------------NDLLLLISN-SGKT-REIVELTQL  target    ARL--KGTRIIVIAC-EYSSTSSKADDAIVVRPGTTPALALGLSHVIMRDKLYDADYVRRWTDLPMLVRTDTLKYLSAED 3etn.1    AHNLNPGLKFIVITGNPDSPLASESDVCLSTGHPAE--------------------------------------------  target    VFGGGPAPL 3etn.1    --------- ``` | | | | | | | | | | | | | | | | | | | | | | | | | | | | | | | | | | | | | | | | | | | | | | | | | |
|  | 2v3v.1.A | PERIPLASMIC NITRATE REDUCTASE  *A NEW CATALYTIC MECHANISM OF PERIPLASMIC NITRATE REDUCTASE FROM DESULFOVIBRIO DESULFURICANS ATCC 27774 FROM CRYSTALLOGRAPHIC AND EPR DATA AND BASED ON DETAILED ANALYSIS OF THE SIXTH LIGAND* | 0.03 |  | 16.36 | 0.14 | 296-351 | X-ray | 1.99 | monomer | 1 x SF4, 1 x MO, 2 x MGD, 4 x LCP | HHblits | 0.26 |
| ``` target    TARREITRRGFLGTAAGAGFAAFVVSATRAWGLEAIENPLARYPDREWERVYRDLWRYDSKFTFLCAPNDTHNCLLDAYV 2v3v.1    --------------------------------------------------------------------------------  target    RSGVMTRIGPTMRYGEARDLDGNRASARWDPRVCQKGLALTRRFYGDRRLRHCMVRAGFKRWVDEGFPRGEDGKPPKEYF 2v3v.1    --------------------------------------------------------------------------------  target    QRARDEWVRASHDEAAAVVAATLANIAATYSGEEGAQRLRDQGYEEETIEAMGGAGVQAMKFRGGMPLLGMTRVFGLYRM 2v3v.1    --------------------------------------------------------------------------------  target    ANSMALLDAKVRGVGPDEARGARGFDNYSWHTDLPPGHPMVTGQQTVDFDLNSVELAKNVVVWGMNWITTKMPDAHWLTE 2v3v.1    -------------------------------------------------------DVKCMIICETNPAHTLP-NLNKVHK  target    ARLK-GTRIIVIACEYS-STSSKADDAIVVRPGTTPALALGLSHVIMRDKLYDADYVRRWTDLPMLVRTDTLKYLSAEDV 2v3v.1    AMSHPESFIVCIEAFPDAVTLEYADLVLPPAFW-----------------------------------------------  target    FGGGPAPL 2v3v.1    -------- ``` | | | | | | | | | | | | | | | | | | | | | | | | | | | | | | | | | | | | | | | | | | | | | | | | | |
|  | 1x94.1.A | putative Phosphoheptose isomerase  *Crystal Structure of a Hypothetical protein* | 0.03 |  | 14.55 | 0.14 | 297-353 | X-ray | 2.50 | homo-dimer |  | HHblits | 0.26 |
| ``` target    TARREITRRGFLGTAAGAGFAAFVVSATRAWGLEAIENPLARYPDREWERVYRDLWRYDSKFTFLCAPNDTHNCLLDAYV 1x94.1    --------------------------------------------------------------------------------  target    RSGVMTRIGPTMRYGEARDLDGNRASARWDPRVCQKGLALTRRFYGDRRLRHCMVRAGFKRWVDEGFPRGEDGKPPKEYF 1x94.1    --------------------------------------------------------------------------------  target    QRARDEWVRASHDEAAAVVAATLANIAATYSGEEGAQRLRDQGYEEETIEAMGGAGVQAMKFRGGMPLLGMTRVFGLYRM 1x94.1    --------------------------------------------------------------------------------  target    ANSMALLDAKVRGVGPDEARGARGFDNYSWHTDLPPGHPMVTGQQTVDFDLNSVELAKNVVVWGMNWITTKMPDAHWLTE 1x94.1    --------------------------------------------------------GDVLFGLSTSG-NS-GNILKAIEA  target    ARLKGTRIIVIAC-EYSSTSSKADDAIVVRPGTTPALALGLSHVIMRDKLYDADYVRRWTDLPMLVRTDTLKYLSAEDVF 1x94.1    AKAKGMKTIALTGKDGGKMAGLADVEIRVPHFGY----------------------------------------------  target    GGGPAPL 1x94.1    ------- ``` | | | | | | | | | | | | | | | | | | | | | | | | | | | | | | | | | | | | | | | | | | | | | | | | | |
|  | 1x94.1.B | putative Phosphoheptose isomerase  *Crystal Structure of a Hypothetical protein* | 0.03 |  | 14.55 | 0.14 | 297-353 | X-ray | 2.50 | homo-dimer |  | HHblits | 0.26 |
| ``` target    TARREITRRGFLGTAAGAGFAAFVVSATRAWGLEAIENPLARYPDREWERVYRDLWRYDSKFTFLCAPNDTHNCLLDAYV 1x94.1    --------------------------------------------------------------------------------  target    RSGVMTRIGPTMRYGEARDLDGNRASARWDPRVCQKGLALTRRFYGDRRLRHCMVRAGFKRWVDEGFPRGEDGKPPKEYF 1x94.1    --------------------------------------------------------------------------------  target    QRARDEWVRASHDEAAAVVAATLANIAATYSGEEGAQRLRDQGYEEETIEAMGGAGVQAMKFRGGMPLLGMTRVFGLYRM 1x94.1    --------------------------------------------------------------------------------  target    ANSMALLDAKVRGVGPDEARGARGFDNYSWHTDLPPGHPMVTGQQTVDFDLNSVELAKNVVVWGMNWITTKMPDAHWLTE 1x94.1    --------------------------------------------------------GDVLFGLSTSG-NS-GNILKAIEA  target    ARLKGTRIIVIAC-EYSSTSSKADDAIVVRPGTTPALALGLSHVIMRDKLYDADYVRRWTDLPMLVRTDTLKYLSAEDVF 1x94.1    AKAKGMKTIALTGKDGGKMAGLADVEIRVPHFGY----------------------------------------------  target    GGGPAPL 1x94.1    ------- ``` | | | | | | | | | | | | | | | | | | | | | | | | | | | | | | | | | | | | | | | | | | | | | | | | | |
|  | 7q5y.1.A | NADH dehydrogenase I chain G  *Structure of NADH:ubichinon oxidoreductase (complex I) of the hyperthermophilic eubacterium Aquifex aeolicus* | 0.03 |  | 16.98 | 0.13 | 296-350 | X-ray | 2.70 | hetero-1-1-1-1-1-1-… | 8 x SF4, 2 x FES, 1 x FMN | HHblits | 0.29 |
| ``` target    TARREITRRGFLGTAAGAGFAAFVVSATRAWGLEAIENPLARYPDREWERVYRDLWRYDSKFTFLCAPNDTHNCLLDAYV 7q5y.1    --------------------------------------------------------------------------------  target    RSGVMTRIGPTMRYGEARDLDGNRASARWDPRVCQKGLALTRRFYGDRRLRHCMVRAGFKRWVDEGFPRGEDGKPPKEYF 7q5y.1    --------------------------------------------------------------------------------  target    QRARDEWVRASHDEAAAVVAATLANIAATYSGEEGAQRLRDQGYEEETIEAMGGAGVQAMKFRGGMPLLGMTRVFGLYRM 7q5y.1    --------------------------------------------------------------------------------  target    ANSMALLDAKVRGVGPDEARGARGFDNYSWHTDLPPGHPMVTGQQTVDFDLNSVELAKNVVVWGMNWITTKMPDAHWLTE 7q5y.1    -------------------------------------------------------DIENLIIFGEDILEFYE-D-KVFEE  target    ARLKGTRIIVIACEYSSTSSKADDAIVVRPGTTPALALGLSHVIMRDKLYDADYVRRWTDLPMLVRTDTLKYLSAEDVFG 7q5y.1    LKEKLEHLVVVSPYEDGLSEYAHIKIPMSL--------------------------------------------------  target    GGPAPL 7q5y.1    ------ ``` | | | | | | | | | | | | | | | | | | | | | | | | | | | | | | | | | | | | | | | | | | | | | | | | | |
|  | 1aa6.1.A | FORMATE DEHYDROGENASE H  *REDUCED FORM OF FORMATE DEHYDROGENASE H FROM E. COLI* | 0.04 |  | 16.67 | 0.13 | 296-350 | X-ray | 2.30 | monomer | 1 x SF4, 2 x MGD, 1 x 4MO | HHblits | 0.27 |
| ``` target    TARREITRRGFLGTAAGAGFAAFVVSATRAWGLEAIENPLARYPDREWERVYRDLWRYDSKFTFLCAPNDTHNCLLDAYV 1aa6.1    --------------------------------------------------------------------------------  target    RSGVMTRIGPTMRYGEARDLDGNRASARWDPRVCQKGLALTRRFYGDRRLRHCMVRAGFKRWVDEGFPRGEDGKPPKEYF 1aa6.1    --------------------------------------------------------------------------------  target    QRARDEWVRASHDEAAAVVAATLANIAATYSGEEGAQRLRDQGYEEETIEAMGGAGVQAMKFRGGMPLLGMTRVFGLYRM 1aa6.1    --------------------------------------------------------------------------------  target    ANSMALLDAKVRGVGPDEARGARGFDNYSWHTDLPPGHPMVTGQQTVDFDLNSVELAKNVVVWGMNWITTKMPDAHWLTE 1aa6.1    -------------------------------------------------------EVRAAYIMGEDPLQTDA-ELSAVRK  target    ARLKGTRIIVIACEYSSTSSKADDAIVVRPGTTPALALGLSHVIMRDKLYDADYVRRWTDLPMLVRTDTLKYLSAEDVFG 1aa6.1    AFEDLELVIVQDIFMTKTASAADVILPSTS--------------------------------------------------  target    GGPAPL 1aa6.1    ------ ``` | | | | | | | | | | | | | | | | | | | | | | | | | | | | | | | | | | | | | | | | | | | | | | | | | |
|  | 1fdo.1.A | FORMATE DEHYDROGENASE H  *OXIDIZED FORM OF FORMATE DEHYDROGENASE H FROM E. COLI* | 0.04 |  | 16.67 | 0.13 | 296-350 | X-ray | 2.80 | monomer | 1 x SF4, 2 x MGD, 1 x 6MO | HHblits | 0.27 |
| ``` target    TARREITRRGFLGTAAGAGFAAFVVSATRAWGLEAIENPLARYPDREWERVYRDLWRYDSKFTFLCAPNDTHNCLLDAYV 1fdo.1    --------------------------------------------------------------------------------  target    RSGVMTRIGPTMRYGEARDLDGNRASARWDPRVCQKGLALTRRFYGDRRLRHCMVRAGFKRWVDEGFPRGEDGKPPKEYF 1fdo.1    --------------------------------------------------------------------------------  target    QRARDEWVRASHDEAAAVVAATLANIAATYSGEEGAQRLRDQGYEEETIEAMGGAGVQAMKFRGGMPLLGMTRVFGLYRM 1fdo.1    --------------------------------------------------------------------------------  target    ANSMALLDAKVRGVGPDEARGARGFDNYSWHTDLPPGHPMVTGQQTVDFDLNSVELAKNVVVWGMNWITTKMPDAHWLTE 1fdo.1    -------------------------------------------------------EVRAAYIMGEDPLQTDA-ELSAVRK  target    ARLKGTRIIVIACEYSSTSSKADDAIVVRPGTTPALALGLSHVIMRDKLYDADYVRRWTDLPMLVRTDTLKYLSAEDVFG 1fdo.1    AFEDLELVIVQDIFMTKTASAADVILPSTS--------------------------------------------------  target    GGPAPL 1fdo.1    ------ ``` | | | | | | | | | | | | | | | | | | | | | | | | | | | | | | | | | | | | | | | | | | | | | | | | | |
|  | 2iv2.1.A | Formate dehydrogenase H  *Reinterpretation of reduced form of formate dehydrogenase H from E. coli* | 0.04 |  | 16.67 | 0.13 | 296-350 | X-ray | 2.27 | monomer | 1 x SF4, 1 x 2MD, 1 x MGD | HHblits | 0.27 |
| ``` target    TARREITRRGFLGTAAGAGFAAFVVSATRAWGLEAIENPLARYPDREWERVYRDLWRYDSKFTFLCAPNDTHNCLLDAYV 2iv2.1    --------------------------------------------------------------------------------  target    RSGVMTRIGPTMRYGEARDLDGNRASARWDPRVCQKGLALTRRFYGDRRLRHCMVRAGFKRWVDEGFPRGEDGKPPKEYF 2iv2.1    --------------------------------------------------------------------------------  target    QRARDEWVRASHDEAAAVVAATLANIAATYSGEEGAQRLRDQGYEEETIEAMGGAGVQAMKFRGGMPLLGMTRVFGLYRM 2iv2.1    --------------------------------------------------------------------------------  target    ANSMALLDAKVRGVGPDEARGARGFDNYSWHTDLPPGHPMVTGQQTVDFDLNSVELAKNVVVWGMNWITTKMPDAHWLTE 2iv2.1    -------------------------------------------------------EVRAAYIMGEDPLQTDA-ELSAVRK  target    ARLKGTRIIVIACEYSSTSSKADDAIVVRPGTTPALALGLSHVIMRDKLYDADYVRRWTDLPMLVRTDTLKYLSAEDVFG 2iv2.1    AFEDLELVIVQDIFMTKTASAADVILPSTS--------------------------------------------------  target    GGPAPL 2iv2.1    ------ ``` | | | | | | | | | | | | | | | | | | | | | | | | | | | | | | | | | | | | | | | | | | | | | | | | | |
|  | 7z0t.1.G | Formate dehydrogenase H  *Structure of the Escherichia coli formate hydrogenlyase complex (aerobic preparation, composite structure)* | 0.03 |  | 16.67 | 0.13 | 296-350 | EM | 0.00 | hetero-1-1-1-1-1-1-… | 1 x NI, 1 x FCO, 8 x SF4, 1 x FE, 2 x MGD, 1 x 6MO | HHblits | 0.27 |
| ``` target    TARREITRRGFLGTAAGAGFAAFVVSATRAWGLEAIENPLARYPDREWERVYRDLWRYDSKFTFLCAPNDTHNCLLDAYV 7z0t.1    --------------------------------------------------------------------------------  target    RSGVMTRIGPTMRYGEARDLDGNRASARWDPRVCQKGLALTRRFYGDRRLRHCMVRAGFKRWVDEGFPRGEDGKPPKEYF 7z0t.1    --------------------------------------------------------------------------------  target    QRARDEWVRASHDEAAAVVAATLANIAATYSGEEGAQRLRDQGYEEETIEAMGGAGVQAMKFRGGMPLLGMTRVFGLYRM 7z0t.1    --------------------------------------------------------------------------------  target    ANSMALLDAKVRGVGPDEARGARGFDNYSWHTDLPPGHPMVTGQQTVDFDLNSVELAKNVVVWGMNWITTKMPDAHWLTE 7z0t.1    -------------------------------------------------------EVRAAYIMGEDPLQTDA-ELSAVRK  target    ARLKGTRIIVIACEYSSTSSKADDAIVVRPGTTPALALGLSHVIMRDKLYDADYVRRWTDLPMLVRTDTLKYLSAEDVFG 7z0t.1    AFEDLELVIVQDIFMTKTASAADVILPSTS--------------------------------------------------  target    GGPAPL 7z0t.1    ------ ``` | | | | | | | | | | | | | | | | | | | | | | | | | | | | | | | | | | | | | | | | | | | | | | | | | |
|  | 5xhs.1.A | NAD-dependent protein deacylase sirtuin-5, mitochondrial  *Crystal structure of SIRT5 complexed with a fluorogenic small-molecule substrate SuBKA* | 0.03 |  | 11.11 | 0.13 | 292-347 | X-ray | 2.19 | monomer | 1 x PHQ, 1 x SLL, 1 x MCM, 1 x ZN | HHblits | 0.27 |
| ``` target    TARREITRRGFLGTAAGAGFAAFVVSATRAWGLEAIENPLARYPDREWERVYRDLWRYDSKFTFLCAPNDTHNCLLDAYV 5xhs.1    --------------------------------------------------------------------------------  target    RSGVMTRIGPTMRYGEARDLDGNRASARWDPRVCQKGLALTRRFYGDRRLRHCMVRAGFKRWVDEGFPRGEDGKPPKEYF 5xhs.1    --------------------------------------------------------------------------------  target    QRARDEWVRASHDEAAAVVAATLANIAATYSGEEGAQRLRDQGYEEETIEAMGGAGVQAMKFRGGMPLLGMTRVFGLYRM 5xhs.1    --------------------------------------------------------------------------------  target    ANSMALLDAKVRGVGPDEARGARGFDNYSWHTDLPPGHPMVTGQQTVDFDLNSVELAKNVVVWGMNWITTKMPDAHWLTE 5xhs.1    ---------------------------------------------------RELAHCDLCLVVGTSSVV--YPAAMFAPQ  target    ARLKGTRIIVIACEYSSTSSKADDAIVVRPGTTPALALGLSHVIMRDKLYDADYVRRWTDLPMLVRTDTLKYLSAEDVFG 5xhs.1    VAARGVPVAEFNTETTPATNRFRFHFQ-----------------------------------------------------  target    GGPAPL 5xhs.1    ------ ``` | | | | | | | | | | | | | | | | | | | | | | | | | | | | | | | | | | | | | | | | | | | | | | | | | |
|  | 6ljm.1.A | NAD-dependent protein deacylase sirtuin-5, mitochondrial  *Crystal structure of human Sirt5 in complex with the fluorogenic tetrapeptide substrate P13* | 0.03 |  | 11.11 | 0.13 | 292-347 | X-ray | 1.78 | monomer | 1 x ZN, 1 x SIN, 1 x MCM, 1 x SER-LEU-GLY-LYS | HHblits | 0.27 |
| ``` target    TARREITRRGFLGTAAGAGFAAFVVSATRAWGLEAIENPLARYPDREWERVYRDLWRYDSKFTFLCAPNDTHNCLLDAYV 6ljm.1    --------------------------------------------------------------------------------  target    RSGVMTRIGPTMRYGEARDLDGNRASARWDPRVCQKGLALTRRFYGDRRLRHCMVRAGFKRWVDEGFPRGEDGKPPKEYF 6ljm.1    --------------------------------------------------------------------------------  target    QRARDEWVRASHDEAAAVVAATLANIAATYSGEEGAQRLRDQGYEEETIEAMGGAGVQAMKFRGGMPLLGMTRVFGLYRM 6ljm.1    --------------------------------------------------------------------------------  target    ANSMALLDAKVRGVGPDEARGARGFDNYSWHTDLPPGHPMVTGQQTVDFDLNSVELAKNVVVWGMNWITTKMPDAHWLTE 6ljm.1    ---------------------------------------------------RELAHCDLCLVVGTSSVV--YPAAMFAPQ  target    ARLKGTRIIVIACEYSSTSSKADDAIVVRPGTTPALALGLSHVIMRDKLYDADYVRRWTDLPMLVRTDTLKYLSAEDVFG 6ljm.1    VAARGVPVAEFNTETTPATNRFRFHFQ-----------------------------------------------------  target    GGPAPL 6ljm.1    ------ ``` | | | | | | | | | | | | | | | | | | | | | | | | | | | | | | | | | | | | | | | | | | | | | | | | | |
|  | 6ljk.1.A | NAD-dependent protein deacylase sirtuin-5, mitochondrial  *Crystal structure of human Sirt5 in complex with an internally quenched fluorescent substrate GluIQF* | 0.03 |  | 11.11 | 0.13 | 292-347 | X-ray | 1.39 | monomer | 1 x ZN, 1 x GUA, 1 x BE2-SER-ALA-ILE-LYS-SER-NIY-GLY-SET | HHblits | 0.27 |
| ``` target    TARREITRRGFLGTAAGAGFAAFVVSATRAWGLEAIENPLARYPDREWERVYRDLWRYDSKFTFLCAPNDTHNCLLDAYV 6ljk.1    --------------------------------------------------------------------------------  target    RSGVMTRIGPTMRYGEARDLDGNRASARWDPRVCQKGLALTRRFYGDRRLRHCMVRAGFKRWVDEGFPRGEDGKPPKEYF 6ljk.1    --------------------------------------------------------------------------------  target    QRARDEWVRASHDEAAAVVAATLANIAATYSGEEGAQRLRDQGYEEETIEAMGGAGVQAMKFRGGMPLLGMTRVFGLYRM 6ljk.1    --------------------------------------------------------------------------------  target    ANSMALLDAKVRGVGPDEARGARGFDNYSWHTDLPPGHPMVTGQQTVDFDLNSVELAKNVVVWGMNWITTKMPDAHWLTE 6ljk.1    ---------------------------------------------------RELAHCDLCLVVGTSSVV--YPAAMFAPQ  target    ARLKGTRIIVIACEYSSTSSKADDAIVVRPGTTPALALGLSHVIMRDKLYDADYVRRWTDLPMLVRTDTLKYLSAEDVFG 6ljk.1    VAARGVPVAEFNTETTPATNRFRFHFQ-----------------------------------------------------  target    GGPAPL 6ljk.1    ------ ``` | | | | | | | | | | | | | | | | | | | | | | | | | | | | | | | | | | | | | | | | | | | | | | | | | |
|  | 3riy.2.A | NAD-dependent deacetylase sirtuin-5  *Sirt5 is an NAD-dependent protein lysine demalonylase and desuccinylase* | 0.03 |  | 11.11 | 0.13 | 292-347 | X-ray | 1.55 | hetero-oligomer | 1 x ZN, 1 x NAD | HHblits | 0.27 |
| ``` target    TARREITRRGFLGTAAGAGFAAFVVSATRAWGLEAIENPLARYPDREWERVYRDLWRYDSKFTFLCAPNDTHNCLLDAYV 3riy.2    --------------------------------------------------------------------------------  target    RSGVMTRIGPTMRYGEARDLDGNRASARWDPRVCQKGLALTRRFYGDRRLRHCMVRAGFKRWVDEGFPRGEDGKPPKEYF 3riy.2    --------------------------------------------------------------------------------  target    QRARDEWVRASHDEAAAVVAATLANIAATYSGEEGAQRLRDQGYEEETIEAMGGAGVQAMKFRGGMPLLGMTRVFGLYRM 3riy.2    --------------------------------------------------------------------------------  target    ANSMALLDAKVRGVGPDEARGARGFDNYSWHTDLPPGHPMVTGQQTVDFDLNSVELAKNVVVWGMNWITTKMPDAHWLTE 3riy.2    ---------------------------------------------------RELAHCDLCLVVGTSSVV--YPAAMFAPQ  target    ARLKGTRIIVIACEYSSTSSKADDAIVVRPGTTPALALGLSHVIMRDKLYDADYVRRWTDLPMLVRTDTLKYLSAEDVFG 3riy.2    VAARGVPVAEFNTETTPATNRFRFHFQ-----------------------------------------------------  target    GGPAPL 3riy.2    ------ ``` | | | | | | | | | | | | | | | | | | | | | | | | | | | | | | | | | | | | | | | | | | | | | | | | | |
|  | 6acp.1.A | NAD-dependent protein deacylase sirtuin-5, mitochondrial  *histone lysine desuccinylase Sirt5 in complex with succinyl peptide H4K91* | 0.03 |  | 11.11 | 0.13 | 292-347 | X-ray | 2.30 | monomer | 1 x ZN, 1 x TYR-ALA-LEU-SLL-ARG-GLN-GLY | HHblits | 0.27 |
| ``` target    TARREITRRGFLGTAAGAGFAAFVVSATRAWGLEAIENPLARYPDREWERVYRDLWRYDSKFTFLCAPNDTHNCLLDAYV 6acp.1    --------------------------------------------------------------------------------  target    RSGVMTRIGPTMRYGEARDLDGNRASARWDPRVCQKGLALTRRFYGDRRLRHCMVRAGFKRWVDEGFPRGEDGKPPKEYF 6acp.1    --------------------------------------------------------------------------------  target    QRARDEWVRASHDEAAAVVAATLANIAATYSGEEGAQRLRDQGYEEETIEAMGGAGVQAMKFRGGMPLLGMTRVFGLYRM 6acp.1    --------------------------------------------------------------------------------  target    ANSMALLDAKVRGVGPDEARGARGFDNYSWHTDLPPGHPMVTGQQTVDFDLNSVELAKNVVVWGMNWITTKMPDAHWLTE 6acp.1    ---------------------------------------------------RELAHCDLCLVVGTSSVV--YPAAMFAPQ  target    ARLKGTRIIVIACEYSSTSSKADDAIVVRPGTTPALALGLSHVIMRDKLYDADYVRRWTDLPMLVRTDTLKYLSAEDVFG 6acp.1    VAARGVPVAEFNTETTPATNRFRFHFQ-----------------------------------------------------  target    GGPAPL 6acp.1    ------ ``` | | | | | | | | | | | | | | | | | | | | | | | | | | | | | | | | | | | | | | | | | | | | | | | | | |
|  | 4g1c.2.A | NAD-dependent protein deacylase sirtuin-5, mitochondrial  *Human SIRT5 bound to Succ-IDH2 and Carba-NAD* | 0.03 |  | 11.11 | 0.13 | 292-347 | X-ray | 1.94 | monomer | 1 x ZN, 1 x ACE-ALA-VAL-SLL-CYS-ALA-NH2 | HHblits | 0.27 |
| ``` target    TARREITRRGFLGTAAGAGFAAFVVSATRAWGLEAIENPLARYPDREWERVYRDLWRYDSKFTFLCAPNDTHNCLLDAYV 4g1c.2    --------------------------------------------------------------------------------  target    RSGVMTRIGPTMRYGEARDLDGNRASARWDPRVCQKGLALTRRFYGDRRLRHCMVRAGFKRWVDEGFPRGEDGKPPKEYF 4g1c.2    --------------------------------------------------------------------------------  target    QRARDEWVRASHDEAAAVVAATLANIAATYSGEEGAQRLRDQGYEEETIEAMGGAGVQAMKFRGGMPLLGMTRVFGLYRM 4g1c.2    --------------------------------------------------------------------------------  target    ANSMALLDAKVRGVGPDEARGARGFDNYSWHTDLPPGHPMVTGQQTVDFDLNSVELAKNVVVWGMNWITTKMPDAHWLTE 4g1c.2    ---------------------------------------------------RELAHCDLCLVVGTSSVV--YPAAMFAPQ  target    ARLKGTRIIVIACEYSSTSSKADDAIVVRPGTTPALALGLSHVIMRDKLYDADYVRRWTDLPMLVRTDTLKYLSAEDVFG 4g1c.2    VAARGVPVAEFNTETTPATNRFRFHFQ-----------------------------------------------------  target    GGPAPL 4g1c.2    ------ ``` | | | | | | | | | | | | | | | | | | | | | | | | | | | | | | | | | | | | | | | | | | | | | | | | | |
|  | 4g1c.1.A | NAD-dependent protein deacylase sirtuin-5, mitochondrial  *Human SIRT5 bound to Succ-IDH2 and Carba-NAD* | 0.03 |  | 11.11 | 0.13 | 292-347 | X-ray | 1.94 | monomer | 1 x ZN, 1 x CNA, 1 x ACE-ALA-VAL-SLL-CYS-ALA-NH2 | HHblits | 0.27 |
| ``` target    TARREITRRGFLGTAAGAGFAAFVVSATRAWGLEAIENPLARYPDREWERVYRDLWRYDSKFTFLCAPNDTHNCLLDAYV 4g1c.1    --------------------------------------------------------------------------------  target    RSGVMTRIGPTMRYGEARDLDGNRASARWDPRVCQKGLALTRRFYGDRRLRHCMVRAGFKRWVDEGFPRGEDGKPPKEYF 4g1c.1    --------------------------------------------------------------------------------  target    QRARDEWVRASHDEAAAVVAATLANIAATYSGEEGAQRLRDQGYEEETIEAMGGAGVQAMKFRGGMPLLGMTRVFGLYRM 4g1c.1    --------------------------------------------------------------------------------  target    ANSMALLDAKVRGVGPDEARGARGFDNYSWHTDLPPGHPMVTGQQTVDFDLNSVELAKNVVVWGMNWITTKMPDAHWLTE 4g1c.1    ---------------------------------------------------RELAHCDLCLVVGTSSVV--YPAAMFAPQ  target    ARLKGTRIIVIACEYSSTSSKADDAIVVRPGTTPALALGLSHVIMRDKLYDADYVRRWTDLPMLVRTDTLKYLSAEDVFG 4g1c.1    VAARGVPVAEFNTETTPATNRFRFHFQ-----------------------------------------------------  target    GGPAPL 4g1c.1    ------ ``` | | | | | | | | | | | | | | | | | | | | | | | | | | | | | | | | | | | | | | | | | | | | | | | | | |
|  | 5bwl.1.A | NAD-dependent protein deacylase sirtuin-5, mitochondrial  *Crystal Structure of SIRT5 in Complex with a Coumarin-Labelled Succinyl Peptide* | 0.03 |  | 11.11 | 0.13 | 292-347 | X-ray | 1.55 | monomer | 1 x ZN, 1 x MCM, 1 x LEU-GLY-SLL | HHblits | 0.27 |
| ``` target    TARREITRRGFLGTAAGAGFAAFVVSATRAWGLEAIENPLARYPDREWERVYRDLWRYDSKFTFLCAPNDTHNCLLDAYV 5bwl.1    --------------------------------------------------------------------------------  target    RSGVMTRIGPTMRYGEARDLDGNRASARWDPRVCQKGLALTRRFYGDRRLRHCMVRAGFKRWVDEGFPRGEDGKPPKEYF 5bwl.1    --------------------------------------------------------------------------------  target    QRARDEWVRASHDEAAAVVAATLANIAATYSGEEGAQRLRDQGYEEETIEAMGGAGVQAMKFRGGMPLLGMTRVFGLYRM 5bwl.1    --------------------------------------------------------------------------------  target    ANSMALLDAKVRGVGPDEARGARGFDNYSWHTDLPPGHPMVTGQQTVDFDLNSVELAKNVVVWGMNWITTKMPDAHWLTE 5bwl.1    ---------------------------------------------------RELAHCDLCLVVGTSSVV--YPAAMFAPQ  target    ARLKGTRIIVIACEYSSTSSKADDAIVVRPGTTPALALGLSHVIMRDKLYDADYVRRWTDLPMLVRTDTLKYLSAEDVFG 5bwl.1    VAARGVPVAEFNTETTPATNRFRFHFQ-----------------------------------------------------  target    GGPAPL 5bwl.1    ------ ``` | | | | | | | | | | | | | | | | | | | | | | | | | | | | | | | | | | | | | | | | | | | | | | | | | |
|  | 3sho.1.A | Transcriptional regulator, RpiR family  *Crystal structure of RpiR transcription factor from Sphaerobacter thermophilus (sugar isomerase domain)* | 0.03 |  | 12.73 | 0.14 | 297-353 | X-ray | 1.80 | homo-tetramer |  | HHblits | 0.26 |
| ``` target    TARREITRRGFLGTAAGAGFAAFVVSATRAWGLEAIENPLARYPDREWERVYRDLWRYDSKFTFLCAPNDTHNCLLDAYV 3sho.1    --------------------------------------------------------------------------------  target    RSGVMTRIGPTMRYGEARDLDGNRASARWDPRVCQKGLALTRRFYGDRRLRHCMVRAGFKRWVDEGFPRGEDGKPPKEYF 3sho.1    --------------------------------------------------------------------------------  target    QRARDEWVRASHDEAAAVVAATLANIAATYSGEEGAQRLRDQGYEEETIEAMGGAGVQAMKFRGGMPLLGMTRVFGLYRM 3sho.1    --------------------------------------------------------------------------------  target    ANSMALLDAKVRGVGPDEARGARGFDNYSWHTDLPPGHPMVTGQQTVDFDLNSVELAKNVVVWGMNWITTKMPDAHWLTE 3sho.1    --------------------------------------------------------TDLMIGVSVWRYL--RDTVAALAG  target    ARLKGTRIIVIACE-YSSTSSKADDAIVVRPGTTPALALGLSHVIMRDKLYDADYVRRWTDLPMLVRTDTLKYLSAEDVF 3sho.1    AAERGVPTMALTDSSVSPPARIADHVLVAATRGV----------------------------------------------  target    GGGPAPL 3sho.1    ------- ``` | | | | | | | | | | | | | | | | | | | | | | | | | | | | | | | | | | | | | | | | | | | | | | | | | |
|  | 3sho.1.C | Transcriptional regulator, RpiR family  *Crystal structure of RpiR transcription factor from Sphaerobacter thermophilus (sugar isomerase domain)* | 0.03 |  | 12.73 | 0.14 | 297-353 | X-ray | 1.80 | homo-tetramer |  | HHblits | 0.26 |
| ``` target    TARREITRRGFLGTAAGAGFAAFVVSATRAWGLEAIENPLARYPDREWERVYRDLWRYDSKFTFLCAPNDTHNCLLDAYV 3sho.1    --------------------------------------------------------------------------------  target    RSGVMTRIGPTMRYGEARDLDGNRASARWDPRVCQKGLALTRRFYGDRRLRHCMVRAGFKRWVDEGFPRGEDGKPPKEYF 3sho.1    --------------------------------------------------------------------------------  target    QRARDEWVRASHDEAAAVVAATLANIAATYSGEEGAQRLRDQGYEEETIEAMGGAGVQAMKFRGGMPLLGMTRVFGLYRM 3sho.1    --------------------------------------------------------------------------------  target    ANSMALLDAKVRGVGPDEARGARGFDNYSWHTDLPPGHPMVTGQQTVDFDLNSVELAKNVVVWGMNWITTKMPDAHWLTE 3sho.1    --------------------------------------------------------TDLMIGVSVWRYL--RDTVAALAG  target    ARLKGTRIIVIACE-YSSTSSKADDAIVVRPGTTPALALGLSHVIMRDKLYDADYVRRWTDLPMLVRTDTLKYLSAEDVF 3sho.1    AAERGVPTMALTDSSVSPPARIADHVLVAATRGV----------------------------------------------  target    GGGPAPL 3sho.1    ------- ``` | | | | | | | | | | | | | | | | | | | | | | | | | | | | | | | | | | | | | | | | | | | | | | | | | |
|  | 6fky.2.A | NAD-dependent protein deacylase sirtuin-5, mitochondrial  *Crystal structure of zebrafish Sirtuin 5 in complex with 3-(benzylthio)succinyl-CPS1 peptide* | 0.03 |  | 15.09 | 0.13 | 292-346 | X-ray | 2.98 | monomer | 1 x ZN | HHblits | 0.28 |
| ``` target    TARREITRRGFLGTAAGAGFAAFVVSATRAWGLEAIENPLARYPDREWERVYRDLWRYDSKFTFLCAPNDTHNCLLDAYV 6fky.2    --------------------------------------------------------------------------------  target    RSGVMTRIGPTMRYGEARDLDGNRASARWDPRVCQKGLALTRRFYGDRRLRHCMVRAGFKRWVDEGFPRGEDGKPPKEYF 6fky.2    --------------------------------------------------------------------------------  target    QRARDEWVRASHDEAAAVVAATLANIAATYSGEEGAQRLRDQGYEEETIEAMGGAGVQAMKFRGGMPLLGMTRVFGLYRM 6fky.2    --------------------------------------------------------------------------------  target    ANSMALLDAKVRGVGPDEARGARGFDNYSWHTDLPPGHPMVTGQQTVDFDLNSVELAKNVVVWGMNWITTKMPDAHWLTE 6fky.2    ---------------------------------------------------RELEKCDLCLVVGTSSIV--YPAAMFAPQ  target    ARLKGTRIIVIACEYSSTSSKADDAIVVRPGTTPALALGLSHVIMRDKLYDADYVRRWTDLPMLVRTDTLKYLSAEDVFG 6fky.2    VASRGVPVAEFNMECTPATQRFKYHF------------------------------------------------------  target    GGPAPL 6fky.2    ------ ``` | | | | | | | | | | | | | | | | | | | | | | | | | | | | | | | | | | | | | | | | | | | | | | | | | |
|  | 6fky.1.A | NAD-dependent protein deacylase sirtuin-5, mitochondrial  *Crystal structure of zebrafish Sirtuin 5 in complex with 3-(benzylthio)succinyl-CPS1 peptide* | 0.03 |  | 15.09 | 0.13 | 292-346 | X-ray | 2.98 | monomer | 1 x ZN, 1 x E9N, 1 x DZK, 2 x GZB-VAL-LEU-LYS-GLU-TYR-GLY-VAL | HHblits | 0.28 |
| ``` target    TARREITRRGFLGTAAGAGFAAFVVSATRAWGLEAIENPLARYPDREWERVYRDLWRYDSKFTFLCAPNDTHNCLLDAYV 6fky.1    --------------------------------------------------------------------------------  target    RSGVMTRIGPTMRYGEARDLDGNRASARWDPRVCQKGLALTRRFYGDRRLRHCMVRAGFKRWVDEGFPRGEDGKPPKEYF 6fky.1    --------------------------------------------------------------------------------  target    QRARDEWVRASHDEAAAVVAATLANIAATYSGEEGAQRLRDQGYEEETIEAMGGAGVQAMKFRGGMPLLGMTRVFGLYRM 6fky.1    --------------------------------------------------------------------------------  target    ANSMALLDAKVRGVGPDEARGARGFDNYSWHTDLPPGHPMVTGQQTVDFDLNSVELAKNVVVWGMNWITTKMPDAHWLTE 6fky.1    ---------------------------------------------------RELEKCDLCLVVGTSSIV--YPAAMFAPQ  target    ARLKGTRIIVIACEYSSTSSKADDAIVVRPGTTPALALGLSHVIMRDKLYDADYVRRWTDLPMLVRTDTLKYLSAEDVFG 6fky.1    VASRGVPVAEFNMECTPATQRFKYHF------------------------------------------------------  target    GGPAPL 6fky.1    ------ ``` | | | | | | | | | | | | | | | | | | | | | | | | | | | | | | | | | | | | | | | | | | | | | | | | | |
|  | 6flg.1.A | NAD-dependent protein deacylase sirtuin-5, mitochondrial  *Crystal structure of zebrafish Sirtuin 5 in complex with 3(S)-(naphthylthio)succinyl-CPS1 peptide* | 0.03 |  | 15.09 | 0.13 | 292-346 | X-ray | 2.50 | monomer | 1 x ZN, 1 x GZB-VAL-LEU-DQK-GLU-TYR-GLY-VAL | HHblits | 0.28 |
| ``` target    TARREITRRGFLGTAAGAGFAAFVVSATRAWGLEAIENPLARYPDREWERVYRDLWRYDSKFTFLCAPNDTHNCLLDAYV 6flg.1    --------------------------------------------------------------------------------  target    RSGVMTRIGPTMRYGEARDLDGNRASARWDPRVCQKGLALTRRFYGDRRLRHCMVRAGFKRWVDEGFPRGEDGKPPKEYF 6flg.1    --------------------------------------------------------------------------------  target    QRARDEWVRASHDEAAAVVAATLANIAATYSGEEGAQRLRDQGYEEETIEAMGGAGVQAMKFRGGMPLLGMTRVFGLYRM 6flg.1    --------------------------------------------------------------------------------  target    ANSMALLDAKVRGVGPDEARGARGFDNYSWHTDLPPGHPMVTGQQTVDFDLNSVELAKNVVVWGMNWITTKMPDAHWLTE 6flg.1    ---------------------------------------------------RELEKCDLCLVVGTSSIV--YPAAMFAPQ  target    ARLKGTRIIVIACEYSSTSSKADDAIVVRPGTTPALALGLSHVIMRDKLYDADYVRRWTDLPMLVRTDTLKYLSAEDVFG 6flg.1    VASRGVPVAEFNMECTPATQRFKYHF------------------------------------------------------  target    GGPAPL 6flg.1    ------ ``` | | | | | | | | | | | | | | | | | | | | | | | | | | | | | | | | | | | | | | | | | | | | | | | | | |
|  | 6eo0.1.A | NAD-dependent protein deacylase sirtuin-5, mitochondrial  *Zebrafish Sirt5 in complex with stalled peptidylimidate and bicyclic intermediate of inhibitory compound 29* | 0.03 |  | 15.09 | 0.13 | 292-346 | X-ray | 2.40 | monomer | 1 x ZN, 1 x BV8, 1 x BVT | HHblits | 0.28 |
| ``` target    TARREITRRGFLGTAAGAGFAAFVVSATRAWGLEAIENPLARYPDREWERVYRDLWRYDSKFTFLCAPNDTHNCLLDAYV 6eo0.1    --------------------------------------------------------------------------------  target    RSGVMTRIGPTMRYGEARDLDGNRASARWDPRVCQKGLALTRRFYGDRRLRHCMVRAGFKRWVDEGFPRGEDGKPPKEYF 6eo0.1    --------------------------------------------------------------------------------  target    QRARDEWVRASHDEAAAVVAATLANIAATYSGEEGAQRLRDQGYEEETIEAMGGAGVQAMKFRGGMPLLGMTRVFGLYRM 6eo0.1    --------------------------------------------------------------------------------  target    ANSMALLDAKVRGVGPDEARGARGFDNYSWHTDLPPGHPMVTGQQTVDFDLNSVELAKNVVVWGMNWITTKMPDAHWLTE 6eo0.1    ---------------------------------------------------RELEKCDLCLVVGTSSIV--YPAAMFAPQ  target    ARLKGTRIIVIACEYSSTSSKADDAIVVRPGTTPALALGLSHVIMRDKLYDADYVRRWTDLPMLVRTDTLKYLSAEDVFG 6eo0.1    VASRGVPVAEFNMECTPATQRFKYHF------------------------------------------------------  target    GGPAPL 6eo0.1    ------ ``` | | | | | | | | | | | | | | | | | | | | | | | | | | | | | | | | | | | | | | | | | | | | | | | | | |
|  | 2h4h.1.A | NAD-dependent deacetylase  *Sir2 H116Y mutant-p53 peptide-NAD* | 0.03 |  | 12.96 | 0.13 | 292-347 | X-ray | 1.99 | hetero-1-1-mer | 1 x ZN, 1 x NAD | HHblits | 0.27 |
| ``` target    TARREITRRGFLGTAAGAGFAAFVVSATRAWGLEAIENPLARYPDREWERVYRDLWRYDSKFTFLCAPNDTHNCLLDAYV 2h4h.1    --------------------------------------------------------------------------------  target    RSGVMTRIGPTMRYGEARDLDGNRASARWDPRVCQKGLALTRRFYGDRRLRHCMVRAGFKRWVDEGFPRGEDGKPPKEYF 2h4h.1    --------------------------------------------------------------------------------  target    QRARDEWVRASHDEAAAVVAATLANIAATYSGEEGAQRLRDQGYEEETIEAMGGAGVQAMKFRGGMPLLGMTRVFGLYRM 2h4h.1    --------------------------------------------------------------------------------  target    ANSMALLDAKVRGVGPDEARGARGFDNYSWHTDLPPGHPMVTGQQTVDFDLNSVELAKNVVVWGMNWITTKMPDAHWLTE 2h4h.1    ---------------------------------------------------GLSSRASLMIVLGSSLVV--YPAAELPLI  target    ARLKGTRIIVIACEYSSTSSKADDAIVVRPGTTPALALGLSHVIMRDKLYDADYVRRWTDLPMLVRTDTLKYLSAEDVFG 2h4h.1    TVRSGGKLVIVNLGETPFDDIATLKYN-----------------------------------------------------  target    GGPAPL 2h4h.1    ------ ``` | | | | | | | | | | | | | | | | | | | | | | | | | | | | | | | | | | | | | | | | | | | | | | | | | |
|  | 2ivf.1.A | ETHYLBENZENE DEHYDROGENASE ALPHA-SUBUNIT  *ETHYLBENZENE DEHYDROGENASE FROM AROMATOLEUM AROMATICUM* | 0.04 |  | 16.67 | 0.13 | 297-350 | X-ray | 1.88 | hetero-oligomer | 1 x MES, 4 x SF4, 1 x MO, 1 x MGD, 1 x MD1, 1 x F3S, 1 x HEM | HHblits | 0.27 |
| ``` target    TARREITRRGFLGTAAGAGFAAFVVSATRAWGLEAIENPLARYPDREWERVYRDLWRYDSKFTFLCAPNDTHNCLLDAYV 2ivf.1    --------------------------------------------------------------------------------  target    RSGVMTRIGPTMRYGEARDLDGNRASARWDPRVCQKGLALTRRFYGDRRLRHCMVRAGFKRWVDEGFPRGEDGKPPKEYF 2ivf.1    --------------------------------------------------------------------------------  target    QRARDEWVRASHDEAAAVVAATLANIAATYSGEEGAQRLRDQGYEEETIEAMGGAGVQAMKFRGGMPLLGMTRVFGLYRM 2ivf.1    --------------------------------------------------------------------------------  target    ANSMALLDAKVRGVGPDEARGARGFDNYSWHTDLPPGHPMVTGQQTVDFDLNSVELAKNVVVWGMNWITTKMPDAHWL-T 2ivf.1    --------------------------------------------------------PQVYMLLSQNPMRRKRSGAKMFPD  target    EARLKGTRIIVIACEYSSTSSKADDAIVVRPGTTPALALGLSHVIMRDKLYDADYVRRWTDLPMLVRTDTLKYLSAEDVF 2ivf.1    VLFPKLKMIFALETRMSSSAMYADIVLPCAW-------------------------------------------------  target    GGGPAPL 2ivf.1    ------- ``` | | | | | | | | | | | | | | | | | | | | | | | | | | | | | | | | | | | | | | | | | | | | | | | | | |
|  | 5nqd.1.A | AroA  *Arsenite oxidase AioAB from Rhizobium sp. str. NT-26 mutant AioBF108A* | 0.02 |  | 9.09 | 0.14 | 297-351 | X-ray | 2.20 | hetero-2-2-mer | 4 x MGD, 2 x O, 2 x 4MO, 2 x F3S, 2 x FES | HHblits | 0.25 |
| ``` target    TARREITRRGFLGTAAGAGFAAFVVSATRAWGLEAIENPLARYPDREWERVYRDLWRYDSKFTFLCAPNDTHNCLLDAYV 5nqd.1    --------------------------------------------------------------------------------  target    RSGVMTRIGPTMRYGEARDLDGNRASARWDPRVCQKGLALTRRFYGDRRLRHCMVRAGFKRWVDEGFPRGEDGKPPKEYF 5nqd.1    --------------------------------------------------------------------------------  target    QRARDEWVRASHDEAAAVVAATLANIAATYSGEEGAQRLRDQGYEEETIEAMGGAGVQAMKFRGGMPLLGMTRVFGLYRM 5nqd.1    --------------------------------------------------------------------------------  target    ANSMALLDAKVRGVGPDEARGARGFDNYSWHTDLPPGHPMVTGQQTVDFDLNSVELAKNVVVWGMNWITTK-----MPD- 5nqd.1    --------------------------------------------------------FKRVYKKRTDMVKDAMSAAPYGDR  target    ---AHWLTEARLKG-TRIIVIACEYSSTSSKADDAIVVRPGTTPALALGLSHVIMRDKLYDADYVRRWTDLPMLVRTDTL 5nqd.1    EAMVNAIVDAINQGGLFAVNVDIIPTKIGEACHVILPAATS---------------------------------------  target    KYLSAEDVFGGGPAPL 5nqd.1    ---------------- ``` | | | | | | | | | | | | | | | | | | | | | | | | | | | | | | | | | | | | | | | | | | | | | | | | | |
|  | 3u31.1.A | Transcriptional regulatory protein sir2 homologue  *Plasmodium falciparum Sir2A preferentially hydrolyzes medium and long chain fatty acyl lysine* | 0.03 |  | 12.96 | 0.13 | 292-347 | X-ray | 2.20 | hetero-oligomer | 1 x NAD, 1 x ZN | HHblits | 0.27 |
| ``` target    TARREITRRGFLGTAAGAGFAAFVVSATRAWGLEAIENPLARYPDREWERVYRDLWRYDSKFTFLCAPNDTHNCLLDAYV 3u31.1    --------------------------------------------------------------------------------  target    RSGVMTRIGPTMRYGEARDLDGNRASARWDPRVCQKGLALTRRFYGDRRLRHCMVRAGFKRWVDEGFPRGEDGKPPKEYF 3u31.1    --------------------------------------------------------------------------------  target    QRARDEWVRASHDEAAAVVAATLANIAATYSGEEGAQRLRDQGYEEETIEAMGGAGVQAMKFRGGMPLLGMTRVFGLYRM 3u31.1    --------------------------------------------------------------------------------  target    ANSMALLDAKVRGVGPDEARGARGFDNYSWHTDLPPGHPMVTGQQTVDFDLNSVELAKNVVVWGMNWITTKMPDAHWLTE 3u31.1    ---------------------------------------------------EEIAKCDLLLVIGTSSTV--STATNLCHF  target    ARLKGTRIIVIACEYSSTSS-KADDAIVVRPGTTPALALGLSHVIMRDKLYDADYVRRWTDLPMLVRTDTLKYLSAEDVF 3u31.1    ACKKKKKIVEINISKTYITNKMSDYHVC----------------------------------------------------  target    GGGPAPL 3u31.1    ------- ``` | | | | | | | | | | | | | | | | | | | | | | | | | | | | | | | | | | | | | | | | | | | | | | | | | |
|  | 3jwp.1.A | Transcriptional regulatory protein sir2 homologue  *Crystal structure of Plasmodium falciparum SIR2A (PF13\_0152) in complex with AMP* | 0.03 |  | 12.96 | 0.13 | 292-347 | X-ray | 2.65 | homo-trimer | 3 x AMP, 3 x ZN | HHblits | 0.27 |
| ``` target    TARREITRRGFLGTAAGAGFAAFVVSATRAWGLEAIENPLARYPDREWERVYRDLWRYDSKFTFLCAPNDTHNCLLDAYV 3jwp.1    --------------------------------------------------------------------------------  target    RSGVMTRIGPTMRYGEARDLDGNRASARWDPRVCQKGLALTRRFYGDRRLRHCMVRAGFKRWVDEGFPRGEDGKPPKEYF 3jwp.1    --------------------------------------------------------------------------------  target    QRARDEWVRASHDEAAAVVAATLANIAATYSGEEGAQRLRDQGYEEETIEAMGGAGVQAMKFRGGMPLLGMTRVFGLYRM 3jwp.1    --------------------------------------------------------------------------------  target    ANSMALLDAKVRGVGPDEARGARGFDNYSWHTDLPPGHPMVTGQQTVDFDLNSVELAKNVVVWGMNWITTKMPDAHWLTE 3jwp.1    ---------------------------------------------------EEIAKCDLLLVIGTSSTV--STATNLCHF  target    ARLKGTRIIVIACEYSSTSS-KADDAIVVRPGTTPALALGLSHVIMRDKLYDADYVRRWTDLPMLVRTDTLKYLSAEDVF 3jwp.1    ACKKKKKIVEINISKTYITNKMSDYHVC----------------------------------------------------  target    GGGPAPL 3jwp.1    ------- ``` | | | | | | | | | | | | | | | | | | | | | | | | | | | | | | | | | | | | | | | | | | | | | | | | | |
|  | 2i2w.1.A | Phosphoheptose isomerase  *Crystal Structure of Escherichia Coli Phosphoheptose Isomerase* | 0.03 |  | 16.67 | 0.13 | 296-351 | X-ray | 1.95 | homo-dimer |  | HHblits | 0.27 |
| ``` target    TARREITRRGFLGTAAGAGFAAFVVSATRAWGLEAIENPLARYPDREWERVYRDLWRYDSKFTFLCAPNDTHNCLLDAYV 2i2w.1    --------------------------------------------------------------------------------  target    RSGVMTRIGPTMRYGEARDLDGNRASARWDPRVCQKGLALTRRFYGDRRLRHCMVRAGFKRWVDEGFPRGEDGKPPKEYF 2i2w.1    --------------------------------------------------------------------------------  target    QRARDEWVRASHDEAAAVVAATLANIAATYSGEEGAQRLRDQGYEEETIEAMGGAGVQAMKFRGGMPLLGMTRVFGLYRM 2i2w.1    --------------------------------------------------------------------------------  target    ANSMALLDAKVRGVGPDEARGARGFDNYSWHTDLPPGHPMVTGQQTVDFDLNSVELAKNVVVWGMNWITTKMPDAHWLTE 2i2w.1    -------------------------------------------------------EGDVLLGISTSGNS--ANVIKAIAA  target    ARLKGTRIIVIACE-YSSTSSKADDAIVVRPGTTPALALGLSHVIMRDKLYDADYVRRWTDLPMLVRTDTLKYLSAEDVF 2i2w.1    AREKGMKVITLTGKDGGKMAGTADIEIRVPHF------------------------------------------------  target    GGGPAPL 2i2w.1    ------- ``` | | | | | | | | | | | | | | | | | | | | | | | | | | | | | | | | | | | | | | | | | | | | | | | | | |
|  | 2i2w.2.B | Phosphoheptose isomerase  *Crystal Structure of Escherichia Coli Phosphoheptose Isomerase* | 0.03 |  | 16.67 | 0.13 | 296-351 | X-ray | 1.95 | homo-dimer |  | HHblits | 0.27 |
| ``` target    TARREITRRGFLGTAAGAGFAAFVVSATRAWGLEAIENPLARYPDREWERVYRDLWRYDSKFTFLCAPNDTHNCLLDAYV 2i2w.2    --------------------------------------------------------------------------------  target    RSGVMTRIGPTMRYGEARDLDGNRASARWDPRVCQKGLALTRRFYGDRRLRHCMVRAGFKRWVDEGFPRGEDGKPPKEYF 2i2w.2    --------------------------------------------------------------------------------  target    QRARDEWVRASHDEAAAVVAATLANIAATYSGEEGAQRLRDQGYEEETIEAMGGAGVQAMKFRGGMPLLGMTRVFGLYRM 2i2w.2    --------------------------------------------------------------------------------  target    ANSMALLDAKVRGVGPDEARGARGFDNYSWHTDLPPGHPMVTGQQTVDFDLNSVELAKNVVVWGMNWITTKMPDAHWLTE 2i2w.2    -------------------------------------------------------EGDVLLGISTSGNS--ANVIKAIAA  target    ARLKGTRIIVIACE-YSSTSSKADDAIVVRPGTTPALALGLSHVIMRDKLYDADYVRRWTDLPMLVRTDTLKYLSAEDVF 2i2w.2    AREKGMKVITLTGKDGGKMAGTADIEIRVPHF------------------------------------------------  target    GGGPAPL 2i2w.2    ------- ``` | | | | | | | | | | | | | | | | | | | | | | | | | | | | | | | | | | | | | | | | | | | | | | | | | |
|  | 7p61.1.C | NADH-quinone oxidoreductase  *Complex I from E. coli, DDM-purified, with NADH, Resting state* | 0.03 |  | 18.87 | 0.13 | 296-350 | EM | 0.00 | hetero-1-1-1-1-1-1-… | 7 x SF4, 1 x FMN, 1 x NAI, 2 x FES, 1 x CA, 2 x 3PE, 1 x UQ8 | HHblits | 0.28 |
| ``` target    TARREITRRGFLGTAAGAGFAAFVVSATRAWGLEAIENPLARYPDREWERVYRDLWRYDSKFTFLCAPNDTHNCLLDAYV 7p61.1    --------------------------------------------------------------------------------  target    RSGVMTRIGPTMRYGEARDLDGNRASARWDPRVCQKGLALTRRFYGDRRLRHCMVRAGFKRWVDEGFPRGEDGKPPKEYF 7p61.1    --------------------------------------------------------------------------------  target    QRARDEWVRASHDEAAAVVAATLANIAATYSGEEGAQRLRDQGYEEETIEAMGGAGVQAMKFRGGMPLLGMTRVFGLYRM 7p61.1    --------------------------------------------------------------------------------  target    ANSMALLDAKVRGVGPDEARGARGFDNYSWHTDLPPGHPMVTGQQTVDFDLNSVELAKNVVVWGMNWITTKMPDAHWLTE 7p61.1    -------------------------------------------------------RADAVVVLE-NDLHRH-ASATRVNA  target    ARLKGTRIIVIACEYSSTSSKADDAIVVRPGTTPALALGLSHVIMRDKLYDADYVRRWTDLPMLVRTDTLKYLSAEDVFG 7p61.1    ALAKAPLVMVVDHQRTAIMENAHLVLSAAS--------------------------------------------------  target    GGPAPL 7p61.1    ------ ``` | | | | | | | | | | | | | | | | | | | | | | | | | | | | | | | | | | | | | | | | | | | | | | | | | |
|  | 8e9g.1.G | NADH-quinone oxidoreductase subunit G  *Mycobacterial respiratory complex I with both quinone positions modelled* | 0.03 |  | 19.23 | 0.13 | 297-350 | EM | 0.00 | hetero-1-1-1-1-1-1-… |  | HHblits | 0.29 |
| ``` target    TARREITRRGFLGTAAGAGFAAFVVSATRAWGLEAIENPLARYPDREWERVYRDLWRYDSKFTFLCAPNDTHNCLLDAYV 8e9g.1    --------------------------------------------------------------------------------  target    RSGVMTRIGPTMRYGEARDLDGNRASARWDPRVCQKGLALTRRFYGDRRLRHCMVRAGFKRWVDEGFPRGEDGKPPKEYF 8e9g.1    --------------------------------------------------------------------------------  target    QRARDEWVRASHDEAAAVVAATLANIAATYSGEEGAQRLRDQGYEEETIEAMGGAGVQAMKFRGGMPLLGMTRVFGLYRM 8e9g.1    --------------------------------------------------------------------------------  target    ANSMALLDAKVRGVGPDEARGARGFDNYSWHTDLPPGHPMVTGQQTVDFDLNSVELAKNVVVWGMNWITTKMPDAHWLTE 8e9g.1    --------------------------------------------------------LAALLVGG-VELGD-LPDPELAVA  target    ARLKGTRIIVIACEYSSTSSKADDAIVVRPGTTPALALGLSHVIMRDKLYDADYVRRWTDLPMLVRTDTLKYLSAEDVFG 8e9g.1    AVRTTPFVVSLELRESAVTELADVVFPVAP--------------------------------------------------  target    GGPAPL 8e9g.1    ------ ``` | | | | | | | | | | | | | | | | | | | | | | | | | | | | | | | | | | | | | | | | | | | | | | | | | |
|  | 7qv7.1.L | Hydrogen dependent carbon dioxide reductase subunit FdhF  *Cryo-EM structure of Hydrogen-dependent CO2 reductase.* | 0.03 |  | 18.87 | 0.13 | 296-349 | EM | 0.00 | hetero-2-6-6-2-mer | 52 x SF4, 6 x 402 | HHblits | 0.28 |
| ``` target    TARREITRRGFLGTAAGAGFAAFVVSATRAWGLEAIENPLARYPDREWERVYRDLWRYDSKFTFLCAPNDTHNCLLDAYV 7qv7.1    --------------------------------------------------------------------------------  target    RSGVMTRIGPTMRYGEARDLDGNRASARWDPRVCQKGLALTRRFYGDRRLRHCMVRAGFKRWVDEGFPRGEDGKPPKEYF 7qv7.1    --------------------------------------------------------------------------------  target    QRARDEWVRASHDEAAAVVAATLANIAATYSGEEGAQRLRDQGYEEETIEAMGGAGVQAMKFRGGMPLLGMTRVFGLYRM 7qv7.1    --------------------------------------------------------------------------------  target    ANSMALLDAKVRGVGPDEARGARGFDNYSWHTDLPPGHPMVTGQQTVDFDLNSVELAKNVVVWGMNWITTKMPDAHWLTE 7qv7.1    -------------------------------------------------------RVRALYIFGENPIMSDP-DSDHLRH  target    ARLKGTRIIVIACEYSSTSSKADDAIVVRPGTTPALALGLSHVIMRDKLYDADYVRRWTDLPMLVRTDTLKYLSAEDVFG 7qv7.1    ALEHLDLLIVQDIFLTETARLAHVVLPAA---------------------------------------------------  target    GGPAPL 7qv7.1    ------ ``` | | | | | | | | | | | | | | | | | | | | | | | | | | | | | | | | | | | | | | | | | | | | | | | | | |
|  | 7qv7.1.O | Hydrogen dependent carbon dioxide reductase subunit FdhF  *Cryo-EM structure of Hydrogen-dependent CO2 reductase.* | 0.03 |  | 18.87 | 0.13 | 296-349 | EM | 0.00 | hetero-2-6-6-2-mer | 52 x SF4, 6 x 402 | HHblits | 0.28 |
| ``` target    TARREITRRGFLGTAAGAGFAAFVVSATRAWGLEAIENPLARYPDREWERVYRDLWRYDSKFTFLCAPNDTHNCLLDAYV 7qv7.1    --------------------------------------------------------------------------------  target    RSGVMTRIGPTMRYGEARDLDGNRASARWDPRVCQKGLALTRRFYGDRRLRHCMVRAGFKRWVDEGFPRGEDGKPPKEYF 7qv7.1    --------------------------------------------------------------------------------  target    QRARDEWVRASHDEAAAVVAATLANIAATYSGEEGAQRLRDQGYEEETIEAMGGAGVQAMKFRGGMPLLGMTRVFGLYRM 7qv7.1    --------------------------------------------------------------------------------  target    ANSMALLDAKVRGVGPDEARGARGFDNYSWHTDLPPGHPMVTGQQTVDFDLNSVELAKNVVVWGMNWITTKMPDAHWLTE 7qv7.1    -------------------------------------------------------RVRALYIFGENPIMSDP-DSDHLRH  target    ARLKGTRIIVIACEYSSTSSKADDAIVVRPGTTPALALGLSHVIMRDKLYDADYVRRWTDLPMLVRTDTLKYLSAEDVFG 7qv7.1    ALEHLDLLIVQDIFLTETARLAHVVLPAA---------------------------------------------------  target    GGPAPL 7qv7.1    ------ ``` | | | | | | | | | | | | | | | | | | | | | | | | | | | | | | | | | | | | | | | | | | | | | | | | | |
|  | 5lu5.1.A | Phosphoheptose isomerase  *A quantum half-site enzyme* | 0.03 |  | 10.91 | 0.14 | 296-352 | X-ray | 1.55 | homo-tetramer | 4 x M7P | HHblits | 0.25 |
| ``` target    TARREITRRGFLGTAAGAGFAAFVVSATRAWGLEAIENPLARYPDREWERVYRDLWRYDSKFTFLCAPNDTHNCLLDAYV 5lu5.1    --------------------------------------------------------------------------------  target    RSGVMTRIGPTMRYGEARDLDGNRASARWDPRVCQKGLALTRRFYGDRRLRHCMVRAGFKRWVDEGFPRGEDGKPPKEYF 5lu5.1    --------------------------------------------------------------------------------  target    QRARDEWVRASHDEAAAVVAATLANIAATYSGEEGAQRLRDQGYEEETIEAMGGAGVQAMKFRGGMPLLGMTRVFGLYRM 5lu5.1    --------------------------------------------------------------------------------  target    ANSMALLDAKVRGVGPDEARGARGFDNYSWHTDLPPGHPMVTGQQTVDFDLNSVELAKNVVVWGMNWITTKMPDAHWLTE 5lu5.1    -------------------------------------------------------EGDVLIGYSTSGK--SPNILAAFRE  target    ARLKGTRIIVIAC-EYSSTSSKADDAIVVRPGTTPALALGLSHVIMRDKLYDADYVRRWTDLPMLVRTDTLKYLSAEDVF 5lu5.1    AKAKGMTCVGFTGNRGGEMRELCDLLLEVPSAD-----------------------------------------------  target    GGGPAPL 5lu5.1    ------- ``` | | | | | | | | | | | | | | | | | | | | | | | | | | | | | | | | | | | | | | | | | | | | | | | | | |
|  | 6cz7.1.A | ArrA  *The arsenate respiratory reductase (Arr) complex from Shewanella sp. ANA-3* | 0.04 |  | 18.52 | 0.13 | 296-350 | X-ray | 1.62 | hetero-1-1-mer | 5 x SF4, 2 x MGD, 1 x MO, 1 x PG5 | HHblits | 0.26 |
| ``` target    TARREITRRGFLGTAAGAGFAAFVVSATRAWGLEAIENPLARYPDREWERVYRDLWRYDSKFTFLCAPNDTHNCLLDAYV 6cz7.1    --------------------------------------------------------------------------------  target    RSGVMTRIGPTMRYGEARDLDGNRASARWDPRVCQKGLALTRRFYGDRRLRHCMVRAGFKRWVDEGFPRGEDGKPPKEYF 6cz7.1    --------------------------------------------------------------------------------  target    QRARDEWVRASHDEAAAVVAATLANIAATYSGEEGAQRLRDQGYEEETIEAMGGAGVQAMKFRGGMPLLGMTRVFGLYRM 6cz7.1    --------------------------------------------------------------------------------  target    ANSMALLDAKVRGVGPDEARGARGFDNYSWHTDLPPGHPMVTGQQTVDFDLNSVELAKNVVVWGMNWITTKMPDAHWLTE 6cz7.1    -------------------------------------------------------EIKVMLAYFNNFNFSNP-EGQRWDE  target    ARLKGTRIIVIACEYSSTSSKADDAIVVRPGTTPALALGLSHVIMRDKLYDADYVRRWTDLPMLVRTDTLKYLSAEDVFG 6cz7.1    ALSKVDFMAHITTNVSEFSWFADVLLPSSH--------------------------------------------------  target    GGPAPL 6cz7.1    ------ ``` | | | | | | | | | | | | | | | | | | | | | | | | | | | | | | | | | | | | | | | | | | | | | | | | | |
|  | 6tg9.1.A | Formate dehydrogenase subunit alpha  *Cryo-EM Structure of NADH reduced form of NAD+-dependent Formate Dehydrogenase from Rhodobacter capsulatus* | 0.03 |  | 14.81 | 0.13 | 296-350 | EM | 3.24 | hetero-2-2-2-2-mer | 4 x MGD, 2 x 6MO, 4 x FES, 10 x SF4, 2 x H2S, 2 x FMN, 2 x NAI | HHblits | 0.26 |
| ``` target    TARREITRRGFLGTAAGAGFAAFVVSATRAWGLEAIENPLARYPDREWERVYRDLWRYDSKFTFLCAPNDTHNCLLDAYV 6tg9.1    --------------------------------------------------------------------------------  target    RSGVMTRIGPTMRYGEARDLDGNRASARWDPRVCQKGLALTRRFYGDRRLRHCMVRAGFKRWVDEGFPRGEDGKPPKEYF 6tg9.1    --------------------------------------------------------------------------------  target    QRARDEWVRASHDEAAAVVAATLANIAATYSGEEGAQRLRDQGYEEETIEAMGGAGVQAMKFRGGMPLLGMTRVFGLYRM 6tg9.1    --------------------------------------------------------------------------------  target    ANSMALLDAKVRGVGPDEARGARGFDNYSWHTDLPPGHPMVTGQQTVDFDLNSVELAKNVVVWGMNWITTKMPDAHWLTE 6tg9.1    -------------------------------------------------------RFKALYVQGEDILQSDP-DTRHVSA  target    ARLKGTRIIVIACEYSSTSSKADDAIVVRPGTTPALALGLSHVIMRDKLYDADYVRRWTDLPMLVRTDTLKYLSAEDVFG 6tg9.1    GLAAMDLVIVHDLFLNETANYAHVFLPGST--------------------------------------------------  target    GGPAPL 6tg9.1    ------ ``` | | | | | | | | | | | | | | | | | | | | | | | | | | | | | | | | | | | | | | | | | | | | | | | | | |
|  | 2v45.1.A | PERIPLASMIC NITRATE REDUCTASE  *A NEW CATALYTIC MECHANISM OF PERIPLASMIC NITRATE REDUCTASE FROM DESULFOVIBRIO DESULFURICANS ATCC 27774 FROM CRYSTALLOGRAPHIC AND EPR DATA AND BASED ON DETAILED ANALYSIS OF THE SIXTH LIGAND* | 0.03 |  | 16.67 | 0.13 | 296-350 | X-ray | 2.40 | monomer | 1 x SF4, 1 x MO, 2 x MGD, 1 x LCP | HHblits | 0.26 |
| ``` target    TARREITRRGFLGTAAGAGFAAFVVSATRAWGLEAIENPLARYPDREWERVYRDLWRYDSKFTFLCAPNDTHNCLLDAYV 2v45.1    --------------------------------------------------------------------------------  target    RSGVMTRIGPTMRYGEARDLDGNRASARWDPRVCQKGLALTRRFYGDRRLRHCMVRAGFKRWVDEGFPRGEDGKPPKEYF 2v45.1    --------------------------------------------------------------------------------  target    QRARDEWVRASHDEAAAVVAATLANIAATYSGEEGAQRLRDQGYEEETIEAMGGAGVQAMKFRGGMPLLGMTRVFGLYRM 2v45.1    --------------------------------------------------------------------------------  target    ANSMALLDAKVRGVGPDEARGARGFDNYSWHTDLPPGHPMVTGQQTVDFDLNSVELAKNVVVWGMNWITTKMPDAHWLTE 2v45.1    -------------------------------------------------------DVKCMIICETNPAHTLP-NLNKVHK  target    ARLKG-TRIIVIACEYS-STSSKADDAIVVRPGTTPALALGLSHVIMRDKLYDADYVRRWTDLPMLVRTDTLKYLSAEDV 2v45.1    AMSHPESFIVCIEAFPDAVTLEYADLVLPPAF------------------------------------------------  target    FGGGPAPL 2v45.1    -------- ``` | | | | | | | | | | | | | | | | | | | | | | | | | | | | | | | | | | | | | | | | | | | | | | | | | |
|  | 4s12.1.A | N-acetylmuramic acid 6-phosphate etherase  *1.55 Angstrom Crystal Structure of N-acetylmuramic acid 6-phosphate Etherase from Yersinia enterocolitica.* | 0.03 |  | 24.07 | 0.13 | 297-352 | X-ray | 1.55 | homo-dimer |  | HHblits | 0.26 |
| ``` target    TARREITRRGFLGTAAGAGFAAFVVSATRAWGLEAIENPLARYPDREWERVYRDLWRYDSKFTFLCAPNDTHNCLLDAYV 4s12.1    --------------------------------------------------------------------------------  target    RSGVMTRIGPTMRYGEARDLDGNRASARWDPRVCQKGLALTRRFYGDRRLRHCMVRAGFKRWVDEGFPRGEDGKPPKEYF 4s12.1    --------------------------------------------------------------------------------  target    QRARDEWVRASHDEAAAVVAATLANIAATYSGEEGAQRLRDQGYEEETIEAMGGAGVQAMKFRGGMPLLGMTRVFGLYRM 4s12.1    --------------------------------------------------------------------------------  target    ANSMALLDAKVRGVGPDEARGARGFDNYSWHTDLPPGHPMVTGQQTVDFDLNSVELAKNVVVWGMNWITTKMPDAHWLTE 4s12.1    --------------------------------------------------------TDMVVGLAAS-GRT-PYVIGALRF  target    ARLKGTRIIVIA-CEYSSTSSKADDAIVVRPGTTPALALGLSHVIMRDKLYDADYVRRWTDLPMLVRTDTLKYLSAEDVF 4s12.1    ARQLGCPTAAISCNPDSPIAQEALVAISPVVGP-----------------------------------------------  target    GGGPAPL 4s12.1    ------- ``` | | | | | | | | | | | | | | | | | | | | | | | | | | | | | | | | | | | | | | | | | | | | | | | | | |
|  | 4s12.2.A | N-acetylmuramic acid 6-phosphate etherase  *1.55 Angstrom Crystal Structure of N-acetylmuramic acid 6-phosphate Etherase from Yersinia enterocolitica.* | 0.03 |  | 24.07 | 0.13 | 297-352 | X-ray | 1.55 | homo-dimer |  | HHblits | 0.26 |
| ``` target    TARREITRRGFLGTAAGAGFAAFVVSATRAWGLEAIENPLARYPDREWERVYRDLWRYDSKFTFLCAPNDTHNCLLDAYV 4s12.2    --------------------------------------------------------------------------------  target    RSGVMTRIGPTMRYGEARDLDGNRASARWDPRVCQKGLALTRRFYGDRRLRHCMVRAGFKRWVDEGFPRGEDGKPPKEYF 4s12.2    --------------------------------------------------------------------------------  target    QRARDEWVRASHDEAAAVVAATLANIAATYSGEEGAQRLRDQGYEEETIEAMGGAGVQAMKFRGGMPLLGMTRVFGLYRM 4s12.2    --------------------------------------------------------------------------------  target    ANSMALLDAKVRGVGPDEARGARGFDNYSWHTDLPPGHPMVTGQQTVDFDLNSVELAKNVVVWGMNWITTKMPDAHWLTE 4s12.2    --------------------------------------------------------TDMVVGLAAS-GRT-PYVIGALRF  target    ARLKGTRIIVIA-CEYSSTSSKADDAIVVRPGTTPALALGLSHVIMRDKLYDADYVRRWTDLPMLVRTDTLKYLSAEDVF 4s12.2    ARQLGCPTAAISCNPDSPIAQEALVAISPVVGP-----------------------------------------------  target    GGGPAPL 4s12.2    ------- ``` | | | | | | | | | | | | | | | | | | | | | | | | | | | | | | | | | | | | | | | | | | | | | | | | | |
|  | 4s12.2.B | N-acetylmuramic acid 6-phosphate etherase  *1.55 Angstrom Crystal Structure of N-acetylmuramic acid 6-phosphate Etherase from Yersinia enterocolitica.* | 0.03 |  | 24.07 | 0.13 | 297-352 | X-ray | 1.55 | homo-dimer |  | HHblits | 0.26 |
| ``` target    TARREITRRGFLGTAAGAGFAAFVVSATRAWGLEAIENPLARYPDREWERVYRDLWRYDSKFTFLCAPNDTHNCLLDAYV 4s12.2    --------------------------------------------------------------------------------  target    RSGVMTRIGPTMRYGEARDLDGNRASARWDPRVCQKGLALTRRFYGDRRLRHCMVRAGFKRWVDEGFPRGEDGKPPKEYF 4s12.2    --------------------------------------------------------------------------------  target    QRARDEWVRASHDEAAAVVAATLANIAATYSGEEGAQRLRDQGYEEETIEAMGGAGVQAMKFRGGMPLLGMTRVFGLYRM 4s12.2    --------------------------------------------------------------------------------  target    ANSMALLDAKVRGVGPDEARGARGFDNYSWHTDLPPGHPMVTGQQTVDFDLNSVELAKNVVVWGMNWITTKMPDAHWLTE 4s12.2    --------------------------------------------------------TDMVVGLAAS-GRT-PYVIGALRF  target    ARLKGTRIIVIA-CEYSSTSSKADDAIVVRPGTTPALALGLSHVIMRDKLYDADYVRRWTDLPMLVRTDTLKYLSAEDVF 4s12.2    ARQLGCPTAAISCNPDSPIAQEALVAISPVVGP-----------------------------------------------  target    GGGPAPL 4s12.2    ------- ``` | | | | | | | | | | | | | | | | | | | | | | | | | | | | | | | | | | | | | | | | | | | | | | | | | |
|  | 6aco.1.A | NAD-dependent protein deacylase sirtuin-5, mitochondrial  *histone lysine desuccinylase Sirt5 in complex with succinyl peptide H2BK120* | 0.03 |  | 11.32 | 0.13 | 292-346 | X-ray | 1.71 | monomer | 1 x ZN, 1 x ALA-VAL-THR-SLL-TYR-THR-SER | HHblits | 0.27 |
| ``` target    TARREITRRGFLGTAAGAGFAAFVVSATRAWGLEAIENPLARYPDREWERVYRDLWRYDSKFTFLCAPNDTHNCLLDAYV 6aco.1    --------------------------------------------------------------------------------  target    RSGVMTRIGPTMRYGEARDLDGNRASARWDPRVCQKGLALTRRFYGDRRLRHCMVRAGFKRWVDEGFPRGEDGKPPKEYF 6aco.1    --------------------------------------------------------------------------------  target    QRARDEWVRASHDEAAAVVAATLANIAATYSGEEGAQRLRDQGYEEETIEAMGGAGVQAMKFRGGMPLLGMTRVFGLYRM 6aco.1    --------------------------------------------------------------------------------  target    ANSMALLDAKVRGVGPDEARGARGFDNYSWHTDLPPGHPMVTGQQTVDFDLNSVELAKNVVVWGMNWITTKMPDAHWLTE 6aco.1    ---------------------------------------------------RELAHCDLCLVVGTSSVV--YPAAMFAPQ  target    ARLKGTRIIVIACEYSSTSSKADDAIVVRPGTTPALALGLSHVIMRDKLYDADYVRRWTDLPMLVRTDTLKYLSAEDVFG 6aco.1    VAARGVPVAEFNTETTPATNRFRFHF------------------------------------------------------  target    GGPAPL 6aco.1    ------ ``` | | | | | | | | | | | | | | | | | | | | | | | | | | | | | | | | | | | | | | | | | | | | | | | | | |
|  | 6eqs.3.A | NAD-dependent protein deacylase sirtuin-5, mitochondrial  *Human Sirt5 in complex with stalled peptidylimidate intermediate of inhibitory compound 29* | 0.03 |  | 11.32 | 0.13 | 292-346 | X-ray | 1.32 | monomer | 1 x ZN, 1 x BV8, 1 x BU2 | HHblits | 0.27 |
| ``` target    TARREITRRGFLGTAAGAGFAAFVVSATRAWGLEAIENPLARYPDREWERVYRDLWRYDSKFTFLCAPNDTHNCLLDAYV 6eqs.3    --------------------------------------------------------------------------------  target    RSGVMTRIGPTMRYGEARDLDGNRASARWDPRVCQKGLALTRRFYGDRRLRHCMVRAGFKRWVDEGFPRGEDGKPPKEYF 6eqs.3    --------------------------------------------------------------------------------  target    QRARDEWVRASHDEAAAVVAATLANIAATYSGEEGAQRLRDQGYEEETIEAMGGAGVQAMKFRGGMPLLGMTRVFGLYRM 6eqs.3    --------------------------------------------------------------------------------  target    ANSMALLDAKVRGVGPDEARGARGFDNYSWHTDLPPGHPMVTGQQTVDFDLNSVELAKNVVVWGMNWITTKMPDAHWLTE 6eqs.3    ---------------------------------------------------RELAHCDLCLVVGTSSVV--YPAAMFAPQ  target    ARLKGTRIIVIACEYSSTSSKADDAIVVRPGTTPALALGLSHVIMRDKLYDADYVRRWTDLPMLVRTDTLKYLSAEDVFG 6eqs.3    VAARGVPVAEFNTETTPATNRFRFHF------------------------------------------------------  target    GGPAPL 6eqs.3    ------ ``` | | | | | | | | | | | | | | | | | | | | | | | | | | | | | | | | | | | | | | | | | | | | | | | | | |
|  | 4hda.1.A | NAD-dependent protein deacylase sirtuin-5, mitochondrial  *Crystal structure of human Sirt5 in complex with Fluor-de-Lys peptide and resveratrol* | 0.03 |  | 11.32 | 0.13 | 292-346 | X-ray | 2.60 | monomer | 1 x ZN | HHblits | 0.27 |
| ``` target    TARREITRRGFLGTAAGAGFAAFVVSATRAWGLEAIENPLARYPDREWERVYRDLWRYDSKFTFLCAPNDTHNCLLDAYV 4hda.1    --------------------------------------------------------------------------------  target    RSGVMTRIGPTMRYGEARDLDGNRASARWDPRVCQKGLALTRRFYGDRRLRHCMVRAGFKRWVDEGFPRGEDGKPPKEYF 4hda.1    --------------------------------------------------------------------------------  target    QRARDEWVRASHDEAAAVVAATLANIAATYSGEEGAQRLRDQGYEEETIEAMGGAGVQAMKFRGGMPLLGMTRVFGLYRM 4hda.1    --------------------------------------------------------------------------------  target    ANSMALLDAKVRGVGPDEARGARGFDNYSWHTDLPPGHPMVTGQQTVDFDLNSVELAKNVVVWGMNWITTKMPDAHWLTE 4hda.1    ---------------------------------------------------RELAHCDLCLVVGTSSVV--YPAAMFAPQ  target    ARLKGTRIIVIACEYSSTSSKADDAIVVRPGTTPALALGLSHVIMRDKLYDADYVRRWTDLPMLVRTDTLKYLSAEDVFG 4hda.1    VAARGVPVAEFNTETTPATNRFRFHF------------------------------------------------------  target    GGPAPL 4hda.1    ------ ``` | | | | | | | | | | | | | | | | | | | | | | | | | | | | | | | | | | | | | | | | | | | | | | | | | |
|  | 4hda.2.A | NAD-dependent protein deacylase sirtuin-5, mitochondrial  *Crystal structure of human Sirt5 in complex with Fluor-de-Lys peptide and resveratrol* | 0.03 |  | 11.32 | 0.13 | 292-346 | X-ray | 2.60 | monomer | 1 x ZN, 1 x STL, 1 x HIS-LYS-FDL | HHblits | 0.27 |
| ``` target    TARREITRRGFLGTAAGAGFAAFVVSATRAWGLEAIENPLARYPDREWERVYRDLWRYDSKFTFLCAPNDTHNCLLDAYV 4hda.2    --------------------------------------------------------------------------------  target    RSGVMTRIGPTMRYGEARDLDGNRASARWDPRVCQKGLALTRRFYGDRRLRHCMVRAGFKRWVDEGFPRGEDGKPPKEYF 4hda.2    --------------------------------------------------------------------------------  target    QRARDEWVRASHDEAAAVVAATLANIAATYSGEEGAQRLRDQGYEEETIEAMGGAGVQAMKFRGGMPLLGMTRVFGLYRM 4hda.2    --------------------------------------------------------------------------------  target    ANSMALLDAKVRGVGPDEARGARGFDNYSWHTDLPPGHPMVTGQQTVDFDLNSVELAKNVVVWGMNWITTKMPDAHWLTE 4hda.2    ---------------------------------------------------RELAHCDLCLVVGTSSVV--YPAAMFAPQ  target    ARLKGTRIIVIACEYSSTSSKADDAIVVRPGTTPALALGLSHVIMRDKLYDADYVRRWTDLPMLVRTDTLKYLSAEDVFG 4hda.2    VAARGVPVAEFNTETTPATNRFRFHF------------------------------------------------------  target    GGPAPL 4hda.2    ------ ``` | | | | | | | | | | | | | | | | | | | | | | | | | | | | | | | | | | | | | | | | | | | | | | | | | |
|  | 2nyr.1.A | NAD-dependent deacetylase sirtuin-5  *Crystal Structure of Human Sirtuin Homolog 5 in Complex with Suramin* | 0.02 |  | 11.32 | 0.13 | 292-346 | X-ray | 2.06 | homo-dimer | 1 x SVR, 2 x ZN | HHblits | 0.27 |
| ``` target    TARREITRRGFLGTAAGAGFAAFVVSATRAWGLEAIENPLARYPDREWERVYRDLWRYDSKFTFLCAPNDTHNCLLDAYV 2nyr.1    --------------------------------------------------------------------------------  target    RSGVMTRIGPTMRYGEARDLDGNRASARWDPRVCQKGLALTRRFYGDRRLRHCMVRAGFKRWVDEGFPRGEDGKPPKEYF 2nyr.1    --------------------------------------------------------------------------------  target    QRARDEWVRASHDEAAAVVAATLANIAATYSGEEGAQRLRDQGYEEETIEAMGGAGVQAMKFRGGMPLLGMTRVFGLYRM 2nyr.1    --------------------------------------------------------------------------------  target    ANSMALLDAKVRGVGPDEARGARGFDNYSWHTDLPPGHPMVTGQQTVDFDLNSVELAKNVVVWGMNWITTKMPDAHWLTE 2nyr.1    ---------------------------------------------------RELAHCDLCLVVGTSSVV--YPAAMFAPQ  target    ARLKGTRIIVIACEYSSTSSKADDAIVVRPGTTPALALGLSHVIMRDKLYDADYVRRWTDLPMLVRTDTLKYLSAEDVFG 2nyr.1    VAARGVPVAEFNTETTPATNRFRFHF------------------------------------------------------  target    GGPAPL 2nyr.1    ------ ``` | | | | | | | | | | | | | | | | | | | | | | | | | | | | | | | | | | | | | | | | | | | | | | | | | |
|  | 2nyr.1.B | NAD-dependent deacetylase sirtuin-5  *Crystal Structure of Human Sirtuin Homolog 5 in Complex with Suramin* | 0.03 |  | 11.32 | 0.13 | 292-346 | X-ray | 2.06 | homo-dimer | 1 x SVR, 2 x ZN | HHblits | 0.27 |
| ``` target    TARREITRRGFLGTAAGAGFAAFVVSATRAWGLEAIENPLARYPDREWERVYRDLWRYDSKFTFLCAPNDTHNCLLDAYV 2nyr.1    --------------------------------------------------------------------------------  target    RSGVMTRIGPTMRYGEARDLDGNRASARWDPRVCQKGLALTRRFYGDRRLRHCMVRAGFKRWVDEGFPRGEDGKPPKEYF 2nyr.1    --------------------------------------------------------------------------------  target    QRARDEWVRASHDEAAAVVAATLANIAATYSGEEGAQRLRDQGYEEETIEAMGGAGVQAMKFRGGMPLLGMTRVFGLYRM 2nyr.1    --------------------------------------------------------------------------------  target    ANSMALLDAKVRGVGPDEARGARGFDNYSWHTDLPPGHPMVTGQQTVDFDLNSVELAKNVVVWGMNWITTKMPDAHWLTE 2nyr.1    ---------------------------------------------------RELAHCDLCLVVGTSSVV--YPAAMFAPQ  target    ARLKGTRIIVIACEYSSTSSKADDAIVVRPGTTPALALGLSHVIMRDKLYDADYVRRWTDLPMLVRTDTLKYLSAEDVFG 2nyr.1    VAARGVPVAEFNTETTPATNRFRFHF------------------------------------------------------  target    GGPAPL 2nyr.1    ------ ``` | | | | | | | | | | | | | | | | | | | | | | | | | | | | | | | | | | | | | | | | | | | | | | | | | |
|  | 2b4y.1.A | NAD-dependent deacetylase sirtuin-5  *Crystal Structure of Human Sirtuin homolog 5* | 0.03 |  | 11.32 | 0.13 | 292-346 | X-ray | 1.90 | monomer | 1 x ZN, 1 x APR | HHblits | 0.27 |
| ``` target    TARREITRRGFLGTAAGAGFAAFVVSATRAWGLEAIENPLARYPDREWERVYRDLWRYDSKFTFLCAPNDTHNCLLDAYV 2b4y.1    --------------------------------------------------------------------------------  target    RSGVMTRIGPTMRYGEARDLDGNRASARWDPRVCQKGLALTRRFYGDRRLRHCMVRAGFKRWVDEGFPRGEDGKPPKEYF 2b4y.1    --------------------------------------------------------------------------------  target    QRARDEWVRASHDEAAAVVAATLANIAATYSGEEGAQRLRDQGYEEETIEAMGGAGVQAMKFRGGMPLLGMTRVFGLYRM 2b4y.1    --------------------------------------------------------------------------------  target    ANSMALLDAKVRGVGPDEARGARGFDNYSWHTDLPPGHPMVTGQQTVDFDLNSVELAKNVVVWGMNWITTKMPDAHWLTE 2b4y.1    ---------------------------------------------------RELAHCDLCLVVGTSSVV--YPAAMFAPQ  target    ARLKGTRIIVIACEYSSTSSKADDAIVVRPGTTPALALGLSHVIMRDKLYDADYVRRWTDLPMLVRTDTLKYLSAEDVFG 2b4y.1    VAARGVPVAEFNTETTPATNRFRFHF------------------------------------------------------  target    GGPAPL 2b4y.1    ------ ``` | | | | | | | | | | | | | | | | | | | | | | | | | | | | | | | | | | | | | | | | | | | | | | | | | |
|  | 2b4y.3.A | NAD-dependent deacetylase sirtuin-5  *Crystal Structure of Human Sirtuin homolog 5* | 0.03 |  | 11.32 | 0.13 | 292-346 | X-ray | 1.90 | monomer | 1 x ZN, 1 x APR | HHblits | 0.27 |
| ``` target    TARREITRRGFLGTAAGAGFAAFVVSATRAWGLEAIENPLARYPDREWERVYRDLWRYDSKFTFLCAPNDTHNCLLDAYV 2b4y.3    --------------------------------------------------------------------------------  target    RSGVMTRIGPTMRYGEARDLDGNRASARWDPRVCQKGLALTRRFYGDRRLRHCMVRAGFKRWVDEGFPRGEDGKPPKEYF 2b4y.3    --------------------------------------------------------------------------------  target    QRARDEWVRASHDEAAAVVAATLANIAATYSGEEGAQRLRDQGYEEETIEAMGGAGVQAMKFRGGMPLLGMTRVFGLYRM 2b4y.3    --------------------------------------------------------------------------------  target    ANSMALLDAKVRGVGPDEARGARGFDNYSWHTDLPPGHPMVTGQQTVDFDLNSVELAKNVVVWGMNWITTKMPDAHWLTE 2b4y.3    ---------------------------------------------------RELAHCDLCLVVGTSSVV--YPAAMFAPQ  target    ARLKGTRIIVIACEYSSTSSKADDAIVVRPGTTPALALGLSHVIMRDKLYDADYVRRWTDLPMLVRTDTLKYLSAEDVFG 2b4y.3    VAARGVPVAEFNTETTPATNRFRFHF------------------------------------------------------  target    GGPAPL 2b4y.3    ------ ``` | | | | | | | | | | | | | | | | | | | | | | | | | | | | | | | | | | | | | | | | | | | | | | | | | |
|  | 2e7z.1.A | Acetylene hydratase Ahy  *Acetylene Hydratase from Pelobacter acetylenicus* | 0.04 |  | 9.09 | 0.14 | 296-351 | X-ray | 1.26 | monomer | 1 x SF4, 2 x MGD, 1 x W | HHblits | 0.24 |
| ``` target    TARREITRRGFLGTAAGAGFAAFVVSATRAWGLEAIENPLARYPDREWERVYRDLWRYDSKFTFLCAPNDTHNCLLDAYV 2e7z.1    --------------------------------------------------------------------------------  target    RSGVMTRIGPTMRYGEARDLDGNRASARWDPRVCQKGLALTRRFYGDRRLRHCMVRAGFKRWVDEGFPRGEDGKPPKEYF 2e7z.1    --------------------------------------------------------------------------------  target    QRARDEWVRASHDEAAAVVAATLANIAATYSGEEGAQRLRDQGYEEETIEAMGGAGVQAMKFRGGMPLLGMTRVFGLYRM 2e7z.1    --------------------------------------------------------------------------------  target    ANSMALLDAKVRGVGPDEARGARGFDNYSWHTDLPPGHPMVTGQQTVDFDLNSVELAKNVVVWGMNWITTKMPDAHWLTE 2e7z.1    -------------------------------------------------------PVKAFFALASNALMGYA-NQQNALK  target    ARLKGTRIIVIACEYSSTSSKADDAIVVRPGTTPALALGLSHVIMRDKLYDADYVRRWTDLPMLVRTDTLKYLSAEDVFG 2e7z.1    GLMNQDLVVCYDQFMTPTAQLADYVLPGDHW-------------------------------------------------  target    GGPAPL 2e7z.1    ------ ``` | | | | | | | | | | | | | | | | | | | | | | | | | | | | | | | | | | | | | | | | | | | | | | | | | |
|  | 5ojn.1.A | NAD-dependent protein deacylase  *Sirtuin 4 from Xenopus tropicalis in complex with thioacetyl-ADP-ribose* | 0.03 |  | 9.26 | 0.13 | 292-347 | X-ray | 1.80 | monomer | 1 x ZN, 1 x 9X8 | HHblits | 0.26 |
| ``` target    TARREITRRGFLGTAAGAGFAAFVVSATRAWGLEAIENPLARYPDREWERVYRDLWRYDSKFTFLCAPNDTHNCLLDAYV 5ojn.1    --------------------------------------------------------------------------------  target    RSGVMTRIGPTMRYGEARDLDGNRASARWDPRVCQKGLALTRRFYGDRRLRHCMVRAGFKRWVDEGFPRGEDGKPPKEYF 5ojn.1    --------------------------------------------------------------------------------  target    QRARDEWVRASHDEAAAVVAATLANIAATYSGEEGAQRLRDQGYEEETIEAMGGAGVQAMKFRGGMPLLGMTRVFGLYRM 5ojn.1    --------------------------------------------------------------------------------  target    ANSMALLDAKVRGVGPDEARGARGFDNYSWHTDLPPGHPMVTGQQTVDFDLNSVELAKNVVVWGMNWITTKMPDAHWLTE 5ojn.1    ---------------------------------------------------EQMKQADAMLIVGSSLQV--YSGYRFALN  target    ARLKGTRIIVIACEYSSTSSKADDAIVVRPGTTPALALGLSHVIMRDKLYDADYVRRWTDLPMLVRTDTLKYLSAEDVFG 5ojn.1    AKELHLPIAILNIGPTRADHLAKVKVS-----------------------------------------------------  target    GGPAPL 5ojn.1    ------ ``` | | | | | | | | | | | | | | | | | | | | | | | | | | | | | | | | | | | | | | | | | | | | | | | | | |
|  | 5oj7.1.A | NAD-dependent protein deacylase  *Sirtuin 4 orthologue from Xenopus Tropicalis in complex with ADP-ribose* | 0.03 |  | 9.26 | 0.13 | 292-347 | X-ray | 1.58 | monomer | 1 x AR6, 1 x ZN | HHblits | 0.26 |
| ``` target    TARREITRRGFLGTAAGAGFAAFVVSATRAWGLEAIENPLARYPDREWERVYRDLWRYDSKFTFLCAPNDTHNCLLDAYV 5oj7.1    --------------------------------------------------------------------------------  target    RSGVMTRIGPTMRYGEARDLDGNRASARWDPRVCQKGLALTRRFYGDRRLRHCMVRAGFKRWVDEGFPRGEDGKPPKEYF 5oj7.1    --------------------------------------------------------------------------------  target    QRARDEWVRASHDEAAAVVAATLANIAATYSGEEGAQRLRDQGYEEETIEAMGGAGVQAMKFRGGMPLLGMTRVFGLYRM 5oj7.1    --------------------------------------------------------------------------------  target    ANSMALLDAKVRGVGPDEARGARGFDNYSWHTDLPPGHPMVTGQQTVDFDLNSVELAKNVVVWGMNWITTKMPDAHWLTE 5oj7.1    ---------------------------------------------------EQMKQADAMLIVGSSLQV--YSGYRFALN  target    ARLKGTRIIVIACEYSSTSSKADDAIVVRPGTTPALALGLSHVIMRDKLYDADYVRRWTDLPMLVRTDTLKYLSAEDVFG 5oj7.1    AKELHLPIAILNIGPTRADHLAKVKVS-----------------------------------------------------  target    GGPAPL 5oj7.1    ------ ``` | | | | | | | | | | | | | | | | | | | | | | | | | | | | | | | | | | | | | | | | | | | | | | | | | |
|  | 2vpz.1.A | THIOSULFATE REDUCTASE  *POLYSULFIDE REDUCTASE NATIVE STRUCTURE* | 0.03 |  | 14.81 | 0.13 | 297-351 | X-ray | 2.40 | hetero-oligomer | 10 x SF4, 4 x MGD, 2 x MO | HHblits | 0.26 |
| ``` target    TARREITRRGFLGTAAGAGFAAFVVSATRAWGLEAIENPLARYPDREWERVYRDLWRYDSKFTFLCAPNDTHNCLLDAYV 2vpz.1    --------------------------------------------------------------------------------  target    RSGVMTRIGPTMRYGEARDLDGNRASARWDPRVCQKGLALTRRFYGDRRLRHCMVRAGFKRWVDEGFPRGEDGKPPKEYF 2vpz.1    --------------------------------------------------------------------------------  target    QRARDEWVRASHDEAAAVVAATLANIAATYSGEEGAQRLRDQGYEEETIEAMGGAGVQAMKFRGGMPLLGMTRVFGLYRM 2vpz.1    --------------------------------------------------------------------------------  target    ANSMALLDAKVRGVGPDEARGARGFDNYSWHTDLPPGHPMVTGQQTVDFDLNSVELAKNVVVWGMNWITTKMPDAHWLTE 2vpz.1    --------------------------------------------------------IKGLFAYGINLFHSIP-NVPRTKE  target    ARLKGTRIIVIACEYSSTSSKADDAIVVRPGTTPALALGLSHVIMRDKLYDADYVRRWTDLPMLVRTDTLKYLSAEDVFG 2vpz.1    ALKNLDLYVAIDVLPQEHVMWADVILPEATY-------------------------------------------------  target    GGPAPL 2vpz.1    ------ ``` | | | | | | | | | | | | | | | | | | | | | | | | | | | | | | | | | | | | | | | | | | | | | | | | | |
|  | 2vpx.1.D | THIOSULFATE REDUCTASE  *POLYSULFIDE REDUCTASE WITH BOUND QUINONE (UQ1)* | 0.03 |  | 14.81 | 0.13 | 297-351 | X-ray | 3.10 | hetero-oligomer | 10 x SF4, 4 x MGD, 2 x MO, 2 x UQ1 | HHblits | 0.26 |
| ``` target    TARREITRRGFLGTAAGAGFAAFVVSATRAWGLEAIENPLARYPDREWERVYRDLWRYDSKFTFLCAPNDTHNCLLDAYV 2vpx.1    --------------------------------------------------------------------------------  target    RSGVMTRIGPTMRYGEARDLDGNRASARWDPRVCQKGLALTRRFYGDRRLRHCMVRAGFKRWVDEGFPRGEDGKPPKEYF 2vpx.1    --------------------------------------------------------------------------------  target    QRARDEWVRASHDEAAAVVAATLANIAATYSGEEGAQRLRDQGYEEETIEAMGGAGVQAMKFRGGMPLLGMTRVFGLYRM 2vpx.1    --------------------------------------------------------------------------------  target    ANSMALLDAKVRGVGPDEARGARGFDNYSWHTDLPPGHPMVTGQQTVDFDLNSVELAKNVVVWGMNWITTKMPDAHWLTE 2vpx.1    --------------------------------------------------------IKGLFAYGINLFHSIP-NVPRTKE  target    ARLKGTRIIVIACEYSSTSSKADDAIVVRPGTTPALALGLSHVIMRDKLYDADYVRRWTDLPMLVRTDTLKYLSAEDVFG 2vpx.1    ALKNLDLYVAIDVLPQEHVMWADVILPEATY-------------------------------------------------  target    GGPAPL 2vpx.1    ------ ``` | | | | | | | | | | | | | | | | | | | | | | | | | | | | | | | | | | | | | | | | | | | | | | | | | |
|  | 2x3y.1.A | PHOSPHOHEPTOSE ISOMERASE  *Crystal structure of GmhA from Burkholderia pseudomallei* | 0.04 |  | 11.11 | 0.13 | 297-352 | X-ray | 2.40 | homo-tetramer | 4 x ZN | HHblits | 0.25 |
| ``` target    TARREITRRGFLGTAAGAGFAAFVVSATRAWGLEAIENPLARYPDREWERVYRDLWRYDSKFTFLCAPNDTHNCLLDAYV 2x3y.1    --------------------------------------------------------------------------------  target    RSGVMTRIGPTMRYGEARDLDGNRASARWDPRVCQKGLALTRRFYGDRRLRHCMVRAGFKRWVDEGFPRGEDGKPPKEYF 2x3y.1    --------------------------------------------------------------------------------  target    QRARDEWVRASHDEAAAVVAATLANIAATYSGEEGAQRLRDQGYEEETIEAMGGAGVQAMKFRGGMPLLGMTRVFGLYRM 2x3y.1    --------------------------------------------------------------------------------  target    ANSMALLDAKVRGVGPDEARGARGFDNYSWHTDLPPGHPMVTGQQTVDFDLNSVELAKNVVVWGMNWITTKMPDAHWLTE 2x3y.1    --------------------------------------------------------GDVLIGYSTSGK-S-PNILAAFRE  target    ARLKGTRIIVIACE-YSSTSSKADDAIVVRPGTTPALALGLSHVIMRDKLYDADYVRRWTDLPMLVRTDTLKYLSAEDVF 2x3y.1    AKAKGMTCVGFTGNRGGEMRELCDLLLEVPSAD-----------------------------------------------  target    GGGPAPL 2x3y.1    ------- ``` | | | | | | | | | | | | | | | | | | | | | | | | | | | | | | | | | | | | | | | | | | | | | | | | | |
|  | 2h59.1.B | NAD-dependent deacetylase  *Sir2 H116A-deacetylated p53 peptide-3'-o-acetyl ADP ribose* | 0.03 |  | 13.21 | 0.13 | 293-347 | X-ray | 1.90 | hetero-2-2-mer | 2 x ZN, 1 x APR, 1 x 3OD | HHblits | 0.27 |
| ``` target    TARREITRRGFLGTAAGAGFAAFVVSATRAWGLEAIENPLARYPDREWERVYRDLWRYDSKFTFLCAPNDTHNCLLDAYV 2h59.1    --------------------------------------------------------------------------------  target    RSGVMTRIGPTMRYGEARDLDGNRASARWDPRVCQKGLALTRRFYGDRRLRHCMVRAGFKRWVDEGFPRGEDGKPPKEYF 2h59.1    --------------------------------------------------------------------------------  target    QRARDEWVRASHDEAAAVVAATLANIAATYSGEEGAQRLRDQGYEEETIEAMGGAGVQAMKFRGGMPLLGMTRVFGLYRM 2h59.1    --------------------------------------------------------------------------------  target    ANSMALLDAKVRGVGPDEARGARGFDNYSWHTDLPPGHPMVTGQQTVDFDLNSVELAKNVVVWGMNWITTKMPDAHWLTE 2h59.1    ----------------------------------------------------LSSRASLMIVLGSSLVV--YPAAELPLI  target    ARLKGTRIIVIACEYSSTSSKADDAIVVRPGTTPALALGLSHVIMRDKLYDADYVRRWTDLPMLVRTDTLKYLSAEDVFG 2h59.1    TVRSGGKLVIVNLGETPFDDIATLKYN-----------------------------------------------------  target    GGPAPL 2h59.1    ------ ``` | | | | | | | | | | | | | | | | | | | | | | | | | | | | | | | | | | | | | | | | | | | | | | | | | |
|  | 3jr3.1.A | NAD-dependent deacetylase  *Sir2 bound to acetylated peptide* | 0.03 |  | 13.21 | 0.13 | 293-347 | X-ray | 1.50 | hetero-1-1-mer | 1 x ZN | HHblits | 0.27 |
| ``` target    TARREITRRGFLGTAAGAGFAAFVVSATRAWGLEAIENPLARYPDREWERVYRDLWRYDSKFTFLCAPNDTHNCLLDAYV 3jr3.1    --------------------------------------------------------------------------------  target    RSGVMTRIGPTMRYGEARDLDGNRASARWDPRVCQKGLALTRRFYGDRRLRHCMVRAGFKRWVDEGFPRGEDGKPPKEYF 3jr3.1    --------------------------------------------------------------------------------  target    QRARDEWVRASHDEAAAVVAATLANIAATYSGEEGAQRLRDQGYEEETIEAMGGAGVQAMKFRGGMPLLGMTRVFGLYRM 3jr3.1    --------------------------------------------------------------------------------  target    ANSMALLDAKVRGVGPDEARGARGFDNYSWHTDLPPGHPMVTGQQTVDFDLNSVELAKNVVVWGMNWITTKMPDAHWLTE 3jr3.1    ----------------------------------------------------LSSRASLMIVLGSSLVV--YPAAELPLI  target    ARLKGTRIIVIACEYSSTSSKADDAIVVRPGTTPALALGLSHVIMRDKLYDADYVRRWTDLPMLVRTDTLKYLSAEDVFG 3jr3.1    TVRSGGKLVIVNLGETPFDDIATLKYN-----------------------------------------------------  target    GGPAPL 3jr3.1    ------ ``` | | | | | | | | | | | | | | | | | | | | | | | | | | | | | | | | | | | | | | | | | | | | | | | | | |
|  | 7p63.1.C | NADH-quinone oxidoreductase  *Complex I from E. coli, DDM/LMNG-purified, under Turnover at pH 6, Closed state* | 0.03 |  | 19.23 | 0.13 | 296-349 | EM | 0.00 | hetero-1-1-1-1-1-1-… | 7 x SF4, 1 x FMN, 1 x NAI, 2 x FES, 1 x CA, 1 x DCQ, 4 x LFA, 8 x 3PE | HHblits | 0.28 |
| ``` target    TARREITRRGFLGTAAGAGFAAFVVSATRAWGLEAIENPLARYPDREWERVYRDLWRYDSKFTFLCAPNDTHNCLLDAYV 7p63.1    --------------------------------------------------------------------------------  target    RSGVMTRIGPTMRYGEARDLDGNRASARWDPRVCQKGLALTRRFYGDRRLRHCMVRAGFKRWVDEGFPRGEDGKPPKEYF 7p63.1    --------------------------------------------------------------------------------  target    QRARDEWVRASHDEAAAVVAATLANIAATYSGEEGAQRLRDQGYEEETIEAMGGAGVQAMKFRGGMPLLGMTRVFGLYRM 7p63.1    --------------------------------------------------------------------------------  target    ANSMALLDAKVRGVGPDEARGARGFDNYSWHTDLPPGHPMVTGQQTVDFDLNSVELAKNVVVWGMNWITTKMPDAHWLTE 7p63.1    -------------------------------------------------------RADAVVVLE-NDLHRH-ASATRVNA  target    ARLKGTRIIVIACEYSSTSSKADDAIVVRPGTTPALALGLSHVIMRDKLYDADYVRRWTDLPMLVRTDTLKYLSAEDVFG 7p63.1    ALAKAPLVMVVDHQRTAIMENAHLVLSAA---------------------------------------------------  target    GGPAPL 7p63.1    ------ ``` | | | | | | | | | | | | | | | | | | | | | | | | | | | | | | | | | | | | | | | | | | | | | | | | | |
|  | 7nz1.1.E | NADH-quinone oxidoreductase subunit G  *Respiratory complex I from Escherichia coli - focused refinement of cytoplasmic arm* | 0.03 |  | 19.23 | 0.13 | 296-349 | EM | 0.00 | hetero-1-1-1-1-1-1-… | 7 x SF4, 2 x FES, 1 x FMN, 1 x CA | HHblits | 0.28 |
| ``` target    TARREITRRGFLGTAAGAGFAAFVVSATRAWGLEAIENPLARYPDREWERVYRDLWRYDSKFTFLCAPNDTHNCLLDAYV 7nz1.1    --------------------------------------------------------------------------------  target    RSGVMTRIGPTMRYGEARDLDGNRASARWDPRVCQKGLALTRRFYGDRRLRHCMVRAGFKRWVDEGFPRGEDGKPPKEYF 7nz1.1    --------------------------------------------------------------------------------  target    QRARDEWVRASHDEAAAVVAATLANIAATYSGEEGAQRLRDQGYEEETIEAMGGAGVQAMKFRGGMPLLGMTRVFGLYRM 7nz1.1    --------------------------------------------------------------------------------  target    ANSMALLDAKVRGVGPDEARGARGFDNYSWHTDLPPGHPMVTGQQTVDFDLNSVELAKNVVVWGMNWITTKMPDAHWLTE 7nz1.1    -------------------------------------------------------RADAVVVLE-NDLHRH-ASAIRVNA  target    ARLKGTRIIVIACEYSSTSSKADDAIVVRPGTTPALALGLSHVIMRDKLYDADYVRRWTDLPMLVRTDTLKYLSAEDVFG 7nz1.1    ALAKAPLVMVVDHQRTAIMENAHLVLSAA---------------------------------------------------  target    GGPAPL 7nz1.1    ------ ``` | | | | | | | | | | | | | | | | | | | | | | | | | | | | | | | | | | | | | | | | | | | | | | | | | |
|  | 4v4c.1.A | Pyrogallol hydroxytransferase large subunit  *Crystal Structure of Pyrogallol-Phloroglucinol Transhydroxylase from Pelobacter acidigallici* | 0.03 |  | 9.09 | 0.14 | 296-351 | X-ray | 2.35 | hetero-oligomer | 2 x CA, 2 x MGD, 1 x 4MO, 3 x SF4 | HHblits | 0.24 |
| ``` target    TARREITRRGFLGTAAGAGFAAFVVSATRAWGLEAIENPLARYPDREWERVYRDLWRYDSKFTFLCAPNDTHNCLLDAYV 4v4c.1    --------------------------------------------------------------------------------  target    RSGVMTRIGPTMRYGEARDLDGNRASARWDPRVCQKGLALTRRFYGDRRLRHCMVRAGFKRWVDEGFPRGEDGKPPKEYF 4v4c.1    --------------------------------------------------------------------------------  target    QRARDEWVRASHDEAAAVVAATLANIAATYSGEEGAQRLRDQGYEEETIEAMGGAGVQAMKFRGGMPLLGMTRVFGLYRM 4v4c.1    --------------------------------------------------------------------------------  target    ANSMALLDAKVRGVGPDEARGARGFDNYSWHTDLPPGHPMVTGQQTVDFDLNSVELAKNVVVWGMNWITTKMPDAHWLTE 4v4c.1    -------------------------------------------------------KIKMFWKYGGPHLGTMT-ATNRYAK  target    AR--LKGTRIIVIACEYSSTSSKADDAIVVRPGTTPALALGLSHVIMRDKLYDADYVRRWTDLPMLVRTDTLKYLSAEDV 4v4c.1    MYTHDSLEFVVSQSIWFEGEVPFADIILPACTN-----------------------------------------------  target    FGGGPAPL 4v4c.1    -------- ``` | | | | | | | | | | | | | | | | | | | | | | | | | | | | | | | | | | | | | | | | | | | | | | | | | |
|  | 7vw6.1.A | Formate dehydrogenase  *Cryo-EM Structure of Formate Dehydrogenase 1 from Methylorubrum extorquens AM1* | 0.03 |  | 17.31 | 0.13 | 296-348 | EM | 0.00 | hetero-1-1-mer | 4 x SF4, 2 x FES, 2 x MGD, 1 x W, 1 x FMN | HHblits | 0.28 |
| ``` target    TARREITRRGFLGTAAGAGFAAFVVSATRAWGLEAIENPLARYPDREWERVYRDLWRYDSKFTFLCAPNDTHNCLLDAYV 7vw6.1    --------------------------------------------------------------------------------  target    RSGVMTRIGPTMRYGEARDLDGNRASARWDPRVCQKGLALTRRFYGDRRLRHCMVRAGFKRWVDEGFPRGEDGKPPKEYF 7vw6.1    --------------------------------------------------------------------------------  target    QRARDEWVRASHDEAAAVVAATLANIAATYSGEEGAQRLRDQGYEEETIEAMGGAGVQAMKFRGGMPLLGMTRVFGLYRM 7vw6.1    --------------------------------------------------------------------------------  target    ANSMALLDAKVRGVGPDEARGARGFDNYSWHTDLPPGHPMVTGQQTVDFDLNSVELAKNVVVWGMNWITTKMPDAHWLTE 7vw6.1    -------------------------------------------------------EIRGMFVEGENPAMSDP-DLNHARH  target    ARLKGTRIIVIACEYSSTSSKADDAIVVRPGTTPALALGLSHVIMRDKLYDADYVRRWTDLPMLVRTDTLKYLSAEDVFG 7vw6.1    ALAMLDHLVVQDLFLTETAFHADVVLPA----------------------------------------------------  target    GGPAPL 7vw6.1    ------ ``` | | | | | | | | | | | | | | | | | | | | | | | | | | | | | | | | | | | | | | | | | | | | | | | | | |
|  | 7e5z.1.A | Formate dehydrogenase  *Dehydrogenase holoenzyme* | 0.02 |  | 17.31 | 0.13 | 296-348 | EM | 0.00 | hetero-1-1-mer | 1 x W, 2 x MGD, 2 x FES, 4 x SF4, 1 x FMN | HHblits | 0.28 |
| ``` target    TARREITRRGFLGTAAGAGFAAFVVSATRAWGLEAIENPLARYPDREWERVYRDLWRYDSKFTFLCAPNDTHNCLLDAYV 7e5z.1    --------------------------------------------------------------------------------  target    RSGVMTRIGPTMRYGEARDLDGNRASARWDPRVCQKGLALTRRFYGDRRLRHCMVRAGFKRWVDEGFPRGEDGKPPKEYF 7e5z.1    --------------------------------------------------------------------------------  target    QRARDEWVRASHDEAAAVVAATLANIAATYSGEEGAQRLRDQGYEEETIEAMGGAGVQAMKFRGGMPLLGMTRVFGLYRM 7e5z.1    --------------------------------------------------------------------------------  target    ANSMALLDAKVRGVGPDEARGARGFDNYSWHTDLPPGHPMVTGQQTVDFDLNSVELAKNVVVWGMNWITTKMPDAHWLTE 7e5z.1    -------------------------------------------------------EIRGMFVEGENPAMSDP-DLNHARH  target    ARLKGTRIIVIACEYSSTSSKADDAIVVRPGTTPALALGLSHVIMRDKLYDADYVRRWTDLPMLVRTDTLKYLSAEDVFG 7e5z.1    ALAMLDHLVVQDLFLTETAFHADVVLPA----------------------------------------------------  target    GGPAPL 7e5z.1    ------ ``` | | | | | | | | | | | | | | | | | | | | | | | | | | | | | | | | | | | | | | | | | | | | | | | | | |
|  | 6btm.1.B | Alternative Complex III subunit B  *Structure of Alternative Complex III from Flavobacterium johnsoniae (Wild Type)* | 0.03 |  | 11.54 | 0.13 | 296-348 | EM | 3.40 | hetero-1-1-1-1-1-1-… | 6 x HEC, 1 x F3S, 1 x SF4, 2 x E87 | HHblits | 0.27 |
| ``` target    TARREITRRGFLGTAAGAGFAAFVVSATRAWGLEAIENPLARYPDREWERVYRDLWRYDSKFTFLCAPNDTHNCLLDAYV 6btm.1    --------------------------------------------------------------------------------  target    RSGVMTRIGPTMRYGEARDLDGNRASARWDPRVCQKGLALTRRFYGDRRLRHCMVRAGFKRWVDEGFPRGEDGKPPKEYF 6btm.1    --------------------------------------------------------------------------------  target    QRARDEWVRASHDEAAAVVAATLANIAATYSGEEGAQRLRDQGYEEETIEAMGGAGVQAMKFRGGMPLLGMTRVFGLYRM 6btm.1    --------------------------------------------------------------------------------  target    ANSMALLDAKVRGVGPDEARGARGFDNYSWHTDLPPGHPMVTGQQTVDFDLNSVELAKNVVVWGMNWITTKMPDAHWLTE 6btm.1    -------------------------------------------------------SVHTLIMSGVNPVYTLADS-ASFVS  target    ARLKGTRIIVIACEYSSTSSKADDAIVVRPGTTPALALGLSHVIMRDKLYDADYVRRWTDLPMLVRTDTLKYLSAEDVFG 6btm.1    GLKKVKTSVAFSLKEDETAAVSTIAAAA----------------------------------------------------  target    GGPAPL 6btm.1    ------ ``` | | | | | | | | | | | | | | | | | | | | | | | | | | | | | | | | | | | | | | | | | | | | | | | | | |
|  | 1yc5.1.A | NAD-dependent deacetylase  *Sir2-p53 peptide-nicotinamide* | 0.03 |  | 13.46 | 0.13 | 293-346 | X-ray | 1.40 | hetero-oligomer | 1 x ZN, 1 x NCA | HHblits | 0.27 |
| ``` target    TARREITRRGFLGTAAGAGFAAFVVSATRAWGLEAIENPLARYPDREWERVYRDLWRYDSKFTFLCAPNDTHNCLLDAYV 1yc5.1    --------------------------------------------------------------------------------  target    RSGVMTRIGPTMRYGEARDLDGNRASARWDPRVCQKGLALTRRFYGDRRLRHCMVRAGFKRWVDEGFPRGEDGKPPKEYF 1yc5.1    --------------------------------------------------------------------------------  target    QRARDEWVRASHDEAAAVVAATLANIAATYSGEEGAQRLRDQGYEEETIEAMGGAGVQAMKFRGGMPLLGMTRVFGLYRM 1yc5.1    --------------------------------------------------------------------------------  target    ANSMALLDAKVRGVGPDEARGARGFDNYSWHTDLPPGHPMVTGQQTVDFDLNSVELAKNVVVWGMNWITTKMPDAHWLTE 1yc5.1    ----------------------------------------------------LSSRASLMIVLGSSLVV--YPAAELPLI  target    ARLKGTRIIVIACEYSSTSSKADDAIVVRPGTTPALALGLSHVIMRDKLYDADYVRRWTDLPMLVRTDTLKYLSAEDVFG 1yc5.1    TVRSGGKLVIVNLGETPFDDIATLKY------------------------------------------------------  target    GGPAPL 1yc5.1    ------ ``` | | | | | | | | | | | | | | | | | | | | | | | | | | | | | | | | | | | | | | | | | | | | | | | | | |
|  | 2h2i.1.A | NAD-dependent deacetylase  *The Structural basis of Sirtuin Substrate Affinity* | 0.03 |  | 13.46 | 0.13 | 293-346 | X-ray | 1.80 | homo-octamer | 8 x ZN, 8 x ZPG | HHblits | 0.27 |
| ``` target    TARREITRRGFLGTAAGAGFAAFVVSATRAWGLEAIENPLARYPDREWERVYRDLWRYDSKFTFLCAPNDTHNCLLDAYV 2h2i.1    --------------------------------------------------------------------------------  target    RSGVMTRIGPTMRYGEARDLDGNRASARWDPRVCQKGLALTRRFYGDRRLRHCMVRAGFKRWVDEGFPRGEDGKPPKEYF 2h2i.1    --------------------------------------------------------------------------------  target    QRARDEWVRASHDEAAAVVAATLANIAATYSGEEGAQRLRDQGYEEETIEAMGGAGVQAMKFRGGMPLLGMTRVFGLYRM 2h2i.1    --------------------------------------------------------------------------------  target    ANSMALLDAKVRGVGPDEARGARGFDNYSWHTDLPPGHPMVTGQQTVDFDLNSVELAKNVVVWGMNWITTKMPDAHWLTE 2h2i.1    ----------------------------------------------------LSSRASLMIVLGSSLVV--YPAAELPLI  target    ARLKGTRIIVIACEYSSTSSKADDAIVVRPGTTPALALGLSHVIMRDKLYDADYVRRWTDLPMLVRTDTLKYLSAEDVFG 2h2i.1    TVRSGGKLVIVNLGETPFDDIATLKY------------------------------------------------------  target    GGPAPL 2h2i.1    ------ ``` | | | | | | | | | | | | | | | | | | | | | | | | | | | | | | | | | | | | | | | | | | | | | | | | | |
|  | 4bv2.3.A | NAD-DEPENDENT PROTEIN DEACETYLASE  *CRYSTAL STRUCTURE OF SIR2 IN COMPLEX WITH THE INHIBITOR EX-527, 2'-O-ACETYL-ADP-RIBOSE AND DEACETYLATED P53-PEPTIDE* | 0.03 |  | 13.46 | 0.13 | 293-346 | X-ray | 3.30 | hetero-oligomer | 1 x OCZ, 1 x OAD, 1 x ZN | HHblits | 0.27 |
| ``` target    TARREITRRGFLGTAAGAGFAAFVVSATRAWGLEAIENPLARYPDREWERVYRDLWRYDSKFTFLCAPNDTHNCLLDAYV 4bv2.3    --------------------------------------------------------------------------------  target    RSGVMTRIGPTMRYGEARDLDGNRASARWDPRVCQKGLALTRRFYGDRRLRHCMVRAGFKRWVDEGFPRGEDGKPPKEYF 4bv2.3    --------------------------------------------------------------------------------  target    QRARDEWVRASHDEAAAVVAATLANIAATYSGEEGAQRLRDQGYEEETIEAMGGAGVQAMKFRGGMPLLGMTRVFGLYRM 4bv2.3    --------------------------------------------------------------------------------  target    ANSMALLDAKVRGVGPDEARGARGFDNYSWHTDLPPGHPMVTGQQTVDFDLNSVELAKNVVVWGMNWITTKMPDAHWLTE 4bv2.3    ----------------------------------------------------LSSRASLMIVLGSSLVV--YPAAELPLI  target    ARLKGTRIIVIACEYSSTSSKADDAIVVRPGTTPALALGLSHVIMRDKLYDADYVRRWTDLPMLVRTDTLKYLSAEDVFG 4bv2.3    TVRSGGKLVIVNLGETPFDDIATLKY------------------------------------------------------  target    GGPAPL 4bv2.3    ------ ``` | | | | | | | | | | | | | | | | | | | | | | | | | | | | | | | | | | | | | | | | | | | | | | | | | |
|  | 4wd3.1.A | L-amino acid ligase  *Crystal structure of an L-amino acid ligase RizA* | 0.02 |  | 15.38 | 0.13 | 300-355 | X-ray | 2.80 | homo-dimer |  | HHblits | 0.27 |
| ``` target    TARREITRRGFLGTAAGAGFAAFVVSATRAWGLEAIENPLARYPDREWERVYRDLWRYDSKFTFLCAPNDTHNCLLDAYV 4wd3.1    --------------------------------------------------------------------------------  target    RSGVMTRIGPTMRYGEARDLDGNRASARWDPRVCQKGLALTRRFYGDRRLRHCMVRAGFKRWVDEGFPRGEDGKPPKEYF 4wd3.1    --------------------------------------------------------------------------------  target    QRARDEWVRASHDEAAAVVAATLANIAATYSGEEGAQRLRDQGYEEETIEAMGGAGVQAMKFRGGMPLLGMTRVFGLYRM 4wd3.1    --------------------------------------------------------------------------------  target    ANSMALLDAKVRGVGPDEARGARGFDNYSWHTDLPPGHPMVTGQQTVDFDLNSVELAKNVVVWGMNWITTKMPDAHWLTE 4wd3.1    -----------------------------------------------------------ILLINSDKP----EPIQFFQK  target    ARLKG--TRIIVIACEYSST--SSKADDAIVVRPGTTPALALGLSHVIMRDKLYDADYVRRWTDLPMLVRTDTLKYLSAE 4wd3.1    DKETNDSINISVITRSCYAPLYSHWADHVYIVDDVTDLT-----------------------------------------  target    DVFGGGPAPL 4wd3.1    ---------- ``` | | | | | | | | | | | | | | | | | | | | | | | | | | | | | | | | | | | | | | | | | | | | | | | | | |
|  | 6f0k.1.B | Fe-S-cluster-containing hydrogenase  *Alternative complex III* | 0.03 |  | 11.54 | 0.13 | 296-348 | EM | 0.00 | hetero-1-1-1-1-1-1-… | 6 x HEC, 1 x F3S, 3 x SF4 | HHblits | 0.27 |
| ``` target    TARREITRRGFLGTAAGAGFAAFVVSATRAWGLEAIENPLARYPDREWERVYRDLWRYDSKFTFLCAPNDTHNCLLDAYV 6f0k.1    --------------------------------------------------------------------------------  target    RSGVMTRIGPTMRYGEARDLDGNRASARWDPRVCQKGLALTRRFYGDRRLRHCMVRAGFKRWVDEGFPRGEDGKPPKEYF 6f0k.1    --------------------------------------------------------------------------------  target    QRARDEWVRASHDEAAAVVAATLANIAATYSGEEGAQRLRDQGYEEETIEAMGGAGVQAMKFRGGMPLLGMTRVFGLYRM 6f0k.1    --------------------------------------------------------------------------------  target    ANSMALLDAKVRGVGPDEARGARGFDNYSWHTDLPPGHPMVTGQQTVDFDLNSVELAKNVVVWGMNWITTKMPDAHWLTE 6f0k.1    -------------------------------------------------------AVDALLLLNVNPVYDAPA-ALGFAE  target    ARLKGTRIIVIACEYSSTSSKADDAIVVRPGTTPALALGLSHVIMRDKLYDADYVRRWTDLPMLVRTDTLKYLSAEDVFG 6f0k.1    ALAQVPEVIHLGLHVDETARRSTWHLPS----------------------------------------------------  target    GGPAPL 6f0k.1    ------ ``` | | | | | | | | | | | | | | | | | | | | | | | | | | | | | | | | | | | | | | | | | | | | | | | | | |
|  | 1ogy.1.A | PERIPLASMIC NITRATE REDUCTASE  *Crystal structure of the heterodimeric nitrate reductase from Rhodobacter sphaeroides* | 0.03 |  | 14.81 | 0.13 | 296-349 | X-ray | 3.20 | hetero-1-1-mer | 1 x SF4, 1 x MO, 2 x MGD, 2 x HEC | HHblits | 0.24 |
| ``` target    TARREITRRGFLGTAAGAGFAAFVVSATRAWGLEAIENPLARYPDREWERVYRDLWRYDSKFTFLCAPNDTHNCLLDAYV 1ogy.1    --------------------------------------------------------------------------------  target    RSGVMTRIGPTMRYGEARDLDGNRASARWDPRVCQKGLALTRRFYGDRRLRHCMVRAGFKRWVDEGFPRGEDGKPPKEYF 1ogy.1    --------------------------------------------------------------------------------  target    QRARDEWVRASHDEAAAVVAATLANIAATYSGEEGAQRLRDQGYEEETIEAMGGAGVQAMKFRGGMPLLGMTRVFGLYRM 1ogy.1    --------------------------------------------------------------------------------  target    ANSMALLDAKVRGVGPDEARGARGFDNYSWHTDLPPGHPMVTGQQTVDFDLNSVELAKNVVVWGMNWITTKMPDA-HWLT 1ogy.1    -------------------------------------------------------EINFYWVQVNNNMQAAPNIDQETYP  target    EARLKGTRIIVIACEYSSTSSKADDAIVVRPGTTPALALGLSHVIMRDKLYDADYVRRWTDLPMLVRTDTLKYLSAEDVF 1ogy.1    GYRNPENFIVVSDAYPTVTGRAADLVLPAA--------------------------------------------------  target    GGGPAPL 1ogy.1    ------- ``` | | | | | | | | | | | | | | | | | | | | | | | | | | | | | | | | | | | | | | | | | | | | | | | | | |
|  | 3o5a.1.A | Periplasmic nitrate reductase  *Crystal Structure of partially reduced Periplasmic Nitrate Reductase from Cupriavidus necator using Ionic Liquids* | 0.03 |  | 15.09 | 0.13 | 295-350 | X-ray | 1.72 | hetero-oligomer | 1 x SF4, 1 x MOS, 2 x MGD, 2 x HEC | HHblits | 0.25 |
| ``` target    TARREITRRGFLGTAAGAGFAAFVVSATRAWGLEAIENPLARYPDREWERVYRDLWRYDSKFTFLCAPNDTHNCLLDAYV 3o5a.1    --------------------------------------------------------------------------------  target    RSGVMTRIGPTMRYGEARDLDGNRASARWDPRVCQKGLALTRRFYGDRRLRHCMVRAGFKRWVDEGFPRGEDGKPPKEYF 3o5a.1    --------------------------------------------------------------------------------  target    QRARDEWVRASHDEAAAVVAATLANIAATYSGEEGAQRLRDQGYEEETIEAMGGAGVQAMKFRGGMPLLGMTRVFGLYRM 3o5a.1    --------------------------------------------------------------------------------  target    ANSMALLDAKVRGVGPDEARGARGFDNYSWHTDLPPGHPMVTGQQTVDFDLNSVELAKNVVVWGMNWITTKMPDAHWLTE 3o5a.1    ------------------------------------------------------GKLNAYWVQVNNNMQAAAN---LMEE  target    AR----LKGTRIIVIACEYSSTSSKADDAIVVRPGTTPALALGLSHVIMRDKLYDADYVRRWTDLPMLVRTDTLKYLSAE 3o5a.1    GLPGYRNPANFIVVSDAYPTVTALAADLVLPSAM----------------------------------------------  target    DVFGGGPAPL 3o5a.1    ---------- ``` | | | | | | | | | | | | | | | | | | | | | | | | | | | | | | | | | | | | | | | | | | | | | | | | | |
|  | 2nya.1.A | Periplasmic nitrate reductase  *Crystal structure of the periplasmic nitrate reductase (NAP) from Escherichia coli* | 0.03 |  | 12.96 | 0.13 | 296-349 | X-ray | 2.50 | monomer | 1 x SF4, 1 x 6MO, 2 x MGD | HHblits | 0.22 |
| ``` target    TARREITRRGFLGTAAGAGFAAFVVSATRAWGLEAIENPLARYPDREWERVYRDLWRYDSKFTFLCAPNDTHNCLLDAYV 2nya.1    --------------------------------------------------------------------------------  target    RSGVMTRIGPTMRYGEARDLDGNRASARWDPRVCQKGLALTRRFYGDRRLRHCMVRAGFKRWVDEGFPRGEDGKPPKEYF 2nya.1    --------------------------------------------------------------------------------  target    QRARDEWVRASHDEAAAVVAATLANIAATYSGEEGAQRLRDQGYEEETIEAMGGAGVQAMKFRGGMPLLGMTRVFGLYRM 2nya.1    --------------------------------------------------------------------------------  target    ANSMALLDAKVRGVGPDEARGARGFDNYSWHTDLPPGHPMVTGQQTVDFDLNSVELAKNVVVWGMNWITTKMPDA-HWLT 2nya.1    -------------------------------------------------------KLNVYWTMCTNNMQAGPNINEERMP  target    EARLKGTRIIVIACEYSSTSSKADDAIVVRPGTTPALALGLSHVIMRDKLYDADYVRRWTDLPMLVRTDTLKYLSAEDVF 2nya.1    GWRDPRNFIIVSDPYPTVSALAADLILPTA--------------------------------------------------  target    GGGPAPL 2nya.1    ------- ``` | | | | | | | | | | | | | | | | | | | | | | | | | | | | | | | | | | | | | | | | | | | | | | | | | |
|  | 7bkb.1.L | Formylmethanofuran dehydrogenase, subunit B  *Formate dehydrogenase - heterodisulfide reductase - formylmethanofuran dehydrogenase complex from Methanospirillum hungatei (hexameric, composite structure)* | 0.03 |  | 13.21 | 0.13 | 295-351 | EM | 0.00 | hetero-2-2-2-2-2-2-… | 48 x SF4, 4 x FAD, 2 x FES, 4 x 9S8, 4 x ZN, 2 x MO, 4 x MGD | HHblits | 0.24 |
| ``` target    TARREITRRGFLGTAAGAGFAAFVVSATRAWGLEAIENPLARYPDREWERVYRDLWRYDSKFTFLCAPNDTHNCLLDAYV 7bkb.1    --------------------------------------------------------------------------------  target    RSGVMTRIGPTMRYGEARDLDGNRASARWDPRVCQKGLALTRRFYGDRRLRHCMVRAGFKRWVDEGFPRGEDGKPPKEYF 7bkb.1    --------------------------------------------------------------------------------  target    QRARDEWVRASHDEAAAVVAATLANIAATYSGEEGAQRLRDQGYEEETIEAMGGAGVQAMKFRGGMPLLGMTRVFGLYRM 7bkb.1    --------------------------------------------------------------------------------  target    ANSMALLDAKVRGVGPDEARGARGFDNYSWHTDLPPGHPMVTGQQTVDFDLNSVELAKNVVVWGMNWITTKMPDAHWLTE 7bkb.1    ------------------------------------------------------DEVDMFINIGTDAAAHFPIP---AVK  target    ARLKGTRIIVIACEYSSTSSKADDAIVVRPGTTPALALGLSHVIMRDKLYDADYVRRWTDLPMLVRTDTLKYLSAEDVFG 7bkb.1    QL-KKHPWVTIDPSINMASEISDLHIPVCIC-------------------------------------------------  target    GGPAPL 7bkb.1    ------ ``` | | | | | | | | | | | | | | | | | | | | | | | | | | | | | | | | | | | | | | | | | | | | | | | | | |
|  | 6lod.1.B | Fe-S-cluster-containing hydrogenase components 1-like protein  *Cryo-EM structure of the air-oxidized photosynthetic alternative complex III from Roseiflexus castenholzii* | 0.03 |  | 13.46 | 0.13 | 296-348 | EM | 0.00 | hetero-1-1-1-1-1-1-… | 6 x HEC, 2 x EL6, 3 x SF4, 1 x F3S | HHblits | 0.25 |
| ``` target    TARREITRRGFLGTAAGAGFAAFVVSATRAWGLEAIENPLARYPDREWERVYRDLWRYDSKFTFLCAPNDTHNCLLDAYV 6lod.1    --------------------------------------------------------------------------------  target    RSGVMTRIGPTMRYGEARDLDGNRASARWDPRVCQKGLALTRRFYGDRRLRHCMVRAGFKRWVDEGFPRGEDGKPPKEYF 6lod.1    --------------------------------------------------------------------------------  target    QRARDEWVRASHDEAAAVVAATLANIAATYSGEEGAQRLRDQGYEEETIEAMGGAGVQAMKFRGGMPLLGMTRVFGLYRM 6lod.1    --------------------------------------------------------------------------------  target    ANSMALLDAKVRGVGPDEARGARGFDNYSWHTDLPPGHPMVTGQQTVDFDLNSVELAKNVVVWGMNWITTKMPDAHWLTE 6lod.1    -------------------------------------------------------TVEVLLMIESNPVYNA-PADIPFAE  target    ARLKGTRIIVIACEYSSTSSKADDAIVVRPGTTPALALGLSHVIMRDKLYDADYVRRWTDLPMLVRTDTLKYLSAEDVFG 6lod.1    ALAKVPLSMHVGLYRDETAQQSVWHING----------------------------------------------------  target    GGPAPL 6lod.1    ------ ``` | | | | | | | | | | | | | | | | | | | | | | | | | | | | | | | | | | | | | | | | | | | | | | | | | |
|  | 5t5i.1.B | Tungsten formylmethanofuran dehydrogenase subunit B  *TUNGSTEN-CONTAINING FORMYLMETHANOFURAN DEHYDROGENASE FROM METHANOTHERMOBACTER WOLFEII, ORTHORHOMBIC FORM AT 1.9 A* | 0.03 |  | 17.65 | 0.13 | 296-350 | X-ray | 1.90 | hetero-oligomer | 4 x ZN, 2 x MG, 18 x K, 22 x SF4, 2 x W, 4 x MGD, 2 x H2S, 2 x CA | HHblits | 0.25 |
| ``` target    TARREITRRGFLGTAAGAGFAAFVVSATRAWGLEAIENPLARYPDREWERVYRDLWRYDSKFTFLCAPNDTHNCLLDAYV 5t5i.1    --------------------------------------------------------------------------------  target    RSGVMTRIGPTMRYGEARDLDGNRASARWDPRVCQKGLALTRRFYGDRRLRHCMVRAGFKRWVDEGFPRGEDGKPPKEYF 5t5i.1    --------------------------------------------------------------------------------  target    QRARDEWVRASHDEAAAVVAATLANIAATYSGEEGAQRLRDQGYEEETIEAMGGAGVQAMKFRGGMPLLGMTRVFGLYRM 5t5i.1    --------------------------------------------------------------------------------  target    ANSMALLDAKVRGVGPDEARGARGFDNYSWHTDLPPGHPMVTGQQTVDFDLNSVELAKNVVVWGMNWITTKMPDAHWLTE 5t5i.1    -------------------------------------------------------EADAMMVIASDPGAHFPQR---ALE  target    ARLKGTRIIVIACEYSSTSSKADDAIVVRPGTTPALALGLSHVIMRDKLYDADYVRRWTDLPMLVRTDTLKYLSAEDVFG 5t5i.1    RMA-EIPVIAIEPHRTPTTEMADIIIPPAI--------------------------------------------------  target    GGPAPL 5t5i.1    ------ ``` | | | | | | | | | | | | | | | | | | | | | | | | | | | | | | | | | | | | | | | | | | | | | | | | | |
|  | 7aqr.1.F | NADH dehydrogenase [ubiquinone] iron-sulfur protein 1, mitochondrial  *Cryo-EM structure of Arabidopsis thaliana Complex-I (peripheral arm)* | 0.03 |  | 14.89 | 0.12 | 295-350 | EM | 0.00 | hetero-1-1-1-1-1-1-… | 6 x SF4, 2 x FES, 1 x FMN, 1 x NDP, 1 x ZN, 1 x 8Q1 | HHblits | 0.28 |
| ``` target    TARREITRRGFLGTAAGAGFAAFVVSATRAWGLEAIENPLARYPDREWERVYRDLWRYDSKFTFLCAPNDTHNCLLDAYV 7aqr.1    --------------------------------------------------------------------------------  target    RSGVMTRIGPTMRYGEARDLDGNRASARWDPRVCQKGLALTRRFYGDRRLRHCMVRAGFKRWVDEGFPRGEDGKPPKEYF 7aqr.1    --------------------------------------------------------------------------------  target    QRARDEWVRASHDEAAAVVAATLANIAATYSGEEGAQRLRDQGYEEETIEAMGGAGVQAMKFRGGMPLLGMTRVFGLYRM 7aqr.1    --------------------------------------------------------------------------------  target    ANSMALLDAKVRGVGPDEARGARGFDNYSWHTDLPPGHPMVTGQQTVDFDLNSVELAKNVVVWGMNWITTKMPDAHWLTE 7aqr.1    ------------------------------------------------------ESAKFVYLMGADDVN---------VD  target    ARLKGTRIIVIACEYSSTSSKADDAIVVRPGTTPALALGLSHVIMRDKLYDADYVRRWTDLPMLVRTDTLKYLSAEDVFG 7aqr.1    KIPKDAFVVYQGHHGDKAVYRANVILPASA--------------------------------------------------  target    GGPAPL 7aqr.1    ------ ``` | | | | | | | | | | | | | | | | | | | | | | | | | | | | | | | | | | | | | | | | | | | | | | | | | |
|  | 7a23.1.O | 75kDa  *Plant mitochondrial respiratory complex I* | 0.02 |  | 14.89 | 0.12 | 295-350 | EM | 0.00 | hetero-1-1-1-1-1-1-… | 6 x SF4, 1 x FMN, 2 x T7X, 3 x CDL, 1 x U10, 1 x PEV, 2 x FES, 1 x NDP, 2 x ZN | HHblits | 0.28 |
| ``` target    TARREITRRGFLGTAAGAGFAAFVVSATRAWGLEAIENPLARYPDREWERVYRDLWRYDSKFTFLCAPNDTHNCLLDAYV 7a23.1    --------------------------------------------------------------------------------  target    RSGVMTRIGPTMRYGEARDLDGNRASARWDPRVCQKGLALTRRFYGDRRLRHCMVRAGFKRWVDEGFPRGEDGKPPKEYF 7a23.1    --------------------------------------------------------------------------------  target    QRARDEWVRASHDEAAAVVAATLANIAATYSGEEGAQRLRDQGYEEETIEAMGGAGVQAMKFRGGMPLLGMTRVFGLYRM 7a23.1    --------------------------------------------------------------------------------  target    ANSMALLDAKVRGVGPDEARGARGFDNYSWHTDLPPGHPMVTGQQTVDFDLNSVELAKNVVVWGMNWITTKMPDAHWLTE 7a23.1    ------------------------------------------------------ESAKFVYLMGADDVN---------VD  target    ARLKGTRIIVIACEYSSTSSKADDAIVVRPGTTPALALGLSHVIMRDKLYDADYVRRWTDLPMLVRTDTLKYLSAEDVFG 7a23.1    KIPKDAFVVYQGHHGDKAVYRANVILPASA--------------------------------------------------  target    GGPAPL 7a23.1    ------ ``` | | | | | | | | | | | | | | | | | | | | | | | | | | | | | | | | | | | | | | | | | | | | | | | | | |
|  | 7ar8.1.G | NADH dehydrogenase [ubiquinone] iron-sulfur protein 1, mitochondrial  *Cryo-EM structure of Arabidopsis thaliana complex-I (closed conformation)* | 0.02 |  | 14.89 | 0.12 | 295-350 | EM | 0.00 | hetero-1-1-1-1-1-1-… | 6 x SF4, 2 x FES, 1 x FMN, 1 x UQ9, 3 x PTY, 2 x PC7, 1 x PGT, 1 x FE, 1 x NDP, 2 x ZN, 2 x 8Q1, 1 x LMN, 1 x PSF, 1 x T7X | HHblits | 0.28 |
| ``` target    TARREITRRGFLGTAAGAGFAAFVVSATRAWGLEAIENPLARYPDREWERVYRDLWRYDSKFTFLCAPNDTHNCLLDAYV 7ar8.1    --------------------------------------------------------------------------------  target    RSGVMTRIGPTMRYGEARDLDGNRASARWDPRVCQKGLALTRRFYGDRRLRHCMVRAGFKRWVDEGFPRGEDGKPPKEYF 7ar8.1    --------------------------------------------------------------------------------  target    QRARDEWVRASHDEAAAVVAATLANIAATYSGEEGAQRLRDQGYEEETIEAMGGAGVQAMKFRGGMPLLGMTRVFGLYRM 7ar8.1    --------------------------------------------------------------------------------  target    ANSMALLDAKVRGVGPDEARGARGFDNYSWHTDLPPGHPMVTGQQTVDFDLNSVELAKNVVVWGMNWITTKMPDAHWLTE 7ar8.1    ------------------------------------------------------ESAKFVYLMGADDVN---------VD  target    ARLKGTRIIVIACEYSSTSSKADDAIVVRPGTTPALALGLSHVIMRDKLYDADYVRRWTDLPMLVRTDTLKYLSAEDVFG 7ar8.1    KIPKDAFVVYQGHHGDKAVYRANVILPASA--------------------------------------------------  target    GGPAPL 7ar8.1    ------ ``` | | | | | | | | | | | | | | | | | | | | | | | | | | | | | | | | | | | | | | | | | | | | | | | | | |
|  | 1kqf.1.A | FORMATE DEHYDROGENASE, NITRATE-INDUCIBLE, MAJOR SUBUNIT  *FORMATE DEHYDROGENASE N FROM E. COLI* | 0.02 |  | 17.02 | 0.12 | 296-343 | X-ray | 1.60 | hetero-oligomer | 3 x 6MO, 15 x SF4, 6 x MGD, 6 x HEM, 3 x CDL | HHblits | 0.27 |
| ``` target    TARREITRRGFLGTAAGAGFAAFVVSATRAWGLEAIENPLARYPDREWERVYRDLWRYDSKFTFLCAPNDTHNCLLDAYV 1kqf.1    --------------------------------------------------------------------------------  target    RSGVMTRIGPTMRYGEARDLDGNRASARWDPRVCQKGLALTRRFYGDRRLRHCMVRAGFKRWVDEGFPRGEDGKPPKEYF 1kqf.1    --------------------------------------------------------------------------------  target    QRARDEWVRASHDEAAAVVAATLANIAATYSGEEGAQRLRDQGYEEETIEAMGGAGVQAMKFRGGMPLLGMTRVFGLYRM 1kqf.1    --------------------------------------------------------------------------------  target    ANSMALLDAKVRGVGPDEARGARGFDNYSWHTDLPPGHPMVTGQQTVDFDLNSVELAKNVVVWGMNWITTKMPDAHWLTE 1kqf.1    -------------------------------------------------------KVTGYFCQGFNPVASFP-DKNKVVS  target    ARLKGTRIIVIACEYSSTSSKADDAIVVRPGTTPALALGLSHVIMRDKLYDADYVRRWTDLPMLVRTDTLKYLSAEDVFG 1kqf.1    CLSKLKYMVVIDPLVTETSTFWQ---------------------------------------------------------  target    GGPAPL 1kqf.1    ------ ``` | | | | | | | | | | | | | | | | | | | | | | | | | | | | | | | | | | | | | | | | | | | | | | | | | |
|  | 8b9z.1.G | NADH-ubiquinone oxidoreductase 75 kDa subunit, mitochondrial  *Drosophila melanogaster complex I in the Active state (Dm1)* | 0.02 |  | 14.58 | 0.12 | 295-349 | EM | 3.28 | hetero-1-1-1-1-1-1-… | 3 x PC1, 16 x 3PE, 6 x SF4, 4 x CDL, 2 x FES, 1 x FMN, 1 x UQ9, 1 x DGT, 1 x NDP, 1 x ZN, 2 x EHZ | HHblits | 0.26 |
| ``` target    TARREITRRGFLGTAAGAGFAAFVVSATRAWGLEAIENPLARYPDREWERVYRDLWRYDSKFTFLCAPNDTHNCLLDAYV 8b9z.1    --------------------------------------------------------------------------------  target    RSGVMTRIGPTMRYGEARDLDGNRASARWDPRVCQKGLALTRRFYGDRRLRHCMVRAGFKRWVDEGFPRGEDGKPPKEYF 8b9z.1    --------------------------------------------------------------------------------  target    QRARDEWVRASHDEAAAVVAATLANIAATYSGEEGAQRLRDQGYEEETIEAMGGAGVQAMKFRGGMPLLGMTRVFGLYRM 8b9z.1    --------------------------------------------------------------------------------  target    ANSMALLDAKVRGVGPDEARGARGFDNYSWHTDLPPGHPMVTGQQTVDFDLNSVELAKNVVVWGMNWITTKMPDAHWLTE 8b9z.1    ------------------------------------------------------AQPKVLFLLNADAG-------KVTRE  target    ARLKGTRIIVIACEYSSTSSKADDAIVVRPGTTPALALGLSHVIMRDKLYDADYVRRWTDLPMLVRTDTLKYLSAEDVFG 8b9z.1    QLPKDCFVVYIGSHGDNGASIADAVLPGA---------------------------------------------------  target    GGPAPL 8b9z.1    ------ ``` | | | | | | | | | | | | | | | | | | | | | | | | | | | | | | | | | | | | | | | | | | | | | | | | | |
|  | 8ba0.1.G | NADH-ubiquinone oxidoreductase 75 kDa subunit, mitochondrial  *Drosophila melanogaster complex I in the Twisted state (Dm2)* | 0.02 |  | 14.58 | 0.12 | 295-349 | EM | 3.68 | hetero-1-1-1-1-1-1-… | 6 x SF4, 6 x 3PE, 2 x FES, 1 x FMN, 2 x CDL, 1 x DGT, 1 x NDP, 1 x ZN, 2 x EHZ | HHblits | 0.26 |
| ``` target    TARREITRRGFLGTAAGAGFAAFVVSATRAWGLEAIENPLARYPDREWERVYRDLWRYDSKFTFLCAPNDTHNCLLDAYV 8ba0.1    --------------------------------------------------------------------------------  target    RSGVMTRIGPTMRYGEARDLDGNRASARWDPRVCQKGLALTRRFYGDRRLRHCMVRAGFKRWVDEGFPRGEDGKPPKEYF 8ba0.1    --------------------------------------------------------------------------------  target    QRARDEWVRASHDEAAAVVAATLANIAATYSGEEGAQRLRDQGYEEETIEAMGGAGVQAMKFRGGMPLLGMTRVFGLYRM 8ba0.1    --------------------------------------------------------------------------------  target    ANSMALLDAKVRGVGPDEARGARGFDNYSWHTDLPPGHPMVTGQQTVDFDLNSVELAKNVVVWGMNWITTKMPDAHWLTE 8ba0.1    ------------------------------------------------------AQPKVLFLLNADAG-------KVTRE  target    ARLKGTRIIVIACEYSSTSSKADDAIVVRPGTTPALALGLSHVIMRDKLYDADYVRRWTDLPMLVRTDTLKYLSAEDVFG 8ba0.1    QLPKDCFVVYIGSHGDNGASIADAVLPGA---------------------------------------------------  target    GGPAPL 8ba0.1    ------ ``` | | | | | | | | | | | | | | | | | | | | | | | | | | | | | | | | | | | | | | | | | | | | | | | | | |
|  | 1h0h.1.A | FORMATE DEHYDROGENASE SUBUNIT ALPHA  *Tungsten containing Formate Dehydrogenase from Desulfovibrio Gigas* | 0.02 |  | 19.57 | 0.11 | 296-342 | X-ray | 1.80 | hetero-1-1-mer | 1 x W, 1 x 2MD, 1 x MGD, 4 x SF4, 1 x CA | HHblits | 0.29 |
| ``` target    TARREITRRGFLGTAAGAGFAAFVVSATRAWGLEAIENPLARYPDREWERVYRDLWRYDSKFTFLCAPNDTHNCLLDAYV 1h0h.1    --------------------------------------------------------------------------------  target    RSGVMTRIGPTMRYGEARDLDGNRASARWDPRVCQKGLALTRRFYGDRRLRHCMVRAGFKRWVDEGFPRGEDGKPPKEYF 1h0h.1    --------------------------------------------------------------------------------  target    QRARDEWVRASHDEAAAVVAATLANIAATYSGEEGAQRLRDQGYEEETIEAMGGAGVQAMKFRGGMPLLGMTRVFGLYRM 1h0h.1    --------------------------------------------------------------------------------  target    ANSMALLDAKVRGVGPDEARGARGFDNYSWHTDLPPGHPMVTGQQTVDFDLNSVELAKNVVVWGMNWITTKMPDAHWLTE 1h0h.1    -------------------------------------------------------KIKGFFAWGQNPACSGA-NSNKTRE  target    ARLKGTRIIVIACEYSSTSSKADDAIVVRPGTTPALALGLSHVIMRDKLYDADYVRRWTDLPMLVRTDTLKYLSAEDVFG 1h0h.1    ALTKLDWMVNVNIFDNETGSFW----------------------------------------------------------  target    GGPAPL 1h0h.1    ------ ``` | | | | | | | | | | | | | | | | | | | | | | | | | | | | | | | | | | | | | | | | | | | | | | | | | |
|  | 5xtb.1.L | NADH-ubiquinone oxidoreductase 75 kDa subunit, mitochondrial  *Cryo-EM structure of human respiratory complex I matrix arm* | 0.01 |  | 14.29 | 0.12 | 296-351 | EM | 0.00 | hetero-1-1-1-1-1-1-… | 6 x SF4, 1 x FMN, 1 x 8Q1, 1 x NDP, 2 x FES | HHblits | 0.23 |
| ``` target    TARREITRRGFLGTAAGAGFAAFVVSATRAWGLEAIENPLARYPDREWERVYRDLWRYDSKFTFLCAPNDTHNCLLDAYV 5xtb.1    --------------------------------------------------------------------------------  target    RSGVMTRIGPTMRYGEARDLDGNRASARWDPRVCQKGLALTRRFYGDRRLRHCMVRAGFKRWVDEGFPRGEDGKPPKEYF 5xtb.1    --------------------------------------------------------------------------------  target    QRARDEWVRASHDEAAAVVAATLANIAATYSGEEGAQRLRDQGYEEETIEAMGGAGVQAMKFRGGMPLLGMTRVFGLYRM 5xtb.1    --------------------------------------------------------------------------------  target    ANSMALLDAKVRGVGPDEARGARGFDNYSWHTDLPPGHPMVTGQQTVDFDLNSVELAKNVVVWGMNWITTKMPDAHWLTE 5xtb.1    -------------------------------------------------------PPKVLFLLGADGGC-------ITRQ  target    ARLKGTRIIVIACEYSSTSSKADDAIVVRPGTTPALALGLSHVIMRDKLYDADYVRRWTDLPMLVRTDTLKYLSAEDVFG 5xtb.1    DLPKDCFIIYQGHHGDVGAPIADVILPGAAY-------------------------------------------------  target    GGPAPL 5xtb.1    ------ ``` | | | | | | | | | | | | | | | | | | | | | | | | | | | | | | | | | | | | | | | | | | | | | | | | | |
|  | 6qcf.1.C | NADH:ubiquinone oxidoreductase core subunit S1  *Ovine respiratory complex I FRC open class 6* | 0.02 |  | 12.24 | 0.12 | 296-351 | EM | 0.00 | hetero-1-1-1-1-1-1-… | 6 x SF4, 1 x FMN, 2 x FES, 1 x ZN, 1 x NDP, 2 x ZMP | HHblits | 0.23 |
| ``` target    TARREITRRGFLGTAAGAGFAAFVVSATRAWGLEAIENPLARYPDREWERVYRDLWRYDSKFTFLCAPNDTHNCLLDAYV 6qcf.1    --------------------------------------------------------------------------------  target    RSGVMTRIGPTMRYGEARDLDGNRASARWDPRVCQKGLALTRRFYGDRRLRHCMVRAGFKRWVDEGFPRGEDGKPPKEYF 6qcf.1    --------------------------------------------------------------------------------  target    QRARDEWVRASHDEAAAVVAATLANIAATYSGEEGAQRLRDQGYEEETIEAMGGAGVQAMKFRGGMPLLGMTRVFGLYRM 6qcf.1    --------------------------------------------------------------------------------  target    ANSMALLDAKVRGVGPDEARGARGFDNYSWHTDLPPGHPMVTGQQTVDFDLNSVELAKNVVVWGMNWITTKMPDAHWLTE 6qcf.1    -------------------------------------------------------PPKMLFLLGADGGC-------VTRQ  target    ARLKGTRIIVIACEYSSTSSKADDAIVVRPGTTPALALGLSHVIMRDKLYDADYVRRWTDLPMLVRTDTLKYLSAEDVFG 6qcf.1    DLPKDCFIVYQGHHGDVGAPIADVILPGAAY-------------------------------------------------  target    GGPAPL 6qcf.1    ------ ``` | | | | | | | | | | | | | | | | | | | | | | | | | | | | | | | | | | | | | | | | | | | | | | | | | |
|  | 6qc5.1.C | NADH:ubiquinone oxidoreductase core subunit S1  *Ovine respiratory complex I FRC closed class 1* | 0.02 |  | 12.24 | 0.12 | 296-351 | EM | 0.00 | hetero-1-1-1-1-1-1-… | 6 x SF4, 1 x FMN, 2 x FES, 2 x 3PE, 1 x ZN, 1 x NDP, 2 x ZMP, 1 x PC1 | HHblits | 0.23 |
| ``` target    TARREITRRGFLGTAAGAGFAAFVVSATRAWGLEAIENPLARYPDREWERVYRDLWRYDSKFTFLCAPNDTHNCLLDAYV 6qc5.1    --------------------------------------------------------------------------------  target    RSGVMTRIGPTMRYGEARDLDGNRASARWDPRVCQKGLALTRRFYGDRRLRHCMVRAGFKRWVDEGFPRGEDGKPPKEYF 6qc5.1    --------------------------------------------------------------------------------  target    QRARDEWVRASHDEAAAVVAATLANIAATYSGEEGAQRLRDQGYEEETIEAMGGAGVQAMKFRGGMPLLGMTRVFGLYRM 6qc5.1    --------------------------------------------------------------------------------  target    ANSMALLDAKVRGVGPDEARGARGFDNYSWHTDLPPGHPMVTGQQTVDFDLNSVELAKNVVVWGMNWITTKMPDAHWLTE 6qc5.1    -------------------------------------------------------PPKMLFLLGADGGC-------VTRQ  target    ARLKGTRIIVIACEYSSTSSKADDAIVVRPGTTPALALGLSHVIMRDKLYDADYVRRWTDLPMLVRTDTLKYLSAEDVFG 6qc5.1    DLPKDCFIVYQGHHGDVGAPIADVILPGAAY-------------------------------------------------  target    GGPAPL 6qc5.1    ------ ``` | | | | | | | | | | | | | | | | | | | | | | | | | | | | | | | | | | | | | | | | | | | | | | | | | |
|  | 7qsd.1.G | NADH-ubiquinone oxidoreductase 75 kDa subunit, mitochondrial  *Bovine complex I in the active state at 3.1 A* | 0.02 |  | 12.24 | 0.12 | 296-351 | EM | 0.00 | hetero-1-1-1-1-1-1-… | 5 x PC1, 13 x 3PE, 6 x SF4, 2 x FES, 1 x FMN, 4 x CDL, 3 x LMT, 1 x GTP, 1 x MG, 1 x NDP, 1 x ZN, 2 x EHZ | HHblits | 0.23 |
| ``` target    TARREITRRGFLGTAAGAGFAAFVVSATRAWGLEAIENPLARYPDREWERVYRDLWRYDSKFTFLCAPNDTHNCLLDAYV 7qsd.1    --------------------------------------------------------------------------------  target    RSGVMTRIGPTMRYGEARDLDGNRASARWDPRVCQKGLALTRRFYGDRRLRHCMVRAGFKRWVDEGFPRGEDGKPPKEYF 7qsd.1    --------------------------------------------------------------------------------  target    QRARDEWVRASHDEAAAVVAATLANIAATYSGEEGAQRLRDQGYEEETIEAMGGAGVQAMKFRGGMPLLGMTRVFGLYRM 7qsd.1    --------------------------------------------------------------------------------  target    ANSMALLDAKVRGVGPDEARGARGFDNYSWHTDLPPGHPMVTGQQTVDFDLNSVELAKNVVVWGMNWITTKMPDAHWLTE 7qsd.1    -------------------------------------------------------PPKMLFLLGADGGC-------ITRQ  target    ARLKGTRIIVIACEYSSTSSKADDAIVVRPGTTPALALGLSHVIMRDKLYDADYVRRWTDLPMLVRTDTLKYLSAEDVFG 7qsd.1    DLPKDCFIVYQGHHGDVGAPIADVILPGAAY-------------------------------------------------  target    GGPAPL 7qsd.1    ------ ``` | | | | | | | | | | | | | | | | | | | | | | | | | | | | | | | | | | | | | | | | | | | | | | | | | |
|  | 6sdv.1.A | Formate dehydrogenase, alpha subunit, selenocysteine-containing,Formate dehydrogenase, alpha subunit, selenocysteine-containing,W-formate dehydrogenase - alpha subunit  *W-formate dehydrogenase from Desulfovibrio vulgaris - Formate reduced form* | 0.02 |  | 24.44 | 0.11 | 296-341 | X-ray | 1.90 | hetero-1-1-mer | 2 x MGD, 4 x SF4, 1 x W, 1 x H2S | HHblits | 0.30 |
| ``` target    TARREITRRGFLGTAAGAGFAAFVVSATRAWGLEAIENPLARYPDREWERVYRDLWRYDSKFTFLCAPNDTHNCLLDAYV 6sdv.1    --------------------------------------------------------------------------------  target    RSGVMTRIGPTMRYGEARDLDGNRASARWDPRVCQKGLALTRRFYGDRRLRHCMVRAGFKRWVDEGFPRGEDGKPPKEYF 6sdv.1    --------------------------------------------------------------------------------  target    QRARDEWVRASHDEAAAVVAATLANIAATYSGEEGAQRLRDQGYEEETIEAMGGAGVQAMKFRGGMPLLGMTRVFGLYRM 6sdv.1    --------------------------------------------------------------------------------  target    ANSMALLDAKVRGVGPDEARGARGFDNYSWHTDLPPGHPMVTGQQTVDFDLNSVELAKNVVVWGMNWITTKMPDAHWLTE 6sdv.1    -------------------------------------------------------EFKGLFAWGMNPACGGA-NANKNRK  target    ARLKGTRIIVIACEYSSTSSKADDAIVVRPGTTPALALGLSHVIMRDKLYDADYVRRWTDLPMLVRTDTLKYLSAEDVFG 6sdv.1    AMGKLEWLVNVNLFENETSSF-----------------------------------------------------------  target    GGPAPL 6sdv.1    ------ ``` | | | | | | | | | | | | | | | | | | | | | | | | | | | | | | | | | | | | | | | | | | | | | | | | | |
|  | 7ar7.1.G | NADH dehydrogenase [ubiquinone] iron-sulfur protein 1, mitochondrial  *Cryo-EM structure of Arabidopsis thaliana complex-I (open conformation)* | 0.02 |  | 15.22 | 0.11 | 295-349 | EM | 0.00 | hetero-1-1-1-1-1-1-… | 6 x SF4, 2 x FES, 1 x FMN, 1 x UQ9, 3 x PTY, 2 x PC7, 1 x LMN, 1 x NDP, 2 x ZN, 2 x 8Q1, 1 x PGT, 1 x PSF, 1 x T7X | HHblits | 0.28 |
| ``` target    TARREITRRGFLGTAAGAGFAAFVVSATRAWGLEAIENPLARYPDREWERVYRDLWRYDSKFTFLCAPNDTHNCLLDAYV 7ar7.1    --------------------------------------------------------------------------------  target    RSGVMTRIGPTMRYGEARDLDGNRASARWDPRVCQKGLALTRRFYGDRRLRHCMVRAGFKRWVDEGFPRGEDGKPPKEYF 7ar7.1    --------------------------------------------------------------------------------  target    QRARDEWVRASHDEAAAVVAATLANIAATYSGEEGAQRLRDQGYEEETIEAMGGAGVQAMKFRGGMPLLGMTRVFGLYRM 7ar7.1    --------------------------------------------------------------------------------  target    ANSMALLDAKVRGVGPDEARGARGFDNYSWHTDLPPGHPMVTGQQTVDFDLNSVELAKNVVVWGMNWITTKMPDAHWLTE 7ar7.1    ------------------------------------------------------ESAKFVYLMGADDVN---------VD  target    ARLKGTRIIVIACEYSSTSSKADDAIVVRPGTTPALALGLSHVIMRDKLYDADYVRRWTDLPMLVRTDTLKYLSAEDVFG 7ar7.1    KIPKDAFVVYQGHHGDKAVYRANVILPAS---------------------------------------------------  target    GGPAPL 7ar7.1    ------ ``` | | | | | | | | | | | | | | | | | | | | | | | | | | | | | | | | | | | | | | | | | | | | | | | | | |
|  | 6zr2.1.G | NADH-ubiquinone oxidoreductase 75 kDa subunit, mitochondrial  *Cryo-EM structure of respiratory complex I in the active state from Mus musculus at 3.1 A* | 0.02 |  | 12.50 | 0.12 | 296-350 | EM | 3.10 | hetero-1-1-1-1-1-1-… | 6 x SF4, 4 x PC1, 2 x FES, 1 x FMN, 9 x 3PE, 7 x CDL, 1 x ATP, 1 x NDP, 1 x ZN, 2 x EHZ | HHblits | 0.24 |
| ``` target    TARREITRRGFLGTAAGAGFAAFVVSATRAWGLEAIENPLARYPDREWERVYRDLWRYDSKFTFLCAPNDTHNCLLDAYV 6zr2.1    --------------------------------------------------------------------------------  target    RSGVMTRIGPTMRYGEARDLDGNRASARWDPRVCQKGLALTRRFYGDRRLRHCMVRAGFKRWVDEGFPRGEDGKPPKEYF 6zr2.1    --------------------------------------------------------------------------------  target    QRARDEWVRASHDEAAAVVAATLANIAATYSGEEGAQRLRDQGYEEETIEAMGGAGVQAMKFRGGMPLLGMTRVFGLYRM 6zr2.1    --------------------------------------------------------------------------------  target    ANSMALLDAKVRGVGPDEARGARGFDNYSWHTDLPPGHPMVTGQQTVDFDLNSVELAKNVVVWGMNWITTKMPDAHWLTE 6zr2.1    -------------------------------------------------------PPKMLFLLGADGGC-------ITRQ  target    ARLKGTRIIVIACEYSSTSSKADDAIVVRPGTTPALALGLSHVIMRDKLYDADYVRRWTDLPMLVRTDTLKYLSAEDVFG 6zr2.1    DLPKDCFIVYQGHHGDVGAPMADVILPGAA--------------------------------------------------  target    GGPAPL 6zr2.1    ------ ``` | | | | | | | | | | | | | | | | | | | | | | | | | | | | | | | | | | | | | | | | | | | | | | | | | |
|  | 6g72.1.G | NADH-ubiquinone oxidoreductase 75 kDa subunit, mitochondrial  *Mouse mitochondrial complex I in the deactive state* | 0.02 |  | 12.50 | 0.12 | 296-350 | EM | 0.00 | hetero-1-1-1-1-1-1-… | 6 x SF4, 2 x FES, 1 x FMN, 1 x ADP, 1 x NDP, 1 x ZN, 2 x EHZ | HHblits | 0.24 |
| ``` target    TARREITRRGFLGTAAGAGFAAFVVSATRAWGLEAIENPLARYPDREWERVYRDLWRYDSKFTFLCAPNDTHNCLLDAYV 6g72.1    --------------------------------------------------------------------------------  target    RSGVMTRIGPTMRYGEARDLDGNRASARWDPRVCQKGLALTRRFYGDRRLRHCMVRAGFKRWVDEGFPRGEDGKPPKEYF 6g72.1    --------------------------------------------------------------------------------  target    QRARDEWVRASHDEAAAVVAATLANIAATYSGEEGAQRLRDQGYEEETIEAMGGAGVQAMKFRGGMPLLGMTRVFGLYRM 6g72.1    --------------------------------------------------------------------------------  target    ANSMALLDAKVRGVGPDEARGARGFDNYSWHTDLPPGHPMVTGQQTVDFDLNSVELAKNVVVWGMNWITTKMPDAHWLTE 6g72.1    -------------------------------------------------------PPKMLFLLGADGGC-------ITRQ  target    ARLKGTRIIVIACEYSSTSSKADDAIVVRPGTTPALALGLSHVIMRDKLYDADYVRRWTDLPMLVRTDTLKYLSAEDVFG 6g72.1    DLPKDCFIVYQGHHGDVGAPMADVILPGAA--------------------------------------------------  target    GGPAPL 6g72.1    ------ ``` | | | | | | | | | | | | | | | | | | | | | | | | | | | | | | | | | | | | | | | | | | | | | | | | | |
|  | 7ak6.1.G | NADH-ubiquinone oxidoreductase 75 kDa subunit, mitochondrial  *Cryo-EM structure of ND6-P25L mutant respiratory complex I from Mus musculus at 3.8 A* | 0.02 |  | 12.50 | 0.12 | 296-350 | EM | 0.00 | hetero-1-1-1-1-1-1-… | 6 x SF4, 1 x PC1, 2 x FES, 1 x FMN, 4 x 3PE, 2 x CDL, 1 x ATP, 1 x NDP, 1 x ZN, 2 x EHZ | HHblits | 0.24 |
| ``` target    TARREITRRGFLGTAAGAGFAAFVVSATRAWGLEAIENPLARYPDREWERVYRDLWRYDSKFTFLCAPNDTHNCLLDAYV 7ak6.1    --------------------------------------------------------------------------------  target    RSGVMTRIGPTMRYGEARDLDGNRASARWDPRVCQKGLALTRRFYGDRRLRHCMVRAGFKRWVDEGFPRGEDGKPPKEYF 7ak6.1    --------------------------------------------------------------------------------  target    QRARDEWVRASHDEAAAVVAATLANIAATYSGEEGAQRLRDQGYEEETIEAMGGAGVQAMKFRGGMPLLGMTRVFGLYRM 7ak6.1    --------------------------------------------------------------------------------  target    ANSMALLDAKVRGVGPDEARGARGFDNYSWHTDLPPGHPMVTGQQTVDFDLNSVELAKNVVVWGMNWITTKMPDAHWLTE 7ak6.1    -------------------------------------------------------PPKMLFLLGADGGC-------ITRQ  target    ARLKGTRIIVIACEYSSTSSKADDAIVVRPGTTPALALGLSHVIMRDKLYDADYVRRWTDLPMLVRTDTLKYLSAEDVFG 7ak6.1    DLPKDCFIVYQGHHGDVGAPMADVILPGAA--------------------------------------------------  target    GGPAPL 7ak6.1    ------ ``` | | | | | | | | | | | | | | | | | | | | | | | | | | | | | | | | | | | | | | | | | | | | | | | | | |
|  | 7ak5.1.G | NADH-ubiquinone oxidoreductase 75 kDa subunit, mitochondrial  *Cryo-EM structure of respiratory complex I in the deactive state from Mus musculus at 3.2 A* | 0.02 |  | 12.50 | 0.12 | 296-350 | EM | 0.00 | hetero-1-1-1-1-1-1-… | 6 x SF4, 2 x PC1, 2 x FES, 1 x FMN, 8 x 3PE, 4 x CDL, 1 x ATP, 1 x NDP, 1 x ZN, 2 x EHZ | HHblits | 0.24 |
| ``` target    TARREITRRGFLGTAAGAGFAAFVVSATRAWGLEAIENPLARYPDREWERVYRDLWRYDSKFTFLCAPNDTHNCLLDAYV 7ak5.1    --------------------------------------------------------------------------------  target    RSGVMTRIGPTMRYGEARDLDGNRASARWDPRVCQKGLALTRRFYGDRRLRHCMVRAGFKRWVDEGFPRGEDGKPPKEYF 7ak5.1    --------------------------------------------------------------------------------  target    QRARDEWVRASHDEAAAVVAATLANIAATYSGEEGAQRLRDQGYEEETIEAMGGAGVQAMKFRGGMPLLGMTRVFGLYRM 7ak5.1    --------------------------------------------------------------------------------  target    ANSMALLDAKVRGVGPDEARGARGFDNYSWHTDLPPGHPMVTGQQTVDFDLNSVELAKNVVVWGMNWITTKMPDAHWLTE 7ak5.1    -------------------------------------------------------PPKMLFLLGADGGC-------ITRQ  target    ARLKGTRIIVIACEYSSTSSKADDAIVVRPGTTPALALGLSHVIMRDKLYDADYVRRWTDLPMLVRTDTLKYLSAEDVFG 7ak5.1    DLPKDCFIVYQGHHGDVGAPMADVILPGAA--------------------------------------------------  target    GGPAPL 7ak5.1    ------ ``` | | | | | | | | | | | | | | | | | | | | | | | | | | | | | | | | | | | | | | | | | | | | | | | | | |
|  | 5gpn.24.A | NADH-ubiquinone oxidoreductase 75 kDa subunit  *Architecture of mammalian respirasome* | 0.02 |  | 14.58 | 0.12 | 296-350 | EM | 0.00 | monomer |  | HHblits | 0.24 |
| ``` target    TARREITRRGFLGTAAGAGFAAFVVSATRAWGLEAIENPLARYPDREWERVYRDLWRYDSKFTFLCAPNDTHNCLLDAYV 5gpn.24   --------------------------------------------------------------------------------  target    RSGVMTRIGPTMRYGEARDLDGNRASARWDPRVCQKGLALTRRFYGDRRLRHCMVRAGFKRWVDEGFPRGEDGKPPKEYF 5gpn.24   --------------------------------------------------------------------------------  target    QRARDEWVRASHDEAAAVVAATLANIAATYSGEEGAQRLRDQGYEEETIEAMGGAGVQAMKFRGGMPLLGMTRVFGLYRM 5gpn.24   --------------------------------------------------------------------------------  target    ANSMALLDAKVRGVGPDEARGARGFDNYSWHTDLPPGHPMVTGQQTVDFDLNSVELAKNVVVWGMNWITTKMPDAHWLTE 5gpn.24   -------------------------------------------------------PPKVLFLLGADGGC-------ITRQ  target    ARLKGTRIIVIACEYSSTSSKADDAIVVRPGTTPALALGLSHVIMRDKLYDADYVRRWTDLPMLVRTDTLKYLSAEDVFG 5gpn.24   DLPKDCFIIYQGHHGDVGAPMADVILPGAA--------------------------------------------------  target    GGPAPL 5gpn.24   ------ ``` | | | | | | | | | | | | | | | | | | | | | | | | | | | | | | | | | | | | | | | | | | | | | | | | | |
|  | 7vxu.1.L | NADH-ubiquinone oxidoreductase 75 kDa subunit, mitochondrial  *Matrix arm of deactive state CI from Q10 dataset* | 0.02 |  | 14.58 | 0.12 | 296-350 | EM | 0.00 | hetero-1-1-1-1-1-1-… | 6 x SF4, 1 x FMN, 1 x PEE, 1 x PLX, 1 x 8Q1, 1 x NDP, 2 x FES, 1 x MG, 1 x CDL, 1 x ZN | HHblits | 0.24 |
| ``` target    TARREITRRGFLGTAAGAGFAAFVVSATRAWGLEAIENPLARYPDREWERVYRDLWRYDSKFTFLCAPNDTHNCLLDAYV 7vxu.1    --------------------------------------------------------------------------------  target    RSGVMTRIGPTMRYGEARDLDGNRASARWDPRVCQKGLALTRRFYGDRRLRHCMVRAGFKRWVDEGFPRGEDGKPPKEYF 7vxu.1    --------------------------------------------------------------------------------  target    QRARDEWVRASHDEAAAVVAATLANIAATYSGEEGAQRLRDQGYEEETIEAMGGAGVQAMKFRGGMPLLGMTRVFGLYRM 7vxu.1    --------------------------------------------------------------------------------  target    ANSMALLDAKVRGVGPDEARGARGFDNYSWHTDLPPGHPMVTGQQTVDFDLNSVELAKNVVVWGMNWITTKMPDAHWLTE 7vxu.1    -------------------------------------------------------PPKVLFLLGADGGC-------ITRQ  target    ARLKGTRIIVIACEYSSTSSKADDAIVVRPGTTPALALGLSHVIMRDKLYDADYVRRWTDLPMLVRTDTLKYLSAEDVFG 7vxu.1    DLPKDCFIIYQGHHGDVGAPMADVILPGAA--------------------------------------------------  target    GGPAPL 7vxu.1    ------ ``` | | | | | | | | | | | | | | | | | | | | | | | | | | | | | | | | | | | | | | | | | | | | | | | | | |
|  | 7zd6.1.4 | NADH-ubiquinone oxidoreductase 75 kDa subunit, mitochondrial  *Complex I from Ovis aries, at pH7.4, Open state* | 0.02 |  | 12.50 | 0.12 | 296-350 | EM | 0.00 | hetero-1-1-1-1-1-1-… | 6 x PC1, 14 x 3PE, 1 x DCQ, 2 x ZMP, 1 x AMP, 1 x MYR, 6 x SF4, 1 x FMN, 1 x NAI, 2 x FES, 1 x K, 1 x ZN, 1 x NDP | HHblits | 0.24 |
| ``` target    TARREITRRGFLGTAAGAGFAAFVVSATRAWGLEAIENPLARYPDREWERVYRDLWRYDSKFTFLCAPNDTHNCLLDAYV 7zd6.1    --------------------------------------------------------------------------------  target    RSGVMTRIGPTMRYGEARDLDGNRASARWDPRVCQKGLALTRRFYGDRRLRHCMVRAGFKRWVDEGFPRGEDGKPPKEYF 7zd6.1    --------------------------------------------------------------------------------  target    QRARDEWVRASHDEAAAVVAATLANIAATYSGEEGAQRLRDQGYEEETIEAMGGAGVQAMKFRGGMPLLGMTRVFGLYRM 7zd6.1    --------------------------------------------------------------------------------  target    ANSMALLDAKVRGVGPDEARGARGFDNYSWHTDLPPGHPMVTGQQTVDFDLNSVELAKNVVVWGMNWITTKMPDAHWLTE 7zd6.1    -------------------------------------------------------PPKMLFLLGADGGC-------VTRQ  target    ARLKGTRIIVIACEYSSTSSKADDAIVVRPGTTPALALGLSHVIMRDKLYDADYVRRWTDLPMLVRTDTLKYLSAEDVFG 7zd6.1    DLPKDCFIVYQGHHGDVGAPIADVILPGAA--------------------------------------------------  target    GGPAPL 7zd6.1    ------ ``` | | | | | | | | | | | | | | | | | | | | | | | | | | | | | | | | | | | | | | | | | | | | | | | | | |
|  | 6zk9.1.C | NADH:ubiquinone oxidoreductase core subunit S1  *Peripheral domain of open complex I during turnover* | 0.02 |  | 12.50 | 0.12 | 296-350 | EM | 0.00 | hetero-1-1-1-1-1-1-… | 6 x SF4, 1 x FMN, 1 x NAI, 2 x FES, 1 x K, 2 x PC1, 2 x 3PE, 1 x ZN, 1 x NDP, 1 x ZMP, 1 x CDL | HHblits | 0.24 |
| ``` target    TARREITRRGFLGTAAGAGFAAFVVSATRAWGLEAIENPLARYPDREWERVYRDLWRYDSKFTFLCAPNDTHNCLLDAYV 6zk9.1    --------------------------------------------------------------------------------  target    RSGVMTRIGPTMRYGEARDLDGNRASARWDPRVCQKGLALTRRFYGDRRLRHCMVRAGFKRWVDEGFPRGEDGKPPKEYF 6zk9.1    --------------------------------------------------------------------------------  target    QRARDEWVRASHDEAAAVVAATLANIAATYSGEEGAQRLRDQGYEEETIEAMGGAGVQAMKFRGGMPLLGMTRVFGLYRM 6zk9.1    --------------------------------------------------------------------------------  target    ANSMALLDAKVRGVGPDEARGARGFDNYSWHTDLPPGHPMVTGQQTVDFDLNSVELAKNVVVWGMNWITTKMPDAHWLTE 6zk9.1    -------------------------------------------------------PPKMLFLLGADGGC-------VTRQ  target    ARLKGTRIIVIACEYSSTSSKADDAIVVRPGTTPALALGLSHVIMRDKLYDADYVRRWTDLPMLVRTDTLKYLSAEDVFG 6zk9.1    DLPKDCFIVYQGHHGDVGAPIADVILPGAA--------------------------------------------------  target    GGPAPL 6zk9.1    ------ ``` | | | | | | | | | | | | | | | | | | | | | | | | | | | | | | | | | | | | | | | | | | | | | | | | | |
|  | 7dgr.10.A | NADH-ubiquinone oxidoreductase 75 kDa subunit, mitochondrial  *Activity optimized supercomplex state2* | 0.02 |  | 12.50 | 0.12 | 296-350 | EM | 0.00 | monomer |  | HHblits | 0.24 |
| ``` target    TARREITRRGFLGTAAGAGFAAFVVSATRAWGLEAIENPLARYPDREWERVYRDLWRYDSKFTFLCAPNDTHNCLLDAYV 7dgr.10   --------------------------------------------------------------------------------  target    RSGVMTRIGPTMRYGEARDLDGNRASARWDPRVCQKGLALTRRFYGDRRLRHCMVRAGFKRWVDEGFPRGEDGKPPKEYF 7dgr.10   --------------------------------------------------------------------------------  target    QRARDEWVRASHDEAAAVVAATLANIAATYSGEEGAQRLRDQGYEEETIEAMGGAGVQAMKFRGGMPLLGMTRVFGLYRM 7dgr.10   --------------------------------------------------------------------------------  target    ANSMALLDAKVRGVGPDEARGARGFDNYSWHTDLPPGHPMVTGQQTVDFDLNSVELAKNVVVWGMNWITTKMPDAHWLTE 7dgr.10   -------------------------------------------------------PPKMLFLLGADGGC-------ITRQ  target    ARLKGTRIIVIACEYSSTSSKADDAIVVRPGTTPALALGLSHVIMRDKLYDADYVRRWTDLPMLVRTDTLKYLSAEDVFG 7dgr.10   DLPKDCFIVYQGHHGDVGAPIADVILPGAA--------------------------------------------------  target    GGPAPL 7dgr.10   ------ ``` | | | | | | | | | | | | | | | | | | | | | | | | | | | | | | | | | | | | | | | | | | | | | | | | | |
|  | 5o31.1.8 | NADH-ubiquinone oxidoreductase 75 kDa subunit, mitochondrial  *Mitochondrial complex I in the deactive state* | 0.02 |  | 12.50 | 0.12 | 296-350 | EM | 4.13 | hetero-1-1-1-1-1-1-… | 6 x SF4, 2 x FES, 1 x FMN, 1 x NAP, 1 x ZN | HHblits | 0.24 |
| ``` target    TARREITRRGFLGTAAGAGFAAFVVSATRAWGLEAIENPLARYPDREWERVYRDLWRYDSKFTFLCAPNDTHNCLLDAYV 5o31.1    --------------------------------------------------------------------------------  target    RSGVMTRIGPTMRYGEARDLDGNRASARWDPRVCQKGLALTRRFYGDRRLRHCMVRAGFKRWVDEGFPRGEDGKPPKEYF 5o31.1    --------------------------------------------------------------------------------  target    QRARDEWVRASHDEAAAVVAATLANIAATYSGEEGAQRLRDQGYEEETIEAMGGAGVQAMKFRGGMPLLGMTRVFGLYRM 5o31.1    --------------------------------------------------------------------------------  target    ANSMALLDAKVRGVGPDEARGARGFDNYSWHTDLPPGHPMVTGQQTVDFDLNSVELAKNVVVWGMNWITTKMPDAHWLTE 5o31.1    -------------------------------------------------------PPKMLFLLGADGGC-------ITRQ  target    ARLKGTRIIVIACEYSSTSSKADDAIVVRPGTTPALALGLSHVIMRDKLYDADYVRRWTDLPMLVRTDTLKYLSAEDVFG 5o31.1    DLPKDCFIVYQGHHGDVGAPIADVILPGAA--------------------------------------------------  target    GGPAPL 5o31.1    ------ ``` | | | | | | | | | | | | | | | | | | | | | | | | | | | | | | | | | | | | | | | | | | | | | | | | | |
|  | 6x89.1.H | NADH dehydrogenase [ubiquinone] iron-sulfur protein 1, mitochondrial  *Vigna radiata mitochondrial complex I\** | 0.02 |  | 13.04 | 0.11 | 295-349 | EM | 0.00 | hetero-1-1-1-1-1-1-… | 1 x NAP, 6 x PC1, 6 x SF4, 2 x FES, 2 x ZN, 1 x FMN | HHblits | 0.27 |
| ``` target    TARREITRRGFLGTAAGAGFAAFVVSATRAWGLEAIENPLARYPDREWERVYRDLWRYDSKFTFLCAPNDTHNCLLDAYV 6x89.1    --------------------------------------------------------------------------------  target    RSGVMTRIGPTMRYGEARDLDGNRASARWDPRVCQKGLALTRRFYGDRRLRHCMVRAGFKRWVDEGFPRGEDGKPPKEYF 6x89.1    --------------------------------------------------------------------------------  target    QRARDEWVRASHDEAAAVVAATLANIAATYSGEEGAQRLRDQGYEEETIEAMGGAGVQAMKFRGGMPLLGMTRVFGLYRM 6x89.1    --------------------------------------------------------------------------------  target    ANSMALLDAKVRGVGPDEARGARGFDNYSWHTDLPPGHPMVTGQQTVDFDLNSVELAKNVVVWGMNWITTKMPDAHWLTE 6x89.1    ------------------------------------------------------ESAKFVYLMGADDVNL---------D  target    ARLKGTRIIVIACEYSSTSSKADDAIVVRPGTTPALALGLSHVIMRDKLYDADYVRRWTDLPMLVRTDTLKYLSAEDVFG 6x89.1    KIPDDAFVVYQGHHGDKSVYRANVILPTA---------------------------------------------------  target    GGPAPL 6x89.1    ------ ``` | | | | | | | | | | | | | | | | | | | | | | | | | | | | | | | | | | | | | | | | | | | | | | | | | |
|  | 8e73.55.A | NDUS1  *Vigna radiata supercomplex I+III2 (full bridge)* | 0.03 |  | 13.04 | 0.11 | 295-349 | EM | 0.00 | monomer |  | HHblits | 0.27 |
| ``` target    TARREITRRGFLGTAAGAGFAAFVVSATRAWGLEAIENPLARYPDREWERVYRDLWRYDSKFTFLCAPNDTHNCLLDAYV 8e73.55   --------------------------------------------------------------------------------  target    RSGVMTRIGPTMRYGEARDLDGNRASARWDPRVCQKGLALTRRFYGDRRLRHCMVRAGFKRWVDEGFPRGEDGKPPKEYF 8e73.55   --------------------------------------------------------------------------------  target    QRARDEWVRASHDEAAAVVAATLANIAATYSGEEGAQRLRDQGYEEETIEAMGGAGVQAMKFRGGMPLLGMTRVFGLYRM 8e73.55   --------------------------------------------------------------------------------  target    ANSMALLDAKVRGVGPDEARGARGFDNYSWHTDLPPGHPMVTGQQTVDFDLNSVELAKNVVVWGMNWITTKMPDAHWLTE 8e73.55   ------------------------------------------------------ESAKFVYLMGADDVNL---------D  target    ARLKGTRIIVIACEYSSTSSKADDAIVVRPGTTPALALGLSHVIMRDKLYDADYVRRWTDLPMLVRTDTLKYLSAEDVFG 8e73.55   KIPDDAFVVYQGHHGDKSVYRANVILPTA---------------------------------------------------  target    GGPAPL 8e73.55   ------ ``` | | | | | | | | | | | | | | | | | | | | | | | | | | | | | | | | | | | | | | | | | | | | | | | | | |
|  | 7tgh.58.A | NADH-ubiquinone oxidoreductase 75 kDa subunit  *Cryo-EM structure of respiratory super-complex CI+III2 from Tetrahymena thermophila* | 0.02 |  | 15.22 | 0.11 | 295-348 | EM | 0.00 | monomer |  | HHblits | 0.26 |
| ``` target    TARREITRRGFLGTAAGAGFAAFVVSATRAWGLEAIENPLARYPDREWERVYRDLWRYDSKFTFLCAPNDTHNCLLDAYV 7tgh.58   --------------------------------------------------------------------------------  target    RSGVMTRIGPTMRYGEARDLDGNRASARWDPRVCQKGLALTRRFYGDRRLRHCMVRAGFKRWVDEGFPRGEDGKPPKEYF 7tgh.58   --------------------------------------------------------------------------------  target    QRARDEWVRASHDEAAAVVAATLANIAATYSGEEGAQRLRDQGYEEETIEAMGGAGVQAMKFRGGMPLLGMTRVFGLYRM 7tgh.58   --------------------------------------------------------------------------------  target    ANSMALLDAKVRGVGPDEARGARGFDNYSWHTDLPPGHPMVTGQQTVDFDLNSVELAKNVVVWGMNWITTKMPDAHWLTE 7tgh.58   ------------------------------------------------------KNAKLVFILGADNNLRP--------E  target    ARLKGTRIIVIACEYSSTSSKADDAIVVRPGTTPALALGLSHVIMRDKLYDADYVRRWTDLPMLVRTDTLKYLSAEDVFG 7tgh.58   DIPADAFVVYFGTHGDEGAYYADIILPT----------------------------------------------------  target    GGPAPL 7tgh.58   ------ ``` | | | | | | | | | | | | | | | | | | | | | | | | | | | | | | | | | | | | | | | | | | | | | | | | | |
|  | 7v2c.1.L | NADH-ubiquinone oxidoreductase 75 kDa subunit, mitochondrial  *Active state complex I from Q10 dataset* | 0.02 |  | 14.89 | 0.12 | 296-349 | EM | 0.00 | hetero-1-1-1-1-1-2-… | 6 x SF4, 1 x FMN, 10 x PEE, 8 x PLX, 2 x 8Q1, 1 x NDP, 2 x UQ, 11 x CDL, 2 x FES, 1 x MG, 1 x ZN, 1 x ADP | HHblits | 0.24 |
| ``` target    TARREITRRGFLGTAAGAGFAAFVVSATRAWGLEAIENPLARYPDREWERVYRDLWRYDSKFTFLCAPNDTHNCLLDAYV 7v2c.1    --------------------------------------------------------------------------------  target    RSGVMTRIGPTMRYGEARDLDGNRASARWDPRVCQKGLALTRRFYGDRRLRHCMVRAGFKRWVDEGFPRGEDGKPPKEYF 7v2c.1    --------------------------------------------------------------------------------  target    QRARDEWVRASHDEAAAVVAATLANIAATYSGEEGAQRLRDQGYEEETIEAMGGAGVQAMKFRGGMPLLGMTRVFGLYRM 7v2c.1    --------------------------------------------------------------------------------  target    ANSMALLDAKVRGVGPDEARGARGFDNYSWHTDLPPGHPMVTGQQTVDFDLNSVELAKNVVVWGMNWITTKMPDAHWLTE 7v2c.1    -------------------------------------------------------PPKVLFLLGADGGC-------ITRQ  target    ARLKGTRIIVIACEYSSTSSKADDAIVVRPGTTPALALGLSHVIMRDKLYDADYVRRWTDLPMLVRTDTLKYLSAEDVFG 7v2c.1    DLPKDCFIIYQGHHGDVGAPMADVILPGA---------------------------------------------------  target    GGPAPL 7v2c.1    ------ ``` | | | | | | | | | | | | | | | | | | | | | | | | | | | | | | | | | | | | | | | | | | | | | | | | | |
|  | 7arc.1.F | 75 kDa  *Cryo-EM structure of Polytomella Complex-I (peripheral arm)* | 0.02 |  | 15.56 | 0.11 | 296-349 | EM | 0.00 | hetero-1-1-1-1-1-1-… | 6 x SF4, 2 x FES, 1 x FMN, 1 x NDP, 1 x ZN, 1 x 8Q1 | HHblits | 0.26 |
| ``` target    TARREITRRGFLGTAAGAGFAAFVVSATRAWGLEAIENPLARYPDREWERVYRDLWRYDSKFTFLCAPNDTHNCLLDAYV 7arc.1    --------------------------------------------------------------------------------  target    RSGVMTRIGPTMRYGEARDLDGNRASARWDPRVCQKGLALTRRFYGDRRLRHCMVRAGFKRWVDEGFPRGEDGKPPKEYF 7arc.1    --------------------------------------------------------------------------------  target    QRARDEWVRASHDEAAAVVAATLANIAATYSGEEGAQRLRDQGYEEETIEAMGGAGVQAMKFRGGMPLLGMTRVFGLYRM 7arc.1    --------------------------------------------------------------------------------  target    ANSMALLDAKVRGVGPDEARGARGFDNYSWHTDLPPGHPMVTGQQTVDFDLNSVELAKNVVVWGMNWITTKMPDAHWLTE 7arc.1    -------------------------------------------------------PAKVVYLLGSDDFKD---------E  target    ARLKGTRIIVIACEYSSTSSKADDAIVVRPGTTPALALGLSHVIMRDKLYDADYVRRWTDLPMLVRTDTLKYLSAEDVFG 7arc.1    EIPADAFVIYQGHHGDKGAARANVVLPGA---------------------------------------------------  target    GGPAPL 7arc.1    ------ ``` | | | | | | | | | | | | | | | | | | | | | | | | | | | | | | | | | | | | | | | | | | | | | | | | | |
|  | 1eiw.1.A | HYPOTHETICAL PROTEIN MTH538  *Solution structure of hypothetical protein MTH538 from Methanobacterium thermoautotrophicum* | 0.01 |  | 22.73 | 0.11 | 294-337 | NMR | 0.00 | monomer |  | HHblits | 0.27 |
| ``` target    TARREITRRGFLGTAAGAGFAAFVVSATRAWGLEAIENPLARYPDREWERVYRDLWRYDSKFTFLCAPNDTHNCLLDAYV 1eiw.1    --------------------------------------------------------------------------------  target    RSGVMTRIGPTMRYGEARDLDGNRASARWDPRVCQKGLALTRRFYGDRRLRHCMVRAGFKRWVDEGFPRGEDGKPPKEYF 1eiw.1    --------------------------------------------------------------------------------  target    QRARDEWVRASHDEAAAVVAATLANIAATYSGEEGAQRLRDQGYEEETIEAMGGAGVQAMKFRGGMPLLGMTRVFGLYRM 1eiw.1    --------------------------------------------------------------------------------  target    ANSMALLDAKVRGVGPDEARGARGFDNYSWHTDLPPGHPMVTGQQTVDFDLNSVELAKNVVVWGMNWITTKMPDAHWLTE 1eiw.1    -----------------------------------------------------PEDADAVIVLAGLWGTRRDEILGAVDL  target    ARLKGTRIIVIACEYSSTSSKADDAIVVRPGTTPALALGLSHVIMRDKLYDADYVRRWTDLPMLVRTDTLKYLSAEDVFG 1eiw.1    ARKSSKPIITVRPYGLE---------------------------------------------------------------  target    GGPAPL 1eiw.1    ------ ``` | | | | | | | | | | | | | | | | | | | | | | | | | | | | | | | | | | | | | | | | | | | | | | | | | |
|  | 6s6y.1.B | Tungsten-containing formylmethanofuran dehydrogenase, subunit B  *X-ray crystal structure of the formyltransferase/hydrolase complex (FhcABCD) from Methylorubrum extorquens in complex with methylofuran* | 0.01 |  | 13.04 | 0.11 | 296-350 | X-ray | 3.10 | hetero-2-2-2-2-mer | 1 x MFN, 4 x ZN, 4 x CA, 4 x K, 3 x DGL, 2 x GLU, 1 x IAS | HHblits | 0.24 |
| ``` target    TARREITRRGFLGTAAGAGFAAFVVSATRAWGLEAIENPLARYPDREWERVYRDLWRYDSKFTFLCAPNDTHNCLLDAYV 6s6y.1    --------------------------------------------------------------------------------  target    RSGVMTRIGPTMRYGEARDLDGNRASARWDPRVCQKGLALTRRFYGDRRLRHCMVRAGFKRWVDEGFPRGEDGKPPKEYF 6s6y.1    --------------------------------------------------------------------------------  target    QRARDEWVRASHDEAAAVVAATLANIAATYSGEEGAQRLRDQGYEEETIEAMGGAGVQAMKFRGGMPLLGMTRVFGLYRM 6s6y.1    --------------------------------------------------------------------------------  target    ANSMALLDAKVRGVGPDEARGARGFDNYSWHTDLPPGHPMVTGQQTVDFDLNSVELAKNVVVWGMNWITTKMPDAHWLTE 6s6y.1    -------------------------------------------------------EADAALWLASLPAPR--------PA  target    ARLKGTRIIVIA--CEYSSTSSKADDAIVVRPGTTPALALGLSHVIMRDKLYDADYVRRWTDLPMLVRTDTLKYLSAEDV 6s6y.1    WLG-SLPTIAIVGEGSQEAAGETAEVVITVGV------------------------------------------------  target    FGGGPAPL 6s6y.1    -------- ``` | | | | | | | | | | | | | | | | | | | | | | | | | | | | | | | | | | | | | | | | | | | | | | | | | |
|  | 6o1x.1.A | DNA translocase coupling protein  *Structure of pCW3 conjugation coupling protein TcpA monomer form with ATPgS* | 0.01 |  | 8.89 | 0.11 | 294-339 | X-ray | 2.46 | monomer | 2 x BGC, 1 x AGS | HHblits | 0.25 |
| ``` target    TARREITRRGFLGTAAGAGFAAFVVSATRAWGLEAIENPLARYPDREWERVYRDLWRYDSKFTFLCAPNDTHNCLLDAYV 6o1x.1    --------------------------------------------------------------------------------  target    RSGVMTRIGPTMRYGEARDLDGNRASARWDPRVCQKGLALTRRFYGDRRLRHCMVRAGFKRWVDEGFPRGEDGKPPKEYF 6o1x.1    --------------------------------------------------------------------------------  target    QRARDEWVRASHDEAAAVVAATLANIAATYSGEEGAQRLRDQGYEEETIEAMGGAGVQAMKFRGGMPLLGMTRVFGLYRM 6o1x.1    --------------------------------------------------------------------------------  target    ANSMALLDAKVRGVGPDEARGARGFDNYSWHTDLPPGHPMVTGQQTVDFDLNSVELAKNVVVWGMNWITTKMPDAH-WLT 6o1x.1    -----------------------------------------------------LNKSPHILSAGETGSG-KSVILRCILW  target    EARLKGTRIIVIACEYSSTSSKADDAIVVRPGTTPALALGLSHVIMRDKLYDADYVRRWTDLPMLVRTDTLKYLSAEDVF 6o1x.1    QLLKQGAIAYMVDFKGGVEF------------------------------------------------------------  target    GGGPAPL 6o1x.1    ------- ``` | | | | | | | | | | | | | | | | | | | | | | | | | | | | | | | | | | | | | | | | | | | | | | | | | |
|  | 6o1x.2.A | DNA translocase coupling protein  *Structure of pCW3 conjugation coupling protein TcpA monomer form with ATPgS* | 0.01 |  | 8.89 | 0.11 | 294-339 | X-ray | 2.46 | monomer |  | HHblits | 0.25 |
| ``` target    TARREITRRGFLGTAAGAGFAAFVVSATRAWGLEAIENPLARYPDREWERVYRDLWRYDSKFTFLCAPNDTHNCLLDAYV 6o1x.2    --------------------------------------------------------------------------------  target    RSGVMTRIGPTMRYGEARDLDGNRASARWDPRVCQKGLALTRRFYGDRRLRHCMVRAGFKRWVDEGFPRGEDGKPPKEYF 6o1x.2    --------------------------------------------------------------------------------  target    QRARDEWVRASHDEAAAVVAATLANIAATYSGEEGAQRLRDQGYEEETIEAMGGAGVQAMKFRGGMPLLGMTRVFGLYRM 6o1x.2    --------------------------------------------------------------------------------  target    ANSMALLDAKVRGVGPDEARGARGFDNYSWHTDLPPGHPMVTGQQTVDFDLNSVELAKNVVVWGMNWITTKMPDAH-WLT 6o1x.2    -----------------------------------------------------LNKSPHILSAGETGSG-KSVILRCILW  target    EARLKGTRIIVIACEYSSTSSKADDAIVVRPGTTPALALGLSHVIMRDKLYDADYVRRWTDLPMLVRTDTLKYLSAEDVF 6o1x.2    QLLKQGAIAYMVDFKGGVEF------------------------------------------------------------  target    GGGPAPL 6o1x.2    ------- ``` | | | | | | | | | | | | | | | | | | | | | | | | | | | | | | | | | | | | | | | | | | | | | | | | | |
|  | 6o1y.1.A | DNA translocase coupling protein  *Structure of pCW3 conjugation coupling protein TcpA monomeric form with ATP* | 0.01 |  | 8.89 | 0.11 | 294-339 | X-ray | 2.70 | monomer | 1 x ATP, 1 x BGC | HHblits | 0.25 |
| ``` target    TARREITRRGFLGTAAGAGFAAFVVSATRAWGLEAIENPLARYPDREWERVYRDLWRYDSKFTFLCAPNDTHNCLLDAYV 6o1y.1    --------------------------------------------------------------------------------  target    RSGVMTRIGPTMRYGEARDLDGNRASARWDPRVCQKGLALTRRFYGDRRLRHCMVRAGFKRWVDEGFPRGEDGKPPKEYF 6o1y.1    --------------------------------------------------------------------------------  target    QRARDEWVRASHDEAAAVVAATLANIAATYSGEEGAQRLRDQGYEEETIEAMGGAGVQAMKFRGGMPLLGMTRVFGLYRM 6o1y.1    --------------------------------------------------------------------------------  target    ANSMALLDAKVRGVGPDEARGARGFDNYSWHTDLPPGHPMVTGQQTVDFDLNSVELAKNVVVWGMNWITTKMPDAH-WLT 6o1y.1    -----------------------------------------------------LNKSPHILSAGETGSG-KSVILRCILW  target    EARLKGTRIIVIACEYSSTSSKADDAIVVRPGTTPALALGLSHVIMRDKLYDADYVRRWTDLPMLVRTDTLKYLSAEDVF 6o1y.1    QLLKQGAIAYMVDFKGGVEF------------------------------------------------------------  target    GGGPAPL 6o1y.1    ------- ``` | | | | | | | | | | | | | | | | | | | | | | | | | | | | | | | | | | | | | | | | | | | | | | | | | |
|  | 6o1y.2.A | DNA translocase coupling protein  *Structure of pCW3 conjugation coupling protein TcpA monomeric form with ATP* | 0.02 |  | 8.89 | 0.11 | 294-339 | X-ray | 2.70 | monomer |  | HHblits | 0.25 |
| ``` target    TARREITRRGFLGTAAGAGFAAFVVSATRAWGLEAIENPLARYPDREWERVYRDLWRYDSKFTFLCAPNDTHNCLLDAYV 6o1y.2    --------------------------------------------------------------------------------  target    RSGVMTRIGPTMRYGEARDLDGNRASARWDPRVCQKGLALTRRFYGDRRLRHCMVRAGFKRWVDEGFPRGEDGKPPKEYF 6o1y.2    --------------------------------------------------------------------------------  target    QRARDEWVRASHDEAAAVVAATLANIAATYSGEEGAQRLRDQGYEEETIEAMGGAGVQAMKFRGGMPLLGMTRVFGLYRM 6o1y.2    --------------------------------------------------------------------------------  target    ANSMALLDAKVRGVGPDEARGARGFDNYSWHTDLPPGHPMVTGQQTVDFDLNSVELAKNVVVWGMNWITTKMPDAH-WLT 6o1y.2    -----------------------------------------------------LNKSPHILSAGETGSG-KSVILRCILW  target    EARLKGTRIIVIACEYSSTSSKADDAIVVRPGTTPALALGLSHVIMRDKLYDADYVRRWTDLPMLVRTDTLKYLSAEDVF 6o1y.2    QLLKQGAIAYMVDFKGGVEF------------------------------------------------------------  target    GGGPAPL 6o1y.2    ------- ``` | | | | | | | | | | | | | | | | | | | | | | | | | | | | | | | | | | | | | | | | | | | | | | | | | |
|  | 7zm7.1.I | NADH-ubiquinone oxidoreductase-like protein  *CryoEM structure of mitochondrial complex I from Chaetomium thermophilum (inhibited by DDM)* | 0.02 |  | 15.91 | 0.11 | 296-348 | EM | 0.00 | hetero-1-1-1-1-1-1-… | 4 x PC1, 14 x LMT, 5 x CDL, 8 x 3PE, 2 x FES, 6 x SF4, 1 x FMN, 1 x NDP, 1 x ZN, 2 x ZMP | HHblits | 0.25 |
| ``` target    TARREITRRGFLGTAAGAGFAAFVVSATRAWGLEAIENPLARYPDREWERVYRDLWRYDSKFTFLCAPNDTHNCLLDAYV 7zm7.1    --------------------------------------------------------------------------------  target    RSGVMTRIGPTMRYGEARDLDGNRASARWDPRVCQKGLALTRRFYGDRRLRHCMVRAGFKRWVDEGFPRGEDGKPPKEYF 7zm7.1    --------------------------------------------------------------------------------  target    QRARDEWVRASHDEAAAVVAATLANIAATYSGEEGAQRLRDQGYEEETIEAMGGAGVQAMKFRGGMPLLGMTRVFGLYRM 7zm7.1    --------------------------------------------------------------------------------  target    ANSMALLDAKVRGVGPDEARGARGFDNYSWHTDLPPGHPMVTGQQTVDFDLNSVELAKNVVVWGMNWITTKMPDAHWLTE 7zm7.1    -------------------------------------------------------KPKFVWLLGADEFDP---------A  target    ARLKGTRIIVIACEYSSTSSKADDAIVVRPGTTPALALGLSHVIMRDKLYDADYVRRWTDLPMLVRTDTLKYLSAEDVFG 7zm7.1    DVPKDAFIVYQGHHGDRGAEIADIVLPG----------------------------------------------------  target    GGPAPL 7zm7.1    ------ ``` | | | | | | | | | | | | | | | | | | | | | | | | | | | | | | | | | | | | | | | | | | | | | | | | | |
[truncated: 157,149 more chars]
