# Supplementary material for: Metabolic capacity is maintained despite shifts in microbial diversity in estuary sediments
Source: ISME Commun. 2025 Oct 11;5(1):ycaf182. doi: 10.1093/ismeco/ycaf182 (PMC12687941; doi:10.1093/ismeco/ycaf182)
Supplement: Supplementary_Data_1_ycaf182 [file supplementary_data_1_ycaf182.zip › SWISS-MODEL/4_1_Jan_SF_Bin21_scaffold_19364_c121714_1/templates.html]

4\_1\_Jan\_SF\_Bin21\_scaffold\_19364\_c1:2-1714\_1 | Templates


**Export Alignment**
  
FASTA format
Clustal Format
PNG Image

**Secondary Structure**
  
None
DSSP
PSIPRED
SSpro

**Colour Scheme** 


Fade Mismatches
Enhance Mismatches

Confidencegradient
Confidenceclass
Indels
Chain
Unique Chain
Rainbow
2° Structure
Clustal
Hydrophobic
Size
Charged
Polar
Proline
Ser/Thr
Cysteine
Aliphatic
Aromatic
No Colour

Use QMEANBrane values

|  |  |  |  |
| --- | --- | --- | --- |
| Background |  |  |  |

**3D Viewer**  
NGL
PV

FASTA
Multi FASTA
ClustalW
PNG


SWISS-MODEL

### 4\_1\_Jan\_SF\_Bin21\_scaffold\_19364\_c1:2-1714\_1

### Created: March 29, 2023, 5:45 p.m. at 17:45

- Templates
- Models

Models | Name | Description | GMQE | QSQE | Seq Id | Coverage | Range | Method | Resolution | Oligo-state | Ligands | Found by | Seq Similarity || ✓ | 7b04.1.B | Nitrite oxidoreductase subunit A  *Structure of Nitrite oxidoreductase (Nxr) from the anammox bacterium Kuenenia stuttgartiensis.* | 0.71 | 0.00 | 39.29 | 0.93 | 28-567 | X-ray | 2.97 | monomer | 4 x SF4, 1 x F3S, 2 x MD1, 1 x MO, 1 x HEM, 2 x CA | HHblits | 0.39 |
| ``` target    FNGAPQYINENPFDLELDASRPARPRQYWRAESAHFYNHEDHP-----LRVGTRLLTGSTHMPTPTKVMWFANANSILGN 7b04.1    ---------------------------RALDEEVAYWNHSERPLIVNTPKYGRKVFTGKTHMPSPTKVLWFTNVNLINN-  target    VKWHYNTVVNA-LPRMEMIAVHEWWWTGSCEWADVVFGVDSWGELKHPDMTASVTNPFLIVFPKTPIKRIFNTVGDIDVL 7b04.1    -AKHVYQMLKNVNPNIEQIMSTDIEITGSIEYADFAFPANSWVEFQEFEITNSCSNPFIQIWGKTGITPVYESKDDVKIL  target    ALVSSKLAELTGDTRFNDMWKFVREGRTDVYLQRILDASTNTKGYRFTELEAKARE--GIPALMNSRTSPKVVGYDQLAD 7b04.1    AGMASKLGELLRDKRFEDNWKFAIEGRASVYINRLLDGSTTMKGYTCEDILNGKYGEPGVAM-LLFRTYPRHPFWEQVHE  target    STPWYTKSGRLEFYREEDEFIEAGENLPVHREPVDSTFYEPNVIVSPKHEAVRPSGPEDYGVARTDLSCEVRCGRNVVLT 7b04.1    SLPFYTPTGRLQAYNDEPEIIEYGENFIVHREGPEATPYLPNAIVSTN-PY---IRPDDYGIPENAEYWEDRTVRNIKKS  target    WAETRQTQHPLVKQGHKFIFHTPKYRHGSHTTPIDTDMNAVLFGPFGDIYRRDKRSPFVTEGYVDINPTDGLELGLQDGD 7b04.1    WEETKKTKNFLWEKGYHFYCVTPKSRHTVHSQWAVTDWNFIWNNNFGDPYRMDKRMPGVGEHQIHIHPQAARDLGIEDGD  target    YVWIDPDPEDRPFRGWQKNAKDMEFARLLCRARFYPGTPRGVTRMWFNMYGATPGSVRGAKARRDGLAKNPDTNYQAMFR 7b04.1    YVYVDANPADRPYEGWKPNDSFYKVSRLMLRAKYNPAYPYNCTMMKHSAWISSDKTVQAHETRPDGRALSP-SGYQSSFR  target    SGSHQSATRGWLKPTWMTDSLVRKGLFGQGIGKGFLPDVHCPTGAPREAFVKISRAEPGGIGGQGLWRPAALGIRPRHES 7b04.1    YGSQQSITRDWSMPMHQLDSLFHKAKIGMKFIFGFEADNHCINTVPKETLVKITKAENGGMGGKGVWDPVKTGYTAGNEN  target    PAMKRYLAGGFFSGPKE 7b04.1    DFMKKFLNGELIKVD-- ``` | | | | | | | | | | | | | | | | | | | | | | | | | | | | | | | | | | | | | | | | | | | | | | | | | |
|  | 7b04.2.B | Nitrite oxidoreductase subunit A  *Structure of Nitrite oxidoreductase (Nxr) from the anammox bacterium Kuenenia stuttgartiensis.* | 0.69 | 0.00 | 39.29 | 0.93 | 28-567 | X-ray | 2.97 | monomer | 4 x SF4, 1 x F3S, 2 x MD1, 1 x MO, 1 x HEM, 2 x CA | HHblits | 0.39 |
| ``` target    FNGAPQYINENPFDLELDASRPARPRQYWRAESAHFYNHEDHP-----LRVGTRLLTGSTHMPTPTKVMWFANANSILGN 7b04.2    ---------------------------RALDEEVAYWNHSERPLIVNTPKYGRKVFTGKTHMPSPTKVLWFTNVNLINN-  target    VKWHYNTVVNA-LPRMEMIAVHEWWWTGSCEWADVVFGVDSWGELKHPDMTASVTNPFLIVFPKTPIKRIFNTVGDIDVL 7b04.2    -AKHVYQMLKNVNPNIEQIMSTDIEITGSIEYADFAFPANSWVEFQEFEITNSCSNPFIQIWGKTGITPVYESKDDVKIL  target    ALVSSKLAELTGDTRFNDMWKFVREGRTDVYLQRILDASTNTKGYRFTELEAKARE--GIPALMNSRTSPKVVGYDQLAD 7b04.2    AGMASKLGELLRDKRFEDNWKFAIEGRASVYINRLLDGSTTMKGYTCEDILNGKYGEPGVAM-LLFRTYPRHPFWEQVHE  target    STPWYTKSGRLEFYREEDEFIEAGENLPVHREPVDSTFYEPNVIVSPKHEAVRPSGPEDYGVARTDLSCEVRCGRNVVLT 7b04.2    SLPFYTPTGRLQAYNDEPEIIEYGENFIVHREGPEATPYLPNAIVSTN-PY---IRPDDYGIPENAEYWEDRTVRNIKKS  target    WAETRQTQHPLVKQGHKFIFHTPKYRHGSHTTPIDTDMNAVLFGPFGDIYRRDKRSPFVTEGYVDINPTDGLELGLQDGD 7b04.2    WEETKKTKNFLWEKGYHFYCVTPKSRHTVHSQWAVTDWNFIWNNNFGDPYRMDKRMPGVGEHQIHIHPQAARDLGIEDGD  target    YVWIDPDPEDRPFRGWQKNAKDMEFARLLCRARFYPGTPRGVTRMWFNMYGATPGSVRGAKARRDGLAKNPDTNYQAMFR 7b04.2    YVYVDANPADRPYEGWKPNDSFYKVSRLMLRAKYNPAYPYNCTMMKHSAWISSDKTVQAHETRPDGRALSP-SGYQSSFR  target    SGSHQSATRGWLKPTWMTDSLVRKGLFGQGIGKGFLPDVHCPTGAPREAFVKISRAEPGGIGGQGLWRPAALGIRPRHES 7b04.2    YGSQQSITRDWSMPMHQLDSLFHKAKIGMKFIFGFEADNHCINTVPKETLVKITKAENGGMGGKGVWDPVKTGYTAGNEN  target    PAMKRYLAGGFFSGPKE 7b04.2    DFMKKFLNGELIKVD-- ``` | | | | | | | | | | | | | | | | | | | | | | | | | | | | | | | | | | | | | | | | | | | | | | | | | |
|  | 7b04.1.B | Nitrite oxidoreductase subunit A  *Structure of Nitrite oxidoreductase (Nxr) from the anammox bacterium Kuenenia stuttgartiensis.* | 0.70 | 0.00 | 41.41 | 0.92 | 32-561 | X-ray | 2.97 | monomer | 4 x SF4, 1 x F3S, 2 x MD1, 1 x MO, 1 x HEM, 2 x CA | BLAST | 0.40 |
| ``` target    FNGAPQYINENPFDLELDASRPARPRQYWRAESAHFYNHEDHPLRVGT-----RLLTGSTHMPTPTKVMWFANANSILGN 7b04.1    -------------------------------EEVAYWNHSERPLIVNTPKYGRKVFTGKTHMPSPTKVLWFTNVN-LINN  target    VKWHYNTVVNALPRMEMIAVHEWWWTGSCEWADVVFGVDSWGELKHPDMTASVTNPFLIVFPKTPIKRIFNTVGDIDVLA 7b04.1    AKHVYQMLKNVNPNIEQIMSTDIEITGSIEYADFAFPANSWVEFQEFEITNSCSNPFIQIWGKTGITPVYESKDDVKILA  target    LVSSKLAELTGDTRFNDMWKFVREGRTDVYLQRILDASTNTKGYRFTE-LEAKAREGIPALMNSRTSPKVVGYDQLADST 7b04.1    GMASKLGELLRDKRFEDNWKFAIEGRASVYINRLLDGSTTMKGYTCEDILNGKYGEPGVAMLLFRTYPRHPFWEQVHESL  target    PWYTKSGRLEFYREEDEFIEAGENLPVHREPVDSTFYEPNVIVSPKHEAVRPSGPEDYGVARTDLSCEVRCGRNVVLTWA 7b04.1    PFYTPTGRLQAYNDEPEIIEYGENFIVHREGPEATPYLPNAIVS-TNPYIRP---DDYGIPENAEYWEDRTVRNIKKSWE  target    ETRQTQHPLVKQGHKFIFHTPKYRHGSHTTPIDTDMNAVLFGPFGDIYRRDKRSPFVTEGYVDINPTDGLELGLQDGDYV 7b04.1    ETKKTKNFLWEKGYHFYCVTPKSRHTVHSQWAVTDWNFIWNNNFGDPYRMDKRMPGVGEHQIHIHPQAARDLGIEDGDYV  target    WIDPDPEDRPFRGWQKNAKDMEFARLLCRARFYPGTPRGVTRMWFNMYGATPGSVRGAKARRDGLAKNPDTNYQAMFRSG 7b04.1    YVDANPADRPYEGWKPNDSFYKVSRLMLRAKYNPAYPYNCTMMKHSAWISSDKTVQAHETRPDGRALSP-SGYQSSFRYG  target    SHQSATRGWLKPTWMTDSLVRKGLFGQGIGKGFLPDVHCPTGAPREAFVKISRAEPGGIGGQGLWRPAALGIRPRHESPA 7b04.1    SQQSITRDWSMPMHQLDSLFHKAKIGMKFIFGFEADNHCINTVPKETLVKITKAENGGMGGKGVWDPVKTGYTAGNENDF  target    MKRYLAGGFFSGPKE 7b04.1    MKKFLNG-------- ``` | | | | | | | | | | | | | | | | | | | | | | | | | | | | | | | | | | | | | | | | | | | | | | | | | |
|  | 7b04.2.B | Nitrite oxidoreductase subunit A  *Structure of Nitrite oxidoreductase (Nxr) from the anammox bacterium Kuenenia stuttgartiensis.* | 0.69 | 0.00 | 41.41 | 0.92 | 32-561 | X-ray | 2.97 | monomer | 4 x SF4, 1 x F3S, 2 x MD1, 1 x MO, 1 x HEM, 2 x CA | BLAST | 0.40 |
| ``` target    FNGAPQYINENPFDLELDASRPARPRQYWRAESAHFYNHEDHPLRVGT-----RLLTGSTHMPTPTKVMWFANANSILGN 7b04.2    -------------------------------EEVAYWNHSERPLIVNTPKYGRKVFTGKTHMPSPTKVLWFTNVN-LINN  target    VKWHYNTVVNALPRMEMIAVHEWWWTGSCEWADVVFGVDSWGELKHPDMTASVTNPFLIVFPKTPIKRIFNTVGDIDVLA 7b04.2    AKHVYQMLKNVNPNIEQIMSTDIEITGSIEYADFAFPANSWVEFQEFEITNSCSNPFIQIWGKTGITPVYESKDDVKILA  target    LVSSKLAELTGDTRFNDMWKFVREGRTDVYLQRILDASTNTKGYRFTE-LEAKAREGIPALMNSRTSPKVVGYDQLADST 7b04.2    GMASKLGELLRDKRFEDNWKFAIEGRASVYINRLLDGSTTMKGYTCEDILNGKYGEPGVAMLLFRTYPRHPFWEQVHESL  target    PWYTKSGRLEFYREEDEFIEAGENLPVHREPVDSTFYEPNVIVSPKHEAVRPSGPEDYGVARTDLSCEVRCGRNVVLTWA 7b04.2    PFYTPTGRLQAYNDEPEIIEYGENFIVHREGPEATPYLPNAIVS-TNPYIRP---DDYGIPENAEYWEDRTVRNIKKSWE  target    ETRQTQHPLVKQGHKFIFHTPKYRHGSHTTPIDTDMNAVLFGPFGDIYRRDKRSPFVTEGYVDINPTDGLELGLQDGDYV 7b04.2    ETKKTKNFLWEKGYHFYCVTPKSRHTVHSQWAVTDWNFIWNNNFGDPYRMDKRMPGVGEHQIHIHPQAARDLGIEDGDYV  target    WIDPDPEDRPFRGWQKNAKDMEFARLLCRARFYPGTPRGVTRMWFNMYGATPGSVRGAKARRDGLAKNPDTNYQAMFRSG 7b04.2    YVDANPADRPYEGWKPNDSFYKVSRLMLRAKYNPAYPYNCTMMKHSAWISSDKTVQAHETRPDGRALSP-SGYQSSFRYG  target    SHQSATRGWLKPTWMTDSLVRKGLFGQGIGKGFLPDVHCPTGAPREAFVKISRAEPGGIGGQGLWRPAALGIRPRHESPA 7b04.2    SQQSITRDWSMPMHQLDSLFHKAKIGMKFIFGFEADNHCINTVPKETLVKITKAENGGMGGKGVWDPVKTGYTAGNENDF  target    MKRYLAGGFFSGPKE 7b04.2    MKKFLNG-------- ``` | | | | | | | | | | | | | | | | | | | | | | | | | | | | | | | | | | | | | | | | | | | | | | | | | |
|  | 3ir5.1.A | Respiratory nitrate reductase 1 alpha chain  *Crystal structure of NarGHI mutant NarG-H49C* | 0.30 | 0.00 | 20.49 | 0.65 | 59-534 | X-ray | 2.30 | monomer | 2 x MD1, 1 x 6MO, 4 x SF4, 1 x AGA, 1 x F3S, 2 x HEM | HHblits | 0.31 |
| ``` target    FNGAPQYINENPFDLELDASRPARPRQYWRAESAHFYNHEDHPLRVGTRLLTGSTHMPTPTKVMWFANANSILGNVKWHY 3ir5.1    ----------------------------------------------------------NHPRNLFIWRSNLLGS--SGKG  target    NT------------------------------V--VNALPRMEMIAVHEWWWTGSCEWADVVFGVDSWGELKHPDMTASV 3ir5.1    HEFMLKYLLGTEHGIQGKDLGQQGGVKPEEVDWQDNGLEGKLDLVVTLDFRLSSTCLYSDIILPTATWYEK--DDMNTSD  target    TNPFLIVFPKTPIKRIFNTVGDIDVLALVSSKLAELTG----DT----RF----------------NDM----------- 3ir5.1    MHPFIHPLSA-AVDPAWEAKSDWEIYKAIAKKFSEVCVGHLGKETDIVTLPIQHDSAAELAQPLDVKDWKKGECDLIPGK  target    ---------------WKFVRE-G-----------------R-TDVYLQR-------------------------ILDAST 3ir5.1    TAPHIMVVERDYPATYERFTSIGPLMEKIGNGGKGIAWNTQSEMDLLRKLNYTKAEGPAKGQPMLNTAIDAAEMILTLAP  target    NTKGY----RFTELEAKAR-----------EGIPALMN------------------SRTSPKVVGYDQLADSTPWYTKSG 3ir5.1    ETNGQVAVKAWAALSEFTGRDHTHLALNKEDEKIRFRDIQAQPRKIISSPTWSGLEDEHVSYNAGYTNVHELIPWRTLSG  target    RLEFYREEDEFIEAGENLPVHREPVDSTFYEPNVIVSPKHEAVRPSGPEDYGVARTDLSCEVRCGRNVVLTWAETRQTQH 3ir5.1    RQQLYQDHQWMRDFGESLLVYRPPIDTRSV-----------------KEVIGQ-------------------------KS  target    PLVKQGHKFIFHTPKYRHGSHTTPIDTDMNAVLFGPFGDIYRRDKRSPFVTEGYVDINPTDGLELGLQDGDYVWIDPDPE 3ir5.1    NG-NQEKALNFLTPHQKWGIHSTYSDNLLMLT----------LG-----RGGPVVWLSEADAKDLGIADNDWIEVFNSN-  target    DRPFRGWQKNAKDMEFARLLCRARFYPGTPRGVTRMWFNMYGATPGSVRGAKARRDGLAKNPDTNYQAMFRSGSHQSATR 3ir5.1    ----------------GALTARAVVSQRVPAGMTMMYHAQER---------IVN---LPG--SEI--TQQRGGIHNSVTR  target    GWLKPTWMTDSLVRKGLFGQGIGKGFLPDVHCPTGAPREAFVKISRAEPGGIGGQGLWRPAALGIRPRHESPAMKRYLAG 3ir5.1    ITPKPTHMI------GGYA---HLAYGFNYYGTVGSNRDEFVVVRKMKNIDWL---------------------------  target    GFFSGPKE 3ir5.1    -------- ``` | | | | | | | | | | | | | | | | | | | | | | | | | | | | | | | | | | | | | | | | | | | | | | | | | |
|  | 3ir7.1.A | Respiratory nitrate reductase 1 alpha chain  *Crystal structure of NarGHI mutant NarG-R94S* | 0.29 | 0.00 | 20.81 | 0.65 | 60-534 | X-ray | 2.50 | monomer | 2 x MD1, 4 x SF4, 1 x 6MO, 1 x AGA, 1 x F3S, 2 x HEM | HHblits | 0.31 |
| ``` target    FNGAPQYINENPFDLELDASRPARPRQYWRAESAHFYNHEDHPLRVGTRLLTGSTHMPTPTKVMWFANANSILGNVKWHY 3ir7.1    -----------------------------------------------------------HPRNLFIWRSNLLGS--SGKG  target    NT------------------------------V--VNALPRMEMIAVHEWWWTGSCEWADVVFGVDSWGELKHPDMTASV 3ir7.1    HEFMLKYLLGTEHGIQGKDLGQQGGVKPEEVDWQDNGLEGKLDLVVTLDFRLSSTCLYSDIILPTATWYEK--DDMNTSD  target    TNPFLIVFPKTPIKRIFNTVGDIDVLALVSSKLAELTG----DT----RFN---------------DMWK---------- 3ir7.1    MHPFIHPLSA-AVDPAWEAKSDWEIYKAIAKKFSEVCVGHLGKETDIVTLPIQHDSAAELAQPLDVKDWKKGECDLIPGK  target    -----------------FVRE-------------G-----R-TDVYLQRI-------------------------LDAST 3ir7.1    TAPHIMVVERDYPATYERFTSIGPLMEKIGNGGKGIAWNTQSEMDLLRKLNYTKAEGPAKGQPMLNTAIDAAEMILTLAP  target    NTKG----YRFTELEAKARE-----------GIPALMN------------------SRTSPKVVGYDQLADSTPWYTKSG 3ir7.1    ETNGQVAVKAWAALSEFTGRDHTHLALNKEDEKIRFRDIQAQPRKIISSPTWSGLEDEHVSYNAGYTNVHELIPWRTLSG  target    RLEFYREEDEFIEAGENLPVHREPVDSTFYEPNVIVSPKHEAVRPSGPEDYGVARTDLSCEVRCGRNVVLTWAETRQTQH 3ir7.1    RQQLYQDHQWMRDFGESLLVYRPPIDTRSV-----------------KEVIG-------------------------QKS  target    PLVKQGHKFIFHTPKYRHGSHTTPIDTDMNAVLFGPFGDIYRRDKRSPFVTEGYVDINPTDGLELGLQDGDYVWIDPDPE 3ir7.1    NG-NQEKALNFLTPHQKWGIHSTYSDNLLMLT----------L--GRG---GPVVWLSEADAKDLGIADNDWIEVFNSN-  target    DRPFRGWQKNAKDMEFARLLCRARFYPGTPRGVTRMWFNMYGATPGSVRGAKARRDGLAKNPDTNYQAMFRSGSHQSATR 3ir7.1    ----------------GALTARAVVSQRVPAGMTMMYHAQE---------RIV---NL---PGSE-ITQQRGGIHNSVTR  target    GWLKPTWMTDSLVRKGLFGQGIGKGFLPDVHCPTGAPREAFVKISRAEPGGIGGQGLWRPAALGIRPRHESPAMKRYLAG 3ir7.1    ITPKPTHMI------GGYA---HLAYGFNYYGTVGSNRDEFVVVRKMKNIDWL---------------------------  target    GFFSGPKE 3ir7.1    -------- ``` | | | | | | | | | | | | | | | | | | | | | | | | | | | | | | | | | | | | | | | | | | | | | | | | | |
| ✓ | 1q16.1.A | Respiratory nitrate reductase 1 alpha chain  *Crystal structure of Nitrate Reductase A, NarGHI, from Escherichia coli* | 0.31 | 0.00 | 20.54 | 0.65 | 60-534 | X-ray | 1.90 | monomer | 2 x MD1, 1 x 6MO, 2 x HEM, 4 x SF4, 1 x F3S, 1 x AGA, 1 x 3PH | HHblits | 0.31 |
| ``` target    FNGAPQYINENPFDLELDASRPARPRQYWRAESAHFYNHEDHPLRVGTRLLTGSTHMPTPTKVMWFANANSILGNVKWHY 1q16.1    -----------------------------------------------------------HPRNLFIWRSNLLGS--SGKG  target    NT------------------------------V--VNALPRMEMIAVHEWWWTGSCEWADVVFGVDSWGELKHPDMTASV 1q16.1    HEFMLKYLLGTEHGIQGKDLGQQGGVKPEEVDWQDNGLEGKLDLVVTLDFRLSSTCLYSDIILPTATWYEK--DDMNTSD  target    TNPFLIVFPKTPIKRIFNTVGDIDVLALVSSKLAELTG----DT----RF----------------NDMWK--------- 1q16.1    MHPFIHPLSA-AVDPAWEAKSDWEIYKAIAKKFSEVCVGHLGKETDIVTLPIQHDSAAELAQPLDVKDWKKGECDLIPGK  target    -----------------FVRE-G------------------RTDVYLQRI-------------------------LDAST 1q16.1    TAPHIMVVERDYPATYERFTSIGPLMEKIGNGGKGIAWNTQSEMDLLRKLNYTKAEGPAKGQPMLNTAIDAAEMILTLAP  target    NTKG----YRFTELEAKAR-----------EGIPALMN------------------SRTSPKVVGYDQLADSTPWYTKSG 1q16.1    ETNGQVAVKAWAALSEFTGRDHTHLALNKEDEKIRFRDIQAQPRKIISSPTWSGLEDEHVSYNAGYTNVHELIPWRTLSG  target    RLEFYREEDEFIEAGENLPVHREPVDSTFYEPNVIVSPKHEAVRPSGPEDYGVARTDLSCEVRCGRNVVLTWAETRQTQH 1q16.1    RQQLYQDHQWMRDFGESLLVYRPPIDTRSV-----------------KEVIGQ-------------------------KS  target    PLVKQGHKFIFHTPKYRHGSHTTPIDTDMNAVLFGPFGDIYRRDKRSPFVTEGYVDINPTDGLELGLQDGDYVWIDPDPE 1q16.1    NG-NQEKALNFLTPHQKWGIHSTYSDNLLMLT----------LG-----RGGPVVWLSEADAKDLGIADNDWIEVFNSN-  target    DRPFRGWQKNAKDMEFARLLCRARFYPGTPRGVTRMWFNMYGATPGSVRGAKARRDGLAKNPDTNYQAMFRSGSHQSATR 1q16.1    ----------------GALTARAVVSQRVPAGMTMMYHAQE---------RIVNLPGS-----EI--TQQRGGIHNSVTR  target    GWLKPTWMTDSLVRKGLFGQGIGKGFLPDVHCPTGAPREAFVKISRAEPGGIGGQGLWRPAALGIRPRHESPAMKRYLAG 1q16.1    ITPKPTHMI------GGYA---HLAYGFNYYGTVGSNRDEFVVVRKMKNIDWL---------------------------  target    GFFSGPKE 1q16.1    -------- ``` | | | | | | | | | | | | | | | | | | | | | | | | | | | | | | | | | | | | | | | | | | | | | | | | | |
|  | 1r27.4.A | Respiratory nitrate reductase 1 alpha chain  *Crystal Structure of NarGH complex* | 0.30 | 0.00 | 20.60 | 0.65 | 60-533 | X-ray | 2.00 | homo-dimer | 4 x MO, 16 x SF4, 8 x MGD, 4 x F3S | HHblits | 0.31 |
| ``` target    FNGAPQYINENPFDLELDASRPARPRQYWRAESAHFYNHEDHPLRVGTRLLTGSTHMPTPTKVMWFANANSILGNVKWHY 1r27.4    -----------------------------------------------------------HPRNLFIWRSNLLGS--SGKG  target    NT------------------------------V--VNALPRMEMIAVHEWWWTGSCEWADVVFGVDSWGELKHPDMTASV 1r27.4    HEFMLKYLLGTEHGIQGKDLGQQGGVKPEEVDWQDNGLEGKLDLVVTLDFRLSSTCLYSDIILPTATWYEK--DDMNTSD  target    TNPFLIVFPKTPIKRIFNTVGDIDVLALVSSKLAELTG----DT----RFN---------------DMWK---------- 1r27.4    MHPFIHPLSA-AVDPAWEAKSDWEIYKAIAKKFSEVCVGHLGKETDIVTLPIQHDSAAELAQPLDVKDWKKGECDLIPGK  target    -----------------FVRE-G-----------------RT-DVYLQRI-------------------------LDAST 1r27.4    TAPHIMVVERDYPATYERFTSIGPLMEKIGNGGKGIAWNTQSEMDLLRKLNYTKAEGPAKGQPMLNTAIDAAEMILTLAP  target    NTKG----YRFTELEAKARE-----------GIPALMN------------------SRTSPKVVGYDQLADSTPWYTKSG 1r27.4    ETNGQVAVKAWAALSEFTGRDHTHLALNKEDEKIRFRDIQAQPRKIISSPTWSGLEDEHVSYNAGYTNVHELIPWRTLSG  target    RLEFYREEDEFIEAGENLPVHREPVDSTFYEPNVIVSPKHEAVRPSGPEDYGVARTDLSCEVRCGRNVVLTWAETRQTQH 1r27.4    RQQLYQDHQWMRDFGESLLVYRPPIDTRSV-----------------KEVIGQ-------------------------KS  target    PLVKQGHKFIFHTPKYRHGSHTTPIDTDMNAVLFGPFGDIYRRDKRSPFVTEGYVDINPTDGLELGLQDGDYVWIDPDPE 1r27.4    N-GNQEKALNFLTPHQKWGIHSTYSDNLLMLT----------LG-----RGGPVVWLSEADAKDLGIADNDWIEVFNSN-  target    DRPFRGWQKNAKDMEFARLLCRARFYPGTPRGVTRMWFNMYGATPGSVRGAKARRDGLAKNPDTNYQAMFRSGSHQSATR 1r27.4    ----------------GALTARAVVSQRVPAGMTMMYHAQE---------RIVNLPGS-----E--ITQQRGGIHNSVTR  target    GWLKPTWMTDSLVRKGLFGQGIGKGFLPDVHCPTGAPREAFVKISRAEPGGIGGQGLWRPAALGIRPRHESPAMKRYLAG 1r27.4    ITPKPTHMI------GGYA---HLAYGFNYYGTVGSNRDEFVVVRKMKNIDW----------------------------  target    GFFSGPKE 1r27.4    -------- ``` | | | | | | | | | | | | | | | | | | | | | | | | | | | | | | | | | | | | | | | | | | | | | | | | | |
|  | 3egw.1.A | Respiratory nitrate reductase 1 alpha chain  *The crystal structure of the NarGHI mutant NarH - C16A* | 0.30 | 0.06 | 20.65 | 0.65 | 59-531 | X-ray | 1.90 | homo-dimer | 2 x MD1, 2 x MGD, 2 x 6MO, 6 x SF4, 4 x F3S, 2 x 3PH, 4 x HEM, 2 x AGA | HHblits | 0.31 |
| ``` target    FNGAPQYINENPFDLELDASRPARPRQYWRAESAHFYNHEDHPLRVGTRLLTGSTHMPTPTKVMWFANANSILGNVKWHY 3egw.1    ----------------------------------------------------------NHPRNLFIWRSNLLGS--SGKG  target    NT------------------------------V--VNALPRMEMIAVHEWWWTGSCEWADVVFGVDSWGELKHPDMTASV 3egw.1    HEFMLKYLLGTEHGIQGKDLGQQGGVKPEEVDWQDNGLEGKLDLVVTLDFRLSSTCLYSDIILPTATWYEK--DDMNTSD  target    TNPFLIVFPKTPIKRIFNTVGDIDVLALVSSKLAELTG----DT----RFN---------------DMWK---------- 3egw.1    MHPFIHPLSA-AVDPAWEAKSDWEIYKAIAKKFSEVCVGHLGKETDIVTLPIQHDSAAELAQPLDVKDWKKGECDLIPGK  target    -----------------FVRE-------------G-----R-TDVYLQRIL-------------------------DAST 3egw.1    TAPHIMVVERDYPATYERFTSIGPLMEKIGNGGKGIAWNTQSEMDLLRKLNYTKAEGPAKGQPMLNTAIDAAEMILTLAP  target    NTKG----YRFTELEAKAR-----------EGIPALMNS------------------RTSPKVVGYDQLADSTPWYTKSG 3egw.1    ETNGQVAVKAWAALSEFTGRDHTHLALNKEDEKIRFRDIQAQPRKIISSPTWSGLEDEHVSYNAGYTNVHELIPWRTLSG  target    RLEFYREEDEFIEAGENLPVHREPVDSTFYEPNVIVSPKHEAVRPSGPEDYGVARTDLSCEVRCGRNVVLTWAETRQTQH 3egw.1    RQQLYQDHQWMRDFGESLLVYRPPIDTRSV-----------------KEVIGQ-------------------------KS  target    PLVKQGHKFIFHTPKYRHGSHTTPIDTDMNAVLFGPFGDIYRRDKRSPFVTEGYVDINPTDGLELGLQDGDYVWIDPDPE 3egw.1    N-GNQEKALNFLTPHQKWGIHSTYSDNLLMLT----------LG-----RGGPVVWLSEADAKDLGIADNDWIEVFNSN-  target    DRPFRGWQKNAKDMEFARLLCRARFYPGTPRGVTRMWFNMYGATPGSVRGAKARRDGLAKNPDTNYQAMFRSGSHQSATR 3egw.1    ----------------GALTARAVVSQRVPAGMTMMYHAQ---------ERIVNLPGS-----EITQ--QRGGIHNSVTR  target    GWLKPTWMTDSLVRKGLFGQGIGKGFLPDVHCPTGAPREAFVKISRAEPGGIGGQGLWRPAALGIRPRHESPAMKRYLAG 3egw.1    ITPKPTHMI------GGYA---HLAYGFNYYGTVGSNRDEFVVVRKMKNI------------------------------  target    GFFSGPKE 3egw.1    -------- ``` | | | | | | | | | | | | | | | | | | | | | | | | | | | | | | | | | | | | | | | | | | | | | | | | | |
|  | 3ir6.1.A | Respiratory nitrate reductase 1 alpha chain  *Crystal structure of NarGHI mutant NarG-H49S* | 0.30 | 0.00 | 20.44 | 0.64 | 59-530 | X-ray | 2.80 | monomer | 2 x GDP, 1 x AGA, 3 x SF4, 1 x F3S, 2 x HEM | HHblits | 0.31 |
| ``` target    FNGAPQYINENPFDLELDASRPARPRQYWRAESAHFYNHEDHPLRVGTRLLTGSTHMPTPTKVMWFANANSILGNVKWHY 3ir6.1    ----------------------------------------------------------NHPRNLFIWRSNLLGS--SGKG  target    NT------------------------------V--VNALPRMEMIAVHEWWWTGSCEWADVVFGVDSWGELKHPDMTASV 3ir6.1    HEFMLKYLLGTEHGIQGKDLGQQGGVKPEEVDWQDNGLEGKLDLVVTLDFRLSSTCLYSDIILPTATWYEK--DDMNTSD  target    TNPFLIVFPKTPIKRIFNTVGDIDVLALVSSKLAELTG----DT----RF---------------NDMWK---------- 3ir6.1    MHPFIHPLSA-AVDPAWEAKSDWEIYKAIAKKFSEVCVGHLGKETDIVTLPIQHDSAAELAQPLDVKDWKKGECDLIPGK  target    -----------------FVRE-G-----------------RT-DVYLQRI-------------------------LDAST 3ir6.1    TAPHIMVVERDYPATYERFTSIGPLMEKIGNGGKGIAWNTQSEMDLLRKLNYTKAEGPAKGQPMLNTAIDAAEMILTLAP  target    NTKG----YRFTELEAKARE-----------GIPALMN------------------SRTSPKVVGYDQLADSTPWYTKSG 3ir6.1    ETNGQVAVKAWAALSEFTGRDHTHLALNKEDEKIRFRDIQAQPRKIISSPTWSGLEDEHVSYNAGYTNVHELIPWRTLSG  target    RLEFYREEDEFIEAGENLPVHREPVDSTFYEPNVIVSPKHEAVRPSGPEDYGVARTDLSCEVRCGRNVVLTWAETRQTQH 3ir6.1    RQQLYQDHQWMRDFGESLLVYRPPIDTRSV-----------------KEVIGQ-------------------------KS  target    PLVKQGHKFIFHTPKYRHGSHTTPIDTDMNAVLFGPFGDIYRRDKRSPFVTEGYVDINPTDGLELGLQDGDYVWIDPDPE 3ir6.1    NG-NQEKALNFLTPHQKWGIHSTYSDNLLMLT----------LG-----RGGPVVWLSEADAKDLGIADNDWIEVFNSN-  target    DRPFRGWQKNAKDMEFARLLCRARFYPGTPRGVTRMWFNMYGATPGSVRGAKARRDGLAKNPDTNYQAMFRSGSHQSATR 3ir6.1    ----------------GALTARAVVSQRVPAGMTMMYHAQ---------ERIVNL---PGS--EI--TQQRGGIHNSVTR  target    GWLKPTWMTDSLVRKGLFGQGIGKGFLPDVHCPTGAPREAFVKISRAEPGGIGGQGLWRPAALGIRPRHESPAMKRYLAG 3ir6.1    ITPKPTHMI------GGYA---HLAYGFNYYGTVGSNRDEFVVVRKMKN-------------------------------  target    GFFSGPKE 3ir6.1    -------- ``` | | | | | | | | | | | | | | | | | | | | | | | | | | | | | | | | | | | | | | | | | | | | | | | | | |
|  | 5e7o.1.A | DMSO reductase family type II enzyme, molybdopterin subunit  *Crystal structure of the perchlorate reductase PcrAB mutant W461E of PcrA from Azospira suillum PS* | 0.34 | 0.00 | 23.28 | 0.61 | 58-532 | X-ray | 2.40 | monomer | 4 x SF4, 1 x MO, 1 x MGD, 1 x MD1, 1 x F3S | HHblits | 0.33 |
| ``` target    FNGAPQYINENPFDLELDASRPARPRQYWRAESAHFYNHEDHPLRVGTRLLTGSTHMPTPTKVMWFANANSILGNVKWHY 5e7o.1    ---------------------------------------------------------GRDPKVFFVYRGNWLNQ--AKGQ  target    NTVV-NALPRMEMIAVHEWWWTGSCEWADVVFGVDSWGELKHPDMTASVTNPFLIVFPKTPIKRIFNTVGDIDVLALVSS 5e7o.1    KYVLENLWPKLELIVDINIRMDSTALYSDVVLPSAHWYEKL--DLNVTSEHSYINMTEP-AIKPMWESKTDWQIFLALAK  target    KLAELTGDTRFND----M--W-K--------FVREGR---TDVYLQRILDASTNTKGYRFTELEAKAREG--IPALMNSR 5e7o.1    RVEMAAKRKKYEKFNDEKFKWVRDLSNLWNQMTMDGKLAEDEAAAQYILDNAPQSKGITIQMLREKPQRFKSNWTSPLKE  target    TSPKVVGYDQLADSTPWYTKSGRLEFYREEDEFIEAGENLPVHREPVDSTFYEPNVIVSPKHEAVRPSGPEDYGVARTDL 5e7o.1    GVPYTPFQYFVVDKKPWPTLTGRQQFYLDHDTFFDMGVELPTYKAPID--------------------------------  target    SCEVRCGRNVVLTWAETRQTQHPLVKQGHKFIFHTPKYRHGSHTTPIDTDMNAVLFGPFGDIYRRDKRSPFVTEGYVDIN 5e7o.1    -------------------------ADKYPFRFNSPHSRHSVHSTFKDNVLML----------RL--Q---RGGPSIEMS  target    PTDGLELGLQDGDYVWIDPDPEDRPFRGWQKNAKDMEFARLLCRARFYPGTPRGVTRMWFNMYGATPGSVRGAKARRDGL 5e7o.1    PLDAKPLGIKDNDWVEAWNNH-----------------GKVICRVKIRNGEQRGRVSMWHCP------------------  target    AKNPDTNYQAMFRSGSHQSATRGWLKPTWMTDSLVRKGLFGQGIGKGFLPDVHCPTGAPREAFVKISRAEPGGIGGQGLW 5e7o.1    -----ELYMDLL-TGGSQSVCPVRINPTNLV------GNYG---HLFFRPNYYGPAGSQRDVRVNVKRYIGAT-------  target    RPAALGIRPRHESPAMKRYLAGGFFSGPKE 5e7o.1    ------------------------------ ``` | | | | | | | | | | | | | | | | | | | | | | | | | | | | | | | | | | | | | | | | | | | | | | | | | |
|  | 4ydd.1.A | DMSO reductase family type II enzyme, molybdopterin subunit  *Crystal structure of the perchlorate reductase PcrAB from Azospira suillum PS* | 0.36 | 0.00 | 23.05 | 0.61 | 58-531 | X-ray | 1.86 | monomer | 4 x SF4, 1 x MO, 1 x MGD, 1 x MD1, 1 x F3S | HHblits | 0.33 |
| ``` target    FNGAPQYINENPFDLELDASRPARPRQYWRAESAHFYNHEDHPLRVGTRLLTGSTHMPTPTKVMWFANANSILGNVKWHY 4ydd.1    ---------------------------------------------------------GRDPKVFFVYRGNWLNQ--AKGQ  target    NTVV-NALPRMEMIAVHEWWWTGSCEWADVVFGVDSWGELKHPDMTASVTNPFLIVFPKTPIKRIFNTVGDIDVLALVSS 4ydd.1    KYVLENLWPKLELIVDINIRMDSTALYSDVVLPSAHWYEKLDLNV--TSEHSYINMTEP-AIKPMWESKTDWQIFLALAK  target    KLAELTGDTRFND--------------MWK-FVREGR---TDVYLQRILDASTNTKGYRFTELEAKAREGIPALM--NSR 4ydd.1    RVEMAAKRKKYEKFNDEKFKWVRDLSNLWNQMTMDGKLAEDEAAAQYILDNAPQSKGITIQMLREKPQRFKSNWTSPLKE  target    TSPKVVGYDQLADSTPWYTKSGRLEFYREEDEFIEAGENLPVHREPVDSTFYEPNVIVSPKHEAVRPSGPEDYGVARTDL 4ydd.1    GVPYTPFQYFVVDKKPWPTLTGRQQFYLDHDTFFDMGVELPTYKAPID--------------------------------  target    SCEVRCGRNVVLTWAETRQTQHPLVKQGHKFIFHTPKYRHGSHTTPIDTDMNAVLFGPFGDIYRRDKRSPFVTEGYVDIN 4ydd.1    -------------------------ADKYPFRFNSPHSRHSVHSTFKDNVLML----------RL--Q---RGGPSIEMS  target    PTDGLELGLQDGDYVWIDPDPEDRPFRGWQKNAKDMEFARLLCRARFYPGTPRGVTRMWFNMYGATPGSVRGAKARRDGL 4ydd.1    PLDAKPLGIKDNDWVEAWNNH-----------------GKVICRVKIRNGEQRGRVSMWHCP------------------  target    AKNPDTNYQAMFRSGSHQSATRGWLKPTWMTDSLVRKGLFGQGIGKGFLPDVHCPTGAPREAFVKISRAEPGGIGGQGLW 4ydd.1    -----ELYMDLLT-GGSQSVCPVRINPTNLV------GNYG---HLFFRPNYYGPAGSQRDVRVNVKRYIGA--------  target    RPAALGIRPRHESPAMKRYLAGGFFSGPKE 4ydd.1    ------------------------------ ``` | | | | | | | | | | | | | | | | | | | | | | | | | | | | | | | | | | | | | | | | | | | | | | | | | |
|  | 2ivf.1.A | ETHYLBENZENE DEHYDROGENASE ALPHA-SUBUNIT  *ETHYLBENZENE DEHYDROGENASE FROM AROMATOLEUM AROMATICUM* | 0.31 | 0.00 | 22.94 | 0.60 | 58-528 | X-ray | 1.88 | monomer | 1 x MES, 4 x SF4, 1 x MO, 1 x MGD, 1 x MD1, 1 x F3S, 1 x HEM | HHblits | 0.31 |
| ``` target    FNGAPQYINENPFDLELDASRPARPRQYWRAESAHFYNHEDHPLRVGTRLLTGSTHMPTPTKVMWFANANSILGNVKWHY 2ivf.1    ---------------------------------------------------------DKTPQVYMLLSQNPMRR--KRSG  target    NTV--VNALPRMEMIAVHEWWWTGSCEWADVVFGVDSWGELKHPDMTAS-VTNPFLIVFPKTPIKRIFNTVGDIDVLALV 2ivf.1    AKMFPDVLFPKLKMIFALETRMSSSAMYADIVLPCAWYYEKH--EMTTPCSGNPFFTFVDR-SVAPPGECREEWDAIALI  target    SSKLAELTG---DTRF----------NDMWK-FVREG---RTDVYLQRILDASTN----TKGYRFTELEAKAREGIPALM 2ivf.1    LKKVGERAAARGLTEFNDHNGRKRRYDELYKKFTMDGHLLTNEDCLKEMVDINRAVGVFAKDYTYEKFKKE---GQTRFL  target    NSR--------------TSPKVVGYDQLADSTPWYTKSGRLEFYREEDEFIEAGENLPVHREPVDSTFYEPNVIVSPKHE 2ivf.1    SMGTGVSRYAHANEVDVTKPIYPMRWHFDDKKVFPTHTRRAQFYLDHDWYLEAGESLPTHKDTPM---------------  target    AVRPSGPEDYGVARTDLSCEVRCGRNVVLTWAETRQTQHPLVKQGHKFIFHTPKYRHGSHTTPIDTDMNAVLFGPFGDIY 2ivf.1    -----------------------------------------VGGDHPFKITGGHPRVSIHSTHLTNSHLSRL--------  target    RRDKRSPFVTEGYVDINPTDGLELGLQDGDYVWIDPDPEDRPFRGWQKNAKDMEFARLLCRARFYPGTPRGVTRMWFNMY 2ivf.1    --H-----RGQPVVHMNSKDAAELGIKDGDMAKLFNDF-----------------ADCEIMVRTAPNVQPKQCIVYFWD-  target    GATPGSVRGAKARRDGLAKNPDTNYQAMFRS-GSHQSATRGWLKPTWMTDSLVRKGLFGQGIGKGFLPDVHCPTG-APRE 2ivf.1    -----------------------AHQ--YKGWKPYDILLIGMPKPLHLA------GGYE---QFRYYFMNGSPAPVTDRG  target    AFVKISRAEPGGIGGQGLWRPAALGIRPRHESPAMKRYLAGGFFSGPKE 2ivf.1    VRVSIKKA----------------------------------------- ``` | | | | | | | | | | | | | | | | | | | | | | | | | | | | | | | | | | | | | | | | | | | | | | | | | |
|  | 6cz7.1.A | ArrA  *The arsenate respiratory reductase (Arr) complex from Shewanella sp. ANA-3* | 0.26 |  | 19.55 | 0.55 | 47-441 | X-ray | 1.62 | hetero-1-1-mer | 5 x SF4, 2 x MGD, 1 x MO, 1 x PG5 | HHblits | 0.29 |
| ``` target    FNGAPQYINENPFDLELDASRPARPRQYWRAESAHFYNHEDHPLRVGTRLLTGSTHMPTPTKVMWFANANSILGNVKWHY 6cz7.1    ----------------------------------------------ANGIRNQ---DPYEIKVMLAYFNNFNFS--NPEG  target    NTVVNALPRMEMIAVHEWWWTGSCEWADVVFGVDS-WGELKHPDMTASVTN--PFLIVFPKTPIKRIFNTVGD-IDVLAL 6cz7.1    QRWDEALSKVDFMAHITTNVSEFSWFADVLLPSSHHMFEKW--GVLDSIGNGVAQISIQQP-SIKRLWDTRIDESEIPYM  target    VSSKLAELTGDTRFNDMWKFVRE-----------GRTDVYLQRILDASTN-----------TKGYRFTELEAKAREGIPA 6cz7.1    LAKKLADK-GFD---APWRYINEQIVDPETGKPAADEAEFAKLMVRYLTAPLWKEDASKYGDKLSSWDEFVQK---GVWN  target    LMNSRTSPKVVGYDQLADSTPWYTKSGRLEFYREEDEFIEAGENLPVHRE----PVDSTFYEPNVIVSPKHEAVRPSGPE 6cz7.1    SSP------YKLE--A-RWGKFKTETTKFEFYSKTLEKA-----LQSHADKHKVSIDEVMKACDY------QA-------  target    DYGVARTDLSCEVRCGRNVVLTWAETRQTQHPLVKQGHKFIFHTPKYRHGSHTTPIDTDMNAVLFGPFGDIYRRDKRSPF 6cz7.1    -RG-----------HLA-FIPHYEEP--YRFGD-ESEFPLLLVDQKSRLNKEGRTANSPWYYEF----KDVD-PGDV---  target    VTEGYVDINPTDGLELGLQDGDYVWIDPDPEDRPFRGWQKNAKDMEFARLLCRARFYPGTPRGVTRMWFNMYGATPGSVR 6cz7.1    ANEDVAKFNPIDGKKFGLKDGDEIRITSPV-----------------GMLTCKAKLWEGVRPGTVAKCFGQ---------  target    GAKARRDGLAKNPDTNYQAMFRSGSHQSATRGWLKPTWMTDSLVRKGLFGQGIGKGFLPDVHCPTGAPREAFVKISRAEP 6cz7.1    --------------------------------------------------------------------------------  target    GGIGGQGLWRPAALGIRPRHESPAMKRYLAGGFFSGPKE 6cz7.1    --------------------------------------- ``` | | | | | | | | | | | | | | | | | | | | | | | | | | | | | | | | | | | | | | | | | | | | | | | | | |
|  | 1kqf.1.A | FORMATE DEHYDROGENASE, NITRATE-INDUCIBLE, MAJOR SUBUNIT  *FORMATE DEHYDROGENASE N FROM E. COLI* | 0.23 |  | 16.35 | 0.56 | 58-441 | X-ray | 1.60 | hetero-oligomer | 3 x 6MO, 15 x SF4, 6 x MGD, 6 x HEM, 3 x CDL | HHblits | 0.27 |
| ``` target    FNGAPQYINENPFDLELDASRPARPRQYWRAESAHFYNHEDHPLRVGTRLLTGSTHMPTPTKVMWFANANSILGNVKWHY 1kqf.1    ---------------------------------------------------------EGKVTGYFCQGFNPVAS--FPDK  target    NTVVNALPRMEMIAVHEWWWTGSCEWAD-----------------VVFGVDSWGELKHPDMTASVTNPFLIVFPKTPIKR 1kqf.1    NKVVSCLSKLKYMVVIDPLVTETSTFWQNHGESNDVDPASIQTEVFRLPSTCFAEE--DGSIA-NSGRWLQWHWK-GQDA  target    IFNTVGDIDVLALVSSKLAELT---GDTRFNDM----WKFVREG--------R-T-DVYLQRILDASTN------TKGYR 1kqf.1    PGEARNDGEILAGIYHHLRELYQSEGGKGVEPLMKMSWNYKQPHEPQSDEVAKENNGYALEDLYDANGVLIAKKGQLLSS  target    FTELEAKAREGIPA----------------------------------LMNSRTS----------------PKVV----- 1kqf.1    FAHLRDDGTTASSCWIYTGSWTEQGNQMANRDNSDPSGLGNTLGWAWAWPLNRRVLYNRASADINGKPWDPKRMLIQWNG  target    -----GYDQLADSTPWYTKSGRLEFYREEDEFIE----AG-ENLPVHREPVDSTFYEPNVIVSPKHEAVRPSGPEDYGVA 1kqf.1    SKWTGNDIPDFGNAAPGTPTGPFIMQPEGMGRLFAINKMAEGPFPEHYEPIETPLGT-----NPLHPN-------VVSNP  target    RTDLSCEVRCGRNVVLTWAETRQTQHPLVKQGHKFIFHTPKYRHGSHTTPIDTDMNAVLFGPFGDIYRRDKRSPFVTEGY 1kqf.1    V------VR-------LYEQDA-LRMG-KKEQFPYVGTTYRLTEHFHTWTKHALLNA-------------IAQP---EQF  target    VDINPTDGLELGLQDGDYVWIDPDPEDRPFRGWQKNAKDMEFARLLCRARFYPGTPR--------GVTRMWFNMYGATPG 1kqf.1    VEISETLAAAKGINNGDRVTVSSKR-----------------GFIRAVAVVTRRLKPLNVNGQQVETVGIPIHW------  target    SVRGAKARRDGLAKNPDTNYQAMFRSGSHQSATRGWLKPTWMTDSLVRKGLFGQGIGKGFLPDVHCPTGAPREAFVKISR 1kqf.1    --------------------------------------------------------------------------------  target    AEPGGIGGQGLWRPAALGIRPRHESPAMKRYLAGGFFSGPKE 1kqf.1    ------------------------------------------ ``` | | | | | | | | | | | | | | | | | | | | | | | | | | | | | | | | | | | | | | | | | | | | | | | | | |
|  | 7qv7.1.L | Hydrogen dependent carbon dioxide reductase subunit FdhF  *Cryo-EM structure of Hydrogen-dependent CO2 reductase.* | 0.19 |  | 18.43 | 0.51 | 42-441 | EM | 0.00 | hetero-2-6-6-2-mer | 52 x SF4, 6 x 402 | HHblits | 0.30 |
| ``` target    FNGAPQYINENPFDLELDASRPARPRQYWRAESAHFYNHEDHPLRVGTRLLTGSTHMPTPTKVMWFANANSILGNVKWHY 7qv7.1    -----------------------------------------RVTEVPDAILNK------RVRALYIFGENPIMS--DPDS  target    NTVVNALPRMEMIAVHEWWWTGSCEWADVVFGVDSWGELKHPDMTASVTNPFLIVFPKTPIKRIFNTVGDIDVLALVSSK 7qv7.1    DHLRHALEHLDLLIVQDIFLTETARLAHVVLPAACWAEKD--GTF-TNTERRVQRVRK-AVEAPGEAKPDWWIFSQIAER  target    LAELTGDTRFNDMWKFVREGRTDVYLQRILDASTN-TKGYRFTELEAKAREGIPALMNSRTSPKVVGYDQLADSTPWYTK 7qv7.1    MGY----TGM--QYNNV-----QEIWDEVRKIVPEKFGGISYARLEKE--KGLAWPCPT---EDHTGTPILYLGGKFATP  target    SGRLEFYREEDEFIEAGENLPVHREPVDSTFYEPNVIVSPKHEAVRPSGPEDYGVARTDLSCEVRCGRNVVLTWAETRQT 7qv7.1    SGKAQMYPVIFYP-----NTCICDEGAEKQDFNH-----------------------------------VIVGS------  target    QHPLVKQGHKFIFHTPKYRHGSHTTPI--DTDMNAVLFGPFGDIYRRDKRSPFVTEGYVDINPTDGLELGLQDGDYVWID 7qv7.1    IAELPDEEYPFTLTTGRRVYHYHTATMTRKSPVI-------------DQIAP---QELVEINPQDATRLGINDGDFLRVS  target    PDPEDRPFRGWQKNAKDMEFARLLCRARFYPGTPRGVTRMWFNMYGATPGSVRGAKARRDGLAKNPDTNYQAMFRSGSHQ 7qv7.1    TRR-----------------GYVATRAWVTERVPKGTIFMTFHY------------------------------------  target    SATRGWLKPTWMTDSLVRKGLFGQGIGKGFLPDVHCPTGAPREAFVKISRAEPGGIGGQGLWRPAALGIRPRHESPAMKR 7qv7.1    --------------------------------------------------------------------------------  target    YLAGGFFSGPKE 7qv7.1    ------------ ``` | | | | | | | | | | | | | | | | | | | | | | | | | | | | | | | | | | | | | | | | | | | | | | | | | |
|  | 7qv7.1.O | Hydrogen dependent carbon dioxide reductase subunit FdhF  *Cryo-EM structure of Hydrogen-dependent CO2 reductase.* | 0.19 |  | 18.43 | 0.51 | 42-441 | EM | 0.00 | hetero-2-6-6-2-mer | 52 x SF4, 6 x 402 | HHblits | 0.30 |
| ``` target    FNGAPQYINENPFDLELDASRPARPRQYWRAESAHFYNHEDHPLRVGTRLLTGSTHMPTPTKVMWFANANSILGNVKWHY 7qv7.1    -----------------------------------------RVTEVPDAILNK------RVRALYIFGENPIMS--DPDS  target    NTVVNALPRMEMIAVHEWWWTGSCEWADVVFGVDSWGELKHPDMTASVTNPFLIVFPKTPIKRIFNTVGDIDVLALVSSK 7qv7.1    DHLRHALEHLDLLIVQDIFLTETARLAHVVLPAACWAEKD--GTF-TNTERRVQRVRK-AVEAPGEAKPDWWIFSQIAER  target    LAELTGDTRFNDMWKFVREGRTDVYLQRILDASTN-TKGYRFTELEAKAREGIPALMNSRTSPKVVGYDQLADSTPWYTK 7qv7.1    MGY----TGM--QYNNV-----QEIWDEVRKIVPEKFGGISYARLEKE--KGLAWPCPT---EDHTGTPILYLGGKFATP  target    SGRLEFYREEDEFIEAGENLPVHREPVDSTFYEPNVIVSPKHEAVRPSGPEDYGVARTDLSCEVRCGRNVVLTWAETRQT 7qv7.1    SGKAQMYPVIFYP-----NTCICDEGAEKQDFNH-----------------------------------VIVGS------  target    QHPLVKQGHKFIFHTPKYRHGSHTTPI--DTDMNAVLFGPFGDIYRRDKRSPFVTEGYVDINPTDGLELGLQDGDYVWID 7qv7.1    IAELPDEEYPFTLTTGRRVYHYHTATMTRKSPVI-------------DQIAP---QELVEINPQDATRLGINDGDFLRVS  target    PDPEDRPFRGWQKNAKDMEFARLLCRARFYPGTPRGVTRMWFNMYGATPGSVRGAKARRDGLAKNPDTNYQAMFRSGSHQ 7qv7.1    TRR-----------------GYVATRAWVTERVPKGTIFMTFHY------------------------------------  target    SATRGWLKPTWMTDSLVRKGLFGQGIGKGFLPDVHCPTGAPREAFVKISRAEPGGIGGQGLWRPAALGIRPRHESPAMKR 7qv7.1    --------------------------------------------------------------------------------  target    YLAGGFFSGPKE 7qv7.1    ------------ ``` | | | | | | | | | | | | | | | | | | | | | | | | | | | | | | | | | | | | | | | | | | | | | | | | | |
|  | 2nya.1.A | Periplasmic nitrate reductase  *Crystal structure of the periplasmic nitrate reductase (NAP) from Escherichia coli* | 0.22 |  | 15.00 | 0.53 | 58-441 | X-ray | 2.50 | monomer | 1 x SF4, 1 x 6MO, 2 x MGD | HHblits | 0.27 |
| ``` target    FNGAPQYINENPFDLELDASRPARPRQYWRAESAHFYNHEDHPLRVGTRLLTGSTHMPTPTKVMWFANANSILGNVKWHY 2nya.1    ---------------------------------------------------------DGKLNVYWTMCTNNMQA--GPNI  target    N--TVVNALPRMEMIAVHEWWWTGSCEWADVVFGVDSWGELKHPDMTASVTNPFLIVFPKTPIKRIFNTVGDIDVLALVS 2nya.1    NEERMPGWRDPRNFIIVSDPYPTVSALAADLILPTAMWVEKE--GAYG-NAERRTQFWRQ-QVQAPGEAKSDLWQLVQFS  target    SKLAELTGDTRFNDMWKFVREGRTDVYLQRILDASTNTKGYRFTELEAKA------REGIPAL-----------M-NSRT 2nya.1    RRFKTEEVWPED--LLAKKPELRGKTLYEVLYATPE-VSKFPVSELAEDQLNDESRELGFYLQKGLFEEYAWFGRGHGHD  target    SPKVVGYDQLADSTPWYTKSGRLEFYR--EEDE-FIEAGENLPVHREPVDSTFYEPNVIVSPKHEAVRPSGPEDYGVART 2nya.1    LAPFDDYH-KARGLRWPVVNGKETQWRYSEGNDPYVKAGEGYKFYGKPDGKAV---------------------------  target    DLSCEVRCGRNVVLTWAETRQTQHPLVKQGHKFIFHTPKYR--HGSHTTPIDTDMNAVLFGPFGDIYRRDKRSPFVTEGY 2nya.1    ----------IFALPFEP---AAEAP-DEEYDLWLSTGRVLEHWHTGSMTRRVPELH-------------RAFP---EAV  target    VDINPTDGLELGLQDGDYVWIDPDPEDRPFRGWQKNAKDMEFARLLCRARFYPGT--PRGVTRMWFNMYGATPGSVRGAK 2nya.1    LFIHPLDAKARDLRRGDKVKVVSRR-----------------GEVISIVETRGRNRPPQGLVYMPFFD------------  target    ARRDGLAKNPDTNYQAMFRSGSHQSATRGWLKPTWMTDSLVRKGLFGQGIGKGFLPDVHCPTGAPREAFVKISRAEPGGI 2nya.1    --------------------------------------------------------------------------------  target    GGQGLWRPAALGIRPRHESPAMKRYLAGGFFSGPKE 2nya.1    ------------------------------------ ``` | | | | | | | | | | | | | | | | | | | | | | | | | | | | | | | | | | | | | | | | | | | | | | | | | |
|  | 4v4c.1.A | Pyrogallol hydroxytransferase large subunit  *Crystal Structure of Pyrogallol-Phloroglucinol Transhydroxylase from Pelobacter acidigallici* | 0.25 |  | 15.93 | 0.52 | 60-441 | X-ray | 2.35 | hetero-oligomer | 2 x CA, 2 x MGD, 1 x 4MO, 3 x SF4 | HHblits | 0.28 |
| ``` target    FNGAPQYINENPFDLELDASRPARPRQYWRAESAHFYNHEDHPLRVGTRLLTGSTHMPTPTKVMWFANANSILGNVKWHY 4v4c.1    -----------------------------------------------------------KIKMFWKYGGPHLGT--MTAT  target    NTVVNAL--PRMEMIAVHEWWWTGSCEWADVVFGVDSWGELKHPDMTASV-----------TNPFLIVFPKTPIKRIFNT 4v4c.1    NRYAKMYTHDSLEFVVSQSIWFEGEVPFADIILPACTNFERWDISEFANCSGYIPDNYQLCNHRVISLQAK-CIEPVGES  target    VGDIDVLALVSSKLAELTGDTRFNDMWKFVREGRT-DVYLQRILDASTNTKGYRFTELEAKAREGIPALMNSRTSPKVVG 4v4c.1    MSDYEIYRLFAKKLNIEE-------MF---SEGKDELAWCEQYFNATDMPKYMTWDEFFKKGYFVVPDNPNRKKTVALRW  target    YD-----------Q----LADSTPWYTKSGRLEFYREEDEFIE-AG------ENLPVHREPVDSTFYEPNVIVSPKHEAV 4v4c.1    FAEGREKDTPDWGPRLNNQVCRKGLQTTTGKVEFIATSLKNFEEQGYIDEHRPSMHTYVPAWESQ---------------  target    RPSGPEDYGVARTDLSCEVRCGRNVVLTWAETRQTQHPLVKQGHKFIFHTPKYRHGSHTTPID-TDMNAVLFGPFGDIYR 4v4c.1    ----------------------------------KHSPL-AVKYPLGMLSPHPRFSMHTMGDGKNSYMNYI----KDHRV  target    RDKRSPFVTEGYVDINPTDGLELGLQDGDYVWIDPDPEDRPFRGWQKNAKDMEFARLLCRARFYPGTPRGVTRMWFNMYG 4v4c.1    EVDG---YKYWIMRVNSIDAEARGIKNGDLIRAYNDR-----------------GSVILAAQVTECLQPGTVHSYESC--  target    ATPGSVRGAKARRDGLAKNPDTNYQAMFRSGSHQSATRGWLKPTWMTDSLVRKGLFGQGIGKGFLPDVHCPTGAPREAFV 4v4c.1    --------------------------------------------------------------------------------  target    KISRAEPGGIGGQGLWRPAALGIRPRHESPAMKRYLAGGFFSGPKE 4v4c.1    ---------------------------------------------- ``` | | | | | | | | | | | | | | | | | | | | | | | | | | | | | | | | | | | | | | | | | | | | | | | | | |
|  | 1e18.1.A | DMSO REDUCTASE.  *TUNGSTEN-SUSBSTITUTED DMSO REDUCTASE FROM RHODOBACTER CAPSULATUS* | 0.25 | 0.00 | 17.77 | 0.50 | 58-441 | X-ray | 2.00 | monomer | 2 x PGD, 1 x 6WO | HHblits | 0.30 |
| ``` target    FNGAPQYINENPFDLELDASRPARPRQYWRAESAHFYNHEDHPLRVGTRLLTGSTHMPTPTKVMWFANANSILGNVKWHY 1e18.1    ---------------------------------------------------------FPDVKMAYWVGGNPFVH--HQDR  target    NTVVNALPRMEMIAVHEWWWTGSCEWADVVFGVDSWGELKHPDMTASVTNPFLIVFPKTPIKRIFNTVGDIDVLALVSSK 1e18.1    NRMVKAWEKLETFVVHDFQWTPTARHADIVLPATTSYERNDIETIGDYSNTGILAMKK-IVEPLYEARSDYDIFAAVAER  target    LAELTGDTRFNDMWKFVREGR-TDVYLQRILDASTN---TKGYRFTELEAKAREGIPALMNSRTSPK--VVGYDQLADST 1e18.1    LGKGK-------EF---TEGKDEMGWIKSFYDDAAKQGKAAGVEMPAFDAFWAEGIVEFPVTDGADFVRYASFREDPLLN  target    PWYTKSGRLEFYREEDEFIEAGENLP---VHREPVDSTFYEPNVIVSPKHEAVRPSGPEDYGVARTDLSCEVRCGRNVVL 1e18.1    PLGTPTGLIEIYSKNIEKMGY-DDCPAHPTWMEPLERL------------------------------------------  target    TWAETRQTQHPLVKQGHKFIFHTPKYRHGSHTTPIDTDMNAVLFGPFGDIYRRDKRSPFVTEGYVDINPTDGLELGLQDG 1e18.1    ----------DGPGAKYPLHIAASHPFNRLHSQL-NGTVLR-------EGYAV------QGHEPCLMHPDDAAARGIADG  target    DYVWIDPDPEDRPFRGWQKNAKDMEFARLLCRARFYPGTPRGVTRMWFNMYGATPGSVRGAKARRDGLAKNPDTNYQAMF 1e18.1    DVVRVHNDR-----------------GQILTGVKVTDAVMKGVIQIYEGG------------------------------  target    RSGSHQSATRGWLKPTWMTDSLVRKGLFGQGIGKGFLPDVHCPTGAPREAFVKISRAEPGGIGGQGLWRPAALGIRPRHE 1e18.1    --------------------------------------------------------------------------------  target    SPAMKRYLAGGFFSGPKE 1e18.1    ------------------ ``` | | | | | | | | | | | | | | | | | | | | | | | | | | | | | | | | | | | | | | | | | | | | | | | | | |
|  | 4dmr.1.A | DMSO REDUCTASE  *REDUCED DMSO REDUCTASE FROM RHODOBACTER CAPSULATUS WITH BOUND DMSO SUBSTRATE* | 0.25 | 0.00 | 17.01 | 0.51 | 58-441 | X-ray | 1.90 | monomer | 2 x PGD, 1 x 4MO, 1 x O | HHblits | 0.30 |
| ``` target    FNGAPQYINENPFDLELDASRPARPRQYWRAESAHFYNHEDHPLRVGTRLLTGSTHMPTPTKVMWFANANSILGNVKWHY 4dmr.1    ---------------------------------------------------------FPDVKMAYWVGGNPFVH--HQDR  target    NTVVNALPRMEMIAVHEWWWTGSCEWADVVFGVDSWGELKHPDMTASVTNPFLIVFPKTPIKRIFNTVGDIDVLALVSSK 4dmr.1    NRMVKAWEKLETFVVHDFQWTPTARHADIVLPATTSYERNDIETIGDYSNTGILAMKK-IVEPLYEARSDYDIFAAVAER  target    LAELTGDTRFNDMWKFVREGRTDVYLQRILDASTN---TKGYRFTELEAKAREGIPALMNSRTSP--KVVGYDQLADSTP 4dmr.1    LGKGAE-------FTEGK--DEMGWIKSFYDDAAKQGKAAGVQMPAFDAFWAEGIVEFPVTDGADFVRYASFREDPLLNP  target    WYTKSGRLEFYREEDEFIEAGEN---LPVHREPVDSTFYEPNVIVSPKHEAVRPSGPEDYGVARTDLSCEVRCGRNVVLT 4dmr.1    LGTPTGLIEIYSKNIEKMGY-DDCPAHPTWMEPLERL-------------------------------------------  target    WAETRQTQHPLVKQGHKFIFHTPKYRHGSHTTPIDTDMNAVLFGPFGDIYRRDKRSPFVTEGYVDINPTDGLELGLQDGD 4dmr.1    ---------DGPGAKYPLHIAASHPFNRLHSQL-NGTVLR-------EGYA---V---QGHEPCLMHPDDAAARGIADGD  target    YVWIDPDPEDRPFRGWQKNAKDMEFARLLCRARFYPGTPRGVTRMWFNMYGATPGSVRGAKARRDGLAKNPDTNYQAMFR 4dmr.1    VVRVHNDR-----------------GQILTGVKVTDAVMKGVIQIYEGG-------------------------------  target    SGSHQSATRGWLKPTWMTDSLVRKGLFGQGIGKGFLPDVHCPTGAPREAFVKISRAEPGGIGGQGLWRPAALGIRPRHES 4dmr.1    --------------------------------------------------------------------------------  target    PAMKRYLAGGFFSGPKE 4dmr.1    ----------------- ``` | | | | | | | | | | | | | | | | | | | | | | | | | | | | | | | | | | | | | | | | | | | | | | | | | |
|  | 1e5v.2.A | Dimethyl sulfoxide/trimethylamine N-oxide reductase  *OXIDIZED DMSO REDUCTASE EXPOSED TO HEPES BUFFER* | 0.25 | 0.00 | 17.01 | 0.51 | 58-441 | X-ray | 2.40 | monomer | 2 x PGD, 1 x 2MO | HHblits | 0.29 |
| ``` target    FNGAPQYINENPFDLELDASRPARPRQYWRAESAHFYNHEDHPLRVGTRLLTGSTHMPTPTKVMWFANANSILGNVKWHY 1e5v.2    ---------------------------------------------------------FPDVKMAYWVGGNPFVH--HQDR  target    NTVVNALPRMEMIAVHEWWWTGSCEWADVVFGVDSWGELKHPDMTASVTNPFLIVFPKTPIKRIFNTVGDIDVLALVSSK 1e5v.2    NRMVKAWEKLETFVVHDFQWTPTARHADIVLPATTSYERNDIETIGDYSNTGILAMKK-IVEPLYEARSDYDIFAAVAER  target    LAELTGDTRFNDMWKFVREGRTDVYLQRILDASTN---TKGYRFTELEAKAREGIPALMNSRTSPK--VVGYDQLADSTP 1e5v.2    LGKGAE-------FTEGK--DEMGWIKSFYDDAAKQGKAAGVQMPAFDAFWAEGIVEFPVTDGADFVRYASFREDPLLNP  target    WYTKSGRLEFYREEDEFIEAGEN---LPVHREPVDSTFYEPNVIVSPKHEAVRPSGPEDYGVARTDLSCEVRCGRNVVLT 1e5v.2    LGTPTGLIEIYSKNIEKMGY-DDCPAHPTWMEPLERL-------------------------------------------  target    WAETRQTQHPLVKQGHKFIFHTPKYRHGSHTTPIDTDMNAVLFGPFGDIYRRDKRSPFVTEGYVDINPTDGLELGLQDGD 1e5v.2    ---------DGPGAKYPLHIAASHPFNRLHSQL-NGTVLR-------EGYAV------QGHEPCLMHPDDAAARGIADGD  target    YVWIDPDPEDRPFRGWQKNAKDMEFARLLCRARFYPGTPRGVTRMWFNMYGATPGSVRGAKARRDGLAKNPDTNYQAMFR 1e5v.2    VVRVHNDR-----------------GQILTGVKVTDAVMKGVIQIYEGG-------------------------------  target    SGSHQSATRGWLKPTWMTDSLVRKGLFGQGIGKGFLPDVHCPTGAPREAFVKISRAEPGGIGGQGLWRPAALGIRPRHES 1e5v.2    --------------------------------------------------------------------------------  target    PAMKRYLAGGFFSGPKE 1e5v.2    ----------------- ``` | | | | | | | | | | | | | | | | | | | | | | | | | | | | | | | | | | | | | | | | | | | | | | | | | |
|  | 1dms.1.A | DMSO REDUCTASE  *STRUCTURE OF DMSO REDUCTASE* | 0.25 | 0.00 | 17.36 | 0.51 | 58-441 | X-ray | 1.88 | monomer | 2 x PGD, 1 x 2MO | HHblits | 0.29 |
| ``` target    FNGAPQYINENPFDLELDASRPARPRQYWRAESAHFYNHEDHPLRVGTRLLTGSTHMPTPTKVMWFANANSILGNVKWHY 1dms.1    ---------------------------------------------------------FPDVKMAYWVGGNPFVH--HQDR  target    NTVVNALPRMEMIAVHEWWWTGSCEWADVVFGVDSWGELKHPDMTASVTNPFLIVFPKTPIKRIFNTVGDIDVLALVSSK 1dms.1    NRMVKAWEKLETFIVHDFQWTPTARHADIVLPATTSYERNDIETIGDYSNTGILAMKK-IVEPLYEARSDYDIFAAVAER  target    LAELTGDTRFNDMWKFVREGRTDVYLQRILDASTN---TKGYRFTELEAKAREGIPALMNSRTSPKV--VGYDQLADSTP 1dms.1    LGKGKE-------FTEGK--DEMGWIKSFYDDAAKQGKAGGVEMPAFDAFWAEGIVEFPVTDGADFVRYASFREDPLLNP  target    WYTKSGRLEFYREEDEFIEAGEN---LPVHREPVDSTFYEPNVIVSPKHEAVRPSGPEDYGVARTDLSCEVRCGRNVVLT 1dms.1    LGTPTGLIEIYSKNIEKMGY-DDCPAHPTWMEPLERL-------------------------------------------  target    WAETRQTQHPLVKQGHKFIFHTPKYRHGSHTTPIDTDMNAVLFGPFGDIYRRDKRSPFVTEGYVDINPTDGLELGLQDGD 1dms.1    ---------DGPGAKYPLHIAASHPFNRLHSQLNG-TVLR-------EGYA---V---QGHEPCLMHPDDAAARGIADGD  target    YVWIDPDPEDRPFRGWQKNAKDMEFARLLCRARFYPGTPRGVTRMWFNMYGATPGSVRGAKARRDGLAKNPDTNYQAMFR 1dms.1    VVRVHNDR-----------------GQILTGVKVTDAVMKGVIQIYEGG-------------------------------  target    SGSHQSATRGWLKPTWMTDSLVRKGLFGQGIGKGFLPDVHCPTGAPREAFVKISRAEPGGIGGQGLWRPAALGIRPRHES 1dms.1    --------------------------------------------------------------------------------  target    PAMKRYLAGGFFSGPKE 1dms.1    ----------------- ``` | | | | | | | | | | | | | | | | | | | | | | | | | | | | | | | | | | | | | | | | | | | | | | | | | |
|  | 1e60.1.A | Dimethyl sulfoxide/trimethylamine N-oxide reductase  *OXIDIZED DMSO REDUCTASE EXPOSED TO HEPES - Structure II BUFFER* | 0.26 | 0.00 | 17.01 | 0.51 | 58-441 | X-ray | 2.00 | monomer | 2 x PGD, 1 x 2MO | HHblits | 0.29 |
| ``` target    FNGAPQYINENPFDLELDASRPARPRQYWRAESAHFYNHEDHPLRVGTRLLTGSTHMPTPTKVMWFANANSILGNVKWHY 1e60.1    ---------------------------------------------------------FPDVKMAYWVGGNPFVH--HQDR  target    NTVVNALPRMEMIAVHEWWWTGSCEWADVVFGVDSWGELKHPDMTASVTNPFLIVFPKTPIKRIFNTVGDIDVLALVSSK 1e60.1    NRMVKAWEKLETFVVHDFQWTPTARHADIVLPATTSYERNDIETIGDYSNTGILAMKK-IVEPLYEARSDYDIFAAVAER  target    LAELTGDTRFNDMWKFVREGRTDVYLQRILDASTN---TKGYRFTELEAKAREGIPALMNSRTSPK--VVGYDQLADSTP 1e60.1    LGKGAE-------FTEGK--DEMGWIKSFYDDAAKQGKAAGVEMPAFDAFWAEGIVEFPVTDGADFVRYASFREDPLLNP  target    WYTKSGRLEFYREEDEFIEAGEN---LPVHREPVDSTFYEPNVIVSPKHEAVRPSGPEDYGVARTDLSCEVRCGRNVVLT 1e60.1    LGTPTGLIEIYSKNIEKMGY-DDCPAHPTWMEPLERL-------------------------------------------  target    WAETRQTQHPLVKQGHKFIFHTPKYRHGSHTTPIDTDMNAVLFGPFGDIYRRDKRSPFVTEGYVDINPTDGLELGLQDGD 1e60.1    --------DG-PGAKYPLHIAASHPFNRLHSQLN-GTVLR-------EGYA---V---QGHEPCLMHPDDAAARGIADGD  target    YVWIDPDPEDRPFRGWQKNAKDMEFARLLCRARFYPGTPRGVTRMWFNMYGATPGSVRGAKARRDGLAKNPDTNYQAMFR 1e60.1    VVRVHNDR-----------------GQILTGVKVTDAVMKGVIQIYEGG-------------------------------  target    SGSHQSATRGWLKPTWMTDSLVRKGLFGQGIGKGFLPDVHCPTGAPREAFVKISRAEPGGIGGQGLWRPAALGIRPRHES 1e60.1    --------------------------------------------------------------------------------  target    PAMKRYLAGGFFSGPKE 1e60.1    ----------------- ``` | | | | | | | | | | | | | | | | | | | | | | | | | | | | | | | | | | | | | | | | | | | | | | | | | |
|  | 1tmo.1.A | TRIMETHYLAMINE N-OXIDE REDUCTASE  *TRIMETHYLAMINE N-OXIDE REDUCTASE FROM SHEWANELLA MASSILIA* | 0.26 |  | 16.61 | 0.51 | 58-441 | X-ray | 2.50 | monomer | 2 x 2MD, 1 x 2MO | HHblits | 0.29 |
| ``` target    FNGAPQYINENPFDLELDASRPARPRQYWRAESAHFYNHEDHPLRVGTRLLTGSTHMPTPTKVMWFANANSILGNVKWHY 1tmo.1    ---------------------------------------------------------YPDIKMMIFSGNNPWNH--HQDR  target    NTVVNALPRMEMIAVHEWWWTGSCEWADVVFGVDSWGELKHPDMTASVTNPFLIVFPKTPIKRIFNTVGDIDVLALVSSK 1tmo.1    NRMKQAFHKLECVVTVDVNWTATCRFSDIVLPACTTYERNDIDVYGAYANRGILAMQK-MVEPLFDSLSDFEIFTRFAAV  target    LAELTGDTRFNDMWKFVREGRTDVYLQRILDASTNT-----KGYRFTELEAKAREGIPALMNSRTSPKVVGYDQLADSTP 1tmo.1    LGKEKE---Y---TRNM---GEMEWLETLYNECKAANAGKFEMPDFATFWK---QGYVHFGDGEVWTRHADFRNDPEINP  target    WYTKSGRLEFYREEDEFIEAG--ENLPVHREPVDSTFYEPNVIVSPKHEAVRPSGPEDYGVARTDLSCEVRCGRNVVLTW 1tmo.1    LGTPSGLIEIFSRKIDQFGYDDCKGHPTWMEKTERSH-------------------------------------------  target    AETRQTQHPLVKQGHKFIFHTPKYRHGSHTTPIDTDMNAVLFGPFGDIYRRDKRSPFVTEGYVDINPTDGLELGLQDGDY 1tmo.1    ------GGPG-SDKHPIWLQSCHPDKRLHSQMCESREYRET----------YAV---NGREPVYISPVDAKARGIKDGDI  target    VWIDPDPEDRPFRGWQKNAKDMEFARLLCRARFYPGTPRGVTRMWFNMYGATPGSVRGAKARRDGLAKNPDTNYQAMFRS 1tmo.1    VRVFNDR-----------------GQLLAGAVVSDNFPKGIVRIHEGA--------------------------------  target    GSHQSATRGWLKPTWMTDSLVRKGLFGQGIGKGFLPDVHCPTGAPREAFVKISRAEPGGIGGQGLWRPAALGIRPRHESP 1tmo.1    --------------------------------------------------------------------------------  target    AMKRYLAGGFFSGPKE 1tmo.1    ---------------- ``` | | | | | | | | | | | | | | | | | | | | | | | | | | | | | | | | | | | | | | | | | | | | | | | | | |
|  | 1eu1.1.A | DIMETHYL SULFOXIDE REDUCTASE  *THE CRYSTAL STRUCTURE OF RHODOBACTER SPHAEROIDES DIMETHYLSULFOXIDE REDUCTASE REVEALS TWO DISTINCT MOLYBDENUM COORDINATION ENVIRONMENTS.* | 0.26 |  | 19.37 | 0.50 | 58-441 | X-ray | 1.30 | monomer | 3 x GLC, 1 x CD, 2 x MGD, 1 x 6MO, 2 x O | HHblits | 0.30 |
| ``` target    FNGAPQYINENPFDLELDASRPARPRQYWRAESAHFYNHEDHPLRVGTRLLTGSTHMPTPTKVMWFANANSILGNVKWHY 1eu1.1    ---------------------------------------------------------YPDVKLAYWAGGNPFAH--HQDR  target    NTVVNALPRMEMIAVHEWWWTGSCEWADVVFGVDSWGELKHPDMTASVTNPFLIVFPKTPIKRIFNTVGDIDVLALVSSK 1eu1.1    NRMLKAWEKLETFIVQDFQWTATARHADIVLPATTSYERNDIESVGDYSNRAILAMKK-VVDPLYEARSDYDIFAALAER  target    LAELTGDTRFNDMWKFVREGR-TDVYLQRILDASTN------TKGYRFTELEAKAREGIPALMNSRTSPKV--VGYDQLA 1eu1.1    LGKGAEFT----------EGRDEMGWISSFYEAAVKQAEFKNVAMPSFEDFWS---EGIVEFPITEGANFVRYADFREDP  target    DSTPWYTKSGRLEFYREEDEFIEAGENL---PVHREPVDSTFYEPNVIVSPKHEAVRPSGPEDYGVARTDLSCEVRCGRN 1eu1.1    LFNPLGTPSGLIEIYSKNIEKMGY-DDCPAHPTWMEPAER-----------------------LG---------------  target    VVLTWAETRQTQHPLVKQGHKFIFHTPKYRHGSHTTPIDTDMNAVLFGPFGDIYRRDKRSPFVTEGYVDINPTDGLELGL 1eu1.1    -------------G-AGAKYPLHVVASHPKSRLHSQLNGTS-LR-------D---LYAV---AGHEPCLINPADAAARGI  target    QDGDYVWIDPDPEDRPFRGWQKNAKDMEFARLLCRARFYPGTPRGVTRMWFNMYGATPGSVRGAKARRDGLAKNPDTNYQ 1eu1.1    ADGDVLRVFNDR-----------------GQILVGAKVSDAVMPGAIQIYEGG---------------------------  target    AMFRSGSHQSATRGWLKPTWMTDSLVRKGLFGQGIGKGFLPDVHCPTGAPREAFVKISRAEPGGIGGQGLWRPAALGIRP 1eu1.1    --------------------------------------------------------------------------------  target    RHESPAMKRYLAGGFFSGPKE 1eu1.1    --------------------- ``` | | | | | | | | | | | | | | | | | | | | | | | | | | | | | | | | | | | | | | | | | | | | | | | | | |
|  | 7l5i.1.A | Trimethylamine-N-oxide reductase  *Crystal Structure of Haemophilus influenzae MtsZ at pH 7.0* | 0.25 |  | 17.42 | 0.50 | 60-441 | X-ray | 1.73 | monomer | 2 x MGD, 1 x MO, 1 x O | HHblits | 0.29 |
| ``` target    FNGAPQYINENPFDLELDASRPARPRQYWRAESAHFYNHEDHPLRVGTRLLTGSTHMPTPTKVMWFANANSILGNVKWHY 7l5i.1    -----------------------------------------------------------DIKAVYWAGGNPFVH--HQDT  target    NTVVNALPRMEMIAVHEWWWTGSCEWADVVFGVDSWGELKHPDMTASVTNPFLIVFPKTPIKRIFNTVGDIDVLALVSSK 7l5i.1    NTLVKAFQKPDVVIVNEVNWTPTARMADIVLPATTSYERNDLTMAGDYSMMSVYPMKQ-VVPPQFEAKNDYDIFVELAKR  target    LAELTGDTRFNDMWKFVREGRTDVYLQRILDASTNT---KGYRFTELEAKAREG--IPA--LMNSRTSPKVVGYDQLADS 7l5i.1    AGVEEQYT----EGKT-----EMEWLEEFYNAAFSAARANRVAMPRFDKFWAENKPLSFEAGEAAKKWVRYGEFREDPLL  target    TPWYTKSGRLEFYREEDEFIEAG--ENLPVHREPVDSTFYEPNVIVSPKHEAVRPSGPEDYGVARTDLSCEVRCGRNVVL 7l5i.1    NPLGTPSGKIEIFSDVVEKMNYNDCKGHPSWMEPEEFA------------------------------------------  target    TWAETRQTQHPLVKQGHKFIFHTPKYRHGSHTTPIDTDMNAVLFGPFGDIYRRDKRSPFVTEGYVDINPTDGLELGLQDG 7l5i.1    ---------GNV-TEEYPLALVTPHPYYRLHSQLAHTSLRQKY-----------AV---NDREPVMIHPEDAAARGIKDG  target    DYVWIDPDPEDRPFRGWQKNAKDMEFARLLCRARFYPGTPRGVTRMWFNMYGATPGSVRGAKARRDGLAKNPDTNYQAMF 7l5i.1    DIVRIHSKR-----------------GQVLAGAAVTENIIKGTVALHEGA------------------------------  target    RSGSHQSATRGWLKPTWMTDSLVRKGLFGQGIGKGFLPDVHCPTGAPREAFVKISRAEPGGIGGQGLWRPAALGIRPRHE 7l5i.1    --------------------------------------------------------------------------------  target    SPAMKRYLAGGFFSGPKE 7l5i.1    ------------------ ``` | | | | | | | | | | | | | | | | | | | | | | | | | | | | | | | | | | | | | | | | | | | | | | | | | |
|  | 7l5s.1.A | Trimethylamine-N-oxide reductase  *Crystal Structure of Haemophilus influenzae MtsZ at pH 5.5* | 0.25 |  | 17.42 | 0.50 | 60-441 | X-ray | 2.09 | monomer | 1 x O, 2 x MGD, 1 x MO | HHblits | 0.29 |
| ``` target    FNGAPQYINENPFDLELDASRPARPRQYWRAESAHFYNHEDHPLRVGTRLLTGSTHMPTPTKVMWFANANSILGNVKWHY 7l5s.1    -----------------------------------------------------------DIKAVYWAGGNPFVH--HQDT  target    NTVVNALPRMEMIAVHEWWWTGSCEWADVVFGVDSWGELKHPDMTASVTNPFLIVFPKTPIKRIFNTVGDIDVLALVSSK 7l5s.1    NTLVKAFQKPDVVIVNEVNWTPTARMADIVLPATTSYERNDLTMAGDYSMMSVYPMKQ-VVPPQFEAKNDYDIFVELAKR  target    LAELTGDTRFNDMWKFVREGRTDVYLQRILDASTNT---KGYRFTELEAKAREG--IPA--LMNSRTSPKVVGYDQLADS 7l5s.1    AGVEEQYT----EGKT-----EMEWLEEFYNAAFSAARANRVAMPRFDKFWAENKPLSFEAGEAAKKWVRYGEFREDPLL  target    TPWYTKSGRLEFYREEDEFIEAG--ENLPVHREPVDSTFYEPNVIVSPKHEAVRPSGPEDYGVARTDLSCEVRCGRNVVL 7l5s.1    NPLGTPSGKIEIFSDVVEKMNYNDCKGHPSWMEPEEFA------------------------------------------  target    TWAETRQTQHPLVKQGHKFIFHTPKYRHGSHTTPIDTDMNAVLFGPFGDIYRRDKRSPFVTEGYVDINPTDGLELGLQDG 7l5s.1    ---------GNV-TEEYPLALVTPHPYYRLHSQLAHTSLRQKY-----------AV---NDREPVMIHPEDAAARGIKDG  target    DYVWIDPDPEDRPFRGWQKNAKDMEFARLLCRARFYPGTPRGVTRMWFNMYGATPGSVRGAKARRDGLAKNPDTNYQAMF 7l5s.1    DIVRIHSKR-----------------GQVLAGAAVTENIIKGTVALHEGA------------------------------  target    RSGSHQSATRGWLKPTWMTDSLVRKGLFGQGIGKGFLPDVHCPTGAPREAFVKISRAEPGGIGGQGLWRPAALGIRPRHE 7l5s.1    --------------------------------------------------------------------------------  target    SPAMKRYLAGGFFSGPKE 7l5s.1    ------------------ ``` | | | | | | | | | | | | | | | | | | | | | | | | | | | | | | | | | | | | | | | | | | | | | | | | | |
|  | 2e7z.1.A | Acetylene hydratase Ahy  *Acetylene Hydratase from Pelobacter acetylenicus* | 0.25 |  | 16.90 | 0.50 | 43-441 | X-ray | 1.26 | monomer | 1 x SF4, 2 x MGD, 1 x W | HHblits | 0.30 |
| ``` target    FNGAPQYINENPFDLELDASRPARPRQYWRAESAHFYNHEDHPLRVGTRLLTGSTHMPTPTKVMWFANANSILGNVKWHY 2e7z.1    ------------------------------------------PTALFTAMATEK---PYPVKAFFALASNALMG--YANQ  target    NTVVNALPRMEMIAVHEWWWTGSCEWADVVFGVDSWGELKHPDMTASVTNPFLIVFPKTPIKRIFNTVGDIDVLALVSSK 2e7z.1    QNALKGLMNQDLVVCYDQFMTPTAQLADYVLPGDHWLERPVVQPN-WEGIPFGNTSQQ-VVEPAGEAKDEYYFIRELAVR  target    LAELTGDTRFNDMWKFVREGRTDVYLQRILDASTNTKGYRFTELEAKAREGIPALMNSRTSPKVVGYDQLADSTPWYTKS 2e7z.1    MGLEEHF-----PWKD-----RLELINYRISP----TGMEWEEYQKQY--TYMS-KL----PD---YF-GPEGVGVATPS  target    GRLEFYREEDEFIEAGENLPVHREPVDSTFYEPNVIVSPKHEAVRPSGPEDYGVARTDLSCEVRCGRNVVLTWAETRQTQ 2e7z.1    GKVELYSSVFEKLGY-DPLPYYHEPLQTEISD------------------------------------------------  target    HPLVKQGHKFIFHTPKYR-HGSHTTPIDTDMNAVLFGPFGDIYRRDKRSPFVTEGYVDINPTDGLELGLQDGDYVWIDPD 2e7z.1    -PELAKEYPLILFAGLREDSNFQSCYHQPGIL-------------RDAEP---DPVALLHPKTAQSLGLPSGEWIWVETT  target    PEDRPFRGWQKNAKDMEFARLLCRARFYPGTPRGVTRMWFNMYGATPGSVRGAKARRDGLAKNPDTNYQAMFRSGSHQSA 2e7z.1    H-----------------GRLKLLLKHDGAQPEGTIRIPHGR--------------------------------------  target    TRGWLKPTWMTDSLVRKGLFGQGIGKGFLPDVHCPTGAPREAFVKISRAEPGGIGGQGLWRPAALGIRPRHESPAMKRYL 2e7z.1    --------------------------------------------------------------------------------  target    AGGFFSGPKE 2e7z.1    ---------- ``` | | | | | | | | | | | | | | | | | | | | | | | | | | | | | | | | | | | | | | | | | | | | | | | | | |
|  | 4aay.1.A | AROA  *Crystal Structure of the arsenite oxidase protein complex from Rhizobium species strain NT-26* | 0.23 |  | 17.89 | 0.50 | 57-441 | X-ray | 2.70 | hetero-oligomer | 4 x MGD, 2 x O, 2 x 4MO, 2 x F3S, 2 x FES | HHblits | 0.29 |
| ``` target    FNGAPQYINENPFDLELDASRPARPRQYWRAESAHFYNHEDHPLRVGTRLLTGSTHMPTPTKVMWFANANSILG------ 4aay.1    --------------------------------------------------------NAHEFKRVYKKRTDMVKDAMSAAP  target    --NVKWHYNTVVNALPRM-EMIAVHEWWWTGSCEWADVVFGVDSWGELKHPDMTASVTNPFLIVFPKTPIKRIFNTVGDI 4aay.1    YGDREAMVNAIVDAINQGGLFAVNVDIIPTKIGEACHVILPAATSGEMN--LTS-MNGERRMRLTER-YMDPPGQSMPDC  target    DVLALVSSKLAELT---GDTRFNDMWKFVREGRTDV-YLQRILDASTNTKGYRFTELEAKAREGIPALMNSRTSPKVVGY 4aay.1    LIAARLANTMERVLTEMGDVGYAAQFKGFDWQTEEDAFMDGYNKNAHGGEFVTYERLSAMGTNGFQEPATGFTDGKIEGT  target    DQLADSTPWYTKSGRLEFYREEDEFIEAGENLPVHREPVDSTFYEPNVIVSPKHEAVRPSGPEDYGVARTDLSCEVRCGR 4aay.1    QRLYTDGVFSTDDGKARFMDAPWR--G----LQ---APGKQ---------------------------------------  target    NVVLTWAETRQTQHPLVKQGHKFIFHTPKYRHGSHTTPID--TDMNAVLFGPFGDIYRRDKRSPFVTEGYVDINPTDGLE 4aay.1    ---------------QQKDSHKYLINNGRANVVWQSAYLDQENDFV-------------MDRFP---YPFIEMNPEDMAE  target    LGLQDGDYVWIDPDPEDRPFRGWQKNAKDMEFARLLCRARFYPGTPRGVTRMWFNMYGATPGSVRGAKARRDGLAKNPDT 4aay.1    AGLKEGDLVEIYNDA-----------------GATQAMAYPTPTARRGETFMLFGF------------------------  target    NYQAMFRSGSHQSATRGWLKPTWMTDSLVRKGLFGQGIGKGFLPDVHCPTGAPREAFVKISRAEPGGIGGQGLWRPAALG 4aay.1    --------------------------------------------------------------------------------  target    IRPRHESPAMKRYLAGGFFSGPKE 4aay.1    ------------------------ ``` | | | | | | | | | | | | | | | | | | | | | | | | | | | | | | | | | | | | | | | | | | | | | | | | | |
|  | 5nqd.1.A | AroA  *Arsenite oxidase AioAB from Rhizobium sp. str. NT-26 mutant AioBF108A* | 0.23 |  | 18.02 | 0.50 | 58-441 | X-ray | 2.20 | hetero-2-2-mer | 4 x MGD, 2 x O, 2 x 4MO, 2 x F3S, 2 x FES | HHblits | 0.29 |
| ``` target    FNGAPQYINENPFDLELDASRPARPRQYWRAESAHFYNHEDHPLRVGTRLLTGSTHMPTPTKVMWFANANSILG------ 5nqd.1    ---------------------------------------------------------AHEFKRVYKKRTDMVKDAMSAAP  target    --NVKWHYNTVVNALPRM-EMIAVHEWWWTGSCEWADVVFGVDSWGELKHPDMTASVTNPFLIVFPKTPIKRIFNTVGDI 5nqd.1    YGDREAMVNAIVDAINQGGLFAVNVDIIPTKIGEACHVILPAATSGEMN--LTS-MNGERRMRLTER-YMDPPGQSMPDC  target    DVLALVSSKLAELT---GDTRFNDMWKFVREGRTDV-YLQRILDASTNTKGYRFTELEAKAREGIPALMNSR-TSPKVVG 5nqd.1    LIAARLANTMERVLTEMGDVGYAAQFKGFDWQTEEDAFMDGYNKNAHGGEFVTYERLSAMGTNGFQE-PATGFTDGKIEG  target    YDQLADSTPWYTKSGRLEFYREEDEFIEAGENLPVHREPVDSTFYEPNVIVSPKHEAVRPSGPEDYGVARTDLSCEVRCG 5nqd.1    TQRLYTDGVFSTDDGKARFMDAPWR------GLQ---APGKQ--------------------------------------  target    RNVVLTWAETRQTQHPLVKQGHKFIFHTPKYRHGSHTTPID--TDMNAVLFGPFGDIYRRDKRSPFVTEGYVDINPTDGL 5nqd.1    ----------------QQKDSHKYLINNGRANVVWQSAYLDQENDFV-------------MDRFP---YPFIEMNPEDMA  target    ELGLQDGDYVWIDPDPEDRPFRGWQKNAKDMEFARLLCRARFYPGTPRGVTRMWFNMYGATPGSVRGAKARRDGLAKNPD 5nqd.1    EAGLKEGDLVEIYNDA-----------------GATQAMAYPTPTARRGETFMLFGF-----------------------  target    TNYQAMFRSGSHQSATRGWLKPTWMTDSLVRKGLFGQGIGKGFLPDVHCPTGAPREAFVKISRAEPGGIGGQGLWRPAAL 5nqd.1    --------------------------------------------------------------------------------  target    GIRPRHESPAMKRYLAGGFFSGPKE 5nqd.1    ------------------------- ``` | | | | | | | | | | | | | | | | | | | | | | | | | | | | | | | | | | | | | | | | | | | | | | | | | |
|  | 2vpz.1.A | THIOSULFATE REDUCTASE  *POLYSULFIDE REDUCTASE NATIVE STRUCTURE* | 0.23 |  | 16.79 | 0.49 | 43-441 | X-ray | 2.40 | hetero-oligomer | 10 x SF4, 4 x MGD, 2 x MO | HHblits | 0.28 |
| ``` target    FNGAPQYINENPFDLELDASRPARPRQYWRAESAHFYNHEDHPLRVGTRLLTGSTHMPTPTKVMWFANANSILGNVKWHY 2vpz.1    ------------------------------------------IQELIEPMITGE---PYPIKGLFAYGINLFHS--IPNV  target    NTVVNALPRMEMIAVHEWWWTGSCEWADVVFGVDSWGELKHPDMTASVTNPFLIVFPKTPIKRIFNTVGDIDVLALVSSK 2vpz.1    PRTKEALKNLDLYVAIDVLPQEHVMWADVILPEATYLERYDDFVLVAHKTPFIQLRTP-AHEPLFDTKPGWWIARELGLR  target    LAELTGDTRFNDMWKFVREGRTDVYLQRILDASTNTKGYRFTELEAKAREGIPALMNSRTSPKVVGYDQLADSTPWYTKS 2vpz.1    LGL----EQ---YFPW---KTIEEYLETRLQSL----GLDLETMKGMGT---LVQR---GKPWLEDWE-KEGRLPFGTAS  target    GRLEFYREEDEFIEAG-ENLPVHREPVDSTFYEPNVIVSPKHEAVRPSGPEDYGVARTDLSCEVRCGRNVVLTWAETRQT 2vpz.1    GKIELYCQRFK--EAGHQPLPVFTPPEEP---------------------------------------------------  target    QHPLVKQGHKFIFHTPKYRHGSHTTPIDTDMNAVLFGPFGDIYRRDKRSPFVTEGYVDINPTDGLELGLQDGDYVWIDPD 2vpz.1    ------PEGFYRLLYGRSPVHTFARTQNNWVLM-------------EMDP---ENEVWIHKEEAKRLGLKEGDYVMLVNQ  target    PEDRPFRGWQKNAKDMEFARLLCR--ARFYPGTPRGVTRMWFNMYGATPGSVRGAKARRDGLAKNPDTNYQAMFRSGSHQ 2vpz.1    D-----------------GVKEGPVRVKPTARIRKDCVYIVHGF------------------------------------  target    SATRGWLKPTWMTDSLVRKGLFGQGIGKGFLPDVHCPTGAPREAFVKISRAEPGGIGGQGLWRPAALGIRPRHESPAMKR 2vpz.1    --------------------------------------------------------------------------------  target    YLAGGFFSGPKE 2vpz.1    ------------ ``` | | | | | | | | | | | | | | | | | | | | | | | | | | | | | | | | | | | | | | | | | | | | | | | | | |
|  | 2vpx.1.D | THIOSULFATE REDUCTASE  *POLYSULFIDE REDUCTASE WITH BOUND QUINONE (UQ1)* | 0.22 |  | 16.79 | 0.49 | 43-441 | X-ray | 3.10 | hetero-oligomer | 10 x SF4, 4 x MGD, 2 x MO, 2 x UQ1 | HHblits | 0.28 |
| ``` target    FNGAPQYINENPFDLELDASRPARPRQYWRAESAHFYNHEDHPLRVGTRLLTGSTHMPTPTKVMWFANANSILGNVKWHY 2vpx.1    ------------------------------------------IQELIEPMITGE---PYPIKGLFAYGINLFHS--IPNV  target    NTVVNALPRMEMIAVHEWWWTGSCEWADVVFGVDSWGELKHPDMTASVTNPFLIVFPKTPIKRIFNTVGDIDVLALVSSK 2vpx.1    PRTKEALKNLDLYVAIDVLPQEHVMWADVILPEATYLERYDDFVLVAHKTPFIQLRTP-AHEPLFDTKPGWWIARELGLR  target    LAELTGDTRFNDMWKFVREGRTDVYLQRILDASTNTKGYRFTELEAKAREGIPALMNSRTSPKVVGYDQLADSTPWYTKS 2vpx.1    LGL----EQ---YFPW---KTIEEYLETRLQSL----GLDLETMKGMGT---LVQR---GKPWLEDWE-KEGRLPFGTAS  target    GRLEFYREEDEFIEAG-ENLPVHREPVDSTFYEPNVIVSPKHEAVRPSGPEDYGVARTDLSCEVRCGRNVVLTWAETRQT 2vpx.1    GKIELYCQRFK--EAGHQPLPVFTPPEEP---------------------------------------------------  target    QHPLVKQGHKFIFHTPKYRHGSHTTPIDTDMNAVLFGPFGDIYRRDKRSPFVTEGYVDINPTDGLELGLQDGDYVWIDPD 2vpx.1    ------PEGFYRLLYGRSPVHTFARTQNNWVLM-------------EMDP---ENEVWIHKEEAKRLGLKEGDYVMLVNQ  target    PEDRPFRGWQKNAKDMEFARLLCR--ARFYPGTPRGVTRMWFNMYGATPGSVRGAKARRDGLAKNPDTNYQAMFRSGSHQ 2vpx.1    D-----------------GVKEGPVRVKPTARIRKDCVYIVHGF------------------------------------  target    SATRGWLKPTWMTDSLVRKGLFGQGIGKGFLPDVHCPTGAPREAFVKISRAEPGGIGGQGLWRPAALGIRPRHESPAMKR 2vpx.1    --------------------------------------------------------------------------------  target    YLAGGFFSGPKE 2vpx.1    ------------ ``` | | | | | | | | | | | | | | | | | | | | | | | | | | | | | | | | | | | | | | | | | | | | | | | | | |
|  | 1ogy.1.A | PERIPLASMIC NITRATE REDUCTASE  *Crystal structure of the heterodimeric nitrate reductase from Rhodobacter sphaeroides* | 0.22 |  | 15.49 | 0.50 | 58-441 | X-ray | 3.20 | hetero-1-1-mer | 1 x SF4, 1 x MO, 2 x MGD, 2 x HEC | HHblits | 0.27 |
| ``` target    FNGAPQYINENPFDLELDASRPARPRQYWRAESAHFYNHEDHPLRVGTRLLTGSTHMPTPTKVMWFANANSILGNVKWHY 1ogy.1    ---------------------------------------------------------DGEINFYWVQVNNNMQA--APNI  target    N--TVVNALPRMEMIAVHEWWWTGSCEWADVVFGVDSWGELKHPDMTASVTNPFLIVFPKTPIKRIFNTVGDIDVLALVS 1ogy.1    DQETYPGYRNPENFIVVSDAYPTVTGRAADLVLPAAMWVEKE--GAYG-NAERRTHFWHQ-LVEAPGEARSDLWQLMEFS  target    SKLAELTGDT-RFNDMWKFVREGRTDVYLQRILDAST-----------------------NTKGYRFTELEAKAR----- 1ogy.1    KRFTTDEVWPEEILSAAPAY-RGK--TLFEVLFANGSVDRFPASDVNPDHANHEAALFGFYPQKGLFEEYAAFGRGHGHD  target    ----------EGIPALMNSRTSPKVVGYDQLADSTPWYTKSGRLEFYREEDEFIEAGENLPVHREPVDSTFYEPNVIVSP 1ogy.1    LAPFDTYHEVRGLHWPVV-EGE--ETRWRYREGFDPYVKPGEGLRFYGKPDGRAVI-LGVP-YEPPAE------------  target    KHEAVRPSGPEDYGVARTDLSCEVRCGRNVVLTWAETRQTQHPLVKQGHKFIFHTPKYRHGSHTTPIDTDMNAVLFGPFG 1ogy.1    -----------------------------------------SP--DEEFGFWLVTGRVLEHWHSGSMTLRWPE-------  target    DIYRRDKRSPFVTEGYVDINPTDGLELGLQDGDYVWIDPDPEDRPFRGWQKNAKDMEFARLLCRARF--YPGTPRGVTRM 1ogy.1    ----LYKAFP---GAVCFMHPEDARSRGLNRGSEVRVISRR-----------------GEIRTRLETRGRNRMPRGVVFV  target    WFNMYGATPGSVRGAKARRDGLAKNPDTNYQAMFRSGSHQSATRGWLKPTWMTDSLVRKGLFGQGIGKGFLPDVHCPTGA 1ogy.1    PWFD----------------------------------------------------------------------------  target    PREAFVKISRAEPGGIGGQGLWRPAALGIRPRHESPAMKRYLAGGFFSGPKE 1ogy.1    ---------------------------------------------------- ``` | | | | | | | | | | | | | | | | | | | | | | | | | | | | | | | | | | | | | | | | | | | | | | | | | |
|  | 1g8k.1.A | ARSENITE OXIDASE  *CRYSTAL STRUCTURE ANALYSIS OF ARSENITE OXIDASE FROM ALCALIGENES FAECALIS* | 0.23 |  | 16.01 | 0.49 | 60-441 | X-ray | 1.64 | hetero-1-1-mer | 3 x HG, 2 x CA, 2 x MGD, 1 x O, 1 x 4MO, 1 x F3S, 1 x FES | HHblits | 0.28 |
| ``` target    FNGAPQYINENPFDLELDASRPARPRQYWRAESAHFYNHEDHPLRVGTRLLTGSTHMPTPTKVMWFANANSILGNVKWHY 1g8k.1    -----------------------------------------------------------KGRIMTWWGCNNFQT--SNNA  target    NTVVNA------------------------------LPR-MEMIAVHEWWWTGSCEWADVVFGVDSWGELKHPDMTASVT 1g8k.1    QALREAILQRSAIVKQAMQKARGATTEEMVDVIYEATQNGGLFVTSINLYPTKLAEAAHLMLPAAHPGEMN--LTS-MNG  target    NPFLIVFPKTPIKRIFNTVGDIDVLALVSSKLAELTG---DTRFNDMWKFVREGRTDVYLQRILDASTN----------- 1g8k.1    ERRIRLSEK-FMDPPGTAMADCLIAARIANALRDMYQKDGKAEMAAQFEGFDWKTEEDAFNDGFRRAGQPGAPAIDSQGG  target    --TKGYRFTELEAKAREGIPALMNSRT-SPKVVGYDQLADSTPWYTKSGRLEFYREEDEFIEAGENLPVHREPVDSTFYE 1g8k.1    STGHLVTYDRLRKSGNNGVQLPVVSWDESKGLVGTEMLYTEGKFDTDDGKAHFKPAPW------NGLPATVQQ-------  target    PNVIVSPKHEAVRPSGPEDYGVARTDLSCEVRCGRNVVLTWAETRQTQHPLVKQGHKFIFHTPKYRHGSHTTPIDTDMNA 1g8k.1    ---------------------------------------------------QKDKYRFWLNNGRNNEVWQTAYHDQY-NS  target    VLFGPFGDIYRRDKRSPFVTEGYVDINPTDGLELGLQDGDYVWIDPDPEDRPFRGWQKNAKDMEFARLLCRARFYPGTPR 1g8k.1    ----------LMQERYP---MAYIEMNPDDCKQLDVTGGDIVEVYNDF-----------------GSTFAMVYPVAEIKR  target    GVTRMWFNMYGATPGSVRGAKARRDGLAKNPDTNYQAMFRSGSHQSATRGWLKPTWMTDSLVRKGLFGQGIGKGFLPDVH 1g8k.1    GQTFMLFGY-----------------------------------------------------------------------  target    CPTGAPREAFVKISRAEPGGIGGQGLWRPAALGIRPRHESPAMKRYLAGGFFSGPKE 1g8k.1    --------------------------------------------------------- ``` | | | | | | | | | | | | | | | | | | | | | | | | | | | | | | | | | | | | | | | | | | | | | | | | | |
|  | 7p61.1.C | NADH-quinone oxidoreductase  *Complex I from E. coli, DDM-purified, with NADH, Resting state* | 0.22 | 0.00 | 15.88 | 0.49 | 60-441 | EM | 0.00 | monomer | 7 x SF4, 1 x FMN, 1 x NAI, 2 x FES, 1 x CA, 2 x 3PE, 1 x UQ8 | HHblits | 0.27 |
| ``` target    FNGAPQYINENPFDLELDASRPARPRQYWRAESAHFYNHEDHPLRVGTRLLTGSTHMPTPTKVMWFANANSILGNVKWHY 7p61.1    -----------------------------------------------------------RADAVVVLE-NDLHR--HASA  target    NTVVNALPRMEMIAVHEWWWTGSCEWADVVFGVDSWGELKHPDMTASVTNPFLIVFPKTPIKRIF-----NTVGDIDVLA 7p61.1    TRVNAALAKAPLVMVVDHQRTAIMENAHLVLSAASFAESDGTVI---NNEGRAQRFFQ-VYDPAYYDSKTVMLESWRWLH  target    LVSSKLAELTGDTRFNDMWKFVREGRTDVYLQRILDASTNTKGYRFTELEAK----A-----------------R-EGIP 7p61.1    SLHSTLLSR----EV--DWTQL-----DHVIDAVVAKIPELAGIKDAAPDATFRIRGQKLAREPHRYSGRTAMRANISVH  target    A---------LM--------------NSRTSPKVVGYD-QLADSTPWYTKSGRLEFYREEDEFIEAG-ENLPVHREPVDS 7p61.1    EPRQPQDIDTMFTFSMEGNNQPTAHRSQVPFAWAPGWNSPQAWNKFQDEVGGKLRFGDPGVRLFETSENGLDYFTSVPAR  target    TFYEPNVIVSPKHEAVRPSGPEDYGVARTDLSCEVRCGRNVVLTWAETRQTQHPLVKQGHKFIFHTPKYRHGSHTTPIDT 7p61.1    ------------------------------------------------------FQPQDGKWRIAPYYHLFGSDELSQRA  target    DMNAVLFGPFGDIYRRDKRSPFVTEGYVDINPTDGLELGLQDGDYVWIDPDPEDRPFRGWQKNAKDMEFARLLCRARFYP 7p61.1    PVFQ-------------SRMP---QPYIKLNPADAAKLGVNAGTRVSFSYDG-----------------NTVTLPVEIAE  target    GTPRGVTRMWFNMYGATPGSVRGAKARRDGLAKNPDTNYQAMFRSGSHQSATRGWLKPTWMTDSLVRKGLFGQGIGKGFL 7p61.1    GLTAGQVGLPMGM-------------------------------------------------------------------  target    PDVHCPTGAPREAFVKISRAEPGGIGGQGLWRPAALGIRPRHESPAMKRYLAGGFFSGPKE 7p61.1    ------------------------------------------------------------- ``` | | | | | | | | | | | | | | | | | | | | | | | | | | | | | | | | | | | | | | | | | | | | | | | | | |
| ✓ | 7p63.1.C | NADH-quinone oxidoreductase  *Complex I from E. coli, DDM/LMNG-purified, under Turnover at pH 6, Closed state* | 0.23 | 0.00 | 16.25 | 0.49 | 60-441 | EM | 0.00 | monomer | 7 x SF4, 1 x FMN, 1 x NAI, 2 x FES, 1 x CA, 1 x DCQ, 4 x LFA, 8 x 3PE | HHblits | 0.27 |
| ``` target    FNGAPQYINENPFDLELDASRPARPRQYWRAESAHFYNHEDHPLRVGTRLLTGSTHMPTPTKVMWFANANSILGNVKWHY 7p63.1    -----------------------------------------------------------RADAVVVLE-NDLHR--HASA  target    NTVVNALPRMEMIAVHEWWWTGSCEWADVVFGVDSWGELKHPDMTASVTNPFLIVFPKTPIKRIF-----NTVGDIDVLA 7p63.1    TRVNAALAKAPLVMVVDHQRTAIMENAHLVLSAASFAESD--GTVI-NNEGRAQRFFQ-VYDPAYYDSKTVMLESWRWLH  target    LVSSKLAELTGDTRFNDMWKFVREGRTDVYLQRILDASTNTKGYRFTELEAKAR----------------------EGIP 7p63.1    SLHSTLLS----REV--DWTQL-----DHVIDAVVAKIPELAGIKDAAPDATFRIRGQKLAREPHRYSGRTAMRANISVH  target    ALMN--------------------S---RTSPKVVGYDQL-ADSTPWYTKSGRLEFYREEDEFIEAG-ENLPVHREPVDS 7p63.1    EPRQPQDIDTMFTFSMEGNNQPTAHRSQVPFAWAPGWNSPQAWNKFQDEVGGKLRFGDPGVRLFETSENGLDYFTSVPAR  target    TFYEPNVIVSPKHEAVRPSGPEDYGVARTDLSCEVRCGRNVVLTWAETRQTQHPLVKQGHKFIFHTPKYRHGSHTTPIDT 7p63.1    ------------------------------------------------------FQPQDGKWRIAPYYHLFGSDELSQRA  target    DMNAVLFGPFGDIYRRDKRSPFVTEGYVDINPTDGLELGLQDGDYVWIDPDPEDRPFRGWQKNAKDMEFARLLCRARFYP 7p63.1    PVFQ-------------SRMP---QPYIKLNPADAAKLGVNAGTRVSFSYDG-----------------NTVTLPVEIAE  target    GTPRGVTRMWFNMYGATPGSVRGAKARRDGLAKNPDTNYQAMFRSGSHQSATRGWLKPTWMTDSLVRKGLFGQGIGKGFL 7p63.1    GLTAGQVGLPMGM-------------------------------------------------------------------  target    PDVHCPTGAPREAFVKISRAEPGGIGGQGLWRPAALGIRPRHESPAMKRYLAGGFFSGPKE 7p63.1    ------------------------------------------------------------- ``` | | | | | | | | | | | | | | | | | | | | | | | | | | | | | | | | | | | | | | | | | | | | | | | | | |
|  | 7nz1.1.E | NADH-quinone oxidoreductase subunit G  *Respiratory complex I from Escherichia coli - focused refinement of cytoplasmic arm* | 0.22 | 0.00 | 15.94 | 0.49 | 60-441 | EM | 0.00 | monomer | 7 x SF4, 2 x FES, 1 x FMN, 1 x CA | HHblits | 0.27 |
| ``` target    FNGAPQYINENPFDLELDASRPARPRQYWRAESAHFYNHEDHPLRVGTRLLTGSTHMPTPTKVMWFANANSILGNVKWHY 7nz1.1    -----------------------------------------------------------RADAVVVLE-NDLHR--HASA  target    NTVVNALPRMEMIAVHEWWWTGSCEWADVVFGVDSWGELKHPDMTASVTNPFLIVFPKTPIKRIF-----NTVGDIDVLA 7nz1.1    IRVNAALAKAPLVMVVDHQRTAIMENAHLVLSAASFAESDGT--VI-NNEGRAQRFFQ-VYDPAYYDSKTVMLESWRWLH  target    LVSSKLAELTGDTRFNDMWKFVREGRTDVYLQRILDASTNTKGYRFTELEA---------------------KARE-GIP 7nz1.1    SLHSTLLSR----EV--DWTQL-----DHVIDAVVAKIPELAGIKDAAPDATFRIRGQKLAREPHRYSGRTAMRANISVH  target    AL---------MN-----------S-RTSP--KVVGYDQLA--DSTPWYTKSGRLEFYREEDEFIEAG-ENLPVHREPVD 7nz1.1    EPRQPQDIDTMFTFSMEGNNQPTAHRSQVPFAWAPGWN-SPQAWNKFQDEVGGKLRFGDPGVRLFETSENGLDYFTSVPA  target    STFYEPNVIVSPKHEAVRPSGPEDYGVARTDLSCEVRCGRNVVLTWAETRQTQHPLVKQGHKFIFHTPKYRHGSHTTPID 7nz1.1    R------------------------------------------------------FQPQDGKWRIAPYYHLFGSDELSQR  target    TDMNAVLFGPFGDIYRRDKRSPFVTEGYVDINPTDGLELGLQDGDYVWIDPDPEDRPFRGWQKNAKDMEFARLLCRARFY 7nz1.1    APVFQ-------------SRMP---QPYIKLNPADAAKLGVNAGTRVSFSYDG-----------------NTVTLPVEIA  target    PGTPRGVTRMWFNMYGATPGSVRGAKARRDGLAKNPDTNYQAMFRSGSHQSATRGWLKPTWMTDSLVRKGLFGQGIGKGF 7nz1.1    EGLTAGQVGLPMGM------------------------------------------------------------------  target    LPDVHCPTGAPREAFVKISRAEPGGIGGQGLWRPAALGIRPRHESPAMKRYLAGGFFSGPKE 7nz1.1    -------------------------------------------------------------- ``` | | | | | | | | | | | | | | | | | | | | | | | | | | | | | | | | | | | | | | | | | | | | | | | | | |
|  | 2v45.1.A | PERIPLASMIC NITRATE REDUCTASE  *A NEW CATALYTIC MECHANISM OF PERIPLASMIC NITRATE REDUCTASE FROM DESULFOVIBRIO DESULFURICANS ATCC 27774 FROM CRYSTALLOGRAPHIC AND EPR DATA AND BASED ON DETAILED ANALYSIS OF THE SIXTH LIGAND* | 0.23 |  | 16.36 | 0.48 | 59-441 | X-ray | 2.40 | monomer | 1 x SF4, 1 x MO, 2 x MGD, 1 x LCP | HHblits | 0.27 |
| ``` target    FNGAPQYINENPFDLELDASRPARPRQYWRAESAHFYNHEDHPLRVGTRLLTGSTHMPTPTKVMWFANANSILGNVKWHY 2v45.1    ----------------------------------------------------------GDVKCMIICETNPAHT--LPNL  target    NTVVNALPRME-MIAVHEWWWT-GSCEWADVVFGVDSWGELKHPDMTASVTNPFLIVFPKTPIKRIFNTVGDIDVLALVS 2v45.1    NKVHKAMSHPESFIVCIEAFPDAVTLEYADLVLPPAFWCERD--GVY-GCGERRYSLTEK-AVDPPGQCRPTVNTLVEFA  target    SKLAELTGDTRFNDMWKFVREGRTDVYLQRILDAST----NTKGYRFTELEAKAREGIPALMNSRTSPK--VVGYDQLAD 2v45.1    RRAGVDPQLVN----FRNA-----EDVWNEWRMVSKGTTYDFWGMTRERLRKE--SGLIWPCPSEDHPGTSLRYVR-GQD  target    STPWYTKSGRLEFYREEDEFIEAGENLPVHREPVDSTFYEPNVIVSPKHEAVRPSGPEDYGVARTDLSCEVRCGRNVVLT 2v45.1    PCVPADHPDRFFFYGKPDG------RAVIWMRPAKGA-------------------------------------------  target    WAETRQTQHPLVKQGHKFIFHTPKYRHGSHTTPI--DTDMNAVLFGPFGDIYRRDKRSPFVTEGYVDINPTDGLELGLQD 2v45.1    --------AEEPDAEYPLYLTSMRVIDHWHTATMTGKVPEL-------------QKANP---IAFVEINEEDAARTGIKH  target    GDYVWIDPDPEDRPFRGWQKNAKDMEFARLLCRARFYPGTPRGVTRMWFNMYGATPGSVRGAKARRDGLAKNPDTNYQAM 2v45.1    GDSVIVETRR-----------------DAMELPARVSDVCRPGLIAVPFFD-----------------------------  target    FRSGSHQSATRGWLKPTWMTDSLVRKGLFGQGIGKGFLPDVHCPTGAPREAFVKISRAEPGGIGGQGLWRPAALGIRPRH 2v45.1    --------------------------------------------------------------------------------  target    ESPAMKRYLAGGFFSGPKE 2v45.1    ------------------- ``` | | | | | | | | | | | | | | | | | | | | | | | | | | | | | | | | | | | | | | | | | | | | | | | | | |
|  | 7vw6.1.A | Formate dehydrogenase  *Cryo-EM Structure of Formate Dehydrogenase 1 from Methylorubrum extorquens AM1* | 0.24 |  | 16.04 | 0.47 | 44-441 | EM | 0.00 | hetero-1-1-mer | 4 x SF4, 2 x FES, 2 x MGD, 1 x W, 1 x FMN | HHblits | 0.28 |
| ``` target    FNGAPQYINENPFDLELDASRPARPRQYWRAESAHFYNHEDHPLRVGTRLLTGSTHMPTPTKVMWFANANSILGNVKWHY 7vw6.1    -------------------------------------------VEIMRAIHAG------EIRGMFVEGENPAMS--DPDL  target    NTVVNALPRMEMIAVHEWWWTGSCEWADVVFGVDSWGELKHPDMTASVTNPFLIVFPKTPIKRIFNTVGDIDVLALVSSK 7vw6.1    NHARHALAMLDHLVVQDLFLTETAFHADVVLPASAFAEKAG--TF-TNTDRRVQIAQP-VVAPPGDARQDWWIIQELARR  target    LAELTGDTRFNDMWKFVREGRTDVYLQRILDASTNTKGYRFTELEAKAREGIPALMNSRTSPKVVGYDQLADSTPWYTKS 7vw6.1    LDLDWNYG-------G-----PADIFAEMAQVMPSLNNITWERLERE---GAVTYPVD--APDQPGNE-IIFYAGFPTES  target    GRLEFYREEDEFIEAGENLPVHREPVDSTFYEPNVIVSPKHEAVRPSGPEDYGVARTDLSCEVRCGRNVVLTWAETRQTQ 7vw6.1    GRAKIVPAAIV--------P----PDEV----------------------------------------------------  target    HPLVKQGHKFIFHTPKYRHGSHTT--PIDTDMNAVLFGPFGDIYRRDKRSPFVTEGYVDINPTDGLELGLQDGDYVWIDP 7vw6.1    ---PDDEFPMVLSTGRVLEHWHTGSMTRRAGVL-------------DALEP---EAVAFMAPKELYRLGLRPGGSMRLET  target    DPEDRPFRGWQKNAKDMEFARLLCRARFYPGTPRGVTRMWFNMYGATPGSVRGAKARRDGLAKNPDTNYQAMFRSGSHQS 7vw6.1    RR-----------------GAVVLKVRSDRDVPIGMIFMPFCY-------------------------------------  target    ATRGWLKPTWMTDSLVRKGLFGQGIGKGFLPDVHCPTGAPREAFVKISRAEPGGIGGQGLWRPAALGIRPRHESPAMKRY 7vw6.1    --------------------------------------------------------------------------------  target    LAGGFFSGPKE 7vw6.1    ----------- ``` | | | | | | | | | | | | | | | | | | | | | | | | | | | | | | | | | | | | | | | | | | | | | | | | | |
|  | 1aa6.1.A | FORMATE DEHYDROGENASE H  *REDUCED FORM OF FORMATE DEHYDROGENASE H FROM E. COLI* | 0.23 | 0.00 | 15.97 | 0.46 | 58-441 | X-ray | 2.30 | monomer | 1 x SF4, 2 x MGD, 1 x 4MO | HHblits | 0.29 |
| ``` target    FNGAPQYINENPFDLELDASRPARPRQYWRAESAHFYNHEDHPLRVGTRLLTGSTHMPTPTKVMWFANANSILGNVKWHY 1aa6.1    ---------------------------------------------------------HGEVRAAYIMGEDPLQ--TDAEL  target    NTVVNALPRMEMIAVHEWWWTGSCEWADVVFGVDSWGELKHPDMTASVTNPFLIVFPKTPIKRIFNTVGDIDVLALVSSK 1aa6.1    SAVRKAFEDLELVIVQDIFMTKTASAADVILPSTSWGEHE--GVFT-AADRGFQRFFK-AVEPKWDLKTDWQIISEIATR  target    LAELTGDTRFNDMWKFVREGRTDVYLQRILDASTNTKGYRFTELEAKAREGIPALMNSRTSPKVVGYDQLADSTPWYTKS 1aa6.1    MGYPMH-------YNN-----TQEIWDELRHLCPDFYGATYEKMGEL---GFIQWPCRDTSDADQG-TSYLFKEKFDTPN  target    GRLEFYREEDEFIEAGENLPVHREPVDSTFYEPNVIVSPKHEAVRPSGPEDYGVARTDLSCEVRCGRNVVLTWAETRQTQ 1aa6.1    GLAQFFTCDWV-----A-------PIDK------------------------------------------------L---  target    HPLVKQGHKFIFHTPKYR--HGSHTTPIDTDMNAVLFGPFGDIYRRDKRSPFVTEGYVDINPTDGLELGLQDGDYVWIDP 1aa6.1    ----TDEYPMVLSTVREVGHYSCRSMTGNCAALA----------ALADE-----PGYAQINTEDAKRLGIEDEALVWVHS  target    DPEDRPFRGWQKNAKDMEFARLLCRARFYPGTPRGVTRMWFNMYGATPGSVRGAKARRDGLAKNPDTNYQAMFRSGSHQS 1aa6.1    RK-----------------GKIITRAQVSDRPNKGAIYMTYQW-------------------------------------  target    ATRGWLKPTWMTDSLVRKGLFGQGIGKGFLPDVHCPTGAPREAFVKISRAEPGGIGGQGLWRPAALGIRPRHESPAMKRY 1aa6.1    --------------------------------------------------------------------------------  target    LAGGFFSGPKE 1aa6.1    ----------- ``` | | | | | | | | | | | | | | | | | | | | | | | | | | | | | | | | | | | | | | | | | | | | | | | | | |
|  | 1fdo.1.A | FORMATE DEHYDROGENASE H  *OXIDIZED FORM OF FORMATE DEHYDROGENASE H FROM E. COLI* | 0.23 | 0.00 | 15.97 | 0.46 | 58-441 | X-ray | 2.80 | monomer | 1 x SF4, 2 x MGD, 1 x 6MO | HHblits | 0.29 |
| ``` target    FNGAPQYINENPFDLELDASRPARPRQYWRAESAHFYNHEDHPLRVGTRLLTGSTHMPTPTKVMWFANANSILGNVKWHY 1fdo.1    ---------------------------------------------------------HGEVRAAYIMGEDPLQ--TDAEL  target    NTVVNALPRMEMIAVHEWWWTGSCEWADVVFGVDSWGELKHPDMTASVTNPFLIVFPKTPIKRIFNTVGDIDVLALVSSK 1fdo.1    SAVRKAFEDLELVIVQDIFMTKTASAADVILPSTSWGEHE--GVFT-AADRGFQRFFK-AVEPKWDLKTDWQIISEIATR  target    LAELTGDTRFNDMWKFVREGRTDVYLQRILDASTNTKGYRFTELEAKAREGIPALMNSRTSPKVVGYDQLADSTPWYTKS 1fdo.1    MGYPMH-------YNN-----TQEIWDELRHLCPDFYGATYEKMGEL---GFIQWPCRDTSDADQG-TSYLFKEKFDTPN  target    GRLEFYREEDEFIEAGENLPVHREPVDSTFYEPNVIVSPKHEAVRPSGPEDYGVARTDLSCEVRCGRNVVLTWAETRQTQ 1fdo.1    GLAQFFTCDWV-----A-------PIDK------------------------------------------------L---  target    HPLVKQGHKFIFHTPKYR--HGSHTTPIDTDMNAVLFGPFGDIYRRDKRSPFVTEGYVDINPTDGLELGLQDGDYVWIDP 1fdo.1    ----TDEYPMVLSTVREVGHYSCRSMTGNCAALA----------ALADE-----PGYAQINTEDAKRLGIEDEALVWVHS  target    DPEDRPFRGWQKNAKDMEFARLLCRARFYPGTPRGVTRMWFNMYGATPGSVRGAKARRDGLAKNPDTNYQAMFRSGSHQS 1fdo.1    RK-----------------GKIITRAQVSDRPNKGAIYMTYQW-------------------------------------  target    ATRGWLKPTWMTDSLVRKGLFGQGIGKGFLPDVHCPTGAPREAFVKISRAEPGGIGGQGLWRPAALGIRPRHESPAMKRY 1fdo.1    --------------------------------------------------------------------------------  target    LAGGFFSGPKE 1fdo.1    ----------- ``` | | | | | | | | | | | | | | | | | | | | | | | | | | | | | | | | | | | | | | | | | | | | | | | | | |
|  | 2iv2.1.A | Formate dehydrogenase H  *Reinterpretation of reduced form of formate dehydrogenase H from E. coli* | 0.24 | 0.00 | 15.97 | 0.46 | 58-441 | X-ray | 2.27 | monomer | 1 x SF4, 1 x 2MD, 1 x MGD | HHblits | 0.29 |
| ``` target    FNGAPQYINENPFDLELDASRPARPRQYWRAESAHFYNHEDHPLRVGTRLLTGSTHMPTPTKVMWFANANSILGNVKWHY 2iv2.1    ---------------------------------------------------------HGEVRAAYIMGEDPLQ--TDAEL  target    NTVVNALPRMEMIAVHEWWWTGSCEWADVVFGVDSWGELKHPDMTASVTNPFLIVFPKTPIKRIFNTVGDIDVLALVSSK 2iv2.1    SAVRKAFEDLELVIVQDIFMTKTASAADVILPSTSWGEHE--GVFT-AADRGFQRFFK-AVEPKWDLKTDWQIISEIATR  target    LAELTGDTRFNDMWKFVREGRTDVYLQRILDASTNTKGYRFTELEAKAREGIPALMNSRTSPKVVGYDQLADSTPWYTKS 2iv2.1    MGYPMH-------YNN-----TQEIWDELRHLCPDFYGATYEKMGEL---GFIQWPCRDTSDADQG-TSYLFKEKFDTPN  target    GRLEFYREEDEFIEAGENLPVHREPVDSTFYEPNVIVSPKHEAVRPSGPEDYGVARTDLSCEVRCGRNVVLTWAETRQTQ 2iv2.1    GLAQFFTCDWV-----A-------PIDK------------------------------------------------L---  target    HPLVKQGHKFIFHTPKYR--HGSHTTPIDTDMNAVLFGPFGDIYRRDKRSPFVTEGYVDINPTDGLELGLQDGDYVWIDP 2iv2.1    ----TDEYPMVLSTVREVGHYSCRSMTGNCAALA----------ALADE-----PGYAQINTEDAKRLGIEDEALVWVHS  target    DPEDRPFRGWQKNAKDMEFARLLCRARFYPGTPRGVTRMWFNMYGATPGSVRGAKARRDGLAKNPDTNYQAMFRSGSHQS 2iv2.1    RK-----------------GKIITRAQVSDRPNKGAIYMTYQW-------------------------------------  target    ATRGWLKPTWMTDSLVRKGLFGQGIGKGFLPDVHCPTGAPREAFVKISRAEPGGIGGQGLWRPAALGIRPRHESPAMKRY 2iv2.1    --------------------------------------------------------------------------------  target    LAGGFFSGPKE 2iv2.1    ----------- ``` | | | | | | | | | | | | | | | | | | | | | | | | | | | | | | | | | | | | | | | | | | | | | | | | | |
|  | 7z0t.1.G | Formate dehydrogenase H  *Structure of the Escherichia coli formate hydrogenlyase complex (aerobic preparation, composite structure)* | 0.23 | 0.00 | 15.97 | 0.46 | 58-441 | EM | 0.00 | monomer | 1 x NI, 1 x FCO, 8 x SF4, 1 x FE, 2 x MGD, 1 x 6MO | HHblits | 0.29 |
| ``` target    FNGAPQYINENPFDLELDASRPARPRQYWRAESAHFYNHEDHPLRVGTRLLTGSTHMPTPTKVMWFANANSILGNVKWHY 7z0t.1    ---------------------------------------------------------HGEVRAAYIMGEDPLQ--TDAEL  target    NTVVNALPRMEMIAVHEWWWTGSCEWADVVFGVDSWGELKHPDMTASVTNPFLIVFPKTPIKRIFNTVGDIDVLALVSSK 7z0t.1    SAVRKAFEDLELVIVQDIFMTKTASAADVILPSTSWGEHE--GVFT-AADRGFQRFFK-AVEPKWDLKTDWQIISEIATR  target    LAELTGDTRFNDMWKFVREGRTDVYLQRILDASTNTKGYRFTELEAKAREGIPALMNSRTSPKVVGYDQLADSTPWYTKS 7z0t.1    MGYPMH-------YNN-----TQEIWDELRHLCPDFYGATYEKMGEL---GFIQWPCRDTSDADQG-TSYLFKEKFDTPN  target    GRLEFYREEDEFIEAGENLPVHREPVDSTFYEPNVIVSPKHEAVRPSGPEDYGVARTDLSCEVRCGRNVVLTWAETRQTQ 7z0t.1    GLAQFFTCDWV-----A-------PIDK------------------------------------------------L---  target    HPLVKQGHKFIFHTPKYR--HGSHTTPIDTDMNAVLFGPFGDIYRRDKRSPFVTEGYVDINPTDGLELGLQDGDYVWIDP 7z0t.1    ----TDEYPMVLSTVREVGHYSCRSMTGNCAALA----------ALADE-----PGYAQINTEDAKRLGIEDEALVWVHS  target    DPEDRPFRGWQKNAKDMEFARLLCRARFYPGTPRGVTRMWFNMYGATPGSVRGAKARRDGLAKNPDTNYQAMFRSGSHQS 7z0t.1    RK-----------------GKIITRAQVSDRPNKGAIYMTYQW-------------------------------------  target    ATRGWLKPTWMTDSLVRKGLFGQGIGKGFLPDVHCPTGAPREAFVKISRAEPGGIGGQGLWRPAALGIRPRHESPAMKRY 7z0t.1    --------------------------------------------------------------------------------  target    LAGGFFSGPKE 7z0t.1    ----------- ``` | | | | | | | | | | | | | | | | | | | | | | | | | | | | | | | | | | | | | | | | | | | | | | | | | |
|  | 7e5z.1.A | Formate dehydrogenase  *Dehydrogenase holoenzyme* | 0.16 |  | 15.67 | 0.47 | 44-441 | EM | 0.00 | hetero-1-1-mer | 1 x W, 2 x MGD, 2 x FES, 4 x SF4, 1 x FMN | HHblits | 0.28 |
| ``` target    FNGAPQYINENPFDLELDASRPARPRQYWRAESAHFYNHEDHPLRVGTRLLTGSTHMPTPTKVMWFANANSILGNVKWHY 7e5z.1    -------------------------------------------VEIMRAIHAG------EIRGMFVEGENPAMS--DPDL  target    NTVVNALPRMEMIAVHEWWWTGSCEWADVVFGVDSWGELKHPDMTASVTNPFLIVFPKTPIKRIFNTVGDIDVLALVSSK 7e5z.1    NHARHALAMLDHLVVQDLFLTETAFHADVVLPASAFAEKAG--TF-TNTDRRVQIAQP-VVAPPGDARQDWWIIQELARR  target    LAELTGDTRFNDMWKFVREGRTDVYLQRILDASTNTKGYRFTELEAKAREGIPALMNSRTSPKVVGYDQLADSTPWYTKS 7e5z.1    LDLDWNYG-------G-----PADIFAEMAQVMPSLNNITWERLERE---GAVTYPVD--APDQPGNE-IIFYAGFPTES  target    GRLEFYREEDEFIEAGENLPVHREPVDSTFYEPNVIVSPKHEAVRPSGPEDYGVARTDLSCEVRCGRNVVLTWAETRQTQ 7e5z.1    GRAKIVPAAIV------P------PDEV----------------------------------------------------  target    HPLVKQGHKFIFHTPKYRHGSHTT--PIDTDMNAVLFGPFGDIYRRDKRSPFVTEGYVDINPTDGLELGLQDGDYVWIDP 7e5z.1    ---PDDEFPMVLSTGRVLEHWHTGSMTRRAGVL-------------DALEP---EAVAFMAPKELYRLGLRPGGSMRLET  target    DPEDRPFRGWQKNAKDMEFARLLCRARFYPGTPRGVTRMWFNMYGATPGSVRGAKARRDGLAKNPDTNYQAMFRSGSHQS 7e5z.1    RR-----------------GAVVLKVRSDRDVPIGMIFMPFCY-------------------------------------  target    ATRGWLKPTWMTDSLVRKGLFGQGIGKGFLPDVHCPTGAPREAFVKISRAEPGGIGGQGLWRPAALGIRPRHESPAMKRY 7e5z.1    --------------------------------------------------------------------------------  target    LAGGFFSGPKE 7e5z.1    ----------- ``` | | | | | | | | | | | | | | | | | | | | | | | | | | | | | | | | | | | | | | | | | | | | | | | | | |
|  | 7bkb.1.F | Formate dehydrogenase  *Formate dehydrogenase - heterodisulfide reductase - formylmethanofuran dehydrogenase complex from Methanospirillum hungatei (hexameric, composite structure)* | 0.17 |  | 17.49 | 0.46 | 58-441 | EM | 0.00 | hetero-2-2-2-2-2-2-… | 48 x SF4, 4 x FAD, 2 x FES, 4 x 9S8, 4 x ZN, 2 x MO, 4 x MGD | HHblits | 0.29 |
| ``` target    FNGAPQYINENPFDLELDASRPARPRQYWRAESAHFYNHEDHPLRVGTRLLTGSTHMPTPTKVMWFANANSILGNVKWHY 7bkb.1    ---------------------------------------------------------GDEIKGMYILGLNPVVT--YPSS  target    NTVVNALPRMEMIAVHEWWWTGSCEWADVVFGVDSWGELKHPDMTASVTNPFLIVFPKTPIKRIFNTVGDIDVLALVSSK 7bkb.1    NHVKAQLEKLDFLVVQDIFFTETCQYADVILPGACFAEKD--GTF-TSGERRINRVRK-AVNPPGQAKEDIHIISELAAK  target    LAELTGDTRFNDMWKFVREGRTDVYLQRILDASTNTKGYRFTELEAKAREGIPALMNSRTSPKVVGYDQLADSTPWYTKS 7bkb.1    MGF----KGF--ELPT-----AKDVWDDMRAVTPSMFGATYEKLERP--EGICWPCPTEEHPGTPIL----HREKFATAD  target    GRLEFYREEDEFIEAGENLPVHREPVDSTFYEPNVIVSPKHEAVRPSGPEDYGVARTDLSCEVRCGRNVVLTWAETRQTQ 7bkb.1    GKGNLFGID------------YRPPAEV----------------------------------------------------  target    HPLVKQGHKFIFHTPKYRHGSHTTPIDTDMNAVLFGPFGDIYRRDKRSPFVTEGYVDINPTDGLELGLQDGDYVWIDPDP 7bkb.1    ---ADAEYPFTLMTGRLIFHYHSRTQTDRA-----------ADLHREVP---ESYAQINIEDARRLGIKNNEYIKLKSRR  target    EDRPFRGWQKNAKDMEFARLLCRARFYPGTPRGVTRMWFNMYGATPGSVRGAKARRDGLAKNPDTNYQAMFRSGSHQSAT 7bkb.1    -----------------GETTTLARVTDEVAPGVVYMTMHF---------------------------------------  target    RGWLKPTWMTDSLVRKGLFGQGIGKGFLPDVHCPTGAPREAFVKISRAEPGGIGGQGLWRPAALGIRPRHESPAMKRYLA 7bkb.1    --------------------------------------------------------------------------------  target    GGFFSGPKE 7bkb.1    --------- ``` | | | | | | | | | | | | | | | | | | | | | | | | | | | | | | | | | | | | | | | | | | | | | | | | | |
|  | 6tg9.1.A | Formate dehydrogenase subunit alpha  *Cryo-EM Structure of NADH reduced form of NAD+-dependent Formate Dehydrogenase from Rhodobacter capsulatus* | 0.22 |  | 15.09 | 0.47 | 44-441 | EM | 3.24 | hetero-2-2-2-2-mer | 4 x MGD, 2 x 6MO, 4 x FES, 10 x SF4, 2 x H2S, 2 x FMN, 2 x NAI | HHblits | 0.28 |
| ``` target    FNGAPQYINENPFDLELDASRPARPRQYWRAESAHFYNHEDHPLRVGTRLLTGSTHMPTPTKVMWFANANSILGNVKWHY 6tg9.1    -------------------------------------------PNMLDAAVEG------RFKALYVQGEDILQS--DPDT  target    NTVVNALPRMEMIAVHEWWWTGSCEWADVVFGVDSWGELKHPDMTASVTNPFLIVFPKTPIKRIFNTVGDIDVLALVSSK 6tg9.1    RHVSAGLAAMDLVIVHDLFLNETANYAHVFLPGSTFLEKDG--TF-TNAERRINRVRR-VMAPKA-GFADWEVTQMLANA  target    LAELTGDTRFNDMWKFVREGRTDVYLQRILDASTNTKGYRFTELEAKAREGIPALMNSRTSPKVVGYDQLADSTPWYTKS 6tg9.1    LGA--GW-----HYTH-----PSEIMAEIAATTPGFAAVTYEMLDAR---GSVQWPCN---------E----KAPEGSPI  target    GRLEFYREEDE-FIEAGENLPVHREPVDSTFYEPNVIVSPKHEAVRPSGPEDYGVARTDLSCEVRCGRNVVLTWAETRQT 6tg9.1    MHVEGFVRGKGRFIRT-AYLPTD----E----------------------------------------------------  target    QHPLVKQGHKFIFHTPKYRHGSHTTPIDTDMNAVLFGPFGDIYRRDKRSPFVTEGYVDINPTDGLELGLQDGDYVWIDPD 6tg9.1    --K-TGPRFPLLLTTGRILSQYNVGAQTRR-----------T-ENT---VWHGEDRLEIHPTDAETRGIRDGDWVRLASR  target    PEDRPFRGWQKNAKDMEFARLLCRARFYPGTPRGVTRMWFNMYGATPGSVRGAKARRDGLAKNPDTNYQAMFRSGSHQSA 6tg9.1    A-----------------GETTLRATVTDRVSPGVVYTTFHH--------------------------------------  target    TRGWLKPTWMTDSLVRKGLFGQGIGKGFLPDVHCPTGAPREAFVKISRAEPGGIGGQGLWRPAALGIRPRHESPAMKRYL 6tg9.1    --------------------------------------------------------------------------------  target    AGGFFSGPKE 6tg9.1    ---------- ``` | | | | | | | | | | | | | | | | | | | | | | | | | | | | | | | | | | | | | | | | | | | | | | | | | |
|  | 2v3v.1.A | PERIPLASMIC NITRATE REDUCTASE  *A NEW CATALYTIC MECHANISM OF PERIPLASMIC NITRATE REDUCTASE FROM DESULFOVIBRIO DESULFURICANS ATCC 27774 FROM CRYSTALLOGRAPHIC AND EPR DATA AND BASED ON DETAILED ANALYSIS OF THE SIXTH LIGAND* | 0.23 |  | 15.30 | 0.47 | 59-441 | X-ray | 1.99 | monomer | 1 x SF4, 1 x MO, 2 x MGD, 4 x LCP | HHblits | 0.27 |
| ``` target    FNGAPQYINENPFDLELDASRPARPRQYWRAESAHFYNHEDHPLRVGTRLLTGSTHMPTPTKVMWFANANSILGNVKWHY 2v3v.1    ----------------------------------------------------------GDVKCMIICETNPAHT--LPNL  target    NTVVNALPRME-MIAVHEWWWT-GSCEWADVVFGVDSWGELKHPDMTASVTNPFLIVFPKTPIKRIFNTVGDIDVLALVS 2v3v.1    NKVHKAMSHPESFIVCIEAFPDAVTLEYADLVLPPAFWCERD--GVY-GCGERRYSLTEK-AVDPPGQCRPTVNTLVEFA  target    SKLAELTGDTRFNDMWKFVREGRTDVYLQRILDAST----NTKGYRFTELEAKAREGIPALMNSRTSPK--VVGYDQ--- 2v3v.1    RRAGVDPQLVN----FRNA-----EDVWNEWRMVSKGTTYDFWGMTRERLRKE--SGLIWPCPSEDHPGTSLRYVRGQDP  target    -----LA-DSTPWYTKSGRLEFYREEDEFIEAGENLPVHREPVDSTFYEPNVIVSPKHEAVRPSGPEDYGVARTDLSCEV 2v3v.1    CVPADHPDRFFFYGKPDGRAVIWMRPAK---------GA-----A-----------------------------------  target    RCGRNVVLTWAETRQTQHPLVKQGHKFIFHTPKYRHGSHTTPIDTDMNAVLFGPFGDIYRRDKRSPFVTEGYVDINPTDG 2v3v.1    -----------------EE-PDAEYPLYLTSMRVIDHWHTATMTGK-----------VPELQKANP---IAFVEINEEDA  target    LELGLQDGDYVWIDPDPEDRPFRGWQKNAKDMEFARLLCRARFYPGTPRGVTRMWFNMYGATPGSVRGAKARRDGLAKNP 2v3v.1    ARTGIKHGDSVIVETRR-----------------DAMELPARVSDVCRPGLIAVPFFD----------------------  target    DTNYQAMFRSGSHQSATRGWLKPTWMTDSLVRKGLFGQGIGKGFLPDVHCPTGAPREAFVKISRAEPGGIGGQGLWRPAA 2v3v.1    --------------------------------------------------------------------------------  target    LGIRPRHESPAMKRYLAGGFFSGPKE 2v3v.1    -------------------------- ``` | | | | | | | | | | | | | | | | | | | | | | | | | | | | | | | | | | | | | | | | | | | | | | | | | |
|  | 1g8j.1.A | ARSENITE OXIDASE  *CRYSTAL STRUCTURE ANALYSIS OF ARSENITE OXIDASE FROM ALCALIGENES FAECALIS* | 0.21 |  | 15.89 | 0.45 | 82-441 | X-ray | 2.03 | hetero-oligomer | 2 x MGD, 1 x O, 1 x 4MO, 1 x F3S, 1 x FES | HHblits | 0.28 |
| ``` target    FNGAPQYINENPFDLELDASRPARPRQYWRAESAHFYNHEDHPLRVGTRLLTGSTHMPTPTKVMWFANANSILGNVKWHY 1g8j.1    --------------------------------------------------------------------------------  target    NTVVNALPRME-MIAVHEWWWTGSCEWADVVFGVDSWGELKHPDMTASVTNPFLIVFPKTPIKRIFNTVGDIDVLALVSS 1g8j.1    -VIYEATQNGGLFVTSINLYPTKLAEAAHLMLPAAHPGEMN--LTS-MNGERRIRLSEK-FMDPPGTAMADCLIAARIAN  target    KLAELTGDT---RFNDMWKFVREGRTDVYLQRILDASTN-------------TKGYRFTELEAKAREGIPALMNS--RTS 1g8j.1    ALRDMYQKDGKAEMAAQFEGFDWKTEEDAFNDGFRRAGQPGAPAIDSQGGSTGHLVTYDRLRKSGNNGVQL-PVVSWDES  target    PKVVGYDQLADSTPWYTKSGRLEFYREEDEFIEAGENLPVHREPVDSTFYEPNVIVSPKHEAVRPSGPEDYGVARTDLSC 1g8j.1    KGLVGTEMLYTEGKFDTDDGKAHFKPAPW------NGLPATVQQ------------------------------------  target    EVRCGRNVVLTWAETRQTQHPLVKQGHKFIFHTPKYRHGSHTTPID--TDMNAVLFGPFGDIYRRDKRSPFVTEGYVDIN 1g8j.1    ----------------------QKDKYRFWLNNGRNNEVWQTAYHDQYNSLM-------------QERYP---MAYIEMN  target    PTDGLELGLQDGDYVWIDPDPEDRPFRGWQKNAKDMEFARLLCRARFYPGTPRGVTRMWFNMYGATPGSVRGAKARRDGL 1g8j.1    PDDCKQLDVTGGDIVEVYNDF-----------------GSTFAMVYPVAEIKRGQTFMLFGY------------------  target    AKNPDTNYQAMFRSGSHQSATRGWLKPTWMTDSLVRKGLFGQGIGKGFLPDVHCPTGAPREAFVKISRAEPGGIGGQGLW 1g8j.1    --------------------------------------------------------------------------------  target    RPAALGIRPRHESPAMKRYLAGGFFSGPKE 1g8j.1    ------------------------------ ``` | | | | | | | | | | | | | | | | | | | | | | | | | | | | | | | | | | | | | | | | | | | | | | | | | |
|  | 6f0k.1.B | Fe-S-cluster-containing hydrogenase  *Alternative complex III* | 0.20 |  | 13.78 | 0.45 | 44-441 | EM | 0.00 | hetero-1-1-1-1-1-1-… | 6 x HEC, 1 x F3S, 3 x SF4 | HHblits | 0.26 |
| ``` target    FNGAPQYINENPFDLELDASRPARPRQYWRAESAHFYNHEDHPLRVGTRLLTGSTHMPTPTKVMWFANANSILGNVKWHY 6f0k.1    -------------------------------------------AELVQAMQAG------AVDALLLLNVNPVYD--APAA  target    NTVVNALPRMEMIAVHEWWWTGSCEWADVVFGVDSWGELKHPDMTASVTNPFLIVFPKTPIKRIFNT-VGDIDVLALVSS 6f0k.1    LGFAEALAQVPEVIHLGLHVDETARRSTWHLPSTHYLEAWG--DGRAY-DGTLSVIQP-LIAPLYEAAHSPLEVLALLAT  target    KLAELTGDTRFNDMWKFVREGRTDVYLQRILDASTNTKGYRFTELEAKAREGIPALMNSRTSPKVVGYDQLADSTPWYTK 6f0k.1    GEEQ----S----AYDLV-----RNTWRRLLAGR----G-AFEQ---AWQR-VLH----------DGFLP---DSGYPTV  target    SGRLEFYREEDEFIEAGENLPVHREPVDSTFYEPNVIVSPKHEAVRPSGPEDYGVARTDLSCEVRCGRNVVLTWAETRQT 6f0k.1    SLRPNR-----------QALADWPQA-----------------------------------------------------A  target    QHPLVKQGHKFIFHTPKYRHGSHTTPIDTDMNAVLFGPFGDIYRRDKRSPFVTEGYVDINPTDGLELGLQD--------G 6f0k.1    -------EGGLEVVFRLDPTVLDGSFANNAWAQELPDP------ITKI---VWDNVAILSPKTAAALGVKAEYHKGVYIA  target    DYVWIDPDPEDRPFRGWQKNAKDMEFARLLCRARFYPGTPRGVTRMWFNMYGATPGSVRGAKARRDGLAKNPDTNYQAMF 6f0k.1    DVIELSLDG-----------------RAVELPVWVLPGHPDDSITVYLGY------------------------------  target    RSGSHQSATRGWLKPTWMTDSLVRKGLFGQGIGKGFLPDVHCPTGAPREAFVKISRAEPGGIGGQGLWRPAALGIRPRHE 6f0k.1    --------------------------------------------------------------------------------  target    SPAMKRYLAGGFFSGPKE 6f0k.1    ------------------ ``` | | | | | | | | | | | | | | | | | | | | | | | | | | | | | | | | | | | | | | | | | | | | | | | | | |
|  | 5e7o.1.A | DMSO reductase family type II enzyme, molybdopterin subunit  *Crystal structure of the perchlorate reductase PcrAB mutant W461E of PcrA from Azospira suillum PS* | 0.14 | 0.00 | 26.37 | 0.35 | 64-271 | X-ray | 2.40 | monomer | 4 x SF4, 1 x MO, 1 x MGD, 1 x MD1, 1 x F3S | BLAST | 0.34 |
| ``` target    FNGAPQYINENPFDLELDASRPARPRQYWRAESAHFYNHEDHPLRVGTRLLTGSTHMPTPTKVMWFANANSILGNVKWHY 5e7o.1    ---------------------------------------------------------------VFFVYRGNWLNQAKGQK  target    NTVVNALPRMEMIAVHEWWWTGSCEWADVVFGVDSWGELKHPDMTASVTNPFLIVFPKTPIKRIFNTVGDIDVLALVSSK 5e7o.1    YVLENLWPKLELIVDINIRMDSTALYSDVVLPSAHWYEKLDLNVTSEHS---YINMTEPAIKPMWESKTDWQIFLALAKR  target    L---AELTGDTRFND-MWKFVRE--------------GRTDVYLQRILDASTNTKGYRFTELEAKAREGIPALMNSRTSP 5e7o.1    VEMAAKRKKYEKFNDEKFKWVRDLSNLWNQMTMDGKLAEDEAAAQYILDNAPQSKGITIQML----REKPQRFKSNWTSP  target    KVVGYDQ------LADSTPWYTKSGRLEFYREEDEFIEAGENLPVHREPVDSTFYEPNVIVSPKHEAVRPSGPEDYGVAR 5e7o.1    LKEGVPYTPFQYFVVDKKPWPTLTGRQQFYLDHDTFFDMGVELPTYKAPIDADKY-------------------------  target    TDLSCEVRCGRNVVLTWAETRQTQHPLVKQGHKFIFHTPKYRHGSHTTPIDTDMNAVLFGPFGDIYRRDKRSPFVTEGYV 5e7o.1    --------------------------------------------------------------------------------  target    DINPTDGLELGLQDGDYVWIDPDPEDRPFRGWQKNAKDMEFARLLCRARFYPGTPRGVTRMWFNMYGATPGSVRGAKARR 5e7o.1    --------------------------------------------------------------------------------  target    DGLAKNPDTNYQAMFRSGSHQSATRGWLKPTWMTDSLVRKGLFGQGIGKGFLPDVHCPTGAPREAFVKISRAEPGGIGGQ 5e7o.1    --------------------------------------------------------------------------------  target    GLWRPAALGIRPRHESPAMKRYLAGGFFSGPKE 5e7o.1    --------------------------------- ``` | | | | | | | | | | | | | | | | | | | | | | | | | | | | | | | | | | | | | | | | | | | | | | | | | |
|  | 4ydd.1.A | DMSO reductase family type II enzyme, molybdopterin subunit  *Crystal structure of the perchlorate reductase PcrAB from Azospira suillum PS* | 0.15 | 0.00 | 26.37 | 0.35 | 64-271 | X-ray | 1.86 | monomer | 4 x SF4, 1 x MO, 1 x MGD, 1 x MD1, 1 x F3S | BLAST | 0.34 |
| ``` target    FNGAPQYINENPFDLELDASRPARPRQYWRAESAHFYNHEDHPLRVGTRLLTGSTHMPTPTKVMWFANANSILGNVKWHY 4ydd.1    ---------------------------------------------------------------VFFVYRGNWLNQAKGQK  target    NTVVNALPRMEMIAVHEWWWTGSCEWADVVFGVDSWGELKHPDMTASVTNPFLIVFPKTPIKRIFNTVGDIDVLALVSSK 4ydd.1    YVLENLWPKLELIVDINIRMDSTALYSDVVLPSAHWYEKLDLNVTSEHS---YINMTEPAIKPMWESKTDWQIFLALAKR  target    L---AELTGDTRFND-MWKFVRE--------------GRTDVYLQRILDASTNTKGYRFTELEAKAREGIPALMNSRTSP 4ydd.1    VEMAAKRKKYEKFNDEKFKWVRDLSNLWNQMTMDGKLAEDEAAAQYILDNAPQSKGITIQML----REKPQRFKSNWTSP  target    KVVGYDQ------LADSTPWYTKSGRLEFYREEDEFIEAGENLPVHREPVDSTFYEPNVIVSPKHEAVRPSGPEDYGVAR 4ydd.1    LKEGVPYTPFQYFVVDKKPWPTLTGRQQFYLDHDTFFDMGVELPTYKAPIDADKY-------------------------  target    TDLSCEVRCGRNVVLTWAETRQTQHPLVKQGHKFIFHTPKYRHGSHTTPIDTDMNAVLFGPFGDIYRRDKRSPFVTEGYV 4ydd.1    --------------------------------------------------------------------------------  target    DINPTDGLELGLQDGDYVWIDPDPEDRPFRGWQKNAKDMEFARLLCRARFYPGTPRGVTRMWFNMYGATPGSVRGAKARR 4ydd.1    --------------------------------------------------------------------------------  target    DGLAKNPDTNYQAMFRSGSHQSATRGWLKPTWMTDSLVRKGLFGQGIGKGFLPDVHCPTGAPREAFVKISRAEPGGIGGQ 4ydd.1    --------------------------------------------------------------------------------  target    GLWRPAALGIRPRHESPAMKRYLAGGFFSGPKE 4ydd.1    --------------------------------- ``` | | | | | | | | | | | | | | | | | | | | | | | | | | | | | | | | | | | | | | | | | | | | | | | | | |
|  | 6lod.1.B | Fe-S-cluster-containing hydrogenase components 1-like protein  *Cryo-EM structure of the air-oxidized photosynthetic alternative complex III from Roseiflexus castenholzii* | 0.07 |  | 11.21 | 0.19 | 44-162 | EM | 0.00 | hetero-1-1-1-1-1-1-… | 6 x HEC, 2 x EL6, 3 x SF4, 1 x F3S | HHblits | 0.25 |
| ``` target    FNGAPQYINENPFDLELDASRPARPRQYWRAESAHFYNHEDHPLRVGTRLLTGSTHMPTPTKVMWFANANSILGNVKWHY 6lod.1    -------------------------------------------SALTQEMNAG------TVEVLLMIESNPVYN--APAD  target    NTVVNALPRMEMIAVHEWWWTGSCEWADVVFGVDSWGELKHPDMTASVTNPFLIVFPKTPIKRIFNTVGDIDVLALVSSK 6lod.1    IPFAEALAKVPLSMHVGLYRDETAQQSVWHINGAHFLEAWG--DVRA-FDGTTTIVQP-LIAPLYNGKSAIEVLNVLLGK  target    LAELTGDTRFNDMWKFVREGRTDVYLQRILDASTNTKGYRFTELEAKAREGIPALMNSRTSPKVVGYDQLADSTPWYTKS 6lod.1    PQ------------------------------------------------------------------------------  target    GRLEFYREEDEFIEAGENLPVHREPVDSTFYEPNVIVSPKHEAVRPSGPEDYGVARTDLSCEVRCGRNVVLTWAETRQTQ 6lod.1    --------------------------------------------------------------------------------  target    HPLVKQGHKFIFHTPKYRHGSHTTPIDTDMNAVLFGPFGDIYRRDKRSPFVTEGYVDINPTDGLELGLQDGDYVWIDPDP 6lod.1    --------------------------------------------------------------------------------  target    EDRPFRGWQKNAKDMEFARLLCRARFYPGTPRGVTRMWFNMYGATPGSVRGAKARRDGLAKNPDTNYQAMFRSGSHQSAT 6lod.1    --------------------------------------------------------------------------------  target    RGWLKPTWMTDSLVRKGLFGQGIGKGFLPDVHCPTGAPREAFVKISRAEPGGIGGQGLWRPAALGIRPRHESPAMKRYLA 6lod.1    --------------------------------------------------------------------------------  target    GGFFSGPKE 6lod.1    --------- ``` | | | | | | | | | | | | | | | | | | | | | | | | | | | | | | | | | | | | | | | | | | | | | | | | | |
|  | 6sdr.1.A | Formate dehydrogenase, alpha subunit, selenocysteine-containing  *W-formate dehydrogenase from Desulfovibrio vulgaris - Oxidized form* | 0.07 | 0.00 | 14.85 | 0.18 | 58-164 | X-ray | 2.10 | monomer | 2 x MGD, 4 x SF4, 1 x H2S, 1 x W | HHblits | 0.26 |
| ``` target    FNGAPQYINENPFDLELDASRPARPRQYWRAESAHFYNHEDHPLRVGTRLLTGSTHMPTPTKVMWFANANSILGNVKWHY 6sdr.1    ---------------------------------------------------------KGEFKGLFAWGMNPAC--GGANA  target    NTVVNALPRMEMIAVHEWWWTGSCEW--------AD-----VVFGVDSWGELKHPDMTASVTNPFLIVFPKTPIKRIFNT 6sdr.1    NKNRKAMGKLEWLVNVNLFENETSSFWKGPGMNPAEIGTEVFFLPCCVSIEKE--GSVA-NSGRWMQWRYR-GPKPYAET  target    VGDIDVLALVSSKLAELTGDTRFNDMWKFVREGRTDVYLQRILDASTNTKGYRFTELEAKAREGIPALMNSRTSPKVVGY 6sdr.1    KPDGDIMLDMFKKVREL---------------------------------------------------------------  target    DQLADSTPWYTKSGRLEFYREEDEFIEAGENLPVHREPVDSTFYEPNVIVSPKHEAVRPSGPEDYGVARTDLSCEVRCGR 6sdr.1    --------------------------------------------------------------------------------  target    NVVLTWAETRQTQHPLVKQGHKFIFHTPKYRHGSHTTPIDTDMNAVLFGPFGDIYRRDKRSPFVTEGYVDINPTDGLELG 6sdr.1    --------------------------------------------------------------------------------  target    LQDGDYVWIDPDPEDRPFRGWQKNAKDMEFARLLCRARFYPGTPRGVTRMWFNMYGATPGSVRGAKARRDGLAKNPDTNY 6sdr.1    --------------------------------------------------------------------------------  target    QAMFRSGSHQSATRGWLKPTWMTDSLVRKGLFGQGIGKGFLPDVHCPTGAPREAFVKISRAEPGGIGGQGLWRPAALGIR 6sdr.1    --------------------------------------------------------------------------------  target    PRHESPAMKRYLAGGFFSGPKE 6sdr.1    ---------------------- ``` | | | | | | | | | | | | | | | | | | | | | | | | | | | | | | | | | | | | | | | | | | | | | | | | | |
|  | 3o5a.1.A | Periplasmic nitrate reductase  *Crystal Structure of partially reduced Periplasmic Nitrate Reductase from Cupriavidus necator using Ionic Liquids* | 0.06 |  | 15.15 | 0.17 | 57-163 | X-ray | 1.72 | hetero-oligomer | 1 x SF4, 1 x MOS, 2 x MGD, 2 x HEC | HHblits | 0.27 |
| ``` target    FNGAPQYINENPFDLELDASRPARPRQYWRAESAHFYNHEDHPLRVGTRLLTGSTHMPTPTKVMWFANANSILGNVKWHY 3o5a.1    --------------------------------------------------------KDGKLNAYWVQVNNNMQAA----A  target    NTVVNALP----RMEMIAVHEWWWTGSCEWADVVFGVDSWGELKHPDMTASVTNPFLIVFPKTPIKRIFNTVGDIDVLAL 3o5a.1    NLMEEGLPGYRNPANFIVVSDAYPTVTALAADLVLPSAMWVEKE--GAYG-NAERRTQFWHQ-LVDAPGEARSDLWQLVE  target    VSSKLAELTGDTRFNDMWKFVREGRTDVYLQRILDASTNTKGYRFTELEAKAREGIPALMNSRTSPKVVGYDQLADSTPW 3o5a.1    FAKRFKV-------------------------------------------------------------------------  target    YTKSGRLEFYREEDEFIEAGENLPVHREPVDSTFYEPNVIVSPKHEAVRPSGPEDYGVARTDLSCEVRCGRNVVLTWAET 3o5a.1    --------------------------------------------------------------------------------  target    RQTQHPLVKQGHKFIFHTPKYRHGSHTTPIDTDMNAVLFGPFGDIYRRDKRSPFVTEGYVDINPTDGLELGLQDGDYVWI 3o5a.1    --------------------------------------------------------------------------------  target    DPDPEDRPFRGWQKNAKDMEFARLLCRARFYPGTPRGVTRMWFNMYGATPGSVRGAKARRDGLAKNPDTNYQAMFRSGSH 3o5a.1    --------------------------------------------------------------------------------  target    QSATRGWLKPTWMTDSLVRKGLFGQGIGKGFLPDVHCPTGAPREAFVKISRAEPGGIGGQGLWRPAALGIRPRHESPAMK 3o5a.1    --------------------------------------------------------------------------------  target    RYLAGGFFSGPKE 3o5a.1    ------------- ``` | | | | | | | | | | | | | | | | | | | | | | | | | | | | | | | | | | | | | | | | | | | | | | | | | |
|  | 6sdv.1.A | Formate dehydrogenase, alpha subunit, selenocysteine-containing,Formate dehydrogenase, alpha subunit, selenocysteine-containing,W-formate dehydrogenase - alpha subunit  *W-formate dehydrogenase from Desulfovibrio vulgaris - Formate reduced form* | 0.07 | 0.00 | 14.00 | 0.18 | 58-163 | X-ray | 1.90 | monomer | 2 x MGD, 4 x SF4, 1 x W, 1 x H2S | HHblits | 0.26 |
| ``` target    FNGAPQYINENPFDLELDASRPARPRQYWRAESAHFYNHEDHPLRVGTRLLTGSTHMPTPTKVMWFANANSILGNVKWHY 6sdv.1    ---------------------------------------------------------KGEFKGLFAWGMNPAC--GGANA  target    NTVVNALPRMEMIAVHEWWWTGSCEW--------AD-----VVFGVDSWGELKHPDMTASVTNPFLIVFPKTPIKRIFNT 6sdv.1    NKNRKAMGKLEWLVNVNLFENETSSFWKGPGMNPAEIGTEVFFLPCCVSIEKE--GSVA-NSGRWMQWRYR-GPKPYAET  target    VGDIDVLALVSSKLAELTGDTRFNDMWKFVREGRTDVYLQRILDASTNTKGYRFTELEAKAREGIPALMNSRTSPKVVGY 6sdv.1    KPDGDIMLDMFKKVRE----------------------------------------------------------------  target    DQLADSTPWYTKSGRLEFYREEDEFIEAGENLPVHREPVDSTFYEPNVIVSPKHEAVRPSGPEDYGVARTDLSCEVRCGR 6sdv.1    --------------------------------------------------------------------------------  target    NVVLTWAETRQTQHPLVKQGHKFIFHTPKYRHGSHTTPIDTDMNAVLFGPFGDIYRRDKRSPFVTEGYVDINPTDGLELG 6sdv.1    --------------------------------------------------------------------------------  target    LQDGDYVWIDPDPEDRPFRGWQKNAKDMEFARLLCRARFYPGTPRGVTRMWFNMYGATPGSVRGAKARRDGLAKNPDTNY 6sdv.1    --------------------------------------------------------------------------------  target    QAMFRSGSHQSATRGWLKPTWMTDSLVRKGLFGQGIGKGFLPDVHCPTGAPREAFVKISRAEPGGIGGQGLWRPAALGIR 6sdv.1    --------------------------------------------------------------------------------  target    PRHESPAMKRYLAGGFFSGPKE 6sdv.1    ---------------------- ``` | | | | | | | | | | | | | | | | | | | | | | | | | | | | | | | | | | | | | | | | | | | | | | | | | |
|  | 8bqg.1.A | Formate dehydrogenase, alpha subunit, selenocysteine-containing  *W-formate dehydrogenase from Desulfovibrio vulgaris - Soaking with Formate 1 min* | 0.07 | 0.00 | 14.00 | 0.18 | 58-163 | X-ray | 1.95 | monomer | 2 x MGD, 4 x SF4, 1 x H2S, 1 x W | HHblits | 0.26 |
| ``` target    FNGAPQYINENPFDLELDASRPARPRQYWRAESAHFYNHEDHPLRVGTRLLTGSTHMPTPTKVMWFANANSILGNVKWHY 8bqg.1    ---------------------------------------------------------KGEFKGLFAWGMNPAC--GGANA  target    NTVVNALPRMEMIAVHEWWWTGSCEW--------AD-----VVFGVDSWGELKHPDMTASVTNPFLIVFPKTPIKRIFNT 8bqg.1    NKNRKAMGKLEWLVNVNLFENETSSFWKGPGMNPAEIGTEVFFLPCCVSIEKE--GSVA-NSGRWMQWRYR-GPKPYAET  target    VGDIDVLALVSSKLAELTGDTRFNDMWKFVREGRTDVYLQRILDASTNTKGYRFTELEAKAREGIPALMNSRTSPKVVGY 8bqg.1    KPDGDIMLDMFKKVRE----------------------------------------------------------------  target    DQLADSTPWYTKSGRLEFYREEDEFIEAGENLPVHREPVDSTFYEPNVIVSPKHEAVRPSGPEDYGVARTDLSCEVRCGR 8bqg.1    --------------------------------------------------------------------------------  target    NVVLTWAETRQTQHPLVKQGHKFIFHTPKYRHGSHTTPIDTDMNAVLFGPFGDIYRRDKRSPFVTEGYVDINPTDGLELG 8bqg.1    --------------------------------------------------------------------------------  target    LQDGDYVWIDPDPEDRPFRGWQKNAKDMEFARLLCRARFYPGTPRGVTRMWFNMYGATPGSVRGAKARRDGLAKNPDTNY 8bqg.1    --------------------------------------------------------------------------------  target    QAMFRSGSHQSATRGWLKPTWMTDSLVRKGLFGQGIGKGFLPDVHCPTGAPREAFVKISRAEPGGIGGQGLWRPAALGIR 8bqg.1    --------------------------------------------------------------------------------  target    PRHESPAMKRYLAGGFFSGPKE 8bqg.1    ---------------------- ``` | | | | | | | | | | | | | | | | | | | | | | | | | | | | | | | | | | | | | | | | | | | | | | | | | |
|  | 1h0h.1.A | FORMATE DEHYDROGENASE SUBUNIT ALPHA  *Tungsten containing Formate Dehydrogenase from Desulfovibrio Gigas* | 0.07 |  | 9.00 | 0.18 | 58-163 | X-ray | 1.80 | hetero-1-1-mer | 1 x W, 1 x 2MD, 1 x MGD, 4 x SF4, 1 x CA | HHblits | 0.24 |
| ``` target    FNGAPQYINENPFDLELDASRPARPRQYWRAESAHFYNHEDHPLRVGTRLLTGSTHMPTPTKVMWFANANSILGNVKWHY 1h0h.1    ---------------------------------------------------------QGKIKGFFAWGQNPACS--GANS  target    NTVVNALPRMEMIAVHEWWWTGSCEWA-------------DVVFGVDSWGELKHPDMTASVTNPFLIVFPKTPIKRIFNT 1h0h.1    NKTREALTKLDWMVNVNIFDNETGSFWRGPDMDPKKIKTEVFFLPCAVAIEKE--GSIS-NSGRWMQWRYV-GPEPRKNA  target    VGDIDVLALVSSKLAELTGDTRFNDMWKFVREGRTDVYLQRILDASTNTKGYRFTELEAKAREGIPALMNSRTSPKVVGY 1h0h.1    IPDGDLIVELAKRVQK----------------------------------------------------------------  target    DQLADSTPWYTKSGRLEFYREEDEFIEAGENLPVHREPVDSTFYEPNVIVSPKHEAVRPSGPEDYGVARTDLSCEVRCGR 1h0h.1    --------------------------------------------------------------------------------  target    NVVLTWAETRQTQHPLVKQGHKFIFHTPKYRHGSHTTPIDTDMNAVLFGPFGDIYRRDKRSPFVTEGYVDINPTDGLELG 1h0h.1    --------------------------------------------------------------------------------  target    LQDGDYVWIDPDPEDRPFRGWQKNAKDMEFARLLCRARFYPGTPRGVTRMWFNMYGATPGSVRGAKARRDGLAKNPDTNY 1h0h.1    --------------------------------------------------------------------------------  target    QAMFRSGSHQSATRGWLKPTWMTDSLVRKGLFGQGIGKGFLPDVHCPTGAPREAFVKISRAEPGGIGGQGLWRPAALGIR 1h0h.1    --------------------------------------------------------------------------------  target    PRHESPAMKRYLAGGFFSGPKE 1h0h.1    ---------------------- ``` | | | | | | | | | | | | | | | | | | | | | | | | | | | | | | | | | | | | | | | | | | | | | | | | | |
|  | 6btm.1.B | Alternative Complex III subunit B  *Structure of Alternative Complex III from Flavobacterium johnsoniae (Wild Type)* | 0.06 |  | 12.24 | 0.17 | 58-161 | EM | 3.40 | hetero-1-1-1-1-1-1-… | 6 x HEC, 1 x F3S, 1 x SF4, 2 x E87 | HHblits | 0.25 |
| ``` target    FNGAPQYINENPFDLELDASRPARPRQYWRAESAHFYNHEDHPLRVGTRLLTGSTHMPTPTKVMWFANANSILGNVKWHY 6btm.1    ---------------------------------------------------------AGSVHTLIMSGVNPVYT--LADS  target    NTVVNALPRMEMIAVHEWWWTGSCEWADVVFGVDSWGELKHPDMTASVTNPFLIVFPKTPIKRIFNTVGDIDVLALVSSK 6btm.1    ASFVSGLKKVKTSVAFSLKEDETAAVSTIAAAAPHYLESWGDVE--IT-KGTYSLTQP-TIRPIFDTKQFQDVLLSVNGT  target    LAELTGDTRFNDMWKFVREGRTDVYLQRILDASTNTKGYRFTELEAKAREGIPALMNSRTSPKVVGYDQLADSTPWYTKS 6btm.1    P-------------------------------------------------------------------------------  target    GRLEFYREEDEFIEAGENLPVHREPVDSTFYEPNVIVSPKHEAVRPSGPEDYGVARTDLSCEVRCGRNVVLTWAETRQTQ 6btm.1    --------------------------------------------------------------------------------  target    HPLVKQGHKFIFHTPKYRHGSHTTPIDTDMNAVLFGPFGDIYRRDKRSPFVTEGYVDINPTDGLELGLQDGDYVWIDPDP 6btm.1    --------------------------------------------------------------------------------  target    EDRPFRGWQKNAKDMEFARLLCRARFYPGTPRGVTRMWFNMYGATPGSVRGAKARRDGLAKNPDTNYQAMFRSGSHQSAT 6btm.1    --------------------------------------------------------------------------------  target    RGWLKPTWMTDSLVRKGLFGQGIGKGFLPDVHCPTGAPREAFVKISRAEPGGIGGQGLWRPAALGIRPRHESPAMKRYLA 6btm.1    --------------------------------------------------------------------------------  target    GGFFSGPKE 6btm.1    --------- ``` | | | | | | | | | | | | | | | | | | | | | | | | | | | | | | | | | | | | | | | | | | | | | | | | | |
|  | 8e9g.1.G | NADH-quinone oxidoreductase subunit G  *Mycobacterial respiratory complex I with both quinone positions modelled* | 0.07 |  | 16.67 | 0.17 | 59-163 | EM | 0.00 | hetero-1-1-1-1-1-1-… |  | HHblits | 0.26 |
| ``` target    FNGAPQYINENPFDLELDASRPARPRQYWRAESAHFYNHEDHPLRVGTRLLTGSTHMPTPTKVMWFANANSILGNVKWHY 8e9g.1    ----------------------------------------------------------GHLAALLVGG-VELG--DLPDP  target    NTVVNALPRMEMIAVHEWWWTGSCEWADVVFGVDSWGELKHPDMTASVTNPFLIVFPKTPIKRIFNTVGDIDVLALVSSK 8e9g.1    ELAVAAVRTTPFVVSLELRESAVTELADVVFPVAPVVEKA--GSFLNW-EGRPRPFAP-SL--KTNAIPDLRVLHYLADE  target    LAELTGDTRFNDMWKFVREGRTDVYLQRILDASTNTKGYRFTELEAKAREGIPALMNSRTSPKVVGYDQLADSTPWYTKS 8e9g.1    IGV-----------------------------------------------------------------------------  target    GRLEFYREEDEFIEAGENLPVHREPVDSTFYEPNVIVSPKHEAVRPSGPEDYGVARTDLSCEVRCGRNVVLTWAETRQTQ 8e9g.1    --------------------------------------------------------------------------------  target    HPLVKQGHKFIFHTPKYRHGSHTTPIDTDMNAVLFGPFGDIYRRDKRSPFVTEGYVDINPTDGLELGLQDGDYVWIDPDP 8e9g.1    --------------------------------------------------------------------------------  target    EDRPFRGWQKNAKDMEFARLLCRARFYPGTPRGVTRMWFNMYGATPGSVRGAKARRDGLAKNPDTNYQAMFRSGSHQSAT 8e9g.1    --------------------------------------------------------------------------------  target    RGWLKPTWMTDSLVRKGLFGQGIGKGFLPDVHCPTGAPREAFVKISRAEPGGIGGQGLWRPAALGIRPRHESPAMKRYLA 8e9g.1    --------------------------------------------------------------------------------  target    GGFFSGPKE 8e9g.1    --------- ``` | | | | | | | | | | | | | | | | | | | | | | | | | | | | | | | | | | | | | | | | | | | | | | | | | |
|  | 5t5i.1.B | Tungsten formylmethanofuran dehydrogenase subunit B  *TUNGSTEN-CONTAINING FORMYLMETHANOFURAN DEHYDROGENASE FROM METHANOTHERMOBACTER WOLFEII, ORTHORHOMBIC FORM AT 1.9 A* | 0.06 |  | 13.68 | 0.17 | 58-163 | X-ray | 1.90 | hetero-oligomer | 4 x ZN, 2 x MG, 18 x K, 22 x SF4, 2 x W, 4 x MGD, 2 x H2S, 2 x CA | HHblits | 0.27 |
| ``` target    FNGAPQYINENPFDLELDASRPARPRQYWRAESAHFYNHEDHPLRVGTRLLTGSTHMPTPTKVMWFANANSILGNVKWHY 5t5i.1    ---------------------------------------------------------NREADAMMVIASDPGAH--FP--  target    NTVVNALPRMEMIAVHEWWWTGSCEWADVVFGVDS-WGELKHPDMTASVTNPFLIVFPKTPIKRIFNTVGDIDVLALVSS 5t5i.1    QRALERMAEIP-VIAIEPHRTPTTEMADIIIPPAIVGMEAEGTAY---RMEGVPIRMKK-VVDS--DLLSDREILERLLE  target    KLAELTGDTRFNDMWKFVREGRTDVYLQRILDASTNTKGYRFTELEAKAREGIPALMNSRTSPKVVGYDQLADSTPWYTK 5t5i.1    KVRE----------------------------------------------------------------------------  target    SGRLEFYREEDEFIEAGENLPVHREPVDSTFYEPNVIVSPKHEAVRPSGPEDYGVARTDLSCEVRCGRNVVLTWAETRQT 5t5i.1    --------------------------------------------------------------------------------  target    QHPLVKQGHKFIFHTPKYRHGSHTTPIDTDMNAVLFGPFGDIYRRDKRSPFVTEGYVDINPTDGLELGLQDGDYVWIDPD 5t5i.1    --------------------------------------------------------------------------------  target    PEDRPFRGWQKNAKDMEFARLLCRARFYPGTPRGVTRMWFNMYGATPGSVRGAKARRDGLAKNPDTNYQAMFRSGSHQSA 5t5i.1    --------------------------------------------------------------------------------  target    TRGWLKPTWMTDSLVRKGLFGQGIGKGFLPDVHCPTGAPREAFVKISRAEPGGIGGQGLWRPAALGIRPRHESPAMKRYL 5t5i.1    --------------------------------------------------------------------------------  target    AGGFFSGPKE 5t5i.1    ---------- ``` | | | | | | | | | | | | | | | | | | | | | | | | | | | | | | | | | | | | | | | | | | | | | | | | | |
|  | 7q5y.1.A | NADH dehydrogenase I chain G  *Structure of NADH:ubichinon oxidoreductase (complex I) of the hyperthermophilic eubacterium Aquifex aeolicus* | 0.06 |  | 11.34 | 0.17 | 60-167 | X-ray | 2.70 | hetero-1-1-1-1-1-1-… | 8 x SF4, 2 x FES, 1 x FMN | HHblits | 0.25 |
| ``` target    FNGAPQYINENPFDLELDASRPARPRQYWRAESAHFYNHEDHPLRVGTRLLTGSTHMPTPTKVMWFANANSILGNVKWHY 7q5y.1    -----------------------------------------------------------DIENLIIFGEDILEF--YED-  target    NTVVNALPRMEMIAVHEWWWTGSCEWADVVFGVDSWGELKHPDMTASVTNPFLIVFPKTPIKRIFNTVGDIDVLALVSSK 7q5y.1    KVFEELKEKLEHLVVVSPYEDGLSEYAHIKIPMSLMGENEGTYK---TFFGEVKG--K-KFLP--WAFDDLAFWKYLGEN  target    LAELTGDTRFNDMWKFVREGRTDVYLQRILDASTNTKGYRFTELEAKAREGIPALMNSRTSPKVVGYDQLADSTPWYTKS 7q5y.1    FKEEKGL-------------------------------------------------------------------------  target    GRLEFYREEDEFIEAGENLPVHREPVDSTFYEPNVIVSPKHEAVRPSGPEDYGVARTDLSCEVRCGRNVVLTWAETRQTQ 7q5y.1    --------------------------------------------------------------------------------  target    HPLVKQGHKFIFHTPKYRHGSHTTPIDTDMNAVLFGPFGDIYRRDKRSPFVTEGYVDINPTDGLELGLQDGDYVWIDPDP 7q5y.1    --------------------------------------------------------------------------------  target    EDRPFRGWQKNAKDMEFARLLCRARFYPGTPRGVTRMWFNMYGATPGSVRGAKARRDGLAKNPDTNYQAMFRSGSHQSAT 7q5y.1    --------------------------------------------------------------------------------  target    RGWLKPTWMTDSLVRKGLFGQGIGKGFLPDVHCPTGAPREAFVKISRAEPGGIGGQGLWRPAALGIRPRHESPAMKRYLA 7q5y.1    --------------------------------------------------------------------------------  target    GGFFSGPKE 7q5y.1    --------- ``` | | | | | | | | | | | | | | | | | | | | | | | | | | | | | | | | | | | | | | | | | | | | | | | | | |
|  | 7bkb.1.L | Formylmethanofuran dehydrogenase, subunit B  *Formate dehydrogenase - heterodisulfide reductase - formylmethanofuran dehydrogenase complex from Methanospirillum hungatei (hexameric, composite structure)* | 0.06 |  | 12.50 | 0.17 | 59-163 | EM | 0.00 | hetero-2-2-2-2-2-2-… | 48 x SF4, 4 x FAD, 2 x FES, 4 x 9S8, 4 x ZN, 2 x MO, 4 x MGD | HHblits | 0.25 |
| ``` target    FNGAPQYINENPFDLELDASRPARPRQYWRAESAHFYNHEDHPLRVGTRLLTGSTHMPTPTKVMWFANANSILGNVKWHY 7bkb.1    ----------------------------------------------------------DEVDMFINIGTDAAAH--FPIP  target    NTVVNALPRMEMIAVHEWWWTGSCEWADVVFGVDSW-GELKHPDMTASVTNPFLIVFPKTPIKRIFNTVGDIDVLALVSS 7bkb.1    --AVKQLKKHPW-VTIDPSINMASEISDLHIPVCICGVDVGGIVY--R-MDNVPIQFRK-VIEPPEGVMDDETLLNKIAD  target    KLAELTGDTRFNDMWKFVREGRTDVYLQRILDASTNTKGYRFTELEAKAREGIPALMNSRTSPKVVGYDQLADSTPWYTK 7bkb.1    RMEE----------------------------------------------------------------------------  target    SGRLEFYREEDEFIEAGENLPVHREPVDSTFYEPNVIVSPKHEAVRPSGPEDYGVARTDLSCEVRCGRNVVLTWAETRQT 7bkb.1    --------------------------------------------------------------------------------  target    QHPLVKQGHKFIFHTPKYRHGSHTTPIDTDMNAVLFGPFGDIYRRDKRSPFVTEGYVDINPTDGLELGLQDGDYVWIDPD 7bkb.1    --------------------------------------------------------------------------------  target    PEDRPFRGWQKNAKDMEFARLLCRARFYPGTPRGVTRMWFNMYGATPGSVRGAKARRDGLAKNPDTNYQAMFRSGSHQSA 7bkb.1    --------------------------------------------------------------------------------  target    TRGWLKPTWMTDSLVRKGLFGQGIGKGFLPDVHCPTGAPREAFVKISRAEPGGIGGQGLWRPAALGIRPRHESPAMKRYL 7bkb.1    --------------------------------------------------------------------------------  target    AGGFFSGPKE 7bkb.1    ---------- ``` | | | | | | | | | | | | | | | | | | | | | | | | | | | | | | | | | | | | | | | | | | | | | | | | | |
|  | 8b9z.1.G | NADH-ubiquinone oxidoreductase 75 kDa subunit, mitochondrial  *Drosophila melanogaster complex I in the Active state (Dm1)* | 0.05 |  | 14.89 | 0.17 | 58-163 | EM | 3.28 | hetero-1-1-1-1-1-1-… | 3 x PC1, 16 x 3PE, 6 x SF4, 4 x CDL, 2 x FES, 1 x FMN, 1 x UQ9, 1 x DGT, 1 x NDP, 1 x ZN, 2 x EHZ | HHblits | 0.26 |
| ``` target    FNGAPQYINENPFDLELDASRPARPRQYWRAESAHFYNHEDHPLRVGTRLLTGSTHMPTPTKVMWFANANSILGNVKWHY 8b9z.1    ---------------------------------------------------------KAQPKVLFLLNADAG--------  target    NTVVNALPRMEMIAVHEWWWTGSCEWADVVFGVDSWGELKHPDMTASVTNPFLIVFPKTPIKRIFNTVGDIDVLALVSSK 8b9z.1    KVTREQLPKDCFVVYIGSHGDNGASIADAVLPGAAYTEKQG--IYV-NTEGRPQQTLP-GVSPPGMAREDWKILRALSEV  target    LAELTGDTRFNDMWKFVREGRTDVYLQRILDASTNTKGYRFTELEAKAREGIPALMNSRTSPKVVGYDQLADSTPWYTKS 8b9z.1    VGK-----------------------------------------------------------------------------  target    GRLEFYREEDEFIEAGENLPVHREPVDSTFYEPNVIVSPKHEAVRPSGPEDYGVARTDLSCEVRCGRNVVLTWAETRQTQ 8b9z.1    --------------------------------------------------------------------------------  target    HPLVKQGHKFIFHTPKYRHGSHTTPIDTDMNAVLFGPFGDIYRRDKRSPFVTEGYVDINPTDGLELGLQDGDYVWIDPDP 8b9z.1    --------------------------------------------------------------------------------  target    EDRPFRGWQKNAKDMEFARLLCRARFYPGTPRGVTRMWFNMYGATPGSVRGAKARRDGLAKNPDTNYQAMFRSGSHQSAT 8b9z.1    --------------------------------------------------------------------------------  target    RGWLKPTWMTDSLVRKGLFGQGIGKGFLPDVHCPTGAPREAFVKISRAEPGGIGGQGLWRPAALGIRPRHESPAMKRYLA 8b9z.1    --------------------------------------------------------------------------------  target    GGFFSGPKE 8b9z.1    --------- ``` | | | | | | | | | | | | | | | | | | | | | | | | | | | | | | | | | | | | | | | | | | | | | | | | | |
|  | 8ba0.1.G | NADH-ubiquinone oxidoreductase 75 kDa subunit, mitochondrial  *Drosophila melanogaster complex I in the Twisted state (Dm2)* | 0.05 |  | 14.89 | 0.17 | 58-163 | EM | 3.68 | hetero-1-1-1-1-1-1-… | 6 x SF4, 6 x 3PE, 2 x FES, 1 x FMN, 2 x CDL, 1 x DGT, 1 x NDP, 1 x ZN, 2 x EHZ | HHblits | 0.26 |
| ``` target    FNGAPQYINENPFDLELDASRPARPRQYWRAESAHFYNHEDHPLRVGTRLLTGSTHMPTPTKVMWFANANSILGNVKWHY 8ba0.1    ---------------------------------------------------------KAQPKVLFLLNADAG--------  target    NTVVNALPRMEMIAVHEWWWTGSCEWADVVFGVDSWGELKHPDMTASVTNPFLIVFPKTPIKRIFNTVGDIDVLALVSSK 8ba0.1    KVTREQLPKDCFVVYIGSHGDNGASIADAVLPGAAYTEKQG--IYV-NTEGRPQQTLP-GVSPPGMAREDWKILRALSEV  target    LAELTGDTRFNDMWKFVREGRTDVYLQRILDASTNTKGYRFTELEAKAREGIPALMNSRTSPKVVGYDQLADSTPWYTKS 8ba0.1    VGK-----------------------------------------------------------------------------  target    GRLEFYREEDEFIEAGENLPVHREPVDSTFYEPNVIVSPKHEAVRPSGPEDYGVARTDLSCEVRCGRNVVLTWAETRQTQ 8ba0.1    --------------------------------------------------------------------------------  target    HPLVKQGHKFIFHTPKYRHGSHTTPIDTDMNAVLFGPFGDIYRRDKRSPFVTEGYVDINPTDGLELGLQDGDYVWIDPDP 8ba0.1    --------------------------------------------------------------------------------  target    EDRPFRGWQKNAKDMEFARLLCRARFYPGTPRGVTRMWFNMYGATPGSVRGAKARRDGLAKNPDTNYQAMFRSGSHQSAT 8ba0.1    --------------------------------------------------------------------------------  target    RGWLKPTWMTDSLVRKGLFGQGIGKGFLPDVHCPTGAPREAFVKISRAEPGGIGGQGLWRPAALGIRPRHESPAMKRYLA 8ba0.1    --------------------------------------------------------------------------------  target    GGFFSGPKE 8ba0.1    --------- ``` | | | | | | | | | | | | | | | | | | | | | | | | | | | | | | | | | | | | | | | | | | | | | | | | | |
|  | 7arc.1.F | 75 kDa  *Cryo-EM structure of Polytomella Complex-I (peripheral arm)* | 0.06 |  | 13.04 | 0.16 | 58-163 | EM | 0.00 | hetero-1-1-1-1-1-1-… | 6 x SF4, 2 x FES, 1 x FMN, 1 x NDP, 1 x ZN, 1 x 8Q1 | HHblits | 0.28 |
| ``` target    FNGAPQYINENPFDLELDASRPARPRQYWRAESAHFYNHEDHPLRVGTRLLTGSTHMPTPTKVMWFANANSILGNVKWHY 7arc.1    ---------------------------------------------------------PVPAKVVYLLGSDDFKD------  target    NTVVNALPRMEMIAVHEWWWTGSCEWADVVFGVDSWGELKHPDMTASVTNPFLIVFPKTPIKRIFNTVGDIDVLALVSSK 7arc.1    ----EEIPADAFVIYQGHHGDKGAARANVVLPGAAYTEKA--SLF-ANTEGRVQTTRT-AVPVLGDAREDWKIIRALSEV  target    LAELTGDTRFNDMWKFVREGRTDVYLQRILDASTNTKGYRFTELEAKAREGIPALMNSRTSPKVVGYDQLADSTPWYTKS 7arc.1    VGQ-----------------------------------------------------------------------------  target    GRLEFYREEDEFIEAGENLPVHREPVDSTFYEPNVIVSPKHEAVRPSGPEDYGVARTDLSCEVRCGRNVVLTWAETRQTQ 7arc.1    --------------------------------------------------------------------------------  target    HPLVKQGHKFIFHTPKYRHGSHTTPIDTDMNAVLFGPFGDIYRRDKRSPFVTEGYVDINPTDGLELGLQDGDYVWIDPDP 7arc.1    --------------------------------------------------------------------------------  target    EDRPFRGWQKNAKDMEFARLLCRARFYPGTPRGVTRMWFNMYGATPGSVRGAKARRDGLAKNPDTNYQAMFRSGSHQSAT 7arc.1    --------------------------------------------------------------------------------  target    RGWLKPTWMTDSLVRKGLFGQGIGKGFLPDVHCPTGAPREAFVKISRAEPGGIGGQGLWRPAALGIRPRHESPAMKRYLA 7arc.1    --------------------------------------------------------------------------------  target    GGFFSGPKE 7arc.1    --------- ``` | | | | | | | | | | | | | | | | | | | | | | | | | | | | | | | | | | | | | | | | | | | | | | | | | |
|  | 6yj4.1.G | Subunit NUAM of NADH:Ubiquinone Oxidoreductase (Complex I)  *Structure of Yarrowia lipolytica complex I at 2.7 A* | 0.05 | 0.00 | 17.39 | 0.16 | 58-163 | EM | 0.00 | monomer | 18 x 3PE, 6 x SF4, 5 x LMT, 8 x PLC, 2 x FES, 1 x FMN, 6 x CDL, 1 x NDP, 1 x ZN, 2 x EHZ | HHblits | 0.28 |
| ``` target    FNGAPQYINENPFDLELDASRPARPRQYWRAESAHFYNHEDHPLRVGTRLLTGSTHMPTPTKVMWFANANSILGNVKWHY 6yj4.1    ---------------------------------------------------------KTTPKMVWLLGADEVAA------  target    NTVVNALPRMEMIAVHEWWWTGSCEWADVVFGVDSWGELKHPDMTASVTNPFLIVFPKTPIKRIFNTVGDIDVLALVSSK 6yj4.1    ----SDIPADAFVVYQGHNGDVGAQFADVVLPGAAYTEKAG--TY-VNTEGRSQISRA-ATGPPGGAREDWKILRAVSEY  target    LAELTGDTRFNDMWKFVREGRTDVYLQRILDASTNTKGYRFTELEAKAREGIPALMNSRTSPKVVGYDQLADSTPWYTKS 6yj4.1    LGV-----------------------------------------------------------------------------  target    GRLEFYREEDEFIEAGENLPVHREPVDSTFYEPNVIVSPKHEAVRPSGPEDYGVARTDLSCEVRCGRNVVLTWAETRQTQ 6yj4.1    --------------------------------------------------------------------------------  target    HPLVKQGHKFIFHTPKYRHGSHTTPIDTDMNAVLFGPFGDIYRRDKRSPFVTEGYVDINPTDGLELGLQDGDYVWIDPDP 6yj4.1    --------------------------------------------------------------------------------  target    EDRPFRGWQKNAKDMEFARLLCRARFYPGTPRGVTRMWFNMYGATPGSVRGAKARRDGLAKNPDTNYQAMFRSGSHQSAT 6yj4.1    --------------------------------------------------------------------------------  target    RGWLKPTWMTDSLVRKGLFGQGIGKGFLPDVHCPTGAPREAFVKISRAEPGGIGGQGLWRPAALGIRPRHESPAMKRYLA 6yj4.1    --------------------------------------------------------------------------------  target    GGFFSGPKE 6yj4.1    --------- ``` | | | | | | | | | | | | | | | | | | | | | | | | | | | | | | | | | | | | | | | | | | | | | | | | | |
|  | 6rfs.1.A | Subunit NUAM of NADH:Ubiquinone Oxidoreductase (Complex I)  *Cryo-EM structure of a respiratory complex I mutant lacking NDUFS4* | 0.05 | 0.00 | 17.39 | 0.16 | 58-163 | EM | 4.04 | monomer | 6 x SF4, 2 x FES, 1 x FMN, 1 x NDP, 1 x ZN, 1 x ZMP | HHblits | 0.28 |
| ``` target    FNGAPQYINENPFDLELDASRPARPRQYWRAESAHFYNHEDHPLRVGTRLLTGSTHMPTPTKVMWFANANSILGNVKWHY 6rfs.1    ---------------------------------------------------------KTTPKMVWLLGADEVAA------  target    NTVVNALPRMEMIAVHEWWWTGSCEWADVVFGVDSWGELKHPDMTASVTNPFLIVFPKTPIKRIFNTVGDIDVLALVSSK 6rfs.1    ----SDIPADAFVVYQGHNGDVGAQFADVVLPGAAYTEKAG--TY-VNTEGRSQISRA-ATGPPGGAREDWKILRAVSEY  target    LAELTGDTRFNDMWKFVREGRTDVYLQRILDASTNTKGYRFTELEAKAREGIPALMNSRTSPKVVGYDQLADSTPWYTKS 6rfs.1    LGV-----------------------------------------------------------------------------  target    GRLEFYREEDEFIEAGENLPVHREPVDSTFYEPNVIVSPKHEAVRPSGPEDYGVARTDLSCEVRCGRNVVLTWAETRQTQ 6rfs.1    --------------------------------------------------------------------------------  target    HPLVKQGHKFIFHTPKYRHGSHTTPIDTDMNAVLFGPFGDIYRRDKRSPFVTEGYVDINPTDGLELGLQDGDYVWIDPDP 6rfs.1    --------------------------------------------------------------------------------  target    EDRPFRGWQKNAKDMEFARLLCRARFYPGTPRGVTRMWFNMYGATPGSVRGAKARRDGLAKNPDTNYQAMFRSGSHQSAT 6rfs.1    --------------------------------------------------------------------------------  target    RGWLKPTWMTDSLVRKGLFGQGIGKGFLPDVHCPTGAPREAFVKISRAEPGGIGGQGLWRPAALGIRPRHESPAMKRYLA 6rfs.1    --------------------------------------------------------------------------------  target    GGFFSGPKE 6rfs.1    --------- ``` | | | | | | | | | | | | | | | | | | | | | | | | | | | | | | | | | | | | | | | | | | | | | | | | | |
|  | 6rfq.1.A | Subunit NUAM of NADH:Ubiquinone Oxidoreductase (Complex I)  *Cryo-EM structure of a respiratory complex I assembly intermediate with NDUFAF2* | 0.05 | 0.00 | 17.39 | 0.16 | 58-163 | EM | 3.30 | monomer | 6 x SF4, 2 x FES, 1 x FMN, 1 x NDP, 10 x 3PE, 2 x LMN, 4 x CDL, 2 x ZMP, 4 x PLC, 3 x T7X, 1 x CPL | HHblits | 0.28 |
| ``` target    FNGAPQYINENPFDLELDASRPARPRQYWRAESAHFYNHEDHPLRVGTRLLTGSTHMPTPTKVMWFANANSILGNVKWHY 6rfq.1    ---------------------------------------------------------KTTPKMVWLLGADEVAA------  target    NTVVNALPRMEMIAVHEWWWTGSCEWADVVFGVDSWGELKHPDMTASVTNPFLIVFPKTPIKRIFNTVGDIDVLALVSSK 6rfq.1    ----SDIPADAFVVYQGHNGDVGAQFADVVLPGAAYTEKAG--TY-VNTEGRSQISRA-ATGPPGGAREDWKILRAVSEY  target    LAELTGDTRFNDMWKFVREGRTDVYLQRILDASTNTKGYRFTELEAKAREGIPALMNSRTSPKVVGYDQLADSTPWYTKS 6rfq.1    LGV-----------------------------------------------------------------------------  target    GRLEFYREEDEFIEAGENLPVHREPVDSTFYEPNVIVSPKHEAVRPSGPEDYGVARTDLSCEVRCGRNVVLTWAETRQTQ 6rfq.1    --------------------------------------------------------------------------------  target    HPLVKQGHKFIFHTPKYRHGSHTTPIDTDMNAVLFGPFGDIYRRDKRSPFVTEGYVDINPTDGLELGLQDGDYVWIDPDP 6rfq.1    --------------------------------------------------------------------------------  target    EDRPFRGWQKNAKDMEFARLLCRARFYPGTPRGVTRMWFNMYGATPGSVRGAKARRDGLAKNPDTNYQAMFRSGSHQSAT 6rfq.1    --------------------------------------------------------------------------------  target    RGWLKPTWMTDSLVRKGLFGQGIGKGFLPDVHCPTGAPREAFVKISRAEPGGIGGQGLWRPAALGIRPRHESPAMKRYLA 6rfq.1    --------------------------------------------------------------------------------  target    GGFFSGPKE 6rfq.1    --------- ``` | | | | | | | | | | | | | | | | | | | | | | | | | | | | | | | | | | | | | | | | | | | | | | | | | |
|  | 6gcs.1.A | 75-KDA PROTEIN (NUAM)  *Cryo-EM structure of respiratory complex I from Yarrowia lipolytica* | 0.06 | 0.00 | 17.39 | 0.16 | 58-163 | EM | 4.32 | monomer | 6 x SF4, 2 x FES, 1 x FMN, 1 x NDP, 1 x ZN, 1 x ZMP, 1 x CDL, 3 x 3PE | HHblits | 0.28 |
| ``` target    FNGAPQYINENPFDLELDASRPARPRQYWRAESAHFYNHEDHPLRVGTRLLTGSTHMPTPTKVMWFANANSILGNVKWHY 6gcs.1    ---------------------------------------------------------KTTPKMVWLLGADEVAA------  target    NTVVNALPRMEMIAVHEWWWTGSCEWADVVFGVDSWGELKHPDMTASVTNPFLIVFPKTPIKRIFNTVGDIDVLALVSSK 6gcs.1    ----SDIPADAFVVYQGHNGDVGAQFADVVLPGAAYTEKAG--TY-VNTEGRSQISRA-ATGPPGGAREDWKILRAVSEY  target    LAELTGDTRFNDMWKFVREGRTDVYLQRILDASTNTKGYRFTELEAKAREGIPALMNSRTSPKVVGYDQLADSTPWYTKS 6gcs.1    LGV-----------------------------------------------------------------------------  target    GRLEFYREEDEFIEAGENLPVHREPVDSTFYEPNVIVSPKHEAVRPSGPEDYGVARTDLSCEVRCGRNVVLTWAETRQTQ 6gcs.1    --------------------------------------------------------------------------------  target    HPLVKQGHKFIFHTPKYRHGSHTTPIDTDMNAVLFGPFGDIYRRDKRSPFVTEGYVDINPTDGLELGLQDGDYVWIDPDP 6gcs.1    --------------------------------------------------------------------------------  target    EDRPFRGWQKNAKDMEFARLLCRARFYPGTPRGVTRMWFNMYGATPGSVRGAKARRDGLAKNPDTNYQAMFRSGSHQSAT 6gcs.1    --------------------------------------------------------------------------------  target    RGWLKPTWMTDSLVRKGLFGQGIGKGFLPDVHCPTGAPREAFVKISRAEPGGIGGQGLWRPAALGIRPRHESPAMKRYLA 6gcs.1    --------------------------------------------------------------------------------  target    GGFFSGPKE 6gcs.1    --------- ``` | | | | | | | | | | | | | | | | | | | | | | | | | | | | | | | | | | | | | | | | | | | | | | | | | |
|  | 7t2r.1.A | NiFe hydrogenase subunit A  *Structure of electron bifurcating Ni-Fe hydrogenase complex HydABCSL in FMN-free apo state* | 0.05 |  | 9.47 | 0.17 | 60-163 | EM | 0.00 | hetero-2-2-2-2-2-mer | 6 x FES, 12 x SF4, 2 x 3NI, 2 x FCO | HHblits | 0.25 |
| ``` target    FNGAPQYINENPFDLELDASRPARPRQYWRAESAHFYNHEDHPLRVGTRLLTGSTHMPTPTKVMWFANANSILGNVKWHY 7t2r.1    -----------------------------------------------------------RRDFLYVFSTAMV-----PEE  target    NTVVNALPRMEMIAVHEWWWT-GSCEWADVVFGVDSWGELKHPDMTASVTNPFLIVFPKTPIKRIFNTVGDIDVLALVSS 7t2r.1    EEILAAISATRFVVVQTPFKVRPLVNLADILLPAPAWYERSG--HFC-TIEGERRKLNT-IVPPKGEIKSLHYVMDEFAK  target    KLAELTGDTRFNDMWKFVREGRTDVYLQRILDASTNTKGYRFTELEAKAREGIPALMNSRTSPKVVGYDQLADSTPWYTK 7t2r.1    KLGV----------------------------------------------------------------------------  target    SGRLEFYREEDEFIEAGENLPVHREPVDSTFYEPNVIVSPKHEAVRPSGPEDYGVARTDLSCEVRCGRNVVLTWAETRQT 7t2r.1    --------------------------------------------------------------------------------  target    QHPLVKQGHKFIFHTPKYRHGSHTTPIDTDMNAVLFGPFGDIYRRDKRSPFVTEGYVDINPTDGLELGLQDGDYVWIDPD 7t2r.1    --------------------------------------------------------------------------------  target    PEDRPFRGWQKNAKDMEFARLLCRARFYPGTPRGVTRMWFNMYGATPGSVRGAKARRDGLAKNPDTNYQAMFRSGSHQSA 7t2r.1    --------------------------------------------------------------------------------  target    TRGWLKPTWMTDSLVRKGLFGQGIGKGFLPDVHCPTGAPREAFVKISRAEPGGIGGQGLWRPAALGIRPRHESPAMKRYL 7t2r.1    --------------------------------------------------------------------------------  target    AGGFFSGPKE 7t2r.1    ---------- ``` | | | | | | | | | | | | | | | | | | | | | | | | | | | | | | | | | | | | | | | | | | | | | | | | | |
|  | 7t30.1.A | NiFe hydrogenase subunit A  *Structure of electron bifurcating Ni-Fe hydrogenase complex HydABCSL in FMN/NAD(H) bound state* | 0.05 |  | 9.47 | 0.17 | 60-163 | EM | 0.00 | hetero-2-2-2-2-2-mer | 4 x FES, 12 x SF4, 2 x NAD, 2 x FMN, 2 x 3NI, 2 x FCO | HHblits | 0.25 |
| ``` target    FNGAPQYINENPFDLELDASRPARPRQYWRAESAHFYNHEDHPLRVGTRLLTGSTHMPTPTKVMWFANANSILGNVKWHY 7t30.1    -----------------------------------------------------------RRDFLYVFSTAMV-----PEE  target    NTVVNALPRMEMIAVHEWWWT-GSCEWADVVFGVDSWGELKHPDMTASVTNPFLIVFPKTPIKRIFNTVGDIDVLALVSS 7t30.1    EEILAAISATRFVVVQTPFKVRPLVNLADILLPAPAWYERSG--HFC-TIEGERRKLNT-IVPPKGEIKSLHYVMDEFAK  target    KLAELTGDTRFNDMWKFVREGRTDVYLQRILDASTNTKGYRFTELEAKAREGIPALMNSRTSPKVVGYDQLADSTPWYTK 7t30.1    KLGV----------------------------------------------------------------------------  target    SGRLEFYREEDEFIEAGENLPVHREPVDSTFYEPNVIVSPKHEAVRPSGPEDYGVARTDLSCEVRCGRNVVLTWAETRQT 7t30.1    --------------------------------------------------------------------------------  target    QHPLVKQGHKFIFHTPKYRHGSHTTPIDTDMNAVLFGPFGDIYRRDKRSPFVTEGYVDINPTDGLELGLQDGDYVWIDPD 7t30.1    --------------------------------------------------------------------------------  target    PEDRPFRGWQKNAKDMEFARLLCRARFYPGTPRGVTRMWFNMYGATPGSVRGAKARRDGLAKNPDTNYQAMFRSGSHQSA 7t30.1    --------------------------------------------------------------------------------  target    TRGWLKPTWMTDSLVRKGLFGQGIGKGFLPDVHCPTGAPREAFVKISRAEPGGIGGQGLWRPAALGIRPRHESPAMKRYL 7t30.1    --------------------------------------------------------------------------------  target    AGGFFSGPKE 7t30.1    ---------- ``` | | | | | | | | | | | | | | | | | | | | | | | | | | | | | | | | | | | | | | | | | | | | | | | | | |
|  | 7zm7.1.I | NADH-ubiquinone oxidoreductase-like protein  *CryoEM structure of mitochondrial complex I from Chaetomium thermophilum (inhibited by DDM)* | 0.06 |  | 17.39 | 0.16 | 58-163 | EM | 0.00 | hetero-1-1-1-1-1-1-… | 4 x PC1, 14 x LMT, 5 x CDL, 8 x 3PE, 2 x FES, 6 x SF4, 1 x FMN, 1 x NDP, 1 x ZN, 2 x ZMP | HHblits | 0.27 |
| ``` target    FNGAPQYINENPFDLELDASRPARPRQYWRAESAHFYNHEDHPLRVGTRLLTGSTHMPTPTKVMWFANANSILGNVKWHY 7zm7.1    ---------------------------------------------------------ATKPKFVWLLGADEFD---P---  target    NTVVNALPRMEMIAVHEWWWTGSCEWADVVFGVDSWGELKHPDMTASVTNPFLIVFPKTPIKRIFNTVGDIDVLALVSSK 7zm7.1    ----ADVPKDAFIVYQGHHGDRGAEIADIVLPGAAYTEKAG--TY-VNTEGRVQMTRA-ATGLPGAARTDWKIIRAVSEF  target    LAELTGDTRFNDMWKFVREGRTDVYLQRILDASTNTKGYRFTELEAKAREGIPALMNSRTSPKVVGYDQLADSTPWYTKS 7zm7.1    LGV-----------------------------------------------------------------------------  target    GRLEFYREEDEFIEAGENLPVHREPVDSTFYEPNVIVSPKHEAVRPSGPEDYGVARTDLSCEVRCGRNVVLTWAETRQTQ 7zm7.1    --------------------------------------------------------------------------------  target    HPLVKQGHKFIFHTPKYRHGSHTTPIDTDMNAVLFGPFGDIYRRDKRSPFVTEGYVDINPTDGLELGLQDGDYVWIDPDP 7zm7.1    --------------------------------------------------------------------------------  target    EDRPFRGWQKNAKDMEFARLLCRARFYPGTPRGVTRMWFNMYGATPGSVRGAKARRDGLAKNPDTNYQAMFRSGSHQSAT 7zm7.1    --------------------------------------------------------------------------------  target    RGWLKPTWMTDSLVRKGLFGQGIGKGFLPDVHCPTGAPREAFVKISRAEPGGIGGQGLWRPAALGIRPRHESPAMKRYLA 7zm7.1    --------------------------------------------------------------------------------  target    GGFFSGPKE 7zm7.1    --------- ``` | | | | | | | | | | | | | | | | | | | | | | | | | | | | | | | | | | | | | | | | | | | | | | | | | |
|  | 7tgh.58.A | NADH-ubiquinone oxidoreductase 75 kDa subunit  *Cryo-EM structure of respiratory super-complex CI+III2 from Tetrahymena thermophila* | 0.06 |  | 10.75 | 0.16 | 58-163 | EM | 0.00 | monomer |  | HHblits | 0.26 |
| ``` target    FNGAPQYINENPFDLELDASRPARPRQYWRAESAHFYNHEDHPLRVGTRLLTGSTHMPTPTKVMWFANANSILGNVKWHY 7tgh.58   ---------------------------------------------------------NKNAKLVFILGADNNLR------  target    NTVVNALPRMEMIAVHEWWWTGSCEWADVVFGVDSWGELKHPDMTASVTNPFLIVFPKTPIKRIFNTVGDIDVLALVSSK 7tgh.58   ---PEDIPADAFVVYFGTHGDEGAYYADIILPTAAYTEKN--ATWV-NTEGRVQQGRL-VVMPPGDAREDWQIIRALSEE  target    LAELTGDTRFNDMWKFVREGRTDVYLQRILDASTNTKGYRFTELEAKAREGIPALMNSRTSPKVVGYDQLADSTPWYTKS 7tgh.58   AGV-----------------------------------------------------------------------------  target    GRLEFYREEDEFIEAGENLPVHREPVDSTFYEPNVIVSPKHEAVRPSGPEDYGVARTDLSCEVRCGRNVVLTWAETRQTQ 7tgh.58   --------------------------------------------------------------------------------  target    HPLVKQGHKFIFHTPKYRHGSHTTPIDTDMNAVLFGPFGDIYRRDKRSPFVTEGYVDINPTDGLELGLQDGDYVWIDPDP 7tgh.58   --------------------------------------------------------------------------------  target    EDRPFRGWQKNAKDMEFARLLCRARFYPGTPRGVTRMWFNMYGATPGSVRGAKARRDGLAKNPDTNYQAMFRSGSHQSAT 7tgh.58   --------------------------------------------------------------------------------  target    RGWLKPTWMTDSLVRKGLFGQGIGKGFLPDVHCPTGAPREAFVKISRAEPGGIGGQGLWRPAALGIRPRHESPAMKRYLA 7tgh.58   --------------------------------------------------------------------------------  target    GGFFSGPKE 7tgh.58   --------- ``` | | | | | | | | | | | | | | | | | | | | | | | | | | | | | | | | | | | | | | | | | | | | | | | | | |
|  | 7ar7.1.G | NADH dehydrogenase [ubiquinone] iron-sulfur protein 1, mitochondrial  *Cryo-EM structure of Arabidopsis thaliana complex-I (open conformation)* | 0.05 | 0.00 | 12.09 | 0.16 | 59-163 | EM | 0.00 | monomer | 6 x SF4, 2 x FES, 1 x FMN, 1 x UQ9, 3 x PTY, 2 x PC7, 1 x LMN, 1 x NDP, 2 x ZN, 2 x 8Q1, 1 x PGT, 1 x PSF, 1 x T7X | HHblits | 0.27 |
| ``` target    FNGAPQYINENPFDLELDASRPARPRQYWRAESAHFYNHEDHPLRVGTRLLTGSTHMPTPTKVMWFANANSILGNVKWHY 7ar7.1    ----------------------------------------------------------ESAKFVYLMGADDVN-------  target    NTVVNALPRMEMIAVHEWWWTGSCEWADVVFGVDSWGELKHPDMTASVTNPFLIVFPKTPIKRIFNTVGDIDVLALVSSK 7ar7.1    ---VDKIPKDAFVVYQGHHGDKAVYRANVILPASAFTEKEG--TY-ENTEGFTQQTVP-AVPTVGDARDDWKIVRALSEV  target    LAELTGDTRFNDMWKFVREGRTDVYLQRILDASTNTKGYRFTELEAKAREGIPALMNSRTSPKVVGYDQLADSTPWYTKS 7ar7.1    SGV-----------------------------------------------------------------------------  target    GRLEFYREEDEFIEAGENLPVHREPVDSTFYEPNVIVSPKHEAVRPSGPEDYGVARTDLSCEVRCGRNVVLTWAETRQTQ 7ar7.1    --------------------------------------------------------------------------------  target    HPLVKQGHKFIFHTPKYRHGSHTTPIDTDMNAVLFGPFGDIYRRDKRSPFVTEGYVDINPTDGLELGLQDGDYVWIDPDP 7ar7.1    --------------------------------------------------------------------------------  target    EDRPFRGWQKNAKDMEFARLLCRARFYPGTPRGVTRMWFNMYGATPGSVRGAKARRDGLAKNPDTNYQAMFRSGSHQSAT 7ar7.1    --------------------------------------------------------------------------------  target    RGWLKPTWMTDSLVRKGLFGQGIGKGFLPDVHCPTGAPREAFVKISRAEPGGIGGQGLWRPAALGIRPRHESPAMKRYLA 7ar7.1    --------------------------------------------------------------------------------  target    GGFFSGPKE 7ar7.1    --------- ``` | | | | | | | | | | | | | | | | | | | | | | | | | | | | | | | | | | | | | | | | | | | | | | | | | |
|  | 7aqr.1.F | NADH dehydrogenase [ubiquinone] iron-sulfur protein 1, mitochondrial  *Cryo-EM structure of Arabidopsis thaliana Complex-I (peripheral arm)* | 0.06 | 0.00 | 12.09 | 0.16 | 59-163 | EM | 0.00 | monomer | 6 x SF4, 2 x FES, 1 x FMN, 1 x NDP, 1 x ZN, 1 x 8Q1 | HHblits | 0.27 |
| ``` target    FNGAPQYINENPFDLELDASRPARPRQYWRAESAHFYNHEDHPLRVGTRLLTGSTHMPTPTKVMWFANANSILGNVKWHY 7aqr.1    ----------------------------------------------------------ESAKFVYLMGADDVN-------  target    NTVVNALPRMEMIAVHEWWWTGSCEWADVVFGVDSWGELKHPDMTASVTNPFLIVFPKTPIKRIFNTVGDIDVLALVSSK 7aqr.1    ---VDKIPKDAFVVYQGHHGDKAVYRANVILPASAFTEKEG--TY-ENTEGFTQQTVP-AVPTVGDARDDWKIVRALSEV  target    LAELTGDTRFNDMWKFVREGRTDVYLQRILDASTNTKGYRFTELEAKAREGIPALMNSRTSPKVVGYDQLADSTPWYTKS 7aqr.1    SGV-----------------------------------------------------------------------------  target    GRLEFYREEDEFIEAGENLPVHREPVDSTFYEPNVIVSPKHEAVRPSGPEDYGVARTDLSCEVRCGRNVVLTWAETRQTQ 7aqr.1    --------------------------------------------------------------------------------  target    HPLVKQGHKFIFHTPKYRHGSHTTPIDTDMNAVLFGPFGDIYRRDKRSPFVTEGYVDINPTDGLELGLQDGDYVWIDPDP 7aqr.1    --------------------------------------------------------------------------------  target    EDRPFRGWQKNAKDMEFARLLCRARFYPGTPRGVTRMWFNMYGATPGSVRGAKARRDGLAKNPDTNYQAMFRSGSHQSAT 7aqr.1    --------------------------------------------------------------------------------  target    RGWLKPTWMTDSLVRKGLFGQGIGKGFLPDVHCPTGAPREAFVKISRAEPGGIGGQGLWRPAALGIRPRHESPAMKRYLA 7aqr.1    --------------------------------------------------------------------------------  target    GGFFSGPKE 7aqr.1    --------- ``` | | | | | | | | | | | | | | | | | | | | | | | | | | | | | | | | | | | | | | | | | | | | | | | | | |
|  | 7a23.1.O | 75kDa  *Plant mitochondrial respiratory complex I* | 0.05 | 0.00 | 12.09 | 0.16 | 59-163 | EM | 0.00 | monomer | 6 x SF4, 1 x FMN, 2 x T7X, 3 x CDL, 1 x U10, 1 x PEV, 2 x FES, 1 x NDP, 2 x ZN | HHblits | 0.27 |
| ``` target    FNGAPQYINENPFDLELDASRPARPRQYWRAESAHFYNHEDHPLRVGTRLLTGSTHMPTPTKVMWFANANSILGNVKWHY 7a23.1    ----------------------------------------------------------ESAKFVYLMGADDVN-------  target    NTVVNALPRMEMIAVHEWWWTGSCEWADVVFGVDSWGELKHPDMTASVTNPFLIVFPKTPIKRIFNTVGDIDVLALVSSK 7a23.1    ---VDKIPKDAFVVYQGHHGDKAVYRANVILPASAFTEKEG--TY-ENTEGFTQQTVP-AVPTVGDARDDWKIVRALSEV  target    LAELTGDTRFNDMWKFVREGRTDVYLQRILDASTNTKGYRFTELEAKAREGIPALMNSRTSPKVVGYDQLADSTPWYTKS 7a23.1    SGV-----------------------------------------------------------------------------  target    GRLEFYREEDEFIEAGENLPVHREPVDSTFYEPNVIVSPKHEAVRPSGPEDYGVARTDLSCEVRCGRNVVLTWAETRQTQ 7a23.1    --------------------------------------------------------------------------------  target    HPLVKQGHKFIFHTPKYRHGSHTTPIDTDMNAVLFGPFGDIYRRDKRSPFVTEGYVDINPTDGLELGLQDGDYVWIDPDP 7a23.1    --------------------------------------------------------------------------------  target    EDRPFRGWQKNAKDMEFARLLCRARFYPGTPRGVTRMWFNMYGATPGSVRGAKARRDGLAKNPDTNYQAMFRSGSHQSAT 7a23.1    --------------------------------------------------------------------------------  target    RGWLKPTWMTDSLVRKGLFGQGIGKGFLPDVHCPTGAPREAFVKISRAEPGGIGGQGLWRPAALGIRPRHESPAMKRYLA 7a23.1    --------------------------------------------------------------------------------  target    GGFFSGPKE 7a23.1    --------- ``` | | | | | | | | | | | | | | | | | | | | | | | | | | | | | | | | | | | | | | | | | | | | | | | | | |
|  | 7ar8.1.G | NADH dehydrogenase [ubiquinone] iron-sulfur protein 1, mitochondrial  *Cryo-EM structure of Arabidopsis thaliana complex-I (closed conformation)* | 0.05 | 0.00 | 12.09 | 0.16 | 59-163 | EM | 0.00 | monomer | 6 x SF4, 2 x FES, 1 x FMN, 1 x UQ9, 3 x PTY, 2 x PC7, 1 x PGT, 1 x FE, 1 x NDP, 2 x ZN, 2 x 8Q1, 1 x LMN, 1 x PSF, 1 x T7X | HHblits | 0.27 |
| ``` target    FNGAPQYINENPFDLELDASRPARPRQYWRAESAHFYNHEDHPLRVGTRLLTGSTHMPTPTKVMWFANANSILGNVKWHY 7ar8.1    ----------------------------------------------------------ESAKFVYLMGADDVN-------  target    NTVVNALPRMEMIAVHEWWWTGSCEWADVVFGVDSWGELKHPDMTASVTNPFLIVFPKTPIKRIFNTVGDIDVLALVSSK 7ar8.1    ---VDKIPKDAFVVYQGHHGDKAVYRANVILPASAFTEKEG--TY-ENTEGFTQQTVP-AVPTVGDARDDWKIVRALSEV  target    LAELTGDTRFNDMWKFVREGRTDVYLQRILDASTNTKGYRFTELEAKAREGIPALMNSRTSPKVVGYDQLADSTPWYTKS 7ar8.1    SGV-----------------------------------------------------------------------------  target    GRLEFYREEDEFIEAGENLPVHREPVDSTFYEPNVIVSPKHEAVRPSGPEDYGVARTDLSCEVRCGRNVVLTWAETRQTQ 7ar8.1    --------------------------------------------------------------------------------  target    HPLVKQGHKFIFHTPKYRHGSHTTPIDTDMNAVLFGPFGDIYRRDKRSPFVTEGYVDINPTDGLELGLQDGDYVWIDPDP 7ar8.1    --------------------------------------------------------------------------------  target    EDRPFRGWQKNAKDMEFARLLCRARFYPGTPRGVTRMWFNMYGATPGSVRGAKARRDGLAKNPDTNYQAMFRSGSHQSAT 7ar8.1    --------------------------------------------------------------------------------  target    RGWLKPTWMTDSLVRKGLFGQGIGKGFLPDVHCPTGAPREAFVKISRAEPGGIGGQGLWRPAALGIRPRHESPAMKRYLA 7ar8.1    --------------------------------------------------------------------------------  target    GGFFSGPKE 7ar8.1    --------- ``` | | | | | | | | | | | | | | | | | | | | | | | | | | | | | | | | | | | | | | | | | | | | | | | | | |
|  | 5gpn.24.A | NADH-ubiquinone oxidoreductase 75 kDa subunit  *Architecture of mammalian respirasome* | 0.05 | 0.00 | 15.05 | 0.16 | 59-163 | EM | 0.00 | monomer |  | HHblits | 0.26 |
| ``` target    FNGAPQYINENPFDLELDASRPARPRQYWRAESAHFYNHEDHPLRVGTRLLTGSTHMPTPTKVMWFANANSILGNVKWHY 5gpn.24   ----------------------------------------------------------NPPKVLFLLGADGG--------  target    NTVVNALPRMEMIAVHEWWWTGSCEWADVVFGVDSWGELKHPDMTASVTNPFLIVFPKTPIKRIFNTVGDIDVLALVSSK 5gpn.24   CITRQDLPKDCFIIYQGHHGDVGAPMADVILPGAAYTEKSA--TY-VNTEGRAQQTKV-AVTPPGLAREDWKIIRALSEI  target    LAELTGDTRFNDMWKFVREGRTDVYLQRILDASTNTKGYRFTELEAKAREGIPALMNSRTSPKVVGYDQLADSTPWYTKS 5gpn.24   AGM-----------------------------------------------------------------------------  target    GRLEFYREEDEFIEAGENLPVHREPVDSTFYEPNVIVSPKHEAVRPSGPEDYGVARTDLSCEVRCGRNVVLTWAETRQTQ 5gpn.24   --------------------------------------------------------------------------------  target    HPLVKQGHKFIFHTPKYRHGSHTTPIDTDMNAVLFGPFGDIYRRDKRSPFVTEGYVDINPTDGLELGLQDGDYVWIDPDP 5gpn.24   --------------------------------------------------------------------------------  target    EDRPFRGWQKNAKDMEFARLLCRARFYPGTPRGVTRMWFNMYGATPGSVRGAKARRDGLAKNPDTNYQAMFRSGSHQSAT 5gpn.24   --------------------------------------------------------------------------------  target    RGWLKPTWMTDSLVRKGLFGQGIGKGFLPDVHCPTGAPREAFVKISRAEPGGIGGQGLWRPAALGIRPRHESPAMKRYLA 5gpn.24   --------------------------------------------------------------------------------  target    GGFFSGPKE 5gpn.24   --------- ``` | | | | | | | | | | | | | | | | | | | | | | | | | | | | | | | | | | | | | | | | | | | | | | | | | |
|  | 7qsd.1.G | NADH-ubiquinone oxidoreductase 75 kDa subunit, mitochondrial  *Bovine complex I in the active state at 3.1 A* | 0.06 | 0.00 | 13.98 | 0.16 | 59-163 | EM | 0.00 | monomer | 5 x PC1, 13 x 3PE, 6 x SF4, 2 x FES, 1 x FMN, 4 x CDL, 3 x LMT, 1 x GTP, 1 x MG, 1 x NDP, 1 x ZN, 2 x EHZ | HHblits | 0.26 |
| ``` target    FNGAPQYINENPFDLELDASRPARPRQYWRAESAHFYNHEDHPLRVGTRLLTGSTHMPTPTKVMWFANANSILGNVKWHY 7qsd.1    ----------------------------------------------------------NPPKMLFLLGADGGC-------  target    NTVVNALPRMEMIAVHEWWWTGSCEWADVVFGVDSWGELKHPDMTASVTNPFLIVFPKTPIKRIFNTVGDIDVLALVSSK 7qsd.1    -ITRQDLPKDCFIVYQGHHGDVGAPIADVILPGAAYTEKSA--TYV-NTEGRAQQTKV-AVTPPGLAREDWKIIRALSEI  target    LAELTGDTRFNDMWKFVREGRTDVYLQRILDASTNTKGYRFTELEAKAREGIPALMNSRTSPKVVGYDQLADSTPWYTKS 7qsd.1    AGM-----------------------------------------------------------------------------  target    GRLEFYREEDEFIEAGENLPVHREPVDSTFYEPNVIVSPKHEAVRPSGPEDYGVARTDLSCEVRCGRNVVLTWAETRQTQ 7qsd.1    --------------------------------------------------------------------------------  target    HPLVKQGHKFIFHTPKYRHGSHTTPIDTDMNAVLFGPFGDIYRRDKRSPFVTEGYVDINPTDGLELGLQDGDYVWIDPDP 7qsd.1    --------------------------------------------------------------------------------  target    EDRPFRGWQKNAKDMEFARLLCRARFYPGTPRGVTRMWFNMYGATPGSVRGAKARRDGLAKNPDTNYQAMFRSGSHQSAT 7qsd.1    --------------------------------------------------------------------------------  target    RGWLKPTWMTDSLVRKGLFGQGIGKGFLPDVHCPTGAPREAFVKISRAEPGGIGGQGLWRPAALGIRPRHESPAMKRYLA 7qsd.1    --------------------------------------------------------------------------------  target    GGFFSGPKE 7qsd.1    --------- ``` | | | | | | | | | | | | | | | | | | | | | | | | | | | | | | | | | | | | | | | | | | | | | | | | | |
|  | 6qcf.1.C | NADH:ubiquinone oxidoreductase core subunit S1  *Ovine respiratory complex I FRC open class 6* | 0.05 | 0.00 | 13.98 | 0.16 | 59-163 | EM | 0.00 | monomer | 6 x SF4, 1 x FMN, 2 x FES, 1 x ZN, 1 x NDP, 2 x ZMP | HHblits | 0.26 |
| ``` target    FNGAPQYINENPFDLELDASRPARPRQYWRAESAHFYNHEDHPLRVGTRLLTGSTHMPTPTKVMWFANANSILGNVKWHY 6qcf.1    ----------------------------------------------------------NPPKMLFLLGADGGC-------  target    NTVVNALPRMEMIAVHEWWWTGSCEWADVVFGVDSWGELKHPDMTASVTNPFLIVFPKTPIKRIFNTVGDIDVLALVSSK 6qcf.1    -VTRQDLPKDCFIVYQGHHGDVGAPIADVILPGAAYTEKSA--TY-VNTEGRAQQTKV-AVMPPGLAREDWKIIRALSEI  target    LAELTGDTRFNDMWKFVREGRTDVYLQRILDASTNTKGYRFTELEAKAREGIPALMNSRTSPKVVGYDQLADSTPWYTKS 6qcf.1    AGM-----------------------------------------------------------------------------  target    GRLEFYREEDEFIEAGENLPVHREPVDSTFYEPNVIVSPKHEAVRPSGPEDYGVARTDLSCEVRCGRNVVLTWAETRQTQ 6qcf.1    --------------------------------------------------------------------------------  target    HPLVKQGHKFIFHTPKYRHGSHTTPIDTDMNAVLFGPFGDIYRRDKRSPFVTEGYVDINPTDGLELGLQDGDYVWIDPDP 6qcf.1    --------------------------------------------------------------------------------  target    EDRPFRGWQKNAKDMEFARLLCRARFYPGTPRGVTRMWFNMYGATPGSVRGAKARRDGLAKNPDTNYQAMFRSGSHQSAT 6qcf.1    --------------------------------------------------------------------------------  target    RGWLKPTWMTDSLVRKGLFGQGIGKGFLPDVHCPTGAPREAFVKISRAEPGGIGGQGLWRPAALGIRPRHESPAMKRYLA 6qcf.1    --------------------------------------------------------------------------------  target    GGFFSGPKE 6qcf.1    --------- ``` | | | | | | | | | | | | | | | | | | | | | | | | | | | | | | | | | | | | | | | | | | | | | | | | | |
|  | 6qc5.1.C | NADH:ubiquinone oxidoreductase core subunit S1  *Ovine respiratory complex I FRC closed class 1* | 0.05 | 0.00 | 13.98 | 0.16 | 59-163 | EM | 0.00 | monomer | 6 x SF4, 1 x FMN, 2 x FES, 2 x 3PE, 1 x ZN, 1 x NDP, 2 x ZMP, 1 x PC1 | HHblits | 0.26 |
| ``` target    FNGAPQYINENPFDLELDASRPARPRQYWRAESAHFYNHEDHPLRVGTRLLTGSTHMPTPTKVMWFANANSILGNVKWHY 6qc5.1    ----------------------------------------------------------NPPKMLFLLGADGGC-------  target    NTVVNALPRMEMIAVHEWWWTGSCEWADVVFGVDSWGELKHPDMTASVTNPFLIVFPKTPIKRIFNTVGDIDVLALVSSK 6qc5.1    -VTRQDLPKDCFIVYQGHHGDVGAPIADVILPGAAYTEKSA--TY-VNTEGRAQQTKV-AVMPPGLAREDWKIIRALSEI  target    LAELTGDTRFNDMWKFVREGRTDVYLQRILDASTNTKGYRFTELEAKAREGIPALMNSRTSPKVVGYDQLADSTPWYTKS 6qc5.1    AGM-----------------------------------------------------------------------------  target    GRLEFYREEDEFIEAGENLPVHREPVDSTFYEPNVIVSPKHEAVRPSGPEDYGVARTDLSCEVRCGRNVVLTWAETRQTQ 6qc5.1    --------------------------------------------------------------------------------  target    HPLVKQGHKFIFHTPKYRHGSHTTPIDTDMNAVLFGPFGDIYRRDKRSPFVTEGYVDINPTDGLELGLQDGDYVWIDPDP 6qc5.1    --------------------------------------------------------------------------------  target    EDRPFRGWQKNAKDMEFARLLCRARFYPGTPRGVTRMWFNMYGATPGSVRGAKARRDGLAKNPDTNYQAMFRSGSHQSAT 6qc5.1    --------------------------------------------------------------------------------  target    RGWLKPTWMTDSLVRKGLFGQGIGKGFLPDVHCPTGAPREAFVKISRAEPGGIGGQGLWRPAALGIRPRHESPAMKRYLA 6qc5.1    --------------------------------------------------------------------------------  target    GGFFSGPKE 6qc5.1    --------- ``` | | | | | | | | | | | | | | | | | | | | | | | | | | | | | | | | | | | | | | | | | | | | | | | | | |
|  | 7dgr.10.A | NADH-ubiquinone oxidoreductase 75 kDa subunit, mitochondrial  *Activity optimized supercomplex state2* | 0.05 | 0.00 | 13.98 | 0.16 | 59-163 | EM | 0.00 | monomer |  | HHblits | 0.25 |
| ``` target    FNGAPQYINENPFDLELDASRPARPRQYWRAESAHFYNHEDHPLRVGTRLLTGSTHMPTPTKVMWFANANSILGNVKWHY 7dgr.10   ----------------------------------------------------------NPPKMLFLLGADGG--------  target    NTVVNALPRMEMIAVHEWWWTGSCEWADVVFGVDSWGELKHPDMTASVTNPFLIVFPKTPIKRIFNTVGDIDVLALVSSK 7dgr.10   CITRQDLPKDCFIVYQGHHGDVGAPIADVILPGAAYTEKSA--TYV-NTEGRAQQTKV-AVTPPGLAREDWKIIRALSEI  target    LAELTGDTRFNDMWKFVREGRTDVYLQRILDASTNTKGYRFTELEAKAREGIPALMNSRTSPKVVGYDQLADSTPWYTKS 7dgr.10   AGM-----------------------------------------------------------------------------  target    GRLEFYREEDEFIEAGENLPVHREPVDSTFYEPNVIVSPKHEAVRPSGPEDYGVARTDLSCEVRCGRNVVLTWAETRQTQ 7dgr.10   --------------------------------------------------------------------------------  target    HPLVKQGHKFIFHTPKYRHGSHTTPIDTDMNAVLFGPFGDIYRRDKRSPFVTEGYVDINPTDGLELGLQDGDYVWIDPDP 7dgr.10   --------------------------------------------------------------------------------  target    EDRPFRGWQKNAKDMEFARLLCRARFYPGTPRGVTRMWFNMYGATPGSVRGAKARRDGLAKNPDTNYQAMFRSGSHQSAT 7dgr.10   --------------------------------------------------------------------------------  target    RGWLKPTWMTDSLVRKGLFGQGIGKGFLPDVHCPTGAPREAFVKISRAEPGGIGGQGLWRPAALGIRPRHESPAMKRYLA 7dgr.10   --------------------------------------------------------------------------------  target    GGFFSGPKE 7dgr.10   --------- ``` | | | | | | | | | | | | | | | | | | | | | | | | | | | | | | | | | | | | | | | | | | | | | | | | | |
|  | 5o31.1.8 | NADH-ubiquinone oxidoreductase 75 kDa subunit, mitochondrial  *Mitochondrial complex I in the deactive state* | 0.05 | 0.00 | 13.98 | 0.16 | 59-163 | EM | 4.13 | monomer | 6 x SF4, 2 x FES, 1 x FMN, 1 x NAP, 1 x ZN | HHblits | 0.25 |
| ``` target    FNGAPQYINENPFDLELDASRPARPRQYWRAESAHFYNHEDHPLRVGTRLLTGSTHMPTPTKVMWFANANSILGNVKWHY 5o31.1    ----------------------------------------------------------NPPKMLFLLGADGG--------  target    NTVVNALPRMEMIAVHEWWWTGSCEWADVVFGVDSWGELKHPDMTASVTNPFLIVFPKTPIKRIFNTVGDIDVLALVSSK 5o31.1    CITRQDLPKDCFIVYQGHHGDVGAPIADVILPGAAYTEKSA--TYV-NTEGRAQQTKV-AVTPPGLAREDWKIIRALSEI  target    LAELTGDTRFNDMWKFVREGRTDVYLQRILDASTNTKGYRFTELEAKAREGIPALMNSRTSPKVVGYDQLADSTPWYTKS 5o31.1    AGM-----------------------------------------------------------------------------  target    GRLEFYREEDEFIEAGENLPVHREPVDSTFYEPNVIVSPKHEAVRPSGPEDYGVARTDLSCEVRCGRNVVLTWAETRQTQ 5o31.1    --------------------------------------------------------------------------------  target    HPLVKQGHKFIFHTPKYRHGSHTTPIDTDMNAVLFGPFGDIYRRDKRSPFVTEGYVDINPTDGLELGLQDGDYVWIDPDP 5o31.1    --------------------------------------------------------------------------------  target    EDRPFRGWQKNAKDMEFARLLCRARFYPGTPRGVTRMWFNMYGATPGSVRGAKARRDGLAKNPDTNYQAMFRSGSHQSAT 5o31.1    --------------------------------------------------------------------------------  target    RGWLKPTWMTDSLVRKGLFGQGIGKGFLPDVHCPTGAPREAFVKISRAEPGGIGGQGLWRPAALGIRPRHESPAMKRYLA 5o31.1    --------------------------------------------------------------------------------  target    GGFFSGPKE 5o31.1    --------- ``` | | | | | | | | | | | | | | | | | | | | | | | | | | | | | | | | | | | | | | | | | | | | | | | | | |
|  | 7ak5.1.G | NADH-ubiquinone oxidoreductase 75 kDa subunit, mitochondrial  *Cryo-EM structure of respiratory complex I in the deactive state from Mus musculus at 3.2 A* | 0.06 | 0.00 | 13.98 | 0.16 | 59-163 | EM | 0.00 | monomer | 6 x SF4, 2 x PC1, 2 x FES, 1 x FMN, 8 x 3PE, 4 x CDL, 1 x ATP, 1 x NDP, 1 x ZN, 2 x EHZ | HHblits | 0.25 |
| ``` target    FNGAPQYINENPFDLELDASRPARPRQYWRAESAHFYNHEDHPLRVGTRLLTGSTHMPTPTKVMWFANANSILGNVKWHY 7ak5.1    ----------------------------------------------------------NPPKMLFLLGADGG--------  target    NTVVNALPRMEMIAVHEWWWTGSCEWADVVFGVDSWGELKHPDMTASVTNPFLIVFPKTPIKRIFNTVGDIDVLALVSSK 7ak5.1    CITRQDLPKDCFIVYQGHHGDVGAPMADVILPGAAYTEKSA--TY-VNTEGRAQQTKV-AVTPPGLAREDWKIIRALSEI  target    LAELTGDTRFNDMWKFVREGRTDVYLQRILDASTNTKGYRFTELEAKAREGIPALMNSRTSPKVVGYDQLADSTPWYTKS 7ak5.1    AGI-----------------------------------------------------------------------------  target    GRLEFYREEDEFIEAGENLPVHREPVDSTFYEPNVIVSPKHEAVRPSGPEDYGVARTDLSCEVRCGRNVVLTWAETRQTQ 7ak5.1    --------------------------------------------------------------------------------  target    HPLVKQGHKFIFHTPKYRHGSHTTPIDTDMNAVLFGPFGDIYRRDKRSPFVTEGYVDINPTDGLELGLQDGDYVWIDPDP 7ak5.1    --------------------------------------------------------------------------------  target    EDRPFRGWQKNAKDMEFARLLCRARFYPGTPRGVTRMWFNMYGATPGSVRGAKARRDGLAKNPDTNYQAMFRSGSHQSAT 7ak5.1    --------------------------------------------------------------------------------  target    RGWLKPTWMTDSLVRKGLFGQGIGKGFLPDVHCPTGAPREAFVKISRAEPGGIGGQGLWRPAALGIRPRHESPAMKRYLA 7ak5.1    --------------------------------------------------------------------------------  target    GGFFSGPKE 7ak5.1    --------- ``` | | | | | | | | | | | | | | | | | | | | | | | | | | | | | | | | | | | | | | | | | | | | | | | | | |
|  | 6x89.1.H | NADH dehydrogenase [ubiquinone] iron-sulfur protein 1, mitochondrial  *Vigna radiata mitochondrial complex I\** | 0.05 |  | 10.99 | 0.16 | 59-163 | EM | 0.00 | hetero-1-1-1-1-1-1-… | 1 x NAP, 6 x PC1, 6 x SF4, 2 x FES, 2 x ZN, 1 x FMN | HHblits | 0.27 |
| ``` target    FNGAPQYINENPFDLELDASRPARPRQYWRAESAHFYNHEDHPLRVGTRLLTGSTHMPTPTKVMWFANANSILGNVKWHY 6x89.1    ----------------------------------------------------------ESAKFVYLMGADDVN-------  target    NTVVNALPRMEMIAVHEWWWTGSCEWADVVFGVDSWGELKHPDMTASVTNPFLIVFPKTPIKRIFNTVGDIDVLALVSSK 6x89.1    ---LDKIPDDAFVVYQGHHGDKSVYRANVILPTAAFSEKEG--TY-QNTEGCTQQTLP-AVPTVGDSRDDWKIIRALSEV  target    LAELTGDTRFNDMWKFVREGRTDVYLQRILDASTNTKGYRFTELEAKAREGIPALMNSRTSPKVVGYDQLADSTPWYTKS 6x89.1    AGV-----------------------------------------------------------------------------  target    GRLEFYREEDEFIEAGENLPVHREPVDSTFYEPNVIVSPKHEAVRPSGPEDYGVARTDLSCEVRCGRNVVLTWAETRQTQ 6x89.1    --------------------------------------------------------------------------------  target    HPLVKQGHKFIFHTPKYRHGSHTTPIDTDMNAVLFGPFGDIYRRDKRSPFVTEGYVDINPTDGLELGLQDGDYVWIDPDP 6x89.1    --------------------------------------------------------------------------------  target    EDRPFRGWQKNAKDMEFARLLCRARFYPGTPRGVTRMWFNMYGATPGSVRGAKARRDGLAKNPDTNYQAMFRSGSHQSAT 6x89.1    --------------------------------------------------------------------------------  target    RGWLKPTWMTDSLVRKGLFGQGIGKGFLPDVHCPTGAPREAFVKISRAEPGGIGGQGLWRPAALGIRPRHESPAMKRYLA 6x89.1    --------------------------------------------------------------------------------  target    GGFFSGPKE 6x89.1    --------- ``` | | | | | | | | | | | | | | | | | | | | | | | | | | | | | | | | | | | | | | | | | | | | | | | | | |
|  | 8e73.55.A | NDUS1  *Vigna radiata supercomplex I+III2 (full bridge)* | 0.05 |  | 10.99 | 0.16 | 59-163 | EM | 0.00 | monomer |  | HHblits | 0.27 |
| ``` target    FNGAPQYINENPFDLELDASRPARPRQYWRAESAHFYNHEDHPLRVGTRLLTGSTHMPTPTKVMWFANANSILGNVKWHY 8e73.55   ----------------------------------------------------------ESAKFVYLMGADDVN-------  target    NTVVNALPRMEMIAVHEWWWTGSCEWADVVFGVDSWGELKHPDMTASVTNPFLIVFPKTPIKRIFNTVGDIDVLALVSSK 8e73.55   ---LDKIPDDAFVVYQGHHGDKSVYRANVILPTAAFSEKEG--TY-QNTEGCTQQTLP-AVPTVGDSRDDWKIIRALSEV  target    LAELTGDTRFNDMWKFVREGRTDVYLQRILDASTNTKGYRFTELEAKAREGIPALMNSRTSPKVVGYDQLADSTPWYTKS 8e73.55   AGV-----------------------------------------------------------------------------  target    GRLEFYREEDEFIEAGENLPVHREPVDSTFYEPNVIVSPKHEAVRPSGPEDYGVARTDLSCEVRCGRNVVLTWAETRQTQ 8e73.55   --------------------------------------------------------------------------------  target    HPLVKQGHKFIFHTPKYRHGSHTTPIDTDMNAVLFGPFGDIYRRDKRSPFVTEGYVDINPTDGLELGLQDGDYVWIDPDP 8e73.55   --------------------------------------------------------------------------------  target    EDRPFRGWQKNAKDMEFARLLCRARFYPGTPRGVTRMWFNMYGATPGSVRGAKARRDGLAKNPDTNYQAMFRSGSHQSAT 8e73.55   --------------------------------------------------------------------------------  target    RGWLKPTWMTDSLVRKGLFGQGIGKGFLPDVHCPTGAPREAFVKISRAEPGGIGGQGLWRPAALGIRPRHESPAMKRYLA 8e73.55   --------------------------------------------------------------------------------  target    GGFFSGPKE 8e73.55   --------- ``` | | | | | | | | | | | | | | | | | | | | | | | | | | | | | | | | | | | | | | | | | | | | | | | | | |
|  | 7vxu.1.L | NADH-ubiquinone oxidoreductase 75 kDa subunit, mitochondrial  *Matrix arm of deactive state CI from Q10 dataset* | 0.06 | 0.00 | 15.22 | 0.16 | 60-163 | EM | 0.00 | monomer | 6 x SF4, 1 x FMN, 1 x PEE, 1 x PLX, 1 x 8Q1, 1 x NDP, 2 x FES, 1 x MG, 1 x CDL, 1 x ZN | HHblits | 0.26 |
| ``` target    FNGAPQYINENPFDLELDASRPARPRQYWRAESAHFYNHEDHPLRVGTRLLTGSTHMPTPTKVMWFANANSILGNVKWHY 7vxu.1    -----------------------------------------------------------PPKVLFLLGADGG--------  target    NTVVNALPRMEMIAVHEWWWTGSCEWADVVFGVDSWGELKHPDMTASVTNPFLIVFPKTPIKRIFNTVGDIDVLALVSSK 7vxu.1    CITRQDLPKDCFIIYQGHHGDVGAPMADVILPGAAYTEKSA--TYV-NTEGRAQQTKV-AVTPPGLAREDWKIIRALSEI  target    LAELTGDTRFNDMWKFVREGRTDVYLQRILDASTNTKGYRFTELEAKAREGIPALMNSRTSPKVVGYDQLADSTPWYTKS 7vxu.1    AGM-----------------------------------------------------------------------------  target    GRLEFYREEDEFIEAGENLPVHREPVDSTFYEPNVIVSPKHEAVRPSGPEDYGVARTDLSCEVRCGRNVVLTWAETRQTQ 7vxu.1    --------------------------------------------------------------------------------  target    HPLVKQGHKFIFHTPKYRHGSHTTPIDTDMNAVLFGPFGDIYRRDKRSPFVTEGYVDINPTDGLELGLQDGDYVWIDPDP 7vxu.1    --------------------------------------------------------------------------------  target    EDRPFRGWQKNAKDMEFARLLCRARFYPGTPRGVTRMWFNMYGATPGSVRGAKARRDGLAKNPDTNYQAMFRSGSHQSAT 7vxu.1    --------------------------------------------------------------------------------  target    RGWLKPTWMTDSLVRKGLFGQGIGKGFLPDVHCPTGAPREAFVKISRAEPGGIGGQGLWRPAALGIRPRHESPAMKRYLA 7vxu.1    --------------------------------------------------------------------------------  target    GGFFSGPKE 7vxu.1    --------- ``` | | | | | | | | | | | | | | | | | | | | | | | | | | | | | | | | | | | | | | | | | | | | | | | | | |
|  | 7v2c.1.L | NADH-ubiquinone oxidoreductase 75 kDa subunit, mitochondrial  *Active state complex I from Q10 dataset* | 0.06 | 0.00 | 15.22 | 0.16 | 60-163 | EM | 0.00 | monomer | 6 x SF4, 1 x FMN, 10 x PEE, 8 x PLX, 2 x 8Q1, 1 x NDP, 2 x UQ, 11 x CDL, 2 x FES, 1 x MG, 1 x ZN, 1 x ADP | HHblits | 0.26 |
| ``` target    FNGAPQYINENPFDLELDASRPARPRQYWRAESAHFYNHEDHPLRVGTRLLTGSTHMPTPTKVMWFANANSILGNVKWHY 7v2c.1    -----------------------------------------------------------PPKVLFLLGADGG--------  target    NTVVNALPRMEMIAVHEWWWTGSCEWADVVFGVDSWGELKHPDMTASVTNPFLIVFPKTPIKRIFNTVGDIDVLALVSSK 7v2c.1    CITRQDLPKDCFIIYQGHHGDVGAPMADVILPGAAYTEKS--ATYV-NTEGRAQQTKV-AVTPPGLAREDWKIIRALSEI  target    LAELTGDTRFNDMWKFVREGRTDVYLQRILDASTNTKGYRFTELEAKAREGIPALMNSRTSPKVVGYDQLADSTPWYTKS 7v2c.1    AGM-----------------------------------------------------------------------------  target    GRLEFYREEDEFIEAGENLPVHREPVDSTFYEPNVIVSPKHEAVRPSGPEDYGVARTDLSCEVRCGRNVVLTWAETRQTQ 7v2c.1    --------------------------------------------------------------------------------  target    HPLVKQGHKFIFHTPKYRHGSHTTPIDTDMNAVLFGPFGDIYRRDKRSPFVTEGYVDINPTDGLELGLQDGDYVWIDPDP 7v2c.1    --------------------------------------------------------------------------------  target    EDRPFRGWQKNAKDMEFARLLCRARFYPGTPRGVTRMWFNMYGATPGSVRGAKARRDGLAKNPDTNYQAMFRSGSHQSAT 7v2c.1    --------------------------------------------------------------------------------  target    RGWLKPTWMTDSLVRKGLFGQGIGKGFLPDVHCPTGAPREAFVKISRAEPGGIGGQGLWRPAALGIRPRHESPAMKRYLA 7v2c.1    --------------------------------------------------------------------------------  target    GGFFSGPKE 7v2c.1    --------- ``` | | | | | | | | | | | | | | | | | | | | | | | | | | | | | | | | | | | | | | | | | | | | | | | | | |
|  | 5xtb.1.L | NADH-ubiquinone oxidoreductase 75 kDa subunit, mitochondrial  *Cryo-EM structure of human respiratory complex I matrix arm* | 0.05 | 0.00 | 15.22 | 0.16 | 60-163 | EM | 0.00 | monomer | 6 x SF4, 1 x FMN, 1 x 8Q1, 1 x NDP, 2 x FES | HHblits | 0.26 |
| ``` target    FNGAPQYINENPFDLELDASRPARPRQYWRAESAHFYNHEDHPLRVGTRLLTGSTHMPTPTKVMWFANANSILGNVKWHY 5xtb.1    -----------------------------------------------------------PPKVLFLLGADGG--------  target    NTVVNALPRMEMIAVHEWWWTGSCEWADVVFGVDSWGELKHPDMTASVTNPFLIVFPKTPIKRIFNTVGDIDVLALVSSK 5xtb.1    CITRQDLPKDCFIIYQGHHGDVGAPIADVILPGAAYTEKSA--TYV-NTEGRAQQTKV-AVTPPGLAREDWKIIRALSEI  target    LAELTGDTRFNDMWKFVREGRTDVYLQRILDASTNTKGYRFTELEAKAREGIPALMNSRTSPKVVGYDQLADSTPWYTKS 5xtb.1    AGM-----------------------------------------------------------------------------  target    GRLEFYREEDEFIEAGENLPVHREPVDSTFYEPNVIVSPKHEAVRPSGPEDYGVARTDLSCEVRCGRNVVLTWAETRQTQ 5xtb.1    --------------------------------------------------------------------------------  target    HPLVKQGHKFIFHTPKYRHGSHTTPIDTDMNAVLFGPFGDIYRRDKRSPFVTEGYVDINPTDGLELGLQDGDYVWIDPDP 5xtb.1    --------------------------------------------------------------------------------  target    EDRPFRGWQKNAKDMEFARLLCRARFYPGTPRGVTRMWFNMYGATPGSVRGAKARRDGLAKNPDTNYQAMFRSGSHQSAT 5xtb.1    --------------------------------------------------------------------------------  target    RGWLKPTWMTDSLVRKGLFGQGIGKGFLPDVHCPTGAPREAFVKISRAEPGGIGGQGLWRPAALGIRPRHESPAMKRYLA 5xtb.1    --------------------------------------------------------------------------------  target    GGFFSGPKE 5xtb.1    --------- ``` | | | | | | | | | | | | | | | | | | | | | | | | | | | | | | | | | | | | | | | | | | | | | | | | | |
|  | 6zk9.1.C | NADH:ubiquinone oxidoreductase core subunit S1  *Peripheral domain of open complex I during turnover* | 0.06 | 0.00 | 14.13 | 0.16 | 60-163 | EM | 0.00 | monomer | 6 x SF4, 1 x FMN, 1 x NAI, 2 x FES, 1 x K, 2 x PC1, 2 x 3PE, 1 x ZN, 1 x NDP, 1 x ZMP, 1 x CDL | HHblits | 0.26 |
| ``` target    FNGAPQYINENPFDLELDASRPARPRQYWRAESAHFYNHEDHPLRVGTRLLTGSTHMPTPTKVMWFANANSILGNVKWHY 6zk9.1    -----------------------------------------------------------PPKMLFLLGADGGC-------  target    NTVVNALPRMEMIAVHEWWWTGSCEWADVVFGVDSWGELKHPDMTASVTNPFLIVFPKTPIKRIFNTVGDIDVLALVSSK 6zk9.1    -VTRQDLPKDCFIVYQGHHGDVGAPIADVILPGAAYTEKSA--TY-VNTEGRAQQTKV-AVMPPGLAREDWKIIRALSEI  target    LAELTGDTRFNDMWKFVREGRTDVYLQRILDASTNTKGYRFTELEAKAREGIPALMNSRTSPKVVGYDQLADSTPWYTKS 6zk9.1    AGM-----------------------------------------------------------------------------  target    GRLEFYREEDEFIEAGENLPVHREPVDSTFYEPNVIVSPKHEAVRPSGPEDYGVARTDLSCEVRCGRNVVLTWAETRQTQ 6zk9.1    --------------------------------------------------------------------------------  target    HPLVKQGHKFIFHTPKYRHGSHTTPIDTDMNAVLFGPFGDIYRRDKRSPFVTEGYVDINPTDGLELGLQDGDYVWIDPDP 6zk9.1    --------------------------------------------------------------------------------  target    EDRPFRGWQKNAKDMEFARLLCRARFYPGTPRGVTRMWFNMYGATPGSVRGAKARRDGLAKNPDTNYQAMFRSGSHQSAT 6zk9.1    --------------------------------------------------------------------------------  target    RGWLKPTWMTDSLVRKGLFGQGIGKGFLPDVHCPTGAPREAFVKISRAEPGGIGGQGLWRPAALGIRPRHESPAMKRYLA 6zk9.1    --------------------------------------------------------------------------------  target    GGFFSGPKE 6zk9.1    --------- ``` | | | | | | | | | | | | | | | | | | | | | | | | | | | | | | | | | | | | | | | | | | | | | | | | | |
|  | 7zd6.1.4 | NADH-ubiquinone oxidoreductase 75 kDa subunit, mitochondrial  *Complex I from Ovis aries, at pH7.4, Open state* | 0.06 | 0.00 | 14.13 | 0.16 | 60-163 | EM | 0.00 | monomer | 6 x PC1, 14 x 3PE, 1 x DCQ, 2 x ZMP, 1 x AMP, 1 x MYR, 6 x SF4, 1 x FMN, 1 x NAI, 2 x FES, 1 x K, 1 x ZN, 1 x NDP | HHblits | 0.26 |
| ``` target    FNGAPQYINENPFDLELDASRPARPRQYWRAESAHFYNHEDHPLRVGTRLLTGSTHMPTPTKVMWFANANSILGNVKWHY 7zd6.1    -----------------------------------------------------------PPKMLFLLGADGGC-------  target    NTVVNALPRMEMIAVHEWWWTGSCEWADVVFGVDSWGELKHPDMTASVTNPFLIVFPKTPIKRIFNTVGDIDVLALVSSK 7zd6.1    -VTRQDLPKDCFIVYQGHHGDVGAPIADVILPGAAYTEKSA--TY-VNTEGRAQQTKV-AVMPPGLAREDWKIIRALSEI  target    LAELTGDTRFNDMWKFVREGRTDVYLQRILDASTNTKGYRFTELEAKAREGIPALMNSRTSPKVVGYDQLADSTPWYTKS 7zd6.1    AGM-----------------------------------------------------------------------------  target    GRLEFYREEDEFIEAGENLPVHREPVDSTFYEPNVIVSPKHEAVRPSGPEDYGVARTDLSCEVRCGRNVVLTWAETRQTQ 7zd6.1    --------------------------------------------------------------------------------  target    HPLVKQGHKFIFHTPKYRHGSHTTPIDTDMNAVLFGPFGDIYRRDKRSPFVTEGYVDINPTDGLELGLQDGDYVWIDPDP 7zd6.1    --------------------------------------------------------------------------------  target    EDRPFRGWQKNAKDMEFARLLCRARFYPGTPRGVTRMWFNMYGATPGSVRGAKARRDGLAKNPDTNYQAMFRSGSHQSAT 7zd6.1    --------------------------------------------------------------------------------  target    RGWLKPTWMTDSLVRKGLFGQGIGKGFLPDVHCPTGAPREAFVKISRAEPGGIGGQGLWRPAALGIRPRHESPAMKRYLA 7zd6.1    --------------------------------------------------------------------------------  target    GGFFSGPKE 7zd6.1    --------- ``` | | | | | | | | | | | | | | | | | | | | | | | | | | | | | | | | | | | | | | | | | | | | | | | | | |
|  | 6zr2.1.G | NADH-ubiquinone oxidoreductase 75 kDa subunit, mitochondrial  *Cryo-EM structure of respiratory complex I in the active state from Mus musculus at 3.1 A* | 0.05 |  | 14.13 | 0.16 | 60-163 | EM | 3.10 | hetero-1-1-1-1-1-1-… | 6 x SF4, 4 x PC1, 2 x FES, 1 x FMN, 9 x 3PE, 7 x CDL, 1 x ATP, 1 x NDP, 1 x ZN, 2 x EHZ | HHblits | 0.25 |
| ``` target    FNGAPQYINENPFDLELDASRPARPRQYWRAESAHFYNHEDHPLRVGTRLLTGSTHMPTPTKVMWFANANSILGNVKWHY 6zr2.1    -----------------------------------------------------------PPKMLFLLGADGG--------  target    NTVVNALPRMEMIAVHEWWWTGSCEWADVVFGVDSWGELKHPDMTASVTNPFLIVFPKTPIKRIFNTVGDIDVLALVSSK 6zr2.1    CITRQDLPKDCFIVYQGHHGDVGAPMADVILPGAAYTEKSA--TY-VNTEGRAQQTKV-AVTPPGLAREDWKIIRALSEI  target    LAELTGDTRFNDMWKFVREGRTDVYLQRILDASTNTKGYRFTELEAKAREGIPALMNSRTSPKVVGYDQLADSTPWYTKS 6zr2.1    AGI-----------------------------------------------------------------------------  target    GRLEFYREEDEFIEAGENLPVHREPVDSTFYEPNVIVSPKHEAVRPSGPEDYGVARTDLSCEVRCGRNVVLTWAETRQTQ 6zr2.1    --------------------------------------------------------------------------------  target    HPLVKQGHKFIFHTPKYRHGSHTTPIDTDMNAVLFGPFGDIYRRDKRSPFVTEGYVDINPTDGLELGLQDGDYVWIDPDP 6zr2.1    --------------------------------------------------------------------------------  target    EDRPFRGWQKNAKDMEFARLLCRARFYPGTPRGVTRMWFNMYGATPGSVRGAKARRDGLAKNPDTNYQAMFRSGSHQSAT 6zr2.1    --------------------------------------------------------------------------------  target    RGWLKPTWMTDSLVRKGLFGQGIGKGFLPDVHCPTGAPREAFVKISRAEPGGIGGQGLWRPAALGIRPRHESPAMKRYLA 6zr2.1    --------------------------------------------------------------------------------  target    GGFFSGPKE 6zr2.1    --------- ``` | | | | | | | | | | | | | | | | | | | | | | | | | | | | | | | | | | | | | | | | | | | | | | | | | |
|  | 6g72.1.G | NADH-ubiquinone oxidoreductase 75 kDa subunit, mitochondrial  *Mouse mitochondrial complex I in the deactive state* | 0.05 |  | 14.13 | 0.16 | 60-163 | EM | 0.00 | hetero-1-1-1-1-1-1-… | 6 x SF4, 2 x FES, 1 x FMN, 1 x ADP, 1 x NDP, 1 x ZN, 2 x EHZ | HHblits | 0.25 |
| ``` target    FNGAPQYINENPFDLELDASRPARPRQYWRAESAHFYNHEDHPLRVGTRLLTGSTHMPTPTKVMWFANANSILGNVKWHY 6g72.1    -----------------------------------------------------------PPKMLFLLGADGG--------  target    NTVVNALPRMEMIAVHEWWWTGSCEWADVVFGVDSWGELKHPDMTASVTNPFLIVFPKTPIKRIFNTVGDIDVLALVSSK 6g72.1    CITRQDLPKDCFIVYQGHHGDVGAPMADVILPGAAYTEKSA--TY-VNTEGRAQQTKV-AVTPPGLAREDWKIIRALSEI  target    LAELTGDTRFNDMWKFVREGRTDVYLQRILDASTNTKGYRFTELEAKAREGIPALMNSRTSPKVVGYDQLADSTPWYTKS 6g72.1    AGI-----------------------------------------------------------------------------  target    GRLEFYREEDEFIEAGENLPVHREPVDSTFYEPNVIVSPKHEAVRPSGPEDYGVARTDLSCEVRCGRNVVLTWAETRQTQ 6g72.1    --------------------------------------------------------------------------------  target    HPLVKQGHKFIFHTPKYRHGSHTTPIDTDMNAVLFGPFGDIYRRDKRSPFVTEGYVDINPTDGLELGLQDGDYVWIDPDP 6g72.1    --------------------------------------------------------------------------------  target    EDRPFRGWQKNAKDMEFARLLCRARFYPGTPRGVTRMWFNMYGATPGSVRGAKARRDGLAKNPDTNYQAMFRSGSHQSAT 6g72.1    --------------------------------------------------------------------------------  target    RGWLKPTWMTDSLVRKGLFGQGIGKGFLPDVHCPTGAPREAFVKISRAEPGGIGGQGLWRPAALGIRPRHESPAMKRYLA 6g72.1    --------------------------------------------------------------------------------  target    GGFFSGPKE 6g72.1    --------- ``` | | | | | | | | | | | | | | | | | | | | | | | | | | | | | | | | | | | | | | | | | | | | | | | | | |
|  | 7ak6.1.G | NADH-ubiquinone oxidoreductase 75 kDa subunit, mitochondrial  *Cryo-EM structure of ND6-P25L mutant respiratory complex I from Mus musculus at 3.8 A* | 0.06 |  | 14.13 | 0.16 | 60-163 | EM | 0.00 | hetero-1-1-1-1-1-1-… | 6 x SF4, 1 x PC1, 2 x FES, 1 x FMN, 4 x 3PE, 2 x CDL, 1 x ATP, 1 x NDP, 1 x ZN, 2 x EHZ | HHblits | 0.25 |
| ``` target    FNGAPQYINENPFDLELDASRPARPRQYWRAESAHFYNHEDHPLRVGTRLLTGSTHMPTPTKVMWFANANSILGNVKWHY 7ak6.1    -----------------------------------------------------------PPKMLFLLGADGG--------  target    NTVVNALPRMEMIAVHEWWWTGSCEWADVVFGVDSWGELKHPDMTASVTNPFLIVFPKTPIKRIFNTVGDIDVLALVSSK 7ak6.1    CITRQDLPKDCFIVYQGHHGDVGAPMADVILPGAAYTEKSA--TY-VNTEGRAQQTKV-AVTPPGLAREDWKIIRALSEI  target    LAELTGDTRFNDMWKFVREGRTDVYLQRILDASTNTKGYRFTELEAKAREGIPALMNSRTSPKVVGYDQLADSTPWYTKS 7ak6.1    AGI-----------------------------------------------------------------------------  target    GRLEFYREEDEFIEAGENLPVHREPVDSTFYEPNVIVSPKHEAVRPSGPEDYGVARTDLSCEVRCGRNVVLTWAETRQTQ 7ak6.1    --------------------------------------------------------------------------------  target    HPLVKQGHKFIFHTPKYRHGSHTTPIDTDMNAVLFGPFGDIYRRDKRSPFVTEGYVDINPTDGLELGLQDGDYVWIDPDP 7ak6.1    --------------------------------------------------------------------------------  target    EDRPFRGWQKNAKDMEFARLLCRARFYPGTPRGVTRMWFNMYGATPGSVRGAKARRDGLAKNPDTNYQAMFRSGSHQSAT 7ak6.1    --------------------------------------------------------------------------------  target    RGWLKPTWMTDSLVRKGLFGQGIGKGFLPDVHCPTGAPREAFVKISRAEPGGIGGQGLWRPAALGIRPRHESPAMKRYLA 7ak6.1    --------------------------------------------------------------------------------  target    GGFFSGPKE 7ak6.1    --------- ``` | | | | | | | | | | | | | | | | | | | | | | | | | | | | | | | | | | | | | | | | | | | | | | | | | |
|  | 6s6y.1.B | Tungsten-containing formylmethanofuran dehydrogenase, subunit B  *X-ray crystal structure of the formyltransferase/hydrolase complex (FhcABCD) from Methylorubrum extorquens in complex with methylofuran* | 0.05 |  | 15.38 | 0.16 | 60-163 | X-ray | 3.10 | hetero-2-2-2-2-mer | 1 x MFN, 4 x ZN, 4 x CA, 4 x K, 3 x DGL, 2 x GLU, 1 x IAS | HHblits | 0.25 |
| ``` target    FNGAPQYINENPFDLELDASRPARPRQYWRAESAHFYNHEDHPLRVGTRLLTGSTHMPTPTKVMWFANANSILGNVKWHY 6s6y.1    -----------------------------------------------------------EADAALWLASLPAP-------  target    NTVVNALPRMEMIAVHE-WWWTGSCEWADVVFGVDSW-GELKHPDMTASVTNPFLIVFPKTPIKRI---FNTVGDIDVLA 6s6y.1    --RPAWLGSLPTIAIVGEGSQEAAGETAEVVITVGVPGQSVGGALW---NDRRGVIAYAE-ASDPAKTPAETETAAGVLT  target    LVSSKLAELTGDTRFNDMWKFVREGRTDVYLQRILDASTNTKGYRFTELEAKAREGIPALMNSRTSPKVVGYDQLADSTP 6s6y.1    RIRDRLIE------------------------------------------------------------------------  target    WYTKSGRLEFYREEDEFIEAGENLPVHREPVDSTFYEPNVIVSPKHEAVRPSGPEDYGVARTDLSCEVRCGRNVVLTWAE 6s6y.1    --------------------------------------------------------------------------------  target    TRQTQHPLVKQGHKFIFHTPKYRHGSHTTPIDTDMNAVLFGPFGDIYRRDKRSPFVTEGYVDINPTDGLELGLQDGDYVW 6s6y.1    --------------------------------------------------------------------------------  target    IDPDPEDRPFRGWQKNAKDMEFARLLCRARFYPGTPRGVTRMWFNMYGATPGSVRGAKARRDGLAKNPDTNYQAMFRSGS 6s6y.1    --------------------------------------------------------------------------------  target    HQSATRGWLKPTWMTDSLVRKGLFGQGIGKGFLPDVHCPTGAPREAFVKISRAEPGGIGGQGLWRPAALGIRPRHESPAM 6s6y.1    --------------------------------------------------------------------------------  target    KRYLAGGFFSGPKE 6s6y.1    -------------- ``` | | | | | | | | | | | | | | | | | | | | | | | | | | | | | | | | | | | | | | | | | | | | | | | | | |
|  | 6lod.1.B | Fe-S-cluster-containing hydrogenase components 1-like protein  *Cryo-EM structure of the air-oxidized photosynthetic alternative complex III from Roseiflexus castenholzii* | 0.05 |  | 14.77 | 0.15 | 327-442 | EM | 0.00 | hetero-1-1-1-1-1-1-… | 6 x HEC, 2 x EL6, 3 x SF4, 1 x F3S | HHblits | 0.26 |
| ``` target    FNGAPQYINENPFDLELDASRPARPRQYWRAESAHFYNHEDHPLRVGTRLLTGSTHMPTPTKVMWFANANSILGNVKWHY 6lod.1    --------------------------------------------------------------------------------  target    NTVVNALPRMEMIAVHEWWWTGSCEWADVVFGVDSWGELKHPDMTASVTNPFLIVFPKTPIKRIFNTVGDIDVLALVSSK 6lod.1    --------------------------------------------------------------------------------  target    LAELTGDTRFNDMWKFVREGRTDVYLQRILDASTNTKGYRFTELEAKAREGIPALMNSRTSPKVVGYDQLADSTPWYTKS 6lod.1    --------------------------------------------------------------------------------  target    GRLEFYREEDEFIEAGENLPVHREPVDSTFYEPNVIVSPKHEAVRPSGPEDYGVARTDLSCEVRCGRNVVLTWAETRQTQ 6lod.1    --------------------------------------------------------------------------------  target    HPLVKQGHKFIFHTPKYRHGSHTTPIDTDMNAVLFGPFGDIYRRDKRSPFVTEGYVDINPTDGLELGLQDGDYVWIDPDP 6lod.1    ------GLEIVFRPDPS--LWDGAFANNAWLQETPKPY------TKL---TWDNVALMSVRTANALGLKNGDVVRLTYQG  target    EDRPFRGWQKNAKDMEFARLLCRARFYPGTPRGVTRMWFNMYGATPGSVRGAKARRDGLAKNPDTNYQAMFRSGSHQSAT 6lod.1    -----------------RSVDAPVWVQPGHADDSVTVHFGFG--------------------------------------  target    RGWLKPTWMTDSLVRKGLFGQGIGKGFLPDVHCPTGAPREAFVKISRAEPGGIGGQGLWRPAALGIRPRHESPAMKRYLA 6lod.1    --------------------------------------------------------------------------------  target    GGFFSGPKE 6lod.1    --------- ``` | | | | | | | | | | | | | | | | | | | | | | | | | | | | | | | | | | | | | | | | | | | | | | | | | |
|  | 3o5a.1.A | Periplasmic nitrate reductase  *Crystal Structure of partially reduced Periplasmic Nitrate Reductase from Cupriavidus necator using Ionic Liquids* | 0.05 |  | 19.51 | 0.14 | 326-440 | X-ray | 1.72 | hetero-oligomer | 1 x SF4, 1 x MOS, 2 x MGD, 2 x HEC | HHblits | 0.29 |
| ``` target    FNGAPQYINENPFDLELDASRPARPRQYWRAESAHFYNHEDHPLRVGTRLLTGSTHMPTPTKVMWFANANSILGNVKWHY 3o5a.1    --------------------------------------------------------------------------------  target    NTVVNALPRMEMIAVHEWWWTGSCEWADVVFGVDSWGELKHPDMTASVTNPFLIVFPKTPIKRIFNTVGDIDVLALVSSK 3o5a.1    --------------------------------------------------------------------------------  target    LAELTGDTRFNDMWKFVREGRTDVYLQRILDASTNTKGYRFTELEAKAREGIPALMNSRTSPKVVGYDQLADSTPWYTKS 3o5a.1    --------------------------------------------------------------------------------  target    GRLEFYREEDEFIEAGENLPVHREPVDSTFYEPNVIVSPKHEAVRPSGPEDYGVARTDLSCEVRCGRNVVLTWAETRQTQ 3o5a.1    --------------------------------------------------------------------------------  target    HPLVKQGHKFIFHTPKYRHGSHTT--PIDTDMNAVLFGPFGDIYRRDKRSPFVTEGYVDINPTDGLELGLQDGDYVWIDP 3o5a.1    -----KEYPYWLVTGRVLEHWHSGSMTRRVPEL-------------YRSFP---NAVVFMHPEDAKALGLRRGVEVEVVS  target    DPEDRPFRGWQKNAKDMEFARLLCRARFY--PGTPRGVTRMWFNMYGATPGSVRGAKARRDGLAKNPDTNYQAMFRSGSH 3o5a.1    RR-----------------GRMRSRIETRGRDAPPRGLVFVPWF------------------------------------  target    QSATRGWLKPTWMTDSLVRKGLFGQGIGKGFLPDVHCPTGAPREAFVKISRAEPGGIGGQGLWRPAALGIRPRHESPAMK 3o5a.1    --------------------------------------------------------------------------------  target    RYLAGGFFSGPKE 3o5a.1    ------------- ``` | | | | | | | | | | | | | | | | | | | | | | | | | | | | | | | | | | | | | | | | | | | | | | | | | |
|  | 2ki8.1.A | Tungsten formylmethanofuran dehydrogenase, subunit D (FwdD-2)  *Solution NMR structure of tungsten formylmethanofuran dehydrogenase subunit D from Archaeoglobus fulgidus, Northeast Structural Genomics Consortium target AtT7* | 0.05 |  | 18.75 | 0.14 | 327-441 | NMR | 0.00 | monomer |  | HHblits | 0.29 |
| ``` target    FNGAPQYINENPFDLELDASRPARPRQYWRAESAHFYNHEDHPLRVGTRLLTGSTHMPTPTKVMWFANANSILGNVKWHY 2ki8.1    --------------------------------------------------------------------------------  target    NTVVNALPRMEMIAVHEWWWTGSCEWADVVFGVDSWGELKHPDMTASVTNPFLIVFPKTPIKRIFNTVGDIDVLALVSSK 2ki8.1    --------------------------------------------------------------------------------  target    LAELTGDTRFNDMWKFVREGRTDVYLQRILDASTNTKGYRFTELEAKAREGIPALMNSRTSPKVVGYDQLADSTPWYTKS 2ki8.1    --------------------------------------------------------------------------------  target    GRLEFYREEDEFIEAGENLPVHREPVDSTFYEPNVIVSPKHEAVRPSGPEDYGVARTDLSCEVRCGRNVVLTWAETRQTQ 2ki8.1    --------------------------------------------------------------------------------  target    HPLVKQGHKFIFHTPKYRHGSHTTPIDTDMNAVLFGPFGDIYRRDKRSPFVTEGYVDINPTDGLELGLQDGDYVWIDPDP 2ki8.1    ------MLEVEVISGRTLNQGATVE--EKLT------------EEYF---NAVNYAEINEEDWNALGLQEGDRVKVKTEF  target    EDRPFRGWQKNAKDMEFARLLCRARFYPGTPRGVTRMWFNMYGATPGSVRGAKARRDGLAKNPDTNYQAMFRSGSHQSAT 2ki8.1    -----------------GEVVVFAKKG-DVPKGMIFIPMGP---------------------------------------  target    RGWLKPTWMTDSLVRKGLFGQGIGKGFLPDVHCPTGAPREAFVKISRAEPGGIGGQGLWRPAALGIRPRHESPAMKRYLA 2ki8.1    --------------------------------------------------------------------------------  target    GGFFSGPKE 2ki8.1    --------- ``` | | | | | | | | | | | | | | | | | | | | | | | | | | | | | | | | | | | | | | | | | | | | | | | | | |
|  | 7bkb.1.J | Formylmethanofuran dehydrogenase, subunit D  *Formate dehydrogenase - heterodisulfide reductase - formylmethanofuran dehydrogenase complex from Methanospirillum hungatei (hexameric, composite structure)* | 0.04 |  | 12.20 | 0.14 | 327-441 | EM | 0.00 | hetero-2-2-2-2-2-2-… | 48 x SF4, 4 x FAD, 2 x FES, 4 x 9S8, 4 x ZN, 2 x MO, 4 x MGD | HHblits | 0.26 |
| ``` target    FNGAPQYINENPFDLELDASRPARPRQYWRAESAHFYNHEDHPLRVGTRLLTGSTHMPTPTKVMWFANANSILGNVKWHY 7bkb.1    --------------------------------------------------------------------------------  target    NTVVNALPRMEMIAVHEWWWTGSCEWADVVFGVDSWGELKHPDMTASVTNPFLIVFPKTPIKRIFNTVGDIDVLALVSSK 7bkb.1    --------------------------------------------------------------------------------  target    LAELTGDTRFNDMWKFVREGRTDVYLQRILDASTNTKGYRFTELEAKAREGIPALMNSRTSPKVVGYDQLADSTPWYTKS 7bkb.1    --------------------------------------------------------------------------------  target    GRLEFYREEDEFIEAGENLPVHREPVDSTFYEPNVIVSPKHEAVRPSGPEDYGVARTDLSCEVRCGRNVVLTWAETRQTQ 7bkb.1    --------------------------------------------------------------------------------  target    HPLVKQGHKFIFHTPKYRHGSHTTPIDTDMNAVLFGPFGDIYRRDKRSPFVTEGYVDINPTDGLELGLQDGDYVWIDPDP 7bkb.1    ------KKTLNMITQRAVEEGIAMEI-GK--------TSR----QYFDA---CSIIEMNEQDMKELGIMKNTNVRVKSES  target    EDRPFRGWQKNAKDMEFARLLCRARFY-PGTPRGVTRMWFNMYGATPGSVRGAKARRDGLAKNPDTNYQAMFRSGSHQSA 7bkb.1    -----------------GEVVVKAVVGRQTCYPGLCHIRQGV--------------------------------------  target    TRGWLKPTWMTDSLVRKGLFGQGIGKGFLPDVHCPTGAPREAFVKISRAEPGGIGGQGLWRPAALGIRPRHESPAMKRYL 7bkb.1    --------------------------------------------------------------------------------  target    AGGFFSGPKE 7bkb.1    ---------- ``` | | | | | | | | | | | | | | | | | | | | | | | | | | | | | | | | | | | | | | | | | | | | | | | | | |
|  | 8e9g.1.G | NADH-quinone oxidoreductase subunit G  *Mycobacterial respiratory complex I with both quinone positions modelled* | 0.05 |  | 11.11 | 0.14 | 327-441 | EM | 0.00 | hetero-1-1-1-1-1-1-… |  | HHblits | 0.26 |
| ``` target    FNGAPQYINENPFDLELDASRPARPRQYWRAESAHFYNHEDHPLRVGTRLLTGSTHMPTPTKVMWFANANSILGNVKWHY 8e9g.1    --------------------------------------------------------------------------------  target    NTVVNALPRMEMIAVHEWWWTGSCEWADVVFGVDSWGELKHPDMTASVTNPFLIVFPKTPIKRIFNTVGDIDVLALVSSK 8e9g.1    --------------------------------------------------------------------------------  target    LAELTGDTRFNDMWKFVREGRTDVYLQRILDASTNTKGYRFTELEAKAREGIPALMNSRTSPKVVGYDQLADSTPWYTKS 8e9g.1    --------------------------------------------------------------------------------  target    GRLEFYREEDEFIEAGENLPVHREPVDSTFYEPNVIVSPKHEAVRPSGPEDYGVARTDLSCEVRCGRNVVLTWAETRQTQ 8e9g.1    --------------------------------------------------------------------------------  target    HPLVKQGHKFIFHTPKYRHGSHTTPIDTDMNAVLFGPFGDIYRRDKRSPFVTEGYVDINPTDGLELGLQDGDYVWIDPDP 8e9g.1    ------SGQAVLASWRMLLDAGRLQDGEPHLA-------------GTAV---RPVARMSAATAAGIGASDGAPVTVSTER  target    EDRPFRGWQKNAKDMEFARLLCRARFYPGTPRGVTRMWFNMYGATPGSVRGAKARRDGLAKNPDTNYQAMFRSGSHQSAT 8e9g.1    -----------------GAVTLPLAVTD-MPDGVVWLPMNS---------------------------------------  target    RGWLKPTWMTDSLVRKGLFGQGIGKGFLPDVHCPTGAPREAFVKISRAEPGGIGGQGLWRPAALGIRPRHESPAMKRYLA 8e9g.1    --------------------------------------------------------------------------------  target    GGFFSGPKE 8e9g.1    --------- ``` | | | | | | | | | | | | | | | | | | | | | | | | | | | | | | | | | | | | | | | | | | | | | | | | | |
|  | 3m9s.1.C | NADH-quinone oxidoreductase subunit 3  *Crystal structure of respiratory complex I from Thermus thermophilus* | 0.05 |  | 16.46 | 0.14 | 81-163 | X-ray | 4.50 | hetero-oligomer | 7 x SF4, 2 x FES, 1 x FMN | HHblits | 0.27 |
| ``` target    FNGAPQYINENPFDLELDASRPARPRQYWRAESAHFYNHEDHPLRVGTRLLTGSTHMPTPTKVMWFANANSILGNVKWHY 3m9s.1    --------------------------------------------------------------------------------  target    NTVVNALPRMEMIAVHEWWWTGSC-EWADVVFGVDSWGELKHPDMTASVTNPFLIVFPKTPIKRIFNTVGDIDVLALVSS 3m9s.1    VPPEEALKGKRFVVMHLSHLHPLAERYAHVVLPAPTFYEKRGHLV---NLEGRVLPLSP-APIENGEAEGALQVLALLAE  target    KLAELTGDTRFNDMWKFVREGRTDVYLQRILDASTNTKGYRFTELEAKAREGIPALMNSRTSPKVVGYDQLADSTPWYTK 3m9s.1    ALGV----------------------------------------------------------------------------  target    SGRLEFYREEDEFIEAGENLPVHREPVDSTFYEPNVIVSPKHEAVRPSGPEDYGVARTDLSCEVRCGRNVVLTWAETRQT 3m9s.1    --------------------------------------------------------------------------------  target    QHPLVKQGHKFIFHTPKYRHGSHTTPIDTDMNAVLFGPFGDIYRRDKRSPFVTEGYVDINPTDGLELGLQDGDYVWIDPD 3m9s.1    --------------------------------------------------------------------------------  target    PEDRPFRGWQKNAKDMEFARLLCRARFYPGTPRGVTRMWFNMYGATPGSVRGAKARRDGLAKNPDTNYQAMFRSGSHQSA 3m9s.1    --------------------------------------------------------------------------------  target    TRGWLKPTWMTDSLVRKGLFGQGIGKGFLPDVHCPTGAPREAFVKISRAEPGGIGGQGLWRPAALGIRPRHESPAMKRYL 3m9s.1    --------------------------------------------------------------------------------  target    AGGFFSGPKE 3m9s.1    ---------- ``` | | | | | | | | | | | | | | | | | | | | | | | | | | | | | | | | | | | | | | | | | | | | | | | | | |
|  | 2fug.2.C | NADH-quinone oxidoreductase chain 3  *Crystal structure of the hydrophilic domain of respiratory complex I from Thermus thermophilus* | 0.05 |  | 16.46 | 0.14 | 81-163 | X-ray | 3.30 | hetero-1-1-1-1-1-1-… | 7 x SF4, 2 x FES, 1 x FMN | HHblits | 0.27 |
| ``` target    FNGAPQYINENPFDLELDASRPARPRQYWRAESAHFYNHEDHPLRVGTRLLTGSTHMPTPTKVMWFANANSILGNVKWHY 2fug.2    --------------------------------------------------------------------------------  target    NTVVNALPRMEMIAVHEWWWTGSC-EWADVVFGVDSWGELKHPDMTASVTNPFLIVFPKTPIKRIFNTVGDIDVLALVSS 2fug.2    VPPEEALKGKRFVVMHLSHLHPLAERYAHVVLPAPTFYEKRGHLV---NLEGRVLPLSP-APIENGEAEGALQVLALLAE  target    KLAELTGDTRFNDMWKFVREGRTDVYLQRILDASTNTKGYRFTELEAKAREGIPALMNSRTSPKVVGYDQLADSTPWYTK 2fug.2    ALGV----------------------------------------------------------------------------  target    SGRLEFYREEDEFIEAGENLPVHREPVDSTFYEPNVIVSPKHEAVRPSGPEDYGVARTDLSCEVRCGRNVVLTWAETRQT 2fug.2    --------------------------------------------------------------------------------  target    QHPLVKQGHKFIFHTPKYRHGSHTTPIDTDMNAVLFGPFGDIYRRDKRSPFVTEGYVDINPTDGLELGLQDGDYVWIDPD 2fug.2    --------------------------------------------------------------------------------  target    PEDRPFRGWQKNAKDMEFARLLCRARFYPGTPRGVTRMWFNMYGATPGSVRGAKARRDGLAKNPDTNYQAMFRSGSHQSA 2fug.2    --------------------------------------------------------------------------------  target    TRGWLKPTWMTDSLVRKGLFGQGIGKGFLPDVHCPTGAPREAFVKISRAEPGGIGGQGLWRPAALGIRPRHESPAMKRYL 2fug.2    --------------------------------------------------------------------------------  target    AGGFFSGPKE 2fug.2    ---------- ``` | | | | | | | | | | | | | | | | | | | | | | | | | | | | | | | | | | | | | | | | | | | | | | | | | |
|  | 6zjl.1.C | NADH-quinone oxidoreductase subunit 3  *Respiratory complex I from Thermus thermophilus, NAD+ dataset, major state* | 0.05 |  | 16.46 | 0.14 | 81-163 | EM | 0.00 | hetero-1-1-1-1-1-1-… | 7 x SF4, 1 x FMN, 2 x FES | HHblits | 0.27 |
| ``` target    FNGAPQYINENPFDLELDASRPARPRQYWRAESAHFYNHEDHPLRVGTRLLTGSTHMPTPTKVMWFANANSILGNVKWHY 6zjl.1    --------------------------------------------------------------------------------  target    NTVVNALPRMEMIAVHEWWWTGSC-EWADVVFGVDSWGELKHPDMTASVTNPFLIVFPKTPIKRIFNTVGDIDVLALVSS 6zjl.1    VPPEEALKGKRFVVMHLSHLHPLAERYAHVVLPAPTFYEKRGHLV---NLEGRVLPLSP-APIENGEAEGALQVLALLAE  target    KLAELTGDTRFNDMWKFVREGRTDVYLQRILDASTNTKGYRFTELEAKAREGIPALMNSRTSPKVVGYDQLADSTPWYTK 6zjl.1    ALGV----------------------------------------------------------------------------  target    SGRLEFYREEDEFIEAGENLPVHREPVDSTFYEPNVIVSPKHEAVRPSGPEDYGVARTDLSCEVRCGRNVVLTWAETRQT 6zjl.1    --------------------------------------------------------------------------------  target    QHPLVKQGHKFIFHTPKYRHGSHTTPIDTDMNAVLFGPFGDIYRRDKRSPFVTEGYVDINPTDGLELGLQDGDYVWIDPD 6zjl.1    --------------------------------------------------------------------------------  target    PEDRPFRGWQKNAKDMEFARLLCRARFYPGTPRGVTRMWFNMYGATPGSVRGAKARRDGLAKNPDTNYQAMFRSGSHQSA 6zjl.1    --------------------------------------------------------------------------------  target    TRGWLKPTWMTDSLVRKGLFGQGIGKGFLPDVHCPTGAPREAFVKISRAEPGGIGGQGLWRPAALGIRPRHESPAMKRYL 6zjl.1    --------------------------------------------------------------------------------  target    AGGFFSGPKE 6zjl.1    ---------- ``` | | | | | | | | | | | | | | | | | | | | | | | | | | | | | | | | | | | | | | | | | | | | | | | | | |
|  | 6q8o.1.C | NADH-quinone oxidoreductase subunit 3  *Respiratory complex I from Thermus thermophilus with bound Piericidin A* | 0.05 |  | 16.46 | 0.14 | 81-163 | X-ray | 3.61 | hetero-1-1-1-1-1-1-… | 7 x SF4, 1 x FMN, 2 x FES, 1 x HQH | HHblits | 0.27 |
| ``` target    FNGAPQYINENPFDLELDASRPARPRQYWRAESAHFYNHEDHPLRVGTRLLTGSTHMPTPTKVMWFANANSILGNVKWHY 6q8o.1    --------------------------------------------------------------------------------  target    NTVVNALPRMEMIAVHEWWWTGSC-EWADVVFGVDSWGELKHPDMTASVTNPFLIVFPKTPIKRIFNTVGDIDVLALVSS 6q8o.1    VPPEEALKGKRFVVMHLSHLHPLAERYAHVVLPAPTFYEKRGHLV---NLEGRVLPLSP-APIENGEAEGALQVLALLAE  target    KLAELTGDTRFNDMWKFVREGRTDVYLQRILDASTNTKGYRFTELEAKAREGIPALMNSRTSPKVVGYDQLADSTPWYTK 6q8o.1    ALGV----------------------------------------------------------------------------  target    SGRLEFYREEDEFIEAGENLPVHREPVDSTFYEPNVIVSPKHEAVRPSGPEDYGVARTDLSCEVRCGRNVVLTWAETRQT 6q8o.1    --------------------------------------------------------------------------------  target    QHPLVKQGHKFIFHTPKYRHGSHTTPIDTDMNAVLFGPFGDIYRRDKRSPFVTEGYVDINPTDGLELGLQDGDYVWIDPD 6q8o.1    --------------------------------------------------------------------------------  target    PEDRPFRGWQKNAKDMEFARLLCRARFYPGTPRGVTRMWFNMYGATPGSVRGAKARRDGLAKNPDTNYQAMFRSGSHQSA 6q8o.1    --------------------------------------------------------------------------------  target    TRGWLKPTWMTDSLVRKGLFGQGIGKGFLPDVHCPTGAPREAFVKISRAEPGGIGGQGLWRPAALGIRPRHESPAMKRYL 6q8o.1    --------------------------------------------------------------------------------  target    AGGFFSGPKE 6q8o.1    ---------- ``` | | | | | | | | | | | | | | | | | | | | | | | | | | | | | | | | | | | | | | | | | | | | | | | | | |
|  | 6zjy.1.C | NADH-quinone oxidoreductase subunit 3  *Respiratory complex I from Thermus thermophilus, NAD+ dataset, minor state* | 0.05 |  | 16.46 | 0.14 | 81-163 | EM | 0.00 | hetero-1-1-1-1-1-1-… | 7 x SF4, 2 x FES | HHblits | 0.27 |
| ``` target    FNGAPQYINENPFDLELDASRPARPRQYWRAESAHFYNHEDHPLRVGTRLLTGSTHMPTPTKVMWFANANSILGNVKWHY 6zjy.1    --------------------------------------------------------------------------------  target    NTVVNALPRMEMIAVHEWWWTGSC-EWADVVFGVDSWGELKHPDMTASVTNPFLIVFPKTPIKRIFNTVGDIDVLALVSS 6zjy.1    VPPEEALKGKRFVVMHLSHLHPLAERYAHVVLPAPTFYEKRGHLV---NLEGRVLPLSP-APIENGEAEGALQVLALLAE  target    KLAELTGDTRFNDMWKFVREGRTDVYLQRILDASTNTKGYRFTELEAKAREGIPALMNSRTSPKVVGYDQLADSTPWYTK 6zjy.1    ALGV----------------------------------------------------------------------------  target    SGRLEFYREEDEFIEAGENLPVHREPVDSTFYEPNVIVSPKHEAVRPSGPEDYGVARTDLSCEVRCGRNVVLTWAETRQT 6zjy.1    --------------------------------------------------------------------------------  target    QHPLVKQGHKFIFHTPKYRHGSHTTPIDTDMNAVLFGPFGDIYRRDKRSPFVTEGYVDINPTDGLELGLQDGDYVWIDPD 6zjy.1    --------------------------------------------------------------------------------  target    PEDRPFRGWQKNAKDMEFARLLCRARFYPGTPRGVTRMWFNMYGATPGSVRGAKARRDGLAKNPDTNYQAMFRSGSHQSA 6zjy.1    --------------------------------------------------------------------------------  target    TRGWLKPTWMTDSLVRKGLFGQGIGKGFLPDVHCPTGAPREAFVKISRAEPGGIGGQGLWRPAALGIRPRHESPAMKRYL 6zjy.1    --------------------------------------------------------------------------------  target    AGGFFSGPKE 6zjy.1    ---------- ``` | | | | | | | | | | | | | | | | | | | | | | | | | | | | | | | | | | | | | | | | | | | | | | | | | |
|  | 6zjn.1.C | NADH-quinone oxidoreductase subunit 3  *Respiratory complex I from Thermus thermophilus, NADH dataset, minor state* | 0.05 |  | 16.46 | 0.14 | 81-163 | EM | 0.00 | hetero-1-1-1-1-1-1-… | 7 x SF4, 2 x FES | HHblits | 0.27 |
| ``` target    FNGAPQYINENPFDLELDASRPARPRQYWRAESAHFYNHEDHPLRVGTRLLTGSTHMPTPTKVMWFANANSILGNVKWHY 6zjn.1    --------------------------------------------------------------------------------  target    NTVVNALPRMEMIAVHEWWWTGSC-EWADVVFGVDSWGELKHPDMTASVTNPFLIVFPKTPIKRIFNTVGDIDVLALVSS 6zjn.1    VPPEEALKGKRFVVMHLSHLHPLAERYAHVVLPAPTFYEKRGHLV---NLEGRVLPLSP-APIENGEAEGALQVLALLAE  target    KLAELTGDTRFNDMWKFVREGRTDVYLQRILDASTNTKGYRFTELEAKAREGIPALMNSRTSPKVVGYDQLADSTPWYTK 6zjn.1    ALGV----------------------------------------------------------------------------  target    SGRLEFYREEDEFIEAGENLPVHREPVDSTFYEPNVIVSPKHEAVRPSGPEDYGVARTDLSCEVRCGRNVVLTWAETRQT 6zjn.1    --------------------------------------------------------------------------------  target    QHPLVKQGHKFIFHTPKYRHGSHTTPIDTDMNAVLFGPFGDIYRRDKRSPFVTEGYVDINPTDGLELGLQDGDYVWIDPD 6zjn.1    --------------------------------------------------------------------------------  target    PEDRPFRGWQKNAKDMEFARLLCRARFYPGTPRGVTRMWFNMYGATPGSVRGAKARRDGLAKNPDTNYQAMFRSGSHQSA 6zjn.1    --------------------------------------------------------------------------------  target    TRGWLKPTWMTDSLVRKGLFGQGIGKGFLPDVHCPTGAPREAFVKISRAEPGGIGGQGLWRPAALGIRPRHESPAMKRYL 6zjn.1    --------------------------------------------------------------------------------  target    AGGFFSGPKE 6zjn.1    ---------- ``` | | | | | | | | | | | | | | | | | | | | | | | | | | | | | | | | | | | | | | | | | | | | | | | | | |
|  | 6ziy.1.C | NADH-quinone oxidoreductase subunit 3  *Respiratory complex I from Thermus thermophilus, NADH dataset, major state* | 0.05 |  | 16.46 | 0.14 | 81-163 | EM | 0.00 | hetero-1-1-1-1-1-1-… | 7 x SF4, 1 x FMN, 1 x NAI, 2 x FES | HHblits | 0.27 |
| ``` target    FNGAPQYINENPFDLELDASRPARPRQYWRAESAHFYNHEDHPLRVGTRLLTGSTHMPTPTKVMWFANANSILGNVKWHY 6ziy.1    --------------------------------------------------------------------------------  target    NTVVNALPRMEMIAVHEWWWTGSC-EWADVVFGVDSWGELKHPDMTASVTNPFLIVFPKTPIKRIFNTVGDIDVLALVSS 6ziy.1    VPPEEALKGKRFVVMHLSHLHPLAERYAHVVLPAPTFYEKRGHLV---NLEGRVLPLSP-APIENGEAEGALQVLALLAE  target    KLAELTGDTRFNDMWKFVREGRTDVYLQRILDASTNTKGYRFTELEAKAREGIPALMNSRTSPKVVGYDQLADSTPWYTK 6ziy.1    ALGV----------------------------------------------------------------------------  target    SGRLEFYREEDEFIEAGENLPVHREPVDSTFYEPNVIVSPKHEAVRPSGPEDYGVARTDLSCEVRCGRNVVLTWAETRQT 6ziy.1    --------------------------------------------------------------------------------  target    QHPLVKQGHKFIFHTPKYRHGSHTTPIDTDMNAVLFGPFGDIYRRDKRSPFVTEGYVDINPTDGLELGLQDGDYVWIDPD 6ziy.1    --------------------------------------------------------------------------------  target    PEDRPFRGWQKNAKDMEFARLLCRARFYPGTPRGVTRMWFNMYGATPGSVRGAKARRDGLAKNPDTNYQAMFRSGSHQSA 6ziy.1    --------------------------------------------------------------------------------  target    TRGWLKPTWMTDSLVRKGLFGQGIGKGFLPDVHCPTGAPREAFVKISRAEPGGIGGQGLWRPAALGIRPRHESPAMKRYL 6ziy.1    --------------------------------------------------------------------------------  target    AGGFFSGPKE 6ziy.1    ---------- ``` | | | | | | | | | | | | | | | | | | | | | | | | | | | | | | | | | | | | | | | | | | | | | | | | | |
|  | 1h0h.1.A | FORMATE DEHYDROGENASE SUBUNIT ALPHA  *Tungsten containing Formate Dehydrogenase from Desulfovibrio Gigas* | 0.03 |  | 14.29 | 0.14 | 326-435 | X-ray | 1.80 | hetero-1-1-mer | 1 x W, 1 x 2MD, 1 x MGD, 4 x SF4, 1 x CA | HHblits | 0.27 |
| ``` target    FNGAPQYINENPFDLELDASRPARPRQYWRAESAHFYNHEDHPLRVGTRLLTGSTHMPTPTKVMWFANANSILGNVKWHY 1h0h.1    --------------------------------------------------------------------------------  target    NTVVNALPRMEMIAVHEWWWTGSCEWADVVFGVDSWGELKHPDMTASVTNPFLIVFPKTPIKRIFNTVGDIDVLALVSSK 1h0h.1    --------------------------------------------------------------------------------  target    LAELTGDTRFNDMWKFVREGRTDVYLQRILDASTNTKGYRFTELEAKAREGIPALMNSRTSPKVVGYDQLADSTPWYTKS 1h0h.1    --------------------------------------------------------------------------------  target    GRLEFYREEDEFIEAGENLPVHREPVDSTFYEPNVIVSPKHEAVRPSGPEDYGVARTDLSCEVRCGRNVVLTWAETRQTQ 1h0h.1    --------------------------------------------------------------------------------  target    HPLVKQGHKFIFHTPKYR--HGSHTTPIDTDMNAVLFGPFGDIYRRDKRSPFVTEGYVDINPTDGLELGLQDGDYVWIDP 1h0h.1    -----PRYPFICSTYRVTEHWQTGLMTRNTPWLL-------------EAEP---QMFCEMSEELATLRGIKNGDKVILES  target    DPEDRPFRGWQKNAKDMEFARLLCRARFYPGTPRGVTRMWFNMYGATPGSVRGAKARRDGLAKNPDTNYQAMFRSGSHQS 1h0h.1    VR-----------------GKLWAKAIITKRIKPFAI-------------------------------------------  target    ATRGWLKPTWMTDSLVRKGLFGQGIGKGFLPDVHCPTGAPREAFVKISRAEPGGIGGQGLWRPAALGIRPRHESPAMKRY 1h0h.1    --------------------------------------------------------------------------------  target    LAGGFFSGPKE 1h0h.1    ----------- ``` | | | | | | | | | | | | | | | | | | | | | | | | | | | | | | | | | | | | | | | | | | | | | | | | | |
|  | 8bqg.1.A | Formate dehydrogenase, alpha subunit, selenocysteine-containing  *W-formate dehydrogenase from Desulfovibrio vulgaris - Soaking with Formate 1 min* | 0.03 |  | 15.58 | 0.14 | 326-435 | X-ray | 1.95 | hetero-1-1-mer | 2 x MGD, 4 x SF4, 1 x H2S, 1 x W | HHblits | 0.26 |
| ``` target    FNGAPQYINENPFDLELDASRPARPRQYWRAESAHFYNHEDHPLRVGTRLLTGSTHMPTPTKVMWFANANSILGNVKWHY 8bqg.1    --------------------------------------------------------------------------------  target    NTVVNALPRMEMIAVHEWWWTGSCEWADVVFGVDSWGELKHPDMTASVTNPFLIVFPKTPIKRIFNTVGDIDVLALVSSK 8bqg.1    --------------------------------------------------------------------------------  target    LAELTGDTRFNDMWKFVREGRTDVYLQRILDASTNTKGYRFTELEAKAREGIPALMNSRTSPKVVGYDQLADSTPWYTKS 8bqg.1    --------------------------------------------------------------------------------  target    GRLEFYREEDEFIEAGENLPVHREPVDSTFYEPNVIVSPKHEAVRPSGPEDYGVARTDLSCEVRCGRNVVLTWAETRQTQ 8bqg.1    --------------------------------------------------------------------------------  target    HPLVKQGHKFIFHTPKYRHGSHT--TPIDTDMNAVLFGPFGDIYRRDKRSPFVTEGYVDINPTDGLELGLQDGDYVWIDP 8bqg.1    -----PRYPFIGTTYRVTEHWQTGLMTRRCAWLV-------------EAEP---QIFCEISKELAKLRGIGNGDTVKVSS  target    DPEDRPFRGWQKNAKDMEFARLLCRARFYPGTPRGVTRMWFNMYGATPGSVRGAKARRDGLAKNPDTNYQAMFRSGSHQS 8bqg.1    LR-----------------GALEAVAIVTERIRPFKI-------------------------------------------  target    ATRGWLKPTWMTDSLVRKGLFGQGIGKGFLPDVHCPTGAPREAFVKISRAEPGGIGGQGLWRPAALGIRPRHESPAMKRY 8bqg.1    --------------------------------------------------------------------------------  target    LAGGFFSGPKE 8bqg.1    ----------- ``` | | | | | | | | | | | | | | | | | | | | | | | | | | | | | | | | | | | | | | | | | | | | | | | | | |
|  | 6sdv.1.A | Formate dehydrogenase, alpha subunit, selenocysteine-containing,Formate dehydrogenase, alpha subunit, selenocysteine-containing,W-formate dehydrogenase - alpha subunit  *W-formate dehydrogenase from Desulfovibrio vulgaris - Formate reduced form* | 0.03 |  | 14.29 | 0.14 | 326-435 | X-ray | 1.90 | hetero-1-1-mer | 2 x MGD, 4 x SF4, 1 x W, 1 x H2S | HHblits | 0.25 |
| ``` target    FNGAPQYINENPFDLELDASRPARPRQYWRAESAHFYNHEDHPLRVGTRLLTGSTHMPTPTKVMWFANANSILGNVKWHY 6sdv.1    --------------------------------------------------------------------------------  target    NTVVNALPRMEMIAVHEWWWTGSCEWADVVFGVDSWGELKHPDMTASVTNPFLIVFPKTPIKRIFNTVGDIDVLALVSSK 6sdv.1    --------------------------------------------------------------------------------  target    LAELTGDTRFNDMWKFVREGRTDVYLQRILDASTNTKGYRFTELEAKAREGIPALMNSRTSPKVVGYDQLADSTPWYTKS 6sdv.1    --------------------------------------------------------------------------------  target    GRLEFYREEDEFIEAGENLPVHREPVDSTFYEPNVIVSPKHEAVRPSGPEDYGVARTDLSCEVRCGRNVVLTWAETRQTQ 6sdv.1    --------------------------------------------------------------------------------  target    HPLVKQGHKFIFHTPKYRHG--SHTTPIDTDMNAVLFGPFGDIYRRDKRSPFVTEGYVDINPTDGLELGLQDGDYVWIDP 6sdv.1    -----PRYPFIGTTYRVTEHWQTGLMTRRCAWLV-------------EAEP---QIFCEISKELAKLRGIGNGDTVKVSS  target    DPEDRPFRGWQKNAKDMEFARLLCRARFYPGTPRGVTRMWFNMYGATPGSVRGAKARRDGLAKNPDTNYQAMFRSGSHQS 6sdv.1    LR-----------------GALEAVAIVTERIRPFKI-------------------------------------------  target    ATRGWLKPTWMTDSLVRKGLFGQGIGKGFLPDVHCPTGAPREAFVKISRAEPGGIGGQGLWRPAALGIRPRHESPAMKRY 6sdv.1    --------------------------------------------------------------------------------  target    LAGGFFSGPKE 6sdv.1    ----------- ``` | | | | | | | | | | | | | | | | | | | | | | | | | | | | | | | | | | | | | | | | | | | | | | | | | |
|  | 6sdr.1.A | Formate dehydrogenase, alpha subunit, selenocysteine-containing  *W-formate dehydrogenase from Desulfovibrio vulgaris - Oxidized form* | 0.03 |  | 14.29 | 0.14 | 326-435 | X-ray | 2.10 | hetero-1-1-mer | 2 x MGD, 4 x SF4, 1 x H2S, 1 x W | HHblits | 0.25 |
| ``` target    FNGAPQYINENPFDLELDASRPARPRQYWRAESAHFYNHEDHPLRVGTRLLTGSTHMPTPTKVMWFANANSILGNVKWHY 6sdr.1    --------------------------------------------------------------------------------  target    NTVVNALPRMEMIAVHEWWWTGSCEWADVVFGVDSWGELKHPDMTASVTNPFLIVFPKTPIKRIFNTVGDIDVLALVSSK 6sdr.1    --------------------------------------------------------------------------------  target    LAELTGDTRFNDMWKFVREGRTDVYLQRILDASTNTKGYRFTELEAKAREGIPALMNSRTSPKVVGYDQLADSTPWYTKS 6sdr.1    --------------------------------------------------------------------------------  target    GRLEFYREEDEFIEAGENLPVHREPVDSTFYEPNVIVSPKHEAVRPSGPEDYGVARTDLSCEVRCGRNVVLTWAETRQTQ 6sdr.1    --------------------------------------------------------------------------------  target    HPLVKQGHKFIFHTPKYRHG--SHTTPIDTDMNAVLFGPFGDIYRRDKRSPFVTEGYVDINPTDGLELGLQDGDYVWIDP 6sdr.1    -----PRYPFIGTTYRVTEHWQTGLMTRRCAWLV-------------EAEP---QIFCEISKELAKLRGIGNGDTVKVSS  target    DPEDRPFRGWQKNAKDMEFARLLCRARFYPGTPRGVTRMWFNMYGATPGSVRGAKARRDGLAKNPDTNYQAMFRSGSHQS 6sdr.1    LR-----------------GALEAVAIVTERIRPFKI-------------------------------------------  target    ATRGWLKPTWMTDSLVRKGLFGQGIGKGFLPDVHCPTGAPREAFVKISRAEPGGIGGQGLWRPAALGIRPRHESPAMKRY 6sdr.1    --------------------------------------------------------------------------------  target    LAGGFFSGPKE 6sdr.1    ----------- ``` | | | | | | | | | | | | | | | | | | | | | | | | | | | | | | | | | | | | | | | | | | | | | | | | | |
|  | 2e7z.1.A | Acetylene hydratase Ahy  *Acetylene Hydratase from Pelobacter acetylenicus* | 0.02 |  | 21.43 | 0.10 | 60-115 | X-ray | 1.26 | monomer | 1 x SF4, 2 x MGD, 1 x W | HHblits | 0.28 |
| ``` target    FNGAPQYINENPFDLELDASRPARPRQYWRAESAHFYNHEDHPLRVGTRLLTGSTHMPTPTKVMWFANANSILGNVKWHY 2e7z.1    -----------------------------------------------------------DSNCLLFIGKNLSNHNWVSQF  target    NTVVNALPRMEMIAVHEWWWTGSCEWADVVFGVDSWGELKHPDMTASVTNPFLIVFPKTPIKRIFNTVGDIDVLALVSSK 2e7z.1    NDLKAALKRGCKLIVLDPRRTKVAEMADIWLPLRY---------------------------------------------  target    LAELTGDTRFNDMWKFVREGRTDVYLQRILDASTNTKGYRFTELEAKAREGIPALMNSRTSPKVVGYDQLADSTPWYTKS 2e7z.1    --------------------------------------------------------------------------------  target    GRLEFYREEDEFIEAGENLPVHREPVDSTFYEPNVIVSPKHEAVRPSGPEDYGVARTDLSCEVRCGRNVVLTWAETRQTQ 2e7z.1    --------------------------------------------------------------------------------  target    HPLVKQGHKFIFHTPKYRHGSHTTPIDTDMNAVLFGPFGDIYRRDKRSPFVTEGYVDINPTDGLELGLQDGDYVWIDPDP 2e7z.1    --------------------------------------------------------------------------------  target    EDRPFRGWQKNAKDMEFARLLCRARFYPGTPRGVTRMWFNMYGATPGSVRGAKARRDGLAKNPDTNYQAMFRSGSHQSAT 2e7z.1    --------------------------------------------------------------------------------  target    RGWLKPTWMTDSLVRKGLFGQGIGKGFLPDVHCPTGAPREAFVKISRAEPGGIGGQGLWRPAALGIRPRHESPAMKRYLA 2e7z.1    --------------------------------------------------------------------------------  target    GGFFSGPKE 2e7z.1    --------- ``` | | | | | | | | | | | | | | | | | | | | | | | | | | | | | | | | | | | | | | | | | | | | | | | | | |
|  | 1e18.1.A | DMSO REDUCTASE.  *TUNGSTEN-SUSBSTITUTED DMSO REDUCTASE FROM RHODOBACTER CAPSULATUS* | 0.01 |  | 19.30 | 0.10 | 59-115 | X-ray | 2.00 | monomer | 2 x PGD, 1 x 6WO | HHblits | 0.26 |
| ``` target    FNGAPQYINENPFDLELDASRPARPRQYWRAESAHFYNHEDHPLRVGTRLLTGSTHMPTPTKVMWFANANSILGNVKWH- 1e18.1    ----------------------------------------------------------ENTEVMVFWAADPIKTSQIGWV  target    -----YNTVVNALP-RMEMIAVHEWWWTGSCEW-ADVVFGVDSWGELKHPDMTASVTNPFLIVFPKTPIKRIFNTVGDID 1e18.1    IPEHGAYPGLEALKAKGTKVIVIDPVRTKTVEFFGAEHITPKP-------------------------------------  target    VLALVSSKLAELTGDTRFNDMWKFVREGRTDVYLQRILDASTNTKGYRFTELEAKAREGIPALMNSRTSPKVVGYDQLAD 1e18.1    --------------------------------------------------------------------------------  target    STPWYTKSGRLEFYREEDEFIEAGENLPVHREPVDSTFYEPNVIVSPKHEAVRPSGPEDYGVARTDLSCEVRCGRNVVLT 1e18.1    --------------------------------------------------------------------------------  target    WAETRQTQHPLVKQGHKFIFHTPKYRHGSHTTPIDTDMNAVLFGPFGDIYRRDKRSPFVTEGYVDINPTDGLELGLQDGD 1e18.1    --------------------------------------------------------------------------------  target    YVWIDPDPEDRPFRGWQKNAKDMEFARLLCRARFYPGTPRGVTRMWFNMYGATPGSVRGAKARRDGLAKNPDTNYQAMFR 1e18.1    --------------------------------------------------------------------------------  target    SGSHQSATRGWLKPTWMTDSLVRKGLFGQGIGKGFLPDVHCPTGAPREAFVKISRAEPGGIGGQGLWRPAALGIRPRHES 1e18.1    --------------------------------------------------------------------------------  target    PAMKRYLAGGFFSGPKE 1e18.1    ----------------- ``` | | | | | | | | | | | | | | | | | | | | | | | | | | | | | | | | | | | | | | | | | | | | | | | | | |
|  | 1e5v.2.A | Dimethyl sulfoxide/trimethylamine N-oxide reductase  *OXIDIZED DMSO REDUCTASE EXPOSED TO HEPES BUFFER* | 0.01 |  | 19.30 | 0.10 | 59-115 | X-ray | 2.40 | monomer | 2 x PGD, 1 x 2MO | HHblits | 0.26 |
| ``` target    FNGAPQYINENPFDLELDASRPARPRQYWRAESAHFYNHEDHPLRVGTRLLTGSTHMPTPTKVMWFANANSILGNVKWH- 1e5v.2    ----------------------------------------------------------ENTEVMVFWAADPIKTSQIGWV  target    -----YNTVVNALP-RMEMIAVHEWWWTGSCEW-ADVVFGVDSWGELKHPDMTASVTNPFLIVFPKTPIKRIFNTVGDID 1e5v.2    IPEHGAYPGLEALKAKGTKVIVIDPVRTKTVEFFGAEHITPKP-------------------------------------  target    VLALVSSKLAELTGDTRFNDMWKFVREGRTDVYLQRILDASTNTKGYRFTELEAKAREGIPALMNSRTSPKVVGYDQLAD 1e5v.2    --------------------------------------------------------------------------------  target    STPWYTKSGRLEFYREEDEFIEAGENLPVHREPVDSTFYEPNVIVSPKHEAVRPSGPEDYGVARTDLSCEVRCGRNVVLT 1e5v.2    --------------------------------------------------------------------------------  target    WAETRQTQHPLVKQGHKFIFHTPKYRHGSHTTPIDTDMNAVLFGPFGDIYRRDKRSPFVTEGYVDINPTDGLELGLQDGD 1e5v.2    --------------------------------------------------------------------------------  target    YVWIDPDPEDRPFRGWQKNAKDMEFARLLCRARFYPGTPRGVTRMWFNMYGATPGSVRGAKARRDGLAKNPDTNYQAMFR 1e5v.2    --------------------------------------------------------------------------------  target    SGSHQSATRGWLKPTWMTDSLVRKGLFGQGIGKGFLPDVHCPTGAPREAFVKISRAEPGGIGGQGLWRPAALGIRPRHES 1e5v.2    --------------------------------------------------------------------------------  target    PAMKRYLAGGFFSGPKE 1e5v.2    ----------------- ``` | | | | | | | | | | | | | | | | | | | | | | | | | | | | | | | | | | | | | | | | | | | | | | | | | |
|  | 1e60.1.A | Dimethyl sulfoxide/trimethylamine N-oxide reductase  *OXIDIZED DMSO REDUCTASE EXPOSED TO HEPES - Structure II BUFFER* | 0.01 |  | 19.30 | 0.10 | 59-115 | X-ray | 2.00 | monomer | 2 x PGD, 1 x 2MO | HHblits | 0.26 |
| ``` target    FNGAPQYINENPFDLELDASRPARPRQYWRAESAHFYNHEDHPLRVGTRLLTGSTHMPTPTKVMWFANANSILGNVKWH- 1e60.1    ----------------------------------------------------------ENTEVMVFWAADPIKTSQIGWV  target    -----YNTVVNALP-RMEMIAVHEWWWTGSCEW-ADVVFGVDSWGELKHPDMTASVTNPFLIVFPKTPIKRIFNTVGDID 1e60.1    IPEHGAYPGLEALKAKGTKVIVIDPVRTKTVEFFGAEHITPKP-------------------------------------  target    VLALVSSKLAELTGDTRFNDMWKFVREGRTDVYLQRILDASTNTKGYRFTELEAKAREGIPALMNSRTSPKVVGYDQLAD 1e60.1    --------------------------------------------------------------------------------  target    STPWYTKSGRLEFYREEDEFIEAGENLPVHREPVDSTFYEPNVIVSPKHEAVRPSGPEDYGVARTDLSCEVRCGRNVVLT 1e60.1    --------------------------------------------------------------------------------  target    WAETRQTQHPLVKQGHKFIFHTPKYRHGSHTTPIDTDMNAVLFGPFGDIYRRDKRSPFVTEGYVDINPTDGLELGLQDGD 1e60.1    --------------------------------------------------------------------------------  target    YVWIDPDPEDRPFRGWQKNAKDMEFARLLCRARFYPGTPRGVTRMWFNMYGATPGSVRGAKARRDGLAKNPDTNYQAMFR 1e60.1    --------------------------------------------------------------------------------  target    SGSHQSATRGWLKPTWMTDSLVRKGLFGQGIGKGFLPDVHCPTGAPREAFVKISRAEPGGIGGQGLWRPAALGIRPRHES 1e60.1    --------------------------------------------------------------------------------  target    PAMKRYLAGGFFSGPKE 1e60.1    ----------------- ``` | | | | | | | | | | | | | | | | | | | | | | | | | | | | | | | | | | | | | | | | | | | | | | | | | |
|  | 1aa6.1.A | FORMATE DEHYDROGENASE H  *REDUCED FORM OF FORMATE DEHYDROGENASE H FROM E. COLI* | 0.02 |  | 17.86 | 0.10 | 59-115 | X-ray | 2.30 | monomer | 1 x SF4, 2 x MGD, 1 x 4MO | HHblits | 0.27 |
| ``` target    FNGAPQYINENPFDLELDASRPARPRQYWRAESAHFYNHEDHPLRVGTRLLTGSTHMPTPTKVMWFANANSILGNVKWHY 1aa6.1    ----------------------------------------------------------DNTDLVFVFGYNPADSH-PIVA  target    NTVVNALPRMEMIAVHEWWWTGSCEWADVVFGVDSWGELKHPDMTASVTNPFLIVFPKTPIKRIFNTVGDIDVLALVSSK 1aa6.1    NHVINAKRNGAKIIVCDPRKIETARIADMHIALKN---------------------------------------------  target    LAELTGDTRFNDMWKFVREGRTDVYLQRILDASTNTKGYRFTELEAKAREGIPALMNSRTSPKVVGYDQLADSTPWYTKS 1aa6.1    --------------------------------------------------------------------------------  target    GRLEFYREEDEFIEAGENLPVHREPVDSTFYEPNVIVSPKHEAVRPSGPEDYGVARTDLSCEVRCGRNVVLTWAETRQTQ 1aa6.1    --------------------------------------------------------------------------------  target    HPLVKQGHKFIFHTPKYRHGSHTTPIDTDMNAVLFGPFGDIYRRDKRSPFVTEGYVDINPTDGLELGLQDGDYVWIDPDP 1aa6.1    --------------------------------------------------------------------------------  target    EDRPFRGWQKNAKDMEFARLLCRARFYPGTPRGVTRMWFNMYGATPGSVRGAKARRDGLAKNPDTNYQAMFRSGSHQSAT 1aa6.1    --------------------------------------------------------------------------------  target    RGWLKPTWMTDSLVRKGLFGQGIGKGFLPDVHCPTGAPREAFVKISRAEPGGIGGQGLWRPAALGIRPRHESPAMKRYLA 1aa6.1    --------------------------------------------------------------------------------  target    GGFFSGPKE 1aa6.1    --------- ``` | | | | | | | | | | | | | | | | | | | | | | | | | | | | | | | | | | | | | | | | | | | | | | | | | |
|  | 1fdo.1.A | FORMATE DEHYDROGENASE H  *OXIDIZED FORM OF FORMATE DEHYDROGENASE H FROM E. COLI* | 0.02 |  | 17.86 | 0.10 | 59-115 | X-ray | 2.80 | monomer | 1 x SF4, 2 x MGD, 1 x 6MO | HHblits | 0.27 |
| ``` target    FNGAPQYINENPFDLELDASRPARPRQYWRAESAHFYNHEDHPLRVGTRLLTGSTHMPTPTKVMWFANANSILGNVKWHY 1fdo.1    ----------------------------------------------------------DNTDLVFVFGYNPADSH-PIVA  target    NTVVNALPRMEMIAVHEWWWTGSCEWADVVFGVDSWGELKHPDMTASVTNPFLIVFPKTPIKRIFNTVGDIDVLALVSSK 1fdo.1    NHVINAKRNGAKIIVCDPRKIETARIADMHIALKN---------------------------------------------  target    LAELTGDTRFNDMWKFVREGRTDVYLQRILDASTNTKGYRFTELEAKAREGIPALMNSRTSPKVVGYDQLADSTPWYTKS 1fdo.1    --------------------------------------------------------------------------------  target    GRLEFYREEDEFIEAGENLPVHREPVDSTFYEPNVIVSPKHEAVRPSGPEDYGVARTDLSCEVRCGRNVVLTWAETRQTQ 1fdo.1    --------------------------------------------------------------------------------  target    HPLVKQGHKFIFHTPKYRHGSHTTPIDTDMNAVLFGPFGDIYRRDKRSPFVTEGYVDINPTDGLELGLQDGDYVWIDPDP 1fdo.1    --------------------------------------------------------------------------------  target    EDRPFRGWQKNAKDMEFARLLCRARFYPGTPRGVTRMWFNMYGATPGSVRGAKARRDGLAKNPDTNYQAMFRSGSHQSAT 1fdo.1    --------------------------------------------------------------------------------  target    RGWLKPTWMTDSLVRKGLFGQGIGKGFLPDVHCPTGAPREAFVKISRAEPGGIGGQGLWRPAALGIRPRHESPAMKRYLA 1fdo.1    --------------------------------------------------------------------------------  target    GGFFSGPKE 1fdo.1    --------- ``` | | | | | | | | | | | | | | | | | | | | | | | | | | | | | | | | | | | | | | | | | | | | | | | | | |
|  | 2iv2.1.A | Formate dehydrogenase H  *Reinterpretation of reduced form of formate dehydrogenase H from E. coli* | 0.02 |  | 17.86 | 0.10 | 59-115 | X-ray | 2.27 | monomer | 1 x SF4, 1 x 2MD, 1 x MGD | HHblits | 0.27 |
| ``` target    FNGAPQYINENPFDLELDASRPARPRQYWRAESAHFYNHEDHPLRVGTRLLTGSTHMPTPTKVMWFANANSILGNVKWHY 2iv2.1    ----------------------------------------------------------DNTDLVFVFGYNPADSH-PIVA  target    NTVVNALPRMEMIAVHEWWWTGSCEWADVVFGVDSWGELKHPDMTASVTNPFLIVFPKTPIKRIFNTVGDIDVLALVSSK 2iv2.1    NHVINAKRNGAKIIVCDPRKIETARIADMHIALKN---------------------------------------------  target    LAELTGDTRFNDMWKFVREGRTDVYLQRILDASTNTKGYRFTELEAKAREGIPALMNSRTSPKVVGYDQLADSTPWYTKS 2iv2.1    --------------------------------------------------------------------------------  target    GRLEFYREEDEFIEAGENLPVHREPVDSTFYEPNVIVSPKHEAVRPSGPEDYGVARTDLSCEVRCGRNVVLTWAETRQTQ 2iv2.1    --------------------------------------------------------------------------------  target    HPLVKQGHKFIFHTPKYRHGSHTTPIDTDMNAVLFGPFGDIYRRDKRSPFVTEGYVDINPTDGLELGLQDGDYVWIDPDP 2iv2.1    --------------------------------------------------------------------------------  target    EDRPFRGWQKNAKDMEFARLLCRARFYPGTPRGVTRMWFNMYGATPGSVRGAKARRDGLAKNPDTNYQAMFRSGSHQSAT 2iv2.1    --------------------------------------------------------------------------------  target    RGWLKPTWMTDSLVRKGLFGQGIGKGFLPDVHCPTGAPREAFVKISRAEPGGIGGQGLWRPAALGIRPRHESPAMKRYLA 2iv2.1    --------------------------------------------------------------------------------  target    GGFFSGPKE 2iv2.1    --------- ``` | | | | | | | | | | | | | | | | | | | | | | | | | | | | | | | | | | | | | | | | | | | | | | | | | |
|  | 7z0t.1.G | Formate dehydrogenase H  *Structure of the Escherichia coli formate hydrogenlyase complex (aerobic preparation, composite structure)* | 0.02 |  | 17.86 | 0.10 | 59-115 | EM | 0.00 | hetero-1-1-1-1-1-1-… | 1 x NI, 1 x FCO, 8 x SF4, 1 x FE, 2 x MGD, 1 x 6MO | HHblits | 0.27 |
| ``` target    FNGAPQYINENPFDLELDASRPARPRQYWRAESAHFYNHEDHPLRVGTRLLTGSTHMPTPTKVMWFANANSILGNVKWHY 7z0t.1    ----------------------------------------------------------DNTDLVFVFGYNPADSH-PIVA  target    NTVVNALPRMEMIAVHEWWWTGSCEWADVVFGVDSWGELKHPDMTASVTNPFLIVFPKTPIKRIFNTVGDIDVLALVSSK 7z0t.1    NHVINAKRNGAKIIVCDPRKIETARIADMHIALKN---------------------------------------------  target    LAELTGDTRFNDMWKFVREGRTDVYLQRILDASTNTKGYRFTELEAKAREGIPALMNSRTSPKVVGYDQLADSTPWYTKS 7z0t.1    --------------------------------------------------------------------------------  target    GRLEFYREEDEFIEAGENLPVHREPVDSTFYEPNVIVSPKHEAVRPSGPEDYGVARTDLSCEVRCGRNVVLTWAETRQTQ 7z0t.1    --------------------------------------------------------------------------------  target    HPLVKQGHKFIFHTPKYRHGSHTTPIDTDMNAVLFGPFGDIYRRDKRSPFVTEGYVDINPTDGLELGLQDGDYVWIDPDP 7z0t.1    --------------------------------------------------------------------------------  target    EDRPFRGWQKNAKDMEFARLLCRARFYPGTPRGVTRMWFNMYGATPGSVRGAKARRDGLAKNPDTNYQAMFRSGSHQSAT 7z0t.1    --------------------------------------------------------------------------------  target    RGWLKPTWMTDSLVRKGLFGQGIGKGFLPDVHCPTGAPREAFVKISRAEPGGIGGQGLWRPAALGIRPRHESPAMKRYLA 7z0t.1    --------------------------------------------------------------------------------  target    GGFFSGPKE 7z0t.1    --------- ``` | | | | | | | | | | | | | | | | | | | | | | | | | | | | | | | | | | | | | | | | | | | | | | | | | |
|  | 1dms.1.A | DMSO REDUCTASE  *STRUCTURE OF DMSO REDUCTASE* | 0.01 |  | 19.30 | 0.10 | 59-115 | X-ray | 1.88 | monomer | 2 x PGD, 1 x 2MO | HHblits | 0.26 |
| ``` target    FNGAPQYINENPFDLELDASRPARPRQYWRAESAHFYNHEDHPLRVGTRLLTGSTHMPTPTKVMWFANANSILGNVKWH- 1dms.1    ----------------------------------------------------------ENTEVMVFWAADPIKTSQIGWV  target    -----YNTVVNALPRM-EMIAVHEWWWTGSCEW-ADVVFGVDSWGELKHPDMTASVTNPFLIVFPKTPIKRIFNTVGDID 1dms.1    IPEHGAYPGLEALKAKGTKVIVIDPVRTKTVEFFGADHVTPKP-------------------------------------  target    VLALVSSKLAELTGDTRFNDMWKFVREGRTDVYLQRILDASTNTKGYRFTELEAKAREGIPALMNSRTSPKVVGYDQLAD 1dms.1    --------------------------------------------------------------------------------  target    STPWYTKSGRLEFYREEDEFIEAGENLPVHREPVDSTFYEPNVIVSPKHEAVRPSGPEDYGVARTDLSCEVRCGRNVVLT 1dms.1    --------------------------------------------------------------------------------  target    WAETRQTQHPLVKQGHKFIFHTPKYRHGSHTTPIDTDMNAVLFGPFGDIYRRDKRSPFVTEGYVDINPTDGLELGLQDGD 1dms.1    --------------------------------------------------------------------------------  target    YVWIDPDPEDRPFRGWQKNAKDMEFARLLCRARFYPGTPRGVTRMWFNMYGATPGSVRGAKARRDGLAKNPDTNYQAMFR 1dms.1    --------------------------------------------------------------------------------  target    SGSHQSATRGWLKPTWMTDSLVRKGLFGQGIGKGFLPDVHCPTGAPREAFVKISRAEPGGIGGQGLWRPAALGIRPRHES 1dms.1    --------------------------------------------------------------------------------  target    PAMKRYLAGGFFSGPKE 1dms.1    ----------------- ``` | | | | | | | | | | | | | | | | | | | | | | | | | | | | | | | | | | | | | | | | | | | | | | | | | |
|  | 7bkb.1.F | Formate dehydrogenase  *Formate dehydrogenase - heterodisulfide reductase - formylmethanofuran dehydrogenase complex from Methanospirillum hungatei (hexameric, composite structure)* | 0.02 |  | 10.71 | 0.10 | 59-115 | EM | 0.00 | hetero-2-2-2-2-2-2-… | 48 x SF4, 4 x FAD, 2 x FES, 4 x 9S8, 4 x ZN, 2 x MO, 4 x MGD | HHblits | 0.26 |
| ``` target    FNGAPQYINENPFDLELDASRPARPRQYWRAESAHFYNHEDHPLRVGTRLLTGSTHMPTPTKVMWFANANSILGNVKWHY 7bkb.1    ----------------------------------------------------------LNADLILIWGSNAVEAH-PLAG  target    NTVVNALPRMEMIAVHEWWWTGSCEWADVVFGVDSWGELKHPDMTASVTNPFLIVFPKTPIKRIFNTVGDIDVLALVSSK 7bkb.1    RRIAQAKKKGIQIIAVDPRYTMTARLADTYVRFNP---------------------------------------------  target    LAELTGDTRFNDMWKFVREGRTDVYLQRILDASTNTKGYRFTELEAKAREGIPALMNSRTSPKVVGYDQLADSTPWYTKS 7bkb.1    --------------------------------------------------------------------------------  target    GRLEFYREEDEFIEAGENLPVHREPVDSTFYEPNVIVSPKHEAVRPSGPEDYGVARTDLSCEVRCGRNVVLTWAETRQTQ 7bkb.1    --------------------------------------------------------------------------------  target    HPLVKQGHKFIFHTPKYRHGSHTTPIDTDMNAVLFGPFGDIYRRDKRSPFVTEGYVDINPTDGLELGLQDGDYVWIDPDP 7bkb.1    --------------------------------------------------------------------------------  target    EDRPFRGWQKNAKDMEFARLLCRARFYPGTPRGVTRMWFNMYGATPGSVRGAKARRDGLAKNPDTNYQAMFRSGSHQSAT 7bkb.1    --------------------------------------------------------------------------------  target    RGWLKPTWMTDSLVRKGLFGQGIGKGFLPDVHCPTGAPREAFVKISRAEPGGIGGQGLWRPAALGIRPRHESPAMKRYLA 7bkb.1    --------------------------------------------------------------------------------  target    GGFFSGPKE 7bkb.1    --------- ``` | | | | | | | | | | | | | | | | | | | | | | | | | | | | | | | | | | | | | | | | | | | | | | | | | |
|  | 7qv7.1.L | Hydrogen dependent carbon dioxide reductase subunit FdhF  *Cryo-EM structure of Hydrogen-dependent CO2 reductase.* | 0.02 |  | 18.18 | 0.10 | 60-115 | EM | 0.00 | hetero-2-6-6-2-mer | 52 x SF4, 6 x 402 | HHblits | 0.28 |
| ``` target    FNGAPQYINENPFDLELDASRPARPRQYWRAESAHFYNHEDHPLRVGTRLLTGSTHMPTPTKVMWFANANSILGNVKWHY 7qv7.1    -----------------------------------------------------------YSDVIFIIGSNTAECH-PLIA  target    NTVVNALPRMEMIAVHEWWWTGSCEWADVVFGVDSWGELKHPDMTASVTNPFLIVFPKTPIKRIFNTVGDIDVLALVSSK 7qv7.1    AHVIKAKERGAKLIVADPRMNAMVHKADIWLRVPS---------------------------------------------  target    LAELTGDTRFNDMWKFVREGRTDVYLQRILDASTNTKGYRFTELEAKAREGIPALMNSRTSPKVVGYDQLADSTPWYTKS 7qv7.1    --------------------------------------------------------------------------------  target    GRLEFYREEDEFIEAGENLPVHREPVDSTFYEPNVIVSPKHEAVRPSGPEDYGVARTDLSCEVRCGRNVVLTWAETRQTQ 7qv7.1    --------------------------------------------------------------------------------  target    HPLVKQGHKFIFHTPKYRHGSHTTPIDTDMNAVLFGPFGDIYRRDKRSPFVTEGYVDINPTDGLELGLQDGDYVWIDPDP 7qv7.1    --------------------------------------------------------------------------------  target    EDRPFRGWQKNAKDMEFARLLCRARFYPGTPRGVTRMWFNMYGATPGSVRGAKARRDGLAKNPDTNYQAMFRSGSHQSAT 7qv7.1    --------------------------------------------------------------------------------  target    RGWLKPTWMTDSLVRKGLFGQGIGKGFLPDVHCPTGAPREAFVKISRAEPGGIGGQGLWRPAALGIRPRHESPAMKRYLA 7qv7.1    --------------------------------------------------------------------------------  target    GGFFSGPKE 7qv7.1    --------- ``` | | | | | | | | | | | | | | | | | | | | | | | | | | | | | | | | | | | | | | | | | | | | | | | | | |
|  | 7qv7.1.O | Hydrogen dependent carbon dioxide reductase subunit FdhF  *Cryo-EM structure of Hydrogen-dependent CO2 reductase.* | 0.02 |  | 18.18 | 0.10 | 60-115 | EM | 0.00 | hetero-2-6-6-2-mer | 52 x SF4, 6 x 402 | HHblits | 0.28 |
| ``` target    FNGAPQYINENPFDLELDASRPARPRQYWRAESAHFYNHEDHPLRVGTRLLTGSTHMPTPTKVMWFANANSILGNVKWHY 7qv7.1    -----------------------------------------------------------YSDVIFIIGSNTAECH-PLIA  target    NTVVNALPRMEMIAVHEWWWTGSCEWADVVFGVDSWGELKHPDMTASVTNPFLIVFPKTPIKRIFNTVGDIDVLALVSSK 7qv7.1    AHVIKAKERGAKLIVADPRMNAMVHKADIWLRVPS---------------------------------------------  target    LAELTGDTRFNDMWKFVREGRTDVYLQRILDASTNTKGYRFTELEAKAREGIPALMNSRTSPKVVGYDQLADSTPWYTKS 7qv7.1    --------------------------------------------------------------------------------  target    GRLEFYREEDEFIEAGENLPVHREPVDSTFYEPNVIVSPKHEAVRPSGPEDYGVARTDLSCEVRCGRNVVLTWAETRQTQ 7qv7.1    --------------------------------------------------------------------------------  target    HPLVKQGHKFIFHTPKYRHGSHTTPIDTDMNAVLFGPFGDIYRRDKRSPFVTEGYVDINPTDGLELGLQDGDYVWIDPDP 7qv7.1    --------------------------------------------------------------------------------  target    EDRPFRGWQKNAKDMEFARLLCRARFYPGTPRGVTRMWFNMYGATPGSVRGAKARRDGLAKNPDTNYQAMFRSGSHQSAT 7qv7.1    --------------------------------------------------------------------------------  target    RGWLKPTWMTDSLVRKGLFGQGIGKGFLPDVHCPTGAPREAFVKISRAEPGGIGGQGLWRPAALGIRPRHESPAMKRYLA 7qv7.1    --------------------------------------------------------------------------------  target    GGFFSGPKE 7qv7.1    --------- ``` | | | | | | | | | | | | | | | | | | | | | | | | | | | | | | | | | | | | | | | | | | | | | | | | | |
|  | 6f0k.1.B | Fe-S-cluster-containing hydrogenase  *Alternative complex III* | 0.01 |  | 14.29 | 0.10 | 60-116 | EM | 0.00 | hetero-1-1-1-1-1-1-… | 6 x HEC, 1 x F3S, 3 x SF4 | HHblits | 0.26 |
| ``` target    FNGAPQYINENPFDLELDASRPARPRQYWRAESAHFYNHEDHPLRVGTRLLTGSTHMPTPTKVMWFANANSILGNVKWHY 6f0k.1    -----------------------------------------------------------EARVIVSLDADFLGPT-DRNF  target    ---NTVVNAL-------PRMEMIAVHEWWWTGSCEWADVVFGVDSWGELKHPDMTASVTNPFLIVFPKTPIKRIFNTVGD 6f0k.1    VENTREFAASRRMERPEDEISRLYVIESTYTVTGGMADHRLRLRAG----------------------------------  target    IDVLALVSSKLAELTGDTRFNDMWKFVREGRTDVYLQRILDASTNTKGYRFTELEAKAREGIPALMNSRTSPKVVGYDQL 6f0k.1    --------------------------------------------------------------------------------  target    ADSTPWYTKSGRLEFYREEDEFIEAGENLPVHREPVDSTFYEPNVIVSPKHEAVRPSGPEDYGVARTDLSCEVRCGRNVV 6f0k.1    --------------------------------------------------------------------------------  target    LTWAETRQTQHPLVKQGHKFIFHTPKYRHGSHTTPIDTDMNAVLFGPFGDIYRRDKRSPFVTEGYVDINPTDGLELGLQD 6f0k.1    --------------------------------------------------------------------------------  target    GDYVWIDPDPEDRPFRGWQKNAKDMEFARLLCRARFYPGTPRGVTRMWFNMYGATPGSVRGAKARRDGLAKNPDTNYQAM 6f0k.1    --------------------------------------------------------------------------------  target    FRSGSHQSATRGWLKPTWMTDSLVRKGLFGQGIGKGFLPDVHCPTGAPREAFVKISRAEPGGIGGQGLWRPAALGIRPRH 6f0k.1    --------------------------------------------------------------------------------  target    ESPAMKRYLAGGFFSGPKE 6f0k.1    ------------------- ``` | | | | | | | | | | | | | | | | | | | | | | | | | | | | | | | | | | | | | | | | | | | | | | | | | |
|  | 4dmr.1.A | DMSO REDUCTASE  *REDUCED DMSO REDUCTASE FROM RHODOBACTER CAPSULATUS WITH BOUND DMSO SUBSTRATE* | 0.02 |  | 19.64 | 0.10 | 60-115 | X-ray | 1.90 | monomer | 2 x PGD, 1 x 4MO, 1 x O | HHblits | 0.26 |
| ``` target    FNGAPQYINENPFDLELDASRPARPRQYWRAESAHFYNHEDHPLRVGTRLLTGSTHMPTPTKVMWFANANSILGNVKWH- 4dmr.1    -----------------------------------------------------------NTEVMVFWAADPIKTSQIGWV  target    -----YNTVVNALP-RMEMIAVHEWWWTGSCEW-ADVVFGVDSWGELKHPDMTASVTNPFLIVFPKTPIKRIFNTVGDID 4dmr.1    IPEHGAYPGLEALKAKGTKVIVIDPVRTKTVEFFGAEHITPKP-------------------------------------  target    VLALVSSKLAELTGDTRFNDMWKFVREGRTDVYLQRILDASTNTKGYRFTELEAKAREGIPALMNSRTSPKVVGYDQLAD 4dmr.1    --------------------------------------------------------------------------------  target    STPWYTKSGRLEFYREEDEFIEAGENLPVHREPVDSTFYEPNVIVSPKHEAVRPSGPEDYGVARTDLSCEVRCGRNVVLT 4dmr.1    --------------------------------------------------------------------------------  target    WAETRQTQHPLVKQGHKFIFHTPKYRHGSHTTPIDTDMNAVLFGPFGDIYRRDKRSPFVTEGYVDINPTDGLELGLQDGD 4dmr.1    --------------------------------------------------------------------------------  target    YVWIDPDPEDRPFRGWQKNAKDMEFARLLCRARFYPGTPRGVTRMWFNMYGATPGSVRGAKARRDGLAKNPDTNYQAMFR 4dmr.1    --------------------------------------------------------------------------------  target    SGSHQSATRGWLKPTWMTDSLVRKGLFGQGIGKGFLPDVHCPTGAPREAFVKISRAEPGGIGGQGLWRPAALGIRPRHES 4dmr.1    --------------------------------------------------------------------------------  target    PAMKRYLAGGFFSGPKE 4dmr.1    ----------------- ``` | | | | | | | | | | | | | | | | | | | | | | | | | | | | | | | | | | | | | | | | | | | | | | | | | |
|  | 7b04.1.B | Nitrite oxidoreductase subunit A  *Structure of Nitrite oxidoreductase (Nxr) from the anammox bacterium Kuenenia stuttgartiensis.* | 0.02 |  | 18.18 | 0.10 | 59-114 | X-ray | 2.97 | hetero-1-1-1-mer | 4 x SF4, 1 x F3S, 2 x MD1, 1 x MO, 1 x HEM, 2 x CA | HHblits | 0.27 |
| ``` target    FNGAPQYINENPFDLELDASRPARPRQYWRAESAHFYNHEDHPLRVGTRLLTGSTHMPTPTKVMWFANANSILGNVKWHY 7b04.1    ----------------------------------------------------------RFSKLLIQTGKNLIENK-MPEA  target    NTVVNALPRMEMIAVHEWWWTGSCEWADVVFGVDSWGELKHPDMTASVTNPFLIVFPKTPIKRIFNTVGDIDVLALVSSK 7b04.1    HWVTEVMERGGKIVVITPEYSPSAQKADYWIPIR----------------------------------------------  target    LAELTGDTRFNDMWKFVREGRTDVYLQRILDASTNTKGYRFTELEAKAREGIPALMNSRTSPKVVGYDQLADSTPWYTKS 7b04.1    --------------------------------------------------------------------------------  target    GRLEFYREEDEFIEAGENLPVHREPVDSTFYEPNVIVSPKHEAVRPSGPEDYGVARTDLSCEVRCGRNVVLTWAETRQTQ 7b04.1    --------------------------------------------------------------------------------  target    HPLVKQGHKFIFHTPKYRHGSHTTPIDTDMNAVLFGPFGDIYRRDKRSPFVTEGYVDINPTDGLELGLQDGDYVWIDPDP 7b04.1    --------------------------------------------------------------------------------  target    EDRPFRGWQKNAKDMEFARLLCRARFYPGTPRGVTRMWFNMYGATPGSVRGAKARRDGLAKNPDTNYQAMFRSGSHQSAT 7b04.1    --------------------------------------------------------------------------------  target    RGWLKPTWMTDSLVRKGLFGQGIGKGFLPDVHCPTGAPREAFVKISRAEPGGIGGQGLWRPAALGIRPRHESPAMKRYLA 7b04.1    --------------------------------------------------------------------------------  target    GGFFSGPKE 7b04.1    --------- ``` | | | | | | | | | | | | | | | | | | | | | | | | | | | | | | | | | | | | | | | | | | | | | | | | | |
|  | 7b04.2.B | Nitrite oxidoreductase subunit A  *Structure of Nitrite oxidoreductase (Nxr) from the anammox bacterium Kuenenia stuttgartiensis.* | 0.02 |  | 18.18 | 0.10 | 59-114 | X-ray | 2.97 | hetero-1-1-1-mer | 4 x SF4, 1 x F3S, 2 x MD1, 1 x MO, 1 x HEM, 2 x CA | HHblits | 0.27 |
| ``` target    FNGAPQYINENPFDLELDASRPARPRQYWRAESAHFYNHEDHPLRVGTRLLTGSTHMPTPTKVMWFANANSILGNVKWHY 7b04.2    ----------------------------------------------------------RFSKLLIQTGKNLIENK-MPEA  target    NTVVNALPRMEMIAVHEWWWTGSCEWADVVFGVDSWGELKHPDMTASVTNPFLIVFPKTPIKRIFNTVGDIDVLALVSSK 7b04.2    HWVTEVMERGGKIVVITPEYSPSAQKADYWIPIR----------------------------------------------  target    LAELTGDTRFNDMWKFVREGRTDVYLQRILDASTNTKGYRFTELEAKAREGIPALMNSRTSPKVVGYDQLADSTPWYTKS 7b04.2    --------------------------------------------------------------------------------  target    GRLEFYREEDEFIEAGENLPVHREPVDSTFYEPNVIVSPKHEAVRPSGPEDYGVARTDLSCEVRCGRNVVLTWAETRQTQ 7b04.2    --------------------------------------------------------------------------------  target    HPLVKQGHKFIFHTPKYRHGSHTTPIDTDMNAVLFGPFGDIYRRDKRSPFVTEGYVDINPTDGLELGLQDGDYVWIDPDP 7b04.2    --------------------------------------------------------------------------------  target    EDRPFRGWQKNAKDMEFARLLCRARFYPGTPRGVTRMWFNMYGATPGSVRGAKARRDGLAKNPDTNYQAMFRSGSHQSAT 7b04.2    --------------------------------------------------------------------------------  target    RGWLKPTWMTDSLVRKGLFGQGIGKGFLPDVHCPTGAPREAFVKISRAEPGGIGGQGLWRPAALGIRPRHESPAMKRYLA 7b04.2    --------------------------------------------------------------------------------  target    GGFFSGPKE 7b04.2    --------- ``` | | | | | | | | | | | | | | | | | | | | | | | | | | | | | | | | | | | | | | | | | | | | | | | | | |
|  | 2v45.1.A | PERIPLASMIC NITRATE REDUCTASE  *A NEW CATALYTIC MECHANISM OF PERIPLASMIC NITRATE REDUCTASE FROM DESULFOVIBRIO DESULFURICANS ATCC 27774 FROM CRYSTALLOGRAPHIC AND EPR DATA AND BASED ON DETAILED ANALYSIS OF THE SIXTH LIGAND* | 0.02 |  | 10.71 | 0.10 | 59-115 | X-ray | 2.40 | monomer | 1 x SF4, 1 x MO, 2 x MGD, 1 x LCP | HHblits | 0.25 |
| ``` target    FNGAPQYINENPFDLELDASRPARPRQYWRAESAHFYNHEDHPLRVGTRLLTGSTHMPTPTKVMWFANANSILGNVKWHY 2v45.1    ----------------------------------------------------------DQATCFFIIGSNTSEAH-PVLF  target    NTVVNAL--PRMEMIAVHEWWWTGSCEWADVVFGVDSWGELKHPDMTASVTNPFLIVFPKTPIKRIFNTVGDIDVLALVS 2v45.1    RRIARRKQVEPGVKIIVADPRRTNTSRIADMHVAFRP-------------------------------------------  target    SKLAELTGDTRFNDMWKFVREGRTDVYLQRILDASTNTKGYRFTELEAKAREGIPALMNSRTSPKVVGYDQLADSTPWYT 2v45.1    --------------------------------------------------------------------------------  target    KSGRLEFYREEDEFIEAGENLPVHREPVDSTFYEPNVIVSPKHEAVRPSGPEDYGVARTDLSCEVRCGRNVVLTWAETRQ 2v45.1    --------------------------------------------------------------------------------  target    TQHPLVKQGHKFIFHTPKYRHGSHTTPIDTDMNAVLFGPFGDIYRRDKRSPFVTEGYVDINPTDGLELGLQDGDYVWIDP 2v45.1    --------------------------------------------------------------------------------  target    DPEDRPFRGWQKNAKDMEFARLLCRARFYPGTPRGVTRMWFNMYGATPGSVRGAKARRDGLAKNPDTNYQAMFRSGSHQS 2v45.1    --------------------------------------------------------------------------------  target    ATRGWLKPTWMTDSLVRKGLFGQGIGKGFLPDVHCPTGAPREAFVKISRAEPGGIGGQGLWRPAALGIRPRHESPAMKRY 2v45.1    --------------------------------------------------------------------------------  target    LAGGFFSGPKE 2v45.1    ----------- ``` | | | | | | | | | | | | | | | | | | | | | | | | | | | | | | | | | | | | | | | | | | | | | | | | | |
|  | 2ivf.1.A | ETHYLBENZENE DEHYDROGENASE ALPHA-SUBUNIT  *ETHYLBENZENE DEHYDROGENASE FROM AROMATOLEUM AROMATICUM* | 0.02 |  | 12.50 | 0.10 | 59-115 | X-ray | 1.88 | hetero-oligomer | 1 x MES, 4 x SF4, 1 x MO, 1 x MGD, 1 x MD1, 1 x F3S, 1 x HEM | HHblits | 0.25 |
| ``` target    FNGAPQYINENPFDLELDASRPARPRQYWRAESAHFYNHEDHPLRVGTRLLTGSTHMPTPTKVMWFANANSILGNVKWHY 2ivf.1    ----------------------------------------------------------LDAELIFMTCSNWSYTY-PSSY  target    NTVVNALPRMEMIAVHEWWWTGSCEWADVVFGVDSWGELKHPDMTASVTNPFLIVFPKTPIKRIFNTVGDIDVLALVSSK 2ivf.1    HFLSEARYKGAEVVVIAPDFNPTTPAADLHVPVRV---------------------------------------------  target    LAELTGDTRFNDMWKFVREGRTDVYLQRILDASTNTKGYRFTELEAKAREGIPALMNSRTSPKVVGYDQLADSTPWYTKS 2ivf.1    --------------------------------------------------------------------------------  target    GRLEFYREEDEFIEAGENLPVHREPVDSTFYEPNVIVSPKHEAVRPSGPEDYGVARTDLSCEVRCGRNVVLTWAETRQTQ 2ivf.1    --------------------------------------------------------------------------------  target    HPLVKQGHKFIFHTPKYRHGSHTTPIDTDMNAVLFGPFGDIYRRDKRSPFVTEGYVDINPTDGLELGLQDGDYVWIDPDP 2ivf.1    --------------------------------------------------------------------------------  target    EDRPFRGWQKNAKDMEFARLLCRARFYPGTPRGVTRMWFNMYGATPGSVRGAKARRDGLAKNPDTNYQAMFRSGSHQSAT 2ivf.1    --------------------------------------------------------------------------------  target    RGWLKPTWMTDSLVRKGLFGQGIGKGFLPDVHCPTGAPREAFVKISRAEPGGIGGQGLWRPAALGIRPRHESPAMKRYLA 2ivf.1    --------------------------------------------------------------------------------  target    GGFFSGPKE 2ivf.1    --------- ``` | | | | | | | | | | | | | | | | | | | | | | | | | | | | | | | | | | | | | | | | | | | | | | | | | |
|  | 5t5i.1.D | Tungsten formylmethanofuran dehydrogenase subunit fwdD  *TUNGSTEN-CONTAINING FORMYLMETHANOFURAN DEHYDROGENASE FROM METHANOTHERMOBACTER WOLFEII, ORTHORHOMBIC FORM AT 1.9 A* | 0.03 |  | 19.23 | 0.09 | 373-441 | X-ray | 1.90 | hetero-oligomer | 4 x ZN, 2 x MG, 18 x K, 22 x SF4, 2 x W, 4 x MGD, 2 x H2S, 2 x CA | HHblits | 0.31 |
| ``` target    FNGAPQYINENPFDLELDASRPARPRQYWRAESAHFYNHEDHPLRVGTRLLTGSTHMPTPTKVMWFANANSILGNVKWHY 5t5i.1    --------------------------------------------------------------------------------  target    NTVVNALPRMEMIAVHEWWWTGSCEWADVVFGVDSWGELKHPDMTASVTNPFLIVFPKTPIKRIFNTVGDIDVLALVSSK 5t5i.1    --------------------------------------------------------------------------------  target    LAELTGDTRFNDMWKFVREGRTDVYLQRILDASTNTKGYRFTELEAKAREGIPALMNSRTSPKVVGYDQLADSTPWYTKS 5t5i.1    --------------------------------------------------------------------------------  target    GRLEFYREEDEFIEAGENLPVHREPVDSTFYEPNVIVSPKHEAVRPSGPEDYGVARTDLSCEVRCGRNVVLTWAETRQTQ 5t5i.1    --------------------------------------------------------------------------------  target    HPLVKQGHKFIFHTPKYRHGSHTTPIDTDMNAVLFGPFGDIYRRDKRSPFVTEGYVDINPTDGLELGLQDGDYVWIDPDP 5t5i.1    ----------------------------------------------------AAIIQMNPEMMKQLGIAEGDNVKVISEY  target    EDRPFRGWQKNAKDMEFARLLCRAR-FYPGTPRGVTRMWFNMYGATPGSVRGAKARRDGLAKNPDTNYQAMFRSGSHQSA 5t5i.1    -----------------GDVVVKAVEAKEPLPEGMVYIPMGP--------------------------------------  target    TRGWLKPTWMTDSLVRKGLFGQGIGKGFLPDVHCPTGAPREAFVKISRAEPGGIGGQGLWRPAALGIRPRHESPAMKRYL 5t5i.1    --------------------------------------------------------------------------------  target    AGGFFSGPKE 5t5i.1    ---------- ``` | | | | | | | | | | | | | | | | | | | | | | | | | | | | | | | | | | | | | | | | | | | | | | | | | |
|  | 1kqf.1.A | FORMATE DEHYDROGENASE, NITRATE-INDUCIBLE, MAJOR SUBUNIT  *FORMATE DEHYDROGENASE N FROM E. COLI* | 0.02 |  | 12.96 | 0.09 | 60-114 | X-ray | 1.60 | hetero-oligomer | 3 x 6MO, 15 x SF4, 6 x MGD, 6 x HEM, 3 x CDL | HHblits | 0.27 |
| ``` target    FNGAPQYINENPFDLELDASRPARPRQYWRAESAHFYNHEDHPLRVGTRLLTGSTHMPTPTKVMWFANANSILGNVKWHY 1kqf.1    -----------------------------------------------------------NANVVMVMGGNAAEAH-PVGF  target    NTVVNALP-RMEMIAVHEWWWTGSCEWADVVFGVDSWGELKHPDMTASVTNPFLIVFPKTPIKRIFNTVGDIDVLALVSS 1kqf.1    RWAMEAKNNNDATLIVVDPRFTRTASVADIYAPIR---------------------------------------------  target    KLAELTGDTRFNDMWKFVREGRTDVYLQRILDASTNTKGYRFTELEAKAREGIPALMNSRTSPKVVGYDQLADSTPWYTK 1kqf.1    --------------------------------------------------------------------------------  target    SGRLEFYREEDEFIEAGENLPVHREPVDSTFYEPNVIVSPKHEAVRPSGPEDYGVARTDLSCEVRCGRNVVLTWAETRQT 1kqf.1    --------------------------------------------------------------------------------  target    QHPLVKQGHKFIFHTPKYRHGSHTTPIDTDMNAVLFGPFGDIYRRDKRSPFVTEGYVDINPTDGLELGLQDGDYVWIDPD 1kqf.1    --------------------------------------------------------------------------------  target    PEDRPFRGWQKNAKDMEFARLLCRARFYPGTPRGVTRMWFNMYGATPGSVRGAKARRDGLAKNPDTNYQAMFRSGSHQSA 1kqf.1    --------------------------------------------------------------------------------  target    TRGWLKPTWMTDSLVRKGLFGQGIGKGFLPDVHCPTGAPREAFVKISRAEPGGIGGQGLWRPAALGIRPRHESPAMKRYL 1kqf.1    --------------------------------------------------------------------------------  target    AGGFFSGPKE 1kqf.1    ---------- ``` | | | | | | | | | | | | | | | | | | | | | | | | | | | | | | | | | | | | | | | | | | | | | | | | | |
|  | 7bkb.1.L | Formylmethanofuran dehydrogenase, subunit B  *Formate dehydrogenase - heterodisulfide reductase - formylmethanofuran dehydrogenase complex from Methanospirillum hungatei (hexameric, composite structure)* | 0.01 |  | 12.96 | 0.09 | 60-115 | EM | 0.00 | hetero-2-2-2-2-2-2-… | 48 x SF4, 4 x FAD, 2 x FES, 4 x 9S8, 4 x ZN, 2 x MO, 4 x MGD | HHblits | 0.27 |
| ``` target    FNGAPQYINENPFDLELDASRPARPRQYWRAESAHFYNHEDHPLRVGTRLLTGSTHMPTPTKVMWFANANSILGNVKWHY 7bkb.1    -----------------------------------------------------------RADVIVYWGSNPAHAH--PRH  target    -NTV--------VNALPRMEMIAVHEWWWTGSCEWADVVFGVDSWGELKHPDMTASVTNPFLIVFPKTPIKRIFNTVGDI 7bkb.1    MSRYSIFPRGFFTGKGQKKRTVIVIDPRFTDTANVADYHLQVKQ------------------------------------  target    DVLALVSSKLAELTGDTRFNDMWKFVREGRTDVYLQRILDASTNTKGYRFTELEAKAREGIPALMNSRTSPKVVGYDQLA 7bkb.1    --------------------------------------------------------------------------------  target    DSTPWYTKSGRLEFYREEDEFIEAGENLPVHREPVDSTFYEPNVIVSPKHEAVRPSGPEDYGVARTDLSCEVRCGRNVVL 7bkb.1    --------------------------------------------------------------------------------  target    TWAETRQTQHPLVKQGHKFIFHTPKYRHGSHTTPIDTDMNAVLFGPFGDIYRRDKRSPFVTEGYVDINPTDGLELGLQDG 7bkb.1    --------------------------------------------------------------------------------  target    DYVWIDPDPEDRPFRGWQKNAKDMEFARLLCRARFYPGTPRGVTRMWFNMYGATPGSVRGAKARRDGLAKNPDTNYQAMF 7bkb.1    --------------------------------------------------------------------------------  target    RSGSHQSATRGWLKPTWMTDSLVRKGLFGQGIGKGFLPDVHCPTGAPREAFVKISRAEPGGIGGQGLWRPAALGIRPRHE 7bkb.1    --------------------------------------------------------------------------------  target    SPAMKRYLAGGFFSGPKE 7bkb.1    ------------------ ``` | | | | | | | | | | | | | | | | | | | | | | | | | | | | | | | | | | | | | | | | | | | | | | | | | |
|  | 2v3v.1.A | PERIPLASMIC NITRATE REDUCTASE  *A NEW CATALYTIC MECHANISM OF PERIPLASMIC NITRATE REDUCTASE FROM DESULFOVIBRIO DESULFURICANS ATCC 27774 FROM CRYSTALLOGRAPHIC AND EPR DATA AND BASED ON DETAILED ANALYSIS OF THE SIXTH LIGAND* | 0.02 |  | 10.91 | 0.10 | 60-115 | X-ray | 1.99 | monomer | 1 x SF4, 1 x MO, 2 x MGD, 4 x LCP | HHblits | 0.25 |
| ``` target    FNGAPQYINENPFDLELDASRPARPRQYWRAESAHFYNHEDHPLRVGTRLLTGSTHMPTPTKVMWFANANSILGNVKWHY 2v3v.1    -----------------------------------------------------------QATCFFIIGSNTSEAH-PVLF  target    NTVVNAL--PRMEMIAVHEWWWTGSCEWADVVFGVDSWGELKHPDMTASVTNPFLIVFPKTPIKRIFNTVGDIDVLALVS 2v3v.1    RRIARRKQVEPGVKIIVADPRRTNTSRIADMHVAFRP-------------------------------------------  target    SKLAELTGDTRFNDMWKFVREGRTDVYLQRILDASTNTKGYRFTELEAKAREGIPALMNSRTSPKVVGYDQLADSTPWYT 2v3v.1    --------------------------------------------------------------------------------  target    KSGRLEFYREEDEFIEAGENLPVHREPVDSTFYEPNVIVSPKHEAVRPSGPEDYGVARTDLSCEVRCGRNVVLTWAETRQ 2v3v.1    --------------------------------------------------------------------------------  target    TQHPLVKQGHKFIFHTPKYRHGSHTTPIDTDMNAVLFGPFGDIYRRDKRSPFVTEGYVDINPTDGLELGLQDGDYVWIDP 2v3v.1    --------------------------------------------------------------------------------  target    DPEDRPFRGWQKNAKDMEFARLLCRARFYPGTPRGVTRMWFNMYGATPGSVRGAKARRDGLAKNPDTNYQAMFRSGSHQS 2v3v.1    --------------------------------------------------------------------------------  target    ATRGWLKPTWMTDSLVRKGLFGQGIGKGFLPDVHCPTGAPREAFVKISRAEPGGIGGQGLWRPAALGIRPRHESPAMKRY 2v3v.1    --------------------------------------------------------------------------------  target    LAGGFFSGPKE 2v3v.1    ----------- ``` | | | | | | | | | | | | | | | | | | | | | | | | | | | | | | | | | | | | | | | | | | | | | | | | | |
|  | 4v4c.1.A | Pyrogallol hydroxytransferase large subunit  *Crystal Structure of Pyrogallol-Phloroglucinol Transhydroxylase from Pelobacter acidigallici* | 0.01 |  | 10.91 | 0.10 | 59-115 | X-ray | 2.35 | hetero-oligomer | 2 x CA, 2 x MGD, 1 x 4MO, 3 x SF4 | HHblits | 0.25 |
| ``` target    FNGAPQYINENPFDLELDASRPARPRQYWRAESAHFYNHEDHPLRVGTRLLTGSTHMPTPTKVMWFANANSILGNVKWHY 4v4c.1    ----------------------------------------------------------KHAEMIVFWSSDPETN--SGIY  target    N----TVVNA-LPRME-MIAVHEWWWTGSCE-WADVVFGVDSWGELKHPDMTASVTNPFLIVFPKTPIKRIFNTVGDIDV 4v4c.1    AGFESNIRRQWLKDLGVDFVFIDPHMNHTARLVADKWFSPKI--------------------------------------  target    LALVSSKLAELTGDTRFNDMWKFVREGRTDVYLQRILDASTNTKGYRFTELEAKAREGIPALMNSRTSPKVVGYDQLADS 4v4c.1    --------------------------------------------------------------------------------  target    TPWYTKSGRLEFYREEDEFIEAGENLPVHREPVDSTFYEPNVIVSPKHEAVRPSGPEDYGVARTDLSCEVRCGRNVVLTW 4v4c.1    --------------------------------------------------------------------------------  target    AETRQTQHPLVKQGHKFIFHTPKYRHGSHTTPIDTDMNAVLFGPFGDIYRRDKRSPFVTEGYVDINPTDGLELGLQDGDY 4v4c.1    --------------------------------------------------------------------------------  target    VWIDPDPEDRPFRGWQKNAKDMEFARLLCRARFYPGTPRGVTRMWFNMYGATPGSVRGAKARRDGLAKNPDTNYQAMFRS 4v4c.1    --------------------------------------------------------------------------------  target    GSHQSATRGWLKPTWMTDSLVRKGLFGQGIGKGFLPDVHCPTGAPREAFVKISRAEPGGIGGQGLWRPAALGIRPRHESP 4v4c.1    --------------------------------------------------------------------------------  target    AMKRYLAGGFFSGPKE 4v4c.1    ---------------- ``` | | | | | | | | | | | | | | | | | | | | | | | | | | | | | | | | | | | | | | | | | | | | | | | | | |
|  | 7l5i.1.A | Trimethylamine-N-oxide reductase  *Crystal Structure of Haemophilus influenzae MtsZ at pH 7.0* | 0.01 |  | 8.93 | 0.10 | 60-115 | X-ray | 1.73 | monomer | 2 x MGD, 1 x MO, 1 x O | HHblits | 0.24 |
| ``` target    FNGAPQYINENPFDLELDASRPARPRQYWRAESAHFYNHEDHPLRVGTRLLTGSTHMPTPTKVMWFANANSILGNVKW-- 7l5i.1    -----------------------------------------------------------SSDIIVLWSANPLTTMRIAWM  target    -----HYNTVVNALPRMEMIAVHEWWWTGSCEW-ADVVFGVDSWGELKHPDMTASVTNPFLIVFPKTPIKRIFNTVGDID 7l5i.1    STDQKGIEYFKKFQASGKRIICIDPQKSETCQMLNAEWIPVNT-------------------------------------  target    VLALVSSKLAELTGDTRFNDMWKFVREGRTDVYLQRILDASTNTKGYRFTELEAKAREGIPALMNSRTSPKVVGYDQLAD 7l5i.1    --------------------------------------------------------------------------------  target    STPWYTKSGRLEFYREEDEFIEAGENLPVHREPVDSTFYEPNVIVSPKHEAVRPSGPEDYGVARTDLSCEVRCGRNVVLT 7l5i.1    --------------------------------------------------------------------------------  target    WAETRQTQHPLVKQGHKFIFHTPKYRHGSHTTPIDTDMNAVLFGPFGDIYRRDKRSPFVTEGYVDINPTDGLELGLQDGD 7l5i.1    --------------------------------------------------------------------------------  target    YVWIDPDPEDRPFRGWQKNAKDMEFARLLCRARFYPGTPRGVTRMWFNMYGATPGSVRGAKARRDGLAKNPDTNYQAMFR 7l5i.1    --------------------------------------------------------------------------------  target    SGSHQSATRGWLKPTWMTDSLVRKGLFGQGIGKGFLPDVHCPTGAPREAFVKISRAEPGGIGGQGLWRPAALGIRPRHES 7l5i.1    --------------------------------------------------------------------------------  target    PAMKRYLAGGFFSGPKE 7l5i.1    ----------------- ``` | | | | | | | | | | | | | | | | | | | | | | | | | | | | | | | | | | | | | | | | | | | | | | | | | |
|  | 7l5s.1.A | Trimethylamine-N-oxide reductase  *Crystal Structure of Haemophilus influenzae MtsZ at pH 5.5* | 0.01 |  | 8.93 | 0.10 | 60-115 | X-ray | 2.09 | monomer | 1 x O, 2 x MGD, 1 x MO | HHblits | 0.24 |
| ``` target    FNGAPQYINENPFDLELDASRPARPRQYWRAESAHFYNHEDHPLRVGTRLLTGSTHMPTPTKVMWFANANSILGNVKW-- 7l5s.1    -----------------------------------------------------------SSDIIVLWSANPLTTMRIAWM  target    -----HYNTVVNALPRMEMIAVHEWWWTGSCEW-ADVVFGVDSWGELKHPDMTASVTNPFLIVFPKTPIKRIFNTVGDID 7l5s.1    STDQKGIEYFKKFQASGKRIICIDPQKSETCQMLNAEWIPVNT-------------------------------------  target    VLALVSSKLAELTGDTRFNDMWKFVREGRTDVYLQRILDASTNTKGYRFTELEAKAREGIPALMNSRTSPKVVGYDQLAD 7l5s.1    --------------------------------------------------------------------------------  target    STPWYTKSGRLEFYREEDEFIEAGENLPVHREPVDSTFYEPNVIVSPKHEAVRPSGPEDYGVARTDLSCEVRCGRNVVLT 7l5s.1    --------------------------------------------------------------------------------  target    WAETRQTQHPLVKQGHKFIFHTPKYRHGSHTTPIDTDMNAVLFGPFGDIYRRDKRSPFVTEGYVDINPTDGLELGLQDGD 7l5s.1    --------------------------------------------------------------------------------  target    YVWIDPDPEDRPFRGWQKNAKDMEFARLLCRARFYPGTPRGVTRMWFNMYGATPGSVRGAKARRDGLAKNPDTNYQAMFR 7l5s.1    --------------------------------------------------------------------------------  target    SGSHQSATRGWLKPTWMTDSLVRKGLFGQGIGKGFLPDVHCPTGAPREAFVKISRAEPGGIGGQGLWRPAALGIRPRHES 7l5s.1    --------------------------------------------------------------------------------  target    PAMKRYLAGGFFSGPKE 7l5s.1    ----------------- ``` | | | | | | | | | | | | | | | | | | | | | | | | | | | | | | | | | | | | | | | | | | | | | | | | | |
|  | 5t5i.1.B | Tungsten formylmethanofuran dehydrogenase subunit B  *TUNGSTEN-CONTAINING FORMYLMETHANOFURAN DEHYDROGENASE FROM METHANOTHERMOBACTER WOLFEII, ORTHORHOMBIC FORM AT 1.9 A* | 0.02 |  | 14.81 | 0.09 | 60-115 | X-ray | 1.90 | hetero-oligomer | 4 x ZN, 2 x MG, 18 x K, 22 x SF4, 2 x W, 4 x MGD, 2 x H2S, 2 x CA | HHblits | 0.26 |
| ``` target    FNGAPQYINENPFDLELDASRPARPRQYWRAESAHFYNHEDHPLRVGTRLLTGSTHMPTPTKVMWFANANSILGNVKWHY 5t5i.1    -----------------------------------------------------------RADVVVYWGCNPMHA--HPRH  target    NT--------VVNALPRMEMIAVHEWWWTGSCEWADVVFGVDSWGELKHPDMTASVTNPFLIVFPKTPIKRIFNTVGDID 5t5i.1    MSRNVFARGFFRERGRSDRTLIVVDPRKTDSAKLADIHLQLDF-------------------------------------  target    VLALVSSKLAELTGDTRFNDMWKFVREGRTDVYLQRILDASTNTKGYRFTELEAKAREGIPALMNSRTSPKVVGYDQLAD 5t5i.1    --------------------------------------------------------------------------------  target    STPWYTKSGRLEFYREEDEFIEAGENLPVHREPVDSTFYEPNVIVSPKHEAVRPSGPEDYGVARTDLSCEVRCGRNVVLT 5t5i.1    --------------------------------------------------------------------------------  target    WAETRQTQHPLVKQGHKFIFHTPKYRHGSHTTPIDTDMNAVLFGPFGDIYRRDKRSPFVTEGYVDINPTDGLELGLQDGD 5t5i.1    --------------------------------------------------------------------------------  target    YVWIDPDPEDRPFRGWQKNAKDMEFARLLCRARFYPGTPRGVTRMWFNMYGATPGSVRGAKARRDGLAKNPDTNYQAMFR 5t5i.1    --------------------------------------------------------------------------------  target    SGSHQSATRGWLKPTWMTDSLVRKGLFGQGIGKGFLPDVHCPTGAPREAFVKISRAEPGGIGGQGLWRPAALGIRPRHES 5t5i.1    --------------------------------------------------------------------------------  target    PAMKRYLAGGFFSGPKE 5t5i.1    ----------------- ``` | | | | | | | | | | | | | | | | | | | | | | | | | | | | | | | | | | | | | | | | | | | | | | | | | |
|  | 7e5z.1.A | Formate dehydrogenase  *Dehydrogenase holoenzyme* | 0.01 |  | 10.91 | 0.10 | 60-115 | EM | 0.00 | hetero-1-1-mer | 1 x W, 2 x MGD, 2 x FES, 4 x SF4, 1 x FMN | HHblits | 0.25 |
| ``` target    FNGAPQYINENPFDLELDASRPARPRQYWRAESAHFYNHEDHPLRVGTRLLTGSTHMPTPTKVMWFANANSILGNVKWHY 7e5z.1    -----------------------------------------------------------DAEVIVVIGANPTVNH-PVAA  target    NTVVNALPRME-MIAVHEWWWTGSCEWADVVFGVDSWGELKHPDMTASVTNPFLIVFPKTPIKRIFNTVGDIDVLALVSS 7e5z.1    TFLKNAVKQRGAKLIIMDPRRQTLSRHAYRHLAFRP--------------------------------------------  target    KLAELTGDTRFNDMWKFVREGRTDVYLQRILDASTNTKGYRFTELEAKAREGIPALMNSRTSPKVVGYDQLADSTPWYTK 7e5z.1    --------------------------------------------------------------------------------  target    SGRLEFYREEDEFIEAGENLPVHREPVDSTFYEPNVIVSPKHEAVRPSGPEDYGVARTDLSCEVRCGRNVVLTWAETRQT 7e5z.1    --------------------------------------------------------------------------------  target    QHPLVKQGHKFIFHTPKYRHGSHTTPIDTDMNAVLFGPFGDIYRRDKRSPFVTEGYVDINPTDGLELGLQDGDYVWIDPD 7e5z.1    --------------------------------------------------------------------------------  target    PEDRPFRGWQKNAKDMEFARLLCRARFYPGTPRGVTRMWFNMYGATPGSVRGAKARRDGLAKNPDTNYQAMFRSGSHQSA 7e5z.1    --------------------------------------------------------------------------------  target    TRGWLKPTWMTDSLVRKGLFGQGIGKGFLPDVHCPTGAPREAFVKISRAEPGGIGGQGLWRPAALGIRPRHESPAMKRYL 7e5z.1    --------------------------------------------------------------------------------  target    AGGFFSGPKE 7e5z.1    ---------- ``` | | | | | | | | | | | | | | | | | | | | | | | | | | | | | | | | | | | | | | | | | | | | | | | | | |
|  | 7vw6.1.A | Formate dehydrogenase  *Cryo-EM Structure of Formate Dehydrogenase 1 from Methylorubrum extorquens AM1* | 0.02 |  | 10.91 | 0.10 | 60-115 | EM | 0.00 | hetero-1-1-mer | 4 x SF4, 2 x FES, 2 x MGD, 1 x W, 1 x FMN | HHblits | 0.25 |
| ``` target    FNGAPQYINENPFDLELDASRPARPRQYWRAESAHFYNHEDHPLRVGTRLLTGSTHMPTPTKVMWFANANSILGNVKWHY 7vw6.1    -----------------------------------------------------------DAEVIVVIGANPTVNH-PVAA  target    NTVVNALPRME-MIAVHEWWWTGSCEWADVVFGVDSWGELKHPDMTASVTNPFLIVFPKTPIKRIFNTVGDIDVLALVSS 7vw6.1    TFLKNAVKQRGAKLIIMDPRRQTLSRHAYRHLAFRP--------------------------------------------  target    KLAELTGDTRFNDMWKFVREGRTDVYLQRILDASTNTKGYRFTELEAKAREGIPALMNSRTSPKVVGYDQLADSTPWYTK 7vw6.1    --------------------------------------------------------------------------------  target    SGRLEFYREEDEFIEAGENLPVHREPVDSTFYEPNVIVSPKHEAVRPSGPEDYGVARTDLSCEVRCGRNVVLTWAETRQT 7vw6.1    --------------------------------------------------------------------------------  target    QHPLVKQGHKFIFHTPKYRHGSHTTPIDTDMNAVLFGPFGDIYRRDKRSPFVTEGYVDINPTDGLELGLQDGDYVWIDPD 7vw6.1    --------------------------------------------------------------------------------  target    PEDRPFRGWQKNAKDMEFARLLCRARFYPGTPRGVTRMWFNMYGATPGSVRGAKARRDGLAKNPDTNYQAMFRSGSHQSA 7vw6.1    --------------------------------------------------------------------------------  target    TRGWLKPTWMTDSLVRKGLFGQGIGKGFLPDVHCPTGAPREAFVKISRAEPGGIGGQGLWRPAALGIRPRHESPAMKRYL 7vw6.1    --------------------------------------------------------------------------------  target    AGGFFSGPKE 7vw6.1    ---------- ``` | | | | | | | | | | | | | | | | | | | | | | | | | | | | | | | | | | | | | | | | | | | | | | | | | |
|  | 2nya.1.A | Periplasmic nitrate reductase  *Crystal structure of the periplasmic nitrate reductase (NAP) from Escherichia coli* | 0.02 |  | 16.67 | 0.09 | 60-114 | X-ray | 2.50 | monomer | 1 x SF4, 1 x 6MO, 2 x MGD | HHblits | 0.26 |
| ``` target    FNGAPQYINENPFDLELDASRPARPRQYWRAESAHFYNHEDHPLRVGTRLLTGSTHMPTPTKVMWFANANSILGNVKWHY 2nya.1    -----------------------------------------------------------QADAFVLWGANMAEMH-PILW  target    NTVVNALP--RMEMIAVHEWWWTGSCEWADVVFGVDSWGELKHPDMTASVTNPFLIVFPKTPIKRIFNTVGDIDVLALVS 2nya.1    SRITNRRLSNQNVTVAVLSTYQHRSFELADNGIIFT--------------------------------------------  target    SKLAELTGDTRFNDMWKFVREGRTDVYLQRILDASTNTKGYRFTELEAKAREGIPALMNSRTSPKVVGYDQLADSTPWYT 2nya.1    --------------------------------------------------------------------------------  target    KSGRLEFYREEDEFIEAGENLPVHREPVDSTFYEPNVIVSPKHEAVRPSGPEDYGVARTDLSCEVRCGRNVVLTWAETRQ 2nya.1    --------------------------------------------------------------------------------  target    TQHPLVKQGHKFIFHTPKYRHGSHTTPIDTDMNAVLFGPFGDIYRRDKRSPFVTEGYVDINPTDGLELGLQDGDYVWIDP 2nya.1    --------------------------------------------------------------------------------  target    DPEDRPFRGWQKNAKDMEFARLLCRARFYPGTPRGVTRMWFNMYGATPGSVRGAKARRDGLAKNPDTNYQAMFRSGSHQS 2nya.1    --------------------------------------------------------------------------------  target    ATRGWLKPTWMTDSLVRKGLFGQGIGKGFLPDVHCPTGAPREAFVKISRAEPGGIGGQGLWRPAALGIRPRHESPAMKRY 2nya.1    --------------------------------------------------------------------------------  target    LAGGFFSGPKE 2nya.1    ----------- ``` | | | | | | | | | | | | | | | | | | | | | | | | | | | | | | | | | | | | | | | | | | | | | | | | | |
|  | 2vpz.1.A | THIOSULFATE REDUCTASE  *POLYSULFIDE REDUCTASE NATIVE STRUCTURE* | 0.02 |  | 7.14 | 0.10 | 60-115 | X-ray | 2.40 | hetero-oligomer | 10 x SF4, 4 x MGD, 2 x MO | HHblits | 0.23 |
| ``` target    FNGAPQYINENPFDLELDASRPARPRQYWRAESAHFYNHEDHPLRVGTRLLTGSTHMPTPTKVMWFANANSILGNVKWHY 2vpz.1    -----------------------------------------------------------NARYIVLIGHHIGEDTHNTQL  target    NTVVNALPRMEMIAVHEWWWTGSCEWADVVFGVDSWGELKHPDMTASVTNPFLIVFPKTPIKRIFNTVGDIDVLALVSSK 2vpz.1    QDFALALKNGAKVVVVDPRFSTAAAKAHRWLPIKP---------------------------------------------  target    LAELTGDTRFNDMWKFVREGRTDVYLQRILDASTNTKGYRFTELEAKAREGIPALMNSRTSPKVVGYDQLADSTPWYTKS 2vpz.1    --------------------------------------------------------------------------------  target    GRLEFYREEDEFIEAGENLPVHREPVDSTFYEPNVIVSPKHEAVRPSGPEDYGVARTDLSCEVRCGRNVVLTWAETRQTQ 2vpz.1    --------------------------------------------------------------------------------  target    HPLVKQGHKFIFHTPKYRHGSHTTPIDTDMNAVLFGPFGDIYRRDKRSPFVTEGYVDINPTDGLELGLQDGDYVWIDPDP 2vpz.1    --------------------------------------------------------------------------------  target    EDRPFRGWQKNAKDMEFARLLCRARFYPGTPRGVTRMWFNMYGATPGSVRGAKARRDGLAKNPDTNYQAMFRSGSHQSAT 2vpz.1    --------------------------------------------------------------------------------  target    RGWLKPTWMTDSLVRKGLFGQGIGKGFLPDVHCPTGAPREAFVKISRAEPGGIGGQGLWRPAALGIRPRHESPAMKRYLA 2vpz.1    --------------------------------------------------------------------------------  target    GGFFSGPKE 2vpz.1    --------- ``` | | | | | | | | | | | | | | | | | | | | | | | | | | | | | | | | | | | | | | | | | | | | | | | | | |
|  | 2vpx.1.D | THIOSULFATE REDUCTASE  *POLYSULFIDE REDUCTASE WITH BOUND QUINONE (UQ1)* | 0.02 |  | 7.14 | 0.10 | 60-115 | X-ray | 3.10 | hetero-oligomer | 10 x SF4, 4 x MGD, 2 x MO, 2 x UQ1 | HHblits | 0.23 |
| ``` target    FNGAPQYINENPFDLELDASRPARPRQYWRAESAHFYNHEDHPLRVGTRLLTGSTHMPTPTKVMWFANANSILGNVKWHY 2vpx.1    -----------------------------------------------------------NARYIVLIGHHIGEDTHNTQL  target    NTVVNALPRMEMIAVHEWWWTGSCEWADVVFGVDSWGELKHPDMTASVTNPFLIVFPKTPIKRIFNTVGDIDVLALVSSK 2vpx.1    QDFALALKNGAKVVVVDPRFSTAAAKAHRWLPIKP---------------------------------------------  target    LAELTGDTRFNDMWKFVREGRTDVYLQRILDASTNTKGYRFTELEAKAREGIPALMNSRTSPKVVGYDQLADSTPWYTKS 2vpx.1    --------------------------------------------------------------------------------  target    GRLEFYREEDEFIEAGENLPVHREPVDSTFYEPNVIVSPKHEAVRPSGPEDYGVARTDLSCEVRCGRNVVLTWAETRQTQ 2vpx.1    --------------------------------------------------------------------------------  target    HPLVKQGHKFIFHTPKYRHGSHTTPIDTDMNAVLFGPFGDIYRRDKRSPFVTEGYVDINPTDGLELGLQDGDYVWIDPDP 2vpx.1    --------------------------------------------------------------------------------  target    EDRPFRGWQKNAKDMEFARLLCRARFYPGTPRGVTRMWFNMYGATPGSVRGAKARRDGLAKNPDTNYQAMFRSGSHQSAT 2vpx.1    --------------------------------------------------------------------------------  target    RGWLKPTWMTDSLVRKGLFGQGIGKGFLPDVHCPTGAPREAFVKISRAEPGGIGGQGLWRPAALGIRPRHESPAMKRYLA 2vpx.1    --------------------------------------------------------------------------------  target    GGFFSGPKE 2vpx.1    --------- ``` | | | | | | | | | | | | | | | | | | | | | | | | | | | | | | | | | | | | | | | | | | | | | | | | | |
|  | 1eu1.1.A | DIMETHYL SULFOXIDE REDUCTASE  *THE CRYSTAL STRUCTURE OF RHODOBACTER SPHAEROIDES DIMETHYLSULFOXIDE REDUCTASE REVEALS TWO DISTINCT MOLYBDENUM COORDINATION ENVIRONMENTS.* | 0.01 |  | 16.67 | 0.09 | 59-114 | X-ray | 1.30 | monomer | 3 x GLC, 1 x CD, 2 x MGD, 1 x 6MO, 2 x O | HHblits | 0.26 |
| ``` target    FNGAPQYINENPFDLELDASRPARPRQYWRAESAHFYNHEDHPLRVGTRLLTGSTHMPTPTKVMWFANANSILGNVKWHY 1eu1.1    ----------------------------------------------------------ENTDLMVFWAADPMKTN--EIG  target    --------NTVVNAL-PRMEMIAVHEWWWTGSCEWAD-VVFGVDSWGELKHPDMTASVTNPFLIVFPKTPIKRIFNTVGD 1eu1.1    WVIPDHGAYAGMKALKEKGTRVICINPVRTETADYFGADVVSPR------------------------------------  target    IDVLALVSSKLAELTGDTRFNDMWKFVREGRTDVYLQRILDASTNTKGYRFTELEAKAREGIPALMNSRTSPKVVGYDQL 1eu1.1    --------------------------------------------------------------------------------  target    ADSTPWYTKSGRLEFYREEDEFIEAGENLPVHREPVDSTFYEPNVIVSPKHEAVRPSGPEDYGVARTDLSCEVRCGRNVV 1eu1.1    --------------------------------------------------------------------------------  target    LTWAETRQTQHPLVKQGHKFIFHTPKYRHGSHTTPIDTDMNAVLFGPFGDIYRRDKRSPFVTEGYVDINPTDGLELGLQD 1eu1.1    --------------------------------------------------------------------------------  target    GDYVWIDPDPEDRPFRGWQKNAKDMEFARLLCRARFYPGTPRGVTRMWFNMYGATPGSVRGAKARRDGLAKNPDTNYQAM 1eu1.1    --------------------------------------------------------------------------------  target    FRSGSHQSATRGWLKPTWMTDSLVRKGLFGQGIGKGFLPDVHCPTGAPREAFVKISRAEPGGIGGQGLWRPAALGIRPRH 1eu1.1    --------------------------------------------------------------------------------  target    ESPAMKRYLAGGFFSGPKE 1eu1.1    ------------------- ``` | | | | | | | | | | | | | | | | | | | | | | | | | | | | | | | | | | | | | | | | | | | | | | | | | |
|  | 1tmo.1.A | TRIMETHYLAMINE N-OXIDE REDUCTASE  *TRIMETHYLAMINE N-OXIDE REDUCTASE FROM SHEWANELLA MASSILIA* | 0.01 |  | 3.51 | 0.10 | 59-115 | X-ray | 2.50 | monomer | 2 x 2MD, 1 x 2MO | HHblits | 0.21 |
| ``` target    FNGAPQYINENPFDLELDASRPARPRQYWRAESAHFYNHEDHPLRVGTRLLTGSTHMPTPTKVMWFANANSILGNV---- 1tmo.1    ----------------------------------------------------------EHSDTIVLWSNDPYKNLQVGWN  target    ---KWHY---NTVVNALPRME-MIAVHEWWWTGSCE-WADVVFGVDSWGELKHPDMTASVTNPFLIVFPKTPIKRIFNTV 1tmo.1    AETHESFAYLAQLKEKVKQGKIRVISIDPVVTKTQAYLGCEQLYVNP---------------------------------  target    GDIDVLALVSSKLAELTGDTRFNDMWKFVREGRTDVYLQRILDASTNTKGYRFTELEAKAREGIPALMNSRTSPKVVGYD 1tmo.1    --------------------------------------------------------------------------------  target    QLADSTPWYTKSGRLEFYREEDEFIEAGENLPVHREPVDSTFYEPNVIVSPKHEAVRPSGPEDYGVARTDLSCEVRCGRN 1tmo.1    --------------------------------------------------------------------------------  target    VVLTWAETRQTQHPLVKQGHKFIFHTPKYRHGSHTTPIDTDMNAVLFGPFGDIYRRDKRSPFVTEGYVDINPTDGLELGL 1tmo.1    --------------------------------------------------------------------------------  target    QDGDYVWIDPDPEDRPFRGWQKNAKDMEFARLLCRARFYPGTPRGVTRMWFNMYGATPGSVRGAKARRDGLAKNPDTNYQ 1tmo.1    --------------------------------------------------------------------------------  target    AMFRSGSHQSATRGWLKPTWMTDSLVRKGLFGQGIGKGFLPDVHCPTGAPREAFVKISRAEPGGIGGQGLWRPAALGIRP 1tmo.1    --------------------------------------------------------------------------------  target    RHESPAMKRYLAGGFFSGPKE 1tmo.1    --------------------- ``` | | | | | | | | | | | | | | | | | | | | | | | | | | | | | | | | | | | | | | | | | | | | | | | | | |
|  | 1ogy.1.A | PERIPLASMIC NITRATE REDUCTASE  *Crystal structure of the heterodimeric nitrate reductase from Rhodobacter sphaeroides* | 0.02 |  | 9.09 | 0.10 | 60-115 | X-ray | 3.20 | hetero-1-1-mer | 1 x SF4, 1 x MO, 2 x MGD, 2 x HEC | HHblits | 0.24 |
| ``` target    FNGAPQYINENPFDLELDASRPARPRQYWRAESAHFYNHEDHPLRVGTRLLTGSTHMPTPTKVMWFANANSILGNVKWHY 1ogy.1    -----------------------------------------------------------AADAFVLWGSNMAEMH-PILW  target    NTVVNALP--RMEMIAVHEWWWTGSCEWADVVFGVDSWGELKHPDMTASVTNPFLIVFPKTPIKRIFNTVGDIDVLALVS 1ogy.1    SRLTDRRLSHEHVRVAVLSTFTHRSSDLSDTPIIFRP-------------------------------------------  target    SKLAELTGDTRFNDMWKFVREGRTDVYLQRILDASTNTKGYRFTELEAKAREGIPALMNSRTSPKVVGYDQLADSTPWYT 1ogy.1    --------------------------------------------------------------------------------  target    KSGRLEFYREEDEFIEAGENLPVHREPVDSTFYEPNVIVSPKHEAVRPSGPEDYGVARTDLSCEVRCGRNVVLTWAETRQ 1ogy.1    --------------------------------------------------------------------------------  target    TQHPLVKQGHKFIFHTPKYRHGSHTTPIDTDMNAVLFGPFGDIYRRDKRSPFVTEGYVDINPTDGLELGLQDGDYVWIDP 1ogy.1    --------------------------------------------------------------------------------  target    DPEDRPFRGWQKNAKDMEFARLLCRARFYPGTPRGVTRMWFNMYGATPGSVRGAKARRDGLAKNPDTNYQAMFRSGSHQS 1ogy.1    --------------------------------------------------------------------------------  target    ATRGWLKPTWMTDSLVRKGLFGQGIGKGFLPDVHCPTGAPREAFVKISRAEPGGIGGQGLWRPAALGIRPRHESPAMKRY 1ogy.1    --------------------------------------------------------------------------------  target    LAGGFFSGPKE 1ogy.1    ----------- ``` | | | | | | | | | | | | | | | | | | | | | | | | | | | | | | | | | | | | | | | | | | | | | | | | | |
|  | 4ydd.1.A | DMSO reductase family type II enzyme, molybdopterin subunit  *Crystal structure of the perchlorate reductase PcrAB from Azospira suillum PS* | 0.02 |  | 10.71 | 0.10 | 59-115 | X-ray | 1.86 | hetero-oligomer | 4 x SF4, 1 x MO, 1 x MGD, 1 x MD1, 1 x F3S | HHblits | 0.22 |
| ``` target    FNGAPQYINENPFDLELDASRPARPRQYWRAESAHFYNHEDHPLRVGTRLLTGSTHMPTPTKVMWFANANSILGNVKWHY 4ydd.1    ----------------------------------------------------------FNSKYIILWGSNPTQTR-IPDA  target    NTVVNALPRMEMIAVHEWWWTGSCEWADVVFGVDSWGELKHPDMTASVTNPFLIVFPKTPIKRIFNTVGDIDVLALVSSK 4ydd.1    HFLSEAQLNGAKIVSISPDYNSSTIKVDKWIHPQP---------------------------------------------  target    LAELTGDTRFNDMWKFVREGRTDVYLQRILDASTNTKGYRFTELEAKAREGIPALMNSRTSPKVVGYDQLADSTPWYTKS 4ydd.1    --------------------------------------------------------------------------------  target    GRLEFYREEDEFIEAGENLPVHREPVDSTFYEPNVIVSPKHEAVRPSGPEDYGVARTDLSCEVRCGRNVVLTWAETRQTQ 4ydd.1    --------------------------------------------------------------------------------  target    HPLVKQGHKFIFHTPKYRHGSHTTPIDTDMNAVLFGPFGDIYRRDKRSPFVTEGYVDINPTDGLELGLQDGDYVWIDPDP 4ydd.1    --------------------------------------------------------------------------------  target    EDRPFRGWQKNAKDMEFARLLCRARFYPGTPRGVTRMWFNMYGATPGSVRGAKARRDGLAKNPDTNYQAMFRSGSHQSAT 4ydd.1    --------------------------------------------------------------------------------  target    RGWLKPTWMTDSLVRKGLFGQGIGKGFLPDVHCPTGAPREAFVKISRAEPGGIGGQGLWRPAALGIRPRHESPAMKRYLA 4ydd.1    --------------------------------------------------------------------------------  target    GGFFSGPKE 4ydd.1    --------- ``` | | | | | | | | | | | | | | | | | | | | | | | | | | | | | | | | | | | | | | | | | | | | | | | | | |
|  | 5e7o.1.A | DMSO reductase family type II enzyme, molybdopterin subunit  *Crystal structure of the perchlorate reductase PcrAB mutant W461E of PcrA from Azospira suillum PS* | 0.02 |  | 10.71 | 0.10 | 59-115 | X-ray | 2.40 | hetero-oligomer | 4 x SF4, 1 x MO, 1 x MGD, 1 x MD1, 1 x F3S | HHblits | 0.22 |
| ``` target    FNGAPQYINENPFDLELDASRPARPRQYWRAESAHFYNHEDHPLRVGTRLLTGSTHMPTPTKVMWFANANSILGNVKWHY 5e7o.1    ----------------------------------------------------------FNSKYIILWGSNPTQTR-IPDA  target    NTVVNALPRMEMIAVHEWWWTGSCEWADVVFGVDSWGELKHPDMTASVTNPFLIVFPKTPIKRIFNTVGDIDVLALVSSK 5e7o.1    HFLSEAQLNGAKIVSISPDYNSSTIKVDKWIHPQP---------------------------------------------  target    LAELTGDTRFNDMWKFVREGRTDVYLQRILDASTNTKGYRFTELEAKAREGIPALMNSRTSPKVVGYDQLADSTPWYTKS 5e7o.1    --------------------------------------------------------------------------------  target    GRLEFYREEDEFIEAGENLPVHREPVDSTFYEPNVIVSPKHEAVRPSGPEDYGVARTDLSCEVRCGRNVVLTWAETRQTQ 5e7o.1    --------------------------------------------------------------------------------  target    HPLVKQGHKFIFHTPKYRHGSHTTPIDTDMNAVLFGPFGDIYRRDKRSPFVTEGYVDINPTDGLELGLQDGDYVWIDPDP 5e7o.1    --------------------------------------------------------------------------------  target    EDRPFRGWQKNAKDMEFARLLCRARFYPGTPRGVTRMWFNMYGATPGSVRGAKARRDGLAKNPDTNYQAMFRSGSHQSAT 5e7o.1    --------------------------------------------------------------------------------  target    RGWLKPTWMTDSLVRKGLFGQGIGKGFLPDVHCPTGAPREAFVKISRAEPGGIGGQGLWRPAALGIRPRHESPAMKRYLA 5e7o.1    --------------------------------------------------------------------------------  target    GGFFSGPKE 5e7o.1    --------- ``` | | | | | | | | | | | | | | | | | | | | | | | | | | | | | | | | | | | | | | | | | | | | | | | | | |
|  | 7wbb.1.A | AFG2 isoform 1  *Cryo-EM structure of substrate engaged Drg1 hexamer* | 0.02 |  | 19.23 | 0.09 | 373-440 | EM | 0.00 | hetero-6-1-mer | 11 x ATP | HHblits | 0.27 |
| ``` target    FNGAPQYINENPFDLELDASRPARPRQYWRAESAHFYNHEDHPLRVGTRLLTGSTHMPTPTKVMWFANANSILGNVKWHY 7wbb.1    --------------------------------------------------------------------------------  target    NTVVNALPRMEMIAVHEWWWTGSCEWADVVFGVDSWGELKHPDMTASVTNPFLIVFPKTPIKRIFNTVGDIDVLALVSSK 7wbb.1    --------------------------------------------------------------------------------  target    LAELTGDTRFNDMWKFVREGRTDVYLQRILDASTNTKGYRFTELEAKAREGIPALMNSRTSPKVVGYDQLADSTPWYTKS 7wbb.1    --------------------------------------------------------------------------------  target    GRLEFYREEDEFIEAGENLPVHREPVDSTFYEPNVIVSPKHEAVRPSGPEDYGVARTDLSCEVRCGRNVVLTWAETRQTQ 7wbb.1    --------------------------------------------------------------------------------  target    HPLVKQGHKFIFHTPKYRHGSHTTPIDTDMNAVLFGPFGDIYRRDKRSPFVTEGYVDINPTDGLELGLQDGDYVWIDPDP 7wbb.1    ----------------------------------------------------TCTAYIHPNVLSSLEINPGSFCTVGKI-  target    EDRPFRGWQKNAKDMEFARLLCRAR--FYPGTPRGVTRMWFNMYGATPGSVRGAKARRDGLAKNPDTNYQAMFRSGSHQS 7wbb.1    ------G---------ENGILVIARAGDEEVHPVNVITLSTT--------------------------------------  target    ATRGWLKPTWMTDSLVRKGLFGQGIGKGFLPDVHCPTGAPREAFVKISRAEPGGIGGQGLWRPAALGIRPRHESPAMKRY 7wbb.1    --------------------------------------------------------------------------------  target    LAGGFFSGPKE 7wbb.1    ----------- ``` | | | | | | | | | | | | | | | | | | | | | | | | | | | | | | | | | | | | | | | | | | | | | | | | | |
|  | 7wbb.1.B | AFG2 isoform 1  *Cryo-EM structure of substrate engaged Drg1 hexamer* | 0.01 |  | 19.23 | 0.09 | 373-440 | EM | 0.00 | hetero-6-1-mer | 11 x ATP | HHblits | 0.27 |
| ``` target    FNGAPQYINENPFDLELDASRPARPRQYWRAESAHFYNHEDHPLRVGTRLLTGSTHMPTPTKVMWFANANSILGNVKWHY 7wbb.1    --------------------------------------------------------------------------------  target    NTVVNALPRMEMIAVHEWWWTGSCEWADVVFGVDSWGELKHPDMTASVTNPFLIVFPKTPIKRIFNTVGDIDVLALVSSK 7wbb.1    --------------------------------------------------------------------------------  target    LAELTGDTRFNDMWKFVREGRTDVYLQRILDASTNTKGYRFTELEAKAREGIPALMNSRTSPKVVGYDQLADSTPWYTKS 7wbb.1    --------------------------------------------------------------------------------  target    GRLEFYREEDEFIEAGENLPVHREPVDSTFYEPNVIVSPKHEAVRPSGPEDYGVARTDLSCEVRCGRNVVLTWAETRQTQ 7wbb.1    --------------------------------------------------------------------------------  target    HPLVKQGHKFIFHTPKYRHGSHTTPIDTDMNAVLFGPFGDIYRRDKRSPFVTEGYVDINPTDGLELGLQDGDYVWIDPDP 7wbb.1    ----------------------------------------------------TCTAYIHPNVLSSLEINPGSFCTVGKI-  target    EDRPFRGWQKNAKDMEFARLLCRAR--FYPGTPRGVTRMWFNMYGATPGSVRGAKARRDGLAKNPDTNYQAMFRSGSHQS 7wbb.1    ------G---------ENGILVIARAGDEEVHPVNVITLSTT--------------------------------------  target    ATRGWLKPTWMTDSLVRKGLFGQGIGKGFLPDVHCPTGAPREAFVKISRAEPGGIGGQGLWRPAALGIRPRHESPAMKRY 7wbb.1    --------------------------------------------------------------------------------  target    LAGGFFSGPKE 7wbb.1    ----------- ``` | | | | | | | | | | | | | | | | | | | | | | | | | | | | | | | | | | | | | | | | | | | | | | | | | |
|  | 7wbb.1.C | AFG2 isoform 1  *Cryo-EM structure of substrate engaged Drg1 hexamer* | 0.02 |  | 19.23 | 0.09 | 373-440 | EM | 0.00 | hetero-6-1-mer | 11 x ATP | HHblits | 0.27 |
| ``` target    FNGAPQYINENPFDLELDASRPARPRQYWRAESAHFYNHEDHPLRVGTRLLTGSTHMPTPTKVMWFANANSILGNVKWHY 7wbb.1    --------------------------------------------------------------------------------  target    NTVVNALPRMEMIAVHEWWWTGSCEWADVVFGVDSWGELKHPDMTASVTNPFLIVFPKTPIKRIFNTVGDIDVLALVSSK 7wbb.1    --------------------------------------------------------------------------------  target    LAELTGDTRFNDMWKFVREGRTDVYLQRILDASTNTKGYRFTELEAKAREGIPALMNSRTSPKVVGYDQLADSTPWYTKS 7wbb.1    --------------------------------------------------------------------------------  target    GRLEFYREEDEFIEAGENLPVHREPVDSTFYEPNVIVSPKHEAVRPSGPEDYGVARTDLSCEVRCGRNVVLTWAETRQTQ 7wbb.1    --------------------------------------------------------------------------------  target    HPLVKQGHKFIFHTPKYRHGSHTTPIDTDMNAVLFGPFGDIYRRDKRSPFVTEGYVDINPTDGLELGLQDGDYVWIDPDP 7wbb.1    ----------------------------------------------------TCTAYIHPNVLSSLEINPGSFCTVGKI-  target    EDRPFRGWQKNAKDMEFARLLCRAR--FYPGTPRGVTRMWFNMYGATPGSVRGAKARRDGLAKNPDTNYQAMFRSGSHQS 7wbb.1    ------G---------ENGILVIARAGDEEVHPVNVITLSTT--------------------------------------  target    ATRGWLKPTWMTDSLVRKGLFGQGIGKGFLPDVHCPTGAPREAFVKISRAEPGGIGGQGLWRPAALGIRPRHESPAMKRY 7wbb.1    --------------------------------------------------------------------------------  target    LAGGFFSGPKE 7wbb.1    ----------- ``` | | | | | | | | | | | | | | | | | | | | | | | | | | | | | | | | | | | | | | | | | | | | | | | | | |
|  | 7wbb.1.D | AFG2 isoform 1  *Cryo-EM structure of substrate engaged Drg1 hexamer* | 0.02 |  | 19.23 | 0.09 | 373-440 | EM | 0.00 | hetero-6-1-mer | 11 x ATP | HHblits | 0.27 |
| ``` target    FNGAPQYINENPFDLELDASRPARPRQYWRAESAHFYNHEDHPLRVGTRLLTGSTHMPTPTKVMWFANANSILGNVKWHY 7wbb.1    --------------------------------------------------------------------------------  target    NTVVNALPRMEMIAVHEWWWTGSCEWADVVFGVDSWGELKHPDMTASVTNPFLIVFPKTPIKRIFNTVGDIDVLALVSSK 7wbb.1    --------------------------------------------------------------------------------  target    LAELTGDTRFNDMWKFVREGRTDVYLQRILDASTNTKGYRFTELEAKAREGIPALMNSRTSPKVVGYDQLADSTPWYTKS 7wbb.1    --------------------------------------------------------------------------------  target    GRLEFYREEDEFIEAGENLPVHREPVDSTFYEPNVIVSPKHEAVRPSGPEDYGVARTDLSCEVRCGRNVVLTWAETRQTQ 7wbb.1    --------------------------------------------------------------------------------  target    HPLVKQGHKFIFHTPKYRHGSHTTPIDTDMNAVLFGPFGDIYRRDKRSPFVTEGYVDINPTDGLELGLQDGDYVWIDPDP 7wbb.1    ----------------------------------------------------TCTAYIHPNVLSSLEINPGSFCTVGKI-  target    EDRPFRGWQKNAKDMEFARLLCRAR--FYPGTPRGVTRMWFNMYGATPGSVRGAKARRDGLAKNPDTNYQAMFRSGSHQS 7wbb.1    ------G---------ENGILVIARAGDEEVHPVNVITLSTT--------------------------------------  target    ATRGWLKPTWMTDSLVRKGLFGQGIGKGFLPDVHCPTGAPREAFVKISRAEPGGIGGQGLWRPAALGIRPRHESPAMKRY 7wbb.1    --------------------------------------------------------------------------------  target    LAGGFFSGPKE 7wbb.1    ----------- ``` | | | | | | | | | | | | | | | | | | | | | | | | | | | | | | | | | | | | | | | | | | | | | | | | | |
|  | 7wbb.1.E | AFG2 isoform 1  *Cryo-EM structure of substrate engaged Drg1 hexamer* | 0.02 |  | 19.23 | 0.09 | 373-440 | EM | 0.00 | hetero-6-1-mer | 11 x ATP | HHblits | 0.27 |
| ``` target    FNGAPQYINENPFDLELDASRPARPRQYWRAESAHFYNHEDHPLRVGTRLLTGSTHMPTPTKVMWFANANSILGNVKWHY 7wbb.1    --------------------------------------------------------------------------------  target    NTVVNALPRMEMIAVHEWWWTGSCEWADVVFGVDSWGELKHPDMTASVTNPFLIVFPKTPIKRIFNTVGDIDVLALVSSK 7wbb.1    --------------------------------------------------------------------------------  target    LAELTGDTRFNDMWKFVREGRTDVYLQRILDASTNTKGYRFTELEAKAREGIPALMNSRTSPKVVGYDQLADSTPWYTKS 7wbb.1    --------------------------------------------------------------------------------  target    GRLEFYREEDEFIEAGENLPVHREPVDSTFYEPNVIVSPKHEAVRPSGPEDYGVARTDLSCEVRCGRNVVLTWAETRQTQ 7wbb.1    --------------------------------------------------------------------------------  target    HPLVKQGHKFIFHTPKYRHGSHTTPIDTDMNAVLFGPFGDIYRRDKRSPFVTEGYVDINPTDGLELGLQDGDYVWIDPDP 7wbb.1    ----------------------------------------------------TCTAYIHPNVLSSLEINPGSFCTVGKI-  target    EDRPFRGWQKNAKDMEFARLLCRAR--FYPGTPRGVTRMWFNMYGATPGSVRGAKARRDGLAKNPDTNYQAMFRSGSHQS 7wbb.1    ------G---------ENGILVIARAGDEEVHPVNVITLSTT--------------------------------------  target    ATRGWLKPTWMTDSLVRKGLFGQGIGKGFLPDVHCPTGAPREAFVKISRAEPGGIGGQGLWRPAALGIRPRHESPAMKRY 7wbb.1    --------------------------------------------------------------------------------  target    LAGGFFSGPKE 7wbb.1    ----------- ``` | | | | | | | | | | | | | | | | | | | | | | | | | | | | | | | | | | | | | | | | | | | | | | | | | |
|  | 7wbb.1.G | AFG2 isoform 1  *Cryo-EM structure of substrate engaged Drg1 hexamer* | 0.01 |  | 19.23 | 0.09 | 373-440 | EM | 0.00 | hetero-6-1-mer | 11 x ATP | HHblits | 0.27 |
| ``` target    FNGAPQYINENPFDLELDASRPARPRQYWRAESAHFYNHEDHPLRVGTRLLTGSTHMPTPTKVMWFANANSILGNVKWHY 7wbb.1    --------------------------------------------------------------------------------  target    NTVVNALPRMEMIAVHEWWWTGSCEWADVVFGVDSWGELKHPDMTASVTNPFLIVFPKTPIKRIFNTVGDIDVLALVSSK 7wbb.1    --------------------------------------------------------------------------------  target    LAELTGDTRFNDMWKFVREGRTDVYLQRILDASTNTKGYRFTELEAKAREGIPALMNSRTSPKVVGYDQLADSTPWYTKS 7wbb.1    --------------------------------------------------------------------------------  target    GRLEFYREEDEFIEAGENLPVHREPVDSTFYEPNVIVSPKHEAVRPSGPEDYGVARTDLSCEVRCGRNVVLTWAETRQTQ 7wbb.1    --------------------------------------------------------------------------------  target    HPLVKQGHKFIFHTPKYRHGSHTTPIDTDMNAVLFGPFGDIYRRDKRSPFVTEGYVDINPTDGLELGLQDGDYVWIDPDP 7wbb.1    ----------------------------------------------------TCTAYIHPNVLSSLEINPGSFCTVGKI-  target    EDRPFRGWQKNAKDMEFARLLCRAR--FYPGTPRGVTRMWFNMYGATPGSVRGAKARRDGLAKNPDTNYQAMFRSGSHQS 7wbb.1    ------G---------ENGILVIARAGDEEVHPVNVITLSTT--------------------------------------  target    ATRGWLKPTWMTDSLVRKGLFGQGIGKGFLPDVHCPTGAPREAFVKISRAEPGGIGGQGLWRPAALGIRPRHESPAMKRY 7wbb.1    --------------------------------------------------------------------------------  target    LAGGFFSGPKE 7wbb.1    ----------- ``` | | | | | | | | | | | | | | | | | | | | | | | | | | | | | | | | | | | | | | | | | | | | | | | | | |
|  | 6cz7.1.A | ArrA  *The arsenate respiratory reductase (Arr) complex from Shewanella sp. ANA-3* | 0.02 |  | 11.11 | 0.09 | 60-115 | X-ray | 1.62 | hetero-1-1-mer | 5 x SF4, 2 x MGD, 1 x MO, 1 x PG5 | HHblits | 0.24 |
| ``` target    FNGAPQYINENPFDLELDASRPARPRQYWRAESAHFYNHEDHPLRVGTRLLTGSTHMPTPTKVMWFANANSILGNVKWHY 6cz7.1    -----------------------------------------------------------NAKFILSFGADPIAS--NRQV  target    -NTVVN--ALPRMEMIAVHEWWWTGSCEWADVVFGVDSWGELKHPDMTASVTNPFLIVFPKTPIKRIFNTVGDIDVLALV 6cz7.1    SFYSQTWGDSLDHAKVVVVDPRLSASAAKAHKWIPIEP------------------------------------------  target    SSKLAELTGDTRFNDMWKFVREGRTDVYLQRILDASTNTKGYRFTELEAKAREGIPALMNSRTSPKVVGYDQLADSTPWY 6cz7.1    --------------------------------------------------------------------------------  target    TKSGRLEFYREEDEFIEAGENLPVHREPVDSTFYEPNVIVSPKHEAVRPSGPEDYGVARTDLSCEVRCGRNVVLTWAETR 6cz7.1    --------------------------------------------------------------------------------  target    QTQHPLVKQGHKFIFHTPKYRHGSHTTPIDTDMNAVLFGPFGDIYRRDKRSPFVTEGYVDINPTDGLELGLQDGDYVWID 6cz7.1    --------------------------------------------------------------------------------  target    PDPEDRPFRGWQKNAKDMEFARLLCRARFYPGTPRGVTRMWFNMYGATPGSVRGAKARRDGLAKNPDTNYQAMFRSGSHQ 6cz7.1    --------------------------------------------------------------------------------  target    SATRGWLKPTWMTDSLVRKGLFGQGIGKGFLPDVHCPTGAPREAFVKISRAEPGGIGGQGLWRPAALGIRPRHESPAMKR 6cz7.1    --------------------------------------------------------------------------------  target    YLAGGFFSGPKE 6cz7.1    ------------ ``` | | | | | | | | | | | | | | | | | | | | | | | | | | | | | | | | | | | | | | | | | | | | | | | | | |
|  | 7p63.1.C | NADH-quinone oxidoreductase  *Complex I from E. coli, DDM/LMNG-purified, under Turnover at pH 6, Closed state* | 0.01 |  | 9.09 | 0.10 | 59-115 | EM | 0.00 | hetero-1-1-1-1-1-1-… | 7 x SF4, 1 x FMN, 1 x NAI, 2 x FES, 1 x CA, 1 x DCQ, 4 x LFA, 8 x 3PE | HHblits | 0.22 |
| ``` target    FNGAPQYINENPFDLELDASRPARPRQYWRAESAHFYNHEDHPLRVGTRLLTGSTHMPTPTKVMWFANANSILGNVKWHY 7p63.1    ----------------------------------------------------------ESYDAVLVLGEDVTQTG--ARV  target    -NTVVNALPR-ME-------------------------MIAVHEWWWTGSCEWADVVFGVDSWGELKHPDMTASVTNPFL 7p63.1    ALAVRQAVKGKAREMAAAQKVADWQIAAILNIGQRAKHPLFVTNVDDTRLDDIAAWTYRAPV------------------  target    IVFPKTPIKRIFNTVGDIDVLALVSSKLAELTGDTRFNDMWKFVREGRTDVYLQRILDASTNTKGYRFTELEAKAREGIP 7p63.1    --------------------------------------------------------------------------------  target    ALMNSRTSPKVVGYDQLADSTPWYTKSGRLEFYREEDEFIEAGENLPVHREPVDSTFYEPNVIVSPKHEAVRPSGPEDYG 7p63.1    --------------------------------------------------------------------------------  target    VARTDLSCEVRCGRNVVLTWAETRQTQHPLVKQGHKFIFHTPKYRHGSHTTPIDTDMNAVLFGPFGDIYRRDKRSPFVTE 7p63.1    --------------------------------------------------------------------------------  target    GYVDINPTDGLELGLQDGDYVWIDPDPEDRPFRGWQKNAKDMEFARLLCRARFYPGTPRGVTRMWFNMYGATPGSVRGAK 7p63.1    --------------------------------------------------------------------------------  target    ARRDGLAKNPDTNYQAMFRSGSHQSATRGWLKPTWMTDSLVRKGLFGQGIGKGFLPDVHCPTGAPREAFVKISRAEPGGI 7p63.1    --------------------------------------------------------------------------------  target    GGQGLWRPAALGIRPRHESPAMKRYLAGGFFSGPKE 7p63.1    ------------------------------------ ``` | | | | | | | | | | | | | | | | | | | | | | | | | | | | | | | | | | | | | | | | | | | | | | | | | |
|  | 1r27.4.A | Respiratory nitrate reductase 1 alpha chain  *Crystal Structure of NarGH complex* | 0.02 |  | 3.57 | 0.10 | 58-114 | X-ray | 2.00 | hetero-4-4-mer | 4 x MO, 16 x SF4, 8 x MGD, 4 x F3S | HHblits | 0.21 |
| ``` target    FNGAPQYINENPFDLELDASRPARPRQYWRAESAHFYNHEDHPLRVGTRLLTGSTHMPTPTKVMWFANANSILGNVKWHY 1r27.4    ---------------------------------------------------------WYNSSYIIAWGSNVPQTR-TPDA  target    NTVVNALPRMEMIAVHEWWWTGSCEWADVVFGVDSWGELKHPDMTASVTNPFLIVFPKTPIKRIFNTVGDIDVLALVSSK 1r27.4    HFFTEVRYKGTKTVAVTPDYAEIAKLCDLWLAPK----------------------------------------------  target    LAELTGDTRFNDMWKFVREGRTDVYLQRILDASTNTKGYRFTELEAKAREGIPALMNSRTSPKVVGYDQLADSTPWYTKS 1r27.4    --------------------------------------------------------------------------------  target    GRLEFYREEDEFIEAGENLPVHREPVDSTFYEPNVIVSPKHEAVRPSGPEDYGVARTDLSCEVRCGRNVVLTWAETRQTQ 1r27.4    --------------------------------------------------------------------------------  target    HPLVKQGHKFIFHTPKYRHGSHTTPIDTDMNAVLFGPFGDIYRRDKRSPFVTEGYVDINPTDGLELGLQDGDYVWIDPDP 1r27.4    --------------------------------------------------------------------------------  target    EDRPFRGWQKNAKDMEFARLLCRARFYPGTPRGVTRMWFNMYGATPGSVRGAKARRDGLAKNPDTNYQAMFRSGSHQSAT 1r27.4    --------------------------------------------------------------------------------  target    RGWLKPTWMTDSLVRKGLFGQGIGKGFLPDVHCPTGAPREAFVKISRAEPGGIGGQGLWRPAALGIRPRHESPAMKRYLA 1r27.4    --------------------------------------------------------------------------------  target    GGFFSGPKE 1r27.4    --------- ``` | | | | | | | | | | | | | | | | | | | | | | | | | | | | | | | | | | | | | | | | | | | | | | | | | |
|  | 1q16.1.A | Respiratory nitrate reductase 1 alpha chain  *Crystal structure of Nitrate Reductase A, NarGHI, from Escherichia coli* | 0.02 |  | 3.57 | 0.10 | 58-114 | X-ray | 1.90 | hetero-oligomer | 2 x MD1, 1 x 6MO, 2 x HEM, 4 x SF4, 1 x F3S, 1 x AGA, 1 x 3PH | HHblits | 0.21 |
| ``` target    FNGAPQYINENPFDLELDASRPARPRQYWRAESAHFYNHEDHPLRVGTRLLTGSTHMPTPTKVMWFANANSILGNVKWHY 1q16.1    ---------------------------------------------------------WYNSSYIIAWGSNVPQTR-TPDA  target    NTVVNALPRMEMIAVHEWWWTGSCEWADVVFGVDSWGELKHPDMTASVTNPFLIVFPKTPIKRIFNTVGDIDVLALVSSK 1q16.1    HFFTEVRYKGTKTVAVTPDYAEIAKLCDLWLAPK----------------------------------------------  target    LAELTGDTRFNDMWKFVREGRTDVYLQRILDASTNTKGYRFTELEAKAREGIPALMNSRTSPKVVGYDQLADSTPWYTKS 1q16.1    --------------------------------------------------------------------------------  target    GRLEFYREEDEFIEAGENLPVHREPVDSTFYEPNVIVSPKHEAVRPSGPEDYGVARTDLSCEVRCGRNVVLTWAETRQTQ 1q16.1    --------------------------------------------------------------------------------  target    HPLVKQGHKFIFHTPKYRHGSHTTPIDTDMNAVLFGPFGDIYRRDKRSPFVTEGYVDINPTDGLELGLQDGDYVWIDPDP 1q16.1    --------------------------------------------------------------------------------  target    EDRPFRGWQKNAKDMEFARLLCRARFYPGTPRGVTRMWFNMYGATPGSVRGAKARRDGLAKNPDTNYQAMFRSGSHQSAT 1q16.1    --------------------------------------------------------------------------------  target    RGWLKPTWMTDSLVRKGLFGQGIGKGFLPDVHCPTGAPREAFVKISRAEPGGIGGQGLWRPAALGIRPRHESPAMKRYLA 1q16.1    --------------------------------------------------------------------------------  target    GGFFSGPKE 1q16.1    --------- ``` | | | | | | | | | | | | | | | | | | | | | | | | | | | | | | | | | | | | | | | | | | | | | | | | | |
|  | 4kdl.1.A | Transitional endoplasmic reticulum ATPase  *Crystal structure of p97/VCP N in complex with OTU1 UBXL* | 0.02 |  | 19.23 | 0.09 | 374-441 | X-ray | 1.81 | monomer |  | HHblits | 0.26 |
| ``` target    FNGAPQYINENPFDLELDASRPARPRQYWRAESAHFYNHEDHPLRVGTRLLTGSTHMPTPTKVMWFANANSILGNVKWHY 4kdl.1    --------------------------------------------------------------------------------  target    NTVVNALPRMEMIAVHEWWWTGSCEWADVVFGVDSWGELKHPDMTASVTNPFLIVFPKTPIKRIFNTVGDIDVLALVSSK 4kdl.1    --------------------------------------------------------------------------------  target    LAELTGDTRFNDMWKFVREGRTDVYLQRILDASTNTKGYRFTELEAKAREGIPALMNSRTSPKVVGYDQLADSTPWYTKS 4kdl.1    --------------------------------------------------------------------------------  target    GRLEFYREEDEFIEAGENLPVHREPVDSTFYEPNVIVSPKHEAVRPSGPEDYGVARTDLSCEVRCGRNVVLTWAETRQTQ 4kdl.1    --------------------------------------------------------------------------------  target    HPLVKQGHKFIFHTPKYRHGSHTTPIDTDMNAVLFGPFGDIYRRDKRSPFVTEGYVDINPTDGLELGLQDGDYVWIDPDP 4kdl.1    -----------------------------------------------------SVVSLSQPKMDELQLFRGDTVLLKGKK  target    EDRPFRGWQKNAKDMEFARLLCRARFYPGTPRGVTRMWFNMYGATPGSVRGAKARRDGLAKNPDTNYQAMFRSGSHQSAT 4kdl.1    ------R----------REAVCIVLSDDTCSDEKIRMNRVV---------------------------------------  target    RGWLKPTWMTDSLVRKGLFGQGIGKGFLPDVHCPTGAPREAFVKISRAEPGGIGGQGLWRPAALGIRPRHESPAMKRYLA 4kdl.1    --------------------------------------------------------------------------------  target    GGFFSGPKE 4kdl.1    --------- ``` | | | | | | | | | | | | | | | | | | | | | | | | | | | | | | | | | | | | | | | | | | | | | | | | | |
|  | 4kdi.2.A | Transitional endoplasmic reticulum ATPase  *Crystal structure of p97/VCP N in complex with OTU1 UBXL* | 0.02 |  | 19.23 | 0.09 | 374-441 | X-ray | 1.86 | hetero-oligomer |  | HHblits | 0.26 |
| ``` target    FNGAPQYINENPFDLELDASRPARPRQYWRAESAHFYNHEDHPLRVGTRLLTGSTHMPTPTKVMWFANANSILGNVKWHY 4kdi.2    --------------------------------------------------------------------------------  target    NTVVNALPRMEMIAVHEWWWTGSCEWADVVFGVDSWGELKHPDMTASVTNPFLIVFPKTPIKRIFNTVGDIDVLALVSSK 4kdi.2    --------------------------------------------------------------------------------  target    LAELTGDTRFNDMWKFVREGRTDVYLQRILDASTNTKGYRFTELEAKAREGIPALMNSRTSPKVVGYDQLADSTPWYTKS 4kdi.2    --------------------------------------------------------------------------------  target    GRLEFYREEDEFIEAGENLPVHREPVDSTFYEPNVIVSPKHEAVRPSGPEDYGVARTDLSCEVRCGRNVVLTWAETRQTQ 4kdi.2    --------------------------------------------------------------------------------  target    HPLVKQGHKFIFHTPKYRHGSHTTPIDTDMNAVLFGPFGDIYRRDKRSPFVTEGYVDINPTDGLELGLQDGDYVWIDPDP 4kdi.2    -----------------------------------------------------SVVSLSQPKMDELQLFRGDTVLLKGKK  target    EDRPFRGWQKNAKDMEFARLLCRARFYPGTPRGVTRMWFNMYGATPGSVRGAKARRDGLAKNPDTNYQAMFRSGSHQSAT 4kdi.2    ------R----------REAVCIVLSDDTCSDEKIRMNRVV---------------------------------------  target    RGWLKPTWMTDSLVRKGLFGQGIGKGFLPDVHCPTGAPREAFVKISRAEPGGIGGQGLWRPAALGIRPRHESPAMKRYLA 4kdi.2    --------------------------------------------------------------------------------  target    GGFFSGPKE 4kdi.2    --------- ``` | | | | | | | | | | | | | | | | | | | | | | | | | | | | | | | | | | | | | | | | | | | | | | | | | |
|  | 4kdi.1.A | Transitional endoplasmic reticulum ATPase  *Crystal structure of p97/VCP N in complex with OTU1 UBXL* | 0.02 |  | 19.23 | 0.09 | 374-441 | X-ray | 1.86 | hetero-oligomer |  | HHblits | 0.26 |
| ``` target    FNGAPQYINENPFDLELDASRPARPRQYWRAESAHFYNHEDHPLRVGTRLLTGSTHMPTPTKVMWFANANSILGNVKWHY 4kdi.1    --------------------------------------------------------------------------------  target    NTVVNALPRMEMIAVHEWWWTGSCEWADVVFGVDSWGELKHPDMTASVTNPFLIVFPKTPIKRIFNTVGDIDVLALVSSK 4kdi.1    --------------------------------------------------------------------------------  target    LAELTGDTRFNDMWKFVREGRTDVYLQRILDASTNTKGYRFTELEAKAREGIPALMNSRTSPKVVGYDQLADSTPWYTKS 4kdi.1    --------------------------------------------------------------------------------  target    GRLEFYREEDEFIEAGENLPVHREPVDSTFYEPNVIVSPKHEAVRPSGPEDYGVARTDLSCEVRCGRNVVLTWAETRQTQ 4kdi.1    --------------------------------------------------------------------------------  target    HPLVKQGHKFIFHTPKYRHGSHTTPIDTDMNAVLFGPFGDIYRRDKRSPFVTEGYVDINPTDGLELGLQDGDYVWIDPDP 4kdi.1    -----------------------------------------------------SVVSLSQPKMDELQLFRGDTVLLKGKK  target    EDRPFRGWQKNAKDMEFARLLCRARFYPGTPRGVTRMWFNMYGATPGSVRGAKARRDGLAKNPDTNYQAMFRSGSHQSAT 4kdi.1    ------R----------REAVCIVLSDDTCSDEKIRMNRVV---------------------------------------  target    RGWLKPTWMTDSLVRKGLFGQGIGKGFLPDVHCPTGAPREAFVKISRAEPGGIGGQGLWRPAALGIRPRHESPAMKRYLA 4kdi.1    --------------------------------------------------------------------------------  target    GGFFSGPKE 4kdi.1    --------- ``` | | | | | | | | | | | | | | | | | | | | | | | | | | | | | | | | | | | | | | | | | | | | | | | | | |
|  | 7t2r.1.A | NiFe hydrogenase subunit A  *Structure of electron bifurcating Ni-Fe hydrogenase complex HydABCSL in FMN-free apo state* | 0.02 |  | 5.45 | 0.10 | 59-115 | EM | 0.00 | hetero-2-2-2-2-2-mer | 6 x FES, 12 x SF4, 2 x 3NI, 2 x FCO | HHblits | 0.21 |
| ``` target    FNGAPQYINENPFDLELDASRPARPRQYWRAESAHFYNHEDHPLRVGTRLLTGSTHMPTPTKVMWFANANSILGNVKWHY 7t2r.1    ----------------------------------------------------------LDSDLIITMFADPQKE--APVV  target    -NTVVNALPRME-MIAVHEWWWTGSCEWADVVFGVDSWGELKHPDMTASVTNPFLIVFPKTPIKRIFNTVGDIDVLALVS 7t2r.1    ASYIRVACLHRNAKLMNLSYGPSPFPGLVDLDIRLPE-------------------------------------------  target    SKLAELTGDTRFNDMWKFVREGRTDVYLQRILDASTNTKGYRFTELEAKAREGIPALMNSRTSPKVVGYDQLADSTPWYT 7t2r.1    --------------------------------------------------------------------------------  target    KSGRLEFYREEDEFIEAGENLPVHREPVDSTFYEPNVIVSPKHEAVRPSGPEDYGVARTDLSCEVRCGRNVVLTWAETRQ 7t2r.1    --------------------------------------------------------------------------------  target    TQHPLVKQGHKFIFHTPKYRHGSHTTPIDTDMNAVLFGPFGDIYRRDKRSPFVTEGYVDINPTDGLELGLQDGDYVWIDP 7t2r.1    --------------------------------------------------------------------------------  target    DPEDRPFRGWQKNAKDMEFARLLCRARFYPGTPRGVTRMWFNMYGATPGSVRGAKARRDGLAKNPDTNYQAMFRSGSHQS 7t2r.1    --------------------------------------------------------------------------------  target    ATRGWLKPTWMTDSLVRKGLFGQGIGKGFLPDVHCPTGAPREAFVKISRAEPGGIGGQGLWRPAALGIRPRHESPAMKRY 7t2r.1    --------------------------------------------------------------------------------  target    LAGGFFSGPKE 7t2r.1    ----------- ``` | | | | | | | | | | | | | | | | | | | | | | | | | | | | | | | | | | | | | | | | | | | | | | | | | |
|  | 7t30.1.A | NiFe hydrogenase subunit A  *Structure of electron bifurcating Ni-Fe hydrogenase complex HydABCSL in FMN/NAD(H) bound state* | 0.01 |  | 5.45 | 0.10 | 59-115 | EM | 0.00 | hetero-2-2-2-2-2-mer | 4 x FES, 12 x SF4, 2 x NAD, 2 x FMN, 2 x 3NI, 2 x FCO | HHblits | 0.21 |
| ``` target    FNGAPQYINENPFDLELDASRPARPRQYWRAESAHFYNHEDHPLRVGTRLLTGSTHMPTPTKVMWFANANSILGNVKWHY 7t30.1    ----------------------------------------------------------LDSDLIITMFADPQKE--APVV  target    -NTVVNALPRME-MIAVHEWWWTGSCEWADVVFGVDSWGELKHPDMTASVTNPFLIVFPKTPIKRIFNTVGDIDVLALVS 7t30.1    ASYIRVACLHRNAKLMNLSYGPSPFPGLVDLDIRLPE-------------------------------------------  target    SKLAELTGDTRFNDMWKFVREGRTDVYLQRILDASTNTKGYRFTELEAKAREGIPALMNSRTSPKVVGYDQLADSTPWYT 7t30.1    --------------------------------------------------------------------------------  target    KSGRLEFYREEDEFIEAGENLPVHREPVDSTFYEPNVIVSPKHEAVRPSGPEDYGVARTDLSCEVRCGRNVVLTWAETRQ 7t30.1    --------------------------------------------------------------------------------  target    TQHPLVKQGHKFIFHTPKYRHGSHTTPIDTDMNAVLFGPFGDIYRRDKRSPFVTEGYVDINPTDGLELGLQDGDYVWIDP 7t30.1    --------------------------------------------------------------------------------  target    DPEDRPFRGWQKNAKDMEFARLLCRARFYPGTPRGVTRMWFNMYGATPGSVRGAKARRDGLAKNPDTNYQAMFRSGSHQS 7t30.1    --------------------------------------------------------------------------------  target    ATRGWLKPTWMTDSLVRKGLFGQGIGKGFLPDVHCPTGAPREAFVKISRAEPGGIGGQGLWRPAALGIRPRHESPAMKRY 7t30.1    --------------------------------------------------------------------------------  target    LAGGFFSGPKE 7t30.1    ----------- ``` | | | | | | | | | | | | | | | | | | | | | | | | | | | | | | | | | | | | | | | | | | | | | | | | | |
|  | 3ir7.1.A | Respiratory nitrate reductase 1 alpha chain  *Crystal structure of NarGHI mutant NarG-R94S* | 0.02 |  | 3.64 | 0.10 | 59-114 | X-ray | 2.50 | hetero-1-1-1-mer | 2 x MD1, 4 x SF4, 1 x 6MO, 1 x AGA, 1 x F3S, 2 x HEM | HHblits | 0.21 |
| ``` target    FNGAPQYINENPFDLELDASRPARPRQYWRAESAHFYNHEDHPLRVGTRLLTGSTHMPTPTKVMWFANANSILGNVKWHY 3ir7.1    ----------------------------------------------------------YNSSYIIAWGSNVPQTR-TPDA  target    NTVVNALPRMEMIAVHEWWWTGSCEWADVVFGVDSWGELKHPDMTASVTNPFLIVFPKTPIKRIFNTVGDIDVLALVSSK 3ir7.1    HFFTEVRYKGTKTVAVTPDYAEIAKLCDLWLAPK----------------------------------------------  target    LAELTGDTRFNDMWKFVREGRTDVYLQRILDASTNTKGYRFTELEAKAREGIPALMNSRTSPKVVGYDQLADSTPWYTKS 3ir7.1    --------------------------------------------------------------------------------  target    GRLEFYREEDEFIEAGENLPVHREPVDSTFYEPNVIVSPKHEAVRPSGPEDYGVARTDLSCEVRCGRNVVLTWAETRQTQ 3ir7.1    --------------------------------------------------------------------------------  target    HPLVKQGHKFIFHTPKYRHGSHTTPIDTDMNAVLFGPFGDIYRRDKRSPFVTEGYVDINPTDGLELGLQDGDYVWIDPDP 3ir7.1    --------------------------------------------------------------------------------  target    EDRPFRGWQKNAKDMEFARLLCRARFYPGTPRGVTRMWFNMYGATPGSVRGAKARRDGLAKNPDTNYQAMFRSGSHQSAT 3ir7.1    --------------------------------------------------------------------------------  target    RGWLKPTWMTDSLVRKGLFGQGIGKGFLPDVHCPTGAPREAFVKISRAEPGGIGGQGLWRPAALGIRPRHESPAMKRYLA 3ir7.1    --------------------------------------------------------------------------------  target    GGFFSGPKE 3ir7.1    --------- ``` | | | | | | | | | | | | | | | | | | | | | | | | | | | | | | | | | | | | | | | | | | | | | | | | | |
|  | 3egw.1.A | Respiratory nitrate reductase 1 alpha chain  *The crystal structure of the NarGHI mutant NarH - C16A* | 0.02 |  | 3.64 | 0.10 | 59-114 | X-ray | 1.90 | hetero-2-2-2-mer | 2 x MD1, 2 x MGD, 2 x 6MO, 6 x SF4, 4 x F3S, 2 x 3PH, 4 x HEM, 2 x AGA | HHblits | 0.21 |
| ``` target    FNGAPQYINENPFDLELDASRPARPRQYWRAESAHFYNHEDHPLRVGTRLLTGSTHMPTPTKVMWFANANSILGNVKWHY 3egw.1    ----------------------------------------------------------YNSSYIIAWGSNVPQTR-TPDA  target    NTVVNALPRMEMIAVHEWWWTGSCEWADVVFGVDSWGELKHPDMTASVTNPFLIVFPKTPIKRIFNTVGDIDVLALVSSK 3egw.1    HFFTEVRYKGTKTVAVTPDYAEIAKLCDLWLAPK----------------------------------------------  target    LAELTGDTRFNDMWKFVREGRTDVYLQRILDASTNTKGYRFTELEAKAREGIPALMNSRTSPKVVGYDQLADSTPWYTKS 3egw.1    --------------------------------------------------------------------------------  target    GRLEFYREEDEFIEAGENLPVHREPVDSTFYEPNVIVSPKHEAVRPSGPEDYGVARTDLSCEVRCGRNVVLTWAETRQTQ 3egw.1    --------------------------------------------------------------------------------  target    HPLVKQGHKFIFHTPKYRHGSHTTPIDTDMNAVLFGPFGDIYRRDKRSPFVTEGYVDINPTDGLELGLQDGDYVWIDPDP 3egw.1    --------------------------------------------------------------------------------  target    EDRPFRGWQKNAKDMEFARLLCRARFYPGTPRGVTRMWFNMYGATPGSVRGAKARRDGLAKNPDTNYQAMFRSGSHQSAT 3egw.1    --------------------------------------------------------------------------------  target    RGWLKPTWMTDSLVRKGLFGQGIGKGFLPDVHCPTGAPREAFVKISRAEPGGIGGQGLWRPAALGIRPRHESPAMKRYLA 3egw.1    --------------------------------------------------------------------------------  target    GGFFSGPKE 3egw.1    --------- ``` | | | | | | | | | | | | | | | | | | | | | | | | | | | | | | | | | | | | | | | | | | | | | | | | | |
|  | 3ir5.1.A | Respiratory nitrate reductase 1 alpha chain  *Crystal structure of NarGHI mutant NarG-H49C* | 0.02 |  | 3.64 | 0.10 | 59-114 | X-ray | 2.30 | hetero-1-1-1-mer | 2 x MD1, 1 x 6MO, 4 x SF4, 1 x AGA, 1 x F3S, 2 x HEM | HHblits | 0.21 |
| ``` target    FNGAPQYINENPFDLELDASRPARPRQYWRAESAHFYNHEDHPLRVGTRLLTGSTHMPTPTKVMWFANANSILGNVKWHY 3ir5.1    ----------------------------------------------------------YNSSYIIAWGSNVPQTR-TPDA  target    NTVVNALPRMEMIAVHEWWWTGSCEWADVVFGVDSWGELKHPDMTASVTNPFLIVFPKTPIKRIFNTVGDIDVLALVSSK 3ir5.1    HFFTEVRYKGTKTVAVTPDYAEIAKLCDLWLAPK----------------------------------------------  target    LAELTGDTRFNDMWKFVREGRTDVYLQRILDASTNTKGYRFTELEAKAREGIPALMNSRTSPKVVGYDQLADSTPWYTKS 3ir5.1    --------------------------------------------------------------------------------  target    GRLEFYREEDEFIEAGENLPVHREPVDSTFYEPNVIVSPKHEAVRPSGPEDYGVARTDLSCEVRCGRNVVLTWAETRQTQ 3ir5.1    --------------------------------------------------------------------------------  target    HPLVKQGHKFIFHTPKYRHGSHTTPIDTDMNAVLFGPFGDIYRRDKRSPFVTEGYVDINPTDGLELGLQDGDYVWIDPDP 3ir5.1    --------------------------------------------------------------------------------  target    EDRPFRGWQKNAKDMEFARLLCRARFYPGTPRGVTRMWFNMYGATPGSVRGAKARRDGLAKNPDTNYQAMFRSGSHQSAT 3ir5.1    --------------------------------------------------------------------------------  target    RGWLKPTWMTDSLVRKGLFGQGIGKGFLPDVHCPTGAPREAFVKISRAEPGGIGGQGLWRPAALGIRPRHESPAMKRYLA 3ir5.1    --------------------------------------------------------------------------------  target    GGFFSGPKE 3ir5.1    --------- ``` | | | | | | | | | | | | | | | | | | | | | | | | | | | | | | | | | | | | | | | | | | | | | | | | | |
|  | 3ir6.1.A | Respiratory nitrate reductase 1 alpha chain  *Crystal structure of NarGHI mutant NarG-H49S* | 0.02 |  | 3.64 | 0.10 | 59-114 | X-ray | 2.80 | hetero-1-1-1-mer | 2 x GDP, 1 x AGA, 3 x SF4, 1 x F3S, 2 x HEM | HHblits | 0.21 |
| ``` target    FNGAPQYINENPFDLELDASRPARPRQYWRAESAHFYNHEDHPLRVGTRLLTGSTHMPTPTKVMWFANANSILGNVKWHY 3ir6.1    ----------------------------------------------------------YNSSYIIAWGSNVPQTR-TPDA  target    NTVVNALPRMEMIAVHEWWWTGSCEWADVVFGVDSWGELKHPDMTASVTNPFLIVFPKTPIKRIFNTVGDIDVLALVSSK 3ir6.1    HFFTEVRYKGTKTVAVTPDYAEIAKLCDLWLAPK----------------------------------------------  target    LAELTGDTRFNDMWKFVREGRTDVYLQRILDASTNTKGYRFTELEAKAREGIPALMNSRTSPKVVGYDQLADSTPWYTKS 3ir6.1    --------------------------------------------------------------------------------  target    GRLEFYREEDEFIEAGENLPVHREPVDSTFYEPNVIVSPKHEAVRPSGPEDYGVARTDLSCEVRCGRNVVLTWAETRQTQ 3ir6.1    --------------------------------------------------------------------------------  target    HPLVKQGHKFIFHTPKYRHGSHTTPIDTDMNAVLFGPFGDIYRRDKRSPFVTEGYVDINPTDGLELGLQDGDYVWIDPDP 3ir6.1    --------------------------------------------------------------------------------  target    EDRPFRGWQKNAKDMEFARLLCRARFYPGTPRGVTRMWFNMYGATPGSVRGAKARRDGLAKNPDTNYQAMFRSGSHQSAT 3ir6.1    --------------------------------------------------------------------------------  target    RGWLKPTWMTDSLVRKGLFGQGIGKGFLPDVHCPTGAPREAFVKISRAEPGGIGGQGLWRPAALGIRPRHESPAMKRYLA 3ir6.1    --------------------------------------------------------------------------------  target    GGFFSGPKE 3ir6.1    --------- ``` | | | | | | | | | | | | | | | | | | | | | | | | | | | | | | | | | | | | | | | | | | | | | | | | | |
|  | 4rv0.1.A | Transitional endoplasmic reticulum ATPase TER94  *Crystal structure of TN complex* | 0.02 |  | 21.57 | 0.09 | 374-440 | X-ray | 2.00 | hetero-oligomer |  | HHblits | 0.27 |
| ``` target    FNGAPQYINENPFDLELDASRPARPRQYWRAESAHFYNHEDHPLRVGTRLLTGSTHMPTPTKVMWFANANSILGNVKWHY 4rv0.1    --------------------------------------------------------------------------------  target    NTVVNALPRMEMIAVHEWWWTGSCEWADVVFGVDSWGELKHPDMTASVTNPFLIVFPKTPIKRIFNTVGDIDVLALVSSK 4rv0.1    --------------------------------------------------------------------------------  target    LAELTGDTRFNDMWKFVREGRTDVYLQRILDASTNTKGYRFTELEAKAREGIPALMNSRTSPKVVGYDQLADSTPWYTKS 4rv0.1    --------------------------------------------------------------------------------  target    GRLEFYREEDEFIEAGENLPVHREPVDSTFYEPNVIVSPKHEAVRPSGPEDYGVARTDLSCEVRCGRNVVLTWAETRQTQ 4rv0.1    --------------------------------------------------------------------------------  target    HPLVKQGHKFIFHTPKYRHGSHTTPIDTDMNAVLFGPFGDIYRRDKRSPFVTEGYVDINPTDGLELGLQDGDYVWIDPDP 4rv0.1    -----------------------------------------------------SVVSLSQAKMDELQLFRGDTVILKGKR  target    EDRPFRGWQKNAKDMEFARLLCRARFYPGTPRGVTRMWFNMYGATPGSVRGAKARRDGLAKNPDTNYQAMFRSGSHQSAT 4rv0.1    ------R----------KETVCIVLSDDTCPDEKIRMNRV----------------------------------------  target    RGWLKPTWMTDSLVRKGLFGQGIGKGFLPDVHCPTGAPREAFVKISRAEPGGIGGQGLWRPAALGIRPRHESPAMKRYLA 4rv0.1    --------------------------------------------------------------------------------  target    GGFFSGPKE 4rv0.1    --------- ``` | | | | | | | | | | | | | | | | | | | | | | | | | | | | | | | | | | | | | | | | | | | | | | | | | |
|  | 7p61.1.C | NADH-quinone oxidoreductase  *Complex I from E. coli, DDM-purified, with NADH, Resting state* | 0.01 |  | 9.26 | 0.09 | 60-115 | EM | 0.00 | hetero-1-1-1-1-1-1-… | 7 x SF4, 1 x FMN, 1 x NAI, 2 x FES, 1 x CA, 2 x 3PE, 1 x UQ8 | HHblits | 0.22 |
| ``` target    FNGAPQYINENPFDLELDASRPARPRQYWRAESAHFYNHEDHPLRVGTRLLTGSTHMPTPTKVMWFANANSILGNVKWHY 7p61.1    -----------------------------------------------------------SYDAVLVLGEDVTQTG--ARV  target    -NTVVNALPR-M------------------------E-MIAVHEWWWTGSCEWADVVFGVDSWGELKHPDMTASVTNPFL 7p61.1    ALAVRQAVKGKAREMAAAQKVADWQIAAILNIGQRAKHPLFVTNVDDTRLDDIAAWTYRAPV------------------  target    IVFPKTPIKRIFNTVGDIDVLALVSSKLAELTGDTRFNDMWKFVREGRTDVYLQRILDASTNTKGYRFTELEAKAREGIP 7p61.1    --------------------------------------------------------------------------------  target    ALMNSRTSPKVVGYDQLADSTPWYTKSGRLEFYREEDEFIEAGENLPVHREPVDSTFYEPNVIVSPKHEAVRPSGPEDYG 7p61.1    --------------------------------------------------------------------------------  target    VARTDLSCEVRCGRNVVLTWAETRQTQHPLVKQGHKFIFHTPKYRHGSHTTPIDTDMNAVLFGPFGDIYRRDKRSPFVTE 7p61.1    --------------------------------------------------------------------------------  target    GYVDINPTDGLELGLQDGDYVWIDPDPEDRPFRGWQKNAKDMEFARLLCRARFYPGTPRGVTRMWFNMYGATPGSVRGAK 7p61.1    --------------------------------------------------------------------------------  target    ARRDGLAKNPDTNYQAMFRSGSHQSATRGWLKPTWMTDSLVRKGLFGQGIGKGFLPDVHCPTGAPREAFVKISRAEPGGI 7p61.1    --------------------------------------------------------------------------------  target    GGQGLWRPAALGIRPRHESPAMKRYLAGGFFSGPKE 7p61.1    ------------------------------------ ``` | | | | | | | | | | | | | | | | | | | | | | | | | | | | | | | | | | | | | | | | | | | | | | | | | |
|  | 2pjh.1.B | Transitional endoplasmic reticulum ATPase  *Strctural Model of the p97 N domain- npl4 UBD complex* | 0.02 |  | 19.61 | 0.09 | 373-439 | NMR | 0.00 | hetero-1-1-mer |  | HHblits | 0.26 |
| ``` target    FNGAPQYINENPFDLELDASRPARPRQYWRAESAHFYNHEDHPLRVGTRLLTGSTHMPTPTKVMWFANANSILGNVKWHY 2pjh.1    --------------------------------------------------------------------------------  target    NTVVNALPRMEMIAVHEWWWTGSCEWADVVFGVDSWGELKHPDMTASVTNPFLIVFPKTPIKRIFNTVGDIDVLALVSSK 2pjh.1    --------------------------------------------------------------------------------  target    LAELTGDTRFNDMWKFVREGRTDVYLQRILDASTNTKGYRFTELEAKAREGIPALMNSRTSPKVVGYDQLADSTPWYTKS 2pjh.1    --------------------------------------------------------------------------------  target    GRLEFYREEDEFIEAGENLPVHREPVDSTFYEPNVIVSPKHEAVRPSGPEDYGVARTDLSCEVRCGRNVVLTWAETRQTQ 2pjh.1    --------------------------------------------------------------------------------  target    HPLVKQGHKFIFHTPKYRHGSHTTPIDTDMNAVLFGPFGDIYRRDKRSPFVTEGYVDINPTDGLELGLQDGDYVWIDPDP 2pjh.1    ----------------------------------------------------NSVVSLSQPKMDELQLFRGDTVLLKGKK  target    EDRPFRGWQKNAKDMEFARLLCRARFYPGTPRGVTRMWFNMYGATPGSVRGAKARRDGLAKNPDTNYQAMFRSGSHQSAT 2pjh.1    ------R----------REAVCIVLSDDTCSDEKIRMNR-----------------------------------------  target    RGWLKPTWMTDSLVRKGLFGQGIGKGFLPDVHCPTGAPREAFVKISRAEPGGIGGQGLWRPAALGIRPRHESPAMKRYLA 2pjh.1    --------------------------------------------------------------------------------  target    GGFFSGPKE 2pjh.1    --------- ``` | | | | | | | | | | | | | | | | | | | | | | | | | | | | | | | | | | | | | | | | | | | | | | | | | |
|  | 3qwz.1.A | Transitional endoplasmic reticulum ATPase  *Crystal structure of FAF1 UBX-p97N-domain complex* | 0.02 |  | 19.61 | 0.09 | 375-441 | X-ray | 2.00 | hetero-oligomer |  | HHblits | 0.26 |
| ``` target    FNGAPQYINENPFDLELDASRPARPRQYWRAESAHFYNHEDHPLRVGTRLLTGSTHMPTPTKVMWFANANSILGNVKWHY 3qwz.1    --------------------------------------------------------------------------------  target    NTVVNALPRMEMIAVHEWWWTGSCEWADVVFGVDSWGELKHPDMTASVTNPFLIVFPKTPIKRIFNTVGDIDVLALVSSK 3qwz.1    --------------------------------------------------------------------------------  target    LAELTGDTRFNDMWKFVREGRTDVYLQRILDASTNTKGYRFTELEAKAREGIPALMNSRTSPKVVGYDQLADSTPWYTKS 3qwz.1    --------------------------------------------------------------------------------  target    GRLEFYREEDEFIEAGENLPVHREPVDSTFYEPNVIVSPKHEAVRPSGPEDYGVARTDLSCEVRCGRNVVLTWAETRQTQ 3qwz.1    --------------------------------------------------------------------------------  target    HPLVKQGHKFIFHTPKYRHGSHTTPIDTDMNAVLFGPFGDIYRRDKRSPFVTEGYVDINPTDGLELGLQDGDYVWIDPDP 3qwz.1    ------------------------------------------------------VVSLSQPKMDELQLFRGDTVLLKGKK  target    EDRPFRGWQKNAKDMEFARLLCRARFYPGTPRGVTRMWFNMYGATPGSVRGAKARRDGLAKNPDTNYQAMFRSGSHQSAT 3qwz.1    ------R----------REAVCIVLSDDTCSDEKIRMNRVV---------------------------------------  target    RGWLKPTWMTDSLVRKGLFGQGIGKGFLPDVHCPTGAPREAFVKISRAEPGGIGGQGLWRPAALGIRPRHESPAMKRYLA 3qwz.1    --------------------------------------------------------------------------------  target    GGFFSGPKE 3qwz.1    --------- ``` | | | | | | | | | | | | | | | | | | | | | | | | | | | | | | | | | | | | | | | | | | | | | | | | | |
|  | 3qc8.1.A | Transitional endoplasmic reticulum ATPase  *Crystal Structure of FAF1 UBX Domain In Complex with p97/VCP N Domain Reveals The Conserved FcisP Touch-Turn Motif of UBX Domain Suffering Conformational Change* | 0.02 |  | 19.61 | 0.09 | 374-440 | X-ray | 2.20 | hetero-oligomer |  | HHblits | 0.26 |
| ``` target    FNGAPQYINENPFDLELDASRPARPRQYWRAESAHFYNHEDHPLRVGTRLLTGSTHMPTPTKVMWFANANSILGNVKWHY 3qc8.1    --------------------------------------------------------------------------------  target    NTVVNALPRMEMIAVHEWWWTGSCEWADVVFGVDSWGELKHPDMTASVTNPFLIVFPKTPIKRIFNTVGDIDVLALVSSK 3qc8.1    --------------------------------------------------------------------------------  target    LAELTGDTRFNDMWKFVREGRTDVYLQRILDASTNTKGYRFTELEAKAREGIPALMNSRTSPKVVGYDQLADSTPWYTKS 3qc8.1    --------------------------------------------------------------------------------  target    GRLEFYREEDEFIEAGENLPVHREPVDSTFYEPNVIVSPKHEAVRPSGPEDYGVARTDLSCEVRCGRNVVLTWAETRQTQ 3qc8.1    --------------------------------------------------------------------------------  target    HPLVKQGHKFIFHTPKYRHGSHTTPIDTDMNAVLFGPFGDIYRRDKRSPFVTEGYVDINPTDGLELGLQDGDYVWIDPDP 3qc8.1    -----------------------------------------------------SVVSLSQPKMDELQLFRGDTVLLKGKK  target    EDRPFRGWQKNAKDMEFARLLCRARFYPGTPRGVTRMWFNMYGATPGSVRGAKARRDGLAKNPDTNYQAMFRSGSHQSAT 3qc8.1    ------R----------REAVCIVLSDDTCSDEKIRMNRV----------------------------------------  target    RGWLKPTWMTDSLVRKGLFGQGIGKGFLPDVHCPTGAPREAFVKISRAEPGGIGGQGLWRPAALGIRPRHESPAMKRYLA 3qc8.1    --------------------------------------------------------------------------------  target    GGFFSGPKE 3qc8.1    --------- ``` | | | | | | | | | | | | | | | | | | | | | | | | | | | | | | | | | | | | | | | | | | | | | | | | | |
|  | 5x4l.1.A | Transitional endoplasmic reticulum ATPase  *Crystal structure of the UBX domain of human UBXD7 in complex with p97 N domain* | 0.02 |  | 19.61 | 0.09 | 374-440 | X-ray | 2.40 | hetero-oligomer |  | HHblits | 0.26 |
| ``` target    FNGAPQYINENPFDLELDASRPARPRQYWRAESAHFYNHEDHPLRVGTRLLTGSTHMPTPTKVMWFANANSILGNVKWHY 5x4l.1    --------------------------------------------------------------------------------  target    NTVVNALPRMEMIAVHEWWWTGSCEWADVVFGVDSWGELKHPDMTASVTNPFLIVFPKTPIKRIFNTVGDIDVLALVSSK 5x4l.1    --------------------------------------------------------------------------------  target    LAELTGDTRFNDMWKFVREGRTDVYLQRILDASTNTKGYRFTELEAKAREGIPALMNSRTSPKVVGYDQLADSTPWYTKS 5x4l.1    --------------------------------------------------------------------------------  target    GRLEFYREEDEFIEAGENLPVHREPVDSTFYEPNVIVSPKHEAVRPSGPEDYGVARTDLSCEVRCGRNVVLTWAETRQTQ 5x4l.1    --------------------------------------------------------------------------------  target    HPLVKQGHKFIFHTPKYRHGSHTTPIDTDMNAVLFGPFGDIYRRDKRSPFVTEGYVDINPTDGLELGLQDGDYVWIDPDP 5x4l.1    -----------------------------------------------------SVVSLSQPKMDELQLFRGDTVLLKGKK  target    EDRPFRGWQKNAKDMEFARLLCRARFYPGTPRGVTRMWFNMYGATPGSVRGAKARRDGLAKNPDTNYQAMFRSGSHQSAT 5x4l.1    ------R----------REAVCIVLSDDTCSDEKIRMNRV----------------------------------------  target    RGWLKPTWMTDSLVRKGLFGQGIGKGFLPDVHCPTGAPREAFVKISRAEPGGIGGQGLWRPAALGIRPRHESPAMKRYLA 5x4l.1    --------------------------------------------------------------------------------  target    GGFFSGPKE 5x4l.1    --------- ``` | | | | | | | | | | | | | | | | | | | | | | | | | | | | | | | | | | | | | | | | | | | | | | | | | |
|  | 5x4l.2.A | Transitional endoplasmic reticulum ATPase  *Crystal structure of the UBX domain of human UBXD7 in complex with p97 N domain* | 0.02 |  | 19.61 | 0.09 | 374-440 | X-ray | 2.40 | hetero-oligomer |  | HHblits | 0.26 |
| ``` target    FNGAPQYINENPFDLELDASRPARPRQYWRAESAHFYNHEDHPLRVGTRLLTGSTHMPTPTKVMWFANANSILGNVKWHY 5x4l.2    --------------------------------------------------------------------------------  target    NTVVNALPRMEMIAVHEWWWTGSCEWADVVFGVDSWGELKHPDMTASVTNPFLIVFPKTPIKRIFNTVGDIDVLALVSSK 5x4l.2    --------------------------------------------------------------------------------  target    LAELTGDTRFNDMWKFVREGRTDVYLQRILDASTNTKGYRFTELEAKAREGIPALMNSRTSPKVVGYDQLADSTPWYTKS 5x4l.2    --------------------------------------------------------------------------------  target    GRLEFYREEDEFIEAGENLPVHREPVDSTFYEPNVIVSPKHEAVRPSGPEDYGVARTDLSCEVRCGRNVVLTWAETRQTQ 5x4l.2    --------------------------------------------------------------------------------  target    HPLVKQGHKFIFHTPKYRHGSHTTPIDTDMNAVLFGPFGDIYRRDKRSPFVTEGYVDINPTDGLELGLQDGDYVWIDPDP 5x4l.2    -----------------------------------------------------SVVSLSQPKMDELQLFRGDTVLLKGKK  target    EDRPFRGWQKNAKDMEFARLLCRARFYPGTPRGVTRMWFNMYGATPGSVRGAKARRDGLAKNPDTNYQAMFRSGSHQSAT 5x4l.2    ------R----------REAVCIVLSDDTCSDEKIRMNRV----------------------------------------  target    RGWLKPTWMTDSLVRKGLFGQGIGKGFLPDVHCPTGAPREAFVKISRAEPGGIGGQGLWRPAALGIRPRHESPAMKRYLA 5x4l.2    --------------------------------------------------------------------------------  target    GGFFSGPKE 5x4l.2    --------- ``` | | | | | | | | | | | | | | | | | | | | | | | | | | | | | | | | | | | | | | | | | | | | | | | | | |
|  | 3tiw.1.A | Transitional endoplasmic reticulum ATPase  *Crystal structure of p97N in complex with the C-terminus of gp78* | 0.02 |  | 19.61 | 0.09 | 374-440 | X-ray | 1.80 | hetero-oligomer |  | HHblits | 0.26 |
| ``` target    FNGAPQYINENPFDLELDASRPARPRQYWRAESAHFYNHEDHPLRVGTRLLTGSTHMPTPTKVMWFANANSILGNVKWHY 3tiw.1    --------------------------------------------------------------------------------  target    NTVVNALPRMEMIAVHEWWWTGSCEWADVVFGVDSWGELKHPDMTASVTNPFLIVFPKTPIKRIFNTVGDIDVLALVSSK 3tiw.1    --------------------------------------------------------------------------------  target    LAELTGDTRFNDMWKFVREGRTDVYLQRILDASTNTKGYRFTELEAKAREGIPALMNSRTSPKVVGYDQLADSTPWYTKS 3tiw.1    --------------------------------------------------------------------------------  target    GRLEFYREEDEFIEAGENLPVHREPVDSTFYEPNVIVSPKHEAVRPSGPEDYGVARTDLSCEVRCGRNVVLTWAETRQTQ 3tiw.1    --------------------------------------------------------------------------------  target    HPLVKQGHKFIFHTPKYRHGSHTTPIDTDMNAVLFGPFGDIYRRDKRSPFVTEGYVDINPTDGLELGLQDGDYVWIDPDP 3tiw.1    -----------------------------------------------------SVVSLSQPKMDELQLFRGDTVLLKGKK  target    EDRPFRGWQKNAKDMEFARLLCRARFYPGTPRGVTRMWFNMYGATPGSVRGAKARRDGLAKNPDTNYQAMFRSGSHQSAT 3tiw.1    ------R----------REAVCIVLSDDTCSDEKIRMNRV----------------------------------------  target    RGWLKPTWMTDSLVRKGLFGQGIGKGFLPDVHCPTGAPREAFVKISRAEPGGIGGQGLWRPAALGIRPRHESPAMKRYLA 3tiw.1    --------------------------------------------------------------------------------  target    GGFFSGPKE 3tiw.1    --------- ``` | | | | | | | | | | | | | | | | | | | | | | | | | | | | | | | | | | | | | | | | | | | | | | | | | |
|  | 3tiw.2.A | Transitional endoplasmic reticulum ATPase  *Crystal structure of p97N in complex with the C-terminus of gp78* | 0.02 |  | 19.61 | 0.09 | 374-440 | X-ray | 1.80 | hetero-oligomer |  | HHblits | 0.26 |
| ``` target    FNGAPQYINENPFDLELDASRPARPRQYWRAESAHFYNHEDHPLRVGTRLLTGSTHMPTPTKVMWFANANSILGNVKWHY 3tiw.2    --------------------------------------------------------------------------------  target    NTVVNALPRMEMIAVHEWWWTGSCEWADVVFGVDSWGELKHPDMTASVTNPFLIVFPKTPIKRIFNTVGDIDVLALVSSK 3tiw.2    --------------------------------------------------------------------------------  target    LAELTGDTRFNDMWKFVREGRTDVYLQRILDASTNTKGYRFTELEAKAREGIPALMNSRTSPKVVGYDQLADSTPWYTKS 3tiw.2    --------------------------------------------------------------------------------  target    GRLEFYREEDEFIEAGENLPVHREPVDSTFYEPNVIVSPKHEAVRPSGPEDYGVARTDLSCEVRCGRNVVLTWAETRQTQ 3tiw.2    --------------------------------------------------------------------------------  target    HPLVKQGHKFIFHTPKYRHGSHTTPIDTDMNAVLFGPFGDIYRRDKRSPFVTEGYVDINPTDGLELGLQDGDYVWIDPDP 3tiw.2    -----------------------------------------------------SVVSLSQPKMDELQLFRGDTVLLKGKK  target    EDRPFRGWQKNAKDMEFARLLCRARFYPGTPRGVTRMWFNMYGATPGSVRGAKARRDGLAKNPDTNYQAMFRSGSHQSAT 3tiw.2    ------R----------REAVCIVLSDDTCSDEKIRMNRV----------------------------------------  target    RGWLKPTWMTDSLVRKGLFGQGIGKGFLPDVHCPTGAPREAFVKISRAEPGGIGGQGLWRPAALGIRPRHESPAMKRYLA 3tiw.2    --------------------------------------------------------------------------------  target    GGFFSGPKE 3tiw.2    --------- ``` | | | | | | | | | | | | | | | | | | | | | | | | | | | | | | | | | | | | | | | | | | | | | | | | | |
|  | 6yj4.1.G | Subunit NUAM of NADH:Ubiquinone Oxidoreductase (Complex I)  *Structure of Yarrowia lipolytica complex I at 2.7 A* | 0.01 |  | 13.46 | 0.09 | 59-111 | EM | 0.00 | hetero-1-1-1-1-1-1-… | 18 x 3PE, 6 x SF4, 5 x LMT, 8 x PLC, 2 x FES, 1 x FMN, 6 x CDL, 1 x NDP, 1 x ZN, 2 x EHZ | HHblits | 0.24 |
| ``` target    FNGAPQYINENPFDLELDASRPARPRQYWRAESAHFYNHEDHPLRVGTRLLTGSTHMPTPTKVMWFANANSILGNVKWHY 6yj4.1    ----------------------------------------------------------EDADAILLVGTNPRREA-AVMN  target    NTVVNALPRME-MIAVHEWWWTGSCEWADVVFGVDSWGELKHPDMTASVTNPFLIVFPKTPIKRIFNTVGDIDVLALVSS 6yj4.1    ARIRKAWLRQELEIASVGPTLDATFDVAELGN------------------------------------------------  target    KLAELTGDTRFNDMWKFVREGRTDVYLQRILDASTNTKGYRFTELEAKAREGIPALMNSRTSPKVVGYDQLADSTPWYTK 6yj4.1    --------------------------------------------------------------------------------  target    SGRLEFYREEDEFIEAGENLPVHREPVDSTFYEPNVIVSPKHEAVRPSGPEDYGVARTDLSCEVRCGRNVVLTWAETRQT 6yj4.1    --------------------------------------------------------------------------------  target    QHPLVKQGHKFIFHTPKYRHGSHTTPIDTDMNAVLFGPFGDIYRRDKRSPFVTEGYVDINPTDGLELGLQDGDYVWIDPD 6yj4.1    --------------------------------------------------------------------------------  target    PEDRPFRGWQKNAKDMEFARLLCRARFYPGTPRGVTRMWFNMYGATPGSVRGAKARRDGLAKNPDTNYQAMFRSGSHQSA 6yj4.1    --------------------------------------------------------------------------------  target    TRGWLKPTWMTDSLVRKGLFGQGIGKGFLPDVHCPTGAPREAFVKISRAEPGGIGGQGLWRPAALGIRPRHESPAMKRYL 6yj4.1    --------------------------------------------------------------------------------  target    AGGFFSGPKE 6yj4.1    ---------- ``` | | | | | | | | | | | | | | | | | | | | | | | | | | | | | | | | | | | | | | | | | | | | | | | | | |
|  | 6rfs.1.A | Subunit NUAM of NADH:Ubiquinone Oxidoreductase (Complex I)  *Cryo-EM structure of a respiratory complex I mutant lacking NDUFS4* | 0.01 |  | 13.46 | 0.09 | 59-111 | EM | 4.04 | hetero-1-1-1-1-1-1-… | 6 x SF4, 2 x FES, 1 x FMN, 1 x NDP, 1 x ZN, 1 x ZMP | HHblits | 0.24 |
| ``` target    FNGAPQYINENPFDLELDASRPARPRQYWRAESAHFYNHEDHPLRVGTRLLTGSTHMPTPTKVMWFANANSILGNVKWHY 6rfs.1    ----------------------------------------------------------EDADAILLVGTNPRREA-AVMN  target    NTVVNALPRME-MIAVHEWWWTGSCEWADVVFGVDSWGELKHPDMTASVTNPFLIVFPKTPIKRIFNTVGDIDVLALVSS 6rfs.1    ARIRKAWLRQELEIASVGPTLDATFDVAELGN------------------------------------------------  target    KLAELTGDTRFNDMWKFVREGRTDVYLQRILDASTNTKGYRFTELEAKAREGIPALMNSRTSPKVVGYDQLADSTPWYTK 6rfs.1    --------------------------------------------------------------------------------  target    SGRLEFYREEDEFIEAGENLPVHREPVDSTFYEPNVIVSPKHEAVRPSGPEDYGVARTDLSCEVRCGRNVVLTWAETRQT 6rfs.1    --------------------------------------------------------------------------------  target    QHPLVKQGHKFIFHTPKYRHGSHTTPIDTDMNAVLFGPFGDIYRRDKRSPFVTEGYVDINPTDGLELGLQDGDYVWIDPD 6rfs.1    --------------------------------------------------------------------------------  target    PEDRPFRGWQKNAKDMEFARLLCRARFYPGTPRGVTRMWFNMYGATPGSVRGAKARRDGLAKNPDTNYQAMFRSGSHQSA 6rfs.1    --------------------------------------------------------------------------------  target    TRGWLKPTWMTDSLVRKGLFGQGIGKGFLPDVHCPTGAPREAFVKISRAEPGGIGGQGLWRPAALGIRPRHESPAMKRYL 6rfs.1    --------------------------------------------------------------------------------  target    AGGFFSGPKE 6rfs.1    ---------- ``` | | | | | | | | | | | | | | | | | | | | | | | | | | | | | | | | | | | | | | | | | | | | | | | | | |
|  | 6rfq.1.A | Subunit NUAM of NADH:Ubiquinone Oxidoreductase (Complex I)  *Cryo-EM structure of a respiratory complex I assembly intermediate with NDUFAF2* | 0.01 |  | 13.46 | 0.09 | 59-111 | EM | 3.30 | hetero-1-1-1-1-1-1-… | 6 x SF4, 2 x FES, 1 x FMN, 1 x NDP, 10 x 3PE, 2 x LMN, 4 x CDL, 2 x ZMP, 4 x PLC, 3 x T7X, 1 x CPL | HHblits | 0.24 |
| ``` target    FNGAPQYINENPFDLELDASRPARPRQYWRAESAHFYNHEDHPLRVGTRLLTGSTHMPTPTKVMWFANANSILGNVKWHY 6rfq.1    ----------------------------------------------------------EDADAILLVGTNPRREA-AVMN  target    NTVVNALPRME-MIAVHEWWWTGSCEWADVVFGVDSWGELKHPDMTASVTNPFLIVFPKTPIKRIFNTVGDIDVLALVSS 6rfq.1    ARIRKAWLRQELEIASVGPTLDATFDVAELGN------------------------------------------------  target    KLAELTGDTRFNDMWKFVREGRTDVYLQRILDASTNTKGYRFTELEAKAREGIPALMNSRTSPKVVGYDQLADSTPWYTK 6rfq.1    --------------------------------------------------------------------------------  target    SGRLEFYREEDEFIEAGENLPVHREPVDSTFYEPNVIVSPKHEAVRPSGPEDYGVARTDLSCEVRCGRNVVLTWAETRQT 6rfq.1    --------------------------------------------------------------------------------  target    QHPLVKQGHKFIFHTPKYRHGSHTTPIDTDMNAVLFGPFGDIYRRDKRSPFVTEGYVDINPTDGLELGLQDGDYVWIDPD 6rfq.1    --------------------------------------------------------------------------------  target    PEDRPFRGWQKNAKDMEFARLLCRARFYPGTPRGVTRMWFNMYGATPGSVRGAKARRDGLAKNPDTNYQAMFRSGSHQSA 6rfq.1    --------------------------------------------------------------------------------  target    TRGWLKPTWMTDSLVRKGLFGQGIGKGFLPDVHCPTGAPREAFVKISRAEPGGIGGQGLWRPAALGIRPRHESPAMKRYL 6rfq.1    --------------------------------------------------------------------------------  target    AGGFFSGPKE 6rfq.1    ---------- ``` | | | | | | | | | | | | | | | | | | | | | | | | | | | | | | | | | | | | | | | | | | | | | | | | | |
|  | 6gcs.1.A | 75-KDA PROTEIN (NUAM)  *Cryo-EM structure of respiratory complex I from Yarrowia lipolytica* | 0.01 |  | 13.46 | 0.09 | 59-111 | EM | 4.32 | hetero-1-1-1-1-1-1-… | 6 x SF4, 2 x FES, 1 x FMN, 1 x NDP, 1 x ZN, 1 x ZMP, 1 x CDL, 3 x 3PE | HHblits | 0.24 |
| ``` target    FNGAPQYINENPFDLELDASRPARPRQYWRAESAHFYNHEDHPLRVGTRLLTGSTHMPTPTKVMWFANANSILGNVKWHY 6gcs.1    ----------------------------------------------------------EDADAILLVGTNPRREA-AVMN  target    NTVVNALPRME-MIAVHEWWWTGSCEWADVVFGVDSWGELKHPDMTASVTNPFLIVFPKTPIKRIFNTVGDIDVLALVSS 6gcs.1    ARIRKAWLRQELEIASVGPTLDATFDVAELGN------------------------------------------------  target    KLAELTGDTRFNDMWKFVREGRTDVYLQRILDASTNTKGYRFTELEAKAREGIPALMNSRTSPKVVGYDQLADSTPWYTK 6gcs.1    --------------------------------------------------------------------------------  target    SGRLEFYREEDEFIEAGENLPVHREPVDSTFYEPNVIVSPKHEAVRPSGPEDYGVARTDLSCEVRCGRNVVLTWAETRQT 6gcs.1    --------------------------------------------------------------------------------  target    QHPLVKQGHKFIFHTPKYRHGSHTTPIDTDMNAVLFGPFGDIYRRDKRSPFVTEGYVDINPTDGLELGLQDGDYVWIDPD 6gcs.1    --------------------------------------------------------------------------------  target    PEDRPFRGWQKNAKDMEFARLLCRARFYPGTPRGVTRMWFNMYGATPGSVRGAKARRDGLAKNPDTNYQAMFRSGSHQSA 6gcs.1    --------------------------------------------------------------------------------  target    TRGWLKPTWMTDSLVRKGLFGQGIGKGFLPDVHCPTGAPREAFVKISRAEPGGIGGQGLWRPAALGIRPRHESPAMKRYL 6gcs.1    --------------------------------------------------------------------------------  target    AGGFFSGPKE 6gcs.1    ---------- ``` | | | | | | | | | | | | | | | | | | | | | | | | | | | | | | | | | | | | | | | | | | | | | | | | | |
|  | 4ga5.1.A | Putative thymidine phosphorylase  *Crystal structure of AMP phosphorylase C-terminal deletion mutant in the apo-form* | 0.03 |  | 20.00 | 0.09 | 373-439 | X-ray | 3.25 | homo-dimer |  | HHblits | 0.27 |
| ``` target    FNGAPQYINENPFDLELDASRPARPRQYWRAESAHFYNHEDHPLRVGTRLLTGSTHMPTPTKVMWFANANSILGNVKWHY 4ga5.1    --------------------------------------------------------------------------------  target    NTVVNALPRMEMIAVHEWWWTGSCEWADVVFGVDSWGELKHPDMTASVTNPFLIVFPKTPIKRIFNTVGDIDVLALVSSK 4ga5.1    --------------------------------------------------------------------------------  target    LAELTGDTRFNDMWKFVREGRTDVYLQRILDASTNTKGYRFTELEAKAREGIPALMNSRTSPKVVGYDQLADSTPWYTKS 4ga5.1    --------------------------------------------------------------------------------  target    GRLEFYREEDEFIEAGENLPVHREPVDSTFYEPNVIVSPKHEAVRPSGPEDYGVARTDLSCEVRCGRNVVLTWAETRQTQ 4ga5.1    --------------------------------------------------------------------------------  target    HPLVKQGHKFIFHTPKYRHGSHTTPIDTDMNAVLFGPFGDIYRRDKRSPFVTEGYVDINPTDGLELGLQDGDYVWIDPDP 4ga5.1    ----------------------------------------------------RYTVLINEEDAKEAKLHPDDLVKIEAGK  target    EDRPFRGWQKNAKDMEFARLLCRARFYPGTPRGVTRMWFNMYGATPGSVRGAKARRDGLAKNPDTNYQAMFRSGSHQSAT 4ga5.1    -----------------KAVYGSVALSNLVGKGEVGISR-----------------------------------------  target    RGWLKPTWMTDSLVRKGLFGQGIGKGFLPDVHCPTGAPREAFVKISRAEPGGIGGQGLWRPAALGIRPRHESPAMKRYLA 4ga5.1    --------------------------------------------------------------------------------  target    GGFFSGPKE 4ga5.1    --------- ``` | | | | | | | | | | | | | | | | | | | | | | | | | | | | | | | | | | | | | | | | | | | | | | | | | |
|  | 4ga6.1.A | Putative thymidine phosphorylase  *Crystal structure of AMP phosphorylase C-terminal deletion mutant in complex with substrates* | 0.03 |  | 20.00 | 0.09 | 373-439 | X-ray | 2.21 | homo-dimer | 2 x AMP | HHblits | 0.27 |
| ``` target    FNGAPQYINENPFDLELDASRPARPRQYWRAESAHFYNHEDHPLRVGTRLLTGSTHMPTPTKVMWFANANSILGNVKWHY 4ga6.1    --------------------------------------------------------------------------------  target    NTVVNALPRMEMIAVHEWWWTGSCEWADVVFGVDSWGELKHPDMTASVTNPFLIVFPKTPIKRIFNTVGDIDVLALVSSK 4ga6.1    --------------------------------------------------------------------------------  target    LAELTGDTRFNDMWKFVREGRTDVYLQRILDASTNTKGYRFTELEAKAREGIPALMNSRTSPKVVGYDQLADSTPWYTKS 4ga6.1    --------------------------------------------------------------------------------  target    GRLEFYREEDEFIEAGENLPVHREPVDSTFYEPNVIVSPKHEAVRPSGPEDYGVARTDLSCEVRCGRNVVLTWAETRQTQ 4ga6.1    --------------------------------------------------------------------------------  target    HPLVKQGHKFIFHTPKYRHGSHTTPIDTDMNAVLFGPFGDIYRRDKRSPFVTEGYVDINPTDGLELGLQDGDYVWIDPDP 4ga6.1    ----------------------------------------------------RYTVLINEEDAKEAKLHPDDLVKIEAGK  target    EDRPFRGWQKNAKDMEFARLLCRARFYPGTPRGVTRMWFNMYGATPGSVRGAKARRDGLAKNPDTNYQAMFRSGSHQSAT 4ga6.1    -----------------KAVYGSVALSNLVGKGEVGISR-----------------------------------------  target    RGWLKPTWMTDSLVRKGLFGQGIGKGFLPDVHCPTGAPREAFVKISRAEPGGIGGQGLWRPAALGIRPRHESPAMKRYLA 4ga6.1    --------------------------------------------------------------------------------  target    GGFFSGPKE 4ga6.1    --------- ``` | | | | | | | | | | | | | | | | | | | | | | | | | | | | | | | | | | | | | | | | | | | | | | | | | |
|  | 5epp.1.A | Transitional endoplasmic reticulum ATPase  *Structural Insights into the Interaction of p97 N-terminus Domain and VBM Motif in Rhomboid Protease, RHBDL4* | 0.02 |  | 20.00 | 0.09 | 375-440 | X-ray | 1.88 | hetero-oligomer |  | HHblits | 0.26 |
| ``` target    FNGAPQYINENPFDLELDASRPARPRQYWRAESAHFYNHEDHPLRVGTRLLTGSTHMPTPTKVMWFANANSILGNVKWHY 5epp.1    --------------------------------------------------------------------------------  target    NTVVNALPRMEMIAVHEWWWTGSCEWADVVFGVDSWGELKHPDMTASVTNPFLIVFPKTPIKRIFNTVGDIDVLALVSSK 5epp.1    --------------------------------------------------------------------------------  target    LAELTGDTRFNDMWKFVREGRTDVYLQRILDASTNTKGYRFTELEAKAREGIPALMNSRTSPKVVGYDQLADSTPWYTKS 5epp.1    --------------------------------------------------------------------------------  target    GRLEFYREEDEFIEAGENLPVHREPVDSTFYEPNVIVSPKHEAVRPSGPEDYGVARTDLSCEVRCGRNVVLTWAETRQTQ 5epp.1    --------------------------------------------------------------------------------  target    HPLVKQGHKFIFHTPKYRHGSHTTPIDTDMNAVLFGPFGDIYRRDKRSPFVTEGYVDINPTDGLELGLQDGDYVWIDPDP 5epp.1    ------------------------------------------------------VVSLSQPKMDELQLFRGDTVLLKGKK  target    EDRPFRGWQKNAKDMEFARLLCRARFYPGTPRGVTRMWFNMYGATPGSVRGAKARRDGLAKNPDTNYQAMFRSGSHQSAT 5epp.1    ------R----------REAVCIVLSDDTCSDEKIRMNRV----------------------------------------  target    RGWLKPTWMTDSLVRKGLFGQGIGKGFLPDVHCPTGAPREAFVKISRAEPGGIGGQGLWRPAALGIRPRHESPAMKRYLA 5epp.1    --------------------------------------------------------------------------------  target    GGFFSGPKE 5epp.1    --------- ``` | | | | | | | | | | | | | | | | | | | | | | | | | | | | | | | | | | | | | | | | | | | | | | | | | |
|  | 5glf.2.A | Transitional endoplasmic reticulum ATPase  *Structural insights into the interaction of p97 N-terminal domain and SHP motif in Derlin-1 rhomboid pseudoprotease* | 0.02 |  | 20.00 | 0.09 | 375-440 | X-ray | 2.25 | hetero-1-1-mer |  | HHblits | 0.26 |
| ``` target    FNGAPQYINENPFDLELDASRPARPRQYWRAESAHFYNHEDHPLRVGTRLLTGSTHMPTPTKVMWFANANSILGNVKWHY 5glf.2    --------------------------------------------------------------------------------  target    NTVVNALPRMEMIAVHEWWWTGSCEWADVVFGVDSWGELKHPDMTASVTNPFLIVFPKTPIKRIFNTVGDIDVLALVSSK 5glf.2    --------------------------------------------------------------------------------  target    LAELTGDTRFNDMWKFVREGRTDVYLQRILDASTNTKGYRFTELEAKAREGIPALMNSRTSPKVVGYDQLADSTPWYTKS 5glf.2    --------------------------------------------------------------------------------  target    GRLEFYREEDEFIEAGENLPVHREPVDSTFYEPNVIVSPKHEAVRPSGPEDYGVARTDLSCEVRCGRNVVLTWAETRQTQ 5glf.2    --------------------------------------------------------------------------------  target    HPLVKQGHKFIFHTPKYRHGSHTTPIDTDMNAVLFGPFGDIYRRDKRSPFVTEGYVDINPTDGLELGLQDGDYVWIDPDP 5glf.2    ------------------------------------------------------VVSLSQPKMDELQLFRGDTVLLKGKK  target    EDRPFRGWQKNAKDMEFARLLCRARFYPGTPRGVTRMWFNMYGATPGSVRGAKARRDGLAKNPDTNYQAMFRSGSHQSAT 5glf.2    ------R----------REAVCIVLSDDTCSDEKIRMNRV----------------------------------------  target    RGWLKPTWMTDSLVRKGLFGQGIGKGFLPDVHCPTGAPREAFVKISRAEPGGIGGQGLWRPAALGIRPRHESPAMKRYLA 5glf.2    --------------------------------------------------------------------------------  target    GGFFSGPKE 5glf.2    --------- ``` | | | | | | | | | | | | | | | | | | | | | | | | | | | | | | | | | | | | | | | | | | | | | | | | | |
|  | 5glf.3.A | Transitional endoplasmic reticulum ATPase  *Structural insights into the interaction of p97 N-terminal domain and SHP motif in Derlin-1 rhomboid pseudoprotease* | 0.02 |  | 20.00 | 0.09 | 375-440 | X-ray | 2.25 | hetero-1-1-mer |  | HHblits | 0.26 |
| ``` target    FNGAPQYINENPFDLELDASRPARPRQYWRAESAHFYNHEDHPLRVGTRLLTGSTHMPTPTKVMWFANANSILGNVKWHY 5glf.3    --------------------------------------------------------------------------------  target    NTVVNALPRMEMIAVHEWWWTGSCEWADVVFGVDSWGELKHPDMTASVTNPFLIVFPKTPIKRIFNTVGDIDVLALVSSK 5glf.3    --------------------------------------------------------------------------------  target    LAELTGDTRFNDMWKFVREGRTDVYLQRILDASTNTKGYRFTELEAKAREGIPALMNSRTSPKVVGYDQLADSTPWYTKS 5glf.3    --------------------------------------------------------------------------------  target    GRLEFYREEDEFIEAGENLPVHREPVDSTFYEPNVIVSPKHEAVRPSGPEDYGVARTDLSCEVRCGRNVVLTWAETRQTQ 5glf.3    --------------------------------------------------------------------------------  target    HPLVKQGHKFIFHTPKYRHGSHTTPIDTDMNAVLFGPFGDIYRRDKRSPFVTEGYVDINPTDGLELGLQDGDYVWIDPDP 5glf.3    ------------------------------------------------------VVSLSQPKMDELQLFRGDTVLLKGKK  target    EDRPFRGWQKNAKDMEFARLLCRARFYPGTPRGVTRMWFNMYGATPGSVRGAKARRDGLAKNPDTNYQAMFRSGSHQSAT 5glf.3    ------R----------REAVCIVLSDDTCSDEKIRMNRV----------------------------------------  target    RGWLKPTWMTDSLVRKGLFGQGIGKGFLPDVHCPTGAPREAFVKISRAEPGGIGGQGLWRPAALGIRPRHESPAMKRYLA 5glf.3    --------------------------------------------------------------------------------  target    GGFFSGPKE 5glf.3    --------- ``` | | | | | | | | | | | | | | | | | | | | | | | | | | | | | | | | | | | | | | | | | | | | | | | | | |
|  | 5glf.1.A | Transitional endoplasmic reticulum ATPase  *Structural insights into the interaction of p97 N-terminal domain and SHP motif in Derlin-1 rhomboid pseudoprotease* | 0.02 |  | 20.00 | 0.09 | 375-440 | X-ray | 2.25 | hetero-1-1-mer |  | HHblits | 0.26 |
| ``` target    FNGAPQYINENPFDLELDASRPARPRQYWRAESAHFYNHEDHPLRVGTRLLTGSTHMPTPTKVMWFANANSILGNVKWHY 5glf.1    --------------------------------------------------------------------------------  target    NTVVNALPRMEMIAVHEWWWTGSCEWADVVFGVDSWGELKHPDMTASVTNPFLIVFPKTPIKRIFNTVGDIDVLALVSSK 5glf.1    --------------------------------------------------------------------------------  target    LAELTGDTRFNDMWKFVREGRTDVYLQRILDASTNTKGYRFTELEAKAREGIPALMNSRTSPKVVGYDQLADSTPWYTKS 5glf.1    --------------------------------------------------------------------------------  target    GRLEFYREEDEFIEAGENLPVHREPVDSTFYEPNVIVSPKHEAVRPSGPEDYGVARTDLSCEVRCGRNVVLTWAETRQTQ 5glf.1    --------------------------------------------------------------------------------  target    HPLVKQGHKFIFHTPKYRHGSHTTPIDTDMNAVLFGPFGDIYRRDKRSPFVTEGYVDINPTDGLELGLQDGDYVWIDPDP 5glf.1    ------------------------------------------------------VVSLSQPKMDELQLFRGDTVLLKGKK  target    EDRPFRGWQKNAKDMEFARLLCRARFYPGTPRGVTRMWFNMYGATPGSVRGAKARRDGLAKNPDTNYQAMFRSGSHQSAT 5glf.1    ------R----------REAVCIVLSDDTCSDEKIRMNRV----------------------------------------  target    RGWLKPTWMTDSLVRKGLFGQGIGKGFLPDVHCPTGAPREAFVKISRAEPGGIGGQGLWRPAALGIRPRHESPAMKRYLA 5glf.1    --------------------------------------------------------------------------------  target    GGFFSGPKE 5glf.1    --------- ``` | | | | | | | | | | | | | | | | | | | | | | | | | | | | | | | | | | | | | | | | | | | | | | | | | |
|  | 5glf.4.A | Transitional endoplasmic reticulum ATPase  *Structural insights into the interaction of p97 N-terminal domain and SHP motif in Derlin-1 rhomboid pseudoprotease* | 0.02 |  | 20.00 | 0.09 | 375-440 | X-ray | 2.25 | hetero-1-1-mer |  | HHblits | 0.26 |
| ``` target    FNGAPQYINENPFDLELDASRPARPRQYWRAESAHFYNHEDHPLRVGTRLLTGSTHMPTPTKVMWFANANSILGNVKWHY 5glf.4    --------------------------------------------------------------------------------  target    NTVVNALPRMEMIAVHEWWWTGSCEWADVVFGVDSWGELKHPDMTASVTNPFLIVFPKTPIKRIFNTVGDIDVLALVSSK 5glf.4    --------------------------------------------------------------------------------  target    LAELTGDTRFNDMWKFVREGRTDVYLQRILDASTNTKGYRFTELEAKAREGIPALMNSRTSPKVVGYDQLADSTPWYTKS 5glf.4    --------------------------------------------------------------------------------  target    GRLEFYREEDEFIEAGENLPVHREPVDSTFYEPNVIVSPKHEAVRPSGPEDYGVARTDLSCEVRCGRNVVLTWAETRQTQ 5glf.4    --------------------------------------------------------------------------------  target    HPLVKQGHKFIFHTPKYRHGSHTTPIDTDMNAVLFGPFGDIYRRDKRSPFVTEGYVDINPTDGLELGLQDGDYVWIDPDP 5glf.4    ------------------------------------------------------VVSLSQPKMDELQLFRGDTVLLKGKK  target    EDRPFRGWQKNAKDMEFARLLCRARFYPGTPRGVTRMWFNMYGATPGSVRGAKARRDGLAKNPDTNYQAMFRSGSHQSAT 5glf.4    ------R----------REAVCIVLSDDTCSDEKIRMNRV----------------------------------------  target    RGWLKPTWMTDSLVRKGLFGQGIGKGFLPDVHCPTGAPREAFVKISRAEPGGIGGQGLWRPAALGIRPRHESPAMKRYLA 5glf.4    --------------------------------------------------------------------------------  target    GGFFSGPKE 5glf.4    --------- ``` | | | | | | | | | | | | | | | | | | | | | | | | | | | | | | | | | | | | | | | | | | | | | | | | | |
|  | 3qq8.1.A | Transitional endoplasmic reticulum ATPase  *Crystal structure of p97-N in complex with FAF1-UBX* | 0.02 |  | 20.00 | 0.09 | 375-440 | X-ray | 2.00 | hetero-oligomer |  | HHblits | 0.26 |
| ``` target    FNGAPQYINENPFDLELDASRPARPRQYWRAESAHFYNHEDHPLRVGTRLLTGSTHMPTPTKVMWFANANSILGNVKWHY 3qq8.1    --------------------------------------------------------------------------------  target    NTVVNALPRMEMIAVHEWWWTGSCEWADVVFGVDSWGELKHPDMTASVTNPFLIVFPKTPIKRIFNTVGDIDVLALVSSK 3qq8.1    --------------------------------------------------------------------------------  target    LAELTGDTRFNDMWKFVREGRTDVYLQRILDASTNTKGYRFTELEAKAREGIPALMNSRTSPKVVGYDQLADSTPWYTKS 3qq8.1    --------------------------------------------------------------------------------  target    GRLEFYREEDEFIEAGENLPVHREPVDSTFYEPNVIVSPKHEAVRPSGPEDYGVARTDLSCEVRCGRNVVLTWAETRQTQ 3qq8.1    --------------------------------------------------------------------------------  target    HPLVKQGHKFIFHTPKYRHGSHTTPIDTDMNAVLFGPFGDIYRRDKRSPFVTEGYVDINPTDGLELGLQDGDYVWIDPDP 3qq8.1    ------------------------------------------------------VVSLSQPKMDELQLFRGDTVLLKGKK  target    EDRPFRGWQKNAKDMEFARLLCRARFYPGTPRGVTRMWFNMYGATPGSVRGAKARRDGLAKNPDTNYQAMFRSGSHQSAT 3qq8.1    ------R----------REAVCIVLSDDTCSDEKIRMNRV----------------------------------------  target    RGWLKPTWMTDSLVRKGLFGQGIGKGFLPDVHCPTGAPREAFVKISRAEPGGIGGQGLWRPAALGIRPRHESPAMKRYLA 3qq8.1    --------------------------------------------------------------------------------  target    GGFFSGPKE 3qq8.1    --------- ``` | | | | | | | | | | | | | | | | | | | | | | | | | | | | | | | | | | | | | | | | | | | | | | | | | |
|  | 3qq7.1.A | Transitional endoplasmic reticulum ATPase  *Crystal Structure of the p97 N-terminal domain* | 0.02 |  | 20.00 | 0.09 | 375-440 | X-ray | 2.65 | monomer | 1 x HEZ, 1 x CO | HHblits | 0.26 |
| ``` target    FNGAPQYINENPFDLELDASRPARPRQYWRAESAHFYNHEDHPLRVGTRLLTGSTHMPTPTKVMWFANANSILGNVKWHY 3qq7.1    --------------------------------------------------------------------------------  target    NTVVNALPRMEMIAVHEWWWTGSCEWADVVFGVDSWGELKHPDMTASVTNPFLIVFPKTPIKRIFNTVGDIDVLALVSSK 3qq7.1    --------------------------------------------------------------------------------  target    LAELTGDTRFNDMWKFVREGRTDVYLQRILDASTNTKGYRFTELEAKAREGIPALMNSRTSPKVVGYDQLADSTPWYTKS 3qq7.1    --------------------------------------------------------------------------------  target    GRLEFYREEDEFIEAGENLPVHREPVDSTFYEPNVIVSPKHEAVRPSGPEDYGVARTDLSCEVRCGRNVVLTWAETRQTQ 3qq7.1    --------------------------------------------------------------------------------  target    HPLVKQGHKFIFHTPKYRHGSHTTPIDTDMNAVLFGPFGDIYRRDKRSPFVTEGYVDINPTDGLELGLQDGDYVWIDPDP 3qq7.1    ------------------------------------------------------VVSLSQPKMDELQLFRGDTVLLKGKK  target    EDRPFRGWQKNAKDMEFARLLCRARFYPGTPRGVTRMWFNMYGATPGSVRGAKARRDGLAKNPDTNYQAMFRSGSHQSAT 3qq7.1    ------R----------REAVCIVLSDDTCSDEKIRMNRV----------------------------------------  target    RGWLKPTWMTDSLVRKGLFGQGIGKGFLPDVHCPTGAPREAFVKISRAEPGGIGGQGLWRPAALGIRPRHESPAMKRYLA 3qq7.1    --------------------------------------------------------------------------------  target    GGFFSGPKE 3qq7.1    --------- ``` | | | | | | | | | | | | | | | | | | | | | | | | | | | | | | | | | | | | | | | | | | | | | | | | | |
|  | 5g4g.1.A | VCP-LIKE ATPASE  *Structure of the ATPgS-bound VAT complex* | 0.01 |  | 16.33 | 0.09 | 373-437 | EM | 7.80 | homo-hexamer |  | HHblits | 0.27 |
| ``` target    FNGAPQYINENPFDLELDASRPARPRQYWRAESAHFYNHEDHPLRVGTRLLTGSTHMPTPTKVMWFANANSILGNVKWHY 5g4g.1    --------------------------------------------------------------------------------  target    NTVVNALPRMEMIAVHEWWWTGSCEWADVVFGVDSWGELKHPDMTASVTNPFLIVFPKTPIKRIFNTVGDIDVLALVSSK 5g4g.1    --------------------------------------------------------------------------------  target    LAELTGDTRFNDMWKFVREGRTDVYLQRILDASTNTKGYRFTELEAKAREGIPALMNSRTSPKVVGYDQLADSTPWYTKS 5g4g.1    --------------------------------------------------------------------------------  target    GRLEFYREEDEFIEAGENLPVHREPVDSTFYEPNVIVSPKHEAVRPSGPEDYGVARTDLSCEVRCGRNVVLTWAETRQTQ 5g4g.1    --------------------------------------------------------------------------------  target    HPLVKQGHKFIFHTPKYRHGSHTTPIDTDMNAVLFGPFGDIYRRDKRSPFVTEGYVDINPTDGLELGLQDGDYVWIDPDP 5g4g.1    ----------------------------------------------------MSRVRLDESSRRLLDAEIGDVVEIEKVR  target    EDRPFRGWQKNAKDMEFARLLCRARFYPGTPRGVTRMWFNMYGATPGSVRGAKARRDGLAKNPDTNYQAMFRSGSHQSAT 5g4g.1    K----------------TVGRVYRARPEDENKGIVRI-------------------------------------------  target    RGWLKPTWMTDSLVRKGLFGQGIGKGFLPDVHCPTGAPREAFVKISRAEPGGIGGQGLWRPAALGIRPRHESPAMKRYLA 5g4g.1    --------------------------------------------------------------------------------  target    GGFFSGPKE 5g4g.1    --------- ``` | | | | | | | | | | | | | | | | | | | | | | | | | | | | | | | | | | | | | | | | | | | | | | | | | |
|  | 5g4f.1.A | VCP-LIKE ATPASE  *Structure of the ADP-bound VAT complex* | 0.02 |  | 16.33 | 0.09 | 373-437 | EM | 7.00 | homo-hexamer |  | HHblits | 0.27 |
| ``` target    FNGAPQYINENPFDLELDASRPARPRQYWRAESAHFYNHEDHPLRVGTRLLTGSTHMPTPTKVMWFANANSILGNVKWHY 5g4f.1    --------------------------------------------------------------------------------  target    NTVVNALPRMEMIAVHEWWWTGSCEWADVVFGVDSWGELKHPDMTASVTNPFLIVFPKTPIKRIFNTVGDIDVLALVSSK 5g4f.1    --------------------------------------------------------------------------------  target    LAELTGDTRFNDMWKFVREGRTDVYLQRILDASTNTKGYRFTELEAKAREGIPALMNSRTSPKVVGYDQLADSTPWYTKS 5g4f.1    --------------------------------------------------------------------------------  target    GRLEFYREEDEFIEAGENLPVHREPVDSTFYEPNVIVSPKHEAVRPSGPEDYGVARTDLSCEVRCGRNVVLTWAETRQTQ 5g4f.1    --------------------------------------------------------------------------------  target    HPLVKQGHKFIFHTPKYRHGSHTTPIDTDMNAVLFGPFGDIYRRDKRSPFVTEGYVDINPTDGLELGLQDGDYVWIDPDP 5g4f.1    ----------------------------------------------------MSRVRLDESSRRLLDAEIGDVVEIEKVR  target    EDRPFRGWQKNAKDMEFARLLCRARFYPGTPRGVTRMWFNMYGATPGSVRGAKARRDGLAKNPDTNYQAMFRSGSHQSAT 5g4f.1    K----------------TVGRVYRARPEDENKGIVRI-------------------------------------------  target    RGWLKPTWMTDSLVRKGLFGQGIGKGFLPDVHCPTGAPREAFVKISRAEPGGIGGQGLWRPAALGIRPRHESPAMKRYLA 5g4f.1    --------------------------------------------------------------------------------  target    GGFFSGPKE 5g4f.1    --------- ``` | | | | | | | | | | | | | | | | | | | | | | | | | | | | | | | | | | | | | | | | | | | | | | | | | |
|  | 5g4f.1.B | VCP-LIKE ATPASE  *Structure of the ADP-bound VAT complex* | 0.02 |  | 16.33 | 0.09 | 373-437 | EM | 7.00 | homo-hexamer |  | HHblits | 0.27 |
| ``` target    FNGAPQYINENPFDLELDASRPARPRQYWRAESAHFYNHEDHPLRVGTRLLTGSTHMPTPTKVMWFANANSILGNVKWHY 5g4f.1    --------------------------------------------------------------------------------  target    NTVVNALPRMEMIAVHEWWWTGSCEWADVVFGVDSWGELKHPDMTASVTNPFLIVFPKTPIKRIFNTVGDIDVLALVSSK 5g4f.1    --------------------------------------------------------------------------------  target    LAELTGDTRFNDMWKFVREGRTDVYLQRILDASTNTKGYRFTELEAKAREGIPALMNSRTSPKVVGYDQLADSTPWYTKS 5g4f.1    --------------------------------------------------------------------------------  target    GRLEFYREEDEFIEAGENLPVHREPVDSTFYEPNVIVSPKHEAVRPSGPEDYGVARTDLSCEVRCGRNVVLTWAETRQTQ 5g4f.1    --------------------------------------------------------------------------------  target    HPLVKQGHKFIFHTPKYRHGSHTTPIDTDMNAVLFGPFGDIYRRDKRSPFVTEGYVDINPTDGLELGLQDGDYVWIDPDP 5g4f.1    ----------------------------------------------------MSRVRLDESSRRLLDAEIGDVVEIEKVR  target    EDRPFRGWQKNAKDMEFARLLCRARFYPGTPRGVTRMWFNMYGATPGSVRGAKARRDGLAKNPDTNYQAMFRSGSHQSAT 5g4f.1    K----------------TVGRVYRARPEDENKGIVRI-------------------------------------------  target    RGWLKPTWMTDSLVRKGLFGQGIGKGFLPDVHCPTGAPREAFVKISRAEPGGIGGQGLWRPAALGIRPRHESPAMKRYLA 5g4f.1    --------------------------------------------------------------------------------  target    GGFFSGPKE 5g4f.1    --------- ``` | | | | | | | | | | | | | | | | | | | | | | | | | | | | | | | | | | | | | | | | | | | | | | | | | |
|  | 5g4f.1.C | VCP-LIKE ATPASE  *Structure of the ADP-bound VAT complex* | 0.03 |  | 16.33 | 0.09 | 373-437 | EM | 7.00 | homo-hexamer |  | HHblits | 0.27 |
| ``` target    FNGAPQYINENPFDLELDASRPARPRQYWRAESAHFYNHEDHPLRVGTRLLTGSTHMPTPTKVMWFANANSILGNVKWHY 5g4f.1    --------------------------------------------------------------------------------  target    NTVVNALPRMEMIAVHEWWWTGSCEWADVVFGVDSWGELKHPDMTASVTNPFLIVFPKTPIKRIFNTVGDIDVLALVSSK 5g4f.1    --------------------------------------------------------------------------------  target    LAELTGDTRFNDMWKFVREGRTDVYLQRILDASTNTKGYRFTELEAKAREGIPALMNSRTSPKVVGYDQLADSTPWYTKS 5g4f.1    --------------------------------------------------------------------------------  target    GRLEFYREEDEFIEAGENLPVHREPVDSTFYEPNVIVSPKHEAVRPSGPEDYGVARTDLSCEVRCGRNVVLTWAETRQTQ 5g4f.1    --------------------------------------------------------------------------------  target    HPLVKQGHKFIFHTPKYRHGSHTTPIDTDMNAVLFGPFGDIYRRDKRSPFVTEGYVDINPTDGLELGLQDGDYVWIDPDP 5g4f.1    ----------------------------------------------------MSRVRLDESSRRLLDAEIGDVVEIEKVR  target    EDRPFRGWQKNAKDMEFARLLCRARFYPGTPRGVTRMWFNMYGATPGSVRGAKARRDGLAKNPDTNYQAMFRSGSHQSAT 5g4f.1    K----------------TVGRVYRARPEDENKGIVRI-------------------------------------------  target    RGWLKPTWMTDSLVRKGLFGQGIGKGFLPDVHCPTGAPREAFVKISRAEPGGIGGQGLWRPAALGIRPRHESPAMKRYLA 5g4f.1    --------------------------------------------------------------------------------  target    GGFFSGPKE 5g4f.1    --------- ``` | | | | | | | | | | | | | | | | | | | | | | | | | | | | | | | | | | | | | | | | | | | | | | | | | |
|  | 5g4f.1.D | VCP-LIKE ATPASE  *Structure of the ADP-bound VAT complex* | 0.02 |  | 16.33 | 0.09 | 373-437 | EM | 7.00 | homo-hexamer |  | HHblits | 0.27 |
| ``` target    FNGAPQYINENPFDLELDASRPARPRQYWRAESAHFYNHEDHPLRVGTRLLTGSTHMPTPTKVMWFANANSILGNVKWHY 5g4f.1    --------------------------------------------------------------------------------  target    NTVVNALPRMEMIAVHEWWWTGSCEWADVVFGVDSWGELKHPDMTASVTNPFLIVFPKTPIKRIFNTVGDIDVLALVSSK 5g4f.1    --------------------------------------------------------------------------------  target    LAELTGDTRFNDMWKFVREGRTDVYLQRILDASTNTKGYRFTELEAKAREGIPALMNSRTSPKVVGYDQLADSTPWYTKS 5g4f.1    --------------------------------------------------------------------------------  target    GRLEFYREEDEFIEAGENLPVHREPVDSTFYEPNVIVSPKHEAVRPSGPEDYGVARTDLSCEVRCGRNVVLTWAETRQTQ 5g4f.1    --------------------------------------------------------------------------------  target    HPLVKQGHKFIFHTPKYRHGSHTTPIDTDMNAVLFGPFGDIYRRDKRSPFVTEGYVDINPTDGLELGLQDGDYVWIDPDP 5g4f.1    ----------------------------------------------------MSRVRLDESSRRLLDAEIGDVVEIEKVR  target    EDRPFRGWQKNAKDMEFARLLCRARFYPGTPRGVTRMWFNMYGATPGSVRGAKARRDGLAKNPDTNYQAMFRSGSHQSAT 5g4f.1    K----------------TVGRVYRARPEDENKGIVRI-------------------------------------------  target    RGWLKPTWMTDSLVRKGLFGQGIGKGFLPDVHCPTGAPREAFVKISRAEPGGIGGQGLWRPAALGIRPRHESPAMKRYLA 5g4f.1    --------------------------------------------------------------------------------  target    GGFFSGPKE 5g4f.1    --------- ``` | | | | | | | | | | | | | | | | | | | | | | | | | | | | | | | | | | | | | | | | | | | | | | | | | |
|  | 5g4f.1.E | VCP-LIKE ATPASE  *Structure of the ADP-bound VAT complex* | 0.02 |  | 16.33 | 0.09 | 373-437 | EM | 7.00 | homo-hexamer |  | HHblits | 0.27 |
| ``` target    FNGAPQYINENPFDLELDASRPARPRQYWRAESAHFYNHEDHPLRVGTRLLTGSTHMPTPTKVMWFANANSILGNVKWHY 5g4f.1    --------------------------------------------------------------------------------  target    NTVVNALPRMEMIAVHEWWWTGSCEWADVVFGVDSWGELKHPDMTASVTNPFLIVFPKTPIKRIFNTVGDIDVLALVSSK 5g4f.1    --------------------------------------------------------------------------------  target    LAELTGDTRFNDMWKFVREGRTDVYLQRILDASTNTKGYRFTELEAKAREGIPALMNSRTSPKVVGYDQLADSTPWYTKS 5g4f.1    --------------------------------------------------------------------------------  target    GRLEFYREEDEFIEAGENLPVHREPVDSTFYEPNVIVSPKHEAVRPSGPEDYGVARTDLSCEVRCGRNVVLTWAETRQTQ 5g4f.1    --------------------------------------------------------------------------------  target    HPLVKQGHKFIFHTPKYRHGSHTTPIDTDMNAVLFGPFGDIYRRDKRSPFVTEGYVDINPTDGLELGLQDGDYVWIDPDP 5g4f.1    ----------------------------------------------------MSRVRLDESSRRLLDAEIGDVVEIEKVR  target    EDRPFRGWQKNAKDMEFARLLCRARFYPGTPRGVTRMWFNMYGATPGSVRGAKARRDGLAKNPDTNYQAMFRSGSHQSAT 5g4f.1    K----------------TVGRVYRARPEDENKGIVRI-------------------------------------------  target    RGWLKPTWMTDSLVRKGLFGQGIGKGFLPDVHCPTGAPREAFVKISRAEPGGIGGQGLWRPAALGIRPRHESPAMKRYLA 5g4f.1    --------------------------------------------------------------------------------  target    GGFFSGPKE 5g4f.1    --------- ``` | | | | | | | | | | | | | | | | | | | | | | | | | | | | | | | | | | | | | | | | | | | | | | | | | |
|  | 5g4f.1.F | VCP-LIKE ATPASE  *Structure of the ADP-bound VAT complex* | 0.02 |  | 16.33 | 0.09 | 373-437 | EM | 7.00 | homo-hexamer |  | HHblits | 0.27 |
| ``` target    FNGAPQYINENPFDLELDASRPARPRQYWRAESAHFYNHEDHPLRVGTRLLTGSTHMPTPTKVMWFANANSILGNVKWHY 5g4f.1    --------------------------------------------------------------------------------  target    NTVVNALPRMEMIAVHEWWWTGSCEWADVVFGVDSWGELKHPDMTASVTNPFLIVFPKTPIKRIFNTVGDIDVLALVSSK 5g4f.1    --------------------------------------------------------------------------------  target    LAELTGDTRFNDMWKFVREGRTDVYLQRILDASTNTKGYRFTELEAKAREGIPALMNSRTSPKVVGYDQLADSTPWYTKS 5g4f.1    --------------------------------------------------------------------------------  target    GRLEFYREEDEFIEAGENLPVHREPVDSTFYEPNVIVSPKHEAVRPSGPEDYGVARTDLSCEVRCGRNVVLTWAETRQTQ 5g4f.1    --------------------------------------------------------------------------------  target    HPLVKQGHKFIFHTPKYRHGSHTTPIDTDMNAVLFGPFGDIYRRDKRSPFVTEGYVDINPTDGLELGLQDGDYVWIDPDP 5g4f.1    ----------------------------------------------------MSRVRLDESSRRLLDAEIGDVVEIEKVR  target    EDRPFRGWQKNAKDMEFARLLCRARFYPGTPRGVTRMWFNMYGATPGSVRGAKARRDGLAKNPDTNYQAMFRSGSHQSAT 5g4f.1    K----------------TVGRVYRARPEDENKGIVRI-------------------------------------------  target    RGWLKPTWMTDSLVRKGLFGQGIGKGFLPDVHCPTGAPREAFVKISRAEPGGIGGQGLWRPAALGIRPRHESPAMKRYLA 5g4f.1    --------------------------------------------------------------------------------  target    GGFFSGPKE 5g4f.1    --------- ``` | | | | | | | | | | | | | | | | | | | | | | | | | | | | | | | | | | | | | | | | | | | | | | | | | |
|  | 5b6c.1.A | Transitional endoplasmic reticulum ATPase  *Structural Details of Ufd1 binding to p97* | 0.02 |  | 20.41 | 0.09 | 375-439 | X-ray | 1.55 | hetero-oligomer |  | HHblits | 0.26 |
| ``` target    FNGAPQYINENPFDLELDASRPARPRQYWRAESAHFYNHEDHPLRVGTRLLTGSTHMPTPTKVMWFANANSILGNVKWHY 5b6c.1    --------------------------------------------------------------------------------  target    NTVVNALPRMEMIAVHEWWWTGSCEWADVVFGVDSWGELKHPDMTASVTNPFLIVFPKTPIKRIFNTVGDIDVLALVSSK 5b6c.1    --------------------------------------------------------------------------------  target    LAELTGDTRFNDMWKFVREGRTDVYLQRILDASTNTKGYRFTELEAKAREGIPALMNSRTSPKVVGYDQLADSTPWYTKS 5b6c.1    --------------------------------------------------------------------------------  target    GRLEFYREEDEFIEAGENLPVHREPVDSTFYEPNVIVSPKHEAVRPSGPEDYGVARTDLSCEVRCGRNVVLTWAETRQTQ 5b6c.1    --------------------------------------------------------------------------------  target    HPLVKQGHKFIFHTPKYRHGSHTTPIDTDMNAVLFGPFGDIYRRDKRSPFVTEGYVDINPTDGLELGLQDGDYVWIDPDP 5b6c.1    ------------------------------------------------------VVSLSQPKMDELQLFRGDTVLLKGKK  target    EDRPFRGWQKNAKDMEFARLLCRARFYPGTPRGVTRMWFNMYGATPGSVRGAKARRDGLAKNPDTNYQAMFRSGSHQSAT 5b6c.1    ------R----------REAVCIVLSDDTCSDEKIRMNR-----------------------------------------  target    RGWLKPTWMTDSLVRKGLFGQGIGKGFLPDVHCPTGAPREAFVKISRAEPGGIGGQGLWRPAALGIRPRHESPAMKRYLA 5b6c.1    --------------------------------------------------------------------------------  target    GGFFSGPKE 5b6c.1    --------- ``` | | | | | | | | | | | | | | | | | | | | | | | | | | | | | | | | | | | | | | | | | | | | | | | | | |
|  | 1wlf.1.A | Peroxisome biogenesis factor 1  *Structure of the N-terminal domain of PEX1 AAA-ATPase: Characterization of a putative adaptor-binding domain* | 0.00 |  | 40.74 | 0.05 | 373-399 | X-ray | 2.05 | monomer |  | HHblits | 0.39 |
| ``` target    FNGAPQYINENPFDLELDASRPARPRQYWRAESAHFYNHEDHPLRVGTRLLTGSTHMPTPTKVMWFANANSILGNVKWHY 1wlf.1    --------------------------------------------------------------------------------  target    NTVVNALPRMEMIAVHEWWWTGSCEWADVVFGVDSWGELKHPDMTASVTNPFLIVFPKTPIKRIFNTVGDIDVLALVSSK 1wlf.1    --------------------------------------------------------------------------------  target    LAELTGDTRFNDMWKFVREGRTDVYLQRILDASTNTKGYRFTELEAKAREGIPALMNSRTSPKVVGYDQLADSTPWYTKS 1wlf.1    --------------------------------------------------------------------------------  target    GRLEFYREEDEFIEAGENLPVHREPVDSTFYEPNVIVSPKHEAVRPSGPEDYGVARTDLSCEVRCGRNVVLTWAETRQTQ 1wlf.1    --------------------------------------------------------------------------------  target    HPLVKQGHKFIFHTPKYRHGSHTTPIDTDMNAVLFGPFGDIYRRDKRSPFVTEGYVDINPTDGLELGLQDGDYVWIDPDP 1wlf.1    ----------------------------------------------------ENVAEINRQVGQKLGLSSGDQVFLRPC-  target    EDRPFRGWQKNAKDMEFARLLCRARFYPGTPRGVTRMWFNMYGATPGSVRGAKARRDGLAKNPDTNYQAMFRSGSHQSAT 1wlf.1    --------------------------------------------------------------------------------  target    RGWLKPTWMTDSLVRKGLFGQGIGKGFLPDVHCPTGAPREAFVKISRAEPGGIGGQGLWRPAALGIRPRHESPAMKRYLA 1wlf.1    --------------------------------------------------------------------------------  target    GGFFSGPKE 1wlf.1    --------- ``` | | | | | | | | | | | | | | | | | | | | | | | | | | | | | | | | | | | | | | | | | | | | | | | | | |
|  | 7dvc.1.A | reDPBB\_sym1 protein  *Crystal structure of the computationally designed reDPBB\_sym1 protein* | 0.00 |  | 28.57 | 0.05 | 373-400 | X-ray | 1.71 | monomer |  | HHblits | 0.33 |
| ``` target    FNGAPQYINENPFDLELDASRPARPRQYWRAESAHFYNHEDHPLRVGTRLLTGSTHMPTPTKVMWFANANSILGNVKWHY 7dvc.1    --------------------------------------------------------------------------------  target    NTVVNALPRMEMIAVHEWWWTGSCEWADVVFGVDSWGELKHPDMTASVTNPFLIVFPKTPIKRIFNTVGDIDVLALVSSK 7dvc.1    --------------------------------------------------------------------------------  target    LAELTGDTRFNDMWKFVREGRTDVYLQRILDASTNTKGYRFTELEAKAREGIPALMNSRTSPKVVGYDQLADSTPWYTKS 7dvc.1    --------------------------------------------------------------------------------  target    GRLEFYREEDEFIEAGENLPVHREPVDSTFYEPNVIVSPKHEAVRPSGPEDYGVARTDLSCEVRCGRNVVLTWAETRQTQ 7dvc.1    --------------------------------------------------------------------------------  target    HPLVKQGHKFIFHTPKYRHGSHTTPIDTDMNAVLFGPFGDIYRRDKRSPFVTEGYVDINPTDGLELGLQDGDYVWIDPDP 7dvc.1    ----------------------------------------------------KGIVRMDKASRDKLGVSAGDLVEIKGSK  target    EDRPFRGWQKNAKDMEFARLLCRARFYPGTPRGVTRMWFNMYGATPGSVRGAKARRDGLAKNPDTNYQAMFRSGSHQSAT 7dvc.1    --------------------------------------------------------------------------------  target    RGWLKPTWMTDSLVRKGLFGQGIGKGFLPDVHCPTGAPREAFVKISRAEPGGIGGQGLWRPAALGIRPRHESPAMKRYLA 7dvc.1    --------------------------------------------------------------------------------  target    GGFFSGPKE 7dvc.1    --------- ``` | | | | | | | | | | | | | | | | | | | | | | | | | | | | | | | | | | | | | | | | | | | | | | | | | |
|  | 7dvc.5.A | reDPBB\_sym1 protein  *Crystal structure of the computationally designed reDPBB\_sym1 protein* | 0.00 |  | 28.57 | 0.05 | 373-400 | X-ray | 1.71 | monomer |  | HHblits | 0.33 |
| ``` target    FNGAPQYINENPFDLELDASRPARPRQYWRAESAHFYNHEDHPLRVGTRLLTGSTHMPTPTKVMWFANANSILGNVKWHY 7dvc.5    --------------------------------------------------------------------------------  target    NTVVNALPRMEMIAVHEWWWTGSCEWADVVFGVDSWGELKHPDMTASVTNPFLIVFPKTPIKRIFNTVGDIDVLALVSSK 7dvc.5    --------------------------------------------------------------------------------  target    LAELTGDTRFNDMWKFVREGRTDVYLQRILDASTNTKGYRFTELEAKAREGIPALMNSRTSPKVVGYDQLADSTPWYTKS 7dvc.5    --------------------------------------------------------------------------------  target    GRLEFYREEDEFIEAGENLPVHREPVDSTFYEPNVIVSPKHEAVRPSGPEDYGVARTDLSCEVRCGRNVVLTWAETRQTQ 7dvc.5    --------------------------------------------------------------------------------  target    HPLVKQGHKFIFHTPKYRHGSHTTPIDTDMNAVLFGPFGDIYRRDKRSPFVTEGYVDINPTDGLELGLQDGDYVWIDPDP 7dvc.5    ----------------------------------------------------KGIVRMDKASRDKLGVSAGDLVEIKGSK  target    EDRPFRGWQKNAKDMEFARLLCRARFYPGTPRGVTRMWFNMYGATPGSVRGAKARRDGLAKNPDTNYQAMFRSGSHQSAT 7dvc.5    --------------------------------------------------------------------------------  target    RGWLKPTWMTDSLVRKGLFGQGIGKGFLPDVHCPTGAPREAFVKISRAEPGGIGGQGLWRPAALGIRPRHESPAMKRYLA 7dvc.5    --------------------------------------------------------------------------------  target    GGFFSGPKE 7dvc.5    --------- ``` | | | | | | | | | | | | | | | | | | | | | | | | | | | | | | | | | | | | | | | | | | | | | | | | | |
|  | 7du7.1.A | mkDPBB\_sym1 protein  *Crystal structure of the rationally designed mkDPBB\_sym1 protein* | 0.00 |  | 29.63 | 0.05 | 373-399 | X-ray | 1.20 | monomer |  | HHblits | 0.35 |
| ``` target    FNGAPQYINENPFDLELDASRPARPRQYWRAESAHFYNHEDHPLRVGTRLLTGSTHMPTPTKVMWFANANSILGNVKWHY 7du7.1    --------------------------------------------------------------------------------  target    NTVVNALPRMEMIAVHEWWWTGSCEWADVVFGVDSWGELKHPDMTASVTNPFLIVFPKTPIKRIFNTVGDIDVLALVSSK 7du7.1    --------------------------------------------------------------------------------  target    LAELTGDTRFNDMWKFVREGRTDVYLQRILDASTNTKGYRFTELEAKAREGIPALMNSRTSPKVVGYDQLADSTPWYTKS 7du7.1    --------------------------------------------------------------------------------  target    GRLEFYREEDEFIEAGENLPVHREPVDSTFYEPNVIVSPKHEAVRPSGPEDYGVARTDLSCEVRCGRNVVLTWAETRQTQ 7du7.1    --------------------------------------------------------------------------------  target    HPLVKQGHKFIFHTPKYRHGSHTTPIDTDMNAVLFGPFGDIYRRDKRSPFVTEGYVDINPTDGLELGLQDGDYVWIDPDP 7du7.1    ----------------------------------------------------KGIVRMDKASRAKLGVSVGDYVEVKKV-  target    EDRPFRGWQKNAKDMEFARLLCRARFYPGTPRGVTRMWFNMYGATPGSVRGAKARRDGLAKNPDTNYQAMFRSGSHQSAT 7du7.1    --------------------------------------------------------------------------------  target    RGWLKPTWMTDSLVRKGLFGQGIGKGFLPDVHCPTGAPREAFVKISRAEPGGIGGQGLWRPAALGIRPRHESPAMKRYLA 7du7.1    --------------------------------------------------------------------------------  target    GGFFSGPKE 7du7.1    --------- ``` | | | | | | | | | | | | | | | | | | | | | | | | | | | | | | | | | | | | | | | | | | | | | | | | | |
|  | 5cup.1.A | Phosphate propanoyltransferase  *Structure of Rhodopseudomonas palustris PduL - phosphate bound form* | 0.00 |  | 26.92 | 0.05 | 373-398 | X-ray | 2.10 | homo-dimer | 4 x ZN | HHblits | 0.38 |
| ``` target    FNGAPQYINENPFDLELDASRPARPRQYWRAESAHFYNHEDHPLRVGTRLLTGSTHMPTPTKVMWFANANSILGNVKWHY 5cup.1    --------------------------------------------------------------------------------  target    NTVVNALPRMEMIAVHEWWWTGSCEWADVVFGVDSWGELKHPDMTASVTNPFLIVFPKTPIKRIFNTVGDIDVLALVSSK 5cup.1    --------------------------------------------------------------------------------  target    LAELTGDTRFNDMWKFVREGRTDVYLQRILDASTNTKGYRFTELEAKAREGIPALMNSRTSPKVVGYDQLADSTPWYTKS 5cup.1    --------------------------------------------------------------------------------  target    GRLEFYREEDEFIEAGENLPVHREPVDSTFYEPNVIVSPKHEAVRPSGPEDYGVARTDLSCEVRCGRNVVLTWAETRQTQ 5cup.1    --------------------------------------------------------------------------------  target    HPLVKQGHKFIFHTPKYRHGSHTTPIDTDMNAVLFGPFGDIYRRDKRSPFVTEGYVDINPTDGLELGLQDGDYVWIDPDP 5cup.1    ----------------------------------------------------QRHIHMHPSTAAKLGLRNGDEVDVEA--  target    EDRPFRGWQKNAKDMEFARLLCRARFYPGTPRGVTRMWFNMYGATPGSVRGAKARRDGLAKNPDTNYQAMFRSGSHQSAT 5cup.1    --------------------------------------------------------------------------------  target    RGWLKPTWMTDSLVRKGLFGQGIGKGFLPDVHCPTGAPREAFVKISRAEPGGIGGQGLWRPAALGIRPRHESPAMKRYLA 5cup.1    --------------------------------------------------------------------------------  target    GGFFSGPKE 5cup.1    --------- ``` | | | | | | | | | | | | | | | | | | | | | | | | | | | | | | | | | | | | | | | | | | | | | | | | | |
|  | 5cuo.1.A | Phosphate propanoyltransferase  *Structure of Rhodopseudomonas palustris PduL - CoA bound form* | 0.00 |  | 26.92 | 0.05 | 373-398 | X-ray | 1.54 | homo-dimer | 2 x COA, 4 x ZN | HHblits | 0.38 |
| ``` target    FNGAPQYINENPFDLELDASRPARPRQYWRAESAHFYNHEDHPLRVGTRLLTGSTHMPTPTKVMWFANANSILGNVKWHY 5cuo.1    --------------------------------------------------------------------------------  target    NTVVNALPRMEMIAVHEWWWTGSCEWADVVFGVDSWGELKHPDMTASVTNPFLIVFPKTPIKRIFNTVGDIDVLALVSSK 5cuo.1    --------------------------------------------------------------------------------  target    LAELTGDTRFNDMWKFVREGRTDVYLQRILDASTNTKGYRFTELEAKAREGIPALMNSRTSPKVVGYDQLADSTPWYTKS 5cuo.1    --------------------------------------------------------------------------------  target    GRLEFYREEDEFIEAGENLPVHREPVDSTFYEPNVIVSPKHEAVRPSGPEDYGVARTDLSCEVRCGRNVVLTWAETRQTQ 5cuo.1    --------------------------------------------------------------------------------  target    HPLVKQGHKFIFHTPKYRHGSHTTPIDTDMNAVLFGPFGDIYRRDKRSPFVTEGYVDINPTDGLELGLQDGDYVWIDPDP 5cuo.1    ----------------------------------------------------QRHIHMHPSTAAKLGLRNGDEVDVEA--  target    EDRPFRGWQKNAKDMEFARLLCRARFYPGTPRGVTRMWFNMYGATPGSVRGAKARRDGLAKNPDTNYQAMFRSGSHQSAT 5cuo.1    --------------------------------------------------------------------------------  target    RGWLKPTWMTDSLVRKGLFGQGIGKGFLPDVHCPTGAPREAFVKISRAEPGGIGGQGLWRPAALGIRPRHESPAMKRYLA 5cuo.1    --------------------------------------------------------------------------------  target    GGFFSGPKE 5cuo.1    --------- ``` | | | | | | | | | | | | | | | | | | | | | | | | | | | | | | | | | | | | | | | | | | | | | | | | | |
|  | 5e7p.1.A | Cell division control protein Cdc48  *Crystal Structure of MSMEG\_0858 (Uniprot A0QQS4), a AAA ATPase.* | 0.00 |  | 25.00 | 0.05 | 373-400 | X-ray | 2.51 | monomer | 2 x ADP | HHblits | 0.32 |
| ``` target    FNGAPQYINENPFDLELDASRPARPRQYWRAESAHFYNHEDHPLRVGTRLLTGSTHMPTPTKVMWFANANSILGNVKWHY 5e7p.1    --------------------------------------------------------------------------------  target    NTVVNALPRMEMIAVHEWWWTGSCEWADVVFGVDSWGELKHPDMTASVTNPFLIVFPKTPIKRIFNTVGDIDVLALVSSK 5e7p.1    --------------------------------------------------------------------------------  target    LAELTGDTRFNDMWKFVREGRTDVYLQRILDASTNTKGYRFTELEAKAREGIPALMNSRTSPKVVGYDQLADSTPWYTKS 5e7p.1    --------------------------------------------------------------------------------  target    GRLEFYREEDEFIEAGENLPVHREPVDSTFYEPNVIVSPKHEAVRPSGPEDYGVARTDLSCEVRCGRNVVLTWAETRQTQ 5e7p.1    --------------------------------------------------------------------------------  target    HPLVKQGHKFIFHTPKYRHGSHTTPIDTDMNAVLFGPFGDIYRRDKRSPFVTEGYVDINPTDGLELGLQDGDYVWIDPDP 5e7p.1    ----------------------------------------------------RGVVRLHPEVLAALGIREWDAVALTGTR  target    EDRPFRGWQKNAKDMEFARLLCRARFYPGTPRGVTRMWFNMYGATPGSVRGAKARRDGLAKNPDTNYQAMFRSGSHQSAT 5e7p.1    --------------------------------------------------------------------------------  target    RGWLKPTWMTDSLVRKGLFGQGIGKGFLPDVHCPTGAPREAFVKISRAEPGGIGGQGLWRPAALGIRPRHESPAMKRYLA 5e7p.1    --------------------------------------------------------------------------------  target    GGFFSGPKE 5e7p.1    --------- ``` | | | | | | | | | | | | | | | | | | | | | | | | | | | | | | | | | | | | | | | | | | | | | | | | | |
|  | 7dvf.1.A | reDPBB\_sym2 protein  *Crystal structure of the computationally designed reDPBB\_sym2 protein* | 0.00 |  | 29.63 | 0.05 | 373-399 | X-ray | 1.21 | monomer |  | HHblits | 0.33 |
| ``` target    FNGAPQYINENPFDLELDASRPARPRQYWRAESAHFYNHEDHPLRVGTRLLTGSTHMPTPTKVMWFANANSILGNVKWHY 7dvf.1    --------------------------------------------------------------------------------  target    NTVVNALPRMEMIAVHEWWWTGSCEWADVVFGVDSWGELKHPDMTASVTNPFLIVFPKTPIKRIFNTVGDIDVLALVSSK 7dvf.1    --------------------------------------------------------------------------------  target    LAELTGDTRFNDMWKFVREGRTDVYLQRILDASTNTKGYRFTELEAKAREGIPALMNSRTSPKVVGYDQLADSTPWYTKS 7dvf.1    --------------------------------------------------------------------------------  target    GRLEFYREEDEFIEAGENLPVHREPVDSTFYEPNVIVSPKHEAVRPSGPEDYGVARTDLSCEVRCGRNVVLTWAETRQTQ 7dvf.1    --------------------------------------------------------------------------------  target    HPLVKQGHKFIFHTPKYRHGSHTTPIDTDMNAVLFGPFGDIYRRDKRSPFVTEGYVDINPTDGLELGLQDGDYVWIDPDP 7dvf.1    ----------------------------------------------------KGIVRMDKASREKLGVSAGDLVEIKGS-  target    EDRPFRGWQKNAKDMEFARLLCRARFYPGTPRGVTRMWFNMYGATPGSVRGAKARRDGLAKNPDTNYQAMFRSGSHQSAT 7dvf.1    --------------------------------------------------------------------------------  target    RGWLKPTWMTDSLVRKGLFGQGIGKGFLPDVHCPTGAPREAFVKISRAEPGGIGGQGLWRPAALGIRPRHESPAMKRYLA 7dvf.1    --------------------------------------------------------------------------------  target    GGFFSGPKE 7dvf.1    --------- ``` | | | | | | | | | | | | | | | | | | | | | | | | | | | | | | | | | | | | | | | | | | | | | | | | | |
|  | 7dvh.2.A | reDPBB\_sym4 protein  *Crystal structure of the computationally designed reDPBB\_sym4 protein* | 0.00 |  | 30.77 | 0.05 | 373-398 | X-ray | 1.70 | monomer |  | HHblits | 0.35 |
| ``` target    FNGAPQYINENPFDLELDASRPARPRQYWRAESAHFYNHEDHPLRVGTRLLTGSTHMPTPTKVMWFANANSILGNVKWHY 7dvh.2    --------------------------------------------------------------------------------  target    NTVVNALPRMEMIAVHEWWWTGSCEWADVVFGVDSWGELKHPDMTASVTNPFLIVFPKTPIKRIFNTVGDIDVLALVSSK 7dvh.2    --------------------------------------------------------------------------------  target    LAELTGDTRFNDMWKFVREGRTDVYLQRILDASTNTKGYRFTELEAKAREGIPALMNSRTSPKVVGYDQLADSTPWYTKS 7dvh.2    --------------------------------------------------------------------------------  target    GRLEFYREEDEFIEAGENLPVHREPVDSTFYEPNVIVSPKHEAVRPSGPEDYGVARTDLSCEVRCGRNVVLTWAETRQTQ 7dvh.2    --------------------------------------------------------------------------------  target    HPLVKQGHKFIFHTPKYRHGSHTTPIDTDMNAVLFGPFGDIYRRDKRSPFVTEGYVDINPTDGLELGLQDGDYVWIDPDP 7dvh.2    ----------------------------------------------------KGIVRMDKYERQNLGVSVGDYVEVKK--  target    EDRPFRGWQKNAKDMEFARLLCRARFYPGTPRGVTRMWFNMYGATPGSVRGAKARRDGLAKNPDTNYQAMFRSGSHQSAT 7dvh.2    --------------------------------------------------------------------------------  target    RGWLKPTWMTDSLVRKGLFGQGIGKGFLPDVHCPTGAPREAFVKISRAEPGGIGGQGLWRPAALGIRPRHESPAMKRYLA 7dvh.2    --------------------------------------------------------------------------------  target    GGFFSGPKE 7dvh.2    --------- ``` | | | | | | | | | | | | | | | | | | | | | | | | | | | | | | | | | | | | | | | | | | | | | | | | | |
|  | 7dvh.1.A | reDPBB\_sym4 protein  *Crystal structure of the computationally designed reDPBB\_sym4 protein* | 0.00 |  | 30.77 | 0.05 | 373-398 | X-ray | 1.70 | monomer |  | HHblits | 0.35 |
| ``` target    FNGAPQYINENPFDLELDASRPARPRQYWRAESAHFYNHEDHPLRVGTRLLTGSTHMPTPTKVMWFANANSILGNVKWHY 7dvh.1    --------------------------------------------------------------------------------  target    NTVVNALPRMEMIAVHEWWWTGSCEWADVVFGVDSWGELKHPDMTASVTNPFLIVFPKTPIKRIFNTVGDIDVLALVSSK 7dvh.1    --------------------------------------------------------------------------------  target    LAELTGDTRFNDMWKFVREGRTDVYLQRILDASTNTKGYRFTELEAKAREGIPALMNSRTSPKVVGYDQLADSTPWYTKS 7dvh.1    --------------------------------------------------------------------------------  target    GRLEFYREEDEFIEAGENLPVHREPVDSTFYEPNVIVSPKHEAVRPSGPEDYGVARTDLSCEVRCGRNVVLTWAETRQTQ 7dvh.1    --------------------------------------------------------------------------------  target    HPLVKQGHKFIFHTPKYRHGSHTTPIDTDMNAVLFGPFGDIYRRDKRSPFVTEGYVDINPTDGLELGLQDGDYVWIDPDP 7dvh.1    ----------------------------------------------------KGIVRMDKYERQNLGVSVGDYVEVKK--  target    EDRPFRGWQKNAKDMEFARLLCRARFYPGTPRGVTRMWFNMYGATPGSVRGAKARRDGLAKNPDTNYQAMFRSGSHQSAT 7dvh.1    --------------------------------------------------------------------------------  target    RGWLKPTWMTDSLVRKGLFGQGIGKGFLPDVHCPTGAPREAFVKISRAEPGGIGGQGLWRPAALGIRPRHESPAMKRYLA 7dvh.1    --------------------------------------------------------------------------------  target    GGFFSGPKE 7dvh.1    --------- ``` | | | | | | | | | | | | | | | | | | | | | | | | | | | | | | | | | | | | | | | | | | | | | | | | | |
|  | 7dvh.4.A | reDPBB\_sym4 protein  *Crystal structure of the computationally designed reDPBB\_sym4 protein* | 0.00 |  | 30.77 | 0.05 | 373-398 | X-ray | 1.70 | monomer |  | HHblits | 0.35 |
| ``` target    FNGAPQYINENPFDLELDASRPARPRQYWRAESAHFYNHEDHPLRVGTRLLTGSTHMPTPTKVMWFANANSILGNVKWHY 7dvh.4    --------------------------------------------------------------------------------  target    NTVVNALPRMEMIAVHEWWWTGSCEWADVVFGVDSWGELKHPDMTASVTNPFLIVFPKTPIKRIFNTVGDIDVLALVSSK 7dvh.4    --------------------------------------------------------------------------------  target    LAELTGDTRFNDMWKFVREGRTDVYLQRILDASTNTKGYRFTELEAKAREGIPALMNSRTSPKVVGYDQLADSTPWYTKS 7dvh.4    --------------------------------------------------------------------------------  target    GRLEFYREEDEFIEAGENLPVHREPVDSTFYEPNVIVSPKHEAVRPSGPEDYGVARTDLSCEVRCGRNVVLTWAETRQTQ 7dvh.4    --------------------------------------------------------------------------------  target    HPLVKQGHKFIFHTPKYRHGSHTTPIDTDMNAVLFGPFGDIYRRDKRSPFVTEGYVDINPTDGLELGLQDGDYVWIDPDP 7dvh.4    ----------------------------------------------------KGIVRMDKYERQNLGVSVGDYVEVKK--  target    EDRPFRGWQKNAKDMEFARLLCRARFYPGTPRGVTRMWFNMYGATPGSVRGAKARRDGLAKNPDTNYQAMFRSGSHQSAT 7dvh.4    --------------------------------------------------------------------------------  target    RGWLKPTWMTDSLVRKGLFGQGIGKGFLPDVHCPTGAPREAFVKISRAEPGGIGGQGLWRPAALGIRPRHESPAMKRYLA 7dvh.4    --------------------------------------------------------------------------------  target    GGFFSGPKE 7dvh.4    --------- ``` | | | | | | | | | | | | | | | | | | | | | | | | | | | | | | | | | | | | | | | | | | | | | | | | | |
|  | 6hd3.1.A | Cell division control protein 48 homolog A  *Common mode of remodeling AAA ATPases p97/CDC48 by their disassembly cofactors ASPL/PUX1* | 0.00 |  | 25.93 | 0.05 | 374-400 | X-ray | 2.80 | homo-24-mer | 24 x ADP | HHblits | 0.31 |
| ``` target    FNGAPQYINENPFDLELDASRPARPRQYWRAESAHFYNHEDHPLRVGTRLLTGSTHMPTPTKVMWFANANSILGNVKWHY 6hd3.1    --------------------------------------------------------------------------------  target    NTVVNALPRMEMIAVHEWWWTGSCEWADVVFGVDSWGELKHPDMTASVTNPFLIVFPKTPIKRIFNTVGDIDVLALVSSK 6hd3.1    --------------------------------------------------------------------------------  target    LAELTGDTRFNDMWKFVREGRTDVYLQRILDASTNTKGYRFTELEAKAREGIPALMNSRTSPKVVGYDQLADSTPWYTKS 6hd3.1    --------------------------------------------------------------------------------  target    GRLEFYREEDEFIEAGENLPVHREPVDSTFYEPNVIVSPKHEAVRPSGPEDYGVARTDLSCEVRCGRNVVLTWAETRQTQ 6hd3.1    --------------------------------------------------------------------------------  target    HPLVKQGHKFIFHTPKYRHGSHTTPIDTDMNAVLFGPFGDIYRRDKRSPFVTEGYVDINPTDGLELGLQDGDYVWIDPDP 6hd3.1    -----------------------------------------------------SVVSLHPATMEKLQLFRGDTILIKGKK  target    EDRPFRGWQKNAKDMEFARLLCRARFYPGTPRGVTRMWFNMYGATPGSVRGAKARRDGLAKNPDTNYQAMFRSGSHQSAT 6hd3.1    --------------------------------------------------------------------------------  target    RGWLKPTWMTDSLVRKGLFGQGIGKGFLPDVHCPTGAPREAFVKISRAEPGGIGGQGLWRPAALGIRPRHESPAMKRYLA 6hd3.1    --------------------------------------------------------------------------------  target    GGFFSGPKE 6hd3.1    --------- ``` | | | | | | | | | | | | | | | | | | | | | | | | | | | | | | | | | | | | | | | | | | | | | | | | | |
|  | 7du7.1.A | mkDPBB\_sym1 protein  *Crystal structure of the rationally designed mkDPBB\_sym1 protein* | 0.00 |  | 32.00 | 0.04 | 373-397 | X-ray | 1.20 | monomer |  | HHblits | 0.37 |
| ``` target    FNGAPQYINENPFDLELDASRPARPRQYWRAESAHFYNHEDHPLRVGTRLLTGSTHMPTPTKVMWFANANSILGNVKWHY 7du7.1    --------------------------------------------------------------------------------  target    NTVVNALPRMEMIAVHEWWWTGSCEWADVVFGVDSWGELKHPDMTASVTNPFLIVFPKTPIKRIFNTVGDIDVLALVSSK 7du7.1    --------------------------------------------------------------------------------  target    LAELTGDTRFNDMWKFVREGRTDVYLQRILDASTNTKGYRFTELEAKAREGIPALMNSRTSPKVVGYDQLADSTPWYTKS 7du7.1    --------------------------------------------------------------------------------  target    GRLEFYREEDEFIEAGENLPVHREPVDSTFYEPNVIVSPKHEAVRPSGPEDYGVARTDLSCEVRCGRNVVLTWAETRQTQ 7du7.1    --------------------------------------------------------------------------------  target    HPLVKQGHKFIFHTPKYRHGSHTTPIDTDMNAVLFGPFGDIYRRDKRSPFVTEGYVDINPTDGLELGLQDGDYVWIDPDP 7du7.1    ----------------------------------------------------KGIVRMDKASRAKLGVSVGDYVEVK---  target    EDRPFRGWQKNAKDMEFARLLCRARFYPGTPRGVTRMWFNMYGATPGSVRGAKARRDGLAKNPDTNYQAMFRSGSHQSAT 7du7.1    --------------------------------------------------------------------------------  target    RGWLKPTWMTDSLVRKGLFGQGIGKGFLPDVHCPTGAPREAFVKISRAEPGGIGGQGLWRPAALGIRPRHESPAMKRYLA 7du7.1    --------------------------------------------------------------------------------  target    GGFFSGPKE 7du7.1    --------- ``` | | | | | | | | | | | | | | | | | | | | | | | | | | | | | | | | | | | | | | | | | | | | | | | | | |
|  | 7di1.1.A | mkDPBB\_sym\_86 protein  *Crystal structure of the rationally designed mkDPBB\_sym\_86 protein* | 0.00 |  | 32.00 | 0.04 | 373-397 | X-ray | 2.10 | monomer |  | HHblits | 0.37 |
| ``` target    FNGAPQYINENPFDLELDASRPARPRQYWRAESAHFYNHEDHPLRVGTRLLTGSTHMPTPTKVMWFANANSILGNVKWHY 7di1.1    --------------------------------------------------------------------------------  target    NTVVNALPRMEMIAVHEWWWTGSCEWADVVFGVDSWGELKHPDMTASVTNPFLIVFPKTPIKRIFNTVGDIDVLALVSSK 7di1.1    --------------------------------------------------------------------------------  target    LAELTGDTRFNDMWKFVREGRTDVYLQRILDASTNTKGYRFTELEAKAREGIPALMNSRTSPKVVGYDQLADSTPWYTKS 7di1.1    --------------------------------------------------------------------------------  target    GRLEFYREEDEFIEAGENLPVHREPVDSTFYEPNVIVSPKHEAVRPSGPEDYGVARTDLSCEVRCGRNVVLTWAETRQTQ 7di1.1    --------------------------------------------------------------------------------  target    HPLVKQGHKFIFHTPKYRHGSHTTPIDTDMNAVLFGPFGDIYRRDKRSPFVTEGYVDINPTDGLELGLQDGDYVWIDPDP 7di1.1    ----------------------------------------------------KGIVRMDKYERAKLGVSVGDYVEVK---  target    EDRPFRGWQKNAKDMEFARLLCRARFYPGTPRGVTRMWFNMYGATPGSVRGAKARRDGLAKNPDTNYQAMFRSGSHQSAT 7di1.1    --------------------------------------------------------------------------------  target    RGWLKPTWMTDSLVRKGLFGQGIGKGFLPDVHCPTGAPREAFVKISRAEPGGIGGQGLWRPAALGIRPRHESPAMKRYLA 7di1.1    --------------------------------------------------------------------------------  target    GGFFSGPKE 7di1.1    --------- ``` | | | | | | | | | | | | | | | | | | | | | | | | | | | | | | | | | | | | | | | | | | | | | | | | | |
|  | 7dg9.1.A | Cell division control protein 48, AAA family  *DPBB domain of VCP-like ATPase from Aeropyrum pernix* | 0.00 |  | 26.92 | 0.05 | 374-399 | X-ray | 1.60 | monomer | 4 x ZN | HHblits | 0.33 |
| ``` target    FNGAPQYINENPFDLELDASRPARPRQYWRAESAHFYNHEDHPLRVGTRLLTGSTHMPTPTKVMWFANANSILGNVKWHY 7dg9.1    --------------------------------------------------------------------------------  target    NTVVNALPRMEMIAVHEWWWTGSCEWADVVFGVDSWGELKHPDMTASVTNPFLIVFPKTPIKRIFNTVGDIDVLALVSSK 7dg9.1    --------------------------------------------------------------------------------  target    LAELTGDTRFNDMWKFVREGRTDVYLQRILDASTNTKGYRFTELEAKAREGIPALMNSRTSPKVVGYDQLADSTPWYTKS 7dg9.1    --------------------------------------------------------------------------------  target    GRLEFYREEDEFIEAGENLPVHREPVDSTFYEPNVIVSPKHEAVRPSGPEDYGVARTDLSCEVRCGRNVVLTWAETRQTQ 7dg9.1    --------------------------------------------------------------------------------  target    HPLVKQGHKFIFHTPKYRHGSHTTPIDTDMNAVLFGPFGDIYRRDKRSPFVTEGYVDINPTDGLELGLQDGDYVWIDPDP 7dg9.1    -----------------------------------------------------KIVRIDRQTAARLGVEVGDFVKVSKG-  target    EDRPFRGWQKNAKDMEFARLLCRARFYPGTPRGVTRMWFNMYGATPGSVRGAKARRDGLAKNPDTNYQAMFRSGSHQSAT 7dg9.1    --------------------------------------------------------------------------------  target    RGWLKPTWMTDSLVRKGLFGQGIGKGFLPDVHCPTGAPREAFVKISRAEPGGIGGQGLWRPAALGIRPRHESPAMKRYLA 7dg9.1    --------------------------------------------------------------------------------  target    GGFFSGPKE 7dg9.1    --------- ``` | | | | | | | | | | | | | | | | | | | | | | | | | | | | | | | | | | | | | | | | | | | | | | | | | |
|  | 7di1.1.A | mkDPBB\_sym\_86 protein  *Crystal structure of the rationally designed mkDPBB\_sym\_86 protein* | 0.00 |  | 26.92 | 0.05 | 374-399 | X-ray | 2.10 | monomer |  | HHblits | 0.33 |
| ``` target    FNGAPQYINENPFDLELDASRPARPRQYWRAESAHFYNHEDHPLRVGTRLLTGSTHMPTPTKVMWFANANSILGNVKWHY 7di1.1    --------------------------------------------------------------------------------  target    NTVVNALPRMEMIAVHEWWWTGSCEWADVVFGVDSWGELKHPDMTASVTNPFLIVFPKTPIKRIFNTVGDIDVLALVSSK 7di1.1    --------------------------------------------------------------------------------  target    LAELTGDTRFNDMWKFVREGRTDVYLQRILDASTNTKGYRFTELEAKAREGIPALMNSRTSPKVVGYDQLADSTPWYTKS 7di1.1    --------------------------------------------------------------------------------  target    GRLEFYREEDEFIEAGENLPVHREPVDSTFYEPNVIVSPKHEAVRPSGPEDYGVARTDLSCEVRCGRNVVLTWAETRQTQ 7di1.1    --------------------------------------------------------------------------------  target    HPLVKQGHKFIFHTPKYRHGSHTTPIDTDMNAVLFGPFGDIYRRDKRSPFVTEGYVDINPTDGLELGLQDGDYVWIDPDP 7di1.1    -----------------------------------------------------RIVRMDKASRAKLGVSVGDYVEVKKV-  target    EDRPFRGWQKNAKDMEFARLLCRARFYPGTPRGVTRMWFNMYGATPGSVRGAKARRDGLAKNPDTNYQAMFRSGSHQSAT 7di1.1    --------------------------------------------------------------------------------  target    RGWLKPTWMTDSLVRKGLFGQGIGKGFLPDVHCPTGAPREAFVKISRAEPGGIGGQGLWRPAALGIRPRHESPAMKRYLA 7di1.1    --------------------------------------------------------------------------------  target    GGFFSGPKE 7di1.1    --------- ``` | | | | | | | | | | | | | | | | | | | | | | | | | | | | | | | | | | | | | | | | | | | | | | | | | |
|  | 7dxs.1.A | ap1h protein  *Crystal structure of the ap1h peptide homodimer.* | 0.00 |  | 32.00 | 0.04 | 373-397 | X-ray | 2.10 | homo-dimer |  | HHblits | 0.36 |
| ``` target    FNGAPQYINENPFDLELDASRPARPRQYWRAESAHFYNHEDHPLRVGTRLLTGSTHMPTPTKVMWFANANSILGNVKWHY 7dxs.1    --------------------------------------------------------------------------------  target    NTVVNALPRMEMIAVHEWWWTGSCEWADVVFGVDSWGELKHPDMTASVTNPFLIVFPKTPIKRIFNTVGDIDVLALVSSK 7dxs.1    --------------------------------------------------------------------------------  target    LAELTGDTRFNDMWKFVREGRTDVYLQRILDASTNTKGYRFTELEAKAREGIPALMNSRTSPKVVGYDQLADSTPWYTKS 7dxs.1    --------------------------------------------------------------------------------  target    GRLEFYREEDEFIEAGENLPVHREPVDSTFYEPNVIVSPKHEAVRPSGPEDYGVARTDLSCEVRCGRNVVLTWAETRQTQ 7dxs.1    --------------------------------------------------------------------------------  target    HPLVKQGHKFIFHTPKYRHGSHTTPIDTDMNAVLFGPFGDIYRRDKRSPFVTEGYVDINPTDGLELGLQDGDYVWIDPDP 7dxs.1    ----------------------------------------------------RGIVRMDKQTRAKLGVSVGDYVEVK---  target    EDRPFRGWQKNAKDMEFARLLCRARFYPGTPRGVTRMWFNMYGATPGSVRGAKARRDGLAKNPDTNYQAMFRSGSHQSAT 7dxs.1    --------------------------------------------------------------------------------  target    RGWLKPTWMTDSLVRKGLFGQGIGKGFLPDVHCPTGAPREAFVKISRAEPGGIGGQGLWRPAALGIRPRHESPAMKRYLA 7dxs.1    --------------------------------------------------------------------------------  target    GGFFSGPKE 7dxs.1    --------- ``` | | | | | | | | | | | | | | | | | | | | | | | | | | | | | | | | | | | | | | | | | | | | | | | | | |
|  | 7dxs.1.B | ap1h protein  *Crystal structure of the ap1h peptide homodimer.* | 0.00 |  | 32.00 | 0.04 | 373-397 | X-ray | 2.10 | homo-dimer |  | HHblits | 0.36 |
| ``` target    FNGAPQYINENPFDLELDASRPARPRQYWRAESAHFYNHEDHPLRVGTRLLTGSTHMPTPTKVMWFANANSILGNVKWHY 7dxs.1    --------------------------------------------------------------------------------  target    NTVVNALPRMEMIAVHEWWWTGSCEWADVVFGVDSWGELKHPDMTASVTNPFLIVFPKTPIKRIFNTVGDIDVLALVSSK 7dxs.1    --------------------------------------------------------------------------------  target    LAELTGDTRFNDMWKFVREGRTDVYLQRILDASTNTKGYRFTELEAKAREGIPALMNSRTSPKVVGYDQLADSTPWYTKS 7dxs.1    --------------------------------------------------------------------------------  target    GRLEFYREEDEFIEAGENLPVHREPVDSTFYEPNVIVSPKHEAVRPSGPEDYGVARTDLSCEVRCGRNVVLTWAETRQTQ 7dxs.1    --------------------------------------------------------------------------------  target    HPLVKQGHKFIFHTPKYRHGSHTTPIDTDMNAVLFGPFGDIYRRDKRSPFVTEGYVDINPTDGLELGLQDGDYVWIDPDP 7dxs.1    ----------------------------------------------------RGIVRMDKQTRAKLGVSVGDYVEVK---  target    EDRPFRGWQKNAKDMEFARLLCRARFYPGTPRGVTRMWFNMYGATPGSVRGAKARRDGLAKNPDTNYQAMFRSGSHQSAT 7dxs.1    --------------------------------------------------------------------------------  target    RGWLKPTWMTDSLVRKGLFGQGIGKGFLPDVHCPTGAPREAFVKISRAEPGGIGGQGLWRPAALGIRPRHESPAMKRYLA 7dxs.1    --------------------------------------------------------------------------------  target    GGFFSGPKE 7dxs.1    --------- ``` | | | | | | | | | | | | | | | | | | | | | | | | | | | | | | | | | | | | | | | | | | | | | | | | | |
|  | 7dxs.2.A | ap1h protein  *Crystal structure of the ap1h peptide homodimer.* | 0.00 |  | 32.00 | 0.04 | 373-397 | X-ray | 2.10 | homo-dimer |  | HHblits | 0.36 |
| ``` target    FNGAPQYINENPFDLELDASRPARPRQYWRAESAHFYNHEDHPLRVGTRLLTGSTHMPTPTKVMWFANANSILGNVKWHY 7dxs.2    --------------------------------------------------------------------------------  target    NTVVNALPRMEMIAVHEWWWTGSCEWADVVFGVDSWGELKHPDMTASVTNPFLIVFPKTPIKRIFNTVGDIDVLALVSSK 7dxs.2    --------------------------------------------------------------------------------  target    LAELTGDTRFNDMWKFVREGRTDVYLQRILDASTNTKGYRFTELEAKAREGIPALMNSRTSPKVVGYDQLADSTPWYTKS 7dxs.2    --------------------------------------------------------------------------------  target    GRLEFYREEDEFIEAGENLPVHREPVDSTFYEPNVIVSPKHEAVRPSGPEDYGVARTDLSCEVRCGRNVVLTWAETRQTQ 7dxs.2    --------------------------------------------------------------------------------  target    HPLVKQGHKFIFHTPKYRHGSHTTPIDTDMNAVLFGPFGDIYRRDKRSPFVTEGYVDINPTDGLELGLQDGDYVWIDPDP 7dxs.2    ----------------------------------------------------RGIVRMDKQTRAKLGVSVGDYVEVK---  target    EDRPFRGWQKNAKDMEFARLLCRARFYPGTPRGVTRMWFNMYGATPGSVRGAKARRDGLAKNPDTNYQAMFRSGSHQSAT 7dxs.2    --------------------------------------------------------------------------------  target    RGWLKPTWMTDSLVRKGLFGQGIGKGFLPDVHCPTGAPREAFVKISRAEPGGIGGQGLWRPAALGIRPRHESPAMKRYLA 7dxs.2    --------------------------------------------------------------------------------  target    GGFFSGPKE 7dxs.2    --------- ``` | | | | | | | | | | | | | | | | | | | | | | | | | | | | | | | | | | | | | | | | | | | | | | | | | |
|  | 7dxs.2.B | ap1h protein  *Crystal structure of the ap1h peptide homodimer.* | 0.00 |  | 32.00 | 0.04 | 373-397 | X-ray | 2.10 | homo-dimer |  | HHblits | 0.36 |
| ``` target    FNGAPQYINENPFDLELDASRPARPRQYWRAESAHFYNHEDHPLRVGTRLLTGSTHMPTPTKVMWFANANSILGNVKWHY 7dxs.2    --------------------------------------------------------------------------------  target    NTVVNALPRMEMIAVHEWWWTGSCEWADVVFGVDSWGELKHPDMTASVTNPFLIVFPKTPIKRIFNTVGDIDVLALVSSK 7dxs.2    --------------------------------------------------------------------------------  target    LAELTGDTRFNDMWKFVREGRTDVYLQRILDASTNTKGYRFTELEAKAREGIPALMNSRTSPKVVGYDQLADSTPWYTKS 7dxs.2    --------------------------------------------------------------------------------  target    GRLEFYREEDEFIEAGENLPVHREPVDSTFYEPNVIVSPKHEAVRPSGPEDYGVARTDLSCEVRCGRNVVLTWAETRQTQ 7dxs.2    --------------------------------------------------------------------------------  target    HPLVKQGHKFIFHTPKYRHGSHTTPIDTDMNAVLFGPFGDIYRRDKRSPFVTEGYVDINPTDGLELGLQDGDYVWIDPDP 7dxs.2    ----------------------------------------------------RGIVRMDKQTRAKLGVSVGDYVEVK---  target    EDRPFRGWQKNAKDMEFARLLCRARFYPGTPRGVTRMWFNMYGATPGSVRGAKARRDGLAKNPDTNYQAMFRSGSHQSAT 7dxs.2    --------------------------------------------------------------------------------  target    RGWLKPTWMTDSLVRKGLFGQGIGKGFLPDVHCPTGAPREAFVKISRAEPGGIGGQGLWRPAALGIRPRHESPAMKRYLA 7dxs.2    --------------------------------------------------------------------------------  target    GGFFSGPKE 7dxs.2    --------- ``` | | | | | | | | | | | | | | | | | | | | | | | | | | | | | | | | | | | | | | | | | | | | | | | | | |
|  | 3hu1.1.A | Transitional endoplasmic reticulum ATPase  *Structure of p97 N-D1 R95G mutant in complex with ATPgS* | 0.00 |  | 25.93 | 0.05 | 374-400 | X-ray | 2.81 | homo-hexamer | 6 x AGS, 6 x MG | HHblits | 0.29 |
| ``` target    FNGAPQYINENPFDLELDASRPARPRQYWRAESAHFYNHEDHPLRVGTRLLTGSTHMPTPTKVMWFANANSILGNVKWHY 3hu1.1    --------------------------------------------------------------------------------  target    NTVVNALPRMEMIAVHEWWWTGSCEWADVVFGVDSWGELKHPDMTASVTNPFLIVFPKTPIKRIFNTVGDIDVLALVSSK 3hu1.1    --------------------------------------------------------------------------------  target    LAELTGDTRFNDMWKFVREGRTDVYLQRILDASTNTKGYRFTELEAKAREGIPALMNSRTSPKVVGYDQLADSTPWYTKS 3hu1.1    --------------------------------------------------------------------------------  target    GRLEFYREEDEFIEAGENLPVHREPVDSTFYEPNVIVSPKHEAVRPSGPEDYGVARTDLSCEVRCGRNVVLTWAETRQTQ 3hu1.1    --------------------------------------------------------------------------------  target    HPLVKQGHKFIFHTPKYRHGSHTTPIDTDMNAVLFGPFGDIYRRDKRSPFVTEGYVDINPTDGLELGLQDGDYVWIDPDP 3hu1.1    -----------------------------------------------------SVVSLSQPKMDELQLFRGDTVLLKGKK  target    EDRPFRGWQKNAKDMEFARLLCRARFYPGTPRGVTRMWFNMYGATPGSVRGAKARRDGLAKNPDTNYQAMFRSGSHQSAT 3hu1.1    --------------------------------------------------------------------------------  target    RGWLKPTWMTDSLVRKGLFGQGIGKGFLPDVHCPTGAPREAFVKISRAEPGGIGGQGLWRPAALGIRPRHESPAMKRYLA 3hu1.1    --------------------------------------------------------------------------------  target    GGFFSGPKE 3hu1.1    --------- ``` | | | | | | | | | | | | | | | | | | | | | | | | | | | | | | | | | | | | | | | | | | | | | | | | | |
|  | 7di0.1.A | apDPBB\_sym\_79 protein  *Crystal structure of the rationally designed apDPBB\_sym\_79 protein* | 0.00 |  | 26.92 | 0.05 | 374-399 | X-ray | 1.60 | monomer |  | HHblits | 0.32 |
| ``` target    FNGAPQYINENPFDLELDASRPARPRQYWRAESAHFYNHEDHPLRVGTRLLTGSTHMPTPTKVMWFANANSILGNVKWHY 7di0.1    --------------------------------------------------------------------------------  target    NTVVNALPRMEMIAVHEWWWTGSCEWADVVFGVDSWGELKHPDMTASVTNPFLIVFPKTPIKRIFNTVGDIDVLALVSSK 7di0.1    --------------------------------------------------------------------------------  target    LAELTGDTRFNDMWKFVREGRTDVYLQRILDASTNTKGYRFTELEAKAREGIPALMNSRTSPKVVGYDQLADSTPWYTKS 7di0.1    --------------------------------------------------------------------------------  target    GRLEFYREEDEFIEAGENLPVHREPVDSTFYEPNVIVSPKHEAVRPSGPEDYGVARTDLSCEVRCGRNVVLTWAETRQTQ 7di0.1    --------------------------------------------------------------------------------  target    HPLVKQGHKFIFHTPKYRHGSHTTPIDTDMNAVLFGPFGDIYRRDKRSPFVTEGYVDINPTDGLELGLQDGDYVWIDPDP 7di0.1    -----------------------------------------------------KIVRMDKQTRARLGVSVGDYVEVKKV-  target    EDRPFRGWQKNAKDMEFARLLCRARFYPGTPRGVTRMWFNMYGATPGSVRGAKARRDGLAKNPDTNYQAMFRSGSHQSAT 7di0.1    --------------------------------------------------------------------------------  target    RGWLKPTWMTDSLVRKGLFGQGIGKGFLPDVHCPTGAPREAFVKISRAEPGGIGGQGLWRPAALGIRPRHESPAMKRYLA 7di0.1    --------------------------------------------------------------------------------  target    GGFFSGPKE 7di0.1    --------- ``` | | | | | | | | | | | | | | | | | | | | | | | | | | | | | | | | | | | | | | | | | | | | | | | | | |
|  | 7di0.2.A | apDPBB\_sym\_79 protein  *Crystal structure of the rationally designed apDPBB\_sym\_79 protein* | 0.00 |  | 26.92 | 0.05 | 374-399 | X-ray | 1.60 | monomer |  | HHblits | 0.32 |
| ``` target    FNGAPQYINENPFDLELDASRPARPRQYWRAESAHFYNHEDHPLRVGTRLLTGSTHMPTPTKVMWFANANSILGNVKWHY 7di0.2    --------------------------------------------------------------------------------  target    NTVVNALPRMEMIAVHEWWWTGSCEWADVVFGVDSWGELKHPDMTASVTNPFLIVFPKTPIKRIFNTVGDIDVLALVSSK 7di0.2    --------------------------------------------------------------------------------  target    LAELTGDTRFNDMWKFVREGRTDVYLQRILDASTNTKGYRFTELEAKAREGIPALMNSRTSPKVVGYDQLADSTPWYTKS 7di0.2    --------------------------------------------------------------------------------  target    GRLEFYREEDEFIEAGENLPVHREPVDSTFYEPNVIVSPKHEAVRPSGPEDYGVARTDLSCEVRCGRNVVLTWAETRQTQ 7di0.2    --------------------------------------------------------------------------------  target    HPLVKQGHKFIFHTPKYRHGSHTTPIDTDMNAVLFGPFGDIYRRDKRSPFVTEGYVDINPTDGLELGLQDGDYVWIDPDP 7di0.2    -----------------------------------------------------KIVRMDKQTRARLGVSVGDYVEVKKV-  target    EDRPFRGWQKNAKDMEFARLLCRARFYPGTPRGVTRMWFNMYGATPGSVRGAKARRDGLAKNPDTNYQAMFRSGSHQSAT 7di0.2    --------------------------------------------------------------------------------  target    RGWLKPTWMTDSLVRKGLFGQGIGKGFLPDVHCPTGAPREAFVKISRAEPGGIGGQGLWRPAALGIRPRHESPAMKRYLA 7di0.2    --------------------------------------------------------------------------------  target    GGFFSGPKE 7di0.2    --------- ``` | | | | | | | | | | | | | | | | | | | | | | | | | | | | | | | | | | | | | | | | | | | | | | | | | |
|  | 7di0.3.A | apDPBB\_sym\_79 protein  *Crystal structure of the rationally designed apDPBB\_sym\_79 protein* | 0.00 |  | 26.92 | 0.05 | 374-399 | X-ray | 1.60 | monomer |  | HHblits | 0.32 |
| ``` target    FNGAPQYINENPFDLELDASRPARPRQYWRAESAHFYNHEDHPLRVGTRLLTGSTHMPTPTKVMWFANANSILGNVKWHY 7di0.3    --------------------------------------------------------------------------------  target    NTVVNALPRMEMIAVHEWWWTGSCEWADVVFGVDSWGELKHPDMTASVTNPFLIVFPKTPIKRIFNTVGDIDVLALVSSK 7di0.3    --------------------------------------------------------------------------------  target    LAELTGDTRFNDMWKFVREGRTDVYLQRILDASTNTKGYRFTELEAKAREGIPALMNSRTSPKVVGYDQLADSTPWYTKS 7di0.3    --------------------------------------------------------------------------------  target    GRLEFYREEDEFIEAGENLPVHREPVDSTFYEPNVIVSPKHEAVRPSGPEDYGVARTDLSCEVRCGRNVVLTWAETRQTQ 7di0.3    --------------------------------------------------------------------------------  target    HPLVKQGHKFIFHTPKYRHGSHTTPIDTDMNAVLFGPFGDIYRRDKRSPFVTEGYVDINPTDGLELGLQDGDYVWIDPDP 7di0.3    -----------------------------------------------------KIVRMDKQTRARLGVSVGDYVEVKKV-  target    EDRPFRGWQKNAKDMEFARLLCRARFYPGTPRGVTRMWFNMYGATPGSVRGAKARRDGLAKNPDTNYQAMFRSGSHQSAT 7di0.3    --------------------------------------------------------------------------------  target    RGWLKPTWMTDSLVRKGLFGQGIGKGFLPDVHCPTGAPREAFVKISRAEPGGIGGQGLWRPAALGIRPRHESPAMKRYLA 7di0.3    --------------------------------------------------------------------------------  target    GGFFSGPKE 7di0.3    --------- ``` | | | | | | | | | | | | | | | | | | | | | | | | | | | | | | | | | | | | | | | | | | | | | | | | | |
|  | 7dxy.1.A | mk2h\_deltaMILPS  *Crystal structure of the chemically synthesized mk2h\_deltaMILPS peptide homodimer* | 0.00 |  | 24.00 | 0.04 | 373-397 | X-ray | 1.40 | homo-dimer |  | HHblits | 0.34 |
| ``` target    FNGAPQYINENPFDLELDASRPARPRQYWRAESAHFYNHEDHPLRVGTRLLTGSTHMPTPTKVMWFANANSILGNVKWHY 7dxy.1    --------------------------------------------------------------------------------  target    NTVVNALPRMEMIAVHEWWWTGSCEWADVVFGVDSWGELKHPDMTASVTNPFLIVFPKTPIKRIFNTVGDIDVLALVSSK 7dxy.1    --------------------------------------------------------------------------------  target    LAELTGDTRFNDMWKFVREGRTDVYLQRILDASTNTKGYRFTELEAKAREGIPALMNSRTSPKVVGYDQLADSTPWYTKS 7dxy.1    --------------------------------------------------------------------------------  target    GRLEFYREEDEFIEAGENLPVHREPVDSTFYEPNVIVSPKHEAVRPSGPEDYGVARTDLSCEVRCGRNVVLTWAETRQTQ 7dxy.1    --------------------------------------------------------------------------------  target    HPLVKQGHKFIFHTPKYRHGSHTTPIDTDMNAVLFGPFGDIYRRDKRSPFVTEGYVDINPTDGLELGLQDGDYVWIDPDP 7dxy.1    ----------------------------------------------------KRVVRVDKYERAKVGVKVGDYVEVK---  target    EDRPFRGWQKNAKDMEFARLLCRARFYPGTPRGVTRMWFNMYGATPGSVRGAKARRDGLAKNPDTNYQAMFRSGSHQSAT 7dxy.1    --------------------------------------------------------------------------------  target    RGWLKPTWMTDSLVRKGLFGQGIGKGFLPDVHCPTGAPREAFVKISRAEPGGIGGQGLWRPAALGIRPRHESPAMKRYLA 7dxy.1    --------------------------------------------------------------------------------  target    GGFFSGPKE 7dxy.1    --------- ``` | | | | | | | | | | | | | | | | | | | | | | | | | | | | | | | | | | | | | | | | | | | | | | | | | |
|  | 7dxx.1.A | mk2h\_deltaMILPS protein  *Crystal structure of the mk2h\_deltaMILPS peptide homodimer* | 0.00 |  | 24.00 | 0.04 | 373-397 | X-ray | 1.40 | homo-dimer | 1 x MLA | HHblits | 0.34 |
| ``` target    FNGAPQYINENPFDLELDASRPARPRQYWRAESAHFYNHEDHPLRVGTRLLTGSTHMPTPTKVMWFANANSILGNVKWHY 7dxx.1    --------------------------------------------------------------------------------  target    NTVVNALPRMEMIAVHEWWWTGSCEWADVVFGVDSWGELKHPDMTASVTNPFLIVFPKTPIKRIFNTVGDIDVLALVSSK 7dxx.1    --------------------------------------------------------------------------------  target    LAELTGDTRFNDMWKFVREGRTDVYLQRILDASTNTKGYRFTELEAKAREGIPALMNSRTSPKVVGYDQLADSTPWYTKS 7dxx.1    --------------------------------------------------------------------------------  target    GRLEFYREEDEFIEAGENLPVHREPVDSTFYEPNVIVSPKHEAVRPSGPEDYGVARTDLSCEVRCGRNVVLTWAETRQTQ 7dxx.1    --------------------------------------------------------------------------------  target    HPLVKQGHKFIFHTPKYRHGSHTTPIDTDMNAVLFGPFGDIYRRDKRSPFVTEGYVDINPTDGLELGLQDGDYVWIDPDP 7dxx.1    ----------------------------------------------------KRVVRVDKYERAKVGVKVGDYVEVK---  target    EDRPFRGWQKNAKDMEFARLLCRARFYPGTPRGVTRMWFNMYGATPGSVRGAKARRDGLAKNPDTNYQAMFRSGSHQSAT 7dxx.1    --------------------------------------------------------------------------------  target    RGWLKPTWMTDSLVRKGLFGQGIGKGFLPDVHCPTGAPREAFVKISRAEPGGIGGQGLWRPAALGIRPRHESPAMKRYLA 7dxx.1    --------------------------------------------------------------------------------  target    GGFFSGPKE 7dxx.1    --------- ``` | | | | | | | | | | | | | | | | | | | | | | | | | | | | | | | | | | | | | | | | | | | | | | | | | |
|  | 7dxx.1.B | mk2h\_deltaMILPS protein  *Crystal structure of the mk2h\_deltaMILPS peptide homodimer* | 0.00 |  | 24.00 | 0.04 | 373-397 | X-ray | 1.40 | homo-dimer | 1 x MLA | HHblits | 0.34 |
| ``` target    FNGAPQYINENPFDLELDASRPARPRQYWRAESAHFYNHEDHPLRVGTRLLTGSTHMPTPTKVMWFANANSILGNVKWHY 7dxx.1    --------------------------------------------------------------------------------  target    NTVVNALPRMEMIAVHEWWWTGSCEWADVVFGVDSWGELKHPDMTASVTNPFLIVFPKTPIKRIFNTVGDIDVLALVSSK 7dxx.1    --------------------------------------------------------------------------------  target    LAELTGDTRFNDMWKFVREGRTDVYLQRILDASTNTKGYRFTELEAKAREGIPALMNSRTSPKVVGYDQLADSTPWYTKS 7dxx.1    --------------------------------------------------------------------------------  target    GRLEFYREEDEFIEAGENLPVHREPVDSTFYEPNVIVSPKHEAVRPSGPEDYGVARTDLSCEVRCGRNVVLTWAETRQTQ 7dxx.1    --------------------------------------------------------------------------------  target    HPLVKQGHKFIFHTPKYRHGSHTTPIDTDMNAVLFGPFGDIYRRDKRSPFVTEGYVDINPTDGLELGLQDGDYVWIDPDP 7dxx.1    ----------------------------------------------------KRVVRVDKYERAKVGVKVGDYVEVK---  target    EDRPFRGWQKNAKDMEFARLLCRARFYPGTPRGVTRMWFNMYGATPGSVRGAKARRDGLAKNPDTNYQAMFRSGSHQSAT 7dxx.1    --------------------------------------------------------------------------------  target    RGWLKPTWMTDSLVRKGLFGQGIGKGFLPDVHCPTGAPREAFVKISRAEPGGIGGQGLWRPAALGIRPRHESPAMKRYLA 7dxx.1    --------------------------------------------------------------------------------  target    GGFFSGPKE 7dxx.1    --------- ``` | | | | | | | | | | | | | | | | | | | | | | | | | | | | | | | | | | | | | | | | | | | | | | | | | |
|  | 7dxu.1.B | mk2h\_dP protein  *Crystal structure of the mk2h\_deltaP peptide homodimer* | 0.00 |  | 28.00 | 0.04 | 373-397 | X-ray | 2.31 | homo-dimer |  | HHblits | 0.34 |
| ``` target    FNGAPQYINENPFDLELDASRPARPRQYWRAESAHFYNHEDHPLRVGTRLLTGSTHMPTPTKVMWFANANSILGNVKWHY 7dxu.1    --------------------------------------------------------------------------------  target    NTVVNALPRMEMIAVHEWWWTGSCEWADVVFGVDSWGELKHPDMTASVTNPFLIVFPKTPIKRIFNTVGDIDVLALVSSK 7dxu.1    --------------------------------------------------------------------------------  target    LAELTGDTRFNDMWKFVREGRTDVYLQRILDASTNTKGYRFTELEAKAREGIPALMNSRTSPKVVGYDQLADSTPWYTKS 7dxu.1    --------------------------------------------------------------------------------  target    GRLEFYREEDEFIEAGENLPVHREPVDSTFYEPNVIVSPKHEAVRPSGPEDYGVARTDLSCEVRCGRNVVLTWAETRQTQ 7dxu.1    --------------------------------------------------------------------------------  target    HPLVKQGHKFIFHTPKYRHGSHTTPIDTDMNAVLFGPFGDIYRRDKRSPFVTEGYVDINPTDGLELGLQDGDYVWIDPDP 7dxu.1    ----------------------------------------------------KRIVRMDKYERAKLGVSVGDYVEVK---  target    EDRPFRGWQKNAKDMEFARLLCRARFYPGTPRGVTRMWFNMYGATPGSVRGAKARRDGLAKNPDTNYQAMFRSGSHQSAT 7dxu.1    --------------------------------------------------------------------------------  target    RGWLKPTWMTDSLVRKGLFGQGIGKGFLPDVHCPTGAPREAFVKISRAEPGGIGGQGLWRPAALGIRPRHESPAMKRYLA 7dxu.1    --------------------------------------------------------------------------------  target    GGFFSGPKE 7dxu.1    --------- ``` | | | | | | | | | | | | | | | | | | | | | | | | | | | | | | | | | | | | | | | | | | | | | | | | | |
|  | 7dxu.1.A | mk2h\_dP protein  *Crystal structure of the mk2h\_deltaP peptide homodimer* | 0.00 |  | 28.00 | 0.04 | 373-397 | X-ray | 2.31 | homo-dimer |  | HHblits | 0.34 |
| ``` target    FNGAPQYINENPFDLELDASRPARPRQYWRAESAHFYNHEDHPLRVGTRLLTGSTHMPTPTKVMWFANANSILGNVKWHY 7dxu.1    --------------------------------------------------------------------------------  target    NTVVNALPRMEMIAVHEWWWTGSCEWADVVFGVDSWGELKHPDMTASVTNPFLIVFPKTPIKRIFNTVGDIDVLALVSSK 7dxu.1    --------------------------------------------------------------------------------  target    LAELTGDTRFNDMWKFVREGRTDVYLQRILDASTNTKGYRFTELEAKAREGIPALMNSRTSPKVVGYDQLADSTPWYTKS 7dxu.1    --------------------------------------------------------------------------------  target    GRLEFYREEDEFIEAGENLPVHREPVDSTFYEPNVIVSPKHEAVRPSGPEDYGVARTDLSCEVRCGRNVVLTWAETRQTQ 7dxu.1    --------------------------------------------------------------------------------  target    HPLVKQGHKFIFHTPKYRHGSHTTPIDTDMNAVLFGPFGDIYRRDKRSPFVTEGYVDINPTDGLELGLQDGDYVWIDPDP 7dxu.1    ----------------------------------------------------KRIVRMDKYERAKLGVSVGDYVEVK---  target    EDRPFRGWQKNAKDMEFARLLCRARFYPGTPRGVTRMWFNMYGATPGSVRGAKARRDGLAKNPDTNYQAMFRSGSHQSAT 7dxu.1    --------------------------------------------------------------------------------  target    RGWLKPTWMTDSLVRKGLFGQGIGKGFLPDVHCPTGAPREAFVKISRAEPGGIGGQGLWRPAALGIRPRHESPAMKRYLA 7dxu.1    --------------------------------------------------------------------------------  target    GGFFSGPKE 7dxu.1    --------- ``` | | | | | | | | | | | | | | | | | | | | | | | | | | | | | | | | | | | | | | | | | | | | | | | | | |
|  | 7dxu.2.B | mk2h\_dP protein  *Crystal structure of the mk2h\_deltaP peptide homodimer* | 0.00 |  | 28.00 | 0.04 | 373-397 | X-ray | 2.31 | homo-dimer |  | HHblits | 0.34 |
| ``` target    FNGAPQYINENPFDLELDASRPARPRQYWRAESAHFYNHEDHPLRVGTRLLTGSTHMPTPTKVMWFANANSILGNVKWHY 7dxu.2    --------------------------------------------------------------------------------  target    NTVVNALPRMEMIAVHEWWWTGSCEWADVVFGVDSWGELKHPDMTASVTNPFLIVFPKTPIKRIFNTVGDIDVLALVSSK 7dxu.2    --------------------------------------------------------------------------------  target    LAELTGDTRFNDMWKFVREGRTDVYLQRILDASTNTKGYRFTELEAKAREGIPALMNSRTSPKVVGYDQLADSTPWYTKS 7dxu.2    --------------------------------------------------------------------------------  target    GRLEFYREEDEFIEAGENLPVHREPVDSTFYEPNVIVSPKHEAVRPSGPEDYGVARTDLSCEVRCGRNVVLTWAETRQTQ 7dxu.2    --------------------------------------------------------------------------------  target    HPLVKQGHKFIFHTPKYRHGSHTTPIDTDMNAVLFGPFGDIYRRDKRSPFVTEGYVDINPTDGLELGLQDGDYVWIDPDP 7dxu.2    ----------------------------------------------------KRIVRMDKYERAKLGVSVGDYVEVK---  target    EDRPFRGWQKNAKDMEFARLLCRARFYPGTPRGVTRMWFNMYGATPGSVRGAKARRDGLAKNPDTNYQAMFRSGSHQSAT 7dxu.2    --------------------------------------------------------------------------------  target    RGWLKPTWMTDSLVRKGLFGQGIGKGFLPDVHCPTGAPREAFVKISRAEPGGIGGQGLWRPAALGIRPRHESPAMKRYLA 7dxu.2    --------------------------------------------------------------------------------  target    GGFFSGPKE 7dxu.2    --------- ``` | | | | | | | | | | | | | | | | | | | | | | | | | | | | | | | | | | | | | | | | | | | | | | | | | |
|  | 7dxr.1.B | mk2h protein  *Crystal structure of the mk2h peptide homodimer.* | 0.00 |  | 28.00 | 0.04 | 373-397 | X-ray | 1.60 | homo-dimer |  | HHblits | 0.34 |
| ``` target    FNGAPQYINENPFDLELDASRPARPRQYWRAESAHFYNHEDHPLRVGTRLLTGSTHMPTPTKVMWFANANSILGNVKWHY 7dxr.1    --------------------------------------------------------------------------------  target    NTVVNALPRMEMIAVHEWWWTGSCEWADVVFGVDSWGELKHPDMTASVTNPFLIVFPKTPIKRIFNTVGDIDVLALVSSK 7dxr.1    --------------------------------------------------------------------------------  target    LAELTGDTRFNDMWKFVREGRTDVYLQRILDASTNTKGYRFTELEAKAREGIPALMNSRTSPKVVGYDQLADSTPWYTKS 7dxr.1    --------------------------------------------------------------------------------  target    GRLEFYREEDEFIEAGENLPVHREPVDSTFYEPNVIVSPKHEAVRPSGPEDYGVARTDLSCEVRCGRNVVLTWAETRQTQ 7dxr.1    --------------------------------------------------------------------------------  target    HPLVKQGHKFIFHTPKYRHGSHTTPIDTDMNAVLFGPFGDIYRRDKRSPFVTEGYVDINPTDGLELGLQDGDYVWIDPDP 7dxr.1    ----------------------------------------------------KRIVRMDKYERAKLGVSVGDYVEVK---  target    EDRPFRGWQKNAKDMEFARLLCRARFYPGTPRGVTRMWFNMYGATPGSVRGAKARRDGLAKNPDTNYQAMFRSGSHQSAT 7dxr.1    --------------------------------------------------------------------------------  target    RGWLKPTWMTDSLVRKGLFGQGIGKGFLPDVHCPTGAPREAFVKISRAEPGGIGGQGLWRPAALGIRPRHESPAMKRYLA 7dxr.1    --------------------------------------------------------------------------------  target    GGFFSGPKE 7dxr.1    --------- ``` | | | | | | | | | | | | | | | | | | | | | | | | | | | | | | | | | | | | | | | | | | | | | | | | | |
|  | 7dxr.1.A | mk2h protein  *Crystal structure of the mk2h peptide homodimer.* | 0.00 |  | 28.00 | 0.04 | 373-397 | X-ray | 1.60 | homo-dimer |  | HHblits | 0.34 |
| ``` target    FNGAPQYINENPFDLELDASRPARPRQYWRAESAHFYNHEDHPLRVGTRLLTGSTHMPTPTKVMWFANANSILGNVKWHY 7dxr.1    --------------------------------------------------------------------------------  target    NTVVNALPRMEMIAVHEWWWTGSCEWADVVFGVDSWGELKHPDMTASVTNPFLIVFPKTPIKRIFNTVGDIDVLALVSSK 7dxr.1    --------------------------------------------------------------------------------  target    LAELTGDTRFNDMWKFVREGRTDVYLQRILDASTNTKGYRFTELEAKAREGIPALMNSRTSPKVVGYDQLADSTPWYTKS 7dxr.1    --------------------------------------------------------------------------------  target    GRLEFYREEDEFIEAGENLPVHREPVDSTFYEPNVIVSPKHEAVRPSGPEDYGVARTDLSCEVRCGRNVVLTWAETRQTQ 7dxr.1    --------------------------------------------------------------------------------  target    HPLVKQGHKFIFHTPKYRHGSHTTPIDTDMNAVLFGPFGDIYRRDKRSPFVTEGYVDINPTDGLELGLQDGDYVWIDPDP 7dxr.1    ----------------------------------------------------KRIVRMDKYERAKLGVSVGDYVEVK---  target    EDRPFRGWQKNAKDMEFARLLCRARFYPGTPRGVTRMWFNMYGATPGSVRGAKARRDGLAKNPDTNYQAMFRSGSHQSAT 7dxr.1    --------------------------------------------------------------------------------  target    RGWLKPTWMTDSLVRKGLFGQGIGKGFLPDVHCPTGAPREAFVKISRAEPGGIGGQGLWRPAALGIRPRHESPAMKRYLA 7dxr.1    --------------------------------------------------------------------------------  target    GGFFSGPKE 7dxr.1    --------- ``` | | | | | | | | | | | | | | | | | | | | | | | | | | | | | | | | | | | | | | | | | | | | | | | | | |
|  | 7dxr.2.B | mk2h protein  *Crystal structure of the mk2h peptide homodimer.* | 0.00 |  | 28.00 | 0.04 | 373-397 | X-ray | 1.60 | homo-dimer | 1 x CXS | HHblits | 0.34 |
| ``` target    FNGAPQYINENPFDLELDASRPARPRQYWRAESAHFYNHEDHPLRVGTRLLTGSTHMPTPTKVMWFANANSILGNVKWHY 7dxr.2    --------------------------------------------------------------------------------  target    NTVVNALPRMEMIAVHEWWWTGSCEWADVVFGVDSWGELKHPDMTASVTNPFLIVFPKTPIKRIFNTVGDIDVLALVSSK 7dxr.2    --------------------------------------------------------------------------------  target    LAELTGDTRFNDMWKFVREGRTDVYLQRILDASTNTKGYRFTELEAKAREGIPALMNSRTSPKVVGYDQLADSTPWYTKS 7dxr.2    --------------------------------------------------------------------------------  target    GRLEFYREEDEFIEAGENLPVHREPVDSTFYEPNVIVSPKHEAVRPSGPEDYGVARTDLSCEVRCGRNVVLTWAETRQTQ 7dxr.2    --------------------------------------------------------------------------------  target    HPLVKQGHKFIFHTPKYRHGSHTTPIDTDMNAVLFGPFGDIYRRDKRSPFVTEGYVDINPTDGLELGLQDGDYVWIDPDP 7dxr.2    ----------------------------------------------------KRIVRMDKYERAKLGVSVGDYVEVK---  target    EDRPFRGWQKNAKDMEFARLLCRARFYPGTPRGVTRMWFNMYGATPGSVRGAKARRDGLAKNPDTNYQAMFRSGSHQSAT 7dxr.2    --------------------------------------------------------------------------------  target    RGWLKPTWMTDSLVRKGLFGQGIGKGFLPDVHCPTGAPREAFVKISRAEPGGIGGQGLWRPAALGIRPRHESPAMKRYLA 7dxr.2    --------------------------------------------------------------------------------  target    GGFFSGPKE 7dxr.2    --------- ``` | | | | | | | | | | | | | | | | | | | | | | | | | | | | | | | | | | | | | | | | | | | | | | | | | |
|  | 7dxt.1.A | mk2h protein  *Crystal structure of the chemically synthesized mk2h peptide homodimer* | 0.00 |  | 28.00 | 0.04 | 373-397 | X-ray | 1.80 | homo-dimer |  | HHblits | 0.34 |
| ``` target    FNGAPQYINENPFDLELDASRPARPRQYWRAESAHFYNHEDHPLRVGTRLLTGSTHMPTPTKVMWFANANSILGNVKWHY 7dxt.1    --------------------------------------------------------------------------------  target    NTVVNALPRMEMIAVHEWWWTGSCEWADVVFGVDSWGELKHPDMTASVTNPFLIVFPKTPIKRIFNTVGDIDVLALVSSK 7dxt.1    --------------------------------------------------------------------------------  target    LAELTGDTRFNDMWKFVREGRTDVYLQRILDASTNTKGYRFTELEAKAREGIPALMNSRTSPKVVGYDQLADSTPWYTKS 7dxt.1    --------------------------------------------------------------------------------  target    GRLEFYREEDEFIEAGENLPVHREPVDSTFYEPNVIVSPKHEAVRPSGPEDYGVARTDLSCEVRCGRNVVLTWAETRQTQ 7dxt.1    --------------------------------------------------------------------------------  target    HPLVKQGHKFIFHTPKYRHGSHTTPIDTDMNAVLFGPFGDIYRRDKRSPFVTEGYVDINPTDGLELGLQDGDYVWIDPDP 7dxt.1    ----------------------------------------------------KRIVRMDKYERAKLGVSVGDYVEVK---  target    EDRPFRGWQKNAKDMEFARLLCRARFYPGTPRGVTRMWFNMYGATPGSVRGAKARRDGLAKNPDTNYQAMFRSGSHQSAT 7dxt.1    --------------------------------------------------------------------------------  target    RGWLKPTWMTDSLVRKGLFGQGIGKGFLPDVHCPTGAPREAFVKISRAEPGGIGGQGLWRPAALGIRPRHESPAMKRYLA 7dxt.1    --------------------------------------------------------------------------------  target    GGFFSGPKE 7dxt.1    --------- ``` | | | | | | | | | | | | | | | | | | | | | | | | | | | | | | | | | | | | | | | | | | | | | | | | | |
|  | 7du6.1.A | mkDPBB\_sym2 protein  *Crystal structure of the rationally designed mkDPBB\_sym2 protein* | 0.00 |  | 28.00 | 0.04 | 373-397 | X-ray | 1.60 | monomer |  | HHblits | 0.34 |
| ``` target    FNGAPQYINENPFDLELDASRPARPRQYWRAESAHFYNHEDHPLRVGTRLLTGSTHMPTPTKVMWFANANSILGNVKWHY 7du6.1    --------------------------------------------------------------------------------  target    NTVVNALPRMEMIAVHEWWWTGSCEWADVVFGVDSWGELKHPDMTASVTNPFLIVFPKTPIKRIFNTVGDIDVLALVSSK 7du6.1    --------------------------------------------------------------------------------  target    LAELTGDTRFNDMWKFVREGRTDVYLQRILDASTNTKGYRFTELEAKAREGIPALMNSRTSPKVVGYDQLADSTPWYTKS 7du6.1    --------------------------------------------------------------------------------  target    GRLEFYREEDEFIEAGENLPVHREPVDSTFYEPNVIVSPKHEAVRPSGPEDYGVARTDLSCEVRCGRNVVLTWAETRQTQ 7du6.1    --------------------------------------------------------------------------------  target    HPLVKQGHKFIFHTPKYRHGSHTTPIDTDMNAVLFGPFGDIYRRDKRSPFVTEGYVDINPTDGLELGLQDGDYVWIDPDP 7du6.1    ----------------------------------------------------KRIVRMDKYERAKLGVSVGDYVEVK---  target    EDRPFRGWQKNAKDMEFARLLCRARFYPGTPRGVTRMWFNMYGATPGSVRGAKARRDGLAKNPDTNYQAMFRSGSHQSAT 7du6.1    --------------------------------------------------------------------------------  target    RGWLKPTWMTDSLVRKGLFGQGIGKGFLPDVHCPTGAPREAFVKISRAEPGGIGGQGLWRPAALGIRPRHESPAMKRYLA 7du6.1    --------------------------------------------------------------------------------  target    GGFFSGPKE 7du6.1    --------- ``` | | | | | | | | | | | | | | | | | | | | | | | | | | | | | | | | | | | | | | | | | | | | | | | | | |
|  | 7dvf.1.A | reDPBB\_sym2 protein  *Crystal structure of the computationally designed reDPBB\_sym2 protein* | 0.00 |  | 32.00 | 0.04 | 373-397 | X-ray | 1.21 | monomer |  | HHblits | 0.34 |
| ``` target    FNGAPQYINENPFDLELDASRPARPRQYWRAESAHFYNHEDHPLRVGTRLLTGSTHMPTPTKVMWFANANSILGNVKWHY 7dvf.1    --------------------------------------------------------------------------------  target    NTVVNALPRMEMIAVHEWWWTGSCEWADVVFGVDSWGELKHPDMTASVTNPFLIVFPKTPIKRIFNTVGDIDVLALVSSK 7dvf.1    --------------------------------------------------------------------------------  target    LAELTGDTRFNDMWKFVREGRTDVYLQRILDASTNTKGYRFTELEAKAREGIPALMNSRTSPKVVGYDQLADSTPWYTKS 7dvf.1    --------------------------------------------------------------------------------  target    GRLEFYREEDEFIEAGENLPVHREPVDSTFYEPNVIVSPKHEAVRPSGPEDYGVARTDLSCEVRCGRNVVLTWAETRQTQ 7dvf.1    --------------------------------------------------------------------------------  target    HPLVKQGHKFIFHTPKYRHGSHTTPIDTDMNAVLFGPFGDIYRRDKRSPFVTEGYVDINPTDGLELGLQDGDYVWIDPDP 7dvf.1    ----------------------------------------------------KGIVRMDKASREKLGVSAGDLVEIK---  target    EDRPFRGWQKNAKDMEFARLLCRARFYPGTPRGVTRMWFNMYGATPGSVRGAKARRDGLAKNPDTNYQAMFRSGSHQSAT 7dvf.1    --------------------------------------------------------------------------------  target    RGWLKPTWMTDSLVRKGLFGQGIGKGFLPDVHCPTGAPREAFVKISRAEPGGIGGQGLWRPAALGIRPRHESPAMKRYLA 7dvf.1    --------------------------------------------------------------------------------  target    GGFFSGPKE 7dvf.1    --------- ``` | | | | | | | | | | | | | | | | | | | | | | | | | | | | | | | | | | | | | | | | | | | | | | | | | |
|  | 7dxw.1.A | mk2h\_deltaMIL protein  *Crystal structure of the mk2h\_deltaMIL peptide homodimer* | 0.00 |  | 24.00 | 0.04 | 373-397 | X-ray | 1.51 | homo-dimer |  | HHblits | 0.34 |
| ``` target    FNGAPQYINENPFDLELDASRPARPRQYWRAESAHFYNHEDHPLRVGTRLLTGSTHMPTPTKVMWFANANSILGNVKWHY 7dxw.1    --------------------------------------------------------------------------------  target    NTVVNALPRMEMIAVHEWWWTGSCEWADVVFGVDSWGELKHPDMTASVTNPFLIVFPKTPIKRIFNTVGDIDVLALVSSK 7dxw.1    --------------------------------------------------------------------------------  target    LAELTGDTRFNDMWKFVREGRTDVYLQRILDASTNTKGYRFTELEAKAREGIPALMNSRTSPKVVGYDQLADSTPWYTKS 7dxw.1    --------------------------------------------------------------------------------  target    GRLEFYREEDEFIEAGENLPVHREPVDSTFYEPNVIVSPKHEAVRPSGPEDYGVARTDLSCEVRCGRNVVLTWAETRQTQ 7dxw.1    --------------------------------------------------------------------------------  target    HPLVKQGHKFIFHTPKYRHGSHTTPIDTDMNAVLFGPFGDIYRRDKRSPFVTEGYVDINPTDGLELGLQDGDYVWIDPDP 7dxw.1    ----------------------------------------------------KRVVRVDKYERAKVGVSVGDYVEVK---  target    EDRPFRGWQKNAKDMEFARLLCRARFYPGTPRGVTRMWFNMYGATPGSVRGAKARRDGLAKNPDTNYQAMFRSGSHQSAT 7dxw.1    --------------------------------------------------------------------------------  target    RGWLKPTWMTDSLVRKGLFGQGIGKGFLPDVHCPTGAPREAFVKISRAEPGGIGGQGLWRPAALGIRPRHESPAMKRYLA 7dxw.1    --------------------------------------------------------------------------------  target    GGFFSGPKE 7dxw.1    --------- ``` | | | | | | | | | | | | | | | | | | | | | | | | | | | | | | | | | | | | | | | | | | | | | | | | | |
|  | 7dvc.1.A | reDPBB\_sym1 protein  *Crystal structure of the computationally designed reDPBB\_sym1 protein* | 0.00 |  | 32.00 | 0.04 | 373-397 | X-ray | 1.71 | monomer |  | HHblits | 0.34 |
| ``` target    FNGAPQYINENPFDLELDASRPARPRQYWRAESAHFYNHEDHPLRVGTRLLTGSTHMPTPTKVMWFANANSILGNVKWHY 7dvc.1    --------------------------------------------------------------------------------  target    NTVVNALPRMEMIAVHEWWWTGSCEWADVVFGVDSWGELKHPDMTASVTNPFLIVFPKTPIKRIFNTVGDIDVLALVSSK 7dvc.1    --------------------------------------------------------------------------------  target    LAELTGDTRFNDMWKFVREGRTDVYLQRILDASTNTKGYRFTELEAKAREGIPALMNSRTSPKVVGYDQLADSTPWYTKS 7dvc.1    --------------------------------------------------------------------------------  target    GRLEFYREEDEFIEAGENLPVHREPVDSTFYEPNVIVSPKHEAVRPSGPEDYGVARTDLSCEVRCGRNVVLTWAETRQTQ 7dvc.1    --------------------------------------------------------------------------------  target    HPLVKQGHKFIFHTPKYRHGSHTTPIDTDMNAVLFGPFGDIYRRDKRSPFVTEGYVDINPTDGLELGLQDGDYVWIDPDP 7dvc.1    ----------------------------------------------------KGIVRMDKASRDKLGVSAGDLVEIK---  target    EDRPFRGWQKNAKDMEFARLLCRARFYPGTPRGVTRMWFNMYGATPGSVRGAKARRDGLAKNPDTNYQAMFRSGSHQSAT 7dvc.1    --------------------------------------------------------------------------------  target    RGWLKPTWMTDSLVRKGLFGQGIGKGFLPDVHCPTGAPREAFVKISRAEPGGIGGQGLWRPAALGIRPRHESPAMKRYLA 7dvc.1    --------------------------------------------------------------------------------  target    GGFFSGPKE 7dvc.1    --------- ``` | | | | | | | | | | | | | | | | | | | | | | | | | | | | | | | | | | | | | | | | | | | | | | | | | |
|  | 7dvc.5.A | reDPBB\_sym1 protein  *Crystal structure of the computationally designed reDPBB\_sym1 protein* | 0.00 |  | 32.00 | 0.04 | 373-397 | X-ray | 1.71 | monomer |  | HHblits | 0.34 |
[truncated: 50,510 more chars]
