# Supplementary material for: Metabolic capacity is maintained despite shifts in microbial diversity in estuary sediments
Source: ISME Commun. 2025 Oct 11;5(1):ycaf182. doi: 10.1093/ismeco/ycaf182 (PMC12687941; doi:10.1093/ismeco/ycaf182)
Supplement: Supplementary_Data_1_ycaf182 [file supplementary_data_1_ycaf182.zip › SWISS-MODEL/4_1_Jan_SF_Bin21_scaffold_19364_c121714_1/report.html]

4\_1\_Jan\_SF\_Bin21\_scaffold\_19364\_c1:2-1714\_1 | Report


|  |  |  |
| --- | --- | --- |
|  |  | SWISS-MODEL Homology Modelling Report |

## Model Building Report

This document lists the results for the homology modelling project "4\_1\_Jan\_SF\_Bin21\_scaffold\_19364\_c1:2-1714\_1" submitted to SWISS-MODEL workspace
on March 29, 2023, 5:45 p.m..The submitted primary amino acid sequence is given in Table T1.

If you use any results in your research, please cite the relevant publications:

- Waterhouse, A., Bertoni, M., Bienert, S., Studer, G., Tauriello, G., Gumienny, R.,
  Heer, F.T., de Beer, T.A.P., Rempfer, C., Bordoli, L., Lepore, R., Schwede, T.
  SWISS-MODEL: homology modelling of protein structures and complexes.
  Nucleic Acids Res. 46(W1), W296-W303 (2018).
- Bienert, S., Waterhouse, A., de Beer, T.A.P., Tauriello, G., Studer,
  G., Bordoli, L., Schwede, T. The SWISS-MODEL Repository - new features and
  functionality. Nucleic Acids Res. 45, D313-D319 (2017).
- Studer, G., Tauriello, G., Bienert, S.,
  Biasini, M., Johner, N., Schwede, T. ProMod3 - A versatile homology
  modelling toolbox. PLOS Comp. Biol. 17(1), e1008667 (2021).
- Studer, G., Rempfer, C., Waterhouse, A.M.,
  Gumienny, G., Haas, J., Schwede, T. QMEANDisCo - distance constraints
  applied on model quality estimation. Bioinformatics 36, 1765-1771 (2020).
- Bertoni, M., Kiefer, F., Biasini, M., Bordoli, L.,
  Schwede, T. Modeling protein quaternary structure of homo- and
  hetero-oligomers beyond binary interactions by homology. Scientific
  Reports 7 (2017).

## Results

The SWISS-MODEL template library (SMTL version 2023-03-23, PDB release 2023-03-17) was searched with
for evolutionary related structures matching the target sequence in Table T1. For details on the template search, see Materials and Methods. Overall 266 templates were found (Table T2).

## Models

The following models were built (see Materials and Methods "Model Building"):

| Model #01 | File | Built with | Oligo-State | Ligands | GMQE | QMEANDisCo Global |
| --- | --- | --- | --- | --- | --- | --- |
|  | PDB | ProMod3 3.2.1 | monomer | None | 0.71 | 0.69 ± 0.05 |

|  |  |  |
| --- | --- | --- |
|  |  |  |

| Template | Seq Identity | Oligo-state | QSQE | Found by | Method | Resolution | Seq Similarity | Range | Coverage | Description |
| --- | --- | --- | --- | --- | --- | --- | --- | --- | --- | --- |
| 7b04.1.B | 39.29 | monomer | 0.00 | HHblits | X-ray | 2.97Å | 0.39 | 28 - 567 | 0.93 | Nitrite oxidoreductase subunit A |

  

### Excluded ligands

| Ligand Name.Number | Reason for Exclusion | Description |
| --- | --- | --- |
| CA.10 | Binding site not conserved. | CALCIUM ION |
| CA.11 | Binding site not conserved. | CALCIUM ION |
| F3S.4 | Binding site not conserved. | FE3-S4 CLUSTER |
| HEM.9 | Binding site not conserved. | PROTOPORPHYRIN IX CONTAINING FE |
| MD1.5 | Binding site not conserved. | PHOSPHORIC ACID 4-(2-AMINO-4-OXO-3,4,5,6,-TETRAHYDRO-PTERIDIN-6-YL)-2-HYDROXY-3,4-DIMERCAPTO-BUT-3-EN-YL ESTER GUANYLATE ESTER |
| MD1.6 | Binding site not conserved. | PHOSPHORIC ACID 4-(2-AMINO-4-OXO-3,4,5,6,-TETRAHYDRO-PTERIDIN-6-YL)-2-HYDROXY-3,4-DIMERCAPTO-BUT-3-EN-YL ESTER GUANYLATE ESTER |
| MO.7 | Binding site not conserved. | MOLYBDENUM ATOM |
| SF4.1 | Binding site not conserved. | IRON/SULFUR CLUSTER |
| SF4.2 | Binding site not conserved. | IRON/SULFUR CLUSTER |
| SF4.3 | Binding site not conserved. | IRON/SULFUR CLUSTER |
| SF4.8 | Binding site not conserved. | IRON/SULFUR CLUSTER |

  

```
Target    FNGAPQYINENPFDLELDASRPARPRQYWRAESAHFYNHEDHP-----LRVGTRLLTGSTHMPTPTKVMWFANANSILGN  
7b04.1.B  ---------------------------RALDEEVAYWNHSERPLIVNTPKYGRKVFTGKTHMPSPTKVLWFTNVNLINN-  
  
Target    VKWHYNTVVNA-LPRMEMIAVHEWWWTGSCEWADVVFGVDSWGELKHPDMTASVTNPFLIVFPKTPIKRIFNTVGDIDVL  
7b04.1.B  -AKHVYQMLKNVNPNIEQIMSTDIEITGSIEYADFAFPANSWVEFQEFEITNSCSNPFIQIWGKTGITPVYESKDDVKIL  
  
Target    ALVSSKLAELTGDTRFNDMWKFVREGRTDVYLQRILDASTNTKGYRFTELEAKARE--GIPALMNSRTSPKVVGYDQLAD  
7b04.1.B  AGMASKLGELLRDKRFEDNWKFAIEGRASVYINRLLDGSTTMKGYTCEDILNGKYGEPGVAM-LLFRTYPRHPFWEQVHE  
  
Target    STPWYTKSGRLEFYREEDEFIEAGENLPVHREPVDSTFYEPNVIVSPKHEAVRPSGPEDYGVARTDLSCEVRCGRNVVLT  
7b04.1.B  SLPFYTPTGRLQAYNDEPEIIEYGENFIVHREGPEATPYLPNAIVSTN-PY---IRPDDYGIPENAEYWEDRTVRNIKKS  
  
Target    WAETRQTQHPLVKQGHKFIFHTPKYRHGSHTTPIDTDMNAVLFGPFGDIYRRDKRSPFVTEGYVDINPTDGLELGLQDGD  
7b04.1.B  WEETKKTKNFLWEKGYHFYCVTPKSRHTVHSQWAVTDWNFIWNNNFGDPYRMDKRMPGVGEHQIHIHPQAARDLGIEDGD  
  
Target    YVWIDPDPEDRPFRGWQKNAKDMEFARLLCRARFYPGTPRGVTRMWFNMYGATPGSVRGAKARRDGLAKNPDTNYQAMFR  
7b04.1.B  YVYVDANPADRPYEGWKPNDSFYKVSRLMLRAKYNPAYPYNCTMMKHSAWISSDKTVQAHETRPDGRALSP-SGYQSSFR  
  
Target    SGSHQSATRGWLKPTWMTDSLVRKGLFGQGIGKGFLPDVHCPTGAPREAFVKISRAEPGGIGGQGLWRPAALGIRPRHES  
7b04.1.B  YGSQQSITRDWSMPMHQLDSLFHKAKIGMKFIFGFEADNHCINTVPKETLVKITKAENGGMGGKGVWDPVKTGYTAGNEN  
  
Target    PAMKRYLAGGFFSGPKE  
7b04.1.B  DFMKKFLNGELIKVD--
```

  


---

  

| Model #02 | File | Built with | Oligo-State | Ligands | GMQE | QMEANDisCo Global |
| --- | --- | --- | --- | --- | --- | --- |
|  | PDB | ProMod3 3.2.1 | monomer | None | 0.36 | 0.46 ± 0.05 |

|  |  |  |
| --- | --- | --- |
|  |  |  |

| Template | Seq Identity | Oligo-state | QSQE | Found by | Method | Resolution | Seq Similarity | Range | Coverage | Description |
| --- | --- | --- | --- | --- | --- | --- | --- | --- | --- | --- |
| 1q16.1.A | 20.54 | monomer | 0.00 | HHblits | X-ray | 1.90Å | 0.31 | 60 - 534 | 0.65 | Respiratory nitrate reductase 1 alpha chain |

  

### Excluded ligands

| Ligand Name.Number | Reason for Exclusion | Description |
| --- | --- | --- |
| 3PH.12 | Not in contact with model. | 1,2-DIACYL-GLYCEROL-3-SN-PHOSPHATE |
| 6MO.3 | Binding site not conserved. | MOLYBDENUM(VI) ION |
| AGA.11 | Binding site not conserved. | (1S)-2-{[{[(2S)-2,3-DIHYDROXYPROPYL]OXY}(HYDROXY)PHOSPHORYL]OXY}-1-[(PENTANOYLOXY)METHYL]ETHYL OCTANOATE |
| F3S.10 | Binding site not conserved. | FE3-S4 CLUSTER |
| HEM.4 | Binding site not conserved. | PROTOPORPHYRIN IX CONTAINING FE |
| HEM.5 | Binding site not conserved. | PROTOPORPHYRIN IX CONTAINING FE |
| MD1.1 | Binding site not conserved. | PHOSPHORIC ACID 4-(2-AMINO-4-OXO-3,4,5,6,-TETRAHYDRO-PTERIDIN-6-YL)-2-HYDROXY-3,4-DIMERCAPTO-BUT-3-EN-YL ESTER GUANYLATE ESTER |
| MD1.2 | Binding site not conserved. | PHOSPHORIC ACID 4-(2-AMINO-4-OXO-3,4,5,6,-TETRAHYDRO-PTERIDIN-6-YL)-2-HYDROXY-3,4-DIMERCAPTO-BUT-3-EN-YL ESTER GUANYLATE ESTER |
| SF4.6 | Binding site not conserved. | IRON/SULFUR CLUSTER |
| SF4.7 | Binding site not conserved. | IRON/SULFUR CLUSTER |
| SF4.8 | Binding site not conserved. | IRON/SULFUR CLUSTER |
| SF4.9 | Binding site not conserved. | IRON/SULFUR CLUSTER |

  

```
Target    FNGAPQYINENPFDLELDASRPARPRQYWRAESAHFYNHEDHPLRVGTRLLTGSTHMPTPTKVMWFANANSILGNVKWHY  
1q16.1.A  -----------------------------------------------------------HPRNLFIWRSNLLGS--SGKG  
  
Target    NT------------------------------V--VNALPRMEMIAVHEWWWTGSCEWADVVFGVDSWGELKHPDMTASV  
1q16.1.A  HEFMLKYLLGTEHGIQGKDLGQQGGVKPEEVDWQDNGLEGKLDLVVTLDFRLSSTCLYSDIILPTATWYEK--DDMNTSD  
  
Target    TNPFLIVFPKTPIKRIFNTVGDIDVLALVSSKLAELTG----DT----RF----------------NDMWK---------  
1q16.1.A  MHPFIHPLSA-AVDPAWEAKSDWEIYKAIAKKFSEVCVGHLGKETDIVTLPIQHDSAAELAQPLDVKDWKKGECDLIPGK  
  
Target    -----------------FVRE-G------------------RTDVYLQRI-------------------------LDAST  
1q16.1.A  TAPHIMVVERDYPATYERFTSIGPLMEKIGNGGKGIAWNTQSEMDLLRKLNYTKAEGPAKGQPMLNTAIDAAEMILTLAP  
  
Target    NTKG----YRFTELEAKAR-----------EGIPALMN------------------SRTSPKVVGYDQLADSTPWYTKSG  
1q16.1.A  ETNGQVAVKAWAALSEFTGRDHTHLALNKEDEKIRFRDIQAQPRKIISSPTWSGLEDEHVSYNAGYTNVHELIPWRTLSG  
  
Target    RLEFYREEDEFIEAGENLPVHREPVDSTFYEPNVIVSPKHEAVRPSGPEDYGVARTDLSCEVRCGRNVVLTWAETRQTQH  
1q16.1.A  RQQLYQDHQWMRDFGESLLVYRPPIDTRSV-----------------KEVIGQ-------------------------KS  
  
Target    PLVKQGHKFIFHTPKYRHGSHTTPIDTDMNAVLFGPFGDIYRRDKRSPFVTEGYVDINPTDGLELGLQDGDYVWIDPDPE  
1q16.1.A  NG-NQEKALNFLTPHQKWGIHSTYSDNLLMLT----------LG-----RGGPVVWLSEADAKDLGIADNDWIEVFNSN-  
  
Target    DRPFRGWQKNAKDMEFARLLCRARFYPGTPRGVTRMWFNMYGATPGSVRGAKARRDGLAKNPDTNYQAMFRSGSHQSATR  
1q16.1.A  ----------------GALTARAVVSQRVPAGMTMMYHAQE---------RIVNLPGS-----EI--TQQRGGIHNSVTR  
  
Target    GWLKPTWMTDSLVRKGLFGQGIGKGFLPDVHCPTGAPREAFVKISRAEPGGIGGQGLWRPAALGIRPRHESPAMKRYLAG  
1q16.1.A  ITPKPTHMI------GGYA---HLAYGFNYYGTVGSNRDEFVVVRKMKNIDWL---------------------------  
  
Target    GFFSGPKE  
1q16.1.A  --------
```

  


---

  

| Model #03 | File | Built with | Oligo-State | Ligands | GMQE | QMEANDisCo Global |
| --- | --- | --- | --- | --- | --- | --- |
|  | PDB | ProMod3 3.2.1 | monomer | None | 0.26 | 0.43 ± 0.05 |

|  |  |  |
| --- | --- | --- |
|  |  |  |

| Template | Seq Identity | Oligo-state | QSQE | Found by | Method | Resolution | Seq Similarity | Range | Coverage | Description |
| --- | --- | --- | --- | --- | --- | --- | --- | --- | --- | --- |
| 7p63.1.C | 16.25 | monomer | 0.00 | HHblits | EM | - | 0.27 | 60 - 441 | 0.49 | NADH-quinone oxidoreductase |

  

### Excluded ligands

| Ligand Name.Number | Reason for Exclusion | Description |
| --- | --- | --- |
| 3PE.15 | Binding site not conserved. | 1,2-Distearoyl-sn-glycerophosphoethanolamine |
| 3PE.16 | Binding site not conserved. | 1,2-Distearoyl-sn-glycerophosphoethanolamine |
| 3PE.18 | Binding site not conserved. | 1,2-Distearoyl-sn-glycerophosphoethanolamine |
| 3PE.19 | Binding site not conserved. | 1,2-Distearoyl-sn-glycerophosphoethanolamine |
| 3PE.20 | Binding site not conserved. | 1,2-Distearoyl-sn-glycerophosphoethanolamine |
| 3PE.21 | Binding site not conserved. | 1,2-Distearoyl-sn-glycerophosphoethanolamine |
| 3PE.24 | Binding site not conserved. | 1,2-Distearoyl-sn-glycerophosphoethanolamine |
| 3PE.25 | Binding site not conserved. | 1,2-Distearoyl-sn-glycerophosphoethanolamine |
| CA.9 | Binding site not conserved. | CALCIUM ION |
| DCQ.10 | Binding site not conserved. | 2-decyl-5,6-dimethoxy-3-methylcyclohexa-2,5-diene-1,4-dione |
| FES.4 | Binding site not conserved. | FE2/S2 (INORGANIC) CLUSTER |
| FES.8 | Binding site not conserved. | FE2/S2 (INORGANIC) CLUSTER |
| FMN.2 | Binding site not conserved. | FLAVIN MONONUCLEOTIDE |
| LFA.14 | Binding site not conserved. | EICOSANE |
| LFA.17 | Binding site not conserved. | EICOSANE |
| LFA.22 | Binding site not conserved. | EICOSANE |
| LFA.23 | Binding site not conserved. | EICOSANE |
| NAI.3 | Binding site not conserved. | 1,4-DIHYDRONICOTINAMIDE ADENINE DINUCLEOTIDE |
| SF4.1 | Binding site not conserved. | IRON/SULFUR CLUSTER |
| SF4.5 | Binding site not conserved. | IRON/SULFUR CLUSTER |
| SF4.6 | Binding site not conserved. | IRON/SULFUR CLUSTER |
| SF4.7 | Binding site not conserved. | IRON/SULFUR CLUSTER |
| SF4.11 | Binding site not conserved. | IRON/SULFUR CLUSTER |
| SF4.12 | Binding site not conserved. | IRON/SULFUR CLUSTER |
| SF4.13 | Binding site not conserved. | IRON/SULFUR CLUSTER |

  

```
Target    FNGAPQYINENPFDLELDASRPARPRQYWRAESAHFYNHEDHPLRVGTRLLTGSTHMPTPTKVMWFANANSILGNVKWHY  
7p63.1.C  -----------------------------------------------------------RADAVVVLE-NDLHR--HASA  
  
Target    NTVVNALPRMEMIAVHEWWWTGSCEWADVVFGVDSWGELKHPDMTASVTNPFLIVFPKTPIKRIF-----NTVGDIDVLA  
7p63.1.C  TRVNAALAKAPLVMVVDHQRTAIMENAHLVLSAASFAESD--GTVI-NNEGRAQRFFQ-VYDPAYYDSKTVMLESWRWLH  
  
Target    LVSSKLAELTGDTRFNDMWKFVREGRTDVYLQRILDASTNTKGYRFTELEAKAR----------------------EGIP  
7p63.1.C  SLHSTLLS----REV--DWTQL-----DHVIDAVVAKIPELAGIKDAAPDATFRIRGQKLAREPHRYSGRTAMRANISVH  
  
Target    ALMN--------------------S---RTSPKVVGYDQL-ADSTPWYTKSGRLEFYREEDEFIEAG-ENLPVHREPVDS  
7p63.1.C  EPRQPQDIDTMFTFSMEGNNQPTAHRSQVPFAWAPGWNSPQAWNKFQDEVGGKLRFGDPGVRLFETSENGLDYFTSVPAR  
  
Target    TFYEPNVIVSPKHEAVRPSGPEDYGVARTDLSCEVRCGRNVVLTWAETRQTQHPLVKQGHKFIFHTPKYRHGSHTTPIDT  
7p63.1.C  ------------------------------------------------------FQPQDGKWRIAPYYHLFGSDELSQRA  
  
Target    DMNAVLFGPFGDIYRRDKRSPFVTEGYVDINPTDGLELGLQDGDYVWIDPDPEDRPFRGWQKNAKDMEFARLLCRARFYP  
7p63.1.C  PVFQ-------------SRMP---QPYIKLNPADAAKLGVNAGTRVSFSYDG-----------------NTVTLPVEIAE  
  
Target    GTPRGVTRMWFNMYGATPGSVRGAKARRDGLAKNPDTNYQAMFRSGSHQSATRGWLKPTWMTDSLVRKGLFGQGIGKGFL  
7p63.1.C  GLTAGQVGLPMGM-------------------------------------------------------------------  
  
Target    PDVHCPTGAPREAFVKISRAEPGGIGGQGLWRPAALGIRPRHESPAMKRYLAGGFFSGPKE  
7p63.1.C  -------------------------------------------------------------
```

  


---

  

## Materials and Methods

## Template Search

Template search with
has been performed against the SWISS-MODEL template library (SMTL, last update: 2023-03-23, last included PDB release: 2023-03-17).

## Template Selection

For each identified template, the template's quality has been predicted from features of the target-template alignment.
The templates with the highest quality have then been selected for model building.

## Model Building

Models are built based on the target-template alignment using ProMod3 (Studer et al.). Coordinates which are conserved between the target and the template are copied from the template to the model. Insertions and deletions are remodelled using a fragment library. Side chains are then rebuilt. Finally, the geometry of the resulting model is regularized by using a force field.

## Model Quality Estimation

The global and per-residue model quality has been assessed using the QMEAN scoring function (Studer et al.).

## Ligand Modelling

Ligands present in the template structure are transferred by homology to the model when the following criteria are met: (a) The ligands are annotated as biologically relevant in the template library, (b) the ligand is in contact with the model, (c) the ligand is not clashing with the protein, (d) the residues in contact with the ligand are conserved between the target and the template. If any of these four criteria is not satisfied, a certain ligand will not be included in the model. The model summary includes information on why and which ligand has not been included.

## Oligomeric State Conservation

The quaternary structure annotation of the template is used to model the target sequence in its oligomeric form. The method (Bertoni et al.) is based on a supervised machine learning algorithm, Support Vector Machines (SVM), which combines interface conservation, structural clustering, and other template features to provide a quaternary structure quality estimate (QSQE). The QSQE score is a number between 0 and 1, reflecting the expected accuracy of the interchain contacts for a model built based a given alignment and template. Higher numbers indicate higher reliability. This complements the GMQE score which estimates the accuracy of the tertiary structure of the resulting model.

## References

- **BLAST**  
  Camacho, C., Coulouris, G., Avagyan, V., Ma, N., Papadopoulos, J.,
  Bealer, K., Madden, T.L. BLAST+: architecture and applications. BMC
  Bioinformatics 10, 421-430 (2009).
- **HHblits**  
  Steinegger, M., Meier, M., Mirdita, M., Vöhringer, H., Haunsberger,
  S. J., Söding, J. HH-suite3 for fast remote homology detection and
  deep protein annotation. BMC Bioinformatics 20, 473 (2019).

## Table T1:

Primary amino acid sequence for which templates were searched and models were built.

FNGAPQYINENPFDLELDASRPARPRQYWRAESAHFYNHEDHPLRVGTRLLTGSTHMPTPTKVMWFANANSILGNVKWHYNTVVNALPRMEMIAVHEWWW  
TGSCEWADVVFGVDSWGELKHPDMTASVTNPFLIVFPKTPIKRIFNTVGDIDVLALVSSKLAELTGDTRFNDMWKFVREGRTDVYLQRILDASTNTKGYR  
FTELEAKAREGIPALMNSRTSPKVVGYDQLADSTPWYTKSGRLEFYREEDEFIEAGENLPVHREPVDSTFYEPNVIVSPKHEAVRPSGPEDYGVARTDLS  
CEVRCGRNVVLTWAETRQTQHPLVKQGHKFIFHTPKYRHGSHTTPIDTDMNAVLFGPFGDIYRRDKRSPFVTEGYVDINPTDGLELGLQDGDYVWIDPDP  
EDRPFRGWQKNAKDMEFARLLCRARFYPGTPRGVTRMWFNMYGATPGSVRGAKARRDGLAKNPDTNYQAMFRSGSHQSATRGWLKPTWMTDSLVRKGLFG  
QGIGKGFLPDVHCPTGAPREAFVKISRAEPGGIGGQGLWRPAALGIRPRHESPAMKRYLAGGFFSGPKE

## Table T2:

| Template | Seq Identity | Oligo-state | QSQE | Found by | Method | Resolution | Seq Similarity | Coverage | Description |
| --- | --- | --- | --- | --- | --- | --- | --- | --- | --- |
| 7b04.1.B | 39.29 | monomer | - | HHblits | X-ray | 2.97Å | 0.39 | 0.93 | Nitrite oxidoreductase subunit A |
| 7b04.1.B | 41.41 | monomer | - | BLAST | X-ray | 2.97Å | 0.40 | 0.92 | Nitrite oxidoreductase subunit A |
| 7b04.2.B | 39.29 | monomer | - | HHblits | X-ray | 2.97Å | 0.39 | 0.93 | Nitrite oxidoreductase subunit A |
| 7b04.2.B | 41.41 | monomer | - | BLAST | X-ray | 2.97Å | 0.40 | 0.92 | Nitrite oxidoreductase subunit A |
| 4ydd.1.A | 23.05 | monomer | - | HHblits | X-ray | 1.86Å | 0.33 | 0.61 | DMSO reductase family type II enzyme, molybdopterin subunit |
| 5e7o.1.A | 23.28 | monomer | - | HHblits | X-ray | 2.40Å | 0.33 | 0.61 | DMSO reductase family type II enzyme, molybdopterin subunit |
| 2ivf.1.A | 22.94 | monomer | - | HHblits | X-ray | 1.88Å | 0.31 | 0.60 | ETHYLBENZENE DEHYDROGENASE ALPHA-SUBUNIT |
| 1q16.1.A | 20.54 | monomer | - | HHblits | X-ray | 1.90Å | 0.31 | 0.65 | Respiratory nitrate reductase 1 alpha chain |
| 1r27.4.A | 20.60 | homo-dimer | 0.00 | HHblits | X-ray | 2.00Å | 0.31 | 0.65 | Respiratory nitrate reductase 1 alpha chain |
| 3ir5.1.A | 20.49 | monomer | - | HHblits | X-ray | 2.30Å | 0.31 | 0.65 | Respiratory nitrate reductase 1 alpha chain |
| 3egw.1.A | 20.65 | homo-dimer | 0.06 | HHblits | X-ray | 1.90Å | 0.31 | 0.65 | Respiratory nitrate reductase 1 alpha chain |
| 3ir6.1.A | 20.44 | monomer | - | HHblits | X-ray | 2.80Å | 0.31 | 0.64 | Respiratory nitrate reductase 1 alpha chain |
| 3ir7.1.A | 20.81 | monomer | - | HHblits | X-ray | 2.50Å | 0.31 | 0.65 | Respiratory nitrate reductase 1 alpha chain |
| 1e60.1.A | 17.01 | monomer | - | HHblits | X-ray | 2.00Å | 0.29 | 0.51 | Dimethyl sulfoxide/trimethylamine N-oxide reductase |
| 1e5v.2.A | 17.01 | monomer | - | HHblits | X-ray | 2.40Å | 0.29 | 0.51 | Dimethyl sulfoxide/trimethylamine N-oxide reductase |
| 1dms.1.A | 17.36 | monomer | - | HHblits | X-ray | 1.88Å | 0.29 | 0.51 | DMSO REDUCTASE |
| 4dmr.1.A | 17.01 | monomer | - | HHblits | X-ray | 1.90Å | 0.30 | 0.51 | DMSO REDUCTASE |
| 1e18.1.A | 17.77 | monomer | - | HHblits | X-ray | 2.00Å | 0.30 | 0.50 | DMSO REDUCTASE. |
| 2iv2.1.A | 15.97 | monomer | - | HHblits | X-ray | 2.27Å | 0.29 | 0.46 | Formate dehydrogenase H |
| 1aa6.1.A | 15.97 | monomer | - | HHblits | X-ray | 2.30Å | 0.29 | 0.46 | FORMATE DEHYDROGENASE H |
| 7z0t.1.G | 15.97 | monomer | - | HHblits | EM | NA | 0.29 | 0.46 | Formate dehydrogenase H |
| 7p63.1.C | 16.25 | monomer | - | HHblits | EM | NA | 0.27 | 0.49 | NADH-quinone oxidoreductase |
| 1fdo.1.A | 15.97 | monomer | - | HHblits | X-ray | 2.80Å | 0.29 | 0.46 | FORMATE DEHYDROGENASE H |
| 7nz1.1.E | 15.94 | monomer | - | HHblits | EM | NA | 0.27 | 0.49 | NADH-quinone oxidoreductase subunit G |
| 7p61.1.C | 15.88 | monomer | - | HHblits | EM | NA | 0.27 | 0.49 | NADH-quinone oxidoreductase |
| 4ydd.1.A | 26.37 | monomer | - | BLAST | X-ray | 1.86Å | 0.34 | 0.35 | DMSO reductase family type II enzyme, molybdopterin subunit |
| 5e7o.1.A | 26.37 | monomer | - | BLAST | X-ray | 2.40Å | 0.34 | 0.35 | DMSO reductase family type II enzyme, molybdopterin subunit |
| 6sdr.1.A | 14.85 | monomer | - | HHblits | X-ray | 2.10Å | 0.26 | 0.18 | Formate dehydrogenase, alpha subunit, selenocysteine-containing |
| 8bqg.1.A | 14.00 | monomer | - | HHblits | X-ray | 1.95Å | 0.26 | 0.18 | Formate dehydrogenase, alpha subunit, selenocysteine-containing |
| 6sdv.1.A | 14.00 | monomer | - | HHblits | X-ray | 1.90Å | 0.26 | 0.18 | Formate dehydrogenase, alpha subunit, selenocysteine-containing,Formate dehydrogenase, alpha subunit, selenocysteine-containing,W-formate dehydrogenase - alpha subunit |
| 7ak5.1.G | 13.98 | monomer | - | HHblits | EM | NA | 0.25 | 0.16 | NADH-ubiquinone oxidoreductase 75 kDa subunit, mitochondrial |
| 6gcs.1.A | 17.39 | monomer | - | HHblits | EM | 4.32Å | 0.28 | 0.16 | 75-KDA PROTEIN (NUAM) |
| 7qsd.1.G | 13.98 | monomer | - | HHblits | EM | NA | 0.26 | 0.16 | NADH-ubiquinone oxidoreductase 75 kDa subunit, mitochondrial |
| 7aqr.1.F | 12.09 | monomer | - | HHblits | EM | NA | 0.27 | 0.16 | NADH dehydrogenase [ubiquinone] iron-sulfur protein 1, mitochondrial |
| 6zk9.1.C | 14.13 | monomer | - | HHblits | EM | NA | 0.26 | 0.16 | NADH:ubiquinone oxidoreductase core subunit S1 |
| 7zd6.1.4 | 14.13 | monomer | - | HHblits | EM | NA | 0.26 | 0.16 | NADH-ubiquinone oxidoreductase 75 kDa subunit, mitochondrial |
| 7v2c.1.L | 15.22 | monomer | - | HHblits | EM | NA | 0.26 | 0.16 | NADH-ubiquinone oxidoreductase 75 kDa subunit, mitochondrial |
| 7vxu.1.L | 15.22 | monomer | - | HHblits | EM | NA | 0.26 | 0.16 | NADH-ubiquinone oxidoreductase 75 kDa subunit, mitochondrial |
| 6yj4.1.G | 17.39 | monomer | - | HHblits | EM | NA | 0.28 | 0.16 | Subunit NUAM of NADH:Ubiquinone Oxidoreductase (Complex I) |
| 6qc5.1.C | 13.98 | monomer | - | HHblits | EM | NA | 0.26 | 0.16 | NADH:ubiquinone oxidoreductase core subunit S1 |
| 6qcf.1.C | 13.98 | monomer | - | HHblits | EM | NA | 0.26 | 0.16 | NADH:ubiquinone oxidoreductase core subunit S1 |
| 7ar7.1.G | 12.09 | monomer | - | HHblits | EM | NA | 0.27 | 0.16 | NADH dehydrogenase [ubiquinone] iron-sulfur protein 1, mitochondrial |
| 6rfq.1.A | 17.39 | monomer | - | HHblits | EM | 3.30Å | 0.28 | 0.16 | Subunit NUAM of NADH:Ubiquinone Oxidoreductase (Complex I) |
| 6rfs.1.A | 17.39 | monomer | - | HHblits | EM | 4.04Å | 0.28 | 0.16 | Subunit NUAM of NADH:Ubiquinone Oxidoreductase (Complex I) |
| 5o31.1.8 | 13.98 | monomer | - | HHblits | EM | 4.13Å | 0.25 | 0.16 | NADH-ubiquinone oxidoreductase 75 kDa subunit, mitochondrial |
| 5gpn.24.A | 15.05 | monomer | - | HHblits | EM | NA | 0.26 | 0.16 | NADH-ubiquinone oxidoreductase 75 kDa subunit |
| 7a23.1.O | 12.09 | monomer | - | HHblits | EM | NA | 0.27 | 0.16 | 75kDa |
| 7ar8.1.G | 12.09 | monomer | - | HHblits | EM | NA | 0.27 | 0.16 | NADH dehydrogenase [ubiquinone] iron-sulfur protein 1, mitochondrial |
| 5xtb.1.L | 15.22 | monomer | - | HHblits | EM | NA | 0.26 | 0.16 | NADH-ubiquinone oxidoreductase 75 kDa subunit, mitochondrial |
| 7dgr.10.A | 13.98 | monomer | - | HHblits | EM | NA | 0.25 | 0.16 | NADH-ubiquinone oxidoreductase 75 kDa subunit, mitochondrial |

  
The table above shows the top 50 filtered templates. A further 171 templates were found which were considered to be less suitable for modelling than the filtered list.  
1aa6.1.A, 1cz4.1.A, 1cz5.1.A, 1dms.1.A, 1e18.1.A, 1e5v.2.A, 1e60.1.A, 1eu1.1.A, 1fdo.1.A, 1g8j.1.A, 1g8k.1.A, 1h0h.1.A, 1kqf.1.A, 1ogy.1.A, 1q16.1.A, 1r27.4.A, 1tmo.1.A, 1wlf.1.A, 2e7z.1.A, 2fug.2.C, 2iv2.1.A, 2ivf.1.A, 2ki8.1.A, 2nya.1.A, 2pjh.1.B, 2v3v.1.A, 2v45.1.A, 2vpx.1.D, 2vpz.1.A, 3egw.1.A, 3hu1.1.A, 3ir5.1.A, 3ir6.1.A, 3ir7.1.A, 3m9s.1.C, 3o27.1.A, 3o27.1.B, 3o5a.1.A, 3qc8.1.A, 3qq7.1.A, 3qq8.1.A, 3qwz.1.A, 3tiw.1.A, 3tiw.2.A, 4aay.1.A, 4dmr.1.A, 4ga5.1.A, 4ga6.1.A, 4kdi.1.A, 4kdi.2.A, 4kdl.1.A, 4rv0.1.A, 4v4c.1.A, 4ydd.1.A, 5b6c.1.A, 5cuo.1.A, 5cup.1.A, 5e7o.1.A, 5e7p.1.A, 5epp.1.A, 5g4f.1.A, 5g4f.1.B, 5g4f.1.C, 5g4f.1.D, 5g4f.1.E, 5g4f.1.F, 5g4g.1.A, 5glf.1.A, 5glf.2.A, 5glf.3.A, 5glf.4.A, 5nqd.1.A, 5t5i.1.B, 5t5i.1.D, 5x4l.1.A, 5x4l.2.A, 6btm.1.B, 6cz7.1.A, 6f0k.1.B, 6g72.1.G, 6gcs.1.A, 6hd3.1.A, 6lod.1.B, 6q8o.1.C, 6rfq.1.A, 6rfs.1.A, 6s6y.1.B, 6sdr.1.A, 6sdv.1.A, 6tg9.1.A, 6x89.1.H, 6yj4.1.G, 6ziy.1.C, 6zjl.1.C, 6zjn.1.C, 6zjy.1.C, 6zr2.1.G, 7ak6.1.G, 7arc.1.F, 7b04.1.B, 7b04.2.B, 7bkb.1.F, 7bkb.1.J, 7bkb.1.L, 7dbo.1.A, 7dbo.2.A, 7dg7.1.A, 7dg9.1.A, 7di0.1.A, 7di0.2.A, 7di0.3.A, 7di1.1.A, 7du6.1.A, 7du7.1.A, 7dvc.1.A, 7dvc.5.A, 7dvf.1.A, 7dvh.1.A, 7dvh.2.A, 7dvh.4.A, 7dww.1.A, 7dww.2.A, 7dxr.1.A, 7dxr.1.B, 7dxr.2.B, 7dxs.1.A, 7dxs.1.B, 7dxs.2.A, 7dxs.2.B, 7dxt.1.A, 7dxu.1.A, 7dxu.1.B, 7dxu.2.B, 7dxv.1.A, 7dxv.1.B, 7dxw.1.A, 7dxx.1.A, 7dxx.1.B, 7dxy.1.A, 7dxz.1.A, 7dxz.2.A, 7dxz.2.B, 7dxz.3.A, 7dyc.1.A, 7dyc.2.A, 7dyc.3.A, 7e5z.1.A, 7l5i.1.A, 7l5s.1.A, 7p61.1.C, 7p63.1.C, 7q5y.1.A, 7qv7.1.L, 7qv7.1.O, 7t2r.1.A, 7t30.1.A, 7tgh.58.A, 7vw6.1.A, 7wbb.1.A, 7wbb.1.B, 7wbb.1.C, 7wbb.1.D, 7wbb.1.E, 7wbb.1.G, 7z0t.1.G, 7zm7.1.I, 8b9z.1.G, 8ba0.1.G, 8bqg.1.A, 8e73.55.A, 8e9g.1.G

Swiss Institute of Bioinformatics
Contact Us
