# Supplementary material for: Metabolic capacity is maintained despite shifts in microbial diversity in estuary sediments
Source: ISME Commun. 2025 Oct 11;5(1):ycaf182. doi: 10.1093/ismeco/ycaf182 (PMC12687941; doi:10.1093/ismeco/ycaf182)
Supplement: Supplementary_Data_1_ycaf182 [file supplementary_data_1_ycaf182.zip › SWISS-MODEL/4_1_Jan_SF_Bin21_scaffold_19364_c121714_1/models.html]

4\_1\_Jan\_SF\_Bin21\_scaffold\_19364\_c1:2-1714\_1 | Models


**Export Alignment**
  
FASTA format
Clustal Format
PNG Image

**Secondary Structure**
  
None
DSSP
PSIPRED
SSpro

**Colour Scheme** 


Fade Mismatches
Enhance Mismatches

Confidencegradient
Confidenceclass
Indels
Chain
Unique Chain
Rainbow
2° Structure
Clustal
Hydrophobic
Size
Charged
Polar
Proline
Ser/Thr
Cysteine
Aliphatic
Aromatic
No Colour

Use QMEANBrane values

|  |  |  |  |
| --- | --- | --- | --- |
| Background |  |  |  |

**3D Viewer**  
NGL
PV

FASTA
Multi FASTA
ClustalW
PNG


SWISS-MODEL

### 4\_1\_Jan\_SF\_Bin21\_scaffold\_19364\_c1:2-1714\_1

### Created: March 29, 2023, 5:45 p.m. at 17:45

- Templates
- Models

Order by:
GMQE
QMEANDisCo
Oligo State
Ligands
Seq Identity
Similarity
Coverage

Model 01

- PDB Format *(Display)*
- JSON Format *(Display)*
- Model Report *(Display)*

Oligo-State
:   Monomer

GMQE
:   0.71

QMEANDisCo Global:
:   0.69  ± 0.05

Ligands

QMEANDisCo Local

QMEAN Z-Scores

Template

7b04.1.B

Nitrite oxidoreductase subunit A  
Structure of Nitrite oxidoreductase (Nxr) from the anammox bacterium Kuenenia stuttgartiensis.

Seq Identity
:   39.29%

Coverage

|  |  |
| --- | --- |
| **Biounit Oligo State** | Hetero-trimer |
| **QSQE** | 0.00 |
| **Method** | X-ray, 2.97 Å |
| **Seq Similarity** | 0.39 |
| **Coverage** | 0.93 |
| **Range** | 28-567 |

| Ligand | Added to Model | Description |
| --- | --- | --- |
| CA | ✕ - Binding site not conserved. | CALCIUM ION |
| CA | ✕ - Binding site not conserved. | CALCIUM ION |
| F3S | ✕ - Binding site not conserved. | FE3-S4 CLUSTER |
| HEM | ✕ - Binding site not conserved. | PROTOPORPHYRIN IX CONTAINING FE |
| MD1 | ✕ - Binding site not conserved. | PHOSPHORIC ACID 4-(2-AMINO-4-OXO-3,4,5,6,-TETRAHYDRO-PTERIDIN-6-YL)-2-HYDROXY-3,4-DIMERCAPTO-BUT-3-EN-YL ESTER GUANYLATE ESTER |
| MD1 | ✕ - Binding site not conserved. | PHOSPHORIC ACID 4-(2-AMINO-4-OXO-3,4,5,6,-TETRAHYDRO-PTERIDIN-6-YL)-2-HYDROXY-3,4-DIMERCAPTO-BUT-3-EN-YL ESTER GUANYLATE ESTER |
| MO | ✕ - Binding site not conserved. | MOLYBDENUM ATOM |
| SF4 | ✕ - Binding site not conserved. | IRON/SULFUR CLUSTER |
| SF4 | ✕ - Binding site not conserved. | IRON/SULFUR CLUSTER |
| SF4 | ✕ - Binding site not conserved. | IRON/SULFUR CLUSTER |
| SF4 | ✕ - Binding site not conserved. | IRON/SULFUR CLUSTER |

Model-Template Alignment

|  |  |  |
| --- | --- | --- |
|  |  |  |

Model 02

- PDB Format *(Display)*
- JSON Format *(Display)*
- Model Report *(Display)*

Oligo-State
:   Monomer

GMQE
:   0.36

QMEANDisCo Global:
:   0.46  ± 0.05

Ligands

QMEANDisCo Local

QMEAN Z-Scores

Template

1q16.1.A

Respiratory nitrate reductase 1 alpha chain  
Crystal structure of Nitrate Reductase A, NarGHI, from Escherichia coli

Seq Identity
:   20.54%

Coverage

|  |  |
| --- | --- |
| **Biounit Oligo State** | Hetero-trimer |
| **QSQE** | 0.00 |
| **Method** | X-ray, 1.90 Å |
| **Seq Similarity** | 0.31 |
| **Coverage** | 0.65 |
| **Range** | 60-534 |

| Ligand | Added to Model | Description |
| --- | --- | --- |
| 3PH | ✕ - Not in contact with model. | 1,2-DIACYL-GLYCEROL-3-SN-PHOSPHATE |
| 6MO | ✕ - Binding site not conserved. | MOLYBDENUM(VI) ION |
| AGA | ✕ - Binding site not conserved. | (1S)-2-{[{[(2S)-2,3-DIHYDROXYPROPYL]OXY}(HYDROXY)PHOSPHORYL]OXY}-1-[(PENTANOYLOXY)METHYL]ETHYL OCTANOATE |
| F3S | ✕ - Binding site not conserved. | FE3-S4 CLUSTER |
| HEM | ✕ - Binding site not conserved. | PROTOPORPHYRIN IX CONTAINING FE |
| HEM | ✕ - Binding site not conserved. | PROTOPORPHYRIN IX CONTAINING FE |
| MD1 | ✕ - Binding site not conserved. | PHOSPHORIC ACID 4-(2-AMINO-4-OXO-3,4,5,6,-TETRAHYDRO-PTERIDIN-6-YL)-2-HYDROXY-3,4-DIMERCAPTO-BUT-3-EN-YL ESTER GUANYLATE ESTER |
| MD1 | ✕ - Binding site not conserved. | PHOSPHORIC ACID 4-(2-AMINO-4-OXO-3,4,5,6,-TETRAHYDRO-PTERIDIN-6-YL)-2-HYDROXY-3,4-DIMERCAPTO-BUT-3-EN-YL ESTER GUANYLATE ESTER |
| SF4 | ✕ - Binding site not conserved. | IRON/SULFUR CLUSTER |
| SF4 | ✕ - Binding site not conserved. | IRON/SULFUR CLUSTER |
| SF4 | ✕ - Binding site not conserved. | IRON/SULFUR CLUSTER |
| SF4 | ✕ - Binding site not conserved. | IRON/SULFUR CLUSTER |

Model-Template Alignment

|  |  |  |
| --- | --- | --- |
|  |  |  |

Model 03

- PDB Format *(Display)*
- JSON Format *(Display)*
- Model Report *(Display)*

Oligo-State
:   Monomer

GMQE
:   0.26

QMEANDisCo Global:
:   0.43  ± 0.05

Ligands

QMEANDisCo Local

QMEAN Z-Scores

Template

7p63.1.C

NADH-quinone oxidoreductase  
Complex I from E. coli, DDM/LMNG-purified, under Turnover at pH 6, Closed state

Seq Identity
:   16.25%

Coverage

|  |  |
| --- | --- |
| **Biounit Oligo State** | Hetero-13-mer |
| **QSQE** | 0.00 |
| **Method** | EM |
| **Seq Similarity** | 0.27 |
| **Coverage** | 0.49 |
| **Range** | 60-441 |

| Ligand | Added to Model | Description |
| --- | --- | --- |
| 3PE | ✕ - Binding site not conserved. | 1,2-Distearoyl-sn-glycerophosphoethanolamine |
| 3PE | ✕ - Binding site not conserved. | 1,2-Distearoyl-sn-glycerophosphoethanolamine |
| 3PE | ✕ - Binding site not conserved. | 1,2-Distearoyl-sn-glycerophosphoethanolamine |
| 3PE | ✕ - Binding site not conserved. | 1,2-Distearoyl-sn-glycerophosphoethanolamine |
| 3PE | ✕ - Binding site not conserved. | 1,2-Distearoyl-sn-glycerophosphoethanolamine |
| 3PE | ✕ - Binding site not conserved. | 1,2-Distearoyl-sn-glycerophosphoethanolamine |
| 3PE | ✕ - Binding site not conserved. | 1,2-Distearoyl-sn-glycerophosphoethanolamine |
| 3PE | ✕ - Binding site not conserved. | 1,2-Distearoyl-sn-glycerophosphoethanolamine |
| CA | ✕ - Binding site not conserved. | CALCIUM ION |
| DCQ | ✕ - Binding site not conserved. | 2-decyl-5,6-dimethoxy-3-methylcyclohexa-2,5-diene-1,4-dione |
| FES | ✕ - Binding site not conserved. | FE2/S2 (INORGANIC) CLUSTER |
| FES | ✕ - Binding site not conserved. | FE2/S2 (INORGANIC) CLUSTER |
| FMN | ✕ - Binding site not conserved. | FLAVIN MONONUCLEOTIDE |
| LFA | ✕ - Binding site not conserved. | EICOSANE |
| LFA | ✕ - Binding site not conserved. | EICOSANE |
| LFA | ✕ - Binding site not conserved. | EICOSANE |
| LFA | ✕ - Binding site not conserved. | EICOSANE |
| NAI | ✕ - Binding site not conserved. | 1,4-DIHYDRONICOTINAMIDE ADENINE DINUCLEOTIDE |
| SF4 | ✕ - Binding site not conserved. | IRON/SULFUR CLUSTER |
| SF4 | ✕ - Binding site not conserved. | IRON/SULFUR CLUSTER |
| SF4 | ✕ - Binding site not conserved. | IRON/SULFUR CLUSTER |
| SF4 | ✕ - Binding site not conserved. | IRON/SULFUR CLUSTER |
| SF4 | ✕ - Binding site not conserved. | IRON/SULFUR CLUSTER |
| SF4 | ✕ - Binding site not conserved. | IRON/SULFUR CLUSTER |
| SF4 | ✕ - Binding site not conserved. | IRON/SULFUR CLUSTER |

Model-Template Alignment

|  |  |  |
| --- | --- | --- |
|  |  |  |

Apply

Close

Cartoon

- Cartoon
- Tube
- Trace
- Lines
- Ball+Stick
- Licorice
- Hyperball
- Rope
- Surface
- Spacefill
- Outline
- Fog

###### Background

- Transparent

###### Resolution

- Low
- Medium
- High
- Extreme

##### Click model image to view in 3D

##### Click model image to view in 3D

×

### Delete Model - ""

Are you sure you want to delete this model?  
(This really can't be undone!)

Close
Delete Model
