# Supplementary material for: Metabolic capacity is maintained despite shifts in microbial diversity in estuary sediments
Source: ISME Commun. 2025 Oct 11;5(1):ycaf182. doi: 10.1093/ismeco/ycaf182 (PMC12687941; doi:10.1093/ismeco/ycaf182)
Supplement: Supplementary_Data_1_ycaf182 [file supplementary_data_1_ycaf182.zip › SWISS-MODEL/4_1_Oct_SF_Bin32_scaffold_7496_c2_2505_1/templates.html]

4\_1\_Oct\_SF\_Bin32\_scaffold\_7496\_c2\_2-505\_1 | Templates


**Export Alignment**
  
FASTA format
Clustal Format
PNG Image

**Secondary Structure**
  
None
DSSP
PSIPRED
SSpro

**Colour Scheme** 


Fade Mismatches
Enhance Mismatches

Confidencegradient
Confidenceclass
Indels
Chain
Unique Chain
Rainbow
2° Structure
Clustal
Hydrophobic
Size
Charged
Polar
Proline
Ser/Thr
Cysteine
Aliphatic
Aromatic
No Colour

Use QMEANBrane values

|  |  |  |  |
| --- | --- | --- | --- |
| Background |  |  |  |

**3D Viewer**  
NGL
PV

FASTA
Multi FASTA
ClustalW
PNG


SWISS-MODEL

### 4\_1\_Oct\_SF\_Bin32\_scaffold\_7496\_c2\_2-505\_1

### Created: March 29, 2023, 8:50 p.m. at 20:50

- Templates
- Models

Models | Name | Description | GMQE | QSQE | Seq Id | Coverage | Range | Method | Resolution | Oligo-state | Ligands | Found by | Seq Similarity || ✓ | 7b04.1.B | Nitrite oxidoreductase subunit A  *Structure of Nitrite oxidoreductase (Nxr) from the anammox bacterium Kuenenia stuttgartiensis.* | 0.56 | 0.00 | 44.60 | 0.83 | 3-148 | X-ray | 2.97 | monomer | 4 x SF4, 1 x F3S, 2 x MD1, 1 x MO, 1 x HEM, 2 x CA | HHblits | 0.42 |
| ``` target    MAQGVSRRQLLGRALALGSGAALADLLGPARFLSPAGAATAGAVVPGNPLRVMPDRTWEQIYRNQFEDDSTFVFTCAPND 7b04.1    --MKLTRRAFLQVAGATGATLTLAKNAMAFRLL-------KPAVVVDNPLDTYPDRRWESVYRDQYQYDRTFTYCCSPND  target    THNCLLRAHVKNGVVVRISPTYGYGEATDLYGNRASHRWDPRTCQKGLILSRRFYSERRVKAPMIRKGFKDWVEAGYPRN 7b04.1    THACRIRAFVRNNVMMRVEQNYDHQNYSDLYGNKATRNWNPRMCLKGYTFHRRVYGPYRLRYPLIRKG------------  target    DDGTPQM 7b04.1    ------- ``` | | | | | | | | | | | | | | | | | | | | | | | | | | | | | | | | | | | | | | | | | | | | | | | | | |
|  | 7b04.2.B | Nitrite oxidoreductase subunit A  *Structure of Nitrite oxidoreductase (Nxr) from the anammox bacterium Kuenenia stuttgartiensis.* | 0.54 | 0.00 | 44.60 | 0.83 | 3-148 | X-ray | 2.97 | monomer | 4 x SF4, 1 x F3S, 2 x MD1, 1 x MO, 1 x HEM, 2 x CA | HHblits | 0.42 |
| ``` target    MAQGVSRRQLLGRALALGSGAALADLLGPARFLSPAGAATAGAVVPGNPLRVMPDRTWEQIYRNQFEDDSTFVFTCAPND 7b04.2    --MKLTRRAFLQVAGATGATLTLAKNAMAFRLL-------KPAVVVDNPLDTYPDRRWESVYRDQYQYDRTFTYCCSPND  target    THNCLLRAHVKNGVVVRISPTYGYGEATDLYGNRASHRWDPRTCQKGLILSRRFYSERRVKAPMIRKGFKDWVEAGYPRN 7b04.2    THACRIRAFVRNNVMMRVEQNYDHQNYSDLYGNKATRNWNPRMCLKGYTFHRRVYGPYRLRYPLIRKG------------  target    DDGTPQM 7b04.2    ------- ``` | | | | | | | | | | | | | | | | | | | | | | | | | | | | | | | | | | | | | | | | | | | | | | | | | |
|  | 2ivf.1.A | ETHYLBENZENE DEHYDROGENASE ALPHA-SUBUNIT  *ETHYLBENZENE DEHYDROGENASE FROM AROMATOLEUM AROMATICUM* | 0.35 |  | 23.29 | 0.87 | 3-161 | X-ray | 1.88 | hetero-oligomer | 1 x MES, 4 x SF4, 1 x MO, 1 x MGD, 1 x MD1, 1 x F3S, 1 x HEM | HHblits | 0.31 |
| ``` target    MAQGVSRRQLLGRALALGSGAALADLLGPARFLSPAGAATAGAVVPGNPLRVMPDRTWEQIYRNQFEDDSTFVFTCAPND 2ivf.1    --QDQHRRDFLKRSGAAVLSLSLSSLATGV-V--PGFL--K---DAQAGTKAPGYASWEDIYRKEWKWDKVNWGSHLNIC  target    --THNCLLRAHVKNGVVVRISPTYGYGEATDLYGNRASHRWDPRTCQKGLILSRRFYSERRVKAPMIRKG---FKDWVEA 2ivf.1    WPQGSCKFYVYVRNGIVWREEQAAQTPACN-----VDYVDYNPLGCQKGSAFNNNLYGDERVKYPLKRVGKRGEGKWKRV  target    GYPRNDDGTPQM 2ivf.1    SWDEAA------ ``` | | | | | | | | | | | | | | | | | | | | | | | | | | | | | | | | | | | | | | | | | | | | | | | | | |
|  | 7b04.1.B | Nitrite oxidoreductase subunit A  *Structure of Nitrite oxidoreductase (Nxr) from the anammox bacterium Kuenenia stuttgartiensis.* | 0.50 | 0.00 | 47.32 | 0.67 | 48-159 | X-ray | 2.97 | monomer | 4 x SF4, 1 x F3S, 2 x MD1, 1 x MO, 1 x HEM, 2 x CA | BLAST | 0.46 |
| ``` target    MAQGVSRRQLLGRALALGSGAALADLLGPARFLSPAGAATAGAVVPGNPLRVMPDRTWEQIYRNQFEDDSTFVFTCAPND 7b04.1    -----------------------------------------------NPLDTYPDRRWESVYRDQYQYDRTFTYCCSPND  target    THNCLLRAHVKNGVVVRISPTYGYGEATDLYGNRASHRWDPRTCQKGLILSRRFYSERRVKAPMIRKGFKDWVEAGYPRN 7b04.1    THACRIRAFVRNNVMMRVEQNYDHQNYSDLYGNKATRNWNPRMCLKGYTFHRRVYGPYRLRYPLIRKGWKRWADDGFPE-  target    DDGTPQM 7b04.1    ------- ``` | | | | | | | | | | | | | | | | | | | | | | | | | | | | | | | | | | | | | | | | | | | | | | | | | |
|  | 7b04.2.B | Nitrite oxidoreductase subunit A  *Structure of Nitrite oxidoreductase (Nxr) from the anammox bacterium Kuenenia stuttgartiensis.* | 0.50 | 0.00 | 47.32 | 0.67 | 48-159 | X-ray | 2.97 | monomer | 4 x SF4, 1 x F3S, 2 x MD1, 1 x MO, 1 x HEM, 2 x CA | BLAST | 0.46 |
| ``` target    MAQGVSRRQLLGRALALGSGAALADLLGPARFLSPAGAATAGAVVPGNPLRVMPDRTWEQIYRNQFEDDSTFVFTCAPND 7b04.2    -----------------------------------------------NPLDTYPDRRWESVYRDQYQYDRTFTYCCSPND  target    THNCLLRAHVKNGVVVRISPTYGYGEATDLYGNRASHRWDPRTCQKGLILSRRFYSERRVKAPMIRKGFKDWVEAGYPRN 7b04.2    THACRIRAFVRNNVMMRVEQNYDHQNYSDLYGNKATRNWNPRMCLKGYTFHRRVYGPYRLRYPLIRKGWKRWADDGFPE-  target    DDGTPQM 7b04.2    ------- ``` | | | | | | | | | | | | | | | | | | | | | | | | | | | | | | | | | | | | | | | | | | | | | | | | | |
|  | 3ir5.1.A | Respiratory nitrate reductase 1 alpha chain  *Crystal structure of NarGHI mutant NarG-H49C* | 0.45 | 0.00 | 28.21 | 0.70 | 1-148 | X-ray | 2.30 | monomer | 2 x MD1, 1 x 6MO, 4 x SF4, 1 x AGA, 1 x F3S, 2 x HEM | HHblits | 0.34 |
| ``` target    MAQGVSRRQLLGRALALGSGAALADLLGPARFLSPAGAATAGAVVPGNPLRVMPDRTWEQIYRNQFEDDSTFVFTCAPND 3ir5.1    MSKFLDRFRYFKQKGETFADG-HGQL-------------------------LNTNRDWEDGYRQRWQHDKIVRSTCGVNC  target    THNCLLRAHVKNGVVVRISPTYGYGEATDLYGNRASHRWDPRTCQKGLILSRRFYSERRVKAPMIRKGFKDWVEAGYPRN 3ir5.1    TGSCSWKIYVKNGLVTWETQQTDYPRTR-----PDLPNHEPRGCPRGASYSWYLYSANRLKYPMMRKR------------  target    DDGTPQM 3ir5.1    ------- ``` | | | | | | | | | | | | | | | | | | | | | | | | | | | | | | | | | | | | | | | | | | | | | | | | | |
|  | 3ir6.1.A | Respiratory nitrate reductase 1 alpha chain  *Crystal structure of NarGHI mutant NarG-H49S* | 0.41 | 0.00 | 27.35 | 0.70 | 1-148 | X-ray | 2.80 | monomer | 2 x GDP, 1 x AGA, 3 x SF4, 1 x F3S, 2 x HEM | HHblits | 0.33 |
| ``` target    MAQGVSRRQLLGRALALGSGAALADLLGPARFLSPAGAATAGAVVPGNPLRVMPDRTWEQIYRNQFEDDSTFVFTCAPND 3ir6.1    MSKFLDRFRYFKQKGETFADGHGQ-L-------------------------LNTNRDWEDGYRQRWQHDKIVRSTSGVNC  target    THNCLLRAHVKNGVVVRISPTYGYGEATDLYGNRASHRWDPRTCQKGLILSRRFYSERRVKAPMIRKGFKDWVEAGYPRN 3ir6.1    TGSCSWKIYVKNGLVTWETQQTDYPRTR-----PDLPNHEPRGCPRGASYSWYLYSANRLKYPMMRKR------------  target    DDGTPQM 3ir6.1    ------- ``` | | | | | | | | | | | | | | | | | | | | | | | | | | | | | | | | | | | | | | | | | | | | | | | | | |
|  | 1kqf.1.A | FORMATE DEHYDROGENASE, NITRATE-INDUCIBLE, MAJOR SUBUNIT  *FORMATE DEHYDROGENASE N FROM E. COLI* | 0.30 |  | 23.97 | 0.72 | 3-160 | X-ray | 1.60 | hetero-oligomer | 3 x 6MO, 15 x SF4, 6 x MGD, 6 x HEM, 3 x CDL | HHblits | 0.30 |
| ``` target    MAQGVSRRQLLGRALALGSGAALADLLGPARFLSPAGAATAGAVVPGNPLRVMPDRTWEQIYRNQFEDDSTFVFTCAPND 1kqf.1    --MDVSRRQFFKICAGGMAGTTVAAL-G---F-APKQA-----------LA--QARNYK------LLRAKEIRNTC-TYC  target    THNCLLRAHVKNG-------VVVRISPTYGYGEATDLYGNRASHRWDPRTCQKGLILSRRFYSERRVKAPMIRKG-FKDW 1kqf.1    SVGCGLLMYSLGDGAKNAREAIYHIEGDPD------------HPVSRGALCPKGAGLLDYVNSENRLRYPEYRAPGSDKW  target    VEAGYPRNDDGTPQM 1kqf.1    QRISWEEA------- ``` | | | | | | | | | | | | | | | | | | | | | | | | | | | | | | | | | | | | | | | | | | | | | | | | | |
|  | 1q16.1.A | Respiratory nitrate reductase 1 alpha chain  *Crystal structure of Nitrate Reductase A, NarGHI, from Escherichia coli* | 0.44 | 0.00 | 26.50 | 0.70 | 1-148 | X-ray | 1.90 | monomer | 2 x MD1, 1 x 6MO, 2 x HEM, 4 x SF4, 1 x F3S, 1 x AGA, 1 x 3PH | HHblits | 0.33 |
| ``` target    MAQGVSRRQLLGRALALGSGAALADLLGPARFLSPAGAATAGAVVPGNPLRVMPDRTWEQIYRNQFEDDSTFVFTCAPND 1q16.1    MSKFLDRFRYFKQKGETFADGHGQ--------------------------LLNTNRDWEDGYRQRWQHDKIVRSTHGVNC  target    THNCLLRAHVKNGVVVRISPTYGYGEATDLYGNRASHRWDPRTCQKGLILSRRFYSERRVKAPMIRKGFKDWVEAGYPRN 1q16.1    TGSCSWKIYVKNGLVTWETQQTDYPRTR-----PDLPNHEPRGCPRGASYSWYLYSANRLKYPMMRKR------------  target    DDGTPQM 1q16.1    ------- ``` | | | | | | | | | | | | | | | | | | | | | | | | | | | | | | | | | | | | | | | | | | | | | | | | | |
|  | 3ir7.1.A | Respiratory nitrate reductase 1 alpha chain  *Crystal structure of NarGHI mutant NarG-R94S* | 0.42 | 0.00 | 26.50 | 0.70 | 1-148 | X-ray | 2.50 | monomer | 2 x MD1, 4 x SF4, 1 x 6MO, 1 x AGA, 1 x F3S, 2 x HEM | HHblits | 0.32 |
| ``` target    MAQGVSRRQLLGRALALGSGAALADLLGPARFLSPAGAATAGAVVPGNPLRVMPDRTWEQIYRNQFEDDSTFVFTCAPND 3ir7.1    MSKFLDRFRYFKQKGETFADGHGQ--------------------------LLNTNRDWEDGYRQRWQHDKIVRSTHGVNC  target    THNCLLRAHVKNGVVVRISPTYGYGEATDLYGNRASHRWDPRTCQKGLILSRRFYSERRVKAPMIRKGFKDWVEAGYPRN 3ir7.1    TGSCSWKIYVKNGLVTWETQQTDYPRTR-----PDLPNHEPRGCPSGASYSWYLYSANRLKYPMMRKR------------  target    DDGTPQM 3ir7.1    ------- ``` | | | | | | | | | | | | | | | | | | | | | | | | | | | | | | | | | | | | | | | | | | | | | | | | | |
|  | 6sdv.1.A | Formate dehydrogenase, alpha subunit, selenocysteine-containing,Formate dehydrogenase, alpha subunit, selenocysteine-containing,W-formate dehydrogenase - alpha subunit  *W-formate dehydrogenase from Desulfovibrio vulgaris - Formate reduced form* | 0.28 |  | 17.21 | 0.73 | 3-161 | X-ray | 1.90 | hetero-1-1-mer | 2 x MGD, 4 x SF4, 1 x W, 1 x H2S | HHblits | 0.28 |
| ``` target    MAQGVSRRQLLGRALALGSGAALADLLGPARFLSPAGAATAGAVVPGNPLRVMPDRTWEQIYRNQFEDDSTFVFTCAPND 6sdv.1    --MTVTRRHFLKLSAGAAVAGAFTGLG--LSL-APTV----------ARA--------EL-QK--LQWAKQTTSIC-CYC  target    THNCLLRAHVK---NGVVVRISPTYGYGEATDLYGNRASHRWDPRTCQKGLILSRRFYSERRVKAPMIRKG-FKDWVEAG 6sdv.1    AVGCGLIVHTAKDGQGRAVNVEGDPD------------HPINEGSLCPKGASIFQLGENDQRGTQPLYRAPFSDTWKPVT  target    YPRNDDGTPQM 6sdv.1    WDFAL------ ``` | | | | | | | | | | | | | | | | | | | | | | | | | | | | | | | | | | | | | | | | | | | | | | | | | |
|  | 6sdr.1.A | Formate dehydrogenase, alpha subunit, selenocysteine-containing  *W-formate dehydrogenase from Desulfovibrio vulgaris - Oxidized form* | 0.29 |  | 17.36 | 0.72 | 3-160 | X-ray | 2.10 | hetero-1-1-mer | 2 x MGD, 4 x SF4, 1 x H2S, 1 x W | HHblits | 0.28 |
| ``` target    MAQGVSRRQLLGRALALGSGAALADLLGPARFLSPAGAATAGAVVPGNPLRVMPDRTWEQIYRNQFEDDSTFVFTCAPND 6sdr.1    --MTVTRRHFLKLSAGAAVAGAFTGLG--LSL-APTV-----------ARA-------EL-QK--LQWAKQ-TTSICCYC  target    THNCLLRAHVK---NGVVVRISPTYGYGEATDLYGNRASHRWDPRTCQKGLILSRRFYSERRVKAPMIRKGF-KDWVEAG 6sdr.1    AVGCGLIVHTAKDGQGRAVNVEGDPD------------HPINEGSLCPKGASIFQLGENDQRGTQPLYRAPFSDTWKPVT  target    YPRNDDGTPQM 6sdr.1    WDFA------- ``` | | | | | | | | | | | | | | | | | | | | | | | | | | | | | | | | | | | | | | | | | | | | | | | | | |
|  | 1r27.4.A | Respiratory nitrate reductase 1 alpha chain  *Crystal Structure of NarGH complex* | 0.35 | 0.11 | 26.09 | 0.69 | 3-148 | X-ray | 2.00 | homo-dimer | 4 x MO, 16 x SF4, 8 x MGD, 4 x F3S | HHblits | 0.32 |
| ``` target    MAQGVSRRQLLGRALALGSGAALADLLGPARFLSPAGAATAGAVVPGNPLRVMPDRTWEQIYRNQFEDDSTFVFTCAPND 1r27.4    --KFLDRFRYFKQKGETFADGHGQ--------------------------LLNTNRDWEDGYRQRWQHDKIVRSTHGVNC  target    THNCLLRAHVKNGVVVRISPTYGYGEATDLYGNRASHRWDPRTCQKGLILSRRFYSERRVKAPMIRKGFKDWVEAGYPRN 1r27.4    TGSCSWKIYVKNGLVTWETQQTDYPRTR-----PDLPNHEPRGCPRGASYSWYLYSANRLKYPMMRKR------------  target    DDGTPQM 1r27.4    ------- ``` | | | | | | | | | | | | | | | | | | | | | | | | | | | | | | | | | | | | | | | | | | | | | | | | | |
|  | 2vpz.1.A | THIOSULFATE REDUCTASE  *POLYSULFIDE REDUCTASE NATIVE STRUCTURE* | 0.30 |  | 18.26 | 0.69 | 5-160 | X-ray | 2.40 | hetero-oligomer | 10 x SF4, 4 x MGD, 2 x MO | HHblits | 0.29 |
| ``` target    MAQGVSRRQLLGRALALGSGAALADLLGPARFLSPAGAATAGAVVPGNPLRVMPDRTWEQIYRNQFEDDSTFVFTCAPND 2vpz.1    ----MQRREFLKLSALGVGAMALRGS----GPA-------K-----------ALKAPWY------AQEVKSVYQIC-EGC  target    THNCLLRAHVKNGVVVRISPTYGYGEATDLYGNRASHRWDPRTCQKGLILSRRFYSERRVKAPMIRKG-----FKDWVEA 2vpz.1    FWRCGIVAHAVGNRVYKVEGYEA------------NPKSRGRLCPRGQGAPQTTYDPDRLKRPLIRVEGSQRGEGKYRVA  target    GYPRNDDGTPQM 2vpz.1    TWEEA------- ``` | | | | | | | | | | | | | | | | | | | | | | | | | | | | | | | | | | | | | | | | | | | | | | | | | |
|  | 2vpx.1.D | THIOSULFATE REDUCTASE  *POLYSULFIDE REDUCTASE WITH BOUND QUINONE (UQ1)* | 0.28 |  | 18.26 | 0.69 | 5-160 | X-ray | 3.10 | hetero-oligomer | 10 x SF4, 4 x MGD, 2 x MO, 2 x UQ1 | HHblits | 0.29 |
| ``` target    MAQGVSRRQLLGRALALGSGAALADLLGPARFLSPAGAATAGAVVPGNPLRVMPDRTWEQIYRNQFEDDSTFVFTCAPND 2vpx.1    ----MQRREFLKLSALGVGAMALRGS----GPA-------K-----------ALKAPWY------AQEVKSVYQIC-EGC  target    THNCLLRAHVKNGVVVRISPTYGYGEATDLYGNRASHRWDPRTCQKGLILSRRFYSERRVKAPMIRKG-----FKDWVEA 2vpx.1    FWRCGIVAHAVGNRVYKVEGYEA------------NPKSRGRLCPRGQGAPQTTYDPDRLKRPLIRVEGSQRGEGKYRVA  target    GYPRNDDGTPQM 2vpx.1    TWEEA------- ``` | | | | | | | | | | | | | | | | | | | | | | | | | | | | | | | | | | | | | | | | | | | | | | | | | |
|  | 3ir5.1.A | Respiratory nitrate reductase 1 alpha chain  *Crystal structure of NarGHI mutant NarG-H49C* | 0.35 | 0.00 | 35.29 | 0.61 | 55-161 | X-ray | 2.30 | monomer | 2 x MD1, 1 x 6MO, 4 x SF4, 1 x AGA, 1 x F3S, 2 x HEM | BLAST | 0.37 |
| ``` target    MAQGVSRRQLLGRALALGSGAALADLLGPARFLSPAGAATAGAVVPGNPLRVMPDRTWEQIYRNQFEDDSTFVFTCAPND 3ir5.1    ------------------------------------------------------NRDWEDGYRQRWQHDKIVRSTCGVNC  target    THNCLLRAHVKNGVVVRISPTYGYGEATDLYGNRASHRWDPRTCQKGLILSRRFYSERRVKAPMIRKGF-KDWVEAGYPR 3ir5.1    TGSCSWKIYVKNGLVTWETQQTDYPRTRPDLPNH-----EPRGCPRGASYSWYLYSANRLKYPMMRKRLMKMWREAKALH  target    NDDGTPQM 3ir5.1    SD------ ``` | | | | | | | | | | | | | | | | | | | | | | | | | | | | | | | | | | | | | | | | | | | | | | | | | |
|  | 3ir6.1.A | Respiratory nitrate reductase 1 alpha chain  *Crystal structure of NarGHI mutant NarG-H49S* | 0.35 | 0.00 | 34.31 | 0.61 | 55-161 | X-ray | 2.80 | monomer | 2 x GDP, 1 x AGA, 3 x SF4, 1 x F3S, 2 x HEM | BLAST | 0.36 |
| ``` target    MAQGVSRRQLLGRALALGSGAALADLLGPARFLSPAGAATAGAVVPGNPLRVMPDRTWEQIYRNQFEDDSTFVFTCAPND 3ir6.1    ------------------------------------------------------NRDWEDGYRQRWQHDKIVRSTSGVNC  target    THNCLLRAHVKNGVVVRISPTYGYGEATDLYGNRASHRWDPRTCQKGLILSRRFYSERRVKAPMIRKGF-KDWVEAGYPR 3ir6.1    TGSCSWKIYVKNGLVTWETQQTDYPRTRPDLPNH-----EPRGCPRGASYSWYLYSANRLKYPMMRKRLMKMWREAKALH  target    NDDGTPQM 3ir6.1    SD------ ``` | | | | | | | | | | | | | | | | | | | | | | | | | | | | | | | | | | | | | | | | | | | | | | | | | |
|  | 1r27.4.A | Respiratory nitrate reductase 1 alpha chain  *Crystal Structure of NarGH complex* | 0.35 | 0.12 | 34.31 | 0.61 | 55-161 | X-ray | 2.00 | homo-dimer | 4 x MO, 16 x SF4, 8 x MGD, 4 x F3S | BLAST | 0.36 |
| ``` target    MAQGVSRRQLLGRALALGSGAALADLLGPARFLSPAGAATAGAVVPGNPLRVMPDRTWEQIYRNQFEDDSTFVFTCAPND 1r27.4    ------------------------------------------------------NRDWEDGYRQRWQHDKIVRSTHGVNC  target    THNCLLRAHVKNGVVVRISPTYGYGEATDLYGNRASHRWDPRTCQKGLILSRRFYSERRVKAPMIRKGF-KDWVEAGYPR 1r27.4    TGSCSWKIYVKNGLVTWETQQTDYPRTRPDLPNH-----EPRGCPRGASYSWYLYSANRLKYPMMRKRLMKMWREAKALH  target    NDDGTPQM 1r27.4    SD------ ``` | | | | | | | | | | | | | | | | | | | | | | | | | | | | | | | | | | | | | | | | | | | | | | | | | |
|  | 1q16.1.A | Respiratory nitrate reductase 1 alpha chain  *Crystal structure of Nitrate Reductase A, NarGHI, from Escherichia coli* | 0.35 |  | 34.31 | 0.61 | 55-161 | X-ray | 1.90 | hetero-oligomer | 2 x MD1, 1 x 6MO, 2 x HEM, 4 x SF4, 1 x F3S, 1 x AGA, 1 x 3PH | BLAST | 0.36 |
| ``` target    MAQGVSRRQLLGRALALGSGAALADLLGPARFLSPAGAATAGAVVPGNPLRVMPDRTWEQIYRNQFEDDSTFVFTCAPND 1q16.1    ------------------------------------------------------NRDWEDGYRQRWQHDKIVRSTHGVNC  target    THNCLLRAHVKNGVVVRISPTYGYGEATDLYGNRASHRWDPRTCQKGLILSRRFYSERRVKAPMIRKGF-KDWVEAGYPR 1q16.1    TGSCSWKIYVKNGLVTWETQQTDYPRTRPDLPNH-----EPRGCPRGASYSWYLYSANRLKYPMMRKRLMKMWREAKALH  target    NDDGTPQM 1q16.1    SD------ ``` | | | | | | | | | | | | | | | | | | | | | | | | | | | | | | | | | | | | | | | | | | | | | | | | | |
|  | 3egw.1.A | Respiratory nitrate reductase 1 alpha chain  *The crystal structure of the NarGHI mutant NarH - C16A* | 0.36 |  | 34.31 | 0.61 | 55-161 | X-ray | 1.90 | hetero-2-2-2-mer | 2 x MD1, 2 x MGD, 2 x 6MO, 6 x SF4, 4 x F3S, 2 x 3PH, 4 x HEM, 2 x AGA | BLAST | 0.36 |
| ``` target    MAQGVSRRQLLGRALALGSGAALADLLGPARFLSPAGAATAGAVVPGNPLRVMPDRTWEQIYRNQFEDDSTFVFTCAPND 3egw.1    ------------------------------------------------------NRDWEDGYRQRWQHDKIVRSTHGVNC  target    THNCLLRAHVKNGVVVRISPTYGYGEATDLYGNRASHRWDPRTCQKGLILSRRFYSERRVKAPMIRKGF-KDWVEAGYPR 3egw.1    TGSCSWKIYVKNGLVTWETQQTDYPRTRPDLPNH-----EPRGCPRGASYSWYLYSANRLKYPMMRKRLMKMWREAKALH  target    NDDGTPQM 3egw.1    SD------ ``` | | | | | | | | | | | | | | | | | | | | | | | | | | | | | | | | | | | | | | | | | | | | | | | | | |
|  | 3ir7.1.A | Respiratory nitrate reductase 1 alpha chain  *Crystal structure of NarGHI mutant NarG-R94S* | 0.37 |  | 34.31 | 0.61 | 55-161 | X-ray | 2.50 | hetero-1-1-1-mer | 2 x MD1, 4 x SF4, 1 x 6MO, 1 x AGA, 1 x F3S, 2 x HEM | BLAST | 0.36 |
| ``` target    MAQGVSRRQLLGRALALGSGAALADLLGPARFLSPAGAATAGAVVPGNPLRVMPDRTWEQIYRNQFEDDSTFVFTCAPND 3ir7.1    ------------------------------------------------------NRDWEDGYRQRWQHDKIVRSTHGVNC  target    THNCLLRAHVKNGVVVRISPTYGYGEATDLYGNRASHRWDPRTCQKGLILSRRFYSERRVKAPMIRKGF-KDWVEAGYPR 3ir7.1    TGSCSWKIYVKNGLVTWETQQTDYPRTRPDLPNH-----EPRGCPSGASYSWYLYSANRLKYPMMRKRLMKMWREAKALH  target    NDDGTPQM 3ir7.1    SD------ ``` | | | | | | | | | | | | | | | | | | | | | | | | | | | | | | | | | | | | | | | | | | | | | | | | | |
|  | 1e60.1.A | Dimethyl sulfoxide/trimethylamine N-oxide reductase  *OXIDIZED DMSO REDUCTASE EXPOSED TO HEPES - Structure II BUFFER* | 0.24 |  | 22.02 | 0.65 | 1-161 | X-ray | 2.00 | monomer | 2 x PGD, 1 x 2MO | HHblits | 0.30 |
| ``` target    MAQGVSRRQLLGRALALGSGAALADLLGPARFLSPAGAATAGAVVPGNPLRVMPDRTWEQIYRNQFEDDSTFVFTCAPND 1e60.1    LRAELYRRAFLSYSVAPGALGMFGRS-----LL-A---------KGA---------------RAE------ALANGTVMS  target    THNCL-LRAHVKNGVVVRISPTYGYGEATDLYGNRASHRWDPRTCQKGLILSRRFYSERRVKAPMIRK------------ 1e60.1    GSHWGVFTATVENGRATAFTPWEK----------------DPHPSPMLAGVLDSIYSPTRIKYPMVRREFLEKGVNADRS  target    --GFKDWVEAGYPRNDDGTPQM 1e60.1    TRGNGDFVRVSWDQAL------ ``` | | | | | | | | | | | | | | | | | | | | | | | | | | | | | | | | | | | | | | | | | | | | | | | | | |
|  | 1e5v.2.A | Dimethyl sulfoxide/trimethylamine N-oxide reductase  *OXIDIZED DMSO REDUCTASE EXPOSED TO HEPES BUFFER* | 0.24 |  | 21.10 | 0.65 | 1-161 | X-ray | 2.40 | monomer | 2 x PGD, 1 x 2MO | HHblits | 0.30 |
| ``` target    MAQGVSRRQLLGRALALGSGAALADLLGPARFLSPAGAATAGAVVPGNPLRVMPDRTWEQIYRNQFEDDSTFVFTCAPND 1e5v.2    LRAELYRRAFLSYSVAPGALGMFGRS-----L--LA--------K-----GA----------RA-----EAL-ANGTVMS  target    THNCL-LRAHVKNGVVVRISPTYGYGEATDLYGNRASHRWDPRTCQKGLILSRRFYSERRVKAPMIRK------------ 1e5v.2    GSHWGVFTATVENGRATAFTPWEK------------D----PHPSPMLAGVLDSIYSPTRIKYPMVRREFLEKGVNADRS  target    --GFKDWVEAGYPRNDDGTPQM 1e5v.2    TRGNGDFVRVSWDQAL------ ``` | | | | | | | | | | | | | | | | | | | | | | | | | | | | | | | | | | | | | | | | | | | | | | | | | |
|  | 1e18.1.A | DMSO REDUCTASE.  *TUNGSTEN-SUSBSTITUTED DMSO REDUCTASE FROM RHODOBACTER CAPSULATUS* | 0.24 |  | 21.30 | 0.65 | 2-161 | X-ray | 2.00 | monomer | 2 x PGD, 1 x 6WO | HHblits | 0.30 |
| ``` target    MAQGVSRRQLLGRALALGSGAALADLLGPARFLSPAGAATAGAVVPGNPLRVMPDRTWEQIYRNQFEDDSTFVFTCAPND 1e18.1    -RAELYRRAFLSYSVAPGALGMFGRS-----L-LA---------K-----GA----------RA-----EALA-NGTVMS  target    THNCL-LRAHVKNGVVVRISPTYGYGEATDLYGNRASHRWDPRTCQKGLILSRRFYSERRVKAPMIRK------------ 1e18.1    GSHWGVFTATVENGRATAFTPWEK----------------DPHPSPMLAGVLDSIYSPTRIKYPMVRREFLEKGVNADRS  target    --GFKDWVEAGYPRNDDGTPQM 1e18.1    TRGNGDFVRVSWDQAL------ ``` | | | | | | | | | | | | | | | | | | | | | | | | | | | | | | | | | | | | | | | | | | | | | | | | | |
|  | 4ydd.1.A | DMSO reductase family type II enzyme, molybdopterin subunit  *Crystal structure of the perchlorate reductase PcrAB from Azospira suillum PS* | 0.39 |  | 25.96 | 0.62 | 51-160 | X-ray | 1.86 | hetero-oligomer | 4 x SF4, 1 x MO, 1 x MGD, 1 x MD1, 1 x F3S | HHblits | 0.32 |
| ``` target    MAQGVSRRQLLGRALALGSGAALADLLGPARFLSPAGAATAGAVVPGNPLRVMPDRTWEQIYRNQFEDDSTFVFTCAPND 4ydd.1    --------------------------------------------------GAFEYSGWENFHRTQWSWDKKTRGAHLVNC  target    THNCLLRAHVKNGVVVRISPTYGYGEATDLYGNRASHRWDPRTCQKGLILSRRFYSERRVKAPMIRKG---FKDWVEAGY 4ydd.1    TGACPHFVYSKDGVVMREEQSKDIA------PMPNIPEYNPRGCNKGECGHDYMYGPHRIKYPLIRVGERGEGKWRRATW  target    PRNDDGTPQM 4ydd.1    EEA------- ``` | | | | | | | | | | | | | | | | | | | | | | | | | | | | | | | | | | | | | | | | | | | | | | | | | |
|  | 5e7o.1.A | DMSO reductase family type II enzyme, molybdopterin subunit  *Crystal structure of the perchlorate reductase PcrAB mutant W461E of PcrA from Azospira suillum PS* | 0.39 |  | 26.21 | 0.62 | 52-160 | X-ray | 2.40 | hetero-oligomer | 4 x SF4, 1 x MO, 1 x MGD, 1 x MD1, 1 x F3S | HHblits | 0.32 |
| ``` target    MAQGVSRRQLLGRALALGSGAALADLLGPARFLSPAGAATAGAVVPGNPLRVMPDRTWEQIYRNQFEDDSTFVFTCAPND 5e7o.1    ---------------------------------------------------AFEYSGWENFHRTQWSWDKKTRGAHLVNC  target    THNCLLRAHVKNGVVVRISPTYGYGEATDLYGNRASHRWDPRTCQKGLILSRRFYSERRVKAPMIRKG---FKDWVEAGY 5e7o.1    TGACPHFVYSKDGVVMREEQSKDIA------PMPNIPEYNPRGCNKGECGHDYMYGPHRIKYPLIRVGERGEGKWRRATW  target    PRNDDGTPQM 5e7o.1    EEA------- ``` | | | | | | | | | | | | | | | | | | | | | | | | | | | | | | | | | | | | | | | | | | | | | | | | | |
|  | 6f0k.1.B | Fe-S-cluster-containing hydrogenase  *Alternative complex III* | 0.27 |  | 19.82 | 0.66 | 3-147 | EM | 0.00 | hetero-1-1-1-1-1-1-… | 6 x HEC, 1 x F3S, 3 x SF4 | HHblits | 0.26 |
| ``` target    MAQGVSRRQLLGRALALGSGAALADLLGPARFLSPAGAATAGAVVPGNPLRVMPDRTWEQIYRNQFEDDSTFVFTCAPND 6f0k.1    --SGTSRRQFLQIMGASMALAGLTAC----RR--PV--------EKILP---YV-RQPEE----IIPGIPLYYATAMPFR  target    THNCLLRAHVKNGVVVRISPTYGYGEATDLYGNRASHRWDPRTCQKGLILSRRFYSERRVKAPMIRKGFKDWVEAGYPRN 6f0k.1    GSVRPLLVESHEGRPTKIEGNP------------DHPLSRGATGVFEQASLLNLYDPDRSQQVLRKG-------------  target    DDGTPQM 6f0k.1    ------- ``` | | | | | | | | | | | | | | | | | | | | | | | | | | | | | | | | | | | | | | | | | | | | | | | | | |
|  | 3egw.1.A | Respiratory nitrate reductase 1 alpha chain  *The crystal structure of the NarGHI mutant NarH - C16A* | 0.37 |  | 31.18 | 0.56 | 51-148 | X-ray | 1.90 | hetero-2-2-2-mer | 2 x MD1, 2 x MGD, 2 x 6MO, 6 x SF4, 4 x F3S, 2 x 3PH, 4 x HEM, 2 x AGA | HHblits | 0.35 |
| ``` target    MAQGVSRRQLLGRALALGSGAALADLLGPARFLSPAGAATAGAVVPGNPLRVMPDRTWEQIYRNQFEDDSTFVFTCAPND 3egw.1    --------------------------------------------------LLNTNRDWEDGYRQRWQHDKIVRSTHGVNC  target    THNCLLRAHVKNGVVVRISPTYGYGEATDLYGNRASHRWDPRTCQKGLILSRRFYSERRVKAPMIRKGFKDWVEAGYPRN 3egw.1    TGSCSWKIYVKNGLVTWETQQTDYPRTR-----PDLPNHEPRGCPRGASYSWYLYSANRLKYPMMRKR------------  target    DDGTPQM 3egw.1    ------- ``` | | | | | | | | | | | | | | | | | | | | | | | | | | | | | | | | | | | | | | | | | | | | | | | | | |
|  | 4v4c.1.A | Pyrogallol hydroxytransferase large subunit  *Crystal Structure of Pyrogallol-Phloroglucinol Transhydroxylase from Pelobacter acidigallici* | 0.27 |  | 20.69 | 0.52 | 73-161 | X-ray | 2.35 | hetero-oligomer | 2 x CA, 2 x MGD, 1 x 4MO, 3 x SF4 | HHblits | 0.30 |
| ``` target    MAQGVSRRQLLGRALALGSGAALADLLGPARFLSPAGAATAGAVVPGNPLRVMPDRTWEQIYRNQFEDDSTFVFTCAPND 4v4c.1    ------------------------------------------------------------------------VVRLTN-S  target    THNCLLRAHVKNGVVVRISPTYGYGEATDL-------YGNRASHRWDPRTCQKGLILSRRFYSERRVKAPMIRKGF---- 4v4c.1    STGGPVFVYVKDGKIIRMTPMDFDD-AVDAPSWKIEARGKTFTPPRKTSIAPYTAGFKSMIYSDLRIPYPMKRKSFDPNG  target    -----------------KDWVEAGYPRNDDGTPQM 4v4c.1    ERNPQLRGAGLSKQDPWSDYERISWDEAT------ ``` | | | | | | | | | | | | | | | | | | | | | | | | | | | | | | | | | | | | | | | | | | | | | | | | | |
|  | 3o5a.1.A | Periplasmic nitrate reductase  *Crystal Structure of partially reduced Periplasmic Nitrate Reductase from Cupriavidus necator using Ionic Liquids* | 0.26 |  | 18.60 | 0.51 | 62-160 | X-ray | 1.72 | hetero-oligomer | 1 x SF4, 1 x MOS, 2 x MGD, 2 x HEC | HHblits | 0.29 |
| ``` target    MAQGVSRRQLLGRALALGSGAALADLLGPARFLSPAGAATAGAVVPGNPLRVMPDRTWEQIYRNQFEDDSTFVFTCAPND 3o5a.1    -------------------------------------------------------------TDSEVTKLKWSKAPCR-FC  target    THNCLLRAHVKNGVVVRISPTYGYGEATDLYGNRASHRWDPRTCQKGLILSRRFYSERRVKAPMIRKG------FKDWVE 3o5a.1    GTGCGVTVAVKDNKVVATQGDPQ------------AEVNKGLNCVKGYFLSKIMYGQDRLTRPLMRMKNGKYDKNGDFAP  target    AGYPRNDDGTPQM 3o5a.1    VTWDQA------- ``` | | | | | | | | | | | | | | | | | | | | | | | | | | | | | | | | | | | | | | | | | | | | | | | | | |
|  | 8bqg.1.A | Formate dehydrogenase, alpha subunit, selenocysteine-containing  *W-formate dehydrogenase from Desulfovibrio vulgaris - Soaking with Formate 1 min* | 0.28 |  | 14.94 | 0.52 | 59-161 | X-ray | 1.95 | hetero-1-1-mer | 2 x MGD, 4 x SF4, 1 x H2S, 1 x W | HHblits | 0.27 |
| ``` target    MAQGVSRRQLLGRALALGSGAALADLLGPARFLSPAGAATAGAVVPGNPLRVMPDRTWEQIYRNQFEDDSTFVFTCAPND 8bqg.1    ----------------------------------------------------------EL-QKL--QWAKQTT-SICCYC  target    THNCLLRAHVK---NGVVVRISPTYGYGEATDLYGNRASHRWDPRTCQKGLILSRRFYSERRVKAPMIRKG-FKDWVEAG 8bqg.1    AVGCGLIVHTAKDGQGRAVNVEGDPD------------HPINEGSLCPKGASIFQLGENDQRGTQPLYRAPFSDTWKPVT  target    YPRNDDGTPQM 8bqg.1    WDFAL------ ``` | | | | | | | | | | | | | | | | | | | | | | | | | | | | | | | | | | | | | | | | | | | | | | | | | |
|  | 7t2r.1.A | NiFe hydrogenase subunit A  *Structure of electron bifurcating Ni-Fe hydrogenase complex HydABCSL in FMN-free apo state* | 0.28 |  | 24.69 | 0.49 | 66-160 | EM | 0.00 | hetero-2-2-2-2-2-mer | 6 x FES, 12 x SF4, 2 x 3NI, 2 x FCO | HHblits | 0.33 |
| ``` target    MAQGVSRRQLLGRALALGSGAALADLLGPARFLSPAGAATAGAVVPGNPLRVMPDRTWEQIYRNQFEDDSTFVFTCAPND 7t2r.1    -----------------------------------------------------------------SECDAVVESVC-PLC  target    THNCLLRAHVKNGVVVRISPTYGYGEATDLYGNRASHRWDPRTCQKGLILSRRFYSERRVKAPMIRKGFKDWVEAGYPRN 7t2r.1    AVGCKIKTYVRTGSIVRVEGTGV------------EEPDGGQLCHMGRWWLPESTERERVTVPLIREG-ASYREATWEEA  target    DDGTPQM 7t2r.1    ------- ``` | | | | | | | | | | | | | | | | | | | | | | | | | | | | | | | | | | | | | | | | | | | | | | | | | |
|  | 7t30.1.A | NiFe hydrogenase subunit A  *Structure of electron bifurcating Ni-Fe hydrogenase complex HydABCSL in FMN/NAD(H) bound state* | 0.28 |  | 24.69 | 0.49 | 66-160 | EM | 0.00 | hetero-2-2-2-2-2-mer | 4 x FES, 12 x SF4, 2 x NAD, 2 x FMN, 2 x 3NI, 2 x FCO | HHblits | 0.33 |
| ``` target    MAQGVSRRQLLGRALALGSGAALADLLGPARFLSPAGAATAGAVVPGNPLRVMPDRTWEQIYRNQFEDDSTFVFTCAPND 7t30.1    -----------------------------------------------------------------SECDAVVESVC-PLC  target    THNCLLRAHVKNGVVVRISPTYGYGEATDLYGNRASHRWDPRTCQKGLILSRRFYSERRVKAPMIRKGFKDWVEAGYPRN 7t30.1    AVGCKIKTYVRTGSIVRVEGTGV------------EEPDGGQLCHMGRWWLPESTERERVTVPLIREG-ASYREATWEEA  target    DDGTPQM 7t30.1    ------- ``` | | | | | | | | | | | | | | | | | | | | | | | | | | | | | | | | | | | | | | | | | | | | | | | | | |
|  | 4aay.1.A | AROA  *Crystal Structure of the arsenite oxidase protein complex from Rhizobium species strain NT-26* | 0.25 |  | 7.69 | 0.54 | 70-161 | X-ray | 2.70 | hetero-oligomer | 4 x MGD, 2 x O, 2 x 4MO, 2 x F3S, 2 x FES | HHblits | 0.23 |
| ``` target    MAQGVSRRQLLGRALALGSGAALADLLGPARFLSPAGAATAGAVVPGNPLRVMPDRTWEQIYRNQFEDDSTFVFTCAPND 4aay.1    ---------------------------------------------------------------------AKKHNVTCHFC  target    THNCLLRAHVK--------------NGVVVRISPTYGYGEATD---------------LYGNRA--SHRWDPRTCQKGLI 4aay.1    IVGCGYHAYTWPINKQGGTDPQNNIFGVDLSEQQQAESDAWYSPSMYNVVKQDGRDVHVVIKPDHECVVNSGLGSVRGAR  target    LSRRFY------SERRVKAPMIRKGFKDWVEAGYPRNDDGTPQM 4aay.1    MAETSFSEARNTQQQRLTDPLVWRY-GQMQPTSWDDAL------ ``` | | | | | | | | | | | | | | | | | | | | | | | | | | | | | | | | | | | | | | | | | | | | | | | | | |
|  | 1ogy.1.A | PERIPLASMIC NITRATE REDUCTASE  *Crystal structure of the heterodimeric nitrate reductase from Rhodobacter sphaeroides* | 0.25 |  | 17.65 | 0.51 | 63-160 | X-ray | 3.20 | hetero-1-1-mer | 1 x SF4, 1 x MO, 2 x MGD, 2 x HEC | HHblits | 0.28 |
| ``` target    MAQGVSRRQLLGRALALGSGAALADLLGPARFLSPAGAATAGAVVPGNPLRVMPDRTWEQIYRNQFEDDSTFVFTCAPND 1ogy.1    --------------------------------------------------------------GAEALRIRWSKAPC-RFC  target    THNCLLRAHVKNGVVVRISPTYGYGEATDLYGNRASHRWDPRTCQKGLILSRRFYSERRVKAPMIRKG------FKDWVE 1ogy.1    GTGCGVMVGTRDGQVVATHGDTQ------------AEVNRGLNCVKGYFLSKIMYGEDRLTTPLLRMKDGVYHKEGEFAP  target    AGYPRNDDGTPQM 1ogy.1    VSWDEA------- ``` | | | | | | | | | | | | | | | | | | | | | | | | | | | | | | | | | | | | | | | | | | | | | | | | | |
|  | 6cz7.1.A | ArrA  *The arsenate respiratory reductase (Arr) complex from Shewanella sp. ANA-3* | 0.29 |  | 17.07 | 0.49 | 67-161 | X-ray | 1.62 | hetero-1-1-mer | 5 x SF4, 2 x MGD, 1 x MO, 1 x PG5 | HHblits | 0.30 |
| ``` target    MAQGVSRRQLLGRALALGSGAALADLLGPARFLSPAGAATAGAVVPGNPLRVMPDRTWEQIYRNQFEDDSTFVFTCAPND 6cz7.1    ------------------------------------------------------------------GVGEWLATTC-QGC  target    THNCLLRAHVKNGVVVRISPTYGYGEATDLYGNRASHRWDPRTCQKGLILSRRFYSERRVKAPMIRKG-------FKDWV 6cz7.1    TSWCAKQIYVMDGRALKVRGNPN------------SGVHGMSSCPRQHLSLQQVYDPDRLRTPMMRTNPKKGRDQDPKFV  target    EAGYPRNDDGTPQM 6cz7.1    PISWDKAL------ ``` | | | | | | | | | | | | | | | | | | | | | | | | | | | | | | | | | | | | | | | | | | | | | | | | | |
|  | 6tg9.1.A | Formate dehydrogenase subunit alpha  *Cryo-EM Structure of NADH reduced form of NAD+-dependent Formate Dehydrogenase from Rhodobacter capsulatus* | 0.27 |  | 25.00 | 0.48 | 69-161 | EM | 3.24 | hetero-2-2-2-2-mer | 4 x MGD, 2 x 6MO, 4 x FES, 10 x SF4, 2 x H2S, 2 x FMN, 2 x NAI | HHblits | 0.32 |
| ``` target    MAQGVSRRQLLGRALALGSGAALADLLGPARFLSPAGAATAGAVVPGNPLRVMPDRTWEQIYRNQFEDDSTFVFTCAPND 6tg9.1    --------------------------------------------------------------------ERKVVTTC-AYC  target    THNCLLRAHVKNGVVVRISPTYGYGEATDLYGNRASHRWDPRTCQKGLILSRRFYSERRVKAPMIRKG-FKDWVEAGYPR 6tg9.1    GVGCSFEAHMLGDQLVRMVPWKG------------GAANRGHSCVKGRFAYGYATHQDRILKPMIRDKITDPWREVNWTE  target    NDDGTPQM 6tg9.1    AL------ ``` | | | | | | | | | | | | | | | | | | | | | | | | | | | | | | | | | | | | | | | | | | | | | | | | | |
|  | 7bkb.1.F | Formate dehydrogenase  *Formate dehydrogenase - heterodisulfide reductase - formylmethanofuran dehydrogenase complex from Methanospirillum hungatei (hexameric, composite structure)* | 0.27 |  | 26.92 | 0.47 | 70-161 | EM | 0.00 | hetero-2-2-2-2-2-2-… | 48 x SF4, 4 x FAD, 2 x FES, 4 x 9S8, 4 x ZN, 2 x MO, 4 x MGD | HHblits | 0.33 |
| ``` target    MAQGVSRRQLLGRALALGSGAALADLLGPARFLSPAGAATAGAVVPGNPLRVMPDRTWEQIYRNQFEDDSTFVFTCAPND 7bkb.1    ---------------------------------------------------------------------KYVATTCP-YC  target    THNCLLRAHVKNGVVVRISPTYGYGEATDLYGNRASHRWDPRTCQKGLILSRRFYSERRVKAPMIRKGFKDWVEAGYPRN 7bkb.1    GVGCTLNLVVSNGKVVGVEPNQR------------SPINEGKLCPKGVTCWEHIHSPDRLTTPLIKKD-GKFIEASWDEA  target    DDGTPQM 7bkb.1    L------ ``` | | | | | | | | | | | | | | | | | | | | | | | | | | | | | | | | | | | | | | | | | | | | | | | | | |
|  | 2v3v.1.A | PERIPLASMIC NITRATE REDUCTASE  *A NEW CATALYTIC MECHANISM OF PERIPLASMIC NITRATE REDUCTASE FROM DESULFOVIBRIO DESULFURICANS ATCC 27774 FROM CRYSTALLOGRAPHIC AND EPR DATA AND BASED ON DETAILED ANALYSIS OF THE SIXTH LIGAND* | 0.27 |  | 21.52 | 0.47 | 68-160 | X-ray | 1.99 | monomer | 1 x SF4, 1 x MO, 2 x MGD, 4 x LCP | HHblits | 0.30 |
| ``` target    MAQGVSRRQLLGRALALGSGAALADLLGPARFLSPAGAATAGAVVPGNPLRVMPDRTWEQIYRNQFEDDSTFVFTCAPND 2v3v.1    -------------------------------------------------------------------PEKWVKGVC-RYC  target    THNCLLRAHVKNGVVVRISPTYGYGEATDLYGNRASHRWDPRTCQKGLILSRRFYSERRVKAPMIRKG-FKDWVEAGYPR 2v3v.1    GTGCGVLVGVKDGKAVAIQGDPN------------NH-NAGLLCLKGSLLIPVLNSKERVTQPLVRRHKGGKLEPVSWDE  target    NDDGTPQM 2v3v.1    A------- ``` | | | | | | | | | | | | | | | | | | | | | | | | | | | | | | | | | | | | | | | | | | | | | | | | | |
|  | 2v45.1.A | PERIPLASMIC NITRATE REDUCTASE  *A NEW CATALYTIC MECHANISM OF PERIPLASMIC NITRATE REDUCTASE FROM DESULFOVIBRIO DESULFURICANS ATCC 27774 FROM CRYSTALLOGRAPHIC AND EPR DATA AND BASED ON DETAILED ANALYSIS OF THE SIXTH LIGAND* | 0.27 |  | 21.52 | 0.47 | 69-161 | X-ray | 2.40 | monomer | 1 x SF4, 1 x MO, 2 x MGD, 1 x LCP | HHblits | 0.30 |
| ``` target    MAQGVSRRQLLGRALALGSGAALADLLGPARFLSPAGAATAGAVVPGNPLRVMPDRTWEQIYRNQFEDDSTFVFTCAPND 2v45.1    --------------------------------------------------------------------EKWVKGVC-RYC  target    THNCLLRAHVKNGVVVRISPTYGYGEATDLYGNRASHRWDPRTCQKGLILSRRFYSERRVKAPMIRKG-FKDWVEAGYPR 2v45.1    GTGCGVLVGVKDGKAVAIQGNPN------------NH-NAGLLCLKGSLLIPVLNSKERVTQPLVRRHKGGKLEPVSWDE  target    NDDGTPQM 2v45.1    AL------ ``` | | | | | | | | | | | | | | | | | | | | | | | | | | | | | | | | | | | | | | | | | | | | | | | | | |
|  | 7qv7.1.L | Hydrogen dependent carbon dioxide reductase subunit FdhF  *Cryo-EM structure of Hydrogen-dependent CO2 reductase.* | 0.26 |  | 23.38 | 0.46 | 70-160 | EM | 0.00 | hetero-2-6-6-2-mer | 52 x SF4, 6 x 402 | HHblits | 0.32 |
| ``` target    MAQGVSRRQLLGRALALGSGAALADLLGPARFLSPAGAATAGAVVPGNPLRVMPDRTWEQIYRNQFEDDSTFVFTCAPND 7qv7.1    ---------------------------------------------------------------------EKVLTTC-PYC  target    THNCLLRAHVKNGVVVRISPTYGYGEATDLYGNRASHRWDPRTCQKGLILSRRFYSERRVKAPMIRKGFKDWVEAGYPRN 7qv7.1    GTGCGLYLKVENEKIVGVEPDKL------------HPVNQGELCIKGYYGYKYVHDPRRLTSPLIKKN-GKFVPVSWDEA  target    DDGTPQM 7qv7.1    ------- ``` | | | | | | | | | | | | | | | | | | | | | | | | | | | | | | | | | | | | | | | | | | | | | | | | | |
|  | 7qv7.1.O | Hydrogen dependent carbon dioxide reductase subunit FdhF  *Cryo-EM structure of Hydrogen-dependent CO2 reductase.* | 0.27 |  | 23.38 | 0.46 | 70-160 | EM | 0.00 | hetero-2-6-6-2-mer | 52 x SF4, 6 x 402 | HHblits | 0.32 |
| ``` target    MAQGVSRRQLLGRALALGSGAALADLLGPARFLSPAGAATAGAVVPGNPLRVMPDRTWEQIYRNQFEDDSTFVFTCAPND 7qv7.1    ---------------------------------------------------------------------EKVLTTC-PYC  target    THNCLLRAHVKNGVVVRISPTYGYGEATDLYGNRASHRWDPRTCQKGLILSRRFYSERRVKAPMIRKGFKDWVEAGYPRN 7qv7.1    GTGCGLYLKVENEKIVGVEPDKL------------HPVNQGELCIKGYYGYKYVHDPRRLTSPLIKKN-GKFVPVSWDEA  target    DDGTPQM 7qv7.1    ------- ``` | | | | | | | | | | | | | | | | | | | | | | | | | | | | | | | | | | | | | | | | | | | | | | | | | |
|  | 1h0h.1.A | FORMATE DEHYDROGENASE SUBUNIT ALPHA  *Tungsten containing Formate Dehydrogenase from Desulfovibrio Gigas* | 0.22 |  | 18.99 | 0.47 | 70-161 | X-ray | 1.80 | hetero-1-1-mer | 1 x W, 1 x 2MD, 1 x MGD, 4 x SF4, 1 x CA | HHblits | 0.29 |
| ``` target    MAQGVSRRQLLGRALALGSGAALADLLGPARFLSPAGAATAGAVVPGNPLRVMPDRTWEQIYRNQFEDDSTFVFTCAPND 1h0h.1    ---------------------------------------------------------------------KQTTSVC-CYC  target    THNCLLRAHV--KNGVVVRISPTYGYGEATDLYGNRASHRWDPRTCQKGLILSRRFYSERRVKAPMIRKG-FKDWVEAGY 1h0h.1    SVGCGLIVHTDKKTNRAINVEGDPD------------HPINEGSLCAKGASTWQLAENERRPANPLYRAPGSDQWEEKSW  target    PRNDDGTPQM 1h0h.1    DWML------ ``` | | | | | | | | | | | | | | | | | | | | | | | | | | | | | | | | | | | | | | | | | | | | | | | | | |
|  | 7vw6.1.A | Formate dehydrogenase  *Cryo-EM Structure of Formate Dehydrogenase 1 from Methylorubrum extorquens AM1* | 0.26 |  | 21.52 | 0.47 | 69-161 | EM | 0.00 | hetero-1-1-mer | 4 x SF4, 2 x FES, 2 x MGD, 1 x W, 1 x FMN | HHblits | 0.29 |
| ``` target    MAQGVSRRQLLGRALALGSGAALADLLGPARFLSPAGAATAGAVVPGNPLRVMPDRTWEQIYRNQFEDDSTFVFTCAPND 7vw6.1    --------------------------------------------------------------------DREVKSLC-PYC  target    THNCLLRAHVKNGVVVRISPTYGYGEATDLYGNRASHRWDPRTCQKGLILSRRFYSERRVKAPMIRKGF----------- 7vw6.1    GVGCQVSYKVKDERIVYAEGVN-------------GPANQNRLCVKGRFGFDYVHHPHRLTVPLIRLENVPKDANDQVDP  target    ----KDWVEAGYPRNDDGTPQM 7vw6.1    ANPWTHFREATWEEAL------ ``` | | | | | | | | | | | | | | | | | | | | | | | | | | | | | | | | | | | | | | | | | | | | | | | | | |
|  | 7e5z.1.A | Formate dehydrogenase  *Dehydrogenase holoenzyme* | 0.23 |  | 21.52 | 0.47 | 69-161 | EM | 0.00 | hetero-1-1-mer | 1 x W, 2 x MGD, 2 x FES, 4 x SF4, 1 x FMN | HHblits | 0.29 |
| ``` target    MAQGVSRRQLLGRALALGSGAALADLLGPARFLSPAGAATAGAVVPGNPLRVMPDRTWEQIYRNQFEDDSTFVFTCAPND 7e5z.1    --------------------------------------------------------------------DREVKSLC-PYC  target    THNCLLRAHVKNGVVVRISPTYGYGEATDLYGNRASHRWDPRTCQKGLILSRRFYSERRVKAPMIRKGF----------- 7e5z.1    GVGCQVSYKVKDERIVYAEGVN-------------GPANQNRLCVKGRFGFDYVHHPHRLTVPLIRLENVPKDANDQVDP  target    ----KDWVEAGYPRNDDGTPQM 7e5z.1    ANPWTHFREATWEEAL------ ``` | | | | | | | | | | | | | | | | | | | | | | | | | | | | | | | | | | | | | | | | | | | | | | | | | |
|  | 3m9s.1.C | NADH-quinone oxidoreductase subunit 3  *Crystal structure of respiratory complex I from Thermus thermophilus* | 0.25 | 0.00 | 22.08 | 0.46 | 70-161 | X-ray | 4.50 | monomer | 7 x SF4, 2 x FES, 1 x FMN | HHblits | 0.30 |
| ``` target    MAQGVSRRQLLGRALALGSGAALADLLGPARFLSPAGAATAGAVVPGNPLRVMPDRTWEQIYRNQFEDDSTFVFTCAPND 3m9s.1    ---------------------------------------------------------------------EETPTTC-ALC  target    THNCLLRAHVKNGVVVRISPTYGYGEATDLYGNRASHRWDPRTCQKGLILSRRFYSERRVKAPMIRKGFKDWVEAGYPRN 3m9s.1    PVGCGITADTRSGELLRIRAREV------------PEVNEIWICDAGRFGHE-WADQNRLKTPLVRKE-GRLVEATWEEA  target    DDGTPQM 3m9s.1    F------ ``` | | | | | | | | | | | | | | | | | | | | | | | | | | | | | | | | | | | | | | | | | | | | | | | | | |
|  | 2fug.2.C | NADH-quinone oxidoreductase chain 3  *Crystal structure of the hydrophilic domain of respiratory complex I from Thermus thermophilus* | 0.24 | 0.00 | 22.08 | 0.46 | 70-161 | X-ray | 3.30 | monomer | 7 x SF4, 2 x FES, 1 x FMN | HHblits | 0.30 |
| ``` target    MAQGVSRRQLLGRALALGSGAALADLLGPARFLSPAGAATAGAVVPGNPLRVMPDRTWEQIYRNQFEDDSTFVFTCAPND 2fug.2    ---------------------------------------------------------------------EETPTTC-ALC  target    THNCLLRAHVKNGVVVRISPTYGYGEATDLYGNRASHRWDPRTCQKGLILSRRFYSERRVKAPMIRKGFKDWVEAGYPRN 2fug.2    PVGCGITADTRSGELLRIRAREV------------PEVNEIWICDAGRFGHE-WADQNRLKTPLVRKE-GRLVEATWEEA  target    DDGTPQM 2fug.2    F------ ``` | | | | | | | | | | | | | | | | | | | | | | | | | | | | | | | | | | | | | | | | | | | | | | | | | |
|  | 6zjl.1.C | NADH-quinone oxidoreductase subunit 3  *Respiratory complex I from Thermus thermophilus, NAD+ dataset, major state* | 0.25 | 0.00 | 22.08 | 0.46 | 70-161 | EM | 0.00 | monomer | 7 x SF4, 1 x FMN, 2 x FES | HHblits | 0.30 |
| ``` target    MAQGVSRRQLLGRALALGSGAALADLLGPARFLSPAGAATAGAVVPGNPLRVMPDRTWEQIYRNQFEDDSTFVFTCAPND 6zjl.1    ---------------------------------------------------------------------EETPTTC-ALC  target    THNCLLRAHVKNGVVVRISPTYGYGEATDLYGNRASHRWDPRTCQKGLILSRRFYSERRVKAPMIRKGFKDWVEAGYPRN 6zjl.1    PVGCGITADTRSGELLRIRAREV------------PEVNEIWICDAGRFGHE-WADQNRLKTPLVRKE-GRLVEATWEEA  target    DDGTPQM 6zjl.1    F------ ``` | | | | | | | | | | | | | | | | | | | | | | | | | | | | | | | | | | | | | | | | | | | | | | | | | |
|  | 6q8o.1.C | NADH-quinone oxidoreductase subunit 3  *Respiratory complex I from Thermus thermophilus with bound Piericidin A* | 0.25 | 0.00 | 22.08 | 0.46 | 70-161 | X-ray | 3.61 | monomer | 7 x SF4, 1 x FMN, 2 x FES, 1 x HQH | HHblits | 0.30 |
| ``` target    MAQGVSRRQLLGRALALGSGAALADLLGPARFLSPAGAATAGAVVPGNPLRVMPDRTWEQIYRNQFEDDSTFVFTCAPND 6q8o.1    ---------------------------------------------------------------------EETPTTC-ALC  target    THNCLLRAHVKNGVVVRISPTYGYGEATDLYGNRASHRWDPRTCQKGLILSRRFYSERRVKAPMIRKGFKDWVEAGYPRN 6q8o.1    PVGCGITADTRSGELLRIRAREV------------PEVNEIWICDAGRFGHE-WADQNRLKTPLVRKE-GRLVEATWEEA  target    DDGTPQM 6q8o.1    F------ ``` | | | | | | | | | | | | | | | | | | | | | | | | | | | | | | | | | | | | | | | | | | | | | | | | | |
|  | 6zjy.1.C | NADH-quinone oxidoreductase subunit 3  *Respiratory complex I from Thermus thermophilus, NAD+ dataset, minor state* | 0.24 | 0.00 | 22.08 | 0.46 | 70-161 | EM | 0.00 | monomer | 7 x SF4, 2 x FES | HHblits | 0.30 |
| ``` target    MAQGVSRRQLLGRALALGSGAALADLLGPARFLSPAGAATAGAVVPGNPLRVMPDRTWEQIYRNQFEDDSTFVFTCAPND 6zjy.1    ---------------------------------------------------------------------EETPTTC-ALC  target    THNCLLRAHVKNGVVVRISPTYGYGEATDLYGNRASHRWDPRTCQKGLILSRRFYSERRVKAPMIRKGFKDWVEAGYPRN 6zjy.1    PVGCGITADTRSGELLRIRAREV------------PEVNEIWICDAGRFGHE-WADQNRLKTPLVRKE-GRLVEATWEEA  target    DDGTPQM 6zjy.1    F------ ``` | | | | | | | | | | | | | | | | | | | | | | | | | | | | | | | | | | | | | | | | | | | | | | | | | |
|  | 6zjn.1.C | NADH-quinone oxidoreductase subunit 3  *Respiratory complex I from Thermus thermophilus, NADH dataset, minor state* | 0.25 | 0.00 | 22.08 | 0.46 | 70-161 | EM | 0.00 | monomer | 7 x SF4, 2 x FES | HHblits | 0.30 |
| ``` target    MAQGVSRRQLLGRALALGSGAALADLLGPARFLSPAGAATAGAVVPGNPLRVMPDRTWEQIYRNQFEDDSTFVFTCAPND 6zjn.1    ---------------------------------------------------------------------EETPTTC-ALC  target    THNCLLRAHVKNGVVVRISPTYGYGEATDLYGNRASHRWDPRTCQKGLILSRRFYSERRVKAPMIRKGFKDWVEAGYPRN 6zjn.1    PVGCGITADTRSGELLRIRAREV------------PEVNEIWICDAGRFGHE-WADQNRLKTPLVRKE-GRLVEATWEEA  target    DDGTPQM 6zjn.1    F------ ``` | | | | | | | | | | | | | | | | | | | | | | | | | | | | | | | | | | | | | | | | | | | | | | | | | |
|  | 6ziy.1.C | NADH-quinone oxidoreductase subunit 3  *Respiratory complex I from Thermus thermophilus, NADH dataset, major state* | 0.24 | 0.00 | 22.08 | 0.46 | 70-161 | EM | 0.00 | monomer | 7 x SF4, 1 x FMN, 1 x NAI, 2 x FES | HHblits | 0.30 |
| ``` target    MAQGVSRRQLLGRALALGSGAALADLLGPARFLSPAGAATAGAVVPGNPLRVMPDRTWEQIYRNQFEDDSTFVFTCAPND 6ziy.1    ---------------------------------------------------------------------EETPTTC-ALC  target    THNCLLRAHVKNGVVVRISPTYGYGEATDLYGNRASHRWDPRTCQKGLILSRRFYSERRVKAPMIRKGFKDWVEAGYPRN 6ziy.1    PVGCGITADTRSGELLRIRAREV------------PEVNEIWICDAGRFGHE-WADQNRLKTPLVRKE-GRLVEATWEEA  target    DDGTPQM 6ziy.1    F------ ``` | | | | | | | | | | | | | | | | | | | | | | | | | | | | | | | | | | | | | | | | | | | | | | | | | |
|  | 1aa6.1.A | FORMATE DEHYDROGENASE H  *REDUCED FORM OF FORMATE DEHYDROGENASE H FROM E. COLI* | 0.25 |  | 25.00 | 0.46 | 71-160 | X-ray | 2.30 | monomer | 1 x SF4, 2 x MGD, 1 x 4MO | HHblits | 0.31 |
| ``` target    MAQGVSRRQLLGRALALGSGAALADLLGPARFLSPAGAATAGAVVPGNPLRVMPDRTWEQIYRNQFEDDSTFVFTCAPND 1aa6.1    ----------------------------------------------------------------------KVVTVC-PYC  target    THNCLLRAHVKNGVVVRISPTYGYGEATDLYGNRASHRWDPRTCQKGLILSRRFYSE----RRVKAPMIRKG-FKDWVEA 1aa6.1    ASGCKINLVVDNGKIVRAEAAQG-------------KTNQGTLCLKGYYGWDFINDTQILTPRLKTPMIRRQRGGKLEPV  target    GYPRNDDGTPQM 1aa6.1    SWDEA------- ``` | | | | | | | | | | | | | | | | | | | | | | | | | | | | | | | | | | | | | | | | | | | | | | | | | |
|  | 1fdo.1.A | FORMATE DEHYDROGENASE H  *OXIDIZED FORM OF FORMATE DEHYDROGENASE H FROM E. COLI* | 0.24 |  | 25.00 | 0.46 | 71-160 | X-ray | 2.80 | monomer | 1 x SF4, 2 x MGD, 1 x 6MO | HHblits | 0.31 |
| ``` target    MAQGVSRRQLLGRALALGSGAALADLLGPARFLSPAGAATAGAVVPGNPLRVMPDRTWEQIYRNQFEDDSTFVFTCAPND 1fdo.1    ----------------------------------------------------------------------KVVTVC-PYC  target    THNCLLRAHVKNGVVVRISPTYGYGEATDLYGNRASHRWDPRTCQKGLILSRRFYSE----RRVKAPMIRKG-FKDWVEA 1fdo.1    ASGCKINLVVDNGKIVRAEAAQG-------------KTNQGTLCLKGYYGWDFINDTQILTPRLKTPMIRRQRGGKLEPV  target    GYPRNDDGTPQM 1fdo.1    SWDEA------- ``` | | | | | | | | | | | | | | | | | | | | | | | | | | | | | | | | | | | | | | | | | | | | | | | | | |
|  | 2iv2.1.A | Formate dehydrogenase H  *Reinterpretation of reduced form of formate dehydrogenase H from E. coli* | 0.25 |  | 25.00 | 0.46 | 71-160 | X-ray | 2.27 | monomer | 1 x SF4, 1 x 2MD, 1 x MGD | HHblits | 0.31 |
| ``` target    MAQGVSRRQLLGRALALGSGAALADLLGPARFLSPAGAATAGAVVPGNPLRVMPDRTWEQIYRNQFEDDSTFVFTCAPND 2iv2.1    ----------------------------------------------------------------------KVVTVC-PYC  target    THNCLLRAHVKNGVVVRISPTYGYGEATDLYGNRASHRWDPRTCQKGLILSRRFYSE----RRVKAPMIRKG-FKDWVEA 2iv2.1    ASGCKINLVVDNGKIVRAEAAQG-------------KTNQGTLCLKGYYGWDFINDTQILTPRLKTPMIRRQRGGKLEPV  target    GYPRNDDGTPQM 2iv2.1    SWDEA------- ``` | | | | | | | | | | | | | | | | | | | | | | | | | | | | | | | | | | | | | | | | | | | | | | | | | |
|  | 7z0t.1.G | Formate dehydrogenase H  *Structure of the Escherichia coli formate hydrogenlyase complex (aerobic preparation, composite structure)* | 0.24 |  | 25.00 | 0.46 | 71-160 | EM | 0.00 | hetero-1-1-1-1-1-1-… | 1 x NI, 1 x FCO, 8 x SF4, 1 x FE, 2 x MGD, 1 x 6MO | HHblits | 0.31 |
| ``` target    MAQGVSRRQLLGRALALGSGAALADLLGPARFLSPAGAATAGAVVPGNPLRVMPDRTWEQIYRNQFEDDSTFVFTCAPND 7z0t.1    ----------------------------------------------------------------------KVVTVC-PYC  target    THNCLLRAHVKNGVVVRISPTYGYGEATDLYGNRASHRWDPRTCQKGLILSRRFYSE----RRVKAPMIRKG-FKDWVEA 7z0t.1    ASGCKINLVVDNGKIVRAEAAQG-------------KTNQGTLCLKGYYGWDFINDTQILTPRLKTPMIRRQRGGKLEPV  target    GYPRNDDGTPQM 7z0t.1    SWDEA------- ``` | | | | | | | | | | | | | | | | | | | | | | | | | | | | | | | | | | | | | | | | | | | | | | | | | |
|  | 5nqd.1.A | AroA  *Arsenite oxidase AioAB from Rhizobium sp. str. NT-26 mutant AioBF108A* | 0.22 |  | 9.76 | 0.49 | 69-161 | X-ray | 2.20 | hetero-2-2-mer | 4 x MGD, 2 x O, 2 x 4MO, 2 x F3S, 2 x FES | HHblits | 0.25 |
| ``` target    MAQGVSRRQLLGRALALGSGAALADLLGPARFLSPAGAATAGAVVPGNPLRVMPDRTWEQIYRNQFEDDSTFVFTCAPND 5nqd.1    --------------------------------------------------------------------DAKKHNVTCHFC  target    THNCLLRAHV-----------------------------------------KNGVVVRISPTYGYGEATDLYGNRASHRW 5nqd.1    IVGCGYHAYTWPINKQGGTDPQNNIFGVDLSEQQQAESDAWYSPSMYNVVKQDGRDVHVVIKPDH----------ECVVN  target    DPRTCQKGLILSRRFY------SERRVKAPMIRKGFKDWVEAGYPRNDDGTPQM 5nqd.1    SGLGSVRGARMAETSFSEARNTQQQRLTDPLVWRY-GQMQPTSWDDAL------ ``` | | | | | | | | | | | | | | | | | | | | | | | | | | | | | | | | | | | | | | | | | | | | | | | | | |
|  | 1g8k.1.A | ARSENITE OXIDASE  *CRYSTAL STRUCTURE ANALYSIS OF ARSENITE OXIDASE FROM ALCALIGENES FAECALIS* | 0.22 |  | 15.38 | 0.47 | 70-161 | X-ray | 1.64 | hetero-1-1-mer | 3 x HG, 2 x CA, 2 x MGD, 1 x O, 1 x 4MO, 1 x F3S, 1 x FES | HHblits | 0.28 |
| ``` target    MAQGVSRRQLLGRALALGSGAALADLLGPARFLSPAGAATAGAVVPGNPLRVMPDRTWEQIYRNQFEDDSTFVFTCAPND 1g8k.1    ---------------------------------------------------------------------QRTNMT-CHFC  target    THNCLLRAHVKN-----GV--------------------------------------VVRISPTYGYGEATDLYGNRASH 1g8k.1    IVGCGYHVYKWPELEEGGRAPEQNALGLDFRKQLPPLAVTLTPAMTNVVTEHDGARYDIMVVPDK------------ACV  target    RWDPRTCQKGLILSRRFYSE-----RRVKAPMIRKGFKDWVEAGYPRNDDGTPQM 1g8k.1    VNSGLSSTRGGKMASYMYTPTGDGKERLSAPRLYAA-DEWVDTTWDHAM------ ``` | | | | | | | | | | | | | | | | | | | | | | | | | | | | | | | | | | | | | | | | | | | | | | | | | |
|  | 6qcf.1.C | NADH:ubiquinone oxidoreductase core subunit S1  *Ovine respiratory complex I FRC open class 6* | 0.23 | 0.00 | 13.75 | 0.48 | 69-161 | EM | 0.00 | monomer | 6 x SF4, 1 x FMN, 2 x FES, 1 x ZN, 1 x NDP, 2 x ZMP | HHblits | 0.26 |
| ``` target    MAQGVSRRQLLGRALALGSGAALADLLGPARFLSPAGAATAGAVVPGNPLRVMPDRTWEQIYRNQFEDDSTFVFTCAPND 6qcf.1    --------------------------------------------------------------------ETRKTESIDVMD  target    THNCLLRAHVKNGVVVRISPTYGYGEATDLYGNRASHRWDPRTCQKGLILSRRFYSERRVKAPMIRKGFKDWVEAGYPRN 6qcf.1    AVGSNIVVSTRTGEVMRILPRMH------------EDINEEWISDKTRFAYDGLK-RQRLTEPMVRNEKGLLTHTTWEDA  target    DDGTPQM 6qcf.1    L------ ``` | | | | | | | | | | | | | | | | | | | | | | | | | | | | | | | | | | | | | | | | | | | | | | | | | |
|  | 6qc5.1.C | NADH:ubiquinone oxidoreductase core subunit S1  *Ovine respiratory complex I FRC closed class 1* | 0.23 | 0.00 | 13.75 | 0.48 | 69-161 | EM | 0.00 | monomer | 6 x SF4, 1 x FMN, 2 x FES, 2 x 3PE, 1 x ZN, 1 x NDP, 2 x ZMP, 1 x PC1 | HHblits | 0.26 |
| ``` target    MAQGVSRRQLLGRALALGSGAALADLLGPARFLSPAGAATAGAVVPGNPLRVMPDRTWEQIYRNQFEDDSTFVFTCAPND 6qc5.1    --------------------------------------------------------------------ETRKTESIDVMD  target    THNCLLRAHVKNGVVVRISPTYGYGEATDLYGNRASHRWDPRTCQKGLILSRRFYSERRVKAPMIRKGFKDWVEAGYPRN 6qc5.1    AVGSNIVVSTRTGEVMRILPRMH------------EDINEEWISDKTRFAYDGLK-RQRLTEPMVRNEKGLLTHTTWEDA  target    DDGTPQM 6qc5.1    L------ ``` | | | | | | | | | | | | | | | | | | | | | | | | | | | | | | | | | | | | | | | | | | | | | | | | | |
|  | 8e9g.1.G | NADH-quinone oxidoreductase subunit G  *Mycobacterial respiratory complex I with both quinone positions modelled* | 0.25 |  | 19.48 | 0.46 | 71-161 | EM | 0.00 | hetero-1-1-1-1-1-1-… |  | HHblits | 0.28 |
| ``` target    MAQGVSRRQLLGRALALGSGAALADLLGPARFLSPAGAATAGAVVPGNPLRVMPDRTWEQIYRNQFEDDSTFVFTCAPND 8e9g.1    ----------------------------------------------------------------------SSPSVC-EHC  target    THNCLLRAHVKNGVVVRISPTYGYGEATDLYGNRASHRWDPRTCQKGLILSRRFYSERRVKAPMIRKGFKDWVEAGYPRN 8e9g.1    ASGCAQRTDHRRGKVLRRLAGDE------------PEVNEEWNCDKGRWAFTYATVGDRITTPMLRDG-GVLRPASWSEA  target    DDGTPQM 8e9g.1    L------ ``` | | | | | | | | | | | | | | | | | | | | | | | | | | | | | | | | | | | | | | | | | | | | | | | | | |
|  | 7tgh.58.A | NADH-ubiquinone oxidoreductase 75 kDa subunit  *Cryo-EM structure of respiratory super-complex CI+III2 from Tetrahymena thermophila* | 0.23 |  | 12.82 | 0.47 | 71-161 | EM | 0.00 | monomer |  | HHblits | 0.27 |
| ``` target    MAQGVSRRQLLGRALALGSGAALADLLGPARFLSPAGAATAGAVVPGNPLRVMPDRTWEQIYRNQFEDDSTFVFTCAPND 7tgh.58   ----------------------------------------------------------------------KSFYTSDVFD  target    THNCLLRAHVKNGVVVRISPTYGYGEATDLYGNRASHRWDPRTCQKGLILSRRFYSERRVKAPMIRKGFKDWVEAGYPRN 7tgh.58   TLGSAIQVDTRGPEIMRVLPRIH------------EEINEEWISDKTRHAFDGLK-RQRINSPMKRSKDGNYEDIFWEEA  target    DDGTPQM 7tgh.58   I------ ``` | | | | | | | | | | | | | | | | | | | | | | | | | | | | | | | | | | | | | | | | | | | | | | | | | |
|  | 7nz1.1.E | NADH-quinone oxidoreductase subunit G  *Respiratory complex I from Escherichia coli - focused refinement of cytoplasmic arm* | 0.24 |  | 16.88 | 0.46 | 71-161 | EM | 0.00 | hetero-1-1-1-1-1-1-… | 7 x SF4, 2 x FES, 1 x FMN, 1 x CA | HHblits | 0.28 |
| ``` target    MAQGVSRRQLLGRALALGSGAALADLLGPARFLSPAGAATAGAVVPGNPLRVMPDRTWEQIYRNQFEDDSTFVFTCAPND 7nz1.1    ----------------------------------------------------------------------FAPSIC-QQC  target    THNCLLRAHVKNGVVVRISPTYGYGEATDLYGNRASHRWDPRTCQKGLILSRRFYSERRVKAPMIRKGFKDWVEAGYPRN 7nz1.1    SIGCNISPGERYGELRRIENRYN------------GTVNHYFLCDRGRFGYGYVNLKDRPRQPVQRRG-DDFITLNAEQA  target    DDGTPQM 7nz1.1    M------ ``` | | | | | | | | | | | | | | | | | | | | | | | | | | | | | | | | | | | | | | | | | | | | | | | | | |
|  | 2e7z.1.A | Acetylene hydratase Ahy  *Acetylene Hydratase from Pelobacter acetylenicus* | 0.23 |  | 18.42 | 0.46 | 75-161 | X-ray | 1.26 | monomer | 1 x SF4, 2 x MGD, 1 x W | HHblits | 0.29 |
| ``` target    MAQGVSRRQLLGRALALGSGAALADLLGPARFLSPAGAATAGAVVPGNPLRVMPDRTWEQIYRNQFEDDSTFVFTCAPND 2e7z.1    --------------------------------------------------------------------------VVCQSC  target    THNCLLRAHVK-NGVVVRISPTYGYGEATDLYGNRASHRWDPRTCQKGLILSRRFYSERRVKAPMIRKG----FKDWVEA 2e7z.1    DINCVVEAEVKADGKIQTKSISEPHP-----------TTPPNSICMKSVNADTIRTHKDRVLYPLKNVGSKRGEQRWERI  target    GYPRNDDGTPQM 2e7z.1    SWDQAL------ ``` | | | | | | | | | | | | | | | | | | | | | | | | | | | | | | | | | | | | | | | | | | | | | | | | | |
|  | 1g8j.1.A | ARSENITE OXIDASE  *CRYSTAL STRUCTURE ANALYSIS OF ARSENITE OXIDASE FROM ALCALIGENES FAECALIS* | 0.19 |  | 14.47 | 0.46 | 72-160 | X-ray | 2.03 | hetero-oligomer | 2 x MGD, 1 x O, 1 x 4MO, 1 x F3S, 1 x FES | HHblits | 0.28 |
| ``` target    MAQGVSRRQLLGRALALGSGAALADLLGPARFLSPAGAATAGAVVPGNPLRVMPDRTWEQIYRNQFEDDSTFVFTCAPND 1g8j.1    -----------------------------------------------------------------------RTNMTCHFC  target    THNCLLRAHVKN-----GV--------------------------------------VVRISPTYGYGEATDLYGNRASH 1g8j.1    IVGCGYHVYKWPELEEGGRAPEQNALGLDFRKQLPPLASTLTPAMTNVVTEHDGARYDIMVVPDK------------ACV  target    RWDPRTCQKGLILSRRFYSE-----RRVKAPMIRKGFKDWVEAGYPRNDDGTPQM 1g8j.1    VNSGLSSTRGGKMASYMYTPTGDGKERLSAPRLYAA-DEWVDTTWDHA------- ``` | | | | | | | | | | | | | | | | | | | | | | | | | | | | | | | | | | | | | | | | | | | | | | | | | |
|  | 5xtb.1.L | NADH-ubiquinone oxidoreductase 75 kDa subunit, mitochondrial  *Cryo-EM structure of human respiratory complex I matrix arm* | 0.24 | 0.00 | 14.10 | 0.47 | 71-161 | EM | 0.00 | monomer | 6 x SF4, 1 x FMN, 1 x 8Q1, 1 x NDP, 2 x FES | HHblits | 0.26 |
| ``` target    MAQGVSRRQLLGRALALGSGAALADLLGPARFLSPAGAATAGAVVPGNPLRVMPDRTWEQIYRNQFEDDSTFVFTCAPND 5xtb.1    ----------------------------------------------------------------------RKTESIDVMD  target    THNCLLRAHVKNGVVVRISPTYGYGEATDLYGNRASHRWDPRTCQKGLILSRRFYSERRVKAPMIRKGFKDWVEAGYPRN 5xtb.1    AVGSNIVVSTRTGEVMRILPRMH------------EDINEEWISDKTRFAYDGLK-RQRLTEPMVRNEKGLLTYTSWEDA  target    DDGTPQM 5xtb.1    L------ ``` | | | | | | | | | | | | | | | | | | | | | | | | | | | | | | | | | | | | | | | | | | | | | | | | | |
|  | 7dgr.10.A | NADH-ubiquinone oxidoreductase 75 kDa subunit, mitochondrial  *Activity optimized supercomplex state2* | 0.21 | 0.00 | 14.10 | 0.47 | 71-161 | EM | 0.00 | monomer |  | HHblits | 0.26 |
| ``` target    MAQGVSRRQLLGRALALGSGAALADLLGPARFLSPAGAATAGAVVPGNPLRVMPDRTWEQIYRNQFEDDSTFVFTCAPND 7dgr.10   ----------------------------------------------------------------------RKTESIDVMD  target    THNCLLRAHVKNGVVVRISPTYGYGEATDLYGNRASHRWDPRTCQKGLILSRRFYSERRVKAPMIRKGFKDWVEAGYPRN 7dgr.10   AVGSNIVVSTRTGEVMRILPRMH------------EDINEEWISDKTRFAYDGLK-RQRLTEPMVRNEKGLLTHTTWEDA  target    DDGTPQM 7dgr.10   L------ ``` | | | | | | | | | | | | | | | | | | | | | | | | | | | | | | | | | | | | | | | | | | | | | | | | | |
|  | 5o31.1.8 | NADH-ubiquinone oxidoreductase 75 kDa subunit, mitochondrial  *Mitochondrial complex I in the deactive state* | 0.21 | 0.00 | 14.10 | 0.47 | 71-161 | EM | 4.13 | monomer | 6 x SF4, 2 x FES, 1 x FMN, 1 x NAP, 1 x ZN | HHblits | 0.26 |
| ``` target    MAQGVSRRQLLGRALALGSGAALADLLGPARFLSPAGAATAGAVVPGNPLRVMPDRTWEQIYRNQFEDDSTFVFTCAPND 5o31.1    ----------------------------------------------------------------------RKTESIDVMD  target    THNCLLRAHVKNGVVVRISPTYGYGEATDLYGNRASHRWDPRTCQKGLILSRRFYSERRVKAPMIRKGFKDWVEAGYPRN 5o31.1    AVGSNIVVSTRTGEVMRILPRMH------------EDINEEWISDKTRFAYDGLK-RQRLTEPMVRNEKGLLTHTTWEDA  target    DDGTPQM 5o31.1    L------ ``` | | | | | | | | | | | | | | | | | | | | | | | | | | | | | | | | | | | | | | | | | | | | | | | | | |
|  | 7p63.1.C | NADH-quinone oxidoreductase  *Complex I from E. coli, DDM/LMNG-purified, under Turnover at pH 6, Closed state* | 0.23 |  | 15.79 | 0.46 | 73-161 | EM | 0.00 | hetero-1-1-1-1-1-1-… | 7 x SF4, 1 x FMN, 1 x NAI, 2 x FES, 1 x CA, 1 x DCQ, 4 x LFA, 8 x 3PE | HHblits | 0.28 |
| ``` target    MAQGVSRRQLLGRALALGSGAALADLLGPARFLSPAGAATAGAVVPGNPLRVMPDRTWEQIYRNQFEDDSTFVFTCAPND 7p63.1    ------------------------------------------------------------------------APSICQQC  target    THNCLLRAHVKNGVVVRISPTYGYGEATDLYGNRASHRWDPRTCQKGLILSRRFYSERRVKAPMIRKGFKDWVEAGYPRN 7p63.1    SIGCNISPGERYGELRRIENRYN------------GTVNHYFLCDRGRFGYGYVNLKDRPRQPVQRRG-DDFITLNAEQA  target    DDGTPQM 7p63.1    M------ ``` | | | | | | | | | | | | | | | | | | | | | | | | | | | | | | | | | | | | | | | | | | | | | | | | | |
|  | 7p61.1.C | NADH-quinone oxidoreductase  *Complex I from E. coli, DDM-purified, with NADH, Resting state* | 0.22 |  | 15.79 | 0.46 | 73-161 | EM | 0.00 | hetero-1-1-1-1-1-1-… | 7 x SF4, 1 x FMN, 1 x NAI, 2 x FES, 1 x CA, 2 x 3PE, 1 x UQ8 | HHblits | 0.28 |
| ``` target    MAQGVSRRQLLGRALALGSGAALADLLGPARFLSPAGAATAGAVVPGNPLRVMPDRTWEQIYRNQFEDDSTFVFTCAPND 7p61.1    ------------------------------------------------------------------------APSICQQC  target    THNCLLRAHVKNGVVVRISPTYGYGEATDLYGNRASHRWDPRTCQKGLILSRRFYSERRVKAPMIRKGFKDWVEAGYPRN 7p61.1    SIGCNISPGERYGELRRIENRYN------------GTVNHYFLCDRGRFGYGYVNLKDRPRQPVQRRG-DDFITLNAEQA  target    DDGTPQM 7p61.1    M------ ``` | | | | | | | | | | | | | | | | | | | | | | | | | | | | | | | | | | | | | | | | | | | | | | | | | |
|  | 7arc.1.F | 75 kDa  *Cryo-EM structure of Polytomella Complex-I (peripheral arm)* | 0.23 |  | 16.00 | 0.45 | 73-161 | EM | 0.00 | hetero-1-1-1-1-1-1-… | 6 x SF4, 2 x FES, 1 x FMN, 1 x NDP, 1 x ZN, 1 x 8Q1 | HHblits | 0.28 |
| ``` target    MAQGVSRRQLLGRALALGSGAALADLLGPARFLSPAGAATAGAVVPGNPLRVMPDRTWEQIYRNQFEDDSTFVFTCAPND 7arc.1    ------------------------------------------------------------------------TETIDVSD  target    THNCLLRAHVKNGVVVRISPTYGYGEATDLYGNRASHRWDPRTCQKGLILSRRFYSERRVKAPMIRKGFKDWVEAGYPRN 7arc.1    ALGSNIKVDCRGTEVMRITPRLN------------DAINEEWLSDKGRFQYDGLK-RQRLNTPLVKGA-KGLENATWSAA  target    DDGTPQM 7arc.1    F------ ``` | | | | | | | | | | | | | | | | | | | | | | | | | | | | | | | | | | | | | | | | | | | | | | | | | |
|  | 2nya.1.A | Periplasmic nitrate reductase  *Crystal structure of the periplasmic nitrate reductase (NAP) from Escherichia coli* | 0.23 |  | 17.33 | 0.45 | 73-160 | X-ray | 2.50 | monomer | 1 x SF4, 1 x 6MO, 2 x MGD | HHblits | 0.28 |
| ``` target    MAQGVSRRQLLGRALALGSGAALADLLGPARFLSPAGAATAGAVVPGNPLRVMPDRTWEQIYRNQFEDDSTFVFTCAPND 2nya.1    ------------------------------------------------------------------------KAPC-RFC  target    THNCLLRAHVKNGVVVRISPTYGYGEATDLYGNRASHRWDPRTCQKGLILSRRFYSERRVKAPMIRKG------FKDWVE 2nya.1    GTGCGVLVGTQQGRVVACQGDPD------------APVNRGLNCIKGYFLPKIMYGKDRLTQPLLRMKNGKYDKEGEFTP  target    AGYPRNDDGTPQM 2nya.1    ITWDQA------- ``` | | | | | | | | | | | | | | | | | | | | | | | | | | | | | | | | | | | | | | | | | | | | | | | | | |
|  | 7ar7.1.G | NADH dehydrogenase [ubiquinone] iron-sulfur protein 1, mitochondrial  *Cryo-EM structure of Arabidopsis thaliana complex-I (open conformation)* | 0.23 |  | 17.11 | 0.46 | 73-161 | EM | 0.00 | hetero-1-1-1-1-1-1-… | 6 x SF4, 2 x FES, 1 x FMN, 1 x UQ9, 3 x PTY, 2 x PC7, 1 x LMN, 1 x NDP, 2 x ZN, 2 x 8Q1, 1 x PGT, 1 x PSF, 1 x T7X | HHblits | 0.27 |
| ``` target    MAQGVSRRQLLGRALALGSGAALADLLGPARFLSPAGAATAGAVVPGNPLRVMPDRTWEQIYRNQFEDDSTFVFTCAPND 7ar7.1    ------------------------------------------------------------------------TETIDVSD  target    THNCLLRAHVKNGVVVRISPTYGYGEATDLYGNRASHRWDPRTCQKGLILSRRFYSERRVKAPMIRKGFKDWVEAGYPRN 7ar7.1    AVGSNIRVDSRGPEVMRIIPRLN------------EDINEEWISDKTRFCYDGLK-RQRLSDPMIRDSDGRFKAVSWRDA  target    DDGTPQM 7ar7.1    L------ ``` | | | | | | | | | | | | | | | | | | | | | | | | | | | | | | | | | | | | | | | | | | | | | | | | | |
|  | 1dms.1.A | DMSO REDUCTASE  *STRUCTURE OF DMSO REDUCTASE* | 0.21 |  | 25.35 | 0.43 | 75-161 | X-ray | 1.88 | monomer | 2 x PGD, 1 x 2MO | HHblits | 0.32 |
| ``` target    MAQGVSRRQLLGRALALGSGAALADLLGPARFLSPAGAATAGAVVPGNPLRVMPDRTWEQIYRNQFEDDSTFVFTCAPND 1dms.1    --------------------------------------------------------------------------GTVMSG  target    THNCLLRAHVKNGVVVRISPTYGYGEATDLYGNRASHRWDPRTCQKGLILSRRFYSERRVKAPMIRK------------- 1dms.1    SHWGVFTATVENGRATAFTPWEK----------------DPHPTPMLEGVLDSIYSPTRIKYPMVRREFLEKGVNADRST  target    -GFKDWVEAGYPRNDDGTPQM 1dms.1    RGNGDFVRVSWDQAL------ ``` | | | | | | | | | | | | | | | | | | | | | | | | | | | | | | | | | | | | | | | | | | | | | | | | | |
|  | 6yj4.1.G | Subunit NUAM of NADH:Ubiquinone Oxidoreductase (Complex I)  *Structure of Yarrowia lipolytica complex I at 2.7 A* | 0.24 |  | 18.67 | 0.45 | 73-161 | EM | 0.00 | hetero-1-1-1-1-1-1-… | 18 x 3PE, 6 x SF4, 5 x LMT, 8 x PLC, 2 x FES, 1 x FMN, 6 x CDL, 1 x NDP, 1 x ZN, 2 x EHZ | HHblits | 0.28 |
| ``` target    MAQGVSRRQLLGRALALGSGAALADLLGPARFLSPAGAATAGAVVPGNPLRVMPDRTWEQIYRNQFEDDSTFVFTCAPND 6yj4.1    ------------------------------------------------------------------------TESIDVMD  target    THNCLLRAHVKNGVVVRISPTYGYGEATDLYGNRASHRWDPRTCQKGLILSRRFYSERRVKAPMIRKGFKDWVEAGYPRN 6yj4.1    AVGSNIRIDSKGVEVMRVIPRVH------------EDVNEEWINDKSRFACDGLK-TQRLTTPLIRVG-DKFVNATWDDA  target    DDGTPQM 6yj4.1    L------ ``` | | | | | | | | | | | | | | | | | | | | | | | | | | | | | | | | | | | | | | | | | | | | | | | | | |
|  | 6rfs.1.A | Subunit NUAM of NADH:Ubiquinone Oxidoreductase (Complex I)  *Cryo-EM structure of a respiratory complex I mutant lacking NDUFS4* | 0.23 |  | 18.67 | 0.45 | 73-161 | EM | 4.04 | hetero-1-1-1-1-1-1-… | 6 x SF4, 2 x FES, 1 x FMN, 1 x NDP, 1 x ZN, 1 x ZMP | HHblits | 0.28 |
| ``` target    MAQGVSRRQLLGRALALGSGAALADLLGPARFLSPAGAATAGAVVPGNPLRVMPDRTWEQIYRNQFEDDSTFVFTCAPND 6rfs.1    ------------------------------------------------------------------------TESIDVMD  target    THNCLLRAHVKNGVVVRISPTYGYGEATDLYGNRASHRWDPRTCQKGLILSRRFYSERRVKAPMIRKGFKDWVEAGYPRN 6rfs.1    AVGSNIRIDSKGVEVMRVIPRVH------------EDVNEEWINDKSRFACDGLK-TQRLTTPLIRVG-DKFVNATWDDA  target    DDGTPQM 6rfs.1    L------ ``` | | | | | | | | | | | | | | | | | | | | | | | | | | | | | | | | | | | | | | | | | | | | | | | | | |
|  | 6rfq.1.A | Subunit NUAM of NADH:Ubiquinone Oxidoreductase (Complex I)  *Cryo-EM structure of a respiratory complex I assembly intermediate with NDUFAF2* | 0.22 |  | 18.67 | 0.45 | 73-161 | EM | 3.30 | hetero-1-1-1-1-1-1-… | 6 x SF4, 2 x FES, 1 x FMN, 1 x NDP, 10 x 3PE, 2 x LMN, 4 x CDL, 2 x ZMP, 4 x PLC, 3 x T7X, 1 x CPL | HHblits | 0.28 |
| ``` target    MAQGVSRRQLLGRALALGSGAALADLLGPARFLSPAGAATAGAVVPGNPLRVMPDRTWEQIYRNQFEDDSTFVFTCAPND 6rfq.1    ------------------------------------------------------------------------TESIDVMD  target    THNCLLRAHVKNGVVVRISPTYGYGEATDLYGNRASHRWDPRTCQKGLILSRRFYSERRVKAPMIRKGFKDWVEAGYPRN 6rfq.1    AVGSNIRIDSKGVEVMRVIPRVH------------EDVNEEWINDKSRFACDGLK-TQRLTTPLIRVG-DKFVNATWDDA  target    DDGTPQM 6rfq.1    L------ ``` | | | | | | | | | | | | | | | | | | | | | | | | | | | | | | | | | | | | | | | | | | | | | | | | | |
|  | 6gcs.1.A | 75-KDA PROTEIN (NUAM)  *Cryo-EM structure of respiratory complex I from Yarrowia lipolytica* | 0.23 |  | 18.67 | 0.45 | 73-161 | EM | 4.32 | hetero-1-1-1-1-1-1-… | 6 x SF4, 2 x FES, 1 x FMN, 1 x NDP, 1 x ZN, 1 x ZMP, 1 x CDL, 3 x 3PE | HHblits | 0.28 |
| ``` target    MAQGVSRRQLLGRALALGSGAALADLLGPARFLSPAGAATAGAVVPGNPLRVMPDRTWEQIYRNQFEDDSTFVFTCAPND 6gcs.1    ------------------------------------------------------------------------TESIDVMD  target    THNCLLRAHVKNGVVVRISPTYGYGEATDLYGNRASHRWDPRTCQKGLILSRRFYSERRVKAPMIRKGFKDWVEAGYPRN 6gcs.1    AVGSNIRIDSKGVEVMRVIPRVH------------EDVNEEWINDKSRFACDGLK-TQRLTTPLIRVG-DKFVNATWDDA  target    DDGTPQM 6gcs.1    L------ ``` | | | | | | | | | | | | | | | | | | | | | | | | | | | | | | | | | | | | | | | | | | | | | | | | | |
|  | 8b9z.1.G | NADH-ubiquinone oxidoreductase 75 kDa subunit, mitochondrial  *Drosophila melanogaster complex I in the Active state (Dm1)* | 0.23 |  | 15.79 | 0.46 | 73-161 | EM | 3.28 | hetero-1-1-1-1-1-1-… | 3 x PC1, 16 x 3PE, 6 x SF4, 4 x CDL, 2 x FES, 1 x FMN, 1 x UQ9, 1 x DGT, 1 x NDP, 1 x ZN, 2 x EHZ | HHblits | 0.27 |
| ``` target    MAQGVSRRQLLGRALALGSGAALADLLGPARFLSPAGAATAGAVVPGNPLRVMPDRTWEQIYRNQFEDDSTFVFTCAPND 8b9z.1    ------------------------------------------------------------------------VSSIDVLD  target    THNCLLRAHVKNGVVVRISPTYGYGEATDLYGNRASHRWDPRTCQKGLILSRRFYSERRVKAPMIRKGFKDWVEAGYPRN 8b9z.1    AVGSNIVVSTRTNEVLRILPREN------------EDVNEEWLADKSRFACDGLK-RQRLVAPMVRMPNGELQAVEWEGA  target    DDGTPQM 8b9z.1    L------ ``` | | | | | | | | | | | | | | | | | | | | | | | | | | | | | | | | | | | | | | | | | | | | | | | | | |
|  | 8ba0.1.G | NADH-ubiquinone oxidoreductase 75 kDa subunit, mitochondrial  *Drosophila melanogaster complex I in the Twisted state (Dm2)* | 0.22 |  | 15.79 | 0.46 | 73-161 | EM | 3.68 | hetero-1-1-1-1-1-1-… | 6 x SF4, 6 x 3PE, 2 x FES, 1 x FMN, 2 x CDL, 1 x DGT, 1 x NDP, 1 x ZN, 2 x EHZ | HHblits | 0.27 |
| ``` target    MAQGVSRRQLLGRALALGSGAALADLLGPARFLSPAGAATAGAVVPGNPLRVMPDRTWEQIYRNQFEDDSTFVFTCAPND 8ba0.1    ------------------------------------------------------------------------VSSIDVLD  target    THNCLLRAHVKNGVVVRISPTYGYGEATDLYGNRASHRWDPRTCQKGLILSRRFYSERRVKAPMIRKGFKDWVEAGYPRN 8ba0.1    AVGSNIVVSTRTNEVLRILPREN------------EDVNEEWLADKSRFACDGLK-RQRLVAPMVRMPNGELQAVEWEGA  target    DDGTPQM 8ba0.1    L------ ``` | | | | | | | | | | | | | | | | | | | | | | | | | | | | | | | | | | | | | | | | | | | | | | | | | |
|  | 6zk9.1.C | NADH:ubiquinone oxidoreductase core subunit S1  *Peripheral domain of open complex I during turnover* | 0.23 | 0.00 | 14.29 | 0.46 | 72-161 | EM | 0.00 | monomer | 6 x SF4, 1 x FMN, 1 x NAI, 2 x FES, 1 x K, 2 x PC1, 2 x 3PE, 1 x ZN, 1 x NDP, 1 x ZMP, 1 x CDL | HHblits | 0.26 |
| ``` target    MAQGVSRRQLLGRALALGSGAALADLLGPARFLSPAGAATAGAVVPGNPLRVMPDRTWEQIYRNQFEDDSTFVFTCAPND 6zk9.1    -----------------------------------------------------------------------KTESIDVMD  target    THNCLLRAHVKNGVVVRISPTYGYGEATDLYGNRASHRWDPRTCQKGLILSRRFYSERRVKAPMIRKGFKDWVEAGYPRN 6zk9.1    AVGSNIVVSTRTGEVMRILPRMH------------EDINEEWISDKTRFAYDGLK-RQRLTEPMVRNEKGLLTHTTWEDA  target    DDGTPQM 6zk9.1    L------ ``` | | | | | | | | | | | | | | | | | | | | | | | | | | | | | | | | | | | | | | | | | | | | | | | | | |
|  | 7qsd.1.G | NADH-ubiquinone oxidoreductase 75 kDa subunit, mitochondrial  *Bovine complex I in the active state at 3.1 A* | 0.22 | 0.00 | 14.29 | 0.46 | 72-161 | EM | 0.00 | monomer | 5 x PC1, 13 x 3PE, 6 x SF4, 2 x FES, 1 x FMN, 4 x CDL, 3 x LMT, 1 x GTP, 1 x MG, 1 x NDP, 1 x ZN, 2 x EHZ | HHblits | 0.26 |
| ``` target    MAQGVSRRQLLGRALALGSGAALADLLGPARFLSPAGAATAGAVVPGNPLRVMPDRTWEQIYRNQFEDDSTFVFTCAPND 7qsd.1    -----------------------------------------------------------------------KTESIDVMD  target    THNCLLRAHVKNGVVVRISPTYGYGEATDLYGNRASHRWDPRTCQKGLILSRRFYSERRVKAPMIRKGFKDWVEAGYPRN 7qsd.1    AVGSNIVVSTRTGEVMRILPRMH------------EDINEEWISDKTRFAYDGLK-RQRLTEPMVRNEKGLLTHTTWEDA  target    DDGTPQM 7qsd.1    L------ ``` | | | | | | | | | | | | | | | | | | | | | | | | | | | | | | | | | | | | | | | | | | | | | | | | | |
|  | 7v2c.1.L | NADH-ubiquinone oxidoreductase 75 kDa subunit, mitochondrial  *Active state complex I from Q10 dataset* | 0.23 | 0.00 | 15.58 | 0.46 | 72-161 | EM | 0.00 | monomer | 6 x SF4, 1 x FMN, 10 x PEE, 8 x PLX, 2 x 8Q1, 1 x NDP, 2 x UQ, 11 x CDL, 2 x FES, 1 x MG, 1 x ZN, 1 x ADP | HHblits | 0.26 |
| ``` target    MAQGVSRRQLLGRALALGSGAALADLLGPARFLSPAGAATAGAVVPGNPLRVMPDRTWEQIYRNQFEDDSTFVFTCAPND 7v2c.1    -----------------------------------------------------------------------KTESIDVMD  target    THNCLLRAHVKNGVVVRISPTYGYGEATDLYGNRASHRWDPRTCQKGLILSRRFYSERRVKAPMIRKGFKDWVEAGYPRN 7v2c.1    AVGSNIVVSTRTGEVMRILPRMH------------EDINEEWISDKTRFAYDGLK-RQRLTQPMIRNEKGLLTYTTWEDA  target    DDGTPQM 7v2c.1    L------ ``` | | | | | | | | | | | | | | | | | | | | | | | | | | | | | | | | | | | | | | | | | | | | | | | | | |
|  | 7aqr.1.F | NADH dehydrogenase [ubiquinone] iron-sulfur protein 1, mitochondrial  *Cryo-EM structure of Arabidopsis thaliana Complex-I (peripheral arm)* | 0.22 |  | 17.33 | 0.45 | 74-161 | EM | 0.00 | hetero-1-1-1-1-1-1-… | 6 x SF4, 2 x FES, 1 x FMN, 1 x NDP, 1 x ZN, 1 x 8Q1 | HHblits | 0.27 |
| ``` target    MAQGVSRRQLLGRALALGSGAALADLLGPARFLSPAGAATAGAVVPGNPLRVMPDRTWEQIYRNQFEDDSTFVFTCAPND 7aqr.1    -------------------------------------------------------------------------ETIDVSD  target    THNCLLRAHVKNGVVVRISPTYGYGEATDLYGNRASHRWDPRTCQKGLILSRRFYSERRVKAPMIRKGFKDWVEAGYPRN 7aqr.1    AVGSNIRVDSRGPEVMRIIPRLN------------EDINEEWISDKTRFCYDGLK-RQRLSDPMIRDSDGRFKAVSWRDA  target    DDGTPQM 7aqr.1    L------ ``` | | | | | | | | | | | | | | | | | | | | | | | | | | | | | | | | | | | | | | | | | | | | | | | | | |
|  | 7a23.1.O | 75kDa  *Plant mitochondrial respiratory complex I* | 0.22 |  | 17.33 | 0.45 | 74-161 | EM | 0.00 | hetero-1-1-1-1-1-1-… | 6 x SF4, 1 x FMN, 2 x T7X, 3 x CDL, 1 x U10, 1 x PEV, 2 x FES, 1 x NDP, 2 x ZN | HHblits | 0.27 |
| ``` target    MAQGVSRRQLLGRALALGSGAALADLLGPARFLSPAGAATAGAVVPGNPLRVMPDRTWEQIYRNQFEDDSTFVFTCAPND 7a23.1    -------------------------------------------------------------------------ETIDVSD  target    THNCLLRAHVKNGVVVRISPTYGYGEATDLYGNRASHRWDPRTCQKGLILSRRFYSERRVKAPMIRKGFKDWVEAGYPRN 7a23.1    AVGSNIRVDSRGPEVMRIIPRLN------------EDINEEWISDKTRFCYDGLK-RQRLSDPMIRDSDGRFKAVSWRDA  target    DDGTPQM 7a23.1    L------ ``` | | | | | | | | | | | | | | | | | | | | | | | | | | | | | | | | | | | | | | | | | | | | | | | | | |
|  | 7ar8.1.G | NADH dehydrogenase [ubiquinone] iron-sulfur protein 1, mitochondrial  *Cryo-EM structure of Arabidopsis thaliana complex-I (closed conformation)* | 0.22 |  | 17.33 | 0.45 | 74-161 | EM | 0.00 | hetero-1-1-1-1-1-1-… | 6 x SF4, 2 x FES, 1 x FMN, 1 x UQ9, 3 x PTY, 2 x PC7, 1 x PGT, 1 x FE, 1 x NDP, 2 x ZN, 2 x 8Q1, 1 x LMN, 1 x PSF, 1 x T7X | HHblits | 0.27 |
| ``` target    MAQGVSRRQLLGRALALGSGAALADLLGPARFLSPAGAATAGAVVPGNPLRVMPDRTWEQIYRNQFEDDSTFVFTCAPND 7ar8.1    -------------------------------------------------------------------------ETIDVSD  target    THNCLLRAHVKNGVVVRISPTYGYGEATDLYGNRASHRWDPRTCQKGLILSRRFYSERRVKAPMIRKGFKDWVEAGYPRN 7ar8.1    AVGSNIRVDSRGPEVMRIIPRLN------------EDINEEWISDKTRFCYDGLK-RQRLSDPMIRDSDGRFKAVSWRDA  target    DDGTPQM 7ar8.1    L------ ``` | | | | | | | | | | | | | | | | | | | | | | | | | | | | | | | | | | | | | | | | | | | | | | | | | |
|  | 7ak5.1.G | NADH-ubiquinone oxidoreductase 75 kDa subunit, mitochondrial  *Cryo-EM structure of respiratory complex I in the deactive state from Mus musculus at 3.2 A* | 0.23 |  | 14.47 | 0.46 | 73-161 | EM | 0.00 | hetero-1-1-1-1-1-1-… | 6 x SF4, 2 x PC1, 2 x FES, 1 x FMN, 8 x 3PE, 4 x CDL, 1 x ATP, 1 x NDP, 1 x ZN, 2 x EHZ | HHblits | 0.26 |
| ``` target    MAQGVSRRQLLGRALALGSGAALADLLGPARFLSPAGAATAGAVVPGNPLRVMPDRTWEQIYRNQFEDDSTFVFTCAPND 7ak5.1    ------------------------------------------------------------------------TESIDVMD  target    THNCLLRAHVKNGVVVRISPTYGYGEATDLYGNRASHRWDPRTCQKGLILSRRFYSERRVKAPMIRKGFKDWVEAGYPRN 7ak5.1    AVGSNIVVSTRTGEVMRILPRMH------------EDINEEWISDKTRFAYDGLK-RQRLTEPMVRNEKGLLTYTSWEDA  target    DDGTPQM 7ak5.1    L------ ``` | | | | | | | | | | | | | | | | | | | | | | | | | | | | | | | | | | | | | | | | | | | | | | | | | |
|  | 6zr2.1.G | NADH-ubiquinone oxidoreductase 75 kDa subunit, mitochondrial  *Cryo-EM structure of respiratory complex I in the active state from Mus musculus at 3.1 A* | 0.22 |  | 14.47 | 0.46 | 73-161 | EM | 3.10 | hetero-1-1-1-1-1-1-… | 6 x SF4, 4 x PC1, 2 x FES, 1 x FMN, 9 x 3PE, 7 x CDL, 1 x ATP, 1 x NDP, 1 x ZN, 2 x EHZ | HHblits | 0.26 |
| ``` target    MAQGVSRRQLLGRALALGSGAALADLLGPARFLSPAGAATAGAVVPGNPLRVMPDRTWEQIYRNQFEDDSTFVFTCAPND 6zr2.1    ------------------------------------------------------------------------TESIDVMD  target    THNCLLRAHVKNGVVVRISPTYGYGEATDLYGNRASHRWDPRTCQKGLILSRRFYSERRVKAPMIRKGFKDWVEAGYPRN 6zr2.1    AVGSNIVVSTRTGEVMRILPRMH------------EDINEEWISDKTRFAYDGLK-RQRLTEPMVRNEKGLLTYTSWEDA  target    DDGTPQM 6zr2.1    L------ ``` | | | | | | | | | | | | | | | | | | | | | | | | | | | | | | | | | | | | | | | | | | | | | | | | | |
|  | 6g72.1.G | NADH-ubiquinone oxidoreductase 75 kDa subunit, mitochondrial  *Mouse mitochondrial complex I in the deactive state* | 0.22 |  | 14.47 | 0.46 | 73-161 | EM | 0.00 | hetero-1-1-1-1-1-1-… | 6 x SF4, 2 x FES, 1 x FMN, 1 x ADP, 1 x NDP, 1 x ZN, 2 x EHZ | HHblits | 0.26 |
| ``` target    MAQGVSRRQLLGRALALGSGAALADLLGPARFLSPAGAATAGAVVPGNPLRVMPDRTWEQIYRNQFEDDSTFVFTCAPND 6g72.1    ------------------------------------------------------------------------TESIDVMD  target    THNCLLRAHVKNGVVVRISPTYGYGEATDLYGNRASHRWDPRTCQKGLILSRRFYSERRVKAPMIRKGFKDWVEAGYPRN 6g72.1    AVGSNIVVSTRTGEVMRILPRMH------------EDINEEWISDKTRFAYDGLK-RQRLTEPMVRNEKGLLTYTSWEDA  target    DDGTPQM 6g72.1    L------ ``` | | | | | | | | | | | | | | | | | | | | | | | | | | | | | | | | | | | | | | | | | | | | | | | | | |
|  | 7ak6.1.G | NADH-ubiquinone oxidoreductase 75 kDa subunit, mitochondrial  *Cryo-EM structure of ND6-P25L mutant respiratory complex I from Mus musculus at 3.8 A* | 0.21 |  | 14.47 | 0.46 | 73-161 | EM | 0.00 | hetero-1-1-1-1-1-1-… | 6 x SF4, 1 x PC1, 2 x FES, 1 x FMN, 4 x 3PE, 2 x CDL, 1 x ATP, 1 x NDP, 1 x ZN, 2 x EHZ | HHblits | 0.26 |
| ``` target    MAQGVSRRQLLGRALALGSGAALADLLGPARFLSPAGAATAGAVVPGNPLRVMPDRTWEQIYRNQFEDDSTFVFTCAPND 7ak6.1    ------------------------------------------------------------------------TESIDVMD  target    THNCLLRAHVKNGVVVRISPTYGYGEATDLYGNRASHRWDPRTCQKGLILSRRFYSERRVKAPMIRKGFKDWVEAGYPRN 7ak6.1    AVGSNIVVSTRTGEVMRILPRMH------------EDINEEWISDKTRFAYDGLK-RQRLTEPMVRNEKGLLTYTSWEDA  target    DDGTPQM 7ak6.1    L------ ``` | | | | | | | | | | | | | | | | | | | | | | | | | | | | | | | | | | | | | | | | | | | | | | | | | |
|  | 7zd6.1.4 | NADH-ubiquinone oxidoreductase 75 kDa subunit, mitochondrial  *Complex I from Ovis aries, at pH7.4, Open state* | 0.22 |  | 14.47 | 0.46 | 73-161 | EM | 0.00 | hetero-1-1-1-1-1-1-… | 6 x PC1, 14 x 3PE, 1 x DCQ, 2 x ZMP, 1 x AMP, 1 x MYR, 6 x SF4, 1 x FMN, 1 x NAI, 2 x FES, 1 x K, 1 x ZN, 1 x NDP | HHblits | 0.26 |
| ``` target    MAQGVSRRQLLGRALALGSGAALADLLGPARFLSPAGAATAGAVVPGNPLRVMPDRTWEQIYRNQFEDDSTFVFTCAPND 7zd6.1    ------------------------------------------------------------------------TESIDVMD  target    THNCLLRAHVKNGVVVRISPTYGYGEATDLYGNRASHRWDPRTCQKGLILSRRFYSERRVKAPMIRKGFKDWVEAGYPRN 7zd6.1    AVGSNIVVSTRTGEVMRILPRMH------------EDINEEWISDKTRFAYDGLK-RQRLTEPMVRNEKGLLTHTTWEDA  target    DDGTPQM 7zd6.1    L------ ``` | | | | | | | | | | | | | | | | | | | | | | | | | | | | | | | | | | | | | | | | | | | | | | | | | |
|  | 6x89.1.H | NADH dehydrogenase [ubiquinone] iron-sulfur protein 1, mitochondrial  *Vigna radiata mitochondrial complex I\** | 0.22 |  | 17.33 | 0.45 | 73-160 | EM | 0.00 | hetero-1-1-1-1-1-1-… | 1 x NAP, 6 x PC1, 6 x SF4, 2 x FES, 2 x ZN, 1 x FMN | HHblits | 0.27 |
| ``` target    MAQGVSRRQLLGRALALGSGAALADLLGPARFLSPAGAATAGAVVPGNPLRVMPDRTWEQIYRNQFEDDSTFVFTCAPND 6x89.1    ------------------------------------------------------------------------TETIDVTD  target    THNCLLRAHVKNGVVVRISPTYGYGEATDLYGNRASHRWDPRTCQKGLILSRRFYSERRVKAPMIRKGFKDWVEAGYPRN 6x89.1    AVGSNIRIDSRGPEVMRIVPRLN------------EDINEEWISDKTRFCYDGLK-RQRLNDPMIRGPDGRFKAVNWRDA  target    DDGTPQM 6x89.1    ------- ``` | | | | | | | | | | | | | | | | | | | | | | | | | | | | | | | | | | | | | | | | | | | | | | | | | |
|  | 8e73.55.A | NDUS1  *Vigna radiata supercomplex I+III2 (full bridge)* | 0.23 |  | 17.33 | 0.45 | 73-160 | EM | 0.00 | monomer |  | HHblits | 0.27 |
| ``` target    MAQGVSRRQLLGRALALGSGAALADLLGPARFLSPAGAATAGAVVPGNPLRVMPDRTWEQIYRNQFEDDSTFVFTCAPND 8e73.55   ------------------------------------------------------------------------TETIDVTD  target    THNCLLRAHVKNGVVVRISPTYGYGEATDLYGNRASHRWDPRTCQKGLILSRRFYSERRVKAPMIRKGFKDWVEAGYPRN 8e73.55   AVGSNIRIDSRGPEVMRIVPRLN------------EDINEEWISDKTRFCYDGLK-RQRLNDPMIRGPDGRFKAVNWRDA  target    DDGTPQM 8e73.55   ------- ``` | | | | | | | | | | | | | | | | | | | | | | | | | | | | | | | | | | | | | | | | | | | | | | | | | |
|  | 7vxu.1.L | NADH-ubiquinone oxidoreductase 75 kDa subunit, mitochondrial  *Matrix arm of deactive state CI from Q10 dataset* | 0.22 |  | 15.79 | 0.46 | 73-161 | EM | 0.00 | hetero-1-1-1-1-1-1-… | 6 x SF4, 1 x FMN, 1 x PEE, 1 x PLX, 1 x 8Q1, 1 x NDP, 2 x FES, 1 x MG, 1 x CDL, 1 x ZN | HHblits | 0.26 |
| ``` target    MAQGVSRRQLLGRALALGSGAALADLLGPARFLSPAGAATAGAVVPGNPLRVMPDRTWEQIYRNQFEDDSTFVFTCAPND 7vxu.1    ------------------------------------------------------------------------TESIDVMD  target    THNCLLRAHVKNGVVVRISPTYGYGEATDLYGNRASHRWDPRTCQKGLILSRRFYSERRVKAPMIRKGFKDWVEAGYPRN 7vxu.1    AVGSNIVVSTRTGEVMRILPRMH------------EDINEEWISDKTRFAYDGLK-RQRLTQPMIRNEKGLLTYTTWEDA  target    DDGTPQM 7vxu.1    L------ ``` | | | | | | | | | | | | | | | | | | | | | | | | | | | | | | | | | | | | | | | | | | | | | | | | | |
|  | 5gpn.24.A | NADH-ubiquinone oxidoreductase 75 kDa subunit  *Architecture of mammalian respirasome* | 0.22 |  | 15.79 | 0.46 | 73-161 | EM | 0.00 | monomer |  | HHblits | 0.26 |
| ``` target    MAQGVSRRQLLGRALALGSGAALADLLGPARFLSPAGAATAGAVVPGNPLRVMPDRTWEQIYRNQFEDDSTFVFTCAPND 5gpn.24   ------------------------------------------------------------------------TESIDVMD  target    THNCLLRAHVKNGVVVRISPTYGYGEATDLYGNRASHRWDPRTCQKGLILSRRFYSERRVKAPMIRKGFKDWVEAGYPRN 5gpn.24   AVGSNIVVSTRTGEVMRILPRMH------------EDINEEWISDKTRFAYDGLK-RQRLTQPMIRNEKGLLTYTTWEDA  target    DDGTPQM 5gpn.24   L------ ``` | | | | | | | | | | | | | | | | | | | | | | | | | | | | | | | | | | | | | | | | | | | | | | | | | |
|  | 7zm7.1.I | NADH-ubiquinone oxidoreductase-like protein  *CryoEM structure of mitochondrial complex I from Chaetomium thermophilum (inhibited by DDM)* | 0.23 |  | 14.67 | 0.45 | 73-161 | EM | 0.00 | hetero-1-1-1-1-1-1-… | 4 x PC1, 14 x LMT, 5 x CDL, 8 x 3PE, 2 x FES, 6 x SF4, 1 x FMN, 1 x NDP, 1 x ZN, 2 x ZMP | HHblits | 0.26 |
| ``` target    MAQGVSRRQLLGRALALGSGAALADLLGPARFLSPAGAATAGAVVPGNPLRVMPDRTWEQIYRNQFEDDSTFVFTCAPND 7zm7.1    ------------------------------------------------------------------------TESIDVLD  target    THNCLLRAHVKNGVVVRISPTYGYGEATDLYGNRASHRWDPRTCQKGLILSRRFYSERRVKAPMIRKGFKDWVEAGYPRN 7zm7.1    GLGSNIRVDSRGLEVMRILPRLN------------DDVNEEWINDKTRFACDGLK-TQRLTMPLVRRD-GKFEPATWEQA  target    DDGTPQM 7zm7.1    L------ ``` | | | | | | | | | | | | | | | | | | | | | | | | | | | | | | | | | | | | | | | | | | | | | | | | | |
|  | 1eu1.1.A | DIMETHYL SULFOXIDE REDUCTASE  *THE CRYSTAL STRUCTURE OF RHODOBACTER SPHAEROIDES DIMETHYLSULFOXIDE REDUCTASE REVEALS TWO DISTINCT MOLYBDENUM COORDINATION ENVIRONMENTS.* | 0.19 |  | 22.22 | 0.43 | 73-161 | X-ray | 1.30 | monomer | 3 x GLC, 1 x CD, 2 x MGD, 1 x 6MO, 2 x O | HHblits | 0.29 |
| ``` target    MAQGVSRRQLLGRALALGSGAALADLLGPARFLSPAGAATAGAVVPGNPLRVMPDRTWEQIYRNQFEDDSTFVFTCAPND 1eu1.1    ------------------------------------------------------------------------NGEVMSGC  target    THNCLLRAHVKNGVVVRISPTYGYGEATDLYGNRASHRWDPRTCQKGLILSRRFYSERRVKAPMIRK------------- 1eu1.1    HWGV-FKARVENGRAVAFEPWDKD------------PAPSHQLP----GVLDSIYSPTRIKYPMVRREFLEKGVNADRST  target    -GFKDWVEAGYPRNDDGTPQM 1eu1.1    RGNGDFVRVTWDEAL------ ``` | | | | | | | | | | | | | | | | | | | | | | | | | | | | | | | | | | | | | | | | | | | | | | | | | |
|  | 4dmr.1.A | DMSO REDUCTASE  *REDUCED DMSO REDUCTASE FROM RHODOBACTER CAPSULATUS WITH BOUND DMSO SUBSTRATE* | 0.19 |  | 23.19 | 0.41 | 77-161 | X-ray | 1.90 | monomer | 2 x PGD, 1 x 4MO, 1 x O | HHblits | 0.31 |
| ``` target    MAQGVSRRQLLGRALALGSGAALADLLGPARFLSPAGAATAGAVVPGNPLRVMPDRTWEQIYRNQFEDDSTFVFTCAPND 4dmr.1    ----------------------------------------------------------------------------TVMS  target    THNCL-LRAHVKNGVVVRISPTYGYGEATDLYGNRASHRWDPRTCQKGLILSRRFYSERRVKAPMIRK------------ 4dmr.1    GSHWGVFTATVENGRATAFTPWEKD----------------PHPSPMLAGVLDSIYSPTRIKYPMVRREFLEKGVNADRS  target    --GFKDWVEAGYPRNDDGTPQM 4dmr.1    TRGNGDFVRVSWDQAL------ ``` | | | | | | | | | | | | | | | | | | | | | | | | | | | | | | | | | | | | | | | | | | | | | | | | | |
|  | 6btm.1.B | Alternative Complex III subunit B  *Structure of Alternative Complex III from Flavobacterium johnsoniae (Wild Type)* | 0.17 |  | 12.33 | 0.44 | 70-155 | EM | 3.40 | hetero-1-1-1-1-1-1-… | 6 x HEC, 1 x F3S, 1 x SF4, 2 x E87 | HHblits | 0.26 |
| ``` target    MAQGVSRRQLLGRALALGSGAALADLLGPARFLSPAGAATAGAVVPGNPLRVMPDRTWEQIYRNQFEDDSTFVFTCAPND 6btm.1    ---------------------------------------------------------------------ADYYATTVFDG  target    THNCLLRAHVKNGVVVRISPTYGYGEATDLYGNRASHRWDPRTCQKGLILSRRFYSERRVKAPMIRKGFKDWVEAGYPRN 6btm.1    FDFANLLVKTREGRPIKIENNTI------------AGA-KFSANARIHASILGLYDSMRLKEPKLDGKNSSWSAV-----  target    DDGTPQM 6btm.1    ------- ``` | | | | | | | | | | | | | | | | | | | | | | | | | | | | | | | | | | | | | | | | | | | | | | | | | |
|  | 6lod.1.B | Fe-S-cluster-containing hydrogenase components 1-like protein  *Cryo-EM structure of the air-oxidized photosynthetic alternative complex III from Roseiflexus castenholzii* | 0.20 |  | 15.07 | 0.44 | 72-161 | EM | 0.00 | hetero-1-1-1-1-1-1-… | 6 x HEC, 2 x EL6, 3 x SF4, 1 x F3S | HHblits | 0.24 |
| ``` target    MAQGVSRRQLLGRALALGSGAALADLLGPARFLSPAGAATAGAVVPGNPLRVMPDRTWEQIYRNQFEDDSTFVFTCAPND 6lod.1    -----------------------------------------------------------------------FFATAVTFA  target    THNCLLRAHVKNGVVVRISPTYGYGEATDLYGNRASHRWDPRTCQKGLILSRRFYSERRVKAPMIRKGFKDWVEAGYPRN 6lod.1    GFGVGLLVESHEGRPTKIEGNP------------DHPASLGSTDLITQAMILTMYDPDRSQAPTNA-----GQETTWDAF  target    DDGTPQM 6lod.1    V------ ``` | | | | | | | | | | | | | | | | | | | | | | | | | | | | | | | | | | | | | | | | | | | | | | | | | |
|  | 7bkb.1.L | Formylmethanofuran dehydrogenase, subunit B  *Formate dehydrogenase - heterodisulfide reductase - formylmethanofuran dehydrogenase complex from Methanospirillum hungatei (hexameric, composite structure)* | 0.15 |  | 15.94 | 0.41 | 73-161 | EM | 0.00 | hetero-2-2-2-2-2-2-… | 48 x SF4, 4 x FAD, 2 x FES, 4 x 9S8, 4 x ZN, 2 x MO, 4 x MGD | HHblits | 0.27 |
| ``` target    MAQGVSRRQLLGRALALGSGAALADLLGPARFLSPAGAATAGAVVPGNPLRVMPDRTWEQIYRNQFEDDSTFVFTCAPND 7bkb.1    ------------------------------------------------------------------------ENVGCPYC  target    THNCL-LRAHVKNGVVVRISPTYGYGEATDLYGNRASHRWDPRTCQKGLILSRRFYSERRVKAPMIRKGFKDWVEAGYPR 7bkb.1    GCSCDDVRITVSDDGKD--------------------ILEVENVCAIGTEIFKHGCSKDRIRLPRMRQPDGSMKDISYEE  target    NDDGTPQM 7bkb.1    AI------ ``` | | | | | | | | | | | | | | | | | | | | | | | | | | | | | | | | | | | | | | | | | | | | | | | | | |
|  | 7l5i.1.A | Trimethylamine-N-oxide reductase  *Crystal Structure of Haemophilus influenzae MtsZ at pH 7.0* | 0.18 |  | 29.03 | 0.37 | 83-160 | X-ray | 1.73 | monomer | 2 x MGD, 1 x MO, 1 x O | HHblits | 0.34 |
| ``` target    MAQGVSRRQLLGRALALGSGAALADLLGPARFLSPAGAATAGAVVPGNPLRVMPDRTWEQIYRNQFEDDSTFVFTCAPND 7l5i.1    --------------------------------------------------------------------------------  target    THNCLLRAHVKNGVVVRISPTYGYGEATDLYGNRASHRWDPRTCQKGLILSRRFYSERRVKAPMIRKG------------ 7l5i.1    --WGSIGVVVQDGKVVKSGPAIE-------------PAVPNELQ---TVVADQLYSEARVKCPMVRKGFLANPGKSDTTM  target    --FKDWVEAGYPRNDDGTPQM 7l5i.1    RGRDEWVRVSWDEA------- ``` | | | | | | | | | | | | | | | | | | | | | | | | | | | | | | | | | | | | | | | | | | | | | | | | | |
|  | 7l5s.1.A | Trimethylamine-N-oxide reductase  *Crystal Structure of Haemophilus influenzae MtsZ at pH 5.5* | 0.18 |  | 29.03 | 0.37 | 83-160 | X-ray | 2.09 | monomer | 1 x O, 2 x MGD, 1 x MO | HHblits | 0.34 |
| ``` target    MAQGVSRRQLLGRALALGSGAALADLLGPARFLSPAGAATAGAVVPGNPLRVMPDRTWEQIYRNQFEDDSTFVFTCAPND 7l5s.1    --------------------------------------------------------------------------------  target    THNCLLRAHVKNGVVVRISPTYGYGEATDLYGNRASHRWDPRTCQKGLILSRRFYSERRVKAPMIRKG------------ 7l5s.1    --WGSIGVVVQDGKVVKSGPAIE-------------PAVPNELQ---TVVADQLYSEARVKCPMVRKGFLANPGKSDTTM  target    --FKDWVEAGYPRNDDGTPQM 7l5s.1    RGRDEWVRVSWDEA------- ``` | | | | | | | | | | | | | | | | | | | | | | | | | | | | | | | | | | | | | | | | | | | | | | | | | |
|  | 5t5i.1.B | Tungsten formylmethanofuran dehydrogenase subunit B  *TUNGSTEN-CONTAINING FORMYLMETHANOFURAN DEHYDROGENASE FROM METHANOTHERMOBACTER WOLFEII, ORTHORHOMBIC FORM AT 1.9 A* | 0.16 |  | 23.44 | 0.38 | 74-160 | X-ray | 1.90 | hetero-oligomer | 4 x ZN, 2 x MG, 18 x K, 22 x SF4, 2 x W, 4 x MGD, 2 x H2S, 2 x CA | HHblits | 0.31 |
| ``` target    MAQGVSRRQLLGRALALGSGAALADLLGPARFLSPAGAATAGAVVPGNPLRVMPDRTWEQIYRNQFEDDSTFVFTCAPND 5t5i.1    -------------------------------------------------------------------------NVVCPFC  target    THNCL-LRAHVKNGVVVRISPTYGYGEATDLYGNRASHRWDPRTCQKGLILSRRFYSERRVKAPMIRKGFKDWVEAGYPR 5t5i.1    GTLCDDIICKVEGNEIVGTI----------------------NACRIGHSKFVHAEGAMRYKKPLIRKN-GEFVEVSYDE  target    NDDGTPQM 5t5i.1    A------- ``` | | | | | | | | | | | | | | | | | | | | | | | | | | | | | | | | | | | | | | | | | | | | | | | | | |
|  | 1tmo.1.A | TRIMETHYLAMINE N-OXIDE REDUCTASE  *TRIMETHYLAMINE N-OXIDE REDUCTASE FROM SHEWANELLA MASSILIA* | 0.17 |  | 22.95 | 0.37 | 84-160 | X-ray | 2.50 | monomer | 2 x 2MD, 1 x 2MO | HHblits | 0.31 |
| ``` target    MAQGVSRRQLLGRALALGSGAALADLLGPARFLSPAGAATAGAVVPGNPLRVMPDRTWEQIYRNQFEDDSTFVFTCAPND 1tmo.1    --------------------------------------------------------------------------------  target    THNCLLRAHVKNGVVVRISPTYGYGEATDLYGNRASHRWDPRTCQKGLILSRRFYSERRVKAPMIRKG------------ 1tmo.1    ---GAFKMKRKNGVIAEVKPFDLD--------------KYPTDMING--IRGMVYNPSRVRYPMVRLDFLLKGHKSNTHQ  target    --FKDWVEAGYPRNDDGTPQM 1tmo.1    RGDFRFVRVTWDKA------- ``` | | | | | | | | | | | | | | | | | | | | | | | | | | | | | | | | | | | | | | | | | | | | | | | | | |
|  | 7q5y.1.A | NADH dehydrogenase I chain G  *Structure of NADH:ubichinon oxidoreductase (complex I) of the hyperthermophilic eubacterium Aquifex aeolicus* | 0.11 |  | 19.30 | 0.34 | 73-146 | X-ray | 2.70 | hetero-1-1-1-1-1-1-… | 8 x SF4, 2 x FES, 1 x FMN | HHblits | 0.28 |
| ``` target    MAQGVSRRQLLGRALALGSGAALADLLGPARFLSPAGAATAGAVVPGNPLRVMPDRTWEQIYRNQFEDDSTFVFTCAPND 7q5y.1    ------------------------------------------------------------------------GRTVCNLC  target    THNCLLRAHVKNG------VVVRISPTYGYGEATDLYGNRASHRWDPRTCQKGLILSRRFYSERRVKAPMIRKGFKDWVE 7q5y.1    PVGCEIQIEYGVGDWRSKRKVYRTKP-----------------TDELNICAKGFFGYDSINHKRLLKTKVGK--------  target    AGYPRNDDGTPQM 7q5y.1    ------------- ``` | | | | | | | | | | | | | | | | | | | | | | | | | | | | | | | | | | | | | | | | | | | | | | | | | |
|  | 4rku.1.H | Photosystem I reaction center subunit VI, chloroplastic  *Crystal structure of plant Photosystem I at 3 Angstrom resolution* | 0.02 |  | 38.10 | 0.13 | 3-23 | X-ray | 3.00 | hetero-oligomer | 2 x PQN, 21 x BCR, 5 x LHG, 152 x CLA, 3 x SF4, 1 x CL0, 1 x DGD, 2 x LMG, 6 x LUT, 1 x NEX, 1 x G3P | HHblits | 0.36 |
| ``` target    MAQGVSRRQLLGRALALGSGAALADLLGPARFLSPAGAATAGAVVPGNPLRVMPDRTWEQIYRNQFEDDSTFVFTCAPND 4rku.1    --APFTKRGLLLKFLILGGGSTL---------------------------------------------------------  target    THNCLLRAHVKNGVVVRISPTYGYGEATDLYGNRASHRWDPRTCQKGLILSRRFYSERRVKAPMIRKGFKDWVEAGYPRN 4rku.1    --------------------------------------------------------------------------------  target    DDGTPQM 4rku.1    ------- ``` | | | | | | | | | | | | | | | | | | | | | | | | | | | | | | | | | | | | | | | | | | | | | | | | | |
|  | 2pq4.1.B | Periplasmic nitrate reductase precursor  *NMR solution structure of NapD in complex with NapA1-35 signal peptide* | 0.02 |  | 22.73 | 0.13 | 3-24 | NMR | 0.00 | hetero-1-1-mer |  | HHblits | 0.32 |
| ``` target    MAQGVSRRQLLGRALALGSGAALADLLGPARFLSPAGAATAGAVVPGNPLRVMPDRTWEQIYRNQFEDDSTFVFTCAPND 2pq4.1    --MKLSRRSFMKANAVAAAAAAAG--------------------------------------------------------  target    THNCLLRAHVKNGVVVRISPTYGYGEATDLYGNRASHRWDPRTCQKGLILSRRFYSERRVKAPMIRKGFKDWVEAGYPRN 2pq4.1    --------------------------------------------------------------------------------  target    DDGTPQM 2pq4.1    ------- ``` | | | | | | | | | | | | | | | | | | | | | | | | | | | | | | | | | | | | | | | | | | | | | | | | | |
|  | 3lw5.1.H | Putative uncharacterized protein  *Improved model of plant photosystem I* | 0.00 |  | 40.00 | 0.12 | 4-23 | X-ray | 3.30 | hetero-1-1-1-1-1-1-… | 151 x CLA, 2 x PQN, 18 x BCR, 15 x LMU, 3 x SF4, 1 x LMG | HHblits | 0.37 |
| ``` target    MAQGVSRRQLLGRALALGSGAALADLLGPARFLSPAGAATAGAVVPGNPLRVMPDRTWEQIYRNQFEDDSTFVFTCAPND 3lw5.1    ---PFTKRGLLLKFLILGGGSTL---------------------------------------------------------  target    THNCLLRAHVKNGVVVRISPTYGYGEATDLYGNRASHRWDPRTCQKGLILSRRFYSERRVKAPMIRKGFKDWVEAGYPRN 3lw5.1    --------------------------------------------------------------------------------  target    DDGTPQM 3lw5.1    ------- ``` | | | | | | | | | | | | | | | | | | | | | | | | | | | | | | | | | | | | | | | | | | | | | | | | | |
|  | 2o01.1.H | Photosystem I reaction center subunit VI, chloroplast  *The Structure of a plant photosystem I supercomplex at 3.4 Angstrom resolution* | 0.00 |  | 40.00 | 0.12 | 4-23 | X-ray | 3.40 | hetero-oligomer | 145 x CLA, 3 x SF4, 2 x PQN, 5 x BCR | HHblits | 0.37 |
| ``` target    MAQGVSRRQLLGRALALGSGAALADLLGPARFLSPAGAATAGAVVPGNPLRVMPDRTWEQIYRNQFEDDSTFVFTCAPND 2o01.1    ---PFTKRGLLLKFLILGGGSLL---------------------------------------------------------  target    THNCLLRAHVKNGVVVRISPTYGYGEATDLYGNRASHRWDPRTCQKGLILSRRFYSERRVKAPMIRKGFKDWVEAGYPRN 2o01.1    --------------------------------------------------------------------------------  target    DDGTPQM 2o01.1    ------- ``` | | | | | | | | | | | | | | | | | | | | | | | | | | | | | | | | | | | | | | | | | | | | | | | | | |
|  | 7ewk.1.G | Photosystem I reaction center subunit H  *Barley photosystem I-LHCI-Lhca6 supercomplex* | 0.02 |  | 30.00 | 0.12 | 4-23 | EM | 0.00 | hetero-1-1-1-1-1-1-… | 1 x CL0, 136 x CLA, 2 x PQN, 5 x LHG, 24 x BCR, 3 x SF4, 2 x DGD, 2 x LMG, 6 x LUT, 11 x CHL, 2 x XAT | HHblits | 0.35 |
| ``` target    MAQGVSRRQLLGRALALGSGAALADLLGPARFLSPAGAATAGAVVPGNPLRVMPDRTWEQIYRNQFEDDSTFVFTCAPND 7ewk.1    ---PFTKRGLLLKFLLIGGGSLV---------------------------------------------------------  target    THNCLLRAHVKNGVVVRISPTYGYGEATDLYGNRASHRWDPRTCQKGLILSRRFYSERRVKAPMIRKGFKDWVEAGYPRN 7ewk.1    --------------------------------------------------------------------------------  target    DDGTPQM 7ewk.1    ------- ``` | | | | | | | | | | | | | | | | | | | | | | | | | | | | | | | | | | | | | | | | | | | | | | | | | |
|  | 6rqf.1.D | Cytochrome b6-f complex iron-sulfur subunit, chloroplastic  *3.6 Angstrom cryo-EM structure of the dimeric cytochrome b6f complex from Spinacia oleracea with natively bound thylakoid lipids and plastoquinone molecules* | 0.03 |  | 13.64 | 0.13 | 1-22 | EM | 0.00 | hetero-2-2-2-2-2-2-… | 4 x HEM, 4 x HEC, 2 x CLA, 2 x BCR, 3 x PL9, 3 x 6PL, 2 x LMG, 4 x PGV, 2 x FES, 3 x SQD | HHblits | 0.27 |
| ``` target    MAQGVSRRQLLGRALALGSGAALADLLGPARFLSPAGAATAGAVVPGNPLRVMPDRTWEQIYRNQFEDDSTFVFTCAPND 6rqf.1    NVPDMQKRETLNLLLLGALSLP----------------------------------------------------------  target    THNCLLRAHVKNGVVVRISPTYGYGEATDLYGNRASHRWDPRTCQKGLILSRRFYSERRVKAPMIRKGFKDWVEAGYPRN 6rqf.1    --------------------------------------------------------------------------------  target    DDGTPQM 6rqf.1    ------- ``` | | | | | | | | | | | | | | | | | | | | | | | | | | | | | | | | | | | | | | | | | | | | | | | | | |
|  | 6rqf.1.L | Cytochrome b6-f complex iron-sulfur subunit, chloroplastic  *3.6 Angstrom cryo-EM structure of the dimeric cytochrome b6f complex from Spinacia oleracea with natively bound thylakoid lipids and plastoquinone molecules* | 0.02 |  | 13.64 | 0.13 | 1-22 | EM | 0.00 | hetero-2-2-2-2-2-2-… | 4 x HEM, 4 x HEC, 2 x CLA, 2 x BCR, 3 x PL9, 3 x 6PL, 2 x LMG, 4 x PGV, 2 x FES, 3 x SQD | HHblits | 0.27 |
| ``` target    MAQGVSRRQLLGRALALGSGAALADLLGPARFLSPAGAATAGAVVPGNPLRVMPDRTWEQIYRNQFEDDSTFVFTCAPND 6rqf.1    NVPDMQKRETLNLLLLGALSLP----------------------------------------------------------  target    THNCLLRAHVKNGVVVRISPTYGYGEATDLYGNRASHRWDPRTCQKGLILSRRFYSERRVKAPMIRKGFKDWVEAGYPRN 6rqf.1    --------------------------------------------------------------------------------  target    DDGTPQM 6rqf.1    ------- ``` | | | | | | | | | | | | | | | | | | | | | | | | | | | | | | | | | | | | | | | | | | | | | | | | | |
|  | 1q90.1.E | Cytochrome B6-F complex iron-sulfur subunit  *Structure of the cytochrome b6f (plastohydroquinone : plastocyanin oxidoreductase) from Chlamydomonas reinhardtii* | 0.02 |  | 26.32 | 0.11 | 2-20 | X-ray | 3.10 | hetero-2-2-2-2-2-2-… | 8 x HEC, 2 x BCR, 2 x LFA, 2 x FES, 2 x CLA, 2 x TDS, 4 x LMG, 2 x SQD | HHblits | 0.35 |
| ``` target    MAQGVSRRQLLGRALALGSGAALADLLGPARFLSPAGAATAGAVVPGNPLRVMPDRTWEQIYRNQFEDDSTFVFTCAPND 1q90.1    -VPDMNKRNIMNLILAGGAG------------------------------------------------------------  target    THNCLLRAHVKNGVVVRISPTYGYGEATDLYGNRASHRWDPRTCQKGLILSRRFYSERRVKAPMIRKGFKDWVEAGYPRN 1q90.1    --------------------------------------------------------------------------------  target    DDGTPQM 1q90.1    ------- ``` | | | | | | | | | | | | | | | | | | | | | | | | | | | | | | | | | | | | | | | | | | | | | | | | | |
|  | 7qrm.1.D | Cytochrome b6-f complex iron-sulfur subunit, chloroplastic  *Cryo-EM structure of catalytically active Spinacia oleracea cytochrome b6f in complex with endogenous plastoquinones at 2.7 A resolution* | 0.02 |  | 14.29 | 0.13 | 3-23 | EM | 0.00 | hetero-2-2-2-2-2-2-… | 4 x HEM, 4 x HEC, 2 x CLA, 7 x PL9, 10 x UMQ, 4 x PGT, 2 x SQD, 2 x FES, 2 x BCR | HHblits | 0.27 |
| ``` target    MAQGVSRRQLLGRALALGSGAALADLLGPARFLSPAGAATAGAVVPGNPLRVMPDRTWEQIYRNQFEDDSTFVFTCAPND 7qrm.1    --PDMQKRETLNLLLLGALSLPT---------------------------------------------------------  target    THNCLLRAHVKNGVVVRISPTYGYGEATDLYGNRASHRWDPRTCQKGLILSRRFYSERRVKAPMIRKGFKDWVEAGYPRN 7qrm.1    --------------------------------------------------------------------------------  target    DDGTPQM 7qrm.1    ------- ``` | | | | | | | | | | | | | | | | | | | | | | | | | | | | | | | | | | | | | | | | | | | | | | | | | |
|  | 6adq.1.L | Rieske iron-sulfur protein QcrA  *Respiratory Complex CIII2CIV2SOD2 from Mycobacterium smegmatis* | 0.02 |  | 33.33 | 0.11 | 3-20 | EM | 0.00 | hetero-2-2-2-2-2-2-… | 8 x CU, 4 x HEA, 18 x CDL, 8 x 9Y0, 4 x PLM, 4 x 9XX, 8 x 9YF, 4 x HEM, 10 x MQ9, 4 x HEC, 2 x FES | HHblits | 0.39 |
| ``` target    MAQGVSRRQLLGRALALGSGAALADLLGPARFLSPAGAATAGAVVPGNPLRVMPDRTWEQIYRNQFEDDSTFVFTCAPND 6adq.1    --STLKRRKVIGLSLGIGLG------------------------------------------------------------  target    THNCLLRAHVKNGVVVRISPTYGYGEATDLYGNRASHRWDPRTCQKGLILSRRFYSERRVKAPMIRKGFKDWVEAGYPRN 6adq.1    --------------------------------------------------------------------------------  target    DDGTPQM 6adq.1    ------- ``` | | | | | | | | | | | | | | | | | | | | | | | | | | | | | | | | | | | | | | | | | | | | | | | | | |
|  | 2fyn.1.C | Ubiquinol-cytochrome c reductase iron-sulfur subunit  *Crystal Structure Analysis of the double mutant Rhodobacter Sphaeroides bc1 complex* | 0.02 |  | 25.00 | 0.12 | 2-21 | X-ray | 3.20 | hetero-2-2-2-mer | 6 x HEM, 2 x SMA, 2 x LOP, 2 x FES | HHblits | 0.29 |
| ``` target    MAQGVSRRQLLGRALALGSGAALADLLGPARFLSPAGAATAGAVVPGNPLRVMPDRTWEQIYRNQFEDDSTFVFTCAPND 2fyn.1    -DHAGTRRDFLYYATAGAGAV-----------------------------------------------------------  target    THNCLLRAHVKNGVVVRISPTYGYGEATDLYGNRASHRWDPRTCQKGLILSRRFYSERRVKAPMIRKGFKDWVEAGYPRN 2fyn.1    --------------------------------------------------------------------------------  target    DDGTPQM 2fyn.1    ------- ``` | | | | | | | | | | | | | | | | | | | | | | | | | | | | | | | | | | | | | | | | | | | | | | | | | |
|  | 2fyn.2.C | Ubiquinol-cytochrome c reductase iron-sulfur subunit  *Crystal Structure Analysis of the double mutant Rhodobacter Sphaeroides bc1 complex* | 0.02 |  | 25.00 | 0.12 | 2-21 | X-ray | 3.20 | hetero-2-2-2-mer | 6 x HEM, 2 x SMA, 2 x LOP, 2 x FES | HHblits | 0.29 |
| ``` target    MAQGVSRRQLLGRALALGSGAALADLLGPARFLSPAGAATAGAVVPGNPLRVMPDRTWEQIYRNQFEDDSTFVFTCAPND 2fyn.2    -DHAGTRRDFLYYATAGAGAV-----------------------------------------------------------  target    THNCLLRAHVKNGVVVRISPTYGYGEATDLYGNRASHRWDPRTCQKGLILSRRFYSERRVKAPMIRKGFKDWVEAGYPRN 2fyn.2    --------------------------------------------------------------------------------  target    DDGTPQM 2fyn.2    ------- ``` | | | | | | | | | | | | | | | | | | | | | | | | | | | | | | | | | | | | | | | | | | | | | | | | | |
|  | 2qjy.3.F | Ubiquinol-cytochrome c reductase iron-sulfur subunit  *Crystal structure of rhodobacter sphaeroides double mutant with stigmatellin and UQ2* | 0.02 |  | 25.00 | 0.12 | 2-21 | X-ray | 2.40 | hetero-2-2-2-mer | 6 x HEM, 2 x SMA, 2 x LOP, 2 x UQ2, 2 x BGL, 2 x FES | HHblits | 0.29 |
| ``` target    MAQGVSRRQLLGRALALGSGAALADLLGPARFLSPAGAATAGAVVPGNPLRVMPDRTWEQIYRNQFEDDSTFVFTCAPND 2qjy.3    -DHAGTRRDFLYYATAGAGAV-----------------------------------------------------------  target    THNCLLRAHVKNGVVVRISPTYGYGEATDLYGNRASHRWDPRTCQKGLILSRRFYSERRVKAPMIRKGFKDWVEAGYPRN 2qjy.3    --------------------------------------------------------------------------------  target    DDGTPQM 2qjy.3    ------- ``` | | | | | | | | | | | | | | | | | | | | | | | | | | | | | | | | | | | | | | | | | | | | | | | | | |
|  | 6nin.1.C | Ubiquinol-cytochrome c reductase iron-sulfur subunit  *Rhodobacter sphaeroides bc1 with STIGMATELLIN A* | 0.02 |  | 25.00 | 0.12 | 2-21 | X-ray | 3.60 | hetero-2-2-2-mer | 4 x HEM, 2 x SMA, 2 x 6PE, 2 x HEC, 2 x BOG, 2 x FES | HHblits | 0.29 |
| ``` target    MAQGVSRRQLLGRALALGSGAALADLLGPARFLSPAGAATAGAVVPGNPLRVMPDRTWEQIYRNQFEDDSTFVFTCAPND 6nin.1    -DHAGTRRDFLYYATAGAGAV-----------------------------------------------------------  target    THNCLLRAHVKNGVVVRISPTYGYGEATDLYGNRASHRWDPRTCQKGLILSRRFYSERRVKAPMIRKGFKDWVEAGYPRN 6nin.1    --------------------------------------------------------------------------------  target    DDGTPQM 6nin.1    ------- ``` | | | | | | | | | | | | | | | | | | | | | | | | | | | | | | | | | | | | | | | | | | | | | | | | | |
|  | 7o37.1.E | Cytochrome b-c1 complex subunit Rieske, mitochondrial  *Murine supercomplex CIII2CIV in the assembled locked conformation* | 0.02 |  | 9.52 | 0.13 | 2-22 | EM | 0.00 | hetero-2-2-2-2-2-2-… | 16 x 3PE, 6 x CDL, 4 x HEM, 2 x HEC, 2 x FES, 5 x PC1, 1 x TGL, 1 x CU, 2 x HEA, 1 x MG, 1 x CUA, 1 x ZN | HHblits | 0.25 |
| ``` target    MAQGVSRRQLLGRALALGSGAALADLLGPARFLSPAGAATAGAVVPGNPLRVMPDRTWEQIYRNQFEDDSTFVFTCAPND 7o37.1    -ESSEARKGFSYLVTATTTVGV----------------------------------------------------------  target    THNCLLRAHVKNGVVVRISPTYGYGEATDLYGNRASHRWDPRTCQKGLILSRRFYSERRVKAPMIRKGFKDWVEAGYPRN 7o37.1    --------------------------------------------------------------------------------  target    DDGTPQM 7o37.1    ------- ``` | | | | | | | | | | | | | | | | | | | | | | | | | | | | | | | | | | | | | | | | | | | | | | | | | |
|  | 7o37.1.O | Cytochrome b-c1 complex subunit Rieske, mitochondrial  *Murine supercomplex CIII2CIV in the assembled locked conformation* | 0.02 |  | 9.52 | 0.13 | 2-22 | EM | 0.00 | hetero-2-2-2-2-2-2-… | 16 x 3PE, 6 x CDL, 4 x HEM, 2 x HEC, 2 x FES, 5 x PC1, 1 x TGL, 1 x CU, 2 x HEA, 1 x MG, 1 x CUA, 1 x ZN | HHblits | 0.25 |
| ``` target    MAQGVSRRQLLGRALALGSGAALADLLGPARFLSPAGAATAGAVVPGNPLRVMPDRTWEQIYRNQFEDDSTFVFTCAPND 7o37.1    -ESSEARKGFSYLVTATTTVGV----------------------------------------------------------  target    THNCLLRAHVKNGVVVRISPTYGYGEATDLYGNRASHRWDPRTCQKGLILSRRFYSERRVKAPMIRKGFKDWVEAGYPRN 7o37.1    --------------------------------------------------------------------------------  target    DDGTPQM 7o37.1    ------- ``` | | | | | | | | | | | | | | | | | | | | | | | | | | | | | | | | | | | | | | | | | | | | | | | | | |
|  | 7o3c.1.E | Cytochrome b-c1 complex subunit Rieske, mitochondrial  *Murine supercomplex CIII2CIV in the mature unlocked conformation* | 0.02 |  | 9.52 | 0.13 | 2-22 | EM | 0.00 | hetero-2-2-2-2-2-2-… | 15 x 3PE, 7 x CDL, 4 x HEM, 2 x HEC, 2 x FES, 3 x PC1, 1 x CU, 2 x HEA, 1 x MG, 1 x CUA, 1 x ZN, 1 x TGL | HHblits | 0.25 |
| ``` target    MAQGVSRRQLLGRALALGSGAALADLLGPARFLSPAGAATAGAVVPGNPLRVMPDRTWEQIYRNQFEDDSTFVFTCAPND 7o3c.1    -ESSEARKGFSYLVTATTTVGV----------------------------------------------------------  target    THNCLLRAHVKNGVVVRISPTYGYGEATDLYGNRASHRWDPRTCQKGLILSRRFYSERRVKAPMIRKGFKDWVEAGYPRN 7o3c.1    --------------------------------------------------------------------------------  target    DDGTPQM 7o3c.1    ------- ``` | | | | | | | | | | | | | | | | | | | | | | | | | | | | | | | | | | | | | | | | | | | | | | | | | |
|  | 7o3c.1.O | Cytochrome b-c1 complex subunit Rieske, mitochondrial  *Murine supercomplex CIII2CIV in the mature unlocked conformation* | 0.02 |  | 9.52 | 0.13 | 2-22 | EM | 0.00 | hetero-2-2-2-2-2-2-… | 15 x 3PE, 7 x CDL, 4 x HEM, 2 x HEC, 2 x FES, 3 x PC1, 1 x CU, 2 x HEA, 1 x MG, 1 x CUA, 1 x ZN, 1 x TGL | HHblits | 0.25 |
| ``` target    MAQGVSRRQLLGRALALGSGAALADLLGPARFLSPAGAATAGAVVPGNPLRVMPDRTWEQIYRNQFEDDSTFVFTCAPND 7o3c.1    -ESSEARKGFSYLVTATTTVGV----------------------------------------------------------  target    THNCLLRAHVKNGVVVRISPTYGYGEATDLYGNRASHRWDPRTCQKGLILSRRFYSERRVKAPMIRKGFKDWVEAGYPRN 7o3c.1    --------------------------------------------------------------------------------  target    DDGTPQM 7o3c.1    ------- ``` | | | | | | | | | | | | | | | | | | | | | | | | | | | | | | | | | | | | | | | | | | | | | | | | | |
|  | 7o3h.1.E | Cytochrome b-c1 complex subunit Rieske, mitochondrial  *Murine CIII2 focus-refined from supercomplex CICIII2* | 0.02 |  | 9.52 | 0.13 | 2-22 | EM | 0.00 | hetero-2-2-2-2-2-2-… | 8 x 3PE, 6 x CDL, 4 x HEM, 2 x HEC, 2 x FES, 2 x PC1 | HHblits | 0.25 |
| ``` target    MAQGVSRRQLLGRALALGSGAALADLLGPARFLSPAGAATAGAVVPGNPLRVMPDRTWEQIYRNQFEDDSTFVFTCAPND 7o3h.1    -ESSEARKGFSYLVTATTTVGV----------------------------------------------------------  target    THNCLLRAHVKNGVVVRISPTYGYGEATDLYGNRASHRWDPRTCQKGLILSRRFYSERRVKAPMIRKGFKDWVEAGYPRN 7o3h.1    --------------------------------------------------------------------------------  target    DDGTPQM 7o3h.1    ------- ``` | | | | | | | | | | | | | | | | | | | | | | | | | | | | | | | | | | | | | | | | | | | | | | | | | |
|  | 7o3h.1.O | Cytochrome b-c1 complex subunit Rieske, mitochondrial  *Murine CIII2 focus-refined from supercomplex CICIII2* | 0.02 |  | 9.52 | 0.13 | 2-22 | EM | 0.00 | hetero-2-2-2-2-2-2-… | 8 x 3PE, 6 x CDL, 4 x HEM, 2 x HEC, 2 x FES, 2 x PC1 | HHblits | 0.25 |
| ``` target    MAQGVSRRQLLGRALALGSGAALADLLGPARFLSPAGAATAGAVVPGNPLRVMPDRTWEQIYRNQFEDDSTFVFTCAPND 7o3h.1    -ESSEARKGFSYLVTATTTVGV----------------------------------------------------------  target    THNCLLRAHVKNGVVVRISPTYGYGEATDLYGNRASHRWDPRTCQKGLILSRRFYSERRVKAPMIRKGFKDWVEAGYPRN 7o3h.1    --------------------------------------------------------------------------------  target    DDGTPQM 7o3h.1    ------- ``` | | | | | | | | | | | | | | | | | | | | | | | | | | | | | | | | | | | | | | | | | | | | | | | | | |
|  | 5kli.1.C | Ubiquinol-cytochrome c reductase iron-sulfur subunit  *Rhodobacter sphaeroides bc1 with stigmatellin and antimycin* | 0.02 |  | 26.32 | 0.11 | 2-20 | X-ray | 3.00 | hetero-oligomer | 4 x HEM, 2 x SMA, 2 x ANJ, 2 x LOP, 2 x HEC, 2 x FES | HHblits | 0.29 |
| ``` target    MAQGVSRRQLLGRALALGSGAALADLLGPARFLSPAGAATAGAVVPGNPLRVMPDRTWEQIYRNQFEDDSTFVFTCAPND 5kli.1    -DHAGTRRDFLYYATAGAGA------------------------------------------------------------  target    THNCLLRAHVKNGVVVRISPTYGYGEATDLYGNRASHRWDPRTCQKGLILSRRFYSERRVKAPMIRKGFKDWVEAGYPRN 5kli.1    --------------------------------------------------------------------------------  target    DDGTPQM 5kli.1    ------- ``` | | | | | | | | | | | | | | | | | | | | | | | | | | | | | | | | | | | | | | | | | | | | | | | | | |
|  | 5kkz.1.C | Ubiquinol-cytochrome c reductase iron-sulfur subunit  *Rhodobacter sphaeroides bc1 with famoxadone* | 0.02 |  | 26.32 | 0.11 | 2-20 | X-ray | 2.97 | hetero-2-2-2-mer | 4 x HEM, 2 x FMX, 2 x ASC, 2 x LOP, 2 x HEC, 2 x BOG, 2 x FES | HHblits | 0.29 |
| ``` target    MAQGVSRRQLLGRALALGSGAALADLLGPARFLSPAGAATAGAVVPGNPLRVMPDRTWEQIYRNQFEDDSTFVFTCAPND 5kkz.1    -DHAGTRRDFLYYATAGAGA------------------------------------------------------------  target    THNCLLRAHVKNGVVVRISPTYGYGEATDLYGNRASHRWDPRTCQKGLILSRRFYSERRVKAPMIRKGFKDWVEAGYPRN 5kkz.1    --------------------------------------------------------------------------------  target    DDGTPQM 5kkz.1    ------- ``` | | | | | | | | | | | | | | | | | | | | | | | | | | | | | | | | | | | | | | | | | | | | | | | | | |
|  | 7tce.2.F | Ubiquinol-cytochrome c reductase iron-sulfur subunit  *Crystal structure of delta sub IV Rhodobacter Sphaeroides bc1 with the antimalarial drug atovaquone.* | 0.01 |  | 26.32 | 0.11 | 2-20 | X-ray | 3.85 | hetero-2-2-2-mer | 4 x HEM, 2 x AOQ, 2 x 6PE, 2 x BOG, 2 x HEC, 2 x FES | HHblits | 0.29 |
| ``` target    MAQGVSRRQLLGRALALGSGAALADLLGPARFLSPAGAATAGAVVPGNPLRVMPDRTWEQIYRNQFEDDSTFVFTCAPND 7tce.2    -DHAGTRRDFLYYATAGAGA------------------------------------------------------------  target    THNCLLRAHVKNGVVVRISPTYGYGEATDLYGNRASHRWDPRTCQKGLILSRRFYSERRVKAPMIRKGFKDWVEAGYPRN 7tce.2    --------------------------------------------------------------------------------  target    DDGTPQM 7tce.2    ------- ``` | | | | | | | | | | | | | | | | | | | | | | | | | | | | | | | | | | | | | | | | | | | | | | | | | |
|  | 7tlj.1.C | Ubiquinol-cytochrome c reductase iron-sulfur subunit  *Rhodobacter sphaeroides Mitochondrial respiratory chain complex* | 0.02 |  | 26.32 | 0.11 | 2-20 | EM | 0.00 | hetero-2-2-2-2-mer | 4 x HEM, 2 x PQU, 2 x LOP, 2 x HEC, 2 x FES | HHblits | 0.29 |
| ``` target    MAQGVSRRQLLGRALALGSGAALADLLGPARFLSPAGAATAGAVVPGNPLRVMPDRTWEQIYRNQFEDDSTFVFTCAPND 7tlj.1    -DHAGTRRDFLYYATAGAGA------------------------------------------------------------  target    THNCLLRAHVKNGVVVRISPTYGYGEATDLYGNRASHRWDPRTCQKGLILSRRFYSERRVKAPMIRKGFKDWVEAGYPRN 7tlj.1    --------------------------------------------------------------------------------  target    DDGTPQM 7tlj.1    ------- ``` | | | | | | | | | | | | | | | | | | | | | | | | | | | | | | | | | | | | | | | | | | | | | | | | | |
|  | 8asi.1.E | Ubiquinol-cytochrome c reductase iron-sulfur subunit  *Four subunit cytochrome b-c1 complex from Rhodobacter sphaeroides in native nanodiscs - consensus refinement in the b-b conformation* | 0.02 |  | 26.32 | 0.11 | 2-20 | EM | 0.00 | hetero-2-2-2-2-mer | 2 x FES, 12 x PEE, 4 x HEM, 2 x HEC, 1 x U10 | HHblits | 0.29 |
| ``` target    MAQGVSRRQLLGRALALGSGAALADLLGPARFLSPAGAATAGAVVPGNPLRVMPDRTWEQIYRNQFEDDSTFVFTCAPND 8asi.1    -DHAGTRRDFLYYATAGAGA------------------------------------------------------------  target    THNCLLRAHVKNGVVVRISPTYGYGEATDLYGNRASHRWDPRTCQKGLILSRRFYSERRVKAPMIRKGFKDWVEAGYPRN 8asi.1    --------------------------------------------------------------------------------  target    DDGTPQM 8asi.1    ------- ``` | | | | | | | | | | | | | | | | | | | | | | | | | | | | | | | | | | | | | | | | | | | | | | | | | |
|  | 8asj.1.E | Ubiquinol-cytochrome c reductase iron-sulfur subunit  *Four subunit cytochrome b-c1 complex from Rhodobacter sphaeroides in native nanodiscs - focussed refinement in the b-c conformation* | 0.02 |  | 26.32 | 0.11 | 2-20 | EM | 0.00 | hetero-2-2-2-2-mer | 2 x FES, 12 x PEE, 4 x HEM, 2 x HEC, 1 x U10 | HHblits | 0.29 |
| ``` target    MAQGVSRRQLLGRALALGSGAALADLLGPARFLSPAGAATAGAVVPGNPLRVMPDRTWEQIYRNQFEDDSTFVFTCAPND 8asj.1    -DHAGTRRDFLYYATAGAGA------------------------------------------------------------  target    THNCLLRAHVKNGVVVRISPTYGYGEATDLYGNRASHRWDPRTCQKGLILSRRFYSERRVKAPMIRKGFKDWVEAGYPRN 8asj.1    --------------------------------------------------------------------------------  target    DDGTPQM 8asj.1    ------- ``` | | | | | | | | | | | | | | | | | | | | | | | | | | | | | | | | | | | | | | | | | | | | | | | | | |
|  | 8asi.1.A | Ubiquinol-cytochrome c reductase iron-sulfur subunit  *Four subunit cytochrome b-c1 complex from Rhodobacter sphaeroides in native nanodiscs - consensus refinement in the b-b conformation* | 0.02 |  | 26.32 | 0.11 | 2-20 | EM | 0.00 | hetero-2-2-2-2-mer | 2 x FES, 12 x PEE, 4 x HEM, 2 x HEC, 1 x U10 | HHblits | 0.29 |
| ``` target    MAQGVSRRQLLGRALALGSGAALADLLGPARFLSPAGAATAGAVVPGNPLRVMPDRTWEQIYRNQFEDDSTFVFTCAPND 8asi.1    -DHAGTRRDFLYYATAGAGA------------------------------------------------------------  target    THNCLLRAHVKNGVVVRISPTYGYGEATDLYGNRASHRWDPRTCQKGLILSRRFYSERRVKAPMIRKGFKDWVEAGYPRN 8asi.1    --------------------------------------------------------------------------------  target    DDGTPQM 8asi.1    ------- ``` | | | | | | | | | | | | | | | | | | | | | | | | | | | | | | | | | | | | | | | | | | | | | | | | | |
|  | 7e1v.1.P | Cytochrome bc1 complex Rieske iron-sulfur subunit  *Cryo-EM structure of apo hybrid respiratory supercomplex consisting of Mycobacterium tuberculosis complexIII and Mycobacterium smegmatis complexIV* | 0.02 |  | 29.41 | 0.10 | 3-19 | EM | 2.68 | hetero-2-2-2-2-2-2-… | 8 x CU, 17 x CDL, 4 x PLM, 4 x HEA, 2 x 9Y0, 4 x HEM, 10 x MQ9, 2 x FES, 4 x 9YF, 4 x HEC | HHblits | 0.38 |
| ``` target    MAQGVSRRQLLGRALALGSGAALADLLGPARFLSPAGAATAGAVVPGNPLRVMPDRTWEQIYRNQFEDDSTFVFTCAPND 7e1v.1    --STIRRRKLIGLSFGVGM-------------------------------------------------------------  target    THNCLLRAHVKNGVVVRISPTYGYGEATDLYGNRASHRWDPRTCQKGLILSRRFYSERRVKAPMIRKGFKDWVEAGYPRN 7e1v.1    --------------------------------------------------------------------------------  target    DDGTPQM 7e1v.1    ------- ``` | | | | | | | | | | | | | | | | | | | | | | | | | | | | | | | | | | | | | | | | | | | | | | | | | |
|  | 7rh5.1.V | Cytochrome bc1 complex Rieske iron-sulfur subunit  *Mycobacterial CIII2CIV2 supercomplex, Inhibitor free* | 0.02 |  | 29.41 | 0.10 | 3-19 | EM | 0.00 | hetero-2-2-2-2-2-2-… | 6 x CU, 4 x HEA, 16 x CDL, 4 x HEM, 8 x MQ9, 6 x 9Y0, 4 x PLM, 4 x 9XX, 4 x HEC, 8 x 9YF, 2 x FES | HHblits | 0.38 |
| ``` target    MAQGVSRRQLLGRALALGSGAALADLLGPARFLSPAGAATAGAVVPGNPLRVMPDRTWEQIYRNQFEDDSTFVFTCAPND 7rh5.1    --STLKRRKVIGLSLGIGL-------------------------------------------------------------  target    THNCLLRAHVKNGVVVRISPTYGYGEATDLYGNRASHRWDPRTCQKGLILSRRFYSERRVKAPMIRKGFKDWVEAGYPRN 7rh5.1    --------------------------------------------------------------------------------  target    DDGTPQM 7rh5.1    ------- ``` | | | | | | | | | | | | | | | | | | | | | | | | | | | | | | | | | | | | | | | | | | | | | | | | | |
|  | 6hwh.1.A | Ubiquinol-cytochrome c reductase iron-sulfur subunit  *Structure of a functional obligate respiratory supercomplex from Mycobacterium smegmatis* | 0.02 |  | 29.41 | 0.10 | 3-19 | EM | 0.00 | hetero-2-2-2-2-2-4-… | 2 x FES, 8 x CDL, 4 x MQ9, 6 x CU, 4 x HAS, 4 x HEC, 4 x HEM | HHblits | 0.38 |
| ``` target    MAQGVSRRQLLGRALALGSGAALADLLGPARFLSPAGAATAGAVVPGNPLRVMPDRTWEQIYRNQFEDDSTFVFTCAPND 6hwh.1    --STLKRRKVIGLSLGIGL-------------------------------------------------------------  target    THNCLLRAHVKNGVVVRISPTYGYGEATDLYGNRASHRWDPRTCQKGLILSRRFYSERRVKAPMIRKGFKDWVEAGYPRN 6hwh.1    --------------------------------------------------------------------------------  target    DDGTPQM 6hwh.1    ------- ``` | | | | | | | | | | | | | | | | | | | | | | | | | | | | | | | | | | | | | | | | | | | | | | | | | |
|  | 2ybb.1.b | CYTOCHROME B-C1 COMPLEX SUBUNIT RIESKE, MITOCHONDRIAL  *Fitted model for bovine mitochondrial supercomplex I1III2IV1 by single particle cryo-EM (EMD-1876)* | 0.02 |  | 10.00 | 0.12 | 2-21 | EM | 19.00 | hetero-oligomer | 7 x SF4, 1 x FMN, 1 x NAI, 7 x MG, 4 x FES, 1 x CA, 5 x HEM, 2 x SMA, 2 x UQ1, 2 x HEC, 4 x CDL, 2 x HEA, 3 x CU, 1 x ZN | HHblits | 0.25 |
| ``` target    MAQGVSRRQLLGRALALGSGAALADLLGPARFLSPAGAATAGAVVPGNPLRVMPDRTWEQIYRNQFEDDSTFVFTCAPND 2ybb.1    -ESSEARKGFSYLVTATTTVG-----------------------------------------------------------  target    THNCLLRAHVKNGVVVRISPTYGYGEATDLYGNRASHRWDPRTCQKGLILSRRFYSERRVKAPMIRKGFKDWVEAGYPRN 2ybb.1    --------------------------------------------------------------------------------  target    DDGTPQM 2ybb.1    ------- ``` | | | | | | | | | | | | | | | | | | | | | | | | | | | | | | | | | | | | | | | | | | | | | | | | | |
|  | 1be3.1.E | CYTOCHROME BC1 COMPLEX  *CYTOCHROME BC1 COMPLEX FROM BOVINE* | 0.02 |  | 10.00 | 0.12 | 2-21 | X-ray | 3.00 | hetero-oligomer | 2 x HEM, 1 x HEC, 1 x FES | HHblits | 0.25 |
| ``` target    MAQGVSRRQLLGRALALGSGAALADLLGPARFLSPAGAATAGAVVPGNPLRVMPDRTWEQIYRNQFEDDSTFVFTCAPND 1be3.1    -ESSEARKGFSYLVTATTTVG-----------------------------------------------------------  target    THNCLLRAHVKNGVVVRISPTYGYGEATDLYGNRASHRWDPRTCQKGLILSRRFYSERRVKAPMIRKGFKDWVEAGYPRN 1be3.1    --------------------------------------------------------------------------------  target    DDGTPQM 1be3.1    ------- ``` | | | | | | | | | | | | | | | | | | | | | | | | | | | | | | | | | | | | | | | | | | | | | | | | | |
|  | 1bgy.1.P | CYTOCHROME BC1 COMPLEX  *CYTOCHROME BC1 COMPLEX FROM BOVINE* | 0.02 |  | 10.00 | 0.12 | 2-21 | X-ray | 3.00 | hetero-oligomer | 4 x HEM, 2 x HEC, 1 x FES | HHblits | 0.25 |
| ``` target    MAQGVSRRQLLGRALALGSGAALADLLGPARFLSPAGAATAGAVVPGNPLRVMPDRTWEQIYRNQFEDDSTFVFTCAPND 1bgy.1    -ESSEARKGFSYLVTATTTVG-----------------------------------------------------------  target    THNCLLRAHVKNGVVVRISPTYGYGEATDLYGNRASHRWDPRTCQKGLILSRRFYSERRVKAPMIRKGFKDWVEAGYPRN 1bgy.1    --------------------------------------------------------------------------------  target    DDGTPQM 1bgy.1    ------- ``` | | | | | | | | | | | | | | | | | | | | | | | | | | | | | | | | | | | | | | | | | | | | | | | | | |
|  | 1l0n.1.E | UBIQUINOL-CYTOCHROME C REDUCTASE IRON-SULFUR SUBUNIT  *native structure of bovine mitochondrial cytochrome bc1 complex* | 0.02 |  | 10.00 | 0.12 | 2-21 | X-ray | 2.60 | hetero-oligomer | 3 x HEM, 1 x FES | HHblits | 0.25 |
| ``` target    MAQGVSRRQLLGRALALGSGAALADLLGPARFLSPAGAATAGAVVPGNPLRVMPDRTWEQIYRNQFEDDSTFVFTCAPND 1l0n.1    -ESSEARKGFSYLVTATTTVG-----------------------------------------------------------  target    THNCLLRAHVKNGVVVRISPTYGYGEATDLYGNRASHRWDPRTCQKGLILSRRFYSERRVKAPMIRKGFKDWVEAGYPRN 1l0n.1    --------------------------------------------------------------------------------  target    DDGTPQM 1l0n.1    ------- ``` | | | | | | | | | | | | | | | | | | | | | | | | | | | | | | | | | | | | | | | | | | | | | | | | | |
|  | 1l0l.1.E | UBIQUINOL-CYTOCHROME C REDUCTASE IRON-SULFUR SUBUNIT  *structure of bovine mitochondrial cytochrome bc1 complex with a bound fungicide famoxadone* | 0.02 |  | 10.00 | 0.12 | 2-21 | X-ray | 2.35 | hetero-oligomer | 3 x HEM, 1 x FMX, 1 x FES | HHblits | 0.25 |
| ``` target    MAQGVSRRQLLGRALALGSGAALADLLGPARFLSPAGAATAGAVVPGNPLRVMPDRTWEQIYRNQFEDDSTFVFTCAPND 1l0l.1    -ESSEARKGFSYLVTATTTVG-----------------------------------------------------------  target    THNCLLRAHVKNGVVVRISPTYGYGEATDLYGNRASHRWDPRTCQKGLILSRRFYSERRVKAPMIRKGFKDWVEAGYPRN 1l0l.1    --------------------------------------------------------------------------------  target    DDGTPQM 1l0l.1    ------- ``` | | | | | | | | | | | | | | | | | | | | | | | | | | | | | | | | | | | | | | | | | | | | | | | | | |
|  | 1ntk.1.E | UBIQUINOL-CYTOCHROME C REDUCTASE IRON-SULFUR SUBUNIT, MITOCHONDRIAL  *Crystal Structure of Mitochondrial Cytochrome bc1 in Complex with Antimycin A1* | 0.02 |  | 10.00 | 0.12 | 2-21 | X-ray | 2.60 | hetero-oligomer | 6 x HEM, 2 x AY1, 2 x FES | HHblits | 0.25 |
| ``` target    MAQGVSRRQLLGRALALGSGAALADLLGPARFLSPAGAATAGAVVPGNPLRVMPDRTWEQIYRNQFEDDSTFVFTCAPND 1ntk.1    -ESSEARKGFSYLVTATTTVG-----------------------------------------------------------  target    THNCLLRAHVKNGVVVRISPTYGYGEATDLYGNRASHRWDPRTCQKGLILSRRFYSERRVKAPMIRKGFKDWVEAGYPRN 1ntk.1    --------------------------------------------------------------------------------  target    DDGTPQM 1ntk.1    ------- ``` | | | | | | | | | | | | | | | | | | | | | | | | | | | | | | | | | | | | | | | | | | | | | | | | | |
|  | 1sqb.1.E | Ubiquinol-cytochrome c reductase iron-sulfur subunit  *Crystal Structure Analysis of Bovine Bc1 with Azoxystrobin* | 0.02 |  | 10.00 | 0.12 | 2-21 | X-ray | 2.69 | hetero-oligomer | 6 x HEM, 2 x FES, 2 x AZO | HHblits | 0.25 |
| ``` target    MAQGVSRRQLLGRALALGSGAALADLLGPARFLSPAGAATAGAVVPGNPLRVMPDRTWEQIYRNQFEDDSTFVFTCAPND 1sqb.1    -ESSEARKGFSYLVTATTTVG-----------------------------------------------------------  target    THNCLLRAHVKNGVVVRISPTYGYGEATDLYGNRASHRWDPRTCQKGLILSRRFYSERRVKAPMIRKGFKDWVEAGYPRN 1sqb.1    --------------------------------------------------------------------------------  target    DDGTPQM 1sqb.1    ------- ``` | | | | | | | | | | | | | | | | | | | | | | | | | | | | | | | | | | | | | | | | | | | | | | | | | |
|  | 2fyu.1.E | Ubiquinol-cytochrome c reductase iron-sulfur subunit, mitochondrial  *Crystal structure of bovine heart mitochondrial bc1 with jg144 inhibitor* | 0.02 |  | 10.00 | 0.12 | 2-21 | X-ray | 2.26 | hetero-oligomer | 6 x HEM, 2 x FDN, 2 x FES | HHblits | 0.25 |
| ``` target    MAQGVSRRQLLGRALALGSGAALADLLGPARFLSPAGAATAGAVVPGNPLRVMPDRTWEQIYRNQFEDDSTFVFTCAPND 2fyu.1    -ESSEARKGFSYLVTATTTVG-----------------------------------------------------------  target    THNCLLRAHVKNGVVVRISPTYGYGEATDLYGNRASHRWDPRTCQKGLILSRRFYSERRVKAPMIRKGFKDWVEAGYPRN 2fyu.1    --------------------------------------------------------------------------------  target    DDGTPQM 2fyu.1    ------- ``` | | | | | | | | | | | | | | | | | | | | | | | | | | | | | | | | | | | | | | | | | | | | | | | | | |
|  | 1ntm.1.E | UBIQUINOL-CYTOCHROME C REDUCTASE IRON-SULFUR SUBUNIT, mitochondrial  *Crystal Structure of Mitochondrial Cytochrome bc1 Complex at 2.4 Angstrom* | 0.02 |  | 10.00 | 0.12 | 2-21 | X-ray | 2.40 | hetero-oligomer | 6 x HEM, 2 x FES | HHblits | 0.25 |
| ``` target    MAQGVSRRQLLGRALALGSGAALADLLGPARFLSPAGAATAGAVVPGNPLRVMPDRTWEQIYRNQFEDDSTFVFTCAPND 1ntm.1    -ESSEARKGFSYLVTATTTVG-----------------------------------------------------------  target    THNCLLRAHVKNGVVVRISPTYGYGEATDLYGNRASHRWDPRTCQKGLILSRRFYSERRVKAPMIRKGFKDWVEAGYPRN 1ntm.1    --------------------------------------------------------------------------------  target    DDGTPQM 1ntm.1    ------- ``` | | | | | | | | | | | | | | | | | | | | | | | | | | | | | | | | | | | | | | | | | | | | | | | | | |
|  | 5klv.1.P | Cytochrome b-c1 complex subunit Rieske, mitochondrial  *Structure of bos taurus cytochrome bc1 with fenamidone inhibited* | 0.02 |  | 10.00 | 0.12 | 2-21 | X-ray | 2.65 | hetero-oligomer | 4 x 6PE, 6 x CDL, 4 x HEM, 2 x FNM, 2 x 8PE, 2 x HEC, 2 x PEF, 2 x FES, 2 x PX4 | HHblits | 0.25 |
| ``` target    MAQGVSRRQLLGRALALGSGAALADLLGPARFLSPAGAATAGAVVPGNPLRVMPDRTWEQIYRNQFEDDSTFVFTCAPND 5klv.1    -ESSEARKGFSYLVTATTTVG-----------------------------------------------------------  target    THNCLLRAHVKNGVVVRISPTYGYGEATDLYGNRASHRWDPRTCQKGLILSRRFYSERRVKAPMIRKGFKDWVEAGYPRN 5klv.1    --------------------------------------------------------------------------------  target    DDGTPQM 5klv.1    ------- ``` | | | | | | | | | | | | | | | | | | | | | | | | | | | | | | | | | | | | | | | | | | | | | | | | | |
|  | 5okd.1.E | Cytochrome b-c1 complex subunit Rieske, mitochondrial  *Crystal structure of bovine Cytochrome bc1 in complex with inhibitor SCR0911.* | 0.02 |  | 10.00 | 0.12 | 2-21 | X-ray | 3.10 | hetero-1-1-1-1-1-1-… | 1 x 6PE, 4 x CDL, 2 x HEM, 1 x 9XE, 1 x LMT, 2 x PEE, 1 x HEC, 1 x FES, 1 x PX4 | HHblits | 0.25 |
| ``` target    MAQGVSRRQLLGRALALGSGAALADLLGPARFLSPAGAATAGAVVPGNPLRVMPDRTWEQIYRNQFEDDSTFVFTCAPND 5okd.1    -ESSEARKGFSYLVTATTTVG-----------------------------------------------------------  target    THNCLLRAHVKNGVVVRISPTYGYGEATDLYGNRASHRWDPRTCQKGLILSRRFYSERRVKAPMIRKGFKDWVEAGYPRN 5okd.1    --------------------------------------------------------------------------------  target    DDGTPQM 5okd.1    ------- ``` | | | | | | | | | | | | | | | | | | | | | | | | | | | | | | | | | | | | | | | | | | | | | | | | | |
|  | 6nhg.1.E | Cytochrome b-c1 complex subunit Rieske, mitochondrial  *Rhodobacter sphaeroides Mitochondrial respiratory chain complex* | 0.02 |  | 10.00 | 0.12 | 2-21 | X-ray | 2.80 | hetero-2-2-2-2-2-2-… | 4 x 6PE, 6 x CDL, 4 x HEM, 2 x AZO, 2 x 8PE, 2 x HEC, 2 x FES, 2 x MC3 | HHblits | 0.25 |
| ``` target    MAQGVSRRQLLGRALALGSGAALADLLGPARFLSPAGAATAGAVVPGNPLRVMPDRTWEQIYRNQFEDDSTFVFTCAPND 6nhg.1    -ESSEARKGFSYLVTATTTVG-----------------------------------------------------------  target    THNCLLRAHVKNGVVVRISPTYGYGEATDLYGNRASHRWDPRTCQKGLILSRRFYSERRVKAPMIRKGFKDWVEAGYPRN 6nhg.1    --------------------------------------------------------------------------------  target    DDGTPQM 6nhg.1    ------- ``` | | | | | | | | | | | | | | | | | | | | | | | | | | | | | | | | | | | | | | | | | | | | | | | | | |
|  | 1sqp.1.P | Ubiquinol-cytochrome c reductase iron-sulfur subunit, mitochondrial precursor (EC 1.10.2.2) (Rieske iron-sulfur protein) (RISP) [Contains: Ubiquinol-cytochrome c reductase 8 kDa protein (Complex III subunit IX)]  *Crystal Structure Analysis of Bovine Bc1 with Myxothiazol* | 0.02 |  | 10.00 | 0.12 | 2-21 | X-ray | 2.70 | hetero-2-2-2-2-2-2-… | 6 x CDL, 6 x PEE, 6 x HEC, 2 x MYX, 2 x FES, 2 x PLX | HHblits | 0.25 |
| ``` target    MAQGVSRRQLLGRALALGSGAALADLLGPARFLSPAGAATAGAVVPGNPLRVMPDRTWEQIYRNQFEDDSTFVFTCAPND 1sqp.1    -ESSEARKGFSYLVTATTTVG-----------------------------------------------------------  target    THNCLLRAHVKNGVVVRISPTYGYGEATDLYGNRASHRWDPRTCQKGLILSRRFYSERRVKAPMIRKGFKDWVEAGYPRN 1sqp.1    --------------------------------------------------------------------------------  target    DDGTPQM 1sqp.1    ------- ``` | | | | | | | | | | | | | | | | | | | | | | | | | | | | | | | | | | | | | | | | | | | | | | | | | |
|  | 1sqq.1.P | Ubiquinol-cytochrome c reductase iron-sulfur subunit, mitochondrial precursor (EC 1.10.2.2) (Rieske iron-sulfur protein) (RISP) [Contains: Ubiquinol-cytochrome c reductase 8 kDa protein (Complex III subunit IX)]  *Crystal Structure Analysis of Bovine Bc1 with Methoxy Acrylate Stilbene (MOAS)* | 0.02 |  | 10.00 | 0.12 | 2-21 | X-ray | 3.00 | hetero-2-2-2-2-2-2-… | 6 x HEC, 2 x UQ2, 2 x OST, 2 x FES | HHblits | 0.25 |
| ``` target    MAQGVSRRQLLGRALALGSGAALADLLGPARFLSPAGAATAGAVVPGNPLRVMPDRTWEQIYRNQFEDDSTFVFTCAPND 1sqq.1    -ESSEARKGFSYLVTATTTVG-----------------------------------------------------------  target    THNCLLRAHVKNGVVVRISPTYGYGEATDLYGNRASHRWDPRTCQKGLILSRRFYSERRVKAPMIRKGFKDWVEAGYPRN 1sqq.1    --------------------------------------------------------------------------------  target    DDGTPQM 1sqq.1    ------- ``` | | | | | | | | | | | | | | | | | | | | | | | | | | | | | | | | | | | | | | | | | | | | | | | | | |
|  | 1sqv.1.E | Ubiquinol-cytochrome c reductase iron-sulfur subunit  *Crystal Structure Analysis of Bovine Bc1 with UHDBT* | 0.02 |  | 10.00 | 0.12 | 2-21 | X-ray | 2.85 | hetero-2-2-2-2-2-2-… | 6 x HEC, 2 x UHD, 2 x UQ2, 2 x FES | HHblits | 0.25 |
| ``` target    MAQGVSRRQLLGRALALGSGAALADLLGPARFLSPAGAATAGAVVPGNPLRVMPDRTWEQIYRNQFEDDSTFVFTCAPND 1sqv.1    -ESSEARKGFSYLVTATTTVG-----------------------------------------------------------  target    THNCLLRAHVKNGVVVRISPTYGYGEATDLYGNRASHRWDPRTCQKGLILSRRFYSERRVKAPMIRKGFKDWVEAGYPRN 1sqv.1    --------------------------------------------------------------------------------  target    DDGTPQM 1sqv.1    ------- ``` | | | | | | | | | | | | | | | | | | | | | | | | | | | | | | | | | | | | | | | | | | | | | | | | | |
|  | 7dgs.60.A | Cytochrome b-c1 complex subunit Rieske, mitochondrial  *Activity optimized supercomplex state3* | 0.01 |  | 10.00 | 0.12 | 2-21 | EM | 0.00 | monomer |  | HHblits | 0.25 |
| ``` target    MAQGVSRRQLLGRALALGSGAALADLLGPARFLSPAGAATAGAVVPGNPLRVMPDRTWEQIYRNQFEDDSTFVFTCAPND 7dgs.60   -ESSEARKGFSYLVTATTTVG-----------------------------------------------------------  target    THNCLLRAHVKNGVVVRISPTYGYGEATDLYGNRASHRWDPRTCQKGLILSRRFYSERRVKAPMIRKGFKDWVEAGYPRN 7dgs.60   --------------------------------------------------------------------------------  target    DDGTPQM 7dgs.60   ------- ``` | | | | | | | | | | | | | | | | | | | | | | | | | | | | | | | | | | | | | | | | | | | | | | | | | |
|  | 7dgs.50.A | Cytochrome b-c1 complex subunit Rieske, mitochondrial  *Activity optimized supercomplex state3* | 0.02 |  | 10.00 | 0.12 | 2-21 | EM | 0.00 | monomer |  | HHblits | 0.25 |
| ``` target    MAQGVSRRQLLGRALALGSGAALADLLGPARFLSPAGAATAGAVVPGNPLRVMPDRTWEQIYRNQFEDDSTFVFTCAPND 7dgs.50   -ESSEARKGFSYLVTATTTVG-----------------------------------------------------------  target    THNCLLRAHVKNGVVVRISPTYGYGEATDLYGNRASHRWDPRTCQKGLILSRRFYSERRVKAPMIRKGFKDWVEAGYPRN 7dgs.50   --------------------------------------------------------------------------------  target    DDGTPQM 7dgs.50   ------- ``` | | | | | | | | | | | | | | | | | | | | | | | | | | | | | | | | | | | | | | | | | | | | | | | | | |
|  | 7dgr.60.A | Cytochrome b-c1 complex subunit Rieske, mitochondrial  *Activity optimized supercomplex state2* | 0.02 |  | 10.00 | 0.12 | 2-21 | EM | 0.00 | monomer |  | HHblits | 0.25 |
| ``` target    MAQGVSRRQLLGRALALGSGAALADLLGPARFLSPAGAATAGAVVPGNPLRVMPDRTWEQIYRNQFEDDSTFVFTCAPND 7dgr.60   -ESSEARKGFSYLVTATTTVG-----------------------------------------------------------  target    THNCLLRAHVKNGVVVRISPTYGYGEATDLYGNRASHRWDPRTCQKGLILSRRFYSERRVKAPMIRKGFKDWVEAGYPRN 7dgr.60   --------------------------------------------------------------------------------  target    DDGTPQM 7dgr.60   ------- ``` | | | | | | | | | | | | | | | | | | | | | | | | | | | | | | | | | | | | | | | | | | | | | | | | | |
|  | 7tz6.1.P | Cytochrome b-c1 complex subunit Rieske, mitochondrial  *Structure of mitochondrial bc1 in complex with ck-2-68* | 0.02 |  | 10.00 | 0.12 | 2-21 | EM | 0.00 | hetero-2-2-2-2-2-2-… | 4 x HEM, 2 x JHB, 2 x HEC, 2 x FES | HHblits | 0.25 |
| ``` target    MAQGVSRRQLLGRALALGSGAALADLLGPARFLSPAGAATAGAVVPGNPLRVMPDRTWEQIYRNQFEDDSTFVFTCAPND 7tz6.1    -ESSEARKGFSYLVTATTTVG-----------------------------------------------------------  target    THNCLLRAHVKNGVVVRISPTYGYGEATDLYGNRASHRWDPRTCQKGLILSRRFYSERRVKAPMIRKGFKDWVEAGYPRN 7tz6.1    --------------------------------------------------------------------------------  target    DDGTPQM 7tz6.1    ------- ``` | | | | | | | | | | | | | | | | | | | | | | | | | | | | | | | | | | | | | | | | | | | | | | | | | |
|  | 7tz6.1.E | Cytochrome b-c1 complex subunit Rieske, mitochondrial  *Structure of mitochondrial bc1 in complex with ck-2-68* | 0.02 |  | 10.00 | 0.12 | 2-21 | EM | 0.00 | hetero-2-2-2-2-2-2-… | 4 x HEM, 2 x JHB, 2 x HEC, 2 x FES | HHblits | 0.25 |
| ``` target    MAQGVSRRQLLGRALALGSGAALADLLGPARFLSPAGAATAGAVVPGNPLRVMPDRTWEQIYRNQFEDDSTFVFTCAPND 7tz6.1    -ESSEARKGFSYLVTATTTVG-----------------------------------------------------------  target    THNCLLRAHVKNGVVVRISPTYGYGEATDLYGNRASHRWDPRTCQKGLILSRRFYSERRVKAPMIRKGFKDWVEAGYPRN 7tz6.1    --------------------------------------------------------------------------------  target    DDGTPQM 7tz6.1    ------- ``` | | | | | | | | | | | | | | | | | | | | | | | | | | | | | | | | | | | | | | | | | | | | | | | | | |
|  | 8bpx.51.A | Cytochrome b-c1 complex subunit Rieske-1, mitochondrial  *Cryo-EM structure of the Arabidopsis thaliana I+III2 supercomplex (Complete composition)* | 0.02 |  | 21.05 | 0.11 | 3-21 | EM | 0.00 | monomer |  | HHblits | 0.27 |
| ``` target    MAQGVSRRQLLGRALALGSGAALADLLGPARFLSPAGAATAGAVVPGNPLRVMPDRTWEQIYRNQFEDDSTFVFTCAPND 8bpx.51   --GDPSKRAFAYFVLSGGRFV-----------------------------------------------------------  target    THNCLLRAHVKNGVVVRISPTYGYGEATDLYGNRASHRWDPRTCQKGLILSRRFYSERRVKAPMIRKGFKDWVEAGYPRN 8bpx.51   --------------------------------------------------------------------------------  target    DDGTPQM 8bpx.51   ------- ``` | | | | | | | | | | | | | | | | | | | | | | | | | | | | | | | | | | | | | | | | | | | | | | | | | |
|  | 8bel.1.I | Cytochrome b-c1 complex subunit Rieske-1, mitochondrial  *Cryo-EM structure of the Arabidopsis thaliana I+III2 supercomplex (CIII membrane domain)* | 0.02 |  | 21.05 | 0.11 | 3-21 | EM | 0.00 | hetero-2-2-2-2-2-2-… | 6 x HEM, 3 x UQ5, 6 x 3PH, 4 x PGT, 7 x CDL, 5 x PC7, 2 x FES, 3 x PTY, 1 x UQ7, 2 x Q7G | HHblits | 0.27 |
| ``` target    MAQGVSRRQLLGRALALGSGAALADLLGPARFLSPAGAATAGAVVPGNPLRVMPDRTWEQIYRNQFEDDSTFVFTCAPND 8bel.1    --GDPSKRAFAYFVLSGGRFV-----------------------------------------------------------  target    THNCLLRAHVKNGVVVRISPTYGYGEATDLYGNRASHRWDPRTCQKGLILSRRFYSERRVKAPMIRKGFKDWVEAGYPRN 8bel.1    --------------------------------------------------------------------------------  target    DDGTPQM 8bel.1    ------- ``` | | | | | | | | | | | | | | | | | | | | | | | | | | | | | | | | | | | | | | | | | | | | | | | | | |
|  | 8bel.1.B | Cytochrome b-c1 complex subunit Rieske-1, mitochondrial  *Cryo-EM structure of the Arabidopsis thaliana I+III2 supercomplex (CIII membrane domain)* | 0.02 |  | 21.05 | 0.11 | 3-21 | EM | 0.00 | hetero-2-2-2-2-2-2-… | 6 x HEM, 3 x UQ5, 6 x 3PH, 4 x PGT, 7 x CDL, 5 x PC7, 2 x FES, 3 x PTY, 1 x UQ7, 2 x Q7G | HHblits | 0.27 |
| ``` target    MAQGVSRRQLLGRALALGSGAALADLLGPARFLSPAGAATAGAVVPGNPLRVMPDRTWEQIYRNQFEDDSTFVFTCAPND 8bel.1    --GDPSKRAFAYFVLSGGRFV-----------------------------------------------------------  target    THNCLLRAHVKNGVVVRISPTYGYGEATDLYGNRASHRWDPRTCQKGLILSRRFYSERRVKAPMIRKGFKDWVEAGYPRN 8bel.1    --------------------------------------------------------------------------------  target    DDGTPQM 8bel.1    ------- ``` | | | | | | | | | | | | | | | | | | | | | | | | | | | | | | | | | | | | | | | | | | | | | | | | | |
|  | 3bcc.1.E | UBIQUINOL CYTOCHROME C OXIDOREDUCTASE  *STIGMATELLIN AND ANTIMYCIN BOUND CYTOCHROME BC1 COMPLEX FROM CHICKEN* | 0.02 |  | 15.79 | 0.11 | 2-20 | X-ray | 3.70 | hetero-2-2-2-2-2-2-… | 6 x HEM, 2 x SIG, 2 x AMY, 2 x FES | HHblits | 0.26 |
| ``` target    MAQGVSRRQLLGRALALGSGAALADLLGPARFLSPAGAATAGAVVPGNPLRVMPDRTWEQIYRNQFEDDSTFVFTCAPND 3bcc.1    -ESDPSRKGFSYLVTAVTTL------------------------------------------------------------  target    THNCLLRAHVKNGVVVRISPTYGYGEATDLYGNRASHRWDPRTCQKGLILSRRFYSERRVKAPMIRKGFKDWVEAGYPRN 3bcc.1    --------------------------------------------------------------------------------  target    DDGTPQM 3bcc.1    ------- ``` | | | | | | | | | | | | | | | | | | | | | | | | | | | | | | | | | | | | | | | | | | | | | | | | | |
|  | 1bcc.1.O | UBIQUINOL CYTOCHROME C OXIDOREDUCTASE  *CYTOCHROME BC1 COMPLEX FROM CHICKEN* | 0.01 |  | 15.79 | 0.11 | 2-20 | X-ray | 3.16 | hetero-2-2-2-2-2-2-… | 6 x HEM, 2 x U10, 4 x PEE, 2 x BOG, 2 x FES | HHblits | 0.26 |
| ``` target    MAQGVSRRQLLGRALALGSGAALADLLGPARFLSPAGAATAGAVVPGNPLRVMPDRTWEQIYRNQFEDDSTFVFTCAPND 1bcc.1    -ESDPSRKGFSYLVTAVTTL------------------------------------------------------------  target    THNCLLRAHVKNGVVVRISPTYGYGEATDLYGNRASHRWDPRTCQKGLILSRRFYSERRVKAPMIRKGFKDWVEAGYPRN 1bcc.1    --------------------------------------------------------------------------------  target    DDGTPQM 1bcc.1    ------- ``` | | | | | | | | | | | | | | | | | | | | | | | | | | | | | | | | | | | | | | | | | | | | | | | | | |
|  | 5j8k.55.A | Cytochrome b-c1 complex subunit Rieske, mitochondrial  *Architecture of supercomplex I-III2* | 0.02 |  | 10.53 | 0.11 | 3-21 | EM | 0.00 | monomer |  | HHblits | 0.25 |
| ``` target    MAQGVSRRQLLGRALALGSGAALADLLGPARFLSPAGAATAGAVVPGNPLRVMPDRTWEQIYRNQFEDDSTFVFTCAPND 5j8k.55   --SSEARKGFSYLITATTTVG-----------------------------------------------------------  target    THNCLLRAHVKNGVVVRISPTYGYGEATDLYGNRASHRWDPRTCQKGLILSRRFYSERRVKAPMIRKGFKDWVEAGYPRN 5j8k.55   --------------------------------------------------------------------------------  target    DDGTPQM 5j8k.55   ------- ``` | | | | | | | | | | | | | | | | | | | | | | | | | | | | | | | | | | | | | | | | | | | | | | | | | |
|  | 6q9e.1.E | Cytochrome b-c1 complex subunit Rieske, mitochondrial  *Complex III2 focused refinement from Ovine respiratory supercomplex I+III2* | 0.02 |  | 10.53 | 0.11 | 3-21 | EM | 0.00 | hetero-2-2-2-2-2-2-… | 4 x HEM, 4 x 3PE, 5 x CDL, 3 x U10, 2 x HEC, 2 x FES | HHblits | 0.25 |
| ``` target    MAQGVSRRQLLGRALALGSGAALADLLGPARFLSPAGAATAGAVVPGNPLRVMPDRTWEQIYRNQFEDDSTFVFTCAPND 6q9e.1    --SSEARKGFSYLITATTTVG-----------------------------------------------------------  target    THNCLLRAHVKNGVVVRISPTYGYGEATDLYGNRASHRWDPRTCQKGLILSRRFYSERRVKAPMIRKGFKDWVEAGYPRN 6q9e.1    --------------------------------------------------------------------------------  target    DDGTPQM 6q9e.1    ------- ``` | | | | | | | | | | | | | | | | | | | | | | | | | | | | | | | | | | | | | | | | | | | | | | | | | |
|  | 6q9e.1.O | Cytochrome b-c1 complex subunit Rieske, mitochondrial  *Complex III2 focused refinement from Ovine respiratory supercomplex I+III2* | 0.02 |  | 10.53 | 0.11 | 3-21 | EM | 0.00 | hetero-2-2-2-2-2-2-… | 4 x HEM, 4 x 3PE, 5 x CDL, 3 x U10, 2 x HEC, 2 x FES | HHblits | 0.25 |
| ``` target    MAQGVSRRQLLGRALALGSGAALADLLGPARFLSPAGAATAGAVVPGNPLRVMPDRTWEQIYRNQFEDDSTFVFTCAPND 6q9e.1    --SSEARKGFSYLITATTTVG-----------------------------------------------------------  target    THNCLLRAHVKNGVVVRISPTYGYGEATDLYGNRASHRWDPRTCQKGLILSRRFYSERRVKAPMIRKGFKDWVEAGYPRN 6q9e.1    --------------------------------------------------------------------------------  target    DDGTPQM 6q9e.1    ------- ``` | | | | | | | | | | | | | | | | | | | | | | | | | | | | | | | | | | | | | | | | | | | | | | | | | |
|  | 6qc2.43.A | Cytochrome b-c1 complex subunit Rieske, mitochondrial  *Ovine respiratory supercomplex I+III2 open class 2* | 0.02 |  | 10.53 | 0.11 | 3-21 | EM | 0.00 | monomer |  | HHblits | 0.25 |
| ``` target    MAQGVSRRQLLGRALALGSGAALADLLGPARFLSPAGAATAGAVVPGNPLRVMPDRTWEQIYRNQFEDDSTFVFTCAPND 6qc2.43   --SSEARKGFSYLITATTTVG-----------------------------------------------------------  target    THNCLLRAHVKNGVVVRISPTYGYGEATDLYGNRASHRWDPRTCQKGLILSRRFYSERRVKAPMIRKGFKDWVEAGYPRN 6qc2.43   --------------------------------------------------------------------------------  target    DDGTPQM 6qc2.43   ------- ``` | | | | | | | | | | | | | | | | | | | | | | | | | | | | | | | | | | | | | | | | | | | | | | | | | |
|  | 6qc2.33.A | Cytochrome b-c1 complex subunit Rieske, mitochondrial  *Ovine respiratory supercomplex I+III2 open class 2* | 0.02 |  | 10.53 | 0.11 | 3-21 | EM | 0.00 | monomer |  | HHblits | 0.25 |
| ``` target    MAQGVSRRQLLGRALALGSGAALADLLGPARFLSPAGAATAGAVVPGNPLRVMPDRTWEQIYRNQFEDDSTFVFTCAPND 6qc2.33   --SSEARKGFSYLITATTTVG-----------------------------------------------------------  target    THNCLLRAHVKNGVVVRISPTYGYGEATDLYGNRASHRWDPRTCQKGLILSRRFYSERRVKAPMIRKGFKDWVEAGYPRN 6qc2.33   --------------------------------------------------------------------------------  target    DDGTPQM 6qc2.33   ------- ``` | | | | | | | | | | | | | | | | | | | | | | | | | | | | | | | | | | | | | | | | | | | | | | | | | |
|  | 6qbx.15.A | Cytochrome b-c1 complex subunit Rieske, mitochondrial  *Ovine respiratory supercomplex I+III2 closed class.* | 0.02 |  | 10.53 | 0.11 | 3-21 | EM | 0.00 | monomer |  | HHblits | 0.25 |
| ``` target    MAQGVSRRQLLGRALALGSGAALADLLGPARFLSPAGAATAGAVVPGNPLRVMPDRTWEQIYRNQFEDDSTFVFTCAPND 6qbx.15   --SSEARKGFSYLITATTTVG-----------------------------------------------------------  target    THNCLLRAHVKNGVVVRISPTYGYGEATDLYGNRASHRWDPRTCQKGLILSRRFYSERRVKAPMIRKGFKDWVEAGYPRN 6qbx.15   --------------------------------------------------------------------------------  target    DDGTPQM 6qbx.15   ------- ``` | | | | | | | | | | | | | | | | | | | | | | | | | | | | | | | | | | | | | | | | | | | | | | | | | |
|  | 6qbx.5.A | Cytochrome b-c1 complex subunit Rieske, mitochondrial  *Ovine respiratory supercomplex I+III2 closed class.* | 0.02 |  | 10.53 | 0.11 | 3-21 | EM | 0.00 | monomer |  | HHblits | 0.25 |
| ``` target    MAQGVSRRQLLGRALALGSGAALADLLGPARFLSPAGAATAGAVVPGNPLRVMPDRTWEQIYRNQFEDDSTFVFTCAPND 6qbx.5    --SSEARKGFSYLITATTTVG-----------------------------------------------------------  target    THNCLLRAHVKNGVVVRISPTYGYGEATDLYGNRASHRWDPRTCQKGLILSRRFYSERRVKAPMIRKGFKDWVEAGYPRN 6qbx.5    --------------------------------------------------------------------------------  target    DDGTPQM 6qbx.5    ------- ``` | | | | | | | | | | | | | | | | | | | | | | | | | | | | | | | | | | | | | | | | | | | | | | | | | |
|  | 6qc4.5.A | Cytochrome b-c1 complex subunit Rieske, mitochondrial  *Ovine respiratory supercomplex I+III2 open class 3* | 0.02 |  | 10.53 | 0.11 | 3-21 | EM | 0.00 | monomer |  | HHblits | 0.25 |
| ``` target    MAQGVSRRQLLGRALALGSGAALADLLGPARFLSPAGAATAGAVVPGNPLRVMPDRTWEQIYRNQFEDDSTFVFTCAPND 6qc4.5    --SSEARKGFSYLITATTTVG-----------------------------------------------------------  target    THNCLLRAHVKNGVVVRISPTYGYGEATDLYGNRASHRWDPRTCQKGLILSRRFYSERRVKAPMIRKGFKDWVEAGYPRN 6qc4.5    --------------------------------------------------------------------------------  target    DDGTPQM 6qc4.5    ------- ``` | | | | | | | | | | | | | | | | | | | | | | | | | | | | | | | | | | | | | | | | | | | | | | | | | |
|  | 6qc4.15.A | Cytochrome b-c1 complex subunit Rieske, mitochondrial  *Ovine respiratory supercomplex I+III2 open class 3* | 0.02 |  | 10.53 | 0.11 | 3-21 | EM | 0.00 | monomer |  | HHblits | 0.25 |
| ``` target    MAQGVSRRQLLGRALALGSGAALADLLGPARFLSPAGAATAGAVVPGNPLRVMPDRTWEQIYRNQFEDDSTFVFTCAPND 6qc4.15   --SSEARKGFSYLITATTTVG-----------------------------------------------------------  target    THNCLLRAHVKNGVVVRISPTYGYGEATDLYGNRASHRWDPRTCQKGLILSRRFYSERRVKAPMIRKGFKDWVEAGYPRN 6qc4.15   --------------------------------------------------------------------------------  target    DDGTPQM 6qc4.15   ------- ``` | | | | | | | | | | | | | | | | | | | | | | | | | | | | | | | | | | | | | | | | | | | | | | | | | |
|  | 6qc3.15.A | Cytochrome b-c1 complex subunit Rieske, mitochondrial  *Ovine respiratory supercomplex I+III2 open class 1* | 0.02 |  | 10.53 | 0.11 | 3-21 | EM | 0.00 | monomer |  | HHblits | 0.25 |
| ``` target    MAQGVSRRQLLGRALALGSGAALADLLGPARFLSPAGAATAGAVVPGNPLRVMPDRTWEQIYRNQFEDDSTFVFTCAPND 6qc3.15   --SSEARKGFSYLITATTTVG-----------------------------------------------------------  target    THNCLLRAHVKNGVVVRISPTYGYGEATDLYGNRASHRWDPRTCQKGLILSRRFYSERRVKAPMIRKGFKDWVEAGYPRN 6qc3.15   --------------------------------------------------------------------------------  target    DDGTPQM 6qc3.15   ------- ``` | | | | | | | | | | | | | | | | | | | | | | | | | | | | | | | | | | | | | | | | | | | | | | | | | |
|  | 6qc3.5.A | Cytochrome b-c1 complex subunit Rieske, mitochondrial  *Ovine respiratory supercomplex I+III2 open class 1* | 0.02 |  | 10.53 | 0.11 | 3-21 | EM | 0.00 | monomer |  | HHblits | 0.25 |
| ``` target    MAQGVSRRQLLGRALALGSGAALADLLGPARFLSPAGAATAGAVVPGNPLRVMPDRTWEQIYRNQFEDDSTFVFTCAPND 6qc3.5    --SSEARKGFSYLITATTTVG-----------------------------------------------------------  target    THNCLLRAHVKNGVVVRISPTYGYGEATDLYGNRASHRWDPRTCQKGLILSRRFYSERRVKAPMIRKGFKDWVEAGYPRN 6qc3.5    --------------------------------------------------------------------------------  target    DDGTPQM 6qc3.5    ------- ``` | | | | | | | | | | | | | | | | | | | | | | | | | | | | | | | | | | | | | | | | | | | | | | | | | |
|  | 5xte.1.C | Cytochrome b-c1 complex subunit Rieske, mitochondrial  *Cryo-EM structure of human respiratory complex III (cytochrome bc1 complex)* | 0.01 |  | 5.26 | 0.11 | 2-20 | EM | 0.00 | hetero-2-2-2-2-2-2-… | 9 x CDL, 2 x FES, 6 x PEE, 2 x HEC, 4 x HEM, 3 x PLX | HHblits | 0.24 |
| ``` target    MAQGVSRRQLLGRALALGSGAALADLLGPARFLSPAGAATAGAVVPGNPLRVMPDRTWEQIYRNQFEDDSTFVFTCAPND 5xte.1    -ESSEARKGFSYLVTGVTTV------------------------------------------------------------  target    THNCLLRAHVKNGVVVRISPTYGYGEATDLYGNRASHRWDPRTCQKGLILSRRFYSERRVKAPMIRKGFKDWVEAGYPRN 5xte.1    --------------------------------------------------------------------------------  target    DDGTPQM 5xte.1    ------- ``` | | | | | | | | | | | | | | | | | | | | | | | | | | | | | | | | | | | | | | | | | | | | | | | | | |
|  | 7rjb.1.I | Cytochrome b-c1 complex subunit Rieske, mitochondrial  *Complex III2 from Candida albicans, inhibitor free, Rieske head domain in b position* | 0.02 |  | 10.53 | 0.11 | 2-20 | EM | 0.00 | hetero-1-1-1-1-1-1-… | 2 x HEM, 2 x U10, 1 x HEC, 1 x FES | HHblits | 0.24 |
| ``` target    MAQGVSRRQLLGRALALGSGAALADLLGPARFLSPAGAATAGAVVPGNPLRVMPDRTWEQIYRNQFEDDSTFVFTCAPND 7rjb.1    -KSGQGSRNFTYFMVGSMGL------------------------------------------------------------  target    THNCLLRAHVKNGVVVRISPTYGYGEATDLYGNRASHRWDPRTCQKGLILSRRFYSERRVKAPMIRKGFKDWVEAGYPRN 7rjb.1    --------------------------------------------------------------------------------  target    DDGTPQM 7rjb.1    ------- ``` | | | | | | | | | | | | | | | | | | | | | | | | | | | | | | | | | | | | | | | | | | | | | | | | | |
|  | 7rja.1.H | Cytochrome b-c1 complex subunit Rieske, mitochondrial  *Complex III2 from Candida albicans, inhibitor free* | 0.02 |  | 10.53 | 0.11 | 2-20 | EM | 0.00 | hetero-2-2-2-2-2-2-… | 4 x HEM, 4 x U10, 2 x HEC, 2 x FES | HHblits | 0.24 |
| ``` target    MAQGVSRRQLLGRALALGSGAALADLLGPARFLSPAGAATAGAVVPGNPLRVMPDRTWEQIYRNQFEDDSTFVFTCAPND 7rja.1    -KSGQGSRNFTYFMVGSMGL------------------------------------------------------------  target    THNCLLRAHVKNGVVVRISPTYGYGEATDLYGNRASHRWDPRTCQKGLILSRRFYSERRVKAPMIRKGFKDWVEAGYPRN 7rja.1    --------------------------------------------------------------------------------  target    DDGTPQM 7rja.1    ------- ``` | | | | | | | | | | | | | | | | | | | | | | | | | | | | | | | | | | | | | | | | | | | | | | | | | |
|  | 1zrt.1.F | Ubiquinol-cytochrome c reductase iron-sulfur subunit  *Rhodobacter capsulatus cytochrome bc1 complex with stigmatellin bound* | 0.00 |  | 29.41 | 0.10 | 3-19 | X-ray | 3.51 | hetero-2-2-2-mer | 4 x HEM, 2 x SMA, 2 x UNL, 2 x HEC, 2 x FES, 1 x PG6 | HHblits | 0.32 |
| ``` target    MAQGVSRRQLLGRALALGSGAALADLLGPARFLSPAGAATAGAVVPGNPLRVMPDRTWEQIYRNQFEDDSTFVFTCAPND 1zrt.1    --NAGTRRDFLYHATAATG-------------------------------------------------------------  target    THNCLLRAHVKNGVVVRISPTYGYGEATDLYGNRASHRWDPRTCQKGLILSRRFYSERRVKAPMIRKGFKDWVEAGYPRN 1zrt.1    --------------------------------------------------------------------------------  target    DDGTPQM 1zrt.1    ------- ``` | | | | | | | | | | | | | | | | | | | | | | | | | | | | | | | | | | | | | | | | | | | | | | | | | |
|  | 1zrt.1.C | Ubiquinol-cytochrome c reductase iron-sulfur subunit  *Rhodobacter capsulatus cytochrome bc1 complex with stigmatellin bound* | 0.00 |  | 29.41 | 0.10 | 3-19 | X-ray | 3.51 | hetero-2-2-2-mer | 4 x HEM, 2 x SMA, 2 x UNL, 2 x HEC, 2 x FES, 1 x PG6 | HHblits | 0.32 |
| ``` target    MAQGVSRRQLLGRALALGSGAALADLLGPARFLSPAGAATAGAVVPGNPLRVMPDRTWEQIYRNQFEDDSTFVFTCAPND 1zrt.1    --NAGTRRDFLYHATAATG-------------------------------------------------------------  target    THNCLLRAHVKNGVVVRISPTYGYGEATDLYGNRASHRWDPRTCQKGLILSRRFYSERRVKAPMIRKGFKDWVEAGYPRN 1zrt.1    --------------------------------------------------------------------------------  target    DDGTPQM 1zrt.1    ------- ``` | | | | | | | | | | | | | | | | | | | | | | | | | | | | | | | | | | | | | | | | | | | | | | | | | |
|  | 7q21.1.G | Cytochrome bc1 complex Rieske iron-sulfur subunit  *III2-IV2 respiratory supercomplex from Corynebacterium glutamicum* | 0.02 |  | 23.53 | 0.10 | 3-19 | EM | 3.00 | hetero-2-2-2-2-2-2-… | 14 x CDL, 16 x 7PH, 17 x TRD, 4 x 9XX, 2 x TWT, 6 x CU, 2 x MG, 4 x HAS, 2 x CA, 2 x FES, 6 x MQ9, 4 x 9YF, 4 x PLM, 4 x HEM, 4 x HEC | HHblits | 0.31 |
| ``` target    MAQGVSRRQLLGRALALGSGAALADLLGPARFLSPAGAATAGAVVPGNPLRVMPDRTWEQIYRNQFEDDSTFVFTCAPND 7q21.1    --STLGRRKLIMGLAGGGA-------------------------------------------------------------  target    THNCLLRAHVKNGVVVRISPTYGYGEATDLYGNRASHRWDPRTCQKGLILSRRFYSERRVKAPMIRKGFKDWVEAGYPRN 7q21.1    --------------------------------------------------------------------------------  target    DDGTPQM 7q21.1    ------- ``` | | | | | | | | | | | | | | | | | | | | | | | | | | | | | | | | | | | | | | | | | | | | | | | | | |
|  | 7qhm.1.A | Cytochrome bc1 complex Rieske iron-sulfur subunit  *Cytochrome bcc-aa3 supercomplex (respiratory supercomplex III2/IV2) from Corynebacterium glutamicum (stigmatellin and azide bound)* | 0.02 |  | 23.53 | 0.10 | 3-19 | EM | 0.00 | hetero-2-2-2-2-2-2-… | 2 x FES, 2 x IZL, 4 x 9YF, 4 x MQ9, 17 x 3PE, 2 x SMA, 4 x HEM, 16 x CDL, 2 x LYC, 2 x LMT, 4 x HEC, 4 x HAS, 2 x CU, 2 x MN, 2 x CA, 2 x AZI, 4 x CUA, 6 x DGA, 4 x PLM, 2 x IX7, 1 x OXY | HHblits | 0.31 |
| ``` target    MAQGVSRRQLLGRALALGSGAALADLLGPARFLSPAGAATAGAVVPGNPLRVMPDRTWEQIYRNQFEDDSTFVFTCAPND 7qhm.1    --STLGRRKLIMGLAGGGA-------------------------------------------------------------  target    THNCLLRAHVKNGVVVRISPTYGYGEATDLYGNRASHRWDPRTCQKGLILSRRFYSERRVKAPMIRKGFKDWVEAGYPRN 7qhm.1    --------------------------------------------------------------------------------  target    DDGTPQM 7qhm.1    ------- ``` | | | | | | | | | | | | | | | | | | | | | | | | | | | | | | | | | | | | | | | | | | | | | | | | | |
|  | 7qho.1.A | Cytochrome bc1 complex Rieske iron-sulfur subunit  *Cytochrome bcc-aa3 supercomplex (respiratory supercomplex III2/IV2) from Corynebacterium glutamicum (as isolated)* | 0.02 |  | 23.53 | 0.10 | 3-19 | EM | 0.00 | hetero-2-2-2-2-2-2-… | 2 x FES, 2 x IZL, 4 x 9YF, 6 x MQ9, 10 x 3PE, 4 x HEM, 14 x CDL, 2 x LYC, 2 x LMT, 4 x HEC, 2 x 3PH, 4 x HAS, 2 x CU, 2 x CA, 2 x MN, 4 x CUA, 6 x DGA, 4 x PLM, 2 x IX7 | HHblits | 0.31 |
| ``` target    MAQGVSRRQLLGRALALGSGAALADLLGPARFLSPAGAATAGAVVPGNPLRVMPDRTWEQIYRNQFEDDSTFVFTCAPND 7qho.1    --STLGRRKLIMGLAGGGA-------------------------------------------------------------  target    THNCLLRAHVKNGVVVRISPTYGYGEATDLYGNRASHRWDPRTCQKGLILSRRFYSERRVKAPMIRKGFKDWVEAGYPRN 7qho.1    --------------------------------------------------------------------------------  target    DDGTPQM 7qho.1    ------- ``` | | | | | | | | | | | | | | | | | | | | | | | | | | | | | | | | | | | | | | | | | | | | | | | | | |
|  | 7qhm.1.N | Cytochrome bc1 complex Rieske iron-sulfur subunit  *Cytochrome bcc-aa3 supercomplex (respiratory supercomplex III2/IV2) from Corynebacterium glutamicum (stigmatellin and azide bound)* | 0.02 |  | 23.53 | 0.10 | 3-19 | EM | 0.00 | hetero-2-2-2-2-2-2-… | 2 x FES, 2 x IZL, 4 x 9YF, 4 x MQ9, 17 x 3PE, 2 x SMA, 4 x HEM, 16 x CDL, 2 x LYC, 2 x LMT, 4 x HEC, 4 x HAS, 2 x CU, 2 x MN, 2 x CA, 2 x AZI, 4 x CUA, 6 x DGA, 4 x PLM, 2 x IX7, 1 x OXY | HHblits | 0.31 |
| ``` target    MAQGVSRRQLLGRALALGSGAALADLLGPARFLSPAGAATAGAVVPGNPLRVMPDRTWEQIYRNQFEDDSTFVFTCAPND 7qhm.1    --STLGRRKLIMGLAGGGA-------------------------------------------------------------  target    THNCLLRAHVKNGVVVRISPTYGYGEATDLYGNRASHRWDPRTCQKGLILSRRFYSERRVKAPMIRKGFKDWVEAGYPRN 7qhm.1    --------------------------------------------------------------------------------  target    DDGTPQM 7qhm.1    ------- ``` | | | | | | | | | | | | | | | | | | | | | | | | | | | | | | | | | | | | | | | | | | | | | | | | | |
|  | 2qjk.1.C | Ubiquinol-cytochrome c reductase iron-sulfur subunit  *Crystal Structure Analysis of mutant rhodobacter sphaeroides bc1 with stigmatellin and antimycin* | 0.02 |  | 29.41 | 0.10 | 5-21 | X-ray | 3.10 | hetero-2-2-2-mer | 2 x BGL, 6 x HEM, 2 x SMA, 2 x LOP, 2 x ANJ, 2 x FES | HHblits | 0.31 |
| ``` target    MAQGVSRRQLLGRALALGSGAALADLLGPARFLSPAGAATAGAVVPGNPLRVMPDRTWEQIYRNQFEDDSTFVFTCAPND 2qjk.1    ----GTRRDFLYYATAGAGAV-----------------------------------------------------------  target    THNCLLRAHVKNGVVVRISPTYGYGEATDLYGNRASHRWDPRTCQKGLILSRRFYSERRVKAPMIRKGFKDWVEAGYPRN 2qjk.1    --------------------------------------------------------------------------------  target    DDGTPQM 2qjk.1    ------- ``` | | | | | | | | | | | | | | | | | | | | | | | | | | | | | | | | | | | | | | | | | | | | | | | | | |
|  | 2qjp.1.C | Ubiquinol-cytochrome c reductase iron-sulfur subunit  *Crystal structure of wild type rhodobacter sphaeroides with stigmatellin and antimycin inhibited* | 0.02 |  | 29.41 | 0.10 | 5-21 | X-ray | 2.60 | hetero-2-2-2-mer | 2 x BGL, 6 x HEM, 2 x SMA, 2 x LOP, 2 x ANJ, 2 x FES | HHblits | 0.31 |
| ``` target    MAQGVSRRQLLGRALALGSGAALADLLGPARFLSPAGAATAGAVVPGNPLRVMPDRTWEQIYRNQFEDDSTFVFTCAPND 2qjp.1    ----GTRRDFLYYATAGAGAV-----------------------------------------------------------  target    THNCLLRAHVKNGVVVRISPTYGYGEATDLYGNRASHRWDPRTCQKGLILSRRFYSERRVKAPMIRKGFKDWVEAGYPRN 2qjp.1    --------------------------------------------------------------------------------  target    DDGTPQM 2qjp.1    ------- ``` | | | | | | | | | | | | | | | | | | | | | | | | | | | | | | | | | | | | | | | | | | | | | | | | | |
|  | 1vf5.1.D | RIESKE IRON-SULFUR PROTEIN  *Crystal Structure of Cytochrome b6f Complex from M.laminosus* | 0.00 |  | 17.65 | 0.10 | 2-18 | X-ray | 3.00 | hetero-oligomer | 8 x HEM, 2 x TDS, 2 x PL9, 4 x OPC, 2 x CLA, 2 x FES, 2 x BCR | HHblits | 0.28 |
| ``` target    MAQGVSRRQLLGRALALGSGAALADLLGPARFLSPAGAATAGAVVPGNPLRVMPDRTWEQIYRNQFEDDSTFVFTCAPND 1vf5.1    -VPDMGRRQFMNLLAFGT--------------------------------------------------------------  target    THNCLLRAHVKNGVVVRISPTYGYGEATDLYGNRASHRWDPRTCQKGLILSRRFYSERRVKAPMIRKGFKDWVEAGYPRN 1vf5.1    --------------------------------------------------------------------------------  target    DDGTPQM 1vf5.1    ------- ``` | | | | | | | | | | | | | | | | | | | | | | | | | | | | | | | | | | | | | | | | | | | | | | | | | |
|  | 1vf5.1.L | RIESKE IRON-SULFUR PROTEIN  *Crystal Structure of Cytochrome b6f Complex from M.laminosus* | 0.00 |  | 17.65 | 0.10 | 2-18 | X-ray | 3.00 | hetero-oligomer | 8 x HEM, 2 x TDS, 2 x PL9, 4 x OPC, 2 x CLA, 2 x FES, 2 x BCR | HHblits | 0.28 |
| ``` target    MAQGVSRRQLLGRALALGSGAALADLLGPARFLSPAGAATAGAVVPGNPLRVMPDRTWEQIYRNQFEDDSTFVFTCAPND 1vf5.1    -VPDMGRRQFMNLLAFGT--------------------------------------------------------------  target    THNCLLRAHVKNGVVVRISPTYGYGEATDLYGNRASHRWDPRTCQKGLILSRRFYSERRVKAPMIRKGFKDWVEAGYPRN 1vf5.1    --------------------------------------------------------------------------------  target    DDGTPQM 1vf5.1    ------- ``` | | | | | | | | | | | | | | | | | | | | | | | | | | | | | | | | | | | | | | | | | | | | | | | | | |
|  | 2d2c.1.D | Cytochrome b6-f complex iron-sulfur subunit  *Crystal Structure Of Cytochrome B6F Complex with DBMIB From M. Laminosus* | 0.00 |  | 17.65 | 0.10 | 2-18 | X-ray | 3.80 | hetero-oligomer | 6 x HEM, 2 x HEC, 4 x OPC, 2 x BNT, 2 x CLA, 2 x FES, 2 x BCR | HHblits | 0.28 |
| ``` target    MAQGVSRRQLLGRALALGSGAALADLLGPARFLSPAGAATAGAVVPGNPLRVMPDRTWEQIYRNQFEDDSTFVFTCAPND 2d2c.1    -VPDMGRRQFMNLLAFGT--------------------------------------------------------------  target    THNCLLRAHVKNGVVVRISPTYGYGEATDLYGNRASHRWDPRTCQKGLILSRRFYSERRVKAPMIRKGFKDWVEAGYPRN 2d2c.1    --------------------------------------------------------------------------------  target    DDGTPQM 2d2c.1    ------- ``` | | | | | | | | | | | | | | | | | | | | | | | | | | | | | | | | | | | | | | | | | | | | | | | | | |
|  | 2d2c.1.L | Cytochrome b6-f complex iron-sulfur subunit  *Crystal Structure Of Cytochrome B6F Complex with DBMIB From M. Laminosus* | 0.00 |  | 17.65 | 0.10 | 2-18 | X-ray | 3.80 | hetero-oligomer | 6 x HEM, 2 x HEC, 4 x OPC, 2 x BNT, 2 x CLA, 2 x FES, 2 x BCR | HHblits | 0.28 |
| ``` target    MAQGVSRRQLLGRALALGSGAALADLLGPARFLSPAGAATAGAVVPGNPLRVMPDRTWEQIYRNQFEDDSTFVFTCAPND 2d2c.1    -VPDMGRRQFMNLLAFGT--------------------------------------------------------------  target    THNCLLRAHVKNGVVVRISPTYGYGEATDLYGNRASHRWDPRTCQKGLILSRRFYSERRVKAPMIRKGFKDWVEAGYPRN 2d2c.1    --------------------------------------------------------------------------------  target    DDGTPQM 2d2c.1    ------- ``` | | | | | | | | | | | | | | | | | | | | | | | | | | | | | | | | | | | | | | | | | | | | | | | | | |
|  | 7zxy.1.D | Cytochrome b6-f complex iron-sulfur subunit 2  *3.15 Angstrom cryo-EM structure of the dimeric cytochrome b6f complex from Synechocystis sp. PCC 6803 with natively bound plastoquinone and lipid molecules.* | 0.01 |  | 17.65 | 0.10 | 2-18 | EM | 0.00 | hetero-2-2-2-2-2-2-… | 2 x ECH, 4 x HEM, 4 x HEC, 2 x CLA, 7 x PGV, 2 x FES, 1 x LFA | HHblits | 0.28 |
| ``` target    MAQGVSRRQLLGRALALGSGAALADLLGPARFLSPAGAATAGAVVPGNPLRVMPDRTWEQIYRNQFEDDSTFVFTCAPND 7zxy.1    -VPDLGRRQFMNLLTFGT--------------------------------------------------------------  target    THNCLLRAHVKNGVVVRISPTYGYGEATDLYGNRASHRWDPRTCQKGLILSRRFYSERRVKAPMIRKGFKDWVEAGYPRN 7zxy.1    --------------------------------------------------------------------------------  target    DDGTPQM 7zxy.1    ------- ``` | | | | | | | | | | | | | | | | | | | | | | | | | | | | | | | | | | | | | | | | | | | | | | | | | |
|  | 7zxy.1.L | Cytochrome b6-f complex iron-sulfur subunit 2  *3.15 Angstrom cryo-EM structure of the dimeric cytochrome b6f complex from Synechocystis sp. PCC 6803 with natively bound plastoquinone and lipid molecules.* | 0.01 |  | 17.65 | 0.10 | 2-18 | EM | 0.00 | hetero-2-2-2-2-2-2-… | 2 x ECH, 4 x HEM, 4 x HEC, 2 x CLA, 7 x PGV, 2 x FES, 1 x LFA | HHblits | 0.28 |
| ``` target    MAQGVSRRQLLGRALALGSGAALADLLGPARFLSPAGAATAGAVVPGNPLRVMPDRTWEQIYRNQFEDDSTFVFTCAPND 7zxy.1    -VPDLGRRQFMNLLTFGT--------------------------------------------------------------  target    THNCLLRAHVKNGVVVRISPTYGYGEATDLYGNRASHRWDPRTCQKGLILSRRFYSERRVKAPMIRKGFKDWVEAGYPRN 7zxy.1    --------------------------------------------------------------------------------  target    DDGTPQM 7zxy.1    ------- ``` | | | | | | | | | | | | | | | | | | | | | | | | | | | | | | | | | | | | | | | | | | | | | | | | | |
|  | 2e75.1.D | Cytochrome b6-f complex iron-sulfur subunit  *Crystal Structure of the Cytochrome b6f Complex with 2-nonyl-4-hydroxyquinoline N-oxide (NQNO) from M.laminosus* | 0.01 |  | 17.65 | 0.10 | 2-18 | X-ray | 3.55 | hetero-oligomer | 4 x CD, 8 x HEM, 4 x OPC, 8 x UMQ, 2 x QNO, 2 x CLA, 2 x FES, 2 x SQD, 2 x BCR | HHblits | 0.28 |
| ``` target    MAQGVSRRQLLGRALALGSGAALADLLGPARFLSPAGAATAGAVVPGNPLRVMPDRTWEQIYRNQFEDDSTFVFTCAPND 2e75.1    -VPDMGRRQFMNLLAFGT--------------------------------------------------------------  target    THNCLLRAHVKNGVVVRISPTYGYGEATDLYGNRASHRWDPRTCQKGLILSRRFYSERRVKAPMIRKGFKDWVEAGYPRN 2e75.1    --------------------------------------------------------------------------------  target    DDGTPQM 2e75.1    ------- ``` | | | | | | | | | | | | | | | | | | | | | | | | | | | | | | | | | | | | | | | | | | | | | | | | | |
|  | 2e76.1.D | Cytochrome b6-f complex iron-sulfur subunit  *Crystal Structure of the Cytochrome b6f Complex with tridecyl-stigmatellin (TDS) from M.laminosus* | 0.01 |  | 17.65 | 0.10 | 2-18 | X-ray | 3.41 | hetero-oligomer | 2 x CD, 8 x HEM, 4 x OPC, 8 x UMQ, 2 x CLA, 4 x TDS, 2 x FES, 2 x SQD, 2 x BCR | HHblits | 0.28 |
| ``` target    MAQGVSRRQLLGRALALGSGAALADLLGPARFLSPAGAATAGAVVPGNPLRVMPDRTWEQIYRNQFEDDSTFVFTCAPND 2e76.1    -VPDMGRRQFMNLLAFGT--------------------------------------------------------------  target    THNCLLRAHVKNGVVVRISPTYGYGEATDLYGNRASHRWDPRTCQKGLILSRRFYSERRVKAPMIRKGFKDWVEAGYPRN 2e76.1    --------------------------------------------------------------------------------  target    DDGTPQM 2e76.1    ------- ``` | | | | | | | | | | | | | | | | | | | | | | | | | | | | | | | | | | | | | | | | | | | | | | | | | |
|  | 4pv1.1.L | Cytochrome b6-f complex iron-sulfur subunit  *Cytochrome B6F structure from M. laminosus with the quinone analog inhibitor stigmatellin* | 0.01 |  | 17.65 | 0.10 | 2-18 | X-ray | 3.00 | hetero-2-2-2-2-2-2-… | 2 x MYS, 4 x CD, 8 x HEC, 6 x UMQ, 2 x SMA, 2 x 7PH, 2 x 8K6, 2 x CLA, 6 x OPC, 2 x FES, 2 x SQD, 2 x BCR | HHblits | 0.28 |
| ``` target    MAQGVSRRQLLGRALALGSGAALADLLGPARFLSPAGAATAGAVVPGNPLRVMPDRTWEQIYRNQFEDDSTFVFTCAPND 4pv1.1    -VPDMGRRQFMNLLAFGT--------------------------------------------------------------  target    THNCLLRAHVKNGVVVRISPTYGYGEATDLYGNRASHRWDPRTCQKGLILSRRFYSERRVKAPMIRKGFKDWVEAGYPRN 4pv1.1    --------------------------------------------------------------------------------  target    DDGTPQM 4pv1.1    ------- ``` | | | | | | | | | | | | | | | | | | | | | | | | | | | | | | | | | | | | | | | | | | | | | | | | | |
|  | 7jrg.1.E | Cytochrome b-c1 complex subunit Rieske, mitochondrial  *Plant Mitochondrial complex III2 from Vigna radiata* | 0.01 |  | 23.53 | 0.10 | 3-19 | EM | 0.00 | hetero-2-2-2-2-2-2-… | 2 x ZN, 3 x PC1, 9 x CDL, 4 x HEM, 17 x 3PE, 2 x HEC | HHblits | 0.28 |
| ``` target    MAQGVSRRQLLGRALALGSGAALADLLGPARFLSPAGAATAGAVVPGNPLRVMPDRTWEQIYRNQFEDDSTFVFTCAPND 7jrg.1    --GDPSKRAFAYFVLTGGR-------------------------------------------------------------  target    THNCLLRAHVKNGVVVRISPTYGYGEATDLYGNRASHRWDPRTCQKGLILSRRFYSERRVKAPMIRKGFKDWVEAGYPRN 7jrg.1    --------------------------------------------------------------------------------  target    DDGTPQM 7jrg.1    ------- ``` | | | | | | | | | | | | | | | | | | | | | | | | | | | | | | | | | | | | | | | | | | | | | | | | | |
|  | 5gpn.17.A | Cytochrome b-c1 complex subunit Rieske, mitochondrial  *Architecture of mammalian respirasome* | 0.01 |  | 11.76 | 0.10 | 3-19 | EM | 0.00 | monomer |  | HHblits | 0.26 |
| ``` target    MAQGVSRRQLLGRALALGSGAALADLLGPARFLSPAGAATAGAVVPGNPLRVMPDRTWEQIYRNQFEDDSTFVFTCAPND 5gpn.17   --SSEARKGFSYLVTATTT-------------------------------------------------------------  target    THNCLLRAHVKNGVVVRISPTYGYGEATDLYGNRASHRWDPRTCQKGLILSRRFYSERRVKAPMIRKGFKDWVEAGYPRN 5gpn.17   --------------------------------------------------------------------------------  target    DDGTPQM 5gpn.17   ------- ``` | | | | | | | | | | | | | | | | | | | | | | | | | | | | | | | | | | | | | | | | | | | | | | | | | |
|  | 5gpn.5.A | Cytochrome b-c1 complex subunit Rieske, mitochondrial  *Architecture of mammalian respirasome* | 0.01 |  | 11.76 | 0.10 | 3-19 | EM | 0.00 | monomer |  | HHblits | 0.26 |
| ``` target    MAQGVSRRQLLGRALALGSGAALADLLGPARFLSPAGAATAGAVVPGNPLRVMPDRTWEQIYRNQFEDDSTFVFTCAPND 5gpn.5    --SSEARKGFSYLVTATTT-------------------------------------------------------------  target    THNCLLRAHVKNGVVVRISPTYGYGEATDLYGNRASHRWDPRTCQKGLILSRRFYSERRVKAPMIRKGFKDWVEAGYPRN 5gpn.5    --------------------------------------------------------------------------------  target    DDGTPQM 5gpn.5    ------- ``` | | | | | | | | | | | | | | | | | | | | | | | | | | | | | | | | | | | | | | | | | | | | | | | | | |
|  | 4d6t.1.E | CYTOCHROME B-C1 COMPLEX SUBUNIT RIESKE, MITOCHONDRIAL  *Cytochrome bc1 bound to the 4(1H)-pyridone GW844520* | 0.01 |  | 11.76 | 0.10 | 3-19 | X-ray | 3.57 | hetero-oligomer | 2 x HEM, 1 x 4X9, 2 x PEE, 1 x HEC, 2 x CDL | HHblits | 0.26 |
| ``` target    MAQGVSRRQLLGRALALGSGAALADLLGPARFLSPAGAATAGAVVPGNPLRVMPDRTWEQIYRNQFEDDSTFVFTCAPND 4d6t.1    --SSEARKGFSYLVTATTT-------------------------------------------------------------  target    THNCLLRAHVKNGVVVRISPTYGYGEATDLYGNRASHRWDPRTCQKGLILSRRFYSERRVKAPMIRKGFKDWVEAGYPRN 4d6t.1    --------------------------------------------------------------------------------  target    DDGTPQM 4d6t.1    ------- ``` | | | | | | | | | | | | | | | | | | | | | | | | | | | | | | | | | | | | | | | | | | | | | | | | | |
|  | 5nmi.1.E | Cytochrome b-c1 complex subunit Rieske, mitochondrial  *Cytochrome bc1 bound to the inhibitor MJM170* | 0.01 |  | 11.76 | 0.10 | 3-19 | X-ray | 3.50 | hetero-2-2-2-2-4-2-… | 4 x HEM, 2 x MJM, 6 x PEE, 6 x CDL, 2 x HEC, 1 x FES | HHblits | 0.26 |
| ``` target    MAQGVSRRQLLGRALALGSGAALADLLGPARFLSPAGAATAGAVVPGNPLRVMPDRTWEQIYRNQFEDDSTFVFTCAPND 5nmi.1    --SSEARKGFSYLVTATTT-------------------------------------------------------------  target    THNCLLRAHVKNGVVVRISPTYGYGEATDLYGNRASHRWDPRTCQKGLILSRRFYSERRVKAPMIRKGFKDWVEAGYPRN 5nmi.1    --------------------------------------------------------------------------------  target    DDGTPQM 5nmi.1    ------- ``` | | | | | | | | | | | | | | | | | | | | | | | | | | | | | | | | | | | | | | | | | | | | | | | | | |
|  | 6fo6.1.J | Cytochrome b-c1 complex subunit Rieske, mitochondrial  *CryoEM structure of bovine cytochrome bc1 in complex with the anti-malarial inhibitor SCR0911* | 0.01 |  | 11.76 | 0.10 | 3-19 | EM | 0.00 | hetero-2-2-2-2-2-2-… | 4 x HEM, 2 x DY2, 2 x HEC | HHblits | 0.26 |
| ``` target    MAQGVSRRQLLGRALALGSGAALADLLGPARFLSPAGAATAGAVVPGNPLRVMPDRTWEQIYRNQFEDDSTFVFTCAPND 6fo6.1    --SSEARKGFSYLVTATTT-------------------------------------------------------------  target    THNCLLRAHVKNGVVVRISPTYGYGEATDLYGNRASHRWDPRTCQKGLILSRRFYSERRVKAPMIRKGFKDWVEAGYPRN 6fo6.1    --------------------------------------------------------------------------------  target    DDGTPQM 6fo6.1    ------- ``` | | | | | | | | | | | | | | | | | | | | | | | | | | | | | | | | | | | | | | | | | | | | | | | | | |
|  | 6fo0.1.E | Cytochrome b-c1 complex subunit Rieske, mitochondrial  *CryoEM structure of bovine cytochrome bc1 in complex with the anti-malarial compound GSK932121* | 0.01 |  | 11.76 | 0.10 | 3-19 | EM | 0.00 | hetero-2-2-2-2-2-2-… | 4 x HEM, 2 x G8U, 2 x HEC | HHblits | 0.26 |
| ``` target    MAQGVSRRQLLGRALALGSGAALADLLGPARFLSPAGAATAGAVVPGNPLRVMPDRTWEQIYRNQFEDDSTFVFTCAPND 6fo0.1    --SSEARKGFSYLVTATTT-------------------------------------------------------------  target    THNCLLRAHVKNGVVVRISPTYGYGEATDLYGNRASHRWDPRTCQKGLILSRRFYSERRVKAPMIRKGFKDWVEAGYPRN 6fo0.1    --------------------------------------------------------------------------------  target    DDGTPQM 6fo0.1    ------- ``` | | | | | | | | | | | | | | | | | | | | | | | | | | | | | | | | | | | | | | | | | | | | | | | | | |
|  | 3h1h.1.O | Cytochrome b-c1 complex subunit Rieske, mitochondrial  *Cytochrome bc1 complex from chicken* | 0.01 |  | 11.76 | 0.10 | 3-19 | X-ray | 3.16 | hetero-2-2-2-2-2-2-… | 4 x HEM, 2 x UQ, 4 x CDL, 6 x PEE, 2 x HEC, 5 x BOG, 2 x FES | HHblits | 0.25 |
| ``` target    MAQGVSRRQLLGRALALGSGAALADLLGPARFLSPAGAATAGAVVPGNPLRVMPDRTWEQIYRNQFEDDSTFVFTCAPND 3h1h.1    --SSEDRKGFSYLVTATAC-------------------------------------------------------------  target    THNCLLRAHVKNGVVVRISPTYGYGEATDLYGNRASHRWDPRTCQKGLILSRRFYSERRVKAPMIRKGFKDWVEAGYPRN 3h1h.1    --------------------------------------------------------------------------------  target    DDGTPQM 3h1h.1    ------- ``` | | | | | | | | | | | | | | | | | | | | | | | | | | | | | | | | | | | | | | | | | | | | | | | | | |
|  | 3h1i.1.E | Cytochrome b-c1 complex subunit Rieske, mitochondrial  *Stigmatellin and antimycin bound cytochrome bc1 complex from chicken* | 0.01 |  | 11.76 | 0.10 | 3-19 | X-ray | 3.53 | hetero-oligomer | 4 x HEM, 2 x HEC, 2 x FES, 4 x CDL, 6 x PEE, 1 x PLC, 7 x UNL, 1 x SMA, 1 x ANY, 1 x GOL | HHblits | 0.25 |
| ``` target    MAQGVSRRQLLGRALALGSGAALADLLGPARFLSPAGAATAGAVVPGNPLRVMPDRTWEQIYRNQFEDDSTFVFTCAPND 3h1i.1    --SSEDRKGFSYLVTATAC-------------------------------------------------------------  target    THNCLLRAHVKNGVVVRISPTYGYGEATDLYGNRASHRWDPRTCQKGLILSRRFYSERRVKAPMIRKGFKDWVEAGYPRN 3h1i.1    --------------------------------------------------------------------------------  target    DDGTPQM 3h1i.1    ------- ``` | | | | | | | | | | | | | | | | | | | | | | | | | | | | | | | | | | | | | | | | | | | | | | | | | |
|  | 3h1h.1.E | Cytochrome b-c1 complex subunit Rieske, mitochondrial  *Cytochrome bc1 complex from chicken* | 0.01 |  | 11.76 | 0.10 | 3-19 | X-ray | 3.16 | hetero-2-2-2-2-2-2-… | 4 x HEM, 2 x UQ, 4 x CDL, 6 x PEE, 2 x HEC, 5 x BOG, 2 x FES | HHblits | 0.25 |
| ``` target    MAQGVSRRQLLGRALALGSGAALADLLGPARFLSPAGAATAGAVVPGNPLRVMPDRTWEQIYRNQFEDDSTFVFTCAPND 3h1h.1    --SSEDRKGFSYLVTATAC-------------------------------------------------------------  target    THNCLLRAHVKNGVVVRISPTYGYGEATDLYGNRASHRWDPRTCQKGLILSRRFYSERRVKAPMIRKGFKDWVEAGYPRN 3h1h.1    --------------------------------------------------------------------------------  target    DDGTPQM 3h1h.1    ------- ``` | | | | | | | | | | | | | | | | | | | | | | | | | | | | | | | | | | | | | | | | | | | | | | | | | |
|  | 3cwb.1.E | MITOCHONDRIAL UBIQUINOL-CYTOCHROME C REDUCTASE IRON-SULFUR PROTEIN  *Chicken Cytochrome BC1 Complex inhibited by an iodinated analogue of the polyketide Crocacin-D* | 0.01 |  | 11.76 | 0.10 | 3-19 | X-ray | 3.51 | hetero-2-2-2-2-2-2-… | 6 x PEE, 6 x BOG, 2 x AZI, 4 x HEM, 2 x ICX, 2 x UQ, 2 x HEC, 4 x CDL, 2 x FES, 2 x UNL | HHblits | 0.25 |
| ``` target    MAQGVSRRQLLGRALALGSGAALADLLGPARFLSPAGAATAGAVVPGNPLRVMPDRTWEQIYRNQFEDDSTFVFTCAPND 3cwb.1    --SSEDRKGFSYLVTATAC-------------------------------------------------------------  target    THNCLLRAHVKNGVVVRISPTYGYGEATDLYGNRASHRWDPRTCQKGLILSRRFYSERRVKAPMIRKGFKDWVEAGYPRN 3cwb.1    --------------------------------------------------------------------------------  target    DDGTPQM 3cwb.1    ------- ``` | | | | | | | | | | | | | | | | | | | | | | | | | | | | | | | | | | | | | | | | | | | | | | | | | |
|  | 3l75.1.O | CYTOCHROME B-C1 COMPLEX SUBUNIT 5, RIESKE IRONSULFUR PROTEIN, MITOCHONDRIAL  *Cytochrome BC1 complex from chicken with fenamidone bound* | 0.01 |  | 11.76 | 0.10 | 3-19 | X-ray | 2.79 | hetero-2-2-2-2-2-2-… | 6 x PEE, 4 x HEM, 2 x FNM, 2 x UQ, 2 x AZI, 6 x BOG, 2 x HEC, 4 x CDL, 2 x FES | HHblits | 0.25 |
| ``` target    MAQGVSRRQLLGRALALGSGAALADLLGPARFLSPAGAATAGAVVPGNPLRVMPDRTWEQIYRNQFEDDSTFVFTCAPND 3l75.1    --SSEDRKGFSYLVTATAC-------------------------------------------------------------  target    THNCLLRAHVKNGVVVRISPTYGYGEATDLYGNRASHRWDPRTCQKGLILSRRFYSERRVKAPMIRKGFKDWVEAGYPRN 3l75.1    --------------------------------------------------------------------------------  target    DDGTPQM 3l75.1    ------- ``` | | | | | | | | | | | | | | | | | | | | | | | | | | | | | | | | | | | | | | | | | | | | | | | | | |
|  | 3l75.1.E | CYTOCHROME B-C1 COMPLEX SUBUNIT 5, RIESKE IRONSULFUR PROTEIN, MITOCHONDRIAL  *Cytochrome BC1 complex from chicken with fenamidone bound* | 0.01 |  | 11.76 | 0.10 | 3-19 | X-ray | 2.79 | hetero-2-2-2-2-2-2-… | 6 x PEE, 4 x HEM, 2 x FNM, 2 x UQ, 2 x AZI, 6 x BOG, 2 x HEC, 4 x CDL, 2 x FES | HHblits | 0.25 |
| ``` target    MAQGVSRRQLLGRALALGSGAALADLLGPARFLSPAGAATAGAVVPGNPLRVMPDRTWEQIYRNQFEDDSTFVFTCAPND 3l75.1    --SSEDRKGFSYLVTATAC-------------------------------------------------------------  target    THNCLLRAHVKNGVVVRISPTYGYGEATDLYGNRASHRWDPRTCQKGLILSRRFYSERRVKAPMIRKGFKDWVEAGYPRN 3l75.1    --------------------------------------------------------------------------------  target    DDGTPQM 3l75.1    ------- ``` | | | | | | | | | | | | | | | | | | | | | | | | | | | | | | | | | | | | | | | | | | | | | | | | | |
|  | 4u3f.1.E | Cytochrome b-c1 complex subunit Rieske, mitochondrial  *Cytochrome bc1 complex from chicken with designed inhibitor bound* | 0.01 |  | 11.76 | 0.10 | 3-19 | X-ray | 3.23 | hetero-2-2-2-2-2-2-… | 14 x PEE, 4 x HEM, 2 x Y52, 2 x U10, 1 x MES, 2 x HEC, 4 x CDL, 3 x BOG, 2 x FES | HHblits | 0.25 |
| ``` target    MAQGVSRRQLLGRALALGSGAALADLLGPARFLSPAGAATAGAVVPGNPLRVMPDRTWEQIYRNQFEDDSTFVFTCAPND 4u3f.1    --SSEDRKGFSYLVTATAC-------------------------------------------------------------  target    THNCLLRAHVKNGVVVRISPTYGYGEATDLYGNRASHRWDPRTCQKGLILSRRFYSERRVKAPMIRKGFKDWVEAGYPRN 4u3f.1    --------------------------------------------------------------------------------  target    DDGTPQM 4u3f.1    ------- ``` | | | | | | | | | | | | | | | | | | | | | | | | | | | | | | | | | | | | | | | | | | | | | | | | | |
|  | 1kb9.1.E | UBIQUINOL-CYTOCHROME C REDUCTASE IRON-SULFUR SUBUNIT  *YEAST CYTOCHROME BC1 COMPLEX* | 0.02 |  | 5.56 | 0.11 | 3-20 | X-ray | 2.30 | hetero-oligomer | 3 x HEM, 1 x FES, 1 x SMA, 1 x UQ6, 1 x PIE, 2 x PEF, 1 x CDL, 1 x PCF, 1 x UMQ | HHblits | 0.21 |
| ``` target    MAQGVSRRQLLGRALALGSGAALADLLGPARFLSPAGAATAGAVVPGNPLRVMPDRTWEQIYRNQFEDDSTFVFTCAPND 1kb9.1    --DADKGRSYAYFMVGAMGL------------------------------------------------------------  target    THNCLLRAHVKNGVVVRISPTYGYGEATDLYGNRASHRWDPRTCQKGLILSRRFYSERRVKAPMIRKGFKDWVEAGYPRN 1kb9.1    --------------------------------------------------------------------------------  target    DDGTPQM 1kb9.1    ------- ``` | | | | | | | | | | | | | | | | | | | | | | | | | | | | | | | | | | | | | | | | | | | | | | | | | |
|  | 6hu9.1.E | Cytochrome b-c1 complex subunit Rieske, mitochondrial  *III2-IV2 mitochondrial respiratory supercomplex from S. cerevisiae* | 0.02 |  | 5.56 | 0.11 | 3-20 | EM | 0.00 | hetero-2-2-2-2-2-2-… | 28 x PEF, 4 x HEM, 1 x UQ6, 8 x CDL, 8 x PCF, 2 x HEC, 2 x FES, 2 x CU, 4 x HEA, 2 x CA, 2 x MG, 4 x CUA, 2 x ZN | HHblits | 0.21 |
| ``` target    MAQGVSRRQLLGRALALGSGAALADLLGPARFLSPAGAATAGAVVPGNPLRVMPDRTWEQIYRNQFEDDSTFVFTCAPND 6hu9.1    --DADKGRSYAYFMVGAMGL------------------------------------------------------------  target    THNCLLRAHVKNGVVVRISPTYGYGEATDLYGNRASHRWDPRTCQKGLILSRRFYSERRVKAPMIRKGFKDWVEAGYPRN 6hu9.1    --------------------------------------------------------------------------------  target    DDGTPQM 6hu9.1    ------- ``` | | | | | | | | | | | | | | | | | | | | | | | | | | | | | | | | | | | | | | | | | | | | | | | | | |
|  | 6t0b.1.O | Cytochrome b-c1 complex subunit Rieske, mitochondrial  *The III2-IV(5B)2 respiratory supercomplex from S. cerevisiae* | 0.02 |  | 5.56 | 0.11 | 3-20 | EM | 0.00 | hetero-2-2-2-2-2-2-… | 8 x CDL, 26 x PEF, 4 x HEM, 7 x PCF, 2 x HEC, 2 x FES, 2 x CU, 4 x HEA, 2 x CA, 2 x MG, 2 x CUA, 2 x ZN | HHblits | 0.21 |
| ``` target    MAQGVSRRQLLGRALALGSGAALADLLGPARFLSPAGAATAGAVVPGNPLRVMPDRTWEQIYRNQFEDDSTFVFTCAPND 6t0b.1    --DADKGRSYAYFMVGAMGL------------------------------------------------------------  target    THNCLLRAHVKNGVVVRISPTYGYGEATDLYGNRASHRWDPRTCQKGLILSRRFYSERRVKAPMIRKGFKDWVEAGYPRN 6t0b.1    --------------------------------------------------------------------------------  target    DDGTPQM 6t0b.1    ------- ``` | | | | | | | | | | | | | | | | | | | | | | | | | | | | | | | | | | | | | | | | | | | | | | | | | |
|  | 6t0b.1.E | Cytochrome b-c1 complex subunit Rieske, mitochondrial  *The III2-IV(5B)2 respiratory supercomplex from S. cerevisiae* | 0.02 |  | 5.56 | 0.11 | 3-20 | EM | 0.00 | hetero-2-2-2-2-2-2-… | 8 x CDL, 26 x PEF, 4 x HEM, 7 x PCF, 2 x HEC, 2 x FES, 2 x CU, 4 x HEA, 2 x CA, 2 x MG, 2 x CUA, 2 x ZN | HHblits | 0.21 |
| ``` target    MAQGVSRRQLLGRALALGSGAALADLLGPARFLSPAGAATAGAVVPGNPLRVMPDRTWEQIYRNQFEDDSTFVFTCAPND 6t0b.1    --DADKGRSYAYFMVGAMGL------------------------------------------------------------  target    THNCLLRAHVKNGVVVRISPTYGYGEATDLYGNRASHRWDPRTCQKGLILSRRFYSERRVKAPMIRKGFKDWVEAGYPRN 6t0b.1    --------------------------------------------------------------------------------  target    DDGTPQM 6t0b.1    ------- ``` | | | | | | | | | | | | | | | | | | | | | | | | | | | | | | | | | | | | | | | | | | | | | | | | | |
|  | 6t15.1.E | CYTOCHROME B-C1 COMPLEX SUBUNIT RIESKE, MITOCHONDRIAL; SYNONYM: COMPLEX III SUBUNIT 5, RIESKE IRON-SULFUR PROTEIN, RISP, UBIQUINOL-CYTOCHROME C REDUCTASE IRON-SULFUR SUBUNIT  *The III2-IV(5B)1 respiratory supercomplex from S. cerevisiae* | 0.02 |  | 5.56 | 0.11 | 3-20 | EM | 0.00 | hetero-2-2-2-2-2-2-… | 18 x PEF, 4 x HEM, 7 x CDL, 6 x PCF, 2 x HEC, 2 x FES, 1 x CU, 2 x HEA, 1 x MG, 1 x CUA, 1 x ZN | HHblits | 0.21 |
| ``` target    MAQGVSRRQLLGRALALGSGAALADLLGPARFLSPAGAATAGAVVPGNPLRVMPDRTWEQIYRNQFEDDSTFVFTCAPND 6t15.1    --DADKGRSYAYFMVGAMGL------------------------------------------------------------  target    THNCLLRAHVKNGVVVRISPTYGYGEATDLYGNRASHRWDPRTCQKGLILSRRFYSERRVKAPMIRKGFKDWVEAGYPRN 6t15.1    --------------------------------------------------------------------------------  target    DDGTPQM 6t15.1    ------- ``` | | | | | | | | | | | | | | | | | | | | | | | | | | | | | | | | | | | | | | | | | | | | | | | | | |
|  | 6t15.1.O | CYTOCHROME B-C1 COMPLEX SUBUNIT RIESKE, MITOCHONDRIAL; SYNONYM: COMPLEX III SUBUNIT 5, RIESKE IRON-SULFUR PROTEIN, RISP, UBIQUINOL-CYTOCHROME C REDUCTASE IRON-SULFUR SUBUNIT  *The III2-IV(5B)1 respiratory supercomplex from S. cerevisiae* | 0.02 |  | 5.56 | 0.11 | 3-20 | EM | 0.00 | hetero-2-2-2-2-2-2-… | 18 x PEF, 4 x HEM, 7 x CDL, 6 x PCF, 2 x HEC, 2 x FES, 1 x CU, 2 x HEA, 1 x MG, 1 x CUA, 1 x ZN | HHblits | 0.21 |
| ``` target    MAQGVSRRQLLGRALALGSGAALADLLGPARFLSPAGAATAGAVVPGNPLRVMPDRTWEQIYRNQFEDDSTFVFTCAPND 6t15.1    --DADKGRSYAYFMVGAMGL------------------------------------------------------------  target    THNCLLRAHVKNGVVVRISPTYGYGEATDLYGNRASHRWDPRTCQKGLILSRRFYSERRVKAPMIRKGFKDWVEAGYPRN 6t15.1    --------------------------------------------------------------------------------  target    DDGTPQM 6t15.1    ------- ``` | | | | | | | | | | | | | | | | | | | | | | | | | | | | | | | | | | | | | | | | | | | | | | | | | |
|  | 6ymx.1.Z | Cytochrome b-c1 complex subunit Rieske, mitochondrial  *CIII2/CIV respiratory supercomplex from Saccharomyces cerevisiae* | 0.00 |  | 5.56 | 0.11 | 3-20 | EM | 0.00 | hetero-1-1-1-1-1-1-… | 1 x CU, 2 x HEA, 7 x PTY, 2 x CN3, 1 x CUA, 5 x PCF, 1 x ZN, 2 x 6PH, 6 x HEM, 2 x 8PE, 1 x CN5, 2 x UQ6, 2 x 9PE, 2 x 7PH, 2 x FES | HHblits | 0.21 |
| ``` target    MAQGVSRRQLLGRALALGSGAALADLLGPARFLSPAGAATAGAVVPGNPLRVMPDRTWEQIYRNQFEDDSTFVFTCAPND 6ymx.1    --DADKGRSYAYFMVGAMGL------------------------------------------------------------  target    THNCLLRAHVKNGVVVRISPTYGYGEATDLYGNRASHRWDPRTCQKGLILSRRFYSERRVKAPMIRKGFKDWVEAGYPRN 6ymx.1    --------------------------------------------------------------------------------  target    DDGTPQM 6ymx.1    ------- ``` | | | | | | | | | | | | | | | | | | | | | | | | | | | | | | | | | | | | | | | | | | | | | | | | | |
|  | 6ymx.1.Q | Cytochrome b-c1 complex subunit Rieske, mitochondrial  *CIII2/CIV respiratory supercomplex from Saccharomyces cerevisiae* | 0.02 |  | 5.56 | 0.11 | 3-20 | EM | 0.00 | hetero-1-1-1-1-1-1-… | 1 x CU, 2 x HEA, 7 x PTY, 2 x CN3, 1 x CUA, 5 x PCF, 1 x ZN, 2 x 6PH, 6 x HEM, 2 x 8PE, 1 x CN5, 2 x UQ6, 2 x 9PE, 2 x 7PH, 2 x FES | HHblits | 0.21 |
| ``` target    MAQGVSRRQLLGRALALGSGAALADLLGPARFLSPAGAATAGAVVPGNPLRVMPDRTWEQIYRNQFEDDSTFVFTCAPND 6ymx.1    --DADKGRSYAYFMVGAMGL------------------------------------------------------------  target    THNCLLRAHVKNGVVVRISPTYGYGEATDLYGNRASHRWDPRTCQKGLILSRRFYSERRVKAPMIRKGFKDWVEAGYPRN 6ymx.1    --------------------------------------------------------------------------------  target    DDGTPQM 6ymx.1    ------- ``` | | | | | | | | | | | | | | | | | | | | | | | | | | | | | | | | | | | | | | | | | | | | | | | | | |
|  | 5gup.54.A | Cytochrome b-c1 complex subunit Rieske, mitochondrial  *Cryo-EM structure of mammalian respiratory supercomplex I1III2IV1* | 0.01 |  | 11.76 | 0.10 | 3-19 | EM | 0.00 | monomer |  | HHblits | 0.25 |
| ``` target    MAQGVSRRQLLGRALALGSGAALADLLGPARFLSPAGAATAGAVVPGNPLRVMPDRTWEQIYRNQFEDDSTFVFTCAPND 5gup.54   --SSDARKGFSYLITATTT-------------------------------------------------------------  target    THNCLLRAHVKNGVVVRISPTYGYGEATDLYGNRASHRWDPRTCQKGLILSRRFYSERRVKAPMIRKGFKDWVEAGYPRN 5gup.54   --------------------------------------------------------------------------------  target    DDGTPQM 5gup.54   ------- ``` | | | | | | | | | | | | | | | | | | | | | | | | | | | | | | | | | | | | | | | | | | | | | | | | | |
|  | 5gup.56.A | Cytochrome b-c1 complex subunit Rieske, mitochondrial  *Cryo-EM structure of mammalian respiratory supercomplex I1III2IV1* | 0.01 |  | 11.76 | 0.10 | 3-19 | EM | 0.00 | monomer |  | HHblits | 0.25 |
| ``` target    MAQGVSRRQLLGRALALGSGAALADLLGPARFLSPAGAATAGAVVPGNPLRVMPDRTWEQIYRNQFEDDSTFVFTCAPND 5gup.56   --SSDARKGFSYLITATTT-------------------------------------------------------------  target    THNCLLRAHVKNGVVVRISPTYGYGEATDLYGNRASHRWDPRTCQKGLILSRRFYSERRVKAPMIRKGFKDWVEAGYPRN 5gup.56   --------------------------------------------------------------------------------  target    DDGTPQM 5gup.56   ------- ``` | | | | | | | | | | | | | | | | | | | | | | | | | | | | | | | | | | | | | | | | | | | | | | | | | |
|  | 7r0w.1.L | Rieske domain, PetC  *2.8 Angstrom cryo-EM structure of the dimeric cytochrome b6f-PetP complex from Synechocystis sp. PCC 6803 with natively bound lipids and plastoquinone molecules* | 0.01 |  | 18.75 | 0.10 | 3-18 | EM | 0.00 | hetero-2-2-2-2-2-2-… | 6 x HEM, 4 x PGV, 2 x ECH, 2 x PL9, 2 x CLA, 2 x HEC, 1 x LMG, 2 x 6PL, 1 x 2WA, 2 x FES, 2 x SQD, 1 x LFA | HHblits | 0.28 |
| ``` target    MAQGVSRRQLLGRALALGSGAALADLLGPARFLSPAGAATAGAVVPGNPLRVMPDRTWEQIYRNQFEDDSTFVFTCAPND 7r0w.1    --PDLGRRQFMNLLTFGT--------------------------------------------------------------  target    THNCLLRAHVKNGVVVRISPTYGYGEATDLYGNRASHRWDPRTCQKGLILSRRFYSERRVKAPMIRKGFKDWVEAGYPRN 7r0w.1    --------------------------------------------------------------------------------  target    DDGTPQM 7r0w.1    ------- ``` | | | | | | | | | | | | | | | | | | | | | | | | | | | | | | | | | | | | | | | | | | | | | | | | | |
|  | 7r0w.1.Q | Rieske domain, PetC  *2.8 Angstrom cryo-EM structure of the dimeric cytochrome b6f-PetP complex from Synechocystis sp. PCC 6803 with natively bound lipids and plastoquinone molecules* | 0.01 |  | 18.75 | 0.10 | 3-18 | EM | 0.00 | hetero-2-2-2-2-2-2-… | 6 x HEM, 4 x PGV, 2 x ECH, 2 x PL9, 2 x CLA, 2 x HEC, 1 x LMG, 2 x 6PL, 1 x 2WA, 2 x FES, 2 x SQD, 1 x LFA | HHblits | 0.28 |
| ``` target    MAQGVSRRQLLGRALALGSGAALADLLGPARFLSPAGAATAGAVVPGNPLRVMPDRTWEQIYRNQFEDDSTFVFTCAPND 7r0w.1    --PDLGRRQFMNLLTFGT--------------------------------------------------------------  target    THNCLLRAHVKNGVVVRISPTYGYGEATDLYGNRASHRWDPRTCQKGLILSRRFYSERRVKAPMIRKGFKDWVEAGYPRN 7r0w.1    --------------------------------------------------------------------------------  target    DDGTPQM 7r0w.1    ------- ``` | | | | | | | | | | | | | | | | | | | | | | | | | | | | | | | | | | | | | | | | | | | | | | | | | |
|  | 6giq.1.E | Cytochrome b-c1 complex subunit Rieske, mitochondrial  *Saccharomyces cerevisiae respiratory supercomplex III2IV* | 0.02 |  | 5.88 | 0.10 | 3-19 | EM | 0.00 | hetero-2-2-2-2-2-2-… | 2 x 6PH, 6 x HEM, 2 x 8PE, 1 x CN5, 2 x UQ6, 2 x 7PH, 2 x FES, 2 x PCF, 2 x 9PE, 1 x CN3, 1 x CU, 2 x HEA, 1 x CUA | HHblits | 0.22 |
| ``` target    MAQGVSRRQLLGRALALGSGAALADLLGPARFLSPAGAATAGAVVPGNPLRVMPDRTWEQIYRNQFEDDSTFVFTCAPND 6giq.1    --DADKGRSYAYFMVGAMG-------------------------------------------------------------  target    THNCLLRAHVKNGVVVRISPTYGYGEATDLYGNRASHRWDPRTCQKGLILSRRFYSERRVKAPMIRKGFKDWVEAGYPRN 6giq.1    --------------------------------------------------------------------------------  target    DDGTPQM 6giq.1    ------- ``` | | | | | | | | | | | | | | | | | | | | | | | | | | | | | | | | | | | | | | | | | | | | | | | | | |
|  | 4h44.1.D | Cytochrome b6-f complex iron-sulfur subunit 1  *2.70 A Cytochrome b6f Complex Structure From Nostoc PCC 7120* | 0.01 |  | 20.00 | 0.09 | 2-16 | X-ray | 2.70 | hetero-oligomer | 8 x HEM, 10 x UMQ, 2 x MYS, 2 x 8K6, 2 x CLA, 4 x OPC, 2 x 7PH, 2 x SQD, 2 x CD, 2 x FES, 2 x OCT, 2 x BCR | HHblits | 0.31 |
| ``` target    MAQGVSRRQLLGRALALGSGAALADLLGPARFLSPAGAATAGAVVPGNPLRVMPDRTWEQIYRNQFEDDSTFVFTCAPND 4h44.1    -VPDMGRRQFMNLLTF----------------------------------------------------------------  target    THNCLLRAHVKNGVVVRISPTYGYGEATDLYGNRASHRWDPRTCQKGLILSRRFYSERRVKAPMIRKGFKDWVEAGYPRN 4h44.1    --------------------------------------------------------------------------------  target    DDGTPQM 4h44.1    ------- ``` | | | | | | | | | | | | | | | | | | | | | | | | | | | | | | | | | | | | | | | | | | | | | | | | | |
|  | 4ogq.1.L | Cytochrome b6-f complex iron-sulfur subunit 1  *Internal Lipid Architecture of the Hetero-Oligomeric Cytochrome b6f Complex* | 0.00 |  | 20.00 | 0.09 | 2-16 | X-ray | 2.50 | hetero-2-2-2-2-2-2-… | 8 x HEC, 10 x UMQ, 10 x 7PH, 8 x 8K6, 2 x 2WM, 2 x CLA, 2 x OPC, 2 x CD, 2 x MYS, 2 x SQD, 2 x FES, 2 x 2WD, 2 x 3WM, 2 x 2WA, 2 x OCT, 2 x 1O2, 2 x BCR | HHblits | 0.31 |
| ``` target    MAQGVSRRQLLGRALALGSGAALADLLGPARFLSPAGAATAGAVVPGNPLRVMPDRTWEQIYRNQFEDDSTFVFTCAPND 4ogq.1    -VPDMGRRQFMNLLTF----------------------------------------------------------------  target    THNCLLRAHVKNGVVVRISPTYGYGEATDLYGNRASHRWDPRTCQKGLILSRRFYSERRVKAPMIRKGFKDWVEAGYPRN 4ogq.1    --------------------------------------------------------------------------------  target    DDGTPQM 4ogq.1    ------- ``` | | | | | | | | | | | | | | | | | | | | | | | | | | | | | | | | | | | | | | | | | | | | | | | | | |
