# Supplementary material for: Metabolic capacity is maintained despite shifts in microbial diversity in estuary sediments
Source: ISME Commun. 2025 Oct 11;5(1):ycaf182. doi: 10.1093/ismeco/ycaf182 (PMC12687941; doi:10.1093/ismeco/ycaf182)
Supplement: Supplementary_Data_1_ycaf182 [file supplementary_data_1_ycaf182.zip › SWISS-MODEL/4_1_Jan_SF_Bin61_scaffold_2525_c13704_1/templates.html]

4\_1\_Jan\_SF\_Bin61\_scaffold\_2525\_c1:3-704\_1 | Templates


**Export Alignment**
  
FASTA format
Clustal Format
PNG Image

**Secondary Structure**
  
None
DSSP
PSIPRED
SSpro

**Colour Scheme** 


Fade Mismatches
Enhance Mismatches

Confidencegradient
Confidenceclass
Indels
Chain
Unique Chain
Rainbow
2° Structure
Clustal
Hydrophobic
Size
Charged
Polar
Proline
Ser/Thr
Cysteine
Aliphatic
Aromatic
No Colour

Use QMEANBrane values

|  |  |  |  |
| --- | --- | --- | --- |
| Background |  |  |  |

**3D Viewer**  
NGL
PV

FASTA
Multi FASTA
ClustalW
PNG


SWISS-MODEL

### 4\_1\_Jan\_SF\_Bin61\_scaffold\_2525\_c1:3-704\_1

### Created: March 29, 2023, 6:31 p.m. at 18:31

- Templates
- Models

Models | Name | Description | GMQE | QSQE | Seq Id | Coverage | Range | Method | Resolution | Oligo-state | Ligands | Found by | Seq Similarity ||  | 7b04.1.B | Nitrite oxidoreductase subunit A  *Structure of Nitrite oxidoreductase (Nxr) from the anammox bacterium Kuenenia stuttgartiensis.* | 0.58 | 0.00 | 40.51 | 0.84 | 3-204 | X-ray | 2.97 | monomer | 4 x SF4, 1 x F3S, 2 x MD1, 1 x MO, 1 x HEM, 2 x CA | HHblits | 0.41 |
| ``` target    MAQGVSRRQLLGRALALGSGAALADLLGPARFLSPAGAATAGAVVPGNPLRVMPDRTWEQIYRNQFEDDSTFVFTCAPND 7b04.1    --MKLTRRAFLQVAGATGATLTLAKNAMAFRLLKP-------AVVVDNPLDTYPDRRWESVYRDQYQYDRTFTYCCSPND  target    THNCLLRAHVKNGVVVRISPTYGYGEATDLYGNRASHRWDPRTCQKGLILSRRFYSERRVKAPMIRKGFKDWVEAGYPRN 7b04.1    THACRIRAFVRNNVMMRVEQNYDHQNYSDLYGNKATRNWNPRMCLKGYTFHRRVYGPYRLRYPLIRKGWKRWADDGFPEL  target    DDGTP-QMDVTLRGSDDWIRISWDEATTIAAKTMEDVARTF-NGDEGARKLLAQGYHPEMVEVMHGAGVQALKLR 7b04.1    TPENKTKYMFDNRGNDELLRASWDEAFTYASKGIIHITKKYSGPEG----------------------------- ``` | | | | | | | | | | | | | | | | | | | | | | | | | | | | | | | | | | | | | | | | | | | | | | | | | |
|  | 7b04.2.B | Nitrite oxidoreductase subunit A  *Structure of Nitrite oxidoreductase (Nxr) from the anammox bacterium Kuenenia stuttgartiensis.* | 0.55 | 0.00 | 40.51 | 0.84 | 3-204 | X-ray | 2.97 | monomer | 4 x SF4, 1 x F3S, 2 x MD1, 1 x MO, 1 x HEM, 2 x CA | HHblits | 0.41 |
| ``` target    MAQGVSRRQLLGRALALGSGAALADLLGPARFLSPAGAATAGAVVPGNPLRVMPDRTWEQIYRNQFEDDSTFVFTCAPND 7b04.2    --MKLTRRAFLQVAGATGATLTLAKNAMAFRLLKP-------AVVVDNPLDTYPDRRWESVYRDQYQYDRTFTYCCSPND  target    THNCLLRAHVKNGVVVRISPTYGYGEATDLYGNRASHRWDPRTCQKGLILSRRFYSERRVKAPMIRKGFKDWVEAGYPRN 7b04.2    THACRIRAFVRNNVMMRVEQNYDHQNYSDLYGNKATRNWNPRMCLKGYTFHRRVYGPYRLRYPLIRKGWKRWADDGFPEL  target    DDGTP-QMDVTLRGSDDWIRISWDEATTIAAKTMEDVARTF-NGDEGARKLLAQGYHPEMVEVMHGAGVQALKLR 7b04.2    TPENKTKYMFDNRGNDELLRASWDEAFTYASKGIIHITKKYSGPEG----------------------------- ``` | | | | | | | | | | | | | | | | | | | | | | | | | | | | | | | | | | | | | | | | | | | | | | | | | |
| ✓ | 7b04.1.B | Nitrite oxidoreductase subunit A  *Structure of Nitrite oxidoreductase (Nxr) from the anammox bacterium Kuenenia stuttgartiensis.* | 0.60 | 0.00 | 46.45 | 0.79 | 48-233 | X-ray | 2.97 | monomer | 4 x SF4, 1 x F3S, 2 x MD1, 1 x MO, 1 x HEM, 2 x CA | BLAST | 0.45 |
| ``` target    MAQGVSRRQLLGRALALGSGAALADLLGPARFLSPAGAATAGAVVPGNPLRVMPDRTWEQIYRNQFEDDSTFVFTCAPND 7b04.1    -----------------------------------------------NPLDTYPDRRWESVYRDQYQYDRTFTYCCSPND  target    THNCLLRAHVKNGVVVRISPTYGYGEATDLYGNRASHRWDPRTCQKGLILSRRFYSERRVKAPMIRKGFKDWVEAGYPRN 7b04.1    THACRIRAFVRNNVMMRVEQNYDHQNYSDLYGNKATRNWNPRMCLKGYTFHRRVYGPYRLRYPLIRKGWKRWADDGFP--  target    DDGTPQMDVTL----RGSDDWIRISWDEATTIAAKTMEDVARTFNGDEGARKLLAQGYHPEMVEVMHGAGVQALKLR 7b04.1    -ELTPENKTKYMFDNRGNDELLRASWDEAFTYASKGIIHITKKYSGPEGAQKLIDQGYPKEMVDRMQGAGTRTFKGR ``` | | | | | | | | | | | | | | | | | | | | | | | | | | | | | | | | | | | | | | | | | | | | | | | | | |
|  | 7b04.2.B | Nitrite oxidoreductase subunit A  *Structure of Nitrite oxidoreductase (Nxr) from the anammox bacterium Kuenenia stuttgartiensis.* | 0.60 | 0.00 | 46.45 | 0.79 | 48-233 | X-ray | 2.97 | monomer | 4 x SF4, 1 x F3S, 2 x MD1, 1 x MO, 1 x HEM, 2 x CA | BLAST | 0.45 |
| ``` target    MAQGVSRRQLLGRALALGSGAALADLLGPARFLSPAGAATAGAVVPGNPLRVMPDRTWEQIYRNQFEDDSTFVFTCAPND 7b04.2    -----------------------------------------------NPLDTYPDRRWESVYRDQYQYDRTFTYCCSPND  target    THNCLLRAHVKNGVVVRISPTYGYGEATDLYGNRASHRWDPRTCQKGLILSRRFYSERRVKAPMIRKGFKDWVEAGYPRN 7b04.2    THACRIRAFVRNNVMMRVEQNYDHQNYSDLYGNKATRNWNPRMCLKGYTFHRRVYGPYRLRYPLIRKGWKRWADDGFP--  target    DDGTPQMDVTL----RGSDDWIRISWDEATTIAAKTMEDVARTFNGDEGARKLLAQGYHPEMVEVMHGAGVQALKLR 7b04.2    -ELTPENKTKYMFDNRGNDELLRASWDEAFTYASKGIIHITKKYSGPEGAQKLIDQGYPKEMVDRMQGAGTRTFKGR ``` | | | | | | | | | | | | | | | | | | | | | | | | | | | | | | | | | | | | | | | | | | | | | | | | | |
|  | 3ir5.1.A | Respiratory nitrate reductase 1 alpha chain  *Crystal structure of NarGHI mutant NarG-H49C* | 0.50 | 0.00 | 26.02 | 0.84 | 1-231 | X-ray | 2.30 | monomer | 2 x MD1, 1 x 6MO, 4 x SF4, 1 x AGA, 1 x F3S, 2 x HEM | HHblits | 0.33 |
| ``` target    MAQGVSRRQLLGRALALGSGAALADLLGPARFLSPAGAATAGAVVPGNPLRVMPDRTWEQIYRNQFEDDSTFVFTCAPND 3ir5.1    MSKFLDRFRYFKQKGETFADG-HGQ-------------------------LLNTNRDWEDGYRQRWQHDKIVRSTCGVNC  target    THNCLLRAHVKNGVVVRISPTYGYGEATDLYGNRASHRWDPRTCQKGLILSRRFYSERRVKAPMIRKGF-KDWVEAGY-- 3ir5.1    TGSCSWKIYVKNGLVTWETQQTDYPR-----TRPDLPNHEPRGCPRGASYSWYLYSANRLKYPMMRKRLMKMWREAKALH  target    --PR-------NDDGTPQMDVTLRGSDDWIRISWDEATTIAAKTMEDVARTFNGDEGARKLLAQGYHPEMVEVMHGAGVQ 3ir5.1    SDPVEAWASIIEDADKAKSFKQARGRGGFVRSSWQEVNELIAASNVYTIKNYGPDR----VAGFSPIPAMSMVSYASGAR  target    ALKLR 3ir5.1    YLS-- ``` | | | | | | | | | | | | | | | | | | | | | | | | | | | | | | | | | | | | | | | | | | | | | | | | | |
|  | 3ir6.1.A | Respiratory nitrate reductase 1 alpha chain  *Crystal structure of NarGHI mutant NarG-H49S* | 0.46 | 0.00 | 25.64 | 0.84 | 1-230 | X-ray | 2.80 | monomer | 2 x GDP, 1 x AGA, 3 x SF4, 1 x F3S, 2 x HEM | HHblits | 0.33 |
| ``` target    MAQGVSRRQLLGRALALGSGAALADLLGPARFLSPAGAATAGAVVPGNPLRVMPDRTWEQIYRNQFEDDSTFVFTCAPND 3ir6.1    MSKFLDRFRYFKQKGETFADGHGQ--------------------------LLNTNRDWEDGYRQRWQHDKIVRSTSGVNC  target    THNCLLRAHVKNGVVVRISPTYGYGEATDLYGNRASHRWDPRTCQKGLILSRRFYSERRVKAPMIRKGF-KDWVEAGY-- 3ir6.1    TGSCSWKIYVKNGLVTWETQQTDYPR-----TRPDLPNHEPRGCPRGASYSWYLYSANRLKYPMMRKRLMKMWREAKALH  target    --PR-------NDDGTPQMDVTLRGSDDWIRISWDEATTIAAKTMEDVARTFNGDEGARKLLAQGYHPEMVEVMHGAGVQ 3ir6.1    SDPVEAWASIIEDADKAKSFKQARGRGGFVRSSWQEVNELIAASNVYTIKNYGPDR----VAGFSPIPAMSMVSYASGAR  target    ALKLR 3ir6.1    YL--- ``` | | | | | | | | | | | | | | | | | | | | | | | | | | | | | | | | | | | | | | | | | | | | | | | | | |
| ✓ | 1q16.1.A | Respiratory nitrate reductase 1 alpha chain  *Crystal structure of Nitrate Reductase A, NarGHI, from Escherichia coli* | 0.50 | 0.00 | 25.64 | 0.84 | 1-230 | X-ray | 1.90 | monomer | 2 x MD1, 1 x 6MO, 2 x HEM, 4 x SF4, 1 x F3S, 1 x AGA, 1 x 3PH | HHblits | 0.32 |
| ``` target    MAQGVSRRQLLGRALALGSGAALADLLGPARFLSPAGAATAGAVVPGNPLRVMPDRTWEQIYRNQFEDDSTFVFTCAPND 1q16.1    MSKFLDRFRYFKQKGETFADGH--------------------------GQLLNTNRDWEDGYRQRWQHDKIVRSTHGVNC  target    THNCLLRAHVKNGVVVRISPTYGYGEATDLYGNRASHRWDPRTCQKGLILSRRFYSERRVKAPMIRKGF-KDWVEAGY-- 1q16.1    TGSCSWKIYVKNGLVTWETQQTDYPR-----TRPDLPNHEPRGCPRGASYSWYLYSANRLKYPMMRKRLMKMWREAKALH  target    --PR-------NDDGTPQMDVTLRGSDDWIRISWDEATTIAAKTMEDVARTFNGDEGARKLLAQGYHPEMVEVMHGAGVQ 1q16.1    SDPVEAWASIIEDADKAKSFKQARGRGGFVRSSWQEVNELIAASNVYTIKNYGPDR----VAGFSPIPAMSMVSYASGAR  target    ALKLR 1q16.1    YL--- ``` | | | | | | | | | | | | | | | | | | | | | | | | | | | | | | | | | | | | | | | | | | | | | | | | | |
|  | 3ir7.1.A | Respiratory nitrate reductase 1 alpha chain  *Crystal structure of NarGHI mutant NarG-R94S* | 0.48 | 0.00 | 25.64 | 0.84 | 1-230 | X-ray | 2.50 | monomer | 2 x MD1, 4 x SF4, 1 x 6MO, 1 x AGA, 1 x F3S, 2 x HEM | HHblits | 0.32 |
| ``` target    MAQGVSRRQLLGRALALGSGAALADLLGPARFLSPAGAATAGAVVPGNPLRVMPDRTWEQIYRNQFEDDSTFVFTCAPND 3ir7.1    MSKFLDRFRYFKQKGETFADGHG--------------------------QLLNTNRDWEDGYRQRWQHDKIVRSTHGVNC  target    THNCLLRAHVKNGVVVRISPTYGYGEATDLYGNRASHRWDPRTCQKGLILSRRFYSERRVKAPMIRKGF-KDWVEAGY-- 3ir7.1    TGSCSWKIYVKNGLVTWETQQTDYPR-----TRPDLPNHEPRGCPSGASYSWYLYSANRLKYPMMRKRLMKMWREAKALH  target    --PR-------NDDGTPQMDVTLRGSDDWIRISWDEATTIAAKTMEDVARTFNGDEGARKLLAQGYHPEMVEVMHGAGVQ 3ir7.1    SDPVEAWASIIEDADKAKSFKQARGRGGFVRSSWQEVNELIAASNVYTIKNYGPDR----VAGFSPIPAMSMVSYASGAR  target    ALKLR 3ir7.1    YL--- ``` | | | | | | | | | | | | | | | | | | | | | | | | | | | | | | | | | | | | | | | | | | | | | | | | | |
|  | 1r27.4.A | Respiratory nitrate reductase 1 alpha chain  *Crystal Structure of NarGH complex* | 0.47 | 0.11 | 25.39 | 0.83 | 3-230 | X-ray | 2.00 | homo-dimer | 4 x MO, 16 x SF4, 8 x MGD, 4 x F3S | HHblits | 0.32 |
| ``` target    MAQGVSRRQLLGRALALGSGAALADLLGPARFLSPAGAATAGAVVPGNPLRVMPDRTWEQIYRNQFEDDSTFVFTCAPND 1r27.4    --KFLDRFRYFKQKGETFADGHG--------------------------QLLNTNRDWEDGYRQRWQHDKIVRSTHGVNC  target    THNCLLRAHVKNGVVVRISPTYGYGEATDLYGNRASHRWDPRTCQKGLILSRRFYSERRVKAPMIRKGF-KDWVEAGY-- 1r27.4    TGSCSWKIYVKNGLVTWETQQTDYPR-----TRPDLPNHEPRGCPRGASYSWYLYSANRLKYPMMRKRLMKMWREAKALH  target    --PR-------NDDGTPQMDVTLRGSDDWIRISWDEATTIAAKTMEDVARTFNGDEGARKLLAQGYHPEMVEVMHGAGVQ 1r27.4    SDPVEAWASIIEDADKAKSFKQARGRGGFVRSSWQEVNELIAASNVYTIKNYGPDR----VAGFSPIPAMSMVSYASGAR  target    ALKLR 1r27.4    YL--- ``` | | | | | | | | | | | | | | | | | | | | | | | | | | | | | | | | | | | | | | | | | | | | | | | | | |
|  | 3egw.1.A | Respiratory nitrate reductase 1 alpha chain  *The crystal structure of the NarGHI mutant NarH - C16A* | 0.49 | 0.13 | 27.68 | 0.76 | 45-230 | X-ray | 1.90 | homo-dimer | 2 x MD1, 2 x MGD, 2 x 6MO, 6 x SF4, 4 x F3S, 2 x 3PH, 4 x HEM, 2 x AGA | HHblits | 0.34 |
| ``` target    MAQGVSRRQLLGRALALGSGAALADLLGPARFLSPAGAATAGAVVPGNPLRVMPDRTWEQIYRNQFEDDSTFVFTCAPND 3egw.1    --------------------------------------------ADGHGQLLNTNRDWEDGYRQRWQHDKIVRSTHGVNC  target    THNCLLRAHVKNGVVVRISPTYGYGEATDLYGNRASHRWDPRTCQKGLILSRRFYSERRVKAPMIRKGF-KDWVEAGY-- 3egw.1    TGSCSWKIYVKNGLVTWETQQTDYPR-----TRPDLPNHEPRGCPRGASYSWYLYSANRLKYPMMRKRLMKMWREAKALH  target    --PR-------NDDGTPQMDVTLRGSDDWIRISWDEATTIAAKTMEDVARTFNGDEGARKLLAQGYHPEMVEVMHGAGVQ 3egw.1    SDPVEAWASIIEDADKAKSFKQARGRGGFVRSSWQEVNELIAASNVYTIKNYGPDR----VAGFSPIPAMSMVSYASGAR  target    ALKLR 3egw.1    YL--- ``` | | | | | | | | | | | | | | | | | | | | | | | | | | | | | | | | | | | | | | | | | | | | | | | | | |
|  | 3ir5.1.A | Respiratory nitrate reductase 1 alpha chain  *Crystal structure of NarGHI mutant NarG-H49C* | 0.44 | 0.00 | 31.14 | 0.72 | 55-232 | X-ray | 2.30 | monomer | 2 x MD1, 1 x 6MO, 4 x SF4, 1 x AGA, 1 x F3S, 2 x HEM | BLAST | 0.36 |
| ``` target    MAQGVSRRQLLGRALALGSGAALADLLGPARFLSPAGAATAGAVVPGNPLRVMPDRTWEQIYRNQFEDDSTFVFTCAPND 3ir5.1    ------------------------------------------------------NRDWEDGYRQRWQHDKIVRSTCGVNC  target    THNCLLRAHVKNGVVVRISPTYGYGEATDLYGNRASHRWDPRTCQKGLILSRRFYSERRVKAPMIRKGF-KDWVEAGYPR 3ir5.1    TGSCSWKIYVKNGLVTWETQQTDYPRTRPDLPNH-----EPRGCPRGASYSWYLYSANRLKYPMMRKRLMKMWREAKALH  target    NDDGTPQMDVT-----------LRGSDDWIRISWDEATTIAAKTMEDVARTFNGDEGARKLLAQGYH--PEMVEVMHGAG 3ir5.1    SDPVEAWASIIEDADKAKSFKQARGRGGFVRSSWQEVNELIAASNVYTIKNYGPDRVA------GFSPIPAMSMVSYASG  target    VQALKLR 3ir5.1    ARYLSL- ``` | | | | | | | | | | | | | | | | | | | | | | | | | | | | | | | | | | | | | | | | | | | | | | | | | |
|  | 3ir6.1.A | Respiratory nitrate reductase 1 alpha chain  *Crystal structure of NarGHI mutant NarG-H49S* | 0.43 | 0.00 | 30.54 | 0.72 | 55-232 | X-ray | 2.80 | monomer | 2 x GDP, 1 x AGA, 3 x SF4, 1 x F3S, 2 x HEM | BLAST | 0.35 |
| ``` target    MAQGVSRRQLLGRALALGSGAALADLLGPARFLSPAGAATAGAVVPGNPLRVMPDRTWEQIYRNQFEDDSTFVFTCAPND 3ir6.1    ------------------------------------------------------NRDWEDGYRQRWQHDKIVRSTSGVNC  target    THNCLLRAHVKNGVVVRISPTYGYGEATDLYGNRASHRWDPRTCQKGLILSRRFYSERRVKAPMIRKGF-KDWVEAGYPR 3ir6.1    TGSCSWKIYVKNGLVTWETQQTDYPRTRPDLPNH-----EPRGCPRGASYSWYLYSANRLKYPMMRKRLMKMWREAKALH  target    NDDGTPQMDVT-----------LRGSDDWIRISWDEATTIAAKTMEDVARTFNGDEGARKLLAQGYH--PEMVEVMHGAG 3ir6.1    SDPVEAWASIIEDADKAKSFKQARGRGGFVRSSWQEVNELIAASNVYTIKNYGPDRVA------GFSPIPAMSMVSYASG  target    VQALKLR 3ir6.1    ARYLSL- ``` | | | | | | | | | | | | | | | | | | | | | | | | | | | | | | | | | | | | | | | | | | | | | | | | | |
|  | 1r27.4.A | Respiratory nitrate reductase 1 alpha chain  *Crystal Structure of NarGH complex* | 0.43 | 0.13 | 30.54 | 0.72 | 55-232 | X-ray | 2.00 | homo-dimer | 4 x MO, 16 x SF4, 8 x MGD, 4 x F3S | BLAST | 0.35 |
| ``` target    MAQGVSRRQLLGRALALGSGAALADLLGPARFLSPAGAATAGAVVPGNPLRVMPDRTWEQIYRNQFEDDSTFVFTCAPND 1r27.4    ------------------------------------------------------NRDWEDGYRQRWQHDKIVRSTHGVNC  target    THNCLLRAHVKNGVVVRISPTYGYGEATDLYGNRASHRWDPRTCQKGLILSRRFYSERRVKAPMIRKGF-KDWVEAGYPR 1r27.4    TGSCSWKIYVKNGLVTWETQQTDYPRTRPDLPNH-----EPRGCPRGASYSWYLYSANRLKYPMMRKRLMKMWREAKALH  target    NDDGTPQMDVT-----------LRGSDDWIRISWDEATTIAAKTMEDVARTFNGDEGARKLLAQGYH--PEMVEVMHGAG 1r27.4    SDPVEAWASIIEDADKAKSFKQARGRGGFVRSSWQEVNELIAASNVYTIKNYGPDRVA------GFSPIPAMSMVSYASG  target    VQALKLR 1r27.4    ARYLSL- ``` | | | | | | | | | | | | | | | | | | | | | | | | | | | | | | | | | | | | | | | | | | | | | | | | | |
|  | 1q16.1.A | Respiratory nitrate reductase 1 alpha chain  *Crystal structure of Nitrate Reductase A, NarGHI, from Escherichia coli* | 0.43 | 0.00 | 30.54 | 0.72 | 55-232 | X-ray | 1.90 | monomer | 2 x MD1, 1 x 6MO, 2 x HEM, 4 x SF4, 1 x F3S, 1 x AGA, 1 x 3PH | BLAST | 0.35 |
| ``` target    MAQGVSRRQLLGRALALGSGAALADLLGPARFLSPAGAATAGAVVPGNPLRVMPDRTWEQIYRNQFEDDSTFVFTCAPND 1q16.1    ------------------------------------------------------NRDWEDGYRQRWQHDKIVRSTHGVNC  target    THNCLLRAHVKNGVVVRISPTYGYGEATDLYGNRASHRWDPRTCQKGLILSRRFYSERRVKAPMIRKGF-KDWVEAGYPR 1q16.1    TGSCSWKIYVKNGLVTWETQQTDYPRTRPDLPNH-----EPRGCPRGASYSWYLYSANRLKYPMMRKRLMKMWREAKALH  target    NDDGTPQMDVT-----------LRGSDDWIRISWDEATTIAAKTMEDVARTFNGDEGARKLLAQGYH--PEMVEVMHGAG 1q16.1    SDPVEAWASIIEDADKAKSFKQARGRGGFVRSSWQEVNELIAASNVYTIKNYGPDRVA------GFSPIPAMSMVSYASG  target    VQALKLR 1q16.1    ARYLSL- ``` | | | | | | | | | | | | | | | | | | | | | | | | | | | | | | | | | | | | | | | | | | | | | | | | | |
|  | 3egw.1.A | Respiratory nitrate reductase 1 alpha chain  *The crystal structure of the NarGHI mutant NarH - C16A* | 0.43 | 0.13 | 30.54 | 0.72 | 55-232 | X-ray | 1.90 | homo-dimer | 2 x MD1, 2 x MGD, 2 x 6MO, 6 x SF4, 4 x F3S, 2 x 3PH, 4 x HEM, 2 x AGA | BLAST | 0.35 |
| ``` target    MAQGVSRRQLLGRALALGSGAALADLLGPARFLSPAGAATAGAVVPGNPLRVMPDRTWEQIYRNQFEDDSTFVFTCAPND 3egw.1    ------------------------------------------------------NRDWEDGYRQRWQHDKIVRSTHGVNC  target    THNCLLRAHVKNGVVVRISPTYGYGEATDLYGNRASHRWDPRTCQKGLILSRRFYSERRVKAPMIRKGF-KDWVEAGYPR 3egw.1    TGSCSWKIYVKNGLVTWETQQTDYPRTRPDLPNH-----EPRGCPRGASYSWYLYSANRLKYPMMRKRLMKMWREAKALH  target    NDDGTPQMDVT-----------LRGSDDWIRISWDEATTIAAKTMEDVARTFNGDEGARKLLAQGYH--PEMVEVMHGAG 3egw.1    SDPVEAWASIIEDADKAKSFKQARGRGGFVRSSWQEVNELIAASNVYTIKNYGPDRVA------GFSPIPAMSMVSYASG  target    VQALKLR 3egw.1    ARYLSL- ``` | | | | | | | | | | | | | | | | | | | | | | | | | | | | | | | | | | | | | | | | | | | | | | | | | |
|  | 3ir7.1.A | Respiratory nitrate reductase 1 alpha chain  *Crystal structure of NarGHI mutant NarG-R94S* | 0.44 | 0.00 | 30.54 | 0.72 | 55-232 | X-ray | 2.50 | monomer | 2 x MD1, 4 x SF4, 1 x 6MO, 1 x AGA, 1 x F3S, 2 x HEM | BLAST | 0.35 |
| ``` target    MAQGVSRRQLLGRALALGSGAALADLLGPARFLSPAGAATAGAVVPGNPLRVMPDRTWEQIYRNQFEDDSTFVFTCAPND 3ir7.1    ------------------------------------------------------NRDWEDGYRQRWQHDKIVRSTHGVNC  target    THNCLLRAHVKNGVVVRISPTYGYGEATDLYGNRASHRWDPRTCQKGLILSRRFYSERRVKAPMIRKGF-KDWVEAGYPR 3ir7.1    TGSCSWKIYVKNGLVTWETQQTDYPRTRPDLPNH-----EPRGCPSGASYSWYLYSANRLKYPMMRKRLMKMWREAKALH  target    NDDGTPQMDVT-----------LRGSDDWIRISWDEATTIAAKTMEDVARTFNGDEGARKLLAQGYH--PEMVEVMHGAG 3ir7.1    SDPVEAWASIIEDADKAKSFKQARGRGGFVRSSWQEVNELIAASNVYTIKNYGPDRVA------GFSPIPAMSMVSYASG  target    VQALKLR 3ir7.1    ARYLSL- ``` | | | | | | | | | | | | | | | | | | | | | | | | | | | | | | | | | | | | | | | | | | | | | | | | | |
|  | 2ivf.1.A | ETHYLBENZENE DEHYDROGENASE ALPHA-SUBUNIT  *ETHYLBENZENE DEHYDROGENASE FROM AROMATOLEUM AROMATICUM* | 0.34 | 0.00 | 26.95 | 0.72 | 3-204 | X-ray | 1.88 | monomer | 1 x MES, 4 x SF4, 1 x MO, 1 x MGD, 1 x MD1, 1 x F3S, 1 x HEM | HHblits | 0.32 |
| ``` target    MAQGVSRRQLLGRALALGSGAALADLLGPARFLSPAGAATAGAVVPGNPLRVMPDRTWEQIYRNQFEDDSTFVFTCAPND 2ivf.1    --QDQHRRDFLKRSGAAVLSLSLSSLATG-VVPGFL----K---DAQAGTKAPGYASWEDIYRKEWKWDKVNWGSHLNIC  target    T--HNCLLRAHVKNGVVVRISPTYGYGEATDLYGNRASHRWDPRTCQKGLILSRRFYSERRVKAPMIRKGFKDWVEAGYP 2ivf.1    WPQGSCKFYVYVRNGIVWREEQAAQTPA-----CNVDYVDYNPLGCQKGSAFNNNLYGDERVKYPLKRVG----------  target    RNDDGTPQMDVTLRGSDDWIRISWDEATTIAAKTMEDVARTFNGDEGARKLLAQGYHPEMVEVMHGAGVQALKLR 2ivf.1    ------------KRGEGKWKRVSWDEAAGDIADSIIDSFEAQGSDG----------------------------- ``` | | | | | | | | | | | | | | | | | | | | | | | | | | | | | | | | | | | | | | | | | | | | | | | | | |
|  | 2ivf.1.A | ETHYLBENZENE DEHYDROGENASE ALPHA-SUBUNIT  *ETHYLBENZENE DEHYDROGENASE FROM AROMATOLEUM AROMATICUM* | 0.34 | 0.00 | 31.47 | 0.61 | 57-232 | X-ray | 1.88 | monomer | 1 x MES, 4 x SF4, 1 x MO, 1 x MGD, 1 x MD1, 1 x F3S, 1 x HEM | BLAST | 0.37 |
| ``` target    MAQGVSRRQLLGRALALGSGAALADLLGPARFLSPAGAATAGAVVPGNPLRVMPDRTWEQIYRNQFEDDS----TFVFTC 2ivf.1    --------------------------------------------------------SWEDIYRKEWKWDKVNWGSHLNIC  target    APNDTHNCLLRAHVKNGVVVRISPTYGYGEATDLYGNRASHRWDPRTCQKGLILSRRFYSERRVKAPMIRKGFKDWVEAG 2ivf.1    WPQGS--CKFYVYVRNGIVWREEQA-----AQTPACNVDYVDYNPLGCQKGSAFNNNLYGDERVKYPLKRVG--------  target    YPRNDDGTPQMDVTLRGSDDWIRISWDEATTIAAKTMEDVARTFNGDEGARKLLAQGYHPEMVEVMHGAGVQALKLR 2ivf.1    --------------KRGEGKWKRVSWDEA---AGDIADSIIDSFEA-QGSDGFILDAPHVHAGSIAWGAGFRMTYL- ``` | | | | | | | | | | | | | | | | | | | | | | | | | | | | | | | | | | | | | | | | | | | | | | | | | |
|  | 1kqf.1.A | FORMATE DEHYDROGENASE, NITRATE-INDUCIBLE, MAJOR SUBUNIT  *FORMATE DEHYDROGENASE N FROM E. COLI* | 0.29 | 0.11 | 28.17 | 0.61 | 3-205 | X-ray | 1.60 | homo-trimer | 3 x 6MO, 15 x SF4, 6 x MGD, 6 x HEM, 3 x CDL | HHblits | 0.32 |
| ``` target    MAQGVSRRQLLGRALALGSGAALADLLGPARFLSPAGAATAGAVVPGNPLRVMPDRTWEQIYRNQFEDDSTFVFTCAPND 1kqf.1    --MDVSRRQFFKICAGGMAGTTVAAL-G---F-APKQA-----------LA--QARNYK------LLRA-KEIRNTCTYC  target    THNCLLRAHVKN-G------VVVRISPTYGYGEATDLYGNRASHRWDPRTCQKGLILSRRFYSERRVKAPMIRKGFKDWV 1kqf.1    SVGCGLLMYSLGDGAKNAREAIYHIEG------------DPDHPVSRGALCPKGAGLLDYVNSENRLRYPEYRA------  target    EAGYPRNDDGTPQMDVTLRGSDDWIRISWDEATTIAAKTMEDVARTFNGDEGARKLLAQGYHPEMVEVMHGAGVQALKLR 1kqf.1    ------------------PGSDKWQRISWEEAFSRIAKLMKADRDANFIEKN---------------------------- ``` | | | | | | | | | | | | | | | | | | | | | | | | | | | | | | | | | | | | | | | | | | | | | | | | | |
|  | 1e60.1.A | Dimethyl sulfoxide/trimethylamine N-oxide reductase  *OXIDIZED DMSO REDUCTASE EXPOSED TO HEPES - Structure II BUFFER* | 0.27 | 0.00 | 24.11 | 0.61 | 1-204 | X-ray | 2.00 | monomer | 2 x PGD, 1 x 2MO | HHblits | 0.32 |
| ``` target    MAQGVSRRQLLGRALALGSGAALADLLGPARFLSPAGAATAGAVVPGNPLRVMPDRTWEQIYRNQFEDDSTFVFTCAPND 1e60.1    LRAELYRRAFLSYSVAPGALGMFGRS-----LL-AK---------GA---------------RA------EALANGTVMS  target    THNCL-LRAHVKNGVVVRISPTYGYGEATDLYGNRASHRWDPRTCQKGLILSRRFYSERRVKAPMIRKGFKDWVEAGYPR 1e60.1    GSHWGVFTATVENGRATAFTPWE------------KDP----HPSPMLAGVLDSIYSPTRIKYPMVRREF---LEKGV--  target    NDDGTPQMDVTLRGSDDWIRISWDEATTIAAKTMEDVARTFNGDEGARKLLAQGYHPEMVEVMHGAGVQALKLR 1e60.1    ------NADRSTRGNGDFVRVSWDQALDLVAAEVKRVEETYGPEG----------------------------- ``` | | | | | | | | | | | | | | | | | | | | | | | | | | | | | | | | | | | | | | | | | | | | | | | | | |
|  | 4dmr.1.A | DMSO REDUCTASE  *REDUCED DMSO REDUCTASE FROM RHODOBACTER CAPSULATUS WITH BOUND DMSO SUBSTRATE* | 0.27 | 0.00 | 24.11 | 0.61 | 1-204 | X-ray | 1.90 | monomer | 2 x PGD, 1 x 4MO, 1 x O | HHblits | 0.32 |
| ``` target    MAQGVSRRQLLGRALALGSGAALADLLGPARFLSPAGAATAGAVVPGNPLRVMPDRTWEQIYRNQFEDDSTFVFTCAPND 4dmr.1    LRAELYRRAFLSYSVAPGALGMFGRS-----LL-AK---------GA---------------RA------EALANGTVMS  target    THNCL-LRAHVKNGVVVRISPTYGYGEATDLYGNRASHRWDPRTCQKGLILSRRFYSERRVKAPMIRKGFKDWVEAGYPR 4dmr.1    GSHWGVFTATVENGRATAFTPWE------------KDPH----PSPMLAGVLDSIYSPTRIKYPMVRREFL---EKGV--  target    NDDGTPQMDVTLRGSDDWIRISWDEATTIAAKTMEDVARTFNGDEGARKLLAQGYHPEMVEVMHGAGVQALKLR 4dmr.1    ------NADRSTRGNGDFVRVSWDQALDLVAAEVKRVEETYGPSG----------------------------- ``` | | | | | | | | | | | | | | | | | | | | | | | | | | | | | | | | | | | | | | | | | | | | | | | | | |
|  | 1e5v.2.A | Dimethyl sulfoxide/trimethylamine N-oxide reductase  *OXIDIZED DMSO REDUCTASE EXPOSED TO HEPES BUFFER* | 0.27 | 0.00 | 22.14 | 0.60 | 1-204 | X-ray | 2.40 | monomer | 2 x PGD, 1 x 2MO | HHblits | 0.31 |
| ``` target    MAQGVSRRQLLGRALALGSGAALADLLGPARFLSPAGAATAGAVVPGNPLRVMPDRTWEQIYRNQFEDDSTFVFTCAPND 1e5v.2    LRAELYRRAFLSYSVAPGALGMFGRS-----L-LAK---------GARA--------------------E-ALANGTVMS  target    THNCL-LRAHVKNGVVVRISPTYGYGEATDLYGNRASHRWDPRTCQKGLILSRRFYSERRVKAPMIRKGF-KDWVEAGYP 1e5v.2    GSHWGVFTATVENGRATAFTPWE------------KDPH----PSPMLAGVLDSIYSPTRIKYPMVRREFLEKGVN----  target    RNDDGTPQMDVTLRGSDDWIRISWDEATTIAAKTMEDVARTFNGDEGARKLLAQGYHPEMVEVMHGAGVQALKLR 1e5v.2    --------ADRSTRGNGDFVRVSWDQALDLVAAEVKRVEETYGPEG----------------------------- ``` | | | | | | | | | | | | | | | | | | | | | | | | | | | | | | | | | | | | | | | | | | | | | | | | | |
|  | 1e18.1.A | DMSO REDUCTASE.  *TUNGSTEN-SUSBSTITUTED DMSO REDUCTASE FROM RHODOBACTER CAPSULATUS* | 0.27 | 0.00 | 23.02 | 0.60 | 2-204 | X-ray | 2.00 | monomer | 2 x PGD, 1 x 6WO | HHblits | 0.31 |
| ``` target    MAQGVSRRQLLGRALALGSGAALADLLGPARFLSPAGAATAGAVVPGNPLRVMPDRTWEQIYRNQFEDDSTFVFTCAPND 1e18.1    -RAELYRRAFLSYSVAPGALGMFGRS-----L-LAK--------------GARA----------------EALANGTVMS  target    THNCL-LRAHVKNGVVVRISPTYGYGEATDLYGNRASHRWDPRTCQKGLILSRRFYSERRVKAPMIRKGF-KDWVEAGYP 1e18.1    GSHWGVFTATVENGRATAFTPWE------------KDPH----PSPMLAGVLDSIYSPTRIKYPMVRREFLEKGVNA---  target    RNDDGTPQMDVTLRGSDDWIRISWDEATTIAAKTMEDVARTFNGDEGARKLLAQGYHPEMVEVMHGAGVQALKLR 1e18.1    ---------DRSTRGNGDFVRVSWDQALDLVAAEVKRVEETYGPQG----------------------------- ``` | | | | | | | | | | | | | | | | | | | | | | | | | | | | | | | | | | | | | | | | | | | | | | | | | |
|  | 6sdv.1.A | Formate dehydrogenase, alpha subunit, selenocysteine-containing,Formate dehydrogenase, alpha subunit, selenocysteine-containing,W-formate dehydrogenase - alpha subunit  *W-formate dehydrogenase from Desulfovibrio vulgaris - Formate reduced form* | 0.27 | 0.00 | 20.57 | 0.61 | 3-204 | X-ray | 1.90 | monomer | 2 x MGD, 4 x SF4, 1 x W, 1 x H2S | HHblits | 0.29 |
| ``` target    MAQGVSRRQLLGRALALGSGAALADLLGPARFLSPAGAATAGAVVPGNPLRVMPDRTWEQIYRNQFEDDSTFVFTCAPND 6sdv.1    --MTVTRRHFLKLSAGAAVAGAFTGLGL--S-LAPTV----------ARA--------EL-QK--LQWAK-QTTSICCYC  target    THNCLLRAHVK---NGVVVRISPTYGYGEATDLYGNRASHRWDPRTCQKGLILSRRFYSERRVKAPMIRKGFKDWVEAGY 6sdv.1    AVGCGLIVHTAKDGQGRAVNVEGDP------------DHPINEGSLCPKGASIFQLGENDQRGTQPLYRAP---------  target    PRNDDGTPQMDVTLRGSDDWIRISWDEATTIAAKTMEDVARTFNGDEGARKLLAQGYHPEMVEVMHGAGVQALKLR 6sdv.1    ---------------FSDTWKPVTWDFALTEIAKRIKKTRDASFTEK----------------------------- ``` | | | | | | | | | | | | | | | | | | | | | | | | | | | | | | | | | | | | | | | | | | | | | | | | | |
|  | 6sdr.1.A | Formate dehydrogenase, alpha subunit, selenocysteine-containing  *W-formate dehydrogenase from Desulfovibrio vulgaris - Oxidized form* | 0.26 | 0.00 | 20.57 | 0.61 | 3-204 | X-ray | 2.10 | monomer | 2 x MGD, 4 x SF4, 1 x H2S, 1 x W | HHblits | 0.29 |
| ``` target    MAQGVSRRQLLGRALALGSGAALADLLGPARFLSPAGAATAGAVVPGNPLRVMPDRTWEQIYRNQFEDDSTFVFTCAPND 6sdr.1    --MTVTRRHFLKLSAGAAVAGAFTGLGL--SL-APTVA-----------RA-------EL-QKL--QWA-KQTTSICCYC  target    THNCLLRAHVK---NGVVVRISPTYGYGEATDLYGNRASHRWDPRTCQKGLILSRRFYSERRVKAPMIRKGFKDWVEAGY 6sdr.1    AVGCGLIVHTAKDGQGRAVNVEGDP------------DHPINEGSLCPKGASIFQLGENDQRGTQPLYRAP---------  target    PRNDDGTPQMDVTLRGSDDWIRISWDEATTIAAKTMEDVARTFNGDEGARKLLAQGYHPEMVEVMHGAGVQALKLR 6sdr.1    ---------------FSDTWKPVTWDFALTEIAKRIKKTRDASFTEK----------------------------- ``` | | | | | | | | | | | | | | | | | | | | | | | | | | | | | | | | | | | | | | | | | | | | | | | | | |
|  | 5e7o.1.A | DMSO reductase family type II enzyme, molybdopterin subunit  *Crystal structure of the perchlorate reductase PcrAB mutant W461E of PcrA from Azospira suillum PS* | 0.37 |  | 28.80 | 0.54 | 52-204 | X-ray | 2.40 | hetero-oligomer | 4 x SF4, 1 x MO, 1 x MGD, 1 x MD1, 1 x F3S | HHblits | 0.34 |
| ``` target    MAQGVSRRQLLGRALALGSGAALADLLGPARFLSPAGAATAGAVVPGNPLRVMPDRTWEQIYRNQFEDDSTFVFTCAPND 5e7o.1    ---------------------------------------------------AFEYSGWENFHRTQWSWDKKTRGAHLVNC  target    THNCLLRAHVKNGVVVRISPTYGYGEATDLYGNRASHRWDPRTCQKGLILSRRFYSERRVKAPMIRKGFKDWVEAGYPRN 5e7o.1    TGACPHFVYSKDGVVMREEQSK------DIAPMPNIPEYNPRGCNKGECGHDYMYGPHRIKYPLIRVG------------  target    DDGTPQMDVTLRGSDDWIRISWDEATTIAAKTMEDVARTFNGDEGARKLLAQGYHPEMVEVMHGAGVQALKLR 5e7o.1    ----------ERGEGKWRRATWEEALDMIADKCVDTIKNHAPDC----------------------------- ``` | | | | | | | | | | | | | | | | | | | | | | | | | | | | | | | | | | | | | | | | | | | | | | | | | |
|  | 4ydd.1.A | DMSO reductase family type II enzyme, molybdopterin subunit  *Crystal structure of the perchlorate reductase PcrAB from Azospira suillum PS* | 0.36 |  | 28.80 | 0.54 | 52-204 | X-ray | 1.86 | hetero-oligomer | 4 x SF4, 1 x MO, 1 x MGD, 1 x MD1, 1 x F3S | HHblits | 0.34 |
| ``` target    MAQGVSRRQLLGRALALGSGAALADLLGPARFLSPAGAATAGAVVPGNPLRVMPDRTWEQIYRNQFEDDSTFVFTCAPND 4ydd.1    ---------------------------------------------------AFEYSGWENFHRTQWSWDKKTRGAHLVNC  target    THNCLLRAHVKNGVVVRISPTYGYGEATDLYGNRASHRWDPRTCQKGLILSRRFYSERRVKAPMIRKGFKDWVEAGYPRN 4ydd.1    TGACPHFVYSKDGVVMREEQSK------DIAPMPNIPEYNPRGCNKGECGHDYMYGPHRIKYPLIRVG------------  target    DDGTPQMDVTLRGSDDWIRISWDEATTIAAKTMEDVARTFNGDEGARKLLAQGYHPEMVEVMHGAGVQALKLR 4ydd.1    ----------ERGEGKWRRATWEEALDMIADKCVDTIKNHAPDC----------------------------- ``` | | | | | | | | | | | | | | | | | | | | | | | | | | | | | | | | | | | | | | | | | | | | | | | | | |
|  | 6f0k.1.B | Fe-S-cluster-containing hydrogenase  *Alternative complex III* | 0.26 |  | 20.90 | 0.58 | 3-204 | EM | 0.00 | hetero-1-1-1-1-1-1-… | 6 x HEC, 1 x F3S, 3 x SF4 | HHblits | 0.27 |
| ``` target    MAQGVSRRQLLGRALALGSGAALADLLGPARFLSPAGAATAGAVVPGNPLRVMPDRTWEQIYRNQFEDDSTFVFTCAPND 6f0k.1    --SGTSRRQFLQIMGASMALAGLTAC----R--RPV-EK-------ILPYV----RQPEE----IIPGIPLYYATAMPFR  target    THNCLLRAHVKNGVVVRISPTYGYGEATDLYGNRASHRWDPRTCQKGLILSRRFYSERRVKAPMIRKGFKDWVEAGYPRN 6f0k.1    GSVRPLLVESHEGRPTKIE------------GNPDHPLSRGATGVFEQASLLNLYDPDRSQQVLRK-G------------  target    DDGTPQMDVTLRGSDDWIRISWDEATTIAAKTMEDVARTFNGDEGARKLLAQGYHPEMVEVMHGAGVQALKLR 6f0k.1    -----------------EPASWGDFVQFARSLA----AEAGTKR----------------------------- ``` | | | | | | | | | | | | | | | | | | | | | | | | | | | | | | | | | | | | | | | | | | | | | | | | | |
|  | 4v4c.1.A | Pyrogallol hydroxytransferase large subunit  *Crystal Structure of Pyrogallol-Phloroglucinol Transhydroxylase from Pelobacter acidigallici* | 0.28 |  | 26.27 | 0.51 | 73-204 | X-ray | 2.35 | hetero-oligomer | 2 x CA, 2 x MGD, 1 x 4MO, 3 x SF4 | HHblits | 0.33 |
| ``` target    MAQGVSRRQLLGRALALGSGAALADLLGPARFLSPAGAATAGAVVPGNPLRVMPDRTWEQIYRNQFEDDSTFVFTCAPND 4v4c.1    ------------------------------------------------------------------------VVRLTN-S  target    THNCLLRAHVKNGVVVRISPTYGYGEATD-------LYGNRASHRWDPRTCQKGLILSRRFYSERRVKAPMIRKGFKDWV 4v4c.1    STGGPVFVYVKDGKIIRMTPMDFDD-AVDAPSWKIEARGKTFTPPRKTSIAPYTAGFKSMIYSDLRIPYPMKRKSFD---  target    EAGYPRNDDGTPQMDVTLRGSD--------DWIRISWDEATTIAAKTMEDVARTFNGDEGARKLLAQGYHPEMVEVMHGA 4v4c.1    PNGE---------RNPQLRGAGLSKQDPWSDYERISWDEATDIVVAEINRIKHAYGPSA---------------------  target    GVQALKLR 4v4c.1    -------- ``` | | | | | | | | | | | | | | | | | | | | | | | | | | | | | | | | | | | | | | | | | | | | | | | | | |
|  | 7l5i.1.A | Trimethylamine-N-oxide reductase  *Crystal Structure of Haemophilus influenzae MtsZ at pH 7.0* | 0.26 | 0.00 | 31.37 | 0.44 | 75-204 | X-ray | 1.73 | monomer | 2 x MGD, 1 x MO, 1 x O | HHblits | 0.36 |
| ``` target    MAQGVSRRQLLGRALALGSGAALADLLGPARFLSPAGAATAGAVVPGNPLRVMPDRTWEQIYRNQFEDDSTFVFTCAPND 7l5i.1    --------------------------------------------------------------------------TVVTAA  target    THNCLLRAHVKNGVVVRISPTYGYGEATDLYGNRASHRWDPRTCQKGLILSRRFYSERRVKAPMIRKGFKDWVEAGYPRN 7l5i.1    H-WGSIGVVVQDGKVVKSGPAIE-------------PAVPNELQT---VVADQLYSEARVKCPMVRKGFLA---N-----  target    DDGTPQMDVTLRGSDDWIRISWDEATTIAAKTMEDVARTFNGDEGARKLLAQGYHPEMVEVMHGAGVQALKLR 7l5i.1    -PG--KSDTTMRGRDEWVRVSWDEALDLVHNQLKRVRDEHGSTG----------------------------- ``` | | | | | | | | | | | | | | | | | | | | | | | | | | | | | | | | | | | | | | | | | | | | | | | | | |
|  | 7l5s.1.A | Trimethylamine-N-oxide reductase  *Crystal Structure of Haemophilus influenzae MtsZ at pH 5.5* | 0.27 | 0.00 | 31.37 | 0.44 | 75-204 | X-ray | 2.09 | monomer | 1 x O, 2 x MGD, 1 x MO | HHblits | 0.36 |
| ``` target    MAQGVSRRQLLGRALALGSGAALADLLGPARFLSPAGAATAGAVVPGNPLRVMPDRTWEQIYRNQFEDDSTFVFTCAPND 7l5s.1    --------------------------------------------------------------------------TVVTAA  target    THNCLLRAHVKNGVVVRISPTYGYGEATDLYGNRASHRWDPRTCQKGLILSRRFYSERRVKAPMIRKGFKDWVEAGYPRN 7l5s.1    H-WGSIGVVVQDGKVVKSGPAIE-------------PAVPNELQT---VVADQLYSEARVKCPMVRKGFLA---N-----  target    DDGTPQMDVTLRGSDDWIRISWDEATTIAAKTMEDVARTFNGDEGARKLLAQGYHPEMVEVMHGAGVQALKLR 7l5s.1    -PG--KSDTTMRGRDEWVRVSWDEALDLVHNQLKRVRDEHGSTG----------------------------- ``` | | | | | | | | | | | | | | | | | | | | | | | | | | | | | | | | | | | | | | | | | | | | | | | | | |
|  | 1dms.1.A | DMSO REDUCTASE  *STRUCTURE OF DMSO REDUCTASE* | 0.24 |  | 28.16 | 0.44 | 74-204 | X-ray | 1.88 | monomer | 2 x PGD, 1 x 2MO | HHblits | 0.34 |
| ``` target    MAQGVSRRQLLGRALALGSGAALADLLGPARFLSPAGAATAGAVVPGNPLRVMPDRTWEQIYRNQFEDDSTFVFTCAPND 1dms.1    -------------------------------------------------------------------------NGTVMSG  target    THNCLLRAHVKNGVVVRISPTYGYGEATDLYGNRASHRWDPRTCQKGLILSRRFYSERRVKAPMIRKGF-KDWVEAGYPR 1dms.1    SHWGVFTATVENGRATAFTPWE------------KD----PHPTPMLEGVLDSIYSPTRIKYPMVRREFLEKGVNA----  target    NDDGTPQMDVTLRGSDDWIRISWDEATTIAAKTMEDVARTFNGDEGARKLLAQGYHPEMVEVMHGAGVQALKLR 1dms.1    --------DRSTRGNGDFVRVSWDQALDLVAAEVKRVEETYGPQG----------------------------- ``` | | | | | | | | | | | | | | | | | | | | | | | | | | | | | | | | | | | | | | | | | | | | | | | | | |
|  | 1tmo.1.A | TRIMETHYLAMINE N-OXIDE REDUCTASE  *TRIMETHYLAMINE N-OXIDE REDUCTASE FROM SHEWANELLA MASSILIA* | 0.25 |  | 23.08 | 0.45 | 70-204 | X-ray | 2.50 | monomer | 2 x 2MD, 1 x 2MO | HHblits | 0.33 |
| ``` target    MAQGVSRRQLLGRALALGSGAALADLLGPARFLSPAGAATAGAVVPGNPLRVMPDRTWEQIYRNQFEDDSTFVFTCAPND 1tmo.1    ---------------------------------------------------------------------DEWLTTGSH-F  target    THNCLLRAHVKNGVVVRISPTYGYGEATDLYGNRASHRWDPRTCQKGLILSRRFYSERRVKAPMIRKGFKDWVEAGYPRN 1tmo.1    ---GAFKMKRKNGVIAEVKPFDL------------DKY--PTDMING--IRGMVYNPSRVRYPMVRLDF---LLKGH---  target    DDGTPQMDVTLRGSDDWIRISWDEATTIAAKTMEDVARTFNGDEGARKLLAQGYHPEMVEVMHGAGVQALKLR 1tmo.1    -----KSNTHQRGDFRFVRVTWDKALTLFKHSLDEVQTQYGPSG----------------------------- ``` | | | | | | | | | | | | | | | | | | | | | | | | | | | | | | | | | | | | | | | | | | | | | | | | | |
|  | 1eu1.1.A | DIMETHYL SULFOXIDE REDUCTASE  *THE CRYSTAL STRUCTURE OF RHODOBACTER SPHAEROIDES DIMETHYLSULFOXIDE REDUCTASE REVEALS TWO DISTINCT MOLYBDENUM COORDINATION ENVIRONMENTS.* | 0.24 |  | 26.21 | 0.44 | 73-204 | X-ray | 1.30 | monomer | 3 x GLC, 1 x CD, 2 x MGD, 1 x 6MO, 2 x O | HHblits | 0.34 |
| ``` target    MAQGVSRRQLLGRALALGSGAALADLLGPARFLSPAGAATAGAVVPGNPLRVMPDRTWEQIYRNQFEDDSTFVFTCAPND 1eu1.1    ------------------------------------------------------------------------NGEVMSGC  target    THNCLLRAHVKNGVVVRISPTYGYGEATDLYGNRASHRWDPRTCQKGLILSRRFYSERRVKAPMIRKGF-KDWVEAGYPR 1eu1.1    HWGV-FKARVENGRAVAFEPW------------DKDPAPSHQLPG----VLDSIYSPTRIKYPMVRREFLEKGVNA----  target    NDDGTPQMDVTLRGSDDWIRISWDEATTIAAKTMEDVARTFNGDEGARKLLAQGYHPEMVEVMHGAGVQALKLR 1eu1.1    --------DRSTRGNGDFVRVTWDEALDLVARELKRVQESYGPTG----------------------------- ``` | | | | | | | | | | | | | | | | | | | | | | | | | | | | | | | | | | | | | | | | | | | | | | | | | |
|  | 7e5z.1.A | Formate dehydrogenase  *Dehydrogenase holoenzyme* | 0.20 |  | 18.18 | 0.47 | 72-204 | EM | 0.00 | hetero-1-1-mer | 1 x W, 2 x MGD, 2 x FES, 4 x SF4, 1 x FMN | HHblits | 0.28 |
| ``` target    MAQGVSRRQLLGRALALGSGAALADLLGPARFLSPAGAATAGAVVPGNPLRVMPDRTWEQIYRNQFEDDSTFVFTCAPND 7e5z.1    -----------------------------------------------------------------------EVKSLCPYC  target    THNCLLRAHVKNGVVVRISPTYGYGEATDLYGNRASHRWDPRTCQKGLILSRRFYSERRVKAPMIRKGFKDWVEAGYPRN 7e5z.1    GVGCQVSYKVKDERIVYAEGV-------------NGPANQNRLCVKGRFGFDYVHHPHRLTVPLIRLENV---PKDA---  target    DDGTPQMDVTLRGSDDWIRISWDEATTIAAKTMEDVARTFNGDEGARKLLAQGYHPEMVEVMHGAGVQALKLR 7e5z.1    --N--DQVDPANPWTHFREATWEEALDRAAGGLKAIRDTNGRKA----------------------------- ``` | | | | | | | | | | | | | | | | | | | | | | | | | | | | | | | | | | | | | | | | | | | | | | | | | |
|  | 4aay.1.A | AROA  *Crystal Structure of the arsenite oxidase protein complex from Rhizobium species strain NT-26* | 0.23 |  | 12.61 | 0.48 | 68-204 | X-ray | 2.70 | hetero-oligomer | 4 x MGD, 2 x O, 2 x 4MO, 2 x F3S, 2 x FES | HHblits | 0.26 |
| ``` target    MAQGVSRRQLLGRALALGSGAALADLLGPARFLSPAGAATAGAVVPGNPLRVMPDRTWEQIYRNQFEDDSTFVFTCAPND 4aay.1    -------------------------------------------------------------------ADAKKHNVTCHFC  target    THNCLLRAHVK--------------NGVVVRISPTYGYGE---------------ATDLYGNRA--SHRWDPRTCQKGLI 4aay.1    IVGCGYHAYTWPINKQGGTDPQNNIFGVDLSEQQQAESDAWYSPSMYNVVKQDGRDVHVVIKPDHECVVNSGLGSVRGAR  target    LSRRFY------SERRVKAPMIRKGFKDWVEAGYPRNDDGTPQMDVTLRGSDDWIRISWDEATTIAAKTMEDVARTFNGD 4aay.1    MAETSFSEARNTQQQRLTDPLVWRY--------------------------GQMQPTSWDDALDLVARVTAKIVKEKGED  target    EGARKLLAQGYHPEMVEVMHGAGVQALKLR 4aay.1    A----------------------------- ``` | | | | | | | | | | | | | | | | | | | | | | | | | | | | | | | | | | | | | | | | | | | | | | | | | |
|  | 7vw6.1.A | Formate dehydrogenase  *Cryo-EM Structure of Formate Dehydrogenase 1 from Methylorubrum extorquens AM1* | 0.26 |  | 18.35 | 0.47 | 73-204 | EM | 0.00 | hetero-1-1-mer | 4 x SF4, 2 x FES, 2 x MGD, 1 x W, 1 x FMN | HHblits | 0.28 |
| ``` target    MAQGVSRRQLLGRALALGSGAALADLLGPARFLSPAGAATAGAVVPGNPLRVMPDRTWEQIYRNQFEDDSTFVFTCAPND 7vw6.1    ------------------------------------------------------------------------VKSLCPYC  target    THNCLLRAHVKNGVVVRISPTYGYGEATDLYGNRASHRWDPRTCQKGLILSRRFYSERRVKAPMIRKGFKDWVEAGYPRN 7vw6.1    GVGCQVSYKVKDERIVYAEGV-------------NGPANQNRLCVKGRFGFDYVHHPHRLTVPLIRLENV---PKDA---  target    DDGTPQMDVTLRGSDDWIRISWDEATTIAAKTMEDVARTFNGDEGARKLLAQGYHPEMVEVMHGAGVQALKLR 7vw6.1    --N--DQVDPANPWTHFREATWEEALDRAAGGLKAIRDTNGRKA----------------------------- ``` | | | | | | | | | | | | | | | | | | | | | | | | | | | | | | | | | | | | | | | | | | | | | | | | | |
|  | 8bqg.1.A | Formate dehydrogenase, alpha subunit, selenocysteine-containing  *W-formate dehydrogenase from Desulfovibrio vulgaris - Soaking with Formate 1 min* | 0.27 | 0.00 | 19.81 | 0.45 | 59-204 | X-ray | 1.95 | monomer | 2 x MGD, 4 x SF4, 1 x H2S, 1 x W | HHblits | 0.29 |
| ``` target    MAQGVSRRQLLGRALALGSGAALADLLGPARFLSPAGAATAGAVVPGNPLRVMPDRTWEQIYRNQFEDDSTFVFTCAPND 8bqg.1    ----------------------------------------------------------EL-QKL--QWA-KQTTSICCYC  target    THNCLLRAHVK---NGVVVRISPTYGYGEATDLYGNRASHRWDPRTCQKGLILSRRFYSERRVKAPMIRKGFKDWVEAGY 8bqg.1    AVGCGLIVHTAKDGQGRAVNVEGD------------PDHPINEGSLCPKGASIFQLGENDQRGTQPLYRAP---------  target    PRNDDGTPQMDVTLRGSDDWIRISWDEATTIAAKTMEDVARTFNGDEGARKLLAQGYHPEMVEVMHGAGVQALKLR 8bqg.1    ---------------FSDTWKPVTWDFALTEIAKRIKKTRDASFTEK----------------------------- ``` | | | | | | | | | | | | | | | | | | | | | | | | | | | | | | | | | | | | | | | | | | | | | | | | | |
|  | 6cz7.1.A | ArrA  *The arsenate respiratory reductase (Arr) complex from Shewanella sp. ANA-3* | 0.24 |  | 19.42 | 0.44 | 72-204 | X-ray | 1.62 | hetero-1-1-mer | 5 x SF4, 2 x MGD, 1 x MO, 1 x PG5 | HHblits | 0.30 |
| ``` target    MAQGVSRRQLLGRALALGSGAALADLLGPARFLSPAGAATAGAVVPGNPLRVMPDRTWEQIYRNQFEDDSTFVFTCAPND 6cz7.1    -----------------------------------------------------------------------WLATTCQGC  target    THNCLLRAHVKNGVVVRISPTYGYGEATDLYGNRASHRWDPRTCQKGLILSRRFYSERRVKAPMIRKGFKDWVEAGYPRN 6cz7.1    TSWCAKQIYVMDGRALKVRG------------NPNSGVHGMSSCPRQHLSLQQVYDPDRLRTPMMRTNPK----------  target    DDGTPQMDVTLRGSDDWIRISWDEATTIAAKTMEDVARTFNGDEGARKLLAQGYHPEMVEVMHGAGVQALKLR 6cz7.1    --------KGRDQDPKFVPISWDKALDMLADKIIALRVANEPHK----------------------------- ``` | | | | | | | | | | | | | | | | | | | | | | | | | | | | | | | | | | | | | | | | | | | | | | | | | |
|  | 1ogy.1.A | PERIPLASMIC NITRATE REDUCTASE  *Crystal structure of the heterodimeric nitrate reductase from Rhodobacter sphaeroides* | 0.24 |  | 20.39 | 0.44 | 71-204 | X-ray | 3.20 | hetero-1-1-mer | 1 x SF4, 1 x MO, 2 x MGD, 2 x HEC | HHblits | 0.30 |
| ``` target    MAQGVSRRQLLGRALALGSGAALADLLGPARFLSPAGAATAGAVVPGNPLRVMPDRTWEQIYRNQFEDDSTFVFTCAPND 1ogy.1    ----------------------------------------------------------------------RWSKAPCRFC  target    THNCLLRAHVKNGVVVRISPTYGYGEATDLYGNRASHRWDPRTCQKGLILSRRFYSERRVKAPMIRKGFKDWVEAGYPRN 1ogy.1    GTGCGVMVGTRDGQVVATHGD------------TQAEVNRGLNCVKGYFLSKIMYGEDRLTTPLLRMKDG----------  target    DDGTPQMDVTLRGSDDWIRISWDEATTIAAKTMEDVARTFNGDEGARKLLAQGYHPEMVEVMHGAGVQALKLR 1ogy.1    ---------VYHKEGEFAPVSWDEAFDVMAAQAKLVLKEKAPEA----------------------------- ``` | | | | | | | | | | | | | | | | | | | | | | | | | | | | | | | | | | | | | | | | | | | | | | | | | |
|  | 7bkb.1.F | Formate dehydrogenase  *Formate dehydrogenase - heterodisulfide reductase - formylmethanofuran dehydrogenase complex from Methanospirillum hungatei (hexameric, composite structure)* | 0.24 |  | 28.87 | 0.42 | 70-204 | EM | 0.00 | hetero-2-2-2-2-2-2-… | 48 x SF4, 4 x FAD, 2 x FES, 4 x 9S8, 4 x ZN, 2 x MO, 4 x MGD | HHblits | 0.34 |
| ``` target    MAQGVSRRQLLGRALALGSGAALADLLGPARFLSPAGAATAGAVVPGNPLRVMPDRTWEQIYRNQFEDDSTFVFTCAPND 7bkb.1    ---------------------------------------------------------------------MKYVATTCPYC  target    THNCLLRAHVKNGVVVRISPTYGYGEATDLYGNRASHRWDPRTCQKGLILSRRFYSERRVKAPMIRKGFKDWVEAGYPRN 7bkb.1    GVGCTLNLVVSNGKVVGVEPN------------QRSPINEGKLCPKGVTCWEHIHSPDRLTTPLIKKD------------  target    DDGTPQMDVTLRGSDDWIRISWDEATTIAAKTMEDVARTFNGDEGARKLLAQGYHPEMVEVMHGAGVQALKLR 7bkb.1    --------------GKFIEASWDEALDLVAKNLKVIYDKHGPKG----------------------------- ``` | | | | | | | | | | | | | | | | | | | | | | | | | | | | | | | | | | | | | | | | | | | | | | | | | |
|  | 2vpz.1.A | THIOSULFATE REDUCTASE  *POLYSULFIDE REDUCTASE NATIVE STRUCTURE* | 0.25 |  | 23.00 | 0.43 | 73-204 | X-ray | 2.40 | hetero-oligomer | 10 x SF4, 4 x MGD, 2 x MO | HHblits | 0.32 |
| ``` target    MAQGVSRRQLLGRALALGSGAALADLLGPARFLSPAGAATAGAVVPGNPLRVMPDRTWEQIYRNQFEDDSTFVFTCAPND 2vpz.1    ------------------------------------------------------------------------VYQICEGC  target    THNCLLRAHVKNGVVVRISPTYGYGEATDLYGNRASHRWDPRTCQKGLILSRRFYSERRVKAPMIRKGFKDWVEAGYPRN 2vpz.1    FWRCGIVAHAVGNRVYKVEG------------YEANPKSRGRLCPRGQGAPQTTYDPDRLKRPLIRVEGS----------  target    DDGTPQMDVTLRGSDDWIRISWDEATTIAAKTMEDVARTFNGDEGARKLLAQGYHPEMVEVMHGAGVQALKLR 2vpz.1    ----------QRGEGKYRVATWEEALDHIAKKMLEIREKYGPEA----------------------------- ``` | | | | | | | | | | | | | | | | | | | | | | | | | | | | | | | | | | | | | | | | | | | | | | | | | |
|  | 2vpx.1.D | THIOSULFATE REDUCTASE  *POLYSULFIDE REDUCTASE WITH BOUND QUINONE (UQ1)* | 0.24 |  | 23.00 | 0.43 | 73-204 | X-ray | 3.10 | hetero-oligomer | 10 x SF4, 4 x MGD, 2 x MO, 2 x UQ1 | HHblits | 0.32 |
| ``` target    MAQGVSRRQLLGRALALGSGAALADLLGPARFLSPAGAATAGAVVPGNPLRVMPDRTWEQIYRNQFEDDSTFVFTCAPND 2vpx.1    ------------------------------------------------------------------------VYQICEGC  target    THNCLLRAHVKNGVVVRISPTYGYGEATDLYGNRASHRWDPRTCQKGLILSRRFYSERRVKAPMIRKGFKDWVEAGYPRN 2vpx.1    FWRCGIVAHAVGNRVYKVEG------------YEANPKSRGRLCPRGQGAPQTTYDPDRLKRPLIRVEGS----------  target    DDGTPQMDVTLRGSDDWIRISWDEATTIAAKTMEDVARTFNGDEGARKLLAQGYHPEMVEVMHGAGVQALKLR 2vpx.1    ----------QRGEGKYRVATWEEALDHIAKKMLEIREKYGPEA----------------------------- ``` | | | | | | | | | | | | | | | | | | | | | | | | | | | | | | | | | | | | | | | | | | | | | | | | | |
|  | 2e7z.1.A | Acetylene hydratase Ahy  *Acetylene Hydratase from Pelobacter acetylenicus* | 0.23 |  | 23.23 | 0.42 | 73-204 | X-ray | 1.26 | monomer | 1 x SF4, 2 x MGD, 1 x W | HHblits | 0.32 |
| ``` target    MAQGVSRRQLLGRALALGSGAALADLLGPARFLSPAGAATAGAVVPGNPLRVMPDRTWEQIYRNQFEDDSTFVFTCAPND 2e7z.1    ------------------------------------------------------------------------KHVVCQSC  target    THNCLLRAHVK-NGVVVRISPTYGYGEATDLYGNRASHRW-DPRTCQKGLILSRRFYSERRVKAPMIRKGFKDWVEAGYP 2e7z.1    DINCVVEAEVKADGKIQTKSISE------------PHPTTPPNSICMKSVNADTIRTHKDRVLYPLKNVGS---------  target    RNDDGTPQMDVTLRGSDDWIRISWDEATTIAAKTMEDVARTFNGDEGARKLLAQGYHPEMVEVMHGAGVQALKLR 2e7z.1    ------------KRGEQRWERISWDQALDEIAEKLKKIIAKYGPES----------------------------- ``` | | | | | | | | | | | | | | | | | | | | | | | | | | | | | | | | | | | | | | | | | | | | | | | | | |
|  | 3o5a.1.A | Periplasmic nitrate reductase  *Crystal Structure of partially reduced Periplasmic Nitrate Reductase from Cupriavidus necator using Ionic Liquids* | 0.24 |  | 18.63 | 0.44 | 72-204 | X-ray | 1.72 | hetero-oligomer | 1 x SF4, 1 x MOS, 2 x MGD, 2 x HEC | HHblits | 0.29 |
| ``` target    MAQGVSRRQLLGRALALGSGAALADLLGPARFLSPAGAATAGAVVPGNPLRVMPDRTWEQIYRNQFEDDSTFVFTCAPND 3o5a.1    -----------------------------------------------------------------------WSKAPCRFC  target    THNCLLRAHVKNGVVVRISPTYGYGEATDLYGNRASHRWDPRTCQKGLILSRRFYSERRVKAPMIRKGFKDWVEAGYPRN 3o5a.1    GTGCGVTVAVKDNKVVATQG------------DPQAEVNKGLNCVKGYFLSKIMYGQDRLTRPLMRMKNG----------  target    DDGTPQMDVTLRGSDDWIRISWDEATTIAAKTMEDVARTFNGDEGARKLLAQGYHPEMVEVMHGAGVQALKLR 3o5a.1    ---------KYDKNGDFAPVTWDQAFDEMERQFKRVLKEKGPTA----------------------------- ``` | | | | | | | | | | | | | | | | | | | | | | | | | | | | | | | | | | | | | | | | | | | | | | | | | |
|  | 6tg9.1.A | Formate dehydrogenase subunit alpha  *Cryo-EM Structure of NADH reduced form of NAD+-dependent Formate Dehydrogenase from Rhodobacter capsulatus* | 0.23 |  | 23.71 | 0.42 | 72-204 | EM | 3.24 | hetero-2-2-2-2-mer | 4 x MGD, 2 x 6MO, 4 x FES, 10 x SF4, 2 x H2S, 2 x FMN, 2 x NAI | HHblits | 0.32 |
| ``` target    MAQGVSRRQLLGRALALGSGAALADLLGPARFLSPAGAATAGAVVPGNPLRVMPDRTWEQIYRNQFEDDSTFVFTCAPND 6tg9.1    -----------------------------------------------------------------------KVVTTCAYC  target    THNCLLRAHVKNGVVVRISPTYGYGEATDLYGNRASHRWDPRTCQKGLILSRRFYSERRVKAPMIRKGFKDWVEAGYPRN 6tg9.1    GVGCSFEAHMLGDQLVRMVPW------------KGGAANRGHSCVKGRFAYGYATHQDRILKPMIRD-------------  target    DDGTPQMDVTLRGSDDWIRISWDEATTIAAKTMEDVARTFNGDEGARKLLAQGYHPEMVEVMHGAGVQALKLR 6tg9.1    -----------KITDPWREVNWTEALDFTATRLRALRDSHGADA----------------------------- ``` | | | | | | | | | | | | | | | | | | | | | | | | | | | | | | | | | | | | | | | | | | | | | | | | | |
|  | 2nya.1.A | Periplasmic nitrate reductase  *Crystal structure of the periplasmic nitrate reductase (NAP) from Escherichia coli* | 0.24 |  | 15.69 | 0.44 | 72-204 | X-ray | 2.50 | monomer | 1 x SF4, 1 x 6MO, 2 x MGD | HHblits | 0.28 |
| ``` target    MAQGVSRRQLLGRALALGSGAALADLLGPARFLSPAGAATAGAVVPGNPLRVMPDRTWEQIYRNQFEDDSTFVFTCAPND 2nya.1    -----------------------------------------------------------------------WDKAPCRFC  target    THNCLLRAHVKNGVVVRISPTYGYGEATDLYGNRASHRWDPRTCQKGLILSRRFYSERRVKAPMIRKGFKDWVEAGYPRN 2nya.1    GTGCGVLVGTQQGRVVACQG------------DPDAPVNRGLNCIKGYFLPKIMYGKDRLTQPLLRMKNG----------  target    DDGTPQMDVTLRGSDDWIRISWDEATTIAAKTMEDVARTFNGDEGARKLLAQGYHPEMVEVMHGAGVQALKLR 2nya.1    ---------KYDKEGEFTPITWDQAFDVMEEKFKTALKEKGPES----------------------------- ``` | | | | | | | | | | | | | | | | | | | | | | | | | | | | | | | | | | | | | | | | | | | | | | | | | |
|  | 7qv7.1.L | Hydrogen dependent carbon dioxide reductase subunit FdhF  *Cryo-EM structure of Hydrogen-dependent CO2 reductase.* | 0.22 |  | 25.26 | 0.41 | 72-204 | EM | 0.00 | hetero-2-6-6-2-mer | 52 x SF4, 6 x 402 | HHblits | 0.34 |
| ``` target    MAQGVSRRQLLGRALALGSGAALADLLGPARFLSPAGAATAGAVVPGNPLRVMPDRTWEQIYRNQFEDDSTFVFTCAPND 7qv7.1    -----------------------------------------------------------------------KVLTTCPYC  target    THNCLLRAHVKNGVVVRISPTYGYGEATDLYGNRASHRWDPRTCQKGLILSRRFYSERRVKAPMIRKGFKDWVEAGYPRN 7qv7.1    GTGCGLYLKVENEKIVGVEPD------------KLHPVNQGELCIKGYYGYKYVHDPRRLTSPLIKKN------------  target    DDGTPQMDVTLRGSDDWIRISWDEATTIAAKTMEDVARTFNGDEGARKLLAQGYHPEMVEVMHGAGVQALKLR 7qv7.1    --------------GKFVPVSWDEALNFIANGLKKIKSEYGSDA----------------------------- ``` | | | | | | | | | | | | | | | | | | | | | | | | | | | | | | | | | | | | | | | | | | | | | | | | | |
|  | 7qv7.1.O | Hydrogen dependent carbon dioxide reductase subunit FdhF  *Cryo-EM structure of Hydrogen-dependent CO2 reductase.* | 0.23 |  | 25.26 | 0.41 | 72-204 | EM | 0.00 | hetero-2-6-6-2-mer | 52 x SF4, 6 x 402 | HHblits | 0.34 |
| ``` target    MAQGVSRRQLLGRALALGSGAALADLLGPARFLSPAGAATAGAVVPGNPLRVMPDRTWEQIYRNQFEDDSTFVFTCAPND 7qv7.1    -----------------------------------------------------------------------KVLTTCPYC  target    THNCLLRAHVKNGVVVRISPTYGYGEATDLYGNRASHRWDPRTCQKGLILSRRFYSERRVKAPMIRKGFKDWVEAGYPRN 7qv7.1    GTGCGLYLKVENEKIVGVEPD------------KLHPVNQGELCIKGYYGYKYVHDPRRLTSPLIKKN------------  target    DDGTPQMDVTLRGSDDWIRISWDEATTIAAKTMEDVARTFNGDEGARKLLAQGYHPEMVEVMHGAGVQALKLR 7qv7.1    --------------GKFVPVSWDEALNFIANGLKKIKSEYGSDA----------------------------- ``` | | | | | | | | | | | | | | | | | | | | | | | | | | | | | | | | | | | | | | | | | | | | | | | | | |
|  | 1aa6.1.A | FORMATE DEHYDROGENASE H  *REDUCED FORM OF FORMATE DEHYDROGENASE H FROM E. COLI* | 0.23 | 0.00 | 27.37 | 0.41 | 73-204 | X-ray | 2.30 | monomer | 1 x SF4, 2 x MGD, 1 x 4MO | HHblits | 0.33 |
| ``` target    MAQGVSRRQLLGRALALGSGAALADLLGPARFLSPAGAATAGAVVPGNPLRVMPDRTWEQIYRNQFEDDSTFVFTCAPND 1aa6.1    ------------------------------------------------------------------------VVTVCPYC  target    THNCLLRAHVKNGVVVRISPTYGYGEATDLYGNRASHRWDPRTCQKGLILSRRFYSE----RRVKAPMIRKGFKDWVEAG 1aa6.1    ASGCKINLVVDNGKIVRAEAAQ-------------GKTNQGTLCLKGYYGWDFINDTQILTPRLKTPMIRRQ--------  target    YPRNDDGTPQMDVTLRGSDDWIRISWDEATTIAAKTMEDVARTFNGDEGARKLLAQGYHPEMVEVMHGAGVQALKLR 1aa6.1    ---------------R-GGKLEPVSWDEALNYVAERLSAIKEKYGPDA----------------------------- ``` | | | | | | | | | | | | | | | | | | | | | | | | | | | | | | | | | | | | | | | | | | | | | | | | | |
|  | 1fdo.1.A | FORMATE DEHYDROGENASE H  *OXIDIZED FORM OF FORMATE DEHYDROGENASE H FROM E. COLI* | 0.23 | 0.00 | 27.37 | 0.41 | 73-204 | X-ray | 2.80 | monomer | 1 x SF4, 2 x MGD, 1 x 6MO | HHblits | 0.33 |
| ``` target    MAQGVSRRQLLGRALALGSGAALADLLGPARFLSPAGAATAGAVVPGNPLRVMPDRTWEQIYRNQFEDDSTFVFTCAPND 1fdo.1    ------------------------------------------------------------------------VVTVCPYC  target    THNCLLRAHVKNGVVVRISPTYGYGEATDLYGNRASHRWDPRTCQKGLILSRRFYSE----RRVKAPMIRKGFKDWVEAG 1fdo.1    ASGCKINLVVDNGKIVRAEAAQ-------------GKTNQGTLCLKGYYGWDFINDTQILTPRLKTPMIRRQ--------  target    YPRNDDGTPQMDVTLRGSDDWIRISWDEATTIAAKTMEDVARTFNGDEGARKLLAQGYHPEMVEVMHGAGVQALKLR 1fdo.1    ---------------R-GGKLEPVSWDEALNYVAERLSAIKEKYGPDA----------------------------- ``` | | | | | | | | | | | | | | | | | | | | | | | | | | | | | | | | | | | | | | | | | | | | | | | | | |
|  | 2iv2.1.A | Formate dehydrogenase H  *Reinterpretation of reduced form of formate dehydrogenase H from E. coli* | 0.23 | 0.00 | 27.37 | 0.41 | 73-204 | X-ray | 2.27 | monomer | 1 x SF4, 1 x 2MD, 1 x MGD | HHblits | 0.33 |
| ``` target    MAQGVSRRQLLGRALALGSGAALADLLGPARFLSPAGAATAGAVVPGNPLRVMPDRTWEQIYRNQFEDDSTFVFTCAPND 2iv2.1    ------------------------------------------------------------------------VVTVCPYC  target    THNCLLRAHVKNGVVVRISPTYGYGEATDLYGNRASHRWDPRTCQKGLILSRRFYSE----RRVKAPMIRKGFKDWVEAG 2iv2.1    ASGCKINLVVDNGKIVRAEAAQ-------------GKTNQGTLCLKGYYGWDFINDTQILTPRLKTPMIRRQ--------  target    YPRNDDGTPQMDVTLRGSDDWIRISWDEATTIAAKTMEDVARTFNGDEGARKLLAQGYHPEMVEVMHGAGVQALKLR 2iv2.1    ---------------R-GGKLEPVSWDEALNYVAERLSAIKEKYGPDA----------------------------- ``` | | | | | | | | | | | | | | | | | | | | | | | | | | | | | | | | | | | | | | | | | | | | | | | | | |
|  | 7z0t.1.G | Formate dehydrogenase H  *Structure of the Escherichia coli formate hydrogenlyase complex (aerobic preparation, composite structure)* | 0.23 | 0.00 | 27.37 | 0.41 | 73-204 | EM | 0.00 | monomer | 1 x NI, 1 x FCO, 8 x SF4, 1 x FE, 2 x MGD, 1 x 6MO | HHblits | 0.33 |
| ``` target    MAQGVSRRQLLGRALALGSGAALADLLGPARFLSPAGAATAGAVVPGNPLRVMPDRTWEQIYRNQFEDDSTFVFTCAPND 7z0t.1    ------------------------------------------------------------------------VVTVCPYC  target    THNCLLRAHVKNGVVVRISPTYGYGEATDLYGNRASHRWDPRTCQKGLILSRRFYSE----RRVKAPMIRKGFKDWVEAG 7z0t.1    ASGCKINLVVDNGKIVRAEAAQ-------------GKTNQGTLCLKGYYGWDFINDTQILTPRLKTPMIRRQ--------  target    YPRNDDGTPQMDVTLRGSDDWIRISWDEATTIAAKTMEDVARTFNGDEGARKLLAQGYHPEMVEVMHGAGVQALKLR 7z0t.1    ---------------R-GGKLEPVSWDEALNYVAERLSAIKEKYGPDA----------------------------- ``` | | | | | | | | | | | | | | | | | | | | | | | | | | | | | | | | | | | | | | | | | | | | | | | | | |
|  | 1h0h.1.A | FORMATE DEHYDROGENASE SUBUNIT ALPHA  *Tungsten containing Formate Dehydrogenase from Desulfovibrio Gigas* | 0.22 |  | 20.41 | 0.42 | 70-203 | X-ray | 1.80 | hetero-1-1-mer | 1 x W, 1 x 2MD, 1 x MGD, 4 x SF4, 1 x CA | HHblits | 0.30 |
| ``` target    MAQGVSRRQLLGRALALGSGAALADLLGPARFLSPAGAATAGAVVPGNPLRVMPDRTWEQIYRNQFEDDSTFVFTCAPND 1h0h.1    ---------------------------------------------------------------------AKQTTSVCCYC  target    THNCLLRAHV--KNGVVVRISPTYGYGEATDLYGNRASHRWDPRTCQKGLILSRRFYSERRVKAPMIRKGFKDWVEAGYP 1h0h.1    SVGCGLIVHTDKKTNRAINVEGD------------PDHPINEGSLCAKGASTWQLAENERRPANPLYRA-----------  target    RNDDGTPQMDVTLRGSDDWIRISWDEATTIAAKTMEDVARTFNGDEGARKLLAQGYHPEMVEVMHGAGVQALKLR 1h0h.1    -------------PGSDQWEEKSWDWMLDTIAERVAKTREATFVT------------------------------ ``` | | | | | | | | | | | | | | | | | | | | | | | | | | | | | | | | | | | | | | | | | | | | | | | | | |
|  | 2v45.1.A | PERIPLASMIC NITRATE REDUCTASE  *A NEW CATALYTIC MECHANISM OF PERIPLASMIC NITRATE REDUCTASE FROM DESULFOVIBRIO DESULFURICANS ATCC 27774 FROM CRYSTALLOGRAPHIC AND EPR DATA AND BASED ON DETAILED ANALYSIS OF THE SIXTH LIGAND* | 0.23 |  | 23.96 | 0.41 | 72-204 | X-ray | 2.40 | monomer | 1 x SF4, 1 x MO, 2 x MGD, 1 x LCP | HHblits | 0.31 |
| ``` target    MAQGVSRRQLLGRALALGSGAALADLLGPARFLSPAGAATAGAVVPGNPLRVMPDRTWEQIYRNQFEDDSTFVFTCAPND 2v45.1    -----------------------------------------------------------------------WVKGVCRYC  target    THNCLLRAHVKNGVVVRISPTYGYGEATDLYGNRASHRWDPRTCQKGLILSRRFYSERRVKAPMIRKGFKDWVEAGYPRN 2v45.1    GTGCGVLVGVKDGKAVAIQGNP------------NNH-NAGLLCLKGSLLIPVLNSKERVTQPLVRRH------------  target    DDGTPQMDVTLRGSDDWIRISWDEATTIAAKTMEDVARTFNGDEGARKLLAQGYHPEMVEVMHGAGVQALKLR 2v45.1    ------------KGGKLEPVSWDEALDLMASRFRSSIDMYGPNS----------------------------- ``` | | | | | | | | | | | | | | | | | | | | | | | | | | | | | | | | | | | | | | | | | | | | | | | | | |
|  | 2v3v.1.A | PERIPLASMIC NITRATE REDUCTASE  *A NEW CATALYTIC MECHANISM OF PERIPLASMIC NITRATE REDUCTASE FROM DESULFOVIBRIO DESULFURICANS ATCC 27774 FROM CRYSTALLOGRAPHIC AND EPR DATA AND BASED ON DETAILED ANALYSIS OF THE SIXTH LIGAND* | 0.23 |  | 23.96 | 0.41 | 72-204 | X-ray | 1.99 | monomer | 1 x SF4, 1 x MO, 2 x MGD, 4 x LCP | HHblits | 0.31 |
| ``` target    MAQGVSRRQLLGRALALGSGAALADLLGPARFLSPAGAATAGAVVPGNPLRVMPDRTWEQIYRNQFEDDSTFVFTCAPND 2v3v.1    -----------------------------------------------------------------------WVKGVCRYC  target    THNCLLRAHVKNGVVVRISPTYGYGEATDLYGNRASHRWDPRTCQKGLILSRRFYSERRVKAPMIRKGFKDWVEAGYPRN 2v3v.1    GTGCGVLVGVKDGKAVAIQGDP------------NNH-NAGLLCLKGSLLIPVLNSKERVTQPLVRRH------------  target    DDGTPQMDVTLRGSDDWIRISWDEATTIAAKTMEDVARTFNGDEGARKLLAQGYHPEMVEVMHGAGVQALKLR 2v3v.1    ------------KGGKLEPVSWDEALDLMASRFRSSIDMYGPNS----------------------------- ``` | | | | | | | | | | | | | | | | | | | | | | | | | | | | | | | | | | | | | | | | | | | | | | | | | |
|  | 5nqd.1.A | AroA  *Arsenite oxidase AioAB from Rhizobium sp. str. NT-26 mutant AioBF108A* | 0.21 |  | 14.14 | 0.42 | 68-204 | X-ray | 2.20 | hetero-2-2-mer | 4 x MGD, 2 x O, 2 x 4MO, 2 x F3S, 2 x FES | HHblits | 0.28 |
| ``` target    MAQGVSRRQLLGRALALGSGAALADLLGPARFLSPAGAATAGAVVPGNPLRVMPDRTWEQIYRNQFEDDSTFVFTCAPND 5nqd.1    -------------------------------------------------------------------ADAKKHNVTCHFC  target    THNCLLRAHV-----------------------------------------KNGVVVRISPTYGYGEATDLYGNRA--SH 5nqd.1    IVGCGYHAYTWPINKQGGTDPQNNIFGVDLSEQQQAESDAWYSPSMYNVVKQDGRDVHVVI------------KPDHECV  target    RWDPRTCQKGLILSRRFY------SERRVKAPMIRKGFKDWVEAGYPRNDDGTPQMDVTLRGSDDWIRISWDEATTIAAK 5nqd.1    VNSGLGSVRGARMAETSFSEARNTQQQRLTDPLVWRY--------------------------GQMQPTSWDDALDLVAR  target    TMEDVARTFNGDEGARKLLAQGYHPEMVEVMHGAGVQALKLR 5nqd.1    VTAKIVKEKGEDA----------------------------- ``` | | | | | | | | | | | | | | | | | | | | | | | | | | | | | | | | | | | | | | | | | | | | | | | | | |
|  | 7t2r.1.A | NiFe hydrogenase subunit A  *Structure of electron bifurcating Ni-Fe hydrogenase complex HydABCSL in FMN-free apo state* | 0.20 |  | 21.51 | 0.40 | 72-202 | EM | 0.00 | hetero-2-2-2-2-2-mer | 6 x FES, 12 x SF4, 2 x 3NI, 2 x FCO | HHblits | 0.32 |
| ``` target    MAQGVSRRQLLGRALALGSGAALADLLGPARFLSPAGAATAGAVVPGNPLRVMPDRTWEQIYRNQFEDDSTFVFTCAPND 7t2r.1    -----------------------------------------------------------------------VVESVCPLC  target    THNCLLRAHVKNGVVVRISPTYGYGEATDLYGNRASHRWDPRTCQKGLILSRRFYSERRVKAPMIRKGFKDWVEAGYPRN 7t2r.1    AVGCKIKTYVRTGSIVRVEGT------------GVEEPDGGQLCHMGRWWLPESTERERVTVPLIREG------------  target    DDGTPQMDVTLRGSDDWIRISWDEATTIAAKTMEDVARTFNGDEGARKLLAQGYHPEMVEVMHGAGVQALKLR 7t2r.1    --------------ASYREATWEEALALASAEFKKAYDQEKA------------------------------- ``` | | | | | | | | | | | | | | | | | | | | | | | | | | | | | | | | | | | | | | | | | | | | | | | | | |
|  | 7t30.1.A | NiFe hydrogenase subunit A  *Structure of electron bifurcating Ni-Fe hydrogenase complex HydABCSL in FMN/NAD(H) bound state* | 0.21 |  | 21.51 | 0.40 | 72-202 | EM | 0.00 | hetero-2-2-2-2-2-mer | 4 x FES, 12 x SF4, 2 x NAD, 2 x FMN, 2 x 3NI, 2 x FCO | HHblits | 0.32 |
| ``` target    MAQGVSRRQLLGRALALGSGAALADLLGPARFLSPAGAATAGAVVPGNPLRVMPDRTWEQIYRNQFEDDSTFVFTCAPND 7t30.1    -----------------------------------------------------------------------VVESVCPLC  target    THNCLLRAHVKNGVVVRISPTYGYGEATDLYGNRASHRWDPRTCQKGLILSRRFYSERRVKAPMIRKGFKDWVEAGYPRN 7t30.1    AVGCKIKTYVRTGSIVRVEGT------------GVEEPDGGQLCHMGRWWLPESTERERVTVPLIREG------------  target    DDGTPQMDVTLRGSDDWIRISWDEATTIAAKTMEDVARTFNGDEGARKLLAQGYHPEMVEVMHGAGVQALKLR 7t30.1    --------------ASYREATWEEALALASAEFKKAYDQEKA------------------------------- ``` | | | | | | | | | | | | | | | | | | | | | | | | | | | | | | | | | | | | | | | | | | | | | | | | | |
|  | 1g8j.1.A | ARSENITE OXIDASE  *CRYSTAL STRUCTURE ANALYSIS OF ARSENITE OXIDASE FROM ALCALIGENES FAECALIS* | 0.20 |  | 14.43 | 0.42 | 70-204 | X-ray | 2.03 | hetero-oligomer | 2 x MGD, 1 x O, 1 x 4MO, 1 x F3S, 1 x FES | HHblits | 0.29 |
| ``` target    MAQGVSRRQLLGRALALGSGAALADLLGPARFLSPAGAATAGAVVPGNPLRVMPDRTWEQIYRNQFEDDSTFVFTCAPND 1g8j.1    ---------------------------------------------------------------------AQRTNMTCHFC  target    THNCLLRAHVKN-----GV--------------------------------------VVRISPTYGYGEATDLYGNRASH 1g8j.1    IVGCGYHVYKWPELEEGGRAPEQNALGLDFRKQLPPLASTLTPAMTNVVTEHDGARYDIMVV------------PDKACV  target    RWDPRTCQKGLILSRRFYSE-----RRVKAPMIRKGFKDWVEAGYPRNDDGTPQMDVTLRGSDDWIRISWDEATTIAAKT 1g8j.1    VNSGLSSTRGGKMASYMYTPTGDGKERLSAPRLYAA--------------------------DEWVDTTWDHAMALYAGL  target    MEDVARTFNGDEGARKLLAQGYHPEMVEVMHGAGVQALKLR 1g8j.1    IKKTLDSDGPQG----------------------------- ``` | | | | | | | | | | | | | | | | | | | | | | | | | | | | | | | | | | | | | | | | | | | | | | | | | |
|  | 1g8k.1.A | ARSENITE OXIDASE  *CRYSTAL STRUCTURE ANALYSIS OF ARSENITE OXIDASE FROM ALCALIGENES FAECALIS* | 0.21 |  | 14.43 | 0.42 | 70-204 | X-ray | 1.64 | hetero-1-1-mer | 3 x HG, 2 x CA, 2 x MGD, 1 x O, 1 x 4MO, 1 x F3S, 1 x FES | HHblits | 0.28 |
| ``` target    MAQGVSRRQLLGRALALGSGAALADLLGPARFLSPAGAATAGAVVPGNPLRVMPDRTWEQIYRNQFEDDSTFVFTCAPND 1g8k.1    ---------------------------------------------------------------------AQRTNMTCHFC  target    THNCLLRAHVKN-----GV--------------------------------------VVRISPTYGYGEATDLYGNRASH 1g8k.1    IVGCGYHVYKWPELEEGGRAPEQNALGLDFRKQLPPLAVTLTPAMTNVVTEHDGARYDIMVV------------PDKACV  target    RWDPRTCQKGLILSRRFYSE-----RRVKAPMIRKGFKDWVEAGYPRNDDGTPQMDVTLRGSDDWIRISWDEATTIAAKT 1g8k.1    VNSGLSSTRGGKMASYMYTPTGDGKERLSAPRLYAA--------------------------DEWVDTTWDHAMALYAGL  target    MEDVARTFNGDEGARKLLAQGYHPEMVEVMHGAGVQALKLR 1g8k.1    IKKTLDKDGPQG----------------------------- ``` | | | | | | | | | | | | | | | | | | | | | | | | | | | | | | | | | | | | | | | | | | | | | | | | | |
|  | 7nz1.1.E | NADH-quinone oxidoreductase subunit G  *Respiratory complex I from Escherichia coli - focused refinement of cytoplasmic arm* | 0.18 | 0.00 | 18.95 | 0.41 | 69-201 | EM | 0.00 | monomer | 7 x SF4, 2 x FES, 1 x FMN, 1 x CA | HHblits | 0.29 |
| ``` target    MAQGVSRRQLLGRALALGSGAALADLLGPARFLSPAGAATAGAVVPGNPLRVMPDRTWEQIYRNQFEDDSTFVFTCAPND 7nz1.1    --------------------------------------------------------------------DMQFAPSICQQC  target    THNCLLRAHVKNGVVVRISPTYGYGEATDLYGNRASHRWDPRTCQKGLILSRRFYSERRVKAPMIRKGFKDWVEAGYPRN 7nz1.1    SIGCNISPGERYGELRRIENR------------YNGTVNHYFLCDRGRFGYGYVNLKDRPRQPVQRRG------------  target    DDGTPQMDVTLRGSDDWIRISWDEATTIAAKTMEDVARTFNGDEGARKLLAQGYHPEMVEVMHGAGVQALKLR 7nz1.1    --------------DDFITLNAEQAMQGAADILRQSKKVIG-------------------------------- ``` | | | | | | | | | | | | | | | | | | | | | | | | | | | | | | | | | | | | | | | | | | | | | | | | | |
|  | 7p63.1.C | NADH-quinone oxidoreductase  *Complex I from E. coli, DDM/LMNG-purified, under Turnover at pH 6, Closed state* | 0.18 | 0.00 | 18.95 | 0.41 | 69-201 | EM | 0.00 | monomer | 7 x SF4, 1 x FMN, 1 x NAI, 2 x FES, 1 x CA, 1 x DCQ, 4 x LFA, 8 x 3PE | HHblits | 0.29 |
| ``` target    MAQGVSRRQLLGRALALGSGAALADLLGPARFLSPAGAATAGAVVPGNPLRVMPDRTWEQIYRNQFEDDSTFVFTCAPND 7p63.1    --------------------------------------------------------------------DMQFAPSICQQC  target    THNCLLRAHVKNGVVVRISPTYGYGEATDLYGNRASHRWDPRTCQKGLILSRRFYSERRVKAPMIRKGFKDWVEAGYPRN 7p63.1    SIGCNISPGERYGELRRIENR------------YNGTVNHYFLCDRGRFGYGYVNLKDRPRQPVQRRG------------  target    DDGTPQMDVTLRGSDDWIRISWDEATTIAAKTMEDVARTFNGDEGARKLLAQGYHPEMVEVMHGAGVQALKLR 7p63.1    --------------DDFITLNAEQAMQGAADILRQSKKVIG-------------------------------- ``` | | | | | | | | | | | | | | | | | | | | | | | | | | | | | | | | | | | | | | | | | | | | | | | | | |
|  | 7p61.1.C | NADH-quinone oxidoreductase  *Complex I from E. coli, DDM-purified, with NADH, Resting state* | 0.18 | 0.00 | 19.15 | 0.40 | 69-200 | EM | 0.00 | monomer | 7 x SF4, 1 x FMN, 1 x NAI, 2 x FES, 1 x CA, 2 x 3PE, 1 x UQ8 | HHblits | 0.29 |
| ``` target    MAQGVSRRQLLGRALALGSGAALADLLGPARFLSPAGAATAGAVVPGNPLRVMPDRTWEQIYRNQFEDDSTFVFTCAPND 7p61.1    --------------------------------------------------------------------DMQFAPSICQQC  target    THNCLLRAHVKNGVVVRISPTYGYGEATDLYGNRASHRWDPRTCQKGLILSRRFYSERRVKAPMIRKGFKDWVEAGYPRN 7p61.1    SIGCNISPGERYGELRRIENR------------YNGTVNHYFLCDRGRFGYGYVNLKDRPRQPVQRRG------------  target    DDGTPQMDVTLRGSDDWIRISWDEATTIAAKTMEDVARTFNGDEGARKLLAQGYHPEMVEVMHGAGVQALKLR 7p61.1    --------------DDFITLNAEQAMQGAADILRQSKKVI--------------------------------- ``` | | | | | | | | | | | | | | | | | | | | | | | | | | | | | | | | | | | | | | | | | | | | | | | | | |
|  | 7zm7.1.I | NADH-ubiquinone oxidoreductase-like protein  *CryoEM structure of mitochondrial complex I from Chaetomium thermophilum (inhibited by DDM)* | 0.20 |  | 17.02 | 0.40 | 70-202 | EM | 0.00 | hetero-1-1-1-1-1-1-… | 4 x PC1, 14 x LMT, 5 x CDL, 8 x 3PE, 2 x FES, 6 x SF4, 1 x FMN, 1 x NDP, 1 x ZN, 2 x ZMP | HHblits | 0.28 |
| ``` target    MAQGVSRRQLLGRALALGSGAALADLLGPARFLSPAGAATAGAVVPGNPLRVMPDRTWEQIYRNQFEDDSTFVFTCAPND 7zm7.1    ---------------------------------------------------------------------LKRTESIDVLD  target    THNCLLRAHVKNGVVVRISPTYGYGEATDLYGNRASHRWDPRTCQKGLILSRRFYSERRVKAPMIRKGFKDWVEAGYPRN 7zm7.1    GLGSNIRVDSRGLEVMRILPRL------------NDDVNEEWINDKTRFACDGLK-TQRLTMPLVRRD------------  target    DDGTPQMDVTLRGSDDWIRISWDEATTIAAKTMEDVARTFNGDEGARKLLAQGYHPEMVEVMHGAGVQALKLR 7zm7.1    --------------GKFEPATWEQALTEIAHAYQTLAPKENE------------------------------- ``` | | | | | | | | | | | | | | | | | | | | | | | | | | | | | | | | | | | | | | | | | | | | | | | | | |
|  | 8e9g.1.G | NADH-quinone oxidoreductase subunit G  *Mycobacterial respiratory complex I with both quinone positions modelled* | 0.21 |  | 24.18 | 0.39 | 69-197 | EM | 0.00 | hetero-1-1-1-1-1-1-… |  | HHblits | 0.30 |
| ``` target    MAQGVSRRQLLGRALALGSGAALADLLGPARFLSPAGAATAGAVVPGNPLRVMPDRTWEQIYRNQFEDDSTFVFTCAPND 8e9g.1    --------------------------------------------------------------------DLVSSPSVCEHC  target    THNCLLRAHVKNGVVVRISPTYGYGEATDLYGNRASHRWDPRTCQKGLILSRRFYSERRVKAPMIRKGFKDWVEAGYPRN 8e9g.1    ASGCAQRTDHRRGKVLRRLAG------------DEPEVNEEWNCDKGRWAFTYATVGDRITTPMLRDG------------  target    DDGTPQMDVTLRGSDDWIRISWDEATTIAAKTMEDVARTFNGDEGARKLLAQGYHPEMVEVMHGAGVQALKLR 8e9g.1    --------------GVLRPASWSEALTVAAAGLLTAA------------------------------------ ``` | | | | | | | | | | | | | | | | | | | | | | | | | | | | | | | | | | | | | | | | | | | | | | | | | |
|  | 3m9s.1.C | NADH-quinone oxidoreductase subunit 3  *Crystal structure of respiratory complex I from Thermus thermophilus* | 0.19 | 0.00 | 17.78 | 0.39 | 69-197 | X-ray | 4.50 | monomer | 7 x SF4, 2 x FES, 1 x FMN | HHblits | 0.31 |
| ``` target    MAQGVSRRQLLGRALALGSGAALADLLGPARFLSPAGAATAGAVVPGNPLRVMPDRTWEQIYRNQFEDDSTFVFTCAPND 3m9s.1    --------------------------------------------------------------------EMEETPTTCALC  target    THNCLLRAHVKNGVVVRISPTYGYGEATDLYGNRASHRWDPRTCQKGLILSRRFYSERRVKAPMIRKGFKDWVEAGYPRN 3m9s.1    PVGCGITADTRSGELLRIRAR------------EVPEVNEIWICDAGRFGHE-WADQNRLKTPLVRKE------------  target    DDGTPQMDVTLRGSDDWIRISWDEATTIAAKTMEDVARTFNGDEGARKLLAQGYHPEMVEVMHGAGVQALKLR 3m9s.1    --------------GRLVEATWEEAFLALKEGLKEAR------------------------------------ ``` | | | | | | | | | | | | | | | | | | | | | | | | | | | | | | | | | | | | | | | | | | | | | | | | | |
|  | 2fug.2.C | NADH-quinone oxidoreductase chain 3  *Crystal structure of the hydrophilic domain of respiratory complex I from Thermus thermophilus* | 0.20 | 0.00 | 17.78 | 0.39 | 69-197 | X-ray | 3.30 | monomer | 7 x SF4, 2 x FES, 1 x FMN | HHblits | 0.31 |
| ``` target    MAQGVSRRQLLGRALALGSGAALADLLGPARFLSPAGAATAGAVVPGNPLRVMPDRTWEQIYRNQFEDDSTFVFTCAPND 2fug.2    --------------------------------------------------------------------EMEETPTTCALC  target    THNCLLRAHVKNGVVVRISPTYGYGEATDLYGNRASHRWDPRTCQKGLILSRRFYSERRVKAPMIRKGFKDWVEAGYPRN 2fug.2    PVGCGITADTRSGELLRIRAR------------EVPEVNEIWICDAGRFGHE-WADQNRLKTPLVRKE------------  target    DDGTPQMDVTLRGSDDWIRISWDEATTIAAKTMEDVARTFNGDEGARKLLAQGYHPEMVEVMHGAGVQALKLR 2fug.2    --------------GRLVEATWEEAFLALKEGLKEAR------------------------------------ ``` | | | | | | | | | | | | | | | | | | | | | | | | | | | | | | | | | | | | | | | | | | | | | | | | | |
|  | 6zjl.1.C | NADH-quinone oxidoreductase subunit 3  *Respiratory complex I from Thermus thermophilus, NAD+ dataset, major state* | 0.17 | 0.00 | 17.78 | 0.39 | 69-197 | EM | 0.00 | monomer | 7 x SF4, 1 x FMN, 2 x FES | HHblits | 0.31 |
| ``` target    MAQGVSRRQLLGRALALGSGAALADLLGPARFLSPAGAATAGAVVPGNPLRVMPDRTWEQIYRNQFEDDSTFVFTCAPND 6zjl.1    --------------------------------------------------------------------EMEETPTTCALC  target    THNCLLRAHVKNGVVVRISPTYGYGEATDLYGNRASHRWDPRTCQKGLILSRRFYSERRVKAPMIRKGFKDWVEAGYPRN 6zjl.1    PVGCGITADTRSGELLRIRAR------------EVPEVNEIWICDAGRFGHE-WADQNRLKTPLVRKE------------  target    DDGTPQMDVTLRGSDDWIRISWDEATTIAAKTMEDVARTFNGDEGARKLLAQGYHPEMVEVMHGAGVQALKLR 6zjl.1    --------------GRLVEATWEEAFLALKEGLKEAR------------------------------------ ``` | | | | | | | | | | | | | | | | | | | | | | | | | | | | | | | | | | | | | | | | | | | | | | | | | |
|  | 6q8o.1.C | NADH-quinone oxidoreductase subunit 3  *Respiratory complex I from Thermus thermophilus with bound Piericidin A* | 0.19 | 0.00 | 17.78 | 0.39 | 69-197 | X-ray | 3.61 | monomer | 7 x SF4, 1 x FMN, 2 x FES, 1 x HQH | HHblits | 0.31 |
| ``` target    MAQGVSRRQLLGRALALGSGAALADLLGPARFLSPAGAATAGAVVPGNPLRVMPDRTWEQIYRNQFEDDSTFVFTCAPND 6q8o.1    --------------------------------------------------------------------EMEETPTTCALC  target    THNCLLRAHVKNGVVVRISPTYGYGEATDLYGNRASHRWDPRTCQKGLILSRRFYSERRVKAPMIRKGFKDWVEAGYPRN 6q8o.1    PVGCGITADTRSGELLRIRAR------------EVPEVNEIWICDAGRFGHE-WADQNRLKTPLVRKE------------  target    DDGTPQMDVTLRGSDDWIRISWDEATTIAAKTMEDVARTFNGDEGARKLLAQGYHPEMVEVMHGAGVQALKLR 6q8o.1    --------------GRLVEATWEEAFLALKEGLKEAR------------------------------------ ``` | | | | | | | | | | | | | | | | | | | | | | | | | | | | | | | | | | | | | | | | | | | | | | | | | |
|  | 6zjy.1.C | NADH-quinone oxidoreductase subunit 3  *Respiratory complex I from Thermus thermophilus, NAD+ dataset, minor state* | 0.17 | 0.00 | 17.78 | 0.39 | 69-197 | EM | 0.00 | monomer | 7 x SF4, 2 x FES | HHblits | 0.31 |
| ``` target    MAQGVSRRQLLGRALALGSGAALADLLGPARFLSPAGAATAGAVVPGNPLRVMPDRTWEQIYRNQFEDDSTFVFTCAPND 6zjy.1    --------------------------------------------------------------------EMEETPTTCALC  target    THNCLLRAHVKNGVVVRISPTYGYGEATDLYGNRASHRWDPRTCQKGLILSRRFYSERRVKAPMIRKGFKDWVEAGYPRN 6zjy.1    PVGCGITADTRSGELLRIRAR------------EVPEVNEIWICDAGRFGHE-WADQNRLKTPLVRKE------------  target    DDGTPQMDVTLRGSDDWIRISWDEATTIAAKTMEDVARTFNGDEGARKLLAQGYHPEMVEVMHGAGVQALKLR 6zjy.1    --------------GRLVEATWEEAFLALKEGLKEAR------------------------------------ ``` | | | | | | | | | | | | | | | | | | | | | | | | | | | | | | | | | | | | | | | | | | | | | | | | | |
|  | 6zjn.1.C | NADH-quinone oxidoreductase subunit 3  *Respiratory complex I from Thermus thermophilus, NADH dataset, minor state* | 0.18 | 0.00 | 17.78 | 0.39 | 69-197 | EM | 0.00 | monomer | 7 x SF4, 2 x FES | HHblits | 0.31 |
| ``` target    MAQGVSRRQLLGRALALGSGAALADLLGPARFLSPAGAATAGAVVPGNPLRVMPDRTWEQIYRNQFEDDSTFVFTCAPND 6zjn.1    --------------------------------------------------------------------EMEETPTTCALC  target    THNCLLRAHVKNGVVVRISPTYGYGEATDLYGNRASHRWDPRTCQKGLILSRRFYSERRVKAPMIRKGFKDWVEAGYPRN 6zjn.1    PVGCGITADTRSGELLRIRAR------------EVPEVNEIWICDAGRFGHE-WADQNRLKTPLVRKE------------  target    DDGTPQMDVTLRGSDDWIRISWDEATTIAAKTMEDVARTFNGDEGARKLLAQGYHPEMVEVMHGAGVQALKLR 6zjn.1    --------------GRLVEATWEEAFLALKEGLKEAR------------------------------------ ``` | | | | | | | | | | | | | | | | | | | | | | | | | | | | | | | | | | | | | | | | | | | | | | | | | |
|  | 6ziy.1.C | NADH-quinone oxidoreductase subunit 3  *Respiratory complex I from Thermus thermophilus, NADH dataset, major state* | 0.17 | 0.00 | 17.78 | 0.39 | 69-197 | EM | 0.00 | monomer | 7 x SF4, 1 x FMN, 1 x NAI, 2 x FES | HHblits | 0.31 |
| ``` target    MAQGVSRRQLLGRALALGSGAALADLLGPARFLSPAGAATAGAVVPGNPLRVMPDRTWEQIYRNQFEDDSTFVFTCAPND 6ziy.1    --------------------------------------------------------------------EMEETPTTCALC  target    THNCLLRAHVKNGVVVRISPTYGYGEATDLYGNRASHRWDPRTCQKGLILSRRFYSERRVKAPMIRKGFKDWVEAGYPRN 6ziy.1    PVGCGITADTRSGELLRIRAR------------EVPEVNEIWICDAGRFGHE-WADQNRLKTPLVRKE------------  target    DDGTPQMDVTLRGSDDWIRISWDEATTIAAKTMEDVARTFNGDEGARKLLAQGYHPEMVEVMHGAGVQALKLR 6ziy.1    --------------GRLVEATWEEAFLALKEGLKEAR------------------------------------ ``` | | | | | | | | | | | | | | | | | | | | | | | | | | | | | | | | | | | | | | | | | | | | | | | | | |
|  | 6yj4.1.G | Subunit NUAM of NADH:Ubiquinone Oxidoreductase (Complex I)  *Structure of Yarrowia lipolytica complex I at 2.7 A* | 0.21 | 0.00 | 21.11 | 0.39 | 69-197 | EM | 0.00 | monomer | 18 x 3PE, 6 x SF4, 5 x LMT, 8 x PLC, 2 x FES, 1 x FMN, 6 x CDL, 1 x NDP, 1 x ZN, 2 x EHZ | HHblits | 0.31 |
| ``` target    MAQGVSRRQLLGRALALGSGAALADLLGPARFLSPAGAATAGAVVPGNPLRVMPDRTWEQIYRNQFEDDSTFVFTCAPND 6yj4.1    --------------------------------------------------------------------ELKKTESIDVMD  target    THNCLLRAHVKNGVVVRISPTYGYGEATDLYGNRASHRWDPRTCQKGLILSRRFYSERRVKAPMIRKGFKDWVEAGYPRN 6yj4.1    AVGSNIRIDSKGVEVMRVIPRV------------HEDVNEEWINDKSRFACDGLK-TQRLTTPLIRVG------------  target    DDGTPQMDVTLRGSDDWIRISWDEATTIAAKTMEDVARTFNGDEGARKLLAQGYHPEMVEVMHGAGVQALKLR 6yj4.1    --------------DKFVNATWDDALSTIAKAYQQKA------------------------------------ ``` | | | | | | | | | | | | | | | | | | | | | | | | | | | | | | | | | | | | | | | | | | | | | | | | | |
|  | 6rfs.1.A | Subunit NUAM of NADH:Ubiquinone Oxidoreductase (Complex I)  *Cryo-EM structure of a respiratory complex I mutant lacking NDUFS4* | 0.22 | 0.00 | 21.11 | 0.39 | 69-197 | EM | 4.04 | monomer | 6 x SF4, 2 x FES, 1 x FMN, 1 x NDP, 1 x ZN, 1 x ZMP | HHblits | 0.31 |
| ``` target    MAQGVSRRQLLGRALALGSGAALADLLGPARFLSPAGAATAGAVVPGNPLRVMPDRTWEQIYRNQFEDDSTFVFTCAPND 6rfs.1    --------------------------------------------------------------------ELKKTESIDVMD  target    THNCLLRAHVKNGVVVRISPTYGYGEATDLYGNRASHRWDPRTCQKGLILSRRFYSERRVKAPMIRKGFKDWVEAGYPRN 6rfs.1    AVGSNIRIDSKGVEVMRVIPRV------------HEDVNEEWINDKSRFACDGLK-TQRLTTPLIRVG------------  target    DDGTPQMDVTLRGSDDWIRISWDEATTIAAKTMEDVARTFNGDEGARKLLAQGYHPEMVEVMHGAGVQALKLR 6rfs.1    --------------DKFVNATWDDALSTIAKAYQQKA------------------------------------ ``` | | | | | | | | | | | | | | | | | | | | | | | | | | | | | | | | | | | | | | | | | | | | | | | | | |
|  | 6rfq.1.A | Subunit NUAM of NADH:Ubiquinone Oxidoreductase (Complex I)  *Cryo-EM structure of a respiratory complex I assembly intermediate with NDUFAF2* | 0.20 | 0.00 | 21.11 | 0.39 | 69-197 | EM | 3.30 | monomer | 6 x SF4, 2 x FES, 1 x FMN, 1 x NDP, 10 x 3PE, 2 x LMN, 4 x CDL, 2 x ZMP, 4 x PLC, 3 x T7X, 1 x CPL | HHblits | 0.31 |
| ``` target    MAQGVSRRQLLGRALALGSGAALADLLGPARFLSPAGAATAGAVVPGNPLRVMPDRTWEQIYRNQFEDDSTFVFTCAPND 6rfq.1    --------------------------------------------------------------------ELKKTESIDVMD  target    THNCLLRAHVKNGVVVRISPTYGYGEATDLYGNRASHRWDPRTCQKGLILSRRFYSERRVKAPMIRKGFKDWVEAGYPRN 6rfq.1    AVGSNIRIDSKGVEVMRVIPRV------------HEDVNEEWINDKSRFACDGLK-TQRLTTPLIRVG------------  target    DDGTPQMDVTLRGSDDWIRISWDEATTIAAKTMEDVARTFNGDEGARKLLAQGYHPEMVEVMHGAGVQALKLR 6rfq.1    --------------DKFVNATWDDALSTIAKAYQQKA------------------------------------ ``` | | | | | | | | | | | | | | | | | | | | | | | | | | | | | | | | | | | | | | | | | | | | | | | | | |
|  | 6gcs.1.A | 75-KDA PROTEIN (NUAM)  *Cryo-EM structure of respiratory complex I from Yarrowia lipolytica* | 0.21 | 0.00 | 21.11 | 0.39 | 69-197 | EM | 4.32 | monomer | 6 x SF4, 2 x FES, 1 x FMN, 1 x NDP, 1 x ZN, 1 x ZMP, 1 x CDL, 3 x 3PE | HHblits | 0.31 |
| ``` target    MAQGVSRRQLLGRALALGSGAALADLLGPARFLSPAGAATAGAVVPGNPLRVMPDRTWEQIYRNQFEDDSTFVFTCAPND 6gcs.1    --------------------------------------------------------------------ELKKTESIDVMD  target    THNCLLRAHVKNGVVVRISPTYGYGEATDLYGNRASHRWDPRTCQKGLILSRRFYSERRVKAPMIRKGFKDWVEAGYPRN 6gcs.1    AVGSNIRIDSKGVEVMRVIPRV------------HEDVNEEWINDKSRFACDGLK-TQRLTTPLIRVG------------  target    DDGTPQMDVTLRGSDDWIRISWDEATTIAAKTMEDVARTFNGDEGARKLLAQGYHPEMVEVMHGAGVQALKLR 6gcs.1    --------------DKFVNATWDDALSTIAKAYQQKA------------------------------------ ``` | | | | | | | | | | | | | | | | | | | | | | | | | | | | | | | | | | | | | | | | | | | | | | | | | |
|  | 7tgh.58.A | NADH-ubiquinone oxidoreductase 75 kDa subunit  *Cryo-EM structure of respiratory super-complex CI+III2 from Tetrahymena thermophila* | 0.20 |  | 16.48 | 0.39 | 69-197 | EM | 0.00 | monomer |  | HHblits | 0.29 |
| ``` target    MAQGVSRRQLLGRALALGSGAALADLLGPARFLSPAGAATAGAVVPGNPLRVMPDRTWEQIYRNQFEDDSTFVFTCAPND 7tgh.58   --------------------------------------------------------------------ELKSFYTSDVFD  target    THNCLLRAHVKNGVVVRISPTYGYGEATDLYGNRASHRWDPRTCQKGLILSRRFYSERRVKAPMIRKGFKDWVEAGYPRN 7tgh.58   TLGSAIQVDTRGPEIMRVLPR------------IHEEINEEWISDKTRHAFDGLK-RQRINSPMKRSK------------  target    DDGTPQMDVTLRGSDDWIRISWDEATTIAAKTMEDVARTFNGDEGARKLLAQGYHPEMVEVMHGAGVQALKLR 7tgh.58   -------------DGNYEDIFWEEAIQTISKKCLNTP------------------------------------ ``` | | | | | | | | | | | | | | | | | | | | | | | | | | | | | | | | | | | | | | | | | | | | | | | | | |
|  | 7ar7.1.G | NADH dehydrogenase [ubiquinone] iron-sulfur protein 1, mitochondrial  *Cryo-EM structure of Arabidopsis thaliana complex-I (open conformation)* | 0.21 | 0.00 | 18.89 | 0.39 | 69-196 | EM | 0.00 | monomer | 6 x SF4, 2 x FES, 1 x FMN, 1 x UQ9, 3 x PTY, 2 x PC7, 1 x LMN, 1 x NDP, 2 x ZN, 2 x 8Q1, 1 x PGT, 1 x PSF, 1 x T7X | HHblits | 0.29 |
| ``` target    MAQGVSRRQLLGRALALGSGAALADLLGPARFLSPAGAATAGAVVPGNPLRVMPDRTWEQIYRNQFEDDSTFVFTCAPND 7ar7.1    --------------------------------------------------------------------ELKATETIDVSD  target    THNCLLRAHVKNGVVVRISPTYGYGEATDLYGNRASHRWDPRTCQKGLILSRRFYSERRVKAPMIRKGFKDWVEAGYPRN 7ar7.1    AVGSNIRVDSRGPEVMRIIPRL------------NEDINEEWISDKTRFCYDGLK-RQRLSDPMIRDS------------  target    DDGTPQMDVTLRGSDDWIRISWDEATTIAAKTMEDVARTFNGDEGARKLLAQGYHPEMVEVMHGAGVQALKLR 7ar7.1    -------------DGRFKAVSWRDALAVVGDIIHQV------------------------------------- ``` | | | | | | | | | | | | | | | | | | | | | | | | | | | | | | | | | | | | | | | | | | | | | | | | | |
|  | 6lod.1.B | Fe-S-cluster-containing hydrogenase components 1-like protein  *Cryo-EM structure of the air-oxidized photosynthetic alternative complex III from Roseiflexus castenholzii* | 0.18 |  | 17.20 | 0.40 | 69-203 | EM | 0.00 | hetero-1-1-1-1-1-1-… | 6 x HEC, 2 x EL6, 3 x SF4, 1 x F3S | HHblits | 0.27 |
| ``` target    MAQGVSRRQLLGRALALGSGAALADLLGPARFLSPAGAATAGAVVPGNPLRVMPDRTWEQIYRNQFEDDSTFVFTCAPND 6lod.1    --------------------------------------------------------------------RPLFFATAVTFA  target    THNCLLRAHVKNGVVVRISPTYGYGEATDLYGNRASHRWDPRTCQKGLILSRRFYSERRVKAPMIRKGFKDWVEAGYPRN 6lod.1    GFGVGLLVESHEGRPTKIE------------GNPDHPASLGSTDLITQAMILTMYDPDRSQAPTNA--------------  target    DDGTPQMDVTLRGSDDWIRISWDEATTIAAKTMEDVARTFNGDEGARKLLAQGYHPEMVEVMHGAGVQALKLR 6lod.1    ----------------GQETTWDAFVAAATAAMQAQTAKQGAG------------------------------ ``` | | | | | | | | | | | | | | | | | | | | | | | | | | | | | | | | | | | | | | | | | | | | | | | | | |
|  | 6zr2.1.G | NADH-ubiquinone oxidoreductase 75 kDa subunit, mitochondrial  *Cryo-EM structure of respiratory complex I in the active state from Mus musculus at 3.1 A* | 0.20 | 0.00 | 16.48 | 0.39 | 68-196 | EM | 3.10 | monomer | 6 x SF4, 4 x PC1, 2 x FES, 1 x FMN, 9 x 3PE, 7 x CDL, 1 x ATP, 1 x NDP, 1 x ZN, 2 x EHZ | HHblits | 0.28 |
| ``` target    MAQGVSRRQLLGRALALGSGAALADLLGPARFLSPAGAATAGAVVPGNPLRVMPDRTWEQIYRNQFEDDSTFVFTCAPND 6zr2.1    -------------------------------------------------------------------WETRKTESIDVMD  target    THNCLLRAHVKNGVVVRISPTYGYGEATDLYGNRASHRWDPRTCQKGLILSRRFYSERRVKAPMIRKGFKDWVEAGYPRN 6zr2.1    AVGSNIVVSTRTGEVMRILPR------------MHEDINEEWISDKTRFAYDGLK-RQRLTEPMVRNE------------  target    DDGTPQMDVTLRGSDDWIRISWDEATTIAAKTMEDVARTFNGDEGARKLLAQGYHPEMVEVMHGAGVQALKLR 6zr2.1    -------------KGLLTYTSWEDALSRVAGMLQNF------------------------------------- ``` | | | | | | | | | | | | | | | | | | | | | | | | | | | | | | | | | | | | | | | | | | | | | | | | | |
|  | 6g72.1.G | NADH-ubiquinone oxidoreductase 75 kDa subunit, mitochondrial  *Mouse mitochondrial complex I in the deactive state* | 0.20 | 0.00 | 16.48 | 0.39 | 68-196 | EM | 0.00 | monomer | 6 x SF4, 2 x FES, 1 x FMN, 1 x ADP, 1 x NDP, 1 x ZN, 2 x EHZ | HHblits | 0.28 |
| ``` target    MAQGVSRRQLLGRALALGSGAALADLLGPARFLSPAGAATAGAVVPGNPLRVMPDRTWEQIYRNQFEDDSTFVFTCAPND 6g72.1    -------------------------------------------------------------------WETRKTESIDVMD  target    THNCLLRAHVKNGVVVRISPTYGYGEATDLYGNRASHRWDPRTCQKGLILSRRFYSERRVKAPMIRKGFKDWVEAGYPRN 6g72.1    AVGSNIVVSTRTGEVMRILPR------------MHEDINEEWISDKTRFAYDGLK-RQRLTEPMVRNE------------  target    DDGTPQMDVTLRGSDDWIRISWDEATTIAAKTMEDVARTFNGDEGARKLLAQGYHPEMVEVMHGAGVQALKLR 6g72.1    -------------KGLLTYTSWEDALSRVAGMLQNF------------------------------------- ``` | | | | | | | | | | | | | | | | | | | | | | | | | | | | | | | | | | | | | | | | | | | | | | | | | |
|  | 7ak6.1.G | NADH-ubiquinone oxidoreductase 75 kDa subunit, mitochondrial  *Cryo-EM structure of ND6-P25L mutant respiratory complex I from Mus musculus at 3.8 A* | 0.19 | 0.00 | 16.48 | 0.39 | 68-196 | EM | 0.00 | monomer | 6 x SF4, 1 x PC1, 2 x FES, 1 x FMN, 4 x 3PE, 2 x CDL, 1 x ATP, 1 x NDP, 1 x ZN, 2 x EHZ | HHblits | 0.28 |
| ``` target    MAQGVSRRQLLGRALALGSGAALADLLGPARFLSPAGAATAGAVVPGNPLRVMPDRTWEQIYRNQFEDDSTFVFTCAPND 7ak6.1    -------------------------------------------------------------------WETRKTESIDVMD  target    THNCLLRAHVKNGVVVRISPTYGYGEATDLYGNRASHRWDPRTCQKGLILSRRFYSERRVKAPMIRKGFKDWVEAGYPRN 7ak6.1    AVGSNIVVSTRTGEVMRILPR------------MHEDINEEWISDKTRFAYDGLK-RQRLTEPMVRNE------------  target    DDGTPQMDVTLRGSDDWIRISWDEATTIAAKTMEDVARTFNGDEGARKLLAQGYHPEMVEVMHGAGVQALKLR 7ak6.1    -------------KGLLTYTSWEDALSRVAGMLQNF------------------------------------- ``` | | | | | | | | | | | | | | | | | | | | | | | | | | | | | | | | | | | | | | | | | | | | | | | | | |
|  | 8b9z.1.G | NADH-ubiquinone oxidoreductase 75 kDa subunit, mitochondrial  *Drosophila melanogaster complex I in the Active state (Dm1)* | 0.21 |  | 17.78 | 0.39 | 69-196 | EM | 3.28 | hetero-1-1-1-1-1-1-… | 3 x PC1, 16 x 3PE, 6 x SF4, 4 x CDL, 2 x FES, 1 x FMN, 1 x UQ9, 1 x DGT, 1 x NDP, 1 x ZN, 2 x EHZ | HHblits | 0.29 |
| ``` target    MAQGVSRRQLLGRALALGSGAALADLLGPARFLSPAGAATAGAVVPGNPLRVMPDRTWEQIYRNQFEDDSTFVFTCAPND 8b9z.1    --------------------------------------------------------------------EIRKVSSIDVLD  target    THNCLLRAHVKNGVVVRISPTYGYGEATDLYGNRASHRWDPRTCQKGLILSRRFYSERRVKAPMIRKGFKDWVEAGYPRN 8b9z.1    AVGSNIVVSTRTNEVLRILPRE------------NEDVNEEWLADKSRFACDGLK-RQRLVAPMVRMP------------  target    DDGTPQMDVTLRGSDDWIRISWDEATTIAAKTMEDVARTFNGDEGARKLLAQGYHPEMVEVMHGAGVQALKLR 8b9z.1    -------------NGELQAVEWEGALIAVAKAIKAA------------------------------------- ``` | | | | | | | | | | | | | | | | | | | | | | | | | | | | | | | | | | | | | | | | | | | | | | | | | |
|  | 8ba0.1.G | NADH-ubiquinone oxidoreductase 75 kDa subunit, mitochondrial  *Drosophila melanogaster complex I in the Twisted state (Dm2)* | 0.20 |  | 17.78 | 0.39 | 69-196 | EM | 3.68 | hetero-1-1-1-1-1-1-… | 6 x SF4, 6 x 3PE, 2 x FES, 1 x FMN, 2 x CDL, 1 x DGT, 1 x NDP, 1 x ZN, 2 x EHZ | HHblits | 0.29 |
| ``` target    MAQGVSRRQLLGRALALGSGAALADLLGPARFLSPAGAATAGAVVPGNPLRVMPDRTWEQIYRNQFEDDSTFVFTCAPND 8ba0.1    --------------------------------------------------------------------EIRKVSSIDVLD  target    THNCLLRAHVKNGVVVRISPTYGYGEATDLYGNRASHRWDPRTCQKGLILSRRFYSERRVKAPMIRKGFKDWVEAGYPRN 8ba0.1    AVGSNIVVSTRTNEVLRILPRE------------NEDVNEEWLADKSRFACDGLK-RQRLVAPMVRMP------------  target    DDGTPQMDVTLRGSDDWIRISWDEATTIAAKTMEDVARTFNGDEGARKLLAQGYHPEMVEVMHGAGVQALKLR 8ba0.1    -------------NGELQAVEWEGALIAVAKAIKAA------------------------------------- ``` | | | | | | | | | | | | | | | | | | | | | | | | | | | | | | | | | | | | | | | | | | | | | | | | | |
|  | 5xtb.1.L | NADH-ubiquinone oxidoreductase 75 kDa subunit, mitochondrial  *Cryo-EM structure of human respiratory complex I matrix arm* | 0.20 |  | 16.48 | 0.39 | 68-196 | EM | 0.00 | hetero-1-1-1-1-1-1-… | 6 x SF4, 1 x FMN, 1 x 8Q1, 1 x NDP, 2 x FES | HHblits | 0.28 |
| ``` target    MAQGVSRRQLLGRALALGSGAALADLLGPARFLSPAGAATAGAVVPGNPLRVMPDRTWEQIYRNQFEDDSTFVFTCAPND 5xtb.1    -------------------------------------------------------------------WETRKTESIDVMD  target    THNCLLRAHVKNGVVVRISPTYGYGEATDLYGNRASHRWDPRTCQKGLILSRRFYSERRVKAPMIRKGFKDWVEAGYPRN 5xtb.1    AVGSNIVVSTRTGEVMRILPRM------------HEDINEEWISDKTRFAYDGLK-RQRLTEPMVRNE------------  target    DDGTPQMDVTLRGSDDWIRISWDEATTIAAKTMEDVARTFNGDEGARKLLAQGYHPEMVEVMHGAGVQALKLR 5xtb.1    -------------KGLLTYTSWEDALSRVAGMLQSF------------------------------------- ``` | | | | | | | | | | | | | | | | | | | | | | | | | | | | | | | | | | | | | | | | | | | | | | | | | |
|  | 6qcf.1.C | NADH:ubiquinone oxidoreductase core subunit S1  *Ovine respiratory complex I FRC open class 6* | 0.20 |  | 15.38 | 0.39 | 68-196 | EM | 0.00 | hetero-1-1-1-1-1-1-… | 6 x SF4, 1 x FMN, 2 x FES, 1 x ZN, 1 x NDP, 2 x ZMP | HHblits | 0.28 |
| ``` target    MAQGVSRRQLLGRALALGSGAALADLLGPARFLSPAGAATAGAVVPGNPLRVMPDRTWEQIYRNQFEDDSTFVFTCAPND 6qcf.1    -------------------------------------------------------------------WETRKTESIDVMD  target    THNCLLRAHVKNGVVVRISPTYGYGEATDLYGNRASHRWDPRTCQKGLILSRRFYSERRVKAPMIRKGFKDWVEAGYPRN 6qcf.1    AVGSNIVVSTRTGEVMRILPRM------------HEDINEEWISDKTRFAYDGLK-RQRLTEPMVRNE------------  target    DDGTPQMDVTLRGSDDWIRISWDEATTIAAKTMEDVARTFNGDEGARKLLAQGYHPEMVEVMHGAGVQALKLR 6qcf.1    -------------KGLLTHTTWEDALSRVAGMLQSC------------------------------------- ``` | | | | | | | | | | | | | | | | | | | | | | | | | | | | | | | | | | | | | | | | | | | | | | | | | |
|  | 6qc5.1.C | NADH:ubiquinone oxidoreductase core subunit S1  *Ovine respiratory complex I FRC closed class 1* | 0.19 |  | 15.38 | 0.39 | 68-196 | EM | 0.00 | hetero-1-1-1-1-1-1-… | 6 x SF4, 1 x FMN, 2 x FES, 2 x 3PE, 1 x ZN, 1 x NDP, 2 x ZMP, 1 x PC1 | HHblits | 0.28 |
| ``` target    MAQGVSRRQLLGRALALGSGAALADLLGPARFLSPAGAATAGAVVPGNPLRVMPDRTWEQIYRNQFEDDSTFVFTCAPND 6qc5.1    -------------------------------------------------------------------WETRKTESIDVMD  target    THNCLLRAHVKNGVVVRISPTYGYGEATDLYGNRASHRWDPRTCQKGLILSRRFYSERRVKAPMIRKGFKDWVEAGYPRN 6qc5.1    AVGSNIVVSTRTGEVMRILPRM------------HEDINEEWISDKTRFAYDGLK-RQRLTEPMVRNE------------  target    DDGTPQMDVTLRGSDDWIRISWDEATTIAAKTMEDVARTFNGDEGARKLLAQGYHPEMVEVMHGAGVQALKLR 6qc5.1    -------------KGLLTHTTWEDALSRVAGMLQSC------------------------------------- ``` | | | | | | | | | | | | | | | | | | | | | | | | | | | | | | | | | | | | | | | | | | | | | | | | | |
|  | 7dgr.10.A | NADH-ubiquinone oxidoreductase 75 kDa subunit, mitochondrial  *Activity optimized supercomplex state2* | 0.17 |  | 15.38 | 0.39 | 68-196 | EM | 0.00 | monomer |  | HHblits | 0.28 |
| ``` target    MAQGVSRRQLLGRALALGSGAALADLLGPARFLSPAGAATAGAVVPGNPLRVMPDRTWEQIYRNQFEDDSTFVFTCAPND 7dgr.10   -------------------------------------------------------------------WETRKTESIDVMD  target    THNCLLRAHVKNGVVVRISPTYGYGEATDLYGNRASHRWDPRTCQKGLILSRRFYSERRVKAPMIRKGFKDWVEAGYPRN 7dgr.10   AVGSNIVVSTRTGEVMRILPRM------------HEDINEEWISDKTRFAYDGLK-RQRLTEPMVRNE------------  target    DDGTPQMDVTLRGSDDWIRISWDEATTIAAKTMEDVARTFNGDEGARKLLAQGYHPEMVEVMHGAGVQALKLR 7dgr.10   -------------KGLLTHTTWEDALSRVAGMLQSF------------------------------------- ``` | | | | | | | | | | | | | | | | | | | | | | | | | | | | | | | | | | | | | | | | | | | | | | | | | |
|  | 5o31.1.8 | NADH-ubiquinone oxidoreductase 75 kDa subunit, mitochondrial  *Mitochondrial complex I in the deactive state* | 0.19 |  | 15.38 | 0.39 | 68-196 | EM | 4.13 | hetero-1-1-1-1-1-1-… | 6 x SF4, 2 x FES, 1 x FMN, 1 x NAP, 1 x ZN | HHblits | 0.28 |
| ``` target    MAQGVSRRQLLGRALALGSGAALADLLGPARFLSPAGAATAGAVVPGNPLRVMPDRTWEQIYRNQFEDDSTFVFTCAPND 5o31.1    -------------------------------------------------------------------WETRKTESIDVMD  target    THNCLLRAHVKNGVVVRISPTYGYGEATDLYGNRASHRWDPRTCQKGLILSRRFYSERRVKAPMIRKGFKDWVEAGYPRN 5o31.1    AVGSNIVVSTRTGEVMRILPRM------------HEDINEEWISDKTRFAYDGLK-RQRLTEPMVRNE------------  target    DDGTPQMDVTLRGSDDWIRISWDEATTIAAKTMEDVARTFNGDEGARKLLAQGYHPEMVEVMHGAGVQALKLR 5o31.1    -------------KGLLTHTTWEDALSRVAGMLQSF------------------------------------- ``` | | | | | | | | | | | | | | | | | | | | | | | | | | | | | | | | | | | | | | | | | | | | | | | | | |
|  | 6zk9.1.C | NADH:ubiquinone oxidoreductase core subunit S1  *Peripheral domain of open complex I during turnover* | 0.21 |  | 15.38 | 0.39 | 68-196 | EM | 0.00 | hetero-1-1-1-1-1-1-… | 6 x SF4, 1 x FMN, 1 x NAI, 2 x FES, 1 x K, 2 x PC1, 2 x 3PE, 1 x ZN, 1 x NDP, 1 x ZMP, 1 x CDL | HHblits | 0.28 |
| ``` target    MAQGVSRRQLLGRALALGSGAALADLLGPARFLSPAGAATAGAVVPGNPLRVMPDRTWEQIYRNQFEDDSTFVFTCAPND 6zk9.1    -------------------------------------------------------------------WETRKTESIDVMD  target    THNCLLRAHVKNGVVVRISPTYGYGEATDLYGNRASHRWDPRTCQKGLILSRRFYSERRVKAPMIRKGFKDWVEAGYPRN 6zk9.1    AVGSNIVVSTRTGEVMRILPRM------------HEDINEEWISDKTRFAYDGLK-RQRLTEPMVRNE------------  target    DDGTPQMDVTLRGSDDWIRISWDEATTIAAKTMEDVARTFNGDEGARKLLAQGYHPEMVEVMHGAGVQALKLR 6zk9.1    -------------KGLLTHTTWEDALSRVAGMLQSC------------------------------------- ``` | | | | | | | | | | | | | | | | | | | | | | | | | | | | | | | | | | | | | | | | | | | | | | | | | |
|  | 7zd6.1.4 | NADH-ubiquinone oxidoreductase 75 kDa subunit, mitochondrial  *Complex I from Ovis aries, at pH7.4, Open state* | 0.21 |  | 15.38 | 0.39 | 68-196 | EM | 0.00 | hetero-1-1-1-1-1-1-… | 6 x PC1, 14 x 3PE, 1 x DCQ, 2 x ZMP, 1 x AMP, 1 x MYR, 6 x SF4, 1 x FMN, 1 x NAI, 2 x FES, 1 x K, 1 x ZN, 1 x NDP | HHblits | 0.28 |
| ``` target    MAQGVSRRQLLGRALALGSGAALADLLGPARFLSPAGAATAGAVVPGNPLRVMPDRTWEQIYRNQFEDDSTFVFTCAPND 7zd6.1    -------------------------------------------------------------------WETRKTESIDVMD  target    THNCLLRAHVKNGVVVRISPTYGYGEATDLYGNRASHRWDPRTCQKGLILSRRFYSERRVKAPMIRKGFKDWVEAGYPRN 7zd6.1    AVGSNIVVSTRTGEVMRILPRM------------HEDINEEWISDKTRFAYDGLK-RQRLTEPMVRNE------------  target    DDGTPQMDVTLRGSDDWIRISWDEATTIAAKTMEDVARTFNGDEGARKLLAQGYHPEMVEVMHGAGVQALKLR 7zd6.1    -------------KGLLTHTTWEDALSRVAGMLQSC------------------------------------- ``` | | | | | | | | | | | | | | | | | | | | | | | | | | | | | | | | | | | | | | | | | | | | | | | | | |
|  | 7ak5.1.G | NADH-ubiquinone oxidoreductase 75 kDa subunit, mitochondrial  *Cryo-EM structure of respiratory complex I in the deactive state from Mus musculus at 3.2 A* | 0.20 |  | 16.67 | 0.39 | 69-196 | EM | 0.00 | hetero-1-1-1-1-1-1-… | 6 x SF4, 2 x PC1, 2 x FES, 1 x FMN, 8 x 3PE, 4 x CDL, 1 x ATP, 1 x NDP, 1 x ZN, 2 x EHZ | HHblits | 0.29 |
| ``` target    MAQGVSRRQLLGRALALGSGAALADLLGPARFLSPAGAATAGAVVPGNPLRVMPDRTWEQIYRNQFEDDSTFVFTCAPND 7ak5.1    --------------------------------------------------------------------ETRKTESIDVMD  target    THNCLLRAHVKNGVVVRISPTYGYGEATDLYGNRASHRWDPRTCQKGLILSRRFYSERRVKAPMIRKGFKDWVEAGYPRN 7ak5.1    AVGSNIVVSTRTGEVMRILPRM------------HEDINEEWISDKTRFAYDGLK-RQRLTEPMVRNE------------  target    DDGTPQMDVTLRGSDDWIRISWDEATTIAAKTMEDVARTFNGDEGARKLLAQGYHPEMVEVMHGAGVQALKLR 7ak5.1    -------------KGLLTYTSWEDALSRVAGMLQNF------------------------------------- ``` | | | | | | | | | | | | | | | | | | | | | | | | | | | | | | | | | | | | | | | | | | | | | | | | | |
|  | 7qsd.1.G | NADH-ubiquinone oxidoreductase 75 kDa subunit, mitochondrial  *Bovine complex I in the active state at 3.1 A* | 0.20 |  | 15.56 | 0.39 | 69-196 | EM | 0.00 | hetero-1-1-1-1-1-1-… | 5 x PC1, 13 x 3PE, 6 x SF4, 2 x FES, 1 x FMN, 4 x CDL, 3 x LMT, 1 x GTP, 1 x MG, 1 x NDP, 1 x ZN, 2 x EHZ | HHblits | 0.28 |
| ``` target    MAQGVSRRQLLGRALALGSGAALADLLGPARFLSPAGAATAGAVVPGNPLRVMPDRTWEQIYRNQFEDDSTFVFTCAPND 7qsd.1    --------------------------------------------------------------------ETRKTESIDVMD  target    THNCLLRAHVKNGVVVRISPTYGYGEATDLYGNRASHRWDPRTCQKGLILSRRFYSERRVKAPMIRKGFKDWVEAGYPRN 7qsd.1    AVGSNIVVSTRTGEVMRILPRM------------HEDINEEWISDKTRFAYDGLK-RQRLTEPMVRNE------------  target    DDGTPQMDVTLRGSDDWIRISWDEATTIAAKTMEDVARTFNGDEGARKLLAQGYHPEMVEVMHGAGVQALKLR 7qsd.1    -------------KGLLTHTTWEDALSRVAGMLQSF------------------------------------- ``` | | | | | | | | | | | | | | | | | | | | | | | | | | | | | | | | | | | | | | | | | | | | | | | | | |
|  | 7vxu.1.L | NADH-ubiquinone oxidoreductase 75 kDa subunit, mitochondrial  *Matrix arm of deactive state CI from Q10 dataset* | 0.20 |  | 16.67 | 0.39 | 69-196 | EM | 0.00 | hetero-1-1-1-1-1-1-… | 6 x SF4, 1 x FMN, 1 x PEE, 1 x PLX, 1 x 8Q1, 1 x NDP, 2 x FES, 1 x MG, 1 x CDL, 1 x ZN | HHblits | 0.28 |
| ``` target    MAQGVSRRQLLGRALALGSGAALADLLGPARFLSPAGAATAGAVVPGNPLRVMPDRTWEQIYRNQFEDDSTFVFTCAPND 7vxu.1    --------------------------------------------------------------------ETRKTESIDVMD  target    THNCLLRAHVKNGVVVRISPTYGYGEATDLYGNRASHRWDPRTCQKGLILSRRFYSERRVKAPMIRKGFKDWVEAGYPRN 7vxu.1    AVGSNIVVSTRTGEVMRILPRM------------HEDINEEWISDKTRFAYDGLK-RQRLTQPMIRNE------------  target    DDGTPQMDVTLRGSDDWIRISWDEATTIAAKTMEDVARTFNGDEGARKLLAQGYHPEMVEVMHGAGVQALKLR 7vxu.1    -------------KGLLTYTTWEDALSRVAGMLQSF------------------------------------- ``` | | | | | | | | | | | | | | | | | | | | | | | | | | | | | | | | | | | | | | | | | | | | | | | | | |
|  | 7v2c.1.L | NADH-ubiquinone oxidoreductase 75 kDa subunit, mitochondrial  *Active state complex I from Q10 dataset* | 0.20 |  | 16.67 | 0.39 | 69-196 | EM | 0.00 | hetero-1-1-1-1-1-2-… | 6 x SF4, 1 x FMN, 10 x PEE, 8 x PLX, 2 x 8Q1, 1 x NDP, 2 x UQ, 11 x CDL, 2 x FES, 1 x MG, 1 x ZN, 1 x ADP | HHblits | 0.28 |
| ``` target    MAQGVSRRQLLGRALALGSGAALADLLGPARFLSPAGAATAGAVVPGNPLRVMPDRTWEQIYRNQFEDDSTFVFTCAPND 7v2c.1    --------------------------------------------------------------------ETRKTESIDVMD  target    THNCLLRAHVKNGVVVRISPTYGYGEATDLYGNRASHRWDPRTCQKGLILSRRFYSERRVKAPMIRKGFKDWVEAGYPRN 7v2c.1    AVGSNIVVSTRTGEVMRILPRM------------HEDINEEWISDKTRFAYDGLK-RQRLTQPMIRNE------------  target    DDGTPQMDVTLRGSDDWIRISWDEATTIAAKTMEDVARTFNGDEGARKLLAQGYHPEMVEVMHGAGVQALKLR 7v2c.1    -------------KGLLTYTTWEDALSRVAGMLQSF------------------------------------- ``` | | | | | | | | | | | | | | | | | | | | | | | | | | | | | | | | | | | | | | | | | | | | | | | | | |
|  | 6x89.1.H | NADH dehydrogenase [ubiquinone] iron-sulfur protein 1, mitochondrial  *Vigna radiata mitochondrial complex I\** | 0.20 |  | 19.10 | 0.38 | 70-196 | EM | 0.00 | hetero-1-1-1-1-1-1-… | 1 x NAP, 6 x PC1, 6 x SF4, 2 x FES, 2 x ZN, 1 x FMN | HHblits | 0.29 |
| ``` target    MAQGVSRRQLLGRALALGSGAALADLLGPARFLSPAGAATAGAVVPGNPLRVMPDRTWEQIYRNQFEDDSTFVFTCAPND 6x89.1    ---------------------------------------------------------------------LKGTETIDVTD  target    THNCLLRAHVKNGVVVRISPTYGYGEATDLYGNRASHRWDPRTCQKGLILSRRFYSERRVKAPMIRKGFKDWVEAGYPRN 6x89.1    AVGSNIRIDSRGPEVMRIVPRL------------NEDINEEWISDKTRFCYDGLK-RQRLNDPMIRGP------------  target    DDGTPQMDVTLRGSDDWIRISWDEATTIAAKTMEDVARTFNGDEGARKLLAQGYHPEMVEVMHGAGVQALKLR 6x89.1    -------------DGRFKAVNWRDALSVIADIAHQV------------------------------------- ``` | | | | | | | | | | | | | | | | | | | | | | | | | | | | | | | | | | | | | | | | | | | | | | | | | |
|  | 8e73.55.A | NDUS1  *Vigna radiata supercomplex I+III2 (full bridge)* | 0.21 |  | 19.10 | 0.38 | 70-196 | EM | 0.00 | monomer |  | HHblits | 0.29 |
| ``` target    MAQGVSRRQLLGRALALGSGAALADLLGPARFLSPAGAATAGAVVPGNPLRVMPDRTWEQIYRNQFEDDSTFVFTCAPND 8e73.55   ---------------------------------------------------------------------LKGTETIDVTD  target    THNCLLRAHVKNGVVVRISPTYGYGEATDLYGNRASHRWDPRTCQKGLILSRRFYSERRVKAPMIRKGFKDWVEAGYPRN 8e73.55   AVGSNIRIDSRGPEVMRIVPRL------------NEDINEEWISDKTRFCYDGLK-RQRLNDPMIRGP------------  target    DDGTPQMDVTLRGSDDWIRISWDEATTIAAKTMEDVARTFNGDEGARKLLAQGYHPEMVEVMHGAGVQALKLR 8e73.55   -------------DGRFKAVNWRDALSVIADIAHQV------------------------------------- ``` | | | | | | | | | | | | | | | | | | | | | | | | | | | | | | | | | | | | | | | | | | | | | | | | | |
|  | 7aqr.1.F | NADH dehydrogenase [ubiquinone] iron-sulfur protein 1, mitochondrial  *Cryo-EM structure of Arabidopsis thaliana Complex-I (peripheral arm)* | 0.19 |  | 19.32 | 0.38 | 71-196 | EM | 0.00 | hetero-1-1-1-1-1-1-… | 6 x SF4, 2 x FES, 1 x FMN, 1 x NDP, 1 x ZN, 1 x 8Q1 | HHblits | 0.29 |
| ``` target    MAQGVSRRQLLGRALALGSGAALADLLGPARFLSPAGAATAGAVVPGNPLRVMPDRTWEQIYRNQFEDDSTFVFTCAPND 7aqr.1    ----------------------------------------------------------------------KATETIDVSD  target    THNCLLRAHVKNGVVVRISPTYGYGEATDLYGNRASHRWDPRTCQKGLILSRRFYSERRVKAPMIRKGFKDWVEAGYPRN 7aqr.1    AVGSNIRVDSRGPEVMRIIPRL------------NEDINEEWISDKTRFCYDGLK-RQRLSDPMIRDS------------  target    DDGTPQMDVTLRGSDDWIRISWDEATTIAAKTMEDVARTFNGDEGARKLLAQGYHPEMVEVMHGAGVQALKLR 7aqr.1    -------------DGRFKAVSWRDALAVVGDIIHQV------------------------------------- ``` | | | | | | | | | | | | | | | | | | | | | | | | | | | | | | | | | | | | | | | | | | | | | | | | | |
|  | 7a23.1.O | 75kDa  *Plant mitochondrial respiratory complex I* | 0.20 |  | 19.32 | 0.38 | 71-196 | EM | 0.00 | hetero-1-1-1-1-1-1-… | 6 x SF4, 1 x FMN, 2 x T7X, 3 x CDL, 1 x U10, 1 x PEV, 2 x FES, 1 x NDP, 2 x ZN | HHblits | 0.29 |
| ``` target    MAQGVSRRQLLGRALALGSGAALADLLGPARFLSPAGAATAGAVVPGNPLRVMPDRTWEQIYRNQFEDDSTFVFTCAPND 7a23.1    ----------------------------------------------------------------------KATETIDVSD  target    THNCLLRAHVKNGVVVRISPTYGYGEATDLYGNRASHRWDPRTCQKGLILSRRFYSERRVKAPMIRKGFKDWVEAGYPRN 7a23.1    AVGSNIRVDSRGPEVMRIIPRL------------NEDINEEWISDKTRFCYDGLK-RQRLSDPMIRDS------------  target    DDGTPQMDVTLRGSDDWIRISWDEATTIAAKTMEDVARTFNGDEGARKLLAQGYHPEMVEVMHGAGVQALKLR 7a23.1    -------------DGRFKAVSWRDALAVVGDIIHQV------------------------------------- ``` | | | | | | | | | | | | | | | | | | | | | | | | | | | | | | | | | | | | | | | | | | | | | | | | | |
|  | 7ar8.1.G | NADH dehydrogenase [ubiquinone] iron-sulfur protein 1, mitochondrial  *Cryo-EM structure of Arabidopsis thaliana complex-I (closed conformation)* | 0.20 |  | 19.32 | 0.38 | 71-196 | EM | 0.00 | hetero-1-1-1-1-1-1-… | 6 x SF4, 2 x FES, 1 x FMN, 1 x UQ9, 3 x PTY, 2 x PC7, 1 x PGT, 1 x FE, 1 x NDP, 2 x ZN, 2 x 8Q1, 1 x LMN, 1 x PSF, 1 x T7X | HHblits | 0.29 |
| ``` target    MAQGVSRRQLLGRALALGSGAALADLLGPARFLSPAGAATAGAVVPGNPLRVMPDRTWEQIYRNQFEDDSTFVFTCAPND 7ar8.1    ----------------------------------------------------------------------KATETIDVSD  target    THNCLLRAHVKNGVVVRISPTYGYGEATDLYGNRASHRWDPRTCQKGLILSRRFYSERRVKAPMIRKGFKDWVEAGYPRN 7ar8.1    AVGSNIRVDSRGPEVMRIIPRL------------NEDINEEWISDKTRFCYDGLK-RQRLSDPMIRDS------------  target    DDGTPQMDVTLRGSDDWIRISWDEATTIAAKTMEDVARTFNGDEGARKLLAQGYHPEMVEVMHGAGVQALKLR 7ar8.1    -------------DGRFKAVSWRDALAVVGDIIHQV------------------------------------- ``` | | | | | | | | | | | | | | | | | | | | | | | | | | | | | | | | | | | | | | | | | | | | | | | | | |
|  | 5gpn.24.A | NADH-ubiquinone oxidoreductase 75 kDa subunit  *Architecture of mammalian respirasome* | 0.19 |  | 16.85 | 0.38 | 70-196 | EM | 0.00 | monomer |  | HHblits | 0.28 |
| ``` target    MAQGVSRRQLLGRALALGSGAALADLLGPARFLSPAGAATAGAVVPGNPLRVMPDRTWEQIYRNQFEDDSTFVFTCAPND 5gpn.24   ---------------------------------------------------------------------TRKTESIDVMD  target    THNCLLRAHVKNGVVVRISPTYGYGEATDLYGNRASHRWDPRTCQKGLILSRRFYSERRVKAPMIRKGFKDWVEAGYPRN 5gpn.24   AVGSNIVVSTRTGEVMRILPRM------------HEDINEEWISDKTRFAYDGLK-RQRLTQPMIRNE------------  target    DDGTPQMDVTLRGSDDWIRISWDEATTIAAKTMEDVARTFNGDEGARKLLAQGYHPEMVEVMHGAGVQALKLR 5gpn.24   -------------KGLLTYTTWEDALSRVAGMLQSF------------------------------------- ``` | | | | | | | | | | | | | | | | | | | | | | | | | | | | | | | | | | | | | | | | | | | | | | | | | |
|  | 7arc.1.F | 75 kDa  *Cryo-EM structure of Polytomella Complex-I (peripheral arm)* | 0.18 |  | 13.64 | 0.38 | 71-197 | EM | 0.00 | hetero-1-1-1-1-1-1-… | 6 x SF4, 2 x FES, 1 x FMN, 1 x NDP, 1 x ZN, 1 x 8Q1 | HHblits | 0.28 |
| ``` target    MAQGVSRRQLLGRALALGSGAALADLLGPARFLSPAGAATAGAVVPGNPLRVMPDRTWEQIYRNQFEDDSTFVFTCAPND 7arc.1    ----------------------------------------------------------------------KGTETIDVSD  target    THNCLLRAHVKNGVVVRISPTYGYGEATDLYGNRASHRWDPRTCQKGLILSRRFYSERRVKAPMIRKGFKDWVEAGYPRN 7arc.1    ALGSNIKVDCRGTEVMRITPRL------------NDAINEEWLSDKGRFQYDGLK-RQRLNTPLVKGA------------  target    DDGTPQMDVTLRGSDDWIRISWDEATTIAAKTMEDVARTFNGDEGARKLLAQGYHPEMVEVMHGAGVQALKLR 7arc.1    --------------KGLENATWSAAFDAIRTAIAGAK------------------------------------ ``` | | | | | | | | | | | | | | | | | | | | | | | | | | | | | | | | | | | | | | | | | | | | | | | | | |
|  | 6btm.1.B | Alternative Complex III subunit B  *Structure of Alternative Complex III from Flavobacterium johnsoniae (Wild Type)* | 0.17 |  | 13.64 | 0.38 | 68-198 | EM | 3.40 | hetero-1-1-1-1-1-1-… | 6 x HEC, 1 x F3S, 1 x SF4, 2 x E87 | HHblits | 0.27 |
| ``` target    MAQGVSRRQLLGRALALGSGAALADLLGPARFLSPAGAATAGAVVPGNPLRVMPDRTWEQIYRNQFEDDSTFVFTCAPND 6btm.1    -------------------------------------------------------------------GVADYYATTVFDG  target    THNCLLRAHVKNGVVVRISPTYGYGEATDLYGNRASHRWDPRTCQKGLILSRRFYSERRVKAPMIRKGFKDWVEAGYPRN 6btm.1    FDFANLLVKTREGRPIKIE------------NNTIAGAK-FSANARIHASILGLYDSMRLKEPKLDG-------------  target    DDGTPQMDVTLRGSDDWIRISWDEATTIAAKTMEDVARTFNGDEGARKLLAQGYHPEMVEVMHGAGVQALKLR 6btm.1    -----------------KNSSWSAVDLKIKSSLADAKA----------------------------------- ``` | | | | | | | | | | | | | | | | | | | | | | | | | | | | | | | | | | | | | | | | | | | | | | | | | |
|  | 5t5i.1.B | Tungsten formylmethanofuran dehydrogenase subunit B  *TUNGSTEN-CONTAINING FORMYLMETHANOFURAN DEHYDROGENASE FROM METHANOTHERMOBACTER WOLFEII, ORTHORHOMBIC FORM AT 1.9 A* | 0.14 |  | 24.69 | 0.35 | 71-199 | X-ray | 1.90 | hetero-oligomer | 4 x ZN, 2 x MG, 18 x K, 22 x SF4, 2 x W, 4 x MGD, 2 x H2S, 2 x CA | HHblits | 0.31 |
| ``` target    MAQGVSRRQLLGRALALGSGAALADLLGPARFLSPAGAATAGAVVPGNPLRVMPDRTWEQIYRNQFEDDSTFVFTCAPND 5t5i.1    ----------------------------------------------------------------------YVKNVVCPFC  target    THNCL-LRAHVKNGVVVRISPTYGYGEATDLYGNRASHRWDPRTCQKGLILSRRFYSERRVKAPMIRKGFKDWVEAGYPR 5t5i.1    GTLCDDIICKVEGNEIVGT----------------------INACRIGHSKFVHAEGAMRYKKPLIRKN-----------  target    NDDGTPQMDVTLRGSDDWIRISWDEATTIAAKTMEDVARTFNGDEGARKLLAQGYHPEMVEVMHGAGVQALKLR 5t5i.1    ---------------GEFVEVSYDEAIDKAAKILAESKRP---------------------------------- ``` | | | | | | | | | | | | | | | | | | | | | | | | | | | | | | | | | | | | | | | | | | | | | | | | | |
|  | 7bkb.1.L | Formylmethanofuran dehydrogenase, subunit B  *Formate dehydrogenase - heterodisulfide reductase - formylmethanofuran dehydrogenase complex from Methanospirillum hungatei (hexameric, composite structure)* | 0.14 |  | 17.86 | 0.36 | 70-198 | EM | 0.00 | hetero-2-2-2-2-2-2-… | 48 x SF4, 4 x FAD, 2 x FES, 4 x 9S8, 4 x ZN, 2 x MO, 4 x MGD | HHblits | 0.29 |
| ``` target    MAQGVSRRQLLGRALALGSGAALADLLGPARFLSPAGAATAGAVVPGNPLRVMPDRTWEQIYRNQFEDDSTFVFTCAPND 7bkb.1    ---------------------------------------------------------------------KVIENVGCPYC  target    THNCL-LRAHVKNGVVVRISPTYGYGEATDLYGNRASHRWDPRTCQKGLILSRRFYSERRVKAPMIRKGFKDWVEAGYPR 7bkb.1    GCSCDDVRITVSDDGK--------------------DILEVENVCAIGTEIFKHGCSKDRIRLPRMRQP-----------  target    NDDGTPQMDVTLRGSDDWIRISWDEATTIAAKTMEDVARTFNGDEGARKLLAQGYHPEMVEVMHGAGVQALKLR 7bkb.1    --------------DGSMKDISYEEAIDWTARHLLKAKK----------------------------------- ``` | | | | | | | | | | | | | | | | | | | | | | | | | | | | | | | | | | | | | | | | | | | | | | | | | |
|  | 7q5y.1.A | NADH dehydrogenase I chain G  *Structure of NADH:ubichinon oxidoreductase (complex I) of the hyperthermophilic eubacterium Aquifex aeolicus* | 0.12 |  | 12.99 | 0.33 | 71-195 | X-ray | 2.70 | hetero-1-1-1-1-1-1-… | 8 x SF4, 2 x FES, 1 x FMN | HHblits | 0.26 |
| ``` target    MAQGVSRRQLLGRALALGSGAALADLLGPARFLSPAGAATAGAVVPGNPLRVMPDRTWEQIYRNQFEDDSTFVFTCAPND 7q5y.1    ----------------------------------------------------------------------EKGRTVCNLC  target    THNCLLRAHVKNGV------VVRISPTYGYGEATDLYGNRASHRWDPRTCQKGLILSRRFYSERRVKAPMIRKGFKDWVE 7q5y.1    PVGCEIQIEYGVGDWRSKRKVYRTK-----------------PTDELNICAKGFFGYDSINHKRLLKTKVGKR-------  target    AGYPRNDDGTPQMDVTLRGSDDWIRISWDEATTIAAKTMEDVARTFNGDEGARKLLAQGYHPEMVEVMHGAGVQALKLR 7q5y.1    ------------------------EETPGNVVNLLTTILTE-------------------------------------- ``` | | | | | | | | | | | | | | | | | | | | | | | | | | | | | | | | | | | | | | | | | | | | | | | | | |
|  | 7l5i.1.A | Trimethylamine-N-oxide reductase  *Crystal Structure of Haemophilus influenzae MtsZ at pH 7.0* | 0.11 |  | 45.61 | 0.24 | 129-196 | X-ray | 1.73 | monomer | 2 x MGD, 1 x MO, 1 x O | BLAST | 0.45 |
| ``` target    MAQGVSRRQLLGRALALGSGAALADLLGPARFLSPAGAATAGAVVPGNPLRVMPDRTWEQIYRNQFEDDSTFVFTCAPND 7l5i.1    --------------------------------------------------------------------------------  target    THNCLLRAHVKNGVVVRISPTYGYGEATDLYGNRASHRWDPRTCQKGLILSRRFYSERRVKAPMIRKGFKDWVEAGYPRN 7l5i.1    ------------------------------------------------VVADQLYSEARVKCPMVRKGF---------LA  target    DDGTPQMDVTLRGSDDWIRISWDEATTIAAKTMEDVARTFNGDEGARKLLAQGYHPEMVEVMHGAGVQALKLR 7l5i.1    NPG--KSDTTMRGRDEWVRVSWDEALDLVHNQLKRV------------------------------------- ``` | | | | | | | | | | | | | | | | | | | | | | | | | | | | | | | | | | | | | | | | | | | | | | | | | |
|  | 7l5s.1.A | Trimethylamine-N-oxide reductase  *Crystal Structure of Haemophilus influenzae MtsZ at pH 5.5* | 0.11 |  | 45.61 | 0.24 | 129-196 | X-ray | 2.09 | monomer | 1 x O, 2 x MGD, 1 x MO | BLAST | 0.45 |
| ``` target    MAQGVSRRQLLGRALALGSGAALADLLGPARFLSPAGAATAGAVVPGNPLRVMPDRTWEQIYRNQFEDDSTFVFTCAPND 7l5s.1    --------------------------------------------------------------------------------  target    THNCLLRAHVKNGVVVRISPTYGYGEATDLYGNRASHRWDPRTCQKGLILSRRFYSERRVKAPMIRKGFKDWVEAGYPRN 7l5s.1    ------------------------------------------------VVADQLYSEARVKCPMVRKGF---------LA  target    DDGTPQMDVTLRGSDDWIRISWDEATTIAAKTMEDVARTFNGDEGARKLLAQGYHPEMVEVMHGAGVQALKLR 7l5s.1    NPG--KSDTTMRGRDEWVRVSWDEALDLVHNQLKRV------------------------------------- ``` | | | | | | | | | | | | | | | | | | | | | | | | | | | | | | | | | | | | | | | | | | | | | | | | | |
|  | 6nr8.1.C | Prefoldin subunit 3  *hTRiC-hPFD Class6* | 0.03 |  | 17.24 | 0.12 | 172-200 | EM | 0.00 | hetero-1-1-1-1-1-1-… |  | HHblits | 0.32 |
| ``` target    MAQGVSRRQLLGRALALGSGAALADLLGPARFLSPAGAATAGAVVPGNPLRVMPDRTWEQIYRNQFEDDSTFVFTCAPND 6nr8.1    --------------------------------------------------------------------------------  target    THNCLLRAHVKNGVVVRISPTYGYGEATDLYGNRASHRWDPRTCQKGLILSRRFYSERRVKAPMIRKGFKDWVEAGYPRN 6nr8.1    --------------------------------------------------------------------------------  target    DDGTPQMDVTLRGSDDWIRISWDEATTIAAKTMEDVARTFNGDEGARKLLAQGYHPEMVEVMHGAGVQALKLR 6nr8.1    -----------LGANVMLEYDIDEAQALLEKNLSTATKNL--------------------------------- ``` | | | | | | | | | | | | | | | | | | | | | | | | | | | | | | | | | | | | | | | | | | | | | | | | | |
|  | 6nr9.1.S | Prefoldin subunit 3  *hTRiC-hPFD Class5* | 0.02 |  | 17.24 | 0.12 | 172-200 | EM | 0.00 | hetero-2-2-2-2-2-2-… |  | HHblits | 0.32 |
| ``` target    MAQGVSRRQLLGRALALGSGAALADLLGPARFLSPAGAATAGAVVPGNPLRVMPDRTWEQIYRNQFEDDSTFVFTCAPND 6nr9.1    --------------------------------------------------------------------------------  target    THNCLLRAHVKNGVVVRISPTYGYGEATDLYGNRASHRWDPRTCQKGLILSRRFYSERRVKAPMIRKGFKDWVEAGYPRN 6nr9.1    --------------------------------------------------------------------------------  target    DDGTPQMDVTLRGSDDWIRISWDEATTIAAKTMEDVARTFNGDEGARKLLAQGYHPEMVEVMHGAGVQALKLR 6nr9.1    -----------LGANVMLEYDIDEAQALLEKNLSTATKNL--------------------------------- ``` | | | | | | | | | | | | | | | | | | | | | | | | | | | | | | | | | | | | | | | | | | | | | | | | | |
|  | 6nrb.1.S | Prefoldin subunit 3  *hTRiC-hPFD Class2* | 0.03 |  | 17.24 | 0.12 | 172-200 | EM | 0.00 | hetero-2-2-2-2-2-2-… |  | HHblits | 0.32 |
| ``` target    MAQGVSRRQLLGRALALGSGAALADLLGPARFLSPAGAATAGAVVPGNPLRVMPDRTWEQIYRNQFEDDSTFVFTCAPND 6nrb.1    --------------------------------------------------------------------------------  target    THNCLLRAHVKNGVVVRISPTYGYGEATDLYGNRASHRWDPRTCQKGLILSRRFYSERRVKAPMIRKGFKDWVEAGYPRN 6nrb.1    --------------------------------------------------------------------------------  target    DDGTPQMDVTLRGSDDWIRISWDEATTIAAKTMEDVARTFNGDEGARKLLAQGYHPEMVEVMHGAGVQALKLR 6nrb.1    -----------LGANVMLEYDIDEAQALLEKNLSTATKNL--------------------------------- ``` | | | | | | | | | | | | | | | | | | | | | | | | | | | | | | | | | | | | | | | | | | | | | | | | | |
|  | 6nrc.1.C | Prefoldin subunit 3  *hTRiC-hPFD Class3* | 0.03 |  | 17.24 | 0.12 | 172-200 | EM | 0.00 | hetero-1-1-1-1-1-1-… |  | HHblits | 0.32 |
| ``` target    MAQGVSRRQLLGRALALGSGAALADLLGPARFLSPAGAATAGAVVPGNPLRVMPDRTWEQIYRNQFEDDSTFVFTCAPND 6nrc.1    --------------------------------------------------------------------------------  target    THNCLLRAHVKNGVVVRISPTYGYGEATDLYGNRASHRWDPRTCQKGLILSRRFYSERRVKAPMIRKGFKDWVEAGYPRN 6nrc.1    --------------------------------------------------------------------------------  target    DDGTPQMDVTLRGSDDWIRISWDEATTIAAKTMEDVARTFNGDEGARKLLAQGYHPEMVEVMHGAGVQALKLR 6nrc.1    -----------LGANVMLEYDIDEAQALLEKNLSTATKNL--------------------------------- ``` | | | | | | | | | | | | | | | | | | | | | | | | | | | | | | | | | | | | | | | | | | | | | | | | | |
|  | 6nrd.1.S | Prefoldin subunit 3  *hTRiC-hPFD Class4* | 0.02 |  | 17.24 | 0.12 | 172-200 | EM | 0.00 | hetero-2-2-2-2-2-2-… |  | HHblits | 0.32 |
| ``` target    MAQGVSRRQLLGRALALGSGAALADLLGPARFLSPAGAATAGAVVPGNPLRVMPDRTWEQIYRNQFEDDSTFVFTCAPND 6nrd.1    --------------------------------------------------------------------------------  target    THNCLLRAHVKNGVVVRISPTYGYGEATDLYGNRASHRWDPRTCQKGLILSRRFYSERRVKAPMIRKGFKDWVEAGYPRN 6nrd.1    --------------------------------------------------------------------------------  target    DDGTPQMDVTLRGSDDWIRISWDEATTIAAKTMEDVARTFNGDEGARKLLAQGYHPEMVEVMHGAGVQALKLR 6nrd.1    -----------LGANVMLEYDIDEAQALLEKNLSTATKNL--------------------------------- ``` | | | | | | | | | | | | | | | | | | | | | | | | | | | | | | | | | | | | | | | | | | | | | | | | | |
|  | 2zdi.1.C | Prefoldin subunit alpha  *Crystal structure of Prefoldin from Pyrococcus horikoshii OT3* | 0.03 |  | 24.14 | 0.12 | 172-200 | X-ray | 3.00 | hetero-oligomer |  | HHblits | 0.31 |
| ``` target    MAQGVSRRQLLGRALALGSGAALADLLGPARFLSPAGAATAGAVVPGNPLRVMPDRTWEQIYRNQFEDDSTFVFTCAPND 2zdi.1    --------------------------------------------------------------------------------  target    THNCLLRAHVKNGVVVRISPTYGYGEATDLYGNRASHRWDPRTCQKGLILSRRFYSERRVKAPMIRKGFKDWVEAGYPRN 2zdi.1    --------------------------------------------------------------------------------  target    DDGTPQMDVTLRGSDDWIRISWDEATTIAAKTMEDVARTFNGDEGARKLLAQGYHPEMVEVMHGAGVQALKLR 2zdi.1    -----------VGSGYAVERSIDEAISFLEKRLKEYDEAI--------------------------------- ``` | | | | | | | | | | | | | | | | | | | | | | | | | | | | | | | | | | | | | | | | | | | | | | | | | |
|  | 6nr8.1.E | Prefoldin subunit 5  *hTRiC-hPFD Class6* | 0.03 |  | 6.67 | 0.13 | 172-201 | EM | 0.00 | hetero-1-1-1-1-1-1-… |  | HHblits | 0.28 |
| ``` target    MAQGVSRRQLLGRALALGSGAALADLLGPARFLSPAGAATAGAVVPGNPLRVMPDRTWEQIYRNQFEDDSTFVFTCAPND 6nr8.1    --------------------------------------------------------------------------------  target    THNCLLRAHVKNGVVVRISPTYGYGEATDLYGNRASHRWDPRTCQKGLILSRRFYSERRVKAPMIRKGFKDWVEAGYPRN 6nr8.1    --------------------------------------------------------------------------------  target    DDGTPQMDVTLRGSDDWIRISWDEATTIAAKTMEDVARTFNGDEGARKLLAQGYHPEMVEVMHGAGVQALKLR 6nr8.1    -----------VGTGYYVEKTAEDAKDFFKRKIDFLTKQME-------------------------------- ``` | | | | | | | | | | | | | | | | | | | | | | | | | | | | | | | | | | | | | | | | | | | | | | | | | |
|  | 6nr9.1.U | Prefoldin subunit 5  *hTRiC-hPFD Class5* | 0.03 |  | 6.67 | 0.13 | 172-201 | EM | 0.00 | hetero-2-2-2-2-2-2-… |  | HHblits | 0.28 |
| ``` target    MAQGVSRRQLLGRALALGSGAALADLLGPARFLSPAGAATAGAVVPGNPLRVMPDRTWEQIYRNQFEDDSTFVFTCAPND 6nr9.1    --------------------------------------------------------------------------------  target    THNCLLRAHVKNGVVVRISPTYGYGEATDLYGNRASHRWDPRTCQKGLILSRRFYSERRVKAPMIRKGFKDWVEAGYPRN 6nr9.1    --------------------------------------------------------------------------------  target    DDGTPQMDVTLRGSDDWIRISWDEATTIAAKTMEDVARTFNGDEGARKLLAQGYHPEMVEVMHGAGVQALKLR 6nr9.1    -----------VGTGYYVEKTAEDAKDFFKRKIDFLTKQME-------------------------------- ``` | | | | | | | | | | | | | | | | | | | | | | | | | | | | | | | | | | | | | | | | | | | | | | | | | |
|  | 6nrb.1.U | Prefoldin subunit 5  *hTRiC-hPFD Class2* | 0.02 |  | 6.67 | 0.13 | 172-201 | EM | 0.00 | hetero-2-2-2-2-2-2-… |  | HHblits | 0.28 |
| ``` target    MAQGVSRRQLLGRALALGSGAALADLLGPARFLSPAGAATAGAVVPGNPLRVMPDRTWEQIYRNQFEDDSTFVFTCAPND 6nrb.1    --------------------------------------------------------------------------------  target    THNCLLRAHVKNGVVVRISPTYGYGEATDLYGNRASHRWDPRTCQKGLILSRRFYSERRVKAPMIRKGFKDWVEAGYPRN 6nrb.1    --------------------------------------------------------------------------------  target    DDGTPQMDVTLRGSDDWIRISWDEATTIAAKTMEDVARTFNGDEGARKLLAQGYHPEMVEVMHGAGVQALKLR 6nrb.1    -----------VGTGYYVEKTAEDAKDFFKRKIDFLTKQME-------------------------------- ``` | | | | | | | | | | | | | | | | | | | | | | | | | | | | | | | | | | | | | | | | | | | | | | | | | |
|  | 6nrc.1.E | Prefoldin subunit 5  *hTRiC-hPFD Class3* | 0.03 |  | 6.67 | 0.13 | 172-201 | EM | 0.00 | hetero-1-1-1-1-1-1-… |  | HHblits | 0.28 |
| ``` target    MAQGVSRRQLLGRALALGSGAALADLLGPARFLSPAGAATAGAVVPGNPLRVMPDRTWEQIYRNQFEDDSTFVFTCAPND 6nrc.1    --------------------------------------------------------------------------------  target    THNCLLRAHVKNGVVVRISPTYGYGEATDLYGNRASHRWDPRTCQKGLILSRRFYSERRVKAPMIRKGFKDWVEAGYPRN 6nrc.1    --------------------------------------------------------------------------------  target    DDGTPQMDVTLRGSDDWIRISWDEATTIAAKTMEDVARTFNGDEGARKLLAQGYHPEMVEVMHGAGVQALKLR 6nrc.1    -----------VGTGYYVEKTAEDAKDFFKRKIDFLTKQME-------------------------------- ``` | | | | | | | | | | | | | | | | | | | | | | | | | | | | | | | | | | | | | | | | | | | | | | | | | |
|  | 6nrd.1.U | Prefoldin subunit 5  *hTRiC-hPFD Class4* | 0.03 |  | 6.67 | 0.13 | 172-201 | EM | 0.00 | hetero-2-2-2-2-2-2-… |  | HHblits | 0.28 |
| ``` target    MAQGVSRRQLLGRALALGSGAALADLLGPARFLSPAGAATAGAVVPGNPLRVMPDRTWEQIYRNQFEDDSTFVFTCAPND 6nrd.1    --------------------------------------------------------------------------------  target    THNCLLRAHVKNGVVVRISPTYGYGEATDLYGNRASHRWDPRTCQKGLILSRRFYSERRVKAPMIRKGFKDWVEAGYPRN 6nrd.1    --------------------------------------------------------------------------------  target    DDGTPQMDVTLRGSDDWIRISWDEATTIAAKTMEDVARTFNGDEGARKLLAQGYHPEMVEVMHGAGVQALKLR 6nrd.1    -----------VGTGYYVEKTAEDAKDFFKRKIDFLTKQME-------------------------------- ``` | | | | | | | | | | | | | | | | | | | | | | | | | | | | | | | | | | | | | | | | | | | | | | | | | |
|  | 6vy1.1.A | Prefoldin subunit alpha 2  *Cryo-EM structure of filamentous PFD from Methanocaldococcus jannaschii* | 0.03 |  | 10.00 | 0.13 | 172-201 | EM | 0.00 | homo-14-mer |  | HHblits | 0.26 |
| ``` target    MAQGVSRRQLLGRALALGSGAALADLLGPARFLSPAGAATAGAVVPGNPLRVMPDRTWEQIYRNQFEDDSTFVFTCAPND 6vy1.1    --------------------------------------------------------------------------------  target    THNCLLRAHVKNGVVVRISPTYGYGEATDLYGNRASHRWDPRTCQKGLILSRRFYSERRVKAPMIRKGFKDWVEAGYPRN 6vy1.1    --------------------------------------------------------------------------------  target    DDGTPQMDVTLRGSDDWIRISWDEATTIAAKTMEDVARTFNGDEGARKLLAQGYHPEMVEVMHGAGVQALKLR 6vy1.1    -----------VGQNISAELEYEEALKYIEDEIKKLLTFRL-------------------------------- ``` | | | | | | | | | | | | | | | | | | | | | | | | | | | | | | | | | | | | | | | | | | | | | | | | | |
|  | 6vy1.1.G | Prefoldin subunit alpha 2  *Cryo-EM structure of filamentous PFD from Methanocaldococcus jannaschii* | 0.03 |  | 10.00 | 0.13 | 172-201 | EM | 0.00 | homo-14-mer |  | HHblits | 0.26 |
| ``` target    MAQGVSRRQLLGRALALGSGAALADLLGPARFLSPAGAATAGAVVPGNPLRVMPDRTWEQIYRNQFEDDSTFVFTCAPND 6vy1.1    --------------------------------------------------------------------------------  target    THNCLLRAHVKNGVVVRISPTYGYGEATDLYGNRASHRWDPRTCQKGLILSRRFYSERRVKAPMIRKGFKDWVEAGYPRN 6vy1.1    --------------------------------------------------------------------------------  target    DDGTPQMDVTLRGSDDWIRISWDEATTIAAKTMEDVARTFNGDEGARKLLAQGYHPEMVEVMHGAGVQALKLR 6vy1.1    -----------VGQNISAELEYEEALKYIEDEIKKLLTFRL-------------------------------- ``` | | | | | | | | | | | | | | | | | | | | | | | | | | | | | | | | | | | | | | | | | | | | | | | | | |
|  | 6vy1.1.M | Prefoldin subunit alpha 2  *Cryo-EM structure of filamentous PFD from Methanocaldococcus jannaschii* | 0.03 |  | 10.00 | 0.13 | 172-201 | EM | 0.00 | homo-14-mer |  | HHblits | 0.26 |
| ``` target    MAQGVSRRQLLGRALALGSGAALADLLGPARFLSPAGAATAGAVVPGNPLRVMPDRTWEQIYRNQFEDDSTFVFTCAPND 6vy1.1    --------------------------------------------------------------------------------  target    THNCLLRAHVKNGVVVRISPTYGYGEATDLYGNRASHRWDPRTCQKGLILSRRFYSERRVKAPMIRKGFKDWVEAGYPRN 6vy1.1    --------------------------------------------------------------------------------  target    DDGTPQMDVTLRGSDDWIRISWDEATTIAAKTMEDVARTFNGDEGARKLLAQGYHPEMVEVMHGAGVQALKLR 6vy1.1    -----------VGQNISAELEYEEALKYIEDEIKKLLTFRL-------------------------------- ``` | | | | | | | | | | | | | | | | | | | | | | | | | | | | | | | | | | | | | | | | | | | | | | | | | |
|  | 1fxk.1.C | PROTEIN (PREFOLDIN)  *CRYSTAL STRUCTURE OF ARCHAEAL PREFOLDIN (GIMC).* | 0.03 |  | 13.79 | 0.12 | 172-200 | X-ray | 2.30 | hetero-oligomer |  | HHblits | 0.29 |
| ``` target    MAQGVSRRQLLGRALALGSGAALADLLGPARFLSPAGAATAGAVVPGNPLRVMPDRTWEQIYRNQFEDDSTFVFTCAPND 1fxk.1    --------------------------------------------------------------------------------  target    THNCLLRAHVKNGVVVRISPTYGYGEATDLYGNRASHRWDPRTCQKGLILSRRFYSERRVKAPMIRKGFKDWVEAGYPRN 1fxk.1    --------------------------------------------------------------------------------  target    DDGTPQMDVTLRGSDDWIRISWDEATTIAAKTMEDVARTFNGDEGARKLLAQGYHPEMVEVMHGAGVQALKLR 1fxk.1    -----------VGAGVAIKKNFEDAMESIKSQKNELESTL--------------------------------- ``` | | | | | | | | | | | | | | | | | | | | | | | | | | | | | | | | | | | | | | | | | | | | | | | | | |
|  | 7wu7.1.E | Prefoldin subunit 5  *Prefoldin-tubulin-TRiC complex* | 0.03 |  | 6.90 | 0.12 | 172-200 | EM | 0.00 | hetero-1-1-1-1-1-1-… | 4 x ADP | HHblits | 0.28 |
| ``` target    MAQGVSRRQLLGRALALGSGAALADLLGPARFLSPAGAATAGAVVPGNPLRVMPDRTWEQIYRNQFEDDSTFVFTCAPND 7wu7.1    --------------------------------------------------------------------------------  target    THNCLLRAHVKNGVVVRISPTYGYGEATDLYGNRASHRWDPRTCQKGLILSRRFYSERRVKAPMIRKGFKDWVEAGYPRN 7wu7.1    --------------------------------------------------------------------------------  target    DDGTPQMDVTLRGSDDWIRISWDEATTIAAKTMEDVARTFNGDEGARKLLAQGYHPEMVEVMHGAGVQALKLR 7wu7.1    -----------VGTGYYVEKTAEDAKDFFKRKIDFLTKQM--------------------------------- ``` | | | | | | | | | | | | | | | | | | | | | | | | | | | | | | | | | | | | | | | | | | | | | | | | | |
|  | 3lw5.1.H | Putative uncharacterized protein  *Improved model of plant photosystem I* | 0.00 |  | 38.10 | 0.09 | 3-23 | X-ray | 3.30 | hetero-1-1-1-1-1-1-… | 151 x CLA, 2 x PQN, 18 x BCR, 15 x LMU, 3 x SF4, 1 x LMG | HHblits | 0.36 |
| ``` target    MAQGVSRRQLLGRALALGSGAALADLLGPARFLSPAGAATAGAVVPGNPLRVMPDRTWEQIYRNQFEDDSTFVFTCAPND 3lw5.1    --APFTKRGLLLKFLILGGGSTL---------------------------------------------------------  target    THNCLLRAHVKNGVVVRISPTYGYGEATDLYGNRASHRWDPRTCQKGLILSRRFYSERRVKAPMIRKGFKDWVEAGYPRN 3lw5.1    --------------------------------------------------------------------------------  target    DDGTPQMDVTLRGSDDWIRISWDEATTIAAKTMEDVARTFNGDEGARKLLAQGYHPEMVEVMHGAGVQALKLR 3lw5.1    ------------------------------------------------------------------------- ``` | | | | | | | | | | | | | | | | | | | | | | | | | | | | | | | | | | | | | | | | | | | | | | | | | |
|  | 2o01.1.H | Photosystem I reaction center subunit VI, chloroplast  *The Structure of a plant photosystem I supercomplex at 3.4 Angstrom resolution* | 0.00 |  | 38.10 | 0.09 | 3-23 | X-ray | 3.40 | hetero-oligomer | 145 x CLA, 3 x SF4, 2 x PQN, 5 x BCR | HHblits | 0.36 |
| ``` target    MAQGVSRRQLLGRALALGSGAALADLLGPARFLSPAGAATAGAVVPGNPLRVMPDRTWEQIYRNQFEDDSTFVFTCAPND 2o01.1    --APFTKRGLLLKFLILGGGSLL---------------------------------------------------------  target    THNCLLRAHVKNGVVVRISPTYGYGEATDLYGNRASHRWDPRTCQKGLILSRRFYSERRVKAPMIRKGFKDWVEAGYPRN 2o01.1    --------------------------------------------------------------------------------  target    DDGTPQMDVTLRGSDDWIRISWDEATTIAAKTMEDVARTFNGDEGARKLLAQGYHPEMVEVMHGAGVQALKLR 2o01.1    ------------------------------------------------------------------------- ``` | | | | | | | | | | | | | | | | | | | | | | | | | | | | | | | | | | | | | | | | | | | | | | | | | |
|  | 2pq4.1.B | Periplasmic nitrate reductase precursor  *NMR solution structure of NapD in complex with NapA1-35 signal peptide* | 0.01 |  | 23.81 | 0.09 | 3-23 | NMR | 0.00 | hetero-1-1-mer |  | HHblits | 0.32 |
| ``` target    MAQGVSRRQLLGRALALGSGAALADLLGPARFLSPAGAATAGAVVPGNPLRVMPDRTWEQIYRNQFEDDSTFVFTCAPND 2pq4.1    --MKLSRRSFMKANAVAAAAAAA---------------------------------------------------------  target    THNCLLRAHVKNGVVVRISPTYGYGEATDLYGNRASHRWDPRTCQKGLILSRRFYSERRVKAPMIRKGFKDWVEAGYPRN 2pq4.1    --------------------------------------------------------------------------------  target    DDGTPQMDVTLRGSDDWIRISWDEATTIAAKTMEDVARTFNGDEGARKLLAQGYHPEMVEVMHGAGVQALKLR 2pq4.1    ------------------------------------------------------------------------- ``` | | | | | | | | | | | | | | | | | | | | | | | | | | | | | | | | | | | | | | | | | | | | | | | | | |
|  | 1q90.1.E | Cytochrome B6-F complex iron-sulfur subunit  *Structure of the cytochrome b6f (plastohydroquinone : plastocyanin oxidoreductase) from Chlamydomonas reinhardtii* | 0.01 |  | 26.32 | 0.08 | 2-20 | X-ray | 3.10 | hetero-2-2-2-2-2-2-… | 8 x HEC, 2 x BCR, 2 x LFA, 2 x FES, 2 x CLA, 2 x TDS, 4 x LMG, 2 x SQD | HHblits | 0.35 |
| ``` target    MAQGVSRRQLLGRALALGSGAALADLLGPARFLSPAGAATAGAVVPGNPLRVMPDRTWEQIYRNQFEDDSTFVFTCAPND 1q90.1    -VPDMNKRNIMNLILAGGAG------------------------------------------------------------  target    THNCLLRAHVKNGVVVRISPTYGYGEATDLYGNRASHRWDPRTCQKGLILSRRFYSERRVKAPMIRKGFKDWVEAGYPRN 1q90.1    --------------------------------------------------------------------------------  target    DDGTPQMDVTLRGSDDWIRISWDEATTIAAKTMEDVARTFNGDEGARKLLAQGYHPEMVEVMHGAGVQALKLR 1q90.1    ------------------------------------------------------------------------- ``` | | | | | | | | | | | | | | | | | | | | | | | | | | | | | | | | | | | | | | | | | | | | | | | | | |
|  | 6rqf.1.D | Cytochrome b6-f complex iron-sulfur subunit, chloroplastic  *3.6 Angstrom cryo-EM structure of the dimeric cytochrome b6f complex from Spinacia oleracea with natively bound thylakoid lipids and plastoquinone molecules* | 0.02 |  | 14.29 | 0.09 | 1-21 | EM | 0.00 | hetero-2-2-2-2-2-2-… | 4 x HEM, 4 x HEC, 2 x CLA, 2 x BCR, 3 x PL9, 3 x 6PL, 2 x LMG, 4 x PGV, 2 x FES, 3 x SQD | HHblits | 0.27 |
| ``` target    MAQGVSRRQLLGRALALGSGAALADLLGPARFLSPAGAATAGAVVPGNPLRVMPDRTWEQIYRNQFEDDSTFVFTCAPND 6rqf.1    NVPDMQKRETLNLLLLGALSL-----------------------------------------------------------  target    THNCLLRAHVKNGVVVRISPTYGYGEATDLYGNRASHRWDPRTCQKGLILSRRFYSERRVKAPMIRKGFKDWVEAGYPRN 6rqf.1    --------------------------------------------------------------------------------  target    DDGTPQMDVTLRGSDDWIRISWDEATTIAAKTMEDVARTFNGDEGARKLLAQGYHPEMVEVMHGAGVQALKLR 6rqf.1    ------------------------------------------------------------------------- ``` | | | | | | | | | | | | | | | | | | | | | | | | | | | | | | | | | | | | | | | | | | | | | | | | | |
|  | 6rqf.1.L | Cytochrome b6-f complex iron-sulfur subunit, chloroplastic  *3.6 Angstrom cryo-EM structure of the dimeric cytochrome b6f complex from Spinacia oleracea with natively bound thylakoid lipids and plastoquinone molecules* | 0.01 |  | 14.29 | 0.09 | 1-21 | EM | 0.00 | hetero-2-2-2-2-2-2-… | 4 x HEM, 4 x HEC, 2 x CLA, 2 x BCR, 3 x PL9, 3 x 6PL, 2 x LMG, 4 x PGV, 2 x FES, 3 x SQD | HHblits | 0.27 |
| ``` target    MAQGVSRRQLLGRALALGSGAALADLLGPARFLSPAGAATAGAVVPGNPLRVMPDRTWEQIYRNQFEDDSTFVFTCAPND 6rqf.1    NVPDMQKRETLNLLLLGALSL-----------------------------------------------------------  target    THNCLLRAHVKNGVVVRISPTYGYGEATDLYGNRASHRWDPRTCQKGLILSRRFYSERRVKAPMIRKGFKDWVEAGYPRN 6rqf.1    --------------------------------------------------------------------------------  target    DDGTPQMDVTLRGSDDWIRISWDEATTIAAKTMEDVARTFNGDEGARKLLAQGYHPEMVEVMHGAGVQALKLR 6rqf.1    ------------------------------------------------------------------------- ``` | | | | | | | | | | | | | | | | | | | | | | | | | | | | | | | | | | | | | | | | | | | | | | | | | |
|  | 7qrm.1.D | Cytochrome b6-f complex iron-sulfur subunit, chloroplastic  *Cryo-EM structure of catalytically active Spinacia oleracea cytochrome b6f in complex with endogenous plastoquinones at 2.7 A resolution* | 0.00 |  | 14.29 | 0.09 | 3-23 | EM | 0.00 | hetero-2-2-2-2-2-2-… | 4 x HEM, 4 x HEC, 2 x CLA, 7 x PL9, 10 x UMQ, 4 x PGT, 2 x SQD, 2 x FES, 2 x BCR | HHblits | 0.27 |
| ``` target    MAQGVSRRQLLGRALALGSGAALADLLGPARFLSPAGAATAGAVVPGNPLRVMPDRTWEQIYRNQFEDDSTFVFTCAPND 7qrm.1    --PDMQKRETLNLLLLGALSLPT---------------------------------------------------------  target    THNCLLRAHVKNGVVVRISPTYGYGEATDLYGNRASHRWDPRTCQKGLILSRRFYSERRVKAPMIRKGFKDWVEAGYPRN 7qrm.1    --------------------------------------------------------------------------------  target    DDGTPQMDVTLRGSDDWIRISWDEATTIAAKTMEDVARTFNGDEGARKLLAQGYHPEMVEVMHGAGVQALKLR 7qrm.1    ------------------------------------------------------------------------- ``` | | | | | | | | | | | | | | | | | | | | | | | | | | | | | | | | | | | | | | | | | | | | | | | | | |
|  | 6adq.1.L | Rieske iron-sulfur protein QcrA  *Respiratory Complex CIII2CIV2SOD2 from Mycobacterium smegmatis* | 0.01 |  | 33.33 | 0.08 | 3-20 | EM | 0.00 | hetero-2-2-2-2-2-2-… | 8 x CU, 4 x HEA, 18 x CDL, 8 x 9Y0, 4 x PLM, 4 x 9XX, 8 x 9YF, 4 x HEM, 10 x MQ9, 4 x HEC, 2 x FES | HHblits | 0.39 |
| ``` target    MAQGVSRRQLLGRALALGSGAALADLLGPARFLSPAGAATAGAVVPGNPLRVMPDRTWEQIYRNQFEDDSTFVFTCAPND 6adq.1    --STLKRRKVIGLSLGIGLG------------------------------------------------------------  target    THNCLLRAHVKNGVVVRISPTYGYGEATDLYGNRASHRWDPRTCQKGLILSRRFYSERRVKAPMIRKGFKDWVEAGYPRN 6adq.1    --------------------------------------------------------------------------------  target    DDGTPQMDVTLRGSDDWIRISWDEATTIAAKTMEDVARTFNGDEGARKLLAQGYHPEMVEVMHGAGVQALKLR 6adq.1    ------------------------------------------------------------------------- ``` | | | | | | | | | | | | | | | | | | | | | | | | | | | | | | | | | | | | | | | | | | | | | | | | | |
|  | 2fyn.1.C | Ubiquinol-cytochrome c reductase iron-sulfur subunit  *Crystal Structure Analysis of the double mutant Rhodobacter Sphaeroides bc1 complex* | 0.01 |  | 25.00 | 0.09 | 2-21 | X-ray | 3.20 | hetero-2-2-2-mer | 6 x HEM, 2 x SMA, 2 x LOP, 2 x FES | HHblits | 0.29 |
| ``` target    MAQGVSRRQLLGRALALGSGAALADLLGPARFLSPAGAATAGAVVPGNPLRVMPDRTWEQIYRNQFEDDSTFVFTCAPND 2fyn.1    -DHAGTRRDFLYYATAGAGAV-----------------------------------------------------------  target    THNCLLRAHVKNGVVVRISPTYGYGEATDLYGNRASHRWDPRTCQKGLILSRRFYSERRVKAPMIRKGFKDWVEAGYPRN 2fyn.1    --------------------------------------------------------------------------------  target    DDGTPQMDVTLRGSDDWIRISWDEATTIAAKTMEDVARTFNGDEGARKLLAQGYHPEMVEVMHGAGVQALKLR 2fyn.1    ------------------------------------------------------------------------- ``` | | | | | | | | | | | | | | | | | | | | | | | | | | | | | | | | | | | | | | | | | | | | | | | | | |
|  | 2fyn.2.C | Ubiquinol-cytochrome c reductase iron-sulfur subunit  *Crystal Structure Analysis of the double mutant Rhodobacter Sphaeroides bc1 complex* | 0.01 |  | 25.00 | 0.09 | 2-21 | X-ray | 3.20 | hetero-2-2-2-mer | 6 x HEM, 2 x SMA, 2 x LOP, 2 x FES | HHblits | 0.29 |
| ``` target    MAQGVSRRQLLGRALALGSGAALADLLGPARFLSPAGAATAGAVVPGNPLRVMPDRTWEQIYRNQFEDDSTFVFTCAPND 2fyn.2    -DHAGTRRDFLYYATAGAGAV-----------------------------------------------------------  target    THNCLLRAHVKNGVVVRISPTYGYGEATDLYGNRASHRWDPRTCQKGLILSRRFYSERRVKAPMIRKGFKDWVEAGYPRN 2fyn.2    --------------------------------------------------------------------------------  target    DDGTPQMDVTLRGSDDWIRISWDEATTIAAKTMEDVARTFNGDEGARKLLAQGYHPEMVEVMHGAGVQALKLR 2fyn.2    ------------------------------------------------------------------------- ``` | | | | | | | | | | | | | | | | | | | | | | | | | | | | | | | | | | | | | | | | | | | | | | | | | |
|  | 2qjy.3.F | Ubiquinol-cytochrome c reductase iron-sulfur subunit  *Crystal structure of rhodobacter sphaeroides double mutant with stigmatellin and UQ2* | 0.00 |  | 25.00 | 0.09 | 2-21 | X-ray | 2.40 | hetero-2-2-2-mer | 6 x HEM, 2 x SMA, 2 x LOP, 2 x UQ2, 2 x BGL, 2 x FES | HHblits | 0.29 |
| ``` target    MAQGVSRRQLLGRALALGSGAALADLLGPARFLSPAGAATAGAVVPGNPLRVMPDRTWEQIYRNQFEDDSTFVFTCAPND 2qjy.3    -DHAGTRRDFLYYATAGAGAV-----------------------------------------------------------  target    THNCLLRAHVKNGVVVRISPTYGYGEATDLYGNRASHRWDPRTCQKGLILSRRFYSERRVKAPMIRKGFKDWVEAGYPRN 2qjy.3    --------------------------------------------------------------------------------  target    DDGTPQMDVTLRGSDDWIRISWDEATTIAAKTMEDVARTFNGDEGARKLLAQGYHPEMVEVMHGAGVQALKLR 2qjy.3    ------------------------------------------------------------------------- ``` | | | | | | | | | | | | | | | | | | | | | | | | | | | | | | | | | | | | | | | | | | | | | | | | | |
|  | 6nin.1.C | Ubiquinol-cytochrome c reductase iron-sulfur subunit  *Rhodobacter sphaeroides bc1 with STIGMATELLIN A* | 0.01 |  | 25.00 | 0.09 | 2-21 | X-ray | 3.60 | hetero-2-2-2-mer | 4 x HEM, 2 x SMA, 2 x 6PE, 2 x HEC, 2 x BOG, 2 x FES | HHblits | 0.29 |
| ``` target    MAQGVSRRQLLGRALALGSGAALADLLGPARFLSPAGAATAGAVVPGNPLRVMPDRTWEQIYRNQFEDDSTFVFTCAPND 6nin.1    -DHAGTRRDFLYYATAGAGAV-----------------------------------------------------------  target    THNCLLRAHVKNGVVVRISPTYGYGEATDLYGNRASHRWDPRTCQKGLILSRRFYSERRVKAPMIRKGFKDWVEAGYPRN 6nin.1    --------------------------------------------------------------------------------  target    DDGTPQMDVTLRGSDDWIRISWDEATTIAAKTMEDVARTFNGDEGARKLLAQGYHPEMVEVMHGAGVQALKLR 6nin.1    ------------------------------------------------------------------------- ``` | | | | | | | | | | | | | | | | | | | | | | | | | | | | | | | | | | | | | | | | | | | | | | | | | |
|  | 7o37.1.E | Cytochrome b-c1 complex subunit Rieske, mitochondrial  *Murine supercomplex CIII2CIV in the assembled locked conformation* | 0.01 |  | 9.52 | 0.09 | 2-22 | EM | 0.00 | hetero-2-2-2-2-2-2-… | 16 x 3PE, 6 x CDL, 4 x HEM, 2 x HEC, 2 x FES, 5 x PC1, 1 x TGL, 1 x CU, 2 x HEA, 1 x MG, 1 x CUA, 1 x ZN | HHblits | 0.25 |
| ``` target    MAQGVSRRQLLGRALALGSGAALADLLGPARFLSPAGAATAGAVVPGNPLRVMPDRTWEQIYRNQFEDDSTFVFTCAPND 7o37.1    -ESSEARKGFSYLVTATTTVGV----------------------------------------------------------  target    THNCLLRAHVKNGVVVRISPTYGYGEATDLYGNRASHRWDPRTCQKGLILSRRFYSERRVKAPMIRKGFKDWVEAGYPRN 7o37.1    --------------------------------------------------------------------------------  target    DDGTPQMDVTLRGSDDWIRISWDEATTIAAKTMEDVARTFNGDEGARKLLAQGYHPEMVEVMHGAGVQALKLR 7o37.1    ------------------------------------------------------------------------- ``` | | | | | | | | | | | | | | | | | | | | | | | | | | | | | | | | | | | | | | | | | | | | | | | | | |
|  | 7o37.1.O | Cytochrome b-c1 complex subunit Rieske, mitochondrial  *Murine supercomplex CIII2CIV in the assembled locked conformation* | 0.01 |  | 9.52 | 0.09 | 2-22 | EM | 0.00 | hetero-2-2-2-2-2-2-… | 16 x 3PE, 6 x CDL, 4 x HEM, 2 x HEC, 2 x FES, 5 x PC1, 1 x TGL, 1 x CU, 2 x HEA, 1 x MG, 1 x CUA, 1 x ZN | HHblits | 0.25 |
| ``` target    MAQGVSRRQLLGRALALGSGAALADLLGPARFLSPAGAATAGAVVPGNPLRVMPDRTWEQIYRNQFEDDSTFVFTCAPND 7o37.1    -ESSEARKGFSYLVTATTTVGV----------------------------------------------------------  target    THNCLLRAHVKNGVVVRISPTYGYGEATDLYGNRASHRWDPRTCQKGLILSRRFYSERRVKAPMIRKGFKDWVEAGYPRN 7o37.1    --------------------------------------------------------------------------------  target    DDGTPQMDVTLRGSDDWIRISWDEATTIAAKTMEDVARTFNGDEGARKLLAQGYHPEMVEVMHGAGVQALKLR 7o37.1    ------------------------------------------------------------------------- ``` | | | | | | | | | | | | | | | | | | | | | | | | | | | | | | | | | | | | | | | | | | | | | | | | | |
|  | 7o3c.1.E | Cytochrome b-c1 complex subunit Rieske, mitochondrial  *Murine supercomplex CIII2CIV in the mature unlocked conformation* | 0.01 |  | 9.52 | 0.09 | 2-22 | EM | 0.00 | hetero-2-2-2-2-2-2-… | 15 x 3PE, 7 x CDL, 4 x HEM, 2 x HEC, 2 x FES, 3 x PC1, 1 x CU, 2 x HEA, 1 x MG, 1 x CUA, 1 x ZN, 1 x TGL | HHblits | 0.25 |
| ``` target    MAQGVSRRQLLGRALALGSGAALADLLGPARFLSPAGAATAGAVVPGNPLRVMPDRTWEQIYRNQFEDDSTFVFTCAPND 7o3c.1    -ESSEARKGFSYLVTATTTVGV----------------------------------------------------------  target    THNCLLRAHVKNGVVVRISPTYGYGEATDLYGNRASHRWDPRTCQKGLILSRRFYSERRVKAPMIRKGFKDWVEAGYPRN 7o3c.1    --------------------------------------------------------------------------------  target    DDGTPQMDVTLRGSDDWIRISWDEATTIAAKTMEDVARTFNGDEGARKLLAQGYHPEMVEVMHGAGVQALKLR 7o3c.1    ------------------------------------------------------------------------- ``` | | | | | | | | | | | | | | | | | | | | | | | | | | | | | | | | | | | | | | | | | | | | | | | | | |
|  | 7o3c.1.O | Cytochrome b-c1 complex subunit Rieske, mitochondrial  *Murine supercomplex CIII2CIV in the mature unlocked conformation* | 0.01 |  | 9.52 | 0.09 | 2-22 | EM | 0.00 | hetero-2-2-2-2-2-2-… | 15 x 3PE, 7 x CDL, 4 x HEM, 2 x HEC, 2 x FES, 3 x PC1, 1 x CU, 2 x HEA, 1 x MG, 1 x CUA, 1 x ZN, 1 x TGL | HHblits | 0.25 |
| ``` target    MAQGVSRRQLLGRALALGSGAALADLLGPARFLSPAGAATAGAVVPGNPLRVMPDRTWEQIYRNQFEDDSTFVFTCAPND 7o3c.1    -ESSEARKGFSYLVTATTTVGV----------------------------------------------------------  target    THNCLLRAHVKNGVVVRISPTYGYGEATDLYGNRASHRWDPRTCQKGLILSRRFYSERRVKAPMIRKGFKDWVEAGYPRN 7o3c.1    --------------------------------------------------------------------------------  target    DDGTPQMDVTLRGSDDWIRISWDEATTIAAKTMEDVARTFNGDEGARKLLAQGYHPEMVEVMHGAGVQALKLR 7o3c.1    ------------------------------------------------------------------------- ``` | | | | | | | | | | | | | | | | | | | | | | | | | | | | | | | | | | | | | | | | | | | | | | | | | |
|  | 7o3h.1.E | Cytochrome b-c1 complex subunit Rieske, mitochondrial  *Murine CIII2 focus-refined from supercomplex CICIII2* | 0.01 |  | 9.52 | 0.09 | 2-22 | EM | 0.00 | hetero-2-2-2-2-2-2-… | 8 x 3PE, 6 x CDL, 4 x HEM, 2 x HEC, 2 x FES, 2 x PC1 | HHblits | 0.25 |
| ``` target    MAQGVSRRQLLGRALALGSGAALADLLGPARFLSPAGAATAGAVVPGNPLRVMPDRTWEQIYRNQFEDDSTFVFTCAPND 7o3h.1    -ESSEARKGFSYLVTATTTVGV----------------------------------------------------------  target    THNCLLRAHVKNGVVVRISPTYGYGEATDLYGNRASHRWDPRTCQKGLILSRRFYSERRVKAPMIRKGFKDWVEAGYPRN 7o3h.1    --------------------------------------------------------------------------------  target    DDGTPQMDVTLRGSDDWIRISWDEATTIAAKTMEDVARTFNGDEGARKLLAQGYHPEMVEVMHGAGVQALKLR 7o3h.1    ------------------------------------------------------------------------- ``` | | | | | | | | | | | | | | | | | | | | | | | | | | | | | | | | | | | | | | | | | | | | | | | | | |
|  | 7o3h.1.O | Cytochrome b-c1 complex subunit Rieske, mitochondrial  *Murine CIII2 focus-refined from supercomplex CICIII2* | 0.01 |  | 9.52 | 0.09 | 2-22 | EM | 0.00 | hetero-2-2-2-2-2-2-… | 8 x 3PE, 6 x CDL, 4 x HEM, 2 x HEC, 2 x FES, 2 x PC1 | HHblits | 0.25 |
| ``` target    MAQGVSRRQLLGRALALGSGAALADLLGPARFLSPAGAATAGAVVPGNPLRVMPDRTWEQIYRNQFEDDSTFVFTCAPND 7o3h.1    -ESSEARKGFSYLVTATTTVGV----------------------------------------------------------  target    THNCLLRAHVKNGVVVRISPTYGYGEATDLYGNRASHRWDPRTCQKGLILSRRFYSERRVKAPMIRKGFKDWVEAGYPRN 7o3h.1    --------------------------------------------------------------------------------  target    DDGTPQMDVTLRGSDDWIRISWDEATTIAAKTMEDVARTFNGDEGARKLLAQGYHPEMVEVMHGAGVQALKLR 7o3h.1    ------------------------------------------------------------------------- ``` | | | | | | | | | | | | | | | | | | | | | | | | | | | | | | | | | | | | | | | | | | | | | | | | | |
|  | 6s6y.1.B | Tungsten-containing formylmethanofuran dehydrogenase, subunit B  *X-ray crystal structure of the formyltransferase/hydrolase complex (FhcABCD) from Methylorubrum extorquens in complex with methylofuran* | 0.00 |  | 20.00 | 0.09 | 178-197 | X-ray | 3.10 | hetero-2-2-2-2-mer | 1 x MFN, 4 x ZN, 4 x CA, 4 x K, 3 x DGL, 2 x GLU, 1 x IAS | HHblits | 0.27 |
| ``` target    MAQGVSRRQLLGRALALGSGAALADLLGPARFLSPAGAATAGAVVPGNPLRVMPDRTWEQIYRNQFEDDSTFVFTCAPND 6s6y.1    --------------------------------------------------------------------------------  target    THNCLLRAHVKNGVVVRISPTYGYGEATDLYGNRASHRWDPRTCQKGLILSRRFYSERRVKAPMIRKGFKDWVEAGYPRN 6s6y.1    --------------------------------------------------------------------------------  target    DDGTPQMDVTLRGSDDWIRISWDEATTIAAKTMEDVARTFNGDEGARKLLAQGYHPEMVEVMHGAGVQALKLR 6s6y.1    -----------------GAADVDAAVEAAADLLAASR------------------------------------ ``` | | | | | | | | | | | | | | | | | | | | | | | | | | | | | | | | | | | | | | | | | | | | | | | | | |
|  | 5kli.1.C | Ubiquinol-cytochrome c reductase iron-sulfur subunit  *Rhodobacter sphaeroides bc1 with stigmatellin and antimycin* | 0.00 |  | 26.32 | 0.08 | 2-20 | X-ray | 3.00 | hetero-oligomer | 4 x HEM, 2 x SMA, 2 x ANJ, 2 x LOP, 2 x HEC, 2 x FES | HHblits | 0.29 |
| ``` target    MAQGVSRRQLLGRALALGSGAALADLLGPARFLSPAGAATAGAVVPGNPLRVMPDRTWEQIYRNQFEDDSTFVFTCAPND 5kli.1    -DHAGTRRDFLYYATAGAGA------------------------------------------------------------  target    THNCLLRAHVKNGVVVRISPTYGYGEATDLYGNRASHRWDPRTCQKGLILSRRFYSERRVKAPMIRKGFKDWVEAGYPRN 5kli.1    --------------------------------------------------------------------------------  target    DDGTPQMDVTLRGSDDWIRISWDEATTIAAKTMEDVARTFNGDEGARKLLAQGYHPEMVEVMHGAGVQALKLR 5kli.1    ------------------------------------------------------------------------- ``` | | | | | | | | | | | | | | | | | | | | | | | | | | | | | | | | | | | | | | | | | | | | | | | | | |
|  | 5kkz.1.C | Ubiquinol-cytochrome c reductase iron-sulfur subunit  *Rhodobacter sphaeroides bc1 with famoxadone* | 0.00 |  | 26.32 | 0.08 | 2-20 | X-ray | 2.97 | hetero-2-2-2-mer | 4 x HEM, 2 x FMX, 2 x ASC, 2 x LOP, 2 x HEC, 2 x BOG, 2 x FES | HHblits | 0.29 |
| ``` target    MAQGVSRRQLLGRALALGSGAALADLLGPARFLSPAGAATAGAVVPGNPLRVMPDRTWEQIYRNQFEDDSTFVFTCAPND 5kkz.1    -DHAGTRRDFLYYATAGAGA------------------------------------------------------------  target    THNCLLRAHVKNGVVVRISPTYGYGEATDLYGNRASHRWDPRTCQKGLILSRRFYSERRVKAPMIRKGFKDWVEAGYPRN 5kkz.1    --------------------------------------------------------------------------------  target    DDGTPQMDVTLRGSDDWIRISWDEATTIAAKTMEDVARTFNGDEGARKLLAQGYHPEMVEVMHGAGVQALKLR 5kkz.1    ------------------------------------------------------------------------- ``` | | | | | | | | | | | | | | | | | | | | | | | | | | | | | | | | | | | | | | | | | | | | | | | | | |
|  | 7tce.2.F | Ubiquinol-cytochrome c reductase iron-sulfur subunit  *Crystal structure of delta sub IV Rhodobacter Sphaeroides bc1 with the antimalarial drug atovaquone.* | 0.00 |  | 26.32 | 0.08 | 2-20 | X-ray | 3.85 | hetero-2-2-2-mer | 4 x HEM, 2 x AOQ, 2 x 6PE, 2 x BOG, 2 x HEC, 2 x FES | HHblits | 0.29 |
| ``` target    MAQGVSRRQLLGRALALGSGAALADLLGPARFLSPAGAATAGAVVPGNPLRVMPDRTWEQIYRNQFEDDSTFVFTCAPND 7tce.2    -DHAGTRRDFLYYATAGAGA------------------------------------------------------------  target    THNCLLRAHVKNGVVVRISPTYGYGEATDLYGNRASHRWDPRTCQKGLILSRRFYSERRVKAPMIRKGFKDWVEAGYPRN 7tce.2    --------------------------------------------------------------------------------  target    DDGTPQMDVTLRGSDDWIRISWDEATTIAAKTMEDVARTFNGDEGARKLLAQGYHPEMVEVMHGAGVQALKLR 7tce.2    ------------------------------------------------------------------------- ``` | | | | | | | | | | | | | | | | | | | | | | | | | | | | | | | | | | | | | | | | | | | | | | | | | |
|  | 7tlj.1.C | Ubiquinol-cytochrome c reductase iron-sulfur subunit  *Rhodobacter sphaeroides Mitochondrial respiratory chain complex* | 0.00 |  | 26.32 | 0.08 | 2-20 | EM | 0.00 | hetero-2-2-2-2-mer | 4 x HEM, 2 x PQU, 2 x LOP, 2 x HEC, 2 x FES | HHblits | 0.29 |
| ``` target    MAQGVSRRQLLGRALALGSGAALADLLGPARFLSPAGAATAGAVVPGNPLRVMPDRTWEQIYRNQFEDDSTFVFTCAPND 7tlj.1    -DHAGTRRDFLYYATAGAGA------------------------------------------------------------  target    THNCLLRAHVKNGVVVRISPTYGYGEATDLYGNRASHRWDPRTCQKGLILSRRFYSERRVKAPMIRKGFKDWVEAGYPRN 7tlj.1    --------------------------------------------------------------------------------  target    DDGTPQMDVTLRGSDDWIRISWDEATTIAAKTMEDVARTFNGDEGARKLLAQGYHPEMVEVMHGAGVQALKLR 7tlj.1    ------------------------------------------------------------------------- ``` | | | | | | | | | | | | | | | | | | | | | | | | | | | | | | | | | | | | | | | | | | | | | | | | | |
|  | 8asi.1.E | Ubiquinol-cytochrome c reductase iron-sulfur subunit  *Four subunit cytochrome b-c1 complex from Rhodobacter sphaeroides in native nanodiscs - consensus refinement in the b-b conformation* | 0.01 |  | 26.32 | 0.08 | 2-20 | EM | 0.00 | hetero-2-2-2-2-mer | 2 x FES, 12 x PEE, 4 x HEM, 2 x HEC, 1 x U10 | HHblits | 0.29 |
| ``` target    MAQGVSRRQLLGRALALGSGAALADLLGPARFLSPAGAATAGAVVPGNPLRVMPDRTWEQIYRNQFEDDSTFVFTCAPND 8asi.1    -DHAGTRRDFLYYATAGAGA------------------------------------------------------------  target    THNCLLRAHVKNGVVVRISPTYGYGEATDLYGNRASHRWDPRTCQKGLILSRRFYSERRVKAPMIRKGFKDWVEAGYPRN 8asi.1    --------------------------------------------------------------------------------  target    DDGTPQMDVTLRGSDDWIRISWDEATTIAAKTMEDVARTFNGDEGARKLLAQGYHPEMVEVMHGAGVQALKLR 8asi.1    ------------------------------------------------------------------------- ``` | | | | | | | | | | | | | | | | | | | | | | | | | | | | | | | | | | | | | | | | | | | | | | | | | |
|  | 8asj.1.E | Ubiquinol-cytochrome c reductase iron-sulfur subunit  *Four subunit cytochrome b-c1 complex from Rhodobacter sphaeroides in native nanodiscs - focussed refinement in the b-c conformation* | 0.01 |  | 26.32 | 0.08 | 2-20 | EM | 0.00 | hetero-2-2-2-2-mer | 2 x FES, 12 x PEE, 4 x HEM, 2 x HEC, 1 x U10 | HHblits | 0.29 |
| ``` target    MAQGVSRRQLLGRALALGSGAALADLLGPARFLSPAGAATAGAVVPGNPLRVMPDRTWEQIYRNQFEDDSTFVFTCAPND 8asj.1    -DHAGTRRDFLYYATAGAGA------------------------------------------------------------  target    THNCLLRAHVKNGVVVRISPTYGYGEATDLYGNRASHRWDPRTCQKGLILSRRFYSERRVKAPMIRKGFKDWVEAGYPRN 8asj.1    --------------------------------------------------------------------------------  target    DDGTPQMDVTLRGSDDWIRISWDEATTIAAKTMEDVARTFNGDEGARKLLAQGYHPEMVEVMHGAGVQALKLR 8asj.1    ------------------------------------------------------------------------- ``` | | | | | | | | | | | | | | | | | | | | | | | | | | | | | | | | | | | | | | | | | | | | | | | | | |
|  | 8asi.1.A | Ubiquinol-cytochrome c reductase iron-sulfur subunit  *Four subunit cytochrome b-c1 complex from Rhodobacter sphaeroides in native nanodiscs - consensus refinement in the b-b conformation* | 0.01 |  | 26.32 | 0.08 | 2-20 | EM | 0.00 | hetero-2-2-2-2-mer | 2 x FES, 12 x PEE, 4 x HEM, 2 x HEC, 1 x U10 | HHblits | 0.29 |
| ``` target    MAQGVSRRQLLGRALALGSGAALADLLGPARFLSPAGAATAGAVVPGNPLRVMPDRTWEQIYRNQFEDDSTFVFTCAPND 8asi.1    -DHAGTRRDFLYYATAGAGA------------------------------------------------------------  target    THNCLLRAHVKNGVVVRISPTYGYGEATDLYGNRASHRWDPRTCQKGLILSRRFYSERRVKAPMIRKGFKDWVEAGYPRN 8asi.1    --------------------------------------------------------------------------------  target    DDGTPQMDVTLRGSDDWIRISWDEATTIAAKTMEDVARTFNGDEGARKLLAQGYHPEMVEVMHGAGVQALKLR 8asi.1    ------------------------------------------------------------------------- ``` | | | | | | | | | | | | | | | | | | | | | | | | | | | | | | | | | | | | | | | | | | | | | | | | | |
|  | 7e1v.1.P | Cytochrome bc1 complex Rieske iron-sulfur subunit  *Cryo-EM structure of apo hybrid respiratory supercomplex consisting of Mycobacterium tuberculosis complexIII and Mycobacterium smegmatis complexIV* | 0.01 |  | 29.41 | 0.07 | 3-19 | EM | 2.68 | hetero-2-2-2-2-2-2-… | 8 x CU, 17 x CDL, 4 x PLM, 4 x HEA, 2 x 9Y0, 4 x HEM, 10 x MQ9, 2 x FES, 4 x 9YF, 4 x HEC | HHblits | 0.38 |
| ``` target    MAQGVSRRQLLGRALALGSGAALADLLGPARFLSPAGAATAGAVVPGNPLRVMPDRTWEQIYRNQFEDDSTFVFTCAPND 7e1v.1    --STIRRRKLIGLSFGVGM-------------------------------------------------------------  target    THNCLLRAHVKNGVVVRISPTYGYGEATDLYGNRASHRWDPRTCQKGLILSRRFYSERRVKAPMIRKGFKDWVEAGYPRN 7e1v.1    --------------------------------------------------------------------------------  target    DDGTPQMDVTLRGSDDWIRISWDEATTIAAKTMEDVARTFNGDEGARKLLAQGYHPEMVEVMHGAGVQALKLR 7e1v.1    ------------------------------------------------------------------------- ``` | | | | | | | | | | | | | | | | | | | | | | | | | | | | | | | | | | | | | | | | | | | | | | | | | |
|  | 7rh5.1.V | Cytochrome bc1 complex Rieske iron-sulfur subunit  *Mycobacterial CIII2CIV2 supercomplex, Inhibitor free* | 0.01 |  | 29.41 | 0.07 | 3-19 | EM | 0.00 | hetero-2-2-2-2-2-2-… | 6 x CU, 4 x HEA, 16 x CDL, 4 x HEM, 8 x MQ9, 6 x 9Y0, 4 x PLM, 4 x 9XX, 4 x HEC, 8 x 9YF, 2 x FES | HHblits | 0.38 |
| ``` target    MAQGVSRRQLLGRALALGSGAALADLLGPARFLSPAGAATAGAVVPGNPLRVMPDRTWEQIYRNQFEDDSTFVFTCAPND 7rh5.1    --STLKRRKVIGLSLGIGL-------------------------------------------------------------  target    THNCLLRAHVKNGVVVRISPTYGYGEATDLYGNRASHRWDPRTCQKGLILSRRFYSERRVKAPMIRKGFKDWVEAGYPRN 7rh5.1    --------------------------------------------------------------------------------  target    DDGTPQMDVTLRGSDDWIRISWDEATTIAAKTMEDVARTFNGDEGARKLLAQGYHPEMVEVMHGAGVQALKLR 7rh5.1    ------------------------------------------------------------------------- ``` | | | | | | | | | | | | | | | | | | | | | | | | | | | | | | | | | | | | | | | | | | | | | | | | | |
|  | 6hwh.1.A | Ubiquinol-cytochrome c reductase iron-sulfur subunit  *Structure of a functional obligate respiratory supercomplex from Mycobacterium smegmatis* | 0.01 |  | 29.41 | 0.07 | 3-19 | EM | 0.00 | hetero-2-2-2-2-2-4-… | 2 x FES, 8 x CDL, 4 x MQ9, 6 x CU, 4 x HAS, 4 x HEC, 4 x HEM | HHblits | 0.38 |
| ``` target    MAQGVSRRQLLGRALALGSGAALADLLGPARFLSPAGAATAGAVVPGNPLRVMPDRTWEQIYRNQFEDDSTFVFTCAPND 6hwh.1    --STLKRRKVIGLSLGIGL-------------------------------------------------------------  target    THNCLLRAHVKNGVVVRISPTYGYGEATDLYGNRASHRWDPRTCQKGLILSRRFYSERRVKAPMIRKGFKDWVEAGYPRN 6hwh.1    --------------------------------------------------------------------------------  target    DDGTPQMDVTLRGSDDWIRISWDEATTIAAKTMEDVARTFNGDEGARKLLAQGYHPEMVEVMHGAGVQALKLR 6hwh.1    ------------------------------------------------------------------------- ``` | | | | | | | | | | | | | | | | | | | | | | | | | | | | | | | | | | | | | | | | | | | | | | | | | |
|  | 8bpx.51.A | Cytochrome b-c1 complex subunit Rieske-1, mitochondrial  *Cryo-EM structure of the Arabidopsis thaliana I+III2 supercomplex (Complete composition)* | 0.00 |  | 21.05 | 0.08 | 3-21 | EM | 0.00 | monomer |  | HHblits | 0.27 |
| ``` target    MAQGVSRRQLLGRALALGSGAALADLLGPARFLSPAGAATAGAVVPGNPLRVMPDRTWEQIYRNQFEDDSTFVFTCAPND 8bpx.51   --GDPSKRAFAYFVLSGGRFV-----------------------------------------------------------  target    THNCLLRAHVKNGVVVRISPTYGYGEATDLYGNRASHRWDPRTCQKGLILSRRFYSERRVKAPMIRKGFKDWVEAGYPRN 8bpx.51   --------------------------------------------------------------------------------  target    DDGTPQMDVTLRGSDDWIRISWDEATTIAAKTMEDVARTFNGDEGARKLLAQGYHPEMVEVMHGAGVQALKLR 8bpx.51   ------------------------------------------------------------------------- ``` | | | | | | | | | | | | | | | | | | | | | | | | | | | | | | | | | | | | | | | | | | | | | | | | | |
|  | 8bel.1.I | Cytochrome b-c1 complex subunit Rieske-1, mitochondrial  *Cryo-EM structure of the Arabidopsis thaliana I+III2 supercomplex (CIII membrane domain)* | 0.00 |  | 21.05 | 0.08 | 3-21 | EM | 0.00 | hetero-2-2-2-2-2-2-… | 6 x HEM, 3 x UQ5, 6 x 3PH, 4 x PGT, 7 x CDL, 5 x PC7, 2 x FES, 3 x PTY, 1 x UQ7, 2 x Q7G | HHblits | 0.27 |
| ``` target    MAQGVSRRQLLGRALALGSGAALADLLGPARFLSPAGAATAGAVVPGNPLRVMPDRTWEQIYRNQFEDDSTFVFTCAPND 8bel.1    --GDPSKRAFAYFVLSGGRFV-----------------------------------------------------------  target    THNCLLRAHVKNGVVVRISPTYGYGEATDLYGNRASHRWDPRTCQKGLILSRRFYSERRVKAPMIRKGFKDWVEAGYPRN 8bel.1    --------------------------------------------------------------------------------  target    DDGTPQMDVTLRGSDDWIRISWDEATTIAAKTMEDVARTFNGDEGARKLLAQGYHPEMVEVMHGAGVQALKLR 8bel.1    ------------------------------------------------------------------------- ``` | | | | | | | | | | | | | | | | | | | | | | | | | | | | | | | | | | | | | | | | | | | | | | | | | |
|  | 8bel.1.B | Cytochrome b-c1 complex subunit Rieske-1, mitochondrial  *Cryo-EM structure of the Arabidopsis thaliana I+III2 supercomplex (CIII membrane domain)* | 0.00 |  | 21.05 | 0.08 | 3-21 | EM | 0.00 | hetero-2-2-2-2-2-2-… | 6 x HEM, 3 x UQ5, 6 x 3PH, 4 x PGT, 7 x CDL, 5 x PC7, 2 x FES, 3 x PTY, 1 x UQ7, 2 x Q7G | HHblits | 0.27 |
| ``` target    MAQGVSRRQLLGRALALGSGAALADLLGPARFLSPAGAATAGAVVPGNPLRVMPDRTWEQIYRNQFEDDSTFVFTCAPND 8bel.1    --GDPSKRAFAYFVLSGGRFV-----------------------------------------------------------  target    THNCLLRAHVKNGVVVRISPTYGYGEATDLYGNRASHRWDPRTCQKGLILSRRFYSERRVKAPMIRKGFKDWVEAGYPRN 8bel.1    --------------------------------------------------------------------------------  target    DDGTPQMDVTLRGSDDWIRISWDEATTIAAKTMEDVARTFNGDEGARKLLAQGYHPEMVEVMHGAGVQALKLR 8bel.1    ------------------------------------------------------------------------- ``` | | | | | | | | | | | | | | | | | | | | | | | | | | | | | | | | | | | | | | | | | | | | | | | | | |
|  | 3bcc.1.E | UBIQUINOL CYTOCHROME C OXIDOREDUCTASE  *STIGMATELLIN AND ANTIMYCIN BOUND CYTOCHROME BC1 COMPLEX FROM CHICKEN* | 0.01 |  | 15.79 | 0.08 | 2-20 | X-ray | 3.70 | hetero-2-2-2-2-2-2-… | 6 x HEM, 2 x SIG, 2 x AMY, 2 x FES | HHblits | 0.26 |
| ``` target    MAQGVSRRQLLGRALALGSGAALADLLGPARFLSPAGAATAGAVVPGNPLRVMPDRTWEQIYRNQFEDDSTFVFTCAPND 3bcc.1    -ESDPSRKGFSYLVTAVTTL------------------------------------------------------------  target    THNCLLRAHVKNGVVVRISPTYGYGEATDLYGNRASHRWDPRTCQKGLILSRRFYSERRVKAPMIRKGFKDWVEAGYPRN 3bcc.1    --------------------------------------------------------------------------------  target    DDGTPQMDVTLRGSDDWIRISWDEATTIAAKTMEDVARTFNGDEGARKLLAQGYHPEMVEVMHGAGVQALKLR 3bcc.1    ------------------------------------------------------------------------- ``` | | | | | | | | | | | | | | | | | | | | | | | | | | | | | | | | | | | | | | | | | | | | | | | | | |
|  | 1bcc.1.O | UBIQUINOL CYTOCHROME C OXIDOREDUCTASE  *CYTOCHROME BC1 COMPLEX FROM CHICKEN* | 0.00 |  | 15.79 | 0.08 | 2-20 | X-ray | 3.16 | hetero-2-2-2-2-2-2-… | 6 x HEM, 2 x U10, 4 x PEE, 2 x BOG, 2 x FES | HHblits | 0.26 |
| ``` target    MAQGVSRRQLLGRALALGSGAALADLLGPARFLSPAGAATAGAVVPGNPLRVMPDRTWEQIYRNQFEDDSTFVFTCAPND 1bcc.1    -ESDPSRKGFSYLVTAVTTL------------------------------------------------------------  target    THNCLLRAHVKNGVVVRISPTYGYGEATDLYGNRASHRWDPRTCQKGLILSRRFYSERRVKAPMIRKGFKDWVEAGYPRN 1bcc.1    --------------------------------------------------------------------------------  target    DDGTPQMDVTLRGSDDWIRISWDEATTIAAKTMEDVARTFNGDEGARKLLAQGYHPEMVEVMHGAGVQALKLR 1bcc.1    ------------------------------------------------------------------------- ``` | | | | | | | | | | | | | | | | | | | | | | | | | | | | | | | | | | | | | | | | | | | | | | | | | |
|  | 2ybb.1.b | CYTOCHROME B-C1 COMPLEX SUBUNIT RIESKE, MITOCHONDRIAL  *Fitted model for bovine mitochondrial supercomplex I1III2IV1 by single particle cryo-EM (EMD-1876)* | 0.00 |  | 10.53 | 0.08 | 3-21 | EM | 19.00 | hetero-oligomer | 7 x SF4, 1 x FMN, 1 x NAI, 7 x MG, 4 x FES, 1 x CA, 5 x HEM, 2 x SMA, 2 x UQ1, 2 x HEC, 4 x CDL, 2 x HEA, 3 x CU, 1 x ZN | HHblits | 0.25 |
| ``` target    MAQGVSRRQLLGRALALGSGAALADLLGPARFLSPAGAATAGAVVPGNPLRVMPDRTWEQIYRNQFEDDSTFVFTCAPND 2ybb.1    --SSEARKGFSYLVTATTTVG-----------------------------------------------------------  target    THNCLLRAHVKNGVVVRISPTYGYGEATDLYGNRASHRWDPRTCQKGLILSRRFYSERRVKAPMIRKGFKDWVEAGYPRN 2ybb.1    --------------------------------------------------------------------------------  target    DDGTPQMDVTLRGSDDWIRISWDEATTIAAKTMEDVARTFNGDEGARKLLAQGYHPEMVEVMHGAGVQALKLR 2ybb.1    ------------------------------------------------------------------------- ``` | | | | | | | | | | | | | | | | | | | | | | | | | | | | | | | | | | | | | | | | | | | | | | | | | |
|  | 1be3.1.E | CYTOCHROME BC1 COMPLEX  *CYTOCHROME BC1 COMPLEX FROM BOVINE* | 0.00 |  | 10.53 | 0.08 | 3-21 | X-ray | 3.00 | hetero-oligomer | 2 x HEM, 1 x HEC, 1 x FES | HHblits | 0.25 |
| ``` target    MAQGVSRRQLLGRALALGSGAALADLLGPARFLSPAGAATAGAVVPGNPLRVMPDRTWEQIYRNQFEDDSTFVFTCAPND 1be3.1    --SSEARKGFSYLVTATTTVG-----------------------------------------------------------  target    THNCLLRAHVKNGVVVRISPTYGYGEATDLYGNRASHRWDPRTCQKGLILSRRFYSERRVKAPMIRKGFKDWVEAGYPRN 1be3.1    --------------------------------------------------------------------------------  target    DDGTPQMDVTLRGSDDWIRISWDEATTIAAKTMEDVARTFNGDEGARKLLAQGYHPEMVEVMHGAGVQALKLR 1be3.1    ------------------------------------------------------------------------- ``` | | | | | | | | | | | | | | | | | | | | | | | | | | | | | | | | | | | | | | | | | | | | | | | | | |
|  | 1bgy.1.P | CYTOCHROME BC1 COMPLEX  *CYTOCHROME BC1 COMPLEX FROM BOVINE* | 0.00 |  | 10.53 | 0.08 | 3-21 | X-ray | 3.00 | hetero-oligomer | 4 x HEM, 2 x HEC, 1 x FES | HHblits | 0.25 |
| ``` target    MAQGVSRRQLLGRALALGSGAALADLLGPARFLSPAGAATAGAVVPGNPLRVMPDRTWEQIYRNQFEDDSTFVFTCAPND 1bgy.1    --SSEARKGFSYLVTATTTVG-----------------------------------------------------------  target    THNCLLRAHVKNGVVVRISPTYGYGEATDLYGNRASHRWDPRTCQKGLILSRRFYSERRVKAPMIRKGFKDWVEAGYPRN 1bgy.1    --------------------------------------------------------------------------------  target    DDGTPQMDVTLRGSDDWIRISWDEATTIAAKTMEDVARTFNGDEGARKLLAQGYHPEMVEVMHGAGVQALKLR 1bgy.1    ------------------------------------------------------------------------- ``` | | | | | | | | | | | | | | | | | | | | | | | | | | | | | | | | | | | | | | | | | | | | | | | | | |
|  | 1l0n.1.E | UBIQUINOL-CYTOCHROME C REDUCTASE IRON-SULFUR SUBUNIT  *native structure of bovine mitochondrial cytochrome bc1 complex* | 0.00 |  | 10.53 | 0.08 | 3-21 | X-ray | 2.60 | hetero-oligomer | 3 x HEM, 1 x FES | HHblits | 0.25 |
| ``` target    MAQGVSRRQLLGRALALGSGAALADLLGPARFLSPAGAATAGAVVPGNPLRVMPDRTWEQIYRNQFEDDSTFVFTCAPND 1l0n.1    --SSEARKGFSYLVTATTTVG-----------------------------------------------------------  target    THNCLLRAHVKNGVVVRISPTYGYGEATDLYGNRASHRWDPRTCQKGLILSRRFYSERRVKAPMIRKGFKDWVEAGYPRN 1l0n.1    --------------------------------------------------------------------------------  target    DDGTPQMDVTLRGSDDWIRISWDEATTIAAKTMEDVARTFNGDEGARKLLAQGYHPEMVEVMHGAGVQALKLR 1l0n.1    ------------------------------------------------------------------------- ``` | | | | | | | | | | | | | | | | | | | | | | | | | | | | | | | | | | | | | | | | | | | | | | | | | |
|  | 1l0l.1.E | UBIQUINOL-CYTOCHROME C REDUCTASE IRON-SULFUR SUBUNIT  *structure of bovine mitochondrial cytochrome bc1 complex with a bound fungicide famoxadone* | 0.00 |  | 10.53 | 0.08 | 3-21 | X-ray | 2.35 | hetero-oligomer | 3 x HEM, 1 x FMX, 1 x FES | HHblits | 0.25 |
| ``` target    MAQGVSRRQLLGRALALGSGAALADLLGPARFLSPAGAATAGAVVPGNPLRVMPDRTWEQIYRNQFEDDSTFVFTCAPND 1l0l.1    --SSEARKGFSYLVTATTTVG-----------------------------------------------------------  target    THNCLLRAHVKNGVVVRISPTYGYGEATDLYGNRASHRWDPRTCQKGLILSRRFYSERRVKAPMIRKGFKDWVEAGYPRN 1l0l.1    --------------------------------------------------------------------------------  target    DDGTPQMDVTLRGSDDWIRISWDEATTIAAKTMEDVARTFNGDEGARKLLAQGYHPEMVEVMHGAGVQALKLR 1l0l.1    ------------------------------------------------------------------------- ``` | | | | | | | | | | | | | | | | | | | | | | | | | | | | | | | | | | | | | | | | | | | | | | | | | |
|  | 1ntk.1.E | UBIQUINOL-CYTOCHROME C REDUCTASE IRON-SULFUR SUBUNIT, MITOCHONDRIAL  *Crystal Structure of Mitochondrial Cytochrome bc1 in Complex with Antimycin A1* | 0.01 |  | 10.53 | 0.08 | 3-21 | X-ray | 2.60 | hetero-oligomer | 6 x HEM, 2 x AY1, 2 x FES | HHblits | 0.25 |
| ``` target    MAQGVSRRQLLGRALALGSGAALADLLGPARFLSPAGAATAGAVVPGNPLRVMPDRTWEQIYRNQFEDDSTFVFTCAPND 1ntk.1    --SSEARKGFSYLVTATTTVG-----------------------------------------------------------  target    THNCLLRAHVKNGVVVRISPTYGYGEATDLYGNRASHRWDPRTCQKGLILSRRFYSERRVKAPMIRKGFKDWVEAGYPRN 1ntk.1    --------------------------------------------------------------------------------  target    DDGTPQMDVTLRGSDDWIRISWDEATTIAAKTMEDVARTFNGDEGARKLLAQGYHPEMVEVMHGAGVQALKLR 1ntk.1    ------------------------------------------------------------------------- ``` | | | | | | | | | | | | | | | | | | | | | | | | | | | | | | | | | | | | | | | | | | | | | | | | | |
|  | 1sqb.1.E | Ubiquinol-cytochrome c reductase iron-sulfur subunit  *Crystal Structure Analysis of Bovine Bc1 with Azoxystrobin* | 0.00 |  | 10.53 | 0.08 | 3-21 | X-ray | 2.69 | hetero-oligomer | 6 x HEM, 2 x FES, 2 x AZO | HHblits | 0.25 |
| ``` target    MAQGVSRRQLLGRALALGSGAALADLLGPARFLSPAGAATAGAVVPGNPLRVMPDRTWEQIYRNQFEDDSTFVFTCAPND 1sqb.1    --SSEARKGFSYLVTATTTVG-----------------------------------------------------------  target    THNCLLRAHVKNGVVVRISPTYGYGEATDLYGNRASHRWDPRTCQKGLILSRRFYSERRVKAPMIRKGFKDWVEAGYPRN 1sqb.1    --------------------------------------------------------------------------------  target    DDGTPQMDVTLRGSDDWIRISWDEATTIAAKTMEDVARTFNGDEGARKLLAQGYHPEMVEVMHGAGVQALKLR 1sqb.1    ------------------------------------------------------------------------- ``` | | | | | | | | | | | | | | | | | | | | | | | | | | | | | | | | | | | | | | | | | | | | | | | | | |
|  | 2fyu.1.E | Ubiquinol-cytochrome c reductase iron-sulfur subunit, mitochondrial  *Crystal structure of bovine heart mitochondrial bc1 with jg144 inhibitor* | 0.00 |  | 10.53 | 0.08 | 3-21 | X-ray | 2.26 | hetero-oligomer | 6 x HEM, 2 x FDN, 2 x FES | HHblits | 0.25 |
| ``` target    MAQGVSRRQLLGRALALGSGAALADLLGPARFLSPAGAATAGAVVPGNPLRVMPDRTWEQIYRNQFEDDSTFVFTCAPND 2fyu.1    --SSEARKGFSYLVTATTTVG-----------------------------------------------------------  target    THNCLLRAHVKNGVVVRISPTYGYGEATDLYGNRASHRWDPRTCQKGLILSRRFYSERRVKAPMIRKGFKDWVEAGYPRN 2fyu.1    --------------------------------------------------------------------------------  target    DDGTPQMDVTLRGSDDWIRISWDEATTIAAKTMEDVARTFNGDEGARKLLAQGYHPEMVEVMHGAGVQALKLR 2fyu.1    ------------------------------------------------------------------------- ``` | | | | | | | | | | | | | | | | | | | | | | | | | | | | | | | | | | | | | | | | | | | | | | | | | |
|  | 1ntm.1.E | UBIQUINOL-CYTOCHROME C REDUCTASE IRON-SULFUR SUBUNIT, mitochondrial  *Crystal Structure of Mitochondrial Cytochrome bc1 Complex at 2.4 Angstrom* | 0.00 |  | 10.53 | 0.08 | 3-21 | X-ray | 2.40 | hetero-oligomer | 6 x HEM, 2 x FES | HHblits | 0.25 |
| ``` target    MAQGVSRRQLLGRALALGSGAALADLLGPARFLSPAGAATAGAVVPGNPLRVMPDRTWEQIYRNQFEDDSTFVFTCAPND 1ntm.1    --SSEARKGFSYLVTATTTVG-----------------------------------------------------------  target    THNCLLRAHVKNGVVVRISPTYGYGEATDLYGNRASHRWDPRTCQKGLILSRRFYSERRVKAPMIRKGFKDWVEAGYPRN 1ntm.1    --------------------------------------------------------------------------------  target    DDGTPQMDVTLRGSDDWIRISWDEATTIAAKTMEDVARTFNGDEGARKLLAQGYHPEMVEVMHGAGVQALKLR 1ntm.1    ------------------------------------------------------------------------- ``` | | | | | | | | | | | | | | | | | | | | | | | | | | | | | | | | | | | | | | | | | | | | | | | | | |
|  | 5klv.1.P | Cytochrome b-c1 complex subunit Rieske, mitochondrial  *Structure of bos taurus cytochrome bc1 with fenamidone inhibited* | 0.00 |  | 10.53 | 0.08 | 3-21 | X-ray | 2.65 | hetero-oligomer | 4 x 6PE, 6 x CDL, 4 x HEM, 2 x FNM, 2 x 8PE, 2 x HEC, 2 x PEF, 2 x FES, 2 x PX4 | HHblits | 0.25 |
| ``` target    MAQGVSRRQLLGRALALGSGAALADLLGPARFLSPAGAATAGAVVPGNPLRVMPDRTWEQIYRNQFEDDSTFVFTCAPND 5klv.1    --SSEARKGFSYLVTATTTVG-----------------------------------------------------------  target    THNCLLRAHVKNGVVVRISPTYGYGEATDLYGNRASHRWDPRTCQKGLILSRRFYSERRVKAPMIRKGFKDWVEAGYPRN 5klv.1    --------------------------------------------------------------------------------  target    DDGTPQMDVTLRGSDDWIRISWDEATTIAAKTMEDVARTFNGDEGARKLLAQGYHPEMVEVMHGAGVQALKLR 5klv.1    ------------------------------------------------------------------------- ``` | | | | | | | | | | | | | | | | | | | | | | | | | | | | | | | | | | | | | | | | | | | | | | | | | |
|  | 5okd.1.E | Cytochrome b-c1 complex subunit Rieske, mitochondrial  *Crystal structure of bovine Cytochrome bc1 in complex with inhibitor SCR0911.* | 0.00 |  | 10.53 | 0.08 | 3-21 | X-ray | 3.10 | hetero-1-1-1-1-1-1-… | 1 x 6PE, 4 x CDL, 2 x HEM, 1 x 9XE, 1 x LMT, 2 x PEE, 1 x HEC, 1 x FES, 1 x PX4 | HHblits | 0.25 |
| ``` target    MAQGVSRRQLLGRALALGSGAALADLLGPARFLSPAGAATAGAVVPGNPLRVMPDRTWEQIYRNQFEDDSTFVFTCAPND 5okd.1    --SSEARKGFSYLVTATTTVG-----------------------------------------------------------  target    THNCLLRAHVKNGVVVRISPTYGYGEATDLYGNRASHRWDPRTCQKGLILSRRFYSERRVKAPMIRKGFKDWVEAGYPRN 5okd.1    --------------------------------------------------------------------------------  target    DDGTPQMDVTLRGSDDWIRISWDEATTIAAKTMEDVARTFNGDEGARKLLAQGYHPEMVEVMHGAGVQALKLR 5okd.1    ------------------------------------------------------------------------- ``` | | | | | | | | | | | | | | | | | | | | | | | | | | | | | | | | | | | | | | | | | | | | | | | | | |
|  | 6nhg.1.E | Cytochrome b-c1 complex subunit Rieske, mitochondrial  *Rhodobacter sphaeroides Mitochondrial respiratory chain complex* | 0.00 |  | 10.53 | 0.08 | 3-21 | X-ray | 2.80 | hetero-2-2-2-2-2-2-… | 4 x 6PE, 6 x CDL, 4 x HEM, 2 x AZO, 2 x 8PE, 2 x HEC, 2 x FES, 2 x MC3 | HHblits | 0.25 |
| ``` target    MAQGVSRRQLLGRALALGSGAALADLLGPARFLSPAGAATAGAVVPGNPLRVMPDRTWEQIYRNQFEDDSTFVFTCAPND 6nhg.1    --SSEARKGFSYLVTATTTVG-----------------------------------------------------------  target    THNCLLRAHVKNGVVVRISPTYGYGEATDLYGNRASHRWDPRTCQKGLILSRRFYSERRVKAPMIRKGFKDWVEAGYPRN 6nhg.1    --------------------------------------------------------------------------------  target    DDGTPQMDVTLRGSDDWIRISWDEATTIAAKTMEDVARTFNGDEGARKLLAQGYHPEMVEVMHGAGVQALKLR 6nhg.1    ------------------------------------------------------------------------- ``` | | | | | | | | | | | | | | | | | | | | | | | | | | | | | | | | | | | | | | | | | | | | | | | | | |
|  | 1sqp.1.P | Ubiquinol-cytochrome c reductase iron-sulfur subunit, mitochondrial precursor (EC 1.10.2.2) (Rieske iron-sulfur protein) (RISP) [Contains: Ubiquinol-cytochrome c reductase 8 kDa protein (Complex III subunit IX)]  *Crystal Structure Analysis of Bovine Bc1 with Myxothiazol* | 0.00 |  | 10.53 | 0.08 | 3-21 | X-ray | 2.70 | hetero-2-2-2-2-2-2-… | 6 x CDL, 6 x PEE, 6 x HEC, 2 x MYX, 2 x FES, 2 x PLX | HHblits | 0.25 |
| ``` target    MAQGVSRRQLLGRALALGSGAALADLLGPARFLSPAGAATAGAVVPGNPLRVMPDRTWEQIYRNQFEDDSTFVFTCAPND 1sqp.1    --SSEARKGFSYLVTATTTVG-----------------------------------------------------------  target    THNCLLRAHVKNGVVVRISPTYGYGEATDLYGNRASHRWDPRTCQKGLILSRRFYSERRVKAPMIRKGFKDWVEAGYPRN 1sqp.1    --------------------------------------------------------------------------------  target    DDGTPQMDVTLRGSDDWIRISWDEATTIAAKTMEDVARTFNGDEGARKLLAQGYHPEMVEVMHGAGVQALKLR 1sqp.1    ------------------------------------------------------------------------- ``` | | | | | | | | | | | | | | | | | | | | | | | | | | | | | | | | | | | | | | | | | | | | | | | | | |
|  | 1sqq.1.P | Ubiquinol-cytochrome c reductase iron-sulfur subunit, mitochondrial precursor (EC 1.10.2.2) (Rieske iron-sulfur protein) (RISP) [Contains: Ubiquinol-cytochrome c reductase 8 kDa protein (Complex III subunit IX)]  *Crystal Structure Analysis of Bovine Bc1 with Methoxy Acrylate Stilbene (MOAS)* | 0.00 |  | 10.53 | 0.08 | 3-21 | X-ray | 3.00 | hetero-2-2-2-2-2-2-… | 6 x HEC, 2 x UQ2, 2 x OST, 2 x FES | HHblits | 0.25 |
| ``` target    MAQGVSRRQLLGRALALGSGAALADLLGPARFLSPAGAATAGAVVPGNPLRVMPDRTWEQIYRNQFEDDSTFVFTCAPND 1sqq.1    --SSEARKGFSYLVTATTTVG-----------------------------------------------------------  target    THNCLLRAHVKNGVVVRISPTYGYGEATDLYGNRASHRWDPRTCQKGLILSRRFYSERRVKAPMIRKGFKDWVEAGYPRN 1sqq.1    --------------------------------------------------------------------------------  target    DDGTPQMDVTLRGSDDWIRISWDEATTIAAKTMEDVARTFNGDEGARKLLAQGYHPEMVEVMHGAGVQALKLR 1sqq.1    ------------------------------------------------------------------------- ``` | | | | | | | | | | | | | | | | | | | | | | | | | | | | | | | | | | | | | | | | | | | | | | | | | |
|  | 1sqv.1.E | Ubiquinol-cytochrome c reductase iron-sulfur subunit  *Crystal Structure Analysis of Bovine Bc1 with UHDBT* | 0.00 |  | 10.53 | 0.08 | 3-21 | X-ray | 2.85 | hetero-2-2-2-2-2-2-… | 6 x HEC, 2 x UHD, 2 x UQ2, 2 x FES | HHblits | 0.25 |
| ``` target    MAQGVSRRQLLGRALALGSGAALADLLGPARFLSPAGAATAGAVVPGNPLRVMPDRTWEQIYRNQFEDDSTFVFTCAPND 1sqv.1    --SSEARKGFSYLVTATTTVG-----------------------------------------------------------  target    THNCLLRAHVKNGVVVRISPTYGYGEATDLYGNRASHRWDPRTCQKGLILSRRFYSERRVKAPMIRKGFKDWVEAGYPRN 1sqv.1    --------------------------------------------------------------------------------  target    DDGTPQMDVTLRGSDDWIRISWDEATTIAAKTMEDVARTFNGDEGARKLLAQGYHPEMVEVMHGAGVQALKLR 1sqv.1    ------------------------------------------------------------------------- ``` | | | | | | | | | | | | | | | | | | | | | | | | | | | | | | | | | | | | | | | | | | | | | | | | | |
|  | 7dgs.60.A | Cytochrome b-c1 complex subunit Rieske, mitochondrial  *Activity optimized supercomplex state3* | 0.00 |  | 10.53 | 0.08 | 3-21 | EM | 0.00 | monomer |  | HHblits | 0.25 |
| ``` target    MAQGVSRRQLLGRALALGSGAALADLLGPARFLSPAGAATAGAVVPGNPLRVMPDRTWEQIYRNQFEDDSTFVFTCAPND 7dgs.60   --SSEARKGFSYLVTATTTVG-----------------------------------------------------------  target    THNCLLRAHVKNGVVVRISPTYGYGEATDLYGNRASHRWDPRTCQKGLILSRRFYSERRVKAPMIRKGFKDWVEAGYPRN 7dgs.60   --------------------------------------------------------------------------------  target    DDGTPQMDVTLRGSDDWIRISWDEATTIAAKTMEDVARTFNGDEGARKLLAQGYHPEMVEVMHGAGVQALKLR 7dgs.60   ------------------------------------------------------------------------- ``` | | | | | | | | | | | | | | | | | | | | | | | | | | | | | | | | | | | | | | | | | | | | | | | | | |
|  | 7dgs.50.A | Cytochrome b-c1 complex subunit Rieske, mitochondrial  *Activity optimized supercomplex state3* | 0.00 |  | 10.53 | 0.08 | 3-21 | EM | 0.00 | monomer |  | HHblits | 0.25 |
| ``` target    MAQGVSRRQLLGRALALGSGAALADLLGPARFLSPAGAATAGAVVPGNPLRVMPDRTWEQIYRNQFEDDSTFVFTCAPND 7dgs.50   --SSEARKGFSYLVTATTTVG-----------------------------------------------------------  target    THNCLLRAHVKNGVVVRISPTYGYGEATDLYGNRASHRWDPRTCQKGLILSRRFYSERRVKAPMIRKGFKDWVEAGYPRN 7dgs.50   --------------------------------------------------------------------------------  target    DDGTPQMDVTLRGSDDWIRISWDEATTIAAKTMEDVARTFNGDEGARKLLAQGYHPEMVEVMHGAGVQALKLR 7dgs.50   ------------------------------------------------------------------------- ``` | | | | | | | | | | | | | | | | | | | | | | | | | | | | | | | | | | | | | | | | | | | | | | | | | |
|  | 7dgr.60.A | Cytochrome b-c1 complex subunit Rieske, mitochondrial  *Activity optimized supercomplex state2* | 0.00 |  | 10.53 | 0.08 | 3-21 | EM | 0.00 | monomer |  | HHblits | 0.25 |
| ``` target    MAQGVSRRQLLGRALALGSGAALADLLGPARFLSPAGAATAGAVVPGNPLRVMPDRTWEQIYRNQFEDDSTFVFTCAPND 7dgr.60   --SSEARKGFSYLVTATTTVG-----------------------------------------------------------  target    THNCLLRAHVKNGVVVRISPTYGYGEATDLYGNRASHRWDPRTCQKGLILSRRFYSERRVKAPMIRKGFKDWVEAGYPRN 7dgr.60   --------------------------------------------------------------------------------  target    DDGTPQMDVTLRGSDDWIRISWDEATTIAAKTMEDVARTFNGDEGARKLLAQGYHPEMVEVMHGAGVQALKLR 7dgr.60   ------------------------------------------------------------------------- ``` | | | | | | | | | | | | | | | | | | | | | | | | | | | | | | | | | | | | | | | | | | | | | | | | | |
|  | 7tz6.1.P | Cytochrome b-c1 complex subunit Rieske, mitochondrial  *Structure of mitochondrial bc1 in complex with ck-2-68* | 0.01 |  | 10.53 | 0.08 | 3-21 | EM | 0.00 | hetero-2-2-2-2-2-2-… | 4 x HEM, 2 x JHB, 2 x HEC, 2 x FES | HHblits | 0.25 |
| ``` target    MAQGVSRRQLLGRALALGSGAALADLLGPARFLSPAGAATAGAVVPGNPLRVMPDRTWEQIYRNQFEDDSTFVFTCAPND 7tz6.1    --SSEARKGFSYLVTATTTVG-----------------------------------------------------------  target    THNCLLRAHVKNGVVVRISPTYGYGEATDLYGNRASHRWDPRTCQKGLILSRRFYSERRVKAPMIRKGFKDWVEAGYPRN 7tz6.1    --------------------------------------------------------------------------------  target    DDGTPQMDVTLRGSDDWIRISWDEATTIAAKTMEDVARTFNGDEGARKLLAQGYHPEMVEVMHGAGVQALKLR 7tz6.1    ------------------------------------------------------------------------- ``` | | | | | | | | | | | | | | | | | | | | | | | | | | | | | | | | | | | | | | | | | | | | | | | | | |
|  | 7tz6.1.E | Cytochrome b-c1 complex subunit Rieske, mitochondrial  *Structure of mitochondrial bc1 in complex with ck-2-68* | 0.00 |  | 10.53 | 0.08 | 3-21 | EM | 0.00 | hetero-2-2-2-2-2-2-… | 4 x HEM, 2 x JHB, 2 x HEC, 2 x FES | HHblits | 0.25 |
| ``` target    MAQGVSRRQLLGRALALGSGAALADLLGPARFLSPAGAATAGAVVPGNPLRVMPDRTWEQIYRNQFEDDSTFVFTCAPND 7tz6.1    --SSEARKGFSYLVTATTTVG-----------------------------------------------------------  target    THNCLLRAHVKNGVVVRISPTYGYGEATDLYGNRASHRWDPRTCQKGLILSRRFYSERRVKAPMIRKGFKDWVEAGYPRN 7tz6.1    --------------------------------------------------------------------------------  target    DDGTPQMDVTLRGSDDWIRISWDEATTIAAKTMEDVARTFNGDEGARKLLAQGYHPEMVEVMHGAGVQALKLR 7tz6.1    ------------------------------------------------------------------------- ``` | | | | | | | | | | | | | | | | | | | | | | | | | | | | | | | | | | | | | | | | | | | | | | | | | |
|  | 5j8k.55.A | Cytochrome b-c1 complex subunit Rieske, mitochondrial  *Architecture of supercomplex I-III2* | 0.00 |  | 10.53 | 0.08 | 3-21 | EM | 0.00 | monomer |  | HHblits | 0.25 |
| ``` target    MAQGVSRRQLLGRALALGSGAALADLLGPARFLSPAGAATAGAVVPGNPLRVMPDRTWEQIYRNQFEDDSTFVFTCAPND 5j8k.55   --SSEARKGFSYLITATTTVG-----------------------------------------------------------  target    THNCLLRAHVKNGVVVRISPTYGYGEATDLYGNRASHRWDPRTCQKGLILSRRFYSERRVKAPMIRKGFKDWVEAGYPRN 5j8k.55   --------------------------------------------------------------------------------  target    DDGTPQMDVTLRGSDDWIRISWDEATTIAAKTMEDVARTFNGDEGARKLLAQGYHPEMVEVMHGAGVQALKLR 5j8k.55   ------------------------------------------------------------------------- ``` | | | | | | | | | | | | | | | | | | | | | | | | | | | | | | | | | | | | | | | | | | | | | | | | | |
|  | 6q9e.1.E | Cytochrome b-c1 complex subunit Rieske, mitochondrial  *Complex III2 focused refinement from Ovine respiratory supercomplex I+III2* | 0.00 |  | 10.53 | 0.08 | 3-21 | EM | 0.00 | hetero-2-2-2-2-2-2-… | 4 x HEM, 4 x 3PE, 5 x CDL, 3 x U10, 2 x HEC, 2 x FES | HHblits | 0.25 |
| ``` target    MAQGVSRRQLLGRALALGSGAALADLLGPARFLSPAGAATAGAVVPGNPLRVMPDRTWEQIYRNQFEDDSTFVFTCAPND 6q9e.1    --SSEARKGFSYLITATTTVG-----------------------------------------------------------  target    THNCLLRAHVKNGVVVRISPTYGYGEATDLYGNRASHRWDPRTCQKGLILSRRFYSERRVKAPMIRKGFKDWVEAGYPRN 6q9e.1    --------------------------------------------------------------------------------  target    DDGTPQMDVTLRGSDDWIRISWDEATTIAAKTMEDVARTFNGDEGARKLLAQGYHPEMVEVMHGAGVQALKLR 6q9e.1    ------------------------------------------------------------------------- ``` | | | | | | | | | | | | | | | | | | | | | | | | | | | | | | | | | | | | | | | | | | | | | | | | | |
|  | 6q9e.1.O | Cytochrome b-c1 complex subunit Rieske, mitochondrial  *Complex III2 focused refinement from Ovine respiratory supercomplex I+III2* | 0.01 |  | 10.53 | 0.08 | 3-21 | EM | 0.00 | hetero-2-2-2-2-2-2-… | 4 x HEM, 4 x 3PE, 5 x CDL, 3 x U10, 2 x HEC, 2 x FES | HHblits | 0.25 |
| ``` target    MAQGVSRRQLLGRALALGSGAALADLLGPARFLSPAGAATAGAVVPGNPLRVMPDRTWEQIYRNQFEDDSTFVFTCAPND 6q9e.1    --SSEARKGFSYLITATTTVG-----------------------------------------------------------  target    THNCLLRAHVKNGVVVRISPTYGYGEATDLYGNRASHRWDPRTCQKGLILSRRFYSERRVKAPMIRKGFKDWVEAGYPRN 6q9e.1    --------------------------------------------------------------------------------  target    DDGTPQMDVTLRGSDDWIRISWDEATTIAAKTMEDVARTFNGDEGARKLLAQGYHPEMVEVMHGAGVQALKLR 6q9e.1    ------------------------------------------------------------------------- ``` | | | | | | | | | | | | | | | | | | | | | | | | | | | | | | | | | | | | | | | | | | | | | | | | | |
|  | 6qc2.43.A | Cytochrome b-c1 complex subunit Rieske, mitochondrial  *Ovine respiratory supercomplex I+III2 open class 2* | 0.00 |  | 10.53 | 0.08 | 3-21 | EM | 0.00 | monomer |  | HHblits | 0.25 |
| ``` target    MAQGVSRRQLLGRALALGSGAALADLLGPARFLSPAGAATAGAVVPGNPLRVMPDRTWEQIYRNQFEDDSTFVFTCAPND 6qc2.43   --SSEARKGFSYLITATTTVG-----------------------------------------------------------  target    THNCLLRAHVKNGVVVRISPTYGYGEATDLYGNRASHRWDPRTCQKGLILSRRFYSERRVKAPMIRKGFKDWVEAGYPRN 6qc2.43   --------------------------------------------------------------------------------  target    DDGTPQMDVTLRGSDDWIRISWDEATTIAAKTMEDVARTFNGDEGARKLLAQGYHPEMVEVMHGAGVQALKLR 6qc2.43   ------------------------------------------------------------------------- ``` | | | | | | | | | | | | | | | | | | | | | | | | | | | | | | | | | | | | | | | | | | | | | | | | | |
|  | 6qc2.33.A | Cytochrome b-c1 complex subunit Rieske, mitochondrial  *Ovine respiratory supercomplex I+III2 open class 2* | 0.00 |  | 10.53 | 0.08 | 3-21 | EM | 0.00 | monomer |  | HHblits | 0.25 |
| ``` target    MAQGVSRRQLLGRALALGSGAALADLLGPARFLSPAGAATAGAVVPGNPLRVMPDRTWEQIYRNQFEDDSTFVFTCAPND 6qc2.33   --SSEARKGFSYLITATTTVG-----------------------------------------------------------  target    THNCLLRAHVKNGVVVRISPTYGYGEATDLYGNRASHRWDPRTCQKGLILSRRFYSERRVKAPMIRKGFKDWVEAGYPRN 6qc2.33   --------------------------------------------------------------------------------  target    DDGTPQMDVTLRGSDDWIRISWDEATTIAAKTMEDVARTFNGDEGARKLLAQGYHPEMVEVMHGAGVQALKLR 6qc2.33   ------------------------------------------------------------------------- ``` | | | | | | | | | | | | | | | | | | | | | | | | | | | | | | | | | | | | | | | | | | | | | | | | | |
|  | 6qbx.15.A | Cytochrome b-c1 complex subunit Rieske, mitochondrial  *Ovine respiratory supercomplex I+III2 closed class.* | 0.00 |  | 10.53 | 0.08 | 3-21 | EM | 0.00 | monomer |  | HHblits | 0.25 |
| ``` target    MAQGVSRRQLLGRALALGSGAALADLLGPARFLSPAGAATAGAVVPGNPLRVMPDRTWEQIYRNQFEDDSTFVFTCAPND 6qbx.15   --SSEARKGFSYLITATTTVG-----------------------------------------------------------  target    THNCLLRAHVKNGVVVRISPTYGYGEATDLYGNRASHRWDPRTCQKGLILSRRFYSERRVKAPMIRKGFKDWVEAGYPRN 6qbx.15   --------------------------------------------------------------------------------  target    DDGTPQMDVTLRGSDDWIRISWDEATTIAAKTMEDVARTFNGDEGARKLLAQGYHPEMVEVMHGAGVQALKLR 6qbx.15   ------------------------------------------------------------------------- ``` | | | | | | | | | | | | | | | | | | | | | | | | | | | | | | | | | | | | | | | | | | | | | | | | | |
|  | 6qbx.5.A | Cytochrome b-c1 complex subunit Rieske, mitochondrial  *Ovine respiratory supercomplex I+III2 closed class.* | 0.00 |  | 10.53 | 0.08 | 3-21 | EM | 0.00 | monomer |  | HHblits | 0.25 |
| ``` target    MAQGVSRRQLLGRALALGSGAALADLLGPARFLSPAGAATAGAVVPGNPLRVMPDRTWEQIYRNQFEDDSTFVFTCAPND 6qbx.5    --SSEARKGFSYLITATTTVG-----------------------------------------------------------  target    THNCLLRAHVKNGVVVRISPTYGYGEATDLYGNRASHRWDPRTCQKGLILSRRFYSERRVKAPMIRKGFKDWVEAGYPRN 6qbx.5    --------------------------------------------------------------------------------  target    DDGTPQMDVTLRGSDDWIRISWDEATTIAAKTMEDVARTFNGDEGARKLLAQGYHPEMVEVMHGAGVQALKLR 6qbx.5    ------------------------------------------------------------------------- ``` | | | | | | | | | | | | | | | | | | | | | | | | | | | | | | | | | | | | | | | | | | | | | | | | | |
|  | 6qc4.5.A | Cytochrome b-c1 complex subunit Rieske, mitochondrial  *Ovine respiratory supercomplex I+III2 open class 3* | 0.00 |  | 10.53 | 0.08 | 3-21 | EM | 0.00 | monomer |  | HHblits | 0.25 |
| ``` target    MAQGVSRRQLLGRALALGSGAALADLLGPARFLSPAGAATAGAVVPGNPLRVMPDRTWEQIYRNQFEDDSTFVFTCAPND 6qc4.5    --SSEARKGFSYLITATTTVG-----------------------------------------------------------  target    THNCLLRAHVKNGVVVRISPTYGYGEATDLYGNRASHRWDPRTCQKGLILSRRFYSERRVKAPMIRKGFKDWVEAGYPRN 6qc4.5    --------------------------------------------------------------------------------  target    DDGTPQMDVTLRGSDDWIRISWDEATTIAAKTMEDVARTFNGDEGARKLLAQGYHPEMVEVMHGAGVQALKLR 6qc4.5    ------------------------------------------------------------------------- ``` | | | | | | | | | | | | | | | | | | | | | | | | | | | | | | | | | | | | | | | | | | | | | | | | | |
|  | 6qc4.15.A | Cytochrome b-c1 complex subunit Rieske, mitochondrial  *Ovine respiratory supercomplex I+III2 open class 3* | 0.00 |  | 10.53 | 0.08 | 3-21 | EM | 0.00 | monomer |  | HHblits | 0.25 |
| ``` target    MAQGVSRRQLLGRALALGSGAALADLLGPARFLSPAGAATAGAVVPGNPLRVMPDRTWEQIYRNQFEDDSTFVFTCAPND 6qc4.15   --SSEARKGFSYLITATTTVG-----------------------------------------------------------  target    THNCLLRAHVKNGVVVRISPTYGYGEATDLYGNRASHRWDPRTCQKGLILSRRFYSERRVKAPMIRKGFKDWVEAGYPRN 6qc4.15   --------------------------------------------------------------------------------  target    DDGTPQMDVTLRGSDDWIRISWDEATTIAAKTMEDVARTFNGDEGARKLLAQGYHPEMVEVMHGAGVQALKLR 6qc4.15   ------------------------------------------------------------------------- ``` | | | | | | | | | | | | | | | | | | | | | | | | | | | | | | | | | | | | | | | | | | | | | | | | | |
|  | 6qc3.15.A | Cytochrome b-c1 complex subunit Rieske, mitochondrial  *Ovine respiratory supercomplex I+III2 open class 1* | 0.00 |  | 10.53 | 0.08 | 3-21 | EM | 0.00 | monomer |  | HHblits | 0.25 |
| ``` target    MAQGVSRRQLLGRALALGSGAALADLLGPARFLSPAGAATAGAVVPGNPLRVMPDRTWEQIYRNQFEDDSTFVFTCAPND 6qc3.15   --SSEARKGFSYLITATTTVG-----------------------------------------------------------  target    THNCLLRAHVKNGVVVRISPTYGYGEATDLYGNRASHRWDPRTCQKGLILSRRFYSERRVKAPMIRKGFKDWVEAGYPRN 6qc3.15   --------------------------------------------------------------------------------  target    DDGTPQMDVTLRGSDDWIRISWDEATTIAAKTMEDVARTFNGDEGARKLLAQGYHPEMVEVMHGAGVQALKLR 6qc3.15   ------------------------------------------------------------------------- ``` | | | | | | | | | | | | | | | | | | | | | | | | | | | | | | | | | | | | | | | | | | | | | | | | | |
|  | 6qc3.5.A | Cytochrome b-c1 complex subunit Rieske, mitochondrial  *Ovine respiratory supercomplex I+III2 open class 1* | 0.01 |  | 10.53 | 0.08 | 3-21 | EM | 0.00 | monomer |  | HHblits | 0.25 |
| ``` target    MAQGVSRRQLLGRALALGSGAALADLLGPARFLSPAGAATAGAVVPGNPLRVMPDRTWEQIYRNQFEDDSTFVFTCAPND 6qc3.5    --SSEARKGFSYLITATTTVG-----------------------------------------------------------  target    THNCLLRAHVKNGVVVRISPTYGYGEATDLYGNRASHRWDPRTCQKGLILSRRFYSERRVKAPMIRKGFKDWVEAGYPRN 6qc3.5    --------------------------------------------------------------------------------  target    DDGTPQMDVTLRGSDDWIRISWDEATTIAAKTMEDVARTFNGDEGARKLLAQGYHPEMVEVMHGAGVQALKLR 6qc3.5    ------------------------------------------------------------------------- ``` | | | | | | | | | | | | | | | | | | | | | | | | | | | | | | | | | | | | | | | | | | | | | | | | | |
|  | 5xte.1.C | Cytochrome b-c1 complex subunit Rieske, mitochondrial  *Cryo-EM structure of human respiratory complex III (cytochrome bc1 complex)* | 0.00 |  | 5.26 | 0.08 | 2-20 | EM | 0.00 | hetero-2-2-2-2-2-2-… | 9 x CDL, 2 x FES, 6 x PEE, 2 x HEC, 4 x HEM, 3 x PLX | HHblits | 0.24 |
| ``` target    MAQGVSRRQLLGRALALGSGAALADLLGPARFLSPAGAATAGAVVPGNPLRVMPDRTWEQIYRNQFEDDSTFVFTCAPND 5xte.1    -ESSEARKGFSYLVTGVTTV------------------------------------------------------------  target    THNCLLRAHVKNGVVVRISPTYGYGEATDLYGNRASHRWDPRTCQKGLILSRRFYSERRVKAPMIRKGFKDWVEAGYPRN 5xte.1    --------------------------------------------------------------------------------  target    DDGTPQMDVTLRGSDDWIRISWDEATTIAAKTMEDVARTFNGDEGARKLLAQGYHPEMVEVMHGAGVQALKLR 5xte.1    ------------------------------------------------------------------------- ``` | | | | | | | | | | | | | | | | | | | | | | | | | | | | | | | | | | | | | | | | | | | | | | | | | |
|  | 1zrt.1.F | Ubiquinol-cytochrome c reductase iron-sulfur subunit  *Rhodobacter capsulatus cytochrome bc1 complex with stigmatellin bound* | 0.00 |  | 29.41 | 0.07 | 3-19 | X-ray | 3.51 | hetero-2-2-2-mer | 4 x HEM, 2 x SMA, 2 x UNL, 2 x HEC, 2 x FES, 1 x PG6 | HHblits | 0.32 |
| ``` target    MAQGVSRRQLLGRALALGSGAALADLLGPARFLSPAGAATAGAVVPGNPLRVMPDRTWEQIYRNQFEDDSTFVFTCAPND 1zrt.1    --NAGTRRDFLYHATAATG-------------------------------------------------------------  target    THNCLLRAHVKNGVVVRISPTYGYGEATDLYGNRASHRWDPRTCQKGLILSRRFYSERRVKAPMIRKGFKDWVEAGYPRN 1zrt.1    --------------------------------------------------------------------------------  target    DDGTPQMDVTLRGSDDWIRISWDEATTIAAKTMEDVARTFNGDEGARKLLAQGYHPEMVEVMHGAGVQALKLR 1zrt.1    ------------------------------------------------------------------------- ``` | | | | | | | | | | | | | | | | | | | | | | | | | | | | | | | | | | | | | | | | | | | | | | | | | |
|  | 1zrt.1.C | Ubiquinol-cytochrome c reductase iron-sulfur subunit  *Rhodobacter capsulatus cytochrome bc1 complex with stigmatellin bound* | 0.00 |  | 29.41 | 0.07 | 3-19 | X-ray | 3.51 | hetero-2-2-2-mer | 4 x HEM, 2 x SMA, 2 x UNL, 2 x HEC, 2 x FES, 1 x PG6 | HHblits | 0.32 |
| ``` target    MAQGVSRRQLLGRALALGSGAALADLLGPARFLSPAGAATAGAVVPGNPLRVMPDRTWEQIYRNQFEDDSTFVFTCAPND 1zrt.1    --NAGTRRDFLYHATAATG-------------------------------------------------------------  target    THNCLLRAHVKNGVVVRISPTYGYGEATDLYGNRASHRWDPRTCQKGLILSRRFYSERRVKAPMIRKGFKDWVEAGYPRN 1zrt.1    --------------------------------------------------------------------------------  target    DDGTPQMDVTLRGSDDWIRISWDEATTIAAKTMEDVARTFNGDEGARKLLAQGYHPEMVEVMHGAGVQALKLR 1zrt.1    ------------------------------------------------------------------------- ``` | | | | | | | | | | | | | | | | | | | | | | | | | | | | | | | | | | | | | | | | | | | | | | | | | |
|  | 7q21.1.G | Cytochrome bc1 complex Rieske iron-sulfur subunit  *III2-IV2 respiratory supercomplex from Corynebacterium glutamicum* | 0.01 |  | 23.53 | 0.07 | 3-19 | EM | 3.00 | hetero-2-2-2-2-2-2-… | 14 x CDL, 16 x 7PH, 17 x TRD, 4 x 9XX, 2 x TWT, 6 x CU, 2 x MG, 4 x HAS, 2 x CA, 2 x FES, 6 x MQ9, 4 x 9YF, 4 x PLM, 4 x HEM, 4 x HEC | HHblits | 0.31 |
| ``` target    MAQGVSRRQLLGRALALGSGAALADLLGPARFLSPAGAATAGAVVPGNPLRVMPDRTWEQIYRNQFEDDSTFVFTCAPND 7q21.1    --STLGRRKLIMGLAGGGA-------------------------------------------------------------  target    THNCLLRAHVKNGVVVRISPTYGYGEATDLYGNRASHRWDPRTCQKGLILSRRFYSERRVKAPMIRKGFKDWVEAGYPRN 7q21.1    --------------------------------------------------------------------------------  target    DDGTPQMDVTLRGSDDWIRISWDEATTIAAKTMEDVARTFNGDEGARKLLAQGYHPEMVEVMHGAGVQALKLR 7q21.1    ------------------------------------------------------------------------- ``` | | | | | | | | | | | | | | | | | | | | | | | | | | | | | | | | | | | | | | | | | | | | | | | | | |
|  | 7qhm.1.A | Cytochrome bc1 complex Rieske iron-sulfur subunit  *Cytochrome bcc-aa3 supercomplex (respiratory supercomplex III2/IV2) from Corynebacterium glutamicum (stigmatellin and azide bound)* | 0.01 |  | 23.53 | 0.07 | 3-19 | EM | 0.00 | hetero-2-2-2-2-2-2-… | 2 x FES, 2 x IZL, 4 x 9YF, 4 x MQ9, 17 x 3PE, 2 x SMA, 4 x HEM, 16 x CDL, 2 x LYC, 2 x LMT, 4 x HEC, 4 x HAS, 2 x CU, 2 x MN, 2 x CA, 2 x AZI, 4 x CUA, 6 x DGA, 4 x PLM, 2 x IX7, 1 x OXY | HHblits | 0.31 |
| ``` target    MAQGVSRRQLLGRALALGSGAALADLLGPARFLSPAGAATAGAVVPGNPLRVMPDRTWEQIYRNQFEDDSTFVFTCAPND 7qhm.1    --STLGRRKLIMGLAGGGA-------------------------------------------------------------  target    THNCLLRAHVKNGVVVRISPTYGYGEATDLYGNRASHRWDPRTCQKGLILSRRFYSERRVKAPMIRKGFKDWVEAGYPRN 7qhm.1    --------------------------------------------------------------------------------  target    DDGTPQMDVTLRGSDDWIRISWDEATTIAAKTMEDVARTFNGDEGARKLLAQGYHPEMVEVMHGAGVQALKLR 7qhm.1    ------------------------------------------------------------------------- ``` | | | | | | | | | | | | | | | | | | | | | | | | | | | | | | | | | | | | | | | | | | | | | | | | | |
|  | 7qho.1.A | Cytochrome bc1 complex Rieske iron-sulfur subunit  *Cytochrome bcc-aa3 supercomplex (respiratory supercomplex III2/IV2) from Corynebacterium glutamicum (as isolated)* | 0.01 |  | 23.53 | 0.07 | 3-19 | EM | 0.00 | hetero-2-2-2-2-2-2-… | 2 x FES, 2 x IZL, 4 x 9YF, 6 x MQ9, 10 x 3PE, 4 x HEM, 14 x CDL, 2 x LYC, 2 x LMT, 4 x HEC, 2 x 3PH, 4 x HAS, 2 x CU, 2 x CA, 2 x MN, 4 x CUA, 6 x DGA, 4 x PLM, 2 x IX7 | HHblits | 0.31 |
| ``` target    MAQGVSRRQLLGRALALGSGAALADLLGPARFLSPAGAATAGAVVPGNPLRVMPDRTWEQIYRNQFEDDSTFVFTCAPND 7qho.1    --STLGRRKLIMGLAGGGA-------------------------------------------------------------  target    THNCLLRAHVKNGVVVRISPTYGYGEATDLYGNRASHRWDPRTCQKGLILSRRFYSERRVKAPMIRKGFKDWVEAGYPRN 7qho.1    --------------------------------------------------------------------------------  target    DDGTPQMDVTLRGSDDWIRISWDEATTIAAKTMEDVARTFNGDEGARKLLAQGYHPEMVEVMHGAGVQALKLR 7qho.1    ------------------------------------------------------------------------- ``` | | | | | | | | | | | | | | | | | | | | | | | | | | | | | | | | | | | | | | | | | | | | | | | | | |
|  | 7qhm.1.N | Cytochrome bc1 complex Rieske iron-sulfur subunit  *Cytochrome bcc-aa3 supercomplex (respiratory supercomplex III2/IV2) from Corynebacterium glutamicum (stigmatellin and azide bound)* | 0.01 |  | 23.53 | 0.07 | 3-19 | EM | 0.00 | hetero-2-2-2-2-2-2-… | 2 x FES, 2 x IZL, 4 x 9YF, 4 x MQ9, 17 x 3PE, 2 x SMA, 4 x HEM, 16 x CDL, 2 x LYC, 2 x LMT, 4 x HEC, 4 x HAS, 2 x CU, 2 x MN, 2 x CA, 2 x AZI, 4 x CUA, 6 x DGA, 4 x PLM, 2 x IX7, 1 x OXY | HHblits | 0.31 |
| ``` target    MAQGVSRRQLLGRALALGSGAALADLLGPARFLSPAGAATAGAVVPGNPLRVMPDRTWEQIYRNQFEDDSTFVFTCAPND 7qhm.1    --STLGRRKLIMGLAGGGA-------------------------------------------------------------  target    THNCLLRAHVKNGVVVRISPTYGYGEATDLYGNRASHRWDPRTCQKGLILSRRFYSERRVKAPMIRKGFKDWVEAGYPRN 7qhm.1    --------------------------------------------------------------------------------  target    DDGTPQMDVTLRGSDDWIRISWDEATTIAAKTMEDVARTFNGDEGARKLLAQGYHPEMVEVMHGAGVQALKLR 7qhm.1    ------------------------------------------------------------------------- ``` | | | | | | | | | | | | | | | | | | | | | | | | | | | | | | | | | | | | | | | | | | | | | | | | | |
|  | 2qjk.1.C | Ubiquinol-cytochrome c reductase iron-sulfur subunit  *Crystal Structure Analysis of mutant rhodobacter sphaeroides bc1 with stigmatellin and antimycin* | 0.01 |  | 29.41 | 0.07 | 5-21 | X-ray | 3.10 | hetero-2-2-2-mer | 2 x BGL, 6 x HEM, 2 x SMA, 2 x LOP, 2 x ANJ, 2 x FES | HHblits | 0.31 |
| ``` target    MAQGVSRRQLLGRALALGSGAALADLLGPARFLSPAGAATAGAVVPGNPLRVMPDRTWEQIYRNQFEDDSTFVFTCAPND 2qjk.1    ----GTRRDFLYYATAGAGAV-----------------------------------------------------------  target    THNCLLRAHVKNGVVVRISPTYGYGEATDLYGNRASHRWDPRTCQKGLILSRRFYSERRVKAPMIRKGFKDWVEAGYPRN 2qjk.1    --------------------------------------------------------------------------------  target    DDGTPQMDVTLRGSDDWIRISWDEATTIAAKTMEDVARTFNGDEGARKLLAQGYHPEMVEVMHGAGVQALKLR 2qjk.1    ------------------------------------------------------------------------- ``` | | | | | | | | | | | | | | | | | | | | | | | | | | | | | | | | | | | | | | | | | | | | | | | | | |
|  | 2qjp.1.C | Ubiquinol-cytochrome c reductase iron-sulfur subunit  *Crystal structure of wild type rhodobacter sphaeroides with stigmatellin and antimycin inhibited* | 0.01 |  | 29.41 | 0.07 | 5-21 | X-ray | 2.60 | hetero-2-2-2-mer | 2 x BGL, 6 x HEM, 2 x SMA, 2 x LOP, 2 x ANJ, 2 x FES | HHblits | 0.31 |
| ``` target    MAQGVSRRQLLGRALALGSGAALADLLGPARFLSPAGAATAGAVVPGNPLRVMPDRTWEQIYRNQFEDDSTFVFTCAPND 2qjp.1    ----GTRRDFLYYATAGAGAV-----------------------------------------------------------  target    THNCLLRAHVKNGVVVRISPTYGYGEATDLYGNRASHRWDPRTCQKGLILSRRFYSERRVKAPMIRKGFKDWVEAGYPRN 2qjp.1    --------------------------------------------------------------------------------  target    DDGTPQMDVTLRGSDDWIRISWDEATTIAAKTMEDVARTFNGDEGARKLLAQGYHPEMVEVMHGAGVQALKLR 2qjp.1    ------------------------------------------------------------------------- ``` | | | | | | | | | | | | | | | | | | | | | | | | | | | | | | | | | | | | | | | | | | | | | | | | | |
|  | 1vf5.1.D | RIESKE IRON-SULFUR PROTEIN  *Crystal Structure of Cytochrome b6f Complex from M.laminosus* | 0.00 |  | 17.65 | 0.07 | 2-18 | X-ray | 3.00 | hetero-oligomer | 8 x HEM, 2 x TDS, 2 x PL9, 4 x OPC, 2 x CLA, 2 x FES, 2 x BCR | HHblits | 0.28 |
| ``` target    MAQGVSRRQLLGRALALGSGAALADLLGPARFLSPAGAATAGAVVPGNPLRVMPDRTWEQIYRNQFEDDSTFVFTCAPND 1vf5.1    -VPDMGRRQFMNLLAFGT--------------------------------------------------------------  target    THNCLLRAHVKNGVVVRISPTYGYGEATDLYGNRASHRWDPRTCQKGLILSRRFYSERRVKAPMIRKGFKDWVEAGYPRN 1vf5.1    --------------------------------------------------------------------------------  target    DDGTPQMDVTLRGSDDWIRISWDEATTIAAKTMEDVARTFNGDEGARKLLAQGYHPEMVEVMHGAGVQALKLR 1vf5.1    ------------------------------------------------------------------------- ``` | | | | | | | | | | | | | | | | | | | | | | | | | | | | | | | | | | | | | | | | | | | | | | | | | |
|  | 1vf5.1.L | RIESKE IRON-SULFUR PROTEIN  *Crystal Structure of Cytochrome b6f Complex from M.laminosus* | 0.00 |  | 17.65 | 0.07 | 2-18 | X-ray | 3.00 | hetero-oligomer | 8 x HEM, 2 x TDS, 2 x PL9, 4 x OPC, 2 x CLA, 2 x FES, 2 x BCR | HHblits | 0.28 |
| ``` target    MAQGVSRRQLLGRALALGSGAALADLLGPARFLSPAGAATAGAVVPGNPLRVMPDRTWEQIYRNQFEDDSTFVFTCAPND 1vf5.1    -VPDMGRRQFMNLLAFGT--------------------------------------------------------------  target    THNCLLRAHVKNGVVVRISPTYGYGEATDLYGNRASHRWDPRTCQKGLILSRRFYSERRVKAPMIRKGFKDWVEAGYPRN 1vf5.1    --------------------------------------------------------------------------------  target    DDGTPQMDVTLRGSDDWIRISWDEATTIAAKTMEDVARTFNGDEGARKLLAQGYHPEMVEVMHGAGVQALKLR 1vf5.1    ------------------------------------------------------------------------- ``` | | | | | | | | | | | | | | | | | | | | | | | | | | | | | | | | | | | | | | | | | | | | | | | | | |
|  | 2d2c.1.D | Cytochrome b6-f complex iron-sulfur subunit  *Crystal Structure Of Cytochrome B6F Complex with DBMIB From M. Laminosus* | 0.00 |  | 17.65 | 0.07 | 2-18 | X-ray | 3.80 | hetero-oligomer | 6 x HEM, 2 x HEC, 4 x OPC, 2 x BNT, 2 x CLA, 2 x FES, 2 x BCR | HHblits | 0.28 |
| ``` target    MAQGVSRRQLLGRALALGSGAALADLLGPARFLSPAGAATAGAVVPGNPLRVMPDRTWEQIYRNQFEDDSTFVFTCAPND 2d2c.1    -VPDMGRRQFMNLLAFGT--------------------------------------------------------------  target    THNCLLRAHVKNGVVVRISPTYGYGEATDLYGNRASHRWDPRTCQKGLILSRRFYSERRVKAPMIRKGFKDWVEAGYPRN 2d2c.1    --------------------------------------------------------------------------------  target    DDGTPQMDVTLRGSDDWIRISWDEATTIAAKTMEDVARTFNGDEGARKLLAQGYHPEMVEVMHGAGVQALKLR 2d2c.1    ------------------------------------------------------------------------- ``` | | | | | | | | | | | | | | | | | | | | | | | | | | | | | | | | | | | | | | | | | | | | | | | | | |
|  | 2d2c.1.L | Cytochrome b6-f complex iron-sulfur subunit  *Crystal Structure Of Cytochrome B6F Complex with DBMIB From M. Laminosus* | 0.00 |  | 17.65 | 0.07 | 2-18 | X-ray | 3.80 | hetero-oligomer | 6 x HEM, 2 x HEC, 4 x OPC, 2 x BNT, 2 x CLA, 2 x FES, 2 x BCR | HHblits | 0.28 |
| ``` target    MAQGVSRRQLLGRALALGSGAALADLLGPARFLSPAGAATAGAVVPGNPLRVMPDRTWEQIYRNQFEDDSTFVFTCAPND 2d2c.1    -VPDMGRRQFMNLLAFGT--------------------------------------------------------------  target    THNCLLRAHVKNGVVVRISPTYGYGEATDLYGNRASHRWDPRTCQKGLILSRRFYSERRVKAPMIRKGFKDWVEAGYPRN 2d2c.1    --------------------------------------------------------------------------------  target    DDGTPQMDVTLRGSDDWIRISWDEATTIAAKTMEDVARTFNGDEGARKLLAQGYHPEMVEVMHGAGVQALKLR 2d2c.1    ------------------------------------------------------------------------- ``` | | | | | | | | | | | | | | | | | | | | | | | | | | | | | | | | | | | | | | | | | | | | | | | | | |
|  | 7zxy.1.D | Cytochrome b6-f complex iron-sulfur subunit 2  *3.15 Angstrom cryo-EM structure of the dimeric cytochrome b6f complex from Synechocystis sp. PCC 6803 with natively bound plastoquinone and lipid molecules.* | 0.00 |  | 17.65 | 0.07 | 2-18 | EM | 0.00 | hetero-2-2-2-2-2-2-… | 2 x ECH, 4 x HEM, 4 x HEC, 2 x CLA, 7 x PGV, 2 x FES, 1 x LFA | HHblits | 0.28 |
| ``` target    MAQGVSRRQLLGRALALGSGAALADLLGPARFLSPAGAATAGAVVPGNPLRVMPDRTWEQIYRNQFEDDSTFVFTCAPND 7zxy.1    -VPDLGRRQFMNLLTFGT--------------------------------------------------------------  target    THNCLLRAHVKNGVVVRISPTYGYGEATDLYGNRASHRWDPRTCQKGLILSRRFYSERRVKAPMIRKGFKDWVEAGYPRN 7zxy.1    --------------------------------------------------------------------------------  target    DDGTPQMDVTLRGSDDWIRISWDEATTIAAKTMEDVARTFNGDEGARKLLAQGYHPEMVEVMHGAGVQALKLR 7zxy.1    ------------------------------------------------------------------------- ``` | | | | | | | | | | | | | | | | | | | | | | | | | | | | | | | | | | | | | | | | | | | | | | | | | |
|  | 7zxy.1.L | Cytochrome b6-f complex iron-sulfur subunit 2  *3.15 Angstrom cryo-EM structure of the dimeric cytochrome b6f complex from Synechocystis sp. PCC 6803 with natively bound plastoquinone and lipid molecules.* | 0.00 |  | 17.65 | 0.07 | 2-18 | EM | 0.00 | hetero-2-2-2-2-2-2-… | 2 x ECH, 4 x HEM, 4 x HEC, 2 x CLA, 7 x PGV, 2 x FES, 1 x LFA | HHblits | 0.28 |
| ``` target    MAQGVSRRQLLGRALALGSGAALADLLGPARFLSPAGAATAGAVVPGNPLRVMPDRTWEQIYRNQFEDDSTFVFTCAPND 7zxy.1    -VPDLGRRQFMNLLTFGT--------------------------------------------------------------  target    THNCLLRAHVKNGVVVRISPTYGYGEATDLYGNRASHRWDPRTCQKGLILSRRFYSERRVKAPMIRKGFKDWVEAGYPRN 7zxy.1    --------------------------------------------------------------------------------  target    DDGTPQMDVTLRGSDDWIRISWDEATTIAAKTMEDVARTFNGDEGARKLLAQGYHPEMVEVMHGAGVQALKLR 7zxy.1    ------------------------------------------------------------------------- ``` | | | | | | | | | | | | | | | | | | | | | | | | | | | | | | | | | | | | | | | | | | | | | | | | | |
|  | 2e75.1.D | Cytochrome b6-f complex iron-sulfur subunit  *Crystal Structure of the Cytochrome b6f Complex with 2-nonyl-4-hydroxyquinoline N-oxide (NQNO) from M.laminosus* | 0.00 |  | 17.65 | 0.07 | 2-18 | X-ray | 3.55 | hetero-oligomer | 4 x CD, 8 x HEM, 4 x OPC, 8 x UMQ, 2 x QNO, 2 x CLA, 2 x FES, 2 x SQD, 2 x BCR | HHblits | 0.28 |
| ``` target    MAQGVSRRQLLGRALALGSGAALADLLGPARFLSPAGAATAGAVVPGNPLRVMPDRTWEQIYRNQFEDDSTFVFTCAPND 2e75.1    -VPDMGRRQFMNLLAFGT--------------------------------------------------------------  target    THNCLLRAHVKNGVVVRISPTYGYGEATDLYGNRASHRWDPRTCQKGLILSRRFYSERRVKAPMIRKGFKDWVEAGYPRN 2e75.1    --------------------------------------------------------------------------------  target    DDGTPQMDVTLRGSDDWIRISWDEATTIAAKTMEDVARTFNGDEGARKLLAQGYHPEMVEVMHGAGVQALKLR 2e75.1    ------------------------------------------------------------------------- ``` | | | | | | | | | | | | | | | | | | | | | | | | | | | | | | | | | | | | | | | | | | | | | | | | | |
|  | 2e76.1.D | Cytochrome b6-f complex iron-sulfur subunit  *Crystal Structure of the Cytochrome b6f Complex with tridecyl-stigmatellin (TDS) from M.laminosus* | 0.00 |  | 17.65 | 0.07 | 2-18 | X-ray | 3.41 | hetero-oligomer | 2 x CD, 8 x HEM, 4 x OPC, 8 x UMQ, 2 x CLA, 4 x TDS, 2 x FES, 2 x SQD, 2 x BCR | HHblits | 0.28 |
| ``` target    MAQGVSRRQLLGRALALGSGAALADLLGPARFLSPAGAATAGAVVPGNPLRVMPDRTWEQIYRNQFEDDSTFVFTCAPND 2e76.1    -VPDMGRRQFMNLLAFGT--------------------------------------------------------------  target    THNCLLRAHVKNGVVVRISPTYGYGEATDLYGNRASHRWDPRTCQKGLILSRRFYSERRVKAPMIRKGFKDWVEAGYPRN 2e76.1    --------------------------------------------------------------------------------  target    DDGTPQMDVTLRGSDDWIRISWDEATTIAAKTMEDVARTFNGDEGARKLLAQGYHPEMVEVMHGAGVQALKLR 2e76.1    ------------------------------------------------------------------------- ``` | | | | | | | | | | | | | | | | | | | | | | | | | | | | | | | | | | | | | | | | | | | | | | | | | |
|  | 4pv1.1.L | Cytochrome b6-f complex iron-sulfur subunit  *Cytochrome B6F structure from M. laminosus with the quinone analog inhibitor stigmatellin* | 0.00 |  | 17.65 | 0.07 | 2-18 | X-ray | 3.00 | hetero-2-2-2-2-2-2-… | 2 x MYS, 4 x CD, 8 x HEC, 6 x UMQ, 2 x SMA, 2 x 7PH, 2 x 8K6, 2 x CLA, 6 x OPC, 2 x FES, 2 x SQD, 2 x BCR | HHblits | 0.28 |
| ``` target    MAQGVSRRQLLGRALALGSGAALADLLGPARFLSPAGAATAGAVVPGNPLRVMPDRTWEQIYRNQFEDDSTFVFTCAPND 4pv1.1    -VPDMGRRQFMNLLAFGT--------------------------------------------------------------  target    THNCLLRAHVKNGVVVRISPTYGYGEATDLYGNRASHRWDPRTCQKGLILSRRFYSERRVKAPMIRKGFKDWVEAGYPRN 4pv1.1    --------------------------------------------------------------------------------  target    DDGTPQMDVTLRGSDDWIRISWDEATTIAAKTMEDVARTFNGDEGARKLLAQGYHPEMVEVMHGAGVQALKLR 4pv1.1    ------------------------------------------------------------------------- ``` | | | | | | | | | | | | | | | | | | | | | | | | | | | | | | | | | | | | | | | | | | | | | | | | | |
|  | 7rjb.1.I | Cytochrome b-c1 complex subunit Rieske, mitochondrial  *Complex III2 from Candida albicans, inhibitor free, Rieske head domain in b position* | 0.00 |  | 11.11 | 0.08 | 3-20 | EM | 0.00 | hetero-1-1-1-1-1-1-… | 2 x HEM, 2 x U10, 1 x HEC, 1 x FES | HHblits | 0.24 |
| ``` target    MAQGVSRRQLLGRALALGSGAALADLLGPARFLSPAGAATAGAVVPGNPLRVMPDRTWEQIYRNQFEDDSTFVFTCAPND 7rjb.1    --SGQGSRNFTYFMVGSMGL------------------------------------------------------------  target    THNCLLRAHVKNGVVVRISPTYGYGEATDLYGNRASHRWDPRTCQKGLILSRRFYSERRVKAPMIRKGFKDWVEAGYPRN 7rjb.1    --------------------------------------------------------------------------------  target    DDGTPQMDVTLRGSDDWIRISWDEATTIAAKTMEDVARTFNGDEGARKLLAQGYHPEMVEVMHGAGVQALKLR 7rjb.1    ------------------------------------------------------------------------- ``` | | | | | | | | | | | | | | | | | | | | | | | | | | | | | | | | | | | | | | | | | | | | | | | | | |
|  | 7rja.1.H | Cytochrome b-c1 complex subunit Rieske, mitochondrial  *Complex III2 from Candida albicans, inhibitor free* | 0.01 |  | 11.11 | 0.08 | 3-20 | EM | 0.00 | hetero-2-2-2-2-2-2-… | 4 x HEM, 4 x U10, 2 x HEC, 2 x FES | HHblits | 0.24 |
| ``` target    MAQGVSRRQLLGRALALGSGAALADLLGPARFLSPAGAATAGAVVPGNPLRVMPDRTWEQIYRNQFEDDSTFVFTCAPND 7rja.1    --SGQGSRNFTYFMVGSMGL------------------------------------------------------------  target    THNCLLRAHVKNGVVVRISPTYGYGEATDLYGNRASHRWDPRTCQKGLILSRRFYSERRVKAPMIRKGFKDWVEAGYPRN 7rja.1    --------------------------------------------------------------------------------  target    DDGTPQMDVTLRGSDDWIRISWDEATTIAAKTMEDVARTFNGDEGARKLLAQGYHPEMVEVMHGAGVQALKLR 7rja.1    ------------------------------------------------------------------------- ``` | | | | | | | | | | | | | | | | | | | | | | | | | | | | | | | | | | | | | | | | | | | | | | | | | |
|  | 7jrg.1.E | Cytochrome b-c1 complex subunit Rieske, mitochondrial  *Plant Mitochondrial complex III2 from Vigna radiata* | 0.00 |  | 23.53 | 0.07 | 3-19 | EM | 0.00 | hetero-2-2-2-2-2-2-… | 2 x ZN, 3 x PC1, 9 x CDL, 4 x HEM, 17 x 3PE, 2 x HEC | HHblits | 0.28 |
| ``` target    MAQGVSRRQLLGRALALGSGAALADLLGPARFLSPAGAATAGAVVPGNPLRVMPDRTWEQIYRNQFEDDSTFVFTCAPND 7jrg.1    --GDPSKRAFAYFVLTGGR-------------------------------------------------------------  target    THNCLLRAHVKNGVVVRISPTYGYGEATDLYGNRASHRWDPRTCQKGLILSRRFYSERRVKAPMIRKGFKDWVEAGYPRN 7jrg.1    --------------------------------------------------------------------------------  target    DDGTPQMDVTLRGSDDWIRISWDEATTIAAKTMEDVARTFNGDEGARKLLAQGYHPEMVEVMHGAGVQALKLR 7jrg.1    ------------------------------------------------------------------------- ``` | | | | | | | | | | | | | | | | | | | | | | | | | | | | | | | | | | | | | | | | | | | | | | | | | |
|  | 5gpn.17.A | Cytochrome b-c1 complex subunit Rieske, mitochondrial  *Architecture of mammalian respirasome* | 0.00 |  | 11.76 | 0.07 | 3-19 | EM | 0.00 | monomer |  | HHblits | 0.26 |
| ``` target    MAQGVSRRQLLGRALALGSGAALADLLGPARFLSPAGAATAGAVVPGNPLRVMPDRTWEQIYRNQFEDDSTFVFTCAPND 5gpn.17   --SSEARKGFSYLVTATTT-------------------------------------------------------------  target    THNCLLRAHVKNGVVVRISPTYGYGEATDLYGNRASHRWDPRTCQKGLILSRRFYSERRVKAPMIRKGFKDWVEAGYPRN 5gpn.17   --------------------------------------------------------------------------------  target    DDGTPQMDVTLRGSDDWIRISWDEATTIAAKTMEDVARTFNGDEGARKLLAQGYHPEMVEVMHGAGVQALKLR 5gpn.17   ------------------------------------------------------------------------- ``` | | | | | | | | | | | | | | | | | | | | | | | | | | | | | | | | | | | | | | | | | | | | | | | | | |
|  | 5gpn.5.A | Cytochrome b-c1 complex subunit Rieske, mitochondrial  *Architecture of mammalian respirasome* | 0.00 |  | 11.76 | 0.07 | 3-19 | EM | 0.00 | monomer |  | HHblits | 0.26 |
| ``` target    MAQGVSRRQLLGRALALGSGAALADLLGPARFLSPAGAATAGAVVPGNPLRVMPDRTWEQIYRNQFEDDSTFVFTCAPND 5gpn.5    --SSEARKGFSYLVTATTT-------------------------------------------------------------  target    THNCLLRAHVKNGVVVRISPTYGYGEATDLYGNRASHRWDPRTCQKGLILSRRFYSERRVKAPMIRKGFKDWVEAGYPRN 5gpn.5    --------------------------------------------------------------------------------  target    DDGTPQMDVTLRGSDDWIRISWDEATTIAAKTMEDVARTFNGDEGARKLLAQGYHPEMVEVMHGAGVQALKLR 5gpn.5    ------------------------------------------------------------------------- ``` | | | | | | | | | | | | | | | | | | | | | | | | | | | | | | | | | | | | | | | | | | | | | | | | | |
|  | 4d6t.1.E | CYTOCHROME B-C1 COMPLEX SUBUNIT RIESKE, MITOCHONDRIAL  *Cytochrome bc1 bound to the 4(1H)-pyridone GW844520* | 0.00 |  | 11.76 | 0.07 | 3-19 | X-ray | 3.57 | hetero-oligomer | 2 x HEM, 1 x 4X9, 2 x PEE, 1 x HEC, 2 x CDL | HHblits | 0.26 |
| ``` target    MAQGVSRRQLLGRALALGSGAALADLLGPARFLSPAGAATAGAVVPGNPLRVMPDRTWEQIYRNQFEDDSTFVFTCAPND 4d6t.1    --SSEARKGFSYLVTATTT-------------------------------------------------------------  target    THNCLLRAHVKNGVVVRISPTYGYGEATDLYGNRASHRWDPRTCQKGLILSRRFYSERRVKAPMIRKGFKDWVEAGYPRN 4d6t.1    --------------------------------------------------------------------------------  target    DDGTPQMDVTLRGSDDWIRISWDEATTIAAKTMEDVARTFNGDEGARKLLAQGYHPEMVEVMHGAGVQALKLR 4d6t.1    ------------------------------------------------------------------------- ``` | | | | | | | | | | | | | | | | | | | | | | | | | | | | | | | | | | | | | | | | | | | | | | | | | |
|  | 5nmi.1.E | Cytochrome b-c1 complex subunit Rieske, mitochondrial  *Cytochrome bc1 bound to the inhibitor MJM170* | 0.00 |  | 11.76 | 0.07 | 3-19 | X-ray | 3.50 | hetero-2-2-2-2-4-2-… | 4 x HEM, 2 x MJM, 6 x PEE, 6 x CDL, 2 x HEC, 1 x FES | HHblits | 0.26 |
| ``` target    MAQGVSRRQLLGRALALGSGAALADLLGPARFLSPAGAATAGAVVPGNPLRVMPDRTWEQIYRNQFEDDSTFVFTCAPND 5nmi.1    --SSEARKGFSYLVTATTT-------------------------------------------------------------  target    THNCLLRAHVKNGVVVRISPTYGYGEATDLYGNRASHRWDPRTCQKGLILSRRFYSERRVKAPMIRKGFKDWVEAGYPRN 5nmi.1    --------------------------------------------------------------------------------  target    DDGTPQMDVTLRGSDDWIRISWDEATTIAAKTMEDVARTFNGDEGARKLLAQGYHPEMVEVMHGAGVQALKLR 5nmi.1    ------------------------------------------------------------------------- ``` | | | | | | | | | | | | | | | | | | | | | | | | | | | | | | | | | | | | | | | | | | | | | | | | | |
|  | 6fo6.1.J | Cytochrome b-c1 complex subunit Rieske, mitochondrial  *CryoEM structure of bovine cytochrome bc1 in complex with the anti-malarial inhibitor SCR0911* | 0.00 |  | 11.76 | 0.07 | 3-19 | EM | 0.00 | hetero-2-2-2-2-2-2-… | 4 x HEM, 2 x DY2, 2 x HEC | HHblits | 0.26 |
| ``` target    MAQGVSRRQLLGRALALGSGAALADLLGPARFLSPAGAATAGAVVPGNPLRVMPDRTWEQIYRNQFEDDSTFVFTCAPND 6fo6.1    --SSEARKGFSYLVTATTT-------------------------------------------------------------  target    THNCLLRAHVKNGVVVRISPTYGYGEATDLYGNRASHRWDPRTCQKGLILSRRFYSERRVKAPMIRKGFKDWVEAGYPRN 6fo6.1    --------------------------------------------------------------------------------  target    DDGTPQMDVTLRGSDDWIRISWDEATTIAAKTMEDVARTFNGDEGARKLLAQGYHPEMVEVMHGAGVQALKLR 6fo6.1    ------------------------------------------------------------------------- ``` | | | | | | | | | | | | | | | | | | | | | | | | | | | | | | | | | | | | | | | | | | | | | | | | | |
|  | 6fo0.1.E | Cytochrome b-c1 complex subunit Rieske, mitochondrial  *CryoEM structure of bovine cytochrome bc1 in complex with the anti-malarial compound GSK932121* | 0.00 |  | 11.76 | 0.07 | 3-19 | EM | 0.00 | hetero-2-2-2-2-2-2-… | 4 x HEM, 2 x G8U, 2 x HEC | HHblits | 0.26 |
| ``` target    MAQGVSRRQLLGRALALGSGAALADLLGPARFLSPAGAATAGAVVPGNPLRVMPDRTWEQIYRNQFEDDSTFVFTCAPND 6fo0.1    --SSEARKGFSYLVTATTT-------------------------------------------------------------  target    THNCLLRAHVKNGVVVRISPTYGYGEATDLYGNRASHRWDPRTCQKGLILSRRFYSERRVKAPMIRKGFKDWVEAGYPRN 6fo0.1    --------------------------------------------------------------------------------  target    DDGTPQMDVTLRGSDDWIRISWDEATTIAAKTMEDVARTFNGDEGARKLLAQGYHPEMVEVMHGAGVQALKLR 6fo0.1    ------------------------------------------------------------------------- ``` | | | | | | | | | | | | | | | | | | | | | | | | | | | | | | | | | | | | | | | | | | | | | | | | | |
|  | 3h1h.1.O | Cytochrome b-c1 complex subunit Rieske, mitochondrial  *Cytochrome bc1 complex from chicken* | 0.00 |  | 11.76 | 0.07 | 3-19 | X-ray | 3.16 | hetero-2-2-2-2-2-2-… | 4 x HEM, 2 x UQ, 4 x CDL, 6 x PEE, 2 x HEC, 5 x BOG, 2 x FES | HHblits | 0.25 |
| ``` target    MAQGVSRRQLLGRALALGSGAALADLLGPARFLSPAGAATAGAVVPGNPLRVMPDRTWEQIYRNQFEDDSTFVFTCAPND 3h1h.1    --SSEDRKGFSYLVTATAC-------------------------------------------------------------  target    THNCLLRAHVKNGVVVRISPTYGYGEATDLYGNRASHRWDPRTCQKGLILSRRFYSERRVKAPMIRKGFKDWVEAGYPRN 3h1h.1    --------------------------------------------------------------------------------  target    DDGTPQMDVTLRGSDDWIRISWDEATTIAAKTMEDVARTFNGDEGARKLLAQGYHPEMVEVMHGAGVQALKLR 3h1h.1    ------------------------------------------------------------------------- ``` | | | | | | | | | | | | | | | | | | | | | | | | | | | | | | | | | | | | | | | | | | | | | | | | | |
|  | 3h1i.1.E | Cytochrome b-c1 complex subunit Rieske, mitochondrial  *Stigmatellin and antimycin bound cytochrome bc1 complex from chicken* | 0.00 |  | 11.76 | 0.07 | 3-19 | X-ray | 3.53 | hetero-oligomer | 4 x HEM, 2 x HEC, 2 x FES, 4 x CDL, 6 x PEE, 1 x PLC, 7 x UNL, 1 x SMA, 1 x ANY, 1 x GOL | HHblits | 0.25 |
| ``` target    MAQGVSRRQLLGRALALGSGAALADLLGPARFLSPAGAATAGAVVPGNPLRVMPDRTWEQIYRNQFEDDSTFVFTCAPND 3h1i.1    --SSEDRKGFSYLVTATAC-------------------------------------------------------------  target    THNCLLRAHVKNGVVVRISPTYGYGEATDLYGNRASHRWDPRTCQKGLILSRRFYSERRVKAPMIRKGFKDWVEAGYPRN 3h1i.1    --------------------------------------------------------------------------------  target    DDGTPQMDVTLRGSDDWIRISWDEATTIAAKTMEDVARTFNGDEGARKLLAQGYHPEMVEVMHGAGVQALKLR 3h1i.1    ------------------------------------------------------------------------- ``` | | | | | | | | | | | | | | | | | | | | | | | | | | | | | | | | | | | | | | | | | | | | | | | | | |
|  | 3h1h.1.E | Cytochrome b-c1 complex subunit Rieske, mitochondrial  *Cytochrome bc1 complex from chicken* | 0.00 |  | 11.76 | 0.07 | 3-19 | X-ray | 3.16 | hetero-2-2-2-2-2-2-… | 4 x HEM, 2 x UQ, 4 x CDL, 6 x PEE, 2 x HEC, 5 x BOG, 2 x FES | HHblits | 0.25 |
| ``` target    MAQGVSRRQLLGRALALGSGAALADLLGPARFLSPAGAATAGAVVPGNPLRVMPDRTWEQIYRNQFEDDSTFVFTCAPND 3h1h.1    --SSEDRKGFSYLVTATAC-------------------------------------------------------------  target    THNCLLRAHVKNGVVVRISPTYGYGEATDLYGNRASHRWDPRTCQKGLILSRRFYSERRVKAPMIRKGFKDWVEAGYPRN 3h1h.1    --------------------------------------------------------------------------------  target    DDGTPQMDVTLRGSDDWIRISWDEATTIAAKTMEDVARTFNGDEGARKLLAQGYHPEMVEVMHGAGVQALKLR 3h1h.1    ------------------------------------------------------------------------- ``` | | | | | | | | | | | | | | | | | | | | | | | | | | | | | | | | | | | | | | | | | | | | | | | | | |
|  | 3cwb.1.E | MITOCHONDRIAL UBIQUINOL-CYTOCHROME C REDUCTASE IRON-SULFUR PROTEIN  *Chicken Cytochrome BC1 Complex inhibited by an iodinated analogue of the polyketide Crocacin-D* | 0.00 |  | 11.76 | 0.07 | 3-19 | X-ray | 3.51 | hetero-2-2-2-2-2-2-… | 6 x PEE, 6 x BOG, 2 x AZI, 4 x HEM, 2 x ICX, 2 x UQ, 2 x HEC, 4 x CDL, 2 x FES, 2 x UNL | HHblits | 0.25 |
| ``` target    MAQGVSRRQLLGRALALGSGAALADLLGPARFLSPAGAATAGAVVPGNPLRVMPDRTWEQIYRNQFEDDSTFVFTCAPND 3cwb.1    --SSEDRKGFSYLVTATAC-------------------------------------------------------------  target    THNCLLRAHVKNGVVVRISPTYGYGEATDLYGNRASHRWDPRTCQKGLILSRRFYSERRVKAPMIRKGFKDWVEAGYPRN 3cwb.1    --------------------------------------------------------------------------------  target    DDGTPQMDVTLRGSDDWIRISWDEATTIAAKTMEDVARTFNGDEGARKLLAQGYHPEMVEVMHGAGVQALKLR 3cwb.1    ------------------------------------------------------------------------- ``` | | | | | | | | | | | | | | | | | | | | | | | | | | | | | | | | | | | | | | | | | | | | | | | | | |
|  | 3l75.1.O | CYTOCHROME B-C1 COMPLEX SUBUNIT 5, RIESKE IRONSULFUR PROTEIN, MITOCHONDRIAL  *Cytochrome BC1 complex from chicken with fenamidone bound* | 0.00 |  | 11.76 | 0.07 | 3-19 | X-ray | 2.79 | hetero-2-2-2-2-2-2-… | 6 x PEE, 4 x HEM, 2 x FNM, 2 x UQ, 2 x AZI, 6 x BOG, 2 x HEC, 4 x CDL, 2 x FES | HHblits | 0.25 |
| ``` target    MAQGVSRRQLLGRALALGSGAALADLLGPARFLSPAGAATAGAVVPGNPLRVMPDRTWEQIYRNQFEDDSTFVFTCAPND 3l75.1    --SSEDRKGFSYLVTATAC-------------------------------------------------------------  target    THNCLLRAHVKNGVVVRISPTYGYGEATDLYGNRASHRWDPRTCQKGLILSRRFYSERRVKAPMIRKGFKDWVEAGYPRN 3l75.1    --------------------------------------------------------------------------------  target    DDGTPQMDVTLRGSDDWIRISWDEATTIAAKTMEDVARTFNGDEGARKLLAQGYHPEMVEVMHGAGVQALKLR 3l75.1    ------------------------------------------------------------------------- ``` | | | | | | | | | | | | | | | | | | | | | | | | | | | | | | | | | | | | | | | | | | | | | | | | | |
|  | 3l75.1.E | CYTOCHROME B-C1 COMPLEX SUBUNIT 5, RIESKE IRONSULFUR PROTEIN, MITOCHONDRIAL  *Cytochrome BC1 complex from chicken with fenamidone bound* | 0.00 |  | 11.76 | 0.07 | 3-19 | X-ray | 2.79 | hetero-2-2-2-2-2-2-… | 6 x PEE, 4 x HEM, 2 x FNM, 2 x UQ, 2 x AZI, 6 x BOG, 2 x HEC, 4 x CDL, 2 x FES | HHblits | 0.25 |
| ``` target    MAQGVSRRQLLGRALALGSGAALADLLGPARFLSPAGAATAGAVVPGNPLRVMPDRTWEQIYRNQFEDDSTFVFTCAPND 3l75.1    --SSEDRKGFSYLVTATAC-------------------------------------------------------------  target    THNCLLRAHVKNGVVVRISPTYGYGEATDLYGNRASHRWDPRTCQKGLILSRRFYSERRVKAPMIRKGFKDWVEAGYPRN 3l75.1    --------------------------------------------------------------------------------  target    DDGTPQMDVTLRGSDDWIRISWDEATTIAAKTMEDVARTFNGDEGARKLLAQGYHPEMVEVMHGAGVQALKLR 3l75.1    ------------------------------------------------------------------------- ``` | | | | | | | | | | | | | | | | | | | | | | | | | | | | | | | | | | | | | | | | | | | | | | | | | |
|  | 4u3f.1.E | Cytochrome b-c1 complex subunit Rieske, mitochondrial  *Cytochrome bc1 complex from chicken with designed inhibitor bound* | 0.00 |  | 11.76 | 0.07 | 3-19 | X-ray | 3.23 | hetero-2-2-2-2-2-2-… | 14 x PEE, 4 x HEM, 2 x Y52, 2 x U10, 1 x MES, 2 x HEC, 4 x CDL, 3 x BOG, 2 x FES | HHblits | 0.25 |
| ``` target    MAQGVSRRQLLGRALALGSGAALADLLGPARFLSPAGAATAGAVVPGNPLRVMPDRTWEQIYRNQFEDDSTFVFTCAPND 4u3f.1    --SSEDRKGFSYLVTATAC-------------------------------------------------------------  target    THNCLLRAHVKNGVVVRISPTYGYGEATDLYGNRASHRWDPRTCQKGLILSRRFYSERRVKAPMIRKGFKDWVEAGYPRN 4u3f.1    --------------------------------------------------------------------------------  target    DDGTPQMDVTLRGSDDWIRISWDEATTIAAKTMEDVARTFNGDEGARKLLAQGYHPEMVEVMHGAGVQALKLR 4u3f.1    ------------------------------------------------------------------------- ``` | | | | | | | | | | | | | | | | | | | | | | | | | | | | | | | | | | | | | | | | | | | | | | | | | |
|  | 1kb9.1.E | UBIQUINOL-CYTOCHROME C REDUCTASE IRON-SULFUR SUBUNIT  *YEAST CYTOCHROME BC1 COMPLEX* | 0.00 |  | 5.56 | 0.08 | 3-20 | X-ray | 2.30 | hetero-oligomer | 3 x HEM, 1 x FES, 1 x SMA, 1 x UQ6, 1 x PIE, 2 x PEF, 1 x CDL, 1 x PCF, 1 x UMQ | HHblits | 0.21 |
| ``` target    MAQGVSRRQLLGRALALGSGAALADLLGPARFLSPAGAATAGAVVPGNPLRVMPDRTWEQIYRNQFEDDSTFVFTCAPND 1kb9.1    --DADKGRSYAYFMVGAMGL------------------------------------------------------------  target    THNCLLRAHVKNGVVVRISPTYGYGEATDLYGNRASHRWDPRTCQKGLILSRRFYSERRVKAPMIRKGFKDWVEAGYPRN 1kb9.1    --------------------------------------------------------------------------------  target    DDGTPQMDVTLRGSDDWIRISWDEATTIAAKTMEDVARTFNGDEGARKLLAQGYHPEMVEVMHGAGVQALKLR 1kb9.1    ------------------------------------------------------------------------- ``` | | | | | | | | | | | | | | | | | | | | | | | | | | | | | | | | | | | | | | | | | | | | | | | | | |
|  | 6hu9.1.E | Cytochrome b-c1 complex subunit Rieske, mitochondrial  *III2-IV2 mitochondrial respiratory supercomplex from S. cerevisiae* | 0.00 |  | 5.56 | 0.08 | 3-20 | EM | 0.00 | hetero-2-2-2-2-2-2-… | 28 x PEF, 4 x HEM, 1 x UQ6, 8 x CDL, 8 x PCF, 2 x HEC, 2 x FES, 2 x CU, 4 x HEA, 2 x CA, 2 x MG, 4 x CUA, 2 x ZN | HHblits | 0.21 |
| ``` target    MAQGVSRRQLLGRALALGSGAALADLLGPARFLSPAGAATAGAVVPGNPLRVMPDRTWEQIYRNQFEDDSTFVFTCAPND 6hu9.1    --DADKGRSYAYFMVGAMGL------------------------------------------------------------  target    THNCLLRAHVKNGVVVRISPTYGYGEATDLYGNRASHRWDPRTCQKGLILSRRFYSERRVKAPMIRKGFKDWVEAGYPRN 6hu9.1    --------------------------------------------------------------------------------  target    DDGTPQMDVTLRGSDDWIRISWDEATTIAAKTMEDVARTFNGDEGARKLLAQGYHPEMVEVMHGAGVQALKLR 6hu9.1    ------------------------------------------------------------------------- ``` | | | | | | | | | | | | | | | | | | | | | | | | | | | | | | | | | | | | | | | | | | | | | | | | | |
|  | 6t0b.1.O | Cytochrome b-c1 complex subunit Rieske, mitochondrial  *The III2-IV(5B)2 respiratory supercomplex from S. cerevisiae* | 0.00 |  | 5.56 | 0.08 | 3-20 | EM | 0.00 | hetero-2-2-2-2-2-2-… | 8 x CDL, 26 x PEF, 4 x HEM, 7 x PCF, 2 x HEC, 2 x FES, 2 x CU, 4 x HEA, 2 x CA, 2 x MG, 2 x CUA, 2 x ZN | HHblits | 0.21 |
| ``` target    MAQGVSRRQLLGRALALGSGAALADLLGPARFLSPAGAATAGAVVPGNPLRVMPDRTWEQIYRNQFEDDSTFVFTCAPND 6t0b.1    --DADKGRSYAYFMVGAMGL------------------------------------------------------------  target    THNCLLRAHVKNGVVVRISPTYGYGEATDLYGNRASHRWDPRTCQKGLILSRRFYSERRVKAPMIRKGFKDWVEAGYPRN 6t0b.1    --------------------------------------------------------------------------------  target    DDGTPQMDVTLRGSDDWIRISWDEATTIAAKTMEDVARTFNGDEGARKLLAQGYHPEMVEVMHGAGVQALKLR 6t0b.1    ------------------------------------------------------------------------- ``` | | | | | | | | | | | | | | | | | | | | | | | | | | | | | | | | | | | | | | | | | | | | | | | | | |
|  | 6t0b.1.E | Cytochrome b-c1 complex subunit Rieske, mitochondrial  *The III2-IV(5B)2 respiratory supercomplex from S. cerevisiae* | 0.00 |  | 5.56 | 0.08 | 3-20 | EM | 0.00 | hetero-2-2-2-2-2-2-… | 8 x CDL, 26 x PEF, 4 x HEM, 7 x PCF, 2 x HEC, 2 x FES, 2 x CU, 4 x HEA, 2 x CA, 2 x MG, 2 x CUA, 2 x ZN | HHblits | 0.21 |
| ``` target    MAQGVSRRQLLGRALALGSGAALADLLGPARFLSPAGAATAGAVVPGNPLRVMPDRTWEQIYRNQFEDDSTFVFTCAPND 6t0b.1    --DADKGRSYAYFMVGAMGL------------------------------------------------------------  target    THNCLLRAHVKNGVVVRISPTYGYGEATDLYGNRASHRWDPRTCQKGLILSRRFYSERRVKAPMIRKGFKDWVEAGYPRN 6t0b.1    --------------------------------------------------------------------------------  target    DDGTPQMDVTLRGSDDWIRISWDEATTIAAKTMEDVARTFNGDEGARKLLAQGYHPEMVEVMHGAGVQALKLR 6t0b.1    ------------------------------------------------------------------------- ``` | | | | | | | | | | | | | | | | | | | | | | | | | | | | | | | | | | | | | | | | | | | | | | | | | |
|  | 6t15.1.E | CYTOCHROME B-C1 COMPLEX SUBUNIT RIESKE, MITOCHONDRIAL; SYNONYM: COMPLEX III SUBUNIT 5, RIESKE IRON-SULFUR PROTEIN, RISP, UBIQUINOL-CYTOCHROME C REDUCTASE IRON-SULFUR SUBUNIT  *The III2-IV(5B)1 respiratory supercomplex from S. cerevisiae* | 0.00 |  | 5.56 | 0.08 | 3-20 | EM | 0.00 | hetero-2-2-2-2-2-2-… | 18 x PEF, 4 x HEM, 7 x CDL, 6 x PCF, 2 x HEC, 2 x FES, 1 x CU, 2 x HEA, 1 x MG, 1 x CUA, 1 x ZN | HHblits | 0.21 |
| ``` target    MAQGVSRRQLLGRALALGSGAALADLLGPARFLSPAGAATAGAVVPGNPLRVMPDRTWEQIYRNQFEDDSTFVFTCAPND 6t15.1    --DADKGRSYAYFMVGAMGL------------------------------------------------------------  target    THNCLLRAHVKNGVVVRISPTYGYGEATDLYGNRASHRWDPRTCQKGLILSRRFYSERRVKAPMIRKGFKDWVEAGYPRN 6t15.1    --------------------------------------------------------------------------------  target    DDGTPQMDVTLRGSDDWIRISWDEATTIAAKTMEDVARTFNGDEGARKLLAQGYHPEMVEVMHGAGVQALKLR 6t15.1    ------------------------------------------------------------------------- ``` | | | | | | | | | | | | | | | | | | | | | | | | | | | | | | | | | | | | | | | | | | | | | | | | | |
|  | 6t15.1.O | CYTOCHROME B-C1 COMPLEX SUBUNIT RIESKE, MITOCHONDRIAL; SYNONYM: COMPLEX III SUBUNIT 5, RIESKE IRON-SULFUR PROTEIN, RISP, UBIQUINOL-CYTOCHROME C REDUCTASE IRON-SULFUR SUBUNIT  *The III2-IV(5B)1 respiratory supercomplex from S. cerevisiae* | 0.00 |  | 5.56 | 0.08 | 3-20 | EM | 0.00 | hetero-2-2-2-2-2-2-… | 18 x PEF, 4 x HEM, 7 x CDL, 6 x PCF, 2 x HEC, 2 x FES, 1 x CU, 2 x HEA, 1 x MG, 1 x CUA, 1 x ZN | HHblits | 0.21 |
| ``` target    MAQGVSRRQLLGRALALGSGAALADLLGPARFLSPAGAATAGAVVPGNPLRVMPDRTWEQIYRNQFEDDSTFVFTCAPND 6t15.1    --DADKGRSYAYFMVGAMGL------------------------------------------------------------  target    THNCLLRAHVKNGVVVRISPTYGYGEATDLYGNRASHRWDPRTCQKGLILSRRFYSERRVKAPMIRKGFKDWVEAGYPRN 6t15.1    --------------------------------------------------------------------------------  target    DDGTPQMDVTLRGSDDWIRISWDEATTIAAKTMEDVARTFNGDEGARKLLAQGYHPEMVEVMHGAGVQALKLR 6t15.1    ------------------------------------------------------------------------- ``` | | | | | | | | | | | | | | | | | | | | | | | | | | | | | | | | | | | | | | | | | | | | | | | | | |
|  | 6ymx.1.Z | Cytochrome b-c1 complex subunit Rieske, mitochondrial  *CIII2/CIV respiratory supercomplex from Saccharomyces cerevisiae* | 0.00 |  | 5.56 | 0.08 | 3-20 | EM | 0.00 | hetero-1-1-1-1-1-1-… | 1 x CU, 2 x HEA, 7 x PTY, 2 x CN3, 1 x CUA, 5 x PCF, 1 x ZN, 2 x 6PH, 6 x HEM, 2 x 8PE, 1 x CN5, 2 x UQ6, 2 x 9PE, 2 x 7PH, 2 x FES | HHblits | 0.21 |
| ``` target    MAQGVSRRQLLGRALALGSGAALADLLGPARFLSPAGAATAGAVVPGNPLRVMPDRTWEQIYRNQFEDDSTFVFTCAPND 6ymx.1    --DADKGRSYAYFMVGAMGL------------------------------------------------------------  target    THNCLLRAHVKNGVVVRISPTYGYGEATDLYGNRASHRWDPRTCQKGLILSRRFYSERRVKAPMIRKGFKDWVEAGYPRN 6ymx.1    --------------------------------------------------------------------------------  target    DDGTPQMDVTLRGSDDWIRISWDEATTIAAKTMEDVARTFNGDEGARKLLAQGYHPEMVEVMHGAGVQALKLR 6ymx.1    ------------------------------------------------------------------------- ``` | | | | | | | | | | | | | | | | | | | | | | | | | | | | | | | | | | | | | | | | | | | | | | | | | |
|  | 6ymx.1.Q | Cytochrome b-c1 complex subunit Rieske, mitochondrial  *CIII2/CIV respiratory supercomplex from Saccharomyces cerevisiae* | 0.01 |  | 5.56 | 0.08 | 3-20 | EM | 0.00 | hetero-1-1-1-1-1-1-… | 1 x CU, 2 x HEA, 7 x PTY, 2 x CN3, 1 x CUA, 5 x PCF, 1 x ZN, 2 x 6PH, 6 x HEM, 2 x 8PE, 1 x CN5, 2 x UQ6, 2 x 9PE, 2 x 7PH, 2 x FES | HHblits | 0.21 |
| ``` target    MAQGVSRRQLLGRALALGSGAALADLLGPARFLSPAGAATAGAVVPGNPLRVMPDRTWEQIYRNQFEDDSTFVFTCAPND 6ymx.1    --DADKGRSYAYFMVGAMGL------------------------------------------------------------  target    THNCLLRAHVKNGVVVRISPTYGYGEATDLYGNRASHRWDPRTCQKGLILSRRFYSERRVKAPMIRKGFKDWVEAGYPRN 6ymx.1    --------------------------------------------------------------------------------  target    DDGTPQMDVTLRGSDDWIRISWDEATTIAAKTMEDVARTFNGDEGARKLLAQGYHPEMVEVMHGAGVQALKLR 6ymx.1    ------------------------------------------------------------------------- ``` | | | | | | | | | | | | | | | | | | | | | | | | | | | | | | | | | | | | | | | | | | | | | | | | | |
|  | 5gup.54.A | Cytochrome b-c1 complex subunit Rieske, mitochondrial  *Cryo-EM structure of mammalian respiratory supercomplex I1III2IV1* | 0.00 |  | 11.76 | 0.07 | 3-19 | EM | 0.00 | monomer |  | HHblits | 0.25 |
| ``` target    MAQGVSRRQLLGRALALGSGAALADLLGPARFLSPAGAATAGAVVPGNPLRVMPDRTWEQIYRNQFEDDSTFVFTCAPND 5gup.54   --SSDARKGFSYLITATTT-------------------------------------------------------------  target    THNCLLRAHVKNGVVVRISPTYGYGEATDLYGNRASHRWDPRTCQKGLILSRRFYSERRVKAPMIRKGFKDWVEAGYPRN 5gup.54   --------------------------------------------------------------------------------  target    DDGTPQMDVTLRGSDDWIRISWDEATTIAAKTMEDVARTFNGDEGARKLLAQGYHPEMVEVMHGAGVQALKLR 5gup.54   ------------------------------------------------------------------------- ``` | | | | | | | | | | | | | | | | | | | | | | | | | | | | | | | | | | | | | | | | | | | | | | | | | |
|  | 5gup.56.A | Cytochrome b-c1 complex subunit Rieske, mitochondrial  *Cryo-EM structure of mammalian respiratory supercomplex I1III2IV1* | 0.00 |  | 11.76 | 0.07 | 3-19 | EM | 0.00 | monomer |  | HHblits | 0.25 |
| ``` target    MAQGVSRRQLLGRALALGSGAALADLLGPARFLSPAGAATAGAVVPGNPLRVMPDRTWEQIYRNQFEDDSTFVFTCAPND 5gup.56   --SSDARKGFSYLITATTT-------------------------------------------------------------  target    THNCLLRAHVKNGVVVRISPTYGYGEATDLYGNRASHRWDPRTCQKGLILSRRFYSERRVKAPMIRKGFKDWVEAGYPRN 5gup.56   --------------------------------------------------------------------------------  target    DDGTPQMDVTLRGSDDWIRISWDEATTIAAKTMEDVARTFNGDEGARKLLAQGYHPEMVEVMHGAGVQALKLR 5gup.56   ------------------------------------------------------------------------- ``` | | | | | | | | | | | | | | | | | | | | | | | | | | | | | | | | | | | | | | | | | | | | | | | | | |
|  | 7r0w.1.L | Rieske domain, PetC  *2.8 Angstrom cryo-EM structure of the dimeric cytochrome b6f-PetP complex from Synechocystis sp. PCC 6803 with natively bound lipids and plastoquinone molecules* | 0.00 |  | 18.75 | 0.07 | 3-18 | EM | 0.00 | hetero-2-2-2-2-2-2-… | 6 x HEM, 4 x PGV, 2 x ECH, 2 x PL9, 2 x CLA, 2 x HEC, 1 x LMG, 2 x 6PL, 1 x 2WA, 2 x FES, 2 x SQD, 1 x LFA | HHblits | 0.28 |
| ``` target    MAQGVSRRQLLGRALALGSGAALADLLGPARFLSPAGAATAGAVVPGNPLRVMPDRTWEQIYRNQFEDDSTFVFTCAPND 7r0w.1    --PDLGRRQFMNLLTFGT--------------------------------------------------------------  target    THNCLLRAHVKNGVVVRISPTYGYGEATDLYGNRASHRWDPRTCQKGLILSRRFYSERRVKAPMIRKGFKDWVEAGYPRN 7r0w.1    --------------------------------------------------------------------------------  target    DDGTPQMDVTLRGSDDWIRISWDEATTIAAKTMEDVARTFNGDEGARKLLAQGYHPEMVEVMHGAGVQALKLR 7r0w.1    ------------------------------------------------------------------------- ``` | | | | | | | | | | | | | | | | | | | | | | | | | | | | | | | | | | | | | | | | | | | | | | | | | |
|  | 7r0w.1.Q | Rieske domain, PetC  *2.8 Angstrom cryo-EM structure of the dimeric cytochrome b6f-PetP complex from Synechocystis sp. PCC 6803 with natively bound lipids and plastoquinone molecules* | 0.00 |  | 18.75 | 0.07 | 3-18 | EM | 0.00 | hetero-2-2-2-2-2-2-… | 6 x HEM, 4 x PGV, 2 x ECH, 2 x PL9, 2 x CLA, 2 x HEC, 1 x LMG, 2 x 6PL, 1 x 2WA, 2 x FES, 2 x SQD, 1 x LFA | HHblits | 0.28 |
| ``` target    MAQGVSRRQLLGRALALGSGAALADLLGPARFLSPAGAATAGAVVPGNPLRVMPDRTWEQIYRNQFEDDSTFVFTCAPND 7r0w.1    --PDLGRRQFMNLLTFGT--------------------------------------------------------------  target    THNCLLRAHVKNGVVVRISPTYGYGEATDLYGNRASHRWDPRTCQKGLILSRRFYSERRVKAPMIRKGFKDWVEAGYPRN 7r0w.1    --------------------------------------------------------------------------------  target    DDGTPQMDVTLRGSDDWIRISWDEATTIAAKTMEDVARTFNGDEGARKLLAQGYHPEMVEVMHGAGVQALKLR 7r0w.1    ------------------------------------------------------------------------- ``` | | | | | | | | | | | | | | | | | | | | | | | | | | | | | | | | | | | | | | | | | | | | | | | | | |
|  | 6giq.1.E | Cytochrome b-c1 complex subunit Rieske, mitochondrial  *Saccharomyces cerevisiae respiratory supercomplex III2IV* | 0.00 |  | 5.88 | 0.07 | 3-19 | EM | 0.00 | hetero-2-2-2-2-2-2-… | 2 x 6PH, 6 x HEM, 2 x 8PE, 1 x CN5, 2 x UQ6, 2 x 7PH, 2 x FES, 2 x PCF, 2 x 9PE, 1 x CN3, 1 x CU, 2 x HEA, 1 x CUA | HHblits | 0.22 |
| ``` target    MAQGVSRRQLLGRALALGSGAALADLLGPARFLSPAGAATAGAVVPGNPLRVMPDRTWEQIYRNQFEDDSTFVFTCAPND 6giq.1    --DADKGRSYAYFMVGAMG-------------------------------------------------------------  target    THNCLLRAHVKNGVVVRISPTYGYGEATDLYGNRASHRWDPRTCQKGLILSRRFYSERRVKAPMIRKGFKDWVEAGYPRN 6giq.1    --------------------------------------------------------------------------------  target    DDGTPQMDVTLRGSDDWIRISWDEATTIAAKTMEDVARTFNGDEGARKLLAQGYHPEMVEVMHGAGVQALKLR 6giq.1    ------------------------------------------------------------------------- ``` | | | | | | | | | | | | | | | | | | | | | | | | | | | | | | | | | | | | | | | | | | | | | | | | | |
|  | 4h44.1.D | Cytochrome b6-f complex iron-sulfur subunit 1  *2.70 A Cytochrome b6f Complex Structure From Nostoc PCC 7120* | 0.00 |  | 20.00 | 0.06 | 2-16 | X-ray | 2.70 | hetero-oligomer | 8 x HEM, 10 x UMQ, 2 x MYS, 2 x 8K6, 2 x CLA, 4 x OPC, 2 x 7PH, 2 x SQD, 2 x CD, 2 x FES, 2 x OCT, 2 x BCR | HHblits | 0.31 |
| ``` target    MAQGVSRRQLLGRALALGSGAALADLLGPARFLSPAGAATAGAVVPGNPLRVMPDRTWEQIYRNQFEDDSTFVFTCAPND 4h44.1    -VPDMGRRQFMNLLTF----------------------------------------------------------------  target    THNCLLRAHVKNGVVVRISPTYGYGEATDLYGNRASHRWDPRTCQKGLILSRRFYSERRVKAPMIRKGFKDWVEAGYPRN 4h44.1    --------------------------------------------------------------------------------  target    DDGTPQMDVTLRGSDDWIRISWDEATTIAAKTMEDVARTFNGDEGARKLLAQGYHPEMVEVMHGAGVQALKLR 4h44.1    ------------------------------------------------------------------------- ``` | | | | | | | | | | | | | | | | | | | | | | | | | | | | | | | | | | | | | | | | | | | | | | | | | |
|  | 4ogq.1.L | Cytochrome b6-f complex iron-sulfur subunit 1  *Internal Lipid Architecture of the Hetero-Oligomeric Cytochrome b6f Complex* | 0.00 |  | 20.00 | 0.06 | 2-16 | X-ray | 2.50 | hetero-2-2-2-2-2-2-… | 8 x HEC, 10 x UMQ, 10 x 7PH, 8 x 8K6, 2 x 2WM, 2 x CLA, 2 x OPC, 2 x CD, 2 x MYS, 2 x SQD, 2 x FES, 2 x 2WD, 2 x 3WM, 2 x 2WA, 2 x OCT, 2 x 1O2, 2 x BCR | HHblits | 0.31 |
| ``` target    MAQGVSRRQLLGRALALGSGAALADLLGPARFLSPAGAATAGAVVPGNPLRVMPDRTWEQIYRNQFEDDSTFVFTCAPND 4ogq.1    -VPDMGRRQFMNLLTF----------------------------------------------------------------  target    THNCLLRAHVKNGVVVRISPTYGYGEATDLYGNRASHRWDPRTCQKGLILSRRFYSERRVKAPMIRKGFKDWVEAGYPRN 4ogq.1    --------------------------------------------------------------------------------  target    DDGTPQMDVTLRGSDDWIRISWDEATTIAAKTMEDVARTFNGDEGARKLLAQGYHPEMVEVMHGAGVQALKLR 4ogq.1    ------------------------------------------------------------------------- ``` | | | | | | | | | | | | | | | | | | | | | | | | | | | | | | | | | | | | | | | | | | | | | | | | | |
