# Supplementary material for: Metabolic capacity is maintained despite shifts in microbial diversity in estuary sediments
Source: ISME Commun. 2025 Oct 11;5(1):ycaf182. doi: 10.1093/ismeco/ycaf182 (PMC12687941; doi:10.1093/ismeco/ycaf182)
Supplement: Supplementary_Data_1_ycaf182 [file supplementary_data_1_ycaf182.zip › SWISS-MODEL/4_1_Oct_SF_Bin14_scaffold_2633_c1_6664172_1/report.html]

4\_1\_Oct\_SF\_Bin14\_scaffold\_2633\_c1\_666-4172\_1 | Report


|  |  |  |
| --- | --- | --- |
|  |  | SWISS-MODEL Homology Modelling Report |

## Model Building Report

This document lists the results for the homology modelling project "4\_1\_Oct\_SF\_Bin14\_scaffold\_2633\_c1\_666-4172\_1" submitted to SWISS-MODEL workspace
on March 29, 2023, 10:15 p.m..The submitted primary amino acid sequence is given in Table T1.

If you use any results in your research, please cite the relevant publications:

- Waterhouse, A., Bertoni, M., Bienert, S., Studer, G., Tauriello, G., Gumienny, R.,
  Heer, F.T., de Beer, T.A.P., Rempfer, C., Bordoli, L., Lepore, R., Schwede, T.
  SWISS-MODEL: homology modelling of protein structures and complexes.
  Nucleic Acids Res. 46(W1), W296-W303 (2018).
- Bienert, S., Waterhouse, A., de Beer, T.A.P., Tauriello, G., Studer,
  G., Bordoli, L., Schwede, T. The SWISS-MODEL Repository - new features and
  functionality. Nucleic Acids Res. 45, D313-D319 (2017).
- Studer, G., Tauriello, G., Bienert, S.,
  Biasini, M., Johner, N., Schwede, T. ProMod3 - A versatile homology
  modelling toolbox. PLOS Comp. Biol. 17(1), e1008667 (2021).
- Studer, G., Rempfer, C., Waterhouse, A.M.,
  Gumienny, G., Haas, J., Schwede, T. QMEANDisCo - distance constraints
  applied on model quality estimation. Bioinformatics 36, 1765-1771 (2020).
- Bertoni, M., Kiefer, F., Biasini, M., Bordoli, L.,
  Schwede, T. Modeling protein quaternary structure of homo- and
  hetero-oligomers beyond binary interactions by homology. Scientific
  Reports 7 (2017).

## Results

The SWISS-MODEL template library (SMTL version 2023-03-23, PDB release 2023-03-17) was searched with
for evolutionary related structures matching the target sequence in Table T1. For details on the template search, see Materials and Methods. Overall 513 templates were found (Table T2).

## Models

The following models were built (see Materials and Methods "Model Building"):

| Model #01 | File | Built with | Oligo-State | Ligands | GMQE | QMEANDisCo Global |
| --- | --- | --- | --- | --- | --- | --- |
|  | PDB | ProMod3 3.2.1 | monomer | 1 x MO: MOLYBDENUM ATOM; | 0.70 | 0.71 ± 0.05 |

|  |  |  |
| --- | --- | --- |
|  |  |  |

| Template | Seq Identity | Oligo-state | QSQE | Found by | Method | Resolution | Seq Similarity | Range | Coverage | Description |
| --- | --- | --- | --- | --- | --- | --- | --- | --- | --- | --- |
| 7b04.1.B | 40.77 | monomer | 0.00 | BLAST | X-ray | 2.97Å | 0.41 | 28 - 1160 | 0.96 | Nitrite oxidoreductase subunit A |

  

### Included Ligands

| Ligand | Description |
| --- | --- |
| 1 x MO | MOLYBDENUM ATOM |

  

### Excluded ligands

| Ligand Name.Number | Reason for Exclusion | Description |
| --- | --- | --- |
| CA.10 | Binding site not conserved. | CALCIUM ION |
| CA.11 | Binding site not conserved. | CALCIUM ION |
| F3S.4 | Binding site not conserved. | FE3-S4 CLUSTER |
| HEM.9 | Binding site not conserved. | PROTOPORPHYRIN IX CONTAINING FE |
| MD1.5 | Binding site not conserved. | PHOSPHORIC ACID 4-(2-AMINO-4-OXO-3,4,5,6,-TETRAHYDRO-PTERIDIN-6-YL)-2-HYDROXY-3,4-DIMERCAPTO-BUT-3-EN-YL ESTER GUANYLATE ESTER |
| MD1.6 | Binding site not conserved. | PHOSPHORIC ACID 4-(2-AMINO-4-OXO-3,4,5,6,-TETRAHYDRO-PTERIDIN-6-YL)-2-HYDROXY-3,4-DIMERCAPTO-BUT-3-EN-YL ESTER GUANYLATE ESTER |
| SF4.1 | Binding site not conserved. | IRON/SULFUR CLUSTER |
| SF4.2 | Binding site not conserved. | IRON/SULFUR CLUSTER |
| SF4.3 | Binding site not conserved. | IRON/SULFUR CLUSTER |
| SF4.8 | Binding site not conserved. | IRON/SULFUR CLUSTER |

  

```
Target    SDLSRRELLKRAVVVGTGAGLAELFLPAQFLSSASAQSEPQAVAIANPLAQMPDRSWERIYRDQFAEEDSFVFTCAPNDT  
7b04.1.B  --LTRRAFLQVAGATGATLTLAKNAMAFRLLKPA--------VVVDNPLDTYPDRRWESVYRDQYQYDRTFTYCCSPNDT  
  
Target    HNCLLRAHVKNGVIVRISPTYGYGKATDLAGNQASHRWDPRICQKGLILGRRIYGDRRVKAPMIRKGFKEWADAGFPRHD  
7b04.1.B  HACRIRAFVRNNVMMRVEQNYDHQNYSDLYGNKATRNWNPRMCLKGYTFHRRVYGPYRLRYPLIRKGWKRWADDGFPELT  
  
Target    -DGTPRADMEKRGYDEWLQIPWDEALAIAAKTLQNVAETYKGEDGAGKLLEQGYEPAMVEAMHGAGVQAIKMRGGMPLLG  
7b04.1.B  PENKTKYMFDNRGNDELLRASWDEAFTYASKGIIHITKKYSGPEGAQKLIDQGYPKEMVDRMQGAGTRTFKGRGGMGLLG  
  
Target    AGRVFGFYRFANMLALLDGKLRPEAPPEEIVGSRAFDNYAWHTDLPPGHPMVSGSQTVDFDLFAAEHSKLLVLIGMNWIC  
7b04.1.B  VIGKYGMYRFNNCLAIVDAHNRGVGP-DQALGGRNWSNYTWHGDQAPGHPFSHGLQTSDVDMNDVRFSKLLIQTGKNLIE  
  
Target    TKMPDAHWIGDARLKGTRVVVISADYMPTANKADEIVILRPGTDTAFLLGVARELITKKLYDRDAVIQRTDLPLLVRLDT  
7b04.1.B  NKMPEAHWVTEVMERGGKIVVITPEYSPSAQKADYWIPIRNNTDTALFLGITKILIDNKWYDADYVKKFTDFPLLIRTDT  
  
Target    GERLSARDVFEGYRQAPLENYVALKTEEELAAPPSPPFTADKQVVPTELREEWGDFVYWDRATNGPAAVNRDEIG---AK  
7b04.1.B  LKRVSPKDIIPNYKL------------QDISDGPSYHI----QGLKDEQREIIGDFVVWDAKSKGPKAITRDDVGETLVK  
  
Target    FAGDPALLGAFDVTLVDGTNVKARTAFSLLKEYLDENFDVQTTSEVCNVDPAAVRSLARQLAANKGNALLAAGMGPNHYF  
7b04.1.B  KGIDPVLEGSFKLKTIDGKEIEVMTLLEMYKIHL-RDYDIDSVVSMTNSPKDLIERLAKDIATIKPVAIHYGE-GVNHYF  
  
Target    NADLFGRVHFLVAALTDNIGHFSGNVGSYAGNYRGSLFQA-------MGQWIAENPFDQEADLTKPAR---VKRYFKSES  
7b04.1.B  HATLMNRSYYLPVMLTGNVGYFGSGSHTWAGNYKAGNFQASKWSGPGFYGWVAEDVFKPNLDPYASAKDLNIKGRALDEE  
  
Target    AHYWNYGDRPLVSPS-----EIITGKSHMPTPTKLIWFGNSNSLLGNAKWSFDVVKNTLPKQDAVFCNEWHWTSSCEYSD  
7b04.1.B  VAYWNHSERPLIVNTPKYGRKVFTGKTHMPSPTKVLWFTNVN-LINNAKHVYQMLKNVNPNIEQIMSTDIEITGSIEYAD  
  
Target    LVFPADSWAEFKLPDMTASCTNPFLLAFPKTPLARIHNTRSDYEILAGVAAALADLVDEPRMKTYWKGILDGDPTPYLQR  
7b04.1.B  FAFPANSWVEFQEFEITNSCSNPFIQIWGKTGITPVYESKDDVKILAGMASKLGELLRDKRFEDNWKFAIEGRASVYINR  
  
Target    VLSGSNATRGILYEDLHASS--AKGVPLLMNARTYPRHAGWEQRQEDKPWYTPTGRLEFYRPEPEWQAAGESLPIWREPV  
7b04.1.B  LLDGSTTMKGYTCEDILNGKYGEPGVAMLL-FRTYPRHPFWEQVHESLPFYTPTGRLQAYNDEPEIIEYGENFIVHREGP  
  
Target    DATFYEPNAILANSKHPSINPRAPEDYGVPESQMDVETRQYRNVVRTWQELKLSKHPLTEKDPAYRFVFQTPKYRWGAHS  
7b04.1.B  EATPYLPNAIV--STNPYIR---PDDYGIPENAEYWEDRTVRNIKKSWEETKKTKNFLWEK--GYHFYCVTPKSRHTVHS  
  
Target    TAVDSDWIAMLFGPFGDPYRRDSRTPWTGEAYAEINPRDAKELGLKDGDYIWLDADPEDRPYRGADSSDEFYDVARAMMR  
7b04.1.B  QWAVTDWNFIWNNNFGDPYRMDKRMPGVGEHQIHIHPQAARDLGIEDGDYVYVDANPADRPYEGWKPNDSFYKVSRLMLR  
  
Target    VRIYSGMPRRVIRTWFNMYAATPGTVQAQKDVPGGPAQNQDTGYVALFRHGSHQSGTRAYLRPTQMTDSMNRKAYFGQTI  
7b04.1.B  AKYNPAYPYNCTMMKHSAWISSDKTVQAHETRPDGRALSP-SGYQSSFRYGSQQSITRDWSMPMHQLDSLFHKAKIGMKF  
  
Target    GKGFEADVHSPSGAPKEGYVKVEKAEDGGDEGVGEWRPVTLGLRPDDPSEAMQAYLAGEFVTRKRKGS  
7b04.1.B  IFGFEADNHCINTVPKETLVKITKAENGGMGGKGVWDPVKTGYTAGNENDFMKKFLNGELI-------
```

  


---

  

| Model #02 | File | Built with | Oligo-State | Ligands | GMQE | QMEANDisCo Global |
| --- | --- | --- | --- | --- | --- | --- |
|  | PDB | ProMod3 3.2.1 | monomer | None | 0.41 | 0.51 ± 0.05 |

|  |  |  |
| --- | --- | --- |
|  |  |  |

| Template | Seq Identity | Oligo-state | QSQE | Found by | Method | Resolution | Seq Similarity | Range | Coverage | Description |
| --- | --- | --- | --- | --- | --- | --- | --- | --- | --- | --- |
| 3ir5.1.A | 24.12 | monomer | 0.00 | HHblits | X-ray | 2.30Å | 0.32 | 54 - 1130 | 0.78 | Respiratory nitrate reductase 1 alpha chain |

  

### Excluded ligands

| Ligand Name.Number | Reason for Exclusion | Description |
| --- | --- | --- |
| 6MO.3 | Clashing with protein. | MOLYBDENUM(VI) ION |
| AGA.5 | Binding site not conserved. | (1S)-2-{[{[(2S)-2,3-DIHYDROXYPROPYL]OXY}(HYDROXY)PHOSPHORYL]OXY}-1-[(PENTANOYLOXY)METHYL]ETHYL OCTANOATE |
| F3S.9 | Binding site not conserved. | FE3-S4 CLUSTER |
| HEM.10 | Binding site not conserved. | PROTOPORPHYRIN IX CONTAINING FE |
| HEM.11 | Binding site not conserved. | PROTOPORPHYRIN IX CONTAINING FE |
| MD1.1 | Binding site not conserved. | PHOSPHORIC ACID 4-(2-AMINO-4-OXO-3,4,5,6,-TETRAHYDRO-PTERIDIN-6-YL)-2-HYDROXY-3,4-DIMERCAPTO-BUT-3-EN-YL ESTER GUANYLATE ESTER |
| MD1.2 | Binding site not conserved. | PHOSPHORIC ACID 4-(2-AMINO-4-OXO-3,4,5,6,-TETRAHYDRO-PTERIDIN-6-YL)-2-HYDROXY-3,4-DIMERCAPTO-BUT-3-EN-YL ESTER GUANYLATE ESTER |
| SF4.4 | Binding site not conserved. | IRON/SULFUR CLUSTER |
| SF4.6 | Binding site not conserved. | IRON/SULFUR CLUSTER |
| SF4.7 | Binding site not conserved. | IRON/SULFUR CLUSTER |
| SF4.8 | Binding site not conserved. | IRON/SULFUR CLUSTER |

  

```
Target    SDLSRRELLKRAVVVGTGAGLAELFLPAQFLSSASAQSEPQAVAIANPLAQMPDRSWERIYRDQFAEEDSFVFTCAPNDT  
3ir5.1.A  -----------------------------------------------------NRDWEDGYRQRWQHDKIVRSTCGVNCT  
  
Target    HNCLLRAHVKNGVIVRISPTYGYGKATDLAGNQASHRWDPRICQKGLILGRRIYGDRRVKAPMIRKGFKE-WADAGFP--  
3ir5.1.A  GSCSWKIYVKNGLVTWETQQTDYPR-----TRPDLPNHEPRGCPRGASYSWYLYSANRLKYPMMRKRLMKMWREAKALHS  
  
Target    -------R-HD-DGTPRADMEKRGYDEWLQIPWDEALAIAAKTLQNVAETYKGEDGAGKLLEQGYEPAMVEAMHGAGVQA  
3ir5.1.A  DPVEAWASIIEDADKAKSFKQARGRGGFVRSSWQEVNELIAASNVYTIKNYGPDR----VAG----------FSPIPAMS  
  
Target    IKMRGGMPLLGAGRVFGFYRFANMLALLDGKLRPEAPPEEIVGSRAFDNYAWHTDLPPGHPMVSGSQTVDFDLFAAEHSK  
3ir5.1.A  M-----------VSYASGARYLS-----------------LIGGTCLSFYDWYCDLPPASPQTWGEQTDVPESADWYNSS  
  
Target    LLVLIGMNWICTKMPDAHWIGDARLKGTRVVVISADYMPTANKADEIVILRPGTDTAFLLGVARELITKKL------YDR  
3ir5.1.A  YIIAWGSNVPQTRTPDAHFFTEVRYKGTKTVAVTPDYAEIAKLCDLWLAPKQGTDAAMALAMGHVMLREFHLDNPSQYFT  
  
Target    DAVIQRTDLPLLVRLD-------TGERLSARDVFEGYRQAPL--ENYVALKTEEELAAPPSPPFTADKQVVPT-ELREEW  
3ir5.1.A  DYVRRYTDMPMLVMLEERDGYYAAGRMLRAADLVDALGQENNPEWKTVAFNT-------------NGEMVAPNGSIGFRW  
  
Target    GDFVYWDR-----ATNGPAAVNR------DE----IGAKFA-----------GDPALLG---AFDVTLVDGTNVKARTAF  
3ir5.1.A  GEKGKWNLEQRDGKTGEETELQLSLLGSQDEIAEVGFPYFGGDGTEHFNKVELENVLLHKLPVKRLQLADGSTALVTTVY  
  
Target    SLLK------------------EYLDENFDVQTTSEVCNVDPAAVRSLARQLAANK----GNALLAAGMGPNHYFNADLF  
3ir5.1.A  DLTLANYGLERGLNDVNCATSYDDV-KAYTPAWAEQITGVSRSQIIRIAREFADNADKTHGRSMIIVGAGLNHWYHLDMN  
  
Target    GRVHFLVAALTDNIGHFSGNVGSYAGNYRGSLFQAMGQ--------------------------WIAENPFDQEADLTKP  
3ir5.1.A  YRGLINMLIFCGCVGQSGGGWAHYVGQEKLRPQTGWQPLAFALDWQRPARHMNSTSYFYNHSSQWRYETVTAEE--LLSP  
  
Target    A-RVKRYFK--------SESAHYW----NYGDRPL----------V-----SPSEIITGKS--------HMPTPTKLIWF  
3ir5.1.A  MADKSRYTGHLIDFNVRAERMGWLPSAPQLGTNPLTIAGEAEKAGMNPVDYTVKSLKEGSIRFAAEQPENGKNHPRNLFI  
  
Target    GNSNSLLGNAKWSFDV------------------------------VKNTLPKQDAVFCNEWHWTSSCEYSDLVFPADSW  
3ir5.1.A  WRSNLLGSSGKGHEFMLKYLLGTEHGIQGKDLGQQGGVKPEEVDWQDNGLEGKLDLVVTLDFRLSSTCLYSDIILPTATW  
  
Target    AEFKLPDMTASCTNPFLLAFPKTPLARIHNTRSDYEILAGVAAALADLVDEP------------------------RMKT  
3ir5.1.A  YEK--DDMNTSDMHPFIHPLSA-AVDPAWEAKSDWEIYKAIAKKFSEVCVGHLGKETDIVTLPIQHDSAAELAQPLDVKD  
  
Target    YWKGILD---G------------------------------------------DPTPYLQ--------------------  
3ir5.1.A  WKKGECDLIPGKTAPHIMVVERDYPATYERFTSIGPLMEKIGNGGKGIAWNTQSEMDLLRKLNYTKAEGPAKGQPMLNTA  
  
Target    -----RVLSGSNATRGI----LYEDLHASSA-----------KGVPLLMNA------------------RTYPRHAGWEQ  
3ir5.1.A  IDAAEMILTLAPETNGQVAVKAWAALSEFTGRDHTHLALNKEDEKIRFRDIQAQPRKIISSPTWSGLEDEHVSYNAGYTN  
  
Target    RQEDKPWYTPTGRLEFYRPEPEWQAAGESLPIWREPVDATFYEPNAILANSKHPSINPRAPEDYGVPESQMDVETRQYRN  
3ir5.1.A  VHELIPWRTLSGRQQLYQDHQWMRDFGESLLVYRPPIDTRSVKE-------------------V----------------  
  
Target    VVRTWQELKLSKHPLTEKDPAYRFVFQTPKYRWGAHSTAVDSDWIAMLFGPFGDPYRRDSRTPWTGEAYAEINPRDAKEL  
3ir5.1.A  -----I------GQKSNGNQEKALNFLTPHQKWGIHSTYSDNLLMLTLG---------------RGGPVVWLSEADAKDL  
  
Target    GLKDGDYIWLDADPEDRPYRGADSSDEFYDVARAMMRVRIYSGMPRRVIRTWFNMYAATPGTVQAQKDVPGGPAQNQDTG  
3ir5.1.A  GIADNDWIEVFNS-----------------NGALTARAVVSQRVPAGMTMMYHAQERIVN--------LPGSEI------  
  
Target    YVALFRHGSHQSGTRAYLRPTQMTDSMNRKAYFGQTIGKGFEADVHSPSGAPKEGYVKVEKAEDGGDEGVGEWRPVTLGL  
3ir5.1.A  --TQQRGGIHNSVTRITPKPTHMIGGYAHLAY-------G--FNYYGTVGSNRDEFVVVRKMKNIDWL------------  
  
Target    RPDDPSEAMQAYLAGEFVTRKRKGS  
3ir5.1.A  -------------------------
```

  


---

  

| Model #03 | File | Built with | Oligo-State | Ligands | GMQE | QMEANDisCo Global |
| --- | --- | --- | --- | --- | --- | --- |
|  | PDB | ProMod3 3.2.1 | monomer | None | 0.27 | 0.47 ± 0.05 |

|  |  |  |
| --- | --- | --- |
|  |  |  |

| Template | Seq Identity | Oligo-state | QSQE | Found by | Method | Resolution | Seq Similarity | Range | Coverage | Description |
| --- | --- | --- | --- | --- | --- | --- | --- | --- | --- | --- |
| 1h0h.1.A | 17.09 | monomer | 0.00 | HHblits | X-ray | 1.80Å | 0.28 | 69 - 756 | 0.47 | FORMATE DEHYDROGENASE SUBUNIT ALPHA |

  

### Excluded ligands

| Ligand Name.Number | Reason for Exclusion | Description |
| --- | --- | --- |
| 2MD.2 | Binding site not conserved. | GUANYLATE-O'-PHOSPHORIC ACID MONO-(2-AMINO-5,6-DIMERCAPTO-4-OXO-3,5,6,7,8A,9,10,10A-OCTAHYDRO-4H-8-OXA-1,3,9,10-TETRAAZA-ANTHRACEN-7-YLMETHYL) ESTER |
| CA.6 | Binding site not conserved. | CALCIUM ION |
| EPE.7 | Not biologically relevant. | 4-(2-HYDROXYETHYL)-1-PIPERAZINE ETHANESULFONIC ACID |
| MGD.3 | Binding site not conserved. | 2-AMINO-5,6-DIMERCAPTO-7-METHYL-3,7,8A,9-TETRAHYDRO-8-OXA-1,3,9,10-TETRAAZA-ANTHRACEN-4-ONE GUANOSINE DINUCLEOTIDE |
| SF4.5 | Binding site not conserved. | IRON/SULFUR CLUSTER |
| SF4.8 | Binding site not conserved. | IRON/SULFUR CLUSTER |
| SF4.9 | Binding site not conserved. | IRON/SULFUR CLUSTER |
| SF4.10 | Binding site not conserved. | IRON/SULFUR CLUSTER |
| UNX.4 | Not biologically relevant. | UNKNOWN ATOM OR ION |
| W.1 | Not in contact with model. | TUNGSTEN ION |

  

```
Target    SDLSRRELLKRAVVVGTGAGLAELFLPAQFLSSASAQSEPQAVAIANPLAQMPDRSWERIYRDQFAEEDSFVFTCAPNDT  
1h0h.1.A  --------------------------------------------------------------------AKQTTSVCCYCS  
  
Target    HNCLLRAHV--KNGVIVRISPTYGYGKATDLAGNQASHRWDPRICQKGLILGRRIYGDRRVKAPMIRKGFKEWADAGFPR  
1h0h.1.A  VGCGLIVHTDKKTNRAINVEGD------------PDHPINEGSLCAKGASTWQLAENERRPANPLYRA------------  
  
Target    HDDGTPRADMEKRGYDEWLQIPWDEALAIAAKTLQNVAETYKGEDGAG-KLLEQGYEPAMVEAMHGAGVQAIKMRGGMPL  
1h0h.1.A  ------------PGSDQWEEKSWDWMLDTIAERVAKTREATFVTKNAKGQVVNRC--------------DGIA-------  
  
Target    LGAGRVFGFYRFANMLALLDGKLRPEAPPEEIVGSRAFDNYAWHTDLP--PGHPMVSGSQTVDFDLFAAEHSKLLVLIGM  
1h0h.1.A  -----SVGSAAMDNEECWIYQAWLR------SLGLFYIEHQARIUHSATVAALAESYGRGAMTNHWIDLKNSDVILMMGS  
  
Target    NWICTKMPDAHWIGDARLKGTRVVVISADYMPTANKADEIVILRPGTDTAFLLGVARELITKKLYDRDAVIQRTDLPLLV  
1h0h.1.A  NPAENHPISFKWVMRAKDKGATLIHVDPRYTRTSTKCDLYAPLRSGSDIAFLNGMTKYILEKELYFKDYVVNYTNASFIV  
  
Target    RLDTGERLSARDVFEGYRQAPLENYVALKTEEELAAPPSPPFTADKQVVPTELREEWGDFVYWDRATNGPAAVNRDEIGA  
1h0h.1.A  GEGF---AFEEGLF----------------------------------------------AGYNKETRKYDKSKW-----  
  
Target    KFAGDPALLGAFDVTLVDGTNVKARTAFSLLKEYLDENFDVQTTSEVCNVDPAAVRSLARQLAANK---GNALLAAGMGP  
1h0h.1.A  --GFERDENGNP---KRDETLKHPRCVFQIMKKHYE-RYDLDKISAICGTPKELILKVYDAYCATGKPDKAGTIMYAMGW  
  
Target    NHYFNADLFGRVHFLVAALTDNIGHFSGNVGSYAGNY--RGSLFQAMGQWIAENPFDQE---------A----DL----T  
1h0h.1.A  TQHTVGVQNIRAMSINQLLLGNIGVAGGGVNALRGEANVQGST--DHGLLMHIYPGYLGTARASIPTYEEYTKKFTPVSK  
  
Target    KPARVKRYFKS------ESAHYWNYGDR-------------PLVSPSEIITGKSHMPTPTKLIWFGNSNSLLGNAKWSFD  
1h0h.1.A  DPQSANWWSNFPKYSASYIKSMWPDADLNEAYGYLPKGEDGKDYSWLTLFDDM--FQGKIKGFFAWGQNPACSGAN--SN  
  
Target    VVKNTLPKQDAVFCNEWHWTSSCEYS-------------DLVFPADSWAEFKLPDMTASCTNPFLLAFPKTPLARIHNTR  
1h0h.1.A  KTREALTKLDWMVNVNIFDNETGSFWRGPDMDPKKIKTEVFFLPCAVAIEKE--GSI-SNSGRWMQWRYV-GPEPRKNAI  
  
Target    SDYEILAGVAAALADLVDEPRMKTYWKGILDGDPTPYLQRVLSGSNATRGILYEDLHASSAKGVPLLMNARTYPRHAGWE  
1h0h.1.A  PDGDLIVELAKRVQK-----------------------------------------------------------------  
  
Target    QRQEDKPWYTPTGRLEFYRPEPEWQAAGESLPIWREPVDATFYEPNAILANSKHPSINPRAPEDYGVPESQMDVETRQYR  
1h0h.1.A  --------------------------------------------------------------------------------  
  
Target    NVVRTWQELKLSKHPLTEKDPAYRFVFQTPKYRWGAHSTAVDSDWIAMLFGPFGDPYRRDSRTPWTGEAYAEINPRDAKE  
1h0h.1.A  --------------------------------------------------------------------------------  
  
Target    LGLKDGDYIWLDADPEDRPYRGADSSDEFYDVARAMMRVRIYSGMPRRVIRTWFNMYAATPGTVQAQKDVPGGPAQNQDT  
1h0h.1.A  --------------------------------------------------------------------------------  
  
Target    GYVALFRHGSHQSGTRAYLRPTQMTDSMNRKAYFGQTIGKGFEADVHSPSGAPKEGYVKVEKAEDGGDEGVGEWRPVTLG  
1h0h.1.A  --------------------------------------------------------------------------------  
  
Target    LRPDDPSEAMQAYLAGEFVTRKRKGS  
1h0h.1.A  --------------------------
```

  


---

  

| Model #04 | File | Built with | Oligo-State | Ligands | GMQE | QMEANDisCo Global |
| --- | --- | --- | --- | --- | --- | --- |
|  | PDB | ProMod3 3.2.1 | monomer | None | 0.14 | 0.44 ± 0.05 |

|  |  |  |
| --- | --- | --- |
|  |  |  |

| Template | Seq Identity | Oligo-state | QSQE | Found by | Method | Resolution | Seq Similarity | Range | Coverage | Description |
| --- | --- | --- | --- | --- | --- | --- | --- | --- | --- | --- |
| 3ir7.1.A | 30.59 | monomer | 0.00 | BLAST | X-ray | 2.50Å | 0.35 | 54 - 432 | 0.29 | Respiratory nitrate reductase 1 alpha chain |

  

### Excluded ligands

| Ligand Name.Number | Reason for Exclusion | Description |
| --- | --- | --- |
| 6MO.4 | Binding site not conserved. | MOLYBDENUM(VI) ION |
| AGA.5 | Binding site not conserved. | (1S)-2-{[{[(2S)-2,3-DIHYDROXYPROPYL]OXY}(HYDROXY)PHOSPHORYL]OXY}-1-[(PENTANOYLOXY)METHYL]ETHYL OCTANOATE |
| F3S.9 | Binding site not conserved. | FE3-S4 CLUSTER |
| HEM.10 | Binding site not conserved. | PROTOPORPHYRIN IX CONTAINING FE |
| HEM.11 | Binding site not conserved. | PROTOPORPHYRIN IX CONTAINING FE |
| MD1.1 | Binding site not conserved. | PHOSPHORIC ACID 4-(2-AMINO-4-OXO-3,4,5,6,-TETRAHYDRO-PTERIDIN-6-YL)-2-HYDROXY-3,4-DIMERCAPTO-BUT-3-EN-YL ESTER GUANYLATE ESTER |
| MD1.2 | Binding site not conserved. | PHOSPHORIC ACID 4-(2-AMINO-4-OXO-3,4,5,6,-TETRAHYDRO-PTERIDIN-6-YL)-2-HYDROXY-3,4-DIMERCAPTO-BUT-3-EN-YL ESTER GUANYLATE ESTER |
| SF4.3 | Binding site not conserved. | IRON/SULFUR CLUSTER |
| SF4.6 | Binding site not conserved. | IRON/SULFUR CLUSTER |
| SF4.7 | Binding site not conserved. | IRON/SULFUR CLUSTER |
| SF4.8 | Binding site not conserved. | IRON/SULFUR CLUSTER |

  

```
Target    SDLSRRELLKRAVVVGTGAGLAELFLPAQFLSSASAQSEPQAVAIANPLAQMPDRSWERIYRDQFAEEDSFVFTCAPNDT  
3ir7.1.A  -----------------------------------------------------NRDWEDGYRQRWQHDKIVRSTHGVNCT  
  
Target    HNCLLRAHVKNGVIVRISPTYGYGKAT-DLAGNQASHRWDPRICQKGLILGRRIYGDRRVKAPMIRKGF-KEWADAGFPR  
3ir7.1.A  GSCSWKIYVKNGLVTWETQQTDYPRTRPDLPNHE------PRGCPSGASYSWYLYSANRLKYPMMRKRLMKMWREAKALH  
  
Target    HDDGTPRADMEKRGYDEWLQIPWDEALAIAAKTLQNVAETYKGEDGAGKLLEQGYEPAMVEAMHGAGVQAIKMRGGMPLL  
3ir7.1.A  SDP-----------VEAWASIIED----------ADKAKSFKQARGRGGFVRSSWQ-EVNELIAASNVYTIKN------Y  
  
Target    GAGRVFGFYRF--ANMLALLDGKLRPEAPPEEIVGSRAFDNYAWHTDLPPGHPMVSGSQTVDFDLFAAEHSKLLVLIGMN  
3ir7.1.A  GPDRVAGFSPIPAMSMVSYASG-----ARYLSLIGGTCLSFYDWYCDLPPASPQTWGEQTDVPESADWYNSSYIIAWGSN  
  
Target    WICTKMPDAHWIGDARLKGTRVVVISADYMPTANKADEIVILRPGTDTAFLLGVARELITK------KLYDRDAVIQRTD  
3ir7.1.A  VPQTRTPDAHFFTEVRYKGTKTVAVTPDYAEIAKLCDLWLAPKQGTDAAMALAMGHVMLREFHLDNPSQYFTDYVRRYTD  
  
Target    LPLLVRLD-------TGERLSARDVFE--GYRQAPLENYVALKTEEELAAPPSPPFTADKQVVPTELREEWGDFVYWDRA  
3ir7.1.A  MPMLVMLEERDGYYAAGRMLRAADLVDALGQENNPEWKTVAFNTNGEMVAP-----------------------------  
  
Target    TNGPAAVNRDEIGAKFAGDPALLGAFDVTLVDGTNVKARTAFSLLKEYLDENFDVQTTSEVCNVDPAAVRSLARQLAANK  
3ir7.1.A  --------------------------------------------------------------------------------  
  
Target    GNALLAAGMGPNHYFNADLFGRVHFLVAALTDNIGHFSGNVGSYAGNYRGSLFQAMGQWIAENPFDQEADLTKPARVKRY  
3ir7.1.A  --------------------------------------------------------------------------------  
  
Target    FKSESAHYWNYGDRPLVSPSEIITGKSHMPTPTKLIWFGNSNSLLGNAKWSFDVVKNTLPKQDAVFCNEWHWTSSCEYSD  
3ir7.1.A  --------------------------------------------------------------------------------  
  
Target    LVFPADSWAEFKLPDMTASCTNPFLLAFPKTPLARIHNTRSDYEILAGVAAALADLVDEPRMKTYWKGILDGDPTPYLQR  
3ir7.1.A  --------------------------------------------------------------------------------  
  
Target    VLSGSNATRGILYEDLHASSAKGVPLLMNARTYPRHAGWEQRQEDKPWYTPTGRLEFYRPEPEWQAAGESLPIWREPVDA  
3ir7.1.A  --------------------------------------------------------------------------------  
  
Target    TFYEPNAILANSKHPSINPRAPEDYGVPESQMDVETRQYRNVVRTWQELKLSKHPLTEKDPAYRFVFQTPKYRWGAHSTA  
3ir7.1.A  --------------------------------------------------------------------------------  
  
Target    VDSDWIAMLFGPFGDPYRRDSRTPWTGEAYAEINPRDAKELGLKDGDYIWLDADPEDRPYRGADSSDEFYDVARAMMRVR  
3ir7.1.A  --------------------------------------------------------------------------------  
  
Target    IYSGMPRRVIRTWFNMYAATPGTVQAQKDVPGGPAQNQDTGYVALFRHGSHQSGTRAYLRPTQMTDSMNRKAYFGQTIGK  
3ir7.1.A  --------------------------------------------------------------------------------  
  
Target    GFEADVHSPSGAPKEGYVKVEKAEDGGDEGVGEWRPVTLGLRPDDPSEAMQAYLAGEFVTRKRKGS  
3ir7.1.A  ------------------------------------------------------------------
```

  


---

  

| Model #05 | File | Built with | Oligo-State | Ligands | GMQE | QMEANDisCo Global |
| --- | --- | --- | --- | --- | --- | --- |
|  | PDB | ProMod3 3.2.1 | monomer | None | 0.11 | 0.38 ± 0.05 |

|  |  |  |
| --- | --- | --- |
|  |  |  |

| Template | Seq Identity | Oligo-state | QSQE | Found by | Method | Resolution | Seq Similarity | Range | Coverage | Description |
| --- | --- | --- | --- | --- | --- | --- | --- | --- | --- | --- |
| 6sdv.1.A | 26.79 | monomer | 0.00 | BLAST | X-ray | 1.90Å | 0.33 | 76 - 415 | 0.24 | Formate dehydrogenase, alpha subunit, selenocysteine-containing,Formate dehydrogenase, alpha subunit, selenocysteine-containing,W-formate dehydrogenase - alpha subunit |

  

### Excluded ligands

| Ligand Name.Number | Reason for Exclusion | Description |
| --- | --- | --- |
| GOL.6 | Not biologically relevant. | GLYCEROL |
| GOL.7 | Not biologically relevant. | GLYCEROL |
| GOL.8 | Not biologically relevant. | GLYCEROL |
| GOL.9 | Not biologically relevant. | GLYCEROL |
| GOL.10 | Not biologically relevant. | GLYCEROL |
| GOL.11 | Not biologically relevant. | GLYCEROL |
| GOL.12 | Not biologically relevant. | GLYCEROL |
| GOL.13 | Not biologically relevant. | GLYCEROL |
| GOL.14 | Not biologically relevant. | GLYCEROL |
| GOL.15 | Not biologically relevant. | GLYCEROL |
| H2S.5 | Binding site not conserved. | HYDROSULFURIC ACID |
| MGD.1 | Binding site not conserved. | 2-AMINO-5,6-DIMERCAPTO-7-METHYL-3,7,8A,9-TETRAHYDRO-8-OXA-1,3,9,10-TETRAAZA-ANTHRACEN-4-ONE GUANOSINE DINUCLEOTIDE |
| MGD.2 | Binding site not conserved. | 2-AMINO-5,6-DIMERCAPTO-7-METHYL-3,7,8A,9-TETRAHYDRO-8-OXA-1,3,9,10-TETRAAZA-ANTHRACEN-4-ONE GUANOSINE DINUCLEOTIDE |
| NO3.16 | Not biologically relevant. | NITRATE ION |
| NO3.17 | Not biologically relevant. | NITRATE ION |
| PEG.18 | Not biologically relevant. | DI(HYDROXYETHYL)ETHER |
| SF4.3 | Binding site not conserved. | IRON/SULFUR CLUSTER |
| SF4.19 | Binding site not conserved. | IRON/SULFUR CLUSTER |
| SF4.20 | Binding site not conserved. | IRON/SULFUR CLUSTER |
| SF4.21 | Binding site not conserved. | IRON/SULFUR CLUSTER |
| W.4 | Not in contact with model. | TUNGSTEN ION |

  

```
Target    SDLSRRELLKRAVVVGTGAGLAELFLPAQFLSSASAQSEPQAVAIANPLAQMPDRSWERIYRDQFAEEDSFVFTCAPNDT  
6sdv.1.A  ---------------------------------------------------------------------------AKQTT  
  
Target    HNCLLRAHVKNGVIVRISPTYGYGKATDLAGNQASHRWDPRICQKGLILGRRIYGDRRVKAPMIRKGFKEWADAGFPRHD  
6sdv.1.A  SICCYCA-VGCGLIVHTAKD-GQGRAVNVEGDPDHPINEGSLCPKGASIFQLGENDQRGTQPLYRAPFS-----------  
  
Target    DGTPRADMEKRGYDEWLQIPWDEALAIAAKTLQNVAE-TYKGEDGAGKLLEQGYEPAMVEAMHGAGVQAIKMRGGMPLLG  
6sdv.1.A  -------------DTWKPVTWDFALTEIAKRIKKTRDASFTEKNAAGDLVNR------TEAIASFGSAAMDNEECWAYGN  
  
Target    AGRVFGFYRFANMLALLDGKLRPEAPPEEIVGSRAFDNYAWHTDLPPGHPMVSGSQTVDFDLFAAEHSKLLVLIGMNWIC  
6sdv.1.A  ILRSLG-------LVYIEHQARIUHSPTVPALAESFGR---------------GAMTNHWNDLA--NSDCILIMGSNAAE  
  
Target    TKMPDAHWIGDARLKGTRVVVISADYMPTANKADEIVILRPGTDTAFLLGVARELITKKLYDRDAVIQRTDLPLLVRLDT  
6sdv.1.A  NHPIAFKWVLRAKDKGATLIHVDPRFTRTSARCDVYAPIRSGADIPFLGGLIKYILDNKLYFTDYVREYTNASLIV----  
  
Target    GERLSARD-VFEGYRQAPLENYVALKTEEELAAPPSPPFTADKQVVPTELREEWGDFVYWDRATNGPAAVNRDEIGAKFA  
6sdv.1.A  GEKFSFKDGLFSGYDAA---------------------------------------------------------------  
  
Target    GDPALLGAFDVTLVDGTNVKARTAFSLLKEYLDENFDVQTTSEVCNVDPAAVRSLARQLAANKGNALLAAGMGPNHYFNA  
6sdv.1.A  --------------------------------------------------------------------------------  
  
Target    DLFGRVHFLVAALTDNIGHFSGNVGSYAGNYRGSLFQAMGQWIAENPFDQEADLTKPARVKRYFKSESAHYWNYGDRPLV  
6sdv.1.A  --------------------------------------------------------------------------------  
  
Target    SPSEIITGKSHMPTPTKLIWFGNSNSLLGNAKWSFDVVKNTLPKQDAVFCNEWHWTSSCEYSDLVFPADSWAEFKLPDMT  
6sdv.1.A  --------------------------------------------------------------------------------  
  
Target    ASCTNPFLLAFPKTPLARIHNTRSDYEILAGVAAALADLVDEPRMKTYWKGILDGDPTPYLQRVLSGSNATRGILYEDLH  
6sdv.1.A  --------------------------------------------------------------------------------  
  
Target    ASSAKGVPLLMNARTYPRHAGWEQRQEDKPWYTPTGRLEFYRPEPEWQAAGESLPIWREPVDATFYEPNAILANSKHPSI  
6sdv.1.A  --------------------------------------------------------------------------------  
  
Target    NPRAPEDYGVPESQMDVETRQYRNVVRTWQELKLSKHPLTEKDPAYRFVFQTPKYRWGAHSTAVDSDWIAMLFGPFGDPY  
6sdv.1.A  --------------------------------------------------------------------------------  
  
Target    RRDSRTPWTGEAYAEINPRDAKELGLKDGDYIWLDADPEDRPYRGADSSDEFYDVARAMMRVRIYSGMPRRVIRTWFNMY  
6sdv.1.A  --------------------------------------------------------------------------------  
  
Target    AATPGTVQAQKDVPGGPAQNQDTGYVALFRHGSHQSGTRAYLRPTQMTDSMNRKAYFGQTIGKGFEADVHSPSGAPKEGY  
6sdv.1.A  --------------------------------------------------------------------------------  
  
Target    VKVEKAEDGGDEGVGEWRPVTLGLRPDDPSEAMQAYLAGEFVTRKRKGS  
6sdv.1.A  -------------------------------------------------
```

  


---

  

| Model #06 | File | Built with | Oligo-State | Ligands | GMQE | QMEANDisCo Global |
| --- | --- | --- | --- | --- | --- | --- |
|  | PDB | ProMod3 3.2.1 | monomer | None | 0.08 | 0.40 ± 0.05 |

|  |  |  |
| --- | --- | --- |
|  |  |  |

| Template | Seq Identity | Oligo-state | QSQE | Found by | Method | Resolution | Seq Similarity | Range | Coverage | Description |
| --- | --- | --- | --- | --- | --- | --- | --- | --- | --- | --- |
| 7l5i.1.A | 24.11 | monomer | 0.00 | BLAST | X-ray | 1.73Å | 0.34 | 128 - 389 | 0.19 | Trimethylamine-N-oxide reductase |

  

### Excluded ligands

| Ligand Name.Number | Reason for Exclusion | Description |
| --- | --- | --- |
| CL.6 | Not biologically relevant. | CHLORIDE ION |
| EPE.1 | Not biologically relevant. | 4-(2-HYDROXYETHYL)-1-PIPERAZINE ETHANESULFONIC ACID |
| EPE.2 | Not biologically relevant. | 4-(2-HYDROXYETHYL)-1-PIPERAZINE ETHANESULFONIC ACID |
| EPE.7 | Not biologically relevant. | 4-(2-HYDROXYETHYL)-1-PIPERAZINE ETHANESULFONIC ACID |
| MGD.3 | Binding site not conserved. | 2-AMINO-5,6-DIMERCAPTO-7-METHYL-3,7,8A,9-TETRAHYDRO-8-OXA-1,3,9,10-TETRAAZA-ANTHRACEN-4-ONE GUANOSINE DINUCLEOTIDE |
| MGD.4 | Binding site not conserved. | 2-AMINO-5,6-DIMERCAPTO-7-METHYL-3,7,8A,9-TETRAHYDRO-8-OXA-1,3,9,10-TETRAAZA-ANTHRACEN-4-ONE GUANOSINE DINUCLEOTIDE |
| MO.5 | Binding site not conserved. | MOLYBDENUM ATOM |
| O.8 | Binding site not conserved. | OXYGEN ATOM |

  

```
Target    SDLSRRELLKRAVVVGTGAGLAELFLPAQFLSSASAQSEPQAVAIANPLAQMPDRSWERIYRDQFAEEDSFVFTCAPNDT  
7l5i.1.A  --------------------------------------------------------------------------------  
  
Target    HNCLLRAHVKNGVIVRISPTYGYGKATDLAGNQASHRWDPRICQKGLILGRRIYGDRRVKAPMIRKGFKEWADAGFPRHD  
7l5i.1.A  -----------------------------------------------VVADQLYSEARVKCPMVRKGF--LANPG-----  
  
Target    DGTPRADMEKRGYDEWLQIPWDEALAIAAKTLQNVAETYKGEDGAGKLLEQGYEPAMVEAMHGAGVQAIKMRGGMPLLGA  
7l5i.1.A  ----KSDTTMRGRDEWVRVSWDEALDLVHNQLKRV----RDEHGSTGIFAGSYGWFSCGSLH-----------------A  
  
Target    GRVFGFYRFANMLALLDG-KLRPEAPPEEIVGSRAFDNYAWHTDLPPGHPMVSGSQTVDFDLFAAEHSKLLVLIGMNWIC  
7l5i.1.A  SRTL-LQRYMNATGGFVGHKGDYSTGAAQVIMPHVLGTIEVYEQQTSWESILESS-----DIIVLWSANPLTTMRIAWMS  
  
Target    TKMPDAHWIGDARLKGTRVVVISADYMPTANKAD-EIVILRPGTDTAFLLGVARELITKKLYDRDAVIQRTDLPLLVRLD  
7l5i.1.A  TDQKGIEYFKKFQASGKRIICIDPQKSETCQMLNAEWIPVNTATDVPLMLGIAHTLVEQGKHDKDFLKKYT---------  
  
Target    TGERLSARDVFEGYRQAPLENYVALKTEEELAAPPSPPFTADKQVVPTELREEWGDFVYWDRATNGPAAVNRDEIGAKFA  
7l5i.1.A  --------------------------------------------------------------------------------  
  
Target    GDPALLGAFDVTLVDGTNVKARTAFSLLKEYLDENFDVQTTSEVCNVDPAAVRSLARQLAANKGNALLAAGMGPNHYFNA  
7l5i.1.A  --------------------------------------------------------------------------------  
  
Target    DLFGRVHFLVAALTDNIGHFSGNVGSYAGNYRGSLFQAMGQWIAENPFDQEADLTKPARVKRYFKSESAHYWNYGDRPLV  
7l5i.1.A  --------------------------------------------------------------------------------  
  
Target    SPSEIITGKSHMPTPTKLIWFGNSNSLLGNAKWSFDVVKNTLPKQDAVFCNEWHWTSSCEYSDLVFPADSWAEFKLPDMT  
7l5i.1.A  --------------------------------------------------------------------------------  
  
Target    ASCTNPFLLAFPKTPLARIHNTRSDYEILAGVAAALADLVDEPRMKTYWKGILDGDPTPYLQRVLSGSNATRGILYEDLH  
7l5i.1.A  --------------------------------------------------------------------------------  
  
Target    ASSAKGVPLLMNARTYPRHAGWEQRQEDKPWYTPTGRLEFYRPEPEWQAAGESLPIWREPVDATFYEPNAILANSKHPSI  
7l5i.1.A  --------------------------------------------------------------------------------  
  
Target    NPRAPEDYGVPESQMDVETRQYRNVVRTWQELKLSKHPLTEKDPAYRFVFQTPKYRWGAHSTAVDSDWIAMLFGPFGDPY  
7l5i.1.A  --------------------------------------------------------------------------------  
  
Target    RRDSRTPWTGEAYAEINPRDAKELGLKDGDYIWLDADPEDRPYRGADSSDEFYDVARAMMRVRIYSGMPRRVIRTWFNMY  
7l5i.1.A  --------------------------------------------------------------------------------  
  
Target    AATPGTVQAQKDVPGGPAQNQDTGYVALFRHGSHQSGTRAYLRPTQMTDSMNRKAYFGQTIGKGFEADVHSPSGAPKEGY  
7l5i.1.A  --------------------------------------------------------------------------------  
  
Target    VKVEKAEDGGDEGVGEWRPVTLGLRPDDPSEAMQAYLAGEFVTRKRKGS  
7l5i.1.A  -------------------------------------------------
```

  


---

  

| Model #07 | File | Built with | Oligo-State | Ligands | GMQE | QMEANDisCo Global |
| --- | --- | --- | --- | --- | --- | --- |
|  | PDB | ProMod3 3.2.1 | monomer | None | 0.07 | 0.36 ± 0.05 |

|  |  |  |
| --- | --- | --- |
|  |  |  |

| Template | Seq Identity | Oligo-state | QSQE | Found by | Method | Resolution | Seq Similarity | Range | Coverage | Description |
| --- | --- | --- | --- | --- | --- | --- | --- | --- | --- | --- |
| 1eu1.1.A | 33.04 | monomer | 0.00 | BLAST | X-ray | 1.30Å | 0.36 | 133 - 401 | 0.19 | DIMETHYL SULFOXIDE REDUCTASE |

  

### Excluded ligands

| Ligand Name.Number | Reason for Exclusion | Description |
| --- | --- | --- |
| 6MO.8 | Binding site not conserved. | MOLYBDENUM(VI) ION |
| CD.5 | Binding site not conserved. | CADMIUM ION |
| EPE.11 | Not biologically relevant. | 4-(2-HYDROXYETHYL)-1-PIPERAZINE ETHANESULFONIC ACID |
| GLC.1 | Not in contact with model. | alpha-D-glucopyranose |
| GLC.2 | Binding site not conserved. | alpha-D-glucopyranose |
| GLC.3 | Binding site not conserved. | alpha-D-glucopyranose |
| MGD.6 | Binding site not conserved. | 2-AMINO-5,6-DIMERCAPTO-7-METHYL-3,7,8A,9-TETRAHYDRO-8-OXA-1,3,9,10-TETRAAZA-ANTHRACEN-4-ONE GUANOSINE DINUCLEOTIDE |
| MGD.7 | Binding site not conserved. | 2-AMINO-5,6-DIMERCAPTO-7-METHYL-3,7,8A,9-TETRAHYDRO-8-OXA-1,3,9,10-TETRAAZA-ANTHRACEN-4-ONE GUANOSINE DINUCLEOTIDE |
| O.9 | Binding site not conserved. | OXYGEN ATOM |
| O.10 | Binding site not conserved. | OXYGEN ATOM |
| SO4.4 | Not biologically relevant. | SULFATE ION |

  

```
Target    SDLSRRELLKRAVVVGTGAGLAELFLPAQFLSSASAQSEPQAVAIANPLAQMPDRSWERIYRDQFAEEDSFVFTCAPNDT  
1eu1.1.A  --------------------------------------------------------------------------------  
  
Target    HNCLLRAHVKNGVIVRISPTYGYGKATDLAGNQASHRWDPRICQKGLILGRRIYGDRRVKAPMIRKGFKEWADAGFPRHD  
1eu1.1.A  ----------------------------------------------------IYSPTRIKYPMVRREFLEKG--------  
  
Target    DGTPRADMEKRGYDEWLQIPWDEALAIAAKTLQNVAETYKGEDGAGKLLEQGYEPAMVEAMHGAGVQAIKMRGGMPLLGA  
1eu1.1.A  ---VNADRSTRGNGDFVRVTWDEALDLVARELKRVQESY----GPTGTFGGSYGWKSPGRLHNCQVL---MRRALNLAG-  
  
Target    GRVFGFYRFANMLALLDGKLRPEAPPEEIVGSRAFDNYAWHTDLPPGHPMVSGSQTVDFDLFAAEHSKLLVL-----IGM  
1eu1.1.A  GFVNSSGDYSTAAAQI---IMP-----HVMGT--LEVYEQQTAWP----------------VVVENTDLMVFWAADPMKT  
  
Target    NWICTKMPD-AHWIGDARLK--GTRVVVISADYMPTANK-ADEIVILRPGTDTAFLLGVARELITKKLYDRDAVIQ-RTD  
1eu1.1.A  NEIGWVIPDHGAYAGMKALKEKGTRVICINPVRTETADYFGADVVSPRPQTDVALMLGMAHTLYSEDLHDKDFLENCTTG  
  
Target    LPLLVRLDTGERLSARDVFEGYRQAPLENYVALKTEEELAAPPSPPFTADKQVVPTELREEWGDFVYWDRATNGPAAVNR  
1eu1.1.A  FDLFAAYLTGE---------------------------------------------------------------------  
  
Target    DEIGAKFAGDPALLGAFDVTLVDGTNVKARTAFSLLKEYLDENFDVQTTSEVCNVDPAAVRSLARQLAANKGNALLAAGM  
1eu1.1.A  --------------------------------------------------------------------------------  
  
Target    GPNHYFNADLFGRVHFLVAALTDNIGHFSGNVGSYAGNYRGSLFQAMGQWIAENPFDQEADLTKPARVKRYFKSESAHYW  
1eu1.1.A  --------------------------------------------------------------------------------  
  
Target    NYGDRPLVSPSEIITGKSHMPTPTKLIWFGNSNSLLGNAKWSFDVVKNTLPKQDAVFCNEWHWTSSCEYSDLVFPADSWA  
1eu1.1.A  --------------------------------------------------------------------------------  
  
Target    EFKLPDMTASCTNPFLLAFPKTPLARIHNTRSDYEILAGVAAALADLVDEPRMKTYWKGILDGDPTPYLQRVLSGSNATR  
1eu1.1.A  --------------------------------------------------------------------------------  
  
Target    GILYEDLHASSAKGVPLLMNARTYPRHAGWEQRQEDKPWYTPTGRLEFYRPEPEWQAAGESLPIWREPVDATFYEPNAIL  
1eu1.1.A  --------------------------------------------------------------------------------  
  
Target    ANSKHPSINPRAPEDYGVPESQMDVETRQYRNVVRTWQELKLSKHPLTEKDPAYRFVFQTPKYRWGAHSTAVDSDWIAML  
1eu1.1.A  --------------------------------------------------------------------------------  
  
Target    FGPFGDPYRRDSRTPWTGEAYAEINPRDAKELGLKDGDYIWLDADPEDRPYRGADSSDEFYDVARAMMRVRIYSGMPRRV  
1eu1.1.A  --------------------------------------------------------------------------------  
  
Target    IRTWFNMYAATPGTVQAQKDVPGGPAQNQDTGYVALFRHGSHQSGTRAYLRPTQMTDSMNRKAYFGQTIGKGFEADVHSP  
1eu1.1.A  --------------------------------------------------------------------------------  
  
Target    SGAPKEGYVKVEKAEDGGDEGVGEWRPVTLGLRPDDPSEAMQAYLAGEFVTRKRKGS  
1eu1.1.A  ---------------------------------------------------------
```

  


---

  

## Materials and Methods

## Template Search

Template search with
has been performed against the SWISS-MODEL template library (SMTL, last update: 2023-03-23, last included PDB release: 2023-03-17).

## Template Selection

For each identified template, the template's quality has been predicted from features of the target-template alignment.
The templates with the highest quality have then been selected for model building.

## Model Building

Models are built based on the target-template alignment using ProMod3 (Studer et al.). Coordinates which are conserved between the target and the template are copied from the template to the model. Insertions and deletions are remodelled using a fragment library. Side chains are then rebuilt. Finally, the geometry of the resulting model is regularized by using a force field.

## Model Quality Estimation

The global and per-residue model quality has been assessed using the QMEAN scoring function (Studer et al.).

## Ligand Modelling

Ligands present in the template structure are transferred by homology to the model when the following criteria are met: (a) The ligands are annotated as biologically relevant in the template library, (b) the ligand is in contact with the model, (c) the ligand is not clashing with the protein, (d) the residues in contact with the ligand are conserved between the target and the template. If any of these four criteria is not satisfied, a certain ligand will not be included in the model. The model summary includes information on why and which ligand has not been included.

## Oligomeric State Conservation

The quaternary structure annotation of the template is used to model the target sequence in its oligomeric form. The method (Bertoni et al.) is based on a supervised machine learning algorithm, Support Vector Machines (SVM), which combines interface conservation, structural clustering, and other template features to provide a quaternary structure quality estimate (QSQE). The QSQE score is a number between 0 and 1, reflecting the expected accuracy of the interchain contacts for a model built based a given alignment and template. Higher numbers indicate higher reliability. This complements the GMQE score which estimates the accuracy of the tertiary structure of the resulting model.

## References

- **BLAST**  
  Camacho, C., Coulouris, G., Avagyan, V., Ma, N., Papadopoulos, J.,
  Bealer, K., Madden, T.L. BLAST+: architecture and applications. BMC
  Bioinformatics 10, 421-430 (2009).
- **HHblits**  
  Steinegger, M., Meier, M., Mirdita, M., Vöhringer, H., Haunsberger,
  S. J., Söding, J. HH-suite3 for fast remote homology detection and
  deep protein annotation. BMC Bioinformatics 20, 473 (2019).

## Table T1:

Primary amino acid sequence for which templates were searched and models were built.

SDLSRRELLKRAVVVGTGAGLAELFLPAQFLSSASAQSEPQAVAIANPLAQMPDRSWERIYRDQFAEEDSFVFTCAPNDTHNCLLRAHVKNGVIVRISPT  
YGYGKATDLAGNQASHRWDPRICQKGLILGRRIYGDRRVKAPMIRKGFKEWADAGFPRHDDGTPRADMEKRGYDEWLQIPWDEALAIAAKTLQNVAETYK  
GEDGAGKLLEQGYEPAMVEAMHGAGVQAIKMRGGMPLLGAGRVFGFYRFANMLALLDGKLRPEAPPEEIVGSRAFDNYAWHTDLPPGHPMVSGSQTVDFD  
LFAAEHSKLLVLIGMNWICTKMPDAHWIGDARLKGTRVVVISADYMPTANKADEIVILRPGTDTAFLLGVARELITKKLYDRDAVIQRTDLPLLVRLDTG  
ERLSARDVFEGYRQAPLENYVALKTEEELAAPPSPPFTADKQVVPTELREEWGDFVYWDRATNGPAAVNRDEIGAKFAGDPALLGAFDVTLVDGTNVKAR  
TAFSLLKEYLDENFDVQTTSEVCNVDPAAVRSLARQLAANKGNALLAAGMGPNHYFNADLFGRVHFLVAALTDNIGHFSGNVGSYAGNYRGSLFQAMGQW  
IAENPFDQEADLTKPARVKRYFKSESAHYWNYGDRPLVSPSEIITGKSHMPTPTKLIWFGNSNSLLGNAKWSFDVVKNTLPKQDAVFCNEWHWTSSCEYS  
DLVFPADSWAEFKLPDMTASCTNPFLLAFPKTPLARIHNTRSDYEILAGVAAALADLVDEPRMKTYWKGILDGDPTPYLQRVLSGSNATRGILYEDLHAS  
SAKGVPLLMNARTYPRHAGWEQRQEDKPWYTPTGRLEFYRPEPEWQAAGESLPIWREPVDATFYEPNAILANSKHPSINPRAPEDYGVPESQMDVETRQY  
RNVVRTWQELKLSKHPLTEKDPAYRFVFQTPKYRWGAHSTAVDSDWIAMLFGPFGDPYRRDSRTPWTGEAYAEINPRDAKELGLKDGDYIWLDADPEDRP  
YRGADSSDEFYDVARAMMRVRIYSGMPRRVIRTWFNMYAATPGTVQAQKDVPGGPAQNQDTGYVALFRHGSHQSGTRAYLRPTQMTDSMNRKAYFGQTIG  
KGFEADVHSPSGAPKEGYVKVEKAEDGGDEGVGEWRPVTLGLRPDDPSEAMQAYLAGEFVTRKRKGS

## Table T2:

| Template | Seq Identity | Oligo-state | QSQE | Found by | Method | Resolution | Seq Similarity | Coverage | Description |
| --- | --- | --- | --- | --- | --- | --- | --- | --- | --- |
| 7b04.1.B | 40.77 | monomer | - | BLAST | X-ray | 2.97Å | 0.41 | 0.96 | Nitrite oxidoreductase subunit A |
| 7b04.1.B | 39.64 | monomer | - | HHblits | X-ray | 2.97Å | 0.40 | 0.96 | Nitrite oxidoreductase subunit A |
| 7b04.2.B | 40.77 | monomer | - | BLAST | X-ray | 2.97Å | 0.41 | 0.96 | Nitrite oxidoreductase subunit A |
| 7b04.2.B | 39.64 | monomer | - | HHblits | X-ray | 2.97Å | 0.40 | 0.96 | Nitrite oxidoreductase subunit A |
| 4ydd.1.A | 25.48 | monomer | - | HHblits | X-ray | 1.86Å | 0.33 | 0.71 | DMSO reductase family type II enzyme, molybdopterin subunit |
| 5e7o.1.A | 25.82 | monomer | - | HHblits | X-ray | 2.40Å | 0.33 | 0.71 | DMSO reductase family type II enzyme, molybdopterin subunit |
| 3ir5.1.A | 24.12 | monomer | - | HHblits | X-ray | 2.30Å | 0.32 | 0.78 | Respiratory nitrate reductase 1 alpha chain |
| 1r27.4.A | 23.96 | homo-dimer | 0.14 | HHblits | X-ray | 2.00Å | 0.32 | 0.80 | Respiratory nitrate reductase 1 alpha chain |
| 2ivf.1.A | 24.43 | monomer | - | HHblits | X-ray | 1.88Å | 0.32 | 0.75 | ETHYLBENZENE DEHYDROGENASE ALPHA-SUBUNIT |
| 3egw.1.A | 24.12 | homo-dimer | 0.12 | HHblits | X-ray | 1.90Å | 0.32 | 0.78 | Respiratory nitrate reductase 1 alpha chain |
| 3ir7.1.A | 23.85 | monomer | - | HHblits | X-ray | 2.50Å | 0.32 | 0.80 | Respiratory nitrate reductase 1 alpha chain |
| 1q16.1.A | 24.04 | monomer | - | HHblits | X-ray | 1.90Å | 0.32 | 0.78 | Respiratory nitrate reductase 1 alpha chain |
| 3ir6.1.A | 23.85 | monomer | - | HHblits | X-ray | 2.80Å | 0.32 | 0.80 | Respiratory nitrate reductase 1 alpha chain |
| 5e7o.1.A | 28.33 | monomer | - | BLAST | X-ray | 2.40Å | 0.34 | 0.60 | DMSO reductase family type II enzyme, molybdopterin subunit |
| 4ydd.1.A | 28.33 | monomer | - | BLAST | X-ray | 1.86Å | 0.35 | 0.60 | DMSO reductase family type II enzyme, molybdopterin subunit |
| 1e60.1.A | 20.00 | monomer | - | HHblits | X-ray | 2.00Å | 0.30 | 0.63 | Dimethyl sulfoxide/trimethylamine N-oxide reductase |
| 1e18.1.A | 19.86 | monomer | - | HHblits | X-ray | 2.00Å | 0.30 | 0.63 | DMSO REDUCTASE. |
| 1e5v.2.A | 19.84 | monomer | - | HHblits | X-ray | 2.40Å | 0.30 | 0.63 | Dimethyl sulfoxide/trimethylamine N-oxide reductase |
| 4dmr.1.A | 20.06 | monomer | - | HHblits | X-ray | 1.90Å | 0.30 | 0.59 | DMSO REDUCTASE |
| 1dms.1.A | 20.64 | monomer | - | HHblits | X-ray | 1.88Å | 0.30 | 0.59 | DMSO REDUCTASE |
| 1h0h.1.A | 17.09 | monomer | - | HHblits | X-ray | 1.80Å | 0.28 | 0.47 | FORMATE DEHYDROGENASE SUBUNIT ALPHA |
| 2ivf.1.A | 36.10 | monomer | - | BLAST | X-ray | 1.88Å | 0.38 | 0.38 | ETHYLBENZENE DEHYDROGENASE ALPHA-SUBUNIT |
| 6q8o.1.C | 17.17 | monomer | - | HHblits | X-ray | 3.61Å | 0.27 | 0.42 | NADH-quinone oxidoreductase subunit 3 |
| 3m9s.1.C | 17.17 | monomer | - | HHblits | X-ray | 4.50Å | 0.27 | 0.42 | NADH-quinone oxidoreductase subunit 3 |
| 6ziy.1.C | 17.17 | monomer | - | HHblits | EM | NA | 0.27 | 0.42 | NADH-quinone oxidoreductase subunit 3 |
| 6zjl.1.C | 17.17 | monomer | - | HHblits | EM | NA | 0.27 | 0.42 | NADH-quinone oxidoreductase subunit 3 |
| 6zjn.1.C | 17.17 | monomer | - | HHblits | EM | NA | 0.27 | 0.42 | NADH-quinone oxidoreductase subunit 3 |
| 2fug.2.C | 17.17 | monomer | - | HHblits | X-ray | 3.30Å | 0.27 | 0.42 | NADH-quinone oxidoreductase chain 3 |
| 6zjy.1.C | 17.17 | monomer | - | HHblits | EM | NA | 0.27 | 0.42 | NADH-quinone oxidoreductase subunit 3 |
| 6zk9.1.C | 14.65 | monomer | - | HHblits | EM | NA | 0.27 | 0.33 | NADH:ubiquinone oxidoreductase core subunit S1 |
| 7zd6.1.4 | 14.40 | monomer | - | HHblits | EM | NA | 0.27 | 0.33 | NADH-ubiquinone oxidoreductase 75 kDa subunit, mitochondrial |
| 6qcf.1.C | 14.40 | monomer | - | HHblits | EM | NA | 0.27 | 0.33 | NADH:ubiquinone oxidoreductase core subunit S1 |
| 6qc5.1.C | 14.40 | monomer | - | HHblits | EM | NA | 0.27 | 0.33 | NADH:ubiquinone oxidoreductase core subunit S1 |
| 7qsd.1.G | 14.65 | monomer | - | HHblits | EM | NA | 0.27 | 0.33 | NADH-ubiquinone oxidoreductase 75 kDa subunit, mitochondrial |
| 5o31.1.8 | 14.91 | monomer | - | HHblits | EM | 4.13Å | 0.27 | 0.33 | NADH-ubiquinone oxidoreductase 75 kDa subunit, mitochondrial |
| 7dgr.10.A | 14.91 | monomer | - | HHblits | EM | NA | 0.27 | 0.33 | NADH-ubiquinone oxidoreductase 75 kDa subunit, mitochondrial |
| 3ir7.1.A | 30.59 | monomer | - | BLAST | X-ray | 2.50Å | 0.35 | 0.29 | Respiratory nitrate reductase 1 alpha chain |
| 3ir5.1.A | 30.88 | monomer | - | BLAST | X-ray | 2.30Å | 0.36 | 0.29 | Respiratory nitrate reductase 1 alpha chain |
| 1r27.4.A | 30.59 | homo-dimer | - | BLAST | X-ray | 2.00Å | 0.36 | 0.29 | Respiratory nitrate reductase 1 alpha chain |
| 1q16.1.A | 30.59 | monomer | - | BLAST | X-ray | 1.90Å | 0.36 | 0.29 | Respiratory nitrate reductase 1 alpha chain |
| 3egw.1.A | 30.59 | homo-dimer | - | BLAST | X-ray | 1.90Å | 0.35 | 0.29 | Respiratory nitrate reductase 1 alpha chain |
| 3ir6.1.A | 30.59 | monomer | - | BLAST | X-ray | 2.80Å | 0.36 | 0.29 | Respiratory nitrate reductase 1 alpha chain |
| 6sdv.1.A | 26.79 | monomer | - | BLAST | X-ray | 1.90Å | 0.33 | 0.24 | Formate dehydrogenase, alpha subunit, selenocysteine-containing,Formate dehydrogenase, alpha subunit, selenocysteine-containing,W-formate dehydrogenase - alpha subunit |
| 6sdr.1.A | 26.79 | monomer | - | BLAST | X-ray | 2.10Å | 0.33 | 0.24 | Formate dehydrogenase, alpha subunit, selenocysteine-containing |
| 8bqg.1.A | 26.79 | monomer | - | BLAST | X-ray | 1.95Å | 0.33 | 0.24 | Formate dehydrogenase, alpha subunit, selenocysteine-containing |
| 7l5i.1.A | 24.11 | monomer | - | BLAST | X-ray | 1.73Å | 0.34 | 0.19 | Trimethylamine-N-oxide reductase |
| 7l5s.1.A | 24.11 | monomer | - | BLAST | X-ray | 2.09Å | 0.34 | 0.19 | Trimethylamine-N-oxide reductase |
| 4dmr.1.A | 29.09 | monomer | - | BLAST | X-ray | 1.90Å | 0.35 | 0.19 | DMSO REDUCTASE |
| 1e18.1.A | 29.09 | monomer | - | BLAST | X-ray | 2.00Å | 0.35 | 0.19 | DMSO REDUCTASE. |
| 1eu1.1.A | 33.04 | monomer | - | BLAST | X-ray | 1.30Å | 0.36 | 0.19 | DIMETHYL SULFOXIDE REDUCTASE |

  
The table above shows the top 50 filtered templates. A further 372 templates were found which were considered to be less suitable for modelling than the filtered list.  
1aa6.1.A, 1be3.1.E, 1bgy.1.P, 1cz4.1.A, 1cz5.1.A, 1dms.1.A, 1e18.1.A, 1e5v.2.A, 1e60.1.A, 1eu1.1.A, 1fdo.1.A, 1g8j.1.A, 1g8k.1.A, 1h0h.1.A, 1ici.1.A, 1jeo.1.A, 1kb9.1.E, 1kqf.1.A, 1l0l.1.E, 1l0n.1.E, 1m2g.1.A, 1m2h.1.A, 1m2j.1.A, 1m2k.1.A, 1m2n.1.A, 1m2n.1.B, 1ma3.1.A, 1ntk.1.E, 1ntm.1.E, 1ogy.1.A, 1q16.1.A, 1q90.1.E, 1r27.4.A, 1s5p.1.A, 1s7g.1.A, 1s7g.1.B, 1s7g.1.C, 1s7g.1.D, 1s7g.1.E, 1sqb.1.E, 1sqp.1.P, 1sqq.1.P, 1sqv.1.E, 1tmo.1.A, 1uhd.1.B, 1uhe.1.B, 1vf5.1.D, 1vf5.1.L, 1wlf.1.A, 1x94.1.A, 1x94.1.B, 1yc5.1.A, 1zrt.1.C, 1zrt.1.F, 2a3n.1.A, 2b4y.1.A, 2b4y.3.A, 2d2c.1.D, 2d2c.1.L, 2e75.1.D, 2e76.1.D, 2e7z.1.A, 2fyn.1.C, 2fyn.2.C, 2fyu.1.E, 2h2i.1.A, 2h4h.1.A, 2h59.1.B, 2i2w.1.A, 2i2w.2.B, 2iv2.1.A, 2ivf.1.A, 2ki8.1.A, 2nya.1.A, 2nyr.1.A, 2nyr.1.B, 2pjh.1.B, 2pq4.1.B, 2qjk.1.C, 2qjp.1.C, 2qjy.3.F, 2v3v.1.A, 2v45.1.A, 2vpx.1.D, 2vpz.1.A, 2x3y.1.A, 2ybb.1.b, 3cwb.1.E, 3egw.1.A, 3etn.1.A, 3eua.1.A, 3fj1.1.A, 3h1h.1.E, 3h1h.1.O, 3h1i.1.E, 3ir5.1.A, 3ir6.1.A, 3ir7.1.A, 3jr3.1.A, 3k35.1.A, 3l75.1.E, 3l75.1.O, 3o27.1.A, 3o27.1.B, 3o5a.1.A, 3pki.1.A, 3plx.1.B, 3qc8.1.A, 3qq7.1.A, 3qq8.1.A, 3qwz.1.A, 3riy.2.A, 3sho.1.A, 3sho.1.C, 3tiw.1.A, 3tiw.2.A, 3zg6.1.A, 4aay.1.A, 4aok.1.B, 4aok.1.D, 4bv2.3.A, 4dmr.1.A, 4g1c.1.A, 4g1c.2.A, 4ga5.1.A, 4ga6.1.A, 4h44.1.D, 4hda.1.A, 4hda.2.A, 4ivn.1.A, 4kdi.1.A, 4kdi.2.A, 4kdl.1.A, 4ogq.1.L, 4pv1.1.L, 4rv0.1.A, 4twi.1.A, 4twj.1.A, 4u3f.1.E, 4utn.1.A, 4utn.2.A, 4v4c.1.A, 4wd3.1.A, 4ydd.1.A, 5b6c.1.A, 5bwl.1.A, 5cuo.1.A, 5cup.1.A, 5e7o.1.A, 5e7p.1.A, 5epp.1.A, 5g4f.1.A, 5g4f.1.B, 5g4f.1.C, 5g4f.1.D, 5g4f.1.E, 5g4f.1.F, 5glf.1.A, 5glf.2.A, 5glf.3.A, 5glf.4.A, 5gpn.24.A, 5j8k.55.A, 5kkz.1.C, 5kli.1.C, 5klv.1.P, 5ls7.1.L, 5ltz.1.A, 5lu5.1.A, 5lu6.1.A, 5lu7.1.A, 5mf6.1.A, 5nqd.1.A, 5o31.1.8, 5oj7.1.A, 5ojn.1.A, 5okd.1.E, 5t5i.1.B, 5t5i.1.D, 5x16.1.A, 5x4l.1.A, 5x4l.2.A, 5xhs.1.A, 5xtb.1.L, 5xte.1.C, 5y2f.1.A, 6aco.1.A, 6acp.1.A, 6adq.1.L, 6btm.1.B, 6cz7.1.A, 6enx.1.A, 6eo0.1.A, 6eqs.3.A, 6f0k.1.B, 6fky.1.A, 6fky.2.A, 6flg.1.A, 6g72.1.G, 6gcs.1.A, 6giq.1.E, 6hd3.1.A, 6hu9.1.E, 6hwh.1.A, 6ljk.1.A, 6ljm.1.A, 6lod.1.B, 6nhg.1.E, 6nin.1.C, 6q9e.1.E, 6q9e.1.O, 6qbx.15.A, 6qbx.5.A, 6qc2.33.A, 6qc2.43.A, 6qc3.15.A, 6qc3.5.A, 6qc4.15.A, 6qc4.5.A, 6qc5.1.C, 6qcf.1.C, 6rfq.1.A, 6rfs.1.A, 6rqf.1.D, 6rqf.1.L, 6rxj.1.A, 6rxm.1.A, 6rxm.2.A, 6rxm.3.A, 6rxm.4.A, 6rxm.5.A, 6rxm.6.A, 6rxo.1.A, 6rxo.2.A, 6rxp.2.A, 6rxq.4.A, 6rxs.1.A, 6s6y.1.B, 6sdr.1.A, 6sdv.1.A, 6t0b.1.E, 6t0b.1.O, 6t15.1.E, 6t15.1.O, 6tg9.1.A, 6x89.1.H, 6xvg.3.A, 6yj4.1.G, 6ymx.1.Q, 6ymx.1.Z, 6zk9.1.C, 6zr2.1.G, 7a23.1.O, 7ak5.1.G, 7ak6.1.G, 7aqr.1.F, 7ar7.1.G, 7ar8.1.G, 7arc.1.F, 7b04.1.B, 7b04.2.B, 7bkb.1.F, 7bkb.1.J, 7bkb.1.L, 7cl0.1.A, 7dbo.1.A, 7dbo.2.A, 7dg7.1.A, 7dg9.1.A, 7dgr.10.A, 7dgr.60.A, 7dgs.50.A, 7dgs.60.A, 7di0.1.A, 7di0.2.A, 7di0.3.A, 7di1.1.A, 7du6.1.A, 7du7.1.A, 7dvc.1.A, 7dvc.5.A, 7dvf.1.A, 7dvh.1.A, 7dvh.2.A, 7dvh.4.A, 7dww.1.A, 7dww.2.A, 7dxr.1.A, 7dxr.1.B, 7dxr.2.B, 7dxs.1.A, 7dxs.1.B, 7dxs.2.A, 7dxs.2.B, 7dxt.1.A, 7dxu.1.A, 7dxu.1.B, 7dxu.2.B, 7dxv.1.A, 7dxv.1.B, 7dxw.1.A, 7dxx.1.A, 7dxx.1.B, 7dxy.1.A, 7dxz.1.A, 7dxz.2.A, 7dxz.2.B, 7dxz.3.A, 7dyc.1.A, 7dyc.2.A, 7dyc.3.A, 7e1v.1.P, 7e5z.1.A, 7en5.1.A, 7en6.1.A, 7en6.1.B, 7en6.1.C, 7en6.1.D, 7jrg.1.E, 7l5i.1.A, 7l5s.1.A, 7nz1.1.E, 7o37.1.E, 7o37.1.O, 7o3c.1.E, 7o3c.1.O, 7o3h.1.E, 7o3h.1.O, 7p61.1.C, 7p63.1.C, 7q5y.1.A, 7qrm.1.D, 7qsd.1.G, 7qv7.1.L, 7qv7.1.O, 7r0w.1.L, 7r0w.1.Q, 7rh5.1.V, 7rja.1.H, 7rjb.1.I, 7t2r.1.A, 7t30.1.A, 7tce.2.F, 7tgh.58.A, 7tlj.1.C, 7tz6.1.E, 7tz6.1.P, 7v2c.1.L, 7vw6.1.A, 7vxu.1.L, 7wbb.1.A, 7wbb.1.B, 7wbb.1.C, 7wbb.1.D, 7wbb.1.E, 7wbb.1.G, 7z0t.1.G, 7zd6.1.4, 7zm7.1.I, 7zxy.1.D, 7zxy.1.L, 8asi.1.A, 8asi.1.E, 8asj.1.E, 8b9z.1.G, 8ba0.1.G, 8bel.1.B, 8bel.1.I, 8bpx.51.A, 8bqg.1.A, 8e73.55.A, 8e9g.1.G

Swiss Institute of Bioinformatics
Contact Us
