# Supplementary material for: Metabolic capacity is maintained despite shifts in microbial diversity in estuary sediments
Source: ISME Commun. 2025 Oct 11;5(1):ycaf182. doi: 10.1093/ismeco/ycaf182 (PMC12687941; doi:10.1093/ismeco/ycaf182)
Supplement: Supplementary_Data_1_ycaf182 [file supplementary_data_1_ycaf182.zip › SWISS-MODEL/21_Jan_SF_Bin67_scaffold_32672_c132330_1/templates.html]

21\_Jan\_SF\_Bin67\_scaffold\_32672\_c1:3-2330\_1 | Templates


**Export Alignment**
  
FASTA format
Clustal Format
PNG Image

**Secondary Structure**
  
None
DSSP
PSIPRED
SSpro

**Colour Scheme** 


Fade Mismatches
Enhance Mismatches

Confidencegradient
Confidenceclass
Indels
Chain
Unique Chain
Rainbow
2° Structure
Clustal
Hydrophobic
Size
Charged
Polar
Proline
Ser/Thr
Cysteine
Aliphatic
Aromatic
No Colour

Use QMEANBrane values

|  |  |  |  |
| --- | --- | --- | --- |
| Background |  |  |  |

**3D Viewer**  
NGL
PV

FASTA
Multi FASTA
ClustalW
PNG


SWISS-MODEL

### 21\_Jan\_SF\_Bin67\_scaffold\_32672\_c1:3-2330\_1

### Created: March 29, 2023, 6:30 p.m. at 18:30

- Templates
- Models

Models | Name | Description | GMQE | QSQE | Seq Id | Coverage | Range | Method | Resolution | Oligo-state | Ligands | Found by | Seq Similarity || ✓ | 7b04.1.B | Nitrite oxidoreductase subunit A  *Structure of Nitrite oxidoreductase (Nxr) from the anammox bacterium Kuenenia stuttgartiensis.* | 0.88 | 0.00 | 53.57 | 1.00 | 1-772 | X-ray | 2.97 | monomer | 4 x SF4, 1 x F3S, 2 x MD1, 1 x MO, 1 x HEM, 2 x CA | HHblits | 0.46 |
| ``` target    RPFLERFTDMPLLVRLDTLQRLRADEVFADYSSDLDVDGPSFTLHGMTEEQHERNGDRVVFDDASGALRAINREDVGDRL 7b04.1    ADYVKKFTDFPLLIRTDTLKRVSPKDIIPNYKLQDISDGPSYHIQGLKDEQREIIGDFVVWDAKSKGPKAITRDDVGETL  target    DDKGIDPALDYQGTVTLVDGSTVEVMSVLSMYREHLADYDIDSVVDMTGAPRNLIEQLLDDMTTLSPVAFHVGEGVNHYF 7b04.1    VKKGIDPVLEGSFKLKTIDGKEIEVMTLLEMYKIHLRDYDIDSVVSMTNSPKDLIERLAKDIATIKPVAIHYGEGVNHYF  target    HATLHNRATYLVGMLLGSVGVSGGGVSTWAGNYKGGVFQAAPWFGPGVGGFVNEDPFHPLTDPSARYSAETARHLVHGED 7b04.1    HATLMNRSYYLPVMLTGNVGYFGSGSHTWAGNYKAGNFQASKWSGPGFYGWVAEDVFKPNLDPYASAKDLNIKGRALDEE  target    TSYWGFGDRPLVVDTPEDGRKVFTGTTHMPTPTKALWYNNANLINQAKWHYELVKNVNPKVDLIVDQQIEWTGSAEFADI 7b04.1    VAYWNHSERPLIVNTPKYGRKVFTGKTHMPSPTKVLWFTNVNLINNAKHVYQMLKNVNPNIEQIMSTDIEITGSIEYADF  target    VLPANSWMEAETWEMGASCSNPFLQVWK-GGIEPLNDTRDDIAIFAGVANALTELTGDERFSQAFMF--ADRPEVYLDRV 7b04.1    AFPANSWVEFQEFEITNSCSNPFIQIWGKTGITPVYESKDDVKILAGMASKLGELLRDKRFEDNWKFAIEGRASVYINRL  target    LAGSFTTEGYTVEDLTAGRYGPPGGALMQYRSYPRIPFKEQIEDSLPFYTDTGRMHGYVDIPEAIEYGENLIVHREAVEA 7b04.1    LDGSTTMKGYTCEDILNGKYGEPGVAMLLFRTYPRHPFWEQVHESLPFYTPTGRLQAYNDEPEIIEYGENFIVHREGPEA  target    TPYLPNVIVSTSPYLRPRDYGIAPEELDGDARSVRNIMMSWAEVKETENPLFAAGYNYLCLTPKSRHAVHSSWAVTDWHW 7b04.1    TPYLPNAIVSTNPYIRPDDYGIPENAEYWEDRTVRNIKKSWEETKKTKNFLWEKGYHFYCVTPKSRHTVHSQWAVTDWNF  target    LWSSSFSDPYRVETRAPGVGEPAIHLNPDDARSLGIRNGDYVWVDSNPKDRPYRDADVDESFLDVARLLVRVTYNPAYPP 7b04.1    IWNNNFGDPYRMDKRMPGVGEHQIHIHPQAARDLGIEDGDYVYVDANPADRPYEGWKPNDSFYKVSRLMLRAKYNPAYPY  target    GVTMLKHAFYMATPRTFRAAQERSDGRALAETTGYQSSFRSGSHQSITRGWAPPMHQTDSLFHKRAGVFGFTYGFDVDNH 7b04.1    NCTMMKHSAWISSDKTVQAHETRPDGRALSP-SGYQSSFRYGSQQSITRDWSMPMHQLDSLFHKAKIGMKFIFGFEADNH  target    AINTVPKETVVRITKAEDGGVGGSGAWTRGRPGSMPGDEDDAMQAYLAGELTVVRRT 7b04.1    CINTVPKETLVKITKAENGGMGGKGVWDPVKTGYTAGNENDFMKKFLNGELIKVD-- ``` | | | | | | | | | | | | | | | | | | | | | | | | | | | | | | | | | | | | | | | | | | | | | | | | | |
|  | 7b04.2.B | Nitrite oxidoreductase subunit A  *Structure of Nitrite oxidoreductase (Nxr) from the anammox bacterium Kuenenia stuttgartiensis.* | 0.86 | 0.00 | 53.57 | 1.00 | 1-772 | X-ray | 2.97 | monomer | 4 x SF4, 1 x F3S, 2 x MD1, 1 x MO, 1 x HEM, 2 x CA | HHblits | 0.46 |
| ``` target    RPFLERFTDMPLLVRLDTLQRLRADEVFADYSSDLDVDGPSFTLHGMTEEQHERNGDRVVFDDASGALRAINREDVGDRL 7b04.2    ADYVKKFTDFPLLIRTDTLKRVSPKDIIPNYKLQDISDGPSYHIQGLKDEQREIIGDFVVWDAKSKGPKAITRDDVGETL  target    DDKGIDPALDYQGTVTLVDGSTVEVMSVLSMYREHLADYDIDSVVDMTGAPRNLIEQLLDDMTTLSPVAFHVGEGVNHYF 7b04.2    VKKGIDPVLEGSFKLKTIDGKEIEVMTLLEMYKIHLRDYDIDSVVSMTNSPKDLIERLAKDIATIKPVAIHYGEGVNHYF  target    HATLHNRATYLVGMLLGSVGVSGGGVSTWAGNYKGGVFQAAPWFGPGVGGFVNEDPFHPLTDPSARYSAETARHLVHGED 7b04.2    HATLMNRSYYLPVMLTGNVGYFGSGSHTWAGNYKAGNFQASKWSGPGFYGWVAEDVFKPNLDPYASAKDLNIKGRALDEE  target    TSYWGFGDRPLVVDTPEDGRKVFTGTTHMPTPTKALWYNNANLINQAKWHYELVKNVNPKVDLIVDQQIEWTGSAEFADI 7b04.2    VAYWNHSERPLIVNTPKYGRKVFTGKTHMPSPTKVLWFTNVNLINNAKHVYQMLKNVNPNIEQIMSTDIEITGSIEYADF  target    VLPANSWMEAETWEMGASCSNPFLQVWK-GGIEPLNDTRDDIAIFAGVANALTELTGDERFSQAFMF--ADRPEVYLDRV 7b04.2    AFPANSWVEFQEFEITNSCSNPFIQIWGKTGITPVYESKDDVKILAGMASKLGELLRDKRFEDNWKFAIEGRASVYINRL  target    LAGSFTTEGYTVEDLTAGRYGPPGGALMQYRSYPRIPFKEQIEDSLPFYTDTGRMHGYVDIPEAIEYGENLIVHREAVEA 7b04.2    LDGSTTMKGYTCEDILNGKYGEPGVAMLLFRTYPRHPFWEQVHESLPFYTPTGRLQAYNDEPEIIEYGENFIVHREGPEA  target    TPYLPNVIVSTSPYLRPRDYGIAPEELDGDARSVRNIMMSWAEVKETENPLFAAGYNYLCLTPKSRHAVHSSWAVTDWHW 7b04.2    TPYLPNAIVSTNPYIRPDDYGIPENAEYWEDRTVRNIKKSWEETKKTKNFLWEKGYHFYCVTPKSRHTVHSQWAVTDWNF  target    LWSSSFSDPYRVETRAPGVGEPAIHLNPDDARSLGIRNGDYVWVDSNPKDRPYRDADVDESFLDVARLLVRVTYNPAYPP 7b04.2    IWNNNFGDPYRMDKRMPGVGEHQIHIHPQAARDLGIEDGDYVYVDANPADRPYEGWKPNDSFYKVSRLMLRAKYNPAYPY  target    GVTMLKHAFYMATPRTFRAAQERSDGRALAETTGYQSSFRSGSHQSITRGWAPPMHQTDSLFHKRAGVFGFTYGFDVDNH 7b04.2    NCTMMKHSAWISSDKTVQAHETRPDGRALSP-SGYQSSFRYGSQQSITRDWSMPMHQLDSLFHKAKIGMKFIFGFEADNH  target    AINTVPKETVVRITKAEDGGVGGSGAWTRGRPGSMPGDEDDAMQAYLAGELTVVRRT 7b04.2    CINTVPKETLVKITKAENGGMGGKGVWDPVKTGYTAGNENDFMKKFLNGELIKVD-- ``` | | | | | | | | | | | | | | | | | | | | | | | | | | | | | | | | | | | | | | | | | | | | | | | | | |
|  | 7b04.1.B | Nitrite oxidoreductase subunit A  *Structure of Nitrite oxidoreductase (Nxr) from the anammox bacterium Kuenenia stuttgartiensis.* | 0.88 | 0.00 | 54.04 | 0.99 | 3-771 | X-ray | 2.97 | monomer | 4 x SF4, 1 x F3S, 2 x MD1, 1 x MO, 1 x HEM, 2 x CA | BLAST | 0.46 |
| ``` target    RPFLERFTDMPLLVRLDTLQRLRADEVFADYSSDLDVDGPSFTLHGMTEEQHERNGDRVVFDDASGALRAINREDVGDRL 7b04.1    --YVKKFTDFPLLIRTDTLKRVSPKDIIPNYKLQDISDGPSYHIQGLKDEQREIIGDFVVWDAKSKGPKAITRDDVGETL  target    DDKGIDPALDYQGTVTLVDGSTVEVMSVLSMYREHLADYDIDSVVDMTGAPRNLIEQLLDDMTTLSPVAFHVGEGVNHYF 7b04.1    VKKGIDPVLEGSFKLKTIDGKEIEVMTLLEMYKIHLRDYDIDSVVSMTNSPKDLIERLAKDIATIKPVAIHYGEGVNHYF  target    HATLHNRATYLVGMLLGSVGVSGGGVSTWAGNYKGGVFQAAPWFGPGVGGFVNEDPFHPLTDPSARYSAETARHLVHGED 7b04.1    HATLMNRSYYLPVMLTGNVGYFGSGSHTWAGNYKAGNFQASKWSGPGFYGWVAEDVFKPNLDPYASAKDLNIKGRALDEE  target    TSYWGFGDRPLVVDTPEDGRKVFTGTTHMPTPTKALWYNNANLINQAKWHYELVKNVNPKVDLIVDQQIEWTGSAEFADI 7b04.1    VAYWNHSERPLIVNTPKYGRKVFTGKTHMPSPTKVLWFTNVNLINNAKHVYQMLKNVNPNIEQIMSTDIEITGSIEYADF  target    VLPANSWMEAETWEMGASCSNPFLQVW-KGGIEPLNDTRDDIAIFAGVANALTELTGDERFSQAFMFA--DRPEVYLDRV 7b04.1    AFPANSWVEFQEFEITNSCSNPFIQIWGKTGITPVYESKDDVKILAGMASKLGELLRDKRFEDNWKFAIEGRASVYINRL  target    LAGSFTTEGYTVEDLTAGRYGPPGGALMQYRSYPRIPFKEQIEDSLPFYTDTGRMHGYVDIPEAIEYGENLIVHREAVEA 7b04.1    LDGSTTMKGYTCEDILNGKYGEPGVAMLLFRTYPRHPFWEQVHESLPFYTPTGRLQAYNDEPEIIEYGENFIVHREGPEA  target    TPYLPNVIVSTSPYLRPRDYGIAPEELDGDARSVRNIMMSWAEVKETENPLFAAGYNYLCLTPKSRHAVHSSWAVTDWHW 7b04.1    TPYLPNAIVSTNPYIRPDDYGIPENAEYWEDRTVRNIKKSWEETKKTKNFLWEKGYHFYCVTPKSRHTVHSQWAVTDWNF  target    LWSSSFSDPYRVETRAPGVGEPAIHLNPDDARSLGIRNGDYVWVDSNPKDRPYRDADVDESFLDVARLLVRVTYNPAYPP 7b04.1    IWNNNFGDPYRMDKRMPGVGEHQIHIHPQAARDLGIEDGDYVYVDANPADRPYEGWKPNDSFYKVSRLMLRAKYNPAYPY  target    GVTMLKHAFYMATPRTFRAAQERSDGRALAETTGYQSSFRSGSHQSITRGWAPPMHQTDSLFHKRAGVFGFTYGFDVDNH 7b04.1    NCTMMKHSAWISSDKTVQAHETRPDGRALS-PSGYQSSFRYGSQQSITRDWSMPMHQLDSLFHKAKIGMKFIFGFEADNH  target    AINTVPKETVVRITKAEDGGVGGSGAWTRGRPGSMPGDEDDAMQAYLAGELTVVRRT 7b04.1    CINTVPKETLVKITKAENGGMGGKGVWDPVKTGYTAGNENDFMKKFLNGELIKV--- ``` | | | | | | | | | | | | | | | | | | | | | | | | | | | | | | | | | | | | | | | | | | | | | | | | | |
|  | 7b04.2.B | Nitrite oxidoreductase subunit A  *Structure of Nitrite oxidoreductase (Nxr) from the anammox bacterium Kuenenia stuttgartiensis.* | 0.86 | 0.00 | 54.04 | 0.99 | 3-771 | X-ray | 2.97 | monomer | 4 x SF4, 1 x F3S, 2 x MD1, 1 x MO, 1 x HEM, 2 x CA | BLAST | 0.46 |
| ``` target    RPFLERFTDMPLLVRLDTLQRLRADEVFADYSSDLDVDGPSFTLHGMTEEQHERNGDRVVFDDASGALRAINREDVGDRL 7b04.2    --YVKKFTDFPLLIRTDTLKRVSPKDIIPNYKLQDISDGPSYHIQGLKDEQREIIGDFVVWDAKSKGPKAITRDDVGETL  target    DDKGIDPALDYQGTVTLVDGSTVEVMSVLSMYREHLADYDIDSVVDMTGAPRNLIEQLLDDMTTLSPVAFHVGEGVNHYF 7b04.2    VKKGIDPVLEGSFKLKTIDGKEIEVMTLLEMYKIHLRDYDIDSVVSMTNSPKDLIERLAKDIATIKPVAIHYGEGVNHYF  target    HATLHNRATYLVGMLLGSVGVSGGGVSTWAGNYKGGVFQAAPWFGPGVGGFVNEDPFHPLTDPSARYSAETARHLVHGED 7b04.2    HATLMNRSYYLPVMLTGNVGYFGSGSHTWAGNYKAGNFQASKWSGPGFYGWVAEDVFKPNLDPYASAKDLNIKGRALDEE  target    TSYWGFGDRPLVVDTPEDGRKVFTGTTHMPTPTKALWYNNANLINQAKWHYELVKNVNPKVDLIVDQQIEWTGSAEFADI 7b04.2    VAYWNHSERPLIVNTPKYGRKVFTGKTHMPSPTKVLWFTNVNLINNAKHVYQMLKNVNPNIEQIMSTDIEITGSIEYADF  target    VLPANSWMEAETWEMGASCSNPFLQVW-KGGIEPLNDTRDDIAIFAGVANALTELTGDERFSQAFMFA--DRPEVYLDRV 7b04.2    AFPANSWVEFQEFEITNSCSNPFIQIWGKTGITPVYESKDDVKILAGMASKLGELLRDKRFEDNWKFAIEGRASVYINRL  target    LAGSFTTEGYTVEDLTAGRYGPPGGALMQYRSYPRIPFKEQIEDSLPFYTDTGRMHGYVDIPEAIEYGENLIVHREAVEA 7b04.2    LDGSTTMKGYTCEDILNGKYGEPGVAMLLFRTYPRHPFWEQVHESLPFYTPTGRLQAYNDEPEIIEYGENFIVHREGPEA  target    TPYLPNVIVSTSPYLRPRDYGIAPEELDGDARSVRNIMMSWAEVKETENPLFAAGYNYLCLTPKSRHAVHSSWAVTDWHW 7b04.2    TPYLPNAIVSTNPYIRPDDYGIPENAEYWEDRTVRNIKKSWEETKKTKNFLWEKGYHFYCVTPKSRHTVHSQWAVTDWNF  target    LWSSSFSDPYRVETRAPGVGEPAIHLNPDDARSLGIRNGDYVWVDSNPKDRPYRDADVDESFLDVARLLVRVTYNPAYPP 7b04.2    IWNNNFGDPYRMDKRMPGVGEHQIHIHPQAARDLGIEDGDYVYVDANPADRPYEGWKPNDSFYKVSRLMLRAKYNPAYPY  target    GVTMLKHAFYMATPRTFRAAQERSDGRALAETTGYQSSFRSGSHQSITRGWAPPMHQTDSLFHKRAGVFGFTYGFDVDNH 7b04.2    NCTMMKHSAWISSDKTVQAHETRPDGRALS-PSGYQSSFRYGSQQSITRDWSMPMHQLDSLFHKAKIGMKFIFGFEADNH  target    AINTVPKETVVRITKAEDGGVGGSGAWTRGRPGSMPGDEDDAMQAYLAGELTVVRRT 7b04.2    CINTVPKETLVKITKAENGGMGGKGVWDPVKTGYTAGNENDFMKKFLNGELIKV--- ``` | | | | | | | | | | | | | | | | | | | | | | | | | | | | | | | | | | | | | | | | | | | | | | | | | |
|  | 1r27.4.A | Respiratory nitrate reductase 1 alpha chain  *Crystal Structure of NarGH complex* | 0.39 |  | 22.74 | 0.80 | 1-739 | X-ray | 2.00 | homo-dimer | 4 x MO, 16 x SF4, 8 x MGD, 4 x F3S | HHblits | 0.31 |
| ``` target    RPFLERFTDMPLLVRLD-------TLQRLRADEVFADYSSDLDVDGPSFTLHGMTEEQHERNGDRVVFDDASGALRAINR 1r27.4    TDYVRRYTDMPMLVMLEERDGYYAAGRMLRAADLVDAL-------GQ----------ENNPEWKTVAFNT-NGEMVAPNG  target    EDVGD--------------------RLD---DKGI-------DP-------------ALD----Y---QGTVTLVDGSTV 1r27.4    SIGFRWGEKGKWNLEQRDGKTGEETELQLSLLGSQDEIAEVGFPYFGGDGTEHFNKVELENVLLHKLPVKRLQLADGSTA  target    EVMSVLSMYR------------------EHLADYDIDSVVDMTGAPRNLIEQLLDDMTTL-----SPVAFHVGEGVNHYF 1r27.4    LVTTVYDLTLANYGLERGLNDVNCATSYDDVKAYTPAWAEQITGVSRSQIIRIAREFADNADKTHGRSMIIVGAGLNHWY  target    HATLHNRATYLVGMLLGSVGVSGGGVSTWAGNYKGGV---FQ----AAPWFGPGVGGFVNEDPFHPLTDPSARYSA---- 1r27.4    HLDMNYRGLINMLIFCGCVGQSGGGWAHYVGQEKLRPQTGWQPLAFALDWQRPA--RHMNSTSYFYNHSSQWRYETVTAE  target    --------------ETAR----HLVHGEDTSYWGFGDRPL----------------VVDTPEDGRKVF--TGTTHMPTPT 1r27.4    ELLSPMADKSRYTGHLIDFNVRAERMGWLPSAPQLGTNPLTIAGEAEKAGMNPVDYTVKSLKEGSIRFAAEQPENGKNHP  target    KALWYNNANLINQAKWHYE-L------------------------------VKNVNPKVDLIVDQQIEWTGSAEFADIVL 1r27.4    RNLFIWRSNLLGSSGKGHEFMLKYLLGTEHGIQGKDLGQQGGVKPEEVDWQDNGLEGKLDLVVTLDFRLSSTCLYSDIIL  target    PANSWMEAETWEMGASCSNPFLQVWKGGIEPLNDTRDDIAIFAGVANALTELTGDERFSQ-----AFM-FADRP------ 1r27.4    PTATWYEKDDMN--TSDMHPFIHPLSAAVDPAWEAKSDWEIYKAIAKKFSEVCVGH-LGKETDIVTLPIQHDSAAELAQP  target    ---EVY---------------------------------------------------------LDRV------------- 1r27.4    LDVKDWKKGECDLIPGKTAPHIMVVERDYPATYERFTSIGPLMEKIGNGGKGIAWNTQSEMDLLRKLNYTKAEGPAKGQP  target    ------------LAGSFTTEG----YTVEDLTAGRY-G----PP--GGALMQY---------------------RSYPRI 1r27.4    MLNTAIDAAEMILTLAPETNGQVAVKAWAALSEFTGRDHTHLALNKEDEKIRFRDIQAQPRKIISSPTWSGLEDEHVSYN  target    PFKEQIEDSLPFYTDTGRMHGYVDIPEAIEYGENLIVHREAVEATPYLPNVIVSTSPYLRPRDYGIAPEELDGDARSVRN 1r27.4    AGYTNVHELIPWRTLSGRQQLYQDHQWMRDFGESLLVYRPPIDTRSVK-E------------------------------  target    IMMSWAEVKETENPLFAAGYNYLCLTPKSRHAVHSSWAVTDWHWLWSSSFSDPYRVETRAPGVGEPAIHLNPDDARSLGI 1r27.4    ----V---IG-QKSNGNQEKALNFLTPHQKWGIHSTYSDNLLM------------LTL---GRGGPVVWLSEADAKDLGI  target    RNGDYVWVDSNPKDRPYRDADVDESFLDVARLLVRVTYNPAYPPGVTMLKHAFYMATPRTFRAAQERSDGRALAETTGYQ 1r27.4    ADNDWIEVFNS-----------------NGALTARAVVSQRVPAGMTMMYHAQERIVN------------LPGSEI----  target    SSFRSGSHQSITRGWAPPMHQTDSLFHKRAGVFGFTYGFDVDNHAINTVPKETVVRITKAEDGGVGGSGAWTRGRPGSMP 1r27.4    TQQRGGIHNSVTRITPKPTHMI-------GGYAHLAYGFNYYG--TVGSNRDEFVVVRKMKNIDWL--------------  target    GDEDDAMQAYLAGELTVVRRT 1r27.4    --------------------- ``` | | | | | | | | | | | | | | | | | | | | | | | | | | | | | | | | | | | | | | | | | | | | | | | | | |
|  | 1q16.1.A | Respiratory nitrate reductase 1 alpha chain  *Crystal structure of Nitrate Reductase A, NarGHI, from Escherichia coli* | 0.39 | 0.00 | 22.74 | 0.80 | 1-739 | X-ray | 1.90 | monomer | 2 x MD1, 1 x 6MO, 2 x HEM, 4 x SF4, 1 x F3S, 1 x AGA, 1 x 3PH | HHblits | 0.31 |
| ``` target    RPFLERFTDMPLLVRLD-------TLQRLRADEVFADYSSDLDVDGPSFTLHGMTEEQHERNGDRVVFDDASGALRAINR 1q16.1    TDYVRRYTDMPMLVMLEERDGYYAAGRMLRAADLVDAL-------GQ----------ENNPEWKTVAFNT-NGEMVAPNG  target    EDVGD--------------------RLD---DKGI-------DP-------------ALD----Y---QGTVTLVDGSTV 1q16.1    SIGFRWGEKGKWNLEQRDGKTGEETELQLSLLGSQDEIAEVGFPYFGGDGTEHFNKVELENVLLHKLPVKRLQLADGSTA  target    EVMSVLSMYR------------------EHLADYDIDSVVDMTGAPRNLIEQLLDDMTTL-----SPVAFHVGEGVNHYF 1q16.1    LVTTVYDLTLANYGLERGLNDVNCATSYDDVKAYTPAWAEQITGVSRSQIIRIAREFADNADKTHGRSMIIVGAGLNHWY  target    HATLHNRATYLVGMLLGSVGVSGGGVSTWAGNYKGGV---FQ----AAPWFGPGVGGFVNEDPFHPLTDPSARYS----- 1q16.1    HLDMNYRGLINMLIFCGCVGQSGGGWAHYVGQEKLRPQTGWQPLAFALDWQRPA--RHMNSTSYFYNHSSQWRYETVTAE  target    -------------AETAR----HLVHGEDTSYWGFGDRPL----------------VVDTPEDGRKVF--TGTTHMPTPT 1q16.1    ELLSPMADKSRYTGHLIDFNVRAERMGWLPSAPQLGTNPLTIAGEAEKAGMNPVDYTVKSLKEGSIRFAAEQPENGKNHP  target    KALWYNNANLINQAKWHYEL-------------------------------VKNVNPKVDLIVDQQIEWTGSAEFADIVL 1q16.1    RNLFIWRSNLLGSSGKGHEFMLKYLLGTEHGIQGKDLGQQGGVKPEEVDWQDNGLEGKLDLVVTLDFRLSSTCLYSDIIL  target    PANSWMEAETWEMGASCSNPFLQVWKGGIEPLNDTRDDIAIFAGVANALTELTGDE---R---FSQAFMFADR------- 1q16.1    PTATWYEKDDMN--TSDMHPFIHPLSAAVDPAWEAKSDWEIYKAIAKKFSEVCVGHLGKETDIVTLPI-QHDSAAELAQP  target    --PEVY---------------------------------------------------------LDR-------------- 1q16.1    LDVKDWKKGECDLIPGKTAPHIMVVERDYPATYERFTSIGPLMEKIGNGGKGIAWNTQSEMDLLRKLNYTKAEGPAKGQP  target    -----------VLAGSFTTEG----YTVEDLTAGRY-GP----P--GGALMQY---------------------RSYPRI 1q16.1    MLNTAIDAAEMILTLAPETNGQVAVKAWAALSEFTGRDHTHLALNKEDEKIRFRDIQAQPRKIISSPTWSGLEDEHVSYN  target    PFKEQIEDSLPFYTDTGRMHGYVDIPEAIEYGENLIVHREAVEATPYLPNVIVSTSPYLRPRDYGIAPEELDGDARSVRN 1q16.1    AGYTNVHELIPWRTLSGRQQLYQDHQWMRDFGESLLVYRPPIDTRSV-KE------------------------------  target    IMMSWAEVKETENPLFAAGYNYLCLTPKSRHAVHSSWAVTDWHWLWSSSFSDPYRVETRAPGVGEPAIHLNPDDARSLGI 1q16.1    ----VIG----QKSNGNQEKALNFLTPHQKWGIHSTYSDNLLM------------LTL---GRGGPVVWLSEADAKDLGI  target    RNGDYVWVDSNPKDRPYRDADVDESFLDVARLLVRVTYNPAYPPGVTMLKHAFYMATPRTFRAAQERSDGRALAETTGYQ 1q16.1    ADNDWIEVFNS-----------------NGALTARAVVSQRVPAGMTMMYHAQERIVN------------LPGSEI----  target    SSFRSGSHQSITRGWAPPMHQTDSLFHKRAGVFGFTYGFDVDNHAINTVPKETVVRITKAEDGGVGGSGAWTRGRPGSMP 1q16.1    TQQRGGIHNSVTRITPKPTHMI-------GGYAHLAYGFNYYGT--VGSNRDEFVVVRKMKNIDWL--------------  target    GDEDDAMQAYLAGELTVVRRT 1q16.1    --------------------- ``` | | | | | | | | | | | | | | | | | | | | | | | | | | | | | | | | | | | | | | | | | | | | | | | | | |
| ✓ | 3ir7.1.A | Respiratory nitrate reductase 1 alpha chain  *Crystal structure of NarGHI mutant NarG-R94S* | 0.40 | 0.00 | 22.74 | 0.80 | 1-739 | X-ray | 2.50 | monomer | 2 x MD1, 4 x SF4, 1 x 6MO, 1 x AGA, 1 x F3S, 2 x HEM | HHblits | 0.31 |
| ``` target    RPFLERFTDMPLLVRLD-------TLQRLRADEVFADYSSDLDVDGPSFTLHGMTEEQHERNGDRVVFDDASGALRAINR 3ir7.1    TDYVRRYTDMPMLVMLEERDGYYAAGRMLRAADLVDAL-------GQ----------ENNPEWKTVAFNT-NGEMVAPNG  target    EDVGD--------------------RLD---DK------------------------GIDPALDY---QGTVTLVDGSTV 3ir7.1    SIGFRWGEKGKWNLEQRDGKTGEETELQLSLLGSQDEIAEVGFPYFGGDGTEHFNKVELENVLLHKLPVKRLQLADGSTA  target    EVMSVLSMYR------------------EHLADYDIDSVVDMTGAPRNLIEQLLDDMTTL-----SPVAFHVGEGVNHYF 3ir7.1    LVTTVYDLTLANYGLERGLNDVNCATSYDDVKAYTPAWAEQITGVSRSQIIRIAREFADNADKTHGRSMIIVGAGLNHWY  target    HATLHNRATYLVGMLLGSVGVSGGGVSTWAGNYKGGV---FQ----AAPWFGPGVGGFVNEDPFHPLTDPSARYSA---- 3ir7.1    HLDMNYRGLINMLIFCGCVGQSGGGWAHYVGQEKLRPQTGWQPLAFALDWQRPA--RHMNSTSYFYNHSSQWRYETVTAE  target    --------------ETAR----HLVHGEDTSYWGFGDRPL----------------VVDTPEDGRKVF--TGTTHMPTPT 3ir7.1    ELLSPMADKSRYTGHLIDFNVRAERMGWLPSAPQLGTNPLTIAGEAEKAGMNPVDYTVKSLKEGSIRFAAEQPENGKNHP  target    KALWYNNANLINQAKWHYEL-------------------------------VKNVNPKVDLIVDQQIEWTGSAEFADIVL 3ir7.1    RNLFIWRSNLLGSSGKGHEFMLKYLLGTEHGIQGKDLGQQGGVKPEEVDWQDNGLEGKLDLVVTLDFRLSSTCLYSDIIL  target    PANSWMEAETWEMGASCSNPFLQVWKGGIEPLNDTRDDIAIFAGVANALTELTGDE---R---FSQAFMFADRP------ 3ir7.1    PTATWYEKDDMN--TSDMHPFIHPLSAAVDPAWEAKSDWEIYKAIAKKFSEVCVGHLGKETDIVTLPI-QHDSAAELAQP  target    ---EVY---------------------------------------------------------LDRV------------- 3ir7.1    LDVKDWKKGECDLIPGKTAPHIMVVERDYPATYERFTSIGPLMEKIGNGGKGIAWNTQSEMDLLRKLNYTKAEGPAKGQP  target    ------------LAGSFTTEG----YTVEDLTAGRYG-----PP--GGALMQYR---------------------SYPRI 3ir7.1    MLNTAIDAAEMILTLAPETNGQVAVKAWAALSEFTGRDHTHLALNKEDEKIRFRDIQAQPRKIISSPTWSGLEDEHVSYN  target    PFKEQIEDSLPFYTDTGRMHGYVDIPEAIEYGENLIVHREAVEATPYLPNVIVSTSPYLRPRDYGIAPEELDGDARSVRN 3ir7.1    AGYTNVHELIPWRTLSGRQQLYQDHQWMRDFGESLLVYRPPIDTRSV-KE------------------------------  target    IMMSWAEVKETENPLFAAGYNYLCLTPKSRHAVHSSWAVTDWHWLWSSSFSDPYRVETRAPGVGEPAIHLNPDDARSLGI 3ir7.1    ----VI---G-QKSNGNQEKALNFLTPHQKWGIHSTYSDNLLM------------LTL---GRGGPVVWLSEADAKDLGI  target    RNGDYVWVDSNPKDRPYRDADVDESFLDVARLLVRVTYNPAYPPGVTMLKHAFYMATPRTFRAAQERSDGRALAETTGYQ 3ir7.1    ADNDWIEVFNS-----------------NGALTARAVVSQRVPAGMTMMYHAQERIVN------------LPGSEI----  target    SSFRSGSHQSITRGWAPPMHQTDSLFHKRAGVFGFTYGFDVDNHAINTVPKETVVRITKAEDGGVGGSGAWTRGRPGSMP 3ir7.1    TQQRGGIHNSVTRITPKPTHMI-------GGYAHLAYGFNYYGT--VGSNRDEFVVVRKMKNIDWL--------------  target    GDEDDAMQAYLAGELTVVRRT 3ir7.1    --------------------- ``` | | | | | | | | | | | | | | | | | | | | | | | | | | | | | | | | | | | | | | | | | | | | | | | | | |
|  | 3ir5.1.A | Respiratory nitrate reductase 1 alpha chain  *Crystal structure of NarGHI mutant NarG-H49C* | 0.39 | 0.00 | 22.58 | 0.80 | 1-739 | X-ray | 2.30 | monomer | 2 x MD1, 1 x 6MO, 4 x SF4, 1 x AGA, 1 x F3S, 2 x HEM | HHblits | 0.31 |
| ``` target    RPFLERFTDMPLLVRLD-------TLQRLRADEVFADYSSDLDVDGPSFTLHGMTEEQHERNGDRVVFDDASGALRAINR 3ir5.1    TDYVRRYTDMPMLVMLEERDGYYAAGRMLRAADLVDAL-------GQ----------ENNPEWKTVAFNT-NGEMVAPNG  target    EDVGD--------------------RLD---DKGI-------D-------------PALD----Y---QGTVTLVDGSTV 3ir5.1    SIGFRWGEKGKWNLEQRDGKTGEETELQLSLLGSQDEIAEVGFPYFGGDGTEHFNKVELENVLLHKLPVKRLQLADGSTA  target    EVMSVLSMYR------------------EHLADYDIDSVVDMTGAPRNLIEQLLDDMTTL-----SPVAFHVGEGVNHYF 3ir5.1    LVTTVYDLTLANYGLERGLNDVNCATSYDDVKAYTPAWAEQITGVSRSQIIRIAREFADNADKTHGRSMIIVGAGLNHWY  target    HATLHNRATYLVGMLLGSVGVSGGGVSTWAGNYKGGV---F----QAAPWFGPGVGGFVNEDPFHPLTDPSARYSA---- 3ir5.1    HLDMNYRGLINMLIFCGCVGQSGGGWAHYVGQEKLRPQTGWQPLAFALDWQRPA--RHMNSTSYFYNHSSQWRYETVTAE  target    --------------ETAR----HLVHGEDTSYWGFGDRPL----------------VVDTPEDGRKVF--TGTTHMPTPT 3ir5.1    ELLSPMADKSRYTGHLIDFNVRAERMGWLPSAPQLGTNPLTIAGEAEKAGMNPVDYTVKSLKEGSIRFAAEQPENGKNHP  target    KALWYNNANLINQAKWHYE-L------------------------------VKNVNPKVDLIVDQQIEWTGSAEFADIVL 3ir5.1    RNLFIWRSNLLGSSGKGHEFMLKYLLGTEHGIQGKDLGQQGGVKPEEVDWQDNGLEGKLDLVVTLDFRLSSTCLYSDIIL  target    PANSWMEAETWEMGASCSNPFLQVWKGGIEPLNDTRDDIAIFAGVANALTELTGDE---R---FSQAFMFADRP------ 3ir5.1    PTATWYEKDDMN--TSDMHPFIHPLSAAVDPAWEAKSDWEIYKAIAKKFSEVCVGHLGKETDIVTLPI-QHDSAAELAQP  target    ---EVY---------------------------------------------------------LDR-------------- 3ir5.1    LDVKDWKKGECDLIPGKTAPHIMVVERDYPATYERFTSIGPLMEKIGNGGKGIAWNTQSEMDLLRKLNYTKAEGPAKGQP  target    -----------VLAGSFTTEGY----TVEDLTAGRYG-----PP--GGALMQYR---------------------SYPRI 3ir5.1    MLNTAIDAAEMILTLAPETNGQVAVKAWAALSEFTGRDHTHLALNKEDEKIRFRDIQAQPRKIISSPTWSGLEDEHVSYN  target    PFKEQIEDSLPFYTDTGRMHGYVDIPEAIEYGENLIVHREAVEATPYLPNVIVSTSPYLRPRDYGIAPEELDGDARSVRN 3ir5.1    AGYTNVHELIPWRTLSGRQQLYQDHQWMRDFGESLLVYRPPIDTRSV-KE------------------------------  target    IMMSWAEVKETENPLFAAGYNYLCLTPKSRHAVHSSWAVTDWHWLWSSSFSDPYRVETRAPGVGEPAIHLNPDDARSLGI 3ir5.1    ----VIGQ----KSNGNQEKALNFLTPHQKWGIHSTYSDNLLM------------LTL---GRGGPVVWLSEADAKDLGI  target    RNGDYVWVDSNPKDRPYRDADVDESFLDVARLLVRVTYNPAYPPGVTMLKHAFYMATPRTFRAAQERSDGRALAETTGYQ 3ir5.1    ADNDWIEVFNS-----------------NGALTARAVVSQRVPAGMTMMYHAQERIVN------------LPGSEI----  target    SSFRSGSHQSITRGWAPPMHQTDSLFHKRAGVFGFTYGFDVDNHAINTVPKETVVRITKAEDGGVGGSGAWTRGRPGSMP 3ir5.1    TQQRGGIHNSVTRITPKPTHMI-------GGYAHLAYGFNYYGTV--GSNRDEFVVVRKMKNIDWL--------------  target    GDEDDAMQAYLAGELTVVRRT 3ir5.1    --------------------- ``` | | | | | | | | | | | | | | | | | | | | | | | | | | | | | | | | | | | | | | | | | | | | | | | | | |
|  | 3egw.1.A | Respiratory nitrate reductase 1 alpha chain  *The crystal structure of the NarGHI mutant NarH - C16A* | 0.40 | 0.06 | 22.85 | 0.80 | 1-736 | X-ray | 1.90 | homo-dimer | 2 x MD1, 2 x MGD, 2 x 6MO, 6 x SF4, 4 x F3S, 2 x 3PH, 4 x HEM, 2 x AGA | HHblits | 0.31 |
| ``` target    RPFLERFTDMPLLVRLD-------TLQRLRADEVFADYSSDLDVDGPSFTLHGMTEEQHERNGDRVVFDDASGALRAINR 3egw.1    TDYVRRYTDMPMLVMLEERDGYYAAGRMLRAADLVAAL-------GQ----------ENNPEWKTVAFNT-NGEMVAPNG  target    EDVGD--------------------R---------------------------LDDKGIDPALDY---QGTVTLVDGSTV 3egw.1    SIGFRWGEKGKWNLEQRDGKTGEETELQLSLLGSQDEIAEVGFPYFGGDGTEHFNKVELENVLLHKLPVKRLQLADGSTA  target    EVMSVLSMYR------------------EHLADYDIDSVVDMTGAPRNLIEQLLDDMTTL-----SPVAFHVGEGVNHYF 3egw.1    LVTTVYDLTLANYGLERGLNDVNCATSYDDVKAYTPAWAEQITGVSRSQIIRIAREFADNADKTHGRSMIIVGAGLNHWY  target    HATLHNRATYLVGMLLGSVGVSGGGVSTWAGNYKGGV---FQ----AAPWFGPGVGGFVNEDPFHPLTDPSARYS----- 3egw.1    HLDMNYRGLINMLIFCGCVGQSGGGWAHYVGQEKLRPQTGWQPLAFALDWQRPA--RHMNSTSYFYNHSSQWRYETVTAE  target    -------------AETARH----LVHGEDTSYWGFGDRPL----------------VVDTPEDGRKVF--TGTTHMPTPT 3egw.1    ELLSPMADKSRYTGHLIDFNVRAERMGWLPSAPQLGTNPLTIAGEAEKAGMNPVDYTVKSLKEGSIRFAAEQPENGKNHP  target    KALWYNNANLINQAKWHYEL-------------------------------VKNVNPKVDLIVDQQIEWTGSAEFADIVL 3egw.1    RNLFIWRSNLLGSSGKGHEFMLKYLLGTEHGIQGKDLGQQGGVKPEEVDWQDNGLEGKLDLVVTLDFRLSSTCLYSDIIL  target    PANSWMEAETWEMGASCSNPFLQVWKGGIEPLNDTRDDIAIFAGVANALTELTGDE---R---FSQAFMFADR------- 3egw.1    PTATWYEKDDMN--TSDMHPFIHPLSAAVDPAWEAKSDWEIYKAIAKKFSEVCVGHLGKETDIVTLPI-QHDSAAELAQP  target    --PEVY---------------------------------------------------------LDRV------------- 3egw.1    LDVKDWKKGECDLIPGKTAPHIMVVERDYPATYERFTSIGPLMEKIGNGGKGIAWNTQSEMDLLRKLNYTKAEGPAKGQP  target    ------------LAGSFTTEG----YTVEDLTAGRY-G----PP--GGALMQYR---------------------SYPRI 3egw.1    MLNTAIDAAEMILTLAPETNGQVAVKAWAALSEFTGRDHTHLALNKEDEKIRFRDIQAQPRKIISSPTWSGLEDEHVSYN  target    PFKEQIEDSLPFYTDTGRMHGYVDIPEAIEYGENLIVHREAVEATPYLPNVIVSTSPYLRPRDYGIAPEELDGDARSVRN 3egw.1    AGYTNVHELIPWRTLSGRQQLYQDHQWMRDFGESLLVYRPPIDTRSVK-E------------------------------  target    IMMSWAEVKETENPLFAAGYNYLCLTPKSRHAVHSSWAVTDWHWLWSSSFSDPYRVETRAPGVGEPAIHLNPDDARSLGI 3egw.1    ----VIGQ----KSNGNQEKALNFLTPHQKWGIHSTYSDNLLM------------LTL---GRGGPVVWLSEADAKDLGI  target    RNGDYVWVDSNPKDRPYRDADVDESFLDVARLLVRVTYNPAYPPGVTMLKHAFYMATPRTFRAAQERSDGRALAETTGYQ 3egw.1    ADNDWIEVFNS-----------------NGALTARAVVSQRVPAGMTMMYHAQERIVN------------LPGSEI----  target    SSFRSGSHQSITRGWAPPMHQTDSLFHKRAGVFGFTYGFDVDNHAINTVPKETVVRITKAEDGGVGGSGAWTRGRPGSMP 3egw.1    TQQRGGIHNSVTRITPKPTHMI-------GGYAHLAYGFNYY--GTVGSNRDEFVVVRKMKNI-----------------  target    GDEDDAMQAYLAGELTVVRRT 3egw.1    --------------------- ``` | | | | | | | | | | | | | | | | | | | | | | | | | | | | | | | | | | | | | | | | | | | | | | | | | |
|  | 3ir6.1.A | Respiratory nitrate reductase 1 alpha chain  *Crystal structure of NarGHI mutant NarG-H49S* | 0.39 | 0.00 | 23.05 | 0.80 | 1-735 | X-ray | 2.80 | monomer | 2 x GDP, 1 x AGA, 3 x SF4, 1 x F3S, 2 x HEM | HHblits | 0.32 |
| ``` target    RPFLERFTDMPLLVRLD-------TLQRLRADEVFADYSSDLDVDGPSFTLHGMTEEQHERNGDRVVFDDASGALRAINR 3ir6.1    TDYVRRYTDMPMLVMLEERDGYYAAGRMLRAADLVDAL-------GQ----------ENNPEWKTVAFNT-NGEMVAPNG  target    EDVGD--------------------RLD---DKGI-------DPA-------------LD----Y---QGTVTLVDGSTV 3ir6.1    SIGFRWGEKGKWNLEQRDGKTGEETELQLSLLGSQDEIAEVGFPYFGGDGTEHFNKVELENVLLHKLPVKRLQLADGSTA  target    EVMSVLSMYR------------------EHLADYDIDSVVDMTGAPRNLIEQLLDDMTTL-----SPVAFHVGEGVNHYF 3ir6.1    LVTTVYDLTLANYGLERGLNDVNCATSYDDVKAYTPAWAEQITGVSRSQIIRIAREFADNADKTHGRSMIIVGAGLNHWY  target    HATLHNRATYLVGMLLGSVGVSGGGVSTWAGNYKGGV---FQ----AAPWFGPGVGGFVNEDPFHPLTDPSARYS----- 3ir6.1    HLDMNYRGLINMLIFCGCVGQSGGGWAHYVGQEKLRPQTGWQPLAFALDWQRPA--RHMNSTSYFYNHSSQWRYETVTAE  target    -------------AETAR----HLVHGEDTSYWGFGDRPL----------------VVDTPEDGRKVF--TGTTHMPTPT 3ir6.1    ELLSPMADKSRYTGHLIDFNVRAERMGWLPSAPQLGTNPLTIAGEAEKAGMNPVDYTVKSLKEGSIRFAAEQPENGKNHP  target    KALWYNNANLINQAKWHYEL-------------------------------VKNVNPKVDLIVDQQIEWTGSAEFADIVL 3ir6.1    RNLFIWRSNLLGSSGKGHEFMLKYLLGTEHGIQGKDLGQQGGVKPEEVDWQDNGLEGKLDLVVTLDFRLSSTCLYSDIIL  target    PANSWMEAETWEMGASCSNPFLQVWKGGIEPLNDTRDDIAIFAGVANALTELTGDE------RFSQAFMFADR------- 3ir6.1    PTATWYEKDDMN--TSDMHPFIHPLSAAVDPAWEAKSDWEIYKAIAKKFSEVCVGHLGKETDIVTLPI-QHDSAAELAQP  target    --PEV---------------------------------------------------------YLDRV------------- 3ir6.1    LDVKDWKKGECDLIPGKTAPHIMVVERDYPATYERFTSIGPLMEKIGNGGKGIAWNTQSEMDLLRKLNYTKAEGPAKGQP  target    ------------LAGSFTTEG----YTVEDLTAGR-YGPP------GGALMQYR---------------------SYPRI 3ir6.1    MLNTAIDAAEMILTLAPETNGQVAVKAWAALSEFTGRDHTHLALNKEDEKIRFRDIQAQPRKIISSPTWSGLEDEHVSYN  target    PFKEQIEDSLPFYTDTGRMHGYVDIPEAIEYGENLIVHREAVEATPYLPNVIVSTSPYLRPRDYGIAPEELDGDARSVRN 3ir6.1    AGYTNVHELIPWRTLSGRQQLYQDHQWMRDFGESLLVYRPPIDTRSV-KE------------------------------  target    IMMSWAEVKETENPLFAAGYNYLCLTPKSRHAVHSSWAVTDWHWLWSSSFSDPYRVETRAPGVGEPAIHLNPDDARSLGI 3ir6.1    ----VIG----QKSNGNQEKALNFLTPHQKWGIHSTYSDNLLM------------LTL---GRGGPVVWLSEADAKDLGI  target    RNGDYVWVDSNPKDRPYRDADVDESFLDVARLLVRVTYNPAYPPGVTMLKHAFYMATPRTFRAAQERSDGRALAETTGYQ 3ir6.1    ADNDWIEVFNS-----------------NGALTARAVVSQRVPAGMTMMYHAQERIVN------------LPGSEI----  target    SSFRSGSHQSITRGWAPPMHQTDSLFHKRAGVFGFTYGFDVDNHAINTVPKETVVRITKAEDGGVGGSGAWTRGRPGSMP 3ir6.1    TQQRGGIHNSVTRITPKPTHMI-------GGYAHLAYGFNYYGTV--GSNRDEFVVVRKMKN------------------  target    GDEDDAMQAYLAGELTVVRRT 3ir6.1    --------------------- ``` | | | | | | | | | | | | | | | | | | | | | | | | | | | | | | | | | | | | | | | | | | | | | | | | | |
|  | 2ivf.1.A | ETHYLBENZENE DEHYDROGENASE ALPHA-SUBUNIT  *ETHYLBENZENE DEHYDROGENASE FROM AROMATOLEUM AROMATICUM* | 0.42 |  | 23.04 | 0.76 | 1-733 | X-ray | 1.88 | hetero-oligomer | 1 x MES, 4 x SF4, 1 x MO, 1 x MGD, 1 x MD1, 1 x F3S, 1 x HEM | HHblits | 0.31 |
| ``` target    RPFLERFTDMPLLVRLDTLQRLRADEVFADYSSDLDVDGPSFTLHGMTEEQHERNGDRVVFDDASGALRAINREDVGDRL 2ivf.1    RQFVCEQTDLPLLVRMDTGKFLSAEDVDG-----------------------GEAKQFYFFDEKAGSVRKASRGTLK---  target    DDKGIDPALDYQGTVTLVDGSTVEVMSVLSMYREHLADYDIDSVVDMTGAPRNLIEQLLDDMTTLSPVAFHVGEGVNHYF 2ivf.1    --LDFMPALEGTFSARLKNGKTIQVRTVFEGLREHLKDYTPEKASAKCGVPVSLIRELGRKVAKK-RTCSYIGFSSAKSY  target    HATLHNRATYLVGMLLGSVGVSGGGVSTWAGNYKGGVF---QAAPWFGPGVGGFVN-EDPFHP--L-TDPSARYSAETAR 2ivf.1    HGDLMERSLFLAMALSGNWGKPGTGAFAWAYSDDNMVYLGVMSKPTAQGGMDELHQMAEGFNKRTLEADPTSTDEMGNIE  target    HL-----VHGEDTS------------YWGFG--DRPLVV--------DTPEDGRKVFT-GTTHMPTPTKALWYNNANLIN 2ivf.1    FMKVVTSAVGLVPPAMWLYYHVGYDQLWNNKAWTDPALKKSFGAYLDEAKEKGWWTNDHIRPAPDKTPQVYMLLSQNPMR  target    QAKWHYELV-KNVNPKVDLIVDQQIEWTGSAEFADIVLPANSWMEAETWEMGASCSNPFLQVWKGGIEPLNDTRDDIAIF 2ivf.1    RKRSGAKMFPDVLFPKLKMIFALETRMSSSAMYADIVLPCAWYYEKHEMTTP-CSGNPFFTFVDRSVAPPGECREEWDAI  target    AGVANALTELTGDERFS-------------QAFM-F-----ADRPEVYLDRVLAGSFT----TEGYTVEDLTAGRYGPP- 2ivf.1    ALILKKVGERAAARGLTEFNDHNGRKRRYDELYKKFTMDGHLLTNEDCLKEMVDINRAVGVFAKDYTYEKFKKEGQTRFL  target    --GGALMQYR-------SYPRIPFKEQIEDSLPFYTDTGRMHGYVDIPEAIEYGENLIVHREAVEATPYLPNVIVSTSPY 2ivf.1    SMGTGVSRYAHANEVDVTKPIYPMRWHFDDKKVFPTHTRRAQFYLDHDWYLEAGESLPTHKDT-----------------  target    LRPRDYGIAPEELDGDARSVRNIMMSWAEVKETENPLFAAGYNYLCLTPKSRHAVHSSWAVTDWHWLWSSSFSDPYRVET 2ivf.1    ------------------------------P-----MVGGDHPFKITGGHPRVSIHSTHLTNSH--L----------SRL  target    RAPGVGEPAIHLNPDDARSLGIRNGDYVWVDSNPKDRPYRDADVDESFLDVARLLVRVTYNPAYPPGVTMLKHAFYMATP 2ivf.1    ---HRGQPVVHMNSKDAAELGIKDGDMAKLFND-----------------FADCEIMVRTAPNVQPKQCIVYFWDAHQY-  target    RTFRAAQERSDGRALAETTGYQSSFRSGSHQSITRGWAPPMHQTDSLFHKRAGVFGFTYGFDVDNHAINTVPKETVVRIT 2ivf.1    ----------------------K-GWK-PYDILLIGMPKPLHLA-------GGYEQFRYYFMNGSPAP-VTDRGVRVSIK  target    KAEDGGVGGSGAWTRGRPGSMPGDEDDAMQAYLAGELTVVRRT 2ivf.1    KA----------------------------------------- ``` | | | | | | | | | | | | | | | | | | | | | | | | | | | | | | | | | | | | | | | | | | | | | | | | | |
|  | 5e7o.1.A | DMSO reductase family type II enzyme, molybdopterin subunit  *Crystal structure of the perchlorate reductase PcrAB mutant W461E of PcrA from Azospira suillum PS* | 0.44 |  | 23.10 | 0.75 | 1-737 | X-ray | 2.40 | hetero-oligomer | 4 x SF4, 1 x MO, 1 x MGD, 1 x MD1, 1 x F3S | HHblits | 0.32 |
| ``` target    RPFLERFTDMPLLVRLDTLQRLRADEVFADYSSDLDVDGPSFTLHGMTEEQHERNGDRVVFDDASGALRAINREDV---- 5e7o.1    AHSLKEQTDLSYLVRSDTKRFLREADVVAG----------------------GSKDKFYFWNAKTGKPVIPKGSWGDQPE  target    -----------------GDRLDDKGIDPALDYQGTVTLVDGSTVEVMSVLSMYREHL-ADYDIDSVVDMTGAPRNLIEQL 5e7o.1    KKGSPVGFLGRNTFAFPKGYIDLGDLDPALEGKFNMQLLDGKTVEVRPVFEILKSRLMADNTPEKAAKITGVTAKAITEL  target    LDDMTTLSPVAFHVGEGVNHYFHATLHNRATYLVGMLLGSVGVSGGGVSTWAGNYKGGVFQAAPWFGPGVGGFVNEDPFH 5e7o.1    AREFATAKPSMIICGGGTQHWYYSDVLLRAMHLLTALTGTEGTNGGGMNHYIGQEKPAFVAG--LVAL---A-------F  target    PLTDPSARYSAETARHLVHGEDT-SY--WGFGDRPLVVDTPEDGRKVFTGTTHMPTPTKALWYNNANLINQAKWHYELVK 5e7o.1    PEGVNKQRFCQTTIWTYIHAEVNDEIISSDIDTEKYLRDSITTGQM--PNMPEQGRDPKVFFVYRGNWLNQAKGQKYVLE  target    NVNPKVDLIVDQQIEWTGSAEFADIVLPANSWMEAETWEMGASCSNPFLQVWKGGIEPLNDTRDDIAIFAGVANALTELT 5e7o.1    NLWPKLELIVDINIRMDSTALYSDVVLPSAHWYEKLDLN--VTSEHSYINMTEPAIKPMWESKTDWQIFLALAKRVEMAA  target    GDER---FSQ-AFMFA----------------DRPEVYLDRVLAGSFTTEGYTVEDLTAGRYGPPGGALM--QYRSYPRI 5e7o.1    KRKKYEKFNDEKFKWVRDLSNLWNQMTMDGKLAEDEAAAQYILDNAPQSKGITIQMLREKPQRFKS-NWTSPLKEGVPYT  target    PFKEQIEDSLPFYTDTGRMHGYVDIPEAIEYGENLIVHREAVEATPYLPNVIVSTSPYLRPRDYGIAPEELDGDARSVRN 5e7o.1    PFQYFVVDKKPWPTLTGRQQFYLDHDTFFDMGVELPTYKA----------------------------------------  target    IMMSWAEVKETENPLFAAGYNYLCLTPKSRHAVHSSWAVTDWHWLWSSSFSDPYRVETRAPGVGEPAIHLNPDDARSLGI 5e7o.1    -------PI------DADKYPFRFNSPHSRHSVHSTFKDNVL------------MLRL---QRGGPSIEMSPLDAKPLGI  target    RNGDYVWVDSNPKDRPYRDADVDESFLDVARLLVRVTYNPAYPPGVTMLKHAFYMATPRTFRAAQERSDGRALAETTGYQ 5e7o.1    KDNDWVEAWNN-----------------HGKVICRVKIRNGEQRGRVSMWHCPEL-----------------------YM  target    SSFRSGSHQSITRGWAPPMHQTDSLFHKRAGVFGFTYGFDVDNHAINTVPKETVVRITKAEDGGVGGSGAWTRGRPGSMP 5e7o.1    D-LLTGGSQSVCPVRINPTNLV-------GNYGHLFFRPNY--YGPAGSQRDVRVNVKRYIGAT----------------  target    GDEDDAMQAYLAGELTVVRRT 5e7o.1    --------------------- ``` | | | | | | | | | | | | | | | | | | | | | | | | | | | | | | | | | | | | | | | | | | | | | | | | | |
|  | 4ydd.1.A | DMSO reductase family type II enzyme, molybdopterin subunit  *Crystal structure of the perchlorate reductase PcrAB from Azospira suillum PS* | 0.44 |  | 23.14 | 0.75 | 1-736 | X-ray | 1.86 | hetero-oligomer | 4 x SF4, 1 x MO, 1 x MGD, 1 x MD1, 1 x F3S | HHblits | 0.32 |
| ``` target    RPFLERFTDMPLLVRLDTLQRLRADEVFADYSSDLDVDGPSFTLHGMTEEQHERNGDRVVFDDASGALRAINREDV---- 4ydd.1    AHSLKEQTDLSYLVRSDTKRFLREADVVAGG----------------------SKDKFYFWNAKTGKPVIPKGSWGDQPE  target    -----------------GDRLDDKGIDPALDYQGTVTLVDGSTVEVMSVLSMYREHL-ADYDIDSVVDMTGAPRNLIEQL 4ydd.1    KKGSPVGFLGRNTFAFPKGYIDLGDLDPALEGKFNMQLLDGKTVEVRPVFEILKSRLMADNTPEKAAKITGVTAKAITEL  target    LDDMTTLSPVAFHVGEGVNHYFHATLHNRATYLVGMLLGSVGVSGGGVSTWAGNYKGGVFQAAPWFGPGVGGFVNEDPFH 4ydd.1    AREFATAKPSMIICGGGTQHWYYSDVLLRAMHLLTALTGTEGTNGGGMNHYIGQWKPAFVAGL--VAL---AF-------  target    PLTDPSARYSAETARHLVHGEDTS-YW--GFGDRPLVVDTPEDGRKVFTGTTHMPTPTKALWYNNANLINQAKWHYELVK 4ydd.1    PEGVNKQRFCQTTIWTYIHAEVNDEIISSDIDTEKYLRDSITTGQM--PNMPEQGRDPKVFFVYRGNWLNQAKGQKYVLE  target    NVNPKVDLIVDQQIEWTGSAEFADIVLPANSWMEAETWEMGASCSNPFLQVWKGGIEPLNDTRDDIAIFAGVANALTELT 4ydd.1    NLWPKLELIVDINIRMDSTALYSDVVLPSAHWYEKLDLN--VTSEHSYINMTEPAIKPMWESKTDWQIFLALAKRVEMAA  target    GDERFSQ----AFMFA----------------DRPEVYLDRVLAGSFTTEGYTVEDLTAGRYGPPGGALM--QYRSYPRI 4ydd.1    KRKKYEKFNDEKFKWVRDLSNLWNQMTMDGKLAEDEAAAQYILDNAPQSKGITIQMLREKPQRFK-SNWTSPLKEGVPYT  target    PFKEQIEDSLPFYTDTGRMHGYVDIPEAIEYGENLIVHREAVEATPYLPNVIVSTSPYLRPRDYGIAPEELDGDARSVRN 4ydd.1    PFQYFVVDKKPWPTLTGRQQFYLDHDTFFDMGVELPTYKA----------------------------------------  target    IMMSWAEVKETENPLFAAGYNYLCLTPKSRHAVHSSWAVTDWHWLWSSSFSDPYRVETRAPGVGEPAIHLNPDDARSLGI 4ydd.1    -------PI------DADKYPFRFNSPHSRHSVHSTFKDNVL------------MLRL---QRGGPSIEMSPLDAKPLGI  target    RNGDYVWVDSNPKDRPYRDADVDESFLDVARLLVRVTYNPAYPPGVTMLKHAFYMATPRTFRAAQERSDGRALAETTGYQ 4ydd.1    KDNDWVEAWNN-----------------HGKVICRVKIRNGEQRGRVSMWHCPEL-----------------------YM  target    SSFRSGSHQSITRGWAPPMHQTDSLFHKRAGVFGFTYGFDVDNHAINTVPKETVVRITKAEDGGVGGSGAWTRGRPGSMP 4ydd.1    D-LLTGGSQSVCPVRINPTNLV-------GNYGHLFFRPN--YYGPAGSQRDVRVNVKRYIGA-----------------  target    GDEDDAMQAYLAGELTVVRRT 4ydd.1    --------------------- ``` | | | | | | | | | | | | | | | | | | | | | | | | | | | | | | | | | | | | | | | | | | | | | | | | | |
|  | 4ydd.1.A | DMSO reductase family type II enzyme, molybdopterin subunit  *Crystal structure of the perchlorate reductase PcrAB from Azospira suillum PS* | 0.31 |  | 29.65 | 0.67 | 4-599 | X-ray | 1.86 | hetero-oligomer | 4 x SF4, 1 x MO, 1 x MGD, 1 x MD1, 1 x F3S | BLAST | 0.35 |
| ``` target    RPFLERFTDMPLLVRLDTLQRLRADEVFADYSSDL-----DVDGPSFTLHGMTEEQHERNGDRVVFDDASGALRAINRED 4ydd.1    ---LKEQTDLSYLVRSDTKRFLREADVVAGGSKDKFYFWNAKTGKPVIPKGSWGDQPEKKGSPVGFLGRNTFAFPKGYID  target    VGDRLDDKGIDPALDYQGTVTLVDGSTVEVMSVLSMYREHL-ADYDIDSVVDMTGAPRNLIEQLLDDMTTLSPVAFHVGE 4ydd.1    LGD------LDPALEGKFNMQLLDGKTVEVRPVFEILKSRLMADNTPEKAAKITGVTAKAITELAREFATAKPSMIICGG  target    GVNHYFHATLHNRATYLVGMLLGSVGVSGGGVSTWAGNYKGGVFQAAPWFGPGVGGFVNEDPFHPLTDPSARYSAETARH 4ydd.1    GTQHWYYSDVLLRAMHLLTALTGTEGTNGGGMNHYIGQWK-------PAFVAGLVALA-----FPEGVNKQRFCQTTIWT  target    LVHGEDTSYWGFGDRPLVVDTPEDGRKVFTGTTHMPT------PTKALWYNNANLINQAKWHYELVKNVNPKVDLIVDQQ 4ydd.1    YIHAEVNDEIISSD----IDTEKYLRDSIT-TGQMPNMPEQGRDPKVFFVYRGNWLNQAKGQKYVLENLWPKLELIVDIN  target    IEWTGSAEFADIVLPANSWMEAETWEMGASCSNPFLQVWKGGIEPLNDTRDDIAIFAGVANALTELTGDERFSQAFMFAD 4ydd.1    IRMDSTALYSDVVLPSAHWYEK--LDLNVTSEHSYINMTEPAIKPMWESKTDWQIFLALAKRVEMAAKRKKYEK---FND  target    RPEVYLDRV--LAGSFTTEGYTVEDLTAGRY------GPPGGALMQYRSYPRIPFKEQIEDSLPFYTDTGRMHGYVDIPE 4ydd.1    EKFKWVRDLSNLWNQMTMDGKLAEDEAAAQYILDNAPQSKGITIQMLREKPQ-RFKSNWTSPL-----------------  target    AIEYGENLIVHREAVEATPYLPNVIVSTSPYLRPRDYGIAPEELDGDARSVRNIMMSWAEVKETENPLFAAGYNYLCLTP 4ydd.1    -----------KEGVPYTPF-QYFVVDKKPW--PTLTGRQQFYLDHDTFFDMGV-----ELPTYKAPIDADKYPFRFNSP  target    KSRHAVHSSWAVTDWHWLWSSSFSDPYRVETRAPGVGEPAIHLNPDDARSLGIRNGDYVWVDSNPKDRPYRDADVDESFL 4ydd.1    HSRHSVHST-------------FKD--NVLMLRLQRGGPSIEMSPLDAKPLGIKDNDWV---------------------  target    DVARLLVRVTYNPAYPPGVTMLKHAFYMATPRTFRAAQERSDGRALAETTGYQSSFRSGSHQSITRGWAPPMHQTDSLFH 4ydd.1    --------------------------------------------------------------------------------  target    KRAGVFGFTYGFDVDNHAINTVPKETVVRITKAEDGGVGGSGAWTRGRPGSMPGDEDDAMQAYLAGELTVVRRT 4ydd.1    -------------------------------------------------------------------------- ``` | | | | | | | | | | | | | | | | | | | | | | | | | | | | | | | | | | | | | | | | | | | | | | | | | |
|  | 5e7o.1.A | DMSO reductase family type II enzyme, molybdopterin subunit  *Crystal structure of the perchlorate reductase PcrAB mutant W461E of PcrA from Azospira suillum PS* | 0.31 |  | 29.65 | 0.67 | 4-599 | X-ray | 2.40 | hetero-oligomer | 4 x SF4, 1 x MO, 1 x MGD, 1 x MD1, 1 x F3S | BLAST | 0.35 |
| ``` target    RPFLERFTDMPLLVRLDTLQRLRADEVFADYSSDL-----DVDGPSFTLHGMTEEQHERNGDRVVFDDASGALRAINRED 5e7o.1    ---LKEQTDLSYLVRSDTKRFLREADVVAGGSKDKFYFWNAKTGKPVIPKGSWGDQPEKKGSPVGFLGRNTFAFPKGYID  target    VGDRLDDKGIDPALDYQGTVTLVDGSTVEVMSVLSMYREHL-ADYDIDSVVDMTGAPRNLIEQLLDDMTTLSPVAFHVGE 5e7o.1    LGD------LDPALEGKFNMQLLDGKTVEVRPVFEILKSRLMADNTPEKAAKITGVTAKAITELAREFATAKPSMIICGG  target    GVNHYFHATLHNRATYLVGMLLGSVGVSGGGVSTWAGNYKGGVFQAAPWFGPGVGGFVNEDPFHPLTDPSARYSAETARH 5e7o.1    GTQHWYYSDVLLRAMHLLTALTGTEGTNGGGMNHYIG-------QEKPAFVAGLVALA-----FPEGVNKQRFCQTTIWT  target    LVHGEDTSYWGFGDRPLVVDTPEDGRKVFTGTTHMPT------PTKALWYNNANLINQAKWHYELVKNVNPKVDLIVDQQ 5e7o.1    YIHAEVNDEIISSD----IDTEKYLRDSIT-TGQMPNMPEQGRDPKVFFVYRGNWLNQAKGQKYVLENLWPKLELIVDIN  target    IEWTGSAEFADIVLPANSWMEAETWEMGASCSNPFLQVWKGGIEPLNDTRDDIAIFAGVANALTELTGDERFSQAFMFAD 5e7o.1    IRMDSTALYSDVVLPSAHWYEK--LDLNVTSEHSYINMTEPAIKPMWESKTDWQIFLALAKRVEMAAKRKKYEK---FND  target    RPEVYLDRV--LAGSFTTEGYTVEDLTAGRY------GPPGGALMQYRSYPRIPFKEQIEDSLPFYTDTGRMHGYVDIPE 5e7o.1    EKFKWVRDLSNLWNQMTMDGKLAEDEAAAQYILDNAPQSKGITIQMLREKPQ-RFKSNWTSPL-----------------  target    AIEYGENLIVHREAVEATPYLPNVIVSTSPYLRPRDYGIAPEELDGDARSVRNIMMSWAEVKETENPLFAAGYNYLCLTP 5e7o.1    -----------KEGVPYTPF-QYFVVDKKPW--PTLTGRQQFYLDHDTFFDMGV-----ELPTYKAPIDADKYPFRFNSP  target    KSRHAVHSSWAVTDWHWLWSSSFSDPYRVETRAPGVGEPAIHLNPDDARSLGIRNGDYVWVDSNPKDRPYRDADVDESFL 5e7o.1    HSRHSVHST-------------FKD--NVLMLRLQRGGPSIEMSPLDAKPLGIKDNDWV---------------------  target    DVARLLVRVTYNPAYPPGVTMLKHAFYMATPRTFRAAQERSDGRALAETTGYQSSFRSGSHQSITRGWAPPMHQTDSLFH 5e7o.1    --------------------------------------------------------------------------------  target    KRAGVFGFTYGFDVDNHAINTVPKETVVRITKAEDGGVGGSGAWTRGRPGSMPGDEDDAMQAYLAGELTVVRRT 5e7o.1    -------------------------------------------------------------------------- ``` | | | | | | | | | | | | | | | | | | | | | | | | | | | | | | | | | | | | | | | | | | | | | | | | | |
|  | 4v4c.1.A | Pyrogallol hydroxytransferase large subunit  *Crystal Structure of Pyrogallol-Phloroglucinol Transhydroxylase from Pelobacter acidigallici* | 0.30 |  | 16.35 | 0.61 | 99-647 | X-ray | 2.35 | hetero-oligomer | 2 x CA, 2 x MGD, 1 x 4MO, 3 x SF4 | HHblits | 0.29 |
| ``` target    RPFLERFTDMPLLVRLDTLQRLRADEVFADYSSDLDVDGPSFTLHGMTEEQHERNGDRVVFDDASGALRAINREDVGDRL 4v4c.1    --------------------------------------------------------------------------------  target    DDKGIDPALDYQGTVTLVDGSTVEV-MSVLSMYREHLAD------YDIDSVVDMTGAPRNLIEQLLDDMTTLSPVAFHVG 4v4c.1    ------------------DKEYVAANAHGFEEWADYVLGKTDGTPKTCEWAEEESGVPACEIRALARQWAKKNTYLAAGG  target    E----GVNHYFHATLHNRATYLVGMLLGSVGVSGGGVSTWAGNYKGGVFQAAPWFGPGVGGF-VNEDPFHPLTDPSARYS 4v4c.1    LGGWGGACRASHGIEWARGMIALATMQG-MGKPGSNMWSTTQGVPLD-Y---EFYFPGYAEGGISGDCENSA--AGFKFA  target    AETA---RHLVHGEDTSYWGFGDRPL--VVDTPEDG-----------RKVFTGTTH------MPTPTKALWYNNANLINQ 4v4c.1    WRMFDGKTTFPSPSNLNTSAGQHIPRLKIPECIMGGKFQWSGKGFAGGDISHQLHQYEYPAPGYSKIKMFWKYGGPHLGT  target    AKWHYELVKNVN--PKVDLIVDQQIEWTGSAEFADIVLPANSWMEAETWEMGAS-----------CSNPFLQVWKGGIEP 4v4c.1    MTATNRY-AKMYTHDSLEFVVSQSIWFEGEVPFADIILPACTNFERWDISEFANCSGYIPDNYQLCNHRVISLQAKCIEP  target    LNDTRDDIAIFAGVANALTELTGDERFSQAFMFADRPEVYLDRVLAGSFTTEGYTVEDLTAGRYGP-PG-------GALM 4v4c.1    VGESMSDYEIYRLFAKKLNIEE-------MFSEGKDELAWCEQYFNATDMPKYMTWDEFFKKGYFVVPDNPNRKKTVALR  target    QYRSY---PRIPFKEQ---IEDSLPFYTDTGRMHGYVDIPEAIE-YGENLIVHREAVEATPYLPNVIVSTSPYLRPRDYG 4v4c.1    WFAEGREKDTPDWGPRLNNQVCRKGLQTTTGKVEFIATSLKNFEEQG-----YID--EHRPSMHTY--------------  target    IAPEELDGDARSVRNIMMSWAEVKETENPLFAAGYNYLCLTPKSRHAVHSSWAVTDWHWLWSSSFSDPYRVETRAPGVGE 4v4c.1    ----------------VPAWESQKH--SP-LAVKYPLGMLSPHPRFSMHTMGDGKN-SYMNY--IKDHR-VEV--DGYKY  target    PAIHLNPDDARSLGIRNGDYVWVDSNPKDRPYRDADVDESFLDVARLLVRVTYNPAYPPGVTMLKHAFYMATPRTFRAAQ 4v4c.1    WIMRVNSIDAEARGIKNGDLIRAYND-----------------RGSVILAAQVTECLQPGTVHSYESCA-----------  target    ERSDGRALAETTGYQSSFRSGSHQSITRGWAPPMHQTDSLFHKRAGVFGFTYGFDVDNHAINTVPKETVVRITKAEDGGV 4v4c.1    --------------------------------------------------------------------------------  target    GGSGAWTRGRPGSMPGDEDDAMQAYLAGELTVVRRT 4v4c.1    ------------------------------------ ``` | | | | | | | | | | | | | | | | | | | | | | | | | | | | | | | | | | | | | | | | | | | | | | | | | |
|  | 1tmo.1.A | TRIMETHYLAMINE N-OXIDE REDUCTASE  *TRIMETHYLAMINE N-OXIDE REDUCTASE FROM SHEWANELLA MASSILIA* | 0.29 |  | 17.37 | 0.58 | 98-649 | X-ray | 2.50 | monomer | 2 x 2MD, 1 x 2MO | HHblits | 0.29 |
| ``` target    RPFLERFTDMPLLVRLDTLQRLRADEVFADYSSDLDVDGPSFTLHGMTEEQHERNGDRVVFDDASGALRAINREDVGDRL 1tmo.1    --------------------------------------------------------------------------------  target    DDKGIDPALDYQGTVTLVDGSTVEVMS-VLSMYREHLA------DYDIDSVVDMTGAPRNLIEQLLDDMTTLSPVAFHVG 1tmo.1    -----------------YDDKFIQGYSLGFEEFVPYVMGTKDGVAKTPEWAAPICGVEAHVIRDLAKTLVKGR-TQFMMG  target    EGVNHYFHATLHNRATYLVGMLLGSVGVSGGGVSTWAGNYKGGVFQAAPWFGPGVGGFVNEDPFHPLTDPSARYSAETAR 1tmo.1    WCIQRQQHGEQPYWMAAVLATMIGQIGLPGGGISYGHHYSSIG-VPSSGAAAP--GAFPR--NLDEN---QKPL-FDSS-  target    HLVHGEDTSYWGFGDRPLVVDTP-EDGRKVFTGTTH-MPTPTKALWYNNANLINQAKWHYELVKNVNPKVDLIVDQQIEW 1tmo.1    DFK-GASS---TIP-VARWIDAILEPGKTIDANGSKVVYPDIKMMIFSGNNPWNHHQDRNR-MKQAFHKLECVVTVDVNW  target    TGSAEFADIVLPANSWMEAETWEMGASCSNPFLQVWKGGIEPLNDTRDDIAIFAGVANALTELTGDERFSQAFMFADRPE 1tmo.1    TATCRFSDIVLPACTTYERNDIDVYGAYANRGILAMQKMVEPLFDSLSDFEIFTRFAAVLGKE-------KEYTRNMGEM  target    VYLDRVLAGSFT-----TEGYTVEDLTAGRYGPPGGALMQYRSYPRIPFKEQIEDSLPFYTDTGRMHGYVDIPEAIEYGE 1tmo.1    EWLETLYNECKAANAGKFEMPDFATFWKQGYVHFGDGEV-WTRH--ADFRND-PEINPLGTPSGLIEIFSRKIDQFGYD-  target    NLIVHREAVEATPYLPNVIVSTSPYLRPRDYGIAPEELDGDARSVRNIMMSWAEVKETE-NPLFAAGYNYLCLTPKSRHA 1tmo.1    ----------DCKGHPT----------------------------------WMEKTERSHGGPGSDKHPIWLQSCHPDKR  target    VHSSWAVTDWHWLWSSSFSDPYRVETRAPGVGEPAIHLNPDDARSLGIRNGDYVWVDSNPKDRPYRDADVDESFLDVARL 1tmo.1    LHSQMCESREY--RE----------TY-AVNGREPVYISPVDAKARGIKDGDIVRVFND-----------------RGQL  target    LVRVTYNPAYPPGVTMLKHAFYMATPRTFRAAQERSDGRALAETTGYQSSFRSGSHQSITRGWAPPMHQTDSLFHKRAGV 1tmo.1    LAGAVVSDNFPKGIVRIHEGAWYG--------------------------------------------------------  target    FGFTYGFDVDNHAINTVPKETVVRITKAEDGGVGGSGAWTRGRPGSMPGDEDDAMQAYLAGELTVVRRT 1tmo.1    --------------------------------------------------------------------- ``` | | | | | | | | | | | | | | | | | | | | | | | | | | | | | | | | | | | | | | | | | | | | | | | | | |
| ✓ | 2ivf.1.A | ETHYLBENZENE DEHYDROGENASE ALPHA-SUBUNIT  *ETHYLBENZENE DEHYDROGENASE FROM AROMATOLEUM AROMATICUM* | 0.20 | 0.00 | 32.76 | 0.53 | 1-473 | X-ray | 1.88 | monomer | 1 x MES, 4 x SF4, 1 x MO, 1 x MGD, 1 x MD1, 1 x F3S, 1 x HEM | BLAST | 0.36 |
| ``` target    RPFLERFTDMPLLVRLDTLQRLRADEVFADYSSDLDVDGPSFTLHGMTEEQHERNGDRVVFDDASGALRAINREDVGDRL 2ivf.1    RQFVCEQTDLPLLVRMDTGKFLSAE----------DVDG------GEAKQFY-------FFDEKAGSVRKASRGTL--KL  target    DDKGIDPALDYQGTVTLVDGSTVEVMSVLSMYREHLADYDIDSVVDMTGAPRNLIEQLLDDMTTLSPVAFHVGEGVNHYF 2ivf.1    D---FMPALEGTFSARLKNGKTIQVRTVFEGLREHLKDYTPEKASAKCGVPVSLIRELGRKVAKKRTCSY-IGFSSAKSY  target    HATLHNRATYLVGMLLGSVGVSGGGVSTWAGNYKGGVFQAAPWFGPGVGGFV------NEDPFHPLTDPSARYSAETARH 2ivf.1    HGDLMERSLFLAMAL--------------SGN----------WGKPGTGAFAWAYSDDNMVYLGVMSKPTAQGGMDELHQ  target    LVHGEDTSYWGFGDRPLVVD---TPEDGR----KVFTGTTHMPTPTKALWYNNA--NLINQAKW---------------- 2ivf.1    MAE-------GFNKRTLEADPTSTDEMGNIEFMKVVTSAVGLVPPAMWLYYHVGYDQLWNNKAWTDPALKKSFGAYLDEA  target    ---------HYELVKNVNPKVDLIVDQ---------------------------QIEWTGSAEFADIVLPANSWMEAETW 2ivf.1    KEKGWWTNDHIRPAPDKTPQVYMLLSQNPMRRKRSGAKMFPDVLFPKLKMIFALETRMSSSAMYADIVLPCAWYYEKH--  target    EMGASCS-NPFLQVWKGGIEPLNDTR---DDIAIFA------GVANALTELTG--------DERFSQAFMFAD------- 2ivf.1    EMTTPCSGNPFFTFVDRSVAPPGECREEWDAIALILKKVGERAAARGLTEFNDHNGRKRRYDELYKKFTMDGHLLTNEDC  target    -RPEVYLDR---VLAGSFTTEGYTVEDLTAGRYGPPGGALMQYR-------SYPRIPFKEQIEDSLPFYTDTGRMHGYVD 2ivf.1    LKEMVDINRAVGVFAKDYTYEKFKKEGQT--RFLSMGTGVSRYAHANEVDVTKPIYPMRWHFDDKKVFPTHTRRAQFYLD  target    IPEAIEYGENLIVHREAVEATPYLPNVIVSTSPYLRPRDYGIAPEELDGDARSVRNIMMSWAEVKETENPLFAAGYNYLC 2ivf.1    HDWYLEAGESLPTHKD----------------------------------------------------------------  target    LTPKSRHAVHSSWAVTDWHWLWSSSFSDPYRVETRAPGVGEPAIHLNPDDARSLGIRNGDYVWVDSNPKDRPYRDADVDE 2ivf.1    --------------------------------------------------------------------------------  target    SFLDVARLLVRVTYNPAYPPGVTMLKHAFYMATPRTFRAAQERSDGRALAETTGYQSSFRSGSHQSITRGWAPPMHQTDS 2ivf.1    --------------------------------------------------------------------------------  target    LFHKRAGVFGFTYGFDVDNHAINTVPKETVVRITKAEDGGVGGSGAWTRGRPGSMPGDEDDAMQAYLAGELTVVRRT 2ivf.1    ----------------------------------------------------------------------------- ``` | | | | | | | | | | | | | | | | | | | | | | | | | | | | | | | | | | | | | | | | | | | | | | | | | |
|  | 4dmr.1.A | DMSO REDUCTASE  *REDUCED DMSO REDUCTASE FROM RHODOBACTER CAPSULATUS WITH BOUND DMSO SUBSTRATE* | 0.29 | 0.00 | 21.80 | 0.57 | 97-648 | X-ray | 1.90 | monomer | 2 x PGD, 1 x 4MO, 1 x O | HHblits | 0.30 |
| ``` target    RPFLERFTDMPLLVRLDTLQRLRADEVFADYSSDLDVDGPSFTLHGMTEEQHERNGDRVVFDDASGALRAINREDVGDRL 4dmr.1    --------------------------------------------------------------------------------  target    DDKGIDPALDYQGTVTLVDGSTVEVMS-VLSMYREHLA------DYDIDSVVDMTGAPRNLIEQLLDDMTTLSPVAFHVG 4dmr.1    ----------------LYDKDFIANYTSGFDKFLPYLDGETDSTPKTAEWAEGISGVPAETIKELARLFES-KRTMLAAG  target    EGVNHYFHATLHNRATYLVGMLLGSVGVSGGGVSTWAGNYKGGVFQAAPWFGPGVGGFVNEDPFHPLTDPSARYSAETAR 4dmr.1    WSMQRMHHGEQAHWMLVTLASMLGQIGLPGGGFGLSYHYSGGGTPS---TSGPALAGITDG--GAATKGPEWLAAS-GAS  target    HLVHGEDTSYWGFGDRPLVVDTPEDGRKV--FTGTTHMPTPTKALWYNNANLINQAKWHYELVKNVNPKVDLIVDQQIEW 4dmr.1    VI--------PV----ARVVDMLENPGAEFDFNGTRSKFPDVKMAYWVGGNPFVHHQDRNR-MVKAWEKLETFVVHDFQW  target    TGSAEFADIVLPANSWMEAETWEMGASCSNPFLQVWKGGIEPLNDTRDDIAIFAGVANALTELTGDERFSQAFMFADRPE 4dmr.1    TPTARHADIVLPATTSYERNDIETIGDYSNTGILAMKKIVEPLYEARSDYDIFAAVAERLGKGA---EFT----EGKDEM  target    VYLDRVLAGSF---TTEGY---TVEDLTAGRYGPPGG-ALMQYRSYPRIPFKEQIEDSLPFYTDTGRMHGYVDIPEAIEY 4dmr.1    GWIKSFYDDAAKQGKAAGVQMPAFDAFWAEGIVEFPVTDGADFVRY--ASFRED-PLLNPLGTPTGLIEIYSKNIEKMGY  target    GENLIVHREAVEATPYLPNVIVSTSPYLRPRDYGIAPEELDGDARSVRNIMMSWAEVKETENPLFAAGYNYLCLTPKSRH 4dmr.1    D-----------DCPAH----------------------------------PTWMEPLERL-DGPGAKYPLHIAASHPFN  target    AVHSSWAVTDWHWLWSSSFSDPYRVETRAPGVGEPAIHLNPDDARSLGIRNGDYVWVDSNPKDRPYRDADVDESFLDVAR 4dmr.1    RLHSQLNG-T--VLRE----------GY-AVQGHEPCLMHPDDAAARGIADGDVVRVHND-----------------RGQ  target    LLVRVTYNPAYPPGVTMLKHAFYMATPRTFRAAQERSDGRALAETTGYQSSFRSGSHQSITRGWAPPMHQTDSLFHKRAG 4dmr.1    ILTGVKVTDAVMKGVIQIYEGGWY--------------------------------------------------------  target    VFGFTYGFDVDNHAINTVPKETVVRITKAEDGGVGGSGAWTRGRPGSMPGDEDDAMQAYLAGELTVVRRT 4dmr.1    ---------------------------------------------------------------------- ``` | | | | | | | | | | | | | | | | | | | | | | | | | | | | | | | | | | | | | | | | | | | | | | | | | |
|  | 1dms.1.A | DMSO REDUCTASE  *STRUCTURE OF DMSO REDUCTASE* | 0.30 | 0.00 | 21.85 | 0.57 | 98-648 | X-ray | 1.88 | monomer | 2 x PGD, 1 x 2MO | HHblits | 0.30 |
| ``` target    RPFLERFTDMPLLVRLDTLQRLRADEVFADYSSDLDVDGPSFTLHGMTEEQHERNGDRVVFDDASGALRAINREDVGDRL 1dms.1    --------------------------------------------------------------------------------  target    DDKGIDPALDYQGTVTLVDGSTVEVMS-VLSMYREHLA------DYDIDSVVDMTGAPRNLIEQLLDDMTTLSPVAFHVG 1dms.1    -----------------YDKDFIANYTSGFDKFLPYLMGETDSTPKTAEWASDISGVPAETIKELARLFKS-KRTMLAAG  target    EGVNHYFHATLHNRATYLVGMLLGSVGVSGGGVSTWAGNYKGGVFQAAPWFGPGVGGFVNEDPFHPLTDPSARYSAETAR 1dms.1    WSMQRMHHGEQAHWMLVTLASMLGQIGLPGGGFGLSYHYSGGGTPS---SSGPALSGITDG--GAATKGPE------WLA  target    HLVHGEDTSYWGFGDRPLVVDTPEDGRKVF--TGTTHMPTPTKALWYNNANLINQAKWHYELVKNVNPKVDLIVDQQIEW 1dms.1    A--SG-ASVIPVA----RVVDMLENPGAEFDFNGTRSKFPDVKMAYWVGGNPFVHHQDRNR-MVKAWEKLETFIVHDFQW  target    TGSAEFADIVLPANSWMEAETWEMGASCSNPFLQVWKGGIEPLNDTRDDIAIFAGVANALTELTGDERFSQAFMFADRPE 1dms.1    TPTARHADIVLPATTSYERNDIETIGDYSNTGILAMKKIVEPLYEARSDYDIFAAVAERLGKGK---EF----TEGKDEM  target    VYLDRVLAGSF---TTEGY---TVEDLTAGRYGPPGG-ALMQYRSYPRIPFKEQIEDSLPFYTDTGRMHGYVDIPEAIEY 1dms.1    GWIKSFYDDAAKQGKAGGVEMPAFDAFWAEGIVEFPVTDGADF--VRYASFRED-PLLNPLGTPTGLIEIYSKNIEKMGY  target    GENLIVHREAVEATPYLPNVIVSTSPYLRPRDYGIAPEELDGDARSVRNIMMSWAEVKETENPLFAAGYNYLCLTPKSRH 1dms.1    D-----------DCPAHP----------------------------------TWMEPLERL-DGPGAKYPLHIAASHPFN  target    AVHSSWAVTDWHWLWSSSFSDPYRVETRAPGVGEPAIHLNPDDARSLGIRNGDYVWVDSNPKDRPYRDADVDESFLDVAR 1dms.1    RLHSQLNGT---VLRE----------GY-AVQGHEPCLMHPDDAAARGIADGDVVRVHND-----------------RGQ  target    LLVRVTYNPAYPPGVTMLKHAFYMATPRTFRAAQERSDGRALAETTGYQSSFRSGSHQSITRGWAPPMHQTDSLFHKRAG 1dms.1    ILTGVKVTDAVMKGVIQIYEGGWY--------------------------------------------------------  target    VFGFTYGFDVDNHAINTVPKETVVRITKAEDGGVGGSGAWTRGRPGSMPGDEDDAMQAYLAGELTVVRRT 1dms.1    ---------------------------------------------------------------------- ``` | | | | | | | | | | | | | | | | | | | | | | | | | | | | | | | | | | | | | | | | | | | | | | | | | |
|  | 7l5i.1.A | Trimethylamine-N-oxide reductase  *Crystal Structure of Haemophilus influenzae MtsZ at pH 7.0* | 0.29 |  | 19.86 | 0.57 | 98-648 | X-ray | 1.73 | monomer | 2 x MGD, 1 x MO, 1 x O | HHblits | 0.30 |
| ``` target    RPFLERFTDMPLLVRLDTLQRLRADEVFADYSSDLDVDGPSFTLHGMTEEQHERNGDRVVFDDASGALRAINREDVGDRL 7l5i.1    --------------------------------------------------------------------------------  target    DDKGIDPALDYQGTVTLVDGSTVEVMS-VLSMYREHL------ADYDIDSVVDMTGAPRNLIEQLLDDMTTLSPVAFHVG 7l5i.1    -----------------HDKDFLKKYTSGYAKFEEYLLGKTDGQPKTAEWAAKICGVPAETIKQLAADFAS-KRTMLMGG  target    EGVNHYFHATLHNRATYLVGMLLGSVGVSGGGVSTWAGNYKGGVFQAAPWFGPGVGGFVNEDPFHPLTDPSA-RYS-AET 7l5i.1    WGMQRQRHGEQTHWMLVTLASMLGQIGLPGGGFGLSYHYSNGGVPTATG-GII--GSITA----SPSGKAGAKTWLDDTS  target    ARHLVHGEDTSYWGFGDRPLVVDTP-EDGRKVFTGTTHMP-TPTKALWYNNANLINQAKWHYELVKNVNPKVDLIVDQQI 7l5i.1    KSAF-----PLARI-------ADVLLHPGKKIQYNGTEITYPDIKAVYWAGGNPFVHHQDTNTL-VKAFQKPDVVIVNEV  target    EWTGSAEFADIVLPANSWMEAETWEMGASCSNPFLQVWKGGIEPLNDTRDDIAIFAGVANALTELTGDERFSQAFMFADR 7l5i.1    NWTPTARMADIVLPATTSYERNDLTMAGDYSMMSVYPMKQVVPPQFEAKNDYDIFVELAKRAGVEE-------QYTEGKT  target    PEVYLDRVLAGSFT----T--EGYTVEDLTAGRYGPPGGALMQY-RSYPRIPFKEQI--EDSLPFYTDTGRMHGYVDIPE 7l5i.1    EMEWLEEFYNAAFSAARANRVAMPRFDKFWAENKPL---SFEAGEAAKKWVRYGEFREDPLLNPLGTPSGKIEIFSDVVE  target    AIEYGENLIVHREAVEATPYLPNVIVSTSPYLRPRDYGIAPEELDGDARSVRNIMMSWAEVKETENPLFAAGYNYLCLTP 7l5i.1    K--MNYN------D---CKGHP----------------------------------SWMEPEEFA-GNVTEEYPLALVTP  target    KSRHAVHSSWAVTDWHWLWSSSFSDPYRVETRAPGVGEPAIHLNPDDARSLGIRNGDYVWVDSNPKDRPYRDADVDESFL 7l5i.1    HPYYRLHSQLAHTSLR------------QKY--AVNDREPVMIHPEDAAARGIKDGDIVRIHSK----------------  target    DVARLLVRVTYNPAYPPGVTMLKHAFYMATPRTFRAAQERSDGRALAETTGYQSSFRSGSHQSITRGWAPPMHQTDSLFH 7l5i.1    -RGQVLAGAAVTENIIKGTVALHEGAWY----------------------------------------------------  target    KRAGVFGFTYGFDVDNHAINTVPKETVVRITKAEDGGVGGSGAWTRGRPGSMPGDEDDAMQAYLAGELTVVRRT 7l5i.1    -------------------------------------------------------------------------- ``` | | | | | | | | | | | | | | | | | | | | | | | | | | | | | | | | | | | | | | | | | | | | | | | | | |
|  | 7l5s.1.A | Trimethylamine-N-oxide reductase  *Crystal Structure of Haemophilus influenzae MtsZ at pH 5.5* | 0.29 |  | 19.86 | 0.57 | 98-648 | X-ray | 2.09 | monomer | 1 x O, 2 x MGD, 1 x MO | HHblits | 0.30 |
| ``` target    RPFLERFTDMPLLVRLDTLQRLRADEVFADYSSDLDVDGPSFTLHGMTEEQHERNGDRVVFDDASGALRAINREDVGDRL 7l5s.1    --------------------------------------------------------------------------------  target    DDKGIDPALDYQGTVTLVDGSTVEVMS-VLSMYREHL------ADYDIDSVVDMTGAPRNLIEQLLDDMTTLSPVAFHVG 7l5s.1    -----------------HDKDFLKKYTSGYAKFEEYLLGKTDGQPKTAEWAAKICGVPAETIKQLAADFAS-KRTMLMGG  target    EGVNHYFHATLHNRATYLVGMLLGSVGVSGGGVSTWAGNYKGGVFQAAPWFGPGVGGFVNEDPFHPLTDPSA-RYS-AET 7l5s.1    WGMQRQRHGEQTHWMLVTLASMLGQIGLPGGGFGLSYHYSNGGVPTATG-GII--GSITA----SPSGKAGAKTWLDDTS  target    ARHLVHGEDTSYWGFGDRPLVVDTP-EDGRKVFTGTTHMP-TPTKALWYNNANLINQAKWHYELVKNVNPKVDLIVDQQI 7l5s.1    KSAF-----PLARI-------ADVLLHPGKKIQYNGTEITYPDIKAVYWAGGNPFVHHQDTNTL-VKAFQKPDVVIVNEV  target    EWTGSAEFADIVLPANSWMEAETWEMGASCSNPFLQVWKGGIEPLNDTRDDIAIFAGVANALTELTGDERFSQAFMFADR 7l5s.1    NWTPTARMADIVLPATTSYERNDLTMAGDYSMMSVYPMKQVVPPQFEAKNDYDIFVELAKRAGVEE-------QYTEGKT  target    PEVYLDRVLAGSFT----T--EGYTVEDLTAGRYGPPGGALMQY-RSYPRIPFKEQI--EDSLPFYTDTGRMHGYVDIPE 7l5s.1    EMEWLEEFYNAAFSAARANRVAMPRFDKFWAENKPL---SFEAGEAAKKWVRYGEFREDPLLNPLGTPSGKIEIFSDVVE  target    AIEYGENLIVHREAVEATPYLPNVIVSTSPYLRPRDYGIAPEELDGDARSVRNIMMSWAEVKETENPLFAAGYNYLCLTP 7l5s.1    K--MNYN------D---CKGHP----------------------------------SWMEPEEFA-GNVTEEYPLALVTP  target    KSRHAVHSSWAVTDWHWLWSSSFSDPYRVETRAPGVGEPAIHLNPDDARSLGIRNGDYVWVDSNPKDRPYRDADVDESFL 7l5s.1    HPYYRLHSQLAHTSLR------------QKY--AVNDREPVMIHPEDAAARGIKDGDIVRIHSK----------------  target    DVARLLVRVTYNPAYPPGVTMLKHAFYMATPRTFRAAQERSDGRALAETTGYQSSFRSGSHQSITRGWAPPMHQTDSLFH 7l5s.1    -RGQVLAGAAVTENIIKGTVALHEGAWY----------------------------------------------------  target    KRAGVFGFTYGFDVDNHAINTVPKETVVRITKAEDGGVGGSGAWTRGRPGSMPGDEDDAMQAYLAGELTVVRRT 7l5s.1    -------------------------------------------------------------------------- ``` | | | | | | | | | | | | | | | | | | | | | | | | | | | | | | | | | | | | | | | | | | | | | | | | | |
|  | 1e18.1.A | DMSO REDUCTASE.  *TUNGSTEN-SUSBSTITUTED DMSO REDUCTASE FROM RHODOBACTER CAPSULATUS* | 0.30 | 0.00 | 22.12 | 0.57 | 97-648 | X-ray | 2.00 | monomer | 2 x PGD, 1 x 6WO | HHblits | 0.30 |
| ``` target    RPFLERFTDMPLLVRLDTLQRLRADEVFADYSSDLDVDGPSFTLHGMTEEQHERNGDRVVFDDASGALRAINREDVGDRL 1e18.1    --------------------------------------------------------------------------------  target    DDKGIDPALDYQGTVTLVDGSTVEVMS-VLSMYREHLA------DYDIDSVVDMTGAPRNLIEQLLDDMTTLSPVAFHVG 1e18.1    ----------------LYDKDFIANYTSGFDKFLPYLDGETDSTPKTAEWAEGISGVPAETIKELARLFES-KRTMLAAG  target    EGVNHYFHATLHNRATYLVGMLLGSVGVSGGGVSTWAGNYKGGVFQAAPWFGPGVGGFVNEDPFHPLTDPSARYSAETAR 1e18.1    WSMQRMHHGEQAHWMLVTLASMLGQIGLPGGGFGLSYHYSGGGTPS---TSGPALAGITDG--GAATKGPEWL-------  target    HLVHGEDTSYWGFGDRPL--VVDTPEDGRKV--FTGTTHMPTPTKALWYNNANLINQAKWHYELVKNVNPKVDLIVDQQI 1e18.1    A--------ASGASVIPVARVVDMLENPGAEFDFNGTRSKFPDVKMAYWVGGNPFVHHQDRNR-MVKAWEKLETFVVHDF  target    EWTGSAEFADIVLPANSWMEAETWEMGASCSNPFLQVWKGGIEPLNDTRDDIAIFAGVANALTELTGDERFSQAFMFADR 1e18.1    QWTPTARHADIVLPATTSYERNDIETIGDYSNTGILAMKKIVEPLYEARSDYDIFAAVAERLGKGK---EF----TEGKD  target    PEVYLDRVLAGSF---TTEG---YTVEDLTAGRYGPPGG-ALMQYRSYPRIPFKEQIEDSLPFYTDTGRMHGYVDIPEAI 1e18.1    EMGWIKSFYDDAAKQGKAAGVEMPAFDAFWAEGIVEFPVTDGADFVRY--ASFREDP-LLNPLGTPTGLIEIYSKNIEKM  target    EYGENLIVHREAVEATPYLPNVIVSTSPYLRPRDYGIAPEELDGDARSVRNIMMSWAEVKETENPLFAAGYNYLCLTPKS 1e18.1    GYD-----------DCPAH----------------------------------PTWMEPLERLD-GPGAKYPLHIAASHP  target    RHAVHSSWAVTDWHWLWSSSFSDPYRVETRAPGVGEPAIHLNPDDARSLGIRNGDYVWVDSNPKDRPYRDADVDESFLDV 1e18.1    FNRLHSQLNG-T--VLRE----------GY-AVQGHEPCLMHPDDAAARGIADGDVVRVHND-----------------R  target    ARLLVRVTYNPAYPPGVTMLKHAFYMATPRTFRAAQERSDGRALAETTGYQSSFRSGSHQSITRGWAPPMHQTDSLFHKR 1e18.1    GQILTGVKVTDAVMKGVIQIYEGGWY------------------------------------------------------  target    AGVFGFTYGFDVDNHAINTVPKETVVRITKAEDGGVGGSGAWTRGRPGSMPGDEDDAMQAYLAGELTVVRRT 1e18.1    ------------------------------------------------------------------------ ``` | | | | | | | | | | | | | | | | | | | | | | | | | | | | | | | | | | | | | | | | | | | | | | | | | |
|  | 1e60.1.A | Dimethyl sulfoxide/trimethylamine N-oxide reductase  *OXIDIZED DMSO REDUCTASE EXPOSED TO HEPES - Structure II BUFFER* | 0.30 | 0.00 | 21.95 | 0.57 | 98-648 | X-ray | 2.00 | monomer | 2 x PGD, 1 x 2MO | HHblits | 0.30 |
| ``` target    RPFLERFTDMPLLVRLDTLQRLRADEVFADYSSDLDVDGPSFTLHGMTEEQHERNGDRVVFDDASGALRAINREDVGDRL 1e60.1    --------------------------------------------------------------------------------  target    DDKGIDPALDYQGTVTLVDGSTVEVMS-VLSMYREHLA------DYDIDSVVDMTGAPRNLIEQLLDDMTTLSPVAFHVG 1e60.1    -----------------YDKDFIANYTSGFDKFLPYLDGETDSTPKTAEWAEGISGVPAETIKELARLFES-KRTMLAAG  target    EGVNHYFHATLHNRATYLVGMLLGSVGVSGGGVSTWAGNYKGGVFQAAPWFGPGVGGFVNEDPFHPLTDPSARYSAETAR 1e60.1    WSMQRMHHGEQAHWMLVTLASMLGQIGLPGGGFGLSYHYSGGGTPS---TSGPALAGITDG--GAATKGPEWL-------  target    HLVHGEDTSYWGFGDRPL--VVDTPEDGRKV--FTGTTHMPTPTKALWYNNANLINQAKWHYELVKNVNPKVDLIVDQQI 1e60.1    --A------ASGASVIPVARVVDMLENPGAEFDFNGTRSKFPDVKMAYWVGGNPFVHHQDRNR-MVKAWEKLETFVVHDF  target    EWTGSAEFADIVLPANSWMEAETWEMGASCSNPFLQVWKGGIEPLNDTRDDIAIFAGVANALTELTGDERFSQAFMFADR 1e60.1    QWTPTARHADIVLPATTSYERNDIETIGDYSNTGILAMKKIVEPLYEARSDYDIFAAVAERLGKGA---EFT----EGKD  target    PEVYLDRVLAGSF---TTEGY---TVEDLTAGRYGPPGGA-LMQYRSYPRIPFKEQIEDSLPFYTDTGRMHGYVDIPEAI 1e60.1    EMGWIKSFYDDAAKQGKAAGVEMPAFDAFWAEGIVEFPVTDGADFVRY--ASFRED-PLLNPLGTPTGLIEIYSKNIEKM  target    EYGENLIVHREAVEATPYLPNVIVSTSPYLRPRDYGIAPEELDGDARSVRNIMMSWAEVKETENPLFAAGYNYLCLTPKS 1e60.1    GYD-----------DCPAH----------------------------------PTWMEPLERL-DGPGAKYPLHIAASHP  target    RHAVHSSWAVTDWHWLWSSSFSDPYRVETRAPGVGEPAIHLNPDDARSLGIRNGDYVWVDSNPKDRPYRDADVDESFLDV 1e60.1    FNRLHSQLNG-T--VLRE----------GY-AVQGHEPCLMHPDDAAARGIADGDVVRVHND-----------------R  target    ARLLVRVTYNPAYPPGVTMLKHAFYMATPRTFRAAQERSDGRALAETTGYQSSFRSGSHQSITRGWAPPMHQTDSLFHKR 1e60.1    GQILTGVKVTDAVMKGVIQIYEGGWY------------------------------------------------------  target    AGVFGFTYGFDVDNHAINTVPKETVVRITKAEDGGVGGSGAWTRGRPGSMPGDEDDAMQAYLAGELTVVRRT 1e60.1    ------------------------------------------------------------------------ ``` | | | | | | | | | | | | | | | | | | | | | | | | | | | | | | | | | | | | | | | | | | | | | | | | | |
|  | 1eu1.1.A | DIMETHYL SULFOXIDE REDUCTASE  *THE CRYSTAL STRUCTURE OF RHODOBACTER SPHAEROIDES DIMETHYLSULFOXIDE REDUCTASE REVEALS TWO DISTINCT MOLYBDENUM COORDINATION ENVIRONMENTS.* | 0.30 |  | 21.14 | 0.57 | 98-647 | X-ray | 1.30 | monomer | 3 x GLC, 1 x CD, 2 x MGD, 1 x 6MO, 2 x O | HHblits | 0.30 |
| ``` target    RPFLERFTDMPLLVRLDTLQRLRADEVFADYSSDLDVDGPSFTLHGMTEEQHERNGDRVVFDDASGALRAINREDVGDRL 1eu1.1    --------------------------------------------------------------------------------  target    DDKGIDPALDYQGTVTLVDGSTVEVM-SVLSMYREHLA------DYDIDSVVDMTGAPRNLIEQLLDDMTTLSPVAFHVG 1eu1.1    -----------------HDKDFLENCTTGFDLFAAYLTGESDGTPKTAEWAAEICGLPAEQIRELARSFVAG-RTMLAAG  target    EGVNHYFHATLHNRATYLVGMLLGSVGVSGGGVSTWAGNYKGGVFQAAPWFGPGVGGFVNEDPFHPLTDPSARYSAETAR 1eu1.1    WSIQRMHHGEQAHWMLVTLASMIGQIGLPGGGFGLSYHYSNGGS-PTSD--GPALGGISDG--GKAVEGA--A-------  target    HLVHGEDTSYWGFGDRP--LVVDTPEDGRKV--FTGTTHMPTPTKALWYNNANLINQAKWHYELVKNVNPKVDLIVDQQI 1eu1.1    WL------SESGATSIPCARVVDMLLNPGGEFQFNGATATYPDVKLAYWAGGNPFAHHQDRNRM-LKAWEKLETFIVQDF  target    EWTGSAEFADIVLPANSWMEAETWEMGASCSNPFLQVWKGGIEPLNDTRDDIAIFAGVANALTELTGDERFSQAFMFADR 1eu1.1    QWTATARHADIVLPATTSYERNDIESVGDYSNRAILAMKKVVDPLYEARSDYDIFAALAERLGKGA---EFT----EGRD  target    PEVYLDRVLAGSF------TTEGYTVEDLTAGRYGPPGGALMQYRSYPRIPFKEQIE--DSLPFYTDTGRMHGYVDIPEA 1eu1.1    EMGWISSFYEAAVKQAEFKNVAMPSFEDFWSEGIVEFP----ITEGANFVRYADFREDPLFNPLGTPSGLIEIYSKNIEK  target    IEYGENLIVHREAVEATPYLPNVIVSTSPYLRPRDYGIAPEELDGDARSVRNIMMSWAEVKETENPLFAAGYNYLCLTPK 1eu1.1    MGYD-----------DCPAH----------------------------------PTWMEPAERL-GGAGAKYPLHVVASH  target    SRHAVHSSWAVTDWHWLWSSSFSDPYRVETRAPGVGEPAIHLNPDDARSLGIRNGDYVWVDSNPKDRPYRDADVDESFLD 1eu1.1    PKSRLHSQLNGTSLRDLY--------------AVAGHEPCLINPADAAARGIADGDVLRVFND-----------------  target    VARLLVRVTYNPAYPPGVTMLKHAFYMATPRTFRAAQERSDGRALAETTGYQSSFRSGSHQSITRGWAPPMHQTDSLFHK 1eu1.1    RGQILVGAKVSDAVMPGAIQIYEGGW------------------------------------------------------  target    RAGVFGFTYGFDVDNHAINTVPKETVVRITKAEDGGVGGSGAWTRGRPGSMPGDEDDAMQAYLAGELTVVRRT 1eu1.1    ------------------------------------------------------------------------- ``` | | | | | | | | | | | | | | | | | | | | | | | | | | | | | | | | | | | | | | | | | | | | | | | | | |
|  | 1e5v.2.A | Dimethyl sulfoxide/trimethylamine N-oxide reductase  *OXIDIZED DMSO REDUCTASE EXPOSED TO HEPES BUFFER* | 0.30 | 0.00 | 21.95 | 0.57 | 98-648 | X-ray | 2.40 | monomer | 2 x PGD, 1 x 2MO | HHblits | 0.30 |
| ``` target    RPFLERFTDMPLLVRLDTLQRLRADEVFADYSSDLDVDGPSFTLHGMTEEQHERNGDRVVFDDASGALRAINREDVGDRL 1e5v.2    --------------------------------------------------------------------------------  target    DDKGIDPALDYQGTVTLVDGSTVEVMS-VLSMYREHLA------DYDIDSVVDMTGAPRNLIEQLLDDMTTLSPVAFHVG 1e5v.2    -----------------YDKDFIANYTSGFDKFLPYLDGETDSTPKTAEWAEGISGVPAETIKELARLFES-KRTMLAAG  target    EGVNHYFHATLHNRATYLVGMLLGSVGVSGGGVSTWAGNYKGGVFQAAPWFGPGVGGFVNEDPFHPLTDPSARYSAETAR 1e5v.2    WSMQRMHHGEQAHWMLVTLASMLGQIGLPGGGFGLSYHYSGGGTPS---TSGPALAGITDG--GAATKGPEWL-------  target    HLVHGEDTSYWGFGDRP--LVVDTPEDGRKVF--TGTTHMPTPTKALWYNNANLINQAKWHYELVKNVNPKVDLIVDQQI 1e5v.2    --------AASGASVIPVARVVDMLENPGAEFDFNGTRSKFPDVKMAYWVGGNPFVHHQDRNR-MVKAWEKLETFVVHDF  target    EWTGSAEFADIVLPANSWMEAETWEMGASCSNPFLQVWKGGIEPLNDTRDDIAIFAGVANALTELTGDERFSQAFMFADR 1e5v.2    QWTPTARHADIVLPATTSYERNDIETIGDYSNTGILAMKKIVEPLYEARSDYDIFAAVAERLGKGA---EFT----EGKD  target    PEVYLDRVLAGSF---TTEGY---TVEDLTAGRYGPPGGA-LMQYRSYPRIPFKEQIEDSLPFYTDTGRMHGYVDIPEAI 1e5v.2    EMGWIKSFYDDAAKQGKAAGVQMPAFDAFWAEGIVEFPVTDGADFVRY--ASFRED-PLLNPLGTPTGLIEIYSKNIEKM  target    EYGENLIVHREAVEATPYLPNVIVSTSPYLRPRDYGIAPEELDGDARSVRNIMMSWAEVKETENPLFAAGYNYLCLTPKS 1e5v.2    GYD-----------DCPAH----------------------------------PTWMEPLERL-DGPGAKYPLHIAASHP  target    RHAVHSSWAVTDWHWLWSSSFSDPYRVETRAPGVGEPAIHLNPDDARSLGIRNGDYVWVDSNPKDRPYRDADVDESFLDV 1e5v.2    FNRLHSQLNG---TVLRE----------GY-AVQGHEPCLMHPDDAAARGIADGDVVRVHND-----------------R  target    ARLLVRVTYNPAYPPGVTMLKHAFYMATPRTFRAAQERSDGRALAETTGYQSSFRSGSHQSITRGWAPPMHQTDSLFHKR 1e5v.2    GQILTGVKVTDAVMKGVIQIYEGGWY------------------------------------------------------  target    AGVFGFTYGFDVDNHAINTVPKETVVRITKAEDGGVGGSGAWTRGRPGSMPGDEDDAMQAYLAGELTVVRRT 1e5v.2    ------------------------------------------------------------------------ ``` | | | | | | | | | | | | | | | | | | | | | | | | | | | | | | | | | | | | | | | | | | | | | | | | | |
|  | 6cz7.1.A | ArrA  *The arsenate respiratory reductase (Arr) complex from Shewanella sp. ANA-3* | 0.28 |  | 16.22 | 0.58 | 109-647 | X-ray | 1.62 | hetero-1-1-mer | 5 x SF4, 2 x MGD, 1 x MO, 1 x PG5 | HHblits | 0.28 |
| ``` target    RPFLERFTDMPLLVRLDTLQRLRADEVFADYSSDLDVDGPSFTLHGMTEEQHERNGDRVVFDDASGALRAINREDVGDRL 6cz7.1    --------------------------------------------------------------------------------  target    DDKGIDPALDYQGTVTLVDGSTVEVMSVLSMYREHLADYDIDSVVDMTGAPRNLIEQLLDDMTTLSPVA-FHVGEGVNHY 6cz7.1    ----------------------------VEWWNQALKDYTPEWASKITGIDPKTIIAIAKDMGAAAPAVQVWTSRGAVMQ  target    FHATLHNRATYLVGMLLGSVGVSGGGVSTWAGNYKGGVFQAAPWFGPGVGGFVNEDPFHPLTDPSARYSAETARHLVHGE 6cz7.1    ARGTYTSISCHALNGLFGGIDSKGGLFPGNKTPL----LKE----YPEAKAYMDEIAAKGVK-K-EKI-----DQRGRLE  target    DTSYWGFG-DRPLVVDTPEDGRKVFTGTTHMPTPTKALWYNNANLINQAKWHYELVKNVNPKVDLIVDQQIEWTGSAEFA 6cz7.1    FPALAKGKSGGGVITANAANG-----IRNQDPYEIKVMLAYFNNFNFSNPEGQR-WDEALSKVDFMAHITTNVSEFSWFA  target    DIVLPANS-WMEAETWEMGASCSNPFLQVWKGGIEPLNDTRDD-IAIFAGVANALTELTGDERFSQAFMFA--------- 6cz7.1    DVLLPSSHHMFEKWGVLDSIGNGVAQISIQQPSIKRLWDTRIDESEIPYMLAKKLADK----GFDAPWRYINEQIVDPET  target    ----DRPEVYLDRVLAGSF-----------TTEGYTVEDLTAGRYGPPGGALMQYRSYPRIPFKEQIEDSLPFYTDTGRM 6cz7.1    GKPAADEAEFAKLMVRYLTAPLWKEDASKYGDKLSSWDEFVQKGVWNS-------SPYK-LE-----ARWGKFKTETTKF  target    HGYVDIPEAIEYGENLIVHREAVEATPYLPNVIVSTSPYLRPRDYGIAPEELDGDARSVRNIMMSWAEVKETENPLFAAG 6cz7.1    EFYSKTLEKA-----LQSHADK---HKVSIDE------VMKACDYQ---------ARGHLAFIPHYEEPYRFG---DESE  target    YNYLCLTPKSRHAVHSSWAVTDWHWLWSSSFSDPYRVETRAPGVGEPAIHLNPDDARSLGIRNGDYVWVDSNPKDRPYRD 6cz7.1    FPLLLVDQKSRLNKEGRTANSPW--YYEF--KDVDP----GDVANEDVAKFNPIDGKKFGLKDGDEIRITSP--------  target    ADVDESFLDVARLLVRVTYNPAYPPGVTMLKHAFYMATPRTFRAAQERSDGRALAETTGYQSSFRSGSHQSITRGWAPPM 6cz7.1    ---------VGMLTCKAKLWEGVRPGTVAKCFGQG---------------------------------------------  target    HQTDSLFHKRAGVFGFTYGFDVDNHAINTVPKETVVRITKAEDGGVGGSGAWTRGRPGSMPGDEDDAMQAYLAGELTVVR 6cz7.1    --------------------------------------------------------------------------------  target    RT 6cz7.1    -- ``` | | | | | | | | | | | | | | | | | | | | | | | | | | | | | | | | | | | | | | | | | | | | | | | | | |
|  | 2e7z.1.A | Acetylene hydratase Ahy  *Acetylene Hydratase from Pelobacter acetylenicus* | 0.29 |  | 18.22 | 0.57 | 99-650 | X-ray | 1.26 | monomer | 1 x SF4, 2 x MGD, 1 x W | HHblits | 0.29 |
| ``` target    RPFLERFTDMPLLVRLDTLQRLRADEVFADYSSDLDVDGPSFTLHGMTEEQHERNGDRVVFDDASGALRAINREDVGDRL 2e7z.1    --------------------------------------------------------------------------------  target    DDKGIDPALDYQGTVTLVDGSTVEVM-SVLSMYREHLADYDIDSVVDMTGAPRNLIEQLLDDMTTLSPVAFHVGEGVNHY 2e7z.1    ------------------DKEFVENWCVGFEELKERVQEYPLDKVAEITGCDAGEIRKAAVMFATESPASIPWAVSTDMQ  target    FHATLHNRATYLVGMLLGSVGVSGGGVSTWAGNYKGGVFQAA-PWFGPGV-GGFVNEDPFHPLTDPSARYSAETARHLVH 2e7z.1    KNSCSAIRAQCILRAIVGSFVN-GAEILGAPHSDLVPISKIQMHEALPEEKKKLQLGTETYPFLTYTGMS--ALEEP---  target    GEDTSYWGFGDRPLVVDTPEDGRKVFT-GTTHMPTPTKALWYNNANLINQAKWHYELVKNVNPKVDLIVDQQIEWTGSAE 2e7z.1    --SERVYGVKYFHNMGAFMANPTALFTAMATEKPYPVKAFFALASNALMGYANQQN-ALKGLMNQDLVVCYDQFMTPTAQ  target    FADIVLPANSWMEAETWEMGASCSNPFLQVWKGGIEPLNDTRDDIAIFAGVANALTELTGDERFSQAFMFADRPEVYLDR 2e7z.1    LADYVLPGDHWLERPVVQPNW-EGIPFGNTSQQVVEPAGEAKDEYYFIRELAVRMGLE-------EHFPW-KDRLELINY  target    VLAGSFTTEGYTVEDLTAGRYGPPGGALMQYRSYPRIPFKEQIEDSLPFYTDTGRMHGYVDIPEAIEYGENLIVHREAVE 2e7z.1    RISP----TGMEWEEYQKQYTYM-----SKLPDY--FG-----PEGVGVATPSGKVELYSSVFEKLGY-DPLPYYHEPLQ  target    ATPYLPNVIVSTSPYLRPRDYGIAPEELDGDARSVRNIMMSWAEVKETENPLFAAGYNYLCLTPKSR-HAVHSSWAVTDW 2e7z.1    T---------------------------------------------EISDPELAKEYPLILFAGLREDSNFQSCYHQPGI  target    HWLWSSSFSDPYRVETRAPGVGEPAIHLNPDDARSLGIRNGDYVWVDSNPKDRPYRDADVDESFLDVARLLVRVTYNPAY 2e7z.1    --L----------R----DAEPDPVALLHPKTAQSLGLPSGEWIWVETT-----------------HGRLKLLLKHDGAQ  target    PPGVTMLKHAFYMATPRTFRAAQERSDGRALAETTGYQSSFRSGSHQSITRGWAPPMHQTDSLFHKRAGVFGFTYGFDVD 2e7z.1    PEGTIRIPHGRWCPE-----------------------------------------------------------------  target    NHAINTVPKETVVRITKAEDGGVGGSGAWTRGRPGSMPGDEDDAMQAYLAGELTVVRRT 2e7z.1    ----------------------------------------------------------- ``` | | | | | | | | | | | | | | | | | | | | | | | | | | | | | | | | | | | | | | | | | | | | | | | | | |
|  | 1ogy.1.A | PERIPLASMIC NITRATE REDUCTASE  *Crystal structure of the heterodimeric nitrate reductase from Rhodobacter sphaeroides* | 0.26 |  | 19.40 | 0.56 | 103-645 | X-ray | 3.20 | hetero-1-1-mer | 1 x SF4, 1 x MO, 2 x MGD, 2 x HEC | HHblits | 0.29 |
| ``` target    RPFLERFTDMPLLVRLDTLQRLRADEVFADYSSDLDVDGPSFTLHGMTEEQHERNGDRVVFDDASGALRAINREDVGDRL 1ogy.1    --------------------------------------------------------------------------------  target    DDKGIDPALDYQGTVTLVDGSTVEVMSVLSMYREHLADYDIDSVVDMTGAPRNLIEQLLDDMTTLSP-VAFHVGEGVNHY 1ogy.1    ----------------------AMTPTDFETFAALVSEYTLEKAAEISGVEPALLEELAELYADPDRKWMSLWTMGFNQH  target    FHATLHNRATYLVGMLLGSVGVSGGGVSTWAGNYKGGVFQAAPWFGPGVGGFVNEDPFH-PLTDPSARYSAETARHLVHG 1ogy.1    VRGVWANHMVYNLHLLTGKISEPGNSPFSLTGQPFAC-GTARE-----VGTFAHRLPADMVVTNPEHRAHAEE-------  target    EDTSYWGFGDRPLVVDTP-EDGRKVFTGTTHMPTPTKALWYNNANLINQAKWHY-ELVKNVNPKVDLIVDQQIEWTGSAE 1ogy.1    ----IWKLPA-GLLPDWVGAHAVEQD--RKLHDGEINFYWVQVNNNMQAAPNIDQETYPGYRNPENFIVVSDAYPTVTGR  target    FADIVLPANSWMEAETWEMGASCSNPFLQVWKGGIEPLNDTRDDIAIFAGVANALTELTGDE-RFSQAFMFADRPEVYLD 1ogy.1    AADLVLPAAMWVEKEGAY---GNAERRTHFWHQLVEAPGEARSDLWQLMEFSKRFTTDEVWPEEILSAAP-AYRGKTLFE  target    RVLAGSFTTEGY------------------------TVEDLTAGRYGPPGGALMQYRSY--------PR----IPFKEQI 1ogy.1    VLFANG-SVDRFPASDVNPDHANHEAALFGFYPQKGLFEEYAAFGRGH-GHDLAPFDTYHEVRGLHWPVVEGEETRWRYR  target    EDSLPFYTDTGRMHGYVDIPEAIEYGENLIVHREAVEATPYLPNVIVSTSPYLRPRDYGIAPEELDGDARSVRNIMMSWA 1ogy.1    EGFDPYVKPGEGLRFYGKPDGRAVI------L-----GVP-----------Y---------------------------E  target    EVKETENPLFAAGYNYLCLTPKSRHAVHSSWAVTDWHWLWSSSFSDPYRVETRAPGVGEPAIHLNPDDARSLGIRNGDYV 1ogy.1    PPAE--SP--DEEFGFWLVTGRVLEHWHSGSMTLRWPELYK--------------AFPGAVCFMHPEDARSRGLNRGSEV  target    WVDSNPKDRPYRDADVDESFLDVARLLVRVTY--NPAYPPGVTMLKHAFYMATPRTFRAAQERSDGRALAETTGYQSSFR 1ogy.1    RVISR-----------------RGEIRTRLETRGRNRMPRGVVFVPWF--------------------------------  target    SGSHQSITRGWAPPMHQTDSLFHKRAGVFGFTYGFDVDNHAINTVPKETVVRITKAEDGGVGGSGAWTRGRPGSMPGDED 1ogy.1    --------------------------------------------------------------------------------  target    DAMQAYLAGELTVVRRT 1ogy.1    ----------------- ``` | | | | | | | | | | | | | | | | | | | | | | | | | | | | | | | | | | | | | | | | | | | | | | | | | |
|  | 7qv7.1.L | Hydrogen dependent carbon dioxide reductase subunit FdhF  *Cryo-EM structure of Hydrogen-dependent CO2 reductase.* | 0.24 |  | 17.82 | 0.56 | 99-647 | EM | 0.00 | hetero-2-6-6-2-mer | 52 x SF4, 6 x 402 | HHblits | 0.28 |
| ``` target    RPFLERFTDMPLLVRLDTLQRLRADEVFADYSSDLDVDGPSFTLHGMTEEQHERNGDRVVFDDASGALRAINREDVGDRL 7qv7.1    --------------------------------------------------------------------------------  target    DDKGIDPALDYQGTVTLVDGSTVEVM-SVLSMYREHLADYDIDSVVDMTGAPRNLIEQLLDDMTTLSPVAFHVGEGVNHY 7qv7.1    ------------------KTDFVKNHAVGFEEMAKAVEKYTPEYVEELTGIPKKDLIKAARFYGQAQAAAILYSMGVTQF  target    FHATLHNRATYLVGMLLGSVGVSGGGVSTWAGNYKG-GVF--QAAPWFGPGVGGFVNEDPFHPLTDPSARYSAETARHLV 7qv7.1    SHGTGNVVSLANLAVITGNLGRPGAGICPLRGQNNVQGACDVGALPNVLPG---------YLDVTKEQNR------ER--  target    HGEDTSYWGFGDRPLVVDTPEDGRKVFTGTTHMPTPTKALWYNNANLINQAKWHYELVKNVNPKVDLIVDQQIEWTGSAE 7qv7.1    ---FEKVWGVKL-PS--NIGLRVTEVPD--AILNKRVRALYIFGENPIMSDPDSDH-LRHALEHLDLLIVQDIFLTETAR  target    FADIVLPANSWMEAETWEMGASCSNPFLQVWKGGIEPLNDTRDDIAIFAGVANALTELTGDERFSQAFMFADRPEVYLDR 7qv7.1    LAHVVLPAACWAEKDGTF---TNTERRVQRVRKAVEAPGEAKPDWWIFSQIAERMGYT----GM----QY-NNVQEIWDE  target    VLAGSF-TTEGYTVEDLTAGRYGPPGGALMQYRSYPRIPFKEQIEDSLPFYTDTGRMHGYVDIPEAIEYGENLIVHREAV 7qv7.1    VRKIVPEKFGGISYARLEKEKGLA------WPCPTEDHTGTPILYLGGKFATPSGKAQMYPVIFYPN-----TCICDEG-  target    EATPYLPNVIVSTSPYLRPRDYGIAPEELDGDARSVRNIMMSWAEVKETENPLFAAGYNYLCLTPKSRHAVHSSWAVTDW 7qv7.1    ---AEKQD--------FNH-----VI----------------VG--SI--AELPDEEYPFTLTTGRRVYHYHTATMTRKS  target    HWLWSSSFSDPYRVETRAPGVGEPAIHLNPDDARSLGIRNGDYVWVDSNPKDRPYRDADVDESFLDVARLLVRVTYNPAY 7qv7.1    PVID----------Q----IAPQELVEINPQDATRLGINDGDFLRVSTR-----------------RGYVATRAWVTERV  target    PPGVTMLKHAFYMATPRTFRAAQERSDGRALAETTGYQSSFRSGSHQSITRGWAPPMHQTDSLFHKRAGVFGFTYGFDVD 7qv7.1    PKGTIFMTFHYW--------------------------------------------------------------------  target    NHAINTVPKETVVRITKAEDGGVGGSGAWTRGRPGSMPGDEDDAMQAYLAGELTVVRRT 7qv7.1    ----------------------------------------------------------- ``` | | | | | | | | | | | | | | | | | | | | | | | | | | | | | | | | | | | | | | | | | | | | | | | | | |
|  | 7qv7.1.O | Hydrogen dependent carbon dioxide reductase subunit FdhF  *Cryo-EM structure of Hydrogen-dependent CO2 reductase.* | 0.23 |  | 17.82 | 0.56 | 99-647 | EM | 0.00 | hetero-2-6-6-2-mer | 52 x SF4, 6 x 402 | HHblits | 0.28 |
| ``` target    RPFLERFTDMPLLVRLDTLQRLRADEVFADYSSDLDVDGPSFTLHGMTEEQHERNGDRVVFDDASGALRAINREDVGDRL 7qv7.1    --------------------------------------------------------------------------------  target    DDKGIDPALDYQGTVTLVDGSTVEVM-SVLSMYREHLADYDIDSVVDMTGAPRNLIEQLLDDMTTLSPVAFHVGEGVNHY 7qv7.1    ------------------KTDFVKNHAVGFEEMAKAVEKYTPEYVEELTGIPKKDLIKAARFYGQAQAAAILYSMGVTQF  target    FHATLHNRATYLVGMLLGSVGVSGGGVSTWAGNYKG-GVF--QAAPWFGPGVGGFVNEDPFHPLTDPSARYSAETARHLV 7qv7.1    SHGTGNVVSLANLAVITGNLGRPGAGICPLRGQNNVQGACDVGALPNVLPG---------YLDVTKEQNR------ER--  target    HGEDTSYWGFGDRPLVVDTPEDGRKVFTGTTHMPTPTKALWYNNANLINQAKWHYELVKNVNPKVDLIVDQQIEWTGSAE 7qv7.1    ---FEKVWGVKL-PS--NIGLRVTEVPD--AILNKRVRALYIFGENPIMSDPDSDH-LRHALEHLDLLIVQDIFLTETAR  target    FADIVLPANSWMEAETWEMGASCSNPFLQVWKGGIEPLNDTRDDIAIFAGVANALTELTGDERFSQAFMFADRPEVYLDR 7qv7.1    LAHVVLPAACWAEKDGTF---TNTERRVQRVRKAVEAPGEAKPDWWIFSQIAERMGYT----GM----QY-NNVQEIWDE  target    VLAGSF-TTEGYTVEDLTAGRYGPPGGALMQYRSYPRIPFKEQIEDSLPFYTDTGRMHGYVDIPEAIEYGENLIVHREAV 7qv7.1    VRKIVPEKFGGISYARLEKEKGLA------WPCPTEDHTGTPILYLGGKFATPSGKAQMYPVIFYPN-----TCICDEG-  target    EATPYLPNVIVSTSPYLRPRDYGIAPEELDGDARSVRNIMMSWAEVKETENPLFAAGYNYLCLTPKSRHAVHSSWAVTDW 7qv7.1    ---AEKQD--------FNH-----VI----------------VG--SI--AELPDEEYPFTLTTGRRVYHYHTATMTRKS  target    HWLWSSSFSDPYRVETRAPGVGEPAIHLNPDDARSLGIRNGDYVWVDSNPKDRPYRDADVDESFLDVARLLVRVTYNPAY 7qv7.1    PVID----------Q----IAPQELVEINPQDATRLGINDGDFLRVSTR-----------------RGYVATRAWVTERV  target    PPGVTMLKHAFYMATPRTFRAAQERSDGRALAETTGYQSSFRSGSHQSITRGWAPPMHQTDSLFHKRAGVFGFTYGFDVD 7qv7.1    PKGTIFMTFHYW--------------------------------------------------------------------  target    NHAINTVPKETVVRITKAEDGGVGGSGAWTRGRPGSMPGDEDDAMQAYLAGELTVVRRT 7qv7.1    ----------------------------------------------------------- ``` | | | | | | | | | | | | | | | | | | | | | | | | | | | | | | | | | | | | | | | | | | | | | | | | | |
|  | 2vpz.1.A | THIOSULFATE REDUCTASE  *POLYSULFIDE REDUCTASE NATIVE STRUCTURE* | 0.28 |  | 21.51 | 0.55 | 99-648 | X-ray | 2.40 | hetero-oligomer | 10 x SF4, 4 x MGD, 2 x MO | HHblits | 0.29 |
| ``` target    RPFLERFTDMPLLVRLDTLQRLRADEVFADYSSDLDVDGPSFTLHGMTEEQHERNGDRVVFDDASGALRAINREDVGDRL 2vpz.1    --------------------------------------------------------------------------------  target    DDKGIDPALDYQGTVTLVDGSTVEV-MSVLSMYREHLADYDIDSVVDMTGAPRNLIEQLLDDMTTLSPVAFHVGEGVN-H 2vpz.1    ------------------DKEYVAKYTVGFEELKAHVKDFTPEWAEKHTEIPAQVIREVAREMAAHKPRAVLPPTRHNVW  target    YFHATLHNRATYLVGMLLGSVGVSGGGVSTWAGNYKGGVFQAAPWFGPGVGGFVNEDPFHPLTDPSARYSAETARHLVHG 2vpz.1    YGDDTYRVMALLYVNVLLGNYGRPGGFYIAQSPYLEKYPLPPLP-LEPAAGGC-SGPSGGDHEPEGFKPRADK------G  target    EDTSYWGFGDRPLVVDTPEDGRKVFTGTTHMPTPTKALWYNNANLINQAKWHYELVKNVNPKVDLIVDQQIEWTGSAEFA 2vpz.1    ---KFFA---RSTAIQEL--IE---PMITGEPYPIKGLFAYGINLFHSIPNVPR-TKEALKNLDLYVAIDVLPQEHVMWA  target    DIVLPANSWMEAETWEMGASCSNPFLQVWKGGIEPLNDTRDDIAIFAGVANALTELTGDERFSQAFMFADRPEVYLDRVL 2vpz.1    DVILPEATYLERYDDFVLVAHKTPFIQLRTPAHEPLFDTKPGWWIARELGLRLGLE-------QYFPW-KTIEEYLETRL  target    AGSFTTEGYTVEDLTAGRYGPPGGALMQYRSYPRIPFKEQIEDSLPFYTDTGRMHGYVDIPEAIEYGENLIVHREAVEAT 2vpz.1    QS----LGLDLETMKGMGTLVQRG-KPWLE-----DWE--KEGRLPFGTASGKIELYCQRFKEAGH-QPLPV--------  target    PYLPNVIVSTSPYLRPRDYGIAPEELDGDARSVRNIMMSWAEVKETENPLFAAGYNYLCLTPKSRHAVHSSWAVTDWHWL 2vpz.1    ---------------------------------------FTPPEE------PPEGFYRLLYGRSPVHTFARTQNNWV--L  target    WSSSFSDPYRVETRAPGVGEPAIHLNPDDARSLGIRNGDYVWVDSNPKDRPYRDADVDESFLDVARLL--VRVTYNPAYP 2vpz.1    ----------ME----MDPENEVWIHKEEAKRLGLKEGDYVMLVNQ-----------------DGVKEGPVRVKPTARIR  target    PGVTMLKHAFYMATPRTFRAAQERSDGRALAETTGYQSSFRSGSHQSITRGWAPPMHQTDSLFHKRAGVFGFTYGFDVDN 2vpz.1    KDCVYIVHGFGH--------------------------------------------------------------------  target    HAINTVPKETVVRITKAEDGGVGGSGAWTRGRPGSMPGDEDDAMQAYLAGELTVVRRT 2vpz.1    ---------------------------------------------------------- ``` | | | | | | | | | | | | | | | | | | | | | | | | | | | | | | | | | | | | | | | | | | | | | | | | | |
|  | 2vpx.1.D | THIOSULFATE REDUCTASE  *POLYSULFIDE REDUCTASE WITH BOUND QUINONE (UQ1)* | 0.28 |  | 21.51 | 0.55 | 99-648 | X-ray | 3.10 | hetero-oligomer | 10 x SF4, 4 x MGD, 2 x MO, 2 x UQ1 | HHblits | 0.29 |
| ``` target    RPFLERFTDMPLLVRLDTLQRLRADEVFADYSSDLDVDGPSFTLHGMTEEQHERNGDRVVFDDASGALRAINREDVGDRL 2vpx.1    --------------------------------------------------------------------------------  target    DDKGIDPALDYQGTVTLVDGSTVEV-MSVLSMYREHLADYDIDSVVDMTGAPRNLIEQLLDDMTTLSPVAFHVGEGVN-H 2vpx.1    ------------------DKEYVAKYTVGFEELKAHVKDFTPEWAEKHTEIPAQVIREVAREMAAHKPRAVLPPTRHNVW  target    YFHATLHNRATYLVGMLLGSVGVSGGGVSTWAGNYKGGVFQAAPWFGPGVGGFVNEDPFHPLTDPSARYSAETARHLVHG 2vpx.1    YGDDTYRVMALLYVNVLLGNYGRPGGFYIAQSPYLEKYPLPPLP-LEPAAGGC-SGPSGGDHEPEGFKPRADK------G  target    EDTSYWGFGDRPLVVDTPEDGRKVFTGTTHMPTPTKALWYNNANLINQAKWHYELVKNVNPKVDLIVDQQIEWTGSAEFA 2vpx.1    ---KFFA---RSTAIQEL--IE---PMITGEPYPIKGLFAYGINLFHSIPNVPR-TKEALKNLDLYVAIDVLPQEHVMWA  target    DIVLPANSWMEAETWEMGASCSNPFLQVWKGGIEPLNDTRDDIAIFAGVANALTELTGDERFSQAFMFADRPEVYLDRVL 2vpx.1    DVILPEATYLERYDDFVLVAHKTPFIQLRTPAHEPLFDTKPGWWIARELGLRLGLE-------QYFPW-KTIEEYLETRL  target    AGSFTTEGYTVEDLTAGRYGPPGGALMQYRSYPRIPFKEQIEDSLPFYTDTGRMHGYVDIPEAIEYGENLIVHREAVEAT 2vpx.1    QS----LGLDLETMKGMGTLVQRG-KPWLE-----DWE--KEGRLPFGTASGKIELYCQRFKEAGH-QPLPV--------  target    PYLPNVIVSTSPYLRPRDYGIAPEELDGDARSVRNIMMSWAEVKETENPLFAAGYNYLCLTPKSRHAVHSSWAVTDWHWL 2vpx.1    ---------------------------------------FTPPEE------PPEGFYRLLYGRSPVHTFARTQNNWV--L  target    WSSSFSDPYRVETRAPGVGEPAIHLNPDDARSLGIRNGDYVWVDSNPKDRPYRDADVDESFLDVARLL--VRVTYNPAYP 2vpx.1    ----------ME----MDPENEVWIHKEEAKRLGLKEGDYVMLVNQ-----------------DGVKEGPVRVKPTARIR  target    PGVTMLKHAFYMATPRTFRAAQERSDGRALAETTGYQSSFRSGSHQSITRGWAPPMHQTDSLFHKRAGVFGFTYGFDVDN 2vpx.1    KDCVYIVHGFGH--------------------------------------------------------------------  target    HAINTVPKETVVRITKAEDGGVGGSGAWTRGRPGSMPGDEDDAMQAYLAGELTVVRRT 2vpx.1    ---------------------------------------------------------- ``` | | | | | | | | | | | | | | | | | | | | | | | | | | | | | | | | | | | | | | | | | | | | | | | | | |
|  | 2v45.1.A | PERIPLASMIC NITRATE REDUCTASE  *A NEW CATALYTIC MECHANISM OF PERIPLASMIC NITRATE REDUCTASE FROM DESULFOVIBRIO DESULFURICANS ATCC 27774 FROM CRYSTALLOGRAPHIC AND EPR DATA AND BASED ON DETAILED ANALYSIS OF THE SIXTH LIGAND* | 0.27 |  | 15.13 | 0.55 | 106-647 | X-ray | 2.40 | monomer | 1 x SF4, 1 x MO, 2 x MGD, 1 x LCP | HHblits | 0.27 |
| ``` target    RPFLERFTDMPLLVRLDTLQRLRADEVFADYSSDLDVDGPSFTLHGMTEEQHERNGDRVVFDDASGALRAINREDVGDRL 2v45.1    --------------------------------------------------------------------------------  target    DDKGIDPALDYQGTVTLVDGSTVEVMSVLSMYREHLADYDIDSVVDMTGAPRNLIEQLLDDMTTLSPVAFHVGEGVNHYF 2v45.1    -------------------------PSDFEGYKAFLENYRPEKVAEICRVPVEQIYGAARAFAESAATMSLWCMGINQRV  target    HATLHNRATYLVGMLLGSVGVSGGGVSTWAGNYK--GGVFQAAPWFGPGVGGFVNEDPFHPLTDPSARYSAETARHLVHG 2v45.1    QGVFANNLIHNLHLITGQICRPGATSFSLTGQPNACGGVRDGG--ALS---HLLPA--GRAIP--NAKHRAEM-------  target    EDTSYWGFGDRPLVVDTPEDGRKVFTGTTHMPTPTKALWYNNANLINQAKWHYELVKNVNPKVD-LIVDQQIEWT-GSAE 2v45.1    --EKLWGLPEGRIAPEPGYHTVALFE--ALGRGDVKCMIICETNPAHTLPNLNKV-HKAMSHPESFIVCIEAFPDAVTLE  target    FADIVLPANSWMEAETWEMGASCSNPFLQVWKGGIEPLNDTRDDIAIFAGVANALTELTGDERFSQAFMFADRPEVYLDR 2v45.1    YADLVLPPAFWCERDGV---YGCGERRYSLTEKAVDPPGQCRPTVNTLVEFARRAGVDPQL------VNF-RNAEDVWNE  target    VLAGSF----TTEGYTVEDLTAGRYGPPGGALMQYRSYPR--IPFKEQIEDSLPFYTDTGRMHGYVDIPEAIEYGENLIV 2v45.1    WRMVSKGTTYDFWGMTRERLRKESGLIWP---CPSEDHPGTSLRYVRG-QDPCVPADHPDRFFFYGKPDGRA------VI  target    HREAVEATPYLPNVIVSTSPYLRPRDYGIAPEELDGDARSVRNIMMSWAEVKETENPLFAAGYNYLCLTPKSRHAVHSSW 2v45.1    WMRP-----------------------------------------------AKGAAEEPDAEYPLYLTSMRVIDHWHTAT  target    AVTDWHWLWSSSFSDPYRVETRAPGVGEPAIHLNPDDARSLGIRNGDYVWVDSNPKDRPYRDADVDESFLDVARLLVRVT 2v45.1    MTGKVPELQ--------------KANPIAFVEINEEDAARTGIKHGDSVIVETR-----------------RDAMELPAR  target    YNPAYPPGVTMLKHAFYMATPRTFRAAQERSDGRALAETTGYQSSFRSGSHQSITRGWAPPMHQTDSLFHKRAGVFGFTY 2v45.1    VSDVCRPGLIAVPFFDP---------------------------------------------------------------  target    GFDVDNHAINTVPKETVVRITKAEDGGVGGSGAWTRGRPGSMPGDEDDAMQAYLAGELTVVRRT 2v45.1    ---------------------------------------------------------------- ``` | | | | | | | | | | | | | | | | | | | | | | | | | | | | | | | | | | | | | | | | | | | | | | | | | |
|  | 1aa6.1.A | FORMATE DEHYDROGENASE H  *REDUCED FORM OF FORMATE DEHYDROGENASE H FROM E. COLI* | 0.27 |  | 18.40 | 0.53 | 98-647 | X-ray | 2.30 | monomer | 1 x SF4, 2 x MGD, 1 x 4MO | HHblits | 0.29 |
| ``` target    RPFLERFTDMPLLVRLDTLQRLRADEVFADYSSDLDVDGPSFTLHGMTEEQHERNGDRVVFDDASGALRAINREDVGDRL 1aa6.1    --------------------------------------------------------------------------------  target    DDKGIDPALDYQGTVTLVDGSTVEVMS-VLSMYREHLADYDIDSVVDMTGAPRNLIEQLLDDMTTLSPVAFHVGEGVNHY 1aa6.1    -----------------YDKAFVASRTEGFEEYRKIVEGYTPESVEDITGVSASEIRQAARMYAQAKSAAILWGMGVTQF  target    FHATLHNRATYLVGMLLGSVGVSGGGVSTWAGNYKG-GVFQAAPWFGPGVGGFVNEDPFHPLTDPSARYSAETARHLVHG 1aa6.1    YQGVETVRSLTSLAMLTGNLGKPHAGVNPVRGQNNVQGACDM-G-ALPD--TY---PGYQYVKDPANRE------KF---  target    EDTSYWGFGDRPLVVDTPEDGRKVFTGTTHMPTPTKALWYNNANLINQAKWHYELVKNVNPKVDLIVDQQIEWTGSAEFA 1aa6.1    --AKAWGVESLPAHT-GYR-ISEL--PHRAAHGEVRAAYIMGEDPLQTDAELSAV-RKAFEDLELVIVQDIFMTKTASAA  target    DIVLPANSWMEAETWEMGASCSNPFLQVWKGGIEPLNDTRDDIAIFAGVANALTELTGDERFSQAFMFADRPEVYLDRVL 1aa6.1    DVILPSTSWGEHEGVF---TAADRGFQRFFKAVEPKWDLKTDWQIISEIATRMGYP-----M----HY-NNTQEIWDELR  target    AGSFTTEGYTVEDLTAGRYGPPGGA-LMQYRSYPRIPFKEQIEDSLPFYTDTGRMHGYVDIPEAIEYGENLIVHREAVEA 1aa6.1    HLCPDFYGATYEKMGELGFIQWPCRDTSDADQGTSYL------FKEKFDTPNGLAQFFTCDWV------------AP---  target    TPYLPNVIVSTSPYLRPRDYGIAPEELDGDARSVRNIMMSWAEVKETENPLFAAGYNYLCLTPKSR--HAVHSSWAVTDW 1aa6.1    I------------------------------------------------DKLTDEYPMVLSTVREVGHYSCRSMTGNCAA  target    HWLWSSSFSDPYRVETRAPGVGEPAIHLNPDDARSLGIRNGDYVWVDSNPKDRPYRDADVDESFLDVARLLVRVTYNPAY 1aa6.1    --LA----------AL---ADEPGYAQINTEDAKRLGIEDEALVWVHSR-----------------KGKIITRAQVSDRP  target    PPGVTMLKHAFYMATPRTFRAAQERSDGRALAETTGYQSSFRSGSHQSITRGWAPPMHQTDSLFHKRAGVFGFTYGFDVD 1aa6.1    NKGAIYMTYQWW--------------------------------------------------------------------  target    NHAINTVPKETVVRITKAEDGGVGGSGAWTRGRPGSMPGDEDDAMQAYLAGELTVVRRT 1aa6.1    ----------------------------------------------------------- ``` | | | | | | | | | | | | | | | | | | | | | | | | | | | | | | | | | | | | | | | | | | | | | | | | | |
|  | 1fdo.1.A | FORMATE DEHYDROGENASE H  *OXIDIZED FORM OF FORMATE DEHYDROGENASE H FROM E. COLI* | 0.28 |  | 18.40 | 0.53 | 98-647 | X-ray | 2.80 | monomer | 1 x SF4, 2 x MGD, 1 x 6MO | HHblits | 0.29 |
| ``` target    RPFLERFTDMPLLVRLDTLQRLRADEVFADYSSDLDVDGPSFTLHGMTEEQHERNGDRVVFDDASGALRAINREDVGDRL 1fdo.1    --------------------------------------------------------------------------------  target    DDKGIDPALDYQGTVTLVDGSTVEVMS-VLSMYREHLADYDIDSVVDMTGAPRNLIEQLLDDMTTLSPVAFHVGEGVNHY 1fdo.1    -----------------YDKAFVASRTEGFEEYRKIVEGYTPESVEDITGVSASEIRQAARMYAQAKSAAILWGMGVTQF  target    FHATLHNRATYLVGMLLGSVGVSGGGVSTWAGNYKG-GVFQAAPWFGPGVGGFVNEDPFHPLTDPSARYSAETARHLVHG 1fdo.1    YQGVETVRSLTSLAMLTGNLGKPHAGVNPVRGQNNVQGACDM-G-ALPD--TY---PGYQYVKDPANRE------KF---  target    EDTSYWGFGDRPLVVDTPEDGRKVFTGTTHMPTPTKALWYNNANLINQAKWHYELVKNVNPKVDLIVDQQIEWTGSAEFA 1fdo.1    --AKAWGVESLPAHT-GYR-ISEL--PHRAAHGEVRAAYIMGEDPLQTDAELSAV-RKAFEDLELVIVQDIFMTKTASAA  target    DIVLPANSWMEAETWEMGASCSNPFLQVWKGGIEPLNDTRDDIAIFAGVANALTELTGDERFSQAFMFADRPEVYLDRVL 1fdo.1    DVILPSTSWGEHEGVF---TAADRGFQRFFKAVEPKWDLKTDWQIISEIATRMGYP-----M----HY-NNTQEIWDELR  target    AGSFTTEGYTVEDLTAGRYGPPGGA-LMQYRSYPRIPFKEQIEDSLPFYTDTGRMHGYVDIPEAIEYGENLIVHREAVEA 1fdo.1    HLCPDFYGATYEKMGELGFIQWPCRDTSDADQGTSYL------FKEKFDTPNGLAQFFTCDWV------------AP---  target    TPYLPNVIVSTSPYLRPRDYGIAPEELDGDARSVRNIMMSWAEVKETENPLFAAGYNYLCLTPKSR--HAVHSSWAVTDW 1fdo.1    I------------------------------------------------DKLTDEYPMVLSTVREVGHYSCRSMTGNCAA  target    HWLWSSSFSDPYRVETRAPGVGEPAIHLNPDDARSLGIRNGDYVWVDSNPKDRPYRDADVDESFLDVARLLVRVTYNPAY 1fdo.1    --LA----------AL---ADEPGYAQINTEDAKRLGIEDEALVWVHSR-----------------KGKIITRAQVSDRP  target    PPGVTMLKHAFYMATPRTFRAAQERSDGRALAETTGYQSSFRSGSHQSITRGWAPPMHQTDSLFHKRAGVFGFTYGFDVD 1fdo.1    NKGAIYMTYQWW--------------------------------------------------------------------  target    NHAINTVPKETVVRITKAEDGGVGGSGAWTRGRPGSMPGDEDDAMQAYLAGELTVVRRT 1fdo.1    ----------------------------------------------------------- ``` | | | | | | | | | | | | | | | | | | | | | | | | | | | | | | | | | | | | | | | | | | | | | | | | | |
|  | 2iv2.1.A | Formate dehydrogenase H  *Reinterpretation of reduced form of formate dehydrogenase H from E. coli* | 0.27 |  | 18.40 | 0.53 | 98-647 | X-ray | 2.27 | monomer | 1 x SF4, 1 x 2MD, 1 x MGD | HHblits | 0.29 |
| ``` target    RPFLERFTDMPLLVRLDTLQRLRADEVFADYSSDLDVDGPSFTLHGMTEEQHERNGDRVVFDDASGALRAINREDVGDRL 2iv2.1    --------------------------------------------------------------------------------  target    DDKGIDPALDYQGTVTLVDGSTVEVMS-VLSMYREHLADYDIDSVVDMTGAPRNLIEQLLDDMTTLSPVAFHVGEGVNHY 2iv2.1    -----------------YDKAFVASRTEGFEEYRKIVEGYTPESVEDITGVSASEIRQAARMYAQAKSAAILWGMGVTQF  target    FHATLHNRATYLVGMLLGSVGVSGGGVSTWAGNYKG-GVFQAAPWFGPGVGGFVNEDPFHPLTDPSARYSAETARHLVHG 2iv2.1    YQGVETVRSLTSLAMLTGNLGKPHAGVNPVRGQNNVQGACDM-G-ALPD--TY---PGYQYVKDPANRE------KF---  target    EDTSYWGFGDRPLVVDTPEDGRKVFTGTTHMPTPTKALWYNNANLINQAKWHYELVKNVNPKVDLIVDQQIEWTGSAEFA 2iv2.1    --AKAWGVESLPAHT-GYR-ISEL--PHRAAHGEVRAAYIMGEDPLQTDAELSAV-RKAFEDLELVIVQDIFMTKTASAA  target    DIVLPANSWMEAETWEMGASCSNPFLQVWKGGIEPLNDTRDDIAIFAGVANALTELTGDERFSQAFMFADRPEVYLDRVL 2iv2.1    DVILPSTSWGEHEGVF---TAADRGFQRFFKAVEPKWDLKTDWQIISEIATRMGYP-----M----HY-NNTQEIWDELR  target    AGSFTTEGYTVEDLTAGRYGPPGGA-LMQYRSYPRIPFKEQIEDSLPFYTDTGRMHGYVDIPEAIEYGENLIVHREAVEA 2iv2.1    HLCPDFYGATYEKMGELGFIQWPCRDTSDADQGTSYL------FKEKFDTPNGLAQFFTCDWV------------AP---  target    TPYLPNVIVSTSPYLRPRDYGIAPEELDGDARSVRNIMMSWAEVKETENPLFAAGYNYLCLTPKSR--HAVHSSWAVTDW 2iv2.1    I------------------------------------------------DKLTDEYPMVLSTVREVGHYSCRSMTGNCAA  target    HWLWSSSFSDPYRVETRAPGVGEPAIHLNPDDARSLGIRNGDYVWVDSNPKDRPYRDADVDESFLDVARLLVRVTYNPAY 2iv2.1    --LA----------AL---ADEPGYAQINTEDAKRLGIEDEALVWVHSR-----------------KGKIITRAQVSDRP  target    PPGVTMLKHAFYMATPRTFRAAQERSDGRALAETTGYQSSFRSGSHQSITRGWAPPMHQTDSLFHKRAGVFGFTYGFDVD 2iv2.1    NKGAIYMTYQWW--------------------------------------------------------------------  target    NHAINTVPKETVVRITKAEDGGVGGSGAWTRGRPGSMPGDEDDAMQAYLAGELTVVRRT 2iv2.1    ----------------------------------------------------------- ``` | | | | | | | | | | | | | | | | | | | | | | | | | | | | | | | | | | | | | | | | | | | | | | | | | |
|  | 7z0t.1.G | Formate dehydrogenase H  *Structure of the Escherichia coli formate hydrogenlyase complex (aerobic preparation, composite structure)* | 0.28 |  | 18.40 | 0.53 | 98-647 | EM | 0.00 | hetero-1-1-1-1-1-1-… | 1 x NI, 1 x FCO, 8 x SF4, 1 x FE, 2 x MGD, 1 x 6MO | HHblits | 0.29 |
| ``` target    RPFLERFTDMPLLVRLDTLQRLRADEVFADYSSDLDVDGPSFTLHGMTEEQHERNGDRVVFDDASGALRAINREDVGDRL 7z0t.1    --------------------------------------------------------------------------------  target    DDKGIDPALDYQGTVTLVDGSTVEVMS-VLSMYREHLADYDIDSVVDMTGAPRNLIEQLLDDMTTLSPVAFHVGEGVNHY 7z0t.1    -----------------YDKAFVASRTEGFEEYRKIVEGYTPESVEDITGVSASEIRQAARMYAQAKSAAILWGMGVTQF  target    FHATLHNRATYLVGMLLGSVGVSGGGVSTWAGNYKG-GVFQAAPWFGPGVGGFVNEDPFHPLTDPSARYSAETARHLVHG 7z0t.1    YQGVETVRSLTSLAMLTGNLGKPHAGVNPVRGQNNVQGACDM-G-ALPD--TY---PGYQYVKDPANRE------KF---  target    EDTSYWGFGDRPLVVDTPEDGRKVFTGTTHMPTPTKALWYNNANLINQAKWHYELVKNVNPKVDLIVDQQIEWTGSAEFA 7z0t.1    --AKAWGVESLPAHT-GYR-ISEL--PHRAAHGEVRAAYIMGEDPLQTDAELSAV-RKAFEDLELVIVQDIFMTKTASAA  target    DIVLPANSWMEAETWEMGASCSNPFLQVWKGGIEPLNDTRDDIAIFAGVANALTELTGDERFSQAFMFADRPEVYLDRVL 7z0t.1    DVILPSTSWGEHEGVF---TAADRGFQRFFKAVEPKWDLKTDWQIISEIATRMGYP-----M----HY-NNTQEIWDELR  target    AGSFTTEGYTVEDLTAGRYGPPGGA-LMQYRSYPRIPFKEQIEDSLPFYTDTGRMHGYVDIPEAIEYGENLIVHREAVEA 7z0t.1    HLCPDFYGATYEKMGELGFIQWPCRDTSDADQGTSYL------FKEKFDTPNGLAQFFTCDWV------------AP---  target    TPYLPNVIVSTSPYLRPRDYGIAPEELDGDARSVRNIMMSWAEVKETENPLFAAGYNYLCLTPKSR--HAVHSSWAVTDW 7z0t.1    I------------------------------------------------DKLTDEYPMVLSTVREVGHYSCRSMTGNCAA  target    HWLWSSSFSDPYRVETRAPGVGEPAIHLNPDDARSLGIRNGDYVWVDSNPKDRPYRDADVDESFLDVARLLVRVTYNPAY 7z0t.1    --LA----------AL---ADEPGYAQINTEDAKRLGIEDEALVWVHSR-----------------KGKIITRAQVSDRP  target    PPGVTMLKHAFYMATPRTFRAAQERSDGRALAETTGYQSSFRSGSHQSITRGWAPPMHQTDSLFHKRAGVFGFTYGFDVD 7z0t.1    NKGAIYMTYQWW--------------------------------------------------------------------  target    NHAINTVPKETVVRITKAEDGGVGGSGAWTRGRPGSMPGDEDDAMQAYLAGELTVVRRT 7z0t.1    ----------------------------------------------------------- ``` | | | | | | | | | | | | | | | | | | | | | | | | | | | | | | | | | | | | | | | | | | | | | | | | | |
|  | 5nqd.1.A | AroA  *Arsenite oxidase AioAB from Rhizobium sp. str. NT-26 mutant AioBF108A* | 0.26 |  | 17.56 | 0.53 | 108-647 | X-ray | 2.20 | hetero-2-2-mer | 4 x MGD, 2 x O, 2 x 4MO, 2 x F3S, 2 x FES | HHblits | 0.29 |
| ``` target    RPFLERFTDMPLLVRLDTLQRLRADEVFADYSSDLDVDGPSFTLHGMTEEQHERNGDRVVFDDASGALRAINREDVGDRL 5nqd.1    --------------------------------------------------------------------------------  target    DDKGIDPALDYQGTVTLVDGSTVEVMSVLSMYREHLA--DYDIDSVVDMTGAPRNLIEQLLDDMTTLS------PVAFHV 5nqd.1    ---------------------------HLSSFEDAVEGCRMSIEEAAEITGLDAAQIIKAAEWIGMPKEGGKRRRVMFGY  target    GEGVNHYFHATLHNRATYLVGMLLGSVGVSGGGVSTWAGNYKGGVFQAAPWFGPGVGGFVNEDPFHPLTDPSARYSAETA 5nqd.1    EKGLIWGNDNYRTNGALVNLALATGNIGRPGGGVVRLGGHQEGY-------VRPSDAH--VGRP-AAYVDQ-LLIG-GQ-  target    RHLVHGEDTSYWGFGDRPLVVDTPEDGRKVFTGTTHMPTPTKALWYNNANLINQ------AKWH---YELVKNVNPKV-D 5nqd.1    -----GGVHHIWGCDH--Y-----K------T--TLNAHEFKRVYKKRTDMVKDAMSAAPYGDREAMVNAIVDAINQGGL  target    LIVDQQIEWTGSAEFADIVLPANSWMEAETWEMGASCSNPFLQVWKGGIEPLNDTRDDIAIFAGVANALTELTGDE---R 5nqd.1    FAVNVDIIPTKIGEACHVILPAATSGEMNLT---SMNGERRMRLTERYMDPPGQSMPDCLIAARLANTMERVLTEMGDVG  target    FS---QAFMFADRPEVYLDRVLAGSFTTEGYTVEDLTAGR---YGPPGGALMQYRSYPRIPFKEQIEDSLPFYTDTGRMH 5nqd.1    YAAQFKGFDWQTEEDAFMDGYNKNAHGGEFVTYERLSAMGTNGFQEPATG---FT-DGKIEGTQRLYTDGVFSTDDGKAR  target    GYVDIPEAIEYGENLIVHREAVEATPYLPNVIVSTSPYLRPRDYGIAPEELDGDARSVRNIMMSWAEVKETENPLFAAGY 5nqd.1    FMDAPWR---------GLQ-----AP---G---------------------------------------K---QQQKDSH  target    NYLCLTPKSRHAVHSSWAVTDWHWLWSSSFSDPYRVETRAPGVGEPAIHLNPDDARSLGIRNGDYVWVDSNPKDRPYRDA 5nqd.1    KYLINNGRANVVWQSAYLDQENDFVMD--------------RFPYPFIEMNPEDMAEAGLKEGDLVEIYND---------  target    DVDESFLDVARLLVRVTYNPAYPPGVTMLKHAFYMATPRTFRAAQERSDGRALAETTGYQSSFRSGSHQSITRGWAPPMH 5nqd.1    --------AGATQAMAYPTPTARRGETFMLFGFP----------------------------------------------  target    QTDSLFHKRAGVFGFTYGFDVDNHAINTVPKETVVRITKAEDGGVGGSGAWTRGRPGSMPGDEDDAMQAYLAGELTVVRR 5nqd.1    --------------------------------------------------------------------------------  target    T 5nqd.1    - ``` | | | | | | | | | | | | | | | | | | | | | | | | | | | | | | | | | | | | | | | | | | | | | | | | | |
|  | 4aay.1.A | AROA  *Crystal Structure of the arsenite oxidase protein complex from Rhizobium species strain NT-26* | 0.26 |  | 16.99 | 0.53 | 108-647 | X-ray | 2.70 | hetero-oligomer | 4 x MGD, 2 x O, 2 x 4MO, 2 x F3S, 2 x FES | HHblits | 0.28 |
| ``` target    RPFLERFTDMPLLVRLDTLQRLRADEVFADYSSDLDVDGPSFTLHGMTEEQHERNGDRVVFDDASGALRAINREDVGDRL 4aay.1    --------------------------------------------------------------------------------  target    DDKGIDPALDYQGTVTLVDGSTVEVMSVLSMYREHLA--DYDIDSVVDMTGAPRNLIEQLLDDMTTLS------PVAFHV 4aay.1    ---------------------------HLSSFEDAVEGCRMSIEEAAEITGLDAAQIIKAAEWIGMPKEGGKRRRVMFGY  target    GEGVNHYFHATLHNRATYLVGMLLGSVGVSGGGVSTWAGNYKGGVFQAAPWFGPGVGGFVNEDPFHPLTDPSARYSAETA 4aay.1    EKGLIWGNDNYRTNGALVNLALATGNIGRPGGGVVRLGGHQEGY--V-----RPSDAHV--G---RP----A-AYVDQLL  target    RHLVHGEDTSYWGFGDRPLVVDTPEDGRKVFTGTTHMPTPTKALWYNNANLINQ------AKW---HYELVKNVNPKV-D 4aay.1    IG-GQGGVHHIWGCDH-------Y-------K-TTLNAHEFKRVYKKRTDMVKDAMSAAPYGDREAMVNAIVDAINQGGL  target    LIVDQQIEWTGSAEFADIVLPANSWMEAETWEMGASCSNPFLQVWKGGIEPLNDTRDDIAIFAGVANALTELTGDER--- 4aay.1    FAVNVDIIPTKIGEACHVILPAATSGEMNLT---SMNGERRMRLTERYMDPPGQSMPDCLIAARLANTMERVLTEMGDVG  target    FS---QAFMFADRPEVYLDRVLAGSFTTEGYTVEDLTAGRYGPPGGALMQYR-SYPRIPFKEQIEDSLPFYTDTGRMHGY 4aay.1    YAAQFKGFDWQTEEDAFMDGYNKNAHGGEFVTYERLSAMGTNGF--QEPATGFTDGKIEGTQRLYTDGVFSTDDGKARFM  target    VDIPEAIEYGENLIVHREAVEATPYLPNVIVSTSPYLRPRDYGIAPEELDGDARSVRNIMMSWAEVKETENPLFAAGYNY 4aay.1    DAPWRG---------LQAP--------G---------------------------------------K---QQQKDSHKY  target    LCLTPKSRHAVHSSWAVTDWHWLWSSSFSDPYRVETRAPGVGEPAIHLNPDDARSLGIRNGDYVWVDSNPKDRPYRDADV 4aay.1    LINNGRANVVWQSAYLDQENDFVMD--------------RFPYPFIEMNPEDMAEAGLKEGDLVEIYND-----------  target    DESFLDVARLLVRVTYNPAYPPGVTMLKHAFYMATPRTFRAAQERSDGRALAETTGYQSSFRSGSHQSITRGWAPPMHQT 4aay.1    ------AGATQAMAYPTPTARRGETFMLFGFP------------------------------------------------  target    DSLFHKRAGVFGFTYGFDVDNHAINTVPKETVVRITKAEDGGVGGSGAWTRGRPGSMPGDEDDAMQAYLAGELTVVRRT 4aay.1    ------------------------------------------------------------------------------- ``` | | | | | | | | | | | | | | | | | | | | | | | | | | | | | | | | | | | | | | | | | | | | | | | | | |
|  | 6tg9.1.A | Formate dehydrogenase subunit alpha  *Cryo-EM Structure of NADH reduced form of NAD+-dependent Formate Dehydrogenase from Rhodobacter capsulatus* | 0.27 |  | 19.16 | 0.53 | 97-647 | EM | 3.24 | hetero-2-2-2-2-mer | 4 x MGD, 2 x 6MO, 4 x FES, 10 x SF4, 2 x H2S, 2 x FMN, 2 x NAI | HHblits | 0.29 |
| ``` target    RPFLERFTDMPLLVRLDTLQRLRADEVFADYSSDLDVDGPSFTLHGMTEEQHERNGDRVVFDDASGALRAINREDVGDRL 6tg9.1    --------------------------------------------------------------------------------  target    DDKGIDPALDYQGTVTLVDGSTVEVMSVLSMYR---EHL--ADYDIDSVVDMTGAPRNLIEQLLDDMTTLSPVAFHVGEG 6tg9.1    ----------------IFDKRFIGDRCDWDEWADYAEFVANPEYAPEAVESLTGVPAGLLRQAARAYAAAPNAAIYYGLG  target    VNHYFHATLHNRATYLVGMLLGSVGVSGGGVSTWAGNYKG-GV--FQAAPWFGPGVGGFVNEDPFHPLTDPSARYSAETA 6tg9.1    VTEHSQGSTTVIAIANLAMMTGNIGRPGVGVNPLRGQNNVQGSCDMGSFPHEFPG---------YRHVSDDATR------  target    RHLVHGEDTSYWGFGDRPLVVDTPEDGRKVFTGTTHMPTPTKALWYNNANLINQAKWHYELVKNVNPKVDLIVDQQIEWT 6tg9.1    -----GLFERTWGVTLSSE-P-GLR-IPNMLD--AAVEGRFKALYVQGEDILQSDPDTR-HVSAGLAAMDLVIVHDLFLN  target    GSAEFADIVLPANSWMEAETWEMGASCSNPFLQVWKGGIEPLNDTRDDIAIFAGVANALTELTGDERFSQAFMFADRPEV 6tg9.1    ETANYAHVFLPGSTFLEKDGT---FTNAERRINRVRRVMAPKA-GFADWEVTQMLANALGAG---------WHY-THPSE  target    YLDRVLAGSFTTEGYTVEDLTAGRYGPPGGALMQYRSYPRIPFKEQIEDSLPFYTDTGRMHGYVDIPEAIEYGENLIVHR 6tg9.1    IMAEIAATTPGFAAVTYEMLDARGSVQ------WPC-NEK----------APEGSPIMHVEGFVRGK-----G-RF----  target    EAVEATPYLPNVIVSTSPYLRPRDYGIAPEELDGDARSVRNIMMSWAEVKETENPLFAAGYNYLCLTPKSRHAVHSSWAV 6tg9.1    -----IR----------TAYLP--------------------------TDE----KTGPRFPLLLTTGRILSQYNVGAQT  target    TDWHWLWSSSFSDPYRVETRAPGVGEPAIHLNPDDARSLGIRNGDYVWVDSNPKDRPYRDADVDESFLDVARLLVRVTYN 6tg9.1    RRT----------E-NT----VWHGEDRLEIHPTDAETRGIRDGDWVRLASR-----------------AGETTLRATVT  target    PAYPPGVTMLKHAFYMATPRTFRAAQERSDGRALAETTGYQSSFRSGSHQSITRGWAPPMHQTDSLFHKRAGVFGFTYGF 6tg9.1    DRVSPGVVYTTFHHP-----------------------------------------------------------------  target    DVDNHAINTVPKETVVRITKAEDGGVGGSGAWTRGRPGSMPGDEDDAMQAYLAGELTVVRRT 6tg9.1    -------------------------------------------------------------- ``` | | | | | | | | | | | | | | | | | | | | | | | | | | | | | | | | | | | | | | | | | | | | | | | | | |
|  | 7e5z.1.A | Formate dehydrogenase  *Dehydrogenase holoenzyme* | 0.23 |  | 17.36 | 0.53 | 98-647 | EM | 0.00 | hetero-1-1-mer | 1 x W, 2 x MGD, 2 x FES, 4 x SF4, 1 x FMN | HHblits | 0.29 |
| ``` target    RPFLERFTDMPLLVRLDTLQRLRADEVFADYSSDLDVDGPSFTLHGMTEEQHERNGDRVVFDDASGALRAINREDVGDRL 7e5z.1    --------------------------------------------------------------------------------  target    DDKGIDPALDYQGTVTLVDGSTVEVMS-VLSMYREHLADYDIDSVVDMTGAPRNLIEQLLDDMTTLSPVAFHVGEGVNHY 7e5z.1    -----------------YDEQYIAGYTENFEALREKIVDFTPEKMASVCGIDAETLREVARLYARAKSSLIFWGMGVSQH  target    FHATLHNRATYLVGMLLGSVGVSGGGVSTWAGNYKG-GVFQAAPWFGPGVGGFVNEDPFHPLTDPSARYSAETARHLVHG 7e5z.1    VHGTDNSRCLIALALITGQIGRPGTGLHPLRGQNNVQGASDA--GLIPM---VY--PDYQSVEKDAVRE------L----  target    EDTSYWGFGDRPLVVDTPEDGR---KVFTGTTHMPTPTKALWYNNANLINQAKWHYELVKNVNPKVDLIVDQQIEWTGSA 7e5z.1    -FEEFWGQSLD------PQKGLTVVEIMR--AIHAGEIRGMFVEGENPAMSDPDLNH-ARHALAMLDHLVVQDLFLTETA  target    EFADIVLPANSWMEAETWEMGASCSNPFLQVWKGGIEPLNDTRDDIAIFAGVANALTELTGDERFSQAFMFADRPEVYLD 7e5z.1    FHADVVLPASAFAEKAGT---FTNTDRRVQIAQPVVAPPGDARQDWWIIQELARRLDLD-----W----NY-GGPADIFA  target    RVLAGSFTTEGYTVEDLTAGRYGPPGGALMQYRSYPRIPFKEQIEDSLPFYTDTGRMHGYVDIPEAIEYGENLIVHREAV 7e5z.1    EMAQVMPSLNNITWERLEREGAVTYP-----VDA-PDQPGNE-IIFYAGFPTESGRAKIVPAAIV---------------  target    EATPYLPNVIVSTSPYLRPRDYGIAPEELDGDARSVRNIMMSWAEVKETENPLFAAGYNYLCLTPKSRHAVHSSWAVTDW 7e5z.1    ---P--------------P---------------------------D----EVPDDEFPMVLSTGRVLEHWHTGSMTRRA  target    HWLWSSSFSDPYRVETRAPGVGEPAIHLNPDDARSLGIRNGDYVWVDSNPKDRPYRDADVDESFLDVARLLVRVTYNPAY 7e5z.1    GVL----------D----ALEPEAVAFMAPKELYRLGLRPGGSMRLETR-----------------RGAVVLKVRSDRDV  target    PPGVTMLKHAFYMATPRTFRAAQERSDGRALAETTGYQSSFRSGSHQSITRGWAPPMHQTDSLFHKRAGVFGFTYGFDVD 7e5z.1    PIGMIFMPFCYA--------------------------------------------------------------------  target    NHAINTVPKETVVRITKAEDGGVGGSGAWTRGRPGSMPGDEDDAMQAYLAGELTVVRRT 7e5z.1    ----------------------------------------------------------- ``` | | | | | | | | | | | | | | | | | | | | | | | | | | | | | | | | | | | | | | | | | | | | | | | | | |
|  | 7vw6.1.A | Formate dehydrogenase  *Cryo-EM Structure of Formate Dehydrogenase 1 from Methylorubrum extorquens AM1* | 0.27 |  | 17.44 | 0.53 | 98-647 | EM | 0.00 | hetero-1-1-mer | 4 x SF4, 2 x FES, 2 x MGD, 1 x W, 1 x FMN | HHblits | 0.29 |
| ``` target    RPFLERFTDMPLLVRLDTLQRLRADEVFADYSSDLDVDGPSFTLHGMTEEQHERNGDRVVFDDASGALRAINREDVGDRL 7vw6.1    --------------------------------------------------------------------------------  target    DDKGIDPALDYQGTVTLVDGSTVEVMS-VLSMYREHLADYDIDSVVDMTGAPRNLIEQLLDDMTTLSPVAFHVGEGVNHY 7vw6.1    -----------------YDEQYIAGYTENFEALREKIVDFTPEKMASVCGIDAETLREVARLYARAKSSLIFWGMGVSQH  target    FHATLHNRATYLVGMLLGSVGVSGGGVSTWAGNYKG-GV--FQAAPWFGPGVGGFVNEDPFHPLTDPSARYSAETARHLV 7vw6.1    VHGTDNSRCLIALALITGQIGRPGTGLHPLRGQNNVQGASDAGLIPMVYPD---------YQSVEKDAVRE------L--  target    HGEDTSYWGFGDRPLVVDTPEDGR---KVFTGTTHMPTPTKALWYNNANLINQAKWHYELVKNVNPKVDLIVDQQIEWTG 7vw6.1    ---FEEFWGQSLD------PQKGLTVVEIMR--AIHAGEIRGMFVEGENPAMSDPDLNH-ARHALAMLDHLVVQDLFLTE  target    SAEFADIVLPANSWMEAETWEMGASCSNPFLQVWKGGIEPLNDTRDDIAIFAGVANALTELTGDERFSQAFMFADRPEVY 7vw6.1    TAFHADVVLPASAFAEKAGT---FTNTDRRVQIAQPVVAPPGDARQDWWIIQELARRLDLD-----W----NY-GGPADI  target    LDRVLAGSFTTEGYTVEDLTAGRYGPPGGALMQYRSYPRIPFKEQIEDSLPFYTDTGRMHGYVDIPEAIEYGENLIVHRE 7vw6.1    FAEMAQVMPSLNNITWERLEREGAVTYP-----VDA-PDQPGNE-IIFYAGFPTESGRAKIVPAAIV-------------  target    AVEATPYLPNVIVSTSPYLRPRDYGIAPEELDGDARSVRNIMMSWAEVKETENPLFAAGYNYLCLTPKSRHAVHSSWAVT 7vw6.1    -----P--------------P---------------------------D----EVPDDEFPMVLSTGRVLEHWHTGSMTR  target    DWHWLWSSSFSDPYRVETRAPGVGEPAIHLNPDDARSLGIRNGDYVWVDSNPKDRPYRDADVDESFLDVARLLVRVTYNP 7vw6.1    RAGVL-----------D---ALEPEAVAFMAPKELYRLGLRPGGSMRLETR-----------------RGAVVLKVRSDR  target    AYPPGVTMLKHAFYMATPRTFRAAQERSDGRALAETTGYQSSFRSGSHQSITRGWAPPMHQTDSLFHKRAGVFGFTYGFD 7vw6.1    DVPIGMIFMPFCYA------------------------------------------------------------------  target    VDNHAINTVPKETVVRITKAEDGGVGGSGAWTRGRPGSMPGDEDDAMQAYLAGELTVVRRT 7vw6.1    ------------------------------------------------------------- ``` | | | | | | | | | | | | | | | | | | | | | | | | | | | | | | | | | | | | | | | | | | | | | | | | | |
|  | 1g8k.1.A | ARSENITE OXIDASE  *CRYSTAL STRUCTURE ANALYSIS OF ARSENITE OXIDASE FROM ALCALIGENES FAECALIS* | 0.26 |  | 14.42 | 0.54 | 106-647 | X-ray | 1.64 | hetero-1-1-mer | 3 x HG, 2 x CA, 2 x MGD, 1 x O, 1 x 4MO, 1 x F3S, 1 x FES | HHblits | 0.27 |
| ``` target    RPFLERFTDMPLLVRLDTLQRLRADEVFADYSSDLDVDGPSFTLHGMTEEQHERNGDRVVFDDASGALRAINREDVGDRL 1g8k.1    --------------------------------------------------------------------------------  target    DDKGIDPALDYQGTVTLVDGSTVEVMSVLSMYREHLADYDIDSVVDMTGAPRNLIEQLLDDMTTLS------PVAFHVGE 1g8k.1    -------------------------TKGFDDAVK-TNRLSLDECSNITGVPVDMLKRAAEWSYKPKASGQAPRTMHAYEK  target    GVNHYFHATLHNRATYLVGMLLGSVGVSGGGVSTWAGNYKGGVFQAAPWFGPGVGGFVNEDPFHPLTDPSARYSAETARH 1g8k.1    GIIWGNDNYVIQSALLDLVIATHNVGRRGTGCVRMGGHQEGYTRP----PYPGDKK------I-YIDQ-E--LIKGKGRI  target    LVHGEDTSYWGFGDRPLVVDTPEDGRKVFTG-TTHMPTPTKALWYNNANLINQAKWHYELVKNVNPK-VDLIVDQQIEWT 1g8k.1    M------TWWG--CNNFQT-SN-NAQALREAILQRSAIVKQAMQKARGATTEE--M-VDVIYEATQNGGLFVTSINLYPT  target    GSAEFADIVLPANSWMEAETWEMGASCSNPFLQVWKGGIEPLNDTRDDIAIFAGVANALTELTGDE---RFS---QAFMF 1g8k.1    KLAEAAHLMLPAAHPGEMNLT---SMNGERRIRLSEKFMDPPGTAMADCLIAARIANALRDMYQKDGKAEMAAQFEGFDW  target    ADRPEVYLDRVLAGSFT-------------TEGYTVEDLTAGRYG---PPGGALMQYRSYPRIPFKEQIEDSLPFYTDTG 1g8k.1    -KTEEDAFNDGFRRAGQPGAPAIDSQGGSTGHLVTYDRLRKSGNNGVQLPVV--SWDE-SKGLVGTEMLYTEGKFDTDDG  target    RMHGYVDIPEAIEYGENLIVHREAVEATPYLPNVIVSTSPYLRPRDYGIAPEELDGDARSVRNIMMSWAEVKETENPLFA 1g8k.1    KAHFKPAPWN------GLPAT-----------------------------------------------VQ-------QQK  target    AGYNYLCLTPKSRHAVHSSWAVTDWHWLWSSSFSDPYRVETRAPGVGEPAIHLNPDDARSLGIRNGDYVWVDSNPKDRPY 1g8k.1    DKYRFWLNNGRNNEVWQTAYHDQYNSLMQ--------------ERYPMAYIEMNPDDCKQLDVTGGDIVEVYND------  target    RDADVDESFLDVARLLVRVTYNPAYPPGVTMLKHAFYMATPRTFRAAQERSDGRALAETTGYQSSFRSGSHQSITRGWAP 1g8k.1    -----------FGSTFAMVYPVAEIKRGQTFMLFGYV-------------------------------------------  target    PMHQTDSLFHKRAGVFGFTYGFDVDNHAINTVPKETVVRITKAEDGGVGGSGAWTRGRPGSMPGDEDDAMQAYLAGELTV 1g8k.1    --------------------------------------------------------------------------------  target    VRRT 1g8k.1    ---- ``` | | | | | | | | | | | | | | | | | | | | | | | | | | | | | | | | | | | | | | | | | | | | | | | | | |
|  | 1g8j.1.A | ARSENITE OXIDASE  *CRYSTAL STRUCTURE ANALYSIS OF ARSENITE OXIDASE FROM ALCALIGENES FAECALIS* | 0.26 |  | 14.66 | 0.54 | 106-647 | X-ray | 2.03 | hetero-oligomer | 2 x MGD, 1 x O, 1 x 4MO, 1 x F3S, 1 x FES | HHblits | 0.27 |
| ``` target    RPFLERFTDMPLLVRLDTLQRLRADEVFADYSSDLDVDGPSFTLHGMTEEQHERNGDRVVFDDASGALRAINREDVGDRL 1g8j.1    --------------------------------------------------------------------------------  target    DDKGIDPALDYQGTVTLVDGSTVEVMSVLSMYREHLADYDIDSVVDMTGAPRNLIEQLLDDMTTL------SPVAFHVGE 1g8j.1    -------------------------TKGFDDAVK-TNRLSLDECSNITGVPVDMLKRAAEWSYKPKASGQAPRTMHAYEK  target    GVNHYFHATLHNRATYLVGMLLGSVGVSGGGVSTWAGNYKGGVFQAAPWFGPGVGGFVNEDPFHPLTDPSARYSAETARH 1g8j.1    GIIWGNDNYVIQSALLDLVIATHNVGRRGTGCVRMGGHQEGY--T--RPPYPGDKK------I-YIDQE---LIKGKGRI  target    LVHGEDTSYWGFGDRPLVVDTPEDGRKVFT-GTTHMPTPTKALWYNNANLINQAKWHYELVKNVNPKVD-LIVDQQIEWT 1g8j.1    M------TWWG--CNNFQT-SN-NAQALREAILQRSAIVKQAMQKARGATTEEM---VDVIYEATQNGGLFVTSINLYPT  target    GSAEFADIVLPANSWMEAETWEMGASCSNPFLQVWKGGIEPLNDTRDDIAIFAGVANALTELTGDER---FS---QAFMF 1g8j.1    KLAEAAHLMLPAAHPGEMNLTS---MNGERRIRLSEKFMDPPGTAMADCLIAARIANALRDMYQKDGKAEMAAQFEGFDW  target    ADRPEVYLDRVLAGSFT-------------TEGYTVEDLTAGRY---GPPGGALMQYRSYPRIPFKEQIEDSLPFYTDTG 1g8j.1    -KTEEDAFNDGFRRAGQPGAPAIDSQGGSTGHLVTYDRLRKSGNNGVQLPVV--SWDE-SKGLVGTEMLYTEGKFDTDDG  target    RMHGYVDIPEAIEYGENLIVHREAVEATPYLPNVIVSTSPYLRPRDYGIAPEELDGDARSVRNIMMSWAEVKETENPLFA 1g8j.1    KAHFKPAPWN------GLPA-----------------------------------------------TVQ-----Q--QK  target    AGYNYLCLTPKSRHAVHSSWAVTDWHWLWSSSFSDPYRVETRAPGVGEPAIHLNPDDARSLGIRNGDYVWVDSNPKDRPY 1g8j.1    DKYRFWLNNGRNNEVWQTAYHDQYNSLMQ----------E----RYPMAYIEMNPDDCKQLDVTGGDIVEVYND------  target    RDADVDESFLDVARLLVRVTYNPAYPPGVTMLKHAFYMATPRTFRAAQERSDGRALAETTGYQSSFRSGSHQSITRGWAP 1g8j.1    -----------FGSTFAMVYPVAEIKRGQTFMLFGYV-------------------------------------------  target    PMHQTDSLFHKRAGVFGFTYGFDVDNHAINTVPKETVVRITKAEDGGVGGSGAWTRGRPGSMPGDEDDAMQAYLAGELTV 1g8j.1    --------------------------------------------------------------------------------  target    VRRT 1g8j.1    ---- ``` | | | | | | | | | | | | | | | | | | | | | | | | | | | | | | | | | | | | | | | | | | | | | | | | | |
|  | 2v3v.1.A | PERIPLASMIC NITRATE REDUCTASE  *A NEW CATALYTIC MECHANISM OF PERIPLASMIC NITRATE REDUCTASE FROM DESULFOVIBRIO DESULFURICANS ATCC 27774 FROM CRYSTALLOGRAPHIC AND EPR DATA AND BASED ON DETAILED ANALYSIS OF THE SIXTH LIGAND* | 0.26 |  | 15.14 | 0.54 | 106-647 | X-ray | 1.99 | monomer | 1 x SF4, 1 x MO, 2 x MGD, 4 x LCP | HHblits | 0.27 |
| ``` target    RPFLERFTDMPLLVRLDTLQRLRADEVFADYSSDLDVDGPSFTLHGMTEEQHERNGDRVVFDDASGALRAINREDVGDRL 2v3v.1    --------------------------------------------------------------------------------  target    DDKGIDPALDYQGTVTLVDGSTVEVMSVLSMYREHLADYDIDSVVDMTGAPRNLIEQLLDDMTTLSPVAFHVGEGVNHYF 2v3v.1    -------------------------PSDFEGYKAFLENYRPEKVAEICRVPVEQIYGAARAFAESAATMSLWCMGINQRV  target    HATLHNRATYLVGMLLGSVGVSGGGVSTWAGNYKG--GVFQAAPWFGPGVGGFVNEDPFHPLTDPSARYSAETARHLVHG 2v3v.1    QGVFANNLIHNLHLITGQICRPGATSFSLTGQPNACGGVRDG-G-AL---SHLLP--AGRAIPNAKHRA---EM------  target    EDTSYWGFGDRPLVVDTPEDGRKVFT-GTTHMPTPTKALWYNNANLINQAKWHYELVKNVNPKVD-LIVDQQIEWT-GSA 2v3v.1    --EKLWGLPEGRIA---PEPGYHTVALFEALGRGDVKCMIICETNPAHTLPNLNKV-HKAMSHPESFIVCIEAFPDAVTL  target    EFADIVLPANSWMEAETWEMGASCSNPFLQVWKGGIEPLNDTRDDIAIFAGVANALTELTGDERFSQAFMFADRPEVYLD 2v3v.1    EYADLVLPPAFWCERDGV---YGCGERRYSLTEKAVDPPGQCRPTVNTLVEFARRAGVDPQ---L---VNF-RNAEDVWN  target    RVLAGSF----TTEGYTVEDLTAGR-YGPPGGAL-MQYRSYPRIPFK------EQIEDSLPFYTDTGRMHGYVDIPEAIE 2v3v.1    EWRMVSKGTTYDFWGMTRERLRKESGLIWPCPSEDHPGTSLRYVRGQDPCVPADHPDRFFFYGKPDGRAVIWMRPA----  target    YGENLIVHREAVEATPYLPNVIVSTSPYLRPRDYGIAPEELDGDARSVRNIMMSWAEVKETENPLFAAGYNYLCLTPKSR 2v3v.1    -----------------------------------------------------------KGAAEEPDAEYPLYLTSMRVI  target    HAVHSSWAVTDWHWLWSSSFSDPYRVETRAPGVGEPAIHLNPDDARSLGIRNGDYVWVDSNPKDRPYRDADVDESFLDVA 2v3v.1    DHWHTATMTGKVPELQ--------------KANPIAFVEINEEDAARTGIKHGDSVIVETR-----------------RD  target    RLLVRVTYNPAYPPGVTMLKHAFYMATPRTFRAAQERSDGRALAETTGYQSSFRSGSHQSITRGWAPPMHQTDSLFHKRA 2v3v.1    AMELPARVSDVCRPGLIAVPFFDP--------------------------------------------------------  target    GVFGFTYGFDVDNHAINTVPKETVVRITKAEDGGVGGSGAWTRGRPGSMPGDEDDAMQAYLAGELTVVRRT 2v3v.1    ----------------------------------------------------------------------- ``` | | | | | | | | | | | | | | | | | | | | | | | | | | | | | | | | | | | | | | | | | | | | | | | | | |
|  | 7bkb.1.F | Formate dehydrogenase  *Formate dehydrogenase - heterodisulfide reductase - formylmethanofuran dehydrogenase complex from Methanospirillum hungatei (hexameric, composite structure)* | 0.24 |  | 18.86 | 0.52 | 106-647 | EM | 0.00 | hetero-2-2-2-2-2-2-… | 48 x SF4, 4 x FAD, 2 x FES, 4 x 9S8, 4 x ZN, 2 x MO, 4 x MGD | HHblits | 0.29 |
| ``` target    RPFLERFTDMPLLVRLDTLQRLRADEVFADYSSDLDVDGPSFTLHGMTEEQHERNGDRVVFDDASGALRAINREDVGDRL 7bkb.1    --------------------------------------------------------------------------------  target    DDKGIDPALDYQGTVTLVDGSTVEVMSVLSMYREHLADYDIDSVVDMTGAPRNLIEQLLDDMTTLSPVAFHVGEGVNHYF 7bkb.1    -------------------------VNGFEDLKKTVENY--ADAEAIHGVPLDVVKDIAFRYAKAKNAVIIYCLGITELT  target    HATLHNRATYLVGMLLGSVGVSGGGVSTWAGNYKG-GVFQAAPWFGPGVGGFVNEDPFHPLTDPSARYSAETARHLVHGE 7bkb.1    TGTDNVRSMGNLALLTGNVGREGVGVNPLRGQNNVQGACDMGA--YPN---VY--SGYQKCEVAENRA------KM----  target    DTSYWGFGDRPLVVDTPEDGRKVFTGTTHMPTPTKALWYNNANLINQAKWHYELVKNVNPKVDLIVDQQIEWTGSAEFAD 7bkb.1    -EKAWSVTNLPD-----WYGATLTEQINQCGDEIKGMYILGLNPVVTYPSSNH-VKAQLEKLDFLVVQDIFFTETCQYAD  target    IVLPANSWMEAETWEMGASCSNPFLQVWKGGIEPLNDTRDDIAIFAGVANALTELTGDERFSQAFMFADRPEVYLDRVLA 7bkb.1    VILPGACFAEKDGTF---TSGERRINRVRKAVNPPGQAKEDIHIISELAAKMGFK----GF----EL-PTAKDVWDDMRA  target    GSFTTEGYTVEDLTAGRYGPPGGALMQYRSYPRIPFKEQIEDSLPFYTDTGRMHGYVDIPEAIEYGENLIVHREAVEATP 7bkb.1    VTPSMFGATYEKLERPEGICWP---CPTEEHPGTPI----LHREKFATADGKGNLFGID------------YRPP-----  target    YLPNVIVSTSPYLRPRDYGIAPEELDGDARSVRNIMMSWAEVKETENPLFAAGYNYLCLTPKSRHAVHSSWAVTDWHWLW 7bkb.1    ------------------------------------------AE--V--ADAEYPFTLMTGRLIFHYHSRTQTDRAADL-  target    SSSFSDPYRVETRAPGVGEPAIHLNPDDARSLGIRNGDYVWVDSNPKDRPYRDADVDESFLDVARLLVRVTYNPAYPPGV 7bkb.1    ---------H----REVPESYAQINIEDARRLGIKNNEYIKLKSR-----------------RGETTTLARVTDEVAPGV  target    TMLKHAFYMATPRTFRAAQERSDGRALAETTGYQSSFRSGSHQSITRGWAPPMHQTDSLFHKRAGVFGFTYGFDVDNHAI 7bkb.1    VYMTMHFA------------------------------------------------------------------------  target    NTVPKETVVRITKAEDGGVGGSGAWTRGRPGSMPGDEDDAMQAYLAGELTVVRRT 7bkb.1    ------------------------------------------------------- ``` | | | | | | | | | | | | | | | | | | | | | | | | | | | | | | | | | | | | | | | | | | | | | | | | | |
|  | 7nz1.1.E | NADH-quinone oxidoreductase subunit G  *Respiratory complex I from Escherichia coli - focused refinement of cytoplasmic arm* | 0.21 |  | 14.06 | 0.49 | 116-648 | EM | 0.00 | hetero-1-1-1-1-1-1-… | 7 x SF4, 2 x FES, 1 x FMN, 1 x CA | HHblits | 0.25 |
| ``` target    RPFLERFTDMPLLVRLDTLQRLRADEVFADYSSDLDVDGPSFTLHGMTEEQHERNGDRVVFDDASGALRAINREDVGDRL 7nz1.1    --------------------------------------------------------------------------------  target    DDKGIDPALDYQGTVTLVDGSTVEVMSVLSMYREHLADYDIDSVVDMTGAPR---NLIEQLLDDMTTLSPVAFHVGEGVN 7nz1.1    -----------------------------------IAHALDNSAPAVDGIEPELQSKIDVIVQALAGAKKPLIISGTNAG  target    HYFHATLHNRATYLVGMLLGSVGVSGGGVSTWAGNYKGGVFQAAPWFGPGVGGFVNEDPFHPLTDPSARYSAETARHLVH 7nz1.1    ----SLEVIQAAANVAKALKGRGADVGITMIARS-V-----N-----SM---GL------GIM-----------------  target    GEDTSYWGFGDRPLVVDTPEDGRKVFTGTTHMPTPTKALWYNNANLINQAKWHYELVKNVNPKVDLIVDQQIEWTGSAEF 7nz1.1    G------GG-SLEE----------ALTE--LETGRADAVVVLE-NDLHRHASA-IRVNAALAKAPLVMVVDHQRTAIMEN  target    ADIVLPANSWMEAETWEMGASCSNPFLQVWKGGIEPLN-----DTRDDIAIFAGVANALTELTGDERFSQAFMFADRPEV 7nz1.1    AHLVLSAASFAESDGTV---INNEGRAQRFFQVYDPAYYDSKTVMLESWRWLHSLHSTLLSR----EV----DW-TQLDH  target    YLDRVLAGSFTTEGYTVEDL------------------------TAGR-YGPPG-----GALMQY--------R------ 7nz1.1    VIDAVVAKIPELAGIKDAAPDATFRIRGQKLAREPHRYSGRTAMRANISVHEPRQPQDIDTMFTFSMEGNNQPTAHRSQV  target    SYPRI-PFKEQIEDSLPFYTDTGRMHGYVDIPEAIEYGENLIVHREAVEATPYLPNVIVSTSPYLRPRDYGIAPEELDGD 7nz1.1    PFAWAPGWNSPQAWNKFQDEVGGKLRFGDPGVRLFETS---------ENGLD---Y------------------------  target    ARSVRNIMMSWAEVKETENPLFAAGYNYLCLTPKSRHAVHSSWAVTDWHWLWSSSFSDPYRVETRAPGVGEPAIHLNPDD 7nz1.1    ----------FTSVPAR---FQPQDGKWRIAPYYHLFGSDELSQRAPV--F----------QS----RMPQPYIKLNPAD  target    ARSLGIRNGDYVWVDSNPKDRPYRDADVDESFLDVARLLVRVTYNPAYPPGVTMLKHAFYMATPRTFRAAQERSDGRALA 7nz1.1    AAKLGVNAGTRVSFSYD-----------------GNTVTLPVEIAEGLTAGQVGLPMGMSG-------------------  target    ETTGYQSSFRSGSHQSITRGWAPPMHQTDSLFHKRAGVFGFTYGFDVDNHAINTVPKETVVRITKAEDGGVGGSGAWTRG 7nz1.1    --------------------------------------------------------------------------------  target    RPGSMPGDEDDAMQAYLAGELTVVRRT 7nz1.1    --------------------------- ``` | | | | | | | | | | | | | | | | | | | | | | | | | | | | | | | | | | | | | | | | | | | | | | | | | |
|  | 7p63.1.C | NADH-quinone oxidoreductase  *Complex I from E. coli, DDM/LMNG-purified, under Turnover at pH 6, Closed state* | 0.22 |  | 14.63 | 0.49 | 117-648 | EM | 0.00 | hetero-1-1-1-1-1-1-… | 7 x SF4, 1 x FMN, 1 x NAI, 2 x FES, 1 x CA, 1 x DCQ, 4 x LFA, 8 x 3PE | HHblits | 0.25 |
| ``` target    RPFLERFTDMPLLVRLDTLQRLRADEVFADYSSDLDVDGPSFTLHGMTEEQHERNGDRVVFDDASGALRAINREDVGDRL 7p63.1    --------------------------------------------------------------------------------  target    DDKGIDPALDYQGTVTLVDGSTVEVMSVLSMYREHLADYDIDSVVDMTGAPR---NLIEQLLDDMTTLSPVAFHVGEGVN 7p63.1    ------------------------------------AHALDNSAPAVDGIEPELQSKIDVIVQALAGAKKPLIISGTNAG  target    HYFHATLHNRATYLVGMLLGSVGVSGGGVSTWAGNYKGGVFQAAPWFGPGVGGFVNEDPFHPLTDPSARYSAETARHLVH 7p63.1    ----SLEVIQAAANVAKALKGRGADVGITMIARSV------N-----SM---G------LGIM-----------------  target    GEDTSYWGFGDRPLVVDTPEDGRKVFTGTTHMPTPTKALWYNNANLINQAKWHYELVKNVNPKVDLIVDQQIEWTGSAEF 7p63.1    G------GG-SLEE----------ALT--ELETGRADAVVVLE-NDLHRHASA-TRVNAALAKAPLVMVVDHQRTAIMEN  target    ADIVLPANSWMEAETWEMGASCSNPFLQVWKGGIEPLN-----DTRDDIAIFAGVANALTELTGDERFSQAFMFADRPEV 7p63.1    AHLVLSAASFAESDGTV---INNEGRAQRFFQVYDPAYYDSKTVMLESWRWLHSLHSTLLSR----E----VDW-TQLDH  target    YLDRVLAGSFTTEGYTVEDLT-----------AGR--------------YGPPGG-----ALMQ-----------YRS-- 7p63.1    VIDAVVAKIPELAGIKDAAPDATFRIRGQKLAREPHRYSGRTAMRANISVHEPRQPQDIDTMFTFSMEGNNQPTAHRSQV  target    -YPRI-PFKEQIEDSLPFYTDTGRMHGYVDIPEAIEYGENLIVHREAVEATPYLPNVIVSTSPYLRPRDYGIAPEELDGD 7p63.1    PFAWAPGWNSPQAWNKFQDEVGGKLRFGDPGVRLFETS---------ENGLDY-----------FTS-------------  target    ARSVRNIMMSWAEVKETENPLFAAGYNYLCLTPKSRHAVHSSWAVTDWHWLWSSSFSDPYRVETRAPGVGEPAIHLNPDD 7p63.1    -------------VPAR---FQPQDGKWRIAPYYHLFGSDELSQRAPV--F----------QS----RMPQPYIKLNPAD  target    ARSLGIRNGDYVWVDSNPKDRPYRDADVDESFLDVARLLVRVTYNPAYPPGVTMLKHAFYMATPRTFRAAQERSDGRALA 7p63.1    AAKLGVNAGTRVSFSYD-----------------GNTVTLPVEIAEGLTAGQVGLPMGMSG-------------------  target    ETTGYQSSFRSGSHQSITRGWAPPMHQTDSLFHKRAGVFGFTYGFDVDNHAINTVPKETVVRITKAEDGGVGGSGAWTRG 7p63.1    --------------------------------------------------------------------------------  target    RPGSMPGDEDDAMQAYLAGELTVVRRT 7p63.1    --------------------------- ``` | | | | | | | | | | | | | | | | | | | | | | | | | | | | | | | | | | | | | | | | | | | | | | | | | |
|  | 7p61.1.C | NADH-quinone oxidoreductase  *Complex I from E. coli, DDM-purified, with NADH, Resting state* | 0.22 |  | 13.87 | 0.48 | 118-648 | EM | 0.00 | hetero-1-1-1-1-1-1-… | 7 x SF4, 1 x FMN, 1 x NAI, 2 x FES, 1 x CA, 2 x 3PE, 1 x UQ8 | HHblits | 0.25 |
| ``` target    RPFLERFTDMPLLVRLDTLQRLRADEVFADYSSDLDVDGPSFTLHGMTEEQHERNGDRVVFDDASGALRAINREDVGDRL 7p61.1    --------------------------------------------------------------------------------  target    DDKGIDPALDYQGTVTLVDGSTVEVMSVLSMYREHLADYDIDSVVDMTGAP---RNLIEQLLDDMTTLSPVAFHVGEGVN 7p61.1    -------------------------------------HALDNSAPAVDGIEPELQSKIDVIVQALAGAKKPLIISGTNAG  target    HYFHATLHNRATYLVGMLLGSVGVSGGGVSTWAGNYKGGVFQAAPWFGPGVGGFVNEDPFHPLTDPSARYSAETARHLVH 7p61.1    ----SLEVIQAAANVAKALKGRGADVGITMIARSV------N-----SM---GL------GIM-----------------  target    GEDTSYWGFGDRPLVVDTPEDGRKVFTGTTHMPTPTKALWYNNANLINQAKWHYELVKNVNPKVDLIVDQQIEWTGSAEF 7p61.1    G------GG-SLE----E------ALTE--LETGRADAVVVLE-NDLHRHASA-TRVNAALAKAPLVMVVDHQRTAIMEN  target    ADIVLPANSWMEAETWEMGASCSNPFLQVWKGGIEPLN-----DTRDDIAIFAGVANALTELTGDERFSQAFMFADRPEV 7p61.1    AHLVLSAASFAESDGTV---INNEGRAQRFFQVYDPAYYDSKTVMLESWRWLHSLHSTLLSR----EV----DW-TQLDH  target    YLDRVLAGSFTTEGYTVEDLT-----------AGR--------------YGPPGG-----ALMQY--------RS---YP 7p61.1    VIDAVVAKIPELAGIKDAAPDATFRIRGQKLAREPHRYSGRTAMRANISVHEPRQPQDIDTMFTFSMEGNNQPTAHRSQV  target    RIPFKE----QIEDSLPFYTDTGRMHGYVDIPEAIEYGENLIVHREAVEATPYLPNVIVSTSPYLRPRDYGIAPEELDGD 7p61.1    PFAWAPGWNSPQAWNKFQDEVGGKLRFGDPGVRLFETS---------ENGLDY-----------FT--------------  target    ARSVRNIMMSWAEVKETENPLFAAGYNYLCLTPKSRHAVHSSWAVTDWHWLWSSSFSDPYRVETRAPGVGEPAIHLNPDD 7p61.1    ------------SVPAR---FQPQDGKWRIAPYYHLFGSDELSQRAPV--F----------QS----RMPQPYIKLNPAD  target    ARSLGIRNGDYVWVDSNPKDRPYRDADVDESFLDVARLLVRVTYNPAYPPGVTMLKHAFYMATPRTFRAAQERSDGRALA 7p61.1    AAKLGVNAGTRVSFSYD-----------------GNTVTLPVEIAEGLTAGQVGLPMGMSG-------------------  target    ETTGYQSSFRSGSHQSITRGWAPPMHQTDSLFHKRAGVFGFTYGFDVDNHAINTVPKETVVRITKAEDGGVGGSGAWTRG 7p61.1    --------------------------------------------------------------------------------  target    RPGSMPGDEDDAMQAYLAGELTVVRRT 7p61.1    --------------------------- ``` | | | | | | | | | | | | | | | | | | | | | | | | | | | | | | | | | | | | | | | | | | | | | | | | | |
|  | 1h0h.1.A | FORMATE DEHYDROGENASE SUBUNIT ALPHA  *Tungsten containing Formate Dehydrogenase from Desulfovibrio Gigas* | 0.20 |  | 17.65 | 0.42 | 1-373 | X-ray | 1.80 | hetero-1-1-mer | 1 x W, 1 x 2MD, 1 x MGD, 4 x SF4, 1 x CA | HHblits | 0.28 |
| ``` target    RPFLERFTDMPLLVRLDTLQRLRADEVFADYSSDLDVDGPSFTLHGMTEEQHERNGDRVVFDDASGALRAINREDVGDRL 1h0h.1    KDYVVNYTNASFIVGEG---FAFEE------------------------------GLFAGYNKETRKYDKSKW-------  target    DDKGIDPALDYQGTVTLVDGSTVEVMSVLSMYREHLADYDIDSVVDMTGAPRNLIEQLLDDMTTL----SPVAFHVGEGV 1h0h.1    ---GFERDENGN--PK-RDETLKHPRCVFQIMKKHYERYDLDKISAICGTPKELILKVYDAYCATGKPDKAGTIMYAMGW  target    NHYFHATLHNRATYLVGMLLGSVGVSGGGVSTWAGNYKG-GVF--QAAPWFGPGVG-----GFVNEDPFHPLTDPSARYS 1h0h.1    TQHTVGVQNIRAMSINQLLLGNIGVAGGGVNALRGEANVQGSTDHGLLMHIYPGYLGTARASIPTYEEYTKKFTPVSKDP  target    AE--TARHLVH---GEDTSYWGFGDRPLVVDTP---EDG--RKVFT-GTTHMPTPTKALWYNNANLINQAKWHYELVKNV 1h0h.1    QSANWWSNFPKYSASYIKSMWPDADLNEAYGYLPKGEDGKDYSWLTLFDDMFQGKIKGFFAWGQNPACSGANSNKT-REA  target    NPKVDLIVDQQIEWTGSAEFA-------------DIVLPANSWMEAETWEMGASCSNPFLQVWKGGIEPLNDTRDDIAIF 1h0h.1    LTKLDWMVNVNIFDNETGSFWRGPDMDPKKIKTEVFFLPCAVAIEKEGSIS---NSGRWMQWRYVGPEPRKNAIPDGDLI  target    AGVANALTELTGDERFSQAFMFADRPEVYLDRVLAGSFTTEGYTVEDLTAGRYGPPGGALMQYRSYPRIPFKEQIEDSLP 1h0h.1    VELAKRVQK-----------------------------------------------------------------------  target    FYTDTGRMHGYVDIPEAIEYGENLIVHREAVEATPYLPNVIVSTSPYLRPRDYGIAPEELDGDARSVRNIMMSWAEVKET 1h0h.1    --------------------------------------------------------------------------------  target    ENPLFAAGYNYLCLTPKSRHAVHSSWAVTDWHWLWSSSFSDPYRVETRAPGVGEPAIHLNPDDARSLGIRNGDYVWVDSN 1h0h.1    --------------------------------------------------------------------------------  target    PKDRPYRDADVDESFLDVARLLVRVTYNPAYPPGVTMLKHAFYMATPRTFRAAQERSDGRALAETTGYQSSFRSGSHQSI 1h0h.1    --------------------------------------------------------------------------------  target    TRGWAPPMHQTDSLFHKRAGVFGFTYGFDVDNHAINTVPKETVVRITKAEDGGVGGSGAWTRGRPGSMPGDEDDAMQAYL 1h0h.1    --------------------------------------------------------------------------------  target    AGELTVVRRT 1h0h.1    ---------- ``` | | | | | | | | | | | | | | | | | | | | | | | | | | | | | | | | | | | | | | | | | | | | | | | | | |
|  | 1kqf.1.A | FORMATE DEHYDROGENASE, NITRATE-INDUCIBLE, MAJOR SUBUNIT  *FORMATE DEHYDROGENASE N FROM E. COLI* | 0.16 |  | 18.87 | 0.34 | 98-373 | X-ray | 1.60 | hetero-oligomer | 3 x 6MO, 15 x SF4, 6 x MGD, 6 x HEM, 3 x CDL | HHblits | 0.29 |
| ``` target    RPFLERFTDMPLLVRLDTLQRLRADEVFADYSSDLDVDGPSFTLHGMTEEQHERNGDRVVFDDASGALRAINREDVGDRL 1kqf.1    --------------------------------------------------------------------------------  target    DDKGIDPALDYQGTVTLVDGSTVEVMSVLSMYREHLADYDIDSVVDMTGAPRNLIEQLLDDMTTL----SPVAFHVGEGV 1kqf.1    -----------------RDETLTHPRCVWNLLKEHVSRYTPDVVENICGTPKADFLKVCEVLASTSAPDRTTTFLYALGW  target    NHYFHATLHNRATYLVGMLLGSVGVSGGGVSTWAGNYKG-GV--FQAAPWFGPGVGGFVNED-----PFHPLTDPSARYS 1kqf.1    TQHTVGAQNIRTMAMIQLLLGNMGMAGGGVNALRGHSNIQGLTDLGLLSTSLPGYLTLPSEKQVDLQSYLEANTPKATLA  target    AETARHLVHGEDTS------------------YWGFGDRPLVVDTPEDGRKVFT-GTTHMPTPTKALWYNNANLINQAKW 1kqf.1    DQV--N-YWSNYPKFFVSLMKSFYGDAAQKENNWGYDWLPK----WDQTYDVIKYFNMMDEGKVTGYFCQGFNPVASFPD  target    HYELVKNVNPKVDLIVDQQIEWTGSAEFAD-----------------IVLPANSWMEAETWEMGASCSNPFLQVWKGGIE 1kqf.1    KNKV-VSCLSKLKYMVVIDPLVTETSTFWQNHGESNDVDPASIQTEVFRLPSTCFAEEDGSI---ANSGRWLQWHWKGQD  target    PLNDTRDDIAIFAGVANALTELTGDERFSQAFMFADRPEVYLDRVLAGSFTTEGYTVEDLTAGRYGPPGGALMQYRSYPR 1kqf.1    APGEARNDGEILAGIYHHLRE-----------------------------------------------------------  target    IPFKEQIEDSLPFYTDTGRMHGYVDIPEAIEYGENLIVHREAVEATPYLPNVIVSTSPYLRPRDYGIAPEELDGDARSVR 1kqf.1    --------------------------------------------------------------------------------  target    NIMMSWAEVKETENPLFAAGYNYLCLTPKSRHAVHSSWAVTDWHWLWSSSFSDPYRVETRAPGVGEPAIHLNPDDARSLG 1kqf.1    --------------------------------------------------------------------------------  target    IRNGDYVWVDSNPKDRPYRDADVDESFLDVARLLVRVTYNPAYPPGVTMLKHAFYMATPRTFRAAQERSDGRALAETTGY 1kqf.1    --------------------------------------------------------------------------------  target    QSSFRSGSHQSITRGWAPPMHQTDSLFHKRAGVFGFTYGFDVDNHAINTVPKETVVRITKAEDGGVGGSGAWTRGRPGSM 1kqf.1    --------------------------------------------------------------------------------  target    PGDEDDAMQAYLAGELTVVRRT 1kqf.1    ---------------------- ``` | | | | | | | | | | | | | | | | | | | | | | | | | | | | | | | | | | | | | | | | | | | | | | | | | |
|  | 6sdr.1.A | Formate dehydrogenase, alpha subunit, selenocysteine-containing  *W-formate dehydrogenase from Desulfovibrio vulgaris - Oxidized form* | 0.17 |  | 19.08 | 0.34 | 103-373 | X-ray | 2.10 | hetero-1-1-mer | 2 x MGD, 4 x SF4, 1 x H2S, 1 x W | HHblits | 0.30 |
| ``` target    RPFLERFTDMPLLVRLDTLQRLRADEVFADYSSDLDVDGPSFTLHGMTEEQHERNGDRVVFDDASGALRAINREDVGDRL 6sdr.1    --------------------------------------------------------------------------------  target    DDKGIDPALDYQGTVTLVDGSTVEVMSVLSMYREHLADYDIDSVVDMTGAPRNLIEQLLDDMTTL----SPVAFHVGEGV 6sdr.1    ----------------------KHPRCVINLLKKHYERYNLDKVAAITGTSKEQLQQVYKAYAATGKPDKAGTIMYAMGW  target    NHYFHATLHNRATYLVGMLLGSVGVSGGGVSTWAGNYKG-GV--FQAAPWFGPGVGGFVNED--PF--HPLTDPSARYSA 6sdr.1    TQHSVGVQNIRAMAMIQLLLGNIGVAGGGVNALRGESNVQGSTDQGLLAHIWPGYNPVPNSKAATLELYNAATPQSKDPM  target    ETARHLVHGEDTSYWG------FG-DRP----LVVDTPEDGR--------KVFTGTTHMPTPTKALWYNNANLINQAKWH 6sdr.1    SV-NW--WQNRPKYVASYLKALYPDEEPAAAYDYLPRIDAGRKLTDYFWLNIF--EKMDKGEFKGLFAWGMNPACGGANA  target    YELVKNVNPKVDLIVDQQIEWTGSAEF--------AD-----IVLPANSWMEAETWEMGASCSNPFLQVWKGGIEPLNDT 6sdr.1    NKN-RKAMGKLEWLVNVNLFENETSSFWKGPGMNPAEIGTEVFFLPCCVSIEKEGSVA---NSGRWMQWRYRGPKPYAET  target    RDDIAIFAGVANALTELTGDERFSQAFMFADRPEVYLDRVLAGSFTTEGYTVEDLTAGRYGPPGGALMQYRSYPRIPFKE 6sdr.1    KPDGDIMLDMFKKVRE----------------------------------------------------------------  target    QIEDSLPFYTDTGRMHGYVDIPEAIEYGENLIVHREAVEATPYLPNVIVSTSPYLRPRDYGIAPEELDGDARSVRNIMMS 6sdr.1    --------------------------------------------------------------------------------  target    WAEVKETENPLFAAGYNYLCLTPKSRHAVHSSWAVTDWHWLWSSSFSDPYRVETRAPGVGEPAIHLNPDDARSLGIRNGD 6sdr.1    --------------------------------------------------------------------------------  target    YVWVDSNPKDRPYRDADVDESFLDVARLLVRVTYNPAYPPGVTMLKHAFYMATPRTFRAAQERSDGRALAETTGYQSSFR 6sdr.1    --------------------------------------------------------------------------------  target    SGSHQSITRGWAPPMHQTDSLFHKRAGVFGFTYGFDVDNHAINTVPKETVVRITKAEDGGVGGSGAWTRGRPGSMPGDED 6sdr.1    --------------------------------------------------------------------------------  target    DAMQAYLAGELTVVRRT 6sdr.1    ----------------- ``` | | | | | | | | | | | | | | | | | | | | | | | | | | | | | | | | | | | | | | | | | | | | | | | | | |
|  | 6sdv.1.A | Formate dehydrogenase, alpha subunit, selenocysteine-containing,Formate dehydrogenase, alpha subunit, selenocysteine-containing,W-formate dehydrogenase - alpha subunit  *W-formate dehydrogenase from Desulfovibrio vulgaris - Formate reduced form* | 0.17 |  | 18.56 | 0.34 | 103-373 | X-ray | 1.90 | hetero-1-1-mer | 2 x MGD, 4 x SF4, 1 x W, 1 x H2S | HHblits | 0.29 |
| ``` target    RPFLERFTDMPLLVRLDTLQRLRADEVFADYSSDLDVDGPSFTLHGMTEEQHERNGDRVVFDDASGALRAINREDVGDRL 6sdv.1    --------------------------------------------------------------------------------  target    DDKGIDPALDYQGTVTLVDGSTVEVMSVLSMYREHLADYDIDSVVDMTGAPRNLIEQLLDDMTTL----SPVAFHVGEGV 6sdv.1    ----------------------KHPRCVINLLKKHYERYNLDKVAAITGTSKEQLQQVYKAYAATGKPDKAGTIMYAMGW  target    NHYFHATLHNRATYLVGMLLGSVGVSGGGVSTWAGNYKG-GVF--QAAPWFGPGVGGFVNE-DP-FHPL--TDPSARYSA 6sdv.1    TQHSVGVQNIRAMAMIQLLLGNIGVAGGGVNALRGESNVQGSTDQGLLAHIWPGYNPVPNSKAATLELYNAATPQSKDPM  target    ETARHLVH-GED------TSYWGF-GD-RPLVVDTPEDGR--------KVFTGTTHMPTPTKALWYNNANLINQAKWHYE 6sdv.1    SV-NWWQNRPKYVASYLKALYPDEEPAAAYDYLPRIDAGRKLTDYFWLNIF--EKMDKGEFKGLFAWGMNPACGGANANK  target    LVKNVNPKVDLIVDQQIEWTGSAEF--------AD-----IVLPANSWMEAETWEMGASCSNPFLQVWKGGIEPLNDTRD 6sdv.1    N-RKAMGKLEWLVNVNLFENETSSFWKGPGMNPAEIGTEVFFLPCCVSIEKEGSVA---NSGRWMQWRYRGPKPYAETKP  target    DIAIFAGVANALTELTGDERFSQAFMFADRPEVYLDRVLAGSFTTEGYTVEDLTAGRYGPPGGALMQYRSYPRIPFKEQI 6sdv.1    DGDIMLDMFKKVRE------------------------------------------------------------------  target    EDSLPFYTDTGRMHGYVDIPEAIEYGENLIVHREAVEATPYLPNVIVSTSPYLRPRDYGIAPEELDGDARSVRNIMMSWA 6sdv.1    --------------------------------------------------------------------------------  target    EVKETENPLFAAGYNYLCLTPKSRHAVHSSWAVTDWHWLWSSSFSDPYRVETRAPGVGEPAIHLNPDDARSLGIRNGDYV 6sdv.1    --------------------------------------------------------------------------------  target    WVDSNPKDRPYRDADVDESFLDVARLLVRVTYNPAYPPGVTMLKHAFYMATPRTFRAAQERSDGRALAETTGYQSSFRSG 6sdv.1    --------------------------------------------------------------------------------  target    SHQSITRGWAPPMHQTDSLFHKRAGVFGFTYGFDVDNHAINTVPKETVVRITKAEDGGVGGSGAWTRGRPGSMPGDEDDA 6sdv.1    --------------------------------------------------------------------------------  target    MQAYLAGELTVVRRT 6sdv.1    --------------- ``` | | | | | | | | | | | | | | | | | | | | | | | | | | | | | | | | | | | | | | | | | | | | | | | | | |
|  | 8bqg.1.A | Formate dehydrogenase, alpha subunit, selenocysteine-containing  *W-formate dehydrogenase from Desulfovibrio vulgaris - Soaking with Formate 1 min* | 0.17 |  | 18.25 | 0.34 | 103-373 | X-ray | 1.95 | hetero-1-1-mer | 2 x MGD, 4 x SF4, 1 x H2S, 1 x W | HHblits | 0.29 |
| ``` target    RPFLERFTDMPLLVRLDTLQRLRADEVFADYSSDLDVDGPSFTLHGMTEEQHERNGDRVVFDDASGALRAINREDVGDRL 8bqg.1    --------------------------------------------------------------------------------  target    DDKGIDPALDYQGTVTLVDGSTVEVMSVLSMYREHLADYDIDSVVDMTGAPRNLIEQLLDDMTTL----SPVAFHVGEGV 8bqg.1    ----------------------KHPRCVINLLKKHYERYNLDKVAAITGTSKEQLQQVYKAYAATGKPDKAGTIMYAMGW  target    NHYFHATLHNRATYLVGMLLGSVGVSGGGVSTWAGNYKG-GVF--QAAPWFGPGVGGFVNED-P---FHPLTDPSARYSA 8bqg.1    TQHSVGVQNIRAMAMIQLLLGNIGVAGGGVNALRGESNVQGSTDQGLLAHIWPGYNPVPNSKAATLELYNAATPQSKDPM  target    E--T-------ARHLVHGEDTS-YWGFGDRPLVVDTPEDGR--------KVFTGTTHMPTPTKALWYNNANLINQAKWHY 8bqg.1    SVNWWQNRPKYVASYLKALYPDEEPAA-AYDY-LPRIDAGRKLTDYFWLNIFE--KMDKGEFKGLFAWGMNPACGGANAN  target    ELVKNVNPKVDLIVDQQIEWTGSAEF--------AD-----IVLPANSWMEAETWEMGASCSNPFLQVWKGGIEPLNDTR 8bqg.1    KN-RKAMGKLEWLVNVNLFENETSSFWKGPGMNPAEIGTEVFFLPCCVSIEKEGSVA---NSGRWMQWRYRGPKPYAETK  target    DDIAIFAGVANALTELTGDERFSQAFMFADRPEVYLDRVLAGSFTTEGYTVEDLTAGRYGPPGGALMQYRSYPRIPFKEQ 8bqg.1    PDGDIMLDMFKKVRE-----------------------------------------------------------------  target    IEDSLPFYTDTGRMHGYVDIPEAIEYGENLIVHREAVEATPYLPNVIVSTSPYLRPRDYGIAPEELDGDARSVRNIMMSW 8bqg.1    --------------------------------------------------------------------------------  target    AEVKETENPLFAAGYNYLCLTPKSRHAVHSSWAVTDWHWLWSSSFSDPYRVETRAPGVGEPAIHLNPDDARSLGIRNGDY 8bqg.1    --------------------------------------------------------------------------------  target    VWVDSNPKDRPYRDADVDESFLDVARLLVRVTYNPAYPPGVTMLKHAFYMATPRTFRAAQERSDGRALAETTGYQSSFRS 8bqg.1    --------------------------------------------------------------------------------  target    GSHQSITRGWAPPMHQTDSLFHKRAGVFGFTYGFDVDNHAINTVPKETVVRITKAEDGGVGGSGAWTRGRPGSMPGDEDD 8bqg.1    --------------------------------------------------------------------------------  target    AMQAYLAGELTVVRRT 8bqg.1    ---------------- ``` | | | | | | | | | | | | | | | | | | | | | | | | | | | | | | | | | | | | | | | | | | | | | | | | | |
|  | 2nya.1.A | Periplasmic nitrate reductase  *Crystal structure of the periplasmic nitrate reductase (NAP) from Escherichia coli* | 0.15 |  | 17.27 | 0.32 | 102-373 | X-ray | 2.50 | monomer | 1 x SF4, 1 x 6MO, 2 x MGD | HHblits | 0.29 |
| ``` target    RPFLERFTDMPLLVRLDTLQRLRADEVFADYSSDLDVDGPSFTLHGMTEEQHERNGDRVVFDDASGALRAINREDVGDRL 2nya.1    --------------------------------------------------------------------------------  target    DDKGIDPALDYQGTVTLVDGSTVEVMSVLSMYREHLADYDIDSVVDMTGAPRNLIEQLLDDMTTLSPV-AFHVGEGVNHY 2nya.1    ---------------------DASEPMSFEDYKAFVAEYTLEKTAEMTGVPKDQLEQLAQLYADPNKKVISYWTMGFNQH  target    FHATLHNRATYLVGMLLGSVGVSGGGVSTWAGNYKGGVFQAAPWFGPGVGGFVNEDPF-HPLTDPSARYSAETARHLVHG 2nya.1    TRGVWANNLVYNLHLLTGKISQPGCGPFSLTGQPSACGTA---REV---GTFAHRLPADMVVTNEKHRD------IC---  target    EDTSYWGFGDRPLVVDTPEDGRKVFT-GTTHMPTPTKALWYNNANLINQAKWHYE-LVKNVNPKVDLIVDQQIEWTGSAE 2nya.1    --EKKWNIPSGTI-P--AKIGLHAVAQDRALKDGKLNVYWTMCTNNMQAGPNINEERMPGWRDPRNFIIVSDPYPTVSAL  target    FADIVLPANSWMEAETWEMGASCSNPFLQVWKGGIEPLNDTRDDIAIFAGVANALTELTGDERFSQAFMFADRPEVYLDR 2nya.1    AADLILPTAMWVEKEGAY---GNAERRTQFWRQQVQAPGEAKSDLWQLVQFSRRFKT-----------------------  target    VLAGSFTTEGYTVEDLTAGRYGPPGGALMQYRSYPRIPFKEQIEDSLPFYTDTGRMHGYVDIPEAIEYGENLIVHREAVE 2nya.1    --------------------------------------------------------------------------------  target    ATPYLPNVIVSTSPYLRPRDYGIAPEELDGDARSVRNIMMSWAEVKETENPLFAAGYNYLCLTPKSRHAVHSSWAVTDWH 2nya.1    --------------------------------------------------------------------------------  target    WLWSSSFSDPYRVETRAPGVGEPAIHLNPDDARSLGIRNGDYVWVDSNPKDRPYRDADVDESFLDVARLLVRVTYNPAYP 2nya.1    --------------------------------------------------------------------------------  target    PGVTMLKHAFYMATPRTFRAAQERSDGRALAETTGYQSSFRSGSHQSITRGWAPPMHQTDSLFHKRAGVFGFTYGFDVDN 2nya.1    --------------------------------------------------------------------------------  target    HAINTVPKETVVRITKAEDGGVGGSGAWTRGRPGSMPGDEDDAMQAYLAGELTVVRRT 2nya.1    ---------------------------------------------------------- ``` | | | | | | | | | | | | | | | | | | | | | | | | | | | | | | | | | | | | | | | | | | | | | | | | | |
|  | 3o5a.1.A | Periplasmic nitrate reductase  *Crystal Structure of partially reduced Periplasmic Nitrate Reductase from Cupriavidus necator using Ionic Liquids* | 0.15 |  | 18.95 | 0.32 | 104-373 | X-ray | 1.72 | hetero-oligomer | 1 x SF4, 1 x MOS, 2 x MGD, 2 x HEC | HHblits | 0.29 |
| ``` target    RPFLERFTDMPLLVRLDTLQRLRADEVFADYSSDLDVDGPSFTLHGMTEEQHERNGDRVVFDDASGALRAINREDVGDRL 3o5a.1    --------------------------------------------------------------------------------  target    DDKGIDPALDYQGTVTLVDGSTVEVMSVLSMYREHLADYDIDSVVDMTGAPRNLIEQLLDDMTTLS-PVAFHVGEGVNHY 3o5a.1    -----------------------AKVITFDEFAKFVSKYDADYVSKLSAVPKAKLDQLAELYADPNIKVMSLWTMGFNQH  target    FHATLHNRATYLVGMLLGSVGVSGGGVSTWAGNYKGG-VFQAAPWFGPGVGGFVNEDPFHPLTDPSARYSAETARHLVHG 3o5a.1    TRGTWANNMVYNLHLLTGKIATPGNSPFSLTGQPSACGTAREVG-TF---SHRLP--ADMVVTNPKHRE---EA------  target    EDTSYWGFGDRPLVVDTPEDGRKVFTGTTHMPTPTKALWYNNANLINQAKWH-YELVKNVNPKVDLIVDQQIEWTGSAEF 3o5a.1    --ERIWKLPPG-TIPDKPGY-DAVLQNRMLKDGKLNAYWVQVNNNMQAAANLMEEGLPGYRNPANFIVVSDAYPTVTALA  target    ADIVLPANSWMEAETWEMGASCSNPFLQVWKGGIEPLNDTRDDIAIFAGVANALTELTGDERFSQAFMFADRPEVYLDRV 3o5a.1    ADLVLPSAMWVEKEGAY---GNAERRTQFWHQLVDAPGEARSDLWQLVEFAKRFKV------------------------  target    LAGSFTTEGYTVEDLTAGRYGPPGGALMQYRSYPRIPFKEQIEDSLPFYTDTGRMHGYVDIPEAIEYGENLIVHREAVEA 3o5a.1    --------------------------------------------------------------------------------  target    TPYLPNVIVSTSPYLRPRDYGIAPEELDGDARSVRNIMMSWAEVKETENPLFAAGYNYLCLTPKSRHAVHSSWAVTDWHW 3o5a.1    --------------------------------------------------------------------------------  target    LWSSSFSDPYRVETRAPGVGEPAIHLNPDDARSLGIRNGDYVWVDSNPKDRPYRDADVDESFLDVARLLVRVTYNPAYPP 3o5a.1    --------------------------------------------------------------------------------  target    GVTMLKHAFYMATPRTFRAAQERSDGRALAETTGYQSSFRSGSHQSITRGWAPPMHQTDSLFHKRAGVFGFTYGFDVDNH 3o5a.1    --------------------------------------------------------------------------------  target    AINTVPKETVVRITKAEDGGVGGSGAWTRGRPGSMPGDEDDAMQAYLAGELTVVRRT 3o5a.1    --------------------------------------------------------- ``` | | | | | | | | | | | | | | | | | | | | | | | | | | | | | | | | | | | | | | | | | | | | | | | | | |
|  | 6f0k.1.B | Fe-S-cluster-containing hydrogenase  *Alternative complex III* | 0.15 |  | 18.70 | 0.32 | 270-650 | EM | 0.00 | hetero-1-1-1-1-1-1-… | 6 x HEC, 1 x F3S, 3 x SF4 | HHblits | 0.28 |
| ``` target    RPFLERFTDMPLLVRLDTLQRLRADEVFADYSSDLDVDGPSFTLHGMTEEQHERNGDRVVFDDASGALRAINREDVGDRL 6f0k.1    --------------------------------------------------------------------------------  target    DDKGIDPALDYQGTVTLVDGSTVEVMSVLSMYREHLADYDIDSVVDMTGAPRNLIEQLLDDMTTLSPVAFHVGEGVNHYF 6f0k.1    --------------------------------------------------------------------------------  target    HATLHNRATYLVGMLLGSVGVSGGGVSTWAGNYKGGVFQAAPWFGPGVGGFVNEDPFHPLTDPSARYSAETARHLVHGED 6f0k.1    --------------------------------------------------------------------------------  target    TSYWGFGDRPLVVDTPEDGRKVFTGTTHMPTPTKALWYNNANLINQAKWHYELVKNVNPKVDLIVDQQIEWTGSAEFADI 6f0k.1    -----------------------------AGAVDALLLLNVNPVYDAPAALGF-AEALAQVPEVIHLGLHVDETARRSTW  target    VLPANSWMEAETWEMGASCSNPFLQVWKGGIEPLNDT-RDDIAIFAGVANALTELTGDERFSQAFMFADRPEVYLDRVLA 6f0k.1    HLPSTHYLEAWGDGR---AYDGTLSVIQPLIAPLYEAAHSPLEVLALLATGEEQS-----------AYDLVRNTWRRLLA  target    GSFTTEGYTVED----LTAGRYGPPGGALMQYRSYPRIPFKEQIEDSLPFYTDTGRMHGYVDIPEAIEYGENLIVHREAV 6f0k.1    GR-----GAFEQAWQRVLHDGFLP----------------------DSGYPTVSLRPNR-----------QALAD-----  target    EATPYLPNVIVSTSPYLRPRDYGIAPEELDGDARSVRNIMMSWAEVKETENPLFAAGYNYLCLTPKSRHAVHSSWAVTDW 6f0k.1    ------------------------------------------WPQ---------AAEGGLEVVFRLDPTVLDGSFANNAW  target    HWLWSSSFSDPYRVETRAPGVGEPAIHLNPDDARSLGIRN--------GDYVWVDSNPKDRPYRDADVDESFLDVARLLV 6f0k.1    --AQEL--PDPITK-----IVWDNVAILSPKTAAALGVKAEYHKGVYIADVIELSLD-----------------GRAVEL  target    RVTYNPAYPPGVTMLKHAFYMATPRTFRAAQERSDGRALAETTGYQSSFRSGSHQSITRGWAPPMHQTDSLFHKRAGVFG 6f0k.1    PVWVLPGHPDDSITVYLGYGREI---------------------------------------------------------  target    FTYGFDVDNHAINTVPKETVVRITKAEDGGVGGSGAWTRGRPGSMPGDEDDAMQAYLAGELTVVRRT 6f0k.1    ------------------------------------------------------------------- ``` | | | | | | | | | | | | | | | | | | | | | | | | | | | | | | | | | | | | | | | | | | | | | | | | | |
|  | 7bkb.1.L | Formylmethanofuran dehydrogenase, subunit B  *Formate dehydrogenase - heterodisulfide reductase - formylmethanofuran dehydrogenase complex from Methanospirillum hungatei (hexameric, composite structure)* | 0.11 |  | 14.56 | 0.27 | 112-373 | EM | 0.00 | hetero-2-2-2-2-2-2-… | 48 x SF4, 4 x FAD, 2 x FES, 4 x 9S8, 4 x ZN, 2 x MO, 4 x MGD | HHblits | 0.27 |
| ``` target    RPFLERFTDMPLLVRLDTLQRLRADEVFADYSSDLDVDGPSFTLHGMTEEQHERNGDRVVFDDASGALRAINREDVGDRL 7bkb.1    --------------------------------------------------------------------------------  target    DDKGIDPALDYQGTVTLVDGSTVEVMSVLSMYREHLADYDIDSVVDMTGAPRNLIEQLLDDMTTLSPVAFHVGEGVNHYF 7bkb.1    -------------------------------FRMVIHGHGKDLPDEVAGIKKETILEVAEIMKNARFGTTFFGMGLTHTD  target    HATLHNRATY------------LVGMLLGSVGVSGGGVSTWAGNYKGGVFQAAPWFGPGVGGFVNEDPFHPLTDPSARYS 7bkb.1    GRNHNIDIAISLTRDLNKISKWTIMAMRGHYNIAGPGVVWSWT-------FG----FPYCL---------DLTKQN----  target    AETARHLVHGEDTSYWGFGDRPLVVDTPEDGRKVFTGTTHMPTPTKALWYNNANLINQAKWHYELVKNVNPKVDLIVDQQ 7bkb.1    -----H------A-HMN----PGETS-------SVD--MAMRDEVDMFINIGTDAAAHFPIP---AVKQLKKHPW-VTID  target    IEWTGSAEFADIVLPANSW-MEAETWEMGASCSNPFLQVWKGGIEPLNDTRDDIAIFAGVANALTELTGDERFSQAFMFA 7bkb.1    PSINMASEISDLHIPVCICGVDVGGIVY---RMDNVPIQFRKVIEPPEGVMDDETLLNKIADRMEE--------------  target    DRPEVYLDRVLAGSFTTEGYTVEDLTAGRYGPPGGALMQYRSYPRIPFKEQIEDSLPFYTDTGRMHGYVDIPEAIEYGEN 7bkb.1    --------------------------------------------------------------------------------  target    LIVHREAVEATPYLPNVIVSTSPYLRPRDYGIAPEELDGDARSVRNIMMSWAEVKETENPLFAAGYNYLCLTPKSRHAVH 7bkb.1    --------------------------------------------------------------------------------  target    SSWAVTDWHWLWSSSFSDPYRVETRAPGVGEPAIHLNPDDARSLGIRNGDYVWVDSNPKDRPYRDADVDESFLDVARLLV 7bkb.1    --------------------------------------------------------------------------------  target    RVTYNPAYPPGVTMLKHAFYMATPRTFRAAQERSDGRALAETTGYQSSFRSGSHQSITRGWAPPMHQTDSLFHKRAGVFG 7bkb.1    --------------------------------------------------------------------------------  target    FTYGFDVDNHAINTVPKETVVRITKAEDGGVGGSGAWTRGRPGSMPGDEDDAMQAYLAGELTVVRRT 7bkb.1    ------------------------------------------------------------------- ``` | | | | | | | | | | | | | | | | | | | | | | | | | | | | | | | | | | | | | | | | | | | | | | | | | |
|  | 5t5i.1.B | Tungsten formylmethanofuran dehydrogenase subunit B  *TUNGSTEN-CONTAINING FORMYLMETHANOFURAN DEHYDROGENASE FROM METHANOTHERMOBACTER WOLFEII, ORTHORHOMBIC FORM AT 1.9 A* | 0.11 |  | 14.63 | 0.26 | 122-373 | X-ray | 1.90 | hetero-oligomer | 4 x ZN, 2 x MG, 18 x K, 22 x SF4, 2 x W, 4 x MGD, 2 x H2S, 2 x CA | HHblits | 0.27 |
| ``` target    RPFLERFTDMPLLVRLDTLQRLRADEVFADYSSDLDVDGPSFTLHGMTEEQHERNGDRVVFDDASGALRAINREDVGDRL 5t5i.1    --------------------------------------------------------------------------------  target    DDKGIDPALDYQGTVTLVDGSTVEVMSVLSMYREHLADYDIDSVVDMTGAPRNLIEQLLDDMTTLSPVAFHVGEGVNHYF 5t5i.1    -----------------------------------------ILYDEVAGVPREQIEEAVEVLKNAQFGILFFGMGITHSR  target    HATLHNRATYLVGMLLGSVGVSGGGVSTWAGNYKGGVFQAAPWFGPGVGGFVNEDPFHPLTDPSARYSAETARHLVHGED 5t5i.1    GKHRNIDTAIMMVQDLNDY--AKWTLIPMRGHYNV--TGFNQ-V----CTW---ESGYPYCV---D-------F-SGG-E  target    TSYWGFGDRPLVVDTPEDGRKVFTGTTHMPTPTKALWYNNANLINQAKWHYELVKNVNPKVDLIVDQQIEWTGSAEFADI 5t5i.1    P---RY--NPGETG-A-------N-DLLQNREADAMMVIASDPGAHFPQR---ALERMAEIP-VIAIEPHRTPTTEMADI  target    VLPANS-WMEAETWEMGASCSNPFLQVWKGGIEPLNDTRDDIAIFAGVANALTELTGDERFSQAFMFADRPEVYLDRVLA 5t5i.1    IIPPAIVGMEAEGTA---YRMEGVPIRMKKVVDS--DLLSDREILERLLEKVRE--------------------------  target    GSFTTEGYTVEDLTAGRYGPPGGALMQYRSYPRIPFKEQIEDSLPFYTDTGRMHGYVDIPEAIEYGENLIVHREAVEATP 5t5i.1    --------------------------------------------------------------------------------  target    YLPNVIVSTSPYLRPRDYGIAPEELDGDARSVRNIMMSWAEVKETENPLFAAGYNYLCLTPKSRHAVHSSWAVTDWHWLW 5t5i.1    --------------------------------------------------------------------------------  target    SSSFSDPYRVETRAPGVGEPAIHLNPDDARSLGIRNGDYVWVDSNPKDRPYRDADVDESFLDVARLLVRVTYNPAYPPGV 5t5i.1    --------------------------------------------------------------------------------  target    TMLKHAFYMATPRTFRAAQERSDGRALAETTGYQSSFRSGSHQSITRGWAPPMHQTDSLFHKRAGVFGFTYGFDVDNHAI 5t5i.1    --------------------------------------------------------------------------------  target    NTVPKETVVRITKAEDGGVGGSGAWTRGRPGSMPGDEDDAMQAYLAGELTVVRRT 5t5i.1    ------------------------------------------------------- ``` | | | | | | | | | | | | | | | | | | | | | | | | | | | | | | | | | | | | | | | | | | | | | | | | | |
|  | 3m9s.1.C | NADH-quinone oxidoreductase subunit 3  *Crystal structure of respiratory complex I from Thermus thermophilus* | 0.12 | 0.00 | 16.58 | 0.25 | 296-650 | X-ray | 4.50 | monomer | 7 x SF4, 2 x FES, 1 x FMN | HHblits | 0.27 |
| ``` target    RPFLERFTDMPLLVRLDTLQRLRADEVFADYSSDLDVDGPSFTLHGMTEEQHERNGDRVVFDDASGALRAINREDVGDRL 3m9s.1    --------------------------------------------------------------------------------  target    DDKGIDPALDYQGTVTLVDGSTVEVMSVLSMYREHLADYDIDSVVDMTGAPRNLIEQLLDDMTTLSPVAFHVGEGVNHYF 3m9s.1    --------------------------------------------------------------------------------  target    HATLHNRATYLVGMLLGSVGVSGGGVSTWAGNYKGGVFQAAPWFGPGVGGFVNEDPFHPLTDPSARYSAETARHLVHGED 3m9s.1    --------------------------------------------------------------------------------  target    TSYWGFGDRPLVVDTPEDGRKVFTGTTHMPTPTKALWYNNANLINQAKWHYELVKNVNPKVDLIVDQQIEWTGSA-EFAD 3m9s.1    -------------------------------------------------------EALKGKRFVVMHLSHLHPLAERYAH  target    IVLPANSWMEAETWEMGASCSNPFLQVWKGGIEPLNDTRDDIAIFAGVANALTELTGDERFSQAFMFADRPEVYLDRVLA 3m9s.1    VVLPAPTFYEKRGH---LVNLEGRVLPLSPAPIENGEAEGALQVLALLAEALGVR-------P--PFR-LHLEA------  target    GSFTTEGYTVEDLTAGRYGPPGGALMQYRSYPRIPFKEQIEDSLPFYTDTGRMHGYVDIPEAIEYGENLIVHREAVEATP 3m9s.1    ---------QKALKA----------------------------RKVPEAMGRLSFRLKELR------------------P  target    YLPNVIVSTSPYLRPRDYGIAPEELDGDARSVRNIMMSWAEVKETENPLFAAGYNYLCLTPKSRHAVHSSWAVTDWHWLW 3m9s.1    -------------------------------------------------KERKGAFYLRPTMW--KAHQ---AVGK--A-  target    SSSFSDPYRVETRAPGVGEPAIHLNPDDARSLGIRNGDYVWVDSNPKDRPYRDADVDESFLDVARLLVRVTYNPAYPPGV 3m9s.1    ---------Q-----EAARAELWAHPETARAEALPEGAQVAVETP-----------------FGRVEARVVHREDVPKGH  target    TMLKHAFYMATPRTFRAAQERSDGRALAETTGYQSSFRSGSHQSITRGWAPPMHQTDSLFHKRAGVFGFTYGFDVDNHAI 3m9s.1    LYLSALGPAAG---------------------------------------------------------------------  target    NTVPKETVVRITKAEDGGVGGSGAWTRGRPGSMPGDEDDAMQAYLAGELTVVRRT 3m9s.1    ------------------------------------------------------- ``` | | | | | | | | | | | | | | | | | | | | | | | | | | | | | | | | | | | | | | | | | | | | | | | | | |
| ✓ | 2fug.2.C | NADH-quinone oxidoreductase chain 3  *Crystal structure of the hydrophilic domain of respiratory complex I from Thermus thermophilus* | 0.11 | 0.00 | 16.58 | 0.25 | 296-650 | X-ray | 3.30 | monomer | 7 x SF4, 2 x FES, 1 x FMN | HHblits | 0.27 |
| ``` target    RPFLERFTDMPLLVRLDTLQRLRADEVFADYSSDLDVDGPSFTLHGMTEEQHERNGDRVVFDDASGALRAINREDVGDRL 2fug.2    --------------------------------------------------------------------------------  target    DDKGIDPALDYQGTVTLVDGSTVEVMSVLSMYREHLADYDIDSVVDMTGAPRNLIEQLLDDMTTLSPVAFHVGEGVNHYF 2fug.2    --------------------------------------------------------------------------------  target    HATLHNRATYLVGMLLGSVGVSGGGVSTWAGNYKGGVFQAAPWFGPGVGGFVNEDPFHPLTDPSARYSAETARHLVHGED 2fug.2    --------------------------------------------------------------------------------  target    TSYWGFGDRPLVVDTPEDGRKVFTGTTHMPTPTKALWYNNANLINQAKWHYELVKNVNPKVDLIVDQQIEWTGSA-EFAD 2fug.2    -------------------------------------------------------EALKGKRFVVMHLSHLHPLAERYAH  target    IVLPANSWMEAETWEMGASCSNPFLQVWKGGIEPLNDTRDDIAIFAGVANALTELTGDERFSQAFMFADRPEVYLDRVLA 2fug.2    VVLPAPTFYEKRGH---LVNLEGRVLPLSPAPIENGEAEGALQVLALLAEALGVR-------P--PFR-LHLEA------  target    GSFTTEGYTVEDLTAGRYGPPGGALMQYRSYPRIPFKEQIEDSLPFYTDTGRMHGYVDIPEAIEYGENLIVHREAVEATP 2fug.2    ---------QKALKA----------------------------RKVPEAMGRLSFRLKELR------------------P  target    YLPNVIVSTSPYLRPRDYGIAPEELDGDARSVRNIMMSWAEVKETENPLFAAGYNYLCLTPKSRHAVHSSWAVTDWHWLW 2fug.2    -------------------------------------------------KERKGAFYLRPTMW--KAHQ---AVGK--A-  target    SSSFSDPYRVETRAPGVGEPAIHLNPDDARSLGIRNGDYVWVDSNPKDRPYRDADVDESFLDVARLLVRVTYNPAYPPGV 2fug.2    ---------Q-----EAARAELWAHPETARAEALPEGAQVAVETP-----------------FGRVEARVVHREDVPKGH  target    TMLKHAFYMATPRTFRAAQERSDGRALAETTGYQSSFRSGSHQSITRGWAPPMHQTDSLFHKRAGVFGFTYGFDVDNHAI 2fug.2    LYLSALGPAAG---------------------------------------------------------------------  target    NTVPKETVVRITKAEDGGVGGSGAWTRGRPGSMPGDEDDAMQAYLAGELTVVRRT 2fug.2    ------------------------------------------------------- ``` | | | | | | | | | | | | | | | | | | | | | | | | | | | | | | | | | | | | | | | | | | | | | | | | | |
|  | 6zjl.1.C | NADH-quinone oxidoreductase subunit 3  *Respiratory complex I from Thermus thermophilus, NAD+ dataset, major state* | 0.12 | 0.00 | 16.58 | 0.25 | 296-650 | EM | 0.00 | monomer | 7 x SF4, 1 x FMN, 2 x FES | HHblits | 0.27 |
| ``` target    RPFLERFTDMPLLVRLDTLQRLRADEVFADYSSDLDVDGPSFTLHGMTEEQHERNGDRVVFDDASGALRAINREDVGDRL 6zjl.1    --------------------------------------------------------------------------------  target    DDKGIDPALDYQGTVTLVDGSTVEVMSVLSMYREHLADYDIDSVVDMTGAPRNLIEQLLDDMTTLSPVAFHVGEGVNHYF 6zjl.1    --------------------------------------------------------------------------------  target    HATLHNRATYLVGMLLGSVGVSGGGVSTWAGNYKGGVFQAAPWFGPGVGGFVNEDPFHPLTDPSARYSAETARHLVHGED 6zjl.1    --------------------------------------------------------------------------------  target    TSYWGFGDRPLVVDTPEDGRKVFTGTTHMPTPTKALWYNNANLINQAKWHYELVKNVNPKVDLIVDQQIEWTGSA-EFAD 6zjl.1    -------------------------------------------------------EALKGKRFVVMHLSHLHPLAERYAH  target    IVLPANSWMEAETWEMGASCSNPFLQVWKGGIEPLNDTRDDIAIFAGVANALTELTGDERFSQAFMFADRPEVYLDRVLA 6zjl.1    VVLPAPTFYEKRGH---LVNLEGRVLPLSPAPIENGEAEGALQVLALLAEALGVR-------P--PFR-LHLEA------  target    GSFTTEGYTVEDLTAGRYGPPGGALMQYRSYPRIPFKEQIEDSLPFYTDTGRMHGYVDIPEAIEYGENLIVHREAVEATP 6zjl.1    ---------QKALKA----------------------------RKVPEAMGRLSFRLKELR------------------P  target    YLPNVIVSTSPYLRPRDYGIAPEELDGDARSVRNIMMSWAEVKETENPLFAAGYNYLCLTPKSRHAVHSSWAVTDWHWLW 6zjl.1    -------------------------------------------------KERKGAFYLRPTMW--KAHQ---AVGK--A-  target    SSSFSDPYRVETRAPGVGEPAIHLNPDDARSLGIRNGDYVWVDSNPKDRPYRDADVDESFLDVARLLVRVTYNPAYPPGV 6zjl.1    ---------Q-----EAARAELWAHPETARAEALPEGAQVAVETP-----------------FGRVEARVVHREDVPKGH  target    TMLKHAFYMATPRTFRAAQERSDGRALAETTGYQSSFRSGSHQSITRGWAPPMHQTDSLFHKRAGVFGFTYGFDVDNHAI 6zjl.1    LYLSALGPAAG---------------------------------------------------------------------  target    NTVPKETVVRITKAEDGGVGGSGAWTRGRPGSMPGDEDDAMQAYLAGELTVVRRT 6zjl.1    ------------------------------------------------------- ``` | | | | | | | | | | | | | | | | | | | | | | | | | | | | | | | | | | | | | | | | | | | | | | | | | |
|  | 6q8o.1.C | NADH-quinone oxidoreductase subunit 3  *Respiratory complex I from Thermus thermophilus with bound Piericidin A* | 0.12 | 0.00 | 16.58 | 0.25 | 296-650 | X-ray | 3.61 | monomer | 7 x SF4, 1 x FMN, 2 x FES, 1 x HQH | HHblits | 0.27 |
| ``` target    RPFLERFTDMPLLVRLDTLQRLRADEVFADYSSDLDVDGPSFTLHGMTEEQHERNGDRVVFDDASGALRAINREDVGDRL 6q8o.1    --------------------------------------------------------------------------------  target    DDKGIDPALDYQGTVTLVDGSTVEVMSVLSMYREHLADYDIDSVVDMTGAPRNLIEQLLDDMTTLSPVAFHVGEGVNHYF 6q8o.1    --------------------------------------------------------------------------------  target    HATLHNRATYLVGMLLGSVGVSGGGVSTWAGNYKGGVFQAAPWFGPGVGGFVNEDPFHPLTDPSARYSAETARHLVHGED 6q8o.1    --------------------------------------------------------------------------------  target    TSYWGFGDRPLVVDTPEDGRKVFTGTTHMPTPTKALWYNNANLINQAKWHYELVKNVNPKVDLIVDQQIEWTGSA-EFAD 6q8o.1    -------------------------------------------------------EALKGKRFVVMHLSHLHPLAERYAH  target    IVLPANSWMEAETWEMGASCSNPFLQVWKGGIEPLNDTRDDIAIFAGVANALTELTGDERFSQAFMFADRPEVYLDRVLA 6q8o.1    VVLPAPTFYEKRGH---LVNLEGRVLPLSPAPIENGEAEGALQVLALLAEALGVR-------P--PFR-LHLEA------  target    GSFTTEGYTVEDLTAGRYGPPGGALMQYRSYPRIPFKEQIEDSLPFYTDTGRMHGYVDIPEAIEYGENLIVHREAVEATP 6q8o.1    ---------QKALKA----------------------------RKVPEAMGRLSFRLKELR------------------P  target    YLPNVIVSTSPYLRPRDYGIAPEELDGDARSVRNIMMSWAEVKETENPLFAAGYNYLCLTPKSRHAVHSSWAVTDWHWLW 6q8o.1    -------------------------------------------------KERKGAFYLRPTMW--KAHQ---AVGK--A-  target    SSSFSDPYRVETRAPGVGEPAIHLNPDDARSLGIRNGDYVWVDSNPKDRPYRDADVDESFLDVARLLVRVTYNPAYPPGV 6q8o.1    ---------Q-----EAARAELWAHPETARAEALPEGAQVAVETP-----------------FGRVEARVVHREDVPKGH  target    TMLKHAFYMATPRTFRAAQERSDGRALAETTGYQSSFRSGSHQSITRGWAPPMHQTDSLFHKRAGVFGFTYGFDVDNHAI 6q8o.1    LYLSALGPAAG---------------------------------------------------------------------  target    NTVPKETVVRITKAEDGGVGGSGAWTRGRPGSMPGDEDDAMQAYLAGELTVVRRT 6q8o.1    ------------------------------------------------------- ``` | | | | | | | | | | | | | | | | | | | | | | | | | | | | | | | | | | | | | | | | | | | | | | | | | |
|  | 6zjy.1.C | NADH-quinone oxidoreductase subunit 3  *Respiratory complex I from Thermus thermophilus, NAD+ dataset, minor state* | 0.12 | 0.00 | 16.58 | 0.25 | 296-650 | EM | 0.00 | monomer | 7 x SF4, 2 x FES | HHblits | 0.27 |
| ``` target    RPFLERFTDMPLLVRLDTLQRLRADEVFADYSSDLDVDGPSFTLHGMTEEQHERNGDRVVFDDASGALRAINREDVGDRL 6zjy.1    --------------------------------------------------------------------------------  target    DDKGIDPALDYQGTVTLVDGSTVEVMSVLSMYREHLADYDIDSVVDMTGAPRNLIEQLLDDMTTLSPVAFHVGEGVNHYF 6zjy.1    --------------------------------------------------------------------------------  target    HATLHNRATYLVGMLLGSVGVSGGGVSTWAGNYKGGVFQAAPWFGPGVGGFVNEDPFHPLTDPSARYSAETARHLVHGED 6zjy.1    --------------------------------------------------------------------------------  target    TSYWGFGDRPLVVDTPEDGRKVFTGTTHMPTPTKALWYNNANLINQAKWHYELVKNVNPKVDLIVDQQIEWTGSA-EFAD 6zjy.1    -------------------------------------------------------EALKGKRFVVMHLSHLHPLAERYAH  target    IVLPANSWMEAETWEMGASCSNPFLQVWKGGIEPLNDTRDDIAIFAGVANALTELTGDERFSQAFMFADRPEVYLDRVLA 6zjy.1    VVLPAPTFYEKRGH---LVNLEGRVLPLSPAPIENGEAEGALQVLALLAEALGVR-------P--PFR-LHLEA------  target    GSFTTEGYTVEDLTAGRYGPPGGALMQYRSYPRIPFKEQIEDSLPFYTDTGRMHGYVDIPEAIEYGENLIVHREAVEATP 6zjy.1    ---------QKALKA----------------------------RKVPEAMGRLSFRLKELR------------------P  target    YLPNVIVSTSPYLRPRDYGIAPEELDGDARSVRNIMMSWAEVKETENPLFAAGYNYLCLTPKSRHAVHSSWAVTDWHWLW 6zjy.1    -------------------------------------------------KERKGAFYLRPTMW--KAHQ---AVGK--A-  target    SSSFSDPYRVETRAPGVGEPAIHLNPDDARSLGIRNGDYVWVDSNPKDRPYRDADVDESFLDVARLLVRVTYNPAYPPGV 6zjy.1    ---------Q-----EAARAELWAHPETARAEALPEGAQVAVETP-----------------FGRVEARVVHREDVPKGH  target    TMLKHAFYMATPRTFRAAQERSDGRALAETTGYQSSFRSGSHQSITRGWAPPMHQTDSLFHKRAGVFGFTYGFDVDNHAI 6zjy.1    LYLSALGPAAG---------------------------------------------------------------------  target    NTVPKETVVRITKAEDGGVGGSGAWTRGRPGSMPGDEDDAMQAYLAGELTVVRRT 6zjy.1    ------------------------------------------------------- ``` | | | | | | | | | | | | | | | | | | | | | | | | | | | | | | | | | | | | | | | | | | | | | | | | | |
|  | 6zjn.1.C | NADH-quinone oxidoreductase subunit 3  *Respiratory complex I from Thermus thermophilus, NADH dataset, minor state* | 0.12 | 0.00 | 16.58 | 0.25 | 296-650 | EM | 0.00 | monomer | 7 x SF4, 2 x FES | HHblits | 0.27 |
| ``` target    RPFLERFTDMPLLVRLDTLQRLRADEVFADYSSDLDVDGPSFTLHGMTEEQHERNGDRVVFDDASGALRAINREDVGDRL 6zjn.1    --------------------------------------------------------------------------------  target    DDKGIDPALDYQGTVTLVDGSTVEVMSVLSMYREHLADYDIDSVVDMTGAPRNLIEQLLDDMTTLSPVAFHVGEGVNHYF 6zjn.1    --------------------------------------------------------------------------------  target    HATLHNRATYLVGMLLGSVGVSGGGVSTWAGNYKGGVFQAAPWFGPGVGGFVNEDPFHPLTDPSARYSAETARHLVHGED 6zjn.1    --------------------------------------------------------------------------------  target    TSYWGFGDRPLVVDTPEDGRKVFTGTTHMPTPTKALWYNNANLINQAKWHYELVKNVNPKVDLIVDQQIEWTGSA-EFAD 6zjn.1    -------------------------------------------------------EALKGKRFVVMHLSHLHPLAERYAH  target    IVLPANSWMEAETWEMGASCSNPFLQVWKGGIEPLNDTRDDIAIFAGVANALTELTGDERFSQAFMFADRPEVYLDRVLA 6zjn.1    VVLPAPTFYEKRGH---LVNLEGRVLPLSPAPIENGEAEGALQVLALLAEALGVR-------P--PFR-LHLEA------  target    GSFTTEGYTVEDLTAGRYGPPGGALMQYRSYPRIPFKEQIEDSLPFYTDTGRMHGYVDIPEAIEYGENLIVHREAVEATP 6zjn.1    ---------QKALKA----------------------------RKVPEAMGRLSFRLKELR------------------P  target    YLPNVIVSTSPYLRPRDYGIAPEELDGDARSVRNIMMSWAEVKETENPLFAAGYNYLCLTPKSRHAVHSSWAVTDWHWLW 6zjn.1    -------------------------------------------------KERKGAFYLRPTMW--KAHQ---AVGK--A-  target    SSSFSDPYRVETRAPGVGEPAIHLNPDDARSLGIRNGDYVWVDSNPKDRPYRDADVDESFLDVARLLVRVTYNPAYPPGV 6zjn.1    ---------Q-----EAARAELWAHPETARAEALPEGAQVAVETP-----------------FGRVEARVVHREDVPKGH  target    TMLKHAFYMATPRTFRAAQERSDGRALAETTGYQSSFRSGSHQSITRGWAPPMHQTDSLFHKRAGVFGFTYGFDVDNHAI 6zjn.1    LYLSALGPAAG---------------------------------------------------------------------  target    NTVPKETVVRITKAEDGGVGGSGAWTRGRPGSMPGDEDDAMQAYLAGELTVVRRT 6zjn.1    ------------------------------------------------------- ``` | | | | | | | | | | | | | | | | | | | | | | | | | | | | | | | | | | | | | | | | | | | | | | | | | |
|  | 6ziy.1.C | NADH-quinone oxidoreductase subunit 3  *Respiratory complex I from Thermus thermophilus, NADH dataset, major state* | 0.12 | 0.00 | 16.58 | 0.25 | 296-650 | EM | 0.00 | monomer | 7 x SF4, 1 x FMN, 1 x NAI, 2 x FES | HHblits | 0.27 |
| ``` target    RPFLERFTDMPLLVRLDTLQRLRADEVFADYSSDLDVDGPSFTLHGMTEEQHERNGDRVVFDDASGALRAINREDVGDRL 6ziy.1    --------------------------------------------------------------------------------  target    DDKGIDPALDYQGTVTLVDGSTVEVMSVLSMYREHLADYDIDSVVDMTGAPRNLIEQLLDDMTTLSPVAFHVGEGVNHYF 6ziy.1    --------------------------------------------------------------------------------  target    HATLHNRATYLVGMLLGSVGVSGGGVSTWAGNYKGGVFQAAPWFGPGVGGFVNEDPFHPLTDPSARYSAETARHLVHGED 6ziy.1    --------------------------------------------------------------------------------  target    TSYWGFGDRPLVVDTPEDGRKVFTGTTHMPTPTKALWYNNANLINQAKWHYELVKNVNPKVDLIVDQQIEWTGSA-EFAD 6ziy.1    -------------------------------------------------------EALKGKRFVVMHLSHLHPLAERYAH  target    IVLPANSWMEAETWEMGASCSNPFLQVWKGGIEPLNDTRDDIAIFAGVANALTELTGDERFSQAFMFADRPEVYLDRVLA 6ziy.1    VVLPAPTFYEKRGH---LVNLEGRVLPLSPAPIENGEAEGALQVLALLAEALGVR-------P--PFR-LHLEA------  target    GSFTTEGYTVEDLTAGRYGPPGGALMQYRSYPRIPFKEQIEDSLPFYTDTGRMHGYVDIPEAIEYGENLIVHREAVEATP 6ziy.1    ---------QKALKA----------------------------RKVPEAMGRLSFRLKELR------------------P  target    YLPNVIVSTSPYLRPRDYGIAPEELDGDARSVRNIMMSWAEVKETENPLFAAGYNYLCLTPKSRHAVHSSWAVTDWHWLW 6ziy.1    -------------------------------------------------KERKGAFYLRPTMW--KAHQ---AVGK--A-  target    SSSFSDPYRVETRAPGVGEPAIHLNPDDARSLGIRNGDYVWVDSNPKDRPYRDADVDESFLDVARLLVRVTYNPAYPPGV 6ziy.1    ---------Q-----EAARAELWAHPETARAEALPEGAQVAVETP-----------------FGRVEARVVHREDVPKGH  target    TMLKHAFYMATPRTFRAAQERSDGRALAETTGYQSSFRSGSHQSITRGWAPPMHQTDSLFHKRAGVFGFTYGFDVDNHAI 6ziy.1    LYLSALGPAAG---------------------------------------------------------------------  target    NTVPKETVVRITKAEDGGVGGSGAWTRGRPGSMPGDEDDAMQAYLAGELTVVRRT 6ziy.1    ------------------------------------------------------- ``` | | | | | | | | | | | | | | | | | | | | | | | | | | | | | | | | | | | | | | | | | | | | | | | | | |
|  | 7arc.1.F | 75 kDa  *Cryo-EM structure of Polytomella Complex-I (peripheral arm)* | 0.10 |  | 16.76 | 0.24 | 119-372 | EM | 0.00 | hetero-1-1-1-1-1-1-… | 6 x SF4, 2 x FES, 1 x FMN, 1 x NDP, 1 x ZN, 1 x 8Q1 | HHblits | 0.28 |
| ``` target    RPFLERFTDMPLLVRLDTLQRLRADEVFADYSSDLDVDGPSFTLHGMTEEQHERNGDRVVFDDASGALRAINREDVGDRL 7arc.1    --------------------------------------------------------------------------------  target    DDKGIDPALDYQGTVTLVDGSTVEVMSVLSMYREHLADYDIDSVVDMTGAPRNLIEQLL-------DDMTTLSPVAFHVG 7arc.1    --------------------------------------VDLTYAYQHLGADVAALESLASGKGAFFEALKGAKNPVVIVG  target    EGVNHYFHATLHNRATYLVGMLLGSVGVSGGGVSTWAGNYKGGVFQAAPWFGPGVGGFVNEDPFHPLTDPSARYSAETAR 7arc.1    SSVLRRDDREAVLKTVNDLVDAAGVVKEGWNGFNVLHDNASR------------VAA-------LD--------------  target    HLVHGEDTSYWGFGDRPLVVDTPEDGRKVFTGTTHMPTPTKALWYNNANLINQAKWHYELVKNVNPKVDLIVDQQIEWTG 7arc.1    ---IGFVP---------S---A-S------A--RTNPVPAKVVYLLGSDDFKD---------EEIPADAFVIYQGHHGDK  target    SAEFADIVLPANSWMEAETWEMGASCSNPFLQVWKGGIEPLNDTRDDIAIFAGVANALTELTGDERFSQAFMFADRPEVY 7arc.1    GAARANVVLPGAAYTEKASLF---ANTEGRVQTTRTAVPVLGDAREDWKIIRALSEVVG---------------------  target    LDRVLAGSFTTEGYTVEDLTAGRYGPPGGALMQYRSYPRIPFKEQIEDSLPFYTDTGRMHGYVDIPEAIEYGENLIVHRE 7arc.1    --------------------------------------------------------------------------------  target    AVEATPYLPNVIVSTSPYLRPRDYGIAPEELDGDARSVRNIMMSWAEVKETENPLFAAGYNYLCLTPKSRHAVHSSWAVT 7arc.1    --------------------------------------------------------------------------------  target    DWHWLWSSSFSDPYRVETRAPGVGEPAIHLNPDDARSLGIRNGDYVWVDSNPKDRPYRDADVDESFLDVARLLVRVTYNP 7arc.1    --------------------------------------------------------------------------------  target    AYPPGVTMLKHAFYMATPRTFRAAQERSDGRALAETTGYQSSFRSGSHQSITRGWAPPMHQTDSLFHKRAGVFGFTYGFD 7arc.1    --------------------------------------------------------------------------------  target    VDNHAINTVPKETVVRITKAEDGGVGGSGAWTRGRPGSMPGDEDDAMQAYLAGELTVVRRT 7arc.1    ------------------------------------------------------------- ``` | | | | | | | | | | | | | | | | | | | | | | | | | | | | | | | | | | | | | | | | | | | | | | | | | |
|  | 6lod.1.B | Fe-S-cluster-containing hydrogenase components 1-like protein  *Cryo-EM structure of the air-oxidized photosynthetic alternative complex III from Roseiflexus castenholzii* | 0.11 |  | 12.23 | 0.24 | 116-372 | EM | 0.00 | hetero-1-1-1-1-1-1-… | 6 x HEC, 2 x EL6, 3 x SF4, 1 x F3S | HHblits | 0.26 |
| ``` target    RPFLERFTDMPLLVRLDTLQRLRADEVFADYSSDLDVDGPSFTLHGMTEEQHERNGDRVVFDDASGALRAINREDVGDRL 6lod.1    --------------------------------------------------------------------------------  target    DDKGIDPALDYQGTVTLVDGSTVEVMSVLSMYREHLADYDIDSVVDMTGAPRNLIEQLLDDMTTLSPVAFHVGEGVNHYF 6lod.1    -----------------------------------VGVPNVAAGAPLSDTEKKWVEAAAKDLQANRGACVVLV-GESQP-  target    HATLHNRATYLVGMLLGSVGVSGGGVSTWAGNYKGGVFQAAPWFGPGVGGFVNEDPFHPLTDPSARYSAETARHLVHGED 6lod.1    --PVVHALGHAINAQLGNVGST---VVYTE--------PV-E-DD-------------PS--G-----------------  target    TSYWGFGDRPLVVDTPEDGRKVFTGTTHMPTPTKALWYNNANLINQAKWHYELVKNVNPKVDLIVDQQIEWTGSAEFADI 6lod.1    ----GIAALSALTQE------------MNAGTVEVLLMIESNPVYNAPADIP-FAEALAKVPLSMHVGLYRDETAQQSVW  target    VLPANSWMEAETWEMGASCSNPFLQVWKGGIEPLNDTRDDIAIFAGVANALTELTGDERFSQAFMFADRPEVYLDRVLAG 6lod.1    HINGAHFLEAWGDVR---AFDGTTTIVQPLIAPLYNGKSAIEVLNVLLGKPQ----------------------------  target    SFTTEGYTVEDLTAGRYGPPGGALMQYRSYPRIPFKEQIEDSLPFYTDTGRMHGYVDIPEAIEYGENLIVHREAVEATPY 6lod.1    --------------------------------------------------------------------------------  target    LPNVIVSTSPYLRPRDYGIAPEELDGDARSVRNIMMSWAEVKETENPLFAAGYNYLCLTPKSRHAVHSSWAVTDWHWLWS 6lod.1    --------------------------------------------------------------------------------  target    SSFSDPYRVETRAPGVGEPAIHLNPDDARSLGIRNGDYVWVDSNPKDRPYRDADVDESFLDVARLLVRVTYNPAYPPGVT 6lod.1    --------------------------------------------------------------------------------  target    MLKHAFYMATPRTFRAAQERSDGRALAETTGYQSSFRSGSHQSITRGWAPPMHQTDSLFHKRAGVFGFTYGFDVDNHAIN 6lod.1    --------------------------------------------------------------------------------  target    TVPKETVVRITKAEDGGVGGSGAWTRGRPGSMPGDEDDAMQAYLAGELTVVRRT 6lod.1    ------------------------------------------------------ ``` | | | | | | | | | | | | | | | | | | | | | | | | | | | | | | | | | | | | | | | | | | | | | | | | | |
|  | 7t2r.1.A | NiFe hydrogenase subunit A  *Structure of electron bifurcating Ni-Fe hydrogenase complex HydABCSL in FMN-free apo state* | 0.10 |  | 14.05 | 0.24 | 120-373 | EM | 0.00 | hetero-2-2-2-2-2-mer | 6 x FES, 12 x SF4, 2 x 3NI, 2 x FCO | HHblits | 0.26 |
| ``` target    RPFLERFTDMPLLVRLDTLQRLRADEVFADYSSDLDVDGPSFTLHGMTEEQHERNGDRVVFDDASGALRAINREDVGDRL 7t2r.1    --------------------------------------------------------------------------------  target    DDKGIDPALDYQGTVTLVDGSTVEVMSVLSMYREHLADYDIDSVVDMTGAPRNLIEQLLDDMTTLSPVAFHVGEGVNHYF 7t2r.1    ---------------------------------------SIEESARAMGLDPKIAEEVALMLISARRPIFIIGGRA---T  target    HATLHNRATYLVGMLLGSVGVSGGGVSTWAGNYKGGVFQAAPWFGPGVGGFVNEDPFHPLTDPSARYSAETARHLVHGED 7t2r.1    KSHELVTAACNLAVASKAFFEDGLGVVPLLVSA-----N-----S--LGA-------RN--------------T---V--  target    TSYWGFGDRPLVVDTPEDGRKVFTGTTHMPTPTKALWYNNANLINQAKWHYELVKNVNPKVDLIVDQQIEWT-GSAEFAD 7t2r.1    -----VSENPW------------L----GRERRDFLYVFSTAMVPE---EEE-ILAAISATRFVVVQTPFKVRPLVNLAD  target    IVLPANSWMEAETWEMGASCSNPFLQVWKGGIEPLNDTRDDIAIFAGVANALTELTGDERFSQAFMFADRPEVYLDRVLA 7t2r.1    ILLPAPAWYERSGH---FCTIEGERRKLNTIVPPKGEIKSLHYVMDEFAKKLGV--------------------------  target    GSFTTEGYTVEDLTAGRYGPPGGALMQYRSYPRIPFKEQIEDSLPFYTDTGRMHGYVDIPEAIEYGENLIVHREAVEATP 7t2r.1    --------------------------------------------------------------------------------  target    YLPNVIVSTSPYLRPRDYGIAPEELDGDARSVRNIMMSWAEVKETENPLFAAGYNYLCLTPKSRHAVHSSWAVTDWHWLW 7t2r.1    --------------------------------------------------------------------------------  target    SSSFSDPYRVETRAPGVGEPAIHLNPDDARSLGIRNGDYVWVDSNPKDRPYRDADVDESFLDVARLLVRVTYNPAYPPGV 7t2r.1    --------------------------------------------------------------------------------  target    TMLKHAFYMATPRTFRAAQERSDGRALAETTGYQSSFRSGSHQSITRGWAPPMHQTDSLFHKRAGVFGFTYGFDVDNHAI 7t2r.1    --------------------------------------------------------------------------------  target    NTVPKETVVRITKAEDGGVGGSGAWTRGRPGSMPGDEDDAMQAYLAGELTVVRRT 7t2r.1    ------------------------------------------------------- ``` | | | | | | | | | | | | | | | | | | | | | | | | | | | | | | | | | | | | | | | | | | | | | | | | | |
|  | 7t30.1.A | NiFe hydrogenase subunit A  *Structure of electron bifurcating Ni-Fe hydrogenase complex HydABCSL in FMN/NAD(H) bound state* | 0.10 |  | 14.05 | 0.24 | 120-373 | EM | 0.00 | hetero-2-2-2-2-2-mer | 4 x FES, 12 x SF4, 2 x NAD, 2 x FMN, 2 x 3NI, 2 x FCO | HHblits | 0.26 |
| ``` target    RPFLERFTDMPLLVRLDTLQRLRADEVFADYSSDLDVDGPSFTLHGMTEEQHERNGDRVVFDDASGALRAINREDVGDRL 7t30.1    --------------------------------------------------------------------------------  target    DDKGIDPALDYQGTVTLVDGSTVEVMSVLSMYREHLADYDIDSVVDMTGAPRNLIEQLLDDMTTLSPVAFHVGEGVNHYF 7t30.1    ---------------------------------------SIEESARAMGLDPKIAEEVALMLISARRPIFIIGGRA---T  target    HATLHNRATYLVGMLLGSVGVSGGGVSTWAGNYKGGVFQAAPWFGPGVGGFVNEDPFHPLTDPSARYSAETARHLVHGED 7t30.1    KSHELVTAACNLAVASKAFFEDGLGVVPLLVSA-----N-----S--LGA-------RN--------------T---V--  target    TSYWGFGDRPLVVDTPEDGRKVFTGTTHMPTPTKALWYNNANLINQAKWHYELVKNVNPKVDLIVDQQIEWT-GSAEFAD 7t30.1    -----VSENPW------------L----GRERRDFLYVFSTAMVPE---EEE-ILAAISATRFVVVQTPFKVRPLVNLAD  target    IVLPANSWMEAETWEMGASCSNPFLQVWKGGIEPLNDTRDDIAIFAGVANALTELTGDERFSQAFMFADRPEVYLDRVLA 7t30.1    ILLPAPAWYERSGH---FCTIEGERRKLNTIVPPKGEIKSLHYVMDEFAKKLGV--------------------------  target    GSFTTEGYTVEDLTAGRYGPPGGALMQYRSYPRIPFKEQIEDSLPFYTDTGRMHGYVDIPEAIEYGENLIVHREAVEATP 7t30.1    --------------------------------------------------------------------------------  target    YLPNVIVSTSPYLRPRDYGIAPEELDGDARSVRNIMMSWAEVKETENPLFAAGYNYLCLTPKSRHAVHSSWAVTDWHWLW 7t30.1    --------------------------------------------------------------------------------  target    SSSFSDPYRVETRAPGVGEPAIHLNPDDARSLGIRNGDYVWVDSNPKDRPYRDADVDESFLDVARLLVRVTYNPAYPPGV 7t30.1    --------------------------------------------------------------------------------  target    TMLKHAFYMATPRTFRAAQERSDGRALAETTGYQSSFRSGSHQSITRGWAPPMHQTDSLFHKRAGVFGFTYGFDVDNHAI 7t30.1    --------------------------------------------------------------------------------  target    NTVPKETVVRITKAEDGGVGGSGAWTRGRPGSMPGDEDDAMQAYLAGELTVVRRT 7t30.1    ------------------------------------------------------- ``` | | | | | | | | | | | | | | | | | | | | | | | | | | | | | | | | | | | | | | | | | | | | | | | | | |
|  | 7tgh.58.A | NADH-ubiquinone oxidoreductase 75 kDa subunit  *Cryo-EM structure of respiratory super-complex CI+III2 from Tetrahymena thermophila* | 0.09 |  | 12.37 | 0.24 | 117-372 | EM | 0.00 | monomer |  | HHblits | 0.26 |
| ``` target    RPFLERFTDMPLLVRLDTLQRLRADEVFADYSSDLDVDGPSFTLHGMTEEQHERNGDRVVFDDASGALRAINREDVGDRL 7tgh.58   --------------------------------------------------------------------------------  target    DDKGIDPALDYQGTVTLVDGSTVEVMSVLSMYREHLADYDIDSVVDMTGAPRNLIEQLLDDMTTLSPVAFHVGEGVNHYF 7tgh.58   ------------------------------------KNVHLGNSTKVLKEIADGTHPFAERLKKAKLPMIMVGASALERE  target    HATLHNRATYLVGMLLGSVGVSGGGVSTWAGNYKGGVFQAAPWFGPGVGGFVNEDPFHPLTDPSARYSAETARHLVHGED 7tgh.58   DGAELYNTLKVISNKTGVISEEKSWNGFNILHK-----------EM-----------GRIN---------A---------  target    TSYWGFGDRPLVVDTPEDGRKVFTGTTHMPTPTKALWYNNANLINQAKWHYELVKNVNPKVDLIVDQQIEWTGSAEFADI 7tgh.58   ---------------LELGI---N-PTSVNKNAKLVFILGADNNLRP--------EDIPADAFVVYFGTHGDEGAYYADI  target    VLPANSWMEAETWEMGASCSNPFLQVWKGGIEPLNDTRDDIAIFAGVANALTELTGDERFSQAFMFADRPEVYLDRVLAG 7tgh.58   ILPTAAYTEKNATW---VNTEGRVQQGRLVVMPPGDAREDWQIIRALSEEAG----------------------------  target    SFTTEGYTVEDLTAGRYGPPGGALMQYRSYPRIPFKEQIEDSLPFYTDTGRMHGYVDIPEAIEYGENLIVHREAVEATPY 7tgh.58   --------------------------------------------------------------------------------  target    LPNVIVSTSPYLRPRDYGIAPEELDGDARSVRNIMMSWAEVKETENPLFAAGYNYLCLTPKSRHAVHSSWAVTDWHWLWS 7tgh.58   --------------------------------------------------------------------------------  target    SSFSDPYRVETRAPGVGEPAIHLNPDDARSLGIRNGDYVWVDSNPKDRPYRDADVDESFLDVARLLVRVTYNPAYPPGVT 7tgh.58   --------------------------------------------------------------------------------  target    MLKHAFYMATPRTFRAAQERSDGRALAETTGYQSSFRSGSHQSITRGWAPPMHQTDSLFHKRAGVFGFTYGFDVDNHAIN 7tgh.58   --------------------------------------------------------------------------------  target    TVPKETVVRITKAEDGGVGGSGAWTRGRPGSMPGDEDDAMQAYLAGELTVVRRT 7tgh.58   ------------------------------------------------------ ``` | | | | | | | | | | | | | | | | | | | | | | | | | | | | | | | | | | | | | | | | | | | | | | | | | |
|  | 5xtb.1.L | NADH-ubiquinone oxidoreductase 75 kDa subunit, mitochondrial  *Cryo-EM structure of human respiratory complex I matrix arm* | 0.09 |  | 15.52 | 0.22 | 134-372 | EM | 0.00 | hetero-1-1-1-1-1-1-… | 6 x SF4, 1 x FMN, 1 x 8Q1, 1 x NDP, 2 x FES | HHblits | 0.27 |
| ``` target    RPFLERFTDMPLLVRLDTLQRLRADEVFADYSSDLDVDGPSFTLHGMTEEQHERNGDRVVFDDASGALRAINREDVGDRL 5xtb.1    --------------------------------------------------------------------------------  target    DDKGIDPALDYQGTVTLVDGSTVEVMSVLSMYREHLADYDIDSVVDMTGAPRNLIEQLLDDMTTLSPVAFHVGEGVNHYF 5xtb.1    -----------------------------------------------------GSHPFSQVLKEAKKPMVVLGSSALQRN  target    HATLHNRATYLVGMLLGSVGVSGGGVSTWAGNYKGGVFQAAPWFGPGVGGFVNEDPFHPLTDPSARYSAETARHLVHGED 5xtb.1    DGAAILAAVSSIAQKIRMTSGVTGDWKVMNI------LH------RIA----S-----QVA---------A---LD----  target    TSYWGFGDRPLVVDTPEDGRKVFTGTTHMPTPTKALWYNNANLINQAKWHYELVKNVNPKVDLIVDQQIEWTGSAEFADI 5xtb.1    ---LGY--KPG-VEA------------IRKNPPKVLFLLGADGGCI-------TRQDLPKDCFIIYQGHHGDVGAPIADV  target    VLPANSWMEAETWEMGASCSNPFLQVWKGGIEPLNDTRDDIAIFAGVANALTELTGDERFSQAFMFADRPEVYLDRVLAG 5xtb.1    ILPGAAYTEKSATY---VNTEGRAQQTKVAVTPPGLAREDWKIIRALSEIAG----------------------------  target    SFTTEGYTVEDLTAGRYGPPGGALMQYRSYPRIPFKEQIEDSLPFYTDTGRMHGYVDIPEAIEYGENLIVHREAVEATPY 5xtb.1    --------------------------------------------------------------------------------  target    LPNVIVSTSPYLRPRDYGIAPEELDGDARSVRNIMMSWAEVKETENPLFAAGYNYLCLTPKSRHAVHSSWAVTDWHWLWS 5xtb.1    --------------------------------------------------------------------------------  target    SSFSDPYRVETRAPGVGEPAIHLNPDDARSLGIRNGDYVWVDSNPKDRPYRDADVDESFLDVARLLVRVTYNPAYPPGVT 5xtb.1    --------------------------------------------------------------------------------  target    MLKHAFYMATPRTFRAAQERSDGRALAETTGYQSSFRSGSHQSITRGWAPPMHQTDSLFHKRAGVFGFTYGFDVDNHAIN 5xtb.1    --------------------------------------------------------------------------------  target    TVPKETVVRITKAEDGGVGGSGAWTRGRPGSMPGDEDDAMQAYLAGELTVVRRT 5xtb.1    ------------------------------------------------------ ``` | | | | | | | | | | | | | | | | | | | | | | | | | | | | | | | | | | | | | | | | | | | | | | | | | |
|  | 7qsd.1.G | NADH-ubiquinone oxidoreductase 75 kDa subunit, mitochondrial  *Bovine complex I in the active state at 3.1 A* | 0.09 |  | 16.76 | 0.22 | 135-372 | EM | 0.00 | hetero-1-1-1-1-1-1-… | 5 x PC1, 13 x 3PE, 6 x SF4, 2 x FES, 1 x FMN, 4 x CDL, 3 x LMT, 1 x GTP, 1 x MG, 1 x NDP, 1 x ZN, 2 x EHZ | HHblits | 0.27 |
| ``` target    RPFLERFTDMPLLVRLDTLQRLRADEVFADYSSDLDVDGPSFTLHGMTEEQHERNGDRVVFDDASGALRAINREDVGDRL 7qsd.1    --------------------------------------------------------------------------------  target    DDKGIDPALDYQGTVTLVDGSTVEVMSVLSMYREHLADYDIDSVVDMTGAPRNLIEQLLDDMTTLSPVAFHVGEGVNHYF 7qsd.1    ------------------------------------------------------SHPFSQVLQEAKKPMVILGSSALQRN  target    HATLHNRATYLVGMLLGSVGVSGGGVSTWAGNYKGGVFQAAPWFGPGVGGFVNEDPFHPLTDPSARYSAETARHLVHGED 7qsd.1    DGAAILAAVSNIAQKIRTSSGVTGDWKVMNI------LH----------RIAS-----QV----AA--------LD----  target    TSYWGFGDRPLVVDTPEDGRKVFTGTTHMPTPTKALWYNNANLINQAKWHYELVKNVNPKVDLIVDQQIEWTGSAEFADI 7qsd.1    ---LGY--KP--------GV---E--AIQKNPPKMLFLLGADGGCI-------TRQDLPKDCFIVYQGHHGDVGAPIADV  target    VLPANSWMEAETWEMGASCSNPFLQVWKGGIEPLNDTRDDIAIFAGVANALTELTGDERFSQAFMFADRPEVYLDRVLAG 7qsd.1    ILPGAAYTEKSAT---YVNTEGRAQQTKVAVTPPGLAREDWKIIRALSEIAG----------------------------  target    SFTTEGYTVEDLTAGRYGPPGGALMQYRSYPRIPFKEQIEDSLPFYTDTGRMHGYVDIPEAIEYGENLIVHREAVEATPY 7qsd.1    --------------------------------------------------------------------------------  target    LPNVIVSTSPYLRPRDYGIAPEELDGDARSVRNIMMSWAEVKETENPLFAAGYNYLCLTPKSRHAVHSSWAVTDWHWLWS 7qsd.1    --------------------------------------------------------------------------------  target    SSFSDPYRVETRAPGVGEPAIHLNPDDARSLGIRNGDYVWVDSNPKDRPYRDADVDESFLDVARLLVRVTYNPAYPPGVT 7qsd.1    --------------------------------------------------------------------------------  target    MLKHAFYMATPRTFRAAQERSDGRALAETTGYQSSFRSGSHQSITRGWAPPMHQTDSLFHKRAGVFGFTYGFDVDNHAIN 7qsd.1    --------------------------------------------------------------------------------  target    TVPKETVVRITKAEDGGVGGSGAWTRGRPGSMPGDEDDAMQAYLAGELTVVRRT 7qsd.1    ------------------------------------------------------ ``` | | | | | | | | | | | | | | | | | | | | | | | | | | | | | | | | | | | | | | | | | | | | | | | | | |
|  | 7dgr.10.A | NADH-ubiquinone oxidoreductase 75 kDa subunit, mitochondrial  *Activity optimized supercomplex state2* | 0.09 | 0.00 | 16.18 | 0.22 | 135-372 | EM | 0.00 | monomer |  | HHblits | 0.27 |
| ``` target    RPFLERFTDMPLLVRLDTLQRLRADEVFADYSSDLDVDGPSFTLHGMTEEQHERNGDRVVFDDASGALRAINREDVGDRL 7dgr.10   --------------------------------------------------------------------------------  target    DDKGIDPALDYQGTVTLVDGSTVEVMSVLSMYREHLADYDIDSVVDMTGAPRNLIEQLLDDMTTLSPVAFHVGEGVNHYF 7dgr.10   ------------------------------------------------------SHPFSQVLQEAKKPMVILGSSALQRN  target    HATLHNRATYLVGMLLGSVGVSGGGVSTWAGNYKGGVFQAAPWFGPGVGGFVNEDPFHPLTDPSARYSAETARHLVHGED 7dgr.10   DGAAILAAVSNIAQKIRTSSGVTGDWKVMNI------LH----------RIASQ--VAAL---------------D----  target    TSYWGFGDRPLVVDTPEDGRKVFTGTTHMPTPTKALWYNNANLINQAKWHYELVKNVNPKVDLIVDQQIEWTGSAEFADI 7dgr.10   ---LGY--KP--------GV---E--AIQKNPPKMLFLLGADGGCI-------TRQDLPKDCFIVYQGHHGDVGAPIADV  target    VLPANSWMEAETWEMGASCSNPFLQVWKGGIEPLNDTRDDIAIFAGVANALTELTGDERFSQAFMFADRPEVYLDRVLAG 7dgr.10   ILPGAAYTEKSAT---YVNTEGRAQQTKVAVTPPGLAREDWKIIRALSEIAG----------------------------  target    SFTTEGYTVEDLTAGRYGPPGGALMQYRSYPRIPFKEQIEDSLPFYTDTGRMHGYVDIPEAIEYGENLIVHREAVEATPY 7dgr.10   --------------------------------------------------------------------------------  target    LPNVIVSTSPYLRPRDYGIAPEELDGDARSVRNIMMSWAEVKETENPLFAAGYNYLCLTPKSRHAVHSSWAVTDWHWLWS 7dgr.10   --------------------------------------------------------------------------------  target    SSFSDPYRVETRAPGVGEPAIHLNPDDARSLGIRNGDYVWVDSNPKDRPYRDADVDESFLDVARLLVRVTYNPAYPPGVT 7dgr.10   --------------------------------------------------------------------------------  target    MLKHAFYMATPRTFRAAQERSDGRALAETTGYQSSFRSGSHQSITRGWAPPMHQTDSLFHKRAGVFGFTYGFDVDNHAIN 7dgr.10   --------------------------------------------------------------------------------  target    TVPKETVVRITKAEDGGVGGSGAWTRGRPGSMPGDEDDAMQAYLAGELTVVRRT 7dgr.10   ------------------------------------------------------ ``` | | | | | | | | | | | | | | | | | | | | | | | | | | | | | | | | | | | | | | | | | | | | | | | | | |
|  | 5o31.1.8 | NADH-ubiquinone oxidoreductase 75 kDa subunit, mitochondrial  *Mitochondrial complex I in the deactive state* | 0.09 | 0.00 | 16.18 | 0.22 | 135-372 | EM | 4.13 | monomer | 6 x SF4, 2 x FES, 1 x FMN, 1 x NAP, 1 x ZN | HHblits | 0.27 |
| ``` target    RPFLERFTDMPLLVRLDTLQRLRADEVFADYSSDLDVDGPSFTLHGMTEEQHERNGDRVVFDDASGALRAINREDVGDRL 5o31.1    --------------------------------------------------------------------------------  target    DDKGIDPALDYQGTVTLVDGSTVEVMSVLSMYREHLADYDIDSVVDMTGAPRNLIEQLLDDMTTLSPVAFHVGEGVNHYF 5o31.1    ------------------------------------------------------SHPFSQVLQEAKKPMVILGSSALQRN  target    HATLHNRATYLVGMLLGSVGVSGGGVSTWAGNYKGGVFQAAPWFGPGVGGFVNEDPFHPLTDPSARYSAETARHLVHGED 5o31.1    DGAAILAAVSNIAQKIRTSSGVTGDWKVMNI------LH----------RIASQ--VAAL---------------D----  target    TSYWGFGDRPLVVDTPEDGRKVFTGTTHMPTPTKALWYNNANLINQAKWHYELVKNVNPKVDLIVDQQIEWTGSAEFADI 5o31.1    ---LGY--KP--------GV---E--AIQKNPPKMLFLLGADGGCI-------TRQDLPKDCFIVYQGHHGDVGAPIADV  target    VLPANSWMEAETWEMGASCSNPFLQVWKGGIEPLNDTRDDIAIFAGVANALTELTGDERFSQAFMFADRPEVYLDRVLAG 5o31.1    ILPGAAYTEKSAT---YVNTEGRAQQTKVAVTPPGLAREDWKIIRALSEIAG----------------------------  target    SFTTEGYTVEDLTAGRYGPPGGALMQYRSYPRIPFKEQIEDSLPFYTDTGRMHGYVDIPEAIEYGENLIVHREAVEATPY 5o31.1    --------------------------------------------------------------------------------  target    LPNVIVSTSPYLRPRDYGIAPEELDGDARSVRNIMMSWAEVKETENPLFAAGYNYLCLTPKSRHAVHSSWAVTDWHWLWS 5o31.1    --------------------------------------------------------------------------------  target    SSFSDPYRVETRAPGVGEPAIHLNPDDARSLGIRNGDYVWVDSNPKDRPYRDADVDESFLDVARLLVRVTYNPAYPPGVT 5o31.1    --------------------------------------------------------------------------------  target    MLKHAFYMATPRTFRAAQERSDGRALAETTGYQSSFRSGSHQSITRGWAPPMHQTDSLFHKRAGVFGFTYGFDVDNHAIN 5o31.1    --------------------------------------------------------------------------------  target    TVPKETVVRITKAEDGGVGGSGAWTRGRPGSMPGDEDDAMQAYLAGELTVVRRT 5o31.1    ------------------------------------------------------ ``` | | | | | | | | | | | | | | | | | | | | | | | | | | | | | | | | | | | | | | | | | | | | | | | | | |
|  | 6zr2.1.G | NADH-ubiquinone oxidoreductase 75 kDa subunit, mitochondrial  *Cryo-EM structure of respiratory complex I in the active state from Mus musculus at 3.1 A* | 0.09 |  | 15.52 | 0.22 | 134-372 | EM | 3.10 | hetero-1-1-1-1-1-1-… | 6 x SF4, 4 x PC1, 2 x FES, 1 x FMN, 9 x 3PE, 7 x CDL, 1 x ATP, 1 x NDP, 1 x ZN, 2 x EHZ | HHblits | 0.27 |
| ``` target    RPFLERFTDMPLLVRLDTLQRLRADEVFADYSSDLDVDGPSFTLHGMTEEQHERNGDRVVFDDASGALRAINREDVGDRL 6zr2.1    --------------------------------------------------------------------------------  target    DDKGIDPALDYQGTVTLVDGSTVEVMSVLSMYREHLADYDIDSVVDMTGAPRNLIEQLLDDMTTLSPVAFHVGEGVNHYF 6zr2.1    -----------------------------------------------------GRHSFCEVLKDAKKPMVVLGSSALQRD  target    HATLHNRATYLVGMLLGSVGVSGGGVSTWAGNYKGGVFQAAPWFGPGVGGFVNEDPFHPLTDPSARYSAETARHLVHGED 6zr2.1    DGAAILVAVSNMVQKIRVTTGVAAEWKVM---------NIL---H----RIAS-----QV----A-----A---------  target    TSYWGFGDRPLVVDTPEDGRKVFTGTTHMPTPTKALWYNNANLINQAKWHYELVKNVNPKVDLIVDQQIEWTGSAEFADI 6zr2.1    ---LDLGYKP--------GV---E--AIRKNPPKMLFLLGADGGC-------ITRQDLPKDCFIVYQGHHGDVGAPMADV  target    VLPANSWMEAETWEMGASCSNPFLQVWKGGIEPLNDTRDDIAIFAGVANALTELTGDERFSQAFMFADRPEVYLDRVLAG 6zr2.1    ILPGAAYTEKSAT---YVNTEGRAQQTKVAVTPPGLAREDWKIIRALSEIAG----------------------------  target    SFTTEGYTVEDLTAGRYGPPGGALMQYRSYPRIPFKEQIEDSLPFYTDTGRMHGYVDIPEAIEYGENLIVHREAVEATPY 6zr2.1    --------------------------------------------------------------------------------  target    LPNVIVSTSPYLRPRDYGIAPEELDGDARSVRNIMMSWAEVKETENPLFAAGYNYLCLTPKSRHAVHSSWAVTDWHWLWS 6zr2.1    --------------------------------------------------------------------------------  target    SSFSDPYRVETRAPGVGEPAIHLNPDDARSLGIRNGDYVWVDSNPKDRPYRDADVDESFLDVARLLVRVTYNPAYPPGVT 6zr2.1    --------------------------------------------------------------------------------  target    MLKHAFYMATPRTFRAAQERSDGRALAETTGYQSSFRSGSHQSITRGWAPPMHQTDSLFHKRAGVFGFTYGFDVDNHAIN 6zr2.1    --------------------------------------------------------------------------------  target    TVPKETVVRITKAEDGGVGGSGAWTRGRPGSMPGDEDDAMQAYLAGELTVVRRT 6zr2.1    ------------------------------------------------------ ``` | | | | | | | | | | | | | | | | | | | | | | | | | | | | | | | | | | | | | | | | | | | | | | | | | |
|  | 6g72.1.G | NADH-ubiquinone oxidoreductase 75 kDa subunit, mitochondrial  *Mouse mitochondrial complex I in the deactive state* | 0.09 |  | 15.52 | 0.22 | 134-372 | EM | 0.00 | hetero-1-1-1-1-1-1-… | 6 x SF4, 2 x FES, 1 x FMN, 1 x ADP, 1 x NDP, 1 x ZN, 2 x EHZ | HHblits | 0.27 |
| ``` target    RPFLERFTDMPLLVRLDTLQRLRADEVFADYSSDLDVDGPSFTLHGMTEEQHERNGDRVVFDDASGALRAINREDVGDRL 6g72.1    --------------------------------------------------------------------------------  target    DDKGIDPALDYQGTVTLVDGSTVEVMSVLSMYREHLADYDIDSVVDMTGAPRNLIEQLLDDMTTLSPVAFHVGEGVNHYF 6g72.1    -----------------------------------------------------GRHSFCEVLKDAKKPMVVLGSSALQRD  target    HATLHNRATYLVGMLLGSVGVSGGGVSTWAGNYKGGVFQAAPWFGPGVGGFVNEDPFHPLTDPSARYSAETARHLVHGED 6g72.1    DGAAILVAVSNMVQKIRVTTGVAAEWKVM---------NIL---H----RIAS-----QV----A-----A---------  target    TSYWGFGDRPLVVDTPEDGRKVFTGTTHMPTPTKALWYNNANLINQAKWHYELVKNVNPKVDLIVDQQIEWTGSAEFADI 6g72.1    ---LDLGYKP--------GV---E--AIRKNPPKMLFLLGADGGC-------ITRQDLPKDCFIVYQGHHGDVGAPMADV  target    VLPANSWMEAETWEMGASCSNPFLQVWKGGIEPLNDTRDDIAIFAGVANALTELTGDERFSQAFMFADRPEVYLDRVLAG 6g72.1    ILPGAAYTEKSAT---YVNTEGRAQQTKVAVTPPGLAREDWKIIRALSEIAG----------------------------  target    SFTTEGYTVEDLTAGRYGPPGGALMQYRSYPRIPFKEQIEDSLPFYTDTGRMHGYVDIPEAIEYGENLIVHREAVEATPY 6g72.1    --------------------------------------------------------------------------------  target    LPNVIVSTSPYLRPRDYGIAPEELDGDARSVRNIMMSWAEVKETENPLFAAGYNYLCLTPKSRHAVHSSWAVTDWHWLWS 6g72.1    --------------------------------------------------------------------------------  target    SSFSDPYRVETRAPGVGEPAIHLNPDDARSLGIRNGDYVWVDSNPKDRPYRDADVDESFLDVARLLVRVTYNPAYPPGVT 6g72.1    --------------------------------------------------------------------------------  target    MLKHAFYMATPRTFRAAQERSDGRALAETTGYQSSFRSGSHQSITRGWAPPMHQTDSLFHKRAGVFGFTYGFDVDNHAIN 6g72.1    --------------------------------------------------------------------------------  target    TVPKETVVRITKAEDGGVGGSGAWTRGRPGSMPGDEDDAMQAYLAGELTVVRRT 6g72.1    ------------------------------------------------------ ``` | | | | | | | | | | | | | | | | | | | | | | | | | | | | | | | | | | | | | | | | | | | | | | | | | |
|  | 7ak6.1.G | NADH-ubiquinone oxidoreductase 75 kDa subunit, mitochondrial  *Cryo-EM structure of ND6-P25L mutant respiratory complex I from Mus musculus at 3.8 A* | 0.09 |  | 15.52 | 0.22 | 134-372 | EM | 0.00 | hetero-1-1-1-1-1-1-… | 6 x SF4, 1 x PC1, 2 x FES, 1 x FMN, 4 x 3PE, 2 x CDL, 1 x ATP, 1 x NDP, 1 x ZN, 2 x EHZ | HHblits | 0.27 |
| ``` target    RPFLERFTDMPLLVRLDTLQRLRADEVFADYSSDLDVDGPSFTLHGMTEEQHERNGDRVVFDDASGALRAINREDVGDRL 7ak6.1    --------------------------------------------------------------------------------  target    DDKGIDPALDYQGTVTLVDGSTVEVMSVLSMYREHLADYDIDSVVDMTGAPRNLIEQLLDDMTTLSPVAFHVGEGVNHYF 7ak6.1    -----------------------------------------------------GRHSFCEVLKDAKKPMVVLGSSALQRD  target    HATLHNRATYLVGMLLGSVGVSGGGVSTWAGNYKGGVFQAAPWFGPGVGGFVNEDPFHPLTDPSARYSAETARHLVHGED 7ak6.1    DGAAILVAVSNMVQKIRVTTGVAAEWKVM---------NIL---H----RIAS-----QV----A-----A---------  target    TSYWGFGDRPLVVDTPEDGRKVFTGTTHMPTPTKALWYNNANLINQAKWHYELVKNVNPKVDLIVDQQIEWTGSAEFADI 7ak6.1    ---LDLGYKP--------GV---E--AIRKNPPKMLFLLGADGGC-------ITRQDLPKDCFIVYQGHHGDVGAPMADV  target    VLPANSWMEAETWEMGASCSNPFLQVWKGGIEPLNDTRDDIAIFAGVANALTELTGDERFSQAFMFADRPEVYLDRVLAG 7ak6.1    ILPGAAYTEKSAT---YVNTEGRAQQTKVAVTPPGLAREDWKIIRALSEIAG----------------------------  target    SFTTEGYTVEDLTAGRYGPPGGALMQYRSYPRIPFKEQIEDSLPFYTDTGRMHGYVDIPEAIEYGENLIVHREAVEATPY 7ak6.1    --------------------------------------------------------------------------------  target    LPNVIVSTSPYLRPRDYGIAPEELDGDARSVRNIMMSWAEVKETENPLFAAGYNYLCLTPKSRHAVHSSWAVTDWHWLWS 7ak6.1    --------------------------------------------------------------------------------  target    SSFSDPYRVETRAPGVGEPAIHLNPDDARSLGIRNGDYVWVDSNPKDRPYRDADVDESFLDVARLLVRVTYNPAYPPGVT 7ak6.1    --------------------------------------------------------------------------------  target    MLKHAFYMATPRTFRAAQERSDGRALAETTGYQSSFRSGSHQSITRGWAPPMHQTDSLFHKRAGVFGFTYGFDVDNHAIN 7ak6.1    --------------------------------------------------------------------------------  target    TVPKETVVRITKAEDGGVGGSGAWTRGRPGSMPGDEDDAMQAYLAGELTVVRRT 7ak6.1    ------------------------------------------------------ ``` | | | | | | | | | | | | | | | | | | | | | | | | | | | | | | | | | | | | | | | | | | | | | | | | | |
|  | 7vxu.1.L | NADH-ubiquinone oxidoreductase 75 kDa subunit, mitochondrial  *Matrix arm of deactive state CI from Q10 dataset* | 0.09 |  | 16.18 | 0.22 | 135-372 | EM | 0.00 | hetero-1-1-1-1-1-1-… | 6 x SF4, 1 x FMN, 1 x PEE, 1 x PLX, 1 x 8Q1, 1 x NDP, 2 x FES, 1 x MG, 1 x CDL, 1 x ZN | HHblits | 0.27 |
| ``` target    RPFLERFTDMPLLVRLDTLQRLRADEVFADYSSDLDVDGPSFTLHGMTEEQHERNGDRVVFDDASGALRAINREDVGDRL 7vxu.1    --------------------------------------------------------------------------------  target    DDKGIDPALDYQGTVTLVDGSTVEVMSVLSMYREHLADYDIDSVVDMTGAPRNLIEQLLDDMTTLSPVAFHVGEGVNHYF 7vxu.1    ------------------------------------------------------NHPFSQILKEAKKPMVVLGSSALQRS  target    HATLHNRATYLVGMLLGSVGVSGGGVSTWAGNYKGGVFQAAPWFGPGVGGFVNEDPFHPLTDPSARYSAETARHLVHGED 7vxu.1    DGTAILAAVSNIAQNIRLSSGVTGDWKVMN---------ILHRIA-------S-----QV----AAL--------D----  target    TSYWGFGDRPLVVDTPEDGRKVFTGTTHMPTPTKALWYNNANLINQAKWHYELVKNVNPKVDLIVDQQIEWTGSAEFADI 7vxu.1    ---LGY--KPG-VEA------------IRKNPPKVLFLLGADGGCI-------TRQDLPKDCFIIYQGHHGDVGAPMADV  target    VLPANSWMEAETWEMGASCSNPFLQVWKGGIEPLNDTRDDIAIFAGVANALTELTGDERFSQAFMFADRPEVYLDRVLAG 7vxu.1    ILPGAAYTEKSAT---YVNTEGRAQQTKVAVTPPGLAREDWKIIRALSEIAG----------------------------  target    SFTTEGYTVEDLTAGRYGPPGGALMQYRSYPRIPFKEQIEDSLPFYTDTGRMHGYVDIPEAIEYGENLIVHREAVEATPY 7vxu.1    --------------------------------------------------------------------------------  target    LPNVIVSTSPYLRPRDYGIAPEELDGDARSVRNIMMSWAEVKETENPLFAAGYNYLCLTPKSRHAVHSSWAVTDWHWLWS 7vxu.1    --------------------------------------------------------------------------------  target    SSFSDPYRVETRAPGVGEPAIHLNPDDARSLGIRNGDYVWVDSNPKDRPYRDADVDESFLDVARLLVRVTYNPAYPPGVT 7vxu.1    --------------------------------------------------------------------------------  target    MLKHAFYMATPRTFRAAQERSDGRALAETTGYQSSFRSGSHQSITRGWAPPMHQTDSLFHKRAGVFGFTYGFDVDNHAIN 7vxu.1    --------------------------------------------------------------------------------  target    TVPKETVVRITKAEDGGVGGSGAWTRGRPGSMPGDEDDAMQAYLAGELTVVRRT 7vxu.1    ------------------------------------------------------ ``` | | | | | | | | | | | | | | | | | | | | | | | | | | | | | | | | | | | | | | | | | | | | | | | | | |
|  | 7zd6.1.4 | NADH-ubiquinone oxidoreductase 75 kDa subunit, mitochondrial  *Complex I from Ovis aries, at pH7.4, Open state* | 0.09 | 0.00 | 16.18 | 0.22 | 135-372 | EM | 0.00 | monomer | 6 x PC1, 14 x 3PE, 1 x DCQ, 2 x ZMP, 1 x AMP, 1 x MYR, 6 x SF4, 1 x FMN, 1 x NAI, 2 x FES, 1 x K, 1 x ZN, 1 x NDP | HHblits | 0.27 |
| ``` target    RPFLERFTDMPLLVRLDTLQRLRADEVFADYSSDLDVDGPSFTLHGMTEEQHERNGDRVVFDDASGALRAINREDVGDRL 7zd6.1    --------------------------------------------------------------------------------  target    DDKGIDPALDYQGTVTLVDGSTVEVMSVLSMYREHLADYDIDSVVDMTGAPRNLIEQLLDDMTTLSPVAFHVGEGVNHYF 7zd6.1    ------------------------------------------------------SHPFSQVLQEAKKPMVVLGSSALQRN  target    HATLHNRATYLVGMLLGSVGVSGGGVSTWAGNYKGGVFQAAPWFGPGVGGFVNEDPFHPLTDPSARYSAETARHLVHGED 7zd6.1    DGAAILAAVSNIAQKIRTSSGVTGDWKVMNI------LH----------RIAS-----QV----A-----A---------  target    TSYWGFGDRPLVVDTPEDGRKVFTGTTHMPTPTKALWYNNANLINQAKWHYELVKNVNPKVDLIVDQQIEWTGSAEFADI 7zd6.1    ---LDLGYKPG-VEA------------IRKNPPKMLFLLGADGGCV-------TRQDLPKDCFIVYQGHHGDVGAPIADV  target    VLPANSWMEAETWEMGASCSNPFLQVWKGGIEPLNDTRDDIAIFAGVANALTELTGDERFSQAFMFADRPEVYLDRVLAG 7zd6.1    ILPGAAYTEKSAT---YVNTEGRAQQTKVAVMPPGLAREDWKIIRALSEIAG----------------------------  target    SFTTEGYTVEDLTAGRYGPPGGALMQYRSYPRIPFKEQIEDSLPFYTDTGRMHGYVDIPEAIEYGENLIVHREAVEATPY 7zd6.1    --------------------------------------------------------------------------------  target    LPNVIVSTSPYLRPRDYGIAPEELDGDARSVRNIMMSWAEVKETENPLFAAGYNYLCLTPKSRHAVHSSWAVTDWHWLWS 7zd6.1    --------------------------------------------------------------------------------  target    SSFSDPYRVETRAPGVGEPAIHLNPDDARSLGIRNGDYVWVDSNPKDRPYRDADVDESFLDVARLLVRVTYNPAYPPGVT 7zd6.1    --------------------------------------------------------------------------------  target    MLKHAFYMATPRTFRAAQERSDGRALAETTGYQSSFRSGSHQSITRGWAPPMHQTDSLFHKRAGVFGFTYGFDVDNHAIN 7zd6.1    --------------------------------------------------------------------------------  target    TVPKETVVRITKAEDGGVGGSGAWTRGRPGSMPGDEDDAMQAYLAGELTVVRRT 7zd6.1    ------------------------------------------------------ ``` | | | | | | | | | | | | | | | | | | | | | | | | | | | | | | | | | | | | | | | | | | | | | | | | | |
|  | 7v2c.1.L | NADH-ubiquinone oxidoreductase 75 kDa subunit, mitochondrial  *Active state complex I from Q10 dataset* | 0.09 |  | 16.18 | 0.22 | 135-372 | EM | 0.00 | hetero-1-1-1-1-1-2-… | 6 x SF4, 1 x FMN, 10 x PEE, 8 x PLX, 2 x 8Q1, 1 x NDP, 2 x UQ, 11 x CDL, 2 x FES, 1 x MG, 1 x ZN, 1 x ADP | HHblits | 0.27 |
| ``` target    RPFLERFTDMPLLVRLDTLQRLRADEVFADYSSDLDVDGPSFTLHGMTEEQHERNGDRVVFDDASGALRAINREDVGDRL 7v2c.1    --------------------------------------------------------------------------------  target    DDKGIDPALDYQGTVTLVDGSTVEVMSVLSMYREHLADYDIDSVVDMTGAPRNLIEQLLDDMTTLSPVAFHVGEGVNHYF 7v2c.1    ------------------------------------------------------NHPFSQILKEAKKPMVVLGSSALQRS  target    HATLHNRATYLVGMLLGSVGVSGGGVSTWAGNYKGGVFQAAPWFGPGVGGFVNEDPFHPLTDPSARYSAETARHLVHGED 7v2c.1    DGTAILAAVSNIAQNIRLSSGVTGDWKVM---------NIL---H----RIAS-----QV----A-----A---------  target    TSYWGFGDRPLVVDTPEDGRKVFTGTTHMPTPTKALWYNNANLINQAKWHYELVKNVNPKVDLIVDQQIEWTGSAEFADI 7v2c.1    ---LDLGYKPG-V----------E--AIRKNPPKVLFLLGADGGCI-------TRQDLPKDCFIIYQGHHGDVGAPMADV  target    VLPANSWMEAETWEMGASCSNPFLQVWKGGIEPLNDTRDDIAIFAGVANALTELTGDERFSQAFMFADRPEVYLDRVLAG 7v2c.1    ILPGAAYTEKSAT---YVNTEGRAQQTKVAVTPPGLAREDWKIIRALSEIAG----------------------------  target    SFTTEGYTVEDLTAGRYGPPGGALMQYRSYPRIPFKEQIEDSLPFYTDTGRMHGYVDIPEAIEYGENLIVHREAVEATPY 7v2c.1    --------------------------------------------------------------------------------  target    LPNVIVSTSPYLRPRDYGIAPEELDGDARSVRNIMMSWAEVKETENPLFAAGYNYLCLTPKSRHAVHSSWAVTDWHWLWS 7v2c.1    --------------------------------------------------------------------------------  target    SSFSDPYRVETRAPGVGEPAIHLNPDDARSLGIRNGDYVWVDSNPKDRPYRDADVDESFLDVARLLVRVTYNPAYPPGVT 7v2c.1    --------------------------------------------------------------------------------  target    MLKHAFYMATPRTFRAAQERSDGRALAETTGYQSSFRSGSHQSITRGWAPPMHQTDSLFHKRAGVFGFTYGFDVDNHAIN 7v2c.1    --------------------------------------------------------------------------------  target    TVPKETVVRITKAEDGGVGGSGAWTRGRPGSMPGDEDDAMQAYLAGELTVVRRT 7v2c.1    ------------------------------------------------------ ``` | | | | | | | | | | | | | | | | | | | | | | | | | | | | | | | | | | | | | | | | | | | | | | | | | |
|  | 7ak5.1.G | NADH-ubiquinone oxidoreductase 75 kDa subunit, mitochondrial  *Cryo-EM structure of respiratory complex I in the deactive state from Mus musculus at 3.2 A* | 0.09 |  | 14.94 | 0.22 | 134-372 | EM | 0.00 | hetero-1-1-1-1-1-1-… | 6 x SF4, 2 x PC1, 2 x FES, 1 x FMN, 8 x 3PE, 4 x CDL, 1 x ATP, 1 x NDP, 1 x ZN, 2 x EHZ | HHblits | 0.26 |
| ``` target    RPFLERFTDMPLLVRLDTLQRLRADEVFADYSSDLDVDGPSFTLHGMTEEQHERNGDRVVFDDASGALRAINREDVGDRL 7ak5.1    --------------------------------------------------------------------------------  target    DDKGIDPALDYQGTVTLVDGSTVEVMSVLSMYREHLADYDIDSVVDMTGAPRNLIEQLLDDMTTLSPVAFHVGEGVNHYF 7ak5.1    -----------------------------------------------------GRHSFCEVLKDAKKPMVVLGSSALQRD  target    HATLHNRATYLVGMLLGSVGVSGGGVSTWAGNYKGGVFQAAPWFGPGVGGFVNEDPFHPLTDPSARYSAETARHLVHGED 7ak5.1    DGAAILVAVSNMVQKIRVTTGVAAEWKV---------MNIL---H----RIAS-----QVA---------A---------  target    TSYWGFGDRPLVVDTPEDGRKVFTGTTHMPTPTKALWYNNANLINQAKWHYELVKNVNPKVDLIVDQQIEWTGSAEFADI 7ak5.1    ---LDLGYKP--------GV---E--AIRKNPPKMLFLLGADGGC-------ITRQDLPKDCFIVYQGHHGDVGAPMADV  target    VLPANSWMEAETWEMGASCSNPFLQVWKGGIEPLNDTRDDIAIFAGVANALTELTGDERFSQAFMFADRPEVYLDRVLAG 7ak5.1    ILPGAAYTEKSAT---YVNTEGRAQQTKVAVTPPGLAREDWKIIRALSEIAG----------------------------  target    SFTTEGYTVEDLTAGRYGPPGGALMQYRSYPRIPFKEQIEDSLPFYTDTGRMHGYVDIPEAIEYGENLIVHREAVEATPY 7ak5.1    --------------------------------------------------------------------------------  target    LPNVIVSTSPYLRPRDYGIAPEELDGDARSVRNIMMSWAEVKETENPLFAAGYNYLCLTPKSRHAVHSSWAVTDWHWLWS 7ak5.1    --------------------------------------------------------------------------------  target    SSFSDPYRVETRAPGVGEPAIHLNPDDARSLGIRNGDYVWVDSNPKDRPYRDADVDESFLDVARLLVRVTYNPAYPPGVT 7ak5.1    --------------------------------------------------------------------------------  target    MLKHAFYMATPRTFRAAQERSDGRALAETTGYQSSFRSGSHQSITRGWAPPMHQTDSLFHKRAGVFGFTYGFDVDNHAIN 7ak5.1    --------------------------------------------------------------------------------  target    TVPKETVVRITKAEDGGVGGSGAWTRGRPGSMPGDEDDAMQAYLAGELTVVRRT 7ak5.1    ------------------------------------------------------ ``` | | | | | | | | | | | | | | | | | | | | | | | | | | | | | | | | | | | | | | | | | | | | | | | | | |
|  | 5gpn.24.A | NADH-ubiquinone oxidoreductase 75 kDa subunit  *Architecture of mammalian respirasome* | 0.08 |  | 16.18 | 0.22 | 135-372 | EM | 0.00 | monomer |  | HHblits | 0.27 |
| ``` target    RPFLERFTDMPLLVRLDTLQRLRADEVFADYSSDLDVDGPSFTLHGMTEEQHERNGDRVVFDDASGALRAINREDVGDRL 5gpn.24   --------------------------------------------------------------------------------  target    DDKGIDPALDYQGTVTLVDGSTVEVMSVLSMYREHLADYDIDSVVDMTGAPRNLIEQLLDDMTTLSPVAFHVGEGVNHYF 5gpn.24   ------------------------------------------------------NHPFSQILKEAKKPMVVLGSSALQRS  target    HATLHNRATYLVGMLLGSVGVSGGGVSTWAGNYKGGVFQAAPWFGPGVGGFVNEDPFHPLTDPSARYSAETARHLVHGED 5gpn.24   DGTAILAAVSNIAQNIRLSSGVTGDWKVMN---------IL---HRI----AS-----QV----AAL--------D----  target    TSYWGFGDRPLVVDTPEDGRKVFTGTTHMPTPTKALWYNNANLINQAKWHYELVKNVNPKVDLIVDQQIEWTGSAEFADI 5gpn.24   ---LGY--KPG-V----------E--AIRKNPPKVLFLLGADGGCI-------TRQDLPKDCFIIYQGHHGDVGAPMADV  target    VLPANSWMEAETWEMGASCSNPFLQVWKGGIEPLNDTRDDIAIFAGVANALTELTGDERFSQAFMFADRPEVYLDRVLAG 5gpn.24   ILPGAAYTEKSAT---YVNTEGRAQQTKVAVTPPGLAREDWKIIRALSEIAG----------------------------  target    SFTTEGYTVEDLTAGRYGPPGGALMQYRSYPRIPFKEQIEDSLPFYTDTGRMHGYVDIPEAIEYGENLIVHREAVEATPY 5gpn.24   --------------------------------------------------------------------------------  target    LPNVIVSTSPYLRPRDYGIAPEELDGDARSVRNIMMSWAEVKETENPLFAAGYNYLCLTPKSRHAVHSSWAVTDWHWLWS 5gpn.24   --------------------------------------------------------------------------------  target    SSFSDPYRVETRAPGVGEPAIHLNPDDARSLGIRNGDYVWVDSNPKDRPYRDADVDESFLDVARLLVRVTYNPAYPPGVT 5gpn.24   --------------------------------------------------------------------------------  target    MLKHAFYMATPRTFRAAQERSDGRALAETTGYQSSFRSGSHQSITRGWAPPMHQTDSLFHKRAGVFGFTYGFDVDNHAIN 5gpn.24   --------------------------------------------------------------------------------  target    TVPKETVVRITKAEDGGVGGSGAWTRGRPGSMPGDEDDAMQAYLAGELTVVRRT 5gpn.24   ------------------------------------------------------ ``` | | | | | | | | | | | | | | | | | | | | | | | | | | | | | | | | | | | | | | | | | | | | | | | | | |
|  | 6zk9.1.C | NADH:ubiquinone oxidoreductase core subunit S1  *Peripheral domain of open complex I during turnover* | 0.09 | 0.00 | 16.18 | 0.22 | 135-372 | EM | 0.00 | monomer | 6 x SF4, 1 x FMN, 1 x NAI, 2 x FES, 1 x K, 2 x PC1, 2 x 3PE, 1 x ZN, 1 x NDP, 1 x ZMP, 1 x CDL | HHblits | 0.27 |
| ``` target    RPFLERFTDMPLLVRLDTLQRLRADEVFADYSSDLDVDGPSFTLHGMTEEQHERNGDRVVFDDASGALRAINREDVGDRL 6zk9.1    --------------------------------------------------------------------------------  target    DDKGIDPALDYQGTVTLVDGSTVEVMSVLSMYREHLADYDIDSVVDMTGAPRNLIEQLLDDMTTLSPVAFHVGEGVNHYF 6zk9.1    ------------------------------------------------------SHPFSQVLQEAKKPMVVLGSSALQRN  target    HATLHNRATYLVGMLLGSVGVSGGGVSTWAGNYKGGVFQAAPWFGPGVGGFVNEDPFHPLTDPSARYSAETARHLVHGED 6zk9.1    DGAAILAAVSNIAQKIRTSSGVTGDWKVMNI------LH------RI----AS-----QV----AA--------------  target    TSYWGFGDRPLVVDTPEDGRKVFTGTTHMPTPTKALWYNNANLINQAKWHYELVKNVNPKVDLIVDQQIEWTGSAEFADI 6zk9.1    ---LDLGYKPG-VEA------------IRKNPPKMLFLLGADGGCV-------TRQDLPKDCFIVYQGHHGDVGAPIADV  target    VLPANSWMEAETWEMGASCSNPFLQVWKGGIEPLNDTRDDIAIFAGVANALTELTGDERFSQAFMFADRPEVYLDRVLAG 6zk9.1    ILPGAAYTEKSAT---YVNTEGRAQQTKVAVMPPGLAREDWKIIRALSEIAG----------------------------  target    SFTTEGYTVEDLTAGRYGPPGGALMQYRSYPRIPFKEQIEDSLPFYTDTGRMHGYVDIPEAIEYGENLIVHREAVEATPY 6zk9.1    --------------------------------------------------------------------------------  target    LPNVIVSTSPYLRPRDYGIAPEELDGDARSVRNIMMSWAEVKETENPLFAAGYNYLCLTPKSRHAVHSSWAVTDWHWLWS 6zk9.1    --------------------------------------------------------------------------------  target    SSFSDPYRVETRAPGVGEPAIHLNPDDARSLGIRNGDYVWVDSNPKDRPYRDADVDESFLDVARLLVRVTYNPAYPPGVT 6zk9.1    --------------------------------------------------------------------------------  target    MLKHAFYMATPRTFRAAQERSDGRALAETTGYQSSFRSGSHQSITRGWAPPMHQTDSLFHKRAGVFGFTYGFDVDNHAIN 6zk9.1    --------------------------------------------------------------------------------  target    TVPKETVVRITKAEDGGVGGSGAWTRGRPGSMPGDEDDAMQAYLAGELTVVRRT 6zk9.1    ------------------------------------------------------ ``` | | | | | | | | | | | | | | | | | | | | | | | | | | | | | | | | | | | | | | | | | | | | | | | | | |
|  | 6qcf.1.C | NADH:ubiquinone oxidoreductase core subunit S1  *Ovine respiratory complex I FRC open class 6* | 0.09 | 0.00 | 15.61 | 0.22 | 135-372 | EM | 0.00 | monomer | 6 x SF4, 1 x FMN, 2 x FES, 1 x ZN, 1 x NDP, 2 x ZMP | HHblits | 0.27 |
| ``` target    RPFLERFTDMPLLVRLDTLQRLRADEVFADYSSDLDVDGPSFTLHGMTEEQHERNGDRVVFDDASGALRAINREDVGDRL 6qcf.1    --------------------------------------------------------------------------------  target    DDKGIDPALDYQGTVTLVDGSTVEVMSVLSMYREHLADYDIDSVVDMTGAPRNLIEQLLDDMTTLSPVAFHVGEGVNHYF 6qcf.1    ------------------------------------------------------SHPFSQVLQEAKKPMVVLGSSALQRN  target    HATLHNRATYLVGMLLGSVGVSGGGVSTWAGNYKGGVFQAAPWFGPGVGGFVNEDPFHPLTDPSARYSAETARHLVHGED 6qcf.1    DGAAILAAVSNIAQKIRTSSGVTGDWKVMNI------LH----------RIASQ--VAA---------------------  target    TSYWGFGDRPLVVDTPEDGRKVFTGTTHMPTPTKALWYNNANLINQAKWHYELVKNVNPKVDLIVDQQIEWTGSAEFADI 6qcf.1    ---LDLGYKPG-V----------E--AIRKNPPKMLFLLGADGGCV-------TRQDLPKDCFIVYQGHHGDVGAPIADV  target    VLPANSWMEAETWEMGASCSNPFLQVWKGGIEPLNDTRDDIAIFAGVANALTELTGDERFSQAFMFADRPEVYLDRVLAG 6qcf.1    ILPGAAYTEKSAT---YVNTEGRAQQTKVAVMPPGLAREDWKIIRALSEIAG----------------------------  target    SFTTEGYTVEDLTAGRYGPPGGALMQYRSYPRIPFKEQIEDSLPFYTDTGRMHGYVDIPEAIEYGENLIVHREAVEATPY 6qcf.1    --------------------------------------------------------------------------------  target    LPNVIVSTSPYLRPRDYGIAPEELDGDARSVRNIMMSWAEVKETENPLFAAGYNYLCLTPKSRHAVHSSWAVTDWHWLWS 6qcf.1    --------------------------------------------------------------------------------  target    SSFSDPYRVETRAPGVGEPAIHLNPDDARSLGIRNGDYVWVDSNPKDRPYRDADVDESFLDVARLLVRVTYNPAYPPGVT 6qcf.1    --------------------------------------------------------------------------------  target    MLKHAFYMATPRTFRAAQERSDGRALAETTGYQSSFRSGSHQSITRGWAPPMHQTDSLFHKRAGVFGFTYGFDVDNHAIN 6qcf.1    --------------------------------------------------------------------------------  target    TVPKETVVRITKAEDGGVGGSGAWTRGRPGSMPGDEDDAMQAYLAGELTVVRRT 6qcf.1    ------------------------------------------------------ ``` | | | | | | | | | | | | | | | | | | | | | | | | | | | | | | | | | | | | | | | | | | | | | | | | | |
|  | 6qc5.1.C | NADH:ubiquinone oxidoreductase core subunit S1  *Ovine respiratory complex I FRC closed class 1* | 0.09 | 0.00 | 15.61 | 0.22 | 135-372 | EM | 0.00 | monomer | 6 x SF4, 1 x FMN, 2 x FES, 2 x 3PE, 1 x ZN, 1 x NDP, 2 x ZMP, 1 x PC1 | HHblits | 0.27 |
| ``` target    RPFLERFTDMPLLVRLDTLQRLRADEVFADYSSDLDVDGPSFTLHGMTEEQHERNGDRVVFDDASGALRAINREDVGDRL 6qc5.1    --------------------------------------------------------------------------------  target    DDKGIDPALDYQGTVTLVDGSTVEVMSVLSMYREHLADYDIDSVVDMTGAPRNLIEQLLDDMTTLSPVAFHVGEGVNHYF 6qc5.1    ------------------------------------------------------SHPFSQVLQEAKKPMVVLGSSALQRN  target    HATLHNRATYLVGMLLGSVGVSGGGVSTWAGNYKGGVFQAAPWFGPGVGGFVNEDPFHPLTDPSARYSAETARHLVHGED 6qc5.1    DGAAILAAVSNIAQKIRTSSGVTGDWKVMNI------LH----------RIASQ--VAA---------------------  target    TSYWGFGDRPLVVDTPEDGRKVFTGTTHMPTPTKALWYNNANLINQAKWHYELVKNVNPKVDLIVDQQIEWTGSAEFADI 6qc5.1    ---LDLGYKPG-V----------E--AIRKNPPKMLFLLGADGGCV-------TRQDLPKDCFIVYQGHHGDVGAPIADV  target    VLPANSWMEAETWEMGASCSNPFLQVWKGGIEPLNDTRDDIAIFAGVANALTELTGDERFSQAFMFADRPEVYLDRVLAG 6qc5.1    ILPGAAYTEKSAT---YVNTEGRAQQTKVAVMPPGLAREDWKIIRALSEIAG----------------------------  target    SFTTEGYTVEDLTAGRYGPPGGALMQYRSYPRIPFKEQIEDSLPFYTDTGRMHGYVDIPEAIEYGENLIVHREAVEATPY 6qc5.1    --------------------------------------------------------------------------------  target    LPNVIVSTSPYLRPRDYGIAPEELDGDARSVRNIMMSWAEVKETENPLFAAGYNYLCLTPKSRHAVHSSWAVTDWHWLWS 6qc5.1    --------------------------------------------------------------------------------  target    SSFSDPYRVETRAPGVGEPAIHLNPDDARSLGIRNGDYVWVDSNPKDRPYRDADVDESFLDVARLLVRVTYNPAYPPGVT 6qc5.1    --------------------------------------------------------------------------------  target    MLKHAFYMATPRTFRAAQERSDGRALAETTGYQSSFRSGSHQSITRGWAPPMHQTDSLFHKRAGVFGFTYGFDVDNHAIN 6qc5.1    --------------------------------------------------------------------------------  target    TVPKETVVRITKAEDGGVGGSGAWTRGRPGSMPGDEDDAMQAYLAGELTVVRRT 6qc5.1    ------------------------------------------------------ ``` | | | | | | | | | | | | | | | | | | | | | | | | | | | | | | | | | | | | | | | | | | | | | | | | | |
|  | 6yj4.1.G | Subunit NUAM of NADH:Ubiquinone Oxidoreductase (Complex I)  *Structure of Yarrowia lipolytica complex I at 2.7 A* | 0.09 |  | 19.76 | 0.22 | 135-372 | EM | 0.00 | hetero-1-1-1-1-1-1-… | 18 x 3PE, 6 x SF4, 5 x LMT, 8 x PLC, 2 x FES, 1 x FMN, 6 x CDL, 1 x NDP, 1 x ZN, 2 x EHZ | HHblits | 0.29 |
| ``` target    RPFLERFTDMPLLVRLDTLQRLRADEVFADYSSDLDVDGPSFTLHGMTEEQHERNGDRVVFDDASGALRAINREDVGDRL 6yj4.1    --------------------------------------------------------------------------------  target    DDKGIDPALDYQGTVTLVDGSTVEVMSVLSMYREHLADYDIDSVVDMTGAPRNLIEQLLDDMTTLSPVAFHVGEGVNHYF 6yj4.1    ------------------------------------------------------SGEFGEVLKNAKNPLIIVGSGITDRE  target    HATLHNRATYLVGMLLGSV-GVSGGGVSTWAGNYKGGVFQAAPWFGPGVGGFVNEDPFHPLTDPSARYSAETARHLVHGE 6yj4.1    DAGAFFNTIGKFVESTPSVLNENWNGYNVLQRSA-----S-----RA--GA-------YD--------------------  target    DTSYWGFGDRPLVVDTPEDGRKVFTGTTHMPTPTKALWYNNANLINQAKWHYELVKNVNPKVDLIVDQQIEWTGSAEFAD 6yj4.1    ----IGF--TPS--------------DEASKTTPKMVWLLGADEVAAS---------DIPADAFVVYQGHNGDVGAQFAD  target    IVLPANSWMEAETWEMGASCSNPFLQVWKGGIEPLNDTRDDIAIFAGVANALTELTGDERFSQAFMFADRPEVYLDRVLA 6yj4.1    VVLPGAAYTEKAGT---YVNTEGRSQISRAATGPPGGAREDWKILRAVSEYLG---------------------------  target    GSFTTEGYTVEDLTAGRYGPPGGALMQYRSYPRIPFKEQIEDSLPFYTDTGRMHGYVDIPEAIEYGENLIVHREAVEATP 6yj4.1    --------------------------------------------------------------------------------  target    YLPNVIVSTSPYLRPRDYGIAPEELDGDARSVRNIMMSWAEVKETENPLFAAGYNYLCLTPKSRHAVHSSWAVTDWHWLW 6yj4.1    --------------------------------------------------------------------------------  target    SSSFSDPYRVETRAPGVGEPAIHLNPDDARSLGIRNGDYVWVDSNPKDRPYRDADVDESFLDVARLLVRVTYNPAYPPGV 6yj4.1    --------------------------------------------------------------------------------  target    TMLKHAFYMATPRTFRAAQERSDGRALAETTGYQSSFRSGSHQSITRGWAPPMHQTDSLFHKRAGVFGFTYGFDVDNHAI 6yj4.1    --------------------------------------------------------------------------------  target    NTVPKETVVRITKAEDGGVGGSGAWTRGRPGSMPGDEDDAMQAYLAGELTVVRRT 6yj4.1    ------------------------------------------------------- ``` | | | | | | | | | | | | | | | | | | | | | | | | | | | | | | | | | | | | | | | | | | | | | | | | | |
|  | 6rfs.1.A | Subunit NUAM of NADH:Ubiquinone Oxidoreductase (Complex I)  *Cryo-EM structure of a respiratory complex I mutant lacking NDUFS4* | 0.09 |  | 19.76 | 0.22 | 135-372 | EM | 4.04 | hetero-1-1-1-1-1-1-… | 6 x SF4, 2 x FES, 1 x FMN, 1 x NDP, 1 x ZN, 1 x ZMP | HHblits | 0.29 |
| ``` target    RPFLERFTDMPLLVRLDTLQRLRADEVFADYSSDLDVDGPSFTLHGMTEEQHERNGDRVVFDDASGALRAINREDVGDRL 6rfs.1    --------------------------------------------------------------------------------  target    DDKGIDPALDYQGTVTLVDGSTVEVMSVLSMYREHLADYDIDSVVDMTGAPRNLIEQLLDDMTTLSPVAFHVGEGVNHYF 6rfs.1    ------------------------------------------------------SGEFGEVLKNAKNPLIIVGSGITDRE  target    HATLHNRATYLVGMLLGSV-GVSGGGVSTWAGNYKGGVFQAAPWFGPGVGGFVNEDPFHPLTDPSARYSAETARHLVHGE 6rfs.1    DAGAFFNTIGKFVESTPSVLNENWNGYNVLQRSA-----S-----RA--GA-------YD--------------------  target    DTSYWGFGDRPLVVDTPEDGRKVFTGTTHMPTPTKALWYNNANLINQAKWHYELVKNVNPKVDLIVDQQIEWTGSAEFAD 6rfs.1    ----IGF--TPS--------------DEASKTTPKMVWLLGADEVAAS---------DIPADAFVVYQGHNGDVGAQFAD  target    IVLPANSWMEAETWEMGASCSNPFLQVWKGGIEPLNDTRDDIAIFAGVANALTELTGDERFSQAFMFADRPEVYLDRVLA 6rfs.1    VVLPGAAYTEKAGT---YVNTEGRSQISRAATGPPGGAREDWKILRAVSEYLG---------------------------  target    GSFTTEGYTVEDLTAGRYGPPGGALMQYRSYPRIPFKEQIEDSLPFYTDTGRMHGYVDIPEAIEYGENLIVHREAVEATP 6rfs.1    --------------------------------------------------------------------------------  target    YLPNVIVSTSPYLRPRDYGIAPEELDGDARSVRNIMMSWAEVKETENPLFAAGYNYLCLTPKSRHAVHSSWAVTDWHWLW 6rfs.1    --------------------------------------------------------------------------------  target    SSSFSDPYRVETRAPGVGEPAIHLNPDDARSLGIRNGDYVWVDSNPKDRPYRDADVDESFLDVARLLVRVTYNPAYPPGV 6rfs.1    --------------------------------------------------------------------------------  target    TMLKHAFYMATPRTFRAAQERSDGRALAETTGYQSSFRSGSHQSITRGWAPPMHQTDSLFHKRAGVFGFTYGFDVDNHAI 6rfs.1    --------------------------------------------------------------------------------  target    NTVPKETVVRITKAEDGGVGGSGAWTRGRPGSMPGDEDDAMQAYLAGELTVVRRT 6rfs.1    ------------------------------------------------------- ``` | | | | | | | | | | | | | | | | | | | | | | | | | | | | | | | | | | | | | | | | | | | | | | | | | |
|  | 6rfq.1.A | Subunit NUAM of NADH:Ubiquinone Oxidoreductase (Complex I)  *Cryo-EM structure of a respiratory complex I assembly intermediate with NDUFAF2* | 0.09 |  | 19.76 | 0.22 | 135-372 | EM | 3.30 | hetero-1-1-1-1-1-1-… | 6 x SF4, 2 x FES, 1 x FMN, 1 x NDP, 10 x 3PE, 2 x LMN, 4 x CDL, 2 x ZMP, 4 x PLC, 3 x T7X, 1 x CPL | HHblits | 0.29 |
| ``` target    RPFLERFTDMPLLVRLDTLQRLRADEVFADYSSDLDVDGPSFTLHGMTEEQHERNGDRVVFDDASGALRAINREDVGDRL 6rfq.1    --------------------------------------------------------------------------------  target    DDKGIDPALDYQGTVTLVDGSTVEVMSVLSMYREHLADYDIDSVVDMTGAPRNLIEQLLDDMTTLSPVAFHVGEGVNHYF 6rfq.1    ------------------------------------------------------SGEFGEVLKNAKNPLIIVGSGITDRE  target    HATLHNRATYLVGMLLGSV-GVSGGGVSTWAGNYKGGVFQAAPWFGPGVGGFVNEDPFHPLTDPSARYSAETARHLVHGE 6rfq.1    DAGAFFNTIGKFVESTPSVLNENWNGYNVLQRSA-----S-----RA--GA-------YD--------------------  target    DTSYWGFGDRPLVVDTPEDGRKVFTGTTHMPTPTKALWYNNANLINQAKWHYELVKNVNPKVDLIVDQQIEWTGSAEFAD 6rfq.1    ----IGF--TPS--------------DEASKTTPKMVWLLGADEVAAS---------DIPADAFVVYQGHNGDVGAQFAD  target    IVLPANSWMEAETWEMGASCSNPFLQVWKGGIEPLNDTRDDIAIFAGVANALTELTGDERFSQAFMFADRPEVYLDRVLA 6rfq.1    VVLPGAAYTEKAGT---YVNTEGRSQISRAATGPPGGAREDWKILRAVSEYLG---------------------------  target    GSFTTEGYTVEDLTAGRYGPPGGALMQYRSYPRIPFKEQIEDSLPFYTDTGRMHGYVDIPEAIEYGENLIVHREAVEATP 6rfq.1    --------------------------------------------------------------------------------  target    YLPNVIVSTSPYLRPRDYGIAPEELDGDARSVRNIMMSWAEVKETENPLFAAGYNYLCLTPKSRHAVHSSWAVTDWHWLW 6rfq.1    --------------------------------------------------------------------------------  target    SSSFSDPYRVETRAPGVGEPAIHLNPDDARSLGIRNGDYVWVDSNPKDRPYRDADVDESFLDVARLLVRVTYNPAYPPGV 6rfq.1    --------------------------------------------------------------------------------  target    TMLKHAFYMATPRTFRAAQERSDGRALAETTGYQSSFRSGSHQSITRGWAPPMHQTDSLFHKRAGVFGFTYGFDVDNHAI 6rfq.1    --------------------------------------------------------------------------------  target    NTVPKETVVRITKAEDGGVGGSGAWTRGRPGSMPGDEDDAMQAYLAGELTVVRRT 6rfq.1    ------------------------------------------------------- ``` | | | | | | | | | | | | | | | | | | | | | | | | | | | | | | | | | | | | | | | | | | | | | | | | | |
|  | 6gcs.1.A | 75-KDA PROTEIN (NUAM)  *Cryo-EM structure of respiratory complex I from Yarrowia lipolytica* | 0.09 |  | 19.76 | 0.22 | 135-372 | EM | 4.32 | hetero-1-1-1-1-1-1-… | 6 x SF4, 2 x FES, 1 x FMN, 1 x NDP, 1 x ZN, 1 x ZMP, 1 x CDL, 3 x 3PE | HHblits | 0.29 |
| ``` target    RPFLERFTDMPLLVRLDTLQRLRADEVFADYSSDLDVDGPSFTLHGMTEEQHERNGDRVVFDDASGALRAINREDVGDRL 6gcs.1    --------------------------------------------------------------------------------  target    DDKGIDPALDYQGTVTLVDGSTVEVMSVLSMYREHLADYDIDSVVDMTGAPRNLIEQLLDDMTTLSPVAFHVGEGVNHYF 6gcs.1    ------------------------------------------------------SGEFGEVLKNAKNPLIIVGSGITDRE  target    HATLHNRATYLVGMLLGSV-GVSGGGVSTWAGNYKGGVFQAAPWFGPGVGGFVNEDPFHPLTDPSARYSAETARHLVHGE 6gcs.1    DAGAFFNTIGKFVESTPSVLNENWNGYNVLQRSA-----S-----RA--GA-------YD--------------------  target    DTSYWGFGDRPLVVDTPEDGRKVFTGTTHMPTPTKALWYNNANLINQAKWHYELVKNVNPKVDLIVDQQIEWTGSAEFAD 6gcs.1    ----IGF--TPS--------------DEASKTTPKMVWLLGADEVAAS---------DIPADAFVVYQGHNGDVGAQFAD  target    IVLPANSWMEAETWEMGASCSNPFLQVWKGGIEPLNDTRDDIAIFAGVANALTELTGDERFSQAFMFADRPEVYLDRVLA 6gcs.1    VVLPGAAYTEKAGT---YVNTEGRSQISRAATGPPGGAREDWKILRAVSEYLG---------------------------  target    GSFTTEGYTVEDLTAGRYGPPGGALMQYRSYPRIPFKEQIEDSLPFYTDTGRMHGYVDIPEAIEYGENLIVHREAVEATP 6gcs.1    --------------------------------------------------------------------------------  target    YLPNVIVSTSPYLRPRDYGIAPEELDGDARSVRNIMMSWAEVKETENPLFAAGYNYLCLTPKSRHAVHSSWAVTDWHWLW 6gcs.1    --------------------------------------------------------------------------------  target    SSSFSDPYRVETRAPGVGEPAIHLNPDDARSLGIRNGDYVWVDSNPKDRPYRDADVDESFLDVARLLVRVTYNPAYPPGV 6gcs.1    --------------------------------------------------------------------------------  target    TMLKHAFYMATPRTFRAAQERSDGRALAETTGYQSSFRSGSHQSITRGWAPPMHQTDSLFHKRAGVFGFTYGFDVDNHAI 6gcs.1    --------------------------------------------------------------------------------  target    NTVPKETVVRITKAEDGGVGGSGAWTRGRPGSMPGDEDDAMQAYLAGELTVVRRT 6gcs.1    ------------------------------------------------------- ``` | | | | | | | | | | | | | | | | | | | | | | | | | | | | | | | | | | | | | | | | | | | | | | | | | |
|  | 7ar7.1.G | NADH dehydrogenase [ubiquinone] iron-sulfur protein 1, mitochondrial  *Cryo-EM structure of Arabidopsis thaliana complex-I (open conformation)* | 0.09 |  | 15.88 | 0.22 | 132-372 | EM | 0.00 | hetero-1-1-1-1-1-1-… | 6 x SF4, 2 x FES, 1 x FMN, 1 x UQ9, 3 x PTY, 2 x PC7, 1 x LMN, 1 x NDP, 2 x ZN, 2 x 8Q1, 1 x PGT, 1 x PSF, 1 x T7X | HHblits | 0.27 |
| ``` target    RPFLERFTDMPLLVRLDTLQRLRADEVFADYSSDLDVDGPSFTLHGMTEEQHERNGDRVVFDDASGALRAINREDVGDRL 7ar7.1    --------------------------------------------------------------------------------  target    DDKGIDPALDYQGTVTLVDGSTVEVMSVLSMYREHLADYDIDSVVDMTGAPRNLIEQLLDDMTTLSPVAFHVGEGVNHYF 7ar7.1    ---------------------------------------------------AEGRHPFCTALKNAKNPAIIVGAGLFNRT  target    HATLHNRATYLVGMLLGSVGVSGGGVSTWAGNYKGGVFQAAPWFGPGVGGFVNEDPFHPLTDPSARYSAETARHLVHGED 7ar7.1    DKNAILSSVESIAQANNVVRPDWNGLNFLLQYAA----------QA--AA-------LD-----------------LG--  target    TSYWGFGDRPLVVDTPEDGRKVFTGTTHMPTPTKALWYNNANLINQAKWHYELVKNVNPKVDLIVDQQIEWTGSAEFADI 7ar7.1    -----L--IQ----Q----------SAKALESAKFVYLMGADDVN---------VDKIPKDAFVVYQGHHGDKAVYRANV  target    VLPANSWMEAETWEMGASCSNPFLQVWKGGIEPLNDTRDDIAIFAGVANALTELTGDERFSQAFMFADRPEVYLDRVLAG 7ar7.1    ILPASAFTEKEGT---YENTEGFTQQTVPAVPTVGDARDDWKIVRALSEVSG----------------------------  target    SFTTEGYTVEDLTAGRYGPPGGALMQYRSYPRIPFKEQIEDSLPFYTDTGRMHGYVDIPEAIEYGENLIVHREAVEATPY 7ar7.1    --------------------------------------------------------------------------------  target    LPNVIVSTSPYLRPRDYGIAPEELDGDARSVRNIMMSWAEVKETENPLFAAGYNYLCLTPKSRHAVHSSWAVTDWHWLWS 7ar7.1    --------------------------------------------------------------------------------  target    SSFSDPYRVETRAPGVGEPAIHLNPDDARSLGIRNGDYVWVDSNPKDRPYRDADVDESFLDVARLLVRVTYNPAYPPGVT 7ar7.1    --------------------------------------------------------------------------------  target    MLKHAFYMATPRTFRAAQERSDGRALAETTGYQSSFRSGSHQSITRGWAPPMHQTDSLFHKRAGVFGFTYGFDVDNHAIN 7ar7.1    --------------------------------------------------------------------------------  target    TVPKETVVRITKAEDGGVGGSGAWTRGRPGSMPGDEDDAMQAYLAGELTVVRRT 7ar7.1    ------------------------------------------------------ ``` | | | | | | | | | | | | | | | | | | | | | | | | | | | | | | | | | | | | | | | | | | | | | | | | | |
|  | 7aqr.1.F | NADH dehydrogenase [ubiquinone] iron-sulfur protein 1, mitochondrial  *Cryo-EM structure of Arabidopsis thaliana Complex-I (peripheral arm)* | 0.09 |  | 15.88 | 0.22 | 132-372 | EM | 0.00 | hetero-1-1-1-1-1-1-… | 6 x SF4, 2 x FES, 1 x FMN, 1 x NDP, 1 x ZN, 1 x 8Q1 | HHblits | 0.27 |
| ``` target    RPFLERFTDMPLLVRLDTLQRLRADEVFADYSSDLDVDGPSFTLHGMTEEQHERNGDRVVFDDASGALRAINREDVGDRL 7aqr.1    --------------------------------------------------------------------------------  target    DDKGIDPALDYQGTVTLVDGSTVEVMSVLSMYREHLADYDIDSVVDMTGAPRNLIEQLLDDMTTLSPVAFHVGEGVNHYF 7aqr.1    ---------------------------------------------------AEGRHPFCTALKNAKNPAIIVGAGLFNRT  target    HATLHNRATYLVGMLLGSVGVSGGGVSTWAGNYKGGVFQAAPWFGPGVGGFVNEDPFHPLTDPSARYSAETARHLVHGED 7aqr.1    DKNAILSSVESIAQANNVVRPDWNGLNFLLQYA-----A-----QA--AA-------LD-----------------LG--  target    TSYWGFGDRPLVVDTPEDGRKVFTGTTHMPTPTKALWYNNANLINQAKWHYELVKNVNPKVDLIVDQQIEWTGSAEFADI 7aqr.1    -----LI--Q---QS-----------AKALESAKFVYLMGADDVNV---------DKIPKDAFVVYQGHHGDKAVYRANV  target    VLPANSWMEAETWEMGASCSNPFLQVWKGGIEPLNDTRDDIAIFAGVANALTELTGDERFSQAFMFADRPEVYLDRVLAG 7aqr.1    ILPASAFTEKEGT---YENTEGFTQQTVPAVPTVGDARDDWKIVRALSEVSG----------------------------  target    SFTTEGYTVEDLTAGRYGPPGGALMQYRSYPRIPFKEQIEDSLPFYTDTGRMHGYVDIPEAIEYGENLIVHREAVEATPY 7aqr.1    --------------------------------------------------------------------------------  target    LPNVIVSTSPYLRPRDYGIAPEELDGDARSVRNIMMSWAEVKETENPLFAAGYNYLCLTPKSRHAVHSSWAVTDWHWLWS 7aqr.1    --------------------------------------------------------------------------------  target    SSFSDPYRVETRAPGVGEPAIHLNPDDARSLGIRNGDYVWVDSNPKDRPYRDADVDESFLDVARLLVRVTYNPAYPPGVT 7aqr.1    --------------------------------------------------------------------------------  target    MLKHAFYMATPRTFRAAQERSDGRALAETTGYQSSFRSGSHQSITRGWAPPMHQTDSLFHKRAGVFGFTYGFDVDNHAIN 7aqr.1    --------------------------------------------------------------------------------  target    TVPKETVVRITKAEDGGVGGSGAWTRGRPGSMPGDEDDAMQAYLAGELTVVRRT 7aqr.1    ------------------------------------------------------ ``` | | | | | | | | | | | | | | | | | | | | | | | | | | | | | | | | | | | | | | | | | | | | | | | | | |
|  | 7a23.1.O | 75kDa  *Plant mitochondrial respiratory complex I* | 0.09 |  | 15.88 | 0.22 | 132-372 | EM | 0.00 | hetero-1-1-1-1-1-1-… | 6 x SF4, 1 x FMN, 2 x T7X, 3 x CDL, 1 x U10, 1 x PEV, 2 x FES, 1 x NDP, 2 x ZN | HHblits | 0.27 |
| ``` target    RPFLERFTDMPLLVRLDTLQRLRADEVFADYSSDLDVDGPSFTLHGMTEEQHERNGDRVVFDDASGALRAINREDVGDRL 7a23.1    --------------------------------------------------------------------------------  target    DDKGIDPALDYQGTVTLVDGSTVEVMSVLSMYREHLADYDIDSVVDMTGAPRNLIEQLLDDMTTLSPVAFHVGEGVNHYF 7a23.1    ---------------------------------------------------AEGRHPFCTALKNAKNPAIIVGAGLFNRT  target    HATLHNRATYLVGMLLGSVGVSGGGVSTWAGNYKGGVFQAAPWFGPGVGGFVNEDPFHPLTDPSARYSAETARHLVHGED 7a23.1    DKNAILSSVESIAQANNVVRPDWNGLNFLLQYA-----A-----QA--AA-------LD-----------------LG--  target    TSYWGFGDRPLVVDTPEDGRKVFTGTTHMPTPTKALWYNNANLINQAKWHYELVKNVNPKVDLIVDQQIEWTGSAEFADI 7a23.1    -----LI--Q---QS-----------AKALESAKFVYLMGADDVNV---------DKIPKDAFVVYQGHHGDKAVYRANV  target    VLPANSWMEAETWEMGASCSNPFLQVWKGGIEPLNDTRDDIAIFAGVANALTELTGDERFSQAFMFADRPEVYLDRVLAG 7a23.1    ILPASAFTEKEGT---YENTEGFTQQTVPAVPTVGDARDDWKIVRALSEVSG----------------------------  target    SFTTEGYTVEDLTAGRYGPPGGALMQYRSYPRIPFKEQIEDSLPFYTDTGRMHGYVDIPEAIEYGENLIVHREAVEATPY 7a23.1    --------------------------------------------------------------------------------  target    LPNVIVSTSPYLRPRDYGIAPEELDGDARSVRNIMMSWAEVKETENPLFAAGYNYLCLTPKSRHAVHSSWAVTDWHWLWS 7a23.1    --------------------------------------------------------------------------------  target    SSFSDPYRVETRAPGVGEPAIHLNPDDARSLGIRNGDYVWVDSNPKDRPYRDADVDESFLDVARLLVRVTYNPAYPPGVT 7a23.1    --------------------------------------------------------------------------------  target    MLKHAFYMATPRTFRAAQERSDGRALAETTGYQSSFRSGSHQSITRGWAPPMHQTDSLFHKRAGVFGFTYGFDVDNHAIN 7a23.1    --------------------------------------------------------------------------------  target    TVPKETVVRITKAEDGGVGGSGAWTRGRPGSMPGDEDDAMQAYLAGELTVVRRT 7a23.1    ------------------------------------------------------ ``` | | | | | | | | | | | | | | | | | | | | | | | | | | | | | | | | | | | | | | | | | | | | | | | | | |
|  | 7ar8.1.G | NADH dehydrogenase [ubiquinone] iron-sulfur protein 1, mitochondrial  *Cryo-EM structure of Arabidopsis thaliana complex-I (closed conformation)* | 0.09 |  | 15.88 | 0.22 | 132-372 | EM | 0.00 | hetero-1-1-1-1-1-1-… | 6 x SF4, 2 x FES, 1 x FMN, 1 x UQ9, 3 x PTY, 2 x PC7, 1 x PGT, 1 x FE, 1 x NDP, 2 x ZN, 2 x 8Q1, 1 x LMN, 1 x PSF, 1 x T7X | HHblits | 0.27 |
| ``` target    RPFLERFTDMPLLVRLDTLQRLRADEVFADYSSDLDVDGPSFTLHGMTEEQHERNGDRVVFDDASGALRAINREDVGDRL 7ar8.1    --------------------------------------------------------------------------------  target    DDKGIDPALDYQGTVTLVDGSTVEVMSVLSMYREHLADYDIDSVVDMTGAPRNLIEQLLDDMTTLSPVAFHVGEGVNHYF 7ar8.1    ---------------------------------------------------AEGRHPFCTALKNAKNPAIIVGAGLFNRT  target    HATLHNRATYLVGMLLGSVGVSGGGVSTWAGNYKGGVFQAAPWFGPGVGGFVNEDPFHPLTDPSARYSAETARHLVHGED 7ar8.1    DKNAILSSVESIAQANNVVRPDWNGLNFLLQYA-----A-----QA--AA-------LD-----------------LG--  target    TSYWGFGDRPLVVDTPEDGRKVFTGTTHMPTPTKALWYNNANLINQAKWHYELVKNVNPKVDLIVDQQIEWTGSAEFADI 7ar8.1    -----LI--Q---QS-----------AKALESAKFVYLMGADDVNV---------DKIPKDAFVVYQGHHGDKAVYRANV  target    VLPANSWMEAETWEMGASCSNPFLQVWKGGIEPLNDTRDDIAIFAGVANALTELTGDERFSQAFMFADRPEVYLDRVLAG 7ar8.1    ILPASAFTEKEGT---YENTEGFTQQTVPAVPTVGDARDDWKIVRALSEVSG----------------------------  target    SFTTEGYTVEDLTAGRYGPPGGALMQYRSYPRIPFKEQIEDSLPFYTDTGRMHGYVDIPEAIEYGENLIVHREAVEATPY 7ar8.1    --------------------------------------------------------------------------------  target    LPNVIVSTSPYLRPRDYGIAPEELDGDARSVRNIMMSWAEVKETENPLFAAGYNYLCLTPKSRHAVHSSWAVTDWHWLWS 7ar8.1    --------------------------------------------------------------------------------  target    SSFSDPYRVETRAPGVGEPAIHLNPDDARSLGIRNGDYVWVDSNPKDRPYRDADVDESFLDVARLLVRVTYNPAYPPGVT 7ar8.1    --------------------------------------------------------------------------------  target    MLKHAFYMATPRTFRAAQERSDGRALAETTGYQSSFRSGSHQSITRGWAPPMHQTDSLFHKRAGVFGFTYGFDVDNHAIN 7ar8.1    --------------------------------------------------------------------------------  target    TVPKETVVRITKAEDGGVGGSGAWTRGRPGSMPGDEDDAMQAYLAGELTVVRRT 7ar8.1    ------------------------------------------------------ ``` | | | | | | | | | | | | | | | | | | | | | | | | | | | | | | | | | | | | | | | | | | | | | | | | | |
|  | 6x89.1.H | NADH dehydrogenase [ubiquinone] iron-sulfur protein 1, mitochondrial  *Vigna radiata mitochondrial complex I\** | 0.09 |  | 15.98 | 0.22 | 134-373 | EM | 0.00 | hetero-1-1-1-1-1-1-… | 1 x NAP, 6 x PC1, 6 x SF4, 2 x FES, 2 x ZN, 1 x FMN | HHblits | 0.27 |
| ``` target    RPFLERFTDMPLLVRLDTLQRLRADEVFADYSSDLDVDGPSFTLHGMTEEQHERNGDRVVFDDASGALRAINREDVGDRL 6x89.1    --------------------------------------------------------------------------------  target    DDKGIDPALDYQGTVTLVDGSTVEVMSVLSMYREHLADYDIDSVVDMTGAPRNLIEQLLDDMTTLSPVAFHVGEGVNHYF 6x89.1    -----------------------------------------------------GRHPFFKTLSDAKNPVIIVGAGVFERK  target    HATLHNRATYLVGMLLGSVGVSGGGVSTWAGNYKGGVFQAAPWFGPGVGGFVNEDPFHPLTDPSARYSAETARHLVHGED 6x89.1    DQDAIFAAVETIAQKANVVRPDWNGLNVLLLHAA----------QA--AA-------LD-----------------LG--  target    TSYWGFGDRPLVVDTPEDGRKVFTGTTHMPTPTKALWYNNANLINQAKWHYELVKNVNPKVDLIVDQQIEWTGSAEFADI 6x89.1    -----L--VPQ---S-----------EKSLESAKFVYLMGADDVNL---------DKIPDDAFVVYQGHHGDKSVYRANV  target    VLPANSWMEAETWEMGASCSNPFLQVWKGGIEPLNDTRDDIAIFAGVANALTELTGDERFSQAFMFADRPEVYLDRVLAG 6x89.1    ILPTAAFSEKEGT---YQNTEGCTQQTLPAVPTVGDSRDDWKIIRALSEVAGV---------------------------  target    SFTTEGYTVEDLTAGRYGPPGGALMQYRSYPRIPFKEQIEDSLPFYTDTGRMHGYVDIPEAIEYGENLIVHREAVEATPY 6x89.1    --------------------------------------------------------------------------------  target    LPNVIVSTSPYLRPRDYGIAPEELDGDARSVRNIMMSWAEVKETENPLFAAGYNYLCLTPKSRHAVHSSWAVTDWHWLWS 6x89.1    --------------------------------------------------------------------------------  target    SSFSDPYRVETRAPGVGEPAIHLNPDDARSLGIRNGDYVWVDSNPKDRPYRDADVDESFLDVARLLVRVTYNPAYPPGVT 6x89.1    --------------------------------------------------------------------------------  target    MLKHAFYMATPRTFRAAQERSDGRALAETTGYQSSFRSGSHQSITRGWAPPMHQTDSLFHKRAGVFGFTYGFDVDNHAIN 6x89.1    --------------------------------------------------------------------------------  target    TVPKETVVRITKAEDGGVGGSGAWTRGRPGSMPGDEDDAMQAYLAGELTVVRRT 6x89.1    ------------------------------------------------------ ``` | | | | | | | | | | | | | | | | | | | | | | | | | | | | | | | | | | | | | | | | | | | | | | | | | |
|  | 8e73.55.A | NDUS1  *Vigna radiata supercomplex I+III2 (full bridge)* | 0.09 |  | 15.98 | 0.22 | 134-373 | EM | 0.00 | monomer |  | HHblits | 0.27 |
| ``` target    RPFLERFTDMPLLVRLDTLQRLRADEVFADYSSDLDVDGPSFTLHGMTEEQHERNGDRVVFDDASGALRAINREDVGDRL 8e73.55   --------------------------------------------------------------------------------  target    DDKGIDPALDYQGTVTLVDGSTVEVMSVLSMYREHLADYDIDSVVDMTGAPRNLIEQLLDDMTTLSPVAFHVGEGVNHYF 8e73.55   -----------------------------------------------------GRHPFFKTLSDAKNPVIIVGAGVFERK  target    HATLHNRATYLVGMLLGSVGVSGGGVSTWAGNYKGGVFQAAPWFGPGVGGFVNEDPFHPLTDPSARYSAETARHLVHGED 8e73.55   DQDAIFAAVETIAQKANVVRPDWNGLNVLLLHAA----------QA--AA-------LD-----------------LG--  target    TSYWGFGDRPLVVDTPEDGRKVFTGTTHMPTPTKALWYNNANLINQAKWHYELVKNVNPKVDLIVDQQIEWTGSAEFADI 8e73.55   -----L--VPQ---S-----------EKSLESAKFVYLMGADDVNL---------DKIPDDAFVVYQGHHGDKSVYRANV  target    VLPANSWMEAETWEMGASCSNPFLQVWKGGIEPLNDTRDDIAIFAGVANALTELTGDERFSQAFMFADRPEVYLDRVLAG 8e73.55   ILPTAAFSEKEGT---YQNTEGCTQQTLPAVPTVGDSRDDWKIIRALSEVAGV---------------------------  target    SFTTEGYTVEDLTAGRYGPPGGALMQYRSYPRIPFKEQIEDSLPFYTDTGRMHGYVDIPEAIEYGENLIVHREAVEATPY 8e73.55   --------------------------------------------------------------------------------  target    LPNVIVSTSPYLRPRDYGIAPEELDGDARSVRNIMMSWAEVKETENPLFAAGYNYLCLTPKSRHAVHSSWAVTDWHWLWS 8e73.55   --------------------------------------------------------------------------------  target    SSFSDPYRVETRAPGVGEPAIHLNPDDARSLGIRNGDYVWVDSNPKDRPYRDADVDESFLDVARLLVRVTYNPAYPPGVT 8e73.55   --------------------------------------------------------------------------------  target    MLKHAFYMATPRTFRAAQERSDGRALAETTGYQSSFRSGSHQSITRGWAPPMHQTDSLFHKRAGVFGFTYGFDVDNHAIN 8e73.55   --------------------------------------------------------------------------------  target    TVPKETVVRITKAEDGGVGGSGAWTRGRPGSMPGDEDDAMQAYLAGELTVVRRT 8e73.55   ------------------------------------------------------ ``` | | | | | | | | | | | | | | | | | | | | | | | | | | | | | | | | | | | | | | | | | | | | | | | | | |
|  | 6btm.1.B | Alternative Complex III subunit B  *Structure of Alternative Complex III from Flavobacterium johnsoniae (Wild Type)* | 0.09 |  | 12.28 | 0.22 | 127-371 | EM | 3.40 | hetero-1-1-1-1-1-1-… | 6 x HEC, 1 x F3S, 1 x SF4, 2 x E87 | HHblits | 0.25 |
| ``` target    RPFLERFTDMPLLVRLDTLQRLRADEVFADYSSDLDVDGPSFTLHGMTEEQHERNGDRVVFDDASGALRAINREDVGDRL 6btm.1    --------------------------------------------------------------------------------  target    DDKGIDPALDYQGTVTLVDGSTVEVMSVLSMYREHLADYDIDSVVDMTGAPRNLIEQLLDDMTTLSP-VAFHVGEGVNHY 6btm.1    ----------------------------------------------LDAKFKAEVVKAAQQLKAAGTKGILVSGIED---  target    FHATLHNRATYLVGMLLGSVGVSGGGVSTWAGNYKGGVFQAAPWFGPGVGGFVNEDPFHPLTDPSARYSAETARHLVHGE 6btm.1    ---KNAQLLVLAINQALASEAFSTAGTRQ----------I-----RKG--S------NAVV-------------------  target    DTSYWGFGDRPLVVDTPEDGRKVFTGTTHMPTPTKALWYNNANLINQAKWHYELVKNVNPKVDLIVDQQIEWTGSAEFAD 6btm.1    ---------AQL-I------------KDMNAGSVHTLIMSGVNPVYTLADSAS-FVSGLKKVKTSVAFSLKEDETAAVST  target    IVLPANSWMEAETWEMGASCSNPFLQVWKGGIEPLNDTRDDIAIFAGVANALTELTGDERFSQAFMFADRPEVYLDRVLA 6btm.1    IAAAAPHYLESWGDVE---ITKGTYSLTQPTIRPIFDTKQFQDVLLSVNGTP----------------------------  target    GSFTTEGYTVEDLTAGRYGPPGGALMQYRSYPRIPFKEQIEDSLPFYTDTGRMHGYVDIPEAIEYGENLIVHREAVEATP 6btm.1    --------------------------------------------------------------------------------  target    YLPNVIVSTSPYLRPRDYGIAPEELDGDARSVRNIMMSWAEVKETENPLFAAGYNYLCLTPKSRHAVHSSWAVTDWHWLW 6btm.1    --------------------------------------------------------------------------------  target    SSSFSDPYRVETRAPGVGEPAIHLNPDDARSLGIRNGDYVWVDSNPKDRPYRDADVDESFLDVARLLVRVTYNPAYPPGV 6btm.1    --------------------------------------------------------------------------------  target    TMLKHAFYMATPRTFRAAQERSDGRALAETTGYQSSFRSGSHQSITRGWAPPMHQTDSLFHKRAGVFGFTYGFDVDNHAI 6btm.1    --------------------------------------------------------------------------------  target    NTVPKETVVRITKAEDGGVGGSGAWTRGRPGSMPGDEDDAMQAYLAGELTVVRRT 6btm.1    ------------------------------------------------------- ``` | | | | | | | | | | | | | | | | | | | | | | | | | | | | | | | | | | | | | | | | | | | | | | | | | |
|  | 7zm7.1.I | NADH-ubiquinone oxidoreductase-like protein  *CryoEM structure of mitochondrial complex I from Chaetomium thermophilum (inhibited by DDM)* | 0.09 |  | 20.61 | 0.21 | 138-372 | EM | 0.00 | hetero-1-1-1-1-1-1-… | 4 x PC1, 14 x LMT, 5 x CDL, 8 x 3PE, 2 x FES, 6 x SF4, 1 x FMN, 1 x NDP, 1 x ZN, 2 x ZMP | HHblits | 0.28 |
| ``` target    RPFLERFTDMPLLVRLDTLQRLRADEVFADYSSDLDVDGPSFTLHGMTEEQHERNGDRVVFDDASGALRAINREDVGDRL 7zm7.1    --------------------------------------------------------------------------------  target    DDKGIDPALDYQGTVTLVDGSTVEVMSVLSMYREHLADYDIDSVVDMTGAPRNLIEQLLDDMTTLSPVAFHVGEGVNHYF 7zm7.1    ---------------------------------------------------------FGEKLKKAKRPMIIVGSGVTEHP  target    HATLHNRATYLVGMLLGS--VGVSGGGVSTWAGNYKGGVFQAAPWFGPGVGGFVNEDPFHPLTDPSARYSAETARHLVHG 7zm7.1    DAKAFYETVWSFVEKNASNFLTEEWCGYNVLQRAA-------------------SRA--G------------A---FEVG  target    EDTSYWGFGDRPLVVDTPEDGRKVFTGTTHMPTPTKALWYNNANLINQAKWHYELVKNVNPKVDLIVDQQIEWTGSAEFA 7zm7.1    -------FVV-------PS--------PEVAATKPKFVWLLGADEFDP---------ADVPKDAFIVYQGHHGDRGAEIA  target    DIVLPANSWMEAETWEMGASCSNPFLQVWKGGIEPLNDTRDDIAIFAGVANALTELTGDERFSQAFMFADRPEVYLDRVL 7zm7.1    DIVLPGAAYTEKAGT---YVNTEGRVQMTRAATGLPGAARTDWKIIRAVSEFLG--------------------------  target    AGSFTTEGYTVEDLTAGRYGPPGGALMQYRSYPRIPFKEQIEDSLPFYTDTGRMHGYVDIPEAIEYGENLIVHREAVEAT 7zm7.1    --------------------------------------------------------------------------------  target    PYLPNVIVSTSPYLRPRDYGIAPEELDGDARSVRNIMMSWAEVKETENPLFAAGYNYLCLTPKSRHAVHSSWAVTDWHWL 7zm7.1    --------------------------------------------------------------------------------  target    WSSSFSDPYRVETRAPGVGEPAIHLNPDDARSLGIRNGDYVWVDSNPKDRPYRDADVDESFLDVARLLVRVTYNPAYPPG 7zm7.1    --------------------------------------------------------------------------------  target    VTMLKHAFYMATPRTFRAAQERSDGRALAETTGYQSSFRSGSHQSITRGWAPPMHQTDSLFHKRAGVFGFTYGFDVDNHA 7zm7.1    --------------------------------------------------------------------------------  target    INTVPKETVVRITKAEDGGVGGSGAWTRGRPGSMPGDEDDAMQAYLAGELTVVRRT 7zm7.1    -------------------------------------------------------- ``` | | | | | | | | | | | | | | | | | | | | | | | | | | | | | | | | | | | | | | | | | | | | | | | | | |
|  | 8b9z.1.G | NADH-ubiquinone oxidoreductase 75 kDa subunit, mitochondrial  *Drosophila melanogaster complex I in the Active state (Dm1)* | 0.09 |  | 15.57 | 0.22 | 135-372 | EM | 3.28 | hetero-1-1-1-1-1-1-… | 3 x PC1, 16 x 3PE, 6 x SF4, 4 x CDL, 2 x FES, 1 x FMN, 1 x UQ9, 1 x DGT, 1 x NDP, 1 x ZN, 2 x EHZ | HHblits | 0.27 |
| ``` target    RPFLERFTDMPLLVRLDTLQRLRADEVFADYSSDLDVDGPSFTLHGMTEEQHERNGDRVVFDDASGALRAINREDVGDRL 8b9z.1    --------------------------------------------------------------------------------  target    DDKGIDPALDYQGTVTLVDGSTVEVMSVLSMYREHLADYDIDSVVDMTGAPRNLIEQLLDDMTTLSPVAFHVGEGVNHYF 8b9z.1    ------------------------------------------------------AHAFSKVLEGAKKPAIIIGADLLERA  target    HATLHNRATYLVGMLLGSVGVSGGGVST-WAGNYKGGVFQAAPWFGPGVGGFVNEDPFHPLTDPSARYSAETARHLVHGE 8b9z.1    DGAAIHATV---AEYCKKLKKPNWNPFNVLQTNA-----A-----QV--GA-------LD-----------------VG-  target    DTSYWGFGDRPLVVDTPEDGRKVFTGTTHMPTPTKALWYNNANLINQAKWHYELVKNVNPKVDLIVDQQIEWTGSAEFAD 8b9z.1    ------Y--KAG----A---------QTAVKAQPKVLFLLNADAGK-------VTREQLPKDCFVVYIGSHGDNGASIAD  target    IVLPANSWMEAETWEMGASCSNPFLQVWKGGIEPLNDTRDDIAIFAGVANALTELTGDERFSQAFMFADRPEVYLDRVLA 8b9z.1    AVLPGAAYTEKQGI---YVNTEGRPQQTLPGVSPPGMAREDWKILRALSEVVG---------------------------  target    GSFTTEGYTVEDLTAGRYGPPGGALMQYRSYPRIPFKEQIEDSLPFYTDTGRMHGYVDIPEAIEYGENLIVHREAVEATP 8b9z.1    --------------------------------------------------------------------------------  target    YLPNVIVSTSPYLRPRDYGIAPEELDGDARSVRNIMMSWAEVKETENPLFAAGYNYLCLTPKSRHAVHSSWAVTDWHWLW 8b9z.1    --------------------------------------------------------------------------------  target    SSSFSDPYRVETRAPGVGEPAIHLNPDDARSLGIRNGDYVWVDSNPKDRPYRDADVDESFLDVARLLVRVTYNPAYPPGV 8b9z.1    --------------------------------------------------------------------------------  target    TMLKHAFYMATPRTFRAAQERSDGRALAETTGYQSSFRSGSHQSITRGWAPPMHQTDSLFHKRAGVFGFTYGFDVDNHAI 8b9z.1    --------------------------------------------------------------------------------  target    NTVPKETVVRITKAEDGGVGGSGAWTRGRPGSMPGDEDDAMQAYLAGELTVVRRT 8b9z.1    ------------------------------------------------------- ``` | | | | | | | | | | | | | | | | | | | | | | | | | | | | | | | | | | | | | | | | | | | | | | | | | |
|  | 8ba0.1.G | NADH-ubiquinone oxidoreductase 75 kDa subunit, mitochondrial  *Drosophila melanogaster complex I in the Twisted state (Dm2)* | 0.08 |  | 15.57 | 0.22 | 135-372 | EM | 3.68 | hetero-1-1-1-1-1-1-… | 6 x SF4, 6 x 3PE, 2 x FES, 1 x FMN, 2 x CDL, 1 x DGT, 1 x NDP, 1 x ZN, 2 x EHZ | HHblits | 0.27 |
| ``` target    RPFLERFTDMPLLVRLDTLQRLRADEVFADYSSDLDVDGPSFTLHGMTEEQHERNGDRVVFDDASGALRAINREDVGDRL 8ba0.1    --------------------------------------------------------------------------------  target    DDKGIDPALDYQGTVTLVDGSTVEVMSVLSMYREHLADYDIDSVVDMTGAPRNLIEQLLDDMTTLSPVAFHVGEGVNHYF 8ba0.1    ------------------------------------------------------AHAFSKVLEGAKKPAIIIGADLLERA  target    HATLHNRATYLVGMLLGSVGVSGGGVST-WAGNYKGGVFQAAPWFGPGVGGFVNEDPFHPLTDPSARYSAETARHLVHGE 8ba0.1    DGAAIHATV---AEYCKKLKKPNWNPFNVLQTNA-----A-----QV--GA-------LD-----------------VG-  target    DTSYWGFGDRPLVVDTPEDGRKVFTGTTHMPTPTKALWYNNANLINQAKWHYELVKNVNPKVDLIVDQQIEWTGSAEFAD 8ba0.1    ------Y--KAG----A---------QTAVKAQPKVLFLLNADAGK-------VTREQLPKDCFVVYIGSHGDNGASIAD  target    IVLPANSWMEAETWEMGASCSNPFLQVWKGGIEPLNDTRDDIAIFAGVANALTELTGDERFSQAFMFADRPEVYLDRVLA 8ba0.1    AVLPGAAYTEKQGI---YVNTEGRPQQTLPGVSPPGMAREDWKILRALSEVVG---------------------------  target    GSFTTEGYTVEDLTAGRYGPPGGALMQYRSYPRIPFKEQIEDSLPFYTDTGRMHGYVDIPEAIEYGENLIVHREAVEATP 8ba0.1    --------------------------------------------------------------------------------  target    YLPNVIVSTSPYLRPRDYGIAPEELDGDARSVRNIMMSWAEVKETENPLFAAGYNYLCLTPKSRHAVHSSWAVTDWHWLW 8ba0.1    --------------------------------------------------------------------------------  target    SSSFSDPYRVETRAPGVGEPAIHLNPDDARSLGIRNGDYVWVDSNPKDRPYRDADVDESFLDVARLLVRVTYNPAYPPGV 8ba0.1    --------------------------------------------------------------------------------  target    TMLKHAFYMATPRTFRAAQERSDGRALAETTGYQSSFRSGSHQSITRGWAPPMHQTDSLFHKRAGVFGFTYGFDVDNHAI 8ba0.1    --------------------------------------------------------------------------------  target    NTVPKETVVRITKAEDGGVGGSGAWTRGRPGSMPGDEDDAMQAYLAGELTVVRRT 8ba0.1    ------------------------------------------------------- ``` | | | | | | | | | | | | | | | | | | | | | | | | | | | | | | | | | | | | | | | | | | | | | | | | | |
|  | 8e9g.1.G | NADH-quinone oxidoreductase subunit G  *Mycobacterial respiratory complex I with both quinone positions modelled* | 0.05 |  | 12.50 | 0.12 | 270-372 | EM | 0.00 | hetero-1-1-1-1-1-1-… |  | HHblits | 0.26 |
| ``` target    RPFLERFTDMPLLVRLDTLQRLRADEVFADYSSDLDVDGPSFTLHGMTEEQHERNGDRVVFDDASGALRAINREDVGDRL 8e9g.1    --------------------------------------------------------------------------------  target    DDKGIDPALDYQGTVTLVDGSTVEVMSVLSMYREHLADYDIDSVVDMTGAPRNLIEQLLDDMTTLSPVAFHVGEGVNHYF 8e9g.1    --------------------------------------------------------------------------------  target    HATLHNRATYLVGMLLGSVGVSGGGVSTWAGNYKGGVFQAAPWFGPGVGGFVNEDPFHPLTDPSARYSAETARHLVHGED 8e9g.1    --------------------------------------------------------------------------------  target    TSYWGFGDRPLVVDTPEDGRKVFTGTTHMPTPTKALWYNNANLINQAKWHYELVKNVNPKVDLIVDQQIEWTGSAEFADI 8e9g.1    -----------------------------SGHLAALLVGG-VELGDLPDPE-LAVAAVRTTPFVVSLELRESAVTELADV  target    VLPANSWMEAETWEMGASCSNPFLQVWKGGIEPLNDTRDDIAIFAGVANALTELTGDERFSQAFMFADRPEVYLDRVLAG 8e9g.1    VFPVAPVVEKAGSF---LNWEGRPRPFAPSLK--TNAIPDLRVLHYLADEIG----------------------------  target    SFTTEGYTVEDLTAGRYGPPGGALMQYRSYPRIPFKEQIEDSLPFYTDTGRMHGYVDIPEAIEYGENLIVHREAVEATPY 8e9g.1    --------------------------------------------------------------------------------  target    LPNVIVSTSPYLRPRDYGIAPEELDGDARSVRNIMMSWAEVKETENPLFAAGYNYLCLTPKSRHAVHSSWAVTDWHWLWS 8e9g.1    --------------------------------------------------------------------------------  target    SSFSDPYRVETRAPGVGEPAIHLNPDDARSLGIRNGDYVWVDSNPKDRPYRDADVDESFLDVARLLVRVTYNPAYPPGVT 8e9g.1    --------------------------------------------------------------------------------  target    MLKHAFYMATPRTFRAAQERSDGRALAETTGYQSSFRSGSHQSITRGWAPPMHQTDSLFHKRAGVFGFTYGFDVDNHAIN 8e9g.1    --------------------------------------------------------------------------------  target    TVPKETVVRITKAEDGGVGGSGAWTRGRPGSMPGDEDDAMQAYLAGELTVVRRT 8e9g.1    ------------------------------------------------------ ``` | | | | | | | | | | | | | | | | | | | | | | | | | | | | | | | | | | | | | | | | | | | | | | | | | |
|  | 7q5y.1.A | NADH dehydrogenase I chain G  *Structure of NADH:ubichinon oxidoreductase (complex I) of the hyperthermophilic eubacterium Aquifex aeolicus* | 0.04 |  | 17.02 | 0.12 | 271-373 | X-ray | 2.70 | hetero-1-1-1-1-1-1-… | 8 x SF4, 2 x FES, 1 x FMN | HHblits | 0.28 |
| ``` target    RPFLERFTDMPLLVRLDTLQRLRADEVFADYSSDLDVDGPSFTLHGMTEEQHERNGDRVVFDDASGALRAINREDVGDRL 7q5y.1    --------------------------------------------------------------------------------  target    DDKGIDPALDYQGTVTLVDGSTVEVMSVLSMYREHLADYDIDSVVDMTGAPRNLIEQLLDDMTTLSPVAFHVGEGVNHYF 7q5y.1    --------------------------------------------------------------------------------  target    HATLHNRATYLVGMLLGSVGVSGGGVSTWAGNYKGGVFQAAPWFGPGVGGFVNEDPFHPLTDPSARYSAETARHLVHGED 7q5y.1    --------------------------------------------------------------------------------  target    TSYWGFGDRPLVVDTPEDGRKVFTGTTHMPTPTKALWYNNANLINQAKWHYELVKNVNPKVDLIVDQQIEWTGSAEFADI 7q5y.1    ------------------------------GDIENLIIFGEDILEFYED--KVFEELKEKLEHLVVVSPYEDGLSEYAHI  target    VLPANSWMEAETWEMGASCSNPFLQVWKGGIEPLNDTRDDIAIFAGVANALTELTGDERFSQAFMFADRPEVYLDRVLAG 7q5y.1    KIPMSLMGENEGT---YKTFFGEVKGKK--FLP--WAFDDLAFWKYLGENFKE---------------------------  target    SFTTEGYTVEDLTAGRYGPPGGALMQYRSYPRIPFKEQIEDSLPFYTDTGRMHGYVDIPEAIEYGENLIVHREAVEATPY 7q5y.1    --------------------------------------------------------------------------------  target    LPNVIVSTSPYLRPRDYGIAPEELDGDARSVRNIMMSWAEVKETENPLFAAGYNYLCLTPKSRHAVHSSWAVTDWHWLWS 7q5y.1    --------------------------------------------------------------------------------  target    SSFSDPYRVETRAPGVGEPAIHLNPDDARSLGIRNGDYVWVDSNPKDRPYRDADVDESFLDVARLLVRVTYNPAYPPGVT 7q5y.1    --------------------------------------------------------------------------------  target    MLKHAFYMATPRTFRAAQERSDGRALAETTGYQSSFRSGSHQSITRGWAPPMHQTDSLFHKRAGVFGFTYGFDVDNHAIN 7q5y.1    --------------------------------------------------------------------------------  target    TVPKETVVRITKAEDGGVGGSGAWTRGRPGSMPGDEDDAMQAYLAGELTVVRRT 7q5y.1    ------------------------------------------------------ ``` | | | | | | | | | | | | | | | | | | | | | | | | | | | | | | | | | | | | | | | | | | | | | | | | | |
|  | 6lod.1.B | Fe-S-cluster-containing hydrogenase components 1-like protein  *Cryo-EM structure of the air-oxidized photosynthetic alternative complex III from Roseiflexus castenholzii* | 0.03 |  | 17.39 | 0.12 | 531-650 | EM | 0.00 | hetero-1-1-1-1-1-1-… | 6 x HEC, 2 x EL6, 3 x SF4, 1 x F3S | HHblits | 0.29 |
| ``` target    RPFLERFTDMPLLVRLDTLQRLRADEVFADYSSDLDVDGPSFTLHGMTEEQHERNGDRVVFDDASGALRAINREDVGDRL 6lod.1    --------------------------------------------------------------------------------  target    DDKGIDPALDYQGTVTLVDGSTVEVMSVLSMYREHLADYDIDSVVDMTGAPRNLIEQLLDDMTTLSPVAFHVGEGVNHYF 6lod.1    --------------------------------------------------------------------------------  target    HATLHNRATYLVGMLLGSVGVSGGGVSTWAGNYKGGVFQAAPWFGPGVGGFVNEDPFHPLTDPSARYSAETARHLVHGED 6lod.1    --------------------------------------------------------------------------------  target    TSYWGFGDRPLVVDTPEDGRKVFTGTTHMPTPTKALWYNNANLINQAKWHYELVKNVNPKVDLIVDQQIEWTGSAEFADI 6lod.1    --------------------------------------------------------------------------------  target    VLPANSWMEAETWEMGASCSNPFLQVWKGGIEPLNDTRDDIAIFAGVANALTELTGDERFSQAFMFADRPEVYLDRVLAG 6lod.1    --------------------------------------------------------------------------------  target    SFTTEGYTVEDLTAGRYGPPGGALMQYRSYPRIPFKEQIEDSLPFYTDTGRMHGYVDIPEAIEYGENLIVHREAVEATPY 6lod.1    --------------------------------------------------------------------------------  target    LPNVIVSTSPYLRPRDYGIAPEELDGDARSVRNIMMSWAEVKETENPLFAAGYNYLCLTPKSRHAVHSSWAVTDWHWLWS 6lod.1    --------------------------------------------------QGLEIVFRPD--PSLWDGAFANN--AWLQE  target    SSFSDPYRVETRAPGVGEPAIHLNPDDARSLGIRNGDYVWVDSNPKDRPYRDADVDESFLDVARLLVRVTYNPAYPPGVT 6lod.1    TP--KPYTK-----LTWDNVALMSVRTANALGLKNGDVVRLTYQ-----------------GRSVDAPVWVQPGHADDSV  target    MLKHAFYMATPRTFRAAQERSDGRALAETTGYQSSFRSGSHQSITRGWAPPMHQTDSLFHKRAGVFGFTYGFDVDNHAIN 6lod.1    TVHFGFGRTA----------------------------------------------------------------------  target    TVPKETVVRITKAEDGGVGGSGAWTRGRPGSMPGDEDDAMQAYLAGELTVVRRT 6lod.1    ------------------------------------------------------ ``` | | | | | | | | | | | | | | | | | | | | | | | | | | | | | | | | | | | | | | | | | | | | | | | | | |
|  | 6s6y.1.B | Tungsten-containing formylmethanofuran dehydrogenase, subunit B  *X-ray crystal structure of the formyltransferase/hydrolase complex (FhcABCD) from Methylorubrum extorquens in complex with methylofuran* | 0.04 |  | 9.78 | 0.12 | 271-373 | X-ray | 3.10 | hetero-2-2-2-2-mer | 1 x MFN, 4 x ZN, 4 x CA, 4 x K, 3 x DGL, 2 x GLU, 1 x IAS | HHblits | 0.25 |
| ``` target    RPFLERFTDMPLLVRLDTLQRLRADEVFADYSSDLDVDGPSFTLHGMTEEQHERNGDRVVFDDASGALRAINREDVGDRL 6s6y.1    --------------------------------------------------------------------------------  target    DDKGIDPALDYQGTVTLVDGSTVEVMSVLSMYREHLADYDIDSVVDMTGAPRNLIEQLLDDMTTLSPVAFHVGEGVNHYF 6s6y.1    --------------------------------------------------------------------------------  target    HATLHNRATYLVGMLLGSVGVSGGGVSTWAGNYKGGVFQAAPWFGPGVGGFVNEDPFHPLTDPSARYSAETARHLVHGED 6s6y.1    --------------------------------------------------------------------------------  target    TSYWGFGDRPLVVDTPEDGRKVFTGTTHMPTPTKALWYNNANLINQAKWHYELVKNVNPKVDLIVDQQ-IEWTGSAEFAD 6s6y.1    ------------------------------GEADAALWLASLPAPR--------PAWLGSLPTIAIVGEGSQEAAGETAE  target    IVLPANSW-MEAETWEMGASCSNPFLQVWKGGIEPL---NDTRDDIAIFAGVANALTELTGDERFSQAFMFADRPEVYLD 6s6y.1    VVITVGVPGQSVGGA---LWNDRRGVIAYAEASDPAKTPAETETAAGVLTRIRDRLIE----------------------  target    RVLAGSFTTEGYTVEDLTAGRYGPPGGALMQYRSYPRIPFKEQIEDSLPFYTDTGRMHGYVDIPEAIEYGENLIVHREAV 6s6y.1    --------------------------------------------------------------------------------  target    EATPYLPNVIVSTSPYLRPRDYGIAPEELDGDARSVRNIMMSWAEVKETENPLFAAGYNYLCLTPKSRHAVHSSWAVTDW 6s6y.1    --------------------------------------------------------------------------------  target    HWLWSSSFSDPYRVETRAPGVGEPAIHLNPDDARSLGIRNGDYVWVDSNPKDRPYRDADVDESFLDVARLLVRVTYNPAY 6s6y.1    --------------------------------------------------------------------------------  target    PPGVTMLKHAFYMATPRTFRAAQERSDGRALAETTGYQSSFRSGSHQSITRGWAPPMHQTDSLFHKRAGVFGFTYGFDVD 6s6y.1    --------------------------------------------------------------------------------  target    NHAINTVPKETVVRITKAEDGGVGGSGAWTRGRPGSMPGDEDDAMQAYLAGELTVVRRT 6s6y.1    ----------------------------------------------------------- ``` | | | | | | | | | | | | | | | | | | | | | | | | | | | | | | | | | | | | | | | | | | | | | | | | | |
|  | 1h0h.1.A | FORMATE DEHYDROGENASE SUBUNIT ALPHA  *Tungsten containing Formate Dehydrogenase from Desulfovibrio Gigas* | 0.03 |  | 20.00 | 0.11 | 530-647 | X-ray | 1.80 | hetero-1-1-mer | 1 x W, 1 x 2MD, 1 x MGD, 4 x SF4, 1 x CA | HHblits | 0.31 |
| ``` target    RPFLERFTDMPLLVRLDTLQRLRADEVFADYSSDLDVDGPSFTLHGMTEEQHERNGDRVVFDDASGALRAINREDVGDRL 1h0h.1    --------------------------------------------------------------------------------  target    DDKGIDPALDYQGTVTLVDGSTVEVMSVLSMYREHLADYDIDSVVDMTGAPRNLIEQLLDDMTTLSPVAFHVGEGVNHYF 1h0h.1    --------------------------------------------------------------------------------  target    HATLHNRATYLVGMLLGSVGVSGGGVSTWAGNYKGGVFQAAPWFGPGVGGFVNEDPFHPLTDPSARYSAETARHLVHGED 1h0h.1    --------------------------------------------------------------------------------  target    TSYWGFGDRPLVVDTPEDGRKVFTGTTHMPTPTKALWYNNANLINQAKWHYELVKNVNPKVDLIVDQQIEWTGSAEFADI 1h0h.1    --------------------------------------------------------------------------------  target    VLPANSWMEAETWEMGASCSNPFLQVWKGGIEPLNDTRDDIAIFAGVANALTELTGDERFSQAFMFADRPEVYLDRVLAG 1h0h.1    --------------------------------------------------------------------------------  target    SFTTEGYTVEDLTAGRYGPPGGALMQYRSYPRIPFKEQIEDSLPFYTDTGRMHGYVDIPEAIEYGENLIVHREAVEATPY 1h0h.1    --------------------------------------------------------------------------------  target    LPNVIVSTSPYLRPRDYGIAPEELDGDARSVRNIMMSWAEVKETENPLFAAGYNYLCLTPKSRHAVHS--SWAVTDWHWL 1h0h.1    -------------------------------------------------DPRYPFICSTYRVTEHWQTGLMTRNTPW--L  target    WSSSFSDPYRVETRAPGVGEPAIHLNPDDARSLGIRNGDYVWVDSNPKDRPYRDADVDESFLDVARLLVRVTYNPAYPPG 1h0h.1    L----------E----AEPQMFCEMSEELATLRGIKNGDKVILESV-----------------RGKLWAKAIITKRIKPF  target    --------VTMLK--HAFYMATPRTFRAAQERSDGRALAETTGYQSSFRSGSHQSITRGWAPPMHQTDSLFHKRAGVFGF 1h0h.1    AIQGQQVHMVGIPWHYGWS-------------------------------------------------------------  target    TYGFDVDNHAINTVPKETVVRITKAEDGGVGGSGAWTRGRPGSMPGDEDDAMQAYLAGELTVVRRT 1h0h.1    ------------------------------------------------------------------ ``` | | | | | | | | | | | | | | | | | | | | | | | | | | | | | | | | | | | | | | | | | | | | | | | | | |
|  | 3o5a.1.A | Periplasmic nitrate reductase  *Crystal Structure of partially reduced Periplasmic Nitrate Reductase from Cupriavidus necator using Ionic Liquids* | 0.03 |  | 25.30 | 0.11 | 531-644 | X-ray | 1.72 | hetero-oligomer | 1 x SF4, 1 x MOS, 2 x MGD, 2 x HEC | HHblits | 0.32 |
| ``` target    RPFLERFTDMPLLVRLDTLQRLRADEVFADYSSDLDVDGPSFTLHGMTEEQHERNGDRVVFDDASGALRAINREDVGDRL 3o5a.1    --------------------------------------------------------------------------------  target    DDKGIDPALDYQGTVTLVDGSTVEVMSVLSMYREHLADYDIDSVVDMTGAPRNLIEQLLDDMTTLSPVAFHVGEGVNHYF 3o5a.1    --------------------------------------------------------------------------------  target    HATLHNRATYLVGMLLGSVGVSGGGVSTWAGNYKGGVFQAAPWFGPGVGGFVNEDPFHPLTDPSARYSAETARHLVHGED 3o5a.1    --------------------------------------------------------------------------------  target    TSYWGFGDRPLVVDTPEDGRKVFTGTTHMPTPTKALWYNNANLINQAKWHYELVKNVNPKVDLIVDQQIEWTGSAEFADI 3o5a.1    --------------------------------------------------------------------------------  target    VLPANSWMEAETWEMGASCSNPFLQVWKGGIEPLNDTRDDIAIFAGVANALTELTGDERFSQAFMFADRPEVYLDRVLAG 3o5a.1    --------------------------------------------------------------------------------  target    SFTTEGYTVEDLTAGRYGPPGGALMQYRSYPRIPFKEQIEDSLPFYTDTGRMHGYVDIPEAIEYGENLIVHREAVEATPY 3o5a.1    --------------------------------------------------------------------------------  target    LPNVIVSTSPYLRPRDYGIAPEELDGDARSVRNIMMSWAEVKETENPLFAAGYNYLCLTPKSRHAVHSSWAVTDWHWLWS 3o5a.1    --------------------------------------------------KEYPYWLVTGRVLEHWHSGSMTRRVPELYR  target    SSFSDPYRVETRAPGVGEPAIHLNPDDARSLGIRNGDYVWVDSNPKDRPYRDADVDESFLDVARLLVRVTYN--PAYPPG 3o5a.1    --------------SFPNAVVFMHPEDAKALGLRRGVEVEVVSR-----------------RGRMRSRIETRGRDAPPRG  target    VTMLKHAFYMATPRTFRAAQERSDGRALAETTGYQSSFRSGSHQSITRGWAPPMHQTDSLFHKRAGVFGFTYGFDVDNHA 3o5a.1    LVFVPW--------------------------------------------------------------------------  target    INTVPKETVVRITKAEDGGVGGSGAWTRGRPGSMPGDEDDAMQAYLAGELTVVRRT 3o5a.1    -------------------------------------------------------- ``` | | | | | | | | | | | | | | | | | | | | | | | | | | | | | | | | | | | | | | | | | | | | | | | | | |
|  | 7bkb.1.J | Formylmethanofuran dehydrogenase, subunit D  *Formate dehydrogenase - heterodisulfide reductase - formylmethanofuran dehydrogenase complex from Methanospirillum hungatei (hexameric, composite structure)* | 0.02 |  | 16.67 | 0.11 | 531-647 | EM | 0.00 | hetero-2-2-2-2-2-2-… | 48 x SF4, 4 x FAD, 2 x FES, 4 x 9S8, 4 x ZN, 2 x MO, 4 x MGD | HHblits | 0.28 |
| ``` target    RPFLERFTDMPLLVRLDTLQRLRADEVFADYSSDLDVDGPSFTLHGMTEEQHERNGDRVVFDDASGALRAINREDVGDRL 7bkb.1    --------------------------------------------------------------------------------  target    DDKGIDPALDYQGTVTLVDGSTVEVMSVLSMYREHLADYDIDSVVDMTGAPRNLIEQLLDDMTTLSPVAFHVGEGVNHYF 7bkb.1    --------------------------------------------------------------------------------  target    HATLHNRATYLVGMLLGSVGVSGGGVSTWAGNYKGGVFQAAPWFGPGVGGFVNEDPFHPLTDPSARYSAETARHLVHGED 7bkb.1    --------------------------------------------------------------------------------  target    TSYWGFGDRPLVVDTPEDGRKVFTGTTHMPTPTKALWYNNANLINQAKWHYELVKNVNPKVDLIVDQQIEWTGSAEFADI 7bkb.1    --------------------------------------------------------------------------------  target    VLPANSWMEAETWEMGASCSNPFLQVWKGGIEPLNDTRDDIAIFAGVANALTELTGDERFSQAFMFADRPEVYLDRVLAG 7bkb.1    --------------------------------------------------------------------------------  target    SFTTEGYTVEDLTAGRYGPPGGALMQYRSYPRIPFKEQIEDSLPFYTDTGRMHGYVDIPEAIEYGENLIVHREAVEATPY 7bkb.1    --------------------------------------------------------------------------------  target    LPNVIVSTSPYLRPRDYGIAPEELDGDARSVRNIMMSWAEVKETENPLFAAGYNYLCLTPKSRHAVHSSWAVTDWHWLWS 7bkb.1    --------------------------------------------------AKKTLNMITQRAVEEGIAMEI-GKT-----  target    SSFSDPYRVETRAPGVGEPAIHLNPDDARSLGIRNGDYVWVDSNPKDRPYRDADVDESFLDVARLLVRVTYN-PAYPPGV 7bkb.1    -------SRQY---FDACSIIEMNEQDMKELGIMKNTNVRVKSE-----------------SGEVVVKAVVGRQTCYPGL  target    TMLKHAFYMATPRTFRAAQERSDGRALAETTGYQSSFRSGSHQSITRGWAPPMHQTDSLFHKRAGVFGFTYGFDVDNHAI 7bkb.1    CHIRQGVW------------------------------------------------------------------------  target    NTVPKETVVRITKAEDGGVGGSGAWTRGRPGSMPGDEDDAMQAYLAGELTVVRRT 7bkb.1    ------------------------------------------------------- ``` | | | | | | | | | | | | | | | | | | | | | | | | | | | | | | | | | | | | | | | | | | | | | | | | | |
|  | 1kqf.1.A | FORMATE DEHYDROGENASE, NITRATE-INDUCIBLE, MAJOR SUBUNIT  *FORMATE DEHYDROGENASE N FROM E. COLI* | 0.03 |  | 16.47 | 0.11 | 530-647 | X-ray | 1.60 | hetero-oligomer | 3 x 6MO, 15 x SF4, 6 x MGD, 6 x HEM, 3 x CDL | HHblits | 0.27 |
| ``` target    RPFLERFTDMPLLVRLDTLQRLRADEVFADYSSDLDVDGPSFTLHGMTEEQHERNGDRVVFDDASGALRAINREDVGDRL 1kqf.1    --------------------------------------------------------------------------------  target    DDKGIDPALDYQGTVTLVDGSTVEVMSVLSMYREHLADYDIDSVVDMTGAPRNLIEQLLDDMTTLSPVAFHVGEGVNHYF 1kqf.1    --------------------------------------------------------------------------------  target    HATLHNRATYLVGMLLGSVGVSGGGVSTWAGNYKGGVFQAAPWFGPGVGGFVNEDPFHPLTDPSARYSAETARHLVHGED 1kqf.1    --------------------------------------------------------------------------------  target    TSYWGFGDRPLVVDTPEDGRKVFTGTTHMPTPTKALWYNNANLINQAKWHYELVKNVNPKVDLIVDQQIEWTGSAEFADI 1kqf.1    --------------------------------------------------------------------------------  target    VLPANSWMEAETWEMGASCSNPFLQVWKGGIEPLNDTRDDIAIFAGVANALTELTGDERFSQAFMFADRPEVYLDRVLAG 1kqf.1    --------------------------------------------------------------------------------  target    SFTTEGYTVEDLTAGRYGPPGGALMQYRSYPRIPFKEQIEDSLPFYTDTGRMHGYVDIPEAIEYGENLIVHREAVEATPY 1kqf.1    --------------------------------------------------------------------------------  target    LPNVIVSTSPYLRPRDYGIAPEELDGDARSVRNIMMSWAEVKETENPLFAAGYNYLCLTPKSRHAVHSSWAVTDWHWLWS 1kqf.1    -------------------------------------------------KEQFPYVGTTYRLTEHFHTWTKHALLN----  target    SSFSDPYRVETRAPGVGEPAIHLNPDDARSLGIRNGDYVWVDSNPKDRPYRDADVDESFLDVARLLVRVTYNPAYPP--- 1kqf.1    --------A----IAQPEQFVEISETLAAAKGINNGDRVTVSSK-----------------RGFIRAVAVVTRRLKPLNV  target    -----GVTMLKHAFYMATPRTFRAAQERSDGRALAETTGYQSSFRSGSHQSITRGWAPPMHQTDSLFHKRAGVFGFTYGF 1kqf.1    NGQQVETVGIPIHWG-----------------------------------------------------------------  target    DVDNHAINTVPKETVVRITKAEDGGVGGSGAWTRGRPGSMPGDEDDAMQAYLAGELTVVRRT 1kqf.1    -------------------------------------------------------------- ``` | | | | | | | | | | | | | | | | | | | | | | | | | | | | | | | | | | | | | | | | | | | | | | | | | |
|  | 2nya.1.A | Periplasmic nitrate reductase  *Crystal structure of the periplasmic nitrate reductase (NAP) from Escherichia coli* | 0.03 |  | 21.95 | 0.11 | 531-645 | X-ray | 2.50 | monomer | 1 x SF4, 1 x 6MO, 2 x MGD | HHblits | 0.30 |
| ``` target    RPFLERFTDMPLLVRLDTLQRLRADEVFADYSSDLDVDGPSFTLHGMTEEQHERNGDRVVFDDASGALRAINREDVGDRL 2nya.1    --------------------------------------------------------------------------------  target    DDKGIDPALDYQGTVTLVDGSTVEVMSVLSMYREHLADYDIDSVVDMTGAPRNLIEQLLDDMTTLSPVAFHVGEGVNHYF 2nya.1    --------------------------------------------------------------------------------  target    HATLHNRATYLVGMLLGSVGVSGGGVSTWAGNYKGGVFQAAPWFGPGVGGFVNEDPFHPLTDPSARYSAETARHLVHGED 2nya.1    --------------------------------------------------------------------------------  target    TSYWGFGDRPLVVDTPEDGRKVFTGTTHMPTPTKALWYNNANLINQAKWHYELVKNVNPKVDLIVDQQIEWTGSAEFADI 2nya.1    --------------------------------------------------------------------------------  target    VLPANSWMEAETWEMGASCSNPFLQVWKGGIEPLNDTRDDIAIFAGVANALTELTGDERFSQAFMFADRPEVYLDRVLAG 2nya.1    --------------------------------------------------------------------------------  target    SFTTEGYTVEDLTAGRYGPPGGALMQYRSYPRIPFKEQIEDSLPFYTDTGRMHGYVDIPEAIEYGENLIVHREAVEATPY 2nya.1    --------------------------------------------------------------------------------  target    LPNVIVSTSPYLRPRDYGIAPEELDGDARSVRNIMMSWAEVKETENPLFAAGYNYLCLTPKSRHAVH--SSWAVTDWHWL 2nya.1    --------------------------------------------------EEYDLWLSTGRVLEHWHTGSMTRRVPE--L  target    WSSSFSDPYRVETRAPGVGEPAIHLNPDDARSLGIRNGDYVWVDSNPKDRPYRDADVDESFLDVARLLVRVTYNPA--YP 2nya.1    ----------HR----AFPEAVLFIHPLDAKARDLRRGDKVKVVSR-----------------RGEVISIVETRGRNRPP  target    PGVTMLKHAFYMATPRTFRAAQERSDGRALAETTGYQSSFRSGSHQSITRGWAPPMHQTDSLFHKRAGVFGFTYGFDVDN 2nya.1    QGLVYMPFF-----------------------------------------------------------------------  target    HAINTVPKETVVRITKAEDGGVGGSGAWTRGRPGSMPGDEDDAMQAYLAGELTVVRRT 2nya.1    ---------------------------------------------------------- ``` | | | | | | | | | | | | | | | | | | | | | | | | | | | | | | | | | | | | | | | | | | | | | | | | | |
|  | 5t5i.1.D | Tungsten formylmethanofuran dehydrogenase subunit fwdD  *TUNGSTEN-CONTAINING FORMYLMETHANOFURAN DEHYDROGENASE FROM METHANOTHERMOBACTER WOLFEII, ORTHORHOMBIC FORM AT 1.9 A* | 0.02 |  | 19.75 | 0.10 | 534-647 | X-ray | 1.90 | hetero-oligomer | 4 x ZN, 2 x MG, 18 x K, 22 x SF4, 2 x W, 4 x MGD, 2 x H2S, 2 x CA | HHblits | 0.29 |
| ``` target    RPFLERFTDMPLLVRLDTLQRLRADEVFADYSSDLDVDGPSFTLHGMTEEQHERNGDRVVFDDASGALRAINREDVGDRL 5t5i.1    --------------------------------------------------------------------------------  target    DDKGIDPALDYQGTVTLVDGSTVEVMSVLSMYREHLADYDIDSVVDMTGAPRNLIEQLLDDMTTLSPVAFHVGEGVNHYF 5t5i.1    --------------------------------------------------------------------------------  target    HATLHNRATYLVGMLLGSVGVSGGGVSTWAGNYKGGVFQAAPWFGPGVGGFVNEDPFHPLTDPSARYSAETARHLVHGED 5t5i.1    --------------------------------------------------------------------------------  target    TSYWGFGDRPLVVDTPEDGRKVFTGTTHMPTPTKALWYNNANLINQAKWHYELVKNVNPKVDLIVDQQIEWTGSAEFADI 5t5i.1    --------------------------------------------------------------------------------  target    VLPANSWMEAETWEMGASCSNPFLQVWKGGIEPLNDTRDDIAIFAGVANALTELTGDERFSQAFMFADRPEVYLDRVLAG 5t5i.1    --------------------------------------------------------------------------------  target    SFTTEGYTVEDLTAGRYGPPGGALMQYRSYPRIPFKEQIEDSLPFYTDTGRMHGYVDIPEAIEYGENLIVHREAVEATPY 5t5i.1    --------------------------------------------------------------------------------  target    LPNVIVSTSPYLRPRDYGIAPEELDGDARSVRNIMMSWAEVKETENPLFAAGYNYLCLTPKSRHAVHSSWAVTDWHWLWS 5t5i.1    -----------------------------------------------------RVILNTGRTIWQGQAIESGKDL-----  target    SSFSDPYRVETRAPGVGEPAIHLNPDDARSLGIRNGDYVWVDSNPKDRPYRDADVDESFLDVARLLVRVT-YNPAYPPGV 5t5i.1    --------KM---YVDAAAIIQMNPEMMKQLGIAEGDNVKVISE-----------------YGDVVVKAVEAKEPLPEGM  target    TMLKHAFYMATPRTFRAAQERSDGRALAETTGYQSSFRSGSHQSITRGWAPPMHQTDSLFHKRAGVFGFTYGFDVDNHAI 5t5i.1    VYIPMGPW------------------------------------------------------------------------  target    NTVPKETVVRITKAEDGGVGGSGAWTRGRPGSMPGDEDDAMQAYLAGELTVVRRT 5t5i.1    ------------------------------------------------------- ``` | | | | | | | | | | | | | | | | | | | | | | | | | | | | | | | | | | | | | | | | | | | | | | | | | |
|  | 8e9g.1.G | NADH-quinone oxidoreductase subunit G  *Mycobacterial respiratory complex I with both quinone positions modelled* | 0.03 |  | 13.10 | 0.11 | 531-648 | EM | 0.00 | hetero-1-1-1-1-1-1-… |  | HHblits | 0.26 |
| ``` target    RPFLERFTDMPLLVRLDTLQRLRADEVFADYSSDLDVDGPSFTLHGMTEEQHERNGDRVVFDDASGALRAINREDVGDRL 8e9g.1    --------------------------------------------------------------------------------  target    DDKGIDPALDYQGTVTLVDGSTVEVMSVLSMYREHLADYDIDSVVDMTGAPRNLIEQLLDDMTTLSPVAFHVGEGVNHYF 8e9g.1    --------------------------------------------------------------------------------  target    HATLHNRATYLVGMLLGSVGVSGGGVSTWAGNYKGGVFQAAPWFGPGVGGFVNEDPFHPLTDPSARYSAETARHLVHGED 8e9g.1    --------------------------------------------------------------------------------  target    TSYWGFGDRPLVVDTPEDGRKVFTGTTHMPTPTKALWYNNANLINQAKWHYELVKNVNPKVDLIVDQQIEWTGSAEFADI 8e9g.1    --------------------------------------------------------------------------------  target    VLPANSWMEAETWEMGASCSNPFLQVWKGGIEPLNDTRDDIAIFAGVANALTELTGDERFSQAFMFADRPEVYLDRVLAG 8e9g.1    --------------------------------------------------------------------------------  target    SFTTEGYTVEDLTAGRYGPPGGALMQYRSYPRIPFKEQIEDSLPFYTDTGRMHGYVDIPEAIEYGENLIVHREAVEATPY 8e9g.1    --------------------------------------------------------------------------------  target    LPNVIVSTSPYLRPRDYGIAPEELDGDARSVRNIMMSWAEVKETENPLFAAGYNYLCLTPKSRHAVHSSWAVTDWHWLWS 8e9g.1    --------------------------------------------------GSGQAVLASWRMLLDAGRLQDGEPH--L--  target    SSFSDPYRVETRAPGVGEPAIHLNPDDARSLGIRNGDYVWVDSNPKDRPYRDADVDESFLDVARLLVRVTYNPAYPPGVT 8e9g.1    --------AG----TAVRPVARMSAATAAGIGASDGAPVTVSTE-----------------RGAVTLPLAVTD-MPDGVV  target    MLKHAFYMATPRTFRAAQERSDGRALAETTGYQSSFRSGSHQSITRGWAPPMHQTDSLFHKRAGVFGFTYGFDVDNHAIN 8e9g.1    WLPMNSPG------------------------------------------------------------------------  target    TVPKETVVRITKAEDGGVGGSGAWTRGRPGSMPGDEDDAMQAYLAGELTVVRRT 8e9g.1    ------------------------------------------------------ ``` | | | | | | | | | | | | | | | | | | | | | | | | | | | | | | | | | | | | | | | | | | | | | | | | | |
|  | 2ki8.1.A | Tungsten formylmethanofuran dehydrogenase, subunit D (FwdD-2)  *Solution NMR structure of tungsten formylmethanofuran dehydrogenase subunit D from Archaeoglobus fulgidus, Northeast Structural Genomics Consortium target AtT7* | 0.03 |  | 15.00 | 0.10 | 532-646 | NMR | 0.00 | monomer |  | HHblits | 0.28 |
| ``` target    RPFLERFTDMPLLVRLDTLQRLRADEVFADYSSDLDVDGPSFTLHGMTEEQHERNGDRVVFDDASGALRAINREDVGDRL 2ki8.1    --------------------------------------------------------------------------------  target    DDKGIDPALDYQGTVTLVDGSTVEVMSVLSMYREHLADYDIDSVVDMTGAPRNLIEQLLDDMTTLSPVAFHVGEGVNHYF 2ki8.1    --------------------------------------------------------------------------------  target    HATLHNRATYLVGMLLGSVGVSGGGVSTWAGNYKGGVFQAAPWFGPGVGGFVNEDPFHPLTDPSARYSAETARHLVHGED 2ki8.1    --------------------------------------------------------------------------------  target    TSYWGFGDRPLVVDTPEDGRKVFTGTTHMPTPTKALWYNNANLINQAKWHYELVKNVNPKVDLIVDQQIEWTGSAEFADI 2ki8.1    --------------------------------------------------------------------------------  target    VLPANSWMEAETWEMGASCSNPFLQVWKGGIEPLNDTRDDIAIFAGVANALTELTGDERFSQAFMFADRPEVYLDRVLAG 2ki8.1    --------------------------------------------------------------------------------  target    SFTTEGYTVEDLTAGRYGPPGGALMQYRSYPRIPFKEQIEDSLPFYTDTGRMHGYVDIPEAIEYGENLIVHREAVEATPY 2ki8.1    --------------------------------------------------------------------------------  target    LPNVIVSTSPYLRPRDYGIAPEELDGDARSVRNIMMSWAEVKETENPLFAAGYNYLCLTPKSRHAVHSSWAVTDWHWLWS 2ki8.1    ---------------------------------------------------MLEVEVISGRTLNQGATVE--EKL-----  target    SSFSDPYRVETRAPGVGEPAIHLNPDDARSLGIRNGDYVWVDSNPKDRPYRDADVDESFLDVARLLVRVTYNPAYPPGVT 2ki8.1    -------TEE---YFNAVNYAEINEEDWNALGLQEGDRVKVKTE-----------------FGEVVVFAKKG-DVPKGMI  target    MLKHAFYMATPRTFRAAQERSDGRALAETTGYQSSFRSGSHQSITRGWAPPMHQTDSLFHKRAGVFGFTYGFDVDNHAIN 2ki8.1    FIPMGP--------------------------------------------------------------------------  target    TVPKETVVRITKAEDGGVGGSGAWTRGRPGSMPGDEDDAMQAYLAGELTVVRRT 2ki8.1    ------------------------------------------------------ ``` | | | | | | | | | | | | | | | | | | | | | | | | | | | | | | | | | | | | | | | | | | | | | | | | | |
|  | 6sdr.1.A | Formate dehydrogenase, alpha subunit, selenocysteine-containing  *W-formate dehydrogenase from Desulfovibrio vulgaris - Oxidized form* | 0.02 |  | 20.25 | 0.10 | 531-640 | X-ray | 2.10 | hetero-1-1-mer | 2 x MGD, 4 x SF4, 1 x H2S, 1 x W | HHblits | 0.28 |
| ``` target    RPFLERFTDMPLLVRLDTLQRLRADEVFADYSSDLDVDGPSFTLHGMTEEQHERNGDRVVFDDASGALRAINREDVGDRL 6sdr.1    --------------------------------------------------------------------------------  target    DDKGIDPALDYQGTVTLVDGSTVEVMSVLSMYREHLADYDIDSVVDMTGAPRNLIEQLLDDMTTLSPVAFHVGEGVNHYF 6sdr.1    --------------------------------------------------------------------------------  target    HATLHNRATYLVGMLLGSVGVSGGGVSTWAGNYKGGVFQAAPWFGPGVGGFVNEDPFHPLTDPSARYSAETARHLVHGED 6sdr.1    --------------------------------------------------------------------------------  target    TSYWGFGDRPLVVDTPEDGRKVFTGTTHMPTPTKALWYNNANLINQAKWHYELVKNVNPKVDLIVDQQIEWTGSAEFADI 6sdr.1    --------------------------------------------------------------------------------  target    VLPANSWMEAETWEMGASCSNPFLQVWKGGIEPLNDTRDDIAIFAGVANALTELTGDERFSQAFMFADRPEVYLDRVLAG 6sdr.1    --------------------------------------------------------------------------------  target    SFTTEGYTVEDLTAGRYGPPGGALMQYRSYPRIPFKEQIEDSLPFYTDTGRMHGYVDIPEAIEYGENLIVHREAVEATPY 6sdr.1    --------------------------------------------------------------------------------  target    LPNVIVSTSPYLRPRDYGIAPEELDGDARSVRNIMMSWAEVKETENPLFAAGYNYLCLTPKSRHAVHSSWAVTDWHWLWS 6sdr.1    --------------------------------------------------PRYPFIGTTYRVTEHWQTGLMTRRCAWLV-  target    SSFSDPYRVETRAPGVGEPAIHLNPDDARSLGIRNGDYVWVDSNPKDRPYRDADVDESFLDVARLLVRVTYNPAYPPGVT 6sdr.1    ---------E----AEPQIFCEISKELAKLRGIGNGDTVKVSSL-----------------RGALEAVAIVTERIRPFKI  target    MLKHAFYMATPRTFRAAQERSDGRALAETTGYQSSFRSGSHQSITRGWAPPMHQTDSLFHKRAGVFGFTYGFDVDNHAIN 6sdr.1    --------------------------------------------------------------------------------  target    TVPKETVVRITKAEDGGVGGSGAWTRGRPGSMPGDEDDAMQAYLAGELTVVRRT 6sdr.1    ------------------------------------------------------ ``` | | | | | | | | | | | | | | | | | | | | | | | | | | | | | | | | | | | | | | | | | | | | | | | | | |
|  | 6sdv.1.A | Formate dehydrogenase, alpha subunit, selenocysteine-containing,Formate dehydrogenase, alpha subunit, selenocysteine-containing,W-formate dehydrogenase - alpha subunit  *W-formate dehydrogenase from Desulfovibrio vulgaris - Formate reduced form* | 0.02 |  | 18.99 | 0.10 | 531-640 | X-ray | 1.90 | hetero-1-1-mer | 2 x MGD, 4 x SF4, 1 x W, 1 x H2S | HHblits | 0.28 |
| ``` target    RPFLERFTDMPLLVRLDTLQRLRADEVFADYSSDLDVDGPSFTLHGMTEEQHERNGDRVVFDDASGALRAINREDVGDRL 6sdv.1    --------------------------------------------------------------------------------  target    DDKGIDPALDYQGTVTLVDGSTVEVMSVLSMYREHLADYDIDSVVDMTGAPRNLIEQLLDDMTTLSPVAFHVGEGVNHYF 6sdv.1    --------------------------------------------------------------------------------  target    HATLHNRATYLVGMLLGSVGVSGGGVSTWAGNYKGGVFQAAPWFGPGVGGFVNEDPFHPLTDPSARYSAETARHLVHGED 6sdv.1    --------------------------------------------------------------------------------  target    TSYWGFGDRPLVVDTPEDGRKVFTGTTHMPTPTKALWYNNANLINQAKWHYELVKNVNPKVDLIVDQQIEWTGSAEFADI 6sdv.1    --------------------------------------------------------------------------------  target    VLPANSWMEAETWEMGASCSNPFLQVWKGGIEPLNDTRDDIAIFAGVANALTELTGDERFSQAFMFADRPEVYLDRVLAG 6sdv.1    --------------------------------------------------------------------------------  target    SFTTEGYTVEDLTAGRYGPPGGALMQYRSYPRIPFKEQIEDSLPFYTDTGRMHGYVDIPEAIEYGENLIVHREAVEATPY 6sdv.1    --------------------------------------------------------------------------------  target    LPNVIVSTSPYLRPRDYGIAPEELDGDARSVRNIMMSWAEVKETENPLFAAGYNYLCLTPKSRHAVHSSWAVTDWHWLWS 6sdv.1    --------------------------------------------------PRYPFIGTTYRVTEHWQTGLMTRRCAWLVE  target    SSFSDPYRVETRAPGVGEPAIHLNPDDARSLGIRNGDYVWVDSNPKDRPYRDADVDESFLDVARLLVRVTYNPAYPPGVT 6sdv.1    --------------AEPQIFCEISKELAKLRGIGNGDTVKVSSL-----------------RGALEAVAIVTERIRPFKI  target    MLKHAFYMATPRTFRAAQERSDGRALAETTGYQSSFRSGSHQSITRGWAPPMHQTDSLFHKRAGVFGFTYGFDVDNHAIN 6sdv.1    --------------------------------------------------------------------------------  target    TVPKETVVRITKAEDGGVGGSGAWTRGRPGSMPGDEDDAMQAYLAGELTVVRRT 6sdv.1    ------------------------------------------------------ ``` | | | | | | | | | | | | | | | | | | | | | | | | | | | | | | | | | | | | | | | | | | | | | | | | | |
|  | 8bqg.1.A | Formate dehydrogenase, alpha subunit, selenocysteine-containing  *W-formate dehydrogenase from Desulfovibrio vulgaris - Soaking with Formate 1 min* | 0.02 |  | 18.99 | 0.10 | 530-639 | X-ray | 1.95 | hetero-1-1-mer | 2 x MGD, 4 x SF4, 1 x H2S, 1 x W | HHblits | 0.28 |
| ``` target    RPFLERFTDMPLLVRLDTLQRLRADEVFADYSSDLDVDGPSFTLHGMTEEQHERNGDRVVFDDASGALRAINREDVGDRL 8bqg.1    --------------------------------------------------------------------------------  target    DDKGIDPALDYQGTVTLVDGSTVEVMSVLSMYREHLADYDIDSVVDMTGAPRNLIEQLLDDMTTLSPVAFHVGEGVNHYF 8bqg.1    --------------------------------------------------------------------------------  target    HATLHNRATYLVGMLLGSVGVSGGGVSTWAGNYKGGVFQAAPWFGPGVGGFVNEDPFHPLTDPSARYSAETARHLVHGED 8bqg.1    --------------------------------------------------------------------------------  target    TSYWGFGDRPLVVDTPEDGRKVFTGTTHMPTPTKALWYNNANLINQAKWHYELVKNVNPKVDLIVDQQIEWTGSAEFADI 8bqg.1    --------------------------------------------------------------------------------  target    VLPANSWMEAETWEMGASCSNPFLQVWKGGIEPLNDTRDDIAIFAGVANALTELTGDERFSQAFMFADRPEVYLDRVLAG 8bqg.1    --------------------------------------------------------------------------------  target    SFTTEGYTVEDLTAGRYGPPGGALMQYRSYPRIPFKEQIEDSLPFYTDTGRMHGYVDIPEAIEYGENLIVHREAVEATPY 8bqg.1    --------------------------------------------------------------------------------  target    LPNVIVSTSPYLRPRDYGIAPEELDGDARSVRNIMMSWAEVKETENPLFAAGYNYLCLTPKSRHAVHSSWAVTDWHWLWS 8bqg.1    -------------------------------------------------DPRYPFIGTTYRVTEHWQTGLMTRRCAWLVE  target    SSFSDPYRVETRAPGVGEPAIHLNPDDARSLGIRNGDYVWVDSNPKDRPYRDADVDESFLDVARLLVRVTYNPAYPPGVT 8bqg.1    --------------AEPQIFCEISKELAKLRGIGNGDTVKVSSL-----------------RGALEAVAIVTERIRPFK-  target    MLKHAFYMATPRTFRAAQERSDGRALAETTGYQSSFRSGSHQSITRGWAPPMHQTDSLFHKRAGVFGFTYGFDVDNHAIN 8bqg.1    --------------------------------------------------------------------------------  target    TVPKETVVRITKAEDGGVGGSGAWTRGRPGSMPGDEDDAMQAYLAGELTVVRRT 8bqg.1    ------------------------------------------------------ ``` | | | | | | | | | | | | | | | | | | | | | | | | | | | | | | | | | | | | | | | | | | | | | | | | | |
|  | 6f0k.1.B | Fe-S-cluster-containing hydrogenase  *Alternative complex III* | 0.00 |  | 8.62 | 0.07 | 272-329 | EM | 0.00 | hetero-1-1-1-1-1-1-… | 6 x HEC, 1 x F3S, 3 x SF4 | HHblits | 0.26 |
| ``` target    RPFLERFTDMPLLVRLDTLQRLRADEVFADYSSDLDVDGPSFTLHGMTEEQHERNGDRVVFDDASGALRAINREDVGDRL 6f0k.1    --------------------------------------------------------------------------------  target    DDKGIDPALDYQGTVTLVDGSTVEVMSVLSMYREHLADYDIDSVVDMTGAPRNLIEQLLDDMTTLSPVAFHVGEGVNHYF 6f0k.1    --------------------------------------------------------------------------------  target    HATLHNRATYLVGMLLGSVGVSGGGVSTWAGNYKGGVFQAAPWFGPGVGGFVNEDPFHPLTDPSARYSAETARHLVHGED 6f0k.1    --------------------------------------------------------------------------------  target    TSYWGFGDRPLVVDTPEDGRKVFTGTTHMPTPTKALWYNNANLINQA-KWHY---------ELVKNVNPKVDLIVDQQIE 6f0k.1    -------------------------------EARVIVSLDADFLGPTDRNFVENTREFAASRRMERPEDEISRLYVIEST  target    WTGSAEFADIVLPANSWMEAETWEMGASCSNPFLQVWKGGIEPLNDTRDDIAIFAGVANALTELTGDERFSQAFMFADRP 6f0k.1    YTVTGGMADHRLRLRAGDI-------------------------------------------------------------  target    EVYLDRVLAGSFTTEGYTVEDLTAGRYGPPGGALMQYRSYPRIPFKEQIEDSLPFYTDTGRMHGYVDIPEAIEYGENLIV 6f0k.1    --------------------------------------------------------------------------------  target    HREAVEATPYLPNVIVSTSPYLRPRDYGIAPEELDGDARSVRNIMMSWAEVKETENPLFAAGYNYLCLTPKSRHAVHSSW 6f0k.1    --------------------------------------------------------------------------------  target    AVTDWHWLWSSSFSDPYRVETRAPGVGEPAIHLNPDDARSLGIRNGDYVWVDSNPKDRPYRDADVDESFLDVARLLVRVT 6f0k.1    --------------------------------------------------------------------------------  target    YNPAYPPGVTMLKHAFYMATPRTFRAAQERSDGRALAETTGYQSSFRSGSHQSITRGWAPPMHQTDSLFHKRAGVFGFTY 6f0k.1    --------------------------------------------------------------------------------  target    GFDVDNHAINTVPKETVVRITKAEDGGVGGSGAWTRGRPGSMPGDEDDAMQAYLAGELTVVRRT 6f0k.1    ---------------------------------------------------------------- ``` | | | | | | | | | | | | | | | | | | | | | | | | | | | | | | | | | | | | | | | | | | | | | | | | | |
|  | 2e7z.1.A | Acetylene hydratase Ahy  *Acetylene Hydratase from Pelobacter acetylenicus* | 0.01 |  | 23.64 | 0.07 | 272-326 | X-ray | 1.26 | monomer | 1 x SF4, 2 x MGD, 1 x W | HHblits | 0.29 |
| ``` target    RPFLERFTDMPLLVRLDTLQRLRADEVFADYSSDLDVDGPSFTLHGMTEEQHERNGDRVVFDDASGALRAINREDVGDRL 2e7z.1    --------------------------------------------------------------------------------  target    DDKGIDPALDYQGTVTLVDGSTVEVMSVLSMYREHLADYDIDSVVDMTGAPRNLIEQLLDDMTTLSPVAFHVGEGVNHYF 2e7z.1    --------------------------------------------------------------------------------  target    HATLHNRATYLVGMLLGSVGVSGGGVSTWAGNYKGGVFQAAPWFGPGVGGFVNEDPFHPLTDPSARYSAETARHLVHGED 2e7z.1    --------------------------------------------------------------------------------  target    TSYWGFGDRPLVVDTPEDGRKVFTGTTHMPTPTKALWYNNANLINQAKWH-YELVKNVNPKVDLIVDQQIEWTGSAEFAD 2e7z.1    -------------------------------DSNCLLFIGKNLSNHNWVSQFNDLKAALKRGCKLIVLDPRRTKVAEMAD  target    IVLPANSWMEAETWEMGASCSNPFLQVWKGGIEPLNDTRDDIAIFAGVANALTELTGDERFSQAFMFADRPEVYLDRVLA 2e7z.1    IWLPLRY-------------------------------------------------------------------------  target    GSFTTEGYTVEDLTAGRYGPPGGALMQYRSYPRIPFKEQIEDSLPFYTDTGRMHGYVDIPEAIEYGENLIVHREAVEATP 2e7z.1    --------------------------------------------------------------------------------  target    YLPNVIVSTSPYLRPRDYGIAPEELDGDARSVRNIMMSWAEVKETENPLFAAGYNYLCLTPKSRHAVHSSWAVTDWHWLW 2e7z.1    --------------------------------------------------------------------------------  target    SSSFSDPYRVETRAPGVGEPAIHLNPDDARSLGIRNGDYVWVDSNPKDRPYRDADVDESFLDVARLLVRVTYNPAYPPGV 2e7z.1    --------------------------------------------------------------------------------  target    TMLKHAFYMATPRTFRAAQERSDGRALAETTGYQSSFRSGSHQSITRGWAPPMHQTDSLFHKRAGVFGFTYGFDVDNHAI 2e7z.1    --------------------------------------------------------------------------------  target    NTVPKETVVRITKAEDGGVGGSGAWTRGRPGSMPGDEDDAMQAYLAGELTVVRRT 2e7z.1    ------------------------------------------------------- ``` | | | | | | | | | | | | | | | | | | | | | | | | | | | | | | | | | | | | | | | | | | | | | | | | | |
|  | 7b04.1.B | Nitrite oxidoreductase subunit A  *Structure of Nitrite oxidoreductase (Nxr) from the anammox bacterium Kuenenia stuttgartiensis.* | 0.01 |  | 27.78 | 0.07 | 272-325 | X-ray | 2.97 | hetero-1-1-1-mer | 4 x SF4, 1 x F3S, 2 x MD1, 1 x MO, 1 x HEM, 2 x CA | HHblits | 0.30 |
| ``` target    RPFLERFTDMPLLVRLDTLQRLRADEVFADYSSDLDVDGPSFTLHGMTEEQHERNGDRVVFDDASGALRAINREDVGDRL 7b04.1    --------------------------------------------------------------------------------  target    DDKGIDPALDYQGTVTLVDGSTVEVMSVLSMYREHLADYDIDSVVDMTGAPRNLIEQLLDDMTTLSPVAFHVGEGVNHYF 7b04.1    --------------------------------------------------------------------------------  target    HATLHNRATYLVGMLLGSVGVSGGGVSTWAGNYKGGVFQAAPWFGPGVGGFVNEDPFHPLTDPSARYSAETARHLVHGED 7b04.1    --------------------------------------------------------------------------------  target    TSYWGFGDRPLVVDTPEDGRKVFTGTTHMPTPTKALWYNNANLINQAKWHYELVKNVNPKVDLIVDQQIEWTGSAEFADI 7b04.1    -------------------------------FSKLLIQTGKNLIENKMPEAHWVTEVMERGGKIVVITPEYSPSAQKADY  target    VLPANSWMEAETWEMGASCSNPFLQVWKGGIEPLNDTRDDIAIFAGVANALTELTGDERFSQAFMFADRPEVYLDRVLAG 7b04.1    WIPIR---------------------------------------------------------------------------  target    SFTTEGYTVEDLTAGRYGPPGGALMQYRSYPRIPFKEQIEDSLPFYTDTGRMHGYVDIPEAIEYGENLIVHREAVEATPY 7b04.1    --------------------------------------------------------------------------------  target    LPNVIVSTSPYLRPRDYGIAPEELDGDARSVRNIMMSWAEVKETENPLFAAGYNYLCLTPKSRHAVHSSWAVTDWHWLWS 7b04.1    --------------------------------------------------------------------------------  target    SSFSDPYRVETRAPGVGEPAIHLNPDDARSLGIRNGDYVWVDSNPKDRPYRDADVDESFLDVARLLVRVTYNPAYPPGVT 7b04.1    --------------------------------------------------------------------------------  target    MLKHAFYMATPRTFRAAQERSDGRALAETTGYQSSFRSGSHQSITRGWAPPMHQTDSLFHKRAGVFGFTYGFDVDNHAIN 7b04.1    --------------------------------------------------------------------------------  target    TVPKETVVRITKAEDGGVGGSGAWTRGRPGSMPGDEDDAMQAYLAGELTVVRRT 7b04.1    ------------------------------------------------------ ``` | | | | | | | | | | | | | | | | | | | | | | | | | | | | | | | | | | | | | | | | | | | | | | | | | |
|  | 7b04.2.B | Nitrite oxidoreductase subunit A  *Structure of Nitrite oxidoreductase (Nxr) from the anammox bacterium Kuenenia stuttgartiensis.* | 0.01 |  | 27.78 | 0.07 | 272-325 | X-ray | 2.97 | hetero-1-1-1-mer | 4 x SF4, 1 x F3S, 2 x MD1, 1 x MO, 1 x HEM, 2 x CA | HHblits | 0.30 |
| ``` target    RPFLERFTDMPLLVRLDTLQRLRADEVFADYSSDLDVDGPSFTLHGMTEEQHERNGDRVVFDDASGALRAINREDVGDRL 7b04.2    --------------------------------------------------------------------------------  target    DDKGIDPALDYQGTVTLVDGSTVEVMSVLSMYREHLADYDIDSVVDMTGAPRNLIEQLLDDMTTLSPVAFHVGEGVNHYF 7b04.2    --------------------------------------------------------------------------------  target    HATLHNRATYLVGMLLGSVGVSGGGVSTWAGNYKGGVFQAAPWFGPGVGGFVNEDPFHPLTDPSARYSAETARHLVHGED 7b04.2    --------------------------------------------------------------------------------  target    TSYWGFGDRPLVVDTPEDGRKVFTGTTHMPTPTKALWYNNANLINQAKWHYELVKNVNPKVDLIVDQQIEWTGSAEFADI 7b04.2    -------------------------------FSKLLIQTGKNLIENKMPEAHWVTEVMERGGKIVVITPEYSPSAQKADY  target    VLPANSWMEAETWEMGASCSNPFLQVWKGGIEPLNDTRDDIAIFAGVANALTELTGDERFSQAFMFADRPEVYLDRVLAG 7b04.2    WIPIR---------------------------------------------------------------------------  target    SFTTEGYTVEDLTAGRYGPPGGALMQYRSYPRIPFKEQIEDSLPFYTDTGRMHGYVDIPEAIEYGENLIVHREAVEATPY 7b04.2    --------------------------------------------------------------------------------  target    LPNVIVSTSPYLRPRDYGIAPEELDGDARSVRNIMMSWAEVKETENPLFAAGYNYLCLTPKSRHAVHSSWAVTDWHWLWS 7b04.2    --------------------------------------------------------------------------------  target    SSFSDPYRVETRAPGVGEPAIHLNPDDARSLGIRNGDYVWVDSNPKDRPYRDADVDESFLDVARLLVRVTYNPAYPPGVT 7b04.2    --------------------------------------------------------------------------------  target    MLKHAFYMATPRTFRAAQERSDGRALAETTGYQSSFRSGSHQSITRGWAPPMHQTDSLFHKRAGVFGFTYGFDVDNHAIN 7b04.2    --------------------------------------------------------------------------------  target    TVPKETVVRITKAEDGGVGGSGAWTRGRPGSMPGDEDDAMQAYLAGELTVVRRT 7b04.2    ------------------------------------------------------ ``` | | | | | | | | | | | | | | | | | | | | | | | | | | | | | | | | | | | | | | | | | | | | | | | | | |
|  | 5t5i.1.B | Tungsten formylmethanofuran dehydrogenase subunit B  *TUNGSTEN-CONTAINING FORMYLMETHANOFURAN DEHYDROGENASE FROM METHANOTHERMOBACTER WOLFEII, ORTHORHOMBIC FORM AT 1.9 A* | 0.01 |  | 18.18 | 0.07 | 272-326 | X-ray | 1.90 | hetero-oligomer | 4 x ZN, 2 x MG, 18 x K, 22 x SF4, 2 x W, 4 x MGD, 2 x H2S, 2 x CA | HHblits | 0.28 |
| ``` target    RPFLERFTDMPLLVRLDTLQRLRADEVFADYSSDLDVDGPSFTLHGMTEEQHERNGDRVVFDDASGALRAINREDVGDRL 5t5i.1    --------------------------------------------------------------------------------  target    DDKGIDPALDYQGTVTLVDGSTVEVMSVLSMYREHLADYDIDSVVDMTGAPRNLIEQLLDDMTTLSPVAFHVGEGVNHYF 5t5i.1    --------------------------------------------------------------------------------  target    HATLHNRATYLVGMLLGSVGVSGGGVSTWAGNYKGGVFQAAPWFGPGVGGFVNEDPFHPLTDPSARYSAETARHLVHGED 5t5i.1    --------------------------------------------------------------------------------  target    TSYWGFGDRPLVVDTPEDGRKVFTGTTHMPTPTKALWYNNANLINQAKWHYE-------LVKNVNPKVDLIVDQQIEWTG 5t5i.1    -------------------------------RADVVVYWGCNPMHAHPRHMSRNVFARGFFRERGRSDRTLIVVDPRKTD  target    SAEFADIVLPANSWMEAETWEMGASCSNPFLQVWKGGIEPLNDTRDDIAIFAGVANALTELTGDERFSQAFMFADRPEVY 5t5i.1    SAKLADIHLQLDF-------------------------------------------------------------------  target    LDRVLAGSFTTEGYTVEDLTAGRYGPPGGALMQYRSYPRIPFKEQIEDSLPFYTDTGRMHGYVDIPEAIEYGENLIVHRE 5t5i.1    --------------------------------------------------------------------------------  target    AVEATPYLPNVIVSTSPYLRPRDYGIAPEELDGDARSVRNIMMSWAEVKETENPLFAAGYNYLCLTPKSRHAVHSSWAVT 5t5i.1    --------------------------------------------------------------------------------  target    DWHWLWSSSFSDPYRVETRAPGVGEPAIHLNPDDARSLGIRNGDYVWVDSNPKDRPYRDADVDESFLDVARLLVRVTYNP 5t5i.1    --------------------------------------------------------------------------------  target    AYPPGVTMLKHAFYMATPRTFRAAQERSDGRALAETTGYQSSFRSGSHQSITRGWAPPMHQTDSLFHKRAGVFGFTYGFD 5t5i.1    --------------------------------------------------------------------------------  target    VDNHAINTVPKETVVRITKAEDGGVGGSGAWTRGRPGSMPGDEDDAMQAYLAGELTVVRRT 5t5i.1    ------------------------------------------------------------- ``` | | | | | | | | | | | | | | | | | | | | | | | | | | | | | | | | | | | | | | | | | | | | | | | | | |
|  | 4ga5.1.A | Putative thymidine phosphorylase  *Crystal structure of AMP phosphorylase C-terminal deletion mutant in the apo-form* | 0.02 |  | 14.81 | 0.07 | 577-647 | X-ray | 3.25 | homo-dimer |  | HHblits | 0.29 |
| ``` target    RPFLERFTDMPLLVRLDTLQRLRADEVFADYSSDLDVDGPSFTLHGMTEEQHERNGDRVVFDDASGALRAINREDVGDRL 4ga5.1    --------------------------------------------------------------------------------  target    DDKGIDPALDYQGTVTLVDGSTVEVMSVLSMYREHLADYDIDSVVDMTGAPRNLIEQLLDDMTTLSPVAFHVGEGVNHYF 4ga5.1    --------------------------------------------------------------------------------  target    HATLHNRATYLVGMLLGSVGVSGGGVSTWAGNYKGGVFQAAPWFGPGVGGFVNEDPFHPLTDPSARYSAETARHLVHGED 4ga5.1    --------------------------------------------------------------------------------  target    TSYWGFGDRPLVVDTPEDGRKVFTGTTHMPTPTKALWYNNANLINQAKWHYELVKNVNPKVDLIVDQQIEWTGSAEFADI 4ga5.1    --------------------------------------------------------------------------------  target    VLPANSWMEAETWEMGASCSNPFLQVWKGGIEPLNDTRDDIAIFAGVANALTELTGDERFSQAFMFADRPEVYLDRVLAG 4ga5.1    --------------------------------------------------------------------------------  target    SFTTEGYTVEDLTAGRYGPPGGALMQYRSYPRIPFKEQIEDSLPFYTDTGRMHGYVDIPEAIEYGENLIVHREAVEATPY 4ga5.1    --------------------------------------------------------------------------------  target    LPNVIVSTSPYLRPRDYGIAPEELDGDARSVRNIMMSWAEVKETENPLFAAGYNYLCLTPKSRHAVHSSWAVTDWHWLWS 4ga5.1    --------------------------------------------------------------------------------  target    SSFSDPYRVETRAPGVGEPAIHLNPDDARSLGIRNGDYVWVDSNPKDRPYRDADVDESFLDVARLLVRVTYNPAYPPGVT 4ga5.1    ----------------GRYTVLINEEDAKEAKLHPDDLVKIEAG-----------------KKAVYGSVALSNLVGKGEV  target    MLKHAFYMATPRTFRAAQERSDGRALAETTGYQSSFRSGSHQSITRGWAPPMHQTDSLFHKRAGVFGFTYGFDVDNHAIN 4ga5.1    GISRDVL-------------------------------------------------------------------------  target    TVPKETVVRITKAEDGGVGGSGAWTRGRPGSMPGDEDDAMQAYLAGELTVVRRT 4ga5.1    ------------------------------------------------------ ``` | | | | | | | | | | | | | | | | | | | | | | | | | | | | | | | | | | | | | | | | | | | | | | | | | |
|  | 4ga6.1.A | Putative thymidine phosphorylase  *Crystal structure of AMP phosphorylase C-terminal deletion mutant in complex with substrates* | 0.02 |  | 14.81 | 0.07 | 577-647 | X-ray | 2.21 | homo-dimer | 2 x AMP | HHblits | 0.29 |
| ``` target    RPFLERFTDMPLLVRLDTLQRLRADEVFADYSSDLDVDGPSFTLHGMTEEQHERNGDRVVFDDASGALRAINREDVGDRL 4ga6.1    --------------------------------------------------------------------------------  target    DDKGIDPALDYQGTVTLVDGSTVEVMSVLSMYREHLADYDIDSVVDMTGAPRNLIEQLLDDMTTLSPVAFHVGEGVNHYF 4ga6.1    --------------------------------------------------------------------------------  target    HATLHNRATYLVGMLLGSVGVSGGGVSTWAGNYKGGVFQAAPWFGPGVGGFVNEDPFHPLTDPSARYSAETARHLVHGED 4ga6.1    --------------------------------------------------------------------------------  target    TSYWGFGDRPLVVDTPEDGRKVFTGTTHMPTPTKALWYNNANLINQAKWHYELVKNVNPKVDLIVDQQIEWTGSAEFADI 4ga6.1    --------------------------------------------------------------------------------  target    VLPANSWMEAETWEMGASCSNPFLQVWKGGIEPLNDTRDDIAIFAGVANALTELTGDERFSQAFMFADRPEVYLDRVLAG 4ga6.1    --------------------------------------------------------------------------------  target    SFTTEGYTVEDLTAGRYGPPGGALMQYRSYPRIPFKEQIEDSLPFYTDTGRMHGYVDIPEAIEYGENLIVHREAVEATPY 4ga6.1    --------------------------------------------------------------------------------  target    LPNVIVSTSPYLRPRDYGIAPEELDGDARSVRNIMMSWAEVKETENPLFAAGYNYLCLTPKSRHAVHSSWAVTDWHWLWS 4ga6.1    --------------------------------------------------------------------------------  target    SSFSDPYRVETRAPGVGEPAIHLNPDDARSLGIRNGDYVWVDSNPKDRPYRDADVDESFLDVARLLVRVTYNPAYPPGVT 4ga6.1    ----------------GRYTVLINEEDAKEAKLHPDDLVKIEAG-----------------KKAVYGSVALSNLVGKGEV  target    MLKHAFYMATPRTFRAAQERSDGRALAETTGYQSSFRSGSHQSITRGWAPPMHQTDSLFHKRAGVFGFTYGFDVDNHAIN 4ga6.1    GISRDVL-------------------------------------------------------------------------  target    TVPKETVVRITKAEDGGVGGSGAWTRGRPGSMPGDEDDAMQAYLAGELTVVRRT 4ga6.1    ------------------------------------------------------ ``` | | | | | | | | | | | | | | | | | | | | | | | | | | | | | | | | | | | | | | | | | | | | | | | | | |
|  | 2ivf.1.A | ETHYLBENZENE DEHYDROGENASE ALPHA-SUBUNIT  *ETHYLBENZENE DEHYDROGENASE FROM AROMATOLEUM AROMATICUM* | 0.01 |  | 12.73 | 0.07 | 272-326 | X-ray | 1.88 | hetero-oligomer | 1 x MES, 4 x SF4, 1 x MO, 1 x MGD, 1 x MD1, 1 x F3S, 1 x HEM | HHblits | 0.27 |
| ``` target    RPFLERFTDMPLLVRLDTLQRLRADEVFADYSSDLDVDGPSFTLHGMTEEQHERNGDRVVFDDASGALRAINREDVGDRL 2ivf.1    --------------------------------------------------------------------------------  target    DDKGIDPALDYQGTVTLVDGSTVEVMSVLSMYREHLADYDIDSVVDMTGAPRNLIEQLLDDMTTLSPVAFHVGEGVNHYF 2ivf.1    --------------------------------------------------------------------------------  target    HATLHNRATYLVGMLLGSVGVSGGGVSTWAGNYKGGVFQAAPWFGPGVGGFVNEDPFHPLTDPSARYSAETARHLVHGED 2ivf.1    --------------------------------------------------------------------------------  target    TSYWGFGDRPLVVDTPEDGRKVFTGTTHMPTPTKALWYNNANLINQAKWHYELVKNVNPKVDLIVDQQIEWTGSAEFADI 2ivf.1    -------------------------------DAELIFMTCSNWSYTYPSSYHFLSEARYKGAEVVVIAPDFNPTTPAADL  target    VLPANSWMEAETWEMGASCSNPFLQVWKGGIEPLNDTRDDIAIFAGVANALTELTGDERFSQAFMFADRPEVYLDRVLAG 2ivf.1    HVPVRV--------------------------------------------------------------------------  target    SFTTEGYTVEDLTAGRYGPPGGALMQYRSYPRIPFKEQIEDSLPFYTDTGRMHGYVDIPEAIEYGENLIVHREAVEATPY 2ivf.1    --------------------------------------------------------------------------------  target    LPNVIVSTSPYLRPRDYGIAPEELDGDARSVRNIMMSWAEVKETENPLFAAGYNYLCLTPKSRHAVHSSWAVTDWHWLWS 2ivf.1    --------------------------------------------------------------------------------  target    SSFSDPYRVETRAPGVGEPAIHLNPDDARSLGIRNGDYVWVDSNPKDRPYRDADVDESFLDVARLLVRVTYNPAYPPGVT 2ivf.1    --------------------------------------------------------------------------------  target    MLKHAFYMATPRTFRAAQERSDGRALAETTGYQSSFRSGSHQSITRGWAPPMHQTDSLFHKRAGVFGFTYGFDVDNHAIN 2ivf.1    --------------------------------------------------------------------------------  target    TVPKETVVRITKAEDGGVGGSGAWTRGRPGSMPGDEDDAMQAYLAGELTVVRRT 2ivf.1    ------------------------------------------------------ ``` | | | | | | | | | | | | | | | | | | | | | | | | | | | | | | | | | | | | | | | | | | | | | | | | | |
|  | 1e5v.2.A | Dimethyl sulfoxide/trimethylamine N-oxide reductase  *OXIDIZED DMSO REDUCTASE EXPOSED TO HEPES BUFFER* | 0.00 | 0.00 | 14.55 | 0.07 | 272-326 | X-ray | 2.40 | monomer | 2 x PGD, 1 x 2MO | HHblits | 0.27 |
| ``` target    RPFLERFTDMPLLVRLDTLQRLRADEVFADYSSDLDVDGPSFTLHGMTEEQHERNGDRVVFDDASGALRAINREDVGDRL 1e5v.2    --------------------------------------------------------------------------------  target    DDKGIDPALDYQGTVTLVDGSTVEVMSVLSMYREHLADYDIDSVVDMTGAPRNLIEQLLDDMTTLSPVAFHVGEGVNHYF 1e5v.2    --------------------------------------------------------------------------------  target    HATLHNRATYLVGMLLGSVGVSGGGVSTWAGNYKGGVFQAAPWFGPGVGGFVNEDPFHPLTDPSARYSAETARHLVHGED 1e5v.2    --------------------------------------------------------------------------------  target    TSYWGFGDRPLVVDTPEDGRKVFTGTTHMPTPTKALWYNNANLINQAKWH--------YELVKNVNPKVDLIVDQQIEWT 1e5v.2    -------------------------------NTEVMVFWAADPIKTSQIGWVIPEHGAYPGLEALKAKGTKVIVIDPVRT  target    GSAEF-ADIVLPANSWMEAETWEMGASCSNPFLQVWKGGIEPLNDTRDDIAIFAGVANALTELTGDERFSQAFMFADRPE 1e5v.2    KTVEFFGAEHITPKP-----------------------------------------------------------------  target    VYLDRVLAGSFTTEGYTVEDLTAGRYGPPGGALMQYRSYPRIPFKEQIEDSLPFYTDTGRMHGYVDIPEAIEYGENLIVH 1e5v.2    --------------------------------------------------------------------------------  target    REAVEATPYLPNVIVSTSPYLRPRDYGIAPEELDGDARSVRNIMMSWAEVKETENPLFAAGYNYLCLTPKSRHAVHSSWA 1e5v.2    --------------------------------------------------------------------------------  target    VTDWHWLWSSSFSDPYRVETRAPGVGEPAIHLNPDDARSLGIRNGDYVWVDSNPKDRPYRDADVDESFLDVARLLVRVTY 1e5v.2    --------------------------------------------------------------------------------  target    NPAYPPGVTMLKHAFYMATPRTFRAAQERSDGRALAETTGYQSSFRSGSHQSITRGWAPPMHQTDSLFHKRAGVFGFTYG 1e5v.2    --------------------------------------------------------------------------------  target    FDVDNHAINTVPKETVVRITKAEDGGVGGSGAWTRGRPGSMPGDEDDAMQAYLAGELTVVRRT 1e5v.2    --------------------------------------------------------------- ``` | | | | | | | | | | | | | | | | | | | | | | | | | | | | | | | | | | | | | | | | | | | | | | | | | |
|  | 1e18.1.A | DMSO REDUCTASE.  *TUNGSTEN-SUSBSTITUTED DMSO REDUCTASE FROM RHODOBACTER CAPSULATUS* | 0.00 | 0.00 | 14.55 | 0.07 | 272-326 | X-ray | 2.00 | monomer | 2 x PGD, 1 x 6WO | HHblits | 0.27 |
| ``` target    RPFLERFTDMPLLVRLDTLQRLRADEVFADYSSDLDVDGPSFTLHGMTEEQHERNGDRVVFDDASGALRAINREDVGDRL 1e18.1    --------------------------------------------------------------------------------  target    DDKGIDPALDYQGTVTLVDGSTVEVMSVLSMYREHLADYDIDSVVDMTGAPRNLIEQLLDDMTTLSPVAFHVGEGVNHYF 1e18.1    --------------------------------------------------------------------------------  target    HATLHNRATYLVGMLLGSVGVSGGGVSTWAGNYKGGVFQAAPWFGPGVGGFVNEDPFHPLTDPSARYSAETARHLVHGED 1e18.1    --------------------------------------------------------------------------------  target    TSYWGFGDRPLVVDTPEDGRKVFTGTTHMPTPTKALWYNNANLINQAKWH--------YELVKNVNPKVDLIVDQQIEWT 1e18.1    -------------------------------NTEVMVFWAADPIKTSQIGWVIPEHGAYPGLEALKAKGTKVIVIDPVRT  target    GSAEF-ADIVLPANSWMEAETWEMGASCSNPFLQVWKGGIEPLNDTRDDIAIFAGVANALTELTGDERFSQAFMFADRPE 1e18.1    KTVEFFGAEHITPKP-----------------------------------------------------------------  target    VYLDRVLAGSFTTEGYTVEDLTAGRYGPPGGALMQYRSYPRIPFKEQIEDSLPFYTDTGRMHGYVDIPEAIEYGENLIVH 1e18.1    --------------------------------------------------------------------------------  target    REAVEATPYLPNVIVSTSPYLRPRDYGIAPEELDGDARSVRNIMMSWAEVKETENPLFAAGYNYLCLTPKSRHAVHSSWA 1e18.1    --------------------------------------------------------------------------------  target    VTDWHWLWSSSFSDPYRVETRAPGVGEPAIHLNPDDARSLGIRNGDYVWVDSNPKDRPYRDADVDESFLDVARLLVRVTY 1e18.1    --------------------------------------------------------------------------------  target    NPAYPPGVTMLKHAFYMATPRTFRAAQERSDGRALAETTGYQSSFRSGSHQSITRGWAPPMHQTDSLFHKRAGVFGFTYG 1e18.1    --------------------------------------------------------------------------------  target    FDVDNHAINTVPKETVVRITKAEDGGVGGSGAWTRGRPGSMPGDEDDAMQAYLAGELTVVRRT 1e18.1    --------------------------------------------------------------- ``` | | | | | | | | | | | | | | | | | | | | | | | | | | | | | | | | | | | | | | | | | | | | | | | | | |
|  | 1e60.1.A | Dimethyl sulfoxide/trimethylamine N-oxide reductase  *OXIDIZED DMSO REDUCTASE EXPOSED TO HEPES - Structure II BUFFER* | 0.00 | 0.00 | 14.55 | 0.07 | 272-326 | X-ray | 2.00 | monomer | 2 x PGD, 1 x 2MO | HHblits | 0.27 |
| ``` target    RPFLERFTDMPLLVRLDTLQRLRADEVFADYSSDLDVDGPSFTLHGMTEEQHERNGDRVVFDDASGALRAINREDVGDRL 1e60.1    --------------------------------------------------------------------------------  target    DDKGIDPALDYQGTVTLVDGSTVEVMSVLSMYREHLADYDIDSVVDMTGAPRNLIEQLLDDMTTLSPVAFHVGEGVNHYF 1e60.1    --------------------------------------------------------------------------------  target    HATLHNRATYLVGMLLGSVGVSGGGVSTWAGNYKGGVFQAAPWFGPGVGGFVNEDPFHPLTDPSARYSAETARHLVHGED 1e60.1    --------------------------------------------------------------------------------  target    TSYWGFGDRPLVVDTPEDGRKVFTGTTHMPTPTKALWYNNANLINQAKWH--------YELVKNVNPKVDLIVDQQIEWT 1e60.1    -------------------------------NTEVMVFWAADPIKTSQIGWVIPEHGAYPGLEALKAKGTKVIVIDPVRT  target    GSAEF-ADIVLPANSWMEAETWEMGASCSNPFLQVWKGGIEPLNDTRDDIAIFAGVANALTELTGDERFSQAFMFADRPE 1e60.1    KTVEFFGAEHITPKP-----------------------------------------------------------------  target    VYLDRVLAGSFTTEGYTVEDLTAGRYGPPGGALMQYRSYPRIPFKEQIEDSLPFYTDTGRMHGYVDIPEAIEYGENLIVH 1e60.1    --------------------------------------------------------------------------------  target    REAVEATPYLPNVIVSTSPYLRPRDYGIAPEELDGDARSVRNIMMSWAEVKETENPLFAAGYNYLCLTPKSRHAVHSSWA 1e60.1    --------------------------------------------------------------------------------  target    VTDWHWLWSSSFSDPYRVETRAPGVGEPAIHLNPDDARSLGIRNGDYVWVDSNPKDRPYRDADVDESFLDVARLLVRVTY 1e60.1    --------------------------------------------------------------------------------  target    NPAYPPGVTMLKHAFYMATPRTFRAAQERSDGRALAETTGYQSSFRSGSHQSITRGWAPPMHQTDSLFHKRAGVFGFTYG 1e60.1    --------------------------------------------------------------------------------  target    FDVDNHAINTVPKETVVRITKAEDGGVGGSGAWTRGRPGSMPGDEDDAMQAYLAGELTVVRRT 1e60.1    --------------------------------------------------------------- ``` | | | | | | | | | | | | | | | | | | | | | | | | | | | | | | | | | | | | | | | | | | | | | | | | | |
|  | 7qv7.1.L | Hydrogen dependent carbon dioxide reductase subunit FdhF  *Cryo-EM structure of Hydrogen-dependent CO2 reductase.* | 0.01 |  | 12.50 | 0.07 | 272-327 | EM | 0.00 | hetero-2-6-6-2-mer | 52 x SF4, 6 x 402 | HHblits | 0.25 |
| ``` target    RPFLERFTDMPLLVRLDTLQRLRADEVFADYSSDLDVDGPSFTLHGMTEEQHERNGDRVVFDDASGALRAINREDVGDRL 7qv7.1    --------------------------------------------------------------------------------  target    DDKGIDPALDYQGTVTLVDGSTVEVMSVLSMYREHLADYDIDSVVDMTGAPRNLIEQLLDDMTTLSPVAFHVGEGVNHYF 7qv7.1    --------------------------------------------------------------------------------  target    HATLHNRATYLVGMLLGSVGVSGGGVSTWAGNYKGGVFQAAPWFGPGVGGFVNEDPFHPLTDPSARYSAETARHLVHGED 7qv7.1    --------------------------------------------------------------------------------  target    TSYWGFGDRPLVVDTPEDGRKVFTGTTHMPTPTKALWYNNANLINQAKWHYELVKNVNPKVDLIVDQQIEWTGSAEFADI 7qv7.1    -------------------------------YSDVIFIIGSNTAECHPLIAAHVIKAKERGAKLIVADPRMNAMVHKADI  target    VLPANSWMEAETWEMGASCSNPFLQVWKGGIEPLNDTRDDIAIFAGVANALTELTGDERFSQAFMFADRPEVYLDRVLAG 7qv7.1    WLRVPSG-------------------------------------------------------------------------  target    SFTTEGYTVEDLTAGRYGPPGGALMQYRSYPRIPFKEQIEDSLPFYTDTGRMHGYVDIPEAIEYGENLIVHREAVEATPY 7qv7.1    --------------------------------------------------------------------------------  target    LPNVIVSTSPYLRPRDYGIAPEELDGDARSVRNIMMSWAEVKETENPLFAAGYNYLCLTPKSRHAVHSSWAVTDWHWLWS 7qv7.1    --------------------------------------------------------------------------------  target    SSFSDPYRVETRAPGVGEPAIHLNPDDARSLGIRNGDYVWVDSNPKDRPYRDADVDESFLDVARLLVRVTYNPAYPPGVT 7qv7.1    --------------------------------------------------------------------------------  target    MLKHAFYMATPRTFRAAQERSDGRALAETTGYQSSFRSGSHQSITRGWAPPMHQTDSLFHKRAGVFGFTYGFDVDNHAIN 7qv7.1    --------------------------------------------------------------------------------  target    TVPKETVVRITKAEDGGVGGSGAWTRGRPGSMPGDEDDAMQAYLAGELTVVRRT 7qv7.1    ------------------------------------------------------ ``` | | | | | | | | | | | | | | | | | | | | | | | | | | | | | | | | | | | | | | | | | | | | | | | | | |
|  | 7qv7.1.O | Hydrogen dependent carbon dioxide reductase subunit FdhF  *Cryo-EM structure of Hydrogen-dependent CO2 reductase.* | 0.01 |  | 12.50 | 0.07 | 272-327 | EM | 0.00 | hetero-2-6-6-2-mer | 52 x SF4, 6 x 402 | HHblits | 0.25 |
| ``` target    RPFLERFTDMPLLVRLDTLQRLRADEVFADYSSDLDVDGPSFTLHGMTEEQHERNGDRVVFDDASGALRAINREDVGDRL 7qv7.1    --------------------------------------------------------------------------------  target    DDKGIDPALDYQGTVTLVDGSTVEVMSVLSMYREHLADYDIDSVVDMTGAPRNLIEQLLDDMTTLSPVAFHVGEGVNHYF 7qv7.1    --------------------------------------------------------------------------------  target    HATLHNRATYLVGMLLGSVGVSGGGVSTWAGNYKGGVFQAAPWFGPGVGGFVNEDPFHPLTDPSARYSAETARHLVHGED 7qv7.1    --------------------------------------------------------------------------------  target    TSYWGFGDRPLVVDTPEDGRKVFTGTTHMPTPTKALWYNNANLINQAKWHYELVKNVNPKVDLIVDQQIEWTGSAEFADI 7qv7.1    -------------------------------YSDVIFIIGSNTAECHPLIAAHVIKAKERGAKLIVADPRMNAMVHKADI  target    VLPANSWMEAETWEMGASCSNPFLQVWKGGIEPLNDTRDDIAIFAGVANALTELTGDERFSQAFMFADRPEVYLDRVLAG 7qv7.1    WLRVPSG-------------------------------------------------------------------------  target    SFTTEGYTVEDLTAGRYGPPGGALMQYRSYPRIPFKEQIEDSLPFYTDTGRMHGYVDIPEAIEYGENLIVHREAVEATPY 7qv7.1    --------------------------------------------------------------------------------  target    LPNVIVSTSPYLRPRDYGIAPEELDGDARSVRNIMMSWAEVKETENPLFAAGYNYLCLTPKSRHAVHSSWAVTDWHWLWS 7qv7.1    --------------------------------------------------------------------------------  target    SSFSDPYRVETRAPGVGEPAIHLNPDDARSLGIRNGDYVWVDSNPKDRPYRDADVDESFLDVARLLVRVTYNPAYPPGVT 7qv7.1    --------------------------------------------------------------------------------  target    MLKHAFYMATPRTFRAAQERSDGRALAETTGYQSSFRSGSHQSITRGWAPPMHQTDSLFHKRAGVFGFTYGFDVDNHAIN 7qv7.1    --------------------------------------------------------------------------------  target    TVPKETVVRITKAEDGGVGGSGAWTRGRPGSMPGDEDDAMQAYLAGELTVVRRT 7qv7.1    ------------------------------------------------------ ``` | | | | | | | | | | | | | | | | | | | | | | | | | | | | | | | | | | | | | | | | | | | | | | | | | |
|  | 4dmr.1.A | DMSO REDUCTASE  *REDUCED DMSO REDUCTASE FROM RHODOBACTER CAPSULATUS WITH BOUND DMSO SUBSTRATE* | 0.00 | 0.00 | 14.81 | 0.07 | 272-325 | X-ray | 1.90 | monomer | 2 x PGD, 1 x 4MO, 1 x O | HHblits | 0.27 |
| ``` target    RPFLERFTDMPLLVRLDTLQRLRADEVFADYSSDLDVDGPSFTLHGMTEEQHERNGDRVVFDDASGALRAINREDVGDRL 4dmr.1    --------------------------------------------------------------------------------  target    DDKGIDPALDYQGTVTLVDGSTVEVMSVLSMYREHLADYDIDSVVDMTGAPRNLIEQLLDDMTTLSPVAFHVGEGVNHYF 4dmr.1    --------------------------------------------------------------------------------  target    HATLHNRATYLVGMLLGSVGVSGGGVSTWAGNYKGGVFQAAPWFGPGVGGFVNEDPFHPLTDPSARYSAETARHLVHGED 4dmr.1    --------------------------------------------------------------------------------  target    TSYWGFGDRPLVVDTPEDGRKVFTGTTHMPTPTKALWYNNANLINQAKWH--------YELVKNVNPKVDLIVDQQIEWT 4dmr.1    -------------------------------NTEVMVFWAADPIKTSQIGWVIPEHGAYPGLEALKAKGTKVIVIDPVRT  target    GSAEF-ADIVLPANSWMEAETWEMGASCSNPFLQVWKGGIEPLNDTRDDIAIFAGVANALTELTGDERFSQAFMFADRPE 4dmr.1    KTVEFFGAEHITPK------------------------------------------------------------------  target    VYLDRVLAGSFTTEGYTVEDLTAGRYGPPGGALMQYRSYPRIPFKEQIEDSLPFYTDTGRMHGYVDIPEAIEYGENLIVH 4dmr.1    --------------------------------------------------------------------------------  target    REAVEATPYLPNVIVSTSPYLRPRDYGIAPEELDGDARSVRNIMMSWAEVKETENPLFAAGYNYLCLTPKSRHAVHSSWA 4dmr.1    --------------------------------------------------------------------------------  target    VTDWHWLWSSSFSDPYRVETRAPGVGEPAIHLNPDDARSLGIRNGDYVWVDSNPKDRPYRDADVDESFLDVARLLVRVTY 4dmr.1    --------------------------------------------------------------------------------  target    NPAYPPGVTMLKHAFYMATPRTFRAAQERSDGRALAETTGYQSSFRSGSHQSITRGWAPPMHQTDSLFHKRAGVFGFTYG 4dmr.1    --------------------------------------------------------------------------------  target    FDVDNHAINTVPKETVVRITKAEDGGVGGSGAWTRGRPGSMPGDEDDAMQAYLAGELTVVRRT 4dmr.1    --------------------------------------------------------------- ``` | | | | | | | | | | | | | | | | | | | | | | | | | | | | | | | | | | | | | | | | | | | | | | | | | |
|  | 1dms.1.A | DMSO REDUCTASE  *STRUCTURE OF DMSO REDUCTASE* | 0.00 | 0.00 | 14.81 | 0.07 | 272-325 | X-ray | 1.88 | monomer | 2 x PGD, 1 x 2MO | HHblits | 0.27 |
| ``` target    RPFLERFTDMPLLVRLDTLQRLRADEVFADYSSDLDVDGPSFTLHGMTEEQHERNGDRVVFDDASGALRAINREDVGDRL 1dms.1    --------------------------------------------------------------------------------  target    DDKGIDPALDYQGTVTLVDGSTVEVMSVLSMYREHLADYDIDSVVDMTGAPRNLIEQLLDDMTTLSPVAFHVGEGVNHYF 1dms.1    --------------------------------------------------------------------------------  target    HATLHNRATYLVGMLLGSVGVSGGGVSTWAGNYKGGVFQAAPWFGPGVGGFVNEDPFHPLTDPSARYSAETARHLVHGED 1dms.1    --------------------------------------------------------------------------------  target    TSYWGFGDRPLVVDTPEDGRKVFTGTTHMPTPTKALWYNNANLINQAKWH--------YELVKNVNPKVDLIVDQQIEWT 1dms.1    -------------------------------NTEVMVFWAADPIKTSQIGWVIPEHGAYPGLEALKAKGTKVIVIDPVRT  target    GSAEF-ADIVLPANSWMEAETWEMGASCSNPFLQVWKGGIEPLNDTRDDIAIFAGVANALTELTGDERFSQAFMFADRPE 1dms.1    KTVEFFGADHVTPK------------------------------------------------------------------  target    VYLDRVLAGSFTTEGYTVEDLTAGRYGPPGGALMQYRSYPRIPFKEQIEDSLPFYTDTGRMHGYVDIPEAIEYGENLIVH 1dms.1    --------------------------------------------------------------------------------  target    REAVEATPYLPNVIVSTSPYLRPRDYGIAPEELDGDARSVRNIMMSWAEVKETENPLFAAGYNYLCLTPKSRHAVHSSWA 1dms.1    --------------------------------------------------------------------------------  target    VTDWHWLWSSSFSDPYRVETRAPGVGEPAIHLNPDDARSLGIRNGDYVWVDSNPKDRPYRDADVDESFLDVARLLVRVTY 1dms.1    --------------------------------------------------------------------------------  target    NPAYPPGVTMLKHAFYMATPRTFRAAQERSDGRALAETTGYQSSFRSGSHQSITRGWAPPMHQTDSLFHKRAGVFGFTYG 1dms.1    --------------------------------------------------------------------------------  target    FDVDNHAINTVPKETVVRITKAEDGGVGGSGAWTRGRPGSMPGDEDDAMQAYLAGELTVVRRT 1dms.1    --------------------------------------------------------------- ``` | | | | | | | | | | | | | | | | | | | | | | | | | | | | | | | | | | | | | | | | | | | | | | | | | |
|  | 7l5i.1.A | Trimethylamine-N-oxide reductase  *Crystal Structure of Haemophilus influenzae MtsZ at pH 7.0* | 0.00 |  | 12.96 | 0.07 | 272-325 | X-ray | 1.73 | monomer | 2 x MGD, 1 x MO, 1 x O | HHblits | 0.27 |
| ``` target    RPFLERFTDMPLLVRLDTLQRLRADEVFADYSSDLDVDGPSFTLHGMTEEQHERNGDRVVFDDASGALRAINREDVGDRL 7l5i.1    --------------------------------------------------------------------------------  target    DDKGIDPALDYQGTVTLVDGSTVEVMSVLSMYREHLADYDIDSVVDMTGAPRNLIEQLLDDMTTLSPVAFHVGEGVNHYF 7l5i.1    --------------------------------------------------------------------------------  target    HATLHNRATYLVGMLLGSVGVSGGGVSTWAGNYKGGVFQAAPWFGPGVGGFVNEDPFHPLTDPSARYSAETARHLVHGED 7l5i.1    --------------------------------------------------------------------------------  target    TSYWGFGDRPLVVDTPEDGRKVFTGTTHMPTPTKALWYNNANLINQAKWH--------YELVKNVNPKVDLIVDQQIEWT 7l5i.1    -------------------------------SSDIIVLWSANPLTTMRIAWMSTDQKGIEYFKKFQASGKRIICIDPQKS  target    GSAEF-ADIVLPANSWMEAETWEMGASCSNPFLQVWKGGIEPLNDTRDDIAIFAGVANALTELTGDERFSQAFMFADRPE 7l5i.1    ETCQMLNAEWIPVN------------------------------------------------------------------  target    VYLDRVLAGSFTTEGYTVEDLTAGRYGPPGGALMQYRSYPRIPFKEQIEDSLPFYTDTGRMHGYVDIPEAIEYGENLIVH 7l5i.1    --------------------------------------------------------------------------------  target    REAVEATPYLPNVIVSTSPYLRPRDYGIAPEELDGDARSVRNIMMSWAEVKETENPLFAAGYNYLCLTPKSRHAVHSSWA 7l5i.1    --------------------------------------------------------------------------------  target    VTDWHWLWSSSFSDPYRVETRAPGVGEPAIHLNPDDARSLGIRNGDYVWVDSNPKDRPYRDADVDESFLDVARLLVRVTY 7l5i.1    --------------------------------------------------------------------------------  target    NPAYPPGVTMLKHAFYMATPRTFRAAQERSDGRALAETTGYQSSFRSGSHQSITRGWAPPMHQTDSLFHKRAGVFGFTYG 7l5i.1    --------------------------------------------------------------------------------  target    FDVDNHAINTVPKETVVRITKAEDGGVGGSGAWTRGRPGSMPGDEDDAMQAYLAGELTVVRRT 7l5i.1    --------------------------------------------------------------- ``` | | | | | | | | | | | | | | | | | | | | | | | | | | | | | | | | | | | | | | | | | | | | | | | | | |
|  | 7l5s.1.A | Trimethylamine-N-oxide reductase  *Crystal Structure of Haemophilus influenzae MtsZ at pH 5.5* | 0.00 |  | 12.96 | 0.07 | 272-325 | X-ray | 2.09 | monomer | 1 x O, 2 x MGD, 1 x MO | HHblits | 0.27 |
| ``` target    RPFLERFTDMPLLVRLDTLQRLRADEVFADYSSDLDVDGPSFTLHGMTEEQHERNGDRVVFDDASGALRAINREDVGDRL 7l5s.1    --------------------------------------------------------------------------------  target    DDKGIDPALDYQGTVTLVDGSTVEVMSVLSMYREHLADYDIDSVVDMTGAPRNLIEQLLDDMTTLSPVAFHVGEGVNHYF 7l5s.1    --------------------------------------------------------------------------------  target    HATLHNRATYLVGMLLGSVGVSGGGVSTWAGNYKGGVFQAAPWFGPGVGGFVNEDPFHPLTDPSARYSAETARHLVHGED 7l5s.1    --------------------------------------------------------------------------------  target    TSYWGFGDRPLVVDTPEDGRKVFTGTTHMPTPTKALWYNNANLINQAKWH--------YELVKNVNPKVDLIVDQQIEWT 7l5s.1    -------------------------------SSDIIVLWSANPLTTMRIAWMSTDQKGIEYFKKFQASGKRIICIDPQKS  target    GSAEF-ADIVLPANSWMEAETWEMGASCSNPFLQVWKGGIEPLNDTRDDIAIFAGVANALTELTGDERFSQAFMFADRPE 7l5s.1    ETCQMLNAEWIPVN------------------------------------------------------------------  target    VYLDRVLAGSFTTEGYTVEDLTAGRYGPPGGALMQYRSYPRIPFKEQIEDSLPFYTDTGRMHGYVDIPEAIEYGENLIVH 7l5s.1    --------------------------------------------------------------------------------  target    REAVEATPYLPNVIVSTSPYLRPRDYGIAPEELDGDARSVRNIMMSWAEVKETENPLFAAGYNYLCLTPKSRHAVHSSWA 7l5s.1    --------------------------------------------------------------------------------  target    VTDWHWLWSSSFSDPYRVETRAPGVGEPAIHLNPDDARSLGIRNGDYVWVDSNPKDRPYRDADVDESFLDVARLLVRVTY 7l5s.1    --------------------------------------------------------------------------------  target    NPAYPPGVTMLKHAFYMATPRTFRAAQERSDGRALAETTGYQSSFRSGSHQSITRGWAPPMHQTDSLFHKRAGVFGFTYG 7l5s.1    --------------------------------------------------------------------------------  target    FDVDNHAINTVPKETVVRITKAEDGGVGGSGAWTRGRPGSMPGDEDDAMQAYLAGELTVVRRT 7l5s.1    --------------------------------------------------------------- ``` | | | | | | | | | | | | | | | | | | | | | | | | | | | | | | | | | | | | | | | | | | | | | | | | | |
|  | 1eu1.1.A | DIMETHYL SULFOXIDE REDUCTASE  *THE CRYSTAL STRUCTURE OF RHODOBACTER SPHAEROIDES DIMETHYLSULFOXIDE REDUCTASE REVEALS TWO DISTINCT MOLYBDENUM COORDINATION ENVIRONMENTS.* | 0.00 |  | 14.81 | 0.07 | 272-325 | X-ray | 1.30 | monomer | 3 x GLC, 1 x CD, 2 x MGD, 1 x 6MO, 2 x O | HHblits | 0.27 |
| ``` target    RPFLERFTDMPLLVRLDTLQRLRADEVFADYSSDLDVDGPSFTLHGMTEEQHERNGDRVVFDDASGALRAINREDVGDRL 1eu1.1    --------------------------------------------------------------------------------  target    DDKGIDPALDYQGTVTLVDGSTVEVMSVLSMYREHLADYDIDSVVDMTGAPRNLIEQLLDDMTTLSPVAFHVGEGVNHYF 1eu1.1    --------------------------------------------------------------------------------  target    HATLHNRATYLVGMLLGSVGVSGGGVSTWAGNYKGGVFQAAPWFGPGVGGFVNEDPFHPLTDPSARYSAETARHLVHGED 1eu1.1    --------------------------------------------------------------------------------  target    TSYWGFGDRPLVVDTPEDGRKVFTGTTHMPTPTKALWYNNANLINQAKWH--------YELVKNVNPKVDLIVDQQIEWT 1eu1.1    -------------------------------NTDLMVFWAADPMKTNEIGWVIPDHGAYAGMKALKEKGTRVICINPVRT  target    GSAEFAD-IVLPANSWMEAETWEMGASCSNPFLQVWKGGIEPLNDTRDDIAIFAGVANALTELTGDERFSQAFMFADRPE 1eu1.1    ETADYFGADVVSPR------------------------------------------------------------------  target    VYLDRVLAGSFTTEGYTVEDLTAGRYGPPGGALMQYRSYPRIPFKEQIEDSLPFYTDTGRMHGYVDIPEAIEYGENLIVH 1eu1.1    --------------------------------------------------------------------------------  target    REAVEATPYLPNVIVSTSPYLRPRDYGIAPEELDGDARSVRNIMMSWAEVKETENPLFAAGYNYLCLTPKSRHAVHSSWA 1eu1.1    --------------------------------------------------------------------------------  target    VTDWHWLWSSSFSDPYRVETRAPGVGEPAIHLNPDDARSLGIRNGDYVWVDSNPKDRPYRDADVDESFLDVARLLVRVTY 1eu1.1    --------------------------------------------------------------------------------  target    NPAYPPGVTMLKHAFYMATPRTFRAAQERSDGRALAETTGYQSSFRSGSHQSITRGWAPPMHQTDSLFHKRAGVFGFTYG 1eu1.1    --------------------------------------------------------------------------------  target    FDVDNHAINTVPKETVVRITKAEDGGVGGSGAWTRGRPGSMPGDEDDAMQAYLAGELTVVRRT 1eu1.1    --------------------------------------------------------------- ``` | | | | | | | | | | | | | | | | | | | | | | | | | | | | | | | | | | | | | | | | | | | | | | | | | |
|  | 2v45.1.A | PERIPLASMIC NITRATE REDUCTASE  *A NEW CATALYTIC MECHANISM OF PERIPLASMIC NITRATE REDUCTASE FROM DESULFOVIBRIO DESULFURICANS ATCC 27774 FROM CRYSTALLOGRAPHIC AND EPR DATA AND BASED ON DETAILED ANALYSIS OF THE SIXTH LIGAND* | 0.01 |  | 9.09 | 0.07 | 272-326 | X-ray | 2.40 | monomer | 1 x SF4, 1 x MO, 2 x MGD, 1 x LCP | HHblits | 0.25 |
| ``` target    RPFLERFTDMPLLVRLDTLQRLRADEVFADYSSDLDVDGPSFTLHGMTEEQHERNGDRVVFDDASGALRAINREDVGDRL 2v45.1    --------------------------------------------------------------------------------  target    DDKGIDPALDYQGTVTLVDGSTVEVMSVLSMYREHLADYDIDSVVDMTGAPRNLIEQLLDDMTTLSPVAFHVGEGVNHYF 2v45.1    --------------------------------------------------------------------------------  target    HATLHNRATYLVGMLLGSVGVSGGGVSTWAGNYKGGVFQAAPWFGPGVGGFVNEDPFHPLTDPSARYSAETARHLVHGED 2v45.1    --------------------------------------------------------------------------------  target    TSYWGFGDRPLVVDTPEDGRKVFTGTTHMPTPTKALWYNNANLINQAKWHYELVKNVN--PKVDLIVDQQIEWTGSAEFA 2v45.1    -------------------------------QATCFFIIGSNTSEAHPVLFRRIARRKQVEPGVKIIVADPRRTNTSRIA  target    DIVLPANSWMEAETWEMGASCSNPFLQVWKGGIEPLNDTRDDIAIFAGVANALTELTGDERFSQAFMFADRPEVYLDRVL 2v45.1    DMHVAFRP------------------------------------------------------------------------  target    AGSFTTEGYTVEDLTAGRYGPPGGALMQYRSYPRIPFKEQIEDSLPFYTDTGRMHGYVDIPEAIEYGENLIVHREAVEAT 2v45.1    --------------------------------------------------------------------------------  target    PYLPNVIVSTSPYLRPRDYGIAPEELDGDARSVRNIMMSWAEVKETENPLFAAGYNYLCLTPKSRHAVHSSWAVTDWHWL 2v45.1    --------------------------------------------------------------------------------  target    WSSSFSDPYRVETRAPGVGEPAIHLNPDDARSLGIRNGDYVWVDSNPKDRPYRDADVDESFLDVARLLVRVTYNPAYPPG 2v45.1    --------------------------------------------------------------------------------  target    VTMLKHAFYMATPRTFRAAQERSDGRALAETTGYQSSFRSGSHQSITRGWAPPMHQTDSLFHKRAGVFGFTYGFDVDNHA 2v45.1    --------------------------------------------------------------------------------  target    INTVPKETVVRITKAEDGGVGGSGAWTRGRPGSMPGDEDDAMQAYLAGELTVVRRT 2v45.1    -------------------------------------------------------- ``` | | | | | | | | | | | | | | | | | | | | | | | | | | | | | | | | | | | | | | | | | | | | | | | | | |
|  | 7bkb.1.F | Formate dehydrogenase  *Formate dehydrogenase - heterodisulfide reductase - formylmethanofuran dehydrogenase complex from Methanospirillum hungatei (hexameric, composite structure)* | 0.01 |  | 15.09 | 0.07 | 273-325 | EM | 0.00 | hetero-2-2-2-2-2-2-… | 48 x SF4, 4 x FAD, 2 x FES, 4 x 9S8, 4 x ZN, 2 x MO, 4 x MGD | HHblits | 0.28 |
| ``` target    RPFLERFTDMPLLVRLDTLQRLRADEVFADYSSDLDVDGPSFTLHGMTEEQHERNGDRVVFDDASGALRAINREDVGDRL 7bkb.1    --------------------------------------------------------------------------------  target    DDKGIDPALDYQGTVTLVDGSTVEVMSVLSMYREHLADYDIDSVVDMTGAPRNLIEQLLDDMTTLSPVAFHVGEGVNHYF 7bkb.1    --------------------------------------------------------------------------------  target    HATLHNRATYLVGMLLGSVGVSGGGVSTWAGNYKGGVFQAAPWFGPGVGGFVNEDPFHPLTDPSARYSAETARHLVHGED 7bkb.1    --------------------------------------------------------------------------------  target    TSYWGFGDRPLVVDTPEDGRKVFTGTTHMPTPTKALWYNNANLINQAKWHYELVKNVNPKVDLIVDQQIEWTGSAEFADI 7bkb.1    --------------------------------ADLILIWGSNAVEAHPLAGRRIAQAKKKGIQIIAVDPRYTMTARLADT  target    VLPANSWMEAETWEMGASCSNPFLQVWKGGIEPLNDTRDDIAIFAGVANALTELTGDERFSQAFMFADRPEVYLDRVLAG 7bkb.1    YVRFN---------------------------------------------------------------------------  target    SFTTEGYTVEDLTAGRYGPPGGALMQYRSYPRIPFKEQIEDSLPFYTDTGRMHGYVDIPEAIEYGENLIVHREAVEATPY 7bkb.1    --------------------------------------------------------------------------------  target    LPNVIVSTSPYLRPRDYGIAPEELDGDARSVRNIMMSWAEVKETENPLFAAGYNYLCLTPKSRHAVHSSWAVTDWHWLWS 7bkb.1    --------------------------------------------------------------------------------  target    SSFSDPYRVETRAPGVGEPAIHLNPDDARSLGIRNGDYVWVDSNPKDRPYRDADVDESFLDVARLLVRVTYNPAYPPGVT 7bkb.1    --------------------------------------------------------------------------------  target    MLKHAFYMATPRTFRAAQERSDGRALAETTGYQSSFRSGSHQSITRGWAPPMHQTDSLFHKRAGVFGFTYGFDVDNHAIN 7bkb.1    --------------------------------------------------------------------------------  target    TVPKETVVRITKAEDGGVGGSGAWTRGRPGSMPGDEDDAMQAYLAGELTVVRRT 7bkb.1    ------------------------------------------------------ ``` | | | | | | | | | | | | | | | | | | | | | | | | | | | | | | | | | | | | | | | | | | | | | | | | | |
|  | 6cz7.1.A | ArrA  *The arsenate respiratory reductase (Arr) complex from Shewanella sp. ANA-3* | 0.01 |  | 14.55 | 0.07 | 272-326 | X-ray | 1.62 | hetero-1-1-mer | 5 x SF4, 2 x MGD, 1 x MO, 1 x PG5 | HHblits | 0.24 |
| ``` target    RPFLERFTDMPLLVRLDTLQRLRADEVFADYSSDLDVDGPSFTLHGMTEEQHERNGDRVVFDDASGALRAINREDVGDRL 6cz7.1    --------------------------------------------------------------------------------  target    DDKGIDPALDYQGTVTLVDGSTVEVMSVLSMYREHLADYDIDSVVDMTGAPRNLIEQLLDDMTTLSPVAFHVGEGVNHYF 6cz7.1    --------------------------------------------------------------------------------  target    HATLHNRATYLVGMLLGSVGVSGGGVSTWAGNYKGGVFQAAPWFGPGVGGFVNEDPFHPLTDPSARYSAETARHLVHGED 6cz7.1    --------------------------------------------------------------------------------  target    TSYWGFGDRPLVVDTPEDGRKVFTGTTHMPTPTKALWYNNANLINQAKWHYELVKN--VNPKVDLIVDQQIEWTGSAEFA 6cz7.1    -------------------------------NAKFILSFGADPIASNRQVSFYSQTWGDSLDHAKVVVVDPRLSASAAKA  target    DIVLPANSWMEAETWEMGASCSNPFLQVWKGGIEPLNDTRDDIAIFAGVANALTELTGDERFSQAFMFADRPEVYLDRVL 6cz7.1    HKWIPIEP------------------------------------------------------------------------  target    AGSFTTEGYTVEDLTAGRYGPPGGALMQYRSYPRIPFKEQIEDSLPFYTDTGRMHGYVDIPEAIEYGENLIVHREAVEAT 6cz7.1    --------------------------------------------------------------------------------  target    PYLPNVIVSTSPYLRPRDYGIAPEELDGDARSVRNIMMSWAEVKETENPLFAAGYNYLCLTPKSRHAVHSSWAVTDWHWL 6cz7.1    --------------------------------------------------------------------------------  target    WSSSFSDPYRVETRAPGVGEPAIHLNPDDARSLGIRNGDYVWVDSNPKDRPYRDADVDESFLDVARLLVRVTYNPAYPPG 6cz7.1    --------------------------------------------------------------------------------  target    VTMLKHAFYMATPRTFRAAQERSDGRALAETTGYQSSFRSGSHQSITRGWAPPMHQTDSLFHKRAGVFGFTYGFDVDNHA 6cz7.1    --------------------------------------------------------------------------------  target    INTVPKETVVRITKAEDGGVGGSGAWTRGRPGSMPGDEDDAMQAYLAGELTVVRRT 6cz7.1    -------------------------------------------------------- ``` | | | | | | | | | | | | | | | | | | | | | | | | | | | | | | | | | | | | | | | | | | | | | | | | | |
|  | 5e7o.1.A | DMSO reductase family type II enzyme, molybdopterin subunit  *Crystal structure of the perchlorate reductase PcrAB mutant W461E of PcrA from Azospira suillum PS* | 0.01 |  | 10.91 | 0.07 | 272-326 | X-ray | 2.40 | hetero-oligomer | 4 x SF4, 1 x MO, 1 x MGD, 1 x MD1, 1 x F3S | HHblits | 0.24 |
| ``` target    RPFLERFTDMPLLVRLDTLQRLRADEVFADYSSDLDVDGPSFTLHGMTEEQHERNGDRVVFDDASGALRAINREDVGDRL 5e7o.1    --------------------------------------------------------------------------------  target    DDKGIDPALDYQGTVTLVDGSTVEVMSVLSMYREHLADYDIDSVVDMTGAPRNLIEQLLDDMTTLSPVAFHVGEGVNHYF 5e7o.1    --------------------------------------------------------------------------------  target    HATLHNRATYLVGMLLGSVGVSGGGVSTWAGNYKGGVFQAAPWFGPGVGGFVNEDPFHPLTDPSARYSAETARHLVHGED 5e7o.1    --------------------------------------------------------------------------------  target    TSYWGFGDRPLVVDTPEDGRKVFTGTTHMPTPTKALWYNNANLINQAKWHYELVKNVNPKVDLIVDQQIEWTGSAEFADI 5e7o.1    -------------------------------NSKYIILWGSNPTQTRIPDAHFLSEAQLNGAKIVSISPDYNSSTIKVDK  target    VLPANSWMEAETWEMGASCSNPFLQVWKGGIEPLNDTRDDIAIFAGVANALTELTGDERFSQAFMFADRPEVYLDRVLAG 5e7o.1    WIHPQP--------------------------------------------------------------------------  target    SFTTEGYTVEDLTAGRYGPPGGALMQYRSYPRIPFKEQIEDSLPFYTDTGRMHGYVDIPEAIEYGENLIVHREAVEATPY 5e7o.1    --------------------------------------------------------------------------------  target    LPNVIVSTSPYLRPRDYGIAPEELDGDARSVRNIMMSWAEVKETENPLFAAGYNYLCLTPKSRHAVHSSWAVTDWHWLWS 5e7o.1    --------------------------------------------------------------------------------  target    SSFSDPYRVETRAPGVGEPAIHLNPDDARSLGIRNGDYVWVDSNPKDRPYRDADVDESFLDVARLLVRVTYNPAYPPGVT 5e7o.1    --------------------------------------------------------------------------------  target    MLKHAFYMATPRTFRAAQERSDGRALAETTGYQSSFRSGSHQSITRGWAPPMHQTDSLFHKRAGVFGFTYGFDVDNHAIN 5e7o.1    --------------------------------------------------------------------------------  target    TVPKETVVRITKAEDGGVGGSGAWTRGRPGSMPGDEDDAMQAYLAGELTVVRRT 5e7o.1    ------------------------------------------------------ ``` | | | | | | | | | | | | | | | | | | | | | | | | | | | | | | | | | | | | | | | | | | | | | | | | | |
|  | 3egw.1.A | Respiratory nitrate reductase 1 alpha chain  *The crystal structure of the NarGHI mutant NarH - C16A* | 0.01 | 0.00 | 12.96 | 0.07 | 272-325 | X-ray | 1.90 | monomer | 2 x MD1, 2 x MGD, 2 x 6MO, 6 x SF4, 4 x F3S, 2 x 3PH, 4 x HEM, 2 x AGA | HHblits | 0.25 |
| ``` target    RPFLERFTDMPLLVRLDTLQRLRADEVFADYSSDLDVDGPSFTLHGMTEEQHERNGDRVVFDDASGALRAINREDVGDRL 3egw.1    --------------------------------------------------------------------------------  target    DDKGIDPALDYQGTVTLVDGSTVEVMSVLSMYREHLADYDIDSVVDMTGAPRNLIEQLLDDMTTLSPVAFHVGEGVNHYF 3egw.1    --------------------------------------------------------------------------------  target    HATLHNRATYLVGMLLGSVGVSGGGVSTWAGNYKGGVFQAAPWFGPGVGGFVNEDPFHPLTDPSARYSAETARHLVHGED 3egw.1    --------------------------------------------------------------------------------  target    TSYWGFGDRPLVVDTPEDGRKVFTGTTHMPTPTKALWYNNANLINQAKWHYELVKNVNPKVDLIVDQQIEWTGSAEFADI 3egw.1    -------------------------------NSSYIIAWGSNVPQTRTPDAHFFTEVRYKGTKTVAVTPDYAEIAKLCDL  target    VLPANSWMEAETWEMGASCSNPFLQVWKGGIEPLNDTRDDIAIFAGVANALTELTGDERFSQAFMFADRPEVYLDRVLAG 3egw.1    WLAPK---------------------------------------------------------------------------  target    SFTTEGYTVEDLTAGRYGPPGGALMQYRSYPRIPFKEQIEDSLPFYTDTGRMHGYVDIPEAIEYGENLIVHREAVEATPY 3egw.1    --------------------------------------------------------------------------------  target    LPNVIVSTSPYLRPRDYGIAPEELDGDARSVRNIMMSWAEVKETENPLFAAGYNYLCLTPKSRHAVHSSWAVTDWHWLWS 3egw.1    --------------------------------------------------------------------------------  target    SSFSDPYRVETRAPGVGEPAIHLNPDDARSLGIRNGDYVWVDSNPKDRPYRDADVDESFLDVARLLVRVTYNPAYPPGVT 3egw.1    --------------------------------------------------------------------------------  target    MLKHAFYMATPRTFRAAQERSDGRALAETTGYQSSFRSGSHQSITRGWAPPMHQTDSLFHKRAGVFGFTYGFDVDNHAIN 3egw.1    --------------------------------------------------------------------------------  target    TVPKETVVRITKAEDGGVGGSGAWTRGRPGSMPGDEDDAMQAYLAGELTVVRRT 3egw.1    ------------------------------------------------------ ``` | | | | | | | | | | | | | | | | | | | | | | | | | | | | | | | | | | | | | | | | | | | | | | | | | |
|  | 1r27.4.A | Respiratory nitrate reductase 1 alpha chain  *Crystal Structure of NarGH complex* | 0.01 | 0.00 | 12.96 | 0.07 | 272-325 | X-ray | 2.00 | monomer | 4 x MO, 16 x SF4, 8 x MGD, 4 x F3S | HHblits | 0.25 |
| ``` target    RPFLERFTDMPLLVRLDTLQRLRADEVFADYSSDLDVDGPSFTLHGMTEEQHERNGDRVVFDDASGALRAINREDVGDRL 1r27.4    --------------------------------------------------------------------------------  target    DDKGIDPALDYQGTVTLVDGSTVEVMSVLSMYREHLADYDIDSVVDMTGAPRNLIEQLLDDMTTLSPVAFHVGEGVNHYF 1r27.4    --------------------------------------------------------------------------------  target    HATLHNRATYLVGMLLGSVGVSGGGVSTWAGNYKGGVFQAAPWFGPGVGGFVNEDPFHPLTDPSARYSAETARHLVHGED 1r27.4    --------------------------------------------------------------------------------  target    TSYWGFGDRPLVVDTPEDGRKVFTGTTHMPTPTKALWYNNANLINQAKWHYELVKNVNPKVDLIVDQQIEWTGSAEFADI 1r27.4    -------------------------------NSSYIIAWGSNVPQTRTPDAHFFTEVRYKGTKTVAVTPDYAEIAKLCDL  target    VLPANSWMEAETWEMGASCSNPFLQVWKGGIEPLNDTRDDIAIFAGVANALTELTGDERFSQAFMFADRPEVYLDRVLAG 1r27.4    WLAPK---------------------------------------------------------------------------  target    SFTTEGYTVEDLTAGRYGPPGGALMQYRSYPRIPFKEQIEDSLPFYTDTGRMHGYVDIPEAIEYGENLIVHREAVEATPY 1r27.4    --------------------------------------------------------------------------------  target    LPNVIVSTSPYLRPRDYGIAPEELDGDARSVRNIMMSWAEVKETENPLFAAGYNYLCLTPKSRHAVHSSWAVTDWHWLWS 1r27.4    --------------------------------------------------------------------------------  target    SSFSDPYRVETRAPGVGEPAIHLNPDDARSLGIRNGDYVWVDSNPKDRPYRDADVDESFLDVARLLVRVTYNPAYPPGVT 1r27.4    --------------------------------------------------------------------------------  target    MLKHAFYMATPRTFRAAQERSDGRALAETTGYQSSFRSGSHQSITRGWAPPMHQTDSLFHKRAGVFGFTYGFDVDNHAIN 1r27.4    --------------------------------------------------------------------------------  target    TVPKETVVRITKAEDGGVGGSGAWTRGRPGSMPGDEDDAMQAYLAGELTVVRRT 1r27.4    ------------------------------------------------------ ``` | | | | | | | | | | | | | | | | | | | | | | | | | | | | | | | | | | | | | | | | | | | | | | | | | |
|  | 1q16.1.A | Respiratory nitrate reductase 1 alpha chain  *Crystal structure of Nitrate Reductase A, NarGHI, from Escherichia coli* | 0.01 | 0.00 | 12.96 | 0.07 | 272-325 | X-ray | 1.90 | monomer | 2 x MD1, 1 x 6MO, 2 x HEM, 4 x SF4, 1 x F3S, 1 x AGA, 1 x 3PH | HHblits | 0.25 |
| ``` target    RPFLERFTDMPLLVRLDTLQRLRADEVFADYSSDLDVDGPSFTLHGMTEEQHERNGDRVVFDDASGALRAINREDVGDRL 1q16.1    --------------------------------------------------------------------------------  target    DDKGIDPALDYQGTVTLVDGSTVEVMSVLSMYREHLADYDIDSVVDMTGAPRNLIEQLLDDMTTLSPVAFHVGEGVNHYF 1q16.1    --------------------------------------------------------------------------------  target    HATLHNRATYLVGMLLGSVGVSGGGVSTWAGNYKGGVFQAAPWFGPGVGGFVNEDPFHPLTDPSARYSAETARHLVHGED 1q16.1    --------------------------------------------------------------------------------  target    TSYWGFGDRPLVVDTPEDGRKVFTGTTHMPTPTKALWYNNANLINQAKWHYELVKNVNPKVDLIVDQQIEWTGSAEFADI 1q16.1    -------------------------------NSSYIIAWGSNVPQTRTPDAHFFTEVRYKGTKTVAVTPDYAEIAKLCDL  target    VLPANSWMEAETWEMGASCSNPFLQVWKGGIEPLNDTRDDIAIFAGVANALTELTGDERFSQAFMFADRPEVYLDRVLAG 1q16.1    WLAPK---------------------------------------------------------------------------  target    SFTTEGYTVEDLTAGRYGPPGGALMQYRSYPRIPFKEQIEDSLPFYTDTGRMHGYVDIPEAIEYGENLIVHREAVEATPY 1q16.1    --------------------------------------------------------------------------------  target    LPNVIVSTSPYLRPRDYGIAPEELDGDARSVRNIMMSWAEVKETENPLFAAGYNYLCLTPKSRHAVHSSWAVTDWHWLWS 1q16.1    --------------------------------------------------------------------------------  target    SSFSDPYRVETRAPGVGEPAIHLNPDDARSLGIRNGDYVWVDSNPKDRPYRDADVDESFLDVARLLVRVTYNPAYPPGVT 1q16.1    --------------------------------------------------------------------------------  target    MLKHAFYMATPRTFRAAQERSDGRALAETTGYQSSFRSGSHQSITRGWAPPMHQTDSLFHKRAGVFGFTYGFDVDNHAIN 1q16.1    --------------------------------------------------------------------------------  target    TVPKETVVRITKAEDGGVGGSGAWTRGRPGSMPGDEDDAMQAYLAGELTVVRRT 1q16.1    ------------------------------------------------------ ``` | | | | | | | | | | | | | | | | | | | | | | | | | | | | | | | | | | | | | | | | | | | | | | | | | |
|  | 3ir5.1.A | Respiratory nitrate reductase 1 alpha chain  *Crystal structure of NarGHI mutant NarG-H49C* | 0.01 | 0.00 | 12.96 | 0.07 | 272-325 | X-ray | 2.30 | monomer | 2 x MD1, 1 x 6MO, 4 x SF4, 1 x AGA, 1 x F3S, 2 x HEM | HHblits | 0.25 |
| ``` target    RPFLERFTDMPLLVRLDTLQRLRADEVFADYSSDLDVDGPSFTLHGMTEEQHERNGDRVVFDDASGALRAINREDVGDRL 3ir5.1    --------------------------------------------------------------------------------  target    DDKGIDPALDYQGTVTLVDGSTVEVMSVLSMYREHLADYDIDSVVDMTGAPRNLIEQLLDDMTTLSPVAFHVGEGVNHYF 3ir5.1    --------------------------------------------------------------------------------  target    HATLHNRATYLVGMLLGSVGVSGGGVSTWAGNYKGGVFQAAPWFGPGVGGFVNEDPFHPLTDPSARYSAETARHLVHGED 3ir5.1    --------------------------------------------------------------------------------  target    TSYWGFGDRPLVVDTPEDGRKVFTGTTHMPTPTKALWYNNANLINQAKWHYELVKNVNPKVDLIVDQQIEWTGSAEFADI 3ir5.1    -------------------------------NSSYIIAWGSNVPQTRTPDAHFFTEVRYKGTKTVAVTPDYAEIAKLCDL  target    VLPANSWMEAETWEMGASCSNPFLQVWKGGIEPLNDTRDDIAIFAGVANALTELTGDERFSQAFMFADRPEVYLDRVLAG 3ir5.1    WLAPK---------------------------------------------------------------------------  target    SFTTEGYTVEDLTAGRYGPPGGALMQYRSYPRIPFKEQIEDSLPFYTDTGRMHGYVDIPEAIEYGENLIVHREAVEATPY 3ir5.1    --------------------------------------------------------------------------------  target    LPNVIVSTSPYLRPRDYGIAPEELDGDARSVRNIMMSWAEVKETENPLFAAGYNYLCLTPKSRHAVHSSWAVTDWHWLWS 3ir5.1    --------------------------------------------------------------------------------  target    SSFSDPYRVETRAPGVGEPAIHLNPDDARSLGIRNGDYVWVDSNPKDRPYRDADVDESFLDVARLLVRVTYNPAYPPGVT 3ir5.1    --------------------------------------------------------------------------------  target    MLKHAFYMATPRTFRAAQERSDGRALAETTGYQSSFRSGSHQSITRGWAPPMHQTDSLFHKRAGVFGFTYGFDVDNHAIN 3ir5.1    --------------------------------------------------------------------------------  target    TVPKETVVRITKAEDGGVGGSGAWTRGRPGSMPGDEDDAMQAYLAGELTVVRRT 3ir5.1    ------------------------------------------------------ ``` | | | | | | | | | | | | | | | | | | | | | | | | | | | | | | | | | | | | | | | | | | | | | | | | | |
|  | 3ir6.1.A | Respiratory nitrate reductase 1 alpha chain  *Crystal structure of NarGHI mutant NarG-H49S* | 0.01 | 0.00 | 12.96 | 0.07 | 272-325 | X-ray | 2.80 | monomer | 2 x GDP, 1 x AGA, 3 x SF4, 1 x F3S, 2 x HEM | HHblits | 0.25 |
| ``` target    RPFLERFTDMPLLVRLDTLQRLRADEVFADYSSDLDVDGPSFTLHGMTEEQHERNGDRVVFDDASGALRAINREDVGDRL 3ir6.1    --------------------------------------------------------------------------------  target    DDKGIDPALDYQGTVTLVDGSTVEVMSVLSMYREHLADYDIDSVVDMTGAPRNLIEQLLDDMTTLSPVAFHVGEGVNHYF 3ir6.1    --------------------------------------------------------------------------------  target    HATLHNRATYLVGMLLGSVGVSGGGVSTWAGNYKGGVFQAAPWFGPGVGGFVNEDPFHPLTDPSARYSAETARHLVHGED 3ir6.1    --------------------------------------------------------------------------------  target    TSYWGFGDRPLVVDTPEDGRKVFTGTTHMPTPTKALWYNNANLINQAKWHYELVKNVNPKVDLIVDQQIEWTGSAEFADI 3ir6.1    -------------------------------NSSYIIAWGSNVPQTRTPDAHFFTEVRYKGTKTVAVTPDYAEIAKLCDL  target    VLPANSWMEAETWEMGASCSNPFLQVWKGGIEPLNDTRDDIAIFAGVANALTELTGDERFSQAFMFADRPEVYLDRVLAG 3ir6.1    WLAPK---------------------------------------------------------------------------  target    SFTTEGYTVEDLTAGRYGPPGGALMQYRSYPRIPFKEQIEDSLPFYTDTGRMHGYVDIPEAIEYGENLIVHREAVEATPY 3ir6.1    --------------------------------------------------------------------------------  target    LPNVIVSTSPYLRPRDYGIAPEELDGDARSVRNIMMSWAEVKETENPLFAAGYNYLCLTPKSRHAVHSSWAVTDWHWLWS 3ir6.1    --------------------------------------------------------------------------------  target    SSFSDPYRVETRAPGVGEPAIHLNPDDARSLGIRNGDYVWVDSNPKDRPYRDADVDESFLDVARLLVRVTYNPAYPPGVT 3ir6.1    --------------------------------------------------------------------------------  target    MLKHAFYMATPRTFRAAQERSDGRALAETTGYQSSFRSGSHQSITRGWAPPMHQTDSLFHKRAGVFGFTYGFDVDNHAIN 3ir6.1    --------------------------------------------------------------------------------  target    TVPKETVVRITKAEDGGVGGSGAWTRGRPGSMPGDEDDAMQAYLAGELTVVRRT 3ir6.1    ------------------------------------------------------ ``` | | | | | | | | | | | | | | | | | | | | | | | | | | | | | | | | | | | | | | | | | | | | | | | | | |
|  | 1aa6.1.A | FORMATE DEHYDROGENASE H  *REDUCED FORM OF FORMATE DEHYDROGENASE H FROM E. COLI* | 0.01 |  | 14.81 | 0.07 | 272-325 | X-ray | 2.30 | monomer | 1 x SF4, 2 x MGD, 1 x 4MO | HHblits | 0.25 |
| ``` target    RPFLERFTDMPLLVRLDTLQRLRADEVFADYSSDLDVDGPSFTLHGMTEEQHERNGDRVVFDDASGALRAINREDVGDRL 1aa6.1    --------------------------------------------------------------------------------  target    DDKGIDPALDYQGTVTLVDGSTVEVMSVLSMYREHLADYDIDSVVDMTGAPRNLIEQLLDDMTTLSPVAFHVGEGVNHYF 1aa6.1    --------------------------------------------------------------------------------  target    HATLHNRATYLVGMLLGSVGVSGGGVSTWAGNYKGGVFQAAPWFGPGVGGFVNEDPFHPLTDPSARYSAETARHLVHGED 1aa6.1    --------------------------------------------------------------------------------  target    TSYWGFGDRPLVVDTPEDGRKVFTGTTHMPTPTKALWYNNANLINQAKWHYELVKNVNPKVDLIVDQQIEWTGSAEFADI 1aa6.1    -------------------------------NTDLVFVFGYNPADSHPIVANHVINAKRNGAKIIVCDPRKIETARIADM  target    VLPANSWMEAETWEMGASCSNPFLQVWKGGIEPLNDTRDDIAIFAGVANALTELTGDERFSQAFMFADRPEVYLDRVLAG 1aa6.1    HIALK---------------------------------------------------------------------------  target    SFTTEGYTVEDLTAGRYGPPGGALMQYRSYPRIPFKEQIEDSLPFYTDTGRMHGYVDIPEAIEYGENLIVHREAVEATPY 1aa6.1    --------------------------------------------------------------------------------  target    LPNVIVSTSPYLRPRDYGIAPEELDGDARSVRNIMMSWAEVKETENPLFAAGYNYLCLTPKSRHAVHSSWAVTDWHWLWS 1aa6.1    --------------------------------------------------------------------------------  target    SSFSDPYRVETRAPGVGEPAIHLNPDDARSLGIRNGDYVWVDSNPKDRPYRDADVDESFLDVARLLVRVTYNPAYPPGVT 1aa6.1    --------------------------------------------------------------------------------  target    MLKHAFYMATPRTFRAAQERSDGRALAETTGYQSSFRSGSHQSITRGWAPPMHQTDSLFHKRAGVFGFTYGFDVDNHAIN 1aa6.1    --------------------------------------------------------------------------------  target    TVPKETVVRITKAEDGGVGGSGAWTRGRPGSMPGDEDDAMQAYLAGELTVVRRT 1aa6.1    ------------------------------------------------------ ``` | | | | | | | | | | | | | | | | | | | | | | | | | | | | | | | | | | | | | | | | | | | | | | | | | |
|  | 1fdo.1.A | FORMATE DEHYDROGENASE H  *OXIDIZED FORM OF FORMATE DEHYDROGENASE H FROM E. COLI* | 0.01 |  | 14.81 | 0.07 | 272-325 | X-ray | 2.80 | monomer | 1 x SF4, 2 x MGD, 1 x 6MO | HHblits | 0.25 |
| ``` target    RPFLERFTDMPLLVRLDTLQRLRADEVFADYSSDLDVDGPSFTLHGMTEEQHERNGDRVVFDDASGALRAINREDVGDRL 1fdo.1    --------------------------------------------------------------------------------  target    DDKGIDPALDYQGTVTLVDGSTVEVMSVLSMYREHLADYDIDSVVDMTGAPRNLIEQLLDDMTTLSPVAFHVGEGVNHYF 1fdo.1    --------------------------------------------------------------------------------  target    HATLHNRATYLVGMLLGSVGVSGGGVSTWAGNYKGGVFQAAPWFGPGVGGFVNEDPFHPLTDPSARYSAETARHLVHGED 1fdo.1    --------------------------------------------------------------------------------  target    TSYWGFGDRPLVVDTPEDGRKVFTGTTHMPTPTKALWYNNANLINQAKWHYELVKNVNPKVDLIVDQQIEWTGSAEFADI 1fdo.1    -------------------------------NTDLVFVFGYNPADSHPIVANHVINAKRNGAKIIVCDPRKIETARIADM  target    VLPANSWMEAETWEMGASCSNPFLQVWKGGIEPLNDTRDDIAIFAGVANALTELTGDERFSQAFMFADRPEVYLDRVLAG 1fdo.1    HIALK---------------------------------------------------------------------------  target    SFTTEGYTVEDLTAGRYGPPGGALMQYRSYPRIPFKEQIEDSLPFYTDTGRMHGYVDIPEAIEYGENLIVHREAVEATPY 1fdo.1    --------------------------------------------------------------------------------  target    LPNVIVSTSPYLRPRDYGIAPEELDGDARSVRNIMMSWAEVKETENPLFAAGYNYLCLTPKSRHAVHSSWAVTDWHWLWS 1fdo.1    --------------------------------------------------------------------------------  target    SSFSDPYRVETRAPGVGEPAIHLNPDDARSLGIRNGDYVWVDSNPKDRPYRDADVDESFLDVARLLVRVTYNPAYPPGVT 1fdo.1    --------------------------------------------------------------------------------  target    MLKHAFYMATPRTFRAAQERSDGRALAETTGYQSSFRSGSHQSITRGWAPPMHQTDSLFHKRAGVFGFTYGFDVDNHAIN 1fdo.1    --------------------------------------------------------------------------------  target    TVPKETVVRITKAEDGGVGGSGAWTRGRPGSMPGDEDDAMQAYLAGELTVVRRT 1fdo.1    ------------------------------------------------------ ``` | | | | | | | | | | | | | | | | | | | | | | | | | | | | | | | | | | | | | | | | | | | | | | | | | |
|  | 2iv2.1.A | Formate dehydrogenase H  *Reinterpretation of reduced form of formate dehydrogenase H from E. coli* | 0.01 |  | 14.81 | 0.07 | 272-325 | X-ray | 2.27 | monomer | 1 x SF4, 1 x 2MD, 1 x MGD | HHblits | 0.25 |
| ``` target    RPFLERFTDMPLLVRLDTLQRLRADEVFADYSSDLDVDGPSFTLHGMTEEQHERNGDRVVFDDASGALRAINREDVGDRL 2iv2.1    --------------------------------------------------------------------------------  target    DDKGIDPALDYQGTVTLVDGSTVEVMSVLSMYREHLADYDIDSVVDMTGAPRNLIEQLLDDMTTLSPVAFHVGEGVNHYF 2iv2.1    --------------------------------------------------------------------------------  target    HATLHNRATYLVGMLLGSVGVSGGGVSTWAGNYKGGVFQAAPWFGPGVGGFVNEDPFHPLTDPSARYSAETARHLVHGED 2iv2.1    --------------------------------------------------------------------------------  target    TSYWGFGDRPLVVDTPEDGRKVFTGTTHMPTPTKALWYNNANLINQAKWHYELVKNVNPKVDLIVDQQIEWTGSAEFADI 2iv2.1    -------------------------------NTDLVFVFGYNPADSHPIVANHVINAKRNGAKIIVCDPRKIETARIADM  target    VLPANSWMEAETWEMGASCSNPFLQVWKGGIEPLNDTRDDIAIFAGVANALTELTGDERFSQAFMFADRPEVYLDRVLAG 2iv2.1    HIALK---------------------------------------------------------------------------  target    SFTTEGYTVEDLTAGRYGPPGGALMQYRSYPRIPFKEQIEDSLPFYTDTGRMHGYVDIPEAIEYGENLIVHREAVEATPY 2iv2.1    --------------------------------------------------------------------------------  target    LPNVIVSTSPYLRPRDYGIAPEELDGDARSVRNIMMSWAEVKETENPLFAAGYNYLCLTPKSRHAVHSSWAVTDWHWLWS 2iv2.1    --------------------------------------------------------------------------------  target    SSFSDPYRVETRAPGVGEPAIHLNPDDARSLGIRNGDYVWVDSNPKDRPYRDADVDESFLDVARLLVRVTYNPAYPPGVT 2iv2.1    --------------------------------------------------------------------------------  target    MLKHAFYMATPRTFRAAQERSDGRALAETTGYQSSFRSGSHQSITRGWAPPMHQTDSLFHKRAGVFGFTYGFDVDNHAIN 2iv2.1    --------------------------------------------------------------------------------  target    TVPKETVVRITKAEDGGVGGSGAWTRGRPGSMPGDEDDAMQAYLAGELTVVRRT 2iv2.1    ------------------------------------------------------ ``` | | | | | | | | | | | | | | | | | | | | | | | | | | | | | | | | | | | | | | | | | | | | | | | | | |
|  | 7z0t.1.G | Formate dehydrogenase H  *Structure of the Escherichia coli formate hydrogenlyase complex (aerobic preparation, composite structure)* | 0.01 |  | 14.81 | 0.07 | 272-325 | EM | 0.00 | hetero-1-1-1-1-1-1-… | 1 x NI, 1 x FCO, 8 x SF4, 1 x FE, 2 x MGD, 1 x 6MO | HHblits | 0.25 |
| ``` target    RPFLERFTDMPLLVRLDTLQRLRADEVFADYSSDLDVDGPSFTLHGMTEEQHERNGDRVVFDDASGALRAINREDVGDRL 7z0t.1    --------------------------------------------------------------------------------  target    DDKGIDPALDYQGTVTLVDGSTVEVMSVLSMYREHLADYDIDSVVDMTGAPRNLIEQLLDDMTTLSPVAFHVGEGVNHYF 7z0t.1    --------------------------------------------------------------------------------  target    HATLHNRATYLVGMLLGSVGVSGGGVSTWAGNYKGGVFQAAPWFGPGVGGFVNEDPFHPLTDPSARYSAETARHLVHGED 7z0t.1    --------------------------------------------------------------------------------  target    TSYWGFGDRPLVVDTPEDGRKVFTGTTHMPTPTKALWYNNANLINQAKWHYELVKNVNPKVDLIVDQQIEWTGSAEFADI 7z0t.1    -------------------------------NTDLVFVFGYNPADSHPIVANHVINAKRNGAKIIVCDPRKIETARIADM  target    VLPANSWMEAETWEMGASCSNPFLQVWKGGIEPLNDTRDDIAIFAGVANALTELTGDERFSQAFMFADRPEVYLDRVLAG 7z0t.1    HIALK---------------------------------------------------------------------------  target    SFTTEGYTVEDLTAGRYGPPGGALMQYRSYPRIPFKEQIEDSLPFYTDTGRMHGYVDIPEAIEYGENLIVHREAVEATPY 7z0t.1    --------------------------------------------------------------------------------  target    LPNVIVSTSPYLRPRDYGIAPEELDGDARSVRNIMMSWAEVKETENPLFAAGYNYLCLTPKSRHAVHSSWAVTDWHWLWS 7z0t.1    --------------------------------------------------------------------------------  target    SSFSDPYRVETRAPGVGEPAIHLNPDDARSLGIRNGDYVWVDSNPKDRPYRDADVDESFLDVARLLVRVTYNPAYPPGVT 7z0t.1    --------------------------------------------------------------------------------  target    MLKHAFYMATPRTFRAAQERSDGRALAETTGYQSSFRSGSHQSITRGWAPPMHQTDSLFHKRAGVFGFTYGFDVDNHAIN 7z0t.1    --------------------------------------------------------------------------------  target    TVPKETVVRITKAEDGGVGGSGAWTRGRPGSMPGDEDDAMQAYLAGELTVVRRT 7z0t.1    ------------------------------------------------------ ``` | | | | | | | | | | | | | | | | | | | | | | | | | | | | | | | | | | | | | | | | | | | | | | | | | |
|  | 2v3v.1.A | PERIPLASMIC NITRATE REDUCTASE  *A NEW CATALYTIC MECHANISM OF PERIPLASMIC NITRATE REDUCTASE FROM DESULFOVIBRIO DESULFURICANS ATCC 27774 FROM CRYSTALLOGRAPHIC AND EPR DATA AND BASED ON DETAILED ANALYSIS OF THE SIXTH LIGAND* | 0.01 |  | 9.26 | 0.07 | 272-325 | X-ray | 1.99 | monomer | 1 x SF4, 1 x MO, 2 x MGD, 4 x LCP | HHblits | 0.25 |
| ``` target    RPFLERFTDMPLLVRLDTLQRLRADEVFADYSSDLDVDGPSFTLHGMTEEQHERNGDRVVFDDASGALRAINREDVGDRL 2v3v.1    --------------------------------------------------------------------------------  target    DDKGIDPALDYQGTVTLVDGSTVEVMSVLSMYREHLADYDIDSVVDMTGAPRNLIEQLLDDMTTLSPVAFHVGEGVNHYF 2v3v.1    --------------------------------------------------------------------------------  target    HATLHNRATYLVGMLLGSVGVSGGGVSTWAGNYKGGVFQAAPWFGPGVGGFVNEDPFHPLTDPSARYSAETARHLVHGED 2v3v.1    --------------------------------------------------------------------------------  target    TSYWGFGDRPLVVDTPEDGRKVFTGTTHMPTPTKALWYNNANLINQAKWHYELVKNVN--PKVDLIVDQQIEWTGSAEFA 2v3v.1    -------------------------------QATCFFIIGSNTSEAHPVLFRRIARRKQVEPGVKIIVADPRRTNTSRIA  target    DIVLPANSWMEAETWEMGASCSNPFLQVWKGGIEPLNDTRDDIAIFAGVANALTELTGDERFSQAFMFADRPEVYLDRVL 2v3v.1    DMHVAFR-------------------------------------------------------------------------  target    AGSFTTEGYTVEDLTAGRYGPPGGALMQYRSYPRIPFKEQIEDSLPFYTDTGRMHGYVDIPEAIEYGENLIVHREAVEAT 2v3v.1    --------------------------------------------------------------------------------  target    PYLPNVIVSTSPYLRPRDYGIAPEELDGDARSVRNIMMSWAEVKETENPLFAAGYNYLCLTPKSRHAVHSSWAVTDWHWL 2v3v.1    --------------------------------------------------------------------------------  target    WSSSFSDPYRVETRAPGVGEPAIHLNPDDARSLGIRNGDYVWVDSNPKDRPYRDADVDESFLDVARLLVRVTYNPAYPPG 2v3v.1    --------------------------------------------------------------------------------  target    VTMLKHAFYMATPRTFRAAQERSDGRALAETTGYQSSFRSGSHQSITRGWAPPMHQTDSLFHKRAGVFGFTYGFDVDNHA 2v3v.1    --------------------------------------------------------------------------------  target    INTVPKETVVRITKAEDGGVGGSGAWTRGRPGSMPGDEDDAMQAYLAGELTVVRRT 2v3v.1    -------------------------------------------------------- ``` | | | | | | | | | | | | | | | | | | | | | | | | | | | | | | | | | | | | | | | | | | | | | | | | | |
|  | 2vpz.1.A | THIOSULFATE REDUCTASE  *POLYSULFIDE REDUCTASE NATIVE STRUCTURE* | 0.01 |  | 9.09 | 0.07 | 272-326 | X-ray | 2.40 | hetero-oligomer | 10 x SF4, 4 x MGD, 2 x MO | HHblits | 0.23 |
| ``` target    RPFLERFTDMPLLVRLDTLQRLRADEVFADYSSDLDVDGPSFTLHGMTEEQHERNGDRVVFDDASGALRAINREDVGDRL 2vpz.1    --------------------------------------------------------------------------------  target    DDKGIDPALDYQGTVTLVDGSTVEVMSVLSMYREHLADYDIDSVVDMTGAPRNLIEQLLDDMTTLSPVAFHVGEGVNHYF 2vpz.1    --------------------------------------------------------------------------------  target    HATLHNRATYLVGMLLGSVGVSGGGVSTWAGNYKGGVFQAAPWFGPGVGGFVNEDPFHPLTDPSARYSAETARHLVHGED 2vpz.1    --------------------------------------------------------------------------------  target    TSYWGFGDRPLVVDTPEDGRKVFTGTTHMPTPTKALWYNNANLINQ-AKWHYELVKNVNPKVDLIVDQQIEWTGSAEFAD 2vpz.1    -------------------------------NARYIVLIGHHIGEDTHNTQLQDFALALKNGAKVVVVDPRFSTAAAKAH  target    IVLPANSWMEAETWEMGASCSNPFLQVWKGGIEPLNDTRDDIAIFAGVANALTELTGDERFSQAFMFADRPEVYLDRVLA 2vpz.1    RWLPIKP-------------------------------------------------------------------------  target    GSFTTEGYTVEDLTAGRYGPPGGALMQYRSYPRIPFKEQIEDSLPFYTDTGRMHGYVDIPEAIEYGENLIVHREAVEATP 2vpz.1    --------------------------------------------------------------------------------  target    YLPNVIVSTSPYLRPRDYGIAPEELDGDARSVRNIMMSWAEVKETENPLFAAGYNYLCLTPKSRHAVHSSWAVTDWHWLW 2vpz.1    --------------------------------------------------------------------------------  target    SSSFSDPYRVETRAPGVGEPAIHLNPDDARSLGIRNGDYVWVDSNPKDRPYRDADVDESFLDVARLLVRVTYNPAYPPGV 2vpz.1    --------------------------------------------------------------------------------  target    TMLKHAFYMATPRTFRAAQERSDGRALAETTGYQSSFRSGSHQSITRGWAPPMHQTDSLFHKRAGVFGFTYGFDVDNHAI 2vpz.1    --------------------------------------------------------------------------------  target    NTVPKETVVRITKAEDGGVGGSGAWTRGRPGSMPGDEDDAMQAYLAGELTVVRRT 2vpz.1    ------------------------------------------------------- ``` | | | | | | | | | | | | | | | | | | | | | | | | | | | | | | | | | | | | | | | | | | | | | | | | | |
|  | 2vpx.1.D | THIOSULFATE REDUCTASE  *POLYSULFIDE REDUCTASE WITH BOUND QUINONE (UQ1)* | 0.01 |  | 9.09 | 0.07 | 272-326 | X-ray | 3.10 | hetero-oligomer | 10 x SF4, 4 x MGD, 2 x MO, 2 x UQ1 | HHblits | 0.23 |
| ``` target    RPFLERFTDMPLLVRLDTLQRLRADEVFADYSSDLDVDGPSFTLHGMTEEQHERNGDRVVFDDASGALRAINREDVGDRL 2vpx.1    --------------------------------------------------------------------------------  target    DDKGIDPALDYQGTVTLVDGSTVEVMSVLSMYREHLADYDIDSVVDMTGAPRNLIEQLLDDMTTLSPVAFHVGEGVNHYF 2vpx.1    --------------------------------------------------------------------------------  target    HATLHNRATYLVGMLLGSVGVSGGGVSTWAGNYKGGVFQAAPWFGPGVGGFVNEDPFHPLTDPSARYSAETARHLVHGED 2vpx.1    --------------------------------------------------------------------------------  target    TSYWGFGDRPLVVDTPEDGRKVFTGTTHMPTPTKALWYNNANLINQ-AKWHYELVKNVNPKVDLIVDQQIEWTGSAEFAD 2vpx.1    -------------------------------NARYIVLIGHHIGEDTHNTQLQDFALALKNGAKVVVVDPRFSTAAAKAH  target    IVLPANSWMEAETWEMGASCSNPFLQVWKGGIEPLNDTRDDIAIFAGVANALTELTGDERFSQAFMFADRPEVYLDRVLA 2vpx.1    RWLPIKP-------------------------------------------------------------------------  target    GSFTTEGYTVEDLTAGRYGPPGGALMQYRSYPRIPFKEQIEDSLPFYTDTGRMHGYVDIPEAIEYGENLIVHREAVEATP 2vpx.1    --------------------------------------------------------------------------------  target    YLPNVIVSTSPYLRPRDYGIAPEELDGDARSVRNIMMSWAEVKETENPLFAAGYNYLCLTPKSRHAVHSSWAVTDWHWLW 2vpx.1    --------------------------------------------------------------------------------  target    SSSFSDPYRVETRAPGVGEPAIHLNPDDARSLGIRNGDYVWVDSNPKDRPYRDADVDESFLDVARLLVRVTYNPAYPPGV 2vpx.1    --------------------------------------------------------------------------------  target    TMLKHAFYMATPRTFRAAQERSDGRALAETTGYQSSFRSGSHQSITRGWAPPMHQTDSLFHKRAGVFGFTYGFDVDNHAI 2vpx.1    --------------------------------------------------------------------------------  target    NTVPKETVVRITKAEDGGVGGSGAWTRGRPGSMPGDEDDAMQAYLAGELTVVRRT 2vpx.1    ------------------------------------------------------- ``` | | | | | | | | | | | | | | | | | | | | | | | | | | | | | | | | | | | | | | | | | | | | | | | | | |
|  | 5x4l.1.A | Transitional endoplasmic reticulum ATPase  *Crystal structure of the UBX domain of human UBXD7 in complex with p97 N domain* | 0.01 | 0.00 | 11.32 | 0.07 | 579-647 | X-ray | 2.40 | monomer |  | HHblits | 0.26 |
| ``` target    RPFLERFTDMPLLVRLDTLQRLRADEVFADYSSDLDVDGPSFTLHGMTEEQHERNGDRVVFDDASGALRAINREDVGDRL 5x4l.1    --------------------------------------------------------------------------------  target    DDKGIDPALDYQGTVTLVDGSTVEVMSVLSMYREHLADYDIDSVVDMTGAPRNLIEQLLDDMTTLSPVAFHVGEGVNHYF 5x4l.1    --------------------------------------------------------------------------------  target    HATLHNRATYLVGMLLGSVGVSGGGVSTWAGNYKGGVFQAAPWFGPGVGGFVNEDPFHPLTDPSARYSAETARHLVHGED 5x4l.1    --------------------------------------------------------------------------------  target    TSYWGFGDRPLVVDTPEDGRKVFTGTTHMPTPTKALWYNNANLINQAKWHYELVKNVNPKVDLIVDQQIEWTGSAEFADI 5x4l.1    --------------------------------------------------------------------------------  target    VLPANSWMEAETWEMGASCSNPFLQVWKGGIEPLNDTRDDIAIFAGVANALTELTGDERFSQAFMFADRPEVYLDRVLAG 5x4l.1    --------------------------------------------------------------------------------  target    SFTTEGYTVEDLTAGRYGPPGGALMQYRSYPRIPFKEQIEDSLPFYTDTGRMHGYVDIPEAIEYGENLIVHREAVEATPY 5x4l.1    --------------------------------------------------------------------------------  target    LPNVIVSTSPYLRPRDYGIAPEELDGDARSVRNIMMSWAEVKETENPLFAAGYNYLCLTPKSRHAVHSSWAVTDWHWLWS 5x4l.1    --------------------------------------------------------------------------------  target    SSFSDPYRVETRAPGVGEPAIHLNPDDARSLGIRNGDYVWVDSNPKDRPYRDADVDESFLDVARLLVRVTYNPAYPPGVT 5x4l.1    ------------------SVVSLSQPKMDELQLFRGDTVLLKGKK----------------RREAVCIVLSDDTCSDEKI  target    MLKHAFYMATPRTFRAAQERSDGRALAETTGYQSSFRSGSHQSITRGWAPPMHQTDSLFHKRAGVFGFTYGFDVDNHAIN 5x4l.1    RMNRVVR-------------------------------------------------------------------------  target    TVPKETVVRITKAEDGGVGGSGAWTRGRPGSMPGDEDDAMQAYLAGELTVVRRT 5x4l.1    ------------------------------------------------------ ``` | | | | | | | | | | | | | | | | | | | | | | | | | | | | | | | | | | | | | | | | | | | | | | | | | |
|  | 5x4l.2.A | Transitional endoplasmic reticulum ATPase  *Crystal structure of the UBX domain of human UBXD7 in complex with p97 N domain* | 0.01 | 0.00 | 11.32 | 0.07 | 579-647 | X-ray | 2.40 | monomer |  | HHblits | 0.26 |
| ``` target    RPFLERFTDMPLLVRLDTLQRLRADEVFADYSSDLDVDGPSFTLHGMTEEQHERNGDRVVFDDASGALRAINREDVGDRL 5x4l.2    --------------------------------------------------------------------------------  target    DDKGIDPALDYQGTVTLVDGSTVEVMSVLSMYREHLADYDIDSVVDMTGAPRNLIEQLLDDMTTLSPVAFHVGEGVNHYF 5x4l.2    --------------------------------------------------------------------------------  target    HATLHNRATYLVGMLLGSVGVSGGGVSTWAGNYKGGVFQAAPWFGPGVGGFVNEDPFHPLTDPSARYSAETARHLVHGED 5x4l.2    --------------------------------------------------------------------------------  target    TSYWGFGDRPLVVDTPEDGRKVFTGTTHMPTPTKALWYNNANLINQAKWHYELVKNVNPKVDLIVDQQIEWTGSAEFADI 5x4l.2    --------------------------------------------------------------------------------  target    VLPANSWMEAETWEMGASCSNPFLQVWKGGIEPLNDTRDDIAIFAGVANALTELTGDERFSQAFMFADRPEVYLDRVLAG 5x4l.2    --------------------------------------------------------------------------------  target    SFTTEGYTVEDLTAGRYGPPGGALMQYRSYPRIPFKEQIEDSLPFYTDTGRMHGYVDIPEAIEYGENLIVHREAVEATPY 5x4l.2    --------------------------------------------------------------------------------  target    LPNVIVSTSPYLRPRDYGIAPEELDGDARSVRNIMMSWAEVKETENPLFAAGYNYLCLTPKSRHAVHSSWAVTDWHWLWS 5x4l.2    --------------------------------------------------------------------------------  target    SSFSDPYRVETRAPGVGEPAIHLNPDDARSLGIRNGDYVWVDSNPKDRPYRDADVDESFLDVARLLVRVTYNPAYPPGVT 5x4l.2    ------------------SVVSLSQPKMDELQLFRGDTVLLKGKK----------------RREAVCIVLSDDTCSDEKI  target    MLKHAFYMATPRTFRAAQERSDGRALAETTGYQSSFRSGSHQSITRGWAPPMHQTDSLFHKRAGVFGFTYGFDVDNHAIN 5x4l.2    RMNRVVR-------------------------------------------------------------------------  target    TVPKETVVRITKAEDGGVGGSGAWTRGRPGSMPGDEDDAMQAYLAGELTVVRRT 5x4l.2    ------------------------------------------------------ ``` | | | | | | | | | | | | | | | | | | | | | | | | | | | | | | | | | | | | | | | | | | | | | | | | | |
|  | 4ydd.1.A | DMSO reductase family type II enzyme, molybdopterin subunit  *Crystal structure of the perchlorate reductase PcrAB from Azospira suillum PS* | 0.01 |  | 11.11 | 0.07 | 272-325 | X-ray | 1.86 | hetero-oligomer | 4 x SF4, 1 x MO, 1 x MGD, 1 x MD1, 1 x F3S | HHblits | 0.24 |
| ``` target    RPFLERFTDMPLLVRLDTLQRLRADEVFADYSSDLDVDGPSFTLHGMTEEQHERNGDRVVFDDASGALRAINREDVGDRL 4ydd.1    --------------------------------------------------------------------------------  target    DDKGIDPALDYQGTVTLVDGSTVEVMSVLSMYREHLADYDIDSVVDMTGAPRNLIEQLLDDMTTLSPVAFHVGEGVNHYF 4ydd.1    --------------------------------------------------------------------------------  target    HATLHNRATYLVGMLLGSVGVSGGGVSTWAGNYKGGVFQAAPWFGPGVGGFVNEDPFHPLTDPSARYSAETARHLVHGED 4ydd.1    --------------------------------------------------------------------------------  target    TSYWGFGDRPLVVDTPEDGRKVFTGTTHMPTPTKALWYNNANLINQAKWHYELVKNVNPKVDLIVDQQIEWTGSAEFADI 4ydd.1    -------------------------------NSKYIILWGSNPTQTRIPDAHFLSEAQLNGAKIVSISPDYNSSTIKVDK  target    VLPANSWMEAETWEMGASCSNPFLQVWKGGIEPLNDTRDDIAIFAGVANALTELTGDERFSQAFMFADRPEVYLDRVLAG 4ydd.1    WIHPQ---------------------------------------------------------------------------  target    SFTTEGYTVEDLTAGRYGPPGGALMQYRSYPRIPFKEQIEDSLPFYTDTGRMHGYVDIPEAIEYGENLIVHREAVEATPY 4ydd.1    --------------------------------------------------------------------------------  target    LPNVIVSTSPYLRPRDYGIAPEELDGDARSVRNIMMSWAEVKETENPLFAAGYNYLCLTPKSRHAVHSSWAVTDWHWLWS 4ydd.1    --------------------------------------------------------------------------------  target    SSFSDPYRVETRAPGVGEPAIHLNPDDARSLGIRNGDYVWVDSNPKDRPYRDADVDESFLDVARLLVRVTYNPAYPPGVT 4ydd.1    --------------------------------------------------------------------------------  target    MLKHAFYMATPRTFRAAQERSDGRALAETTGYQSSFRSGSHQSITRGWAPPMHQTDSLFHKRAGVFGFTYGFDVDNHAIN 4ydd.1    --------------------------------------------------------------------------------  target    TVPKETVVRITKAEDGGVGGSGAWTRGRPGSMPGDEDDAMQAYLAGELTVVRRT 4ydd.1    ------------------------------------------------------ ``` | | | | | | | | | | | | | | | | | | | | | | | | | | | | | | | | | | | | | | | | | | | | | | | | | |
|  | 3ir7.1.A | Respiratory nitrate reductase 1 alpha chain  *Crystal structure of NarGHI mutant NarG-R94S* | 0.01 | 0.00 | 13.21 | 0.07 | 272-324 | X-ray | 2.50 | monomer | 2 x MD1, 4 x SF4, 1 x 6MO, 1 x AGA, 1 x F3S, 2 x HEM | HHblits | 0.25 |
| ``` target    RPFLERFTDMPLLVRLDTLQRLRADEVFADYSSDLDVDGPSFTLHGMTEEQHERNGDRVVFDDASGALRAINREDVGDRL 3ir7.1    --------------------------------------------------------------------------------  target    DDKGIDPALDYQGTVTLVDGSTVEVMSVLSMYREHLADYDIDSVVDMTGAPRNLIEQLLDDMTTLSPVAFHVGEGVNHYF 3ir7.1    --------------------------------------------------------------------------------  target    HATLHNRATYLVGMLLGSVGVSGGGVSTWAGNYKGGVFQAAPWFGPGVGGFVNEDPFHPLTDPSARYSAETARHLVHGED 3ir7.1    --------------------------------------------------------------------------------  target    TSYWGFGDRPLVVDTPEDGRKVFTGTTHMPTPTKALWYNNANLINQAKWHYELVKNVNPKVDLIVDQQIEWTGSAEFADI 3ir7.1    -------------------------------NSSYIIAWGSNVPQTRTPDAHFFTEVRYKGTKTVAVTPDYAEIAKLCDL  target    VLPANSWMEAETWEMGASCSNPFLQVWKGGIEPLNDTRDDIAIFAGVANALTELTGDERFSQAFMFADRPEVYLDRVLAG 3ir7.1    WLAP----------------------------------------------------------------------------  target    SFTTEGYTVEDLTAGRYGPPGGALMQYRSYPRIPFKEQIEDSLPFYTDTGRMHGYVDIPEAIEYGENLIVHREAVEATPY 3ir7.1    --------------------------------------------------------------------------------  target    LPNVIVSTSPYLRPRDYGIAPEELDGDARSVRNIMMSWAEVKETENPLFAAGYNYLCLTPKSRHAVHSSWAVTDWHWLWS 3ir7.1    --------------------------------------------------------------------------------  target    SSFSDPYRVETRAPGVGEPAIHLNPDDARSLGIRNGDYVWVDSNPKDRPYRDADVDESFLDVARLLVRVTYNPAYPPGVT 3ir7.1    --------------------------------------------------------------------------------  target    MLKHAFYMATPRTFRAAQERSDGRALAETTGYQSSFRSGSHQSITRGWAPPMHQTDSLFHKRAGVFGFTYGFDVDNHAIN 3ir7.1    --------------------------------------------------------------------------------  target    TVPKETVVRITKAEDGGVGGSGAWTRGRPGSMPGDEDDAMQAYLAGELTVVRRT 3ir7.1    ------------------------------------------------------ ``` | | | | | | | | | | | | | | | | | | | | | | | | | | | | | | | | | | | | | | | | | | | | | | | | | |
|  | 7e5z.1.A | Formate dehydrogenase  *Dehydrogenase holoenzyme* | 0.00 |  | 11.11 | 0.07 | 272-325 | EM | 0.00 | hetero-1-1-mer | 1 x W, 2 x MGD, 2 x FES, 4 x SF4, 1 x FMN | HHblits | 0.24 |
| ``` target    RPFLERFTDMPLLVRLDTLQRLRADEVFADYSSDLDVDGPSFTLHGMTEEQHERNGDRVVFDDASGALRAINREDVGDRL 7e5z.1    --------------------------------------------------------------------------------  target    DDKGIDPALDYQGTVTLVDGSTVEVMSVLSMYREHLADYDIDSVVDMTGAPRNLIEQLLDDMTTLSPVAFHVGEGVNHYF 7e5z.1    --------------------------------------------------------------------------------  target    HATLHNRATYLVGMLLGSVGVSGGGVSTWAGNYKGGVFQAAPWFGPGVGGFVNEDPFHPLTDPSARYSAETARHLVHGED 7e5z.1    --------------------------------------------------------------------------------  target    TSYWGFGDRPLVVDTPEDGRKVFTGTTHMPTPTKALWYNNANLINQAKWHYELVKNVNP-KVDLIVDQQIEWTGSAEFAD 7e5z.1    -------------------------------DAEVIVVIGANPTVNHPVAATFLKNAVKQRGAKLIIMDPRRQTLSRHAY  target    IVLPANSWMEAETWEMGASCSNPFLQVWKGGIEPLNDTRDDIAIFAGVANALTELTGDERFSQAFMFADRPEVYLDRVLA 7e5z.1    RHLAFR--------------------------------------------------------------------------  target    GSFTTEGYTVEDLTAGRYGPPGGALMQYRSYPRIPFKEQIEDSLPFYTDTGRMHGYVDIPEAIEYGENLIVHREAVEATP 7e5z.1    --------------------------------------------------------------------------------  target    YLPNVIVSTSPYLRPRDYGIAPEELDGDARSVRNIMMSWAEVKETENPLFAAGYNYLCLTPKSRHAVHSSWAVTDWHWLW 7e5z.1    --------------------------------------------------------------------------------  target    SSSFSDPYRVETRAPGVGEPAIHLNPDDARSLGIRNGDYVWVDSNPKDRPYRDADVDESFLDVARLLVRVTYNPAYPPGV 7e5z.1    --------------------------------------------------------------------------------  target    TMLKHAFYMATPRTFRAAQERSDGRALAETTGYQSSFRSGSHQSITRGWAPPMHQTDSLFHKRAGVFGFTYGFDVDNHAI 7e5z.1    --------------------------------------------------------------------------------  target    NTVPKETVVRITKAEDGGVGGSGAWTRGRPGSMPGDEDDAMQAYLAGELTVVRRT 7e5z.1    ------------------------------------------------------- ``` | | | | | | | | | | | | | | | | | | | | | | | | | | | | | | | | | | | | | | | | | | | | | | | | | |
|  | 7vw6.1.A | Formate dehydrogenase  *Cryo-EM Structure of Formate Dehydrogenase 1 from Methylorubrum extorquens AM1* | 0.01 |  | 11.11 | 0.07 | 272-325 | EM | 0.00 | hetero-1-1-mer | 4 x SF4, 2 x FES, 2 x MGD, 1 x W, 1 x FMN | HHblits | 0.24 |
| ``` target    RPFLERFTDMPLLVRLDTLQRLRADEVFADYSSDLDVDGPSFTLHGMTEEQHERNGDRVVFDDASGALRAINREDVGDRL 7vw6.1    --------------------------------------------------------------------------------  target    DDKGIDPALDYQGTVTLVDGSTVEVMSVLSMYREHLADYDIDSVVDMTGAPRNLIEQLLDDMTTLSPVAFHVGEGVNHYF 7vw6.1    --------------------------------------------------------------------------------  target    HATLHNRATYLVGMLLGSVGVSGGGVSTWAGNYKGGVFQAAPWFGPGVGGFVNEDPFHPLTDPSARYSAETARHLVHGED 7vw6.1    --------------------------------------------------------------------------------  target    TSYWGFGDRPLVVDTPEDGRKVFTGTTHMPTPTKALWYNNANLINQAKWHYELVKNVNP-KVDLIVDQQIEWTGSAEFAD 7vw6.1    -------------------------------DAEVIVVIGANPTVNHPVAATFLKNAVKQRGAKLIIMDPRRQTLSRHAY  target    IVLPANSWMEAETWEMGASCSNPFLQVWKGGIEPLNDTRDDIAIFAGVANALTELTGDERFSQAFMFADRPEVYLDRVLA 7vw6.1    RHLAFR--------------------------------------------------------------------------  target    GSFTTEGYTVEDLTAGRYGPPGGALMQYRSYPRIPFKEQIEDSLPFYTDTGRMHGYVDIPEAIEYGENLIVHREAVEATP 7vw6.1    --------------------------------------------------------------------------------  target    YLPNVIVSTSPYLRPRDYGIAPEELDGDARSVRNIMMSWAEVKETENPLFAAGYNYLCLTPKSRHAVHSSWAVTDWHWLW 7vw6.1    --------------------------------------------------------------------------------  target    SSSFSDPYRVETRAPGVGEPAIHLNPDDARSLGIRNGDYVWVDSNPKDRPYRDADVDESFLDVARLLVRVTYNPAYPPGV 7vw6.1    --------------------------------------------------------------------------------  target    TMLKHAFYMATPRTFRAAQERSDGRALAETTGYQSSFRSGSHQSITRGWAPPMHQTDSLFHKRAGVFGFTYGFDVDNHAI 7vw6.1    --------------------------------------------------------------------------------  target    NTVPKETVVRITKAEDGGVGGSGAWTRGRPGSMPGDEDDAMQAYLAGELTVVRRT 7vw6.1    ------------------------------------------------------- ``` | | | | | | | | | | | | | | | | | | | | | | | | | | | | | | | | | | | | | | | | | | | | | | | | | |
|  | 1tmo.1.A | TRIMETHYLAMINE N-OXIDE REDUCTASE  *TRIMETHYLAMINE N-OXIDE REDUCTASE FROM SHEWANELLA MASSILIA* | 0.00 |  | 7.41 | 0.07 | 272-325 | X-ray | 2.50 | monomer | 2 x 2MD, 1 x 2MO | HHblits | 0.24 |
| ``` target    RPFLERFTDMPLLVRLDTLQRLRADEVFADYSSDLDVDGPSFTLHGMTEEQHERNGDRVVFDDASGALRAINREDVGDRL 1tmo.1    --------------------------------------------------------------------------------  target    DDKGIDPALDYQGTVTLVDGSTVEVMSVLSMYREHLADYDIDSVVDMTGAPRNLIEQLLDDMTTLSPVAFHVGEGVNHYF 1tmo.1    --------------------------------------------------------------------------------  target    HATLHNRATYLVGMLLGSVGVSGGGVSTWAGNYKGGVFQAAPWFGPGVGGFVNEDPFHPLTDPSARYSAETARHLVHGED 1tmo.1    --------------------------------------------------------------------------------  target    TSYWGFGDRPLVVDTPEDGRKVFTGTTHMPTPTKALWYNNANLINQAK-----------WHYELVKNVNPK-VDLIVDQQ 1tmo.1    -------------------------------HSDTIVLWSNDPYKNLQVGWNAETHESFAYLAQLKEKVKQGKIRVISID  target    IEWTGSAEF-ADIVLPANSWMEAETWEMGASCSNPFLQVWKGGIEPLNDTRDDIAIFAGVANALTELTGDERFSQAFMFA 1tmo.1    PVVTKTQAYLGCEQLYVN--------------------------------------------------------------  target    DRPEVYLDRVLAGSFTTEGYTVEDLTAGRYGPPGGALMQYRSYPRIPFKEQIEDSLPFYTDTGRMHGYVDIPEAIEYGEN 1tmo.1    --------------------------------------------------------------------------------  target    LIVHREAVEATPYLPNVIVSTSPYLRPRDYGIAPEELDGDARSVRNIMMSWAEVKETENPLFAAGYNYLCLTPKSRHAVH 1tmo.1    --------------------------------------------------------------------------------  target    SSWAVTDWHWLWSSSFSDPYRVETRAPGVGEPAIHLNPDDARSLGIRNGDYVWVDSNPKDRPYRDADVDESFLDVARLLV 1tmo.1    --------------------------------------------------------------------------------  target    RVTYNPAYPPGVTMLKHAFYMATPRTFRAAQERSDGRALAETTGYQSSFRSGSHQSITRGWAPPMHQTDSLFHKRAGVFG 1tmo.1    --------------------------------------------------------------------------------  target    FTYGFDVDNHAINTVPKETVVRITKAEDGGVGGSGAWTRGRPGSMPGDEDDAMQAYLAGELTVVRRT 1tmo.1    ------------------------------------------------------------------- ``` | | | | | | | | | | | | | | | | | | | | | | | | | | | | | | | | | | | | | | | | | | | | | | | | | |
|  | 2pjh.1.B | Transitional endoplasmic reticulum ATPase  *Strctural Model of the p97 N domain- npl4 UBD complex* | 0.02 | 0.00 | 11.54 | 0.07 | 578-645 | NMR | 0.00 | monomer |  | HHblits | 0.26 |
| ``` target    RPFLERFTDMPLLVRLDTLQRLRADEVFADYSSDLDVDGPSFTLHGMTEEQHERNGDRVVFDDASGALRAINREDVGDRL 2pjh.1    --------------------------------------------------------------------------------  target    DDKGIDPALDYQGTVTLVDGSTVEVMSVLSMYREHLADYDIDSVVDMTGAPRNLIEQLLDDMTTLSPVAFHVGEGVNHYF 2pjh.1    --------------------------------------------------------------------------------  target    HATLHNRATYLVGMLLGSVGVSGGGVSTWAGNYKGGVFQAAPWFGPGVGGFVNEDPFHPLTDPSARYSAETARHLVHGED 2pjh.1    --------------------------------------------------------------------------------  target    TSYWGFGDRPLVVDTPEDGRKVFTGTTHMPTPTKALWYNNANLINQAKWHYELVKNVNPKVDLIVDQQIEWTGSAEFADI 2pjh.1    --------------------------------------------------------------------------------  target    VLPANSWMEAETWEMGASCSNPFLQVWKGGIEPLNDTRDDIAIFAGVANALTELTGDERFSQAFMFADRPEVYLDRVLAG 2pjh.1    --------------------------------------------------------------------------------  target    SFTTEGYTVEDLTAGRYGPPGGALMQYRSYPRIPFKEQIEDSLPFYTDTGRMHGYVDIPEAIEYGENLIVHREAVEATPY 2pjh.1    --------------------------------------------------------------------------------  target    LPNVIVSTSPYLRPRDYGIAPEELDGDARSVRNIMMSWAEVKETENPLFAAGYNYLCLTPKSRHAVHSSWAVTDWHWLWS 2pjh.1    --------------------------------------------------------------------------------  target    SSFSDPYRVETRAPGVGEPAIHLNPDDARSLGIRNGDYVWVDSNPKDRPYRDADVDESFLDVARLLVRVTYNPAYPPGVT 2pjh.1    -----------------NSVVSLSQPKMDELQLFRGDTVLLKGKK----------------RREAVCIVLSDDTCSDEKI  target    MLKHAFYMATPRTFRAAQERSDGRALAETTGYQSSFRSGSHQSITRGWAPPMHQTDSLFHKRAGVFGFTYGFDVDNHAIN 2pjh.1    RMNRV---------------------------------------------------------------------------  target    TVPKETVVRITKAEDGGVGGSGAWTRGRPGSMPGDEDDAMQAYLAGELTVVRRT 2pjh.1    ------------------------------------------------------ ``` | | | | | | | | | | | | | | | | | | | | | | | | | | | | | | | | | | | | | | | | | | | | | | | | | |
|  | 5b6c.1.A | Transitional endoplasmic reticulum ATPase  *Structural Details of Ufd1 binding to p97* | 0.01 | 0.00 | 11.54 | 0.07 | 580-647 | X-ray | 1.55 | monomer |  | HHblits | 0.26 |
| ``` target    RPFLERFTDMPLLVRLDTLQRLRADEVFADYSSDLDVDGPSFTLHGMTEEQHERNGDRVVFDDASGALRAINREDVGDRL 5b6c.1    --------------------------------------------------------------------------------  target    DDKGIDPALDYQGTVTLVDGSTVEVMSVLSMYREHLADYDIDSVVDMTGAPRNLIEQLLDDMTTLSPVAFHVGEGVNHYF 5b6c.1    --------------------------------------------------------------------------------  target    HATLHNRATYLVGMLLGSVGVSGGGVSTWAGNYKGGVFQAAPWFGPGVGGFVNEDPFHPLTDPSARYSAETARHLVHGED 5b6c.1    --------------------------------------------------------------------------------  target    TSYWGFGDRPLVVDTPEDGRKVFTGTTHMPTPTKALWYNNANLINQAKWHYELVKNVNPKVDLIVDQQIEWTGSAEFADI 5b6c.1    --------------------------------------------------------------------------------  target    VLPANSWMEAETWEMGASCSNPFLQVWKGGIEPLNDTRDDIAIFAGVANALTELTGDERFSQAFMFADRPEVYLDRVLAG 5b6c.1    --------------------------------------------------------------------------------  target    SFTTEGYTVEDLTAGRYGPPGGALMQYRSYPRIPFKEQIEDSLPFYTDTGRMHGYVDIPEAIEYGENLIVHREAVEATPY 5b6c.1    --------------------------------------------------------------------------------  target    LPNVIVSTSPYLRPRDYGIAPEELDGDARSVRNIMMSWAEVKETENPLFAAGYNYLCLTPKSRHAVHSSWAVTDWHWLWS 5b6c.1    --------------------------------------------------------------------------------  target    SSFSDPYRVETRAPGVGEPAIHLNPDDARSLGIRNGDYVWVDSNPKDRPYRDADVDESFLDVARLLVRVTYNPAYPPGVT 5b6c.1    -------------------VVSLSQPKMDELQLFRGDTVLLKGKK----------------RREAVCIVLSDDTCSDEKI  target    MLKHAFYMATPRTFRAAQERSDGRALAETTGYQSSFRSGSHQSITRGWAPPMHQTDSLFHKRAGVFGFTYGFDVDNHAIN 5b6c.1    RMNRVVR-------------------------------------------------------------------------  target    TVPKETVVRITKAEDGGVGGSGAWTRGRPGSMPGDEDDAMQAYLAGELTVVRRT 5b6c.1    ------------------------------------------------------ ``` | | | | | | | | | | | | | | | | | | | | | | | | | | | | | | | | | | | | | | | | | | | | | | | | | |
|  | 4kdl.1.A | Transitional endoplasmic reticulum ATPase  *Crystal structure of p97/VCP N in complex with OTU1 UBXL* | 0.01 | 0.00 | 11.54 | 0.07 | 580-647 | X-ray | 1.81 | monomer |  | HHblits | 0.26 |
| ``` target    RPFLERFTDMPLLVRLDTLQRLRADEVFADYSSDLDVDGPSFTLHGMTEEQHERNGDRVVFDDASGALRAINREDVGDRL 4kdl.1    --------------------------------------------------------------------------------  target    DDKGIDPALDYQGTVTLVDGSTVEVMSVLSMYREHLADYDIDSVVDMTGAPRNLIEQLLDDMTTLSPVAFHVGEGVNHYF 4kdl.1    --------------------------------------------------------------------------------  target    HATLHNRATYLVGMLLGSVGVSGGGVSTWAGNYKGGVFQAAPWFGPGVGGFVNEDPFHPLTDPSARYSAETARHLVHGED 4kdl.1    --------------------------------------------------------------------------------  target    TSYWGFGDRPLVVDTPEDGRKVFTGTTHMPTPTKALWYNNANLINQAKWHYELVKNVNPKVDLIVDQQIEWTGSAEFADI 4kdl.1    --------------------------------------------------------------------------------  target    VLPANSWMEAETWEMGASCSNPFLQVWKGGIEPLNDTRDDIAIFAGVANALTELTGDERFSQAFMFADRPEVYLDRVLAG 4kdl.1    --------------------------------------------------------------------------------  target    SFTTEGYTVEDLTAGRYGPPGGALMQYRSYPRIPFKEQIEDSLPFYTDTGRMHGYVDIPEAIEYGENLIVHREAVEATPY 4kdl.1    --------------------------------------------------------------------------------  target    LPNVIVSTSPYLRPRDYGIAPEELDGDARSVRNIMMSWAEVKETENPLFAAGYNYLCLTPKSRHAVHSSWAVTDWHWLWS 4kdl.1    --------------------------------------------------------------------------------  target    SSFSDPYRVETRAPGVGEPAIHLNPDDARSLGIRNGDYVWVDSNPKDRPYRDADVDESFLDVARLLVRVTYNPAYPPGVT 4kdl.1    -------------------VVSLSQPKMDELQLFRGDTVLLKGKK----------------RREAVCIVLSDDTCSDEKI  target    MLKHAFYMATPRTFRAAQERSDGRALAETTGYQSSFRSGSHQSITRGWAPPMHQTDSLFHKRAGVFGFTYGFDVDNHAIN 4kdl.1    RMNRVVR-------------------------------------------------------------------------  target    TVPKETVVRITKAEDGGVGGSGAWTRGRPGSMPGDEDDAMQAYLAGELTVVRRT 4kdl.1    ------------------------------------------------------ ``` | | | | | | | | | | | | | | | | | | | | | | | | | | | | | | | | | | | | | | | | | | | | | | | | | |
|  | 4kdi.2.A | Transitional endoplasmic reticulum ATPase  *Crystal structure of p97/VCP N in complex with OTU1 UBXL* | 0.01 | 0.00 | 11.54 | 0.07 | 580-647 | X-ray | 1.86 | monomer |  | HHblits | 0.26 |
| ``` target    RPFLERFTDMPLLVRLDTLQRLRADEVFADYSSDLDVDGPSFTLHGMTEEQHERNGDRVVFDDASGALRAINREDVGDRL 4kdi.2    --------------------------------------------------------------------------------  target    DDKGIDPALDYQGTVTLVDGSTVEVMSVLSMYREHLADYDIDSVVDMTGAPRNLIEQLLDDMTTLSPVAFHVGEGVNHYF 4kdi.2    --------------------------------------------------------------------------------  target    HATLHNRATYLVGMLLGSVGVSGGGVSTWAGNYKGGVFQAAPWFGPGVGGFVNEDPFHPLTDPSARYSAETARHLVHGED 4kdi.2    --------------------------------------------------------------------------------  target    TSYWGFGDRPLVVDTPEDGRKVFTGTTHMPTPTKALWYNNANLINQAKWHYELVKNVNPKVDLIVDQQIEWTGSAEFADI 4kdi.2    --------------------------------------------------------------------------------  target    VLPANSWMEAETWEMGASCSNPFLQVWKGGIEPLNDTRDDIAIFAGVANALTELTGDERFSQAFMFADRPEVYLDRVLAG 4kdi.2    --------------------------------------------------------------------------------  target    SFTTEGYTVEDLTAGRYGPPGGALMQYRSYPRIPFKEQIEDSLPFYTDTGRMHGYVDIPEAIEYGENLIVHREAVEATPY 4kdi.2    --------------------------------------------------------------------------------  target    LPNVIVSTSPYLRPRDYGIAPEELDGDARSVRNIMMSWAEVKETENPLFAAGYNYLCLTPKSRHAVHSSWAVTDWHWLWS 4kdi.2    --------------------------------------------------------------------------------  target    SSFSDPYRVETRAPGVGEPAIHLNPDDARSLGIRNGDYVWVDSNPKDRPYRDADVDESFLDVARLLVRVTYNPAYPPGVT 4kdi.2    -------------------VVSLSQPKMDELQLFRGDTVLLKGKK----------------RREAVCIVLSDDTCSDEKI  target    MLKHAFYMATPRTFRAAQERSDGRALAETTGYQSSFRSGSHQSITRGWAPPMHQTDSLFHKRAGVFGFTYGFDVDNHAIN 4kdi.2    RMNRVVR-------------------------------------------------------------------------  target    TVPKETVVRITKAEDGGVGGSGAWTRGRPGSMPGDEDDAMQAYLAGELTVVRRT 4kdi.2    ------------------------------------------------------ ``` | | | | | | | | | | | | | | | | | | | | | | | | | | | | | | | | | | | | | | | | | | | | | | | | | |
|  | 4kdi.1.A | Transitional endoplasmic reticulum ATPase  *Crystal structure of p97/VCP N in complex with OTU1 UBXL* | 0.01 | 0.00 | 11.54 | 0.07 | 580-647 | X-ray | 1.86 | monomer |  | HHblits | 0.26 |
| ``` target    RPFLERFTDMPLLVRLDTLQRLRADEVFADYSSDLDVDGPSFTLHGMTEEQHERNGDRVVFDDASGALRAINREDVGDRL 4kdi.1    --------------------------------------------------------------------------------  target    DDKGIDPALDYQGTVTLVDGSTVEVMSVLSMYREHLADYDIDSVVDMTGAPRNLIEQLLDDMTTLSPVAFHVGEGVNHYF 4kdi.1    --------------------------------------------------------------------------------  target    HATLHNRATYLVGMLLGSVGVSGGGVSTWAGNYKGGVFQAAPWFGPGVGGFVNEDPFHPLTDPSARYSAETARHLVHGED 4kdi.1    --------------------------------------------------------------------------------  target    TSYWGFGDRPLVVDTPEDGRKVFTGTTHMPTPTKALWYNNANLINQAKWHYELVKNVNPKVDLIVDQQIEWTGSAEFADI 4kdi.1    --------------------------------------------------------------------------------  target    VLPANSWMEAETWEMGASCSNPFLQVWKGGIEPLNDTRDDIAIFAGVANALTELTGDERFSQAFMFADRPEVYLDRVLAG 4kdi.1    --------------------------------------------------------------------------------  target    SFTTEGYTVEDLTAGRYGPPGGALMQYRSYPRIPFKEQIEDSLPFYTDTGRMHGYVDIPEAIEYGENLIVHREAVEATPY 4kdi.1    --------------------------------------------------------------------------------  target    LPNVIVSTSPYLRPRDYGIAPEELDGDARSVRNIMMSWAEVKETENPLFAAGYNYLCLTPKSRHAVHSSWAVTDWHWLWS 4kdi.1    --------------------------------------------------------------------------------  target    SSFSDPYRVETRAPGVGEPAIHLNPDDARSLGIRNGDYVWVDSNPKDRPYRDADVDESFLDVARLLVRVTYNPAYPPGVT 4kdi.1    -------------------VVSLSQPKMDELQLFRGDTVLLKGKK----------------RREAVCIVLSDDTCSDEKI  target    MLKHAFYMATPRTFRAAQERSDGRALAETTGYQSSFRSGSHQSITRGWAPPMHQTDSLFHKRAGVFGFTYGFDVDNHAIN 4kdi.1    RMNRVVR-------------------------------------------------------------------------  target    TVPKETVVRITKAEDGGVGGSGAWTRGRPGSMPGDEDDAMQAYLAGELTVVRRT 4kdi.1    ------------------------------------------------------ ``` | | | | | | | | | | | | | | | | | | | | | | | | | | | | | | | | | | | | | | | | | | | | | | | | | |
|  | 1ogy.1.A | PERIPLASMIC NITRATE REDUCTASE  *Crystal structure of the heterodimeric nitrate reductase from Rhodobacter sphaeroides* | 0.01 |  | 7.55 | 0.07 | 273-325 | X-ray | 3.20 | hetero-1-1-mer | 1 x SF4, 1 x MO, 2 x MGD, 2 x HEC | HHblits | 0.24 |
| ``` target    RPFLERFTDMPLLVRLDTLQRLRADEVFADYSSDLDVDGPSFTLHGMTEEQHERNGDRVVFDDASGALRAINREDVGDRL 1ogy.1    --------------------------------------------------------------------------------  target    DDKGIDPALDYQGTVTLVDGSTVEVMSVLSMYREHLADYDIDSVVDMTGAPRNLIEQLLDDMTTLSPVAFHVGEGVNHYF 1ogy.1    --------------------------------------------------------------------------------  target    HATLHNRATYLVGMLLGSVGVSGGGVSTWAGNYKGGVFQAAPWFGPGVGGFVNEDPFHPLTDPSARYSAETARHLVHGED 1ogy.1    --------------------------------------------------------------------------------  target    TSYWGFGDRPLVVDTPEDGRKVFTGTTHMPTPTKALWYNNANLINQAKWHYELVKNVN--PKVDLIVDQQIEWTGSAEFA 1ogy.1    --------------------------------ADAFVLWGSNMAEMHPILWSRLTDRRLSHEHVRVAVLSTFTHRSSDLS  target    DIVLPANSWMEAETWEMGASCSNPFLQVWKGGIEPLNDTRDDIAIFAGVANALTELTGDERFSQAFMFADRPEVYLDRVL 1ogy.1    DTPIIFR-------------------------------------------------------------------------  target    AGSFTTEGYTVEDLTAGRYGPPGGALMQYRSYPRIPFKEQIEDSLPFYTDTGRMHGYVDIPEAIEYGENLIVHREAVEAT 1ogy.1    --------------------------------------------------------------------------------  target    PYLPNVIVSTSPYLRPRDYGIAPEELDGDARSVRNIMMSWAEVKETENPLFAAGYNYLCLTPKSRHAVHSSWAVTDWHWL 1ogy.1    --------------------------------------------------------------------------------  target    WSSSFSDPYRVETRAPGVGEPAIHLNPDDARSLGIRNGDYVWVDSNPKDRPYRDADVDESFLDVARLLVRVTYNPAYPPG 1ogy.1    --------------------------------------------------------------------------------  target    VTMLKHAFYMATPRTFRAAQERSDGRALAETTGYQSSFRSGSHQSITRGWAPPMHQTDSLFHKRAGVFGFTYGFDVDNHA 1ogy.1    --------------------------------------------------------------------------------  target    INTVPKETVVRITKAEDGGVGGSGAWTRGRPGSMPGDEDDAMQAYLAGELTVVRRT 1ogy.1    -------------------------------------------------------- ``` | | | | | | | | | | | | | | | | | | | | | | | | | | | | | | | | | | | | | | | | | | | | | | | | | |
|  | 4rv0.1.A | Transitional endoplasmic reticulum ATPase TER94  *Crystal structure of TN complex* | 0.01 |  | 13.73 | 0.07 | 580-646 | X-ray | 2.00 | hetero-oligomer |  | HHblits | 0.27 |
| ``` target    RPFLERFTDMPLLVRLDTLQRLRADEVFADYSSDLDVDGPSFTLHGMTEEQHERNGDRVVFDDASGALRAINREDVGDRL 4rv0.1    --------------------------------------------------------------------------------  target    DDKGIDPALDYQGTVTLVDGSTVEVMSVLSMYREHLADYDIDSVVDMTGAPRNLIEQLLDDMTTLSPVAFHVGEGVNHYF 4rv0.1    --------------------------------------------------------------------------------  target    HATLHNRATYLVGMLLGSVGVSGGGVSTWAGNYKGGVFQAAPWFGPGVGGFVNEDPFHPLTDPSARYSAETARHLVHGED 4rv0.1    --------------------------------------------------------------------------------  target    TSYWGFGDRPLVVDTPEDGRKVFTGTTHMPTPTKALWYNNANLINQAKWHYELVKNVNPKVDLIVDQQIEWTGSAEFADI 4rv0.1    --------------------------------------------------------------------------------  target    VLPANSWMEAETWEMGASCSNPFLQVWKGGIEPLNDTRDDIAIFAGVANALTELTGDERFSQAFMFADRPEVYLDRVLAG 4rv0.1    --------------------------------------------------------------------------------  target    SFTTEGYTVEDLTAGRYGPPGGALMQYRSYPRIPFKEQIEDSLPFYTDTGRMHGYVDIPEAIEYGENLIVHREAVEATPY 4rv0.1    --------------------------------------------------------------------------------  target    LPNVIVSTSPYLRPRDYGIAPEELDGDARSVRNIMMSWAEVKETENPLFAAGYNYLCLTPKSRHAVHSSWAVTDWHWLWS 4rv0.1    --------------------------------------------------------------------------------  target    SSFSDPYRVETRAPGVGEPAIHLNPDDARSLGIRNGDYVWVDSNPKDRPYRDADVDESFLDVARLLVRVTYNPAYPPGVT 4rv0.1    -------------------VVSLSQAKMDELQLFRGDTVILKGKR----------------RKETVCIVLSDDTCPDEKI  target    MLKHAFYMATPRTFRAAQERSDGRALAETTGYQSSFRSGSHQSITRGWAPPMHQTDSLFHKRAGVFGFTYGFDVDNHAIN 4rv0.1    RMNRVV--------------------------------------------------------------------------  target    TVPKETVVRITKAEDGGVGGSGAWTRGRPGSMPGDEDDAMQAYLAGELTVVRRT 4rv0.1    ------------------------------------------------------ ``` | | | | | | | | | | | | | | | | | | | | | | | | | | | | | | | | | | | | | | | | | | | | | | | | | |
|  | 3qc8.1.A | Transitional endoplasmic reticulum ATPase  *Crystal Structure of FAF1 UBX Domain In Complex with p97/VCP N Domain Reveals The Conserved FcisP Touch-Turn Motif of UBX Domain Suffering Conformational Change* | 0.01 | 0.00 | 11.76 | 0.07 | 579-645 | X-ray | 2.20 | monomer |  | HHblits | 0.26 |
| ``` target    RPFLERFTDMPLLVRLDTLQRLRADEVFADYSSDLDVDGPSFTLHGMTEEQHERNGDRVVFDDASGALRAINREDVGDRL 3qc8.1    --------------------------------------------------------------------------------  target    DDKGIDPALDYQGTVTLVDGSTVEVMSVLSMYREHLADYDIDSVVDMTGAPRNLIEQLLDDMTTLSPVAFHVGEGVNHYF 3qc8.1    --------------------------------------------------------------------------------  target    HATLHNRATYLVGMLLGSVGVSGGGVSTWAGNYKGGVFQAAPWFGPGVGGFVNEDPFHPLTDPSARYSAETARHLVHGED 3qc8.1    --------------------------------------------------------------------------------  target    TSYWGFGDRPLVVDTPEDGRKVFTGTTHMPTPTKALWYNNANLINQAKWHYELVKNVNPKVDLIVDQQIEWTGSAEFADI 3qc8.1    --------------------------------------------------------------------------------  target    VLPANSWMEAETWEMGASCSNPFLQVWKGGIEPLNDTRDDIAIFAGVANALTELTGDERFSQAFMFADRPEVYLDRVLAG 3qc8.1    --------------------------------------------------------------------------------  target    SFTTEGYTVEDLTAGRYGPPGGALMQYRSYPRIPFKEQIEDSLPFYTDTGRMHGYVDIPEAIEYGENLIVHREAVEATPY 3qc8.1    --------------------------------------------------------------------------------  target    LPNVIVSTSPYLRPRDYGIAPEELDGDARSVRNIMMSWAEVKETENPLFAAGYNYLCLTPKSRHAVHSSWAVTDWHWLWS 3qc8.1    --------------------------------------------------------------------------------  target    SSFSDPYRVETRAPGVGEPAIHLNPDDARSLGIRNGDYVWVDSNPKDRPYRDADVDESFLDVARLLVRVTYNPAYPPGVT 3qc8.1    ------------------SVVSLSQPKMDELQLFRGDTVLLKGKK----------------RREAVCIVLSDDTCSDEKI  target    MLKHAFYMATPRTFRAAQERSDGRALAETTGYQSSFRSGSHQSITRGWAPPMHQTDSLFHKRAGVFGFTYGFDVDNHAIN 3qc8.1    RMNRV---------------------------------------------------------------------------  target    TVPKETVVRITKAEDGGVGGSGAWTRGRPGSMPGDEDDAMQAYLAGELTVVRRT 3qc8.1    ------------------------------------------------------ ``` | | | | | | | | | | | | | | | | | | | | | | | | | | | | | | | | | | | | | | | | | | | | | | | | | |
|  | 3qwz.1.A | Transitional endoplasmic reticulum ATPase  *Crystal structure of FAF1 UBX-p97N-domain complex* | 0.01 |  | 11.76 | 0.07 | 580-646 | X-ray | 2.00 | hetero-oligomer |  | HHblits | 0.26 |
| ``` target    RPFLERFTDMPLLVRLDTLQRLRADEVFADYSSDLDVDGPSFTLHGMTEEQHERNGDRVVFDDASGALRAINREDVGDRL 3qwz.1    --------------------------------------------------------------------------------  target    DDKGIDPALDYQGTVTLVDGSTVEVMSVLSMYREHLADYDIDSVVDMTGAPRNLIEQLLDDMTTLSPVAFHVGEGVNHYF 3qwz.1    --------------------------------------------------------------------------------  target    HATLHNRATYLVGMLLGSVGVSGGGVSTWAGNYKGGVFQAAPWFGPGVGGFVNEDPFHPLTDPSARYSAETARHLVHGED 3qwz.1    --------------------------------------------------------------------------------  target    TSYWGFGDRPLVVDTPEDGRKVFTGTTHMPTPTKALWYNNANLINQAKWHYELVKNVNPKVDLIVDQQIEWTGSAEFADI 3qwz.1    --------------------------------------------------------------------------------  target    VLPANSWMEAETWEMGASCSNPFLQVWKGGIEPLNDTRDDIAIFAGVANALTELTGDERFSQAFMFADRPEVYLDRVLAG 3qwz.1    --------------------------------------------------------------------------------  target    SFTTEGYTVEDLTAGRYGPPGGALMQYRSYPRIPFKEQIEDSLPFYTDTGRMHGYVDIPEAIEYGENLIVHREAVEATPY 3qwz.1    --------------------------------------------------------------------------------  target    LPNVIVSTSPYLRPRDYGIAPEELDGDARSVRNIMMSWAEVKETENPLFAAGYNYLCLTPKSRHAVHSSWAVTDWHWLWS 3qwz.1    --------------------------------------------------------------------------------  target    SSFSDPYRVETRAPGVGEPAIHLNPDDARSLGIRNGDYVWVDSNPKDRPYRDADVDESFLDVARLLVRVTYNPAYPPGVT 3qwz.1    -------------------VVSLSQPKMDELQLFRGDTVLLKGKK----------------RREAVCIVLSDDTCSDEKI  target    MLKHAFYMATPRTFRAAQERSDGRALAETTGYQSSFRSGSHQSITRGWAPPMHQTDSLFHKRAGVFGFTYGFDVDNHAIN 3qwz.1    RMNRVV--------------------------------------------------------------------------  target    TVPKETVVRITKAEDGGVGGSGAWTRGRPGSMPGDEDDAMQAYLAGELTVVRRT 3qwz.1    ------------------------------------------------------ ``` | | | | | | | | | | | | | | | | | | | | | | | | | | | | | | | | | | | | | | | | | | | | | | | | | |
|  | 7t2r.1.A | NiFe hydrogenase subunit A  *Structure of electron bifurcating Ni-Fe hydrogenase complex HydABCSL in FMN-free apo state* | 0.01 |  | 5.66 | 0.07 | 272-325 | EM | 0.00 | hetero-2-2-2-2-2-mer | 6 x FES, 12 x SF4, 2 x 3NI, 2 x FCO | HHblits | 0.23 |
| ``` target    RPFLERFTDMPLLVRLDTLQRLRADEVFADYSSDLDVDGPSFTLHGMTEEQHERNGDRVVFDDASGALRAINREDVGDRL 7t2r.1    --------------------------------------------------------------------------------  target    DDKGIDPALDYQGTVTLVDGSTVEVMSVLSMYREHLADYDIDSVVDMTGAPRNLIEQLLDDMTTLSPVAFHVGEGVNHYF 7t2r.1    --------------------------------------------------------------------------------  target    HATLHNRATYLVGMLLGSVGVSGGGVSTWAGNYKGGVFQAAPWFGPGVGGFVNEDPFHPLTDPSARYSAETARHLVHGED 7t2r.1    --------------------------------------------------------------------------------  target    TSYWGFGDRPLVVDTPEDGRKVFTGTTHMPTPTKALWYNNANLINQAKWHYELVKNVN--PKVDLIVDQQIEWTGSAEFA 7t2r.1    -------------------------------DSDLIITMFADPQKEAPVVASYIRVACLHRNAK-LMNLSYGPSPFPGLV  target    DIVLPANSWMEAETWEMGASCSNPFLQVWKGGIEPLNDTRDDIAIFAGVANALTELTGDERFSQAFMFADRPEVYLDRVL 7t2r.1    DLDIRLP-------------------------------------------------------------------------  target    AGSFTTEGYTVEDLTAGRYGPPGGALMQYRSYPRIPFKEQIEDSLPFYTDTGRMHGYVDIPEAIEYGENLIVHREAVEAT 7t2r.1    --------------------------------------------------------------------------------  target    PYLPNVIVSTSPYLRPRDYGIAPEELDGDARSVRNIMMSWAEVKETENPLFAAGYNYLCLTPKSRHAVHSSWAVTDWHWL 7t2r.1    --------------------------------------------------------------------------------  target    WSSSFSDPYRVETRAPGVGEPAIHLNPDDARSLGIRNGDYVWVDSNPKDRPYRDADVDESFLDVARLLVRVTYNPAYPPG 7t2r.1    --------------------------------------------------------------------------------  target    VTMLKHAFYMATPRTFRAAQERSDGRALAETTGYQSSFRSGSHQSITRGWAPPMHQTDSLFHKRAGVFGFTYGFDVDNHA 7t2r.1    --------------------------------------------------------------------------------  target    INTVPKETVVRITKAEDGGVGGSGAWTRGRPGSMPGDEDDAMQAYLAGELTVVRRT 7t2r.1    -------------------------------------------------------- ``` | | | | | | | | | | | | | | | | | | | | | | | | | | | | | | | | | | | | | | | | | | | | | | | | | |
|  | 7t30.1.A | NiFe hydrogenase subunit A  *Structure of electron bifurcating Ni-Fe hydrogenase complex HydABCSL in FMN/NAD(H) bound state* | 0.01 |  | 5.66 | 0.07 | 272-325 | EM | 0.00 | hetero-2-2-2-2-2-mer | 4 x FES, 12 x SF4, 2 x NAD, 2 x FMN, 2 x 3NI, 2 x FCO | HHblits | 0.23 |
| ``` target    RPFLERFTDMPLLVRLDTLQRLRADEVFADYSSDLDVDGPSFTLHGMTEEQHERNGDRVVFDDASGALRAINREDVGDRL 7t30.1    --------------------------------------------------------------------------------  target    DDKGIDPALDYQGTVTLVDGSTVEVMSVLSMYREHLADYDIDSVVDMTGAPRNLIEQLLDDMTTLSPVAFHVGEGVNHYF 7t30.1    --------------------------------------------------------------------------------  target    HATLHNRATYLVGMLLGSVGVSGGGVSTWAGNYKGGVFQAAPWFGPGVGGFVNEDPFHPLTDPSARYSAETARHLVHGED 7t30.1    --------------------------------------------------------------------------------  target    TSYWGFGDRPLVVDTPEDGRKVFTGTTHMPTPTKALWYNNANLINQAKWHYELVKNVN--PKVDLIVDQQIEWTGSAEFA 7t30.1    -------------------------------DSDLIITMFADPQKEAPVVASYIRVACLHRNAK-LMNLSYGPSPFPGLV  target    DIVLPANSWMEAETWEMGASCSNPFLQVWKGGIEPLNDTRDDIAIFAGVANALTELTGDERFSQAFMFADRPEVYLDRVL 7t30.1    DLDIRLP-------------------------------------------------------------------------  target    AGSFTTEGYTVEDLTAGRYGPPGGALMQYRSYPRIPFKEQIEDSLPFYTDTGRMHGYVDIPEAIEYGENLIVHREAVEAT 7t30.1    --------------------------------------------------------------------------------  target    PYLPNVIVSTSPYLRPRDYGIAPEELDGDARSVRNIMMSWAEVKETENPLFAAGYNYLCLTPKSRHAVHSSWAVTDWHWL 7t30.1    --------------------------------------------------------------------------------  target    WSSSFSDPYRVETRAPGVGEPAIHLNPDDARSLGIRNGDYVWVDSNPKDRPYRDADVDESFLDVARLLVRVTYNPAYPPG 7t30.1    --------------------------------------------------------------------------------  target    VTMLKHAFYMATPRTFRAAQERSDGRALAETTGYQSSFRSGSHQSITRGWAPPMHQTDSLFHKRAGVFGFTYGFDVDNHA 7t30.1    --------------------------------------------------------------------------------  target    INTVPKETVVRITKAEDGGVGGSGAWTRGRPGSMPGDEDDAMQAYLAGELTVVRRT 7t30.1    -------------------------------------------------------- ``` | | | | | | | | | | | | | | | | | | | | | | | | | | | | | | | | | | | | | | | | | | | | | | | | | |
|  | 5epp.1.A | Transitional endoplasmic reticulum ATPase  *Structural Insights into the Interaction of p97 N-terminus Domain and VBM Motif in Rhomboid Protease, RHBDL4* | 0.01 |  | 12.00 | 0.06 | 580-645 | X-ray | 1.88 | hetero-oligomer |  | HHblits | 0.26 |
| ``` target    RPFLERFTDMPLLVRLDTLQRLRADEVFADYSSDLDVDGPSFTLHGMTEEQHERNGDRVVFDDASGALRAINREDVGDRL 5epp.1    --------------------------------------------------------------------------------  target    DDKGIDPALDYQGTVTLVDGSTVEVMSVLSMYREHLADYDIDSVVDMTGAPRNLIEQLLDDMTTLSPVAFHVGEGVNHYF 5epp.1    --------------------------------------------------------------------------------  target    HATLHNRATYLVGMLLGSVGVSGGGVSTWAGNYKGGVFQAAPWFGPGVGGFVNEDPFHPLTDPSARYSAETARHLVHGED 5epp.1    --------------------------------------------------------------------------------  target    TSYWGFGDRPLVVDTPEDGRKVFTGTTHMPTPTKALWYNNANLINQAKWHYELVKNVNPKVDLIVDQQIEWTGSAEFADI 5epp.1    --------------------------------------------------------------------------------  target    VLPANSWMEAETWEMGASCSNPFLQVWKGGIEPLNDTRDDIAIFAGVANALTELTGDERFSQAFMFADRPEVYLDRVLAG 5epp.1    --------------------------------------------------------------------------------  target    SFTTEGYTVEDLTAGRYGPPGGALMQYRSYPRIPFKEQIEDSLPFYTDTGRMHGYVDIPEAIEYGENLIVHREAVEATPY 5epp.1    --------------------------------------------------------------------------------  target    LPNVIVSTSPYLRPRDYGIAPEELDGDARSVRNIMMSWAEVKETENPLFAAGYNYLCLTPKSRHAVHSSWAVTDWHWLWS 5epp.1    --------------------------------------------------------------------------------  target    SSFSDPYRVETRAPGVGEPAIHLNPDDARSLGIRNGDYVWVDSNPKDRPYRDADVDESFLDVARLLVRVTYNPAYPPGVT 5epp.1    -------------------VVSLSQPKMDELQLFRGDTVLLKGKK----------------RREAVCIVLSDDTCSDEKI  target    MLKHAFYMATPRTFRAAQERSDGRALAETTGYQSSFRSGSHQSITRGWAPPMHQTDSLFHKRAGVFGFTYGFDVDNHAIN 5epp.1    RMNRV---------------------------------------------------------------------------  target    TVPKETVVRITKAEDGGVGGSGAWTRGRPGSMPGDEDDAMQAYLAGELTVVRRT 5epp.1    ------------------------------------------------------ ``` | | | | | | | | | | | | | | | | | | | | | | | | | | | | | | | | | | | | | | | | | | | | | | | | | |
|  | 5glf.2.A | Transitional endoplasmic reticulum ATPase  *Structural insights into the interaction of p97 N-terminal domain and SHP motif in Derlin-1 rhomboid pseudoprotease* | 0.01 |  | 12.00 | 0.06 | 580-645 | X-ray | 2.25 | hetero-1-1-mer |  | HHblits | 0.26 |
| ``` target    RPFLERFTDMPLLVRLDTLQRLRADEVFADYSSDLDVDGPSFTLHGMTEEQHERNGDRVVFDDASGALRAINREDVGDRL 5glf.2    --------------------------------------------------------------------------------  target    DDKGIDPALDYQGTVTLVDGSTVEVMSVLSMYREHLADYDIDSVVDMTGAPRNLIEQLLDDMTTLSPVAFHVGEGVNHYF 5glf.2    --------------------------------------------------------------------------------  target    HATLHNRATYLVGMLLGSVGVSGGGVSTWAGNYKGGVFQAAPWFGPGVGGFVNEDPFHPLTDPSARYSAETARHLVHGED 5glf.2    --------------------------------------------------------------------------------  target    TSYWGFGDRPLVVDTPEDGRKVFTGTTHMPTPTKALWYNNANLINQAKWHYELVKNVNPKVDLIVDQQIEWTGSAEFADI 5glf.2    --------------------------------------------------------------------------------  target    VLPANSWMEAETWEMGASCSNPFLQVWKGGIEPLNDTRDDIAIFAGVANALTELTGDERFSQAFMFADRPEVYLDRVLAG 5glf.2    --------------------------------------------------------------------------------  target    SFTTEGYTVEDLTAGRYGPPGGALMQYRSYPRIPFKEQIEDSLPFYTDTGRMHGYVDIPEAIEYGENLIVHREAVEATPY 5glf.2    --------------------------------------------------------------------------------  target    LPNVIVSTSPYLRPRDYGIAPEELDGDARSVRNIMMSWAEVKETENPLFAAGYNYLCLTPKSRHAVHSSWAVTDWHWLWS 5glf.2    --------------------------------------------------------------------------------  target    SSFSDPYRVETRAPGVGEPAIHLNPDDARSLGIRNGDYVWVDSNPKDRPYRDADVDESFLDVARLLVRVTYNPAYPPGVT 5glf.2    -------------------VVSLSQPKMDELQLFRGDTVLLKGKK----------------RREAVCIVLSDDTCSDEKI  target    MLKHAFYMATPRTFRAAQERSDGRALAETTGYQSSFRSGSHQSITRGWAPPMHQTDSLFHKRAGVFGFTYGFDVDNHAIN 5glf.2    RMNRV---------------------------------------------------------------------------  target    TVPKETVVRITKAEDGGVGGSGAWTRGRPGSMPGDEDDAMQAYLAGELTVVRRT 5glf.2    ------------------------------------------------------ ``` | | | | | | | | | | | | | | | | | | | | | | | | | | | | | | | | | | | | | | | | | | | | | | | | | |
|  | 5glf.3.A | Transitional endoplasmic reticulum ATPase  *Structural insights into the interaction of p97 N-terminal domain and SHP motif in Derlin-1 rhomboid pseudoprotease* | 0.01 |  | 12.00 | 0.06 | 580-645 | X-ray | 2.25 | hetero-1-1-mer |  | HHblits | 0.26 |
| ``` target    RPFLERFTDMPLLVRLDTLQRLRADEVFADYSSDLDVDGPSFTLHGMTEEQHERNGDRVVFDDASGALRAINREDVGDRL 5glf.3    --------------------------------------------------------------------------------  target    DDKGIDPALDYQGTVTLVDGSTVEVMSVLSMYREHLADYDIDSVVDMTGAPRNLIEQLLDDMTTLSPVAFHVGEGVNHYF 5glf.3    --------------------------------------------------------------------------------  target    HATLHNRATYLVGMLLGSVGVSGGGVSTWAGNYKGGVFQAAPWFGPGVGGFVNEDPFHPLTDPSARYSAETARHLVHGED 5glf.3    --------------------------------------------------------------------------------  target    TSYWGFGDRPLVVDTPEDGRKVFTGTTHMPTPTKALWYNNANLINQAKWHYELVKNVNPKVDLIVDQQIEWTGSAEFADI 5glf.3    --------------------------------------------------------------------------------  target    VLPANSWMEAETWEMGASCSNPFLQVWKGGIEPLNDTRDDIAIFAGVANALTELTGDERFSQAFMFADRPEVYLDRVLAG 5glf.3    --------------------------------------------------------------------------------  target    SFTTEGYTVEDLTAGRYGPPGGALMQYRSYPRIPFKEQIEDSLPFYTDTGRMHGYVDIPEAIEYGENLIVHREAVEATPY 5glf.3    --------------------------------------------------------------------------------  target    LPNVIVSTSPYLRPRDYGIAPEELDGDARSVRNIMMSWAEVKETENPLFAAGYNYLCLTPKSRHAVHSSWAVTDWHWLWS 5glf.3    --------------------------------------------------------------------------------  target    SSFSDPYRVETRAPGVGEPAIHLNPDDARSLGIRNGDYVWVDSNPKDRPYRDADVDESFLDVARLLVRVTYNPAYPPGVT 5glf.3    -------------------VVSLSQPKMDELQLFRGDTVLLKGKK----------------RREAVCIVLSDDTCSDEKI  target    MLKHAFYMATPRTFRAAQERSDGRALAETTGYQSSFRSGSHQSITRGWAPPMHQTDSLFHKRAGVFGFTYGFDVDNHAIN 5glf.3    RMNRV---------------------------------------------------------------------------  target    TVPKETVVRITKAEDGGVGGSGAWTRGRPGSMPGDEDDAMQAYLAGELTVVRRT 5glf.3    ------------------------------------------------------ ``` | | | | | | | | | | | | | | | | | | | | | | | | | | | | | | | | | | | | | | | | | | | | | | | | | |
|  | 5glf.1.A | Transitional endoplasmic reticulum ATPase  *Structural insights into the interaction of p97 N-terminal domain and SHP motif in Derlin-1 rhomboid pseudoprotease* | 0.01 |  | 12.00 | 0.06 | 580-645 | X-ray | 2.25 | hetero-1-1-mer |  | HHblits | 0.26 |
| ``` target    RPFLERFTDMPLLVRLDTLQRLRADEVFADYSSDLDVDGPSFTLHGMTEEQHERNGDRVVFDDASGALRAINREDVGDRL 5glf.1    --------------------------------------------------------------------------------  target    DDKGIDPALDYQGTVTLVDGSTVEVMSVLSMYREHLADYDIDSVVDMTGAPRNLIEQLLDDMTTLSPVAFHVGEGVNHYF 5glf.1    --------------------------------------------------------------------------------  target    HATLHNRATYLVGMLLGSVGVSGGGVSTWAGNYKGGVFQAAPWFGPGVGGFVNEDPFHPLTDPSARYSAETARHLVHGED 5glf.1    --------------------------------------------------------------------------------  target    TSYWGFGDRPLVVDTPEDGRKVFTGTTHMPTPTKALWYNNANLINQAKWHYELVKNVNPKVDLIVDQQIEWTGSAEFADI 5glf.1    --------------------------------------------------------------------------------  target    VLPANSWMEAETWEMGASCSNPFLQVWKGGIEPLNDTRDDIAIFAGVANALTELTGDERFSQAFMFADRPEVYLDRVLAG 5glf.1    --------------------------------------------------------------------------------  target    SFTTEGYTVEDLTAGRYGPPGGALMQYRSYPRIPFKEQIEDSLPFYTDTGRMHGYVDIPEAIEYGENLIVHREAVEATPY 5glf.1    --------------------------------------------------------------------------------  target    LPNVIVSTSPYLRPRDYGIAPEELDGDARSVRNIMMSWAEVKETENPLFAAGYNYLCLTPKSRHAVHSSWAVTDWHWLWS 5glf.1    --------------------------------------------------------------------------------  target    SSFSDPYRVETRAPGVGEPAIHLNPDDARSLGIRNGDYVWVDSNPKDRPYRDADVDESFLDVARLLVRVTYNPAYPPGVT 5glf.1    -------------------VVSLSQPKMDELQLFRGDTVLLKGKK----------------RREAVCIVLSDDTCSDEKI  target    MLKHAFYMATPRTFRAAQERSDGRALAETTGYQSSFRSGSHQSITRGWAPPMHQTDSLFHKRAGVFGFTYGFDVDNHAIN 5glf.1    RMNRV---------------------------------------------------------------------------  target    TVPKETVVRITKAEDGGVGGSGAWTRGRPGSMPGDEDDAMQAYLAGELTVVRRT 5glf.1    ------------------------------------------------------ ``` | | | | | | | | | | | | | | | | | | | | | | | | | | | | | | | | | | | | | | | | | | | | | | | | | |
|  | 5glf.4.A | Transitional endoplasmic reticulum ATPase  *Structural insights into the interaction of p97 N-terminal domain and SHP motif in Derlin-1 rhomboid pseudoprotease* | 0.01 |  | 12.00 | 0.06 | 580-645 | X-ray | 2.25 | hetero-1-1-mer |  | HHblits | 0.26 |
| ``` target    RPFLERFTDMPLLVRLDTLQRLRADEVFADYSSDLDVDGPSFTLHGMTEEQHERNGDRVVFDDASGALRAINREDVGDRL 5glf.4    --------------------------------------------------------------------------------  target    DDKGIDPALDYQGTVTLVDGSTVEVMSVLSMYREHLADYDIDSVVDMTGAPRNLIEQLLDDMTTLSPVAFHVGEGVNHYF 5glf.4    --------------------------------------------------------------------------------  target    HATLHNRATYLVGMLLGSVGVSGGGVSTWAGNYKGGVFQAAPWFGPGVGGFVNEDPFHPLTDPSARYSAETARHLVHGED 5glf.4    --------------------------------------------------------------------------------  target    TSYWGFGDRPLVVDTPEDGRKVFTGTTHMPTPTKALWYNNANLINQAKWHYELVKNVNPKVDLIVDQQIEWTGSAEFADI 5glf.4    --------------------------------------------------------------------------------  target    VLPANSWMEAETWEMGASCSNPFLQVWKGGIEPLNDTRDDIAIFAGVANALTELTGDERFSQAFMFADRPEVYLDRVLAG 5glf.4    --------------------------------------------------------------------------------  target    SFTTEGYTVEDLTAGRYGPPGGALMQYRSYPRIPFKEQIEDSLPFYTDTGRMHGYVDIPEAIEYGENLIVHREAVEATPY 5glf.4    --------------------------------------------------------------------------------  target    LPNVIVSTSPYLRPRDYGIAPEELDGDARSVRNIMMSWAEVKETENPLFAAGYNYLCLTPKSRHAVHSSWAVTDWHWLWS 5glf.4    --------------------------------------------------------------------------------  target    SSFSDPYRVETRAPGVGEPAIHLNPDDARSLGIRNGDYVWVDSNPKDRPYRDADVDESFLDVARLLVRVTYNPAYPPGVT 5glf.4    -------------------VVSLSQPKMDELQLFRGDTVLLKGKK----------------RREAVCIVLSDDTCSDEKI  target    MLKHAFYMATPRTFRAAQERSDGRALAETTGYQSSFRSGSHQSITRGWAPPMHQTDSLFHKRAGVFGFTYGFDVDNHAIN 5glf.4    RMNRV---------------------------------------------------------------------------  target    TVPKETVVRITKAEDGGVGGSGAWTRGRPGSMPGDEDDAMQAYLAGELTVVRRT 5glf.4    ------------------------------------------------------ ``` | | | | | | | | | | | | | | | | | | | | | | | | | | | | | | | | | | | | | | | | | | | | | | | | | |
|  | 3tiw.1.A | Transitional endoplasmic reticulum ATPase  *Crystal structure of p97N in complex with the C-terminus of gp78* | 0.01 |  | 12.00 | 0.06 | 580-645 | X-ray | 1.80 | hetero-oligomer |  | HHblits | 0.26 |
| ``` target    RPFLERFTDMPLLVRLDTLQRLRADEVFADYSSDLDVDGPSFTLHGMTEEQHERNGDRVVFDDASGALRAINREDVGDRL 3tiw.1    --------------------------------------------------------------------------------  target    DDKGIDPALDYQGTVTLVDGSTVEVMSVLSMYREHLADYDIDSVVDMTGAPRNLIEQLLDDMTTLSPVAFHVGEGVNHYF 3tiw.1    --------------------------------------------------------------------------------  target    HATLHNRATYLVGMLLGSVGVSGGGVSTWAGNYKGGVFQAAPWFGPGVGGFVNEDPFHPLTDPSARYSAETARHLVHGED 3tiw.1    --------------------------------------------------------------------------------  target    TSYWGFGDRPLVVDTPEDGRKVFTGTTHMPTPTKALWYNNANLINQAKWHYELVKNVNPKVDLIVDQQIEWTGSAEFADI 3tiw.1    --------------------------------------------------------------------------------  target    VLPANSWMEAETWEMGASCSNPFLQVWKGGIEPLNDTRDDIAIFAGVANALTELTGDERFSQAFMFADRPEVYLDRVLAG 3tiw.1    --------------------------------------------------------------------------------  target    SFTTEGYTVEDLTAGRYGPPGGALMQYRSYPRIPFKEQIEDSLPFYTDTGRMHGYVDIPEAIEYGENLIVHREAVEATPY 3tiw.1    --------------------------------------------------------------------------------  target    LPNVIVSTSPYLRPRDYGIAPEELDGDARSVRNIMMSWAEVKETENPLFAAGYNYLCLTPKSRHAVHSSWAVTDWHWLWS 3tiw.1    --------------------------------------------------------------------------------  target    SSFSDPYRVETRAPGVGEPAIHLNPDDARSLGIRNGDYVWVDSNPKDRPYRDADVDESFLDVARLLVRVTYNPAYPPGVT 3tiw.1    -------------------VVSLSQPKMDELQLFRGDTVLLKGKK----------------RREAVCIVLSDDTCSDEKI  target    MLKHAFYMATPRTFRAAQERSDGRALAETTGYQSSFRSGSHQSITRGWAPPMHQTDSLFHKRAGVFGFTYGFDVDNHAIN 3tiw.1    RMNRV---------------------------------------------------------------------------  target    TVPKETVVRITKAEDGGVGGSGAWTRGRPGSMPGDEDDAMQAYLAGELTVVRRT 3tiw.1    ------------------------------------------------------ ``` | | | | | | | | | | | | | | | | | | | | | | | | | | | | | | | | | | | | | | | | | | | | | | | | | |
|  | 3tiw.2.A | Transitional endoplasmic reticulum ATPase  *Crystal structure of p97N in complex with the C-terminus of gp78* | 0.01 |  | 12.00 | 0.06 | 580-645 | X-ray | 1.80 | hetero-oligomer |  | HHblits | 0.26 |
| ``` target    RPFLERFTDMPLLVRLDTLQRLRADEVFADYSSDLDVDGPSFTLHGMTEEQHERNGDRVVFDDASGALRAINREDVGDRL 3tiw.2    --------------------------------------------------------------------------------  target    DDKGIDPALDYQGTVTLVDGSTVEVMSVLSMYREHLADYDIDSVVDMTGAPRNLIEQLLDDMTTLSPVAFHVGEGVNHYF 3tiw.2    --------------------------------------------------------------------------------  target    HATLHNRATYLVGMLLGSVGVSGGGVSTWAGNYKGGVFQAAPWFGPGVGGFVNEDPFHPLTDPSARYSAETARHLVHGED 3tiw.2    --------------------------------------------------------------------------------  target    TSYWGFGDRPLVVDTPEDGRKVFTGTTHMPTPTKALWYNNANLINQAKWHYELVKNVNPKVDLIVDQQIEWTGSAEFADI 3tiw.2    --------------------------------------------------------------------------------  target    VLPANSWMEAETWEMGASCSNPFLQVWKGGIEPLNDTRDDIAIFAGVANALTELTGDERFSQAFMFADRPEVYLDRVLAG 3tiw.2    --------------------------------------------------------------------------------  target    SFTTEGYTVEDLTAGRYGPPGGALMQYRSYPRIPFKEQIEDSLPFYTDTGRMHGYVDIPEAIEYGENLIVHREAVEATPY 3tiw.2    --------------------------------------------------------------------------------  target    LPNVIVSTSPYLRPRDYGIAPEELDGDARSVRNIMMSWAEVKETENPLFAAGYNYLCLTPKSRHAVHSSWAVTDWHWLWS 3tiw.2    --------------------------------------------------------------------------------  target    SSFSDPYRVETRAPGVGEPAIHLNPDDARSLGIRNGDYVWVDSNPKDRPYRDADVDESFLDVARLLVRVTYNPAYPPGVT 3tiw.2    -------------------VVSLSQPKMDELQLFRGDTVLLKGKK----------------RREAVCIVLSDDTCSDEKI  target    MLKHAFYMATPRTFRAAQERSDGRALAETTGYQSSFRSGSHQSITRGWAPPMHQTDSLFHKRAGVFGFTYGFDVDNHAIN 3tiw.2    RMNRV---------------------------------------------------------------------------  target    TVPKETVVRITKAEDGGVGGSGAWTRGRPGSMPGDEDDAMQAYLAGELTVVRRT 3tiw.2    ------------------------------------------------------ ``` | | | | | | | | | | | | | | | | | | | | | | | | | | | | | | | | | | | | | | | | | | | | | | | | | |
|  | 3qq8.1.A | Transitional endoplasmic reticulum ATPase  *Crystal structure of p97-N in complex with FAF1-UBX* | 0.01 |  | 12.24 | 0.06 | 580-644 | X-ray | 2.00 | hetero-oligomer |  | HHblits | 0.26 |
| ``` target    RPFLERFTDMPLLVRLDTLQRLRADEVFADYSSDLDVDGPSFTLHGMTEEQHERNGDRVVFDDASGALRAINREDVGDRL 3qq8.1    --------------------------------------------------------------------------------  target    DDKGIDPALDYQGTVTLVDGSTVEVMSVLSMYREHLADYDIDSVVDMTGAPRNLIEQLLDDMTTLSPVAFHVGEGVNHYF 3qq8.1    --------------------------------------------------------------------------------  target    HATLHNRATYLVGMLLGSVGVSGGGVSTWAGNYKGGVFQAAPWFGPGVGGFVNEDPFHPLTDPSARYSAETARHLVHGED 3qq8.1    --------------------------------------------------------------------------------  target    TSYWGFGDRPLVVDTPEDGRKVFTGTTHMPTPTKALWYNNANLINQAKWHYELVKNVNPKVDLIVDQQIEWTGSAEFADI 3qq8.1    --------------------------------------------------------------------------------  target    VLPANSWMEAETWEMGASCSNPFLQVWKGGIEPLNDTRDDIAIFAGVANALTELTGDERFSQAFMFADRPEVYLDRVLAG 3qq8.1    --------------------------------------------------------------------------------  target    SFTTEGYTVEDLTAGRYGPPGGALMQYRSYPRIPFKEQIEDSLPFYTDTGRMHGYVDIPEAIEYGENLIVHREAVEATPY 3qq8.1    --------------------------------------------------------------------------------  target    LPNVIVSTSPYLRPRDYGIAPEELDGDARSVRNIMMSWAEVKETENPLFAAGYNYLCLTPKSRHAVHSSWAVTDWHWLWS 3qq8.1    --------------------------------------------------------------------------------  target    SSFSDPYRVETRAPGVGEPAIHLNPDDARSLGIRNGDYVWVDSNPKDRPYRDADVDESFLDVARLLVRVTYNPAYPPGVT 3qq8.1    -------------------VVSLSQPKMDELQLFRGDTVLLKGKK----------------RREAVCIVLSDDTCSDEKI  target    MLKHAFYMATPRTFRAAQERSDGRALAETTGYQSSFRSGSHQSITRGWAPPMHQTDSLFHKRAGVFGFTYGFDVDNHAIN 3qq8.1    RMNR----------------------------------------------------------------------------  target    TVPKETVVRITKAEDGGVGGSGAWTRGRPGSMPGDEDDAMQAYLAGELTVVRRT 3qq8.1    ------------------------------------------------------ ``` | | | | | | | | | | | | | | | | | | | | | | | | | | | | | | | | | | | | | | | | | | | | | | | | | |
|  | 3qq7.1.A | Transitional endoplasmic reticulum ATPase  *Crystal Structure of the p97 N-terminal domain* | 0.01 |  | 12.24 | 0.06 | 580-644 | X-ray | 2.65 | monomer | 1 x HEZ, 1 x CO | HHblits | 0.26 |
| ``` target    RPFLERFTDMPLLVRLDTLQRLRADEVFADYSSDLDVDGPSFTLHGMTEEQHERNGDRVVFDDASGALRAINREDVGDRL 3qq7.1    --------------------------------------------------------------------------------  target    DDKGIDPALDYQGTVTLVDGSTVEVMSVLSMYREHLADYDIDSVVDMTGAPRNLIEQLLDDMTTLSPVAFHVGEGVNHYF 3qq7.1    --------------------------------------------------------------------------------  target    HATLHNRATYLVGMLLGSVGVSGGGVSTWAGNYKGGVFQAAPWFGPGVGGFVNEDPFHPLTDPSARYSAETARHLVHGED 3qq7.1    --------------------------------------------------------------------------------  target    TSYWGFGDRPLVVDTPEDGRKVFTGTTHMPTPTKALWYNNANLINQAKWHYELVKNVNPKVDLIVDQQIEWTGSAEFADI 3qq7.1    --------------------------------------------------------------------------------  target    VLPANSWMEAETWEMGASCSNPFLQVWKGGIEPLNDTRDDIAIFAGVANALTELTGDERFSQAFMFADRPEVYLDRVLAG 3qq7.1    --------------------------------------------------------------------------------  target    SFTTEGYTVEDLTAGRYGPPGGALMQYRSYPRIPFKEQIEDSLPFYTDTGRMHGYVDIPEAIEYGENLIVHREAVEATPY 3qq7.1    --------------------------------------------------------------------------------  target    LPNVIVSTSPYLRPRDYGIAPEELDGDARSVRNIMMSWAEVKETENPLFAAGYNYLCLTPKSRHAVHSSWAVTDWHWLWS 3qq7.1    --------------------------------------------------------------------------------  target    SSFSDPYRVETRAPGVGEPAIHLNPDDARSLGIRNGDYVWVDSNPKDRPYRDADVDESFLDVARLLVRVTYNPAYPPGVT 3qq7.1    -------------------VVSLSQPKMDELQLFRGDTVLLKGKK----------------RREAVCIVLSDDTCSDEKI  target    MLKHAFYMATPRTFRAAQERSDGRALAETTGYQSSFRSGSHQSITRGWAPPMHQTDSLFHKRAGVFGFTYGFDVDNHAIN 3qq7.1    RMNR----------------------------------------------------------------------------  target    TVPKETVVRITKAEDGGVGGSGAWTRGRPGSMPGDEDDAMQAYLAGELTVVRRT 3qq7.1    ------------------------------------------------------ ``` | | | | | | | | | | | | | | | | | | | | | | | | | | | | | | | | | | | | | | | | | | | | | | | | | |
|  | 2lnb.1.A | Z-DNA-binding protein 1  *Solution NMR structure of N-terminal domain (6-74) of human ZBP1 protein, Northeast Structural Genomics Consortium Target HR8174A.* | 0.00 |  | 19.51 | 0.05 | 105-145 | NMR | 0.00 | monomer |  | HHblits | 0.29 |
| ``` target    RPFLERFTDMPLLVRLDTLQRLRADEVFADYSSDLDVDGPSFTLHGMTEEQHERNGDRVVFDDASGALRAINREDVGDRL 2lnb.1    --------------------------------------------------------------------------------  target    DDKGIDPALDYQGTVTLVDGSTVEVMSVLSMYREHLADYDIDSVVDMTGAPRNLIEQLLDDMTTLSPVAFHVGEGVNHYF 2lnb.1    ------------------------EQRILQVLTEAGSPVKLAQLVKECQAPKRELNQVLYRMKKE---------------  target    HATLHNRATYLVGMLLGSVGVSGGGVSTWAGNYKGGVFQAAPWFGPGVGGFVNEDPFHPLTDPSARYSAETARHLVHGED 2lnb.1    --------------------------------------------------------------------------------  target    TSYWGFGDRPLVVDTPEDGRKVFTGTTHMPTPTKALWYNNANLINQAKWHYELVKNVNPKVDLIVDQQIEWTGSAEFADI 2lnb.1    --------------------------------------------------------------------------------  target    VLPANSWMEAETWEMGASCSNPFLQVWKGGIEPLNDTRDDIAIFAGVANALTELTGDERFSQAFMFADRPEVYLDRVLAG 2lnb.1    --------------------------------------------------------------------------------  target    SFTTEGYTVEDLTAGRYGPPGGALMQYRSYPRIPFKEQIEDSLPFYTDTGRMHGYVDIPEAIEYGENLIVHREAVEATPY 2lnb.1    --------------------------------------------------------------------------------  target    LPNVIVSTSPYLRPRDYGIAPEELDGDARSVRNIMMSWAEVKETENPLFAAGYNYLCLTPKSRHAVHSSWAVTDWHWLWS 2lnb.1    --------------------------------------------------------------------------------  target    SSFSDPYRVETRAPGVGEPAIHLNPDDARSLGIRNGDYVWVDSNPKDRPYRDADVDESFLDVARLLVRVTYNPAYPPGVT 2lnb.1    --------------------------------------------------------------------------------  target    MLKHAFYMATPRTFRAAQERSDGRALAETTGYQSSFRSGSHQSITRGWAPPMHQTDSLFHKRAGVFGFTYGFDVDNHAIN 2lnb.1    --------------------------------------------------------------------------------  target    TVPKETVVRITKAEDGGVGGSGAWTRGRPGSMPGDEDDAMQAYLAGELTVVRRT 2lnb.1    ------------------------------------------------------ ``` | | | | | | | | | | | | | | | | | | | | | | | | | | | | | | | | | | | | | | | | | | | | | | | | | |
|  | 1j75.1.A | Tumor Stroma and Activated Macrophage Protein DLM-1  *Crystal Structure of the DNA-Binding Domain Zalpha of DLM-1 Bound to Z-DNA* | 0.00 |  | 14.63 | 0.05 | 106-146 | X-ray | 1.85 | homo-dimer | 2 x DT-DC-DG-DC-DG-DC-DG | HHblits | 0.29 |
| ``` target    RPFLERFTDMPLLVRLDTLQRLRADEVFADYSSDLDVDGPSFTLHGMTEEQHERNGDRVVFDDASGALRAINREDVGDRL 1j75.1    --------------------------------------------------------------------------------  target    DDKGIDPALDYQGTVTLVDGSTVEVMSVLSMYREHLADYDIDSVVDMTGAPRNLIEQLLDDMTTLSPVAFHVGEGVNHYF 1j75.1    -------------------------QKILQVLSDDGGPVKIGQLVKKCQVPKKTLNQVLYRLKKED--------------  target    HATLHNRATYLVGMLLGSVGVSGGGVSTWAGNYKGGVFQAAPWFGPGVGGFVNEDPFHPLTDPSARYSAETARHLVHGED 1j75.1    --------------------------------------------------------------------------------  target    TSYWGFGDRPLVVDTPEDGRKVFTGTTHMPTPTKALWYNNANLINQAKWHYELVKNVNPKVDLIVDQQIEWTGSAEFADI 1j75.1    --------------------------------------------------------------------------------  target    VLPANSWMEAETWEMGASCSNPFLQVWKGGIEPLNDTRDDIAIFAGVANALTELTGDERFSQAFMFADRPEVYLDRVLAG 1j75.1    --------------------------------------------------------------------------------  target    SFTTEGYTVEDLTAGRYGPPGGALMQYRSYPRIPFKEQIEDSLPFYTDTGRMHGYVDIPEAIEYGENLIVHREAVEATPY 1j75.1    --------------------------------------------------------------------------------  target    LPNVIVSTSPYLRPRDYGIAPEELDGDARSVRNIMMSWAEVKETENPLFAAGYNYLCLTPKSRHAVHSSWAVTDWHWLWS 1j75.1    --------------------------------------------------------------------------------  target    SSFSDPYRVETRAPGVGEPAIHLNPDDARSLGIRNGDYVWVDSNPKDRPYRDADVDESFLDVARLLVRVTYNPAYPPGVT 1j75.1    --------------------------------------------------------------------------------  target    MLKHAFYMATPRTFRAAQERSDGRALAETTGYQSSFRSGSHQSITRGWAPPMHQTDSLFHKRAGVFGFTYGFDVDNHAIN 1j75.1    --------------------------------------------------------------------------------  target    TVPKETVVRITKAEDGGVGGSGAWTRGRPGSMPGDEDDAMQAYLAGELTVVRRT 1j75.1    ------------------------------------------------------ ``` | | | | | | | | | | | | | | | | | | | | | | | | | | | | | | | | | | | | | | | | | | | | | | | | | |
|  | 2heo.1.A | Z-DNA binding protein 1  *General Structure-Based Approach to the Design of Protein Ligands: Application to the Design of Kv1.2 Potassium Channel Blockers.* | 0.00 |  | 15.00 | 0.05 | 107-146 | X-ray | 1.70 | homo-dimer | 2 x DC-DG-DC-DG-DC-DG | HHblits | 0.29 |
| ``` target    RPFLERFTDMPLLVRLDTLQRLRADEVFADYSSDLDVDGPSFTLHGMTEEQHERNGDRVVFDDASGALRAINREDVGDRL 2heo.1    --------------------------------------------------------------------------------  target    DDKGIDPALDYQGTVTLVDGSTVEVMSVLSMYREHLADYDIDSVVDMTGAPRNLIEQLLDDMTTLSPVAFHVGEGVNHYF 2heo.1    --------------------------KILQVLSDDGGPVAIFQLVKKCQVPKKTLNQVLYRLKKED--------------  target    HATLHNRATYLVGMLLGSVGVSGGGVSTWAGNYKGGVFQAAPWFGPGVGGFVNEDPFHPLTDPSARYSAETARHLVHGED 2heo.1    --------------------------------------------------------------------------------  target    TSYWGFGDRPLVVDTPEDGRKVFTGTTHMPTPTKALWYNNANLINQAKWHYELVKNVNPKVDLIVDQQIEWTGSAEFADI 2heo.1    --------------------------------------------------------------------------------  target    VLPANSWMEAETWEMGASCSNPFLQVWKGGIEPLNDTRDDIAIFAGVANALTELTGDERFSQAFMFADRPEVYLDRVLAG 2heo.1    --------------------------------------------------------------------------------  target    SFTTEGYTVEDLTAGRYGPPGGALMQYRSYPRIPFKEQIEDSLPFYTDTGRMHGYVDIPEAIEYGENLIVHREAVEATPY 2heo.1    --------------------------------------------------------------------------------  target    LPNVIVSTSPYLRPRDYGIAPEELDGDARSVRNIMMSWAEVKETENPLFAAGYNYLCLTPKSRHAVHSSWAVTDWHWLWS 2heo.1    --------------------------------------------------------------------------------  target    SSFSDPYRVETRAPGVGEPAIHLNPDDARSLGIRNGDYVWVDSNPKDRPYRDADVDESFLDVARLLVRVTYNPAYPPGVT 2heo.1    --------------------------------------------------------------------------------  target    MLKHAFYMATPRTFRAAQERSDGRALAETTGYQSSFRSGSHQSITRGWAPPMHQTDSLFHKRAGVFGFTYGFDVDNHAIN 2heo.1    --------------------------------------------------------------------------------  target    TVPKETVVRITKAEDGGVGGSGAWTRGRPGSMPGDEDDAMQAYLAGELTVVRRT 2heo.1    ------------------------------------------------------ ``` | | | | | | | | | | | | | | | | | | | | | | | | | | | | | | | | | | | | | | | | | | | | | | | | | |
|  | 2heo.1.B | Z-DNA binding protein 1  *General Structure-Based Approach to the Design of Protein Ligands: Application to the Design of Kv1.2 Potassium Channel Blockers.* | 0.00 |  | 15.00 | 0.05 | 107-146 | X-ray | 1.70 | homo-dimer | 2 x DC-DG-DC-DG-DC-DG | HHblits | 0.29 |
| ``` target    RPFLERFTDMPLLVRLDTLQRLRADEVFADYSSDLDVDGPSFTLHGMTEEQHERNGDRVVFDDASGALRAINREDVGDRL 2heo.1    --------------------------------------------------------------------------------  target    DDKGIDPALDYQGTVTLVDGSTVEVMSVLSMYREHLADYDIDSVVDMTGAPRNLIEQLLDDMTTLSPVAFHVGEGVNHYF 2heo.1    --------------------------KILQVLSDDGGPVAIFQLVKKCQVPKKTLNQVLYRLKKED--------------  target    HATLHNRATYLVGMLLGSVGVSGGGVSTWAGNYKGGVFQAAPWFGPGVGGFVNEDPFHPLTDPSARYSAETARHLVHGED 2heo.1    --------------------------------------------------------------------------------  target    TSYWGFGDRPLVVDTPEDGRKVFTGTTHMPTPTKALWYNNANLINQAKWHYELVKNVNPKVDLIVDQQIEWTGSAEFADI 2heo.1    --------------------------------------------------------------------------------  target    VLPANSWMEAETWEMGASCSNPFLQVWKGGIEPLNDTRDDIAIFAGVANALTELTGDERFSQAFMFADRPEVYLDRVLAG 2heo.1    --------------------------------------------------------------------------------  target    SFTTEGYTVEDLTAGRYGPPGGALMQYRSYPRIPFKEQIEDSLPFYTDTGRMHGYVDIPEAIEYGENLIVHREAVEATPY 2heo.1    --------------------------------------------------------------------------------  target    LPNVIVSTSPYLRPRDYGIAPEELDGDARSVRNIMMSWAEVKETENPLFAAGYNYLCLTPKSRHAVHSSWAVTDWHWLWS 2heo.1    --------------------------------------------------------------------------------  target    SSFSDPYRVETRAPGVGEPAIHLNPDDARSLGIRNGDYVWVDSNPKDRPYRDADVDESFLDVARLLVRVTYNPAYPPGVT 2heo.1    --------------------------------------------------------------------------------  target    MLKHAFYMATPRTFRAAQERSDGRALAETTGYQSSFRSGSHQSITRGWAPPMHQTDSLFHKRAGVFGFTYGFDVDNHAIN 2heo.1    --------------------------------------------------------------------------------  target    TVPKETVVRITKAEDGGVGGSGAWTRGRPGSMPGDEDDAMQAYLAGELTVVRRT 2heo.1    ------------------------------------------------------ ``` | | | | | | | | | | | | | | | | | | | | | | | | | | | | | | | | | | | | | | | | | | | | | | | | | |
|  | 2xvc.1.A | ESCRT-III  *Molecular and structural basis of ESCRT-III recruitment to membranes during archaeal cell division* | 0.00 |  | 15.79 | 0.05 | 109-146 | X-ray | 2.15 | hetero-1-1-mer | 2 x CD | HHblits | 0.29 |
| ``` target    RPFLERFTDMPLLVRLDTLQRLRADEVFADYSSDLDVDGPSFTLHGMTEEQHERNGDRVVFDDASGALRAINREDVGDRL 2xvc.1    --------------------------------------------------------------------------------  target    DDKGIDPALDYQGTVTLVDGSTVEVMSVLSMYREHLADYDIDSVVDMTGAPRNLIEQLLDDMTTLSPVAFHVGEGVNHYF 2xvc.1    ----------------------------LDYIVNNGGFLDIEHFSKVYGVEKQEVVKLLEALKNKG--------------  target    HATLHNRATYLVGMLLGSVGVSGGGVSTWAGNYKGGVFQAAPWFGPGVGGFVNEDPFHPLTDPSARYSAETARHLVHGED 2xvc.1    --------------------------------------------------------------------------------  target    TSYWGFGDRPLVVDTPEDGRKVFTGTTHMPTPTKALWYNNANLINQAKWHYELVKNVNPKVDLIVDQQIEWTGSAEFADI 2xvc.1    --------------------------------------------------------------------------------  target    VLPANSWMEAETWEMGASCSNPFLQVWKGGIEPLNDTRDDIAIFAGVANALTELTGDERFSQAFMFADRPEVYLDRVLAG 2xvc.1    --------------------------------------------------------------------------------  target    SFTTEGYTVEDLTAGRYGPPGGALMQYRSYPRIPFKEQIEDSLPFYTDTGRMHGYVDIPEAIEYGENLIVHREAVEATPY 2xvc.1    --------------------------------------------------------------------------------  target    LPNVIVSTSPYLRPRDYGIAPEELDGDARSVRNIMMSWAEVKETENPLFAAGYNYLCLTPKSRHAVHSSWAVTDWHWLWS 2xvc.1    --------------------------------------------------------------------------------  target    SSFSDPYRVETRAPGVGEPAIHLNPDDARSLGIRNGDYVWVDSNPKDRPYRDADVDESFLDVARLLVRVTYNPAYPPGVT 2xvc.1    --------------------------------------------------------------------------------  target    MLKHAFYMATPRTFRAAQERSDGRALAETTGYQSSFRSGSHQSITRGWAPPMHQTDSLFHKRAGVFGFTYGFDVDNHAIN 2xvc.1    --------------------------------------------------------------------------------  target    TVPKETVVRITKAEDGGVGGSGAWTRGRPGSMPGDEDDAMQAYLAGELTVVRRT 2xvc.1    ------------------------------------------------------ ``` | | | | | | | | | | | | | | | | | | | | | | | | | | | | | | | | | | | | | | | | | | | | | | | | | |
|  | 2kz3.1.A | Putative uncharacterized protein RAD51L3  *Backbone 1H, 13C, and 15N Chemical Shift Assignments for human Rad51D from 1 to 83* | 0.00 |  | 13.51 | 0.05 | 104-145 | NMR | 0.00 | monomer |  | HHblits | 0.29 |
[truncated: 208,253 more chars]
