# Supplementary material for: Metabolic capacity is maintained despite shifts in microbial diversity in estuary sediments
Source: ISME Commun. 2025 Oct 11;5(1):ycaf182. doi: 10.1093/ismeco/ycaf182 (PMC12687941; doi:10.1093/ismeco/ycaf182)
Supplement: Supplementary_Data_1_ycaf182 [file supplementary_data_1_ycaf182.zip › SWISS-MODEL/21_Jan_SF_Bin67_scaffold_32672_c132330_1/report.html]

21\_Jan\_SF\_Bin67\_scaffold\_32672\_c1:3-2330\_1 | Report


|  |  |  |
| --- | --- | --- |
|  |  | SWISS-MODEL Homology Modelling Report |

## Model Building Report

This document lists the results for the homology modelling project "21\_Jan\_SF\_Bin67\_scaffold\_32672\_c1:3-2330\_1" submitted to SWISS-MODEL workspace
on March 29, 2023, 6:30 p.m..The submitted primary amino acid sequence is given in Table T1.

If you use any results in your research, please cite the relevant publications:

- Waterhouse, A., Bertoni, M., Bienert, S., Studer, G., Tauriello, G., Gumienny, R.,
  Heer, F.T., de Beer, T.A.P., Rempfer, C., Bordoli, L., Lepore, R., Schwede, T.
  SWISS-MODEL: homology modelling of protein structures and complexes.
  Nucleic Acids Res. 46(W1), W296-W303 (2018).
- Bienert, S., Waterhouse, A., de Beer, T.A.P., Tauriello, G., Studer,
  G., Bordoli, L., Schwede, T. The SWISS-MODEL Repository - new features and
  functionality. Nucleic Acids Res. 45, D313-D319 (2017).
- Studer, G., Tauriello, G., Bienert, S.,
  Biasini, M., Johner, N., Schwede, T. ProMod3 - A versatile homology
  modelling toolbox. PLOS Comp. Biol. 17(1), e1008667 (2021).
- Studer, G., Rempfer, C., Waterhouse, A.M.,
  Gumienny, G., Haas, J., Schwede, T. QMEANDisCo - distance constraints
  applied on model quality estimation. Bioinformatics 36, 1765-1771 (2020).
- Bertoni, M., Kiefer, F., Biasini, M., Bordoli, L.,
  Schwede, T. Modeling protein quaternary structure of homo- and
  hetero-oligomers beyond binary interactions by homology. Scientific
  Reports 7 (2017).

## Results

The SWISS-MODEL template library (SMTL version 2023-03-23, PDB release 2023-03-17) was searched with
for evolutionary related structures matching the target sequence in Table T1. For details on the template search, see Materials and Methods. Overall 282 templates were found (Table T2).

## Models

The following models were built (see Materials and Methods "Model Building"):

| Model #01 | File | Built with | Oligo-State | Ligands | GMQE | QMEANDisCo Global |
| --- | --- | --- | --- | --- | --- | --- |
|  | PDB | ProMod3 3.2.1 | monomer | None | 0.81 | 0.78 ± 0.05 |

|  |  |  |
| --- | --- | --- |
|  |  |  |

| Template | Seq Identity | Oligo-state | QSQE | Found by | Method | Resolution | Seq Similarity | Range | Coverage | Description |
| --- | --- | --- | --- | --- | --- | --- | --- | --- | --- | --- |
| 7b04.1.B | 53.57 | monomer | 0.00 | HHblits | X-ray | 2.97Å | 0.46 | 1 - 772 | 1.00 | Nitrite oxidoreductase subunit A |

  

### Excluded ligands

| Ligand Name.Number | Reason for Exclusion | Description |
| --- | --- | --- |
| CA.10 | Binding site not conserved. | CALCIUM ION |
| CA.11 | Binding site not conserved. | CALCIUM ION |
| F3S.4 | Binding site not conserved. | FE3-S4 CLUSTER |
| HEM.9 | Binding site not conserved. | PROTOPORPHYRIN IX CONTAINING FE |
| MD1.5 | Binding site not conserved. | PHOSPHORIC ACID 4-(2-AMINO-4-OXO-3,4,5,6,-TETRAHYDRO-PTERIDIN-6-YL)-2-HYDROXY-3,4-DIMERCAPTO-BUT-3-EN-YL ESTER GUANYLATE ESTER |
| MD1.6 | Binding site not conserved. | PHOSPHORIC ACID 4-(2-AMINO-4-OXO-3,4,5,6,-TETRAHYDRO-PTERIDIN-6-YL)-2-HYDROXY-3,4-DIMERCAPTO-BUT-3-EN-YL ESTER GUANYLATE ESTER |
| MO.7 | Binding site not conserved. | MOLYBDENUM ATOM |
| SF4.1 | Binding site not conserved. | IRON/SULFUR CLUSTER |
| SF4.2 | Binding site not conserved. | IRON/SULFUR CLUSTER |
| SF4.3 | Binding site not conserved. | IRON/SULFUR CLUSTER |
| SF4.8 | Binding site not conserved. | IRON/SULFUR CLUSTER |

  

```
Target    RPFLERFTDMPLLVRLDTLQRLRADEVFADYSSDLDVDGPSFTLHGMTEEQHERNGDRVVFDDASGALRAINREDVGDRL  
7b04.1.B  ADYVKKFTDFPLLIRTDTLKRVSPKDIIPNYKLQDISDGPSYHIQGLKDEQREIIGDFVVWDAKSKGPKAITRDDVGETL  
  
Target    DDKGIDPALDYQGTVTLVDGSTVEVMSVLSMYREHLADYDIDSVVDMTGAPRNLIEQLLDDMTTLSPVAFHVGEGVNHYF  
7b04.1.B  VKKGIDPVLEGSFKLKTIDGKEIEVMTLLEMYKIHLRDYDIDSVVSMTNSPKDLIERLAKDIATIKPVAIHYGEGVNHYF  
  
Target    HATLHNRATYLVGMLLGSVGVSGGGVSTWAGNYKGGVFQAAPWFGPGVGGFVNEDPFHPLTDPSARYSAETARHLVHGED  
7b04.1.B  HATLMNRSYYLPVMLTGNVGYFGSGSHTWAGNYKAGNFQASKWSGPGFYGWVAEDVFKPNLDPYASAKDLNIKGRALDEE  
  
Target    TSYWGFGDRPLVVDTPEDGRKVFTGTTHMPTPTKALWYNNANLINQAKWHYELVKNVNPKVDLIVDQQIEWTGSAEFADI  
7b04.1.B  VAYWNHSERPLIVNTPKYGRKVFTGKTHMPSPTKVLWFTNVNLINNAKHVYQMLKNVNPNIEQIMSTDIEITGSIEYADF  
  
Target    VLPANSWMEAETWEMGASCSNPFLQVWK-GGIEPLNDTRDDIAIFAGVANALTELTGDERFSQAFMF--ADRPEVYLDRV  
7b04.1.B  AFPANSWVEFQEFEITNSCSNPFIQIWGKTGITPVYESKDDVKILAGMASKLGELLRDKRFEDNWKFAIEGRASVYINRL  
  
Target    LAGSFTTEGYTVEDLTAGRYGPPGGALMQYRSYPRIPFKEQIEDSLPFYTDTGRMHGYVDIPEAIEYGENLIVHREAVEA  
7b04.1.B  LDGSTTMKGYTCEDILNGKYGEPGVAMLLFRTYPRHPFWEQVHESLPFYTPTGRLQAYNDEPEIIEYGENFIVHREGPEA  
  
Target    TPYLPNVIVSTSPYLRPRDYGIAPEELDGDARSVRNIMMSWAEVKETENPLFAAGYNYLCLTPKSRHAVHSSWAVTDWHW  
7b04.1.B  TPYLPNAIVSTNPYIRPDDYGIPENAEYWEDRTVRNIKKSWEETKKTKNFLWEKGYHFYCVTPKSRHTVHSQWAVTDWNF  
  
Target    LWSSSFSDPYRVETRAPGVGEPAIHLNPDDARSLGIRNGDYVWVDSNPKDRPYRDADVDESFLDVARLLVRVTYNPAYPP  
7b04.1.B  IWNNNFGDPYRMDKRMPGVGEHQIHIHPQAARDLGIEDGDYVYVDANPADRPYEGWKPNDSFYKVSRLMLRAKYNPAYPY  
  
Target    GVTMLKHAFYMATPRTFRAAQERSDGRALAETTGYQSSFRSGSHQSITRGWAPPMHQTDSLFHKRAGVFGFTYGFDVDNH  
7b04.1.B  NCTMMKHSAWISSDKTVQAHETRPDGRALSP-SGYQSSFRYGSQQSITRDWSMPMHQLDSLFHKAKIGMKFIFGFEADNH  
  
Target    AINTVPKETVVRITKAEDGGVGGSGAWTRGRPGSMPGDEDDAMQAYLAGELTVVRRT  
7b04.1.B  CINTVPKETLVKITKAENGGMGGKGVWDPVKTGYTAGNENDFMKKFLNGELIKVD--
```

  


---

  

| Model #02 | File | Built with | Oligo-State | Ligands | GMQE | QMEANDisCo Global |
| --- | --- | --- | --- | --- | --- | --- |
|  | PDB | ProMod3 3.2.1 | monomer | None | 0.40 | 0.45 ± 0.05 |

|  |  |  |
| --- | --- | --- |
|  |  |  |

| Template | Seq Identity | Oligo-state | QSQE | Found by | Method | Resolution | Seq Similarity | Range | Coverage | Description |
| --- | --- | --- | --- | --- | --- | --- | --- | --- | --- | --- |
| 3ir7.1.A | 22.74 | monomer | 0.00 | HHblits | X-ray | 2.50Å | 0.31 | 1 - 739 | 0.80 | Respiratory nitrate reductase 1 alpha chain |

  

### Excluded ligands

| Ligand Name.Number | Reason for Exclusion | Description |
| --- | --- | --- |
| 6MO.4 | Binding site not conserved. | MOLYBDENUM(VI) ION |
| AGA.5 | Binding site not conserved. | (1S)-2-{[{[(2S)-2,3-DIHYDROXYPROPYL]OXY}(HYDROXY)PHOSPHORYL]OXY}-1-[(PENTANOYLOXY)METHYL]ETHYL OCTANOATE |
| F3S.9 | Binding site not conserved. | FE3-S4 CLUSTER |
| HEM.10 | Binding site not conserved. | PROTOPORPHYRIN IX CONTAINING FE |
| HEM.11 | Binding site not conserved. | PROTOPORPHYRIN IX CONTAINING FE |
| MD1.1 | Binding site not conserved. | PHOSPHORIC ACID 4-(2-AMINO-4-OXO-3,4,5,6,-TETRAHYDRO-PTERIDIN-6-YL)-2-HYDROXY-3,4-DIMERCAPTO-BUT-3-EN-YL ESTER GUANYLATE ESTER |
| MD1.2 | Binding site not conserved. | PHOSPHORIC ACID 4-(2-AMINO-4-OXO-3,4,5,6,-TETRAHYDRO-PTERIDIN-6-YL)-2-HYDROXY-3,4-DIMERCAPTO-BUT-3-EN-YL ESTER GUANYLATE ESTER |
| SF4.3 | Binding site not conserved. | IRON/SULFUR CLUSTER |
| SF4.6 | Binding site not conserved. | IRON/SULFUR CLUSTER |
| SF4.7 | Binding site not conserved. | IRON/SULFUR CLUSTER |
| SF4.8 | Binding site not conserved. | IRON/SULFUR CLUSTER |

  

```
Target    RPFLERFTDMPLLVRLD-------TLQRLRADEVFADYSSDLDVDGPSFTLHGMTEEQHERNGDRVVFDDASGALRAINR  
3ir7.1.A  TDYVRRYTDMPMLVMLEERDGYYAAGRMLRAADLVDAL-------GQ----------ENNPEWKTVAFNT-NGEMVAPNG  
  
Target    EDVGD--------------------RLD---DK------------------------GIDPALDY---QGTVTLVDGSTV  
3ir7.1.A  SIGFRWGEKGKWNLEQRDGKTGEETELQLSLLGSQDEIAEVGFPYFGGDGTEHFNKVELENVLLHKLPVKRLQLADGSTA  
  
Target    EVMSVLSMYR------------------EHLADYDIDSVVDMTGAPRNLIEQLLDDMTTL-----SPVAFHVGEGVNHYF  
3ir7.1.A  LVTTVYDLTLANYGLERGLNDVNCATSYDDVKAYTPAWAEQITGVSRSQIIRIAREFADNADKTHGRSMIIVGAGLNHWY  
  
Target    HATLHNRATYLVGMLLGSVGVSGGGVSTWAGNYKGGV---FQ----AAPWFGPGVGGFVNEDPFHPLTDPSARYSA----  
3ir7.1.A  HLDMNYRGLINMLIFCGCVGQSGGGWAHYVGQEKLRPQTGWQPLAFALDWQRPA--RHMNSTSYFYNHSSQWRYETVTAE  
  
Target    --------------ETAR----HLVHGEDTSYWGFGDRPL----------------VVDTPEDGRKVF--TGTTHMPTPT  
3ir7.1.A  ELLSPMADKSRYTGHLIDFNVRAERMGWLPSAPQLGTNPLTIAGEAEKAGMNPVDYTVKSLKEGSIRFAAEQPENGKNHP  
  
Target    KALWYNNANLINQAKWHYEL-------------------------------VKNVNPKVDLIVDQQIEWTGSAEFADIVL  
3ir7.1.A  RNLFIWRSNLLGSSGKGHEFMLKYLLGTEHGIQGKDLGQQGGVKPEEVDWQDNGLEGKLDLVVTLDFRLSSTCLYSDIIL  
  
Target    PANSWMEAETWEMGASCSNPFLQVWKGGIEPLNDTRDDIAIFAGVANALTELTGDE---R---FSQAFMFADRP------  
3ir7.1.A  PTATWYEKDDMN--TSDMHPFIHPLSAAVDPAWEAKSDWEIYKAIAKKFSEVCVGHLGKETDIVTLPI-QHDSAAELAQP  
  
Target    ---EVY---------------------------------------------------------LDRV-------------  
3ir7.1.A  LDVKDWKKGECDLIPGKTAPHIMVVERDYPATYERFTSIGPLMEKIGNGGKGIAWNTQSEMDLLRKLNYTKAEGPAKGQP  
  
Target    ------------LAGSFTTEG----YTVEDLTAGRYG-----PP--GGALMQYR---------------------SYPRI  
3ir7.1.A  MLNTAIDAAEMILTLAPETNGQVAVKAWAALSEFTGRDHTHLALNKEDEKIRFRDIQAQPRKIISSPTWSGLEDEHVSYN  
  
Target    PFKEQIEDSLPFYTDTGRMHGYVDIPEAIEYGENLIVHREAVEATPYLPNVIVSTSPYLRPRDYGIAPEELDGDARSVRN  
3ir7.1.A  AGYTNVHELIPWRTLSGRQQLYQDHQWMRDFGESLLVYRPPIDTRSV-KE------------------------------  
  
Target    IMMSWAEVKETENPLFAAGYNYLCLTPKSRHAVHSSWAVTDWHWLWSSSFSDPYRVETRAPGVGEPAIHLNPDDARSLGI  
3ir7.1.A  ----VI---G-QKSNGNQEKALNFLTPHQKWGIHSTYSDNLLM------------LTL---GRGGPVVWLSEADAKDLGI  
  
Target    RNGDYVWVDSNPKDRPYRDADVDESFLDVARLLVRVTYNPAYPPGVTMLKHAFYMATPRTFRAAQERSDGRALAETTGYQ  
3ir7.1.A  ADNDWIEVFNS-----------------NGALTARAVVSQRVPAGMTMMYHAQERIVN------------LPGSEI----  
  
Target    SSFRSGSHQSITRGWAPPMHQTDSLFHKRAGVFGFTYGFDVDNHAINTVPKETVVRITKAEDGGVGGSGAWTRGRPGSMP  
3ir7.1.A  TQQRGGIHNSVTRITPKPTHMI-------GGYAHLAYGFNYYGT--VGSNRDEFVVVRKMKNIDWL--------------  
  
Target    GDEDDAMQAYLAGELTVVRRT  
3ir7.1.A  ---------------------
```

  


---

  

| Model #03 | File | Built with | Oligo-State | Ligands | GMQE | QMEANDisCo Global |
| --- | --- | --- | --- | --- | --- | --- |
|  | PDB | ProMod3 3.2.1 | monomer | None | 0.21 | 0.37 ± 0.05 |

|  |  |  |
| --- | --- | --- |
|  |  |  |

| Template | Seq Identity | Oligo-state | QSQE | Found by | Method | Resolution | Seq Similarity | Range | Coverage | Description |
| --- | --- | --- | --- | --- | --- | --- | --- | --- | --- | --- |
| 2ivf.1.A | 32.76 | monomer | 0.00 | BLAST | X-ray | 1.88Å | 0.36 | 1 - 473 | 0.53 | ETHYLBENZENE DEHYDROGENASE ALPHA-SUBUNIT |

  

### Excluded ligands

| Ligand Name.Number | Reason for Exclusion | Description |
| --- | --- | --- |
| ACT.2 | Not biologically relevant. | ACETATE ION |
| ACT.7 | Not biologically relevant. | ACETATE ION |
| F3S.15 | Binding site not conserved. | FE3-S4 CLUSTER |
| GOL.3 | Not biologically relevant. | GLYCEROL |
| GOL.4 | Not biologically relevant. | GLYCEROL |
| GOL.5 | Not biologically relevant. | GLYCEROL |
| GOL.6 | Not biologically relevant. | GLYCEROL |
| GOL.12 | Not biologically relevant. | GLYCEROL |
| GOL.14 | Not biologically relevant. | GLYCEROL |
| GOL.19 | Not biologically relevant. | GLYCEROL |
| GOL.20 | Not biologically relevant. | GLYCEROL |
| HEM.21 | Binding site not conserved. | PROTOPORPHYRIN IX CONTAINING FE |
| MD1.11 | Binding site not conserved. | PHOSPHORIC ACID 4-(2-AMINO-4-OXO-3,4,5,6,-TETRAHYDRO-PTERIDIN-6-YL)-2-HYDROXY-3,4-DIMERCAPTO-BUT-3-EN-YL ESTER GUANYLATE ESTER |
| MES.1 | Binding site not conserved. | 2-(N-MORPHOLINO)-ETHANESULFONIC ACID |
| MGD.10 | Binding site not conserved. | 2-AMINO-5,6-DIMERCAPTO-7-METHYL-3,7,8A,9-TETRAHYDRO-8-OXA-1,3,9,10-TETRAAZA-ANTHRACEN-4-ONE GUANOSINE DINUCLEOTIDE |
| MO.9 | Binding site not conserved. | MOLYBDENUM ATOM |
| PO4.13 | Not biologically relevant. | PHOSPHATE ION |
| SF4.8 | Binding site not conserved. | IRON/SULFUR CLUSTER |
| SF4.16 | Binding site not conserved. | IRON/SULFUR CLUSTER |
| SF4.17 | Binding site not conserved. | IRON/SULFUR CLUSTER |
| SF4.18 | Binding site not conserved. | IRON/SULFUR CLUSTER |

  

```
Target    RPFLERFTDMPLLVRLDTLQRLRADEVFADYSSDLDVDGPSFTLHGMTEEQHERNGDRVVFDDASGALRAINREDVGDRL  
2ivf.1.A  RQFVCEQTDLPLLVRMDTGKFLSAE----------DVDG------GEAKQFY-------FFDEKAGSVRKASRGTL--KL  
  
Target    DDKGIDPALDYQGTVTLVDGSTVEVMSVLSMYREHLADYDIDSVVDMTGAPRNLIEQLLDDMTTLSPVAFHVGEGVNHYF  
2ivf.1.A  D---FMPALEGTFSARLKNGKTIQVRTVFEGLREHLKDYTPEKASAKCGVPVSLIRELGRKVAKKRTCSY-IGFSSAKSY  
  
Target    HATLHNRATYLVGMLLGSVGVSGGGVSTWAGNYKGGVFQAAPWFGPGVGGFV------NEDPFHPLTDPSARYSAETARH  
2ivf.1.A  HGDLMERSLFLAMAL--------------SGN----------WGKPGTGAFAWAYSDDNMVYLGVMSKPTAQGGMDELHQ  
  
Target    LVHGEDTSYWGFGDRPLVVD---TPEDGR----KVFTGTTHMPTPTKALWYNNA--NLINQAKW----------------  
2ivf.1.A  MAE-------GFNKRTLEADPTSTDEMGNIEFMKVVTSAVGLVPPAMWLYYHVGYDQLWNNKAWTDPALKKSFGAYLDEA  
  
Target    ---------HYELVKNVNPKVDLIVDQ---------------------------QIEWTGSAEFADIVLPANSWMEAETW  
2ivf.1.A  KEKGWWTNDHIRPAPDKTPQVYMLLSQNPMRRKRSGAKMFPDVLFPKLKMIFALETRMSSSAMYADIVLPCAWYYEKH--  
  
Target    EMGASCS-NPFLQVWKGGIEPLNDTR---DDIAIFA------GVANALTELTG--------DERFSQAFMFAD-------  
2ivf.1.A  EMTTPCSGNPFFTFVDRSVAPPGECREEWDAIALILKKVGERAAARGLTEFNDHNGRKRRYDELYKKFTMDGHLLTNEDC  
  
Target    -RPEVYLDR---VLAGSFTTEGYTVEDLTAGRYGPPGGALMQYR-------SYPRIPFKEQIEDSLPFYTDTGRMHGYVD  
2ivf.1.A  LKEMVDINRAVGVFAKDYTYEKFKKEGQT--RFLSMGTGVSRYAHANEVDVTKPIYPMRWHFDDKKVFPTHTRRAQFYLD  
  
Target    IPEAIEYGENLIVHREAVEATPYLPNVIVSTSPYLRPRDYGIAPEELDGDARSVRNIMMSWAEVKETENPLFAAGYNYLC  
2ivf.1.A  HDWYLEAGESLPTHKD----------------------------------------------------------------  
  
Target    LTPKSRHAVHSSWAVTDWHWLWSSSFSDPYRVETRAPGVGEPAIHLNPDDARSLGIRNGDYVWVDSNPKDRPYRDADVDE  
2ivf.1.A  --------------------------------------------------------------------------------  
  
Target    SFLDVARLLVRVTYNPAYPPGVTMLKHAFYMATPRTFRAAQERSDGRALAETTGYQSSFRSGSHQSITRGWAPPMHQTDS  
2ivf.1.A  --------------------------------------------------------------------------------  
  
Target    LFHKRAGVFGFTYGFDVDNHAINTVPKETVVRITKAEDGGVGGSGAWTRGRPGSMPGDEDDAMQAYLAGELTVVRRT  
2ivf.1.A  -----------------------------------------------------------------------------
```

  


---

  

| Model #04 | File | Built with | Oligo-State | Ligands | GMQE | QMEANDisCo Global |
| --- | --- | --- | --- | --- | --- | --- |
|  | PDB | ProMod3 3.2.1 | monomer | None | 0.12 | 0.33 ± 0.05 |

|  |  |  |
| --- | --- | --- |
|  |  |  |

| Template | Seq Identity | Oligo-state | QSQE | Found by | Method | Resolution | Seq Similarity | Range | Coverage | Description |
| --- | --- | --- | --- | --- | --- | --- | --- | --- | --- | --- |
| 2fug.2.C | 16.58 | monomer | 0.00 | HHblits | X-ray | 3.30Å | 0.27 | 296 - 649 | 0.25 | NADH-quinone oxidoreductase chain 3 |

  

### Excluded ligands

| Ligand Name.Number | Reason for Exclusion | Description |
| --- | --- | --- |
| FES.2 | Binding site not conserved. | FE2/S2 (INORGANIC) CLUSTER |
| FES.6 | Binding site not conserved. | FE2/S2 (INORGANIC) CLUSTER |
| FMN.10 | Binding site not conserved. | FLAVIN MONONUCLEOTIDE |
| SF4.1 | Binding site not conserved. | IRON/SULFUR CLUSTER |
| SF4.3 | Binding site not conserved. | IRON/SULFUR CLUSTER |
| SF4.4 | Binding site not conserved. | IRON/SULFUR CLUSTER |
| SF4.5 | Binding site not conserved. | IRON/SULFUR CLUSTER |
| SF4.7 | Binding site not conserved. | IRON/SULFUR CLUSTER |
| SF4.8 | Binding site not conserved. | IRON/SULFUR CLUSTER |
| SF4.9 | Binding site not conserved. | IRON/SULFUR CLUSTER |

  

```
Target    RPFLERFTDMPLLVRLDTLQRLRADEVFADYSSDLDVDGPSFTLHGMTEEQHERNGDRVVFDDASGALRAINREDVGDRL  
2fug.2.C  --------------------------------------------------------------------------------  
  
Target    DDKGIDPALDYQGTVTLVDGSTVEVMSVLSMYREHLADYDIDSVVDMTGAPRNLIEQLLDDMTTLSPVAFHVGEGVNHYF  
2fug.2.C  --------------------------------------------------------------------------------  
  
Target    HATLHNRATYLVGMLLGSVGVSGGGVSTWAGNYKGGVFQAAPWFGPGVGGFVNEDPFHPLTDPSARYSAETARHLVHGED  
2fug.2.C  --------------------------------------------------------------------------------  
  
Target    TSYWGFGDRPLVVDTPEDGRKVFTGTTHMPTPTKALWYNNANLINQAKWHYELVKNVNPKVDLIVDQQIEWTGSA-EFAD  
2fug.2.C  -------------------------------------------------------EALKGKRFVVMHLSHLHPLAERYAH  
  
Target    IVLPANSWMEAETWEMGASCSNPFLQVWKGGIEPLNDTRDDIAIFAGVANALTELTGDERFSQAFMFADRPEVYLDRVLA  
2fug.2.C  VVLPAPTFYEKRGH---LVNLEGRVLPLSPAPIENGEAEGALQVLALLAEALGVR-------P--PFR-LHLEA------  
  
Target    GSFTTEGYTVEDLTAGRYGPPGGALMQYRSYPRIPFKEQIEDSLPFYTDTGRMHGYVDIPEAIEYGENLIVHREAVEATP  
2fug.2.C  ---------QKALKA----------------------------RKVPEAMGRLSFRLKELR------------------P  
  
Target    YLPNVIVSTSPYLRPRDYGIAPEELDGDARSVRNIMMSWAEVKETENPLFAAGYNYLCLTPKSRHAVHSSWAVTDWHWLW  
2fug.2.C  -------------------------------------------------KERKGAFYLRPTMW--KAHQ---AVGK--A-  
  
Target    SSSFSDPYRVETRAPGVGEPAIHLNPDDARSLGIRNGDYVWVDSNPKDRPYRDADVDESFLDVARLLVRVTYNPAYPPGV  
2fug.2.C  ---------Q-----EAARAELWAHPETARAEALPEGAQVAVETP-----------------FGRVEARVVHREDVPKGH  
  
Target    TMLKHAFYMATPRTFRAAQERSDGRALAETTGYQSSFRSGSHQSITRGWAPPMHQTDSLFHKRAGVFGFTYGFDVDNHAI  
2fug.2.C  LYLSALGPAAG---------------------------------------------------------------------  
  
Target    NTVPKETVVRITKAEDGGVGGSGAWTRGRPGSMPGDEDDAMQAYLAGELTVVRRT  
2fug.2.C  -------------------------------------------------------
```

  


---

  

## Materials and Methods

## Template Search

Template search with
has been performed against the SWISS-MODEL template library (SMTL, last update: 2023-03-23, last included PDB release: 2023-03-17).

## Template Selection

For each identified template, the template's quality has been predicted from features of the target-template alignment.
The templates with the highest quality have then been selected for model building.

## Model Building

Models are built based on the target-template alignment using ProMod3 (Studer et al.). Coordinates which are conserved between the target and the template are copied from the template to the model. Insertions and deletions are remodelled using a fragment library. Side chains are then rebuilt. Finally, the geometry of the resulting model is regularized by using a force field.

## Model Quality Estimation

The global and per-residue model quality has been assessed using the QMEAN scoring function (Studer et al.).

## Ligand Modelling

Ligands present in the template structure are transferred by homology to the model when the following criteria are met: (a) The ligands are annotated as biologically relevant in the template library, (b) the ligand is in contact with the model, (c) the ligand is not clashing with the protein, (d) the residues in contact with the ligand are conserved between the target and the template. If any of these four criteria is not satisfied, a certain ligand will not be included in the model. The model summary includes information on why and which ligand has not been included.

## Oligomeric State Conservation

The quaternary structure annotation of the template is used to model the target sequence in its oligomeric form. The method (Bertoni et al.) is based on a supervised machine learning algorithm, Support Vector Machines (SVM), which combines interface conservation, structural clustering, and other template features to provide a quaternary structure quality estimate (QSQE). The QSQE score is a number between 0 and 1, reflecting the expected accuracy of the interchain contacts for a model built based a given alignment and template. Higher numbers indicate higher reliability. This complements the GMQE score which estimates the accuracy of the tertiary structure of the resulting model.

## References

- **BLAST**  
  Camacho, C., Coulouris, G., Avagyan, V., Ma, N., Papadopoulos, J.,
  Bealer, K., Madden, T.L. BLAST+: architecture and applications. BMC
  Bioinformatics 10, 421-430 (2009).
- **HHblits**  
  Steinegger, M., Meier, M., Mirdita, M., Vöhringer, H., Haunsberger,
  S. J., Söding, J. HH-suite3 for fast remote homology detection and
  deep protein annotation. BMC Bioinformatics 20, 473 (2019).

## Table T1:

Primary amino acid sequence for which templates were searched and models were built.

RPFLERFTDMPLLVRLDTLQRLRADEVFADYSSDLDVDGPSFTLHGMTEEQHERNGDRVVFDDASGALRAINREDVGDRLDDKGIDPALDYQGTVTLVDG  
STVEVMSVLSMYREHLADYDIDSVVDMTGAPRNLIEQLLDDMTTLSPVAFHVGEGVNHYFHATLHNRATYLVGMLLGSVGVSGGGVSTWAGNYKGGVFQA  
APWFGPGVGGFVNEDPFHPLTDPSARYSAETARHLVHGEDTSYWGFGDRPLVVDTPEDGRKVFTGTTHMPTPTKALWYNNANLINQAKWHYELVKNVNPK  
VDLIVDQQIEWTGSAEFADIVLPANSWMEAETWEMGASCSNPFLQVWKGGIEPLNDTRDDIAIFAGVANALTELTGDERFSQAFMFADRPEVYLDRVLAG  
SFTTEGYTVEDLTAGRYGPPGGALMQYRSYPRIPFKEQIEDSLPFYTDTGRMHGYVDIPEAIEYGENLIVHREAVEATPYLPNVIVSTSPYLRPRDYGIA  
PEELDGDARSVRNIMMSWAEVKETENPLFAAGYNYLCLTPKSRHAVHSSWAVTDWHWLWSSSFSDPYRVETRAPGVGEPAIHLNPDDARSLGIRNGDYVW  
VDSNPKDRPYRDADVDESFLDVARLLVRVTYNPAYPPGVTMLKHAFYMATPRTFRAAQERSDGRALAETTGYQSSFRSGSHQSITRGWAPPMHQTDSLFH  
KRAGVFGFTYGFDVDNHAINTVPKETVVRITKAEDGGVGGSGAWTRGRPGSMPGDEDDAMQAYLAGELTVVRRT

## Table T2:

| Template | Seq Identity | Oligo-state | QSQE | Found by | Method | Resolution | Seq Similarity | Coverage | Description |
| --- | --- | --- | --- | --- | --- | --- | --- | --- | --- |
| 7b04.1.B | 53.57 | monomer | - | HHblits | X-ray | 2.97Å | 0.46 | 1.00 | Nitrite oxidoreductase subunit A |
| 7b04.1.B | 54.04 | monomer | - | BLAST | X-ray | 2.97Å | 0.46 | 0.99 | Nitrite oxidoreductase subunit A |
| 7b04.2.B | 53.57 | monomer | - | HHblits | X-ray | 2.97Å | 0.46 | 1.00 | Nitrite oxidoreductase subunit A |
| 7b04.2.B | 54.04 | monomer | - | BLAST | X-ray | 2.97Å | 0.46 | 0.99 | Nitrite oxidoreductase subunit A |
| 3ir7.1.A | 22.74 | monomer | - | HHblits | X-ray | 2.50Å | 0.31 | 0.80 | Respiratory nitrate reductase 1 alpha chain |
| 3egw.1.A | 22.85 | homo-dimer | 0.06 | HHblits | X-ray | 1.90Å | 0.31 | 0.80 | Respiratory nitrate reductase 1 alpha chain |
| 3ir6.1.A | 23.05 | monomer | - | HHblits | X-ray | 2.80Å | 0.32 | 0.80 | Respiratory nitrate reductase 1 alpha chain |
| 1q16.1.A | 22.74 | monomer | - | HHblits | X-ray | 1.90Å | 0.31 | 0.80 | Respiratory nitrate reductase 1 alpha chain |
| 3ir5.1.A | 22.58 | monomer | - | HHblits | X-ray | 2.30Å | 0.31 | 0.80 | Respiratory nitrate reductase 1 alpha chain |
| 1r27.4.A | 22.74 | homo-dimer | - | HHblits | X-ray | 2.00Å | 0.31 | 0.80 | Respiratory nitrate reductase 1 alpha chain |
| 1dms.1.A | 21.85 | monomer | - | HHblits | X-ray | 1.88Å | 0.30 | 0.57 | DMSO REDUCTASE |
| 1e60.1.A | 21.95 | monomer | - | HHblits | X-ray | 2.00Å | 0.30 | 0.57 | Dimethyl sulfoxide/trimethylamine N-oxide reductase |
| 1e5v.2.A | 21.95 | monomer | - | HHblits | X-ray | 2.40Å | 0.30 | 0.57 | Dimethyl sulfoxide/trimethylamine N-oxide reductase |
| 1e18.1.A | 22.12 | monomer | - | HHblits | X-ray | 2.00Å | 0.30 | 0.57 | DMSO REDUCTASE. |
| 4dmr.1.A | 21.80 | monomer | - | HHblits | X-ray | 1.90Å | 0.30 | 0.57 | DMSO REDUCTASE |
| 2ivf.1.A | 32.76 | monomer | - | BLAST | X-ray | 1.88Å | 0.36 | 0.53 | ETHYLBENZENE DEHYDROGENASE ALPHA-SUBUNIT |
| 6q8o.1.C | 16.58 | monomer | - | HHblits | X-ray | 3.61Å | 0.27 | 0.25 | NADH-quinone oxidoreductase subunit 3 |
| 6zjl.1.C | 16.58 | monomer | - | HHblits | EM | NA | 0.27 | 0.25 | NADH-quinone oxidoreductase subunit 3 |
| 6ziy.1.C | 16.58 | monomer | - | HHblits | EM | NA | 0.27 | 0.25 | NADH-quinone oxidoreductase subunit 3 |
| 6zjn.1.C | 16.58 | monomer | - | HHblits | EM | NA | 0.27 | 0.25 | NADH-quinone oxidoreductase subunit 3 |
| 3m9s.1.C | 16.58 | monomer | - | HHblits | X-ray | 4.50Å | 0.27 | 0.25 | NADH-quinone oxidoreductase subunit 3 |
| 6zjy.1.C | 16.58 | monomer | - | HHblits | EM | NA | 0.27 | 0.25 | NADH-quinone oxidoreductase subunit 3 |
| 2fug.2.C | 16.58 | monomer | - | HHblits | X-ray | 3.30Å | 0.27 | 0.25 | NADH-quinone oxidoreductase chain 3 |
| 6zk9.1.C | 16.18 | monomer | - | HHblits | EM | NA | 0.27 | 0.22 | NADH:ubiquinone oxidoreductase core subunit S1 |
| 7zd6.1.4 | 16.18 | monomer | - | HHblits | EM | NA | 0.27 | 0.22 | NADH-ubiquinone oxidoreductase 75 kDa subunit, mitochondrial |
| 7dgr.10.A | 16.18 | monomer | - | HHblits | EM | NA | 0.27 | 0.22 | NADH-ubiquinone oxidoreductase 75 kDa subunit, mitochondrial |
| 6qcf.1.C | 15.61 | monomer | - | HHblits | EM | NA | 0.27 | 0.22 | NADH:ubiquinone oxidoreductase core subunit S1 |
| 6qc5.1.C | 15.61 | monomer | - | HHblits | EM | NA | 0.27 | 0.22 | NADH:ubiquinone oxidoreductase core subunit S1 |
| 5o31.1.8 | 16.18 | monomer | - | HHblits | EM | 4.13Å | 0.27 | 0.22 | NADH-ubiquinone oxidoreductase 75 kDa subunit, mitochondrial |
| 2pjh.1.B | 11.54 | monomer | - | HHblits | NMR | NA | 0.26 | 0.07 | Transitional endoplasmic reticulum ATPase |
| 5x4l.2.A | 11.32 | monomer | - | HHblits | X-ray | 2.40Å | 0.26 | 0.07 | Transitional endoplasmic reticulum ATPase |
| 3ir5.1.A | 12.96 | monomer | - | HHblits | X-ray | 2.30Å | 0.25 | 0.07 | Respiratory nitrate reductase 1 alpha chain |
| 5x4l.1.A | 11.32 | monomer | - | HHblits | X-ray | 2.40Å | 0.26 | 0.07 | Transitional endoplasmic reticulum ATPase |
| 4kdl.1.A | 11.54 | monomer | - | HHblits | X-ray | 1.81Å | 0.26 | 0.07 | Transitional endoplasmic reticulum ATPase |
| 5b6c.1.A | 11.54 | monomer | - | HHblits | X-ray | 1.55Å | 0.26 | 0.07 | Transitional endoplasmic reticulum ATPase |
| 3qc8.1.A | 11.76 | monomer | - | HHblits | X-ray | 2.20Å | 0.26 | 0.07 | Transitional endoplasmic reticulum ATPase |
| 4kdi.1.A | 11.54 | monomer | - | HHblits | X-ray | 1.86Å | 0.26 | 0.07 | Transitional endoplasmic reticulum ATPase |
| 3ir6.1.A | 12.96 | monomer | - | HHblits | X-ray | 2.80Å | 0.25 | 0.07 | Respiratory nitrate reductase 1 alpha chain |
| 4kdi.2.A | 11.54 | monomer | - | HHblits | X-ray | 1.86Å | 0.26 | 0.07 | Transitional endoplasmic reticulum ATPase |
| 1r27.4.A | 12.96 | monomer | - | HHblits | X-ray | 2.00Å | 0.25 | 0.07 | Respiratory nitrate reductase 1 alpha chain |
| 3ir7.1.A | 13.21 | monomer | - | HHblits | X-ray | 2.50Å | 0.25 | 0.07 | Respiratory nitrate reductase 1 alpha chain |
| 3egw.1.A | 12.96 | monomer | - | HHblits | X-ray | 1.90Å | 0.25 | 0.07 | Respiratory nitrate reductase 1 alpha chain |
| 1q16.1.A | 12.96 | monomer | - | HHblits | X-ray | 1.90Å | 0.25 | 0.07 | Respiratory nitrate reductase 1 alpha chain |
| 1e60.1.A | 14.55 | monomer | - | HHblits | X-ray | 2.00Å | 0.27 | 0.07 | Dimethyl sulfoxide/trimethylamine N-oxide reductase |
| 1e18.1.A | 14.55 | monomer | - | HHblits | X-ray | 2.00Å | 0.27 | 0.07 | DMSO REDUCTASE. |
| 1e5v.2.A | 14.55 | monomer | - | HHblits | X-ray | 2.40Å | 0.27 | 0.07 | Dimethyl sulfoxide/trimethylamine N-oxide reductase |
| 4dmr.1.A | 14.81 | monomer | - | HHblits | X-ray | 1.90Å | 0.27 | 0.07 | DMSO REDUCTASE |
| 1dms.1.A | 14.81 | monomer | - | HHblits | X-ray | 1.88Å | 0.27 | 0.07 | DMSO REDUCTASE |
| 5cup.1.A | 46.15 | monomer | - | HHblits | X-ray | 2.10Å | 0.43 | 0.03 | Phosphate propanoyltransferase |
| 5cuo.1.A | 46.15 | monomer | - | HHblits | X-ray | 1.54Å | 0.43 | 0.03 | Phosphate propanoyltransferase |

  
The table above shows the top 50 filtered templates. A further 179 templates were found which were considered to be less suitable for modelling than the filtered list.  
1aa6.1.A, 1cz4.1.A, 1cz5.1.A, 1eu1.1.A, 1fdo.1.A, 1g8j.1.A, 1g8k.1.A, 1h0h.1.A, 1j75.1.A, 1kqf.1.A, 1ogy.1.A, 1tmo.1.A, 1uhd.1.B, 1uhe.1.B, 1wlf.1.A, 2d9r.1.A, 2e7z.1.A, 2heo.1.A, 2heo.1.B, 2iv2.1.A, 2ivf.1.A, 2ki8.1.A, 2kz3.1.A, 2l1p.1.A, 2lnb.1.A, 2mrn.1.A, 2mru.1.A, 2mru.1.B, 2nya.1.A, 2o3f.1.A, 2v3v.1.A, 2v45.1.A, 2vpx.1.D, 2vpz.1.A, 2xvc.1.A, 3hu1.1.A, 3hu2.1.A, 3iwf.1.A, 3nzl.1.A, 3o27.1.A, 3o27.1.B, 3o5a.1.A, 3plx.1.B, 3qq7.1.A, 3qq8.1.A, 3qwz.1.A, 3tiw.1.A, 3tiw.2.A, 4aay.1.A, 4ga5.1.A, 4ga6.1.A, 4rv0.1.A, 4v4c.1.A, 4ydd.1.A, 5cuo.1.A, 5cup.1.A, 5e7o.1.A, 5e7p.1.A, 5epp.1.A, 5g4f.1.A, 5g4f.1.B, 5g4f.1.C, 5g4f.1.D, 5g4f.1.E, 5g4f.1.F, 5g4g.1.A, 5glf.1.A, 5glf.2.A, 5glf.3.A, 5glf.4.A, 5gpn.24.A, 5itm.1.A, 5itm.1.B, 5itm.1.E, 5nqd.1.A, 5t5i.1.B, 5t5i.1.D, 5xtb.1.L, 6btm.1.B, 6cz7.1.A, 6f0k.1.B, 6f49.1.A, 6g72.1.G, 6gcs.1.A, 6hd3.1.A, 6lod.1.B, 6rfq.1.A, 6rfs.1.A, 6s6y.1.B, 6sdr.1.A, 6sdv.1.A, 6tg9.1.A, 6x89.1.H, 6yj4.1.G, 6zr2.1.G, 7a23.1.O, 7ak5.1.G, 7ak6.1.G, 7aqr.1.F, 7ar7.1.G, 7ar8.1.G, 7arc.1.F, 7b04.1.B, 7b04.2.B, 7bkb.1.F, 7bkb.1.J, 7bkb.1.L, 7dbo.1.A, 7dbo.2.A, 7dg7.1.A, 7dg9.1.A, 7di0.1.A, 7di0.2.A, 7di0.3.A, 7di1.1.A, 7du6.1.A, 7du7.1.A, 7dvc.1.A, 7dvc.5.A, 7dvf.1.A, 7dvh.1.A, 7dvh.2.A, 7dvh.4.A, 7dww.1.A, 7dww.2.A, 7dxr.1.A, 7dxr.1.B, 7dxr.2.B, 7dxs.1.A, 7dxs.1.B, 7dxs.2.A, 7dxs.2.B, 7dxt.1.A, 7dxu.1.A, 7dxu.1.B, 7dxu.2.B, 7dxv.1.A, 7dxv.1.B, 7dxw.1.A, 7dxx.1.A, 7dxx.1.B, 7dxy.1.A, 7dxz.1.A, 7dxz.2.A, 7dxz.2.B, 7dxz.3.A, 7dyc.1.A, 7dyc.2.A, 7dyc.3.A, 7e5z.1.A, 7l5i.1.A, 7l5s.1.A, 7nz1.1.E, 7p61.1.C, 7p63.1.C, 7q5y.1.A, 7qsd.1.G, 7qv7.1.L, 7qv7.1.O, 7t2r.1.A, 7t30.1.A, 7tgh.58.A, 7ur8.1.A, 7v2c.1.L, 7vw6.1.A, 7vxu.1.L, 7wbb.1.A, 7wbb.1.B, 7wbb.1.C, 7wbb.1.D, 7wbb.1.E, 7wbb.1.G, 7z0t.1.G, 7zm7.1.I, 8b9z.1.G, 8ba0.1.G, 8bqg.1.A, 8e73.55.A, 8e9g.1.G

Swiss Institute of Bioinformatics
Contact Us
