# Supplementary material for: Metabolic capacity is maintained despite shifts in microbial diversity in estuary sediments
Source: ISME Commun. 2025 Oct 11;5(1):ycaf182. doi: 10.1093/ismeco/ycaf182 (PMC12687941; doi:10.1093/ismeco/ycaf182)
Supplement: Supplementary_Data_1_ycaf182 [file supplementary_data_1_ycaf182.zip › SWISS-MODEL/4_1_May_SF_Bin55_scaffold_648_c1_10064512_1/templates.html]

4\_1\_May\_SF\_Bin55\_scaffold\_648\_c1\_1006-4512\_1 | Templates


**Export Alignment**
  
FASTA format
Clustal Format
PNG Image

**Secondary Structure**
  
None
DSSP
PSIPRED
SSpro

**Colour Scheme** 


Fade Mismatches
Enhance Mismatches

Confidencegradient
Confidenceclass
Indels
Chain
Unique Chain
Rainbow
2° Structure
Clustal
Hydrophobic
Size
Charged
Polar
Proline
Ser/Thr
Cysteine
Aliphatic
Aromatic
No Colour

Use QMEANBrane values

|  |  |  |  |
| --- | --- | --- | --- |
| Background |  |  |  |

**3D Viewer**  
NGL
PV

FASTA
Multi FASTA
ClustalW
PNG


SWISS-MODEL

### 4\_1\_May\_SF\_Bin55\_scaffold\_648\_c1\_1006-4512\_1

### Created: March 29, 2023, 8:51 p.m. at 20:51

- Templates
- Models

Models | Name | Description | GMQE | QSQE | Seq Id | Coverage | Range | Method | Resolution | Oligo-state | Ligands | Found by | Seq Similarity || ✓ | 7b04.1.B | Nitrite oxidoreductase subunit A  *Structure of Nitrite oxidoreductase (Nxr) from the anammox bacterium Kuenenia stuttgartiensis.* | 0.75 | 0.00 | 40.77 | 0.96 | 3-1160 | X-ray | 2.97 | monomer | 4 x SF4, 1 x F3S, 2 x MD1, 1 x MO, 1 x HEM, 2 x CA | BLAST | 0.41 |
| ``` target    SDLSRRELLKRAVVVGTGAGLAELFLPAQFLSSASAQSEPQAVAIANPLAQMPDRSWERIYRDQFAEEDSFVFTCAPNDT 7b04.1    --LTRRAFLQVAGATGATLTLAKNAMAFRLLKPA--------VVVDNPLDTYPDRRWESVYRDQYQYDRTFTYCCSPNDT  target    HNCLLRAHVKNGVIVRISPTYGYGKATDLAGNQASHRWDPRICQKGLILGRRIYGDRRVKAPMIRKGFKEWADAGFPRHD 7b04.1    HACRIRAFVRNNVMMRVEQNYDHQNYSDLYGNKATRNWNPRMCLKGYTFHRRVYGPYRLRYPLIRKGWKRWADDGFPELT  target    -DGTPRADMEKRGYDEWLQIPWDEALAIAAKTLQNVAETYKGEDGAGKLLEQGYEPAMVEAMHGAGVQAIKMRGGMPLLG 7b04.1    PENKTKYMFDNRGNDELLRASWDEAFTYASKGIIHITKKYSGPEGAQKLIDQGYPKEMVDRMQGAGTRTFKGRGGMGLLG  target    AGRVFGFYRFANMLALLDGKLRPEAPPEEIVGSRAFDNYAWHTDLPPGHPMVSGSQTVDFDLFAAEHSKLLVLIGMNWIC 7b04.1    VIGKYGMYRFNNCLAIVDAHNRGVGP-DQALGGRNWSNYTWHGDQAPGHPFSHGLQTSDVDMNDVRFSKLLIQTGKNLIE  target    TKMPDAHWIGDARLKGTRVVVISADYMPTANKADEIVILRPGTDTAFLLGVARELITKKLYDRDAVIQRTDLPLLVRLDT 7b04.1    NKMPEAHWVTEVMERGGKIVVITPEYSPSAQKADYWIPIRNNTDTALFLGITKILIDNKWYDADYVKKFTDFPLLIRTDT  target    GERLSARDVFEGYRQAPLENYVALKTEEELAAPPSPPFTADKQVVPTELREEWGDFVYWDRATNGPAAVNRDEIG---AK 7b04.1    LKRVSPKDIIPNYKL------------QDISDGPSYHI----QGLKDEQREIIGDFVVWDAKSKGPKAITRDDVGETLVK  target    FAGDPALLGAFDVTLVDGTNVKARTAFSLLKEYLDENFDVQTTSEVCNVDPAAVRSLARQLAANKGNALLAAGMGPNHYF 7b04.1    KGIDPVLEGSFKLKTIDGKEIEVMTLLEMYKIHL-RDYDIDSVVSMTNSPKDLIERLAKDIATIKPVAIHYGE-GVNHYF  target    NADLFGRVHFLVAALTDNIGHFSGNVGSYAGNYRGSLFQA-------MGQWIAENPFDQEADLTKPAR---VKRYFKSES 7b04.1    HATLMNRSYYLPVMLTGNVGYFGSGSHTWAGNYKAGNFQASKWSGPGFYGWVAEDVFKPNLDPYASAKDLNIKGRALDEE  target    AHYWNYGDRPLVSPS-----EIITGKSHMPTPTKLIWFGNSNSLLGNAKWSFDVVKNTLPKQDAVFCNEWHWTSSCEYSD 7b04.1    VAYWNHSERPLIVNTPKYGRKVFTGKTHMPSPTKVLWFTNVN-LINNAKHVYQMLKNVNPNIEQIMSTDIEITGSIEYAD  target    LVFPADSWAEFKLPDMTASCTNPFLLAFPKTPLARIHNTRSDYEILAGVAAALADLVDEPRMKTYWKGILDGDPTPYLQR 7b04.1    FAFPANSWVEFQEFEITNSCSNPFIQIWGKTGITPVYESKDDVKILAGMASKLGELLRDKRFEDNWKFAIEGRASVYINR  target    VLSGSNATRGILYEDLHASS--AKGVPLLMNARTYPRHAGWEQRQEDKPWYTPTGRLEFYRPEPEWQAAGESLPIWREPV 7b04.1    LLDGSTTMKGYTCEDILNGKYGEPGVAMLL-FRTYPRHPFWEQVHESLPFYTPTGRLQAYNDEPEIIEYGENFIVHREGP  target    DATFYEPNAILANSKHPSINPRAPEDYGVPESQMDVETRQYRNVVRTWQELKLSKHPLTEKDPAYRFVFQTPKYRWGAHS 7b04.1    EATPYLPNAIV--STNPYIR---PDDYGIPENAEYWEDRTVRNIKKSWEETKKTKNFLWEK--GYHFYCVTPKSRHTVHS  target    TAVDSDWIAMLFGPFGDPYRRDSRTPWTGEAYAEINPRDAKELGLKDGDYIWLDADPEDRPYRGADSSDEFYDVARAMMR 7b04.1    QWAVTDWNFIWNNNFGDPYRMDKRMPGVGEHQIHIHPQAARDLGIEDGDYVYVDANPADRPYEGWKPNDSFYKVSRLMLR  target    VRIYSGMPRRVIRTWFNMYAATPGTVQAQKDVPGGPAQNQDTGYVALFRHGSHQSGTRAYLRPTQMTDSMNRKAYFGQTI 7b04.1    AKYNPAYPYNCTMMKHSAWISSDKTVQAHETRPDGRALSP-SGYQSSFRYGSQQSITRDWSMPMHQLDSLFHKAKIGMKF  target    GKGFEADVHSPSGAPKEGYVKVEKAEDGGDEGVGEWRPVTLGLRPDDPSEAMQAYLAGEFVTRKRKGS 7b04.1    IFGFEADNHCINTVPKETLVKITKAENGGMGGKGVWDPVKTGYTAGNENDFMKKFLNGELI------- ``` | | | | | | | | | | | | | | | | | | | | | | | | | | | | | | | | | | | | | | | | | | | | | | | | | |
|  | 7b04.2.B | Nitrite oxidoreductase subunit A  *Structure of Nitrite oxidoreductase (Nxr) from the anammox bacterium Kuenenia stuttgartiensis.* | 0.72 | 0.00 | 40.77 | 0.96 | 3-1160 | X-ray | 2.97 | monomer | 4 x SF4, 1 x F3S, 2 x MD1, 1 x MO, 1 x HEM, 2 x CA | BLAST | 0.41 |
| ``` target    SDLSRRELLKRAVVVGTGAGLAELFLPAQFLSSASAQSEPQAVAIANPLAQMPDRSWERIYRDQFAEEDSFVFTCAPNDT 7b04.2    --LTRRAFLQVAGATGATLTLAKNAMAFRLLKPA--------VVVDNPLDTYPDRRWESVYRDQYQYDRTFTYCCSPNDT  target    HNCLLRAHVKNGVIVRISPTYGYGKATDLAGNQASHRWDPRICQKGLILGRRIYGDRRVKAPMIRKGFKEWADAGFPRHD 7b04.2    HACRIRAFVRNNVMMRVEQNYDHQNYSDLYGNKATRNWNPRMCLKGYTFHRRVYGPYRLRYPLIRKGWKRWADDGFPELT  target    -DGTPRADMEKRGYDEWLQIPWDEALAIAAKTLQNVAETYKGEDGAGKLLEQGYEPAMVEAMHGAGVQAIKMRGGMPLLG 7b04.2    PENKTKYMFDNRGNDELLRASWDEAFTYASKGIIHITKKYSGPEGAQKLIDQGYPKEMVDRMQGAGTRTFKGRGGMGLLG  target    AGRVFGFYRFANMLALLDGKLRPEAPPEEIVGSRAFDNYAWHTDLPPGHPMVSGSQTVDFDLFAAEHSKLLVLIGMNWIC 7b04.2    VIGKYGMYRFNNCLAIVDAHNRGVGP-DQALGGRNWSNYTWHGDQAPGHPFSHGLQTSDVDMNDVRFSKLLIQTGKNLIE  target    TKMPDAHWIGDARLKGTRVVVISADYMPTANKADEIVILRPGTDTAFLLGVARELITKKLYDRDAVIQRTDLPLLVRLDT 7b04.2    NKMPEAHWVTEVMERGGKIVVITPEYSPSAQKADYWIPIRNNTDTALFLGITKILIDNKWYDADYVKKFTDFPLLIRTDT  target    GERLSARDVFEGYRQAPLENYVALKTEEELAAPPSPPFTADKQVVPTELREEWGDFVYWDRATNGPAAVNRDEIG---AK 7b04.2    LKRVSPKDIIPNYKL------------QDISDGPSYHI----QGLKDEQREIIGDFVVWDAKSKGPKAITRDDVGETLVK  target    FAGDPALLGAFDVTLVDGTNVKARTAFSLLKEYLDENFDVQTTSEVCNVDPAAVRSLARQLAANKGNALLAAGMGPNHYF 7b04.2    KGIDPVLEGSFKLKTIDGKEIEVMTLLEMYKIHL-RDYDIDSVVSMTNSPKDLIERLAKDIATIKPVAIHYGE-GVNHYF  target    NADLFGRVHFLVAALTDNIGHFSGNVGSYAGNYRGSLFQA-------MGQWIAENPFDQEADLTKPAR---VKRYFKSES 7b04.2    HATLMNRSYYLPVMLTGNVGYFGSGSHTWAGNYKAGNFQASKWSGPGFYGWVAEDVFKPNLDPYASAKDLNIKGRALDEE  target    AHYWNYGDRPLVSPS-----EIITGKSHMPTPTKLIWFGNSNSLLGNAKWSFDVVKNTLPKQDAVFCNEWHWTSSCEYSD 7b04.2    VAYWNHSERPLIVNTPKYGRKVFTGKTHMPSPTKVLWFTNVN-LINNAKHVYQMLKNVNPNIEQIMSTDIEITGSIEYAD  target    LVFPADSWAEFKLPDMTASCTNPFLLAFPKTPLARIHNTRSDYEILAGVAAALADLVDEPRMKTYWKGILDGDPTPYLQR 7b04.2    FAFPANSWVEFQEFEITNSCSNPFIQIWGKTGITPVYESKDDVKILAGMASKLGELLRDKRFEDNWKFAIEGRASVYINR  target    VLSGSNATRGILYEDLHASS--AKGVPLLMNARTYPRHAGWEQRQEDKPWYTPTGRLEFYRPEPEWQAAGESLPIWREPV 7b04.2    LLDGSTTMKGYTCEDILNGKYGEPGVAMLL-FRTYPRHPFWEQVHESLPFYTPTGRLQAYNDEPEIIEYGENFIVHREGP  target    DATFYEPNAILANSKHPSINPRAPEDYGVPESQMDVETRQYRNVVRTWQELKLSKHPLTEKDPAYRFVFQTPKYRWGAHS 7b04.2    EATPYLPNAIV--STNPYIR---PDDYGIPENAEYWEDRTVRNIKKSWEETKKTKNFLWEK--GYHFYCVTPKSRHTVHS  target    TAVDSDWIAMLFGPFGDPYRRDSRTPWTGEAYAEINPRDAKELGLKDGDYIWLDADPEDRPYRGADSSDEFYDVARAMMR 7b04.2    QWAVTDWNFIWNNNFGDPYRMDKRMPGVGEHQIHIHPQAARDLGIEDGDYVYVDANPADRPYEGWKPNDSFYKVSRLMLR  target    VRIYSGMPRRVIRTWFNMYAATPGTVQAQKDVPGGPAQNQDTGYVALFRHGSHQSGTRAYLRPTQMTDSMNRKAYFGQTI 7b04.2    AKYNPAYPYNCTMMKHSAWISSDKTVQAHETRPDGRALSP-SGYQSSFRYGSQQSITRDWSMPMHQLDSLFHKAKIGMKF  target    GKGFEADVHSPSGAPKEGYVKVEKAEDGGDEGVGEWRPVTLGLRPDDPSEAMQAYLAGEFVTRKRKGS 7b04.2    IFGFEADNHCINTVPKETLVKITKAENGGMGGKGVWDPVKTGYTAGNENDFMKKFLNGELI------- ``` | | | | | | | | | | | | | | | | | | | | | | | | | | | | | | | | | | | | | | | | | | | | | | | | | |
|  | 7b04.1.B | Nitrite oxidoreductase subunit A  *Structure of Nitrite oxidoreductase (Nxr) from the anammox bacterium Kuenenia stuttgartiensis.* | 0.75 | 0.00 | 39.64 | 0.96 | 1-1163 | X-ray | 2.97 | monomer | 4 x SF4, 1 x F3S, 2 x MD1, 1 x MO, 1 x HEM, 2 x CA | HHblits | 0.40 |
| ``` target    SDLSRRELLKRAVVVGTGAGLAELFLPAQFLSSASAQSEPQAVAIANPLAQMPDRSWERIYRDQFAEEDSFVFTCAPNDT 7b04.1    MKLTRRAFLQVAGATGATLTLAKNAMAFRLLK--------PAVVVDNPLDTYPDRRWESVYRDQYQYDRTFTYCCSPNDT  target    HNCLLRAHVKNGVIVRISPTYGYGKATDLAGNQASHRWDPRICQKGLILGRRIYGDRRVKAPMIRKGFKEWADAGFPRHD 7b04.1    HACRIRAFVRNNVMMRVEQNYDHQNYSDLYGNKATRNWNPRMCLKGYTFHRRVYGPYRLRYPLIRKGWKRWADDGFPELT  target    D-GTPRADMEKRGYDEWLQIPWDEALAIAAKTLQNVAETY-KGEDGAGKLLEQGYEPAMVEAMHGAGVQAIKMRGGMPLL 7b04.1    PENKTKYMFDNRGNDELLRASWDEAFTYASKGIIHITKKYSGPEGA-QKLIDQGYPKEMVDRMQGAGTRTFKGRGGMGLL  target    GAGRVFGFYRFANMLALLDGKLRPEAPPEEIVGSRAFDNYAWHTDLPPGHPMVSGSQTVDFDLFAAEHSKLLVLIGMNWI 7b04.1    GVIGKYGMYRFNNCLAIVDAHNRG-VGPDQALGGRNWSNYTWHGDQAPGHPFSHGLQTSDVDMNDVRFSKLLIQTGKNLI  target    CTKMPDAHWIGDARLKGTRVVVISADYMPTANKADEIVILRPGTDTAFLLGVARELITKKLYDRDAVIQRTDLPLLVRLD 7b04.1    ENKMPEAHWVTEVMERGGKIVVITPEYSPSAQKADYWIPIRNNTDTALFLGITKILIDNKWYDADYVKKFTDFPLLIRTD  target    TGERLSARDVFEGYRQAPLENYVALKTEEELAAPPSPPFTADKQVVPTELREEWGDFVYWDRATNGPAAVNRDEIG---A 7b04.1    TLKRVSPKDIIPNYKLQDISD--------------GPSY--HIQGLKDEQREIIGDFVVWDAKSKGPKAITRDDVGETLV  target    KFAGDPALLGAFDVTLVDGTNVKARTAFSLLKEYLDENFDVQTTSEVCNVDPAAVRSLARQLAANKGNALLAAGMGPNHY 7b04.1    KKGIDPVLEGSFKLKTIDGKEIEVMTLLEMYKIHLR-DYDIDSVVSMTNSPKDLIERLAKDIATIKP-VAIHYGEGVNHY  target    FNADLFGRVHFLVAALTDNIGHFSGNVGSYAGNYRGSLFQA---MG-Q---WIAENPFDQEAD-LTKP--ARVKRYFKSE 7b04.1    FHATLMNRSYYLPVMLTGNVGYFGSGSHTWAGNYKAGNFQASKWSGPGFYGWVAEDVFKPNLDPYASAKDLNIKGRALDE  target    SAHYWNYGDRPLV-----SPSEIITGKSHMPTPTKLIWFGNSNSLLGNAKWSFDVVKNTLPKQDAVFCNEWHWTSSCEYS 7b04.1    EVAYWNHSERPLIVNTPKYGRKVFTGKTHMPSPTKVLWFTNVNLINNAKHV-YQMLKNVNPNIEQIMSTDIEITGSIEYA  target    DLVFPADSWAEFKLPDMTASCTNPFLLAFPKTPLARIHNTRSDYEILAGVAAALADLVDEPRMKTYWKGILDGDPTPYLQ 7b04.1    DFAFPANSWVEFQEFEITNSCSNPFIQIWGKTGITPVYESKDDVKILAGMASKLGELLRDKRFEDNWKFAIEGRASVYIN  target    RVLSGSNATRGILYEDLHASSA--KGVPLLMNARTYPRHAGWEQRQEDKPWYTPTGRLEFYRPEPEWQAAGESLPIWREP 7b04.1    RLLDGSTTMKGYTCEDILNGKYGEPGVAM-LLFRTYPRHPFWEQVHESLPFYTPTGRLQAYNDEPEIIEYGENFIVHREG  target    VDATFYEPNAILANSKHPSINPRAPEDYGVPESQMDVETRQYRNVVRTWQELKLSKHPLTEKDPAYRFVFQTPKYRWGAH 7b04.1    PEATPYLPNAIVST--NPYI---RPDDYGIPENAEYWEDRTVRNIKKSWEETKKTKNFLW--EKGYHFYCVTPKSRHTVH  target    STAVDSDWIAMLFGPFGDPYRRDSRTPWTGEAYAEINPRDAKELGLKDGDYIWLDADPEDRPYRGADSSDEFYDVARAMM 7b04.1    SQWAVTDWNFIWNNNFGDPYRMDKRMPGVGEHQIHIHPQAARDLGIEDGDYVYVDANPADRPYEGWKPNDSFYKVSRLML  target    RVRIYSGMPRRVIRTWFNMYAATPGTVQAQKDVPGGPAQNQDTGYVALFRHGSHQSGTRAYLRPTQMTDSMNRKAYFGQT 7b04.1    RAKYNPAYPYNCTMMKHSAWISSDKTVQAHETRPDGRAL-SPSGYQSSFRYGSQQSITRDWSMPMHQLDSLFHKAKIGMK  target    IGKGFEADVHSPSGAPKEGYVKVEKAEDGGDEGVGEWRPVTLGLRPDDPSEAMQAYLAGEFVTRKRKGS 7b04.1    FIFGFEADNHCINTVPKETLVKITKAENGGMGGKGVWDPVKTGYTAGNENDFMKKFLNGELIKVD---- ``` | | | | | | | | | | | | | | | | | | | | | | | | | | | | | | | | | | | | | | | | | | | | | | | | | |
|  | 7b04.2.B | Nitrite oxidoreductase subunit A  *Structure of Nitrite oxidoreductase (Nxr) from the anammox bacterium Kuenenia stuttgartiensis.* | 0.72 | 0.00 | 39.64 | 0.96 | 1-1163 | X-ray | 2.97 | monomer | 4 x SF4, 1 x F3S, 2 x MD1, 1 x MO, 1 x HEM, 2 x CA | HHblits | 0.40 |
| ``` target    SDLSRRELLKRAVVVGTGAGLAELFLPAQFLSSASAQSEPQAVAIANPLAQMPDRSWERIYRDQFAEEDSFVFTCAPNDT 7b04.2    MKLTRRAFLQVAGATGATLTLAKNAMAFRLLK--------PAVVVDNPLDTYPDRRWESVYRDQYQYDRTFTYCCSPNDT  target    HNCLLRAHVKNGVIVRISPTYGYGKATDLAGNQASHRWDPRICQKGLILGRRIYGDRRVKAPMIRKGFKEWADAGFPRHD 7b04.2    HACRIRAFVRNNVMMRVEQNYDHQNYSDLYGNKATRNWNPRMCLKGYTFHRRVYGPYRLRYPLIRKGWKRWADDGFPELT  target    D-GTPRADMEKRGYDEWLQIPWDEALAIAAKTLQNVAETY-KGEDGAGKLLEQGYEPAMVEAMHGAGVQAIKMRGGMPLL 7b04.2    PENKTKYMFDNRGNDELLRASWDEAFTYASKGIIHITKKYSGPEGA-QKLIDQGYPKEMVDRMQGAGTRTFKGRGGMGLL  target    GAGRVFGFYRFANMLALLDGKLRPEAPPEEIVGSRAFDNYAWHTDLPPGHPMVSGSQTVDFDLFAAEHSKLLVLIGMNWI 7b04.2    GVIGKYGMYRFNNCLAIVDAHNRG-VGPDQALGGRNWSNYTWHGDQAPGHPFSHGLQTSDVDMNDVRFSKLLIQTGKNLI  target    CTKMPDAHWIGDARLKGTRVVVISADYMPTANKADEIVILRPGTDTAFLLGVARELITKKLYDRDAVIQRTDLPLLVRLD 7b04.2    ENKMPEAHWVTEVMERGGKIVVITPEYSPSAQKADYWIPIRNNTDTALFLGITKILIDNKWYDADYVKKFTDFPLLIRTD  target    TGERLSARDVFEGYRQAPLENYVALKTEEELAAPPSPPFTADKQVVPTELREEWGDFVYWDRATNGPAAVNRDEIG---A 7b04.2    TLKRVSPKDIIPNYKLQDISD--------------GPSY--HIQGLKDEQREIIGDFVVWDAKSKGPKAITRDDVGETLV  target    KFAGDPALLGAFDVTLVDGTNVKARTAFSLLKEYLDENFDVQTTSEVCNVDPAAVRSLARQLAANKGNALLAAGMGPNHY 7b04.2    KKGIDPVLEGSFKLKTIDGKEIEVMTLLEMYKIHLR-DYDIDSVVSMTNSPKDLIERLAKDIATIKP-VAIHYGEGVNHY  target    FNADLFGRVHFLVAALTDNIGHFSGNVGSYAGNYRGSLFQA---MG-Q---WIAENPFDQEAD-LTKP--ARVKRYFKSE 7b04.2    FHATLMNRSYYLPVMLTGNVGYFGSGSHTWAGNYKAGNFQASKWSGPGFYGWVAEDVFKPNLDPYASAKDLNIKGRALDE  target    SAHYWNYGDRPLV-----SPSEIITGKSHMPTPTKLIWFGNSNSLLGNAKWSFDVVKNTLPKQDAVFCNEWHWTSSCEYS 7b04.2    EVAYWNHSERPLIVNTPKYGRKVFTGKTHMPSPTKVLWFTNVNLINNAKHV-YQMLKNVNPNIEQIMSTDIEITGSIEYA  target    DLVFPADSWAEFKLPDMTASCTNPFLLAFPKTPLARIHNTRSDYEILAGVAAALADLVDEPRMKTYWKGILDGDPTPYLQ 7b04.2    DFAFPANSWVEFQEFEITNSCSNPFIQIWGKTGITPVYESKDDVKILAGMASKLGELLRDKRFEDNWKFAIEGRASVYIN  target    RVLSGSNATRGILYEDLHASSA--KGVPLLMNARTYPRHAGWEQRQEDKPWYTPTGRLEFYRPEPEWQAAGESLPIWREP 7b04.2    RLLDGSTTMKGYTCEDILNGKYGEPGVAM-LLFRTYPRHPFWEQVHESLPFYTPTGRLQAYNDEPEIIEYGENFIVHREG  target    VDATFYEPNAILANSKHPSINPRAPEDYGVPESQMDVETRQYRNVVRTWQELKLSKHPLTEKDPAYRFVFQTPKYRWGAH 7b04.2    PEATPYLPNAIVST--NPYI---RPDDYGIPENAEYWEDRTVRNIKKSWEETKKTKNFLW--EKGYHFYCVTPKSRHTVH  target    STAVDSDWIAMLFGPFGDPYRRDSRTPWTGEAYAEINPRDAKELGLKDGDYIWLDADPEDRPYRGADSSDEFYDVARAMM 7b04.2    SQWAVTDWNFIWNNNFGDPYRMDKRMPGVGEHQIHIHPQAARDLGIEDGDYVYVDANPADRPYEGWKPNDSFYKVSRLML  target    RVRIYSGMPRRVIRTWFNMYAATPGTVQAQKDVPGGPAQNQDTGYVALFRHGSHQSGTRAYLRPTQMTDSMNRKAYFGQT 7b04.2    RAKYNPAYPYNCTMMKHSAWISSDKTVQAHETRPDGRAL-SPSGYQSSFRYGSQQSITRDWSMPMHQLDSLFHKAKIGMK  target    IGKGFEADVHSPSGAPKEGYVKVEKAEDGGDEGVGEWRPVTLGLRPDDPSEAMQAYLAGEFVTRKRKGS 7b04.2    FIFGFEADNHCINTVPKETLVKITKAENGGMGGKGVWDPVKTGYTAGNENDFMKKFLNGELIKVD---- ``` | | | | | | | | | | | | | | | | | | | | | | | | | | | | | | | | | | | | | | | | | | | | | | | | | |
|  | 1r27.4.A | Respiratory nitrate reductase 1 alpha chain  *Crystal Structure of NarGH complex* | 0.42 | 0.14 | 23.96 | 0.80 | 2-1130 | X-ray | 2.00 | homo-dimer | 4 x MO, 16 x SF4, 8 x MGD, 4 x F3S | HHblits | 0.32 |
| ``` target    SDLSRRELLKRAVVVGTGAGLAELFLPAQFLSSASAQSEPQAVAIANPLAQMPDRSWERIYRDQFAEEDSFVFTCAPNDT 1r27.4    -FLDRFRYFKQKGETFADGHG----------Q-----------------LLNTNRDWEDGYRQRWQHDKIVRSTHGVNCT  target    HNCLLRAHVKNGVIVRISPTYGYGKATDLAGNQASHRWDPRICQKGLILGRRIYGDRRVKAPMIRKGFKE-WADAGF--- 1r27.4    GSCSWKIYVKNGLVTWETQQTDYPR-----TRPDLPNHEPRGCPRGASYSWYLYSANRLKYPMMRKRLMKMWREAKALHS  target    -P-------RHDDGTPRADMEKRGYDEWLQIPWDEALAIAAKTLQNVAETYKGEDGAGKLLEQGYEPAMVEAMHGAGVQA 1r27.4    DPVEAWASIIEDADKAKSFKQARGRGGFVRSSWQEVNELIAASNVYTIKNYGPDR----VAG----------FSPIPAMS  target    IKMRGGMPLLGAGRVFGFYRFANMLALLDGKLRPEAPPEEIVGSRAFDNYAWHTDLPPGHPMVSGSQTVDFDLFAAEHSK 1r27.4    M-----------VSYASGARYLS-----------------LIGGTCLSFYDWYCDLPPASPQTWGEQTDVPESADWYNSS  target    LLVLIGMNWICTKMPDAHWIGDARLKGTRVVVISADYMPTANKADEIVILRPGTDTAFLLGVARELITKKL------YDR 1r27.4    YIIAWGSNVPQTRTPDAHFFTEVRYKGTKTVAVTPDYAEIAKLCDLWLAPKQGTDAAMALAMGHVMLREFHLDNPSQYFT  target    DAVIQRTDLPLLVRLDT-------GERLSARDVFEGYRQAPLE--NYVALKTEEELAAPPSPPFTADKQVVPT-ELREEW 1r27.4    DYVRRYTDMPMLVMLEERDGYYAAGRMLRAADLVDALGQENNPEWKTVAFNT-------------NGEMVAPNGSIGFRW  target    GDFVYWDR-----ATNGPAAVNR------DEI----GAKFAG-------DPAL----LG---AFDVTLVDGTNVKARTAF 1r27.4    GEKGKWNLEQRDGKTGEETELQLSLLGSQDEIAEVGFPYFGGDGTEHFNKVELENVLLHKLPVKRLQLADGSTALVTTVY  target    SLLK------------------EYLDENFDVQTTSEVCNVDPAAVRSLARQLAANK----GNALLAAGMGPNHYFNADLF 1r27.4    DLTLANYGLERGLNDVNCATSYDDV-KAYTPAWAEQITGVSRSQIIRIAREFADNADKTHGRSMIIVGAGLNHWYHLDMN  target    GRVHFLVAALTDNIGHFSGNVGSYAGNYRGSLFQAMGQWIAENPFD--Q-----E------------A-----DLTKPA- 1r27.4    YRGLINMLIFCGCVGQSGGGWAHYVGQEKLRPQTGWQPLAFALDWQRPARHMNSTSYFYNHSSQWRYETVTAEELLSPMA  target    RVKRYFK--------SESAHYW----NYGDRPL----------V-----SPSEIITGKS--------HMPTPTKLIWFGN 1r27.4    DKSRYTGHLIDFNVRAERMGWLPSAPQLGTNPLTIAGEAEKAGMNPVDYTVKSLKEGSIRFAAEQPENGKNHPRNLFIWR  target    SNSLLGNAKWSFDV------------------------------VKNTLPKQDAVFCNEWHWTSSCEYSDLVFPADSWAE 1r27.4    SNLLGSSGKGHEFMLKYLLGTEHGIQGKDLGQQGGVKPEEVDWQDNGLEGKLDLVVTLDFRLSSTCLYSDIILPTATWYE  target    FKLPDMTASCTNPFLLAFPKTPLARIHNTRSDYEILAGVAAALADLVDEP------------------------RMKTYW 1r27.4    K--DDMNTSDMHPFIHPLSA-AVDPAWEAKSDWEIYKAIAKKFSEVCVGHLGKETDIVTLPIQHDSAAELAQPLDVKDWK  target    KGILD--------------G-------------------------------DPTPYLQR--------------------- 1r27.4    KGECDLIPGKTAPHIMVVERDYPATYERFTSIGPLMEKIGNGGKGIAWNTQSEMDLLRKLNYTKAEGPAKGQPMLNTAID  target    ----VLSGSNATRGI----LYEDLHASSAK-----------GVPLLMNA------------------RTYPRHAGWEQRQ 1r27.4    AAEMILTLAPETNGQVAVKAWAALSEFTGRDHTHLALNKEDEKIRFRDIQAQPRKIISSPTWSGLEDEHVSYNAGYTNVH  target    EDKPWYTPTGRLEFYRPEPEWQAAGESLPIWREPVDATFYEPNAILANSKHPSINPRAPEDYGVPESQMDVETRQYRNVV 1r27.4    ELIPWRTLSGRQQLYQDHQWMRDFGESLLVYRPPIDTRSVK------------------EVIG-----------------  target    RTWQELKLSKHPLTEKDPAYRFVFQTPKYRWGAHSTAVDSDWIAMLFGPFGDPYRRDSRTPWTGEAYAEINPRDAKELGL 1r27.4    -----------QKSNGNQEKALNFLTPHQKWGIHSTYSDNLLMLTLG---------------RGGPVVWLSEADAKDLGI  target    KDGDYIWLDADPEDRPYRGADSSDEFYDVARAMMRVRIYSGMPRRVIRTWFNMYAATPGTVQAQKDVPGGPAQNQDTGYV 1r27.4    ADNDWIEVFNS-----------------NGALTARAVVSQRVPAGMTMMYHAQERI----V----NLPGSEIT-------  target    ALFRHGSHQSGTRAYLRPTQMTDSMNRKAYFGQTIGKGFEADVHSPSGAPKEGYVKVEKAEDGGDEGVGEWRPVTLGLRP 1r27.4    -QQRGGIHNSVTRITPKPTHMIGGYAHLAY-------G--FNYYGTVGSNRDEFVVVRKMKNIDWL--------------  target    DDPSEAMQAYLAGEFVTRKRKGS 1r27.4    ----------------------- ``` | | | | | | | | | | | | | | | | | | | | | | | | | | | | | | | | | | | | | | | | | | | | | | | | | |
|  | 3ir7.1.A | Respiratory nitrate reductase 1 alpha chain  *Crystal structure of NarGHI mutant NarG-R94S* | 0.42 | 0.00 | 23.85 | 0.80 | 2-1130 | X-ray | 2.50 | monomer | 2 x MD1, 4 x SF4, 1 x 6MO, 1 x AGA, 1 x F3S, 2 x HEM | HHblits | 0.32 |
| ``` target    SDLSRRELLKRAVVVGTGAGLAELFLPAQFLSSASAQSEPQAVAIANPLAQMPDRSWERIYRDQFAEEDSFVFTCAPNDT 3ir7.1    -FLDRFRYFKQKGETFADGHG----------Q-----------------LLNTNRDWEDGYRQRWQHDKIVRSTHGVNCT  target    HNCLLRAHVKNGVIVRISPTYGYGKATDLAGNQASHRWDPRICQKGLILGRRIYGDRRVKAPMIRKGFKE-WADAGFP-- 3ir7.1    GSCSWKIYVKNGLVTWETQQTDYPR-----TRPDLPNHEPRGCPSGASYSWYLYSANRLKYPMMRKRLMKMWREAKALHS  target    --------RHDD-GTPRADMEKRGYDEWLQIPWDEALAIAAKTLQNVAETYKGEDGAGKLLEQGYEPAMVEAMHGAGVQA 3ir7.1    DPVEAWASIIEDADKAKSFKQARGRGGFVRSSWQEVNELIAASNVYTIKNYGPDR----VAG----------FSPIPAMS  target    IKMRGGMPLLGAGRVFGFYRFANMLALLDGKLRPEAPPEEIVGSRAFDNYAWHTDLPPGHPMVSGSQTVDFDLFAAEHSK 3ir7.1    M-----------VSYASGARYLS-----------------LIGGTCLSFYDWYCDLPPASPQTWGEQTDVPESADWYNSS  target    LLVLIGMNWICTKMPDAHWIGDARLKGTRVVVISADYMPTANKADEIVILRPGTDTAFLLGVARELITKKL------YDR 3ir7.1    YIIAWGSNVPQTRTPDAHFFTEVRYKGTKTVAVTPDYAEIAKLCDLWLAPKQGTDAAMALAMGHVMLREFHLDNPSQYFT  target    DAVIQRTDLPLLVRLDT-------GERLSARDVFEGYRQAPLE--NYVALKTEEELAAPPSPPFTADKQVVPT-ELREEW 3ir7.1    DYVRRYTDMPMLVMLEERDGYYAAGRMLRAADLVDALGQENNPEWKTVAFNT-------------NGEMVAPNGSIGFRW  target    GDFVYWDRATN-----GPAAVNR------DEI----GAKF-----------AGDPALLG---AFDVTLVDGTNVKARTAF 3ir7.1    GEKGKWNLEQRDGKTGEETELQLSLLGSQDEIAEVGFPYFGGDGTEHFNKVELENVLLHKLPVKRLQLADGSTALVTTVY  target    SLLK------------------EYLDENFDVQTTSEVCNVDPAAVRSLARQLAANK----GNALLAAGMGPNHYFNADLF 3ir7.1    DLTLANYGLERGLNDVNCATSYDDV-KAYTPAWAEQITGVSRSQIIRIAREFADNADKTHGRSMIIVGAGLNHWYHLDMN  target    GRVHFLVAALTDNIGHFSGNVGSYAGNYRGSLFQAMGQWIAENPFDQEA------------------------DLTKPA- 3ir7.1    YRGLINMLIFCGCVGQSGGGWAHYVGQEKLRPQTGWQPLAFALDWQRPARHMNSTSYFYNHSSQWRYETVTAEELLSPMA  target    RVKRYF--------KSESAHYW----NYGDRPL----------V-----SPSEIITGKS--------HMPTPTKLIWFGN 3ir7.1    DKSRYTGHLIDFNVRAERMGWLPSAPQLGTNPLTIAGEAEKAGMNPVDYTVKSLKEGSIRFAAEQPENGKNHPRNLFIWR  target    SNSLLGNAKWSFDV------------------------------VKNTLPKQDAVFCNEWHWTSSCEYSDLVFPADSWAE 3ir7.1    SNLLGSSGKGHEFMLKYLLGTEHGIQGKDLGQQGGVKPEEVDWQDNGLEGKLDLVVTLDFRLSSTCLYSDIILPTATWYE  target    FKLPDMTASCTNPFLLAFPKTPLARIHNTRSDYEILAGVAAALADLVDEP------------------------RMKTYW 3ir7.1    K--DDMNTSDMHPFIHPLSA-AVDPAWEAKSDWEIYKAIAKKFSEVCVGHLGKETDIVTLPIQHDSAAELAQPLDVKDWK  target    KGILD---G-----------D-------------------------------PTPYLQR--------------------- 3ir7.1    KGECDLIPGKTAPHIMVVERDYPATYERFTSIGPLMEKIGNGGKGIAWNTQSEMDLLRKLNYTKAEGPAKGQPMLNTAID  target    ----VLSGSNATRGI----LYEDLHASSA-----------KGVPLLMNA------------------RTYPRHAGWEQRQ 3ir7.1    AAEMILTLAPETNGQVAVKAWAALSEFTGRDHTHLALNKEDEKIRFRDIQAQPRKIISSPTWSGLEDEHVSYNAGYTNVH  target    EDKPWYTPTGRLEFYRPEPEWQAAGESLPIWREPVDATFYEPNAILANSKHPSINPRAPEDYGVPESQMDVETRQYRNVV 3ir7.1    ELIPWRTLSGRQQLYQDHQWMRDFGESLLVYRPPIDTRSVK------------------EVI------------------  target    RTWQELKLSKHPLTEKDPAYRFVFQTPKYRWGAHSTAVDSDWIAMLFGPFGDPYRRDSRTPWTGEAYAEINPRDAKELGL 3ir7.1    ----------GQKSNGNQEKALNFLTPHQKWGIHSTYSDNLLMLTLG---------------RGGPVVWLSEADAKDLGI  target    KDGDYIWLDADPEDRPYRGADSSDEFYDVARAMMRVRIYSGMPRRVIRTWFNMYAATPGTVQAQKDVPGGPAQNQDTGYV 3ir7.1    ADNDWIEVFNS-----------------NGALTARAVVSQRVPAGMTMMYHAQERI----V----NLPGSEI--------  target    ALFRHGSHQSGTRAYLRPTQMTDSMNRKAYFGQTIGKGFEADVHSPSGAPKEGYVKVEKAEDGGDEGVGEWRPVTLGLRP 3ir7.1    TQQRGGIHNSVTRITPKPTHMIGGYAHLAY-------G--FNYYGTVGSNRDEFVVVRKMKNIDWL--------------  target    DDPSEAMQAYLAGEFVTRKRKGS 3ir7.1    ----------------------- ``` | | | | | | | | | | | | | | | | | | | | | | | | | | | | | | | | | | | | | | | | | | | | | | | | | |
|  | 3ir6.1.A | Respiratory nitrate reductase 1 alpha chain  *Crystal structure of NarGHI mutant NarG-H49S* | 0.41 | 0.00 | 23.85 | 0.80 | 2-1126 | X-ray | 2.80 | monomer | 2 x GDP, 1 x AGA, 3 x SF4, 1 x F3S, 2 x HEM | HHblits | 0.32 |
| ``` target    SDLSRRELLKRAVVVGTGAGLAELFLPAQFLSSASAQSEPQAVAIANPLAQMPDRSWERIYRDQFAEEDSFVFTCAPNDT 3ir6.1    -FLDRFRYFKQKGETFADGH--GQ-------------------------LLNTNRDWEDGYRQRWQHDKIVRSTSGVNCT  target    HNCLLRAHVKNGVIVRISPTYGYGKATDLAGNQASHRWDPRICQKGLILGRRIYGDRRVKAPMIRKGFKE-WADAGF--- 3ir6.1    GSCSWKIYVKNGLVTWETQQTDYPR-----TRPDLPNHEPRGCPRGASYSWYLYSANRLKYPMMRKRLMKMWREAKALHS  target    -P------RHD-DGTPRADMEKRGYDEWLQIPWDEALAIAAKTLQNVAETYKGEDGAGKLLEQGYEPAMVEAMHGAGVQA 3ir6.1    DPVEAWASIIEDADKAKSFKQARGRGGFVRSSWQEVNELIAASNVYTIKNYGPDR----VAG----------FSPIPAMS  target    IKMRGGMPLLGAGRVFGFYRFANMLALLDGKLRPEAPPEEIVGSRAFDNYAWHTDLPPGHPMVSGSQTVDFDLFAAEHSK 3ir6.1    MV-----------SYASGARYLS-----------------LIGGTCLSFYDWYCDLPPASPQTWGEQTDVPESADWYNSS  target    LLVLIGMNWICTKMPDAHWIGDARLKGTRVVVISADYMPTANKADEIVILRPGTDTAFLLGVARELITKKL------YDR 3ir6.1    YIIAWGSNVPQTRTPDAHFFTEVRYKGTKTVAVTPDYAEIAKLCDLWLAPKQGTDAAMALAMGHVMLREFHLDNPSQYFT  target    DAVIQRTDLPLLVRLD-------TGERLSARDVFEGYRQAPLE--NYVALKTEEELAAPPSPPFTADKQVVPT-ELREEW 3ir6.1    DYVRRYTDMPMLVMLEERDGYYAAGRMLRAADLVDALGQENNPEWKTVAFNT-------------NGEMVAPNGSIGFRW  target    GDFVYWDR-----ATNGPAAVNR------DEI----GAKFAG-------DPAL----LG---AFDVTLVDGTNVKARTAF 3ir6.1    GEKGKWNLEQRDGKTGEETELQLSLLGSQDEIAEVGFPYFGGDGTEHFNKVELENVLLHKLPVKRLQLADGSTALVTTVY  target    SLLK------------------EYLDENFDVQTTSEVCNVDPAAVRSLARQLAANK----GNALLAAGMGPNHYFNADLF 3ir6.1    DLTLANYGLERGLNDVNCATSYDDV-KAYTPAWAEQITGVSRSQIIRIAREFADNADKTHGRSMIIVGAGLNHWYHLDMN  target    GRVHFLVAALTDNIGHFSGNVGSYAGNYRGSLFQAMGQWIAENPF--DQE----A------------------DLTKPA- 3ir6.1    YRGLINMLIFCGCVGQSGGGWAHYVGQEKLRPQTGWQPLAFALDWQRPARHMNSTSYFYNHSSQWRYETVTAEELLSPMA  target    RVKRYFK--------SESAHYW----NYGDRPL----------V-----SPSEIITGKS--------HMPTPTKLIWFGN 3ir6.1    DKSRYTGHLIDFNVRAERMGWLPSAPQLGTNPLTIAGEAEKAGMNPVDYTVKSLKEGSIRFAAEQPENGKNHPRNLFIWR  target    SNSLLGNAKWSFD----------------------------V--VKNTLPKQDAVFCNEWHWTSSCEYSDLVFPADSWAE 3ir6.1    SNLLGSSGKGHEFMLKYLLGTEHGIQGKDLGQQGGVKPEEVDWQDNGLEGKLDLVVTLDFRLSSTCLYSDIILPTATWYE  target    FKLPDMTASCTNPFLLAFPKTPLARIHNTRSDYEILAGVAAALADLVDEP------------------------RMKTYW 3ir6.1    K--DDMNTSDMHPFIHPLSA-AVDPAWEAKSDWEIYKAIAKKFSEVCVGHLGKETDIVTLPIQHDSAAELAQPLDVKDWK  target    KGILDG---------------------------------------------DPTPYLQR--------------------- 3ir6.1    KGECDLIPGKTAPHIMVVERDYPATYERFTSIGPLMEKIGNGGKGIAWNTQSEMDLLRKLNYTKAEGPAKGQPMLNTAID  target    ----VLSGSNATRGI----LYEDLHASSA-----------KGVPLLMNA------------------RTYPRHAGWEQRQ 3ir6.1    AAEMILTLAPETNGQVAVKAWAALSEFTGRDHTHLALNKEDEKIRFRDIQAQPRKIISSPTWSGLEDEHVSYNAGYTNVH  target    EDKPWYTPTGRLEFYRPEPEWQAAGESLPIWREPVDATFYEPNAILANSKHPSINPRAPEDYGVPESQMDVETRQYRNVV 3ir6.1    ELIPWRTLSGRQQLYQDHQWMRDFGESLLVYRPPIDTRSVK------------------EVIG-----------------  target    RTWQELKLSKHPLTEKDPAYRFVFQTPKYRWGAHSTAVDSDWIAMLFGPFGDPYRRDSRTPWTGEAYAEINPRDAKELGL 3ir6.1    --------Q---KSNGNQEKALNFLTPHQKWGIHSTYSDNLLMLTLG---------------RGGPVVWLSEADAKDLGI  target    KDGDYIWLDADPEDRPYRGADSSDEFYDVARAMMRVRIYSGMPRRVIRTWFNMYAATPGTVQAQKDVPGGPAQNQDTGYV 3ir6.1    ADNDWIEVFNS-----------------NGALTARAVVSQRVPAGMTMMYHAQERIVN--------LPGSEI--------  target    ALFRHGSHQSGTRAYLRPTQMTDSMNRKAYFGQTIGKGFEADVHSPSGAPKEGYVKVEKAEDGGDEGVGEWRPVTLGLRP 3ir6.1    TQQRGGIHNSVTRITPKPTHMIGGYAHLAY-------G--FNYYGTVGSNRDEFVVVRKMKN------------------  target    DDPSEAMQAYLAGEFVTRKRKGS 3ir6.1    ----------------------- ``` | | | | | | | | | | | | | | | | | | | | | | | | | | | | | | | | | | | | | | | | | | | | | | | | | |
|  | 1q16.1.A | Respiratory nitrate reductase 1 alpha chain  *Crystal structure of Nitrate Reductase A, NarGHI, from Escherichia coli* | 0.42 | 0.00 | 24.04 | 0.78 | 53-1130 | X-ray | 1.90 | monomer | 2 x MD1, 1 x 6MO, 2 x HEM, 4 x SF4, 1 x F3S, 1 x AGA, 1 x 3PH | HHblits | 0.32 |
| ``` target    SDLSRRELLKRAVVVGTGAGLAELFLPAQFLSSASAQSEPQAVAIANPLAQMPDRSWERIYRDQFAEEDSFVFTCAPNDT 1q16.1    ----------------------------------------------------TNRDWEDGYRQRWQHDKIVRSTHGVNCT  target    HNCLLRAHVKNGVIVRISPTYGYGKATDLAGNQASHRWDPRICQKGLILGRRIYGDRRVKAPMIRKGFKE-WADAGF--- 1q16.1    GSCSWKIYVKNGLVTWETQQTDYPR-----TRPDLPNHEPRGCPRGASYSWYLYSANRLKYPMMRKRLMKMWREAKALHS  target    -P------R-HDDGTPRADMEKRGYDEWLQIPWDEALAIAAKTLQNVAETYKGEDGAGKLLEQGYEPAMVEAMHGAGVQA 1q16.1    DPVEAWASIIEDADKAKSFKQARGRGGFVRSSWQEVNELIAASNVYTIKNYGPDR----VAG----------FSPIPAMS  target    IKMRGGMPLLGAGRVFGFYRFANMLALLDGKLRPEAPPEEIVGSRAFDNYAWHTDLPPGHPMVSGSQTVDFDLFAAEHSK 1q16.1    M-----------VSYASGARYLS-----------------LIGGTCLSFYDWYCDLPPASPQTWGEQTDVPESADWYNSS  target    LLVLIGMNWICTKMPDAHWIGDARLKGTRVVVISADYMPTANKADEIVILRPGTDTAFLLGVARELITKKL------YDR 1q16.1    YIIAWGSNVPQTRTPDAHFFTEVRYKGTKTVAVTPDYAEIAKLCDLWLAPKQGTDAAMALAMGHVMLREFHLDNPSQYFT  target    DAVIQRTDLPLLVRLDT-------GERLSARDVFEGYRQAPLE--NYVALKTEEELAAPPSPPFTADKQVVPT-ELREEW 1q16.1    DYVRRYTDMPMLVMLEERDGYYAAGRMLRAADLVDALGQENNPEWKTVAFNT-------------NGEMVAPNGSIGFRW  target    GDFVYWDRA-----TNGPAAVNR------DEI----GAKFAG-------DPAL----LG---AFDVTLVDGTNVKARTAF 1q16.1    GEKGKWNLEQRDGKTGEETELQLSLLGSQDEIAEVGFPYFGGDGTEHFNKVELENVLLHKLPVKRLQLADGSTALVTTVY  target    SLLK------------------EYLDENFDVQTTSEVCNVDPAAVRSLARQLAANK----GNALLAAGMGPNHYFNADLF 1q16.1    DLTLANYGLERGLNDVNCATSYDDV-KAYTPAWAEQITGVSRSQIIRIAREFADNADKTHGRSMIIVGAGLNHWYHLDMN  target    GRVHFLVAALTDNIGHFSGNVGSYAGNYRGSLFQAMGQWIAENPFD--QE-----------------A-----DLTKPA- 1q16.1    YRGLINMLIFCGCVGQSGGGWAHYVGQEKLRPQTGWQPLAFALDWQRPARHMNSTSYFYNHSSQWRYETVTAEELLSPMA  target    RVKRYFK--------SESAHYW----NYGDRPL----------V-----SPSEIITGKS--------HMPTPTKLIWFGN 1q16.1    DKSRYTGHLIDFNVRAERMGWLPSAPQLGTNPLTIAGEAEKAGMNPVDYTVKSLKEGSIRFAAEQPENGKNHPRNLFIWR  target    SNSLLGNAKWSFDV------------------------------VKNTLPKQDAVFCNEWHWTSSCEYSDLVFPADSWAE 1q16.1    SNLLGSSGKGHEFMLKYLLGTEHGIQGKDLGQQGGVKPEEVDWQDNGLEGKLDLVVTLDFRLSSTCLYSDIILPTATWYE  target    FKLPDMTASCTNPFLLAFPKTPLARIHNTRSDYEILAGVAAALADLVDEP------------------------RMKTYW 1q16.1    K--DDMNTSDMHPFIHPLSA-AVDPAWEAKSDWEIYKAIAKKFSEVCVGHLGKETDIVTLPIQHDSAAELAQPLDVKDWK  target    KGILDG---------------------------------------------DPTPYLQR--------------------- 1q16.1    KGECDLIPGKTAPHIMVVERDYPATYERFTSIGPLMEKIGNGGKGIAWNTQSEMDLLRKLNYTKAEGPAKGQPMLNTAID  target    ----VLSGSNATRGI----LYEDLHASSA-----------KGVPLLMNA------------------RTYPRHAGWEQRQ 1q16.1    AAEMILTLAPETNGQVAVKAWAALSEFTGRDHTHLALNKEDEKIRFRDIQAQPRKIISSPTWSGLEDEHVSYNAGYTNVH  target    EDKPWYTPTGRLEFYRPEPEWQAAGESLPIWREPVDATFYEPNAILANSKHPSINPRAPEDYGVPESQMDVETRQYRNVV 1q16.1    ELIPWRTLSGRQQLYQDHQWMRDFGESLLVYRPPIDTRSVK------------------EVI------------------  target    RTWQELKLSKHPLTEKDPAYRFVFQTPKYRWGAHSTAVDSDWIAMLFGPFGDPYRRDSRTPWTGEAYAEINPRDAKELGL 1q16.1    ----------GQKSNGNQEKALNFLTPHQKWGIHSTYSDNLLMLTLG---------------RGGPVVWLSEADAKDLGI  target    KDGDYIWLDADPEDRPYRGADSSDEFYDVARAMMRVRIYSGMPRRVIRTWFNMYAATPGTVQAQKDVPGGPAQNQDTGYV 1q16.1    ADNDWIEVFNS-----------------NGALTARAVVSQRVPAGMTMMYHAQERIVN--------LPGSEI--------  target    ALFRHGSHQSGTRAYLRPTQMTDSMNRKAYFGQTIGKGFEADVHSPSGAPKEGYVKVEKAEDGGDEGVGEWRPVTLGLRP 1q16.1    TQQRGGIHNSVTRITPKPTHMIGGYAHLAY-------G--FNYYGTVGSNRDEFVVVRKMKNIDWL--------------  target    DDPSEAMQAYLAGEFVTRKRKGS 1q16.1    ----------------------- ``` | | | | | | | | | | | | | | | | | | | | | | | | | | | | | | | | | | | | | | | | | | | | | | | | | |
|  | 3egw.1.A | Respiratory nitrate reductase 1 alpha chain  *The crystal structure of the NarGHI mutant NarH - C16A* | 0.42 | 0.12 | 24.12 | 0.78 | 53-1127 | X-ray | 1.90 | homo-dimer | 2 x MD1, 2 x MGD, 2 x 6MO, 6 x SF4, 4 x F3S, 2 x 3PH, 4 x HEM, 2 x AGA | HHblits | 0.32 |
| ``` target    SDLSRRELLKRAVVVGTGAGLAELFLPAQFLSSASAQSEPQAVAIANPLAQMPDRSWERIYRDQFAEEDSFVFTCAPNDT 3egw.1    ----------------------------------------------------TNRDWEDGYRQRWQHDKIVRSTHGVNCT  target    HNCLLRAHVKNGVIVRISPTYGYGKATDLAGNQASHRWDPRICQKGLILGRRIYGDRRVKAPMIRKGFKE-WADAGF--- 3egw.1    GSCSWKIYVKNGLVTWETQQTDYPR-----TRPDLPNHEPRGCPRGASYSWYLYSANRLKYPMMRKRLMKMWREAKALHS  target    -P-----RH--DDGTPRADMEKRGYDEWLQIPWDEALAIAAKTLQNVAETYKGEDGAGKLLEQGYEPAMVEAMHGAGVQA 3egw.1    DPVEAWASIIEDADKAKSFKQARGRGGFVRSSWQEVNELIAASNVYTIKNYGPDR----VAG----------FSPIPAMS  target    IKMRGGMPLLGAGRVFGFYRFANMLALLDGKLRPEAPPEEIVGSRAFDNYAWHTDLPPGHPMVSGSQTVDFDLFAAEHSK 3egw.1    MV-----------SYASGARYLS-----------------LIGGTCLSFYDWYCDLPPASPQTWGEQTDVPESADWYNSS  target    LLVLIGMNWICTKMPDAHWIGDARLKGTRVVVISADYMPTANKADEIVILRPGTDTAFLLGVARELITKKL------YDR 3egw.1    YIIAWGSNVPQTRTPDAHFFTEVRYKGTKTVAVTPDYAEIAKLCDLWLAPKQGTDAAMALAMGHVMLREFHLDNPSQYFT  target    DAVIQRTDLPLLVRLD-------TGERLSARDVFEGYRQAPLE--NYVALKTEEELAAPPSPPFTADKQVVPT-ELREEW 3egw.1    DYVRRYTDMPMLVMLEERDGYYAAGRMLRAADLVAALGQENNPEWKTVAFNT-------------NGEMVAPNGSIGFRW  target    GDFVYWDRA-----TNGPAAVNR------DEI----GAKF-----------AGDPALLG---AFDVTLVDGTNVKARTAF 3egw.1    GEKGKWNLEQRDGKTGEETELQLSLLGSQDEIAEVGFPYFGGDGTEHFNKVELENVLLHKLPVKRLQLADGSTALVTTVY  target    SLLK------------------EYLDENFDVQTTSEVCNVDPAAVRSLARQLAANK----GNALLAAGMGPNHYFNADLF 3egw.1    DLTLANYGLERGLNDVNCATSYDDV-KAYTPAWAEQITGVSRSQIIRIAREFADNADKTHGRSMIIVGAGLNHWYHLDMN  target    GRVHFLVAALTDNIGHFSGNVGSYAGNYRGSLFQAMGQW--IA--ENPFD---QE-------------A----DLTKPA- 3egw.1    YRGLINMLIFCGCVGQSGGGWAHYVGQEKLRPQTGWQPLAFALDWQRPARHMNSTSYFYNHSSQWRYETVTAEELLSPMA  target    RVKRYFK--------SESAHYW----NYGDRPL----------V-----SPSEIITGKS--------HMPTPTKLIWFGN 3egw.1    DKSRYTGHLIDFNVRAERMGWLPSAPQLGTNPLTIAGEAEKAGMNPVDYTVKSLKEGSIRFAAEQPENGKNHPRNLFIWR  target    SNSLLGNAKWSFDV------------------------------VKNTLPKQDAVFCNEWHWTSSCEYSDLVFPADSWAE 3egw.1    SNLLGSSGKGHEFMLKYLLGTEHGIQGKDLGQQGGVKPEEVDWQDNGLEGKLDLVVTLDFRLSSTCLYSDIILPTATWYE  target    FKLPDMTASCTNPFLLAFPKTPLARIHNTRSDYEILAGVAAALADLVDE----P--------------------RMKTYW 3egw.1    K--DDMNTSDMHPFIHPLSA-AVDPAWEAKSDWEIYKAIAKKFSEVCVGHLGKETDIVTLPIQHDSAAELAQPLDVKDWK  target    KGILD--------------G-------------------------------DPTPYLQRV-------------------- 3egw.1    KGECDLIPGKTAPHIMVVERDYPATYERFTSIGPLMEKIGNGGKGIAWNTQSEMDLLRKLNYTKAEGPAKGQPMLNTAID  target    -----LSGSNATRGI----LYEDLHASSA-----------KGVPLLMNA------------------RTYPRHAGWEQRQ 3egw.1    AAEMILTLAPETNGQVAVKAWAALSEFTGRDHTHLALNKEDEKIRFRDIQAQPRKIISSPTWSGLEDEHVSYNAGYTNVH  target    EDKPWYTPTGRLEFYRPEPEWQAAGESLPIWREPVDATFYEPNAILANSKHPSINPRAPEDYGVPESQMDVETRQYRNVV 3egw.1    ELIPWRTLSGRQQLYQDHQWMRDFGESLLVYRPPIDTRSVKE------------------VIG-----------------  target    RTWQELKLSKHPLTEKDPAYRFVFQTPKYRWGAHSTAVDSDWIAMLFGPFGDPYRRDSRTPWTGEAYAEINPRDAKELGL 3egw.1    -----------QKSNGNQEKALNFLTPHQKWGIHSTYSDNLLMLTLG---------------RGGPVVWLSEADAKDLGI  target    KDGDYIWLDADPEDRPYRGADSSDEFYDVARAMMRVRIYSGMPRRVIRTWFNMYAATPGTVQAQKDVPGGPAQNQDTGYV 3egw.1    ADNDWIEVFNS-----------------NGALTARAVVSQRVPAGMTMMYHAQERIVN--------LPGSEI--------  target    ALFRHGSHQSGTRAYLRPTQMTDSMNRKAYFGQTIGKGFEADVHSPSGAPKEGYVKVEKAEDGGDEGVGEWRPVTLGLRP 3egw.1    TQQRGGIHNSVTRITPKPTHMIGGYAHLAY-------G--FNYYGTVGSNRDEFVVVRKMKNI-----------------  target    DDPSEAMQAYLAGEFVTRKRKGS 3egw.1    ----------------------- ``` | | | | | | | | | | | | | | | | | | | | | | | | | | | | | | | | | | | | | | | | | | | | | | | | | |
| ✓ | 3ir5.1.A | Respiratory nitrate reductase 1 alpha chain  *Crystal structure of NarGHI mutant NarG-H49C* | 0.43 | 0.00 | 24.12 | 0.78 | 54-1130 | X-ray | 2.30 | monomer | 2 x MD1, 1 x 6MO, 4 x SF4, 1 x AGA, 1 x F3S, 2 x HEM | HHblits | 0.32 |
| ``` target    SDLSRRELLKRAVVVGTGAGLAELFLPAQFLSSASAQSEPQAVAIANPLAQMPDRSWERIYRDQFAEEDSFVFTCAPNDT 3ir5.1    -----------------------------------------------------NRDWEDGYRQRWQHDKIVRSTCGVNCT  target    HNCLLRAHVKNGVIVRISPTYGYGKATDLAGNQASHRWDPRICQKGLILGRRIYGDRRVKAPMIRKGFKE-WADAGFP-- 3ir5.1    GSCSWKIYVKNGLVTWETQQTDYPR-----TRPDLPNHEPRGCPRGASYSWYLYSANRLKYPMMRKRLMKMWREAKALHS  target    -------R-HD-DGTPRADMEKRGYDEWLQIPWDEALAIAAKTLQNVAETYKGEDGAGKLLEQGYEPAMVEAMHGAGVQA 3ir5.1    DPVEAWASIIEDADKAKSFKQARGRGGFVRSSWQEVNELIAASNVYTIKNYGPDR----VAG----------FSPIPAMS  target    IKMRGGMPLLGAGRVFGFYRFANMLALLDGKLRPEAPPEEIVGSRAFDNYAWHTDLPPGHPMVSGSQTVDFDLFAAEHSK 3ir5.1    M-----------VSYASGARYLS-----------------LIGGTCLSFYDWYCDLPPASPQTWGEQTDVPESADWYNSS  target    LLVLIGMNWICTKMPDAHWIGDARLKGTRVVVISADYMPTANKADEIVILRPGTDTAFLLGVARELITKKL------YDR 3ir5.1    YIIAWGSNVPQTRTPDAHFFTEVRYKGTKTVAVTPDYAEIAKLCDLWLAPKQGTDAAMALAMGHVMLREFHLDNPSQYFT  target    DAVIQRTDLPLLVRLD-------TGERLSARDVFEGYRQAPL--ENYVALKTEEELAAPPSPPFTADKQVVPT-ELREEW 3ir5.1    DYVRRYTDMPMLVMLEERDGYYAAGRMLRAADLVDALGQENNPEWKTVAFNT-------------NGEMVAPNGSIGFRW  target    GDFVYWDR-----ATNGPAAVNR------DE----IGAKFA-----------GDPALLG---AFDVTLVDGTNVKARTAF 3ir5.1    GEKGKWNLEQRDGKTGEETELQLSLLGSQDEIAEVGFPYFGGDGTEHFNKVELENVLLHKLPVKRLQLADGSTALVTTVY  target    SLLK------------------EYLDENFDVQTTSEVCNVDPAAVRSLARQLAANK----GNALLAAGMGPNHYFNADLF 3ir5.1    DLTLANYGLERGLNDVNCATSYDDV-KAYTPAWAEQITGVSRSQIIRIAREFADNADKTHGRSMIIVGAGLNHWYHLDMN  target    GRVHFLVAALTDNIGHFSGNVGSYAGNYRGSLFQAMGQ--------------------------WIAENPFDQEADLTKP 3ir5.1    YRGLINMLIFCGCVGQSGGGWAHYVGQEKLRPQTGWQPLAFALDWQRPARHMNSTSYFYNHSSQWRYETVTAEE--LLSP  target    A-RVKRYFK--------SESAHYW----NYGDRPL----------V-----SPSEIITGKS--------HMPTPTKLIWF 3ir5.1    MADKSRYTGHLIDFNVRAERMGWLPSAPQLGTNPLTIAGEAEKAGMNPVDYTVKSLKEGSIRFAAEQPENGKNHPRNLFI  target    GNSNSLLGNAKWSFDV------------------------------VKNTLPKQDAVFCNEWHWTSSCEYSDLVFPADSW 3ir5.1    WRSNLLGSSGKGHEFMLKYLLGTEHGIQGKDLGQQGGVKPEEVDWQDNGLEGKLDLVVTLDFRLSSTCLYSDIILPTATW  target    AEFKLPDMTASCTNPFLLAFPKTPLARIHNTRSDYEILAGVAAALADLVDEP------------------------RMKT 3ir5.1    YEK--DDMNTSDMHPFIHPLSA-AVDPAWEAKSDWEIYKAIAKKFSEVCVGHLGKETDIVTLPIQHDSAAELAQPLDVKD  target    YWKGILD---G------------------------------------------DPTPYLQ-------------------- 3ir5.1    WKKGECDLIPGKTAPHIMVVERDYPATYERFTSIGPLMEKIGNGGKGIAWNTQSEMDLLRKLNYTKAEGPAKGQPMLNTA  target    -----RVLSGSNATRGI----LYEDLHASSA-----------KGVPLLMNA------------------RTYPRHAGWEQ 3ir5.1    IDAAEMILTLAPETNGQVAVKAWAALSEFTGRDHTHLALNKEDEKIRFRDIQAQPRKIISSPTWSGLEDEHVSYNAGYTN  target    RQEDKPWYTPTGRLEFYRPEPEWQAAGESLPIWREPVDATFYEPNAILANSKHPSINPRAPEDYGVPESQMDVETRQYRN 3ir5.1    VHELIPWRTLSGRQQLYQDHQWMRDFGESLLVYRPPIDTRSVKE-------------------V----------------  target    VVRTWQELKLSKHPLTEKDPAYRFVFQTPKYRWGAHSTAVDSDWIAMLFGPFGDPYRRDSRTPWTGEAYAEINPRDAKEL 3ir5.1    -----I------GQKSNGNQEKALNFLTPHQKWGIHSTYSDNLLMLTLG---------------RGGPVVWLSEADAKDL  target    GLKDGDYIWLDADPEDRPYRGADSSDEFYDVARAMMRVRIYSGMPRRVIRTWFNMYAATPGTVQAQKDVPGGPAQNQDTG 3ir5.1    GIADNDWIEVFNS-----------------NGALTARAVVSQRVPAGMTMMYHAQERIVN--------LPGSEI------  target    YVALFRHGSHQSGTRAYLRPTQMTDSMNRKAYFGQTIGKGFEADVHSPSGAPKEGYVKVEKAEDGGDEGVGEWRPVTLGL 3ir5.1    --TQQRGGIHNSVTRITPKPTHMIGGYAHLAY-------G--FNYYGTVGSNRDEFVVVRKMKNIDWL------------  target    RPDDPSEAMQAYLAGEFVTRKRKGS 3ir5.1    ------------------------- ``` | | | | | | | | | | | | | | | | | | | | | | | | | | | | | | | | | | | | | | | | | | | | | | | | | |
|  | 2ivf.1.A | ETHYLBENZENE DEHYDROGENASE ALPHA-SUBUNIT  *ETHYLBENZENE DEHYDROGENASE FROM AROMATOLEUM AROMATICUM* | 0.42 | 0.00 | 24.43 | 0.75 | 1-1124 | X-ray | 1.88 | monomer | 1 x MES, 4 x SF4, 1 x MO, 1 x MGD, 1 x MD1, 1 x F3S, 1 x HEM | HHblits | 0.32 |
| ``` target    SDLSRRELLKRAVVVGTGAGLAELFLPAQFLSSASAQSEPQAVAIANPLAQMPDRSWERIYRDQFAEEDSFVFTCAPND- 2ivf.1    QDQHRRDFLKRSGAAVLSLSLSSLATGVVPGFL---KD--AQAGT----KAPGYASWEDIYRKEWKWDKVNWGSHLNICW  target    -THNCLLRAHVKNGVIVRISPTYGYGKATDLAGNQASHRWDPRICQKGLILGRRIYGDRRVKAPMIRKGFKEWADAGFPR 2ivf.1    PQGSCKFYVYVRNGIVWREEQAAQTPA-----CNVDYVDYNPLGCQKGSAFNNNLYGDERVKYPLKRVG-----------  target    HDDGTPRADMEKRGYDEWLQIPWDEALAIAAKTLQNVAETYKGEDGAGKLLEQGYEPAMVEAMHGAGVQAIKMRGGMPLL 2ivf.1    -----------KRGEGKWKRVSWDEAAGDIADSIIDSFEAQGSDG----FIL----------DAPHVHA-----GSIA--  target    GAGRVFGFYRFANMLALLDGKLRPEAPPEEIVGSRAFDNYAWHTDLPPGHPMVSGSQTVDFDLFAAEHSKLLVLIGMNWI 2ivf.1    ----WGAGFRMTYLM----DGV---------SPDINVD----IGDTYMGAFHTFGKMHMGYSADNLLDAELIFMTCSNWS  target    CTKMPDAHWIGDARLKGTRVVVISADYMPTANKADEIVILRPGTDTAFLLGVARELITKKLYDRDAVIQRTDLPLLVRLD 2ivf.1    YTYPSSYHFLSEARYKGAEVVVIAPDFNPTTPAADLHVPVRVGSDAAFWLGLSQVMIDEKLFDRQFVCEQTDLPLLVRMD  target    TGERLSARDVFEGYRQAPLENYVALKTEEELAAPPSPPFTADKQVVPTELREEWGDFVYWDRATNGPAAVNRDEIGAKFA 2ivf.1    TGKFLSAEDVDGG---------------------------------------EAKQFYFFDEKAGSVRKASRGTLKL--D  target    GDPALLGAFDVTLVDGTNVKARTAFSLLKEYLDENFDVQTTSEVCNVDPAAVRSLARQLAANKGNALLAAGMGPNHYFNA 2ivf.1    FMPALEGTFSARLKNGKTIQVRTVFEGLREHLK-DYTPEKASAKCGVPVSLIRELGRKVAKKR--TCSYIGFSSAKSYHG  target    DLFGRVHFLVAALTDNIGHFSGNVGSYAGNYRGS-------LFQAMGQWIA----ENPFDQEADLTKPAR-----VKRY- 2ivf.1    DLMERSLFLAMALSGNWGKPGTGAFAWAYSDDNMVYLGVMSKPTAQGGMDELHQMAEGFNKRTLEADPTSTDEMGNIEFM  target    -----------------FKSESAHYWNYG--DRPLV--SPSEII-----TG-------KSHMPTPTKLIWFGNSNSLLGN 2ivf.1    KVVTSAVGLVPPAMWLYYHVGYDQLWNNKAWTDPALKKSFGAYLDEAKEKGWWTNDHIRPAPDKTPQVYMLLSQNPMRRK  target    AKWSFDVV--KNTLPKQDAVFCNEWHWTSSCEYSDLVFPADSWAEFKLPDMTAS-CTNPFLLAFPKTPLARIHNTRSDYE 2ivf.1    RSG--AKMFPDVLFPKLKMIFALETRMSSSAMYADIVLPCAWYYEKHE--MTTPCSGNPFFTFVDR-SVAPPGECREEWD  target    ILAGVAAALADLVDEPRMK-------------TYWKGI-L---DGDPTPYLQRVLSGSNA----TRGILYEDLHASSAKG 2ivf.1    AIALILKKVGERAAARGLTEFNDHNGRKRRYDELYKKFTMDGHLLTNEDCLKEMVDINRAVGVFAKDYTYEKFKKEGQTR  target    VPLLMNAR-----------TYPRHAGWEQRQEDKPWYTPTGRLEFYRPEPEWQAAGESLPIWREPVDATFYEPNAILANS 2ivf.1    FLSMGTGVSRYAHANEVDVTKPIYPMRWHFDDKKVFPTHTRRAQFYLDHDWYLEAGESLPTHKDTPM-------------  target    KHPSINPRAPEDYGVPESQMDVETRQYRNVVRTWQELKLSKHPLTEKDPAYRFVFQTPKYRWGAHSTAVDSDWIAMLFGP 2ivf.1    ----------------------------------------------VGGDHPFKITGGHPRVSIHSTHLTNSHLSRLH--  target    FGDPYRRDSRTPWTGEAYAEINPRDAKELGLKDGDYIWLDADPEDRPYRGADSSDEFYDVARAMMRVRIYSGMPRRVIRT 2ivf.1    -------------RGQPVVHMNSKDAAELGIKDGDMAKLFND-----------------FADCEIMVRTAPNVQPKQCIV  target    WFNMYAATPGTVQAQKDVPGGPAQNQDTGYVALFRHGSHQSGTRAYLRPTQMTDSMNRKAYFGQTIGKGFEADVHSPSG- 2ivf.1    YFWDAHQ---------------Y----KG------WKPYDILLIGMPKPLHLAGGYEQFRY-------Y--FMNGSPAPV  target    APKEGYVKVEKAEDGGDEGVGEWRPVTLGLRPDDPSEAMQAYLAGEFVTRKRKGS 2ivf.1    TDRGVRVSIKKA------------------------------------------- ``` | | | | | | | | | | | | | | | | | | | | | | | | | | | | | | | | | | | | | | | | | | | | | | | | | |
|  | 4ydd.1.A | DMSO reductase family type II enzyme, molybdopterin subunit  *Crystal structure of the perchlorate reductase PcrAB from Azospira suillum PS* | 0.45 | 0.00 | 25.48 | 0.71 | 52-1127 | X-ray | 1.86 | monomer | 4 x SF4, 1 x MO, 1 x MGD, 1 x MD1, 1 x F3S | HHblits | 0.33 |
| ``` target    SDLSRRELLKRAVVVGTGAGLAELFLPAQFLSSASAQSEPQAVAIANPLAQMPDRSWERIYRDQFAEEDSFVFTCAPNDT 4ydd.1    ---------------------------------------------------FEYSGWENFHRTQWSWDKKTRGAHLVNCT  target    HNCLLRAHVKNGVIVRISPTYGYGKATDLAGNQASHRWDPRICQKGLILGRRIYGDRRVKAPMIRKGFKEWADAGFPRHD 4ydd.1    GACPHFVYSKDGVVMREEQSKD------IAPMPNIPEYNPRGCNKGECGHDYMYGPHRIKYPLIRVG-------------  target    DGTPRADMEKRGYDEWLQIPWDEALAIAAKTLQNVAETYKGEDGAGKLLEQGYEPAMVEAMHGAGVQAIKMRGGMPLLGA 4ydd.1    ---------ERGEGKWRRATWEEALDMIADKCVDTIKNHAPDCIS--VYS-------------PV-PAVSP----V----  target    GRVFGFYRFANMLALLDGKLRPEAPPEEIVGSRAFDNYAWHTDLPPGHPMVSGSQTVDFDLFAAEHSKLLVLIGMNWICT 4ydd.1    -SFSAGHRFAHY-----------------IGAHAHTFYDWYGDHPTGQTQTCGVQGDTCETADWFNSKYIILWGSNPTQT  target    KMPDAHWIGDARLKGTRVVVISADYMPTANKADEIVILRPGTDTAFLLGVARELITKKLYDRDAVIQRTDLPLLVRLDTG 4ydd.1    RIPDAHFLSEAQLNGAKIVSISPDYNSSTIKVDKWIHPQPGTDGALAMAMAHVIIKEKLYDAHSLKEQTDLSYLVRSDTK  target    ERLSARDVFEGYRQAPLENYVALKTEEELAAPPSPPFTADKQVVPTELREEWGDFVYWDRATNGPAAVNRDEI------- 4ydd.1    RFLREADVVAGG--------------------------------------SKDKFYFWNAKTGKPVIPKGSWGDQPEKKG  target    -----------------GAKFAGDPALLGAFDVTLVDGTNVKARTAFSLLKEYLDENFDVQTTSEVCNVDPAAVRSLARQ 4ydd.1    SPVGFLGRNTFAFPKGYIDLGDLDPALEGKFNMQLLDGKTVEVRPVFEILKSRLMADNTPEKAAKITGVTAKAITELARE  target    LAANKGNALLAAGMGPNHYFNADLFGRVHFLVAALTDNIGHFSGNVGSYAGNYRGSLFQAMGQWIAENPFDQEADLTKPA 4ydd.1    FATAKP-SMIICGGGTQHWYYSDVLLRAMHLLTALTGTEGTNGGGMNHYIGQWKPAFVAGLVA--LAFPEGV-----NKQ  target    RVKRYFKSESAHYW--NYGD------RPLV--SPSEIITGK----SHMPTPTKLIWFGNSNSLLGNAKWSFDVVKNTLPK 4ydd.1    RFC---QTTIWTYIHAEVNDEIISSDIDTEKYLRDSITTGQMPNMPEQGRDPKVFFVYRGNWLNQAKGQ-KYVLENLWPK  target    QDAVFCNEWHWTSSCEYSDLVFPADSWAEFKLPDMTASCTNPFLLAFPKTPLARIHNTRSDYEILAGVAAALADLVDEPR 4ydd.1    LELIVDINIRMDSTALYSDVVLPSAHWYEKL--DLNVTSEHSYINMTEP-AIKPMWESKTDWQIFLALAKRVEMAAKRKK  target    MKTY--------------WKGIL----DGDPTPYLQRVLSGSNATRGILYEDLHASSAKGVPLLMN--ARTYPRHAGWEQ 4ydd.1    YEKFNDEKFKWVRDLSNLWNQMTMDGKLAEDEAAAQYILDNAPQSKGITIQMLREKPQRFKSNWTSPLKEGVPYTPFQYF  target    RQEDKPWYTPTGRLEFYRPEPEWQAAGESLPIWREPVDATFYEPNAILANSKHPSINPRAPEDYGVPESQMDVETRQYRN 4ydd.1    VVDKKPWPTLTGRQQFYLDHDTFFDMGVELPTYKAPID------------------------------------------  target    VVRTWQELKLSKHPLTEKDPAYRFVFQTPKYRWGAHSTAVDSDWIAMLFGPFGDPYRRDSRTPWTGEAYAEINPRDAKEL 4ydd.1    ------------------ADKYPFRFNSPHSRHSVHSTFKDNVLMLRLQ---------------RGGPSIEMSPLDAKPL  target    GLKDGDYIWLDADPEDRPYRGADSSDEFYDVARAMMRVRIYSGMPRRVIRTWFNMYAATPGTVQAQKDVPGGPAQNQDTG 4ydd.1    GIKDNDWVEAWNN-----------------HGKVICRVKIRNGEQRGRVSMWHCPELYMD--------------------  target    YVALFRHGSHQSGTRAYLRPTQMTDSMNRKAYFGQTIGKGFEADVHSPSGAPKEGYVKVEKAEDGGDEGVGEWRPVTLGL 4ydd.1    ----LLTGGSQSVCPVRINPTNLVGNYGHLFF---------RPNYYGPAGSQRDVRVNVKRYIGA---------------  target    RPDDPSEAMQAYLAGEFVTRKRKGS 4ydd.1    ------------------------- ``` | | | | | | | | | | | | | | | | | | | | | | | | | | | | | | | | | | | | | | | | | | | | | | | | | |
|  | 5e7o.1.A | DMSO reductase family type II enzyme, molybdopterin subunit  *Crystal structure of the perchlorate reductase PcrAB mutant W461E of PcrA from Azospira suillum PS* | 0.44 | 0.00 | 25.82 | 0.71 | 52-1127 | X-ray | 2.40 | monomer | 4 x SF4, 1 x MO, 1 x MGD, 1 x MD1, 1 x F3S | HHblits | 0.33 |
| ``` target    SDLSRRELLKRAVVVGTGAGLAELFLPAQFLSSASAQSEPQAVAIANPLAQMPDRSWERIYRDQFAEEDSFVFTCAPNDT 5e7o.1    ---------------------------------------------------FEYSGWENFHRTQWSWDKKTRGAHLVNCT  target    HNCLLRAHVKNGVIVRISPTYGYGKATDLAGNQASHRWDPRICQKGLILGRRIYGDRRVKAPMIRKGFKEWADAGFPRHD 5e7o.1    GACPHFVYSKDGVVMREEQSKD------IAPMPNIPEYNPRGCNKGECGHDYMYGPHRIKYPLIRVG-------------  target    DGTPRADMEKRGYDEWLQIPWDEALAIAAKTLQNVAETYKGEDGAGKLLEQGYEPAMVEAMHGAGVQAIKMRGGMPLLGA 5e7o.1    ---------ERGEGKWRRATWEEALDMIADKCVDTIKNHAPDCIS--VY------------S-PVPAV-SP----V----  target    GRVFGFYRFANMLALLDGKLRPEAPPEEIVGSRAFDNYAWHTDLPPGHPMVSGSQTVDFDLFAAEHSKLLVLIGMNWICT 5e7o.1    -SFSAGHRFAHY-----------------IGAHAHTFYDWYGDHPTGQTQTCGVQGDTCETADWFNSKYIILWGSNPTQT  target    KMPDAHWIGDARLKGTRVVVISADYMPTANKADEIVILRPGTDTAFLLGVARELITKKLYDRDAVIQRTDLPLLVRLDTG 5e7o.1    RIPDAHFLSEAQLNGAKIVSISPDYNSSTIKVDKWIHPQPGTDGALAMAMAHVIIKEKLYDAHSLKEQTDLSYLVRSDTK  target    ERLSARDVFEGYRQAPLENYVALKTEEELAAPPSPPFTADKQVVPTELREEWGDFVYWDRATNGPAAVNRDEI------- 5e7o.1    RFLREADVVAGG--------------------------------------SKDKFYFWNAKTGKPVIPKGSWGDQPEKKG  target    -----------------GAKFAGDPALLGAFDVTLVDGTNVKARTAFSLLKEYLDENFDVQTTSEVCNVDPAAVRSLARQ 5e7o.1    SPVGFLGRNTFAFPKGYIDLGDLDPALEGKFNMQLLDGKTVEVRPVFEILKSRLMADNTPEKAAKITGVTAKAITELARE  target    LAANKGNALLAAGMGPNHYFNADLFGRVHFLVAALTDNIGHFSGNVGSYAGNYRGSLFQAMGQWIAENPFDQEADLTKPA 5e7o.1    FATAKP-SMIICGGGTQHWYYSDVLLRAMHLLTALTGTEGTNGGGMNHYIGQEKPAFVA--GLVA--LAFPEG--V-NKQ  target    RVKRYFKSESAHYWNYG-----D---RPLV-----SPSEIITGK----SHMPTPTKLIWFGNSNSLLGNAKWSFDVVKNT 5e7o.1    RFC---Q---TTIWTYIHAEVNDEIISSDIDTEKYLRDSITTGQMPNMPEQGRDPKVFFVYRGNWLNQAKGQ-KYVLENL  target    LPKQDAVFCNEWHWTSSCEYSDLVFPADSWAEFKLPDMTASCTNPFLLAFPKTPLARIHNTRSDYEILAGVAAALADLVD 5e7o.1    WPKLELIVDINIRMDSTALYSDVVLPSAHWYEKL--DLNVTSEHSYINMTEP-AIKPMWESKTDWQIFLALAKRVEMAAK  target    EPRMKTY------WKGIL------------DGDPTPYLQRVLSGSNATRGILYEDLHASSAKGVPLLMNA--RTYPRHAG 5e7o.1    RKKYEKFNDEKFKWVRDLSNLWNQMTMDGKLAEDEAAAQYILDNAPQSKGITIQMLREKPQRFKSNWTSPLKEGVPYTPF  target    WEQRQEDKPWYTPTGRLEFYRPEPEWQAAGESLPIWREPVDATFYEPNAILANSKHPSINPRAPEDYGVPESQMDVETRQ 5e7o.1    QYFVVDKKPWPTLTGRQQFYLDHDTFFDMGVELPTYKAPID---------------------------------------  target    YRNVVRTWQELKLSKHPLTEKDPAYRFVFQTPKYRWGAHSTAVDSDWIAMLFGPFGDPYRRDSRTPWTGEAYAEINPRDA 5e7o.1    ---------------------ADKYPFRFNSPHSRHSVHSTFKDNVLMLRL---------------QRGGPSIEMSPLDA  target    KELGLKDGDYIWLDADPEDRPYRGADSSDEFYDVARAMMRVRIYSGMPRRVIRTWFNMYAATPGTVQAQKDVPGGPAQNQ 5e7o.1    KPLGIKDNDWVEAWNN-----------------HGKVICRVKIRNGEQRGRVSMWHCPELYMD-----------------  target    DTGYVALFRHGSHQSGTRAYLRPTQMTDSMNRKAYFGQTIGKGFEADVHSPSGAPKEGYVKVEKAEDGGDEGVGEWRPVT 5e7o.1    -------LLTGGSQSVCPVRINPTNLVGNYGHLFF---------RPNYYGPAGSQRDVRVNVKRYIGA------------  target    LGLRPDDPSEAMQAYLAGEFVTRKRKGS 5e7o.1    ---------------------------- ``` | | | | | | | | | | | | | | | | | | | | | | | | | | | | | | | | | | | | | | | | | | | | | | | | | |
|  | 6sdv.1.A | Formate dehydrogenase, alpha subunit, selenocysteine-containing,Formate dehydrogenase, alpha subunit, selenocysteine-containing,W-formate dehydrogenase - alpha subunit  *W-formate dehydrogenase from Desulfovibrio vulgaris - Formate reduced form* | 0.32 |  | 18.25 | 0.69 | 1-1031 | X-ray | 1.90 | hetero-1-1-mer | 2 x MGD, 4 x SF4, 1 x W, 1 x H2S | HHblits | 0.29 |
| ``` target    SDLSRRELLKRAVVVGTGAGLAELFLPAQFLSSASAQSEPQAVAIANPLAQMPDRSWERIYRDQFAEEDSFVFTCAPNDT 6sdv.1    MTVTRRHFLKLSAGAAVAGAFTGLGLSL------------------APTVARAEL-----Q--KLQWA-KQTTSICCYCA  target    HNCLLRAHVK---NGVIVRISPTYGYGKATDLAGNQASHRWDPRICQKGLILGRRIYGDRRVKAPMIRKGFKEWADAGFP 6sdv.1    VGCGLIVHTAKDGQGRAVNVEGD------------PDHPINEGSLCPKGASIFQLGENDQRGTQPLYRAP----------  target    RHDDGTPRADMEKRGYDEWLQIPWDEALAIAAKTLQNVAETYKGEDGAGKLLEQGYEPAMVEAMHGAGVQAIKMRGGMPL 6sdv.1    --------------FSDTWKPVTWDFALTEIAKRIKKTRDASFTEKNAAGDLVN-------------RTEAIA-------  target    LGAGRVFGFYRFANMLALLDGKLRPEAPPEEIVGSRAFDNYAWHTDL--PPGHPMVSGSQTVDFDLFAAEHSKLLVLIGM 6sdv.1    -----SFGSAAMDNEECWAYGNILR------SLGLVYIEHQARIUHSPTVPALAESFGRGAMTNHWNDLANSDCILIMGS  target    NWICTKMPDAHWIGDARLKGTRVVVISADYMPTANKADEIVILRPGTDTAFLLGVARELITKKLYDRDAVIQRTDLPLLV 6sdv.1    NAAENHPIAFKWVLRAKDKGATLIHVDPRFTRTSARCDVYAPIRSGADIPFLGGLIKYILDNKLYFTDYVREYTNASLIV  target    RLDTGERLSARDVFEGYRQAPLENYVALKTEEELAAPPSPPFTADKQVVPTELREEWGDFVYWDRATNGPAAVNRDEIGA 6sdv.1    GEKFS---FKDGLFSGYDA---------------------------------------ANKKYDKSM-------------  target    KFAGDPALLGAFD-VTLVDGTNVKARTAFSLLKEYLDENFDVQTTSEVCNVDPAAVRSLARQLAANK---GNALLAAGMG 6sdv.1    -----WAFELDANGVPKRDPALKHPRCVINLLKKHYE-RYNLDKVAAITGTSKEQLQQVYKAYAATGKPDKAGTIMYAMG  target    PNHYFNADLFGRVHFLVAALTDNIGHFSGNVGSYAGNY--RGSLFQA-MGQ-WIAENPFDQEA--D--L--------TKP 6sdv.1    WTQHSVGVQNIRAMAMIQLLLGNIGVAGGGVNALRGESNVQGSTDQGLLAHIWPGYNPVPNSKAATLELYNAATPQSKDP  target    ARVKRYFKSESAHYWN------YGD-RP---------------L--VSPSEIITGKSHMPTPTKLIWFGNSNSLLGNAKW 6sdv.1    MSVNW-WQNR-PKYVASYLKALYPDEEPAAAYDYLPRIDAGRKLTDYFWLNIFEK--MDKGEFKGLFAWGMNPACGGAN-  target    SFDVVKNTLPKQDAVFCNEWHWTSSCEY--------SD-----LVFPADSWAEFKLPDMTASCTNPFLLAFPKTPLARIH 6sdv.1    -ANKNRKAMGKLEWLVNVNLFENETSSFWKGPGMNPAEIGTEVFFLPCCVSIEKE--GSVA-NSGRWMQWRYR-GPKPYA  target    NTRSDYEILAGVAAALADLVDEP-R-M--------KTYWKGILDGDPTPYLQRVLSGSNATRGILYEDLHASSAKGVPLL 6sdv.1    ETKPDGDIMLDMFKKVRELYAKEGGAYPAPIAKLNIADWEEHNEFSPTKVAKLMNGYFLKDTEVGGKQFKKGQ--QVPSF  target    M---------------------------------------------NARTYPR-----HAGWEQRQEDKPWYTPTGRLEF 6sdv.1    AFLTADGSTCSGNWLHAGSFTDAGNLMARRDKTQTPEQARIGLFPNWSFCWPVNRRILYNRASVDKTGKPWNPAKAVIEW  target    YRPEPEW-----------------Q-----------AAGESLPIWREPVDATFYEPNAILANSKHPSINPRAPEDYGVPE 6sdv.1    KDGKWVGDVVDGGGDPGTKHPFIMQTHGFGALYGPGREEGPFPEHYEPLECPVSKN---------P-----FS-------  target    SQMDVETRQYRNVVRTWQELKLSKHPLTEKDPAYRFVFQTPKYRWG--AHSTAVDSDWIAMLFGPFGDPYRRDSRTPWTG 6sdv.1    -------KQLHNPVAFQIEG----EKKAVCDPRYPFIGTTYRVTEHWQTGLMTRRCAWLVEAE----------------P  target    EAYAEINPRDAKELGLKDGDYIWLDADPEDRPYRGADSSDEFYDVARAMMRVRIYSGMPRRVIRTWFNMYAATPGTVQAQ 6sdv.1    QIFCEISKELAKLRGIGNGDTVKVSSL-----------------RGALEAVAIVTERIRPFKI-----------------  target    KDVPGGPAQNQDTGYVALFRHGSHQSGTRAYLRPTQMTDSMNRKAYFGQTIGKGFEADVHSPSGAPKEGYVKVEKAEDGG 6sdv.1    --------------------------------------------------------------------------------  target    DEGVGEWRPVTLGLRPDDPSEAMQAYLAGEFVTRKRKGS 6sdv.1    --------------------------------------- ``` | | | | | | | | | | | | | | | | | | | | | | | | | | | | | | | | | | | | | | | | | | | | | | | | | |
|  | 1kqf.1.A | FORMATE DEHYDROGENASE, NITRATE-INDUCIBLE, MAJOR SUBUNIT  *FORMATE DEHYDROGENASE N FROM E. COLI* | 0.34 |  | 18.05 | 0.66 | 70-1037 | X-ray | 1.60 | hetero-oligomer | 3 x 6MO, 15 x SF4, 6 x MGD, 6 x HEM, 3 x CDL | HHblits | 0.28 |
| ``` target    SDLSRRELLKRAVVVGTGAGLAELFLPAQFLSSASAQSEPQAVAIANPLAQMPDRSWERIYRDQFAEEDSFVFTCAPNDT 1kqf.1    ---------------------------------------------------------------------KEIRNTCTYCS  target    HNCLLRAHVKNG-------VIVRISPTYGYGKATDLAGNQASHRWDPRICQKGLILGRRIYGDRRVKAPMIRKGFKEWAD 1kqf.1    VGCGLLMYSLGDGAKNAREAIYHIEG------------DPDHPVSRGALCPKGAGLLDYVNSENRLRYPEYRAP------  target    AGFPRHDDGTPRADMEKRGYDEWLQIPWDEALAIAAKTLQNVAETYKGEDGAGKLLEQGYEPAMVEAMHGAGVQAIKMRG 1kqf.1    ------------------GSDKWQRISWEEAFSRIAKLMKADRDANFIEKNEQGVTVNRW--LSTGMLCASGAS------  target    GMPLLGAGRVFGFYRFANMLALLDGKLRPEAPPEEIVGSRAFDNYAWHTDL--PPGHPMVSGSQTVDFDLFAAEHSKLLV 1kqf.1    ------NETGMLTQKFARS-----------------LGMLAVDNQARVUHGPTVASLAPTFGRGAMTNHWVDIKNANVVM  target    LIGMNWICTKMPDAHWIGDARL-KGTRVVVISADYMPTANKADEIVILRPGTDTAFLLGVARELITKKLYDRDAVIQRTD 1kqf.1    VMGGNAAEAHPVGFRWAMEAKNNNDATLIVVDPRFTRTASVADIYAPIRSGTDITFLSGVLRYLIENNKINAEYVKHYTN  target    LPLLVRLDTGERLSARDVFEGYRQAPLENYVALKTEEELAAPPSPPFTADKQVVPTELREEWGDFVYWDRATNGPAAVNR 1kqf.1    ASLLVRDDF-AFEDGLF--SGYDAEK---------------------------------RQY-DKSSWNYQLDE------  target    DEIGAKFAGDPALLGAFDVTLVDGTNVKARTAFSLLKEYLDENFDVQTTSEVCNVDPAAVRSLARQLAANK---GNALLA 1kqf.1    -------------NG---YAKRDETLTHPRCVWNLLKEHVS-RYTPDVVENICGTPKADFLKVCEVLASTSAPDRTTTFL  target    AGMGPNHYFNADLFGRVHFLVAALTDNIGHFSGNVGSYAGNYRGSLFQAMGQWIAEN----PFDQEAD------------ 1kqf.1    YALGWTQHTVGAQNIRTMAMIQLLLGNMGMAGGGVNALRGHSNIQGLTDLGLLSTSLPGYLTLPSEKQVDLQSYLEANTP  target    ---LTK--------PARVKRYFKS------ESAHYWNYGDRP----LVSPSEIITGKSHMPTPTKLIWFGNSNSLLGNAK 1kqf.1    KATLADQVNYWSNYPKFFVSLMKSFYGDAAQKENNWGYDWLPKWDQTYDVIKYFNM--MDEGKVTGYFCQGFNPVASFPD  target    WSFDVVKNTLPKQDAVFCNEWHWTSSCEYSD-----------------LVFPADSWAEFKLPDMTASCTNPFLLAFPKTP 1kqf.1    --KNKVVSCLSKLKYMVVIDPLVTETSTFWQNHGESNDVDPASIQTEVFRLPSTCFAEED--GSI-ANSGRWLQWHWK-G  target    LARIHNTRSDYEILAGVAAALADLVDEPR---MKTYWKGILD-GDPT-------------PYLQRVLSGS------NATR 1kqf.1    QDAPGEARNDGEILAGIYHHLRELYQSEGGKGVEPLMKMSWNYKQPHEPQSDEVAKENNGYALEDLYDANGVLIAKKGQL  target    GILYEDLHASSAKGV--PLLMN--------------------------ARTYPR----------------------HA-- 1kqf.1    LSSFAHLRDDGTTASSCWIYTGSWTEQGNQMANRDNSDPSGLGNTLGWAWAWPLNRRVLYNRASADINGKPWDPKRMLIQ  target    --------GWEQRQEDKPWYTPTGRLEFYRPEPEWQ-----AAGESLPIWREPVDATFYEPNAILANSKHPSINPRAPED 1kqf.1    WNGSKWTGNDIPDFGNAAPGTPTGPFIMQPEGMGRLFAINKMAEGPFPEHYEPIETPLGT---------NP-LHPN--VV  target    YGVPESQMDVETRQYRNVVRTWQELKLSKHPLTEKDPAYRFVFQTPKYRWGAHSTAVDSDWIAMLFGPFGDPYRRDSRTP 1kqf.1    SN-------PV-------VRLYEQD---ALR-MGKKEQFPYVGTTYRLTEHFHTWTKHALLNAIA---------------  target    WTGEAYAEINPRDAKELGLKDGDYIWLDADPEDRPYRGADSSDEFYDVARAMMRVRIYSGMPR--------RVIRTWFNM 1kqf.1    -QPEQFVEISETLAAAKGINNGDRVTVSSK-----------------RGFIRAVAVVTRRLKPLNVNGQQVETVGIPIHW  target    YAATPGTVQAQKDVPGGPAQNQDTGYVALFRHGSHQSGTRAYLRPTQMTDSMNRKAYFGQTIGKGFEADVHSPSGAPKEG 1kqf.1    --------------------------------------------------------------------------------  target    YVKVEKAEDGGDEGVGEWRPVTLGLRPDDPSEAMQAYLAGEFVTRKRKGS 1kqf.1    -------------------------------------------------- ``` | | | | | | | | | | | | | | | | | | | | | | | | | | | | | | | | | | | | | | | | | | | | | | | | | |
|  | 4ydd.1.A | DMSO reductase family type II enzyme, molybdopterin subunit  *Crystal structure of the perchlorate reductase PcrAB from Azospira suillum PS* | 0.32 | 0.00 | 28.33 | 0.60 | 57-864 | X-ray | 1.86 | monomer | 4 x SF4, 1 x MO, 1 x MGD, 1 x MD1, 1 x F3S | BLAST | 0.35 |
| ``` target    SDLSRRELLKRAVVVGTGAGLAELFLPAQFLSSASAQSEPQAVAIANPLAQMPDRSWERIYRDQFAEEDSFVFTCAPNDT 4ydd.1    --------------------------------------------------------WENFHRTQWSWDKKTRGAHLVNCT  target    HNCLLRAHVKNGVIVRISPTYGYGKATDLAGNQASHRWDPRICQKGLILGRRIYGDRRVKAPMIRKGFKEWADAGFPRHD 4ydd.1    GACPHFVYSKDGVVMREE------QSKDIAPMPNIPEYNPRGCNKGECGHDYMYGPHRIKYPLIRVG-------------  target    DGTPRADMEKRGYDEWLQIPWDEALAIAAKTLQNVAETYKGEDGAGKLLEQGYEPAMVEAMHGAGVQAIKMRGGMPLLGA 4ydd.1    ---------ERGEGKWRRATWEEALDMIADKC-------------------------VDTIKNHAPDCISVYSPVPAVSP  target    GRVFGFYRFANMLALLDGKLRPEAPPEEIVGSRAFDNYAWHTDLPPGHPMVSGSQTVDFDLFAAEHSKLLVLIGMNWICT 4ydd.1    VSFSAGHRFAHY-----------------IGAHAHTFYDWYGDHPTGQTQTCGVQGDTCETADWFNSKYIILWGSNPTQT  target    KMPDAHWIGDARLKGTRVVVISADYMPTANKADEIVILRPGTDTAFLLGVARELITKKLYDRDAVIQRTDLPLLVRLDTG 4ydd.1    RIPDAHFLSEAQLNGAKIVSISPDYNSSTIKVDKWIHPQPGTDGALAMAMAHVIIKEKLYDAHSLKEQTDLSYLVRSDTK  target    ERLSARDVFEGYRQAPLENYVALKTEEELAAPPSPPFTADKQVVPTELREEWGDFVYWDRATNGPAAVNRDEIGAKFAG- 4ydd.1    RFLREADVVAGGSKDKFYFWNA---------------KTGKPVIP---KGSWGDQPEKKGSPVGFLGRNTFAFPKGYIDL  target    ---DPALLGAFDVTLVDGTNVKARTAFSLLKEYLDENFDVQTTSEVCNVDPAAVRSLARQLAANKGNALLAAGMGPNHYF 4ydd.1    GDLDPALEGKFNMQLLDGKTVEVRPVFEILKSRLMADNTPEKAAKITGVTAKAITELAREFATAKPSMIICGG-GTQHWY  target    NADLFGRVHFLVAALTDNIGHFSGNVGSYAGNYR-----GSLFQAMGQWIAENPFDQEADLTK-PARVKRYFKSESAHYW 4ydd.1    YSDVLLRAMHLLTALTGTEGTNGGGMNHYIGQWKPAFVAGLVALAFPEGVNKQRFCQTTIWTYIHAEVNDEIISSDI---  target    NYGDRPLVSPSEIITGKS-HMPTPTK--LIWFGNSNSLLGNAKWSFDVVKNTLPKQDAVFCNEWHWTSSCEYSDLVFPAD 4ydd.1    ---DTEKYLRDSITTGQMPNMPEQGRDPKVFFVYRGNWLNQAKGQKYVLENLWPKLELIVDINIRMDSTALYSDVVLPSA  target    SWAEFKLPDMTASCTNPFLLAFPKTPLARIHNTRSDYEILAGVAAALADLVDEPRMKTY--------------WKGI-LD 4ydd.1    HWYE-KL-DLNVTSEHSY-INMTEPAIKPMWESKTDWQIFLALAKRVEMAAKRKKYEKFNDEKFKWVRDLSNLWNQMTMD  target    G---DPTPYLQRVLSGSNATRGILYEDLHASSAKGVPLLMNARTYPRHAG--WEQRQ----EDKPWYTPTGRLEFYRPEP 4ydd.1    GKLAEDEAAAQYILDNAPQSKGITIQMLREKPQR----FKSNWTSPLKEGVPYTPFQYFVVDKKPWPTLTGRQQFYLDHD  target    EWQAAGESLPIWREPVDATFYEPNAILANSKHPSINPRAPEDYGVPESQMDVETRQYRNVVRTWQELKLSKHPLTEKDPA 4ydd.1    TFFDMGVELPTYKAPIDADKY-----------------------------------------------------------  target    YRFVFQTPKYRWGAHSTAVDSDWIAMLFGPFGDPYRRDSRTPWTGEAYAEINPRDAKELGLKDGDYIWLDADPEDRPYRG 4ydd.1    --------------------------------------------------------------------------------  target    ADSSDEFYDVARAMMRVRIYSGMPRRVIRTWFNMYAATPGTVQAQKDVPGGPAQNQDTGYVALFRHGSHQSGTRAYLRPT 4ydd.1    --------------------------------------------------------------------------------  target    QMTDSMNRKAYFGQTIGKGFEADVHSPSGAPKEGYVKVEKAEDGGDEGVGEWRPVTLGLRPDDPSEAMQAYLAGEFVTRK 4ydd.1    --------------------------------------------------------------------------------  target    RKGS 4ydd.1    ---- ``` | | | | | | | | | | | | | | | | | | | | | | | | | | | | | | | | | | | | | | | | | | | | | | | | | |
|  | 5e7o.1.A | DMSO reductase family type II enzyme, molybdopterin subunit  *Crystal structure of the perchlorate reductase PcrAB mutant W461E of PcrA from Azospira suillum PS* | 0.32 | 0.00 | 28.33 | 0.60 | 57-864 | X-ray | 2.40 | monomer | 4 x SF4, 1 x MO, 1 x MGD, 1 x MD1, 1 x F3S | BLAST | 0.34 |
| ``` target    SDLSRRELLKRAVVVGTGAGLAELFLPAQFLSSASAQSEPQAVAIANPLAQMPDRSWERIYRDQFAEEDSFVFTCAPNDT 5e7o.1    --------------------------------------------------------WENFHRTQWSWDKKTRGAHLVNCT  target    HNCLLRAHVKNGVIVRISPTYGYGKATDLAGNQASHRWDPRICQKGLILGRRIYGDRRVKAPMIRKGFKEWADAGFPRHD 5e7o.1    GACPHFVYSKDGVVMREE------QSKDIAPMPNIPEYNPRGCNKGECGHDYMYGPHRIKYPLIRVG-------------  target    DGTPRADMEKRGYDEWLQIPWDEALAIAAKTLQNVAETYKGEDGAGKLLEQGYEPAMVEAMHGAGVQAIKMRGGMPLLGA 5e7o.1    ---------ERGEGKWRRATWEEALDMIADKC-------------------------VDTIKNHAPDCISVYSPVPAVSP  target    GRVFGFYRFANMLALLDGKLRPEAPPEEIVGSRAFDNYAWHTDLPPGHPMVSGSQTVDFDLFAAEHSKLLVLIGMNWICT 5e7o.1    VSFSAGHRFAHY-----------------IGAHAHTFYDWYGDHPTGQTQTCGVQGDTCETADWFNSKYIILWGSNPTQT  target    KMPDAHWIGDARLKGTRVVVISADYMPTANKADEIVILRPGTDTAFLLGVARELITKKLYDRDAVIQRTDLPLLVRLDTG 5e7o.1    RIPDAHFLSEAQLNGAKIVSISPDYNSSTIKVDKWIHPQPGTDGALAMAMAHVIIKEKLYDAHSLKEQTDLSYLVRSDTK  target    ERLSARDVFEGYRQAPLENYVALKTEEELAAPPSPPFTADKQVVPTELREEWGDFVYWDRATNGPAAVNRDEIGAKFAG- 5e7o.1    RFLREADVVAGGSKDKFYFWNA---------------KTGKPVIP---KGSWGDQPEKKGSPVGFLGRNTFAFPKGYIDL  target    ---DPALLGAFDVTLVDGTNVKARTAFSLLKEYLDENFDVQTTSEVCNVDPAAVRSLARQLAANKGNALLAAGMGPNHYF 5e7o.1    GDLDPALEGKFNMQLLDGKTVEVRPVFEILKSRLMADNTPEKAAKITGVTAKAITELAREFATAKPSMIICGG-GTQHWY  target    NADLFGRVHFLVAALTDNIGHFSGNVGSYAGNYR-----GSLFQAMGQWIAENPFDQEADLTK-PARVKRYFKSESAHYW 5e7o.1    YSDVLLRAMHLLTALTGTEGTNGGGMNHYIGQEKPAFVAGLVALAFPEGVNKQRFCQTTIWTYIHAEVNDEIISSDI---  target    NYGDRPLVSPSEIITGKS-HMPTPTK--LIWFGNSNSLLGNAKWSFDVVKNTLPKQDAVFCNEWHWTSSCEYSDLVFPAD 5e7o.1    ---DTEKYLRDSITTGQMPNMPEQGRDPKVFFVYRGNWLNQAKGQKYVLENLWPKLELIVDINIRMDSTALYSDVVLPSA  target    SWAEFKLPDMTASCTNPFLLAFPKTPLARIHNTRSDYEILAGVAAALADLVDEPRMKTY--------------WKGI-LD 5e7o.1    HWYE-KL-DLNVTSEHSY-INMTEPAIKPMWESKTDWQIFLALAKRVEMAAKRKKYEKFNDEKFKWVRDLSNLWNQMTMD  target    G---DPTPYLQRVLSGSNATRGILYEDLHASSAKGVPLLMNARTYPRHAG--WEQRQ----EDKPWYTPTGRLEFYRPEP 5e7o.1    GKLAEDEAAAQYILDNAPQSKGITIQMLREKPQR----FKSNWTSPLKEGVPYTPFQYFVVDKKPWPTLTGRQQFYLDHD  target    EWQAAGESLPIWREPVDATFYEPNAILANSKHPSINPRAPEDYGVPESQMDVETRQYRNVVRTWQELKLSKHPLTEKDPA 5e7o.1    TFFDMGVELPTYKAPIDADKY-----------------------------------------------------------  target    YRFVFQTPKYRWGAHSTAVDSDWIAMLFGPFGDPYRRDSRTPWTGEAYAEINPRDAKELGLKDGDYIWLDADPEDRPYRG 5e7o.1    --------------------------------------------------------------------------------  target    ADSSDEFYDVARAMMRVRIYSGMPRRVIRTWFNMYAATPGTVQAQKDVPGGPAQNQDTGYVALFRHGSHQSGTRAYLRPT 5e7o.1    --------------------------------------------------------------------------------  target    QMTDSMNRKAYFGQTIGKGFEADVHSPSGAPKEGYVKVEKAEDGGDEGVGEWRPVTLGLRPDDPSEAMQAYLAGEFVTRK 5e7o.1    --------------------------------------------------------------------------------  target    RKGS 5e7o.1    ---- ``` | | | | | | | | | | | | | | | | | | | | | | | | | | | | | | | | | | | | | | | | | | | | | | | | | |
|  | 7l5i.1.A | Trimethylamine-N-oxide reductase  *Crystal Structure of Haemophilus influenzae MtsZ at pH 7.0* | 0.31 |  | 19.32 | 0.63 | 74-1130 | X-ray | 1.73 | monomer | 2 x MGD, 1 x MO, 1 x O | HHblits | 0.30 |
| ``` target    SDLSRRELLKRAVVVGTGAGLAELFLPAQFLSSASAQSEPQAVAIANPLAQMPDRSWERIYRDQFAEEDSFVFTCAPNDT 7l5i.1    -------------------------------------------------------------------------TVVTAAH  target    HNCLLRAHVKNGVIVRISPTYGYGKATDLAGNQASHRWDPRICQKGLILGRRIYGDRRVKAPMIRKGFKEWADAGFPRHD 7l5i.1    -WGSIGVVVQDGKVVKSGPAIE-------------PAVPNELQT---VVADQLYSEARVKCPMVRKGFLA----------  target    DGTPRADMEKRGYDEWLQIPWDEALAIAAKTLQNVAETYKGEDGAGKLLEQGYEPAMVEAMHGAGVQAIKMRGGMPLLGA 7l5i.1    N-PGKSDTTMRGRDEWVRVSWDEALDLVHNQLKRVRDEHGSTG----IFAGSYG------WFSCGSLH------------  target    GRVFGFYRFANMLALLDGKLRPEAPPEEIVGSRAFDNYAWHTDLPPGHPMVSGSQT---VDFDL-FAAEHSKLLVLIGMN 7l5i.1    ASRTLLQRYMNATGG---FV----------GHK--GDYSTGA-AQVIMPHVLGTIEVYEQQTSWESILESSDIIVLWSAN  target    WICTKMPD--------AHWIGDARLKGTRVVVISADYMPTANK-ADEIVILRPGTDTAFLLGVARELITKKLYDRDAVIQ 7l5i.1    PLTTMRIAWMSTDQKGIEYFKKFQASGKRIICIDPQKSETCQMLNAEWIPVNTATDVPLMLGIAHTLVEQGKHDKDFLKK  target    RTDLPLLVRLDTGERLSARDVFEGYRQAPLENYVALKTEEELAAPPSPPFTADKQVVPTELREEWGDFVYWDRATNGPAA 7l5i.1    YTSGYAK---------------------------------------------------------FEEYL-----------  target    VNRDEIGAKFAGDPALLGAFDVTLVDGTNVKARTAFSLLKEYLDENFDVQTTSEVCNVDPAAVRSLARQLAANKGNALLA 7l5i.1    ----------------LGK-----TDG-----------------QPKTAEWAAKICGVPAETIKQLAADFAS-KR-TMLM  target    AGMGPNHYFNADLFGRVHFLVAALTDNIGHFSGNVGSYAGNYRGSLFQAMGQWIA---ENPFD--QEADLTKPARVKRYF 7l5i.1    GGWGMQRQRHGEQTHWMLVTLASMLGQIGLPGGGFGLSYHYSNGGVPTATGGIIGSITASPSGKAGAKTWLDDTSKSA-F  target    KSESAHYWNYGDRPLVSPSEIITGKSHMPTPTKLIWFGNSNSLLGNAKWSFDVVKNTLPKQDAVFCNEWHWTSSCEYSDL 7l5i.1    --PLARIADVLLHPGKKIQYNGTEI--TYPDIKAVYWAGGNPFVHHQD--TNTLVKAFQKPDVVIVNEVNWTPTARMADI  target    VFPADSWAEFKLPDMTASCTNPFLLAFPKTPLARIHNTRSDYEILAGVAAALADLVDEPRMKTYWKGILDGDPTPYLQRV 7l5i.1    VLPATTSYERNDLTMAGDYSMMSVYPMKQ-VVPPQFEAKNDYDIFVELAKRAGVEEQYT----EGK-----TEMEWLEEF  target    LSG----SNA--TRGILYEDLHASSAKGVPL--LMNARTYPRHAGWEQRQEDKPWYTPTGRLEFYRPEPEWQA--AGESL 7l5i.1    YNAAFSAARANRVAMPRFDKFWAEN-KPLSFEAGEAAKKWVRYGEFREDPLLNPLGTPSGKIEIFSDVVEKMNYNDCKGH  target    PIWREPVDATFYEPNAILANSKHPSINPRAPEDYGVPESQMDVETRQYRNVVRTWQELKLSKHPLTEKDPAYRFVFQTPK 7l5i.1    PSWMEPEEFA-------------------------------------------------------GNVTEEYPLALVTPH  target    YRWGAHSTAVDSDWIAMLFGPFGDPYRRDSRTPWTGEAYAEINPRDAKELGLKDGDYIWLDADPEDRPYRGADSSDEFYD 7l5i.1    PYYRLHSQLAHTSLRQKYA--------------VNDREPVMIHPEDAAARGIKDGDIVRIHSK-----------------  target    VARAMMRVRIYSGMPRRVIRTWFNMYAATPGTVQAQKDVPGGPAQNQDTGYVALFRHGSHQSGTRAYLRPTQMTDSMNRK 7l5i.1    RGQVLAGAAVTENIIKGTVALHEGAWYDPMYLGE--------SE----KP---LCKNGCANVLTRDEG-TSKLAQG----  target    AYFGQTIGKGFEADVHSPSGAPKEGYVKVEKAEDGGDEGVGEWRPVTLGLRPDDPSEAMQAYLAGEFVTRKRKGS 7l5i.1    -------------------NSPNTCIVQIEKFIGVAPE------------------------------------- ``` | | | | | | | | | | | | | | | | | | | | | | | | | | | | | | | | | | | | | | | | | | | | | | | | | |
|  | 7l5s.1.A | Trimethylamine-N-oxide reductase  *Crystal Structure of Haemophilus influenzae MtsZ at pH 5.5* | 0.30 |  | 19.32 | 0.63 | 74-1130 | X-ray | 2.09 | monomer | 1 x O, 2 x MGD, 1 x MO | HHblits | 0.30 |
| ``` target    SDLSRRELLKRAVVVGTGAGLAELFLPAQFLSSASAQSEPQAVAIANPLAQMPDRSWERIYRDQFAEEDSFVFTCAPNDT 7l5s.1    -------------------------------------------------------------------------TVVTAAH  target    HNCLLRAHVKNGVIVRISPTYGYGKATDLAGNQASHRWDPRICQKGLILGRRIYGDRRVKAPMIRKGFKEWADAGFPRHD 7l5s.1    -WGSIGVVVQDGKVVKSGPAIE-------------PAVPNELQT---VVADQLYSEARVKCPMVRKGFLA----------  target    DGTPRADMEKRGYDEWLQIPWDEALAIAAKTLQNVAETYKGEDGAGKLLEQGYEPAMVEAMHGAGVQAIKMRGGMPLLGA 7l5s.1    N-PGKSDTTMRGRDEWVRVSWDEALDLVHNQLKRVRDEHGSTG----IFAGSYG------WFSCGSLH------------  target    GRVFGFYRFANMLALLDGKLRPEAPPEEIVGSRAFDNYAWHTDLPPGHPMVSGSQT---VDFDL-FAAEHSKLLVLIGMN 7l5s.1    ASRTLLQRYMNATGG---FV----------GHK--GDYSTGA-AQVIMPHVLGTIEVYEQQTSWESILESSDIIVLWSAN  target    WICTKMPD--------AHWIGDARLKGTRVVVISADYMPTANK-ADEIVILRPGTDTAFLLGVARELITKKLYDRDAVIQ 7l5s.1    PLTTMRIAWMSTDQKGIEYFKKFQASGKRIICIDPQKSETCQMLNAEWIPVNTATDVPLMLGIAHTLVEQGKHDKDFLKK  target    RTDLPLLVRLDTGERLSARDVFEGYRQAPLENYVALKTEEELAAPPSPPFTADKQVVPTELREEWGDFVYWDRATNGPAA 7l5s.1    YTSGYAK---------------------------------------------------------FEEYL-----------  target    VNRDEIGAKFAGDPALLGAFDVTLVDGTNVKARTAFSLLKEYLDENFDVQTTSEVCNVDPAAVRSLARQLAANKGNALLA 7l5s.1    ----------------LGK-----TDG-----------------QPKTAEWAAKICGVPAETIKQLAADFAS-KR-TMLM  target    AGMGPNHYFNADLFGRVHFLVAALTDNIGHFSGNVGSYAGNYRGSLFQAMGQWIA---ENPFD--QEADLTKPARVKRYF 7l5s.1    GGWGMQRQRHGEQTHWMLVTLASMLGQIGLPGGGFGLSYHYSNGGVPTATGGIIGSITASPSGKAGAKTWLDDTSKSA-F  target    KSESAHYWNYGDRPLVSPSEIITGKSHMPTPTKLIWFGNSNSLLGNAKWSFDVVKNTLPKQDAVFCNEWHWTSSCEYSDL 7l5s.1    --PLARIADVLLHPGKKIQYNGTEI--TYPDIKAVYWAGGNPFVHHQD--TNTLVKAFQKPDVVIVNEVNWTPTARMADI  target    VFPADSWAEFKLPDMTASCTNPFLLAFPKTPLARIHNTRSDYEILAGVAAALADLVDEPRMKTYWKGILDGDPTPYLQRV 7l5s.1    VLPATTSYERNDLTMAGDYSMMSVYPMKQ-VVPPQFEAKNDYDIFVELAKRAGVEEQYT----EGK-----TEMEWLEEF  target    LSG----SNA--TRGILYEDLHASSAKGVPL--LMNARTYPRHAGWEQRQEDKPWYTPTGRLEFYRPEPEWQA--AGESL 7l5s.1    YNAAFSAARANRVAMPRFDKFWAEN-KPLSFEAGEAAKKWVRYGEFREDPLLNPLGTPSGKIEIFSDVVEKMNYNDCKGH  target    PIWREPVDATFYEPNAILANSKHPSINPRAPEDYGVPESQMDVETRQYRNVVRTWQELKLSKHPLTEKDPAYRFVFQTPK 7l5s.1    PSWMEPEEFA-------------------------------------------------------GNVTEEYPLALVTPH  target    YRWGAHSTAVDSDWIAMLFGPFGDPYRRDSRTPWTGEAYAEINPRDAKELGLKDGDYIWLDADPEDRPYRGADSSDEFYD 7l5s.1    PYYRLHSQLAHTSLRQKYA--------------VNDREPVMIHPEDAAARGIKDGDIVRIHSK-----------------  target    VARAMMRVRIYSGMPRRVIRTWFNMYAATPGTVQAQKDVPGGPAQNQDTGYVALFRHGSHQSGTRAYLRPTQMTDSMNRK 7l5s.1    RGQVLAGAAVTENIIKGTVALHEGAWYDPMYLGE--------SE----KP---LCKNGCANVLTRDEG-TSKLAQG----  target    AYFGQTIGKGFEADVHSPSGAPKEGYVKVEKAEDGGDEGVGEWRPVTLGLRPDDPSEAMQAYLAGEFVTRKRKGS 7l5s.1    -------------------NSPNTCIVQIEKFIGVAPE------------------------------------- ``` | | | | | | | | | | | | | | | | | | | | | | | | | | | | | | | | | | | | | | | | | | | | | | | | | |
|  | 1e60.1.A | Dimethyl sulfoxide/trimethylamine N-oxide reductase  *OXIDIZED DMSO REDUCTASE EXPOSED TO HEPES - Structure II BUFFER* | 0.32 | 0.00 | 20.00 | 0.63 | 73-1133 | X-ray | 2.00 | monomer | 2 x PGD, 1 x 2MO | HHblits | 0.30 |
| ``` target    SDLSRRELLKRAVVVGTGAGLAELFLPAQFLSSASAQSEPQAVAIANPLAQMPDRSWERIYRDQFAEEDSFVFTCAPNDT 1e60.1    ------------------------------------------------------------------------ANGTVMSG  target    HNCL-LRAHVKNGVIVRISPTYGYGKATDLAGNQASHRWDPRICQKGLILGRRIYGDRRVKAPMIRKGFKEWADAGFPRH 1e60.1    SHWGVFTATVENGRATAFTPWE------------KDP----HPSPMLAGVLDSIYSPTRIKYPMVRREFLEK--------  target    DDGTPRADMEKRGYDEWLQIPWDEALAIAAKTLQNVAETYKGEDGAGKLLEQGYEPAMVEAMHGAGVQAIKMRGGMPLLG 1e60.1    ---GVNADRSTRGNGDFVRVSWDQALDLVAAEVKRVEETYGPEG----VFGGSYGWK------SPGRLH-----------  target    AGRVFGFYRFANMLALLDGKLRPEAPPEEIVGSRAFDNYAWHTDLPPGHPMVSGSQTVDFDLFAAEHSKLLVLIGMNWIC 1e60.1    NCT-TLLRRMLTLA---GGYV-------NGAGDYSTGAAQVIMPHVVGTLEVYEQQ--TAWPVLAENTEVMVFWAADPIK  target    TKMPDA--------HWIGDARLKGTRVVVISADYMPTANK-ADEIVILRPGTDTAFLLGVARELITKKLYDRDAVIQRTD 1e60.1    TSQIGWVIPEHGAYPGLEALKAKGTKVIVIDPVRTKTVEFFGAEHITPKPQTDVAIMLGMAHTLVAEDLYDKDFIANYTS  target    LPLLVRLDTGERLSARDVFEGYRQAPLENYVALKTEEELAAPPSPPFTADKQVVPTELREEWGDFVYWDRATNGPAAVNR 1e60.1    GF-----------------DKF----------------------------------------LPY---------------  target    DEIGAKFAGDPALLGAFDVTLVDGTNVKARTAFSLLKEYLDENFDVQTTSEVCNVDPAAVRSLARQLAANKGNALLAAGM 1e60.1    ------------LDGETD---------------------S-TPKTAEWAEGISGVPAETIKELARLFESK--RTMLAAGW  target    GPNHYFNADLFGRVHFLVAALTDNIGHFSGNVGSYAGNYRGSLFQAMGQWIAENPFDQEADLT-KPARVKRYFKSESAHY 1e60.1    SMQRMHHGEQAHWMLVTLASMLGQIGLPGGGFGLSYHYSGGGTPSTSGP--ALAGITDGGAATKGPEWLA---------A  target    WNYGDRPLVSPSEIIT---------GKSHMPTPTKLIWFGNSNSLLGNAKWSFDVVKNTLPKQDAVFCNEWHWTSSCEYS 1e60.1    SGASVIPVARVVDMLENPGAEFDFNGTRSKFPDVKMAYWVGGNPFVHHQD--RNRMVKAWEKLETFVVHDFQWTPTARHA  target    DLVFPADSWAEFKLPDMTASCTNPFLLAFPKTPLARIHNTRSDYEILAGVAAALADLVDEPRMKTYWKGILDGDPTPYLQ 1e60.1    DIVLPATTSYERNDIETIGDYSNTGILAMKK-IVEPLYEARSDYDIFAAVAERLGKGAE------FTE---GKDEMGWIK  target    RVLSGSN---ATRGI---LYEDLHASSAKGVPLLMN--ARTYPRHAGWEQRQEDKPWYTPTGRLEFYRPEPEWQAA--GE 1e60.1    SFYDDAAKQGKAAGVEMPAFDAFWAE---GIVEFPVTDGADFVRYASFREDPLLNPLGTPTGLIEIYSKNIEKMGYDDCP  target    SLPIWREPVDATFYEPNAILANSKHPSINPRAPEDYGVPESQMDVETRQYRNVVRTWQELKLSKHPLTEKDPAYRFVFQT 1e60.1    AHPTWMEPLERL-------------------------------------------------------DGPGAKYPLHIAA  target    PKYRWGAHSTAVDSDWIAMLFGPFGDPYRRDSRTPWTGEAYAEINPRDAKELGLKDGDYIWLDADPEDRPYRGADSSDEF 1e60.1    SHPFNRLHSQLN-GTVLREGYA-------------VQGHEPCLMHPDDAAARGIADGDVVRVHND---------------  target    YDVARAMMRVRIYSGMPRRVIRTWFNMYAATPGTVQAQKDVPGGPAQNQDTGYVALFRHGSHQSGTRAYLRPTQMTDSMN 1e60.1    --RGQILTGVKVTDAVMKGVIQIYEGGWYDPSDVT--------EPG-----T---LDKYGDVNVLSADI-GTSKLAQG--  target    RKAYFGQTIGKGFEADVHSPSGAPKEGYVKVEKAEDGGDEGVGEWRPVTLGLRPDDPSEAMQAYLAGEFVTRKRKGS 1e60.1    ---------------------NCGQTVLAEVEKYTGPAVTLTG---------------------------------- ``` | | | | | | | | | | | | | | | | | | | | | | | | | | | | | | | | | | | | | | | | | | | | | | | | | |
|  | 1e5v.2.A | Dimethyl sulfoxide/trimethylamine N-oxide reductase  *OXIDIZED DMSO REDUCTASE EXPOSED TO HEPES BUFFER* | 0.31 | 0.00 | 19.84 | 0.63 | 74-1130 | X-ray | 2.40 | monomer | 2 x PGD, 1 x 2MO | HHblits | 0.30 |
| ``` target    SDLSRRELLKRAVVVGTGAGLAELFLPAQFLSSASAQSEPQAVAIANPLAQMPDRSWERIYRDQFAEEDSFVFTCAPNDT 1e5v.2    -------------------------------------------------------------------------NGTVMSG  target    HNCL-LRAHVKNGVIVRISPTYGYGKATDLAGNQASHRWDPRICQKGLILGRRIYGDRRVKAPMIRKGFKEWADAGFPRH 1e5v.2    SHWGVFTATVENGRATAFTPWE------------KDP----HPSPMLAGVLDSIYSPTRIKYPMVRREFLEK--------  target    DDGTPRADMEKRGYDEWLQIPWDEALAIAAKTLQNVAETYKGEDGAGKLLEQGYEPAMVEAMHGAGVQAIKMRGGMPLLG 1e5v.2    ---GVNADRSTRGNGDFVRVSWDQALDLVAAEVKRVEETYGPEG----VFGGSYGW------KSPGRLH-----------  target    AGRVFGFYRFANMLALLDGKLRPEAPPEEIVGSRAFDNYAWHTDLPPGHPMVSGSQTVDFDLFAAEHSKLLVLIGMNWIC 1e5v.2    NC-TTLLRRMLTLA---GGYV-------NGAGDYSTGAAQVIMPHVVGTLEVYEQQT--AWPVLAENTEVMVFWAADPIK  target    TKMPDA--------HWIGDARLKGTRVVVISADYMPTANK-ADEIVILRPGTDTAFLLGVARELITKKLYDRDAVIQRTD 1e5v.2    TSQIGWVIPEHGAYPGLEALKAKGTKVIVIDPVRTKTVEFFGAEHITPKPQTDVAIMLGMAHTLVAEDLYDKDFIANYTS  target    LPLLVRLDTGERLSARDVFEGYRQAPLENYVALKTEEELAAPPSPPFTADKQVVPTELREEWGDFVYWDRATNGPAAVNR 1e5v.2    GF-----------------DKF----------------------------------------LPY---------------  target    DEIGAKFAGDPALLGAFDVTLVDGTNVKARTAFSLLKEYLDENFDVQTTSEVCNVDPAAVRSLARQLAANKGNALLAAGM 1e5v.2    ------------LDGETD---------------------S-TPKTAEWAEGISGVPAETIKELARLFESK--RTMLAAGW  target    GPNHYFNADLFGRVHFLVAALTDNIGHFSGNVGSYAGNYRGSLFQAMGQWIAENPFDQEADLT-KPARVKRYFKSESAHY 1e5v.2    SMQRMHHGEQAHWMLVTLASMLGQIGLPGGGFGLSYHYSGGGTPSTSGPAL--AGITDGGAATKGPEWLA---------A  target    WNYGDRPLVSPSEIIT---------GKSHMPTPTKLIWFGNSNSLLGNAKWSFDVVKNTLPKQDAVFCNEWHWTSSCEYS 1e5v.2    SGASVIPVARVVDMLENPGAEFDFNGTRSKFPDVKMAYWVGGNPFVHHQD--RNRMVKAWEKLETFVVHDFQWTPTARHA  target    DLVFPADSWAEFKLPDMTASCTNPFLLAFPKTPLARIHNTRSDYEILAGVAAALADLVDEPRMKTYWKGILDGDPTPYLQ 1e5v.2    DIVLPATTSYERNDIETIGDYSNTGILAMK-KIVEPLYEARSDYDIFAAVAERLGKGAE------FTE---GKDEMGWIK  target    RVLSGSN---ATRGI---LYEDLHASSAKGVPLLMN--ARTYPRHAGWEQRQEDKPWYTPTGRLEFYRPEPEWQAA--GE 1e5v.2    SFYDDAAKQGKAAGVQMPAFDAFWAE---GIVEFPVTDGADFVRYASFREDPLLNPLGTPTGLIEIYSKNIEKMGYDDCP  target    SLPIWREPVDATFYEPNAILANSKHPSINPRAPEDYGVPESQMDVETRQYRNVVRTWQELKLSKHPLTEKDPAYRFVFQT 1e5v.2    AHPTWMEPLERL-------------------------------------------------------DGPGAKYPLHIAA  target    PKYRWGAHSTAVDSDWIAMLFGPFGDPYRRDSRTPWTGEAYAEINPRDAKELGLKDGDYIWLDADPEDRPYRGADSSDEF 1e5v.2    SHPFNRLHSQL-NGTVLREGYA-------------VQGHEPCLMHPDDAAARGIADGDVVRVHND---------------  target    YDVARAMMRVRIYSGMPRRVIRTWFNMYAATPGTVQAQKDVPGGPAQNQDTGYVALFRHGSHQSGTRAYLRPTQMTDSMN 1e5v.2    --RGQILTGVKVTDAVMKGVIQIYEGGWYDPSDVT--------EPG-----T---LDKYGDVNVLSADI-GTSKLAQG--  target    RKAYFGQTIGKGFEADVHSPSGAPKEGYVKVEKAEDGGDEGVGEWRPVTLGLRPDDPSEAMQAYLAGEFVTRKRKGS 1e5v.2    ---------------------NCGQTVLAEVEKYTGPAVT------------------------------------- ``` | | | | | | | | | | | | | | | | | | | | | | | | | | | | | | | | | | | | | | | | | | | | | | | | | |
|  | 1e18.1.A | DMSO REDUCTASE.  *TUNGSTEN-SUSBSTITUTED DMSO REDUCTASE FROM RHODOBACTER CAPSULATUS* | 0.31 | 0.00 | 19.86 | 0.63 | 74-1129 | X-ray | 2.00 | monomer | 2 x PGD, 1 x 6WO | HHblits | 0.30 |
| ``` target    SDLSRRELLKRAVVVGTGAGLAELFLPAQFLSSASAQSEPQAVAIANPLAQMPDRSWERIYRDQFAEEDSFVFTCAPNDT 1e18.1    -------------------------------------------------------------------------NGTVMSG  target    HNCL-LRAHVKNGVIVRISPTYGYGKATDLAGNQASHRWDPRICQKGLILGRRIYGDRRVKAPMIRKGFKEWADAGFPRH 1e18.1    SHWGVFTATVENGRATAFTPWE------------KDP----HPSPMLAGVLDSIYSPTRIKYPMVRREFLEK--------  target    DDGTPRADMEKRGYDEWLQIPWDEALAIAAKTLQNVAETYKGEDGAGKLLEQGYEPAMVEAMHGAGVQAIKMRGGMPLLG 1e18.1    ---GVNADRSTRGNGDFVRVSWDQALDLVAAEVKRVEETYGPQG----VFGGSYGWK------SPGRL-----------H  target    AGRVFGFYRFANMLALLDGKLRPEAPPEEIVGSRAFDNYAWHTDLPPGHPMVSGSQTVDFDLFAAEHSKLLVLIGMNWIC 1e18.1    NCT-TLLRRMLTLA---GGYV-------NGAGDYSTGAAQVIMPHVVGTLEVYEQQ--TAWPVLAENTEVMVFWAADPIK  target    TKMPDA--------HWIGDARLKGTRVVVISADYMPTANK-ADEIVILRPGTDTAFLLGVARELITKKLYDRDAVIQRTD 1e18.1    TSQIGWVIPEHGAYPGLEALKAKGTKVIVIDPVRTKTVEFFGAEHITPKPQTDVAIMLGMAHTLVAEDLYDKDFIANYTS  target    LPLLVRLDTGERLSARDVFEGYRQAPLENYVALKTEEELAAPPSPPFTADKQVVPTELREEWGDFVYWDRATNGPAAVNR 1e18.1    GF-----------------DKF----------------------------------------LPY---------------  target    DEIGAKFAGDPALLGAFDVTLVDGTNVKARTAFSLLKEYLDENFDVQTTSEVCNVDPAAVRSLARQLAANKGNALLAAGM 1e18.1    ------------LDGETD---------------------S-TPKTAEWAEGISGVPAETIKELARLFESK--RTMLAAGW  target    GPNHYFNADLFGRVHFLVAALTDNIGHFSGNVGSYAGNYRGSLFQAMGQWIAENPFDQEADLT-KPARVKRYFKSESAHY 1e18.1    SMQRMHHGEQAHWMLVTLASMLGQIGLPGGGFGLSYHYSGGGTPSTSGP--ALAGITDGGAATKGPEWL----A-----A  target    WNYGDRPLVSPSEIIT---------GKSHMPTPTKLIWFGNSNSLLGNAKWSFDVVKNTLPKQDAVFCNEWHWTSSCEYS 1e18.1    SGASVIPVARVVDMLENPGAEFDFNGTRSKFPDVKMAYWVGGNPFVHHQD--RNRMVKAWEKLETFVVHDFQWTPTARHA  target    DLVFPADSWAEFKLPDMTASCTNPFLLAFPKTPLARIHNTRSDYEILAGVAAALADLVDEPRMKTYWKGILDGDPTPYLQ 1e18.1    DIVLPATTSYERNDIETIGDYSNTGILAMKK-IVEPLYEARSDYDIFAAVAERLGKGKE------FTE---GKDEMGWIK  target    RVLSGSN---ATRGI---LYEDLHASSAKGVPLLMNA--RTYPRHAGWEQRQEDKPWYTPTGRLEFYRPEPEWQAA--GE 1e18.1    SFYDDAAKQGKAAGVEMPAFDAFWAE---GIVEFPVTDGADFVRYASFREDPLLNPLGTPTGLIEIYSKNIEKMGYDDCP  target    SLPIWREPVDATFYEPNAILANSKHPSINPRAPEDYGVPESQMDVETRQYRNVVRTWQELKLSKHPLTEKDPAYRFVFQT 1e18.1    AHPTWMEPLERL-------------------------------------------------------DGPGAKYPLHIAA  target    PKYRWGAHSTAVDSDWIAMLFGPFGDPYRRDSRTPWTGEAYAEINPRDAKELGLKDGDYIWLDADPEDRPYRGADSSDEF 1e18.1    SHPFNRLHSQLN-GTVLREGYA-------------VQGHEPCLMHPDDAAARGIADGDVVRVHND---------------  target    YDVARAMMRVRIYSGMPRRVIRTWFNMYAATPGTVQAQKDVPGGPAQNQDTGYVALFRHGSHQSGTRAYLRPTQMTDSMN 1e18.1    --RGQILTGVKVTDAVMKGVIQIYEGGWYDPSDVT--------EP-----GT---LDKYGDVNVLSADI-GTSKLAQG--  target    RKAYFGQTIGKGFEADVHSPSGAPKEGYVKVEKAEDGGDEGVGEWRPVTLGLRPDDPSEAMQAYLAGEFVTRKRKGS 1e18.1    ---------------------NCGQTVLAEVEKYTGPAV-------------------------------------- ``` | | | | | | | | | | | | | | | | | | | | | | | | | | | | | | | | | | | | | | | | | | | | | | | | | |
|  | 7qv7.1.L | Hydrogen dependent carbon dioxide reductase subunit FdhF  *Cryo-EM structure of Hydrogen-dependent CO2 reductase.* | 0.28 |  | 18.95 | 0.62 | 71-1127 | EM | 0.00 | hetero-2-6-6-2-mer | 52 x SF4, 6 x 402 | HHblits | 0.29 |
| ``` target    SDLSRRELLKRAVVVGTGAGLAELFLPAQFLSSASAQSEPQAVAIANPLAQMPDRSWERIYRDQFAEEDSFVFTCAPNDT 7qv7.1    ----------------------------------------------------------------------KVLTTCPYCG  target    HNCLLRAHVKNGVIVRISPTYGYGKATDLAGNQASHRWDPRICQKGLILGRRIYGDRRVKAPMIRKGFKEWADAGFPRHD 7qv7.1    TGCGLYLKVENEKIVGVEPD------------KLHPVNQGELCIKGYYGYKYVHDPRRLTSPLIKKN-------------  target    DGTPRADMEKRGYDEWLQIPWDEALAIAAKTLQNVAETYKGEDGAGKLLEQGYEPAMVEAMHGAGVQAIKMRGGMPLLGA 7qv7.1    -------------GKFVPVSWDEALNFIANGLKKIKSEYGSDAFA--MF------------CS-----------------  target    GRVFGFYRFANMLALLDGKLRPEAPPEEIVGSRAFDNYAWHTDLP--PGHPMVSGSQTVDFDLFAAE-HSKLLVLIGMNW 7qv7.1    ------ARATNEDNYAAQKFAR-----AVIGINNVDHCARLCHAPTVAGLAMTLGSGAMTNSIPEISTYSDVIFIIGSNT  target    ICTKMPDAHWIGDARLKGTRVVVISADYMPTANKADEIVILRPGTDTAFLLGVARELITKKLYDRDAVIQRTDLPLLVRL 7qv7.1    AECHPLIAAHVIKAKERGAKLIVADPRMNAMVHKADIWLRVPSGYNIPLINGMIHIIIKEGLVKTDFVKNHAV-------  target    DTGERLSARDVFEGYRQAPLENYVALKTEEELAAPPSPPFTADKQVVPTELREEWGDFVYWDRATNGPAAVNRDEIGAKF 7qv7.1    --------------------------------------------------------------------------------  target    AGDPALLGAFDVTLVDGTNVKARTAFSLLKEYLDENFDVQTTSEVCNVDPAAVRSLARQLAANKGNALLAAGMGPNHYFN 7qv7.1    ------------------------GFEEMAKAVE-KYTPEYVEELTGIPKKDLIKAARFYGQAQA-AAILYSMGVTQFSH  target    ADLFGRVHFLVAALTDNIGHFSGNVGSYAGN--YRGSLFQAMGQWIAENPFDQEADLTKPARVKRYFKSESAHYWNY--G 7qv7.1    GTGNVVSLANLAVITGNLGRPGAGICPLRGQNNVQGAC--DVGALPNVLPGY--LDVTKEQNRER-----FEKVWGVKLP  target    DRPLVSPSEIITGKSHMPTPTKLIWFGNSNSLLGNAKWSFDVVKNTLPKQDAVFCNEWHWTSSCEYSDLVFPADSWAEFK 7qv7.1    SNIGLRVTEVPDAI--LNKRVRALYIFGENPIMSDPD--SDHLRHALEHLDLLIVQDIFLTETARLAHVVLPAACWAEKD  target    LPDMTASCTNPFLLAFPKTPLARIHNTRSDYEILAGVAAALADLVDEPRMKTYWKGILDGDPTPYLQRVLSGSN-ATRGI 7qv7.1    GT---FTNTERRVQRVRK-AVEAPGEAKPDWWIFSQIAERMGYTG------MQY-----NNVQEIWDEVRKIVPEKFGGI  target    LYEDLHASSAKGVPLLMNARTYPRHAGWEQRQEDKPWYTPTGRLEFYRPEPEWQAAGESLPIWREPVDATFYEPNAILAN 7qv7.1    SYARLEKE--KGLAWPCPTED---HTGTPILYLGGKFATPSGKAQMYPVIFYP-----NTCICDEGAEKQD---------  target    SKHPSINPRAPEDYGVPESQMDVETRQYRNVVRTWQELKLSKHPLTEKDPAYRFVFQTPKYRWGAHSTA--VDSDWIAML 7qv7.1    -----FNH-VIV---------------------GS--------IAELPDEEYPFTLTTGRRVYHYHTATMTRKSPVIDQI  target    FGPFGDPYRRDSRTPWTGEAYAEINPRDAKELGLKDGDYIWLDADPEDRPYRGADSSDEFYDVARAMMRVRIYSGMPRRV 7qv7.1    ----------------APQELVEINPQDATRLGINDGDFLRVSTR-----------------RGYVATRAWVTERVPKGT  target    IRTWFNMYAATPGTVQAQKDVPGGPAQNQDTGYVALFRHGS-HQSGTRAYLRPTQMTDSMN-RKAYFGQTIGKGFEADVH 7qv7.1    IFMTFHYWEACCNELT-------NTAS--------DAICCIPEFKVAAAKVEKISQVEAQAILKEK-----IEK----YQ  target    SPSGAPKEGYVKVEKAEDGGDEGVGEWRPVTLGLRPDDPSEAMQAYLAGEFVTRKRKGS 7qv7.1    --VELEKDVANMLAKEKGG---------------------------------------- ``` | | | | | | | | | | | | | | | | | | | | | | | | | | | | | | | | | | | | | | | | | | | | | | | | | |
|  | 7qv7.1.O | Hydrogen dependent carbon dioxide reductase subunit FdhF  *Cryo-EM structure of Hydrogen-dependent CO2 reductase.* | 0.28 |  | 18.95 | 0.62 | 71-1127 | EM | 0.00 | hetero-2-6-6-2-mer | 52 x SF4, 6 x 402 | HHblits | 0.29 |
| ``` target    SDLSRRELLKRAVVVGTGAGLAELFLPAQFLSSASAQSEPQAVAIANPLAQMPDRSWERIYRDQFAEEDSFVFTCAPNDT 7qv7.1    ----------------------------------------------------------------------KVLTTCPYCG  target    HNCLLRAHVKNGVIVRISPTYGYGKATDLAGNQASHRWDPRICQKGLILGRRIYGDRRVKAPMIRKGFKEWADAGFPRHD 7qv7.1    TGCGLYLKVENEKIVGVEPD------------KLHPVNQGELCIKGYYGYKYVHDPRRLTSPLIKKN-------------  target    DGTPRADMEKRGYDEWLQIPWDEALAIAAKTLQNVAETYKGEDGAGKLLEQGYEPAMVEAMHGAGVQAIKMRGGMPLLGA 7qv7.1    -------------GKFVPVSWDEALNFIANGLKKIKSEYGSDAFA--MF------------CS-----------------  target    GRVFGFYRFANMLALLDGKLRPEAPPEEIVGSRAFDNYAWHTDLP--PGHPMVSGSQTVDFDLFAAE-HSKLLVLIGMNW 7qv7.1    ------ARATNEDNYAAQKFAR-----AVIGINNVDHCARLCHAPTVAGLAMTLGSGAMTNSIPEISTYSDVIFIIGSNT  target    ICTKMPDAHWIGDARLKGTRVVVISADYMPTANKADEIVILRPGTDTAFLLGVARELITKKLYDRDAVIQRTDLPLLVRL 7qv7.1    AECHPLIAAHVIKAKERGAKLIVADPRMNAMVHKADIWLRVPSGYNIPLINGMIHIIIKEGLVKTDFVKNHAV-------  target    DTGERLSARDVFEGYRQAPLENYVALKTEEELAAPPSPPFTADKQVVPTELREEWGDFVYWDRATNGPAAVNRDEIGAKF 7qv7.1    --------------------------------------------------------------------------------  target    AGDPALLGAFDVTLVDGTNVKARTAFSLLKEYLDENFDVQTTSEVCNVDPAAVRSLARQLAANKGNALLAAGMGPNHYFN 7qv7.1    ------------------------GFEEMAKAVE-KYTPEYVEELTGIPKKDLIKAARFYGQAQA-AAILYSMGVTQFSH  target    ADLFGRVHFLVAALTDNIGHFSGNVGSYAGN--YRGSLFQAMGQWIAENPFDQEADLTKPARVKRYFKSESAHYWNY--G 7qv7.1    GTGNVVSLANLAVITGNLGRPGAGICPLRGQNNVQGAC--DVGALPNVLPGY--LDVTKEQNRER-----FEKVWGVKLP  target    DRPLVSPSEIITGKSHMPTPTKLIWFGNSNSLLGNAKWSFDVVKNTLPKQDAVFCNEWHWTSSCEYSDLVFPADSWAEFK 7qv7.1    SNIGLRVTEVPDAI--LNKRVRALYIFGENPIMSDPD--SDHLRHALEHLDLLIVQDIFLTETARLAHVVLPAACWAEKD  target    LPDMTASCTNPFLLAFPKTPLARIHNTRSDYEILAGVAAALADLVDEPRMKTYWKGILDGDPTPYLQRVLSGSN-ATRGI 7qv7.1    GT---FTNTERRVQRVRK-AVEAPGEAKPDWWIFSQIAERMGYTG------MQY-----NNVQEIWDEVRKIVPEKFGGI  target    LYEDLHASSAKGVPLLMNARTYPRHAGWEQRQEDKPWYTPTGRLEFYRPEPEWQAAGESLPIWREPVDATFYEPNAILAN 7qv7.1    SYARLEKE--KGLAWPCPTED---HTGTPILYLGGKFATPSGKAQMYPVIFYP-----NTCICDEGAEKQD---------  target    SKHPSINPRAPEDYGVPESQMDVETRQYRNVVRTWQELKLSKHPLTEKDPAYRFVFQTPKYRWGAHSTA--VDSDWIAML 7qv7.1    -----FNH-VIV---------------------GS--------IAELPDEEYPFTLTTGRRVYHYHTATMTRKSPVIDQI  target    FGPFGDPYRRDSRTPWTGEAYAEINPRDAKELGLKDGDYIWLDADPEDRPYRGADSSDEFYDVARAMMRVRIYSGMPRRV 7qv7.1    ----------------APQELVEINPQDATRLGINDGDFLRVSTR-----------------RGYVATRAWVTERVPKGT  target    IRTWFNMYAATPGTVQAQKDVPGGPAQNQDTGYVALFRHGS-HQSGTRAYLRPTQMTDSMN-RKAYFGQTIGKGFEADVH 7qv7.1    IFMTFHYWEACCNELT-------NTAS--------DAICCIPEFKVAAAKVEKISQVEAQAILKEK-----IEK----YQ  target    SPSGAPKEGYVKVEKAEDGGDEGVGEWRPVTLGLRPDDPSEAMQAYLAGEFVTRKRKGS 7qv7.1    --VELEKDVANMLAKEKGG---------------------------------------- ``` | | | | | | | | | | | | | | | | | | | | | | | | | | | | | | | | | | | | | | | | | | | | | | | | | |
|  | 4v4c.1.A | Pyrogallol hydroxytransferase large subunit  *Crystal Structure of Pyrogallol-Phloroglucinol Transhydroxylase from Pelobacter acidigallici* | 0.30 |  | 18.12 | 0.62 | 72-1037 | X-ray | 2.35 | hetero-oligomer | 2 x CA, 2 x MGD, 1 x 4MO, 3 x SF4 | HHblits | 0.29 |
| ``` target    SDLSRRELLKRAVVVGTGAGLAELFLPAQFLSSASAQSEPQAVAIANPLAQMPDRSWERIYRDQFAEEDSFVFTCAPNDT 4v4c.1    -----------------------------------------------------------------------VVRLTN-SS  target    HNCLLRAHVKNGVIVRISPTYGYGK------ATDLAGNQASHRWDPRICQKGLILGRRIYGDRRVKAPMIRKGFKEWADA 4v4c.1    TGGPVFVYVKDGKIIRMTPMDFDDAVDAPSWKIEARGKTFTPPRKTSIAPYTAGFKSMIYSDLRIPYPMKRKSFDPN---  target    GFPRHDDGTPRADMEKRGYD--------EWLQIPWDEALAIAAKTLQNVAETYKGEDGAGKLLEQGYEPAMVEAMHGAGV 4v4c.1    ---------GERNPQLRGAGLSKQDPWSDYERISWDEATDIVVAEINRIKHAYGPSA----ILS----------TPS--S  target    QAIKMRGGMPLLGAGRVFGFYRFANMLALLDGKLRPEAPPEEIVGSRAFDNYAWHTDLPPGHPMVSGSQTVDFD-LFAAE 4v4c.1    HHMW--GN---VGYR-HSTYFRFMNMMGFTYA----------DHNPDSWEGWHWGGMHMWGFSWRLGNPEQYDLLEDGLK  target    HSKLLVLIGMNWICTKMPDAHW-----IGDARLKGTRVVVISADYMPTAN-KADEIVILRPGTDTAFLLGVARELITKKL 4v4c.1    HAEMIVFWSSDPETNSGIYAGFESNIRRQWLKDLGVDFVFIDPHMNHTARLVADKWFSPKIGTDHALSFAIAYTWLKEDS  target    YDRDAVIQRTDLPLLVRLDTGERLSARDVFEGYRQAPLENYVALKTEEELAAPPSPPFTADKQVVPTELREEWGDFVYWD 4v4c.1    YDKEYVAANAHG--------------------FE-------------------------------------EWADYVL--  target    RATNGPAAVNRDEIGAKFAGDPALLGAFDVTLVDGTNVKARTAFSLLKEYLDENFDVQTTSEVCNVDPAAVRSLARQLAA 4v4c.1    -------------------------GK-----TDG-----------------TPKTCEWAEEESGVPACEIRALARQWAK  target    NKGNALLAAGM----GPNHYFNADLFGRVHFLVAALTDNIGHFSGNVGSYAGNYRGS---LFQAM---G----------- 4v4c.1    KNT-YLAAGGLGGWGGACRASHGIEWARGMIALATMQG-MGKPGSNMWSTTQGVPLDYEFYFPGYAEGGISGDCENSAAG  target    ---QWIA-----ENPFDQE-----ADLTKPARVKRYFKSESAHYWNYGDRPLVSPSEIITGKSH---MPTPTKLIWFGNS 4v4c.1    FKFAWRMFDGKTTFPSPSNLNTSAGQHIPRLKIPECIMGG-KFQWSGKGFAGGDISHQLHQYEYPAPGYSKIKMFWKYGG  target    NSLLGNAKWSFDVVKNTL--PKQDAVFCNEWHWTSSCEYSDLVFPADSWAEFKLPDMTAS-----------CTNPFLLAF 4v4c.1    PHLGTMTA--TNRYAKMYTHDSLEFVVSQSIWFEGEVPFADIILPACTNFERWDISEFANCSGYIPDNYQLCNHRVISLQ  target    PKTPLARIHNTRSDYEILAGVAAALADLVDEPRMKTYWKGILDGDPTPYLQRVLSGSNATRGILYEDLHASSAKGVPLLM 4v4c.1    AK-CIEPVGESMSDYEIYRLFAKKLNIEE-------MFSE--GKDELAWCEQYFNATDMPKYMTWDEFFKKGYFVVPDNP  target    NA---RTYPR--------HAGWE----QRQEDKPWYTPTGRLEFYRPEPEWQ-------AAGESLPIWREPVDATFYEPN 4v4c.1    NRKKTVALRWFAEGREKDTPDWGPRLNNQVCRKGLQTTTGKVEFIATSLKNFEEQGYIDEHRPSMHTYVPAWES------  target    AILANSKHPSINPRAPEDYGVPESQMDVETRQYRNVVRTWQELKLSKHPLTEKDPAYRFVFQTPKYRWGAHSTAVD-SDW 4v4c.1    ------------------------------------------QK--HSP---LAVKYPLGMLSPHPRFSMHTMGDGKNSY  target    IAMLFGPFGDPYRRDSRTPWTGEAYAEINPRDAKELGLKDGDYIWLDADPEDRPYRGADSSDEFYDVARAMMRVRIYSGM 4v4c.1    MNYIKDH-----RV--EVDGYKYWIMRVNSIDAEARGIKNGDLIRAYND-----------------RGSVILAAQVTECL  target    PRRVIRTWFNMYAATPGTVQAQKDVPGGPAQNQDTGYVALFRHGSHQSGTRAYLRPTQMTDSMNRKAYFGQTIGKGFEAD 4v4c.1    QPGTVHSYESC---------------------------------------------------------------------  target    VHSPSGAPKEGYVKVEKAEDGGDEGVGEWRPVTLGLRPDDPSEAMQAYLAGEFVTRKRKGS 4v4c.1    ------------------------------------------------------------- ``` | | | | | | | | | | | | | | | | | | | | | | | | | | | | | | | | | | | | | | | | | | | | | | | | | |
|  | 6cz7.1.A | ArrA  *The arsenate respiratory reductase (Arr) complex from Shewanella sp. ANA-3* | 0.30 |  | 16.60 | 0.61 | 69-1037 | X-ray | 1.62 | hetero-1-1-mer | 5 x SF4, 2 x MGD, 1 x MO, 1 x PG5 | HHblits | 0.29 |
| ``` target    SDLSRRELLKRAVVVGTGAGLAELFLPAQFLSSASAQSEPQAVAIANPLAQMPDRSWERIYRDQFAEEDSFVFTCAPNDT 6cz7.1    --------------------------------------------------------------------GEWLATTCQGCT  target    HNCLLRAHVKNGVIVRISPTYGYGKATDLAGNQASHRWDPRICQKGLILGRRIYGDRRVKAPMIRKGFKEWADAGFPRHD 6cz7.1    SWCAKQIYVMDGRALKVRGN------------PNSGVHGMSSCPRQHLSLQQVYDPDRLRTPMMRTNPKK----------  target    DGTPRADMEKRGYDEWLQIPWDEALAIAAKTLQNVAETYKGEDGAGKLLEQGYEPAMVEAMHGAGVQAIKMRGGMPLLGA 6cz7.1    --------GRDQDPKFVPISWDKALDMLADKIIALRVANEPHKY--ALLR--------------GRYSHI--N-------  target    GRVFGFYRFANMLALLDGKLRPEAPPEEIVGSRAFDNYAWHTDLP--PGHPMVSGSQTVDFDLFAAEHSKLLVLIGMNWI 6cz7.1    --DLLYKKMTNL-----------------IGSPNNISHSSVCAEAHKMGPYYLDGN--WGYNQYDVKNAKFILSFGADPI  target    CTKMPDAHWIG--DARLKGTRVVVISADYMPTANKADEIVILRPGTDTAFLLGVARELITKKLYDRDAVIQRTDLPLLVR 6cz7.1    ASNRQVSFYSQTWGDSLDHAKVVVVDPRLSASAAKAHKWIPIEPGQDSVLALAIAHVALVEGVWHKPFVGDFIEGKNLFK  target    LDTGERLSARDVFEGYRQAPLENYVALKTEEELAAPPSPPFTADKQVVPTELREEWGDFVYWDRATNGPAAVNRDEIGAK 6cz7.1    A--GKTV-----------------------------------------------SVESFK--------------------  target    FAGDPALLGAFDVTLVDGTNVKARTAFSLLKEYLDENFDVQTTSEVCNVDPAAVRSLARQLAANKGNALLAAGMGPNHYF 6cz7.1    -------------------ETHTYGLVEWWNQALK-DYTPEWASKITGIDPKTIIAIAKDMGAAAPAVQVWTSRGAVMQA  target    NADLFGRVHFLVAALTDNIGHFSGNVGSYAGNYRGSLFQAMGQWIAENPFDQEADLTKPARVKRYFKSESAHYWNYG--- 6cz7.1    RGTYTSISCHALNGLFGGIDSKGGLFPGNKTPLL-KEYPEAKAYMD--EIAA-KGV-KKEKIDQRGRLEFPALAKGKSGG  target    DRP-LVSPSEIITGKSHMPTPTKLIWFGNSNSLLGNAKWSFDVVKNTLPKQDAVFCNEWHWTSSCEYSDLVFPADS-WAE 6cz7.1    GVITANAANGIRNQ---DPYEIKVMLAYFNNFNFSNPE--GQRWDEALSKVDFMAHITTNVSEFSWFADVLLPSSHHMFE  target    FKLPDMTASCTNP--FLLAFPKTPLARIHNTRSD-YEILAGVAAALADLVDEPRMKTYWKGIL-----------DGDPTP 6cz7.1    KW--GVLDSIGNGVAQISIQQ-PSIKRLWDTRIDESEIPYMLAKKLADKG----FDAPWRYINEQIVDPETGKPAADEAE  target    YLQRVLSGSN-----------ATRGILYEDLHASSAKGVPLLMNARTYPRHAGWEQRQEDKPWYTPTGRLEFYRPEPEWQ 6cz7.1    FAKLMVRYLTAPLWKEDASKYGDKLSSWDEFVQK---GVWNSS---PYK------LEARWGKFKTETTKFEFYSKTLEK-  target    AAGESLPIWREPVDATFYEPNAILANSKHPSINPRAPEDYGVPESQMDVETRQYRNVVRTWQELKLSKHPLTEKDPAYRF 6cz7.1    ----ALQSHADKHKV---SIDEVM---KAC--------DYQ---------ARGHLAFIPHYEEPY--RF---GDESEFPL  target    VFQTPKYRWGAHSTAVDSDWIAMLFGPFGDPYRRDSRTPWTGEAYAEINPRDAKELGLKDGDYIWLDADPEDRPYRGADS 6cz7.1    LLVDQKSRLNKEGRTANSPWYYEFKDV----D----PGDVANEDVAKFNPIDGKKFGLKDGDEIRITSP-----------  target    SDEFYDVARAMMRVRIYSGMPRRVIRTWFNMYAATPGTVQAQKDVPGGPAQNQDTGYVALFRHGSHQSGTRAYLRPTQMT 6cz7.1    ------VGMLTCKAKLWEGVRPGTVAKCFGQ-------------------------------------------------  target    DSMNRKAYFGQTIGKGFEADVHSPSGAPKEGYVKVEKAEDGGDEGVGEWRPVTLGLRPDDPSEAMQAYLAGEFVTRKRKG 6cz7.1    --------------------------------------------------------------------------------  target    S 6cz7.1    - ``` | | | | | | | | | | | | | | | | | | | | | | | | | | | | | | | | | | | | | | | | | | | | | | | | | |
|  | 2nya.1.A | Periplasmic nitrate reductase  *Crystal structure of the periplasmic nitrate reductase (NAP) from Escherichia coli* | 0.28 |  | 16.18 | 0.61 | 72-1036 | X-ray | 2.50 | monomer | 1 x SF4, 1 x 6MO, 2 x MGD | HHblits | 0.27 |
| ``` target    SDLSRRELLKRAVVVGTGAGLAELFLPAQFLSSASAQSEPQAVAIANPLAQMPDRSWERIYRDQFAEEDSFVFTCAPNDT 2nya.1    -----------------------------------------------------------------------DKAPCRFCG  target    HNCLLRAHVKNGVIVRISPTYGYGKATDLAGNQASHRWDPRICQKGLILGRRIYGDRRVKAPMIRKGFKEWADAGFPRHD 2nya.1    TGCGVLVGTQQGRVVACQG------------DPDAPVNRGLNCIKGYFLPKIMYGKDRLTQPLLRMKNG-----------  target    DGTPRADMEKRGYDEWLQIPWDEALAIAAKTLQNVAETYKGEDGAGKLLEQGYEPAMVEAMHGAGVQAIKMRGGMPLLGA 2nya.1    --------KYDKEGEFTPITWDQAFDVMEEKFKTALKEKGPESIG--MF-------------GSGQWTIW----------  target    GRVFGFYRFANMLALLDGKLRPEAPPEEIVGSRAFDNYAWHT--DLPPGHPMVSGSQTVDFDLFAAEHSKLLVLIGMNWI 2nya.1    -EGYAASKLFKA----------------GFRSNNIDPNARHCMASAVVGFMRTFGMDEPMGCYDDIEQADAFVLWGANMA  target    CTKMPDAHWIGDAR--LKGTRVVVISADYMPTANKADEIVILRPGTDTAFLLGVARELITKKLYDRDAVIQRTDLPLLVR 2nya.1    EMHPILWSRITNRRLSNQNVTVAVLSTYQHRSFELADNGIIFTPQSDLVILNYIANYIIQNNAINQDFFSKHVNLRKGAT  target    LDTGERLSARDVFEGYRQAPLENYVALKTEEELAAPPSPPFTADKQVVPTELREEWGDFVYWDRATNGPAAVNRDEIGAK 2nya.1    D-IGYGLRPTHPLEKA----------------------------------------------------------------  target    FAGDPALLGAFDVTLVDGTNVKARTAFSLLKEYLDENFDVQTTSEVCNVDPAAVRSLARQLAANKGNALLAAGMGPNHYF 2nya.1    --------A-----KNPGSDASEPMSFEDYKAFVA-EYTLEKTAEMTGVPKDQLEQLAQLYADPNKKVISYWTMGFNQHT  target    NADLFGRVHFLVAALTDNIGHFSGNVGSYAGNY--RGSLFQAMGQWIAENPFDQEADLTKPARVKRYFKSESAHYWNY-- 2nya.1    RGVWANNLVYNLHLLTGKISQPGCGPFSLTGQPSACGTA-REVGTFAHRLPADMV--VTNEK-HRDIC----EKKWNIPS  target    ---GDRPLVSPSEIITGKSHMPTPTKLIWFGNSNSLLGNAKWSFDVVKNTLPKQDAVFCNEWHWTSSCEYSDLVFPADSW 2nya.1    GTIPAKIGLHAVAQDRA--LKDGKLNVYWTMCTNNMQAGPNINEERMPGWRDPRNFIIVSDPYPTVSALAADLILPTAMW  target    AEFKLPDMTASCTNPFLLAFPKTPLARIHNTRSDYEILAGVAAALADLVDEP-RMKTYWKGILDGDPTPYLQRVLSGSNA 2nya.1    VEKEG--AY-GNAERRTQFWRQ-QVQAPGEAKSDLWQLVQFSRRFKTEEVWPEDLLAKKPE-L--RGKTLYEVLYAT-PE  target    TRGILYEDLHASS------AKGVPLLM------------NARTYPRHAGWEQRQEDKPWYTPTGRLEFYRPE---PEWQA 2nya.1    VSKFPVSELAEDQLNDESRELGFYLQKGLFEEYAWFGRGHGHDLAPFDDY-HKARGLRWPVVNGKETQWRYSEGNDPYVK  target    AGESLPIWREPVDATFYEPNAILANSKHPSINPRAPEDYGVPESQMDVETRQYRNVVRTWQELKLSKHPLTEKDPAYRFV 2nya.1    AGEGYKFYGKPDGK---------------------AVIFA-----------------LPFEP-----AAE-APDEEYDLW  target    FQTPKYRWGA--HSTAVDSDWIAMLFGPFGDPYRRDSRTPWTGEAYAEINPRDAKELGLKDGDYIWLDADPEDRPYRGAD 2nya.1    LSTGRVLEHWHTGSMTRRVPELHRAF----------------PEAVLFIHPLDAKARDLRRGDKVKVVSR----------  target    SSDEFYDVARAMMRVRIYSGM--PRRVIRTWFNMYAATPGTVQAQKDVPGGPAQNQDTGYVALFRHGSHQSGTRAYLRPT 2nya.1    -------RGEVISIVETRGRNRPPQGLVYMPFF-----------------------------------------------  target    QMTDSMNRKAYFGQTIGKGFEADVHSPSGAPKEGYVKVEKAEDGGDEGVGEWRPVTLGLRPDDPSEAMQAYLAGEFVTRK 2nya.1    --------------------------------------------------------------------------------  target    RKGS 2nya.1    ---- ``` | | | | | | | | | | | | | | | | | | | | | | | | | | | | | | | | | | | | | | | | | | | | | | | | | |
|  | 1dms.1.A | DMSO REDUCTASE  *STRUCTURE OF DMSO REDUCTASE* | 0.29 | 0.00 | 20.64 | 0.59 | 72-1041 | X-ray | 1.88 | monomer | 2 x PGD, 1 x 2MO | HHblits | 0.30 |
| ``` target    SDLSRRELLKRAVVVGTGAGLAELFLPAQFLSSASAQSEPQAVAIANPLAQMPDRSWERIYRDQFAEEDSFVFTCAPNDT 1dms.1    -----------------------------------------------------------------------ANGTVMSGS  target    HNCLLRAHVKNGVIVRISPTYGYGKATDLAGNQASHRWDPRICQKGLILGRRIYGDRRVKAPMIRKGFKE-WADAGFPRH 1dms.1    HWGVFTATVENGRATAFTPWE------------KDP----HPTPMLEGVLDSIYSPTRIKYPMVRREFLEKG--------  target    DDGTPRADMEKRGYDEWLQIPWDEALAIAAKTLQNVAETYKGEDGAGKLLEQGYEPAMVEAMHGAGVQAIKMRGGMPLLG 1dms.1    ----VNADRSTRGNGDFVRVSWDQALDLVAAEVKRVEETYGPQG----VFGGSYGWK------SPGRLH-----------  target    AGRVFGFYRFANMLALLDGKLRPEAPPEEIVGSRAFDNYAWHTDLPPGHPMVSGSQTVDFDLFAAEHSKLLVLIGMNWIC 1dms.1    NC-TTLLRRMLTLA---GGYV-------NGAGDYSTGAAQVIMPHVVGTLEVYEQQ-T-AWPVLAENTEVMVFWAADPIK  target    TKMPDA--------HWIGDARLKGTRVVVISADYMPTANK-ADEIVILRPGTDTAFLLGVARELITKKLYDRDAVIQRTD 1dms.1    TSQIGWVIPEHGAYPGLEALKAKGTKVIVIDPVRTKTVEFFGADHVTPKPQTDVAIMLGMAHTLVAEDLYDKDFIANYTS  target    LPLLVRLDTGERLSARDVFEGYRQAPLENYVALKTEEELAAPPSPPFTADKQVVPTELREEWGDFVYWDRATNGPAAVNR 1dms.1    G--------------------FDK-------------------------------------FLPY---------------  target    DEIGAKFAGDPALLGAFDVTLVDGTNVKARTAFSLLKEYLDENFDVQTTSEVCNVDPAAVRSLARQLAANKGNALLAAGM 1dms.1    ------------LMGET-----D----------------S-TPKTAEWASDISGVPAETIKELARLFKSK-R-TMLAAGW  target    GPNHYFNADLFGRVHFLVAALTDNIGHFSGNVGSYAGNYRGSLFQAMGQWIAENPFDQEADLT-KPARVKRYFKSESAHY 1dms.1    SMQRMHHGEQAHWMLVTLASMLGQIGLPGGGFGLSYHYSGGGTPSSSG--PALSGITDGGAATKGPEWLA---------A  target    WNYGDRPLVSPSEIIT---------GKSHMPTPTKLIWFGNSNSLLGNAKWSFDVVKNTLPKQDAVFCNEWHWTSSCEYS 1dms.1    SGASVIPVARVVDMLENPGAEFDFNGTRSKFPDVKMAYWVGGNPFVHHQD--RNRMVKAWEKLETFIVHDFQWTPTARHA  target    DLVFPADSWAEFKLPDMTASCTNPFLLAFPKTPLARIHNTRSDYEILAGVAAALADLVDEPRMKTYWKGILDGDPTPYLQ 1dms.1    DIVLPATTSYERNDIETIGDYSNTGILAMKK-IVEPLYEARSDYDIFAAVAERLGKGKE------FTE---GKDEMGWIK  target    RVLSGSN---ATRGI---LYEDLHASSAKGVPLLMNA--RTYPRHAGWEQRQEDKPWYTPTGRLEFYRPEPEWQAA--GE 1dms.1    SFYDDAAKQGKAGGVEMPAFDAFWAE---GIVEFPVTDGADFVRYASFREDPLLNPLGTPTGLIEIYSKNIEKMGYDDCP  target    SLPIWREPVDATFYEPNAILANSKHPSINPRAPEDYGVPESQMDVETRQYRNVVRTWQELKLSKHPLTEKDPAYRFVFQT 1dms.1    AHPTWMEPLERL-------------------------------------------------------DGPGAKYPLHIAA  target    PKYRWGAHSTAVDSDWIAMLFGPFGDPYRRDSRTPWTGEAYAEINPRDAKELGLKDGDYIWLDADPEDRPYRGADSSDEF 1dms.1    SHPFNRLHSQLNG-TVLREGY-------------AVQGHEPCLMHPDDAAARGIADGDVVRVHND---------------  target    YDVARAMMRVRIYSGMPRRVIRTWFNMYAATPGTVQAQKDVPGGPAQNQDTGYVALFRHGSHQSGTRAYLRPTQMTDSMN 1dms.1    --RGQILTGVKVTDAVMKGVIQIYEGGWYDP-------------------------------------------------  target    RKAYFGQTIGKGFEADVHSPSGAPKEGYVKVEKAEDGGDEGVGEWRPVTLGLRPDDPSEAMQAYLAGEFVTRKRKGS 1dms.1    ----------------------------------------------------------------------------- ``` | | | | | | | | | | | | | | | | | | | | | | | | | | | | | | | | | | | | | | | | | | | | | | | | | |
|  | 4dmr.1.A | DMSO REDUCTASE  *REDUCED DMSO REDUCTASE FROM RHODOBACTER CAPSULATUS WITH BOUND DMSO SUBSTRATE* | 0.29 | 0.00 | 20.06 | 0.59 | 74-1042 | X-ray | 1.90 | monomer | 2 x PGD, 1 x 4MO, 1 x O | HHblits | 0.30 |
| ``` target    SDLSRRELLKRAVVVGTGAGLAELFLPAQFLSSASAQSEPQAVAIANPLAQMPDRSWERIYRDQFAEEDSFVFTCAPNDT 4dmr.1    -------------------------------------------------------------------------NGTVMSG  target    HNCL-LRAHVKNGVIVRISPTYGYGKATDLAGNQASHRWDPRICQKGLILGRRIYGDRRVKAPMIRKGFKEWADAGFPRH 4dmr.1    SHWGVFTATVENGRATAFTPWE------------KDPH----PSPMLAGVLDSIYSPTRIKYPMVRREFLEK--------  target    DDGTPRADMEKRGYDEWLQIPWDEALAIAAKTLQNVAETYKGEDGAGKLLEQGYEPAMVEAMHGAGVQAIKMRGGMPLLG 4dmr.1    ---GVNADRSTRGNGDFVRVSWDQALDLVAAEVKRVEETYGPSG----VFGGSYGWK------SPGRL-----------H  target    AGRVFGFYRFANMLALLDGKLRPEAPPEEIVGSRAFDNYAWHTDLPPGHPMVSGSQTVDFDLFAAEHSKLLVLIGMNWIC 4dmr.1    N-CTTLLRRMLTLA---GGYV-------NGAGDYSTGAAQVIMPHVVGTLEVYEQQ--TAWPVLAENTEVMVFWAADPIK  target    TKMPDA--------HWIGDARLKGTRVVVISADYMPTANK-ADEIVILRPGTDTAFLLGVARELITKKLYDRDAVIQRTD 4dmr.1    TSQIGWVIPEHGAYPGLEALKAKGTKVIVIDPVRTKTVEFFGAEHITPKPQTDVAIMLGMAHTLVAEDLYDKDFIANYTS  target    LPLLVRLDTGERLSARDVFEGYRQAPLENYVALKTEEELAAPPSPPFTADKQVVPTELREEWGDFVYWDRATNGPAAVNR 4dmr.1    GF-----------------DKF----------------------------------------LPY---------------  target    DEIGAKFAGDPALLGAFDVTLVDGTNVKARTAFSLLKEYLDENFDVQTTSEVCNVDPAAVRSLARQLAANKGNALLAAGM 4dmr.1    ------------LDGET---------------------DS-TPKTAEWAEGISGVPAETIKELARLFESK-R-TMLAAGW  target    GPNHYFNADLFGRVHFLVAALTDNIGHFSGNVGSYAGNYRGSLFQAMGQWIAENPFDQEADLT-KPARVKRYFKSESAHY 4dmr.1    SMQRMHHGEQAHWMLVTLASMLGQIGLPGGGFGLSYHYSGGGTPSTSGPA--LAGITDGGAATKGPEWLA---------A  target    WNYGDRPLVSPSEIIT---------GKSHMPTPTKLIWFGNSNSLLGNAKWSFDVVKNTLPKQDAVFCNEWHWTSSCEYS 4dmr.1    SGASVIPVARVVDMLENPGAEFDFNGTRSKFPDVKMAYWVGGNPFVHHQD--RNRMVKAWEKLETFVVHDFQWTPTARHA  target    DLVFPADSWAEFKLPDMTASCTNPFLLAFPKTPLARIHNTRSDYEILAGVAAALADLVDEPRMKTYWKGILDGDPTPYLQ 4dmr.1    DIVLPATTSYERNDIETIGDYSNTGILAMKK-IVEPLYEARSDYDIFAAVAERLGKGAE------FTE---GKDEMGWIK  target    RVLSGSN---ATRGI---LYEDLHASSAKGVPLLMN--ARTYPRHAGWEQRQEDKPWYTPTGRLEFYRPEPEWQAA--GE 4dmr.1    SFYDDAAKQGKAAGVQMPAFDAFWA---EGIVEFPVTDGADFVRYASFREDPLLNPLGTPTGLIEIYSKNIEKMGYDDCP  target    SLPIWREPVDATFYEPNAILANSKHPSINPRAPEDYGVPESQMDVETRQYRNVVRTWQELKLSKHPLTEKDPAYRFVFQT 4dmr.1    AHPTWMEPLERL-------------------------------------------------------DGPGAKYPLHIAA  target    PKYRWGAHSTAVDSDWIAMLFGPFGDPYRRDSRTPWTGEAYAEINPRDAKELGLKDGDYIWLDADPEDRPYRGADSSDEF 4dmr.1    SHPFNRLHSQLN-GTVLREGYA-------------VQGHEPCLMHPDDAAARGIADGDVVRVHND---------------  target    YDVARAMMRVRIYSGMPRRVIRTWFNMYAATPGTVQAQKDVPGGPAQNQDTGYVALFRHGSHQSGTRAYLRPTQMTDSMN 4dmr.1    --RGQILTGVKVTDAVMKGVIQIYEGGWYDPS------------------------------------------------  target    RKAYFGQTIGKGFEADVHSPSGAPKEGYVKVEKAEDGGDEGVGEWRPVTLGLRPDDPSEAMQAYLAGEFVTRKRKGS 4dmr.1    ----------------------------------------------------------------------------- ``` | | | | | | | | | | | | | | | | | | | | | | | | | | | | | | | | | | | | | | | | | | | | | | | | | |
|  | 1eu1.1.A | DIMETHYL SULFOXIDE REDUCTASE  *THE CRYSTAL STRUCTURE OF RHODOBACTER SPHAEROIDES DIMETHYLSULFOXIDE REDUCTASE REVEALS TWO DISTINCT MOLYBDENUM COORDINATION ENVIRONMENTS.* | 0.29 |  | 20.94 | 0.59 | 72-1039 | X-ray | 1.30 | monomer | 3 x GLC, 1 x CD, 2 x MGD, 1 x 6MO, 2 x O | HHblits | 0.30 |
| ``` target    SDLSRRELLKRAVVVGTGAGLAELFLPAQFLSSASAQSEPQAVAIANPLAQMPDRSWERIYRDQFAEEDSFVFTCAPNDT 1eu1.1    -----------------------------------------------------------------------NGEVMSGCH  target    HNCLLRAHVKNGVIVRISPTYGYGKATDLAGNQASHRWDPRICQKGLILGRRIYGDRRVKAPMIRKGFK-EWADAGFPRH 1eu1.1    WGV-FKARVENGRAVAFEPW------------DKDPAPSHQLPG----VLDSIYSPTRIKYPMVRREFLEKGV-------  target    DDGTPRADMEKRGYDEWLQIPWDEALAIAAKTLQNVAETYKGEDGAGKLLEQGYEPAMVEAMHGAGVQAIKMRGGMPLLG 1eu1.1    -----NADRSTRGNGDFVRVTWDEALDLVARELKRVQESYGPTG----TFGGSYGW------KSPGRL-----------H  target    AGRVFGFYRFANMLALLDGKLRPEAPPEEIVGSRAFDNYAWHTDLPPGHPMVSGSQ----TVDFDLFAAEHSKLLVLIGM 1eu1.1    NCQVL-MRRALNLA---GGFV-------NSSGDY-----STAA-AQIIMPHVMGTLEVYEQQTAWPVVVENTDLMVFWAA  target    NWICTKMPD--------AHWIGDARLKGTRVVVISADYMPTANKAD-EIVILRPGTDTAFLLGVARELITKKLYDRDAVI 1eu1.1    DPMKTNEIGWVIPDHGAYAGMKALKEKGTRVICINPVRTETADYFGADVVSPRPQTDVALMLGMAHTLYSEDLHDKDFLE  target    QRTDLPLLVRLDTGERLSARDVFEGYRQAPLENYVALKTEEELAAPPSPPFTADKQVVPTELREEWGDFVYWDRATNGPA 1eu1.1    NCTTG--------------------FD-------------------------------------LFAAY-----------  target    AVNRDEIGAKFAGDPALLGAFDVTLVDGTNVKARTAFSLLKEYLDENFDVQTTSEVCNVDPAAVRSLARQLAANKGNALL 1eu1.1    ----------------LTGE-----SDG-----------------TPKTAEWAAEICGLPAEQIRELARSFVAGR--TML  target    AAGMGPNHYFNADLFGRVHFLVAALTDNIGHFSGNVGSYAGNYRGS-LFQAMGQWIAENPFDQEADLTKPARVKRYFKSE 1eu1.1    AAGWSIQRMHHGEQAHWMLVTLASMIGQIGLPGGGFGLSYHYSNGGSPTSDGPALG---GISDG--GKAVEGA-AWLS--  target    SAHYWNYGDRPLVS-PSEIIT--------GKSHMPTPTKLIWFGNSNSLLGNAKWSFDVVKNTLPKQDAVFCNEWHWTSS 1eu1.1    ---ESGATSIPCARVVDMLLNPGGEFQFNGATATYPDVKLAYWAGGNPFAHHQD--RNRMLKAWEKLETFIVQDFQWTAT  target    CEYSDLVFPADSWAEFKLPDMTASCTNPFLLAFPKTPLARIHNTRSDYEILAGVAAALADLVDEPRMKTYWKGILDGDPT 1eu1.1    ARHADIVLPATTSYERNDIESVGDYSNRAILAMKK-VVDPLYEARSDYDIFAALAERLGKGAEF---TEG------RDEM  target    PYLQRVLSGS------NATRGILYEDLHASSAKGVPLLMNARTYPRHAGWEQRQEDKPWYTPTGRLEFYRPEPEWQAA-- 1eu1.1    GWISSFYEAAVKQAEFKNVAMPSFEDFWSEGIVEFPITE-GANFVRYADFREDPLFNPLGTPSGLIEIYSKNIEKMGYDD  target    GESLPIWREPVDATFYEPNAILANSKHPSINPRAPEDYGVPESQMDVETRQYRNVVRTWQELKLSKHPLTEKDPAYRFVF 1eu1.1    CPAHPTWMEPAER------------------------------------------------------L-GGAGAKYPLHV  target    QTPKYRWGAHSTAVDSDWIAMLFGPFGDPYRRDSRTPWTGEAYAEINPRDAKELGLKDGDYIWLDADPEDRPYRGADSSD 1eu1.1    VASHPKSRLHSQLNGT-SLRDLY-------------AVAGHEPCLINPADAAARGIADGDVLRVFND-------------  target    EFYDVARAMMRVRIYSGMPRRVIRTWFNMYAATPGTVQAQKDVPGGPAQNQDTGYVALFRHGSHQSGTRAYLRPTQMTDS 1eu1.1    ----RGQILVGAKVSDAVMPGAIQIYEGGWY-------------------------------------------------  target    MNRKAYFGQTIGKGFEADVHSPSGAPKEGYVKVEKAEDGGDEGVGEWRPVTLGLRPDDPSEAMQAYLAGEFVTRKRKGS 1eu1.1    ------------------------------------------------------------------------------- ``` | | | | | | | | | | | | | | | | | | | | | | | | | | | | | | | | | | | | | | | | | | | | | | | | | |
|  | 1tmo.1.A | TRIMETHYLAMINE N-OXIDE REDUCTASE  *TRIMETHYLAMINE N-OXIDE REDUCTASE FROM SHEWANELLA MASSILIA* | 0.29 |  | 19.65 | 0.59 | 69-1041 | X-ray | 2.50 | monomer | 2 x 2MD, 1 x 2MO | HHblits | 0.30 |
| ``` target    SDLSRRELLKRAVVVGTGAGLAELFLPAQFLSSASAQSEPQAVAIANPLAQMPDRSWERIYRDQFAEEDSFVFTCAPNDT 1tmo.1    --------------------------------------------------------------------DEWLTTGSH-F-  target    HNCLLRAHVKNGVIVRISPTYGYGKATDLAGNQASHRWDPRICQKGLILGRRIYGDRRVKAPMIRKGFKEWADAGFPRHD 1tmo.1    --GAFKMKRKNGVIAEVKPFDL------------DKYP--TDMING--IRGMVYNPSRVRYPMVRLDFLLK---------  target    DGTPRADMEKRGYDEWLQIPWDEALAIAAKTLQNVAETYKGEDGAGKLLEQGYEPAMVEAMHGAGVQAIKMRGGMPLLGA 1tmo.1    --GHKSNTHQRGDFRFVRVTWDKALTLFKHSLDEVQTQYGPSG----LHAGQTGW------RATGQLH------------  target    GRVFGFYRFANMLALLDGKLRPEAPPEEIVGSRAFDNYAWHTDLPPGHPMVSGSQ----TVDFDLFAAEHSKLLVLIGMN 1tmo.1    ---SSTSHMQRAVGMHGNYVK-------KIGDYS-----TGAGQ-TILPYVLGSTEVYAQGTSWPLILEHSDTIVLWSND  target    WICTKMP--------DA---HWIGDARL-KGTRVVVISADYMPTANK-ADEIVILRPGTDTAFLLGVARELITKKLYDRD 1tmo.1    PYKNLQVGWNAETHESFAYLAQLKEKVKQGKIRVISIDPVVTKTQAYLGCEQLYVNPQTDVTLMLAIAHEMISKKLYDDK  target    AVIQRTDLPLLVRLDTGERLSARDVFEGYRQAPLENYVALKTEEELAAPPSPPFTADKQVVPTELREEWGDFVYWDRATN 1tmo.1    FIQGYSLG--------------------FEE-------------------------------------FVP---------  target    GPAAVNRDEIGAKFAGDPALLGAFDVTLVDGTNVKARTAFSLLKEYLDENFDVQTTSEVCNVDPAAVRSLARQLAANKGN 1tmo.1    ------------------YVMGT-----KDG-----------------VAKTPEWAAPICGVEAHVIRDLAKTLVKGR--  target    ALLAAGMGPNHYFNADLFGRVHFLVAALTDNIGHFSGNVGSYAGNYR-GSLFQ---AMGQWIAENPFDQEADLTKPARVK 1tmo.1    TQFMMGWCIQRQQHGEQPYWMAAVLATMIGQIGLPGGGISYGHHYSSIGVPSSGAAAPGAFPRNLDENQKP-LFDSS---  target    RYFKSESAHYWNYGDRPLVS-PSEIIT-G-------KSHMPTPTKLIWFGNSNSLLGNAKWSFDVVKNTLPKQDAVFCNE 1tmo.1    DFKG-------ASSTIPVARWIDAILEPGKTIDANGSKVVYPDIKMMIFSGNNPWNHHQD--RNRMKQAFHKLECVVTVD  target    WHWTSSCEYSDLVFPADSWAEFKLPDMTASCTNPFLLAFPKTPLARIHNTRSDYEILAGVAAALADLVDEPRMKTYWKGI 1tmo.1    VNWTATCRFSDIVLPACTTYERNDIDVYGAYANRGILAMQ-KMVEPLFDSLSDFEIFTRFAAVLGKEKE------YTR--  target    LDGDPTPYLQRVLSGSN-----ATRGILYEDLHASSAKGVPLLMNARTYPRHAGWEQRQEDKPWYTPTGRLEFYRPEPEW 1tmo.1    -NMGEMEWLETLYNECKAANAGKFEMPDFATFWKQ---GYVHFGDGEVWTRHADFRNDPEINPLGTPSGLIEIFSRKIDQ  target    QA--AGESLPIWREPVDATFYEPNAILANSKHPSINPRAPEDYGVPESQMDVETRQYRNVVRTWQELKLSKHPLTEKDPA 1tmo.1    FGYDDCKGHPTWMEKTERSH--------------------------------------------------G---GPGSDK  target    YRFVFQTPKYRWGAHSTAVDSDWIAMLFGPFGDPYRRDSRTPWTGEAYAEINPRDAKELGLKDGDYIWLDADPEDRPYRG 1tmo.1    HPIWLQSCHPDKRLHSQMCESREYRETYA-------------VNGREPVYISPVDAKARGIKDGDIVRVFND--------  target    ADSSDEFYDVARAMMRVRIYSGMPRRVIRTWFNMYAATPGTVQAQKDVPGGPAQNQDTGYVALFRHGSHQSGTRAYLRPT 1tmo.1    ---------RGQLLAGAVVSDNFPKGIVRIHEGAWYGP------------------------------------------  target    QMTDSMNRKAYFGQTIGKGFEADVHSPSGAPKEGYVKVEKAEDGGDEGVGEWRPVTLGLRPDDPSEAMQAYLAGEFVTRK 1tmo.1    --------------------------------------------------------------------------------  target    RKGS 1tmo.1    ---- ``` | | | | | | | | | | | | | | | | | | | | | | | | | | | | | | | | | | | | | | | | | | | | | | | | | |
|  | 4aay.1.A | AROA  *Crystal Structure of the arsenite oxidase protein complex from Rhizobium species strain NT-26* | 0.28 |  | 15.45 | 0.59 | 67-1037 | X-ray | 2.70 | hetero-oligomer | 4 x MGD, 2 x O, 2 x 4MO, 2 x F3S, 2 x FES | HHblits | 0.28 |
| ``` target    SDLSRRELLKRAVVVGTGAGLAELFLPAQFLSSASAQSEPQAVAIANPLAQMPDRSWERIYRDQFAEEDSFVFTCAPNDT 4aay.1    ------------------------------------------------------------------ADAKKHNVTCHFCI  target    HNCLLRAHV-----------------------------------------KNGVIVRISPTYGYGKATDLAGNQASHRWD 4aay.1    VGCGYHAYTWPINKQGGTDPQNNIFGVDLSEQQQAESDAWYSPSMYNVVKQDGRDVHVVIKPD----------HECVVNS  target    PRICQKGLILGRRIY------GDRRVKAPMIRKGFKEWADAGFPRHDDGTPRADMEKRGYDEWLQIPWDEALAIAAKTLQ 4aay.1    GLGSVRGARMAETSFSEARNTQQQRLTDPLVWRY--------------------------GQMQPTSWDDALDLVARVTA  target    NVAETYKGEDGAGKLLEQGYEPAMVEAMHGAGVQAIKMRGGMPLLGAGRVFGFYRFANMLALLDGKLRPEAPPEEIVGSR 4aay.1    KIVKEKGEDA----LIVSAFD--------HGGAG---------------------GGYENTWGTGKLY-----FEAMKVK  target    AFDNYAWHTDLPPG-HPMVSGSQTVDFDLFAAEHSKLLVLIGMNWICTKMPDA--HWIG---------------DARLKG 4aay.1    NIRIHNRPAYNSEVHGTRDMGVGELNNCYEDAELADTIVAVGTNALETQTNYFLNHWIPNLRGESLGKKKELMPEEPHEA  target    TRVVVISADYMPTAN------KAD--EIVILRPGTDTAFLLGVARELITKKLYDRDAVIQRTDLPLLVRLDTGERLSARD 4aay.1    GRIIIVDPRRTVTVNACEQTAGADNVLHLAINSGTDLALFNALFTYIADKGWVDRDFIDKSTLREGTARP----------  target    VFEGYRQAPLENYVALKTEEELAAPPSPPFTADKQVVPTELREEWGDFVYWDRATNGPAAVNRDEIGAKFAGDPALLGAF 4aay.1    ----------------------------------------------P--LYPAR-GV-----------------------  target    DVTLVDGTNVKARTAFSLLKEYLD-ENFDVQTTSEVCNVDPAAVRSLARQLAANKG-----NALLAAGMGPNHYFNADLF 4aay.1    ----SEAN----PGHLSSFEDAVEGCRMSIEEAAEITGLDAAQIIKAAEWIGMPKEGGKRRRVMFGYEKGLIWGNDNYRT  target    GRVHFLVAALTDNIGHFSGNVGSYAGNYRGSLFQAMGQWIAENPFDQEADLTKPARVKRYFKSESAHYWNYGDRPLVSPS 4aay.1    NGALVNLALATGNIGRPGGGVVRLGGHQEGYVRPSDAH--VGRPAAYVDQLLIGG------QGGVHHIWGCD--------  target    EIITGKSHMPTPTKLIWFGNSNSLLG------NAK--WSFDVVKNTLPKQ-DAVFCNEWHWTSSCEYSDLVFPADSWAEF 4aay.1    HYKTT--LNAHEFKRVYKKRTDMVKDAMSAAPYGDREAMVNAIVDAINQGGLFAVNVDIIPTKIGEACHVILPAATSGEM  target    KLPDMTASCTNPFLLAFPKTPLARIHNTRSDYEILAGVAAALADLVDEPR---MKTYWKGILDGDPTPYL-QRVLSGSNA 4aay.1    NL---TSMNGERRMRLTER-YMDPPGQSMPDCLIAARLANTMERVLTEMGDVGYAAQFKGFDWQTEEDAFMDGYNKNAHG  target    TRGILYEDLHASSAKGVPLLMNARTYPRHAGWEQRQEDKPWYTPTGRLEFYRPEPEWQAAGESLPIWREPVDATFYEPNA 4aay.1    GEFVTYERLSAMGTNGFQEPATGFTDGKIEGTQRLYTDGVFSTDDGKARFMDAPWR------G---LQAPGKQ-------  target    ILANSKHPSINPRAPEDYGVPESQMDVETRQYRNVVRTWQELKLSKHPLTEKDPAYRFVFQTPKYRWGAHSTAV--DSDW 4aay.1    --------------------------------------------------QQKDSHKYLINNGRANVVWQSAYLDQENDF  target    IAMLFGPFGDPYRRDSRTPWTGEAYAEINPRDAKELGLKDGDYIWLDADPEDRPYRGADSSDEFYDVARAMMRVRIYSGM 4aay.1    VMDR----------------FPYPFIEMNPEDMAEAGLKEGDLVEIYND-----------------AGATQAMAYPTPTA  target    PRRVIRTWFNMYAATPGTVQAQKDVPGGPAQNQDTGYVALFRHGSHQSGTRAYLRPTQMTDSMNRKAYFGQTIGKGFEAD 4aay.1    RRGETFMLFGF---------------------------------------------------------------------  target    VHSPSGAPKEGYVKVEKAEDGGDEGVGEWRPVTLGLRPDDPSEAMQAYLAGEFVTRKRKGS 4aay.1    ------------------------------------------------------------- ``` | | | | | | | | | | | | | | | | | | | | | | | | | | | | | | | | | | | | | | | | | | | | | | | | | |
|  | 5nqd.1.A | AroA  *Arsenite oxidase AioAB from Rhizobium sp. str. NT-26 mutant AioBF108A* | 0.27 |  | 15.45 | 0.59 | 67-1038 | X-ray | 2.20 | hetero-2-2-mer | 4 x MGD, 2 x O, 2 x 4MO, 2 x F3S, 2 x FES | HHblits | 0.28 |
| ``` target    SDLSRRELLKRAVVVGTGAGLAELFLPAQFLSSASAQSEPQAVAIANPLAQMPDRSWERIYRDQFAEEDSFVFTCAPNDT 5nqd.1    ------------------------------------------------------------------ADAKKHNVTCHFCI  target    HNCLLRAHV-----------------------------------------KNGVIVRISPTYGYGKATDLAGNQASHRWD 5nqd.1    VGCGYHAYTWPINKQGGTDPQNNIFGVDLSEQQQAESDAWYSPSMYNVVKQDGRDVHVVIKPD----------HECVVNS  target    PRICQKGLILGRRIY------GDRRVKAPMIRKGFKEWADAGFPRHDDGTPRADMEKRGYDEWLQIPWDEALAIAAKTLQ 5nqd.1    GLGSVRGARMAETSFSEARNTQQQRLTDPLVWRY--------------------------GQMQPTSWDDALDLVARVTA  target    NVAETYKGEDGAGKLLEQGYEPAMVEAMHGAGVQAIKMRGGMPLLGAGRVFGFYRFANMLALLDGKLRPEAPPEEIVGSR 5nqd.1    KIVKEKGEDA----LIVSAFD--------HGGA---------------------GGGYENTWGTGKLY-----FEAMKVK  target    AFDNYAWHTDLPP--GHPMVSGSQTVDFDLFAAEHSKLLVLIGMNWICTKMPDA--HWIG---------------DARLK 5nqd.1    NIRIHNRPAYNSEVHGT-RDMGVGELNNCYEDAELADTIVAVGTNALETQTNYFLNHWIPNLRGESLGKKKELMPEEPHE  target    GTRVVVISADYMPTAN------KAD--EIVILRPGTDTAFLLGVARELITKKLYDRDAVIQRTDLPLLVRLDTGERLSAR 5nqd.1    AGRIIIVDPRRTVTVNACEQTAGADNVLHLAINSGTDLALFNALFTYIADKGWVDRDFIDKSTLREGTARP---------  target    DVFEGYRQAPLENYVALKTEEELAAPPSPPFTADKQVVPTELREEWGDFVYWDRATNGPAAVNRDEIGAKFAGDPALLGA 5nqd.1    -----------------------------------------------P--LYPAR-G-----------------------  target    FDVTLVDGTNVKARTAFSLLKEYLD-ENFDVQTTSEVCNVDPAAVRSLARQLAANKG-----NALLAAGMGPNHYFNADL 5nqd.1    --------VSEANPGHLSSFEDAVEGCRMSIEEAAEITGLDAAQIIKAAEWIGMPKEGGKRRRVMFGYEKGLIWGNDNYR  target    FGRVHFLVAALTDNIGHFSGNVGSYAGNYRGSLFQAMGQWIAENPFDQEADLTKPARVKRYFKSESAHYWNYGDRPLVSP 5nqd.1    TNGALVNLALATGNIGRPGGGVVRLGGHQEGYVRP--SDAHVGRPAAYVDQLLIGGQ------GGVHHIWGCDH--Y---  target    SEIITGKSHMPTPTKLIWFGNSNSLL------GNAK--WSFDVVKNTLPKQ-DAVFCNEWHWTSSCEYSDLVFPADSWAE 5nqd.1    KTTL-----NAHEFKRVYKKRTDMVKDAMSAAPYGDREAMVNAIVDAINQGGLFAVNVDIIPTKIGEACHVILPAATSGE  target    FKLPDMTASCTNPFLLAFPKTPLARIHNTRSDYEILAGVAAALADLVDEPR---MKTYWKGILDGDPTPYL-QRVLSGSN 5nqd.1    MNL---TSMNGERRMRLTER-YMDPPGQSMPDCLIAARLANTMERVLTEMGDVGYAAQFKGFDWQTEEDAFMDGYNKNAH  target    ATRGILYEDLHASSAKGVPLLMNARTYPRHAGWEQRQEDKPWYTPTGRLEFYRPEPEWQAAGESLPIWREPVDATFYEPN 5nqd.1    GGEFVTYERLSAMGTNGFQEPATGFTDGKIEGTQRLYTDGVFSTDDGKARFMDAPWR---------GLQAPGK-------  target    AILANSKHPSINPRAPEDYGVPESQMDVETRQYRNVVRTWQELKLSKHPLTEKDPAYRFVFQTPKYRWGAHSTAV--DSD 5nqd.1    ---------------Q-----------------------------------QQKDSHKYLINNGRANVVWQSAYLDQEND  target    WIAMLFGPFGDPYRRDSRTPWTGEAYAEINPRDAKELGLKDGDYIWLDADPEDRPYRGADSSDEFYDVARAMMRVRIYSG 5nqd.1    FVMDR----------------FPYPFIEMNPEDMAEAGLKEGDLVEIYND-----------------AGATQAMAYPTPT  target    MPRRVIRTWFNMYAATPGTVQAQKDVPGGPAQNQDTGYVALFRHGSHQSGTRAYLRPTQMTDSMNRKAYFGQTIGKGFEA 5nqd.1    ARRGETFMLFGFP-------------------------------------------------------------------  target    DVHSPSGAPKEGYVKVEKAEDGGDEGVGEWRPVTLGLRPDDPSEAMQAYLAGEFVTRKRKGS 5nqd.1    -------------------------------------------------------------- ``` | | | | | | | | | | | | | | | | | | | | | | | | | | | | | | | | | | | | | | | | | | | | | | | | | |
|  | 2e7z.1.A | Acetylene hydratase Ahy  *Acetylene Hydratase from Pelobacter acetylenicus* | 0.29 |  | 20.97 | 0.56 | 72-1041 | X-ray | 1.26 | monomer | 1 x SF4, 2 x MGD, 1 x W | HHblits | 0.30 |
| ``` target    SDLSRRELLKRAVVVGTGAGLAELFLPAQFLSSASAQSEPQAVAIANPLAQMPDRSWERIYRDQFAEEDSFVFTCAPNDT 2e7z.1    -----------------------------------------------------------------------KHVVCQSCD  target    HNCLLRAHVK-NGVIVRISPTYGYGKATDLAGNQASHRW-DPRICQKGLILGRRIYGDRRVKAPMIRKGFKEWADAGFPR 2e7z.1    INCVVEAEVKADGKIQTKSISE------------PHPTTPPNSICMKSVNADTIRTHKDRVLYPLKNVGS----------  target    HDDGTPRADMEKRGYDEWLQIPWDEALAIAAKTLQNVAETYKGEDGAGKLLEQGYEPAMVEAMHGAGVQAIKMRGGMPLL 2e7z.1    -----------KRGEQRWERISWDQALDEIAEKLKKIIAKYGPESLG--VS------------QT-EINQQ---------  target    GAGRVFGFYRFANMLALLDGKLRPEAPPEEIVGSRAFDNYAWHTDLP--PGHPMVSGSQTVDFDLFAAEHSKLLVLIGMN 2e7z.1    --SEYGTLRRFMNL-----------------LGSPNWTSAMYMCIGNTAGVHRVTHGS----YSFASFADSNCLLFIGKN  target    WICTKMPD-AHWIGDARLKGTRVVVISADYMPTANKADEIVILRPGTDTAFLLGVARELITKKLYDRDAVIQRTDLPLLV 2e7z.1    LSNHNWVSQFNDLKAALKRGCKLIVLDPRRTKVAEMADIWLPLRYGTDAALFLGMINVIINEQLYDKEFVENWCV-----  target    RLDTGERLSARDVFEGYRQAPLENYVALKTEEELAAPPSPPFTADKQVVPTELREEWGDFVYWDRATNGPAAVNRDEIGA 2e7z.1    --------------------------------------------------------------------------------  target    KFAGDPALLGAFDVTLVDGTNVKARTAFSLLKEYLDENFDVQTTSEVCNVDPAAVRSLARQLAANKGNALLAAGMGPNHY 2e7z.1    --------------------------GFEELKERVQ-EYPLDKVAEITGCDAGEIRKAAVMFATESP-ASIPWAVSTDMQ  target    FNADLFGRVHFLVAALTDNIGHFSGNVGSYAGNYR-GSLFQAMGQWIAE-NPFDQEA-DLT--KPARVKRYFKSESAHYW 2e7z.1    KNSCSAIRAQCILRAIVGSFVNGAEILGAPHSDLVPISKIQMHEALPEEKKKLQLGTETYPFLTYTGMSA-LEEPSERVY  target    NY------G---DRPLVSPSEIITGKSHMPTPTKLIWFGNSNSLLGNAKWSFDVVKNTLPKQDAVFCNEWHWTSSCEYSD 2e7z.1    GVKYFHNMGAFMANPTALFTAMATE---KPYPVKAFFALASNALMGYAN--QQNALKGLMNQDLVVCYDQFMTPTAQLAD  target    LVFPADSWAEFKLPDMTASCTNPFLLAFPKTPLARIHNTRSDYEILAGVAAALADLVDEPRMKTYWKGILDGDPTPYLQR 2e7z.1    YVLPGDHWLERPVVQPN-WEGIPFGNTSQ-QVVEPAGEAKDEYYFIRELAVRMGLEEH---F--PW-----KDRLELINY  target    VLSGSNATRGILYEDLHASSAKGVPLLMNARTYPRHAGWEQRQEDKPWYTPTGRLEFYRPEPEWQAAGESLPIWREPVDA 2e7z.1    RISP----TGMEWEEYQKQYT--Y-MS----KLPD---Y-FGPEGVGVATPSGKVELYSSVFE-KLGYDPLPYYHEPLQT  target    TFYEPNAILANSKHPSINPRAPEDYGVPESQMDVETRQYRNVVRTWQELKLSKHPLTEKDPAYRFVFQTPKYR-WGAHST 2e7z.1    EI--------------S------------------------------------D--PELAKEYPLILFAGLREDSNFQSC  target    AVDSDWIAMLFGPFGDPYRRDSRTPWTGEAYAEINPRDAKELGLKDGDYIWLDADPEDRPYRGADSSDEFYDVARAMMRV 2e7z.1    YHQPGILRDA----------------EPDPVALLHPKTAQSLGLPSGEWIWVETT-----------------HGRLKLLL  target    RIYSGMPRRVIRTWFNMYAATPGTVQAQKDVPGGPAQNQDTGYVALFRHGSHQSGTRAYLRPTQMTDSMNRKAYFGQTIG 2e7z.1    KHDGAQPEGTIRIPHGRWCPE-----------------------------------------------------------  target    KGFEADVHSPSGAPKEGYVKVEKAEDGGDEGVGEWRPVTLGLRPDDPSEAMQAYLAGEFVTRKRKGS 2e7z.1    ------------------------------------------------------------------- ``` | | | | | | | | | | | | | | | | | | | | | | | | | | | | | | | | | | | | | | | | | | | | | | | | | |
|  | 2vpz.1.A | THIOSULFATE REDUCTASE  *POLYSULFIDE REDUCTASE NATIVE STRUCTURE* | 0.29 |  | 20.31 | 0.56 | 70-1040 | X-ray | 2.40 | hetero-oligomer | 10 x SF4, 4 x MGD, 2 x MO | HHblits | 0.30 |
| ``` target    SDLSRRELLKRAVVVGTGAGLAELFLPAQFLSSASAQSEPQAVAIANPLAQMPDRSWERIYRDQFAEEDSFVFTCAPNDT 2vpz.1    ---------------------------------------------------------------------KSVYQICEGCF  target    HNCLLRAHVKNGVIVRISPTYGYGKATDLAGNQASHRWDPRICQKGLILGRRIYGDRRVKAPMIRKGFKEWADAGFPRHD 2vpz.1    WRCGIVAHAVGNRVYKVEG------------YEANPKSRGRLCPRGQGAPQTTYDPDRLKRPLIRVEGS-----------  target    DGTPRADMEKRGYDEWLQIPWDEALAIAAKTLQNVAETYKGEDGAGKLLEQGYEPAMVEAMHGAGVQAIKMRGGMPLLGA 2vpz.1    ---------QRGEGKYRVATWEEALDHIAKKMLEIREKYGPEA----IAF-----------FGHGTGD------------  target    GRVFGFYRFANMLALLDGKLRPEAPPEEIVGSRAFDNY-AWHTD--LPPGHPMVSGSQTVDFDLFAAEHSKLLVLIGMNW 2vpz.1    ------YWFVDFLP-------------AAWGSPNAAKPSVSLCTAPREVASQWVFGRPIGGHEPIDWENARYIVLIGHHI  target    ICTK-MPDAHWIGDARLKGTRVVVISADYMPTANKADEIVILRPGTDTAFLLGVARELITKKLYDRDAVIQRTDLPLLVR 2vpz.1    GEDTHNTQLQDFALALKNGAKVVVVDPRFSTAAAKAHRWLPIKPGTDTALLLAWIHVLIYEDLYDKEYVAKYTVG-----  target    LDTGERLSARDVFEGYRQAPLENYVALKTEEELAAPPSPPFTADKQVVPTELREEWGDFVYWDRATNGPAAVNRDEIGAK 2vpz.1    --------------------------------------------------------------------------------  target    FAGDPALLGAFDVTLVDGTNVKARTAFSLLKEYLDENFDVQTTSEVCNVDPAAVRSLARQLAANKGNALLAAGMGPN-HY 2vpz.1    --------------------------FEELKAHVK-DFTPEWAEKHTEIPAQVIREVAREMAAHKPRAVL-PPTRHNVWY  target    FNADLFGRVHFLVAALTDNIGHFSGNVGSYAGNYRGSLFQAMGQWIAENPFDQEADLT-KPARV-KRYFK-SESAHYWNY 2vpz.1    GDDTYRVMALLYVNVLLGNYGRPGGFYIAQSPYLEKYPLPPLPL----EPAAGGCSGPSGGDHEPEGFKPRADKGKFFAR  target    GDRPLVSPSEIITGKSHMPTPTKLIWFGNSNSLLGNAKWSFDVVKNTLPKQDAVFCNEWHWTSSCEYSDLVFPADSWAEF 2vpz.1    STAIQELIEPMITGE---PYPIKGLFAYGINLFHSIPN--VPRTKEALKNLDLYVAIDVLPQEHVMWADVILPEATYLER  target    KLPDMTASCTNPFLLAFPKTPLARIHNTRSDYEILAGVAAALADLVDEPRMKTYWKGILDGDPTPYLQRVLSGSNATRGI 2vpz.1    YDDFVLVAHKTPFIQLRTP-AHEPLFDTKPGWWIARELGLRLGLEQ-------YFP---WKTIEEYLETRLQS----LGL  target    LYEDLHASSAKGVPLLMNARTYPRHAGWEQRQEDKPWYTPTGRLEFYRPEPEWQAAGESLPIWREPVDATFYEPNAILAN 2vpz.1    DLETMKGMG---TLVQ-R--GKPWLEDWE-KEGRLPFGTASGKIELYCQRFK-EAGHQPLPVFTPPEE------------  target    SKHPSINPRAPEDYGVPESQMDVETRQYRNVVRTWQELKLSKHPLTEKDPAYRFVFQTPKYRWGAHSTAVDSDWIAMLFG 2vpz.1    ------------------------------------------------PPEGFYRLLYGRSPVHTFARTQNNWVLMEM--  target    PFGDPYRRDSRTPWTGEAYAEINPRDAKELGLKDGDYIWLDADPEDRPYRGADSSDEFYDVARAM--MRVRIYSGMPRRV 2vpz.1    --------------DPENEVWIHKEEAKRLGLKEGDYVMLVNQ-----------------DGVKEGPVRVKPTARIRKDC  target    IRTWFNMYAATPGTVQAQKDVPGGPAQNQDTGYVALFRHGSHQSGTRAYLRPTQMTDSMNRKAYFGQTIGKGFEADVHSP 2vpz.1    VYIVHGFGHK----------------------------------------------------------------------  target    SGAPKEGYVKVEKAEDGGDEGVGEWRPVTLGLRPDDPSEAMQAYLAGEFVTRKRKGS 2vpz.1    --------------------------------------------------------- ``` | | | | | | | | | | | | | | | | | | | | | | | | | | | | | | | | | | | | | | | | | | | | | | | | | |
|  | 2vpx.1.D | THIOSULFATE REDUCTASE  *POLYSULFIDE REDUCTASE WITH BOUND QUINONE (UQ1)* | 0.28 |  | 20.31 | 0.56 | 70-1040 | X-ray | 3.10 | hetero-oligomer | 10 x SF4, 4 x MGD, 2 x MO, 2 x UQ1 | HHblits | 0.30 |
| ``` target    SDLSRRELLKRAVVVGTGAGLAELFLPAQFLSSASAQSEPQAVAIANPLAQMPDRSWERIYRDQFAEEDSFVFTCAPNDT 2vpx.1    ---------------------------------------------------------------------KSVYQICEGCF  target    HNCLLRAHVKNGVIVRISPTYGYGKATDLAGNQASHRWDPRICQKGLILGRRIYGDRRVKAPMIRKGFKEWADAGFPRHD 2vpx.1    WRCGIVAHAVGNRVYKVEG------------YEANPKSRGRLCPRGQGAPQTTYDPDRLKRPLIRVEGS-----------  target    DGTPRADMEKRGYDEWLQIPWDEALAIAAKTLQNVAETYKGEDGAGKLLEQGYEPAMVEAMHGAGVQAIKMRGGMPLLGA 2vpx.1    ---------QRGEGKYRVATWEEALDHIAKKMLEIREKYGPEA----IAF-----------FGHGTGD------------  target    GRVFGFYRFANMLALLDGKLRPEAPPEEIVGSRAFDNY-AWHTD--LPPGHPMVSGSQTVDFDLFAAEHSKLLVLIGMNW 2vpx.1    ------YWFVDFLP-------------AAWGSPNAAKPSVSLCTAPREVASQWVFGRPIGGHEPIDWENARYIVLIGHHI  target    ICTK-MPDAHWIGDARLKGTRVVVISADYMPTANKADEIVILRPGTDTAFLLGVARELITKKLYDRDAVIQRTDLPLLVR 2vpx.1    GEDTHNTQLQDFALALKNGAKVVVVDPRFSTAAAKAHRWLPIKPGTDTALLLAWIHVLIYEDLYDKEYVAKYTVG-----  target    LDTGERLSARDVFEGYRQAPLENYVALKTEEELAAPPSPPFTADKQVVPTELREEWGDFVYWDRATNGPAAVNRDEIGAK 2vpx.1    --------------------------------------------------------------------------------  target    FAGDPALLGAFDVTLVDGTNVKARTAFSLLKEYLDENFDVQTTSEVCNVDPAAVRSLARQLAANKGNALLAAGMGPN-HY 2vpx.1    --------------------------FEELKAHVK-DFTPEWAEKHTEIPAQVIREVAREMAAHKPRAVL-PPTRHNVWY  target    FNADLFGRVHFLVAALTDNIGHFSGNVGSYAGNYRGSLFQAMGQWIAENPFDQEADLT-KPARV-KRYFK-SESAHYWNY 2vpx.1    GDDTYRVMALLYVNVLLGNYGRPGGFYIAQSPYLEKYPLPPLPL----EPAAGGCSGPSGGDHEPEGFKPRADKGKFFAR  target    GDRPLVSPSEIITGKSHMPTPTKLIWFGNSNSLLGNAKWSFDVVKNTLPKQDAVFCNEWHWTSSCEYSDLVFPADSWAEF 2vpx.1    STAIQELIEPMITGE---PYPIKGLFAYGINLFHSIPN--VPRTKEALKNLDLYVAIDVLPQEHVMWADVILPEATYLER  target    KLPDMTASCTNPFLLAFPKTPLARIHNTRSDYEILAGVAAALADLVDEPRMKTYWKGILDGDPTPYLQRVLSGSNATRGI 2vpx.1    YDDFVLVAHKTPFIQLRTP-AHEPLFDTKPGWWIARELGLRLGLEQ-------YFP---WKTIEEYLETRLQS----LGL  target    LYEDLHASSAKGVPLLMNARTYPRHAGWEQRQEDKPWYTPTGRLEFYRPEPEWQAAGESLPIWREPVDATFYEPNAILAN 2vpx.1    DLETMKGMG---TLVQ-R--GKPWLEDWE-KEGRLPFGTASGKIELYCQRFK-EAGHQPLPVFTPPEE------------  target    SKHPSINPRAPEDYGVPESQMDVETRQYRNVVRTWQELKLSKHPLTEKDPAYRFVFQTPKYRWGAHSTAVDSDWIAMLFG 2vpx.1    ------------------------------------------------PPEGFYRLLYGRSPVHTFARTQNNWVLMEM--  target    PFGDPYRRDSRTPWTGEAYAEINPRDAKELGLKDGDYIWLDADPEDRPYRGADSSDEFYDVARAM--MRVRIYSGMPRRV 2vpx.1    --------------DPENEVWIHKEEAKRLGLKEGDYVMLVNQ-----------------DGVKEGPVRVKPTARIRKDC  target    IRTWFNMYAATPGTVQAQKDVPGGPAQNQDTGYVALFRHGSHQSGTRAYLRPTQMTDSMNRKAYFGQTIGKGFEADVHSP 2vpx.1    VYIVHGFGHK----------------------------------------------------------------------  target    SGAPKEGYVKVEKAEDGGDEGVGEWRPVTLGLRPDDPSEAMQAYLAGEFVTRKRKGS 2vpx.1    --------------------------------------------------------- ``` | | | | | | | | | | | | | | | | | | | | | | | | | | | | | | | | | | | | | | | | | | | | | | | | | |
|  | 2v45.1.A | PERIPLASMIC NITRATE REDUCTASE  *A NEW CATALYTIC MECHANISM OF PERIPLASMIC NITRATE REDUCTASE FROM DESULFOVIBRIO DESULFURICANS ATCC 27774 FROM CRYSTALLOGRAPHIC AND EPR DATA AND BASED ON DETAILED ANALYSIS OF THE SIXTH LIGAND* | 0.28 |  | 18.35 | 0.57 | 70-1037 | X-ray | 2.40 | monomer | 1 x SF4, 1 x MO, 2 x MGD, 1 x LCP | HHblits | 0.28 |
| ``` target    SDLSRRELLKRAVVVGTGAGLAELFLPAQFLSSASAQSEPQAVAIANPLAQMPDRSWERIYRDQFAEEDSFVFTCAPNDT 2v45.1    ---------------------------------------------------------------------KWVKGVCRYCG  target    HNCLLRAHVKNGVIVRISPTYGYGKATDLAGNQASHRWDPRICQKGLILGRRIYGDRRVKAPMIRKGFKEWADAGFPRHD 2v45.1    TGCGVLVGVKDGKAVAIQGNP------------NNH-NAGLLCLKGSLLIPVLNSKERVTQPLVRRH-------------  target    DGTPRADMEKRGYDEWLQIPWDEALAIAAKTLQNVAETYKGEDGAGKLLEQGYEPAMVEAMHGAGVQAIKMRGGMPLLGA 2v45.1    -----------KGGKLEPVSWDEALDLMASRFRSSIDMYGPNSVA--WY-------------GSGQ--------------  target    GRVFGFYRFANMLALLDGKLRPEAPPEEIVGSRAFDNYAWHTD--LPPGHPMVSGSQTVDFDLFAAEHSKLLVLIGMNWI 2v45.1    --------CLTEESYVANKIF-----KGGFGTNNVDGNPRLCMASAVGGYVTSFGKDEPMGTYADIDQATCFFIIGSNTS  target    CTKMPDAHWIGDAR--LKGTRVVVISADYMPTANKADEIVILRPGTDTAFLLGVARELITKKLYDRDAVIQRTDLPLLVR 2v45.1    EAHPVLFRRIARRKQVEPGVKIIVADPRRTNTSRIADMHVAFRPGTDLAFMHSMAWVIINEELDNPRFWQRYVNFM----  target    LDTGERLSARDVFEGYRQAPLENYVALKTEEELAAPPSPPFTADKQVVPTELREEWGDFVYWDRATNGPAAVNRDEIGAK 2v45.1    -D---------------A--------------------------------------------------------------  target    FAGDPALLGAFDVTLVDGTNVKARTAFSLLKEYLDENFDVQTTSEVCNVDPAAVRSLARQLAANKGNALLAAGMGPNHYF 2v45.1    ----------------E----GKPSDFEGYKAFLE-NYRPEKVAEICRVPVEQIYGAARAFAESAA-TMSLWCMGINQRV  target    NADLFGRVHFLVAALTDNIGHFSGNVGSYAGN--YRGSLFQAMGQWIAENPFDQEADLTKPARVKRYFKSESAHYWNYG- 2v45.1    QGVFANNLIHNLHLITGQICRPGATSFSLTGQPNACGGVR-DGGALSHLLPAGRA--IPNAKHRAE-----MEKLWGLPE  target    ----DRPLVSPSEIITGKSHMPTPTKLIWFGNSNSLLGNAKWSFDVVKNTLPKQD-AVFCNEWHWT-SSCEYSDLVFPAD 2v45.1    GRIAPEPGYHTVALFEAL--GRGDVKCMIICETNPAHTLPN--LNKVHKAMSHPESFIVCIEAFPDAVTLEYADLVLPPA  target    SWAEFKLPDMTASCTNPFLLAFPKTPLARIHNTRSDYEILAGVAAALADLVDEPRMKTYWKGILDGDPTPYLQRVLSGS- 2v45.1    FWCERDG--V-YGCGERRYSLTEK-AVDPPGQCRPTVNTLVEFARRAGVDPQLVN----F-----RNAEDVWNEWRMVSK  target    ---NATRGILYEDLHASSAKGVPLLMNARTYPRH-AGWEQRQEDKPWYTPTGRLEFYRPEPEWQAAGESLPIWREPVDAT 2v45.1    GTTYDFWGMTRERLRKE--SGLIWPCPSEDHPGTSLRYVRGQDPCVPADHPDRFFFYGKPDG------RAVIWMRPAKG-  target    FYEPNAILANSKHPSINPRAPEDYGVPESQMDVETRQYRNVVRTWQELKLSKHPLTEKDPAYRFVFQTPKYRWGAHSTAV 2v45.1    ----------------------------------------------------AA-EEPDAEYPLYLTSMRVIDHWHTATM  target    --DSDWIAMLFGPFGDPYRRDSRTPWTGEAYAEINPRDAKELGLKDGDYIWLDADPEDRPYRGADSSDEFYDVARAMMRV 2v45.1    TGKVPELQKA----------------NPIAFVEINEEDAARTGIKHGDSVIVETR-----------------RDAMELPA  target    RIYSGMPRRVIRTWFNMYAATPGTVQAQKDVPGGPAQNQDTGYVALFRHGSHQSGTRAYLRPTQMTDSMNRKAYFGQTIG 2v45.1    RVSDVCRPGLIAVPFFD---------------------------------------------------------------  target    KGFEADVHSPSGAPKEGYVKVEKAEDGGDEGVGEWRPVTLGLRPDDPSEAMQAYLAGEFVTRKRKGS 2v45.1    ------------------------------------------------------------------- ``` | | | | | | | | | | | | | | | | | | | | | | | | | | | | | | | | | | | | | | | | | | | | | | | | | |
|  | 7e5z.1.A | Formate dehydrogenase  *Dehydrogenase holoenzyme* | 0.25 |  | 20.98 | 0.56 | 69-1037 | EM | 0.00 | hetero-1-1-mer | 1 x W, 2 x MGD, 2 x FES, 4 x SF4, 1 x FMN | HHblits | 0.30 |
| ``` target    SDLSRRELLKRAVVVGTGAGLAELFLPAQFLSSASAQSEPQAVAIANPLAQMPDRSWERIYRDQFAEEDSFVFTCAPNDT 7e5z.1    --------------------------------------------------------------------DREVKSLCPYCG  target    HNCLLRAHVKNGVIVRISPTYGYGKATDLAGNQASHRWDPRICQKGLILGRRIYGDRRVKAPMIRKGFKEWADAGFPRHD 7e5z.1    VGCQVSYKVKDERIVYAEGV-------------NGPANQNRLCVKGRFGFDYVHHPHRLTVPLIRLENVP-------K--  target    DGTPRADMEKRGYDEWLQIPWDEALAIAAKTLQNVAETYKGEDGAGKLLEQGYEPAMVEAMHGAGVQAIKMRGGMPLLGA 7e5z.1    -DANDQVDPANPWTHFREATWEEALDRAAGGLKAIRDTNGRKALA--GF-------------------------------  target    GRVFGFYRFANMLALLDGKLRPEAPPEEIVGSRAFDNYAWHTDLPP--GHPMVSGSQTVDFDLFAAEHSKLLVLIGMNWI 7e5z.1    ----GSAKGSNEEAYLFQKLVR-----LGFGTNNVDHCTRLCHASSVAALMEGLNSGAVTAPFSAALDAEVIVVIGANPT  target    CTKMPDAHWIGDAR-LKGTRVVVISADYMPTANKADEIVILRPGTDTAFLLGVARELITKKLYDRDAVIQRTDLPLLVRL 7e5z.1    VNHPVAATFLKNAVKQRGAKLIIMDPRRQTLSRHAYRHLAFRPGSDVAMLNAMLNVIVTEGLYDEQYIAGYTEN------  target    DTGERLSARDVFEGYRQAPLENYVALKTEEELAAPPSPPFTADKQVVPTELREEWGDFVYWDRATNGPAAVNRDEIGAKF 7e5z.1    --------------------------------------------------------------------------------  target    AGDPALLGAFDVTLVDGTNVKARTAFSLLKEYLDENFDVQTTSEVCNVDPAAVRSLARQLAANKGNALLAAGMGPNHYFN 7e5z.1    -------------------------FEALREKIV-DFTPEKMASVCGIDAETLREVARLYARAKS-SLIFWGMGVSQHVH  target    ADLFGRVHFLVAALTDNIGHFSGNVGSYAG--NYRGSLFQAMGQWIAENPFDQEADLTKPARVKRYFKSESAHYWNY--G 7e5z.1    GTDNSRCLIALALITGQIGRPGTGLHPLRGQNNVQGAS--DAGLIPMVYPDYQ--SVEKD-AVREL----FEEFWGQSLD  target    DRPLVSPSEIITGKSHMPTPTKLIWFGNSNSLLGNAKWSFDVVKNTLPKQDAVFCNEWHWTSSCEYSDLVFPADSWAEFK 7e5z.1    PQKGLTVVEIMRAI--HAGEIRGMFVEGENPAMSDPD--LNHARHALAMLDHLVVQDLFLTETAFHADVVLPASAFAEKA  target    LPDMTASCTNPFLLAFPKTPLARIHNTRSDYEILAGVAAALADLVDEPRMKTYWKGILDGDPTPYLQRVLSGSNATRGIL 7e5z.1    G---TFTNTDRRVQIAQP-VVAPPGDARQDWWIIQELARRLDLDWN-------Y-----GGPADIFAEMAQVMPSLNNIT  target    YEDLHASSAKGVPLLMNARTYPRHAGWEQRQEDKPWYTPTGRLEFYRPEPEWQAAGESLPIWREPVDATFYEPNAILANS 7e5z.1    WERLERE---GAVTYPVD-A-PDQPGN-EIIFYAGFPTESGRAKIVPAAIV------------PPDE-------------  target    KHPSINPRAPEDYGVPESQMDVETRQYRNVVRTWQELKLSKHPLTEKDPAYRFVFQTPKYRWGAHS--TAVDSDWIAMLF 7e5z.1    ---------------------------------------------VPDDEFPMVLSTGRVLEHWHTGSMTRRAGVLDAL-  target    GPFGDPYRRDSRTPWTGEAYAEINPRDAKELGLKDGDYIWLDADPEDRPYRGADSSDEFYDVARAMMRVRIYSGMPRRVI 7e5z.1    ---------------EPEAVAFMAPKELYRLGLRPGGSMRLETR-----------------RGAVVLKVRSDRDVPIGMI  target    RTWFNMYAATPGTVQAQKDVPGGPAQNQDTGYVALFRHGSHQSGTRAYLRPTQMTDSMNRKAYFGQTIGKGFEADVHSPS 7e5z.1    FMPFCY--------------------------------------------------------------------------  target    GAPKEGYVKVEKAEDGGDEGVGEWRPVTLGLRPDDPSEAMQAYLAGEFVTRKRKGS 7e5z.1    -------------------------------------------------------- ``` | | | | | | | | | | | | | | | | | | | | | | | | | | | | | | | | | | | | | | | | | | | | | | | | | |
|  | 7vw6.1.A | Formate dehydrogenase  *Cryo-EM Structure of Formate Dehydrogenase 1 from Methylorubrum extorquens AM1* | 0.29 |  | 20.98 | 0.56 | 69-1037 | EM | 0.00 | hetero-1-1-mer | 4 x SF4, 2 x FES, 2 x MGD, 1 x W, 1 x FMN | HHblits | 0.30 |
| ``` target    SDLSRRELLKRAVVVGTGAGLAELFLPAQFLSSASAQSEPQAVAIANPLAQMPDRSWERIYRDQFAEEDSFVFTCAPNDT 7vw6.1    --------------------------------------------------------------------DREVKSLCPYCG  target    HNCLLRAHVKNGVIVRISPTYGYGKATDLAGNQASHRWDPRICQKGLILGRRIYGDRRVKAPMIRKGFKEWADAGFPRHD 7vw6.1    VGCQVSYKVKDERIVYAEGV-------------NGPANQNRLCVKGRFGFDYVHHPHRLTVPLIRLENVP-------KDA  target    DGTPRADMEKRGYDEWLQIPWDEALAIAAKTLQNVAETYKGEDGAGKLLEQGYEPAMVEAMHGAGVQAIKMRGGMPLLGA 7vw6.1    ---NDQVDPANPWTHFREATWEEALDRAAGGLKAIRDTNGRKALA--GF-------------------------------  target    GRVFGFYRFANMLALLDGKLRPEAPPEEIVGSRAFDNYAWHTDLP--PGHPMVSGSQTVDFDLFAAEHSKLLVLIGMNWI 7vw6.1    ----GSAKGSNEEAYLFQKLVR-----LGFGTNNVDHCTRLCHASSVAALMEGLNSGAVTAPFSAALDAEVIVVIGANPT  target    CTKMPDAHWIGDAR-LKGTRVVVISADYMPTANKADEIVILRPGTDTAFLLGVARELITKKLYDRDAVIQRTDLPLLVRL 7vw6.1    VNHPVAATFLKNAVKQRGAKLIIMDPRRQTLSRHAYRHLAFRPGSDVAMLNAMLNVIVTEGLYDEQYIAGYTEN------  target    DTGERLSARDVFEGYRQAPLENYVALKTEEELAAPPSPPFTADKQVVPTELREEWGDFVYWDRATNGPAAVNRDEIGAKF 7vw6.1    --------------------------------------------------------------------------------  target    AGDPALLGAFDVTLVDGTNVKARTAFSLLKEYLDENFDVQTTSEVCNVDPAAVRSLARQLAANKGNALLAAGMGPNHYFN 7vw6.1    -------------------------FEALREKIV-DFTPEKMASVCGIDAETLREVARLYARAKS-SLIFWGMGVSQHVH  target    ADLFGRVHFLVAALTDNIGHFSGNVGSYAGN--YRGSLFQAMGQWIAENPFDQEADLTKPARVKRYFKSESAHYWNY--G 7vw6.1    GTDNSRCLIALALITGQIGRPGTGLHPLRGQNNVQGAS--DAGLIPMVYPDYQ--SVEK-DAVREL----FEEFWGQSLD  target    DRPLVSPSEIITGKSHMPTPTKLIWFGNSNSLLGNAKWSFDVVKNTLPKQDAVFCNEWHWTSSCEYSDLVFPADSWAEFK 7vw6.1    PQKGLTVVEIMRAI--HAGEIRGMFVEGENPAMSDPD--LNHARHALAMLDHLVVQDLFLTETAFHADVVLPASAFAEKA  target    LPDMTASCTNPFLLAFPKTPLARIHNTRSDYEILAGVAAALADLVDEPRMKTYWKGILDGDPTPYLQRVLSGSNATRGIL 7vw6.1    G---TFTNTDRRVQIAQP-VVAPPGDARQDWWIIQELARRLDLDWN-------Y-----GGPADIFAEMAQVMPSLNNIT  target    YEDLHASSAKGVPLLMNARTYPRHAGWEQRQEDKPWYTPTGRLEFYRPEPEWQAAGESLPIWREPVDATFYEPNAILANS 7vw6.1    WERLERE---GAVTYPVD-A-PDQPGNE-IIFYAGFPTESGRAKIVPAAIV------------PPDE-------------  target    KHPSINPRAPEDYGVPESQMDVETRQYRNVVRTWQELKLSKHPLTEKDPAYRFVFQTPKYRWGAHS--TAVDSDWIAMLF 7vw6.1    ---------------------------------------------VPDDEFPMVLSTGRVLEHWHTGSMTRRAGVLDAL-  target    GPFGDPYRRDSRTPWTGEAYAEINPRDAKELGLKDGDYIWLDADPEDRPYRGADSSDEFYDVARAMMRVRIYSGMPRRVI 7vw6.1    ---------------EPEAVAFMAPKELYRLGLRPGGSMRLETR-----------------RGAVVLKVRSDRDVPIGMI  target    RTWFNMYAATPGTVQAQKDVPGGPAQNQDTGYVALFRHGSHQSGTRAYLRPTQMTDSMNRKAYFGQTIGKGFEADVHSPS 7vw6.1    FMPFCY--------------------------------------------------------------------------  target    GAPKEGYVKVEKAEDGGDEGVGEWRPVTLGLRPDDPSEAMQAYLAGEFVTRKRKGS 7vw6.1    -------------------------------------------------------- ``` | | | | | | | | | | | | | | | | | | | | | | | | | | | | | | | | | | | | | | | | | | | | | | | | | |
|  | 1aa6.1.A | FORMATE DEHYDROGENASE H  *REDUCED FORM OF FORMATE DEHYDROGENASE H FROM E. COLI* | 0.30 |  | 20.68 | 0.55 | 72-1037 | X-ray | 2.30 | monomer | 1 x SF4, 2 x MGD, 1 x 4MO | HHblits | 0.31 |
| ``` target    SDLSRRELLKRAVVVGTGAGLAELFLPAQFLSSASAQSEPQAVAIANPLAQMPDRSWERIYRDQFAEEDSFVFTCAPNDT 1aa6.1    -----------------------------------------------------------------------VVTVCPYCA  target    HNCLLRAHVKNGVIVRISPTYGYGKATDLAGNQASHRWDPRICQKGLILGRRIYGD----RRVKAPMIRKGFKEWADAGF 1aa6.1    SGCKINLVVDNGKIVRAEAAQ-------------GKTNQGTLCLKGYYGWDFINDTQILTPRLKTPMIRRQ---------  target    PRHDDGTPRADMEKRGYDEWLQIPWDEALAIAAKTLQNVAETYKGEDGAGKLLEQGYEPAMVEAMHGAGVQAIKMRGGMP 1aa6.1    ---------------RGGKLEPVSWDEALNYVAERLSAIKEKYGPDA----IQT----------TGSSRGTGN-------  target    LLGAGRVFGFYRFANMLALLDGKLRPEAPPEEIVGSRAFDNYAWHTDLP--PGHPMVSGSQTVDFDLFAAEHSKLLVLIG 1aa6.1    ----ETNYVMQKFARA----------------VIGTNNVDCCARVUHGPSVAGLHQSVGNGAMSNAINEIDNTDLVFVFG  target    MNWICTKMPDAHWIGDARLKGTRVVVISADYMPTANKADEIVILRPGTDTAFLLGVARELITKKLYDRDAVIQRTDLPLL 1aa6.1    YNPADSHPIVANHVINAKRNGAKIIVCDPRKIETARIADMHIALKNGSNIALLNAMGHVIIEENLYDKAFVASRTEG---  target    VRLDTGERLSARDVFEGYRQAPLENYVALKTEEELAAPPSPPFTADKQVVPTELREEWGDFVYWDRATNGPAAVNRDEIG 1aa6.1    --------------------------------------------------------------------------------  target    AKFAGDPALLGAFDVTLVDGTNVKARTAFSLLKEYLDENFDVQTTSEVCNVDPAAVRSLARQLAANKGNALLAAGMGPNH 1aa6.1    ----------------------------FEEYRKIVE-GYTPESVEDITGVSASEIRQAARMYAQAKS-AAILWGMGVTQ  target    YFNADLFGRVHFLVAALTDNIGHFSGNVGSYAG--NYRGSLFQAMGQWIAENPFDQEADLTKPARVKRYFKSESAHYWNY 1aa6.1    FYQGVETVRSLTSLAMLTGNLGKPHAGVNPVRGQNNVQGAC--DMGALPDTYPGYQ--YVKDPANREKFA--KAWGVESL  target    GDRPLVSPSEIITGKSHMPTPTKLIWFGNSNSLLGNAKWSFDVVKNTLPKQDAVFCNEWHWTSSCEYSDLVFPADSWAEF 1aa6.1    PAHTGYRISELPHRA--AHGEVRAAYIMGEDPLQTDAE--LSAVRKAFEDLELVIVQDIFMTKTASAADVILPSTSWGEH  target    KLPDMTASCTNPFLLAFPKTPLARIHNTRSDYEILAGVAAALADLVDEPRMKTYWKGILDGDPTPYLQRVLSGSNATRGI 1aa6.1    EG--VFT-AADRGFQRFFK-AVEPKWDLKTDWQIISEIATRMGYPMH-------YN-----NTQEIWDELRHLCPDFYGA  target    LYEDLHASSAKGVPLLMNARTYPRHAGWEQRQEDKPWYTPTGRLEFYRPEPEWQAAGESLPIWREPVDATFYEPNAILAN 1aa6.1    TYEKMGEL---GFIQWPCRDTSDADQ-GTSYLFKEKFDTPNGLAQFFTCDW------------VAPID------------  target    SKHPSINPRAPEDYGVPESQMDVETRQYRNVVRTWQELKLSKHPLTEKDPAYRFVFQTPKYR--WGAHSTAVDSDWIAML 1aa6.1    ----------------------------------------------KLTDEYPMVLSTVREVGHYSCRSMTGNCAALAAL  target    FGPFGDPYRRDSRTPWTGEAYAEINPRDAKELGLKDGDYIWLDADPEDRPYRGADSSDEFYDVARAMMRVRIYSGMPRRV 1aa6.1    A---------------DEPGYAQINTEDAKRLGIEDEALVWVHSR-----------------KGKIITRAQVSDRPNKGA  target    IRTWFNMYAATPGTVQAQKDVPGGPAQNQDTGYVALFRHGSHQSGTRAYLRPTQMTDSMNRKAYFGQTIGKGFEADVHSP 1aa6.1    IYMTYQW-------------------------------------------------------------------------  target    SGAPKEGYVKVEKAEDGGDEGVGEWRPVTLGLRPDDPSEAMQAYLAGEFVTRKRKGS 1aa6.1    --------------------------------------------------------- ``` | | | | | | | | | | | | | | | | | | | | | | | | | | | | | | | | | | | | | | | | | | | | | | | | | |
|  | 1fdo.1.A | FORMATE DEHYDROGENASE H  *OXIDIZED FORM OF FORMATE DEHYDROGENASE H FROM E. COLI* | 0.29 |  | 20.68 | 0.55 | 72-1037 | X-ray | 2.80 | monomer | 1 x SF4, 2 x MGD, 1 x 6MO | HHblits | 0.31 |
| ``` target    SDLSRRELLKRAVVVGTGAGLAELFLPAQFLSSASAQSEPQAVAIANPLAQMPDRSWERIYRDQFAEEDSFVFTCAPNDT 1fdo.1    -----------------------------------------------------------------------VVTVCPYCA  target    HNCLLRAHVKNGVIVRISPTYGYGKATDLAGNQASHRWDPRICQKGLILGRRIYGD----RRVKAPMIRKGFKEWADAGF 1fdo.1    SGCKINLVVDNGKIVRAEAAQ-------------GKTNQGTLCLKGYYGWDFINDTQILTPRLKTPMIRRQ---------  target    PRHDDGTPRADMEKRGYDEWLQIPWDEALAIAAKTLQNVAETYKGEDGAGKLLEQGYEPAMVEAMHGAGVQAIKMRGGMP 1fdo.1    ---------------RGGKLEPVSWDEALNYVAERLSAIKEKYGPDA----IQT----------TGSSRGTGN-------  target    LLGAGRVFGFYRFANMLALLDGKLRPEAPPEEIVGSRAFDNYAWHTDLP--PGHPMVSGSQTVDFDLFAAEHSKLLVLIG 1fdo.1    ----ETNYVMQKFARA----------------VIGTNNVDCCARVUHGPSVAGLHQSVGNGAMSNAINEIDNTDLVFVFG  target    MNWICTKMPDAHWIGDARLKGTRVVVISADYMPTANKADEIVILRPGTDTAFLLGVARELITKKLYDRDAVIQRTDLPLL 1fdo.1    YNPADSHPIVANHVINAKRNGAKIIVCDPRKIETARIADMHIALKNGSNIALLNAMGHVIIEENLYDKAFVASRTEG---  target    VRLDTGERLSARDVFEGYRQAPLENYVALKTEEELAAPPSPPFTADKQVVPTELREEWGDFVYWDRATNGPAAVNRDEIG 1fdo.1    --------------------------------------------------------------------------------  target    AKFAGDPALLGAFDVTLVDGTNVKARTAFSLLKEYLDENFDVQTTSEVCNVDPAAVRSLARQLAANKGNALLAAGMGPNH 1fdo.1    ----------------------------FEEYRKIVE-GYTPESVEDITGVSASEIRQAARMYAQAKS-AAILWGMGVTQ  target    YFNADLFGRVHFLVAALTDNIGHFSGNVGSYAG--NYRGSLFQAMGQWIAENPFDQEADLTKPARVKRYFKSESAHYWNY 1fdo.1    FYQGVETVRSLTSLAMLTGNLGKPHAGVNPVRGQNNVQGAC--DMGALPDTYPGYQ--YVKDPANREKFA--KAWGVESL  target    GDRPLVSPSEIITGKSHMPTPTKLIWFGNSNSLLGNAKWSFDVVKNTLPKQDAVFCNEWHWTSSCEYSDLVFPADSWAEF 1fdo.1    PAHTGYRISELPHRA--AHGEVRAAYIMGEDPLQTDAE--LSAVRKAFEDLELVIVQDIFMTKTASAADVILPSTSWGEH  target    KLPDMTASCTNPFLLAFPKTPLARIHNTRSDYEILAGVAAALADLVDEPRMKTYWKGILDGDPTPYLQRVLSGSNATRGI 1fdo.1    EG--VFT-AADRGFQRFFK-AVEPKWDLKTDWQIISEIATRMGYPMH-------YN-----NTQEIWDELRHLCPDFYGA  target    LYEDLHASSAKGVPLLMNARTYPRHAGWEQRQEDKPWYTPTGRLEFYRPEPEWQAAGESLPIWREPVDATFYEPNAILAN 1fdo.1    TYEKMGEL---GFIQWPCRDTSDADQ-GTSYLFKEKFDTPNGLAQFFTCDW------------VAPID------------  target    SKHPSINPRAPEDYGVPESQMDVETRQYRNVVRTWQELKLSKHPLTEKDPAYRFVFQTPKYR--WGAHSTAVDSDWIAML 1fdo.1    ----------------------------------------------KLTDEYPMVLSTVREVGHYSCRSMTGNCAALAAL  target    FGPFGDPYRRDSRTPWTGEAYAEINPRDAKELGLKDGDYIWLDADPEDRPYRGADSSDEFYDVARAMMRVRIYSGMPRRV 1fdo.1    A---------------DEPGYAQINTEDAKRLGIEDEALVWVHSR-----------------KGKIITRAQVSDRPNKGA  target    IRTWFNMYAATPGTVQAQKDVPGGPAQNQDTGYVALFRHGSHQSGTRAYLRPTQMTDSMNRKAYFGQTIGKGFEADVHSP 1fdo.1    IYMTYQW-------------------------------------------------------------------------  target    SGAPKEGYVKVEKAEDGGDEGVGEWRPVTLGLRPDDPSEAMQAYLAGEFVTRKRKGS 1fdo.1    --------------------------------------------------------- ``` | | | | | | | | | | | | | | | | | | | | | | | | | | | | | | | | | | | | | | | | | | | | | | | | | |
|  | 2iv2.1.A | Formate dehydrogenase H  *Reinterpretation of reduced form of formate dehydrogenase H from E. coli* | 0.30 |  | 20.68 | 0.55 | 72-1037 | X-ray | 2.27 | monomer | 1 x SF4, 1 x 2MD, 1 x MGD | HHblits | 0.31 |
| ``` target    SDLSRRELLKRAVVVGTGAGLAELFLPAQFLSSASAQSEPQAVAIANPLAQMPDRSWERIYRDQFAEEDSFVFTCAPNDT 2iv2.1    -----------------------------------------------------------------------VVTVCPYCA  target    HNCLLRAHVKNGVIVRISPTYGYGKATDLAGNQASHRWDPRICQKGLILGRRIYGD----RRVKAPMIRKGFKEWADAGF 2iv2.1    SGCKINLVVDNGKIVRAEAAQ-------------GKTNQGTLCLKGYYGWDFINDTQILTPRLKTPMIRRQ---------  target    PRHDDGTPRADMEKRGYDEWLQIPWDEALAIAAKTLQNVAETYKGEDGAGKLLEQGYEPAMVEAMHGAGVQAIKMRGGMP 2iv2.1    ---------------RGGKLEPVSWDEALNYVAERLSAIKEKYGPDA----IQT----------TGSSRGTGN-------  target    LLGAGRVFGFYRFANMLALLDGKLRPEAPPEEIVGSRAFDNYAWHTDLP--PGHPMVSGSQTVDFDLFAAEHSKLLVLIG 2iv2.1    ----ETNYVMQKFARA----------------VIGTNNVDCCARVUHGPSVAGLHQSVGNGAMSNAINEIDNTDLVFVFG  target    MNWICTKMPDAHWIGDARLKGTRVVVISADYMPTANKADEIVILRPGTDTAFLLGVARELITKKLYDRDAVIQRTDLPLL 2iv2.1    YNPADSHPIVANHVINAKRNGAKIIVCDPRKIETARIADMHIALKNGSNIALLNAMGHVIIEENLYDKAFVASRTEG---  target    VRLDTGERLSARDVFEGYRQAPLENYVALKTEEELAAPPSPPFTADKQVVPTELREEWGDFVYWDRATNGPAAVNRDEIG 2iv2.1    --------------------------------------------------------------------------------  target    AKFAGDPALLGAFDVTLVDGTNVKARTAFSLLKEYLDENFDVQTTSEVCNVDPAAVRSLARQLAANKGNALLAAGMGPNH 2iv2.1    ----------------------------FEEYRKIVE-GYTPESVEDITGVSASEIRQAARMYAQAKS-AAILWGMGVTQ  target    YFNADLFGRVHFLVAALTDNIGHFSGNVGSYAG--NYRGSLFQAMGQWIAENPFDQEADLTKPARVKRYFKSESAHYWNY 2iv2.1    FYQGVETVRSLTSLAMLTGNLGKPHAGVNPVRGQNNVQGAC--DMGALPDTYPGYQ--YVKDPANREKFA--KAWGVESL  target    GDRPLVSPSEIITGKSHMPTPTKLIWFGNSNSLLGNAKWSFDVVKNTLPKQDAVFCNEWHWTSSCEYSDLVFPADSWAEF 2iv2.1    PAHTGYRISELPHRA--AHGEVRAAYIMGEDPLQTDAE--LSAVRKAFEDLELVIVQDIFMTKTASAADVILPSTSWGEH  target    KLPDMTASCTNPFLLAFPKTPLARIHNTRSDYEILAGVAAALADLVDEPRMKTYWKGILDGDPTPYLQRVLSGSNATRGI 2iv2.1    EG--VFT-AADRGFQRFFK-AVEPKWDLKTDWQIISEIATRMGYPMH-------YN-----NTQEIWDELRHLCPDFYGA  target    LYEDLHASSAKGVPLLMNARTYPRHAGWEQRQEDKPWYTPTGRLEFYRPEPEWQAAGESLPIWREPVDATFYEPNAILAN 2iv2.1    TYEKMGEL---GFIQWPCRDTSDADQ-GTSYLFKEKFDTPNGLAQFFTCDW------------VAPID------------  target    SKHPSINPRAPEDYGVPESQMDVETRQYRNVVRTWQELKLSKHPLTEKDPAYRFVFQTPKYR--WGAHSTAVDSDWIAML 2iv2.1    ----------------------------------------------KLTDEYPMVLSTVREVGHYSCRSMTGNCAALAAL  target    FGPFGDPYRRDSRTPWTGEAYAEINPRDAKELGLKDGDYIWLDADPEDRPYRGADSSDEFYDVARAMMRVRIYSGMPRRV 2iv2.1    A---------------DEPGYAQINTEDAKRLGIEDEALVWVHSR-----------------KGKIITRAQVSDRPNKGA  target    IRTWFNMYAATPGTVQAQKDVPGGPAQNQDTGYVALFRHGSHQSGTRAYLRPTQMTDSMNRKAYFGQTIGKGFEADVHSP 2iv2.1    IYMTYQW-------------------------------------------------------------------------  target    SGAPKEGYVKVEKAEDGGDEGVGEWRPVTLGLRPDDPSEAMQAYLAGEFVTRKRKGS 2iv2.1    --------------------------------------------------------- ``` | | | | | | | | | | | | | | | | | | | | | | | | | | | | | | | | | | | | | | | | | | | | | | | | | |
|  | 7z0t.1.G | Formate dehydrogenase H  *Structure of the Escherichia coli formate hydrogenlyase complex (aerobic preparation, composite structure)* | 0.30 |  | 20.68 | 0.55 | 72-1037 | EM | 0.00 | hetero-1-1-1-1-1-1-… | 1 x NI, 1 x FCO, 8 x SF4, 1 x FE, 2 x MGD, 1 x 6MO | HHblits | 0.31 |
| ``` target    SDLSRRELLKRAVVVGTGAGLAELFLPAQFLSSASAQSEPQAVAIANPLAQMPDRSWERIYRDQFAEEDSFVFTCAPNDT 7z0t.1    -----------------------------------------------------------------------VVTVCPYCA  target    HNCLLRAHVKNGVIVRISPTYGYGKATDLAGNQASHRWDPRICQKGLILGRRIYGD----RRVKAPMIRKGFKEWADAGF 7z0t.1    SGCKINLVVDNGKIVRAEAAQ-------------GKTNQGTLCLKGYYGWDFINDTQILTPRLKTPMIRRQ---------  target    PRHDDGTPRADMEKRGYDEWLQIPWDEALAIAAKTLQNVAETYKGEDGAGKLLEQGYEPAMVEAMHGAGVQAIKMRGGMP 7z0t.1    ---------------RGGKLEPVSWDEALNYVAERLSAIKEKYGPDA----IQT----------TGSSRGTGN-------  target    LLGAGRVFGFYRFANMLALLDGKLRPEAPPEEIVGSRAFDNYAWHTDLP--PGHPMVSGSQTVDFDLFAAEHSKLLVLIG 7z0t.1    ----ETNYVMQKFARA----------------VIGTNNVDCCARVUHGPSVAGLHQSVGNGAMSNAINEIDNTDLVFVFG  target    MNWICTKMPDAHWIGDARLKGTRVVVISADYMPTANKADEIVILRPGTDTAFLLGVARELITKKLYDRDAVIQRTDLPLL 7z0t.1    YNPADSHPIVANHVINAKRNGAKIIVCDPRKIETARIADMHIALKNGSNIALLNAMGHVIIEENLYDKAFVASRTEG---  target    VRLDTGERLSARDVFEGYRQAPLENYVALKTEEELAAPPSPPFTADKQVVPTELREEWGDFVYWDRATNGPAAVNRDEIG 7z0t.1    --------------------------------------------------------------------------------  target    AKFAGDPALLGAFDVTLVDGTNVKARTAFSLLKEYLDENFDVQTTSEVCNVDPAAVRSLARQLAANKGNALLAAGMGPNH 7z0t.1    ----------------------------FEEYRKIVE-GYTPESVEDITGVSASEIRQAARMYAQAKS-AAILWGMGVTQ  target    YFNADLFGRVHFLVAALTDNIGHFSGNVGSYAG--NYRGSLFQAMGQWIAENPFDQEADLTKPARVKRYFKSESAHYWNY 7z0t.1    FYQGVETVRSLTSLAMLTGNLGKPHAGVNPVRGQNNVQGAC--DMGALPDTYPGYQ--YVKDPANREKFA--KAWGVESL  target    GDRPLVSPSEIITGKSHMPTPTKLIWFGNSNSLLGNAKWSFDVVKNTLPKQDAVFCNEWHWTSSCEYSDLVFPADSWAEF 7z0t.1    PAHTGYRISELPHRA--AHGEVRAAYIMGEDPLQTDAE--LSAVRKAFEDLELVIVQDIFMTKTASAADVILPSTSWGEH  target    KLPDMTASCTNPFLLAFPKTPLARIHNTRSDYEILAGVAAALADLVDEPRMKTYWKGILDGDPTPYLQRVLSGSNATRGI 7z0t.1    EG--VFT-AADRGFQRFFK-AVEPKWDLKTDWQIISEIATRMGYPMH-------YN-----NTQEIWDELRHLCPDFYGA  target    LYEDLHASSAKGVPLLMNARTYPRHAGWEQRQEDKPWYTPTGRLEFYRPEPEWQAAGESLPIWREPVDATFYEPNAILAN 7z0t.1    TYEKMGEL---GFIQWPCRDTSDADQ-GTSYLFKEKFDTPNGLAQFFTCDW------------VAPID------------  target    SKHPSINPRAPEDYGVPESQMDVETRQYRNVVRTWQELKLSKHPLTEKDPAYRFVFQTPKYR--WGAHSTAVDSDWIAML 7z0t.1    ----------------------------------------------KLTDEYPMVLSTVREVGHYSCRSMTGNCAALAAL  target    FGPFGDPYRRDSRTPWTGEAYAEINPRDAKELGLKDGDYIWLDADPEDRPYRGADSSDEFYDVARAMMRVRIYSGMPRRV 7z0t.1    A---------------DEPGYAQINTEDAKRLGIEDEALVWVHSR-----------------KGKIITRAQVSDRPNKGA  target    IRTWFNMYAATPGTVQAQKDVPGGPAQNQDTGYVALFRHGSHQSGTRAYLRPTQMTDSMNRKAYFGQTIGKGFEADVHSP 7z0t.1    IYMTYQW-------------------------------------------------------------------------  target    SGAPKEGYVKVEKAEDGGDEGVGEWRPVTLGLRPDDPSEAMQAYLAGEFVTRKRKGS 7z0t.1    --------------------------------------------------------- ``` | | | | | | | | | | | | | | | | | | | | | | | | | | | | | | | | | | | | | | | | | | | | | | | | | |
|  | 7bkb.1.F | Formate dehydrogenase  *Formate dehydrogenase - heterodisulfide reductase - formylmethanofuran dehydrogenase complex from Methanospirillum hungatei (hexameric, composite structure)* | 0.27 |  | 21.63 | 0.55 | 69-1037 | EM | 0.00 | hetero-2-2-2-2-2-2-… | 48 x SF4, 4 x FAD, 2 x FES, 4 x 9S8, 4 x ZN, 2 x MO, 4 x MGD | HHblits | 0.31 |
| ``` target    SDLSRRELLKRAVVVGTGAGLAELFLPAQFLSSASAQSEPQAVAIANPLAQMPDRSWERIYRDQFAEEDSFVFTCAPNDT 7bkb.1    --------------------------------------------------------------------MKYVATTCPYCG  target    HNCLLRAHVKNGVIVRISPTYGYGKATDLAGNQASHRWDPRICQKGLILGRRIYGDRRVKAPMIRKGFKEWADAGFPRHD 7bkb.1    VGCTLNLVVSNGKVVGVEPN------------QRSPINEGKLCPKGVTCWEHIHSPDRLTTPLIKKD-------------  target    DGTPRADMEKRGYDEWLQIPWDEALAIAAKTLQNVAETYKGEDGAGKLLEQGYEPAMVEAMHGAGVQAIKMRGGMPLLGA 7bkb.1    -------------GKFIEASWDEALDLVAKNLKVIYDKHGPKGLG--FQT------------S-----------------  target    GRVFGFYRFANMLALLDGKLRPEAPPEEIVGSRAFDNYAWHTDLP--PGHPMVSGSQTVDFDLFAAEHSKLLVLIGMNWI 7bkb.1    ------CRTVNEDCYIFQKFAR-----VGFKTNNVDNCARICHGPSVAGLSLSFGSGAATNGFEDALNADLILIWGSNAV  target    CTKMPDAHWIGDARLKGTRVVVISADYMPTANKADEIVILRPGTDTAFLLGVARELITKKLYDRDAVIQRTDLPLLVRLD 7bkb.1    EAHPLAGRRIAQAKKKGIQIIAVDPRYTMTARLADTYVRFNPSTHIALANSMMYWIIKEGLEDKKFIQDRVNG-------  target    TGERLSARDVFEGYRQAPLENYVALKTEEELAAPPSPPFTADKQVVPTELREEWGDFVYWDRATNGPAAVNRDEIGAKFA 7bkb.1    --------------------------------------------------------------------------------  target    GDPALLGAFDVTLVDGTNVKARTAFSLLKEYLDENFDVQTTSEVCNVDPAAVRSLARQLAANKGNALLAAGMGPNHYFNA 7bkb.1    ------------------------FEDLKKTVE-NY--ADAEAIHGVPLDVVKDIAFRYAKAKN-AVIIYCLGITELTTG  target    DLFGRVHFLVAALTDNIGHFSGNVGSYAGN--YRGSLFQAMGQWIAENPFDQEADLTKPARVKRYFKSESAHYWNYGDR- 7bkb.1    TDNVRSMGNLALLTGNVGREGVGVNPLRGQNNVQGA--CDMGAYPNVYSGYQKCEV-AENRA------KMEKAWSVTNLP  target    --PLVSPSEIITGKSHMPTPTKLIWFGNSNSLLGNAKWSFDVVKNTLPKQDAVFCNEWHWTSSCEYSDLVFPADSWAEFK 7bkb.1    DWYGATLTEQINQ---CGDEIKGMYILGLNPVVTYPS--SNHVKAQLEKLDFLVVQDIFFTETCQYADVILPGACFAEKD  target    LPDMTASCTNPFLLAFPKTPLARIHNTRSDYEILAGVAAALADLVDEPRMKTYWKGILDGDPTPYLQRVLSGSNATRGIL 7bkb.1    G--T-FTSGERRINRVRK-AVNPPGQAKEDIHIISELAAKMGFKG--------FE---LPTAKDVWDDMRAVTPSMFGAT  target    YEDLHASSAKGVPLLMNARTYPRHAGWEQRQEDKPWYTPTGRLEFYRPEPEWQAAGESLPIWREPVDATFYEPNAILANS 7bkb.1    YEKLERP--EGICWPCPTEEHPGTP----ILHREKFATADGKGNLFGID------------YRPPAEV------------  target    KHPSINPRAPEDYGVPESQMDVETRQYRNVVRTWQELKLSKHPLTEKDPAYRFVFQTPKYRWGAHSTAVDSDWIAMLFGP 7bkb.1    ----------------------------------------------ADAEYPFTLMTGRLIFHYHSRTQTDR-AADLH--  target    FGDPYRRDSRTPWTGEAYAEINPRDAKELGLKDGDYIWLDADPEDRPYRGADSSDEFYDVARAMMRVRIYSGMPRRVIRT 7bkb.1    -----------REVPESYAQINIEDARRLGIKNNEYIKLKSR-----------------RGETTTLARVTDEVAPGVVYM  target    WFNMYAATPGTVQAQKDVPGGPAQNQDTGYVALFRHGSHQSGTRAYLRPTQMTDSMNRKAYFGQTIGKGFEADVHSPSGA 7bkb.1    TMHF----------------------------------------------------------------------------  target    PKEGYVKVEKAEDGGDEGVGEWRPVTLGLRPDDPSEAMQAYLAGEFVTRKRKGS 7bkb.1    ------------------------------------------------------ ``` | | | | | | | | | | | | | | | | | | | | | | | | | | | | | | | | | | | | | | | | | | | | | | | | | |
|  | 6tg9.1.A | Formate dehydrogenase subunit alpha  *Cryo-EM Structure of NADH reduced form of NAD+-dependent Formate Dehydrogenase from Rhodobacter capsulatus* | 0.27 |  | 20.19 | 0.55 | 70-1037 | EM | 3.24 | hetero-2-2-2-2-mer | 4 x MGD, 2 x 6MO, 4 x FES, 10 x SF4, 2 x H2S, 2 x FMN, 2 x NAI | HHblits | 0.30 |
| ``` target    SDLSRRELLKRAVVVGTGAGLAELFLPAQFLSSASAQSEPQAVAIANPLAQMPDRSWERIYRDQFAEEDSFVFTCAPNDT 6tg9.1    ---------------------------------------------------------------------RKVVTTCAYCG  target    HNCLLRAHVKNGVIVRISPTYGYGKATDLAGNQASHRWDPRICQKGLILGRRIYGDRRVKAPMIRKGFKEWADAGFPRHD 6tg9.1    VGCSFEAHMLGDQLVRMVPW------------KGGAANRGHSCVKGRFAYGYATHQDRILKPMIRDK-------------  target    DGTPRADMEKRGYDEWLQIPWDEALAIAAKTLQNVAETYKGEDGAGKLLEQGYEPAMVEAMHGAGVQAIKMRGGMPLLGA 6tg9.1    -----------ITDPWREVNWTEALDFTATRLRALRDSHGADALG--VI-------------------------------  target    GRVFGFYRFANMLALLDGKLRPEAPPEEIVGSRAFDNYAWHTDLP--PGHPMVSGSQTVDFDLFAAEHSKLLVLIGMNWI 6tg9.1    ----TSSRCTNEETYLVQKLAR-----AVFGTNNTDTCARVCHSPTGYGLKQTFGTSAGTQDFDSVEETDLALVIGANPT  target    CTKMPDAHWIGDARLKGTRVVVISADYMPTA----NKADEIVILRPGTDTAFLLGVARELITKKLYDRDAVIQRTDLPLL 6tg9.1    DGHPVFASRLRKRLRAGAKLIVVDPRRIDLLNTPHRGEAWHLQLKPGTNVAVMTAMAHVIVTEQIFDKRFIGDRCDW---  target    VRLDTGERLSARDVFEGYRQAPLENYVALKTEEELAAPPSPPFTADKQVVPTELREEWGDFVYWDRATNGPAAVNRDEIG 6tg9.1    ---------------DEW----------------------------------------AD--------------------  target    AKFAGDPALLGAFDVTLVDGTNVKARTAFSLLKEYLD-ENFDVQTTSEVCNVDPAAVRSLARQLAANKGNALLAAGMGPN 6tg9.1    -------------------------------YAEFVANPEYAPEAVESLTGVPAGLLRQAARAYAAAPN-AAIYYGLGVT  target    HYFNADLFGRVHFLVAALTDNIGHFSGNVGSYAGN--YRGSLFQAMGQWIAENPFDQEADLTKPARVKRYFKSESAHYWN 6tg9.1    EHSQGSTTVIAIANLAMMTGNIGRPGVGVNPLRGQNNVQGSC--DMGSFPHEFPGYRH--VSDDATR-----GLFERTWG  target    Y--GDRPLVSPSEIITGKSHMPTPTKLIWFGNSNSLLGNAKWSFDVVKNTLPKQDAVFCNEWHWTSSCEYSDLVFPADSW 6tg9.1    VTLSSEPGLRIPNMLDAA--VEGRFKALYVQGEDILQSDPD--TRHVSAGLAAMDLVIVHDLFLNETANYAHVFLPGSTF  target    AEFKLPDMTASCTNPFLLAFPKTPLARIHNTRSDYEILAGVAAALADLVDEPRMKTYWKGILDGDPTPYLQRVLSGSNAT 6tg9.1    LEKDG---TFTNAERRINRVRR-VMAPKA-GFADWEVTQMLANALGAGW-------HY-----THPSEIMAEIAATTPGF  target    RGILYEDLHASSAKGVPLLMNARTYPRHAGWEQRQEDKPWYTPTGRLEFYRPEPEWQAAGESL-PIWREPVDATFYEPNA 6tg9.1    AAVTYEMLDARG--SVQ-------WPCN-------EKAPEGSPIMHVEGFVRGKG-----RFIRTAYLPT----------  target    ILANSKHPSINPRAPEDYGVPESQMDVETRQYRNVVRTWQELKLSKHPLTEKDPAYRFVFQTPKYRWGAHSTAVDSDWIA 6tg9.1    --------------------------------------D----------EKTGPRFPLLLTTGRILSQYNVGAQTRRT-E  target    MLFGPFGDPYRRDSRTPWTGEAYAEINPRDAKELGLKDGDYIWLDADPEDRPYRGADSSDEFYDVARAMMRVRIYSGMPR 6tg9.1    --------------NTVWHGEDRLEIHPTDAETRGIRDGDWVRLASR-----------------AGETTLRATVTDRVSP  target    RVIRTWFNMYAATPGTVQAQKDVPGGPAQNQDTGYVALFRHGSHQSGTRAYLRPTQMTDSMNRKAYFGQTIGKGFEADVH 6tg9.1    GVVYTTFHH-----------------------------------------------------------------------  target    SPSGAPKEGYVKVEKAEDGGDEGVGEWRPVTLGLRPDDPSEAMQAYLAGEFVTRKRKGS 6tg9.1    ----------------------------------------------------------- ``` | | | | | | | | | | | | | | | | | | | | | | | | | | | | | | | | | | | | | | | | | | | | | | | | | |
|  | 1g8k.1.A | ARSENITE OXIDASE  *CRYSTAL STRUCTURE ANALYSIS OF ARSENITE OXIDASE FROM ALCALIGENES FAECALIS* | 0.28 |  | 16.57 | 0.56 | 68-1037 | X-ray | 1.64 | hetero-1-1-mer | 3 x HG, 2 x CA, 2 x MGD, 1 x O, 1 x 4MO, 1 x F3S, 1 x FES | HHblits | 0.28 |
| ``` target    SDLSRRELLKRAVVVGTGAGLAELFLPAQFLSSASAQSEPQAVAIANPLAQMPDRSWERIYRDQFAEEDSFVFTCAPNDT 1g8k.1    -------------------------------------------------------------------NAQRTNMTCHFCI  target    HNCLLRAHVKN-----GV--------------------------------------IVRISPTYGYGKATDLAGNQASHR 1g8k.1    VGCGYHVYKWPELEEGGRAPEQNALGLDFRKQLPPLAVTLTPAMTNVVTEHDGARYDIMVVP------------DKACVV  target    WDPRICQKGLILGRRIYGD-----RRVKAPMIRKGFKEWADAGFPRHDDGTPRADMEKRGYDEWLQIPWDEALAIAAKTL 1g8k.1    NSGLSSTRGGKMASYMYTPTGDGKERLSAPRLYAA--------------------------DEWVDTTWDHAMALYAGLI  target    QNVAETYKGEDGAGKLLEQGYEPAMVEAMHGAGVQAIKMRGGMPLLGAGRVFGFYRFANMLALLDGKLRPEAPPEEIVGS 1g8k.1    KKTLDKDGPQG----VFFSCFD--------HGGA---------------------GGGFENTWGTGKLM-----FSAIQT  target    RAFDNYAW--HTDLPPGHPMVSGSQTVDFDLFAAEHSKLLVLIGMNWICTKMPD--AHWI---------------GDARL 1g8k.1    PMVRIHNRPAYNSECHA-TREMGIGELNNAYEDAQLADVIWSIGNNPYESQTNYFLNHWLPNLQGATTSKKKERFPNENF  target    KGTRVVVISADYMPTANKA--------DEIVILRPGTDTAFLLGVARELITKKLYDRDAVIQRTDLPLLVRLDTGERLSA 1g8k.1    PQARIIFVDPRETPSVAIARHVAGNDRVLHLAIEPGTDTALFNGLFTYVVEQGWIDKPFIEAHTKG--------------  target    RDVFEGYRQAPLENYVALKTEEELAAPPSPPFTADKQVVPTELREEWGDFVYWDRATNGPAAVNRDEIGAKFAGDPALLG 1g8k.1    --------------------------------------------------------------------------------  target    AFDVTLVDGTNVKARTAFSLLKEYLDENFDVQTTSEVCNVDPAAVRSLARQLAANK-----GNALLAAGMGPNHYFNADL 1g8k.1    -----------------FDDAVK-T-NRLSLDECSNITGVPVDMLKRAAEWSYKPKASGQAPRTMHAYEKGIIWGNDNYV  target    FGRVHFLVAALTDNIGHFSGNVGSYAGNYRGSLFQAMGQWIAENPFDQEADLTKPARVKRYFKSESAHYWNYGDRPLVS- 1g8k.1    IQSALLDLVIATHNVGRRGTGCVRMGGHQEGYTRP---PYPGDKKIYIDQEL-IKGKGR------IMTWW--GCNNFQTS  target    -----PSEIITGKSHMPTPTKLIWFGNSNSLLGNAKWSFDVVKNTLPK-QDAVFCNEWHWTSSCEYSDLVFPADSWAEFK 1g8k.1    NNAQALREAILQR--SAIVKQAMQKARGATTEEM----VDVIYEATQNGGLFVTSINLYPTKLAEAAHLMLPAAHPGEMN  target    LPDMTASCTNPFLLAFPKTPLARIHNTRSDYEILAGVAAALADLVDEP---RMKTYWKGILDGDPTPYLQRVLSGSNA-- 1g8k.1    L---TSMNGERRIRLSEK-FMDPPGTAMADCLIAARIANALRDMYQKDGKAEMAAQFEGFDWKTEEDAFNDGFRRAGQPG  target    -----------TRGILYEDLHASSAKGVPLLMNARTY-PRHAGWEQRQEDKPWYTPTGRLEFYRPEPEWQAAGESLPIWR 1g8k.1    APAIDSQGGSTGHLVTYDRLRKSGNNGVQLPVVSWDESKGLVGTEMLYTEGKFDTDDGKAHFKPAPW------NGLPATV  target    EPVDATFYEPNAILANSKHPSINPRAPEDYGVPESQMDVETRQYRNVVRTWQELKLSKHPLTEKDPAYRFVFQTPKYRWG 1g8k.1    QQ-------------------------------------------------------------QKDKYRFWLNNGRNNEV  target    AHSTAVD--SDWIAMLFGPFGDPYRRDSRTPWTGEAYAEINPRDAKELGLKDGDYIWLDADPEDRPYRGADSSDEFYDVA 1g8k.1    WQTAYHDQYNSLMQER----------------YPMAYIEMNPDDCKQLDVTGGDIVEVYND-----------------FG  target    RAMMRVRIYSGMPRRVIRTWFNMYAATPGTVQAQKDVPGGPAQNQDTGYVALFRHGSHQSGTRAYLRPTQMTDSMNRKAY 1g8k.1    STFAMVYPVAEIKRGQTFMLFGY---------------------------------------------------------  target    FGQTIGKGFEADVHSPSGAPKEGYVKVEKAEDGGDEGVGEWRPVTLGLRPDDPSEAMQAYLAGEFVTRKRKGS 1g8k.1    ------------------------------------------------------------------------- ``` | | | | | | | | | | | | | | | | | | | | | | | | | | | | | | | | | | | | | | | | | | | | | | | | | |
|  | 2v3v.1.A | PERIPLASMIC NITRATE REDUCTASE  *A NEW CATALYTIC MECHANISM OF PERIPLASMIC NITRATE REDUCTASE FROM DESULFOVIBRIO DESULFURICANS ATCC 27774 FROM CRYSTALLOGRAPHIC AND EPR DATA AND BASED ON DETAILED ANALYSIS OF THE SIXTH LIGAND* | 0.28 |  | 18.02 | 0.56 | 70-1037 | X-ray | 1.99 | monomer | 1 x SF4, 1 x MO, 2 x MGD, 4 x LCP | HHblits | 0.28 |
| ``` target    SDLSRRELLKRAVVVGTGAGLAELFLPAQFLSSASAQSEPQAVAIANPLAQMPDRSWERIYRDQFAEEDSFVFTCAPNDT 2v3v.1    ---------------------------------------------------------------------KWVKGVCRYCG  target    HNCLLRAHVKNGVIVRISPTYGYGKATDLAGNQASHRWDPRICQKGLILGRRIYGDRRVKAPMIRKGFKEWADAGFPRHD 2v3v.1    TGCGVLVGVKDGKAVAIQGDP------------NNH-NAGLLCLKGSLLIPVLNSKERVTQPLVRRH-------------  target    DGTPRADMEKRGYDEWLQIPWDEALAIAAKTLQNVAETYKGEDGAGKLLEQGYEPAMVEAMHGAGVQAIKMRGGMPLLGA 2v3v.1    -----------KGGKLEPVSWDEALDLMASRFRSSIDMYGPNS----VAW-----------YGSGQ--------------  target    GRVFGFYRFANMLALLDGKLRPEAPPEEIVGSRAFDNYAWHTD--LPPGHPMVSGSQTVDFDLFAAEHSKLLVLIGMNWI 2v3v.1    --------CLTEESYVANKIF-----KGGFGTNNVDGNPRLCMASAVGGYVTSFGKDEPMGTYADIDQATCFFIIGSNTS  target    CTKMPDAHWIGDAR--LKGTRVVVISADYMPTANKADEIVILRPGTDTAFLLGVARELITKKLYDRDAVIQRTDLPLLVR 2v3v.1    EAHPVLFRRIARRKQVEPGVKIIVADPRRTNTSRIADMHVAFRPGTDLAFMHSMAWVIINEELDNPRFWQRYVNFM----  target    LDTGERLSARDVFEGYRQAPLENYVALKTEEELAAPPSPPFTADKQVVPTELREEWGDFVYWDRATNGPAAVNRDEIGAK 2v3v.1    -DA-----------------------------------------------------------------------------  target    FAGDPALLGAFDVTLVDGTNVKARTAFSLLKEYLDENFDVQTTSEVCNVDPAAVRSLARQLAANKGNALLAAGMGPNHYF 2v3v.1    ----------------EG----KPSDFEGYKAFLE-NYRPEKVAEICRVPVEQIYGAARAFAESAA-TMSLWCMGINQRV  target    NADLFGRVHFLVAALTDNIGHFSGNVGSYAGN--YRGSLFQAMGQWIAENPFDQEADLTKPARVKRYFKSESAHYWNYG- 2v3v.1    QGVFANNLIHNLHLITGQICRPGATSFSLTGQPNACGGVR-DGGALSHLLPAGR--AIPNAKHRAE-----MEKLWGLPE  target    ----DRPLVSPSEIITGKSHMPTPTKLIWFGNSNSLLGNAKWSFDVVKNTLPKQD-AVFCNEWHWT-SSCEYSDLVFPAD 2v3v.1    GRIAPEPGYHTVALFEAL--GRGDVKCMIICETNPAHTLPN--LNKVHKAMSHPESFIVCIEAFPDAVTLEYADLVLPPA  target    SWAEFKLPDMTASCTNPFLLAFPKTPLARIHNTRSDYEILAGVAAALADLVDEPRMKTYWKGILDGDPTPYLQRVLSGSN 2v3v.1    FWCERDG--V-YGCGERRYSLTEK-AVDPPGQCRPTVNTLVEFARRAGVDPQ------LVN---FRNAEDVWNEWRMVSK  target    ----ATRGILYEDLHASSAKGVPLLMNARTYPR--HAGWE---------QRQEDKPWYTPTGRLEFYRPEPEWQAAGESL 2v3v.1    GTTYDFWGMTRERLRKE--SGLIWPCPSEDHPGTSLRYVRGQDPCVPADHPDRFFFYGKPDGRAVIWMRPA---------  target    PIWREPVDATFYEPNAILANSKHPSINPRAPEDYGVPESQMDVETRQYRNVVRTWQELKLSKHPLTEKDPAYRFVFQTPK 2v3v.1    -------KG-----------------------------------------------------AA-EEPDAEYPLYLTSMR  target    YRWGAHSTAV--DSDWIAMLFGPFGDPYRRDSRTPWTGEAYAEINPRDAKELGLKDGDYIWLDADPEDRPYRGADSSDEF 2v3v.1    VIDHWHTATMTGKVPELQKA----------------NPIAFVEINEEDAARTGIKHGDSVIVETR---------------  target    YDVARAMMRVRIYSGMPRRVIRTWFNMYAATPGTVQAQKDVPGGPAQNQDTGYVALFRHGSHQSGTRAYLRPTQMTDSMN 2v3v.1    --RDAMELPARVSDVCRPGLIAVPFFD-----------------------------------------------------  target    RKAYFGQTIGKGFEADVHSPSGAPKEGYVKVEKAEDGGDEGVGEWRPVTLGLRPDDPSEAMQAYLAGEFVTRKRKGS 2v3v.1    ----------------------------------------------------------------------------- ``` | | | | | | | | | | | | | | | | | | | | | | | | | | | | | | | | | | | | | | | | | | | | | | | | | |
|  | 1g8j.1.A | ARSENITE OXIDASE  *CRYSTAL STRUCTURE ANALYSIS OF ARSENITE OXIDASE FROM ALCALIGENES FAECALIS* | 0.27 |  | 16.06 | 0.56 | 68-1037 | X-ray | 2.03 | hetero-oligomer | 2 x MGD, 1 x O, 1 x 4MO, 1 x F3S, 1 x FES | HHblits | 0.28 |
| ``` target    SDLSRRELLKRAVVVGTGAGLAELFLPAQFLSSASAQSEPQAVAIANPLAQMPDRSWERIYRDQFAEEDSFVFTCAPNDT 1g8j.1    -------------------------------------------------------------------NAQRTNMTCHFCI  target    HNCLLRAHVKN-----GV--------------------------------------IVRISPTYGYGKATDLAGNQASHR 1g8j.1    VGCGYHVYKWPELEEGGRAPEQNALGLDFRKQLPPLASTLTPAMTNVVTEHDGARYDIMVVP------------DKACVV  target    WDPRICQKGLILGRRIYGD-----RRVKAPMIRKGFKEWADAGFPRHDDGTPRADMEKRGYDEWLQIPWDEALAIAAKTL 1g8j.1    NSGLSSTRGGKMASYMYTPTGDGKERLSAPRLYAA--------------------------DEWVDTTWDHAMALYAGLI  target    QNVAETYKGEDGAGKLLEQGYEPAMVEAMHGAGVQAIKMRGGMPLLGAGRVFGFYRFANMLALLDGKLRPEAPPEEIVGS 1g8j.1    KKTLDSDGPQG----VFFSCF--------DHGGAGG-GF---------ENTWGTGKLMF----------------SAIQT  target    RAFDN-----YAWHTDLPPGHPMVSGSQTVDFDLFAAEHSKLLVLIGMNWICTKMPDA--HWI---------------GD 1g8j.1    PMVRIHNRPAYNSECHA----TREMGIGELNNAYEDAQLADVIWSIGNNPYESQTNYFLNHWLPNLQGATTSKKKERFPN  target    ARLKGTRVVVISADYMPTANKA--------DEIVILRPGTDTAFLLGVARELITKKLYDRDAVIQRTDLPLLVRLDTGER 1g8j.1    ENFPQARIIFVDPRETPSVAIARHVAGNDRVLHLAIEPGTDTALFNGLFTYVVEQGWIDKPFIEAHTKG-----------  target    LSARDVFEGYRQAPLENYVALKTEEELAAPPSPPFTADKQVVPTELREEWGDFVYWDRATNGPAAVNRDEIGAKFAGDPA 1g8j.1    --------------------------------------------------------------------------------  target    LLGAFDVTLVDGTNVKARTAFSLLKEYLDENFDVQTTSEVCNVDPAAVRSLARQLAANK-----GNALLAAGMGPNHYFN 1g8j.1    --------------------FDDAVK-T-NRLSLDECSNITGVPVDMLKRAAEWSYKPKASGQAPRTMHAYEKGIIWGND  target    ADLFGRVHFLVAALTDNIGHFSGNVGSYAGNYRGSLFQAMGQWIAENPFDQEADLTKPARVKRYFKSESAHYWNYGDRPL 1g8j.1    NYVIQSALLDLVIATHNVGRRGTGCVRMGGHQEGYTRPPYPGD---KKIYIDQEL-IKGKGRI------MTWWG--CNNF  target    VS-------PSEIITGKSHMPTPTKLIWFGNSNSLLGNAKWSFDVVKNTLPKQD-AVFCNEWHWTSSCEYSDLVFPADSW 1g8j.1    QTSNNAQALREAILQR---SAIVKQAMQKARGATTEEM----VDVIYEATQNGGLFVTSINLYPTKLAEAAHLMLPAAHP  target    AEFKLPDMTASCTNPFLLAFPKTPLARIHNTRSDYEILAGVAAALADLVDEPR---MKTYWKGILDGDPTPYLQRVLSGS 1g8j.1    GEMNL---TSMNGERRIRLSEK-FMDPPGTAMADCLIAARIANALRDMYQKDGKAEMAAQFEGFDWKTEEDAFNDGFRRA  target    NA-------------TRGILYEDLHASSAKGVPLLMNAR-TYPRHAGWEQRQEDKPWYTPTGRLEFYRPEPEWQAAGESL 1g8j.1    GQPGAPAIDSQGGSTGHLVTYDRLRKSGNNGVQLPVVSWDESKGLVGTEMLYTEGKFDTDDGKAHFKPAPW------NGL  target    PIWREPVDATFYEPNAILANSKHPSINPRAPEDYGVPESQMDVETRQYRNVVRTWQELKLSKHPLTEKDPAYRFVFQTPK 1g8j.1    PATVQQ-------------------------------------------------------------QKDKYRFWLNNGR  target    YRWGAHSTAV--DSDWIAMLFGPFGDPYRRDSRTPWTGEAYAEINPRDAKELGLKDGDYIWLDADPEDRPYRGADSSDEF 1g8j.1    NNEVWQTAYHDQYNSLMQER----------------YPMAYIEMNPDDCKQLDVTGGDIVEVYND---------------  target    YDVARAMMRVRIYSGMPRRVIRTWFNMYAATPGTVQAQKDVPGGPAQNQDTGYVALFRHGSHQSGTRAYLRPTQMTDSMN 1g8j.1    --FGSTFAMVYPVAEIKRGQTFMLFGY-----------------------------------------------------  target    RKAYFGQTIGKGFEADVHSPSGAPKEGYVKVEKAEDGGDEGVGEWRPVTLGLRPDDPSEAMQAYLAGEFVTRKRKGS 1g8j.1    ----------------------------------------------------------------------------- ``` | | | | | | | | | | | | | | | | | | | | | | | | | | | | | | | | | | | | | | | | | | | | | | | | | |
|  | 6sdr.1.A | Formate dehydrogenase, alpha subunit, selenocysteine-containing  *W-formate dehydrogenase from Desulfovibrio vulgaris - Oxidized form* | 0.27 |  | 17.88 | 0.51 | 1-756 | X-ray | 2.10 | hetero-1-1-mer | 2 x MGD, 4 x SF4, 1 x H2S, 1 x W | HHblits | 0.29 |
| ``` target    SDLSRRELLKRAVVVGTGAGLAELFLPAQFLSSASAQSEPQAVAIANPLAQMPDRSWERIYRDQFAEEDSFVFTCAPNDT 6sdr.1    MTVTRRHFLKLSAGAAVAGAFTGLGLSL------------------APTVARAEL-----QK--LQWA-KQTTSICCYCA  target    HNCLLRAHVK---NGVIVRISPTYGYGKATDLAGNQASHRWDPRICQKGLILGRRIYGDRRVKAPMIRKGFKEWADAGFP 6sdr.1    VGCGLIVHTAKDGQGRAVNVEGD------------PDHPINEGSLCPKGASIFQLGENDQRGTQPLYRAP----------  target    RHDDGTPRADMEKRGYDEWLQIPWDEALAIAAKTLQNVAETYKGEDGAGKLLEQGYEPAMVEAMHGAGVQAIKMRGGMPL 6sdr.1    --------------FSDTWKPVTWDFALTEIAKRIKKTRDASFTEKNAAGDLVNR--TEAIAS-----------------  target    LGAGRVFGFYRFANMLALLDGKLRPEAPPEEIVGSRAFDNYAWHTDL--PPGHPMVSGSQTVDFDLFAAEHSKLLVLIGM 6sdr.1    ------FGSAAMDNEECWAYGNILR------SLGLVYIEHQARIUHSPTVPALAESFGRGAMTNHWNDLANSDCILIMGS  target    NWICTKMPDAHWIGDARLKGTRVVVISADYMPTANKADEIVILRPGTDTAFLLGVARELITKKLYDRDAVIQRTDLPLLV 6sdr.1    NAAENHPIAFKWVLRAKDKGATLIHVDPRFTRTSARCDVYAPIRSGADIPFLGGLIKYILDNKLYFTDYVREYTNASLIV  target    RLDTGERLSARDVFEGYRQAPLENYVALKTEEELAAPPSPPFTADKQVVPTELREEWGDFVYWDRATNGPAAVNRDEIGA 6sdr.1    GEKFS---FKDGLFSGYDAA-N--------------------------------KKYDK-SMWAFEL-------------  target    KFAGDPALLGAFDVTLVDGTNVKARTAFSLLKEYLDENFDVQTTSEVCNVDPAAVRSLARQLAANK---GNALLAAGMGP 6sdr.1    ------DANG---VPKRDPALKHPRCVINLLKKHYE-RYNLDKVAAITGTSKEQLQQVYKAYAATGKPDKAGTIMYAMGW  target    NHYFNADLFGRVHFLVAALTDNIGHFSGNVGSYAGNYRGSLFQAMGQWI----AENPFDQEA-----DL-------TKPA 6sdr.1    TQHSVGVQNIRAMAMIQLLLGNIGVAGGGVNALRGESNVQGSTDQGLLAHIWPGYNPVPNSKAATLELYNAATPQSKDPM  target    RVKRYFKSESAHYWN------YG-DRP---------------L--VSPSEIITGKSHMPTPTKLIWFGNSNSLLGNAKWS 6sdr.1    SVN-WWQ-NRPKYVASYLKALYPDEEPAAAYDYLPRIDAGRKLTDYFWLNIFEK--MDKGEFKGLFAWGMNPACGGAN--  target    FDVVKNTLPKQDAVFCNEWHWTSSCEY--------SD-----LVFPADSWAEFKLPDMTASCTNPFLLAFPKTPLARIHN 6sdr.1    ANKNRKAMGKLEWLVNVNLFENETSSFWKGPGMNPAEIGTEVFFLPCCVSIEKE--GSVA-NSGRWMQWRYR-GPKPYAE  target    TRSDYEILAGVAAALADLVDEPRMKTYWKGILDGDPTPYLQRVLSGSNATRGILYEDLHASSAKGVPLLMNARTYPRHAG 6sdr.1    TKPDGDIMLDMFKKVRE---------------------------------------------------------------  target    WEQRQEDKPWYTPTGRLEFYRPEPEWQAAGESLPIWREPVDATFYEPNAILANSKHPSINPRAPEDYGVPESQMDVETRQ 6sdr.1    --------------------------------------------------------------------------------  target    YRNVVRTWQELKLSKHPLTEKDPAYRFVFQTPKYRWGAHSTAVDSDWIAMLFGPFGDPYRRDSRTPWTGEAYAEINPRDA 6sdr.1    --------------------------------------------------------------------------------  target    KELGLKDGDYIWLDADPEDRPYRGADSSDEFYDVARAMMRVRIYSGMPRRVIRTWFNMYAATPGTVQAQKDVPGGPAQNQ 6sdr.1    --------------------------------------------------------------------------------  target    DTGYVALFRHGSHQSGTRAYLRPTQMTDSMNRKAYFGQTIGKGFEADVHSPSGAPKEGYVKVEKAEDGGDEGVGEWRPVT 6sdr.1    --------------------------------------------------------------------------------  target    LGLRPDDPSEAMQAYLAGEFVTRKRKGS 6sdr.1    ---------------------------- ``` | | | | | | | | | | | | | | | | | | | | | | | | | | | | | | | | | | | | | | | | | | | | | | | | | |
|  | 6f0k.1.B | Fe-S-cluster-containing hydrogenase  *Alternative complex III* | 0.21 |  | 16.47 | 0.51 | 2-1041 | EM | 0.00 | hetero-1-1-1-1-1-1-… | 6 x HEC, 1 x F3S, 3 x SF4 | HHblits | 0.27 |
| ``` target    SDLSRRELLKRAVVVGTGAGLAELFLPAQFLSSASAQSEPQAVAIANPLAQMPDRSWERIYRDQFAEEDSFVFTCAPNDT 6f0k.1    -GTSRRQFLQIMGASMALAGLTACRRPVE---------------KILPYVRQ----PEE----IIPGIPLYYATAMPFRG  target    HNCLLRAHVKNGVIVRISPTYGYGKATDLAGNQASHRWDPRICQKGLILGRRIYGDRRVKAPMIRKGFKEWADAGFPRHD 6f0k.1    SVRPLLVESHEGRPTKIEG------------NPDHPLSRGATGVFEQASLLNLYDPDRSQQVLRK-G-------------  target    DGTPRADMEKRGYDEWLQIPWDEALAIAAKTLQNVAETYKGEDGAGKLLEQGYEPAMVEAMHGAGVQAIKMRGGMPLLGA 6f0k.1    ----------------EPASWGDFVQFARSLA----AEAGTKRL--AVL------------CEPSSS--------PTLA-  target    GRVFGFYRFANMLALLDGKLRPEAPPEEIVGSRAFDNYAWHTD--LPPGHPMVSGSQTVDFDLFAAEHSKLLVLIGMNWI 6f0k.1    ---ALRRELERRY-----------------AQVRWVTYRPEGDDHEALGLQQAFGRPVR--ARYRFSEARVIVSLDADFL  target    CTK-MPD---A------HWIGDARLKGTRVVVISADYMPTANKADEIVILRPGTDTAFLLGVARELITKKLYDRDAVIQR 6f0k.1    GPTDRNFVENTREFAASRRMERPEDEISRLYVIESTYTVTGGMADHRLRLRAGDIPAFAAALAAELGVGELRE-------  target    TDLPLLVRLDTGERLSARDVFEGYRQAPLENYVALKTEEELAAPPSPPFTADKQVVPTELREEWGDFVYWDRATNGPAAV 6f0k.1    --------------------------------------------------------------------------------  target    NRDEIGAKFAGDPALLGAFDVTLVDGTNVKARTAFSLLKEYLDENFDVQTTSEVCNVDPAAVRSLARQLAANKGNALLAA 6f0k.1    -------------------------------------------------AGARFAGH--PYVVEIARDLRAAGARGVVLA  target    GMGPNHYFNADLFGRVHFLVAALTDNIGHFSGNVGSYAGNYRGSLFQAMGQWIAENPFDQEADLTKPARVKRYFKSESAH 6f0k.1    GETQ---PP--AVHALCAVINDLLGSLGRTVILH-AL------------D----EPATA------Q---H----------  target    YWNYGDRPLVSPSEIITGKSHMPTPTKLIWFGNSNSLLGNAKWSFDVVKNTLPKQDAVFCNEWHWTSSCEYSDLVFPADS 6f0k.1    ---------AALAELVQAM--QAGAVDALLLLNVNPVYDAPA--ALGFAEALAQVPEVIHLGLHVDETARRSTWHLPSTH  target    WAEFKLPDMTASCTNPFLLAFPKTPLARIHNT-RSDYEILAGVAAALADLVDEPRMKTYWKGILDGDPTPYLQRVLSGSN 6f0k.1    YLEAWGD--GRAY-DGTLSV-IQPLIAPLYEAAHSPLEVLALLATGEEQSA--------YD-----LVRNTWRRLLAGRG  target    ATRGILYEDLHASSAKGVPLLMNARTYPRHAGWEQRQEDKPWYTPTGRLEFYRPEPEWQAAGESLPIWREPVDATFYEPN 6f0k.1    A-FEQAWQRVLH---DGFL------------------PDSGYPTVSLRPNR-----------QALADWPQ----------  target    AILANSKHPSINPRAPEDYGVPESQMDVETRQYRNVVRTWQELKLSKHPLTEKDPAYRFVFQTPKYRWGAHSTAVDSDWI 6f0k.1    -----------------------------------------------------AAEGGLEVVFRLDPTVLDGSFANNAWA  target    AMLFGPFGDPYRRDSRTPWTGEAYAEINPRDAKELGLKD--------GDYIWLDADPEDRPYRGADSSDEFYDVARAMMR 6f0k.1    QELPDPIT---------KIVWDNVAILSPKTAAALGVKAEYHKGVYIADVIELSLD-----------------GRAVELP  target    VRIYSGMPRRVIRTWFNMYAATPGTVQAQKDVPGGPAQNQDTGYVALFRHGSHQSGTRAYLRPTQMTDSMNRKAYFGQTI 6f0k.1    VWVLPGHPDDSITVYLGYGREI----------------------------------------------------------  target    GKGFEADVHSPSGAPKEGYVKVEKAEDGGDEGVGEWRPVTLGLRPDDPSEAMQAYLAGEFVTRKRKGS 6f0k.1    -------------------------------------------------------------------- ``` | | | | | | | | | | | | | | | | | | | | | | | | | | | | | | | | | | | | | | | | | | | | | | | | | |
|  | 7p61.1.C | NADH-quinone oxidoreductase  *Complex I from E. coli, DDM-purified, with NADH, Resting state* | 0.25 |  | 16.27 | 0.51 | 67-1039 | EM | 0.00 | hetero-1-1-1-1-1-1-… | 7 x SF4, 1 x FMN, 1 x NAI, 2 x FES, 1 x CA, 2 x 3PE, 1 x UQ8 | HHblits | 0.27 |
| ``` target    SDLSRRELLKRAVVVGTGAGLAELFLPAQFLSSASAQSEPQAVAIANPLAQMPDRSWERIYRDQFAEEDSFVFTCAPNDT 7p61.1    ------------------------------------------------------------------WDMQFAPSICQQCS  target    HNCLLRAHVKNGVIVRISPTYGYGKATDLAGNQASHRWDPRICQKGLILGRRIYGDRRVKAPMIRKGFKEWADAGFPRHD 7p61.1    IGCNISPGERYGELRRIENR------------YNGTVNHYFLCDRGRFGYGYVNLKDRPRQPVQRRG-------------  target    DGTPRADMEKRGYDEWLQIPWDEALAIAAKTLQNVAETYKGEDGAGKLLEQGYEPAMVEAMHGAGVQAIKMRGGMPLLGA 7p61.1    -------------DDFITLNAEQAMQGAADILRQSKKVIGIG--------S-----------------------------  target    GRVFGFYRFANMLALLDGKLRPEAPPEEIVGSRAFDNYAWHTDL---PPGHPMVSGSQTVDFDLFAAEHSKLLVLIGMNW 7p61.1    ------PRASVESNFALREL---------VGEENFYTGIAHGEQERLQLALKVLREGGIYTPALREIESYDAVLVLGEDV  target    ICTKMPDAHWIGDARLKGTR--------------------------VVVISADYMPTANKADEIVILRPGTDTAFLLGVA 7p61.1    TQTGARVALAVRQAVKGKAREMAAAQKVADWQIAAILNIGQRAKHPLFVTNVDDTRLDDIAAWTYRAPVEDQARLGFAIA  target    RELITKKLYDRDAVIQRTDLPLLVRLDTGERLSARDVFEGYRQAPLENYVALKTEEELAAPPSPPFTADKQVVPTELREE 7p61.1    HALDNSAP------------------------------------------------------------------------  target    WGDFVYWDRATNGPAAVNRDEIGAKFAGDPALLGAFDVTLVDGTNVKARTAFSLLKEYLDENFDVQTTSEVCNVDPAAVR 7p61.1    ---------------------------------------------------------AV-DGIEPEL--------QSKID  target    SLARQLAANKGNALLAAGMGPNHYFNADLFGRVHFLVAALTDNIGHFSGNVGSYAG-NYRGSLFQAMGQWIAENPFDQEA 7p61.1    VIVQALAGAKK-PLIISGTNAG----SLEVIQAAANVAKALKGRGADVGITMIARSVNSMGL-----------GIMG---  target    DLTKPARVKRYFKSESAHYWNYGDRPLVSPSEIITGKSHMPTPTKLIWFGNSNSLLGNAKWSFDVVKNTLPKQDAVFCNE 7p61.1    --------------------------GGSLEEALTEL--ETGRADAVVVLE-NDLHRHAS--ATRVNAALAKAPLVMVVD  target    WHWTSSCEYSDLVFPADSWAEFKLPDMTASCTNPFLLAFPKTPLARIH-----NTRSDYEILAGVAAALADLVDEPRMKT 7p61.1    HQRTAIMENAHLVLSAASFAESDGTV---INNEGRAQRFFQ-VYDPAYYDSKTVMLESWRWLHSLHSTLLSRE------V  target    YWKGILDGDPTPYLQRVLSGSNATRGILYEDLHAS----S------------------AKGV--PLLM------------ 7p61.1    DWT-----QLDHVIDAVVAKIPELAGIKDAAPDATFRIRGQKLAREPHRYSGRTAMRANISVHEPRQPQDIDTMFTFSME  target    ------NA-RTYP--RHAGWE-QRQEDKPWYTPTGRLEFYRPEPEWQA-AGESLPIWREPVDATFYEPNAILANSKHPSI 7p61.1    GNNQPTAHRSQVPFAWAPGWNSPQAWNKFQDEVGGKLRFGDPGVRLFETSENGLDYFTSVPA------------------  target    NPRAPEDYGVPESQMDVETRQYRNVVRTWQELKLSKHPLTEKDPAYRFVFQTPKYRWGAHSTAVDSDWIAMLFGPFGDPY 7p61.1    ---------------------------------------RFQPQDGKWRIAPYYHLFGSDELSQRAPVFQSRM-------  target    RRDSRTPWTGEAYAEINPRDAKELGLKDGDYIWLDADPEDRPYRGADSSDEFYDVARAMMRVRIYSGMPRRVIRTWFNMY 7p61.1    ---------PQPYIKLNPADAAKLGVNAGTRVSFSYD-----------------GNTVTLPVEIAEGLTAGQVGLPMGMS  target    AATPGTVQAQKDVPGGPAQNQDTGYVALFRHGSHQSGTRAYLRPTQMTDSMNRKAYFGQTIGKGFEADVHSPSGAPKEGY 7p61.1    G-------------------------------------------------------------------------------  target    VKVEKAEDGGDEGVGEWRPVTLGLRPDDPSEAMQAYLAGEFVTRKRKGS 7p61.1    ------------------------------------------------- ``` | | | | | | | | | | | | | | | | | | | | | | | | | | | | | | | | | | | | | | | | | | | | | | | | | |
|  | 7nz1.1.E | NADH-quinone oxidoreductase subunit G  *Respiratory complex I from Escherichia coli - focused refinement of cytoplasmic arm* | 0.25 |  | 16.13 | 0.50 | 68-1039 | EM | 0.00 | hetero-1-1-1-1-1-1-… | 7 x SF4, 2 x FES, 1 x FMN, 1 x CA | HHblits | 0.27 |
| ``` target    SDLSRRELLKRAVVVGTGAGLAELFLPAQFLSSASAQSEPQAVAIANPLAQMPDRSWERIYRDQFAEEDSFVFTCAPNDT 7nz1.1    -------------------------------------------------------------------DMQFAPSICQQCS  target    HNCLLRAHVKNGVIVRISPTYGYGKATDLAGNQASHRWDPRICQKGLILGRRIYGDRRVKAPMIRKGFKEWADAGFPRHD 7nz1.1    IGCNISPGERYGELRRIENR------------YNGTVNHYFLCDRGRFGYGYVNLKDRPRQPVQRRG-------------  target    DGTPRADMEKRGYDEWLQIPWDEALAIAAKTLQNVAETYKGEDGAGKLLEQGYEPAMVEAMHGAGVQAIKMRGGMPLLGA 7nz1.1    -------------DDFITLNAEQAMQGAADILRQSKKVIGIGS-------------------------------------  target    GRVFGFYRFANMLALLDGKLRPEAPPEEIVGSRAFDNYAWHTD---LPPGHPMVSGSQTVDFDLFAAEHSKLLVLIGMNW 7nz1.1    ------PRASVESNFALREL---------VGEENFYTGIAHGEQERLQLALKVLREGGIYTPALREIESYDAVLVLGEDV  target    ICTKMPDAHWIGDARLKGTR--------------------------VVVISADYMPTANKADEIVILRPGTDTAFLLGVA 7nz1.1    TQTGARVALAVRQAVKGKAREMAAAQKVADWQIAAILNIGQRAKHPLFVTNVDDTRLDDIAAWTYRAPVEDQARLGFAIA  target    RELITKKLYDRDAVIQRTDLPLLVRLDTGERLSARDVFEGYRQAPLENYVALKTEEELAAPPSPPFTADKQVVPTELREE 7nz1.1    HALDNSAP------------------------------------------------------------------------  target    WGDFVYWDRATNGPAAVNRDEIGAKFAGDPALLGAFDVTLVDGTNVKARTAFSLLKEYLDENFDVQTTSEVCNVDPAAVR 7nz1.1    ---------------------------------------------------------AV-DGIEPEL--------QSKID  target    SLARQLAANKGNALLAAGMGPNHYFNADLFGRVHFLVAALTDNIGHFSGNVGSYAG-NYRGSLFQAMGQWIAENPFDQEA 7nz1.1    VIVQALAGAKK-PLIISGTNAG----SLEVIQAAANVAKALKGRGADVGITMIARSVNSMGLG-----------IMG---  target    DLTKPARVKRYFKSESAHYWNYGDRPLVSPSEIITGKSHMPTPTKLIWFGNSNSLLGNAKWSFDVVKNTLPKQDAVFCNE 7nz1.1    --------------------------GGSLEEALTEL--ETGRADAVVVLE-NDLHRHAS--AIRVNAALAKAPLVMVVD  target    WHWTSSCEYSDLVFPADSWAEFKLPDMTASCTNPFLLAFPKTPLARIH-----NTRSDYEILAGVAAALADLVDEPRMKT 7nz1.1    HQRTAIMENAHLVLSAASFAESDGTV---INNEGRAQRFFQ-VYDPAYYDSKTVMLESWRWLHSLHSTLLSREV------  target    YWKGILDGDPTPYLQRVLSGSNATRGILYEDL------------------------HASSAKGVPLL-------MN---- 7nz1.1    DWT-----QLDHVIDAVVAKIPELAGIKDAAPDATFRIRGQKLAREPHRYSGRTAMRANISVHEPRQPQDIDTMFTFSME  target    --------A--RTYPRHAGWE-QRQEDKPWYTPTGRLEFYRPEPEWQ-AAGESLPIWREPVDATFYEPNAILANSKHPSI 7nz1.1    GNNQPTAHRSQVPFAWAPGWNSPQAWNKFQDEVGGKLRFGDPGVRLFETSENGLDYFTSVPAR-----------------  target    NPRAPEDYGVPESQMDVETRQYRNVVRTWQELKLSKHPLTEKDPAYRFVFQTPKYRWGAHSTAVDSDWIAMLFGPFGDPY 7nz1.1    ----------------------------------------FQPQDGKWRIAPYYHLFGSDELSQRAPVFQSRM-------  target    RRDSRTPWTGEAYAEINPRDAKELGLKDGDYIWLDADPEDRPYRGADSSDEFYDVARAMMRVRIYSGMPRRVIRTWFNMY 7nz1.1    ---------PQPYIKLNPADAAKLGVNAGTRVSFSYD-----------------GNTVTLPVEIAEGLTAGQVGLPMGMS  target    AATPGTVQAQKDVPGGPAQNQDTGYVALFRHGSHQSGTRAYLRPTQMTDSMNRKAYFGQTIGKGFEADVHSPSGAPKEGY 7nz1.1    G-------------------------------------------------------------------------------  target    VKVEKAEDGGDEGVGEWRPVTLGLRPDDPSEAMQAYLAGEFVTRKRKGS 7nz1.1    ------------------------------------------------- ``` | | | | | | | | | | | | | | | | | | | | | | | | | | | | | | | | | | | | | | | | | | | | | | | | | |
|  | 7p63.1.C | NADH-quinone oxidoreductase  *Complex I from E. coli, DDM/LMNG-purified, under Turnover at pH 6, Closed state* | 0.25 |  | 16.13 | 0.50 | 68-1039 | EM | 0.00 | hetero-1-1-1-1-1-1-… | 7 x SF4, 1 x FMN, 1 x NAI, 2 x FES, 1 x CA, 1 x DCQ, 4 x LFA, 8 x 3PE | HHblits | 0.27 |
| ``` target    SDLSRRELLKRAVVVGTGAGLAELFLPAQFLSSASAQSEPQAVAIANPLAQMPDRSWERIYRDQFAEEDSFVFTCAPNDT 7p63.1    -------------------------------------------------------------------DMQFAPSICQQCS  target    HNCLLRAHVKNGVIVRISPTYGYGKATDLAGNQASHRWDPRICQKGLILGRRIYGDRRVKAPMIRKGFKEWADAGFPRHD 7p63.1    IGCNISPGERYGELRRIENR------------YNGTVNHYFLCDRGRFGYGYVNLKDRPRQPVQRRG-------------  target    DGTPRADMEKRGYDEWLQIPWDEALAIAAKTLQNVAETYKGEDGAGKLLEQGYEPAMVEAMHGAGVQAIKMRGGMPLLGA 7p63.1    -------------DDFITLNAEQAMQGAADILRQSKKVIGIGS-------------------------------------  target    GRVFGFYRFANMLALLDGKLRPEAPPEEIVGSRAFDNYAWHTDL---PPGHPMVSGSQTVDFDLFAAEHSKLLVLIGMNW 7p63.1    ------PRASVESNFALREL---------VGEENFYTGIAHGEQERLQLALKVLREGGIYTPALREIESYDAVLVLGEDV  target    ICTKMPDAHWIGDARLKGTR--------------------------VVVISADYMPTANKADEIVILRPGTDTAFLLGVA 7p63.1    TQTGARVALAVRQAVKGKAREMAAAQKVADWQIAAILNIGQRAKHPLFVTNVDDTRLDDIAAWTYRAPVEDQARLGFAIA  target    RELITKKLYDRDAVIQRTDLPLLVRLDTGERLSARDVFEGYRQAPLENYVALKTEEELAAPPSPPFTADKQVVPTELREE 7p63.1    HALDNSAP------------------------------------------------------------------------  target    WGDFVYWDRATNGPAAVNRDEIGAKFAGDPALLGAFDVTLVDGTNVKARTAFSLLKEYLDENFDVQTTSEVCNVDPAAVR 7p63.1    ---------------------------------------------------------AV-DGIEPEL--------QSKID  target    SLARQLAANKGNALLAAGMGPNHYFNADLFGRVHFLVAALTDNIGHFSGNVGSYAG-NYRGSLFQAMGQWIAENPFDQEA 7p63.1    VIVQALAGAKKP-LIISGTNAG----SLEVIQAAANVAKALKGRGADVGITMIARSVNSMGLG-----------IMG---  target    DLTKPARVKRYFKSESAHYWNYGDRPLVSPSEIITGKSHMPTPTKLIWFGNSNSLLGNAKWSFDVVKNTLPKQDAVFCNE 7p63.1    --------------------------GGSLEEALTEL--ETGRADAVVVLE-NDLHRHAS--ATRVNAALAKAPLVMVVD  target    WHWTSSCEYSDLVFPADSWAEFKLPDMTASCTNPFLLAFPKTPLARIH-----NTRSDYEILAGVAAALADLVDEPRMKT 7p63.1    HQRTAIMENAHLVLSAASFAESDGT---VINNEGRAQRFFQ-VYDPAYYDSKTVMLESWRWLHSLHSTLLSRE------V  target    YWKGILDGDPTPYLQRVLSGSNATRGILYEDLHAS-----------S-----------AKGV--PLLMN----------- 7p63.1    DW-----TQLDHVIDAVVAKIPELAGIKDAAPDATFRIRGQKLAREPHRYSGRTAMRANISVHEPRQPQDIDTMFTFSME  target    ----------ARTYPRHAGWEQR-QEDKPWYTPTGRLEFYRPEPEWQA-AGESLPIWREPVDATFYEPNAILANSKHPSI 7p63.1    GNNQPTAHRSQVPFAWAPGWNSPQAWNKFQDEVGGKLRFGDPGVRLFETSENGLDYFTSVPA------------------  target    NPRAPEDYGVPESQMDVETRQYRNVVRTWQELKLSKHPLTEKDPAYRFVFQTPKYRWGAHSTAVDSDWIAMLFGPFGDPY 7p63.1    ---------------------------------------RFQPQDGKWRIAPYYHLFGSDELSQRAPVFQSRM-------  target    RRDSRTPWTGEAYAEINPRDAKELGLKDGDYIWLDADPEDRPYRGADSSDEFYDVARAMMRVRIYSGMPRRVIRTWFNMY 7p63.1    ---------PQPYIKLNPADAAKLGVNAGTRVSFSYD-----------------GNTVTLPVEIAEGLTAGQVGLPMGMS  target    AATPGTVQAQKDVPGGPAQNQDTGYVALFRHGSHQSGTRAYLRPTQMTDSMNRKAYFGQTIGKGFEADVHSPSGAPKEGY 7p63.1    G-------------------------------------------------------------------------------  target    VKVEKAEDGGDEGVGEWRPVTLGLRPDDPSEAMQAYLAGEFVTRKRKGS 7p63.1    ------------------------------------------------- ``` | | | | | | | | | | | | | | | | | | | | | | | | | | | | | | | | | | | | | | | | | | | | | | | | | |
|  | 8bqg.1.A | Formate dehydrogenase, alpha subunit, selenocysteine-containing  *W-formate dehydrogenase from Desulfovibrio vulgaris - Soaking with Formate 1 min* | 0.26 |  | 17.24 | 0.48 | 54-756 | X-ray | 1.95 | hetero-1-1-mer | 2 x MGD, 4 x SF4, 1 x H2S, 1 x W | HHblits | 0.28 |
| ``` target    SDLSRRELLKRAVVVGTGAGLAELFLPAQFLSSASAQSEPQAVAIANPLAQMPDRSWERIYRDQFAEEDSFVFTCAPNDT 8bqg.1    -----------------------------------------------------EL-----QKL--QWA-KQTTSICCYCA  target    HNCLLRAHVK---NGVIVRISPTYGYGKATDLAGNQASHRWDPRICQKGLILGRRIYGDRRVKAPMIRKGFKEWADAGFP 8bqg.1    VGCGLIVHTAKDGQGRAVNVEGD------------PDHPINEGSLCPKGASIFQLGENDQRGTQPLYRAP----------  target    RHDDGTPRADMEKRGYDEWLQIPWDEALAIAAKTLQNVAETYKGEDGAGKLLEQGYEPAMVEAMHGAGVQAIKMRGGMPL 8bqg.1    --------------FSDTWKPVTWDFALTEIAKRIKKTRDASFTEKNAAGDLVNR--TEAIASF----------------  target    LGAGRVFGFYRFANMLALLDGKLRPEAPPEEIVGSRAFDNYAWHTDL--PPGHPMVSGSQTVDFDLFAAEHSKLLVLIGM 8bqg.1    -------GSAAMDNEECWAYGNILR------SLGLVYIEHQARIUHSPTVPALAESFGRGAMTNHWNDLANSDCILIMGS  target    NWICTKMPDAHWIGDARLKGTRVVVISADYMPTANKADEIVILRPGTDTAFLLGVARELITKKLYDRDAVIQRTDLPLLV 8bqg.1    NAAENHPIAFKWVLRAKDKGATLIHVDPRFTRTSARCDVYAPIRSGADIPFLGGLIKYILDNKLYFTDYVREYTNASLIV  target    RLDTGERLSARDVFEGYRQAPLENYVALKTEEELAAPPSPPFTADKQVVPTELREEWGDF-VYWDRATNGPAAVNRDEIG 8bqg.1    GEKFS---FKDGLFSGYDAA---------------------------------NKKYDKSMWAFELDAN-----------  target    AKFAGDPALLGAFDVTLVDGTNVKARTAFSLLKEYLDENFDVQTTSEVCNVDPAAVRSLARQLAANK---GNALLAAGMG 8bqg.1    ----------G---VPKRDPALKHPRCVINLLKKHYE-RYNLDKVAAITGTSKEQLQQVYKAYAATGKPDKAGTIMYAMG  target    PNHYFNADLFGRVHFLVAALTDNIGHFSGNVGSYAGNYR--GSLFQAMGQWI----AENPFDQEAD-----L--TKPAR- 8bqg.1    WTQHSVGVQNIRAMAMIQLLLGNIGVAGGGVNALRGESNVQGST--DQGLLAHIWPGYNPVPNSKAATLELYNAATPQSK  target    ---VKRYFKSES-------AHYWNY--G-----DRP---------LVSPSEIITGKSHMPTPTKLIWFGNSNSLLGNAKW 8bqg.1    DPMSVNWWQNRPKYVASYLKALYPDEEPAAAYDYLPRIDAGRKLTDYFWLNIFEKM--DKGEFKGLFAWGMNPACGGAN-  target    SFDVVKNTLPKQDAVFCNEWHWTSSCEY--------SD-----LVFPADSWAEFKLPDMTASCTNPFLLAFPKTPLARIH 8bqg.1    -ANKNRKAMGKLEWLVNVNLFENETSSFWKGPGMNPAEIGTEVFFLPCCVSIEKE--GSVA-NSGRWMQWRYR-GPKPYA  target    NTRSDYEILAGVAAALADLVDEPRMKTYWKGILDGDPTPYLQRVLSGSNATRGILYEDLHASSAKGVPLLMNARTYPRHA 8bqg.1    ETKPDGDIMLDMFKKVRE--------------------------------------------------------------  target    GWEQRQEDKPWYTPTGRLEFYRPEPEWQAAGESLPIWREPVDATFYEPNAILANSKHPSINPRAPEDYGVPESQMDVETR 8bqg.1    --------------------------------------------------------------------------------  target    QYRNVVRTWQELKLSKHPLTEKDPAYRFVFQTPKYRWGAHSTAVDSDWIAMLFGPFGDPYRRDSRTPWTGEAYAEINPRD 8bqg.1    --------------------------------------------------------------------------------  target    AKELGLKDGDYIWLDADPEDRPYRGADSSDEFYDVARAMMRVRIYSGMPRRVIRTWFNMYAATPGTVQAQKDVPGGPAQN 8bqg.1    --------------------------------------------------------------------------------  target    QDTGYVALFRHGSHQSGTRAYLRPTQMTDSMNRKAYFGQTIGKGFEADVHSPSGAPKEGYVKVEKAEDGGDEGVGEWRPV 8bqg.1    --------------------------------------------------------------------------------  target    TLGLRPDDPSEAMQAYLAGEFVTRKRKGS 8bqg.1    ----------------------------- ``` | | | | | | | | | | | | | | | | | | | | | | | | | | | | | | | | | | | | | | | | | | | | | | | | | |
| ✓ | 1h0h.1.A | FORMATE DEHYDROGENASE SUBUNIT ALPHA  *Tungsten containing Formate Dehydrogenase from Desulfovibrio Gigas* | 0.25 | 0.00 | 17.09 | 0.47 | 69-756 | X-ray | 1.80 | monomer | 1 x W, 1 x 2MD, 1 x MGD, 4 x SF4, 1 x CA | HHblits | 0.28 |
| ``` target    SDLSRRELLKRAVVVGTGAGLAELFLPAQFLSSASAQSEPQAVAIANPLAQMPDRSWERIYRDQFAEEDSFVFTCAPNDT 1h0h.1    --------------------------------------------------------------------AKQTTSVCCYCS  target    HNCLLRAHV--KNGVIVRISPTYGYGKATDLAGNQASHRWDPRICQKGLILGRRIYGDRRVKAPMIRKGFKEWADAGFPR 1h0h.1    VGCGLIVHTDKKTNRAINVEGD------------PDHPINEGSLCAKGASTWQLAENERRPANPLYRA------------  target    HDDGTPRADMEKRGYDEWLQIPWDEALAIAAKTLQNVAETYKGEDGAG-KLLEQGYEPAMVEAMHGAGVQAIKMRGGMPL 1h0h.1    ------------PGSDQWEEKSWDWMLDTIAERVAKTREATFVTKNAKGQVVNRC--------------DGIA-------  target    LGAGRVFGFYRFANMLALLDGKLRPEAPPEEIVGSRAFDNYAWHTDLP--PGHPMVSGSQTVDFDLFAAEHSKLLVLIGM 1h0h.1    -----SVGSAAMDNEECWIYQAWLR------SLGLFYIEHQARIUHSATVAALAESYGRGAMTNHWIDLKNSDVILMMGS  target    NWICTKMPDAHWIGDARLKGTRVVVISADYMPTANKADEIVILRPGTDTAFLLGVARELITKKLYDRDAVIQRTDLPLLV 1h0h.1    NPAENHPISFKWVMRAKDKGATLIHVDPRYTRTSTKCDLYAPLRSGSDIAFLNGMTKYILEKELYFKDYVVNYTNASFIV  target    RLDTGERLSARDVFEGYRQAPLENYVALKTEEELAAPPSPPFTADKQVVPTELREEWGDFVYWDRATNGPAAVNRDEIGA 1h0h.1    GEGF---AFEEGLF----------------------------------------------AGYNKETRKYDKSKW-----  target    KFAGDPALLGAFDVTLVDGTNVKARTAFSLLKEYLDENFDVQTTSEVCNVDPAAVRSLARQLAANK---GNALLAAGMGP 1h0h.1    --GFERDENGNP---KRDETLKHPRCVFQIMKKHYE-RYDLDKISAICGTPKELILKVYDAYCATGKPDKAGTIMYAMGW  target    NHYFNADLFGRVHFLVAALTDNIGHFSGNVGSYAGNY--RGSLFQAMGQWIAENPFDQE---------A----DL----T 1h0h.1    TQHTVGVQNIRAMSINQLLLGNIGVAGGGVNALRGEANVQGST--DHGLLMHIYPGYLGTARASIPTYEEYTKKFTPVSK  target    KPARVKRYFKS------ESAHYWNYGDR-------------PLVSPSEIITGKSHMPTPTKLIWFGNSNSLLGNAKWSFD 1h0h.1    DPQSANWWSNFPKYSASYIKSMWPDADLNEAYGYLPKGEDGKDYSWLTLFDDM--FQGKIKGFFAWGQNPACSGAN--SN  target    VVKNTLPKQDAVFCNEWHWTSSCEYS-------------DLVFPADSWAEFKLPDMTASCTNPFLLAFPKTPLARIHNTR 1h0h.1    KTREALTKLDWMVNVNIFDNETGSFWRGPDMDPKKIKTEVFFLPCAVAIEKE--GSI-SNSGRWMQWRYV-GPEPRKNAI  target    SDYEILAGVAAALADLVDEPRMKTYWKGILDGDPTPYLQRVLSGSNATRGILYEDLHASSAKGVPLLMNARTYPRHAGWE 1h0h.1    PDGDLIVELAKRVQK-----------------------------------------------------------------  target    QRQEDKPWYTPTGRLEFYRPEPEWQAAGESLPIWREPVDATFYEPNAILANSKHPSINPRAPEDYGVPESQMDVETRQYR 1h0h.1    --------------------------------------------------------------------------------  target    NVVRTWQELKLSKHPLTEKDPAYRFVFQTPKYRWGAHSTAVDSDWIAMLFGPFGDPYRRDSRTPWTGEAYAEINPRDAKE 1h0h.1    --------------------------------------------------------------------------------  target    LGLKDGDYIWLDADPEDRPYRGADSSDEFYDVARAMMRVRIYSGMPRRVIRTWFNMYAATPGTVQAQKDVPGGPAQNQDT 1h0h.1    --------------------------------------------------------------------------------  target    GYVALFRHGSHQSGTRAYLRPTQMTDSMNRKAYFGQTIGKGFEADVHSPSGAPKEGYVKVEKAEDGGDEGVGEWRPVTLG 1h0h.1    --------------------------------------------------------------------------------  target    LRPDDPSEAMQAYLAGEFVTRKRKGS 1h0h.1    -------------------------- ``` | | | | | | | | | | | | | | | | | | | | | | | | | | | | | | | | | | | | | | | | | | | | | | | | | |
|  | 6lod.1.B | Fe-S-cluster-containing hydrogenase components 1-like protein  *Cryo-EM structure of the air-oxidized photosynthetic alternative complex III from Roseiflexus castenholzii* | 0.23 |  | 14.44 | 0.47 | 70-1041 | EM | 0.00 | hetero-1-1-1-1-1-1-… | 6 x HEC, 2 x EL6, 3 x SF4, 1 x F3S | HHblits | 0.27 |
| ``` target    SDLSRRELLKRAVVVGTGAGLAELFLPAQFLSSASAQSEPQAVAIANPLAQMPDRSWERIYRDQFAEEDSFVFTCAPNDT 6lod.1    ---------------------------------------------------------------------LFFATAVTFAG  target    HNCLLRAHVKNGVIVRISPTYGYGKATDLAGNQASHRWDPRICQKGLILGRRIYGDRRVKAPMIRKGFKEWADAGFPRHD 6lod.1    FGVGLLVESHEGRPTKIEG------------NPDHPASLGSTDLITQAMILTMYDPDRSQAPTNA---------------  target    DGTPRADMEKRGYDEWLQIPWDEALAIAAKTLQNVAETYKGEDGAGKLLEQGYEPAMVEAMHGAGVQAIKMRGGMPLLGA 6lod.1    ---------------GQETTWDAFVAAATAAMQAQTAKQGAGL---RVLSGS------------L---------------  target    GRVFGFYRFANMLALLDGKLRPEAPPEEIVGSRAFDNYAWH--TDLPPGHPMVSGSQTVDFDLFAAEHSKLLVLIGMNWI 6lod.1    ----TSPTLIAQKQQLLTQF----------PQAKWYEYEPVGRDNANAGARLAFGAD--VHTIYRLDTAKVIVGFDADFT  target    CTKMPD---AHWIGDAR------LKGTRVVVISADYMPTANKADEIVILRPGTDTAFLLGVARELITKKLYDRDAVIQRT 6lod.1    APSPTGVRMARQLADGRRIRKGTKEVNRLYLAESTPSITGLLADHRLPVRSSQIEHLVRALATLVGVPNVA---------  target    DLPLLVRLDTGERLSARDVFEGYRQAPLENYVALKTEEELAAPPSPPFTADKQVVPTELREEWGDFVYWDRATNGPAAVN 6lod.1    --------------------------------------------------------------------------------  target    RDEIGAKFAGDPALLGAFDVTLVDGTNVKARTAFSLLKEYLDENFDVQTTSEVCNVDPAAVRSLARQLAANKGNALLAAG 6lod.1    ------------------------------------------------AGAPLSDTEKKWVEAAAKDLQANRGACVVLVG  target    MGPNHYFNADLFGRVHFLVAALTDNIGHFSGNVGSYAGNYRGSLFQAMGQWIAENPFDQEADLTKPARVKRYFKSESAHY 6lod.1    ES--QPP---VVHALGHAINAQLGNVGST---VV-YTE-----------------PVED---DPSG-GI-----------  target    WNYGDRPLVSPSEIITGKSHMPTPTKLIWFGNSNSLLGNAKWSFDVVKNTLPKQDAVFCNEWHWTSSCEYSDLVFPADSW 6lod.1    --------AALSALTQEM--NAGTVEVLLMIESNPVYNAPA--DIPFAEALAKVPLSMHVGLYRDETAQQSVWHINGAHF  target    AEFKLPDMTASCTNPFLLAFPKTPLARIHNTRSDYEILAGVAAALADLVDEPRMKTYWKGILDGDPTPYLQRVLSGSNAT 6lod.1    LEAWGDV-R-A-FDGTTTI-VQPLIAPLYNGKSAIEVLNVLLGKPQETG-YQ------------TLTAYWQTQDAS--GN  target    RGILYEDLHASSAKGVPLLMNARTYPRHAGWEQRQEDKPWYTPTGRLEFYRPEPEWQAAGESLPIWREPVDATFYEPNAI 6lod.1    FRVFWNTALH---DGVITA---------------TQ-----ARSRQVTLQ----------QGFADAAPP-----------  target    LANSKHPSINPRAPEDYGVPESQMDVETRQYRNVVRTWQELKLSKHPLTEKDPAYRFVFQTPKYRWGAHSTAVDSDWIAM 6lod.1    -------------------------------------------------APTQGLEIVFRPDP--SLWDGAFANNAWLQE  target    LFGPFGDPYRRDSRTPWTGEAYAEINPRDAKELGLKDGDYIWLDADPEDRPYRGADSSDEFYDVARAMMRVRIYSGMPRR 6lod.1    TPKPYT---------KLTWDNVALMSVRTANALGLKNGDVVRLTYQ-----------------GRSVDAPVWVQPGHADD  target    VIRTWFNMYAATPGTVQAQKDVPGGPAQNQDTGYVALFRHGSHQSGTRAYLRPTQMTDSMNRKAYFGQTIGKGFEADVHS 6lod.1    SVTVHFGFGRTA--------------------------------------------------------------------  target    PSGAPKEGYVKVEKAEDGGDEGVGEWRPVTLGLRPDDPSEAMQAYLAGEFVTRKRKGS 6lod.1    ---------------------------------------------------------- ``` | | | | | | | | | | | | | | | | | | | | | | | | | | | | | | | | | | | | | | | | | | | | | | | | | |
|  | 6btm.1.B | Alternative Complex III subunit B  *Structure of Alternative Complex III from Flavobacterium johnsoniae (Wild Type)* | 0.22 |  | 12.68 | 0.47 | 69-1041 | EM | 3.40 | hetero-1-1-1-1-1-1-… | 6 x HEC, 1 x F3S, 1 x SF4, 2 x E87 | HHblits | 0.26 |
| ``` target    SDLSRRELLKRAVVVGTGAGLAELFLPAQFLSSASAQSEPQAVAIANPLAQMPDRSWERIYRDQFAEEDSFVFTCAPNDT 6btm.1    --------------------------------------------------------------------ADYYATTVFDGF  target    HNCLLRAHVKNGVIVRISPTYGYGKATDLAGNQASHRWDPRICQKGLILGRRIYGDRRVKAPMIRKGFKEWADAGFPRHD 6btm.1    DFANLLVKTREGRPIKIEN------------NTIAGAK-FSANARIHASILGLYDSMRLKEPKLDG--------------  target    DGTPRADMEKRGYDEWLQIPWDEALAIAAKTLQNVAETYKGEDGAGKLLEQGYEPAMVEAMHGAGVQAIKMRGGMPLLGA 6btm.1    ----------------KNSSWSAVDLKIKSSLADAKAK-GGQ---VVLLTNTL---------ASPTTE------------  target    GRVFGFYRFANMLALLDGKLRPEAPPEEIVGSRAFDNYAWHTD--LPPGHPMVSGSQTVDFDLFAAEHSKLLVLIGMNWI 6btm.1    ---KLIGEFIAK-----------------NPNAKHVVYDAVSSSDALDAFETVYGER--ALVDYDFSKASLIVSVGADFL  target    CTKMPD--AHWIGDARL----KGTRVVVISADYMPTANKADEIVILRPGTDTAFLLGVARELITKKLYDRDAVIQRTDLP 6btm.1    GDWQGGGYDAGYAKGRIPQNGKMSRHFQFESNMTLSGAAADKRVPMTTADQKQALVQIYNIVVGASVP------------  target    LLVRLDTGERLSARDVFEGYRQAPLENYVALKTEEELAAPPSPPFTADKQVVPTELREEWGDFVYWDRATNGPAAVNRDE 6btm.1    --------------------------------------------------------------------------------  target    IGAKFAGDPALLGAFDVTLVDGTNVKARTAFSLLKEYLDENFDVQTTSEVCNVDPAAVRSLARQLAANKGNALLAAGMGP 6btm.1    -----------------------------------------V------SLDAKFKAEVVKAAQQLKAAGTKGILVSGIED  target    NHYFNADLFGRVHFLVAALTDNIGHFSGNVGSYAGNYRGSLFQAMGQWIAENPFDQEADLTKPARVKRYFKSESAHYWNY 6btm.1    ------KNAQLLVLAINQALASEAFSTAGTRQI-------------------RKGS------NA----------------  target    GDRPLVSPSEIITGKSHMPTPTKLIWFGNSNSLLGNAKWSFDVVKNTLPKQDAVFCNEWHWTSSCEYSDLVFPADSWAEF 6btm.1    ------VVAQLIKDM--NAGSVHTLIMSGVNPVYTLAD--SASFVSGLKKVKTSVAFSLKEDETAAVSTIAAAAPHYLES  target    KLPDMTASCTNPFLLAFPKTPLARIHNTRSDYEILAGVAAALADLVDEPRMKTYWKGILDGDPTPYLQRVLSGSNATRGI 6btm.1    WGDVEIT---KGTYS-LTQPTIRPIFDTKQFQDVLLSVNGTPGN------FY------------DYLKANSGAI--IAGS  target    LYEDLHASSAKGVPLLMNARTYPRHAGWEQRQEDKPWYTPTGRLEFYRPEPEWQAAGESLPIWREPVDATFYEPNAILAN 6btm.1    SWNKVLH---DGIFVVG------------------SAALAGGSYDFAGAA--------SL------LS------------  target    SKHPSINPRAPEDYGVPESQMDVETRQYRNVVRTWQELKLSKHPLTEKDPAYRFVFQTPKYRWGAHSTAVDSDWIAMLFG 6btm.1    -------K---------------------------------------AKSSGELELVLYTKTGMGDGQHANNPWLQEFPD  target    PFGDPYRRDSRTPWTGEAYAEINPRDAKELGLKD---------GDYIWLDADPEDRPYRGADSSDEFYDVAR--AMMRVR 6btm.1    PI---T----RV--SWDNYVTVSNADAKKFNLSNEIVANGGLNGSYATITTA-----------------DGNKLENVPVI  target    IYSGMPRRVIRTWFNMYAATPGTVQAQKDVPGGPAQNQDTGYVALFRHGSHQSGTRAYLRPTQMTDSMNRKAYFGQTIGK 6btm.1    VQPGQAVGTVGLAVGYGRKA------------------------------------------------------------  target    GFEADVHSPSGAPKEGYVKVEKAEDGGDEGVGEWRPVTLGLRPDDPSEAMQAYLAGEFVTRKRKGS 6btm.1    ------------------------------------------------------------------ ``` | | | | | | | | | | | | | | | | | | | | | | | | | | | | | | | | | | | | | | | | | | | | | | | | | |
|  | 8e9g.1.G | NADH-quinone oxidoreductase subunit G  *Mycobacterial respiratory complex I with both quinone positions modelled* | 0.21 |  | 16.48 | 0.46 | 68-1040 | EM | 0.00 | hetero-1-1-1-1-1-1-… |  | HHblits | 0.26 |
| ``` target    SDLSRRELLKRAVVVGTGAGLAELFLPAQFLSSASAQSEPQAVAIANPLAQMPDRSWERIYRDQFAEEDSFVFTCAPNDT 8e9g.1    -------------------------------------------------------------------DLVSSPSVCEHCA  target    HNCLLRAHVKNGVIVRISPTYGYGKATDLAGNQASHRWDPRICQKGLILGRRIYGDRRVKAPMIRKGFKEWADAGFPRHD 8e9g.1    SGCAQRTDHRRGKVLRRLAG------------DEPEVNEEWNCDKGRWAFTYATVGDRITTPMLRDG-------------  target    DGTPRADMEKRGYDEWLQIPWDEALAIAAKTLQNVAETYKGEDGAGKLLEQGYEPAMVEAMHGAGVQAIKMRGGMPLLGA 8e9g.1    -------------GVLRPASWSEALTVAAAGLLTAAGST-------GVLVG-----------------------------  target    GRVFGFYRFANMLALLDGKLRPEAPPEEIVGSRAFDNYAWHTDLPP--GHPMVSGSQTVDFDLFAAEHSKLLVLIGMNWI 8e9g.1    ------GRCTVEDAYAYAKFAR-----MVLNTNDVDFRARPHSAEEAEFLAAHVAGQTMGLRYAELENAPTVLLAGFEPE  target    CTKMPDAHWIGDA-RLKGTRVVVISADYMP-TANKADEIVILRPGTDTAFLLGVARELITKKLYDRDAVIQRTDLPLLVR 8e9g.1    EESPIVFLRLRKGVRKNGVQVVAVAPWASRGLTKLAGTVVPTVPGDEPAALDGMHD---------DDRLRR---------  target    LDTGERLSARDVFEGYRQAPLENYVALKTEEELAAPPSPPFTADKQVVPTELREEWGDFVYWDRATNGPAAVNRDEIGAK 8e9g.1    --------------------------------------------------------------------------------  target    FAGDPALLGAFDVTLVDGTNVKARTAFSLLKEYLDENFDVQTTSEVCNVDPAAVRSLARQLAANKGNALLAAGMGPNHYF 8e9g.1    ------------------------------------PGAVILVGERLATSPGALSAAVRLAAATGAR-LAWIP----RRA  target    NADLFGRVHFLVAALTDNIGHFSGNVGSYAGNYRGSLFQAMGQWIAENPFDQEADLTKPARVKRYFKSESAHYWNYGDRP 8e9g.1    GERGAIEAGALPNLLPGGRPVDDADAR--------AEV--ARAWY---------------------------ISALPEAP  target    LVSPSEIITGKSHMPTPTKLIWFGNSNSLLGNAKWSFDVVKNTLPKQDAVFCNEWHWTSSCEYSDLVFPADSWAEFKLPD 8e9g.1    GRDTAAILSTA--ASGHLAALLVGG-VELGDLPD--PELAVAAVRTTPFVVSLELRESAVTELADVVFPVAPVVEKAGSF  target    MTASCTNPFLLAFPKTPLARIHNTRSDYEILAGVAAALADLVDEPRMKTYWKGILDGDPTPYLQRVLSGSNATRGILYED 8e9g.1    ---LNWEGRPRPFAP---SLKTNAIPDLRVLHYLADEIGVDLA-------LP-----TAEAAD---------------AE  target    LHASSAKGVPLLMNARTYPRHAGWEQRQEDKPWYTPTGRLEFYRPEPEWQAAGESLPIWREPVDATFYEPNAILANSKHP 8e9g.1    LAQL---GT-W-------G----------GARPPAPT---------------------A-PPTAR---------------  target    SINPRAPEDYGVPESQMDVETRQYRNVVRTWQELKLSKHPLTEKDPAYRFVFQTPKYRWGAHSTAVDSDWIAMLFGPFGD 8e9g.1    ---P---------------------------------------EAGSGQAVLASWRMLLDAGRLQDGEPHLAGTA-----  target    PYRRDSRTPWTGEAYAEINPRDAKELGLKDGDYIWLDADPEDRPYRGADSSDEFYDVARAMMRVRIYSGMPRRVIRTWFN 8e9g.1    -----------VRPVARMSAATAAGIGASDGAPVTVSTE-----------------RGAVTLPLAVTD-MPDGVVWLPMN  target    MYAATPGTVQAQKDVPGGPAQNQDTGYVALFRHGSHQSGTRAYLRPTQMTDSMNRKAYFGQTIGKGFEADVHSPSGAPKE 8e9g.1    SPGS----------------------------------------------------------------------------  target    GYVKVEKAEDGGDEGVGEWRPVTLGLRPDDPSEAMQAYLAGEFVTRKRKGS 8e9g.1    --------------------------------------------------- ``` | | | | | | | | | | | | | | | | | | | | | | | | | | | | | | | | | | | | | | | | | | | | | | | | | |
|  | 1ogy.1.A | PERIPLASMIC NITRATE REDUCTASE  *Crystal structure of the heterodimeric nitrate reductase from Rhodobacter sphaeroides* | 0.24 |  | 18.55 | 0.45 | 70-756 | X-ray | 3.20 | hetero-1-1-mer | 1 x SF4, 1 x MO, 2 x MGD, 2 x HEC | HHblits | 0.28 |
| ``` target    SDLSRRELLKRAVVVGTGAGLAELFLPAQFLSSASAQSEPQAVAIANPLAQMPDRSWERIYRDQFAEEDSFVFTCAPNDT 1ogy.1    ---------------------------------------------------------------------RWSKAPCRFCG  target    HNCLLRAHVKNGVIVRISPTYGYGKATDLAGNQASHRWDPRICQKGLILGRRIYGDRRVKAPMIRKGFKEWADAGFPRHD 1ogy.1    TGCGVMVGTRDGQVVATHGD------------TQAEVNRGLNCVKGYFLSKIMYGEDRLTTPLLRMKDG-----------  target    DGTPRADMEKRGYDEWLQIPWDEALAIAAKTLQNVAETYKGEDGAGKLLEQGYEPAMVEAMHGAGVQAIKMRGGMPLLGA 1ogy.1    --------VYHKEGEFAPVSWDEAFDVMAAQAKLVLKEKAPEA----VGM----------F-GSGQWTIW----------  target    GRVFGFYRFANMLALLDGKLRPEAPPEEIVGSRAFDNYAWHT--DLPPGHPMVSGSQTVDFDLFAAEHSKLLVLIGMNWI 1ogy.1    -EGYAASKLMRA----------------GFRSNNLDPNARHCMASAATAFMRTFGMDEPMGCYDDFEAADAFVLWGSNMA  target    CTKMPDAHWIGDAR--LKGTRVVVISADYMPTANKADEIVILRPGTDTAFLLGVARELITKKLYDRDAVIQRTDLPLLVR 1ogy.1    EMHPILWSRLTDRRLSHEHVRVAVLSTFTHRSSDLSDTPIIFRPGTDRAILNYIAHHIISTGRVNRDFVDRHTNFALGAT  target    LDTGERLSARDVFEGYRQAPLENYVALKTEEELAAPPSPPFTADKQVVPTELREEWGDFVYWDRATNGPAAVNRDEIGAK 1ogy.1    D-IGYGLRPEH-----------------------------------------------QLQLAAK---------------  target    FAGDPALLGAFDVTLVDGTNVKARTAFSLLKEYLDENFDVQTTSEVCNVDPAAVRSLARQLAANKGNALLAAGMGPNHYF 1ogy.1    --------G------AADAGAMTPTDFETFAALVS-EYTLEKAAEISGVEPALLEELAELYADPDRKWMSLWTMGFNQHV  target    NADLFGRVHFLVAALTDNIGHFSGNVGSYAGNYRGSL-FQAMGQWIAENPFDQEADLTKPARVKRYFKSESAHYWNY--- 1ogy.1    RGVWANHMVYNLHLLTGKISEPGNSPFSLTGQPFACGTAREVGTFAHRLPADMV--VTNPEHRAH-----AEEIWKLPAG  target    --GDRPLVSPSEIITGKSHMPTPTKLIWFGNSNSLLGNAKWSFDVVKNTLPKQDAVFCNEWHWTSSCEYSDLVFPADSWA 1ogy.1    LLPDWVGAHAVEQDRKL--HDGEINFYWVQVNNNMQAAPNIDQETYPGYRNPENFIVVSDAYPTVTGRAADLVLPAAMWV  target    EFKLPDMTASCTNPFLLAFPKTPLARIHNTRSDYEILAGVAAALADLVDEPRMKTYWKGILDGDPTPYLQRVLSGSNATR 1ogy.1    EKEG--AY-GNAERRTHFWHQ-LVEAPGEARSDLWQLMEFSKRFTT----------------------------------  target    GILYEDLHASSAKGVPLLMNARTYPRHAGWEQRQEDKPWYTPTGRLEFYRPEPEWQAAGESLPIWREPVDATFYEPNAIL 1ogy.1    --------------------------------------------------------------------------------  target    ANSKHPSINPRAPEDYGVPESQMDVETRQYRNVVRTWQELKLSKHPLTEKDPAYRFVFQTPKYRWGAHSTAVDSDWIAML 1ogy.1    --------------------------------------------------------------------------------  target    FGPFGDPYRRDSRTPWTGEAYAEINPRDAKELGLKDGDYIWLDADPEDRPYRGADSSDEFYDVARAMMRVRIYSGMPRRV 1ogy.1    --------------------------------------------------------------------------------  target    IRTWFNMYAATPGTVQAQKDVPGGPAQNQDTGYVALFRHGSHQSGTRAYLRPTQMTDSMNRKAYFGQTIGKGFEADVHSP 1ogy.1    --------------------------------------------------------------------------------  target    SGAPKEGYVKVEKAEDGGDEGVGEWRPVTLGLRPDDPSEAMQAYLAGEFVTRKRKGS 1ogy.1    --------------------------------------------------------- ``` | | | | | | | | | | | | | | | | | | | | | | | | | | | | | | | | | | | | | | | | | | | | | | | | | |
|  | 3o5a.1.A | Periplasmic nitrate reductase  *Crystal Structure of partially reduced Periplasmic Nitrate Reductase from Cupriavidus necator using Ionic Liquids* | 0.24 |  | 17.37 | 0.45 | 71-756 | X-ray | 1.72 | hetero-oligomer | 1 x SF4, 1 x MOS, 2 x MGD, 2 x HEC | HHblits | 0.28 |
| ``` target    SDLSRRELLKRAVVVGTGAGLAELFLPAQFLSSASAQSEPQAVAIANPLAQMPDRSWERIYRDQFAEEDSFVFTCAPNDT 3o5a.1    ----------------------------------------------------------------------WSKAPCRFCG  target    HNCLLRAHVKNGVIVRISPTYGYGKATDLAGNQASHRWDPRICQKGLILGRRIYGDRRVKAPMIRKGFKEWADAGFPRHD 3o5a.1    TGCGVTVAVKDNKVVATQG------------DPQAEVNKGLNCVKGYFLSKIMYGQDRLTRPLMRMKNG-----------  target    DGTPRADMEKRGYDEWLQIPWDEALAIAAKTLQNVAETYKGEDGAGKLLEQGYEPAMVEAMHGAGVQAIKMRGGMPLLGA 3o5a.1    --------KYDKNGDFAPVTWDQAFDEMERQFKRVLKEKGPTA----VGM-----------FGSGQWTVW----------  target    GRVFGFYRFANMLALLDGKLRPEAPPEEIVGSRAFDNYAWHTD--LPPGHPMVSGSQTVDFDLFAAEHSKLLVLIGMNWI 3o5a.1    -EGYAAAKLYK----------------AGFRSNNIDPNARHCMASAAAGFMRTFGMDEPMGCYDDFEAADAFVLWGSNMA  target    CTKMPDAHWIGDAR--LKGTRVVVISADYMPTANKADEIVILRPGTDTAFLLGVARELITKKLYDRDAVIQRTDLPLLVR 3o5a.1    EMHPILWTRVTDRRLSHPKTRVVVLSTFTHRCFDLADIGIIFKPQTDLAMLNYIANYIIRNNKVNKDFVNKHTVFKEGVT  target    LDTGERLSARDVFEGYRQAPLENYVALKTEEELAAPPSPPFTADKQVVPTELREEWGDFVYWDRATNGPAAVNRDEIGAK 3o5a.1    DIG-YGLRPDHPLQKA-------------------------------------------------AK-------------  target    FAGDPALLGAFDVTLVDGTNVKARTAFSLLKEYLDENFDVQTTSEVCNVDPAAVRSLARQLAANKGNALLAAGMGPNHYF 3o5a.1    --------------NASDPGAAKVITFDEFAKFVS-KYDADYVSKLSAVPKAKLDQLAELYADPNIKVMSLWTMGFNQHT  target    NADLFGRVHFLVAALTDNIGHFSGNVGSYAGNYRGSL-FQAMGQWIAENPFDQEADLTKPARVKRYFKSESAHYWNYG-- 3o5a.1    RGTWANNMVYNLHLLTGKIATPGNSPFSLTGQPSACGTAREVGTFSHRLPADM--VVTNPKHREE-----AERIWKLPPG  target    DRPLV-SPSEIITGKSHMPTPTKLIWFGNSNSLLGNAKWSFDVVKNTLPKQDAVFCNEWHWTSSCEYSDLVFPADSWAEF 3o5a.1    TIPDKPGYDAVLQNRMLKDGKLNAYWVQVNNNMQAAANLMEEGLPGYRNPANFIVVSDAYPTVTALAADLVLPSAMWVEK  target    KLPDMTASCTNPFLLAFPKTPLARIHNTRSDYEILAGVAAALADLVDEPRMKTYWKGILDGDPTPYLQRVLSGSNATRGI 3o5a.1    E--GAY-GNAERRTQFWHQ-LVDAPGEARSDLWQLVEFAKRFKV------------------------------------  target    LYEDLHASSAKGVPLLMNARTYPRHAGWEQRQEDKPWYTPTGRLEFYRPEPEWQAAGESLPIWREPVDATFYEPNAILAN 3o5a.1    --------------------------------------------------------------------------------  target    SKHPSINPRAPEDYGVPESQMDVETRQYRNVVRTWQELKLSKHPLTEKDPAYRFVFQTPKYRWGAHSTAVDSDWIAMLFG 3o5a.1    --------------------------------------------------------------------------------  target    PFGDPYRRDSRTPWTGEAYAEINPRDAKELGLKDGDYIWLDADPEDRPYRGADSSDEFYDVARAMMRVRIYSGMPRRVIR 3o5a.1    --------------------------------------------------------------------------------  target    TWFNMYAATPGTVQAQKDVPGGPAQNQDTGYVALFRHGSHQSGTRAYLRPTQMTDSMNRKAYFGQTIGKGFEADVHSPSG 3o5a.1    --------------------------------------------------------------------------------  target    APKEGYVKVEKAEDGGDEGVGEWRPVTLGLRPDDPSEAMQAYLAGEFVTRKRKGS 3o5a.1    ------------------------------------------------------- ``` | | | | | | | | | | | | | | | | | | | | | | | | | | | | | | | | | | | | | | | | | | | | | | | | | |
|  | 2ivf.1.A | ETHYLBENZENE DEHYDROGENASE ALPHA-SUBUNIT  *ETHYLBENZENE DEHYDROGENASE FROM AROMATOLEUM AROMATICUM* | 0.23 | 0.00 | 36.10 | 0.38 | 2-576 | X-ray | 1.88 | monomer | 1 x MES, 4 x SF4, 1 x MO, 1 x MGD, 1 x MD1, 1 x F3S, 1 x HEM | BLAST | 0.38 |
| ``` target    SDLSRRELLKR---AVVVGTGAGLAELFLPAQFLSSASAQSEPQAVAIANPLAQMPDRSWERIYRDQFAEED----SFVF 2ivf.1    -DQHRRDFLKRSGAAVLSLSLSSLATGVVPG-FLKDAQAGTKAPGYA-----------SWEDIYRKEWKWDKVNWGSHLN  target    TCAPNDTHNCLLRAHVKNGVIVRISPTYGYGKATDLAGNQASHRWDPRICQKGLILGRRIYGDRRVKAPMIRKGFKEWAD 2ivf.1    ICWPQGS--CKFYVYVRNGIVWREEQA-----AQTPACNVDYVDYNPLGCQKGSAFNNNLYGDERVKYPLKRVG------  target    AGFPRHDDGTPRADMEKRGYDEWLQIPWDEALAIAAKTLQNVAETYKGEDGAGKLLEQGYEPAMVEAMHGAGVQAIKMRG 2ivf.1    ----------------KRGEGKWKRVSWDEAAGDIADSIIDSFEA------------QGSDGFILDAPH--------VHA  target    GMPLLGAGRVFGFYRFANMLALLDGKLRPEAPPEEIVGSRAFDNY--AWHTDLPPGHPMVSGSQTVDFDLFAAEHSKLLV 2ivf.1    GSIAWGAG--------FRMTYLMDG-VSPD------INVDIGDTYMGAFHT-FGKMHMGYSADNLLD--------AELIF  target    LIGMNWICTKMPDAHWIGDARLKGTRVVVISADYMPTANKADEIVILRPGTDTAFLLGVARELITKKLYDRDAVIQRTDL 2ivf.1    MTCSNWSYTYPSSYHFLSEARYKGAEVVVIAPDFNPTTPAADLHVPVRVGSDAAFWLGLSQVMIDEKLFDRQFVCEQTDL  target    PLLVRLDTGERLSARDVFEGYRQAPLENYVALKTEEELAAPPSPPFTADKQVVPTELREEWGDFVYWDRATNGPAAVNRD 2ivf.1    PLLVRMDTGKFLSAEDVDGG---------------------------------------EAKQFYFFDEKAGSVRKASRG  target    EIGAKFAGDPALLGAFDVTLVDGTNVKARTAFSLLKEYLDENFDVQTTSEVCNVDPAAVRSLARQLAANKGNALLAAGMG 2ivf.1    TLKLDFM--PALEGTFSARLKNGKTIQVRTVFEGLREHL-KDYTPEKASAKCGVPVSLIRELGRKVAKKRTCSYIGFSSA  target    PNHYFNADLFGRVHFLVAALTDNIGHFSGNVGSYAGNYRGSLFQAMGQWIAENPFDQEADLTKPARVKRYFKSESAHYWN 2ivf.1    KS--YHGDLMERSLFLAMALSGNWG-------------------------------------------------------  target    YGDRPLVSPSEIITGKSHMPTPTKLIWFGNSNSLLGNAKWSFDVVKNTLPKQDAVFCNEWHWTSSCEYSDLVFPADSWAE 2ivf.1    --------------------------------------------------------------------------------  target    FKLPDMTASCTNPFLLAFPKTPLARIHNTRSDYEILAGVAAALADLVDEPRMKTYWKGILDGDPTPYLQRVLSGSNATRG 2ivf.1    --------------------------------------------------------------------------------  target    ILYEDLHASSAKGVPLLMNARTYPRHAGWEQRQEDKPWYTPTGRLEFYRPEPEWQAAGESLPIWREPVDATFYEPNAILA 2ivf.1    --------------------------------------------------------------------------------  target    NSKHPSINPRAPEDYGVPESQMDVETRQYRNVVRTWQELKLSKHPLTEKDPAYRFVFQTPKYRWGAHSTAVDSDWIAMLF 2ivf.1    --------------------------------------------------------------------------------  target    GPFGDPYRRDSRTPWTGEAYAEINPRDAKELGLKDGDYIWLDADPEDRPYRGADSSDEFYDVARAMMRVRIYSGMPRRVI 2ivf.1    --------------------------------------------------------------------------------  target    RTWFNMYAATPGTVQAQKDVPGGPAQNQDTGYVALFRHGSHQSGTRAYLRPTQMTDSMNRKAYFGQTIGKGFEADVHSPS 2ivf.1    --------------------------------------------------------------------------------  target    GAPKEGYVKVEKAEDGGDEGVGEWRPVTLGLRPDDPSEAMQAYLAGEFVTRKRKGS 2ivf.1    -------------------------------------------------------- ``` | | | | | | | | | | | | | | | | | | | | | | | | | | | | | | | | | | | | | | | | | | | | | | | | | |
|  | 3m9s.1.C | NADH-quinone oxidoreductase subunit 3  *Crystal structure of respiratory complex I from Thermus thermophilus* | 0.21 | 0.00 | 17.17 | 0.42 | 67-1042 | X-ray | 4.50 | monomer | 7 x SF4, 2 x FES, 1 x FMN | HHblits | 0.27 |
| ``` target    SDLSRRELLKRAVVVGTGAGLAELFLPAQFLSSASAQSEPQAVAIANPLAQMPDRSWERIYRDQFAEEDSFVFTCAPNDT 3m9s.1    ------------------------------------------------------------------WEMEETPTTCALCP  target    HNCLLRAHVKNGVIVRISPTYGYGKATDLAGNQASHRWDPRICQKGLILGRRIYGDRRVKAPMIRKGFKEWADAGFPRHD 3m9s.1    VGCGITADTRSGELLRIRAR------------EVPEVNEIWICDAGRFGHEW-ADQNRLKTPLVRKE-------------  target    DGTPRADMEKRGYDEWLQIPWDEALAIAAKTLQNVAETYKGEDGAGKLLEQGYEPAMVEAMHGAGVQAIKMRGGMPLLGA 3m9s.1    -------------GRLVEATWEEAFLALKEGLKEARG----EEV--GLY-------------------------------  target    GRVFGFYRFANMLALLDGKLRPEAPPEEIVGSRAFDNYAWHTDLPPGHPMVSGSQTVDFDLFAAEHSKLLVLIGMNWICT 3m9s.1    ----LAHDATLEEGLLASELAK------ALKTPHLDFQGRTAAPA--------SLFPPASLEDLLQADFALVLG-DPTEE  target    KMPDAHWIGD-------------------------ARLKGTRVVVISADYMPTANKADEIVILRPGTDTAFLLGVARELI 3m9s.1    APILHLRLSEFVRDLKPPHRYNHGTPFADLQIKERMPRRTDKMALFAPYRAPLMKWAAIHEVHRPGEEREILLALLGDKE  target    TKKLYDRDAVIQRTDLPLLVRLDTGERLSARDVFEGYRQAPLENYVALKTEEELAAPPSPPFTADKQVVPTELREEWGDF 3m9s.1    --------------------------------------------------------------------------------  target    VYWDRATNGPAAVNRDEIGAKFAGDPALLGAFDVTLVDGTNVKARTAFSLLKEYLDENFDVQTTSEVCNVDPAAVRSLAR 3m9s.1    ----------------------------------------------------------------------GSEMVAKAKE  target    QLAANKGNALLAAGMGPNHYFNADLFGRVHFLVAALTDNIGHFSGNVGSYAG--NYRGSLFQAMGQWIAENPFDQEADLT 3m9s.1    AWEKAKN-PVLILGAGVLQDTVAAERARL------LA---ERKGAKVLAMTPAANARGLE--AMGVL-------------  target    KPARVKRYFKSESAHYWNYGDRPLVSPSEIITGKSHMPTPTKLIWFGNSNSLLGNAKWSFDVVKNTLPKQDAVFCNEWHW 3m9s.1    -PG---------------------A--KGASWDE---PGALYAY--YGFV-----------PPEEALKGKRFVVMHLSHL  target    TSSC-EYSDLVFPADSWAEFKLPDMTASCTNPFLLAFPKTPLARIHNTRSDYEILAGVAAALADLVDEPRMKTYWKGILD 3m9s.1    HPLAERYAHVVLPAPTFYEKRGH---LVNLEGRVLPLSP-APIENGEAEGALQVLALLAEALGVRPP-------FR----  target    GDPTPYLQRVLSGSNATRGILYEDLHASSAKGVPLLMNARTYPRHAGWEQRQEDKPWYTPTGRLEFYRPEPEWQAAGESL 3m9s.1    -LHLEA---------------QKALK---------------------------ARKVPEAMGRLSFRLKELR--------  target    PIWREPVDATFYEPNAILANSKHPSINPRAPEDYGVPESQMDVETRQYRNVVRTWQELKLSKHPLTEKDPAYRFVFQTPK 3m9s.1    -----PK-------------------------------------------------------------ERKGAFYLRPTM  target    YRWGAHSTAVDSDWIAMLFGPFGDPYRRDSRTPWTGEAYAEINPRDAKELGLKDGDYIWLDADPEDRPYRGADSSDEFYD 3m9s.1    WKAHQA-----VGKAQE-----------------AARAELWAHPETARAEALPEGAQVAVETP-----------------  target    VARAMMRVRIYSGMPRRVIRTWFNMYAATPGTVQAQKDVPGGPAQNQDTGYVALFRHGSHQSGTRAYLRPTQMTDSMNRK 3m9s.1    FGRVEARVVHREDVPKGHLYLSALGPAAGL--------------------------------------------------  target    AYFGQTIGKGFEADVHSPSGAPKEGYVKVEKAEDGGDEGVGEWRPVTLGLRPDDPSEAMQAYLAGEFVTRKRKGS 3m9s.1    --------------------------------------------------------------------------- ``` | | | | | | | | | | | | | | | | | | | | | | | | | | | | | | | | | | | | | | | | | | | | | | | | | |
|  | 2fug.2.C | NADH-quinone oxidoreductase chain 3  *Crystal structure of the hydrophilic domain of respiratory complex I from Thermus thermophilus* | 0.20 | 0.00 | 17.17 | 0.42 | 67-1042 | X-ray | 3.30 | monomer | 7 x SF4, 2 x FES, 1 x FMN | HHblits | 0.27 |
| ``` target    SDLSRRELLKRAVVVGTGAGLAELFLPAQFLSSASAQSEPQAVAIANPLAQMPDRSWERIYRDQFAEEDSFVFTCAPNDT 2fug.2    ------------------------------------------------------------------WEMEETPTTCALCP  target    HNCLLRAHVKNGVIVRISPTYGYGKATDLAGNQASHRWDPRICQKGLILGRRIYGDRRVKAPMIRKGFKEWADAGFPRHD 2fug.2    VGCGITADTRSGELLRIRAR------------EVPEVNEIWICDAGRFGHEW-ADQNRLKTPLVRKE-------------  target    DGTPRADMEKRGYDEWLQIPWDEALAIAAKTLQNVAETYKGEDGAGKLLEQGYEPAMVEAMHGAGVQAIKMRGGMPLLGA 2fug.2    -------------GRLVEATWEEAFLALKEGLKEARG----EEV--GLY-------------------------------  target    GRVFGFYRFANMLALLDGKLRPEAPPEEIVGSRAFDNYAWHTDLPPGHPMVSGSQTVDFDLFAAEHSKLLVLIGMNWICT 2fug.2    ----LAHDATLEEGLLASELAK------ALKTPHLDFQGRTAAPA--------SLFPPASLEDLLQADFALVLG-DPTEE  target    KMPDAHWIGD-------------------------ARLKGTRVVVISADYMPTANKADEIVILRPGTDTAFLLGVARELI 2fug.2    APILHLRLSEFVRDLKPPHRYNHGTPFADLQIKERMPRRTDKMALFAPYRAPLMKWAAIHEVHRPGEEREILLALLGDKE  target    TKKLYDRDAVIQRTDLPLLVRLDTGERLSARDVFEGYRQAPLENYVALKTEEELAAPPSPPFTADKQVVPTELREEWGDF 2fug.2    --------------------------------------------------------------------------------  target    VYWDRATNGPAAVNRDEIGAKFAGDPALLGAFDVTLVDGTNVKARTAFSLLKEYLDENFDVQTTSEVCNVDPAAVRSLAR 2fug.2    ----------------------------------------------------------------------GSEMVAKAKE  target    QLAANKGNALLAAGMGPNHYFNADLFGRVHFLVAALTDNIGHFSGNVGSYAG--NYRGSLFQAMGQWIAENPFDQEADLT 2fug.2    AWEKAKN-PVLILGAGVLQDTVAAERARL------LA---ERKGAKVLAMTPAANARGLE--AMGVL-------------  target    KPARVKRYFKSESAHYWNYGDRPLVSPSEIITGKSHMPTPTKLIWFGNSNSLLGNAKWSFDVVKNTLPKQDAVFCNEWHW 2fug.2    -PG---------------------A--KGASWDE---PGALYAY--YGFV-----------PPEEALKGKRFVVMHLSHL  target    TSSC-EYSDLVFPADSWAEFKLPDMTASCTNPFLLAFPKTPLARIHNTRSDYEILAGVAAALADLVDEPRMKTYWKGILD 2fug.2    HPLAERYAHVVLPAPTFYEKRGH---LVNLEGRVLPLSP-APIENGEAEGALQVLALLAEALGVRPP-------FR----  target    GDPTPYLQRVLSGSNATRGILYEDLHASSAKGVPLLMNARTYPRHAGWEQRQEDKPWYTPTGRLEFYRPEPEWQAAGESL 2fug.2    -LHLEA---------------QKALK---------------------------ARKVPEAMGRLSFRLKELR--------  target    PIWREPVDATFYEPNAILANSKHPSINPRAPEDYGVPESQMDVETRQYRNVVRTWQELKLSKHPLTEKDPAYRFVFQTPK 2fug.2    -----PK-------------------------------------------------------------ERKGAFYLRPTM  target    YRWGAHSTAVDSDWIAMLFGPFGDPYRRDSRTPWTGEAYAEINPRDAKELGLKDGDYIWLDADPEDRPYRGADSSDEFYD 2fug.2    WKAHQA-----VGKAQE-----------------AARAELWAHPETARAEALPEGAQVAVETP-----------------  target    VARAMMRVRIYSGMPRRVIRTWFNMYAATPGTVQAQKDVPGGPAQNQDTGYVALFRHGSHQSGTRAYLRPTQMTDSMNRK 2fug.2    FGRVEARVVHREDVPKGHLYLSALGPAAGL--------------------------------------------------  target    AYFGQTIGKGFEADVHSPSGAPKEGYVKVEKAEDGGDEGVGEWRPVTLGLRPDDPSEAMQAYLAGEFVTRKRKGS 2fug.2    --------------------------------------------------------------------------- ``` | | | | | | | | | | | | | | | | | | | | | | | | | | | | | | | | | | | | | | | | | | | | | | | | | |
|  | 6zjl.1.C | NADH-quinone oxidoreductase subunit 3  *Respiratory complex I from Thermus thermophilus, NAD+ dataset, major state* | 0.21 | 0.00 | 17.17 | 0.42 | 67-1042 | EM | 0.00 | monomer | 7 x SF4, 1 x FMN, 2 x FES | HHblits | 0.27 |
| ``` target    SDLSRRELLKRAVVVGTGAGLAELFLPAQFLSSASAQSEPQAVAIANPLAQMPDRSWERIYRDQFAEEDSFVFTCAPNDT 6zjl.1    ------------------------------------------------------------------WEMEETPTTCALCP  target    HNCLLRAHVKNGVIVRISPTYGYGKATDLAGNQASHRWDPRICQKGLILGRRIYGDRRVKAPMIRKGFKEWADAGFPRHD 6zjl.1    VGCGITADTRSGELLRIRAR------------EVPEVNEIWICDAGRFGHEW-ADQNRLKTPLVRKE-------------  target    DGTPRADMEKRGYDEWLQIPWDEALAIAAKTLQNVAETYKGEDGAGKLLEQGYEPAMVEAMHGAGVQAIKMRGGMPLLGA 6zjl.1    -------------GRLVEATWEEAFLALKEGLKEARG----EEV--GLY-------------------------------  target    GRVFGFYRFANMLALLDGKLRPEAPPEEIVGSRAFDNYAWHTDLPPGHPMVSGSQTVDFDLFAAEHSKLLVLIGMNWICT 6zjl.1    ----LAHDATLEEGLLASELAK------ALKTPHLDFQGRTAAPA--------SLFPPASLEDLLQADFALVLG-DPTEE  target    KMPDAHWIGD-------------------------ARLKGTRVVVISADYMPTANKADEIVILRPGTDTAFLLGVARELI 6zjl.1    APILHLRLSEFVRDLKPPHRYNHGTPFADLQIKERMPRRTDKMALFAPYRAPLMKWAAIHEVHRPGEEREILLALLGDKE  target    TKKLYDRDAVIQRTDLPLLVRLDTGERLSARDVFEGYRQAPLENYVALKTEEELAAPPSPPFTADKQVVPTELREEWGDF 6zjl.1    --------------------------------------------------------------------------------  target    VYWDRATNGPAAVNRDEIGAKFAGDPALLGAFDVTLVDGTNVKARTAFSLLKEYLDENFDVQTTSEVCNVDPAAVRSLAR 6zjl.1    ----------------------------------------------------------------------GSEMVAKAKE  target    QLAANKGNALLAAGMGPNHYFNADLFGRVHFLVAALTDNIGHFSGNVGSYAG--NYRGSLFQAMGQWIAENPFDQEADLT 6zjl.1    AWEKAKN-PVLILGAGVLQDTVAAERARL------LA---ERKGAKVLAMTPAANARGLE--AMGVL-------------  target    KPARVKRYFKSESAHYWNYGDRPLVSPSEIITGKSHMPTPTKLIWFGNSNSLLGNAKWSFDVVKNTLPKQDAVFCNEWHW 6zjl.1    -PG---------------------A--KGASWDE---PGALYAY--YGFV-----------PPEEALKGKRFVVMHLSHL  target    TSSC-EYSDLVFPADSWAEFKLPDMTASCTNPFLLAFPKTPLARIHNTRSDYEILAGVAAALADLVDEPRMKTYWKGILD 6zjl.1    HPLAERYAHVVLPAPTFYEKRGH---LVNLEGRVLPLSP-APIENGEAEGALQVLALLAEALGVRPP-------FR----  target    GDPTPYLQRVLSGSNATRGILYEDLHASSAKGVPLLMNARTYPRHAGWEQRQEDKPWYTPTGRLEFYRPEPEWQAAGESL 6zjl.1    -LHLEA---------------QKALK---------------------------ARKVPEAMGRLSFRLKELR--------  target    PIWREPVDATFYEPNAILANSKHPSINPRAPEDYGVPESQMDVETRQYRNVVRTWQELKLSKHPLTEKDPAYRFVFQTPK 6zjl.1    -----PK-------------------------------------------------------------ERKGAFYLRPTM  target    YRWGAHSTAVDSDWIAMLFGPFGDPYRRDSRTPWTGEAYAEINPRDAKELGLKDGDYIWLDADPEDRPYRGADSSDEFYD 6zjl.1    WKAHQA-----VGKAQE-----------------AARAELWAHPETARAEALPEGAQVAVETP-----------------  target    VARAMMRVRIYSGMPRRVIRTWFNMYAATPGTVQAQKDVPGGPAQNQDTGYVALFRHGSHQSGTRAYLRPTQMTDSMNRK 6zjl.1    FGRVEARVVHREDVPKGHLYLSALGPAAGL--------------------------------------------------  target    AYFGQTIGKGFEADVHSPSGAPKEGYVKVEKAEDGGDEGVGEWRPVTLGLRPDDPSEAMQAYLAGEFVTRKRKGS 6zjl.1    --------------------------------------------------------------------------- ``` | | | | | | | | | | | | | | | | | | | | | | | | | | | | | | | | | | | | | | | | | | | | | | | | | |
|  | 6q8o.1.C | NADH-quinone oxidoreductase subunit 3  *Respiratory complex I from Thermus thermophilus with bound Piericidin A* | 0.22 | 0.00 | 17.17 | 0.42 | 67-1042 | X-ray | 3.61 | monomer | 7 x SF4, 1 x FMN, 2 x FES, 1 x HQH | HHblits | 0.27 |
| ``` target    SDLSRRELLKRAVVVGTGAGLAELFLPAQFLSSASAQSEPQAVAIANPLAQMPDRSWERIYRDQFAEEDSFVFTCAPNDT 6q8o.1    ------------------------------------------------------------------WEMEETPTTCALCP  target    HNCLLRAHVKNGVIVRISPTYGYGKATDLAGNQASHRWDPRICQKGLILGRRIYGDRRVKAPMIRKGFKEWADAGFPRHD 6q8o.1    VGCGITADTRSGELLRIRAR------------EVPEVNEIWICDAGRFGHEW-ADQNRLKTPLVRKE-------------  target    DGTPRADMEKRGYDEWLQIPWDEALAIAAKTLQNVAETYKGEDGAGKLLEQGYEPAMVEAMHGAGVQAIKMRGGMPLLGA 6q8o.1    -------------GRLVEATWEEAFLALKEGLKEARG----EEV--GLY-------------------------------  target    GRVFGFYRFANMLALLDGKLRPEAPPEEIVGSRAFDNYAWHTDLPPGHPMVSGSQTVDFDLFAAEHSKLLVLIGMNWICT 6q8o.1    ----LAHDATLEEGLLASELAK------ALKTPHLDFQGRTAAPA--------SLFPPASLEDLLQADFALVLG-DPTEE  target    KMPDAHWIGD-------------------------ARLKGTRVVVISADYMPTANKADEIVILRPGTDTAFLLGVARELI 6q8o.1    APILHLRLSEFVRDLKPPHRYNHGTPFADLQIKERMPRRTDKMALFAPYRAPLMKWAAIHEVHRPGEEREILLALLGDKE  target    TKKLYDRDAVIQRTDLPLLVRLDTGERLSARDVFEGYRQAPLENYVALKTEEELAAPPSPPFTADKQVVPTELREEWGDF 6q8o.1    --------------------------------------------------------------------------------  target    VYWDRATNGPAAVNRDEIGAKFAGDPALLGAFDVTLVDGTNVKARTAFSLLKEYLDENFDVQTTSEVCNVDPAAVRSLAR 6q8o.1    ----------------------------------------------------------------------GSEMVAKAKE  target    QLAANKGNALLAAGMGPNHYFNADLFGRVHFLVAALTDNIGHFSGNVGSYAG--NYRGSLFQAMGQWIAENPFDQEADLT 6q8o.1    AWEKAKN-PVLILGAGVLQDTVAAERARL------LA---ERKGAKVLAMTPAANARGLE--AMGVL-------------  target    KPARVKRYFKSESAHYWNYGDRPLVSPSEIITGKSHMPTPTKLIWFGNSNSLLGNAKWSFDVVKNTLPKQDAVFCNEWHW 6q8o.1    -PG---------------------A--KGASWDE---PGALYAY--YGFV-----------PPEEALKGKRFVVMHLSHL  target    TSSC-EYSDLVFPADSWAEFKLPDMTASCTNPFLLAFPKTPLARIHNTRSDYEILAGVAAALADLVDEPRMKTYWKGILD 6q8o.1    HPLAERYAHVVLPAPTFYEKRGH---LVNLEGRVLPLSP-APIENGEAEGALQVLALLAEALGVRPP-------FR----  target    GDPTPYLQRVLSGSNATRGILYEDLHASSAKGVPLLMNARTYPRHAGWEQRQEDKPWYTPTGRLEFYRPEPEWQAAGESL 6q8o.1    -LHLEA---------------QKALK---------------------------ARKVPEAMGRLSFRLKELR--------  target    PIWREPVDATFYEPNAILANSKHPSINPRAPEDYGVPESQMDVETRQYRNVVRTWQELKLSKHPLTEKDPAYRFVFQTPK 6q8o.1    -----PK-------------------------------------------------------------ERKGAFYLRPTM  target    YRWGAHSTAVDSDWIAMLFGPFGDPYRRDSRTPWTGEAYAEINPRDAKELGLKDGDYIWLDADPEDRPYRGADSSDEFYD 6q8o.1    WKAHQA-----VGKAQE-----------------AARAELWAHPETARAEALPEGAQVAVETP-----------------  target    VARAMMRVRIYSGMPRRVIRTWFNMYAATPGTVQAQKDVPGGPAQNQDTGYVALFRHGSHQSGTRAYLRPTQMTDSMNRK 6q8o.1    FGRVEARVVHREDVPKGHLYLSALGPAAGL--------------------------------------------------  target    AYFGQTIGKGFEADVHSPSGAPKEGYVKVEKAEDGGDEGVGEWRPVTLGLRPDDPSEAMQAYLAGEFVTRKRKGS 6q8o.1    --------------------------------------------------------------------------- ``` | | | | | | | | | | | | | | | | | | | | | | | | | | | | | | | | | | | | | | | | | | | | | | | | | |
|  | 6zjy.1.C | NADH-quinone oxidoreductase subunit 3  *Respiratory complex I from Thermus thermophilus, NAD+ dataset, minor state* | 0.20 | 0.00 | 17.17 | 0.42 | 67-1042 | EM | 0.00 | monomer | 7 x SF4, 2 x FES | HHblits | 0.27 |
| ``` target    SDLSRRELLKRAVVVGTGAGLAELFLPAQFLSSASAQSEPQAVAIANPLAQMPDRSWERIYRDQFAEEDSFVFTCAPNDT 6zjy.1    ------------------------------------------------------------------WEMEETPTTCALCP  target    HNCLLRAHVKNGVIVRISPTYGYGKATDLAGNQASHRWDPRICQKGLILGRRIYGDRRVKAPMIRKGFKEWADAGFPRHD 6zjy.1    VGCGITADTRSGELLRIRAR------------EVPEVNEIWICDAGRFGHEW-ADQNRLKTPLVRKE-------------  target    DGTPRADMEKRGYDEWLQIPWDEALAIAAKTLQNVAETYKGEDGAGKLLEQGYEPAMVEAMHGAGVQAIKMRGGMPLLGA 6zjy.1    -------------GRLVEATWEEAFLALKEGLKEARG----EEV--GLY-------------------------------  target    GRVFGFYRFANMLALLDGKLRPEAPPEEIVGSRAFDNYAWHTDLPPGHPMVSGSQTVDFDLFAAEHSKLLVLIGMNWICT 6zjy.1    ----LAHDATLEEGLLASELAK------ALKTPHLDFQGRTAAPA--------SLFPPASLEDLLQADFALVLG-DPTEE  target    KMPDAHWIGD-------------------------ARLKGTRVVVISADYMPTANKADEIVILRPGTDTAFLLGVARELI 6zjy.1    APILHLRLSEFVRDLKPPHRYNHGTPFADLQIKERMPRRTDKMALFAPYRAPLMKWAAIHEVHRPGEEREILLALLGDKE  target    TKKLYDRDAVIQRTDLPLLVRLDTGERLSARDVFEGYRQAPLENYVALKTEEELAAPPSPPFTADKQVVPTELREEWGDF 6zjy.1    --------------------------------------------------------------------------------  target    VYWDRATNGPAAVNRDEIGAKFAGDPALLGAFDVTLVDGTNVKARTAFSLLKEYLDENFDVQTTSEVCNVDPAAVRSLAR 6zjy.1    ----------------------------------------------------------------------GSEMVAKAKE  target    QLAANKGNALLAAGMGPNHYFNADLFGRVHFLVAALTDNIGHFSGNVGSYAG--NYRGSLFQAMGQWIAENPFDQEADLT 6zjy.1    AWEKAKN-PVLILGAGVLQDTVAAERARL------LA---ERKGAKVLAMTPAANARGLE--AMGVL-------------  target    KPARVKRYFKSESAHYWNYGDRPLVSPSEIITGKSHMPTPTKLIWFGNSNSLLGNAKWSFDVVKNTLPKQDAVFCNEWHW 6zjy.1    -PG---------------------A--KGASWDE---PGALYAY--YGFV-----------PPEEALKGKRFVVMHLSHL  target    TSSC-EYSDLVFPADSWAEFKLPDMTASCTNPFLLAFPKTPLARIHNTRSDYEILAGVAAALADLVDEPRMKTYWKGILD 6zjy.1    HPLAERYAHVVLPAPTFYEKRGH---LVNLEGRVLPLSP-APIENGEAEGALQVLALLAEALGVRPP-------FR----  target    GDPTPYLQRVLSGSNATRGILYEDLHASSAKGVPLLMNARTYPRHAGWEQRQEDKPWYTPTGRLEFYRPEPEWQAAGESL 6zjy.1    -LHLEA---------------QKALK---------------------------ARKVPEAMGRLSFRLKELR--------  target    PIWREPVDATFYEPNAILANSKHPSINPRAPEDYGVPESQMDVETRQYRNVVRTWQELKLSKHPLTEKDPAYRFVFQTPK 6zjy.1    -----PK-------------------------------------------------------------ERKGAFYLRPTM  target    YRWGAHSTAVDSDWIAMLFGPFGDPYRRDSRTPWTGEAYAEINPRDAKELGLKDGDYIWLDADPEDRPYRGADSSDEFYD 6zjy.1    WKAHQA-----VGKAQE-----------------AARAELWAHPETARAEALPEGAQVAVETP-----------------  target    VARAMMRVRIYSGMPRRVIRTWFNMYAATPGTVQAQKDVPGGPAQNQDTGYVALFRHGSHQSGTRAYLRPTQMTDSMNRK 6zjy.1    FGRVEARVVHREDVPKGHLYLSALGPAAGL--------------------------------------------------  target    AYFGQTIGKGFEADVHSPSGAPKEGYVKVEKAEDGGDEGVGEWRPVTLGLRPDDPSEAMQAYLAGEFVTRKRKGS 6zjy.1    --------------------------------------------------------------------------- ``` | | | | | | | | | | | | | | | | | | | | | | | | | | | | | | | | | | | | | | | | | | | | | | | | | |
|  | 6zjn.1.C | NADH-quinone oxidoreductase subunit 3  *Respiratory complex I from Thermus thermophilus, NADH dataset, minor state* | 0.21 | 0.00 | 17.17 | 0.42 | 67-1042 | EM | 0.00 | monomer | 7 x SF4, 2 x FES | HHblits | 0.27 |
| ``` target    SDLSRRELLKRAVVVGTGAGLAELFLPAQFLSSASAQSEPQAVAIANPLAQMPDRSWERIYRDQFAEEDSFVFTCAPNDT 6zjn.1    ------------------------------------------------------------------WEMEETPTTCALCP  target    HNCLLRAHVKNGVIVRISPTYGYGKATDLAGNQASHRWDPRICQKGLILGRRIYGDRRVKAPMIRKGFKEWADAGFPRHD 6zjn.1    VGCGITADTRSGELLRIRAR------------EVPEVNEIWICDAGRFGHEW-ADQNRLKTPLVRKE-------------  target    DGTPRADMEKRGYDEWLQIPWDEALAIAAKTLQNVAETYKGEDGAGKLLEQGYEPAMVEAMHGAGVQAIKMRGGMPLLGA 6zjn.1    -------------GRLVEATWEEAFLALKEGLKEARG----EEV--GLY-------------------------------  target    GRVFGFYRFANMLALLDGKLRPEAPPEEIVGSRAFDNYAWHTDLPPGHPMVSGSQTVDFDLFAAEHSKLLVLIGMNWICT 6zjn.1    ----LAHDATLEEGLLASELAK------ALKTPHLDFQGRTAAPA--------SLFPPASLEDLLQADFALVLG-DPTEE  target    KMPDAHWIGD-------------------------ARLKGTRVVVISADYMPTANKADEIVILRPGTDTAFLLGVARELI 6zjn.1    APILHLRLSEFVRDLKPPHRYNHGTPFADLQIKERMPRRTDKMALFAPYRAPLMKWAAIHEVHRPGEEREILLALLGDKE  target    TKKLYDRDAVIQRTDLPLLVRLDTGERLSARDVFEGYRQAPLENYVALKTEEELAAPPSPPFTADKQVVPTELREEWGDF 6zjn.1    --------------------------------------------------------------------------------  target    VYWDRATNGPAAVNRDEIGAKFAGDPALLGAFDVTLVDGTNVKARTAFSLLKEYLDENFDVQTTSEVCNVDPAAVRSLAR 6zjn.1    ----------------------------------------------------------------------GSEMVAKAKE  target    QLAANKGNALLAAGMGPNHYFNADLFGRVHFLVAALTDNIGHFSGNVGSYAG--NYRGSLFQAMGQWIAENPFDQEADLT 6zjn.1    AWEKAKN-PVLILGAGVLQDTVAAERARL------LA---ERKGAKVLAMTPAANARGLE--AMGVL-------------  target    KPARVKRYFKSESAHYWNYGDRPLVSPSEIITGKSHMPTPTKLIWFGNSNSLLGNAKWSFDVVKNTLPKQDAVFCNEWHW 6zjn.1    -PG---------------------A--KGASWDE---PGALYAY--YGFV-----------PPEEALKGKRFVVMHLSHL  target    TSSC-EYSDLVFPADSWAEFKLPDMTASCTNPFLLAFPKTPLARIHNTRSDYEILAGVAAALADLVDEPRMKTYWKGILD 6zjn.1    HPLAERYAHVVLPAPTFYEKRGH---LVNLEGRVLPLSP-APIENGEAEGALQVLALLAEALGVRPP-------FR----  target    GDPTPYLQRVLSGSNATRGILYEDLHASSAKGVPLLMNARTYPRHAGWEQRQEDKPWYTPTGRLEFYRPEPEWQAAGESL 6zjn.1    -LHLEA---------------QKALK---------------------------ARKVPEAMGRLSFRLKELR--------  target    PIWREPVDATFYEPNAILANSKHPSINPRAPEDYGVPESQMDVETRQYRNVVRTWQELKLSKHPLTEKDPAYRFVFQTPK 6zjn.1    -----PK-------------------------------------------------------------ERKGAFYLRPTM  target    YRWGAHSTAVDSDWIAMLFGPFGDPYRRDSRTPWTGEAYAEINPRDAKELGLKDGDYIWLDADPEDRPYRGADSSDEFYD 6zjn.1    WKAHQA-----VGKAQE-----------------AARAELWAHPETARAEALPEGAQVAVETP-----------------  target    VARAMMRVRIYSGMPRRVIRTWFNMYAATPGTVQAQKDVPGGPAQNQDTGYVALFRHGSHQSGTRAYLRPTQMTDSMNRK 6zjn.1    FGRVEARVVHREDVPKGHLYLSALGPAAGL--------------------------------------------------  target    AYFGQTIGKGFEADVHSPSGAPKEGYVKVEKAEDGGDEGVGEWRPVTLGLRPDDPSEAMQAYLAGEFVTRKRKGS 6zjn.1    --------------------------------------------------------------------------- ``` | | | | | | | | | | | | | | | | | | | | | | | | | | | | | | | | | | | | | | | | | | | | | | | | | |
|  | 6ziy.1.C | NADH-quinone oxidoreductase subunit 3  *Respiratory complex I from Thermus thermophilus, NADH dataset, major state* | 0.21 | 0.00 | 17.17 | 0.42 | 67-1042 | EM | 0.00 | monomer | 7 x SF4, 1 x FMN, 1 x NAI, 2 x FES | HHblits | 0.27 |
| ``` target    SDLSRRELLKRAVVVGTGAGLAELFLPAQFLSSASAQSEPQAVAIANPLAQMPDRSWERIYRDQFAEEDSFVFTCAPNDT 6ziy.1    ------------------------------------------------------------------WEMEETPTTCALCP  target    HNCLLRAHVKNGVIVRISPTYGYGKATDLAGNQASHRWDPRICQKGLILGRRIYGDRRVKAPMIRKGFKEWADAGFPRHD 6ziy.1    VGCGITADTRSGELLRIRAR------------EVPEVNEIWICDAGRFGHEW-ADQNRLKTPLVRKE-------------  target    DGTPRADMEKRGYDEWLQIPWDEALAIAAKTLQNVAETYKGEDGAGKLLEQGYEPAMVEAMHGAGVQAIKMRGGMPLLGA 6ziy.1    -------------GRLVEATWEEAFLALKEGLKEARG----EEV--GLY-------------------------------  target    GRVFGFYRFANMLALLDGKLRPEAPPEEIVGSRAFDNYAWHTDLPPGHPMVSGSQTVDFDLFAAEHSKLLVLIGMNWICT 6ziy.1    ----LAHDATLEEGLLASELAK------ALKTPHLDFQGRTAAPA--------SLFPPASLEDLLQADFALVLG-DPTEE  target    KMPDAHWIGD-------------------------ARLKGTRVVVISADYMPTANKADEIVILRPGTDTAFLLGVARELI 6ziy.1    APILHLRLSEFVRDLKPPHRYNHGTPFADLQIKERMPRRTDKMALFAPYRAPLMKWAAIHEVHRPGEEREILLALLGDKE  target    TKKLYDRDAVIQRTDLPLLVRLDTGERLSARDVFEGYRQAPLENYVALKTEEELAAPPSPPFTADKQVVPTELREEWGDF 6ziy.1    --------------------------------------------------------------------------------  target    VYWDRATNGPAAVNRDEIGAKFAGDPALLGAFDVTLVDGTNVKARTAFSLLKEYLDENFDVQTTSEVCNVDPAAVRSLAR 6ziy.1    ----------------------------------------------------------------------GSEMVAKAKE  target    QLAANKGNALLAAGMGPNHYFNADLFGRVHFLVAALTDNIGHFSGNVGSYAG--NYRGSLFQAMGQWIAENPFDQEADLT 6ziy.1    AWEKAKN-PVLILGAGVLQDTVAAERARL------LA---ERKGAKVLAMTPAANARGLE--AMGVL-------------  target    KPARVKRYFKSESAHYWNYGDRPLVSPSEIITGKSHMPTPTKLIWFGNSNSLLGNAKWSFDVVKNTLPKQDAVFCNEWHW 6ziy.1    -PG---------------------A--KGASWDE---PGALYAY--YGFV-----------PPEEALKGKRFVVMHLSHL  target    TSSC-EYSDLVFPADSWAEFKLPDMTASCTNPFLLAFPKTPLARIHNTRSDYEILAGVAAALADLVDEPRMKTYWKGILD 6ziy.1    HPLAERYAHVVLPAPTFYEKRGH---LVNLEGRVLPLSP-APIENGEAEGALQVLALLAEALGVRPP-------FR----  target    GDPTPYLQRVLSGSNATRGILYEDLHASSAKGVPLLMNARTYPRHAGWEQRQEDKPWYTPTGRLEFYRPEPEWQAAGESL 6ziy.1    -LHLEA---------------QKALK---------------------------ARKVPEAMGRLSFRLKELR--------  target    PIWREPVDATFYEPNAILANSKHPSINPRAPEDYGVPESQMDVETRQYRNVVRTWQELKLSKHPLTEKDPAYRFVFQTPK 6ziy.1    -----PK-------------------------------------------------------------ERKGAFYLRPTM  target    YRWGAHSTAVDSDWIAMLFGPFGDPYRRDSRTPWTGEAYAEINPRDAKELGLKDGDYIWLDADPEDRPYRGADSSDEFYD 6ziy.1    WKAHQA-----VGKAQE-----------------AARAELWAHPETARAEALPEGAQVAVETP-----------------  target    VARAMMRVRIYSGMPRRVIRTWFNMYAATPGTVQAQKDVPGGPAQNQDTGYVALFRHGSHQSGTRAYLRPTQMTDSMNRK 6ziy.1    FGRVEARVVHREDVPKGHLYLSALGPAAGL--------------------------------------------------  target    AYFGQTIGKGFEADVHSPSGAPKEGYVKVEKAEDGGDEGVGEWRPVTLGLRPDDPSEAMQAYLAGEFVTRKRKGS 6ziy.1    --------------------------------------------------------------------------- ``` | | | | | | | | | | | | | | | | | | | | | | | | | | | | | | | | | | | | | | | | | | | | | | | | | |
|  | 7t2r.1.A | NiFe hydrogenase subunit A  *Structure of electron bifurcating Ni-Fe hydrogenase complex HydABCSL in FMN-free apo state* | 0.18 |  | 15.14 | 0.37 | 70-756 | EM | 0.00 | hetero-2-2-2-2-2-mer | 6 x FES, 12 x SF4, 2 x 3NI, 2 x FCO | HHblits | 0.27 |
| ``` target    SDLSRRELLKRAVVVGTGAGLAELFLPAQFLSSASAQSEPQAVAIANPLAQMPDRSWERIYRDQFAEEDSFVFTCAPNDT 7t2r.1    ---------------------------------------------------------------------AVVESVCPLCA  target    HNCLLRAHVKNGVIVRISPTYGYGKATDLAGNQASHRWDPRICQKGLILGRRIYGDRRVKAPMIRKGFKEWADAGFPRHD 7t2r.1    VGCKIKTYVRTGSIVRVEGT------------GVEEPDGGQLCHMGRWWLPESTERERVTVPLIREG-------------  target    DGTPRADMEKRGYDEWLQIPWDEALAIAAKTLQNVAETYKGEDGAGKLLEQGYEPAMVEAMHGAGVQAIKMRGGMPLLGA 7t2r.1    -------------ASYREATWEEALALASAEFKKAYDQEKAG----AILS------------------------------  target    GRVFGFYRFANMLALLDGKLRPEAPPEEIVGSRAFDNYAWHTD--LPPGH-PM-VSGSQTVDFDLFAAEHSKLLVLIGMN 7t2r.1    ------SLCTDEELTLFSALFR-----NALKMKHIDTFDGDIIRGFFKGFMPFREQGVR-PFTAAHHILDSDLIITMFAD  target    WICTKMPDAHWIGDAR-LKGTRVVVISADYMPTANKADEIVILRPGTDTAFLLGVARELITKKLYDRDAVIQRTDLPLLV 7t2r.1    PQKEAPVVASYIRVACLHRNAKLMNLSYGPSPFPGLVDLDIRLPEGQAVPKALSNLAEIIGKISLGPSDMASFGE-----  target    RLDTGERLSARDVFEGYRQAPLENYVALKTEEELAAPPSPPFTADKQVVPTELREEWGDFVYWDRATNGPAAVNRDEIGA 7t2r.1    ------------------------F-----------------------------------E-------------------  target    KFAGDPALLGAFDVTLVDGTNVKARTAFSLLKEYLDENFDVQTTSEVCNVDPAAVRSLARQLAANKGNALLAAGMGPNHY 7t2r.1    -----------------AG--------AGKALSSY--RESIEESARAMGLDPKIAEEVALMLISARRPIFI-IGGR---A  target    FNADLFGRVHFLVAALTDNIGHFSGNVGSYAGNYRGSLFQAMGQWIAENPFDQEADLTKPARVKRYFKSESAHYWNYGDR 7t2r.1    TKSHELVTAACNLAVASKAFFEDGLGVVPLLVSAN-----SLGAR------N----T----VV--------------SEN  target    PLVSPSEIITGKSHMPTPTKLIWFGNSNSLLGNAKWSFDVVKNTLPKQDAVFCNEWHWT-SSCEYSDLVFPADSWAEFKL 7t2r.1    PW-------LG----RERRDFLYVFSTAMV---PE--EEEILAAISATRFVVVQTPFKVRPLVNLADILLPAPAWYERSG  target    PDMTASCTNPFLLAFPKTPLARIHNTRSDYEILAGVAAALADLVDEPRMKTYWKGILDGDPTPYLQRVLSGSNATRGILY 7t2r.1    HF---CTIEGERRKLNT-IVPPKGEIKSLHYVMDEFAKKLGV--------------------------------------  target    EDLHASSAKGVPLLMNARTYPRHAGWEQRQEDKPWYTPTGRLEFYRPEPEWQAAGESLPIWREPVDATFYEPNAILANSK 7t2r.1    --------------------------------------------------------------------------------  target    HPSINPRAPEDYGVPESQMDVETRQYRNVVRTWQELKLSKHPLTEKDPAYRFVFQTPKYRWGAHSTAVDSDWIAMLFGPF 7t2r.1    --------------------------------------------------------------------------------  target    GDPYRRDSRTPWTGEAYAEINPRDAKELGLKDGDYIWLDADPEDRPYRGADSSDEFYDVARAMMRVRIYSGMPRRVIRTW 7t2r.1    --------------------------------------------------------------------------------  target    FNMYAATPGTVQAQKDVPGGPAQNQDTGYVALFRHGSHQSGTRAYLRPTQMTDSMNRKAYFGQTIGKGFEADVHSPSGAP 7t2r.1    --------------------------------------------------------------------------------  target    KEGYVKVEKAEDGGDEGVGEWRPVTLGLRPDDPSEAMQAYLAGEFVTRKRKGS 7t2r.1    ----------------------------------------------------- ``` | | | | | | | | | | | | | | | | | | | | | | | | | | | | | | | | | | | | | | | | | | | | | | | | | |
|  | 7t30.1.A | NiFe hydrogenase subunit A  *Structure of electron bifurcating Ni-Fe hydrogenase complex HydABCSL in FMN/NAD(H) bound state* | 0.18 |  | 15.14 | 0.37 | 70-756 | EM | 0.00 | hetero-2-2-2-2-2-mer | 4 x FES, 12 x SF4, 2 x NAD, 2 x FMN, 2 x 3NI, 2 x FCO | HHblits | 0.27 |
| ``` target    SDLSRRELLKRAVVVGTGAGLAELFLPAQFLSSASAQSEPQAVAIANPLAQMPDRSWERIYRDQFAEEDSFVFTCAPNDT 7t30.1    ---------------------------------------------------------------------AVVESVCPLCA  target    HNCLLRAHVKNGVIVRISPTYGYGKATDLAGNQASHRWDPRICQKGLILGRRIYGDRRVKAPMIRKGFKEWADAGFPRHD 7t30.1    VGCKIKTYVRTGSIVRVEGT------------GVEEPDGGQLCHMGRWWLPESTERERVTVPLIREG-------------  target    DGTPRADMEKRGYDEWLQIPWDEALAIAAKTLQNVAETYKGEDGAGKLLEQGYEPAMVEAMHGAGVQAIKMRGGMPLLGA 7t30.1    -------------ASYREATWEEALALASAEFKKAYDQEKAG----AILS------------------------------  target    GRVFGFYRFANMLALLDGKLRPEAPPEEIVGSRAFDNYAWHTD--LPPGH-PM-VSGSQTVDFDLFAAEHSKLLVLIGMN 7t30.1    ------SLCTDEELTLFSALFR-----NALKMKHIDTFDGDIIRGFFKGFMPFREQGVR-PFTAAHHILDSDLIITMFAD  target    WICTKMPDAHWIGDAR-LKGTRVVVISADYMPTANKADEIVILRPGTDTAFLLGVARELITKKLYDRDAVIQRTDLPLLV 7t30.1    PQKEAPVVASYIRVACLHRNAKLMNLSYGPSPFPGLVDLDIRLPEGQAVPKALSNLAEIIGKISLGPSDMASFGE-----  target    RLDTGERLSARDVFEGYRQAPLENYVALKTEEELAAPPSPPFTADKQVVPTELREEWGDFVYWDRATNGPAAVNRDEIGA 7t30.1    ------------------------F-----------------------------------E-------------------  target    KFAGDPALLGAFDVTLVDGTNVKARTAFSLLKEYLDENFDVQTTSEVCNVDPAAVRSLARQLAANKGNALLAAGMGPNHY 7t30.1    -----------------AG--------AGKALSSY--RESIEESARAMGLDPKIAEEVALMLISARRPIFI-IGGR---A  target    FNADLFGRVHFLVAALTDNIGHFSGNVGSYAGNYRGSLFQAMGQWIAENPFDQEADLTKPARVKRYFKSESAHYWNYGDR 7t30.1    TKSHELVTAACNLAVASKAFFEDGLGVVPLLVSAN-----SLGAR------N----T----VV--------------SEN  target    PLVSPSEIITGKSHMPTPTKLIWFGNSNSLLGNAKWSFDVVKNTLPKQDAVFCNEWHWT-SSCEYSDLVFPADSWAEFKL 7t30.1    PW-------LG----RERRDFLYVFSTAMV---PE--EEEILAAISATRFVVVQTPFKVRPLVNLADILLPAPAWYERSG  target    PDMTASCTNPFLLAFPKTPLARIHNTRSDYEILAGVAAALADLVDEPRMKTYWKGILDGDPTPYLQRVLSGSNATRGILY 7t30.1    HF---CTIEGERRKLNT-IVPPKGEIKSLHYVMDEFAKKLGV--------------------------------------  target    EDLHASSAKGVPLLMNARTYPRHAGWEQRQEDKPWYTPTGRLEFYRPEPEWQAAGESLPIWREPVDATFYEPNAILANSK 7t30.1    --------------------------------------------------------------------------------  target    HPSINPRAPEDYGVPESQMDVETRQYRNVVRTWQELKLSKHPLTEKDPAYRFVFQTPKYRWGAHSTAVDSDWIAMLFGPF 7t30.1    --------------------------------------------------------------------------------  target    GDPYRRDSRTPWTGEAYAEINPRDAKELGLKDGDYIWLDADPEDRPYRGADSSDEFYDVARAMMRVRIYSGMPRRVIRTW 7t30.1    --------------------------------------------------------------------------------  target    FNMYAATPGTVQAQKDVPGGPAQNQDTGYVALFRHGSHQSGTRAYLRPTQMTDSMNRKAYFGQTIGKGFEADVHSPSGAP 7t30.1    --------------------------------------------------------------------------------  target    KEGYVKVEKAEDGGDEGVGEWRPVTLGLRPDDPSEAMQAYLAGEFVTRKRKGS 7t30.1    ----------------------------------------------------- ``` | | | | | | | | | | | | | | | | | | | | | | | | | | | | | | | | | | | | | | | | | | | | | | | | | |
|  | 5t5i.1.B | Tungsten formylmethanofuran dehydrogenase subunit B  *TUNGSTEN-CONTAINING FORMYLMETHANOFURAN DEHYDROGENASE FROM METHANOTHERMOBACTER WOLFEII, ORTHORHOMBIC FORM AT 1.9 A* | 0.17 |  | 17.52 | 0.35 | 70-756 | X-ray | 1.90 | hetero-oligomer | 4 x ZN, 2 x MG, 18 x K, 22 x SF4, 2 x W, 4 x MGD, 2 x H2S, 2 x CA | HHblits | 0.28 |
| ``` target    SDLSRRELLKRAVVVGTGAGLAELFLPAQFLSSASAQSEPQAVAIANPLAQMPDRSWERIYRDQFAEEDSFVFTCAPNDT 5t5i.1    ---------------------------------------------------------------------YVKNVVCPFCG  target    HNCL-LRAHVKNGVIVRISPTYGYGKATDLAGNQASHRWDPRICQKGLILGRRIYGDRRVKAPMIRKGFKEWADAGFPRH 5t5i.1    TLCDDIICKVEGNEIVGT----------------------INACRIGHSKFVHAEGAMRYKKPLIRKN------------  target    DDGTPRADMEKRGYDEWLQIPWDEALAIAAKTLQNVAETYKGEDGAGKLLEQGYEPAMVEAMHGAGVQAIKMRGGMPLLG 5t5i.1    --------------GEFVEVSYDEAIDKAAKILAESKRP--------LMYG-------------WSCTECE---------  target    AGRVFGFYRFANMLALLDGKLRPEAPPEEIVGSRAFDNYAWHTDLP--PGHPMVSGSQTVDFDLFAA-EHSKLLVLIGMN 5t5i.1    --AQAVGVELAEEA-----------------GA-VIDNTASVCHGPSVLALQ-DVGYPI--CTFGEVKNRADVVVYWGCN  target    WICTKMPDAHW-------IGDARLKGTRVVVISADYMPTANKADEIVILRPGTDTAFLLGVARELITKKLYDRDAVIQRT 5t5i.1    PMHAHPRHMSRNVFARGFFRERGRSDRTLIVVDPRKTDSAKLADIHLQLDFDRDYELLDAMRACLLGHE-----------  target    DLPLLVRLDTGERLSARDVFEGYRQAPLENYVALKTEEELAAPPSPPFTADKQVVPTELREEWGDFVYWDRATNGPAAVN 5t5i.1    --------------------------------------------------------------------------------  target    RDEIGAKFAGDPALLGAFDVTLVDGTNVKARTAFSLLKEYLDENFDVQTTSEVCNVDPAAVRSLARQLAANKGNALLAAG 5t5i.1    -----------------------------------------------ILYDEVAGVPREQIEEAVEVLKNAQF-GILFFG  target    MGPNHYFNADLFGRVHFLVAALTDNIGHFSGNVGSYAGN--YRGSLFQAMGQWIAENPFDQEADLTKPARVKRYFKSESA 5t5i.1    MGITHSRGKHRNIDTAIMMVQDLNDY--AKWTLIPMRGHYNVTGFNQ--VCTWESGYPYC--VDFSGG---------E-P  target    HYWNYGDRPLVSPSEIITGKSHMPTPTKLIWFGNSNSLLGNAKWSFDVVKNTLPKQDAVFCNEWHWTSSCEYSDLVFPAD 5t5i.1    RY-N-P--GETGANDLL-----QNREADAMMVIASDPGAHFPQ----RALERMAEIP-VIAIEPHRTPTTEMADIIIPPA  target    S-WAEFKLPDMTASCTNPFLLAFPKTPLARIHNTRSDYEILAGVAAALADLVDEPRMKTYWKGILDGDPTPYLQRVLSGS 5t5i.1    IVGMEAEGTAY---RMEGVPIRMKK-VVDS--DLLSDREILERLLEKVRE------------------------------  target    NATRGILYEDLHASSAKGVPLLMNARTYPRHAGWEQRQEDKPWYTPTGRLEFYRPEPEWQAAGESLPIWREPVDATFYEP 5t5i.1    --------------------------------------------------------------------------------  target    NAILANSKHPSINPRAPEDYGVPESQMDVETRQYRNVVRTWQELKLSKHPLTEKDPAYRFVFQTPKYRWGAHSTAVDSDW 5t5i.1    --------------------------------------------------------------------------------  target    IAMLFGPFGDPYRRDSRTPWTGEAYAEINPRDAKELGLKDGDYIWLDADPEDRPYRGADSSDEFYDVARAMMRVRIYSGM 5t5i.1    --------------------------------------------------------------------------------  target    PRRVIRTWFNMYAATPGTVQAQKDVPGGPAQNQDTGYVALFRHGSHQSGTRAYLRPTQMTDSMNRKAYFGQTIGKGFEAD 5t5i.1    --------------------------------------------------------------------------------  target    VHSPSGAPKEGYVKVEKAEDGGDEGVGEWRPVTLGLRPDDPSEAMQAYLAGEFVTRKRKGS 5t5i.1    ------------------------------------------------------------- ``` | | | | | | | | | | | | | | | | | | | | | | | | | | | | | | | | | | | | | | | | | | | | | | | | | |
|  | 7bkb.1.L | Formylmethanofuran dehydrogenase, subunit B  *Formate dehydrogenase - heterodisulfide reductase - formylmethanofuran dehydrogenase complex from Methanospirillum hungatei (hexameric, composite structure)* | 0.16 |  | 16.02 | 0.35 | 69-757 | EM | 0.00 | hetero-2-2-2-2-2-2-… | 48 x SF4, 4 x FAD, 2 x FES, 4 x 9S8, 4 x ZN, 2 x MO, 4 x MGD | HHblits | 0.27 |
| ``` target    SDLSRRELLKRAVVVGTGAGLAELFLPAQFLSSASAQSEPQAVAIANPLAQMPDRSWERIYRDQFAEEDSFVFTCAPNDT 7bkb.1    --------------------------------------------------------------------KVIENVGCPYCG  target    HNCL-LRAHVKNGVIVRISPTYGYGKATDLAGNQASHRWDPRICQKGLILGRRIYGDRRVKAPMIRKGFKEWADAGFPRH 7bkb.1    CSCDDVRITVSDDGK--------------------DILEVENVCAIGTEIFKHGCSKDRIRLPRMRQP------------  target    DDGTPRADMEKRGYDEWLQIPWDEALAIAAKTLQNVAETYKGEDGAGKLLEQGYEPAMVEAMHGAGVQAIKMRGGMPLLG 7bkb.1    -------------DGSMKDISYEEAIDWTARHLLKAKKP--------LMYG-------------FGSTNCE---------  target    AGRVFGFYRFANMLALLDGKLRPEAPPEEIVGSRAFDNYAWHTDLPPGHPMVSGSQTVDFDLFAA-EHSKLLVLIGMNWI 7bkb.1    --GQAAAARVMEIA-----------------GG-MLDNCATICHGP-SFLAIFDNGYPSCTLGEVKNRADVIVYWGSNPA  target    CTKMPDAHWI--------GDARLKGTRVVVISADYMPTANKADEIVILRPGTDTAFLLGVARELITKKLYDRDAVIQRTD 7bkb.1    HAHPRHMSRYSIFPRGFFTGKGQKKRTVIVIDPRFTDTANVADYHLQVKQGHDYELFNAFRMVIHGHG------------  target    LPLLVRLDTGERLSARDVFEGYRQAPLENYVALKTEEELAAPPSPPFTADKQVVPTELREEWGDFVYWDRATNGPAAVNR 7bkb.1    --------------------------------------------------------------------------------  target    DEIGAKFAGDPALLGAFDVTLVDGTNVKARTAFSLLKEYLDENFDVQTTSEVCNVDPAAVRSLARQLAANKGNALLAAGM 7bkb.1    ---------------------------------------------KDLPDEVAGIKKETILEVAEIMKNARF-GTTFFGM  target    GPNHYFNADLFGRVHF------------LVAALTDNIGHFSGNVGSYAGNYRGSLFQAMGQWIAENPFDQEADLTKPARV 7bkb.1    GLTHTDGRNHNIDIAISLTRDLNKISKWTIMAMRGHYNIAGPGVVWSWTF----------GF----PYCL--DLTKQN--  target    KRYFKSESAHYWNYGDRPLVSPSEIITGKSHMPTPTKLIWFGNSNSLLGNAKWSFDVVKNTLPKQDAVFCNEWHWTSSCE 7bkb.1    --------HAHMN----PG--ETSSVDM--AMRDEVDMFINIGTDAAAHFPI---P-AVKQLKKHPW-VTIDPSINMASE  target    YSDLVFPADSW-AEFKLPDMTASCTNPFLLAFPKTPLARIHNTRSDYEILAGVAAALADLVDEPRMKTYWKGILDGDPTP 7bkb.1    ISDLHIPVCICGVDVGGIV---YRMDNVPIQFRK-VIEPPEGVMDDETLLNKIADRMEEL--------------------  target    YLQRVLSGSNATRGILYEDLHASSAKGVPLLMNARTYPRHAGWEQRQEDKPWYTPTGRLEFYRPEPEWQAAGESLPIWRE 7bkb.1    --------------------------------------------------------------------------------  target    PVDATFYEPNAILANSKHPSINPRAPEDYGVPESQMDVETRQYRNVVRTWQELKLSKHPLTEKDPAYRFVFQTPKYRWGA 7bkb.1    --------------------------------------------------------------------------------  target    HSTAVDSDWIAMLFGPFGDPYRRDSRTPWTGEAYAEINPRDAKELGLKDGDYIWLDADPEDRPYRGADSSDEFYDVARAM 7bkb.1    --------------------------------------------------------------------------------  target    MRVRIYSGMPRRVIRTWFNMYAATPGTVQAQKDVPGGPAQNQDTGYVALFRHGSHQSGTRAYLRPTQMTDSMNRKAYFGQ 7bkb.1    --------------------------------------------------------------------------------  target    TIGKGFEADVHSPSGAPKEGYVKVEKAEDGGDEGVGEWRPVTLGLRPDDPSEAMQAYLAGEFVTRKRKGS 7bkb.1    ---------------------------------------------------------------------- ``` | | | | | | | | | | | | | | | | | | | | | | | | | | | | | | | | | | | | | | | | | | | | | | | | | |
|  | 6yj4.1.G | Subunit NUAM of NADH:Ubiquinone Oxidoreductase (Complex I)  *Structure of Yarrowia lipolytica complex I at 2.7 A* | 0.17 |  | 19.63 | 0.33 | 68-756 | EM | 0.00 | hetero-1-1-1-1-1-1-… | 18 x 3PE, 6 x SF4, 5 x LMT, 8 x PLC, 2 x FES, 1 x FMN, 6 x CDL, 1 x NDP, 1 x ZN, 2 x EHZ | HHblits | 0.29 |
| ``` target    SDLSRRELLKRAVVVGTGAGLAELFLPAQFLSSASAQSEPQAVAIANPLAQMPDRSWERIYRDQFAEEDSFVFTCAPNDT 6yj4.1    -------------------------------------------------------------------ELKKTESIDVMDA  target    HNCLLRAHVKNGVIVRISPTYGYGKATDLAGNQASHRWDPRICQKGLILGRRIYGDRRVKAPMIRKGFKEWADAGFPRHD 6yj4.1    VGSNIRIDSKGVEVMRVIPRV------------HEDVNEEWINDKSRFACDGLK-TQRLTTPLIRVG-------------  target    DGTPRADMEKRGYDEWLQIPWDEALAIAAKTLQNVAETYKGEDGAGKLLEQGYEPAMVEAMHGAGVQAIKMRGGMPLLGA 6yj4.1    -------------DKFVNATWDDALSTIAKAYQQKAP--KGDEF--KAV------------AGALVEV------------  target    GRVFGFYRFANMLALLDGKLRPEAPPEEIVGSRAFDNYAWHTDLPPGHPMVSGSQ-TVDFDLFAAEHSKLLVLIGMNWIC 6yj4.1    ESMVALKDMTNAL-----------------GSENTTTDTPNGNSAPAHGITFRSNYLFNSSIAGIEDADAILLVGTNPRR  target    TKMPDAHWIGDAR-LKGTRVVVISADYMPTANKADEIVILRPGTDTAFLLGVARELITKKLYDRDAVIQRTDLPLLVRLD 6yj4.1    EAAVMNARIRKAWLRQELEIASVGPTLDATFDVAEL--------------GNT---------------------------  target    TGERLSARDVFEGYRQAPLENYVALKTEEELAAPPSPPFTADKQVVPTELREEWGDFVYWDRATNGPAAVNRDEIGAKFA 6yj4.1    --------------------------------------------------------------------------------  target    GDPALLGAFDVTLVDGTNVKARTAFSLLKEYLDENFDVQTTSEVCNVDPAAVRSLARQLAANKGNALLAAGMGPNHYFNA 6yj4.1    ------------------------HADLEKALS-------------------GEFGEVLKNAKN-PLIIVGSGITDREDA  target    DLFGRVHFLVAALTDNI-GHFSGNVGSYAGNYRGSLFQAMGQWIAENPFDQEADLTKPARVKRYFKSESAHYWNYGDRPL 6yj4.1    GAFFNTIGKFVESTPSVLNENWNGYNVLQRSAS-----RAGA------Y----DI--------------------GFTPS  target    VSPSEIITGKSHMPTPTKLIWFGNSNSLLGNAKWSFDVVKNTLPKQDAVFCNEWHWTSSCEYSDLVFPADSWAEFKLPDM 6yj4.1    ---DEA------SKTTPKMVWLLGADEVAAS----------DIPADAFVVYQGHNGDVGAQFADVVLPGAAYTEKAGT--  target    TASCTNPFLLAFPKTPLARIHNTRSDYEILAGVAAALADLVDEPRMKTYWKGILDGDPTPYLQRVLSGSNATRGILYEDL 6yj4.1    -YVNTEGRSQISRA-ATGPPGGAREDWKILRAVSEYLGV-----------------------------------------  target    HASSAKGVPLLMNARTYPRHAGWEQRQEDKPWYTPTGRLEFYRPEPEWQAAGESLPIWREPVDATFYEPNAILANSKHPS 6yj4.1    --------------------------------------------------------------------------------  target    INPRAPEDYGVPESQMDVETRQYRNVVRTWQELKLSKHPLTEKDPAYRFVFQTPKYRWGAHSTAVDSDWIAMLFGPFGDP 6yj4.1    --------------------------------------------------------------------------------  target    YRRDSRTPWTGEAYAEINPRDAKELGLKDGDYIWLDADPEDRPYRGADSSDEFYDVARAMMRVRIYSGMPRRVIRTWFNM 6yj4.1    --------------------------------------------------------------------------------  target    YAATPGTVQAQKDVPGGPAQNQDTGYVALFRHGSHQSGTRAYLRPTQMTDSMNRKAYFGQTIGKGFEADVHSPSGAPKEG 6yj4.1    --------------------------------------------------------------------------------  target    YVKVEKAEDGGDEGVGEWRPVTLGLRPDDPSEAMQAYLAGEFVTRKRKGS 6yj4.1    -------------------------------------------------- ``` | | | | | | | | | | | | | | | | | | | | | | | | | | | | | | | | | | | | | | | | | | | | | | | | | |
|  | 6rfs.1.A | Subunit NUAM of NADH:Ubiquinone Oxidoreductase (Complex I)  *Cryo-EM structure of a respiratory complex I mutant lacking NDUFS4* | 0.17 |  | 19.63 | 0.33 | 68-756 | EM | 4.04 | hetero-1-1-1-1-1-1-… | 6 x SF4, 2 x FES, 1 x FMN, 1 x NDP, 1 x ZN, 1 x ZMP | HHblits | 0.29 |
| ``` target    SDLSRRELLKRAVVVGTGAGLAELFLPAQFLSSASAQSEPQAVAIANPLAQMPDRSWERIYRDQFAEEDSFVFTCAPNDT 6rfs.1    -------------------------------------------------------------------ELKKTESIDVMDA  target    HNCLLRAHVKNGVIVRISPTYGYGKATDLAGNQASHRWDPRICQKGLILGRRIYGDRRVKAPMIRKGFKEWADAGFPRHD 6rfs.1    VGSNIRIDSKGVEVMRVIPRV------------HEDVNEEWINDKSRFACDGLK-TQRLTTPLIRVG-------------  target    DGTPRADMEKRGYDEWLQIPWDEALAIAAKTLQNVAETYKGEDGAGKLLEQGYEPAMVEAMHGAGVQAIKMRGGMPLLGA 6rfs.1    -------------DKFVNATWDDALSTIAKAYQQKAP--KGDEF--KAV------------AGALVEV------------  target    GRVFGFYRFANMLALLDGKLRPEAPPEEIVGSRAFDNYAWHTDLPPGHPMVSGSQ-TVDFDLFAAEHSKLLVLIGMNWIC 6rfs.1    ESMVALKDMTNAL-----------------GSENTTTDTPNGNSAPAHGITFRSNYLFNSSIAGIEDADAILLVGTNPRR  target    TKMPDAHWIGDAR-LKGTRVVVISADYMPTANKADEIVILRPGTDTAFLLGVARELITKKLYDRDAVIQRTDLPLLVRLD 6rfs.1    EAAVMNARIRKAWLRQELEIASVGPTLDATFDVAEL--------------GNT---------------------------  target    TGERLSARDVFEGYRQAPLENYVALKTEEELAAPPSPPFTADKQVVPTELREEWGDFVYWDRATNGPAAVNRDEIGAKFA 6rfs.1    --------------------------------------------------------------------------------  target    GDPALLGAFDVTLVDGTNVKARTAFSLLKEYLDENFDVQTTSEVCNVDPAAVRSLARQLAANKGNALLAAGMGPNHYFNA 6rfs.1    ------------------------HADLEKALS-------------------GEFGEVLKNAKN-PLIIVGSGITDREDA  target    DLFGRVHFLVAALTDNI-GHFSGNVGSYAGNYRGSLFQAMGQWIAENPFDQEADLTKPARVKRYFKSESAHYWNYGDRPL 6rfs.1    GAFFNTIGKFVESTPSVLNENWNGYNVLQRSAS-----RAGA------Y----DI--------------------GFTPS  target    VSPSEIITGKSHMPTPTKLIWFGNSNSLLGNAKWSFDVVKNTLPKQDAVFCNEWHWTSSCEYSDLVFPADSWAEFKLPDM 6rfs.1    ---DEA------SKTTPKMVWLLGADEVAAS----------DIPADAFVVYQGHNGDVGAQFADVVLPGAAYTEKAGT--  target    TASCTNPFLLAFPKTPLARIHNTRSDYEILAGVAAALADLVDEPRMKTYWKGILDGDPTPYLQRVLSGSNATRGILYEDL 6rfs.1    -YVNTEGRSQISRA-ATGPPGGAREDWKILRAVSEYLGV-----------------------------------------  target    HASSAKGVPLLMNARTYPRHAGWEQRQEDKPWYTPTGRLEFYRPEPEWQAAGESLPIWREPVDATFYEPNAILANSKHPS 6rfs.1    --------------------------------------------------------------------------------  target    INPRAPEDYGVPESQMDVETRQYRNVVRTWQELKLSKHPLTEKDPAYRFVFQTPKYRWGAHSTAVDSDWIAMLFGPFGDP 6rfs.1    --------------------------------------------------------------------------------  target    YRRDSRTPWTGEAYAEINPRDAKELGLKDGDYIWLDADPEDRPYRGADSSDEFYDVARAMMRVRIYSGMPRRVIRTWFNM 6rfs.1    --------------------------------------------------------------------------------  target    YAATPGTVQAQKDVPGGPAQNQDTGYVALFRHGSHQSGTRAYLRPTQMTDSMNRKAYFGQTIGKGFEADVHSPSGAPKEG 6rfs.1    --------------------------------------------------------------------------------  target    YVKVEKAEDGGDEGVGEWRPVTLGLRPDDPSEAMQAYLAGEFVTRKRKGS 6rfs.1    -------------------------------------------------- ``` | | | | | | | | | | | | | | | | | | | | | | | | | | | | | | | | | | | | | | | | | | | | | | | | | |
|  | 6rfq.1.A | Subunit NUAM of NADH:Ubiquinone Oxidoreductase (Complex I)  *Cryo-EM structure of a respiratory complex I assembly intermediate with NDUFAF2* | 0.17 |  | 19.63 | 0.33 | 68-756 | EM | 3.30 | hetero-1-1-1-1-1-1-… | 6 x SF4, 2 x FES, 1 x FMN, 1 x NDP, 10 x 3PE, 2 x LMN, 4 x CDL, 2 x ZMP, 4 x PLC, 3 x T7X, 1 x CPL | HHblits | 0.29 |
| ``` target    SDLSRRELLKRAVVVGTGAGLAELFLPAQFLSSASAQSEPQAVAIANPLAQMPDRSWERIYRDQFAEEDSFVFTCAPNDT 6rfq.1    -------------------------------------------------------------------ELKKTESIDVMDA  target    HNCLLRAHVKNGVIVRISPTYGYGKATDLAGNQASHRWDPRICQKGLILGRRIYGDRRVKAPMIRKGFKEWADAGFPRHD 6rfq.1    VGSNIRIDSKGVEVMRVIPRV------------HEDVNEEWINDKSRFACDGLK-TQRLTTPLIRVG-------------  target    DGTPRADMEKRGYDEWLQIPWDEALAIAAKTLQNVAETYKGEDGAGKLLEQGYEPAMVEAMHGAGVQAIKMRGGMPLLGA 6rfq.1    -------------DKFVNATWDDALSTIAKAYQQKAP--KGDEF--KAV------------AGALVEV------------  target    GRVFGFYRFANMLALLDGKLRPEAPPEEIVGSRAFDNYAWHTDLPPGHPMVSGSQ-TVDFDLFAAEHSKLLVLIGMNWIC 6rfq.1    ESMVALKDMTNAL-----------------GSENTTTDTPNGNSAPAHGITFRSNYLFNSSIAGIEDADAILLVGTNPRR  target    TKMPDAHWIGDAR-LKGTRVVVISADYMPTANKADEIVILRPGTDTAFLLGVARELITKKLYDRDAVIQRTDLPLLVRLD 6rfq.1    EAAVMNARIRKAWLRQELEIASVGPTLDATFDVAEL--------------GNT---------------------------  target    TGERLSARDVFEGYRQAPLENYVALKTEEELAAPPSPPFTADKQVVPTELREEWGDFVYWDRATNGPAAVNRDEIGAKFA 6rfq.1    --------------------------------------------------------------------------------  target    GDPALLGAFDVTLVDGTNVKARTAFSLLKEYLDENFDVQTTSEVCNVDPAAVRSLARQLAANKGNALLAAGMGPNHYFNA 6rfq.1    ------------------------HADLEKALS-------------------GEFGEVLKNAKN-PLIIVGSGITDREDA  target    DLFGRVHFLVAALTDNI-GHFSGNVGSYAGNYRGSLFQAMGQWIAENPFDQEADLTKPARVKRYFKSESAHYWNYGDRPL 6rfq.1    GAFFNTIGKFVESTPSVLNENWNGYNVLQRSAS-----RAGA------Y----DI--------------------GFTPS  target    VSPSEIITGKSHMPTPTKLIWFGNSNSLLGNAKWSFDVVKNTLPKQDAVFCNEWHWTSSCEYSDLVFPADSWAEFKLPDM 6rfq.1    ---DEA------SKTTPKMVWLLGADEVAAS----------DIPADAFVVYQGHNGDVGAQFADVVLPGAAYTEKAGT--  target    TASCTNPFLLAFPKTPLARIHNTRSDYEILAGVAAALADLVDEPRMKTYWKGILDGDPTPYLQRVLSGSNATRGILYEDL 6rfq.1    -YVNTEGRSQISRA-ATGPPGGAREDWKILRAVSEYLGV-----------------------------------------  target    HASSAKGVPLLMNARTYPRHAGWEQRQEDKPWYTPTGRLEFYRPEPEWQAAGESLPIWREPVDATFYEPNAILANSKHPS 6rfq.1    --------------------------------------------------------------------------------  target    INPRAPEDYGVPESQMDVETRQYRNVVRTWQELKLSKHPLTEKDPAYRFVFQTPKYRWGAHSTAVDSDWIAMLFGPFGDP 6rfq.1    --------------------------------------------------------------------------------  target    YRRDSRTPWTGEAYAEINPRDAKELGLKDGDYIWLDADPEDRPYRGADSSDEFYDVARAMMRVRIYSGMPRRVIRTWFNM 6rfq.1    --------------------------------------------------------------------------------  target    YAATPGTVQAQKDVPGGPAQNQDTGYVALFRHGSHQSGTRAYLRPTQMTDSMNRKAYFGQTIGKGFEADVHSPSGAPKEG 6rfq.1    --------------------------------------------------------------------------------  target    YVKVEKAEDGGDEGVGEWRPVTLGLRPDDPSEAMQAYLAGEFVTRKRKGS 6rfq.1    -------------------------------------------------- ``` | | | | | | | | | | | | | | | | | | | | | | | | | | | | | | | | | | | | | | | | | | | | | | | | | |
|  | 6gcs.1.A | 75-KDA PROTEIN (NUAM)  *Cryo-EM structure of respiratory complex I from Yarrowia lipolytica* | 0.17 |  | 19.63 | 0.33 | 68-756 | EM | 4.32 | hetero-1-1-1-1-1-1-… | 6 x SF4, 2 x FES, 1 x FMN, 1 x NDP, 1 x ZN, 1 x ZMP, 1 x CDL, 3 x 3PE | HHblits | 0.29 |
| ``` target    SDLSRRELLKRAVVVGTGAGLAELFLPAQFLSSASAQSEPQAVAIANPLAQMPDRSWERIYRDQFAEEDSFVFTCAPNDT 6gcs.1    -------------------------------------------------------------------ELKKTESIDVMDA  target    HNCLLRAHVKNGVIVRISPTYGYGKATDLAGNQASHRWDPRICQKGLILGRRIYGDRRVKAPMIRKGFKEWADAGFPRHD 6gcs.1    VGSNIRIDSKGVEVMRVIPRV------------HEDVNEEWINDKSRFACDGLK-TQRLTTPLIRVG-------------  target    DGTPRADMEKRGYDEWLQIPWDEALAIAAKTLQNVAETYKGEDGAGKLLEQGYEPAMVEAMHGAGVQAIKMRGGMPLLGA 6gcs.1    -------------DKFVNATWDDALSTIAKAYQQKAP--KGDEF--KAV------------AGALVEV------------  target    GRVFGFYRFANMLALLDGKLRPEAPPEEIVGSRAFDNYAWHTDLPPGHPMVSGSQ-TVDFDLFAAEHSKLLVLIGMNWIC 6gcs.1    ESMVALKDMTNAL-----------------GSENTTTDTPNGNSAPAHGITFRSNYLFNSSIAGIEDADAILLVGTNPRR  target    TKMPDAHWIGDAR-LKGTRVVVISADYMPTANKADEIVILRPGTDTAFLLGVARELITKKLYDRDAVIQRTDLPLLVRLD 6gcs.1    EAAVMNARIRKAWLRQELEIASVGPTLDATFDVAEL--------------GNT---------------------------  target    TGERLSARDVFEGYRQAPLENYVALKTEEELAAPPSPPFTADKQVVPTELREEWGDFVYWDRATNGPAAVNRDEIGAKFA 6gcs.1    --------------------------------------------------------------------------------  target    GDPALLGAFDVTLVDGTNVKARTAFSLLKEYLDENFDVQTTSEVCNVDPAAVRSLARQLAANKGNALLAAGMGPNHYFNA 6gcs.1    ------------------------HADLEKALS-------------------GEFGEVLKNAKN-PLIIVGSGITDREDA  target    DLFGRVHFLVAALTDNI-GHFSGNVGSYAGNYRGSLFQAMGQWIAENPFDQEADLTKPARVKRYFKSESAHYWNYGDRPL 6gcs.1    GAFFNTIGKFVESTPSVLNENWNGYNVLQRSAS-----RAGA------Y----DI--------------------GFTPS  target    VSPSEIITGKSHMPTPTKLIWFGNSNSLLGNAKWSFDVVKNTLPKQDAVFCNEWHWTSSCEYSDLVFPADSWAEFKLPDM 6gcs.1    ---DEA------SKTTPKMVWLLGADEVAAS----------DIPADAFVVYQGHNGDVGAQFADVVLPGAAYTEKAGT--  target    TASCTNPFLLAFPKTPLARIHNTRSDYEILAGVAAALADLVDEPRMKTYWKGILDGDPTPYLQRVLSGSNATRGILYEDL 6gcs.1    -YVNTEGRSQISRA-ATGPPGGAREDWKILRAVSEYLGV-----------------------------------------  target    HASSAKGVPLLMNARTYPRHAGWEQRQEDKPWYTPTGRLEFYRPEPEWQAAGESLPIWREPVDATFYEPNAILANSKHPS 6gcs.1    --------------------------------------------------------------------------------  target    INPRAPEDYGVPESQMDVETRQYRNVVRTWQELKLSKHPLTEKDPAYRFVFQTPKYRWGAHSTAVDSDWIAMLFGPFGDP 6gcs.1    --------------------------------------------------------------------------------  target    YRRDSRTPWTGEAYAEINPRDAKELGLKDGDYIWLDADPEDRPYRGADSSDEFYDVARAMMRVRIYSGMPRRVIRTWFNM 6gcs.1    --------------------------------------------------------------------------------  target    YAATPGTVQAQKDVPGGPAQNQDTGYVALFRHGSHQSGTRAYLRPTQMTDSMNRKAYFGQTIGKGFEADVHSPSGAPKEG 6gcs.1    --------------------------------------------------------------------------------  target    YVKVEKAEDGGDEGVGEWRPVTLGLRPDDPSEAMQAYLAGEFVTRKRKGS 6gcs.1    -------------------------------------------------- ``` | | | | | | | | | | | | | | | | | | | | | | | | | | | | | | | | | | | | | | | | | | | | | | | | | |
|  | 5xtb.1.L | NADH-ubiquinone oxidoreductase 75 kDa subunit, mitochondrial  *Cryo-EM structure of human respiratory complex I matrix arm* | 0.16 |  | 15.42 | 0.33 | 67-756 | EM | 0.00 | hetero-1-1-1-1-1-1-… | 6 x SF4, 1 x FMN, 1 x 8Q1, 1 x NDP, 2 x FES | HHblits | 0.27 |
| ``` target    SDLSRRELLKRAVVVGTGAGLAELFLPAQFLSSASAQSEPQAVAIANPLAQMPDRSWERIYRDQFAEEDSFVFTCAPNDT 5xtb.1    ------------------------------------------------------------------WETRKTESIDVMDA  target    HNCLLRAHVKNGVIVRISPTYGYGKATDLAGNQASHRWDPRICQKGLILGRRIYGDRRVKAPMIRKGFKEWADAGFPRHD 5xtb.1    VGSNIVVSTRTGEVMRILPRM------------HEDINEEWISDKTRFAYDGLK-RQRLTEPMVRNE-------------  target    DGTPRADMEKRGYDEWLQIPWDEALAIAAKTLQNVAETYKGEDGAGKLLEQGYEPAMVEAMHGAGVQAIKMRGGMPLLGA 5xtb.1    ------------KGLLTYTSWEDALSRVAGMLQSF----QGKDV--AAIA------------GGLV-D-----------A  target    GRVFGFYRFANMLALLDGKLRPEAPPEEIVGSRAFDNYAWHTDLPPGHPMVSGSQTVDFDLFAAEHSKLLVLIGMNWICT 5xtb.1    EALVALKDLLNRV-----------------DSDTLCTEEVFPTAGAGTDLRSN-YLLNTTIAGVEEADVVLLVGTNPRFE  target    KMPDAHWIGDARL-KGTRVVVISADYMPTANKADEIVILRPGTDTAFLLGVARELITKKLYDRDAVIQRTDLPLLVRLDT 5xtb.1    APLFNARIRKSWLHNDLKVALIGSPVDLTYTYD--H----LGDSPKILQD------------------------------  target    GERLSARDVFEGYRQAPLENYVALKTEEELAAPPSPPFTADKQVVPTELREEWGDFVYWDRATNGPAAVNRDEIGAKFAG 5xtb.1    --------------------------------------------------------------------------------  target    DPALLGAFDVTLVDGTNVKARTAFSLLKEYLDENFDVQTTSEVCNVDPAAVRSLARQLAANKGNALLAAGMGPNHYFNAD 5xtb.1    ------------------------------IA----------------SGSHPFSQVLKEAKK-PMVVLGSSALQRNDGA  target    LFGRVHFLVAALTDNIGHFSGNVGSYAGNYRGSLFQAMGQWIAENPFDQEADLTKPARVKRYFKSESAHYWNYGDRPLVS 5xtb.1    AILAAVSSIAQKIRMTSGVTGDWKVMNILHRI-----------ASQVA---------AL------------DLGYKPG--  target    PSEIITGKSHMPTPTKLIWFGNSNSLLGNAKWSFDVVKNTLPKQDAVFCNEWHWTSSCEYSDLVFPADSWAEFKLPDMTA 5xtb.1    VEAIR------KNPPKVLFLLGADGGC--------ITRQDLPKDCFIIYQGHHGDVGAPIADVILPGAAYTEKSATY---  target    SCTNPFLLAFPKTPLARIHNTRSDYEILAGVAAALADLVDEPRMKTYWKGILDGDPTPYLQRVLSGSNATRGILYEDLHA 5xtb.1    VNTEGRAQQTKV-AVTPPGLAREDWKIIRALSEIAGM-------------------------------------------  target    SSAKGVPLLMNARTYPRHAGWEQRQEDKPWYTPTGRLEFYRPEPEWQAAGESLPIWREPVDATFYEPNAILANSKHPSIN 5xtb.1    --------------------------------------------------------------------------------  target    PRAPEDYGVPESQMDVETRQYRNVVRTWQELKLSKHPLTEKDPAYRFVFQTPKYRWGAHSTAVDSDWIAMLFGPFGDPYR 5xtb.1    --------------------------------------------------------------------------------  target    RDSRTPWTGEAYAEINPRDAKELGLKDGDYIWLDADPEDRPYRGADSSDEFYDVARAMMRVRIYSGMPRRVIRTWFNMYA 5xtb.1    --------------------------------------------------------------------------------  target    ATPGTVQAQKDVPGGPAQNQDTGYVALFRHGSHQSGTRAYLRPTQMTDSMNRKAYFGQTIGKGFEADVHSPSGAPKEGYV 5xtb.1    --------------------------------------------------------------------------------  target    KVEKAEDGGDEGVGEWRPVTLGLRPDDPSEAMQAYLAGEFVTRKRKGS 5xtb.1    ------------------------------------------------ ``` | | | | | | | | | | | | | | | | | | | | | | | | | | | | | | | | | | | | | | | | | | | | | | | | | |
|  | 7dgr.10.A | NADH-ubiquinone oxidoreductase 75 kDa subunit, mitochondrial  *Activity optimized supercomplex state2* | 0.14 | 0.00 | 14.91 | 0.33 | 67-756 | EM | 0.00 | monomer |  | HHblits | 0.27 |
| ``` target    SDLSRRELLKRAVVVGTGAGLAELFLPAQFLSSASAQSEPQAVAIANPLAQMPDRSWERIYRDQFAEEDSFVFTCAPNDT 7dgr.10   ------------------------------------------------------------------WETRKTESIDVMDA  target    HNCLLRAHVKNGVIVRISPTYGYGKATDLAGNQASHRWDPRICQKGLILGRRIYGDRRVKAPMIRKGFKEWADAGFPRHD 7dgr.10   VGSNIVVSTRTGEVMRILPRM------------HEDINEEWISDKTRFAYDGLK-RQRLTEPMVRNE-------------  target    DGTPRADMEKRGYDEWLQIPWDEALAIAAKTLQNVAETYKGEDGAGKLLEQGYEPAMVEAMHGAGVQAIKMRGGMPLLGA 7dgr.10   ------------KGLLTHTTWEDALSRVAGMLQSF----QGNDV--AAIA------------G-GLVD-----------A  target    GRVFGFYRFANMLALLDGKLRPEAPPEEIVGSRAFDNYAWHTDLPPGHPMVSGSQTVDFDLFAAEHSKLLVLIGMNWICT 7dgr.10   EALIALKDLLNRV-----------------DSDTLCTEEVFPTAGAGTDLR-SNYLLNTTIAGVEEADVVLLVGTNPRFE  target    KMPDAHWIGDARL-KGTRVVVISADYMPTANKADEIVILRPGTDTAFLLGVARELITKKLYDRDAVIQRTDLPLLVRLDT 7dgr.10   APLFNARIRKSWLHNDLKVALIGSPVDLTYRYDHLGDSPKILQDIAS---------------------------------  target    GERLSARDVFEGYRQAPLENYVALKTEEELAAPPSPPFTADKQVVPTELREEWGDFVYWDRATNGPAAVNRDEIGAKFAG 7dgr.10   --------------------------------------------------------------------------------  target    DPALLGAFDVTLVDGTNVKARTAFSLLKEYLDENFDVQTTSEVCNVDPAAVRSLARQLAANKGNALLAAGMGPNHYFNAD 7dgr.10   -------------------------------------------------GSHPFSQVLQEAKKP-MVILGSSALQRNDGA  target    LFGRVHFLVAALTDNIGHFSGNVGSYAGNYRGSLFQAMGQWIAENPFDQEADLTKPARVKRYFKSESAHYWNYGDRPLVS 7dgr.10   AILAAVSNIAQKIRTSSGVTGDWKVMN--I----LHRIA-----SQVA-A--------L------------DLGYKPG--  target    PSEIITGKSHMPTPTKLIWFGNSNSLLGNAKWSFDVVKNTLPKQDAVFCNEWHWTSSCEYSDLVFPADSWAEFKLPDMTA 7dgr.10   VEAIQ------KNPPKMLFLLGADGGC--------ITRQDLPKDCFIVYQGHHGDVGAPIADVILPGAAYTEKSAT---Y  target    SCTNPFLLAFPKTPLARIHNTRSDYEILAGVAAALADLVDEPRMKTYWKGILDGDPTPYLQRVLSGSNATRGILYEDLHA 7dgr.10   VNTEGRAQQTKV-AVTPPGLAREDWKIIRALSEIAGM-------------------------------------------  target    SSAKGVPLLMNARTYPRHAGWEQRQEDKPWYTPTGRLEFYRPEPEWQAAGESLPIWREPVDATFYEPNAILANSKHPSIN 7dgr.10   --------------------------------------------------------------------------------  target    PRAPEDYGVPESQMDVETRQYRNVVRTWQELKLSKHPLTEKDPAYRFVFQTPKYRWGAHSTAVDSDWIAMLFGPFGDPYR 7dgr.10   --------------------------------------------------------------------------------  target    RDSRTPWTGEAYAEINPRDAKELGLKDGDYIWLDADPEDRPYRGADSSDEFYDVARAMMRVRIYSGMPRRVIRTWFNMYA 7dgr.10   --------------------------------------------------------------------------------  target    ATPGTVQAQKDVPGGPAQNQDTGYVALFRHGSHQSGTRAYLRPTQMTDSMNRKAYFGQTIGKGFEADVHSPSGAPKEGYV 7dgr.10   --------------------------------------------------------------------------------  target    KVEKAEDGGDEGVGEWRPVTLGLRPDDPSEAMQAYLAGEFVTRKRKGS 7dgr.10   ------------------------------------------------ ``` | | | | | | | | | | | | | | | | | | | | | | | | | | | | | | | | | | | | | | | | | | | | | | | | | |
|  | 5o31.1.8 | NADH-ubiquinone oxidoreductase 75 kDa subunit, mitochondrial  *Mitochondrial complex I in the deactive state* | 0.15 | 0.00 | 14.91 | 0.33 | 67-756 | EM | 4.13 | monomer | 6 x SF4, 2 x FES, 1 x FMN, 1 x NAP, 1 x ZN | HHblits | 0.27 |
| ``` target    SDLSRRELLKRAVVVGTGAGLAELFLPAQFLSSASAQSEPQAVAIANPLAQMPDRSWERIYRDQFAEEDSFVFTCAPNDT 5o31.1    ------------------------------------------------------------------WETRKTESIDVMDA  target    HNCLLRAHVKNGVIVRISPTYGYGKATDLAGNQASHRWDPRICQKGLILGRRIYGDRRVKAPMIRKGFKEWADAGFPRHD 5o31.1    VGSNIVVSTRTGEVMRILPRM------------HEDINEEWISDKTRFAYDGLK-RQRLTEPMVRNE-------------  target    DGTPRADMEKRGYDEWLQIPWDEALAIAAKTLQNVAETYKGEDGAGKLLEQGYEPAMVEAMHGAGVQAIKMRGGMPLLGA 5o31.1    ------------KGLLTHTTWEDALSRVAGMLQSF----QGNDV--AAIA------------G-GLVD-----------A  target    GRVFGFYRFANMLALLDGKLRPEAPPEEIVGSRAFDNYAWHTDLPPGHPMVSGSQTVDFDLFAAEHSKLLVLIGMNWICT 5o31.1    EALIALKDLLNRV-----------------DSDTLCTEEVFPTAGAGTDLR-SNYLLNTTIAGVEEADVVLLVGTNPRFE  target    KMPDAHWIGDARL-KGTRVVVISADYMPTANKADEIVILRPGTDTAFLLGVARELITKKLYDRDAVIQRTDLPLLVRLDT 5o31.1    APLFNARIRKSWLHNDLKVALIGSPVDLTYRYDHLGDSPKILQDIAS---------------------------------  target    GERLSARDVFEGYRQAPLENYVALKTEEELAAPPSPPFTADKQVVPTELREEWGDFVYWDRATNGPAAVNRDEIGAKFAG 5o31.1    --------------------------------------------------------------------------------  target    DPALLGAFDVTLVDGTNVKARTAFSLLKEYLDENFDVQTTSEVCNVDPAAVRSLARQLAANKGNALLAAGMGPNHYFNAD 5o31.1    -------------------------------------------------GSHPFSQVLQEAKKP-MVILGSSALQRNDGA  target    LFGRVHFLVAALTDNIGHFSGNVGSYAGNYRGSLFQAMGQWIAENPFDQEADLTKPARVKRYFKSESAHYWNYGDRPLVS 5o31.1    AILAAVSNIAQKIRTSSGVTGDWKVMN--I----LHRIA-----SQVA-A--------L------------DLGYKPG--  target    PSEIITGKSHMPTPTKLIWFGNSNSLLGNAKWSFDVVKNTLPKQDAVFCNEWHWTSSCEYSDLVFPADSWAEFKLPDMTA 5o31.1    VEAIQ------KNPPKMLFLLGADGGC--------ITRQDLPKDCFIVYQGHHGDVGAPIADVILPGAAYTEKSAT---Y  target    SCTNPFLLAFPKTPLARIHNTRSDYEILAGVAAALADLVDEPRMKTYWKGILDGDPTPYLQRVLSGSNATRGILYEDLHA 5o31.1    VNTEGRAQQTKV-AVTPPGLAREDWKIIRALSEIAGM-------------------------------------------  target    SSAKGVPLLMNARTYPRHAGWEQRQEDKPWYTPTGRLEFYRPEPEWQAAGESLPIWREPVDATFYEPNAILANSKHPSIN 5o31.1    --------------------------------------------------------------------------------  target    PRAPEDYGVPESQMDVETRQYRNVVRTWQELKLSKHPLTEKDPAYRFVFQTPKYRWGAHSTAVDSDWIAMLFGPFGDPYR 5o31.1    --------------------------------------------------------------------------------  target    RDSRTPWTGEAYAEINPRDAKELGLKDGDYIWLDADPEDRPYRGADSSDEFYDVARAMMRVRIYSGMPRRVIRTWFNMYA 5o31.1    --------------------------------------------------------------------------------  target    ATPGTVQAQKDVPGGPAQNQDTGYVALFRHGSHQSGTRAYLRPTQMTDSMNRKAYFGQTIGKGFEADVHSPSGAPKEGYV 5o31.1    --------------------------------------------------------------------------------  target    KVEKAEDGGDEGVGEWRPVTLGLRPDDPSEAMQAYLAGEFVTRKRKGS 5o31.1    ------------------------------------------------ ``` | | | | | | | | | | | | | | | | | | | | | | | | | | | | | | | | | | | | | | | | | | | | | | | | | |
|  | 7qsd.1.G | NADH-ubiquinone oxidoreductase 75 kDa subunit, mitochondrial  *Bovine complex I in the active state at 3.1 A* | 0.16 | 0.00 | 14.65 | 0.33 | 67-756 | EM | 0.00 | monomer | 5 x PC1, 13 x 3PE, 6 x SF4, 2 x FES, 1 x FMN, 4 x CDL, 3 x LMT, 1 x GTP, 1 x MG, 1 x NDP, 1 x ZN, 2 x EHZ | HHblits | 0.27 |
| ``` target    SDLSRRELLKRAVVVGTGAGLAELFLPAQFLSSASAQSEPQAVAIANPLAQMPDRSWERIYRDQFAEEDSFVFTCAPNDT 7qsd.1    ------------------------------------------------------------------WETRKTESIDVMDA  target    HNCLLRAHVKNGVIVRISPTYGYGKATDLAGNQASHRWDPRICQKGLILGRRIYGDRRVKAPMIRKGFKEWADAGFPRHD 7qsd.1    VGSNIVVSTRTGEVMRILPRM------------HEDINEEWISDKTRFAYDGLK-RQRLTEPMVRNE-------------  target    DGTPRADMEKRGYDEWLQIPWDEALAIAAKTLQNVAETYKGEDGAGKLLEQGYEPAMVEAMHGAGVQAIKMRGGMPLLGA 7qsd.1    ------------KGLLTHTTWEDALSRVAGMLQSF----QGNDV--AAIA------------G-GLVD-----------A  target    GRVFGFYRFANMLALLDGKLRPEAPPEEIVGSRAFDNYAWHTDLPPGHPMVSGSQTVDFDLFAAEHSKLLVLIGMNWICT 7qsd.1    EALIALKDLLNRV-----------------DSDTLCTEEVFPTAGAGTDLR-SNYLLNTTIAGVEEADVVLLVGTNPRFE  target    KMPDAHWIGDARL-KGTRVVVISADYMPTANKADEIVILRPGTDTAFLLGVARELITKKLYDRDAVIQRTDLPLLVRLDT 7qsd.1    APLFNARIRKSWLHNDLKVALIGSPVDLTYRYDHLGDSPKILQD------------------------IAS---------  target    GERLSARDVFEGYRQAPLENYVALKTEEELAAPPSPPFTADKQVVPTELREEWGDFVYWDRATNGPAAVNRDEIGAKFAG 7qsd.1    --------------------------------------------------------------------------------  target    DPALLGAFDVTLVDGTNVKARTAFSLLKEYLDENFDVQTTSEVCNVDPAAVRSLARQLAANKGNALLAAGMGPNHYFNAD 7qsd.1    -------------------------------------------------GSHPFSQVLQEAKK-PMVILGSSALQRNDGA  target    LFGRVHFLVAALTDNIGHFSGNVGSYAGNYRGSLFQAMGQWIAENPFDQEADLTKPARVKRYFKSESAHYWNYGDRPLVS 7qsd.1    AILAAVSNIAQKIRTSSGVTGDWKVMN--I----LHR-----IASQVA---------AL------------DLGYKPG--  target    PSEIITGKSHMPTPTKLIWFGNSNSLLGNAKWSFDVVKNTLPKQDAVFCNEWHWTSSCEYSDLVFPADSWAEFKLPDMTA 7qsd.1    VEAIQ------KNPPKMLFLLGADGGC--------ITRQDLPKDCFIVYQGHHGDVGAPIADVILPGAAYTEKSAT---Y  target    SCTNPFLLAFPKTPLARIHNTRSDYEILAGVAAALADLVDEPRMKTYWKGILDGDPTPYLQRVLSGSNATRGILYEDLHA 7qsd.1    VNTEGRAQQTKV-AVTPPGLAREDWKIIRALSEIAGM-------------------------------------------  target    SSAKGVPLLMNARTYPRHAGWEQRQEDKPWYTPTGRLEFYRPEPEWQAAGESLPIWREPVDATFYEPNAILANSKHPSIN 7qsd.1    --------------------------------------------------------------------------------  target    PRAPEDYGVPESQMDVETRQYRNVVRTWQELKLSKHPLTEKDPAYRFVFQTPKYRWGAHSTAVDSDWIAMLFGPFGDPYR 7qsd.1    --------------------------------------------------------------------------------  target    RDSRTPWTGEAYAEINPRDAKELGLKDGDYIWLDADPEDRPYRGADSSDEFYDVARAMMRVRIYSGMPRRVIRTWFNMYA 7qsd.1    --------------------------------------------------------------------------------  target    ATPGTVQAQKDVPGGPAQNQDTGYVALFRHGSHQSGTRAYLRPTQMTDSMNRKAYFGQTIGKGFEADVHSPSGAPKEGYV 7qsd.1    --------------------------------------------------------------------------------  target    KVEKAEDGGDEGVGEWRPVTLGLRPDDPSEAMQAYLAGEFVTRKRKGS 7qsd.1    ------------------------------------------------ ``` | | | | | | | | | | | | | | | | | | | | | | | | | | | | | | | | | | | | | | | | | | | | | | | | | |
|  | 6zk9.1.C | NADH:ubiquinone oxidoreductase core subunit S1  *Peripheral domain of open complex I during turnover* | 0.17 | 0.00 | 14.65 | 0.33 | 67-756 | EM | 0.00 | monomer | 6 x SF4, 1 x FMN, 1 x NAI, 2 x FES, 1 x K, 2 x PC1, 2 x 3PE, 1 x ZN, 1 x NDP, 1 x ZMP, 1 x CDL | HHblits | 0.27 |
| ``` target    SDLSRRELLKRAVVVGTGAGLAELFLPAQFLSSASAQSEPQAVAIANPLAQMPDRSWERIYRDQFAEEDSFVFTCAPNDT 6zk9.1    ------------------------------------------------------------------WETRKTESIDVMDA  target    HNCLLRAHVKNGVIVRISPTYGYGKATDLAGNQASHRWDPRICQKGLILGRRIYGDRRVKAPMIRKGFKEWADAGFPRHD 6zk9.1    VGSNIVVSTRTGEVMRILPRM------------HEDINEEWISDKTRFAYDGLK-RQRLTEPMVRNE-------------  target    DGTPRADMEKRGYDEWLQIPWDEALAIAAKTLQNVAETYKGEDGAGKLLEQGYEPAMVEAMHGAGVQAIKMRGGMPLLGA 6zk9.1    ------------KGLLTHTTWEDALSRVAGMLQSC----QGNDV--AAIA------------G--GLV----------DA  target    GRVFGFYRFANMLALLDGKLRPEAPPEEIVGSRAFDNYAWHTDLPPGHPMVSGSQTVDFDLFAAEHSKLLVLIGMNWICT 6zk9.1    EALIALKDLLNRV-----------------DSDTLCTEEVFPTAGAGTDLR-SNYLLNTTIAGVEEADVVLLVGTNPRFE  target    KMPDAHWIGDARL-KGTRVVVISADYMPTANKADEIVILRPGTDTAFLLGVARELITKKLYDRDAVIQRTDLPLLVRLDT 6zk9.1    APLFNARIRKSWLHNDLKVALIGSPVDLTYRYDHLGD------SP------------------KILQDIAS---------  target    GERLSARDVFEGYRQAPLENYVALKTEEELAAPPSPPFTADKQVVPTELREEWGDFVYWDRATNGPAAVNRDEIGAKFAG 6zk9.1    --------------------------------------------------------------------------------  target    DPALLGAFDVTLVDGTNVKARTAFSLLKEYLDENFDVQTTSEVCNVDPAAVRSLARQLAANKGNALLAAGMGPNHYFNAD 6zk9.1    -------------------------------------------------GSHPFSQVLQEAKK-PMVVLGSSALQRNDGA  target    LFGRVHFLVAALTDNIGHFSGNVGSYAGNYRGSLFQAMGQWIAENPFDQEADLTKPARVKRYFKSESAHYWNYGDRPLVS 6zk9.1    AILAAVSNIAQKIRTSSGVTGDWKVMN--I----LHR-----IASQVA---------AL------------DLGYKPG--  target    PSEIITGKSHMPTPTKLIWFGNSNSLLGNAKWSFDVVKNTLPKQDAVFCNEWHWTSSCEYSDLVFPADSWAEFKLPDMTA 6zk9.1    VEAIR------KNPPKMLFLLGADGGC--------VTRQDLPKDCFIVYQGHHGDVGAPIADVILPGAAYTEKSA---TY  target    SCTNPFLLAFPKTPLARIHNTRSDYEILAGVAAALADLVDEPRMKTYWKGILDGDPTPYLQRVLSGSNATRGILYEDLHA 6zk9.1    VNTEGRAQQTKV-AVMPPGLAREDWKIIRALSEIAGM-------------------------------------------  target    SSAKGVPLLMNARTYPRHAGWEQRQEDKPWYTPTGRLEFYRPEPEWQAAGESLPIWREPVDATFYEPNAILANSKHPSIN 6zk9.1    --------------------------------------------------------------------------------  target    PRAPEDYGVPESQMDVETRQYRNVVRTWQELKLSKHPLTEKDPAYRFVFQTPKYRWGAHSTAVDSDWIAMLFGPFGDPYR 6zk9.1    --------------------------------------------------------------------------------  target    RDSRTPWTGEAYAEINPRDAKELGLKDGDYIWLDADPEDRPYRGADSSDEFYDVARAMMRVRIYSGMPRRVIRTWFNMYA 6zk9.1    --------------------------------------------------------------------------------  target    ATPGTVQAQKDVPGGPAQNQDTGYVALFRHGSHQSGTRAYLRPTQMTDSMNRKAYFGQTIGKGFEADVHSPSGAPKEGYV 6zk9.1    --------------------------------------------------------------------------------  target    KVEKAEDGGDEGVGEWRPVTLGLRPDDPSEAMQAYLAGEFVTRKRKGS 6zk9.1    ------------------------------------------------ ``` | | | | | | | | | | | | | | | | | | | | | | | | | | | | | | | | | | | | | | | | | | | | | | | | | |
|  | 7vxu.1.L | NADH-ubiquinone oxidoreductase 75 kDa subunit, mitochondrial  *Matrix arm of deactive state CI from Q10 dataset* | 0.15 |  | 14.95 | 0.33 | 68-756 | EM | 0.00 | hetero-1-1-1-1-1-1-… | 6 x SF4, 1 x FMN, 1 x PEE, 1 x PLX, 1 x 8Q1, 1 x NDP, 2 x FES, 1 x MG, 1 x CDL, 1 x ZN | HHblits | 0.27 |
| ``` target    SDLSRRELLKRAVVVGTGAGLAELFLPAQFLSSASAQSEPQAVAIANPLAQMPDRSWERIYRDQFAEEDSFVFTCAPNDT 7vxu.1    -------------------------------------------------------------------ETRKTESIDVMDA  target    HNCLLRAHVKNGVIVRISPTYGYGKATDLAGNQASHRWDPRICQKGLILGRRIYGDRRVKAPMIRKGFKEWADAGFPRHD 7vxu.1    VGSNIVVSTRTGEVMRILPRM------------HEDINEEWISDKTRFAYDGLK-RQRLTQPMIRNE-------------  target    DGTPRADMEKRGYDEWLQIPWDEALAIAAKTLQNVAETYKGEDGAGKLLEQGYEPAMVEAMHGAGVQAIKMRGGMPLLGA 7vxu.1    ------------KGLLTYTTWEDALSRVAGMLQSF----QGNDV--AAI------------AG-GLVD-----------A  target    GRVFGFYRFANMLALLDGKLRPEAPPEEIVGSRAFDNYAWHTDLPPGHPMVSGSQTVDFDLFAAEHSKLLVLIGMNWICT 7vxu.1    EALVALKDLLNRV-----------------DSDSLCTEEVFPTAGAGTDLRSN-YLLNTTIAGVEEADVILLVGTNPRFE  target    KMPDAHWIGDARL-KGTRVVVISADYMPTANKADEIVILRPGTDTAFLLGVARELITKKLYDRDAVIQRTDLPLLVRLDT 7vxu.1    APLFNARIRKSWLHNDLKVALIGSPVDLTYRYDHLGDSPK------------------------ILQDIAS---------  target    GERLSARDVFEGYRQAPLENYVALKTEEELAAPPSPPFTADKQVVPTELREEWGDFVYWDRATNGPAAVNRDEIGAKFAG 7vxu.1    --------------------------------------------------------------------------------  target    DPALLGAFDVTLVDGTNVKARTAFSLLKEYLDENFDVQTTSEVCNVDPAAVRSLARQLAANKGNALLAAGMGPNHYFNAD 7vxu.1    ---------------------------------G----------------NHPFSQILKEAKK-PMVVLGSSALQRSDGT  target    LFGRVHFLVAALTDNIGHFSGNVGSYAGNYRGSLFQAMGQWIAENPFDQEADLTKPARVKRYFKSESAHYWNYGDRPLVS 7vxu.1    AILAAVSNIAQNIRLSSGVTGDWKVMN--I-------LHR--IASQVA---------AL------------DLGYKPG--  target    PSEIITGKSHMPTPTKLIWFGNSNSLLGNAKWSFDVVKNTLPKQDAVFCNEWHWTSSCEYSDLVFPADSWAEFKLPDMTA 7vxu.1    VEAIR------KNPPKVLFLLGADGGC--------ITRQDLPKDCFIIYQGHHGDVGAPMADVILPGAAYTEKSAT---Y  target    SCTNPFLLAFPKTPLARIHNTRSDYEILAGVAAALADLVDEPRMKTYWKGILDGDPTPYLQRVLSGSNATRGILYEDLHA 7vxu.1    VNTEGRAQQTKV-AVTPPGLAREDWKIIRALSEIAGM-------------------------------------------  target    SSAKGVPLLMNARTYPRHAGWEQRQEDKPWYTPTGRLEFYRPEPEWQAAGESLPIWREPVDATFYEPNAILANSKHPSIN 7vxu.1    --------------------------------------------------------------------------------  target    PRAPEDYGVPESQMDVETRQYRNVVRTWQELKLSKHPLTEKDPAYRFVFQTPKYRWGAHSTAVDSDWIAMLFGPFGDPYR 7vxu.1    --------------------------------------------------------------------------------  target    RDSRTPWTGEAYAEINPRDAKELGLKDGDYIWLDADPEDRPYRGADSSDEFYDVARAMMRVRIYSGMPRRVIRTWFNMYA 7vxu.1    --------------------------------------------------------------------------------  target    ATPGTVQAQKDVPGGPAQNQDTGYVALFRHGSHQSGTRAYLRPTQMTDSMNRKAYFGQTIGKGFEADVHSPSGAPKEGYV 7vxu.1    --------------------------------------------------------------------------------  target    KVEKAEDGGDEGVGEWRPVTLGLRPDDPSEAMQAYLAGEFVTRKRKGS 7vxu.1    ------------------------------------------------ ``` | | | | | | | | | | | | | | | | | | | | | | | | | | | | | | | | | | | | | | | | | | | | | | | | | |
|  | 5gpn.24.A | NADH-ubiquinone oxidoreductase 75 kDa subunit  *Architecture of mammalian respirasome* | 0.15 |  | 14.95 | 0.33 | 68-756 | EM | 0.00 | monomer |  | HHblits | 0.27 |
| ``` target    SDLSRRELLKRAVVVGTGAGLAELFLPAQFLSSASAQSEPQAVAIANPLAQMPDRSWERIYRDQFAEEDSFVFTCAPNDT 5gpn.24   -------------------------------------------------------------------ETRKTESIDVMDA  target    HNCLLRAHVKNGVIVRISPTYGYGKATDLAGNQASHRWDPRICQKGLILGRRIYGDRRVKAPMIRKGFKEWADAGFPRHD 5gpn.24   VGSNIVVSTRTGEVMRILPRM------------HEDINEEWISDKTRFAYDGLK-RQRLTQPMIRNE-------------  target    DGTPRADMEKRGYDEWLQIPWDEALAIAAKTLQNVAETYKGEDGAGKLLEQGYEPAMVEAMHGAGVQAIKMRGGMPLLGA 5gpn.24   ------------KGLLTYTTWEDALSRVAGMLQSF----QGNDV--AAI------------AG-GLVD-----------A  target    GRVFGFYRFANMLALLDGKLRPEAPPEEIVGSRAFDNYAWHTDLPPGHPMVSGSQTVDFDLFAAEHSKLLVLIGMNWICT 5gpn.24   EALVALKDLLNRV-----------------DSDSLCTEEVFPTAGAGTDLRSN-YLLNTTIAGVEEADVILLVGTNPRFE  target    KMPDAHWIGDARL-KGTRVVVISADYMPTANKADEIVILRPGTDTAFLLGVARELITKKLYDRDAVIQRTDLPLLVRLDT 5gpn.24   APLFNARIRKSWLHNDLKVALIGSPVDLTYRYDHLGDSPK------------------------ILQDIAS---------  target    GERLSARDVFEGYRQAPLENYVALKTEEELAAPPSPPFTADKQVVPTELREEWGDFVYWDRATNGPAAVNRDEIGAKFAG 5gpn.24   --------------------------------------------------------------------------------  target    DPALLGAFDVTLVDGTNVKARTAFSLLKEYLDENFDVQTTSEVCNVDPAAVRSLARQLAANKGNALLAAGMGPNHYFNAD 5gpn.24   ---------------------------------G----------------NHPFSQILKEAKK-PMVVLGSSALQRSDGT  target    LFGRVHFLVAALTDNIGHFSGNVGSYAGNYRGSLFQAMGQWIAENPFDQEADLTKPARVKRYFKSESAHYWNYGDRPLVS 5gpn.24   AILAAVSNIAQNIRLSSGVTGDWKVMN--I----LHR-----IASQVA---------AL------------DLGYKPG--  target    PSEIITGKSHMPTPTKLIWFGNSNSLLGNAKWSFDVVKNTLPKQDAVFCNEWHWTSSCEYSDLVFPADSWAEFKLPDMTA 5gpn.24   VEAIR------KNPPKVLFLLGADGGC--------ITRQDLPKDCFIIYQGHHGDVGAPMADVILPGAAYTEKSAT---Y  target    SCTNPFLLAFPKTPLARIHNTRSDYEILAGVAAALADLVDEPRMKTYWKGILDGDPTPYLQRVLSGSNATRGILYEDLHA 5gpn.24   VNTEGRAQQTKV-AVTPPGLAREDWKIIRALSEIAGM-------------------------------------------  target    SSAKGVPLLMNARTYPRHAGWEQRQEDKPWYTPTGRLEFYRPEPEWQAAGESLPIWREPVDATFYEPNAILANSKHPSIN 5gpn.24   --------------------------------------------------------------------------------  target    PRAPEDYGVPESQMDVETRQYRNVVRTWQELKLSKHPLTEKDPAYRFVFQTPKYRWGAHSTAVDSDWIAMLFGPFGDPYR 5gpn.24   --------------------------------------------------------------------------------  target    RDSRTPWTGEAYAEINPRDAKELGLKDGDYIWLDADPEDRPYRGADSSDEFYDVARAMMRVRIYSGMPRRVIRTWFNMYA 5gpn.24   --------------------------------------------------------------------------------  target    ATPGTVQAQKDVPGGPAQNQDTGYVALFRHGSHQSGTRAYLRPTQMTDSMNRKAYFGQTIGKGFEADVHSPSGAPKEGYV 5gpn.24   --------------------------------------------------------------------------------  target    KVEKAEDGGDEGVGEWRPVTLGLRPDDPSEAMQAYLAGEFVTRKRKGS 5gpn.24   ------------------------------------------------ ``` | | | | | | | | | | | | | | | | | | | | | | | | | | | | | | | | | | | | | | | | | | | | | | | | | |
|  | 7zd6.1.4 | NADH-ubiquinone oxidoreductase 75 kDa subunit, mitochondrial  *Complex I from Ovis aries, at pH7.4, Open state* | 0.16 | 0.00 | 14.40 | 0.33 | 67-756 | EM | 0.00 | monomer | 6 x PC1, 14 x 3PE, 1 x DCQ, 2 x ZMP, 1 x AMP, 1 x MYR, 6 x SF4, 1 x FMN, 1 x NAI, 2 x FES, 1 x K, 1 x ZN, 1 x NDP | HHblits | 0.27 |
| ``` target    SDLSRRELLKRAVVVGTGAGLAELFLPAQFLSSASAQSEPQAVAIANPLAQMPDRSWERIYRDQFAEEDSFVFTCAPNDT 7zd6.1    ------------------------------------------------------------------WETRKTESIDVMDA  target    HNCLLRAHVKNGVIVRISPTYGYGKATDLAGNQASHRWDPRICQKGLILGRRIYGDRRVKAPMIRKGFKEWADAGFPRHD 7zd6.1    VGSNIVVSTRTGEVMRILPRM------------HEDINEEWISDKTRFAYDGLK-RQRLTEPMVRNE-------------  target    DGTPRADMEKRGYDEWLQIPWDEALAIAAKTLQNVAETYKGEDGAGKLLEQGYEPAMVEAMHGAGVQAIKMRGGMPLLGA 7zd6.1    ------------KGLLTHTTWEDALSRVAGMLQSC----QGNDV--AAIAGG-------------LVD-----------A  target    GRVFGFYRFANMLALLDGKLRPEAPPEEIVGSRAFDNYAWHTDLPPGHPMVSGSQTVDFDLFAAEHSKLLVLIGMNWICT 7zd6.1    EALIALKDLLNRV-----------------DSDTLCTEEVFPTAGAGTDLR-SNYLLNTTIAGVEEADVVLLVGTNPRFE  target    KMPDAHWIGDARL-KGTRVVVISADYMPTANKADEIVILRPGTDTAFLLGVARELITKKLYDRDAVIQRTDLPLLVRLDT 7zd6.1    APLFNARIRKSWLHNDLKVALIGSPVDLTYRYDHLGDS------P------------------KILQDIAS---------  target    GERLSARDVFEGYRQAPLENYVALKTEEELAAPPSPPFTADKQVVPTELREEWGDFVYWDRATNGPAAVNRDEIGAKFAG 7zd6.1    --------------------------------------------------------------------------------  target    DPALLGAFDVTLVDGTNVKARTAFSLLKEYLDENFDVQTTSEVCNVDPAAVRSLARQLAANKGNALLAAGMGPNHYFNAD 7zd6.1    -------------------------------------------------GSHPFSQVLQEAKKP-MVVLGSSALQRNDGA  target    LFGRVHFLVAALTDNIGHFSGNVGSYAGNYRGSLFQAMGQWIAENPFDQEADLTKPARVKRYFKSESAHYWNYGDRPLVS 7zd6.1    AILAAVSNIAQKIRTSSGVTGDWKVMN--I----LHR---I--ASQVA---------AL------------DLGYKPG--  target    PSEIITGKSHMPTPTKLIWFGNSNSLLGNAKWSFDVVKNTLPKQDAVFCNEWHWTSSCEYSDLVFPADSWAEFKLPDMTA 7zd6.1    VEAIR------KNPPKMLFLLGADGGC--------VTRQDLPKDCFIVYQGHHGDVGAPIADVILPGAAYTEKSAT---Y  target    SCTNPFLLAFPKTPLARIHNTRSDYEILAGVAAALADLVDEPRMKTYWKGILDGDPTPYLQRVLSGSNATRGILYEDLHA 7zd6.1    VNTEGRAQQTKV-AVMPPGLAREDWKIIRALSEIAGM-------------------------------------------  target    SSAKGVPLLMNARTYPRHAGWEQRQEDKPWYTPTGRLEFYRPEPEWQAAGESLPIWREPVDATFYEPNAILANSKHPSIN 7zd6.1    --------------------------------------------------------------------------------  target    PRAPEDYGVPESQMDVETRQYRNVVRTWQELKLSKHPLTEKDPAYRFVFQTPKYRWGAHSTAVDSDWIAMLFGPFGDPYR 7zd6.1    --------------------------------------------------------------------------------  target    RDSRTPWTGEAYAEINPRDAKELGLKDGDYIWLDADPEDRPYRGADSSDEFYDVARAMMRVRIYSGMPRRVIRTWFNMYA 7zd6.1    --------------------------------------------------------------------------------  target    ATPGTVQAQKDVPGGPAQNQDTGYVALFRHGSHQSGTRAYLRPTQMTDSMNRKAYFGQTIGKGFEADVHSPSGAPKEGYV 7zd6.1    --------------------------------------------------------------------------------  target    KVEKAEDGGDEGVGEWRPVTLGLRPDDPSEAMQAYLAGEFVTRKRKGS 7zd6.1    ------------------------------------------------ ``` | | | | | | | | | | | | | | | | | | | | | | | | | | | | | | | | | | | | | | | | | | | | | | | | | |
|  | 6qcf.1.C | NADH:ubiquinone oxidoreductase core subunit S1  *Ovine respiratory complex I FRC open class 6* | 0.16 | 0.00 | 14.40 | 0.33 | 67-756 | EM | 0.00 | monomer | 6 x SF4, 1 x FMN, 2 x FES, 1 x ZN, 1 x NDP, 2 x ZMP | HHblits | 0.27 |
| ``` target    SDLSRRELLKRAVVVGTGAGLAELFLPAQFLSSASAQSEPQAVAIANPLAQMPDRSWERIYRDQFAEEDSFVFTCAPNDT 6qcf.1    ------------------------------------------------------------------WETRKTESIDVMDA  target    HNCLLRAHVKNGVIVRISPTYGYGKATDLAGNQASHRWDPRICQKGLILGRRIYGDRRVKAPMIRKGFKEWADAGFPRHD 6qcf.1    VGSNIVVSTRTGEVMRILPRM------------HEDINEEWISDKTRFAYDGLK-RQRLTEPMVRNE-------------  target    DGTPRADMEKRGYDEWLQIPWDEALAIAAKTLQNVAETYKGEDGAGKLLEQGYEPAMVEAMHGAGVQAIKMRGGMPLLGA 6qcf.1    ------------KGLLTHTTWEDALSRVAGMLQSC----QGNDV--AAIA------------G--GLV----------DA  target    GRVFGFYRFANMLALLDGKLRPEAPPEEIVGSRAFDNYAWHTDLPPGHPMVSGSQTVDFDLFAAEHSKLLVLIGMNWICT 6qcf.1    EALIALKDLLNRV-----------------DSDTLCTEEVFPTAGAGTDLR-SNYLLNTTIAGVEEADVVLLVGTNPRFE  target    KMPDAHWIGDARL-KGTRVVVISADYMPTANKADEIVILRPGTDTAFLLGVARELITKKLYDRDAVIQRTDLPLLVRLDT 6qcf.1    APLFNARIRKSWLHNDLKVALIGSPVDLTYRYDHLGDSP------------------------KILQDIAS---------  target    GERLSARDVFEGYRQAPLENYVALKTEEELAAPPSPPFTADKQVVPTELREEWGDFVYWDRATNGPAAVNRDEIGAKFAG 6qcf.1    --------------------------------------------------------------------------------  target    DPALLGAFDVTLVDGTNVKARTAFSLLKEYLDENFDVQTTSEVCNVDPAAVRSLARQLAANKGNALLAAGMGPNHYFNAD 6qcf.1    -------------------------------------------------GSHPFSQVLQEAKK-PMVVLGSSALQRNDGA  target    LFGRVHFLVAALTDNIGHFSGNVGSYAGNYRGSLFQAMGQWIAENPFDQEADLTKPARVKRYFKSESAHYWNYGDRPLVS 6qcf.1    AILAAVSNIAQKIRTSSGVTGDWKVMN--I----L---HRI--ASQVA---------AL------------DLGYKPG--  target    PSEIITGKSHMPTPTKLIWFGNSNSLLGNAKWSFDVVKNTLPKQDAVFCNEWHWTSSCEYSDLVFPADSWAEFKLPDMTA 6qcf.1    VEAIR------KNPPKMLFLLGADGGC--------VTRQDLPKDCFIVYQGHHGDVGAPIADVILPGAAYTEKSAT---Y  target    SCTNPFLLAFPKTPLARIHNTRSDYEILAGVAAALADLVDEPRMKTYWKGILDGDPTPYLQRVLSGSNATRGILYEDLHA 6qcf.1    VNTEGRAQQTKV-AVMPPGLAREDWKIIRALSEIAGM-------------------------------------------  target    SSAKGVPLLMNARTYPRHAGWEQRQEDKPWYTPTGRLEFYRPEPEWQAAGESLPIWREPVDATFYEPNAILANSKHPSIN 6qcf.1    --------------------------------------------------------------------------------  target    PRAPEDYGVPESQMDVETRQYRNVVRTWQELKLSKHPLTEKDPAYRFVFQTPKYRWGAHSTAVDSDWIAMLFGPFGDPYR 6qcf.1    --------------------------------------------------------------------------------  target    RDSRTPWTGEAYAEINPRDAKELGLKDGDYIWLDADPEDRPYRGADSSDEFYDVARAMMRVRIYSGMPRRVIRTWFNMYA 6qcf.1    --------------------------------------------------------------------------------  target    ATPGTVQAQKDVPGGPAQNQDTGYVALFRHGSHQSGTRAYLRPTQMTDSMNRKAYFGQTIGKGFEADVHSPSGAPKEGYV 6qcf.1    --------------------------------------------------------------------------------  target    KVEKAEDGGDEGVGEWRPVTLGLRPDDPSEAMQAYLAGEFVTRKRKGS 6qcf.1    ------------------------------------------------ ``` | | | | | | | | | | | | | | | | | | | | | | | | | | | | | | | | | | | | | | | | | | | | | | | | | |
|  | 6qc5.1.C | NADH:ubiquinone oxidoreductase core subunit S1  *Ovine respiratory complex I FRC closed class 1* | 0.16 | 0.00 | 14.40 | 0.33 | 67-756 | EM | 0.00 | monomer | 6 x SF4, 1 x FMN, 2 x FES, 2 x 3PE, 1 x ZN, 1 x NDP, 2 x ZMP, 1 x PC1 | HHblits | 0.27 |
| ``` target    SDLSRRELLKRAVVVGTGAGLAELFLPAQFLSSASAQSEPQAVAIANPLAQMPDRSWERIYRDQFAEEDSFVFTCAPNDT 6qc5.1    ------------------------------------------------------------------WETRKTESIDVMDA  target    HNCLLRAHVKNGVIVRISPTYGYGKATDLAGNQASHRWDPRICQKGLILGRRIYGDRRVKAPMIRKGFKEWADAGFPRHD 6qc5.1    VGSNIVVSTRTGEVMRILPRM------------HEDINEEWISDKTRFAYDGLK-RQRLTEPMVRNE-------------  target    DGTPRADMEKRGYDEWLQIPWDEALAIAAKTLQNVAETYKGEDGAGKLLEQGYEPAMVEAMHGAGVQAIKMRGGMPLLGA 6qc5.1    ------------KGLLTHTTWEDALSRVAGMLQSC----QGNDV--AAIA------------G--GLV----------DA  target    GRVFGFYRFANMLALLDGKLRPEAPPEEIVGSRAFDNYAWHTDLPPGHPMVSGSQTVDFDLFAAEHSKLLVLIGMNWICT 6qc5.1    EALIALKDLLNRV-----------------DSDTLCTEEVFPTAGAGTDLR-SNYLLNTTIAGVEEADVVLLVGTNPRFE  target    KMPDAHWIGDARL-KGTRVVVISADYMPTANKADEIVILRPGTDTAFLLGVARELITKKLYDRDAVIQRTDLPLLVRLDT 6qc5.1    APLFNARIRKSWLHNDLKVALIGSPVDLTYRYDHLGDSP------------------------KILQDIAS---------  target    GERLSARDVFEGYRQAPLENYVALKTEEELAAPPSPPFTADKQVVPTELREEWGDFVYWDRATNGPAAVNRDEIGAKFAG 6qc5.1    --------------------------------------------------------------------------------  target    DPALLGAFDVTLVDGTNVKARTAFSLLKEYLDENFDVQTTSEVCNVDPAAVRSLARQLAANKGNALLAAGMGPNHYFNAD 6qc5.1    -------------------------------------------------GSHPFSQVLQEAKK-PMVVLGSSALQRNDGA  target    LFGRVHFLVAALTDNIGHFSGNVGSYAGNYRGSLFQAMGQWIAENPFDQEADLTKPARVKRYFKSESAHYWNYGDRPLVS 6qc5.1    AILAAVSNIAQKIRTSSGVTGDWKVMN--I----L---HRI--ASQVA---------AL------------DLGYKPG--  target    PSEIITGKSHMPTPTKLIWFGNSNSLLGNAKWSFDVVKNTLPKQDAVFCNEWHWTSSCEYSDLVFPADSWAEFKLPDMTA 6qc5.1    VEAIR------KNPPKMLFLLGADGGC--------VTRQDLPKDCFIVYQGHHGDVGAPIADVILPGAAYTEKSAT---Y  target    SCTNPFLLAFPKTPLARIHNTRSDYEILAGVAAALADLVDEPRMKTYWKGILDGDPTPYLQRVLSGSNATRGILYEDLHA 6qc5.1    VNTEGRAQQTKV-AVMPPGLAREDWKIIRALSEIAGM-------------------------------------------  target    SSAKGVPLLMNARTYPRHAGWEQRQEDKPWYTPTGRLEFYRPEPEWQAAGESLPIWREPVDATFYEPNAILANSKHPSIN 6qc5.1    --------------------------------------------------------------------------------  target    PRAPEDYGVPESQMDVETRQYRNVVRTWQELKLSKHPLTEKDPAYRFVFQTPKYRWGAHSTAVDSDWIAMLFGPFGDPYR 6qc5.1    --------------------------------------------------------------------------------  target    RDSRTPWTGEAYAEINPRDAKELGLKDGDYIWLDADPEDRPYRGADSSDEFYDVARAMMRVRIYSGMPRRVIRTWFNMYA 6qc5.1    --------------------------------------------------------------------------------  target    ATPGTVQAQKDVPGGPAQNQDTGYVALFRHGSHQSGTRAYLRPTQMTDSMNRKAYFGQTIGKGFEADVHSPSGAPKEGYV 6qc5.1    --------------------------------------------------------------------------------  target    KVEKAEDGGDEGVGEWRPVTLGLRPDDPSEAMQAYLAGEFVTRKRKGS 6qc5.1    ------------------------------------------------ ``` | | | | | | | | | | | | | | | | | | | | | | | | | | | | | | | | | | | | | | | | | | | | | | | | | |
|  | 7v2c.1.L | NADH-ubiquinone oxidoreductase 75 kDa subunit, mitochondrial  *Active state complex I from Q10 dataset* | 0.16 |  | 14.69 | 0.33 | 68-756 | EM | 0.00 | hetero-1-1-1-1-1-2-… | 6 x SF4, 1 x FMN, 10 x PEE, 8 x PLX, 2 x 8Q1, 1 x NDP, 2 x UQ, 11 x CDL, 2 x FES, 1 x MG, 1 x ZN, 1 x ADP | HHblits | 0.27 |
| ``` target    SDLSRRELLKRAVVVGTGAGLAELFLPAQFLSSASAQSEPQAVAIANPLAQMPDRSWERIYRDQFAEEDSFVFTCAPNDT 7v2c.1    -------------------------------------------------------------------ETRKTESIDVMDA  target    HNCLLRAHVKNGVIVRISPTYGYGKATDLAGNQASHRWDPRICQKGLILGRRIYGDRRVKAPMIRKGFKEWADAGFPRHD 7v2c.1    VGSNIVVSTRTGEVMRILPRM------------HEDINEEWISDKTRFAYDGLK-RQRLTQPMIRNE-------------  target    DGTPRADMEKRGYDEWLQIPWDEALAIAAKTLQNVAETYKGEDGAGKLLEQGYEPAMVEAMHGAGVQAIKMRGGMPLLGA 7v2c.1    ------------KGLLTYTTWEDALSRVAGMLQSF----QGNDV--AAI------------AGGLV-D-----------A  target    GRVFGFYRFANMLALLDGKLRPEAPPEEIVGSRAFDNYAWHTDLPPGHPMVSGSQTVDFDLFAAEHSKLLVLIGMNWICT 7v2c.1    EALVALKDLLNRV-----------------DSDSLCTEEVFPTAGAGTDLR-SNYLLNTTIAGVEEADVILLVGTNPRFE  target    KMPDAHWIGDARL-KGTRVVVISADYMPTANKADEIVILRPGTDTAFLLGVARELITKKLYDRDAVIQRTDLPLLVRLDT 7v2c.1    APLFNARIRKSWLHNDLKVALIGSPVDLTYRYDHLGDSPK------------------------ILQDIAS---------  target    GERLSARDVFEGYRQAPLENYVALKTEEELAAPPSPPFTADKQVVPTELREEWGDFVYWDRATNGPAAVNRDEIGAKFAG 7v2c.1    --------------------------------------------------------------------------------  target    DPALLGAFDVTLVDGTNVKARTAFSLLKEYLDENFDVQTTSEVCNVDPAAVRSLARQLAANKGNALLAAGMGPNHYFNAD 7v2c.1    ---------------------------------G----------------NHPFSQILKEAKK-PMVVLGSSALQRSDGT  target    LFGRVHFLVAALTDNIGHFSGNVGSYAGNYRGSLFQAMGQWIAENPFDQEADLTKPARVKRYFKSESAHYWNYGDRPLVS 7v2c.1    AILAAVSNIAQNIRLSSGVTGDWKVMN--I-------LHR--IASQVA---------------------ALDLGYKPG--  target    PSEIITGKSHMPTPTKLIWFGNSNSLLGNAKWSFDVVKNTLPKQDAVFCNEWHWTSSCEYSDLVFPADSWAEFKLPDMTA 7v2c.1    VEAIR------KNPPKVLFLLGADGGC--------ITRQDLPKDCFIIYQGHHGDVGAPMADVILPGAAYTEKSAT---Y  target    SCTNPFLLAFPKTPLARIHNTRSDYEILAGVAAALADLVDEPRMKTYWKGILDGDPTPYLQRVLSGSNATRGILYEDLHA 7v2c.1    VNTEGRAQQTKV-AVTPPGLAREDWKIIRALSEIAGM-------------------------------------------  target    SSAKGVPLLMNARTYPRHAGWEQRQEDKPWYTPTGRLEFYRPEPEWQAAGESLPIWREPVDATFYEPNAILANSKHPSIN 7v2c.1    --------------------------------------------------------------------------------  target    PRAPEDYGVPESQMDVETRQYRNVVRTWQELKLSKHPLTEKDPAYRFVFQTPKYRWGAHSTAVDSDWIAMLFGPFGDPYR 7v2c.1    --------------------------------------------------------------------------------  target    RDSRTPWTGEAYAEINPRDAKELGLKDGDYIWLDADPEDRPYRGADSSDEFYDVARAMMRVRIYSGMPRRVIRTWFNMYA 7v2c.1    --------------------------------------------------------------------------------  target    ATPGTVQAQKDVPGGPAQNQDTGYVALFRHGSHQSGTRAYLRPTQMTDSMNRKAYFGQTIGKGFEADVHSPSGAPKEGYV 7v2c.1    --------------------------------------------------------------------------------  target    KVEKAEDGGDEGVGEWRPVTLGLRPDDPSEAMQAYLAGEFVTRKRKGS 7v2c.1    ------------------------------------------------ ``` | | | | | | | | | | | | | | | | | | | | | | | | | | | | | | | | | | | | | | | | | | | | | | | | | |
|  | 7tgh.58.A | NADH-ubiquinone oxidoreductase 75 kDa subunit  *Cryo-EM structure of respiratory super-complex CI+III2 from Tetrahymena thermophila* | 0.17 |  | 15.93 | 0.33 | 67-756 | EM | 0.00 | monomer |  | HHblits | 0.28 |
| ``` target    SDLSRRELLKRAVVVGTGAGLAELFLPAQFLSSASAQSEPQAVAIANPLAQMPDRSWERIYRDQFAEEDSFVFTCAPNDT 7tgh.58   ------------------------------------------------------------------WELKSFYTSDVFDT  target    HNCLLRAHVKNGVIVRISPTYGYGKATDLAGNQASHRWDPRICQKGLILGRRIYGDRRVKAPMIRKGFKEWADAGFPRHD 7tgh.58   LGSAIQVDTRGPEIMRVLPR------------IHEEINEEWISDKTRHAFDGLK-RQRINSPMKRSK-------------  target    DGTPRADMEKRGYDEWLQIPWDEALAIAAKTLQNVAETYKGEDGAGKLLEQGYEPAMVEAMHGAGVQAIKMRGGMPLLGA 7tgh.58   ------------DGNYEDIFWEEAIQTISKKCLNTPSDQI------GAIIGEF------------ADI------------  target    GRVFGFYRFANMLALLDGKLRPEAPPEEIVGSRAFDNYAWHTDLPPGHPMVSGSQTVDFDLFAAEHSKLLVLIGMNWICT 7tgh.58   ESITALKDFLNRL-----------------DVDNFEV-RQHGNLKVSPDFR-ANYLMNSKITGVEDADVLLLVGCNPRYE  target    KMPDAHWIGDARLKGTRVVVISADYMPTANKADEIVILRPGTDTAFLLGVARELITKKLYDRDAVIQRTDLPLLVRLDTG 7tgh.58   APVLNARILKSTRKNLKVFNIGTNQDL--NYKNVHL----GNSTKV----------------------------------  target    ERLSARDVFEGYRQAPLENYVALKTEEELAAPPSPPFTADKQVVPTELREEWGDFVYWDRATNGPAAVNRDEIGAKFAGD 7tgh.58   --------------------------------------------------------------------------------  target    PALLGAFDVTLVDGTNVKARTAFSLLKEYLDENFDVQTTSEVCNVDPAAVRSLARQLAANKGNALLAAGMGPNHYFNADL 7tgh.58   ------------------------------------------LKEIADGTHPFAERLKKAKLP-MIMVGASALEREDGAE  target    FGRVHFLVAALTDNIGHFSGNVGSYAGNYRGSLFQAMGQWIAENPFDQEADLTKPARVKRYFKSESAHYWNYGDRPLVSP 7tgh.58   LYNTLKVISNKTGVISEEKSWNGFNILHK------EM------GRINA---------L------------ELG----INP  target    SEIITGKSHMPTPTKLIWFGNSNSLLGNAKWSFDVVKNTLPKQDAVFCNEWHWTSSCEYSDLVFPADSWAEFKLPDMTAS 7tgh.58   T-------SVNKNAKLVFILGADNNLRP---------EDIPADAFVVYFGTHGDEGAYYADIILPTAAYTEKNATW---V  target    CTNPFLLAFPKTPLARIHNTRSDYEILAGVAAALADLVDEPRMKTYWKGILDGDPTPYLQRVLSGSNATRGILYEDLHAS 7tgh.58   NTEGRVQQGRL-VVMPPGDAREDWQIIRALSEEAGV--------------------------------------------  target    SAKGVPLLMNARTYPRHAGWEQRQEDKPWYTPTGRLEFYRPEPEWQAAGESLPIWREPVDATFYEPNAILANSKHPSINP 7tgh.58   --------------------------------------------------------------------------------  target    RAPEDYGVPESQMDVETRQYRNVVRTWQELKLSKHPLTEKDPAYRFVFQTPKYRWGAHSTAVDSDWIAMLFGPFGDPYRR 7tgh.58   --------------------------------------------------------------------------------  target    DSRTPWTGEAYAEINPRDAKELGLKDGDYIWLDADPEDRPYRGADSSDEFYDVARAMMRVRIYSGMPRRVIRTWFNMYAA 7tgh.58   --------------------------------------------------------------------------------  target    TPGTVQAQKDVPGGPAQNQDTGYVALFRHGSHQSGTRAYLRPTQMTDSMNRKAYFGQTIGKGFEADVHSPSGAPKEGYVK 7tgh.58   --------------------------------------------------------------------------------  target    VEKAEDGGDEGVGEWRPVTLGLRPDDPSEAMQAYLAGEFVTRKRKGS 7tgh.58   ----------------------------------------------- ``` | | | | | | | | | | | | | | | | | | | | | | | | | | | | | | | | | | | | | | | | | | | | | | | | | |
|  | 7zm7.1.I | NADH-ubiquinone oxidoreductase-like protein  *CryoEM structure of mitochondrial complex I from Chaetomium thermophilum (inhibited by DDM)* | 0.17 |  | 16.19 | 0.33 | 68-756 | EM | 0.00 | hetero-1-1-1-1-1-1-… | 4 x PC1, 14 x LMT, 5 x CDL, 8 x 3PE, 2 x FES, 6 x SF4, 1 x FMN, 1 x NDP, 1 x ZN, 2 x ZMP | HHblits | 0.28 |
| ``` target    SDLSRRELLKRAVVVGTGAGLAELFLPAQFLSSASAQSEPQAVAIANPLAQMPDRSWERIYRDQFAEEDSFVFTCAPNDT 7zm7.1    -------------------------------------------------------------------ELKRTESIDVLDG  target    HNCLLRAHVKNGVIVRISPTYGYGKATDLAGNQASHRWDPRICQKGLILGRRIYGDRRVKAPMIRKGFKEWADAGFPRHD 7zm7.1    LGSNIRVDSRGLEVMRILPRL------------NDDVNEEWINDKTRFACDGLK-TQRLTMPLVRRD-------------  target    DGTPRADMEKRGYDEWLQIPWDEALAIAAKTLQNVAETYKGEDGAGKLLEQGYEPAMVEAMHGAGVQAIKMRGGMPLLGA 7zm7.1    -------------GKFEPATWEQALTEIAHAYQTLAPKENEF----KVIAGQL------------VE------------V  target    GRVFGFYRFANMLALLDGKLRPEAPPEEIVGSRAFDNYAWHTDLPPGHPMVSGSQ-TVDFDLFAAEHSKLLVLIGMNWIC 7zm7.1    ESLVAMKDLANR-----------------LGSENLALDFPGGSQPLAHGVDIRSNYLFNSKIWGIEEADAILLVGTNPRH  target    TKMPDAHWIGDAR-LKGTRVVVISADYMPTANKADEIVILRPGTDTAFLLGVARELITKKLYDRDAVIQRTDLPLLVRLD 7zm7.1    EAAVLNARIRKQWLRSDLEIAAVGQPWESTFDYEH------LGTDLAALKNALSGPF-----------------------  target    TGERLSARDVFEGYRQAPLENYVALKTEEELAAPPSPPFTADKQVVPTELREEWGDFVYWDRATNGPAAVNRDEIGAKFA 7zm7.1    --------------------------------------------------------------------------------  target    GDPALLGAFDVTLVDGTNVKARTAFSLLKEYLDENFDVQTTSEVCNVDPAAVRSLARQLAANKGNALLAAGMGPNHYFNA 7zm7.1    -------------------------------------------------------GEKLKKAKR-PMIIVGSGVTEHPDA  target    DLFGRVHFLVAALTDN--IGHFSGNVGSYAGNYRGSLFQAMGQWIAENPFDQEADLTKPARVKRYFKSESAHYWNYGDRP 7zm7.1    KAFYETVWSFVEKNASNFLTEEWCGYNVLQRAA--------------SRAG--------AFEV-----------GF--VV  target    LVSPSEIITGKSHMPTPTKLIWFGNSNSLLGNAKWSFDVVKNTLPKQDAVFCNEWHWTSSCEYSDLVFPADSWAEFKLPD 7zm7.1    P--SPEV------AATKPKFVWLLGADEFDP----------ADVPKDAFIVYQGHHGDRGAEIADIVLPGAAYTEKAGT-  target    MTASCTNPFLLAFPKTPLARIHNTRSDYEILAGVAAALADLVDEPRMKTYWKGILDGDPTPYLQRVLSGSNATRGILYED 7zm7.1    --YVNTEGRVQMTRA-ATGLPGAARTDWKIIRAVSEFLGV----------------------------------------  target    LHASSAKGVPLLMNARTYPRHAGWEQRQEDKPWYTPTGRLEFYRPEPEWQAAGESLPIWREPVDATFYEPNAILANSKHP 7zm7.1    --------------------------------------------------------------------------------  target    SINPRAPEDYGVPESQMDVETRQYRNVVRTWQELKLSKHPLTEKDPAYRFVFQTPKYRWGAHSTAVDSDWIAMLFGPFGD 7zm7.1    --------------------------------------------------------------------------------  target    PYRRDSRTPWTGEAYAEINPRDAKELGLKDGDYIWLDADPEDRPYRGADSSDEFYDVARAMMRVRIYSGMPRRVIRTWFN 7zm7.1    --------------------------------------------------------------------------------  target    MYAATPGTVQAQKDVPGGPAQNQDTGYVALFRHGSHQSGTRAYLRPTQMTDSMNRKAYFGQTIGKGFEADVHSPSGAPKE 7zm7.1    --------------------------------------------------------------------------------  target    GYVKVEKAEDGGDEGVGEWRPVTLGLRPDDPSEAMQAYLAGEFVTRKRKGS 7zm7.1    --------------------------------------------------- ``` | | | | | | | | | | | | | | | | | | | | | | | | | | | | | | | | | | | | | | | | | | | | | | | | | |
|  | 7ak5.1.G | NADH-ubiquinone oxidoreductase 75 kDa subunit, mitochondrial  *Cryo-EM structure of respiratory complex I in the deactive state from Mus musculus at 3.2 A* | 0.16 |  | 13.88 | 0.33 | 67-756 | EM | 0.00 | hetero-1-1-1-1-1-1-… | 6 x SF4, 2 x PC1, 2 x FES, 1 x FMN, 8 x 3PE, 4 x CDL, 1 x ATP, 1 x NDP, 1 x ZN, 2 x EHZ | HHblits | 0.26 |
| ``` target    SDLSRRELLKRAVVVGTGAGLAELFLPAQFLSSASAQSEPQAVAIANPLAQMPDRSWERIYRDQFAEEDSFVFTCAPNDT 7ak5.1    ------------------------------------------------------------------WETRKTESIDVMDA  target    HNCLLRAHVKNGVIVRISPTYGYGKATDLAGNQASHRWDPRICQKGLILGRRIYGDRRVKAPMIRKGFKEWADAGFPRHD 7ak5.1    VGSNIVVSTRTGEVMRILPRM------------HEDINEEWISDKTRFAYDGLK-RQRLTEPMVRNE-------------  target    DGTPRADMEKRGYDEWLQIPWDEALAIAAKTLQNVAETYKGEDGAGKLLEQGYEPAMVEAMHGAGVQAIKMRGGMPLLGA 7ak5.1    ------------KGLLTYTSWEDALSRVAGMLQN----FEGNAV--AAIA------------------------------  target    GRVFGFYRFANMLALLDGKLRPEAPPEEIVGSRAFDNYAWHTDLPPGHPMVSGSQTVDFDLFAAEHSKLLVLIGMNWICT 7ak5.1    -----GGLVDAEALVALKDLLN------KVDSDNLCTEEIFPTEGAGTDLRSNY-LLNTTIAGVEEADVVLLVGTNPRFE  target    KMPDAHWIGDAR-LKGTRVVVISADYMPTANKADEIVILRPGTDTAFLLGVARELITKKLYDRDAVIQRTDLPLLVRLDT 7ak5.1    APLFNARIRKSWLHNDLKVALIGSPVDLTYRYDHLGDSP------------------------KILQDIAS---------  target    GERLSARDVFEGYRQAPLENYVALKTEEELAAPPSPPFTADKQVVPTELREEWGDFVYWDRATNGPAAVNRDEIGAKFAG 7ak5.1    --------------------------------------------------------------------------------  target    DPALLGAFDVTLVDGTNVKARTAFSLLKEYLDENFDVQTTSEVCNVDPAAVRSLARQLAANKGNALLAAGMGPNHYFNAD 7ak5.1    -------------------------------------------------GRHSFCEVLKDAKK-PMVVLGSSALQRDDGA  target    LFGRVHFLVAALTDNIGHFSGNVGSYAGNYRGSLFQAMGQWIAENPFDQEADLTKPARVKRYFKSESAHYWNYGDRPLVS 7ak5.1    AILVAVSNMVQKIRVTTGVAAEWKVMN--I-------LHR--IASQVA---------------------ALDLGYKPG--  target    PSEIITGKSHMPTPTKLIWFGNSNSLLGNAKWSFDVVKNTLPKQDAVFCNEWHWTSSCEYSDLVFPADSWAEFKLPDMTA 7ak5.1    VEAIR------KNPPKMLFLLGADGGC--------ITRQDLPKDCFIVYQGHHGDVGAPMADVILPGAAYTEKSA---TY  target    SCTNPFLLAFPKTPLARIHNTRSDYEILAGVAAALADLVDEPRMKTYWKGILDGDPTPYLQRVLSGSNATRGILYEDLHA 7ak5.1    VNTEGRAQQTKV-AVTPPGLAREDWKIIRALSEIAGI-------------------------------------------  target    SSAKGVPLLMNARTYPRHAGWEQRQEDKPWYTPTGRLEFYRPEPEWQAAGESLPIWREPVDATFYEPNAILANSKHPSIN 7ak5.1    --------------------------------------------------------------------------------  target    PRAPEDYGVPESQMDVETRQYRNVVRTWQELKLSKHPLTEKDPAYRFVFQTPKYRWGAHSTAVDSDWIAMLFGPFGDPYR 7ak5.1    --------------------------------------------------------------------------------  target    RDSRTPWTGEAYAEINPRDAKELGLKDGDYIWLDADPEDRPYRGADSSDEFYDVARAMMRVRIYSGMPRRVIRTWFNMYA 7ak5.1    --------------------------------------------------------------------------------  target    ATPGTVQAQKDVPGGPAQNQDTGYVALFRHGSHQSGTRAYLRPTQMTDSMNRKAYFGQTIGKGFEADVHSPSGAPKEGYV 7ak5.1    --------------------------------------------------------------------------------  target    KVEKAEDGGDEGVGEWRPVTLGLRPDDPSEAMQAYLAGEFVTRKRKGS 7ak5.1    ------------------------------------------------ ``` | | | | | | | | | | | | | | | | | | | | | | | | | | | | | | | | | | | | | | | | | | | | | | | | | |
|  | 6zr2.1.G | NADH-ubiquinone oxidoreductase 75 kDa subunit, mitochondrial  *Cryo-EM structure of respiratory complex I in the active state from Mus musculus at 3.1 A* | 0.16 |  | 13.62 | 0.33 | 67-756 | EM | 3.10 | hetero-1-1-1-1-1-1-… | 6 x SF4, 4 x PC1, 2 x FES, 1 x FMN, 9 x 3PE, 7 x CDL, 1 x ATP, 1 x NDP, 1 x ZN, 2 x EHZ | HHblits | 0.26 |
| ``` target    SDLSRRELLKRAVVVGTGAGLAELFLPAQFLSSASAQSEPQAVAIANPLAQMPDRSWERIYRDQFAEEDSFVFTCAPNDT 6zr2.1    ------------------------------------------------------------------WETRKTESIDVMDA  target    HNCLLRAHVKNGVIVRISPTYGYGKATDLAGNQASHRWDPRICQKGLILGRRIYGDRRVKAPMIRKGFKEWADAGFPRHD 6zr2.1    VGSNIVVSTRTGEVMRILPR------------MHEDINEEWISDKTRFAYDGLK-RQRLTEPMVRNE-------------  target    DGTPRADMEKRGYDEWLQIPWDEALAIAAKTLQNVAETYKGEDGAGKLLEQGYEPAMVEAMHGAGVQAIKMRGGMPLLGA 6zr2.1    ------------KGLLTYTSWEDALSRVAGMLQN----FEGNAV--AAI------------A------------------  target    GRVFGFYRFANMLALLDGKLRPEAPPEEIVGSRAFDNYAWHTDLPPGHPMVSGSQTVDFDLFAAEHSKLLVLIGMNWICT 6zr2.1    -----GGLVDAEALVALKDLLN------KVDSDNLCTEEIFPTEGAGTDLRSNY-LLNTTIAGVEEADVVLLVGTNPRFE  target    KMPDAHWIGDAR-LKGTRVVVISADYMPTANKADEIVILRPGTDTAFLLGVARELITKKLYDRDAVIQRTDLPLLVRLDT 6zr2.1    APLFNARIRKSWLHNDLKVALIGSPVDLTYRYDHLGDSP------------------------KILQDIAS---------  target    GERLSARDVFEGYRQAPLENYVALKTEEELAAPPSPPFTADKQVVPTELREEWGDFVYWDRATNGPAAVNRDEIGAKFAG 6zr2.1    --------------------------------------------------------------------------------  target    DPALLGAFDVTLVDGTNVKARTAFSLLKEYLDENFDVQTTSEVCNVDPAAVRSLARQLAANKGNALLAAGMGPNHYFNAD 6zr2.1    ---------------------------------G----------------RHSFCEVLKDAKK-PMVVLGSSALQRDDGA  target    LFGRVHFLVAALTDNIGHFSGNVGSYAGNYRGSLFQAMGQWIAENPFDQEADLTKPARVKRYFKSESAHYWNYGDRPLVS 6zr2.1    AILVAVSNMVQKIRVTTGVAAEWKVMN------ILHR-----IASQVA----------A-----------LDLGYKPG--  target    PSEIITGKSHMPTPTKLIWFGNSNSLLGNAKWSFDVVKNTLPKQDAVFCNEWHWTSSCEYSDLVFPADSWAEFKLPDMTA 6zr2.1    VEAIR------KNPPKMLFLLGADGGC--------ITRQDLPKDCFIVYQGHHGDVGAPMADVILPGAAYTEKSAT---Y  target    SCTNPFLLAFPKTPLARIHNTRSDYEILAGVAAALADLVDEPRMKTYWKGILDGDPTPYLQRVLSGSNATRGILYEDLHA 6zr2.1    VNTEGRAQQTKV-AVTPPGLAREDWKIIRALSEIAGI-------------------------------------------  target    SSAKGVPLLMNARTYPRHAGWEQRQEDKPWYTPTGRLEFYRPEPEWQAAGESLPIWREPVDATFYEPNAILANSKHPSIN 6zr2.1    --------------------------------------------------------------------------------  target    PRAPEDYGVPESQMDVETRQYRNVVRTWQELKLSKHPLTEKDPAYRFVFQTPKYRWGAHSTAVDSDWIAMLFGPFGDPYR 6zr2.1    --------------------------------------------------------------------------------  target    RDSRTPWTGEAYAEINPRDAKELGLKDGDYIWLDADPEDRPYRGADSSDEFYDVARAMMRVRIYSGMPRRVIRTWFNMYA 6zr2.1    --------------------------------------------------------------------------------  target    ATPGTVQAQKDVPGGPAQNQDTGYVALFRHGSHQSGTRAYLRPTQMTDSMNRKAYFGQTIGKGFEADVHSPSGAPKEGYV 6zr2.1    --------------------------------------------------------------------------------  target    KVEKAEDGGDEGVGEWRPVTLGLRPDDPSEAMQAYLAGEFVTRKRKGS 6zr2.1    ------------------------------------------------ ``` | | | | | | | | | | | | | | | | | | | | | | | | | | | | | | | | | | | | | | | | | | | | | | | | | |
|  | 6g72.1.G | NADH-ubiquinone oxidoreductase 75 kDa subunit, mitochondrial  *Mouse mitochondrial complex I in the deactive state* | 0.16 |  | 13.62 | 0.33 | 67-756 | EM | 0.00 | hetero-1-1-1-1-1-1-… | 6 x SF4, 2 x FES, 1 x FMN, 1 x ADP, 1 x NDP, 1 x ZN, 2 x EHZ | HHblits | 0.26 |
| ``` target    SDLSRRELLKRAVVVGTGAGLAELFLPAQFLSSASAQSEPQAVAIANPLAQMPDRSWERIYRDQFAEEDSFVFTCAPNDT 6g72.1    ------------------------------------------------------------------WETRKTESIDVMDA  target    HNCLLRAHVKNGVIVRISPTYGYGKATDLAGNQASHRWDPRICQKGLILGRRIYGDRRVKAPMIRKGFKEWADAGFPRHD 6g72.1    VGSNIVVSTRTGEVMRILPR------------MHEDINEEWISDKTRFAYDGLK-RQRLTEPMVRNE-------------  target    DGTPRADMEKRGYDEWLQIPWDEALAIAAKTLQNVAETYKGEDGAGKLLEQGYEPAMVEAMHGAGVQAIKMRGGMPLLGA 6g72.1    ------------KGLLTYTSWEDALSRVAGMLQN----FEGNAV--AAI------------A------------------  target    GRVFGFYRFANMLALLDGKLRPEAPPEEIVGSRAFDNYAWHTDLPPGHPMVSGSQTVDFDLFAAEHSKLLVLIGMNWICT 6g72.1    -----GGLVDAEALVALKDLLN------KVDSDNLCTEEIFPTEGAGTDLRSNY-LLNTTIAGVEEADVVLLVGTNPRFE  target    KMPDAHWIGDAR-LKGTRVVVISADYMPTANKADEIVILRPGTDTAFLLGVARELITKKLYDRDAVIQRTDLPLLVRLDT 6g72.1    APLFNARIRKSWLHNDLKVALIGSPVDLTYRYDHLGDSP------------------------KILQDIAS---------  target    GERLSARDVFEGYRQAPLENYVALKTEEELAAPPSPPFTADKQVVPTELREEWGDFVYWDRATNGPAAVNRDEIGAKFAG 6g72.1    --------------------------------------------------------------------------------  target    DPALLGAFDVTLVDGTNVKARTAFSLLKEYLDENFDVQTTSEVCNVDPAAVRSLARQLAANKGNALLAAGMGPNHYFNAD 6g72.1    ---------------------------------G----------------RHSFCEVLKDAKK-PMVVLGSSALQRDDGA  target    LFGRVHFLVAALTDNIGHFSGNVGSYAGNYRGSLFQAMGQWIAENPFDQEADLTKPARVKRYFKSESAHYWNYGDRPLVS 6g72.1    AILVAVSNMVQKIRVTTGVAAEWKVMN------ILHR-----IASQVA----------A-----------LDLGYKPG--  target    PSEIITGKSHMPTPTKLIWFGNSNSLLGNAKWSFDVVKNTLPKQDAVFCNEWHWTSSCEYSDLVFPADSWAEFKLPDMTA 6g72.1    VEAIR------KNPPKMLFLLGADGGC--------ITRQDLPKDCFIVYQGHHGDVGAPMADVILPGAAYTEKSAT---Y  target    SCTNPFLLAFPKTPLARIHNTRSDYEILAGVAAALADLVDEPRMKTYWKGILDGDPTPYLQRVLSGSNATRGILYEDLHA 6g72.1    VNTEGRAQQTKV-AVTPPGLAREDWKIIRALSEIAGI-------------------------------------------  target    SSAKGVPLLMNARTYPRHAGWEQRQEDKPWYTPTGRLEFYRPEPEWQAAGESLPIWREPVDATFYEPNAILANSKHPSIN 6g72.1    --------------------------------------------------------------------------------  target    PRAPEDYGVPESQMDVETRQYRNVVRTWQELKLSKHPLTEKDPAYRFVFQTPKYRWGAHSTAVDSDWIAMLFGPFGDPYR 6g72.1    --------------------------------------------------------------------------------  target    RDSRTPWTGEAYAEINPRDAKELGLKDGDYIWLDADPEDRPYRGADSSDEFYDVARAMMRVRIYSGMPRRVIRTWFNMYA 6g72.1    --------------------------------------------------------------------------------  target    ATPGTVQAQKDVPGGPAQNQDTGYVALFRHGSHQSGTRAYLRPTQMTDSMNRKAYFGQTIGKGFEADVHSPSGAPKEGYV 6g72.1    --------------------------------------------------------------------------------  target    KVEKAEDGGDEGVGEWRPVTLGLRPDDPSEAMQAYLAGEFVTRKRKGS 6g72.1    ------------------------------------------------ ``` | | | | | | | | | | | | | | | | | | | | | | | | | | | | | | | | | | | | | | | | | | | | | | | | | |
|  | 7ak6.1.G | NADH-ubiquinone oxidoreductase 75 kDa subunit, mitochondrial  *Cryo-EM structure of ND6-P25L mutant respiratory complex I from Mus musculus at 3.8 A* | 0.16 |  | 13.62 | 0.33 | 67-756 | EM | 0.00 | hetero-1-1-1-1-1-1-… | 6 x SF4, 1 x PC1, 2 x FES, 1 x FMN, 4 x 3PE, 2 x CDL, 1 x ATP, 1 x NDP, 1 x ZN, 2 x EHZ | HHblits | 0.26 |
| ``` target    SDLSRRELLKRAVVVGTGAGLAELFLPAQFLSSASAQSEPQAVAIANPLAQMPDRSWERIYRDQFAEEDSFVFTCAPNDT 7ak6.1    ------------------------------------------------------------------WETRKTESIDVMDA  target    HNCLLRAHVKNGVIVRISPTYGYGKATDLAGNQASHRWDPRICQKGLILGRRIYGDRRVKAPMIRKGFKEWADAGFPRHD 7ak6.1    VGSNIVVSTRTGEVMRILPR------------MHEDINEEWISDKTRFAYDGLK-RQRLTEPMVRNE-------------  target    DGTPRADMEKRGYDEWLQIPWDEALAIAAKTLQNVAETYKGEDGAGKLLEQGYEPAMVEAMHGAGVQAIKMRGGMPLLGA 7ak6.1    ------------KGLLTYTSWEDALSRVAGMLQN----FEGNAV--AAI------------A------------------  target    GRVFGFYRFANMLALLDGKLRPEAPPEEIVGSRAFDNYAWHTDLPPGHPMVSGSQTVDFDLFAAEHSKLLVLIGMNWICT 7ak6.1    -----GGLVDAEALVALKDLLN------KVDSDNLCTEEIFPTEGAGTDLRSNY-LLNTTIAGVEEADVVLLVGTNPRFE  target    KMPDAHWIGDAR-LKGTRVVVISADYMPTANKADEIVILRPGTDTAFLLGVARELITKKLYDRDAVIQRTDLPLLVRLDT 7ak6.1    APLFNARIRKSWLHNDLKVALIGSPVDLTYRYDHLGDSP------------------------KILQDIAS---------  target    GERLSARDVFEGYRQAPLENYVALKTEEELAAPPSPPFTADKQVVPTELREEWGDFVYWDRATNGPAAVNRDEIGAKFAG 7ak6.1    --------------------------------------------------------------------------------  target    DPALLGAFDVTLVDGTNVKARTAFSLLKEYLDENFDVQTTSEVCNVDPAAVRSLARQLAANKGNALLAAGMGPNHYFNAD 7ak6.1    ---------------------------------G----------------RHSFCEVLKDAKK-PMVVLGSSALQRDDGA  target    LFGRVHFLVAALTDNIGHFSGNVGSYAGNYRGSLFQAMGQWIAENPFDQEADLTKPARVKRYFKSESAHYWNYGDRPLVS 7ak6.1    AILVAVSNMVQKIRVTTGVAAEWKVMN------ILHR-----IASQVA----------A-----------LDLGYKPG--  target    PSEIITGKSHMPTPTKLIWFGNSNSLLGNAKWSFDVVKNTLPKQDAVFCNEWHWTSSCEYSDLVFPADSWAEFKLPDMTA 7ak6.1    VEAIR------KNPPKMLFLLGADGGC--------ITRQDLPKDCFIVYQGHHGDVGAPMADVILPGAAYTEKSAT---Y  target    SCTNPFLLAFPKTPLARIHNTRSDYEILAGVAAALADLVDEPRMKTYWKGILDGDPTPYLQRVLSGSNATRGILYEDLHA 7ak6.1    VNTEGRAQQTKV-AVTPPGLAREDWKIIRALSEIAGI-------------------------------------------  target    SSAKGVPLLMNARTYPRHAGWEQRQEDKPWYTPTGRLEFYRPEPEWQAAGESLPIWREPVDATFYEPNAILANSKHPSIN 7ak6.1    --------------------------------------------------------------------------------  target    PRAPEDYGVPESQMDVETRQYRNVVRTWQELKLSKHPLTEKDPAYRFVFQTPKYRWGAHSTAVDSDWIAMLFGPFGDPYR 7ak6.1    --------------------------------------------------------------------------------  target    RDSRTPWTGEAYAEINPRDAKELGLKDGDYIWLDADPEDRPYRGADSSDEFYDVARAMMRVRIYSGMPRRVIRTWFNMYA 7ak6.1    --------------------------------------------------------------------------------  target    ATPGTVQAQKDVPGGPAQNQDTGYVALFRHGSHQSGTRAYLRPTQMTDSMNRKAYFGQTIGKGFEADVHSPSGAPKEGYV 7ak6.1    --------------------------------------------------------------------------------  target    KVEKAEDGGDEGVGEWRPVTLGLRPDDPSEAMQAYLAGEFVTRKRKGS 7ak6.1    ------------------------------------------------ ``` | | | | | | | | | | | | | | | | | | | | | | | | | | | | | | | | | | | | | | | | | | | | | | | | | |
|  | 6x89.1.H | NADH dehydrogenase [ubiquinone] iron-sulfur protein 1, mitochondrial  *Vigna radiata mitochondrial complex I\** | 0.16 |  | 15.26 | 0.33 | 68-756 | EM | 0.00 | hetero-1-1-1-1-1-1-… | 1 x NAP, 6 x PC1, 6 x SF4, 2 x FES, 2 x ZN, 1 x FMN | HHblits | 0.27 |
| ``` target    SDLSRRELLKRAVVVGTGAGLAELFLPAQFLSSASAQSEPQAVAIANPLAQMPDRSWERIYRDQFAEEDSFVFTCAPNDT 6x89.1    -------------------------------------------------------------------ELKGTETIDVTDA  target    HNCLLRAHVKNGVIVRISPTYGYGKATDLAGNQASHRWDPRICQKGLILGRRIYGDRRVKAPMIRKGFKEWADAGFPRHD 6x89.1    VGSNIRIDSRGPEVMRIVPRL------------NEDINEEWISDKTRFCYDGLK-RQRLNDPMIRGP-------------  target    DGTPRADMEKRGYDEWLQIPWDEALAIAAKTLQNVAETYKGEDGAGKLLEQGYEPAMVEAMHGAGVQAIKMRGGMPLLGA 6x89.1    ------------DGRFKAVNWRDALSVIADIAHQV----KPEE----IV----------GVA--GKLS----------DA  target    GRVFGFYRFANMLALLDGKLRPEAPPEEIVGSRAFDNYAWHTDLPPGHPMVSGSQTVDFDLFAAEHSKLLVLIGMNWICT 6x89.1    ESMIALKDFLNRM-----------------GSNDVWGEGIGVNTNADFRS--G-YIMNTSIAGLEKADVFLLVGTQPRVE  target    KMPDAHWIGDARL-KGTRVVVISADYMPTANKADEIVILRPGTDTAFLLGVARELITKKLYDRDAVIQRTDLPLLVRLDT 6x89.1    AAMVNARIRKTVRSNQAKVGYIGPATDFN--YDHKHLGTDPQTLVEIAEGR-----------------------------  target    GERLSARDVFEGYRQAPLENYVALKTEEELAAPPSPPFTADKQVVPTELREEWGDFVYWDRATNGPAAVNRDEIGAKFAG 6x89.1    --------------------------------------------------------------------------------  target    DPALLGAFDVTLVDGTNVKARTAFSLLKEYLDENFDVQTTSEVCNVDPAAVRSLARQLAANKGNALLAAGMGPNHYFNAD 6x89.1    ---------------------------------------------------HPFFKTLSDAKN-PVIIVGAGVFERKDQD  target    LFGRVHFLVAALTDNIGHFSGNVGSYAGNYRGSLFQAMGQWIAENPFDQEADLTKPARVKRYFKSESAHYWNYGDRPLVS 6x89.1    AIFAAVETIAQKANVVRPDWNGLNVLLLHAAQ-----AAA------L----DL--------------------GLVPQ--  target    PSEIITGKSHMPTPTKLIWFGNSNSLLGNAKWSFDVVKNTLPKQDAVFCNEWHWTSSCEYSDLVFPADSWAEFKLPDMTA 6x89.1    ---SEK----SLESAKFVYLMGADDVN----------LDKIPDDAFVVYQGHHGDKSVYRANVILPTAAFSEKEGT---Y  target    SCTNPFLLAFPKTPLARIHNTRSDYEILAGVAAALADLVDEPRMKTYWKGILDGDPTPYLQRVLSGSNATRGILYEDLHA 6x89.1    QNTEGCTQQTLP-AVPTVGDSRDDWKIIRALSEVAGV-------------------------------------------  target    SSAKGVPLLMNARTYPRHAGWEQRQEDKPWYTPTGRLEFYRPEPEWQAAGESLPIWREPVDATFYEPNAILANSKHPSIN 6x89.1    --------------------------------------------------------------------------------  target    PRAPEDYGVPESQMDVETRQYRNVVRTWQELKLSKHPLTEKDPAYRFVFQTPKYRWGAHSTAVDSDWIAMLFGPFGDPYR 6x89.1    --------------------------------------------------------------------------------  target    RDSRTPWTGEAYAEINPRDAKELGLKDGDYIWLDADPEDRPYRGADSSDEFYDVARAMMRVRIYSGMPRRVIRTWFNMYA 6x89.1    --------------------------------------------------------------------------------  target    ATPGTVQAQKDVPGGPAQNQDTGYVALFRHGSHQSGTRAYLRPTQMTDSMNRKAYFGQTIGKGFEADVHSPSGAPKEGYV 6x89.1    --------------------------------------------------------------------------------  target    KVEKAEDGGDEGVGEWRPVTLGLRPDDPSEAMQAYLAGEFVTRKRKGS 6x89.1    ------------------------------------------------ ``` | | | | | | | | | | | | | | | | | | | | | | | | | | | | | | | | | | | | | | | | | | | | | | | | | |
|  | 8e73.55.A | NDUS1  *Vigna radiata supercomplex I+III2 (full bridge)* | 0.17 |  | 15.26 | 0.33 | 68-756 | EM | 0.00 | monomer |  | HHblits | 0.27 |
| ``` target    SDLSRRELLKRAVVVGTGAGLAELFLPAQFLSSASAQSEPQAVAIANPLAQMPDRSWERIYRDQFAEEDSFVFTCAPNDT 8e73.55   -------------------------------------------------------------------ELKGTETIDVTDA  target    HNCLLRAHVKNGVIVRISPTYGYGKATDLAGNQASHRWDPRICQKGLILGRRIYGDRRVKAPMIRKGFKEWADAGFPRHD 8e73.55   VGSNIRIDSRGPEVMRIVPRL------------NEDINEEWISDKTRFCYDGLK-RQRLNDPMIRGP-------------  target    DGTPRADMEKRGYDEWLQIPWDEALAIAAKTLQNVAETYKGEDGAGKLLEQGYEPAMVEAMHGAGVQAIKMRGGMPLLGA 8e73.55   ------------DGRFKAVNWRDALSVIADIAHQV----KPEE----IV----------GVA--GKLS----------DA  target    GRVFGFYRFANMLALLDGKLRPEAPPEEIVGSRAFDNYAWHTDLPPGHPMVSGSQTVDFDLFAAEHSKLLVLIGMNWICT 8e73.55   ESMIALKDFLNRM-----------------GSNDVWGEGIGVNTNADFRS--G-YIMNTSIAGLEKADVFLLVGTQPRVE  target    KMPDAHWIGDARL-KGTRVVVISADYMPTANKADEIVILRPGTDTAFLLGVARELITKKLYDRDAVIQRTDLPLLVRLDT 8e73.55   AAMVNARIRKTVRSNQAKVGYIGPATDFN--YDHKHLGTDPQTLVEIAEGR-----------------------------  target    GERLSARDVFEGYRQAPLENYVALKTEEELAAPPSPPFTADKQVVPTELREEWGDFVYWDRATNGPAAVNRDEIGAKFAG 8e73.55   --------------------------------------------------------------------------------  target    DPALLGAFDVTLVDGTNVKARTAFSLLKEYLDENFDVQTTSEVCNVDPAAVRSLARQLAANKGNALLAAGMGPNHYFNAD 8e73.55   ---------------------------------------------------HPFFKTLSDAKN-PVIIVGAGVFERKDQD  target    LFGRVHFLVAALTDNIGHFSGNVGSYAGNYRGSLFQAMGQWIAENPFDQEADLTKPARVKRYFKSESAHYWNYGDRPLVS 8e73.55   AIFAAVETIAQKANVVRPDWNGLNVLLLHAAQ-----AAA------L----DL--------------------GLVPQ--  target    PSEIITGKSHMPTPTKLIWFGNSNSLLGNAKWSFDVVKNTLPKQDAVFCNEWHWTSSCEYSDLVFPADSWAEFKLPDMTA 8e73.55   ---SEK----SLESAKFVYLMGADDVN----------LDKIPDDAFVVYQGHHGDKSVYRANVILPTAAFSEKEGT---Y  target    SCTNPFLLAFPKTPLARIHNTRSDYEILAGVAAALADLVDEPRMKTYWKGILDGDPTPYLQRVLSGSNATRGILYEDLHA 8e73.55   QNTEGCTQQTLP-AVPTVGDSRDDWKIIRALSEVAGV-------------------------------------------  target    SSAKGVPLLMNARTYPRHAGWEQRQEDKPWYTPTGRLEFYRPEPEWQAAGESLPIWREPVDATFYEPNAILANSKHPSIN 8e73.55   --------------------------------------------------------------------------------  target    PRAPEDYGVPESQMDVETRQYRNVVRTWQELKLSKHPLTEKDPAYRFVFQTPKYRWGAHSTAVDSDWIAMLFGPFGDPYR 8e73.55   --------------------------------------------------------------------------------  target    RDSRTPWTGEAYAEINPRDAKELGLKDGDYIWLDADPEDRPYRGADSSDEFYDVARAMMRVRIYSGMPRRVIRTWFNMYA 8e73.55   --------------------------------------------------------------------------------  target    ATPGTVQAQKDVPGGPAQNQDTGYVALFRHGSHQSGTRAYLRPTQMTDSMNRKAYFGQTIGKGFEADVHSPSGAPKEGYV 8e73.55   --------------------------------------------------------------------------------  target    KVEKAEDGGDEGVGEWRPVTLGLRPDDPSEAMQAYLAGEFVTRKRKGS 8e73.55   ------------------------------------------------ ``` | | | | | | | | | | | | | | | | | | | | | | | | | | | | | | | | | | | | | | | | | | | | | | | | | |
|  | 8b9z.1.G | NADH-ubiquinone oxidoreductase 75 kDa subunit, mitochondrial  *Drosophila melanogaster complex I in the Active state (Dm1)* | 0.16 |  | 16.09 | 0.32 | 68-756 | EM | 3.28 | hetero-1-1-1-1-1-1-… | 3 x PC1, 16 x 3PE, 6 x SF4, 4 x CDL, 2 x FES, 1 x FMN, 1 x UQ9, 1 x DGT, 1 x NDP, 1 x ZN, 2 x EHZ | HHblits | 0.27 |
| ``` target    SDLSRRELLKRAVVVGTGAGLAELFLPAQFLSSASAQSEPQAVAIANPLAQMPDRSWERIYRDQFAEEDSFVFTCAPNDT 8b9z.1    -------------------------------------------------------------------EIRKVSSIDVLDA  target    HNCLLRAHVKNGVIVRISPTYGYGKATDLAGNQASHRWDPRICQKGLILGRRIYGDRRVKAPMIRKGFKEWADAGFPRHD 8b9z.1    VGSNIVVSTRTNEVLRILPRE------------NEDVNEEWLADKSRFACDGLK-RQRLVAPMVRMP-------------  target    DGTPRADMEKRGYDEWLQIPWDEALAIAAKTLQNVAETYKGEDGAGKLLEQGYEPAMVEAMHGAGVQAIKMRGGMPLLGA 8b9z.1    ------------NGELQAVEWEGALIAVAKAIKAAGGQI-------AGIS--------------GQLA-D---------L  target    GRVFGFYRFANMLALLDGKLRPEAPPEEIVGSRAFDNYAWHTDLPPGHPMVSGSQTVDFDLFAAEHSKLLVLIGMNWICT 8b9z.1    EAQVALKDLLNRL-----------------GSEVVATEQGFIAGGTDNR---ANYLLNSTIAGLEEADAVLLVGTNPRYE  target    KMPDAHWIGDAR-LKGTRVVVISADYMPTANKADEIVILRPGTDTAFLLGVARELITKKLYDRDAVIQRTDLPLLVRLDT 8b9z.1    APLVNTRLRKAYVHNELQIASIGPKIDLS------YDHENLGADAALVKDVCSG--------------------------  target    GERLSARDVFEGYRQAPLENYVALKTEEELAAPPSPPFTADKQVVPTELREEWGDFVYWDRATNGPAAVNRDEIGAKFAG 8b9z.1    --------------------------------------------------------------------------------  target    DPALLGAFDVTLVDGTNVKARTAFSLLKEYLDENFDVQTTSEVCNVDPAAVRSLARQLAANKGNALLAAGMGPNHYFNAD 8b9z.1    --------------------------------------------------AHAFSKVLEGAKK-PAIIIGADLLERADGA  target    LFGRVHFLVAALTDNIGHFSGN-VGSYAGNYRGSLFQAMGQWIAENPFDQEADLTKPARVKRYFKSESAHYWNYGDRPLV 8b9z.1    AIHA---TVAEYCKKLKKPNWNPFNVLQTNAA-----QVGAL------D----V--------------------GYKAG-  target    SPSEIITGKSHMPTPTKLIWFGNSNSLLGNAKWSFDVVKNTLPKQDAVFCNEWHWTSSCEYSDLVFPADSWAEFKLPDMT 8b9z.1    -AQTA------VKAQPKVLFLLNADAG--------KVTREQLPKDCFVVYIGSHGDNGASIADAVLPGAAYTEKQGI---  target    ASCTNPFLLAFPKTPLARIHNTRSDYEILAGVAAALADLVDEPRMKTYWKGILDGDPTPYLQRVLSGSNATRGILYEDLH 8b9z.1    YVNTEGRPQQTLP-GVSPPGMAREDWKILRALSEVVGK------------------------------------------  target    ASSAKGVPLLMNARTYPRHAGWEQRQEDKPWYTPTGRLEFYRPEPEWQAAGESLPIWREPVDATFYEPNAILANSKHPSI 8b9z.1    --------------------------------------------------------------------------------  target    NPRAPEDYGVPESQMDVETRQYRNVVRTWQELKLSKHPLTEKDPAYRFVFQTPKYRWGAHSTAVDSDWIAMLFGPFGDPY 8b9z.1    --------------------------------------------------------------------------------  target    RRDSRTPWTGEAYAEINPRDAKELGLKDGDYIWLDADPEDRPYRGADSSDEFYDVARAMMRVRIYSGMPRRVIRTWFNMY 8b9z.1    --------------------------------------------------------------------------------  target    AATPGTVQAQKDVPGGPAQNQDTGYVALFRHGSHQSGTRAYLRPTQMTDSMNRKAYFGQTIGKGFEADVHSPSGAPKEGY 8b9z.1    --------------------------------------------------------------------------------  target    VKVEKAEDGGDEGVGEWRPVTLGLRPDDPSEAMQAYLAGEFVTRKRKGS 8b9z.1    ------------------------------------------------- ``` | | | | | | | | | | | | | | | | | | | | | | | | | | | | | | | | | | | | | | | | | | | | | | | | | |
|  | 8ba0.1.G | NADH-ubiquinone oxidoreductase 75 kDa subunit, mitochondrial  *Drosophila melanogaster complex I in the Twisted state (Dm2)* | 0.16 |  | 16.09 | 0.32 | 68-756 | EM | 3.68 | hetero-1-1-1-1-1-1-… | 6 x SF4, 6 x 3PE, 2 x FES, 1 x FMN, 2 x CDL, 1 x DGT, 1 x NDP, 1 x ZN, 2 x EHZ | HHblits | 0.27 |
| ``` target    SDLSRRELLKRAVVVGTGAGLAELFLPAQFLSSASAQSEPQAVAIANPLAQMPDRSWERIYRDQFAEEDSFVFTCAPNDT 8ba0.1    -------------------------------------------------------------------EIRKVSSIDVLDA  target    HNCLLRAHVKNGVIVRISPTYGYGKATDLAGNQASHRWDPRICQKGLILGRRIYGDRRVKAPMIRKGFKEWADAGFPRHD 8ba0.1    VGSNIVVSTRTNEVLRILPRE------------NEDVNEEWLADKSRFACDGLK-RQRLVAPMVRMP-------------  target    DGTPRADMEKRGYDEWLQIPWDEALAIAAKTLQNVAETYKGEDGAGKLLEQGYEPAMVEAMHGAGVQAIKMRGGMPLLGA 8ba0.1    ------------NGELQAVEWEGALIAVAKAIKAAGGQI-------AGIS--------------GQLA-D---------L  target    GRVFGFYRFANMLALLDGKLRPEAPPEEIVGSRAFDNYAWHTDLPPGHPMVSGSQTVDFDLFAAEHSKLLVLIGMNWICT 8ba0.1    EAQVALKDLLNRL-----------------GSEVVATEQGFIAGGTDNR---ANYLLNSTIAGLEEADAVLLVGTNPRYE  target    KMPDAHWIGDAR-LKGTRVVVISADYMPTANKADEIVILRPGTDTAFLLGVARELITKKLYDRDAVIQRTDLPLLVRLDT 8ba0.1    APLVNTRLRKAYVHNELQIASIGPKIDLS------YDHENLGADAALVKDVCSG--------------------------  target    GERLSARDVFEGYRQAPLENYVALKTEEELAAPPSPPFTADKQVVPTELREEWGDFVYWDRATNGPAAVNRDEIGAKFAG 8ba0.1    --------------------------------------------------------------------------------  target    DPALLGAFDVTLVDGTNVKARTAFSLLKEYLDENFDVQTTSEVCNVDPAAVRSLARQLAANKGNALLAAGMGPNHYFNAD 8ba0.1    --------------------------------------------------AHAFSKVLEGAKK-PAIIIGADLLERADGA  target    LFGRVHFLVAALTDNIGHFSGN-VGSYAGNYRGSLFQAMGQWIAENPFDQEADLTKPARVKRYFKSESAHYWNYGDRPLV 8ba0.1    AIHA---TVAEYCKKLKKPNWNPFNVLQTNAA-----QVGAL------D----V--------------------GYKAG-  target    SPSEIITGKSHMPTPTKLIWFGNSNSLLGNAKWSFDVVKNTLPKQDAVFCNEWHWTSSCEYSDLVFPADSWAEFKLPDMT 8ba0.1    -AQTA------VKAQPKVLFLLNADAG--------KVTREQLPKDCFVVYIGSHGDNGASIADAVLPGAAYTEKQGI---  target    ASCTNPFLLAFPKTPLARIHNTRSDYEILAGVAAALADLVDEPRMKTYWKGILDGDPTPYLQRVLSGSNATRGILYEDLH 8ba0.1    YVNTEGRPQQTLP-GVSPPGMAREDWKILRALSEVVGK------------------------------------------  target    ASSAKGVPLLMNARTYPRHAGWEQRQEDKPWYTPTGRLEFYRPEPEWQAAGESLPIWREPVDATFYEPNAILANSKHPSI 8ba0.1    --------------------------------------------------------------------------------  target    NPRAPEDYGVPESQMDVETRQYRNVVRTWQELKLSKHPLTEKDPAYRFVFQTPKYRWGAHSTAVDSDWIAMLFGPFGDPY 8ba0.1    --------------------------------------------------------------------------------  target    RRDSRTPWTGEAYAEINPRDAKELGLKDGDYIWLDADPEDRPYRGADSSDEFYDVARAMMRVRIYSGMPRRVIRTWFNMY 8ba0.1    --------------------------------------------------------------------------------  target    AATPGTVQAQKDVPGGPAQNQDTGYVALFRHGSHQSGTRAYLRPTQMTDSMNRKAYFGQTIGKGFEADVHSPSGAPKEGY 8ba0.1    --------------------------------------------------------------------------------  target    VKVEKAEDGGDEGVGEWRPVTLGLRPDDPSEAMQAYLAGEFVTRKRKGS 8ba0.1    ------------------------------------------------- ``` | | | | | | | | | | | | | | | | | | | | | | | | | | | | | | | | | | | | | | | | | | | | | | | | | |
|  | 3ir5.1.A | Respiratory nitrate reductase 1 alpha chain  *Crystal structure of NarGHI mutant NarG-H49C* | 0.13 | 0.00 | 30.88 | 0.29 | 54-432 | X-ray | 2.30 | monomer | 2 x MD1, 1 x 6MO, 4 x SF4, 1 x AGA, 1 x F3S, 2 x HEM | BLAST | 0.36 |
| ``` target    SDLSRRELLKRAVVVGTGAGLAELFLPAQFLSSASAQSEPQAVAIANPLAQMPDRSWERIYRDQFAEEDSFVFTCAPNDT 3ir5.1    -----------------------------------------------------NRDWEDGYRQRWQHDKIVRSTCGVNCT  target    HNCLLRAHVKNGVIVRISPTYGYGKAT-DLAGNQASHRWDPRICQKGLILGRRIYGDRRVKAPMIRKGF-KEWADAGFPR 3ir5.1    GSCSWKIYVKNGLVTWETQQTDYPRTRPDLPNHE------PRGCPRGASYSWYLYSANRLKYPMMRKRLMKMWREAKALH  target    HDDGTPRADMEKRGYDEWLQIPWDEALAIAAKTLQNVAETYKGEDGAGKLLEQGYEPAMVEAMHGAGVQAIKMRGGMPLL 3ir5.1    SDP-----------VEAWASIIED----------ADKAKSFKQARGRGGFVRSSWQ-EVNELIAASNVYTIKN------Y  target    GAGRVFGFYRF--ANMLALLDGKLRPEAPPEEIVGSRAFDNYAWHTDLPPGHPMVSGSQTVDFDLFAAEHSKLLVLIGMN 3ir5.1    GPDRVAGFSPIPAMSMVSYASG-----ARYLSLIGGTCLSFYDWYCDLPPASPQTWGEQTDVPESADWYNSSYIIAWGSN  target    WICTKMPDAHWIGDARLKGTRVVVISADYMPTANKADEIVILRPGTDTAFLLGVARELITK------KLYDRDAVIQRTD 3ir5.1    VPQTRTPDAHFFTEVRYKGTKTVAVTPDYAEIAKLCDLWLAPKQGTDAAMALAMGHVMLREFHLDNPSQYFTDYVRRYTD  target    LPLLVRLD-------TGERLSARDVFE--GYRQAPLENYVALKTEEELAAPPSPPFTADKQVVPTELREEWGDFVYWDRA 3ir5.1    MPMLVMLEERDGYYAAGRMLRAADLVDALGQENNPEWKTVAFNTNGEMVAP-----------------------------  target    TNGPAAVNRDEIGAKFAGDPALLGAFDVTLVDGTNVKARTAFSLLKEYLDENFDVQTTSEVCNVDPAAVRSLARQLAANK 3ir5.1    --------------------------------------------------------------------------------  target    GNALLAAGMGPNHYFNADLFGRVHFLVAALTDNIGHFSGNVGSYAGNYRGSLFQAMGQWIAENPFDQEADLTKPARVKRY 3ir5.1    --------------------------------------------------------------------------------  target    FKSESAHYWNYGDRPLVSPSEIITGKSHMPTPTKLIWFGNSNSLLGNAKWSFDVVKNTLPKQDAVFCNEWHWTSSCEYSD 3ir5.1    --------------------------------------------------------------------------------  target    LVFPADSWAEFKLPDMTASCTNPFLLAFPKTPLARIHNTRSDYEILAGVAAALADLVDEPRMKTYWKGILDGDPTPYLQR 3ir5.1    --------------------------------------------------------------------------------  target    VLSGSNATRGILYEDLHASSAKGVPLLMNARTYPRHAGWEQRQEDKPWYTPTGRLEFYRPEPEWQAAGESLPIWREPVDA 3ir5.1    --------------------------------------------------------------------------------  target    TFYEPNAILANSKHPSINPRAPEDYGVPESQMDVETRQYRNVVRTWQELKLSKHPLTEKDPAYRFVFQTPKYRWGAHSTA 3ir5.1    --------------------------------------------------------------------------------  target    VDSDWIAMLFGPFGDPYRRDSRTPWTGEAYAEINPRDAKELGLKDGDYIWLDADPEDRPYRGADSSDEFYDVARAMMRVR 3ir5.1    --------------------------------------------------------------------------------  target    IYSGMPRRVIRTWFNMYAATPGTVQAQKDVPGGPAQNQDTGYVALFRHGSHQSGTRAYLRPTQMTDSMNRKAYFGQTIGK 3ir5.1    --------------------------------------------------------------------------------  target    GFEADVHSPSGAPKEGYVKVEKAEDGGDEGVGEWRPVTLGLRPDDPSEAMQAYLAGEFVTRKRKGS 3ir5.1    ------------------------------------------------------------------ ``` | | | | | | | | | | | | | | | | | | | | | | | | | | | | | | | | | | | | | | | | | | | | | | | | | |
|  | 7ar7.1.G | NADH dehydrogenase [ubiquinone] iron-sulfur protein 1, mitochondrial  *Cryo-EM structure of Arabidopsis thaliana complex-I (open conformation)* | 0.16 |  | 14.47 | 0.33 | 68-756 | EM | 0.00 | hetero-1-1-1-1-1-1-… | 6 x SF4, 2 x FES, 1 x FMN, 1 x UQ9, 3 x PTY, 2 x PC7, 1 x LMN, 1 x NDP, 2 x ZN, 2 x 8Q1, 1 x PGT, 1 x PSF, 1 x T7X | HHblits | 0.27 |
| ``` target    SDLSRRELLKRAVVVGTGAGLAELFLPAQFLSSASAQSEPQAVAIANPLAQMPDRSWERIYRDQFAEEDSFVFTCAPNDT 7ar7.1    -------------------------------------------------------------------ELKATETIDVSDA  target    HNCLLRAHVKNGVIVRISPTYGYGKATDLAGNQASHRWDPRICQKGLILGRRIYGDRRVKAPMIRKGFKEWADAGFPRHD 7ar7.1    VGSNIRVDSRGPEVMRIIPRL------------NEDINEEWISDKTRFCYDGLK-RQRLSDPMIRDS-------------  target    DGTPRADMEKRGYDEWLQIPWDEALAIAAKTLQNVAETYKGEDGAGKLLEQGYEPAMVEAMHGAGVQAIKMRGGMPLLGA 7ar7.1    ------------DGRFKAVSWRDALAVVGDIIHQV----KPDE----IV----------GVAGQLSD------------A  target    GRVFGFYRFANMLALLDGKLRPEAPPEEIVGSRAFDNYAWHTDLPPGHPMVSGSQTVDFDLFAAEHSKLLVLIGMNWICT 7ar7.1    ESMMVLKDFVNR-----------------MGSDNVWCEGTAAGVDADLRYS---YLMNTSISGLENADLFLLIGTQPRVE  target    KMPDAHWIGDAR-LKGTRVVVISADYMPTANKADEIVILRPGTDTAFLLGVARELITKKLYDRDAVIQRTDLPLLVRLDT 7ar7.1    AAMVNARICKTVRASNAKVGYVGPPAEFN--YDCKHLGTGPDTLKEI---------------------------------  target    GERLSARDVFEGYRQAPLENYVALKTEEELAAPPSPPFTADKQVVPTELREEWGDFVYWDRATNGPAAVNRDEIGAKFAG 7ar7.1    --------------------------------------------------------------------------------  target    DPALLGAFDVTLVDGTNVKARTAFSLLKEYLDENFDVQTTSEVCNVDPAAVRSLARQLAANKGNALLAAGMGPNHYFNAD 7ar7.1    -----------------------------------------------AEGRHPFCTALKNAKN-PAIIVGAGLFNRTDKN  target    LFGRVHFLVAALTDNIGHFSGNVGSYAGNYRGSLFQAMGQWIAENPFDQEADLTKPARVKRYFKSESAHYWNYGDRPLVS 7ar7.1    AILSSVESIAQANNVVRPDWNGLNFLLQYAAQ-----AAA------LD------------------------LGL-----  target    PSEIITGKSHMPTPTKLIWFGNSNSLLGNAKWSFDVVKNTLPKQDAVFCNEWHWTSSCEYSDLVFPADSWAEFKLPDMTA 7ar7.1    IQQSAKA----LESAKFVYLMGADDVN----------VDKIPKDAFVVYQGHHGDKAVYRANVILPASAFTEKEGT---Y  target    SCTNPFLLAFPKTPLARIHNTRSDYEILAGVAAALADLVDEPRMKTYWKGILDGDPTPYLQRVLSGSNATRGILYEDLHA 7ar7.1    ENTEGFTQQTVP-AVPTVGDARDDWKIVRALSEVSGV-------------------------------------------  target    SSAKGVPLLMNARTYPRHAGWEQRQEDKPWYTPTGRLEFYRPEPEWQAAGESLPIWREPVDATFYEPNAILANSKHPSIN 7ar7.1    --------------------------------------------------------------------------------  target    PRAPEDYGVPESQMDVETRQYRNVVRTWQELKLSKHPLTEKDPAYRFVFQTPKYRWGAHSTAVDSDWIAMLFGPFGDPYR 7ar7.1    --------------------------------------------------------------------------------  target    RDSRTPWTGEAYAEINPRDAKELGLKDGDYIWLDADPEDRPYRGADSSDEFYDVARAMMRVRIYSGMPRRVIRTWFNMYA 7ar7.1    --------------------------------------------------------------------------------  target    ATPGTVQAQKDVPGGPAQNQDTGYVALFRHGSHQSGTRAYLRPTQMTDSMNRKAYFGQTIGKGFEADVHSPSGAPKEGYV 7ar7.1    --------------------------------------------------------------------------------  target    KVEKAEDGGDEGVGEWRPVTLGLRPDDPSEAMQAYLAGEFVTRKRKGS 7ar7.1    ------------------------------------------------ ``` | | | | | | | | | | | | | | | | | | | | | | | | | | | | | | | | | | | | | | | | | | | | | | | | | |
|  | 7arc.1.F | 75 kDa  *Cryo-EM structure of Polytomella Complex-I (peripheral arm)* | 0.16 |  | 13.42 | 0.33 | 69-756 | EM | 0.00 | hetero-1-1-1-1-1-1-… | 6 x SF4, 2 x FES, 1 x FMN, 1 x NDP, 1 x ZN, 1 x 8Q1 | HHblits | 0.27 |
| ``` target    SDLSRRELLKRAVVVGTGAGLAELFLPAQFLSSASAQSEPQAVAIANPLAQMPDRSWERIYRDQFAEEDSFVFTCAPNDT 7arc.1    --------------------------------------------------------------------LKGTETIDVSDA  target    HNCLLRAHVKNGVIVRISPTYGYGKATDLAGNQASHRWDPRICQKGLILGRRIYGDRRVKAPMIRKGFKEWADAGFPRHD 7arc.1    LGSNIKVDCRGTEVMRITPRL------------NDAINEEWLSDKGRFQYDGLK-RQRLNTPLVKGA-------------  target    DGTPRADMEKRGYDEWLQIPWDEALAIAAKTLQNVAETYKGEDGAGKLLEQGYEPAMVEAMHGAGVQAIKMRGGMPLLGA 7arc.1    -------------KGLENATWSAAFDAIRTAIAGAK---GNE-L--KAIAGKL------------AD------------A  target    GRVFGFYRFANMLALLDGKLRPEAPPEEIVGSRAFDNYAWHTDLPPGHPMVSGSQTVDFDLFAAEHSKLLVLIGMNWICT 7arc.1    ESMIALKDLFNKL-----------------GSGNLIHEDGSATLSADVRSSY---IANTTIASIEKADVILLVGTNPRFE  target    KMPDAHWIGDARLKGTRVVVISADYMPTANKADEIVILRPGTDTAFLLGVARELITKKLYDRDAVIQRTDLPLLVRLDTG 7arc.1    SPVFNARLRKVFLDGAKVGLVGEKVDLT------YAYQHLGADVAALESLASG---------------------------  target    ERLSARDVFEGYRQAPLENYVALKTEEELAAPPSPPFTADKQVVPTELREEWGDFVYWDRATNGPAAVNRDEIGAKFAGD 7arc.1    --------------------------------------------------------------------------------  target    PALLGAFDVTLVDGTNVKARTAFSLLKEYLDENFDVQTTSEVCNVDPAAVRSLARQLAANKGNALLAAGMGPNHYFNADL 7arc.1    -------------------------------------------------KGAFFEALKGAKN-PVVIVGSSVLRRDDREA  target    FGRVHFLVAALTDNIGHFSGNVGSYAGNYRGSLFQAMGQWIAENPFDQEADLTKPARVKRYFKSESAHYWNYGDRPLVSP 7arc.1    VLKTVNDLVDAAGVVKEGWNGFNVLHDNASR-----VAAL----DIG----F-------------------VP-------  target    SEIITGKSHMPTPTKLIWFGNSNSLLGNAKWSFDVVKNTLPKQDAVFCNEWHWTSSCEYSDLVFPADSWAEFKLPDMTAS 7arc.1    SAS-AR--TNPVPAKVVYLLGSDDFKD----------EEIPADAFVIYQGHHGDKGAARANVVLPGAAYTEKASL---FA  target    CTNPFLLAFPKTPLARIHNTRSDYEILAGVAAALADLVDEPRMKTYWKGILDGDPTPYLQRVLSGSNATRGILYEDLHAS 7arc.1    NTEGRVQTTRT-AVPVLGDAREDWKIIRALSEVVGQ--------------------------------------------  target    SAKGVPLLMNARTYPRHAGWEQRQEDKPWYTPTGRLEFYRPEPEWQAAGESLPIWREPVDATFYEPNAILANSKHPSINP 7arc.1    --------------------------------------------------------------------------------  target    RAPEDYGVPESQMDVETRQYRNVVRTWQELKLSKHPLTEKDPAYRFVFQTPKYRWGAHSTAVDSDWIAMLFGPFGDPYRR 7arc.1    --------------------------------------------------------------------------------  target    DSRTPWTGEAYAEINPRDAKELGLKDGDYIWLDADPEDRPYRGADSSDEFYDVARAMMRVRIYSGMPRRVIRTWFNMYAA 7arc.1    --------------------------------------------------------------------------------  target    TPGTVQAQKDVPGGPAQNQDTGYVALFRHGSHQSGTRAYLRPTQMTDSMNRKAYFGQTIGKGFEADVHSPSGAPKEGYVK 7arc.1    --------------------------------------------------------------------------------  target    VEKAEDGGDEGVGEWRPVTLGLRPDDPSEAMQAYLAGEFVTRKRKGS 7arc.1    ----------------------------------------------- ``` | | | | | | | | | | | | | | | | | | | | | | | | | | | | | | | | | | | | | | | | | | | | | | | | | |
|  | 3ir6.1.A | Respiratory nitrate reductase 1 alpha chain  *Crystal structure of NarGHI mutant NarG-H49S* | 0.13 | 0.00 | 30.59 | 0.29 | 54-432 | X-ray | 2.80 | monomer | 2 x GDP, 1 x AGA, 3 x SF4, 1 x F3S, 2 x HEM | BLAST | 0.36 |
| ``` target    SDLSRRELLKRAVVVGTGAGLAELFLPAQFLSSASAQSEPQAVAIANPLAQMPDRSWERIYRDQFAEEDSFVFTCAPNDT 3ir6.1    -----------------------------------------------------NRDWEDGYRQRWQHDKIVRSTSGVNCT  target    HNCLLRAHVKNGVIVRISPTYGYGKAT-DLAGNQASHRWDPRICQKGLILGRRIYGDRRVKAPMIRKGF-KEWADAGFPR 3ir6.1    GSCSWKIYVKNGLVTWETQQTDYPRTRPDLPNHE------PRGCPRGASYSWYLYSANRLKYPMMRKRLMKMWREAKALH  target    HDDGTPRADMEKRGYDEWLQIPWDEALAIAAKTLQNVAETYKGEDGAGKLLEQGYEPAMVEAMHGAGVQAIKMRGGMPLL 3ir6.1    SDP-----------VEAWASIIED----------ADKAKSFKQARGRGGFVRSSWQ-EVNELIAASNVYTIKN------Y  target    GAGRVFGFYRF--ANMLALLDGKLRPEAPPEEIVGSRAFDNYAWHTDLPPGHPMVSGSQTVDFDLFAAEHSKLLVLIGMN 3ir6.1    GPDRVAGFSPIPAMSMVSYASG-----ARYLSLIGGTCLSFYDWYCDLPPASPQTWGEQTDVPESADWYNSSYIIAWGSN  target    WICTKMPDAHWIGDARLKGTRVVVISADYMPTANKADEIVILRPGTDTAFLLGVARELITK------KLYDRDAVIQRTD 3ir6.1    VPQTRTPDAHFFTEVRYKGTKTVAVTPDYAEIAKLCDLWLAPKQGTDAAMALAMGHVMLREFHLDNPSQYFTDYVRRYTD  target    LPLLVRLD-------TGERLSARDVFE--GYRQAPLENYVALKTEEELAAPPSPPFTADKQVVPTELREEWGDFVYWDRA 3ir6.1    MPMLVMLEERDGYYAAGRMLRAADLVDALGQENNPEWKTVAFNTNGEMVAP-----------------------------  target    TNGPAAVNRDEIGAKFAGDPALLGAFDVTLVDGTNVKARTAFSLLKEYLDENFDVQTTSEVCNVDPAAVRSLARQLAANK 3ir6.1    --------------------------------------------------------------------------------  target    GNALLAAGMGPNHYFNADLFGRVHFLVAALTDNIGHFSGNVGSYAGNYRGSLFQAMGQWIAENPFDQEADLTKPARVKRY 3ir6.1    --------------------------------------------------------------------------------  target    FKSESAHYWNYGDRPLVSPSEIITGKSHMPTPTKLIWFGNSNSLLGNAKWSFDVVKNTLPKQDAVFCNEWHWTSSCEYSD 3ir6.1    --------------------------------------------------------------------------------  target    LVFPADSWAEFKLPDMTASCTNPFLLAFPKTPLARIHNTRSDYEILAGVAAALADLVDEPRMKTYWKGILDGDPTPYLQR 3ir6.1    --------------------------------------------------------------------------------  target    VLSGSNATRGILYEDLHASSAKGVPLLMNARTYPRHAGWEQRQEDKPWYTPTGRLEFYRPEPEWQAAGESLPIWREPVDA 3ir6.1    --------------------------------------------------------------------------------  target    TFYEPNAILANSKHPSINPRAPEDYGVPESQMDVETRQYRNVVRTWQELKLSKHPLTEKDPAYRFVFQTPKYRWGAHSTA 3ir6.1    --------------------------------------------------------------------------------  target    VDSDWIAMLFGPFGDPYRRDSRTPWTGEAYAEINPRDAKELGLKDGDYIWLDADPEDRPYRGADSSDEFYDVARAMMRVR 3ir6.1    --------------------------------------------------------------------------------  target    IYSGMPRRVIRTWFNMYAATPGTVQAQKDVPGGPAQNQDTGYVALFRHGSHQSGTRAYLRPTQMTDSMNRKAYFGQTIGK 3ir6.1    --------------------------------------------------------------------------------  target    GFEADVHSPSGAPKEGYVKVEKAEDGGDEGVGEWRPVTLGLRPDDPSEAMQAYLAGEFVTRKRKGS 3ir6.1    ------------------------------------------------------------------ ``` | | | | | | | | | | | | | | | | | | | | | | | | | | | | | | | | | | | | | | | | | | | | | | | | | |
|  | 1r27.4.A | Respiratory nitrate reductase 1 alpha chain  *Crystal Structure of NarGH complex* | 0.13 | 0.00 | 30.59 | 0.29 | 54-432 | X-ray | 2.00 | homo-dimer | 4 x MO, 16 x SF4, 8 x MGD, 4 x F3S | BLAST | 0.36 |
| ``` target    SDLSRRELLKRAVVVGTGAGLAELFLPAQFLSSASAQSEPQAVAIANPLAQMPDRSWERIYRDQFAEEDSFVFTCAPNDT 1r27.4    -----------------------------------------------------NRDWEDGYRQRWQHDKIVRSTHGVNCT  target    HNCLLRAHVKNGVIVRISPTYGYGKAT-DLAGNQASHRWDPRICQKGLILGRRIYGDRRVKAPMIRKGF-KEWADAGFPR 1r27.4    GSCSWKIYVKNGLVTWETQQTDYPRTRPDLPNHE------PRGCPRGASYSWYLYSANRLKYPMMRKRLMKMWREAKALH  target    HDDGTPRADMEKRGYDEWLQIPWDEALAIAAKTLQNVAETYKGEDGAGKLLEQGYEPAMVEAMHGAGVQAIKMRGGMPLL 1r27.4    SDP-----------VEAWASIIED----------ADKAKSFKQARGRGGFVRSSWQ-EVNELIAASNVYTIKN------Y  target    GAGRVFGFYRF--ANMLALLDGKLRPEAPPEEIVGSRAFDNYAWHTDLPPGHPMVSGSQTVDFDLFAAEHSKLLVLIGMN 1r27.4    GPDRVAGFSPIPAMSMVSYASG-----ARYLSLIGGTCLSFYDWYCDLPPASPQTWGEQTDVPESADWYNSSYIIAWGSN  target    WICTKMPDAHWIGDARLKGTRVVVISADYMPTANKADEIVILRPGTDTAFLLGVARELITK------KLYDRDAVIQRTD 1r27.4    VPQTRTPDAHFFTEVRYKGTKTVAVTPDYAEIAKLCDLWLAPKQGTDAAMALAMGHVMLREFHLDNPSQYFTDYVRRYTD  target    LPLLVRLD-------TGERLSARDVFE--GYRQAPLENYVALKTEEELAAPPSPPFTADKQVVPTELREEWGDFVYWDRA 1r27.4    MPMLVMLEERDGYYAAGRMLRAADLVDALGQENNPEWKTVAFNTNGEMVAP-----------------------------  target    TNGPAAVNRDEIGAKFAGDPALLGAFDVTLVDGTNVKARTAFSLLKEYLDENFDVQTTSEVCNVDPAAVRSLARQLAANK 1r27.4    --------------------------------------------------------------------------------  target    GNALLAAGMGPNHYFNADLFGRVHFLVAALTDNIGHFSGNVGSYAGNYRGSLFQAMGQWIAENPFDQEADLTKPARVKRY 1r27.4    --------------------------------------------------------------------------------  target    FKSESAHYWNYGDRPLVSPSEIITGKSHMPTPTKLIWFGNSNSLLGNAKWSFDVVKNTLPKQDAVFCNEWHWTSSCEYSD 1r27.4    --------------------------------------------------------------------------------  target    LVFPADSWAEFKLPDMTASCTNPFLLAFPKTPLARIHNTRSDYEILAGVAAALADLVDEPRMKTYWKGILDGDPTPYLQR 1r27.4    --------------------------------------------------------------------------------  target    VLSGSNATRGILYEDLHASSAKGVPLLMNARTYPRHAGWEQRQEDKPWYTPTGRLEFYRPEPEWQAAGESLPIWREPVDA 1r27.4    --------------------------------------------------------------------------------  target    TFYEPNAILANSKHPSINPRAPEDYGVPESQMDVETRQYRNVVRTWQELKLSKHPLTEKDPAYRFVFQTPKYRWGAHSTA 1r27.4    --------------------------------------------------------------------------------  target    VDSDWIAMLFGPFGDPYRRDSRTPWTGEAYAEINPRDAKELGLKDGDYIWLDADPEDRPYRGADSSDEFYDVARAMMRVR 1r27.4    --------------------------------------------------------------------------------  target    IYSGMPRRVIRTWFNMYAATPGTVQAQKDVPGGPAQNQDTGYVALFRHGSHQSGTRAYLRPTQMTDSMNRKAYFGQTIGK 1r27.4    --------------------------------------------------------------------------------  target    GFEADVHSPSGAPKEGYVKVEKAEDGGDEGVGEWRPVTLGLRPDDPSEAMQAYLAGEFVTRKRKGS 1r27.4    ------------------------------------------------------------------ ``` | | | | | | | | | | | | | | | | | | | | | | | | | | | | | | | | | | | | | | | | | | | | | | | | | |
|  | 1q16.1.A | Respiratory nitrate reductase 1 alpha chain  *Crystal structure of Nitrate Reductase A, NarGHI, from Escherichia coli* | 0.13 | 0.00 | 30.59 | 0.29 | 54-432 | X-ray | 1.90 | monomer | 2 x MD1, 1 x 6MO, 2 x HEM, 4 x SF4, 1 x F3S, 1 x AGA, 1 x 3PH | BLAST | 0.36 |
| ``` target    SDLSRRELLKRAVVVGTGAGLAELFLPAQFLSSASAQSEPQAVAIANPLAQMPDRSWERIYRDQFAEEDSFVFTCAPNDT 1q16.1    -----------------------------------------------------NRDWEDGYRQRWQHDKIVRSTHGVNCT  target    HNCLLRAHVKNGVIVRISPTYGYGKAT-DLAGNQASHRWDPRICQKGLILGRRIYGDRRVKAPMIRKGF-KEWADAGFPR 1q16.1    GSCSWKIYVKNGLVTWETQQTDYPRTRPDLPNHE------PRGCPRGASYSWYLYSANRLKYPMMRKRLMKMWREAKALH  target    HDDGTPRADMEKRGYDEWLQIPWDEALAIAAKTLQNVAETYKGEDGAGKLLEQGYEPAMVEAMHGAGVQAIKMRGGMPLL 1q16.1    SDP-----------VEAWASIIED----------ADKAKSFKQARGRGGFVRSSWQ-EVNELIAASNVYTIKN------Y  target    GAGRVFGFYRF--ANMLALLDGKLRPEAPPEEIVGSRAFDNYAWHTDLPPGHPMVSGSQTVDFDLFAAEHSKLLVLIGMN 1q16.1    GPDRVAGFSPIPAMSMVSYASG-----ARYLSLIGGTCLSFYDWYCDLPPASPQTWGEQTDVPESADWYNSSYIIAWGSN  target    WICTKMPDAHWIGDARLKGTRVVVISADYMPTANKADEIVILRPGTDTAFLLGVARELITK------KLYDRDAVIQRTD 1q16.1    VPQTRTPDAHFFTEVRYKGTKTVAVTPDYAEIAKLCDLWLAPKQGTDAAMALAMGHVMLREFHLDNPSQYFTDYVRRYTD  target    LPLLVRLD-------TGERLSARDVFE--GYRQAPLENYVALKTEEELAAPPSPPFTADKQVVPTELREEWGDFVYWDRA 1q16.1    MPMLVMLEERDGYYAAGRMLRAADLVDALGQENNPEWKTVAFNTNGEMVAP-----------------------------  target    TNGPAAVNRDEIGAKFAGDPALLGAFDVTLVDGTNVKARTAFSLLKEYLDENFDVQTTSEVCNVDPAAVRSLARQLAANK 1q16.1    --------------------------------------------------------------------------------  target    GNALLAAGMGPNHYFNADLFGRVHFLVAALTDNIGHFSGNVGSYAGNYRGSLFQAMGQWIAENPFDQEADLTKPARVKRY 1q16.1    --------------------------------------------------------------------------------  target    FKSESAHYWNYGDRPLVSPSEIITGKSHMPTPTKLIWFGNSNSLLGNAKWSFDVVKNTLPKQDAVFCNEWHWTSSCEYSD 1q16.1    --------------------------------------------------------------------------------  target    LVFPADSWAEFKLPDMTASCTNPFLLAFPKTPLARIHNTRSDYEILAGVAAALADLVDEPRMKTYWKGILDGDPTPYLQR 1q16.1    --------------------------------------------------------------------------------  target    VLSGSNATRGILYEDLHASSAKGVPLLMNARTYPRHAGWEQRQEDKPWYTPTGRLEFYRPEPEWQAAGESLPIWREPVDA 1q16.1    --------------------------------------------------------------------------------  target    TFYEPNAILANSKHPSINPRAPEDYGVPESQMDVETRQYRNVVRTWQELKLSKHPLTEKDPAYRFVFQTPKYRWGAHSTA 1q16.1    --------------------------------------------------------------------------------  target    VDSDWIAMLFGPFGDPYRRDSRTPWTGEAYAEINPRDAKELGLKDGDYIWLDADPEDRPYRGADSSDEFYDVARAMMRVR 1q16.1    --------------------------------------------------------------------------------  target    IYSGMPRRVIRTWFNMYAATPGTVQAQKDVPGGPAQNQDTGYVALFRHGSHQSGTRAYLRPTQMTDSMNRKAYFGQTIGK 1q16.1    --------------------------------------------------------------------------------  target    GFEADVHSPSGAPKEGYVKVEKAEDGGDEGVGEWRPVTLGLRPDDPSEAMQAYLAGEFVTRKRKGS 1q16.1    ------------------------------------------------------------------ ``` | | | | | | | | | | | | | | | | | | | | | | | | | | | | | | | | | | | | | | | | | | | | | | | | | |
|  | 7aqr.1.F | NADH dehydrogenase [ubiquinone] iron-sulfur protein 1, mitochondrial  *Cryo-EM structure of Arabidopsis thaliana Complex-I (peripheral arm)* | 0.16 |  | 14.51 | 0.32 | 69-756 | EM | 0.00 | hetero-1-1-1-1-1-1-… | 6 x SF4, 2 x FES, 1 x FMN, 1 x NDP, 1 x ZN, 1 x 8Q1 | HHblits | 0.27 |
| ``` target    SDLSRRELLKRAVVVGTGAGLAELFLPAQFLSSASAQSEPQAVAIANPLAQMPDRSWERIYRDQFAEEDSFVFTCAPNDT 7aqr.1    --------------------------------------------------------------------LKATETIDVSDA  target    HNCLLRAHVKNGVIVRISPTYGYGKATDLAGNQASHRWDPRICQKGLILGRRIYGDRRVKAPMIRKGFKEWADAGFPRHD 7aqr.1    VGSNIRVDSRGPEVMRIIPRL------------NEDINEEWISDKTRFCYDGLK-RQRLSDPMIRDS-------------  target    DGTPRADMEKRGYDEWLQIPWDEALAIAAKTLQNVAETYKGEDGAGKLLEQGYEPAMVEAMHGAGVQAIKMRGGMPLLGA 7aqr.1    ------------DGRFKAVSWRDALAVVGDIIHQV----KPDE----IV----------GVAGQLSD------------A  target    GRVFGFYRFANMLALLDGKLRPEAPPEEIVGSRAFDNYAWHTDLPPGHPMVSGSQTVDFDLFAAEHSKLLVLIGMNWICT 7aqr.1    ESMMVLKDFVNRM-----------------GSDNVWCEGTAAGVDADLRYSY---LMNTSISGLENADLFLLIGTQPRVE  target    KMPDAHWIGDAR-LKGTRVVVISADYMPTANKADEIVILRPGTDTAFLLGVARELITKKLYDRDAVIQRTDLPLLVRLDT 7aqr.1    AAMVNARICKTVRASNAKVGYVGPPAEFN--YDCKHLGTGPDTLKEI---------------------------------  target    GERLSARDVFEGYRQAPLENYVALKTEEELAAPPSPPFTADKQVVPTELREEWGDFVYWDRATNGPAAVNRDEIGAKFAG 7aqr.1    --------------------------------------------------------------------------------  target    DPALLGAFDVTLVDGTNVKARTAFSLLKEYLDENFDVQTTSEVCNVDPAAVRSLARQLAANKGNALLAAGMGPNHYFNAD 7aqr.1    -----------------------------------------------AEGRHPFCTALKNAKN-PAIIVGAGLFNRTDKN  target    LFGRVHFLVAALTDNIGHFSGNVGSYAGNYRGSLFQAMGQWIAENPFDQEADLTKPARVKRYFKSESAHYWNYGDRPLVS 7aqr.1    AILSSVESIAQANNVVRPDWNGLNFLLQYAAQ-----AAA------LD----L--------------------GLI----  target    PSEIITGKSHMPTPTKLIWFGNSNSLLGNAKWSFDVVKNTLPKQDAVFCNEWHWTSSCEYSDLVFPADSWAEFKLPDMTA 7aqr.1    -QQSAKA----LESAKFVYLMGADDVN----------VDKIPKDAFVVYQGHHGDKAVYRANVILPASAFTEKEGTY---  target    SCTNPFLLAFPKTPLARIHNTRSDYEILAGVAAALADLVDEPRMKTYWKGILDGDPTPYLQRVLSGSNATRGILYEDLHA 7aqr.1    ENTEGFTQQTVP-AVPTVGDARDDWKIVRALSEVSGV-------------------------------------------  target    SSAKGVPLLMNARTYPRHAGWEQRQEDKPWYTPTGRLEFYRPEPEWQAAGESLPIWREPVDATFYEPNAILANSKHPSIN 7aqr.1    --------------------------------------------------------------------------------  target    PRAPEDYGVPESQMDVETRQYRNVVRTWQELKLSKHPLTEKDPAYRFVFQTPKYRWGAHSTAVDSDWIAMLFGPFGDPYR 7aqr.1    --------------------------------------------------------------------------------  target    RDSRTPWTGEAYAEINPRDAKELGLKDGDYIWLDADPEDRPYRGADSSDEFYDVARAMMRVRIYSGMPRRVIRTWFNMYA 7aqr.1    --------------------------------------------------------------------------------  target    ATPGTVQAQKDVPGGPAQNQDTGYVALFRHGSHQSGTRAYLRPTQMTDSMNRKAYFGQTIGKGFEADVHSPSGAPKEGYV 7aqr.1    --------------------------------------------------------------------------------  target    KVEKAEDGGDEGVGEWRPVTLGLRPDDPSEAMQAYLAGEFVTRKRKGS 7aqr.1    ------------------------------------------------ ``` | | | | | | | | | | | | | | | | | | | | | | | | | | | | | | | | | | | | | | | | | | | | | | | | | |
|  | 7a23.1.O | 75kDa  *Plant mitochondrial respiratory complex I* | 0.16 |  | 14.51 | 0.32 | 69-756 | EM | 0.00 | hetero-1-1-1-1-1-1-… | 6 x SF4, 1 x FMN, 2 x T7X, 3 x CDL, 1 x U10, 1 x PEV, 2 x FES, 1 x NDP, 2 x ZN | HHblits | 0.27 |
| ``` target    SDLSRRELLKRAVVVGTGAGLAELFLPAQFLSSASAQSEPQAVAIANPLAQMPDRSWERIYRDQFAEEDSFVFTCAPNDT 7a23.1    --------------------------------------------------------------------LKATETIDVSDA  target    HNCLLRAHVKNGVIVRISPTYGYGKATDLAGNQASHRWDPRICQKGLILGRRIYGDRRVKAPMIRKGFKEWADAGFPRHD 7a23.1    VGSNIRVDSRGPEVMRIIPRL------------NEDINEEWISDKTRFCYDGLK-RQRLSDPMIRDS-------------  target    DGTPRADMEKRGYDEWLQIPWDEALAIAAKTLQNVAETYKGEDGAGKLLEQGYEPAMVEAMHGAGVQAIKMRGGMPLLGA 7a23.1    ------------DGRFKAVSWRDALAVVGDIIHQV----KPDE----IV----------GVAGQLSD------------A  target    GRVFGFYRFANMLALLDGKLRPEAPPEEIVGSRAFDNYAWHTDLPPGHPMVSGSQTVDFDLFAAEHSKLLVLIGMNWICT 7a23.1    ESMMVLKDFVNRM-----------------GSDNVWCEGTAAGVDADLRYSY---LMNTSISGLENADLFLLIGTQPRVE  target    KMPDAHWIGDAR-LKGTRVVVISADYMPTANKADEIVILRPGTDTAFLLGVARELITKKLYDRDAVIQRTDLPLLVRLDT 7a23.1    AAMVNARICKTVRASNAKVGYVGPPAEFN--YDCKHLGTGPDTLKEI---------------------------------  target    GERLSARDVFEGYRQAPLENYVALKTEEELAAPPSPPFTADKQVVPTELREEWGDFVYWDRATNGPAAVNRDEIGAKFAG 7a23.1    --------------------------------------------------------------------------------  target    DPALLGAFDVTLVDGTNVKARTAFSLLKEYLDENFDVQTTSEVCNVDPAAVRSLARQLAANKGNALLAAGMGPNHYFNAD 7a23.1    -----------------------------------------------AEGRHPFCTALKNAKN-PAIIVGAGLFNRTDKN  target    LFGRVHFLVAALTDNIGHFSGNVGSYAGNYRGSLFQAMGQWIAENPFDQEADLTKPARVKRYFKSESAHYWNYGDRPLVS 7a23.1    AILSSVESIAQANNVVRPDWNGLNFLLQYAAQ-----AAA------LD----L--------------------GLI----  target    PSEIITGKSHMPTPTKLIWFGNSNSLLGNAKWSFDVVKNTLPKQDAVFCNEWHWTSSCEYSDLVFPADSWAEFKLPDMTA 7a23.1    -QQSAKA----LESAKFVYLMGADDVN----------VDKIPKDAFVVYQGHHGDKAVYRANVILPASAFTEKEGTY---  target    SCTNPFLLAFPKTPLARIHNTRSDYEILAGVAAALADLVDEPRMKTYWKGILDGDPTPYLQRVLSGSNATRGILYEDLHA 7a23.1    ENTEGFTQQTVP-AVPTVGDARDDWKIVRALSEVSGV-------------------------------------------  target    SSAKGVPLLMNARTYPRHAGWEQRQEDKPWYTPTGRLEFYRPEPEWQAAGESLPIWREPVDATFYEPNAILANSKHPSIN 7a23.1    --------------------------------------------------------------------------------  target    PRAPEDYGVPESQMDVETRQYRNVVRTWQELKLSKHPLTEKDPAYRFVFQTPKYRWGAHSTAVDSDWIAMLFGPFGDPYR 7a23.1    --------------------------------------------------------------------------------  target    RDSRTPWTGEAYAEINPRDAKELGLKDGDYIWLDADPEDRPYRGADSSDEFYDVARAMMRVRIYSGMPRRVIRTWFNMYA 7a23.1    --------------------------------------------------------------------------------  target    ATPGTVQAQKDVPGGPAQNQDTGYVALFRHGSHQSGTRAYLRPTQMTDSMNRKAYFGQTIGKGFEADVHSPSGAPKEGYV 7a23.1    --------------------------------------------------------------------------------  target    KVEKAEDGGDEGVGEWRPVTLGLRPDDPSEAMQAYLAGEFVTRKRKGS 7a23.1    ------------------------------------------------ ``` | | | | | | | | | | | | | | | | | | | | | | | | | | | | | | | | | | | | | | | | | | | | | | | | | |
|  | 7ar8.1.G | NADH dehydrogenase [ubiquinone] iron-sulfur protein 1, mitochondrial  *Cryo-EM structure of Arabidopsis thaliana complex-I (closed conformation)* | 0.16 |  | 14.51 | 0.32 | 69-756 | EM | 0.00 | hetero-1-1-1-1-1-1-… | 6 x SF4, 2 x FES, 1 x FMN, 1 x UQ9, 3 x PTY, 2 x PC7, 1 x PGT, 1 x FE, 1 x NDP, 2 x ZN, 2 x 8Q1, 1 x LMN, 1 x PSF, 1 x T7X | HHblits | 0.27 |
| ``` target    SDLSRRELLKRAVVVGTGAGLAELFLPAQFLSSASAQSEPQAVAIANPLAQMPDRSWERIYRDQFAEEDSFVFTCAPNDT 7ar8.1    --------------------------------------------------------------------LKATETIDVSDA  target    HNCLLRAHVKNGVIVRISPTYGYGKATDLAGNQASHRWDPRICQKGLILGRRIYGDRRVKAPMIRKGFKEWADAGFPRHD 7ar8.1    VGSNIRVDSRGPEVMRIIPRL------------NEDINEEWISDKTRFCYDGLK-RQRLSDPMIRDS-------------  target    DGTPRADMEKRGYDEWLQIPWDEALAIAAKTLQNVAETYKGEDGAGKLLEQGYEPAMVEAMHGAGVQAIKMRGGMPLLGA 7ar8.1    ------------DGRFKAVSWRDALAVVGDIIHQV----KPDE----IV----------GVAGQLSD------------A  target    GRVFGFYRFANMLALLDGKLRPEAPPEEIVGSRAFDNYAWHTDLPPGHPMVSGSQTVDFDLFAAEHSKLLVLIGMNWICT 7ar8.1    ESMMVLKDFVNRM-----------------GSDNVWCEGTAAGVDADLRYSY---LMNTSISGLENADLFLLIGTQPRVE  target    KMPDAHWIGDAR-LKGTRVVVISADYMPTANKADEIVILRPGTDTAFLLGVARELITKKLYDRDAVIQRTDLPLLVRLDT 7ar8.1    AAMVNARICKTVRASNAKVGYVGPPAEFN--YDCKHLGTGPDTLKEI---------------------------------  target    GERLSARDVFEGYRQAPLENYVALKTEEELAAPPSPPFTADKQVVPTELREEWGDFVYWDRATNGPAAVNRDEIGAKFAG 7ar8.1    --------------------------------------------------------------------------------  target    DPALLGAFDVTLVDGTNVKARTAFSLLKEYLDENFDVQTTSEVCNVDPAAVRSLARQLAANKGNALLAAGMGPNHYFNAD 7ar8.1    -----------------------------------------------AEGRHPFCTALKNAKN-PAIIVGAGLFNRTDKN  target    LFGRVHFLVAALTDNIGHFSGNVGSYAGNYRGSLFQAMGQWIAENPFDQEADLTKPARVKRYFKSESAHYWNYGDRPLVS 7ar8.1    AILSSVESIAQANNVVRPDWNGLNFLLQYAAQ-----AAA------LD----L--------------------GLI----  target    PSEIITGKSHMPTPTKLIWFGNSNSLLGNAKWSFDVVKNTLPKQDAVFCNEWHWTSSCEYSDLVFPADSWAEFKLPDMTA 7ar8.1    -QQSAKA----LESAKFVYLMGADDVN----------VDKIPKDAFVVYQGHHGDKAVYRANVILPASAFTEKEGTY---  target    SCTNPFLLAFPKTPLARIHNTRSDYEILAGVAAALADLVDEPRMKTYWKGILDGDPTPYLQRVLSGSNATRGILYEDLHA 7ar8.1    ENTEGFTQQTVP-AVPTVGDARDDWKIVRALSEVSGV-------------------------------------------  target    SSAKGVPLLMNARTYPRHAGWEQRQEDKPWYTPTGRLEFYRPEPEWQAAGESLPIWREPVDATFYEPNAILANSKHPSIN 7ar8.1    --------------------------------------------------------------------------------  target    PRAPEDYGVPESQMDVETRQYRNVVRTWQELKLSKHPLTEKDPAYRFVFQTPKYRWGAHSTAVDSDWIAMLFGPFGDPYR 7ar8.1    --------------------------------------------------------------------------------  target    RDSRTPWTGEAYAEINPRDAKELGLKDGDYIWLDADPEDRPYRGADSSDEFYDVARAMMRVRIYSGMPRRVIRTWFNMYA 7ar8.1    --------------------------------------------------------------------------------  target    ATPGTVQAQKDVPGGPAQNQDTGYVALFRHGSHQSGTRAYLRPTQMTDSMNRKAYFGQTIGKGFEADVHSPSGAPKEGYV 7ar8.1    --------------------------------------------------------------------------------  target    KVEKAEDGGDEGVGEWRPVTLGLRPDDPSEAMQAYLAGEFVTRKRKGS 7ar8.1    ------------------------------------------------ ``` | | | | | | | | | | | | | | | | | | | | | | | | | | | | | | | | | | | | | | | | | | | | | | | | | |
| ✓ | 3ir7.1.A | Respiratory nitrate reductase 1 alpha chain  *Crystal structure of NarGHI mutant NarG-R94S* | 0.13 | 0.00 | 30.59 | 0.29 | 54-432 | X-ray | 2.50 | monomer | 2 x MD1, 4 x SF4, 1 x 6MO, 1 x AGA, 1 x F3S, 2 x HEM | BLAST | 0.35 |
| ``` target    SDLSRRELLKRAVVVGTGAGLAELFLPAQFLSSASAQSEPQAVAIANPLAQMPDRSWERIYRDQFAEEDSFVFTCAPNDT 3ir7.1    -----------------------------------------------------NRDWEDGYRQRWQHDKIVRSTHGVNCT  target    HNCLLRAHVKNGVIVRISPTYGYGKAT-DLAGNQASHRWDPRICQKGLILGRRIYGDRRVKAPMIRKGF-KEWADAGFPR 3ir7.1    GSCSWKIYVKNGLVTWETQQTDYPRTRPDLPNHE------PRGCPSGASYSWYLYSANRLKYPMMRKRLMKMWREAKALH  target    HDDGTPRADMEKRGYDEWLQIPWDEALAIAAKTLQNVAETYKGEDGAGKLLEQGYEPAMVEAMHGAGVQAIKMRGGMPLL 3ir7.1    SDP-----------VEAWASIIED----------ADKAKSFKQARGRGGFVRSSWQ-EVNELIAASNVYTIKN------Y  target    GAGRVFGFYRF--ANMLALLDGKLRPEAPPEEIVGSRAFDNYAWHTDLPPGHPMVSGSQTVDFDLFAAEHSKLLVLIGMN 3ir7.1    GPDRVAGFSPIPAMSMVSYASG-----ARYLSLIGGTCLSFYDWYCDLPPASPQTWGEQTDVPESADWYNSSYIIAWGSN  target    WICTKMPDAHWIGDARLKGTRVVVISADYMPTANKADEIVILRPGTDTAFLLGVARELITK------KLYDRDAVIQRTD 3ir7.1    VPQTRTPDAHFFTEVRYKGTKTVAVTPDYAEIAKLCDLWLAPKQGTDAAMALAMGHVMLREFHLDNPSQYFTDYVRRYTD  target    LPLLVRLD-------TGERLSARDVFE--GYRQAPLENYVALKTEEELAAPPSPPFTADKQVVPTELREEWGDFVYWDRA 3ir7.1    MPMLVMLEERDGYYAAGRMLRAADLVDALGQENNPEWKTVAFNTNGEMVAP-----------------------------  target    TNGPAAVNRDEIGAKFAGDPALLGAFDVTLVDGTNVKARTAFSLLKEYLDENFDVQTTSEVCNVDPAAVRSLARQLAANK 3ir7.1    --------------------------------------------------------------------------------  target    GNALLAAGMGPNHYFNADLFGRVHFLVAALTDNIGHFSGNVGSYAGNYRGSLFQAMGQWIAENPFDQEADLTKPARVKRY 3ir7.1    --------------------------------------------------------------------------------  target    FKSESAHYWNYGDRPLVSPSEIITGKSHMPTPTKLIWFGNSNSLLGNAKWSFDVVKNTLPKQDAVFCNEWHWTSSCEYSD 3ir7.1    --------------------------------------------------------------------------------  target    LVFPADSWAEFKLPDMTASCTNPFLLAFPKTPLARIHNTRSDYEILAGVAAALADLVDEPRMKTYWKGILDGDPTPYLQR 3ir7.1    --------------------------------------------------------------------------------  target    VLSGSNATRGILYEDLHASSAKGVPLLMNARTYPRHAGWEQRQEDKPWYTPTGRLEFYRPEPEWQAAGESLPIWREPVDA 3ir7.1    --------------------------------------------------------------------------------  target    TFYEPNAILANSKHPSINPRAPEDYGVPESQMDVETRQYRNVVRTWQELKLSKHPLTEKDPAYRFVFQTPKYRWGAHSTA 3ir7.1    --------------------------------------------------------------------------------  target    VDSDWIAMLFGPFGDPYRRDSRTPWTGEAYAEINPRDAKELGLKDGDYIWLDADPEDRPYRGADSSDEFYDVARAMMRVR 3ir7.1    --------------------------------------------------------------------------------  target    IYSGMPRRVIRTWFNMYAATPGTVQAQKDVPGGPAQNQDTGYVALFRHGSHQSGTRAYLRPTQMTDSMNRKAYFGQTIGK 3ir7.1    --------------------------------------------------------------------------------  target    GFEADVHSPSGAPKEGYVKVEKAEDGGDEGVGEWRPVTLGLRPDDPSEAMQAYLAGEFVTRKRKGS 3ir7.1    ------------------------------------------------------------------ ``` | | | | | | | | | | | | | | | | | | | | | | | | | | | | | | | | | | | | | | | | | | | | | | | | | |
|  | 3egw.1.A | Respiratory nitrate reductase 1 alpha chain  *The crystal structure of the NarGHI mutant NarH - C16A* | 0.13 | 0.00 | 30.59 | 0.29 | 54-432 | X-ray | 1.90 | homo-dimer | 2 x MD1, 2 x MGD, 2 x 6MO, 6 x SF4, 4 x F3S, 2 x 3PH, 4 x HEM, 2 x AGA | BLAST | 0.35 |
| ``` target    SDLSRRELLKRAVVVGTGAGLAELFLPAQFLSSASAQSEPQAVAIANPLAQMPDRSWERIYRDQFAEEDSFVFTCAPNDT 3egw.1    -----------------------------------------------------NRDWEDGYRQRWQHDKIVRSTHGVNCT  target    HNCLLRAHVKNGVIVRISPTYGYGKAT-DLAGNQASHRWDPRICQKGLILGRRIYGDRRVKAPMIRKGF-KEWADAGFPR 3egw.1    GSCSWKIYVKNGLVTWETQQTDYPRTRPDLPNHE------PRGCPRGASYSWYLYSANRLKYPMMRKRLMKMWREAKALH  target    HDDGTPRADMEKRGYDEWLQIPWDEALAIAAKTLQNVAETYKGEDGAGKLLEQGYEPAMVEAMHGAGVQAIKMRGGMPLL 3egw.1    SDP-----------VEAWASIIED----------ADKAKSFKQARGRGGFVRSSWQ-EVNELIAASNVYTIKN------Y  target    GAGRVFGFYRF--ANMLALLDGKLRPEAPPEEIVGSRAFDNYAWHTDLPPGHPMVSGSQTVDFDLFAAEHSKLLVLIGMN 3egw.1    GPDRVAGFSPIPAMSMVSYASG-----ARYLSLIGGTCLSFYDWYCDLPPASPQTWGEQTDVPESADWYNSSYIIAWGSN  target    WICTKMPDAHWIGDARLKGTRVVVISADYMPTANKADEIVILRPGTDTAFLLGVARELITK------KLYDRDAVIQRTD 3egw.1    VPQTRTPDAHFFTEVRYKGTKTVAVTPDYAEIAKLCDLWLAPKQGTDAAMALAMGHVMLREFHLDNPSQYFTDYVRRYTD  target    LPLLVRLD-------TGERLSARDVFE--GYRQAPLENYVALKTEEELAAPPSPPFTADKQVVPTELREEWGDFVYWDRA 3egw.1    MPMLVMLEERDGYYAAGRMLRAADLVAALGQENNPEWKTVAFNTNGEMVAP-----------------------------  target    TNGPAAVNRDEIGAKFAGDPALLGAFDVTLVDGTNVKARTAFSLLKEYLDENFDVQTTSEVCNVDPAAVRSLARQLAANK 3egw.1    --------------------------------------------------------------------------------  target    GNALLAAGMGPNHYFNADLFGRVHFLVAALTDNIGHFSGNVGSYAGNYRGSLFQAMGQWIAENPFDQEADLTKPARVKRY 3egw.1    --------------------------------------------------------------------------------  target    FKSESAHYWNYGDRPLVSPSEIITGKSHMPTPTKLIWFGNSNSLLGNAKWSFDVVKNTLPKQDAVFCNEWHWTSSCEYSD 3egw.1    --------------------------------------------------------------------------------  target    LVFPADSWAEFKLPDMTASCTNPFLLAFPKTPLARIHNTRSDYEILAGVAAALADLVDEPRMKTYWKGILDGDPTPYLQR 3egw.1    --------------------------------------------------------------------------------  target    VLSGSNATRGILYEDLHASSAKGVPLLMNARTYPRHAGWEQRQEDKPWYTPTGRLEFYRPEPEWQAAGESLPIWREPVDA 3egw.1    --------------------------------------------------------------------------------  target    TFYEPNAILANSKHPSINPRAPEDYGVPESQMDVETRQYRNVVRTWQELKLSKHPLTEKDPAYRFVFQTPKYRWGAHSTA 3egw.1    --------------------------------------------------------------------------------  target    VDSDWIAMLFGPFGDPYRRDSRTPWTGEAYAEINPRDAKELGLKDGDYIWLDADPEDRPYRGADSSDEFYDVARAMMRVR 3egw.1    --------------------------------------------------------------------------------  target    IYSGMPRRVIRTWFNMYAATPGTVQAQKDVPGGPAQNQDTGYVALFRHGSHQSGTRAYLRPTQMTDSMNRKAYFGQTIGK 3egw.1    --------------------------------------------------------------------------------  target    GFEADVHSPSGAPKEGYVKVEKAEDGGDEGVGEWRPVTLGLRPDDPSEAMQAYLAGEFVTRKRKGS 3egw.1    ------------------------------------------------------------------ ``` | | | | | | | | | | | | | | | | | | | | | | | | | | | | | | | | | | | | | | | | | | | | | | | | | |
|  | 8bqg.1.A | Formate dehydrogenase, alpha subunit, selenocysteine-containing  *W-formate dehydrogenase from Desulfovibrio vulgaris - Soaking with Formate 1 min* | 0.10 | 0.00 | 26.79 | 0.24 | 76-415 | X-ray | 1.95 | monomer | 2 x MGD, 4 x SF4, 1 x H2S, 1 x W | BLAST | 0.33 |
| ``` target    SDLSRRELLKRAVVVGTGAGLAELFLPAQFLSSASAQSEPQAVAIANPLAQMPDRSWERIYRDQFAEEDSFVFTCAPNDT 8bqg.1    ---------------------------------------------------------------------------AKQTT  target    HNCLLRAHVKNGVIVRISPTYGYGKATDLAGNQASHRWDPRICQKGLILGRRIYGDRRVKAPMIRKGFKEWADAGFPRHD 8bqg.1    SICCYCA-VGCGLIVHTAKD-GQGRAVNVEGDPDHPINEGSLCPKGASIFQLGENDQRGTQPLYRAPFS-----------  target    DGTPRADMEKRGYDEWLQIPWDEALAIAAKTLQNVAE-TYKGEDGAGKLLEQGYEPAMVEAMHGAGVQAIKMRGGMPLLG 8bqg.1    -------------DTWKPVTWDFALTEIAKRIKKTRDASFTEKNAAGDLVNR------TEAIASFGSAAMDNEECWAYGN  target    AGRVFGFYRFANMLALLDGKLRPEAPPEEIVGSRAFDNYAWHTDLPPGHPMVSGSQTVDFDLFAAEHSKLLVLIGMNWIC 8bqg.1    ILRSLG-------LVYIEHQARIUHSPTVPALAESFGR---------------GAMTNHWNDLA--NSDCILIMGSNAAE  target    TKMPDAHWIGDARLKGTRVVVISADYMPTANKADEIVILRPGTDTAFLLGVARELITKKLYDRDAVIQRTDLPLLVRLDT 8bqg.1    NHPIAFKWVLRAKDKGATLIHVDPRFTRTSARCDVYAPIRSGADIPFLGGLIKYILDNKLYFTDYVREYTNASLIV----  target    GERLSARD-VFEGYRQAPLENYVALKTEEELAAPPSPPFTADKQVVPTELREEWGDFVYWDRATNGPAAVNRDEIGAKFA 8bqg.1    GEKFSFKDGLFSGYDAA---------------------------------------------------------------  target    GDPALLGAFDVTLVDGTNVKARTAFSLLKEYLDENFDVQTTSEVCNVDPAAVRSLARQLAANKGNALLAAGMGPNHYFNA 8bqg.1    --------------------------------------------------------------------------------  target    DLFGRVHFLVAALTDNIGHFSGNVGSYAGNYRGSLFQAMGQWIAENPFDQEADLTKPARVKRYFKSESAHYWNYGDRPLV 8bqg.1    --------------------------------------------------------------------------------  target    SPSEIITGKSHMPTPTKLIWFGNSNSLLGNAKWSFDVVKNTLPKQDAVFCNEWHWTSSCEYSDLVFPADSWAEFKLPDMT 8bqg.1    --------------------------------------------------------------------------------  target    ASCTNPFLLAFPKTPLARIHNTRSDYEILAGVAAALADLVDEPRMKTYWKGILDGDPTPYLQRVLSGSNATRGILYEDLH 8bqg.1    --------------------------------------------------------------------------------  target    ASSAKGVPLLMNARTYPRHAGWEQRQEDKPWYTPTGRLEFYRPEPEWQAAGESLPIWREPVDATFYEPNAILANSKHPSI 8bqg.1    --------------------------------------------------------------------------------  target    NPRAPEDYGVPESQMDVETRQYRNVVRTWQELKLSKHPLTEKDPAYRFVFQTPKYRWGAHSTAVDSDWIAMLFGPFGDPY 8bqg.1    --------------------------------------------------------------------------------  target    RRDSRTPWTGEAYAEINPRDAKELGLKDGDYIWLDADPEDRPYRGADSSDEFYDVARAMMRVRIYSGMPRRVIRTWFNMY 8bqg.1    --------------------------------------------------------------------------------  target    AATPGTVQAQKDVPGGPAQNQDTGYVALFRHGSHQSGTRAYLRPTQMTDSMNRKAYFGQTIGKGFEADVHSPSGAPKEGY 8bqg.1    --------------------------------------------------------------------------------  target    VKVEKAEDGGDEGVGEWRPVTLGLRPDDPSEAMQAYLAGEFVTRKRKGS 8bqg.1    ------------------------------------------------- ``` | | | | | | | | | | | | | | | | | | | | | | | | | | | | | | | | | | | | | | | | | | | | | | | | | |
| ✓ | 6sdv.1.A | Formate dehydrogenase, alpha subunit, selenocysteine-containing,Formate dehydrogenase, alpha subunit, selenocysteine-containing,W-formate dehydrogenase - alpha subunit  *W-formate dehydrogenase from Desulfovibrio vulgaris - Formate reduced form* | 0.10 | 0.00 | 26.79 | 0.24 | 76-415 | X-ray | 1.90 | monomer | 2 x MGD, 4 x SF4, 1 x W, 1 x H2S | BLAST | 0.33 |
| ``` target    SDLSRRELLKRAVVVGTGAGLAELFLPAQFLSSASAQSEPQAVAIANPLAQMPDRSWERIYRDQFAEEDSFVFTCAPNDT 6sdv.1    ---------------------------------------------------------------------------AKQTT  target    HNCLLRAHVKNGVIVRISPTYGYGKATDLAGNQASHRWDPRICQKGLILGRRIYGDRRVKAPMIRKGFKEWADAGFPRHD 6sdv.1    SICCYCA-VGCGLIVHTAKD-GQGRAVNVEGDPDHPINEGSLCPKGASIFQLGENDQRGTQPLYRAPFS-----------  target    DGTPRADMEKRGYDEWLQIPWDEALAIAAKTLQNVAE-TYKGEDGAGKLLEQGYEPAMVEAMHGAGVQAIKMRGGMPLLG 6sdv.1    -------------DTWKPVTWDFALTEIAKRIKKTRDASFTEKNAAGDLVNR------TEAIASFGSAAMDNEECWAYGN  target    AGRVFGFYRFANMLALLDGKLRPEAPPEEIVGSRAFDNYAWHTDLPPGHPMVSGSQTVDFDLFAAEHSKLLVLIGMNWIC 6sdv.1    ILRSLG-------LVYIEHQARIUHSPTVPALAESFGR---------------GAMTNHWNDLA--NSDCILIMGSNAAE  target    TKMPDAHWIGDARLKGTRVVVISADYMPTANKADEIVILRPGTDTAFLLGVARELITKKLYDRDAVIQRTDLPLLVRLDT 6sdv.1    NHPIAFKWVLRAKDKGATLIHVDPRFTRTSARCDVYAPIRSGADIPFLGGLIKYILDNKLYFTDYVREYTNASLIV----  target    GERLSARD-VFEGYRQAPLENYVALKTEEELAAPPSPPFTADKQVVPTELREEWGDFVYWDRATNGPAAVNRDEIGAKFA 6sdv.1    GEKFSFKDGLFSGYDAA---------------------------------------------------------------  target    GDPALLGAFDVTLVDGTNVKARTAFSLLKEYLDENFDVQTTSEVCNVDPAAVRSLARQLAANKGNALLAAGMGPNHYFNA 6sdv.1    --------------------------------------------------------------------------------  target    DLFGRVHFLVAALTDNIGHFSGNVGSYAGNYRGSLFQAMGQWIAENPFDQEADLTKPARVKRYFKSESAHYWNYGDRPLV 6sdv.1    --------------------------------------------------------------------------------  target    SPSEIITGKSHMPTPTKLIWFGNSNSLLGNAKWSFDVVKNTLPKQDAVFCNEWHWTSSCEYSDLVFPADSWAEFKLPDMT 6sdv.1    --------------------------------------------------------------------------------  target    ASCTNPFLLAFPKTPLARIHNTRSDYEILAGVAAALADLVDEPRMKTYWKGILDGDPTPYLQRVLSGSNATRGILYEDLH 6sdv.1    --------------------------------------------------------------------------------  target    ASSAKGVPLLMNARTYPRHAGWEQRQEDKPWYTPTGRLEFYRPEPEWQAAGESLPIWREPVDATFYEPNAILANSKHPSI 6sdv.1    --------------------------------------------------------------------------------  target    NPRAPEDYGVPESQMDVETRQYRNVVRTWQELKLSKHPLTEKDPAYRFVFQTPKYRWGAHSTAVDSDWIAMLFGPFGDPY 6sdv.1    --------------------------------------------------------------------------------  target    RRDSRTPWTGEAYAEINPRDAKELGLKDGDYIWLDADPEDRPYRGADSSDEFYDVARAMMRVRIYSGMPRRVIRTWFNMY 6sdv.1    --------------------------------------------------------------------------------  target    AATPGTVQAQKDVPGGPAQNQDTGYVALFRHGSHQSGTRAYLRPTQMTDSMNRKAYFGQTIGKGFEADVHSPSGAPKEGY 6sdv.1    --------------------------------------------------------------------------------  target    VKVEKAEDGGDEGVGEWRPVTLGLRPDDPSEAMQAYLAGEFVTRKRKGS 6sdv.1    ------------------------------------------------- ``` | | | | | | | | | | | | | | | | | | | | | | | | | | | | | | | | | | | | | | | | | | | | | | | | | |
|  | 6sdr.1.A | Formate dehydrogenase, alpha subunit, selenocysteine-containing  *W-formate dehydrogenase from Desulfovibrio vulgaris - Oxidized form* | 0.10 | 0.00 | 26.79 | 0.24 | 76-415 | X-ray | 2.10 | monomer | 2 x MGD, 4 x SF4, 1 x H2S, 1 x W | BLAST | 0.33 |
| ``` target    SDLSRRELLKRAVVVGTGAGLAELFLPAQFLSSASAQSEPQAVAIANPLAQMPDRSWERIYRDQFAEEDSFVFTCAPNDT 6sdr.1    ---------------------------------------------------------------------------AKQTT  target    HNCLLRAHVKNGVIVRISPTYGYGKATDLAGNQASHRWDPRICQKGLILGRRIYGDRRVKAPMIRKGFKEWADAGFPRHD 6sdr.1    SICCYCA-VGCGLIVHTAKD-GQGRAVNVEGDPDHPINEGSLCPKGASIFQLGENDQRGTQPLYRAPFS-----------  target    DGTPRADMEKRGYDEWLQIPWDEALAIAAKTLQNVAE-TYKGEDGAGKLLEQGYEPAMVEAMHGAGVQAIKMRGGMPLLG 6sdr.1    -------------DTWKPVTWDFALTEIAKRIKKTRDASFTEKNAAGDLVNR------TEAIASFGSAAMDNEECWAYGN  target    AGRVFGFYRFANMLALLDGKLRPEAPPEEIVGSRAFDNYAWHTDLPPGHPMVSGSQTVDFDLFAAEHSKLLVLIGMNWIC 6sdr.1    ILRSLG-------LVYIEHQARIUHSPTVPALAESFGR---------------GAMTNHWNDLA--NSDCILIMGSNAAE  target    TKMPDAHWIGDARLKGTRVVVISADYMPTANKADEIVILRPGTDTAFLLGVARELITKKLYDRDAVIQRTDLPLLVRLDT 6sdr.1    NHPIAFKWVLRAKDKGATLIHVDPRFTRTSARCDVYAPIRSGADIPFLGGLIKYILDNKLYFTDYVREYTNASLIV----  target    GERLSARD-VFEGYRQAPLENYVALKTEEELAAPPSPPFTADKQVVPTELREEWGDFVYWDRATNGPAAVNRDEIGAKFA 6sdr.1    GEKFSFKDGLFSGYDAA---------------------------------------------------------------  target    GDPALLGAFDVTLVDGTNVKARTAFSLLKEYLDENFDVQTTSEVCNVDPAAVRSLARQLAANKGNALLAAGMGPNHYFNA 6sdr.1    --------------------------------------------------------------------------------  target    DLFGRVHFLVAALTDNIGHFSGNVGSYAGNYRGSLFQAMGQWIAENPFDQEADLTKPARVKRYFKSESAHYWNYGDRPLV 6sdr.1    --------------------------------------------------------------------------------  target    SPSEIITGKSHMPTPTKLIWFGNSNSLLGNAKWSFDVVKNTLPKQDAVFCNEWHWTSSCEYSDLVFPADSWAEFKLPDMT 6sdr.1    --------------------------------------------------------------------------------  target    ASCTNPFLLAFPKTPLARIHNTRSDYEILAGVAAALADLVDEPRMKTYWKGILDGDPTPYLQRVLSGSNATRGILYEDLH 6sdr.1    --------------------------------------------------------------------------------  target    ASSAKGVPLLMNARTYPRHAGWEQRQEDKPWYTPTGRLEFYRPEPEWQAAGESLPIWREPVDATFYEPNAILANSKHPSI 6sdr.1    --------------------------------------------------------------------------------  target    NPRAPEDYGVPESQMDVETRQYRNVVRTWQELKLSKHPLTEKDPAYRFVFQTPKYRWGAHSTAVDSDWIAMLFGPFGDPY 6sdr.1    --------------------------------------------------------------------------------  target    RRDSRTPWTGEAYAEINPRDAKELGLKDGDYIWLDADPEDRPYRGADSSDEFYDVARAMMRVRIYSGMPRRVIRTWFNMY 6sdr.1    --------------------------------------------------------------------------------  target    AATPGTVQAQKDVPGGPAQNQDTGYVALFRHGSHQSGTRAYLRPTQMTDSMNRKAYFGQTIGKGFEADVHSPSGAPKEGY 6sdr.1    --------------------------------------------------------------------------------  target    VKVEKAEDGGDEGVGEWRPVTLGLRPDDPSEAMQAYLAGEFVTRKRKGS 6sdr.1    ------------------------------------------------- ``` | | | | | | | | | | | | | | | | | | | | | | | | | | | | | | | | | | | | | | | | | | | | | | | | | |
| ✓ | 1eu1.1.A | DIMETHYL SULFOXIDE REDUCTASE  *THE CRYSTAL STRUCTURE OF RHODOBACTER SPHAEROIDES DIMETHYLSULFOXIDE REDUCTASE REVEALS TWO DISTINCT MOLYBDENUM COORDINATION ENVIRONMENTS.* | 0.07 | 0.00 | 33.04 | 0.19 | 133-401 | X-ray | 1.30 | monomer | 3 x GLC, 1 x CD, 2 x MGD, 1 x 6MO, 2 x O | BLAST | 0.36 |
| ``` target    SDLSRRELLKRAVVVGTGAGLAELFLPAQFLSSASAQSEPQAVAIANPLAQMPDRSWERIYRDQFAEEDSFVFTCAPNDT 1eu1.1    --------------------------------------------------------------------------------  target    HNCLLRAHVKNGVIVRISPTYGYGKATDLAGNQASHRWDPRICQKGLILGRRIYGDRRVKAPMIRKGFKEWADAGFPRHD 1eu1.1    ----------------------------------------------------IYSPTRIKYPMVRREFLEKG--------  target    DGTPRADMEKRGYDEWLQIPWDEALAIAAKTLQNVAETYKGEDGAGKLLEQGYEPAMVEAMHGAGVQAIKMRGGMPLLGA 1eu1.1    ---VNADRSTRGNGDFVRVTWDEALDLVARELKRVQESY----GPTGTFGGSYGWKSPGRLHNCQVL---MRRALNLAG-  target    GRVFGFYRFANMLALLDGKLRPEAPPEEIVGSRAFDNYAWHTDLPPGHPMVSGSQTVDFDLFAAEHSKLLVL-----IGM 1eu1.1    GFVNSSGDYSTAAAQI---IMP-----HVMGT--LEVYEQQTAWP----------------VVVENTDLMVFWAADPMKT  target    NWICTKMPD-AHWIGDARLK--GTRVVVISADYMPTANK-ADEIVILRPGTDTAFLLGVARELITKKLYDRDAVIQ-RTD 1eu1.1    NEIGWVIPDHGAYAGMKALKEKGTRVICINPVRTETADYFGADVVSPRPQTDVALMLGMAHTLYSEDLHDKDFLENCTTG  target    LPLLVRLDTGERLSARDVFEGYRQAPLENYVALKTEEELAAPPSPPFTADKQVVPTELREEWGDFVYWDRATNGPAAVNR 1eu1.1    FDLFAAYLTGE---------------------------------------------------------------------  target    DEIGAKFAGDPALLGAFDVTLVDGTNVKARTAFSLLKEYLDENFDVQTTSEVCNVDPAAVRSLARQLAANKGNALLAAGM 1eu1.1    --------------------------------------------------------------------------------  target    GPNHYFNADLFGRVHFLVAALTDNIGHFSGNVGSYAGNYRGSLFQAMGQWIAENPFDQEADLTKPARVKRYFKSESAHYW 1eu1.1    --------------------------------------------------------------------------------  target    NYGDRPLVSPSEIITGKSHMPTPTKLIWFGNSNSLLGNAKWSFDVVKNTLPKQDAVFCNEWHWTSSCEYSDLVFPADSWA 1eu1.1    --------------------------------------------------------------------------------  target    EFKLPDMTASCTNPFLLAFPKTPLARIHNTRSDYEILAGVAAALADLVDEPRMKTYWKGILDGDPTPYLQRVLSGSNATR 1eu1.1    --------------------------------------------------------------------------------  target    GILYEDLHASSAKGVPLLMNARTYPRHAGWEQRQEDKPWYTPTGRLEFYRPEPEWQAAGESLPIWREPVDATFYEPNAIL 1eu1.1    --------------------------------------------------------------------------------  target    ANSKHPSINPRAPEDYGVPESQMDVETRQYRNVVRTWQELKLSKHPLTEKDPAYRFVFQTPKYRWGAHSTAVDSDWIAML 1eu1.1    --------------------------------------------------------------------------------  target    FGPFGDPYRRDSRTPWTGEAYAEINPRDAKELGLKDGDYIWLDADPEDRPYRGADSSDEFYDVARAMMRVRIYSGMPRRV 1eu1.1    --------------------------------------------------------------------------------  target    IRTWFNMYAATPGTVQAQKDVPGGPAQNQDTGYVALFRHGSHQSGTRAYLRPTQMTDSMNRKAYFGQTIGKGFEADVHSP 1eu1.1    --------------------------------------------------------------------------------  target    SGAPKEGYVKVEKAEDGGDEGVGEWRPVTLGLRPDDPSEAMQAYLAGEFVTRKRKGS 1eu1.1    --------------------------------------------------------- ``` | | | | | | | | | | | | | | | | | | | | | | | | | | | | | | | | | | | | | | | | | | | | | | | | | |
| ✓ | 7l5i.1.A | Trimethylamine-N-oxide reductase  *Crystal Structure of Haemophilus influenzae MtsZ at pH 7.0* | 0.07 | 0.00 | 24.11 | 0.19 | 128-389 | X-ray | 1.73 | monomer | 2 x MGD, 1 x MO, 1 x O | BLAST | 0.34 |
| ``` target    SDLSRRELLKRAVVVGTGAGLAELFLPAQFLSSASAQSEPQAVAIANPLAQMPDRSWERIYRDQFAEEDSFVFTCAPNDT 7l5i.1    --------------------------------------------------------------------------------  target    HNCLLRAHVKNGVIVRISPTYGYGKATDLAGNQASHRWDPRICQKGLILGRRIYGDRRVKAPMIRKGFKEWADAGFPRHD 7l5i.1    -----------------------------------------------VVADQLYSEARVKCPMVRKGF--LANPG-----  target    DGTPRADMEKRGYDEWLQIPWDEALAIAAKTLQNVAETYKGEDGAGKLLEQGYEPAMVEAMHGAGVQAIKMRGGMPLLGA 7l5i.1    ----KSDTTMRGRDEWVRVSWDEALDLVHNQLKRV----RDEHGSTGIFAGSYGWFSCGSLH-----------------A  target    GRVFGFYRFANMLALLDG-KLRPEAPPEEIVGSRAFDNYAWHTDLPPGHPMVSGSQTVDFDLFAAEHSKLLVLIGMNWIC 7l5i.1    SRTL-LQRYMNATGGFVGHKGDYSTGAAQVIMPHVLGTIEVYEQQTSWESILESS-----DIIVLWSANPLTTMRIAWMS  target    TKMPDAHWIGDARLKGTRVVVISADYMPTANKAD-EIVILRPGTDTAFLLGVARELITKKLYDRDAVIQRTDLPLLVRLD 7l5i.1    TDQKGIEYFKKFQASGKRIICIDPQKSETCQMLNAEWIPVNTATDVPLMLGIAHTLVEQGKHDKDFLKKYT---------  target    TGERLSARDVFEGYRQAPLENYVALKTEEELAAPPSPPFTADKQVVPTELREEWGDFVYWDRATNGPAAVNRDEIGAKFA 7l5i.1    --------------------------------------------------------------------------------  target    GDPALLGAFDVTLVDGTNVKARTAFSLLKEYLDENFDVQTTSEVCNVDPAAVRSLARQLAANKGNALLAAGMGPNHYFNA 7l5i.1    --------------------------------------------------------------------------------  target    DLFGRVHFLVAALTDNIGHFSGNVGSYAGNYRGSLFQAMGQWIAENPFDQEADLTKPARVKRYFKSESAHYWNYGDRPLV 7l5i.1    --------------------------------------------------------------------------------  target    SPSEIITGKSHMPTPTKLIWFGNSNSLLGNAKWSFDVVKNTLPKQDAVFCNEWHWTSSCEYSDLVFPADSWAEFKLPDMT 7l5i.1    --------------------------------------------------------------------------------  target    ASCTNPFLLAFPKTPLARIHNTRSDYEILAGVAAALADLVDEPRMKTYWKGILDGDPTPYLQRVLSGSNATRGILYEDLH 7l5i.1    --------------------------------------------------------------------------------  target    ASSAKGVPLLMNARTYPRHAGWEQRQEDKPWYTPTGRLEFYRPEPEWQAAGESLPIWREPVDATFYEPNAILANSKHPSI 7l5i.1    --------------------------------------------------------------------------------  target    NPRAPEDYGVPESQMDVETRQYRNVVRTWQELKLSKHPLTEKDPAYRFVFQTPKYRWGAHSTAVDSDWIAMLFGPFGDPY 7l5i.1    --------------------------------------------------------------------------------  target    RRDSRTPWTGEAYAEINPRDAKELGLKDGDYIWLDADPEDRPYRGADSSDEFYDVARAMMRVRIYSGMPRRVIRTWFNMY 7l5i.1    --------------------------------------------------------------------------------  target    AATPGTVQAQKDVPGGPAQNQDTGYVALFRHGSHQSGTRAYLRPTQMTDSMNRKAYFGQTIGKGFEADVHSPSGAPKEGY 7l5i.1    --------------------------------------------------------------------------------  target    VKVEKAEDGGDEGVGEWRPVTLGLRPDDPSEAMQAYLAGEFVTRKRKGS 7l5i.1    ------------------------------------------------- ``` | | | | | | | | | | | | | | | | | | | | | | | | | | | | | | | | | | | | | | | | | | | | | | | | | |
|  | 7l5s.1.A | Trimethylamine-N-oxide reductase  *Crystal Structure of Haemophilus influenzae MtsZ at pH 5.5* | 0.07 | 0.00 | 24.11 | 0.19 | 128-389 | X-ray | 2.09 | monomer | 1 x O, 2 x MGD, 1 x MO | BLAST | 0.34 |
| ``` target    SDLSRRELLKRAVVVGTGAGLAELFLPAQFLSSASAQSEPQAVAIANPLAQMPDRSWERIYRDQFAEEDSFVFTCAPNDT 7l5s.1    --------------------------------------------------------------------------------  target    HNCLLRAHVKNGVIVRISPTYGYGKATDLAGNQASHRWDPRICQKGLILGRRIYGDRRVKAPMIRKGFKEWADAGFPRHD 7l5s.1    -----------------------------------------------VVADQLYSEARVKCPMVRKGF--LANPG-----  target    DGTPRADMEKRGYDEWLQIPWDEALAIAAKTLQNVAETYKGEDGAGKLLEQGYEPAMVEAMHGAGVQAIKMRGGMPLLGA 7l5s.1    ----KSDTTMRGRDEWVRVSWDEALDLVHNQLKRV----RDEHGSTGIFAGSYGWFSCGSLH-----------------A  target    GRVFGFYRFANMLALLDG-KLRPEAPPEEIVGSRAFDNYAWHTDLPPGHPMVSGSQTVDFDLFAAEHSKLLVLIGMNWIC 7l5s.1    SRTL-LQRYMNATGGFVGHKGDYSTGAAQVIMPHVLGTIEVYEQQTSWESILESS-----DIIVLWSANPLTTMRIAWMS  target    TKMPDAHWIGDARLKGTRVVVISADYMPTANKAD-EIVILRPGTDTAFLLGVARELITKKLYDRDAVIQRTDLPLLVRLD 7l5s.1    TDQKGIEYFKKFQASGKRIICIDPQKSETCQMLNAEWIPVNTATDVPLMLGIAHTLVEQGKHDKDFLKKYT---------  target    TGERLSARDVFEGYRQAPLENYVALKTEEELAAPPSPPFTADKQVVPTELREEWGDFVYWDRATNGPAAVNRDEIGAKFA 7l5s.1    --------------------------------------------------------------------------------  target    GDPALLGAFDVTLVDGTNVKARTAFSLLKEYLDENFDVQTTSEVCNVDPAAVRSLARQLAANKGNALLAAGMGPNHYFNA 7l5s.1    --------------------------------------------------------------------------------  target    DLFGRVHFLVAALTDNIGHFSGNVGSYAGNYRGSLFQAMGQWIAENPFDQEADLTKPARVKRYFKSESAHYWNYGDRPLV 7l5s.1    --------------------------------------------------------------------------------  target    SPSEIITGKSHMPTPTKLIWFGNSNSLLGNAKWSFDVVKNTLPKQDAVFCNEWHWTSSCEYSDLVFPADSWAEFKLPDMT 7l5s.1    --------------------------------------------------------------------------------  target    ASCTNPFLLAFPKTPLARIHNTRSDYEILAGVAAALADLVDEPRMKTYWKGILDGDPTPYLQRVLSGSNATRGILYEDLH 7l5s.1    --------------------------------------------------------------------------------  target    ASSAKGVPLLMNARTYPRHAGWEQRQEDKPWYTPTGRLEFYRPEPEWQAAGESLPIWREPVDATFYEPNAILANSKHPSI 7l5s.1    --------------------------------------------------------------------------------  target    NPRAPEDYGVPESQMDVETRQYRNVVRTWQELKLSKHPLTEKDPAYRFVFQTPKYRWGAHSTAVDSDWIAMLFGPFGDPY 7l5s.1    --------------------------------------------------------------------------------  target    RRDSRTPWTGEAYAEINPRDAKELGLKDGDYIWLDADPEDRPYRGADSSDEFYDVARAMMRVRIYSGMPRRVIRTWFNMY 7l5s.1    --------------------------------------------------------------------------------  target    AATPGTVQAQKDVPGGPAQNQDTGYVALFRHGSHQSGTRAYLRPTQMTDSMNRKAYFGQTIGKGFEADVHSPSGAPKEGY 7l5s.1    --------------------------------------------------------------------------------  target    VKVEKAEDGGDEGVGEWRPVTLGLRPDDPSEAMQAYLAGEFVTRKRKGS 7l5s.1    ------------------------------------------------- ``` | | | | | | | | | | | | | | | | | | | | | | | | | | | | | | | | | | | | | | | | | | | | | | | | | |
|  | 4dmr.1.A | DMSO REDUCTASE  *REDUCED DMSO REDUCTASE FROM RHODOBACTER CAPSULATUS WITH BOUND DMSO SUBSTRATE* | 0.07 | 0.00 | 29.09 | 0.19 | 133-389 | X-ray | 1.90 | monomer | 2 x PGD, 1 x 4MO, 1 x O | BLAST | 0.35 |
| ``` target    SDLSRRELLKRAVVVGTGAGLAELFLPAQFLSSASAQSEPQAVAIANPLAQMPDRSWERIYRDQFAEEDSFVFTCAPNDT 4dmr.1    --------------------------------------------------------------------------------  target    HNCLLRAHVKNGVIVRISPTYGYGKATDLAGNQASHRWDPRICQKGLILGRRIYGDRRVKAPMIRKGFKEWADAGFPRHD 4dmr.1    ----------------------------------------------------IYSPTRIKYPMVRREFLEKG--------  target    DGTPRADMEKRGYDEWLQIPWDEALAIAAKTLQNVAETYKGEDGAGKLLEQGYEPAMVEAMHGAGVQAIKMRGGMPLLGA 4dmr.1    ---VNADRSTRGNGDFVRVSWDQALDLVAAEVKRVEETY----GPSGVFGGSYGWKSPGRLHNCTTLLRRML----TLAG  target    GRVFGFYRFANMLALLDGKLRPEAPPEEIVGSRAFDNYAWHTDLPPGHPMVSGSQTVDFDLFAAEHSKLLVLIGMNWICT 4dmr.1    GYVNGAGDYSTGAAQV---IMP-----HVVGT--LEVYEQQTAWP-----VLAENTEVMVFWAADPIKTSQI---GWVIP  target    KMPDAHWIGDARLKGTRVVVISADYMPTAN-KADEIVILRPGTDTAFLLGVARELITKKLYDRDAVIQRTDLPLLVRLDT 4dmr.1    EHGAYPGLEALKAKGTKVIVIDPVRTKTVEFFGAEHITPKPQTDVAIMLGMAHTLVAEDLYDKDFIANYT----------  target    GERLSARDVFEGYRQAPLENYVALKTEEELAAPPSPPFTADKQVVPTELREEWGDFVYWDRATNGPAAVNRDEIGAKFAG 4dmr.1    --------------------------------------------------------------------------------  target    DPALLGAFDVTLVDGTNVKARTAFSLLKEYLDENFDVQTTSEVCNVDPAAVRSLARQLAANKGNALLAAGMGPNHYFNAD 4dmr.1    --------------------------------------------------------------------------------  target    LFGRVHFLVAALTDNIGHFSGNVGSYAGNYRGSLFQAMGQWIAENPFDQEADLTKPARVKRYFKSESAHYWNYGDRPLVS 4dmr.1    --------------------------------------------------------------------------------  target    PSEIITGKSHMPTPTKLIWFGNSNSLLGNAKWSFDVVKNTLPKQDAVFCNEWHWTSSCEYSDLVFPADSWAEFKLPDMTA 4dmr.1    --------------------------------------------------------------------------------  target    SCTNPFLLAFPKTPLARIHNTRSDYEILAGVAAALADLVDEPRMKTYWKGILDGDPTPYLQRVLSGSNATRGILYEDLHA 4dmr.1    --------------------------------------------------------------------------------  target    SSAKGVPLLMNARTYPRHAGWEQRQEDKPWYTPTGRLEFYRPEPEWQAAGESLPIWREPVDATFYEPNAILANSKHPSIN 4dmr.1    --------------------------------------------------------------------------------  target    PRAPEDYGVPESQMDVETRQYRNVVRTWQELKLSKHPLTEKDPAYRFVFQTPKYRWGAHSTAVDSDWIAMLFGPFGDPYR 4dmr.1    --------------------------------------------------------------------------------  target    RDSRTPWTGEAYAEINPRDAKELGLKDGDYIWLDADPEDRPYRGADSSDEFYDVARAMMRVRIYSGMPRRVIRTWFNMYA 4dmr.1    --------------------------------------------------------------------------------  target    ATPGTVQAQKDVPGGPAQNQDTGYVALFRHGSHQSGTRAYLRPTQMTDSMNRKAYFGQTIGKGFEADVHSPSGAPKEGYV 4dmr.1    --------------------------------------------------------------------------------  target    KVEKAEDGGDEGVGEWRPVTLGLRPDDPSEAMQAYLAGEFVTRKRKGS 4dmr.1    ------------------------------------------------ ``` | | | | | | | | | | | | | | | | | | | | | | | | | | | | | | | | | | | | | | | | | | | | | | | | | |
|  | 1e18.1.A | DMSO REDUCTASE.  *TUNGSTEN-SUSBSTITUTED DMSO REDUCTASE FROM RHODOBACTER CAPSULATUS* | 0.07 | 0.00 | 29.09 | 0.19 | 133-389 | X-ray | 2.00 | monomer | 2 x PGD, 1 x 6WO | BLAST | 0.35 |
| ``` target    SDLSRRELLKRAVVVGTGAGLAELFLPAQFLSSASAQSEPQAVAIANPLAQMPDRSWERIYRDQFAEEDSFVFTCAPNDT 1e18.1    --------------------------------------------------------------------------------  target    HNCLLRAHVKNGVIVRISPTYGYGKATDLAGNQASHRWDPRICQKGLILGRRIYGDRRVKAPMIRKGFKEWADAGFPRHD 1e18.1    ----------------------------------------------------IYSPTRIKYPMVRREFLEKG--------  target    DGTPRADMEKRGYDEWLQIPWDEALAIAAKTLQNVAETYKGEDGAGKLLEQGYEPAMVEAMHGAGVQAIKMRGGMPLLGA 1e18.1    ---VNADRSTRGNGDFVRVSWDQALDLVAAEVKRVEETY----GPQGVFGGSYGWKSPGRLHNCTTLLRRML----TLAG  target    GRVFGFYRFANMLALLDGKLRPEAPPEEIVGSRAFDNYAWHTDLPPGHPMVSGSQTVDFDLFAAEHSKLLVLIGMNWICT 1e18.1    GYVNGAGDYSTGAAQV---IMP-----HVVGT--LEVYEQQTAWP-----VLAENTEVMVFWAADPIKTSQI---GWVIP  target    KMPDAHWIGDARLKGTRVVVISADYMPTAN-KADEIVILRPGTDTAFLLGVARELITKKLYDRDAVIQRTDLPLLVRLDT 1e18.1    EHGAYPGLEALKAKGTKVIVIDPVRTKTVEFFGAEHITPKPQTDVAIMLGMAHTLVAEDLYDKDFIANYT----------  target    GERLSARDVFEGYRQAPLENYVALKTEEELAAPPSPPFTADKQVVPTELREEWGDFVYWDRATNGPAAVNRDEIGAKFAG 1e18.1    --------------------------------------------------------------------------------  target    DPALLGAFDVTLVDGTNVKARTAFSLLKEYLDENFDVQTTSEVCNVDPAAVRSLARQLAANKGNALLAAGMGPNHYFNAD 1e18.1    --------------------------------------------------------------------------------  target    LFGRVHFLVAALTDNIGHFSGNVGSYAGNYRGSLFQAMGQWIAENPFDQEADLTKPARVKRYFKSESAHYWNYGDRPLVS 1e18.1    --------------------------------------------------------------------------------  target    PSEIITGKSHMPTPTKLIWFGNSNSLLGNAKWSFDVVKNTLPKQDAVFCNEWHWTSSCEYSDLVFPADSWAEFKLPDMTA 1e18.1    --------------------------------------------------------------------------------  target    SCTNPFLLAFPKTPLARIHNTRSDYEILAGVAAALADLVDEPRMKTYWKGILDGDPTPYLQRVLSGSNATRGILYEDLHA 1e18.1    --------------------------------------------------------------------------------  target    SSAKGVPLLMNARTYPRHAGWEQRQEDKPWYTPTGRLEFYRPEPEWQAAGESLPIWREPVDATFYEPNAILANSKHPSIN 1e18.1    --------------------------------------------------------------------------------  target    PRAPEDYGVPESQMDVETRQYRNVVRTWQELKLSKHPLTEKDPAYRFVFQTPKYRWGAHSTAVDSDWIAMLFGPFGDPYR 1e18.1    --------------------------------------------------------------------------------  target    RDSRTPWTGEAYAEINPRDAKELGLKDGDYIWLDADPEDRPYRGADSSDEFYDVARAMMRVRIYSGMPRRVIRTWFNMYA 1e18.1    --------------------------------------------------------------------------------  target    ATPGTVQAQKDVPGGPAQNQDTGYVALFRHGSHQSGTRAYLRPTQMTDSMNRKAYFGQTIGKGFEADVHSPSGAPKEGYV 1e18.1    --------------------------------------------------------------------------------  target    KVEKAEDGGDEGVGEWRPVTLGLRPDDPSEAMQAYLAGEFVTRKRKGS 1e18.1    ------------------------------------------------ ``` | | | | | | | | | | | | | | | | | | | | | | | | | | | | | | | | | | | | | | | | | | | | | | | | | |
|  | 1e5v.2.A | Dimethyl sulfoxide/trimethylamine N-oxide reductase  *OXIDIZED DMSO REDUCTASE EXPOSED TO HEPES BUFFER* | 0.07 |  | 29.09 | 0.19 | 133-389 | X-ray | 2.40 | monomer | 2 x PGD, 1 x 2MO | BLAST | 0.35 |
| ``` target    SDLSRRELLKRAVVVGTGAGLAELFLPAQFLSSASAQSEPQAVAIANPLAQMPDRSWERIYRDQFAEEDSFVFTCAPNDT 1e5v.2    --------------------------------------------------------------------------------  target    HNCLLRAHVKNGVIVRISPTYGYGKATDLAGNQASHRWDPRICQKGLILGRRIYGDRRVKAPMIRKGFKEWADAGFPRHD 1e5v.2    ----------------------------------------------------IYSPTRIKYPMVRREFLEKG--------  target    DGTPRADMEKRGYDEWLQIPWDEALAIAAKTLQNVAETYKGEDGAGKLLEQGYEPAMVEAMHGAGVQAIKMRGGMPLLGA 1e5v.2    ---VNADRSTRGNGDFVRVSWDQALDLVAAEVKRVEETY----GPEGVFGGSYGWKSPGRLHNCTTLLRRML----TLAG  target    GRVFGFYRFANMLALLDGKLRPEAPPEEIVGSRAFDNYAWHTDLPPGHPMVSGSQTVDFDLFAAEHSKLLVLIGMNWICT 1e5v.2    GYVNGAGDYSTGAAQV---IMP-----HVVGT--LEVYEQQTAWP-----VLAENTEVMVFWAADPIKTSQI---GWVIP  target    KMPDAHWIGDARLKGTRVVVISADYMPTAN-KADEIVILRPGTDTAFLLGVARELITKKLYDRDAVIQRTDLPLLVRLDT 1e5v.2    EHGAYPGLEALKAKGTKVIVIDPVRTKTVEFFGAEHITPKPQTDVAIMLGMAHTLVAEDLYDKDFIANYT----------  target    GERLSARDVFEGYRQAPLENYVALKTEEELAAPPSPPFTADKQVVPTELREEWGDFVYWDRATNGPAAVNRDEIGAKFAG 1e5v.2    --------------------------------------------------------------------------------  target    DPALLGAFDVTLVDGTNVKARTAFSLLKEYLDENFDVQTTSEVCNVDPAAVRSLARQLAANKGNALLAAGMGPNHYFNAD 1e5v.2    --------------------------------------------------------------------------------  target    LFGRVHFLVAALTDNIGHFSGNVGSYAGNYRGSLFQAMGQWIAENPFDQEADLTKPARVKRYFKSESAHYWNYGDRPLVS 1e5v.2    --------------------------------------------------------------------------------  target    PSEIITGKSHMPTPTKLIWFGNSNSLLGNAKWSFDVVKNTLPKQDAVFCNEWHWTSSCEYSDLVFPADSWAEFKLPDMTA 1e5v.2    --------------------------------------------------------------------------------  target    SCTNPFLLAFPKTPLARIHNTRSDYEILAGVAAALADLVDEPRMKTYWKGILDGDPTPYLQRVLSGSNATRGILYEDLHA 1e5v.2    --------------------------------------------------------------------------------  target    SSAKGVPLLMNARTYPRHAGWEQRQEDKPWYTPTGRLEFYRPEPEWQAAGESLPIWREPVDATFYEPNAILANSKHPSIN 1e5v.2    --------------------------------------------------------------------------------  target    PRAPEDYGVPESQMDVETRQYRNVVRTWQELKLSKHPLTEKDPAYRFVFQTPKYRWGAHSTAVDSDWIAMLFGPFGDPYR 1e5v.2    --------------------------------------------------------------------------------  target    RDSRTPWTGEAYAEINPRDAKELGLKDGDYIWLDADPEDRPYRGADSSDEFYDVARAMMRVRIYSGMPRRVIRTWFNMYA 1e5v.2    --------------------------------------------------------------------------------  target    ATPGTVQAQKDVPGGPAQNQDTGYVALFRHGSHQSGTRAYLRPTQMTDSMNRKAYFGQTIGKGFEADVHSPSGAPKEGYV 1e5v.2    --------------------------------------------------------------------------------  target    KVEKAEDGGDEGVGEWRPVTLGLRPDDPSEAMQAYLAGEFVTRKRKGS 1e5v.2    ------------------------------------------------ ``` | | | | | | | | | | | | | | | | | | | | | | | | | | | | | | | | | | | | | | | | | | | | | | | | | |
|  | 1e60.1.A | Dimethyl sulfoxide/trimethylamine N-oxide reductase  *OXIDIZED DMSO REDUCTASE EXPOSED TO HEPES - Structure II BUFFER* | 0.07 |  | 29.09 | 0.19 | 133-389 | X-ray | 2.00 | monomer | 2 x PGD, 1 x 2MO | BLAST | 0.35 |
| ``` target    SDLSRRELLKRAVVVGTGAGLAELFLPAQFLSSASAQSEPQAVAIANPLAQMPDRSWERIYRDQFAEEDSFVFTCAPNDT 1e60.1    --------------------------------------------------------------------------------  target    HNCLLRAHVKNGVIVRISPTYGYGKATDLAGNQASHRWDPRICQKGLILGRRIYGDRRVKAPMIRKGFKEWADAGFPRHD 1e60.1    ----------------------------------------------------IYSPTRIKYPMVRREFLEKG--------  target    DGTPRADMEKRGYDEWLQIPWDEALAIAAKTLQNVAETYKGEDGAGKLLEQGYEPAMVEAMHGAGVQAIKMRGGMPLLGA 1e60.1    ---VNADRSTRGNGDFVRVSWDQALDLVAAEVKRVEETY----GPEGVFGGSYGWKSPGRLHNCTTLLRRML----TLAG  target    GRVFGFYRFANMLALLDGKLRPEAPPEEIVGSRAFDNYAWHTDLPPGHPMVSGSQTVDFDLFAAEHSKLLVLIGMNWICT 1e60.1    GYVNGAGDYSTGAAQV---IMP-----HVVGT--LEVYEQQTAWP-----VLAENTEVMVFWAADPIKTSQI---GWVIP  target    KMPDAHWIGDARLKGTRVVVISADYMPTAN-KADEIVILRPGTDTAFLLGVARELITKKLYDRDAVIQRTDLPLLVRLDT 1e60.1    EHGAYPGLEALKAKGTKVIVIDPVRTKTVEFFGAEHITPKPQTDVAIMLGMAHTLVAEDLYDKDFIANYT----------  target    GERLSARDVFEGYRQAPLENYVALKTEEELAAPPSPPFTADKQVVPTELREEWGDFVYWDRATNGPAAVNRDEIGAKFAG 1e60.1    --------------------------------------------------------------------------------  target    DPALLGAFDVTLVDGTNVKARTAFSLLKEYLDENFDVQTTSEVCNVDPAAVRSLARQLAANKGNALLAAGMGPNHYFNAD 1e60.1    --------------------------------------------------------------------------------  target    LFGRVHFLVAALTDNIGHFSGNVGSYAGNYRGSLFQAMGQWIAENPFDQEADLTKPARVKRYFKSESAHYWNYGDRPLVS 1e60.1    --------------------------------------------------------------------------------  target    PSEIITGKSHMPTPTKLIWFGNSNSLLGNAKWSFDVVKNTLPKQDAVFCNEWHWTSSCEYSDLVFPADSWAEFKLPDMTA 1e60.1    --------------------------------------------------------------------------------  target    SCTNPFLLAFPKTPLARIHNTRSDYEILAGVAAALADLVDEPRMKTYWKGILDGDPTPYLQRVLSGSNATRGILYEDLHA 1e60.1    --------------------------------------------------------------------------------  target    SSAKGVPLLMNARTYPRHAGWEQRQEDKPWYTPTGRLEFYRPEPEWQAAGESLPIWREPVDATFYEPNAILANSKHPSIN 1e60.1    --------------------------------------------------------------------------------  target    PRAPEDYGVPESQMDVETRQYRNVVRTWQELKLSKHPLTEKDPAYRFVFQTPKYRWGAHSTAVDSDWIAMLFGPFGDPYR 1e60.1    --------------------------------------------------------------------------------  target    RDSRTPWTGEAYAEINPRDAKELGLKDGDYIWLDADPEDRPYRGADSSDEFYDVARAMMRVRIYSGMPRRVIRTWFNMYA 1e60.1    --------------------------------------------------------------------------------  target    ATPGTVQAQKDVPGGPAQNQDTGYVALFRHGSHQSGTRAYLRPTQMTDSMNRKAYFGQTIGKGFEADVHSPSGAPKEGYV 1e60.1    --------------------------------------------------------------------------------  target    KVEKAEDGGDEGVGEWRPVTLGLRPDDPSEAMQAYLAGEFVTRKRKGS 1e60.1    ------------------------------------------------ ``` | | | | | | | | | | | | | | | | | | | | | | | | | | | | | | | | | | | | | | | | | | | | | | | | | |
|  | 1dms.1.A | DMSO REDUCTASE  *STRUCTURE OF DMSO REDUCTASE* | 0.07 |  | 30.56 | 0.19 | 133-389 | X-ray | 1.88 | monomer | 2 x PGD, 1 x 2MO | BLAST | 0.35 |
| ``` target    SDLSRRELLKRAVVVGTGAGLAELFLPAQFLSSASAQSEPQAVAIANPLAQMPDRSWERIYRDQFAEEDSFVFTCAPNDT 1dms.1    --------------------------------------------------------------------------------  target    HNCLLRAHVKNGVIVRISPTYGYGKATDLAGNQASHRWDPRICQKGLILGRRIYGDRRVKAPMIRKGFKEWADAGFPRHD 1dms.1    ----------------------------------------------------IYSPTRIKYPMVRREFLEKG--------  target    DGTPRADMEKRGYDEWLQIPWDEALAIAAKTLQNVAETYKGEDGAGKLLEQGYEPAMVEAMHGAGVQAIKMRGGMPLLGA 1dms.1    ---VNADRSTRGNGDFVRVSWDQALDLVAAEVKRVEETY----GPQGVFGGSYGWKSPGRLHNCTTLLRRML----TLAG  target    GRVFGFYRFANMLALLDGKLRPEAPPEEIVGSRAFDNYAWHTDLPPGHPMVSGSQTVDFDLFAAEHSKLLVLIGMNWICT 1dms.1    GYVNGAGDYSTGAAQV---IMP-----HVVGT--LEVYEQQTAWP-----VLAENTEVMVFWAADPIKTSQI---GWVIP  target    KMPDAHWIGDARLKGTRVVVISADYMPTANKADEI-----VILRPGTDTAFLLGVARELITKKLYDRDAVIQRTDLPLLV 1dms.1    EHGAYPGLEALKAKGTKVIVID----PVRTKTVEFFGADHVTPKPQTDVAIMLGMAHTLVAEDLYDKDFIANYT------  target    RLDTGERLSARDVFEGYRQAPLENYVALKTEEELAAPPSPPFTADKQVVPTELREEWGDFVYWDRATNGPAAVNRDEIGA 1dms.1    --------------------------------------------------------------------------------  target    KFAGDPALLGAFDVTLVDGTNVKARTAFSLLKEYLDENFDVQTTSEVCNVDPAAVRSLARQLAANKGNALLAAGMGPNHY 1dms.1    --------------------------------------------------------------------------------  target    FNADLFGRVHFLVAALTDNIGHFSGNVGSYAGNYRGSLFQAMGQWIAENPFDQEADLTKPARVKRYFKSESAHYWNYGDR 1dms.1    --------------------------------------------------------------------------------  target    PLVSPSEIITGKSHMPTPTKLIWFGNSNSLLGNAKWSFDVVKNTLPKQDAVFCNEWHWTSSCEYSDLVFPADSWAEFKLP 1dms.1    --------------------------------------------------------------------------------  target    DMTASCTNPFLLAFPKTPLARIHNTRSDYEILAGVAAALADLVDEPRMKTYWKGILDGDPTPYLQRVLSGSNATRGILYE 1dms.1    --------------------------------------------------------------------------------  target    DLHASSAKGVPLLMNARTYPRHAGWEQRQEDKPWYTPTGRLEFYRPEPEWQAAGESLPIWREPVDATFYEPNAILANSKH 1dms.1    --------------------------------------------------------------------------------  target    PSINPRAPEDYGVPESQMDVETRQYRNVVRTWQELKLSKHPLTEKDPAYRFVFQTPKYRWGAHSTAVDSDWIAMLFGPFG 1dms.1    --------------------------------------------------------------------------------  target    DPYRRDSRTPWTGEAYAEINPRDAKELGLKDGDYIWLDADPEDRPYRGADSSDEFYDVARAMMRVRIYSGMPRRVIRTWF 1dms.1    --------------------------------------------------------------------------------  target    NMYAATPGTVQAQKDVPGGPAQNQDTGYVALFRHGSHQSGTRAYLRPTQMTDSMNRKAYFGQTIGKGFEADVHSPSGAPK 1dms.1    --------------------------------------------------------------------------------  target    EGYVKVEKAEDGGDEGVGEWRPVTLGLRPDDPSEAMQAYLAGEFVTRKRKGS 1dms.1    ---------------------------------------------------- ``` | | | | | | | | | | | | | | | | | | | | | | | | | | | | | | | | | | | | | | | | | | | | | | | | | |
|  | 1h0h.1.A | FORMATE DEHYDROGENASE SUBUNIT ALPHA  *Tungsten containing Formate Dehydrogenase from Desulfovibrio Gigas* | 0.03 |  | 29.25 | 0.09 | 305-414 | X-ray | 1.80 | hetero-1-1-mer | 1 x W, 1 x 2MD, 1 x MGD, 4 x SF4, 1 x CA | BLAST | 0.36 |
| ``` target    SDLSRRELLKRAVVVGTGAGLAELFLPAQFLSSASAQSEPQAVAIANPLAQMPDRSWERIYRDQFAEEDSFVFTCAPNDT 1h0h.1    --------------------------------------------------------------------------------  target    HNCLLRAHVKNGVIVRISPTYGYGKATDLAGNQASHRWDPRICQKGLILGRRIYGDRRVKAPMIRKGFKEWADAGFPRHD 1h0h.1    --------------------------------------------------------------------------------  target    DGTPRADMEKRGYDEWLQIPWDEALAIAAKTLQNVAETYKGEDGAGKLLEQGYEPAMVEAMHGAGVQAIKMRGGMPLLGA 1h0h.1    --------------------------------------------------------------------------------  target    GRVFGFYRFANMLALLDGKLRPEAPPEEIVGSRAFDNYAWHTDLPPGHPMVSGSQTVDFDLFAAEHSKLLVLIGMNWICT 1h0h.1    ----------------------------------------------------------------KNSDVILMMGSNPAEN  target    KMPDAHWIGDARLKGTRVVVISADYMPTANKADEIVILRPGTDTAFLLGVARELITKKLYDRDAVIQRTDLPLLVRLDTG 1h0h.1    HPISFKWVMRAKDKGATLIHVDPRYTRTSTKCDLYAPLRSGSDIAFLNGMTKYILEKELYFKDYVVNYTNASFIV----G  target    ERLSARD-VFEGYRQAPLENYVALKTEEELAAPPSPPFTADKQVVPTELREEWGDFVYWDRATNGPAAVNRDEIGAKFAG 1h0h.1    EGFAFEEGLFAGYNK-----------------------------------------------------------------  target    DPALLGAFDVTLVDGTNVKARTAFSLLKEYLDENFDVQTTSEVCNVDPAAVRSLARQLAANKGNALLAAGMGPNHYFNAD 1h0h.1    --------------------------------------------------------------------------------  target    LFGRVHFLVAALTDNIGHFSGNVGSYAGNYRGSLFQAMGQWIAENPFDQEADLTKPARVKRYFKSESAHYWNYGDRPLVS 1h0h.1    --------------------------------------------------------------------------------  target    PSEIITGKSHMPTPTKLIWFGNSNSLLGNAKWSFDVVKNTLPKQDAVFCNEWHWTSSCEYSDLVFPADSWAEFKLPDMTA 1h0h.1    --------------------------------------------------------------------------------  target    SCTNPFLLAFPKTPLARIHNTRSDYEILAGVAAALADLVDEPRMKTYWKGILDGDPTPYLQRVLSGSNATRGILYEDLHA 1h0h.1    --------------------------------------------------------------------------------  target    SSAKGVPLLMNARTYPRHAGWEQRQEDKPWYTPTGRLEFYRPEPEWQAAGESLPIWREPVDATFYEPNAILANSKHPSIN 1h0h.1    --------------------------------------------------------------------------------  target    PRAPEDYGVPESQMDVETRQYRNVVRTWQELKLSKHPLTEKDPAYRFVFQTPKYRWGAHSTAVDSDWIAMLFGPFGDPYR 1h0h.1    --------------------------------------------------------------------------------  target    RDSRTPWTGEAYAEINPRDAKELGLKDGDYIWLDADPEDRPYRGADSSDEFYDVARAMMRVRIYSGMPRRVIRTWFNMYA 1h0h.1    --------------------------------------------------------------------------------  target    ATPGTVQAQKDVPGGPAQNQDTGYVALFRHGSHQSGTRAYLRPTQMTDSMNRKAYFGQTIGKGFEADVHSPSGAPKEGYV 1h0h.1    --------------------------------------------------------------------------------  target    KVEKAEDGGDEGVGEWRPVTLGLRPDDPSEAMQAYLAGEFVTRKRKGS 1h0h.1    ------------------------------------------------ ``` | | | | | | | | | | | | | | | | | | | | | | | | | | | | | | | | | | | | | | | | | | | | | | | | | |
|  | 7q5y.1.A | NADH dehydrogenase I chain G  *Structure of NADH:ubichinon oxidoreductase (complex I) of the hyperthermophilic eubacterium Aquifex aeolicus* | 0.03 |  | 10.53 | 0.08 | 652-757 | X-ray | 2.70 | hetero-1-1-1-1-1-1-… | 8 x SF4, 2 x FES, 1 x FMN | HHblits | 0.24 |
| ``` target    SDLSRRELLKRAVVVGTGAGLAELFLPAQFLSSASAQSEPQAVAIANPLAQMPDRSWERIYRDQFAEEDSFVFTCAPNDT 7q5y.1    --------------------------------------------------------------------------------  target    HNCLLRAHVKNGVIVRISPTYGYGKATDLAGNQASHRWDPRICQKGLILGRRIYGDRRVKAPMIRKGFKEWADAGFPRHD 7q5y.1    --------------------------------------------------------------------------------  target    DGTPRADMEKRGYDEWLQIPWDEALAIAAKTLQNVAETYKGEDGAGKLLEQGYEPAMVEAMHGAGVQAIKMRGGMPLLGA 7q5y.1    --------------------------------------------------------------------------------  target    GRVFGFYRFANMLALLDGKLRPEAPPEEIVGSRAFDNYAWHTDLPPGHPMVSGSQTVDFDLFAAEHSKLLVLIGMNWICT 7q5y.1    --------------------------------------------------------------------------------  target    KMPDAHWIGDARLKGTRVVVISADYMPTANKADEIVILRPGTDTAFLLGVARELITKKLYDRDAVIQRTDLPLLVRLDTG 7q5y.1    --------------------------------------------------------------------------------  target    ERLSARDVFEGYRQAPLENYVALKTEEELAAPPSPPFTADKQVVPTELREEWGDFVYWDRATNGPAAVNRDEIGAKFAGD 7q5y.1    --------------------------------------------------------------------------------  target    PALLGAFDVTLVDGTNVKARTAFSLLKEYLDENFDVQTTSEVCNVDPAAVRSLARQLAANKGNALLAAGMGPNHYFNADL 7q5y.1    --------------------------------------------------------------------------------  target    FGRVHFLVAALTDNIGHFSGNVGSYAGNYRGSLFQAMGQWIAENPFDQEADLTKPARVKRYFKSESAHYWNYGDRPLVSP 7q5y.1    --------------------------------------------------------------------------------  target    SEIITGKSHMPTPTKLIWFGNSNSLLGNAKWSFDVVKNTLPKQDAVFCNEWHWTSSCEYSDLVFPADSWAEFKLPDMTAS 7q5y.1    -----------GDIENLIIFGEDILEFYED---KVFEELKEKLEHLVVVSPYEDGLSEYAHIKIPMSLMGENEG---TYK  target    CTNPFLLAFPKTPLARIHNTRSDYEILAGVAAALADLVDEPRMKTYWKGILDGDPTPYLQRVLSGSNATRGILYEDLHAS 7q5y.1    TFFGEVK--GKK-FLP--WAFDDLAFWKYLGENFKEE-------------------------------------------  target    SAKGVPLLMNARTYPRHAGWEQRQEDKPWYTPTGRLEFYRPEPEWQAAGESLPIWREPVDATFYEPNAILANSKHPSINP 7q5y.1    --------------------------------------------------------------------------------  target    RAPEDYGVPESQMDVETRQYRNVVRTWQELKLSKHPLTEKDPAYRFVFQTPKYRWGAHSTAVDSDWIAMLFGPFGDPYRR 7q5y.1    --------------------------------------------------------------------------------  target    DSRTPWTGEAYAEINPRDAKELGLKDGDYIWLDADPEDRPYRGADSSDEFYDVARAMMRVRIYSGMPRRVIRTWFNMYAA 7q5y.1    --------------------------------------------------------------------------------  target    TPGTVQAQKDVPGGPAQNQDTGYVALFRHGSHQSGTRAYLRPTQMTDSMNRKAYFGQTIGKGFEADVHSPSGAPKEGYVK 7q5y.1    --------------------------------------------------------------------------------  target    VEKAEDGGDEGVGEWRPVTLGLRPDDPSEAMQAYLAGEFVTRKRKGS 7q5y.1    ----------------------------------------------- ``` | | | | | | | | | | | | | | | | | | | | | | | | | | | | | | | | | | | | | | | | | | | | | | | | | |
|  | 6s6y.1.B | Tungsten-containing formylmethanofuran dehydrogenase, subunit B  *X-ray crystal structure of the formyltransferase/hydrolase complex (FhcABCD) from Methylorubrum extorquens in complex with methylofuran* | 0.03 |  | 8.70 | 0.08 | 652-756 | X-ray | 3.10 | hetero-2-2-2-2-mer | 1 x MFN, 4 x ZN, 4 x CA, 4 x K, 3 x DGL, 2 x GLU, 1 x IAS | HHblits | 0.23 |
| ``` target    SDLSRRELLKRAVVVGTGAGLAELFLPAQFLSSASAQSEPQAVAIANPLAQMPDRSWERIYRDQFAEEDSFVFTCAPNDT 6s6y.1    --------------------------------------------------------------------------------  target    HNCLLRAHVKNGVIVRISPTYGYGKATDLAGNQASHRWDPRICQKGLILGRRIYGDRRVKAPMIRKGFKEWADAGFPRHD 6s6y.1    --------------------------------------------------------------------------------  target    DGTPRADMEKRGYDEWLQIPWDEALAIAAKTLQNVAETYKGEDGAGKLLEQGYEPAMVEAMHGAGVQAIKMRGGMPLLGA 6s6y.1    --------------------------------------------------------------------------------  target    GRVFGFYRFANMLALLDGKLRPEAPPEEIVGSRAFDNYAWHTDLPPGHPMVSGSQTVDFDLFAAEHSKLLVLIGMNWICT 6s6y.1    --------------------------------------------------------------------------------  target    KMPDAHWIGDARLKGTRVVVISADYMPTANKADEIVILRPGTDTAFLLGVARELITKKLYDRDAVIQRTDLPLLVRLDTG 6s6y.1    --------------------------------------------------------------------------------  target    ERLSARDVFEGYRQAPLENYVALKTEEELAAPPSPPFTADKQVVPTELREEWGDFVYWDRATNGPAAVNRDEIGAKFAGD 6s6y.1    --------------------------------------------------------------------------------  target    PALLGAFDVTLVDGTNVKARTAFSLLKEYLDENFDVQTTSEVCNVDPAAVRSLARQLAANKGNALLAAGMGPNHYFNADL 6s6y.1    --------------------------------------------------------------------------------  target    FGRVHFLVAALTDNIGHFSGNVGSYAGNYRGSLFQAMGQWIAENPFDQEADLTKPARVKRYFKSESAHYWNYGDRPLVSP 6s6y.1    --------------------------------------------------------------------------------  target    SEIITGKSHMPTPTKLIWFGNSNSLLGNAKWSFDVVKNTLPKQDAVFCNE-WHWTSSCEYSDLVFPADSW-AEFKLPDMT 6s6y.1    -----------GEADAALWLASLPAP---------RPAWLGSLPTIAIVGEGSQEAAGETAEVVITVGVPGQSVGG---A  target    ASCTNPFLLAFPKTPLARI---HNTRSDYEILAGVAAALADLVDEPRMKTYWKGILDGDPTPYLQRVLSGSNATRGILYE 6s6y.1    LWNDRRGVIAYAE-ASDPAKTPAETETAAGVLTRIRDRLIE---------------------------------------  target    DLHASSAKGVPLLMNARTYPRHAGWEQRQEDKPWYTPTGRLEFYRPEPEWQAAGESLPIWREPVDATFYEPNAILANSKH 6s6y.1    --------------------------------------------------------------------------------  target    PSINPRAPEDYGVPESQMDVETRQYRNVVRTWQELKLSKHPLTEKDPAYRFVFQTPKYRWGAHSTAVDSDWIAMLFGPFG 6s6y.1    --------------------------------------------------------------------------------  target    DPYRRDSRTPWTGEAYAEINPRDAKELGLKDGDYIWLDADPEDRPYRGADSSDEFYDVARAMMRVRIYSGMPRRVIRTWF 6s6y.1    --------------------------------------------------------------------------------  target    NMYAATPGTVQAQKDVPGGPAQNQDTGYVALFRHGSHQSGTRAYLRPTQMTDSMNRKAYFGQTIGKGFEADVHSPSGAPK 6s6y.1    --------------------------------------------------------------------------------  target    EGYVKVEKAEDGGDEGVGEWRPVTLGLRPDDPSEAMQAYLAGEFVTRKRKGS 6s6y.1    ---------------------------------------------------- ``` | | | | | | | | | | | | | | | | | | | | | | | | | | | | | | | | | | | | | | | | | | | | | | | | | |
|  | 5t5i.1.D | Tungsten formylmethanofuran dehydrogenase subunit fwdD  *TUNGSTEN-CONTAINING FORMYLMETHANOFURAN DEHYDROGENASE FROM METHANOTHERMOBACTER WOLFEII, ORTHORHOMBIC FORM AT 1.9 A* | 0.01 |  | 16.25 | 0.07 | 925-1037 | X-ray | 1.90 | hetero-oligomer | 4 x ZN, 2 x MG, 18 x K, 22 x SF4, 2 x W, 4 x MGD, 2 x H2S, 2 x CA | HHblits | 0.31 |
| ``` target    SDLSRRELLKRAVVVGTGAGLAELFLPAQFLSSASAQSEPQAVAIANPLAQMPDRSWERIYRDQFAEEDSFVFTCAPNDT 5t5i.1    --------------------------------------------------------------------------------  target    HNCLLRAHVKNGVIVRISPTYGYGKATDLAGNQASHRWDPRICQKGLILGRRIYGDRRVKAPMIRKGFKEWADAGFPRHD 5t5i.1    --------------------------------------------------------------------------------  target    DGTPRADMEKRGYDEWLQIPWDEALAIAAKTLQNVAETYKGEDGAGKLLEQGYEPAMVEAMHGAGVQAIKMRGGMPLLGA 5t5i.1    --------------------------------------------------------------------------------  target    GRVFGFYRFANMLALLDGKLRPEAPPEEIVGSRAFDNYAWHTDLPPGHPMVSGSQTVDFDLFAAEHSKLLVLIGMNWICT 5t5i.1    --------------------------------------------------------------------------------  target    KMPDAHWIGDARLKGTRVVVISADYMPTANKADEIVILRPGTDTAFLLGVARELITKKLYDRDAVIQRTDLPLLVRLDTG 5t5i.1    --------------------------------------------------------------------------------  target    ERLSARDVFEGYRQAPLENYVALKTEEELAAPPSPPFTADKQVVPTELREEWGDFVYWDRATNGPAAVNRDEIGAKFAGD 5t5i.1    --------------------------------------------------------------------------------  target    PALLGAFDVTLVDGTNVKARTAFSLLKEYLDENFDVQTTSEVCNVDPAAVRSLARQLAANKGNALLAAGMGPNHYFNADL 5t5i.1    --------------------------------------------------------------------------------  target    FGRVHFLVAALTDNIGHFSGNVGSYAGNYRGSLFQAMGQWIAENPFDQEADLTKPARVKRYFKSESAHYWNYGDRPLVSP 5t5i.1    --------------------------------------------------------------------------------  target    SEIITGKSHMPTPTKLIWFGNSNSLLGNAKWSFDVVKNTLPKQDAVFCNEWHWTSSCEYSDLVFPADSWAEFKLPDMTAS 5t5i.1    --------------------------------------------------------------------------------  target    CTNPFLLAFPKTPLARIHNTRSDYEILAGVAAALADLVDEPRMKTYWKGILDGDPTPYLQRVLSGSNATRGILYEDLHAS 5t5i.1    --------------------------------------------------------------------------------  target    SAKGVPLLMNARTYPRHAGWEQRQEDKPWYTPTGRLEFYRPEPEWQAAGESLPIWREPVDATFYEPNAILANSKHPSINP 5t5i.1    --------------------------------------------------------------------------------  target    RAPEDYGVPESQMDVETRQYRNVVRTWQELKLSKHPLTEKDPAYRFVFQTPKYRWGAHSTAVDSDWIAMLFGPFGDPYRR 5t5i.1    --------------------------------------------RVILNTGRTIWQGQAIESGKDLKMYV----------  target    DSRTPWTGEAYAEINPRDAKELGLKDGDYIWLDADPEDRPYRGADSSDEFYDVARAMMRVR-IYSGMPRRVIRTWFNMYA 5t5i.1    ------DAAAIIQMNPEMMKQLGIAEGDNVKVISE-----------------YGDVVVKAVEAKEPLPEGMVYIPMGP--  target    ATPGTVQAQKDVPGGPAQNQDTGYVALFRHGSHQSGTRAYLRPTQMTDSMNRKAYFGQTIGKGFEADVHSPSGAPKEGYV 5t5i.1    --------------------------------------------------------------------------------  target    KVEKAEDGGDEGVGEWRPVTLGLRPDDPSEAMQAYLAGEFVTRKRKGS 5t5i.1    ------------------------------------------------ ``` | | | | | | | | | | | | | | | | | | | | | | | | | | | | | | | | | | | | | | | | | | | | | | | | | |
|  | 3o5a.1.A | Periplasmic nitrate reductase  *Crystal Structure of partially reduced Periplasmic Nitrate Reductase from Cupriavidus necator using Ionic Liquids* | 0.01 |  | 20.99 | 0.07 | 922-1035 | X-ray | 1.72 | hetero-oligomer | 1 x SF4, 1 x MOS, 2 x MGD, 2 x HEC | HHblits | 0.29 |
| ``` target    SDLSRRELLKRAVVVGTGAGLAELFLPAQFLSSASAQSEPQAVAIANPLAQMPDRSWERIYRDQFAEEDSFVFTCAPNDT 3o5a.1    --------------------------------------------------------------------------------  target    HNCLLRAHVKNGVIVRISPTYGYGKATDLAGNQASHRWDPRICQKGLILGRRIYGDRRVKAPMIRKGFKEWADAGFPRHD 3o5a.1    --------------------------------------------------------------------------------  target    DGTPRADMEKRGYDEWLQIPWDEALAIAAKTLQNVAETYKGEDGAGKLLEQGYEPAMVEAMHGAGVQAIKMRGGMPLLGA 3o5a.1    --------------------------------------------------------------------------------  target    GRVFGFYRFANMLALLDGKLRPEAPPEEIVGSRAFDNYAWHTDLPPGHPMVSGSQTVDFDLFAAEHSKLLVLIGMNWICT 3o5a.1    --------------------------------------------------------------------------------  target    KMPDAHWIGDARLKGTRVVVISADYMPTANKADEIVILRPGTDTAFLLGVARELITKKLYDRDAVIQRTDLPLLVRLDTG 3o5a.1    --------------------------------------------------------------------------------  target    ERLSARDVFEGYRQAPLENYVALKTEEELAAPPSPPFTADKQVVPTELREEWGDFVYWDRATNGPAAVNRDEIGAKFAGD 3o5a.1    --------------------------------------------------------------------------------  target    PALLGAFDVTLVDGTNVKARTAFSLLKEYLDENFDVQTTSEVCNVDPAAVRSLARQLAANKGNALLAAGMGPNHYFNADL 3o5a.1    --------------------------------------------------------------------------------  target    FGRVHFLVAALTDNIGHFSGNVGSYAGNYRGSLFQAMGQWIAENPFDQEADLTKPARVKRYFKSESAHYWNYGDRPLVSP 3o5a.1    --------------------------------------------------------------------------------  target    SEIITGKSHMPTPTKLIWFGNSNSLLGNAKWSFDVVKNTLPKQDAVFCNEWHWTSSCEYSDLVFPADSWAEFKLPDMTAS 3o5a.1    --------------------------------------------------------------------------------  target    CTNPFLLAFPKTPLARIHNTRSDYEILAGVAAALADLVDEPRMKTYWKGILDGDPTPYLQRVLSGSNATRGILYEDLHAS 3o5a.1    --------------------------------------------------------------------------------  target    SAKGVPLLMNARTYPRHAGWEQRQEDKPWYTPTGRLEFYRPEPEWQAAGESLPIWREPVDATFYEPNAILANSKHPSINP 3o5a.1    --------------------------------------------------------------------------------  target    RAPEDYGVPESQMDVETRQYRNVVRTWQELKLSKHPLTEKDPAYRFVFQTPKYRWGAHST--AVDSDWIAMLFGPFGDPY 3o5a.1    -----------------------------------------KEYPYWLVTGRVLEHWHSGSMTRRVPELYRS--------  target    RRDSRTPWTGEAYAEINPRDAKELGLKDGDYIWLDADPEDRPYRGADSSDEFYDVARAMMRVRIY--SGMPRRVIRTWFN 3o5a.1    --------FPNAVVFMHPEDAKALGLRRGVEVEVVSR-----------------RGRMRSRIETRGRDAPPRGLVFVPW-  target    MYAATPGTVQAQKDVPGGPAQNQDTGYVALFRHGSHQSGTRAYLRPTQMTDSMNRKAYFGQTIGKGFEADVHSPSGAPKE 3o5a.1    --------------------------------------------------------------------------------  target    GYVKVEKAEDGGDEGVGEWRPVTLGLRPDDPSEAMQAYLAGEFVTRKRKGS 3o5a.1    --------------------------------------------------- ``` | | | | | | | | | | | | | | | | | | | | | | | | | | | | | | | | | | | | | | | | | | | | | | | | | |
|  | 1ogy.1.A | PERIPLASMIC NITRATE REDUCTASE  *Crystal structure of the heterodimeric nitrate reductase from Rhodobacter sphaeroides* | 0.01 |  | 19.75 | 0.07 | 922-1035 | X-ray | 3.20 | hetero-1-1-mer | 1 x SF4, 1 x MO, 2 x MGD, 2 x HEC | HHblits | 0.29 |
| ``` target    SDLSRRELLKRAVVVGTGAGLAELFLPAQFLSSASAQSEPQAVAIANPLAQMPDRSWERIYRDQFAEEDSFVFTCAPNDT 1ogy.1    --------------------------------------------------------------------------------  target    HNCLLRAHVKNGVIVRISPTYGYGKATDLAGNQASHRWDPRICQKGLILGRRIYGDRRVKAPMIRKGFKEWADAGFPRHD 1ogy.1    --------------------------------------------------------------------------------  target    DGTPRADMEKRGYDEWLQIPWDEALAIAAKTLQNVAETYKGEDGAGKLLEQGYEPAMVEAMHGAGVQAIKMRGGMPLLGA 1ogy.1    --------------------------------------------------------------------------------  target    GRVFGFYRFANMLALLDGKLRPEAPPEEIVGSRAFDNYAWHTDLPPGHPMVSGSQTVDFDLFAAEHSKLLVLIGMNWICT 1ogy.1    --------------------------------------------------------------------------------  target    KMPDAHWIGDARLKGTRVVVISADYMPTANKADEIVILRPGTDTAFLLGVARELITKKLYDRDAVIQRTDLPLLVRLDTG 1ogy.1    --------------------------------------------------------------------------------  target    ERLSARDVFEGYRQAPLENYVALKTEEELAAPPSPPFTADKQVVPTELREEWGDFVYWDRATNGPAAVNRDEIGAKFAGD 1ogy.1    --------------------------------------------------------------------------------  target    PALLGAFDVTLVDGTNVKARTAFSLLKEYLDENFDVQTTSEVCNVDPAAVRSLARQLAANKGNALLAAGMGPNHYFNADL 1ogy.1    --------------------------------------------------------------------------------  target    FGRVHFLVAALTDNIGHFSGNVGSYAGNYRGSLFQAMGQWIAENPFDQEADLTKPARVKRYFKSESAHYWNYGDRPLVSP 1ogy.1    --------------------------------------------------------------------------------  target    SEIITGKSHMPTPTKLIWFGNSNSLLGNAKWSFDVVKNTLPKQDAVFCNEWHWTSSCEYSDLVFPADSWAEFKLPDMTAS 1ogy.1    --------------------------------------------------------------------------------  target    CTNPFLLAFPKTPLARIHNTRSDYEILAGVAAALADLVDEPRMKTYWKGILDGDPTPYLQRVLSGSNATRGILYEDLHAS 1ogy.1    --------------------------------------------------------------------------------  target    SAKGVPLLMNARTYPRHAGWEQRQEDKPWYTPTGRLEFYRPEPEWQAAGESLPIWREPVDATFYEPNAILANSKHPSINP 1ogy.1    --------------------------------------------------------------------------------  target    RAPEDYGVPESQMDVETRQYRNVVRTWQELKLSKHPLTEKDPAYRFVFQTPKYRWGAHSTAV--DSDWIAMLFGPFGDPY 1ogy.1    -----------------------------------------EEFGFWLVTGRVLEHWHSGSMTLRWPELYKA--------  target    RRDSRTPWTGEAYAEINPRDAKELGLKDGDYIWLDADPEDRPYRGADSSDEFYDVARAMMRVRI--YSGMPRRVIRTWFN 1ogy.1    --------FPGAVCFMHPEDARSRGLNRGSEVRVISR-----------------RGEIRTRLETRGRNRMPRGVVFVPW-  target    MYAATPGTVQAQKDVPGGPAQNQDTGYVALFRHGSHQSGTRAYLRPTQMTDSMNRKAYFGQTIGKGFEADVHSPSGAPKE 1ogy.1    --------------------------------------------------------------------------------  target    GYVKVEKAEDGGDEGVGEWRPVTLGLRPDDPSEAMQAYLAGEFVTRKRKGS 1ogy.1    --------------------------------------------------- ``` | | | | | | | | | | | | | | | | | | | | | | | | | | | | | | | | | | | | | | | | | | | | | | | | | |
|  | 7bkb.1.J | Formylmethanofuran dehydrogenase, subunit D  *Formate dehydrogenase - heterodisulfide reductase - formylmethanofuran dehydrogenase complex from Methanospirillum hungatei (hexameric, composite structure)* | 0.01 |  | 9.64 | 0.07 | 922-1037 | EM | 0.00 | hetero-2-2-2-2-2-2-… | 48 x SF4, 4 x FAD, 2 x FES, 4 x 9S8, 4 x ZN, 2 x MO, 4 x MGD | HHblits | 0.26 |
| ``` target    SDLSRRELLKRAVVVGTGAGLAELFLPAQFLSSASAQSEPQAVAIANPLAQMPDRSWERIYRDQFAEEDSFVFTCAPNDT 7bkb.1    --------------------------------------------------------------------------------  target    HNCLLRAHVKNGVIVRISPTYGYGKATDLAGNQASHRWDPRICQKGLILGRRIYGDRRVKAPMIRKGFKEWADAGFPRHD 7bkb.1    --------------------------------------------------------------------------------  target    DGTPRADMEKRGYDEWLQIPWDEALAIAAKTLQNVAETYKGEDGAGKLLEQGYEPAMVEAMHGAGVQAIKMRGGMPLLGA 7bkb.1    --------------------------------------------------------------------------------  target    GRVFGFYRFANMLALLDGKLRPEAPPEEIVGSRAFDNYAWHTDLPPGHPMVSGSQTVDFDLFAAEHSKLLVLIGMNWICT 7bkb.1    --------------------------------------------------------------------------------  target    KMPDAHWIGDARLKGTRVVVISADYMPTANKADEIVILRPGTDTAFLLGVARELITKKLYDRDAVIQRTDLPLLVRLDTG 7bkb.1    --------------------------------------------------------------------------------  target    ERLSARDVFEGYRQAPLENYVALKTEEELAAPPSPPFTADKQVVPTELREEWGDFVYWDRATNGPAAVNRDEIGAKFAGD 7bkb.1    --------------------------------------------------------------------------------  target    PALLGAFDVTLVDGTNVKARTAFSLLKEYLDENFDVQTTSEVCNVDPAAVRSLARQLAANKGNALLAAGMGPNHYFNADL 7bkb.1    --------------------------------------------------------------------------------  target    FGRVHFLVAALTDNIGHFSGNVGSYAGNYRGSLFQAMGQWIAENPFDQEADLTKPARVKRYFKSESAHYWNYGDRPLVSP 7bkb.1    --------------------------------------------------------------------------------  target    SEIITGKSHMPTPTKLIWFGNSNSLLGNAKWSFDVVKNTLPKQDAVFCNEWHWTSSCEYSDLVFPADSWAEFKLPDMTAS 7bkb.1    --------------------------------------------------------------------------------  target    CTNPFLLAFPKTPLARIHNTRSDYEILAGVAAALADLVDEPRMKTYWKGILDGDPTPYLQRVLSGSNATRGILYEDLHAS 7bkb.1    --------------------------------------------------------------------------------  target    SAKGVPLLMNARTYPRHAGWEQRQEDKPWYTPTGRLEFYRPEPEWQAAGESLPIWREPVDATFYEPNAILANSKHPSINP 7bkb.1    --------------------------------------------------------------------------------  target    RAPEDYGVPESQMDVETRQYRNVVRTWQELKLSKHPLTEKDPAYRFVFQTPKYRWGAHSTAVDSDWIAMLFGPFGDPYRR 7bkb.1    -----------------------------------------AKKTLNMITQRAVEEGIAMEI-GKTSRQY----------  target    DSRTPWTGEAYAEINPRDAKELGLKDGDYIWLDADPEDRPYRGADSSDEFYDVARAMMRVRIY-SGMPRRVIRTWFNMYA 7bkb.1    -----FDACSIIEMNEQDMKELGIMKNTNVRVKSE-----------------SGEVVVKAVVGRQTCYPGLCHIRQGV--  target    ATPGTVQAQKDVPGGPAQNQDTGYVALFRHGSHQSGTRAYLRPTQMTDSMNRKAYFGQTIGKGFEADVHSPSGAPKEGYV 7bkb.1    --------------------------------------------------------------------------------  target    KVEKAEDGGDEGVGEWRPVTLGLRPDDPSEAMQAYLAGEFVTRKRKGS 7bkb.1    ------------------------------------------------ ``` | | | | | | | | | | | | | | | | | | | | | | | | | | | | | | | | | | | | | | | | | | | | | | | | | |
|  | 2ki8.1.A | Tungsten formylmethanofuran dehydrogenase, subunit D (FwdD-2)  *Solution NMR structure of tungsten formylmethanofuran dehydrogenase subunit D from Archaeoglobus fulgidus, Northeast Structural Genomics Consortium target AtT7* | 0.02 |  | 16.25 | 0.07 | 923-1037 | NMR | 0.00 | monomer |  | HHblits | 0.28 |
| ``` target    SDLSRRELLKRAVVVGTGAGLAELFLPAQFLSSASAQSEPQAVAIANPLAQMPDRSWERIYRDQFAEEDSFVFTCAPNDT 2ki8.1    --------------------------------------------------------------------------------  target    HNCLLRAHVKNGVIVRISPTYGYGKATDLAGNQASHRWDPRICQKGLILGRRIYGDRRVKAPMIRKGFKEWADAGFPRHD 2ki8.1    --------------------------------------------------------------------------------  target    DGTPRADMEKRGYDEWLQIPWDEALAIAAKTLQNVAETYKGEDGAGKLLEQGYEPAMVEAMHGAGVQAIKMRGGMPLLGA 2ki8.1    --------------------------------------------------------------------------------  target    GRVFGFYRFANMLALLDGKLRPEAPPEEIVGSRAFDNYAWHTDLPPGHPMVSGSQTVDFDLFAAEHSKLLVLIGMNWICT 2ki8.1    --------------------------------------------------------------------------------  target    KMPDAHWIGDARLKGTRVVVISADYMPTANKADEIVILRPGTDTAFLLGVARELITKKLYDRDAVIQRTDLPLLVRLDTG 2ki8.1    --------------------------------------------------------------------------------  target    ERLSARDVFEGYRQAPLENYVALKTEEELAAPPSPPFTADKQVVPTELREEWGDFVYWDRATNGPAAVNRDEIGAKFAGD 2ki8.1    --------------------------------------------------------------------------------  target    PALLGAFDVTLVDGTNVKARTAFSLLKEYLDENFDVQTTSEVCNVDPAAVRSLARQLAANKGNALLAAGMGPNHYFNADL 2ki8.1    --------------------------------------------------------------------------------  target    FGRVHFLVAALTDNIGHFSGNVGSYAGNYRGSLFQAMGQWIAENPFDQEADLTKPARVKRYFKSESAHYWNYGDRPLVSP 2ki8.1    --------------------------------------------------------------------------------  target    SEIITGKSHMPTPTKLIWFGNSNSLLGNAKWSFDVVKNTLPKQDAVFCNEWHWTSSCEYSDLVFPADSWAEFKLPDMTAS 2ki8.1    --------------------------------------------------------------------------------  target    CTNPFLLAFPKTPLARIHNTRSDYEILAGVAAALADLVDEPRMKTYWKGILDGDPTPYLQRVLSGSNATRGILYEDLHAS 2ki8.1    --------------------------------------------------------------------------------  target    SAKGVPLLMNARTYPRHAGWEQRQEDKPWYTPTGRLEFYRPEPEWQAAGESLPIWREPVDATFYEPNAILANSKHPSINP 2ki8.1    --------------------------------------------------------------------------------  target    RAPEDYGVPESQMDVETRQYRNVVRTWQELKLSKHPLTEKDPAYRFVFQTPKYRWGAHSTAVDSDWIAMLFGPFGDPYRR 2ki8.1    ------------------------------------------MLEVEVISGRTLNQGATVE-EKLTEEY-----------  target    DSRTPWTGEAYAEINPRDAKELGLKDGDYIWLDADPEDRPYRGADSSDEFYDVARAMMRVRIYSGMPRRVIRTWFNMYAA 2ki8.1    -----FNAVNYAEINEEDWNALGLQEGDRVKVKTE-----------------FGEVVVFAKKG-DVPKGMIFIPMGP---  target    TPGTVQAQKDVPGGPAQNQDTGYVALFRHGSHQSGTRAYLRPTQMTDSMNRKAYFGQTIGKGFEADVHSPSGAPKEGYVK 2ki8.1    --------------------------------------------------------------------------------  target    VEKAEDGGDEGVGEWRPVTLGLRPDDPSEAMQAYLAGEFVTRKRKGS 2ki8.1    ----------------------------------------------- ``` | | | | | | | | | | | | | | | | | | | | | | | | | | | | | | | | | | | | | | | | | | | | | | | | | |
|  | 1h0h.1.A | FORMATE DEHYDROGENASE SUBUNIT ALPHA  *Tungsten containing Formate Dehydrogenase from Desulfovibrio Gigas* | 0.01 |  | 19.48 | 0.07 | 922-1031 | X-ray | 1.80 | hetero-1-1-mer | 1 x W, 1 x 2MD, 1 x MGD, 4 x SF4, 1 x CA | HHblits | 0.31 |
[truncated: 1,172,946 more chars]
