# Supplementary material for: Metabolic capacity is maintained despite shifts in microbial diversity in estuary sediments
Source: ISME Commun. 2025 Oct 11;5(1):ycaf182. doi: 10.1093/ismeco/ycaf182 (PMC12687941; doi:10.1093/ismeco/ycaf182)
Supplement: Supplementary_Data_1_ycaf182 [file supplementary_data_1_ycaf182.zip › SWISS-MODEL/4_1_May_SF_Bin55_scaffold_648_c1_10064512_1/models.html]

4\_1\_May\_SF\_Bin55\_scaffold\_648\_c1\_1006-4512\_1 | Models


**Export Alignment**
  
FASTA format
Clustal Format
PNG Image

**Secondary Structure**
  
None
DSSP
PSIPRED
SSpro

**Colour Scheme** 


Fade Mismatches
Enhance Mismatches

Confidencegradient
Confidenceclass
Indels
Chain
Unique Chain
Rainbow
2° Structure
Clustal
Hydrophobic
Size
Charged
Polar
Proline
Ser/Thr
Cysteine
Aliphatic
Aromatic
No Colour

Use QMEANBrane values

|  |  |  |  |
| --- | --- | --- | --- |
| Background |  |  |  |

**3D Viewer**  
NGL
PV

FASTA
Multi FASTA
ClustalW
PNG


SWISS-MODEL

### 4\_1\_May\_SF\_Bin55\_scaffold\_648\_c1\_1006-4512\_1

### Created: March 29, 2023, 8:51 p.m. at 20:51

- Templates
- Models

Order by:
GMQE
QMEANDisCo
Oligo State
Ligands
Seq Identity
Similarity
Coverage

Model 01

- PDB Format *(Display)*
- JSON Format *(Display)*
- Model Report *(Display)*

Oligo-State
:   Monomer

GMQE
:   0.70

QMEANDisCo Global:
:   0.71  ± 0.05

Ligands
:   1 x MO


    1 x MOLYBDENUM ATOM

    |  |  |
    | --- | --- |
    | MO.7: | 4 residues within 4Å:   - Chain B:   H.281,   D.283,   G.587,   K.932  Ligand excluded by PLIP |

QMEANDisCo Local

QMEAN Z-Scores

Template

7b04.1.B

Nitrite oxidoreductase subunit A  
Structure of Nitrite oxidoreductase (Nxr) from the anammox bacterium Kuenenia stuttgartiensis.

Seq Identity
:   40.77%

Coverage

|  |  |
| --- | --- |
| **Biounit Oligo State** | Hetero-trimer |
| **QSQE** | 0.00 |
| **Method** | X-ray, 2.97 Å |
| **Seq Similarity** | 0.41 |
| **Coverage** | 0.96 |
| **Range** | 28-1160 |

| Ligand | Added to Model | Description |
| --- | --- | --- |
| MO | ✓ | MOLYBDENUM ATOM |
| CA | ✕ - Binding site not conserved. | CALCIUM ION |
| CA | ✕ - Binding site not conserved. | CALCIUM ION |
| F3S | ✕ - Binding site not conserved. | FE3-S4 CLUSTER |
| HEM | ✕ - Binding site not conserved. | PROTOPORPHYRIN IX CONTAINING FE |
| MD1 | ✕ - Binding site not conserved. | PHOSPHORIC ACID 4-(2-AMINO-4-OXO-3,4,5,6,-TETRAHYDRO-PTERIDIN-6-YL)-2-HYDROXY-3,4-DIMERCAPTO-BUT-3-EN-YL ESTER GUANYLATE ESTER |
| MD1 | ✕ - Binding site not conserved. | PHOSPHORIC ACID 4-(2-AMINO-4-OXO-3,4,5,6,-TETRAHYDRO-PTERIDIN-6-YL)-2-HYDROXY-3,4-DIMERCAPTO-BUT-3-EN-YL ESTER GUANYLATE ESTER |
| SF4 | ✕ - Binding site not conserved. | IRON/SULFUR CLUSTER |
| SF4 | ✕ - Binding site not conserved. | IRON/SULFUR CLUSTER |
| SF4 | ✕ - Binding site not conserved. | IRON/SULFUR CLUSTER |
| SF4 | ✕ - Binding site not conserved. | IRON/SULFUR CLUSTER |

Model-Template Alignment

|  |  |  |
| --- | --- | --- |
|  |  |  |

Model 02

- PDB Format *(Display)*
- JSON Format *(Display)*
- Model Report *(Display)*

Oligo-State
:   Monomer

GMQE
:   0.41

QMEANDisCo Global:
:   0.51  ± 0.05

Ligands

QMEANDisCo Local

QMEAN Z-Scores

Template

3ir5.1.A

Respiratory nitrate reductase 1 alpha chain  
Crystal structure of NarGHI mutant NarG-H49C

Seq Identity
:   24.12%

Coverage

|  |  |
| --- | --- |
| **Biounit Oligo State** | Hetero-trimer |
| **QSQE** | 0.00 |
| **Method** | X-ray, 2.30 Å |
| **Seq Similarity** | 0.32 |
| **Coverage** | 0.78 |
| **Range** | 54-1130 |

| Ligand | Added to Model | Description |
| --- | --- | --- |
| 6MO | ✕ - Clashing with protein. | MOLYBDENUM(VI) ION |
| AGA | ✕ - Binding site not conserved. | (1S)-2-{[{[(2S)-2,3-DIHYDROXYPROPYL]OXY}(HYDROXY)PHOSPHORYL]OXY}-1-[(PENTANOYLOXY)METHYL]ETHYL OCTANOATE |
| F3S | ✕ - Binding site not conserved. | FE3-S4 CLUSTER |
| HEM | ✕ - Binding site not conserved. | PROTOPORPHYRIN IX CONTAINING FE |
| HEM | ✕ - Binding site not conserved. | PROTOPORPHYRIN IX CONTAINING FE |
| MD1 | ✕ - Binding site not conserved. | PHOSPHORIC ACID 4-(2-AMINO-4-OXO-3,4,5,6,-TETRAHYDRO-PTERIDIN-6-YL)-2-HYDROXY-3,4-DIMERCAPTO-BUT-3-EN-YL ESTER GUANYLATE ESTER |
| MD1 | ✕ - Binding site not conserved. | PHOSPHORIC ACID 4-(2-AMINO-4-OXO-3,4,5,6,-TETRAHYDRO-PTERIDIN-6-YL)-2-HYDROXY-3,4-DIMERCAPTO-BUT-3-EN-YL ESTER GUANYLATE ESTER |
| SF4 | ✕ - Binding site not conserved. | IRON/SULFUR CLUSTER |
| SF4 | ✕ - Binding site not conserved. | IRON/SULFUR CLUSTER |
| SF4 | ✕ - Binding site not conserved. | IRON/SULFUR CLUSTER |
| SF4 | ✕ - Binding site not conserved. | IRON/SULFUR CLUSTER |

Model-Template Alignment

|  |  |  |
| --- | --- | --- |
|  |  |  |

Model 03

- PDB Format *(Display)*
- JSON Format *(Display)*
- Model Report *(Display)*

Oligo-State
:   Monomer

GMQE
:   0.27

QMEANDisCo Global:
:   0.47  ± 0.05

Ligands

QMEANDisCo Local

QMEAN Z-Scores

Template

1h0h.1.A

FORMATE DEHYDROGENASE SUBUNIT ALPHA  
Tungsten containing Formate Dehydrogenase from Desulfovibrio Gigas

Seq Identity
:   17.09%

Coverage

|  |  |
| --- | --- |
| **Biounit Oligo State** | Hetero-dimer |
| **QSQE** | 0.00 |
| **Method** | X-ray, 1.80 Å |
| **Seq Similarity** | 0.28 |
| **Coverage** | 0.47 |
| **Range** | 69-756 |

| Ligand | Added to Model | Description |
| --- | --- | --- |
| 2MD | ✕ - Binding site not conserved. | GUANYLATE-O'-PHOSPHORIC ACID MONO-(2-AMINO-5,6-DIMERCAPTO-4-OXO-3,5,6,7,8A,9,10,10A-OCTAHYDRO-4H-8-OXA-1,3,9,10-TETRAAZA-ANTHRACEN-7-YLMETHYL) ESTER |
| CA | ✕ - Binding site not conserved. | CALCIUM ION |
| EPE | ✕ - Not biologically relevant. | 4-(2-HYDROXYETHYL)-1-PIPERAZINE ETHANESULFONIC ACID |
| MGD | ✕ - Binding site not conserved. | 2-AMINO-5,6-DIMERCAPTO-7-METHYL-3,7,8A,9-TETRAHYDRO-8-OXA-1,3,9,10-TETRAAZA-ANTHRACEN-4-ONE GUANOSINE DINUCLEOTIDE |
| SF4 | ✕ - Binding site not conserved. | IRON/SULFUR CLUSTER |
| SF4 | ✕ - Binding site not conserved. | IRON/SULFUR CLUSTER |
| SF4 | ✕ - Binding site not conserved. | IRON/SULFUR CLUSTER |
| SF4 | ✕ - Binding site not conserved. | IRON/SULFUR CLUSTER |
| UNX | ✕ - Not biologically relevant. | UNKNOWN ATOM OR ION |
| W | ✕ - Not in contact with model. | TUNGSTEN ION |

Model-Template Alignment

|  |  |  |
| --- | --- | --- |
|  |  |  |

Model 04

- PDB Format *(Display)*
- JSON Format *(Display)*
- Model Report *(Display)*

Oligo-State
:   Monomer

GMQE
:   0.14

QMEANDisCo Global:
:   0.44  ± 0.05

Ligands

QMEANDisCo Local

QMEAN Z-Scores

Template

3ir7.1.A

Respiratory nitrate reductase 1 alpha chain  
Crystal structure of NarGHI mutant NarG-R94S

Seq Identity
:   30.59%

Coverage

|  |  |
| --- | --- |
| **Biounit Oligo State** | Hetero-trimer |
| **QSQE** | 0.00 |
| **Method** | X-ray, 2.50 Å |
| **Seq Similarity** | 0.35 |
| **Coverage** | 0.29 |
| **Range** | 54-432 |

| Ligand | Added to Model | Description |
| --- | --- | --- |
| 6MO | ✕ - Binding site not conserved. | MOLYBDENUM(VI) ION |
| AGA | ✕ - Binding site not conserved. | (1S)-2-{[{[(2S)-2,3-DIHYDROXYPROPYL]OXY}(HYDROXY)PHOSPHORYL]OXY}-1-[(PENTANOYLOXY)METHYL]ETHYL OCTANOATE |
| F3S | ✕ - Binding site not conserved. | FE3-S4 CLUSTER |
| HEM | ✕ - Binding site not conserved. | PROTOPORPHYRIN IX CONTAINING FE |
| HEM | ✕ - Binding site not conserved. | PROTOPORPHYRIN IX CONTAINING FE |
| MD1 | ✕ - Binding site not conserved. | PHOSPHORIC ACID 4-(2-AMINO-4-OXO-3,4,5,6,-TETRAHYDRO-PTERIDIN-6-YL)-2-HYDROXY-3,4-DIMERCAPTO-BUT-3-EN-YL ESTER GUANYLATE ESTER |
| MD1 | ✕ - Binding site not conserved. | PHOSPHORIC ACID 4-(2-AMINO-4-OXO-3,4,5,6,-TETRAHYDRO-PTERIDIN-6-YL)-2-HYDROXY-3,4-DIMERCAPTO-BUT-3-EN-YL ESTER GUANYLATE ESTER |
| SF4 | ✕ - Binding site not conserved. | IRON/SULFUR CLUSTER |
| SF4 | ✕ - Binding site not conserved. | IRON/SULFUR CLUSTER |
| SF4 | ✕ - Binding site not conserved. | IRON/SULFUR CLUSTER |
| SF4 | ✕ - Binding site not conserved. | IRON/SULFUR CLUSTER |

Model-Template Alignment

|  |  |  |
| --- | --- | --- |
|  |  |  |

Model 05

- PDB Format *(Display)*
- JSON Format *(Display)*
- Model Report *(Display)*

Oligo-State
:   Monomer

GMQE
:   0.11

QMEANDisCo Global:
:   0.38  ± 0.05

Ligands

QMEANDisCo Local

QMEAN Z-Scores

Template

6sdv.1.A

Formate dehydrogenase, alpha subunit, selenocysteine-containing,Formate dehydrogenase, alpha subunit, selenocysteine-containing,W-formate dehydrogenase - alpha subunit  
W-formate dehydrogenase from Desulfovibrio vulgaris - Formate reduced form

Seq Identity
:   26.79%

Coverage

|  |  |
| --- | --- |
| **Biounit Oligo State** | Hetero-dimer |
| **QSQE** | 0.00 |
| **Method** | X-ray, 1.90 Å |
| **Seq Similarity** | 0.33 |
| **Coverage** | 0.24 |
| **Range** | 76-415 |

| Ligand | Added to Model | Description |
| --- | --- | --- |
| GOL | ✕ - Not biologically relevant. | GLYCEROL |
| GOL | ✕ - Not biologically relevant. | GLYCEROL |
| GOL | ✕ - Not biologically relevant. | GLYCEROL |
| GOL | ✕ - Not biologically relevant. | GLYCEROL |
| GOL | ✕ - Not biologically relevant. | GLYCEROL |
| GOL | ✕ - Not biologically relevant. | GLYCEROL |
| GOL | ✕ - Not biologically relevant. | GLYCEROL |
| GOL | ✕ - Not biologically relevant. | GLYCEROL |
| GOL | ✕ - Not biologically relevant. | GLYCEROL |
| GOL | ✕ - Not biologically relevant. | GLYCEROL |
| H2S | ✕ - Binding site not conserved. | HYDROSULFURIC ACID |
| MGD | ✕ - Binding site not conserved. | 2-AMINO-5,6-DIMERCAPTO-7-METHYL-3,7,8A,9-TETRAHYDRO-8-OXA-1,3,9,10-TETRAAZA-ANTHRACEN-4-ONE GUANOSINE DINUCLEOTIDE |
| MGD | ✕ - Binding site not conserved. | 2-AMINO-5,6-DIMERCAPTO-7-METHYL-3,7,8A,9-TETRAHYDRO-8-OXA-1,3,9,10-TETRAAZA-ANTHRACEN-4-ONE GUANOSINE DINUCLEOTIDE |
| NO3 | ✕ - Not biologically relevant. | NITRATE ION |
| NO3 | ✕ - Not biologically relevant. | NITRATE ION |
| PEG | ✕ - Not biologically relevant. | DI(HYDROXYETHYL)ETHER |
| SF4 | ✕ - Binding site not conserved. | IRON/SULFUR CLUSTER |
| SF4 | ✕ - Binding site not conserved. | IRON/SULFUR CLUSTER |
| SF4 | ✕ - Binding site not conserved. | IRON/SULFUR CLUSTER |
| SF4 | ✕ - Binding site not conserved. | IRON/SULFUR CLUSTER |
| W | ✕ - Not in contact with model. | TUNGSTEN ION |

Model-Template Alignment

|  |  |  |
| --- | --- | --- |
|  |  |  |

Model 06

- PDB Format *(Display)*
- JSON Format *(Display)*
- Model Report *(Display)*

Oligo-State
:   Monomer

GMQE
:   0.08

QMEANDisCo Global:
:   0.40  ± 0.05

Ligands

QMEANDisCo Local

QMEAN Z-Scores

Template

7l5i.1.A

Trimethylamine-N-oxide reductase  
Crystal Structure of Haemophilus influenzae MtsZ at pH 7.0

Seq Identity
:   24.11%

Coverage

|  |  |
| --- | --- |
| **Biounit Oligo State** | Monomer |
| **QSQE** | 0.00 |
| **Method** | X-ray, 1.73 Å |
| **Seq Similarity** | 0.34 |
| **Coverage** | 0.19 |
| **Range** | 128-389 |

| Ligand | Added to Model | Description |
| --- | --- | --- |
| CL | ✕ - Not biologically relevant. | CHLORIDE ION |
| EPE | ✕ - Not biologically relevant. | 4-(2-HYDROXYETHYL)-1-PIPERAZINE ETHANESULFONIC ACID |
| EPE | ✕ - Not biologically relevant. | 4-(2-HYDROXYETHYL)-1-PIPERAZINE ETHANESULFONIC ACID |
| EPE | ✕ - Not biologically relevant. | 4-(2-HYDROXYETHYL)-1-PIPERAZINE ETHANESULFONIC ACID |
| MGD | ✕ - Binding site not conserved. | 2-AMINO-5,6-DIMERCAPTO-7-METHYL-3,7,8A,9-TETRAHYDRO-8-OXA-1,3,9,10-TETRAAZA-ANTHRACEN-4-ONE GUANOSINE DINUCLEOTIDE |
| MGD | ✕ - Binding site not conserved. | 2-AMINO-5,6-DIMERCAPTO-7-METHYL-3,7,8A,9-TETRAHYDRO-8-OXA-1,3,9,10-TETRAAZA-ANTHRACEN-4-ONE GUANOSINE DINUCLEOTIDE |
| MO | ✕ - Binding site not conserved. | MOLYBDENUM ATOM |
| O | ✕ - Binding site not conserved. | OXYGEN ATOM |

Model-Template Alignment

|  |  |  |
| --- | --- | --- |
|  |  |  |

Model 07

- PDB Format *(Display)*
- JSON Format *(Display)*
- Model Report *(Display)*

Oligo-State
:   Monomer

GMQE
:   0.07

QMEANDisCo Global:
:   0.36  ± 0.05

Ligands

QMEANDisCo Local

QMEAN Z-Scores

Template

1eu1.1.A

DIMETHYL SULFOXIDE REDUCTASE  
THE CRYSTAL STRUCTURE OF RHODOBACTER SPHAEROIDES DIMETHYLSULFOXIDE REDUCTASE REVEALS TWO DISTINCT MOLYBDENUM COORDINATION ENVIRONMENTS.

Seq Identity
:   33.04%

Coverage

|  |  |
| --- | --- |
| **Biounit Oligo State** | Monomer |
| **QSQE** | 0.00 |
| **Method** | X-ray, 1.30 Å |
| **Seq Similarity** | 0.36 |
| **Coverage** | 0.19 |
| **Range** | 133-401 |

| Ligand | Added to Model | Description |
| --- | --- | --- |
| 6MO | ✕ - Binding site not conserved. | MOLYBDENUM(VI) ION |
| CD | ✕ - Binding site not conserved. | CADMIUM ION |
| EPE | ✕ - Not biologically relevant. | 4-(2-HYDROXYETHYL)-1-PIPERAZINE ETHANESULFONIC ACID |
| GLC | ✕ - Not in contact with model. | alpha-D-glucopyranose |
| GLC | ✕ - Binding site not conserved. | alpha-D-glucopyranose |
| GLC | ✕ - Binding site not conserved. | alpha-D-glucopyranose |
| MGD | ✕ - Binding site not conserved. | 2-AMINO-5,6-DIMERCAPTO-7-METHYL-3,7,8A,9-TETRAHYDRO-8-OXA-1,3,9,10-TETRAAZA-ANTHRACEN-4-ONE GUANOSINE DINUCLEOTIDE |
| MGD | ✕ - Binding site not conserved. | 2-AMINO-5,6-DIMERCAPTO-7-METHYL-3,7,8A,9-TETRAHYDRO-8-OXA-1,3,9,10-TETRAAZA-ANTHRACEN-4-ONE GUANOSINE DINUCLEOTIDE |
| O | ✕ - Binding site not conserved. | OXYGEN ATOM |
| O | ✕ - Binding site not conserved. | OXYGEN ATOM |
| SO4 | ✕ - Not biologically relevant. | SULFATE ION |

Model-Template Alignment

|  |  |  |
| --- | --- | --- |
|  |  |  |

Apply

Close

Cartoon

- Cartoon
- Tube
- Trace
- Lines
- Ball+Stick
- Licorice
- Hyperball
- Rope
- Surface
- Spacefill
- Outline
- Fog

###### Background

- Transparent

###### Resolution

- Low
- Medium
- High
- Extreme

##### Click model image to view in 3D

##### Click model image to view in 3D

×

### Delete Model - ""

Are you sure you want to delete this model?  
(This really can't be undone!)

Close
Delete Model
