# Supplementary material for: Metabolic capacity is maintained despite shifts in microbial diversity in estuary sediments
Source: ISME Commun. 2025 Oct 11;5(1):ycaf182. doi: 10.1093/ismeco/ycaf182 (PMC12687941; doi:10.1093/ismeco/ycaf182)
Supplement: Supplementary_Data_1_ycaf182 [file supplementary_data_1_ycaf182.zip › SWISS-MODEL/13_July_SF_Bin2_scaffold_7244_c1_43985624_1/templates.html]

13\_July\_SF\_Bin2\_scaffold\_7244\_c1\_4398-5624\_1 | Templates


**Export Alignment**
  
FASTA format
Clustal Format
PNG Image

**Secondary Structure**
  
None
DSSP
PSIPRED
SSpro

**Colour Scheme** 


Fade Mismatches
Enhance Mismatches

Confidencegradient
Confidenceclass
Indels
Chain
Unique Chain
Rainbow
2° Structure
Clustal
Hydrophobic
Size
Charged
Polar
Proline
Ser/Thr
Cysteine
Aliphatic
Aromatic
No Colour

Use QMEANBrane values

|  |  |  |  |
| --- | --- | --- | --- |
| Background |  |  |  |

**3D Viewer**  
NGL
PV

FASTA
Multi FASTA
ClustalW
PNG


SWISS-MODEL

### 13\_July\_SF\_Bin2\_scaffold\_7244\_c1\_4398-5624\_1

### Created: March 29, 2023, 7:37 p.m. at 19:37

- Templates
- Models

Models | Name | Description | GMQE | QSQE | Seq Id | Coverage | Range | Method | Resolution | Oligo-state | Ligands | Found by | Seq Similarity || ✓ | 7b04.1.B | Nitrite oxidoreductase subunit A  *Structure of Nitrite oxidoreductase (Nxr) from the anammox bacterium Kuenenia stuttgartiensis.* | 0.74 | 0.00 | 45.15 | 0.96 | 3-396 | X-ray | 2.97 | monomer | 4 x SF4, 1 x F3S, 2 x MD1, 1 x MO, 1 x HEM, 2 x CA | BLAST | 0.43 |
| ``` target    NNVNRREFLQWIGAAGFSTFALSASNAWGLQ------AIENPLAAYPNREWEKTYRDLWKSDASFTFLCAPNDTHNCILN 7b04.1    --LTRRAFLQVAGATG-ATLTL-AKNAMAFRLLKPAVVVDNPLDTYPDRRWESVYRDQYQYDRTFTYCCSPNDTHACRIR  target    AHVRDGVITRIGPTMKYGEATDLYGSKVTHRWDPRVCQKGLALTRRFYGDRRVRYPMVRKGFKAWADKGFPREKDGRPPK 7b04.1    AFVRNNVMMRVEQNYDHQNYSDLYGNKATRNWNPRMCLKGYTFHRRVYGPYRLRYPLIRKGWKRWADDGFPELTPENKTK  target    DYF-NRARDEWLRLTHEEAADLVAAALINIATTYSGDNGQKLLLQQGYEKEIVEATRGAGTQVLKFRGGMPLLGLTRIFG 7b04.1    YMFDNRGNDELLRASWDEAFTYASKGIIHITKKYSGPEGAQKLIDQGYPKEMVDRMQGAGTRTFKGRGGMGLLGVIGKYG  target    LYRMANSMALLDHKIRGVKPEDALGARGWDNYSWHTDLPPGHPMVTGQQTVDFDLHAVEQARIVVVWGMNWVTTKMPDTH 7b04.1    MYRFNNCLAIVDAHNRGVGPDQALGGRNWSNYTWHGDQAPGHPFSHGLQTSDVDMNDVRFSKLLIQTGKNLIENKMPEAH  target    WLTEARLKGTKVVVIACEYSSSSIKADDAIVVRPGTTPALALGLCNVIMREKIYDGDYVRRFSDLPLLVRADNLKLLRAE 7b04.1    WVTEVMERGGKIVVITPEYSPSAQKADYWIPIRNNTDTALFLGITKILIDNKWYDADYVKKFTDFPLLIRTDTLKRVSPK  target    EVFGTPQAALKNQTR 7b04.1    DII------------ ``` | | | | | | | | | | | | | | | | | | | | | | | | | | | | | | | | | | | | | | | | | | | | | | | | | |
|  | 7b04.2.B | Nitrite oxidoreductase subunit A  *Structure of Nitrite oxidoreductase (Nxr) from the anammox bacterium Kuenenia stuttgartiensis.* | 0.74 | 0.00 | 45.15 | 0.96 | 3-396 | X-ray | 2.97 | monomer | 4 x SF4, 1 x F3S, 2 x MD1, 1 x MO, 1 x HEM, 2 x CA | BLAST | 0.43 |
| ``` target    NNVNRREFLQWIGAAGFSTFALSASNAWGLQ------AIENPLAAYPNREWEKTYRDLWKSDASFTFLCAPNDTHNCILN 7b04.2    --LTRRAFLQVAGATG-ATLTL-AKNAMAFRLLKPAVVVDNPLDTYPDRRWESVYRDQYQYDRTFTYCCSPNDTHACRIR  target    AHVRDGVITRIGPTMKYGEATDLYGSKVTHRWDPRVCQKGLALTRRFYGDRRVRYPMVRKGFKAWADKGFPREKDGRPPK 7b04.2    AFVRNNVMMRVEQNYDHQNYSDLYGNKATRNWNPRMCLKGYTFHRRVYGPYRLRYPLIRKGWKRWADDGFPELTPENKTK  target    DYF-NRARDEWLRLTHEEAADLVAAALINIATTYSGDNGQKLLLQQGYEKEIVEATRGAGTQVLKFRGGMPLLGLTRIFG 7b04.2    YMFDNRGNDELLRASWDEAFTYASKGIIHITKKYSGPEGAQKLIDQGYPKEMVDRMQGAGTRTFKGRGGMGLLGVIGKYG  target    LYRMANSMALLDHKIRGVKPEDALGARGWDNYSWHTDLPPGHPMVTGQQTVDFDLHAVEQARIVVVWGMNWVTTKMPDTH 7b04.2    MYRFNNCLAIVDAHNRGVGPDQALGGRNWSNYTWHGDQAPGHPFSHGLQTSDVDMNDVRFSKLLIQTGKNLIENKMPEAH  target    WLTEARLKGTKVVVIACEYSSSSIKADDAIVVRPGTTPALALGLCNVIMREKIYDGDYVRRFSDLPLLVRADNLKLLRAE 7b04.2    WVTEVMERGGKIVVITPEYSPSAQKADYWIPIRNNTDTALFLGITKILIDNKWYDADYVKKFTDFPLLIRTDTLKRVSPK  target    EVFGTPQAALKNQTR 7b04.2    DII------------ ``` | | | | | | | | | | | | | | | | | | | | | | | | | | | | | | | | | | | | | | | | | | | | | | | | | |
|  | 7b04.1.B | Nitrite oxidoreductase subunit A  *Structure of Nitrite oxidoreductase (Nxr) from the anammox bacterium Kuenenia stuttgartiensis.* | 0.72 | 0.00 | 43.98 | 0.94 | 2-384 | X-ray | 2.97 | monomer | 4 x SF4, 1 x F3S, 2 x MD1, 1 x MO, 1 x HEM, 2 x CA | HHblits | 0.42 |
| ``` target    NNVNRREFLQWIGAAGFSTFALSASNAWGL----QAIENPLAAYPNREWEKTYRDLWKSDASFTFLCAPNDTHNCILNAH 7b04.1    -KLTRRAFLQVAGATGATLTLAKNAMAFRLLKPAVVVDNPLDTYPDRRWESVYRDQYQYDRTFTYCCSPNDTHACRIRAF  target    VRDGVITRIGPTMKYGEATDLYGSKVTHRWDPRVCQKGLALTRRFYGDRRVRYPMVRKGFKAWADKGFPREKDGRP-PKD 7b04.1    VRNNVMMRVEQNYDHQNYSDLYGNKATRNWNPRMCLKGYTFHRRVYGPYRLRYPLIRKGWKRWADDGFPELTPENKTKYM  target    YFNRARDEWLRLTHEEAADLVAAALINIATTY-SGDNGQKLLLQQGYEKEIVEATRGAGTQVLKFRGGMPLLGLTRIFGL 7b04.1    FDNRGNDELLRASWDEAFTYASKGIIHITKKYSGPEGAQK-LIDQGYPKEMVDRMQGAGTRTFKGRGGMGLLGVIGKYGM  target    YRMANSMALLDHKIRGVKPEDALGARGWDNYSWHTDLPPGHPMVTGQQTVDFDLHAVEQARIVVVWGMNWVTTKMPDTHW 7b04.1    YRFNNCLAIVDAHNRGVGPDQALGGRNWSNYTWHGDQAPGHPFSHGLQTSDVDMNDVRFSKLLIQTGKNLIENKMPEAHW  target    LTEARLKGTKVVVIACEYSSSSIKADDAIVVRPGTTPALALGLCNVIMREKIYDGDYVRRFSDLPLLVRADNLKLLRAEE 7b04.1    VTEVMERGGKIVVITPEYSPSAQKADYWIPIRNNTDTALFLGITKILIDNKWYDADYVKKFTDFPLLIRT----------  target    VFGTPQAALKNQTR 7b04.1    -------------- ``` | | | | | | | | | | | | | | | | | | | | | | | | | | | | | | | | | | | | | | | | | | | | | | | | | |
|  | 7b04.2.B | Nitrite oxidoreductase subunit A  *Structure of Nitrite oxidoreductase (Nxr) from the anammox bacterium Kuenenia stuttgartiensis.* | 0.71 | 0.00 | 43.98 | 0.94 | 2-384 | X-ray | 2.97 | monomer | 4 x SF4, 1 x F3S, 2 x MD1, 1 x MO, 1 x HEM, 2 x CA | HHblits | 0.42 |
| ``` target    NNVNRREFLQWIGAAGFSTFALSASNAWGL----QAIENPLAAYPNREWEKTYRDLWKSDASFTFLCAPNDTHNCILNAH 7b04.2    -KLTRRAFLQVAGATGATLTLAKNAMAFRLLKPAVVVDNPLDTYPDRRWESVYRDQYQYDRTFTYCCSPNDTHACRIRAF  target    VRDGVITRIGPTMKYGEATDLYGSKVTHRWDPRVCQKGLALTRRFYGDRRVRYPMVRKGFKAWADKGFPREKDGRP-PKD 7b04.2    VRNNVMMRVEQNYDHQNYSDLYGNKATRNWNPRMCLKGYTFHRRVYGPYRLRYPLIRKGWKRWADDGFPELTPENKTKYM  target    YFNRARDEWLRLTHEEAADLVAAALINIATTY-SGDNGQKLLLQQGYEKEIVEATRGAGTQVLKFRGGMPLLGLTRIFGL 7b04.2    FDNRGNDELLRASWDEAFTYASKGIIHITKKYSGPEGAQK-LIDQGYPKEMVDRMQGAGTRTFKGRGGMGLLGVIGKYGM  target    YRMANSMALLDHKIRGVKPEDALGARGWDNYSWHTDLPPGHPMVTGQQTVDFDLHAVEQARIVVVWGMNWVTTKMPDTHW 7b04.2    YRFNNCLAIVDAHNRGVGPDQALGGRNWSNYTWHGDQAPGHPFSHGLQTSDVDMNDVRFSKLLIQTGKNLIENKMPEAHW  target    LTEARLKGTKVVVIACEYSSSSIKADDAIVVRPGTTPALALGLCNVIMREKIYDGDYVRRFSDLPLLVRADNLKLLRAEE 7b04.2    VTEVMERGGKIVVITPEYSPSAQKADYWIPIRNNTDTALFLGITKILIDNKWYDADYVKKFTDFPLLIRT----------  target    VFGTPQAALKNQTR 7b04.2    -------------- ``` | | | | | | | | | | | | | | | | | | | | | | | | | | | | | | | | | | | | | | | | | | | | | | | | | |
|  | 3ir5.1.A | Respiratory nitrate reductase 1 alpha chain  *Crystal structure of NarGHI mutant NarG-H49C* | 0.50 | 0.00 | 28.26 | 0.79 | 2-384 | X-ray | 2.30 | monomer | 2 x MD1, 1 x 6MO, 4 x SF4, 1 x AGA, 1 x F3S, 2 x HEM | HHblits | 0.35 |
| ``` target    NNVNRREFLQWIGAAGFSTFALSASNAWGLQAIENPLAAYPNREWEKTYRDLWKSDASFTFLCAPNDTHNCILNAHVRDG 3ir5.1    -FLDRFRYFKQKGETFAD-G----HGQ--L--------LNTNRDWEDGYRQRWQHDKIVRSTCGVNCTGSCSWKIYVKNG  target    VITRIGPTMKYGEATDLYGSKVTHRWDPRVCQKGLALTRRFYGDRRVRYPMVRKGFK-AWADKGF----PRE------KD 3ir5.1    LVTWETQQTDYPR-----TRPDLPNHEPRGCPRGASYSWYLYSANRLKYPMMRKRLMKMWREAKALHSDPVEAWASIIED  target    -GRPPKDYFNRARDEWLRLTHEEAADLVAAALINIATTYSGDNGQKLLLQQGYEKEIVEATRGAGTQVLKFRGGMPLLGL 3ir5.1    ADKAKSFKQARGRGGFVRSSWQEVNELIAASNVYTIKNYGPDRV---AGFSPIPA---------MSMV-SYA--------  target    TRIFGLYRMANSMALLDHKIRGVKPEDALGARGWDNYSWHTDLPPGHPMVTGQQTVDFDLHAVEQARIVVVWGMNWVTTK 3ir5.1    ----SGARYLS----------------LIGGTCLSFYDWYCDLPPASPQTWGEQTDVPESADWYNSSYIIAWGSNVPQTR  target    MPDTHWLTEARLKGTKVVVIACEYSSSSIKADDAIVVRPGTTPALALGLCNVIMREKI------YDGDYVRRFSDLPLLV 3ir5.1    TPDAHFFTEVRYKGTKTVAVTPDYAEIAKLCDLWLAPKQGTDAAMALAMGHVMLREFHLDNPSQYFTDYVRRYTDMPMLV  target    RADNLKLLRAEEVFGTPQAALKNQTR 3ir5.1    ML------------------------ ``` | | | | | | | | | | | | | | | | | | | | | | | | | | | | | | | | | | | | | | | | | | | | | | | | | |
|  | 1r27.4.A | Respiratory nitrate reductase 1 alpha chain  *Crystal Structure of NarGH complex* | 0.50 | 0.16 | 27.95 | 0.79 | 2-384 | X-ray | 2.00 | homo-dimer | 4 x MO, 16 x SF4, 8 x MGD, 4 x F3S | HHblits | 0.35 |
| ``` target    NNVNRREFLQWIGAAGFSTFALSASNAWGLQAIENPLAAYPNREWEKTYRDLWKSDASFTFLCAPNDTHNCILNAHVRDG 1r27.4    -FLDRFRYFKQKGETFADGH----GQ-----------LLNTNRDWEDGYRQRWQHDKIVRSTHGVNCTGSCSWKIYVKNG  target    VITRIGPTMKYGEATDLYGSKVTHRWDPRVCQKGLALTRRFYGDRRVRYPMVRKGF-KAWADKGF----PRE------KD 1r27.4    LVTWETQQTDYPR-----TRPDLPNHEPRGCPRGASYSWYLYSANRLKYPMMRKRLMKMWREAKALHSDPVEAWASIIED  target    G-RPPKDYFNRARDEWLRLTHEEAADLVAAALINIATTYSGDNGQKLLLQQGYEKEIVEATRGAGTQVLKFRGGMPLLGL 1r27.4    ADKAKSFKQARGRGGFVRSSWQEVNELIAASNVYTIKNYGPDRV---AGFSPIP-AM--------SMV-SYA--------  target    TRIFGLYRMANSMALLDHKIRGVKPEDALGARGWDNYSWHTDLPPGHPMVTGQQTVDFDLHAVEQARIVVVWGMNWVTTK 1r27.4    ----SGARYLS----------------LIGGTCLSFYDWYCDLPPASPQTWGEQTDVPESADWYNSSYIIAWGSNVPQTR  target    MPDTHWLTEARLKGTKVVVIACEYSSSSIKADDAIVVRPGTTPALALGLCNVIMREKI------YDGDYVRRFSDLPLLV 1r27.4    TPDAHFFTEVRYKGTKTVAVTPDYAEIAKLCDLWLAPKQGTDAAMALAMGHVMLREFHLDNPSQYFTDYVRRYTDMPMLV  target    RADNLKLLRAEEVFGTPQAALKNQTR 1r27.4    ML------------------------ ``` | | | | | | | | | | | | | | | | | | | | | | | | | | | | | | | | | | | | | | | | | | | | | | | | | |
|  | 1q16.1.A | Respiratory nitrate reductase 1 alpha chain  *Crystal structure of Nitrate Reductase A, NarGHI, from Escherichia coli* | 0.50 | 0.00 | 27.33 | 0.79 | 2-384 | X-ray | 1.90 | monomer | 2 x MD1, 1 x 6MO, 2 x HEM, 4 x SF4, 1 x F3S, 1 x AGA, 1 x 3PH | HHblits | 0.34 |
| ``` target    NNVNRREFLQWIGAAGFSTFALSASNAWGLQAIENPLAAYPNREWEKTYRDLWKSDASFTFLCAPNDTHNCILNAHVRDG 1q16.1    -FLDRFRYFKQKGETFAD----GHG------Q-----LLNTNRDWEDGYRQRWQHDKIVRSTHGVNCTGSCSWKIYVKNG  target    VITRIGPTMKYGEATDLYGSKVTHRWDPRVCQKGLALTRRFYGDRRVRYPMVRKGFK-AWADKGF----PR------EKD 1q16.1    LVTWETQQTDYPR-----TRPDLPNHEPRGCPRGASYSWYLYSANRLKYPMMRKRLMKMWREAKALHSDPVEAWASIIED  target    G-RPPKDYFNRARDEWLRLTHEEAADLVAAALINIATTYSGDNGQKLLLQQGYEKEIVEATRGAGTQVLKFRGGMPLLGL 1q16.1    ADKAKSFKQARGRGGFVRSSWQEVNELIAASNVYTIKNYGPDRV---AGFSPIP-AM--------SMV-SY---------  target    TRIFGLYRMANSMALLDHKIRGVKPEDALGARGWDNYSWHTDLPPGHPMVTGQQTVDFDLHAVEQARIVVVWGMNWVTTK 1q16.1    ---ASGARYLS----------------LIGGTCLSFYDWYCDLPPASPQTWGEQTDVPESADWYNSSYIIAWGSNVPQTR  target    MPDTHWLTEARLKGTKVVVIACEYSSSSIKADDAIVVRPGTTPALALGLCNVIMREKI------YDGDYVRRFSDLPLLV 1q16.1    TPDAHFFTEVRYKGTKTVAVTPDYAEIAKLCDLWLAPKQGTDAAMALAMGHVMLREFHLDNPSQYFTDYVRRYTDMPMLV  target    RADNLKLLRAEEVFGTPQAALKNQTR 1q16.1    ML------------------------ ``` | | | | | | | | | | | | | | | | | | | | | | | | | | | | | | | | | | | | | | | | | | | | | | | | | |
|  | 3ir7.1.A | Respiratory nitrate reductase 1 alpha chain  *Crystal structure of NarGHI mutant NarG-R94S* | 0.50 | 0.00 | 27.64 | 0.79 | 2-384 | X-ray | 2.50 | monomer | 2 x MD1, 4 x SF4, 1 x 6MO, 1 x AGA, 1 x F3S, 2 x HEM | HHblits | 0.34 |
| ``` target    NNVNRREFLQWIGAAGFSTFALSASNAWGLQAIENPLAAYPNREWEKTYRDLWKSDASFTFLCAPNDTHNCILNAHVRDG 3ir7.1    -FLDRFRYFKQKGETFADGH----GQ-----------LLNTNRDWEDGYRQRWQHDKIVRSTHGVNCTGSCSWKIYVKNG  target    VITRIGPTMKYGEATDLYGSKVTHRWDPRVCQKGLALTRRFYGDRRVRYPMVRKGF-KAWADKGF----P------REKD 3ir7.1    LVTWETQQTDYPR-----TRPDLPNHEPRGCPSGASYSWYLYSANRLKYPMMRKRLMKMWREAKALHSDPVEAWASIIED  target    GRPPK-DYFNRARDEWLRLTHEEAADLVAAALINIATTYSGDNGQKLLLQQGYEKEIVEATRGAGTQVLKFRGGMPLLGL 3ir7.1    ADKAKSFKQARGRGGFVRSSWQEVNELIAASNVYTIKNYGPDRV---AGFSPIPA---------MSM-VSYA--------  target    TRIFGLYRMANSMALLDHKIRGVKPEDALGARGWDNYSWHTDLPPGHPMVTGQQTVDFDLHAVEQARIVVVWGMNWVTTK 3ir7.1    ----SGARYL----------------SLIGGTCLSFYDWYCDLPPASPQTWGEQTDVPESADWYNSSYIIAWGSNVPQTR  target    MPDTHWLTEARLKGTKVVVIACEYSSSSIKADDAIVVRPGTTPALALGLCNVIMREKI------YDGDYVRRFSDLPLLV 3ir7.1    TPDAHFFTEVRYKGTKTVAVTPDYAEIAKLCDLWLAPKQGTDAAMALAMGHVMLREFHLDNPSQYFTDYVRRYTDMPMLV  target    RADNLKLLRAEEVFGTPQAALKNQTR 3ir7.1    ML------------------------ ``` | | | | | | | | | | | | | | | | | | | | | | | | | | | | | | | | | | | | | | | | | | | | | | | | | |
|  | 3ir6.1.A | Respiratory nitrate reductase 1 alpha chain  *Crystal structure of NarGHI mutant NarG-H49S* | 0.46 | 0.00 | 27.02 | 0.79 | 2-384 | X-ray | 2.80 | monomer | 2 x GDP, 1 x AGA, 3 x SF4, 1 x F3S, 2 x HEM | HHblits | 0.34 |
| ``` target    NNVNRREFLQWIGAAGFSTFALSASNAWGLQAIENPLAAYPNREWEKTYRDLWKSDASFTFLCAPNDTHNCILNAHVRDG 3ir6.1    -FLDRFRYFKQKGETFADGH--G--Q-----------LLNTNRDWEDGYRQRWQHDKIVRSTSGVNCTGSCSWKIYVKNG  target    VITRIGPTMKYGEATDLYGSKVTHRWDPRVCQKGLALTRRFYGDRRVRYPMVRKGFK-AWADKGF----PR------EKD 3ir6.1    LVTWETQQTDYPR-----TRPDLPNHEPRGCPRGASYSWYLYSANRLKYPMMRKRLMKMWREAKALHSDPVEAWASIIED  target    GR-PPKDYFNRARDEWLRLTHEEAADLVAAALINIATTYSGDNGQKLLLQQGYEKEIVEATRGAGTQVLKFRGGMPLLGL 3ir6.1    ADKAKSFKQARGRGGFVRSSWQEVNELIAASNVYTIKNYGPDR---VAGFSPIP-AM--------SM-VSYA-------S  target    TRIFGLYRMANSMALLDHKIRGVKPEDALGARGWDNYSWHTDLPPGHPMVTGQQTVDFDLHAVEQARIVVVWGMNWVTTK 3ir6.1    -----GARYLS----------------LIGGTCLSFYDWYCDLPPASPQTWGEQTDVPESADWYNSSYIIAWGSNVPQTR  target    MPDTHWLTEARLKGTKVVVIACEYSSSSIKADDAIVVRPGTTPALALGLCNVIMREKI------YDGDYVRRFSDLPLLV 3ir6.1    TPDAHFFTEVRYKGTKTVAVTPDYAEIAKLCDLWLAPKQGTDAAMALAMGHVMLREFHLDNPSQYFTDYVRRYTDMPMLV  target    RADNLKLLRAEEVFGTPQAALKNQTR 3ir6.1    ML------------------------ ``` | | | | | | | | | | | | | | | | | | | | | | | | | | | | | | | | | | | | | | | | | | | | | | | | | |
| ✓ | 3ir5.1.A | Respiratory nitrate reductase 1 alpha chain  *Crystal structure of NarGHI mutant NarG-H49C* | 0.45 | 0.00 | 30.49 | 0.75 | 42-382 | X-ray | 2.30 | monomer | 2 x MD1, 1 x 6MO, 4 x SF4, 1 x AGA, 1 x F3S, 2 x HEM | BLAST | 0.37 |
| ``` target    NNVNRREFLQWIGAAGFSTFALSASNAWGLQAIENPLAAYPNREWEKTYRDLWKSDASFTFLCAPNDTHNCILNAHVRDG 3ir5.1    -----------------------------------------NRDWEDGYRQRWQHDKIVRSTCGVNCTGSCSWKIYVKNG  target    VITRIGPTMKYGEA-TDLYGSKVTHRWDPRVCQKGLALTRRFYGDRRVRYPMVRKGF-KAWADKGFPREKDGRPPKDYFN 3ir5.1    LVTWETQQTDYPRTRPDLPNH------EPRGCPRGASYSWYLYSANRLKYPMMRKRLMKMW-----------REAKALHS  target    RARDEWLRLTHEEAADLVAAALINIATTYSGDNGQKLLLQQGYEKEIVEATRGAGTQVLKFRGGMPLLGLTRIFGLYRMA 3ir5.1    DPVEAWASII--EDADK--------AKSFKQARGRGGFVRSSWQ-EVNELIAASNVYTIKNYGPDRVAGFSPI-------  target    NSMALLDHKIRGVKPEDALGARGWDNYSWHTDLPPGHPMVTGQQTVDFDLHAVEQARIVVVWGMNWVTTKMPDTHWLTEA 3ir5.1    PAMSMVSYA-SGARYLSLIGGTCLSFYDWYCDLPPASPQTWGEQTDVPESADWYNSSYIIAWGSNVPQTRTPDAHFFTEV  target    RLKGTKVVVIACEYSSSSIKADDAIVVRPGTTPALALGLCNVIMRE------KIYDGDYVRRFSDLPLLVRADNLKLLRA 3ir5.1    RYKGTKTVAVTPDYAEIAKLCDLWLAPKQGTDAAMALAMGHVMLREFHLDNPSQYFTDYVRRYTDMPMLV----------  target    EEVFGTPQAALKNQTR 3ir5.1    ---------------- ``` | | | | | | | | | | | | | | | | | | | | | | | | | | | | | | | | | | | | | | | | | | | | | | | | | |
|  | 3ir6.1.A | Respiratory nitrate reductase 1 alpha chain  *Crystal structure of NarGHI mutant NarG-H49S* | 0.45 | 0.00 | 30.16 | 0.75 | 42-382 | X-ray | 2.80 | monomer | 2 x GDP, 1 x AGA, 3 x SF4, 1 x F3S, 2 x HEM | BLAST | 0.36 |
| ``` target    NNVNRREFLQWIGAAGFSTFALSASNAWGLQAIENPLAAYPNREWEKTYRDLWKSDASFTFLCAPNDTHNCILNAHVRDG 3ir6.1    -----------------------------------------NRDWEDGYRQRWQHDKIVRSTSGVNCTGSCSWKIYVKNG  target    VITRIGPTMKYGEA-TDLYGSKVTHRWDPRVCQKGLALTRRFYGDRRVRYPMVRKGF-KAWADKGFPREKDGRPPKDYFN 3ir6.1    LVTWETQQTDYPRTRPDLPNH------EPRGCPRGASYSWYLYSANRLKYPMMRKRLMKMW-----------REAKALHS  target    RARDEWLRLTHEEAADLVAAALINIATTYSGDNGQKLLLQQGYEKEIVEATRGAGTQVLKFRGGMPLLGLTRIFGLYRMA 3ir6.1    DPVEAWASII--EDADK--------AKSFKQARGRGGFVRSSWQ-EVNELIAASNVYTIKNYGPDRVAGFSPI-------  target    NSMALLDHKIRGVKPEDALGARGWDNYSWHTDLPPGHPMVTGQQTVDFDLHAVEQARIVVVWGMNWVTTKMPDTHWLTEA 3ir6.1    PAMSMVSYA-SGARYLSLIGGTCLSFYDWYCDLPPASPQTWGEQTDVPESADWYNSSYIIAWGSNVPQTRTPDAHFFTEV  target    RLKGTKVVVIACEYSSSSIKADDAIVVRPGTTPALALGLCNVIMRE------KIYDGDYVRRFSDLPLLVRADNLKLLRA 3ir6.1    RYKGTKTVAVTPDYAEIAKLCDLWLAPKQGTDAAMALAMGHVMLREFHLDNPSQYFTDYVRRYTDMPMLV----------  target    EEVFGTPQAALKNQTR 3ir6.1    ---------------- ``` | | | | | | | | | | | | | | | | | | | | | | | | | | | | | | | | | | | | | | | | | | | | | | | | | |
|  | 2ivf.1.A | ETHYLBENZENE DEHYDROGENASE ALPHA-SUBUNIT  *ETHYLBENZENE DEHYDROGENASE FROM AROMATOLEUM AROMATICUM* | 0.45 | 0.00 | 29.84 | 0.77 | 2-384 | X-ray | 1.88 | monomer | 1 x MES, 4 x SF4, 1 x MO, 1 x MGD, 1 x MD1, 1 x F3S, 1 x HEM | HHblits | 0.34 |
| ``` target    NNVNRREFLQWIGAAGFSTFALSASNAW--G-LQAIENPLAAYPNREWEKTYRDLWKSDASFTFLCAPND--THNCILNA 2ivf.1    -DQHRRDFLKRSGAAVLSLSLSSLATGVVPGFLKDAQAGTKAPGYASWEDIYRKEWKWDKVNWGSHLNICWPQGSCKFYV  target    HVRDGVITRIGPTMKYGEATDLYGSKVTHRWDPRVCQKGLALTRRFYGDRRVRYPMVRKGFKAWADKGFPREKDGRPPKD 2ivf.1    YVRNGIVWREEQAAQTPA-----CNVDYVDYNPLGCQKGSAFNNNLYGDERVKYPLKRVG--------------------  target    YFNRARDEWLRLTHEEAADLVAAALINIATTYSGDNGQKLLLQQGYEKEIVEATRGAGTQVLKFRGGMPLLGLTRIFGLY 2ivf.1    --KRGEGKWKRVSWDEAAGDIADSIIDSFEAQGSDG---FILDAPHV-------H-AGSIA--WGAGF---RMTYLMDGV  target    RMANSMALLDHKIRGVKPEDALGARGWDNYSWHTDLPPGHPMVTGQQTVDFDLHAVEQARIVVVWGMNWVTTKMPDTHWL 2ivf.1    ---------------------SPDI---N-VDIGDTYMGAFHTFGKMHMGYSADNLLDAELIFMTCSNWSYTYPSSYHFL  target    TEARLKGTKVVVIACEYSSSSIKADDAIVVRPGTTPALALGLCNVIMREKIYDGDYVRRFSDLPLLVRADNLKLLRAEEV 2ivf.1    SEARYKGAEVVVIAPDFNPTTPAADLHVPVRVGSDAAFWLGLSQVMIDEKLFDRQFVCEQTDLPLLVRM-----------  target    FGTPQAALKNQTR 2ivf.1    ------------- ``` | | | | | | | | | | | | | | | | | | | | | | | | | | | | | | | | | | | | | | | | | | | | | | | | | |
|  | 1r27.4.A | Respiratory nitrate reductase 1 alpha chain  *Crystal Structure of NarGH complex* | 0.45 | 0.13 | 30.16 | 0.75 | 42-382 | X-ray | 2.00 | homo-dimer | 4 x MO, 16 x SF4, 8 x MGD, 4 x F3S | BLAST | 0.36 |
| ``` target    NNVNRREFLQWIGAAGFSTFALSASNAWGLQAIENPLAAYPNREWEKTYRDLWKSDASFTFLCAPNDTHNCILNAHVRDG 1r27.4    -----------------------------------------NRDWEDGYRQRWQHDKIVRSTHGVNCTGSCSWKIYVKNG  target    VITRIGPTMKYGEA-TDLYGSKVTHRWDPRVCQKGLALTRRFYGDRRVRYPMVRKGF-KAWADKGFPREKDGRPPKDYFN 1r27.4    LVTWETQQTDYPRTRPDLPNH------EPRGCPRGASYSWYLYSANRLKYPMMRKRLMKMW-----------REAKALHS  target    RARDEWLRLTHEEAADLVAAALINIATTYSGDNGQKLLLQQGYEKEIVEATRGAGTQVLKFRGGMPLLGLTRIFGLYRMA 1r27.4    DPVEAWASII--EDADK--------AKSFKQARGRGGFVRSSWQ-EVNELIAASNVYTIKNYGPDRVAGFSPI-------  target    NSMALLDHKIRGVKPEDALGARGWDNYSWHTDLPPGHPMVTGQQTVDFDLHAVEQARIVVVWGMNWVTTKMPDTHWLTEA 1r27.4    PAMSMVSYA-SGARYLSLIGGTCLSFYDWYCDLPPASPQTWGEQTDVPESADWYNSSYIIAWGSNVPQTRTPDAHFFTEV  target    RLKGTKVVVIACEYSSSSIKADDAIVVRPGTTPALALGLCNVIMRE------KIYDGDYVRRFSDLPLLVRADNLKLLRA 1r27.4    RYKGTKTVAVTPDYAEIAKLCDLWLAPKQGTDAAMALAMGHVMLREFHLDNPSQYFTDYVRRYTDMPMLV----------  target    EEVFGTPQAALKNQTR 1r27.4    ---------------- ``` | | | | | | | | | | | | | | | | | | | | | | | | | | | | | | | | | | | | | | | | | | | | | | | | | |
|  | 1q16.1.A | Respiratory nitrate reductase 1 alpha chain  *Crystal structure of Nitrate Reductase A, NarGHI, from Escherichia coli* | 0.45 | 0.00 | 30.16 | 0.75 | 42-382 | X-ray | 1.90 | monomer | 2 x MD1, 1 x 6MO, 2 x HEM, 4 x SF4, 1 x F3S, 1 x AGA, 1 x 3PH | BLAST | 0.36 |
| ``` target    NNVNRREFLQWIGAAGFSTFALSASNAWGLQAIENPLAAYPNREWEKTYRDLWKSDASFTFLCAPNDTHNCILNAHVRDG 1q16.1    -----------------------------------------NRDWEDGYRQRWQHDKIVRSTHGVNCTGSCSWKIYVKNG  target    VITRIGPTMKYGEA-TDLYGSKVTHRWDPRVCQKGLALTRRFYGDRRVRYPMVRKGF-KAWADKGFPREKDGRPPKDYFN 1q16.1    LVTWETQQTDYPRTRPDLPNH------EPRGCPRGASYSWYLYSANRLKYPMMRKRLMKMW-----------REAKALHS  target    RARDEWLRLTHEEAADLVAAALINIATTYSGDNGQKLLLQQGYEKEIVEATRGAGTQVLKFRGGMPLLGLTRIFGLYRMA 1q16.1    DPVEAWASII--EDADK--------AKSFKQARGRGGFVRSSWQ-EVNELIAASNVYTIKNYGPDRVAGFSPI-------  target    NSMALLDHKIRGVKPEDALGARGWDNYSWHTDLPPGHPMVTGQQTVDFDLHAVEQARIVVVWGMNWVTTKMPDTHWLTEA 1q16.1    PAMSMVSYA-SGARYLSLIGGTCLSFYDWYCDLPPASPQTWGEQTDVPESADWYNSSYIIAWGSNVPQTRTPDAHFFTEV  target    RLKGTKVVVIACEYSSSSIKADDAIVVRPGTTPALALGLCNVIMRE------KIYDGDYVRRFSDLPLLVRADNLKLLRA 1q16.1    RYKGTKTVAVTPDYAEIAKLCDLWLAPKQGTDAAMALAMGHVMLREFHLDNPSQYFTDYVRRYTDMPMLV----------  target    EEVFGTPQAALKNQTR 1q16.1    ---------------- ``` | | | | | | | | | | | | | | | | | | | | | | | | | | | | | | | | | | | | | | | | | | | | | | | | | |
|  | 3egw.1.A | Respiratory nitrate reductase 1 alpha chain  *The crystal structure of the NarGHI mutant NarH - C16A* | 0.45 | 0.13 | 30.16 | 0.75 | 42-382 | X-ray | 1.90 | homo-dimer | 2 x MD1, 2 x MGD, 2 x 6MO, 6 x SF4, 4 x F3S, 2 x 3PH, 4 x HEM, 2 x AGA | BLAST | 0.36 |
| ``` target    NNVNRREFLQWIGAAGFSTFALSASNAWGLQAIENPLAAYPNREWEKTYRDLWKSDASFTFLCAPNDTHNCILNAHVRDG 3egw.1    -----------------------------------------NRDWEDGYRQRWQHDKIVRSTHGVNCTGSCSWKIYVKNG  target    VITRIGPTMKYGEA-TDLYGSKVTHRWDPRVCQKGLALTRRFYGDRRVRYPMVRKGF-KAWADKGFPREKDGRPPKDYFN 3egw.1    LVTWETQQTDYPRTRPDLPNH------EPRGCPRGASYSWYLYSANRLKYPMMRKRLMKMW-----------REAKALHS  target    RARDEWLRLTHEEAADLVAAALINIATTYSGDNGQKLLLQQGYEKEIVEATRGAGTQVLKFRGGMPLLGLTRIFGLYRMA 3egw.1    DPVEAWASII--EDADK--------AKSFKQARGRGGFVRSSWQ-EVNELIAASNVYTIKNYGPDRVAGFSPI-------  target    NSMALLDHKIRGVKPEDALGARGWDNYSWHTDLPPGHPMVTGQQTVDFDLHAVEQARIVVVWGMNWVTTKMPDTHWLTEA 3egw.1    PAMSMVSYA-SGARYLSLIGGTCLSFYDWYCDLPPASPQTWGEQTDVPESADWYNSSYIIAWGSNVPQTRTPDAHFFTEV  target    RLKGTKVVVIACEYSSSSIKADDAIVVRPGTTPALALGLCNVIMRE------KIYDGDYVRRFSDLPLLVRADNLKLLRA 3egw.1    RYKGTKTVAVTPDYAEIAKLCDLWLAPKQGTDAAMALAMGHVMLREFHLDNPSQYFTDYVRRYTDMPMLV----------  target    EEVFGTPQAALKNQTR 3egw.1    ---------------- ``` | | | | | | | | | | | | | | | | | | | | | | | | | | | | | | | | | | | | | | | | | | | | | | | | | |
|  | 3ir7.1.A | Respiratory nitrate reductase 1 alpha chain  *Crystal structure of NarGHI mutant NarG-R94S* | 0.45 | 0.00 | 30.16 | 0.75 | 42-382 | X-ray | 2.50 | monomer | 2 x MD1, 4 x SF4, 1 x 6MO, 1 x AGA, 1 x F3S, 2 x HEM | BLAST | 0.36 |
| ``` target    NNVNRREFLQWIGAAGFSTFALSASNAWGLQAIENPLAAYPNREWEKTYRDLWKSDASFTFLCAPNDTHNCILNAHVRDG 3ir7.1    -----------------------------------------NRDWEDGYRQRWQHDKIVRSTHGVNCTGSCSWKIYVKNG  target    VITRIGPTMKYGEA-TDLYGSKVTHRWDPRVCQKGLALTRRFYGDRRVRYPMVRKGF-KAWADKGFPREKDGRPPKDYFN 3ir7.1    LVTWETQQTDYPRTRPDLPNH------EPRGCPSGASYSWYLYSANRLKYPMMRKRLMKMW-----------REAKALHS  target    RARDEWLRLTHEEAADLVAAALINIATTYSGDNGQKLLLQQGYEKEIVEATRGAGTQVLKFRGGMPLLGLTRIFGLYRMA 3ir7.1    DPVEAWASII--EDADK--------AKSFKQARGRGGFVRSSWQ-EVNELIAASNVYTIKNYGPDRVAGFSPI-------  target    NSMALLDHKIRGVKPEDALGARGWDNYSWHTDLPPGHPMVTGQQTVDFDLHAVEQARIVVVWGMNWVTTKMPDTHWLTEA 3ir7.1    PAMSMVSYA-SGARYLSLIGGTCLSFYDWYCDLPPASPQTWGEQTDVPESADWYNSSYIIAWGSNVPQTRTPDAHFFTEV  target    RLKGTKVVVIACEYSSSSIKADDAIVVRPGTTPALALGLCNVIMRE------KIYDGDYVRRFSDLPLLVRADNLKLLRA 3ir7.1    RYKGTKTVAVTPDYAEIAKLCDLWLAPKQGTDAAMALAMGHVMLREFHLDNPSQYFTDYVRRYTDMPMLV----------  target    EEVFGTPQAALKNQTR 3ir7.1    ---------------- ``` | | | | | | | | | | | | | | | | | | | | | | | | | | | | | | | | | | | | | | | | | | | | | | | | | |
|  | 1kqf.1.A | FORMATE DEHYDROGENASE, NITRATE-INDUCIBLE, MAJOR SUBUNIT  *FORMATE DEHYDROGENASE N FROM E. COLI* | 0.47 |  | 20.55 | 0.80 | 2-408 | X-ray | 1.60 | hetero-oligomer | 3 x 6MO, 15 x SF4, 6 x MGD, 6 x HEM, 3 x CDL | HHblits | 0.30 |
| ``` target    NNVNRREFLQWIGAAGFSTFALSASNAWGLQAIENPLAAYPNREWEKTYRDLWKSDASFTFLCAPNDTHNCILNAHVRDG 1kqf.1    -DVSRRQFFKICAGGMAGTTVAAL-GFAPKQA----LA--QARNYK------LLRAKEIRNTCT-YCSVGCGLLMYSLGD  target    -------VITRIGPTMKYGEATDLYGSKVTHRWDPRVCQKGLALTRRFYGDRRVRYPMVRKGFKAWADKGFPREKDGRPP 1kqf.1    GAKNAREAIYHIEGDP------------DHPVSRGALCPKGAGLLDYVNSENRLRYPEYRAP------------------  target    KDYFNRARDEWLRLTHEEAADLVAAALINIATTYSGDNGQKL-LLQQGYEKEIVEATRGAGTQVLKFRGGMPLLGLTRIF 1kqf.1    ------GSDKWQRISWEEAFSRIAKLMKADRDANFIEKNEQGVTVNR---WLSTGMLCASG-------------------  target    GLYRMANSMALLDHKIRGVKPEDALGARGWDNYS--WHTDLPPGHPMVTGQQTVDFDLHAVEQARIVVVWGMNWVTTKMP 1kqf.1    ----ASNETGMLTQKFA-----RSLGMLAVDNQARVUHGPTVASLAPTFGRGAMTNHWVDIKNANVVMVMGGNAAEAHPV  target    DTHWLTEAR-LKGTKVVVIACEYSSSSIKADDAIVVRPGTTPALALGLCNVIMREKIYDGDYVRRFSDLPLLVRA----- 1kqf.1    GFRWAMEAKNNNDATLIVVDPRFTRTASVADIYAPIRSGTDITFLSGVLRYLIENNKINAEYVKHYTNASLLVRDDFAFE  target    -------------------------------------------------DNLKLLRAEEVFGTPQAALKNQTR 1kqf.1    DGLFSGYDAEKRQYDKSSWNYQLDENGYAKRDETLTHPRCVWNLLKEHVSRYTPDVVENICGTPKADFLKVCE ``` | | | | | | | | | | | | | | | | | | | | | | | | | | | | | | | | | | | | | | | | | | | | | | | | | |
|  | 6sdr.1.A | Formate dehydrogenase, alpha subunit, selenocysteine-containing  *W-formate dehydrogenase from Desulfovibrio vulgaris - Oxidized form* | 0.46 | 0.00 | 19.27 | 0.80 | 2-408 | X-ray | 2.10 | monomer | 2 x MGD, 4 x SF4, 1 x H2S, 1 x W | HHblits | 0.29 |
| ``` target    NNVNRREFLQWIGAAGFSTFALSASNAWGLQAIENPLAAYPNREWEKTYRDLWKSDASFTFLCAPNDTHNCILNAHVR-- 6sdr.1    -TVTRRHFLKLSAGAAVAGAFTGLGLSLAPT---VARAE-------L-Q--KLQWAKQTTSIC-CYCAVGCGLIVHTAKD  target    -DGVITRIGPTMKYGEATDLYGSKVTHRWDPRVCQKGLALTRRFYGDRRVRYPMVRKGFKAWADKGFPREKDGRPPKDYF 6sdr.1    GQGRAVNVEGDP------------DHPINEGSLCPKGASIFQLGENDQRGTQPLYRAPF---------------------  target    NRARDEWLRLTHEEAADLVAAALINIATTYSGDNGQKLLLQQGYEKEIVEATRGAGTQVLKFRGGMPLLGLTRIFGLYRM 6sdr.1    ---SDTWKPVTWDFALTEIAKRIKKTRDASFTEKNAAGDLVNR--TEAIAS-----------------------FGSAAM  target    ANSMALLDHKIRGVKPEDALGARGWDNY--SWHTDLPPGHPMVTGQQTVDFDLHAVEQARIVVVWGMNWVTTKMPDTHWL 6sdr.1    DNEECWAYGNIL-----RSLGLVYIEHQARIUHSPTVPALAESFGRGAMTNHWNDLANSDCILIMGSNAAENHPIAFKWV  target    TEARLKGTKVVVIACEYSSSSIKADDAIVVRPGTTPALALGLCNVIMREKIYDGDYVRRFSDLPLLVRA----------- 6sdr.1    LRAKDKGATLIHVDPRFTRTSARCDVYAPIRSGADIPFLGGLIKYILDNKLYFTDYVREYTNASLIVGEKFSFKDGLFSG  target    -------------------------------------------DNLKLLRAEEVFGTPQAALKNQTR 6sdr.1    YDAANKKYDKSMWAFELDANGVPKRDPALKHPRCVINLLKKHYERYNLDKVAAITGTSKEQLQQVYK ``` | | | | | | | | | | | | | | | | | | | | | | | | | | | | | | | | | | | | | | | | | | | | | | | | | |
|  | 6sdv.1.A | Formate dehydrogenase, alpha subunit, selenocysteine-containing,Formate dehydrogenase, alpha subunit, selenocysteine-containing,W-formate dehydrogenase - alpha subunit  *W-formate dehydrogenase from Desulfovibrio vulgaris - Formate reduced form* | 0.46 | 0.00 | 18.96 | 0.80 | 2-408 | X-ray | 1.90 | monomer | 2 x MGD, 4 x SF4, 1 x W, 1 x H2S | HHblits | 0.29 |
| ``` target    NNVNRREFLQWIGAAGFSTFALSASNAWGLQAIENPLAAYPNREWEKTYRDLWKSDASFTFLCAPNDTHNCILNAHVR-- 6sdv.1    -TVTRRHFLKLSAGAAVAGAFTGLGLSLAPT---VARAE--------LQ--KLQWAKQTTSIC-CYCAVGCGLIVHTAKD  target    -DGVITRIGPTMKYGEATDLYGSKVTHRWDPRVCQKGLALTRRFYGDRRVRYPMVRKGFKAWADKGFPREKDGRPPKDYF 6sdv.1    GQGRAVNVEGDP------------DHPINEGSLCPKGASIFQLGENDQRGTQPLYRAP----------------------  target    NRARDEWLRLTHEEAADLVAAALINIATTYSGDNGQKLLLQQGYEKEIVEATRGAGTQVLKFRGGMPLLGLTRIFGLYRM 6sdv.1    --FSDTWKPVTWDFALTEIAKRIKKTRDASFTEKNAAGDLVNRT--EAIAS-----------------------FGSAAM  target    ANSMALLDHKIRGVKPEDALGARGWDNYS--WHTDLPPGHPMVTGQQTVDFDLHAVEQARIVVVWGMNWVTTKMPDTHWL 6sdv.1    DNEECWAYGNILR-----SLGLVYIEHQARIUHSPTVPALAESFGRGAMTNHWNDLANSDCILIMGSNAAENHPIAFKWV  target    TEARLKGTKVVVIACEYSSSSIKADDAIVVRPGTTPALALGLCNVIMREKIYDGDYVRRFSDLPLLVRA----------- 6sdv.1    LRAKDKGATLIHVDPRFTRTSARCDVYAPIRSGADIPFLGGLIKYILDNKLYFTDYVREYTNASLIVGEKFSFKDGLFSG  target    -------------------------------------------DNLKLLRAEEVFGTPQAALKNQTR 6sdv.1    YDAANKKYDKSMWAFELDANGVPKRDPALKHPRCVINLLKKHYERYNLDKVAAITGTSKEQLQQVYK ``` | | | | | | | | | | | | | | | | | | | | | | | | | | | | | | | | | | | | | | | | | | | | | | | | | |
|  | 1e60.1.A | Dimethyl sulfoxide/trimethylamine N-oxide reductase  *OXIDIZED DMSO REDUCTASE EXPOSED TO HEPES - Structure II BUFFER* | 0.41 | 0.00 | 24.92 | 0.78 | 2-408 | X-ray | 2.00 | monomer | 2 x PGD, 1 x 2MO | HHblits | 0.31 |
| ``` target    NNVNRREFLQWIGAAGFSTFALSASNAWGLQAIENPLAAYPNREWEKTYRDLWKSDASFTFLCAPNDTHNCI-LNAHVRD 1e60.1    -ELYRRAFLSYSVAPGALGMFGR-SL---L-AK----GARA--EA----------LA---NGT-VMSGSHWGVFTATVEN  target    GVITRIGPTMKYGEATDLYGSKVTHRWDPRVCQKGLALTRRFYGDRRVRYPMVRKGFKAWADKGFPREKDGRPPKDYFNR 1e60.1    GRATAFTPWEK------------D----PHPSPMLAGVLDSIYSPTRIKYPMVRREFL---EKGVNA--------DRSTR  target    ARDEWLRLTHEEAADLVAAALINIATTYSGDNGQKLLLQQGYEKEIVEATRGAGTQVLKFRGGMPLLGLTRIFGLYRMAN 1e60.1    GNGDFVRVSWDQALDLVAAEVKRVEETYGPEG---VFGG-SYGWKSPGRLHNC-T---TLLR-----RMLTLAGGY--VN  target    SMALLDHKIRGVKPEDALGARGWDNYSWHTD-----LPPGHPMVTGQQTVDFDLHAVEQARIVVVWGMNWVTTKMPDT-- 1e60.1    GA---------------------GDYSTGAAQVIMPHVVGTLEVYEQQ--TAWPVLAENTEVMVFWAADPIKTSQIGWVI  target    ------HWLTEARLKGTKVVVIACEYSSSSIK-ADDAIVVRPGTTPALALGLCNVIMREKIYDGDYVRRFSDLP-LLV-R 1e60.1    PEHGAYPGLEALKAKGTKVIVIDPVRTKTVEFFGAEHITPKPQTDVAIMLGMAHTLVAEDLYDKDFIANYTSGFDKFLPY  target    A------DNLKLLRAEEVFGTPQAALKNQTR 1e60.1    LDGETDSTPKTAEWAEGISGVPAETIKELAR ``` | | | | | | | | | | | | | | | | | | | | | | | | | | | | | | | | | | | | | | | | | | | | | | | | | |
| ✓ | 1e5v.2.A | Dimethyl sulfoxide/trimethylamine N-oxide reductase  *OXIDIZED DMSO REDUCTASE EXPOSED TO HEPES BUFFER* | 0.42 | 0.00 | 24.29 | 0.78 | 2-408 | X-ray | 2.40 | monomer | 2 x PGD, 1 x 2MO | HHblits | 0.31 |
| ``` target    NNVNRREFLQWIGAAGFSTFALSASNAWGLQAIENPLAAYPNREWEKTYRDLWKSDASFTFLCAPNDTHNCI-LNAHVRD 1e5v.2    -ELYRRAFLSYSVAPGALGMFGR-SL---LAK-----GARA--EA----------LAN---GT-VMSGSHWGVFTATVEN  target    GVITRIGPTMKYGEATDLYGSKVTHRWDPRVCQKGLALTRRFYGDRRVRYPMVRKGFKAWADKGFPREKDGRPPKDYFNR 1e5v.2    GRATAFTPWEK------------DP----HPSPMLAGVLDSIYSPTRIKYPMVRREFL---EKGVN--------ADRSTR  target    ARDEWLRLTHEEAADLVAAALINIATTYSGDNGQKLLLQQGYEKEIVEATRGAGTQVLKFRGGMPLLGLTRIFGLYRMAN 1e5v.2    GNGDFVRVSWDQALDLVAAEVKRVEETYGPEG---VFGG-SYGWKSPGRLHNCTTLLRRM---------LTLAGGY--VN  target    SMALLDHKIRGVKPEDALGARGWDNYSWH-----TDLPPGHPMVTGQQTVDFDLHAVEQARIVVVWGMNWVTTKMPD--- 1e5v.2    GA---------------------GDYSTGAAQVIMPHVVGTLEVYEQQ--TAWPVLAENTEVMVFWAADPIKTSQIGWVI  target    -----THWLTEARLKGTKVVVIACEYSSSSIK-ADDAIVVRPGTTPALALGLCNVIMREKIYDGDYVRRFSDLPL----L 1e5v.2    PEHGAYPGLEALKAKGTKVIVIDPVRTKTVEFFGAEHITPKPQTDVAIMLGMAHTLVAEDLYDKDFIANYTSGFDKFLPY  target    VR----ADNLKLLRAEEVFGTPQAALKNQTR 1e5v.2    LDGETDSTPKTAEWAEGISGVPAETIKELAR ``` | | | | | | | | | | | | | | | | | | | | | | | | | | | | | | | | | | | | | | | | | | | | | | | | | |
|  | 4dmr.1.A | DMSO REDUCTASE  *REDUCED DMSO REDUCTASE FROM RHODOBACTER CAPSULATUS WITH BOUND DMSO SUBSTRATE* | 0.42 | 0.00 | 24.29 | 0.78 | 2-408 | X-ray | 1.90 | monomer | 2 x PGD, 1 x 4MO, 1 x O | HHblits | 0.31 |
| ``` target    NNVNRREFLQWIGAAGFSTFALSASNAWGLQAIENPLAAYPNREWEKTYRDLWKSDASFTFLCAPNDTHNCI-LNAHVRD 4dmr.1    -ELYRRAFLSYSVAPGALGMFGR-SL---LA-----KGARA--EA----------LAN---GT-VMSGSHWGVFTATVEN  target    GVITRIGPTMKYGEATDLYGSKVTHRWDPRVCQKGLALTRRFYGDRRVRYPMVRKGFKAWADKGFPREKDGRPPKDYFNR 4dmr.1    GRATAFTPWEK------------DP----HPSPMLAGVLDSIYSPTRIKYPMVRREFL---EKGVN--------ADRSTR  target    ARDEWLRLTHEEAADLVAAALINIATTYSGDNGQKLLLQQGYEKEIVEATRGAGTQVLKFRGGMPLLGLTRIFGLYRMAN 4dmr.1    GNGDFVRVSWDQALDLVAAEVKRVEETYGPSG---VFGG-SYGWKSPGRLHNCTTLLRRM---------LTLAGGY--VN  target    SMALLDHKIRGVKPEDALGARGWDNYSWHT-----DLPPGHPMVTGQQTVDFDLHAVEQARIVVVWGMNWVTTKMPDT-- 4dmr.1    GA---------------------GDYSTGAAQVIMPHVVGTLEVYEQQ--TAWPVLAENTEVMVFWAADPIKTSQIGWVI  target    ------HWLTEARLKGTKVVVIACEYSSSSIK-ADDAIVVRPGTTPALALGLCNVIMREKIYDGDYVRRFSDLP-LLV-R 4dmr.1    PEHGAYPGLEALKAKGTKVIVIDPVRTKTVEFFGAEHITPKPQTDVAIMLGMAHTLVAEDLYDKDFIANYTSGFDKFLPY  target    A------DNLKLLRAEEVFGTPQAALKNQTR 4dmr.1    LDGETDSTPKTAEWAEGISGVPAETIKELAR ``` | | | | | | | | | | | | | | | | | | | | | | | | | | | | | | | | | | | | | | | | | | | | | | | | | |
|  | 1e18.1.A | DMSO REDUCTASE.  *TUNGSTEN-SUSBSTITUTED DMSO REDUCTASE FROM RHODOBACTER CAPSULATUS* | 0.42 | 0.00 | 23.66 | 0.78 | 2-408 | X-ray | 2.00 | monomer | 2 x PGD, 1 x 6WO | HHblits | 0.31 |
| ``` target    NNVNRREFLQWIGAAGFSTFALSASNAWGLQAIENPLAAYPNREWEKTYRDLWKSDASFTFLCAPNDTHNCI-LNAHVRD 1e18.1    -ELYRRAFLSYSVAPGALGMFGRSLL---------AKGARA--EA----------LAN---GT-VMSGSHWGVFTATVEN  target    GVITRIGPTMKYGEATDLYGSKVTHRWDPRVCQKGLALTRRFYGDRRVRYPMVRKGFKAWADKGFPREKDGRPPKDYFNR 1e18.1    GRATAFTPWEK------------DP----HPSPMLAGVLDSIYSPTRIKYPMVRREFL---EKGVNA--------DRSTR  target    ARDEWLRLTHEEAADLVAAALINIATTYSGDNGQKLLLQQGYEKEIVEATRGAGTQVLKFRGGMPLLGLTRIFGLYRMAN 1e18.1    GNGDFVRVSWDQALDLVAAEVKRVEETYGPQG---VFGG-SYGWKSPGRLHNCTTLLRRM---------LTLAGGY--VN  target    SMALLDHKIRGVKPEDALGARGWDNYSWHTD-----LPPGHPMVTGQQTVDFDLHAVEQARIVVVWGMNWVTTKMPDT-- 1e18.1    GA---------------------GDYSTGAAQVIMPHVVGTLEVYEQQ--TAWPVLAENTEVMVFWAADPIKTSQIGWVI  target    ------HWLTEARLKGTKVVVIACEYSSSSIK-ADDAIVVRPGTTPALALGLCNVIMREKIYDGDYVRRFSDLP-LLV-R 1e18.1    PEHGAYPGLEALKAKGTKVIVIDPVRTKTVEFFGAEHITPKPQTDVAIMLGMAHTLVAEDLYDKDFIANYTSGFDKFLPY  target    A------DNLKLLRAEEVFGTPQAALKNQTR 1e18.1    LDGETDSTPKTAEWAEGISGVPAETIKELAR ``` | | | | | | | | | | | | | | | | | | | | | | | | | | | | | | | | | | | | | | | | | | | | | | | | | |
|  | 3egw.1.A | Respiratory nitrate reductase 1 alpha chain  *The crystal structure of the NarGHI mutant NarH - C16A* | 0.49 | 0.15 | 29.19 | 0.73 | 41-384 | X-ray | 1.90 | homo-dimer | 2 x MD1, 2 x MGD, 2 x 6MO, 6 x SF4, 4 x F3S, 2 x 3PH, 4 x HEM, 2 x AGA | HHblits | 0.35 |
| ``` target    NNVNRREFLQWIGAAGFSTFALSASNAWGLQAIENPLAAYPNREWEKTYRDLWKSDASFTFLCAPNDTHNCILNAHVRDG 3egw.1    ----------------------------------------TNRDWEDGYRQRWQHDKIVRSTHGVNCTGSCSWKIYVKNG  target    VITRIGPTMKYGEATDLYGSKVTHRWDPRVCQKGLALTRRFYGDRRVRYPMVRKGF-KAWADKGF----PRE------K- 3egw.1    LVTWETQQTDYPR-----TRPDLPNHEPRGCPRGASYSWYLYSANRLKYPMMRKRLMKMWREAKALHSDPVEAWASIIED  target    DGRPPKDYFNRARDEWLRLTHEEAADLVAAALINIATTYSGDNGQKLLLQQGYEKEIVEATRGAGTQVLKFRGGMPLLGL 3egw.1    ADKAKSFKQARGRGGFVRSSWQEVNELIAASNVYTIKNYGPDRV---AGFSPIPA---------MSMV-SYA-------S  target    TRIFGLYRMANSMALLDHKIRGVKPEDALGARGWDNYSWHTDLPPGHPMVTGQQTVDFDLHAVEQARIVVVWGMNWVTTK 3egw.1    -----GARYLS----------------LIGGTCLSFYDWYCDLPPASPQTWGEQTDVPESADWYNSSYIIAWGSNVPQTR  target    MPDTHWLTEARLKGTKVVVIACEYSSSSIKADDAIVVRPGTTPALALGLCNVIMREKI------YDGDYVRRFSDLPLLV 3egw.1    TPDAHFFTEVRYKGTKTVAVTPDYAEIAKLCDLWLAPKQGTDAAMALAMGHVMLREFHLDNPSQYFTDYVRRYTDMPMLV  target    RADNLKLLRAEEVFGTPQAALKNQTR 3egw.1    ML------------------------ ``` | | | | | | | | | | | | | | | | | | | | | | | | | | | | | | | | | | | | | | | | | | | | | | | | | |
|  | 4ydd.1.A | DMSO reductase family type II enzyme, molybdopterin subunit  *Crystal structure of the perchlorate reductase PcrAB from Azospira suillum PS* | 0.54 | 0.00 | 31.49 | 0.71 | 38-395 | X-ray | 1.86 | monomer | 4 x SF4, 1 x MO, 1 x MGD, 1 x MD1, 1 x F3S | BLAST | 0.37 |
| ``` target    NNVNRREFLQWIGAAGFSTFALSASNAWGLQAIENPLAAYPNREWEKTYRDLWKSDASFTFLCAPNDTHNCILNAHVRDG 4ydd.1    -------------------------------------GAFEYSGWENFHRTQWSWDKKTRGAHLVNCTGACPHFVYSKDG  target    VITRIGPTMKYGEATDLYGSKVTHRWDPRVCQKGLALTRRFYGDRRVRYPMVRKGFKAWADKGFPREKDGRPPKDYFNRA 4ydd.1    VV------MREEQSKDIAPMPNIPEYNPRGCNKGECGHDYMYGPHRIKYPLIRVG----------------------ERG  target    RDEWLRLTHEEAADLVAAALINIATTYSGDNGQKLLLQQGYEKEIVEATRGAGTQVLKFRGGMPLLGLTRIFGLYRMANS 4ydd.1    EGKWRRATWEEALDMIADKCVDTIKNHAPD-------------------------CISVYSPVPAVSPVSFSAGHRFAH-  target    MALLDHKIRGVKPEDALGARGWDNYSWHTDLPPGHPMVTGQQTVDFDLHAVEQARIVVVWGMNWVTTKMPDTHWLTEARL 4ydd.1    ---------------YIGAHAHTFYDWYGDHPTGQTQTCGVQGDTCETADWFNSKYIILWGSNPTQTRIPDAHFLSEAQL  target    KGTKVVVIACEYSSSSIKADDAIVVRPGTTPALALGLCNVIMREKIYDGDYVRRFSDLPLLVRADNLKLLRAEEVFGTPQ 4ydd.1    NGAKIVSISPDYNSSTIKVDKWIHPQPGTDGALAMAMAHVIIKEKLYDAHSLKEQTDLSYLVRSDTKRFLREADV-----  target    AALKNQTR 4ydd.1    -------- ``` | | | | | | | | | | | | | | | | | | | | | | | | | | | | | | | | | | | | | | | | | | | | | | | | | |
|  | 5e7o.1.A | DMSO reductase family type II enzyme, molybdopterin subunit  *Crystal structure of the perchlorate reductase PcrAB mutant W461E of PcrA from Azospira suillum PS* | 0.53 | 0.00 | 31.49 | 0.71 | 38-395 | X-ray | 2.40 | monomer | 4 x SF4, 1 x MO, 1 x MGD, 1 x MD1, 1 x F3S | BLAST | 0.37 |
| ``` target    NNVNRREFLQWIGAAGFSTFALSASNAWGLQAIENPLAAYPNREWEKTYRDLWKSDASFTFLCAPNDTHNCILNAHVRDG 5e7o.1    -------------------------------------GAFEYSGWENFHRTQWSWDKKTRGAHLVNCTGACPHFVYSKDG  target    VITRIGPTMKYGEATDLYGSKVTHRWDPRVCQKGLALTRRFYGDRRVRYPMVRKGFKAWADKGFPREKDGRPPKDYFNRA 5e7o.1    VV------MREEQSKDIAPMPNIPEYNPRGCNKGECGHDYMYGPHRIKYPLIRVG----------------------ERG  target    RDEWLRLTHEEAADLVAAALINIATTYSGDNGQKLLLQQGYEKEIVEATRGAGTQVLKFRGGMPLLGLTRIFGLYRMANS 5e7o.1    EGKWRRATWEEALDMIADKCVDTIKNHAPD-------------------------CISVYSPVPAVSPVSFSAGHRFAH-  target    MALLDHKIRGVKPEDALGARGWDNYSWHTDLPPGHPMVTGQQTVDFDLHAVEQARIVVVWGMNWVTTKMPDTHWLTEARL 5e7o.1    ---------------YIGAHAHTFYDWYGDHPTGQTQTCGVQGDTCETADWFNSKYIILWGSNPTQTRIPDAHFLSEAQL  target    KGTKVVVIACEYSSSSIKADDAIVVRPGTTPALALGLCNVIMREKIYDGDYVRRFSDLPLLVRADNLKLLRAEEVFGTPQ 5e7o.1    NGAKIVSISPDYNSSTIKVDKWIHPQPGTDGALAMAMAHVIIKEKLYDAHSLKEQTDLSYLVRSDTKRFLREADV-----  target    AALKNQTR 5e7o.1    -------- ``` | | | | | | | | | | | | | | | | | | | | | | | | | | | | | | | | | | | | | | | | | | | | | | | | | |
|  | 2vpz.1.A | THIOSULFATE REDUCTASE  *POLYSULFIDE REDUCTASE NATIVE STRUCTURE* | 0.44 |  | 20.70 | 0.77 | 3-408 | X-ray | 2.40 | hetero-oligomer | 10 x SF4, 4 x MGD, 2 x MO | HHblits | 0.30 |
| ``` target    NNVNRREFLQWIGAAGFSTFALSASNAWGLQAIENPLAAYPNREWEKTYRDLWKSDASFTFLCAPNDTHNCILNAHVRDG 2vpz.1    --MQRREFLKLSALGVGAMALRGSGP---A----K----ALKAPWYA------QEVKSVYQIC-EGCFWRCGIVAHAVGN  target    VITRIGPTMKYGEATDLYGSKVTHRWDPRVCQKGLALTRRFYGDRRVRYPMVRKGFKAWADKGFPREKDGRPPKDYFNRA 2vpz.1    RVYKVEGYE------------ANPKSRGRLCPRGQGAPQTTYDPDRLKRPLIRVEGS--------------------QRG  target    RDEWLRLTHEEAADLVAAALINIATTYSGDNGQKLLLQQGYEKEIVEATRGAGTQVLKFRGGMPLLGLTRIFGLYRMANS 2vpz.1    EGKYRVATWEEALDHIAKKMLEIREKYGPEA---IAFFGHGT----------GD--YWFVD-----FLPAAWGSPNAAKP  target    MALLDHKIRGVKPEDALGARGWDNYSWHTDLPPGHPMVTGQQTVDFDLHAVEQARIVVVWGMNWVTT-KMPDTHWLTEAR 2vpz.1    SVS----------------------LCTAPREVASQWVFGRPIGGHEPIDWENARYIVLIGHHIGEDTHNTQLQDFALAL  target    LKGTKVVVIACEYSSSSIKADDAIVVRPGTTPALALGLCNVIMREKIYDGDYVRRFSDL-PLLV-RADNLKLLRAEEVFG 2vpz.1    KNGAKVVVVDPRFSTAAAKAHRWLPIKPGTDTALLLAWIHVLIYEDLYDKEYVAKYTVGFEELKAHVKDFTPEWAEKHTE  target    TPQAALKNQTR 2vpz.1    IPAQVIREVAR ``` | | | | | | | | | | | | | | | | | | | | | | | | | | | | | | | | | | | | | | | | | | | | | | | | | |
|  | 2vpx.1.D | THIOSULFATE REDUCTASE  *POLYSULFIDE REDUCTASE WITH BOUND QUINONE (UQ1)* | 0.43 |  | 20.70 | 0.77 | 3-408 | X-ray | 3.10 | hetero-oligomer | 10 x SF4, 4 x MGD, 2 x MO, 2 x UQ1 | HHblits | 0.30 |
| ``` target    NNVNRREFLQWIGAAGFSTFALSASNAWGLQAIENPLAAYPNREWEKTYRDLWKSDASFTFLCAPNDTHNCILNAHVRDG 2vpx.1    --MQRREFLKLSALGVGAMALRGSGP---A----K----ALKAPWYA------QEVKSVYQIC-EGCFWRCGIVAHAVGN  target    VITRIGPTMKYGEATDLYGSKVTHRWDPRVCQKGLALTRRFYGDRRVRYPMVRKGFKAWADKGFPREKDGRPPKDYFNRA 2vpx.1    RVYKVEGYE------------ANPKSRGRLCPRGQGAPQTTYDPDRLKRPLIRVEGS--------------------QRG  target    RDEWLRLTHEEAADLVAAALINIATTYSGDNGQKLLLQQGYEKEIVEATRGAGTQVLKFRGGMPLLGLTRIFGLYRMANS 2vpx.1    EGKYRVATWEEALDHIAKKMLEIREKYGPEA---IAFFGHGT----------GD--YWFVD-----FLPAAWGSPNAAKP  target    MALLDHKIRGVKPEDALGARGWDNYSWHTDLPPGHPMVTGQQTVDFDLHAVEQARIVVVWGMNWVTT-KMPDTHWLTEAR 2vpx.1    SVS----------------------LCTAPREVASQWVFGRPIGGHEPIDWENARYIVLIGHHIGEDTHNTQLQDFALAL  target    LKGTKVVVIACEYSSSSIKADDAIVVRPGTTPALALGLCNVIMREKIYDGDYVRRFSDL-PLLV-RADNLKLLRAEEVFG 2vpx.1    KNGAKVVVVDPRFSTAAAKAHRWLPIKPGTDTALLLAWIHVLIYEDLYDKEYVAKYTVGFEELKAHVKDFTPEWAEKHTE  target    TPQAALKNQTR 2vpx.1    IPAQVIREVAR ``` | | | | | | | | | | | | | | | | | | | | | | | | | | | | | | | | | | | | | | | | | | | | | | | | | |
|  | 2ivf.1.A | ETHYLBENZENE DEHYDROGENASE ALPHA-SUBUNIT  *ETHYLBENZENE DEHYDROGENASE FROM AROMATOLEUM AROMATICUM* | 0.42 | 0.00 | 36.07 | 0.69 | 45-397 | X-ray | 1.88 | monomer | 1 x MES, 4 x SF4, 1 x MO, 1 x MGD, 1 x MD1, 1 x F3S, 1 x HEM | BLAST | 0.38 |
| ``` target    NNVNRREFLQWIGAAGFSTFALSASNAWGLQAIENPLAAYPNREWEKTYRDLWKSD----ASFTFLCAPNDTHNCILNAH 2ivf.1    --------------------------------------------WEDIYRKEWKWDKVNWGSHLNICWPQGS--CKFYVY  target    VRDGVITRIGPTMKYGEATDLYGSKVTH-RWDPRVCQKGLALTRRFYGDRRVRYPMVRKGFKAWADKGFPREKDGRPPKD 2ivf.1    VRNGIVWR------EEQAAQTPACNVDYVDYNPLGCQKGSAFNNNLYGDERVKYPLKRVG--------------------  target    YFNRARDEWLRLTHEEAADLVAAALINIATTYSGDNGQKLLLQQGYEKEIVEATRGAGTQVLKFRGGMPLLGLTRIFGLY 2ivf.1    --KRGEGKWKRVSWDEAAGDIADSIIDS------------FEAQGSDGFILDAPHVHAGSIAWGAG-------------F  target    RMANSMALLDHKIRGVKPEDALGARGWDNY--SWHTDLPPGHPMVTGQQTVDFDLHAVEQARIVVVWGMNWVTTKMPDTH 2ivf.1    RMT---YLMD----GVSPD--INVDIGDTYMGAFHT---------FGKMHMGYSADNLLDAELIFMTCSNWSYTYPSSYH  target    WLTEARLKGTKVVVIACEYSSSSIKADDAIVVRPGTTPALALGLCNVIMREKIYDGDYVRRFSDLPLLVRADNLKLLRAE 2ivf.1    FLSEARYKGAEVVVIAPDFNPTTPAADLHVPVRVGSDAAFWLGLSQVMIDEKLFDRQFVCEQTDLPLLVRMDTGKFLSAE  target    EVFGTPQAALKNQTR 2ivf.1    DVDG----------- ``` | | | | | | | | | | | | | | | | | | | | | | | | | | | | | | | | | | | | | | | | | | | | | | | | | |
|  | 4v4c.1.A | Pyrogallol hydroxytransferase large subunit  *Crystal Structure of Pyrogallol-Phloroglucinol Transhydroxylase from Pelobacter acidigallici* | 0.41 |  | 19.47 | 0.74 | 62-408 | X-ray | 2.35 | hetero-oligomer | 2 x CA, 2 x MGD, 1 x 4MO, 3 x SF4 | HHblits | 0.29 |
| ``` target    NNVNRREFLQWIGAAGFSTFALSASNAWGLQAIENPLAAYPNREWEKTYRDLWKSDASFTFLCAPNDTHNCILNAHVRDG 4v4c.1    -------------------------------------------------------------RLTN-SSTGGPVFVYVKDG  target    VITRIGPTMKYGEAT------DLYGSKVTHRWDPRVCQKGLALTRRFYGDRRVRYPMVRKGFKAWADKGFPREKDGRPPK 4v4c.1    KIIRMTPMDFDDAVDAPSWKIEARGKTFTPPRKTSIAPYTAGFKSMIYSDLRIPYPMKRKSFDP---NGE---------R  target    DYFNRARD--------EWLRLTHEEAADLVAAALINIATTYSGDNGQKLLLQQGYEKEIVEATRGAGTQVLKFRGGMPLL 4v4c.1    NPQLRGAGLSKQDPWSDYERISWDEATDIVVAEINRIKHAYGPSA---ILSTPSSHH-------MWGNVGYRH--S-TYF  target    GLTRIFGLYRMANSMALLDHKIRGVKPEDALGARGWDNYSWHTDLPPGHPMVTGQQTVDFD-LHAVEQARIVVVWGMNWV 4v4c.1    RFMNMMGFTYADH-------NP-----------DSWEGWHWGGMHMWGFSWRLGNPEQYDLLEDGLKHAEMIVFWSSDPE  target    TTKMPDTHW-----LTEARLKGTKVVVIACEYSSSSI-KADDAIVVRPGTTPALALGLCNVIMREKIYDGDYVRRFSDL- 4v4c.1    TNSGIYAGFESNIRRQWLKDLGVDFVFIDPHMNHTARLVADKWFSPKIGTDHALSFAIAYTWLKEDSYDKEYVAANAHGF  target    ---PLLVRA----DNLKLLRAEEVFGTPQAALKNQTR 4v4c.1    EEWADYVLGKTDGTPKTCEWAEEESGVPACEIRALAR ``` | | | | | | | | | | | | | | | | | | | | | | | | | | | | | | | | | | | | | | | | | | | | | | | | | |
|  | 4ydd.1.A | DMSO reductase family type II enzyme, molybdopterin subunit  *Crystal structure of the perchlorate reductase PcrAB from Azospira suillum PS* | 0.50 | 0.00 | 30.22 | 0.68 | 38-384 | X-ray | 1.86 | monomer | 4 x SF4, 1 x MO, 1 x MGD, 1 x MD1, 1 x F3S | HHblits | 0.36 |
| ``` target    NNVNRREFLQWIGAAGFSTFALSASNAWGLQAIENPLAAYPNREWEKTYRDLWKSDASFTFLCAPNDTHNCILNAHVRDG 4ydd.1    -------------------------------------GAFEYSGWENFHRTQWSWDKKTRGAHLVNCTGACPHFVYSKDG  target    VITRIGPTMKYGEATDLYGSKVTHRWDPRVCQKGLALTRRFYGDRRVRYPMVRKGFKAWADKGFPREKDGRPPKDYFNRA 4ydd.1    VVMREEQSKDI------APMPNIPEYNPRGCNKGECGHDYMYGPHRIKYPLIRVG----------------------ERG  target    RDEWLRLTHEEAADLVAAALINIATTYSGDNGQKLLLQQGYEKEIVEATRGAGTQVLKFRGGMPLLGLTRIFGLYRMANS 4ydd.1    EGKWRRATWEEALDMIADKCVDTIKNHAPDCIS---VYSPVP----------AVSPVSFSAG---HRFAHYIGAH-----  target    MALLDHKIRGVKPEDALGARGWDNYSWHTDLPPGHPMVTGQQTVDFDLHAVEQARIVVVWGMNWVTTKMPDTHWLTEARL 4ydd.1    --------------------AHTFYDWYGDHPTGQTQTCGVQGDTCETADWFNSKYIILWGSNPTQTRIPDAHFLSEAQL  target    KGTKVVVIACEYSSSSIKADDAIVVRPGTTPALALGLCNVIMREKIYDGDYVRRFSDLPLLVRADNLKLLRAEEVFGTPQ 4ydd.1    NGAKIVSISPDYNSSTIKVDKWIHPQPGTDGALAMAMAHVIIKEKLYDAHSLKEQTDLSYLVRS----------------  target    AALKNQTR 4ydd.1    -------- ``` | | | | | | | | | | | | | | | | | | | | | | | | | | | | | | | | | | | | | | | | | | | | | | | | | |
|  | 5e7o.1.A | DMSO reductase family type II enzyme, molybdopterin subunit  *Crystal structure of the perchlorate reductase PcrAB mutant W461E of PcrA from Azospira suillum PS* | 0.50 | 0.00 | 29.86 | 0.68 | 38-384 | X-ray | 2.40 | monomer | 4 x SF4, 1 x MO, 1 x MGD, 1 x MD1, 1 x F3S | HHblits | 0.35 |
| ``` target    NNVNRREFLQWIGAAGFSTFALSASNAWGLQAIENPLAAYPNREWEKTYRDLWKSDASFTFLCAPNDTHNCILNAHVRDG 5e7o.1    -------------------------------------GAFEYSGWENFHRTQWSWDKKTRGAHLVNCTGACPHFVYSKDG  target    VITRIGPTMKYGEATDLYGSKVTHRWDPRVCQKGLALTRRFYGDRRVRYPMVRKGFKAWADKGFPREKDGRPPKDYFNRA 5e7o.1    VVMREEQSKDI------APMPNIPEYNPRGCNKGECGHDYMYGPHRIKYPLIRVG----------------------ERG  target    RDEWLRLTHEEAADLVAAALINIATTYSGDNGQKLLLQQGYEKEIVEATRGAGTQVLKFRGGMPLLGLTRIFGLYRMANS 5e7o.1    EGKWRRATWEEALDMIADKCVDTIKNHAPDCIS---VYSPVP----------AVSPVSFSA---GHRFAHYIGAH-----  target    MALLDHKIRGVKPEDALGARGWDNYSWHTDLPPGHPMVTGQQTVDFDLHAVEQARIVVVWGMNWVTTKMPDTHWLTEARL 5e7o.1    --------------------AHTFYDWYGDHPTGQTQTCGVQGDTCETADWFNSKYIILWGSNPTQTRIPDAHFLSEAQL  target    KGTKVVVIACEYSSSSIKADDAIVVRPGTTPALALGLCNVIMREKIYDGDYVRRFSDLPLLVRADNLKLLRAEEVFGTPQ 5e7o.1    NGAKIVSISPDYNSSTIKVDKWIHPQPGTDGALAMAMAHVIIKEKLYDAHSLKEQTDLSYLVRS----------------  target    AALKNQTR 5e7o.1    -------- ``` | | | | | | | | | | | | | | | | | | | | | | | | | | | | | | | | | | | | | | | | | | | | | | | | | |
|  | 8bqg.1.A | Formate dehydrogenase, alpha subunit, selenocysteine-containing  *W-formate dehydrogenase from Desulfovibrio vulgaris - Soaking with Formate 1 min* | 0.45 | 0.00 | 18.43 | 0.72 | 39-408 | X-ray | 1.95 | monomer | 2 x MGD, 4 x SF4, 1 x H2S, 1 x W | HHblits | 0.29 |
| ``` target    NNVNRREFLQWIGAAGFSTFALSASNAWGLQAIENPLAAYPNREWEKTYRDLWKSDASFTFLCAPNDTHNCILNAHVR-- 8bqg.1    --------------------------------------EL--------Q--KLQWAKQTTSIC-CYCAVGCGLIVHTAKD  target    -DGVITRIGPTMKYGEATDLYGSKVTHRWDPRVCQKGLALTRRFYGDRRVRYPMVRKGFKAWADKGFPREKDGRPPKDYF 8bqg.1    GQGRAVNVEGDP------------DHPINEGSLCPKGASIFQLGENDQRGTQPLYRAP----------------------  target    NRARDEWLRLTHEEAADLVAAALINIATTYSGDNGQKLLLQQGYEKEIVEATRGAGTQVLKFRGGMPLLGLTRIFGLYRM 8bqg.1    --FSDTWKPVTWDFALTEIAKRIKKTRDASFTEKNAAGDLVNR--TEAIASF-----------------------GSAAM  target    ANSMALLDHKIRGVKPEDALGARGWDNYS--WHTDLPPGHPMVTGQQTVDFDLHAVEQARIVVVWGMNWVTTKMPDTHWL 8bqg.1    DNEECWAYGNIL-----RSLGLVYIEHQARIUHSPTVPALAESFGRGAMTNHWNDLANSDCILIMGSNAAENHPIAFKWV  target    TEARLKGTKVVVIACEYSSSSIKADDAIVVRPGTTPALALGLCNVIMREKIYDGDYVRRFSDLPLLVRAD---------- 8bqg.1    LRAKDKGATLIHVDPRFTRTSARCDVYAPIRSGADIPFLGGLIKYILDNKLYFTDYVREYTNASLIVGEKFSFKDGLFSG  target    --------------------------------------------NLKLLRAEEVFGTPQAALKNQTR 8bqg.1    YDAANKKYDKSMWAFELDANGVPKRDPALKHPRCVINLLKKHYERYNLDKVAAITGTSKEQLQQVYK ``` | | | | | | | | | | | | | | | | | | | | | | | | | | | | | | | | | | | | | | | | | | | | | | | | | |
|  | 7l5i.1.A | Trimethylamine-N-oxide reductase  *Crystal Structure of Haemophilus influenzae MtsZ at pH 7.0* | 0.41 |  | 21.13 | 0.70 | 56-408 | X-ray | 1.73 | monomer | 2 x MGD, 1 x MO, 1 x O | HHblits | 0.31 |
| ``` target    NNVNRREFLQWIGAAGFSTFALSASNAWGLQAIENPLAAYPNREWEKTYRDLWKSDASFTFLCAPNDTHNCILNAHVRDG 7l5i.1    -------------------------------------------------------MKTVVTAAH-----WGSIGVVVQDG  target    VITRIGPTMKYGEATDLYGSKVTHRWDPRVCQKGLALTRRFYGDRRVRYPMVRKGFKAWADKGFPREKDGRPPKDYFNRA 7l5i.1    KVVKSGPAIE-------------PAVPNELQT---VVADQLYSEARVKCPMVRKGFLA----N------P-GKSDTTMRG  target    RDEWLRLTHEEAADLVAAALINIATTYSGDNGQKLLLQQGYEKEIVEATRGAGTQVLKFRGGMPLLGLTRIFGLYRMANS 7l5i.1    RDEWVRVSWDEALDLVHNQLKRVRDEHGSTGI---FAGS-YGWFSCGSLHASRTLLQRY---------MNATGGFV----  target    MALLDHKIRGVKPEDALGARGWDNYSWHTDLPPGHPMVTGQQ---TVDFDLH-AVEQARIVVVWGMNWVTTKMPD----- 7l5i.1    ----GH-------------K--GDYSTGA-AQVIMPHVLGTIEVYEQQTSWESILESSDIIVLWSANPLTTMRIAWMSTD  target    ---THWLTEARLKGTKVVVIACEYSSSSIK-ADDAIVVRPGTTPALALGLCNVIMREKIYDGDYVRRFSDLPLLVR---- 7l5i.1    QKGIEYFKKFQASGKRIICIDPQKSETCQMLNAEWIPVNTATDVPLMLGIAHTLVEQGKHDKDFLKKYTSGYAKFEEYLL  target    ----ADNLKLLRAEEVFGTPQAALKNQTR 7l5i.1    GKTDGQPKTAEWAAKICGVPAETIKQLAA ``` | | | | | | | | | | | | | | | | | | | | | | | | | | | | | | | | | | | | | | | | | | | | | | | | | |
|  | 7l5s.1.A | Trimethylamine-N-oxide reductase  *Crystal Structure of Haemophilus influenzae MtsZ at pH 5.5* | 0.40 |  | 21.13 | 0.70 | 56-408 | X-ray | 2.09 | monomer | 1 x O, 2 x MGD, 1 x MO | HHblits | 0.31 |
| ``` target    NNVNRREFLQWIGAAGFSTFALSASNAWGLQAIENPLAAYPNREWEKTYRDLWKSDASFTFLCAPNDTHNCILNAHVRDG 7l5s.1    -------------------------------------------------------MKTVVTAAH-----WGSIGVVVQDG  target    VITRIGPTMKYGEATDLYGSKVTHRWDPRVCQKGLALTRRFYGDRRVRYPMVRKGFKAWADKGFPREKDGRPPKDYFNRA 7l5s.1    KVVKSGPAIE-------------PAVPNELQT---VVADQLYSEARVKCPMVRKGFLA----N------P-GKSDTTMRG  target    RDEWLRLTHEEAADLVAAALINIATTYSGDNGQKLLLQQGYEKEIVEATRGAGTQVLKFRGGMPLLGLTRIFGLYRMANS 7l5s.1    RDEWVRVSWDEALDLVHNQLKRVRDEHGSTGI---FAGS-YGWFSCGSLHASRTLLQRY---------MNATGGFV----  target    MALLDHKIRGVKPEDALGARGWDNYSWHTDLPPGHPMVTGQQ---TVDFDLH-AVEQARIVVVWGMNWVTTKMPD----- 7l5s.1    ----GH-------------K--GDYSTGA-AQVIMPHVLGTIEVYEQQTSWESILESSDIIVLWSANPLTTMRIAWMSTD  target    ---THWLTEARLKGTKVVVIACEYSSSSIK-ADDAIVVRPGTTPALALGLCNVIMREKIYDGDYVRRFSDLPLLVR---- 7l5s.1    QKGIEYFKKFQASGKRIICIDPQKSETCQMLNAEWIPVNTATDVPLMLGIAHTLVEQGKHDKDFLKKYTSGYAKFEEYLL  target    ----ADNLKLLRAEEVFGTPQAALKNQTR 7l5s.1    GKTDGQPKTAEWAAKICGVPAETIKQLAA ``` | | | | | | | | | | | | | | | | | | | | | | | | | | | | | | | | | | | | | | | | | | | | | | | | | |
|  | 1dms.1.A | DMSO REDUCTASE  *STRUCTURE OF DMSO REDUCTASE* | 0.39 |  | 23.84 | 0.69 | 62-408 | X-ray | 1.88 | monomer | 2 x PGD, 1 x 2MO | HHblits | 0.32 |
| ``` target    NNVNRREFLQWIGAAGFSTFALSASNAWGLQAIENPLAAYPNREWEKTYRDLWKSDASFTFLCAPNDTHNCILNAHVRDG 1dms.1    -------------------------------------------------------------GTVMSGSHWGVFTATVENG  target    VITRIGPTMKYGEATDLYGSKVTHRWDPRVCQKGLALTRRFYGDRRVRYPMVRKGFKAWADKGFPREKDGRPPKDYFNRA 1dms.1    RATAFTPWEK------------D----PHPTPMLEGVLDSIYSPTRIKYPMVRREFL---EKGV-------N-ADRSTRG  target    RDEWLRLTHEEAADLVAAALINIATTYSGDNGQKLLLQQGYEKEIVEATRGAGTQVLKFRGGMPLLGLTRIF-GLYRMAN 1dms.1    NGDFVRVSWDQALDLVAAEVKRVEETYGPQG---VFGG-SYGWKSPGRLHNCTTLLRRM---------LTLAGGYVNGA-  target    SMALLDHKIRGVKPEDALGARGWDNYSWHTD-----LPPGHPMVTGQQTVDFDLHAVEQARIVVVWGMNWVTTKMPDT-- 1dms.1    -----------------------GDYSTGAAQVIMPHVVGTLEVYEQQ--TAWPVLAENTEVMVFWAADPIKTSQIGWVI  target    ------HWLTEARLKGTKVVVIACEYSSSSIK-ADDAIVVRPGTTPALALGLCNVIMREKIYDGDYVRRFSDLP-----L 1dms.1    PEHGAYPGLEALKAKGTKVIVIDPVRTKTVEFFGADHVTPKPQTDVAIMLGMAHTLVAEDLYDKDFIANYTSGFDKFLPY  target    LVRA---DNLKLLRAEEVFGTPQAALKNQTR 1dms.1    LMGETDSTPKTAEWASDISGVPAETIKELAR ``` | | | | | | | | | | | | | | | | | | | | | | | | | | | | | | | | | | | | | | | | | | | | | | | | | |
|  | 7e5z.1.A | Formate dehydrogenase  *Dehydrogenase holoenzyme* | 0.40 |  | 21.60 | 0.70 | 56-408 | EM | 0.00 | hetero-1-1-mer | 1 x W, 2 x MGD, 2 x FES, 4 x SF4, 1 x FMN | HHblits | 0.30 |
| ``` target    NNVNRREFLQWIGAAGFSTFALSASNAWGLQAIENPLAAYPNREWEKTYRDLWKSDASFTFLCAPNDTHNCILNAHVRDG 7e5z.1    -------------------------------------------------------DREVKSLC-PYCGVGCQVSYKVKDE  target    VITRIGPTMKYGEATDLYGSKVTHRWDPRVCQKGLALTRRFYGDRRVRYPMVRKGFKAWADKGFPREKDGRPPKDYFNRA 7e5z.1    RIVYAEGVN-------------GPANQNRLCVKGRFGFDYVHHPHRLTVPLIRLENVP---KD-------ANDQVDPANP  target    RDEWLRLTHEEAADLVAAALINIATTYSGDNGQKLLLQQGYEKEIVEATRGAGTQVLKFRGGMPLLGLTR-IFGLYRMAN 7e5z.1    WTHFREATWEEALDRAAGGLKAIRDTNGRKALAGFGSAKG-S----------NEEAYLF------QKLVRLGFGTNN---  target    SMALLDHKIRGVKPEDALGARGWDNYS--WHTDLPPGHPMVTGQQTVDFDLHAVEQARIVVVWGMNWVTTKMPDTHWLTE 7e5z.1    ----------------------VDHCTRLCHASSVAALMEGLNSGAVTAPFSAALDAEVIVVIGANPTVNHPVAATFLKN  target    A-RLKGTKVVVIACEYSSSSIKADDAIVVRPGTTPALALGLCNVIMREKIYDGDYVRRFSDL-PLLV-RADNLKLLRAEE 7e5z.1    AVKQRGAKLIIMDPRRQTLSRHAYRHLAFRPGSDVAMLNAMLNVIVTEGLYDEQYIAGYTENFEALREKIVDFTPEKMAS  target    VFGTPQAALKNQTR 7e5z.1    VCGIDAETLREVAR ``` | | | | | | | | | | | | | | | | | | | | | | | | | | | | | | | | | | | | | | | | | | | | | | | | | |
|  | 7vw6.1.A | Formate dehydrogenase  *Cryo-EM Structure of Formate Dehydrogenase 1 from Methylorubrum extorquens AM1* | 0.42 |  | 21.60 | 0.70 | 56-408 | EM | 0.00 | hetero-1-1-mer | 4 x SF4, 2 x FES, 2 x MGD, 1 x W, 1 x FMN | HHblits | 0.30 |
| ``` target    NNVNRREFLQWIGAAGFSTFALSASNAWGLQAIENPLAAYPNREWEKTYRDLWKSDASFTFLCAPNDTHNCILNAHVRDG 7vw6.1    -------------------------------------------------------DREVKSLC-PYCGVGCQVSYKVKDE  target    VITRIGPTMKYGEATDLYGSKVTHRWDPRVCQKGLALTRRFYGDRRVRYPMVRKGFKAWADKGFPREKDGRPPKDYFNRA 7vw6.1    RIVYAEGVN-------------GPANQNRLCVKGRFGFDYVHHPHRLTVPLIRLENVP---KD-------ANDQVDPANP  target    RDEWLRLTHEEAADLVAAALINIATTYSGDNGQKLLLQQGYEKEIVEATRGAGTQVLKFRGGMPLLGLTR-IFGLYRMAN 7vw6.1    WTHFREATWEEALDRAAGGLKAIRDTNGRKALAGFGSAKG-SN----------EEAYLF------QKLVRLGFGTN----  target    SMALLDHKIRGVKPEDALGARGWDNYS--WHTDLPPGHPMVTGQQTVDFDLHAVEQARIVVVWGMNWVTTKMPDTHWLTE 7vw6.1    ---------------------NVDHCTRLCHASSVAALMEGLNSGAVTAPFSAALDAEVIVVIGANPTVNHPVAATFLKN  target    A-RLKGTKVVVIACEYSSSSIKADDAIVVRPGTTPALALGLCNVIMREKIYDGDYVRRFSDL-PLLV-RADNLKLLRAEE 7vw6.1    AVKQRGAKLIIMDPRRQTLSRHAYRHLAFRPGSDVAMLNAMLNVIVTEGLYDEQYIAGYTENFEALREKIVDFTPEKMAS  target    VFGTPQAALKNQTR 7vw6.1    VCGIDAETLREVAR ``` | | | | | | | | | | | | | | | | | | | | | | | | | | | | | | | | | | | | | | | | | | | | | | | | | |
|  | 1eu1.1.A | DIMETHYL SULFOXIDE REDUCTASE  *THE CRYSTAL STRUCTURE OF RHODOBACTER SPHAEROIDES DIMETHYLSULFOXIDE REDUCTASE REVEALS TWO DISTINCT MOLYBDENUM COORDINATION ENVIRONMENTS.* | 0.38 |  | 22.66 | 0.68 | 65-408 | X-ray | 1.30 | monomer | 3 x GLC, 1 x CD, 2 x MGD, 1 x 6MO, 2 x O | HHblits | 0.30 |
| ``` target    NNVNRREFLQWIGAAGFSTFALSASNAWGLQAIENPLAAYPNREWEKTYRDLWKSDASFTFLCAPNDTHNCILNAHVRDG 1eu1.1    ----------------------------------------------------------------SGCHWGV-FKARVENG  target    VITRIGPTMKYGEATDLYGSKVTHRWDPRVCQKGLALTRRFYGDRRVRYPMVRKGFKAWADKGFPREKDGRPPKDYFNRA 1eu1.1    RAVAFEPWD------------KDPAPSHQLPG----VLDSIYSPTRIKYPMVRREFL---EKGVN--------ADRSTRG  target    RDEWLRLTHEEAADLVAAALINIATTYSGDNGQKLLLQQGYEKEIVEATRGAGTQVLKFRGGMPLLGLTRIFGLYR-MAN 1eu1.1    NGDFVRVTWDEALDLVARELKRVQESYGPTGT---FGGS-YGWKSPGRL----HNCQVLM--------RRALNLAGGFVN  target    SMALLDHKIRGVKPEDALGARGWDNYSWHTDLPPGHPMVTGQQ----TVDFDLHAVEQARIVVVWGMNWVTTKMPD---- 1eu1.1    SS---------------------GDYSTAAAQ-IIMPHVMGTLEVYEQQTAWPVVVENTDLMVFWAADPMKTNEIGWVIP  target    ----THWLTEARLKGTKVVVIACEYSSSSIKAD-DAIVVRPGTTPALALGLCNVIMREKIYDGDYVRRFSDLPLLVR--A 1eu1.1    DHGAYAGMKALKEKGTRVICINPVRTETADYFGADVVSPRPQTDVALMLGMAHTLYSEDLHDKDFLENCTTGFDLFAAYL  target    D------NLKLLRAEEVFGTPQAALKNQTR 1eu1.1    TGESDGTPKTAEWAAEICGLPAEQIRELAR ``` | | | | | | | | | | | | | | | | | | | | | | | | | | | | | | | | | | | | | | | | | | | | | | | | | |
|  | 1h0h.1.A | FORMATE DEHYDROGENASE SUBUNIT ALPHA  *Tungsten containing Formate Dehydrogenase from Desulfovibrio Gigas* | 0.43 |  | 16.73 | 0.69 | 60-408 | X-ray | 1.80 | hetero-1-1-mer | 1 x W, 1 x 2MD, 1 x MGD, 4 x SF4, 1 x CA | HHblits | 0.29 |
| ``` target    NNVNRREFLQWIGAAGFSTFALSASNAWGLQAIENPLAAYPNREWEKTYRDLWKSDASFTFLCAPNDTHNCILNAHV--R 1h0h.1    -----------------------------------------------------------TSVC-CYCSVGCGLIVHTDKK  target    DGVITRIGPTMKYGEATDLYGSKVTHRWDPRVCQKGLALTRRFYGDRRVRYPMVRKGFKAWADKGFPREKDGRPPKDYFN 1h0h.1    TNRAINVEGDP------------DHPINEGSLCAKGASTWQLAENERRPANPLYRAP-----------------------  target    RARDEWLRLTHEEAADLVAAALINIATTYSGDNG-QKLLLQQGYEKEIVEATRGAGTQVLKFRGGMPLLGLTRIFGLYRM 1h0h.1    -GSDQWEEKSWDWMLDTIAERVAKTREATFVTKNAKGQVVNR---CDGIA-----------------------SVGSAAM  target    ANSMALLDHKIRGVKPEDALGARGWDNYS--WHTDLPPGHPMVTGQQTVDFDLHAVEQARIVVVWGMNWVTTKMPDTHWL 1h0h.1    DNEECWIYQAWLR-----SLGLFYIEHQARIUHSATVAALAESYGRGAMTNHWIDLKNSDVILMMGSNPAENHPISFKWV  target    TEARLKGTKVVVIACEYSSSSIKADDAIVVRPGTTPALALGLCNVIMREKIYDGDYVRRFSDLPLLVRA----------- 1h0h.1    MRAKDKGATLIHVDPRYTRTSTKCDLYAPLRSGSDIAFLNGMTKYILEKELYFKDYVVNYTNASFIVGEGFAFEEGLFAG  target    -------------------------------------------DNLKLLRAEEVFGTPQAALKNQTR 1h0h.1    YNKETRKYDKSKWGFERDENGNPKRDETLKHPRCVFQIMKKHYERYDLDKISAICGTPKELILKVYD ``` | | | | | | | | | | | | | | | | | | | | | | | | | | | | | | | | | | | | | | | | | | | | | | | | | |
|  | 6cz7.1.A | ArrA  *The arsenate respiratory reductase (Arr) complex from Shewanella sp. ANA-3* | 0.41 |  | 17.99 | 0.68 | 57-408 | X-ray | 1.62 | hetero-1-1-mer | 5 x SF4, 2 x MGD, 1 x MO, 1 x PG5 | HHblits | 0.29 |
| ``` target    NNVNRREFLQWIGAAGFSTFALSASNAWGLQAIENPLAAYPNREWEKTYRDLWKSDASFTFLCAPNDTHNCILNAHVRDG 6cz7.1    --------------------------------------------------------EWLATTC-QGCTSWCAKQIYVMDG  target    VITRIGPTMKYGEATDLYGSKVTHRWDPRVCQKGLALTRRFYGDRRVRYPMVRKGFKAWADKGFPREKDGRPPKDYFNRA 6cz7.1    RALKVRGNP------------NSGVHGMSSCPRQHLSLQQVYDPDRLRTPMMRTNPKK----G--------------RDQ  target    RDEWLRLTHEEAADLVAAALINIATTYSGDNGQKLLLQQGYEKEIVEATRGAGTQVLKFRGGMPLLGLTRIFGLYRMANS 6cz7.1    DPKFVPISWDKALDMLADKIIALRVANEPHKYA--LLRGRYSHI--------NDL--------LYKKMTNLIGSPNNISH  target    MALLDHKIRGVKPEDALGARGWDNYSWHTDLPPGHPMVTGQQTVDFDLHAVEQARIVVVWGMNWVTTKMPDTHWLT--EA 6cz7.1    -SS---------------------VCAEAH-KMGPYYLDGN--WGYNQYDVKNAKFILSFGADPIASNRQVSFYSQTWGD  target    RLKGTKVVVIACEYSSSSIKADDAIVVRPGTTPALALGLCNVIMREKIYDGDYVRRFSDLP------------------- 6cz7.1    SLDHAKVVVVDPRLSASAAKAHKWIPIEPGQDSVLALAIAHVALVEGVWHKPFVGDFIEGKNLFKAGKTVSVESFKETHT  target    -----LLV-RADNLKLLRAEEVFGTPQAALKNQTR 6cz7.1    YGLVEWWNQALKDYTPEWASKITGIDPKTIIAIAK ``` | | | | | | | | | | | | | | | | | | | | | | | | | | | | | | | | | | | | | | | | | | | | | | | | | |
|  | 1ogy.1.A | PERIPLASMIC NITRATE REDUCTASE  *Crystal structure of the heterodimeric nitrate reductase from Rhodobacter sphaeroides* | 0.41 |  | 20.50 | 0.68 | 59-408 | X-ray | 3.20 | hetero-1-1-mer | 1 x SF4, 1 x MO, 2 x MGD, 2 x HEC | HHblits | 0.29 |
| ``` target    NNVNRREFLQWIGAAGFSTFALSASNAWGLQAIENPLAAYPNREWEKTYRDLWKSDASFTFLCAPNDTHNCILNAHVRDG 1ogy.1    ----------------------------------------------------------SKAPC-RFCGTGCGVMVGTRDG  target    VITRIGPTMKYGEATDLYGSKVTHRWDPRVCQKGLALTRRFYGDRRVRYPMVRKGFKAWADKGFPREKDGRPPKDYFNRA 1ogy.1    QVVATHGDT------------QAEVNRGLNCVKGYFLSKIMYGEDRLTTPLLRMKDG-------------------VYHK  target    RDEWLRLTHEEAADLVAAALINIATTYSGDNGQKLLLQQGYEKEIVEATRGAGTQVLKFRGGMPLLGLTR-IFGLYRMAN 1ogy.1    EGEFAPVSWDEAFDVMAAQAKLVLKEKAPEA---VGMFGSGQW----------TIW----EGYAASKLMRAGFRSNNLDP  target    SMALLDHKIRGVKPEDALGARGWDNYSWHTDLPPGHPMVTGQQTVDFDLHAVEQARIVVVWGMNWVTTKMPDTHWLTEAR 1ogy.1    ----------------------NARH-CMASAATAFMRTFGMDEPMGCYDDFEAADAFVLWGSNMAEMHPILWSRLTDRR  target    --LKGTKVVVIACEYSSSSIKADDAIVVRPGTTPALALGLCNVIMREKIYDGDYVRRFSDLPLLV--------------- 1ogy.1    LSHEHVRVAVLSTFTHRSSDLSDTPIIFRPGTDRAILNYIAHHIISTGRVNRDFVDRHTNFALGATDIGYGLRPEHQLQL  target    ---------------------RADNLKLLRAEEVFGTPQAALKNQTR 1ogy.1    AAKGAADAGAMTPTDFETFAALVSEYTLEKAAEISGVEPALLEELAE ``` | | | | | | | | | | | | | | | | | | | | | | | | | | | | | | | | | | | | | | | | | | | | | | | | | |
|  | 2e7z.1.A | Acetylene hydratase Ahy  *Acetylene Hydratase from Pelobacter acetylenicus* | 0.39 |  | 21.85 | 0.66 | 61-408 | X-ray | 1.26 | monomer | 1 x SF4, 2 x MGD, 1 x W | HHblits | 0.31 |
| ``` target    NNVNRREFLQWIGAAGFSTFALSASNAWGLQAIENPLAAYPNREWEKTYRDLWKSDASFTFLCAPNDTHNCILNAHVR-D 2e7z.1    ------------------------------------------------------------VVC-QSCDINCVVEAEVKAD  target    GVITRIGPTMKYGEATDLYGSKVTHRW-DPRVCQKGLALTRRFYGDRRVRYPMVRKGFKAWADKGFPREKDGRPPKDYFN 2e7z.1    GKIQTKSISEP------------HPTTPPNSICMKSVNADTIRTHKDRVLYPLKNVGS---------------------K  target    RARDEWLRLTHEEAADLVAAALINIATTYSGDNGQKLLLQQGYEKEIVEATRGAGTQVLKFRGGMPLLGLTRIFGLYRMA 2e7z.1    RGEQRWERISWDQALDEIAEKLKKIIAKYGPESLG---VSQTEIN---------QQSEYGT-----LRRFMNLLGSPNWT  target    NSMALLDHKIRGVKPEDALGARGWDNYSWHTDLPPGHPMVTGQQTVDFDLHAVEQARIVVVWGMNWVTTKMPD-THWLTE 2e7z.1    SAM-----------------------YMCIGNTAGVHRVTHGS----YSFASFADSNCLLFIGKNLSNHNWVSQFNDLKA  target    ARLKGTKVVVIACEYSSSSIKADDAIVVRPGTTPALALGLCNVIMREKIYDGDYVRRFSDL-PLLV-RADNLKLLRAEEV 2e7z.1    ALKRGCKLIVLDPRRTKVAEMADIWLPLRYGTDAALFLGMINVIINEQLYDKEFVENWCVGFEELKERVQEYPLDKVAEI  target    FGTPQAALKNQTR 2e7z.1    TGCDAGEIRKAAV ``` | | | | | | | | | | | | | | | | | | | | | | | | | | | | | | | | | | | | | | | | | | | | | | | | | |
|  | 6tg9.1.A | Formate dehydrogenase subunit alpha  *Cryo-EM Structure of NADH reduced form of NAD+-dependent Formate Dehydrogenase from Rhodobacter capsulatus* | 0.41 |  | 20.88 | 0.67 | 57-408 | EM | 3.24 | hetero-2-2-2-2-mer | 4 x MGD, 2 x 6MO, 4 x FES, 10 x SF4, 2 x H2S, 2 x FMN, 2 x NAI | HHblits | 0.30 |
| ``` target    NNVNRREFLQWIGAAGFSTFALSASNAWGLQAIENPLAAYPNREWEKTYRDLWKSDASFTFLCAPNDTHNCILNAHVRDG 6tg9.1    --------------------------------------------------------RKVVTTCA-YCGVGCSFEAHMLGD  target    VITRIGPTMKYGEATDLYGSKVTHRWDPRVCQKGLALTRRFYGDRRVRYPMVRKGFKAWADKGFPREKDGRPPKDYFNRA 6tg9.1    QLVRMVPWK------------GGAANRGHSCVKGRFAYGYATHQDRILKPMIRDK------------------------I  target    RDEWLRLTHEEAADLVAAALINIATTYSGDNGQKLLLQQGYEKEIVEATRGAGTQVLKFRGGMPLLGLTR-IFGLYRMAN 6tg9.1    TDPWREVNWTEALDFTATRLRALRDSHGADALGVITSSR-CTNE----------ET--Y----LVQKLARAVFGTNN---  target    SMALLDHKIRGVKPEDALGARGWDNYS--WHTDLPPGHPMVTGQQTVDFDLHAVEQARIVVVWGMNWVTTKMPDTHWLTE 6tg9.1    ----------------------TDTCARVCHSPTGYGLKQTFGTSAGTQDFDSVEETDLALVIGANPTDGHPVFASRLRK  target    ARLKGTKVVVIACEYSSSS----IKADDAIVVRPGTTPALALGLCNVIMREKIYDGDYVRRFSDLPL------LVRADNL 6tg9.1    RLRAGAKLIVVDPRRIDLLNTPHRGEAWHLQLKPGTNVAVMTAMAHVIVTEQIFDKRFIGDRCDWDEWADYAEFVANPEY  target    KLLRAEEVFGTPQAALKNQTR 6tg9.1    APEAVESLTGVPAGLLRQAAR ``` | | | | | | | | | | | | | | | | | | | | | | | | | | | | | | | | | | | | | | | | | | | | | | | | | |
|  | 1tmo.1.A | TRIMETHYLAMINE N-OXIDE REDUCTASE  *TRIMETHYLAMINE N-OXIDE REDUCTASE FROM SHEWANELLA MASSILIA* | 0.36 |  | 20.44 | 0.67 | 71-408 | X-ray | 2.50 | monomer | 2 x 2MD, 1 x 2MO | HHblits | 0.30 |
| ``` target    NNVNRREFLQWIGAAGFSTFALSASNAWGLQAIENPLAAYPNREWEKTYRDLWKSDASFTFLCAPNDTHNCILNAHVRDG 1tmo.1    ----------------------------------------------------------------------GAFKMKRKNG  target    VITRIGPTMKYGEATDLYGSKVTHRWDPRVCQKGLALTRRFYGDRRVRYPMVRKGFKAWADKGFPREKDGRPPKDYFNRA 1tmo.1    VIAEVKPFDLD------------KY--PTDMING--IRGMVYNPSRVRYPMVRLDFL---LKGHK--------SNTHQRG  target    RDEWLRLTHEEAADLVAAALINIATTYSGDNGQKLLLQQGYEKEIVEATRGAGTQVLKFRGGMPLLGLTRIFGLYRMANS 1tmo.1    DFRFVRVTWDKALTLFKHSLDEVQTQYGPSGLH--AGQTGWR--------ATGQLHSS------TSHMQRAVGMHG--N-  target    MALLDHKIRGVKPEDALGARGWDNYSWHTDLPPGHPMVTGQQ----TVDFDLHAVEQARIVVVWGMNWVTTKMP------ 1tmo.1    --YVK---------------KIGDYSTGAG-QTILPYVLGSTEVYAQGTSWPLILEHSDTIVLWSNDPYKNLQVGWNAET  target    --DT---HWLTEARL-KGTKVVVIACEYSSSSIK-ADDAIVVRPGTTPALALGLCNVIMREKIYDGDYVRRFSDL-PLLV 1tmo.1    HESFAYLAQLKEKVKQGKIRVISIDPVVTKTQAYLGCEQLYVNPQTDVTLMLAIAHEMISKKLYDDKFIQGYSLGFEEFV  target    R-AD------NLKLLRAEEVFGTPQAALKNQTR 1tmo.1    PYVMGTKDGVAKTPEWAAPICGVEAHVIRDLAK ``` | | | | | | | | | | | | | | | | | | | | | | | | | | | | | | | | | | | | | | | | | | | | | | | | | |
|  | 7qv7.1.L | Hydrogen dependent carbon dioxide reductase subunit FdhF  *Cryo-EM structure of Hydrogen-dependent CO2 reductase.* | 0.39 |  | 19.70 | 0.66 | 59-408 | EM | 0.00 | hetero-2-6-6-2-mer | 52 x SF4, 6 x 402 | HHblits | 0.30 |
| ``` target    NNVNRREFLQWIGAAGFSTFALSASNAWGLQAIENPLAAYPNREWEKTYRDLWKSDASFTFLCAPNDTHNCILNAHVRDG 7qv7.1    ----------------------------------------------------------VLTTC-PYCGTGCGLYLKVENE  target    VITRIGPTMKYGEATDLYGSKVTHRWDPRVCQKGLALTRRFYGDRRVRYPMVRKGFKAWADKGFPREKDGRPPKDYFNRA 7qv7.1    KIVGVEPDK------------LHPVNQGELCIKGYYGYKYVHDPRRLTSPLIKKN-------------------------  target    RDEWLRLTHEEAADLVAAALINIATTYSGDNGQKLLLQQGYEKEIVEATRGAGTQVLKFRGGMPLLGLTR-IFGLYRMAN 7qv7.1    -GKFVPVSWDEALNFIANGLKKIKSEYGSDAFAMFCSAR-ATNE-------DNYAAQK---------FARAVIGIN----  target    SMALLDHKIRGVKPEDALGARGWDNYS--WHTDLPPGHPMVTGQQTVDFDLHAVE-QARIVVVWGMNWVTTKMPDTHWLT 7qv7.1    ---------------------NVDHCARLCHAPTVAGLAMTLGSGAMTNSIPEISTYSDVIFIIGSNTAECHPLIAAHVI  target    EARLKGTKVVVIACEYSSSSIKADDAIVVRPGTTPALALGLCNVIMREKIYDGDYVRRFSD-LPLLV-RADNLKLLRAEE 7qv7.1    KAKERGAKLIVADPRMNAMVHKADIWLRVPSGYNIPLINGMIHIIIKEGLVKTDFVKNHAVGFEEMAKAVEKYTPEYVEE  target    VFGTPQAALKNQTR 7qv7.1    LTGIPKKDLIKAAR ``` | | | | | | | | | | | | | | | | | | | | | | | | | | | | | | | | | | | | | | | | | | | | | | | | | |
|  | 7qv7.1.O | Hydrogen dependent carbon dioxide reductase subunit FdhF  *Cryo-EM structure of Hydrogen-dependent CO2 reductase.* | 0.39 |  | 19.70 | 0.66 | 59-408 | EM | 0.00 | hetero-2-6-6-2-mer | 52 x SF4, 6 x 402 | HHblits | 0.30 |
| ``` target    NNVNRREFLQWIGAAGFSTFALSASNAWGLQAIENPLAAYPNREWEKTYRDLWKSDASFTFLCAPNDTHNCILNAHVRDG 7qv7.1    ----------------------------------------------------------VLTTC-PYCGTGCGLYLKVENE  target    VITRIGPTMKYGEATDLYGSKVTHRWDPRVCQKGLALTRRFYGDRRVRYPMVRKGFKAWADKGFPREKDGRPPKDYFNRA 7qv7.1    KIVGVEPDK------------LHPVNQGELCIKGYYGYKYVHDPRRLTSPLIKKN-------------------------  target    RDEWLRLTHEEAADLVAAALINIATTYSGDNGQKLLLQQGYEKEIVEATRGAGTQVLKFRGGMPLLGLTR-IFGLYRMAN 7qv7.1    -GKFVPVSWDEALNFIANGLKKIKSEYGSDAFAMFCSAR-ATNE-------DNYAAQK---------FARAVIGIN----  target    SMALLDHKIRGVKPEDALGARGWDNYS--WHTDLPPGHPMVTGQQTVDFDLHAVE-QARIVVVWGMNWVTTKMPDTHWLT 7qv7.1    ---------------------NVDHCARLCHAPTVAGLAMTLGSGAMTNSIPEISTYSDVIFIIGSNTAECHPLIAAHVI  target    EARLKGTKVVVIACEYSSSSIKADDAIVVRPGTTPALALGLCNVIMREKIYDGDYVRRFSD-LPLLV-RADNLKLLRAEE 7qv7.1    KAKERGAKLIVADPRMNAMVHKADIWLRVPSGYNIPLINGMIHIIIKEGLVKTDFVKNHAVGFEEMAKAVEKYTPEYVEE  target    VFGTPQAALKNQTR 7qv7.1    LTGIPKKDLIKAAR ``` | | | | | | | | | | | | | | | | | | | | | | | | | | | | | | | | | | | | | | | | | | | | | | | | | |
|  | 1dms.1.A | DMSO REDUCTASE  *STRUCTURE OF DMSO REDUCTASE* | 0.29 | 0.00 | 27.06 | 0.63 | 107-408 | X-ray | 1.88 | monomer | 2 x PGD, 1 x 2MO | BLAST | 0.34 |
| ``` target    NNVNRREFLQWIGAAGFSTFALSASNAWGLQAIENPLAAYPNREWEKTYRDLWKSDASFTFLCAPNDTHNCILNAHVRDG 1dms.1    --------------------------------------------------------------------------------  target    VITRIGPTMKYGEATDLYGSKVTHRWDPRVCQKGLALTRRFYGDRRVRYPMVRKGFKAWADKGFPREKDGRPPKDYFNRA 1dms.1    --------------------------DPHPTPMLEGVLDSIYSPTRIKYPMVRREF---LEKGVNADRS--------TRG  target    RDEWLRLTHEEAADLVAAALINIATTYSGDNGQKLLLQQGYEKEIVEATRGAGT---QVLKFRGGMPLLGLTRIFGLYRM 1dms.1    NGDFVRVSWDQALDLVAAEVKRVEETY----GPQGVFGGSYGWKSPGRLHNCTTLLRRMLTLAGG-----YVNGAGDYST  target    ANSMALLDHKIRGVKPEDALGARGWDNYSWHTDLPPGHPMVTGQQTVDFDLHAVEQARIVVVWG--------MNWVTTKM 1dms.1    GAAQVIMPHVV-------------------------GTLEVYEQQTAWPVL--AENTEVMVFWAADPIKTSQIGWVIPEH  target    PDTHWLTEARLKGTKVVVI-ACEYSSSSIKADDAIVVRPGTTPALALGLCNVIMREKIYDGDYVRRFSD-----LPLLVR 1dms.1    GAYPGLEALKAKGTKVIVIDPVRTKTVEFFGADHVTPKPQTDVAIMLGMAHTLVAEDLYDKDFIANYTSGFDKFLPYLMG  target    ADNLKLLRAE---EVFGTPQAALKNQTR 1dms.1    ETDSTPKTAEWASDISGVPAETIKELAR ``` | | | | | | | | | | | | | | | | | | | | | | | | | | | | | | | | | | | | | | | | | | | | | | | | | |
|  | 1aa6.1.A | FORMATE DEHYDROGENASE H  *REDUCED FORM OF FORMATE DEHYDROGENASE H FROM E. COLI* | 0.39 | 0.00 | 19.93 | 0.66 | 59-408 | X-ray | 2.30 | monomer | 1 x SF4, 2 x MGD, 1 x 4MO | HHblits | 0.30 |
| ``` target    NNVNRREFLQWIGAAGFSTFALSASNAWGLQAIENPLAAYPNREWEKTYRDLWKSDASFTFLCAPNDTHNCILNAHVRDG 1aa6.1    ----------------------------------------------------------VVTVC-PYCASGCKINLVVDNG  target    VITRIGPTMKYGEATDLYGSKVTHRWDPRVCQKGLALTRRFYGD----RRVRYPMVRKGFKAWADKGFPREKDGRPPKDY 1aa6.1    KIVRAEAAQ-------------GKTNQGTLCLKGYYGWDFINDTQILTPRLKTPMIRRQR--------------------  target    FNRARDEWLRLTHEEAADLVAAALINIATTYSGDNGQKLLLQQGYEKEIVEATRGAGTQVLKFRGGMPLLGLTR-IFGLY 1aa6.1    ----GGKLEPVSWDEALNYVAERLSAIKEKYGPDAI---QTTGSSR--------GTGNETNYV-----MQKFARAVIGTN  target    RMANSMALLDHKIRGVKPEDALGARGWDNYS--WHTDLPPGHPMVTGQQTVDFDLHAVEQARIVVVWGMNWVTTKMPDTH 1aa6.1    N-------------------------VDCCARVUHGPSVAGLHQSVGNGAMSNAINEIDNTDLVFVFGYNPADSHPIVAN  target    WLTEARLKGTKVVVIACEYSSSSIKADDAIVVRPGTTPALALGLCNVIMREKIYDGDYVRRFSDLP-LLV-RADNLKLLR 1aa6.1    HVINAKRNGAKIIVCDPRKIETARIADMHIALKNGSNIALLNAMGHVIIEENLYDKAFVASRTEGFEEYRKIVEGYTPES  target    AEEVFGTPQAALKNQTR 1aa6.1    VEDITGVSASEIRQAAR ``` | | | | | | | | | | | | | | | | | | | | | | | | | | | | | | | | | | | | | | | | | | | | | | | | | |
|  | 1fdo.1.A | FORMATE DEHYDROGENASE H  *OXIDIZED FORM OF FORMATE DEHYDROGENASE H FROM E. COLI* | 0.39 | 0.00 | 19.93 | 0.66 | 59-408 | X-ray | 2.80 | monomer | 1 x SF4, 2 x MGD, 1 x 6MO | HHblits | 0.30 |
| ``` target    NNVNRREFLQWIGAAGFSTFALSASNAWGLQAIENPLAAYPNREWEKTYRDLWKSDASFTFLCAPNDTHNCILNAHVRDG 1fdo.1    ----------------------------------------------------------VVTVC-PYCASGCKINLVVDNG  target    VITRIGPTMKYGEATDLYGSKVTHRWDPRVCQKGLALTRRFYGD----RRVRYPMVRKGFKAWADKGFPREKDGRPPKDY 1fdo.1    KIVRAEAAQ-------------GKTNQGTLCLKGYYGWDFINDTQILTPRLKTPMIRRQR--------------------  target    FNRARDEWLRLTHEEAADLVAAALINIATTYSGDNGQKLLLQQGYEKEIVEATRGAGTQVLKFRGGMPLLGLTR-IFGLY 1fdo.1    ----GGKLEPVSWDEALNYVAERLSAIKEKYGPDAI---QTTGSSR--------GTGNETNYV-----MQKFARAVIGTN  target    RMANSMALLDHKIRGVKPEDALGARGWDNYS--WHTDLPPGHPMVTGQQTVDFDLHAVEQARIVVVWGMNWVTTKMPDTH 1fdo.1    N-------------------------VDCCARVUHGPSVAGLHQSVGNGAMSNAINEIDNTDLVFVFGYNPADSHPIVAN  target    WLTEARLKGTKVVVIACEYSSSSIKADDAIVVRPGTTPALALGLCNVIMREKIYDGDYVRRFSDLP-LLV-RADNLKLLR 1fdo.1    HVINAKRNGAKIIVCDPRKIETARIADMHIALKNGSNIALLNAMGHVIIEENLYDKAFVASRTEGFEEYRKIVEGYTPES  target    AEEVFGTPQAALKNQTR 1fdo.1    VEDITGVSASEIRQAAR ``` | | | | | | | | | | | | | | | | | | | | | | | | | | | | | | | | | | | | | | | | | | | | | | | | | |
|  | 2iv2.1.A | Formate dehydrogenase H  *Reinterpretation of reduced form of formate dehydrogenase H from E. coli* | 0.40 | 0.00 | 19.93 | 0.66 | 59-408 | X-ray | 2.27 | monomer | 1 x SF4, 1 x 2MD, 1 x MGD | HHblits | 0.30 |
| ``` target    NNVNRREFLQWIGAAGFSTFALSASNAWGLQAIENPLAAYPNREWEKTYRDLWKSDASFTFLCAPNDTHNCILNAHVRDG 2iv2.1    ----------------------------------------------------------VVTVC-PYCASGCKINLVVDNG  target    VITRIGPTMKYGEATDLYGSKVTHRWDPRVCQKGLALTRRFYGD----RRVRYPMVRKGFKAWADKGFPREKDGRPPKDY 2iv2.1    KIVRAEAAQ-------------GKTNQGTLCLKGYYGWDFINDTQILTPRLKTPMIRRQR--------------------  target    FNRARDEWLRLTHEEAADLVAAALINIATTYSGDNGQKLLLQQGYEKEIVEATRGAGTQVLKFRGGMPLLGLTR-IFGLY 2iv2.1    ----GGKLEPVSWDEALNYVAERLSAIKEKYGPDAI---QTTGSSR--------GTGNETNYV-----MQKFARAVIGTN  target    RMANSMALLDHKIRGVKPEDALGARGWDNYS--WHTDLPPGHPMVTGQQTVDFDLHAVEQARIVVVWGMNWVTTKMPDTH 2iv2.1    N-------------------------VDCCARVUHGPSVAGLHQSVGNGAMSNAINEIDNTDLVFVFGYNPADSHPIVAN  target    WLTEARLKGTKVVVIACEYSSSSIKADDAIVVRPGTTPALALGLCNVIMREKIYDGDYVRRFSDLP-LLV-RADNLKLLR 2iv2.1    HVINAKRNGAKIIVCDPRKIETARIADMHIALKNGSNIALLNAMGHVIIEENLYDKAFVASRTEGFEEYRKIVEGYTPES  target    AEEVFGTPQAALKNQTR 2iv2.1    VEDITGVSASEIRQAAR ``` | | | | | | | | | | | | | | | | | | | | | | | | | | | | | | | | | | | | | | | | | | | | | | | | | |
|  | 7z0t.1.G | Formate dehydrogenase H  *Structure of the Escherichia coli formate hydrogenlyase complex (aerobic preparation, composite structure)* | 0.40 | 0.00 | 19.93 | 0.66 | 59-408 | EM | 0.00 | monomer | 1 x NI, 1 x FCO, 8 x SF4, 1 x FE, 2 x MGD, 1 x 6MO | HHblits | 0.30 |
| ``` target    NNVNRREFLQWIGAAGFSTFALSASNAWGLQAIENPLAAYPNREWEKTYRDLWKSDASFTFLCAPNDTHNCILNAHVRDG 7z0t.1    ----------------------------------------------------------VVTVC-PYCASGCKINLVVDNG  target    VITRIGPTMKYGEATDLYGSKVTHRWDPRVCQKGLALTRRFYGD----RRVRYPMVRKGFKAWADKGFPREKDGRPPKDY 7z0t.1    KIVRAEAAQ-------------GKTNQGTLCLKGYYGWDFINDTQILTPRLKTPMIRRQR--------------------  target    FNRARDEWLRLTHEEAADLVAAALINIATTYSGDNGQKLLLQQGYEKEIVEATRGAGTQVLKFRGGMPLLGLTR-IFGLY 7z0t.1    ----GGKLEPVSWDEALNYVAERLSAIKEKYGPDAI---QTTGSSR--------GTGNETNYV-----MQKFARAVIGTN  target    RMANSMALLDHKIRGVKPEDALGARGWDNYS--WHTDLPPGHPMVTGQQTVDFDLHAVEQARIVVVWGMNWVTTKMPDTH 7z0t.1    N-------------------------VDCCARVUHGPSVAGLHQSVGNGAMSNAINEIDNTDLVFVFGYNPADSHPIVAN  target    WLTEARLKGTKVVVIACEYSSSSIKADDAIVVRPGTTPALALGLCNVIMREKIYDGDYVRRFSDLP-LLV-RADNLKLLR 7z0t.1    HVINAKRNGAKIIVCDPRKIETARIADMHIALKNGSNIALLNAMGHVIIEENLYDKAFVASRTEGFEEYRKIVEGYTPES  target    AEEVFGTPQAALKNQTR 7z0t.1    VEDITGVSASEIRQAAR ``` | | | | | | | | | | | | | | | | | | | | | | | | | | | | | | | | | | | | | | | | | | | | | | | | | |
|  | 3o5a.1.A | Periplasmic nitrate reductase  *Crystal Structure of partially reduced Periplasmic Nitrate Reductase from Cupriavidus necator using Ionic Liquids* | 0.41 |  | 17.20 | 0.68 | 58-408 | X-ray | 1.72 | hetero-oligomer | 1 x SF4, 1 x MOS, 2 x MGD, 2 x HEC | HHblits | 0.27 |
| ``` target    NNVNRREFLQWIGAAGFSTFALSASNAWGLQAIENPLAAYPNREWEKTYRDLWKSDASFTFLCAPNDTHNCILNAHVRDG 3o5a.1    ---------------------------------------------------------WSKAPCR-FCGTGCGVTVAVKDN  target    VITRIGPTMKYGEATDLYGSKVTHRWDPRVCQKGLALTRRFYGDRRVRYPMVRKGFKAWADKGFPREKDGRPPKDYFNRA 3o5a.1    KVVATQGDP------------QAEVNKGLNCVKGYFLSKIMYGQDRLTRPLMRMKNG-------------------KYDK  target    RDEWLRLTHEEAADLVAAALINIATTYSGDNGQKLLLQQGYEKEIVEATRGAGTQVLKFRGGMPLLGLT-RIFGLYRMAN 3o5a.1    NGDFAPVTWDQAFDEMERQFKRVLKEKGPTA---VGMFGSGQ----------WTVWEGYA----AAKLYKAGFRSNNIDP  target    SMALLDHKIRGVKPEDALGARGWDNYSWHTDLPPGHPMVTGQQTVDFDLHAVEQARIVVVWGMNWVTTKMPDTHWLTEAR 3o5a.1    ----------------------NARHCM-ASAAAGFMRTFGMDEPMGCYDDFEAADAFVLWGSNMAEMHPILWTRVTDRR  target    --LKGTKVVVIACEYSSSSIKADDAIVVRPGTTPALALGLCNVIMREKIYDGDYVRRFSDLPLLVRA------------- 3o5a.1    LSHPKTRVVVLSTFTHRCFDLADIGIIFKPQTDLAMLNYIANYIIRNNKVNKDFVNKHTVFKEGVTDIGYGLRPDHPLQK  target    -----------------------DNLKLLRAEEVFGTPQAALKNQTR 3o5a.1    AAKNASDPGAAKVITFDEFAKFVSKYDADYVSKLSAVPKAKLDQLAE ``` | | | | | | | | | | | | | | | | | | | | | | | | | | | | | | | | | | | | | | | | | | | | | | | | | |
|  | 7bkb.1.F | Formate dehydrogenase  *Formate dehydrogenase - heterodisulfide reductase - formylmethanofuran dehydrogenase complex from Methanospirillum hungatei (hexameric, composite structure)* | 0.42 |  | 20.15 | 0.66 | 59-407 | EM | 0.00 | hetero-2-2-2-2-2-2-… | 48 x SF4, 4 x FAD, 2 x FES, 4 x 9S8, 4 x ZN, 2 x MO, 4 x MGD | HHblits | 0.31 |
| ``` target    NNVNRREFLQWIGAAGFSTFALSASNAWGLQAIENPLAAYPNREWEKTYRDLWKSDASFTFLCAPNDTHNCILNAHVRDG 7bkb.1    ----------------------------------------------------------VATTCP-YCGVGCTLNLVVSNG  target    VITRIGPTMKYGEATDLYGSKVTHRWDPRVCQKGLALTRRFYGDRRVRYPMVRKGFKAWADKGFPREKDGRPPKDYFNRA 7bkb.1    KVVGVEPNQ------------RSPINEGKLCPKGVTCWEHIHSPDRLTTPLIKKD-------------------------  target    RDEWLRLTHEEAADLVAAALINIATTYSGDNGQKLLLQQGYEKEIVEATRGAGTQVLKFRGGMPLLGLTRIFGLYRMANS 7bkb.1    -GKFIEASWDEALDLVAKNLKVIYDKHGPKGL---GFQTSCRT--------VNEDCYIF----------QKFARV-----  target    MALLDHKIRGVKPEDALGARGWDNYSW--HTDLPPGHPMVTGQQTVDFDLHAVEQARIVVVWGMNWVTTKMPDTHWLTEA 7bkb.1    ---------------GFKTNNVDNCARICHGPSVAGLSLSFGSGAATNGFEDALNADLILIWGSNAVEAHPLAGRRIAQA  target    RLKGTKVVVIACEYSSSSIKADDAIVVRPGTTPALALGLCNVIMREKIYDGDYVRRFSDL-PLLVRADNLKLLRAEEVFG 7bkb.1    KKKGIQIIAVDPRYTMTARLADTYVRFNPSTHIALANSMMYWIIKEGLEDKKFIQDRVNGFEDLKKT-VENYADAEAIHG  target    TPQAALKNQTR 7bkb.1    VPLDVVKDIA- ``` | | | | | | | | | | | | | | | | | | | | | | | | | | | | | | | | | | | | | | | | | | | | | | | | | |
|  | 2v45.1.A | PERIPLASMIC NITRATE REDUCTASE  *A NEW CATALYTIC MECHANISM OF PERIPLASMIC NITRATE REDUCTASE FROM DESULFOVIBRIO DESULFURICANS ATCC 27774 FROM CRYSTALLOGRAPHIC AND EPR DATA AND BASED ON DETAILED ANALYSIS OF THE SIXTH LIGAND* | 0.39 |  | 17.52 | 0.67 | 57-407 | X-ray | 2.40 | monomer | 1 x SF4, 1 x MO, 2 x MGD, 1 x LCP | HHblits | 0.28 |
| ``` target    NNVNRREFLQWIGAAGFSTFALSASNAWGLQAIENPLAAYPNREWEKTYRDLWKSDASFTFLCAPNDTHNCILNAHVRDG 2v45.1    --------------------------------------------------------KWVKGVC-RYCGTGCGVLVGVKDG  target    VITRIGPTMKYGEATDLYGSKVTHRWDPRVCQKGLALTRRFYGDRRVRYPMVRKGFKAWADKGFPREKDGRPPKDYFNRA 2v45.1    KAVAIQGNPN------------NH-NAGLLCLKGSLLIPVLNSKERVTQPLVRRH------------------------K  target    RDEWLRLTHEEAADLVAAALINIATTYSGDNGQKLLLQQGYEKEIVEATRGAGTQVLKFRGGMPLLGLTRIFGLYRMANS 2v45.1    GGKLEPVSWDEALDLMASRFRSSIDMYGPNSV---AWYGSGQ----------CLTEESYVAN---KIFKGGFGTNNVDGN  target    MALLDHKIRGVKPEDALGARGWDNYSWHTDLPPGHPMVTGQQTVDFDLHAVEQARIVVVWGMNWVTTKMPDTHWLTEAR- 2v45.1    ----------------------PRL-CMASAVGGYVTSFGKDEPMGTYADIDQATCFFIIGSNTSEAHPVLFRRIARRKQ  target    -LKGTKVVVIACEYSSSSIKADDAIVVRPGTTPALALGLCNVIMREKIYDGDYVRRFSDL----------PLLV-RADNL 2v45.1    VEPGVKIIVADPRRTNTSRIADMHVAFRPGTDLAFMHSMAWVIINEELDNPRFWQRYVNFMDAEGKPSDFEGYKAFLENY  target    KLLRAEEVFGTPQAALKNQTR 2v45.1    RPEKVAEICRVPVEQIYGAA- ``` | | | | | | | | | | | | | | | | | | | | | | | | | | | | | | | | | | | | | | | | | | | | | | | | | |
|  | 2v3v.1.A | PERIPLASMIC NITRATE REDUCTASE  *A NEW CATALYTIC MECHANISM OF PERIPLASMIC NITRATE REDUCTASE FROM DESULFOVIBRIO DESULFURICANS ATCC 27774 FROM CRYSTALLOGRAPHIC AND EPR DATA AND BASED ON DETAILED ANALYSIS OF THE SIXTH LIGAND* | 0.39 |  | 17.52 | 0.67 | 57-407 | X-ray | 1.99 | monomer | 1 x SF4, 1 x MO, 2 x MGD, 4 x LCP | HHblits | 0.28 |
| ``` target    NNVNRREFLQWIGAAGFSTFALSASNAWGLQAIENPLAAYPNREWEKTYRDLWKSDASFTFLCAPNDTHNCILNAHVRDG 2v3v.1    --------------------------------------------------------KWVKGVC-RYCGTGCGVLVGVKDG  target    VITRIGPTMKYGEATDLYGSKVTHRWDPRVCQKGLALTRRFYGDRRVRYPMVRKGFKAWADKGFPREKDGRPPKDYFNRA 2v3v.1    KAVAIQGDPN------------NH-NAGLLCLKGSLLIPVLNSKERVTQPLVRRH------------------------K  target    RDEWLRLTHEEAADLVAAALINIATTYSGDNGQKLLLQQGYEKEIVEATRGAGTQVLKFRGGMPLLGLTRIFGLYRMANS 2v3v.1    GGKLEPVSWDEALDLMASRFRSSIDMYGPNS---VAWYGSGQC--------LTEESYVANK-----IFKGGFGTNNVDG-  target    MALLDHKIRGVKPEDALGARGWDNYSWHTDLPPGHPMVTGQQTVDFDLHAVEQARIVVVWGMNWVTTKMPDTHWLTEAR- 2v3v.1    ---------------------NPRLCMA-SAVGGYVTSFGKDEPMGTYADIDQATCFFIIGSNTSEAHPVLFRRIARRKQ  target    -LKGTKVVVIACEYSSSSIKADDAIVVRPGTTPALALGLCNVIMREKIYDGDYVRRFSDL----------PLLV-RADNL 2v3v.1    VEPGVKIIVADPRRTNTSRIADMHVAFRPGTDLAFMHSMAWVIINEELDNPRFWQRYVNFMDAEGKPSDFEGYKAFLENY  target    KLLRAEEVFGTPQAALKNQTR 2v3v.1    RPEKVAEICRVPVEQIYGAA- ``` | | | | | | | | | | | | | | | | | | | | | | | | | | | | | | | | | | | | | | | | | | | | | | | | | |
|  | 2nya.1.A | Periplasmic nitrate reductase  *Crystal structure of the periplasmic nitrate reductase (NAP) from Escherichia coli* | 0.41 |  | 16.67 | 0.68 | 61-408 | X-ray | 2.50 | monomer | 1 x SF4, 1 x 6MO, 2 x MGD | HHblits | 0.28 |
| ``` target    NNVNRREFLQWIGAAGFSTFALSASNAWGLQAIENPLAAYPNREWEKTYRDLWKSDASFTFLCAPNDTHNCILNAHVRDG 2nya.1    ------------------------------------------------------------APC-RFCGTGCGVLVGTQQG  target    VITRIGPTMKYGEATDLYGSKVTHRWDPRVCQKGLALTRRFYGDRRVRYPMVRKGFKAWADKGFPREKDGRPPKDYFNRA 2nya.1    RVVACQGDP------------DAPVNRGLNCIKGYFLPKIMYGKDRLTQPLLRMKNG-------------------KYDK  target    RDEWLRLTHEEAADLVAAALINIATTYSGDNGQKLLLQQGYEKEIVEATRGAGTQVLKFRGGMPLLGLT-RIFGLYRMAN 2nya.1    EGEFTPITWDQAFDVMEEKFKTALKEKGPESI---GMFGSGQWTI--------WEGYAA------SKLFKAGFRSNNIDP  target    SMALLDHKIRGVKPEDALGARGWDNYSWHTDLPPGHPMVTGQQTVDFDLHAVEQARIVVVWGMNWVTTKMPDTHWLTEAR 2nya.1    N----------------------ARH-CMASAVVGFMRTFGMDEPMGCYDDIEQADAFVLWGANMAEMHPILWSRITNRR  target    --LKGTKVVVIACEYSSSSIKADDAIVVRPGTTPALALGLCNVIMREKIYDGDYVRRFSDLPLLVR-------------- 2nya.1    LSNQNVTVAVLSTYQHRSFELADNGIIFTPQSDLVILNYIANYIIQNNAINQDFFSKHVNLRKGATDIGYGLRPTHPLEK  target    ---------------------ADNLKLLRAEEVFGTPQAALKNQTR 2nya.1    AAKNPGSDASEPMSFEDYKAFVAEYTLEKTAEMTGVPKDQLEQLAQ ``` | | | | | | | | | | | | | | | | | | | | | | | | | | | | | | | | | | | | | | | | | | | | | | | | | |
|  | 4aay.1.A | AROA  *Crystal Structure of the arsenite oxidase protein complex from Rhizobium species strain NT-26* | 0.34 |  | 16.48 | 0.67 | 60-408 | X-ray | 2.70 | hetero-oligomer | 4 x MGD, 2 x O, 2 x 4MO, 2 x F3S, 2 x FES | HHblits | 0.28 |
| ``` target    NNVNRREFLQWIGAAGFSTFALSASNAWGLQAIENPLAAYPNREWEKTYRDLWKSDASFTFLCAPNDTHNCILNAHV--- 4aay.1    -----------------------------------------------------------NVTC-HFCIVGCGYHAYTWPI  target    --------------------------------------RDGVITRIGPTMKYGEATDLYGSKVTHRWDPRVCQKGLALTR 4aay.1    NKQGGTDPQNNIFGVDLSEQQQAESDAWYSPSMYNVVKQDGRDVHVVIKPDH----------ECVVNSGLGSVRGARMAE  target    RFY------GDRRVRYPMVRKGFKAWADKGFPREKDGRPPKDYFNRARDEWLRLTHEEAADLVAAALINIATTYSGDNGQ 4aay.1    TSFSEARNTQQQRLTDPLVWRY--------------------------GQMQPTSWDDALDLVARVTAKIVKEKGEDA--  target    KLLLQQGYEKEIVEATRGAGTQVLKFRGGMPLLGL-TRIFGLYRMANSMALLDHKIRGVKPEDALGARGWDNYS--WHTD 4aay.1    -LIVS-AFDH-----GGAGGGYENTW----GTGKLYFEAMKV-------------------------KNIRIHNRPAYNS  target    LPPGHPMVTGQQTVDFDLHAVEQARIVVVWGMNWVTTKMPDT--HWLT---------------EARLKGTKVVVIACEYS 4aay.1    EVHGT-RDMGVGELNNCYEDAELADTIVAVGTNALETQTNYFLNHWIPNLRGESLGKKKELMPEEPHEAGRIIIVDPRRT  target    SSSI------KAD--DAIVVRPGTTPALALGLCNVIMREKIYDGDYVRRFSD-------------------------LPL 4aay.1    VTVNACEQTAGADNVLHLAINSGTDLALFNALFTYIADKGWVDRDFIDKSTLREGTARPPLYPARGVSEANPGHLSSFED  target    LVRADNLKLLRAEEVFGTPQAALKNQTR 4aay.1    AVEGCRMSIEEAAEITGLDAAQIIKAAE ``` | | | | | | | | | | | | | | | | | | | | | | | | | | | | | | | | | | | | | | | | | | | | | | | | | |
|  | 5nqd.1.A | AroA  *Arsenite oxidase AioAB from Rhizobium sp. str. NT-26 mutant AioBF108A* | 0.34 |  | 16.48 | 0.67 | 60-408 | X-ray | 2.20 | hetero-2-2-mer | 4 x MGD, 2 x O, 2 x 4MO, 2 x F3S, 2 x FES | HHblits | 0.28 |
| ``` target    NNVNRREFLQWIGAAGFSTFALSASNAWGLQAIENPLAAYPNREWEKTYRDLWKSDASFTFLCAPNDTHNCILNAHV--- 5nqd.1    -----------------------------------------------------------NVTC-HFCIVGCGYHAYTWPI  target    --------------------------------------RDGVITRIGPTMKYGEATDLYGSKVTHRWDPRVCQKGLALTR 5nqd.1    NKQGGTDPQNNIFGVDLSEQQQAESDAWYSPSMYNVVKQDGRDVHVVIKPDH----------ECVVNSGLGSVRGARMAE  target    RFY------GDRRVRYPMVRKGFKAWADKGFPREKDGRPPKDYFNRARDEWLRLTHEEAADLVAAALINIATTYSGDNGQ 5nqd.1    TSFSEARNTQQQRLTDPLVWRY--------------------------GQMQPTSWDDALDLVARVTAKIVKEKGEDA--  target    KLLLQQGYEKEIVEATRGAGTQVLKFRGGMPLLGL-TRIFGLYRMANSMALLDHKIRGVKPEDALGARGWDNYS--WHTD 5nqd.1    -LIVSA-FDH-----GGAGGGYENTW----GTGKLYFEAMKV-------------------------KNIRIHNRPAYNS  target    LPPGHPMVTGQQTVDFDLHAVEQARIVVVWGMNWVTTKMPDT--HWLT---------------EARLKGTKVVVIACEYS 5nqd.1    EVHGT-RDMGVGELNNCYEDAELADTIVAVGTNALETQTNYFLNHWIPNLRGESLGKKKELMPEEPHEAGRIIIVDPRRT  target    SSSI------KAD--DAIVVRPGTTPALALGLCNVIMREKIYDGDYVRRFSD-------------------------LPL 5nqd.1    VTVNACEQTAGADNVLHLAINSGTDLALFNALFTYIADKGWVDRDFIDKSTLREGTARPPLYPARGVSEANPGHLSSFED  target    LVRADNLKLLRAEEVFGTPQAALKNQTR 5nqd.1    AVEGCRMSIEEAAEITGLDAAQIIKAAE ``` | | | | | | | | | | | | | | | | | | | | | | | | | | | | | | | | | | | | | | | | | | | | | | | | | |
|  | 1g8j.1.A | ARSENITE OXIDASE  *CRYSTAL STRUCTURE ANALYSIS OF ARSENITE OXIDASE FROM ALCALIGENES FAECALIS* | 0.34 |  | 15.69 | 0.67 | 59-408 | X-ray | 2.03 | hetero-oligomer | 2 x MGD, 1 x O, 1 x 4MO, 1 x F3S, 1 x FES | HHblits | 0.28 |
| ``` target    NNVNRREFLQWIGAAGFSTFALSASNAWGLQAIENPLAAYPNREWEKTYRDLWKSDASFTFLCAPNDTHNCILNAHVRD- 1g8j.1    ----------------------------------------------------------TNMTC-HFCIVGCGYHVYKWPE  target    ----GV--------------------------------------ITRIGPTMKYGEATDLYGSKVTHRWDPRVCQKGLAL 1g8j.1    LEEGGRAPEQNALGLDFRKQLPPLASTLTPAMTNVVTEHDGARYDIMVVPD------------KACVVNSGLSSTRGGKM  target    TRRFYGD-----RRVRYPMVRKGFKAWADKGFPREKDGRPPKDYFNRARDEWLRLTHEEAADLVAAALINIATTYSGDNG 1g8j.1    ASYMYTPTGDGKERLSAPRLYAA--------------------------DEWVDTTWDHAMALYAGLIKKTLDSDGPQG-  target    QKLLLQQGYEKEIVEATRGAGTQVLKFRGGMPLL-GLTRIFGLYRMANSMALLDHKIRGVKPEDALGARGWDNYSWHTDL 1g8j.1    --VFFSCFD---------HGGAGG-GFENTWGTGKLMFSAIQTPMVRI--------------------HNRPAYN---SE  target    PPGHPMVTGQQTVDFDLHAVEQARIVVVWGMNWVTTKMPDT--HWL---------------TEARLKGTKVVVIACEYSS 1g8j.1    CH-ATREMGIGELNNAYEDAQLADVIWSIGNNPYESQTNYFLNHWLPNLQGATTSKKKERFPNENFPQARIIFVDPRETP  target    SSIKA--------DDAIVVRPGTTPALALGLCNVIMREKIYDGDYVRRFSDL-PLLVRADNLKLLRAEEVFGTPQAALKN 1g8j.1    SVAIARHVAGNDRVLHLAIEPGTDTALFNGLFTYVVEQGWIDKPFIEAHTKGFDDAVKTNRLSLDECSNITGVPVDMLKR  target    QTR 1g8j.1    AAE ``` | | | | | | | | | | | | | | | | | | | | | | | | | | | | | | | | | | | | | | | | | | | | | | | | | |
|  | 1g8k.1.A | ARSENITE OXIDASE  *CRYSTAL STRUCTURE ANALYSIS OF ARSENITE OXIDASE FROM ALCALIGENES FAECALIS* | 0.34 |  | 15.44 | 0.67 | 59-408 | X-ray | 1.64 | hetero-1-1-mer | 3 x HG, 2 x CA, 2 x MGD, 1 x O, 1 x 4MO, 1 x F3S, 1 x FES | HHblits | 0.28 |
| ``` target    NNVNRREFLQWIGAAGFSTFALSASNAWGLQAIENPLAAYPNREWEKTYRDLWKSDASFTFLCAPNDTHNCILNAHVRD- 1g8k.1    ----------------------------------------------------------TNMTC-HFCIVGCGYHVYKWPE  target    ----GV--------------------------------------ITRIGPTMKYGEATDLYGSKVTHRWDPRVCQKGLAL 1g8k.1    LEEGGRAPEQNALGLDFRKQLPPLAVTLTPAMTNVVTEHDGARYDIMVVPD------------KACVVNSGLSSTRGGKM  target    TRRFYGD-----RRVRYPMVRKGFKAWADKGFPREKDGRPPKDYFNRARDEWLRLTHEEAADLVAAALINIATTYSGDNG 1g8k.1    ASYMYTPTGDGKERLSAPRLYAA--------------------------DEWVDTTWDHAMALYAGLIKKTLDKDGPQG-  target    QKLLLQQGYEKEIVEATRGAGTQVLKFRGGMPLLG-LTRIFGLYRMANSMALLDHKIRGVKPEDALGARGWDNYS--WHT 1g8k.1    --VFFSCF-DH--------GGAGGG-FENTWGTGKLMFSAIQTPM-------------------------VRIHNRPAYN  target    DLPPGHPMVTGQQTVDFDLHAVEQARIVVVWGMNWVTTKMPDT--HWL---------------TEARLKGTKVVVIACEY 1g8k.1    SECH-ATREMGIGELNNAYEDAQLADVIWSIGNNPYESQTNYFLNHWLPNLQGATTSKKKERFPNENFPQARIIFVDPRE  target    SSSSIKA--------DDAIVVRPGTTPALALGLCNVIMREKIYDGDYVRRFSD-LPLLVRADNLKLLRAEEVFGTPQAAL 1g8k.1    TPSVAIARHVAGNDRVLHLAIEPGTDTALFNGLFTYVVEQGWIDKPFIEAHTKGFDDAVKTNRLSLDECSNITGVPVDML  target    KNQTR 1g8k.1    KRAAE ``` | | | | | | | | | | | | | | | | | | | | | | | | | | | | | | | | | | | | | | | | | | | | | | | | | |
|  | 7t2r.1.A | NiFe hydrogenase subunit A  *Structure of electron bifurcating Ni-Fe hydrogenase complex HydABCSL in FMN-free apo state* | 0.34 |  | 14.81 | 0.66 | 57-408 | EM | 0.00 | hetero-2-2-2-2-2-mer | 6 x FES, 12 x SF4, 2 x 3NI, 2 x FCO | HHblits | 0.27 |
| ``` target    NNVNRREFLQWIGAAGFSTFALSASNAWGLQAIENPLAAYPNREWEKTYRDLWKSDASFTFLCAPNDTHNCILNAHVRDG 7t2r.1    --------------------------------------------------------AVVESVC-PLCAVGCKIKTYVRTG  target    VITRIGPTMKYGEATDLYGSKVTHRWDPRVCQKGLALTRRFYGDRRVRYPMVRKGFKAWADKGFPREKDGRPPKDYFNRA 7t2r.1    SIVRVEGTG------------VEEPDGGQLCHMGRWWLPESTERERVTVPLIREG-------------------------  target    RDEWLRLTHEEAADLVAAALINIATTYSGDNGQKLLLQQGYEKEIVEATRGAGTQVLKFRGGMPLLGLTRIFGLYRMANS 7t2r.1    -ASYREATWEEALALASAEFKKAYDQEKAG---AILSSLCTDEELT--------LFSALFR--------NALKMKHIDTF  target    MALLDHKIRGVKPEDALGARGWDNYSWHTDLPPGHPM--VTGQQTVDFDLHAVEQARIVVVWGMNWVTTKMPDTHWLTEA 7t2r.1    DG-----------------D-----IIRG-FFKGFMPFREQGV-RPFTAAHHILDSDLIITMFADPQKEAPVVASYIRVA  target    -RLKGTKVVVIACEYSSSSIKADDAIVVRPGTTPALALGLCNVIMREKIYDGD---YVRRFSD-LPLLVRADNLKLLRAE 7t2r.1    CLHRNAKLMNLSYGPSPFPGLVDLDIRLPEGQAVPKALSNLAEIIGKISLGPSDMASFGEFEAGAGKALSSYRESIEESA  target    EVFGTPQAALKNQTR 7t2r.1    RAMGLDPKIAEEVAL ``` | | | | | | | | | | | | | | | | | | | | | | | | | | | | | | | | | | | | | | | | | | | | | | | | | |
|  | 7t30.1.A | NiFe hydrogenase subunit A  *Structure of electron bifurcating Ni-Fe hydrogenase complex HydABCSL in FMN/NAD(H) bound state* | 0.35 |  | 14.81 | 0.66 | 57-408 | EM | 0.00 | hetero-2-2-2-2-2-mer | 4 x FES, 12 x SF4, 2 x NAD, 2 x FMN, 2 x 3NI, 2 x FCO | HHblits | 0.27 |
| ``` target    NNVNRREFLQWIGAAGFSTFALSASNAWGLQAIENPLAAYPNREWEKTYRDLWKSDASFTFLCAPNDTHNCILNAHVRDG 7t30.1    --------------------------------------------------------AVVESVC-PLCAVGCKIKTYVRTG  target    VITRIGPTMKYGEATDLYGSKVTHRWDPRVCQKGLALTRRFYGDRRVRYPMVRKGFKAWADKGFPREKDGRPPKDYFNRA 7t30.1    SIVRVEGTG------------VEEPDGGQLCHMGRWWLPESTERERVTVPLIREG-------------------------  target    RDEWLRLTHEEAADLVAAALINIATTYSGDNGQKLLLQQGYEKEIVEATRGAGTQVLKFRGGMPLLGLTRIFGLYRMANS 7t30.1    -ASYREATWEEALALASAEFKKAYDQEKAG---AILSSLCTDEELT--------LFSALFR--------NALKMKHIDTF  target    MALLDHKIRGVKPEDALGARGWDNYSWHTDLPPGHPM--VTGQQTVDFDLHAVEQARIVVVWGMNWVTTKMPDTHWLTEA 7t30.1    DG-----------------D-----IIRG-FFKGFMPFREQGV-RPFTAAHHILDSDLIITMFADPQKEAPVVASYIRVA  target    -RLKGTKVVVIACEYSSSSIKADDAIVVRPGTTPALALGLCNVIMREKIYDGD---YVRRFSD-LPLLVRADNLKLLRAE 7t30.1    CLHRNAKLMNLSYGPSPFPGLVDLDIRLPEGQAVPKALSNLAEIIGKISLGPSDMASFGEFEAGAGKALSSYRESIEESA  target    EVFGTPQAALKNQTR 7t30.1    RAMGLDPKIAEEVAL ``` | | | | | | | | | | | | | | | | | | | | | | | | | | | | | | | | | | | | | | | | | | | | | | | | | |
|  | 6f0k.1.B | Fe-S-cluster-containing hydrogenase  *Alternative complex III* | 0.27 |  | 16.10 | 0.65 | 2-369 | EM | 0.00 | hetero-1-1-1-1-1-1-… | 6 x HEC, 1 x F3S, 3 x SF4 | HHblits | 0.27 |
| ``` target    NNVNRREFLQWIGAAGFSTFALSASNAWGLQAIENPLAAYPNREWEKTYRDLWKSDASFTFLCAPNDTHNCILNAHVRDG 6f0k.1    -GTSRRQFLQIMGASMALAGLTACR---RPVE---KILPYV-RQPEE----IIPGIPLYYATAMPFRGSVRPLLVESHEG  target    VITRIGPTMKYGEATDLYGSKVTHRWDPRVCQKGLALTRRFYGDRRVRYPMVRKGFKAWADKGFPREKDGRPPKDYFNRA 6f0k.1    RPTKIEGN------------PDHPLSRGATGVFEQASLLNLYDPDRSQQVLRK-G-------------------------  target    RDEWLRLTHEEAADLVAAALINIATTYSGDNGQKLLLQQGYEKEIVEATRGAGTQVLKFRGGMPLLGLTRIFGLYRMANS 6f0k.1    ----EPASWGDFVQFARSLA----AEAGTKR---LAVLCEPS--------SSPTLA-AL-----RRELERRYAQVR----  target    MALLDHKIRGVKPEDALGARGWDNYS--WHTDLPPGHPMVTGQQTVDFDLHAVEQARIVVVWGMNWVTTK-MPD---T-- 6f0k.1    ---------------------WVTYRPEGDDHEALGLQQAFGRPVR--ARYRFSEARVIVSLDADFLGPTDRNFVENTRE  target    ----HWLTEARLKGTKVVVIACEYSSSSIKADDAIVVRPGTTPALALGLCNVIMREKIYDGDYVRRFSDLPLLVRADNLK 6f0k.1    FAASRRMERPEDEISRLYVIESTYTVTGGMADHRLRLRAGDIPAFAAALAAELGVGELREA-------------------  target    LLRAEEVFGTPQAALKNQTR 6f0k.1    -------------------- ``` | | | | | | | | | | | | | | | | | | | | | | | | | | | | | | | | | | | | | | | | | | | | | | | | | |
| ✓ | 7l5i.1.A | Trimethylamine-N-oxide reductase  *Crystal Structure of Haemophilus influenzae MtsZ at pH 7.0* | 0.31 | 0.00 | 25.73 | 0.59 | 77-377 | X-ray | 1.73 | monomer | 2 x MGD, 1 x MO, 1 x O | BLAST | 0.35 |
| ``` target    NNVNRREFLQWIGAAGFSTFALSASNAWGLQAIENPLAAYPNREWEKTYRDLWKSDASFTFLCAPNDTHNCILNAHVRDG 7l5i.1    ----------------------------------------------------------------------------VQDG  target    VITRIGPTMKYGEATDLYGSKVTHRWDPRVCQKGLALTRRFYGDRRVRYPMVRKGFKAWADKGFPREKDGRPPKDYFNRA 7l5i.1    KVVKSGPAIEPAVPNELQ----------------TVVADQLYSEARVKCPMVRKGFLANPGK-----------SDTTMRG  target    RDEWLRLTHEEAADLVAAALINIATTYSGDNGQKLLLQQGYEKEIVEATRGAGTQVLKFRGGMPLLGLTRIFGLYRMANS 7l5i.1    RDEWVRVSWDEALDLVHNQLKRVRDEH-GSTGIFAGSYGWFSCGSLHASRTLLQRYMNATGG--FVGHK---GDYSTGAA  target    MALLDHKIRGVKPEDALGARGWDNYSWHTDLPPGHPMVTGQQTVDFDLHAVEQARIVVVWGMN--------WVTTKMPDT 7l5i.1    QVIMPHVLGTIEVYE-------QQTSWESIL--------------------ESSDIIVLWSANPLTTMRIAWMSTDQKGI  target    HWLTEARLKGTKVVVIACEYSSSSIKAD-DAIVVRPGTTPALALGLCNVIMREKIYDGDYVRRFSDLPLLVRADNLKLLR 7l5i.1    EYFKKFQASGKRIICIDPQKSETCQMLNAEWIPVNTATDVPLMLGIAHTLVEQGKHDKDFLKKYTS--------------  target    AEEVFGTPQAALKNQTR 7l5i.1    ----------------- ``` | | | | | | | | | | | | | | | | | | | | | | | | | | | | | | | | | | | | | | | | | | | | | | | | | |
|  | 7l5s.1.A | Trimethylamine-N-oxide reductase  *Crystal Structure of Haemophilus influenzae MtsZ at pH 5.5* | 0.31 | 0.00 | 25.73 | 0.59 | 77-377 | X-ray | 2.09 | monomer | 1 x O, 2 x MGD, 1 x MO | BLAST | 0.35 |
| ``` target    NNVNRREFLQWIGAAGFSTFALSASNAWGLQAIENPLAAYPNREWEKTYRDLWKSDASFTFLCAPNDTHNCILNAHVRDG 7l5s.1    ----------------------------------------------------------------------------VQDG  target    VITRIGPTMKYGEATDLYGSKVTHRWDPRVCQKGLALTRRFYGDRRVRYPMVRKGFKAWADKGFPREKDGRPPKDYFNRA 7l5s.1    KVVKSGPAIEPAVPNELQ----------------TVVADQLYSEARVKCPMVRKGFLANPGK-----------SDTTMRG  target    RDEWLRLTHEEAADLVAAALINIATTYSGDNGQKLLLQQGYEKEIVEATRGAGTQVLKFRGGMPLLGLTRIFGLYRMANS 7l5s.1    RDEWVRVSWDEALDLVHNQLKRVRDEH-GSTGIFAGSYGWFSCGSLHASRTLLQRYMNATGG--FVGHK---GDYSTGAA  target    MALLDHKIRGVKPEDALGARGWDNYSWHTDLPPGHPMVTGQQTVDFDLHAVEQARIVVVWGMN--------WVTTKMPDT 7l5s.1    QVIMPHVLGTIEVYE-------QQTSWESIL--------------------ESSDIIVLWSANPLTTMRIAWMSTDQKGI  target    HWLTEARLKGTKVVVIACEYSSSSIKAD-DAIVVRPGTTPALALGLCNVIMREKIYDGDYVRRFSDLPLLVRADNLKLLR 7l5s.1    EYFKKFQASGKRIICIDPQKSETCQMLNAEWIPVNTATDVPLMLGIAHTLVEQGKHDKDFLKKYTS--------------  target    AEEVFGTPQAALKNQTR 7l5s.1    ----------------- ``` | | | | | | | | | | | | | | | | | | | | | | | | | | | | | | | | | | | | | | | | | | | | | | | | | |
|  | 7bkb.1.L | Formylmethanofuran dehydrogenase, subunit B  *Formate dehydrogenase - heterodisulfide reductase - formylmethanofuran dehydrogenase complex from Methanospirillum hungatei (hexameric, composite structure)* | 0.27 |  | 18.78 | 0.56 | 60-407 | EM | 0.00 | hetero-2-2-2-2-2-2-… | 48 x SF4, 4 x FAD, 2 x FES, 4 x 9S8, 4 x ZN, 2 x MO, 4 x MGD | HHblits | 0.28 |
| ``` target    NNVNRREFLQWIGAAGFSTFALSASNAWGLQAIENPLAAYPNREWEKTYRDLWKSDASFTFLCAPNDTHNCI-LNAHVRD 7bkb.1    -----------------------------------------------------------NVGCP-YCGCSCDDVRITVSD  target    GVITRIGPTMKYGEATDLYGSKVTHRWDPRVCQKGLALTRRFYGDRRVRYPMVRKGFKAWADKGFPREKDGRPPKDYFNR 7bkb.1    DGK--------------------DILEVENVCAIGTEIFKHGCSKDRIRLPRMRQP------------------------  target    ARDEWLRLTHEEAADLVAAALINIATTYSGDNGQKLLLQQGYEKEIVEATRGAGTQVLKFRGGMPLLGLTRIFGLYRMAN 7bkb.1    -DGSMKDISYEEAIDWTARHLLKAKKP-------LMYGFGSTNCEG-------QAA---------AARVMEIAGGM-LDN  target    SMALLDHKIRGVKPEDALGARGWDNYSWHTDLPPGHPMVTGQQTVDFDLHAV-EQARIVVVWGMNWVTTKMPDTHWL--- 7bkb.1    ----------------------CATICHGP----SFLAIFDNGYPSCTLGEVKNRADVIVYWGSNPAHAHPRHMSRYSIF  target    -----TEARLKGTKVVVIACEYSSSSIKADDAIVVRPGTTPALALGLCNVIMREKIYDGDYVRRFSDLPLLVRADNLKLL 7bkb.1    PRGFFTGKGQKKRTVIVIDPRFTDTANVADYHLQVKQGHDYELFNAFRMVIHGHGK-----------------------D  target    RAEEVFGTPQAALKNQTR 7bkb.1    LPDEVAGIKKETILEVA- ``` | | | | | | | | | | | | | | | | | | | | | | | | | | | | | | | | | | | | | | | | | | | | | | | | | |
|  | 8e9g.1.G | NADH-quinone oxidoreductase subunit G  *Mycobacterial respiratory complex I with both quinone positions modelled* | 0.28 |  | 19.72 | 0.53 | 58-359 | EM | 0.00 | hetero-1-1-1-1-1-1-… |  | HHblits | 0.28 |
| ``` target    NNVNRREFLQWIGAAGFSTFALSASNAWGLQAIENPLAAYPNREWEKTYRDLWKSDASFTFLCAPNDTHNCILNAHVRDG 8e9g.1    ---------------------------------------------------------SSPSVC-EHCASGCAQRTDHRRG  target    VITRIGPTMKYGEATDLYGSKVTHRWDPRVCQKGLALTRRFYGDRRVRYPMVRKGFKAWADKGFPREKDGRPPKDYFNRA 8e9g.1    KVLRRLAGD------------EPEVNEEWNCDKGRWAFTYATVGDRITTPMLRDG-------------------------  target    RDEWLRLTHEEAADLVAAALINIATTYSGDNGQKLLLQQGYEKEIVEATRGAGTQVLKFRGGMPLLGLTRI-FGLYRMAN 8e9g.1    -GVLRPASWSEALTVAAAGLLTAAGSTG------VLVGGRCTVED----------AYA------YAKFARMVLNTNDV--  target    SMALLDHKIRGVKPEDALGARGWDNYSWHTDLPPGHPMVTGQQTVDFDLHAVEQARIVVVWGMNWVTTKMPDTHWLTE-A 8e9g.1    -----DF----------------RARPHSAEEAEFLAAHVAGQTMGLRYAELENAPTVLLAGFEPEEESPIVFLRLRKGV  target    RLKGTKVVVIACEYSS-SSIKADDAIVVRPGTTPALALGLCNVIMREKIYDGDYVRRFSDLPLLVRADNLKLLRAEEVFG 8e9g.1    RKNGVQVVAVAPWASRGLTKLAGTVVPTVPGDEPAALDGMHD--------------------------------------  target    TPQAALKNQTR 8e9g.1    ----------- ``` | | | | | | | | | | | | | | | | | | | | | | | | | | | | | | | | | | | | | | | | | | | | | | | | | |
|  | 7p61.1.C | NADH-quinone oxidoreductase  *Complex I from E. coli, DDM-purified, with NADH, Resting state* | 0.26 |  | 14.86 | 0.54 | 57-365 | EM | 0.00 | hetero-1-1-1-1-1-1-… | 7 x SF4, 1 x FMN, 1 x NAI, 2 x FES, 1 x CA, 2 x 3PE, 1 x UQ8 | HHblits | 0.26 |
| ``` target    NNVNRREFLQWIGAAGFSTFALSASNAWGLQAIENPLAAYPNREWEKTYRDLWKSDASFTFLCAPNDTHNCILNAHVRDG 7p61.1    --------------------------------------------------------QFAPSICQ-QCSIGCNISPGERYG  target    VITRIGPTMKYGEATDLYGSKVTHRWDPRVCQKGLALTRRFYGDRRVRYPMVRKGFKAWADKGFPREKDGRPPKDYFNRA 7p61.1    ELRRIENRY------------NGTVNHYFLCDRGRFGYGYVNLKDRPRQPVQRRG-------------------------  target    RDEWLRLTHEEAADLVAAALINIATTYSGDNGQKLLLQQGYEKEIVEATRGAGTQVLKFRGGMPLLGLTRIFGLYRMANS 7p61.1    -DDFITLNAEQAMQGAADILRQSKKVIGI--------GSPRA---------SVESNF---------ALRELVGEENFYTG  target    MALLDHKIRGVKPEDALGARGWDNYSWHTDLPPGHPMVTGQQTVDFDLHAVEQARIVVVWGMNWVTTKMPDTHWLTEARL 7p61.1    IA----------------------HGEQERLQLALKVLREGGIYTPALREIESYDAVLVLGEDVTQTGARVALAVRQAVK  target    KGTK--------------------------VVVIACEYSSSSIKADDAIVVRPGTTPALALGLCNVIMREKIYDGDYVRR 7p61.1    GKAREMAAAQKVADWQIAAILNIGQRAKHPLFVTNVDDTRLDDIAAWTYRAPVEDQARLGFAIAHALDNSA---------  target    FSDLPLLVRADNLKLLRAEEVFGTPQAALKNQTR 7p61.1    ---------------------------------- ``` | | | | | | | | | | | | | | | | | | | | | | | | | | | | | | | | | | | | | | | | | | | | | | | | | |
|  | 7p63.1.C | NADH-quinone oxidoreductase  *Complex I from E. coli, DDM/LMNG-purified, under Turnover at pH 6, Closed state* | 0.27 |  | 15.38 | 0.54 | 57-364 | EM | 0.00 | hetero-1-1-1-1-1-1-… | 7 x SF4, 1 x FMN, 1 x NAI, 2 x FES, 1 x CA, 1 x DCQ, 4 x LFA, 8 x 3PE | HHblits | 0.26 |
| ``` target    NNVNRREFLQWIGAAGFSTFALSASNAWGLQAIENPLAAYPNREWEKTYRDLWKSDASFTFLCAPNDTHNCILNAHVRDG 7p63.1    --------------------------------------------------------QFAPSIC-QQCSIGCNISPGERYG  target    VITRIGPTMKYGEATDLYGSKVTHRWDPRVCQKGLALTRRFYGDRRVRYPMVRKGFKAWADKGFPREKDGRPPKDYFNRA 7p63.1    ELRRIENRY------------NGTVNHYFLCDRGRFGYGYVNLKDRPRQPVQRRG-------------------------  target    RDEWLRLTHEEAADLVAAALINIATTYSGDNGQKLLLQQGYEKEIVEATRGAGTQVLKFRGGMPLLGLTRIFGLYRMANS 7p63.1    -DDFITLNAEQAMQGAADILRQSKKVIGIGS-------PRASVE----------SNF---------ALRELVGEENFYTG  target    MALLDHKIRGVKPEDALGARGWDNYSWHTDLPPGHPMVTGQQTVDFDLHAVEQARIVVVWGMNWVTTKMPDTHWLTEARL 7p63.1    IA----------------------HGEQERLQLALKVLREGGIYTPALREIESYDAVLVLGEDVTQTGARVALAVRQAVK  target    KGTK--------------------------VVVIACEYSSSSIKADDAIVVRPGTTPALALGLCNVIMREKIYDGDYVRR 7p63.1    GKAREMAAAQKVADWQIAAILNIGQRAKHPLFVTNVDDTRLDDIAAWTYRAPVEDQARLGFAIAHALDNS----------  target    FSDLPLLVRADNLKLLRAEEVFGTPQAALKNQTR 7p63.1    ---------------------------------- ``` | | | | | | | | | | | | | | | | | | | | | | | | | | | | | | | | | | | | | | | | | | | | | | | | | |
|  | 7nz1.1.E | NADH-quinone oxidoreductase subunit G  *Respiratory complex I from Escherichia coli - focused refinement of cytoplasmic arm* | 0.27 |  | 15.38 | 0.54 | 57-364 | EM | 0.00 | hetero-1-1-1-1-1-1-… | 7 x SF4, 2 x FES, 1 x FMN, 1 x CA | HHblits | 0.26 |
| ``` target    NNVNRREFLQWIGAAGFSTFALSASNAWGLQAIENPLAAYPNREWEKTYRDLWKSDASFTFLCAPNDTHNCILNAHVRDG 7nz1.1    --------------------------------------------------------QFAPSICQ-QCSIGCNISPGERYG  target    VITRIGPTMKYGEATDLYGSKVTHRWDPRVCQKGLALTRRFYGDRRVRYPMVRKGFKAWADKGFPREKDGRPPKDYFNRA 7nz1.1    ELRRIENRY------------NGTVNHYFLCDRGRFGYGYVNLKDRPRQPVQRRG-------------------------  target    RDEWLRLTHEEAADLVAAALINIATTYSGDNGQKLLLQQGYEKEIVEATRGAGTQVLKFRGGMPLLGLTRIFGLYRMANS 7nz1.1    -DDFITLNAEQAMQGAADILRQSKKVIGIG-------SPRASVES----------NF---------ALRELVGEENFYTG  target    MALLDHKIRGVKPEDALGARGWDNYSWHTDLPPGHPMVTGQQTVDFDLHAVEQARIVVVWGMNWVTTKMPDTHWLTEARL 7nz1.1    IA----------------------HGEQERLQLALKVLREGGIYTPALREIESYDAVLVLGEDVTQTGARVALAVRQAVK  target    KGTK--------------------------VVVIACEYSSSSIKADDAIVVRPGTTPALALGLCNVIMREKIYDGDYVRR 7nz1.1    GKAREMAAAQKVADWQIAAILNIGQRAKHPLFVTNVDDTRLDDIAAWTYRAPVEDQARLGFAIAHALDNS----------  target    FSDLPLLVRADNLKLLRAEEVFGTPQAALKNQTR 7nz1.1    ---------------------------------- ``` | | | | | | | | | | | | | | | | | | | | | | | | | | | | | | | | | | | | | | | | | | | | | | | | | |
|  | 6lod.1.B | Fe-S-cluster-containing hydrogenase components 1-like protein  *Cryo-EM structure of the air-oxidized photosynthetic alternative complex III from Roseiflexus castenholzii* | 0.24 |  | 12.67 | 0.54 | 61-367 | EM | 0.00 | hetero-1-1-1-1-1-1-… | 6 x HEC, 2 x EL6, 3 x SF4, 1 x F3S | HHblits | 0.26 |
| ``` target    NNVNRREFLQWIGAAGFSTFALSASNAWGLQAIENPLAAYPNREWEKTYRDLWKSDASFTFLCAPNDTHNCILNAHVRDG 6lod.1    ------------------------------------------------------------TAV-TFAGFGVGLLVESHEG  target    VITRIGPTMKYGEATDLYGSKVTHRWDPRVCQKGLALTRRFYGDRRVRYPMVRKGFKAWADKGFPREKDGRPPKDYFNRA 6lod.1    RPTKIEGN------------PDHPASLGSTDLITQAMILTMYDPDRSQAPTNA---------------------------  target    RDEWLRLTHEEAADLVAAALINIATTYSGDNGQKLLLQQGYEKEIVEATRGAGTQVLKFRGGMPLLGLTRIFGLYRMANS 6lod.1    ---GQETTWDAFVAAATAAMQAQTAKQGAGL---RVLSGSLTS---------PT-LIAQ-----KQQLLTQFPQAKWYE-  target    MALLDHKIRGVKPEDALGARGWDNYSWHTDLPPGHPMVTGQQTVDFDLHAVEQARIVVVWGMNWVTTKMPD---THWLTE 6lod.1    ---------------------YEPVG-RDNANAGARLAFGAD--VHTIYRLDTAKVIVGFDADFTAPSPTGVRMARQLAD  target    AR------LKGTKVVVIACEYSSSSIKADDAIVVRPGTTPALALGLCNVIMREKIYDGDYVRRFSDLPLLVRADNLKLLR 6lod.1    GRRIRKGTKEVNRLYLAESTPSITGLLADHRLPVRSSQIEHLVRALATLVGVPNVA------------------------  target    AEEVFGTPQAALKNQTR 6lod.1    ----------------- ``` | | | | | | | | | | | | | | | | | | | | | | | | | | | | | | | | | | | | | | | | | | | | | | | | | |
|  | 7zm7.1.I | NADH-ubiquinone oxidoreductase-like protein  *CryoEM structure of mitochondrial complex I from Chaetomium thermophilum (inhibited by DDM)* | 0.27 |  | 15.60 | 0.53 | 59-366 | EM | 0.00 | hetero-1-1-1-1-1-1-… | 4 x PC1, 14 x LMT, 5 x CDL, 8 x 3PE, 2 x FES, 6 x SF4, 1 x FMN, 1 x NDP, 1 x ZN, 2 x ZMP | HHblits | 0.27 |
| ``` target    NNVNRREFLQWIGAAGFSTFALSASNAWGLQAIENPLAAYPNREWEKTYRDLWKSDASFTFLCAPNDTHNCILNAHVRDG 7zm7.1    ----------------------------------------------------------TESID-VLDGLGSNIRVDSRGL  target    VITRIGPTMKYGEATDLYGSKVTHRWDPRVCQKGLALTRRFYGDRRVRYPMVRKGFKAWADKGFPREKDGRPPKDYFNRA 7zm7.1    EVMRILPRLN------------DDVNEEWINDKTRFACDGLK-TQRLTMPLVRRD-------------------------  target    RDEWLRLTHEEAADLVAAALINIATTYSGDNGQKLLLQQGYEKEIVEATRGAGTQVLKFRGGMPLLGLTRIFGLYRMANS 7zm7.1    -GKFEPATWEQALTEIAHAYQTLAPKENEF---KVIAGQLVEVESL----------V------AMKDLANRLGSENL---  target    MALLDHKIRGVKPEDALGARGWDNYSWHTDLPPGHPMVTGQQ-TVDFDLHAVEQARIVVVWGMNWVTTKMPDTHWLTEAR 7zm7.1    ----------------------ALDFPGGSQPLAHGVDIRSNYLFNSKIWGIEEADAILLVGTNPRHEAAVLNARIRKQW  target    -LKGTKVVVIACEYSSSSIKADDAIVVRPGTTPALALGLCNVIMREKIYDGDYVRRFSDLPLLVRADNLKLLRAEEVFGT 7zm7.1    LRSDLEIAAVGQPWESTFDYEH------LGTDLAALKNALSGPFGEKL--------------------------------  target    PQAALKNQTR 7zm7.1    ---------- ``` | | | | | | | | | | | | | | | | | | | | | | | | | | | | | | | | | | | | | | | | | | | | | | | | | |
|  | 6btm.1.B | Alternative Complex III subunit B  *Structure of Alternative Complex III from Flavobacterium johnsoniae (Wild Type)* | 0.24 |  | 12.56 | 0.53 | 61-365 | EM | 3.40 | hetero-1-1-1-1-1-1-… | 6 x HEC, 1 x F3S, 1 x SF4, 2 x E87 | HHblits | 0.26 |
| ``` target    NNVNRREFLQWIGAAGFSTFALSASNAWGLQAIENPLAAYPNREWEKTYRDLWKSDASFTFLCAPNDTHNCILNAHVRDG 6btm.1    ------------------------------------------------------------TTV-FDGFDFANLLVKTREG  target    VITRIGPTMKYGEATDLYGSKVTHRWDPRVCQKGLALTRRFYGDRRVRYPMVRKGFKAWADKGFPREKDGRPPKDYFNRA 6btm.1    RPIKIENNT------------IAGA-KFSANARIHASILGLYDSMRLKEPKLDG--------------------------  target    RDEWLRLTHEEAADLVAAALINIATTYSGDNGQKLLLQQGYEKEIVEATRGAGTQVLKFRGGMPLLGLTRIFGLYRMANS 6btm.1    ----KNSSWSAVDLKIKSSLADAKAK-GGQ---VVLLTNTLA---------SPTTE-KL-----IGEFIAKNP-------  target    MALLDHKIRGVKPEDALGARGWDNYSW--HTDLPPGHPMVTGQQTVDFDLHAVEQARIVVVWGMNWVTTKMPD--THWLT 6btm.1    ------------------NAKHVVYDAVSSSDALDAFETVYGERA--LVDYDFSKASLIVSVGADFLGDWQGGGYDAGYA  target    EARL----KGTKVVVIACEYSSSSIKADDAIVVRPGTTPALALGLCNVIMREKIYDGDYVRRFSDLPLLVRADNLKLLRA 6btm.1    KGRIPQNGKMSRHFQFESNMTLSGAAADKRVPMTTADQKQALVQIYNIVVGAS---------------------------  target    EEVFGTPQAALKNQTR 6btm.1    ---------------- ``` | | | | | | | | | | | | | | | | | | | | | | | | | | | | | | | | | | | | | | | | | | | | | | | | | |
|  | 3m9s.1.C | NADH-quinone oxidoreductase subunit 3  *Crystal structure of respiratory complex I from Thermus thermophilus* | 0.25 | 0.00 | 19.91 | 0.52 | 57-362 | X-ray | 4.50 | monomer | 7 x SF4, 2 x FES, 1 x FMN | HHblits | 0.27 |
| ``` target    NNVNRREFLQWIGAAGFSTFALSASNAWGLQAIENPLAAYPNREWEKTYRDLWKSDASFTFLCAPNDTHNCILNAHVRDG 3m9s.1    --------------------------------------------------------EETPTTC-ALCPVGCGITADTRSG  target    VITRIGPTMKYGEATDLYGSKVTHRWDPRVCQKGLALTRRFYGDRRVRYPMVRKGFKAWADKGFPREKDGRPPKDYFNRA 3m9s.1    ELLRIRARE------------VPEVNEIWICDAGRFGHEW-ADQNRLKTPLVRKE-------------------------  target    RDEWLRLTHEEAADLVAAALINIATTYSGDNGQKLLLQQGYEKEIVEATRGAGTQVLKFRGGMPLLGLTRIFGLYRMANS 3m9s.1    -GRLVEATWEEAFLALKEGLKEARG----EEV-GLYLAHDATLEE----------GL------LASELAKALKT------  target    MALLDHKIRGVKPEDALGARGWDNYSWHTDLPPGHPMVTGQQTVDFDLHAVEQARIVVVWGMNWVTTKMPDTHWLTE--- 3m9s.1    -------------------PHLDFQGRTA---APA-----SLFPPASLEDLLQADFALVLG-DPTEEAPILHLRLSEFVR  target    ----------------------ARLKGTKVVVIACEYSSSSIKADDAIVVRPGTTPALALGLCNVIMREKIYDGDYVRRF 3m9s.1    DLKPPHRYNHGTPFADLQIKERMPRRTDKMALFAPYRAPLMKWAAIHEVHRPGEEREILLALLGDKE-------------  target    SDLPLLVRADNLKLLRAEEVFGTPQAALKNQTR 3m9s.1    --------------------------------- ``` | | | | | | | | | | | | | | | | | | | | | | | | | | | | | | | | | | | | | | | | | | | | | | | | | |
|  | 2fug.2.C | NADH-quinone oxidoreductase chain 3  *Crystal structure of the hydrophilic domain of respiratory complex I from Thermus thermophilus* | 0.24 | 0.00 | 19.91 | 0.52 | 57-362 | X-ray | 3.30 | monomer | 7 x SF4, 2 x FES, 1 x FMN | HHblits | 0.27 |
| ``` target    NNVNRREFLQWIGAAGFSTFALSASNAWGLQAIENPLAAYPNREWEKTYRDLWKSDASFTFLCAPNDTHNCILNAHVRDG 2fug.2    --------------------------------------------------------EETPTTC-ALCPVGCGITADTRSG  target    VITRIGPTMKYGEATDLYGSKVTHRWDPRVCQKGLALTRRFYGDRRVRYPMVRKGFKAWADKGFPREKDGRPPKDYFNRA 2fug.2    ELLRIRARE------------VPEVNEIWICDAGRFGHEW-ADQNRLKTPLVRKE-------------------------  target    RDEWLRLTHEEAADLVAAALINIATTYSGDNGQKLLLQQGYEKEIVEATRGAGTQVLKFRGGMPLLGLTRIFGLYRMANS 2fug.2    -GRLVEATWEEAFLALKEGLKEARG----EEV-GLYLAHDATLEE----------GL------LASELAKALKT------  target    MALLDHKIRGVKPEDALGARGWDNYSWHTDLPPGHPMVTGQQTVDFDLHAVEQARIVVVWGMNWVTTKMPDTHWLTE--- 2fug.2    -------------------PHLDFQGRTA---APA-----SLFPPASLEDLLQADFALVLG-DPTEEAPILHLRLSEFVR  target    ----------------------ARLKGTKVVVIACEYSSSSIKADDAIVVRPGTTPALALGLCNVIMREKIYDGDYVRRF 2fug.2    DLKPPHRYNHGTPFADLQIKERMPRRTDKMALFAPYRAPLMKWAAIHEVHRPGEEREILLALLGDKE-------------  target    SDLPLLVRADNLKLLRAEEVFGTPQAALKNQTR 2fug.2    --------------------------------- ``` | | | | | | | | | | | | | | | | | | | | | | | | | | | | | | | | | | | | | | | | | | | | | | | | | |
|  | 6zjl.1.C | NADH-quinone oxidoreductase subunit 3  *Respiratory complex I from Thermus thermophilus, NAD+ dataset, major state* | 0.24 | 0.00 | 19.91 | 0.52 | 57-362 | EM | 0.00 | monomer | 7 x SF4, 1 x FMN, 2 x FES | HHblits | 0.27 |
| ``` target    NNVNRREFLQWIGAAGFSTFALSASNAWGLQAIENPLAAYPNREWEKTYRDLWKSDASFTFLCAPNDTHNCILNAHVRDG 6zjl.1    --------------------------------------------------------EETPTTC-ALCPVGCGITADTRSG  target    VITRIGPTMKYGEATDLYGSKVTHRWDPRVCQKGLALTRRFYGDRRVRYPMVRKGFKAWADKGFPREKDGRPPKDYFNRA 6zjl.1    ELLRIRARE------------VPEVNEIWICDAGRFGHEW-ADQNRLKTPLVRKE-------------------------  target    RDEWLRLTHEEAADLVAAALINIATTYSGDNGQKLLLQQGYEKEIVEATRGAGTQVLKFRGGMPLLGLTRIFGLYRMANS 6zjl.1    -GRLVEATWEEAFLALKEGLKEARG----EEV-GLYLAHDATLEE----------GL------LASELAKALKT------  target    MALLDHKIRGVKPEDALGARGWDNYSWHTDLPPGHPMVTGQQTVDFDLHAVEQARIVVVWGMNWVTTKMPDTHWLTE--- 6zjl.1    -------------------PHLDFQGRTA---APA-----SLFPPASLEDLLQADFALVLG-DPTEEAPILHLRLSEFVR  target    ----------------------ARLKGTKVVVIACEYSSSSIKADDAIVVRPGTTPALALGLCNVIMREKIYDGDYVRRF 6zjl.1    DLKPPHRYNHGTPFADLQIKERMPRRTDKMALFAPYRAPLMKWAAIHEVHRPGEEREILLALLGDKE-------------  target    SDLPLLVRADNLKLLRAEEVFGTPQAALKNQTR 6zjl.1    --------------------------------- ``` | | | | | | | | | | | | | | | | | | | | | | | | | | | | | | | | | | | | | | | | | | | | | | | | | |
|  | 6q8o.1.C | NADH-quinone oxidoreductase subunit 3  *Respiratory complex I from Thermus thermophilus with bound Piericidin A* | 0.25 | 0.00 | 19.91 | 0.52 | 57-362 | X-ray | 3.61 | monomer | 7 x SF4, 1 x FMN, 2 x FES, 1 x HQH | HHblits | 0.27 |
| ``` target    NNVNRREFLQWIGAAGFSTFALSASNAWGLQAIENPLAAYPNREWEKTYRDLWKSDASFTFLCAPNDTHNCILNAHVRDG 6q8o.1    --------------------------------------------------------EETPTTC-ALCPVGCGITADTRSG  target    VITRIGPTMKYGEATDLYGSKVTHRWDPRVCQKGLALTRRFYGDRRVRYPMVRKGFKAWADKGFPREKDGRPPKDYFNRA 6q8o.1    ELLRIRARE------------VPEVNEIWICDAGRFGHEW-ADQNRLKTPLVRKE-------------------------  target    RDEWLRLTHEEAADLVAAALINIATTYSGDNGQKLLLQQGYEKEIVEATRGAGTQVLKFRGGMPLLGLTRIFGLYRMANS 6q8o.1    -GRLVEATWEEAFLALKEGLKEARG----EEV-GLYLAHDATLEE----------GL------LASELAKALKT------  target    MALLDHKIRGVKPEDALGARGWDNYSWHTDLPPGHPMVTGQQTVDFDLHAVEQARIVVVWGMNWVTTKMPDTHWLTE--- 6q8o.1    -------------------PHLDFQGRTA---APA-----SLFPPASLEDLLQADFALVLG-DPTEEAPILHLRLSEFVR  target    ----------------------ARLKGTKVVVIACEYSSSSIKADDAIVVRPGTTPALALGLCNVIMREKIYDGDYVRRF 6q8o.1    DLKPPHRYNHGTPFADLQIKERMPRRTDKMALFAPYRAPLMKWAAIHEVHRPGEEREILLALLGDKE-------------  target    SDLPLLVRADNLKLLRAEEVFGTPQAALKNQTR 6q8o.1    --------------------------------- ``` | | | | | | | | | | | | | | | | | | | | | | | | | | | | | | | | | | | | | | | | | | | | | | | | | |
|  | 6zjy.1.C | NADH-quinone oxidoreductase subunit 3  *Respiratory complex I from Thermus thermophilus, NAD+ dataset, minor state* | 0.24 | 0.00 | 19.91 | 0.52 | 57-362 | EM | 0.00 | monomer | 7 x SF4, 2 x FES | HHblits | 0.27 |
| ``` target    NNVNRREFLQWIGAAGFSTFALSASNAWGLQAIENPLAAYPNREWEKTYRDLWKSDASFTFLCAPNDTHNCILNAHVRDG 6zjy.1    --------------------------------------------------------EETPTTC-ALCPVGCGITADTRSG  target    VITRIGPTMKYGEATDLYGSKVTHRWDPRVCQKGLALTRRFYGDRRVRYPMVRKGFKAWADKGFPREKDGRPPKDYFNRA 6zjy.1    ELLRIRARE------------VPEVNEIWICDAGRFGHEW-ADQNRLKTPLVRKE-------------------------  target    RDEWLRLTHEEAADLVAAALINIATTYSGDNGQKLLLQQGYEKEIVEATRGAGTQVLKFRGGMPLLGLTRIFGLYRMANS 6zjy.1    -GRLVEATWEEAFLALKEGLKEARG----EEV-GLYLAHDATLEE----------GL------LASELAKALKT------  target    MALLDHKIRGVKPEDALGARGWDNYSWHTDLPPGHPMVTGQQTVDFDLHAVEQARIVVVWGMNWVTTKMPDTHWLTE--- 6zjy.1    -------------------PHLDFQGRTA---APA-----SLFPPASLEDLLQADFALVLG-DPTEEAPILHLRLSEFVR  target    ----------------------ARLKGTKVVVIACEYSSSSIKADDAIVVRPGTTPALALGLCNVIMREKIYDGDYVRRF 6zjy.1    DLKPPHRYNHGTPFADLQIKERMPRRTDKMALFAPYRAPLMKWAAIHEVHRPGEEREILLALLGDKE-------------  target    SDLPLLVRADNLKLLRAEEVFGTPQAALKNQTR 6zjy.1    --------------------------------- ``` | | | | | | | | | | | | | | | | | | | | | | | | | | | | | | | | | | | | | | | | | | | | | | | | | |
|  | 6zjn.1.C | NADH-quinone oxidoreductase subunit 3  *Respiratory complex I from Thermus thermophilus, NADH dataset, minor state* | 0.24 | 0.00 | 19.91 | 0.52 | 57-362 | EM | 0.00 | monomer | 7 x SF4, 2 x FES | HHblits | 0.27 |
| ``` target    NNVNRREFLQWIGAAGFSTFALSASNAWGLQAIENPLAAYPNREWEKTYRDLWKSDASFTFLCAPNDTHNCILNAHVRDG 6zjn.1    --------------------------------------------------------EETPTTC-ALCPVGCGITADTRSG  target    VITRIGPTMKYGEATDLYGSKVTHRWDPRVCQKGLALTRRFYGDRRVRYPMVRKGFKAWADKGFPREKDGRPPKDYFNRA 6zjn.1    ELLRIRARE------------VPEVNEIWICDAGRFGHEW-ADQNRLKTPLVRKE-------------------------  target    RDEWLRLTHEEAADLVAAALINIATTYSGDNGQKLLLQQGYEKEIVEATRGAGTQVLKFRGGMPLLGLTRIFGLYRMANS 6zjn.1    -GRLVEATWEEAFLALKEGLKEARG----EEV-GLYLAHDATLEE----------GL------LASELAKALKT------  target    MALLDHKIRGVKPEDALGARGWDNYSWHTDLPPGHPMVTGQQTVDFDLHAVEQARIVVVWGMNWVTTKMPDTHWLTE--- 6zjn.1    -------------------PHLDFQGRTA---APA-----SLFPPASLEDLLQADFALVLG-DPTEEAPILHLRLSEFVR  target    ----------------------ARLKGTKVVVIACEYSSSSIKADDAIVVRPGTTPALALGLCNVIMREKIYDGDYVRRF 6zjn.1    DLKPPHRYNHGTPFADLQIKERMPRRTDKMALFAPYRAPLMKWAAIHEVHRPGEEREILLALLGDKE-------------  target    SDLPLLVRADNLKLLRAEEVFGTPQAALKNQTR 6zjn.1    --------------------------------- ``` | | | | | | | | | | | | | | | | | | | | | | | | | | | | | | | | | | | | | | | | | | | | | | | | | |
|  | 6ziy.1.C | NADH-quinone oxidoreductase subunit 3  *Respiratory complex I from Thermus thermophilus, NADH dataset, major state* | 0.24 | 0.00 | 19.91 | 0.52 | 57-362 | EM | 0.00 | monomer | 7 x SF4, 1 x FMN, 1 x NAI, 2 x FES | HHblits | 0.27 |
| ``` target    NNVNRREFLQWIGAAGFSTFALSASNAWGLQAIENPLAAYPNREWEKTYRDLWKSDASFTFLCAPNDTHNCILNAHVRDG 6ziy.1    --------------------------------------------------------EETPTTC-ALCPVGCGITADTRSG  target    VITRIGPTMKYGEATDLYGSKVTHRWDPRVCQKGLALTRRFYGDRRVRYPMVRKGFKAWADKGFPREKDGRPPKDYFNRA 6ziy.1    ELLRIRARE------------VPEVNEIWICDAGRFGHEW-ADQNRLKTPLVRKE-------------------------  target    RDEWLRLTHEEAADLVAAALINIATTYSGDNGQKLLLQQGYEKEIVEATRGAGTQVLKFRGGMPLLGLTRIFGLYRMANS 6ziy.1    -GRLVEATWEEAFLALKEGLKEARG----EEV-GLYLAHDATLEE----------GL------LASELAKALKT------  target    MALLDHKIRGVKPEDALGARGWDNYSWHTDLPPGHPMVTGQQTVDFDLHAVEQARIVVVWGMNWVTTKMPDTHWLTE--- 6ziy.1    -------------------PHLDFQGRTA---APA-----SLFPPASLEDLLQADFALVLG-DPTEEAPILHLRLSEFVR  target    ----------------------ARLKGTKVVVIACEYSSSSIKADDAIVVRPGTTPALALGLCNVIMREKIYDGDYVRRF 6ziy.1    DLKPPHRYNHGTPFADLQIKERMPRRTDKMALFAPYRAPLMKWAAIHEVHRPGEEREILLALLGDKE-------------  target    SDLPLLVRADNLKLLRAEEVFGTPQAALKNQTR 6ziy.1    --------------------------------- ``` | | | | | | | | | | | | | | | | | | | | | | | | | | | | | | | | | | | | | | | | | | | | | | | | | |
|  | 5t5i.1.B | Tungsten formylmethanofuran dehydrogenase subunit B  *TUNGSTEN-CONTAINING FORMYLMETHANOFURAN DEHYDROGENASE FROM METHANOTHERMOBACTER WOLFEII, ORTHORHOMBIC FORM AT 1.9 A* | 0.24 |  | 17.87 | 0.51 | 60-365 | X-ray | 1.90 | hetero-oligomer | 4 x ZN, 2 x MG, 18 x K, 22 x SF4, 2 x W, 4 x MGD, 2 x H2S, 2 x CA | HHblits | 0.28 |
| ``` target    NNVNRREFLQWIGAAGFSTFALSASNAWGLQAIENPLAAYPNREWEKTYRDLWKSDASFTFLCAPNDTHNCI-LNAHVRD 5t5i.1    -----------------------------------------------------------NVVC-PFCGTLCDDIICKVEG  target    GVITRIGPTMKYGEATDLYGSKVTHRWDPRVCQKGLALTRRFYGDRRVRYPMVRKGFKAWADKGFPREKDGRPPKDYFNR 5t5i.1    NEIVGT----------------------INACRIGHSKFVHAEGAMRYKKPLIRKN------------------------  target    ARDEWLRLTHEEAADLVAAALINIATTYSGDNGQKLLLQQGYEKEIVEATRGAGTQVLKFRGGMPLLGLTRIFGLYRMAN 5t5i.1    --GEFVEVSYDEAIDKAAKILAESKRP--------LMYGWSCT--ECE----AQAVGVEL---------AEEAGAV-I--  target    SMALLDHKIRGVKPEDALGARGWDNYSWHTDLPPGHPMVTGQQTVDFDLHAV-EQARIVVVWGMNWVTTKMPDTHW---- 5t5i.1    -----DN---------------TASVCHGPSV-LALQ-DVGY--PICTFGEVKNRADVVVYWGCNPMHAHPRHMSRNVFA  target    ---LTEARLKGTKVVVIACEYSSSSIKADDAIVVRPGTTPALALGLCNVIMREKIYDGDYVRRFSDLPLLVRADNLKLLR 5t5i.1    RGFFRERGRSDRTLIVVDPRKTDSAKLADIHLQLDFDRDYELLDAMRACLLGHE--------------------------  target    AEEVFGTPQAALKNQTR 5t5i.1    ----------------- ``` | | | | | | | | | | | | | | | | | | | | | | | | | | | | | | | | | | | | | | | | | | | | | | | | | |
|  | 7tgh.58.A | NADH-ubiquinone oxidoreductase 75 kDa subunit  *Cryo-EM structure of respiratory super-complex CI+III2 from Tetrahymena thermophila* | 0.24 |  | 14.69 | 0.52 | 58-361 | EM | 0.00 | monomer |  | HHblits | 0.27 |
| ``` target    NNVNRREFLQWIGAAGFSTFALSASNAWGLQAIENPLAAYPNREWEKTYRDLWKSDASFTFLCAPNDTHNCILNAHVRDG 7tgh.58   ---------------------------------------------------------SFYTSD-VFDTLGSAIQVDTRGP  target    VITRIGPTMKYGEATDLYGSKVTHRWDPRVCQKGLALTRRFYGDRRVRYPMVRKGFKAWADKGFPREKDGRPPKDYFNRA 7tgh.58   EIMRVLPRI------------HEEINEEWISDKTRHAFDGLK-RQRINSPMKRSK-------------------------  target    RDEWLRLTHEEAADLVAAALINIATTYSGDNGQKLLLQQGYEKEIVEATRGAGTQVLKFRGGMPLLGLTRIFGLYRMANS 7tgh.58   DGNYEDIFWEEAIQTISKKCLNTPSDQI------GAIIGEF----------ADIESI-----TALKDFLNRLDVDNFEV-  target    MALLDHKIRGVKPEDALGARGWDNYSWHTDLPPGHPMVTGQQTVDFDLHAVEQARIVVVWGMNWVTTKMPDTHWLTEARL 7tgh.58   -------------------------RQHGNLKVSPD-FRANYLMNSKITGVEDADVLLLVGCNPRYEAPVLNARILKSTR  target    KGTKVVVIACEYSSSSIKADDAIVVRPGTTPALALGLCNVIMREKIYDGDYVRRFSDLPLLVRADNLKLLRAEEVFGTPQ 7tgh.58   KNLKVFNIGTNQDL--NYKNVHL----GNSTKVLKEIADGT---------------------------------------  target    AALKNQTR 7tgh.58   -------- ``` | | | | | | | | | | | | | | | | | | | | | | | | | | | | | | | | | | | | | | | | | | | | | | | | | |
|  | 5xtb.1.L | NADH-ubiquinone oxidoreductase 75 kDa subunit, mitochondrial  *Cryo-EM structure of human respiratory complex I matrix arm* | 0.23 |  | 16.35 | 0.51 | 59-359 | EM | 0.00 | hetero-1-1-1-1-1-1-… | 6 x SF4, 1 x FMN, 1 x 8Q1, 1 x NDP, 2 x FES | HHblits | 0.27 |
| ``` target    NNVNRREFLQWIGAAGFSTFALSASNAWGLQAIENPLAAYPNREWEKTYRDLWKSDASFTFLCAPNDTHNCILNAHVRDG 5xtb.1    ----------------------------------------------------------TESID-VMDAVGSNIVVSTRTG  target    VITRIGPTMKYGEATDLYGSKVTHRWDPRVCQKGLALTRRFYGDRRVRYPMVRKGFKAWADKGFPREKDGRPPKDYFNRA 5xtb.1    EVMRILPRM------------HEDINEEWISDKTRFAYDGLK-RQRLTEPMVRNE-------------------------  target    RDEWLRLTHEEAADLVAAALINIATTYSGDNGQKLLLQQGY-EKEIVEATRGAGTQVLKFRGGMPLLGLTRIFGLYRMAN 5xtb.1    KGLLTYTSWEDALSRVAGMLQSF----QGKDV--AAIAGGLVDAEA-------LVALK---------DLLNRVDSDTLCT  target    SMALLDHKIRGVKPEDALGARGWDNYSWHTDLPPGHPMVTGQQTVDFDLHAVEQARIVVVWGMNWVTTKMPDTHWLTEAR 5xtb.1    EE-------------------V---FP-TAGAGTDLRSN---YLLNTTIAGVEEADVVLLVGTNPRFEAPLFNARIRKSW  target    L-KGTKVVVIACEYSSSSIKADDAIVVRPGTTPALALGLCNVIMREKIYDGDYVRRFSDLPLLVRADNLKLLRAEEVFGT 5xtb.1    LHNDLKVALIGSPVDLTYTYD------HLGDSPKILQDIAS---------------------------------------  target    PQAALKNQTR 5xtb.1    ---------- ``` | | | | | | | | | | | | | | | | | | | | | | | | | | | | | | | | | | | | | | | | | | | | | | | | | |
|  | 7dgr.10.A | NADH-ubiquinone oxidoreductase 75 kDa subunit, mitochondrial  *Activity optimized supercomplex state2* | 0.23 |  | 16.75 | 0.51 | 59-353 | EM | 0.00 | monomer |  | HHblits | 0.27 |
| ``` target    NNVNRREFLQWIGAAGFSTFALSASNAWGLQAIENPLAAYPNREWEKTYRDLWKSDASFTFLCAPNDTHNCILNAHVRDG 7dgr.10   ----------------------------------------------------------TESID-VMDAVGSNIVVSTRTG  target    VITRIGPTMKYGEATDLYGSKVTHRWDPRVCQKGLALTRRFYGDRRVRYPMVRKGFKAWADKGFPREKDGRPPKDYFNRA 7dgr.10   EVMRILPRM------------HEDINEEWISDKTRFAYDGLK-RQRLTEPMVRNE-------------------------  target    RDEWLRLTHEEAADLVAAALINIATTYSGDNGQKLLLQQGYEKEIVEATRGAGTQVLKFRGGMPLLGLTRIFGLYRMANS 7dgr.10   KGLLTHTTWEDALSRVAGMLQSF----QGND---VAAIAGGL---------VDAEAL-I----ALKDLLNRVDSDTLCTE  target    MALLDHKIRGVKPEDALGARGWDNYSWHTDLPPGHPMVTGQQTVDFDLHAVEQARIVVVWGMNWVTTKMPDTHWLTEARL 7dgr.10   EV----------------------FP-TAGAGTDL---RSNYLLNTTIAGVEEADVVLLVGTNPRFEAPLFNARIRKSWL  target    -KGTKVVVIACEYSSSSIKADDAIVVRPGTTPALALGLCNVIMREKIYDGDYVRRFSDLPLLVRADNLKLLRAEEVFGTP 7dgr.10   HNDLKVALIGSPVDLTYRYDHLGDSPKILQDIAS----------------------------------------------  target    QAALKNQTR 7dgr.10   --------- ``` | | | | | | | | | | | | | | | | | | | | | | | | | | | | | | | | | | | | | | | | | | | | | | | | | |
|  | 5o31.1.8 | NADH-ubiquinone oxidoreductase 75 kDa subunit, mitochondrial  *Mitochondrial complex I in the deactive state* | 0.23 |  | 16.75 | 0.51 | 59-353 | EM | 4.13 | hetero-1-1-1-1-1-1-… | 6 x SF4, 2 x FES, 1 x FMN, 1 x NAP, 1 x ZN | HHblits | 0.27 |
| ``` target    NNVNRREFLQWIGAAGFSTFALSASNAWGLQAIENPLAAYPNREWEKTYRDLWKSDASFTFLCAPNDTHNCILNAHVRDG 5o31.1    ----------------------------------------------------------TESID-VMDAVGSNIVVSTRTG  target    VITRIGPTMKYGEATDLYGSKVTHRWDPRVCQKGLALTRRFYGDRRVRYPMVRKGFKAWADKGFPREKDGRPPKDYFNRA 5o31.1    EVMRILPRM------------HEDINEEWISDKTRFAYDGLK-RQRLTEPMVRNE-------------------------  target    RDEWLRLTHEEAADLVAAALINIATTYSGDNGQKLLLQQGYEKEIVEATRGAGTQVLKFRGGMPLLGLTRIFGLYRMANS 5o31.1    KGLLTHTTWEDALSRVAGMLQSF----QGND---VAAIAGGL---------VDAEAL-I----ALKDLLNRVDSDTLCTE  target    MALLDHKIRGVKPEDALGARGWDNYSWHTDLPPGHPMVTGQQTVDFDLHAVEQARIVVVWGMNWVTTKMPDTHWLTEARL 5o31.1    EV----------------------FP-TAGAGTDL---RSNYLLNTTIAGVEEADVVLLVGTNPRFEAPLFNARIRKSWL  target    -KGTKVVVIACEYSSSSIKADDAIVVRPGTTPALALGLCNVIMREKIYDGDYVRRFSDLPLLVRADNLKLLRAEEVFGTP 5o31.1    HNDLKVALIGSPVDLTYRYDHLGDSPKILQDIAS----------------------------------------------  target    QAALKNQTR 5o31.1    --------- ``` | | | | | | | | | | | | | | | | | | | | | | | | | | | | | | | | | | | | | | | | | | | | | | | | | |
|  | 7qsd.1.G | NADH-ubiquinone oxidoreductase 75 kDa subunit, mitochondrial  *Bovine complex I in the active state at 3.1 A* | 0.24 | 0.00 | 16.91 | 0.51 | 59-352 | EM | 0.00 | monomer | 5 x PC1, 13 x 3PE, 6 x SF4, 2 x FES, 1 x FMN, 4 x CDL, 3 x LMT, 1 x GTP, 1 x MG, 1 x NDP, 1 x ZN, 2 x EHZ | HHblits | 0.27 |
| ``` target    NNVNRREFLQWIGAAGFSTFALSASNAWGLQAIENPLAAYPNREWEKTYRDLWKSDASFTFLCAPNDTHNCILNAHVRDG 7qsd.1    ----------------------------------------------------------TESID-VMDAVGSNIVVSTRTG  target    VITRIGPTMKYGEATDLYGSKVTHRWDPRVCQKGLALTRRFYGDRRVRYPMVRKGFKAWADKGFPREKDGRPPKDYFNRA 7qsd.1    EVMRILPRMH------------EDINEEWISDKTRFAYDGLK-RQRLTEPMVRNE-------------------------  target    RDEWLRLTHEEAADLVAAALINIATTYSGDNGQKLLLQQGY-EKEIVEATRGAGTQVLKFRGGMPLLGLTRIFGLYRMAN 7qsd.1    KGLLTHTTWEDALSRVAGMLQSF----QGNDV--AAIAGGLVDAEA-------LIALK---------DLLNRVDSDTLCT  target    SMALLDHKIRGVKPEDALGARGWDNYSWHTDLPPGHPMVTGQQTVDFDLHAVEQARIVVVWGMNWVTTKMPDTHWLTEAR 7qsd.1    EEV----------------------F-PTAG--AGT-DLRSNYLLNTTIAGVEEADVVLLVGTNPRFEAPLFNARIRKSW  target    L-KGTKVVVIACEYSSSSIKADDAIVVRPGTTPALALGLCNVIMREKIYDGDYVRRFSDLPLLVRADNLKLLRAEEVFGT 7qsd.1    LHNDLKVALIGSPVDLTYRYDHLGDSPKILQDIA----------------------------------------------  target    PQAALKNQTR 7qsd.1    ---------- ``` | | | | | | | | | | | | | | | | | | | | | | | | | | | | | | | | | | | | | | | | | | | | | | | | | |
|  | 8b9z.1.G | NADH-ubiquinone oxidoreductase 75 kDa subunit, mitochondrial  *Drosophila melanogaster complex I in the Active state (Dm1)* | 0.24 |  | 16.83 | 0.51 | 58-360 | EM | 3.28 | hetero-1-1-1-1-1-1-… | 3 x PC1, 16 x 3PE, 6 x SF4, 4 x CDL, 2 x FES, 1 x FMN, 1 x UQ9, 1 x DGT, 1 x NDP, 1 x ZN, 2 x EHZ | HHblits | 0.26 |
| ``` target    NNVNRREFLQWIGAAGFSTFALSASNAWGLQAIENPLAAYPNREWEKTYRDLWKSDASFTFLCAPNDTHNCILNAHVRDG 8b9z.1    ---------------------------------------------------------KVSSID-VLDAVGSNIVVSTRTN  target    VITRIGPTMKYGEATDLYGSKVTHRWDPRVCQKGLALTRRFYGDRRVRYPMVRKGFKAWADKGFPREKDGRPPKDYFNRA 8b9z.1    EVLRILPREN------------EDVNEEWLADKSRFACDGLK-RQRLVAPMVRMP-------------------------  target    RDEWLRLTHEEAADLVAAALINIATTYSGDNGQKLLLQQGYEKEIVEATRGAGTQVLKFRGGMPLLGLTRIFGLYRMANS 8b9z.1    NGELQAVEWEGALIAVAKAIKAAGGQI-------AGISGQLA----------DLEAQ-V----ALKDLLNRLGSEVVATE  target    MALLDHKIRGVKPEDALGARGWDNYSWHTDLPPGHPMVTGQQTVDFDLHAVEQARIVVVWGMNWVTTKMPDTHWLTEAR- 8b9z.1    Q-------------------------GFI---AGGTDNRANYLLNSTIAGLEEADAVLLVGTNPRYEAPLVNTRLRKAYV  target    LKGTKVVVIACEYSSSSIKADDAIVVRPGTTPALALGLCNVIMREKIYDGDYVRRFSDLPLLVRADNLKLLRAEEVFGTP 8b9z.1    HNELQIASIGPKIDLS------YDHENLGADAALVKDVCSG---------------------------------------  target    QAALKNQTR 8b9z.1    --------- ``` | | | | | | | | | | | | | | | | | | | | | | | | | | | | | | | | | | | | | | | | | | | | | | | | | |
|  | 8ba0.1.G | NADH-ubiquinone oxidoreductase 75 kDa subunit, mitochondrial  *Drosophila melanogaster complex I in the Twisted state (Dm2)* | 0.24 |  | 16.83 | 0.51 | 58-360 | EM | 3.68 | hetero-1-1-1-1-1-1-… | 6 x SF4, 6 x 3PE, 2 x FES, 1 x FMN, 2 x CDL, 1 x DGT, 1 x NDP, 1 x ZN, 2 x EHZ | HHblits | 0.26 |
| ``` target    NNVNRREFLQWIGAAGFSTFALSASNAWGLQAIENPLAAYPNREWEKTYRDLWKSDASFTFLCAPNDTHNCILNAHVRDG 8ba0.1    ---------------------------------------------------------KVSSID-VLDAVGSNIVVSTRTN  target    VITRIGPTMKYGEATDLYGSKVTHRWDPRVCQKGLALTRRFYGDRRVRYPMVRKGFKAWADKGFPREKDGRPPKDYFNRA 8ba0.1    EVLRILPREN------------EDVNEEWLADKSRFACDGLK-RQRLVAPMVRMP-------------------------  target    RDEWLRLTHEEAADLVAAALINIATTYSGDNGQKLLLQQGYEKEIVEATRGAGTQVLKFRGGMPLLGLTRIFGLYRMANS 8ba0.1    NGELQAVEWEGALIAVAKAIKAAGGQI-------AGISGQLA----------DLEAQ-V----ALKDLLNRLGSEVVATE  target    MALLDHKIRGVKPEDALGARGWDNYSWHTDLPPGHPMVTGQQTVDFDLHAVEQARIVVVWGMNWVTTKMPDTHWLTEAR- 8ba0.1    Q-------------------------GFI---AGGTDNRANYLLNSTIAGLEEADAVLLVGTNPRYEAPLVNTRLRKAYV  target    LKGTKVVVIACEYSSSSIKADDAIVVRPGTTPALALGLCNVIMREKIYDGDYVRRFSDLPLLVRADNLKLLRAEEVFGTP 8ba0.1    HNELQIASIGPKIDLS------YDHENLGADAALVKDVCSG---------------------------------------  target    QAALKNQTR 8ba0.1    --------- ``` | | | | | | | | | | | | | | | | | | | | | | | | | | | | | | | | | | | | | | | | | | | | | | | | | |
|  | 7arc.1.F | 75 kDa  *Cryo-EM structure of Polytomella Complex-I (peripheral arm)* | 0.24 |  | 14.08 | 0.50 | 59-360 | EM | 0.00 | hetero-1-1-1-1-1-1-… | 6 x SF4, 2 x FES, 1 x FMN, 1 x NDP, 1 x ZN, 1 x 8Q1 | HHblits | 0.27 |
| ``` target    NNVNRREFLQWIGAAGFSTFALSASNAWGLQAIENPLAAYPNREWEKTYRDLWKSDASFTFLCAPNDTHNCILNAHVRDG 7arc.1    ----------------------------------------------------------TETID-VSDALGSNIKVDCRGT  target    VITRIGPTMKYGEATDLYGSKVTHRWDPRVCQKGLALTRRFYGDRRVRYPMVRKGFKAWADKGFPREKDGRPPKDYFNRA 7arc.1    EVMRITPRLN------------DAINEEWLSDKGRFQYDGLK-RQRLNTPLVKGA-------------------------  target    RDEWLRLTHEEAADLVAAALINIATTYSGDNGQKLLLQQGYEKEIVEATRGAGTQVLKFRGGMPLLGLTRIFGLYRMANS 7arc.1    -KGLENATWSAAFDAIRTAIAGAK---G-NE--LKAIAGKLA----------DAESM-I----ALKDLFNKLGSGNLIH-  target    MALLDHKIRGVKPEDALGARGWDNYSWHTDLPPGHPMVTGQ-QTVDFDLHAVEQARIVVVWGMNWVTTKMPDTHWLTEAR 7arc.1    ---------------------------EDGS-ATLSADVRSSYIANTTIASIEKADVILLVGTNPRFESPVFNARLRKVF  target    LKGTKVVVIACEYSSSSIKADDAIVVRPGTTPALALGLCNVIMREKIYDGDYVRRFSDLPLLVRADNLKLLRAEEVFGTP 7arc.1    LDGAKVGLVGEKVDLT------YAYQHLGADVAALESLASG---------------------------------------  target    QAALKNQTR 7arc.1    --------- ``` | | | | | | | | | | | | | | | | | | | | | | | | | | | | | | | | | | | | | | | | | | | | | | | | | |
|  | 7ar7.1.G | NADH dehydrogenase [ubiquinone] iron-sulfur protein 1, mitochondrial  *Cryo-EM structure of Arabidopsis thaliana complex-I (open conformation)* | 0.25 |  | 12.08 | 0.51 | 59-355 | EM | 0.00 | hetero-1-1-1-1-1-1-… | 6 x SF4, 2 x FES, 1 x FMN, 1 x UQ9, 3 x PTY, 2 x PC7, 1 x LMN, 1 x NDP, 2 x ZN, 2 x 8Q1, 1 x PGT, 1 x PSF, 1 x T7X | HHblits | 0.26 |
| ``` target    NNVNRREFLQWIGAAGFSTFALSASNAWGLQAIENPLAAYPNREWEKTYRDLWKSDASFTFLCAPNDTHNCILNAHVRDG 7ar7.1    ----------------------------------------------------------TETID-VSDAVGSNIRVDSRGP  target    VITRIGPTMKYGEATDLYGSKVTHRWDPRVCQKGLALTRRFYGDRRVRYPMVRKGFKAWADKGFPREKDGRPPKDYFNRA 7ar7.1    EVMRIIPRLN------------EDINEEWISDKTRFCYDGLK-RQRLSDPMIRDS-------------------------  target    RDEWLRLTHEEAADLVAAALINIATTYSGDNGQKLLLQQGYEKEIVEATRGAGTQVLKFRGGMPLLGLTRIFGLYRMANS 7ar7.1    DGRFKAVSWRDALAVVGDIIHQV----KPDE---IVGVAGQLS---------DAESM-M----VLKDFVNRMGSDNV---  target    MALLDHKIRGVKPEDALGARGWDNYSWHTDLPPGHPMVTGQQTVDFDLHAVEQARIVVVWGMNWVTTKMPDTHWLTEA-R 7ar7.1    ----------------------W---CEGTAAGVDADLRYSYLMNTSISGLENADLFLLIGTQPRVEAAMVNARICKTVR  target    LKGTKVVVIACEYSSSSIKADDAIVVRPGTTPALALGLCNVIMREKIYDGDYVRRFSDLPLLVRADNLKLLRAEEVFGTP 7ar7.1    ASNAKVGYVGPPAEFN--YDCKHLGTGPDTLKEIAE--------------------------------------------  target    QAALKNQTR 7ar7.1    --------- ``` | | | | | | | | | | | | | | | | | | | | | | | | | | | | | | | | | | | | | | | | | | | | | | | | | |
|  | 7aqr.1.F | NADH dehydrogenase [ubiquinone] iron-sulfur protein 1, mitochondrial  *Cryo-EM structure of Arabidopsis thaliana Complex-I (peripheral arm)* | 0.25 |  | 12.08 | 0.51 | 59-355 | EM | 0.00 | hetero-1-1-1-1-1-1-… | 6 x SF4, 2 x FES, 1 x FMN, 1 x NDP, 1 x ZN, 1 x 8Q1 | HHblits | 0.26 |
| ``` target    NNVNRREFLQWIGAAGFSTFALSASNAWGLQAIENPLAAYPNREWEKTYRDLWKSDASFTFLCAPNDTHNCILNAHVRDG 7aqr.1    ----------------------------------------------------------TETID-VSDAVGSNIRVDSRGP  target    VITRIGPTMKYGEATDLYGSKVTHRWDPRVCQKGLALTRRFYGDRRVRYPMVRKGFKAWADKGFPREKDGRPPKDYFNRA 7aqr.1    EVMRIIPRLN------------EDINEEWISDKTRFCYDGLK-RQRLSDPMIRDS-------------------------  target    RDEWLRLTHEEAADLVAAALINIATTYSGDNGQKLLLQQGYEKEIVEATRGAGTQVLKFRGGMPLLGLTRIFGLYRMANS 7aqr.1    DGRFKAVSWRDALAVVGDIIHQV----KPDE---IVGVAGQLS---------DAESM-----MVLKDFVNRMGSDNVW--  target    MALLDHKIRGVKPEDALGARGWDNYSWHTDLPPGHPMVTGQQTVDFDLHAVEQARIVVVWGMNWVTTKMPDTHWLTEAR- 7aqr.1    --------------------------CEGTAAGVDADLRYSYLMNTSISGLENADLFLLIGTQPRVEAAMVNARICKTVR  target    LKGTKVVVIACEYSSSSIKADDAIVVRPGTTPALALGLCNVIMREKIYDGDYVRRFSDLPLLVRADNLKLLRAEEVFGTP 7aqr.1    ASNAKVGYVGPPAEFN--YDCKHLGTGPDTLKEIAE--------------------------------------------  target    QAALKNQTR 7aqr.1    --------- ``` | | | | | | | | | | | | | | | | | | | | | | | | | | | | | | | | | | | | | | | | | | | | | | | | | |
|  | 7a23.1.O | 75kDa  *Plant mitochondrial respiratory complex I* | 0.26 |  | 12.08 | 0.51 | 59-355 | EM | 0.00 | hetero-1-1-1-1-1-1-… | 6 x SF4, 1 x FMN, 2 x T7X, 3 x CDL, 1 x U10, 1 x PEV, 2 x FES, 1 x NDP, 2 x ZN | HHblits | 0.26 |
| ``` target    NNVNRREFLQWIGAAGFSTFALSASNAWGLQAIENPLAAYPNREWEKTYRDLWKSDASFTFLCAPNDTHNCILNAHVRDG 7a23.1    ----------------------------------------------------------TETID-VSDAVGSNIRVDSRGP  target    VITRIGPTMKYGEATDLYGSKVTHRWDPRVCQKGLALTRRFYGDRRVRYPMVRKGFKAWADKGFPREKDGRPPKDYFNRA 7a23.1    EVMRIIPRLN------------EDINEEWISDKTRFCYDGLK-RQRLSDPMIRDS-------------------------  target    RDEWLRLTHEEAADLVAAALINIATTYSGDNGQKLLLQQGYEKEIVEATRGAGTQVLKFRGGMPLLGLTRIFGLYRMANS 7a23.1    DGRFKAVSWRDALAVVGDIIHQV----KPDE---IVGVAGQLS---------DAESM-----MVLKDFVNRMGSDNVW--  target    MALLDHKIRGVKPEDALGARGWDNYSWHTDLPPGHPMVTGQQTVDFDLHAVEQARIVVVWGMNWVTTKMPDTHWLTEAR- 7a23.1    --------------------------CEGTAAGVDADLRYSYLMNTSISGLENADLFLLIGTQPRVEAAMVNARICKTVR  target    LKGTKVVVIACEYSSSSIKADDAIVVRPGTTPALALGLCNVIMREKIYDGDYVRRFSDLPLLVRADNLKLLRAEEVFGTP 7a23.1    ASNAKVGYVGPPAEFN--YDCKHLGTGPDTLKEIAE--------------------------------------------  target    QAALKNQTR 7a23.1    --------- ``` | | | | | | | | | | | | | | | | | | | | | | | | | | | | | | | | | | | | | | | | | | | | | | | | | |
|  | 7ar8.1.G | NADH dehydrogenase [ubiquinone] iron-sulfur protein 1, mitochondrial  *Cryo-EM structure of Arabidopsis thaliana complex-I (closed conformation)* | 0.24 |  | 12.08 | 0.51 | 59-355 | EM | 0.00 | hetero-1-1-1-1-1-1-… | 6 x SF4, 2 x FES, 1 x FMN, 1 x UQ9, 3 x PTY, 2 x PC7, 1 x PGT, 1 x FE, 1 x NDP, 2 x ZN, 2 x 8Q1, 1 x LMN, 1 x PSF, 1 x T7X | HHblits | 0.26 |
| ``` target    NNVNRREFLQWIGAAGFSTFALSASNAWGLQAIENPLAAYPNREWEKTYRDLWKSDASFTFLCAPNDTHNCILNAHVRDG 7ar8.1    ----------------------------------------------------------TETID-VSDAVGSNIRVDSRGP  target    VITRIGPTMKYGEATDLYGSKVTHRWDPRVCQKGLALTRRFYGDRRVRYPMVRKGFKAWADKGFPREKDGRPPKDYFNRA 7ar8.1    EVMRIIPRLN------------EDINEEWISDKTRFCYDGLK-RQRLSDPMIRDS-------------------------  target    RDEWLRLTHEEAADLVAAALINIATTYSGDNGQKLLLQQGYEKEIVEATRGAGTQVLKFRGGMPLLGLTRIFGLYRMANS 7ar8.1    DGRFKAVSWRDALAVVGDIIHQV----KPDE---IVGVAGQLS---------DAESM-----MVLKDFVNRMGSDNVW--  target    MALLDHKIRGVKPEDALGARGWDNYSWHTDLPPGHPMVTGQQTVDFDLHAVEQARIVVVWGMNWVTTKMPDTHWLTEAR- 7ar8.1    --------------------------CEGTAAGVDADLRYSYLMNTSISGLENADLFLLIGTQPRVEAAMVNARICKTVR  target    LKGTKVVVIACEYSSSSIKADDAIVVRPGTTPALALGLCNVIMREKIYDGDYVRRFSDLPLLVRADNLKLLRAEEVFGTP 7ar8.1    ASNAKVGYVGPPAEFN--YDCKHLGTGPDTLKEIAE--------------------------------------------  target    QAALKNQTR 7ar8.1    --------- ``` | | | | | | | | | | | | | | | | | | | | | | | | | | | | | | | | | | | | | | | | | | | | | | | | | |
|  | 6qcf.1.C | NADH:ubiquinone oxidoreductase core subunit S1  *Ovine respiratory complex I FRC open class 6* | 0.24 | 0.00 | 16.59 | 0.50 | 59-350 | EM | 0.00 | monomer | 6 x SF4, 1 x FMN, 2 x FES, 1 x ZN, 1 x NDP, 2 x ZMP | HHblits | 0.27 |
| ``` target    NNVNRREFLQWIGAAGFSTFALSASNAWGLQAIENPLAAYPNREWEKTYRDLWKSDASFTFLCAPNDTHNCILNAHVRDG 6qcf.1    ----------------------------------------------------------TESID-VMDAVGSNIVVSTRTG  target    VITRIGPTMKYGEATDLYGSKVTHRWDPRVCQKGLALTRRFYGDRRVRYPMVRKGFKAWADKGFPREKDGRPPKDYFNRA 6qcf.1    EVMRILPRM------------HEDINEEWISDKTRFAYDGLK-RQRLTEPMVRNE-------------------------  target    RDEWLRLTHEEAADLVAAALINIATTYSGDNGQKLLLQQGY-EKEIVEATRGAGTQVLKFRGGMPLLGLTRIFGLYRMAN 6qcf.1    KGLLTHTTWEDALSRVAGMLQSC----QGNDV--AAIAGGLVDAEA-------LIALK---------DLLNRVDSDTLCT  target    SMALLDHKIRGVKPEDALGARGWDNYSWHTDLPPGHPMVTGQQTVDFDLHAVEQARIVVVWGMNWVTTKMPDTHWLTEAR 6qcf.1    EEV-------------------F----PTAG--AG-TDLRSNYLLNTTIAGVEEADVVLLVGTNPRFEAPLFNARIRKSW  target    L-KGTKVVVIACEYSSSSIKADDAIVVRPGTTPALALGLCNVIMREKIYDGDYVRRFSDLPLLVRADNLKLLRAEEVFGT 6qcf.1    LHNDLKVALIGSPVDLTYRYDHLGDSPKILQD------------------------------------------------  target    PQAALKNQTR 6qcf.1    ---------- ``` | | | | | | | | | | | | | | | | | | | | | | | | | | | | | | | | | | | | | | | | | | | | | | | | | |
|  | 6qc5.1.C | NADH:ubiquinone oxidoreductase core subunit S1  *Ovine respiratory complex I FRC closed class 1* | 0.24 | 0.00 | 16.59 | 0.50 | 59-350 | EM | 0.00 | monomer | 6 x SF4, 1 x FMN, 2 x FES, 2 x 3PE, 1 x ZN, 1 x NDP, 2 x ZMP, 1 x PC1 | HHblits | 0.27 |
| ``` target    NNVNRREFLQWIGAAGFSTFALSASNAWGLQAIENPLAAYPNREWEKTYRDLWKSDASFTFLCAPNDTHNCILNAHVRDG 6qc5.1    ----------------------------------------------------------TESID-VMDAVGSNIVVSTRTG  target    VITRIGPTMKYGEATDLYGSKVTHRWDPRVCQKGLALTRRFYGDRRVRYPMVRKGFKAWADKGFPREKDGRPPKDYFNRA 6qc5.1    EVMRILPRM------------HEDINEEWISDKTRFAYDGLK-RQRLTEPMVRNE-------------------------  target    RDEWLRLTHEEAADLVAAALINIATTYSGDNGQKLLLQQGY-EKEIVEATRGAGTQVLKFRGGMPLLGLTRIFGLYRMAN 6qc5.1    KGLLTHTTWEDALSRVAGMLQSC----QGNDV--AAIAGGLVDAEA-------LIALK---------DLLNRVDSDTLCT  target    SMALLDHKIRGVKPEDALGARGWDNYSWHTDLPPGHPMVTGQQTVDFDLHAVEQARIVVVWGMNWVTTKMPDTHWLTEAR 6qc5.1    EEV-------------------F----PTAG--AG-TDLRSNYLLNTTIAGVEEADVVLLVGTNPRFEAPLFNARIRKSW  target    L-KGTKVVVIACEYSSSSIKADDAIVVRPGTTPALALGLCNVIMREKIYDGDYVRRFSDLPLLVRADNLKLLRAEEVFGT 6qc5.1    LHNDLKVALIGSPVDLTYRYDHLGDSPKILQD------------------------------------------------  target    PQAALKNQTR 6qc5.1    ---------- ``` | | | | | | | | | | | | | | | | | | | | | | | | | | | | | | | | | | | | | | | | | | | | | | | | | |
|  | 7vxu.1.L | NADH-ubiquinone oxidoreductase 75 kDa subunit, mitochondrial  *Matrix arm of deactive state CI from Q10 dataset* | 0.24 | 0.00 | 15.61 | 0.50 | 59-350 | EM | 0.00 | monomer | 6 x SF4, 1 x FMN, 1 x PEE, 1 x PLX, 1 x 8Q1, 1 x NDP, 2 x FES, 1 x MG, 1 x CDL, 1 x ZN | HHblits | 0.27 |
| ``` target    NNVNRREFLQWIGAAGFSTFALSASNAWGLQAIENPLAAYPNREWEKTYRDLWKSDASFTFLCAPNDTHNCILNAHVRDG 7vxu.1    ----------------------------------------------------------TESID-VMDAVGSNIVVSTRTG  target    VITRIGPTMKYGEATDLYGSKVTHRWDPRVCQKGLALTRRFYGDRRVRYPMVRKGFKAWADKGFPREKDGRPPKDYFNRA 7vxu.1    EVMRILPRMH------------EDINEEWISDKTRFAYDGLK-RQRLTQPMIRNE-------------------------  target    RDEWLRLTHEEAADLVAAALINIATTYSGDNGQKLLLQQGY-EKEIVEATRGAGTQVLKFRGGMPLLGLTRIFGLYRMAN 7vxu.1    KGLLTYTTWEDALSRVAGMLQSF----QGNDV--AAIAGGLVDAEA----------LV------ALKDLLNRVDSDSLCT  target    SMALLDHKIRGVKPEDALGARGWDNYSWHTDLPPGHPMVTGQQTVDFDLHAVEQARIVVVWGMNWVTTKMPDTHWLTEAR 7vxu.1    EE----------------------VFP-TAGAGTDLRSNY---LLNTTIAGVEEADVILLVGTNPRFEAPLFNARIRKSW  target    L-KGTKVVVIACEYSSSSIKADDAIVVRPGTTPALALGLCNVIMREKIYDGDYVRRFSDLPLLVRADNLKLLRAEEVFGT 7vxu.1    LHNDLKVALIGSPVDLTYRYDHLGDSPKILQD------------------------------------------------  target    PQAALKNQTR 7vxu.1    ---------- ``` | | | | | | | | | | | | | | | | | | | | | | | | | | | | | | | | | | | | | | | | | | | | | | | | | |
|  | 6x89.1.H | NADH dehydrogenase [ubiquinone] iron-sulfur protein 1, mitochondrial  *Vigna radiata mitochondrial complex I\** | 0.24 |  | 12.08 | 0.51 | 59-356 | EM | 0.00 | hetero-1-1-1-1-1-1-… | 1 x NAP, 6 x PC1, 6 x SF4, 2 x FES, 2 x ZN, 1 x FMN | HHblits | 0.26 |
| ``` target    NNVNRREFLQWIGAAGFSTFALSASNAWGLQAIENPLAAYPNREWEKTYRDLWKSDASFTFLCAPNDTHNCILNAHVRDG 6x89.1    ----------------------------------------------------------TETID-VTDAVGSNIRIDSRGP  target    VITRIGPTMKYGEATDLYGSKVTHRWDPRVCQKGLALTRRFYGDRRVRYPMVRKGFKAWADKGFPREKDGRPPKDYFNRA 6x89.1    EVMRIVPRLN------------EDINEEWISDKTRFCYDGLK-RQRLNDPMIRGP-------------------------  target    RDEWLRLTHEEAADLVAAALINIATTYSGDNGQKLLLQQGYEKEIVEATRGAGTQVLKFRGGMPLLGLTRIFGLYRMANS 6x89.1    DGRFKAVNWRDALSVIADIAHQV----KPEE---IVGVAGKLS---------DAESM-I----ALKDFLNRMGSNDVWG-  target    MALLDHKIRGVKPEDALGARGWDNYSWHTDLPPGHPMVTGQ-QTVDFDLHAVEQARIVVVWGMNWVTTKMPDTHWLTEAR 6x89.1    ------------------------EGIGVN----TNADFRSGYIMNTSIAGLEKADVFLLVGTQPRVEAAMVNARIRKTV  target    L-KGTKVVVIACEYSSSSIKADDAIVVRPGTTPALALGLCNVIMREKIYDGDYVRRFSDLPLLVRADNLKLLRAEEVFGT 6x89.1    RSNQAKVGYIGPATDFN--YDHKHLGTDPQTLVEIAEG------------------------------------------  target    PQAALKNQTR 6x89.1    ---------- ``` | | | | | | | | | | | | | | | | | | | | | | | | | | | | | | | | | | | | | | | | | | | | | | | | | |
|  | 8e73.55.A | NDUS1  *Vigna radiata supercomplex I+III2 (full bridge)* | 0.24 |  | 12.08 | 0.51 | 59-356 | EM | 0.00 | monomer |  | HHblits | 0.26 |
| ``` target    NNVNRREFLQWIGAAGFSTFALSASNAWGLQAIENPLAAYPNREWEKTYRDLWKSDASFTFLCAPNDTHNCILNAHVRDG 8e73.55   ----------------------------------------------------------TETID-VTDAVGSNIRIDSRGP  target    VITRIGPTMKYGEATDLYGSKVTHRWDPRVCQKGLALTRRFYGDRRVRYPMVRKGFKAWADKGFPREKDGRPPKDYFNRA 8e73.55   EVMRIVPRLN------------EDINEEWISDKTRFCYDGLK-RQRLNDPMIRGP-------------------------  target    RDEWLRLTHEEAADLVAAALINIATTYSGDNGQKLLLQQGYEKEIVEATRGAGTQVLKFRGGMPLLGLTRIFGLYRMANS 8e73.55   DGRFKAVNWRDALSVIADIAHQV----KPEE---IVGVAGKLS---------DAESM-I----ALKDFLNRMGSNDVWG-  target    MALLDHKIRGVKPEDALGARGWDNYSWHTDLPPGHPMVTGQ-QTVDFDLHAVEQARIVVVWGMNWVTTKMPDTHWLTEAR 8e73.55   ------------------------EGIGVN----TNADFRSGYIMNTSIAGLEKADVFLLVGTQPRVEAAMVNARIRKTV  target    L-KGTKVVVIACEYSSSSIKADDAIVVRPGTTPALALGLCNVIMREKIYDGDYVRRFSDLPLLVRADNLKLLRAEEVFGT 8e73.55   RSNQAKVGYIGPATDFN--YDHKHLGTDPQTLVEIAEG------------------------------------------  target    PQAALKNQTR 8e73.55   ---------- ``` | | | | | | | | | | | | | | | | | | | | | | | | | | | | | | | | | | | | | | | | | | | | | | | | | |
|  | 6zr2.1.G | NADH-ubiquinone oxidoreductase 75 kDa subunit, mitochondrial  *Cryo-EM structure of respiratory complex I in the active state from Mus musculus at 3.1 A* | 0.24 | 0.00 | 16.59 | 0.50 | 59-351 | EM | 3.10 | monomer | 6 x SF4, 4 x PC1, 2 x FES, 1 x FMN, 9 x 3PE, 7 x CDL, 1 x ATP, 1 x NDP, 1 x ZN, 2 x EHZ | HHblits | 0.26 |
| ``` target    NNVNRREFLQWIGAAGFSTFALSASNAWGLQAIENPLAAYPNREWEKTYRDLWKSDASFTFLCAPNDTHNCILNAHVRDG 6zr2.1    ----------------------------------------------------------TESID-VMDAVGSNIVVSTRTG  target    VITRIGPTMKYGEATDLYGSKVTHRWDPRVCQKGLALTRRFYGDRRVRYPMVRKGFKAWADKGFPREKDGRPPKDYFNRA 6zr2.1    EVMRILPRM------------HEDINEEWISDKTRFAYDGLK-RQRLTEPMVRNE-------------------------  target    RDEWLRLTHEEAADLVAAALINIATTYSGDNGQKLLLQQGY-EKEIVEATRGAGTQVLKFRGGMPLLGLTRIFGLYRMAN 6zr2.1    KGLLTYTSWEDALSRVAGMLQNF----EGNAVA--AIAGGLVDAEA----------L--V----ALKDLLNKVDSDNLCT  target    SMALLDHKIRGVKPEDALGARGWDNYSWHTDLPPGHPMVTGQ-QTVDFDLHAVEQARIVVVWGMNWVTTKMPDTHWLTEA 6zr2.1    EEI----------------------F-PTE----GAGTDLRSNYLLNTTIAGVEEADVVLLVGTNPRFEAPLFNARIRKS  target    R-LKGTKVVVIACEYSSSSIKADDAIVVRPGTTPALALGLCNVIMREKIYDGDYVRRFSDLPLLVRADNLKLLRAEEVFG 6zr2.1    WLHNDLKVALIGSPVDLTYRYDHLGDSPKILQDI----------------------------------------------  target    TPQAALKNQTR 6zr2.1    ----------- ``` | | | | | | | | | | | | | | | | | | | | | | | | | | | | | | | | | | | | | | | | | | | | | | | | | |
|  | 6g72.1.G | NADH-ubiquinone oxidoreductase 75 kDa subunit, mitochondrial  *Mouse mitochondrial complex I in the deactive state* | 0.24 | 0.00 | 16.59 | 0.50 | 59-351 | EM | 0.00 | monomer | 6 x SF4, 2 x FES, 1 x FMN, 1 x ADP, 1 x NDP, 1 x ZN, 2 x EHZ | HHblits | 0.26 |
| ``` target    NNVNRREFLQWIGAAGFSTFALSASNAWGLQAIENPLAAYPNREWEKTYRDLWKSDASFTFLCAPNDTHNCILNAHVRDG 6g72.1    ----------------------------------------------------------TESID-VMDAVGSNIVVSTRTG  target    VITRIGPTMKYGEATDLYGSKVTHRWDPRVCQKGLALTRRFYGDRRVRYPMVRKGFKAWADKGFPREKDGRPPKDYFNRA 6g72.1    EVMRILPRM------------HEDINEEWISDKTRFAYDGLK-RQRLTEPMVRNE-------------------------  target    RDEWLRLTHEEAADLVAAALINIATTYSGDNGQKLLLQQGY-EKEIVEATRGAGTQVLKFRGGMPLLGLTRIFGLYRMAN 6g72.1    KGLLTYTSWEDALSRVAGMLQNF----EGNAVA--AIAGGLVDAEA----------L--V----ALKDLLNKVDSDNLCT  target    SMALLDHKIRGVKPEDALGARGWDNYSWHTDLPPGHPMVTGQ-QTVDFDLHAVEQARIVVVWGMNWVTTKMPDTHWLTEA 6g72.1    EEI----------------------F-PTE----GAGTDLRSNYLLNTTIAGVEEADVVLLVGTNPRFEAPLFNARIRKS  target    R-LKGTKVVVIACEYSSSSIKADDAIVVRPGTTPALALGLCNVIMREKIYDGDYVRRFSDLPLLVRADNLKLLRAEEVFG 6g72.1    WLHNDLKVALIGSPVDLTYRYDHLGDSPKILQDI----------------------------------------------  target    TPQAALKNQTR 6g72.1    ----------- ``` | | | | | | | | | | | | | | | | | | | | | | | | | | | | | | | | | | | | | | | | | | | | | | | | | |
|  | 7ak6.1.G | NADH-ubiquinone oxidoreductase 75 kDa subunit, mitochondrial  *Cryo-EM structure of ND6-P25L mutant respiratory complex I from Mus musculus at 3.8 A* | 0.24 | 0.00 | 16.59 | 0.50 | 59-351 | EM | 0.00 | monomer | 6 x SF4, 1 x PC1, 2 x FES, 1 x FMN, 4 x 3PE, 2 x CDL, 1 x ATP, 1 x NDP, 1 x ZN, 2 x EHZ | HHblits | 0.26 |
| ``` target    NNVNRREFLQWIGAAGFSTFALSASNAWGLQAIENPLAAYPNREWEKTYRDLWKSDASFTFLCAPNDTHNCILNAHVRDG 7ak6.1    ----------------------------------------------------------TESID-VMDAVGSNIVVSTRTG  target    VITRIGPTMKYGEATDLYGSKVTHRWDPRVCQKGLALTRRFYGDRRVRYPMVRKGFKAWADKGFPREKDGRPPKDYFNRA 7ak6.1    EVMRILPRM------------HEDINEEWISDKTRFAYDGLK-RQRLTEPMVRNE-------------------------  target    RDEWLRLTHEEAADLVAAALINIATTYSGDNGQKLLLQQGY-EKEIVEATRGAGTQVLKFRGGMPLLGLTRIFGLYRMAN 7ak6.1    KGLLTYTSWEDALSRVAGMLQNF----EGNAVA--AIAGGLVDAEA----------L--V----ALKDLLNKVDSDNLCT  target    SMALLDHKIRGVKPEDALGARGWDNYSWHTDLPPGHPMVTGQ-QTVDFDLHAVEQARIVVVWGMNWVTTKMPDTHWLTEA 7ak6.1    EEI----------------------F-PTE----GAGTDLRSNYLLNTTIAGVEEADVVLLVGTNPRFEAPLFNARIRKS  target    R-LKGTKVVVIACEYSSSSIKADDAIVVRPGTTPALALGLCNVIMREKIYDGDYVRRFSDLPLLVRADNLKLLRAEEVFG 7ak6.1    WLHNDLKVALIGSPVDLTYRYDHLGDSPKILQDI----------------------------------------------  target    TPQAALKNQTR 7ak6.1    ----------- ``` | | | | | | | | | | | | | | | | | | | | | | | | | | | | | | | | | | | | | | | | | | | | | | | | | |
|  | 5gpn.24.A | NADH-ubiquinone oxidoreductase 75 kDa subunit  *Architecture of mammalian respirasome* | 0.23 |  | 15.12 | 0.50 | 59-350 | EM | 0.00 | monomer |  | HHblits | 0.26 |
| ``` target    NNVNRREFLQWIGAAGFSTFALSASNAWGLQAIENPLAAYPNREWEKTYRDLWKSDASFTFLCAPNDTHNCILNAHVRDG 5gpn.24   ----------------------------------------------------------TESID-VMDAVGSNIVVSTRTG  target    VITRIGPTMKYGEATDLYGSKVTHRWDPRVCQKGLALTRRFYGDRRVRYPMVRKGFKAWADKGFPREKDGRPPKDYFNRA 5gpn.24   EVMRILPRMH------------EDINEEWISDKTRFAYDGLK-RQRLTQPMIRNE-------------------------  target    RDEWLRLTHEEAADLVAAALINIATTYSGDNGQKLLLQQGY-EKEIVEATRGAGTQVLKFRGGMPLLGLTRIFGLYRMAN 5gpn.24   KGLLTYTTWEDALSRVAGMLQSF----QGNDV--AAIAGGLVDAEA-------LVALKD---------LLNRVDSDSLCT  target    SMALLDHKIRGVKPEDALGARGWDNYSWHTDLPPGHPMVTGQQTVDFDLHAVEQARIVVVWGMNWVTTKMPDTHWLTEAR 5gpn.24   EE----------------------VFPT-AGAGTDLRSNY---LLNTTIAGVEEADVILLVGTNPRFEAPLFNARIRKSW  target    L-KGTKVVVIACEYSSSSIKADDAIVVRPGTTPALALGLCNVIMREKIYDGDYVRRFSDLPLLVRADNLKLLRAEEVFGT 5gpn.24   LHNDLKVALIGSPVDLTYRYDHLGDSPKILQD------------------------------------------------  target    PQAALKNQTR 5gpn.24   ---------- ``` | | | | | | | | | | | | | | | | | | | | | | | | | | | | | | | | | | | | | | | | | | | | | | | | | |
|  | 7v2c.1.L | NADH-ubiquinone oxidoreductase 75 kDa subunit, mitochondrial  *Active state complex I from Q10 dataset* | 0.24 |  | 15.69 | 0.50 | 59-349 | EM | 0.00 | hetero-1-1-1-1-1-2-… | 6 x SF4, 1 x FMN, 10 x PEE, 8 x PLX, 2 x 8Q1, 1 x NDP, 2 x UQ, 11 x CDL, 2 x FES, 1 x MG, 1 x ZN, 1 x ADP | HHblits | 0.27 |
| ``` target    NNVNRREFLQWIGAAGFSTFALSASNAWGLQAIENPLAAYPNREWEKTYRDLWKSDASFTFLCAPNDTHNCILNAHVRDG 7v2c.1    ----------------------------------------------------------TESID-VMDAVGSNIVVSTRTG  target    VITRIGPTMKYGEATDLYGSKVTHRWDPRVCQKGLALTRRFYGDRRVRYPMVRKGFKAWADKGFPREKDGRPPKDYFNRA 7v2c.1    EVMRILPRMH------------EDINEEWISDKTRFAYDGLK-RQRLTQPMIRNE-------------------------  target    RDEWLRLTHEEAADLVAAALINIATTYSGDNGQKLLLQQGY-EKEIVEATRGAGTQVLKFRGGMPLLGLTRIFGLYRMAN 7v2c.1    KGLLTYTTWEDALSRVAGMLQSF----QGNDV--AAIAGGLVDAEA----------LV------ALKDLLNRVDSDSLCT  target    SMALLDHKIRGVKPEDALGARGWDNYSWHTDLPPGHPMVTGQQTVDFDLHAVEQARIVVVWGMNWVTTKMPDTHWLTEAR 7v2c.1    EE-------------------V---FP-TAGAGTDLRSNY---LLNTTIAGVEEADVILLVGTNPRFEAPLFNARIRKSW  target    L-KGTKVVVIACEYSSSSIKADDAIVVRPGTTPALALGLCNVIMREKIYDGDYVRRFSDLPLLVRADNLKLLRAEEVFGT 7v2c.1    LHNDLKVALIGSPVDLTYRYDHLGDSPKILQ-------------------------------------------------  target    PQAALKNQTR 7v2c.1    ---------- ``` | | | | | | | | | | | | | | | | | | | | | | | | | | | | | | | | | | | | | | | | | | | | | | | | | |
|  | 7ak5.1.G | NADH-ubiquinone oxidoreductase 75 kDa subunit, mitochondrial  *Cryo-EM structure of respiratory complex I in the deactive state from Mus musculus at 3.2 A* | 0.24 |  | 16.75 | 0.50 | 59-349 | EM | 0.00 | hetero-1-1-1-1-1-1-… | 6 x SF4, 2 x PC1, 2 x FES, 1 x FMN, 8 x 3PE, 4 x CDL, 1 x ATP, 1 x NDP, 1 x ZN, 2 x EHZ | HHblits | 0.27 |
| ``` target    NNVNRREFLQWIGAAGFSTFALSASNAWGLQAIENPLAAYPNREWEKTYRDLWKSDASFTFLCAPNDTHNCILNAHVRDG 7ak5.1    ----------------------------------------------------------TESID-VMDAVGSNIVVSTRTG  target    VITRIGPTMKYGEATDLYGSKVTHRWDPRVCQKGLALTRRFYGDRRVRYPMVRKGFKAWADKGFPREKDGRPPKDYFNRA 7ak5.1    EVMRILPRM------------HEDINEEWISDKTRFAYDGLK-RQRLTEPMVRNE-------------------------  target    RDEWLRLTHEEAADLVAAALINIATTYSGDNGQKLLLQQGY-EKEIVEATRGAGTQVLKFRGGMPLLGLTRIFGLYRMAN 7ak5.1    KGLLTYTSWEDALSRVAGMLQNF----EGNAVA--AIAGGLVDAEA----------LV------ALKDLLNKVDSDNLCT  target    SMALLDHKIRGVKPEDALGARGWDNYSWHTDLPPGHPMVTGQ-QTVDFDLHAVEQARIVVVWGMNWVTTKMPDTHWLTEA 7ak5.1    EEI----------------------F-PTE----GAGTDLRSNYLLNTTIAGVEEADVVLLVGTNPRFEAPLFNARIRKS  target    R-LKGTKVVVIACEYSSSSIKADDAIVVRPGTTPALALGLCNVIMREKIYDGDYVRRFSDLPLLVRADNLKLLRAEEVFG 7ak5.1    WLHNDLKVALIGSPVDLTYRYDHLGDSPKILQ------------------------------------------------  target    TPQAALKNQTR 7ak5.1    ----------- ``` | | | | | | | | | | | | | | | | | | | | | | | | | | | | | | | | | | | | | | | | | | | | | | | | | |
|  | 6zk9.1.C | NADH:ubiquinone oxidoreductase core subunit S1  *Peripheral domain of open complex I during turnover* | 0.24 |  | 17.33 | 0.50 | 59-347 | EM | 0.00 | hetero-1-1-1-1-1-1-… | 6 x SF4, 1 x FMN, 1 x NAI, 2 x FES, 1 x K, 2 x PC1, 2 x 3PE, 1 x ZN, 1 x NDP, 1 x ZMP, 1 x CDL | HHblits | 0.27 |
| ``` target    NNVNRREFLQWIGAAGFSTFALSASNAWGLQAIENPLAAYPNREWEKTYRDLWKSDASFTFLCAPNDTHNCILNAHVRDG 6zk9.1    ----------------------------------------------------------TESID-VMDAVGSNIVVSTRTG  target    VITRIGPTMKYGEATDLYGSKVTHRWDPRVCQKGLALTRRFYGDRRVRYPMVRKGFKAWADKGFPREKDGRPPKDYFNRA 6zk9.1    EVMRILPRM------------HEDINEEWISDKTRFAYDGLK-RQRLTEPMVRNE-------------------------  target    RDEWLRLTHEEAADLVAAALINIATTYSGDNGQKLLLQQGY-EKEIVEATRGAGTQVLKFRGGMPLLGLTRIFGLYRMAN 6zk9.1    KGLLTHTTWEDALSRVAGMLQSC----QGNDV--AAIAGGLVDAEA-------LIA---------LKDLLNRVDSDTLCT  target    SMALLDHKIRGVKPEDALGARGWDNYSWHTDLPPGHPMVTGQQTVDFDLHAVEQARIVVVWGMNWVTTKMPDTHWLTEAR 6zk9.1    EEV-----------------------FPTAGA--GT-DLRSNYLLNTTIAGVEEADVVLLVGTNPRFEAPLFNARIRKSW  target    L-KGTKVVVIACEYSSSSIKADDAIVVRPGTTPALALGLCNVIMREKIYDGDYVRRFSDLPLLVRADNLKLLRAEEVFGT 6zk9.1    LHNDLKVALIGSPVDLTYRYDHLGDSPKI---------------------------------------------------  target    PQAALKNQTR 6zk9.1    ---------- ``` | | | | | | | | | | | | | | | | | | | | | | | | | | | | | | | | | | | | | | | | | | | | | | | | | |
|  | 7zd6.1.4 | NADH-ubiquinone oxidoreductase 75 kDa subunit, mitochondrial  *Complex I from Ovis aries, at pH7.4, Open state* | 0.24 |  | 16.26 | 0.50 | 59-348 | EM | 0.00 | hetero-1-1-1-1-1-1-… | 6 x PC1, 14 x 3PE, 1 x DCQ, 2 x ZMP, 1 x AMP, 1 x MYR, 6 x SF4, 1 x FMN, 1 x NAI, 2 x FES, 1 x K, 1 x ZN, 1 x NDP | HHblits | 0.26 |
| ``` target    NNVNRREFLQWIGAAGFSTFALSASNAWGLQAIENPLAAYPNREWEKTYRDLWKSDASFTFLCAPNDTHNCILNAHVRDG 7zd6.1    ----------------------------------------------------------TESID-VMDAVGSNIVVSTRTG  target    VITRIGPTMKYGEATDLYGSKVTHRWDPRVCQKGLALTRRFYGDRRVRYPMVRKGFKAWADKGFPREKDGRPPKDYFNRA 7zd6.1    EVMRILPRMH------------EDINEEWISDKTRFAYDGLK-RQRLTEPMVRNE-------------------------  target    RDEWLRLTHEEAADLVAAALINIATTYSGDNGQKLLLQQGYEKEIVEATRGAGTQVLKFRGGMPLLGLTRIFGLYRMANS 7zd6.1    KGLLTHTTWEDALSRVAGMLQSC----QGNDVA-AIAGGLVDAEA-------LIALKD---------LLNRVDSDTLCTE  target    MALLDHKIRGVKPEDALGARGWDNYSWHTDLPPGHPMVTG-QQTVDFDLHAVEQARIVVVWGMNWVTTKMPDTHWLTEAR 7zd6.1    E-------------------------VFPTA--GAGTDLRSNYLLNTTIAGVEEADVVLLVGTNPRFEAPLFNARIRKSW  target    L-KGTKVVVIACEYSSSSIKADDAIVVRPGTTPALALGLCNVIMREKIYDGDYVRRFSDLPLLVRADNLKLLRAEEVFGT 7zd6.1    LHNDLKVALIGSPVDLTYRYDHLGDSPKIL--------------------------------------------------  target    PQAALKNQTR 7zd6.1    ---------- ``` | | | | | | | | | | | | | | | | | | | | | | | | | | | | | | | | | | | | | | | | | | | | | | | | | |
|  | 6yj4.1.G | Subunit NUAM of NADH:Ubiquinone Oxidoreductase (Complex I)  *Structure of Yarrowia lipolytica complex I at 2.7 A* | 0.25 |  | 14.65 | 0.49 | 59-341 | EM | 0.00 | hetero-1-1-1-1-1-1-… | 18 x 3PE, 6 x SF4, 5 x LMT, 8 x PLC, 2 x FES, 1 x FMN, 6 x CDL, 1 x NDP, 1 x ZN, 2 x EHZ | HHblits | 0.28 |
| ``` target    NNVNRREFLQWIGAAGFSTFALSASNAWGLQAIENPLAAYPNREWEKTYRDLWKSDASFTFLCAPNDTHNCILNAHVRDG 6yj4.1    ----------------------------------------------------------TESID-VMDAVGSNIRIDSKGV  target    VITRIGPTMKYGEATDLYGSKVTHRWDPRVCQKGLALTRRFYGDRRVRYPMVRKGFKAWADKGFPREKDGRPPKDYFNRA 6yj4.1    EVMRVIPRVH------------EDVNEEWINDKSRFACDGLK-TQRLTTPLIRVG-------------------------  target    RDEWLRLTHEEAADLVAAALINIATTYSGDNGQKLLLQQGY-EKEIVEATRGAGTQVLKFRGGMPLLGLTRIFGLYRMAN 6yj4.1    -DKFVNATWDDALSTIAKAYQQKAP--KGDE--FKAVAGALVEVES----------MV------ALKDMTNALGSENT--  target    SMALLDHKIRGVKPEDALGARGWDNYSWHTDLPPGHPMVTGQQ-TVDFDLHAVEQARIVVVWGMNWVTTKMPDTHWLTEA 6yj4.1    -----------------------TTDTPNGNSAPAHGITFRSNYLFNSSIAGIEDADAILLVGTNPRREAAVMNARIRKA  target    R-LKGTKVVVIACEYSSSSIKADDAIVVRPGTTPALALGLCNVIMREKIYDGDYVRRFSDLPLLVRADNLKLLRAEEVFG 6yj4.1    WLRQELEIASVGPTLDATFDVAEL--------------------------------------------------------  target    TPQAALKNQTR 6yj4.1    ----------- ``` | | | | | | | | | | | | | | | | | | | | | | | | | | | | | | | | | | | | | | | | | | | | | | | | | |
|  | 6rfs.1.A | Subunit NUAM of NADH:Ubiquinone Oxidoreductase (Complex I)  *Cryo-EM structure of a respiratory complex I mutant lacking NDUFS4* | 0.24 |  | 14.65 | 0.49 | 59-341 | EM | 4.04 | hetero-1-1-1-1-1-1-… | 6 x SF4, 2 x FES, 1 x FMN, 1 x NDP, 1 x ZN, 1 x ZMP | HHblits | 0.28 |
| ``` target    NNVNRREFLQWIGAAGFSTFALSASNAWGLQAIENPLAAYPNREWEKTYRDLWKSDASFTFLCAPNDTHNCILNAHVRDG 6rfs.1    ----------------------------------------------------------TESID-VMDAVGSNIRIDSKGV  target    VITRIGPTMKYGEATDLYGSKVTHRWDPRVCQKGLALTRRFYGDRRVRYPMVRKGFKAWADKGFPREKDGRPPKDYFNRA 6rfs.1    EVMRVIPRVH------------EDVNEEWINDKSRFACDGLK-TQRLTTPLIRVG-------------------------  target    RDEWLRLTHEEAADLVAAALINIATTYSGDNGQKLLLQQGY-EKEIVEATRGAGTQVLKFRGGMPLLGLTRIFGLYRMAN 6rfs.1    -DKFVNATWDDALSTIAKAYQQKAP--KGDE--FKAVAGALVEVES----------MV------ALKDMTNALGSENT--  target    SMALLDHKIRGVKPEDALGARGWDNYSWHTDLPPGHPMVTGQQ-TVDFDLHAVEQARIVVVWGMNWVTTKMPDTHWLTEA 6rfs.1    -----------------------TTDTPNGNSAPAHGITFRSNYLFNSSIAGIEDADAILLVGTNPRREAAVMNARIRKA  target    R-LKGTKVVVIACEYSSSSIKADDAIVVRPGTTPALALGLCNVIMREKIYDGDYVRRFSDLPLLVRADNLKLLRAEEVFG 6rfs.1    WLRQELEIASVGPTLDATFDVAEL--------------------------------------------------------  target    TPQAALKNQTR 6rfs.1    ----------- ``` | | | | | | | | | | | | | | | | | | | | | | | | | | | | | | | | | | | | | | | | | | | | | | | | | |
|  | 6rfq.1.A | Subunit NUAM of NADH:Ubiquinone Oxidoreductase (Complex I)  *Cryo-EM structure of a respiratory complex I assembly intermediate with NDUFAF2* | 0.24 |  | 14.65 | 0.49 | 59-341 | EM | 3.30 | hetero-1-1-1-1-1-1-… | 6 x SF4, 2 x FES, 1 x FMN, 1 x NDP, 10 x 3PE, 2 x LMN, 4 x CDL, 2 x ZMP, 4 x PLC, 3 x T7X, 1 x CPL | HHblits | 0.28 |
| ``` target    NNVNRREFLQWIGAAGFSTFALSASNAWGLQAIENPLAAYPNREWEKTYRDLWKSDASFTFLCAPNDTHNCILNAHVRDG 6rfq.1    ----------------------------------------------------------TESID-VMDAVGSNIRIDSKGV  target    VITRIGPTMKYGEATDLYGSKVTHRWDPRVCQKGLALTRRFYGDRRVRYPMVRKGFKAWADKGFPREKDGRPPKDYFNRA 6rfq.1    EVMRVIPRVH------------EDVNEEWINDKSRFACDGLK-TQRLTTPLIRVG-------------------------  target    RDEWLRLTHEEAADLVAAALINIATTYSGDNGQKLLLQQGY-EKEIVEATRGAGTQVLKFRGGMPLLGLTRIFGLYRMAN 6rfq.1    -DKFVNATWDDALSTIAKAYQQKAP--KGDE--FKAVAGALVEVES----------MV------ALKDMTNALGSENT--  target    SMALLDHKIRGVKPEDALGARGWDNYSWHTDLPPGHPMVTGQQ-TVDFDLHAVEQARIVVVWGMNWVTTKMPDTHWLTEA 6rfq.1    -----------------------TTDTPNGNSAPAHGITFRSNYLFNSSIAGIEDADAILLVGTNPRREAAVMNARIRKA  target    R-LKGTKVVVIACEYSSSSIKADDAIVVRPGTTPALALGLCNVIMREKIYDGDYVRRFSDLPLLVRADNLKLLRAEEVFG 6rfq.1    WLRQELEIASVGPTLDATFDVAEL--------------------------------------------------------  target    TPQAALKNQTR 6rfq.1    ----------- ``` | | | | | | | | | | | | | | | | | | | | | | | | | | | | | | | | | | | | | | | | | | | | | | | | | |
|  | 6gcs.1.A | 75-KDA PROTEIN (NUAM)  *Cryo-EM structure of respiratory complex I from Yarrowia lipolytica* | 0.24 |  | 14.65 | 0.49 | 59-341 | EM | 4.32 | hetero-1-1-1-1-1-1-… | 6 x SF4, 2 x FES, 1 x FMN, 1 x NDP, 1 x ZN, 1 x ZMP, 1 x CDL, 3 x 3PE | HHblits | 0.28 |
| ``` target    NNVNRREFLQWIGAAGFSTFALSASNAWGLQAIENPLAAYPNREWEKTYRDLWKSDASFTFLCAPNDTHNCILNAHVRDG 6gcs.1    ----------------------------------------------------------TESID-VMDAVGSNIRIDSKGV  target    VITRIGPTMKYGEATDLYGSKVTHRWDPRVCQKGLALTRRFYGDRRVRYPMVRKGFKAWADKGFPREKDGRPPKDYFNRA 6gcs.1    EVMRVIPRVH------------EDVNEEWINDKSRFACDGLK-TQRLTTPLIRVG-------------------------  target    RDEWLRLTHEEAADLVAAALINIATTYSGDNGQKLLLQQGY-EKEIVEATRGAGTQVLKFRGGMPLLGLTRIFGLYRMAN 6gcs.1    -DKFVNATWDDALSTIAKAYQQKAP--KGDE--FKAVAGALVEVES----------MV------ALKDMTNALGSENT--  target    SMALLDHKIRGVKPEDALGARGWDNYSWHTDLPPGHPMVTGQQ-TVDFDLHAVEQARIVVVWGMNWVTTKMPDTHWLTEA 6gcs.1    -----------------------TTDTPNGNSAPAHGITFRSNYLFNSSIAGIEDADAILLVGTNPRREAAVMNARIRKA  target    R-LKGTKVVVIACEYSSSSIKADDAIVVRPGTTPALALGLCNVIMREKIYDGDYVRRFSDLPLLVRADNLKLLRAEEVFG 6gcs.1    WLRQELEIASVGPTLDATFDVAEL--------------------------------------------------------  target    TPQAALKNQTR 6gcs.1    ----------- ``` | | | | | | | | | | | | | | | | | | | | | | | | | | | | | | | | | | | | | | | | | | | | | | | | | |
|  | 7q5y.1.A | NADH dehydrogenase I chain G  *Structure of NADH:ubichinon oxidoreductase (complex I) of the hyperthermophilic eubacterium Aquifex aeolicus* | 0.07 |  | 16.00 | 0.18 | 59-182 | X-ray | 2.70 | hetero-1-1-1-1-1-1-… | 8 x SF4, 2 x FES, 1 x FMN | HHblits | 0.28 |
| ``` target    NNVNRREFLQWIGAAGFSTFALSASNAWGLQAIENPLAAYPNREWEKTYRDLWKSDASFTFLCAPNDTHNCILNAHVRDG 7q5y.1    ----------------------------------------------------------GRTVC-NLCPVGCEIQIEYGVG  target    ------VITRIGPTMKYGEATDLYGSKVTHRWDPRVCQKGLALTRRFYGDRRVRYPMVRKGFKAWADKGFPREKDGRPPK 7q5y.1    DWRSKRKVYRTKP-----------------TDELNICAKGFFGYDSINHKRLLKTKVGKR--------------------  target    DYFNRARDEWLRLTHEEAADLVAAALINIATTYSGDNGQKLLLQQGYEKEIVEATRGAGTQVLKFRGGMPLLGLTRIFGL 7q5y.1    -----------EETPGNVVNLLTTILTE----------------------------------------------------  target    YRMANSMALLDHKIRGVKPEDALGARGWDNYSWHTDLPPGHPMVTGQQTVDFDLHAVEQARIVVVWGMNWVTTKMPDTHW 7q5y.1    --------------------------------------------------------------------------------  target    LTEARLKGTKVVVIACEYSSSSIKADDAIVVRPGTTPALALGLCNVIMREKIYDGDYVRRFSDLPLLVRADNLKLLRAEE 7q5y.1    --------------------------------------------------------------------------------  target    VFGTPQAALKNQTR 7q5y.1    -------------- ``` | | | | | | | | | | | | | | | | | | | | | | | | | | | | | | | | | | | | | | | | | | | | | | | | | |
|  | 6s6y.1.B | Tungsten-containing formylmethanofuran dehydrogenase, subunit B  *X-ray crystal structure of the formyltransferase/hydrolase complex (FhcABCD) from Methylorubrum extorquens in complex with methylofuran* | 0.05 |  | 6.67 | 0.18 | 288-364 | X-ray | 3.10 | hetero-2-2-2-2-mer | 1 x MFN, 4 x ZN, 4 x CA, 4 x K, 3 x DGL, 2 x GLU, 1 x IAS | HHblits | 0.22 |
| ``` target    NNVNRREFLQWIGAAGFSTFALSASNAWGLQAIENPLAAYPNREWEKTYRDLWKSDASFTFLCAPNDTHNCILNAHVRDG 6s6y.1    --------------------------------------------------------------------------------  target    VITRIGPTMKYGEATDLYGSKVTHRWDPRVCQKGLALTRRFYGDRRVRYPMVRKGFKAWADKGFPREKDGRPPKDYFNRA 6s6y.1    --------------------------------------------------------------------------------  target    RDEWLRLTHEEAADLVAAALINIATTYSGDNGQKLLLQQGYEKEIVEATRGAGTQVLKFRGGMPLLGLTRIFGLYRMANS 6s6y.1    --------------------------------------------------------------------------------  target    MALLDHKIRGVKPEDALGARGWDNYSWHTDLPPGHPMVTGQQTVDFDLHAV-EQARIVVVWGMNWVTTKMPDTHWLTEA- 6s6y.1    -----------------------------------------------RAETIGRADVILIVGNRPWDGELIAEIAAAAPS  target    ----RLKGTKVVVIACEYSSSSIKADDAIVVRPGTTPALALGLCNVIMREKIYDGDYVRRFSDLPLLVRADNLKLLRAEE 6s6y.1    RGRAAGAERALLSLGGPQNGAIR--HVAYAADAGGLTISLGHLRAFAKGH------------------------------  target    VFGTPQAALKNQTR 6s6y.1    -------------- ``` | | | | | | | | | | | | | | | | | | | | | | | | | | | | | | | | | | | | | | | | | | | | | | | | | |
|  | 1jeo.1.A | HYPOTHETICAL PROTEIN MJ1247  *Crystal Structure of the Hypothetical Protein MJ1247 from Methanococcus jannaschii at 2.0 A Resolution Infers a Molecular Function of 3-Hexulose-6-Phosphate isomerase.* | 0.04 |  | 12.86 | 0.17 | 290-362 | X-ray | 2.00 | monomer |  | HHblits | 0.26 |
| ``` target    NNVNRREFLQWIGAAGFSTFALSASNAWGLQAIENPLAAYPNREWEKTYRDLWKSDASFTFLCAPNDTHNCILNAHVRDG 1jeo.1    --------------------------------------------------------------------------------  target    VITRIGPTMKYGEATDLYGSKVTHRWDPRVCQKGLALTRRFYGDRRVRYPMVRKGFKAWADKGFPREKDGRPPKDYFNRA 1jeo.1    --------------------------------------------------------------------------------  target    RDEWLRLTHEEAADLVAAALINIATTYSGDNGQKLLLQQGYEKEIVEATRGAGTQVLKFRGGMPLLGLTRIFGLYRMANS 1jeo.1    --------------------------------------------------------------------------------  target    MALLDHKIRGVKPEDALGARGWDNYSWHTDLPPGHPMVTGQQTVDFDLHAVEQARIVVVWGMNWVTTKMPDTHWLTE-AR 1jeo.1    -------------------------------------------------RIIKAKKIFIFGVGRSGYI---GRCFAMRLM  target    LKGTKVVVIACEYSSSSIKADDAIVVRPGTTPALALGLCNVIMREKIYDGDYVRRFSDLPLLVRADNLKLLRAEEVFGTP 1jeo.1    HLGFKSYFVGETTTPSYEKDDLLILISGSGRTESVLTVAKKAK-------------------------------------  target    QAALKNQTR 1jeo.1    --------- ``` | | | | | | | | | | | | | | | | | | | | | | | | | | | | | | | | | | | | | | | | | | | | | | | | | |
|  | 7cl0.1.A | NAD-dependent protein deacetylase sirtuin-6  *Crystal structure of human SIRT6* | 0.05 |  | 13.64 | 0.16 | 290-359 | X-ray | 2.53 | monomer | 1 x AR6, 1 x ZN, 1 x G4U, 1 x THR-ALA-ARG-LYS-SER-THR-GLY | HHblits | 0.28 |
| ``` target    NNVNRREFLQWIGAAGFSTFALSASNAWGLQAIENPLAAYPNREWEKTYRDLWKSDASFTFLCAPNDTHNCILNAHVRDG 7cl0.1    --------------------------------------------------------------------------------  target    VITRIGPTMKYGEATDLYGSKVTHRWDPRVCQKGLALTRRFYGDRRVRYPMVRKGFKAWADKGFPREKDGRPPKDYFNRA 7cl0.1    --------------------------------------------------------------------------------  target    RDEWLRLTHEEAADLVAAALINIATTYSGDNGQKLLLQQGYEKEIVEATRGAGTQVLKFRGGMPLLGLTRIFGLYRMANS 7cl0.1    --------------------------------------------------------------------------------  target    MALLDHKIRGVKPEDALGARGWDNYSWHTDLPPGHPMVTGQQTVDFDLHAVEQARIVVVWGMNWVTTKMPDTHWLTEARL 7cl0.1    -------------------------------------------------ASRNADLSITLGTSLQIR--PSGNLPLATKR  target    KGTKVVVIACEYSSSSIKADDAIVVRPGTTPALALGLCNVIMREKIYDGDYVRRFSDLPLLVRADNLKLLRAEEVFGTPQ 7cl0.1    RGGRLVIVNLQPTKHDRHADLRIHG--YVDEVMTRLMKH-----------------------------------------  target    AALKNQTR 7cl0.1    -------- ``` | | | | | | | | | | | | | | | | | | | | | | | | | | | | | | | | | | | | | | | | | | | | | | | | | |
|  | 3pki.1.A | NAD-dependent deacetylase sirtuin-6  *Human SIRT6 crystal structure in complex with ADP ribose* | 0.05 |  | 13.64 | 0.16 | 290-359 | X-ray | 2.04 | monomer | 1 x ZN, 1 x AR6 | HHblits | 0.27 |
| ``` target    NNVNRREFLQWIGAAGFSTFALSASNAWGLQAIENPLAAYPNREWEKTYRDLWKSDASFTFLCAPNDTHNCILNAHVRDG 3pki.1    --------------------------------------------------------------------------------  target    VITRIGPTMKYGEATDLYGSKVTHRWDPRVCQKGLALTRRFYGDRRVRYPMVRKGFKAWADKGFPREKDGRPPKDYFNRA 3pki.1    --------------------------------------------------------------------------------  target    RDEWLRLTHEEAADLVAAALINIATTYSGDNGQKLLLQQGYEKEIVEATRGAGTQVLKFRGGMPLLGLTRIFGLYRMANS 3pki.1    --------------------------------------------------------------------------------  target    MALLDHKIRGVKPEDALGARGWDNYSWHTDLPPGHPMVTGQQTVDFDLHAVEQARIVVVWGMNWVTTKMPDTHWLTEARL 3pki.1    -------------------------------------------------ASRNADLSITLGTSLQIR--PSGNLPLATKR  target    KGTKVVVIACEYSSSSIKADDAIVVRPGTTPALALGLCNVIMREKIYDGDYVRRFSDLPLLVRADNLKLLRAEEVFGTPQ 3pki.1    RGGRLVIVNLQPTKHDRHADLRIHG--YVDEVMTRLMEH-----------------------------------------  target    AALKNQTR 3pki.1    -------- ``` | | | | | | | | | | | | | | | | | | | | | | | | | | | | | | | | | | | | | | | | | | | | | | | | | |
|  | 1jeo.1.A | HYPOTHETICAL PROTEIN MJ1247  *Crystal Structure of the Hypothetical Protein MJ1247 from Methanococcus jannaschii at 2.0 A Resolution Infers a Molecular Function of 3-Hexulose-6-Phosphate isomerase.* | 0.03 |  | 15.25 | 0.14 | 291-351 | X-ray | 2.00 | monomer |  | HHblits | 0.28 |
| ``` target    NNVNRREFLQWIGAAGFSTFALSASNAWGLQAIENPLAAYPNREWEKTYRDLWKSDASFTFLCAPNDTHNCILNAHVRDG 1jeo.1    --------------------------------------------------------------------------------  target    VITRIGPTMKYGEATDLYGSKVTHRWDPRVCQKGLALTRRFYGDRRVRYPMVRKGFKAWADKGFPREKDGRPPKDYFNRA 1jeo.1    --------------------------------------------------------------------------------  target    RDEWLRLTHEEAADLVAAALINIATTYSGDNGQKLLLQQGYEKEIVEATRGAGTQVLKFRGGMPLLGLTRIFGLYRMANS 1jeo.1    --------------------------------------------------------------------------------  target    MALLDHKIRGVKPEDALGARGWDNYSWHTDLPPGHPMVTGQQTVDFDLHAVEQARIVVVWGMNWVTTKMPDTHWLTEARL 1jeo.1    --------------------------------------------------YEKDDLLILISGSGRTE--SVLTVAKKAKN  target    KGTKVVVIACEYSSSSIKADDAIVVRPGTTPALALGLCNVIMREKIYDGDYVRRFSDLPLLVRADNLKLLRAEEVFGTPQ 1jeo.1    INNNIIAIVCECGNVVEFADLTIPLEVKKSK-------------------------------------------------  target    AALKNQTR 1jeo.1    -------- ``` | | | | | | | | | | | | | | | | | | | | | | | | | | | | | | | | | | | | | | | | | | | | | | | | | |
|  | 2a3n.1.A | putative glucosamine-fructose-6-phosphate aminotransferase  *Crystal structure of a putative glucosamine-fructose-6-phosphate aminotransferase (stm4540.s) from salmonella typhimurium lt2 at 1.35 A resolution* | 0.04 |  | 18.97 | 0.14 | 293-352 | X-ray | 1.23 | homo-dimer |  | HHblits | 0.28 |
| ``` target    NNVNRREFLQWIGAAGFSTFALSASNAWGLQAIENPLAAYPNREWEKTYRDLWKSDASFTFLCAPNDTHNCILNAHVRDG 2a3n.1    --------------------------------------------------------------------------------  target    VITRIGPTMKYGEATDLYGSKVTHRWDPRVCQKGLALTRRFYGDRRVRYPMVRKGFKAWADKGFPREKDGRPPKDYFNRA 2a3n.1    --------------------------------------------------------------------------------  target    RDEWLRLTHEEAADLVAAALINIATTYSGDNGQKLLLQQGYEKEIVEATRGAGTQVLKFRGGMPLLGLTRIFGLYRMANS 2a3n.1    --------------------------------------------------------------------------------  target    MALLDHKIRGVKPEDALGARGWDNYSWHTDLPPGHPMVTGQQTVDFDLHAVEQARIVVVWGMNWVTTKMPDTHWLTEARL 2a3n.1    ----------------------------------------------------KDSVVITLSKSGDT--KESVAIAEWCKA  target    KGTKVVVIACE-YSSSSIKADDAIVVRP--GTTPALALGLCNVIMREKIYDGDYVRRFSDLPLLVRADNLKLLRAEEVFG 2a3n.1    QGIRVVAITKNADSPLAQAATWHIPMRHKNGVEYE---------------------------------------------  target    TPQAALKNQTR 2a3n.1    ----------- ``` | | | | | | | | | | | | | | | | | | | | | | | | | | | | | | | | | | | | | | | | | | | | | | | | | |
|  | 7t2r.1.A | NiFe hydrogenase subunit A  *Structure of electron bifurcating Ni-Fe hydrogenase complex HydABCSL in FMN-free apo state* | 0.03 |  | 16.95 | 0.14 | 292-354 | EM | 0.00 | hetero-2-2-2-2-2-mer | 6 x FES, 12 x SF4, 2 x 3NI, 2 x FCO | HHblits | 0.26 |
| ``` target    NNVNRREFLQWIGAAGFSTFALSASNAWGLQAIENPLAAYPNREWEKTYRDLWKSDASFTFLCAPNDTHNCILNAHVRDG 7t2r.1    --------------------------------------------------------------------------------  target    VITRIGPTMKYGEATDLYGSKVTHRWDPRVCQKGLALTRRFYGDRRVRYPMVRKGFKAWADKGFPREKDGRPPKDYFNRA 7t2r.1    --------------------------------------------------------------------------------  target    RDEWLRLTHEEAADLVAAALINIATTYSGDNGQKLLLQQGYEKEIVEATRGAGTQVLKFRGGMPLLGLTRIFGLYRMANS 7t2r.1    --------------------------------------------------------------------------------  target    MALLDHKIRGVKPEDALGARGWDNYSWHTDLPPGHPMVTGQQTVDFDLHAVEQARIVVVWGMNWVTTKMPDTHWLTEARL 7t2r.1    ---------------------------------------------------ERRDFLYVFSTAMV----PEEEEILAAIS  target    KGTKVVVIACEYS-SSSIKADDAIVVRPG--TTPALALGLCNVIMREKIYDGDYVRRFSDLPLLVRADNLKLLRAEEVFG 7t2r.1    ATRFVVVQTPFKVRPLVNLADILLPAPAWYERSGHFC-------------------------------------------  target    TPQAALKNQTR 7t2r.1    ----------- ``` | | | | | | | | | | | | | | | | | | | | | | | | | | | | | | | | | | | | | | | | | | | | | | | | | |
|  | 7t30.1.A | NiFe hydrogenase subunit A  *Structure of electron bifurcating Ni-Fe hydrogenase complex HydABCSL in FMN/NAD(H) bound state* | 0.03 |  | 16.95 | 0.14 | 292-354 | EM | 0.00 | hetero-2-2-2-2-2-mer | 4 x FES, 12 x SF4, 2 x NAD, 2 x FMN, 2 x 3NI, 2 x FCO | HHblits | 0.26 |
| ``` target    NNVNRREFLQWIGAAGFSTFALSASNAWGLQAIENPLAAYPNREWEKTYRDLWKSDASFTFLCAPNDTHNCILNAHVRDG 7t30.1    --------------------------------------------------------------------------------  target    VITRIGPTMKYGEATDLYGSKVTHRWDPRVCQKGLALTRRFYGDRRVRYPMVRKGFKAWADKGFPREKDGRPPKDYFNRA 7t30.1    --------------------------------------------------------------------------------  target    RDEWLRLTHEEAADLVAAALINIATTYSGDNGQKLLLQQGYEKEIVEATRGAGTQVLKFRGGMPLLGLTRIFGLYRMANS 7t30.1    --------------------------------------------------------------------------------  target    MALLDHKIRGVKPEDALGARGWDNYSWHTDLPPGHPMVTGQQTVDFDLHAVEQARIVVVWGMNWVTTKMPDTHWLTEARL 7t30.1    ---------------------------------------------------ERRDFLYVFSTAMV----PEEEEILAAIS  target    KGTKVVVIACEYS-SSSIKADDAIVVRPG--TTPALALGLCNVIMREKIYDGDYVRRFSDLPLLVRADNLKLLRAEEVFG 7t30.1    ATRFVVVQTPFKVRPLVNLADILLPAPAWYERSGHFC-------------------------------------------  target    TPQAALKNQTR 7t30.1    ----------- ``` | | | | | | | | | | | | | | | | | | | | | | | | | | | | | | | | | | | | | | | | | | | | | | | | | |
|  | 1viv.1.A | Hypothetical protein yckF  *Crystal structure of a hypothetical protein* | 0.03 |  | 17.54 | 0.14 | 292-350 | X-ray | 2.60 | homo-dimer |  | HHblits | 0.28 |
| ``` target    NNVNRREFLQWIGAAGFSTFALSASNAWGLQAIENPLAAYPNREWEKTYRDLWKSDASFTFLCAPNDTHNCILNAHVRDG 1viv.1    --------------------------------------------------------------------------------  target    VITRIGPTMKYGEATDLYGSKVTHRWDPRVCQKGLALTRRFYGDRRVRYPMVRKGFKAWADKGFPREKDGRPPKDYFNRA 1viv.1    --------------------------------------------------------------------------------  target    RDEWLRLTHEEAADLVAAALINIATTYSGDNGQKLLLQQGYEKEIVEATRGAGTQVLKFRGGMPLLGLTRIFGLYRMANS 1viv.1    --------------------------------------------------------------------------------  target    MALLDHKIRGVKPEDALGARGWDNYSWHTDLPPGHPMVTGQQTVDFDLHAVEQARIVVVWGMNWVTTKMPDTHWLTEARL 1viv.1    ---------------------------------------------------AEGDLVIIGSGSGETK--SLIHTAAKAKS  target    KGTKVVVIACE-YSSSSIKADDAIVVRPGTTPALALGLCNVIMREKIYDGDYVRRFSDLPLLVRADNLKLLRAEEVFGTP 1viv.1    LHGIVAALTINPESSIGKQADLIIRMPGSPK-------------------------------------------------  target    QAALKNQTR 1viv.1    --------- ``` | | | | | | | | | | | | | | | | | | | | | | | | | | | | | | | | | | | | | | | | | | | | | | | | | |
|  | 4lzj.1.A | N-acetylmuramic acid 6-phosphate etherase  *Crystal Structure of MurQ from H.influenzae with bound inhibitor* | 0.03 |  | 22.81 | 0.14 | 292-350 | X-ray | 2.41 | homo-dimer |  | HHblits | 0.28 |
| ``` target    NNVNRREFLQWIGAAGFSTFALSASNAWGLQAIENPLAAYPNREWEKTYRDLWKSDASFTFLCAPNDTHNCILNAHVRDG 4lzj.1    --------------------------------------------------------------------------------  target    VITRIGPTMKYGEATDLYGSKVTHRWDPRVCQKGLALTRRFYGDRRVRYPMVRKGFKAWADKGFPREKDGRPPKDYFNRA 4lzj.1    --------------------------------------------------------------------------------  target    RDEWLRLTHEEAADLVAAALINIATTYSGDNGQKLLLQQGYEKEIVEATRGAGTQVLKFRGGMPLLGLTRIFGLYRMANS 4lzj.1    --------------------------------------------------------------------------------  target    MALLDHKIRGVKPEDALGARGWDNYSWHTDLPPGHPMVTGQQTVDFDLHAVEQARIVVVWGMNWVTTKMPDTHWLTEARL 4lzj.1    ---------------------------------------------------SKNDVLVGIAASGRT--PYVIAGLQYAKS  target    KGTKVVVIACE-YSSSSIKADDAIVVRPGTTPALALGLCNVIMREKIYDGDYVRRFSDLPLLVRADNLKLLRAEEVFGTP 4lzj.1    LGALTISIASNPKSEMAEIADIAIETIVGPE-------------------------------------------------  target    QAALKNQTR 4lzj.1    --------- ``` | | | | | | | | | | | | | | | | | | | | | | | | | | | | | | | | | | | | | | | | | | | | | | | | | |
|  | 4lzj.1.B | N-acetylmuramic acid 6-phosphate etherase  *Crystal Structure of MurQ from H.influenzae with bound inhibitor* | 0.03 |  | 22.81 | 0.14 | 292-350 | X-ray | 2.41 | homo-dimer |  | HHblits | 0.28 |
| ``` target    NNVNRREFLQWIGAAGFSTFALSASNAWGLQAIENPLAAYPNREWEKTYRDLWKSDASFTFLCAPNDTHNCILNAHVRDG 4lzj.1    --------------------------------------------------------------------------------  target    VITRIGPTMKYGEATDLYGSKVTHRWDPRVCQKGLALTRRFYGDRRVRYPMVRKGFKAWADKGFPREKDGRPPKDYFNRA 4lzj.1    --------------------------------------------------------------------------------  target    RDEWLRLTHEEAADLVAAALINIATTYSGDNGQKLLLQQGYEKEIVEATRGAGTQVLKFRGGMPLLGLTRIFGLYRMANS 4lzj.1    --------------------------------------------------------------------------------  target    MALLDHKIRGVKPEDALGARGWDNYSWHTDLPPGHPMVTGQQTVDFDLHAVEQARIVVVWGMNWVTTKMPDTHWLTEARL 4lzj.1    ---------------------------------------------------SKNDVLVGIAASGRT--PYVIAGLQYAKS  target    KGTKVVVIACE-YSSSSIKADDAIVVRPGTTPALALGLCNVIMREKIYDGDYVRRFSDLPLLVRADNLKLLRAEEVFGTP 4lzj.1    LGALTISIASNPKSEMAEIADIAIETIVGPE-------------------------------------------------  target    QAALKNQTR 4lzj.1    --------- ``` | | | | | | | | | | | | | | | | | | | | | | | | | | | | | | | | | | | | | | | | | | | | | | | | | |
|  | 4lzj.2.B | N-acetylmuramic acid 6-phosphate etherase  *Crystal Structure of MurQ from H.influenzae with bound inhibitor* | 0.03 |  | 22.81 | 0.14 | 292-350 | X-ray | 2.41 | homo-dimer |  | HHblits | 0.28 |
| ``` target    NNVNRREFLQWIGAAGFSTFALSASNAWGLQAIENPLAAYPNREWEKTYRDLWKSDASFTFLCAPNDTHNCILNAHVRDG 4lzj.2    --------------------------------------------------------------------------------  target    VITRIGPTMKYGEATDLYGSKVTHRWDPRVCQKGLALTRRFYGDRRVRYPMVRKGFKAWADKGFPREKDGRPPKDYFNRA 4lzj.2    --------------------------------------------------------------------------------  target    RDEWLRLTHEEAADLVAAALINIATTYSGDNGQKLLLQQGYEKEIVEATRGAGTQVLKFRGGMPLLGLTRIFGLYRMANS 4lzj.2    --------------------------------------------------------------------------------  target    MALLDHKIRGVKPEDALGARGWDNYSWHTDLPPGHPMVTGQQTVDFDLHAVEQARIVVVWGMNWVTTKMPDTHWLTEARL 4lzj.2    ---------------------------------------------------SKNDVLVGIAASGRT--PYVIAGLQYAKS  target    KGTKVVVIACE-YSSSSIKADDAIVVRPGTTPALALGLCNVIMREKIYDGDYVRRFSDLPLLVRADNLKLLRAEEVFGTP 4lzj.2    LGALTISIASNPKSEMAEIADIAIETIVGPE-------------------------------------------------  target    QAALKNQTR 4lzj.2    --------- ``` | | | | | | | | | | | | | | | | | | | | | | | | | | | | | | | | | | | | | | | | | | | | | | | | | |
|  | 4m0d.1.A | N-acetylmuramic acid 6-phosphate etherase  *Crystal structure of MurQ from H.influenzae in apo form* | 0.03 |  | 22.81 | 0.14 | 292-350 | X-ray | 2.58 | homo-dimer |  | HHblits | 0.28 |
| ``` target    NNVNRREFLQWIGAAGFSTFALSASNAWGLQAIENPLAAYPNREWEKTYRDLWKSDASFTFLCAPNDTHNCILNAHVRDG 4m0d.1    --------------------------------------------------------------------------------  target    VITRIGPTMKYGEATDLYGSKVTHRWDPRVCQKGLALTRRFYGDRRVRYPMVRKGFKAWADKGFPREKDGRPPKDYFNRA 4m0d.1    --------------------------------------------------------------------------------  target    RDEWLRLTHEEAADLVAAALINIATTYSGDNGQKLLLQQGYEKEIVEATRGAGTQVLKFRGGMPLLGLTRIFGLYRMANS 4m0d.1    --------------------------------------------------------------------------------  target    MALLDHKIRGVKPEDALGARGWDNYSWHTDLPPGHPMVTGQQTVDFDLHAVEQARIVVVWGMNWVTTKMPDTHWLTEARL 4m0d.1    ---------------------------------------------------SKNDVLVGIAASGRT--PYVIAGLQYAKS  target    KGTKVVVIACE-YSSSSIKADDAIVVRPGTTPALALGLCNVIMREKIYDGDYVRRFSDLPLLVRADNLKLLRAEEVFGTP 4m0d.1    LGALTISIASNPKSEMAEIADIAIETIVGPE-------------------------------------------------  target    QAALKNQTR 4m0d.1    --------- ``` | | | | | | | | | | | | | | | | | | | | | | | | | | | | | | | | | | | | | | | | | | | | | | | | | |
|  | 4m0d.2.A | N-acetylmuramic acid 6-phosphate etherase  *Crystal structure of MurQ from H.influenzae in apo form* | 0.03 |  | 22.81 | 0.14 | 292-350 | X-ray | 2.58 | homo-dimer |  | HHblits | 0.28 |
| ``` target    NNVNRREFLQWIGAAGFSTFALSASNAWGLQAIENPLAAYPNREWEKTYRDLWKSDASFTFLCAPNDTHNCILNAHVRDG 4m0d.2    --------------------------------------------------------------------------------  target    VITRIGPTMKYGEATDLYGSKVTHRWDPRVCQKGLALTRRFYGDRRVRYPMVRKGFKAWADKGFPREKDGRPPKDYFNRA 4m0d.2    --------------------------------------------------------------------------------  target    RDEWLRLTHEEAADLVAAALINIATTYSGDNGQKLLLQQGYEKEIVEATRGAGTQVLKFRGGMPLLGLTRIFGLYRMANS 4m0d.2    --------------------------------------------------------------------------------  target    MALLDHKIRGVKPEDALGARGWDNYSWHTDLPPGHPMVTGQQTVDFDLHAVEQARIVVVWGMNWVTTKMPDTHWLTEARL 4m0d.2    ---------------------------------------------------SKNDVLVGIAASGRT--PYVIAGLQYAKS  target    KGTKVVVIACE-YSSSSIKADDAIVVRPGTTPALALGLCNVIMREKIYDGDYVRRFSDLPLLVRADNLKLLRAEEVFGTP 4m0d.2    LGALTISIASNPKSEMAEIADIAIETIVGPE-------------------------------------------------  target    QAALKNQTR 4m0d.2    --------- ``` | | | | | | | | | | | | | | | | | | | | | | | | | | | | | | | | | | | | | | | | | | | | | | | | | |
|  | 4m0d.2.B | N-acetylmuramic acid 6-phosphate etherase  *Crystal structure of MurQ from H.influenzae in apo form* | 0.03 |  | 22.81 | 0.14 | 292-350 | X-ray | 2.58 | homo-dimer |  | HHblits | 0.28 |
| ``` target    NNVNRREFLQWIGAAGFSTFALSASNAWGLQAIENPLAAYPNREWEKTYRDLWKSDASFTFLCAPNDTHNCILNAHVRDG 4m0d.2    --------------------------------------------------------------------------------  target    VITRIGPTMKYGEATDLYGSKVTHRWDPRVCQKGLALTRRFYGDRRVRYPMVRKGFKAWADKGFPREKDGRPPKDYFNRA 4m0d.2    --------------------------------------------------------------------------------  target    RDEWLRLTHEEAADLVAAALINIATTYSGDNGQKLLLQQGYEKEIVEATRGAGTQVLKFRGGMPLLGLTRIFGLYRMANS 4m0d.2    --------------------------------------------------------------------------------  target    MALLDHKIRGVKPEDALGARGWDNYSWHTDLPPGHPMVTGQQTVDFDLHAVEQARIVVVWGMNWVTTKMPDTHWLTEARL 4m0d.2    ---------------------------------------------------SKNDVLVGIAASGRT--PYVIAGLQYAKS  target    KGTKVVVIACE-YSSSSIKADDAIVVRPGTTPALALGLCNVIMREKIYDGDYVRRFSDLPLLVRADNLKLLRAEEVFGTP 4m0d.2    LGALTISIASNPKSEMAEIADIAIETIVGPE-------------------------------------------------  target    QAALKNQTR 4m0d.2    --------- ``` | | | | | | | | | | | | | | | | | | | | | | | | | | | | | | | | | | | | | | | | | | | | | | | | | |
|  | 1nri.1.A | Hypothetical protein HI0754  *Crystal Structure of Putative Phosphosugar Isomerase HI0754 from Haemophilus influenzae* | 0.03 |  | 22.81 | 0.14 | 292-350 | X-ray | 1.90 | homo-dimer |  | HHblits | 0.28 |
| ``` target    NNVNRREFLQWIGAAGFSTFALSASNAWGLQAIENPLAAYPNREWEKTYRDLWKSDASFTFLCAPNDTHNCILNAHVRDG 1nri.1    --------------------------------------------------------------------------------  target    VITRIGPTMKYGEATDLYGSKVTHRWDPRVCQKGLALTRRFYGDRRVRYPMVRKGFKAWADKGFPREKDGRPPKDYFNRA 1nri.1    --------------------------------------------------------------------------------  target    RDEWLRLTHEEAADLVAAALINIATTYSGDNGQKLLLQQGYEKEIVEATRGAGTQVLKFRGGMPLLGLTRIFGLYRMANS 1nri.1    --------------------------------------------------------------------------------  target    MALLDHKIRGVKPEDALGARGWDNYSWHTDLPPGHPMVTGQQTVDFDLHAVEQARIVVVWGMNWVTTKMPDTHWLTEARL 1nri.1    ---------------------------------------------------SKNDVLVGIAASGRT--PYVIAGLQYAKS  target    KGTKVVVIAC-EYSSSSIKADDAIVVRPGTTPALALGLCNVIMREKIYDGDYVRRFSDLPLLVRADNLKLLRAEEVFGTP 1nri.1    LGALTISIASNPKSEMAEIADIAIETIVGPE-------------------------------------------------  target    QAALKNQTR 1nri.1    --------- ``` | | | | | | | | | | | | | | | | | | | | | | | | | | | | | | | | | | | | | | | | | | | | | | | | | |
|  | 5ltz.1.A | Phosphoheptose isomerase  *GmhA\_mutant Q175E* | 0.03 |  | 15.79 | 0.14 | 293-351 | X-ray | 1.67 | homo-tetramer | 4 x ZN, 4 x I22 | HHblits | 0.28 |
| ``` target    NNVNRREFLQWIGAAGFSTFALSASNAWGLQAIENPLAAYPNREWEKTYRDLWKSDASFTFLCAPNDTHNCILNAHVRDG 5ltz.1    --------------------------------------------------------------------------------  target    VITRIGPTMKYGEATDLYGSKVTHRWDPRVCQKGLALTRRFYGDRRVRYPMVRKGFKAWADKGFPREKDGRPPKDYFNRA 5ltz.1    --------------------------------------------------------------------------------  target    RDEWLRLTHEEAADLVAAALINIATTYSGDNGQKLLLQQGYEKEIVEATRGAGTQVLKFRGGMPLLGLTRIFGLYRMANS 5ltz.1    --------------------------------------------------------------------------------  target    MALLDHKIRGVKPEDALGARGWDNYSWHTDLPPGHPMVTGQQTVDFDLHAVEQARIVVVWGMNWVTTKMPDTHWLTEARL 5ltz.1    ----------------------------------------------------EGDVLIGYSTSGKS--PNILAAFREAKA  target    KGTKVVVIAC-EYSSSSIKADDAIVVRPGTTPALALGLCNVIMREKIYDGDYVRRFSDLPLLVRADNLKLLRAEEVFGTP 5ltz.1    KGMTCVGFTGNRGGEMRELCDLLLEVPSADTP------------------------------------------------  target    QAALKNQTR 5ltz.1    --------- ``` | | | | | | | | | | | | | | | | | | | | | | | | | | | | | | | | | | | | | | | | | | | | | | | | | |
|  | 1vim.1.A | Hypothetical protein AF1796  *Crystal structure of an hypothetical protein* | 0.03 |  | 23.64 | 0.13 | 292-348 | X-ray | 1.36 | homo-tetramer |  | HHblits | 0.30 |
| ``` target    NNVNRREFLQWIGAAGFSTFALSASNAWGLQAIENPLAAYPNREWEKTYRDLWKSDASFTFLCAPNDTHNCILNAHVRDG 1vim.1    --------------------------------------------------------------------------------  target    VITRIGPTMKYGEATDLYGSKVTHRWDPRVCQKGLALTRRFYGDRRVRYPMVRKGFKAWADKGFPREKDGRPPKDYFNRA 1vim.1    --------------------------------------------------------------------------------  target    RDEWLRLTHEEAADLVAAALINIATTYSGDNGQKLLLQQGYEKEIVEATRGAGTQVLKFRGGMPLLGLTRIFGLYRMANS 1vim.1    --------------------------------------------------------------------------------  target    MALLDHKIRGVKPEDALGARGWDNYSWHTDLPPGHPMVTGQQTVDFDLHAVEQARIVVVWGMNWVTTKMPDTHWLTEARL 1vim.1    ---------------------------------------------------TDQDVLVGISGSGETT--SVVNISKKAKD  target    KGTKVVVIACE-YSSSSIKADDAIVVRPGTTPALALGLCNVIMREKIYDGDYVRRFSDLPLLVRADNLKLLRAEEVFGTP 1vim.1    IGSKLVAVTGKRDSSLAKMADVVMVVKGK---------------------------------------------------  target    QAALKNQTR 1vim.1    --------- ``` | | | | | | | | | | | | | | | | | | | | | | | | | | | | | | | | | | | | | | | | | | | | | | | | | |
|  | 1vim.1.D | Hypothetical protein AF1796  *Crystal structure of an hypothetical protein* | 0.03 |  | 23.64 | 0.13 | 292-348 | X-ray | 1.36 | homo-tetramer |  | HHblits | 0.30 |
| ``` target    NNVNRREFLQWIGAAGFSTFALSASNAWGLQAIENPLAAYPNREWEKTYRDLWKSDASFTFLCAPNDTHNCILNAHVRDG 1vim.1    --------------------------------------------------------------------------------  target    VITRIGPTMKYGEATDLYGSKVTHRWDPRVCQKGLALTRRFYGDRRVRYPMVRKGFKAWADKGFPREKDGRPPKDYFNRA 1vim.1    --------------------------------------------------------------------------------  target    RDEWLRLTHEEAADLVAAALINIATTYSGDNGQKLLLQQGYEKEIVEATRGAGTQVLKFRGGMPLLGLTRIFGLYRMANS 1vim.1    --------------------------------------------------------------------------------  target    MALLDHKIRGVKPEDALGARGWDNYSWHTDLPPGHPMVTGQQTVDFDLHAVEQARIVVVWGMNWVTTKMPDTHWLTEARL 1vim.1    ---------------------------------------------------TDQDVLVGISGSGETT--SVVNISKKAKD  target    KGTKVVVIACE-YSSSSIKADDAIVVRPGTTPALALGLCNVIMREKIYDGDYVRRFSDLPLLVRADNLKLLRAEEVFGTP 1vim.1    IGSKLVAVTGKRDSSLAKMADVVMVVKGK---------------------------------------------------  target    QAALKNQTR 1vim.1    --------- ``` | | | | | | | | | | | | | | | | | | | | | | | | | | | | | | | | | | | | | | | | | | | | | | | | | |
|  | 7en6.1.A | HTH-type transcriptional regulator MurR  *The crystal structure of Escherichia coli MurR in apo form* | 0.03 |  | 15.79 | 0.14 | 292-350 | X-ray | 2.28 | homo-tetramer |  | HHblits | 0.27 |
| ``` target    NNVNRREFLQWIGAAGFSTFALSASNAWGLQAIENPLAAYPNREWEKTYRDLWKSDASFTFLCAPNDTHNCILNAHVRDG 7en6.1    --------------------------------------------------------------------------------  target    VITRIGPTMKYGEATDLYGSKVTHRWDPRVCQKGLALTRRFYGDRRVRYPMVRKGFKAWADKGFPREKDGRPPKDYFNRA 7en6.1    --------------------------------------------------------------------------------  target    RDEWLRLTHEEAADLVAAALINIATTYSGDNGQKLLLQQGYEKEIVEATRGAGTQVLKFRGGMPLLGLTRIFGLYRMANS 7en6.1    --------------------------------------------------------------------------------  target    MALLDHKIRGVKPEDALGARGWDNYSWHTDLPPGHPMVTGQQTVDFDLHAVEQARIVVVWGMNWVTTKMPDTHWLTEARL 7en6.1    ---------------------------------------------------KKGDVQIAISYSGSKK--EIVLCAEAARK  target    KGTKVVVIAC-EYSSSSIKADDAIVVRPGTTPALALGLCNVIMREKIYDGDYVRRFSDLPLLVRADNLKLLRAEEVFGTP 7en6.1    QGATVIAITSLTDSPLRRLAHFTLDTVSGET-------------------------------------------------  target    QAALKNQTR 7en6.1    --------- ``` | | | | | | | | | | | | | | | | | | | | | | | | | | | | | | | | | | | | | | | | | | | | | | | | | |
|  | 7en6.1.B | HTH-type transcriptional regulator MurR  *The crystal structure of Escherichia coli MurR in apo form* | 0.03 |  | 15.79 | 0.14 | 292-350 | X-ray | 2.28 | homo-tetramer |  | HHblits | 0.27 |
| ``` target    NNVNRREFLQWIGAAGFSTFALSASNAWGLQAIENPLAAYPNREWEKTYRDLWKSDASFTFLCAPNDTHNCILNAHVRDG 7en6.1    --------------------------------------------------------------------------------  target    VITRIGPTMKYGEATDLYGSKVTHRWDPRVCQKGLALTRRFYGDRRVRYPMVRKGFKAWADKGFPREKDGRPPKDYFNRA 7en6.1    --------------------------------------------------------------------------------  target    RDEWLRLTHEEAADLVAAALINIATTYSGDNGQKLLLQQGYEKEIVEATRGAGTQVLKFRGGMPLLGLTRIFGLYRMANS 7en6.1    --------------------------------------------------------------------------------  target    MALLDHKIRGVKPEDALGARGWDNYSWHTDLPPGHPMVTGQQTVDFDLHAVEQARIVVVWGMNWVTTKMPDTHWLTEARL 7en6.1    ---------------------------------------------------KKGDVQIAISYSGSKK--EIVLCAEAARK  target    KGTKVVVIAC-EYSSSSIKADDAIVVRPGTTPALALGLCNVIMREKIYDGDYVRRFSDLPLLVRADNLKLLRAEEVFGTP 7en6.1    QGATVIAITSLTDSPLRRLAHFTLDTVSGET-------------------------------------------------  target    QAALKNQTR 7en6.1    --------- ``` | | | | | | | | | | | | | | | | | | | | | | | | | | | | | | | | | | | | | | | | | | | | | | | | | |
|  | 7en6.1.C | HTH-type transcriptional regulator MurR  *The crystal structure of Escherichia coli MurR in apo form* | 0.03 |  | 15.79 | 0.14 | 292-350 | X-ray | 2.28 | homo-tetramer |  | HHblits | 0.27 |
| ``` target    NNVNRREFLQWIGAAGFSTFALSASNAWGLQAIENPLAAYPNREWEKTYRDLWKSDASFTFLCAPNDTHNCILNAHVRDG 7en6.1    --------------------------------------------------------------------------------  target    VITRIGPTMKYGEATDLYGSKVTHRWDPRVCQKGLALTRRFYGDRRVRYPMVRKGFKAWADKGFPREKDGRPPKDYFNRA 7en6.1    --------------------------------------------------------------------------------  target    RDEWLRLTHEEAADLVAAALINIATTYSGDNGQKLLLQQGYEKEIVEATRGAGTQVLKFRGGMPLLGLTRIFGLYRMANS 7en6.1    --------------------------------------------------------------------------------  target    MALLDHKIRGVKPEDALGARGWDNYSWHTDLPPGHPMVTGQQTVDFDLHAVEQARIVVVWGMNWVTTKMPDTHWLTEARL 7en6.1    ---------------------------------------------------KKGDVQIAISYSGSKK--EIVLCAEAARK  target    KGTKVVVIAC-EYSSSSIKADDAIVVRPGTTPALALGLCNVIMREKIYDGDYVRRFSDLPLLVRADNLKLLRAEEVFGTP 7en6.1    QGATVIAITSLTDSPLRRLAHFTLDTVSGET-------------------------------------------------  target    QAALKNQTR 7en6.1    --------- ``` | | | | | | | | | | | | | | | | | | | | | | | | | | | | | | | | | | | | | | | | | | | | | | | | | |
|  | 7en6.1.D | HTH-type transcriptional regulator MurR  *The crystal structure of Escherichia coli MurR in apo form* | 0.03 |  | 15.79 | 0.14 | 292-350 | X-ray | 2.28 | homo-tetramer |  | HHblits | 0.27 |
| ``` target    NNVNRREFLQWIGAAGFSTFALSASNAWGLQAIENPLAAYPNREWEKTYRDLWKSDASFTFLCAPNDTHNCILNAHVRDG 7en6.1    --------------------------------------------------------------------------------  target    VITRIGPTMKYGEATDLYGSKVTHRWDPRVCQKGLALTRRFYGDRRVRYPMVRKGFKAWADKGFPREKDGRPPKDYFNRA 7en6.1    --------------------------------------------------------------------------------  target    RDEWLRLTHEEAADLVAAALINIATTYSGDNGQKLLLQQGYEKEIVEATRGAGTQVLKFRGGMPLLGLTRIFGLYRMANS 7en6.1    --------------------------------------------------------------------------------  target    MALLDHKIRGVKPEDALGARGWDNYSWHTDLPPGHPMVTGQQTVDFDLHAVEQARIVVVWGMNWVTTKMPDTHWLTEARL 7en6.1    ---------------------------------------------------KKGDVQIAISYSGSKK--EIVLCAEAARK  target    KGTKVVVIAC-EYSSSSIKADDAIVVRPGTTPALALGLCNVIMREKIYDGDYVRRFSDLPLLVRADNLKLLRAEEVFGTP 7en6.1    QGATVIAITSLTDSPLRRLAHFTLDTVSGET-------------------------------------------------  target    QAALKNQTR 7en6.1    --------- ``` | | | | | | | | | | | | | | | | | | | | | | | | | | | | | | | | | | | | | | | | | | | | | | | | | |
|  | 5lu6.1.A | Phosphoheptose isomerase  *Heptose isomerase mutant - H64Q* | 0.03 |  | 14.04 | 0.14 | 292-350 | X-ray | 1.67 | homo-tetramer | 4 x I22 | HHblits | 0.27 |
| ``` target    NNVNRREFLQWIGAAGFSTFALSASNAWGLQAIENPLAAYPNREWEKTYRDLWKSDASFTFLCAPNDTHNCILNAHVRDG 5lu6.1    --------------------------------------------------------------------------------  target    VITRIGPTMKYGEATDLYGSKVTHRWDPRVCQKGLALTRRFYGDRRVRYPMVRKGFKAWADKGFPREKDGRPPKDYFNRA 5lu6.1    --------------------------------------------------------------------------------  target    RDEWLRLTHEEAADLVAAALINIATTYSGDNGQKLLLQQGYEKEIVEATRGAGTQVLKFRGGMPLLGLTRIFGLYRMANS 5lu6.1    --------------------------------------------------------------------------------  target    MALLDHKIRGVKPEDALGARGWDNYSWHTDLPPGHPMVTGQQTVDFDLHAVEQARIVVVWGMNWVTTKMPDTHWLTEARL 5lu6.1    ---------------------------------------------------NEGDVLIGYSTSGKSP--NILAAFREAKA  target    KGTKVVVIAC-EYSSSSIKADDAIVVRPGTTPALALGLCNVIMREKIYDGDYVRRFSDLPLLVRADNLKLLRAEEVFGTP 5lu6.1    KGMTCVGFTGNRGGEMRELCDLLLEVPSADT-------------------------------------------------  target    QAALKNQTR 5lu6.1    --------- ``` | | | | | | | | | | | | | | | | | | | | | | | | | | | | | | | | | | | | | | | | | | | | | | | | | |
|  | 3etn.1.A | putative phosphosugar isomerase involved in capsule formation  *Crystal structure of putative phosphosugar isomerase involved in capsule formation (YP\_209877.1) from Bacteroides fragilis NCTC 9343 at 1.70 A resolution* | 0.03 |  | 14.04 | 0.14 | 292-350 | X-ray | 1.70 | homo-tetramer | 4 x CMK | HHblits | 0.27 |
| ``` target    NNVNRREFLQWIGAAGFSTFALSASNAWGLQAIENPLAAYPNREWEKTYRDLWKSDASFTFLCAPNDTHNCILNAHVRDG 3etn.1    --------------------------------------------------------------------------------  target    VITRIGPTMKYGEATDLYGSKVTHRWDPRVCQKGLALTRRFYGDRRVRYPMVRKGFKAWADKGFPREKDGRPPKDYFNRA 3etn.1    --------------------------------------------------------------------------------  target    RDEWLRLTHEEAADLVAAALINIATTYSGDNGQKLLLQQGYEKEIVEATRGAGTQVLKFRGGMPLLGLTRIFGLYRMANS 3etn.1    --------------------------------------------------------------------------------  target    MALLDHKIRGVKPEDALGARGWDNYSWHTDLPPGHPMVTGQQTVDFDLHAVEQARIVVVWGMNWVTTKMPDTHWLTEARL 3etn.1    ---------------------------------------------------QENDLLLLISNSGK-T-REIVELTQLAHN  target    --KGTKVVVIACE-YSSSSIKADDAIVVRPGTTPALALGLCNVIMREKIYDGDYVRRFSDLPLLVRADNLKLLRAEEVFG 3etn.1    LNPGLKFIVITGNPDSPLASESDVCLSTGHPAE-----------------------------------------------  target    TPQAALKNQTR 3etn.1    ----------- ``` | | | | | | | | | | | | | | | | | | | | | | | | | | | | | | | | | | | | | | | | | | | | | | | | | |
|  | 1tk9.1.A | Phosphoheptose isomerase 1  *Crystal Structure of Phosphoheptose isomerase 1* | 0.03 |  | 10.34 | 0.14 | 292-351 | X-ray | 2.10 | homo-tetramer |  | HHblits | 0.26 |
| ``` target    NNVNRREFLQWIGAAGFSTFALSASNAWGLQAIENPLAAYPNREWEKTYRDLWKSDASFTFLCAPNDTHNCILNAHVRDG 1tk9.1    --------------------------------------------------------------------------------  target    VITRIGPTMKYGEATDLYGSKVTHRWDPRVCQKGLALTRRFYGDRRVRYPMVRKGFKAWADKGFPREKDGRPPKDYFNRA 1tk9.1    --------------------------------------------------------------------------------  target    RDEWLRLTHEEAADLVAAALINIATTYSGDNGQKLLLQQGYEKEIVEATRGAGTQVLKFRGGMPLLGLTRIFGLYRMANS 1tk9.1    --------------------------------------------------------------------------------  target    MALLDHKIRGVKPEDALGARGWDNYSWHTDLPPGHPMVTGQQTVDFDLHAVEQARIVVVWGMNWVTTKMPDTHWLTEARL 1tk9.1    ---------------------------------------------------NEKDVLIGISTSGKSP--NVLEALKKAKE  target    KGTKVVVIACEY-SSSSIKADDAIVVRPGTTPALALGLCNVIMREKIYDGDYVRRFSDLPLLVRADNLKLLRAEEVFGTP 1tk9.1    LNMLCLGLSGKGGGMMNKLCDHNLVVPSDDTA------------------------------------------------  target    QAALKNQTR 1tk9.1    --------- ``` | | | | | | | | | | | | | | | | | | | | | | | | | | | | | | | | | | | | | | | | | | | | | | | | | |
|  | 7en5.1.A | HTH-type transcriptional regulator MurR  *The crystal structure of Escherichia coli MurR in complex with N-acetylglucosamine-6-phosphate* | 0.03 |  | 15.79 | 0.14 | 292-350 | X-ray | 1.25 | homo-tetramer | 4 x 4QY, 4 x MXE | HHblits | 0.27 |
| ``` target    NNVNRREFLQWIGAAGFSTFALSASNAWGLQAIENPLAAYPNREWEKTYRDLWKSDASFTFLCAPNDTHNCILNAHVRDG 7en5.1    --------------------------------------------------------------------------------  target    VITRIGPTMKYGEATDLYGSKVTHRWDPRVCQKGLALTRRFYGDRRVRYPMVRKGFKAWADKGFPREKDGRPPKDYFNRA 7en5.1    --------------------------------------------------------------------------------  target    RDEWLRLTHEEAADLVAAALINIATTYSGDNGQKLLLQQGYEKEIVEATRGAGTQVLKFRGGMPLLGLTRIFGLYRMANS 7en5.1    --------------------------------------------------------------------------------  target    MALLDHKIRGVKPEDALGARGWDNYSWHTDLPPGHPMVTGQQTVDFDLHAVEQARIVVVWGMNWVTTKMPDTHWLTEARL 7en5.1    ---------------------------------------------------KKGDVQIAISYSGSKK--EIVLCAEAARK  target    KGTKVVVIACE-YSSSSIKADDAIVVRPGTTPALALGLCNVIMREKIYDGDYVRRFSDLPLLVRADNLKLLRAEEVFGTP 7en5.1    QGATVIAITSLTDSPLRRLAHFTLDTVSGET-------------------------------------------------  target    QAALKNQTR 7en5.1    --------- ``` | | | | | | | | | | | | | | | | | | | | | | | | | | | | | | | | | | | | | | | | | | | | | | | | | |
|  | 1tzb.1.A | glucose-6-phosphate isomerase, conjectural  *Crystal structure of native phosphoglucose/phosphomannose isomerase from Pyrobaculum aerophilum* | 0.03 |  | 10.71 | 0.14 | 294-352 | X-ray | 1.16 | homo-dimer |  | HHblits | 0.27 |
| ``` target    NNVNRREFLQWIGAAGFSTFALSASNAWGLQAIENPLAAYPNREWEKTYRDLWKSDASFTFLCAPNDTHNCILNAHVRDG 1tzb.1    --------------------------------------------------------------------------------  target    VITRIGPTMKYGEATDLYGSKVTHRWDPRVCQKGLALTRRFYGDRRVRYPMVRKGFKAWADKGFPREKDGRPPKDYFNRA 1tzb.1    --------------------------------------------------------------------------------  target    RDEWLRLTHEEAADLVAAALINIATTYSGDNGQKLLLQQGYEKEIVEATRGAGTQVLKFRGGMPLLGLTRIFGLYRMANS 1tzb.1    --------------------------------------------------------------------------------  target    MALLDHKIRGVKPEDALGARGWDNYSWHTDLPPGHPMVTGQQTVDFDLHAVEQARIVVVWGMNWVTTKMPDTHWLTEARL 1tzb.1    -----------------------------------------------------DGLLIAVSYSGNT--IETLYTVEYAKR  target    KGTKVVVIACEYSSSSIKADDAIVVRPGTTPALALGLCNVIMREKIYDGDYVRRFSDLPLLVRADNLKLLRAEEVFGTPQ 1tzb.1    RRIPAVAITTG-GRLAQMGVPTVIVPKASAPR------------------------------------------------  target    AALKNQTR 1tzb.1    -------- ``` | | | | | | | | | | | | | | | | | | | | | | | | | | | | | | | | | | | | | | | | | | | | | | | | | |
|  | 1tzb.1.B | glucose-6-phosphate isomerase, conjectural  *Crystal structure of native phosphoglucose/phosphomannose isomerase from Pyrobaculum aerophilum* | 0.03 |  | 10.71 | 0.14 | 294-352 | X-ray | 1.16 | homo-dimer |  | HHblits | 0.27 |
| ``` target    NNVNRREFLQWIGAAGFSTFALSASNAWGLQAIENPLAAYPNREWEKTYRDLWKSDASFTFLCAPNDTHNCILNAHVRDG 1tzb.1    --------------------------------------------------------------------------------  target    VITRIGPTMKYGEATDLYGSKVTHRWDPRVCQKGLALTRRFYGDRRVRYPMVRKGFKAWADKGFPREKDGRPPKDYFNRA 1tzb.1    --------------------------------------------------------------------------------  target    RDEWLRLTHEEAADLVAAALINIATTYSGDNGQKLLLQQGYEKEIVEATRGAGTQVLKFRGGMPLLGLTRIFGLYRMANS 1tzb.1    --------------------------------------------------------------------------------  target    MALLDHKIRGVKPEDALGARGWDNYSWHTDLPPGHPMVTGQQTVDFDLHAVEQARIVVVWGMNWVTTKMPDTHWLTEARL 1tzb.1    -----------------------------------------------------DGLLIAVSYSGNT--IETLYTVEYAKR  target    KGTKVVVIACEYSSSSIKADDAIVVRPGTTPALALGLCNVIMREKIYDGDYVRRFSDLPLLVRADNLKLLRAEEVFGTPQ 1tzb.1    RRIPAVAITTG-GRLAQMGVPTVIVPKASAPR------------------------------------------------  target    AALKNQTR 1tzb.1    -------- ``` | | | | | | | | | | | | | | | | | | | | | | | | | | | | | | | | | | | | | | | | | | | | | | | | | |
|  | 1x94.1.A | putative Phosphoheptose isomerase  *Crystal Structure of a Hypothetical protein* | 0.03 |  | 14.04 | 0.14 | 292-350 | X-ray | 2.50 | homo-dimer |  | HHblits | 0.26 |
| ``` target    NNVNRREFLQWIGAAGFSTFALSASNAWGLQAIENPLAAYPNREWEKTYRDLWKSDASFTFLCAPNDTHNCILNAHVRDG 1x94.1    --------------------------------------------------------------------------------  target    VITRIGPTMKYGEATDLYGSKVTHRWDPRVCQKGLALTRRFYGDRRVRYPMVRKGFKAWADKGFPREKDGRPPKDYFNRA 1x94.1    --------------------------------------------------------------------------------  target    RDEWLRLTHEEAADLVAAALINIATTYSGDNGQKLLLQQGYEKEIVEATRGAGTQVLKFRGGMPLLGLTRIFGLYRMANS 1x94.1    --------------------------------------------------------------------------------  target    MALLDHKIRGVKPEDALGARGWDNYSWHTDLPPGHPMVTGQQTVDFDLHAVEQARIVVVWGMNWVTTKMPDTHWLTEARL 1x94.1    ---------------------------------------------------AKGDVLFGLSTSGNSG--NILKAIEAAKA  target    KGTKVVVIACE-YSSSSIKADDAIVVRPGTTPALALGLCNVIMREKIYDGDYVRRFSDLPLLVRADNLKLLRAEEVFGTP 1x94.1    KGMKTIALTGKDGGKMAGLADVEIRVPHFGY-------------------------------------------------  target    QAALKNQTR 1x94.1    --------- ``` | | | | | | | | | | | | | | | | | | | | | | | | | | | | | | | | | | | | | | | | | | | | | | | | | |
|  | 1x94.1.B | putative Phosphoheptose isomerase  *Crystal Structure of a Hypothetical protein* | 0.03 |  | 14.04 | 0.14 | 292-350 | X-ray | 2.50 | homo-dimer |  | HHblits | 0.26 |
| ``` target    NNVNRREFLQWIGAAGFSTFALSASNAWGLQAIENPLAAYPNREWEKTYRDLWKSDASFTFLCAPNDTHNCILNAHVRDG 1x94.1    --------------------------------------------------------------------------------  target    VITRIGPTMKYGEATDLYGSKVTHRWDPRVCQKGLALTRRFYGDRRVRYPMVRKGFKAWADKGFPREKDGRPPKDYFNRA 1x94.1    --------------------------------------------------------------------------------  target    RDEWLRLTHEEAADLVAAALINIATTYSGDNGQKLLLQQGYEKEIVEATRGAGTQVLKFRGGMPLLGLTRIFGLYRMANS 1x94.1    --------------------------------------------------------------------------------  target    MALLDHKIRGVKPEDALGARGWDNYSWHTDLPPGHPMVTGQQTVDFDLHAVEQARIVVVWGMNWVTTKMPDTHWLTEARL 1x94.1    ---------------------------------------------------AKGDVLFGLSTSGNSG--NILKAIEAAKA  target    KGTKVVVIACE-YSSSSIKADDAIVVRPGTTPALALGLCNVIMREKIYDGDYVRRFSDLPLLVRADNLKLLRAEEVFGTP 1x94.1    KGMKTIALTGKDGGKMAGLADVEIRVPHFGY-------------------------------------------------  target    QAALKNQTR 1x94.1    --------- ``` | | | | | | | | | | | | | | | | | | | | | | | | | | | | | | | | | | | | | | | | | | | | | | | | | |
|  | 4ydd.1.A | DMSO reductase family type II enzyme, molybdopterin subunit  *Crystal structure of the perchlorate reductase PcrAB from Azospira suillum PS* | 0.04 |  | 16.36 | 0.13 | 293-347 | X-ray | 1.86 | hetero-oligomer | 4 x SF4, 1 x MO, 1 x MGD, 1 x MD1, 1 x F3S | HHblits | 0.29 |
| ``` target    NNVNRREFLQWIGAAGFSTFALSASNAWGLQAIENPLAAYPNREWEKTYRDLWKSDASFTFLCAPNDTHNCILNAHVRDG 4ydd.1    --------------------------------------------------------------------------------  target    VITRIGPTMKYGEATDLYGSKVTHRWDPRVCQKGLALTRRFYGDRRVRYPMVRKGFKAWADKGFPREKDGRPPKDYFNRA 4ydd.1    --------------------------------------------------------------------------------  target    RDEWLRLTHEEAADLVAAALINIATTYSGDNGQKLLLQQGYEKEIVEATRGAGTQVLKFRGGMPLLGLTRIFGLYRMANS 4ydd.1    --------------------------------------------------------------------------------  target    MALLDHKIRGVKPEDALGARGWDNYSWHTDLPPGHPMVTGQQTVDFDLHAVEQARIVVVWGMNWVTTKMPDTHWLTEARL 4ydd.1    ----------------------------------------------------DPKVFFVYRGNWLNQAKGQKYVLENLWP  target    KGTKVVVIACEYSSSSIKADDAIVVRPGTTPALALGLCNVIMREKIYDGDYVRRFSDLPLLVRADNLKLLRAEEVFGTPQ 4ydd.1    KLELIVDINIRMDSTALYSDVVLPSAH-----------------------------------------------------  target    AALKNQTR 4ydd.1    -------- ``` | | | | | | | | | | | | | | | | | | | | | | | | | | | | | | | | | | | | | | | | | | | | | | | | | |
|  | 1ici.1.A | TRANSCRIPTIONAL REGULATORY PROTEIN, SIR2 FAMILY  *CRYSTAL STRUCTURE OF A SIR2 HOMOLOG-NAD COMPLEX* | 0.04 |  | 20.37 | 0.13 | 290-345 | X-ray | 2.10 | homo-dimer | 2 x ZN, 2 x NAD | HHblits | 0.30 |
| ``` target    NNVNRREFLQWIGAAGFSTFALSASNAWGLQAIENPLAAYPNREWEKTYRDLWKSDASFTFLCAPNDTHNCILNAHVRDG 1ici.1    --------------------------------------------------------------------------------  target    VITRIGPTMKYGEATDLYGSKVTHRWDPRVCQKGLALTRRFYGDRRVRYPMVRKGFKAWADKGFPREKDGRPPKDYFNRA 1ici.1    --------------------------------------------------------------------------------  target    RDEWLRLTHEEAADLVAAALINIATTYSGDNGQKLLLQQGYEKEIVEATRGAGTQVLKFRGGMPLLGLTRIFGLYRMANS 1ici.1    --------------------------------------------------------------------------------  target    MALLDHKIRGVKPEDALGARGWDNYSWHTDLPPGHPMVTGQQTVDFDLHAVEQARIVVVWGMNWVTTKMPDTHWLTEARL 1ici.1    -------------------------------------------------EVERADVIIVAGTSAVVQ--PAASLPLIVKQ  target    KGTKVVVIACEYSSSSIKADDAIVVRPGTTPALALGLCNVIMREKIYDGDYVRRFSDLPLLVRADNLKLLRAEEVFGTPQ 1ici.1    RGGAIIEINPDETPLTPIADYSLRG-------------------------------------------------------  target    AALKNQTR 1ici.1    -------- ``` | | | | | | | | | | | | | | | | | | | | | | | | | | | | | | | | | | | | | | | | | | | | | | | | | |
|  | 5e7o.1.A | DMSO reductase family type II enzyme, molybdopterin subunit  *Crystal structure of the perchlorate reductase PcrAB mutant W461E of PcrA from Azospira suillum PS* | 0.04 |  | 16.36 | 0.13 | 294-348 | X-ray | 2.40 | hetero-oligomer | 4 x SF4, 1 x MO, 1 x MGD, 1 x MD1, 1 x F3S | HHblits | 0.28 |
| ``` target    NNVNRREFLQWIGAAGFSTFALSASNAWGLQAIENPLAAYPNREWEKTYRDLWKSDASFTFLCAPNDTHNCILNAHVRDG 5e7o.1    --------------------------------------------------------------------------------  target    VITRIGPTMKYGEATDLYGSKVTHRWDPRVCQKGLALTRRFYGDRRVRYPMVRKGFKAWADKGFPREKDGRPPKDYFNRA 5e7o.1    --------------------------------------------------------------------------------  target    RDEWLRLTHEEAADLVAAALINIATTYSGDNGQKLLLQQGYEKEIVEATRGAGTQVLKFRGGMPLLGLTRIFGLYRMANS 5e7o.1    --------------------------------------------------------------------------------  target    MALLDHKIRGVKPEDALGARGWDNYSWHTDLPPGHPMVTGQQTVDFDLHAVEQARIVVVWGMNWVTTKMPDTHWLTEARL 5e7o.1    -----------------------------------------------------PKVFFVYRGNWLNQAKGQKYVLENLWP  target    KGTKVVVIACEYSSSSIKADDAIVVRPGTTPALALGLCNVIMREKIYDGDYVRRFSDLPLLVRADNLKLLRAEEVFGTPQ 5e7o.1    KLELIVDINIRMDSTALYSDVVLPSAHW----------------------------------------------------  target    AALKNQTR 5e7o.1    -------- ``` | | | | | | | | | | | | | | | | | | | | | | | | | | | | | | | | | | | | | | | | | | | | | | | | | |
|  | 7bkb.1.F | Formate dehydrogenase  *Formate dehydrogenase - heterodisulfide reductase - formylmethanofuran dehydrogenase complex from Methanospirillum hungatei (hexameric, composite structure)* | 0.04 |  | 16.07 | 0.14 | 292-348 | EM | 0.00 | hetero-2-2-2-2-2-2-… | 48 x SF4, 4 x FAD, 2 x FES, 4 x 9S8, 4 x ZN, 2 x MO, 4 x MGD | HHblits | 0.27 |
| ``` target    NNVNRREFLQWIGAAGFSTFALSASNAWGLQAIENPLAAYPNREWEKTYRDLWKSDASFTFLCAPNDTHNCILNAHVRDG 7bkb.1    --------------------------------------------------------------------------------  target    VITRIGPTMKYGEATDLYGSKVTHRWDPRVCQKGLALTRRFYGDRRVRYPMVRKGFKAWADKGFPREKDGRPPKDYFNRA 7bkb.1    --------------------------------------------------------------------------------  target    RDEWLRLTHEEAADLVAAALINIATTYSGDNGQKLLLQQGYEKEIVEATRGAGTQVLKFRGGMPLLGLTRIFGLYRMANS 7bkb.1    --------------------------------------------------------------------------------  target    MALLDHKIRGVKPEDALGARGWDNYSWHTDLPPGHPMVTGQQTVDFDLHAVEQARIVVVWGMNWVTTKMPDTHWLTEARL 7bkb.1    ---------------------------------------------------DEIKGMYILGLNPVVTYPS-SNHVKAQLE  target    KGTKVVVIACEYSSSSIKADDAIVVRPGTTPALALGLCNVIMREKIYDGDYVRRFSDLPLLVRADNLKLLRAEEVFGTPQ 7bkb.1    KLDFLVVQDIFFTETCQYADVILPGACF----------------------------------------------------  target    AALKNQTR 7bkb.1    -------- ``` | | | | | | | | | | | | | | | | | | | | | | | | | | | | | | | | | | | | | | | | | | | | | | | | | |
|  | 5lu7.1.A | Phosphoheptose isomerase  *Heptose isomerase GmhA mutant - D61A* | 0.03 |  | 14.29 | 0.14 | 293-350 | X-ray | 1.92 | homo-tetramer | 4 x ZN, 4 x M7P | HHblits | 0.27 |
| ``` target    NNVNRREFLQWIGAAGFSTFALSASNAWGLQAIENPLAAYPNREWEKTYRDLWKSDASFTFLCAPNDTHNCILNAHVRDG 5lu7.1    --------------------------------------------------------------------------------  target    VITRIGPTMKYGEATDLYGSKVTHRWDPRVCQKGLALTRRFYGDRRVRYPMVRKGFKAWADKGFPREKDGRPPKDYFNRA 5lu7.1    --------------------------------------------------------------------------------  target    RDEWLRLTHEEAADLVAAALINIATTYSGDNGQKLLLQQGYEKEIVEATRGAGTQVLKFRGGMPLLGLTRIFGLYRMANS 5lu7.1    --------------------------------------------------------------------------------  target    MALLDHKIRGVKPEDALGARGWDNYSWHTDLPPGHPMVTGQQTVDFDLHAVEQARIVVVWGMNWVTTKMPDTHWLTEARL 5lu7.1    ----------------------------------------------------EGDVLIGYSTSGKS--PNILAAFREAKA  target    KGTKVVVIAC-EYSSSSIKADDAIVVRPGTTPALALGLCNVIMREKIYDGDYVRRFSDLPLLVRADNLKLLRAEEVFGTP 5lu7.1    KGMTCVGFTGNRGGEMRELCDLLLEVPSADT-------------------------------------------------  target    QAALKNQTR 5lu7.1    --------- ``` | | | | | | | | | | | | | | | | | | | | | | | | | | | | | | | | | | | | | | | | | | | | | | | | | |
|  | 2xbl.1.A | PHOSPHOHEPTOSE ISOMERASE  *Crystal structure of GmhA from Burkholderia pseudomallei in complex with product* | 0.03 |  | 14.29 | 0.14 | 293-350 | X-ray | 1.62 | homo-tetramer | 4 x ZN, 4 x M7P | HHblits | 0.27 |
| ``` target    NNVNRREFLQWIGAAGFSTFALSASNAWGLQAIENPLAAYPNREWEKTYRDLWKSDASFTFLCAPNDTHNCILNAHVRDG 2xbl.1    --------------------------------------------------------------------------------  target    VITRIGPTMKYGEATDLYGSKVTHRWDPRVCQKGLALTRRFYGDRRVRYPMVRKGFKAWADKGFPREKDGRPPKDYFNRA 2xbl.1    --------------------------------------------------------------------------------  target    RDEWLRLTHEEAADLVAAALINIATTYSGDNGQKLLLQQGYEKEIVEATRGAGTQVLKFRGGMPLLGLTRIFGLYRMANS 2xbl.1    --------------------------------------------------------------------------------  target    MALLDHKIRGVKPEDALGARGWDNYSWHTDLPPGHPMVTGQQTVDFDLHAVEQARIVVVWGMNWVTTKMPDTHWLTEARL 2xbl.1    ----------------------------------------------------EGDVLIGYSTSGK-S-PNILAAFREAKA  target    KGTKVVVIAC-EYSSSSIKADDAIVVRPGTTPALALGLCNVIMREKIYDGDYVRRFSDLPLLVRADNLKLLRAEEVFGTP 2xbl.1    KGMTCVGFTGNRGGEMRELCDLLLEVPSADT-------------------------------------------------  target    QAALKNQTR 2xbl.1    --------- ``` | | | | | | | | | | | | | | | | | | | | | | | | | | | | | | | | | | | | | | | | | | | | | | | | | |
|  | 7l5i.1.A | Trimethylamine-N-oxide reductase  *Crystal Structure of Haemophilus influenzae MtsZ at pH 7.0* | 0.04 |  | 22.22 | 0.13 | 293-347 | X-ray | 1.73 | monomer | 2 x MGD, 1 x MO, 1 x O | HHblits | 0.30 |
| ``` target    NNVNRREFLQWIGAAGFSTFALSASNAWGLQAIENPLAAYPNREWEKTYRDLWKSDASFTFLCAPNDTHNCILNAHVRDG 7l5i.1    --------------------------------------------------------------------------------  target    VITRIGPTMKYGEATDLYGSKVTHRWDPRVCQKGLALTRRFYGDRRVRYPMVRKGFKAWADKGFPREKDGRPPKDYFNRA 7l5i.1    --------------------------------------------------------------------------------  target    RDEWLRLTHEEAADLVAAALINIATTYSGDNGQKLLLQQGYEKEIVEATRGAGTQVLKFRGGMPLLGLTRIFGLYRMANS 7l5i.1    --------------------------------------------------------------------------------  target    MALLDHKIRGVKPEDALGARGWDNYSWHTDLPPGHPMVTGQQTVDFDLHAVEQARIVVVWGMNWVTTKMPDTHWLTEARL 7l5i.1    ----------------------------------------------------DIKAVYWAGGNPFVHHQ-DTNTLVKAFQ  target    KGTKVVVIACEYSSSSIKADDAIVVRPGTTPALALGLCNVIMREKIYDGDYVRRFSDLPLLVRADNLKLLRAEEVFGTPQ 7l5i.1    KPDVVIVNEVNWTPTARMADIVLPATT-----------------------------------------------------  target    AALKNQTR 7l5i.1    -------- ``` | | | | | | | | | | | | | | | | | | | | | | | | | | | | | | | | | | | | | | | | | | | | | | | | | |
|  | 7l5s.1.A | Trimethylamine-N-oxide reductase  *Crystal Structure of Haemophilus influenzae MtsZ at pH 5.5* | 0.04 |  | 22.22 | 0.13 | 293-347 | X-ray | 2.09 | monomer | 1 x O, 2 x MGD, 1 x MO | HHblits | 0.30 |
| ``` target    NNVNRREFLQWIGAAGFSTFALSASNAWGLQAIENPLAAYPNREWEKTYRDLWKSDASFTFLCAPNDTHNCILNAHVRDG 7l5s.1    --------------------------------------------------------------------------------  target    VITRIGPTMKYGEATDLYGSKVTHRWDPRVCQKGLALTRRFYGDRRVRYPMVRKGFKAWADKGFPREKDGRPPKDYFNRA 7l5s.1    --------------------------------------------------------------------------------  target    RDEWLRLTHEEAADLVAAALINIATTYSGDNGQKLLLQQGYEKEIVEATRGAGTQVLKFRGGMPLLGLTRIFGLYRMANS 7l5s.1    --------------------------------------------------------------------------------  target    MALLDHKIRGVKPEDALGARGWDNYSWHTDLPPGHPMVTGQQTVDFDLHAVEQARIVVVWGMNWVTTKMPDTHWLTEARL 7l5s.1    ----------------------------------------------------DIKAVYWAGGNPFVHHQ-DTNTLVKAFQ  target    KGTKVVVIACEYSSSSIKADDAIVVRPGTTPALALGLCNVIMREKIYDGDYVRRFSDLPLLVRADNLKLLRAEEVFGTPQ 7l5s.1    KPDVVIVNEVNWTPTARMADIVLPATT-----------------------------------------------------  target    AALKNQTR 7l5s.1    -------- ``` | | | | | | | | | | | | | | | | | | | | | | | | | | | | | | | | | | | | | | | | | | | | | | | | | |
|  | 7b04.1.B | Nitrite oxidoreductase subunit A  *Structure of Nitrite oxidoreductase (Nxr) from the anammox bacterium Kuenenia stuttgartiensis.* | 0.04 |  | 12.50 | 0.14 | 293-348 | X-ray | 2.97 | hetero-1-1-1-mer | 4 x SF4, 1 x F3S, 2 x MD1, 1 x MO, 1 x HEM, 2 x CA | HHblits | 0.27 |
| ``` target    NNVNRREFLQWIGAAGFSTFALSASNAWGLQAIENPLAAYPNREWEKTYRDLWKSDASFTFLCAPNDTHNCILNAHVRDG 7b04.1    --------------------------------------------------------------------------------  target    VITRIGPTMKYGEATDLYGSKVTHRWDPRVCQKGLALTRRFYGDRRVRYPMVRKGFKAWADKGFPREKDGRPPKDYFNRA 7b04.1    --------------------------------------------------------------------------------  target    RDEWLRLTHEEAADLVAAALINIATTYSGDNGQKLLLQQGYEKEIVEATRGAGTQVLKFRGGMPLLGLTRIFGLYRMANS 7b04.1    --------------------------------------------------------------------------------  target    MALLDHKIRGVKPEDALGARGWDNYSWHTDLPPGHPMVTGQQTVDFDLHAVEQARIVVVWGMNWVTTKMPDTHWLTEARL 7b04.1    ----------------------------------------------------PTKVLWFTNVNLINNAKHVYQMLKNVNP  target    KGTKVVVIACEYSSSSIKADDAIVVRPGTTPALALGLCNVIMREKIYDGDYVRRFSDLPLLVRADNLKLLRAEEVFGTPQ 7b04.1    NIEQIMSTDIEITGSIEYADFAFPANSW----------------------------------------------------  target    AALKNQTR 7b04.1    -------- ``` | | | | | | | | | | | | | | | | | | | | | | | | | | | | | | | | | | | | | | | | | | | | | | | | | |
|  | 7b04.2.B | Nitrite oxidoreductase subunit A  *Structure of Nitrite oxidoreductase (Nxr) from the anammox bacterium Kuenenia stuttgartiensis.* | 0.03 |  | 12.50 | 0.14 | 293-348 | X-ray | 2.97 | hetero-1-1-1-mer | 4 x SF4, 1 x F3S, 2 x MD1, 1 x MO, 1 x HEM, 2 x CA | HHblits | 0.27 |
| ``` target    NNVNRREFLQWIGAAGFSTFALSASNAWGLQAIENPLAAYPNREWEKTYRDLWKSDASFTFLCAPNDTHNCILNAHVRDG 7b04.2    --------------------------------------------------------------------------------  target    VITRIGPTMKYGEATDLYGSKVTHRWDPRVCQKGLALTRRFYGDRRVRYPMVRKGFKAWADKGFPREKDGRPPKDYFNRA 7b04.2    --------------------------------------------------------------------------------  target    RDEWLRLTHEEAADLVAAALINIATTYSGDNGQKLLLQQGYEKEIVEATRGAGTQVLKFRGGMPLLGLTRIFGLYRMANS 7b04.2    --------------------------------------------------------------------------------  target    MALLDHKIRGVKPEDALGARGWDNYSWHTDLPPGHPMVTGQQTVDFDLHAVEQARIVVVWGMNWVTTKMPDTHWLTEARL 7b04.2    ----------------------------------------------------PTKVLWFTNVNLINNAKHVYQMLKNVNP  target    KGTKVVVIACEYSSSSIKADDAIVVRPGTTPALALGLCNVIMREKIYDGDYVRRFSDLPLLVRADNLKLLRAEEVFGTPQ 7b04.2    NIEQIMSTDIEITGSIEYADFAFPANSW----------------------------------------------------  target    AALKNQTR 7b04.2    -------- ``` | | | | | | | | | | | | | | | | | | | | | | | | | | | | | | | | | | | | | | | | | | | | | | | | | |
|  | 1q16.1.A | Respiratory nitrate reductase 1 alpha chain  *Crystal structure of Nitrate Reductase A, NarGHI, from Escherichia coli* | 0.02 |  | 18.52 | 0.13 | 294-347 | X-ray | 1.90 | hetero-oligomer | 2 x MD1, 1 x 6MO, 2 x HEM, 4 x SF4, 1 x F3S, 1 x AGA, 1 x 3PH | HHblits | 0.29 |
| ``` target    NNVNRREFLQWIGAAGFSTFALSASNAWGLQAIENPLAAYPNREWEKTYRDLWKSDASFTFLCAPNDTHNCILNAHVRDG 1q16.1    --------------------------------------------------------------------------------  target    VITRIGPTMKYGEATDLYGSKVTHRWDPRVCQKGLALTRRFYGDRRVRYPMVRKGFKAWADKGFPREKDGRPPKDYFNRA 1q16.1    --------------------------------------------------------------------------------  target    RDEWLRLTHEEAADLVAAALINIATTYSGDNGQKLLLQQGYEKEIVEATRGAGTQVLKFRGGMPLLGLTRIFGLYRMANS 1q16.1    --------------------------------------------------------------------------------  target    MALLDHKIRGVKPEDALGARGWDNYSWHTDLPPGHPMVTGQQTVDFDLHAVEQARIVVVWGMNWVTTKMPDTH------- 1q16.1    -----------------------------------------------------PRNLFIWRSNLLGSSGKGHEFMLKYLL  target    -----------------------WLT-EARLKGTKVVVIACEYSSSSIKADDAIVVRPGTTPALALGLCNVIMREKIYDG 1q16.1    GTEHGIQGKDLGQQGGVKPEEVDWQDNGLEGKLDLVVTLDFRLSSTCLYSDIILPTAT----------------------  target    DYVRRFSDLPLLVRADNLKLLRAEEVFGTPQAALKNQTR 1q16.1    --------------------------------------- ``` | | | | | | | | | | | | | | | | | | | | | | | | | | | | | | | | | | | | | | | | | | | | | | | | | |
|  | 2zj4.1.A | Glucosamine--fructose-6-phosphate aminotransferase [isomerizing] 1  *Isomerase domain of human glucose:fructose-6-phosphate amidotransferase* | 0.04 |  | 18.18 | 0.13 | 294-350 | X-ray | 2.20 | homo-dimer | 2 x AGP | HHblits | 0.28 |
| ``` target    NNVNRREFLQWIGAAGFSTFALSASNAWGLQAIENPLAAYPNREWEKTYRDLWKSDASFTFLCAPNDTHNCILNAHVRDG 2zj4.1    --------------------------------------------------------------------------------  target    VITRIGPTMKYGEATDLYGSKVTHRWDPRVCQKGLALTRRFYGDRRVRYPMVRKGFKAWADKGFPREKDGRPPKDYFNRA 2zj4.1    --------------------------------------------------------------------------------  target    RDEWLRLTHEEAADLVAAALINIATTYSGDNGQKLLLQQGYEKEIVEATRGAGTQVLKFRGGMPLLGLTRIFGLYRMANS 2zj4.1    --------------------------------------------------------------------------------  target    MALLDHKIRGVKPEDALGARGWDNYSWHTDLPPGHPMVTGQQTVDFDLHAVEQARIVVVWGMNWVTTKMPDTHWLTEARL 2zj4.1    -----------------------------------------------------DDVCFFLSQSGE-T-ADTLMGLRYCKE  target    KGTKVVVIA-CEYSSSSIKADDAIVVRPGTTPALALGLCNVIMREKIYDGDYVRRFSDLPLLVRADNLKLLRAEEVFGTP 2zj4.1    RGALTVGITNTVGSSISRETDCGVHINAGPE-------------------------------------------------  target    QAALKNQTR 2zj4.1    --------- ``` | | | | | | | | | | | | | | | | | | | | | | | | | | | | | | | | | | | | | | | | | | | | | | | | | |
|  | 2i2w.1.A | Phosphoheptose isomerase  *Crystal Structure of Escherichia Coli Phosphoheptose Isomerase* | 0.03 |  | 18.18 | 0.13 | 292-348 | X-ray | 1.95 | homo-dimer |  | HHblits | 0.28 |
| ``` target    NNVNRREFLQWIGAAGFSTFALSASNAWGLQAIENPLAAYPNREWEKTYRDLWKSDASFTFLCAPNDTHNCILNAHVRDG 2i2w.1    --------------------------------------------------------------------------------  target    VITRIGPTMKYGEATDLYGSKVTHRWDPRVCQKGLALTRRFYGDRRVRYPMVRKGFKAWADKGFPREKDGRPPKDYFNRA 2i2w.1    --------------------------------------------------------------------------------  target    RDEWLRLTHEEAADLVAAALINIATTYSGDNGQKLLLQQGYEKEIVEATRGAGTQVLKFRGGMPLLGLTRIFGLYRMANS 2i2w.1    --------------------------------------------------------------------------------  target    MALLDHKIRGVKPEDALGARGWDNYSWHTDLPPGHPMVTGQQTVDFDLHAVEQARIVVVWGMNWVTTKMPDTHWLTEARL 2i2w.1    ---------------------------------------------------REGDVLLGISTSGNSA--NVIKAIAAARE  target    KGTKVVVIACE-YSSSSIKADDAIVVRPGTTPALALGLCNVIMREKIYDGDYVRRFSDLPLLVRADNLKLLRAEEVFGTP 2i2w.1    KGMKVITLTGKDGGKMAGTADIEIRVPHF---------------------------------------------------  target    QAALKNQTR 2i2w.1    --------- ``` | | | | | | | | | | | | | | | | | | | | | | | | | | | | | | | | | | | | | | | | | | | | | | | | | |
|  | 2i2w.2.B | Phosphoheptose isomerase  *Crystal Structure of Escherichia Coli Phosphoheptose Isomerase* | 0.04 |  | 18.18 | 0.13 | 292-348 | X-ray | 1.95 | homo-dimer |  | HHblits | 0.28 |
| ``` target    NNVNRREFLQWIGAAGFSTFALSASNAWGLQAIENPLAAYPNREWEKTYRDLWKSDASFTFLCAPNDTHNCILNAHVRDG 2i2w.2    --------------------------------------------------------------------------------  target    VITRIGPTMKYGEATDLYGSKVTHRWDPRVCQKGLALTRRFYGDRRVRYPMVRKGFKAWADKGFPREKDGRPPKDYFNRA 2i2w.2    --------------------------------------------------------------------------------  target    RDEWLRLTHEEAADLVAAALINIATTYSGDNGQKLLLQQGYEKEIVEATRGAGTQVLKFRGGMPLLGLTRIFGLYRMANS 2i2w.2    --------------------------------------------------------------------------------  target    MALLDHKIRGVKPEDALGARGWDNYSWHTDLPPGHPMVTGQQTVDFDLHAVEQARIVVVWGMNWVTTKMPDTHWLTEARL 2i2w.2    ---------------------------------------------------REGDVLLGISTSGNSA--NVIKAIAAARE  target    KGTKVVVIACE-YSSSSIKADDAIVVRPGTTPALALGLCNVIMREKIYDGDYVRRFSDLPLLVRADNLKLLRAEEVFGTP 2i2w.2    KGMKVITLTGKDGGKMAGTADIEIRVPHF---------------------------------------------------  target    QAALKNQTR 2i2w.2    --------- ``` | | | | | | | | | | | | | | | | | | | | | | | | | | | | | | | | | | | | | | | | | | | | | | | | | |
|  | 1eu1.1.A | DIMETHYL SULFOXIDE REDUCTASE  *THE CRYSTAL STRUCTURE OF RHODOBACTER SPHAEROIDES DIMETHYLSULFOXIDE REDUCTASE REVEALS TWO DISTINCT MOLYBDENUM COORDINATION ENVIRONMENTS.* | 0.04 |  | 14.55 | 0.13 | 293-348 | X-ray | 1.30 | monomer | 3 x GLC, 1 x CD, 2 x MGD, 1 x 6MO, 2 x O | HHblits | 0.28 |
| ``` target    NNVNRREFLQWIGAAGFSTFALSASNAWGLQAIENPLAAYPNREWEKTYRDLWKSDASFTFLCAPNDTHNCILNAHVRDG 1eu1.1    --------------------------------------------------------------------------------  target    VITRIGPTMKYGEATDLYGSKVTHRWDPRVCQKGLALTRRFYGDRRVRYPMVRKGFKAWADKGFPREKDGRPPKDYFNRA 1eu1.1    --------------------------------------------------------------------------------  target    RDEWLRLTHEEAADLVAAALINIATTYSGDNGQKLLLQQGYEKEIVEATRGAGTQVLKFRGGMPLLGLTRIFGLYRMANS 1eu1.1    --------------------------------------------------------------------------------  target    MALLDHKIRGVKPEDALGARGWDNYSWHTDLPPGHPMVTGQQTVDFDLHAVEQARIVVVWGMNWVTTKMPDTHWLTEARL 1eu1.1    ----------------------------------------------------DVKLAYWAGGNPFAHHQ-DRNRMLKAWE  target    KGTKVVVIACEYSSSSIKADDAIVVRPGTTPALALGLCNVIMREKIYDGDYVRRFSDLPLLVRADNLKLLRAEEVFGTPQ 1eu1.1    KLETFIVQDFQWTATARHADIVLPATTS----------------------------------------------------  target    AALKNQTR 1eu1.1    -------- ``` | | | | | | | | | | | | | | | | | | | | | | | | | | | | | | | | | | | | | | | | | | | | | | | | | |
|  | 5lu5.1.A | Phosphoheptose isomerase  *A quantum half-site enzyme* | 0.03 |  | 12.50 | 0.14 | 292-349 | X-ray | 1.55 | homo-tetramer | 4 x M7P | HHblits | 0.26 |
| ``` target    NNVNRREFLQWIGAAGFSTFALSASNAWGLQAIENPLAAYPNREWEKTYRDLWKSDASFTFLCAPNDTHNCILNAHVRDG 5lu5.1    --------------------------------------------------------------------------------  target    VITRIGPTMKYGEATDLYGSKVTHRWDPRVCQKGLALTRRFYGDRRVRYPMVRKGFKAWADKGFPREKDGRPPKDYFNRA 5lu5.1    --------------------------------------------------------------------------------  target    RDEWLRLTHEEAADLVAAALINIATTYSGDNGQKLLLQQGYEKEIVEATRGAGTQVLKFRGGMPLLGLTRIFGLYRMANS 5lu5.1    --------------------------------------------------------------------------------  target    MALLDHKIRGVKPEDALGARGWDNYSWHTDLPPGHPMVTGQQTVDFDLHAVEQARIVVVWGMNWVTTKMPDTHWLTEARL 5lu5.1    ---------------------------------------------------NEGDVLIGYSTSGKS--PNILAAFREAKA  target    KGTKVVVIAC-EYSSSSIKADDAIVVRPGTTPALALGLCNVIMREKIYDGDYVRRFSDLPLLVRADNLKLLRAEEVFGTP 5lu5.1    KGMTCVGFTGNRGGEMRELCDLLLEVPSAD--------------------------------------------------  target    QAALKNQTR 5lu5.1    --------- ``` | | | | | | | | | | | | | | | | | | | | | | | | | | | | | | | | | | | | | | | | | | | | | | | | | |
|  | 1s5p.1.A | NAD-dependent deacetylase  *Structure and substrate binding properties of cobB, a Sir2 homolog protein deacetylase from Eschericia coli.* | 0.03 |  | 22.64 | 0.13 | 290-344 | X-ray | 1.96 | monomer | 1 x ZN, 1 x LYS-GLY-GLY-ALA-ALY-ARG-HIS-ARG | HHblits | 0.30 |
| ``` target    NNVNRREFLQWIGAAGFSTFALSASNAWGLQAIENPLAAYPNREWEKTYRDLWKSDASFTFLCAPNDTHNCILNAHVRDG 1s5p.1    --------------------------------------------------------------------------------  target    VITRIGPTMKYGEATDLYGSKVTHRWDPRVCQKGLALTRRFYGDRRVRYPMVRKGFKAWADKGFPREKDGRPPKDYFNRA 1s5p.1    --------------------------------------------------------------------------------  target    RDEWLRLTHEEAADLVAAALINIATTYSGDNGQKLLLQQGYEKEIVEATRGAGTQVLKFRGGMPLLGLTRIFGLYRMANS 1s5p.1    --------------------------------------------------------------------------------  target    MALLDHKIRGVKPEDALGARGWDNYSWHTDLPPGHPMVTGQQTVDFDLHAVEQARIVVVWGMNWVTTKMPDTHWLTEARL 1s5p.1    -------------------------------------------------ALSMADIFIAIGTSGHV--YPAAGFVHEAKL  target    KGTKVVVIACEYSSSSIKADDAIVVRPGTTPALALGLCNVIMREKIYDGDYVRRFSDLPLLVRADNLKLLRAEEVFGTPQ 1s5p.1    HGAHTVELNLEPSQVGNEFAEKYY--------------------------------------------------------  target    AALKNQTR 1s5p.1    -------- ``` | | | | | | | | | | | | | | | | | | | | | | | | | | | | | | | | | | | | | | | | | | | | | | | | | |
|  | 4s12.1.A | N-acetylmuramic acid 6-phosphate etherase  *1.55 Angstrom Crystal Structure of N-acetylmuramic acid 6-phosphate Etherase from Yersinia enterocolitica.* | 0.03 |  | 25.45 | 0.13 | 293-349 | X-ray | 1.55 | homo-dimer |  | HHblits | 0.28 |
| ``` target    NNVNRREFLQWIGAAGFSTFALSASNAWGLQAIENPLAAYPNREWEKTYRDLWKSDASFTFLCAPNDTHNCILNAHVRDG 4s12.1    --------------------------------------------------------------------------------  target    VITRIGPTMKYGEATDLYGSKVTHRWDPRVCQKGLALTRRFYGDRRVRYPMVRKGFKAWADKGFPREKDGRPPKDYFNRA 4s12.1    --------------------------------------------------------------------------------  target    RDEWLRLTHEEAADLVAAALINIATTYSGDNGQKLLLQQGYEKEIVEATRGAGTQVLKFRGGMPLLGLTRIFGLYRMANS 4s12.1    --------------------------------------------------------------------------------  target    MALLDHKIRGVKPEDALGARGWDNYSWHTDLPPGHPMVTGQQTVDFDLHAVEQARIVVVWGMNWVTTKMPDTHWLTEARL 4s12.1    ----------------------------------------------------ATDMVVGLAASGRT--PYVIGALRFARQ  target    KGTKVVVIAC-EYSSSSIKADDAIVVRPGTTPALALGLCNVIMREKIYDGDYVRRFSDLPLLVRADNLKLLRAEEVFGTP 4s12.1    LGCPTAAISCNPDSPIAQEALVAISPVVGP--------------------------------------------------  target    QAALKNQTR 4s12.1    --------- ``` | | | | | | | | | | | | | | | | | | | | | | | | | | | | | | | | | | | | | | | | | | | | | | | | | |
|  | 4s12.2.A | N-acetylmuramic acid 6-phosphate etherase  *1.55 Angstrom Crystal Structure of N-acetylmuramic acid 6-phosphate Etherase from Yersinia enterocolitica.* | 0.03 |  | 25.45 | 0.13 | 293-349 | X-ray | 1.55 | homo-dimer |  | HHblits | 0.28 |
| ``` target    NNVNRREFLQWIGAAGFSTFALSASNAWGLQAIENPLAAYPNREWEKTYRDLWKSDASFTFLCAPNDTHNCILNAHVRDG 4s12.2    --------------------------------------------------------------------------------  target    VITRIGPTMKYGEATDLYGSKVTHRWDPRVCQKGLALTRRFYGDRRVRYPMVRKGFKAWADKGFPREKDGRPPKDYFNRA 4s12.2    --------------------------------------------------------------------------------  target    RDEWLRLTHEEAADLVAAALINIATTYSGDNGQKLLLQQGYEKEIVEATRGAGTQVLKFRGGMPLLGLTRIFGLYRMANS 4s12.2    --------------------------------------------------------------------------------  target    MALLDHKIRGVKPEDALGARGWDNYSWHTDLPPGHPMVTGQQTVDFDLHAVEQARIVVVWGMNWVTTKMPDTHWLTEARL 4s12.2    ----------------------------------------------------ATDMVVGLAASGRT--PYVIGALRFARQ  target    KGTKVVVIAC-EYSSSSIKADDAIVVRPGTTPALALGLCNVIMREKIYDGDYVRRFSDLPLLVRADNLKLLRAEEVFGTP 4s12.2    LGCPTAAISCNPDSPIAQEALVAISPVVGP--------------------------------------------------  target    QAALKNQTR 4s12.2    --------- ``` | | | | | | | | | | | | | | | | | | | | | | | | | | | | | | | | | | | | | | | | | | | | | | | | | |
|  | 4s12.2.B | N-acetylmuramic acid 6-phosphate etherase  *1.55 Angstrom Crystal Structure of N-acetylmuramic acid 6-phosphate Etherase from Yersinia enterocolitica.* | 0.03 |  | 25.45 | 0.13 | 293-349 | X-ray | 1.55 | homo-dimer |  | HHblits | 0.28 |
| ``` target    NNVNRREFLQWIGAAGFSTFALSASNAWGLQAIENPLAAYPNREWEKTYRDLWKSDASFTFLCAPNDTHNCILNAHVRDG 4s12.2    --------------------------------------------------------------------------------  target    VITRIGPTMKYGEATDLYGSKVTHRWDPRVCQKGLALTRRFYGDRRVRYPMVRKGFKAWADKGFPREKDGRPPKDYFNRA 4s12.2    --------------------------------------------------------------------------------  target    RDEWLRLTHEEAADLVAAALINIATTYSGDNGQKLLLQQGYEKEIVEATRGAGTQVLKFRGGMPLLGLTRIFGLYRMANS 4s12.2    --------------------------------------------------------------------------------  target    MALLDHKIRGVKPEDALGARGWDNYSWHTDLPPGHPMVTGQQTVDFDLHAVEQARIVVVWGMNWVTTKMPDTHWLTEARL 4s12.2    ----------------------------------------------------ATDMVVGLAASGRT--PYVIGALRFARQ  target    KGTKVVVIAC-EYSSSSIKADDAIVVRPGTTPALALGLCNVIMREKIYDGDYVRRFSDLPLLVRADNLKLLRAEEVFGTP 4s12.2    LGCPTAAISCNPDSPIAQEALVAISPVVGP--------------------------------------------------  target    QAALKNQTR 4s12.2    --------- ``` | | | | | | | | | | | | | | | | | | | | | | | | | | | | | | | | | | | | | | | | | | | | | | | | | |
|  | 1tmo.1.A | TRIMETHYLAMINE N-OXIDE REDUCTASE  *TRIMETHYLAMINE N-OXIDE REDUCTASE FROM SHEWANELLA MASSILIA* | 0.03 |  | 14.55 | 0.13 | 293-348 | X-ray | 2.50 | monomer | 2 x 2MD, 1 x 2MO | HHblits | 0.28 |
| ``` target    NNVNRREFLQWIGAAGFSTFALSASNAWGLQAIENPLAAYPNREWEKTYRDLWKSDASFTFLCAPNDTHNCILNAHVRDG 1tmo.1    --------------------------------------------------------------------------------  target    VITRIGPTMKYGEATDLYGSKVTHRWDPRVCQKGLALTRRFYGDRRVRYPMVRKGFKAWADKGFPREKDGRPPKDYFNRA 1tmo.1    --------------------------------------------------------------------------------  target    RDEWLRLTHEEAADLVAAALINIATTYSGDNGQKLLLQQGYEKEIVEATRGAGTQVLKFRGGMPLLGLTRIFGLYRMANS 1tmo.1    --------------------------------------------------------------------------------  target    MALLDHKIRGVKPEDALGARGWDNYSWHTDLPPGHPMVTGQQTVDFDLHAVEQARIVVVWGMNWVTTKMPDTHWLTEARL 1tmo.1    ----------------------------------------------------DIKMMIFSGNNPWNHHQ-DRNRMKQAFH  target    KGTKVVVIACEYSSSSIKADDAIVVRPGTTPALALGLCNVIMREKIYDGDYVRRFSDLPLLVRADNLKLLRAEEVFGTPQ 1tmo.1    KLECVVTVDVNWTATCRFSDIVLPACTT----------------------------------------------------  target    AALKNQTR 1tmo.1    -------- ``` | | | | | | | | | | | | | | | | | | | | | | | | | | | | | | | | | | | | | | | | | | | | | | | | | |
|  | 1aa6.1.A | FORMATE DEHYDROGENASE H  *REDUCED FORM OF FORMATE DEHYDROGENASE H FROM E. COLI* | 0.04 |  | 14.29 | 0.14 | 292-348 | X-ray | 2.30 | monomer | 1 x SF4, 2 x MGD, 1 x 4MO | HHblits | 0.26 |
| ``` target    NNVNRREFLQWIGAAGFSTFALSASNAWGLQAIENPLAAYPNREWEKTYRDLWKSDASFTFLCAPNDTHNCILNAHVRDG 1aa6.1    --------------------------------------------------------------------------------  target    VITRIGPTMKYGEATDLYGSKVTHRWDPRVCQKGLALTRRFYGDRRVRYPMVRKGFKAWADKGFPREKDGRPPKDYFNRA 1aa6.1    --------------------------------------------------------------------------------  target    RDEWLRLTHEEAADLVAAALINIATTYSGDNGQKLLLQQGYEKEIVEATRGAGTQVLKFRGGMPLLGLTRIFGLYRMANS 1aa6.1    --------------------------------------------------------------------------------  target    MALLDHKIRGVKPEDALGARGWDNYSWHTDLPPGHPMVTGQQTVDFDLHAVEQARIVVVWGMNWVTTKMPDTHWLTEARL 1aa6.1    ---------------------------------------------------GEVRAAYIMGEDPLQTDAE-LSAVRKAFE  target    KGTKVVVIACEYSSSSIKADDAIVVRPGTTPALALGLCNVIMREKIYDGDYVRRFSDLPLLVRADNLKLLRAEEVFGTPQ 1aa6.1    DLELVIVQDIFMTKTASAADVILPSTSW----------------------------------------------------  target    AALKNQTR 1aa6.1    -------- ``` | | | | | | | | | | | | | | | | | | | | | | | | | | | | | | | | | | | | | | | | | | | | | | | | | |
|  | 1fdo.1.A | FORMATE DEHYDROGENASE H  *OXIDIZED FORM OF FORMATE DEHYDROGENASE H FROM E. COLI* | 0.04 |  | 14.29 | 0.14 | 292-348 | X-ray | 2.80 | monomer | 1 x SF4, 2 x MGD, 1 x 6MO | HHblits | 0.26 |
| ``` target    NNVNRREFLQWIGAAGFSTFALSASNAWGLQAIENPLAAYPNREWEKTYRDLWKSDASFTFLCAPNDTHNCILNAHVRDG 1fdo.1    --------------------------------------------------------------------------------  target    VITRIGPTMKYGEATDLYGSKVTHRWDPRVCQKGLALTRRFYGDRRVRYPMVRKGFKAWADKGFPREKDGRPPKDYFNRA 1fdo.1    --------------------------------------------------------------------------------  target    RDEWLRLTHEEAADLVAAALINIATTYSGDNGQKLLLQQGYEKEIVEATRGAGTQVLKFRGGMPLLGLTRIFGLYRMANS 1fdo.1    --------------------------------------------------------------------------------  target    MALLDHKIRGVKPEDALGARGWDNYSWHTDLPPGHPMVTGQQTVDFDLHAVEQARIVVVWGMNWVTTKMPDTHWLTEARL 1fdo.1    ---------------------------------------------------GEVRAAYIMGEDPLQTDAE-LSAVRKAFE  target    KGTKVVVIACEYSSSSIKADDAIVVRPGTTPALALGLCNVIMREKIYDGDYVRRFSDLPLLVRADNLKLLRAEEVFGTPQ 1fdo.1    DLELVIVQDIFMTKTASAADVILPSTSW----------------------------------------------------  target    AALKNQTR 1fdo.1    -------- ``` | | | | | | | | | | | | | | | | | | | | | | | | | | | | | | | | | | | | | | | | | | | | | | | | | |
|  | 2iv2.1.A | Formate dehydrogenase H  *Reinterpretation of reduced form of formate dehydrogenase H from E. coli* | 0.04 |  | 14.29 | 0.14 | 292-348 | X-ray | 2.27 | monomer | 1 x SF4, 1 x 2MD, 1 x MGD | HHblits | 0.26 |
| ``` target    NNVNRREFLQWIGAAGFSTFALSASNAWGLQAIENPLAAYPNREWEKTYRDLWKSDASFTFLCAPNDTHNCILNAHVRDG 2iv2.1    --------------------------------------------------------------------------------  target    VITRIGPTMKYGEATDLYGSKVTHRWDPRVCQKGLALTRRFYGDRRVRYPMVRKGFKAWADKGFPREKDGRPPKDYFNRA 2iv2.1    --------------------------------------------------------------------------------  target    RDEWLRLTHEEAADLVAAALINIATTYSGDNGQKLLLQQGYEKEIVEATRGAGTQVLKFRGGMPLLGLTRIFGLYRMANS 2iv2.1    --------------------------------------------------------------------------------  target    MALLDHKIRGVKPEDALGARGWDNYSWHTDLPPGHPMVTGQQTVDFDLHAVEQARIVVVWGMNWVTTKMPDTHWLTEARL 2iv2.1    ---------------------------------------------------GEVRAAYIMGEDPLQTDAE-LSAVRKAFE  target    KGTKVVVIACEYSSSSIKADDAIVVRPGTTPALALGLCNVIMREKIYDGDYVRRFSDLPLLVRADNLKLLRAEEVFGTPQ 2iv2.1    DLELVIVQDIFMTKTASAADVILPSTSW----------------------------------------------------  target    AALKNQTR 2iv2.1    -------- ``` | | | | | | | | | | | | | | | | | | | | | | | | | | | | | | | | | | | | | | | | | | | | | | | | | |
|  | 7z0t.1.G | Formate dehydrogenase H  *Structure of the Escherichia coli formate hydrogenlyase complex (aerobic preparation, composite structure)* | 0.03 |  | 14.29 | 0.14 | 292-348 | EM | 0.00 | hetero-1-1-1-1-1-1-… | 1 x NI, 1 x FCO, 8 x SF4, 1 x FE, 2 x MGD, 1 x 6MO | HHblits | 0.26 |
| ``` target    NNVNRREFLQWIGAAGFSTFALSASNAWGLQAIENPLAAYPNREWEKTYRDLWKSDASFTFLCAPNDTHNCILNAHVRDG 7z0t.1    --------------------------------------------------------------------------------  target    VITRIGPTMKYGEATDLYGSKVTHRWDPRVCQKGLALTRRFYGDRRVRYPMVRKGFKAWADKGFPREKDGRPPKDYFNRA 7z0t.1    --------------------------------------------------------------------------------  target    RDEWLRLTHEEAADLVAAALINIATTYSGDNGQKLLLQQGYEKEIVEATRGAGTQVLKFRGGMPLLGLTRIFGLYRMANS 7z0t.1    --------------------------------------------------------------------------------  target    MALLDHKIRGVKPEDALGARGWDNYSWHTDLPPGHPMVTGQQTVDFDLHAVEQARIVVVWGMNWVTTKMPDTHWLTEARL 7z0t.1    ---------------------------------------------------GEVRAAYIMGEDPLQTDAE-LSAVRKAFE  target    KGTKVVVIACEYSSSSIKADDAIVVRPGTTPALALGLCNVIMREKIYDGDYVRRFSDLPLLVRADNLKLLRAEEVFGTPQ 7z0t.1    DLELVIVQDIFMTKTASAADVILPSTSW----------------------------------------------------  target    AALKNQTR 7z0t.1    -------- ``` | | | | | | | | | | | | | | | | | | | | | | | | | | | | | | | | | | | | | | | | | | | | | | | | | |
|  | 3sho.1.A | Transcriptional regulator, RpiR family  *Crystal structure of RpiR transcription factor from Sphaerobacter thermophilus (sugar isomerase domain)* | 0.03 |  | 12.50 | 0.14 | 293-350 | X-ray | 1.80 | homo-tetramer |  | HHblits | 0.26 |
| ``` target    NNVNRREFLQWIGAAGFSTFALSASNAWGLQAIENPLAAYPNREWEKTYRDLWKSDASFTFLCAPNDTHNCILNAHVRDG 3sho.1    --------------------------------------------------------------------------------  target    VITRIGPTMKYGEATDLYGSKVTHRWDPRVCQKGLALTRRFYGDRRVRYPMVRKGFKAWADKGFPREKDGRPPKDYFNRA 3sho.1    --------------------------------------------------------------------------------  target    RDEWLRLTHEEAADLVAAALINIATTYSGDNGQKLLLQQGYEKEIVEATRGAGTQVLKFRGGMPLLGLTRIFGLYRMANS 3sho.1    --------------------------------------------------------------------------------  target    MALLDHKIRGVKPEDALGARGWDNYSWHTDLPPGHPMVTGQQTVDFDLHAVEQARIVVVWGMNWVTTKMPDTHWLTEARL 3sho.1    ----------------------------------------------------PTDLMIGVSVWRYLR--DTVAALAGAAE  target    KGTKVVVIACE-YSSSSIKADDAIVVRPGTTPALALGLCNVIMREKIYDGDYVRRFSDLPLLVRADNLKLLRAEEVFGTP 3sho.1    RGVPTMALTDSSVSPPARIADHVLVAATRGV-------------------------------------------------  target    QAALKNQTR 3sho.1    --------- ``` | | | | | | | | | | | | | | | | | | | | | | | | | | | | | | | | | | | | | | | | | | | | | | | | | |
|  | 3sho.1.C | Transcriptional regulator, RpiR family  *Crystal structure of RpiR transcription factor from Sphaerobacter thermophilus (sugar isomerase domain)* | 0.03 |  | 12.50 | 0.14 | 293-350 | X-ray | 1.80 | homo-tetramer |  | HHblits | 0.26 |
| ``` target    NNVNRREFLQWIGAAGFSTFALSASNAWGLQAIENPLAAYPNREWEKTYRDLWKSDASFTFLCAPNDTHNCILNAHVRDG 3sho.1    --------------------------------------------------------------------------------  target    VITRIGPTMKYGEATDLYGSKVTHRWDPRVCQKGLALTRRFYGDRRVRYPMVRKGFKAWADKGFPREKDGRPPKDYFNRA 3sho.1    --------------------------------------------------------------------------------  target    RDEWLRLTHEEAADLVAAALINIATTYSGDNGQKLLLQQGYEKEIVEATRGAGTQVLKFRGGMPLLGLTRIFGLYRMANS 3sho.1    --------------------------------------------------------------------------------  target    MALLDHKIRGVKPEDALGARGWDNYSWHTDLPPGHPMVTGQQTVDFDLHAVEQARIVVVWGMNWVTTKMPDTHWLTEARL 3sho.1    ----------------------------------------------------PTDLMIGVSVWRYLR--DTVAALAGAAE  target    KGTKVVVIACE-YSSSSIKADDAIVVRPGTTPALALGLCNVIMREKIYDGDYVRRFSDLPLLVRADNLKLLRAEEVFGTP 3sho.1    RGVPTMALTDSSVSPPARIADHVLVAATRGV-------------------------------------------------  target    QAALKNQTR 3sho.1    --------- ``` | | | | | | | | | | | | | | | | | | | | | | | | | | | | | | | | | | | | | | | | | | | | | | | | | |
|  | 1dms.1.A | DMSO REDUCTASE  *STRUCTURE OF DMSO REDUCTASE* | 0.04 |  | 14.55 | 0.13 | 293-348 | X-ray | 1.88 | monomer | 2 x PGD, 1 x 2MO | HHblits | 0.27 |
| ``` target    NNVNRREFLQWIGAAGFSTFALSASNAWGLQAIENPLAAYPNREWEKTYRDLWKSDASFTFLCAPNDTHNCILNAHVRDG 1dms.1    --------------------------------------------------------------------------------  target    VITRIGPTMKYGEATDLYGSKVTHRWDPRVCQKGLALTRRFYGDRRVRYPMVRKGFKAWADKGFPREKDGRPPKDYFNRA 1dms.1    --------------------------------------------------------------------------------  target    RDEWLRLTHEEAADLVAAALINIATTYSGDNGQKLLLQQGYEKEIVEATRGAGTQVLKFRGGMPLLGLTRIFGLYRMANS 1dms.1    --------------------------------------------------------------------------------  target    MALLDHKIRGVKPEDALGARGWDNYSWHTDLPPGHPMVTGQQTVDFDLHAVEQARIVVVWGMNWVTTKMPDTHWLTEARL 1dms.1    ----------------------------------------------------DVKMAYWVGGNPFVHHQ-DRNRMVKAWE  target    KGTKVVVIACEYSSSSIKADDAIVVRPGTTPALALGLCNVIMREKIYDGDYVRRFSDLPLLVRADNLKLLRAEEVFGTPQ 1dms.1    KLETFIVHDFQWTPTARHADIVLPATTS----------------------------------------------------  target    AALKNQTR 1dms.1    -------- ``` | | | | | | | | | | | | | | | | | | | | | | | | | | | | | | | | | | | | | | | | | | | | | | | | | |
|  | 2v4m.1.A | GLUCOSAMINE--FRUCTOSE-6-PHOSPHATE AMINOTRANSFERASE [ISOMERIZING] 1  *The isomerase domain of human glutamine-fructose-6-phosphate transaminase 1 (GFPT1) in complex with fructose 6-phosphate* | 0.03 |  | 16.36 | 0.13 | 294-350 | X-ray | 2.29 | homo-dimer | 2 x F6R | HHblits | 0.27 |
| ``` target    NNVNRREFLQWIGAAGFSTFALSASNAWGLQAIENPLAAYPNREWEKTYRDLWKSDASFTFLCAPNDTHNCILNAHVRDG 2v4m.1    --------------------------------------------------------------------------------  target    VITRIGPTMKYGEATDLYGSKVTHRWDPRVCQKGLALTRRFYGDRRVRYPMVRKGFKAWADKGFPREKDGRPPKDYFNRA 2v4m.1    --------------------------------------------------------------------------------  target    RDEWLRLTHEEAADLVAAALINIATTYSGDNGQKLLLQQGYEKEIVEATRGAGTQVLKFRGGMPLLGLTRIFGLYRMANS 2v4m.1    --------------------------------------------------------------------------------  target    MALLDHKIRGVKPEDALGARGWDNYSWHTDLPPGHPMVTGQQTVDFDLHAVEQARIVVVWGMNWVTTKMPDTHWLTEARL 2v4m.1    -----------------------------------------------------DDVCFFLSQSG-E-TADTLMGLRYCKE  target    KGTKVVVIA-CEYSSSSIKADDAIVVRPGTTPALALGLCNVIMREKIYDGDYVRRFSDLPLLVRADNLKLLRAEEVFGTP 2v4m.1    RGALTVGITNTVGSSISRETDCGVHINAGPE-------------------------------------------------  target    QAALKNQTR 2v4m.1    --------- ``` | | | | | | | | | | | | | | | | | | | | | | | | | | | | | | | | | | | | | | | | | | | | | | | | | |
|  | 3hba.1.A | Putative phosphosugar isomerase  *Crystal structure of a putative phosphosugar isomerase (sden\_2705) from shewanella denitrificans os217 at 2.00 A resolution* | 0.03 |  | 16.36 | 0.13 | 294-350 | X-ray | 2.00 | homo-dimer |  | HHblits | 0.27 |
| ``` target    NNVNRREFLQWIGAAGFSTFALSASNAWGLQAIENPLAAYPNREWEKTYRDLWKSDASFTFLCAPNDTHNCILNAHVRDG 3hba.1    --------------------------------------------------------------------------------  target    VITRIGPTMKYGEATDLYGSKVTHRWDPRVCQKGLALTRRFYGDRRVRYPMVRKGFKAWADKGFPREKDGRPPKDYFNRA 3hba.1    --------------------------------------------------------------------------------  target    RDEWLRLTHEEAADLVAAALINIATTYSGDNGQKLLLQQGYEKEIVEATRGAGTQVLKFRGGMPLLGLTRIFGLYRMANS 3hba.1    --------------------------------------------------------------------------------  target    MALLDHKIRGVKPEDALGARGWDNYSWHTDLPPGHPMVTGQQTVDFDLHAVEQARIVVVWGMNWVTTKMPDTHWLTEARL 3hba.1    -----------------------------------------------------GGLVIVISQSGRS--PDILAQARMAKN  target    KGTKVVVIACE-YSSSSIKADDAIVVRPGTTPALALGLCNVIMREKIYDGDYVRRFSDLPLLVRADNLKLLRAEEVFGTP 3hba.1    AGAFCVALVNDETAPIKDIVDVVIPLRAGEE-------------------------------------------------  target    QAALKNQTR 3hba.1    --------- ``` | | | | | | | | | | | | | | | | | | | | | | | | | | | | | | | | | | | | | | | | | | | | | | | | | |
|  | 3hba.1.B | Putative phosphosugar isomerase  *Crystal structure of a putative phosphosugar isomerase (sden\_2705) from shewanella denitrificans os217 at 2.00 A resolution* | 0.03 |  | 16.36 | 0.13 | 294-350 | X-ray | 2.00 | homo-dimer |  | HHblits | 0.27 |
| ``` target    NNVNRREFLQWIGAAGFSTFALSASNAWGLQAIENPLAAYPNREWEKTYRDLWKSDASFTFLCAPNDTHNCILNAHVRDG 3hba.1    --------------------------------------------------------------------------------  target    VITRIGPTMKYGEATDLYGSKVTHRWDPRVCQKGLALTRRFYGDRRVRYPMVRKGFKAWADKGFPREKDGRPPKDYFNRA 3hba.1    --------------------------------------------------------------------------------  target    RDEWLRLTHEEAADLVAAALINIATTYSGDNGQKLLLQQGYEKEIVEATRGAGTQVLKFRGGMPLLGLTRIFGLYRMANS 3hba.1    --------------------------------------------------------------------------------  target    MALLDHKIRGVKPEDALGARGWDNYSWHTDLPPGHPMVTGQQTVDFDLHAVEQARIVVVWGMNWVTTKMPDTHWLTEARL 3hba.1    -----------------------------------------------------GGLVIVISQSGRS--PDILAQARMAKN  target    KGTKVVVIACE-YSSSSIKADDAIVVRPGTTPALALGLCNVIMREKIYDGDYVRRFSDLPLLVRADNLKLLRAEEVFGTP 3hba.1    AGAFCVALVNDETAPIKDIVDVVIPLRAGEE-------------------------------------------------  target    QAALKNQTR 3hba.1    --------- ``` | | | | | | | | | | | | | | | | | | | | | | | | | | | | | | | | | | | | | | | | | | | | | | | | | |
|  | 1s7g.1.D | NAD-dependent deacetylase 2  *Structural Basis for the Mechanism and Regulation of Sir2 Enzymes* | 0.03 |  | 18.87 | 0.13 | 290-344 | X-ray | 2.30 | homo-pentamer | 9 x ZN, 3 x NAD, 1 x APR, 1 x 2PE | HHblits | 0.30 |
| ``` target    NNVNRREFLQWIGAAGFSTFALSASNAWGLQAIENPLAAYPNREWEKTYRDLWKSDASFTFLCAPNDTHNCILNAHVRDG 1s7g.1    --------------------------------------------------------------------------------  target    VITRIGPTMKYGEATDLYGSKVTHRWDPRVCQKGLALTRRFYGDRRVRYPMVRKGFKAWADKGFPREKDGRPPKDYFNRA 1s7g.1    --------------------------------------------------------------------------------  target    RDEWLRLTHEEAADLVAAALINIATTYSGDNGQKLLLQQGYEKEIVEATRGAGTQVLKFRGGMPLLGLTRIFGLYRMANS 1s7g.1    --------------------------------------------------------------------------------  target    MALLDHKIRGVKPEDALGARGWDNYSWHTDLPPGHPMVTGQQTVDFDLHAVEQARIVVVWGMNWVTTKMPDTHWLTEARL 1s7g.1    -------------------------------------------------EAKHCDAFMVVGSSLVVY--PAAELPYIAKK  target    KGTKVVVIACEYSSSSIKADDAIVVRPGTTPALALGLCNVIMREKIYDGDYVRRFSDLPLLVRADNLKLLRAEEVFGTPQ 1s7g.1    AGAKMIIVNAEPTMADPIFDVKII--------------------------------------------------------  target    AALKNQTR 1s7g.1    -------- ``` | | | | | | | | | | | | | | | | | | | | | | | | | | | | | | | | | | | | | | | | | | | | | | | | | |
|  | 1ma3.1.A | Transcriptional regulatory protein, Sir2 family  *Structure of a Sir2 enzyme bound to an acetylated p53 peptide* | 0.03 |  | 18.87 | 0.13 | 290-344 | X-ray | 2.00 | hetero-oligomer | 1 x ZN, 1 x MES | HHblits | 0.30 |
| ``` target    NNVNRREFLQWIGAAGFSTFALSASNAWGLQAIENPLAAYPNREWEKTYRDLWKSDASFTFLCAPNDTHNCILNAHVRDG 1ma3.1    --------------------------------------------------------------------------------  target    VITRIGPTMKYGEATDLYGSKVTHRWDPRVCQKGLALTRRFYGDRRVRYPMVRKGFKAWADKGFPREKDGRPPKDYFNRA 1ma3.1    --------------------------------------------------------------------------------  target    RDEWLRLTHEEAADLVAAALINIATTYSGDNGQKLLLQQGYEKEIVEATRGAGTQVLKFRGGMPLLGLTRIFGLYRMANS 1ma3.1    --------------------------------------------------------------------------------  target    MALLDHKIRGVKPEDALGARGWDNYSWHTDLPPGHPMVTGQQTVDFDLHAVEQARIVVVWGMNWVTTKMPDTHWLTEARL 1ma3.1    -------------------------------------------------EAKHCDAFMVVGSSLVVY--PAAELPYIAKK  target    KGTKVVVIACEYSSSSIKADDAIVVRPGTTPALALGLCNVIMREKIYDGDYVRRFSDLPLLVRADNLKLLRAEEVFGTPQ 1ma3.1    AGAKMIIVNAEPTMADPIFDVKII--------------------------------------------------------  target    AALKNQTR 1ma3.1    -------- ``` | | | | | | | | | | | | | | | | | | | | | | | | | | | | | | | | | | | | | | | | | | | | | | | | | |
|  | 1s7g.1.B | NAD-dependent deacetylase 2  *Structural Basis for the Mechanism and Regulation of Sir2 Enzymes* | 0.03 |  | 18.87 | 0.13 | 290-344 | X-ray | 2.30 | homo-pentamer | 9 x ZN, 3 x NAD, 1 x APR, 1 x 2PE | HHblits | 0.30 |
| ``` target    NNVNRREFLQWIGAAGFSTFALSASNAWGLQAIENPLAAYPNREWEKTYRDLWKSDASFTFLCAPNDTHNCILNAHVRDG 1s7g.1    --------------------------------------------------------------------------------  target    VITRIGPTMKYGEATDLYGSKVTHRWDPRVCQKGLALTRRFYGDRRVRYPMVRKGFKAWADKGFPREKDGRPPKDYFNRA 1s7g.1    --------------------------------------------------------------------------------  target    RDEWLRLTHEEAADLVAAALINIATTYSGDNGQKLLLQQGYEKEIVEATRGAGTQVLKFRGGMPLLGLTRIFGLYRMANS 1s7g.1    --------------------------------------------------------------------------------  target    MALLDHKIRGVKPEDALGARGWDNYSWHTDLPPGHPMVTGQQTVDFDLHAVEQARIVVVWGMNWVTTKMPDTHWLTEARL 1s7g.1    -------------------------------------------------EAKHCDAFMVVGSSLVVY--PAAELPYIAKK  target    KGTKVVVIACEYSSSSIKADDAIVVRPGTTPALALGLCNVIMREKIYDGDYVRRFSDLPLLVRADNLKLLRAEEVFGTPQ 1s7g.1    AGAKMIIVNAEPTMADPIFDVKII--------------------------------------------------------  target    AALKNQTR 1s7g.1    -------- ``` | | | | | | | | | | | | | | | | | | | | | | | | | | | | | | | | | | | | | | | | | | | | | | | | | |
|  | 1s7g.1.A | NAD-dependent deacetylase 2  *Structural Basis for the Mechanism and Regulation of Sir2 Enzymes* | 0.03 |  | 18.87 | 0.13 | 290-344 | X-ray | 2.30 | homo-pentamer | 9 x ZN, 3 x NAD, 1 x APR, 1 x 2PE | HHblits | 0.30 |
| ``` target    NNVNRREFLQWIGAAGFSTFALSASNAWGLQAIENPLAAYPNREWEKTYRDLWKSDASFTFLCAPNDTHNCILNAHVRDG 1s7g.1    --------------------------------------------------------------------------------  target    VITRIGPTMKYGEATDLYGSKVTHRWDPRVCQKGLALTRRFYGDRRVRYPMVRKGFKAWADKGFPREKDGRPPKDYFNRA 1s7g.1    --------------------------------------------------------------------------------  target    RDEWLRLTHEEAADLVAAALINIATTYSGDNGQKLLLQQGYEKEIVEATRGAGTQVLKFRGGMPLLGLTRIFGLYRMANS 1s7g.1    --------------------------------------------------------------------------------  target    MALLDHKIRGVKPEDALGARGWDNYSWHTDLPPGHPMVTGQQTVDFDLHAVEQARIVVVWGMNWVTTKMPDTHWLTEARL 1s7g.1    -------------------------------------------------EAKHCDAFMVVGSSLVVY--PAAELPYIAKK  target    KGTKVVVIACEYSSSSIKADDAIVVRPGTTPALALGLCNVIMREKIYDGDYVRRFSDLPLLVRADNLKLLRAEEVFGTPQ 1s7g.1    AGAKMIIVNAEPTMADPIFDVKII--------------------------------------------------------  target    AALKNQTR 1s7g.1    -------- ``` | | | | | | | | | | | | | | | | | | | | | | | | | | | | | | | | | | | | | | | | | | | | | | | | | |
|  | 1s7g.1.C | NAD-dependent deacetylase 2  *Structural Basis for the Mechanism and Regulation of Sir2 Enzymes* | 0.03 |  | 18.87 | 0.13 | 290-344 | X-ray | 2.30 | homo-pentamer | 9 x ZN, 3 x NAD, 1 x APR, 1 x 2PE | HHblits | 0.30 |
| ``` target    NNVNRREFLQWIGAAGFSTFALSASNAWGLQAIENPLAAYPNREWEKTYRDLWKSDASFTFLCAPNDTHNCILNAHVRDG 1s7g.1    --------------------------------------------------------------------------------  target    VITRIGPTMKYGEATDLYGSKVTHRWDPRVCQKGLALTRRFYGDRRVRYPMVRKGFKAWADKGFPREKDGRPPKDYFNRA 1s7g.1    --------------------------------------------------------------------------------  target    RDEWLRLTHEEAADLVAAALINIATTYSGDNGQKLLLQQGYEKEIVEATRGAGTQVLKFRGGMPLLGLTRIFGLYRMANS 1s7g.1    --------------------------------------------------------------------------------  target    MALLDHKIRGVKPEDALGARGWDNYSWHTDLPPGHPMVTGQQTVDFDLHAVEQARIVVVWGMNWVTTKMPDTHWLTEARL 1s7g.1    -------------------------------------------------EAKHCDAFMVVGSSLVVY--PAAELPYIAKK  target    KGTKVVVIACEYSSSSIKADDAIVVRPGTTPALALGLCNVIMREKIYDGDYVRRFSDLPLLVRADNLKLLRAEEVFGTPQ 1s7g.1    AGAKMIIVNAEPTMADPIFDVKII--------------------------------------------------------  target    AALKNQTR 1s7g.1    -------- ``` | | | | | | | | | | | | | | | | | | | | | | | | | | | | | | | | | | | | | | | | | | | | | | | | | |
|  | 1s7g.1.E | NAD-dependent deacetylase 2  *Structural Basis for the Mechanism and Regulation of Sir2 Enzymes* | 0.03 |  | 18.87 | 0.13 | 290-344 | X-ray | 2.30 | homo-pentamer | 9 x ZN, 3 x NAD, 1 x APR, 1 x 2PE | HHblits | 0.30 |
| ``` target    NNVNRREFLQWIGAAGFSTFALSASNAWGLQAIENPLAAYPNREWEKTYRDLWKSDASFTFLCAPNDTHNCILNAHVRDG 1s7g.1    --------------------------------------------------------------------------------  target    VITRIGPTMKYGEATDLYGSKVTHRWDPRVCQKGLALTRRFYGDRRVRYPMVRKGFKAWADKGFPREKDGRPPKDYFNRA 1s7g.1    --------------------------------------------------------------------------------  target    RDEWLRLTHEEAADLVAAALINIATTYSGDNGQKLLLQQGYEKEIVEATRGAGTQVLKFRGGMPLLGLTRIFGLYRMANS 1s7g.1    --------------------------------------------------------------------------------  target    MALLDHKIRGVKPEDALGARGWDNYSWHTDLPPGHPMVTGQQTVDFDLHAVEQARIVVVWGMNWVTTKMPDTHWLTEARL 1s7g.1    -------------------------------------------------EAKHCDAFMVVGSSLVVY--PAAELPYIAKK  target    KGTKVVVIACEYSSSSIKADDAIVVRPGTTPALALGLCNVIMREKIYDGDYVRRFSDLPLLVRADNLKLLRAEEVFGTPQ 1s7g.1    AGAKMIIVNAEPTMADPIFDVKII--------------------------------------------------------  target    AALKNQTR 1s7g.1    -------- ``` | | | | | | | | | | | | | | | | | | | | | | | | | | | | | | | | | | | | | | | | | | | | | | | | | |
|  | 4twj.1.A | NAD-dependent protein deacylase 2  *The structure of Sir2Af2 bound to a myristoylated histone peptide* | 0.03 |  | 18.87 | 0.13 | 290-344 | X-ray | 1.65 | hetero-1-1-mer | 1 x ZN | HHblits | 0.30 |
| ``` target    NNVNRREFLQWIGAAGFSTFALSASNAWGLQAIENPLAAYPNREWEKTYRDLWKSDASFTFLCAPNDTHNCILNAHVRDG 4twj.1    --------------------------------------------------------------------------------  target    VITRIGPTMKYGEATDLYGSKVTHRWDPRVCQKGLALTRRFYGDRRVRYPMVRKGFKAWADKGFPREKDGRPPKDYFNRA 4twj.1    --------------------------------------------------------------------------------  target    RDEWLRLTHEEAADLVAAALINIATTYSGDNGQKLLLQQGYEKEIVEATRGAGTQVLKFRGGMPLLGLTRIFGLYRMANS 4twj.1    --------------------------------------------------------------------------------  target    MALLDHKIRGVKPEDALGARGWDNYSWHTDLPPGHPMVTGQQTVDFDLHAVEQARIVVVWGMNWVTTKMPDTHWLTEARL 4twj.1    -------------------------------------------------EAKHCDAFMVVGSSLVVY--PAAELPYIAKK  target    KGTKVVVIACEYSSSSIKADDAIVVRPGTTPALALGLCNVIMREKIYDGDYVRRFSDLPLLVRADNLKLLRAEEVFGTPQ 4twj.1    AGAKMIIVNAEPTMADPIFDVKII--------------------------------------------------------  target    AALKNQTR 4twj.1    -------- ``` | | | | | | | | | | | | | | | | | | | | | | | | | | | | | | | | | | | | | | | | | | | | | | | | | |
|  | 6cz7.1.A | ArrA  *The arsenate respiratory reductase (Arr) complex from Shewanella sp. ANA-3* | 0.04 |  | 16.36 | 0.13 | 292-347 | X-ray | 1.62 | hetero-1-1-mer | 5 x SF4, 2 x MGD, 1 x MO, 1 x PG5 | HHblits | 0.27 |
| ``` target    NNVNRREFLQWIGAAGFSTFALSASNAWGLQAIENPLAAYPNREWEKTYRDLWKSDASFTFLCAPNDTHNCILNAHVRDG 6cz7.1    --------------------------------------------------------------------------------  target    VITRIGPTMKYGEATDLYGSKVTHRWDPRVCQKGLALTRRFYGDRRVRYPMVRKGFKAWADKGFPREKDGRPPKDYFNRA 6cz7.1    --------------------------------------------------------------------------------  target    RDEWLRLTHEEAADLVAAALINIATTYSGDNGQKLLLQQGYEKEIVEATRGAGTQVLKFRGGMPLLGLTRIFGLYRMANS 6cz7.1    --------------------------------------------------------------------------------  target    MALLDHKIRGVKPEDALGARGWDNYSWHTDLPPGHPMVTGQQTVDFDLHAVEQARIVVVWGMNWVTTKMPDTHWLTEARL 6cz7.1    ---------------------------------------------------YEIKVMLAYFNNFNFSNP-EGQRWDEALS  target    KGTKVVVIACEYSSSSIKADDAIVVRPGTTPALALGLCNVIMREKIYDGDYVRRFSDLPLLVRADNLKLLRAEEVFGTPQ 6cz7.1    KVDFMAHITTNVSEFSWFADVLLPSSH-----------------------------------------------------  target    AALKNQTR 6cz7.1    -------- ``` | | | | | | | | | | | | | | | | | | | | | | | | | | | | | | | | | | | | | | | | | | | | | | | | | |
|  | 4aay.1.A | AROA  *Crystal Structure of the arsenite oxidase protein complex from Rhizobium species strain NT-26* | 0.01 |  | 12.50 | 0.14 | 293-348 | X-ray | 2.70 | hetero-oligomer | 4 x MGD, 2 x O, 2 x 4MO, 2 x F3S, 2 x FES | HHblits | 0.25 |
| ``` target    NNVNRREFLQWIGAAGFSTFALSASNAWGLQAIENPLAAYPNREWEKTYRDLWKSDASFTFLCAPNDTHNCILNAHVRDG 4aay.1    --------------------------------------------------------------------------------  target    VITRIGPTMKYGEATDLYGSKVTHRWDPRVCQKGLALTRRFYGDRRVRYPMVRKGFKAWADKGFPREKDGRPPKDYFNRA 4aay.1    --------------------------------------------------------------------------------  target    RDEWLRLTHEEAADLVAAALINIATTYSGDNGQKLLLQQGYEKEIVEATRGAGTQVLKFRGGMPLLGLTRIFGLYRMANS 4aay.1    --------------------------------------------------------------------------------  target    MALLDHKIRGVKPEDALGARGWDNYSWHTDLPPGHPMVTGQQTVDFDLHAVEQARIVVVWGMNWVTTKM-----PD---- 4aay.1    ----------------------------------------------------EFKRVYKKRTDMVKDAMSAAPYGDREAM  target    THWLTEARLKG-TKVVVIACEYSSSSIKADDAIVVRPGTTPALALGLCNVIMREKIYDGDYVRRFSDLPLLVRADNLKLL 4aay.1    VNAIVDAINQGGLFAVNVDIIPTKIGEACHVILPAATS------------------------------------------  target    RAEEVFGTPQAALKNQTR 4aay.1    ------------------ ``` | | | | | | | | | | | | | | | | | | | | | | | | | | | | | | | | | | | | | | | | | | | | | | | | | |
|  | 3eua.1.A | PUTATIVE FRUCTOSE-AMINOACID-6-PHOSPHATE DEGLYCASE  *CRYSTAL STRUCTURE OF A PUTATIVE PHOSPHOSUGAR ISOMERASE (BSU32610) FROM BACILLUS SUBTILIS AT 1.90 A RESOLUTION* | 0.03 |  | 16.36 | 0.13 | 294-350 | X-ray | 1.90 | homo-dimer |  | HHblits | 0.27 |
| ``` target    NNVNRREFLQWIGAAGFSTFALSASNAWGLQAIENPLAAYPNREWEKTYRDLWKSDASFTFLCAPNDTHNCILNAHVRDG 3eua.1    --------------------------------------------------------------------------------  target    VITRIGPTMKYGEATDLYGSKVTHRWDPRVCQKGLALTRRFYGDRRVRYPMVRKGFKAWADKGFPREKDGRPPKDYFNRA 3eua.1    --------------------------------------------------------------------------------  target    RDEWLRLTHEEAADLVAAALINIATTYSGDNGQKLLLQQGYEKEIVEATRGAGTQVLKFRGGMPLLGLTRIFGLYRMANS 3eua.1    --------------------------------------------------------------------------------  target    MALLDHKIRGVKPEDALGARGWDNYSWHTDLPPGHPMVTGQQTVDFDLHAVEQARIVVVWGMNWVTTKMPDTHWLTEARL 3eua.1    -----------------------------------------------------KSLVILCSHSGNT--PETVKAAAFARG  target    KGTKVVVIAC-EYSSSSIKADDAIVVRPGTTPALALGLCNVIMREKIYDGDYVRRFSDLPLLVRADNLKLLRAEEVFGTP 3eua.1    KGALTIAMTFKPESPLAQEAQYVAQYDWGDE-------------------------------------------------  target    QAALKNQTR 3eua.1    --------- ``` | | | | | | | | | | | | | | | | | | | | | | | | | | | | | | | | | | | | | | | | | | | | | | | | | |
|  | 1e5v.2.A | Dimethyl sulfoxide/trimethylamine N-oxide reductase  *OXIDIZED DMSO REDUCTASE EXPOSED TO HEPES BUFFER* | 0.04 |  | 14.55 | 0.13 | 293-348 | X-ray | 2.40 | monomer | 2 x PGD, 1 x 2MO | HHblits | 0.27 |
| ``` target    NNVNRREFLQWIGAAGFSTFALSASNAWGLQAIENPLAAYPNREWEKTYRDLWKSDASFTFLCAPNDTHNCILNAHVRDG 1e5v.2    --------------------------------------------------------------------------------  target    VITRIGPTMKYGEATDLYGSKVTHRWDPRVCQKGLALTRRFYGDRRVRYPMVRKGFKAWADKGFPREKDGRPPKDYFNRA 1e5v.2    --------------------------------------------------------------------------------  target    RDEWLRLTHEEAADLVAAALINIATTYSGDNGQKLLLQQGYEKEIVEATRGAGTQVLKFRGGMPLLGLTRIFGLYRMANS 1e5v.2    --------------------------------------------------------------------------------  target    MALLDHKIRGVKPEDALGARGWDNYSWHTDLPPGHPMVTGQQTVDFDLHAVEQARIVVVWGMNWVTTKMPDTHWLTEARL 1e5v.2    ----------------------------------------------------DVKMAYWVGGNPFVHHQD-RNRMVKAWE  target    KGTKVVVIACEYSSSSIKADDAIVVRPGTTPALALGLCNVIMREKIYDGDYVRRFSDLPLLVRADNLKLLRAEEVFGTPQ 1e5v.2    KLETFVVHDFQWTPTARHADIVLPATTS----------------------------------------------------  target    AALKNQTR 1e5v.2    -------- ``` | | | | | | | | | | | | | | | | | | | | | | | | | | | | | | | | | | | | | | | | | | | | | | | | | |
|  | 1e18.1.A | DMSO REDUCTASE.  *TUNGSTEN-SUSBSTITUTED DMSO REDUCTASE FROM RHODOBACTER CAPSULATUS* | 0.04 |  | 14.55 | 0.13 | 293-348 | X-ray | 2.00 | monomer | 2 x PGD, 1 x 6WO | HHblits | 0.27 |
| ``` target    NNVNRREFLQWIGAAGFSTFALSASNAWGLQAIENPLAAYPNREWEKTYRDLWKSDASFTFLCAPNDTHNCILNAHVRDG 1e18.1    --------------------------------------------------------------------------------  target    VITRIGPTMKYGEATDLYGSKVTHRWDPRVCQKGLALTRRFYGDRRVRYPMVRKGFKAWADKGFPREKDGRPPKDYFNRA 1e18.1    --------------------------------------------------------------------------------  target    RDEWLRLTHEEAADLVAAALINIATTYSGDNGQKLLLQQGYEKEIVEATRGAGTQVLKFRGGMPLLGLTRIFGLYRMANS 1e18.1    --------------------------------------------------------------------------------  target    MALLDHKIRGVKPEDALGARGWDNYSWHTDLPPGHPMVTGQQTVDFDLHAVEQARIVVVWGMNWVTTKMPDTHWLTEARL 1e18.1    ----------------------------------------------------DVKMAYWVGGNPFVHHQD-RNRMVKAWE  target    KGTKVVVIACEYSSSSIKADDAIVVRPGTTPALALGLCNVIMREKIYDGDYVRRFSDLPLLVRADNLKLLRAEEVFGTPQ 1e18.1    KLETFVVHDFQWTPTARHADIVLPATTS----------------------------------------------------  target    AALKNQTR 1e18.1    -------- ``` | | | | | | | | | | | | | | | | | | | | | | | | | | | | | | | | | | | | | | | | | | | | | | | | | |
|  | 2yva.1.A | DnaA initiator-associating protein diaA  *Crystal structure of Escherichia coli DiaA* | 0.03 |  | 7.02 | 0.14 | 293-351 | X-ray | 1.85 | homo-tetramer |  | HHblits | 0.24 |
| ``` target    NNVNRREFLQWIGAAGFSTFALSASNAWGLQAIENPLAAYPNREWEKTYRDLWKSDASFTFLCAPNDTHNCILNAHVRDG 2yva.1    --------------------------------------------------------------------------------  target    VITRIGPTMKYGEATDLYGSKVTHRWDPRVCQKGLALTRRFYGDRRVRYPMVRKGFKAWADKGFPREKDGRPPKDYFNRA 2yva.1    --------------------------------------------------------------------------------  target    RDEWLRLTHEEAADLVAAALINIATTYSGDNGQKLLLQQGYEKEIVEATRGAGTQVLKFRGGMPLLGLTRIFGLYRMANS 2yva.1    --------------------------------------------------------------------------------  target    MALLDHKIRGVKPEDALGARGWDNYSWHTDLPPGHPMVTGQQTVDFDLHAVEQARIVVVWGMNWVTTKMPDTHWLTEARL 2yva.1    ----------------------------------------------------AGDVLLAISTRGN-S-RDIVKAVEAAVT  target    KGTKVVVIACE-YSSSSIK---ADDAIVVRPGTTPALALGLCNVIMREKIYDGDYVRRFSDLPLLVRADNLKLLRAEEVF 2yva.1    RDMTIVALTGYDGGELAGLLGPQDVEIRIPSHRSA---------------------------------------------  target    GTPQAALKNQTR 2yva.1    ------------ ``` | | | | | | | | | | | | | | | | | | | | | | | | | | | | | | | | | | | | | | | | | | | | | | | | | |
|  | 1m2h.1.A | Silent Information Regulator 2  *Sir2 homologue S24A mutant-ADP ribose complex* | 0.03 |  | 21.15 | 0.13 | 290-343 | X-ray | 1.80 | monomer | 1 x ZN, 1 x APR | HHblits | 0.31 |
| ``` target    NNVNRREFLQWIGAAGFSTFALSASNAWGLQAIENPLAAYPNREWEKTYRDLWKSDASFTFLCAPNDTHNCILNAHVRDG 1m2h.1    --------------------------------------------------------------------------------  target    VITRIGPTMKYGEATDLYGSKVTHRWDPRVCQKGLALTRRFYGDRRVRYPMVRKGFKAWADKGFPREKDGRPPKDYFNRA 1m2h.1    --------------------------------------------------------------------------------  target    RDEWLRLTHEEAADLVAAALINIATTYSGDNGQKLLLQQGYEKEIVEATRGAGTQVLKFRGGMPLLGLTRIFGLYRMANS 1m2h.1    --------------------------------------------------------------------------------  target    MALLDHKIRGVKPEDALGARGWDNYSWHTDLPPGHPMVTGQQTVDFDLHAVEQARIVVVWGMNWVTTKMPDTHWLTEARL 1m2h.1    -------------------------------------------------EVERADVIIVAGTSAVVQ--PAASLPLIVKQ  target    KGTKVVVIACEYSSSSIKADDAIVVRPGTTPALALGLCNVIMREKIYDGDYVRRFSDLPLLVRADNLKLLRAEEVFGTPQ 1m2h.1    RGGAIIEINPDETPLTPIADYSL---------------------------------------------------------  target    AALKNQTR 1m2h.1    -------- ``` | | | | | | | | | | | | | | | | | | | | | | | | | | | | | | | | | | | | | | | | | | | | | | | | | |
|  | 1m2j.1.A | Silent Information Regulator 2  *Sir2 homologue H80N mutant-ADP ribose complex* | 0.03 |  | 21.15 | 0.13 | 290-343 | X-ray | 1.70 | monomer | 1 x ZN, 1 x APR | HHblits | 0.31 |
| ``` target    NNVNRREFLQWIGAAGFSTFALSASNAWGLQAIENPLAAYPNREWEKTYRDLWKSDASFTFLCAPNDTHNCILNAHVRDG 1m2j.1    --------------------------------------------------------------------------------  target    VITRIGPTMKYGEATDLYGSKVTHRWDPRVCQKGLALTRRFYGDRRVRYPMVRKGFKAWADKGFPREKDGRPPKDYFNRA 1m2j.1    --------------------------------------------------------------------------------  target    RDEWLRLTHEEAADLVAAALINIATTYSGDNGQKLLLQQGYEKEIVEATRGAGTQVLKFRGGMPLLGLTRIFGLYRMANS 1m2j.1    --------------------------------------------------------------------------------  target    MALLDHKIRGVKPEDALGARGWDNYSWHTDLPPGHPMVTGQQTVDFDLHAVEQARIVVVWGMNWVTTKMPDTHWLTEARL 1m2j.1    -------------------------------------------------EVERADVIIVAGTSAVVQ--PAASLPLIVKQ  target    KGTKVVVIACEYSSSSIKADDAIVVRPGTTPALALGLCNVIMREKIYDGDYVRRFSDLPLLVRADNLKLLRAEEVFGTPQ 1m2j.1    RGGAIIEINPDETPLTPIADYSL---------------------------------------------------------  target    AALKNQTR 1m2j.1    -------- ``` | | | | | | | | | | | | | | | | | | | | | | | | | | | | | | | | | | | | | | | | | | | | | | | | | |
|  | 1m2g.1.A | Silent Information Regulator 2  *Sir2 homologue-ADP ribose complex* | 0.03 |  | 21.15 | 0.13 | 290-343 | X-ray | 1.70 | monomer | 1 x ZN, 1 x APR | HHblits | 0.31 |
| ``` target    NNVNRREFLQWIGAAGFSTFALSASNAWGLQAIENPLAAYPNREWEKTYRDLWKSDASFTFLCAPNDTHNCILNAHVRDG 1m2g.1    --------------------------------------------------------------------------------  target    VITRIGPTMKYGEATDLYGSKVTHRWDPRVCQKGLALTRRFYGDRRVRYPMVRKGFKAWADKGFPREKDGRPPKDYFNRA 1m2g.1    --------------------------------------------------------------------------------  target    RDEWLRLTHEEAADLVAAALINIATTYSGDNGQKLLLQQGYEKEIVEATRGAGTQVLKFRGGMPLLGLTRIFGLYRMANS 1m2g.1    --------------------------------------------------------------------------------  target    MALLDHKIRGVKPEDALGARGWDNYSWHTDLPPGHPMVTGQQTVDFDLHAVEQARIVVVWGMNWVTTKMPDTHWLTEARL 1m2g.1    -------------------------------------------------EVERADVIIVAGTSAVVQ--PAASLPLIVKQ  target    KGTKVVVIACEYSSSSIKADDAIVVRPGTTPALALGLCNVIMREKIYDGDYVRRFSDLPLLVRADNLKLLRAEEVFGTPQ 1m2g.1    RGGAIIEINPDETPLTPIADYSL---------------------------------------------------------  target    AALKNQTR 1m2g.1    -------- ``` | | | | | | | | | | | | | | | | | | | | | | | | | | | | | | | | | | | | | | | | | | | | | | | | | |
|  | 1m2n.1.A | Silent Information Regulator 2  *Sir2 homologues (D102G/F159A/R170A) mutant-2'-O-acetyl ADP ribose complex* | 0.03 |  | 21.15 | 0.13 | 290-343 | X-ray | 2.60 | homo-dimer | 2 x ZN, 2 x OAD | HHblits | 0.31 |
| ``` target    NNVNRREFLQWIGAAGFSTFALSASNAWGLQAIENPLAAYPNREWEKTYRDLWKSDASFTFLCAPNDTHNCILNAHVRDG 1m2n.1    --------------------------------------------------------------------------------  target    VITRIGPTMKYGEATDLYGSKVTHRWDPRVCQKGLALTRRFYGDRRVRYPMVRKGFKAWADKGFPREKDGRPPKDYFNRA 1m2n.1    --------------------------------------------------------------------------------  target    RDEWLRLTHEEAADLVAAALINIATTYSGDNGQKLLLQQGYEKEIVEATRGAGTQVLKFRGGMPLLGLTRIFGLYRMANS 1m2n.1    --------------------------------------------------------------------------------  target    MALLDHKIRGVKPEDALGARGWDNYSWHTDLPPGHPMVTGQQTVDFDLHAVEQARIVVVWGMNWVTTKMPDTHWLTEARL 1m2n.1    -------------------------------------------------EVERADVIIVAGTSAVVQ--PAASLPLIVKQ  target    KGTKVVVIACEYSSSSIKADDAIVVRPGTTPALALGLCNVIMREKIYDGDYVRRFSDLPLLVRADNLKLLRAEEVFGTPQ 1m2n.1    RGGAIIEINPDETPLTPIADYSL---------------------------------------------------------  target    AALKNQTR 1m2n.1    -------- ``` | | | | | | | | | | | | | | | | | | | | | | | | | | | | | | | | | | | | | | | | | | | | | | | | | |
|  | 1m2n.1.B | Silent Information Regulator 2  *Sir2 homologues (D102G/F159A/R170A) mutant-2'-O-acetyl ADP ribose complex* | 0.03 |  | 21.15 | 0.13 | 290-343 | X-ray | 2.60 | homo-dimer | 2 x ZN, 2 x OAD | HHblits | 0.31 |
| ``` target    NNVNRREFLQWIGAAGFSTFALSASNAWGLQAIENPLAAYPNREWEKTYRDLWKSDASFTFLCAPNDTHNCILNAHVRDG 1m2n.1    --------------------------------------------------------------------------------  target    VITRIGPTMKYGEATDLYGSKVTHRWDPRVCQKGLALTRRFYGDRRVRYPMVRKGFKAWADKGFPREKDGRPPKDYFNRA 1m2n.1    --------------------------------------------------------------------------------  target    RDEWLRLTHEEAADLVAAALINIATTYSGDNGQKLLLQQGYEKEIVEATRGAGTQVLKFRGGMPLLGLTRIFGLYRMANS 1m2n.1    --------------------------------------------------------------------------------  target    MALLDHKIRGVKPEDALGARGWDNYSWHTDLPPGHPMVTGQQTVDFDLHAVEQARIVVVWGMNWVTTKMPDTHWLTEARL 1m2n.1    -------------------------------------------------EVERADVIIVAGTSAVVQ--PAASLPLIVKQ  target    KGTKVVVIACEYSSSSIKADDAIVVRPGTTPALALGLCNVIMREKIYDGDYVRRFSDLPLLVRADNLKLLRAEEVFGTPQ 1m2n.1    RGGAIIEINPDETPLTPIADYSL---------------------------------------------------------  target    AALKNQTR 1m2n.1    -------- ``` | | | | | | | | | | | | | | | | | | | | | | | | | | | | | | | | | | | | | | | | | | | | | | | | | |
|  | 4twi.1.A | NAD-dependent protein deacylase 1  *The structure of Sir2Af1 bound to a succinylated histone peptide* | 0.03 |  | 21.15 | 0.13 | 290-343 | X-ray | 1.79 | hetero-1-1-mer | 1 x ZN | HHblits | 0.31 |
| ``` target    NNVNRREFLQWIGAAGFSTFALSASNAWGLQAIENPLAAYPNREWEKTYRDLWKSDASFTFLCAPNDTHNCILNAHVRDG 4twi.1    --------------------------------------------------------------------------------  target    VITRIGPTMKYGEATDLYGSKVTHRWDPRVCQKGLALTRRFYGDRRVRYPMVRKGFKAWADKGFPREKDGRPPKDYFNRA 4twi.1    --------------------------------------------------------------------------------  target    RDEWLRLTHEEAADLVAAALINIATTYSGDNGQKLLLQQGYEKEIVEATRGAGTQVLKFRGGMPLLGLTRIFGLYRMANS 4twi.1    --------------------------------------------------------------------------------  target    MALLDHKIRGVKPEDALGARGWDNYSWHTDLPPGHPMVTGQQTVDFDLHAVEQARIVVVWGMNWVTTKMPDTHWLTEARL 4twi.1    -------------------------------------------------EVERADVIIVAGTSAVVQ--PAASLPLIVKQ  target    KGTKVVVIACEYSSSSIKADDAIVVRPGTTPALALGLCNVIMREKIYDGDYVRRFSDLPLLVRADNLKLLRAEEVFGTPQ 4twi.1    RGGAIIEINPDETPLTPIADYSL---------------------------------------------------------  target    AALKNQTR 4twi.1    -------- ``` | | | | | | | | | | | | | | | | | | | | | | | | | | | | | | | | | | | | | | | | | | | | | | | | | |
|  | 1m2k.1.A | Silent Information Regulator 2  *Sir2 homologue F159A mutant-ADP ribose complex* | 0.03 |  | 21.15 | 0.13 | 290-343 | X-ray | 1.47 | monomer | 1 x ZN, 1 x APR | HHblits | 0.31 |
| ``` target    NNVNRREFLQWIGAAGFSTFALSASNAWGLQAIENPLAAYPNREWEKTYRDLWKSDASFTFLCAPNDTHNCILNAHVRDG 1m2k.1    --------------------------------------------------------------------------------  target    VITRIGPTMKYGEATDLYGSKVTHRWDPRVCQKGLALTRRFYGDRRVRYPMVRKGFKAWADKGFPREKDGRPPKDYFNRA 1m2k.1    --------------------------------------------------------------------------------  target    RDEWLRLTHEEAADLVAAALINIATTYSGDNGQKLLLQQGYEKEIVEATRGAGTQVLKFRGGMPLLGLTRIFGLYRMANS 1m2k.1    --------------------------------------------------------------------------------  target    MALLDHKIRGVKPEDALGARGWDNYSWHTDLPPGHPMVTGQQTVDFDLHAVEQARIVVVWGMNWVTTKMPDTHWLTEARL 1m2k.1    -------------------------------------------------EVERADVIIVAGTSAVVQ--PAASLPLIVKQ  target    KGTKVVVIACEYSSSSIKADDAIVVRPGTTPALALGLCNVIMREKIYDGDYVRRFSDLPLLVRADNLKLLRAEEVFGTPQ 1m2k.1    RGGAIIEINPDETPLTPIADYSL---------------------------------------------------------  target    AALKNQTR 1m2k.1    -------- ``` | | | | | | | | | | | | | | | | | | | | | | | | | | | | | | | | | | | | | | | | | | | | | | | | | |
|  | 4ivn.1.A | Transcriptional regulator  *The Vibrio vulnificus NanR protein complexed with ManNAc-6P* | 0.03 |  | 16.36 | 0.13 | 292-348 | X-ray | 1.90 | homo-dimer | 2 x BMX | HHblits | 0.26 |
| ``` target    NNVNRREFLQWIGAAGFSTFALSASNAWGLQAIENPLAAYPNREWEKTYRDLWKSDASFTFLCAPNDTHNCILNAHVRDG 4ivn.1    --------------------------------------------------------------------------------  target    VITRIGPTMKYGEATDLYGSKVTHRWDPRVCQKGLALTRRFYGDRRVRYPMVRKGFKAWADKGFPREKDGRPPKDYFNRA 4ivn.1    --------------------------------------------------------------------------------  target    RDEWLRLTHEEAADLVAAALINIATTYSGDNGQKLLLQQGYEKEIVEATRGAGTQVLKFRGGMPLLGLTRIFGLYRMANS 4ivn.1    --------------------------------------------------------------------------------  target    MALLDHKIRGVKPEDALGARGWDNYSWHTDLPPGHPMVTGQQTVDFDLHAVEQARIVVVWGMNWVTTKMPDTHWLTEARL 4ivn.1    ---------------------------------------------------SQGDLWFAVSSSGSTK--EVIHAAGLAYK  target    KGTKVVVIACE-YSSSSIKADDAIVVRPGTTPALALGLCNVIMREKIYDGDYVRRFSDLPLLVRADNLKLLRAEEVFGTP 4ivn.1    RDIPVVSLTNINHSPLSSLSTEMLVAARP---------------------------------------------------  target    QAALKNQTR 4ivn.1    --------- ``` | | | | | | | | | | | | | | | | | | | | | | | | | | | | | | | | | | | | | | | | | | | | | | | | | |
|  | 5mf6.1.A | NAD-dependent protein deacetylase sirtuin-6  *Human Sirt6 in complex with activator UBCS039* | 0.03 |  | 16.98 | 0.13 | 290-344 | X-ray | 1.87 | monomer | 1 x AR6, 1 x ZN, 1 x 7M2 | HHblits | 0.29 |
| ``` target    NNVNRREFLQWIGAAGFSTFALSASNAWGLQAIENPLAAYPNREWEKTYRDLWKSDASFTFLCAPNDTHNCILNAHVRDG 5mf6.1    --------------------------------------------------------------------------------  target    VITRIGPTMKYGEATDLYGSKVTHRWDPRVCQKGLALTRRFYGDRRVRYPMVRKGFKAWADKGFPREKDGRPPKDYFNRA 5mf6.1    --------------------------------------------------------------------------------  target    RDEWLRLTHEEAADLVAAALINIATTYSGDNGQKLLLQQGYEKEIVEATRGAGTQVLKFRGGMPLLGLTRIFGLYRMANS 5mf6.1    --------------------------------------------------------------------------------  target    MALLDHKIRGVKPEDALGARGWDNYSWHTDLPPGHPMVTGQQTVDFDLHAVEQARIVVVWGMNWVTTKMPDTHWLTEARL 5mf6.1    -------------------------------------------------ASRNADLSITLGTSLQI--RPSGNLPLATKR  target    KGTKVVVIACEYSSSSIKADDAIVVRPGTTPALALGLCNVIMREKIYDGDYVRRFSDLPLLVRADNLKLLRAEEVFGTPQ 5mf6.1    RGGRLVIVNLQPTKHDRHADLRIH--------------------------------------------------------  target    AALKNQTR 5mf6.1    -------- ``` | | | | | | | | | | | | | | | | | | | | | | | | | | | | | | | | | | | | | | | | | | | | | | | | | |
|  | 3zg6.1.A | NAD-DEPENDENT PROTEIN DEACETYLASE SIRTUIN-6  *The novel de-long chain fatty acid function of human sirt6* | 0.03 |  | 16.98 | 0.13 | 290-344 | X-ray | 2.20 | hetero-oligomer | 1 x ZN, 1 x APR | HHblits | 0.29 |
| ``` target    NNVNRREFLQWIGAAGFSTFALSASNAWGLQAIENPLAAYPNREWEKTYRDLWKSDASFTFLCAPNDTHNCILNAHVRDG 3zg6.1    --------------------------------------------------------------------------------  target    VITRIGPTMKYGEATDLYGSKVTHRWDPRVCQKGLALTRRFYGDRRVRYPMVRKGFKAWADKGFPREKDGRPPKDYFNRA 3zg6.1    --------------------------------------------------------------------------------  target    RDEWLRLTHEEAADLVAAALINIATTYSGDNGQKLLLQQGYEKEIVEATRGAGTQVLKFRGGMPLLGLTRIFGLYRMANS 3zg6.1    --------------------------------------------------------------------------------  target    MALLDHKIRGVKPEDALGARGWDNYSWHTDLPPGHPMVTGQQTVDFDLHAVEQARIVVVWGMNWVTTKMPDTHWLTEARL 3zg6.1    -------------------------------------------------ASRNADLSITLGTSLQIR--PSGNLPLATKR  target    KGTKVVVIACEYSSSSIKADDAIVVRPGTTPALALGLCNVIMREKIYDGDYVRRFSDLPLLVRADNLKLLRAEEVFGTPQ 3zg6.1    RGGRLVIVNLQPTKHDRHADLRIH--------------------------------------------------------  target    AALKNQTR 3zg6.1    -------- ``` | | | | | | | | | | | | | | | | | | | | | | | | | | | | | | | | | | | | | | | | | | | | | | | | | |
|  | 3k35.1.A | NAD-dependent deacetylase sirtuin-6  *Crystal Structure of Human SIRT6* | 0.03 |  | 16.98 | 0.13 | 290-344 | X-ray | 2.00 | monomer | 1 x ZN, 1 x APR | HHblits | 0.29 |
| ``` target    NNVNRREFLQWIGAAGFSTFALSASNAWGLQAIENPLAAYPNREWEKTYRDLWKSDASFTFLCAPNDTHNCILNAHVRDG 3k35.1    --------------------------------------------------------------------------------  target    VITRIGPTMKYGEATDLYGSKVTHRWDPRVCQKGLALTRRFYGDRRVRYPMVRKGFKAWADKGFPREKDGRPPKDYFNRA 3k35.1    --------------------------------------------------------------------------------  target    RDEWLRLTHEEAADLVAAALINIATTYSGDNGQKLLLQQGYEKEIVEATRGAGTQVLKFRGGMPLLGLTRIFGLYRMANS 3k35.1    --------------------------------------------------------------------------------  target    MALLDHKIRGVKPEDALGARGWDNYSWHTDLPPGHPMVTGQQTVDFDLHAVEQARIVVVWGMNWVTTKMPDTHWLTEARL 3k35.1    -------------------------------------------------ASRNADLSITLGTSLQIR--PSGNLPLATKR  target    KGTKVVVIACEYSSSSIKADDAIVVRPGTTPALALGLCNVIMREKIYDGDYVRRFSDLPLLVRADNLKLLRAEEVFGTPQ 3k35.1    RGGRLVIVNLQPTKHDRHADLRIH--------------------------------------------------------  target    AALKNQTR 3k35.1    -------- ``` | | | | | | | | | | | | | | | | | | | | | | | | | | | | | | | | | | | | | | | | | | | | | | | | | |
|  | 6xvg.3.A | NAD-dependent protein deacetylase sirtuin-6  *Human Sirt6 3-318 in complex with ADP-ribose and the activator MDL-801* | 0.03 |  | 16.98 | 0.13 | 290-344 | X-ray | 2.10 | monomer | 1 x AR6, 1 x ZN, 1 x 8L9 | HHblits | 0.29 |
| ``` target    NNVNRREFLQWIGAAGFSTFALSASNAWGLQAIENPLAAYPNREWEKTYRDLWKSDASFTFLCAPNDTHNCILNAHVRDG 6xvg.3    --------------------------------------------------------------------------------  target    VITRIGPTMKYGEATDLYGSKVTHRWDPRVCQKGLALTRRFYGDRRVRYPMVRKGFKAWADKGFPREKDGRPPKDYFNRA 6xvg.3    --------------------------------------------------------------------------------  target    RDEWLRLTHEEAADLVAAALINIATTYSGDNGQKLLLQQGYEKEIVEATRGAGTQVLKFRGGMPLLGLTRIFGLYRMANS 6xvg.3    --------------------------------------------------------------------------------  target    MALLDHKIRGVKPEDALGARGWDNYSWHTDLPPGHPMVTGQQTVDFDLHAVEQARIVVVWGMNWVTTKMPDTHWLTEARL 6xvg.3    -------------------------------------------------ASRNADLSITLGTSLQIR--PSGNLPLATKR  target    KGTKVVVIACEYSSSSIKADDAIVVRPGTTPALALGLCNVIMREKIYDGDYVRRFSDLPLLVRADNLKLLRAEEVFGTPQ 6xvg.3    RGGRLVIVNLQPTKHDRHADLRIH--------------------------------------------------------  target    AALKNQTR 6xvg.3    -------- ``` | | | | | | | | | | | | | | | | | | | | | | | | | | | | | | | | | | | | | | | | | | | | | | | | | |
|  | 5y2f.1.A | NAD-dependent protein deacetylase sirtuin-6  *Human SIRT6 in complex with allosteric activator MDL-801* | 0.03 |  | 16.98 | 0.13 | 290-344 | X-ray | 2.53 | monomer | 1 x AR6, 1 x 8L9, 1 x ZN, 1 x HDR, 1 x THR-ALA-ARG-LYS-SER-THR-GLY-GLY | HHblits | 0.29 |
| ``` target    NNVNRREFLQWIGAAGFSTFALSASNAWGLQAIENPLAAYPNREWEKTYRDLWKSDASFTFLCAPNDTHNCILNAHVRDG 5y2f.1    --------------------------------------------------------------------------------  target    VITRIGPTMKYGEATDLYGSKVTHRWDPRVCQKGLALTRRFYGDRRVRYPMVRKGFKAWADKGFPREKDGRPPKDYFNRA 5y2f.1    --------------------------------------------------------------------------------  target    RDEWLRLTHEEAADLVAAALINIATTYSGDNGQKLLLQQGYEKEIVEATRGAGTQVLKFRGGMPLLGLTRIFGLYRMANS 5y2f.1    --------------------------------------------------------------------------------  target    MALLDHKIRGVKPEDALGARGWDNYSWHTDLPPGHPMVTGQQTVDFDLHAVEQARIVVVWGMNWVTTKMPDTHWLTEARL 5y2f.1    -------------------------------------------------ASRNADLSITLGTSLQIR--PSGNLPLATKR  target    KGTKVVVIACEYSSSSIKADDAIVVRPGTTPALALGLCNVIMREKIYDGDYVRRFSDLPLLVRADNLKLLRAEEVFGTPQ 5y2f.1    RGGRLVIVNLQPTKHDRHADLRIH--------------------------------------------------------  target    AALKNQTR 5y2f.1    -------- ``` | | | | | | | | | | | | | | | | | | | | | | | | | | | | | | | | | | | | | | | | | | | | | | | | | |
|  | 5x16.1.A | NAD-dependent protein deacetylase sirtuin-6  *Sirt6 apo structure* | 0.03 |  | 16.98 | 0.13 | 290-344 | X-ray | 1.97 | monomer | 1 x AR6, 1 x ZN | HHblits | 0.29 |
| ``` target    NNVNRREFLQWIGAAGFSTFALSASNAWGLQAIENPLAAYPNREWEKTYRDLWKSDASFTFLCAPNDTHNCILNAHVRDG 5x16.1    --------------------------------------------------------------------------------  target    VITRIGPTMKYGEATDLYGSKVTHRWDPRVCQKGLALTRRFYGDRRVRYPMVRKGFKAWADKGFPREKDGRPPKDYFNRA 5x16.1    --------------------------------------------------------------------------------  target    RDEWLRLTHEEAADLVAAALINIATTYSGDNGQKLLLQQGYEKEIVEATRGAGTQVLKFRGGMPLLGLTRIFGLYRMANS 5x16.1    --------------------------------------------------------------------------------  target    MALLDHKIRGVKPEDALGARGWDNYSWHTDLPPGHPMVTGQQTVDFDLHAVEQARIVVVWGMNWVTTKMPDTHWLTEARL 5x16.1    -------------------------------------------------ASRNADLSITLGTSLQIR--PSGNLPLATKR  target    KGTKVVVIACEYSSSSIKADDAIVVRPGTTPALALGLCNVIMREKIYDGDYVRRFSDLPLLVRADNLKLLRAEEVFGTPQ 5x16.1    RGGRLVIVNLQPTKHDRHADLRIH--------------------------------------------------------  target    AALKNQTR 5x16.1    -------- ``` | | | | | | | | | | | | | | | | | | | | | | | | | | | | | | | | | | | | | | | | | | | | | | | | | |
|  | 2v45.1.A | PERIPLASMIC NITRATE REDUCTASE  *A NEW CATALYTIC MECHANISM OF PERIPLASMIC NITRATE REDUCTASE FROM DESULFOVIBRIO DESULFURICANS ATCC 27774 FROM CRYSTALLOGRAPHIC AND EPR DATA AND BASED ON DETAILED ANALYSIS OF THE SIXTH LIGAND* | 0.03 |  | 12.50 | 0.14 | 292-348 | X-ray | 2.40 | monomer | 1 x SF4, 1 x MO, 2 x MGD, 1 x LCP | HHblits | 0.25 |
| ``` target    NNVNRREFLQWIGAAGFSTFALSASNAWGLQAIENPLAAYPNREWEKTYRDLWKSDASFTFLCAPNDTHNCILNAHVRDG 2v45.1    --------------------------------------------------------------------------------  target    VITRIGPTMKYGEATDLYGSKVTHRWDPRVCQKGLALTRRFYGDRRVRYPMVRKGFKAWADKGFPREKDGRPPKDYFNRA 2v45.1    --------------------------------------------------------------------------------  target    RDEWLRLTHEEAADLVAAALINIATTYSGDNGQKLLLQQGYEKEIVEATRGAGTQVLKFRGGMPLLGLTRIFGLYRMANS 2v45.1    --------------------------------------------------------------------------------  target    MALLDHKIRGVKPEDALGARGWDNYSWHTDLPPGHPMVTGQQTVDFDLHAVEQARIVVVWGMNWVTTKMPDTHWLTEARL 2v45.1    ---------------------------------------------------GDVKCMIICETNPAHTLPN-LNKVHKAMS  target    KG-TKVVVIACEYS-SSSIKADDAIVVRPGTTPALALGLCNVIMREKIYDGDYVRRFSDLPLLVRADNLKLLRAEEVFGT 2v45.1    HPESFIVCIEAFPDAVTLEYADLVLPPAFW--------------------------------------------------  target    PQAALKNQTR 2v45.1    ---------- ``` | | | | | | | | | | | | | | | | | | | | | | | | | | | | | | | | | | | | | | | | | | | | | | | | | |
|  | 6rxs.1.A | NAD-dependent protein deacylase  *Crystal structure of CobB Ac3(A76G,Y92A, I131L, V187Y) in complex with H4K16-Acetyl peptide* | 0.03 |  | 23.08 | 0.13 | 290-343 | X-ray | 1.60 | hetero-1-1-mer | 1 x ZN | HHblits | 0.31 |
| ``` target    NNVNRREFLQWIGAAGFSTFALSASNAWGLQAIENPLAAYPNREWEKTYRDLWKSDASFTFLCAPNDTHNCILNAHVRDG 6rxs.1    --------------------------------------------------------------------------------  target    VITRIGPTMKYGEATDLYGSKVTHRWDPRVCQKGLALTRRFYGDRRVRYPMVRKGFKAWADKGFPREKDGRPPKDYFNRA 6rxs.1    --------------------------------------------------------------------------------  target    RDEWLRLTHEEAADLVAAALINIATTYSGDNGQKLLLQQGYEKEIVEATRGAGTQVLKFRGGMPLLGLTRIFGLYRMANS 6rxs.1    --------------------------------------------------------------------------------  target    MALLDHKIRGVKPEDALGARGWDNYSWHTDLPPGHPMVTGQQTVDFDLHAVEQARIVVVWGMNWVTTKMPDTHWLTEARL 6rxs.1    -------------------------------------------------ALSMADIFIAIGTSGHV--YPAAGFVHEAKL  target    KGTKVVVIACEYSSSSIKADDAIVVRPGTTPALALGLCNVIMREKIYDGDYVRRFSDLPLLVRADNLKLLRAEEVFGTPQ 6rxs.1    HGAHTVELNLEPSQVGNEFAEKY---------------------------------------------------------  target    AALKNQTR 6rxs.1    -------- ``` | | | | | | | | | | | | | | | | | | | | | | | | | | | | | | | | | | | | | | | | | | | | | | | | | |
|  | 6rxo.1.A | NAD-dependent protein deacylase  *Crystal structure of CobB Ac2 (A76G, I131C, V162A) in complex with H4K16-Buturyl peptide* | 0.03 |  | 23.08 | 0.13 | 290-343 | X-ray | 1.95 | hetero-1-1-mer | 1 x ZN | HHblits | 0.31 |
| ``` target    NNVNRREFLQWIGAAGFSTFALSASNAWGLQAIENPLAAYPNREWEKTYRDLWKSDASFTFLCAPNDTHNCILNAHVRDG 6rxo.1    --------------------------------------------------------------------------------  target    VITRIGPTMKYGEATDLYGSKVTHRWDPRVCQKGLALTRRFYGDRRVRYPMVRKGFKAWADKGFPREKDGRPPKDYFNRA 6rxo.1    --------------------------------------------------------------------------------  target    RDEWLRLTHEEAADLVAAALINIATTYSGDNGQKLLLQQGYEKEIVEATRGAGTQVLKFRGGMPLLGLTRIFGLYRMANS 6rxo.1    --------------------------------------------------------------------------------  target    MALLDHKIRGVKPEDALGARGWDNYSWHTDLPPGHPMVTGQQTVDFDLHAVEQARIVVVWGMNWVTTKMPDTHWLTEARL 6rxo.1    -------------------------------------------------ALSMADIFIAIGTSGHV--YPAAGFVHEAKL  target    KGTKVVVIACEYSSSSIKADDAIVVRPGTTPALALGLCNVIMREKIYDGDYVRRFSDLPLLVRADNLKLLRAEEVFGTPQ 6rxo.1    HGAHTVELNLEPSQVGNEFAEKY---------------------------------------------------------  target    AALKNQTR 6rxo.1    -------- ``` | | | | | | | | | | | | | | | | | | | | | | | | | | | | | | | | | | | | | | | | | | | | | | | | | |
|  | 6rxm.1.A | NAD-dependent protein deacylase  *Crystal structure of CobB Ac2 (A76G, I131C, V162G) in complex with H4K16-Acetyl peptide* | 0.03 |  | 23.08 | 0.13 | 290-343 | X-ray | 1.92 | hetero-1-1-mer | 1 x ZN | HHblits | 0.31 |
| ``` target    NNVNRREFLQWIGAAGFSTFALSASNAWGLQAIENPLAAYPNREWEKTYRDLWKSDASFTFLCAPNDTHNCILNAHVRDG 6rxm.1    --------------------------------------------------------------------------------  target    VITRIGPTMKYGEATDLYGSKVTHRWDPRVCQKGLALTRRFYGDRRVRYPMVRKGFKAWADKGFPREKDGRPPKDYFNRA 6rxm.1    --------------------------------------------------------------------------------  target    RDEWLRLTHEEAADLVAAALINIATTYSGDNGQKLLLQQGYEKEIVEATRGAGTQVLKFRGGMPLLGLTRIFGLYRMANS 6rxm.1    --------------------------------------------------------------------------------  target    MALLDHKIRGVKPEDALGARGWDNYSWHTDLPPGHPMVTGQQTVDFDLHAVEQARIVVVWGMNWVTTKMPDTHWLTEARL 6rxm.1    -------------------------------------------------ALSMADIFIAIGTSGHV--YPAAGFVHEAKL  target    KGTKVVVIACEYSSSSIKADDAIVVRPGTTPALALGLCNVIMREKIYDGDYVRRFSDLPLLVRADNLKLLRAEEVFGTPQ 6rxm.1    HGAHTVELNLEPSQVGNEFAEKY---------------------------------------------------------  target    AALKNQTR 6rxm.1    -------- ``` | | | | | | | | | | | | | | | | | | | | | | | | | | | | | | | | | | | | | | | | | | | | | | | | | |
|  | 6rxm.3.A | NAD-dependent protein deacylase  *Crystal structure of CobB Ac2 (A76G, I131C, V162G) in complex with H4K16-Acetyl peptide* | 0.03 |  | 23.08 | 0.13 | 290-343 | X-ray | 1.92 | hetero-1-1-mer | 1 x ZN | HHblits | 0.31 |
| ``` target    NNVNRREFLQWIGAAGFSTFALSASNAWGLQAIENPLAAYPNREWEKTYRDLWKSDASFTFLCAPNDTHNCILNAHVRDG 6rxm.3    --------------------------------------------------------------------------------  target    VITRIGPTMKYGEATDLYGSKVTHRWDPRVCQKGLALTRRFYGDRRVRYPMVRKGFKAWADKGFPREKDGRPPKDYFNRA 6rxm.3    --------------------------------------------------------------------------------  target    RDEWLRLTHEEAADLVAAALINIATTYSGDNGQKLLLQQGYEKEIVEATRGAGTQVLKFRGGMPLLGLTRIFGLYRMANS 6rxm.3    --------------------------------------------------------------------------------  target    MALLDHKIRGVKPEDALGARGWDNYSWHTDLPPGHPMVTGQQTVDFDLHAVEQARIVVVWGMNWVTTKMPDTHWLTEARL 6rxm.3    -------------------------------------------------ALSMADIFIAIGTSGHV--YPAAGFVHEAKL  target    KGTKVVVIACEYSSSSIKADDAIVVRPGTTPALALGLCNVIMREKIYDGDYVRRFSDLPLLVRADNLKLLRAEEVFGTPQ 6rxm.3    HGAHTVELNLEPSQVGNEFAEKY---------------------------------------------------------  target    AALKNQTR 6rxm.3    -------- ``` | | | | | | | | | | | | | | | | | | | | | | | | | | | | | | | | | | | | | | | | | | | | | | | | | |
|  | 6rxm.4.A | NAD-dependent protein deacylase  *Crystal structure of CobB Ac2 (A76G, I131C, V162G) in complex with H4K16-Acetyl peptide* | 0.03 |  | 23.08 | 0.13 | 290-343 | X-ray | 1.92 | hetero-1-1-mer | 1 x ZN | HHblits | 0.31 |
| ``` target    NNVNRREFLQWIGAAGFSTFALSASNAWGLQAIENPLAAYPNREWEKTYRDLWKSDASFTFLCAPNDTHNCILNAHVRDG 6rxm.4    --------------------------------------------------------------------------------  target    VITRIGPTMKYGEATDLYGSKVTHRWDPRVCQKGLALTRRFYGDRRVRYPMVRKGFKAWADKGFPREKDGRPPKDYFNRA 6rxm.4    --------------------------------------------------------------------------------  target    RDEWLRLTHEEAADLVAAALINIATTYSGDNGQKLLLQQGYEKEIVEATRGAGTQVLKFRGGMPLLGLTRIFGLYRMANS 6rxm.4    --------------------------------------------------------------------------------  target    MALLDHKIRGVKPEDALGARGWDNYSWHTDLPPGHPMVTGQQTVDFDLHAVEQARIVVVWGMNWVTTKMPDTHWLTEARL 6rxm.4    -------------------------------------------------ALSMADIFIAIGTSGHV--YPAAGFVHEAKL  target    KGTKVVVIACEYSSSSIKADDAIVVRPGTTPALALGLCNVIMREKIYDGDYVRRFSDLPLLVRADNLKLLRAEEVFGTPQ 6rxm.4    HGAHTVELNLEPSQVGNEFAEKY---------------------------------------------------------  target    AALKNQTR 6rxm.4    -------- ``` | | | | | | | | | | | | | | | | | | | | | | | | | | | | | | | | | | | | | | | | | | | | | | | | | |
|  | 6rxm.5.A | NAD-dependent protein deacylase  *Crystal structure of CobB Ac2 (A76G, I131C, V162G) in complex with H4K16-Acetyl peptide* | 0.03 |  | 23.08 | 0.13 | 290-343 | X-ray | 1.92 | hetero-1-1-mer | 1 x ZN | HHblits | 0.31 |
| ``` target    NNVNRREFLQWIGAAGFSTFALSASNAWGLQAIENPLAAYPNREWEKTYRDLWKSDASFTFLCAPNDTHNCILNAHVRDG 6rxm.5    --------------------------------------------------------------------------------  target    VITRIGPTMKYGEATDLYGSKVTHRWDPRVCQKGLALTRRFYGDRRVRYPMVRKGFKAWADKGFPREKDGRPPKDYFNRA 6rxm.5    --------------------------------------------------------------------------------  target    RDEWLRLTHEEAADLVAAALINIATTYSGDNGQKLLLQQGYEKEIVEATRGAGTQVLKFRGGMPLLGLTRIFGLYRMANS 6rxm.5    --------------------------------------------------------------------------------  target    MALLDHKIRGVKPEDALGARGWDNYSWHTDLPPGHPMVTGQQTVDFDLHAVEQARIVVVWGMNWVTTKMPDTHWLTEARL 6rxm.5    -------------------------------------------------ALSMADIFIAIGTSGHV--YPAAGFVHEAKL  target    KGTKVVVIACEYSSSSIKADDAIVVRPGTTPALALGLCNVIMREKIYDGDYVRRFSDLPLLVRADNLKLLRAEEVFGTPQ 6rxm.5    HGAHTVELNLEPSQVGNEFAEKY---------------------------------------------------------  target    AALKNQTR 6rxm.5    -------- ``` | | | | | | | | | | | | | | | | | | | | | | | | | | | | | | | | | | | | | | | | | | | | | | | | | |
|  | 6rxm.6.A | NAD-dependent protein deacylase  *Crystal structure of CobB Ac2 (A76G, I131C, V162G) in complex with H4K16-Acetyl peptide* | 0.03 |  | 23.08 | 0.13 | 290-343 | X-ray | 1.92 | hetero-1-1-mer | 1 x ZN | HHblits | 0.31 |
| ``` target    NNVNRREFLQWIGAAGFSTFALSASNAWGLQAIENPLAAYPNREWEKTYRDLWKSDASFTFLCAPNDTHNCILNAHVRDG 6rxm.6    --------------------------------------------------------------------------------  target    VITRIGPTMKYGEATDLYGSKVTHRWDPRVCQKGLALTRRFYGDRRVRYPMVRKGFKAWADKGFPREKDGRPPKDYFNRA 6rxm.6    --------------------------------------------------------------------------------  target    RDEWLRLTHEEAADLVAAALINIATTYSGDNGQKLLLQQGYEKEIVEATRGAGTQVLKFRGGMPLLGLTRIFGLYRMANS 6rxm.6    --------------------------------------------------------------------------------  target    MALLDHKIRGVKPEDALGARGWDNYSWHTDLPPGHPMVTGQQTVDFDLHAVEQARIVVVWGMNWVTTKMPDTHWLTEARL 6rxm.6    -------------------------------------------------ALSMADIFIAIGTSGHV--YPAAGFVHEAKL  target    KGTKVVVIACEYSSSSIKADDAIVVRPGTTPALALGLCNVIMREKIYDGDYVRRFSDLPLLVRADNLKLLRAEEVFGTPQ 6rxm.6    HGAHTVELNLEPSQVGNEFAEKY---------------------------------------------------------  target    AALKNQTR 6rxm.6    -------- ``` | | | | | | | | | | | | | | | | | | | | | | | | | | | | | | | | | | | | | | | | | | | | | | | | | |
|  | 6rxm.2.A | NAD-dependent protein deacylase  *Crystal structure of CobB Ac2 (A76G, I131C, V162G) in complex with H4K16-Acetyl peptide* | 0.03 |  | 23.08 | 0.13 | 290-343 | X-ray | 1.92 | hetero-1-1-mer | 1 x ZN | HHblits | 0.31 |
| ``` target    NNVNRREFLQWIGAAGFSTFALSASNAWGLQAIENPLAAYPNREWEKTYRDLWKSDASFTFLCAPNDTHNCILNAHVRDG 6rxm.2    --------------------------------------------------------------------------------  target    VITRIGPTMKYGEATDLYGSKVTHRWDPRVCQKGLALTRRFYGDRRVRYPMVRKGFKAWADKGFPREKDGRPPKDYFNRA 6rxm.2    --------------------------------------------------------------------------------  target    RDEWLRLTHEEAADLVAAALINIATTYSGDNGQKLLLQQGYEKEIVEATRGAGTQVLKFRGGMPLLGLTRIFGLYRMANS 6rxm.2    --------------------------------------------------------------------------------  target    MALLDHKIRGVKPEDALGARGWDNYSWHTDLPPGHPMVTGQQTVDFDLHAVEQARIVVVWGMNWVTTKMPDTHWLTEARL 6rxm.2    -------------------------------------------------ALSMADIFIAIGTSGHV--YPAAGFVHEAKL  target    KGTKVVVIACEYSSSSIKADDAIVVRPGTTPALALGLCNVIMREKIYDGDYVRRFSDLPLLVRADNLKLLRAEEVFGTPQ 6rxm.2    HGAHTVELNLEPSQVGNEFAEKY---------------------------------------------------------  target    AALKNQTR 6rxm.2    -------- ``` | | | | | | | | | | | | | | | | | | | | | | | | | | | | | | | | | | | | | | | | | | | | | | | | | |
|  | 6rxo.2.A | NAD-dependent protein deacylase  *Crystal structure of CobB Ac2 (A76G, I131C, V162A) in complex with H4K16-Buturyl peptide* | 0.03 |  | 23.08 | 0.13 | 290-343 | X-ray | 1.95 | hetero-1-1-mer | 1 x ZN | HHblits | 0.31 |
| ``` target    NNVNRREFLQWIGAAGFSTFALSASNAWGLQAIENPLAAYPNREWEKTYRDLWKSDASFTFLCAPNDTHNCILNAHVRDG 6rxo.2    --------------------------------------------------------------------------------  target    VITRIGPTMKYGEATDLYGSKVTHRWDPRVCQKGLALTRRFYGDRRVRYPMVRKGFKAWADKGFPREKDGRPPKDYFNRA 6rxo.2    --------------------------------------------------------------------------------  target    RDEWLRLTHEEAADLVAAALINIATTYSGDNGQKLLLQQGYEKEIVEATRGAGTQVLKFRGGMPLLGLTRIFGLYRMANS 6rxo.2    --------------------------------------------------------------------------------  target    MALLDHKIRGVKPEDALGARGWDNYSWHTDLPPGHPMVTGQQTVDFDLHAVEQARIVVVWGMNWVTTKMPDTHWLTEARL 6rxo.2    -------------------------------------------------ALSMADIFIAIGTSGHV--YPAAGFVHEAKL  target    KGTKVVVIACEYSSSSIKADDAIVVRPGTTPALALGLCNVIMREKIYDGDYVRRFSDLPLLVRADNLKLLRAEEVFGTPQ 6rxo.2    HGAHTVELNLEPSQVGNEFAEKY---------------------------------------------------------  target    AALKNQTR 6rxo.2    -------- ``` | | | | | | | | | | | | | | | | | | | | | | | | | | | | | | | | | | | | | | | | | | | | | | | | | |
|  | 6rxp.2.A | NAD-dependent protein deacylase  *Crystal structure of CobB Ac2 (A76G,I131C,V162A) in complex with H4K16-Crotonyl peptide* | 0.03 |  | 23.08 | 0.13 | 290-343 | X-ray | 1.80 | hetero-1-1-mer | 1 x ZN | HHblits | 0.31 |
| ``` target    NNVNRREFLQWIGAAGFSTFALSASNAWGLQAIENPLAAYPNREWEKTYRDLWKSDASFTFLCAPNDTHNCILNAHVRDG 6rxp.2    --------------------------------------------------------------------------------  target    VITRIGPTMKYGEATDLYGSKVTHRWDPRVCQKGLALTRRFYGDRRVRYPMVRKGFKAWADKGFPREKDGRPPKDYFNRA 6rxp.2    --------------------------------------------------------------------------------  target    RDEWLRLTHEEAADLVAAALINIATTYSGDNGQKLLLQQGYEKEIVEATRGAGTQVLKFRGGMPLLGLTRIFGLYRMANS 6rxp.2    --------------------------------------------------------------------------------  target    MALLDHKIRGVKPEDALGARGWDNYSWHTDLPPGHPMVTGQQTVDFDLHAVEQARIVVVWGMNWVTTKMPDTHWLTEARL 6rxp.2    -------------------------------------------------ALSMADIFIAIGTSGHV--YPAAGFVHEAKL  target    KGTKVVVIACEYSSSSIKADDAIVVRPGTTPALALGLCNVIMREKIYDGDYVRRFSDLPLLVRADNLKLLRAEEVFGTPQ 6rxp.2    HGAHTVELNLEPSQVGNEFAEKY---------------------------------------------------------  target    AALKNQTR 6rxp.2    -------- ``` | | | | | | | | | | | | | | | | | | | | | | | | | | | | | | | | | | | | | | | | | | | | | | | | | |
|  | 6rxq.4.A | NAD-dependent protein deacylase  *Crystal structure of CobB Ac2 (A76G,I131C,V162A) in complex with H4K16Cr-2'OH-ADPr peptide intermediate after soaking* | 0.03 |  | 23.08 | 0.13 | 290-343 | X-ray | 1.70 | hetero-1-1-mer | 1 x KMQ | HHblits | 0.31 |
| ``` target    NNVNRREFLQWIGAAGFSTFALSASNAWGLQAIENPLAAYPNREWEKTYRDLWKSDASFTFLCAPNDTHNCILNAHVRDG 6rxq.4    --------------------------------------------------------------------------------  target    VITRIGPTMKYGEATDLYGSKVTHRWDPRVCQKGLALTRRFYGDRRVRYPMVRKGFKAWADKGFPREKDGRPPKDYFNRA 6rxq.4    --------------------------------------------------------------------------------  target    RDEWLRLTHEEAADLVAAALINIATTYSGDNGQKLLLQQGYEKEIVEATRGAGTQVLKFRGGMPLLGLTRIFGLYRMANS 6rxq.4    --------------------------------------------------------------------------------  target    MALLDHKIRGVKPEDALGARGWDNYSWHTDLPPGHPMVTGQQTVDFDLHAVEQARIVVVWGMNWVTTKMPDTHWLTEARL 6rxq.4    -------------------------------------------------ALSMADIFIAIGTSGHV--YPAAGFVHEAKL  target    KGTKVVVIACEYSSSSIKADDAIVVRPGTTPALALGLCNVIMREKIYDGDYVRRFSDLPLLVRADNLKLLRAEEVFGTPQ 6rxq.4    HGAHTVELNLEPSQVGNEFAEKY---------------------------------------------------------  target    AALKNQTR 6rxq.4    -------- ``` | | | | | | | | | | | | | | | | | | | | | | | | | | | | | | | | | | | | | | | | | | | | | | | | | |
|  | 6rxj.1.A | NAD-dependent protein deacylase  *Crystal structure of CobB wt in complex with H4K16-Acetyl peptide* | 0.03 |  | 23.08 | 0.13 | 290-343 | X-ray | 1.60 | hetero-1-1-mer | 1 x ZN | HHblits | 0.31 |
| ``` target    NNVNRREFLQWIGAAGFSTFALSASNAWGLQAIENPLAAYPNREWEKTYRDLWKSDASFTFLCAPNDTHNCILNAHVRDG 6rxj.1    --------------------------------------------------------------------------------  target    VITRIGPTMKYGEATDLYGSKVTHRWDPRVCQKGLALTRRFYGDRRVRYPMVRKGFKAWADKGFPREKDGRPPKDYFNRA 6rxj.1    --------------------------------------------------------------------------------  target    RDEWLRLTHEEAADLVAAALINIATTYSGDNGQKLLLQQGYEKEIVEATRGAGTQVLKFRGGMPLLGLTRIFGLYRMANS 6rxj.1    --------------------------------------------------------------------------------  target    MALLDHKIRGVKPEDALGARGWDNYSWHTDLPPGHPMVTGQQTVDFDLHAVEQARIVVVWGMNWVTTKMPDTHWLTEARL 6rxj.1    -------------------------------------------------ALSMADIFIAIGTSGHV--YPAAGFVHEAKL  target    KGTKVVVIACEYSSSSIKADDAIVVRPGTTPALALGLCNVIMREKIYDGDYVRRFSDLPLLVRADNLKLLRAEEVFGTPQ 6rxj.1    HGAHTVELNLEPSQVGNEFAEKY---------------------------------------------------------  target    AALKNQTR 6rxj.1    -------- ``` | | | | | | | | | | | | | | | | | | | | | | | | | | | | | | | | | | | | | | | | | | | | | | | | | |
|  | 2x3y.1.A | PHOSPHOHEPTOSE ISOMERASE  *Crystal structure of GmhA from Burkholderia pseudomallei* | 0.03 |  | 12.73 | 0.13 | 293-349 | X-ray | 2.40 | homo-tetramer | 4 x ZN | HHblits | 0.26 |
| ``` target    NNVNRREFLQWIGAAGFSTFALSASNAWGLQAIENPLAAYPNREWEKTYRDLWKSDASFTFLCAPNDTHNCILNAHVRDG 2x3y.1    --------------------------------------------------------------------------------  target    VITRIGPTMKYGEATDLYGSKVTHRWDPRVCQKGLALTRRFYGDRRVRYPMVRKGFKAWADKGFPREKDGRPPKDYFNRA 2x3y.1    --------------------------------------------------------------------------------  target    RDEWLRLTHEEAADLVAAALINIATTYSGDNGQKLLLQQGYEKEIVEATRGAGTQVLKFRGGMPLLGLTRIFGLYRMANS 2x3y.1    --------------------------------------------------------------------------------  target    MALLDHKIRGVKPEDALGARGWDNYSWHTDLPPGHPMVTGQQTVDFDLHAVEQARIVVVWGMNWVTTKMPDTHWLTEARL 2x3y.1    ----------------------------------------------------EGDVLIGYSTSGKS--PNILAAFREAKA  target    KGTKVVVIACE-YSSSSIKADDAIVVRPGTTPALALGLCNVIMREKIYDGDYVRRFSDLPLLVRADNLKLLRAEEVFGTP 2x3y.1    KGMTCVGFTGNRGGEMRELCDLLLEVPSAD--------------------------------------------------  target    QAALKNQTR 2x3y.1    --------- ``` | | | | | | | | | | | | | | | | | | | | | | | | | | | | | | | | | | | | | | | | | | | | | | | | | |
|  | 5nqd.1.A | AroA  *Arsenite oxidase AioAB from Rhizobium sp. str. NT-26 mutant AioBF108A* | 0.02 |  | 10.71 | 0.14 | 293-348 | X-ray | 2.20 | hetero-2-2-mer | 4 x MGD, 2 x O, 2 x 4MO, 2 x F3S, 2 x FES | HHblits | 0.25 |
| ``` target    NNVNRREFLQWIGAAGFSTFALSASNAWGLQAIENPLAAYPNREWEKTYRDLWKSDASFTFLCAPNDTHNCILNAHVRDG 5nqd.1    --------------------------------------------------------------------------------  target    VITRIGPTMKYGEATDLYGSKVTHRWDPRVCQKGLALTRRFYGDRRVRYPMVRKGFKAWADKGFPREKDGRPPKDYFNRA 5nqd.1    --------------------------------------------------------------------------------  target    RDEWLRLTHEEAADLVAAALINIATTYSGDNGQKLLLQQGYEKEIVEATRGAGTQVLKFRGGMPLLGLTRIFGLYRMANS 5nqd.1    --------------------------------------------------------------------------------  target    MALLDHKIRGVKPEDALGARGWDNYSWHTDLPPGHPMVTGQQTVDFDLHAVEQARIVVVWGMNWVTTK-----MPD---- 5nqd.1    ----------------------------------------------------EFKRVYKKRTDMVKDAMSAAPYGDREAM  target    THWLTEARLKG-TKVVVIACEYSSSSIKADDAIVVRPGTTPALALGLCNVIMREKIYDGDYVRRFSDLPLLVRADNLKLL 5nqd.1    VNAIVDAINQGGLFAVNVDIIPTKIGEACHVILPAATS------------------------------------------  target    RAEEVFGTPQAALKNQTR 5nqd.1    ------------------ ``` | | | | | | | | | | | | | | | | | | | | | | | | | | | | | | | | | | | | | | | | | | | | | | | | | |
|  | 2ivf.1.A | ETHYLBENZENE DEHYDROGENASE ALPHA-SUBUNIT  *ETHYLBENZENE DEHYDROGENASE FROM AROMATOLEUM AROMATICUM* | 0.04 |  | 14.81 | 0.13 | 294-347 | X-ray | 1.88 | hetero-oligomer | 1 x MES, 4 x SF4, 1 x MO, 1 x MGD, 1 x MD1, 1 x F3S, 1 x HEM | HHblits | 0.27 |
| ``` target    NNVNRREFLQWIGAAGFSTFALSASNAWGLQAIENPLAAYPNREWEKTYRDLWKSDASFTFLCAPNDTHNCILNAHVRDG 2ivf.1    --------------------------------------------------------------------------------  target    VITRIGPTMKYGEATDLYGSKVTHRWDPRVCQKGLALTRRFYGDRRVRYPMVRKGFKAWADKGFPREKDGRPPKDYFNRA 2ivf.1    --------------------------------------------------------------------------------  target    RDEWLRLTHEEAADLVAAALINIATTYSGDNGQKLLLQQGYEKEIVEATRGAGTQVLKFRGGMPLLGLTRIFGLYRMANS 2ivf.1    --------------------------------------------------------------------------------  target    MALLDHKIRGVKPEDALGARGWDNYSWHTDLPPGHPMVTGQQTVDFDLHAVEQARIVVVWGMNWVTTKMPDTHWL-TEAR 2ivf.1    -----------------------------------------------------PQVYMLLSQNPMRRKRSGAKMFPDVLF  target    LKGTKVVVIACEYSSSSIKADDAIVVRPGTTPALALGLCNVIMREKIYDGDYVRRFSDLPLLVRADNLKLLRAEEVFGTP 2ivf.1    PKLKMIFALETRMSSSAMYADIVLPCAW----------------------------------------------------  target    QAALKNQTR 2ivf.1    --------- ``` | | | | | | | | | | | | | | | | | | | | | | | | | | | | | | | | | | | | | | | | | | | | | | | | | |
|  | 8e9g.1.G | NADH-quinone oxidoreductase subunit G  *Mycobacterial respiratory complex I with both quinone positions modelled* | 0.03 |  | 22.64 | 0.13 | 294-348 | EM | 0.00 | hetero-1-1-1-1-1-1-… |  | HHblits | 0.29 |
| ``` target    NNVNRREFLQWIGAAGFSTFALSASNAWGLQAIENPLAAYPNREWEKTYRDLWKSDASFTFLCAPNDTHNCILNAHVRDG 8e9g.1    --------------------------------------------------------------------------------  target    VITRIGPTMKYGEATDLYGSKVTHRWDPRVCQKGLALTRRFYGDRRVRYPMVRKGFKAWADKGFPREKDGRPPKDYFNRA 8e9g.1    --------------------------------------------------------------------------------  target    RDEWLRLTHEEAADLVAAALINIATTYSGDNGQKLLLQQGYEKEIVEATRGAGTQVLKFRGGMPLLGLTRIFGLYRMANS 8e9g.1    --------------------------------------------------------------------------------  target    MALLDHKIRGVKPEDALGARGWDNYSWHTDLPPGHPMVTGQQTVDFDLHAVEQARIVVVWGMNWVTTKMPDTHWLTEARL 8e9g.1    -----------------------------------------------------LAALLVGG-VELGD-LPDPELAVAAVR  target    KGTKVVVIACEYSSSSIKADDAIVVRPGTTPALALGLCNVIMREKIYDGDYVRRFSDLPLLVRADNLKLLRAEEVFGTPQ 8e9g.1    TTPFVVSLELRESAVTELADVVFPVAPV----------------------------------------------------  target    AALKNQTR 8e9g.1    -------- ``` | | | | | | | | | | | | | | | | | | | | | | | | | | | | | | | | | | | | | | | | | | | | | | | | | |
|  | 6enx.1.A | NAD-dependent protein deacylase sirtuin-5, mitochondrial  *Zebrafish Sirt5 in complex with stalled bicyclic intermediate of inhibitory compound 10* | 0.03 |  | 13.21 | 0.13 | 290-344 | X-ray | 1.95 | monomer | 1 x ZN, 1 x BJW | HHblits | 0.28 |
| ``` target    NNVNRREFLQWIGAAGFSTFALSASNAWGLQAIENPLAAYPNREWEKTYRDLWKSDASFTFLCAPNDTHNCILNAHVRDG 6enx.1    --------------------------------------------------------------------------------  target    VITRIGPTMKYGEATDLYGSKVTHRWDPRVCQKGLALTRRFYGDRRVRYPMVRKGFKAWADKGFPREKDGRPPKDYFNRA 6enx.1    --------------------------------------------------------------------------------  target    RDEWLRLTHEEAADLVAAALINIATTYSGDNGQKLLLQQGYEKEIVEATRGAGTQVLKFRGGMPLLGLTRIFGLYRMANS 6enx.1    --------------------------------------------------------------------------------  target    MALLDHKIRGVKPEDALGARGWDNYSWHTDLPPGHPMVTGQQTVDFDLHAVEQARIVVVWGMNWVTTKMPDTHWLTEARL 6enx.1    -------------------------------------------------ELEKCDLCLVVGTSSIV--YPAAMFAPQVAS  target    KGTKVVVIACEYSSSSIKADDAIVVRPGTTPALALGLCNVIMREKIYDGDYVRRFSDLPLLVRADNLKLLRAEEVFGTPQ 6enx.1    RGVPVAEFNMECTPATQRFKYHFE--------------------------------------------------------  target    AALKNQTR 6enx.1    -------- ``` | | | | | | | | | | | | | | | | | | | | | | | | | | | | | | | | | | | | | | | | | | | | | | | | | |
|  | 4utn.1.A | NAD-DEPENDENT PROTEIN DEACYLASE SIRTUIN-5, MITOCHONDRIAL  *Crystal structure of zebrafish Sirtuin 5 in complex with succinylated CPS1-peptide* | 0.03 |  | 13.21 | 0.13 | 290-344 | X-ray | 3.00 | monomer | 1 x ZN, 1 x BEZ-GLY-VAL-LEU-SLL-GLU-TYR-GLY-VAL | HHblits | 0.28 |
| ``` target    NNVNRREFLQWIGAAGFSTFALSASNAWGLQAIENPLAAYPNREWEKTYRDLWKSDASFTFLCAPNDTHNCILNAHVRDG 4utn.1    --------------------------------------------------------------------------------  target    VITRIGPTMKYGEATDLYGSKVTHRWDPRVCQKGLALTRRFYGDRRVRYPMVRKGFKAWADKGFPREKDGRPPKDYFNRA 4utn.1    --------------------------------------------------------------------------------  target    RDEWLRLTHEEAADLVAAALINIATTYSGDNGQKLLLQQGYEKEIVEATRGAGTQVLKFRGGMPLLGLTRIFGLYRMANS 4utn.1    --------------------------------------------------------------------------------  target    MALLDHKIRGVKPEDALGARGWDNYSWHTDLPPGHPMVTGQQTVDFDLHAVEQARIVVVWGMNWVTTKMPDTHWLTEARL 4utn.1    -------------------------------------------------ELEKCDLCLVVGTSSIV--YPAAMFAPQVAS  target    KGTKVVVIACEYSSSSIKADDAIVVRPGTTPALALGLCNVIMREKIYDGDYVRRFSDLPLLVRADNLKLLRAEEVFGTPQ 4utn.1    RGVPVAEFNMECTPATQRFKYHFE--------------------------------------------------------  target    AALKNQTR 4utn.1    -------- ``` | | | | | | | | | | | | | | | | | | | | | | | | | | | | | | | | | | | | | | | | | | | | | | | | | |
|  | 4utn.2.A | NAD-DEPENDENT PROTEIN DEACYLASE SIRTUIN-5, MITOCHONDRIAL  *Crystal structure of zebrafish Sirtuin 5 in complex with succinylated CPS1-peptide* | 0.03 |  | 13.21 | 0.13 | 290-344 | X-ray | 3.00 | monomer | 1 x ZN | HHblits | 0.28 |
| ``` target    NNVNRREFLQWIGAAGFSTFALSASNAWGLQAIENPLAAYPNREWEKTYRDLWKSDASFTFLCAPNDTHNCILNAHVRDG 4utn.2    --------------------------------------------------------------------------------  target    VITRIGPTMKYGEATDLYGSKVTHRWDPRVCQKGLALTRRFYGDRRVRYPMVRKGFKAWADKGFPREKDGRPPKDYFNRA 4utn.2    --------------------------------------------------------------------------------  target    RDEWLRLTHEEAADLVAAALINIATTYSGDNGQKLLLQQGYEKEIVEATRGAGTQVLKFRGGMPLLGLTRIFGLYRMANS 4utn.2    --------------------------------------------------------------------------------  target    MALLDHKIRGVKPEDALGARGWDNYSWHTDLPPGHPMVTGQQTVDFDLHAVEQARIVVVWGMNWVTTKMPDTHWLTEARL 4utn.2    -------------------------------------------------ELEKCDLCLVVGTSSIV--YPAAMFAPQVAS  target    KGTKVVVIACEYSSSSIKADDAIVVRPGTTPALALGLCNVIMREKIYDGDYVRRFSDLPLLVRADNLKLLRAEEVFGTPQ 4utn.2    RGVPVAEFNMECTPATQRFKYHFE--------------------------------------------------------  target    AALKNQTR 4utn.2    -------- ``` | | | | | | | | | | | | | | | | | | | | | | | | | | | | | | | | | | | | | | | | | | | | | | | | | |
|  | 8bqg.1.A | Formate dehydrogenase, alpha subunit, selenocysteine-containing  *W-formate dehydrogenase from Desulfovibrio vulgaris - Soaking with Formate 1 min* | 0.03 |  | 16.67 | 0.13 | 292-346 | X-ray | 1.95 | hetero-1-1-mer | 2 x MGD, 4 x SF4, 1 x H2S, 1 x W | HHblits | 0.27 |
| ``` target    NNVNRREFLQWIGAAGFSTFALSASNAWGLQAIENPLAAYPNREWEKTYRDLWKSDASFTFLCAPNDTHNCILNAHVRDG 8bqg.1    --------------------------------------------------------------------------------  target    VITRIGPTMKYGEATDLYGSKVTHRWDPRVCQKGLALTRRFYGDRRVRYPMVRKGFKAWADKGFPREKDGRPPKDYFNRA 8bqg.1    --------------------------------------------------------------------------------  target    RDEWLRLTHEEAADLVAAALINIATTYSGDNGQKLLLQQGYEKEIVEATRGAGTQVLKFRGGMPLLGLTRIFGLYRMANS 8bqg.1    --------------------------------------------------------------------------------  target    MALLDHKIRGVKPEDALGARGWDNYSWHTDLPPGHPMVTGQQTVDFDLHAVEQARIVVVWGMNWVTTKMPDTHWLTEARL 8bqg.1    ---------------------------------------------------GEFKGLFAWGMNPACGGA-NANKNRKAMG  target    KGTKVVVIACEYSSSSIK--------AD-----DAIVVRPGTTPALALGLCNVIMREKIYDGDYVRRFSDLPLLVRADNL 8bqg.1    KLEWLVNVNLFENETSSFWKGPGMNPAEIGTEVFFLPCC-----------------------------------------  target    KLLRAEEVFGTPQAALKNQTR 8bqg.1    --------------------- ``` | | | | | | | | | | | | | | | | | | | | | | | | | | | | | | | | | | | | | | | | | | | | | | | | | |
|  | 4dmr.1.A | DMSO REDUCTASE  *REDUCED DMSO REDUCTASE FROM RHODOBACTER CAPSULATUS WITH BOUND DMSO SUBSTRATE* | 0.04 |  | 14.81 | 0.13 | 293-347 | X-ray | 1.90 | monomer | 2 x PGD, 1 x 4MO, 1 x O | HHblits | 0.27 |
| ``` target    NNVNRREFLQWIGAAGFSTFALSASNAWGLQAIENPLAAYPNREWEKTYRDLWKSDASFTFLCAPNDTHNCILNAHVRDG 4dmr.1    --------------------------------------------------------------------------------  target    VITRIGPTMKYGEATDLYGSKVTHRWDPRVCQKGLALTRRFYGDRRVRYPMVRKGFKAWADKGFPREKDGRPPKDYFNRA 4dmr.1    --------------------------------------------------------------------------------  target    RDEWLRLTHEEAADLVAAALINIATTYSGDNGQKLLLQQGYEKEIVEATRGAGTQVLKFRGGMPLLGLTRIFGLYRMANS 4dmr.1    --------------------------------------------------------------------------------  target    MALLDHKIRGVKPEDALGARGWDNYSWHTDLPPGHPMVTGQQTVDFDLHAVEQARIVVVWGMNWVTTKMPDTHWLTEARL 4dmr.1    ----------------------------------------------------DVKMAYWVGGNPFVHHQD-RNRMVKAWE  target    KGTKVVVIACEYSSSSIKADDAIVVRPGTTPALALGLCNVIMREKIYDGDYVRRFSDLPLLVRADNLKLLRAEEVFGTPQ 4dmr.1    KLETFVVHDFQWTPTARHADIVLPATT-----------------------------------------------------  target    AALKNQTR 4dmr.1    -------- ``` | | | | | | | | | | | | | | | | | | | | | | | | | | | | | | | | | | | | | | | | | | | | | | | | | |
|  | 1e60.1.A | Dimethyl sulfoxide/trimethylamine N-oxide reductase  *OXIDIZED DMSO REDUCTASE EXPOSED TO HEPES - Structure II BUFFER* | 0.04 |  | 14.81 | 0.13 | 293-347 | X-ray | 2.00 | monomer | 2 x PGD, 1 x 2MO | HHblits | 0.27 |
| ``` target    NNVNRREFLQWIGAAGFSTFALSASNAWGLQAIENPLAAYPNREWEKTYRDLWKSDASFTFLCAPNDTHNCILNAHVRDG 1e60.1    --------------------------------------------------------------------------------  target    VITRIGPTMKYGEATDLYGSKVTHRWDPRVCQKGLALTRRFYGDRRVRYPMVRKGFKAWADKGFPREKDGRPPKDYFNRA 1e60.1    --------------------------------------------------------------------------------  target    RDEWLRLTHEEAADLVAAALINIATTYSGDNGQKLLLQQGYEKEIVEATRGAGTQVLKFRGGMPLLGLTRIFGLYRMANS 1e60.1    --------------------------------------------------------------------------------  target    MALLDHKIRGVKPEDALGARGWDNYSWHTDLPPGHPMVTGQQTVDFDLHAVEQARIVVVWGMNWVTTKMPDTHWLTEARL 1e60.1    ----------------------------------------------------DVKMAYWVGGNPFVHHQD-RNRMVKAWE  target    KGTKVVVIACEYSSSSIKADDAIVVRPGTTPALALGLCNVIMREKIYDGDYVRRFSDLPLLVRADNLKLLRAEEVFGTPQ 1e60.1    KLETFVVHDFQWTPTARHADIVLPATT-----------------------------------------------------  target    AALKNQTR 1e60.1    -------- ``` | | | | | | | | | | | | | | | | | | | | | | | | | | | | | | | | | | | | | | | | | | | | | | | | | |
|  | 2v3v.1.A | PERIPLASMIC NITRATE REDUCTASE  *A NEW CATALYTIC MECHANISM OF PERIPLASMIC NITRATE REDUCTASE FROM DESULFOVIBRIO DESULFURICANS ATCC 27774 FROM CRYSTALLOGRAPHIC AND EPR DATA AND BASED ON DETAILED ANALYSIS OF THE SIXTH LIGAND* | 0.03 |  | 12.73 | 0.13 | 293-348 | X-ray | 1.99 | monomer | 1 x SF4, 1 x MO, 2 x MGD, 4 x LCP | HHblits | 0.25 |
| ``` target    NNVNRREFLQWIGAAGFSTFALSASNAWGLQAIENPLAAYPNREWEKTYRDLWKSDASFTFLCAPNDTHNCILNAHVRDG 2v3v.1    --------------------------------------------------------------------------------  target    VITRIGPTMKYGEATDLYGSKVTHRWDPRVCQKGLALTRRFYGDRRVRYPMVRKGFKAWADKGFPREKDGRPPKDYFNRA 2v3v.1    --------------------------------------------------------------------------------  target    RDEWLRLTHEEAADLVAAALINIATTYSGDNGQKLLLQQGYEKEIVEATRGAGTQVLKFRGGMPLLGLTRIFGLYRMANS 2v3v.1    --------------------------------------------------------------------------------  target    MALLDHKIRGVKPEDALGARGWDNYSWHTDLPPGHPMVTGQQTVDFDLHAVEQARIVVVWGMNWVTTKMPDTHWLTEARL 2v3v.1    ----------------------------------------------------DVKCMIICETNPAHTLPN-LNKVHKAMS  target    K-GTKVVVIACEYS-SSSIKADDAIVVRPGTTPALALGLCNVIMREKIYDGDYVRRFSDLPLLVRADNLKLLRAEEVFGT 2v3v.1    HPESFIVCIEAFPDAVTLEYADLVLPPAFW--------------------------------------------------  target    PQAALKNQTR 2v3v.1    ---------- ``` | | | | | | | | | | | | | | | | | | | | | | | | | | | | | | | | | | | | | | | | | | | | | | | | | |
|  | 7qv7.1.L | Hydrogen dependent carbon dioxide reductase subunit FdhF  *Cryo-EM structure of Hydrogen-dependent CO2 reductase.* | 0.03 |  | 12.96 | 0.13 | 293-347 | EM | 0.00 | hetero-2-6-6-2-mer | 52 x SF4, 6 x 402 | HHblits | 0.26 |
| ``` target    NNVNRREFLQWIGAAGFSTFALSASNAWGLQAIENPLAAYPNREWEKTYRDLWKSDASFTFLCAPNDTHNCILNAHVRDG 7qv7.1    --------------------------------------------------------------------------------  target    VITRIGPTMKYGEATDLYGSKVTHRWDPRVCQKGLALTRRFYGDRRVRYPMVRKGFKAWADKGFPREKDGRPPKDYFNRA 7qv7.1    --------------------------------------------------------------------------------  target    RDEWLRLTHEEAADLVAAALINIATTYSGDNGQKLLLQQGYEKEIVEATRGAGTQVLKFRGGMPLLGLTRIFGLYRMANS 7qv7.1    --------------------------------------------------------------------------------  target    MALLDHKIRGVKPEDALGARGWDNYSWHTDLPPGHPMVTGQQTVDFDLHAVEQARIVVVWGMNWVTTKMPDTHWLTEARL 7qv7.1    ----------------------------------------------------RVRALYIFGENPIMSDPD-SDHLRHALE  target    KGTKVVVIACEYSSSSIKADDAIVVRPGTTPALALGLCNVIMREKIYDGDYVRRFSDLPLLVRADNLKLLRAEEVFGTPQ 7qv7.1    HLDLLIVQDIFLTETARLAHVVLPAAC-----------------------------------------------------  target    AALKNQTR 7qv7.1    -------- ``` | | | | | | | | | | | | | | | | | | | | | | | | | | | | | | | | | | | | | | | | | | | | | | | | | |
|  | 7qv7.1.O | Hydrogen dependent carbon dioxide reductase subunit FdhF  *Cryo-EM structure of Hydrogen-dependent CO2 reductase.* | 0.03 |  | 12.96 | 0.13 | 293-347 | EM | 0.00 | hetero-2-6-6-2-mer | 52 x SF4, 6 x 402 | HHblits | 0.26 |
| ``` target    NNVNRREFLQWIGAAGFSTFALSASNAWGLQAIENPLAAYPNREWEKTYRDLWKSDASFTFLCAPNDTHNCILNAHVRDG 7qv7.1    --------------------------------------------------------------------------------  target    VITRIGPTMKYGEATDLYGSKVTHRWDPRVCQKGLALTRRFYGDRRVRYPMVRKGFKAWADKGFPREKDGRPPKDYFNRA 7qv7.1    --------------------------------------------------------------------------------  target    RDEWLRLTHEEAADLVAAALINIATTYSGDNGQKLLLQQGYEKEIVEATRGAGTQVLKFRGGMPLLGLTRIFGLYRMANS 7qv7.1    --------------------------------------------------------------------------------  target    MALLDHKIRGVKPEDALGARGWDNYSWHTDLPPGHPMVTGQQTVDFDLHAVEQARIVVVWGMNWVTTKMPDTHWLTEARL 7qv7.1    ----------------------------------------------------RVRALYIFGENPIMSDPD-SDHLRHALE  target    KGTKVVVIACEYSSSSIKADDAIVVRPGTTPALALGLCNVIMREKIYDGDYVRRFSDLPLLVRADNLKLLRAEEVFGTPQ 7qv7.1    HLDLLIVQDIFLTETARLAHVVLPAAC-----------------------------------------------------  target    AALKNQTR 7qv7.1    -------- ``` | | | | | | | | | | | | | | | | | | | | | | | | | | | | | | | | | | | | | | | | | | | | | | | | | |
|  | 5xhs.1.A | NAD-dependent protein deacylase sirtuin-5, mitochondrial  *Crystal structure of SIRT5 complexed with a fluorogenic small-molecule substrate SuBKA* | 0.03 |  | 13.21 | 0.13 | 290-344 | X-ray | 2.19 | monomer | 1 x PHQ, 1 x SLL, 1 x MCM, 1 x ZN | HHblits | 0.28 |
| ``` target    NNVNRREFLQWIGAAGFSTFALSASNAWGLQAIENPLAAYPNREWEKTYRDLWKSDASFTFLCAPNDTHNCILNAHVRDG 5xhs.1    --------------------------------------------------------------------------------  target    VITRIGPTMKYGEATDLYGSKVTHRWDPRVCQKGLALTRRFYGDRRVRYPMVRKGFKAWADKGFPREKDGRPPKDYFNRA 5xhs.1    --------------------------------------------------------------------------------  target    RDEWLRLTHEEAADLVAAALINIATTYSGDNGQKLLLQQGYEKEIVEATRGAGTQVLKFRGGMPLLGLTRIFGLYRMANS 5xhs.1    --------------------------------------------------------------------------------  target    MALLDHKIRGVKPEDALGARGWDNYSWHTDLPPGHPMVTGQQTVDFDLHAVEQARIVVVWGMNWVTTKMPDTHWLTEARL 5xhs.1    -------------------------------------------------ELAHCDLCLVVGTSSVV--YPAAMFAPQVAA  target    KGTKVVVIACEYSSSSIKADDAIVVRPGTTPALALGLCNVIMREKIYDGDYVRRFSDLPLLVRADNLKLLRAEEVFGTPQ 5xhs.1    RGVPVAEFNTETTPATNRFRFHFQ--------------------------------------------------------  target    AALKNQTR 5xhs.1    -------- ``` | | | | | | | | | | | | | | | | | | | | | | | | | | | | | | | | | | | | | | | | | | | | | | | | | |
|  | 6ljm.1.A | NAD-dependent protein deacylase sirtuin-5, mitochondrial  *Crystal structure of human Sirt5 in complex with the fluorogenic tetrapeptide substrate P13* | 0.03 |  | 13.21 | 0.13 | 290-344 | X-ray | 1.78 | monomer | 1 x ZN, 1 x SIN, 1 x MCM, 1 x SER-LEU-GLY-LYS | HHblits | 0.28 |
| ``` target    NNVNRREFLQWIGAAGFSTFALSASNAWGLQAIENPLAAYPNREWEKTYRDLWKSDASFTFLCAPNDTHNCILNAHVRDG 6ljm.1    --------------------------------------------------------------------------------  target    VITRIGPTMKYGEATDLYGSKVTHRWDPRVCQKGLALTRRFYGDRRVRYPMVRKGFKAWADKGFPREKDGRPPKDYFNRA 6ljm.1    --------------------------------------------------------------------------------  target    RDEWLRLTHEEAADLVAAALINIATTYSGDNGQKLLLQQGYEKEIVEATRGAGTQVLKFRGGMPLLGLTRIFGLYRMANS 6ljm.1    --------------------------------------------------------------------------------  target    MALLDHKIRGVKPEDALGARGWDNYSWHTDLPPGHPMVTGQQTVDFDLHAVEQARIVVVWGMNWVTTKMPDTHWLTEARL 6ljm.1    -------------------------------------------------ELAHCDLCLVVGTSSVV--YPAAMFAPQVAA  target    KGTKVVVIACEYSSSSIKADDAIVVRPGTTPALALGLCNVIMREKIYDGDYVRRFSDLPLLVRADNLKLLRAEEVFGTPQ 6ljm.1    RGVPVAEFNTETTPATNRFRFHFQ--------------------------------------------------------  target    AALKNQTR 6ljm.1    -------- ``` | | | | | | | | | | | | | | | | | | | | | | | | | | | | | | | | | | | | | | | | | | | | | | | | | |
|  | 6ljk.1.A | NAD-dependent protein deacylase sirtuin-5, mitochondrial  *Crystal structure of human Sirt5 in complex with an internally quenched fluorescent substrate GluIQF* | 0.03 |  | 13.21 | 0.13 | 290-344 | X-ray | 1.39 | monomer | 1 x ZN, 1 x GUA, 1 x BE2-SER-ALA-ILE-LYS-SER-NIY-GLY-SET | HHblits | 0.28 |
| ``` target    NNVNRREFLQWIGAAGFSTFALSASNAWGLQAIENPLAAYPNREWEKTYRDLWKSDASFTFLCAPNDTHNCILNAHVRDG 6ljk.1    --------------------------------------------------------------------------------  target    VITRIGPTMKYGEATDLYGSKVTHRWDPRVCQKGLALTRRFYGDRRVRYPMVRKGFKAWADKGFPREKDGRPPKDYFNRA 6ljk.1    --------------------------------------------------------------------------------  target    RDEWLRLTHEEAADLVAAALINIATTYSGDNGQKLLLQQGYEKEIVEATRGAGTQVLKFRGGMPLLGLTRIFGLYRMANS 6ljk.1    --------------------------------------------------------------------------------  target    MALLDHKIRGVKPEDALGARGWDNYSWHTDLPPGHPMVTGQQTVDFDLHAVEQARIVVVWGMNWVTTKMPDTHWLTEARL 6ljk.1    -------------------------------------------------ELAHCDLCLVVGTSSVV--YPAAMFAPQVAA  target    KGTKVVVIACEYSSSSIKADDAIVVRPGTTPALALGLCNVIMREKIYDGDYVRRFSDLPLLVRADNLKLLRAEEVFGTPQ 6ljk.1    RGVPVAEFNTETTPATNRFRFHFQ--------------------------------------------------------  target    AALKNQTR 6ljk.1    -------- ``` | | | | | | | | | | | | | | | | | | | | | | | | | | | | | | | | | | | | | | | | | | | | | | | | | |
|  | 6acp.1.A | NAD-dependent protein deacylase sirtuin-5, mitochondrial  *histone lysine desuccinylase Sirt5 in complex with succinyl peptide H4K91* | 0.03 |  | 13.21 | 0.13 | 290-344 | X-ray | 2.30 | monomer | 1 x ZN, 1 x TYR-ALA-LEU-SLL-ARG-GLN-GLY | HHblits | 0.28 |
| ``` target    NNVNRREFLQWIGAAGFSTFALSASNAWGLQAIENPLAAYPNREWEKTYRDLWKSDASFTFLCAPNDTHNCILNAHVRDG 6acp.1    --------------------------------------------------------------------------------  target    VITRIGPTMKYGEATDLYGSKVTHRWDPRVCQKGLALTRRFYGDRRVRYPMVRKGFKAWADKGFPREKDGRPPKDYFNRA 6acp.1    --------------------------------------------------------------------------------  target    RDEWLRLTHEEAADLVAAALINIATTYSGDNGQKLLLQQGYEKEIVEATRGAGTQVLKFRGGMPLLGLTRIFGLYRMANS 6acp.1    --------------------------------------------------------------------------------  target    MALLDHKIRGVKPEDALGARGWDNYSWHTDLPPGHPMVTGQQTVDFDLHAVEQARIVVVWGMNWVTTKMPDTHWLTEARL 6acp.1    -------------------------------------------------ELAHCDLCLVVGTSSVV--YPAAMFAPQVAA  target    KGTKVVVIACEYSSSSIKADDAIVVRPGTTPALALGLCNVIMREKIYDGDYVRRFSDLPLLVRADNLKLLRAEEVFGTPQ 6acp.1    RGVPVAEFNTETTPATNRFRFHFQ--------------------------------------------------------  target    AALKNQTR 6acp.1    -------- ``` | | | | | | | | | | | | | | | | | | | | | | | | | | | | | | | | | | | | | | | | | | | | | | | | | |
|  | 4g1c.2.A | NAD-dependent protein deacylase sirtuin-5, mitochondrial  *Human SIRT5 bound to Succ-IDH2 and Carba-NAD* | 0.03 |  | 13.21 | 0.13 | 290-344 | X-ray | 1.94 | monomer | 1 x ZN, 1 x ACE-ALA-VAL-SLL-CYS-ALA-NH2 | HHblits | 0.28 |
| ``` target    NNVNRREFLQWIGAAGFSTFALSASNAWGLQAIENPLAAYPNREWEKTYRDLWKSDASFTFLCAPNDTHNCILNAHVRDG 4g1c.2    --------------------------------------------------------------------------------  target    VITRIGPTMKYGEATDLYGSKVTHRWDPRVCQKGLALTRRFYGDRRVRYPMVRKGFKAWADKGFPREKDGRPPKDYFNRA 4g1c.2    --------------------------------------------------------------------------------  target    RDEWLRLTHEEAADLVAAALINIATTYSGDNGQKLLLQQGYEKEIVEATRGAGTQVLKFRGGMPLLGLTRIFGLYRMANS 4g1c.2    --------------------------------------------------------------------------------  target    MALLDHKIRGVKPEDALGARGWDNYSWHTDLPPGHPMVTGQQTVDFDLHAVEQARIVVVWGMNWVTTKMPDTHWLTEARL 4g1c.2    -------------------------------------------------ELAHCDLCLVVGTSSVV--YPAAMFAPQVAA  target    KGTKVVVIACEYSSSSIKADDAIVVRPGTTPALALGLCNVIMREKIYDGDYVRRFSDLPLLVRADNLKLLRAEEVFGTPQ 4g1c.2    RGVPVAEFNTETTPATNRFRFHFQ--------------------------------------------------------  target    AALKNQTR 4g1c.2    -------- ``` | | | | | | | | | | | | | | | | | | | | | | | | | | | | | | | | | | | | | | | | | | | | | | | | | |
|  | 4g1c.1.A | NAD-dependent protein deacylase sirtuin-5, mitochondrial  *Human SIRT5 bound to Succ-IDH2 and Carba-NAD* | 0.03 |  | 13.21 | 0.13 | 290-344 | X-ray | 1.94 | monomer | 1 x ZN, 1 x CNA, 1 x ACE-ALA-VAL-SLL-CYS-ALA-NH2 | HHblits | 0.28 |
| ``` target    NNVNRREFLQWIGAAGFSTFALSASNAWGLQAIENPLAAYPNREWEKTYRDLWKSDASFTFLCAPNDTHNCILNAHVRDG 4g1c.1    --------------------------------------------------------------------------------  target    VITRIGPTMKYGEATDLYGSKVTHRWDPRVCQKGLALTRRFYGDRRVRYPMVRKGFKAWADKGFPREKDGRPPKDYFNRA 4g1c.1    --------------------------------------------------------------------------------  target    RDEWLRLTHEEAADLVAAALINIATTYSGDNGQKLLLQQGYEKEIVEATRGAGTQVLKFRGGMPLLGLTRIFGLYRMANS 4g1c.1    --------------------------------------------------------------------------------  target    MALLDHKIRGVKPEDALGARGWDNYSWHTDLPPGHPMVTGQQTVDFDLHAVEQARIVVVWGMNWVTTKMPDTHWLTEARL 4g1c.1    -------------------------------------------------ELAHCDLCLVVGTSSVV--YPAAMFAPQVAA  target    KGTKVVVIACEYSSSSIKADDAIVVRPGTTPALALGLCNVIMREKIYDGDYVRRFSDLPLLVRADNLKLLRAEEVFGTPQ 4g1c.1    RGVPVAEFNTETTPATNRFRFHFQ--------------------------------------------------------  target    AALKNQTR 4g1c.1    -------- ``` | | | | | | | | | | | | | | | | | | | | | | | | | | | | | | | | | | | | | | | | | | | | | | | | | |
|  | 3riy.2.A | NAD-dependent deacetylase sirtuin-5  *Sirt5 is an NAD-dependent protein lysine demalonylase and desuccinylase* | 0.03 |  | 13.21 | 0.13 | 290-344 | X-ray | 1.55 | hetero-oligomer | 1 x ZN, 1 x NAD | HHblits | 0.28 |
| ``` target    NNVNRREFLQWIGAAGFSTFALSASNAWGLQAIENPLAAYPNREWEKTYRDLWKSDASFTFLCAPNDTHNCILNAHVRDG 3riy.2    --------------------------------------------------------------------------------  target    VITRIGPTMKYGEATDLYGSKVTHRWDPRVCQKGLALTRRFYGDRRVRYPMVRKGFKAWADKGFPREKDGRPPKDYFNRA 3riy.2    --------------------------------------------------------------------------------  target    RDEWLRLTHEEAADLVAAALINIATTYSGDNGQKLLLQQGYEKEIVEATRGAGTQVLKFRGGMPLLGLTRIFGLYRMANS 3riy.2    --------------------------------------------------------------------------------  target    MALLDHKIRGVKPEDALGARGWDNYSWHTDLPPGHPMVTGQQTVDFDLHAVEQARIVVVWGMNWVTTKMPDTHWLTEARL 3riy.2    -------------------------------------------------ELAHCDLCLVVGTSSVV--YPAAMFAPQVAA  target    KGTKVVVIACEYSSSSIKADDAIVVRPGTTPALALGLCNVIMREKIYDGDYVRRFSDLPLLVRADNLKLLRAEEVFGTPQ 3riy.2    RGVPVAEFNTETTPATNRFRFHFQ--------------------------------------------------------  target    AALKNQTR 3riy.2    -------- ``` | | | | | | | | | | | | | | | | | | | | | | | | | | | | | | | | | | | | | | | | | | | | | | | | | |
|  | 5bwl.1.A | NAD-dependent protein deacylase sirtuin-5, mitochondrial  *Crystal Structure of SIRT5 in Complex with a Coumarin-Labelled Succinyl Peptide* | 0.03 |  | 13.21 | 0.13 | 290-344 | X-ray | 1.55 | monomer | 1 x ZN, 1 x MCM, 1 x LEU-GLY-SLL | HHblits | 0.28 |
| ``` target    NNVNRREFLQWIGAAGFSTFALSASNAWGLQAIENPLAAYPNREWEKTYRDLWKSDASFTFLCAPNDTHNCILNAHVRDG 5bwl.1    --------------------------------------------------------------------------------  target    VITRIGPTMKYGEATDLYGSKVTHRWDPRVCQKGLALTRRFYGDRRVRYPMVRKGFKAWADKGFPREKDGRPPKDYFNRA 5bwl.1    --------------------------------------------------------------------------------  target    RDEWLRLTHEEAADLVAAALINIATTYSGDNGQKLLLQQGYEKEIVEATRGAGTQVLKFRGGMPLLGLTRIFGLYRMANS 5bwl.1    --------------------------------------------------------------------------------  target    MALLDHKIRGVKPEDALGARGWDNYSWHTDLPPGHPMVTGQQTVDFDLHAVEQARIVVVWGMNWVTTKMPDTHWLTEARL 5bwl.1    -------------------------------------------------ELAHCDLCLVVGTSSVV--YPAAMFAPQVAA  target    KGTKVVVIACEYSSSSIKADDAIVVRPGTTPALALGLCNVIMREKIYDGDYVRRFSDLPLLVRADNLKLLRAEEVFGTPQ 5bwl.1    RGVPVAEFNTETTPATNRFRFHFQ--------------------------------------------------------  target    AALKNQTR 5bwl.1    -------- ``` | | | | | | | | | | | | | | | | | | | | | | | | | | | | | | | | | | | | | | | | | | | | | | | | | |
|  | 4v4c.1.A | Pyrogallol hydroxytransferase large subunit  *Crystal Structure of Pyrogallol-Phloroglucinol Transhydroxylase from Pelobacter acidigallici* | 0.03 |  | 12.73 | 0.13 | 293-348 | X-ray | 2.35 | hetero-oligomer | 2 x CA, 2 x MGD, 1 x 4MO, 3 x SF4 | HHblits | 0.25 |
| ``` target    NNVNRREFLQWIGAAGFSTFALSASNAWGLQAIENPLAAYPNREWEKTYRDLWKSDASFTFLCAPNDTHNCILNAHVRDG 4v4c.1    --------------------------------------------------------------------------------  target    VITRIGPTMKYGEATDLYGSKVTHRWDPRVCQKGLALTRRFYGDRRVRYPMVRKGFKAWADKGFPREKDGRPPKDYFNRA 4v4c.1    --------------------------------------------------------------------------------  target    RDEWLRLTHEEAADLVAAALINIATTYSGDNGQKLLLQQGYEKEIVEATRGAGTQVLKFRGGMPLLGLTRIFGLYRMANS 4v4c.1    --------------------------------------------------------------------------------  target    MALLDHKIRGVKPEDALGARGWDNYSWHTDLPPGHPMVTGQQTVDFDLHAVEQARIVVVWGMNWVTTKMPDTHWLTEAR- 4v4c.1    ----------------------------------------------------KIKMFWKYGGPHLGTMT-ATNRYAKMYT  target    -LKGTKVVVIACEYSSSSIKADDAIVVRPGTTPALALGLCNVIMREKIYDGDYVRRFSDLPLLVRADNLKLLRAEEVFGT 4v4c.1    HDSLEFVVSQSIWFEGEVPFADIILPACTN--------------------------------------------------  target    PQAALKNQTR 4v4c.1    ---------- ``` | | | | | | | | | | | | | | | | | | | | | | | | | | | | | | | | | | | | | | | | | | | | | | | | | |
|  | 2h4h.1.A | NAD-dependent deacetylase  *Sir2 H116Y mutant-p53 peptide-NAD* | 0.03 |  | 16.98 | 0.13 | 290-344 | X-ray | 1.99 | hetero-1-1-mer | 1 x ZN, 1 x NAD | HHblits | 0.27 |
| ``` target    NNVNRREFLQWIGAAGFSTFALSASNAWGLQAIENPLAAYPNREWEKTYRDLWKSDASFTFLCAPNDTHNCILNAHVRDG 2h4h.1    --------------------------------------------------------------------------------  target    VITRIGPTMKYGEATDLYGSKVTHRWDPRVCQKGLALTRRFYGDRRVRYPMVRKGFKAWADKGFPREKDGRPPKDYFNRA 2h4h.1    --------------------------------------------------------------------------------  target    RDEWLRLTHEEAADLVAAALINIATTYSGDNGQKLLLQQGYEKEIVEATRGAGTQVLKFRGGMPLLGLTRIFGLYRMANS 2h4h.1    --------------------------------------------------------------------------------  target    MALLDHKIRGVKPEDALGARGWDNYSWHTDLPPGHPMVTGQQTVDFDLHAVEQARIVVVWGMNWVTTKMPDTHWLTEARL 2h4h.1    -------------------------------------------------LSSRASLMIVLGSSLVV--YPAAELPLITVR  target    KGTKVVVIACEYSSSSIKADDAIVVRPGTTPALALGLCNVIMREKIYDGDYVRRFSDLPLLVRADNLKLLRAEEVFGTPQ 2h4h.1    SGGKLVIVNLGETPFDDIATLKYN--------------------------------------------------------  target    AALKNQTR 2h4h.1    -------- ``` | | | | | | | | | | | | | | | | | | | | | | | | | | | | | | | | | | | | | | | | | | | | | | | | | |
|  | 7p61.1.C | NADH-quinone oxidoreductase  *Complex I from E. coli, DDM-purified, with NADH, Resting state* | 0.03 |  | 18.87 | 0.13 | 293-347 | EM | 0.00 | hetero-1-1-1-1-1-1-… | 7 x SF4, 1 x FMN, 1 x NAI, 2 x FES, 1 x CA, 2 x 3PE, 1 x UQ8 | HHblits | 0.27 |
| ``` target    NNVNRREFLQWIGAAGFSTFALSASNAWGLQAIENPLAAYPNREWEKTYRDLWKSDASFTFLCAPNDTHNCILNAHVRDG 7p61.1    --------------------------------------------------------------------------------  target    VITRIGPTMKYGEATDLYGSKVTHRWDPRVCQKGLALTRRFYGDRRVRYPMVRKGFKAWADKGFPREKDGRPPKDYFNRA 7p61.1    --------------------------------------------------------------------------------  target    RDEWLRLTHEEAADLVAAALINIATTYSGDNGQKLLLQQGYEKEIVEATRGAGTQVLKFRGGMPLLGLTRIFGLYRMANS 7p61.1    --------------------------------------------------------------------------------  target    MALLDHKIRGVKPEDALGARGWDNYSWHTDLPPGHPMVTGQQTVDFDLHAVEQARIVVVWGMNWVTTKMPDTHWLTEARL 7p61.1    ----------------------------------------------------RADAVVVLE-NDLHRHA-SATRVNAALA  target    KGTKVVVIACEYSSSSIKADDAIVVRPGTTPALALGLCNVIMREKIYDGDYVRRFSDLPLLVRADNLKLLRAEEVFGTPQ 7p61.1    KAPLVMVVDHQRTAIMENAHLVLSAAS-----------------------------------------------------  target    AALKNQTR 7p61.1    -------- ``` | | | | | | | | | | | | | | | | | | | | | | | | | | | | | | | | | | | | | | | | | | | | | | | | | |
|  | 6tg9.1.A | Formate dehydrogenase subunit alpha  *Cryo-EM Structure of NADH reduced form of NAD+-dependent Formate Dehydrogenase from Rhodobacter capsulatus* | 0.03 |  | 12.96 | 0.13 | 293-347 | EM | 3.24 | hetero-2-2-2-2-mer | 4 x MGD, 2 x 6MO, 4 x FES, 10 x SF4, 2 x H2S, 2 x FMN, 2 x NAI | HHblits | 0.26 |
| ``` target    NNVNRREFLQWIGAAGFSTFALSASNAWGLQAIENPLAAYPNREWEKTYRDLWKSDASFTFLCAPNDTHNCILNAHVRDG 6tg9.1    --------------------------------------------------------------------------------  target    VITRIGPTMKYGEATDLYGSKVTHRWDPRVCQKGLALTRRFYGDRRVRYPMVRKGFKAWADKGFPREKDGRPPKDYFNRA 6tg9.1    --------------------------------------------------------------------------------  target    RDEWLRLTHEEAADLVAAALINIATTYSGDNGQKLLLQQGYEKEIVEATRGAGTQVLKFRGGMPLLGLTRIFGLYRMANS 6tg9.1    --------------------------------------------------------------------------------  target    MALLDHKIRGVKPEDALGARGWDNYSWHTDLPPGHPMVTGQQTVDFDLHAVEQARIVVVWGMNWVTTKMPDTHWLTEARL 6tg9.1    ----------------------------------------------------RFKALYVQGEDILQSDP-DTRHVSAGLA  target    KGTKVVVIACEYSSSSIKADDAIVVRPGTTPALALGLCNVIMREKIYDGDYVRRFSDLPLLVRADNLKLLRAEEVFGTPQ 6tg9.1    AMDLVIVHDLFLNETANYAHVFLPGST-----------------------------------------------------  target    AALKNQTR 6tg9.1    -------- ``` | | | | | | | | | | | | | | | | | | | | | | | | | | | | | | | | | | | | | | | | | | | | | | | | | |
|  | 7q5y.1.A | NADH dehydrogenase I chain G  *Structure of NADH:ubichinon oxidoreductase (complex I) of the hyperthermophilic eubacterium Aquifex aeolicus* | 0.03 |  | 14.81 | 0.13 | 293-348 | X-ray | 2.70 | hetero-1-1-1-1-1-1-… | 8 x SF4, 2 x FES, 1 x FMN | HHblits | 0.26 |
| ``` target    NNVNRREFLQWIGAAGFSTFALSASNAWGLQAIENPLAAYPNREWEKTYRDLWKSDASFTFLCAPNDTHNCILNAHVRDG 7q5y.1    --------------------------------------------------------------------------------  target    VITRIGPTMKYGEATDLYGSKVTHRWDPRVCQKGLALTRRFYGDRRVRYPMVRKGFKAWADKGFPREKDGRPPKDYFNRA 7q5y.1    --------------------------------------------------------------------------------  target    RDEWLRLTHEEAADLVAAALINIATTYSGDNGQKLLLQQGYEKEIVEATRGAGTQVLKFRGGMPLLGLTRIFGLYRMANS 7q5y.1    --------------------------------------------------------------------------------  target    MALLDHKIRGVKPEDALGARGWDNYSWHTDLPPGHPMVTGQQTVDFDLHAVEQARIVVVWGMNWVTTKMPDTHWLTEARL 7q5y.1    ----------------------------------------------------DIENLIIFGEDILEFYED--KVFEELKE  target    KGTKVVVIACEYSSSSIKADDAIVVRPGTTPALALGLCNVIMREKIYDGDYVRRFSDLPLLVRADNLKLLRAEEVFGTPQ 7q5y.1    KLEHLVVVSPYEDGLSEYAHIKIPMSLM----------------------------------------------------  target    AALKNQTR 7q5y.1    -------- ``` | | | | | | | | | | | | | | | | | | | | | | | | | | | | | | | | | | | | | | | | | | | | | | | | | |
|  | 6eo0.1.A | NAD-dependent protein deacylase sirtuin-5, mitochondrial  *Zebrafish Sirt5 in complex with stalled peptidylimidate and bicyclic intermediate of inhibitory compound 29* | 0.03 |  | 13.46 | 0.13 | 290-343 | X-ray | 2.40 | monomer | 1 x ZN, 1 x BV8, 1 x BVT | HHblits | 0.29 |
| ``` target    NNVNRREFLQWIGAAGFSTFALSASNAWGLQAIENPLAAYPNREWEKTYRDLWKSDASFTFLCAPNDTHNCILNAHVRDG 6eo0.1    --------------------------------------------------------------------------------  target    VITRIGPTMKYGEATDLYGSKVTHRWDPRVCQKGLALTRRFYGDRRVRYPMVRKGFKAWADKGFPREKDGRPPKDYFNRA 6eo0.1    --------------------------------------------------------------------------------  target    RDEWLRLTHEEAADLVAAALINIATTYSGDNGQKLLLQQGYEKEIVEATRGAGTQVLKFRGGMPLLGLTRIFGLYRMANS 6eo0.1    --------------------------------------------------------------------------------  target    MALLDHKIRGVKPEDALGARGWDNYSWHTDLPPGHPMVTGQQTVDFDLHAVEQARIVVVWGMNWVTTKMPDTHWLTEARL 6eo0.1    -------------------------------------------------ELEKCDLCLVVGTSSIV--YPAAMFAPQVAS  target    KGTKVVVIACEYSSSSIKADDAIVVRPGTTPALALGLCNVIMREKIYDGDYVRRFSDLPLLVRADNLKLLRAEEVFGTPQ 6eo0.1    RGVPVAEFNMECTPATQRFKYHF---------------------------------------------------------  target    AALKNQTR 6eo0.1    -------- ``` | | | | | | | | | | | | | | | | | | | | | | | | | | | | | | | | | | | | | | | | | | | | | | | | | |
|  | 6fky.2.A | NAD-dependent protein deacylase sirtuin-5, mitochondrial  *Crystal structure of zebrafish Sirtuin 5 in complex with 3-(benzylthio)succinyl-CPS1 peptide* | 0.03 |  | 13.46 | 0.13 | 290-343 | X-ray | 2.98 | monomer | 1 x ZN | HHblits | 0.29 |
| ``` target    NNVNRREFLQWIGAAGFSTFALSASNAWGLQAIENPLAAYPNREWEKTYRDLWKSDASFTFLCAPNDTHNCILNAHVRDG 6fky.2    --------------------------------------------------------------------------------  target    VITRIGPTMKYGEATDLYGSKVTHRWDPRVCQKGLALTRRFYGDRRVRYPMVRKGFKAWADKGFPREKDGRPPKDYFNRA 6fky.2    --------------------------------------------------------------------------------  target    RDEWLRLTHEEAADLVAAALINIATTYSGDNGQKLLLQQGYEKEIVEATRGAGTQVLKFRGGMPLLGLTRIFGLYRMANS 6fky.2    --------------------------------------------------------------------------------  target    MALLDHKIRGVKPEDALGARGWDNYSWHTDLPPGHPMVTGQQTVDFDLHAVEQARIVVVWGMNWVTTKMPDTHWLTEARL 6fky.2    -------------------------------------------------ELEKCDLCLVVGTSSIV--YPAAMFAPQVAS  target    KGTKVVVIACEYSSSSIKADDAIVVRPGTTPALALGLCNVIMREKIYDGDYVRRFSDLPLLVRADNLKLLRAEEVFGTPQ 6fky.2    RGVPVAEFNMECTPATQRFKYHF---------------------------------------------------------  target    AALKNQTR 6fky.2    -------- ``` | | | | | | | | | | | | | | | | | | | | | | | | | | | | | | | | | | | | | | | | | | | | | | | | | |
|  | 6fky.1.A | NAD-dependent protein deacylase sirtuin-5, mitochondrial  *Crystal structure of zebrafish Sirtuin 5 in complex with 3-(benzylthio)succinyl-CPS1 peptide* | 0.03 |  | 13.46 | 0.13 | 290-343 | X-ray | 2.98 | monomer | 1 x ZN, 1 x E9N, 1 x DZK, 2 x GZB-VAL-LEU-LYS-GLU-TYR-GLY-VAL | HHblits | 0.29 |
| ``` target    NNVNRREFLQWIGAAGFSTFALSASNAWGLQAIENPLAAYPNREWEKTYRDLWKSDASFTFLCAPNDTHNCILNAHVRDG 6fky.1    --------------------------------------------------------------------------------  target    VITRIGPTMKYGEATDLYGSKVTHRWDPRVCQKGLALTRRFYGDRRVRYPMVRKGFKAWADKGFPREKDGRPPKDYFNRA 6fky.1    --------------------------------------------------------------------------------  target    RDEWLRLTHEEAADLVAAALINIATTYSGDNGQKLLLQQGYEKEIVEATRGAGTQVLKFRGGMPLLGLTRIFGLYRMANS 6fky.1    --------------------------------------------------------------------------------  target    MALLDHKIRGVKPEDALGARGWDNYSWHTDLPPGHPMVTGQQTVDFDLHAVEQARIVVVWGMNWVTTKMPDTHWLTEARL 6fky.1    -------------------------------------------------ELEKCDLCLVVGTSSIV--YPAAMFAPQVAS  target    KGTKVVVIACEYSSSSIKADDAIVVRPGTTPALALGLCNVIMREKIYDGDYVRRFSDLPLLVRADNLKLLRAEEVFGTPQ 6fky.1    RGVPVAEFNMECTPATQRFKYHF---------------------------------------------------------  target    AALKNQTR 6fky.1    -------- ``` | | | | | | | | | | | | | | | | | | | | | | | | | | | | | | | | | | | | | | | | | | | | | | | | | |
|  | 6flg.1.A | NAD-dependent protein deacylase sirtuin-5, mitochondrial  *Crystal structure of zebrafish Sirtuin 5 in complex with 3(S)-(naphthylthio)succinyl-CPS1 peptide* | 0.03 |  | 13.46 | 0.13 | 290-343 | X-ray | 2.50 | monomer | 1 x ZN, 1 x GZB-VAL-LEU-DQK-GLU-TYR-GLY-VAL | HHblits | 0.29 |
| ``` target    NNVNRREFLQWIGAAGFSTFALSASNAWGLQAIENPLAAYPNREWEKTYRDLWKSDASFTFLCAPNDTHNCILNAHVRDG 6flg.1    --------------------------------------------------------------------------------  target    VITRIGPTMKYGEATDLYGSKVTHRWDPRVCQKGLALTRRFYGDRRVRYPMVRKGFKAWADKGFPREKDGRPPKDYFNRA 6flg.1    --------------------------------------------------------------------------------  target    RDEWLRLTHEEAADLVAAALINIATTYSGDNGQKLLLQQGYEKEIVEATRGAGTQVLKFRGGMPLLGLTRIFGLYRMANS 6flg.1    --------------------------------------------------------------------------------  target    MALLDHKIRGVKPEDALGARGWDNYSWHTDLPPGHPMVTGQQTVDFDLHAVEQARIVVVWGMNWVTTKMPDTHWLTEARL 6flg.1    -------------------------------------------------ELEKCDLCLVVGTSSIV--YPAAMFAPQVAS  target    KGTKVVVIACEYSSSSIKADDAIVVRPGTTPALALGLCNVIMREKIYDGDYVRRFSDLPLLVRADNLKLLRAEEVFGTPQ 6flg.1    RGVPVAEFNMECTPATQRFKYHF---------------------------------------------------------  target    AALKNQTR 6flg.1    -------- ``` | | | | | | | | | | | | | | | | | | | | | | | | | | | | | | | | | | | | | | | | | | | | | | | | | |
|  | 6sdr.1.A | Formate dehydrogenase, alpha subunit, selenocysteine-containing  *W-formate dehydrogenase from Desulfovibrio vulgaris - Oxidized form* | 0.03 |  | 16.98 | 0.13 | 293-346 | X-ray | 2.10 | hetero-1-1-mer | 2 x MGD, 4 x SF4, 1 x H2S, 1 x W | HHblits | 0.27 |
| ``` target    NNVNRREFLQWIGAAGFSTFALSASNAWGLQAIENPLAAYPNREWEKTYRDLWKSDASFTFLCAPNDTHNCILNAHVRDG 6sdr.1    --------------------------------------------------------------------------------  target    VITRIGPTMKYGEATDLYGSKVTHRWDPRVCQKGLALTRRFYGDRRVRYPMVRKGFKAWADKGFPREKDGRPPKDYFNRA 6sdr.1    --------------------------------------------------------------------------------  target    RDEWLRLTHEEAADLVAAALINIATTYSGDNGQKLLLQQGYEKEIVEATRGAGTQVLKFRGGMPLLGLTRIFGLYRMANS 6sdr.1    --------------------------------------------------------------------------------  target    MALLDHKIRGVKPEDALGARGWDNYSWHTDLPPGHPMVTGQQTVDFDLHAVEQARIVVVWGMNWVTTKMPDTHWLTEARL 6sdr.1    ----------------------------------------------------EFKGLFAWGMNPACGGAN-ANKNRKAMG  target    KGTKVVVIACEYSSSSIK--------AD-----DAIVVRPGTTPALALGLCNVIMREKIYDGDYVRRFSDLPLLVRADNL 6sdr.1    KLEWLVNVNLFENETSSFWKGPGMNPAEIGTEVFFLPCC-----------------------------------------  target    KLLRAEEVFGTPQAALKNQTR 6sdr.1    --------------------- ``` | | | | | | | | | | | | | | | | | | | | | | | | | | | | | | | | | | | | | | | | | | | | | | | | | |
|  | 1ogy.1.A | PERIPLASMIC NITRATE REDUCTASE  *Crystal structure of the heterodimeric nitrate reductase from Rhodobacter sphaeroides* | 0.04 |  | 12.73 | 0.13 | 292-346 | X-ray | 3.20 | hetero-1-1-mer | 1 x SF4, 1 x MO, 2 x MGD, 2 x HEC | HHblits | 0.24 |
| ``` target    NNVNRREFLQWIGAAGFSTFALSASNAWGLQAIENPLAAYPNREWEKTYRDLWKSDASFTFLCAPNDTHNCILNAHVRDG 1ogy.1    --------------------------------------------------------------------------------  target    VITRIGPTMKYGEATDLYGSKVTHRWDPRVCQKGLALTRRFYGDRRVRYPMVRKGFKAWADKGFPREKDGRPPKDYFNRA 1ogy.1    --------------------------------------------------------------------------------  target    RDEWLRLTHEEAADLVAAALINIATTYSGDNGQKLLLQQGYEKEIVEATRGAGTQVLKFRGGMPLLGLTRIFGLYRMANS 1ogy.1    --------------------------------------------------------------------------------  target    MALLDHKIRGVKPEDALGARGWDNYSWHTDLPPGHPMVTGQQTVDFDLHAVEQARIVVVWGMNWVTTKMPDT-HWLTEAR 1ogy.1    ---------------------------------------------------GEINFYWVQVNNNMQAAPNIDQETYPGYR  target    LKGTKVVVIACEYSSSSIKADDAIVVRPGTTPALALGLCNVIMREKIYDGDYVRRFSDLPLLVRADNLKLLRAEEVFGTP 1ogy.1    NPENFIVVSDAYPTVTGRAADLVLPAA-----------------------------------------------------  target    QAALKNQTR 1ogy.1    --------- ``` | | | | | | | | | | | | | | | | | | | | | | | | | | | | | | | | | | | | | | | | | | | | | | | | | |
|  | 3u31.1.A | Transcriptional regulatory protein sir2 homologue  *Plasmodium falciparum Sir2A preferentially hydrolyzes medium and long chain fatty acyl lysine* | 0.03 |  | 17.31 | 0.13 | 290-343 | X-ray | 2.20 | hetero-oligomer | 1 x NAD, 1 x ZN | HHblits | 0.28 |
| ``` target    NNVNRREFLQWIGAAGFSTFALSASNAWGLQAIENPLAAYPNREWEKTYRDLWKSDASFTFLCAPNDTHNCILNAHVRDG 3u31.1    --------------------------------------------------------------------------------  target    VITRIGPTMKYGEATDLYGSKVTHRWDPRVCQKGLALTRRFYGDRRVRYPMVRKGFKAWADKGFPREKDGRPPKDYFNRA 3u31.1    --------------------------------------------------------------------------------  target    RDEWLRLTHEEAADLVAAALINIATTYSGDNGQKLLLQQGYEKEIVEATRGAGTQVLKFRGGMPLLGLTRIFGLYRMANS 3u31.1    --------------------------------------------------------------------------------  target    MALLDHKIRGVKPEDALGARGWDNYSWHTDLPPGHPMVTGQQTVDFDLHAVEQARIVVVWGMNWVTTKMPDTHWLTEARL 3u31.1    -------------------------------------------------EIAKCDLLLVIGTSSTV--STATNLCHFACK  target    KGTKVVVIACEYSSSS-IKADDAIVVRPGTTPALALGLCNVIMREKIYDGDYVRRFSDLPLLVRADNLKLLRAEEVFGTP 3u31.1    KKKKIVEINISKTYITNKMSDYHV--------------------------------------------------------  target    QAALKNQTR 3u31.1    --------- ``` | | | | | | | | | | | | | | | | | | | | | | | | | | | | | | | | | | | | | | | | | | | | | | | | | |
|  | 3jwp.1.A | Transcriptional regulatory protein sir2 homologue  *Crystal structure of Plasmodium falciparum SIR2A (PF13\_0152) in complex with AMP* | 0.03 |  | 17.31 | 0.13 | 290-343 | X-ray | 2.65 | homo-trimer | 3 x AMP, 3 x ZN | HHblits | 0.28 |
| ``` target    NNVNRREFLQWIGAAGFSTFALSASNAWGLQAIENPLAAYPNREWEKTYRDLWKSDASFTFLCAPNDTHNCILNAHVRDG 3jwp.1    --------------------------------------------------------------------------------  target    VITRIGPTMKYGEATDLYGSKVTHRWDPRVCQKGLALTRRFYGDRRVRYPMVRKGFKAWADKGFPREKDGRPPKDYFNRA 3jwp.1    --------------------------------------------------------------------------------  target    RDEWLRLTHEEAADLVAAALINIATTYSGDNGQKLLLQQGYEKEIVEATRGAGTQVLKFRGGMPLLGLTRIFGLYRMANS 3jwp.1    --------------------------------------------------------------------------------  target    MALLDHKIRGVKPEDALGARGWDNYSWHTDLPPGHPMVTGQQTVDFDLHAVEQARIVVVWGMNWVTTKMPDTHWLTEARL 3jwp.1    -------------------------------------------------EIAKCDLLLVIGTSSTV--STATNLCHFACK  target    KGTKVVVIACEYSSSSI-KADDAIVVRPGTTPALALGLCNVIMREKIYDGDYVRRFSDLPLLVRADNLKLLRAEEVFGTP 3jwp.1    KKKKIVEINISKTYITNKMSDYHV--------------------------------------------------------  target    QAALKNQTR 3jwp.1    --------- ``` | | | | | | | | | | | | | | | | | | | | | | | | | | | | | | | | | | | | | | | | | | | | | | | | | |
|  | 2e7z.1.A | Acetylene hydratase Ahy  *Acetylene Hydratase from Pelobacter acetylenicus* | 0.04 |  | 9.09 | 0.13 | 293-348 | X-ray | 1.26 | monomer | 1 x SF4, 2 x MGD, 1 x W | HHblits | 0.24 |
| ``` target    NNVNRREFLQWIGAAGFSTFALSASNAWGLQAIENPLAAYPNREWEKTYRDLWKSDASFTFLCAPNDTHNCILNAHVRDG 2e7z.1    --------------------------------------------------------------------------------  target    VITRIGPTMKYGEATDLYGSKVTHRWDPRVCQKGLALTRRFYGDRRVRYPMVRKGFKAWADKGFPREKDGRPPKDYFNRA 2e7z.1    --------------------------------------------------------------------------------  target    RDEWLRLTHEEAADLVAAALINIATTYSGDNGQKLLLQQGYEKEIVEATRGAGTQVLKFRGGMPLLGLTRIFGLYRMANS 2e7z.1    --------------------------------------------------------------------------------  target    MALLDHKIRGVKPEDALGARGWDNYSWHTDLPPGHPMVTGQQTVDFDLHAVEQARIVVVWGMNWVTTKMPDTHWLTEARL 2e7z.1    ----------------------------------------------------PVKAFFALASNALMGYA-NQQNALKGLM  target    KGTKVVVIACEYSSSSIKADDAIVVRPGTTPALALGLCNVIMREKIYDGDYVRRFSDLPLLVRADNLKLLRAEEVFGTPQ 2e7z.1    NQDLVVCYDQFMTPTAQLADYVLPGDHW----------------------------------------------------  target    AALKNQTR 2e7z.1    -------- ``` | | | | | | | | | | | | | | | | | | | | | | | | | | | | | | | | | | | | | | | | | | | | | | | | | |
|  | 7vw6.1.A | Formate dehydrogenase  *Cryo-EM Structure of Formate Dehydrogenase 1 from Methylorubrum extorquens AM1* | 0.03 |  | 19.23 | 0.13 | 293-345 | EM | 0.00 | hetero-1-1-mer | 4 x SF4, 2 x FES, 2 x MGD, 1 x W, 1 x FMN | HHblits | 0.28 |
| ``` target    NNVNRREFLQWIGAAGFSTFALSASNAWGLQAIENPLAAYPNREWEKTYRDLWKSDASFTFLCAPNDTHNCILNAHVRDG 7vw6.1    --------------------------------------------------------------------------------  target    VITRIGPTMKYGEATDLYGSKVTHRWDPRVCQKGLALTRRFYGDRRVRYPMVRKGFKAWADKGFPREKDGRPPKDYFNRA 7vw6.1    --------------------------------------------------------------------------------  target    RDEWLRLTHEEAADLVAAALINIATTYSGDNGQKLLLQQGYEKEIVEATRGAGTQVLKFRGGMPLLGLTRIFGLYRMANS 7vw6.1    --------------------------------------------------------------------------------  target    MALLDHKIRGVKPEDALGARGWDNYSWHTDLPPGHPMVTGQQTVDFDLHAVEQARIVVVWGMNWVTTKMPDTHWLTEARL 7vw6.1    ----------------------------------------------------EIRGMFVEGENPAMSDP-DLNHARHALA  target    KGTKVVVIACEYSSSSIKADDAIVVRPGTTPALALGLCNVIMREKIYDGDYVRRFSDLPLLVRADNLKLLRAEEVFGTPQ 7vw6.1    MLDHLVVQDLFLTETAFHADVVLPA-------------------------------------------------------  target    AALKNQTR 7vw6.1    -------- ``` | | | | | | | | | | | | | | | | | | | | | | | | | | | | | | | | | | | | | | | | | | | | | | | | | |
|  | 7e5z.1.A | Formate dehydrogenase  *Dehydrogenase holoenzyme* | 0.02 |  | 19.23 | 0.13 | 293-345 | EM | 0.00 | hetero-1-1-mer | 1 x W, 2 x MGD, 2 x FES, 4 x SF4, 1 x FMN | HHblits | 0.28 |
| ``` target    NNVNRREFLQWIGAAGFSTFALSASNAWGLQAIENPLAAYPNREWEKTYRDLWKSDASFTFLCAPNDTHNCILNAHVRDG 7e5z.1    --------------------------------------------------------------------------------  target    VITRIGPTMKYGEATDLYGSKVTHRWDPRVCQKGLALTRRFYGDRRVRYPMVRKGFKAWADKGFPREKDGRPPKDYFNRA 7e5z.1    --------------------------------------------------------------------------------  target    RDEWLRLTHEEAADLVAAALINIATTYSGDNGQKLLLQQGYEKEIVEATRGAGTQVLKFRGGMPLLGLTRIFGLYRMANS 7e5z.1    --------------------------------------------------------------------------------  target    MALLDHKIRGVKPEDALGARGWDNYSWHTDLPPGHPMVTGQQTVDFDLHAVEQARIVVVWGMNWVTTKMPDTHWLTEARL 7e5z.1    ----------------------------------------------------EIRGMFVEGENPAMSDP-DLNHARHALA  target    KGTKVVVIACEYSSSSIKADDAIVVRPGTTPALALGLCNVIMREKIYDGDYVRRFSDLPLLVRADNLKLLRAEEVFGTPQ 7e5z.1    MLDHLVVQDLFLTETAFHADVVLPA-------------------------------------------------------  target    AALKNQTR 7e5z.1    -------- ``` | | | | | | | | | | | | | | | | | | | | | | | | | | | | | | | | | | | | | | | | | | | | | | | | | |
|  | 5ojn.1.A | NAD-dependent protein deacylase  *Sirtuin 4 from Xenopus tropicalis in complex with thioacetyl-ADP-ribose* | 0.03 |  | 9.43 | 0.13 | 290-344 | X-ray | 1.80 | monomer | 1 x ZN, 1 x 9X8 | HHblits | 0.26 |
| ``` target    NNVNRREFLQWIGAAGFSTFALSASNAWGLQAIENPLAAYPNREWEKTYRDLWKSDASFTFLCAPNDTHNCILNAHVRDG 5ojn.1    --------------------------------------------------------------------------------  target    VITRIGPTMKYGEATDLYGSKVTHRWDPRVCQKGLALTRRFYGDRRVRYPMVRKGFKAWADKGFPREKDGRPPKDYFNRA 5ojn.1    --------------------------------------------------------------------------------  target    RDEWLRLTHEEAADLVAAALINIATTYSGDNGQKLLLQQGYEKEIVEATRGAGTQVLKFRGGMPLLGLTRIFGLYRMANS 5ojn.1    --------------------------------------------------------------------------------  target    MALLDHKIRGVKPEDALGARGWDNYSWHTDLPPGHPMVTGQQTVDFDLHAVEQARIVVVWGMNWVTTKMPDTHWLTEARL 5ojn.1    -------------------------------------------------QMKQADAMLIVGSSLQV--YSGYRFALNAKE  target    KGTKVVVIACEYSSSSIKADDAIVVRPGTTPALALGLCNVIMREKIYDGDYVRRFSDLPLLVRADNLKLLRAEEVFGTPQ 5ojn.1    LHLPIAILNIGPTRADHLAKVKVS--------------------------------------------------------  target    AALKNQTR 5ojn.1    -------- ``` | | | | | | | | | | | | | | | | | | | | | | | | | | | | | | | | | | | | | | | | | | | | | | | | | |
|  | 5oj7.1.A | NAD-dependent protein deacylase  *Sirtuin 4 orthologue from Xenopus Tropicalis in complex with ADP-ribose* | 0.03 |  | 9.43 | 0.13 | 290-344 | X-ray | 1.58 | monomer | 1 x AR6, 1 x ZN | HHblits | 0.26 |
| ``` target    NNVNRREFLQWIGAAGFSTFALSASNAWGLQAIENPLAAYPNREWEKTYRDLWKSDASFTFLCAPNDTHNCILNAHVRDG 5oj7.1    --------------------------------------------------------------------------------  target    VITRIGPTMKYGEATDLYGSKVTHRWDPRVCQKGLALTRRFYGDRRVRYPMVRKGFKAWADKGFPREKDGRPPKDYFNRA 5oj7.1    --------------------------------------------------------------------------------  target    RDEWLRLTHEEAADLVAAALINIATTYSGDNGQKLLLQQGYEKEIVEATRGAGTQVLKFRGGMPLLGLTRIFGLYRMANS 5oj7.1    --------------------------------------------------------------------------------  target    MALLDHKIRGVKPEDALGARGWDNYSWHTDLPPGHPMVTGQQTVDFDLHAVEQARIVVVWGMNWVTTKMPDTHWLTEARL 5oj7.1    -------------------------------------------------QMKQADAMLIVGSSLQV--YSGYRFALNAKE  target    KGTKVVVIACEYSSSSIKADDAIVVRPGTTPALALGLCNVIMREKIYDGDYVRRFSDLPLLVRADNLKLLRAEEVFGTPQ 5oj7.1    LHLPIAILNIGPTRADHLAKVKVS--------------------------------------------------------  target    AALKNQTR 5oj7.1    -------- ``` | | | | | | | | | | | | | | | | | | | | | | | | | | | | | | | | | | | | | | | | | | | | | | | | | |
|  | 2h59.1.B | NAD-dependent deacetylase  *Sir2 H116A-deacetylated p53 peptide-3'-o-acetyl ADP ribose* | 0.03 |  | 17.31 | 0.13 | 290-343 | X-ray | 1.90 | hetero-2-2-mer | 2 x ZN, 1 x APR, 1 x 3OD | HHblits | 0.28 |
| ``` target    NNVNRREFLQWIGAAGFSTFALSASNAWGLQAIENPLAAYPNREWEKTYRDLWKSDASFTFLCAPNDTHNCILNAHVRDG 2h59.1    --------------------------------------------------------------------------------  target    VITRIGPTMKYGEATDLYGSKVTHRWDPRVCQKGLALTRRFYGDRRVRYPMVRKGFKAWADKGFPREKDGRPPKDYFNRA 2h59.1    --------------------------------------------------------------------------------  target    RDEWLRLTHEEAADLVAAALINIATTYSGDNGQKLLLQQGYEKEIVEATRGAGTQVLKFRGGMPLLGLTRIFGLYRMANS 2h59.1    --------------------------------------------------------------------------------  target    MALLDHKIRGVKPEDALGARGWDNYSWHTDLPPGHPMVTGQQTVDFDLHAVEQARIVVVWGMNWVTTKMPDTHWLTEARL 2h59.1    -------------------------------------------------LSSRASLMIVLGSSLVV--YPAAELPLITVR  target    KGTKVVVIACEYSSSSIKADDAIVVRPGTTPALALGLCNVIMREKIYDGDYVRRFSDLPLLVRADNLKLLRAEEVFGTPQ 2h59.1    SGGKLVIVNLGETPFDDIATLKY---------------------------------------------------------  target    AALKNQTR 2h59.1    -------- ``` | | | | | | | | | | | | | | | | | | | | | | | | | | | | | | | | | | | | | | | | | | | | | | | | | |
|  | 3jr3.1.A | NAD-dependent deacetylase  *Sir2 bound to acetylated peptide* | 0.03 |  | 17.31 | 0.13 | 290-343 | X-ray | 1.50 | hetero-1-1-mer | 1 x ZN | HHblits | 0.28 |
| ``` target    NNVNRREFLQWIGAAGFSTFALSASNAWGLQAIENPLAAYPNREWEKTYRDLWKSDASFTFLCAPNDTHNCILNAHVRDG 3jr3.1    --------------------------------------------------------------------------------  target    VITRIGPTMKYGEATDLYGSKVTHRWDPRVCQKGLALTRRFYGDRRVRYPMVRKGFKAWADKGFPREKDGRPPKDYFNRA 3jr3.1    --------------------------------------------------------------------------------  target    RDEWLRLTHEEAADLVAAALINIATTYSGDNGQKLLLQQGYEKEIVEATRGAGTQVLKFRGGMPLLGLTRIFGLYRMANS 3jr3.1    --------------------------------------------------------------------------------  target    MALLDHKIRGVKPEDALGARGWDNYSWHTDLPPGHPMVTGQQTVDFDLHAVEQARIVVVWGMNWVTTKMPDTHWLTEARL 3jr3.1    -------------------------------------------------LSSRASLMIVLGSSLVV--YPAAELPLITVR  target    KGTKVVVIACEYSSSSIKADDAIVVRPGTTPALALGLCNVIMREKIYDGDYVRRFSDLPLLVRADNLKLLRAEEVFGTPQ 3jr3.1    SGGKLVIVNLGETPFDDIATLKY---------------------------------------------------------  target    AALKNQTR 3jr3.1    -------- ``` | | | | | | | | | | | | | | | | | | | | | | | | | | | | | | | | | | | | | | | | | | | | | | | | | |
|  | 1yc5.1.A | NAD-dependent deacetylase  *Sir2-p53 peptide-nicotinamide* | 0.03 |  | 17.31 | 0.13 | 290-343 | X-ray | 1.40 | hetero-oligomer | 1 x ZN, 1 x NCA | HHblits | 0.28 |
| ``` target    NNVNRREFLQWIGAAGFSTFALSASNAWGLQAIENPLAAYPNREWEKTYRDLWKSDASFTFLCAPNDTHNCILNAHVRDG 1yc5.1    --------------------------------------------------------------------------------  target    VITRIGPTMKYGEATDLYGSKVTHRWDPRVCQKGLALTRRFYGDRRVRYPMVRKGFKAWADKGFPREKDGRPPKDYFNRA 1yc5.1    --------------------------------------------------------------------------------  target    RDEWLRLTHEEAADLVAAALINIATTYSGDNGQKLLLQQGYEKEIVEATRGAGTQVLKFRGGMPLLGLTRIFGLYRMANS 1yc5.1    --------------------------------------------------------------------------------  target    MALLDHKIRGVKPEDALGARGWDNYSWHTDLPPGHPMVTGQQTVDFDLHAVEQARIVVVWGMNWVTTKMPDTHWLTEARL 1yc5.1    -------------------------------------------------LSSRASLMIVLGSSLVV--YPAAELPLITVR  target    KGTKVVVIACEYSSSSIKADDAIVVRPGTTPALALGLCNVIMREKIYDGDYVRRFSDLPLLVRADNLKLLRAEEVFGTPQ 1yc5.1    SGGKLVIVNLGETPFDDIATLKY---------------------------------------------------------  target    AALKNQTR 1yc5.1    -------- ``` | | | | | | | | | | | | | | | | | | | | | | | | | | | | | | | | | | | | | | | | | | | | | | | | | |
|  | 2h2i.1.A | NAD-dependent deacetylase  *The Structural basis of Sirtuin Substrate Affinity* | 0.03 |  | 17.31 | 0.13 | 290-343 | X-ray | 1.80 | homo-octamer | 8 x ZN, 8 x ZPG | HHblits | 0.28 |
| ``` target    NNVNRREFLQWIGAAGFSTFALSASNAWGLQAIENPLAAYPNREWEKTYRDLWKSDASFTFLCAPNDTHNCILNAHVRDG 2h2i.1    --------------------------------------------------------------------------------  target    VITRIGPTMKYGEATDLYGSKVTHRWDPRVCQKGLALTRRFYGDRRVRYPMVRKGFKAWADKGFPREKDGRPPKDYFNRA 2h2i.1    --------------------------------------------------------------------------------  target    RDEWLRLTHEEAADLVAAALINIATTYSGDNGQKLLLQQGYEKEIVEATRGAGTQVLKFRGGMPLLGLTRIFGLYRMANS 2h2i.1    --------------------------------------------------------------------------------  target    MALLDHKIRGVKPEDALGARGWDNYSWHTDLPPGHPMVTGQQTVDFDLHAVEQARIVVVWGMNWVTTKMPDTHWLTEARL 2h2i.1    -------------------------------------------------LSSRASLMIVLGSSLVV--YPAAELPLITVR  target    KGTKVVVIACEYSSSSIKADDAIVVRPGTTPALALGLCNVIMREKIYDGDYVRRFSDLPLLVRADNLKLLRAEEVFGTPQ 2h2i.1    SGGKLVIVNLGETPFDDIATLKY---------------------------------------------------------  target    AALKNQTR 2h2i.1    -------- ``` | | | | | | | | | | | | | | | | | | | | | | | | | | | | | | | | | | | | | | | | | | | | | | | | | |
|  | 4bv2.3.A | NAD-DEPENDENT PROTEIN DEACETYLASE  *CRYSTAL STRUCTURE OF SIR2 IN COMPLEX WITH THE INHIBITOR EX-527, 2'-O-ACETYL-ADP-RIBOSE AND DEACETYLATED P53-PEPTIDE* | 0.03 |  | 17.31 | 0.13 | 290-343 | X-ray | 3.30 | hetero-oligomer | 1 x OCZ, 1 x OAD, 1 x ZN | HHblits | 0.28 |
| ``` target    NNVNRREFLQWIGAAGFSTFALSASNAWGLQAIENPLAAYPNREWEKTYRDLWKSDASFTFLCAPNDTHNCILNAHVRDG 4bv2.3    --------------------------------------------------------------------------------  target    VITRIGPTMKYGEATDLYGSKVTHRWDPRVCQKGLALTRRFYGDRRVRYPMVRKGFKAWADKGFPREKDGRPPKDYFNRA 4bv2.3    --------------------------------------------------------------------------------  target    RDEWLRLTHEEAADLVAAALINIATTYSGDNGQKLLLQQGYEKEIVEATRGAGTQVLKFRGGMPLLGLTRIFGLYRMANS 4bv2.3    --------------------------------------------------------------------------------  target    MALLDHKIRGVKPEDALGARGWDNYSWHTDLPPGHPMVTGQQTVDFDLHAVEQARIVVVWGMNWVTTKMPDTHWLTEARL 4bv2.3    -------------------------------------------------LSSRASLMIVLGSSLVV--YPAAELPLITVR  target    KGTKVVVIACEYSSSSIKADDAIVVRPGTTPALALGLCNVIMREKIYDGDYVRRFSDLPLLVRADNLKLLRAEEVFGTPQ 4bv2.3    SGGKLVIVNLGETPFDDIATLKY---------------------------------------------------------  target    AALKNQTR 4bv2.3    -------- ``` | | | | | | | | | | | | | | | | | | | | | | | | | | | | | | | | | | | | | | | | | | | | | | | | | |
|  | 6aco.1.A | NAD-dependent protein deacylase sirtuin-5, mitochondrial  *histone lysine desuccinylase Sirt5 in complex with succinyl peptide H2BK120* | 0.03 |  | 13.46 | 0.13 | 290-343 | X-ray | 1.71 | monomer | 1 x ZN, 1 x ALA-VAL-THR-SLL-TYR-THR-SER | HHblits | 0.28 |
| ``` target    NNVNRREFLQWIGAAGFSTFALSASNAWGLQAIENPLAAYPNREWEKTYRDLWKSDASFTFLCAPNDTHNCILNAHVRDG 6aco.1    --------------------------------------------------------------------------------  target    VITRIGPTMKYGEATDLYGSKVTHRWDPRVCQKGLALTRRFYGDRRVRYPMVRKGFKAWADKGFPREKDGRPPKDYFNRA 6aco.1    --------------------------------------------------------------------------------  target    RDEWLRLTHEEAADLVAAALINIATTYSGDNGQKLLLQQGYEKEIVEATRGAGTQVLKFRGGMPLLGLTRIFGLYRMANS 6aco.1    --------------------------------------------------------------------------------  target    MALLDHKIRGVKPEDALGARGWDNYSWHTDLPPGHPMVTGQQTVDFDLHAVEQARIVVVWGMNWVTTKMPDTHWLTEARL 6aco.1    -------------------------------------------------ELAHCDLCLVVGTSSVV--YPAAMFAPQVAA  target    KGTKVVVIACEYSSSSIKADDAIVVRPGTTPALALGLCNVIMREKIYDGDYVRRFSDLPLLVRADNLKLLRAEEVFGTPQ 6aco.1    RGVPVAEFNTETTPATNRFRFHF---------------------------------------------------------  target    AALKNQTR 6aco.1    -------- ``` | | | | | | | | | | | | | | | | | | | | | | | | | | | | | | | | | | | | | | | | | | | | | | | | | |
|  | 6eqs.3.A | NAD-dependent protein deacylase sirtuin-5, mitochondrial  *Human Sirt5 in complex with stalled peptidylimidate intermediate of inhibitory compound 29* | 0.03 |  | 13.46 | 0.13 | 290-343 | X-ray | 1.32 | monomer | 1 x ZN, 1 x BV8, 1 x BU2 | HHblits | 0.28 |
| ``` target    NNVNRREFLQWIGAAGFSTFALSASNAWGLQAIENPLAAYPNREWEKTYRDLWKSDASFTFLCAPNDTHNCILNAHVRDG 6eqs.3    --------------------------------------------------------------------------------  target    VITRIGPTMKYGEATDLYGSKVTHRWDPRVCQKGLALTRRFYGDRRVRYPMVRKGFKAWADKGFPREKDGRPPKDYFNRA 6eqs.3    --------------------------------------------------------------------------------  target    RDEWLRLTHEEAADLVAAALINIATTYSGDNGQKLLLQQGYEKEIVEATRGAGTQVLKFRGGMPLLGLTRIFGLYRMANS 6eqs.3    --------------------------------------------------------------------------------  target    MALLDHKIRGVKPEDALGARGWDNYSWHTDLPPGHPMVTGQQTVDFDLHAVEQARIVVVWGMNWVTTKMPDTHWLTEARL 6eqs.3    -------------------------------------------------ELAHCDLCLVVGTSSVV--YPAAMFAPQVAA  target    KGTKVVVIACEYSSSSIKADDAIVVRPGTTPALALGLCNVIMREKIYDGDYVRRFSDLPLLVRADNLKLLRAEEVFGTPQ 6eqs.3    RGVPVAEFNTETTPATNRFRFHF---------------------------------------------------------  target    AALKNQTR 6eqs.3    -------- ``` | | | | | | | | | | | | | | | | | | | | | | | | | | | | | | | | | | | | | | | | | | | | | | | | | |
|  | 4hda.1.A | NAD-dependent protein deacylase sirtuin-5, mitochondrial  *Crystal structure of human Sirt5 in complex with Fluor-de-Lys peptide and resveratrol* | 0.03 |  | 13.46 | 0.13 | 290-343 | X-ray | 2.60 | monomer | 1 x ZN | HHblits | 0.28 |
| ``` target    NNVNRREFLQWIGAAGFSTFALSASNAWGLQAIENPLAAYPNREWEKTYRDLWKSDASFTFLCAPNDTHNCILNAHVRDG 4hda.1    --------------------------------------------------------------------------------  target    VITRIGPTMKYGEATDLYGSKVTHRWDPRVCQKGLALTRRFYGDRRVRYPMVRKGFKAWADKGFPREKDGRPPKDYFNRA 4hda.1    --------------------------------------------------------------------------------  target    RDEWLRLTHEEAADLVAAALINIATTYSGDNGQKLLLQQGYEKEIVEATRGAGTQVLKFRGGMPLLGLTRIFGLYRMANS 4hda.1    --------------------------------------------------------------------------------  target    MALLDHKIRGVKPEDALGARGWDNYSWHTDLPPGHPMVTGQQTVDFDLHAVEQARIVVVWGMNWVTTKMPDTHWLTEARL 4hda.1    -------------------------------------------------ELAHCDLCLVVGTSSVV--YPAAMFAPQVAA  target    KGTKVVVIACEYSSSSIKADDAIVVRPGTTPALALGLCNVIMREKIYDGDYVRRFSDLPLLVRADNLKLLRAEEVFGTPQ 4hda.1    RGVPVAEFNTETTPATNRFRFHF---------------------------------------------------------  target    AALKNQTR 4hda.1    -------- ``` | | | | | | | | | | | | | | | | | | | | | | | | | | | | | | | | | | | | | | | | | | | | | | | | | |
|  | 4hda.2.A | NAD-dependent protein deacylase sirtuin-5, mitochondrial  *Crystal structure of human Sirt5 in complex with Fluor-de-Lys peptide and resveratrol* | 0.03 |  | 13.46 | 0.13 | 290-343 | X-ray | 2.60 | monomer | 1 x ZN, 1 x STL, 1 x HIS-LYS-FDL | HHblits | 0.28 |
| ``` target    NNVNRREFLQWIGAAGFSTFALSASNAWGLQAIENPLAAYPNREWEKTYRDLWKSDASFTFLCAPNDTHNCILNAHVRDG 4hda.2    --------------------------------------------------------------------------------  target    VITRIGPTMKYGEATDLYGSKVTHRWDPRVCQKGLALTRRFYGDRRVRYPMVRKGFKAWADKGFPREKDGRPPKDYFNRA 4hda.2    --------------------------------------------------------------------------------  target    RDEWLRLTHEEAADLVAAALINIATTYSGDNGQKLLLQQGYEKEIVEATRGAGTQVLKFRGGMPLLGLTRIFGLYRMANS 4hda.2    --------------------------------------------------------------------------------  target    MALLDHKIRGVKPEDALGARGWDNYSWHTDLPPGHPMVTGQQTVDFDLHAVEQARIVVVWGMNWVTTKMPDTHWLTEARL 4hda.2    -------------------------------------------------ELAHCDLCLVVGTSSVV--YPAAMFAPQVAA  target    KGTKVVVIACEYSSSSIKADDAIVVRPGTTPALALGLCNVIMREKIYDGDYVRRFSDLPLLVRADNLKLLRAEEVFGTPQ 4hda.2    RGVPVAEFNTETTPATNRFRFHF---------------------------------------------------------  target    AALKNQTR 4hda.2    -------- ``` | | | | | | | | | | | | | | | | | | | | | | | | | | | | | | | | | | | | | | | | | | | | | | | | | |
|  | 2nyr.1.A | NAD-dependent deacetylase sirtuin-5  *Crystal Structure of Human Sirtuin Homolog 5 in Complex with Suramin* | 0.02 |  | 13.46 | 0.13 | 290-343 | X-ray | 2.06 | homo-dimer | 1 x SVR, 2 x ZN | HHblits | 0.28 |
| ``` target    NNVNRREFLQWIGAAGFSTFALSASNAWGLQAIENPLAAYPNREWEKTYRDLWKSDASFTFLCAPNDTHNCILNAHVRDG 2nyr.1    --------------------------------------------------------------------------------  target    VITRIGPTMKYGEATDLYGSKVTHRWDPRVCQKGLALTRRFYGDRRVRYPMVRKGFKAWADKGFPREKDGRPPKDYFNRA 2nyr.1    --------------------------------------------------------------------------------  target    RDEWLRLTHEEAADLVAAALINIATTYSGDNGQKLLLQQGYEKEIVEATRGAGTQVLKFRGGMPLLGLTRIFGLYRMANS 2nyr.1    --------------------------------------------------------------------------------  target    MALLDHKIRGVKPEDALGARGWDNYSWHTDLPPGHPMVTGQQTVDFDLHAVEQARIVVVWGMNWVTTKMPDTHWLTEARL 2nyr.1    -------------------------------------------------ELAHCDLCLVVGTSSVV--YPAAMFAPQVAA  target    KGTKVVVIACEYSSSSIKADDAIVVRPGTTPALALGLCNVIMREKIYDGDYVRRFSDLPLLVRADNLKLLRAEEVFGTPQ 2nyr.1    RGVPVAEFNTETTPATNRFRFHF---------------------------------------------------------  target    AALKNQTR 2nyr.1    -------- ``` | | | | | | | | | | | | | | | | | | | | | | | | | | | | | | | | | | | | | | | | | | | | | | | | | |
|  | 2nyr.1.B | NAD-dependent deacetylase sirtuin-5  *Crystal Structure of Human Sirtuin Homolog 5 in Complex with Suramin* | 0.02 |  | 13.46 | 0.13 | 290-343 | X-ray | 2.06 | homo-dimer | 1 x SVR, 2 x ZN | HHblits | 0.28 |
| ``` target    NNVNRREFLQWIGAAGFSTFALSASNAWGLQAIENPLAAYPNREWEKTYRDLWKSDASFTFLCAPNDTHNCILNAHVRDG 2nyr.1    --------------------------------------------------------------------------------  target    VITRIGPTMKYGEATDLYGSKVTHRWDPRVCQKGLALTRRFYGDRRVRYPMVRKGFKAWADKGFPREKDGRPPKDYFNRA 2nyr.1    --------------------------------------------------------------------------------  target    RDEWLRLTHEEAADLVAAALINIATTYSGDNGQKLLLQQGYEKEIVEATRGAGTQVLKFRGGMPLLGLTRIFGLYRMANS 2nyr.1    --------------------------------------------------------------------------------  target    MALLDHKIRGVKPEDALGARGWDNYSWHTDLPPGHPMVTGQQTVDFDLHAVEQARIVVVWGMNWVTTKMPDTHWLTEARL 2nyr.1    -------------------------------------------------ELAHCDLCLVVGTSSVV--YPAAMFAPQVAA  target    KGTKVVVIACEYSSSSIKADDAIVVRPGTTPALALGLCNVIMREKIYDGDYVRRFSDLPLLVRADNLKLLRAEEVFGTPQ 2nyr.1    RGVPVAEFNTETTPATNRFRFHF---------------------------------------------------------  target    AALKNQTR 2nyr.1    -------- ``` | | | | | | | | | | | | | | | | | | | | | | | | | | | | | | | | | | | | | | | | | | | | | | | | | |
|  | 2b4y.1.A | NAD-dependent deacetylase sirtuin-5  *Crystal Structure of Human Sirtuin homolog 5* | 0.03 |  | 13.46 | 0.13 | 290-343 | X-ray | 1.90 | monomer | 1 x ZN, 1 x APR | HHblits | 0.28 |
| ``` target    NNVNRREFLQWIGAAGFSTFALSASNAWGLQAIENPLAAYPNREWEKTYRDLWKSDASFTFLCAPNDTHNCILNAHVRDG 2b4y.1    --------------------------------------------------------------------------------  target    VITRIGPTMKYGEATDLYGSKVTHRWDPRVCQKGLALTRRFYGDRRVRYPMVRKGFKAWADKGFPREKDGRPPKDYFNRA 2b4y.1    --------------------------------------------------------------------------------  target    RDEWLRLTHEEAADLVAAALINIATTYSGDNGQKLLLQQGYEKEIVEATRGAGTQVLKFRGGMPLLGLTRIFGLYRMANS 2b4y.1    --------------------------------------------------------------------------------  target    MALLDHKIRGVKPEDALGARGWDNYSWHTDLPPGHPMVTGQQTVDFDLHAVEQARIVVVWGMNWVTTKMPDTHWLTEARL 2b4y.1    -------------------------------------------------ELAHCDLCLVVGTSSVV--YPAAMFAPQVAA  target    KGTKVVVIACEYSSSSIKADDAIVVRPGTTPALALGLCNVIMREKIYDGDYVRRFSDLPLLVRADNLKLLRAEEVFGTPQ 2b4y.1    RGVPVAEFNTETTPATNRFRFHF---------------------------------------------------------  target    AALKNQTR 2b4y.1    -------- ``` | | | | | | | | | | | | | | | | | | | | | | | | | | | | | | | | | | | | | | | | | | | | | | | | | |
|  | 2b4y.3.A | NAD-dependent deacetylase sirtuin-5  *Crystal Structure of Human Sirtuin homolog 5* | 0.03 |  | 13.46 | 0.13 | 290-343 | X-ray | 1.90 | monomer | 1 x ZN, 1 x APR | HHblits | 0.28 |
| ``` target    NNVNRREFLQWIGAAGFSTFALSASNAWGLQAIENPLAAYPNREWEKTYRDLWKSDASFTFLCAPNDTHNCILNAHVRDG 2b4y.3    --------------------------------------------------------------------------------  target    VITRIGPTMKYGEATDLYGSKVTHRWDPRVCQKGLALTRRFYGDRRVRYPMVRKGFKAWADKGFPREKDGRPPKDYFNRA 2b4y.3    --------------------------------------------------------------------------------  target    RDEWLRLTHEEAADLVAAALINIATTYSGDNGQKLLLQQGYEKEIVEATRGAGTQVLKFRGGMPLLGLTRIFGLYRMANS 2b4y.3    --------------------------------------------------------------------------------  target    MALLDHKIRGVKPEDALGARGWDNYSWHTDLPPGHPMVTGQQTVDFDLHAVEQARIVVVWGMNWVTTKMPDTHWLTEARL 2b4y.3    -------------------------------------------------ELAHCDLCLVVGTSSVV--YPAAMFAPQVAA  target    KGTKVVVIACEYSSSSIKADDAIVVRPGTTPALALGLCNVIMREKIYDGDYVRRFSDLPLLVRADNLKLLRAEEVFGTPQ 2b4y.3    RGVPVAEFNTETTPATNRFRFHF---------------------------------------------------------  target    AALKNQTR 2b4y.3    -------- ``` | | | | | | | | | | | | | | | | | | | | | | | | | | | | | | | | | | | | | | | | | | | | | | | | | |
|  | 2vpz.1.A | THIOSULFATE REDUCTASE  *POLYSULFIDE REDUCTASE NATIVE STRUCTURE* | 0.03 |  | 14.81 | 0.13 | 294-348 | X-ray | 2.40 | hetero-oligomer | 10 x SF4, 4 x MGD, 2 x MO | HHblits | 0.25 |
| ``` target    NNVNRREFLQWIGAAGFSTFALSASNAWGLQAIENPLAAYPNREWEKTYRDLWKSDASFTFLCAPNDTHNCILNAHVRDG 2vpz.1    --------------------------------------------------------------------------------  target    VITRIGPTMKYGEATDLYGSKVTHRWDPRVCQKGLALTRRFYGDRRVRYPMVRKGFKAWADKGFPREKDGRPPKDYFNRA 2vpz.1    --------------------------------------------------------------------------------  target    RDEWLRLTHEEAADLVAAALINIATTYSGDNGQKLLLQQGYEKEIVEATRGAGTQVLKFRGGMPLLGLTRIFGLYRMANS 2vpz.1    --------------------------------------------------------------------------------  target    MALLDHKIRGVKPEDALGARGWDNYSWHTDLPPGHPMVTGQQTVDFDLHAVEQARIVVVWGMNWVTTKMPDTHWLTEARL 2vpz.1    -----------------------------------------------------IKGLFAYGINLFHSIPN-VPRTKEALK  target    KGTKVVVIACEYSSSSIKADDAIVVRPGTTPALALGLCNVIMREKIYDGDYVRRFSDLPLLVRADNLKLLRAEEVFGTPQ 2vpz.1    NLDLYVAIDVLPQEHVMWADVILPEATY----------------------------------------------------  target    AALKNQTR 2vpz.1    -------- ``` | | | | | | | | | | | | | | | | | | | | | | | | | | | | | | | | | | | | | | | | | | | | | | | | | |
|  | 2vpx.1.D | THIOSULFATE REDUCTASE  *POLYSULFIDE REDUCTASE WITH BOUND QUINONE (UQ1)* | 0.03 |  | 14.81 | 0.13 | 294-348 | X-ray | 3.10 | hetero-oligomer | 10 x SF4, 4 x MGD, 2 x MO, 2 x UQ1 | HHblits | 0.25 |
| ``` target    NNVNRREFLQWIGAAGFSTFALSASNAWGLQAIENPLAAYPNREWEKTYRDLWKSDASFTFLCAPNDTHNCILNAHVRDG 2vpx.1    --------------------------------------------------------------------------------  target    VITRIGPTMKYGEATDLYGSKVTHRWDPRVCQKGLALTRRFYGDRRVRYPMVRKGFKAWADKGFPREKDGRPPKDYFNRA 2vpx.1    --------------------------------------------------------------------------------  target    RDEWLRLTHEEAADLVAAALINIATTYSGDNGQKLLLQQGYEKEIVEATRGAGTQVLKFRGGMPLLGLTRIFGLYRMANS 2vpx.1    --------------------------------------------------------------------------------  target    MALLDHKIRGVKPEDALGARGWDNYSWHTDLPPGHPMVTGQQTVDFDLHAVEQARIVVVWGMNWVTTKMPDTHWLTEARL 2vpx.1    -----------------------------------------------------IKGLFAYGINLFHSIPN-VPRTKEALK  target    KGTKVVVIACEYSSSSIKADDAIVVRPGTTPALALGLCNVIMREKIYDGDYVRRFSDLPLLVRADNLKLLRAEEVFGTPQ 2vpx.1    NLDLYVAIDVLPQEHVMWADVILPEATY----------------------------------------------------  target    AALKNQTR 2vpx.1    -------- ``` | | | | | | | | | | | | | | | | | | | | | | | | | | | | | | | | | | | | | | | | | | | | | | | | | |
|  | 7p63.1.C | NADH-quinone oxidoreductase  *Complex I from E. coli, DDM/LMNG-purified, under Turnover at pH 6, Closed state* | 0.03 |  | 19.23 | 0.13 | 293-346 | EM | 0.00 | hetero-1-1-1-1-1-1-… | 7 x SF4, 1 x FMN, 1 x NAI, 2 x FES, 1 x CA, 1 x DCQ, 4 x LFA, 8 x 3PE | HHblits | 0.28 |
| ``` target    NNVNRREFLQWIGAAGFSTFALSASNAWGLQAIENPLAAYPNREWEKTYRDLWKSDASFTFLCAPNDTHNCILNAHVRDG 7p63.1    --------------------------------------------------------------------------------  target    VITRIGPTMKYGEATDLYGSKVTHRWDPRVCQKGLALTRRFYGDRRVRYPMVRKGFKAWADKGFPREKDGRPPKDYFNRA 7p63.1    --------------------------------------------------------------------------------  target    RDEWLRLTHEEAADLVAAALINIATTYSGDNGQKLLLQQGYEKEIVEATRGAGTQVLKFRGGMPLLGLTRIFGLYRMANS 7p63.1    --------------------------------------------------------------------------------  target    MALLDHKIRGVKPEDALGARGWDNYSWHTDLPPGHPMVTGQQTVDFDLHAVEQARIVVVWGMNWVTTKMPDTHWLTEARL 7p63.1    ----------------------------------------------------RADAVVVLE-NDLHRHA-SATRVNAALA  target    KGTKVVVIACEYSSSSIKADDAIVVRPGTTPALALGLCNVIMREKIYDGDYVRRFSDLPLLVRADNLKLLRAEEVFGTPQ 7p63.1    KAPLVMVVDHQRTAIMENAHLVLSAA------------------------------------------------------  target    AALKNQTR 7p63.1    -------- ``` | | | | | | | | | | | | | | | | | | | | | | | | | | | | | | | | | | | | | | | | | | | | | | | | | |
|  | 2nya.1.A | Periplasmic nitrate reductase  *Crystal structure of the periplasmic nitrate reductase (NAP) from Escherichia coli* | 0.04 |  | 11.11 | 0.13 | 293-346 | X-ray | 2.50 | monomer | 1 x SF4, 1 x 6MO, 2 x MGD | HHblits | 0.24 |
| ``` target    NNVNRREFLQWIGAAGFSTFALSASNAWGLQAIENPLAAYPNREWEKTYRDLWKSDASFTFLCAPNDTHNCILNAHVRDG 2nya.1    --------------------------------------------------------------------------------  target    VITRIGPTMKYGEATDLYGSKVTHRWDPRVCQKGLALTRRFYGDRRVRYPMVRKGFKAWADKGFPREKDGRPPKDYFNRA 2nya.1    --------------------------------------------------------------------------------  target    RDEWLRLTHEEAADLVAAALINIATTYSGDNGQKLLLQQGYEKEIVEATRGAGTQVLKFRGGMPLLGLTRIFGLYRMANS 2nya.1    --------------------------------------------------------------------------------  target    MALLDHKIRGVKPEDALGARGWDNYSWHTDLPPGHPMVTGQQTVDFDLHAVEQARIVVVWGMNWVTTKMPDTH-WLTEAR 2nya.1    ----------------------------------------------------KLNVYWTMCTNNMQAGPNINEERMPGWR  target    LKGTKVVVIACEYSSSSIKADDAIVVRPGTTPALALGLCNVIMREKIYDGDYVRRFSDLPLLVRADNLKLLRAEEVFGTP 2nya.1    DPRNFIIVSDPYPTVSALAADLILPTA-----------------------------------------------------  target    QAALKNQTR 2nya.1    --------- ``` | | | | | | | | | | | | | | | | | | | | | | | | | | | | | | | | | | | | | | | | | | | | | | | | | |
|  | 6btm.1.B | Alternative Complex III subunit B  *Structure of Alternative Complex III from Flavobacterium johnsoniae (Wild Type)* | 0.03 |  | 13.46 | 0.13 | 293-345 | EM | 3.40 | hetero-1-1-1-1-1-1-… | 6 x HEC, 1 x F3S, 1 x SF4, 2 x E87 | HHblits | 0.27 |
| ``` target    NNVNRREFLQWIGAAGFSTFALSASNAWGLQAIENPLAAYPNREWEKTYRDLWKSDASFTFLCAPNDTHNCILNAHVRDG 6btm.1    --------------------------------------------------------------------------------  target    VITRIGPTMKYGEATDLYGSKVTHRWDPRVCQKGLALTRRFYGDRRVRYPMVRKGFKAWADKGFPREKDGRPPKDYFNRA 6btm.1    --------------------------------------------------------------------------------  target    RDEWLRLTHEEAADLVAAALINIATTYSGDNGQKLLLQQGYEKEIVEATRGAGTQVLKFRGGMPLLGLTRIFGLYRMANS 6btm.1    --------------------------------------------------------------------------------  target    MALLDHKIRGVKPEDALGARGWDNYSWHTDLPPGHPMVTGQQTVDFDLHAVEQARIVVVWGMNWVTTKMPDTHWLTEARL 6btm.1    ----------------------------------------------------SVHTLIMSGVNPVYTLADS-ASFVSGLK  target    KGTKVVVIACEYSSSSIKADDAIVVRPGTTPALALGLCNVIMREKIYDGDYVRRFSDLPLLVRADNLKLLRAEEVFGTPQ 6btm.1    KVKTSVAFSLKEDETAAVSTIAAAA-------------------------------------------------------  target    AALKNQTR 6btm.1    -------- ``` | | | | | | | | | | | | | | | | | | | | | | | | | | | | | | | | | | | | | | | | | | | | | | | | | |
|  | 3o5a.1.A | Periplasmic nitrate reductase  *Crystal Structure of partially reduced Periplasmic Nitrate Reductase from Cupriavidus necator using Ionic Liquids* | 0.03 |  | 13.21 | 0.13 | 292-347 | X-ray | 1.72 | hetero-oligomer | 1 x SF4, 1 x MOS, 2 x MGD, 2 x HEC | HHblits | 0.25 |
| ``` target    NNVNRREFLQWIGAAGFSTFALSASNAWGLQAIENPLAAYPNREWEKTYRDLWKSDASFTFLCAPNDTHNCILNAHVRDG 3o5a.1    --------------------------------------------------------------------------------  target    VITRIGPTMKYGEATDLYGSKVTHRWDPRVCQKGLALTRRFYGDRRVRYPMVRKGFKAWADKGFPREKDGRPPKDYFNRA 3o5a.1    --------------------------------------------------------------------------------  target    RDEWLRLTHEEAADLVAAALINIATTYSGDNGQKLLLQQGYEKEIVEATRGAGTQVLKFRGGMPLLGLTRIFGLYRMANS 3o5a.1    --------------------------------------------------------------------------------  target    MALLDHKIRGVKPEDALGARGWDNYSWHTDLPPGHPMVTGQQTVDFDLHAVEQARIVVVWGMNWVTTKMPDTHWLTEAR- 3o5a.1    ---------------------------------------------------GKLNAYWVQVNNNMQAAAN---LMEEGLP  target    ---LKGTKVVVIACEYSSSSIKADDAIVVRPGTTPALALGLCNVIMREKIYDGDYVRRFSDLPLLVRADNLKLLRAEEVF 3o5a.1    GYRNPANFIVVSDAYPTVTALAADLVLPSAM-------------------------------------------------  target    GTPQAALKNQTR 3o5a.1    ------------ ``` | | | | | | | | | | | | | | | | | | | | | | | | | | | | | | | | | | | | | | | | | | | | | | | | | |
|  | 7nz1.1.E | NADH-quinone oxidoreductase subunit G  *Respiratory complex I from Escherichia coli - focused refinement of cytoplasmic arm* | 0.03 |  | 19.61 | 0.13 | 293-345 | EM | 0.00 | hetero-1-1-1-1-1-1-… | 7 x SF4, 2 x FES, 1 x FMN, 1 x CA | HHblits | 0.28 |
| ``` target    NNVNRREFLQWIGAAGFSTFALSASNAWGLQAIENPLAAYPNREWEKTYRDLWKSDASFTFLCAPNDTHNCILNAHVRDG 7nz1.1    --------------------------------------------------------------------------------  target    VITRIGPTMKYGEATDLYGSKVTHRWDPRVCQKGLALTRRFYGDRRVRYPMVRKGFKAWADKGFPREKDGRPPKDYFNRA 7nz1.1    --------------------------------------------------------------------------------  target    RDEWLRLTHEEAADLVAAALINIATTYSGDNGQKLLLQQGYEKEIVEATRGAGTQVLKFRGGMPLLGLTRIFGLYRMANS 7nz1.1    --------------------------------------------------------------------------------  target    MALLDHKIRGVKPEDALGARGWDNYSWHTDLPPGHPMVTGQQTVDFDLHAVEQARIVVVWGMNWVTTKMPDTHWLTEARL 7nz1.1    ----------------------------------------------------RADAVVVLE-NDLHRHA-SAIRVNAALA  target    KGTKVVVIACEYSSSSIKADDAIVVRPGTTPALALGLCNVIMREKIYDGDYVRRFSDLPLLVRADNLKLLRAEEVFGTPQ 7nz1.1    KAPLVMVVDHQRTAIMENAHLVLSA-------------------------------------------------------  target    AALKNQTR 7nz1.1    -------- ``` | | | | | | | | | | | | | | | | | | | | | | | | | | | | | | | | | | | | | | | | | | | | | | | | | |
|  | 6f0k.1.B | Fe-S-cluster-containing hydrogenase  *Alternative complex III* | 0.03 |  | 9.62 | 0.13 | 293-345 | EM | 0.00 | hetero-1-1-1-1-1-1-… | 6 x HEC, 1 x F3S, 3 x SF4 | HHblits | 0.26 |
| ``` target    NNVNRREFLQWIGAAGFSTFALSASNAWGLQAIENPLAAYPNREWEKTYRDLWKSDASFTFLCAPNDTHNCILNAHVRDG 6f0k.1    --------------------------------------------------------------------------------  target    VITRIGPTMKYGEATDLYGSKVTHRWDPRVCQKGLALTRRFYGDRRVRYPMVRKGFKAWADKGFPREKDGRPPKDYFNRA 6f0k.1    --------------------------------------------------------------------------------  target    RDEWLRLTHEEAADLVAAALINIATTYSGDNGQKLLLQQGYEKEIVEATRGAGTQVLKFRGGMPLLGLTRIFGLYRMANS 6f0k.1    --------------------------------------------------------------------------------  target    MALLDHKIRGVKPEDALGARGWDNYSWHTDLPPGHPMVTGQQTVDFDLHAVEQARIVVVWGMNWVTTKMPDTHWLTEARL 6f0k.1    ----------------------------------------------------AVDALLLLNVNPVYDAPA-ALGFAEALA  target    KGTKVVVIACEYSSSSIKADDAIVVRPGTTPALALGLCNVIMREKIYDGDYVRRFSDLPLLVRADNLKLLRAEEVFGTPQ 6f0k.1    QVPEVIHLGLHVDETARRSTWHLPS-------------------------------------------------------  target    AALKNQTR 6f0k.1    -------- ``` | | | | | | | | | | | | | | | | | | | | | | | | | | | | | | | | | | | | | | | | | | | | | | | | | |
|  | 7bkb.1.L | Formylmethanofuran dehydrogenase, subunit B  *Formate dehydrogenase - heterodisulfide reductase - formylmethanofuran dehydrogenase complex from Methanospirillum hungatei (hexameric, composite structure)* | 0.04 |  | 13.21 | 0.13 | 292-348 | EM | 0.00 | hetero-2-2-2-2-2-2-… | 48 x SF4, 4 x FAD, 2 x FES, 4 x 9S8, 4 x ZN, 2 x MO, 4 x MGD | HHblits | 0.24 |
| ``` target    NNVNRREFLQWIGAAGFSTFALSASNAWGLQAIENPLAAYPNREWEKTYRDLWKSDASFTFLCAPNDTHNCILNAHVRDG 7bkb.1    --------------------------------------------------------------------------------  target    VITRIGPTMKYGEATDLYGSKVTHRWDPRVCQKGLALTRRFYGDRRVRYPMVRKGFKAWADKGFPREKDGRPPKDYFNRA 7bkb.1    --------------------------------------------------------------------------------  target    RDEWLRLTHEEAADLVAAALINIATTYSGDNGQKLLLQQGYEKEIVEATRGAGTQVLKFRGGMPLLGLTRIFGLYRMANS 7bkb.1    --------------------------------------------------------------------------------  target    MALLDHKIRGVKPEDALGARGWDNYSWHTDLPPGHPMVTGQQTVDFDLHAVEQARIVVVWGMNWVTTKMPDTHWLTEARL 7bkb.1    ---------------------------------------------------DEVDMFINIGTDAAAHFPIP---AVKQLK  target    KGTKVVVIACEYSSSSIKADDAIVVRPGTTPALALGLCNVIMREKIYDGDYVRRFSDLPLLVRADNLKLLRAEEVFGTPQ 7bkb.1    -KHPWVTIDPSINMASEISDLHIPVCIC----------------------------------------------------  target    AALKNQTR 7bkb.1    -------- ``` | | | | | | | | | | | | | | | | | | | | | | | | | | | | | | | | | | | | | | | | | | | | | | | | | |
|  | 6lod.1.B | Fe-S-cluster-containing hydrogenase components 1-like protein  *Cryo-EM structure of the air-oxidized photosynthetic alternative complex III from Roseiflexus castenholzii* | 0.03 |  | 11.54 | 0.13 | 293-345 | EM | 0.00 | hetero-1-1-1-1-1-1-… | 6 x HEC, 2 x EL6, 3 x SF4, 1 x F3S | HHblits | 0.24 |
| ``` target    NNVNRREFLQWIGAAGFSTFALSASNAWGLQAIENPLAAYPNREWEKTYRDLWKSDASFTFLCAPNDTHNCILNAHVRDG 6lod.1    --------------------------------------------------------------------------------  target    VITRIGPTMKYGEATDLYGSKVTHRWDPRVCQKGLALTRRFYGDRRVRYPMVRKGFKAWADKGFPREKDGRPPKDYFNRA 6lod.1    --------------------------------------------------------------------------------  target    RDEWLRLTHEEAADLVAAALINIATTYSGDNGQKLLLQQGYEKEIVEATRGAGTQVLKFRGGMPLLGLTRIFGLYRMANS 6lod.1    --------------------------------------------------------------------------------  target    MALLDHKIRGVKPEDALGARGWDNYSWHTDLPPGHPMVTGQQTVDFDLHAVEQARIVVVWGMNWVTTKMPDTHWLTEARL 6lod.1    ----------------------------------------------------TVEVLLMIESNPVYNAP-ADIPFAEALA  target    KGTKVVVIACEYSSSSIKADDAIVVRPGTTPALALGLCNVIMREKIYDGDYVRRFSDLPLLVRADNLKLLRAEEVFGTPQ 6lod.1    KVPLSMHVGLYRDETAQQSVWHING-------------------------------------------------------  target    AALKNQTR 6lod.1    -------- ``` | | | | | | | | | | | | | | | | | | | | | | | | | | | | | | | | | | | | | | | | | | | | | | | | | |
|  | 5t5i.1.B | Tungsten formylmethanofuran dehydrogenase subunit B  *TUNGSTEN-CONTAINING FORMYLMETHANOFURAN DEHYDROGENASE FROM METHANOTHERMOBACTER WOLFEII, ORTHORHOMBIC FORM AT 1.9 A* | 0.03 |  | 15.69 | 0.13 | 293-347 | X-ray | 1.90 | hetero-oligomer | 4 x ZN, 2 x MG, 18 x K, 22 x SF4, 2 x W, 4 x MGD, 2 x H2S, 2 x CA | HHblits | 0.25 |
| ``` target    NNVNRREFLQWIGAAGFSTFALSASNAWGLQAIENPLAAYPNREWEKTYRDLWKSDASFTFLCAPNDTHNCILNAHVRDG 5t5i.1    --------------------------------------------------------------------------------  target    VITRIGPTMKYGEATDLYGSKVTHRWDPRVCQKGLALTRRFYGDRRVRYPMVRKGFKAWADKGFPREKDGRPPKDYFNRA 5t5i.1    --------------------------------------------------------------------------------  target    RDEWLRLTHEEAADLVAAALINIATTYSGDNGQKLLLQQGYEKEIVEATRGAGTQVLKFRGGMPLLGLTRIFGLYRMANS 5t5i.1    --------------------------------------------------------------------------------  target    MALLDHKIRGVKPEDALGARGWDNYSWHTDLPPGHPMVTGQQTVDFDLHAVEQARIVVVWGMNWVTTKMPDTHWLTEARL 5t5i.1    ----------------------------------------------------EADAMMVIASDPGAHFPQR---ALERMA  target    KGTKVVVIACEYSSSSIKADDAIVVRPGTTPALALGLCNVIMREKIYDGDYVRRFSDLPLLVRADNLKLLRAEEVFGTPQ 5t5i.1    -EIPVIAIEPHRTPTTEMADIIIPPAI-----------------------------------------------------  target    AALKNQTR 5t5i.1    -------- ``` | | | | | | | | | | | | | | | | | | | | | | | | | | | | | | | | | | | | | | | | | | | | | | | | | |
|  | 8b9z.1.G | NADH-ubiquinone oxidoreductase 75 kDa subunit, mitochondrial  *Drosophila melanogaster complex I in the Active state (Dm1)* | 0.02 |  | 16.67 | 0.12 | 292-346 | EM | 3.28 | hetero-1-1-1-1-1-1-… | 3 x PC1, 16 x 3PE, 6 x SF4, 4 x CDL, 2 x FES, 1 x FMN, 1 x UQ9, 1 x DGT, 1 x NDP, 1 x ZN, 2 x EHZ | HHblits | 0.27 |
| ``` target    NNVNRREFLQWIGAAGFSTFALSASNAWGLQAIENPLAAYPNREWEKTYRDLWKSDASFTFLCAPNDTHNCILNAHVRDG 8b9z.1    --------------------------------------------------------------------------------  target    VITRIGPTMKYGEATDLYGSKVTHRWDPRVCQKGLALTRRFYGDRRVRYPMVRKGFKAWADKGFPREKDGRPPKDYFNRA 8b9z.1    --------------------------------------------------------------------------------  target    RDEWLRLTHEEAADLVAAALINIATTYSGDNGQKLLLQQGYEKEIVEATRGAGTQVLKFRGGMPLLGLTRIFGLYRMANS 8b9z.1    --------------------------------------------------------------------------------  target    MALLDHKIRGVKPEDALGARGWDNYSWHTDLPPGHPMVTGQQTVDFDLHAVEQARIVVVWGMNWVTTKMPDTHWLTEARL 8b9z.1    ---------------------------------------------------AQPKVLFLLNADAG-------KVTREQLP  target    KGTKVVVIACEYSSSSIKADDAIVVRPGTTPALALGLCNVIMREKIYDGDYVRRFSDLPLLVRADNLKLLRAEEVFGTPQ 8b9z.1    KDCFVVYIGSHGDNGASIADAVLPGA------------------------------------------------------  target    AALKNQTR 8b9z.1    -------- ``` | | | | | | | | | | | | | | | | | | | | | | | | | | | | | | | | | | | | | | | | | | | | | | | | | |
|  | 8ba0.1.G | NADH-ubiquinone oxidoreductase 75 kDa subunit, mitochondrial  *Drosophila melanogaster complex I in the Twisted state (Dm2)* | 0.02 |  | 16.67 | 0.12 | 292-346 | EM | 3.68 | hetero-1-1-1-1-1-1-… | 6 x SF4, 6 x 3PE, 2 x FES, 1 x FMN, 2 x CDL, 1 x DGT, 1 x NDP, 1 x ZN, 2 x EHZ | HHblits | 0.27 |
| ``` target    NNVNRREFLQWIGAAGFSTFALSASNAWGLQAIENPLAAYPNREWEKTYRDLWKSDASFTFLCAPNDTHNCILNAHVRDG 8ba0.1    --------------------------------------------------------------------------------  target    VITRIGPTMKYGEATDLYGSKVTHRWDPRVCQKGLALTRRFYGDRRVRYPMVRKGFKAWADKGFPREKDGRPPKDYFNRA 8ba0.1    --------------------------------------------------------------------------------  target    RDEWLRLTHEEAADLVAAALINIATTYSGDNGQKLLLQQGYEKEIVEATRGAGTQVLKFRGGMPLLGLTRIFGLYRMANS 8ba0.1    --------------------------------------------------------------------------------  target    MALLDHKIRGVKPEDALGARGWDNYSWHTDLPPGHPMVTGQQTVDFDLHAVEQARIVVVWGMNWVTTKMPDTHWLTEARL 8ba0.1    ---------------------------------------------------AQPKVLFLLNADAG-------KVTREQLP  target    KGTKVVVIACEYSSSSIKADDAIVVRPGTTPALALGLCNVIMREKIYDGDYVRRFSDLPLLVRADNLKLLRAEEVFGTPQ 8ba0.1    KDCFVVYIGSHGDNGASIADAVLPGA------------------------------------------------------  target    AALKNQTR 8ba0.1    -------- ``` | | | | | | | | | | | | | | | | | | | | | | | | | | | | | | | | | | | | | | | | | | | | | | | | | |
|  | 7qsd.1.G | NADH-ubiquinone oxidoreductase 75 kDa subunit, mitochondrial  *Bovine complex I in the active state at 3.1 A* | 0.02 |  | 10.00 | 0.12 | 292-348 | EM | 0.00 | hetero-1-1-1-1-1-1-… | 5 x PC1, 13 x 3PE, 6 x SF4, 2 x FES, 1 x FMN, 4 x CDL, 3 x LMT, 1 x GTP, 1 x MG, 1 x NDP, 1 x ZN, 2 x EHZ | HHblits | 0.24 |
| ``` target    NNVNRREFLQWIGAAGFSTFALSASNAWGLQAIENPLAAYPNREWEKTYRDLWKSDASFTFLCAPNDTHNCILNAHVRDG 7qsd.1    --------------------------------------------------------------------------------  target    VITRIGPTMKYGEATDLYGSKVTHRWDPRVCQKGLALTRRFYGDRRVRYPMVRKGFKAWADKGFPREKDGRPPKDYFNRA 7qsd.1    --------------------------------------------------------------------------------  target    RDEWLRLTHEEAADLVAAALINIATTYSGDNGQKLLLQQGYEKEIVEATRGAGTQVLKFRGGMPLLGLTRIFGLYRMANS 7qsd.1    --------------------------------------------------------------------------------  target    MALLDHKIRGVKPEDALGARGWDNYSWHTDLPPGHPMVTGQQTVDFDLHAVEQARIVVVWGMNWVTTKMPDTHWLTEARL 7qsd.1    ---------------------------------------------------NPPKMLFLLGADGGC-------ITRQDLP  target    KGTKVVVIACEYSSSSIKADDAIVVRPGTTPALALGLCNVIMREKIYDGDYVRRFSDLPLLVRADNLKLLRAEEVFGTPQ 7qsd.1    KDCFIVYQGHHGDVGAPIADVILPGAAY----------------------------------------------------  target    AALKNQTR 7qsd.1    -------- ``` | | | | | | | | | | | | | | | | | | | | | | | | | | | | | | | | | | | | | | | | | | | | | | | | | |
|  | 7dgr.10.A | NADH-ubiquinone oxidoreductase 75 kDa subunit, mitochondrial  *Activity optimized supercomplex state2* | 0.02 |  | 10.00 | 0.12 | 292-348 | EM | 0.00 | monomer |  | HHblits | 0.24 |
| ``` target    NNVNRREFLQWIGAAGFSTFALSASNAWGLQAIENPLAAYPNREWEKTYRDLWKSDASFTFLCAPNDTHNCILNAHVRDG 7dgr.10   --------------------------------------------------------------------------------  target    VITRIGPTMKYGEATDLYGSKVTHRWDPRVCQKGLALTRRFYGDRRVRYPMVRKGFKAWADKGFPREKDGRPPKDYFNRA 7dgr.10   --------------------------------------------------------------------------------  target    RDEWLRLTHEEAADLVAAALINIATTYSGDNGQKLLLQQGYEKEIVEATRGAGTQVLKFRGGMPLLGLTRIFGLYRMANS 7dgr.10   --------------------------------------------------------------------------------  target    MALLDHKIRGVKPEDALGARGWDNYSWHTDLPPGHPMVTGQQTVDFDLHAVEQARIVVVWGMNWVTTKMPDTHWLTEARL 7dgr.10   ---------------------------------------------------NPPKMLFLLGADGGC-------ITRQDLP  target    KGTKVVVIACEYSSSSIKADDAIVVRPGTTPALALGLCNVIMREKIYDGDYVRRFSDLPLLVRADNLKLLRAEEVFGTPQ 7dgr.10   KDCFIVYQGHHGDVGAPIADVILPGAAY----------------------------------------------------  target    AALKNQTR 7dgr.10   -------- ``` | | | | | | | | | | | | | | | | | | | | | | | | | | | | | | | | | | | | | | | | | | | | | | | | | |
|  | 5o31.1.8 | NADH-ubiquinone oxidoreductase 75 kDa subunit, mitochondrial  *Mitochondrial complex I in the deactive state* | 0.02 |  | 10.00 | 0.12 | 292-348 | EM | 4.13 | hetero-1-1-1-1-1-1-… | 6 x SF4, 2 x FES, 1 x FMN, 1 x NAP, 1 x ZN | HHblits | 0.24 |
| ``` target    NNVNRREFLQWIGAAGFSTFALSASNAWGLQAIENPLAAYPNREWEKTYRDLWKSDASFTFLCAPNDTHNCILNAHVRDG 5o31.1    --------------------------------------------------------------------------------  target    VITRIGPTMKYGEATDLYGSKVTHRWDPRVCQKGLALTRRFYGDRRVRYPMVRKGFKAWADKGFPREKDGRPPKDYFNRA 5o31.1    --------------------------------------------------------------------------------  target    RDEWLRLTHEEAADLVAAALINIATTYSGDNGQKLLLQQGYEKEIVEATRGAGTQVLKFRGGMPLLGLTRIFGLYRMANS 5o31.1    --------------------------------------------------------------------------------  target    MALLDHKIRGVKPEDALGARGWDNYSWHTDLPPGHPMVTGQQTVDFDLHAVEQARIVVVWGMNWVTTKMPDTHWLTEARL 5o31.1    ---------------------------------------------------NPPKMLFLLGADGGC-------ITRQDLP  target    KGTKVVVIACEYSSSSIKADDAIVVRPGTTPALALGLCNVIMREKIYDGDYVRRFSDLPLLVRADNLKLLRAEEVFGTPQ 5o31.1    KDCFIVYQGHHGDVGAPIADVILPGAAY----------------------------------------------------  target    AALKNQTR 5o31.1    -------- ``` | | | | | | | | | | | | | | | | | | | | | | | | | | | | | | | | | | | | | | | | | | | | | | | | | |
|  | 7ar7.1.G | NADH dehydrogenase [ubiquinone] iron-sulfur protein 1, mitochondrial  *Cryo-EM structure of Arabidopsis thaliana complex-I (open conformation)* | 0.02 |  | 19.15 | 0.12 | 292-347 | EM | 0.00 | hetero-1-1-1-1-1-1-… | 6 x SF4, 2 x FES, 1 x FMN, 1 x UQ9, 3 x PTY, 2 x PC7, 1 x LMN, 1 x NDP, 2 x ZN, 2 x 8Q1, 1 x PGT, 1 x PSF, 1 x T7X | HHblits | 0.29 |
| ``` target    NNVNRREFLQWIGAAGFSTFALSASNAWGLQAIENPLAAYPNREWEKTYRDLWKSDASFTFLCAPNDTHNCILNAHVRDG 7ar7.1    --------------------------------------------------------------------------------  target    VITRIGPTMKYGEATDLYGSKVTHRWDPRVCQKGLALTRRFYGDRRVRYPMVRKGFKAWADKGFPREKDGRPPKDYFNRA 7ar7.1    --------------------------------------------------------------------------------  target    RDEWLRLTHEEAADLVAAALINIATTYSGDNGQKLLLQQGYEKEIVEATRGAGTQVLKFRGGMPLLGLTRIFGLYRMANS 7ar7.1    --------------------------------------------------------------------------------  target    MALLDHKIRGVKPEDALGARGWDNYSWHTDLPPGHPMVTGQQTVDFDLHAVEQARIVVVWGMNWVTTKMPDTHWLTEARL 7ar7.1    ---------------------------------------------------ESAKFVYLMGADDVN---------VDKIP  target    KGTKVVVIACEYSSSSIKADDAIVVRPGTTPALALGLCNVIMREKIYDGDYVRRFSDLPLLVRADNLKLLRAEEVFGTPQ 7ar7.1    KDAFVVYQGHHGDKAVYRANVILPASA-----------------------------------------------------  target    AALKNQTR 7ar7.1    -------- ``` | | | | | | | | | | | | | | | | | | | | | | | | | | | | | | | | | | | | | | | | | | | | | | | | | |
|  | 7aqr.1.F | NADH dehydrogenase [ubiquinone] iron-sulfur protein 1, mitochondrial  *Cryo-EM structure of Arabidopsis thaliana Complex-I (peripheral arm)* | 0.03 |  | 19.15 | 0.12 | 292-347 | EM | 0.00 | hetero-1-1-1-1-1-1-… | 6 x SF4, 2 x FES, 1 x FMN, 1 x NDP, 1 x ZN, 1 x 8Q1 | HHblits | 0.29 |
| ``` target    NNVNRREFLQWIGAAGFSTFALSASNAWGLQAIENPLAAYPNREWEKTYRDLWKSDASFTFLCAPNDTHNCILNAHVRDG 7aqr.1    --------------------------------------------------------------------------------  target    VITRIGPTMKYGEATDLYGSKVTHRWDPRVCQKGLALTRRFYGDRRVRYPMVRKGFKAWADKGFPREKDGRPPKDYFNRA 7aqr.1    --------------------------------------------------------------------------------  target    RDEWLRLTHEEAADLVAAALINIATTYSGDNGQKLLLQQGYEKEIVEATRGAGTQVLKFRGGMPLLGLTRIFGLYRMANS 7aqr.1    --------------------------------------------------------------------------------  target    MALLDHKIRGVKPEDALGARGWDNYSWHTDLPPGHPMVTGQQTVDFDLHAVEQARIVVVWGMNWVTTKMPDTHWLTEARL 7aqr.1    ---------------------------------------------------ESAKFVYLMGADDVN---------VDKIP  target    KGTKVVVIACEYSSSSIKADDAIVVRPGTTPALALGLCNVIMREKIYDGDYVRRFSDLPLLVRADNLKLLRAEEVFGTPQ 7aqr.1    KDAFVVYQGHHGDKAVYRANVILPASA-----------------------------------------------------  target    AALKNQTR 7aqr.1    -------- ``` | | | | | | | | | | | | | | | | | | | | | | | | | | | | | | | | | | | | | | | | | | | | | | | | | |
|  | 7a23.1.O | 75kDa  *Plant mitochondrial respiratory complex I* | 0.02 |  | 19.15 | 0.12 | 292-347 | EM | 0.00 | hetero-1-1-1-1-1-1-… | 6 x SF4, 1 x FMN, 2 x T7X, 3 x CDL, 1 x U10, 1 x PEV, 2 x FES, 1 x NDP, 2 x ZN | HHblits | 0.29 |
| ``` target    NNVNRREFLQWIGAAGFSTFALSASNAWGLQAIENPLAAYPNREWEKTYRDLWKSDASFTFLCAPNDTHNCILNAHVRDG 7a23.1    --------------------------------------------------------------------------------  target    VITRIGPTMKYGEATDLYGSKVTHRWDPRVCQKGLALTRRFYGDRRVRYPMVRKGFKAWADKGFPREKDGRPPKDYFNRA 7a23.1    --------------------------------------------------------------------------------  target    RDEWLRLTHEEAADLVAAALINIATTYSGDNGQKLLLQQGYEKEIVEATRGAGTQVLKFRGGMPLLGLTRIFGLYRMANS 7a23.1    --------------------------------------------------------------------------------  target    MALLDHKIRGVKPEDALGARGWDNYSWHTDLPPGHPMVTGQQTVDFDLHAVEQARIVVVWGMNWVTTKMPDTHWLTEARL 7a23.1    ---------------------------------------------------ESAKFVYLMGADDVN---------VDKIP  target    KGTKVVVIACEYSSSSIKADDAIVVRPGTTPALALGLCNVIMREKIYDGDYVRRFSDLPLLVRADNLKLLRAEEVFGTPQ 7a23.1    KDAFVVYQGHHGDKAVYRANVILPASA-----------------------------------------------------  target    AALKNQTR 7a23.1    -------- ``` | | | | | | | | | | | | | | | | | | | | | | | | | | | | | | | | | | | | | | | | | | | | | | | | | |
|  | 7ar8.1.G | NADH dehydrogenase [ubiquinone] iron-sulfur protein 1, mitochondrial  *Cryo-EM structure of Arabidopsis thaliana complex-I (closed conformation)* | 0.02 |  | 19.15 | 0.12 | 292-347 | EM | 0.00 | hetero-1-1-1-1-1-1-… | 6 x SF4, 2 x FES, 1 x FMN, 1 x UQ9, 3 x PTY, 2 x PC7, 1 x PGT, 1 x FE, 1 x NDP, 2 x ZN, 2 x 8Q1, 1 x LMN, 1 x PSF, 1 x T7X | HHblits | 0.29 |
| ``` target    NNVNRREFLQWIGAAGFSTFALSASNAWGLQAIENPLAAYPNREWEKTYRDLWKSDASFTFLCAPNDTHNCILNAHVRDG 7ar8.1    --------------------------------------------------------------------------------  target    VITRIGPTMKYGEATDLYGSKVTHRWDPRVCQKGLALTRRFYGDRRVRYPMVRKGFKAWADKGFPREKDGRPPKDYFNRA 7ar8.1    --------------------------------------------------------------------------------  target    RDEWLRLTHEEAADLVAAALINIATTYSGDNGQKLLLQQGYEKEIVEATRGAGTQVLKFRGGMPLLGLTRIFGLYRMANS 7ar8.1    --------------------------------------------------------------------------------  target    MALLDHKIRGVKPEDALGARGWDNYSWHTDLPPGHPMVTGQQTVDFDLHAVEQARIVVVWGMNWVTTKMPDTHWLTEARL 7ar8.1    ---------------------------------------------------ESAKFVYLMGADDVN---------VDKIP  target    KGTKVVVIACEYSSSSIKADDAIVVRPGTTPALALGLCNVIMREKIYDGDYVRRFSDLPLLVRADNLKLLRAEEVFGTPQ 7ar8.1    KDAFVVYQGHHGDKAVYRANVILPASA-----------------------------------------------------  target    AALKNQTR 7ar8.1    -------- ``` | | | | | | | | | | | | | | | | | | | | | | | | | | | | | | | | | | | | | | | | | | | | | | | | | |
|  | 6x89.1.H | NADH dehydrogenase [ubiquinone] iron-sulfur protein 1, mitochondrial  *Vigna radiata mitochondrial complex I\** | 0.02 |  | 19.15 | 0.12 | 292-347 | EM | 0.00 | hetero-1-1-1-1-1-1-… | 1 x NAP, 6 x PC1, 6 x SF4, 2 x FES, 2 x ZN, 1 x FMN | HHblits | 0.28 |
| ``` target    NNVNRREFLQWIGAAGFSTFALSASNAWGLQAIENPLAAYPNREWEKTYRDLWKSDASFTFLCAPNDTHNCILNAHVRDG 6x89.1    --------------------------------------------------------------------------------  target    VITRIGPTMKYGEATDLYGSKVTHRWDPRVCQKGLALTRRFYGDRRVRYPMVRKGFKAWADKGFPREKDGRPPKDYFNRA 6x89.1    --------------------------------------------------------------------------------  target    RDEWLRLTHEEAADLVAAALINIATTYSGDNGQKLLLQQGYEKEIVEATRGAGTQVLKFRGGMPLLGLTRIFGLYRMANS 6x89.1    --------------------------------------------------------------------------------  target    MALLDHKIRGVKPEDALGARGWDNYSWHTDLPPGHPMVTGQQTVDFDLHAVEQARIVVVWGMNWVTTKMPDTHWLTEARL 6x89.1    ---------------------------------------------------ESAKFVYLMGADDVNL---------DKIP  target    KGTKVVVIACEYSSSSIKADDAIVVRPGTTPALALGLCNVIMREKIYDGDYVRRFSDLPLLVRADNLKLLRAEEVFGTPQ 6x89.1    DDAFVVYQGHHGDKSVYRANVILPTAA-----------------------------------------------------  target    AALKNQTR 6x89.1    -------- ``` | | | | | | | | | | | | | | | | | | | | | | | | | | | | | | | | | | | | | | | | | | | | | | | | | |
|  | 8e73.55.A | NDUS1  *Vigna radiata supercomplex I+III2 (full bridge)* | 0.03 |  | 19.15 | 0.12 | 292-347 | EM | 0.00 | monomer |  | HHblits | 0.28 |
| ``` target    NNVNRREFLQWIGAAGFSTFALSASNAWGLQAIENPLAAYPNREWEKTYRDLWKSDASFTFLCAPNDTHNCILNAHVRDG 8e73.55   --------------------------------------------------------------------------------  target    VITRIGPTMKYGEATDLYGSKVTHRWDPRVCQKGLALTRRFYGDRRVRYPMVRKGFKAWADKGFPREKDGRPPKDYFNRA 8e73.55   --------------------------------------------------------------------------------  target    RDEWLRLTHEEAADLVAAALINIATTYSGDNGQKLLLQQGYEKEIVEATRGAGTQVLKFRGGMPLLGLTRIFGLYRMANS 8e73.55   --------------------------------------------------------------------------------  target    MALLDHKIRGVKPEDALGARGWDNYSWHTDLPPGHPMVTGQQTVDFDLHAVEQARIVVVWGMNWVTTKMPDTHWLTEARL 8e73.55   ---------------------------------------------------ESAKFVYLMGADDVNL---------DKIP  target    KGTKVVVIACEYSSSSIKADDAIVVRPGTTPALALGLCNVIMREKIYDGDYVRRFSDLPLLVRADNLKLLRAEEVFGTPQ 8e73.55   DDAFVVYQGHHGDKSVYRANVILPTAA-----------------------------------------------------  target    AALKNQTR 8e73.55   -------- ``` | | | | | | | | | | | | | | | | | | | | | | | | | | | | | | | | | | | | | | | | | | | | | | | | | |
|  | 7tgh.58.A | NADH-ubiquinone oxidoreductase 75 kDa subunit  *Cryo-EM structure of respiratory super-complex CI+III2 from Tetrahymena thermophila* | 0.03 |  | 17.02 | 0.12 | 292-346 | EM | 0.00 | monomer |  | HHblits | 0.28 |
| ``` target    NNVNRREFLQWIGAAGFSTFALSASNAWGLQAIENPLAAYPNREWEKTYRDLWKSDASFTFLCAPNDTHNCILNAHVRDG 7tgh.58   --------------------------------------------------------------------------------  target    VITRIGPTMKYGEATDLYGSKVTHRWDPRVCQKGLALTRRFYGDRRVRYPMVRKGFKAWADKGFPREKDGRPPKDYFNRA 7tgh.58   --------------------------------------------------------------------------------  target    RDEWLRLTHEEAADLVAAALINIATTYSGDNGQKLLLQQGYEKEIVEATRGAGTQVLKFRGGMPLLGLTRIFGLYRMANS 7tgh.58   --------------------------------------------------------------------------------  target    MALLDHKIRGVKPEDALGARGWDNYSWHTDLPPGHPMVTGQQTVDFDLHAVEQARIVVVWGMNWVTTKMPDTHWLTEARL 7tgh.58   ---------------------------------------------------KNAKLVFILGADNNLRP--------EDIP  target    KGTKVVVIACEYSSSSIKADDAIVVRPGTTPALALGLCNVIMREKIYDGDYVRRFSDLPLLVRADNLKLLRAEEVFGTPQ 7tgh.58   ADAFVVYFGTHGDEGAYYADIILPTA------------------------------------------------------  target    AALKNQTR 7tgh.58   -------- ``` | | | | | | | | | | | | | | | | | | | | | | | | | | | | | | | | | | | | | | | | | | | | | | | | | |
|  | 5gpn.24.A | NADH-ubiquinone oxidoreductase 75 kDa subunit  *Architecture of mammalian respirasome* | 0.02 |  | 8.16 | 0.12 | 293-348 | EM | 0.00 | monomer |  | HHblits | 0.24 |
| ``` target    NNVNRREFLQWIGAAGFSTFALSASNAWGLQAIENPLAAYPNREWEKTYRDLWKSDASFTFLCAPNDTHNCILNAHVRDG 5gpn.24   --------------------------------------------------------------------------------  target    VITRIGPTMKYGEATDLYGSKVTHRWDPRVCQKGLALTRRFYGDRRVRYPMVRKGFKAWADKGFPREKDGRPPKDYFNRA 5gpn.24   --------------------------------------------------------------------------------  target    RDEWLRLTHEEAADLVAAALINIATTYSGDNGQKLLLQQGYEKEIVEATRGAGTQVLKFRGGMPLLGLTRIFGLYRMANS 5gpn.24   --------------------------------------------------------------------------------  target    MALLDHKIRGVKPEDALGARGWDNYSWHTDLPPGHPMVTGQQTVDFDLHAVEQARIVVVWGMNWVTTKMPDTHWLTEARL 5gpn.24   ----------------------------------------------------PPKVLFLLGADGGC-------ITRQDLP  target    KGTKVVVIACEYSSSSIKADDAIVVRPGTTPALALGLCNVIMREKIYDGDYVRRFSDLPLLVRADNLKLLRAEEVFGTPQ 5gpn.24   KDCFIIYQGHHGDVGAPMADVILPGAAY----------------------------------------------------  target    AALKNQTR 5gpn.24   -------- ``` | | | | | | | | | | | | | | | | | | | | | | | | | | | | | | | | | | | | | | | | | | | | | | | | | |
|  | 6zr2.1.G | NADH-ubiquinone oxidoreductase 75 kDa subunit, mitochondrial  *Cryo-EM structure of respiratory complex I in the active state from Mus musculus at 3.1 A* | 0.02 |  | 10.20 | 0.12 | 293-348 | EM | 3.10 | hetero-1-1-1-1-1-1-… | 6 x SF4, 4 x PC1, 2 x FES, 1 x FMN, 9 x 3PE, 7 x CDL, 1 x ATP, 1 x NDP, 1 x ZN, 2 x EHZ | HHblits | 0.24 |
| ``` target    NNVNRREFLQWIGAAGFSTFALSASNAWGLQAIENPLAAYPNREWEKTYRDLWKSDASFTFLCAPNDTHNCILNAHVRDG 6zr2.1    --------------------------------------------------------------------------------  target    VITRIGPTMKYGEATDLYGSKVTHRWDPRVCQKGLALTRRFYGDRRVRYPMVRKGFKAWADKGFPREKDGRPPKDYFNRA 6zr2.1    --------------------------------------------------------------------------------  target    RDEWLRLTHEEAADLVAAALINIATTYSGDNGQKLLLQQGYEKEIVEATRGAGTQVLKFRGGMPLLGLTRIFGLYRMANS 6zr2.1    --------------------------------------------------------------------------------  target    MALLDHKIRGVKPEDALGARGWDNYSWHTDLPPGHPMVTGQQTVDFDLHAVEQARIVVVWGMNWVTTKMPDTHWLTEARL 6zr2.1    ----------------------------------------------------PPKMLFLLGADGGC-------ITRQDLP  target    KGTKVVVIACEYSSSSIKADDAIVVRPGTTPALALGLCNVIMREKIYDGDYVRRFSDLPLLVRADNLKLLRAEEVFGTPQ 6zr2.1    KDCFIVYQGHHGDVGAPMADVILPGAAY----------------------------------------------------  target    AALKNQTR 6zr2.1    -------- ``` | | | | | | | | | | | | | | | | | | | | | | | | | | | | | | | | | | | | | | | | | | | | | | | | | |
|  | 6g72.1.G | NADH-ubiquinone oxidoreductase 75 kDa subunit, mitochondrial  *Mouse mitochondrial complex I in the deactive state* | 0.02 |  | 10.20 | 0.12 | 293-348 | EM | 0.00 | hetero-1-1-1-1-1-1-… | 6 x SF4, 2 x FES, 1 x FMN, 1 x ADP, 1 x NDP, 1 x ZN, 2 x EHZ | HHblits | 0.24 |
| ``` target    NNVNRREFLQWIGAAGFSTFALSASNAWGLQAIENPLAAYPNREWEKTYRDLWKSDASFTFLCAPNDTHNCILNAHVRDG 6g72.1    --------------------------------------------------------------------------------  target    VITRIGPTMKYGEATDLYGSKVTHRWDPRVCQKGLALTRRFYGDRRVRYPMVRKGFKAWADKGFPREKDGRPPKDYFNRA 6g72.1    --------------------------------------------------------------------------------  target    RDEWLRLTHEEAADLVAAALINIATTYSGDNGQKLLLQQGYEKEIVEATRGAGTQVLKFRGGMPLLGLTRIFGLYRMANS 6g72.1    --------------------------------------------------------------------------------  target    MALLDHKIRGVKPEDALGARGWDNYSWHTDLPPGHPMVTGQQTVDFDLHAVEQARIVVVWGMNWVTTKMPDTHWLTEARL 6g72.1    ----------------------------------------------------PPKMLFLLGADGGC-------ITRQDLP  target    KGTKVVVIACEYSSSSIKADDAIVVRPGTTPALALGLCNVIMREKIYDGDYVRRFSDLPLLVRADNLKLLRAEEVFGTPQ 6g72.1    KDCFIVYQGHHGDVGAPMADVILPGAAY----------------------------------------------------  target    AALKNQTR 6g72.1    -------- ``` | | | | | | | | | | | | | | | | | | | | | | | | | | | | | | | | | | | | | | | | | | | | | | | | | |
|  | 7ak6.1.G | NADH-ubiquinone oxidoreductase 75 kDa subunit, mitochondrial  *Cryo-EM structure of ND6-P25L mutant respiratory complex I from Mus musculus at 3.8 A* | 0.02 |  | 10.20 | 0.12 | 293-348 | EM | 0.00 | hetero-1-1-1-1-1-1-… | 6 x SF4, 1 x PC1, 2 x FES, 1 x FMN, 4 x 3PE, 2 x CDL, 1 x ATP, 1 x NDP, 1 x ZN, 2 x EHZ | HHblits | 0.24 |
| ``` target    NNVNRREFLQWIGAAGFSTFALSASNAWGLQAIENPLAAYPNREWEKTYRDLWKSDASFTFLCAPNDTHNCILNAHVRDG 7ak6.1    --------------------------------------------------------------------------------  target    VITRIGPTMKYGEATDLYGSKVTHRWDPRVCQKGLALTRRFYGDRRVRYPMVRKGFKAWADKGFPREKDGRPPKDYFNRA 7ak6.1    --------------------------------------------------------------------------------  target    RDEWLRLTHEEAADLVAAALINIATTYSGDNGQKLLLQQGYEKEIVEATRGAGTQVLKFRGGMPLLGLTRIFGLYRMANS 7ak6.1    --------------------------------------------------------------------------------  target    MALLDHKIRGVKPEDALGARGWDNYSWHTDLPPGHPMVTGQQTVDFDLHAVEQARIVVVWGMNWVTTKMPDTHWLTEARL 7ak6.1    ----------------------------------------------------PPKMLFLLGADGGC-------ITRQDLP  target    KGTKVVVIACEYSSSSIKADDAIVVRPGTTPALALGLCNVIMREKIYDGDYVRRFSDLPLLVRADNLKLLRAEEVFGTPQ 7ak6.1    KDCFIVYQGHHGDVGAPMADVILPGAAY----------------------------------------------------  target    AALKNQTR 7ak6.1    -------- ``` | | | | | | | | | | | | | | | | | | | | | | | | | | | | | | | | | | | | | | | | | | | | | | | | | |
|  | 7ak5.1.G | NADH-ubiquinone oxidoreductase 75 kDa subunit, mitochondrial  *Cryo-EM structure of respiratory complex I in the deactive state from Mus musculus at 3.2 A* | 0.03 |  | 10.20 | 0.12 | 293-348 | EM | 0.00 | hetero-1-1-1-1-1-1-… | 6 x SF4, 2 x PC1, 2 x FES, 1 x FMN, 8 x 3PE, 4 x CDL, 1 x ATP, 1 x NDP, 1 x ZN, 2 x EHZ | HHblits | 0.24 |
| ``` target    NNVNRREFLQWIGAAGFSTFALSASNAWGLQAIENPLAAYPNREWEKTYRDLWKSDASFTFLCAPNDTHNCILNAHVRDG 7ak5.1    --------------------------------------------------------------------------------  target    VITRIGPTMKYGEATDLYGSKVTHRWDPRVCQKGLALTRRFYGDRRVRYPMVRKGFKAWADKGFPREKDGRPPKDYFNRA 7ak5.1    --------------------------------------------------------------------------------  target    RDEWLRLTHEEAADLVAAALINIATTYSGDNGQKLLLQQGYEKEIVEATRGAGTQVLKFRGGMPLLGLTRIFGLYRMANS 7ak5.1    --------------------------------------------------------------------------------  target    MALLDHKIRGVKPEDALGARGWDNYSWHTDLPPGHPMVTGQQTVDFDLHAVEQARIVVVWGMNWVTTKMPDTHWLTEARL 7ak5.1    ----------------------------------------------------PPKMLFLLGADGGC-------ITRQDLP  target    KGTKVVVIACEYSSSSIKADDAIVVRPGTTPALALGLCNVIMREKIYDGDYVRRFSDLPLLVRADNLKLLRAEEVFGTPQ 7ak5.1    KDCFIVYQGHHGDVGAPMADVILPGAAY----------------------------------------------------  target    AALKNQTR 7ak5.1    -------- ``` | | | | | | | | | | | | | | | | | | | | | | | | | | | | | | | | | | | | | | | | | | | | | | | | | |
|  | 5xtb.1.L | NADH-ubiquinone oxidoreductase 75 kDa subunit, mitochondrial  *Cryo-EM structure of human respiratory complex I matrix arm* | 0.01 |  | 8.16 | 0.12 | 293-348 | EM | 0.00 | hetero-1-1-1-1-1-1-… | 6 x SF4, 1 x FMN, 1 x 8Q1, 1 x NDP, 2 x FES | HHblits | 0.24 |
| ``` target    NNVNRREFLQWIGAAGFSTFALSASNAWGLQAIENPLAAYPNREWEKTYRDLWKSDASFTFLCAPNDTHNCILNAHVRDG 5xtb.1    --------------------------------------------------------------------------------  target    VITRIGPTMKYGEATDLYGSKVTHRWDPRVCQKGLALTRRFYGDRRVRYPMVRKGFKAWADKGFPREKDGRPPKDYFNRA 5xtb.1    --------------------------------------------------------------------------------  target    RDEWLRLTHEEAADLVAAALINIATTYSGDNGQKLLLQQGYEKEIVEATRGAGTQVLKFRGGMPLLGLTRIFGLYRMANS 5xtb.1    --------------------------------------------------------------------------------  target    MALLDHKIRGVKPEDALGARGWDNYSWHTDLPPGHPMVTGQQTVDFDLHAVEQARIVVVWGMNWVTTKMPDTHWLTEARL 5xtb.1    ----------------------------------------------------PPKVLFLLGADGGC-------ITRQDLP  target    KGTKVVVIACEYSSSSIKADDAIVVRPGTTPALALGLCNVIMREKIYDGDYVRRFSDLPLLVRADNLKLLRAEEVFGTPQ 5xtb.1    KDCFIIYQGHHGDVGAPIADVILPGAAY----------------------------------------------------  target    AALKNQTR 5xtb.1    -------- ``` | | | | | | | | | | | | | | | | | | | | | | | | | | | | | | | | | | | | | | | | | | | | | | | | | |
|  | 6qcf.1.C | NADH:ubiquinone oxidoreductase core subunit S1  *Ovine respiratory complex I FRC open class 6* | 0.02 |  | 10.20 | 0.12 | 293-348 | EM | 0.00 | hetero-1-1-1-1-1-1-… | 6 x SF4, 1 x FMN, 2 x FES, 1 x ZN, 1 x NDP, 2 x ZMP | HHblits | 0.24 |
| ``` target    NNVNRREFLQWIGAAGFSTFALSASNAWGLQAIENPLAAYPNREWEKTYRDLWKSDASFTFLCAPNDTHNCILNAHVRDG 6qcf.1    --------------------------------------------------------------------------------  target    VITRIGPTMKYGEATDLYGSKVTHRWDPRVCQKGLALTRRFYGDRRVRYPMVRKGFKAWADKGFPREKDGRPPKDYFNRA 6qcf.1    --------------------------------------------------------------------------------  target    RDEWLRLTHEEAADLVAAALINIATTYSGDNGQKLLLQQGYEKEIVEATRGAGTQVLKFRGGMPLLGLTRIFGLYRMANS 6qcf.1    --------------------------------------------------------------------------------  target    MALLDHKIRGVKPEDALGARGWDNYSWHTDLPPGHPMVTGQQTVDFDLHAVEQARIVVVWGMNWVTTKMPDTHWLTEARL 6qcf.1    ----------------------------------------------------PPKMLFLLGADGGC-------VTRQDLP  target    KGTKVVVIACEYSSSSIKADDAIVVRPGTTPALALGLCNVIMREKIYDGDYVRRFSDLPLLVRADNLKLLRAEEVFGTPQ 6qcf.1    KDCFIVYQGHHGDVGAPIADVILPGAAY----------------------------------------------------  target    AALKNQTR 6qcf.1    -------- ``` | | | | | | | | | | | | | | | | | | | | | | | | | | | | | | | | | | | | | | | | | | | | | | | | | |
|  | 6qc5.1.C | NADH:ubiquinone oxidoreductase core subunit S1  *Ovine respiratory complex I FRC closed class 1* | 0.02 |  | 10.20 | 0.12 | 293-348 | EM | 0.00 | hetero-1-1-1-1-1-1-… | 6 x SF4, 1 x FMN, 2 x FES, 2 x 3PE, 1 x ZN, 1 x NDP, 2 x ZMP, 1 x PC1 | HHblits | 0.24 |
| ``` target    NNVNRREFLQWIGAAGFSTFALSASNAWGLQAIENPLAAYPNREWEKTYRDLWKSDASFTFLCAPNDTHNCILNAHVRDG 6qc5.1    --------------------------------------------------------------------------------  target    VITRIGPTMKYGEATDLYGSKVTHRWDPRVCQKGLALTRRFYGDRRVRYPMVRKGFKAWADKGFPREKDGRPPKDYFNRA 6qc5.1    --------------------------------------------------------------------------------  target    RDEWLRLTHEEAADLVAAALINIATTYSGDNGQKLLLQQGYEKEIVEATRGAGTQVLKFRGGMPLLGLTRIFGLYRMANS 6qc5.1    --------------------------------------------------------------------------------  target    MALLDHKIRGVKPEDALGARGWDNYSWHTDLPPGHPMVTGQQTVDFDLHAVEQARIVVVWGMNWVTTKMPDTHWLTEARL 6qc5.1    ----------------------------------------------------PPKMLFLLGADGGC-------VTRQDLP  target    KGTKVVVIACEYSSSSIKADDAIVVRPGTTPALALGLCNVIMREKIYDGDYVRRFSDLPLLVRADNLKLLRAEEVFGTPQ 6qc5.1    KDCFIVYQGHHGDVGAPIADVILPGAAY----------------------------------------------------  target    AALKNQTR 6qc5.1    -------- ``` | | | | | | | | | | | | | | | | | | | | | | | | | | | | | | | | | | | | | | | | | | | | | | | | | |
|  | 6zk9.1.C | NADH:ubiquinone oxidoreductase core subunit S1  *Peripheral domain of open complex I during turnover* | 0.02 |  | 10.20 | 0.12 | 293-348 | EM | 0.00 | hetero-1-1-1-1-1-1-… | 6 x SF4, 1 x FMN, 1 x NAI, 2 x FES, 1 x K, 2 x PC1, 2 x 3PE, 1 x ZN, 1 x NDP, 1 x ZMP, 1 x CDL | HHblits | 0.24 |
| ``` target    NNVNRREFLQWIGAAGFSTFALSASNAWGLQAIENPLAAYPNREWEKTYRDLWKSDASFTFLCAPNDTHNCILNAHVRDG 6zk9.1    --------------------------------------------------------------------------------  target    VITRIGPTMKYGEATDLYGSKVTHRWDPRVCQKGLALTRRFYGDRRVRYPMVRKGFKAWADKGFPREKDGRPPKDYFNRA 6zk9.1    --------------------------------------------------------------------------------  target    RDEWLRLTHEEAADLVAAALINIATTYSGDNGQKLLLQQGYEKEIVEATRGAGTQVLKFRGGMPLLGLTRIFGLYRMANS 6zk9.1    --------------------------------------------------------------------------------  target    MALLDHKIRGVKPEDALGARGWDNYSWHTDLPPGHPMVTGQQTVDFDLHAVEQARIVVVWGMNWVTTKMPDTHWLTEARL 6zk9.1    ----------------------------------------------------PPKMLFLLGADGGC-------VTRQDLP  target    KGTKVVVIACEYSSSSIKADDAIVVRPGTTPALALGLCNVIMREKIYDGDYVRRFSDLPLLVRADNLKLLRAEEVFGTPQ 6zk9.1    KDCFIVYQGHHGDVGAPIADVILPGAAY----------------------------------------------------  target    AALKNQTR 6zk9.1    -------- ``` | | | | | | | | | | | | | | | | | | | | | | | | | | | | | | | | | | | | | | | | | | | | | | | | | |
|  | 7arc.1.F | 75 kDa  *Cryo-EM structure of Polytomella Complex-I (peripheral arm)* | 0.03 |  | 12.77 | 0.12 | 292-347 | EM | 0.00 | hetero-1-1-1-1-1-1-… | 6 x SF4, 2 x FES, 1 x FMN, 1 x NDP, 1 x ZN, 1 x 8Q1 | HHblits | 0.26 |
| ``` target    NNVNRREFLQWIGAAGFSTFALSASNAWGLQAIENPLAAYPNREWEKTYRDLWKSDASFTFLCAPNDTHNCILNAHVRDG 7arc.1    --------------------------------------------------------------------------------  target    VITRIGPTMKYGEATDLYGSKVTHRWDPRVCQKGLALTRRFYGDRRVRYPMVRKGFKAWADKGFPREKDGRPPKDYFNRA 7arc.1    --------------------------------------------------------------------------------  target    RDEWLRLTHEEAADLVAAALINIATTYSGDNGQKLLLQQGYEKEIVEATRGAGTQVLKFRGGMPLLGLTRIFGLYRMANS 7arc.1    --------------------------------------------------------------------------------  target    MALLDHKIRGVKPEDALGARGWDNYSWHTDLPPGHPMVTGQQTVDFDLHAVEQARIVVVWGMNWVTTKMPDTHWLTEARL 7arc.1    ---------------------------------------------------VPAKVVYLLGSDDFKD---------EEIP  target    KGTKVVVIACEYSSSSIKADDAIVVRPGTTPALALGLCNVIMREKIYDGDYVRRFSDLPLLVRADNLKLLRAEEVFGTPQ 7arc.1    ADAFVIYQGHHGDKGAARANVVLPGAA-----------------------------------------------------  target    AALKNQTR 7arc.1    -------- ``` | | | | | | | | | | | | | | | | | | | | | | | | | | | | | | | | | | | | | | | | | | | | | | | | | |
|  | 7vxu.1.L | NADH-ubiquinone oxidoreductase 75 kDa subunit, mitochondrial  *Matrix arm of deactive state CI from Q10 dataset* | 0.02 |  | 8.33 | 0.12 | 293-347 | EM | 0.00 | hetero-1-1-1-1-1-1-… | 6 x SF4, 1 x FMN, 1 x PEE, 1 x PLX, 1 x 8Q1, 1 x NDP, 2 x FES, 1 x MG, 1 x CDL, 1 x ZN | HHblits | 0.24 |
| ``` target    NNVNRREFLQWIGAAGFSTFALSASNAWGLQAIENPLAAYPNREWEKTYRDLWKSDASFTFLCAPNDTHNCILNAHVRDG 7vxu.1    --------------------------------------------------------------------------------  target    VITRIGPTMKYGEATDLYGSKVTHRWDPRVCQKGLALTRRFYGDRRVRYPMVRKGFKAWADKGFPREKDGRPPKDYFNRA 7vxu.1    --------------------------------------------------------------------------------  target    RDEWLRLTHEEAADLVAAALINIATTYSGDNGQKLLLQQGYEKEIVEATRGAGTQVLKFRGGMPLLGLTRIFGLYRMANS 7vxu.1    --------------------------------------------------------------------------------  target    MALLDHKIRGVKPEDALGARGWDNYSWHTDLPPGHPMVTGQQTVDFDLHAVEQARIVVVWGMNWVTTKMPDTHWLTEARL 7vxu.1    ----------------------------------------------------PPKVLFLLGADGGC-------ITRQDLP  target    KGTKVVVIACEYSSSSIKADDAIVVRPGTTPALALGLCNVIMREKIYDGDYVRRFSDLPLLVRADNLKLLRAEEVFGTPQ 7vxu.1    KDCFIIYQGHHGDVGAPMADVILPGAA-----------------------------------------------------  target    AALKNQTR 7vxu.1    -------- ``` | | | | | | | | | | | | | | | | | | | | | | | | | | | | | | | | | | | | | | | | | | | | | | | | | |
|  | 7v2c.1.L | NADH-ubiquinone oxidoreductase 75 kDa subunit, mitochondrial  *Active state complex I from Q10 dataset* | 0.02 |  | 8.33 | 0.12 | 293-347 | EM | 0.00 | hetero-1-1-1-1-1-2-… | 6 x SF4, 1 x FMN, 10 x PEE, 8 x PLX, 2 x 8Q1, 1 x NDP, 2 x UQ, 11 x CDL, 2 x FES, 1 x MG, 1 x ZN, 1 x ADP | HHblits | 0.24 |
[truncated: 191,966 more chars]
