# Supplementary material for: Metabolic capacity is maintained despite shifts in microbial diversity in estuary sediments
Source: ISME Commun. 2025 Oct 11;5(1):ycaf182. doi: 10.1093/ismeco/ycaf182 (PMC12687941; doi:10.1093/ismeco/ycaf182)
Supplement: Supplementary_Data_1_ycaf182 [file supplementary_data_1_ycaf182.zip › SWISS-MODEL/4_1_Jan_SF_Bin61_scaffold_1987_c132501_1/templates.html]

4\_1\_Jan\_SF\_Bin61\_scaffold\_1987\_c1:3-2501\_1 | Templates


**Export Alignment**
  
FASTA format
Clustal Format
PNG Image

**Secondary Structure**
  
None
DSSP
PSIPRED
SSpro

**Colour Scheme** 


Fade Mismatches
Enhance Mismatches

Confidencegradient
Confidenceclass
Indels
Chain
Unique Chain
Rainbow
2° Structure
Clustal
Hydrophobic
Size
Charged
Polar
Proline
Ser/Thr
Cysteine
Aliphatic
Aromatic
No Colour

Use QMEANBrane values

|  |  |  |  |
| --- | --- | --- | --- |
| Background |  |  |  |

**3D Viewer**  
NGL
PV

FASTA
Multi FASTA
ClustalW
PNG


SWISS-MODEL

### 4\_1\_Jan\_SF\_Bin61\_scaffold\_1987\_c1:3-2501\_1

### Created: March 29, 2023, 6:30 p.m. at 18:30

- Templates
- Models

Models | Name | Description | GMQE | QSQE | Seq Id | Coverage | Range | Method | Resolution | Oligo-state | Ligands | Found by | Seq Similarity || ✓ | 7b04.1.B | Nitrite oxidoreductase subunit A  *Structure of Nitrite oxidoreductase (Nxr) from the anammox bacterium Kuenenia stuttgartiensis.* | 0.71 | 0.00 | 39.26 | 0.95 | 2-824 | X-ray | 2.97 | monomer | 4 x SF4, 1 x F3S, 2 x MD1, 1 x MO, 1 x HEM, 2 x CA | BLAST | 0.40 |
| ``` target    MPTANKADEVIILRPGTDAAFFLGVARELIEKGLYDRAAVIERTDLPLLVRLDTGERLDARDVIPGYELAALTNYVTLKP 7b04.1    -PSAQKADYWIPIRNNTDTALFLGITKILIDNKWYDADYVKKFTDFPLLIRTDTLKRVSPKDIIPNYKLQDISD----GP  target    DAEIKGNPPPPPFTAGGQVVPTELRDAWGDFVWWDRATGRPRPVSRDEVG---ARFDGDPALLGEFEVELVDGSTVPVRP 7b04.1    SYHIQG-------------LKDEQREIIGDFVVWDAKSKGPKAITRDDVGETLVKKGIDPVLEGSFKLKTIDGKEIEVMT  target    AFDLLKQYLDESFDLRTASEVCRVPPQAIQSIARQLAANKRETLLAAGMGPNHYFQNDLFGRVQFLVAALTDNIGHLGGN 7b04.1    LLEMYKIHLRD-YDIDSVVSMTNSPKDLIERLAKDIATIK-PVAIHYGEGVNHYFHATLMNRSYYLPVMLTGNVGYFGSG  target    VGSYAGNYRGSVFQA-------MGQWIAEDPFAIEPDL-----TKPATVKRYYKAESAHYWNYGERPLRAVAKDDEGDLT 7b04.1    SHTWAGNYKAGNFQASKWSGPGFYGWVAEDVF--KPNLDPYASAKDLNIKGRALDEEVAYWNHSERPL-IVNTPKYGR--  target    KGEVLTGKSHMPTPTKLIWFGNSNSLLGNAKWSFDVVKNTLPRQDAVFCNEWHWTSSCEYADLVFPADSWAEFKLPDATA 7b04.1    --KVFTGKTHMPSPTKVLWFTNVN-LINNAKHVYQMLKNVNPNIEQIMSTDIEITGSIEYADFAFPANSWVEFQEFEITN  target    SCTNPFLLAFPTTPLKRLYDTRSDYEALALTAKALGELIDEPRMEQYWRGILDGDPTPYLQRIFSGSNATRGITYDELHE 7b04.1    SCSNPFIQIWGKTGITPVYESKDDVKILAGMASKLGELLRDKRFEDNWKFAIEGRASVYINRLLDGSTTMKGYTCEDILN  target    SS--KRGVPLLMNMRTYPRSGGWEQRQEDKPWYTATGRLEFYRPEPEFQAAGESLPVWREPVDATFYEPNAILSNAAHPS 7b04.1    GKYGEPGVAMLL-FRTYPRHPFWEQVHESLPFYTPTGRLQAYNDEPEIIEYGENFIVHREGPEATPYLPNAIVS--TNPY  target    IAPRAPEDYGVPESQLDVETRQYRNVVRTWAELQQTLHPLQERDPAFRFVFQTPKYRWGAHSTAVDADWISMLFGPFGDP 7b04.1    IRP---DDYGIPENAEYWEDRTVRNIKKSWEETKKTKNFLWEK--GYHFYCVTPKSRHTVHSQWAVTDWNFIWNNNFGDP  target    YRRDPRMPWTGEAYLEINPKDAAELGLADGDYAWVDADPEDRPYRGWKEDDPYYEVARAMMRVRIYTGMSRGVIRTWFNM 7b04.1    YRMDKRMPGVGEHQIHIHPQAARDLGIEDGDYVYVDANPADRPYEGWKPNDSFYKVSRLMLRAKYNPAYPYNCTMMKHSA  target    YAATPATVANQKATPGNPARNEQTRYVALFRYGSHQSGTRAWLRPTQQTDSLVRKGYFGQVIGTGFEADVHSVSGAPKEA 7b04.1    WISSDKTVQAHETRPDGRALSP-SGYQSSFRYGSQQSITRDWSMPMHQLDSLFHKAKIGMKFIFGFEADNHCINTVPKET  target    FVKIEKAEDGGIGAERLWRPLTLGLRPEAPSAALTAYLAGDYSGTKGS 7b04.1    LVKITKAENGGMGGKGVWDPVKTGYTAGNENDFMKKFLNGE------- ``` | | | | | | | | | | | | | | | | | | | | | | | | | | | | | | | | | | | | | | | | | | | | | | | | | |
|  | 7b04.2.B | Nitrite oxidoreductase subunit A  *Structure of Nitrite oxidoreductase (Nxr) from the anammox bacterium Kuenenia stuttgartiensis.* | 0.69 | 0.00 | 39.26 | 0.95 | 2-824 | X-ray | 2.97 | monomer | 4 x SF4, 1 x F3S, 2 x MD1, 1 x MO, 1 x HEM, 2 x CA | BLAST | 0.40 |
| ``` target    MPTANKADEVIILRPGTDAAFFLGVARELIEKGLYDRAAVIERTDLPLLVRLDTGERLDARDVIPGYELAALTNYVTLKP 7b04.2    -PSAQKADYWIPIRNNTDTALFLGITKILIDNKWYDADYVKKFTDFPLLIRTDTLKRVSPKDIIPNYKLQDISD----GP  target    DAEIKGNPPPPPFTAGGQVVPTELRDAWGDFVWWDRATGRPRPVSRDEVG---ARFDGDPALLGEFEVELVDGSTVPVRP 7b04.2    SYHIQG-------------LKDEQREIIGDFVVWDAKSKGPKAITRDDVGETLVKKGIDPVLEGSFKLKTIDGKEIEVMT  target    AFDLLKQYLDESFDLRTASEVCRVPPQAIQSIARQLAANKRETLLAAGMGPNHYFQNDLFGRVQFLVAALTDNIGHLGGN 7b04.2    LLEMYKIHLRD-YDIDSVVSMTNSPKDLIERLAKDIATIK-PVAIHYGEGVNHYFHATLMNRSYYLPVMLTGNVGYFGSG  target    VGSYAGNYRGSVFQA-------MGQWIAEDPFAIEPDL-----TKPATVKRYYKAESAHYWNYGERPLRAVAKDDEGDLT 7b04.2    SHTWAGNYKAGNFQASKWSGPGFYGWVAEDVF--KPNLDPYASAKDLNIKGRALDEEVAYWNHSERPL-IVNTPKYGR--  target    KGEVLTGKSHMPTPTKLIWFGNSNSLLGNAKWSFDVVKNTLPRQDAVFCNEWHWTSSCEYADLVFPADSWAEFKLPDATA 7b04.2    --KVFTGKTHMPSPTKVLWFTNVN-LINNAKHVYQMLKNVNPNIEQIMSTDIEITGSIEYADFAFPANSWVEFQEFEITN  target    SCTNPFLLAFPTTPLKRLYDTRSDYEALALTAKALGELIDEPRMEQYWRGILDGDPTPYLQRIFSGSNATRGITYDELHE 7b04.2    SCSNPFIQIWGKTGITPVYESKDDVKILAGMASKLGELLRDKRFEDNWKFAIEGRASVYINRLLDGSTTMKGYTCEDILN  target    SS--KRGVPLLMNMRTYPRSGGWEQRQEDKPWYTATGRLEFYRPEPEFQAAGESLPVWREPVDATFYEPNAILSNAAHPS 7b04.2    GKYGEPGVAMLL-FRTYPRHPFWEQVHESLPFYTPTGRLQAYNDEPEIIEYGENFIVHREGPEATPYLPNAIVS--TNPY  target    IAPRAPEDYGVPESQLDVETRQYRNVVRTWAELQQTLHPLQERDPAFRFVFQTPKYRWGAHSTAVDADWISMLFGPFGDP 7b04.2    IRP---DDYGIPENAEYWEDRTVRNIKKSWEETKKTKNFLWEK--GYHFYCVTPKSRHTVHSQWAVTDWNFIWNNNFGDP  target    YRRDPRMPWTGEAYLEINPKDAAELGLADGDYAWVDADPEDRPYRGWKEDDPYYEVARAMMRVRIYTGMSRGVIRTWFNM 7b04.2    YRMDKRMPGVGEHQIHIHPQAARDLGIEDGDYVYVDANPADRPYEGWKPNDSFYKVSRLMLRAKYNPAYPYNCTMMKHSA  target    YAATPATVANQKATPGNPARNEQTRYVALFRYGSHQSGTRAWLRPTQQTDSLVRKGYFGQVIGTGFEADVHSVSGAPKEA 7b04.2    WISSDKTVQAHETRPDGRALSP-SGYQSSFRYGSQQSITRDWSMPMHQLDSLFHKAKIGMKFIFGFEADNHCINTVPKET  target    FVKIEKAEDGGIGAERLWRPLTLGLRPEAPSAALTAYLAGDYSGTKGS 7b04.2    LVKITKAENGGMGGKGVWDPVKTGYTAGNENDFMKKFLNGE------- ``` | | | | | | | | | | | | | | | | | | | | | | | | | | | | | | | | | | | | | | | | | | | | | | | | | |
| ✓ | 7b04.1.B | Nitrite oxidoreductase subunit A  *Structure of Nitrite oxidoreductase (Nxr) from the anammox bacterium Kuenenia stuttgartiensis.* | 0.70 | 0.00 | 37.28 | 0.96 | 1-829 | X-ray | 2.97 | monomer | 4 x SF4, 1 x F3S, 2 x MD1, 1 x MO, 1 x HEM, 2 x CA | HHblits | 0.39 |
| ``` target    MPTANKADEVIILRPGTDAAFFLGVARELIEKGLYDRAAVIERTDLPLLVRLDTGERLDARDVIPGYELAALTNYVTLKP 7b04.1    SPSAQKADYWIPIRNNTDTALFLGITKILIDNKWYDADYVKKFTDFPLLIRTDTLKRVSPKDIIPNYKLQDI--------  target    DAEIKGNPPPPPFTAGGQVVPTELRDAWGDFVWWDRATGRPRPVSRDEV---GARFDGDPALLGEFEVELVDGSTVPVRP 7b04.1    --S---DG-PSYHIQG---LKDEQREIIGDFVVWDAKSKGPKAITRDDVGETLVKKGIDPVLEGSFKLKTIDGKEIEVMT  target    AFDLLKQYLDESFDLRTASEVCRVPPQAIQSIARQLAANKRETLLAAGMGPNHYFQNDLFGRVQFLVAALTDNIGHLGGN 7b04.1    LLEMYKIHLR-DYDIDSVVSMTNSPKDLIERLAKDIATIKPVAI-HYGEGVNHYFHATLMNRSYYLPVMLTGNVGYFGSG  target    VGSYAGNYRGSVFQA---MG-QWIAEDPFA-IEPDLTKPAT------VKRYYKAESAHYWNYGERPLRAVAKDDEGDLTK 7b04.1    SHTWAGNYKAGNFQASKWSGPGFYGWVAEDVFKPNL-DPYASAKDLNIKGRALDEEVAYWNHSERPLI-VNT---P-KYG  target    GEVLTGKSHMPTPTKLIWFGNSNSLLGNAKWSFDVVKNTLPRQDAVFCNEWHWTSSCEYADLVFPADSWAEFKLPDATAS 7b04.1    RKVFTGKTHMPSPTKVLWFTNVNLINNAKHV-YQMLKNVNPNIEQIMSTDIEITGSIEYADFAFPANSWVEFQEFEITNS  target    CTNPFLLAFPTTPLKRLYDTRSDYEALALTAKALGELIDEPRMEQYWRGILDGDPTPYLQRIFSGSNATRGITYDELHES 7b04.1    CSNPFIQIWGKTGITPVYESKDDVKILAGMASKLGELLRDKRFEDNWKFAIEGRASVYINRLLDGSTTMKGYTCEDILNG  target    SKR--GVPLLMNMRTYPRSGGWEQRQEDKPWYTATGRLEFYRPEPEFQAAGESLPVWREPVDATFYEPNAILSNAAHPSI 7b04.1    KYGEPGVAM-LLFRTYPRHPFWEQVHESLPFYTPTGRLQAYNDEPEIIEYGENFIVHREGPEATPYLPNAIVST--NPYI  target    APRAPEDYGVPESQLDVETRQYRNVVRTWAELQQTLHPLQERDPAFRFVFQTPKYRWGAHSTAVDADWISMLFGPFGDPY 7b04.1    ---RPDDYGIPENAEYWEDRTVRNIKKSWEETKKTKNFL--WEKGYHFYCVTPKSRHTVHSQWAVTDWNFIWNNNFGDPY  target    RRDPRMPWTGEAYLEINPKDAAELGLADGDYAWVDADPEDRPYRGWKEDDPYYEVARAMMRVRIYTGMSRGVIRTWFNMY 7b04.1    RMDKRMPGVGEHQIHIHPQAARDLGIEDGDYVYVDANPADRPYEGWKPNDSFYKVSRLMLRAKYNPAYPYNCTMMKHSAW  target    AATPATVANQKATPGNPARNEQTRYVALFRYGSHQSGTRAWLRPTQQTDSLVRKGYFGQVIGTGFEADVHSVSGAPKEAF 7b04.1    ISSDKTVQAHETRPDGRALSP-SGYQSSFRYGSQQSITRDWSMPMHQLDSLFHKAKIGMKFIFGFEADNHCINTVPKETL  target    VKIEKAEDGGIGAERLWRPLTLGLRPEAPSAALTAYLAGDYSGTKGS 7b04.1    VKITKAENGGMGGKGVWDPVKTGYTAGNENDFMKKFLNGELIKVD-- ``` | | | | | | | | | | | | | | | | | | | | | | | | | | | | | | | | | | | | | | | | | | | | | | | | | |
|  | 7b04.2.B | Nitrite oxidoreductase subunit A  *Structure of Nitrite oxidoreductase (Nxr) from the anammox bacterium Kuenenia stuttgartiensis.* | 0.67 | 0.00 | 37.28 | 0.96 | 1-829 | X-ray | 2.97 | monomer | 4 x SF4, 1 x F3S, 2 x MD1, 1 x MO, 1 x HEM, 2 x CA | HHblits | 0.39 |
| ``` target    MPTANKADEVIILRPGTDAAFFLGVARELIEKGLYDRAAVIERTDLPLLVRLDTGERLDARDVIPGYELAALTNYVTLKP 7b04.2    SPSAQKADYWIPIRNNTDTALFLGITKILIDNKWYDADYVKKFTDFPLLIRTDTLKRVSPKDIIPNYKLQDI--------  target    DAEIKGNPPPPPFTAGGQVVPTELRDAWGDFVWWDRATGRPRPVSRDEV---GARFDGDPALLGEFEVELVDGSTVPVRP 7b04.2    --S---DG-PSYHIQG---LKDEQREIIGDFVVWDAKSKGPKAITRDDVGETLVKKGIDPVLEGSFKLKTIDGKEIEVMT  target    AFDLLKQYLDESFDLRTASEVCRVPPQAIQSIARQLAANKRETLLAAGMGPNHYFQNDLFGRVQFLVAALTDNIGHLGGN 7b04.2    LLEMYKIHLR-DYDIDSVVSMTNSPKDLIERLAKDIATIKPVAI-HYGEGVNHYFHATLMNRSYYLPVMLTGNVGYFGSG  target    VGSYAGNYRGSVFQA---MG-QWIAEDPFA-IEPDLTKPAT------VKRYYKAESAHYWNYGERPLRAVAKDDEGDLTK 7b04.2    SHTWAGNYKAGNFQASKWSGPGFYGWVAEDVFKPNL-DPYASAKDLNIKGRALDEEVAYWNHSERPLI-VNT---P-KYG  target    GEVLTGKSHMPTPTKLIWFGNSNSLLGNAKWSFDVVKNTLPRQDAVFCNEWHWTSSCEYADLVFPADSWAEFKLPDATAS 7b04.2    RKVFTGKTHMPSPTKVLWFTNVNLINNAKHV-YQMLKNVNPNIEQIMSTDIEITGSIEYADFAFPANSWVEFQEFEITNS  target    CTNPFLLAFPTTPLKRLYDTRSDYEALALTAKALGELIDEPRMEQYWRGILDGDPTPYLQRIFSGSNATRGITYDELHES 7b04.2    CSNPFIQIWGKTGITPVYESKDDVKILAGMASKLGELLRDKRFEDNWKFAIEGRASVYINRLLDGSTTMKGYTCEDILNG  target    SKR--GVPLLMNMRTYPRSGGWEQRQEDKPWYTATGRLEFYRPEPEFQAAGESLPVWREPVDATFYEPNAILSNAAHPSI 7b04.2    KYGEPGVAM-LLFRTYPRHPFWEQVHESLPFYTPTGRLQAYNDEPEIIEYGENFIVHREGPEATPYLPNAIVST--NPYI  target    APRAPEDYGVPESQLDVETRQYRNVVRTWAELQQTLHPLQERDPAFRFVFQTPKYRWGAHSTAVDADWISMLFGPFGDPY 7b04.2    ---RPDDYGIPENAEYWEDRTVRNIKKSWEETKKTKNFL--WEKGYHFYCVTPKSRHTVHSQWAVTDWNFIWNNNFGDPY  target    RRDPRMPWTGEAYLEINPKDAAELGLADGDYAWVDADPEDRPYRGWKEDDPYYEVARAMMRVRIYTGMSRGVIRTWFNMY 7b04.2    RMDKRMPGVGEHQIHIHPQAARDLGIEDGDYVYVDANPADRPYEGWKPNDSFYKVSRLMLRAKYNPAYPYNCTMMKHSAW  target    AATPATVANQKATPGNPARNEQTRYVALFRYGSHQSGTRAWLRPTQQTDSLVRKGYFGQVIGTGFEADVHSVSGAPKEAF 7b04.2    ISSDKTVQAHETRPDGRALSP-SGYQSSFRYGSQQSITRDWSMPMHQLDSLFHKAKIGMKFIFGFEADNHCINTVPKETL  target    VKIEKAEDGGIGAERLWRPLTLGLRPEAPSAALTAYLAGDYSGTKGS 7b04.2    VKITKAENGGMGGKGVWDPVKTGYTAGNENDFMKKFLNGELIKVD-- ``` | | | | | | | | | | | | | | | | | | | | | | | | | | | | | | | | | | | | | | | | | | | | | | | | | |
|  | 3ir7.1.A | Respiratory nitrate reductase 1 alpha chain  *Crystal structure of NarGHI mutant NarG-R94S* | 0.40 | 0.00 | 23.62 | 0.78 | 1-795 | X-ray | 2.50 | monomer | 2 x MD1, 4 x SF4, 1 x 6MO, 1 x AGA, 1 x F3S, 2 x HEM | HHblits | 0.31 |
| ``` target    MPTANKADEVIILRPGTDAAFFLGVARELIEKGL------YDRAAVIERTDLPLLVRLDT-------GERLDARDVIPGY 3ir7.1    AEIAKLCDLWLAPKQGTDAAMALAMGHVMLREFHLDNPSQYFTDYVRRYTDMPMLVMLEERDGYYAAGRMLRAADLVDAL  target    ELAALTNYVTLKPDAEIKGNPPPPPFTAGGQVVPTELRDAWGDFVWWDRATGRPRPVSRDEV------------------ 3ir7.1    GQ---------ENN-------------------------PEWKTVAFNT-NGEMVAPNGSIGFRWGEKGKWNLEQRDGKT  target    --------------------G-ARF-----------DGDPALLG---EFEVELVDGSTVPVRPAFDLLK----------- 3ir7.1    GEETELQLSLLGSQDEIAEVGFPYFGGDGTEHFNKVELENVLLHKLPVKRLQLADGSTALVTTVYDLTLANYGLERGLND  target    -------QYLDESFDLRTASEVCRVPPQAIQSIARQLAAN-----KRETLLAAGMGPNHYFQNDLFGRVQFLVAALTDNI 3ir7.1    VNCATSYDDVK-AYTPAWAEQITGVSRSQIIRIAREFADNADKTHGRS-MIIVGAGLNHWYHLDMNYRGLINMLIFCGCV  target    GHLGGNVGSYAGNYRGSVFQAMGQWIAEDPFAIEP------------------------DLTKPA-TVKRYYKA------ 3ir7.1    GQSGGGWAHYVGQEKLRPQTGWQPLAFALDWQRPARHMNSTSYFYNHSSQWRYETVTAEELLSPMADKSRYTGHLIDFNV  target    --ESAHYW----NYGERPLRAVAKD-----DEGDLTKGEVLTGKS--------HMPTPTKLIWFGNSNSLLGNAKWSFDV 3ir7.1    RAERMGWLPSAPQLGTNPLTIAGEAEKAGMNPVDYTVKSLKEGSIRFAAEQPENGKNHPRNLFIWRSNLLGSSGKGHEFM  target    ------------------------------VKNTLPRQDAVFCNEWHWTSSCEYADLVFPADSWAEFKLPDATASCTNPF 3ir7.1    LKYLLGTEHGIQGKDLGQQGGVKPEEVDWQDNGLEGKLDLVVTLDFRLSSTCLYSDIILPTATWYEK--DDMNTSDMHPF  target    LLAFPTTPLKRLYDTRSDYEALALTAKALGELIDE---------------PR---------MEQYWRGILDG-------- 3ir7.1    IHP-LSAAVDPAWEAKSDWEIYKAIAKKFSEVCVGHLGKETDIVTLPIQHDSAAELAQPLDVKDWKKGECDLIPGKTAPH  target    ------DPTPYLQRIFSGS-------NATRGI------------------------------------------------ 3ir7.1    IMVVERDYPATYERFTSIGPLMEKIGNGGKGIAWNTQSEMDLLRKLNYTKAEGPAKGQPMLNTAIDAAEMILTLAPETNG  target    -----TYDELHESSKR-----------GVPLLMNMR------------------TYPRSGGWEQRQEDKPWYTATGRLEF 3ir7.1    QVAVKAWAALSEFTGRDHTHLALNKEDEKIRFRDIQAQPRKIISSPTWSGLEDEHVSYNAGYTNVHELIPWRTLSGRQQL  target    YRPEPEFQAAGESLPVWREPVDATFYEPNAILSNAAHPSIAPRAPEDYGVPESQLDVETRQYRNVVRTWAELQQTLHPLQ 3ir7.1    YQDHQWMRDFGESLLVYRPPIDTRSV------------------KEVI----------------------------GQKS  target    ERDPAFRFVFQTPKYRWGAHSTAVDADWISMLFGPFGDPYRRDPRMPWTGEAYLEINPKDAAELGLADGDYAWVDADPED 3ir7.1    NGNQEKALNFLTPHQKWGIHSTYSDNLLMLTLG---------------RGGPVVWLSEADAKDLGIADNDWIEVFNS---  target    RPYRGWKEDDPYYEVARAMMRVRIYTGMSRGVIRTWFNMYAATPATVANQKATPGNPARNEQTRYVALFRYGSHQSGTRA 3ir7.1    --------------NGALTARAVVSQRVPAGMTMMYHAQERIVN--------LPGSEIT--------QQRGGIHNSVTRI  target    WLRPTQQTDSLVRKGYFGQVIGTGFEADVHSVSGAPKEAFVKIEKAEDGGIGAERLWRPLTLGLRPEAPSAALTAYLAGD 3ir7.1    TPKPTHMI------GGYAHL-AYGFN--YYGTVGSNRDEFVVVRKMKNIDW-----------------------------  target    YSGTKGS 3ir7.1    ------- ``` | | | | | | | | | | | | | | | | | | | | | | | | | | | | | | | | | | | | | | | | | | | | | | | | | |
|  | 1r27.4.A | Respiratory nitrate reductase 1 alpha chain  *Crystal Structure of NarGH complex* | 0.40 | 0.05 | 23.16 | 0.78 | 1-795 | X-ray | 2.00 | homo-dimer | 4 x MO, 16 x SF4, 8 x MGD, 4 x F3S | HHblits | 0.31 |
| ``` target    MPTANKADEVIILRPGTDAAFFLGVARELIEKGL------YDRAAVIERTDLPLLVRLDT-------GERLDARDVIPGY 1r27.4    AEIAKLCDLWLAPKQGTDAAMALAMGHVMLREFHLDNPSQYFTDYVRRYTDMPMLVMLEERDGYYAAGRMLRAADLVDAL  target    ELAALTNYVTLKPDAEIKGNPPPPPFTAGGQVVPTELRDAWGDFVWWDRATGRPRPVSRDEV------------------ 1r27.4    GQ---------EN-------------------------NPEWKTVAFNT-NGEMVAPNGSIGFRWGEKGKWNLEQRDGKT  target    ---------------------GARFD-------GDPALL----G---EFEVELVDGSTVPVRPAFDLLK----------- 1r27.4    GEETELQLSLLGSQDEIAEVGFPYFGGDGTEHFNKVELENVLLHKLPVKRLQLADGSTALVTTVYDLTLANYGLERGLND  target    -------QYLDESFDLRTASEVCRVPPQAIQSIARQLAAN-----KRETLLAAGMGPNHYFQNDLFGRVQFLVAALTDNI 1r27.4    VNCATSYDDVK-AYTPAWAEQITGVSRSQIIRIAREFADNADKTHGRS-MIIVGAGLNHWYHLDMNYRGLINMLIFCGCV  target    GHLGGNVGSYAGNYRGSVFQAMGQWIAEDPFAI-----------E--------P-----DLTKPA-TVKRYYKA------ 1r27.4    GQSGGGWAHYVGQEKLRPQTGWQPLAFALDWQRPARHMNSTSYFYNHSSQWRYETVTAEELLSPMADKSRYTGHLIDFNV  target    --ESAHY----WNYGERPLRAVAKD-----DEGDLTKGEVLTGKS--------HMPTPTKLIWFGNSNSLLGNAKWSFDV 1r27.4    RAERMGWLPSAPQLGTNPLTIAGEAEKAGMNPVDYTVKSLKEGSIRFAAEQPENGKNHPRNLFIWRSNLLGSSGKGHEFM  target    V------------------------------KNTLPRQDAVFCNEWHWTSSCEYADLVFPADSWAEFKLPDATASCTNPF 1r27.4    LKYLLGTEHGIQGKDLGQQGGVKPEEVDWQDNGLEGKLDLVVTLDFRLSSTCLYSDIILPTATWYEK--DDMNTSDMHPF  target    LLAFPTTPLKRLYDTRSDYEALALTAKALGELIDE---------------PR---------MEQYWRGILDG-------- 1r27.4    IHP-LSAAVDPAWEAKSDWEIYKAIAKKFSEVCVGHLGKETDIVTLPIQHDSAAELAQPLDVKDWKKGECDLIPGKTAPH  target    ------D-------------------------------PTPYLQRI-------------------------FSGSNATRG 1r27.4    IMVVERDYPATYERFTSIGPLMEKIGNGGKGIAWNTQSEMDLLRKLNYTKAEGPAKGQPMLNTAIDAAEMILTLAPETNG  target    ----ITYDELHESSKR-----------GVPLLMNMR------------------TYPRSGGWEQRQEDKPWYTATGRLEF 1r27.4    QVAVKAWAALSEFTGRDHTHLALNKEDEKIRFRDIQAQPRKIISSPTWSGLEDEHVSYNAGYTNVHELIPWRTLSGRQQL  target    YRPEPEFQAAGESLPVWREPVDATFYEPNAILSNAAHPSIAPRAPEDYGVPESQLDVETRQYRNVVRTWAELQQTLHPLQ 1r27.4    YQDHQWMRDFGESLLVYRPPIDTRSV------------------KEVIG----------------------------QKS  target    ERDPAFRFVFQTPKYRWGAHSTAVDADWISMLFGPFGDPYRRDPRMPWTGEAYLEINPKDAAELGLADGDYAWVDADPED 1r27.4    NGNQEKALNFLTPHQKWGIHSTYSDNLLMLTLG---------------RGGPVVWLSEADAKDLGIADNDWIEVFNS---  target    RPYRGWKEDDPYYEVARAMMRVRIYTGMSRGVIRTWFNMYAATPATVANQKATPGNPARNEQTRYVALFRYGSHQSGTRA 1r27.4    --------------NGALTARAVVSQRVPAGMTMMYHAQERIVN--------LPGSEIT--------QQRGGIHNSVTRI  target    WLRPTQQTDSLVRKGYFGQVIGTGFEADVHSVSGAPKEAFVKIEKAEDGGIGAERLWRPLTLGLRPEAPSAALTAYLAGD 1r27.4    TPKPTHMI------GGYAHL-AYGFN--YYGTVGSNRDEFVVVRKMKNIDW-----------------------------  target    YSGTKGS 1r27.4    ------- ``` | | | | | | | | | | | | | | | | | | | | | | | | | | | | | | | | | | | | | | | | | | | | | | | | | |
| ✓ | 3ir5.1.A | Respiratory nitrate reductase 1 alpha chain  *Crystal structure of NarGHI mutant NarG-H49C* | 0.41 | 0.00 | 23.01 | 0.78 | 1-795 | X-ray | 2.30 | monomer | 2 x MD1, 1 x 6MO, 4 x SF4, 1 x AGA, 1 x F3S, 2 x HEM | HHblits | 0.31 |
| ``` target    MPTANKADEVIILRPGTDAAFFLGVARELIEKGL------YDRAAVIERTDLPLLVRLD-------TGERLDARDVIPGY 3ir5.1    AEIAKLCDLWLAPKQGTDAAMALAMGHVMLREFHLDNPSQYFTDYVRRYTDMPMLVMLEERDGYYAAGRMLRAADLVDAL  target    ELAALTNYVTLKPDAEIKGNPPPPPFTAGGQVVPTELRDAWGDFVWWDRATGRPRPVSRDEV------------------ 3ir5.1    GQ---------EN-------------------------NPEWKTVAFNT-NGEMVAPNGSIGFRWGEKGKWNLEQRDGKT  target    ---------------------GARF-----------DGDPALLG---EFEVELVDGSTVPVRPAFDLLK----------- 3ir5.1    GEETELQLSLLGSQDEIAEVGFPYFGGDGTEHFNKVELENVLLHKLPVKRLQLADGSTALVTTVYDLTLANYGLERGLND  target    -------QYLDESFDLRTASEVCRVPPQAIQSIARQLAAN-----KRETLLAAGMGPNHYFQNDLFGRVQFLVAALTDNI 3ir5.1    VNCATSYDDVK-AYTPAWAEQITGVSRSQIIRIAREFADNADKTHGRS-MIIVGAGLNHWYHLDMNYRGLINMLIFCGCV  target    GHLGGNVGSYAGNYRGSVFQAMGQWIAEDPFAI-----------E--------P-----DLTKPA-TVKRYYKA------ 3ir5.1    GQSGGGWAHYVGQEKLRPQTGWQPLAFALDWQRPARHMNSTSYFYNHSSQWRYETVTAEELLSPMADKSRYTGHLIDFNV  target    --ESAHYW----NYGERPLRAVAKD-----DEGDLTKGEVLTGKS--------HMPTPTKLIWFGNSNSLLGNAKWSFDV 3ir5.1    RAERMGWLPSAPQLGTNPLTIAGEAEKAGMNPVDYTVKSLKEGSIRFAAEQPENGKNHPRNLFIWRSNLLGSSGKGHEFM  target    V------------------------------KNTLPRQDAVFCNEWHWTSSCEYADLVFPADSWAEFKLPDATASCTNPF 3ir5.1    LKYLLGTEHGIQGKDLGQQGGVKPEEVDWQDNGLEGKLDLVVTLDFRLSSTCLYSDIILPTATWYEK--DDMNTSDMHPF  target    LLAFPTTPLKRLYDTRSDYEALALTAKALGELIDE---------------PR---------MEQYWRGILDG-------- 3ir5.1    IHP-LSAAVDPAWEAKSDWEIYKAIAKKFSEVCVGHLGKETDIVTLPIQHDSAAELAQPLDVKDWKKGECDLIPGKTAPH  target    ------D-------------------------------PTPYLQRI-------------------------FSGSNATRG 3ir5.1    IMVVERDYPATYERFTSIGPLMEKIGNGGKGIAWNTQSEMDLLRKLNYTKAEGPAKGQPMLNTAIDAAEMILTLAPETNG  target    I----TYDELHESSK-----------RGVPLLMNMR------------------TYPRSGGWEQRQEDKPWYTATGRLEF 3ir5.1    QVAVKAWAALSEFTGRDHTHLALNKEDEKIRFRDIQAQPRKIISSPTWSGLEDEHVSYNAGYTNVHELIPWRTLSGRQQL  target    YRPEPEFQAAGESLPVWREPVDATFYEPNAILSNAAHPSIAPRAPEDYGVPESQLDVETRQYRNVVRTWAELQQTLHPLQ 3ir5.1    YQDHQWMRDFGESLLVYRPPIDTRSV------------------KEVI----------------------G------QKS  target    ERDPAFRFVFQTPKYRWGAHSTAVDADWISMLFGPFGDPYRRDPRMPWTGEAYLEINPKDAAELGLADGDYAWVDADPED 3ir5.1    NGNQEKALNFLTPHQKWGIHSTYSDNLLMLTLG---------------RGGPVVWLSEADAKDLGIADNDWIEVFNS---  target    RPYRGWKEDDPYYEVARAMMRVRIYTGMSRGVIRTWFNMYAATPATVANQKATPGNPARNEQTRYVALFRYGSHQSGTRA 3ir5.1    --------------NGALTARAVVSQRVPAGMTMMYHAQERIVN--------LPGSEIT--------QQRGGIHNSVTRI  target    WLRPTQQTDSLVRKGYFGQVIGTGFEADVHSVSGAPKEAFVKIEKAEDGGIGAERLWRPLTLGLRPEAPSAALTAYLAGD 3ir5.1    TPKPTHMI------GGYAHL-AYGFN--YYGTVGSNRDEFVVVRKMKNIDW-----------------------------  target    YSGTKGS 3ir5.1    ------- ``` | | | | | | | | | | | | | | | | | | | | | | | | | | | | | | | | | | | | | | | | | | | | | | | | | |
|  | 1q16.1.A | Respiratory nitrate reductase 1 alpha chain  *Crystal structure of Nitrate Reductase A, NarGHI, from Escherichia coli* | 0.40 | 0.00 | 23.38 | 0.78 | 1-795 | X-ray | 1.90 | monomer | 2 x MD1, 1 x 6MO, 2 x HEM, 4 x SF4, 1 x F3S, 1 x AGA, 1 x 3PH | HHblits | 0.31 |
| ``` target    MPTANKADEVIILRPGTDAAFFLGVARELIEKGL------YDRAAVIERTDLPLLVRLDT-------GERLDARDVIPGY 1q16.1    AEIAKLCDLWLAPKQGTDAAMALAMGHVMLREFHLDNPSQYFTDYVRRYTDMPMLVMLEERDGYYAAGRMLRAADLVDAL  target    ELAALTNYVTLKPDAEIKGNPPPPPFTAGGQVVPTELRDAWGDFVWWDRATGRPRPVSRDEV------------------ 1q16.1    GQ---------EN-------------------------NPEWKTVAFNT-NGEMVAPNGSIGFRWGEKGKWNLEQRDGKT  target    ---------------------GARFDG-------DPALL----G---EFEVELVDGSTVPVRPAFDLLK----------- 1q16.1    GEETELQLSLLGSQDEIAEVGFPYFGGDGTEHFNKVELENVLLHKLPVKRLQLADGSTALVTTVYDLTLANYGLERGLND  target    -------QYLDESFDLRTASEVCRVPPQAIQSIARQLAAN-----KRETLLAAGMGPNHYFQNDLFGRVQFLVAALTDNI 1q16.1    VNCATSYDDVK-AYTPAWAEQITGVSRSQIIRIAREFADNADKTHGRS-MIIVGAGLNHWYHLDMNYRGLINMLIFCGCV  target    GHLGGNVGSYAGNYRGSVFQAMGQ--------------------------WIAEDPFAIEPDLTKPA-TVKRYYKA---- 1q16.1    GQSGGGWAHYVGQEKLRPQTGWQPLAFALDWQRPARHMNSTSYFYNHSSQWRYETVTA--EELLSPMADKSRYTGHLIDF  target    -----ESA---HYWNYGERPLRAVAKD-----DEGDLTKGEVLTGKS--------HMPTPTKLIWFGNSNSLLGNAKWSF 1q16.1    NVRAERMGWLPSAPQLGTNPLTIAGEAEKAGMNPVDYTVKSLKEGSIRFAAEQPENGKNHPRNLFIWRSNLLGSSGKGHE  target    DVV------------------------------KNTLPRQDAVFCNEWHWTSSCEYADLVFPADSWAEFKLPDATASCTN 1q16.1    FMLKYLLGTEHGIQGKDLGQQGGVKPEEVDWQDNGLEGKLDLVVTLDFRLSSTCLYSDIILPTATWYEK--DDMNTSDMH  target    PFLLAFPTTPLKRLYDTRSDYEALALTAKALGELIDE---------------PR---------MEQYWRGILDG------ 1q16.1    PFIHP-LSAAVDPAWEAKSDWEIYKAIAKKFSEVCVGHLGKETDIVTLPIQHDSAAELAQPLDVKDWKKGECDLIPGKTA  target    --------D-------------------------------PTPYLQRI-------------------------FSGSNAT 1q16.1    PHIMVVERDYPATYERFTSIGPLMEKIGNGGKGIAWNTQSEMDLLRKLNYTKAEGPAKGQPMLNTAIDAAEMILTLAPET  target    RG----ITYDELHESSK-----------RGVPLLMNMR------------------TYPRSGGWEQRQEDKPWYTATGRL 1q16.1    NGQVAVKAWAALSEFTGRDHTHLALNKEDEKIRFRDIQAQPRKIISSPTWSGLEDEHVSYNAGYTNVHELIPWRTLSGRQ  target    EFYRPEPEFQAAGESLPVWREPVDATFYEPNAILSNAAHPSIAPRAPEDYGVPESQLDVETRQYRNVVRTWAELQQTLHP 1q16.1    QLYQDHQWMRDFGESLLVYRPPIDTRSV------------------KEVI----------------------------GQ  target    LQERDPAFRFVFQTPKYRWGAHSTAVDADWISMLFGPFGDPYRRDPRMPWTGEAYLEINPKDAAELGLADGDYAWVDADP 1q16.1    KSNGNQEKALNFLTPHQKWGIHSTYSDNLLMLTLG---------------RGGPVVWLSEADAKDLGIADNDWIEVFNS-  target    EDRPYRGWKEDDPYYEVARAMMRVRIYTGMSRGVIRTWFNMYAATPATVANQKATPGNPARNEQTRYVALFRYGSHQSGT 1q16.1    ----------------NGALTARAVVSQRVPAGMTMMYHAQERIVN--------LPGSEIT--------QQRGGIHNSVT  target    RAWLRPTQQTDSLVRKGYFGQVIGTGFEADVHSVSGAPKEAFVKIEKAEDGGIGAERLWRPLTLGLRPEAPSAALTAYLA 1q16.1    RITPKPTHMI------GGYAHL-AYGFN--YYGTVGSNRDEFVVVRKMKNIDW---------------------------  target    GDYSGTKGS 1q16.1    --------- ``` | | | | | | | | | | | | | | | | | | | | | | | | | | | | | | | | | | | | | | | | | | | | | | | | | |
|  | 3egw.1.A | Respiratory nitrate reductase 1 alpha chain  *The crystal structure of the NarGHI mutant NarH - C16A* | 0.40 | 0.06 | 23.27 | 0.78 | 1-792 | X-ray | 1.90 | homo-dimer | 2 x MD1, 2 x MGD, 2 x 6MO, 6 x SF4, 4 x F3S, 2 x 3PH, 4 x HEM, 2 x AGA | HHblits | 0.32 |
| ``` target    MPTANKADEVIILRPGTDAAFFLGVARELIEKGL------YDRAAVIERTDLPLLVRLDT-------GERLDARDVIPGY 3egw.1    AEIAKLCDLWLAPKQGTDAAMALAMGHVMLREFHLDNPSQYFTDYVRRYTDMPMLVMLEERDGYYAAGRMLRAADLVAAL  target    ELAALTNYVTLKPDAEIKGNPPPPPFTAGGQVVPTELRDAWGDFVWWDRATGRPRPVSRDE------------------- 3egw.1    GQ---------EN-------------------------NPEWKTVAFNT-NGEMVAPNGSIGFRWGEKGKWNLEQRDGKT  target    ----------------V----GARF-----------DGDPALLG---EFEVELVDGSTVPVRPAFDLLK----------- 3egw.1    GEETELQLSLLGSQDEIAEVGFPYFGGDGTEHFNKVELENVLLHKLPVKRLQLADGSTALVTTVYDLTLANYGLERGLND  target    -------QYLDESFDLRTASEVCRVPPQAIQSIARQLAAN-----KRETLLAAGMGPNHYFQNDLFGRVQFLVAALTDNI 3egw.1    VNCATSYDDVK-AYTPAWAEQITGVSRSQIIRIAREFADNADKTHGRS-MIIVGAGLNHWYHLDMNYRGLINMLIFCGCV  target    GHLGGNVGSYAGNYRGSVFQAMGQWIAEDPFAI-----------E-------------PDLTKPA-TVKRYYKA------ 3egw.1    GQSGGGWAHYVGQEKLRPQTGWQPLAFALDWQRPARHMNSTSYFYNHSSQWRYETVTAEELLSPMADKSRYTGHLIDFNV  target    --ESAHYW----NYGERPLRAVAKDD-----EGDLTKGEVLTGKS--------HMPTPTKLIWFGNSNSLLGNAKWSFDV 3egw.1    RAERMGWLPSAPQLGTNPLTIAGEAEKAGMNPVDYTVKSLKEGSIRFAAEQPENGKNHPRNLFIWRSNLLGSSGKGHEFM  target    ------------------------------VKNTLPRQDAVFCNEWHWTSSCEYADLVFPADSWAEFKLPDATASCTNPF 3egw.1    LKYLLGTEHGIQGKDLGQQGGVKPEEVDWQDNGLEGKLDLVVTLDFRLSSTCLYSDIILPTATWYEKD--DMNTSDMHPF  target    LLAFPTTPLKRLYDTRSDYEALALTAKALGELIDE---------------PR---------MEQYWRGILDG-------- 3egw.1    IHP-LSAAVDPAWEAKSDWEIYKAIAKKFSEVCVGHLGKETDIVTLPIQHDSAAELAQPLDVKDWKKGECDLIPGKTAPH  target    ------D-------------------------------PTPYLQRI-------------------------FSGSNATRG 3egw.1    IMVVERDYPATYERFTSIGPLMEKIGNGGKGIAWNTQSEMDLLRKLNYTKAEGPAKGQPMLNTAIDAAEMILTLAPETNG  target    ----ITYDELHESSK-----------RGVPLLMNMRT------------------YPRSGGWEQRQEDKPWYTATGRLEF 3egw.1    QVAVKAWAALSEFTGRDHTHLALNKEDEKIRFRDIQAQPRKIISSPTWSGLEDEHVSYNAGYTNVHELIPWRTLSGRQQL  target    YRPEPEFQAAGESLPVWREPVDATFYEPNAILSNAAHPSIAPRAPEDYGVPESQLDVETRQYRNVVRTWAELQQTLHPLQ 3egw.1    YQDHQWMRDFGESLLVYRPPIDTRSV------------------KEVIG----------------------------QKS  target    ERDPAFRFVFQTPKYRWGAHSTAVDADWISMLFGPFGDPYRRDPRMPWTGEAYLEINPKDAAELGLADGDYAWVDADPED 3egw.1    NGNQEKALNFLTPHQKWGIHSTYSDNLLMLTLG---------------RGGPVVWLSEADAKDLGIADNDWIEVFNS---  target    RPYRGWKEDDPYYEVARAMMRVRIYTGMSRGVIRTWFNMYAATPATVANQKATPGNPARNEQTRYVALFRYGSHQSGTRA 3egw.1    --------------NGALTARAVVSQRVPAGMTMMYHAQERIVN--------LPGSEIT--------QQRGGIHNSVTRI  target    WLRPTQQTDSLVRKGYFGQVIGTGFEADVHSVSGAPKEAFVKIEKAEDGGIGAERLWRPLTLGLRPEAPSAALTAYLAGD 3egw.1    TPKPTHMI------GGYAHL-AYGFN--YYGTVGSNRDEFVVVRKMKN--------------------------------  target    YSGTKGS 3egw.1    ------- ``` | | | | | | | | | | | | | | | | | | | | | | | | | | | | | | | | | | | | | | | | | | | | | | | | | |
|  | 3ir6.1.A | Respiratory nitrate reductase 1 alpha chain  *Crystal structure of NarGHI mutant NarG-H49S* | 0.39 | 0.00 | 23.46 | 0.78 | 2-792 | X-ray | 2.80 | monomer | 2 x GDP, 1 x AGA, 3 x SF4, 1 x F3S, 2 x HEM | HHblits | 0.32 |
| ``` target    MPTANKADEVIILRPGTDAAFFLGVARELIEKGL------YDRAAVIERTDLPLLVRLDT-------GERLDARDVIPGY 3ir6.1    -EIAKLCDLWLAPKQGTDAAMALAMGHVMLREFHLDNPSQYFTDYVRRYTDMPMLVMLEERDGYYAAGRMLRAADLVDAL  target    ELAALTNYVTLKPDAEIKGNPPPPPFTAGGQVVPTELRDAWGDFVWWDRATGRPRPVSRDE------------------- 3ir6.1    GQ---------ENN-------------------------PEWKTVAFNT-NGEMVAPNGSIGFRWGEKGKWNLEQRDGKT  target    ----------------V----GARFDG-------DPALL----G---EFEVELVDGSTVPVRPAFDLLK----------- 3ir6.1    GEETELQLSLLGSQDEIAEVGFPYFGGDGTEHFNKVELENVLLHKLPVKRLQLADGSTALVTTVYDLTLANYGLERGLND  target    -------QYLDESFDLRTASEVCRVPPQAIQSIARQLAAN-----KRETLLAAGMGPNHYFQNDLFGRVQFLVAALTDNI 3ir6.1    VNCATSYDDVK-AYTPAWAEQITGVSRSQIIRIAREFADNADKTHGRS-MIIVGAGLNHWYHLDMNYRGLINMLIFCGCV  target    GHLGGNVGSYAGNYRGSVFQAMGQWIAEDPFAI-----------EP-------------DLTKPA-TVKRYYKA------ 3ir6.1    GQSGGGWAHYVGQEKLRPQTGWQPLAFALDWQRPARHMNSTSYFYNHSSQWRYETVTAEELLSPMADKSRYTGHLIDFNV  target    --ESAHYW----NYGERPLRAVAKD-----DEGDLTKGEVLTGKS--------HMPTPTKLIWFGNSNSLLGNAKWSFDV 3ir6.1    RAERMGWLPSAPQLGTNPLTIAGEAEKAGMNPVDYTVKSLKEGSIRFAAEQPENGKNHPRNLFIWRSNLLGSSGKGHEFM  target    ------------------------------VKNTLPRQDAVFCNEWHWTSSCEYADLVFPADSWAEFKLPDATASCTNPF 3ir6.1    LKYLLGTEHGIQGKDLGQQGGVKPEEVDWQDNGLEGKLDLVVTLDFRLSSTCLYSDIILPTATWYEK--DDMNTSDMHPF  target    LLAFPTTPLKRLYDTRSDYEALALTAKALGELIDE---------------PR---------MEQYWRGILDG-------- 3ir6.1    IHP-LSAAVDPAWEAKSDWEIYKAIAKKFSEVCVGHLGKETDIVTLPIQHDSAAELAQPLDVKDWKKGECDLIPGKTAPH  target    ------D-------------------------------PTPYLQRI-------------------------FSGSNATRG 3ir6.1    IMVVERDYPATYERFTSIGPLMEKIGNGGKGIAWNTQSEMDLLRKLNYTKAEGPAKGQPMLNTAIDAAEMILTLAPETNG  target    ----ITYDELHESSKR-----------GVPLLMNMR------------------TYPRSGGWEQRQEDKPWYTATGRLEF 3ir6.1    QVAVKAWAALSEFTGRDHTHLALNKEDEKIRFRDIQAQPRKIISSPTWSGLEDEHVSYNAGYTNVHELIPWRTLSGRQQL  target    YRPEPEFQAAGESLPVWREPVDATFYEPNAILSNAAHPSIAPRAPEDYGVPESQLDVETRQYRNVVRTWAELQQTLHPLQ 3ir6.1    YQDHQWMRDFGESLLVYRPPIDTRSV------------------KEVIG----------------------------QKS  target    ERDPAFRFVFQTPKYRWGAHSTAVDADWISMLFGPFGDPYRRDPRMPWTGEAYLEINPKDAAELGLADGDYAWVDADPED 3ir6.1    NGNQEKALNFLTPHQKWGIHSTYSDNLLMLTLG---------------RGGPVVWLSEADAKDLGIADNDWIEVFNS---  target    RPYRGWKEDDPYYEVARAMMRVRIYTGMSRGVIRTWFNMYAATPATVANQKATPGNPARNEQTRYVALFRYGSHQSGTRA 3ir6.1    --------------NGALTARAVVSQRVPAGMTMMYHAQERIVN--------LPGSEIT--------QQRGGIHNSVTRI  target    WLRPTQQTDSLVRKGYFGQVIGTGFEADVHSVSGAPKEAFVKIEKAEDGGIGAERLWRPLTLGLRPEAPSAALTAYLAGD 3ir6.1    TPKPTHMI------GGYAHL-AYGFN--YYGTVGSNRDEFVVVRKMKN--------------------------------  target    YSGTKGS 3ir6.1    ------- ``` | | | | | | | | | | | | | | | | | | | | | | | | | | | | | | | | | | | | | | | | | | | | | | | | | |
|  | 4ydd.1.A | DMSO reductase family type II enzyme, molybdopterin subunit  *Crystal structure of the perchlorate reductase PcrAB from Azospira suillum PS* | 0.45 | 0.00 | 26.07 | 0.73 | 2-793 | X-ray | 1.86 | monomer | 4 x SF4, 1 x MO, 1 x MGD, 1 x MD1, 1 x F3S | HHblits | 0.33 |
| ``` target    MPTANKADEVIILRPGTDAAFFLGVARELIEKGLYDRAAVIERTDLPLLVRLDTGERLDARDVIPGYELAALTNYVTLKP 4ydd.1    -SSTIKVDKWIHPQPGTDGALAMAMAHVIIKEKLYDAHSLKEQTDLSYLVRSDTKRFLREADVVAGG-------------  target    DAEIKGNPPPPPFTAGGQVVPTELRDAWGDFVWWDRATGRPRPVSRDEV------------------------GARFDGD 4ydd.1    --------------------------SKDKFYFWNAKTGKPVIPKGSWGDQPEKKGSPVGFLGRNTFAFPKGYIDLGDLD  target    PALLGEFEVELVDGSTVPVRPAFDLLKQYLDESFDLRTASEVCRVPPQAIQSIARQLAANKRETLLAAGMGPNHYFQNDL 4ydd.1    PALEGKFNMQLLDGKTVEVRPVFEILKSRLMADNTPEKAAKITGVTAKAITELAREFATAKPSMI-ICGGGTQHWYYSDV  target    FGRVQFLVAALTDNIGHLGGNVGSYAGNYRGSVFQAMGQWIAEDPFAIEPDLTKPATVKRYYKAESAHYWNYGERPL-RA 4ydd.1    LLRAMHLLTALTGTEGTNGGGMNHYIGQWKPAFV--AGLVA--LAFP---EGVNK---QRFCQ---TTIWTYIHAEVNDE  target    V-AK-DDEGDLTKGEVLTGK----SHMPTPTKLIWFGNSNSLLGNAKWSFDVVKNTLPRQDAVFCNEWHWTSSCEYADLV 4ydd.1    IISSDIDTEKYLRDSITTGQMPNMPEQGRDPKVFFVYRGNWLNQAKGQ-KYVLENLWPKLELIVDINIRMDSTALYSDVV  target    FPADSWAEFKLPDATASCTNPFLLAFPTTPLKRLYDTRSDYEALALTAKALGELIDEPRME--------------QYWRG 4ydd.1    LPSAHWYEKL--DLNVTSEHSYINM-TEPAIKPMWESKTDWQIFLALAKRVEMAAKRKKYEKFNDEKFKWVRDLSNLWNQ  target    ILD----GDPTPYLQRIFSGSNATRGITYDELHESSKRGVPLLMN-M-RTYPRSGGWEQRQEDKPWYTATGRLEFYRPEP 4ydd.1    MTMDGKLAEDEAAAQYILDNAPQSKGITIQMLREKPQRFKSNWTSPLKEGVPYTPFQYFVVDKKPWPTLTGRQQFYLDHD  target    EFQAAGESLPVWREPVDATFYEPNAILSNAAHPSIAPRAPEDYGVPESQLDVETRQYRNVVRTWAELQQTLHPLQERDPA 4ydd.1    TFFDMGVELPTYKAPID------------------------------------------------------------ADK  target    FRFVFQTPKYRWGAHSTAVDADWISMLFGPFGDPYRRDPRMPWTGEAYLEINPKDAAELGLADGDYAWVDADPEDRPYRG 4ydd.1    YPFRFNSPHSRHSVHSTFKDNVLMLRLQ---------------RGGPSIEMSPLDAKPLGIKDNDWVEAWNN--------  target    WKEDDPYYEVARAMMRVRIYTGMSRGVIRTWFNMYAATPATVANQKATPGNPARNEQTRYVALFRYGSHQSGTRAWLRPT 4ydd.1    ---------HGKVICRVKIRNGEQRGRVSMWHCPELYMD------------------------LLTGGSQSVCPVRINPT  target    QQTDSLVRKGYFGQVIGTGFEADVHSVSGAPKEAFVKIEKAEDGGIGAERLWRPLTLGLRPEAPSAALTAYLAGDYSGTK 4ydd.1    NLV------GNYGHL---FFRPNYYGPAGSQRDVRVNVKRYIGA------------------------------------  target    GS 4ydd.1    -- ``` | | | | | | | | | | | | | | | | | | | | | | | | | | | | | | | | | | | | | | | | | | | | | | | | | |
|  | 5e7o.1.A | DMSO reductase family type II enzyme, molybdopterin subunit  *Crystal structure of the perchlorate reductase PcrAB mutant W461E of PcrA from Azospira suillum PS* | 0.45 | 0.00 | 26.27 | 0.73 | 2-793 | X-ray | 2.40 | monomer | 4 x SF4, 1 x MO, 1 x MGD, 1 x MD1, 1 x F3S | HHblits | 0.33 |
| ``` target    MPTANKADEVIILRPGTDAAFFLGVARELIEKGLYDRAAVIERTDLPLLVRLDTGERLDARDVIPGYELAALTNYVTLKP 5e7o.1    -SSTIKVDKWIHPQPGTDGALAMAMAHVIIKEKLYDAHSLKEQTDLSYLVRSDTKRFLREADVVAGG-------------  target    DAEIKGNPPPPPFTAGGQVVPTELRDAWGDFVWWDRATGRPRPVSRDEV------------------------GARFDGD 5e7o.1    --------------------------SKDKFYFWNAKTGKPVIPKGSWGDQPEKKGSPVGFLGRNTFAFPKGYIDLGDLD  target    PALLGEFEVELVDGSTVPVRPAFDLLKQYLDESFDLRTASEVCRVPPQAIQSIARQLAANKRETLLAAGMGPNHYFQNDL 5e7o.1    PALEGKFNMQLLDGKTVEVRPVFEILKSRLMADNTPEKAAKITGVTAKAITELAREFATAKPSMI-ICGGGTQHWYYSDV  target    FGRVQFLVAALTDNIGHLGGNVGSYAGNYRGSVFQAMGQWIAEDPFAIEPDLTKPATVKRYYKAESAHYWNYGERP---- 5e7o.1    LLRAMHLLTALTGTEGTNGGGMNHYIGQEKPAFV--AGLVA--LAF---PEGVNK---QRFCQ---TTIWTYIHAEVNDE  target    -LR---AVAKDDEGDLTKGEVLTGKSHMPTPTKLIWFGNSNSLLGNAKWSFDVVKNTLPRQDAVFCNEWHWTSSCEYADL 5e7o.1    IISSDIDTEKYLRDSITTGQMPN-MPEQGRDPKVFFVYRGNWLNQAKGQ-KYVLENLWPKLELIVDINIRMDSTALYSDV  target    VFPADSWAEFKLPDATASCTNPFLLAFPTTPLKRLYDTRSDYEALALTAKALGELIDEPRMEQ--------------YWR 5e7o.1    VLPSAHWYEKL--DLNVTSEHSYINM-TEPAIKPMWESKTDWQIFLALAKRVEMAAKRKKYEKFNDEKFKWVRDLSNLWN  target    GIL----DGDPTPYLQRIFSGSNATRGITYDELHESSKRGVPLLM-NMR-TYPRSGGWEQRQEDKPWYTATGRLEFYRPE 5e7o.1    QMTMDGKLAEDEAAAQYILDNAPQSKGITIQMLREKPQRFKSNWTSPLKEGVPYTPFQYFVVDKKPWPTLTGRQQFYLDH  target    PEFQAAGESLPVWREPVDATFYEPNAILSNAAHPSIAPRAPEDYGVPESQLDVETRQYRNVVRTWAELQQTLHPLQERDP 5e7o.1    DTFFDMGVELPTYKAPID------------------------------------------------------------AD  target    AFRFVFQTPKYRWGAHSTAVDADWISMLFGPFGDPYRRDPRMPWTGEAYLEINPKDAAELGLADGDYAWVDADPEDRPYR 5e7o.1    KYPFRFNSPHSRHSVHSTFKDNVLMLRLQ---------------RGGPSIEMSPLDAKPLGIKDNDWVEAWNN-------  target    GWKEDDPYYEVARAMMRVRIYTGMSRGVIRTWFNMYAATPATVANQKATPGNPARNEQTRYVALFRYGSHQSGTRAWLRP 5e7o.1    ----------HGKVICRVKIRNGEQRGRVSMWHCPELYMD------------------------LLTGGSQSVCPVRINP  target    TQQTDSLVRKGYFGQVIGTGFEADVHSVSGAPKEAFVKIEKAEDGGIGAERLWRPLTLGLRPEAPSAALTAYLAGDYSGT 5e7o.1    TNLV------GNYGHL---FFRPNYYGPAGSQRDVRVNVKRYIGA-----------------------------------  target    KGS 5e7o.1    --- ``` | | | | | | | | | | | | | | | | | | | | | | | | | | | | | | | | | | | | | | | | | | | | | | | | | |
|  | 2ivf.1.A | ETHYLBENZENE DEHYDROGENASE ALPHA-SUBUNIT  *ETHYLBENZENE DEHYDROGENASE FROM AROMATOLEUM AROMATICUM* | 0.43 | 0.00 | 26.18 | 0.74 | 1-790 | X-ray | 1.88 | monomer | 1 x MES, 4 x SF4, 1 x MO, 1 x MGD, 1 x MD1, 1 x F3S, 1 x HEM | HHblits | 0.32 |
| ``` target    MPTANKADEVIILRPGTDAAFFLGVARELIEKGLYDRAAVIERTDLPLLVRLDTGERLDARDVIPGYELAALTNYVTLKP 2ivf.1    NPTTPAADLHVPVRVGSDAAFWLGLSQVMIDEKLFDRQFVCEQTDLPLLVRMDTGKFLSAEDVDGG--------------  target    DAEIKGNPPPPPFTAGGQVVPTELRDAWGDFVWWDRATGRPRPVSRDEVGARFDGDPALLGEFEVELVDGSTVPVRPAFD 2ivf.1    --------------------------EAKQFYFFDEKAGSVRKASRGTLKL--DFMPALEGTFSARLKNGKTIQVRTVFE  target    LLKQYLDESFDLRTASEVCRVPPQAIQSIARQLAANKRETLLAAGMGPNHYFQNDLFGRVQFLVAALTDNIGHLGGNVGS 2ivf.1    GLREHLK-DYTPEKASAKCGVPVSLIRELGRKVAKKRT--CSYIGFSSAKSYHGDLMERSLFLAMALSGNWGKPGTGAFA  target    YAGNYRGS-------VFQAMGQWI---A-EDPFAI-----EPD------------------LTKPATVKRYYKAESAHYW 2ivf.1    WAYSDDNMVYLGVMSKPTAQGGMDELHQMAEGFNKRTLEADPTSTDEMGNIEFMKVVTSAVGLVPPAMWLYYHVGYDQLW  target    NYG--ERP-----LRAV-AKDDEGDLTKGEVLTGKSHMPTPTKLIWFGNSNSLLGNAKWSFDVVKNTLPRQDAVFCNEWH 2ivf.1    NNKAWTDPALKKSFGAYLDEAKEKGWWTNDHIRP--APDKTPQVYMLLSQNPMRRKRSGAKMFPDVLFPKLKMIFALETR  target    WTSSCEYADLVFPADSWAEFKLPDATAS-CTNPFLLAFPTTPLKRLYDTRSDYEALALTAKALGELIDEPR--------- 2ivf.1    MSSSAMYADIVLPCAWYYEKHE--MTTPCSGNPFFT-FVDRSVAPPGECREEWDAIALILKKVGERAAARGLTEFNDHNG  target    ----MEQYWRGIL----DGDPTPYLQRIFSGSN----ATRGITYDELHESSKRGVPLLMN-MR----------TYPRSGG 2ivf.1    RKRRYDELYKKFTMDGHLLTNEDCLKEMVDINRAVGVFAKDYTYEKFKKEGQTRFLSMGTGVSRYAHANEVDVTKPIYPM  target    WEQRQEDKPWYTATGRLEFYRPEPEFQAAGESLPVWREPVDATFYEPNAILSNAAHPSIAPRAPEDYGVPESQLDVETRQ 2ivf.1    RWHFDDKKVFPTHTRRAQFYLDHDWYLEAGESLPTHKDTPM---------------------------------------  target    YRNVVRTWAELQQTLHPLQERDPAFRFVFQTPKYRWGAHSTAVDADWISMLFGPFGDPYRRDPRMPWTGEAYLEINPKDA 2ivf.1    --------------------VGGDHPFKITGGHPRVSIHSTHLTNSHLSRLH---------------RGQPVVHMNSKDA  target    AELGLADGDYAWVDADPEDRPYRGWKEDDPYYEVARAMMRVRIYTGMSRGVIRTWFNMYAATPATVANQKATPGNPARNE 2ivf.1    AELGIKDGDMAKLFND-----------------FADCEIMVRTAPNVQPKQCIVYFWDAHQYK---------------G-  target    QTRYVALFRYGSHQSGTRAWLRPTQQTDSLVRKGYFGQVIGTGFEADVHSVSG-APKEAFVKIEKAEDGGIGAERLWRPL 2ivf.1    -------W--KPYDILLIGMPKPLHLA------GGYEQF-RYYFM--NGSPAPVTDRGVRVSIKKA--------------  target    TLGLRPEAPSAALTAYLAGDYSGTKGS 2ivf.1    --------------------------- ``` | | | | | | | | | | | | | | | | | | | | | | | | | | | | | | | | | | | | | | | | | | | | | | | | | |
|  | 1kqf.1.A | FORMATE DEHYDROGENASE, NITRATE-INDUCIBLE, MAJOR SUBUNIT  *FORMATE DEHYDROGENASE N FROM E. COLI* | 0.30 |  | 18.13 | 0.68 | 1-703 | X-ray | 1.60 | hetero-oligomer | 3 x 6MO, 15 x SF4, 6 x MGD, 6 x HEM, 3 x CDL | HHblits | 0.28 |
| ``` target    MPTANKADEVIILRPGTDAAFFLGVARELIEKGLYDRAAVIERTDLPLLVRLDTGERLDARDVIPGYELAALTNYVTLKP 1kqf.1    TRTASVADIYAPIRSGTDITFLSGVLRYLIENNKINAEYVKHYTNASLLVRDDF-AFEDGLFS--GYDAE----------  target    DAEIKGNPPPPPFTAGGQVVPTELRDAWGDFVWWDRATGRPRPVSRDEVGARFDGDPALLGEFEVELVDGSTVPVRPAFD 1kqf.1    ------------------------K-RQYDKSSWNYQLDE-------------------NG---YAKRDETLTHPRCVWN  target    LLKQYLDESFDLRTASEVCRVPPQAIQSIARQLAANK---RETLLAAGMGPNHYFQNDLFGRVQFLVAALTDNIGHLGGN 1kqf.1    LLKEHVS-RYTPDVVENICGTPKADFLKVCEVLASTSAPDRTTTFLYALGWTQHTVGAQNIRTMAMIQLLLGNMGMAGGG  target    VGSYAGNYRGSVFQAMGQWIAEDPFAIEPDLTKPAT--VKRYYKA--------ESAHYWN----YGERPLR--------- 1kqf.1    VNALRGHSNIQGLTDLGLLSTSLP--GYLTLPSEKQVDLQSYLEANTPKATLADQVNYWSNYPKFFVSLMKSFYGDAAQK  target    -------AVAKDDEGDLTKGEVLTGKSHMPTPTKLIWFGNSNSLLGNAKWSFDVVKNTLPRQDAVFCNEWHWTSSCEYAD 1kqf.1    ENNWGYDWLPKW-DQTYDVIKYFNM--MDEGKVTGYFCQGFNPVASFPD--KNKVVSCLSKLKYMVVIDPLVTETSTFWQ  target    -----------------LVFPADSWAEFKLPDATASCTNPFLLAFPTTPLKRLYDTRSDYEALALTAKALGELIDEP--- 1kqf.1    NHGESNDVDPASIQTEVFRLPSTCFAEED--GS-IANSGRWLQ-WHWKGQDAPGEARNDGEILAGIYHHLRELYQSEGGK  target    RMEQYWRGI--------------LDGDPTPYLQRIFSGS------NATRGITYDELHESSKRGVPLL--M---------- 1kqf.1    GVEPLMKMSWNYKQPHEPQSDEVAKENNGYALEDLYDANGVLIAKKGQLLSSFAHLRDDGTTASSCWIYTGSWTEQGNQM  target    -------------------NM-----------------RTY--PRSG----------GWEQRQEDKPWYTATGRLEFYRP 1kqf.1    ANRDNSDPSGLGNTLGWAWAWPLNRRVLYNRASADINGKPWDPKRMLIQWNGSKWTGNDIPDFGNAAPGTPTGPFIMQPE  target    EPEFQ-----AAGESLPVWREPVDATFYEPNAILSNAAHPSIAPRAPEDYGVPESQLDVETRQYRNVVRTWAELQQTLHP 1kqf.1    GMGRLFAINKMAEGPFPEHYEPIETPLGT-N------------PLHPNV------------V-SNPVVRLYEQDA---LR  target    LQERDPAFRFVFQTPKYRWGAHSTAVDADWISMLFGPFGDPYRRDPRMPWTGEAYLEINPKDAAELGLADGDYAWVDADP 1kqf.1    -MGKKEQFPYVGTTYRLTEHFHTWTKHALLNAIA----------------QPEQFVEISETLAAAKGINNGDRVTVSSK-  target    EDRPYRGWKEDDPYYEVARAMMRVRIYTGMSR--------GVIRTWFNMYAATPATVANQKATPGNPARNEQTRYVALFR 1kqf.1    ----------------RGFIRAVAVVTRRLKPLNVNGQQVETVGIPIHW-------------------------------  target    YGSHQSGTRAWLRPTQQTDSLVRKGYFGQVIGTGFEADVHSVSGAPKEAFVKIEKAEDGGIGAERLWRPLTLGLRPEAPS 1kqf.1    --------------------------------------------------------------------------------  target    AALTAYLAGDYSGTKGS 1kqf.1    ----------------- ``` | | | | | | | | | | | | | | | | | | | | | | | | | | | | | | | | | | | | | | | | | | | | | | | | | |
|  | 6sdv.1.A | Formate dehydrogenase, alpha subunit, selenocysteine-containing,Formate dehydrogenase, alpha subunit, selenocysteine-containing,W-formate dehydrogenase - alpha subunit  *W-formate dehydrogenase from Desulfovibrio vulgaris - Formate reduced form* | 0.30 |  | 16.88 | 0.67 | 2-697 | X-ray | 1.90 | hetero-1-1-mer | 2 x MGD, 4 x SF4, 1 x W, 1 x H2S | HHblits | 0.28 |
| ``` target    MPTANKADEVIILRPGTDAAFFLGVARELIEKGLYDRAAVIERTDLPLLVRLDTGERLDARDVIPGYELAALTNYVTLKP 6sdv.1    -RTSARCDVYAPIRSGADIPFLGGLIKYILDNKLYFTDYVREYTNASLIVGEKFSF---KDGLFSGYD------------  target    DAEIKGNPPPPPFTAGGQVVPTELRDAWGDFVWWDRATGRPRPVSRDEVGARFDGDPALLGEFEVELVDGSTVPVRPAFD 6sdv.1    ----------------------------AANKKYDKSMW--------------AFELDANG---VPKRDPALKHPRCVIN  target    LLKQYLDESFDLRTASEVCRVPPQAIQSIARQLAANK---RETLLAAGMGPNHYFQNDLFGRVQFLVAALTDNIGHLGGN 6sdv.1    LLKKHYE-RYNLDKVAAITGTSKEQLQQVYKAYAATGKPDKAGTIMYAMGWTQHSVGVQNIRAMAMIQLLLGNIGVAGGG  target    VGSYAGNYR--GSVFQAMGQWIAEDP-FAIEPDLT-------KPATVKRYYKAESAHYWNYGERPL-------------- 6sdv.1    VNALRGESNVQGST--DQGLLAHIWPGYNPVPNSKAATLELYNAATPQSKDPM-SVNWWQNRPKYVASYLKALYPDEEPA  target    ---RAVAKDD-EG---DLTKGEVLTGKSHMPTPTKLIWFGNSNSLLGNAKWSFDVVKNTLPRQDAVFCNEWHWTSSCEY- 6sdv.1    AAYDYLPRIDAGRKLTDYFWLNIFEK--MDKGEFKGLFAWGMNPACGGAN--ANKNRKAMGKLEWLVNVNLFENETSSFW  target    -------AD-----LVFPADSWAEFKLPDATASCTNPFLLAFPTTPLKRLYDTRSDYEALALTAKALGELIDEP-R-M-- 6sdv.1    KGPGMNPAEIGTEVFFLPCCVSIEKE--GSV-ANSGRWMQ-WRYRGPKPYAETKPDGDIMLDMFKKVRELYAKEGGAYPA  target    ------EQYWRGILDGDPTPYLQRIFSGSNATRGITYDELHESSKRGVPLLM---------------------------- 6sdv.1    PIAKLNIADWEEHNEFSPTKVAKLMNGYFLKDTEVGGKQFKKGQ--QVPSFAFLTADGSTCSGNWLHAGSFTDAGNLMAR  target    -----------------NMRTYPR-----SGGWEQRQEDKPWYTATGRLEFYRPEPEF----------------Q----- 6sdv.1    RDKTQTPEQARIGLFPNWSFCWPVNRRILYNRASVDKTGKPWNPAKAVIEWKDGKWVGDVVDGGGDPGTKHPFIMQTHGF  target    -------AAGESLPVWREPVDATFYEPNAILSNAAHPSIAPRAPEDYGVPESQLDVETRQYRNVVRTWAELQQTLHPLQE 6sdv.1    GALYGPGREEGPFPEHYEPLECPVSK--------------NP--------------FSKQLHNPVAFQIEG----EKKAV  target    RDPAFRFVFQTPKYRWG--AHSTAVDADWISMLFGPFGDPYRRDPRMPWTGEAYLEINPKDAAELGLADGDYAWVDADPE 6sdv.1    CDPRYPFIGTTYRVTEHWQTGLMTRRCAWLVEAE----------------PQIFCEISKELAKLRGIGNGDTVKVSSL--  target    DRPYRGWKEDDPYYEVARAMMRVRIYTGMSRGVIRTWFNMYAATPATVANQKATPGNPARNEQTRYVALFRYGSHQSGTR 6sdv.1    ---------------RGALEAVAIVTERIRPFKI----------------------------------------------  target    AWLRPTQQTDSLVRKGYFGQVIGTGFEADVHSVSGAPKEAFVKIEKAEDGGIGAERLWRPLTLGLRPEAPSAALTAYLAG 6sdv.1    --------------------------------------------------------------------------------  target    DYSGTKGS 6sdv.1    -------- ``` | | | | | | | | | | | | | | | | | | | | | | | | | | | | | | | | | | | | | | | | | | | | | | | | | |
|  | 6cz7.1.A | ArrA  *The arsenate respiratory reductase (Arr) complex from Shewanella sp. ANA-3* | 0.31 |  | 17.59 | 0.63 | 2-703 | X-ray | 1.62 | hetero-1-1-mer | 5 x SF4, 2 x MGD, 1 x MO, 1 x PG5 | HHblits | 0.29 |
| ``` target    MPTANKADEVIILRPGTDAAFFLGVARELIEKGLYDRAAVIERTDLPLLVRLDTGERLDARDVIPGYELAALTNYVTLKP 6cz7.1    -ASAAKAHKWIPIEPGQDSVLALAIAHVALVEGVWHKPFVGDFIEGKNLFKA--GKTVS---------------------  target    DAEIKGNPPPPPFTAGGQVVPTELRDAWGDFVWWDRATGRPRPVSRDEVGARFDGDPALLGEFEVELVDGSTVPVRPAFD 6cz7.1    ---------------------------VESF--------------------------------K-E------THTYGLVE  target    LLKQYLDESFDLRTASEVCRVPPQAIQSIARQLAANKRETLLAAGMGPNHYFQNDLFGRVQFLVAALTDNIGHLGGNVGS 6cz7.1    WWNQALK-DYTPEWASKITGIDPKTIIAIAKDMGAAAPAVQVWTSRGAVMQARGTYTSISCHALNGLFGGIDSKGGLFPG  target    YAGNYRGSVFQAMGQWIAEDPFAIEPDLTKPATVKRYYKAESAHYWNYGERPLRAVAKDDEGDLTKGEVLTGKSHMPTPT 6cz7.1    NKTPLL-KEYPEAKAYM--DEIA-AKGVK-KEKIDQRGRLEFPALAKG--KSGGGVI----TANAANGIRNQ---DPYEI  target    KLIWFGNSNSLLGNAKWSFDVVKNTLPRQDAVFCNEWHWTSSCEYADLVFPADS-WAEFKLPDATASCTNP--FLLAFPT 6cz7.1    KVMLAYFNNFNFSNPE--GQRWDEALSKVDFMAHITTNVSEFSWFADVLLPSSHHMFEKW--GVLDSIGNGVAQI-SIQQ  target    TPLKRLYDTRSD-YEALALTAKALGEL-ID--EPRME-QYWRG---ILDGDPTPYLQRIFSG-----------SNATRGI 6cz7.1    PSIKRLWDTRIDESEIPYMLAKKLADKGFDAPWRYINEQIVDPETGKPAADEAEFAKLMVRYLTAPLWKEDASKYGDKLS  target    TYDELHESSKRGVPLLMNMRTYPRSGGWEQRQEDKPWYTATGRLEFYRPEPEFQAAGESLPVWREPVDATFYEPNAILSN 6cz7.1    SWDEFVQK---GVWNSSP---------YKLEARWGKFKTETTKFEFYSKTLEK-----ALQSHADKHKVS---IDEV---  target    AAHPSIAPRAPEDYGVPESQLDVETRQYRNVVRTWAELQQTLHPLQERDPAFRFVFQTPKYRWGAHSTAVDADWISMLFG 6cz7.1    -------MKAC-DY---------QARGHLAFIPHYEEPYR-----FGDESEFPLLLVDQKSRLNKEGRTANSPWYYEFKD  target    PFGDPYRRDPRMPWTGEAYLEINPKDAAELGLADGDYAWVDADPEDRPYRGWKEDDPYYEVARAMMRVRIYTGMSRGVIR 6cz7.1    V----D----PGDVANEDVAKFNPIDGKKFGLKDGDEIRITSP-----------------VGMLTCKAKLWEGVRPGTVA  target    TWFNMYAATPATVANQKATPGNPARNEQTRYVALFRYGSHQSGTRAWLRPTQQTDSLVRKGYFGQVIGTGFEADVHSVSG 6cz7.1    KCFGQ---------------------------------------------------------------------------  target    APKEAFVKIEKAEDGGIGAERLWRPLTLGLRPEAPSAALTAYLAGDYSGTKGS 6cz7.1    ----------------------------------------------------- ``` | | | | | | | | | | | | | | | | | | | | | | | | | | | | | | | | | | | | | | | | | | | | | | | | | |
|  | 4ydd.1.A | DMSO reductase family type II enzyme, molybdopterin subunit  *Crystal structure of the perchlorate reductase PcrAB from Azospira suillum PS* | 0.30 | 0.00 | 31.43 | 0.57 | 6-530 | X-ray | 1.86 | monomer | 4 x SF4, 1 x MO, 1 x MGD, 1 x MD1, 1 x F3S | BLAST | 0.36 |
| ``` target    MPTANKADEVIILRPGTDAAFFLGVARELIEKGLYDRAAVIERTDLPLLVRLDTGERLDARDVIPG--YELAALTNYVTL 4ydd.1    -----KVDKWIHPQPGTDGALAMAMAHVIIKEKLYDAHSLKEQTDLSYLVRSDTKRFLREADVVAGGSKDKFYFWNAKTG  target    KPDAEIKGNPPPPPFTAGGQVVPTELRDAWGDFVWWDRATGRPR----------PVSRDEVGARFDGDPALLGEFEVELV 4ydd.1    KP------------------VIP---KGSWGD---QPEKKGSPVGFLGRNTFAFPKGYIDLG---DLDPALEGKFNMQLL  target    DGSTVPVRPAFDLLKQYLDESFDLRTASEVCRVPPQAIQSIARQLAANKRETLLAAGMGPNHYFQNDLFGRVQFLVAALT 4ydd.1    DGKTVEVRPVFEILKSRLMADNTPEKAAKITGVTAKAITELAREFATAKPSMIICGG-GTQHWYYSDVLLRAMHLLTALT  target    DNIGHLGGNVGSYAGNYR-----GSVFQAMGQWIAEDPFAIEPDLTKPATVKRYYKAESAHYWNYGERPLRAVAKD-DEG 4ydd.1    GTEGTNGGGMNHYIGQWKPAFVAGLVALAFPEGVNKQRFC-------QTTIWTYIHAEVND---------EIISSDIDTE  target    DLTKGEVLTGKS-HMPTPTK--LIWFGNSNSLLGNAKWSFDVVKNTLPRQDAVFCNEWHWTSSCEYADLVFPADSWAEFK 4ydd.1    KYLRDSITTGQMPNMPEQGRDPKVFFVYRGNWLNQAKGQKYVLENLWPKLELIVDINIRMDSTALYSDVVLPSAHWYE-K  target    LPDATASCTNPFLLAFPTTPLKRLYDTRSDYEALALTAKALGELIDEPRMEQY--------------WRGI-LDG---DP 4ydd.1    LDLNVTSEHSYINMTEPA--IKPMWESKTDWQIFLALAKRVEMAAKRKKYEKFNDEKFKWVRDLSNLWNQMTMDGKLAED  target    TPYLQRIFSGSNATRGITYDELHESSKRGVPLLMNMRTYPRSGG--WEQRQ----EDKPWYTATGRLEFYRPEPEFQAAG 4ydd.1    EAAAQYILDNAPQSKGITIQMLREKPQR----FKSNWTSPLKEGVPYTPFQYFVVDKKPWPTLTGRQQFYLDHDTFFDMG  target    ESLPVWREPVDATFYEPNAILSNAAHPSIAPRAPEDYGVPESQLDVETRQYRNVVRTWAELQQTLHPLQERDPAFRFVFQ 4ydd.1    VELPTYKAPIDADKY-----------------------------------------------------------------  target    TPKYRWGAHSTAVDADWISMLFGPFGDPYRRDPRMPWTGEAYLEINPKDAAELGLADGDYAWVDADPEDRPYRGWKEDDP 4ydd.1    --------------------------------------------------------------------------------  target    YYEVARAMMRVRIYTGMSRGVIRTWFNMYAATPATVANQKATPGNPARNEQTRYVALFRYGSHQSGTRAWLRPTQQTDSL 4ydd.1    --------------------------------------------------------------------------------  target    VRKGYFGQVIGTGFEADVHSVSGAPKEAFVKIEKAEDGGIGAERLWRPLTLGLRPEAPSAALTAYLAGDYSGTKGS 4ydd.1    ---------------------------------------------------------------------------- ``` | | | | | | | | | | | | | | | | | | | | | | | | | | | | | | | | | | | | | | | | | | | | | | | | | |
|  | 5e7o.1.A | DMSO reductase family type II enzyme, molybdopterin subunit  *Crystal structure of the perchlorate reductase PcrAB mutant W461E of PcrA from Azospira suillum PS* | 0.29 | 0.00 | 31.43 | 0.57 | 6-530 | X-ray | 2.40 | monomer | 4 x SF4, 1 x MO, 1 x MGD, 1 x MD1, 1 x F3S | BLAST | 0.36 |
| ``` target    MPTANKADEVIILRPGTDAAFFLGVARELIEKGLYDRAAVIERTDLPLLVRLDTGERLDARDVIPG--YELAALTNYVTL 5e7o.1    -----KVDKWIHPQPGTDGALAMAMAHVIIKEKLYDAHSLKEQTDLSYLVRSDTKRFLREADVVAGGSKDKFYFWNAKTG  target    KPDAEIKGNPPPPPFTAGGQVVPTELRDAWGDFVWWDRATGRPR----------PVSRDEVGARFDGDPALLGEFEVELV 5e7o.1    KP------------------VIP---KGSWGD---QPEKKGSPVGFLGRNTFAFPKGYIDLG---DLDPALEGKFNMQLL  target    DGSTVPVRPAFDLLKQYLDESFDLRTASEVCRVPPQAIQSIARQLAANKRETLLAAGMGPNHYFQNDLFGRVQFLVAALT 5e7o.1    DGKTVEVRPVFEILKSRLMADNTPEKAAKITGVTAKAITELAREFATAKPSMIICGG-GTQHWYYSDVLLRAMHLLTALT  target    DNIGHLGGNVGSYAGNYR-----GSVFQAMGQWIAEDPFAIEPDLTKPATVKRYYKAESAHYWNYGERPLRAVAKD-DEG 5e7o.1    GTEGTNGGGMNHYIGQEKPAFVAGLVALAFPEGVNKQRFC-------QTTIWTYIHAEVND---------EIISSDIDTE  target    DLTKGEVLTGKS-HMPTPTK--LIWFGNSNSLLGNAKWSFDVVKNTLPRQDAVFCNEWHWTSSCEYADLVFPADSWAEFK 5e7o.1    KYLRDSITTGQMPNMPEQGRDPKVFFVYRGNWLNQAKGQKYVLENLWPKLELIVDINIRMDSTALYSDVVLPSAHWYE-K  target    LPDATASCTNPFLLAFPTTPLKRLYDTRSDYEALALTAKALGELIDEPRMEQY--------------WRGI-LDG---DP 5e7o.1    LDLNVTSEHSYINMTEPA--IKPMWESKTDWQIFLALAKRVEMAAKRKKYEKFNDEKFKWVRDLSNLWNQMTMDGKLAED  target    TPYLQRIFSGSNATRGITYDELHESSKRGVPLLMNMRTYPRSGG--WEQRQ----EDKPWYTATGRLEFYRPEPEFQAAG 5e7o.1    EAAAQYILDNAPQSKGITIQMLREKPQR----FKSNWTSPLKEGVPYTPFQYFVVDKKPWPTLTGRQQFYLDHDTFFDMG  target    ESLPVWREPVDATFYEPNAILSNAAHPSIAPRAPEDYGVPESQLDVETRQYRNVVRTWAELQQTLHPLQERDPAFRFVFQ 5e7o.1    VELPTYKAPIDADKY-----------------------------------------------------------------  target    TPKYRWGAHSTAVDADWISMLFGPFGDPYRRDPRMPWTGEAYLEINPKDAAELGLADGDYAWVDADPEDRPYRGWKEDDP 5e7o.1    --------------------------------------------------------------------------------  target    YYEVARAMMRVRIYTGMSRGVIRTWFNMYAATPATVANQKATPGNPARNEQTRYVALFRYGSHQSGTRAWLRPTQQTDSL 5e7o.1    --------------------------------------------------------------------------------  target    VRKGYFGQVIGTGFEADVHSVSGAPKEAFVKIEKAEDGGIGAERLWRPLTLGLRPEAPSAALTAYLAGDYSGTKGS 5e7o.1    ---------------------------------------------------------------------------- ``` | | | | | | | | | | | | | | | | | | | | | | | | | | | | | | | | | | | | | | | | | | | | | | | | | |
|  | 2nya.1.A | Periplasmic nitrate reductase  *Crystal structure of the periplasmic nitrate reductase (NAP) from Escherichia coli* | 0.30 |  | 15.93 | 0.63 | 2-703 | X-ray | 2.50 | monomer | 1 x SF4, 1 x 6MO, 2 x MGD | HHblits | 0.28 |
| ``` target    MPTANKADEVIILRPGTDAAFFLGVARELIEKGLYDRAAVIERTDLPLLVRLDTGERLDARDVIPGYELAALTNYVTLKP 2nya.1    -RSFELADNGIIFTPQSDLVILNYIANYIIQNNAINQDFFSKHVNLRKGAT-DIGYGLRPTHPLEK--------------  target    DAEIKGNPPPPPFTAGGQVVPTELRDAWGDFVWWDRATGRPRPVSRDEVGARFDGDPALLGEFEVELVDGSTVPVRPAFD 2nya.1    -----------------------------------A-----------------------------AKNPGSDASEPMSFE  target    LLKQYLDESFDLRTASEVCRVPPQAIQSIARQLAANKRETLLAAGMGPNHYFQNDLFGRVQFLVAALTDNIGHLGGNVGS 2nya.1    DYKAFVA-EYTLEKTAEMTGVPKDQLEQLAQLYADPNKKVISYWTMGFNQHTRGVWANNLVYNLHLLTGKISQPGCGPFS  target    YAGNY--RGSVFQAMGQWIAEDPFAIEPDLTKPATVKRYYKAESAHYWNYGERPLRAVAKDDEGDLTKGEVLTGKSHMPT 2nya.1    LTGQPSACGTAR-EVGTFAHRLP--ADMVVTNEKHRDI-----CEKKWNIPSGTI-PAK----IGLHAVAQDRA--LKDG  target    PTKLIWFGNSNSLLGNAKWSFDVVKNTLPRQDAVFCNEWHWTSSCEYADLVFPADSWAEFKLPDATASCTNPFLLAFPTT 2nya.1    KLNVYWTMCTNNMQAGPNINEERMPGWRDPRNFIIVSDPYPTVSALAADLILPTAMWVEKEG--A-YGNAERRTQFW-RQ  target    PLKRLYDTRSDYEALALTAKALGELIDEPR-MEQYWRGILDGDPTPYLQRIFSGSNATRGITYDELHESS------KRGV 2nya.1    QVQAPGEAKSDLWQLVQFSRRFKTEEVWPEDLLAKKPEL---RGKTLYEVLYAT-PEVSKFPVSELAEDQLNDESRELGF  target    PLLM----NM--------RTYPRSGGWEQRQEDKPWYTATGRLEFYR--PE-PEFQAAGESLPVWREPVDATFYEPNAIL 2nya.1    YLQKGLFEEYAWFGRGHGHDLAPFDDY-HKARGLRWPVVNGKETQWRYSEGNDPYVKAGEGYKFYGKPDGKA--------  target    SNAAHPSIAPRAPEDYGVPESQLDVETRQYRNVVRTWAELQQTLHPLQERDPAFRFVFQTPKYRW--GAHSTAVDADWIS 2nya.1    -------------VIFA-----------------LPFEP-----AA-EAPDEEYDLWLSTGRVLEHWHTGSMTRRVPELH  target    MLFGPFGDPYRRDPRMPWTGEAYLEINPKDAAELGLADGDYAWVDADPEDRPYRGWKEDDPYYEVARAMMRVRIYTGM-- 2nya.1    RAF----------------PEAVLFIHPLDAKARDLRRGDKVKVVSR-----------------RGEVISIVETRGRNRP  target    SRGVIRTWFNMYAATPATVANQKATPGNPARNEQTRYVALFRYGSHQSGTRAWLRPTQQTDSLVRKGYFGQVIGTGFEAD 2nya.1    PQGLVYMPFFD---------------------------------------------------------------------  target    VHSVSGAPKEAFVKIEKAEDGGIGAERLWRPLTLGLRPEAPSAALTAYLAGDYSGTKGS 2nya.1    ----------------------------------------------------------- ``` | | | | | | | | | | | | | | | | | | | | | | | | | | | | | | | | | | | | | | | | | | | | | | | | | |
|  | 1ogy.1.A | PERIPLASMIC NITRATE REDUCTASE  *Crystal structure of the heterodimeric nitrate reductase from Rhodobacter sphaeroides* | 0.29 |  | 19.13 | 0.61 | 2-702 | X-ray | 3.20 | hetero-1-1-mer | 1 x SF4, 1 x MO, 2 x MGD, 2 x HEC | HHblits | 0.29 |
| ``` target    MPTANKADEVIILRPGTDAAFFLGVARELIEKGLYDRAAVIERTDLPLLVRLDTGERLDARDVIPGYELAALTNYVTLKP 1ogy.1    -RSSDLSDTPIIFRPGTDRAILNYIAHHIISTGRVNRDFVDRHTNFALGAT-DIGYGLRPEH------------------  target    DAEIKGNPPPPPFTAGGQVVPTELRDAWGDFVWWDRATGRPRPVSRDEVGARFDGDPALLGEFEVELVDGSTVPVRPAFD 1ogy.1    ------------------------------QLQLAAK-----------------------------GAADAGAMTPTDFE  target    LLKQYLDESFDLRTASEVCRVPPQAIQSIARQLAANKRETLLAAGMGPNHYFQNDLFGRVQFLVAALTDNIGHLGGNVGS 1ogy.1    TFAALVS-EYTLEKAAEISGVEPALLEELAELYADPDRKWMSLWTMGFNQHVRGVWANHMVYNLHLLTGKISEPGNSPFS  target    YAGNYRGSV-FQAMGQWIAEDPFAIEPDLTKPATVKRYYKAESAHYWNYGERPLRAVAKDDEGDLTKGEVLTGKSHMPTP 1ogy.1    LTGQPFACGTAREVGTFAHRLPA--DMVVTNPEHRAHA-----EEIWKLPA-GLLPDW----VGAHAVEQDRKL--HDGE  target    TKLIWFGNSNSLLGNAKWSFDVVKNTLPRQDAVFCNEWHWTSSCEYADLVFPADSWAEFKLPDATASCTNPFLLAFPTTP 1ogy.1    INFYWVQVNNNMQAAPNIDQETYPGYRNPENFIVVSDAYPTVTGRAADLVLPAAMWVEKEG--A-YGNAERRTHF-WHQL  target    LKRLYDTRSDYEALALTAKALGELIDEPRMEQYWRGILDGDPTPYLQRIFSGSNATRGI--------------------- 1ogy.1    VEAPGEARSDLWQLMEFSKRFTTDEVWP--EEILSAAPAYRGKTLFEVLFANG-SVDRFPASDVNPDHANHEAALFGFYP  target    ---TYDELHESSKR-GVPLLMNMRT--------YP----RSGGWEQRQEDKPWYTATGRLEFYRPEPEFQAAGESLPVWR 1ogy.1    QKGLFEEYAAFGRGHGHDL-APFDTYHEVRGLHWPVVEGEETRWRYREGFDPYVKPGEGLRFYGKPDGRAV-ILGVP-YE  target    EPVDATFYEPNAILSNAAHPSIAPRAPEDYGVPESQLDVETRQYRNVVRTWAELQQTLHPLQERDPAFRFVFQTPKYRWG 1ogy.1    PPA----------------------------------------------------------ESPDEEFGFWLVTGRVLEH  target    AHSTAV--DADWISMLFGPFGDPYRRDPRMPWTGEAYLEINPKDAAELGLADGDYAWVDADPEDRPYRGWKEDDPYYEVA 1ogy.1    WHSGSMTLRWPELYKAF----------------PGAVCFMHPEDARSRGLNRGSEVRVISR-----------------RG  target    RAMMRVRI--YTGMSRGVIRTWFNMYAATPATVANQKATPGNPARNEQTRYVALFRYGSHQSGTRAWLRPTQQTDSLVRK 1ogy.1    EIRTRLETRGRNRMPRGVVFVPWF--------------------------------------------------------  target    GYFGQVIGTGFEADVHSVSGAPKEAFVKIEKAEDGGIGAERLWRPLTLGLRPEAPSAALTAYLAGDYSGTKGS 1ogy.1    ------------------------------------------------------------------------- ``` | | | | | | | | | | | | | | | | | | | | | | | | | | | | | | | | | | | | | | | | | | | | | | | | | |
|  | 4v4c.1.A | Pyrogallol hydroxytransferase large subunit  *Crystal Structure of Pyrogallol-Phloroglucinol Transhydroxylase from Pelobacter acidigallici* | 0.30 |  | 16.70 | 0.60 | 2-703 | X-ray | 2.35 | hetero-oligomer | 2 x CA, 2 x MGD, 1 x 4MO, 3 x SF4 | HHblits | 0.28 |
| ``` target    MPTAN-KADEVIILRPGTDAAFFLGVARELIEKGLYDRAAVIERTDLPLLVRLDTGERLDARDVIPGYELAALTNYVTLK 4v4c.1    -HTARLVADKWFSPKIGTDHALSFAIAYTWLKEDSYDKEYVAANAHG--------------------F------------  target    PDAEIKGNPPPPPFTAGGQVVPTELRDAWGDFVWWDRATGRPRPVSRDEVGARFDGDPALLGEFEVELVDGSTVPVRPAF 4v4c.1    --------------------------------------------------------------------------------  target    DLLKQYLD-----ESFDLRTASEVCRVPPQAIQSIARQLAANKRETLLAAGM----GPNHYFQNDLFGRVQFLVAALTDN 4v4c.1    EEWADYVLGKTDGTPKTCEWAEEESGVPACEIRALARQWAKKNTYLA-AGGLGGWGGACRASHGIEWARGMIALATMQG-  target    IGHLGGNVGSYAGNYRGSVFQAMGQWIAEDPFAI-EPD----------------LTKPATVKRYYKAESAHYWNYGE--- 4v4c.1    MGKPGSNMWSTTQGVPLDYEFYFPGYAEGGISGDCENSAAGFKFAWRMFDGKTTFPSPSNLNT-SAGQHIPRLKIPECIM  target    -RPLRAVAKDDEGDLTKGEVLTGKSH---MPTPTKLIWFGNSNSLLGNAKWSFDVVKNTL--PRQDAVFCNEWHWTSSCE 4v4c.1    GGKFQWSGKGFAGGD-ISHQLHQYEYPAPGYSKIKMFWKYGGPHLGTMTA--TNRYAKMYTHDSLEFVVSQSIWFEGEVP  target    YADLVFPADSWAEFKLPDATASC-----------TNPFLLAFPTTPLKRLYDTRSDYEALALTAKALGELIDEPRMEQYW 4v4c.1    FADIILPACTNFERWDISEFANCSGYIPDNYQLCNHRVIS-LQAKCIEPVGESMSDYEIYRLFAKKLNIEEMFSEG----  target    RGILDGDPTPYLQRIFSGSNATRGITYDELHESSKRGVPLLMNMR-----TYPR------SGGWE----QRQEDKPWYTA 4v4c.1    -----KDELAWCEQYFNATDMPKYMTWDEFFKKGYFVVPDNPNRKKTVALRWFAEGREKDTPDWGPRLNNQVCRKGLQTT  target    TGRLEFYRPEPEFQ-------AAGESLPVWREPVDATFYEPNAILSNAAHPSIAPRAPEDYGVPESQLDVETRQYRNVVR 4v4c.1    TGKVEFIATSLKNFEEQGYIDEHRPSMHTYVPAWES--------------------------------------------  target    TWAELQQTLHPLQERDPAFRFVFQTPKYRWGAHSTAVD-ADWISMLFGPFGDPYRRDPRMPWTGEAYLEINPKDAAELGL 4v4c.1    ----QK--HS---PLAVKYPLGMLSPHPRFSMHTMGDGKNSYMNYIKDHR-----V--EVDGYKYWIMRVNSIDAEARGI  target    ADGDYAWVDADPEDRPYRGWKEDDPYYEVARAMMRVRIYTGMSRGVIRTWFNMYAATPATVANQKATPGNPARNEQTRYV 4v4c.1    KNGDLIRAYND-----------------RGSVILAAQVTECLQPGTVHSYESC---------------------------  target    ALFRYGSHQSGTRAWLRPTQQTDSLVRKGYFGQVIGTGFEADVHSVSGAPKEAFVKIEKAEDGGIGAERLWRPLTLGLRP 4v4c.1    --------------------------------------------------------------------------------  target    EAPSAALTAYLAGDYSGTKGS 4v4c.1    --------------------- ``` | | | | | | | | | | | | | | | | | | | | | | | | | | | | | | | | | | | | | | | | | | | | | | | | | |
|  | 7l5i.1.A | Trimethylamine-N-oxide reductase  *Crystal Structure of Haemophilus influenzae MtsZ at pH 7.0* | 0.31 | 0.00 | 19.58 | 0.58 | 2-704 | X-ray | 1.73 | monomer | 2 x MGD, 1 x MO, 1 x O | HHblits | 0.30 |
| ``` target    MPTANK-ADEVIILRPGTDAAFFLGVARELIEKGLYDRAAVIERTDLPLLVRLDTGERLDARDVIPGYELAALTNYVTLK 7l5i.1    -ETCQMLNAEWIPVNTATDVPLMLGIAHTLVEQGKHDKDFLKKYTSG--------------------Y------------  target    PDAEIKGNPPPPPFTAGGQVVPTELRDAWGDFVWWDRATGRPRPVSRDEVGARFDGDPALLGEFEVELVDGSTVPVRPAF 7l5i.1    --------------------------------------------------------------------------------  target    DLLKQYL-----DESFDLRTASEVCRVPPQAIQSIARQLAANKRETLLAAGMGPNHYFQNDLFGRVQFLVAALTDNIGHL 7l5i.1    AKFEEYLLGKTDGQPKTAEWAAKICGVPAETIKQLAADFAS-KRT-MLMGGWGMQRQRHGEQTHWMLVTLASMLGQIGLP  target    GGNVGSYAGNYRGSVFQAMGQWIAEDPFAIEPDLTKPATVKRYYKAESAHYWNYGERPLRAVA-KDDEGDLTKGEVLTGK 7l5i.1    GGGFGLSYHYSNGGVPTATGGIIG-S-ITASPSGK-A-GAKTWLDDTSK--SAF---PLARIADVLLHPGKKIQYNGTEI  target    SHMPTPTKLIWFGNSNSLLGNAKWSFDVVKNTLPRQDAVFCNEWHWTSSCEYADLVFPADSWAEFKLPDATASCTNPFLL 7l5i.1    --TYPDIKAVYWAGGNPFVHHQD--TNTLVKAFQKPDVVIVNEVNWTPTARMADIVLPATTSYERNDLTMAGDYSMMSVY  target    AFPTTPLKRLYDTRSDYEALALTAKALGELIDEPRM---EQYWRGILDGDPTPYLQRIFSGSN--ATRGITYDELHESSK 7l5i.1    P-MKQVVPPQFEAKNDYDIFVELAKRAGVEEQYTEGKTEMEWLE--------EFYNAAFSAARANRVAMPRFDKFWAENK  target    RGVPLL--MNMRTYPRSGGWEQRQEDKPWYTATGRLEFYRPEPEFQA--AGESLPVWREPVDATFYEPNAILSNAAHPSI 7l5i.1    -PLSFEAGEAAKKWVRYGEFREDPLLNPLGTPSGKIEIFSDVVEKMNYNDCKGHPSWMEPEEFA----------------  target    APRAPEDYGVPESQLDVETRQYRNVVRTWAELQQTLHPLQERDPAFRFVFQTPKYRWGAHSTAVDADWISMLFGPFGDPY 7l5i.1    ---------------------------------------GNVTEEYPLALVTPHPYYRLHSQLAHTSLRQKYA-------  target    RRDPRMPWTGEAYLEINPKDAAELGLADGDYAWVDADPEDRPYRGWKEDDPYYEVARAMMRVRIYTGMSRGVIRTWFNMY 7l5i.1    -------VNDREPVMIHPEDAAARGIKDGDIVRIHSK-----------------RGQVLAGAAVTENIIKGTVALHEGAW  target    AATPATVANQKATPGNPARNEQTRYVALFRYGSHQSGTRAWLRPTQQTDSLVRKGYFGQVIGTGFEADVHSVSGAPKEAF 7l5i.1    --------------------------------------------------------------------------------  target    VKIEKAEDGGIGAERLWRPLTLGLRPEAPSAALTAYLAGDYSGTKGS 7l5i.1    ----------------------------------------------- ``` | | | | | | | | | | | | | | | | | | | | | | | | | | | | | | | | | | | | | | | | | | | | | | | | | |
|  | 7l5s.1.A | Trimethylamine-N-oxide reductase  *Crystal Structure of Haemophilus influenzae MtsZ at pH 5.5* | 0.31 | 0.00 | 19.58 | 0.58 | 2-704 | X-ray | 2.09 | monomer | 1 x O, 2 x MGD, 1 x MO | HHblits | 0.30 |
| ``` target    MPTANK-ADEVIILRPGTDAAFFLGVARELIEKGLYDRAAVIERTDLPLLVRLDTGERLDARDVIPGYELAALTNYVTLK 7l5s.1    -ETCQMLNAEWIPVNTATDVPLMLGIAHTLVEQGKHDKDFLKKYTSG--------------------Y------------  target    PDAEIKGNPPPPPFTAGGQVVPTELRDAWGDFVWWDRATGRPRPVSRDEVGARFDGDPALLGEFEVELVDGSTVPVRPAF 7l5s.1    --------------------------------------------------------------------------------  target    DLLKQYL-----DESFDLRTASEVCRVPPQAIQSIARQLAANKRETLLAAGMGPNHYFQNDLFGRVQFLVAALTDNIGHL 7l5s.1    AKFEEYLLGKTDGQPKTAEWAAKICGVPAETIKQLAADFAS-KRT-MLMGGWGMQRQRHGEQTHWMLVTLASMLGQIGLP  target    GGNVGSYAGNYRGSVFQAMGQWIAEDPFAIEPDLTKPATVKRYYKAESAHYWNYGERPLRAVA-KDDEGDLTKGEVLTGK 7l5s.1    GGGFGLSYHYSNGGVPTATGGIIG-S-ITASPSGK-A-GAKTWLDDTSK--SAF---PLARIADVLLHPGKKIQYNGTEI  target    SHMPTPTKLIWFGNSNSLLGNAKWSFDVVKNTLPRQDAVFCNEWHWTSSCEYADLVFPADSWAEFKLPDATASCTNPFLL 7l5s.1    --TYPDIKAVYWAGGNPFVHHQD--TNTLVKAFQKPDVVIVNEVNWTPTARMADIVLPATTSYERNDLTMAGDYSMMSVY  target    AFPTTPLKRLYDTRSDYEALALTAKALGELIDEPRM---EQYWRGILDGDPTPYLQRIFSGSN--ATRGITYDELHESSK 7l5s.1    P-MKQVVPPQFEAKNDYDIFVELAKRAGVEEQYTEGKTEMEWLE--------EFYNAAFSAARANRVAMPRFDKFWAENK  target    RGVPLL--MNMRTYPRSGGWEQRQEDKPWYTATGRLEFYRPEPEFQA--AGESLPVWREPVDATFYEPNAILSNAAHPSI 7l5s.1    -PLSFEAGEAAKKWVRYGEFREDPLLNPLGTPSGKIEIFSDVVEKMNYNDCKGHPSWMEPEEFA----------------  target    APRAPEDYGVPESQLDVETRQYRNVVRTWAELQQTLHPLQERDPAFRFVFQTPKYRWGAHSTAVDADWISMLFGPFGDPY 7l5s.1    ---------------------------------------GNVTEEYPLALVTPHPYYRLHSQLAHTSLRQKYA-------  target    RRDPRMPWTGEAYLEINPKDAAELGLADGDYAWVDADPEDRPYRGWKEDDPYYEVARAMMRVRIYTGMSRGVIRTWFNMY 7l5s.1    -------VNDREPVMIHPEDAAARGIKDGDIVRIHSK-----------------RGQVLAGAAVTENIIKGTVALHEGAW  target    AATPATVANQKATPGNPARNEQTRYVALFRYGSHQSGTRAWLRPTQQTDSLVRKGYFGQVIGTGFEADVHSVSGAPKEAF 7l5s.1    --------------------------------------------------------------------------------  target    VKIEKAEDGGIGAERLWRPLTLGLRPEAPSAALTAYLAGDYSGTKGS 7l5s.1    ----------------------------------------------- ``` | | | | | | | | | | | | | | | | | | | | | | | | | | | | | | | | | | | | | | | | | | | | | | | | | |
|  | 1dms.1.A | DMSO REDUCTASE  *STRUCTURE OF DMSO REDUCTASE* | 0.32 | 0.00 | 19.83 | 0.58 | 2-705 | X-ray | 1.88 | monomer | 2 x PGD, 1 x 2MO | HHblits | 0.30 |
| ``` target    MPTANK-ADEVIILRPGTDAAFFLGVARELIEKGLYDRAAVIERTDLPLLVRLDTGERLDARDVIPGYELAALTNYVTLK 1dms.1    -KTVEFFGADHVTPKPQTDVAIMLGMAHTLVAEDLYDKDFIANYTS--------------------GF------------  target    PDAEIKGNPPPPPFTAGGQVVPTELRDAWGDFVWWDRATGRPRPVSRDEVGARFDGDPALLGEFEVELVDGSTVPVRPAF 1dms.1    --------------------------------------------------------------------------------  target    DLLKQYLD-----ESFDLRTASEVCRVPPQAIQSIARQLAANKRETLLAAGMGPNHYFQNDLFGRVQFLVAALTDNIGHL 1dms.1    DKFLPYLMGETDSTPKTAEWASDISGVPAETIKELARLFKSK-RT-MLAAGWSMQRMHHGEQAHWMLVTLASMLGQIGLP  target    GGNVGSYAGNYRGSVFQAMGQWIAEDPFAIEPDLTKPATVKRYYKAESAHYWNYGERPLR-AVAKDDEGDLTKGEVLTGK 1dms.1    GGGFGLSYHYSGGGTPS--SSGP---ALSGITDGGAATKGPEWLA-----ASGASVIPVARVVDMLENPGAEFDFN--GT  target    SHMPTPTKLIWFGNSNSLLGNAKWSFDVVKNTLPRQDAVFCNEWHWTSSCEYADLVFPADSWAEFKLPDATASCTNPFLL 1dms.1    RSKFPDVKMAYWVGGNPFVHHQD--RNRMVKAWEKLETFIVHDFQWTPTARHADIVLPATTSYERNDIETIGDYSNTGIL  target    AFPTTPLKRLYDTRSDYEALALTAKALGELIDEPRMEQYWRGILDGDPTPYLQRIFSGS---NATRGI---TYDELHESS 1dms.1    -AMKKIVEPLYEARSDYDIFAAVAERLGKGKEFTEGKD---------EMGWIKSFYDDAAKQGKAGGVEMPAFDAFWAEG  target    KRGVPLLMNMRTYPRSGGWEQRQEDKPWYTATGRLEFYRPEPEFQAA--GESLPVWREPVDATFYEPNAILSNAAHPSIA 1dms.1    IVEFPVT-DGADFVRYASFREDPLLNPLGTPTGLIEIYSKNIEKMGYDDCPAHPTWMEPLERL-----------------  target    PRAPEDYGVPESQLDVETRQYRNVVRTWAELQQTLHPLQERDPAFRFVFQTPKYRWGAHSTAVDADWISMLFGPFGDPYR 1dms.1    --------------------------------------DGPGAKYPLHIAASHPFNRLHSQLN-GTVLREGYA-------  target    RDPRMPWTGEAYLEINPKDAAELGLADGDYAWVDADPEDRPYRGWKEDDPYYEVARAMMRVRIYTGMSRGVIRTWFNMYA 1dms.1    ------VQGHEPCLMHPDDAAARGIADGDVVRVHND-----------------RGQILTGVKVTDAVMKGVIQIYEGGWY  target    ATPATVANQKATPGNPARNEQTRYVALFRYGSHQSGTRAWLRPTQQTDSLVRKGYFGQVIGTGFEADVHSVSGAPKEAFV 1dms.1    --------------------------------------------------------------------------------  target    KIEKAEDGGIGAERLWRPLTLGLRPEAPSAALTAYLAGDYSGTKGS 1dms.1    ---------------------------------------------- ``` | | | | | | | | | | | | | | | | | | | | | | | | | | | | | | | | | | | | | | | | | | | | | | | | | |
|  | 1e60.1.A | Dimethyl sulfoxide/trimethylamine N-oxide reductase  *OXIDIZED DMSO REDUCTASE EXPOSED TO HEPES - Structure II BUFFER* | 0.31 | 0.00 | 20.21 | 0.58 | 2-704 | X-ray | 2.00 | monomer | 2 x PGD, 1 x 2MO | HHblits | 0.30 |
| ``` target    MPTANK-ADEVIILRPGTDAAFFLGVARELIEKGLYDRAAVIERTDLPLLVRLDTGERLDARDVIPGYELAALTNYVTLK 1e60.1    -KTVEFFGAEHITPKPQTDVAIMLGMAHTLVAEDLYDKDFIANYTSG--------------------F------------  target    PDAEIKGNPPPPPFTAGGQVVPTELRDAWGDFVWWDRATGRPRPVSRDEVGARFDGDPALLGEFEVELVDGSTVPVRPAF 1e60.1    --------------------------------------------------------------------------------  target    DLLKQY------LDESFDLRTASEVCRVPPQAIQSIARQLAANKRETLLAAGMGPNHYFQNDLFGRVQFLVAALTDNIGH 1e60.1    DKFLPYLDGETDST-PKTAEWAEGISGVPAETIKELARLFESK-RT-MLAAGWSMQRMHHGEQAHWMLVTLASMLGQIGL  target    LGGNVGSYAGNYRGSVFQAMGQWIAEDPFAIEPDLTKPATVKRYYKAESAHYWNYGERPLRA-VAKDDEGDLTKGEVLTG 1e60.1    PGGGFGLSYHYSGGGTPSTSGP--A---LAGITDGGAATKGPEWLA-----ASGASVIPVARVVDMLENPGAEFDFN--G  target    KSHMPTPTKLIWFGNSNSLLGNAKWSFDVVKNTLPRQDAVFCNEWHWTSSCEYADLVFPADSWAEFKLPDATASCTNPFL 1e60.1    TRSKFPDVKMAYWVGGNPFVHHQD--RNRMVKAWEKLETFVVHDFQWTPTARHADIVLPATTSYERNDIETIGDYSNTGI  target    LAFPTTPLKRLYDTRSDYEALALTAKALGELIDEPRMEQYWRGILDGDPTPYLQRIFSGSNATRGI---TYDELHESSKR 1e60.1    L-AMKKIVEPLYEARSDYDIFAAVAERLGKGAEFTEGKDEMGW-----IKSFYDDAAKQ-GKAAGVEMPAFDAFWAEGIV  target    GVPLLMNMRTYPRSGGWEQRQEDKPWYTATGRLEFYRPEPEFQAA--GESLPVWREPVDATFYEPNAILSNAAHPSIAPR 1e60.1    EFPVT-DGADFVRYASFREDPLLNPLGTPTGLIEIYSKNIEKMGYDDCPAHPTWMEPLERL-------------------  target    APEDYGVPESQLDVETRQYRNVVRTWAELQQTLHPLQERDPAFRFVFQTPKYRWGAHSTAVDADWISMLFGPFGDPYRRD 1e60.1    ------------------------------------DGPGAKYPLHIAASHPFNRLHSQL-NGTVLREGYA---------  target    PRMPWTGEAYLEINPKDAAELGLADGDYAWVDADPEDRPYRGWKEDDPYYEVARAMMRVRIYTGMSRGVIRTWFNMYAAT 1e60.1    ----VQGHEPCLMHPDDAAARGIADGDVVRVHND-----------------RGQILTGVKVTDAVMKGVIQIYEGGW---  target    PATVANQKATPGNPARNEQTRYVALFRYGSHQSGTRAWLRPTQQTDSLVRKGYFGQVIGTGFEADVHSVSGAPKEAFVKI 1e60.1    --------------------------------------------------------------------------------  target    EKAEDGGIGAERLWRPLTLGLRPEAPSAALTAYLAGDYSGTKGS 1e60.1    -------------------------------------------- ``` | | | | | | | | | | | | | | | | | | | | | | | | | | | | | | | | | | | | | | | | | | | | | | | | | |
|  | 1e5v.2.A | Dimethyl sulfoxide/trimethylamine N-oxide reductase  *OXIDIZED DMSO REDUCTASE EXPOSED TO HEPES BUFFER* | 0.32 | 0.00 | 20.29 | 0.58 | 2-705 | X-ray | 2.40 | monomer | 2 x PGD, 1 x 2MO | HHblits | 0.30 |
| ``` target    MPTANK-ADEVIILRPGTDAAFFLGVARELIEKGLYDRAAVIERTDLPLLVRLDTGERLDARDVIPGYELAALTNYVTLK 1e5v.2    -KTVEFFGAEHITPKPQTDVAIMLGMAHTLVAEDLYDKDFIANYTSG--------------------F------------  target    PDAEIKGNPPPPPFTAGGQVVPTELRDAWGDFVWWDRATGRPRPVSRDEVGARFDGDPALLGEFEVELVDGSTVPVRPAF 1e5v.2    --------------------------------------------------------------------------------  target    DLLKQY------LDESFDLRTASEVCRVPPQAIQSIARQLAANKRETLLAAGMGPNHYFQNDLFGRVQFLVAALTDNIGH 1e5v.2    DKFLPYLDGETDST-PKTAEWAEGISGVPAETIKELARLFESK-R-TMLAAGWSMQRMHHGEQAHWMLVTLASMLGQIGL  target    LGGNVGSYAGNYRGSVFQAMGQWIAEDPFAIEPDLTKPATVKRYYKAESAHYWNYGERPLR-AVAKDDEGDLTKGEVLTG 1e5v.2    PGGGFGLSYHYSGGGTPSTSGPA-----LAGITDGGAATKGPEWLA-----ASGASVIPVARVVDMLENPGAEFDF--NG  target    KSHMPTPTKLIWFGNSNSLLGNAKWSFDVVKNTLPRQDAVFCNEWHWTSSCEYADLVFPADSWAEFKLPDATASCTNPFL 1e5v.2    TRSKFPDVKMAYWVGGNPFVHHQD--RNRMVKAWEKLETFVVHDFQWTPTARHADIVLPATTSYERNDIETIGDYSNTGI  target    LAFPTTPLKRLYDTRSDYEALALTAKALGELIDEPRMEQYWRGILDGDPTPYLQRIFSGS---NATRGI---TYDELHES 1e5v.2    L-AMKKIVEPLYEARSDYDIFAAVAERLGKGAEFTEGKDE---------MGWIKSFYDDAAKQGKAAGVQMPAFDAFWAE  target    SKRGVPLLMNMRTYPRSGGWEQRQEDKPWYTATGRLEFYRPEPEFQAA--GESLPVWREPVDATFYEPNAILSNAAHPSI 1e5v.2    GIVEFPVT-DGADFVRYASFREDPLLNPLGTPTGLIEIYSKNIEKMGYDDCPAHPTWMEPLERL----------------  target    APRAPEDYGVPESQLDVETRQYRNVVRTWAELQQTLHPLQERDPAFRFVFQTPKYRWGAHSTAVDADWISMLFGPFGDPY 1e5v.2    ---------------------------------------DGPGAKYPLHIAASHPFNRLHSQL-NGTVLREGYA------  target    RRDPRMPWTGEAYLEINPKDAAELGLADGDYAWVDADPEDRPYRGWKEDDPYYEVARAMMRVRIYTGMSRGVIRTWFNMY 1e5v.2    -------VQGHEPCLMHPDDAAARGIADGDVVRVHND-----------------RGQILTGVKVTDAVMKGVIQIYEGGW  target    AATPATVANQKATPGNPARNEQTRYVALFRYGSHQSGTRAWLRPTQQTDSLVRKGYFGQVIGTGFEADVHSVSGAPKEAF 1e5v.2    Y-------------------------------------------------------------------------------  target    VKIEKAEDGGIGAERLWRPLTLGLRPEAPSAALTAYLAGDYSGTKGS 1e5v.2    ----------------------------------------------- ``` | | | | | | | | | | | | | | | | | | | | | | | | | | | | | | | | | | | | | | | | | | | | | | | | | |
|  | 4dmr.1.A | DMSO REDUCTASE  *REDUCED DMSO REDUCTASE FROM RHODOBACTER CAPSULATUS WITH BOUND DMSO SUBSTRATE* | 0.31 | 0.00 | 20.08 | 0.58 | 2-705 | X-ray | 1.90 | monomer | 2 x PGD, 1 x 4MO, 1 x O | HHblits | 0.30 |
| ``` target    MPTANK-ADEVIILRPGTDAAFFLGVARELIEKGLYDRAAVIERTDLPLLVRLDTGERLDARDVIPGYELAALTNYVTLK 4dmr.1    -KTVEFFGAEHITPKPQTDVAIMLGMAHTLVAEDLYDKDFIANYTSG--------------------F------------  target    PDAEIKGNPPPPPFTAGGQVVPTELRDAWGDFVWWDRATGRPRPVSRDEVGARFDGDPALLGEFEVELVDGSTVPVRPAF 4dmr.1    --------------------------------------------------------------------------------  target    DLLKQY------LDESFDLRTASEVCRVPPQAIQSIARQLAANKRETLLAAGMGPNHYFQNDLFGRVQFLVAALTDNIGH 4dmr.1    DKFLPYLDGETDST-PKTAEWAEGISGVPAETIKELARLFESK-RT-MLAAGWSMQRMHHGEQAHWMLVTLASMLGQIGL  target    LGGNVGSYAGNYRGSVFQAMGQWIAEDPFAIEPDLTKPATVKRYYKAESAHYWNYGERPL-RAVAKDDEGDLTKGEVLTG 4dmr.1    PGGGFGLSYHYSGGGTPSTSGPA----LAGITDGGAAT-KGPEWLA-----ASGASVIPVARVVDMLENPGAEFDFN--G  target    KSHMPTPTKLIWFGNSNSLLGNAKWSFDVVKNTLPRQDAVFCNEWHWTSSCEYADLVFPADSWAEFKLPDATASCTNPFL 4dmr.1    TRSKFPDVKMAYWVGGNPFVHHQD--RNRMVKAWEKLETFVVHDFQWTPTARHADIVLPATTSYERNDIETIGDYSNTGI  target    LAFPTTPLKRLYDTRSDYEALALTAKALGELIDEPRMEQYWRGILDGDPTPYLQRIFSGS---NATRGI---TYDELHES 4dmr.1    L-AMKKIVEPLYEARSDYDIFAAVAERLGKGAEFTEGKDE---------MGWIKSFYDDAAKQGKAAGVQMPAFDAFWAE  target    SKRGVPLLMNMRTYPRSGGWEQRQEDKPWYTATGRLEFYRPEPEFQAA--GESLPVWREPVDATFYEPNAILSNAAHPSI 4dmr.1    GIVEFPVT-DGADFVRYASFREDPLLNPLGTPTGLIEIYSKNIEKMGYDDCPAHPTWMEPLERL----------------  target    APRAPEDYGVPESQLDVETRQYRNVVRTWAELQQTLHPLQERDPAFRFVFQTPKYRWGAHSTAVDADWISMLFGPFGDPY 4dmr.1    ---------------------------------------DGPGAKYPLHIAASHPFNRLHSQL-NGTVLREGYA------  target    RRDPRMPWTGEAYLEINPKDAAELGLADGDYAWVDADPEDRPYRGWKEDDPYYEVARAMMRVRIYTGMSRGVIRTWFNMY 4dmr.1    -------VQGHEPCLMHPDDAAARGIADGDVVRVHND-----------------RGQILTGVKVTDAVMKGVIQIYEGGW  target    AATPATVANQKATPGNPARNEQTRYVALFRYGSHQSGTRAWLRPTQQTDSLVRKGYFGQVIGTGFEADVHSVSGAPKEAF 4dmr.1    Y-------------------------------------------------------------------------------  target    VKIEKAEDGGIGAERLWRPLTLGLRPEAPSAALTAYLAGDYSGTKGS 4dmr.1    ----------------------------------------------- ``` | | | | | | | | | | | | | | | | | | | | | | | | | | | | | | | | | | | | | | | | | | | | | | | | | |
|  | 1e18.1.A | DMSO REDUCTASE.  *TUNGSTEN-SUSBSTITUTED DMSO REDUCTASE FROM RHODOBACTER CAPSULATUS* | 0.31 | 0.00 | 20.08 | 0.58 | 2-705 | X-ray | 2.00 | monomer | 2 x PGD, 1 x 6WO | HHblits | 0.30 |
| ``` target    MPTANK-ADEVIILRPGTDAAFFLGVARELIEKGLYDRAAVIERTDLPLLVRLDTGERLDARDVIPGYELAALTNYVTLK 1e18.1    -KTVEFFGAEHITPKPQTDVAIMLGMAHTLVAEDLYDKDFIANYTSG--------------------F------------  target    PDAEIKGNPPPPPFTAGGQVVPTELRDAWGDFVWWDRATGRPRPVSRDEVGARFDGDPALLGEFEVELVDGSTVPVRPAF 1e18.1    --------------------------------------------------------------------------------  target    DLLKQY------LDESFDLRTASEVCRVPPQAIQSIARQLAANKRETLLAAGMGPNHYFQNDLFGRVQFLVAALTDNIGH 1e18.1    DKFLPYLDGETDST-PKTAEWAEGISGVPAETIKELARLFESK-RT-MLAAGWSMQRMHHGEQAHWMLVTLASMLGQIGL  target    LGGNVGSYAGNYRGSVFQAMGQWIAEDPFAIEPDLTKPATVKRYYKAESAHYWNYGERPLRA-VAKDDEGDLTKGEVLTG 1e18.1    PGGGFGLSYHYSGGGTPSTSGPA--LA--GITDGGAAT-KGPEWLA-----ASGASVIPVARVVDMLENPGAEFDFN--G  target    KSHMPTPTKLIWFGNSNSLLGNAKWSFDVVKNTLPRQDAVFCNEWHWTSSCEYADLVFPADSWAEFKLPDATASCTNPFL 1e18.1    TRSKFPDVKMAYWVGGNPFVHHQD--RNRMVKAWEKLETFVVHDFQWTPTARHADIVLPATTSYERNDIETIGDYSNTGI  target    LAFPTTPLKRLYDTRSDYEALALTAKALGELIDEPRMEQYWRGILDGDPTPYLQRIFSGS---NATRG---ITYDELHES 1e18.1    LA-MKKIVEPLYEARSDYDIFAAVAERLGKGKEFTEGKDE---------MGWIKSFYDDAAKQGKAAGVEMPAFDAFWAE  target    SKRGVPLLMNMRTYPRSGGWEQRQEDKPWYTATGRLEFYRPEPEFQAA--GESLPVWREPVDATFYEPNAILSNAAHPSI 1e18.1    GIVEFPVTDG-ADFVRYASFREDPLLNPLGTPTGLIEIYSKNIEKMGYDDCPAHPTWMEPLERL----------------  target    APRAPEDYGVPESQLDVETRQYRNVVRTWAELQQTLHPLQERDPAFRFVFQTPKYRWGAHSTAVDADWISMLFGPFGDPY 1e18.1    ---------------------------------------DGPGAKYPLHIAASHPFNRLHSQL-NGTVLREGYA------  target    RRDPRMPWTGEAYLEINPKDAAELGLADGDYAWVDADPEDRPYRGWKEDDPYYEVARAMMRVRIYTGMSRGVIRTWFNMY 1e18.1    -------VQGHEPCLMHPDDAAARGIADGDVVRVHND-----------------RGQILTGVKVTDAVMKGVIQIYEGGW  target    AATPATVANQKATPGNPARNEQTRYVALFRYGSHQSGTRAWLRPTQQTDSLVRKGYFGQVIGTGFEADVHSVSGAPKEAF 1e18.1    Y-------------------------------------------------------------------------------  target    VKIEKAEDGGIGAERLWRPLTLGLRPEAPSAALTAYLAGDYSGTKGS 1e18.1    ----------------------------------------------- ``` | | | | | | | | | | | | | | | | | | | | | | | | | | | | | | | | | | | | | | | | | | | | | | | | | |
|  | 1eu1.1.A | DIMETHYL SULFOXIDE REDUCTASE  *THE CRYSTAL STRUCTURE OF RHODOBACTER SPHAEROIDES DIMETHYLSULFOXIDE REDUCTASE REVEALS TWO DISTINCT MOLYBDENUM COORDINATION ENVIRONMENTS.* | 0.31 | 0.00 | 20.81 | 0.57 | 2-704 | X-ray | 1.30 | monomer | 3 x GLC, 1 x CD, 2 x MGD, 1 x 6MO, 2 x O | HHblits | 0.31 |
| ``` target    MPTANKAD-EVIILRPGTDAAFFLGVARELIEKGLYDRAAVIERTDLPLLVRLDTGERLDARDVIPGYELAALTNYVTLK 1eu1.1    -ETADYFGADVVSPRPQTDVALMLGMAHTLYSEDLHDKDFLENCTTG--------------------F------------  target    PDAEIKGNPPPPPFTAGGQVVPTELRDAWGDFVWWDRATGRPRPVSRDEVGARFDGDPALLGEFEVELVDGSTVPVRPAF 1eu1.1    --------------------------------------------------------------------------------  target    DLLKQYLD-----ESFDLRTASEVCRVPPQAIQSIARQLAANKRETLLAAGMGPNHYFQNDLFGRVQFLVAALTDNIGHL 1eu1.1    DLFAAYLTGESDGTPKTAEWAAEICGLPAEQIRELARSFVAGRT--MLAAGWSIQRMHHGEQAHWMLVTLASMIGQIGLP  target    GGNVGSYAGNYRGSVFQAMGQWIAEDPFAIEPDLTKPATVKRYYKAESAHYWNYGERPLRAVAKDDEGDLTKGEVLT--- 1eu1.1    GGGFGLSYHYSNGGSP--TSDGP---ALGGISDGGKAVEGAAWLS-----ESGATSIP---CA------RVVDMLLNPGG  target    -----GKSHMPTPTKLIWFGNSNSLLGNAKWSFDVVKNTLPRQDAVFCNEWHWTSSCEYADLVFPADSWAEFKLPDATAS 1eu1.1    EFQFNGATATYPDVKLAYWAGGNPFAHHQD--RNRMLKAWEKLETFIVQDFQWTATARHADIVLPATTSYERNDIESVGD  target    CTNPFLLAFPTTPLKRLYDTRSDYEALALTAKALGELIDEPRMEQYWRGILDGDPTPYLQRIFS----G--SNATRGITY 1eu1.1    YSNRAIL-AMKKVVDPLYEARSDYDIFAALAERLGKGAEFTEGRDE---------MGWISSFYEAAVKQAEFKNVAMPSF  target    DELHESSKRGVPLLMNMRTYPRSGGWEQRQEDKPWYTATGRLEFYRPEPEFQAA--GESLPVWREPVDATFYEPNAILSN 1eu1.1    EDFWSEGIVEFPITEG-ANFVRYADFREDPLFNPLGTPSGLIEIYSKNIEKMGYDDCPAHPTWMEPAERL----------  target    AAHPSIAPRAPEDYGVPESQLDVETRQYRNVVRTWAELQQTLHPLQERDPAFRFVFQTPKYRWGAHSTAVDADWISMLFG 1eu1.1    ---------------------------------------------GGAGAKYPLHVVASHPKSRLHSQLNGT-SLRDLYA  target    PFGDPYRRDPRMPWTGEAYLEINPKDAAELGLADGDYAWVDADPEDRPYRGWKEDDPYYEVARAMMRVRIYTGMSRGVIR 1eu1.1    -------------VAGHEPCLINPADAAARGIADGDVLRVFND-----------------RGQILVGAKVSDAVMPGAIQ  target    TWFNMYAATPATVANQKATPGNPARNEQTRYVALFRYGSHQSGTRAWLRPTQQTDSLVRKGYFGQVIGTGFEADVHSVSG 1eu1.1    IYEGGW--------------------------------------------------------------------------  target    APKEAFVKIEKAEDGGIGAERLWRPLTLGLRPEAPSAALTAYLAGDYSGTKGS 1eu1.1    ----------------------------------------------------- ``` | | | | | | | | | | | | | | | | | | | | | | | | | | | | | | | | | | | | | | | | | | | | | | | | | |
|  | 1tmo.1.A | TRIMETHYLAMINE N-OXIDE REDUCTASE  *TRIMETHYLAMINE N-OXIDE REDUCTASE FROM SHEWANELLA MASSILIA* | 0.31 |  | 17.92 | 0.58 | 2-704 | X-ray | 2.50 | monomer | 2 x 2MD, 1 x 2MO | HHblits | 0.29 |
| ``` target    MPTANK-ADEVIILRPGTDAAFFLGVARELIEKGLYDRAAVIERTDLPLLVRLDTGERLDARDVIPGYELAALTNYVTLK 1tmo.1    -KTQAYLGCEQLYVNPQTDVTLMLAIAHEMISKKLYDDKFIQGYSL--------------------GF------------  target    PDAEIKGNPPPPPFTAGGQVVPTELRDAWGDFVWWDRATGRPRPVSRDEVGARFDGDPALLGEFEVELVDGSTVPVRPAF 1tmo.1    --------------------------------------------------------------------------------  target    DLLKQYLD-----ESFDLRTASEVCRVPPQAIQSIARQLAANKRETLLAAGMGPNHYFQNDLFGRVQFLVAALTDNIGHL 1tmo.1    EEFVPYVMGTKDGVAKTPEWAAPICGVEAHVIRDLAKTLVKGRT--QFMMGWCIQRQQHGEQPYWMAAVLATMIGQIGLP  target    GGNVGSYAGNYRGSVFQAMGQWIAEDPFAI-EPDLTKPATVKRYYKAESAHYWN-YGERPLRAVAK-DDEGDLTKGEVLT 1tmo.1    GGGISYGHHYSSI-GVPSSGAAA-PGAFPRNLDENQKP-----LFD--SSDFKGASSTIPVARWIDAILEPGKTIDANGS  target    GKSHMPTPTKLIWFGNSNSLLGNAKWSFDVVKNTLPRQDAVFCNEWHWTSSCEYADLVFPADSWAEFKLPDATASCTNPF 1tmo.1    K--VVYPDIKMMIFSGNNPWNHHQD--RNRMKQAFHKLECVVTVDVNWTATCRFSDIVLPACTTYERNDIDVYGAYANRG  target    LLAFPTTPLKRLYDTRSDYEALALTAKALGELIDEPRMEQYWRGILDGDPTPYLQRIFSGS-----NATRGITYDELHES 1tmo.1    IL-AMQKMVEPLFDSLSDFEIFTRFAAVLGKEKEYTRN---------MGEMEWLETLYNECKAANAGKFEMPDFATFWKQ  target    SKRGVPLLMNMRTYPRSGGWEQRQEDKPWYTATGRLEFYRPEPEFQA--AGESLPVWREPVDATFYEPNAILSNAAHPSI 1tmo.1    GY---VHFGDGEVWTRHADFRNDPEINPLGTPSGLIEIFSRKIDQFGYDDCKGHPTWMEKTERSH---------------  target    APRAPEDYGVPESQLDVETRQYRNVVRTWAELQQTLHPLQERDPAFRFVFQTPKYRWGAHSTAVDADWISMLFGPFGDPY 1tmo.1    -------------------------------------G-GPGSDKHPIWLQSCHPDKRLHSQMCESREYRETYA------  target    RRDPRMPWTGEAYLEINPKDAAELGLADGDYAWVDADPEDRPYRGWKEDDPYYEVARAMMRVRIYTGMSRGVIRTWFNMY 1tmo.1    -------VNGREPVYISPVDAKARGIKDGDIVRVFND-----------------RGQLLAGAVVSDNFPKGIVRIHEGAW  target    AATPATVANQKATPGNPARNEQTRYVALFRYGSHQSGTRAWLRPTQQTDSLVRKGYFGQVIGTGFEADVHSVSGAPKEAF 1tmo.1    --------------------------------------------------------------------------------  target    VKIEKAEDGGIGAERLWRPLTLGLRPEAPSAALTAYLAGDYSGTKGS 1tmo.1    ----------------------------------------------- ``` | | | | | | | | | | | | | | | | | | | | | | | | | | | | | | | | | | | | | | | | | | | | | | | | | |
|  | 2e7z.1.A | Acetylene hydratase Ahy  *Acetylene Hydratase from Pelobacter acetylenicus* | 0.30 |  | 20.68 | 0.56 | 2-705 | X-ray | 1.26 | monomer | 1 x SF4, 2 x MGD, 1 x W | HHblits | 0.30 |
| ``` target    MPTANKADEVIILRPGTDAAFFLGVARELIEKGLYDRAAVIERTDLPLLVRLDTGERLDARDVIPGYELAALTNYVTLKP 2e7z.1    -KVAEMADIWLPLRYGTDAALFLGMINVIINEQLYDKEFVENWCV-----------------------------------  target    DAEIKGNPPPPPFTAGGQVVPTELRDAWGDFVWWDRATGRPRPVSRDEVGARFDGDPALLGEFEVELVDGSTVPVRPAFD 2e7z.1    -----------------------------------------------------------------------------GFE  target    LLKQYLDESFDLRTASEVCRVPPQAIQSIARQLAANKRETLLAAGMGPNHYFQNDLFGRVQFLVAALTDNIGHLGGNVGS 2e7z.1    ELKERVQ-EYPLDKVAEITGCDAGEIRKAAVMFATESPASI-PWAVSTDMQKNSCSAIRAQCILRAIVGSFVNGAEILGA  target    YAGNYR-GSVFQAMGQWIAED-P--FA--IEPDLTKPATVKRYYKAESAHYWNYGERPLRAVAKDDEGDLTKGEVLTGKS 2e7z.1    PHSDLVPISKIQMHEALPEEKKKLQLGTETYPFLTYT-GMSALEE-PSERVYGVKYFH-NMGAFMANPTALFTAMATE--  target    HMPTPTKLIWFGNSNSLLGNAKWSFDVVKNTLPRQDAVFCNEWHWTSSCEYADLVFPADSWAEFKLPDATASCTNPFLLA 2e7z.1    -KPYPVKAFFALASNALMGYAN--QQNALKGLMNQDLVVCYDQFMTPTAQLADYVLPGDHWLERPVVQPNW-EGIPFGN-  target    FPTTPLKRLYDTRSDYEALALTAKALGELIDEPRMEQYWRGILDGDPTPYLQRIFSGSNATRGITYDELHESSKRGVPLL 2e7z.1    TSQQVVEPAGEAKDEYYFIRELAVRMGLEEHFP-----W-----KDRLELINYRIS----PTGMEWEEYQKQYT--Y-MS  target    MNMRTYPRSGGWEQRQEDKPWYTATGRLEFYRPEPEFQAAGESLPVWREPVDATFYEPNAILSNAAHPSIAPRAPEDYGV 2e7z.1    -KL---PD---Y-FGPEGVGVATPSGKVELYSSVFEK-LGYDPLPYYHEPLQTEIS------------------------  target    PESQLDVETRQYRNVVRTWAELQQTLHPLQERDPAFRFVFQTPKY-RWGAHSTAVDADWISMLFGPFGDPYRRDPRMPWT 2e7z.1    --------------------------D--PELAKEYPLILFAGLREDSNFQSCYHQPGILRDAE----------------  target    GEAYLEINPKDAAELGLADGDYAWVDADPEDRPYRGWKEDDPYYEVARAMMRVRIYTGMSRGVIRTWFNMYAATPATVAN 2e7z.1    PDPVALLHPKTAQSLGLPSGEWIWVETT-----------------HGRLKLLLKHDGAQPEGTIRIPHGRWC--------  target    QKATPGNPARNEQTRYVALFRYGSHQSGTRAWLRPTQQTDSLVRKGYFGQVIGTGFEADVHSVSGAPKEAFVKIEKAEDG 2e7z.1    --------------------------------------------------------------------------------  target    GIGAERLWRPLTLGLRPEAPSAALTAYLAGDYSGTKGS 2e7z.1    -------------------------------------- ``` | | | | | | | | | | | | | | | | | | | | | | | | | | | | | | | | | | | | | | | | | | | | | | | | | |
|  | 2v45.1.A | PERIPLASMIC NITRATE REDUCTASE  *A NEW CATALYTIC MECHANISM OF PERIPLASMIC NITRATE REDUCTASE FROM DESULFOVIBRIO DESULFURICANS ATCC 27774 FROM CRYSTALLOGRAPHIC AND EPR DATA AND BASED ON DETAILED ANALYSIS OF THE SIXTH LIGAND* | 0.30 |  | 19.92 | 0.57 | 2-703 | X-ray | 2.40 | monomer | 1 x SF4, 1 x MO, 2 x MGD, 1 x LCP | HHblits | 0.29 |
| ``` target    MPTANKADEVIILRPGTDAAFFLGVARELIEKGLYDRAAVIERTDLPLLVRLDTGERLDARDVIPGYELAALTNYVTLKP 2v45.1    -NTSRIADMHVAFRPGTDLAFMHSMAWVIINEELDNPRFWQRYVNF--------------------MD------------  target    DAEIKGNPPPPPFTAGGQVVPTELRDAWGDFVWWDRATGRPRPVSRDEVGARFDGDPALLGEFEVELVDGSTVPVRPAFD 2v45.1    -----------------------------------A------------------------------------EGKPSDFE  target    LLKQYLDESFDLRTASEVCRVPPQAIQSIARQLAANKRETLLAAGMGPNHYFQNDLFGRVQFLVAALTDNIGHLGGNVGS 2v45.1    GYKAFLE-NYRPEKVAEICRVPVEQIYGAARAFAESAATMS-LWCMGINQRVQGVFANNLIHNLHLITGQICRPGATSFS  target    YAGNY--RGSVFQAMGQWIAEDPFAIEPDLTKPATVKRYYKAESAHYWNYGERPLRAVAKDDEGDLTKGEVLTGKSHMPT 2v45.1    LTGQPNACGGVR-DGGALSHLLPAG--RAIPNAKHRAE-----MEKLWGLPEGRIAP-----EPGYHTVALFEAL--GRG  target    PTKLIWFGNSNSLLGNAKWSFDVVKNTLPRQD-AVFCNEWHWT-SSCEYADLVFPADSWAEFKLPDATASCTNPFLLAFP 2v45.1    DVKCMIICETNPAHTLPN--LNKVHKAMSHPESFIVCIEAFPDAVTLEYADLVLPPAFWCERD--G-VYGCGERRYSL-T  target    TTPLKRLYDTRSDYEALALTAKALGELIDE---PRMEQYWRGILDGDPTPYLQRIFSG-SNATRGITYDELHESSKRGVP 2v45.1    EKAVDPPGQCRPTVNTLVEFARRAGVDPQLVNFRNAEDVWNEW---------RMVSKGTTYDFWGMTRERLRKES--GLI  target    LLMNMRTYPR-SGGWEQRQEDKPWYTATGRLEFYRPEPEFQAAGESLPVWREPVDATFYEPNAILSNAAHPSIAPRAPED 2v45.1    WPCPSEDHPGTSLRYVRGQDPCVPADHPDRFFFYGKPDG------RAVIWMRPAKGA-----------------------  target    YGVPESQLDVETRQYRNVVRTWAELQQTLHPLQERDPAFRFVFQTPKYRWGAHSTA--VDADWISMLFGPFGDPYRRDPR 2v45.1    ------------------------------A-EEPDAEYPLYLTSMRVIDHWHTATMTGKVPELQKA-------------  target    MPWTGEAYLEINPKDAAELGLADGDYAWVDADPEDRPYRGWKEDDPYYEVARAMMRVRIYTGMSRGVIRTWFNMYAATPA 2v45.1    ---NPIAFVEINEEDAARTGIKHGDSVIVETR-----------------RDAMELPARVSDVCRPGLIAVPFFD------  target    TVANQKATPGNPARNEQTRYVALFRYGSHQSGTRAWLRPTQQTDSLVRKGYFGQVIGTGFEADVHSVSGAPKEAFVKIEK 2v45.1    --------------------------------------------------------------------------------  target    AEDGGIGAERLWRPLTLGLRPEAPSAALTAYLAGDYSGTKGS 2v45.1    ------------------------------------------ ``` | | | | | | | | | | | | | | | | | | | | | | | | | | | | | | | | | | | | | | | | | | | | | | | | | |
|  | 4aay.1.A | AROA  *Crystal Structure of the arsenite oxidase protein complex from Rhizobium species strain NT-26* | 0.28 |  | 16.39 | 0.57 | 6-703 | X-ray | 2.70 | hetero-oligomer | 4 x MGD, 2 x O, 2 x 4MO, 2 x F3S, 2 x FES | HHblits | 0.28 |
| ``` target    MPTANKAD--EVIILRPGTDAAFFLGVARELIEKGLYDRAAVIERTDLPLLVRLDTGERLDARDVIPGYELAALTNYVTL 4aay.1    -----GADNVLHLAINSGTDLALFNALFTYIADKGWVDRDFIDKSTLREGTARP--------------------------  target    KPDAEIKGNPPPPPFTAGGQVVPTELRDAWGDFVWWDRATGRPRPVSRDEVGARFDGDPALLGEFEVELVDGSTVPVRPA 4aay.1    ---------------------------------PLYPAR-GVS---------------------------EA----NPGH  target    FDLLKQYLD-ESFDLRTASEVCRVPPQAIQSIARQLAANKR-----ETLLAAGMGPNHYFQNDLFGRVQFLVAALTDNIG 4aay.1    LSSFEDAVEGCRMSIEEAAEITGLDAAQIIKAAEWIGMPKEGGKRRRVMFGYEKGLIWGNDNYRTNGALVNLALATGNIG  target    HLGGNVGSYAGNYRGSVFQAMGQWIAEDPFAIEPDLTKPATVKRYYKAESAHYWNYGERPLRAVAKDDEGDLTKGEVLTG 4aay.1    RPGGGVVRLGGHQEGYV-----RP-S-DAHVGRPAA-YVDQLLIGGQGGVHHIWGC------------------DHYKTT  target    KSHMPTPTKLIWFGNSNSLLG------NAK--WSFDVVKNTLPRQ-DAVFCNEWHWTSSCEYADLVFPADSWAEFKLPDA 4aay.1    --LNAHEFKRVYKKRTDMVKDAMSAAPYGDREAMVNAIVDAINQGGLFAVNVDIIPTKIGEACHVILPAATSGEMN---L  target    TASCTNPFLLAFPTTPLKRLYDTRSDYEALALTAKALGELIDEPR---MEQYWRGILDGDPTPYLQR-IFSGSNATRGIT 4aay.1    TSMNGERRMRL-TERYMDPPGQSMPDCLIAARLANTMERVLTEMGDVGYAAQFKGFDWQTEEDAFMDGYNKNAHGGEFVT  target    YDELHESSKRGVPLLMNMRTYPRSGGWEQRQEDKPWYTATGRLEFYRPEPEFQAAGESLPVWREPVDATFYEPNAILSNA 4aay.1    YERLSAMGTNGFQEPATGFTDGKIEGTQRLYTDGVFSTDDGKARFMDAPWR---------GLQAPGK-------------  target    AHPSIAPRAPEDYGVPESQLDVETRQYRNVVRTWAELQQTLHPLQERDPAFRFVFQTPKYRWGAHSTAV--DADWISMLF 4aay.1    --------------------------------------------QQQKDSHKYLINNGRANVVWQSAYLDQENDFVMDRF  target    GPFGDPYRRDPRMPWTGEAYLEINPKDAAELGLADGDYAWVDADPEDRPYRGWKEDDPYYEVARAMMRVRIYTGMSRGVI 4aay.1    ----------------PYPFIEMNPEDMAEAGLKEGDLVEIYND-----------------AGATQAMAYPTPTARRGET  target    RTWFNMYAATPATVANQKATPGNPARNEQTRYVALFRYGSHQSGTRAWLRPTQQTDSLVRKGYFGQVIGTGFEADVHSVS 4aay.1    FMLFGF--------------------------------------------------------------------------  target    GAPKEAFVKIEKAEDGGIGAERLWRPLTLGLRPEAPSAALTAYLAGDYSGTKGS 4aay.1    ------------------------------------------------------ ``` | | | | | | | | | | | | | | | | | | | | | | | | | | | | | | | | | | | | | | | | | | | | | | | | | |
|  | 5nqd.1.A | AroA  *Arsenite oxidase AioAB from Rhizobium sp. str. NT-26 mutant AioBF108A* | 0.28 |  | 16.42 | 0.57 | 6-704 | X-ray | 2.20 | hetero-2-2-mer | 4 x MGD, 2 x O, 2 x 4MO, 2 x F3S, 2 x FES | HHblits | 0.28 |
| ``` target    MPTANKAD--EVIILRPGTDAAFFLGVARELIEKGLYDRAAVIERTDLPLLVRLDTGERLDARDVIPGYELAALTNYVTL 5nqd.1    -----GADNVLHLAINSGTDLALFNALFTYIADKGWVDRDFIDKSTLREGTARP--------------------------  target    KPDAEIKGNPPPPPFTAGGQVVPTELRDAWGDFVWWDRATGRPRPVSRDEVGARFDGDPALLGEFEVELVDGSTVPVRPA 5nqd.1    ---------------------------------PLYPA-RGV-------------------------------SEANPGH  target    FDLLKQYLD-ESFDLRTASEVCRVPPQAIQSIARQLAANKR-----ETLLAAGMGPNHYFQNDLFGRVQFLVAALTDNIG 5nqd.1    LSSFEDAVEGCRMSIEEAAEITGLDAAQIIKAAEWIGMPKEGGKRRRVMFGYEKGLIWGNDNYRTNGALVNLALATGNIG  target    HLGGNVGSYAGNYRGSVFQAMGQWIAEDPFAIEPDLTKPATVKRYYK--AESAHYWNYGERPLRAVAKDDEGDLTKGEVL 5nqd.1    RPGGGVVRLGGHQEGYVR----P--S-DAHVGRPAA-YVD--QLLIGGQGGVHHIWGC------------------DHYK  target    TGKSHMPTPTKLIWFGNSNSLL------GNAK--WSFDVVKNTLPRQ-DAVFCNEWHWTSSCEYADLVFPADSWAEFKLP 5nqd.1    TT--LNAHEFKRVYKKRTDMVKDAMSAAPYGDREAMVNAIVDAINQGGLFAVNVDIIPTKIGEACHVILPAATSGEMN--  target    DATASCTNPFLLAFPTTPLKRLYDTRSDYEALALTAKALGELIDEPR---MEQYWRGILDGDPTPYLQR-IFSGSNATRG 5nqd.1    -LTSMNGERRMRL-TERYMDPPGQSMPDCLIAARLANTMERVLTEMGDVGYAAQFKGFDWQTEEDAFMDGYNKNAHGGEF  target    ITYDELHESSKRGVPLLMNMRTYPRSGGWEQRQEDKPWYTATGRLEFYRPEPEFQAAGESLPVWREPVDATFYEPNAILS 5nqd.1    VTYERLSAMGTNGFQEPATGFTDGKIEGTQRLYTDGVFSTDDGKARFMDAPWR---------GLQAPGK-----------  target    NAAHPSIAPRAPEDYGVPESQLDVETRQYRNVVRTWAELQQTLHPLQERDPAFRFVFQTPKYRWGAHSTAV--DADWISM 5nqd.1    ----------------------------------------------QQQKDSHKYLINNGRANVVWQSAYLDQENDFVMD  target    LFGPFGDPYRRDPRMPWTGEAYLEINPKDAAELGLADGDYAWVDADPEDRPYRGWKEDDPYYEVARAMMRVRIYTGMSRG 5nqd.1    RF----------------PYPFIEMNPEDMAEAGLKEGDLVEIYND-----------------AGATQAMAYPTPTARRG  target    VIRTWFNMYAATPATVANQKATPGNPARNEQTRYVALFRYGSHQSGTRAWLRPTQQTDSLVRKGYFGQVIGTGFEADVHS 5nqd.1    ETFMLFGFP-----------------------------------------------------------------------  target    VSGAPKEAFVKIEKAEDGGIGAERLWRPLTLGLRPEAPSAALTAYLAGDYSGTKGS 5nqd.1    -------------------------------------------------------- ``` | | | | | | | | | | | | | | | | | | | | | | | | | | | | | | | | | | | | | | | | | | | | | | | | | |
|  | 7qv7.1.L | Hydrogen dependent carbon dioxide reductase subunit FdhF  *Cryo-EM structure of Hydrogen-dependent CO2 reductase.* | 0.29 |  | 17.17 | 0.56 | 2-705 | EM | 0.00 | hetero-2-6-6-2-mer | 52 x SF4, 6 x 402 | HHblits | 0.29 |
| ``` target    MPTANKADEVIILRPGTDAAFFLGVARELIEKGLYDRAAVIERTDLPLLVRLDTGERLDARDVIPGYELAALTNYVTLKP 7qv7.1    -AMVHKADIWLRVPSGYNIPLINGMIHIIIKEGLVKTDFVKNHAV-----------------------------------  target    DAEIKGNPPPPPFTAGGQVVPTELRDAWGDFVWWDRATGRPRPVSRDEVGARFDGDPALLGEFEVELVDGSTVPVRPAFD 7qv7.1    -----------------------------------------------------------------------------GFE  target    LLKQYLDESFDLRTASEVCRVPPQAIQSIARQLAANKRETLLAAGMGPNHYFQNDLFGRVQFLVAALTDNIGHLGGNVGS 7qv7.1    EMAKAVE-KYTPEYVEELTGIPKKDLIKAARFYGQAQAAAI-LYSMGVTQFSHGTGNVVSLANLAVITGNLGRPGAGICP  target    YAGNY--RGSVFQAMGQWIAEDPFAIEPDLTKPATVKRYYKAESAHYWNYGERPLRAVAKDDEGDLTKGEVLTGKSHMPT 7qv7.1    LRGQNNVQGAC--DVGALPN--VLPGYLDVTKEQNRER-----FEKVWGVKL-P-------SNIGLRVTEVPDAI--LNK  target    PTKLIWFGNSNSLLGNAKWSFDVVKNTLPRQDAVFCNEWHWTSSCEYADLVFPADSWAEFKLPDATASCTNPFLLAFPTT 7qv7.1    RVRALYIFGENPIMSDPD--SDHLRHALEHLDLLIVQDIFLTETARLAHVVLPAACWAEKDG---TFTNTERRVQRV-RK  target    PLKRLYDTRSDYEALALTAKALGELI-DEPRMEQYWRGILDGDPTPYLQRIFSGSN-ATRGITYDELHESSKRGVPLLMN 7qv7.1    AVEAPGEAKPDWWIFSQIAERMGYTGMQYNNVQ------------EIWDEVRKIVPEKFGGISYARLEKEK--GLAWPCP  target    MRTYPRSGGWEQRQEDKPWYTATGRLEFYRPEPEFQAAGESLPVWREPVDATFYEPNAILSNAAHPSIAPRAPEDYGVPE 7qv7.1    TEDHT-G--TPILYLGGKFATPSGKAQMYPVIFYP-----NTCICDEGAEKQDF---------------NHVIV------  target    SQLDVETRQYRNVVRTWAELQQTLHPLQERDPAFRFVFQTPKYRWGAHSTA--VDADWISMLFGPFGDPYRRDPRMPWTG 7qv7.1    ---------------G--------SIAELPDEEYPFTLTTGRRVYHYHTATMTRKSPVIDQIA----------------P  target    EAYLEINPKDAAELGLADGDYAWVDADPEDRPYRGWKEDDPYYEVARAMMRVRIYTGMSRGVIRTWFNMYAATPATVANQ 7qv7.1    QELVEINPQDATRLGINDGDFLRVSTR-----------------RGYVATRAWVTERVPKGTIFMTFHYWE---------  target    KATPGNPARNEQTRYVALFRYGSHQSGTRAWLRPTQQTDSLVRKGYFGQVIGTGFEADVHSVSGAPKEAFVKIEKAEDGG 7qv7.1    --------------------------------------------------------------------------------  target    IGAERLWRPLTLGLRPEAPSAALTAYLAGDYSGTKGS 7qv7.1    ------------------------------------- ``` | | | | | | | | | | | | | | | | | | | | | | | | | | | | | | | | | | | | | | | | | | | | | | | | | |
|  | 7qv7.1.O | Hydrogen dependent carbon dioxide reductase subunit FdhF  *Cryo-EM structure of Hydrogen-dependent CO2 reductase.* | 0.28 |  | 17.17 | 0.56 | 2-705 | EM | 0.00 | hetero-2-6-6-2-mer | 52 x SF4, 6 x 402 | HHblits | 0.29 |
| ``` target    MPTANKADEVIILRPGTDAAFFLGVARELIEKGLYDRAAVIERTDLPLLVRLDTGERLDARDVIPGYELAALTNYVTLKP 7qv7.1    -AMVHKADIWLRVPSGYNIPLINGMIHIIIKEGLVKTDFVKNHAV-----------------------------------  target    DAEIKGNPPPPPFTAGGQVVPTELRDAWGDFVWWDRATGRPRPVSRDEVGARFDGDPALLGEFEVELVDGSTVPVRPAFD 7qv7.1    -----------------------------------------------------------------------------GFE  target    LLKQYLDESFDLRTASEVCRVPPQAIQSIARQLAANKRETLLAAGMGPNHYFQNDLFGRVQFLVAALTDNIGHLGGNVGS 7qv7.1    EMAKAVE-KYTPEYVEELTGIPKKDLIKAARFYGQAQAAAI-LYSMGVTQFSHGTGNVVSLANLAVITGNLGRPGAGICP  target    YAGNY--RGSVFQAMGQWIAEDPFAIEPDLTKPATVKRYYKAESAHYWNYGERPLRAVAKDDEGDLTKGEVLTGKSHMPT 7qv7.1    LRGQNNVQGAC--DVGALPN--VLPGYLDVTKEQNRER-----FEKVWGVKL-P-------SNIGLRVTEVPDAI--LNK  target    PTKLIWFGNSNSLLGNAKWSFDVVKNTLPRQDAVFCNEWHWTSSCEYADLVFPADSWAEFKLPDATASCTNPFLLAFPTT 7qv7.1    RVRALYIFGENPIMSDPD--SDHLRHALEHLDLLIVQDIFLTETARLAHVVLPAACWAEKDG---TFTNTERRVQRV-RK  target    PLKRLYDTRSDYEALALTAKALGELI-DEPRMEQYWRGILDGDPTPYLQRIFSGSN-ATRGITYDELHESSKRGVPLLMN 7qv7.1    AVEAPGEAKPDWWIFSQIAERMGYTGMQYNNVQ------------EIWDEVRKIVPEKFGGISYARLEKEK--GLAWPCP  target    MRTYPRSGGWEQRQEDKPWYTATGRLEFYRPEPEFQAAGESLPVWREPVDATFYEPNAILSNAAHPSIAPRAPEDYGVPE 7qv7.1    TEDHT-G--TPILYLGGKFATPSGKAQMYPVIFYP-----NTCICDEGAEKQDF---------------NHVIV------  target    SQLDVETRQYRNVVRTWAELQQTLHPLQERDPAFRFVFQTPKYRWGAHSTA--VDADWISMLFGPFGDPYRRDPRMPWTG 7qv7.1    ---------------G--------SIAELPDEEYPFTLTTGRRVYHYHTATMTRKSPVIDQIA----------------P  target    EAYLEINPKDAAELGLADGDYAWVDADPEDRPYRGWKEDDPYYEVARAMMRVRIYTGMSRGVIRTWFNMYAATPATVANQ 7qv7.1    QELVEINPQDATRLGINDGDFLRVSTR-----------------RGYVATRAWVTERVPKGTIFMTFHYWE---------  target    KATPGNPARNEQTRYVALFRYGSHQSGTRAWLRPTQQTDSLVRKGYFGQVIGTGFEADVHSVSGAPKEAFVKIEKAEDGG 7qv7.1    --------------------------------------------------------------------------------  target    IGAERLWRPLTLGLRPEAPSAALTAYLAGDYSGTKGS 7qv7.1    ------------------------------------- ``` | | | | | | | | | | | | | | | | | | | | | | | | | | | | | | | | | | | | | | | | | | | | | | | | | |
|  | 2v3v.1.A | PERIPLASMIC NITRATE REDUCTASE  *A NEW CATALYTIC MECHANISM OF PERIPLASMIC NITRATE REDUCTASE FROM DESULFOVIBRIO DESULFURICANS ATCC 27774 FROM CRYSTALLOGRAPHIC AND EPR DATA AND BASED ON DETAILED ANALYSIS OF THE SIXTH LIGAND* | 0.30 |  | 19.65 | 0.56 | 2-703 | X-ray | 1.99 | monomer | 1 x SF4, 1 x MO, 2 x MGD, 4 x LCP | HHblits | 0.29 |
| ``` target    MPTANKADEVIILRPGTDAAFFLGVARELIEKGLYDRAAVIERTDLPLLVRLDTGERLDARDVIPGYELAALTNYVTLKP 2v3v.1    -NTSRIADMHVAFRPGTDLAFMHSMAWVIINEELDNPRFWQRYVNFM--------------DA-----------------  target    DAEIKGNPPPPPFTAGGQVVPTELRDAWGDFVWWDRATGRPRPVSRDEVGARFDGDPALLGEFEVELVDGSTVPVRPAFD 2v3v.1    ------------------------------------------------------------------------EGKPSDFE  target    LLKQYLDESFDLRTASEVCRVPPQAIQSIARQLAANKRETLLAAGMGPNHYFQNDLFGRVQFLVAALTDNIGHLGGNVGS 2v3v.1    GYKAFLE-NYRPEKVAEICRVPVEQIYGAARAFAESAATMS-LWCMGINQRVQGVFANNLIHNLHLITGQICRPGATSFS  target    YAGNY--RGSVFQAMGQWIAEDPFAIEPDLTKPATVKRYYKAESAHYWNYGERPLRAVAKDDEGDLTKGEVLTGKSHMPT 2v3v.1    LTGQPNACGGVR-DGGALSHLLP--AGRAIPNAKHRAE-----MEKLWGLPEGRI--AP---EPGYHTVALFEA--LGRG  target    PTKLIWFGNSNSLLGNAKWSFDVVKNTLPRQD-AVFCNEWHWT-SSCEYADLVFPADSWAEFKLPDATASCTNPFLLAFP 2v3v.1    DVKCMIICETNPAHTLPN--LNKVHKAMSHPESFIVCIEAFPDAVTLEYADLVLPPAFWCERD--G-VYGCGERRYSL-T  target    TTPLKRLYDTRSDYEALALTAKALGELID---EPRMEQYWRGILDGDPTPYLQRIFSGSNATRGITYDELHESSKRGVPL 2v3v.1    EKAVDPPGQCRPTVNTLVEFARRAGVDPQLVNFRNAEDVWNEWRM--------VSKGTTYDFWGMTRERLRKES--GLIW  target    LMNMRTYPR-SGGWEQ----------RQEDKPWYTATGRLEFYRPEPEFQAAGESLPVWREPVDATFYEPNAILSNAAHP 2v3v.1    PCPSEDHPGTSLRYVRGQDPCVPADHPDRFFFYGKPDGRAVIWM----------------RPAKGA--------------  target    SIAPRAPEDYGVPESQLDVETRQYRNVVRTWAELQQTLHPLQERDPAFRFVFQTPKYRWGAHSTA--VDADWISMLFGPF 2v3v.1    ---------------------------------------A-EEPDAEYPLYLTSMRVIDHWHTATMTGKVPELQKA----  target    GDPYRRDPRMPWTGEAYLEINPKDAAELGLADGDYAWVDADPEDRPYRGWKEDDPYYEVARAMMRVRIYTGMSRGVIRTW 2v3v.1    ------------NPIAFVEINEEDAARTGIKHGDSVIVETR-----------------RDAMELPARVSDVCRPGLIAVP  target    FNMYAATPATVANQKATPGNPARNEQTRYVALFRYGSHQSGTRAWLRPTQQTDSLVRKGYFGQVIGTGFEADVHSVSGAP 2v3v.1    FFD-----------------------------------------------------------------------------  target    KEAFVKIEKAEDGGIGAERLWRPLTLGLRPEAPSAALTAYLAGDYSGTKGS 2v3v.1    --------------------------------------------------- ``` | | | | | | | | | | | | | | | | | | | | | | | | | | | | | | | | | | | | | | | | | | | | | | | | | |
|  | 1g8j.1.A | ARSENITE OXIDASE  *CRYSTAL STRUCTURE ANALYSIS OF ARSENITE OXIDASE FROM ALCALIGENES FAECALIS* | 0.28 |  | 18.83 | 0.56 | 2-703 | X-ray | 2.03 | hetero-oligomer | 2 x MGD, 1 x O, 1 x 4MO, 1 x F3S, 1 x FES | HHblits | 0.29 |
| ``` target    MPTANKA--------DEVIILRPGTDAAFFLGVARELIEKGLYDRAAVIERTDLPLLVRLDTGERLDARDVIPGYELAAL 1g8j.1    -PSVAIARHVAGNDRVLHLAIEPGTDTALFNGLFTYVVEQGWIDKPFIEAHTK--------------------G------  target    TNYVTLKPDAEIKGNPPPPPFTAGGQVVPTELRDAWGDFVWWDRATGRPRPVSRDEVGARFDGDPALLGEFEVELVDGST 1g8j.1    --------------------------------------------------------------------------------  target    VPVRPAFDLLKQYLDESFDLRTASEVCRVPPQAIQSIARQLAANKR-----ETLLAAGMGPNHYFQNDLFGRVQFLVAAL 1g8j.1    ------FDDAVK-TN-RLSLDECSNITGVPVDMLKRAAEWSYKPKASGQAPRTMHAYEKGIIWGNDNYVIQSALLDLVIA  target    TDNIGHLGGNVGSYAGNYRGSVFQAMGQWIAEDPFAIEPDLTKPATVKRYYKAESAHYWNYGERPLRAVAKDDEGDLTKG 1g8j.1    THNVGRRGTGCVRMGGHQEGYTR---PPYPGDKKIYIDQEL-IKGKGR------IMTWWG--CNNFQTS---NNAQALRE  target    EVLTGKSHMPTPTKLIWFGNSNSLLGNAKWSFDVVKNTLPRQD-AVFCNEWHWTSSCEYADLVFPADSWAEFKLPDATAS 1g8j.1    AILQR---SAIVKQAMQKARGATTEEM----VDVIYEATQNGGLFVTSINLYPTKLAEAAHLMLPAAHPGEMNL---TSM  target    CTNPFLLAFPTTPLKRLYDTRSDYEALALTAKALGELIDEPRM---EQYWRGILDGDPTPYLQRIFSGSN---------- 1g8j.1    NGERRIRL-SEKFMDPPGTAMADCLIAARIANALRDMYQKDGKAEMAAQFEGFDWKTEEDAFNDGFRRAGQPGAPAIDSQ  target    ---ATRGITYDELHESSKRGVPLLMN-MRTYPRSGGWEQRQEDKPWYTATGRLEFYRPEPEFQAAGESLPVWREPVDATF 1g8j.1    GGSTGHLVTYDRLRKSGNNGVQLPVVSWDESKGLVGTEMLYTEGKFDTDDGKAHFKPAPW------NGLPATVQ------  target    YEPNAILSNAAHPSIAPRAPEDYGVPESQLDVETRQYRNVVRTWAELQQTLHPLQERDPAFRFVFQTPKYRWGAHSTAV- 1g8j.1    -------------------------------------------------------QQKDKYRFWLNNGRNNEVWQTAYHD  target    -DADWISMLFGPFGDPYRRDPRMPWTGEAYLEINPKDAAELGLADGDYAWVDADPEDRPYRGWKEDDPYYEVARAMMRVR 1g8j.1    QYNSLMQERY----------------PMAYIEMNPDDCKQLDVTGGDIVEVYND-----------------FGSTFAMVY  target    IYTGMSRGVIRTWFNMYAATPATVANQKATPGNPARNEQTRYVALFRYGSHQSGTRAWLRPTQQTDSLVRKGYFGQVIGT 1g8j.1    PVAEIKRGQTFMLFGY----------------------------------------------------------------  target    GFEADVHSVSGAPKEAFVKIEKAEDGGIGAERLWRPLTLGLRPEAPSAALTAYLAGDYSGTKGS 1g8j.1    ---------------------------------------------------------------- ``` | | | | | | | | | | | | | | | | | | | | | | | | | | | | | | | | | | | | | | | | | | | | | | | | | |
|  | 1g8k.1.A | ARSENITE OXIDASE  *CRYSTAL STRUCTURE ANALYSIS OF ARSENITE OXIDASE FROM ALCALIGENES FAECALIS* | 0.28 |  | 18.83 | 0.56 | 2-703 | X-ray | 1.64 | hetero-1-1-mer | 3 x HG, 2 x CA, 2 x MGD, 1 x O, 1 x 4MO, 1 x F3S, 1 x FES | HHblits | 0.29 |
| ``` target    MPTANKA--------DEVIILRPGTDAAFFLGVARELIEKGLYDRAAVIERTDLPLLVRLDTGERLDARDVIPGYELAAL 1g8k.1    -PSVAIARHVAGNDRVLHLAIEPGTDTALFNGLFTYVVEQGWIDKPFIEAHTK--------------------G------  target    TNYVTLKPDAEIKGNPPPPPFTAGGQVVPTELRDAWGDFVWWDRATGRPRPVSRDEVGARFDGDPALLGEFEVELVDGST 1g8k.1    --------------------------------------------------------------------------------  target    VPVRPAFDLLKQYLDESFDLRTASEVCRVPPQAIQSIARQLAANKR-----ETLLAAGMGPNHYFQNDLFGRVQFLVAAL 1g8k.1    ------FDDAVK-TN-RLSLDECSNITGVPVDMLKRAAEWSYKPKASGQAPRTMHAYEKGIIWGNDNYVIQSALLDLVIA  target    TDNIGHLGGNVGSYAGNYRGSVFQAMGQWIAEDPFAIEPDLTKPATVKRYYKAESAHYWNYGERPLRAVAKDDEGDLTKG 1g8k.1    THNVGRRGTGCVRMGGHQEGYTR---PPYPGDKKIYIDQELI-KGKGR------IMTWWG--CNNFQTSN---NA-QALR  target    EVLTGKSHMPTPTKLIWFGNSNSLLGNAKWSFDVVKNTLPR-QDAVFCNEWHWTSSCEYADLVFPADSWAEFKLPDATAS 1g8k.1    EAILQ--RSAIVKQAMQKARGATTE--EM--VDVIYEATQNGGLFVTSINLYPTKLAEAAHLMLPAAHPGEMN---LTSM  target    CTNPFLLAFPTTPLKRLYDTRSDYEALALTAKALGELIDEP---RMEQYWRGILDGDPTPYLQRIFSGSN---------- 1g8k.1    NGERRIRL-SEKFMDPPGTAMADCLIAARIANALRDMYQKDGKAEMAAQFEGFDWKTEEDAFNDGFRRAGQPGAPAIDSQ  target    ---ATRGITYDELHESSKRGVPLLMNMRTY-PRSGGWEQRQEDKPWYTATGRLEFYRPEPEFQAAGESLPVWREPVDATF 1g8k.1    GGSTGHLVTYDRLRKSGNNGVQLPVVSWDESKGLVGTEMLYTEGKFDTDDGKAHFKPAPW------NGLPATVQ------  target    YEPNAILSNAAHPSIAPRAPEDYGVPESQLDVETRQYRNVVRTWAELQQTLHPLQERDPAFRFVFQTPKYRWGAHSTAV- 1g8k.1    -------------------------------------------------------QQKDKYRFWLNNGRNNEVWQTAYHD  target    -DADWISMLFGPFGDPYRRDPRMPWTGEAYLEINPKDAAELGLADGDYAWVDADPEDRPYRGWKEDDPYYEVARAMMRVR 1g8k.1    QYNSLMQERY----------------PMAYIEMNPDDCKQLDVTGGDIVEVYND-----------------FGSTFAMVY  target    IYTGMSRGVIRTWFNMYAATPATVANQKATPGNPARNEQTRYVALFRYGSHQSGTRAWLRPTQQTDSLVRKGYFGQVIGT 1g8k.1    PVAEIKRGQTFMLFGY----------------------------------------------------------------  target    GFEADVHSVSGAPKEAFVKIEKAEDGGIGAERLWRPLTLGLRPEAPSAALTAYLAGDYSGTKGS 1g8k.1    ---------------------------------------------------------------- ``` | | | | | | | | | | | | | | | | | | | | | | | | | | | | | | | | | | | | | | | | | | | | | | | | | |
|  | 1aa6.1.A | FORMATE DEHYDROGENASE H  *REDUCED FORM OF FORMATE DEHYDROGENASE H FROM E. COLI* | 0.32 | 0.00 | 19.87 | 0.54 | 2-703 | X-ray | 2.30 | monomer | 1 x SF4, 2 x MGD, 1 x 4MO | HHblits | 0.31 |
| ``` target    MPTANKADEVIILRPGTDAAFFLGVARELIEKGLYDRAAVIERTDLPLLVRLDTGERLDARDVIPGYELAALTNYVTLKP 1aa6.1    -ETARIADMHIALKNGSNIALLNAMGHVIIEENLYDKAFVASRTE--------------------G--------------  target    DAEIKGNPPPPPFTAGGQVVPTELRDAWGDFVWWDRATGRPRPVSRDEVGARFDGDPALLGEFEVELVDGSTVPVRPAFD 1aa6.1    ------------------------------------------------------------------------------FE  target    LLKQYLDESFDLRTASEVCRVPPQAIQSIARQLAANKRETLLAAGMGPNHYFQNDLFGRVQFLVAALTDNIGHLGGNVGS 1aa6.1    EYRKIVE-GYTPESVEDITGVSASEIRQAARMYAQAKSAAI-LWGMGVTQFYQGVETVRSLTSLAMLTGNLGKPHAGVNP  target    YAGNYRGSVFQAMGQWIAEDPFAIEPDLTKPATVKRYYKAESAHYWNYGERPLRAVAKDDEGDLTKGEVLTGKSHMPTPT 1aa6.1    VRGQNNVQGACDMGALPD--TYPGYQYVKDPANREKF-----AKAWGVESLP-------AHTGYRISELPHRA--AHGEV  target    KLIWFGNSNSLLGNAKWSFDVVKNTLPRQDAVFCNEWHWTSSCEYADLVFPADSWAEFKLPDATASCTNPFLLAFPTTPL 1aa6.1    RAAYIMGEDPLQTDAE--LSAVRKAFEDLELVIVQDIFMTKTASAADVILPSTSWGEHE--GV-FTAADRGFQRF-FKAV  target    KRLYDTRSDYEALALTAKALGELIDEPRMEQYWRGILDGDPTPYLQRIFSGSNATRGITYDELHESSKRGVPLLMNMRTY 1aa6.1    EPKWDLKTDWQIISEIATRMGYPMHYNNTQEI------------WDELRHLCPDFYGATYEKMGELGF---IQWPCRDTS  target    PRSGGWEQRQEDKPWYTATGRLEFYRPEPEFQAAGESLPVWREPVDATFYEPNAILSNAAHPSIAPRAPEDYGVPESQLD 1aa6.1    DAD-QGTSYLFKEKFDTPNGLAQFFTCD------------WVAPID----------------------------------  target    VETRQYRNVVRTWAELQQTLHPLQERDPAFRFVFQTPKYR--WGAHSTAVDADWISMLFGPFGDPYRRDPRMPWTGEAYL 1aa6.1    ------------------------KLTDEYPMVLSTVREVGHYSCRSMTGNCAALAALA---------------DEPGYA  target    EINPKDAAELGLADGDYAWVDADPEDRPYRGWKEDDPYYEVARAMMRVRIYTGMSRGVIRTWFNMYAATPATVANQKATP 1aa6.1    QINTEDAKRLGIEDEALVWVHSR-----------------KGKIITRAQVSDRPNKGAIYMTYQW---------------  target    GNPARNEQTRYVALFRYGSHQSGTRAWLRPTQQTDSLVRKGYFGQVIGTGFEADVHSVSGAPKEAFVKIEKAEDGGIGAE 1aa6.1    --------------------------------------------------------------------------------  target    RLWRPLTLGLRPEAPSAALTAYLAGDYSGTKGS 1aa6.1    --------------------------------- ``` | | | | | | | | | | | | | | | | | | | | | | | | | | | | | | | | | | | | | | | | | | | | | | | | | |
|  | 1fdo.1.A | FORMATE DEHYDROGENASE H  *OXIDIZED FORM OF FORMATE DEHYDROGENASE H FROM E. COLI* | 0.32 | 0.00 | 19.87 | 0.54 | 2-703 | X-ray | 2.80 | monomer | 1 x SF4, 2 x MGD, 1 x 6MO | HHblits | 0.31 |
| ``` target    MPTANKADEVIILRPGTDAAFFLGVARELIEKGLYDRAAVIERTDLPLLVRLDTGERLDARDVIPGYELAALTNYVTLKP 1fdo.1    -ETARIADMHIALKNGSNIALLNAMGHVIIEENLYDKAFVASRTE--------------------G--------------  target    DAEIKGNPPPPPFTAGGQVVPTELRDAWGDFVWWDRATGRPRPVSRDEVGARFDGDPALLGEFEVELVDGSTVPVRPAFD 1fdo.1    ------------------------------------------------------------------------------FE  target    LLKQYLDESFDLRTASEVCRVPPQAIQSIARQLAANKRETLLAAGMGPNHYFQNDLFGRVQFLVAALTDNIGHLGGNVGS 1fdo.1    EYRKIVE-GYTPESVEDITGVSASEIRQAARMYAQAKSAAI-LWGMGVTQFYQGVETVRSLTSLAMLTGNLGKPHAGVNP  target    YAGNYRGSVFQAMGQWIAEDPFAIEPDLTKPATVKRYYKAESAHYWNYGERPLRAVAKDDEGDLTKGEVLTGKSHMPTPT 1fdo.1    VRGQNNVQGACDMGALPD--TYPGYQYVKDPANREKF-----AKAWGVESLP-------AHTGYRISELPHRA--AHGEV  target    KLIWFGNSNSLLGNAKWSFDVVKNTLPRQDAVFCNEWHWTSSCEYADLVFPADSWAEFKLPDATASCTNPFLLAFPTTPL 1fdo.1    RAAYIMGEDPLQTDAE--LSAVRKAFEDLELVIVQDIFMTKTASAADVILPSTSWGEHE--GV-FTAADRGFQRF-FKAV  target    KRLYDTRSDYEALALTAKALGELIDEPRMEQYWRGILDGDPTPYLQRIFSGSNATRGITYDELHESSKRGVPLLMNMRTY 1fdo.1    EPKWDLKTDWQIISEIATRMGYPMHYNNTQEI------------WDELRHLCPDFYGATYEKMGELGF---IQWPCRDTS  target    PRSGGWEQRQEDKPWYTATGRLEFYRPEPEFQAAGESLPVWREPVDATFYEPNAILSNAAHPSIAPRAPEDYGVPESQLD 1fdo.1    DAD-QGTSYLFKEKFDTPNGLAQFFTCD------------WVAPID----------------------------------  target    VETRQYRNVVRTWAELQQTLHPLQERDPAFRFVFQTPKYR--WGAHSTAVDADWISMLFGPFGDPYRRDPRMPWTGEAYL 1fdo.1    ------------------------KLTDEYPMVLSTVREVGHYSCRSMTGNCAALAALA---------------DEPGYA  target    EINPKDAAELGLADGDYAWVDADPEDRPYRGWKEDDPYYEVARAMMRVRIYTGMSRGVIRTWFNMYAATPATVANQKATP 1fdo.1    QINTEDAKRLGIEDEALVWVHSR-----------------KGKIITRAQVSDRPNKGAIYMTYQW---------------  target    GNPARNEQTRYVALFRYGSHQSGTRAWLRPTQQTDSLVRKGYFGQVIGTGFEADVHSVSGAPKEAFVKIEKAEDGGIGAE 1fdo.1    --------------------------------------------------------------------------------  target    RLWRPLTLGLRPEAPSAALTAYLAGDYSGTKGS 1fdo.1    --------------------------------- ``` | | | | | | | | | | | | | | | | | | | | | | | | | | | | | | | | | | | | | | | | | | | | | | | | | |
|  | 2iv2.1.A | Formate dehydrogenase H  *Reinterpretation of reduced form of formate dehydrogenase H from E. coli* | 0.32 | 0.00 | 19.87 | 0.54 | 2-703 | X-ray | 2.27 | monomer | 1 x SF4, 1 x 2MD, 1 x MGD | HHblits | 0.31 |
| ``` target    MPTANKADEVIILRPGTDAAFFLGVARELIEKGLYDRAAVIERTDLPLLVRLDTGERLDARDVIPGYELAALTNYVTLKP 2iv2.1    -ETARIADMHIALKNGSNIALLNAMGHVIIEENLYDKAFVASRTE--------------------G--------------  target    DAEIKGNPPPPPFTAGGQVVPTELRDAWGDFVWWDRATGRPRPVSRDEVGARFDGDPALLGEFEVELVDGSTVPVRPAFD 2iv2.1    ------------------------------------------------------------------------------FE  target    LLKQYLDESFDLRTASEVCRVPPQAIQSIARQLAANKRETLLAAGMGPNHYFQNDLFGRVQFLVAALTDNIGHLGGNVGS 2iv2.1    EYRKIVE-GYTPESVEDITGVSASEIRQAARMYAQAKSAAI-LWGMGVTQFYQGVETVRSLTSLAMLTGNLGKPHAGVNP  target    YAGNYRGSVFQAMGQWIAEDPFAIEPDLTKPATVKRYYKAESAHYWNYGERPLRAVAKDDEGDLTKGEVLTGKSHMPTPT 2iv2.1    VRGQNNVQGACDMGALPD--TYPGYQYVKDPANREKF-----AKAWGVESLP-------AHTGYRISELPHRA--AHGEV  target    KLIWFGNSNSLLGNAKWSFDVVKNTLPRQDAVFCNEWHWTSSCEYADLVFPADSWAEFKLPDATASCTNPFLLAFPTTPL 2iv2.1    RAAYIMGEDPLQTDAE--LSAVRKAFEDLELVIVQDIFMTKTASAADVILPSTSWGEHE--GV-FTAADRGFQRF-FKAV  target    KRLYDTRSDYEALALTAKALGELIDEPRMEQYWRGILDGDPTPYLQRIFSGSNATRGITYDELHESSKRGVPLLMNMRTY 2iv2.1    EPKWDLKTDWQIISEIATRMGYPMHYNNTQEI------------WDELRHLCPDFYGATYEKMGELGF---IQWPCRDTS  target    PRSGGWEQRQEDKPWYTATGRLEFYRPEPEFQAAGESLPVWREPVDATFYEPNAILSNAAHPSIAPRAPEDYGVPESQLD 2iv2.1    DAD-QGTSYLFKEKFDTPNGLAQFFTCD------------WVAPID----------------------------------  target    VETRQYRNVVRTWAELQQTLHPLQERDPAFRFVFQTPKYR--WGAHSTAVDADWISMLFGPFGDPYRRDPRMPWTGEAYL 2iv2.1    ------------------------KLTDEYPMVLSTVREVGHYSCRSMTGNCAALAALA---------------DEPGYA  target    EINPKDAAELGLADGDYAWVDADPEDRPYRGWKEDDPYYEVARAMMRVRIYTGMSRGVIRTWFNMYAATPATVANQKATP 2iv2.1    QINTEDAKRLGIEDEALVWVHSR-----------------KGKIITRAQVSDRPNKGAIYMTYQW---------------  target    GNPARNEQTRYVALFRYGSHQSGTRAWLRPTQQTDSLVRKGYFGQVIGTGFEADVHSVSGAPKEAFVKIEKAEDGGIGAE 2iv2.1    --------------------------------------------------------------------------------  target    RLWRPLTLGLRPEAPSAALTAYLAGDYSGTKGS 2iv2.1    --------------------------------- ``` | | | | | | | | | | | | | | | | | | | | | | | | | | | | | | | | | | | | | | | | | | | | | | | | | |
|  | 7z0t.1.G | Formate dehydrogenase H  *Structure of the Escherichia coli formate hydrogenlyase complex (aerobic preparation, composite structure)* | 0.31 | 0.00 | 19.87 | 0.54 | 2-703 | EM | 0.00 | monomer | 1 x NI, 1 x FCO, 8 x SF4, 1 x FE, 2 x MGD, 1 x 6MO | HHblits | 0.31 |
| ``` target    MPTANKADEVIILRPGTDAAFFLGVARELIEKGLYDRAAVIERTDLPLLVRLDTGERLDARDVIPGYELAALTNYVTLKP 7z0t.1    -ETARIADMHIALKNGSNIALLNAMGHVIIEENLYDKAFVASRTE--------------------G--------------  target    DAEIKGNPPPPPFTAGGQVVPTELRDAWGDFVWWDRATGRPRPVSRDEVGARFDGDPALLGEFEVELVDGSTVPVRPAFD 7z0t.1    ------------------------------------------------------------------------------FE  target    LLKQYLDESFDLRTASEVCRVPPQAIQSIARQLAANKRETLLAAGMGPNHYFQNDLFGRVQFLVAALTDNIGHLGGNVGS 7z0t.1    EYRKIVE-GYTPESVEDITGVSASEIRQAARMYAQAKSAAI-LWGMGVTQFYQGVETVRSLTSLAMLTGNLGKPHAGVNP  target    YAGNYRGSVFQAMGQWIAEDPFAIEPDLTKPATVKRYYKAESAHYWNYGERPLRAVAKDDEGDLTKGEVLTGKSHMPTPT 7z0t.1    VRGQNNVQGACDMGALPD--TYPGYQYVKDPANREKF-----AKAWGVESLP-------AHTGYRISELPHRA--AHGEV  target    KLIWFGNSNSLLGNAKWSFDVVKNTLPRQDAVFCNEWHWTSSCEYADLVFPADSWAEFKLPDATASCTNPFLLAFPTTPL 7z0t.1    RAAYIMGEDPLQTDAE--LSAVRKAFEDLELVIVQDIFMTKTASAADVILPSTSWGEHE--GV-FTAADRGFQRF-FKAV  target    KRLYDTRSDYEALALTAKALGELIDEPRMEQYWRGILDGDPTPYLQRIFSGSNATRGITYDELHESSKRGVPLLMNMRTY 7z0t.1    EPKWDLKTDWQIISEIATRMGYPMHYNNTQEI------------WDELRHLCPDFYGATYEKMGELGF---IQWPCRDTS  target    PRSGGWEQRQEDKPWYTATGRLEFYRPEPEFQAAGESLPVWREPVDATFYEPNAILSNAAHPSIAPRAPEDYGVPESQLD 7z0t.1    DAD-QGTSYLFKEKFDTPNGLAQFFTCD------------WVAPID----------------------------------  target    VETRQYRNVVRTWAELQQTLHPLQERDPAFRFVFQTPKYR--WGAHSTAVDADWISMLFGPFGDPYRRDPRMPWTGEAYL 7z0t.1    ------------------------KLTDEYPMVLSTVREVGHYSCRSMTGNCAALAALA---------------DEPGYA  target    EINPKDAAELGLADGDYAWVDADPEDRPYRGWKEDDPYYEVARAMMRVRIYTGMSRGVIRTWFNMYAATPATVANQKATP 7z0t.1    QINTEDAKRLGIEDEALVWVHSR-----------------KGKIITRAQVSDRPNKGAIYMTYQW---------------  target    GNPARNEQTRYVALFRYGSHQSGTRAWLRPTQQTDSLVRKGYFGQVIGTGFEADVHSVSGAPKEAFVKIEKAEDGGIGAE 7z0t.1    --------------------------------------------------------------------------------  target    RLWRPLTLGLRPEAPSAALTAYLAGDYSGTKGS 7z0t.1    --------------------------------- ``` | | | | | | | | | | | | | | | | | | | | | | | | | | | | | | | | | | | | | | | | | | | | | | | | | |
|  | 2vpz.1.A | THIOSULFATE REDUCTASE  *POLYSULFIDE REDUCTASE NATIVE STRUCTURE* | 0.30 |  | 19.69 | 0.55 | 2-705 | X-ray | 2.40 | hetero-oligomer | 10 x SF4, 4 x MGD, 2 x MO | HHblits | 0.29 |
| ``` target    MPTANKADEVIILRPGTDAAFFLGVARELIEKGLYDRAAVIERTDLPLLVRLDTGERLDARDVIPGYELAALTNYVTLKP 2vpz.1    -TAAAKAHRWLPIKPGTDTALLLAWIHVLIYEDLYDKEYVAKYTV-----------------------------------  target    DAEIKGNPPPPPFTAGGQVVPTELRDAWGDFVWWDRATGRPRPVSRDEVGARFDGDPALLGEFEVELVDGSTVPVRPAFD 2vpz.1    -----------------------------------------------------------------------------GFE  target    LLKQYLDESFDLRTASEVCRVPPQAIQSIARQLAANKRETLLAAGMGP-NHYFQNDLFGRVQFLVAALTDNIGHLGGNVG 2vpz.1    ELKAHVK-DFTPEWAEKHTEIPAQVIREVAREMAAHKPRAVL-PPTRHNVWYGDDTYRVMALLYVNVLLGNYGRPGGFYI  target    SYAGNYRGSVFQAMGQWIAEDPFAIEPDLTKPATVKRYYKAESAHYWN-YGERPLRAVAKDDEGDLTKGEVLTGKSHMPT 2vpz.1    AQSPYLEKYPL---PPLPL-EPAAG--GCSGPSG-GDHEP---EGFKPRADKGK--FFARSTAIQELIEPMITG---EPY  target    PTKLIWFGNSNSLLGNAKWSFDVVKNTLPRQDAVFCNEWHWTSSCEYADLVFPADSWAEFKLPDATASCTNPFLLAFPTT 2vpz.1    PIKGLFAYGINLFHSIPN--VPRTKEALKNLDLYVAIDVLPQEHVMWADVILPEATYLERYDDFVLVAHKTPFIQL-RTP  target    PLKRLYDTRSDYEALALTAKALGELIDEPRMEQYWRGILDGDPTPYLQRIFSGSNATRGITYDELHESSKRGVPLLMNMR 2vpz.1    AHEPLFDTKPGWWIARELGLRLGLEQY-------FP---WKTIEEYLETRLQS----LGLDLETMKGMGT--L-VQRG--  target    TYPRSGGWEQRQEDKPWYTATGRLEFYRPEPEFQAAGESLPVWREPVDATFYEPNAILSNAAHPSIAPRAPEDYGVPESQ 2vpz.1    -KPWLEDW-EKEGRLPFGTASGKIELYCQRFKE-AGHQPLPVFTPPEE--------------------------------  target    LDVETRQYRNVVRTWAELQQTLHPLQERDPAFRFVFQTPKYRWGAHSTAVDADWISMLFGPFGDPYRRDPRMPWTGEAYL 2vpz.1    ----------------------------PPEGFYRLLYGRSPVHTFARTQNNWVLMEMD----------------PENEV  target    EINPKDAAELGLADGDYAWVDADPEDRPYRGWKEDDPYYEVARAMM--RVRIYTGMSRGVIRTWFNMYAATPATVANQKA 2vpz.1    WIHKEEAKRLGLKEGDYVMLVNQD-----------------GVKEGPVRVKPTARIRKDCVYIVHGFGH-----------  target    TPGNPARNEQTRYVALFRYGSHQSGTRAWLRPTQQTDSLVRKGYFGQVIGTGFEADVHSVSGAPKEAFVKIEKAEDGGIG 2vpz.1    --------------------------------------------------------------------------------  target    AERLWRPLTLGLRPEAPSAALTAYLAGDYSGTKGS 2vpz.1    ----------------------------------- ``` | | | | | | | | | | | | | | | | | | | | | | | | | | | | | | | | | | | | | | | | | | | | | | | | | |
|  | 2vpx.1.D | THIOSULFATE REDUCTASE  *POLYSULFIDE REDUCTASE WITH BOUND QUINONE (UQ1)* | 0.30 |  | 19.69 | 0.55 | 2-705 | X-ray | 3.10 | hetero-oligomer | 10 x SF4, 4 x MGD, 2 x MO, 2 x UQ1 | HHblits | 0.29 |
| ``` target    MPTANKADEVIILRPGTDAAFFLGVARELIEKGLYDRAAVIERTDLPLLVRLDTGERLDARDVIPGYELAALTNYVTLKP 2vpx.1    -TAAAKAHRWLPIKPGTDTALLLAWIHVLIYEDLYDKEYVAKYTV-----------------------------------  target    DAEIKGNPPPPPFTAGGQVVPTELRDAWGDFVWWDRATGRPRPVSRDEVGARFDGDPALLGEFEVELVDGSTVPVRPAFD 2vpx.1    -----------------------------------------------------------------------------GFE  target    LLKQYLDESFDLRTASEVCRVPPQAIQSIARQLAANKRETLLAAGMGP-NHYFQNDLFGRVQFLVAALTDNIGHLGGNVG 2vpx.1    ELKAHVK-DFTPEWAEKHTEIPAQVIREVAREMAAHKPRAVL-PPTRHNVWYGDDTYRVMALLYVNVLLGNYGRPGGFYI  target    SYAGNYRGSVFQAMGQWIAEDPFAIEPDLTKPATVKRYYKAESAHYWN-YGERPLRAVAKDDEGDLTKGEVLTGKSHMPT 2vpx.1    AQSPYLEKYPL---PPLPL-EPAAG--GCSGPSG-GDHEP---EGFKPRADKGK--FFARSTAIQELIEPMITG---EPY  target    PTKLIWFGNSNSLLGNAKWSFDVVKNTLPRQDAVFCNEWHWTSSCEYADLVFPADSWAEFKLPDATASCTNPFLLAFPTT 2vpx.1    PIKGLFAYGINLFHSIPN--VPRTKEALKNLDLYVAIDVLPQEHVMWADVILPEATYLERYDDFVLVAHKTPFIQL-RTP  target    PLKRLYDTRSDYEALALTAKALGELIDEPRMEQYWRGILDGDPTPYLQRIFSGSNATRGITYDELHESSKRGVPLLMNMR 2vpx.1    AHEPLFDTKPGWWIARELGLRLGLEQY-------FP---WKTIEEYLETRLQS----LGLDLETMKGMGT--L-VQRG--  target    TYPRSGGWEQRQEDKPWYTATGRLEFYRPEPEFQAAGESLPVWREPVDATFYEPNAILSNAAHPSIAPRAPEDYGVPESQ 2vpx.1    -KPWLEDW-EKEGRLPFGTASGKIELYCQRFKE-AGHQPLPVFTPPEE--------------------------------  target    LDVETRQYRNVVRTWAELQQTLHPLQERDPAFRFVFQTPKYRWGAHSTAVDADWISMLFGPFGDPYRRDPRMPWTGEAYL 2vpx.1    ----------------------------PPEGFYRLLYGRSPVHTFARTQNNWVLMEMD----------------PENEV  target    EINPKDAAELGLADGDYAWVDADPEDRPYRGWKEDDPYYEVARAMM--RVRIYTGMSRGVIRTWFNMYAATPATVANQKA 2vpx.1    WIHKEEAKRLGLKEGDYVMLVNQD-----------------GVKEGPVRVKPTARIRKDCVYIVHGFGH-----------  target    TPGNPARNEQTRYVALFRYGSHQSGTRAWLRPTQQTDSLVRKGYFGQVIGTGFEADVHSVSGAPKEAFVKIEKAEDGGIG 2vpx.1    --------------------------------------------------------------------------------  target    AERLWRPLTLGLRPEAPSAALTAYLAGDYSGTKGS 2vpx.1    ----------------------------------- ``` | | | | | | | | | | | | | | | | | | | | | | | | | | | | | | | | | | | | | | | | | | | | | | | | | |
|  | 7bkb.1.F | Formate dehydrogenase  *Formate dehydrogenase - heterodisulfide reductase - formylmethanofuran dehydrogenase complex from Methanospirillum hungatei (hexameric, composite structure)* | 0.29 |  | 19.46 | 0.53 | 2-703 | EM | 0.00 | hetero-2-2-2-2-2-2-… | 48 x SF4, 4 x FAD, 2 x FES, 4 x 9S8, 4 x ZN, 2 x MO, 4 x MGD | HHblits | 0.30 |
| ``` target    MPTANKADEVIILRPGTDAAFFLGVARELIEKGLYDRAAVIERTDLPLLVRLDTGERLDARDVIPGYELAALTNYVTLKP 7bkb.1    -MTARLADTYVRFNPSTHIALANSMMYWIIKEGLEDKKFIQDRVN--------------------G--------------  target    DAEIKGNPPPPPFTAGGQVVPTELRDAWGDFVWWDRATGRPRPVSRDEVGARFDGDPALLGEFEVELVDGSTVPVRPAFD 7bkb.1    ------------------------------------------------------------------------------FE  target    LLKQYLDESFDLRTASEVCRVPPQAIQSIARQLAANKRETLLAAGMGPNHYFQNDLFGRVQFLVAALTDNIGHLGGNVGS 7bkb.1    DLKKTVE-NY--ADAEAIHGVPLDVVKDIAFRYAKAKNAVI-IYCLGITELTTGTDNVRSMGNLALLTGNVGREGVGVNP  target    YAGNYRGSVFQAMGQWIAEDPFAIEPDLTKPATVKRYYKAESAHYWNYGERPLRAVAKDDEGDLTKGEVLTGKSHMPTPT 7bkb.1    LRGQNNVQGACDMGAYPN--VYSGYQKCEVAENRAK-----MEKAWSVTNLP--DW-----YGATLTEQINQ---CGDEI  target    KLIWFGNSNSLLGNAKWSFDVVKNTLPRQDAVFCNEWHWTSSCEYADLVFPADSWAEFKLPDATASCTNPFLLAFPTTPL 7bkb.1    KGMYILGLNPVVTYPS--SNHVKAQLEKLDFLVVQDIFFTETCQYADVILPGACFAEKDG---TFTSGERRINRV-RKAV  target    KRLYDTRSDYEALALTAKALGELI-DEPRMEQYWRGILDGDPTPYLQRIFSGSNATRGITYDELHESSKRGVPLLMNMRT 7bkb.1    NPPGQAKEDIHIISELAAKMGFKGFELPTAK------------DVWDDMRAVTPSMFGATYEKLERPE--GICWPCPTEE  target    YPRSGGWEQRQEDKPWYTATGRLEFYRPEPEFQAAGESLPVWREPVDATFYEPNAILSNAAHPSIAPRAPEDYGVPESQL 7bkb.1    HPGTP----ILHREKFATADGKGNLFGID------------YRPPAE---------------------------------  target    DVETRQYRNVVRTWAELQQTLHPLQERDPAFRFVFQTPKYRWGAHSTAVDA--DWISMLFGPFGDPYRRDPRMPWTGEAY 7bkb.1    -------------------------VADAEYPFTLMTGRLIFHYHSRTQTDRAADLHRE----------------VPESY  target    LEINPKDAAELGLADGDYAWVDADPEDRPYRGWKEDDPYYEVARAMMRVRIYTGMSRGVIRTWFNMYAATPATVANQKAT 7bkb.1    AQINIEDARRLGIKNNEYIKLKSR-----------------RGETTTLARVTDEVAPGVVYMTMHF--------------  target    PGNPARNEQTRYVALFRYGSHQSGTRAWLRPTQQTDSLVRKGYFGQVIGTGFEADVHSVSGAPKEAFVKIEKAEDGGIGA 7bkb.1    --------------------------------------------------------------------------------  target    ERLWRPLTLGLRPEAPSAALTAYLAGDYSGTKGS 7bkb.1    ---------------------------------- ``` | | | | | | | | | | | | | | | | | | | | | | | | | | | | | | | | | | | | | | | | | | | | | | | | | |
|  | 7vw6.1.A | Formate dehydrogenase  *Cryo-EM Structure of Formate Dehydrogenase 1 from Methylorubrum extorquens AM1* | 0.30 |  | 19.46 | 0.53 | 2-703 | EM | 0.00 | hetero-1-1-mer | 4 x SF4, 2 x FES, 2 x MGD, 1 x W, 1 x FMN | HHblits | 0.30 |
| ``` target    MPTANKADEVIILRPGTDAAFFLGVARELIEKGLYDRAAVIERTDLPLLVRLDTGERLDARDVIPGYELAALTNYVTLKP 7vw6.1    -TLSRHAYRHLAFRPGSDVAMLNAMLNVIVTEGLYDEQYIAGYTE-----------------------------------  target    DAEIKGNPPPPPFTAGGQVVPTELRDAWGDFVWWDRATGRPRPVSRDEVGARFDGDPALLGEFEVELVDGSTVPVRPAFD 7vw6.1    -----------------------------------------------------------------------------NFE  target    LLKQYLDESFDLRTASEVCRVPPQAIQSIARQLAANKRETLLAAGMGPNHYFQNDLFGRVQFLVAALTDNIGHLGGNVGS 7vw6.1    ALREKIV-DFTPEKMASVCGIDAETLREVARLYARAKSSLI-FWGMGVSQHVHGTDNSRCLIALALITGQIGRPGTGLHP  target    YAGNY--RGSVFQAMGQWIAEDPFAIEPDLTKPATVKRYYKAESAHYWNYGERPLRAVAKDDEGDLTKGEVLTGKSHMPT 7vw6.1    LRGQNNVQGAS--DAGLIPM--VYPDYQSVEKDAVRE-----LFEEFWGQS---LDP-----QKGLTVVEIMRAI--HAG  target    PTKLIWFGNSNSLLGNAKWSFDVVKNTLPRQDAVFCNEWHWTSSCEYADLVFPADSWAEFKLPDATASCTNPFLLAFPTT 7vw6.1    EIRGMFVEGENPAMSDPD--LNHARHALAMLDHLVVQDLFLTETAFHADVVLPASAFAEKA---GTFTNTDRRVQIA-QP  target    PLKRLYDTRSDYEALALTAKALGELIDEPRMEQYWRGILDGDPTPYLQRIFSGSNATRGITYDELHESSKRGVPLLMNMR 7vw6.1    VVAPPGDARQDWWIIQELARRLDLDWNYGGPA------------DIFAEMAQVMPSLNNITWERLEREGA--VTYPVDAP  target    TYPRSGGWEQRQEDKPWYTATGRLEFYRPEPEFQAAGESLPVWREPVDATFYEPNAILSNAAHPSIAPRAPEDYGVPESQ 7vw6.1    DQPGN-E---IIFYAGFPTESGRAKIVPAAI------------VPPDE--------------------------------  target    LDVETRQYRNVVRTWAELQQTLHPLQERDPAFRFVFQTPKYRWGAHS--TAVDADWISMLFGPFGDPYRRDPRMPWTGEA 7vw6.1    --------------------------VPDDEFPMVLSTGRVLEHWHTGSMTRRAGVLDALE----------------PEA  target    YLEINPKDAAELGLADGDYAWVDADPEDRPYRGWKEDDPYYEVARAMMRVRIYTGMSRGVIRTWFNMYAATPATVANQKA 7vw6.1    VAFMAPKELYRLGLRPGGSMRLETR-----------------RGAVVLKVRSDRDVPIGMIFMPFCY-------------  target    TPGNPARNEQTRYVALFRYGSHQSGTRAWLRPTQQTDSLVRKGYFGQVIGTGFEADVHSVSGAPKEAFVKIEKAEDGGIG 7vw6.1    --------------------------------------------------------------------------------  target    AERLWRPLTLGLRPEAPSAALTAYLAGDYSGTKGS 7vw6.1    ----------------------------------- ``` | | | | | | | | | | | | | | | | | | | | | | | | | | | | | | | | | | | | | | | | | | | | | | | | | |
|  | 7e5z.1.A | Formate dehydrogenase  *Dehydrogenase holoenzyme* | 0.26 |  | 19.46 | 0.53 | 2-703 | EM | 0.00 | hetero-1-1-mer | 1 x W, 2 x MGD, 2 x FES, 4 x SF4, 1 x FMN | HHblits | 0.30 |
| ``` target    MPTANKADEVIILRPGTDAAFFLGVARELIEKGLYDRAAVIERTDLPLLVRLDTGERLDARDVIPGYELAALTNYVTLKP 7e5z.1    -TLSRHAYRHLAFRPGSDVAMLNAMLNVIVTEGLYDEQYIAGYTE-----------------------------------  target    DAEIKGNPPPPPFTAGGQVVPTELRDAWGDFVWWDRATGRPRPVSRDEVGARFDGDPALLGEFEVELVDGSTVPVRPAFD 7e5z.1    -----------------------------------------------------------------------------NFE  target    LLKQYLDESFDLRTASEVCRVPPQAIQSIARQLAANKRETLLAAGMGPNHYFQNDLFGRVQFLVAALTDNIGHLGGNVGS 7e5z.1    ALREKIV-DFTPEKMASVCGIDAETLREVARLYARAKSSLI-FWGMGVSQHVHGTDNSRCLIALALITGQIGRPGTGLHP  target    YAGNY--RGSVFQAMGQWIAEDPFAIEPDLTKPATVKRYYKAESAHYWNYGERPLRAVAKDDEGDLTKGEVLTGKSHMPT 7e5z.1    LRGQNNVQGAS--DAGLIPM--VYPDYQSVEKDAVRE-----LFEEFWGQS---LDP-----QKGLTVVEIMRAI--HAG  target    PTKLIWFGNSNSLLGNAKWSFDVVKNTLPRQDAVFCNEWHWTSSCEYADLVFPADSWAEFKLPDATASCTNPFLLAFPTT 7e5z.1    EIRGMFVEGENPAMSDPD--LNHARHALAMLDHLVVQDLFLTETAFHADVVLPASAFAEKA---GTFTNTDRRVQIA-QP  target    PLKRLYDTRSDYEALALTAKALGELIDEPRMEQYWRGILDGDPTPYLQRIFSGSNATRGITYDELHESSKRGVPLLMNMR 7e5z.1    VVAPPGDARQDWWIIQELARRLDLDWNYGGPA------------DIFAEMAQVMPSLNNITWERLEREGA--VTYPVDAP  target    TYPRSGGWEQRQEDKPWYTATGRLEFYRPEPEFQAAGESLPVWREPVDATFYEPNAILSNAAHPSIAPRAPEDYGVPESQ 7e5z.1    DQPGN-E---IIFYAGFPTESGRAKIVPAAI------------VPPDE--------------------------------  target    LDVETRQYRNVVRTWAELQQTLHPLQERDPAFRFVFQTPKYRWGAHS--TAVDADWISMLFGPFGDPYRRDPRMPWTGEA 7e5z.1    --------------------------VPDDEFPMVLSTGRVLEHWHTGSMTRRAGVLDALE----------------PEA  target    YLEINPKDAAELGLADGDYAWVDADPEDRPYRGWKEDDPYYEVARAMMRVRIYTGMSRGVIRTWFNMYAATPATVANQKA 7e5z.1    VAFMAPKELYRLGLRPGGSMRLETR-----------------RGAVVLKVRSDRDVPIGMIFMPFCY-------------  target    TPGNPARNEQTRYVALFRYGSHQSGTRAWLRPTQQTDSLVRKGYFGQVIGTGFEADVHSVSGAPKEAFVKIEKAEDGGIG 7e5z.1    --------------------------------------------------------------------------------  target    AERLWRPLTLGLRPEAPSAALTAYLAGDYSGTKGS 7e5z.1    ----------------------------------- ``` | | | | | | | | | | | | | | | | | | | | | | | | | | | | | | | | | | | | | | | | | | | | | | | | | |
|  | 6tg9.1.A | Formate dehydrogenase subunit alpha  *Cryo-EM Structure of NADH reduced form of NAD+-dependent Formate Dehydrogenase from Rhodobacter capsulatus* | 0.28 | 0.00 | 20.05 | 0.53 | 5-703 | EM | 3.24 | monomer | 4 x MGD, 2 x 6MO, 4 x FES, 10 x SF4, 2 x H2S, 2 x FMN, 2 x NAI | HHblits | 0.30 |
| ``` target    MPTANKADEVIILRPGTDAAFFLGVARELIEKGLYDRAAVIERTDLPLLVRLDTGERLDARDVIPGYELAALTNYVTLKP 6tg9.1    ----RGEAWHLQLKPGTNVAVMTAMAHVIVTEQIFDKRFIGDRCDW------------------DE--------------  target    DAEIKGNPPPPPFTAGGQVVPTELRDAWGDFVWWDRATGRPRPVSRDEVGARFDGDPALLGEFEVELVDGSTVPVRPAFD 6tg9.1    ------------------------------------------------------------------------------WA  target    LLKQYLD-ESFDLRTASEVCRVPPQAIQSIARQLAANKRETLLAAGMGPNHYFQNDLFGRVQFLVAALTDNIGHLGGNVG 6tg9.1    DYAEFVANPEYAPEAVESLTGVPAGLLRQAARAYAAAPNA-AIYYGLGVTEHSQGSTTVIAIANLAMMTGNIGRPGVGVN  target    SYAGNY--RGSVFQAMGQWIAEDPFAIEPDLTKPATVKRYYKAESAHYWNYGERPLRAVAKDDEGDLTKGEVLTGKSHMP 6tg9.1    PLRGQNNVQGSC--DMGSFPH--EFPGYRHVSDDATRGL-----FERTWGVTL-S-------SEPGLRIPNMLDAA--VE  target    TPTKLIWFGNSNSLLGNAKWSFDVVKNTLPRQDAVFCNEWHWTSSCEYADLVFPADSWAEFKLPDATASCTNPFLLAFPT 6tg9.1    GRFKALYVQGEDILQSDPD--TRHVSAGLAAMDLVIVHDLFLNETANYAHVFLPGSTFLEKD---GTFTNAERRINRV-R  target    TPLKRLYDTRSDYEALALTAKALGELIDEPRMEQYWRGILDGDPTPYLQRIFSGSNATRGITYDELHESSKRGVPLLMNM 6tg9.1    RVMAPKA-GFADWEVTQMLANALGAGWHYTH------------PSEIMAEIAATTPGFAAVTYEMLDARGS--VQW----  target    RTYPRSGGWEQRQEDKPWYTATGRLEFYRPEPEFQAAGESL-PVWREPVDATFYEPNAILSNAAHPSIAPRAPEDYGVPE 6tg9.1    ---PC-------NEKAPEGSPIMHVEGFVRG-----KGRFIRTAYLPTD-------------------------------  target    SQLDVETRQYRNVVRTWAELQQTLHPLQERDPAFRFVFQTPKYRWGAHSTAVDADWISMLFGPFGDPYRRDPRMPWTGEA 6tg9.1    ---------------------------EKTGPRFPLLLTTGRILSQYNVGAQTRRTEN-T--------------VWHGED  target    YLEINPKDAAELGLADGDYAWVDADPEDRPYRGWKEDDPYYEVARAMMRVRIYTGMSRGVIRTWFNMYAATPATVANQKA 6tg9.1    RLEIHPTDAETRGIRDGDWVRLASR-----------------AGETTLRATVTDRVSPGVVYTTFHH-------------  target    TPGNPARNEQTRYVALFRYGSHQSGTRAWLRPTQQTDSLVRKGYFGQVIGTGFEADVHSVSGAPKEAFVKIEKAEDGGIG 6tg9.1    --------------------------------------------------------------------------------  target    AERLWRPLTLGLRPEAPSAALTAYLAGDYSGTKGS 6tg9.1    ----------------------------------- ``` | | | | | | | | | | | | | | | | | | | | | | | | | | | | | | | | | | | | | | | | | | | | | | | | | |
|  | 7p61.1.C | NADH-quinone oxidoreductase  *Complex I from E. coli, DDM-purified, with NADH, Resting state* | 0.20 |  | 18.16 | 0.43 | 183-705 | EM | 0.00 | hetero-1-1-1-1-1-1-… | 7 x SF4, 1 x FMN, 1 x NAI, 2 x FES, 1 x CA, 2 x 3PE, 1 x UQ8 | HHblits | 0.28 |
| ``` target    MPTANKADEVIILRPGTDAAFFLGVARELIEKGLYDRAAVIERTDLPLLVRLDTGERLDARDVIPGYELAALTNYVTLKP 7p61.1    --------------------------------------------------------------------------------  target    DAEIKGNPPPPPFTAGGQVVPTELRDAWGDFVWWDRATGRPRPVSRDEVGARFDGDPALLGEFEVELVDGSTVPVRPAFD 7p61.1    --------------------------------------------------------------------------------  target    LLKQYLDESFDLRTASEVCRVPPQAIQSIARQLAANKRETLLAAGMGPNHYFQNDLFGRVQFLVAALTDNIGHLGGNVGS 7p61.1    ----------------------QSKIDVIVQALAGAKKPLI-ISGTNAG----SLEVIQAAANVAKALKGRGADVGITMI  target    YAGNYRGSVFQAMGQWIAEDPFAIEPDLTKPATVKRYYKAESAHYWNYGERPLRAVAKDDEGDLTKGEVLTGKSHMPTPT 7p61.1    ARSV------NSMGLG----------IMG-G--------------------------------GSLEEALTEL--ETGRA  target    KLIWFGNSNSLLGNAKWSFDVVKNTLPRQDAVFCNEWHWTSSCEYADLVFPADSWAEFKLPDATASCTNPFLLAFPTTPL 7p61.1    DAVVVLE-NDLHRHAS--ATRVNAALAKAPLVMVVDHQRTAIMENAHLVLSAASFAESDG---TVINNEGRAQRF-FQVY  target    KRLY-----DTRSDYEALALTAKALGEL-IDEPRMEQYWRGILDGDPTPYLQRIFSGSNATRGITYD------------- 7p61.1    DPAYYDSKTVMLESWRWLHSLHSTLLSREVDWTQLDHVIDAV------------VAKIPELAGIKDAAPDATFRIRGQKL  target    -----------------------------ELHESSKRGVPLL---MNMRTYPRSGGWE-QRQEDKPWYTATGRLEFYRPE 7p61.1    AREPHRYSGRTAMRANISVHEPRQPQDIDTMFTFSMEGNNQPTAHRSQVPFAWAPGWNSPQAWNKFQDEVGGKLRFGDPG  target    PEFQA-AGESLPVWREPVDATFYEPNAILSNAAHPSIAPRAPEDYGVPESQLDVETRQYRNVVRTWAELQQTLHPLQERD 7p61.1    VRLFETSENGLDYFTSVPA---------------------------------------------------------RFQP  target    PAFRFVFQTPKYRWGAHSTAVDADWISMLFGPFGDPYRRDPRMPWTGEAYLEINPKDAAELGLADGDYAWVDADPEDRPY 7p61.1    QDGKWRIAPYYHLFGSDELSQRAPVFQSRM----------------PQPYIKLNPADAAKLGVNAGTRVSFSYD------  target    RGWKEDDPYYEVARAMMRVRIYTGMSRGVIRTWFNMYAATPATVANQKATPGNPARNEQTRYVALFRYGSHQSGTRAWLR 7p61.1    -----------GNTVTLPVEIAEGLTAGQVGLPMGMSG------------------------------------------  target    PTQQTDSLVRKGYFGQVIGTGFEADVHSVSGAPKEAFVKIEKAEDGGIGAERLWRPLTLGLRPEAPSAALTAYLAGDYSG 7p61.1    --------------------------------------------------------------------------------  target    TKGS 7p61.1    ---- ``` | | | | | | | | | | | | | | | | | | | | | | | | | | | | | | | | | | | | | | | | | | | | | | | | | |
|  | 6sdr.1.A | Formate dehydrogenase, alpha subunit, selenocysteine-containing  *W-formate dehydrogenase from Desulfovibrio vulgaris - Oxidized form* | 0.24 |  | 18.18 | 0.42 | 2-422 | X-ray | 2.10 | hetero-1-1-mer | 2 x MGD, 4 x SF4, 1 x H2S, 1 x W | HHblits | 0.29 |
| ``` target    MPTANKADEVIILRPGTDAAFFLGVARELIEKGLYDRAAVIERTDLPLLVRLDTGERLDARDVIPGYELAALTNYVTLKP 6sdr.1    -RTSARCDVYAPIRSGADIPFLGGLIKYILDNKLYFTDYVREYTNASLIVGEKFSF---KDGLFSGYDA-----------  target    DAEIKGNPPPPPFTAGGQVVPTELRDAWGDFVWWDRATGRPRPVSRDEVGARFDGDPALLGEFEVELVDGSTVPVRPAFD 6sdr.1    -------------AN----------KKYDK-SMWAFE-------------------LDANG---VPKRDPALKHPRCVIN  target    LLKQYLDESFDLRTASEVCRVPPQAIQSIARQLAANK---RETLLAAGMGPNHYFQNDLFGRVQFLVAALTDNIGHLGGN 6sdr.1    LLKKHYE-RYNLDKVAAITGTSKEQLQQVYKAYAATGKPDKAGTIMYAMGWTQHSVGVQNIRAMAMIQLLLGNIGVAGGG  target    VGSYAGNYRGSVFQAMGQWIAEDPFAIEPDLTKPATVKRYYK-------AESAHYWNYG--------------ERP---L 6sdr.1    VNALRGESNVQGSTDQGLLAHIWPGYNPVPNSKAATLELYNAATPQSKDPMSVNWWQNRPKYVASYLKALYPDEEPAAAY  target    RAVAKDDEG----DLTKGEVLTGKSHMPTPTKLIWFGNSNSLLGNAKWSFDVVKNTLPRQDAVFCNEWHWTSSCEY---- 6sdr.1    DYLPRIDAGRKLTDYFWLNIFEK--MDKGEFKGLFAWGMNPACGGAN--ANKNRKAMGKLEWLVNVNLFENETSSFWKGP  target    ----AD-----LVFPADSWAEFKLPDATASCTNPFLLAFPTTPLKRLYDTRSDYEALALTAKALGELIDEPRMEQYWRGI 6sdr.1    GMNPAEIGTEVFFLPCCVSIEKE--GSV-ANSGRWMQW-RYRGPKPYAETKPDGDIMLDMFKKVRE--------------  target    LDGDPTPYLQRIFSGSNATRGITYDELHESSKRGVPLLMNMRTYPRSGGWEQRQEDKPWYTATGRLEFYRPEPEFQAAGE 6sdr.1    --------------------------------------------------------------------------------  target    SLPVWREPVDATFYEPNAILSNAAHPSIAPRAPEDYGVPESQLDVETRQYRNVVRTWAELQQTLHPLQERDPAFRFVFQT 6sdr.1    --------------------------------------------------------------------------------  target    PKYRWGAHSTAVDADWISMLFGPFGDPYRRDPRMPWTGEAYLEINPKDAAELGLADGDYAWVDADPEDRPYRGWKEDDPY 6sdr.1    --------------------------------------------------------------------------------  target    YEVARAMMRVRIYTGMSRGVIRTWFNMYAATPATVANQKATPGNPARNEQTRYVALFRYGSHQSGTRAWLRPTQQTDSLV 6sdr.1    --------------------------------------------------------------------------------  target    RKGYFGQVIGTGFEADVHSVSGAPKEAFVKIEKAEDGGIGAERLWRPLTLGLRPEAPSAALTAYLAGDYSGTKGS 6sdr.1    --------------------------------------------------------------------------- ``` | | | | | | | | | | | | | | | | | | | | | | | | | | | | | | | | | | | | | | | | | | | | | | | | | |
|  | 7nz1.1.E | NADH-quinone oxidoreductase subunit G  *Respiratory complex I from Escherichia coli - focused refinement of cytoplasmic arm* | 0.20 |  | 17.88 | 0.43 | 183-705 | EM | 0.00 | hetero-1-1-1-1-1-1-… | 7 x SF4, 2 x FES, 1 x FMN, 1 x CA | HHblits | 0.28 |
| ``` target    MPTANKADEVIILRPGTDAAFFLGVARELIEKGLYDRAAVIERTDLPLLVRLDTGERLDARDVIPGYELAALTNYVTLKP 7nz1.1    --------------------------------------------------------------------------------  target    DAEIKGNPPPPPFTAGGQVVPTELRDAWGDFVWWDRATGRPRPVSRDEVGARFDGDPALLGEFEVELVDGSTVPVRPAFD 7nz1.1    --------------------------------------------------------------------------------  target    LLKQYLDESFDLRTASEVCRVPPQAIQSIARQLAANKRETLLAAGMGPNHYFQNDLFGRVQFLVAALTDNIGHLGGNVGS 7nz1.1    ----------------------QSKIDVIVQALAGAKKPLI-ISGTNAG----SLEVIQAAANVAKALKGRGADVGITMI  target    YAGNYRGSVFQAMGQWIAEDPFAIEPDLTKPATVKRYYKAESAHYWNYGERPLRAVAKDDEGDLTKGEVLTGKSHMPTPT 7nz1.1    ARSV------NSMGLG----------IMG---------------------------------GGSLEEALTEL--ETGRA  target    KLIWFGNSNSLLGNAKWSFDVVKNTLPRQDAVFCNEWHWTSSCEYADLVFPADSWAEFKLPDATASCTNPFLLAFPTTPL 7nz1.1    DAVVVLE-NDLHRHAS--AIRVNAALAKAPLVMVVDHQRTAIMENAHLVLSAASFAESDG---TVINNEGRAQRF-FQVY  target    KRLY-----DTRSDYEALALTAKALGELI-DEPRMEQYWRGILDGDPTPYLQRIFSGSNATRGITYD------------- 7nz1.1    DPAYYDSKTVMLESWRWLHSLHSTLLSREVDWTQLDHVIDAVV------------AKIPELAGIKDAAPDATFRIRGQKL  target    -----------ELHESSKRGVPL-------LMN------------M--RTYPRSGGWEQ-RQEDKPWYTATGRLEFYRPE 7nz1.1    AREPHRYSGRTAMRANISVHEPRQPQDIDTMFTFSMEGNNQPTAHRSQVPFAWAPGWNSPQAWNKFQDEVGGKLRFGDPG  target    PEFQ-AAGESLPVWREPVDATFYEPNAILSNAAHPSIAPRAPEDYGVPESQLDVETRQYRNVVRTWAELQQTLHPLQERD 7nz1.1    VRLFETSENGLDYFTSVPA---------------------------------------------------------RFQP  target    PAFRFVFQTPKYRWGAHSTAVDADWISMLFGPFGDPYRRDPRMPWTGEAYLEINPKDAAELGLADGDYAWVDADPEDRPY 7nz1.1    QDGKWRIAPYYHLFGSDELSQRAPVFQSRM----------------PQPYIKLNPADAAKLGVNAGTRVSFSYD------  target    RGWKEDDPYYEVARAMMRVRIYTGMSRGVIRTWFNMYAATPATVANQKATPGNPARNEQTRYVALFRYGSHQSGTRAWLR 7nz1.1    -----------GNTVTLPVEIAEGLTAGQVGLPMGMSG------------------------------------------  target    PTQQTDSLVRKGYFGQVIGTGFEADVHSVSGAPKEAFVKIEKAEDGGIGAERLWRPLTLGLRPEAPSAALTAYLAGDYSG 7nz1.1    --------------------------------------------------------------------------------  target    TKGS 7nz1.1    ---- ``` | | | | | | | | | | | | | | | | | | | | | | | | | | | | | | | | | | | | | | | | | | | | | | | | | |
|  | 8bqg.1.A | Formate dehydrogenase, alpha subunit, selenocysteine-containing  *W-formate dehydrogenase from Desulfovibrio vulgaris - Soaking with Formate 1 min* | 0.23 |  | 18.39 | 0.42 | 2-422 | X-ray | 1.95 | hetero-1-1-mer | 2 x MGD, 4 x SF4, 1 x H2S, 1 x W | HHblits | 0.29 |
| ``` target    MPTANKADEVIILRPGTDAAFFLGVARELIEKGLYDRAAVIERTDLPLLVRLDTGERLDARDVIPGYELAALTNYVTLKP 8bqg.1    -RTSARCDVYAPIRSGADIPFLGGLIKYILDNKLYFTDYVREYTNASLIVGEKFSF---KDGLFSGYDA-----------  target    DAEIKGNPPPPPFTAGGQVVPTELRDAWGD-FVWWDRATGRPRPVSRDEVGARFDGDPALLGEFEVELVDGSTVPVRPAF 8bqg.1    ---A----------N----------KKYDKSMWAFELDAN---------------------G---VPKRDPALKHPRCVI  target    DLLKQYLDESFDLRTASEVCRVPPQAIQSIARQLAANK---RETLLAAGMGPNHYFQNDLFGRVQFLVAALTDNIGHLGG 8bqg.1    NLLKKHYE-RYNLDKVAAITGTSKEQLQQVYKAYAATGKPDKAGTIMYAMGWTQHSVGVQNIRAMAMIQLLLGNIGVAGG  target    NVGSYAGNYR--GSVFQAMGQWIAEDPF-AIEPDLTKPATVKRYYK-------AESAHYWNY-------------G-ERP 8bqg.1    GVNALRGESNVQGST--DQGLLAHIWPGYNPVPNSKAA-TLELYNAATPQSKDPMSVNWWQNRPKYVASYLKALYPDEEP  target    ---LRAVAKDDEG----DLTKGEVLTGKSHMPTPTKLIWFGNSNSLLGNAKWSFDVVKNTLPRQDAVFCNEWHWTSSCEY 8bqg.1    AAAYDYLPRIDAGRKLTDYFWLNIFEKM--DKGEFKGLFAWGMNPACGGAN--ANKNRKAMGKLEWLVNVNLFENETSSF  target    --------AD-----LVFPADSWAEFKLPDATASCTNPFLLAFPTTPLKRLYDTRSDYEALALTAKALGELIDEPRMEQY 8bqg.1    WKGPGMNPAEIGTEVFFLPCCVSIEKE--GSV-ANSGRWMQ-WRYRGPKPYAETKPDGDIMLDMFKKVRE----------  target    WRGILDGDPTPYLQRIFSGSNATRGITYDELHESSKRGVPLLMNMRTYPRSGGWEQRQEDKPWYTATGRLEFYRPEPEFQ 8bqg.1    --------------------------------------------------------------------------------  target    AAGESLPVWREPVDATFYEPNAILSNAAHPSIAPRAPEDYGVPESQLDVETRQYRNVVRTWAELQQTLHPLQERDPAFRF 8bqg.1    --------------------------------------------------------------------------------  target    VFQTPKYRWGAHSTAVDADWISMLFGPFGDPYRRDPRMPWTGEAYLEINPKDAAELGLADGDYAWVDADPEDRPYRGWKE 8bqg.1    --------------------------------------------------------------------------------  target    DDPYYEVARAMMRVRIYTGMSRGVIRTWFNMYAATPATVANQKATPGNPARNEQTRYVALFRYGSHQSGTRAWLRPTQQT 8bqg.1    --------------------------------------------------------------------------------  target    DSLVRKGYFGQVIGTGFEADVHSVSGAPKEAFVKIEKAEDGGIGAERLWRPLTLGLRPEAPSAALTAYLAGDYSGTKGS 8bqg.1    ------------------------------------------------------------------------------- ``` | | | | | | | | | | | | | | | | | | | | | | | | | | | | | | | | | | | | | | | | | | | | | | | | | |
|  | 7p63.1.C | NADH-quinone oxidoreductase  *Complex I from E. coli, DDM/LMNG-purified, under Turnover at pH 6, Closed state* | 0.20 |  | 17.98 | 0.43 | 184-705 | EM | 0.00 | hetero-1-1-1-1-1-1-… | 7 x SF4, 1 x FMN, 1 x NAI, 2 x FES, 1 x CA, 1 x DCQ, 4 x LFA, 8 x 3PE | HHblits | 0.27 |
| ``` target    MPTANKADEVIILRPGTDAAFFLGVARELIEKGLYDRAAVIERTDLPLLVRLDTGERLDARDVIPGYELAALTNYVTLKP 7p63.1    --------------------------------------------------------------------------------  target    DAEIKGNPPPPPFTAGGQVVPTELRDAWGDFVWWDRATGRPRPVSRDEVGARFDGDPALLGEFEVELVDGSTVPVRPAFD 7p63.1    --------------------------------------------------------------------------------  target    LLKQYLDESFDLRTASEVCRVPPQAIQSIARQLAANKRETLLAAGMGPNHYFQNDLFGRVQFLVAALTDNIGHLGGNVGS 7p63.1    -----------------------SKIDVIVQALAGAKKPLI-ISGTNAG----SLEVIQAAANVAKALKGRGADVGITMI  target    YAG-NYRGSVFQAMGQWIAEDPFAIEPDLTKPATVKRYYKAESAHYWNYGERPLRAVAKDDEGDLTKGEVLTGKSHMPTP 7p63.1    ARSVNSM-------GLG----------IMGG---------------------------------GSLEEALTEL--ETGR  target    TKLIWFGNSNSLLGNAKWSFDVVKNTLPRQDAVFCNEWHWTSSCEYADLVFPADSWAEFKLPDATASCTNPFLLAFPTTP 7p63.1    ADAVVVLE-NDLHRHAS--ATRVNAALAKAPLVMVVDHQRTAIMENAHLVLSAASFAESDG---TVINNEGRAQRF-FQV  target    LKRLY-----DTRSDYEALALTAKALGELI-DEPRMEQYWRGILDGDPTPYLQRIFSGSNATRGITYDE----------- 7p63.1    YDPAYYDSKTVMLESWRWLHSLHSTLLSREVDWTQLDHVIDA------------VVAKIPELAGIKDAAPDATFRIRGQK  target    -------------LHESSKRGVPLL-------M--------------NMRTYPRSGGWEQR-QEDKPWYTATGRLEFYRP 7p63.1    LAREPHRYSGRTAMRANISVHEPRQPQDIDTMFTFSMEGNNQPTAHRSQVPFAWAPGWNSPQAWNKFQDEVGGKLRFGDP  target    EPEFQA-AGESLPVWREPVDATFYEPNAILSNAAHPSIAPRAPEDYGVPESQLDVETRQYRNVVRTWAELQQTLHPLQER 7p63.1    GVRLFETSENGLDYFTSVPA---------------------------------------------------------RFQ  target    DPAFRFVFQTPKYRWGAHSTAVDADWISMLFGPFGDPYRRDPRMPWTGEAYLEINPKDAAELGLADGDYAWVDADPEDRP 7p63.1    PQDGKWRIAPYYHLFGSDELSQRAPVFQSRM----------------PQPYIKLNPADAAKLGVNAGTRVSFSYD-----  target    YRGWKEDDPYYEVARAMMRVRIYTGMSRGVIRTWFNMYAATPATVANQKATPGNPARNEQTRYVALFRYGSHQSGTRAWL 7p63.1    ------------GNTVTLPVEIAEGLTAGQVGLPMGMSG-----------------------------------------  target    RPTQQTDSLVRKGYFGQVIGTGFEADVHSVSGAPKEAFVKIEKAEDGGIGAERLWRPLTLGLRPEAPSAALTAYLAGDYS 7p63.1    --------------------------------------------------------------------------------  target    GTKGS 7p63.1    ----- ``` | | | | | | | | | | | | | | | | | | | | | | | | | | | | | | | | | | | | | | | | | | | | | | | | | |
|  | 1h0h.1.A | FORMATE DEHYDROGENASE SUBUNIT ALPHA  *Tungsten containing Formate Dehydrogenase from Desulfovibrio Gigas* | 0.23 |  | 18.10 | 0.42 | 2-422 | X-ray | 1.80 | hetero-1-1-mer | 1 x W, 1 x 2MD, 1 x MGD, 4 x SF4, 1 x CA | HHblits | 0.29 |
| ``` target    MPTANKADEVIILRPGTDAAFFLGVARELIEKGLYDRAAVIERTDLPLLVRLDTGERLDARDVIPGYELAALTNYVTLKP 1h0h.1    -RTSTKCDLYAPLRSGSDIAFLNGMTKYILEKELYFKDYVVNYTNASFIVGEGF---AFEEG------------------  target    DAEIKGNPPPPPFTAGGQVVPTELRDAWGDFVWWDRATGRPRPVSRDEVGARFDGDPALLGEFEVELVDGSTVPVRPAFD 1h0h.1    -----------------------------LFAGYNKETRKYDKSK-------WGFERDENGNP---KRDETLKHPRCVFQ  target    LLKQYLDESFDLRTASEVCRVPPQAIQSIARQLAANK---RETLLAAGMGPNHYFQNDLFGRVQFLVAALTDNIGHLGGN 1h0h.1    IMKKHYE-RYDLDKISAICGTPKELILKVYDAYCATGKPDKAGTIMYAMGWTQHTVGVQNIRAMSINQLLLGNIGVAGGG  target    VGSYAGNY--RGSVFQAMGQWIAEDPFAIEPDLTK---PA--TV-KRYYKA----ESAHYWN-YGER--P-LR-AV---A 1h0h.1    VNALRGEANVQGST--DHGLLMH--IYPGYLGTARASIPTYEEYTKKFTPVSKDPQSANWWSNFPKYSASYIKSMWPDAD  target    KD----------DEGDLTKGEVLTGKSHMPTPTKLIWFGNSNSLLGNAKWSFDVVKNTLPRQDAVFCNEWHWTSSCEYA- 1h0h.1    LNEAYGYLPKGEDGKDYSWLTLFDDM--FQGKIKGFFAWGQNPACSGAN--SNKTREALTKLDWMVNVNIFDNETGSFWR  target    ------------DLVFPADSWAEFKLPDATASCTNPFLLAFPTTPLKRLYDTRSDYEALALTAKALGELIDEPRMEQYWR 1h0h.1    GPDMDPKKIKTEVFFLPCAVAIEKE--GS-ISNSGRWMQ-WRYVGPEPRKNAIPDGDLIVELAKRVQK------------  target    GILDGDPTPYLQRIFSGSNATRGITYDELHESSKRGVPLLMNMRTYPRSGGWEQRQEDKPWYTATGRLEFYRPEPEFQAA 1h0h.1    --------------------------------------------------------------------------------  target    GESLPVWREPVDATFYEPNAILSNAAHPSIAPRAPEDYGVPESQLDVETRQYRNVVRTWAELQQTLHPLQERDPAFRFVF 1h0h.1    --------------------------------------------------------------------------------  target    QTPKYRWGAHSTAVDADWISMLFGPFGDPYRRDPRMPWTGEAYLEINPKDAAELGLADGDYAWVDADPEDRPYRGWKEDD 1h0h.1    --------------------------------------------------------------------------------  target    PYYEVARAMMRVRIYTGMSRGVIRTWFNMYAATPATVANQKATPGNPARNEQTRYVALFRYGSHQSGTRAWLRPTQQTDS 1h0h.1    --------------------------------------------------------------------------------  target    LVRKGYFGQVIGTGFEADVHSVSGAPKEAFVKIEKAEDGGIGAERLWRPLTLGLRPEAPSAALTAYLAGDYSGTKGS 1h0h.1    ----------------------------------------------------------------------------- ``` | | | | | | | | | | | | | | | | | | | | | | | | | | | | | | | | | | | | | | | | | | | | | | | | | |
|  | 3o5a.1.A | Periplasmic nitrate reductase  *Crystal Structure of partially reduced Periplasmic Nitrate Reductase from Cupriavidus necator using Ionic Liquids* | 0.21 |  | 16.92 | 0.39 | 2-423 | X-ray | 1.72 | hetero-oligomer | 1 x SF4, 1 x MOS, 2 x MGD, 2 x HEC | HHblits | 0.28 |
| ``` target    MPTANKADEVIILRPGTDAAFFLGVARELIEKGLYDRAAVIERTDLPLLVRLDTGERLDARDVIPGYELAALTNYVTLKP 3o5a.1    -RCFDLADIGIIFKPQTDLAMLNYIANYIIRNNKVNKDFVNKHTVFKEGVTDIG-YGLRPDHPLQKA-------------  target    DAEIKGNPPPPPFTAGGQVVPTELRDAWGDFVWWDRATGRPRPVSRDEVGARFDGDPALLGEFEVELVDGSTVPVRPAFD 3o5a.1    ----------------------------------------AK------------------------NASDPGAAKVITFD  target    LLKQYLDESFDLRTASEVCRVPPQAIQSIARQLAANKRETLLAAGMGPNHYFQNDLFGRVQFLVAALTDNIGHLGGNVGS 3o5a.1    EFAKFVS-KYDADYVSKLSAVPKAKLDQLAELYADPNIKVMSLWTMGFNQHTRGTWANNMVYNLHLLTGKIATPGNSPFS  target    YAGNYRGSV-FQAMGQWIAEDPFAIEPDLTKPATVKRYYKAESAHYWNYGERPLRAVAKDDEGDLTKGEVLTGKSHMPTP 3o5a.1    LTGQPSACGTAREVGTFSH--RLPADMVVTNPKHREE-----AERIWKLPPGTI-PDK----PGY--DAVLQNRMLKDGK  target    TKLIWFGNSNSLLGNAKWSFDVVKNTLPRQDAVFCNEWHWTSSCEYADLVFPADSWAEFKLPDATASCTNPFLLAFPTTP 3o5a.1    LNAYWVQVNNNMQAAANLMEEGLPGYRNPANFIVVSDAYPTVTALAADLVLPSAMWVEKE--GA-YGNAERRTQF-WHQL  target    LKRLYDTRSDYEALALTAKALGELIDEPRMEQYWRGILDGDPTPYLQRIFSGSNATRGITYDELHESSKRGVPLLMNMRT 3o5a.1    VDAPGEARSDLWQLVEFAKRFKVE--------------------------------------------------------  target    YPRSGGWEQRQEDKPWYTATGRLEFYRPEPEFQAAGESLPVWREPVDATFYEPNAILSNAAHPSIAPRAPEDYGVPESQL 3o5a.1    --------------------------------------------------------------------------------  target    DVETRQYRNVVRTWAELQQTLHPLQERDPAFRFVFQTPKYRWGAHSTAVDADWISMLFGPFGDPYRRDPRMPWTGEAYLE 3o5a.1    --------------------------------------------------------------------------------  target    INPKDAAELGLADGDYAWVDADPEDRPYRGWKEDDPYYEVARAMMRVRIYTGMSRGVIRTWFNMYAATPATVANQKATPG 3o5a.1    --------------------------------------------------------------------------------  target    NPARNEQTRYVALFRYGSHQSGTRAWLRPTQQTDSLVRKGYFGQVIGTGFEADVHSVSGAPKEAFVKIEKAEDGGIGAER 3o5a.1    --------------------------------------------------------------------------------  target    LWRPLTLGLRPEAPSAALTAYLAGDYSGTKGS 3o5a.1    -------------------------------- ``` | | | | | | | | | | | | | | | | | | | | | | | | | | | | | | | | | | | | | | | | | | | | | | | | | |
|  | 6f0k.1.B | Fe-S-cluster-containing hydrogenase  *Alternative complex III* | 0.14 |  | 16.06 | 0.30 | 318-707 | EM | 0.00 | hetero-1-1-1-1-1-1-… | 6 x HEC, 1 x F3S, 3 x SF4 | HHblits | 0.27 |
| ``` target    MPTANKADEVIILRPGTDAAFFLGVARELIEKGLYDRAAVIERTDLPLLVRLDTGERLDARDVIPGYELAALTNYVTLKP 6f0k.1    --------------------------------------------------------------------------------  target    DAEIKGNPPPPPFTAGGQVVPTELRDAWGDFVWWDRATGRPRPVSRDEVGARFDGDPALLGEFEVELVDGSTVPVRPAFD 6f0k.1    --------------------------------------------------------------------------------  target    LLKQYLDESFDLRTASEVCRVPPQAIQSIARQLAANKRETLLAAGMGPNHYFQNDLFGRVQFLVAALTDNIGHLGGNVGS 6f0k.1    --------------------------------------------------------------------------------  target    YAGNYRGSVFQAMGQWIAEDPFAIEPDLTKPATVKRYYKAESAHYWNYGERPLRAVAKDDEGDLTKGEVLTGKSHMPTPT 6f0k.1    -----------------------------------------------------------------------------GAV  target    KLIWFGNSNSLLGNAKWSFDVVKNTLPRQDAVFCNEWHWTSSCEYADLVFPADSWAEFKLPDATASCTNPFLLAFPTTPL 6f0k.1    DALLLLNVNPVYDAPA--ALGFAEALAQVPEVIHLGLHVDETARRSTWHLPSTHYLEAWGD--GRAY-DG-TLSVIQPLI  target    KRLYDT-RSDYEALALTAKALGELIDEPRMEQYWRGILDGDPTPYLQRIFSGSNATRGITYDELHESSKRGVPLLMNMRT 6f0k.1    APLYEAAHSPLEVLALLATGEEQSA-YDLVRNTW------------RRLLAGRGA-FEQAWQRVLHD---GF--------  target    YPRSGGWEQRQEDKPWYTATGRLEFYRPEPEFQAAGESLPVWREPVDATFYEPNAILSNAAHPSIAPRAPEDYGVPESQL 6f0k.1    ------L----PDSGYPTVSLRPNR-----------QALADWPQ------------------------------------  target    DVETRQYRNVVRTWAELQQTLHPLQERDPAFRFVFQTPKYRWGAHSTAVDADWISMLFGPFGDPYRRDPRMPWTGEAYLE 6f0k.1    ---------------------------AAEGGLEVVFRLDPTVLDGSFANNAWAQELPDPI---------TKIVWDNVAI  target    INPKDAAELGLAD--------GDYAWVDADPEDRPYRGWKEDDPYYEVARAMMRVRIYTGMSRGVIRTWFNMYAATPATV 6f0k.1    LSPKTAAALGVKAEYHKGVYIADVIELSLD-----------------GRAVELPVWVLPGHPDDSITVYLGYGREI----  target    ANQKATPGNPARNEQTRYVALFRYGSHQSGTRAWLRPTQQTDSLVRKGYFGQVIGTGFEADVHSVSGAPKEAFVKIEKAE 6f0k.1    --------------------------------------------------------------------------------  target    DGGIGAERLWRPLTLGLRPEAPSAALTAYLAGDYSGTKGS 6f0k.1    ---------------------------------------- ``` | | | | | | | | | | | | | | | | | | | | | | | | | | | | | | | | | | | | | | | | | | | | | | | | | |
|  | 6lod.1.B | Fe-S-cluster-containing hydrogenase components 1-like protein  *Cryo-EM structure of the air-oxidized photosynthetic alternative complex III from Roseiflexus castenholzii* | 0.15 |  | 11.69 | 0.30 | 317-706 | EM | 0.00 | hetero-1-1-1-1-1-1-… | 6 x HEC, 2 x EL6, 3 x SF4, 1 x F3S | HHblits | 0.26 |
| ``` target    MPTANKADEVIILRPGTDAAFFLGVARELIEKGLYDRAAVIERTDLPLLVRLDTGERLDARDVIPGYELAALTNYVTLKP 6lod.1    --------------------------------------------------------------------------------  target    DAEIKGNPPPPPFTAGGQVVPTELRDAWGDFVWWDRATGRPRPVSRDEVGARFDGDPALLGEFEVELVDGSTVPVRPAFD 6lod.1    --------------------------------------------------------------------------------  target    LLKQYLDESFDLRTASEVCRVPPQAIQSIARQLAANKRETLLAAGMGPNHYFQNDLFGRVQFLVAALTDNIGHLGGNVGS 6lod.1    --------------------------------------------------------------------------------  target    YAGNYRGSVFQAMGQWIAEDPFAIEPDLTKPATVKRYYKAESAHYWNYGERPLRAVAKDDEGDLTKGEVLTGKSHMPTPT 6lod.1    ----------------------------------------------------------------------------AGTV  target    KLIWFGNSNSLLGNAKWSFDVVKNTLPRQDAVFCNEWHWTSSCEYADLVFPADSWAEFKLPDATASCTNPFLLAFPTTPL 6lod.1    EVLLMIESNPVYNAPA--DIPFAEALAKVPLSMHVGLYRDETAQQSVWHINGAHFLEAWGDVR--A-FDGT-TTIVQPLI  target    KRLYDTRSDYEALALTAKALGELIDEPRMEQYWRGILDGDPTPYLQRIFSGSNATRGITYDELHESSKRGVPLLMNMRTY 6lod.1    APLYNGKSAIEVLNVLLGKPQETG-YQTLTAYWQTQ------------DA--SGNFRVFWNTALHD---GVITA------  target    PRSGGWEQRQEDKPWYTATGRLEFYRPEPEFQAAGESLPVWREPVDATFYEPNAILSNAAHPSIAPRAPEDYGVPESQLD 6lod.1    ---------TQ---A--RSRQVTLQ----------QGFADAAPP------------------------------------  target    VETRQYRNVVRTWAELQQTLHPLQERDPAFRFVFQTPKYRWGAHSTAVDADWISMLFGPFGDPYRRDPRMPWTGEAYLEI 6lod.1    ------------------------APTQGLEIVFRPDP--SLWDGAFANNAWLQETPKPY---------TKLTWDNVALM  target    NPKDAAELGLADGDYAWVDADPEDRPYRGWKEDDPYYEVARAMMRVRIYTGMSRGVIRTWFNMYAATPATVANQKATPGN 6lod.1    SVRTANALGLKNGDVVRLTYQ-----------------GRSVDAPVWVQPGHADDSVTVHFGFGRT--------------  target    PARNEQTRYVALFRYGSHQSGTRAWLRPTQQTDSLVRKGYFGQVIGTGFEADVHSVSGAPKEAFVKIEKAEDGGIGAERL 6lod.1    --------------------------------------------------------------------------------  target    WRPLTLGLRPEAPSAALTAYLAGDYSGTKGS 6lod.1    ------------------------------- ``` | | | | | | | | | | | | | | | | | | | | | | | | | | | | | | | | | | | | | | | | | | | | | | | | | |
|  | 6btm.1.B | Alternative Complex III subunit B  *Structure of Alternative Complex III from Flavobacterium johnsoniae (Wild Type)* | 0.14 |  | 11.89 | 0.29 | 317-705 | EM | 3.40 | hetero-1-1-1-1-1-1-… | 6 x HEC, 1 x F3S, 1 x SF4, 2 x E87 | HHblits | 0.27 |
| ``` target    MPTANKADEVIILRPGTDAAFFLGVARELIEKGLYDRAAVIERTDLPLLVRLDTGERLDARDVIPGYELAALTNYVTLKP 6btm.1    --------------------------------------------------------------------------------  target    DAEIKGNPPPPPFTAGGQVVPTELRDAWGDFVWWDRATGRPRPVSRDEVGARFDGDPALLGEFEVELVDGSTVPVRPAFD 6btm.1    --------------------------------------------------------------------------------  target    LLKQYLDESFDLRTASEVCRVPPQAIQSIARQLAANKRETLLAAGMGPNHYFQNDLFGRVQFLVAALTDNIGHLGGNVGS 6btm.1    --------------------------------------------------------------------------------  target    YAGNYRGSVFQAMGQWIAEDPFAIEPDLTKPATVKRYYKAESAHYWNYGERPLRAVAKDDEGDLTKGEVLTGKSHMPTPT 6btm.1    ----------------------------------------------------------------------------AGSV  target    KLIWFGNSNSLLGNAKWSFDVVKNTLPRQDAVFCNEWHWTSSCEYADLVFPADSWAEFKLPDATASCTNPFLLAFPTTPL 6btm.1    HTLIMSGVNPVYTLAD--SASFVSGLKKVKTSVAFSLKEDETAAVSTIAAAAPHYLESWGDVEITK---G-TYSLTQPTI  target    KRLYDTRSDYEALALTAKALGELIDEPRMEQYWRGILDGDPTPYLQRIFSGSNATRGITYDELHESSKRGVPLLMNMRTY 6btm.1    RPIFDTKQFQDVLLSVNGTPGN------FYDYLK------------ANSGAI--IAGSSWNKVLHD---GIFVVG-----  target    PRSGGWEQRQEDKPWYTATGRLEFYRPEPEFQAAGESLPVWREPVDATFYEPNAILSNAAHPSIAPRAPEDYGVPESQLD 6btm.1    ----------S---AALAGGSYDFAGA--------ASL------LS----------------------------------  target    VETRQYRNVVRTWAELQQTLHPLQERDPAFRFVFQTPKYRWGAHSTAVDADWISMLFGPFGDPYRRDPRMPWTGEAYLEI 6btm.1    ------------------------KAKSSGELELVLYTKTGMGDGQHANNPWLQEFPDP---------ITRVSWDNYVTV  target    NPKDAAELGLAD---------GDYAWVDADPEDRPYRGWKEDDPYYEVAR--AMMRVRIYTGMSRGVIRTWFNMYAATPA 6btm.1    SNADAKKFNLSNEIVANGGLNGSYATITTAD-----------------GNKLENVPVIVQPGQAVGTVGLAVGYGR----  target    TVANQKATPGNPARNEQTRYVALFRYGSHQSGTRAWLRPTQQTDSLVRKGYFGQVIGTGFEADVHSVSGAPKEAFVKIEK 6btm.1    --------------------------------------------------------------------------------  target    AEDGGIGAERLWRPLTLGLRPEAPSAALTAYLAGDYSGTKGS 6btm.1    ------------------------------------------ ``` | | | | | | | | | | | | | | | | | | | | | | | | | | | | | | | | | | | | | | | | | | | | | | | | | |
|  | 2ivf.1.A | ETHYLBENZENE DEHYDROGENASE ALPHA-SUBUNIT  *ETHYLBENZENE DEHYDROGENASE FROM AROMATOLEUM AROMATICUM* | 0.16 | 0.00 | 40.10 | 0.24 | 2-245 | X-ray | 1.88 | monomer | 1 x MES, 4 x SF4, 1 x MO, 1 x MGD, 1 x MD1, 1 x F3S, 1 x HEM | BLAST | 0.39 |
| ``` target    MPTANKADEVIILRPGTDAAFFLGVARELIEKGLYDRAAVIERTDLPLLVRLDTGERLDARDVIPGYELAALTNYVTLKP 2ivf.1    -PTTPAADLHVPVRVGSDAAFWLGLSQVMIDEKLFDRQFVCEQTDLPLLVRMDTGKFLSAEDV-----------------  target    DAEIKGNPPPPPFTAGGQVVPTELRDAWGDFVWWDRATGRPRPVSRDEVGARFDGDPALLGEFEVELVDGSTVPVRPAFD 2ivf.1    --------------DGGEA---------KQFYFFDEKAGSVRKASRGTL--KLDFMPALEGTFSARLKNGKTIQVRTVFE  target    LLKQYLDESFDLRTASEVCRVPPQAIQSIARQLAANKRETLLAAGMGPNHYFQNDLFGRVQFLVAALTDNIGHLGGNVGS 2ivf.1    GLREHLKD-YTPEKASAKCGVPVSLIRELGRKVA--KKRTCSYIGFSSAKSYHGDLMERSLFLAMALSGNWGKPG--TGA  target    YAGNYRGSVFQAMGQWIAEDPFAIEPDLTKPATVKRYYKAESAHYWNYGERPLRAVAKDDEGDLTKGEVLTGKSHMPTPT 2ivf.1    FAWAY---------------------------------------------------------------------------  target    KLIWFGNSNSLLGNAKWSFDVVKNTLPRQDAVFCNEWHWTSSCEYADLVFPADSWAEFKLPDATASCTNPFLLAFPTTPL 2ivf.1    --------------------------------------------------------------------------------  target    KRLYDTRSDYEALALTAKALGELIDEPRMEQYWRGILDGDPTPYLQRIFSGSNATRGITYDELHESSKRGVPLLMNMRTY 2ivf.1    --------------------------------------------------------------------------------  target    PRSGGWEQRQEDKPWYTATGRLEFYRPEPEFQAAGESLPVWREPVDATFYEPNAILSNAAHPSIAPRAPEDYGVPESQLD 2ivf.1    --------------------------------------------------------------------------------  target    VETRQYRNVVRTWAELQQTLHPLQERDPAFRFVFQTPKYRWGAHSTAVDADWISMLFGPFGDPYRRDPRMPWTGEAYLEI 2ivf.1    --------------------------------------------------------------------------------  target    NPKDAAELGLADGDYAWVDADPEDRPYRGWKEDDPYYEVARAMMRVRIYTGMSRGVIRTWFNMYAATPATVANQKATPGN 2ivf.1    --------------------------------------------------------------------------------  target    PARNEQTRYVALFRYGSHQSGTRAWLRPTQQTDSLVRKGYFGQVIGTGFEADVHSVSGAPKEAFVKIEKAEDGGIGAERL 2ivf.1    --------------------------------------------------------------------------------  target    WRPLTLGLRPEAPSAALTAYLAGDYSGTKGS 2ivf.1    ------------------------------- ``` | | | | | | | | | | | | | | | | | | | | | | | | | | | | | | | | | | | | | | | | | | | | | | | | | |
|  | 8e9g.1.G | NADH-quinone oxidoreductase subunit G  *Mycobacterial respiratory complex I with both quinone positions modelled* | 0.14 |  | 16.44 | 0.26 | 317-705 | EM | 0.00 | hetero-1-1-1-1-1-1-… |  | HHblits | 0.27 |
| ``` target    MPTANKADEVIILRPGTDAAFFLGVARELIEKGLYDRAAVIERTDLPLLVRLDTGERLDARDVIPGYELAALTNYVTLKP 8e9g.1    --------------------------------------------------------------------------------  target    DAEIKGNPPPPPFTAGGQVVPTELRDAWGDFVWWDRATGRPRPVSRDEVGARFDGDPALLGEFEVELVDGSTVPVRPAFD 8e9g.1    --------------------------------------------------------------------------------  target    LLKQYLDESFDLRTASEVCRVPPQAIQSIARQLAANKRETLLAAGMGPNHYFQNDLFGRVQFLVAALTDNIGHLGGNVGS 8e9g.1    --------------------------------------------------------------------------------  target    YAGNYRGSVFQAMGQWIAEDPFAIEPDLTKPATVKRYYKAESAHYWNYGERPLRAVAKDDEGDLTKGEVLTGKSHMPTPT 8e9g.1    ----------------------------------------------------------------------------SGHL  target    KLIWFGNSNSLLGNAKWSFDVVKNTLPRQDAVFCNEWHWTSSCEYADLVFPADSWAEFKLPDATASCTNPFLLAFPTTPL 8e9g.1    AALLVGG-VELGDLPD--PELAVAAVRTTPFVVSLELRESAVTELADVVFPVAPVVEKAG---SFLNWEGRPRPF-APSL  target    KRLYDTRSDYEALALTAKALGELIDEPRMEQYWRGILDGDPTPYLQRIFSGSNATRGITYDELHESSKRGVPLLMNMRTY 8e9g.1    --KTNAIPDLRVLHYLADEIGVDLALPTA------------EAA---------------DAELAQL---GT---------  target    PRSGGWEQRQEDKPWYTATGRLEFYRPEPEFQAAGESLPVWREPVDATFYEPNAILSNAAHPSIAPRAPEDYGVPESQLD 8e9g.1    -----WG----GARPPAPT---------------------A-PPTAR---------------------------------  target    VETRQYRNVVRTWAELQQTLHPLQERDPAFRFVFQTPKYRWGAHSTAVDADWISMLFGPFGDPYRRDPRMPWTGEAYLEI 8e9g.1    ------------------------PEAGSGQAVLASWRMLLDAGRLQDGEPHLAGTA----------------VRPVARM  target    NPKDAAELGLADGDYAWVDADPEDRPYRGWKEDDPYYEVARAMMRVRIYTGMSRGVIRTWFNMYAATPATVANQKATPGN 8e9g.1    SAATAAGIGASDGAPVTVSTE-----------------RGAVTLPLAVTD-MPDGVVWLPMNSPG---------------  target    PARNEQTRYVALFRYGSHQSGTRAWLRPTQQTDSLVRKGYFGQVIGTGFEADVHSVSGAPKEAFVKIEKAEDGGIGAERL 8e9g.1    --------------------------------------------------------------------------------  target    WRPLTLGLRPEAPSAALTAYLAGDYSGTKGS 8e9g.1    ------------------------------- ``` | | | | | | | | | | | | | | | | | | | | | | | | | | | | | | | | | | | | | | | | | | | | | | | | | |
|  | 5t5i.1.B | Tungsten formylmethanofuran dehydrogenase subunit B  *TUNGSTEN-CONTAINING FORMYLMETHANOFURAN DEHYDROGENASE FROM METHANOTHERMOBACTER WOLFEII, ORTHORHOMBIC FORM AT 1.9 A* | 0.11 |  | 17.59 | 0.24 | 177-422 | X-ray | 1.90 | hetero-oligomer | 4 x ZN, 2 x MG, 18 x K, 22 x SF4, 2 x W, 4 x MGD, 2 x H2S, 2 x CA | HHblits | 0.28 |
| ``` target    MPTANKADEVIILRPGTDAAFFLGVARELIEKGLYDRAAVIERTDLPLLVRLDTGERLDARDVIPGYELAALTNYVTLKP 5t5i.1    --------------------------------------------------------------------------------  target    DAEIKGNPPPPPFTAGGQVVPTELRDAWGDFVWWDRATGRPRPVSRDEVGARFDGDPALLGEFEVELVDGSTVPVRPAFD 5t5i.1    --------------------------------------------------------------------------------  target    LLKQYLDESFDLRTASEVCRVPPQAIQSIARQLAANKRETLLAAGMGPNHYFQNDLFGRVQFLVAALTDNIGHLGGNVGS 5t5i.1    ----------------EVAGVPREQIEEAVEVLKNAQFGIL-FFGMGITHSRGKHRNIDTAIMMVQDLNDY--AKWTLIP  target    YAGNY--RGSVFQAMGQWIAEDPFAIEPDLTKPATVKRYYKAESAHYWNYGERPLRAVAKDDEGDLTKGEVLTGKSHMPT 5t5i.1    MRGHYNVTGFN--QVCTWE--SGYP---YC------V--------DFSGGE-PRYNP------GETGANDLL-----QNR  target    PTKLIWFGNSNSLLGNAKWSFDVVKNTLPRQDAVFCNEWHWTSSCEYADLVFPADS-WAEFKLPDATASCTNPFLLAFPT 5t5i.1    EADAMMVIASDPGAHFPQ----RALERMAEIP-VIAIEPHRTPTTEMADIIIPPAIVGMEAEG---TAYRMEGVPIRM-K  target    TPLKRLYDTRSDYEALALTAKALGELIDEPRMEQYWRGILDGDPTPYLQRIFSGSNATRGITYDELHESSKRGVPLLMNM 5t5i.1    KVVDS--DLLSDREILERLLEKVRE-------------------------------------------------------  target    RTYPRSGGWEQRQEDKPWYTATGRLEFYRPEPEFQAAGESLPVWREPVDATFYEPNAILSNAAHPSIAPRAPEDYGVPES 5t5i.1    --------------------------------------------------------------------------------  target    QLDVETRQYRNVVRTWAELQQTLHPLQERDPAFRFVFQTPKYRWGAHSTAVDADWISMLFGPFGDPYRRDPRMPWTGEAY 5t5i.1    --------------------------------------------------------------------------------  target    LEINPKDAAELGLADGDYAWVDADPEDRPYRGWKEDDPYYEVARAMMRVRIYTGMSRGVIRTWFNMYAATPATVANQKAT 5t5i.1    --------------------------------------------------------------------------------  target    PGNPARNEQTRYVALFRYGSHQSGTRAWLRPTQQTDSLVRKGYFGQVIGTGFEADVHSVSGAPKEAFVKIEKAEDGGIGA 5t5i.1    --------------------------------------------------------------------------------  target    ERLWRPLTLGLRPEAPSAALTAYLAGDYSGTKGS 5t5i.1    ---------------------------------- ``` | | | | | | | | | | | | | | | | | | | | | | | | | | | | | | | | | | | | | | | | | | | | | | | | | |
|  | 3m9s.1.C | NADH-quinone oxidoreductase subunit 3  *Crystal structure of respiratory complex I from Thermus thermophilus* | 0.11 | 0.00 | 20.00 | 0.23 | 342-707 | X-ray | 4.50 | monomer | 7 x SF4, 2 x FES, 1 x FMN | HHblits | 0.28 |
| ``` target    MPTANKADEVIILRPGTDAAFFLGVARELIEKGLYDRAAVIERTDLPLLVRLDTGERLDARDVIPGYELAALTNYVTLKP 3m9s.1    --------------------------------------------------------------------------------  target    DAEIKGNPPPPPFTAGGQVVPTELRDAWGDFVWWDRATGRPRPVSRDEVGARFDGDPALLGEFEVELVDGSTVPVRPAFD 3m9s.1    --------------------------------------------------------------------------------  target    LLKQYLDESFDLRTASEVCRVPPQAIQSIARQLAANKRETLLAAGMGPNHYFQNDLFGRVQFLVAALTDNIGHLGGNVGS 3m9s.1    --------------------------------------------------------------------------------  target    YAGNYRGSVFQAMGQWIAEDPFAIEPDLTKPATVKRYYKAESAHYWNYGERPLRAVAKDDEGDLTKGEVLTGKSHMPTPT 3m9s.1    --------------------------------------------------------------------------------  target    KLIWFGNSNSLLGNAKWSFDVVKNTLPRQDAVFCNEWHWTSSC-EYADLVFPADSWAEFKLPDATASCTNPFLLAFPTTP 3m9s.1    ---------------------PEEALKGKRFVVMHLSHLHPLAERYAHVVLPAPTFYEKRG---HLVNLEGRVLPL-SPA  target    LKRLYDTRSDYEALALTAKALGELIDEPRMEQYWRGILDGDPTPYLQRIFSGSNATRGITYDELHESSKRGVPLLMNMRT 3m9s.1    PIENGEAEGALQVLALLAEALGVRPPFRL------------HLEA---------------QKALK---------------  target    YPRSGGWEQRQEDKPWYTATGRLEFYRPEPEFQAAGESLPVWREPVDATFYEPNAILSNAAHPSIAPRAPEDYGVPESQL 3m9s.1    ------------ARKVPEAMGRLSFRLKELR-------------P-----------------------------------  target    DVETRQYRNVVRTWAELQQTLHPLQERDPAFRFVFQTPKYRWGAHSTAVDADWISMLFGPFGDPYRRDPRMPWTGEAYLE 3m9s.1    --------------------------KERKGAFYLRPTMWKAHQ-----AVGKAQEA-----------------ARAELW  target    INPKDAAELGLADGDYAWVDADPEDRPYRGWKEDDPYYEVARAMMRVRIYTGMSRGVIRTWFNMYAATPATVANQKATPG 3m9s.1    AHPETARAEALPEGAQVAVETP-----------------FGRVEARVVHREDVPKGHLYLSALGPAAG------------  target    NPARNEQTRYVALFRYGSHQSGTRAWLRPTQQTDSLVRKGYFGQVIGTGFEADVHSVSGAPKEAFVKIEKAEDGGIGAER 3m9s.1    --------------------------------------------------------------------------------  target    LWRPLTLGLRPEAPSAALTAYLAGDYSGTKGS 3m9s.1    -------------------------------- ``` | | | | | | | | | | | | | | | | | | | | | | | | | | | | | | | | | | | | | | | | | | | | | | | | | |
| ✓ | 2fug.2.C | NADH-quinone oxidoreductase chain 3  *Crystal structure of the hydrophilic domain of respiratory complex I from Thermus thermophilus* | 0.10 | 0.00 | 20.00 | 0.23 | 342-707 | X-ray | 3.30 | monomer | 7 x SF4, 2 x FES, 1 x FMN | HHblits | 0.28 |
| ``` target    MPTANKADEVIILRPGTDAAFFLGVARELIEKGLYDRAAVIERTDLPLLVRLDTGERLDARDVIPGYELAALTNYVTLKP 2fug.2    --------------------------------------------------------------------------------  target    DAEIKGNPPPPPFTAGGQVVPTELRDAWGDFVWWDRATGRPRPVSRDEVGARFDGDPALLGEFEVELVDGSTVPVRPAFD 2fug.2    --------------------------------------------------------------------------------  target    LLKQYLDESFDLRTASEVCRVPPQAIQSIARQLAANKRETLLAAGMGPNHYFQNDLFGRVQFLVAALTDNIGHLGGNVGS 2fug.2    --------------------------------------------------------------------------------  target    YAGNYRGSVFQAMGQWIAEDPFAIEPDLTKPATVKRYYKAESAHYWNYGERPLRAVAKDDEGDLTKGEVLTGKSHMPTPT 2fug.2    --------------------------------------------------------------------------------  target    KLIWFGNSNSLLGNAKWSFDVVKNTLPRQDAVFCNEWHWTSSC-EYADLVFPADSWAEFKLPDATASCTNPFLLAFPTTP 2fug.2    ---------------------PEEALKGKRFVVMHLSHLHPLAERYAHVVLPAPTFYEKRG---HLVNLEGRVLPL-SPA  target    LKRLYDTRSDYEALALTAKALGELIDEPRMEQYWRGILDGDPTPYLQRIFSGSNATRGITYDELHESSKRGVPLLMNMRT 2fug.2    PIENGEAEGALQVLALLAEALGVRPPFRL------------HLEA---------------QKALK---------------  target    YPRSGGWEQRQEDKPWYTATGRLEFYRPEPEFQAAGESLPVWREPVDATFYEPNAILSNAAHPSIAPRAPEDYGVPESQL 2fug.2    ------------ARKVPEAMGRLSFRLKELR-------------P-----------------------------------  target    DVETRQYRNVVRTWAELQQTLHPLQERDPAFRFVFQTPKYRWGAHSTAVDADWISMLFGPFGDPYRRDPRMPWTGEAYLE 2fug.2    --------------------------KERKGAFYLRPTMWKAHQ-----AVGKAQEA-----------------ARAELW  target    INPKDAAELGLADGDYAWVDADPEDRPYRGWKEDDPYYEVARAMMRVRIYTGMSRGVIRTWFNMYAATPATVANQKATPG 2fug.2    AHPETARAEALPEGAQVAVETP-----------------FGRVEARVVHREDVPKGHLYLSALGPAAG------------  target    NPARNEQTRYVALFRYGSHQSGTRAWLRPTQQTDSLVRKGYFGQVIGTGFEADVHSVSGAPKEAFVKIEKAEDGGIGAER 2fug.2    --------------------------------------------------------------------------------  target    LWRPLTLGLRPEAPSAALTAYLAGDYSGTKGS 2fug.2    -------------------------------- ``` | | | | | | | | | | | | | | | | | | | | | | | | | | | | | | | | | | | | | | | | | | | | | | | | | |
|  | 6zjl.1.C | NADH-quinone oxidoreductase subunit 3  *Respiratory complex I from Thermus thermophilus, NAD+ dataset, major state* | 0.11 | 0.00 | 20.00 | 0.23 | 342-707 | EM | 0.00 | monomer | 7 x SF4, 1 x FMN, 2 x FES | HHblits | 0.28 |
| ``` target    MPTANKADEVIILRPGTDAAFFLGVARELIEKGLYDRAAVIERTDLPLLVRLDTGERLDARDVIPGYELAALTNYVTLKP 6zjl.1    --------------------------------------------------------------------------------  target    DAEIKGNPPPPPFTAGGQVVPTELRDAWGDFVWWDRATGRPRPVSRDEVGARFDGDPALLGEFEVELVDGSTVPVRPAFD 6zjl.1    --------------------------------------------------------------------------------  target    LLKQYLDESFDLRTASEVCRVPPQAIQSIARQLAANKRETLLAAGMGPNHYFQNDLFGRVQFLVAALTDNIGHLGGNVGS 6zjl.1    --------------------------------------------------------------------------------  target    YAGNYRGSVFQAMGQWIAEDPFAIEPDLTKPATVKRYYKAESAHYWNYGERPLRAVAKDDEGDLTKGEVLTGKSHMPTPT 6zjl.1    --------------------------------------------------------------------------------  target    KLIWFGNSNSLLGNAKWSFDVVKNTLPRQDAVFCNEWHWTSSC-EYADLVFPADSWAEFKLPDATASCTNPFLLAFPTTP 6zjl.1    ---------------------PEEALKGKRFVVMHLSHLHPLAERYAHVVLPAPTFYEKRG---HLVNLEGRVLPL-SPA  target    LKRLYDTRSDYEALALTAKALGELIDEPRMEQYWRGILDGDPTPYLQRIFSGSNATRGITYDELHESSKRGVPLLMNMRT 6zjl.1    PIENGEAEGALQVLALLAEALGVRPPFRL------------HLEA---------------QKALK---------------  target    YPRSGGWEQRQEDKPWYTATGRLEFYRPEPEFQAAGESLPVWREPVDATFYEPNAILSNAAHPSIAPRAPEDYGVPESQL 6zjl.1    ------------ARKVPEAMGRLSFRLKELR-------------P-----------------------------------  target    DVETRQYRNVVRTWAELQQTLHPLQERDPAFRFVFQTPKYRWGAHSTAVDADWISMLFGPFGDPYRRDPRMPWTGEAYLE 6zjl.1    --------------------------KERKGAFYLRPTMWKAHQ-----AVGKAQEA-----------------ARAELW  target    INPKDAAELGLADGDYAWVDADPEDRPYRGWKEDDPYYEVARAMMRVRIYTGMSRGVIRTWFNMYAATPATVANQKATPG 6zjl.1    AHPETARAEALPEGAQVAVETP-----------------FGRVEARVVHREDVPKGHLYLSALGPAAG------------  target    NPARNEQTRYVALFRYGSHQSGTRAWLRPTQQTDSLVRKGYFGQVIGTGFEADVHSVSGAPKEAFVKIEKAEDGGIGAER 6zjl.1    --------------------------------------------------------------------------------  target    LWRPLTLGLRPEAPSAALTAYLAGDYSGTKGS 6zjl.1    -------------------------------- ``` | | | | | | | | | | | | | | | | | | | | | | | | | | | | | | | | | | | | | | | | | | | | | | | | | |
|  | 6q8o.1.C | NADH-quinone oxidoreductase subunit 3  *Respiratory complex I from Thermus thermophilus with bound Piericidin A* | 0.11 | 0.00 | 20.00 | 0.23 | 342-707 | X-ray | 3.61 | monomer | 7 x SF4, 1 x FMN, 2 x FES, 1 x HQH | HHblits | 0.28 |
| ``` target    MPTANKADEVIILRPGTDAAFFLGVARELIEKGLYDRAAVIERTDLPLLVRLDTGERLDARDVIPGYELAALTNYVTLKP 6q8o.1    --------------------------------------------------------------------------------  target    DAEIKGNPPPPPFTAGGQVVPTELRDAWGDFVWWDRATGRPRPVSRDEVGARFDGDPALLGEFEVELVDGSTVPVRPAFD 6q8o.1    --------------------------------------------------------------------------------  target    LLKQYLDESFDLRTASEVCRVPPQAIQSIARQLAANKRETLLAAGMGPNHYFQNDLFGRVQFLVAALTDNIGHLGGNVGS 6q8o.1    --------------------------------------------------------------------------------  target    YAGNYRGSVFQAMGQWIAEDPFAIEPDLTKPATVKRYYKAESAHYWNYGERPLRAVAKDDEGDLTKGEVLTGKSHMPTPT 6q8o.1    --------------------------------------------------------------------------------  target    KLIWFGNSNSLLGNAKWSFDVVKNTLPRQDAVFCNEWHWTSSC-EYADLVFPADSWAEFKLPDATASCTNPFLLAFPTTP 6q8o.1    ---------------------PEEALKGKRFVVMHLSHLHPLAERYAHVVLPAPTFYEKRG---HLVNLEGRVLPL-SPA  target    LKRLYDTRSDYEALALTAKALGELIDEPRMEQYWRGILDGDPTPYLQRIFSGSNATRGITYDELHESSKRGVPLLMNMRT 6q8o.1    PIENGEAEGALQVLALLAEALGVRPPFRL------------HLEA---------------QKALK---------------  target    YPRSGGWEQRQEDKPWYTATGRLEFYRPEPEFQAAGESLPVWREPVDATFYEPNAILSNAAHPSIAPRAPEDYGVPESQL 6q8o.1    ------------ARKVPEAMGRLSFRLKELR-------------P-----------------------------------  target    DVETRQYRNVVRTWAELQQTLHPLQERDPAFRFVFQTPKYRWGAHSTAVDADWISMLFGPFGDPYRRDPRMPWTGEAYLE 6q8o.1    --------------------------KERKGAFYLRPTMWKAHQ-----AVGKAQEA-----------------ARAELW  target    INPKDAAELGLADGDYAWVDADPEDRPYRGWKEDDPYYEVARAMMRVRIYTGMSRGVIRTWFNMYAATPATVANQKATPG 6q8o.1    AHPETARAEALPEGAQVAVETP-----------------FGRVEARVVHREDVPKGHLYLSALGPAAG------------  target    NPARNEQTRYVALFRYGSHQSGTRAWLRPTQQTDSLVRKGYFGQVIGTGFEADVHSVSGAPKEAFVKIEKAEDGGIGAER 6q8o.1    --------------------------------------------------------------------------------  target    LWRPLTLGLRPEAPSAALTAYLAGDYSGTKGS 6q8o.1    -------------------------------- ``` | | | | | | | | | | | | | | | | | | | | | | | | | | | | | | | | | | | | | | | | | | | | | | | | | |
|  | 6zjy.1.C | NADH-quinone oxidoreductase subunit 3  *Respiratory complex I from Thermus thermophilus, NAD+ dataset, minor state* | 0.11 | 0.00 | 20.00 | 0.23 | 342-707 | EM | 0.00 | monomer | 7 x SF4, 2 x FES | HHblits | 0.28 |
| ``` target    MPTANKADEVIILRPGTDAAFFLGVARELIEKGLYDRAAVIERTDLPLLVRLDTGERLDARDVIPGYELAALTNYVTLKP 6zjy.1    --------------------------------------------------------------------------------  target    DAEIKGNPPPPPFTAGGQVVPTELRDAWGDFVWWDRATGRPRPVSRDEVGARFDGDPALLGEFEVELVDGSTVPVRPAFD 6zjy.1    --------------------------------------------------------------------------------  target    LLKQYLDESFDLRTASEVCRVPPQAIQSIARQLAANKRETLLAAGMGPNHYFQNDLFGRVQFLVAALTDNIGHLGGNVGS 6zjy.1    --------------------------------------------------------------------------------  target    YAGNYRGSVFQAMGQWIAEDPFAIEPDLTKPATVKRYYKAESAHYWNYGERPLRAVAKDDEGDLTKGEVLTGKSHMPTPT 6zjy.1    --------------------------------------------------------------------------------  target    KLIWFGNSNSLLGNAKWSFDVVKNTLPRQDAVFCNEWHWTSSC-EYADLVFPADSWAEFKLPDATASCTNPFLLAFPTTP 6zjy.1    ---------------------PEEALKGKRFVVMHLSHLHPLAERYAHVVLPAPTFYEKRG---HLVNLEGRVLPL-SPA  target    LKRLYDTRSDYEALALTAKALGELIDEPRMEQYWRGILDGDPTPYLQRIFSGSNATRGITYDELHESSKRGVPLLMNMRT 6zjy.1    PIENGEAEGALQVLALLAEALGVRPPFRL------------HLEA---------------QKALK---------------  target    YPRSGGWEQRQEDKPWYTATGRLEFYRPEPEFQAAGESLPVWREPVDATFYEPNAILSNAAHPSIAPRAPEDYGVPESQL 6zjy.1    ------------ARKVPEAMGRLSFRLKELR-------------P-----------------------------------  target    DVETRQYRNVVRTWAELQQTLHPLQERDPAFRFVFQTPKYRWGAHSTAVDADWISMLFGPFGDPYRRDPRMPWTGEAYLE 6zjy.1    --------------------------KERKGAFYLRPTMWKAHQ-----AVGKAQEA-----------------ARAELW  target    INPKDAAELGLADGDYAWVDADPEDRPYRGWKEDDPYYEVARAMMRVRIYTGMSRGVIRTWFNMYAATPATVANQKATPG 6zjy.1    AHPETARAEALPEGAQVAVETP-----------------FGRVEARVVHREDVPKGHLYLSALGPAAG------------  target    NPARNEQTRYVALFRYGSHQSGTRAWLRPTQQTDSLVRKGYFGQVIGTGFEADVHSVSGAPKEAFVKIEKAEDGGIGAER 6zjy.1    --------------------------------------------------------------------------------  target    LWRPLTLGLRPEAPSAALTAYLAGDYSGTKGS 6zjy.1    -------------------------------- ``` | | | | | | | | | | | | | | | | | | | | | | | | | | | | | | | | | | | | | | | | | | | | | | | | | |
|  | 6zjn.1.C | NADH-quinone oxidoreductase subunit 3  *Respiratory complex I from Thermus thermophilus, NADH dataset, minor state* | 0.11 | 0.00 | 20.00 | 0.23 | 342-707 | EM | 0.00 | monomer | 7 x SF4, 2 x FES | HHblits | 0.28 |
| ``` target    MPTANKADEVIILRPGTDAAFFLGVARELIEKGLYDRAAVIERTDLPLLVRLDTGERLDARDVIPGYELAALTNYVTLKP 6zjn.1    --------------------------------------------------------------------------------  target    DAEIKGNPPPPPFTAGGQVVPTELRDAWGDFVWWDRATGRPRPVSRDEVGARFDGDPALLGEFEVELVDGSTVPVRPAFD 6zjn.1    --------------------------------------------------------------------------------  target    LLKQYLDESFDLRTASEVCRVPPQAIQSIARQLAANKRETLLAAGMGPNHYFQNDLFGRVQFLVAALTDNIGHLGGNVGS 6zjn.1    --------------------------------------------------------------------------------  target    YAGNYRGSVFQAMGQWIAEDPFAIEPDLTKPATVKRYYKAESAHYWNYGERPLRAVAKDDEGDLTKGEVLTGKSHMPTPT 6zjn.1    --------------------------------------------------------------------------------  target    KLIWFGNSNSLLGNAKWSFDVVKNTLPRQDAVFCNEWHWTSSC-EYADLVFPADSWAEFKLPDATASCTNPFLLAFPTTP 6zjn.1    ---------------------PEEALKGKRFVVMHLSHLHPLAERYAHVVLPAPTFYEKRG---HLVNLEGRVLPL-SPA  target    LKRLYDTRSDYEALALTAKALGELIDEPRMEQYWRGILDGDPTPYLQRIFSGSNATRGITYDELHESSKRGVPLLMNMRT 6zjn.1    PIENGEAEGALQVLALLAEALGVRPPFRL------------HLEA---------------QKALK---------------  target    YPRSGGWEQRQEDKPWYTATGRLEFYRPEPEFQAAGESLPVWREPVDATFYEPNAILSNAAHPSIAPRAPEDYGVPESQL 6zjn.1    ------------ARKVPEAMGRLSFRLKELR-------------P-----------------------------------  target    DVETRQYRNVVRTWAELQQTLHPLQERDPAFRFVFQTPKYRWGAHSTAVDADWISMLFGPFGDPYRRDPRMPWTGEAYLE 6zjn.1    --------------------------KERKGAFYLRPTMWKAHQ-----AVGKAQEA-----------------ARAELW  target    INPKDAAELGLADGDYAWVDADPEDRPYRGWKEDDPYYEVARAMMRVRIYTGMSRGVIRTWFNMYAATPATVANQKATPG 6zjn.1    AHPETARAEALPEGAQVAVETP-----------------FGRVEARVVHREDVPKGHLYLSALGPAAG------------  target    NPARNEQTRYVALFRYGSHQSGTRAWLRPTQQTDSLVRKGYFGQVIGTGFEADVHSVSGAPKEAFVKIEKAEDGGIGAER 6zjn.1    --------------------------------------------------------------------------------  target    LWRPLTLGLRPEAPSAALTAYLAGDYSGTKGS 6zjn.1    -------------------------------- ``` | | | | | | | | | | | | | | | | | | | | | | | | | | | | | | | | | | | | | | | | | | | | | | | | | |
|  | 6ziy.1.C | NADH-quinone oxidoreductase subunit 3  *Respiratory complex I from Thermus thermophilus, NADH dataset, major state* | 0.11 | 0.00 | 20.00 | 0.23 | 342-707 | EM | 0.00 | monomer | 7 x SF4, 1 x FMN, 1 x NAI, 2 x FES | HHblits | 0.28 |
| ``` target    MPTANKADEVIILRPGTDAAFFLGVARELIEKGLYDRAAVIERTDLPLLVRLDTGERLDARDVIPGYELAALTNYVTLKP 6ziy.1    --------------------------------------------------------------------------------  target    DAEIKGNPPPPPFTAGGQVVPTELRDAWGDFVWWDRATGRPRPVSRDEVGARFDGDPALLGEFEVELVDGSTVPVRPAFD 6ziy.1    --------------------------------------------------------------------------------  target    LLKQYLDESFDLRTASEVCRVPPQAIQSIARQLAANKRETLLAAGMGPNHYFQNDLFGRVQFLVAALTDNIGHLGGNVGS 6ziy.1    --------------------------------------------------------------------------------  target    YAGNYRGSVFQAMGQWIAEDPFAIEPDLTKPATVKRYYKAESAHYWNYGERPLRAVAKDDEGDLTKGEVLTGKSHMPTPT 6ziy.1    --------------------------------------------------------------------------------  target    KLIWFGNSNSLLGNAKWSFDVVKNTLPRQDAVFCNEWHWTSSC-EYADLVFPADSWAEFKLPDATASCTNPFLLAFPTTP 6ziy.1    ---------------------PEEALKGKRFVVMHLSHLHPLAERYAHVVLPAPTFYEKRG---HLVNLEGRVLPL-SPA  target    LKRLYDTRSDYEALALTAKALGELIDEPRMEQYWRGILDGDPTPYLQRIFSGSNATRGITYDELHESSKRGVPLLMNMRT 6ziy.1    PIENGEAEGALQVLALLAEALGVRPPFRL------------HLEA---------------QKALK---------------  target    YPRSGGWEQRQEDKPWYTATGRLEFYRPEPEFQAAGESLPVWREPVDATFYEPNAILSNAAHPSIAPRAPEDYGVPESQL 6ziy.1    ------------ARKVPEAMGRLSFRLKELR-------------P-----------------------------------  target    DVETRQYRNVVRTWAELQQTLHPLQERDPAFRFVFQTPKYRWGAHSTAVDADWISMLFGPFGDPYRRDPRMPWTGEAYLE 6ziy.1    --------------------------KERKGAFYLRPTMWKAHQ-----AVGKAQEA-----------------ARAELW  target    INPKDAAELGLADGDYAWVDADPEDRPYRGWKEDDPYYEVARAMMRVRIYTGMSRGVIRTWFNMYAATPATVANQKATPG 6ziy.1    AHPETARAEALPEGAQVAVETP-----------------FGRVEARVVHREDVPKGHLYLSALGPAAG------------  target    NPARNEQTRYVALFRYGSHQSGTRAWLRPTQQTDSLVRKGYFGQVIGTGFEADVHSVSGAPKEAFVKIEKAEDGGIGAER 6ziy.1    --------------------------------------------------------------------------------  target    LWRPLTLGLRPEAPSAALTAYLAGDYSGTKGS 6ziy.1    -------------------------------- ``` | | | | | | | | | | | | | | | | | | | | | | | | | | | | | | | | | | | | | | | | | | | | | | | | | |
|  | 7bkb.1.L | Formylmethanofuran dehydrogenase, subunit B  *Formate dehydrogenase - heterodisulfide reductase - formylmethanofuran dehydrogenase complex from Methanospirillum hungatei (hexameric, composite structure)* | 0.10 |  | 14.36 | 0.23 | 175-423 | EM | 0.00 | hetero-2-2-2-2-2-2-… | 48 x SF4, 4 x FAD, 2 x FES, 4 x 9S8, 4 x ZN, 2 x MO, 4 x MGD | HHblits | 0.26 |
| ``` target    MPTANKADEVIILRPGTDAAFFLGVARELIEKGLYDRAAVIERTDLPLLVRLDTGERLDARDVIPGYELAALTNYVTLKP 7bkb.1    --------------------------------------------------------------------------------  target    DAEIKGNPPPPPFTAGGQVVPTELRDAWGDFVWWDRATGRPRPVSRDEVGARFDGDPALLGEFEVELVDGSTVPVRPAFD 7bkb.1    --------------------------------------------------------------------------------  target    LLKQYLDESFDLRTASEVCRVPPQAIQSIARQLAANKRETLLAAGMGPNHYFQNDLFGRVQF------------LVAALT 7bkb.1    --------------PDEVAGIKKETILEVAEIMKNARFGTT-FFGMGLTHTDGRNHNIDIAISLTRDLNKISKWTIMAMR  target    DNIGHLGGNVGSYAGNYRGSVFQAMGQWIAEDPFAIEPDLTKPATVKRYYKAESAHYWNYGERPLRAVAKDDEGDLTKGE 7bkb.1    GHYNIAGPGVVWSWTF----------GFP------YCLDLTKQ-N------------HA----HMNP-------GE--TS  target    VLTGKSHMPTPTKLIWFGNSNSLLGNAKWSFDVVKNTLPRQDAVFCNEWHWTSSCEYADLVFPADSW-AEFKLPDATASC 7bkb.1    SVDM--AMRDEVDMFINIGTDAAAHFPI----PAVKQLKKHPW-VTIDPSINMASEISDLHIPVCICGVDVGG---IVYR  target    TNPFLLAFPTTPLKRLYDTRSDYEALALTAKALGELIDEPRMEQYWRGILDGDPTPYLQRIFSGSNATRGITYDELHESS 7bkb.1    MDNVPIQF-RKVIEPPEGVMDDETLLNKIADRMEEL--------------------------------------------  target    KRGVPLLMNMRTYPRSGGWEQRQEDKPWYTATGRLEFYRPEPEFQAAGESLPVWREPVDATFYEPNAILSNAAHPSIAPR 7bkb.1    --------------------------------------------------------------------------------  target    APEDYGVPESQLDVETRQYRNVVRTWAELQQTLHPLQERDPAFRFVFQTPKYRWGAHSTAVDADWISMLFGPFGDPYRRD 7bkb.1    --------------------------------------------------------------------------------  target    PRMPWTGEAYLEINPKDAAELGLADGDYAWVDADPEDRPYRGWKEDDPYYEVARAMMRVRIYTGMSRGVIRTWFNMYAAT 7bkb.1    --------------------------------------------------------------------------------  target    PATVANQKATPGNPARNEQTRYVALFRYGSHQSGTRAWLRPTQQTDSLVRKGYFGQVIGTGFEADVHSVSGAPKEAFVKI 7bkb.1    --------------------------------------------------------------------------------  target    EKAEDGGIGAERLWRPLTLGLRPEAPSAALTAYLAGDYSGTKGS 7bkb.1    -------------------------------------------- ``` | | | | | | | | | | | | | | | | | | | | | | | | | | | | | | | | | | | | | | | | | | | | | | | | | |
|  | 7arc.1.F | 75 kDa  *Cryo-EM structure of Polytomella Complex-I (peripheral arm)* | 0.10 |  | 16.04 | 0.23 | 169-422 | EM | 0.00 | hetero-1-1-1-1-1-1-… | 6 x SF4, 2 x FES, 1 x FMN, 1 x NDP, 1 x ZN, 1 x 8Q1 | HHblits | 0.29 |
| ``` target    MPTANKADEVIILRPGTDAAFFLGVARELIEKGLYDRAAVIERTDLPLLVRLDTGERLDARDVIPGYELAALTNYVTLKP 7arc.1    --------------------------------------------------------------------------------  target    DAEIKGNPPPPPFTAGGQVVPTELRDAWGDFVWWDRATGRPRPVSRDEVGARFDGDPALLGEFEVELVDGSTVPVRPAFD 7arc.1    --------------------------------------------------------------------------------  target    LLKQYLDESFDLRTASEVCRVPPQAIQSIA-------RQLAANKRETLLAAGMGPNHYFQNDLFGRVQFLVAALTDNIGH 7arc.1    --------KVDLTYAYQHLGADVAALESLASGKGAFFEALKGAKNPV-VIVGSSVLRRDDREAVLKTVNDLVDAAGVVKE  target    LGGNVGSYAGNYRGSVFQAMGQWIAEDPFAIEPDLTKPATVKRYYKAESAHYWNYGERPLRAVAKDDEGDLTKGEVLTGK 7arc.1    GWNGFNVLHDNAS-----RVAAL----------DI------G---------FV-----P--------------SASA-R-  target    SHMPTPTKLIWFGNSNSLLGNAKWSFDVVKNTLPRQDAVFCNEWHWTSSCEYADLVFPADSWAEFKLPDATASCTNPFLL 7arc.1    -TNPVPAKVVYLLGSDDFKD----------EEIPADAFVIYQGHHGDKGAARANVVLPGAAYTEKAS---LFANTEGRVQ  target    AFPTTPLKRLYDTRSDYEALALTAKALGELIDEPRMEQYWRGILDGDPTPYLQRIFSGSNATRGITYDELHESSKRGVPL 7arc.1    TT-RTAVPVLGDAREDWKIIRALSEVVGQ---------------------------------------------------  target    LMNMRTYPRSGGWEQRQEDKPWYTATGRLEFYRPEPEFQAAGESLPVWREPVDATFYEPNAILSNAAHPSIAPRAPEDYG 7arc.1    --------------------------------------------------------------------------------  target    VPESQLDVETRQYRNVVRTWAELQQTLHPLQERDPAFRFVFQTPKYRWGAHSTAVDADWISMLFGPFGDPYRRDPRMPWT 7arc.1    --------------------------------------------------------------------------------  target    GEAYLEINPKDAAELGLADGDYAWVDADPEDRPYRGWKEDDPYYEVARAMMRVRIYTGMSRGVIRTWFNMYAATPATVAN 7arc.1    --------------------------------------------------------------------------------  target    QKATPGNPARNEQTRYVALFRYGSHQSGTRAWLRPTQQTDSLVRKGYFGQVIGTGFEADVHSVSGAPKEAFVKIEKAEDG 7arc.1    --------------------------------------------------------------------------------  target    GIGAERLWRPLTLGLRPEAPSAALTAYLAGDYSGTKGS 7arc.1    -------------------------------------- ``` | | | | | | | | | | | | | | | | | | | | | | | | | | | | | | | | | | | | | | | | | | | | | | | | | |
|  | 7t2r.1.A | NiFe hydrogenase subunit A  *Structure of electron bifurcating Ni-Fe hydrogenase complex HydABCSL in FMN-free apo state* | 0.10 |  | 13.37 | 0.23 | 170-423 | EM | 0.00 | hetero-2-2-2-2-2-mer | 6 x FES, 12 x SF4, 2 x 3NI, 2 x FCO | HHblits | 0.27 |
| ``` target    MPTANKADEVIILRPGTDAAFFLGVARELIEKGLYDRAAVIERTDLPLLVRLDTGERLDARDVIPGYELAALTNYVTLKP 7t2r.1    --------------------------------------------------------------------------------  target    DAEIKGNPPPPPFTAGGQVVPTELRDAWGDFVWWDRATGRPRPVSRDEVGARFDGDPALLGEFEVELVDGSTVPVRPAFD 7t2r.1    --------------------------------------------------------------------------------  target    LLKQYLDESFDLRTASEVCRVPPQAIQSIARQLAANKRETLLAAGMGPNHYFQNDLFGRVQFLVAALTDNIGHLGGNVGS 7t2r.1    ---------ESIEESARAMGLDPKIAEEVALMLISARRPIFI-IGGRA---TKSHELVTAACNLAVASKAFFEDGLGVVP  target    YAGNYRGSVFQAMGQWIAEDPFAIEPDLTKPATVKRYYKAESAHYWNYGERPLRAVAKDDEGDLTKGEVLTGKSHMPTPT 7t2r.1    LLVSAN-----SLGAR---------NTVVS-------------------ENP---------------WL--G----RERR  target    KLIWFGNSNSLLGNAKWSFDVVKNTLPRQDAVFCNEWHWT-SSCEYADLVFPADSWAEFKLPDATASCTNPFLLAFPTTP 7t2r.1    DFLYVFSTAMV---PE--EEEILAAISATRFVVVQTPFKVRPLVNLADILLPAPAWYERSG---HFCTIEGERRKL-NTI  target    LKRLYDTRSDYEALALTAKALGELIDEPRMEQYWRGILDGDPTPYLQRIFSGSNATRGITYDELHESSKRGVPLLMNMRT 7t2r.1    VPPKGEIKSLHYVMDEFAKKLGVK--------------------------------------------------------  target    YPRSGGWEQRQEDKPWYTATGRLEFYRPEPEFQAAGESLPVWREPVDATFYEPNAILSNAAHPSIAPRAPEDYGVPESQL 7t2r.1    --------------------------------------------------------------------------------  target    DVETRQYRNVVRTWAELQQTLHPLQERDPAFRFVFQTPKYRWGAHSTAVDADWISMLFGPFGDPYRRDPRMPWTGEAYLE 7t2r.1    --------------------------------------------------------------------------------  target    INPKDAAELGLADGDYAWVDADPEDRPYRGWKEDDPYYEVARAMMRVRIYTGMSRGVIRTWFNMYAATPATVANQKATPG 7t2r.1    --------------------------------------------------------------------------------  target    NPARNEQTRYVALFRYGSHQSGTRAWLRPTQQTDSLVRKGYFGQVIGTGFEADVHSVSGAPKEAFVKIEKAEDGGIGAER 7t2r.1    --------------------------------------------------------------------------------  target    LWRPLTLGLRPEAPSAALTAYLAGDYSGTKGS 7t2r.1    -------------------------------- ``` | | | | | | | | | | | | | | | | | | | | | | | | | | | | | | | | | | | | | | | | | | | | | | | | | |
|  | 7t30.1.A | NiFe hydrogenase subunit A  *Structure of electron bifurcating Ni-Fe hydrogenase complex HydABCSL in FMN/NAD(H) bound state* | 0.10 |  | 13.37 | 0.23 | 170-423 | EM | 0.00 | hetero-2-2-2-2-2-mer | 4 x FES, 12 x SF4, 2 x NAD, 2 x FMN, 2 x 3NI, 2 x FCO | HHblits | 0.27 |
| ``` target    MPTANKADEVIILRPGTDAAFFLGVARELIEKGLYDRAAVIERTDLPLLVRLDTGERLDARDVIPGYELAALTNYVTLKP 7t30.1    --------------------------------------------------------------------------------  target    DAEIKGNPPPPPFTAGGQVVPTELRDAWGDFVWWDRATGRPRPVSRDEVGARFDGDPALLGEFEVELVDGSTVPVRPAFD 7t30.1    --------------------------------------------------------------------------------  target    LLKQYLDESFDLRTASEVCRVPPQAIQSIARQLAANKRETLLAAGMGPNHYFQNDLFGRVQFLVAALTDNIGHLGGNVGS 7t30.1    ---------ESIEESARAMGLDPKIAEEVALMLISARRPIFI-IGGRA---TKSHELVTAACNLAVASKAFFEDGLGVVP  target    YAGNYRGSVFQAMGQWIAEDPFAIEPDLTKPATVKRYYKAESAHYWNYGERPLRAVAKDDEGDLTKGEVLTGKSHMPTPT 7t30.1    LLVSAN-----SLGAR---------NTVVS-------------------ENP---------------WL--G----RERR  target    KLIWFGNSNSLLGNAKWSFDVVKNTLPRQDAVFCNEWHWT-SSCEYADLVFPADSWAEFKLPDATASCTNPFLLAFPTTP 7t30.1    DFLYVFSTAMV---PE--EEEILAAISATRFVVVQTPFKVRPLVNLADILLPAPAWYERSG---HFCTIEGERRKL-NTI  target    LKRLYDTRSDYEALALTAKALGELIDEPRMEQYWRGILDGDPTPYLQRIFSGSNATRGITYDELHESSKRGVPLLMNMRT 7t30.1    VPPKGEIKSLHYVMDEFAKKLGVK--------------------------------------------------------  target    YPRSGGWEQRQEDKPWYTATGRLEFYRPEPEFQAAGESLPVWREPVDATFYEPNAILSNAAHPSIAPRAPEDYGVPESQL 7t30.1    --------------------------------------------------------------------------------  target    DVETRQYRNVVRTWAELQQTLHPLQERDPAFRFVFQTPKYRWGAHSTAVDADWISMLFGPFGDPYRRDPRMPWTGEAYLE 7t30.1    --------------------------------------------------------------------------------  target    INPKDAAELGLADGDYAWVDADPEDRPYRGWKEDDPYYEVARAMMRVRIYTGMSRGVIRTWFNMYAATPATVANQKATPG 7t30.1    --------------------------------------------------------------------------------  target    NPARNEQTRYVALFRYGSHQSGTRAWLRPTQQTDSLVRKGYFGQVIGTGFEADVHSVSGAPKEAFVKIEKAEDGGIGAER 7t30.1    --------------------------------------------------------------------------------  target    LWRPLTLGLRPEAPSAALTAYLAGDYSGTKGS 7t30.1    -------------------------------- ``` | | | | | | | | | | | | | | | | | | | | | | | | | | | | | | | | | | | | | | | | | | | | | | | | | |
|  | 7tgh.58.A | NADH-ubiquinone oxidoreductase 75 kDa subunit  *Cryo-EM structure of respiratory super-complex CI+III2 from Tetrahymena thermophila* | 0.09 |  | 15.05 | 0.22 | 170-423 | EM | 0.00 | monomer |  | HHblits | 0.27 |
| ``` target    MPTANKADEVIILRPGTDAAFFLGVARELIEKGLYDRAAVIERTDLPLLVRLDTGERLDARDVIPGYELAALTNYVTLKP 7tgh.58   --------------------------------------------------------------------------------  target    DAEIKGNPPPPPFTAGGQVVPTELRDAWGDFVWWDRATGRPRPVSRDEVGARFDGDPALLGEFEVELVDGSTVPVRPAFD 7tgh.58   --------------------------------------------------------------------------------  target    LLKQYLDESFDLRTASEVCRVPPQAIQSIARQLAANKRETLLAAGMGPNHYFQNDLFGRVQFLVAALTDNIGHLGGNVGS 7tgh.58   ---------VHLGNSTKVLKEIADGTHPFAERLKKAKLPMI-MVGASALEREDGAELYNTLKVISNKTGVISEEKSWNGF  target    YAGNYRGSVFQAMGQWIAEDPFAIEPDLTKPATVKRYYKAESAHYWNYGERPLRAVAKDDEGDLTKGEVLTGKSHMPTPT 7tgh.58   NILHK------EMG------------RIN---A------------L----------E----LGINPT-------SVNKNA  target    KLIWFGNSNSLLGNAKWSFDVVKNTLPRQDAVFCNEWHWTSSCEYADLVFPADSWAEFKLPDATASCTNPFLLAFPTTPL 7tgh.58   KLVFILGADNNLRP---------EDIPADAFVVYFGTHGDEGAYYADIILPTAAYTEKNA---TWVNTEGRVQQ-GRLVV  target    KRLYDTRSDYEALALTAKALGELIDEPRMEQYWRGILDGDPTPYLQRIFSGSNATRGITYDELHESSKRGVPLLMNMRTY 7tgh.58   MPPGDAREDWQIIRALSEEAGVP---------------------------------------------------------  target    PRSGGWEQRQEDKPWYTATGRLEFYRPEPEFQAAGESLPVWREPVDATFYEPNAILSNAAHPSIAPRAPEDYGVPESQLD 7tgh.58   --------------------------------------------------------------------------------  target    VETRQYRNVVRTWAELQQTLHPLQERDPAFRFVFQTPKYRWGAHSTAVDADWISMLFGPFGDPYRRDPRMPWTGEAYLEI 7tgh.58   --------------------------------------------------------------------------------  target    NPKDAAELGLADGDYAWVDADPEDRPYRGWKEDDPYYEVARAMMRVRIYTGMSRGVIRTWFNMYAATPATVANQKATPGN 7tgh.58   --------------------------------------------------------------------------------  target    PARNEQTRYVALFRYGSHQSGTRAWLRPTQQTDSLVRKGYFGQVIGTGFEADVHSVSGAPKEAFVKIEKAEDGGIGAERL 7tgh.58   --------------------------------------------------------------------------------  target    WRPLTLGLRPEAPSAALTAYLAGDYSGTKGS 7tgh.58   ------------------------------- ``` | | | | | | | | | | | | | | | | | | | | | | | | | | | | | | | | | | | | | | | | | | | | | | | | | |
|  | 5xtb.1.L | NADH-ubiquinone oxidoreductase 75 kDa subunit, mitochondrial  *Cryo-EM structure of human respiratory complex I matrix arm* | 0.08 |  | 13.64 | 0.21 | 185-423 | EM | 0.00 | hetero-1-1-1-1-1-1-… | 6 x SF4, 1 x FMN, 1 x 8Q1, 1 x NDP, 2 x FES | HHblits | 0.27 |
| ``` target    MPTANKADEVIILRPGTDAAFFLGVARELIEKGLYDRAAVIERTDLPLLVRLDTGERLDARDVIPGYELAALTNYVTLKP 5xtb.1    --------------------------------------------------------------------------------  target    DAEIKGNPPPPPFTAGGQVVPTELRDAWGDFVWWDRATGRPRPVSRDEVGARFDGDPALLGEFEVELVDGSTVPVRPAFD 5xtb.1    --------------------------------------------------------------------------------  target    LLKQYLDESFDLRTASEVCRVPPQAIQSIARQLAANKRETLLAAGMGPNHYFQNDLFGRVQFLVAALTDNIGHLGGNVGS 5xtb.1    ------------------------GSHPFSQVLKEAKKPMV-VLGSSALQRNDGAAILAAVSSIAQKIRMTSGVTGDWKV  target    YAGNYRGSVFQAMGQWIAEDPFAIEPDLTKPATVKRYYKAESAHYWNYGERPLRAVAKDDEGDLTKGEVLTGKSHMPTPT 5xtb.1    MNILH---------------RIA---S--QVAA------------LDLGYKP----------G--VEAIR------KNPP  target    KLIWFGNSNSLLGNAKWSFDVVKNTLPRQDAVFCNEWHWTSSCEYADLVFPADSWAEFKLPDATASCTNPFLLAFPTTPL 5xtb.1    KVLFLLGADGG--------CITRQDLPKDCFIIYQGHHGDVGAPIADVILPGAAYTEKS---ATYVNTEGRAQQT-KVAV  target    KRLYDTRSDYEALALTAKALGELIDEPRMEQYWRGILDGDPTPYLQRIFSGSNATRGITYDELHESSKRGVPLLMNMRTY 5xtb.1    TPPGLAREDWKIIRALSEIAGMT---------------------------------------------------------  target    PRSGGWEQRQEDKPWYTATGRLEFYRPEPEFQAAGESLPVWREPVDATFYEPNAILSNAAHPSIAPRAPEDYGVPESQLD 5xtb.1    --------------------------------------------------------------------------------  target    VETRQYRNVVRTWAELQQTLHPLQERDPAFRFVFQTPKYRWGAHSTAVDADWISMLFGPFGDPYRRDPRMPWTGEAYLEI 5xtb.1    --------------------------------------------------------------------------------  target    NPKDAAELGLADGDYAWVDADPEDRPYRGWKEDDPYYEVARAMMRVRIYTGMSRGVIRTWFNMYAATPATVANQKATPGN 5xtb.1    --------------------------------------------------------------------------------  target    PARNEQTRYVALFRYGSHQSGTRAWLRPTQQTDSLVRKGYFGQVIGTGFEADVHSVSGAPKEAFVKIEKAEDGGIGAERL 5xtb.1    --------------------------------------------------------------------------------  target    WRPLTLGLRPEAPSAALTAYLAGDYSGTKGS 5xtb.1    ------------------------------- ``` | | | | | | | | | | | | | | | | | | | | | | | | | | | | | | | | | | | | | | | | | | | | | | | | | |
|  | 6qcf.1.C | NADH:ubiquinone oxidoreductase core subunit S1  *Ovine respiratory complex I FRC open class 6* | 0.08 | 0.00 | 13.71 | 0.21 | 186-423 | EM | 0.00 | monomer | 6 x SF4, 1 x FMN, 2 x FES, 1 x ZN, 1 x NDP, 2 x ZMP | HHblits | 0.27 |
| ``` target    MPTANKADEVIILRPGTDAAFFLGVARELIEKGLYDRAAVIERTDLPLLVRLDTGERLDARDVIPGYELAALTNYVTLKP 6qcf.1    --------------------------------------------------------------------------------  target    DAEIKGNPPPPPFTAGGQVVPTELRDAWGDFVWWDRATGRPRPVSRDEVGARFDGDPALLGEFEVELVDGSTVPVRPAFD 6qcf.1    --------------------------------------------------------------------------------  target    LLKQYLDESFDLRTASEVCRVPPQAIQSIARQLAANKRETLLAAGMGPNHYFQNDLFGRVQFLVAALTDNIGHLGGNVGS 6qcf.1    -------------------------SHPFSQVLQEAKKPMV-VLGSSALQRNDGAAILAAVSNIAQKIRTSSGVTGDWKV  target    YAGNYRGSVFQAMGQWIAEDPFAIEPDLTKPATVKRYYKAESAHYWNYGERPLRAVAKDDEGDLTKGEVLTGKSHMPTPT 6qcf.1    MNI---------LH------RIA---SQVA--A------------LDLGYKP----------G--VEAIR------KNPP  target    KLIWFGNSNSLLGNAKWSFDVVKNTLPRQDAVFCNEWHWTSSCEYADLVFPADSWAEFKLPDATASCTNPFLLAFPTTPL 6qcf.1    KMLFLLGADGGC--------VTRQDLPKDCFIVYQGHHGDVGAPIADVILPGAAYTEKS---ATYVNTEGRAQQT-KVAV  target    KRLYDTRSDYEALALTAKALGELIDEPRMEQYWRGILDGDPTPYLQRIFSGSNATRGITYDELHESSKRGVPLLMNMRTY 6qcf.1    MPPGLAREDWKIIRALSEIAGMT---------------------------------------------------------  target    PRSGGWEQRQEDKPWYTATGRLEFYRPEPEFQAAGESLPVWREPVDATFYEPNAILSNAAHPSIAPRAPEDYGVPESQLD 6qcf.1    --------------------------------------------------------------------------------  target    VETRQYRNVVRTWAELQQTLHPLQERDPAFRFVFQTPKYRWGAHSTAVDADWISMLFGPFGDPYRRDPRMPWTGEAYLEI 6qcf.1    --------------------------------------------------------------------------------  target    NPKDAAELGLADGDYAWVDADPEDRPYRGWKEDDPYYEVARAMMRVRIYTGMSRGVIRTWFNMYAATPATVANQKATPGN 6qcf.1    --------------------------------------------------------------------------------  target    PARNEQTRYVALFRYGSHQSGTRAWLRPTQQTDSLVRKGYFGQVIGTGFEADVHSVSGAPKEAFVKIEKAEDGGIGAERL 6qcf.1    --------------------------------------------------------------------------------  target    WRPLTLGLRPEAPSAALTAYLAGDYSGTKGS 6qcf.1    ------------------------------- ``` | | | | | | | | | | | | | | | | | | | | | | | | | | | | | | | | | | | | | | | | | | | | | | | | | |
|  | 6qc5.1.C | NADH:ubiquinone oxidoreductase core subunit S1  *Ovine respiratory complex I FRC closed class 1* | 0.08 | 0.00 | 13.71 | 0.21 | 186-423 | EM | 0.00 | monomer | 6 x SF4, 1 x FMN, 2 x FES, 2 x 3PE, 1 x ZN, 1 x NDP, 2 x ZMP, 1 x PC1 | HHblits | 0.27 |
| ``` target    MPTANKADEVIILRPGTDAAFFLGVARELIEKGLYDRAAVIERTDLPLLVRLDTGERLDARDVIPGYELAALTNYVTLKP 6qc5.1    --------------------------------------------------------------------------------  target    DAEIKGNPPPPPFTAGGQVVPTELRDAWGDFVWWDRATGRPRPVSRDEVGARFDGDPALLGEFEVELVDGSTVPVRPAFD 6qc5.1    --------------------------------------------------------------------------------  target    LLKQYLDESFDLRTASEVCRVPPQAIQSIARQLAANKRETLLAAGMGPNHYFQNDLFGRVQFLVAALTDNIGHLGGNVGS 6qc5.1    -------------------------SHPFSQVLQEAKKPMV-VLGSSALQRNDGAAILAAVSNIAQKIRTSSGVTGDWKV  target    YAGNYRGSVFQAMGQWIAEDPFAIEPDLTKPATVKRYYKAESAHYWNYGERPLRAVAKDDEGDLTKGEVLTGKSHMPTPT 6qc5.1    MNI---------LH------RIA---SQVA--A------------LDLGYKP----------G--VEAIR------KNPP  target    KLIWFGNSNSLLGNAKWSFDVVKNTLPRQDAVFCNEWHWTSSCEYADLVFPADSWAEFKLPDATASCTNPFLLAFPTTPL 6qc5.1    KMLFLLGADGGC--------VTRQDLPKDCFIVYQGHHGDVGAPIADVILPGAAYTEKS---ATYVNTEGRAQQT-KVAV  target    KRLYDTRSDYEALALTAKALGELIDEPRMEQYWRGILDGDPTPYLQRIFSGSNATRGITYDELHESSKRGVPLLMNMRTY 6qc5.1    MPPGLAREDWKIIRALSEIAGMT---------------------------------------------------------  target    PRSGGWEQRQEDKPWYTATGRLEFYRPEPEFQAAGESLPVWREPVDATFYEPNAILSNAAHPSIAPRAPEDYGVPESQLD 6qc5.1    --------------------------------------------------------------------------------  target    VETRQYRNVVRTWAELQQTLHPLQERDPAFRFVFQTPKYRWGAHSTAVDADWISMLFGPFGDPYRRDPRMPWTGEAYLEI 6qc5.1    --------------------------------------------------------------------------------  target    NPKDAAELGLADGDYAWVDADPEDRPYRGWKEDDPYYEVARAMMRVRIYTGMSRGVIRTWFNMYAATPATVANQKATPGN 6qc5.1    --------------------------------------------------------------------------------  target    PARNEQTRYVALFRYGSHQSGTRAWLRPTQQTDSLVRKGYFGQVIGTGFEADVHSVSGAPKEAFVKIEKAEDGGIGAERL 6qc5.1    --------------------------------------------------------------------------------  target    WRPLTLGLRPEAPSAALTAYLAGDYSGTKGS 6qc5.1    ------------------------------- ``` | | | | | | | | | | | | | | | | | | | | | | | | | | | | | | | | | | | | | | | | | | | | | | | | | |
|  | 7zd6.1.4 | NADH-ubiquinone oxidoreductase 75 kDa subunit, mitochondrial  *Complex I from Ovis aries, at pH7.4, Open state* | 0.09 | 0.00 | 13.14 | 0.21 | 186-423 | EM | 0.00 | monomer | 6 x PC1, 14 x 3PE, 1 x DCQ, 2 x ZMP, 1 x AMP, 1 x MYR, 6 x SF4, 1 x FMN, 1 x NAI, 2 x FES, 1 x K, 1 x ZN, 1 x NDP | HHblits | 0.27 |
| ``` target    MPTANKADEVIILRPGTDAAFFLGVARELIEKGLYDRAAVIERTDLPLLVRLDTGERLDARDVIPGYELAALTNYVTLKP 7zd6.1    --------------------------------------------------------------------------------  target    DAEIKGNPPPPPFTAGGQVVPTELRDAWGDFVWWDRATGRPRPVSRDEVGARFDGDPALLGEFEVELVDGSTVPVRPAFD 7zd6.1    --------------------------------------------------------------------------------  target    LLKQYLDESFDLRTASEVCRVPPQAIQSIARQLAANKRETLLAAGMGPNHYFQNDLFGRVQFLVAALTDNIGHLGGNVGS 7zd6.1    -------------------------SHPFSQVLQEAKKPMV-VLGSSALQRNDGAAILAAVSNIAQKIRTSSGVTGDWKV  target    YAGNYRGSVFQAMGQWIAEDPFAIEPDLTKPATVKRYYKAESAHYWNYGERPLRAVAKDDEGDLTKGEVLTGKSHMPTPT 7zd6.1    MN--I----------L------H---RIASQV--A---------ALDLGYKP----------G--VEAIR------KNPP  target    KLIWFGNSNSLLGNAKWSFDVVKNTLPRQDAVFCNEWHWTSSCEYADLVFPADSWAEFKLPDATASCTNPFLLAFPTTPL 7zd6.1    KMLFLLGADGGC--------VTRQDLPKDCFIVYQGHHGDVGAPIADVILPGAAYTEKS---ATYVNTEGRAQQT-KVAV  target    KRLYDTRSDYEALALTAKALGELIDEPRMEQYWRGILDGDPTPYLQRIFSGSNATRGITYDELHESSKRGVPLLMNMRTY 7zd6.1    MPPGLAREDWKIIRALSEIAGMT---------------------------------------------------------  target    PRSGGWEQRQEDKPWYTATGRLEFYRPEPEFQAAGESLPVWREPVDATFYEPNAILSNAAHPSIAPRAPEDYGVPESQLD 7zd6.1    --------------------------------------------------------------------------------  target    VETRQYRNVVRTWAELQQTLHPLQERDPAFRFVFQTPKYRWGAHSTAVDADWISMLFGPFGDPYRRDPRMPWTGEAYLEI 7zd6.1    --------------------------------------------------------------------------------  target    NPKDAAELGLADGDYAWVDADPEDRPYRGWKEDDPYYEVARAMMRVRIYTGMSRGVIRTWFNMYAATPATVANQKATPGN 7zd6.1    --------------------------------------------------------------------------------  target    PARNEQTRYVALFRYGSHQSGTRAWLRPTQQTDSLVRKGYFGQVIGTGFEADVHSVSGAPKEAFVKIEKAEDGGIGAERL 7zd6.1    --------------------------------------------------------------------------------  target    WRPLTLGLRPEAPSAALTAYLAGDYSGTKGS 7zd6.1    ------------------------------- ``` | | | | | | | | | | | | | | | | | | | | | | | | | | | | | | | | | | | | | | | | | | | | | | | | | |
|  | 6zk9.1.C | NADH:ubiquinone oxidoreductase core subunit S1  *Peripheral domain of open complex I during turnover* | 0.09 | 0.00 | 13.14 | 0.21 | 186-423 | EM | 0.00 | monomer | 6 x SF4, 1 x FMN, 1 x NAI, 2 x FES, 1 x K, 2 x PC1, 2 x 3PE, 1 x ZN, 1 x NDP, 1 x ZMP, 1 x CDL | HHblits | 0.27 |
| ``` target    MPTANKADEVIILRPGTDAAFFLGVARELIEKGLYDRAAVIERTDLPLLVRLDTGERLDARDVIPGYELAALTNYVTLKP 6zk9.1    --------------------------------------------------------------------------------  target    DAEIKGNPPPPPFTAGGQVVPTELRDAWGDFVWWDRATGRPRPVSRDEVGARFDGDPALLGEFEVELVDGSTVPVRPAFD 6zk9.1    --------------------------------------------------------------------------------  target    LLKQYLDESFDLRTASEVCRVPPQAIQSIARQLAANKRETLLAAGMGPNHYFQNDLFGRVQFLVAALTDNIGHLGGNVGS 6zk9.1    -------------------------SHPFSQVLQEAKKPMV-VLGSSALQRNDGAAILAAVSNIAQKIRTSSGVTGDWKV  target    YAGNYRGSVFQAMGQWIAEDPFAIEPDLTKPATVKRYYKAESAHYWNYGERPLRAVAKDDEGDLTKGEVLTGKSHMPTPT 6zk9.1    MNI------------L-H--R------IASQV--A---------ALDLGYKP----------G--VEAIR------KNPP  target    KLIWFGNSNSLLGNAKWSFDVVKNTLPRQDAVFCNEWHWTSSCEYADLVFPADSWAEFKLPDATASCTNPFLLAFPTTPL 6zk9.1    KMLFLLGADGGC--------VTRQDLPKDCFIVYQGHHGDVGAPIADVILPGAAYTEKS---ATYVNTEGRAQQT-KVAV  target    KRLYDTRSDYEALALTAKALGELIDEPRMEQYWRGILDGDPTPYLQRIFSGSNATRGITYDELHESSKRGVPLLMNMRTY 6zk9.1    MPPGLAREDWKIIRALSEIAGMT---------------------------------------------------------  target    PRSGGWEQRQEDKPWYTATGRLEFYRPEPEFQAAGESLPVWREPVDATFYEPNAILSNAAHPSIAPRAPEDYGVPESQLD 6zk9.1    --------------------------------------------------------------------------------  target    VETRQYRNVVRTWAELQQTLHPLQERDPAFRFVFQTPKYRWGAHSTAVDADWISMLFGPFGDPYRRDPRMPWTGEAYLEI 6zk9.1    --------------------------------------------------------------------------------  target    NPKDAAELGLADGDYAWVDADPEDRPYRGWKEDDPYYEVARAMMRVRIYTGMSRGVIRTWFNMYAATPATVANQKATPGN 6zk9.1    --------------------------------------------------------------------------------  target    PARNEQTRYVALFRYGSHQSGTRAWLRPTQQTDSLVRKGYFGQVIGTGFEADVHSVSGAPKEAFVKIEKAEDGGIGAERL 6zk9.1    --------------------------------------------------------------------------------  target    WRPLTLGLRPEAPSAALTAYLAGDYSGTKGS 6zk9.1    ------------------------------- ``` | | | | | | | | | | | | | | | | | | | | | | | | | | | | | | | | | | | | | | | | | | | | | | | | | |
|  | 7vxu.1.L | NADH-ubiquinone oxidoreductase 75 kDa subunit, mitochondrial  *Matrix arm of deactive state CI from Q10 dataset* | 0.08 |  | 12.57 | 0.21 | 186-423 | EM | 0.00 | hetero-1-1-1-1-1-1-… | 6 x SF4, 1 x FMN, 1 x PEE, 1 x PLX, 1 x 8Q1, 1 x NDP, 2 x FES, 1 x MG, 1 x CDL, 1 x ZN | HHblits | 0.27 |
| ``` target    MPTANKADEVIILRPGTDAAFFLGVARELIEKGLYDRAAVIERTDLPLLVRLDTGERLDARDVIPGYELAALTNYVTLKP 7vxu.1    --------------------------------------------------------------------------------  target    DAEIKGNPPPPPFTAGGQVVPTELRDAWGDFVWWDRATGRPRPVSRDEVGARFDGDPALLGEFEVELVDGSTVPVRPAFD 7vxu.1    --------------------------------------------------------------------------------  target    LLKQYLDESFDLRTASEVCRVPPQAIQSIARQLAANKRETLLAAGMGPNHYFQNDLFGRVQFLVAALTDNIGHLGGNVGS 7vxu.1    -------------------------NHPFSQILKEAKKPMV-VLGSSALQRSDGTAILAAVSNIAQNIRLSSGVTGDWKV  target    YAGNYRGSVFQAMGQWIAEDPFAIEPDLTKPATVKRYYKAESAHYWNYGERPLRAVAKDDEGDLTKGEVLTGKSHMPTPT 7vxu.1    MNI---------LH------R------IASQV--A---------ALDLGYKP----------G--VEAIR------KNPP  target    KLIWFGNSNSLLGNAKWSFDVVKNTLPRQDAVFCNEWHWTSSCEYADLVFPADSWAEFKLPDATASCTNPFLLAFPTTPL 7vxu.1    KVLFLLGADGG--------CITRQDLPKDCFIIYQGHHGDVGAPMADVILPGAAYTEKS---ATYVNTEGRAQQT-KVAV  target    KRLYDTRSDYEALALTAKALGELIDEPRMEQYWRGILDGDPTPYLQRIFSGSNATRGITYDELHESSKRGVPLLMNMRTY 7vxu.1    TPPGLAREDWKIIRALSEIAGMT---------------------------------------------------------  target    PRSGGWEQRQEDKPWYTATGRLEFYRPEPEFQAAGESLPVWREPVDATFYEPNAILSNAAHPSIAPRAPEDYGVPESQLD 7vxu.1    --------------------------------------------------------------------------------  target    VETRQYRNVVRTWAELQQTLHPLQERDPAFRFVFQTPKYRWGAHSTAVDADWISMLFGPFGDPYRRDPRMPWTGEAYLEI 7vxu.1    --------------------------------------------------------------------------------  target    NPKDAAELGLADGDYAWVDADPEDRPYRGWKEDDPYYEVARAMMRVRIYTGMSRGVIRTWFNMYAATPATVANQKATPGN 7vxu.1    --------------------------------------------------------------------------------  target    PARNEQTRYVALFRYGSHQSGTRAWLRPTQQTDSLVRKGYFGQVIGTGFEADVHSVSGAPKEAFVKIEKAEDGGIGAERL 7vxu.1    --------------------------------------------------------------------------------  target    WRPLTLGLRPEAPSAALTAYLAGDYSGTKGS 7vxu.1    ------------------------------- ``` | | | | | | | | | | | | | | | | | | | | | | | | | | | | | | | | | | | | | | | | | | | | | | | | | |
|  | 7ak5.1.G | NADH-ubiquinone oxidoreductase 75 kDa subunit, mitochondrial  *Cryo-EM structure of respiratory complex I in the deactive state from Mus musculus at 3.2 A* | 0.09 | 0.00 | 11.93 | 0.21 | 185-423 | EM | 0.00 | monomer | 6 x SF4, 2 x PC1, 2 x FES, 1 x FMN, 8 x 3PE, 4 x CDL, 1 x ATP, 1 x NDP, 1 x ZN, 2 x EHZ | HHblits | 0.26 |
| ``` target    MPTANKADEVIILRPGTDAAFFLGVARELIEKGLYDRAAVIERTDLPLLVRLDTGERLDARDVIPGYELAALTNYVTLKP 7ak5.1    --------------------------------------------------------------------------------  target    DAEIKGNPPPPPFTAGGQVVPTELRDAWGDFVWWDRATGRPRPVSRDEVGARFDGDPALLGEFEVELVDGSTVPVRPAFD 7ak5.1    --------------------------------------------------------------------------------  target    LLKQYLDESFDLRTASEVCRVPPQAIQSIARQLAANKRETLLAAGMGPNHYFQNDLFGRVQFLVAALTDNIGHLGGNVGS 7ak5.1    ------------------------GRHSFCEVLKDAKKPMV-VLGSSALQRDDGAAILVAVSNMVQKIRVTTGVAAEWKV  target    YAGNYRGSVFQAMGQWIAEDPFAIEPDLTKPATVKRYYKAESAHYWNYGERPLRAVAKDDEGDLTKGEVLTGKSHMPTPT 7ak5.1    MN------------ILH---R------IASQVA-----------ALDLGYKP----------GV--EAIR------KNPP  target    KLIWFGNSNSLLGNAKWSFDVVKNTLPRQDAVFCNEWHWTSSCEYADLVFPADSWAEFKLPDATASCTNPFLLAFPTTPL 7ak5.1    KMLFLLGADGG--------CITRQDLPKDCFIVYQGHHGDVGAPMADVILPGAAYTEKS---ATYVNTEGRAQQT-KVAV  target    KRLYDTRSDYEALALTAKALGELIDEPRMEQYWRGILDGDPTPYLQRIFSGSNATRGITYDELHESSKRGVPLLMNMRTY 7ak5.1    TPPGLAREDWKIIRALSEIAGIT---------------------------------------------------------  target    PRSGGWEQRQEDKPWYTATGRLEFYRPEPEFQAAGESLPVWREPVDATFYEPNAILSNAAHPSIAPRAPEDYGVPESQLD 7ak5.1    --------------------------------------------------------------------------------  target    VETRQYRNVVRTWAELQQTLHPLQERDPAFRFVFQTPKYRWGAHSTAVDADWISMLFGPFGDPYRRDPRMPWTGEAYLEI 7ak5.1    --------------------------------------------------------------------------------  target    NPKDAAELGLADGDYAWVDADPEDRPYRGWKEDDPYYEVARAMMRVRIYTGMSRGVIRTWFNMYAATPATVANQKATPGN 7ak5.1    --------------------------------------------------------------------------------  target    PARNEQTRYVALFRYGSHQSGTRAWLRPTQQTDSLVRKGYFGQVIGTGFEADVHSVSGAPKEAFVKIEKAEDGGIGAERL 7ak5.1    --------------------------------------------------------------------------------  target    WRPLTLGLRPEAPSAALTAYLAGDYSGTKGS 7ak5.1    ------------------------------- ``` | | | | | | | | | | | | | | | | | | | | | | | | | | | | | | | | | | | | | | | | | | | | | | | | | |
|  | 6zr2.1.G | NADH-ubiquinone oxidoreductase 75 kDa subunit, mitochondrial  *Cryo-EM structure of respiratory complex I in the active state from Mus musculus at 3.1 A* | 0.09 | 0.00 | 11.93 | 0.21 | 185-423 | EM | 3.10 | monomer | 6 x SF4, 4 x PC1, 2 x FES, 1 x FMN, 9 x 3PE, 7 x CDL, 1 x ATP, 1 x NDP, 1 x ZN, 2 x EHZ | HHblits | 0.26 |
| ``` target    MPTANKADEVIILRPGTDAAFFLGVARELIEKGLYDRAAVIERTDLPLLVRLDTGERLDARDVIPGYELAALTNYVTLKP 6zr2.1    --------------------------------------------------------------------------------  target    DAEIKGNPPPPPFTAGGQVVPTELRDAWGDFVWWDRATGRPRPVSRDEVGARFDGDPALLGEFEVELVDGSTVPVRPAFD 6zr2.1    --------------------------------------------------------------------------------  target    LLKQYLDESFDLRTASEVCRVPPQAIQSIARQLAANKRETLLAAGMGPNHYFQNDLFGRVQFLVAALTDNIGHLGGNVGS 6zr2.1    ------------------------GRHSFCEVLKDAKKPMV-VLGSSALQRDDGAAILVAVSNMVQKIRVTTGVAAEWKV  target    YAGNYRGSVFQAMGQWIAEDPFAIEPDLTKPATVKRYYKAESAHYWNYGERPLRAVAKDDEGDLTKGEVLTGKSHMPTPT 6zr2.1    MN------------IL------H---RIASQV--A---------ALDLGYKP----------G--VEAIR------KNPP  target    KLIWFGNSNSLLGNAKWSFDVVKNTLPRQDAVFCNEWHWTSSCEYADLVFPADSWAEFKLPDATASCTNPFLLAFPTTPL 6zr2.1    KMLFLLGADGG--------CITRQDLPKDCFIVYQGHHGDVGAPMADVILPGAAYTEKS---ATYVNTEGRAQQ-TKVAV  target    KRLYDTRSDYEALALTAKALGELIDEPRMEQYWRGILDGDPTPYLQRIFSGSNATRGITYDELHESSKRGVPLLMNMRTY 6zr2.1    TPPGLAREDWKIIRALSEIAGIT---------------------------------------------------------  target    PRSGGWEQRQEDKPWYTATGRLEFYRPEPEFQAAGESLPVWREPVDATFYEPNAILSNAAHPSIAPRAPEDYGVPESQLD 6zr2.1    --------------------------------------------------------------------------------  target    VETRQYRNVVRTWAELQQTLHPLQERDPAFRFVFQTPKYRWGAHSTAVDADWISMLFGPFGDPYRRDPRMPWTGEAYLEI 6zr2.1    --------------------------------------------------------------------------------  target    NPKDAAELGLADGDYAWVDADPEDRPYRGWKEDDPYYEVARAMMRVRIYTGMSRGVIRTWFNMYAATPATVANQKATPGN 6zr2.1    --------------------------------------------------------------------------------  target    PARNEQTRYVALFRYGSHQSGTRAWLRPTQQTDSLVRKGYFGQVIGTGFEADVHSVSGAPKEAFVKIEKAEDGGIGAERL 6zr2.1    --------------------------------------------------------------------------------  target    WRPLTLGLRPEAPSAALTAYLAGDYSGTKGS 6zr2.1    ------------------------------- ``` | | | | | | | | | | | | | | | | | | | | | | | | | | | | | | | | | | | | | | | | | | | | | | | | | |
|  | 6g72.1.G | NADH-ubiquinone oxidoreductase 75 kDa subunit, mitochondrial  *Mouse mitochondrial complex I in the deactive state* | 0.09 | 0.00 | 11.93 | 0.21 | 185-423 | EM | 0.00 | monomer | 6 x SF4, 2 x FES, 1 x FMN, 1 x ADP, 1 x NDP, 1 x ZN, 2 x EHZ | HHblits | 0.26 |
| ``` target    MPTANKADEVIILRPGTDAAFFLGVARELIEKGLYDRAAVIERTDLPLLVRLDTGERLDARDVIPGYELAALTNYVTLKP 6g72.1    --------------------------------------------------------------------------------  target    DAEIKGNPPPPPFTAGGQVVPTELRDAWGDFVWWDRATGRPRPVSRDEVGARFDGDPALLGEFEVELVDGSTVPVRPAFD 6g72.1    --------------------------------------------------------------------------------  target    LLKQYLDESFDLRTASEVCRVPPQAIQSIARQLAANKRETLLAAGMGPNHYFQNDLFGRVQFLVAALTDNIGHLGGNVGS 6g72.1    ------------------------GRHSFCEVLKDAKKPMV-VLGSSALQRDDGAAILVAVSNMVQKIRVTTGVAAEWKV  target    YAGNYRGSVFQAMGQWIAEDPFAIEPDLTKPATVKRYYKAESAHYWNYGERPLRAVAKDDEGDLTKGEVLTGKSHMPTPT 6g72.1    MN------------IL------H---RIASQV--A---------ALDLGYKP----------G--VEAIR------KNPP  target    KLIWFGNSNSLLGNAKWSFDVVKNTLPRQDAVFCNEWHWTSSCEYADLVFPADSWAEFKLPDATASCTNPFLLAFPTTPL 6g72.1    KMLFLLGADGG--------CITRQDLPKDCFIVYQGHHGDVGAPMADVILPGAAYTEKS---ATYVNTEGRAQQ-TKVAV  target    KRLYDTRSDYEALALTAKALGELIDEPRMEQYWRGILDGDPTPYLQRIFSGSNATRGITYDELHESSKRGVPLLMNMRTY 6g72.1    TPPGLAREDWKIIRALSEIAGIT---------------------------------------------------------  target    PRSGGWEQRQEDKPWYTATGRLEFYRPEPEFQAAGESLPVWREPVDATFYEPNAILSNAAHPSIAPRAPEDYGVPESQLD 6g72.1    --------------------------------------------------------------------------------  target    VETRQYRNVVRTWAELQQTLHPLQERDPAFRFVFQTPKYRWGAHSTAVDADWISMLFGPFGDPYRRDPRMPWTGEAYLEI 6g72.1    --------------------------------------------------------------------------------  target    NPKDAAELGLADGDYAWVDADPEDRPYRGWKEDDPYYEVARAMMRVRIYTGMSRGVIRTWFNMYAATPATVANQKATPGN 6g72.1    --------------------------------------------------------------------------------  target    PARNEQTRYVALFRYGSHQSGTRAWLRPTQQTDSLVRKGYFGQVIGTGFEADVHSVSGAPKEAFVKIEKAEDGGIGAERL 6g72.1    --------------------------------------------------------------------------------  target    WRPLTLGLRPEAPSAALTAYLAGDYSGTKGS 6g72.1    ------------------------------- ``` | | | | | | | | | | | | | | | | | | | | | | | | | | | | | | | | | | | | | | | | | | | | | | | | | |
|  | 7ak6.1.G | NADH-ubiquinone oxidoreductase 75 kDa subunit, mitochondrial  *Cryo-EM structure of ND6-P25L mutant respiratory complex I from Mus musculus at 3.8 A* | 0.09 | 0.00 | 11.93 | 0.21 | 185-423 | EM | 0.00 | monomer | 6 x SF4, 1 x PC1, 2 x FES, 1 x FMN, 4 x 3PE, 2 x CDL, 1 x ATP, 1 x NDP, 1 x ZN, 2 x EHZ | HHblits | 0.26 |
| ``` target    MPTANKADEVIILRPGTDAAFFLGVARELIEKGLYDRAAVIERTDLPLLVRLDTGERLDARDVIPGYELAALTNYVTLKP 7ak6.1    --------------------------------------------------------------------------------  target    DAEIKGNPPPPPFTAGGQVVPTELRDAWGDFVWWDRATGRPRPVSRDEVGARFDGDPALLGEFEVELVDGSTVPVRPAFD 7ak6.1    --------------------------------------------------------------------------------  target    LLKQYLDESFDLRTASEVCRVPPQAIQSIARQLAANKRETLLAAGMGPNHYFQNDLFGRVQFLVAALTDNIGHLGGNVGS 7ak6.1    ------------------------GRHSFCEVLKDAKKPMV-VLGSSALQRDDGAAILVAVSNMVQKIRVTTGVAAEWKV  target    YAGNYRGSVFQAMGQWIAEDPFAIEPDLTKPATVKRYYKAESAHYWNYGERPLRAVAKDDEGDLTKGEVLTGKSHMPTPT 7ak6.1    MN------------IL------H---RIASQV--A---------ALDLGYKP----------G--VEAIR------KNPP  target    KLIWFGNSNSLLGNAKWSFDVVKNTLPRQDAVFCNEWHWTSSCEYADLVFPADSWAEFKLPDATASCTNPFLLAFPTTPL 7ak6.1    KMLFLLGADGG--------CITRQDLPKDCFIVYQGHHGDVGAPMADVILPGAAYTEKS---ATYVNTEGRAQQ-TKVAV  target    KRLYDTRSDYEALALTAKALGELIDEPRMEQYWRGILDGDPTPYLQRIFSGSNATRGITYDELHESSKRGVPLLMNMRTY 7ak6.1    TPPGLAREDWKIIRALSEIAGIT---------------------------------------------------------  target    PRSGGWEQRQEDKPWYTATGRLEFYRPEPEFQAAGESLPVWREPVDATFYEPNAILSNAAHPSIAPRAPEDYGVPESQLD 7ak6.1    --------------------------------------------------------------------------------  target    VETRQYRNVVRTWAELQQTLHPLQERDPAFRFVFQTPKYRWGAHSTAVDADWISMLFGPFGDPYRRDPRMPWTGEAYLEI 7ak6.1    --------------------------------------------------------------------------------  target    NPKDAAELGLADGDYAWVDADPEDRPYRGWKEDDPYYEVARAMMRVRIYTGMSRGVIRTWFNMYAATPATVANQKATPGN 7ak6.1    --------------------------------------------------------------------------------  target    PARNEQTRYVALFRYGSHQSGTRAWLRPTQQTDSLVRKGYFGQVIGTGFEADVHSVSGAPKEAFVKIEKAEDGGIGAERL 7ak6.1    --------------------------------------------------------------------------------  target    WRPLTLGLRPEAPSAALTAYLAGDYSGTKGS 7ak6.1    ------------------------------- ``` | | | | | | | | | | | | | | | | | | | | | | | | | | | | | | | | | | | | | | | | | | | | | | | | | |
|  | 5gpn.24.A | NADH-ubiquinone oxidoreductase 75 kDa subunit  *Architecture of mammalian respirasome* | 0.08 |  | 12.57 | 0.21 | 186-423 | EM | 0.00 | monomer |  | HHblits | 0.27 |
| ``` target    MPTANKADEVIILRPGTDAAFFLGVARELIEKGLYDRAAVIERTDLPLLVRLDTGERLDARDVIPGYELAALTNYVTLKP 5gpn.24   --------------------------------------------------------------------------------  target    DAEIKGNPPPPPFTAGGQVVPTELRDAWGDFVWWDRATGRPRPVSRDEVGARFDGDPALLGEFEVELVDGSTVPVRPAFD 5gpn.24   --------------------------------------------------------------------------------  target    LLKQYLDESFDLRTASEVCRVPPQAIQSIARQLAANKRETLLAAGMGPNHYFQNDLFGRVQFLVAALTDNIGHLGGNVGS 5gpn.24   -------------------------NHPFSQILKEAKKPMV-VLGSSALQRSDGTAILAAVSNIAQNIRLSSGVTGDWKV  target    YAGNYRGSVFQAMGQWIAEDPFAIEPDLTKPATVKRYYKAESAHYWNYGERPLRAVAKDDEGDLTKGEVLTGKSHMPTPT 5gpn.24   MNI------------L------H---RIASQV--A---------ALDLGYKP----------G--VEAIR------KNPP  target    KLIWFGNSNSLLGNAKWSFDVVKNTLPRQDAVFCNEWHWTSSCEYADLVFPADSWAEFKLPDATASCTNPFLLAFPTTPL 5gpn.24   KVLFLLGADGG--------CITRQDLPKDCFIIYQGHHGDVGAPMADVILPGAAYTEKS---ATYVNTEGRAQQT-KVAV  target    KRLYDTRSDYEALALTAKALGELIDEPRMEQYWRGILDGDPTPYLQRIFSGSNATRGITYDELHESSKRGVPLLMNMRTY 5gpn.24   TPPGLAREDWKIIRALSEIAGMT---------------------------------------------------------  target    PRSGGWEQRQEDKPWYTATGRLEFYRPEPEFQAAGESLPVWREPVDATFYEPNAILSNAAHPSIAPRAPEDYGVPESQLD 5gpn.24   --------------------------------------------------------------------------------  target    VETRQYRNVVRTWAELQQTLHPLQERDPAFRFVFQTPKYRWGAHSTAVDADWISMLFGPFGDPYRRDPRMPWTGEAYLEI 5gpn.24   --------------------------------------------------------------------------------  target    NPKDAAELGLADGDYAWVDADPEDRPYRGWKEDDPYYEVARAMMRVRIYTGMSRGVIRTWFNMYAATPATVANQKATPGN 5gpn.24   --------------------------------------------------------------------------------  target    PARNEQTRYVALFRYGSHQSGTRAWLRPTQQTDSLVRKGYFGQVIGTGFEADVHSVSGAPKEAFVKIEKAEDGGIGAERL 5gpn.24   --------------------------------------------------------------------------------  target    WRPLTLGLRPEAPSAALTAYLAGDYSGTKGS 5gpn.24   ------------------------------- ``` | | | | | | | | | | | | | | | | | | | | | | | | | | | | | | | | | | | | | | | | | | | | | | | | | |
|  | 7dgr.10.A | NADH-ubiquinone oxidoreductase 75 kDa subunit, mitochondrial  *Activity optimized supercomplex state2* | 0.08 | 0.00 | 12.00 | 0.21 | 186-423 | EM | 0.00 | monomer |  | HHblits | 0.26 |
| ``` target    MPTANKADEVIILRPGTDAAFFLGVARELIEKGLYDRAAVIERTDLPLLVRLDTGERLDARDVIPGYELAALTNYVTLKP 7dgr.10   --------------------------------------------------------------------------------  target    DAEIKGNPPPPPFTAGGQVVPTELRDAWGDFVWWDRATGRPRPVSRDEVGARFDGDPALLGEFEVELVDGSTVPVRPAFD 7dgr.10   --------------------------------------------------------------------------------  target    LLKQYLDESFDLRTASEVCRVPPQAIQSIARQLAANKRETLLAAGMGPNHYFQNDLFGRVQFLVAALTDNIGHLGGNVGS 7dgr.10   -------------------------SHPFSQVLQEAKKPMV-ILGSSALQRNDGAAILAAVSNIAQKIRTSSGVTGDWKV  target    YAGNYRGSVFQAMGQWIAEDPFAIEPDLTKPATVKRYYKAESAHYWNYGERPLRAVAKDDEGDLTKGEVLTGKSHMPTPT 7dgr.10   MNIL---------H------RIA---SQVA--A------------LDL---GYKP-------G--VEAIQ------KNPP  target    KLIWFGNSNSLLGNAKWSFDVVKNTLPRQDAVFCNEWHWTSSCEYADLVFPADSWAEFKLPDATASCTNPFLLAFPTTPL 7dgr.10   KMLFLLGADGG--------CITRQDLPKDCFIVYQGHHGDVGAPIADVILPGAAYTEKS---ATYVNTEGRAQQT-KVAV  target    KRLYDTRSDYEALALTAKALGELIDEPRMEQYWRGILDGDPTPYLQRIFSGSNATRGITYDELHESSKRGVPLLMNMRTY 7dgr.10   TPPGLAREDWKIIRALSEIAGMT---------------------------------------------------------  target    PRSGGWEQRQEDKPWYTATGRLEFYRPEPEFQAAGESLPVWREPVDATFYEPNAILSNAAHPSIAPRAPEDYGVPESQLD 7dgr.10   --------------------------------------------------------------------------------  target    VETRQYRNVVRTWAELQQTLHPLQERDPAFRFVFQTPKYRWGAHSTAVDADWISMLFGPFGDPYRRDPRMPWTGEAYLEI 7dgr.10   --------------------------------------------------------------------------------  target    NPKDAAELGLADGDYAWVDADPEDRPYRGWKEDDPYYEVARAMMRVRIYTGMSRGVIRTWFNMYAATPATVANQKATPGN 7dgr.10   --------------------------------------------------------------------------------  target    PARNEQTRYVALFRYGSHQSGTRAWLRPTQQTDSLVRKGYFGQVIGTGFEADVHSVSGAPKEAFVKIEKAEDGGIGAERL 7dgr.10   --------------------------------------------------------------------------------  target    WRPLTLGLRPEAPSAALTAYLAGDYSGTKGS 7dgr.10   ------------------------------- ``` | | | | | | | | | | | | | | | | | | | | | | | | | | | | | | | | | | | | | | | | | | | | | | | | | |
|  | 5o31.1.8 | NADH-ubiquinone oxidoreductase 75 kDa subunit, mitochondrial  *Mitochondrial complex I in the deactive state* | 0.08 | 0.00 | 12.00 | 0.21 | 186-423 | EM | 4.13 | monomer | 6 x SF4, 2 x FES, 1 x FMN, 1 x NAP, 1 x ZN | HHblits | 0.26 |
| ``` target    MPTANKADEVIILRPGTDAAFFLGVARELIEKGLYDRAAVIERTDLPLLVRLDTGERLDARDVIPGYELAALTNYVTLKP 5o31.1    --------------------------------------------------------------------------------  target    DAEIKGNPPPPPFTAGGQVVPTELRDAWGDFVWWDRATGRPRPVSRDEVGARFDGDPALLGEFEVELVDGSTVPVRPAFD 5o31.1    --------------------------------------------------------------------------------  target    LLKQYLDESFDLRTASEVCRVPPQAIQSIARQLAANKRETLLAAGMGPNHYFQNDLFGRVQFLVAALTDNIGHLGGNVGS 5o31.1    -------------------------SHPFSQVLQEAKKPMV-ILGSSALQRNDGAAILAAVSNIAQKIRTSSGVTGDWKV  target    YAGNYRGSVFQAMGQWIAEDPFAIEPDLTKPATVKRYYKAESAHYWNYGERPLRAVAKDDEGDLTKGEVLTGKSHMPTPT 5o31.1    MNIL---------H------RIA---SQVA--A------------LDL---GYKP-------G--VEAIQ------KNPP  target    KLIWFGNSNSLLGNAKWSFDVVKNTLPRQDAVFCNEWHWTSSCEYADLVFPADSWAEFKLPDATASCTNPFLLAFPTTPL 5o31.1    KMLFLLGADGG--------CITRQDLPKDCFIVYQGHHGDVGAPIADVILPGAAYTEKS---ATYVNTEGRAQQT-KVAV  target    KRLYDTRSDYEALALTAKALGELIDEPRMEQYWRGILDGDPTPYLQRIFSGSNATRGITYDELHESSKRGVPLLMNMRTY 5o31.1    TPPGLAREDWKIIRALSEIAGMT---------------------------------------------------------  target    PRSGGWEQRQEDKPWYTATGRLEFYRPEPEFQAAGESLPVWREPVDATFYEPNAILSNAAHPSIAPRAPEDYGVPESQLD 5o31.1    --------------------------------------------------------------------------------  target    VETRQYRNVVRTWAELQQTLHPLQERDPAFRFVFQTPKYRWGAHSTAVDADWISMLFGPFGDPYRRDPRMPWTGEAYLEI 5o31.1    --------------------------------------------------------------------------------  target    NPKDAAELGLADGDYAWVDADPEDRPYRGWKEDDPYYEVARAMMRVRIYTGMSRGVIRTWFNMYAATPATVANQKATPGN 5o31.1    --------------------------------------------------------------------------------  target    PARNEQTRYVALFRYGSHQSGTRAWLRPTQQTDSLVRKGYFGQVIGTGFEADVHSVSGAPKEAFVKIEKAEDGGIGAERL 5o31.1    --------------------------------------------------------------------------------  target    WRPLTLGLRPEAPSAALTAYLAGDYSGTKGS 5o31.1    ------------------------------- ``` | | | | | | | | | | | | | | | | | | | | | | | | | | | | | | | | | | | | | | | | | | | | | | | | | |
|  | 7qsd.1.G | NADH-ubiquinone oxidoreductase 75 kDa subunit, mitochondrial  *Bovine complex I in the active state at 3.1 A* | 0.09 |  | 11.43 | 0.21 | 186-423 | EM | 0.00 | hetero-1-1-1-1-1-1-… | 5 x PC1, 13 x 3PE, 6 x SF4, 2 x FES, 1 x FMN, 4 x CDL, 3 x LMT, 1 x GTP, 1 x MG, 1 x NDP, 1 x ZN, 2 x EHZ | HHblits | 0.26 |
| ``` target    MPTANKADEVIILRPGTDAAFFLGVARELIEKGLYDRAAVIERTDLPLLVRLDTGERLDARDVIPGYELAALTNYVTLKP 7qsd.1    --------------------------------------------------------------------------------  target    DAEIKGNPPPPPFTAGGQVVPTELRDAWGDFVWWDRATGRPRPVSRDEVGARFDGDPALLGEFEVELVDGSTVPVRPAFD 7qsd.1    --------------------------------------------------------------------------------  target    LLKQYLDESFDLRTASEVCRVPPQAIQSIARQLAANKRETLLAAGMGPNHYFQNDLFGRVQFLVAALTDNIGHLGGNVGS 7qsd.1    -------------------------SHPFSQVLQEAKKPMV-ILGSSALQRNDGAAILAAVSNIAQKIRTSSGVTGDWKV  target    YAGNYRGSVFQAMGQWIAEDPFAIEPDLTKPATVKRYYKAESAHYWNYGERPLRAVAKDDEGDLTKGEVLTGKSHMPTPT 7qsd.1    MNI------------L------H---RIASQV--A---------ALDL---GYKP-------G--VEAIQ------KNPP  target    KLIWFGNSNSLLGNAKWSFDVVKNTLPRQDAVFCNEWHWTSSCEYADLVFPADSWAEFKLPDATASCTNPFLLAFPTTPL 7qsd.1    KMLFLLGADGG--------CITRQDLPKDCFIVYQGHHGDVGAPIADVILPGAAYTEKS---ATYVNTEGRAQQT-KVAV  target    KRLYDTRSDYEALALTAKALGELIDEPRMEQYWRGILDGDPTPYLQRIFSGSNATRGITYDELHESSKRGVPLLMNMRTY 7qsd.1    TPPGLAREDWKIIRALSEIAGMT---------------------------------------------------------  target    PRSGGWEQRQEDKPWYTATGRLEFYRPEPEFQAAGESLPVWREPVDATFYEPNAILSNAAHPSIAPRAPEDYGVPESQLD 7qsd.1    --------------------------------------------------------------------------------  target    VETRQYRNVVRTWAELQQTLHPLQERDPAFRFVFQTPKYRWGAHSTAVDADWISMLFGPFGDPYRRDPRMPWTGEAYLEI 7qsd.1    --------------------------------------------------------------------------------  target    NPKDAAELGLADGDYAWVDADPEDRPYRGWKEDDPYYEVARAMMRVRIYTGMSRGVIRTWFNMYAATPATVANQKATPGN 7qsd.1    --------------------------------------------------------------------------------  target    PARNEQTRYVALFRYGSHQSGTRAWLRPTQQTDSLVRKGYFGQVIGTGFEADVHSVSGAPKEAFVKIEKAEDGGIGAERL 7qsd.1    --------------------------------------------------------------------------------  target    WRPLTLGLRPEAPSAALTAYLAGDYSGTKGS 7qsd.1    ------------------------------- ``` | | | | | | | | | | | | | | | | | | | | | | | | | | | | | | | | | | | | | | | | | | | | | | | | | |
|  | 7ar7.1.G | NADH dehydrogenase [ubiquinone] iron-sulfur protein 1, mitochondrial  *Cryo-EM structure of Arabidopsis thaliana complex-I (open conformation)* | 0.08 |  | 13.37 | 0.21 | 183-423 | EM | 0.00 | hetero-1-1-1-1-1-1-… | 6 x SF4, 2 x FES, 1 x FMN, 1 x UQ9, 3 x PTY, 2 x PC7, 1 x LMN, 1 x NDP, 2 x ZN, 2 x 8Q1, 1 x PGT, 1 x PSF, 1 x T7X | HHblits | 0.28 |
| ``` target    MPTANKADEVIILRPGTDAAFFLGVARELIEKGLYDRAAVIERTDLPLLVRLDTGERLDARDVIPGYELAALTNYVTLKP 7ar7.1    --------------------------------------------------------------------------------  target    DAEIKGNPPPPPFTAGGQVVPTELRDAWGDFVWWDRATGRPRPVSRDEVGARFDGDPALLGEFEVELVDGSTVPVRPAFD 7ar7.1    --------------------------------------------------------------------------------  target    LLKQYLDESFDLRTASEVCRVPPQAIQSIARQLAANKRETLLAAGMGPNHYFQNDLFGRVQFLVAALTDNIGHLGGNVGS 7ar7.1    ----------------------AEGRHPFCTALKNAKNPAI-IVGAGLFNRTDKNAILSSVESIAQANNVVRPDWNGLNF  target    YAGNYRGSVFQAMGQWIAEDPFAIEPDLTKPATVKRYYKAESAHYWNYGERPLRAVAKDDEGDLTKGEVLTGKSHMPTPT 7ar7.1    LLQYAA-----QAAAL----------DL------------------G----------------LI-QQSAK----ALESA  target    KLIWFGNSNSLLGNAKWSFDVVKNTLPRQDAVFCNEWHWTSSCEYADLVFPADSWAEFKLPDATASCTNPFLLAFPTTPL 7ar7.1    KFVYLMGADDVN----------VDKIPKDAFVVYQGHHGDKAVYRANVILPASAFTEKE---GTYENTEGFTQQT-VPAV  target    KRLYDTRSDYEALALTAKALGELIDEPRMEQYWRGILDGDPTPYLQRIFSGSNATRGITYDELHESSKRGVPLLMNMRTY 7ar7.1    PTVGDARDDWKIVRALSEVSGVK---------------------------------------------------------  target    PRSGGWEQRQEDKPWYTATGRLEFYRPEPEFQAAGESLPVWREPVDATFYEPNAILSNAAHPSIAPRAPEDYGVPESQLD 7ar7.1    --------------------------------------------------------------------------------  target    VETRQYRNVVRTWAELQQTLHPLQERDPAFRFVFQTPKYRWGAHSTAVDADWISMLFGPFGDPYRRDPRMPWTGEAYLEI 7ar7.1    --------------------------------------------------------------------------------  target    NPKDAAELGLADGDYAWVDADPEDRPYRGWKEDDPYYEVARAMMRVRIYTGMSRGVIRTWFNMYAATPATVANQKATPGN 7ar7.1    --------------------------------------------------------------------------------  target    PARNEQTRYVALFRYGSHQSGTRAWLRPTQQTDSLVRKGYFGQVIGTGFEADVHSVSGAPKEAFVKIEKAEDGGIGAERL 7ar7.1    --------------------------------------------------------------------------------  target    WRPLTLGLRPEAPSAALTAYLAGDYSGTKGS 7ar7.1    ------------------------------- ``` | | | | | | | | | | | | | | | | | | | | | | | | | | | | | | | | | | | | | | | | | | | | | | | | | |
|  | 7aqr.1.F | NADH dehydrogenase [ubiquinone] iron-sulfur protein 1, mitochondrial  *Cryo-EM structure of Arabidopsis thaliana Complex-I (peripheral arm)* | 0.08 |  | 13.37 | 0.21 | 183-423 | EM | 0.00 | hetero-1-1-1-1-1-1-… | 6 x SF4, 2 x FES, 1 x FMN, 1 x NDP, 1 x ZN, 1 x 8Q1 | HHblits | 0.28 |
| ``` target    MPTANKADEVIILRPGTDAAFFLGVARELIEKGLYDRAAVIERTDLPLLVRLDTGERLDARDVIPGYELAALTNYVTLKP 7aqr.1    --------------------------------------------------------------------------------  target    DAEIKGNPPPPPFTAGGQVVPTELRDAWGDFVWWDRATGRPRPVSRDEVGARFDGDPALLGEFEVELVDGSTVPVRPAFD 7aqr.1    --------------------------------------------------------------------------------  target    LLKQYLDESFDLRTASEVCRVPPQAIQSIARQLAANKRETLLAAGMGPNHYFQNDLFGRVQFLVAALTDNIGHLGGNVGS 7aqr.1    ----------------------AEGRHPFCTALKNAKNPAI-IVGAGLFNRTDKNAILSSVESIAQANNVVRPDWNGLNF  target    YAGNYRGSVFQAMGQWIAEDPFAIEPDLTKPATVKRYYKAESAHYWNYGERPLRAVAKDDEGDLTKGEVLTGKSHMPTPT 7aqr.1    LLQYAA-----QAAAL----------DL------------------G----------------LI-QQSAK----ALESA  target    KLIWFGNSNSLLGNAKWSFDVVKNTLPRQDAVFCNEWHWTSSCEYADLVFPADSWAEFKLPDATASCTNPFLLAFPTTPL 7aqr.1    KFVYLMGADDVN----------VDKIPKDAFVVYQGHHGDKAVYRANVILPASAFTEKE---GTYENTEGFTQQT-VPAV  target    KRLYDTRSDYEALALTAKALGELIDEPRMEQYWRGILDGDPTPYLQRIFSGSNATRGITYDELHESSKRGVPLLMNMRTY 7aqr.1    PTVGDARDDWKIVRALSEVSGVK---------------------------------------------------------  target    PRSGGWEQRQEDKPWYTATGRLEFYRPEPEFQAAGESLPVWREPVDATFYEPNAILSNAAHPSIAPRAPEDYGVPESQLD 7aqr.1    --------------------------------------------------------------------------------  target    VETRQYRNVVRTWAELQQTLHPLQERDPAFRFVFQTPKYRWGAHSTAVDADWISMLFGPFGDPYRRDPRMPWTGEAYLEI 7aqr.1    --------------------------------------------------------------------------------  target    NPKDAAELGLADGDYAWVDADPEDRPYRGWKEDDPYYEVARAMMRVRIYTGMSRGVIRTWFNMYAATPATVANQKATPGN 7aqr.1    --------------------------------------------------------------------------------  target    PARNEQTRYVALFRYGSHQSGTRAWLRPTQQTDSLVRKGYFGQVIGTGFEADVHSVSGAPKEAFVKIEKAEDGGIGAERL 7aqr.1    --------------------------------------------------------------------------------  target    WRPLTLGLRPEAPSAALTAYLAGDYSGTKGS 7aqr.1    ------------------------------- ``` | | | | | | | | | | | | | | | | | | | | | | | | | | | | | | | | | | | | | | | | | | | | | | | | | |
|  | 7a23.1.O | 75kDa  *Plant mitochondrial respiratory complex I* | 0.09 |  | 13.37 | 0.21 | 183-423 | EM | 0.00 | hetero-1-1-1-1-1-1-… | 6 x SF4, 1 x FMN, 2 x T7X, 3 x CDL, 1 x U10, 1 x PEV, 2 x FES, 1 x NDP, 2 x ZN | HHblits | 0.28 |
| ``` target    MPTANKADEVIILRPGTDAAFFLGVARELIEKGLYDRAAVIERTDLPLLVRLDTGERLDARDVIPGYELAALTNYVTLKP 7a23.1    --------------------------------------------------------------------------------  target    DAEIKGNPPPPPFTAGGQVVPTELRDAWGDFVWWDRATGRPRPVSRDEVGARFDGDPALLGEFEVELVDGSTVPVRPAFD 7a23.1    --------------------------------------------------------------------------------  target    LLKQYLDESFDLRTASEVCRVPPQAIQSIARQLAANKRETLLAAGMGPNHYFQNDLFGRVQFLVAALTDNIGHLGGNVGS 7a23.1    ----------------------AEGRHPFCTALKNAKNPAI-IVGAGLFNRTDKNAILSSVESIAQANNVVRPDWNGLNF  target    YAGNYRGSVFQAMGQWIAEDPFAIEPDLTKPATVKRYYKAESAHYWNYGERPLRAVAKDDEGDLTKGEVLTGKSHMPTPT 7a23.1    LLQYAA-----QAAAL----------DL------------------G----------------LI-QQSAK----ALESA  target    KLIWFGNSNSLLGNAKWSFDVVKNTLPRQDAVFCNEWHWTSSCEYADLVFPADSWAEFKLPDATASCTNPFLLAFPTTPL 7a23.1    KFVYLMGADDVN----------VDKIPKDAFVVYQGHHGDKAVYRANVILPASAFTEKE---GTYENTEGFTQQT-VPAV  target    KRLYDTRSDYEALALTAKALGELIDEPRMEQYWRGILDGDPTPYLQRIFSGSNATRGITYDELHESSKRGVPLLMNMRTY 7a23.1    PTVGDARDDWKIVRALSEVSGVK---------------------------------------------------------  target    PRSGGWEQRQEDKPWYTATGRLEFYRPEPEFQAAGESLPVWREPVDATFYEPNAILSNAAHPSIAPRAPEDYGVPESQLD 7a23.1    --------------------------------------------------------------------------------  target    VETRQYRNVVRTWAELQQTLHPLQERDPAFRFVFQTPKYRWGAHSTAVDADWISMLFGPFGDPYRRDPRMPWTGEAYLEI 7a23.1    --------------------------------------------------------------------------------  target    NPKDAAELGLADGDYAWVDADPEDRPYRGWKEDDPYYEVARAMMRVRIYTGMSRGVIRTWFNMYAATPATVANQKATPGN 7a23.1    --------------------------------------------------------------------------------  target    PARNEQTRYVALFRYGSHQSGTRAWLRPTQQTDSLVRKGYFGQVIGTGFEADVHSVSGAPKEAFVKIEKAEDGGIGAERL 7a23.1    --------------------------------------------------------------------------------  target    WRPLTLGLRPEAPSAALTAYLAGDYSGTKGS 7a23.1    ------------------------------- ``` | | | | | | | | | | | | | | | | | | | | | | | | | | | | | | | | | | | | | | | | | | | | | | | | | |
|  | 7ar8.1.G | NADH dehydrogenase [ubiquinone] iron-sulfur protein 1, mitochondrial  *Cryo-EM structure of Arabidopsis thaliana complex-I (closed conformation)* | 0.08 |  | 13.37 | 0.21 | 183-423 | EM | 0.00 | hetero-1-1-1-1-1-1-… | 6 x SF4, 2 x FES, 1 x FMN, 1 x UQ9, 3 x PTY, 2 x PC7, 1 x PGT, 1 x FE, 1 x NDP, 2 x ZN, 2 x 8Q1, 1 x LMN, 1 x PSF, 1 x T7X | HHblits | 0.28 |
| ``` target    MPTANKADEVIILRPGTDAAFFLGVARELIEKGLYDRAAVIERTDLPLLVRLDTGERLDARDVIPGYELAALTNYVTLKP 7ar8.1    --------------------------------------------------------------------------------  target    DAEIKGNPPPPPFTAGGQVVPTELRDAWGDFVWWDRATGRPRPVSRDEVGARFDGDPALLGEFEVELVDGSTVPVRPAFD 7ar8.1    --------------------------------------------------------------------------------  target    LLKQYLDESFDLRTASEVCRVPPQAIQSIARQLAANKRETLLAAGMGPNHYFQNDLFGRVQFLVAALTDNIGHLGGNVGS 7ar8.1    ----------------------AEGRHPFCTALKNAKNPAI-IVGAGLFNRTDKNAILSSVESIAQANNVVRPDWNGLNF  target    YAGNYRGSVFQAMGQWIAEDPFAIEPDLTKPATVKRYYKAESAHYWNYGERPLRAVAKDDEGDLTKGEVLTGKSHMPTPT 7ar8.1    LLQYAA-----QAAAL----------DL------------------G----------------LI-QQSAK----ALESA  target    KLIWFGNSNSLLGNAKWSFDVVKNTLPRQDAVFCNEWHWTSSCEYADLVFPADSWAEFKLPDATASCTNPFLLAFPTTPL 7ar8.1    KFVYLMGADDVN----------VDKIPKDAFVVYQGHHGDKAVYRANVILPASAFTEKE---GTYENTEGFTQQT-VPAV  target    KRLYDTRSDYEALALTAKALGELIDEPRMEQYWRGILDGDPTPYLQRIFSGSNATRGITYDELHESSKRGVPLLMNMRTY 7ar8.1    PTVGDARDDWKIVRALSEVSGVK---------------------------------------------------------  target    PRSGGWEQRQEDKPWYTATGRLEFYRPEPEFQAAGESLPVWREPVDATFYEPNAILSNAAHPSIAPRAPEDYGVPESQLD 7ar8.1    --------------------------------------------------------------------------------  target    VETRQYRNVVRTWAELQQTLHPLQERDPAFRFVFQTPKYRWGAHSTAVDADWISMLFGPFGDPYRRDPRMPWTGEAYLEI 7ar8.1    --------------------------------------------------------------------------------  target    NPKDAAELGLADGDYAWVDADPEDRPYRGWKEDDPYYEVARAMMRVRIYTGMSRGVIRTWFNMYAATPATVANQKATPGN 7ar8.1    --------------------------------------------------------------------------------  target    PARNEQTRYVALFRYGSHQSGTRAWLRPTQQTDSLVRKGYFGQVIGTGFEADVHSVSGAPKEAFVKIEKAEDGGIGAERL 7ar8.1    --------------------------------------------------------------------------------  target    WRPLTLGLRPEAPSAALTAYLAGDYSGTKGS 7ar8.1    ------------------------------- ``` | | | | | | | | | | | | | | | | | | | | | | | | | | | | | | | | | | | | | | | | | | | | | | | | | |
|  | 6yj4.1.G | Subunit NUAM of NADH:Ubiquinone Oxidoreductase (Complex I)  *Structure of Yarrowia lipolytica complex I at 2.7 A* | 0.08 |  | 15.98 | 0.20 | 186-423 | EM | 0.00 | hetero-1-1-1-1-1-1-… | 18 x 3PE, 6 x SF4, 5 x LMT, 8 x PLC, 2 x FES, 1 x FMN, 6 x CDL, 1 x NDP, 1 x ZN, 2 x EHZ | HHblits | 0.29 |
| ``` target    MPTANKADEVIILRPGTDAAFFLGVARELIEKGLYDRAAVIERTDLPLLVRLDTGERLDARDVIPGYELAALTNYVTLKP 6yj4.1    --------------------------------------------------------------------------------  target    DAEIKGNPPPPPFTAGGQVVPTELRDAWGDFVWWDRATGRPRPVSRDEVGARFDGDPALLGEFEVELVDGSTVPVRPAFD 6yj4.1    --------------------------------------------------------------------------------  target    LLKQYLDESFDLRTASEVCRVPPQAIQSIARQLAANKRETLLAAGMGPNHYFQNDLFGRVQFLVAALTDN-IGHLGGNVG 6yj4.1    -------------------------SGEFGEVLKNAKNPL-IIVGSGITDREDAGAFFNTIGKFVESTPSVLNENWNGYN  target    SYAGNYRGSVFQAMGQWIAEDPFAIEPDLTKPATVKRYYKAESAHYWNYGERPLRAVAKDDEGDLTKGEVLTGKSHMPTP 6yj4.1    VLQRSAS-----RAGAY----------DI------------------GF--TP-------------SDEA------SKTT  target    TKLIWFGNSNSLLGNAKWSFDVVKNTLPRQDAVFCNEWHWTSSCEYADLVFPADSWAEFKLPDATASCTNPFLLAFPTTP 6yj4.1    PKMVWLLGADEVAAS----------DIPADAFVVYQGHNGDVGAQFADVVLPGAAYTEKAG---TYVNTEGRSQI-SRAA  target    LKRLYDTRSDYEALALTAKALGELIDEPRMEQYWRGILDGDPTPYLQRIFSGSNATRGITYDELHESSKRGVPLLMNMRT 6yj4.1    TGPPGGAREDWKILRAVSEYLGVA--------------------------------------------------------  target    YPRSGGWEQRQEDKPWYTATGRLEFYRPEPEFQAAGESLPVWREPVDATFYEPNAILSNAAHPSIAPRAPEDYGVPESQL 6yj4.1    --------------------------------------------------------------------------------  target    DVETRQYRNVVRTWAELQQTLHPLQERDPAFRFVFQTPKYRWGAHSTAVDADWISMLFGPFGDPYRRDPRMPWTGEAYLE 6yj4.1    --------------------------------------------------------------------------------  target    INPKDAAELGLADGDYAWVDADPEDRPYRGWKEDDPYYEVARAMMRVRIYTGMSRGVIRTWFNMYAATPATVANQKATPG 6yj4.1    --------------------------------------------------------------------------------  target    NPARNEQTRYVALFRYGSHQSGTRAWLRPTQQTDSLVRKGYFGQVIGTGFEADVHSVSGAPKEAFVKIEKAEDGGIGAER 6yj4.1    --------------------------------------------------------------------------------  target    LWRPLTLGLRPEAPSAALTAYLAGDYSGTKGS 6yj4.1    -------------------------------- ``` | | | | | | | | | | | | | | | | | | | | | | | | | | | | | | | | | | | | | | | | | | | | | | | | | |
|  | 6rfs.1.A | Subunit NUAM of NADH:Ubiquinone Oxidoreductase (Complex I)  *Cryo-EM structure of a respiratory complex I mutant lacking NDUFS4* | 0.08 |  | 15.98 | 0.20 | 186-423 | EM | 4.04 | hetero-1-1-1-1-1-1-… | 6 x SF4, 2 x FES, 1 x FMN, 1 x NDP, 1 x ZN, 1 x ZMP | HHblits | 0.29 |
| ``` target    MPTANKADEVIILRPGTDAAFFLGVARELIEKGLYDRAAVIERTDLPLLVRLDTGERLDARDVIPGYELAALTNYVTLKP 6rfs.1    --------------------------------------------------------------------------------  target    DAEIKGNPPPPPFTAGGQVVPTELRDAWGDFVWWDRATGRPRPVSRDEVGARFDGDPALLGEFEVELVDGSTVPVRPAFD 6rfs.1    --------------------------------------------------------------------------------  target    LLKQYLDESFDLRTASEVCRVPPQAIQSIARQLAANKRETLLAAGMGPNHYFQNDLFGRVQFLVAALTDN-IGHLGGNVG 6rfs.1    -------------------------SGEFGEVLKNAKNPL-IIVGSGITDREDAGAFFNTIGKFVESTPSVLNENWNGYN  target    SYAGNYRGSVFQAMGQWIAEDPFAIEPDLTKPATVKRYYKAESAHYWNYGERPLRAVAKDDEGDLTKGEVLTGKSHMPTP 6rfs.1    VLQRSAS-----RAGAY----------DI------------------GF--TP-------------SDEA------SKTT  target    TKLIWFGNSNSLLGNAKWSFDVVKNTLPRQDAVFCNEWHWTSSCEYADLVFPADSWAEFKLPDATASCTNPFLLAFPTTP 6rfs.1    PKMVWLLGADEVAAS----------DIPADAFVVYQGHNGDVGAQFADVVLPGAAYTEKAG---TYVNTEGRSQI-SRAA  target    LKRLYDTRSDYEALALTAKALGELIDEPRMEQYWRGILDGDPTPYLQRIFSGSNATRGITYDELHESSKRGVPLLMNMRT 6rfs.1    TGPPGGAREDWKILRAVSEYLGVA--------------------------------------------------------  target    YPRSGGWEQRQEDKPWYTATGRLEFYRPEPEFQAAGESLPVWREPVDATFYEPNAILSNAAHPSIAPRAPEDYGVPESQL 6rfs.1    --------------------------------------------------------------------------------  target    DVETRQYRNVVRTWAELQQTLHPLQERDPAFRFVFQTPKYRWGAHSTAVDADWISMLFGPFGDPYRRDPRMPWTGEAYLE 6rfs.1    --------------------------------------------------------------------------------  target    INPKDAAELGLADGDYAWVDADPEDRPYRGWKEDDPYYEVARAMMRVRIYTGMSRGVIRTWFNMYAATPATVANQKATPG 6rfs.1    --------------------------------------------------------------------------------  target    NPARNEQTRYVALFRYGSHQSGTRAWLRPTQQTDSLVRKGYFGQVIGTGFEADVHSVSGAPKEAFVKIEKAEDGGIGAER 6rfs.1    --------------------------------------------------------------------------------  target    LWRPLTLGLRPEAPSAALTAYLAGDYSGTKGS 6rfs.1    -------------------------------- ``` | | | | | | | | | | | | | | | | | | | | | | | | | | | | | | | | | | | | | | | | | | | | | | | | | |
|  | 6rfq.1.A | Subunit NUAM of NADH:Ubiquinone Oxidoreductase (Complex I)  *Cryo-EM structure of a respiratory complex I assembly intermediate with NDUFAF2* | 0.08 |  | 15.98 | 0.20 | 186-423 | EM | 3.30 | hetero-1-1-1-1-1-1-… | 6 x SF4, 2 x FES, 1 x FMN, 1 x NDP, 10 x 3PE, 2 x LMN, 4 x CDL, 2 x ZMP, 4 x PLC, 3 x T7X, 1 x CPL | HHblits | 0.29 |
| ``` target    MPTANKADEVIILRPGTDAAFFLGVARELIEKGLYDRAAVIERTDLPLLVRLDTGERLDARDVIPGYELAALTNYVTLKP 6rfq.1    --------------------------------------------------------------------------------  target    DAEIKGNPPPPPFTAGGQVVPTELRDAWGDFVWWDRATGRPRPVSRDEVGARFDGDPALLGEFEVELVDGSTVPVRPAFD 6rfq.1    --------------------------------------------------------------------------------  target    LLKQYLDESFDLRTASEVCRVPPQAIQSIARQLAANKRETLLAAGMGPNHYFQNDLFGRVQFLVAALTDN-IGHLGGNVG 6rfq.1    -------------------------SGEFGEVLKNAKNPL-IIVGSGITDREDAGAFFNTIGKFVESTPSVLNENWNGYN  target    SYAGNYRGSVFQAMGQWIAEDPFAIEPDLTKPATVKRYYKAESAHYWNYGERPLRAVAKDDEGDLTKGEVLTGKSHMPTP 6rfq.1    VLQRSAS-----RAGAY----------DI------------------GF--TP-------------SDEA------SKTT  target    TKLIWFGNSNSLLGNAKWSFDVVKNTLPRQDAVFCNEWHWTSSCEYADLVFPADSWAEFKLPDATASCTNPFLLAFPTTP 6rfq.1    PKMVWLLGADEVAAS----------DIPADAFVVYQGHNGDVGAQFADVVLPGAAYTEKAG---TYVNTEGRSQI-SRAA  target    LKRLYDTRSDYEALALTAKALGELIDEPRMEQYWRGILDGDPTPYLQRIFSGSNATRGITYDELHESSKRGVPLLMNMRT 6rfq.1    TGPPGGAREDWKILRAVSEYLGVA--------------------------------------------------------  target    YPRSGGWEQRQEDKPWYTATGRLEFYRPEPEFQAAGESLPVWREPVDATFYEPNAILSNAAHPSIAPRAPEDYGVPESQL 6rfq.1    --------------------------------------------------------------------------------  target    DVETRQYRNVVRTWAELQQTLHPLQERDPAFRFVFQTPKYRWGAHSTAVDADWISMLFGPFGDPYRRDPRMPWTGEAYLE 6rfq.1    --------------------------------------------------------------------------------  target    INPKDAAELGLADGDYAWVDADPEDRPYRGWKEDDPYYEVARAMMRVRIYTGMSRGVIRTWFNMYAATPATVANQKATPG 6rfq.1    --------------------------------------------------------------------------------  target    NPARNEQTRYVALFRYGSHQSGTRAWLRPTQQTDSLVRKGYFGQVIGTGFEADVHSVSGAPKEAFVKIEKAEDGGIGAER 6rfq.1    --------------------------------------------------------------------------------  target    LWRPLTLGLRPEAPSAALTAYLAGDYSGTKGS 6rfq.1    -------------------------------- ``` | | | | | | | | | | | | | | | | | | | | | | | | | | | | | | | | | | | | | | | | | | | | | | | | | |
|  | 6gcs.1.A | 75-KDA PROTEIN (NUAM)  *Cryo-EM structure of respiratory complex I from Yarrowia lipolytica* | 0.08 |  | 15.98 | 0.20 | 186-423 | EM | 4.32 | hetero-1-1-1-1-1-1-… | 6 x SF4, 2 x FES, 1 x FMN, 1 x NDP, 1 x ZN, 1 x ZMP, 1 x CDL, 3 x 3PE | HHblits | 0.29 |
| ``` target    MPTANKADEVIILRPGTDAAFFLGVARELIEKGLYDRAAVIERTDLPLLVRLDTGERLDARDVIPGYELAALTNYVTLKP 6gcs.1    --------------------------------------------------------------------------------  target    DAEIKGNPPPPPFTAGGQVVPTELRDAWGDFVWWDRATGRPRPVSRDEVGARFDGDPALLGEFEVELVDGSTVPVRPAFD 6gcs.1    --------------------------------------------------------------------------------  target    LLKQYLDESFDLRTASEVCRVPPQAIQSIARQLAANKRETLLAAGMGPNHYFQNDLFGRVQFLVAALTDN-IGHLGGNVG 6gcs.1    -------------------------SGEFGEVLKNAKNPL-IIVGSGITDREDAGAFFNTIGKFVESTPSVLNENWNGYN  target    SYAGNYRGSVFQAMGQWIAEDPFAIEPDLTKPATVKRYYKAESAHYWNYGERPLRAVAKDDEGDLTKGEVLTGKSHMPTP 6gcs.1    VLQRSAS-----RAGAY----------DI------------------GF--TP-------------SDEA------SKTT  target    TKLIWFGNSNSLLGNAKWSFDVVKNTLPRQDAVFCNEWHWTSSCEYADLVFPADSWAEFKLPDATASCTNPFLLAFPTTP 6gcs.1    PKMVWLLGADEVAAS----------DIPADAFVVYQGHNGDVGAQFADVVLPGAAYTEKAG---TYVNTEGRSQI-SRAA  target    LKRLYDTRSDYEALALTAKALGELIDEPRMEQYWRGILDGDPTPYLQRIFSGSNATRGITYDELHESSKRGVPLLMNMRT 6gcs.1    TGPPGGAREDWKILRAVSEYLGVA--------------------------------------------------------  target    YPRSGGWEQRQEDKPWYTATGRLEFYRPEPEFQAAGESLPVWREPVDATFYEPNAILSNAAHPSIAPRAPEDYGVPESQL 6gcs.1    --------------------------------------------------------------------------------  target    DVETRQYRNVVRTWAELQQTLHPLQERDPAFRFVFQTPKYRWGAHSTAVDADWISMLFGPFGDPYRRDPRMPWTGEAYLE 6gcs.1    --------------------------------------------------------------------------------  target    INPKDAAELGLADGDYAWVDADPEDRPYRGWKEDDPYYEVARAMMRVRIYTGMSRGVIRTWFNMYAATPATVANQKATPG 6gcs.1    --------------------------------------------------------------------------------  target    NPARNEQTRYVALFRYGSHQSGTRAWLRPTQQTDSLVRKGYFGQVIGTGFEADVHSVSGAPKEAFVKIEKAEDGGIGAER 6gcs.1    --------------------------------------------------------------------------------  target    LWRPLTLGLRPEAPSAALTAYLAGDYSGTKGS 6gcs.1    -------------------------------- ``` | | | | | | | | | | | | | | | | | | | | | | | | | | | | | | | | | | | | | | | | | | | | | | | | | |
|  | 6x89.1.H | NADH dehydrogenase [ubiquinone] iron-sulfur protein 1, mitochondrial  *Vigna radiata mitochondrial complex I\** | 0.08 |  | 13.45 | 0.21 | 184-423 | EM | 0.00 | hetero-1-1-1-1-1-1-… | 1 x NAP, 6 x PC1, 6 x SF4, 2 x FES, 2 x ZN, 1 x FMN | HHblits | 0.28 |
| ``` target    MPTANKADEVIILRPGTDAAFFLGVARELIEKGLYDRAAVIERTDLPLLVRLDTGERLDARDVIPGYELAALTNYVTLKP 6x89.1    --------------------------------------------------------------------------------  target    DAEIKGNPPPPPFTAGGQVVPTELRDAWGDFVWWDRATGRPRPVSRDEVGARFDGDPALLGEFEVELVDGSTVPVRPAFD 6x89.1    --------------------------------------------------------------------------------  target    LLKQYLDESFDLRTASEVCRVPPQAIQSIARQLAANKRETLLAAGMGPNHYFQNDLFGRVQFLVAALTDNIGHLGGNVGS 6x89.1    -----------------------EGRHPFFKTLSDAKNPVI-IVGAGVFERKDQDAIFAAVETIAQKANVVRPDWNGLNV  target    YAGNYRGSVFQAMGQWIAEDPFAIEPDLTKPATVKRYYKAESAHYWNYGERPLRAVAKDDEGDLTKGEVLTGKSHMPTPT 6x89.1    LLLHAA-----QAAAL----------DL------------------GL--VP--------------QSE-----KSLESA  target    KLIWFGNSNSLLGNAKWSFDVVKNTLPRQDAVFCNEWHWTSSCEYADLVFPADSWAEFKLPDATASCTNPFLLAFPTTPL 6x89.1    KFVYLMGADDVN----------LDKIPDDAFVVYQGHHGDKSVYRANVILPTAAFSEKE---GTYQNTEGCTQQT-LPAV  target    KRLYDTRSDYEALALTAKALGELIDEPRMEQYWRGILDGDPTPYLQRIFSGSNATRGITYDELHESSKRGVPLLMNMRTY 6x89.1    PTVGDSRDDWKIIRALSEVAGVR---------------------------------------------------------  target    PRSGGWEQRQEDKPWYTATGRLEFYRPEPEFQAAGESLPVWREPVDATFYEPNAILSNAAHPSIAPRAPEDYGVPESQLD 6x89.1    --------------------------------------------------------------------------------  target    VETRQYRNVVRTWAELQQTLHPLQERDPAFRFVFQTPKYRWGAHSTAVDADWISMLFGPFGDPYRRDPRMPWTGEAYLEI 6x89.1    --------------------------------------------------------------------------------  target    NPKDAAELGLADGDYAWVDADPEDRPYRGWKEDDPYYEVARAMMRVRIYTGMSRGVIRTWFNMYAATPATVANQKATPGN 6x89.1    --------------------------------------------------------------------------------  target    PARNEQTRYVALFRYGSHQSGTRAWLRPTQQTDSLVRKGYFGQVIGTGFEADVHSVSGAPKEAFVKIEKAEDGGIGAERL 6x89.1    --------------------------------------------------------------------------------  target    WRPLTLGLRPEAPSAALTAYLAGDYSGTKGS 6x89.1    ------------------------------- ``` | | | | | | | | | | | | | | | | | | | | | | | | | | | | | | | | | | | | | | | | | | | | | | | | | |
|  | 8e73.55.A | NDUS1  *Vigna radiata supercomplex I+III2 (full bridge)* | 0.09 |  | 13.45 | 0.21 | 184-423 | EM | 0.00 | monomer |  | HHblits | 0.28 |
| ``` target    MPTANKADEVIILRPGTDAAFFLGVARELIEKGLYDRAAVIERTDLPLLVRLDTGERLDARDVIPGYELAALTNYVTLKP 8e73.55   --------------------------------------------------------------------------------  target    DAEIKGNPPPPPFTAGGQVVPTELRDAWGDFVWWDRATGRPRPVSRDEVGARFDGDPALLGEFEVELVDGSTVPVRPAFD 8e73.55   --------------------------------------------------------------------------------  target    LLKQYLDESFDLRTASEVCRVPPQAIQSIARQLAANKRETLLAAGMGPNHYFQNDLFGRVQFLVAALTDNIGHLGGNVGS 8e73.55   -----------------------EGRHPFFKTLSDAKNPVI-IVGAGVFERKDQDAIFAAVETIAQKANVVRPDWNGLNV  target    YAGNYRGSVFQAMGQWIAEDPFAIEPDLTKPATVKRYYKAESAHYWNYGERPLRAVAKDDEGDLTKGEVLTGKSHMPTPT 8e73.55   LLLHAA-----QAAAL----------DL------------------GL--VP--------------QSE-----KSLESA  target    KLIWFGNSNSLLGNAKWSFDVVKNTLPRQDAVFCNEWHWTSSCEYADLVFPADSWAEFKLPDATASCTNPFLLAFPTTPL 8e73.55   KFVYLMGADDVN----------LDKIPDDAFVVYQGHHGDKSVYRANVILPTAAFSEKE---GTYQNTEGCTQQT-LPAV  target    KRLYDTRSDYEALALTAKALGELIDEPRMEQYWRGILDGDPTPYLQRIFSGSNATRGITYDELHESSKRGVPLLMNMRTY 8e73.55   PTVGDSRDDWKIIRALSEVAGVR---------------------------------------------------------  target    PRSGGWEQRQEDKPWYTATGRLEFYRPEPEFQAAGESLPVWREPVDATFYEPNAILSNAAHPSIAPRAPEDYGVPESQLD 8e73.55   --------------------------------------------------------------------------------  target    VETRQYRNVVRTWAELQQTLHPLQERDPAFRFVFQTPKYRWGAHSTAVDADWISMLFGPFGDPYRRDPRMPWTGEAYLEI 8e73.55   --------------------------------------------------------------------------------  target    NPKDAAELGLADGDYAWVDADPEDRPYRGWKEDDPYYEVARAMMRVRIYTGMSRGVIRTWFNMYAATPATVANQKATPGN 8e73.55   --------------------------------------------------------------------------------  target    PARNEQTRYVALFRYGSHQSGTRAWLRPTQQTDSLVRKGYFGQVIGTGFEADVHSVSGAPKEAFVKIEKAEDGGIGAERL 8e73.55   --------------------------------------------------------------------------------  target    WRPLTLGLRPEAPSAALTAYLAGDYSGTKGS 8e73.55   ------------------------------- ``` | | | | | | | | | | | | | | | | | | | | | | | | | | | | | | | | | | | | | | | | | | | | | | | | | |
|  | 7v2c.1.L | NADH-ubiquinone oxidoreductase 75 kDa subunit, mitochondrial  *Active state complex I from Q10 dataset* | 0.04 | 0.00 | 15.96 | 0.11 | 318-423 | EM | 0.00 | monomer | 6 x SF4, 1 x FMN, 10 x PEE, 8 x PLX, 2 x 8Q1, 1 x NDP, 2 x UQ, 11 x CDL, 2 x FES, 1 x MG, 1 x ZN, 1 x ADP | HHblits | 0.29 |
| ``` target    MPTANKADEVIILRPGTDAAFFLGVARELIEKGLYDRAAVIERTDLPLLVRLDTGERLDARDVIPGYELAALTNYVTLKP 7v2c.1    --------------------------------------------------------------------------------  target    DAEIKGNPPPPPFTAGGQVVPTELRDAWGDFVWWDRATGRPRPVSRDEVGARFDGDPALLGEFEVELVDGSTVPVRPAFD 7v2c.1    --------------------------------------------------------------------------------  target    LLKQYLDESFDLRTASEVCRVPPQAIQSIARQLAANKRETLLAAGMGPNHYFQNDLFGRVQFLVAALTDNIGHLGGNVGS 7v2c.1    --------------------------------------------------------------------------------  target    YAGNYRGSVFQAMGQWIAEDPFAIEPDLTKPATVKRYYKAESAHYWNYGERPLRAVAKDDEGDLTKGEVLTGKSHMPTPT 7v2c.1    -----------------------------------------------------------------------------NPP  target    KLIWFGNSNSLLGNAKWSFDVVKNTLPRQDAVFCNEWHWTSSCEYADLVFPADSWAEFKLPDATASCTNPFLLAFPTTPL 7v2c.1    KVLFLLGADGG--------CITRQDLPKDCFIIYQGHHGDVGAPMADVILPGAAYTEKS---ATYVNTEGRAQQT-KVAV  target    KRLYDTRSDYEALALTAKALGELIDEPRMEQYWRGILDGDPTPYLQRIFSGSNATRGITYDELHESSKRGVPLLMNMRTY 7v2c.1    TPPGLAREDWKIIRALSEIAGMT---------------------------------------------------------  target    PRSGGWEQRQEDKPWYTATGRLEFYRPEPEFQAAGESLPVWREPVDATFYEPNAILSNAAHPSIAPRAPEDYGVPESQLD 7v2c.1    --------------------------------------------------------------------------------  target    VETRQYRNVVRTWAELQQTLHPLQERDPAFRFVFQTPKYRWGAHSTAVDADWISMLFGPFGDPYRRDPRMPWTGEAYLEI 7v2c.1    --------------------------------------------------------------------------------  target    NPKDAAELGLADGDYAWVDADPEDRPYRGWKEDDPYYEVARAMMRVRIYTGMSRGVIRTWFNMYAATPATVANQKATPGN 7v2c.1    --------------------------------------------------------------------------------  target    PARNEQTRYVALFRYGSHQSGTRAWLRPTQQTDSLVRKGYFGQVIGTGFEADVHSVSGAPKEAFVKIEKAEDGGIGAERL 7v2c.1    --------------------------------------------------------------------------------  target    WRPLTLGLRPEAPSAALTAYLAGDYSGTKGS 7v2c.1    ------------------------------- ``` | | | | | | | | | | | | | | | | | | | | | | | | | | | | | | | | | | | | | | | | | | | | | | | | | |
|  | 8b9z.1.G | NADH-ubiquinone oxidoreductase 75 kDa subunit, mitochondrial  *Drosophila melanogaster complex I in the Active state (Dm1)* | 0.04 |  | 18.09 | 0.11 | 318-423 | EM | 3.28 | hetero-1-1-1-1-1-1-… | 3 x PC1, 16 x 3PE, 6 x SF4, 4 x CDL, 2 x FES, 1 x FMN, 1 x UQ9, 1 x DGT, 1 x NDP, 1 x ZN, 2 x EHZ | HHblits | 0.29 |
| ``` target    MPTANKADEVIILRPGTDAAFFLGVARELIEKGLYDRAAVIERTDLPLLVRLDTGERLDARDVIPGYELAALTNYVTLKP 8b9z.1    --------------------------------------------------------------------------------  target    DAEIKGNPPPPPFTAGGQVVPTELRDAWGDFVWWDRATGRPRPVSRDEVGARFDGDPALLGEFEVELVDGSTVPVRPAFD 8b9z.1    --------------------------------------------------------------------------------  target    LLKQYLDESFDLRTASEVCRVPPQAIQSIARQLAANKRETLLAAGMGPNHYFQNDLFGRVQFLVAALTDNIGHLGGNVGS 8b9z.1    --------------------------------------------------------------------------------  target    YAGNYRGSVFQAMGQWIAEDPFAIEPDLTKPATVKRYYKAESAHYWNYGERPLRAVAKDDEGDLTKGEVLTGKSHMPTPT 8b9z.1    -----------------------------------------------------------------------------AQP  target    KLIWFGNSNSLLGNAKWSFDVVKNTLPRQDAVFCNEWHWTSSCEYADLVFPADSWAEFKLPDATASCTNPFLLAFPTTPL 8b9z.1    KVLFLLNADAG--------KVTREQLPKDCFVVYIGSHGDNGASIADAVLPGAAYTEKQ---GIYVNTEGRPQQT-LPGV  target    KRLYDTRSDYEALALTAKALGELIDEPRMEQYWRGILDGDPTPYLQRIFSGSNATRGITYDELHESSKRGVPLLMNMRTY 8b9z.1    SPPGMAREDWKILRALSEVVGKP---------------------------------------------------------  target    PRSGGWEQRQEDKPWYTATGRLEFYRPEPEFQAAGESLPVWREPVDATFYEPNAILSNAAHPSIAPRAPEDYGVPESQLD 8b9z.1    --------------------------------------------------------------------------------  target    VETRQYRNVVRTWAELQQTLHPLQERDPAFRFVFQTPKYRWGAHSTAVDADWISMLFGPFGDPYRRDPRMPWTGEAYLEI 8b9z.1    --------------------------------------------------------------------------------  target    NPKDAAELGLADGDYAWVDADPEDRPYRGWKEDDPYYEVARAMMRVRIYTGMSRGVIRTWFNMYAATPATVANQKATPGN 8b9z.1    --------------------------------------------------------------------------------  target    PARNEQTRYVALFRYGSHQSGTRAWLRPTQQTDSLVRKGYFGQVIGTGFEADVHSVSGAPKEAFVKIEKAEDGGIGAERL 8b9z.1    --------------------------------------------------------------------------------  target    WRPLTLGLRPEAPSAALTAYLAGDYSGTKGS 8b9z.1    ------------------------------- ``` | | | | | | | | | | | | | | | | | | | | | | | | | | | | | | | | | | | | | | | | | | | | | | | | | |
|  | 8ba0.1.G | NADH-ubiquinone oxidoreductase 75 kDa subunit, mitochondrial  *Drosophila melanogaster complex I in the Twisted state (Dm2)* | 0.04 |  | 18.09 | 0.11 | 318-423 | EM | 3.68 | hetero-1-1-1-1-1-1-… | 6 x SF4, 6 x 3PE, 2 x FES, 1 x FMN, 2 x CDL, 1 x DGT, 1 x NDP, 1 x ZN, 2 x EHZ | HHblits | 0.29 |
| ``` target    MPTANKADEVIILRPGTDAAFFLGVARELIEKGLYDRAAVIERTDLPLLVRLDTGERLDARDVIPGYELAALTNYVTLKP 8ba0.1    --------------------------------------------------------------------------------  target    DAEIKGNPPPPPFTAGGQVVPTELRDAWGDFVWWDRATGRPRPVSRDEVGARFDGDPALLGEFEVELVDGSTVPVRPAFD 8ba0.1    --------------------------------------------------------------------------------  target    LLKQYLDESFDLRTASEVCRVPPQAIQSIARQLAANKRETLLAAGMGPNHYFQNDLFGRVQFLVAALTDNIGHLGGNVGS 8ba0.1    --------------------------------------------------------------------------------  target    YAGNYRGSVFQAMGQWIAEDPFAIEPDLTKPATVKRYYKAESAHYWNYGERPLRAVAKDDEGDLTKGEVLTGKSHMPTPT 8ba0.1    -----------------------------------------------------------------------------AQP  target    KLIWFGNSNSLLGNAKWSFDVVKNTLPRQDAVFCNEWHWTSSCEYADLVFPADSWAEFKLPDATASCTNPFLLAFPTTPL 8ba0.1    KVLFLLNADAG--------KVTREQLPKDCFVVYIGSHGDNGASIADAVLPGAAYTEKQ---GIYVNTEGRPQQT-LPGV  target    KRLYDTRSDYEALALTAKALGELIDEPRMEQYWRGILDGDPTPYLQRIFSGSNATRGITYDELHESSKRGVPLLMNMRTY 8ba0.1    SPPGMAREDWKILRALSEVVGKP---------------------------------------------------------  target    PRSGGWEQRQEDKPWYTATGRLEFYRPEPEFQAAGESLPVWREPVDATFYEPNAILSNAAHPSIAPRAPEDYGVPESQLD 8ba0.1    --------------------------------------------------------------------------------  target    VETRQYRNVVRTWAELQQTLHPLQERDPAFRFVFQTPKYRWGAHSTAVDADWISMLFGPFGDPYRRDPRMPWTGEAYLEI 8ba0.1    --------------------------------------------------------------------------------  target    NPKDAAELGLADGDYAWVDADPEDRPYRGWKEDDPYYEVARAMMRVRIYTGMSRGVIRTWFNMYAATPATVANQKATPGN 8ba0.1    --------------------------------------------------------------------------------  target    PARNEQTRYVALFRYGSHQSGTRAWLRPTQQTDSLVRKGYFGQVIGTGFEADVHSVSGAPKEAFVKIEKAEDGGIGAERL 8ba0.1    --------------------------------------------------------------------------------  target    WRPLTLGLRPEAPSAALTAYLAGDYSGTKGS 8ba0.1    ------------------------------- ``` | | | | | | | | | | | | | | | | | | | | | | | | | | | | | | | | | | | | | | | | | | | | | | | | | |
|  | 7zm7.1.I | NADH-ubiquinone oxidoreductase-like protein  *CryoEM structure of mitochondrial complex I from Chaetomium thermophilum (inhibited by DDM)* | 0.04 | 0.00 | 18.48 | 0.11 | 318-423 | EM | 0.00 | monomer | 4 x PC1, 14 x LMT, 5 x CDL, 8 x 3PE, 2 x FES, 6 x SF4, 1 x FMN, 1 x NDP, 1 x ZN, 2 x ZMP | HHblits | 0.30 |
| ``` target    MPTANKADEVIILRPGTDAAFFLGVARELIEKGLYDRAAVIERTDLPLLVRLDTGERLDARDVIPGYELAALTNYVTLKP 7zm7.1    --------------------------------------------------------------------------------  target    DAEIKGNPPPPPFTAGGQVVPTELRDAWGDFVWWDRATGRPRPVSRDEVGARFDGDPALLGEFEVELVDGSTVPVRPAFD 7zm7.1    --------------------------------------------------------------------------------  target    LLKQYLDESFDLRTASEVCRVPPQAIQSIARQLAANKRETLLAAGMGPNHYFQNDLFGRVQFLVAALTDNIGHLGGNVGS 7zm7.1    --------------------------------------------------------------------------------  target    YAGNYRGSVFQAMGQWIAEDPFAIEPDLTKPATVKRYYKAESAHYWNYGERPLRAVAKDDEGDLTKGEVLTGKSHMPTPT 7zm7.1    -----------------------------------------------------------------------------TKP  target    KLIWFGNSNSLLGNAKWSFDVVKNTLPRQDAVFCNEWHWTSSCEYADLVFPADSWAEFKLPDATASCTNPFLLAFPTTPL 7zm7.1    KFVWLLGADEFDP----------ADVPKDAFIVYQGHHGDRGAEIADIVLPGAAYTEKA---GTYVNTEGRVQM-TRAAT  target    KRLYDTRSDYEALALTAKALGELIDEPRMEQYWRGILDGDPTPYLQRIFSGSNATRGITYDELHESSKRGVPLLMNMRTY 7zm7.1    GLPGAARTDWKIIRAVSEFLGVP---------------------------------------------------------  target    PRSGGWEQRQEDKPWYTATGRLEFYRPEPEFQAAGESLPVWREPVDATFYEPNAILSNAAHPSIAPRAPEDYGVPESQLD 7zm7.1    --------------------------------------------------------------------------------  target    VETRQYRNVVRTWAELQQTLHPLQERDPAFRFVFQTPKYRWGAHSTAVDADWISMLFGPFGDPYRRDPRMPWTGEAYLEI 7zm7.1    --------------------------------------------------------------------------------  target    NPKDAAELGLADGDYAWVDADPEDRPYRGWKEDDPYYEVARAMMRVRIYTGMSRGVIRTWFNMYAATPATVANQKATPGN 7zm7.1    --------------------------------------------------------------------------------  target    PARNEQTRYVALFRYGSHQSGTRAWLRPTQQTDSLVRKGYFGQVIGTGFEADVHSVSGAPKEAFVKIEKAEDGGIGAERL 7zm7.1    --------------------------------------------------------------------------------  target    WRPLTLGLRPEAPSAALTAYLAGDYSGTKGS 7zm7.1    ------------------------------- ``` | | | | | | | | | | | | | | | | | | | | | | | | | | | | | | | | | | | | | | | | | | | | | | | | | |
|  | 7q5y.1.A | NADH dehydrogenase I chain G  *Structure of NADH:ubichinon oxidoreductase (complex I) of the hyperthermophilic eubacterium Aquifex aeolicus* | 0.04 |  | 10.53 | 0.11 | 318-423 | X-ray | 2.70 | hetero-1-1-1-1-1-1-… | 8 x SF4, 2 x FES, 1 x FMN | HHblits | 0.24 |
| ``` target    MPTANKADEVIILRPGTDAAFFLGVARELIEKGLYDRAAVIERTDLPLLVRLDTGERLDARDVIPGYELAALTNYVTLKP 7q5y.1    --------------------------------------------------------------------------------  target    DAEIKGNPPPPPFTAGGQVVPTELRDAWGDFVWWDRATGRPRPVSRDEVGARFDGDPALLGEFEVELVDGSTVPVRPAFD 7q5y.1    --------------------------------------------------------------------------------  target    LLKQYLDESFDLRTASEVCRVPPQAIQSIARQLAANKRETLLAAGMGPNHYFQNDLFGRVQFLVAALTDNIGHLGGNVGS 7q5y.1    --------------------------------------------------------------------------------  target    YAGNYRGSVFQAMGQWIAEDPFAIEPDLTKPATVKRYYKAESAHYWNYGERPLRAVAKDDEGDLTKGEVLTGKSHMPTPT 7q5y.1    -----------------------------------------------------------------------------GDI  target    KLIWFGNSNSLLGNAKWSFDVVKNTLPRQDAVFCNEWHWTSSCEYADLVFPADSWAEFKLPDATASCTNPFLLAFPTTPL 7q5y.1    ENLIIFGEDILEFYED---KVFEELKEKLEHLVVVSPYEDGLSEYAHIKIPMSLMGEN---EGTYKTFFGEVKGK--K-F  target    KRLYDTRSDYEALALTAKALGELIDEPRMEQYWRGILDGDPTPYLQRIFSGSNATRGITYDELHESSKRGVPLLMNMRTY 7q5y.1    LP--WAFDDLAFWKYLGENFKEE---------------------------------------------------------  target    PRSGGWEQRQEDKPWYTATGRLEFYRPEPEFQAAGESLPVWREPVDATFYEPNAILSNAAHPSIAPRAPEDYGVPESQLD 7q5y.1    --------------------------------------------------------------------------------  target    VETRQYRNVVRTWAELQQTLHPLQERDPAFRFVFQTPKYRWGAHSTAVDADWISMLFGPFGDPYRRDPRMPWTGEAYLEI 7q5y.1    --------------------------------------------------------------------------------  target    NPKDAAELGLADGDYAWVDADPEDRPYRGWKEDDPYYEVARAMMRVRIYTGMSRGVIRTWFNMYAATPATVANQKATPGN 7q5y.1    --------------------------------------------------------------------------------  target    PARNEQTRYVALFRYGSHQSGTRAWLRPTQQTDSLVRKGYFGQVIGTGFEADVHSVSGAPKEAFVKIEKAEDGGIGAERL 7q5y.1    --------------------------------------------------------------------------------  target    WRPLTLGLRPEAPSAALTAYLAGDYSGTKGS 7q5y.1    ------------------------------- ``` | | | | | | | | | | | | | | | | | | | | | | | | | | | | | | | | | | | | | | | | | | | | | | | | | |
|  | 6s6y.1.B | Tungsten-containing formylmethanofuran dehydrogenase, subunit B  *X-ray crystal structure of the formyltransferase/hydrolase complex (FhcABCD) from Methylorubrum extorquens in complex with methylofuran* | 0.03 |  | 10.87 | 0.11 | 318-422 | X-ray | 3.10 | hetero-2-2-2-2-mer | 1 x MFN, 4 x ZN, 4 x CA, 4 x K, 3 x DGL, 2 x GLU, 1 x IAS | HHblits | 0.23 |
| ``` target    MPTANKADEVIILRPGTDAAFFLGVARELIEKGLYDRAAVIERTDLPLLVRLDTGERLDARDVIPGYELAALTNYVTLKP 6s6y.1    --------------------------------------------------------------------------------  target    DAEIKGNPPPPPFTAGGQVVPTELRDAWGDFVWWDRATGRPRPVSRDEVGARFDGDPALLGEFEVELVDGSTVPVRPAFD 6s6y.1    --------------------------------------------------------------------------------  target    LLKQYLDESFDLRTASEVCRVPPQAIQSIARQLAANKRETLLAAGMGPNHYFQNDLFGRVQFLVAALTDNIGHLGGNVGS 6s6y.1    --------------------------------------------------------------------------------  target    YAGNYRGSVFQAMGQWIAEDPFAIEPDLTKPATVKRYYKAESAHYWNYGERPLRAVAKDDEGDLTKGEVLTGKSHMPTPT 6s6y.1    -----------------------------------------------------------------------------GEA  target    KLIWFGNSNSLLGNAKWSFDVVKNTLPRQDAVFCNE-WHWTSSCEYADLVFPADSW-AEFKLPDATASCTNPFLLAFPTT 6s6y.1    DAALWLASLPAP---------RPAWLGSLPTIAIVGEGSQEAAGETAEVVITVGVPGQSVG---GALWNDRRGVIAY-AE  target    PLKRL---YDTRSDYEALALTAKALGELIDEPRMEQYWRGILDGDPTPYLQRIFSGSNATRGITYDELHESSKRGVPLLM 6s6y.1    ASDPAKTPAETETAAGVLTRIRDRLIE-----------------------------------------------------  target    NMRTYPRSGGWEQRQEDKPWYTATGRLEFYRPEPEFQAAGESLPVWREPVDATFYEPNAILSNAAHPSIAPRAPEDYGVP 6s6y.1    --------------------------------------------------------------------------------  target    ESQLDVETRQYRNVVRTWAELQQTLHPLQERDPAFRFVFQTPKYRWGAHSTAVDADWISMLFGPFGDPYRRDPRMPWTGE 6s6y.1    --------------------------------------------------------------------------------  target    AYLEINPKDAAELGLADGDYAWVDADPEDRPYRGWKEDDPYYEVARAMMRVRIYTGMSRGVIRTWFNMYAATPATVANQK 6s6y.1    --------------------------------------------------------------------------------  target    ATPGNPARNEQTRYVALFRYGSHQSGTRAWLRPTQQTDSLVRKGYFGQVIGTGFEADVHSVSGAPKEAFVKIEKAEDGGI 6s6y.1    --------------------------------------------------------------------------------  target    GAERLWRPLTLGLRPEAPSAALTAYLAGDYSGTKGS 6s6y.1    ------------------------------------ ``` | | | | | | | | | | | | | | | | | | | | | | | | | | | | | | | | | | | | | | | | | | | | | | | | | |
|  | 5t5i.1.D | Tungsten formylmethanofuran dehydrogenase subunit fwdD  *TUNGSTEN-CONTAINING FORMYLMETHANOFURAN DEHYDROGENASE FROM METHANOTHERMOBACTER WOLFEII, ORTHORHOMBIC FORM AT 1.9 A* | 0.02 | 0.00 | 17.50 | 0.10 | 591-703 | X-ray | 1.90 | homo-dimer | 4 x ZN, 2 x MG, 18 x K, 22 x SF4, 2 x W, 4 x MGD, 2 x H2S, 2 x CA | HHblits | 0.31 |
| ``` target    MPTANKADEVIILRPGTDAAFFLGVARELIEKGLYDRAAVIERTDLPLLVRLDTGERLDARDVIPGYELAALTNYVTLKP 5t5i.1    --------------------------------------------------------------------------------  target    DAEIKGNPPPPPFTAGGQVVPTELRDAWGDFVWWDRATGRPRPVSRDEVGARFDGDPALLGEFEVELVDGSTVPVRPAFD 5t5i.1    --------------------------------------------------------------------------------  target    LLKQYLDESFDLRTASEVCRVPPQAIQSIARQLAANKRETLLAAGMGPNHYFQNDLFGRVQFLVAALTDNIGHLGGNVGS 5t5i.1    --------------------------------------------------------------------------------  target    YAGNYRGSVFQAMGQWIAEDPFAIEPDLTKPATVKRYYKAESAHYWNYGERPLRAVAKDDEGDLTKGEVLTGKSHMPTPT 5t5i.1    --------------------------------------------------------------------------------  target    KLIWFGNSNSLLGNAKWSFDVVKNTLPRQDAVFCNEWHWTSSCEYADLVFPADSWAEFKLPDATASCTNPFLLAFPTTPL 5t5i.1    --------------------------------------------------------------------------------  target    KRLYDTRSDYEALALTAKALGELIDEPRMEQYWRGILDGDPTPYLQRIFSGSNATRGITYDELHESSKRGVPLLMNMRTY 5t5i.1    --------------------------------------------------------------------------------  target    PRSGGWEQRQEDKPWYTATGRLEFYRPEPEFQAAGESLPVWREPVDATFYEPNAILSNAAHPSIAPRAPEDYGVPESQLD 5t5i.1    --------------------------------------------------------------------------------  target    VETRQYRNVVRTWAELQQTLHPLQERDPAFRFVFQTPKYRWGAHSTAVDADWISMLFGPFGDPYRRDPRMPWTGEAYLEI 5t5i.1    ------------------------------RVILNTGRTIWQGQAIESGKDLKMYV----------------DAAAIIQM  target    NPKDAAELGLADGDYAWVDADPEDRPYRGWKEDDPYYEVARAMMRVR-IYTGMSRGVIRTWFNMYAATPATVANQKATPG 5t5i.1    NPEMMKQLGIAEGDNVKVISE-----------------YGDVVVKAVEAKEPLPEGMVYIPMGP----------------  target    NPARNEQTRYVALFRYGSHQSGTRAWLRPTQQTDSLVRKGYFGQVIGTGFEADVHSVSGAPKEAFVKIEKAEDGGIGAER 5t5i.1    --------------------------------------------------------------------------------  target    LWRPLTLGLRPEAPSAALTAYLAGDYSGTKGS 5t5i.1    -------------------------------- ``` | | | | | | | | | | | | | | | | | | | | | | | | | | | | | | | | | | | | | | | | | | | | | | | | | |
|  | 7bkb.1.J | Formylmethanofuran dehydrogenase, subunit D  *Formate dehydrogenase - heterodisulfide reductase - formylmethanofuran dehydrogenase complex from Methanospirillum hungatei (hexameric, composite structure)* | 0.02 |  | 12.05 | 0.10 | 588-703 | EM | 0.00 | hetero-2-2-2-2-2-2-… | 48 x SF4, 4 x FAD, 2 x FES, 4 x 9S8, 4 x ZN, 2 x MO, 4 x MGD | HHblits | 0.27 |
| ``` target    MPTANKADEVIILRPGTDAAFFLGVARELIEKGLYDRAAVIERTDLPLLVRLDTGERLDARDVIPGYELAALTNYVTLKP 7bkb.1    --------------------------------------------------------------------------------  target    DAEIKGNPPPPPFTAGGQVVPTELRDAWGDFVWWDRATGRPRPVSRDEVGARFDGDPALLGEFEVELVDGSTVPVRPAFD 7bkb.1    --------------------------------------------------------------------------------  target    LLKQYLDESFDLRTASEVCRVPPQAIQSIARQLAANKRETLLAAGMGPNHYFQNDLFGRVQFLVAALTDNIGHLGGNVGS 7bkb.1    --------------------------------------------------------------------------------  target    YAGNYRGSVFQAMGQWIAEDPFAIEPDLTKPATVKRYYKAESAHYWNYGERPLRAVAKDDEGDLTKGEVLTGKSHMPTPT 7bkb.1    --------------------------------------------------------------------------------  target    KLIWFGNSNSLLGNAKWSFDVVKNTLPRQDAVFCNEWHWTSSCEYADLVFPADSWAEFKLPDATASCTNPFLLAFPTTPL 7bkb.1    --------------------------------------------------------------------------------  target    KRLYDTRSDYEALALTAKALGELIDEPRMEQYWRGILDGDPTPYLQRIFSGSNATRGITYDELHESSKRGVPLLMNMRTY 7bkb.1    --------------------------------------------------------------------------------  target    PRSGGWEQRQEDKPWYTATGRLEFYRPEPEFQAAGESLPVWREPVDATFYEPNAILSNAAHPSIAPRAPEDYGVPESQLD 7bkb.1    --------------------------------------------------------------------------------  target    VETRQYRNVVRTWAELQQTLHPLQERDPAFRFVFQTPKYRWGAHSTAVDADWISMLFGPFGDPYRRDPRMPWTGEAYLEI 7bkb.1    ---------------------------AKKTLNMITQRAVEEGIAMEI-GKTSRQYF---------------DACSIIEM  target    NPKDAAELGLADGDYAWVDADPEDRPYRGWKEDDPYYEVARAMMRVRIY-TGMSRGVIRTWFNMYAATPATVANQKATPG 7bkb.1    NEQDMKELGIMKNTNVRVKSE-----------------SGEVVVKAVVGRQTCYPGLCHIRQGV----------------  target    NPARNEQTRYVALFRYGSHQSGTRAWLRPTQQTDSLVRKGYFGQVIGTGFEADVHSVSGAPKEAFVKIEKAEDGGIGAER 7bkb.1    --------------------------------------------------------------------------------  target    LWRPLTLGLRPEAPSAALTAYLAGDYSGTKGS 7bkb.1    -------------------------------- ``` | | | | | | | | | | | | | | | | | | | | | | | | | | | | | | | | | | | | | | | | | | | | | | | | | |
|  | 1h0h.1.A | FORMATE DEHYDROGENASE SUBUNIT ALPHA  *Tungsten containing Formate Dehydrogenase from Desulfovibrio Gigas* | 0.02 | 0.00 | 16.67 | 0.09 | 587-697 | X-ray | 1.80 | monomer | 1 x W, 1 x 2MD, 1 x MGD, 4 x SF4, 1 x CA | HHblits | 0.30 |
| ``` target    MPTANKADEVIILRPGTDAAFFLGVARELIEKGLYDRAAVIERTDLPLLVRLDTGERLDARDVIPGYELAALTNYVTLKP 1h0h.1    --------------------------------------------------------------------------------  target    DAEIKGNPPPPPFTAGGQVVPTELRDAWGDFVWWDRATGRPRPVSRDEVGARFDGDPALLGEFEVELVDGSTVPVRPAFD 1h0h.1    --------------------------------------------------------------------------------  target    LLKQYLDESFDLRTASEVCRVPPQAIQSIARQLAANKRETLLAAGMGPNHYFQNDLFGRVQFLVAALTDNIGHLGGNVGS 1h0h.1    --------------------------------------------------------------------------------  target    YAGNYRGSVFQAMGQWIAEDPFAIEPDLTKPATVKRYYKAESAHYWNYGERPLRAVAKDDEGDLTKGEVLTGKSHMPTPT 1h0h.1    --------------------------------------------------------------------------------  target    KLIWFGNSNSLLGNAKWSFDVVKNTLPRQDAVFCNEWHWTSSCEYADLVFPADSWAEFKLPDATASCTNPFLLAFPTTPL 1h0h.1    --------------------------------------------------------------------------------  target    KRLYDTRSDYEALALTAKALGELIDEPRMEQYWRGILDGDPTPYLQRIFSGSNATRGITYDELHESSKRGVPLLMNMRTY 1h0h.1    --------------------------------------------------------------------------------  target    PRSGGWEQRQEDKPWYTATGRLEFYRPEPEFQAAGESLPVWREPVDATFYEPNAILSNAAHPSIAPRAPEDYGVPESQLD 1h0h.1    --------------------------------------------------------------------------------  target    VETRQYRNVVRTWAELQQTLHPLQERDPAFRFVFQTPKYR--WGAHSTAVDADWISMLFGPFGDPYRRDPRMPWTGEAYL 1h0h.1    --------------------------DPRYPFICSTYRVTEHWQTGLMTRNTPWLLEAE----------------PQMFC  target    EINPKDAAELGLADGDYAWVDADPEDRPYRGWKEDDPYYEVARAMMRVRIYTGMSRGVIRTWFNMYAATPATVANQKATP 1h0h.1    EMSEELATLRGIKNGDKVILESV-----------------RGKLWAKAIITKRIKPFAI---------------------  target    GNPARNEQTRYVALFRYGSHQSGTRAWLRPTQQTDSLVRKGYFGQVIGTGFEADVHSVSGAPKEAFVKIEKAEDGGIGAE 1h0h.1    --------------------------------------------------------------------------------  target    RLWRPLTLGLRPEAPSAALTAYLAGDYSGTKGS 1h0h.1    --------------------------------- ``` | | | | | | | | | | | | | | | | | | | | | | | | | | | | | | | | | | | | | | | | | | | | | | | | | |
|  | 2ki8.1.A | Tungsten formylmethanofuran dehydrogenase, subunit D (FwdD-2)  *Solution NMR structure of tungsten formylmethanofuran dehydrogenase subunit D from Archaeoglobus fulgidus, Northeast Structural Genomics Consortium target AtT7* | 0.03 |  | 16.25 | 0.10 | 589-703 | NMR | 0.00 | monomer |  | HHblits | 0.28 |
| ``` target    MPTANKADEVIILRPGTDAAFFLGVARELIEKGLYDRAAVIERTDLPLLVRLDTGERLDARDVIPGYELAALTNYVTLKP 2ki8.1    --------------------------------------------------------------------------------  target    DAEIKGNPPPPPFTAGGQVVPTELRDAWGDFVWWDRATGRPRPVSRDEVGARFDGDPALLGEFEVELVDGSTVPVRPAFD 2ki8.1    --------------------------------------------------------------------------------  target    LLKQYLDESFDLRTASEVCRVPPQAIQSIARQLAANKRETLLAAGMGPNHYFQNDLFGRVQFLVAALTDNIGHLGGNVGS 2ki8.1    --------------------------------------------------------------------------------  target    YAGNYRGSVFQAMGQWIAEDPFAIEPDLTKPATVKRYYKAESAHYWNYGERPLRAVAKDDEGDLTKGEVLTGKSHMPTPT 2ki8.1    --------------------------------------------------------------------------------  target    KLIWFGNSNSLLGNAKWSFDVVKNTLPRQDAVFCNEWHWTSSCEYADLVFPADSWAEFKLPDATASCTNPFLLAFPTTPL 2ki8.1    --------------------------------------------------------------------------------  target    KRLYDTRSDYEALALTAKALGELIDEPRMEQYWRGILDGDPTPYLQRIFSGSNATRGITYDELHESSKRGVPLLMNMRTY 2ki8.1    --------------------------------------------------------------------------------  target    PRSGGWEQRQEDKPWYTATGRLEFYRPEPEFQAAGESLPVWREPVDATFYEPNAILSNAAHPSIAPRAPEDYGVPESQLD 2ki8.1    --------------------------------------------------------------------------------  target    VETRQYRNVVRTWAELQQTLHPLQERDPAFRFVFQTPKYRWGAHSTAVDADWISMLFGPFGDPYRRDPRMPWTGEAYLEI 2ki8.1    ----------------------------MLEVEVISGRTLNQGATVE-EKLTEEYF----------------NAVNYAEI  target    NPKDAAELGLADGDYAWVDADPEDRPYRGWKEDDPYYEVARAMMRVRIYTGMSRGVIRTWFNMYAATPATVANQKATPGN 2ki8.1    NEEDWNALGLQEGDRVKVKTE-----------------FGEVVVFAKKG-DVPKGMIFIPMGP-----------------  target    PARNEQTRYVALFRYGSHQSGTRAWLRPTQQTDSLVRKGYFGQVIGTGFEADVHSVSGAPKEAFVKIEKAEDGGIGAERL 2ki8.1    --------------------------------------------------------------------------------  target    WRPLTLGLRPEAPSAALTAYLAGDYSGTKGS 2ki8.1    ------------------------------- ``` | | | | | | | | | | | | | | | | | | | | | | | | | | | | | | | | | | | | | | | | | | | | | | | | | |
|  | 8bqg.1.A | Formate dehydrogenase, alpha subunit, selenocysteine-containing  *W-formate dehydrogenase from Desulfovibrio vulgaris - Soaking with Formate 1 min* | 0.02 |  | 16.67 | 0.09 | 587-697 | X-ray | 1.95 | hetero-1-1-mer | 2 x MGD, 4 x SF4, 1 x H2S, 1 x W | HHblits | 0.28 |
| ``` target    MPTANKADEVIILRPGTDAAFFLGVARELIEKGLYDRAAVIERTDLPLLVRLDTGERLDARDVIPGYELAALTNYVTLKP 8bqg.1    --------------------------------------------------------------------------------  target    DAEIKGNPPPPPFTAGGQVVPTELRDAWGDFVWWDRATGRPRPVSRDEVGARFDGDPALLGEFEVELVDGSTVPVRPAFD 8bqg.1    --------------------------------------------------------------------------------  target    LLKQYLDESFDLRTASEVCRVPPQAIQSIARQLAANKRETLLAAGMGPNHYFQNDLFGRVQFLVAALTDNIGHLGGNVGS 8bqg.1    --------------------------------------------------------------------------------  target    YAGNYRGSVFQAMGQWIAEDPFAIEPDLTKPATVKRYYKAESAHYWNYGERPLRAVAKDDEGDLTKGEVLTGKSHMPTPT 8bqg.1    --------------------------------------------------------------------------------  target    KLIWFGNSNSLLGNAKWSFDVVKNTLPRQDAVFCNEWHWTSSCEYADLVFPADSWAEFKLPDATASCTNPFLLAFPTTPL 8bqg.1    --------------------------------------------------------------------------------  target    KRLYDTRSDYEALALTAKALGELIDEPRMEQYWRGILDGDPTPYLQRIFSGSNATRGITYDELHESSKRGVPLLMNMRTY 8bqg.1    --------------------------------------------------------------------------------  target    PRSGGWEQRQEDKPWYTATGRLEFYRPEPEFQAAGESLPVWREPVDATFYEPNAILSNAAHPSIAPRAPEDYGVPESQLD 8bqg.1    --------------------------------------------------------------------------------  target    VETRQYRNVVRTWAELQQTLHPLQERDPAFRFVFQTPKYRWGAHS--TAVDADWISMLFGPFGDPYRRDPRMPWTGEAYL 8bqg.1    --------------------------DPRYPFIGTTYRVTEHWQTGLMTRRCAWLVEAE----------------PQIFC  target    EINPKDAAELGLADGDYAWVDADPEDRPYRGWKEDDPYYEVARAMMRVRIYTGMSRGVIRTWFNMYAATPATVANQKATP 8bqg.1    EISKELAKLRGIGNGDTVKVSSL-----------------RGALEAVAIVTERIRPFKI---------------------  target    GNPARNEQTRYVALFRYGSHQSGTRAWLRPTQQTDSLVRKGYFGQVIGTGFEADVHSVSGAPKEAFVKIEKAEDGGIGAE 8bqg.1    --------------------------------------------------------------------------------  target    RLWRPLTLGLRPEAPSAALTAYLAGDYSGTKGS 8bqg.1    --------------------------------- ``` | | | | | | | | | | | | | | | | | | | | | | | | | | | | | | | | | | | | | | | | | | | | | | | | | |
|  | 6sdr.1.A | Formate dehydrogenase, alpha subunit, selenocysteine-containing  *W-formate dehydrogenase from Desulfovibrio vulgaris - Oxidized form* | 0.02 |  | 16.67 | 0.09 | 587-697 | X-ray | 2.10 | hetero-1-1-mer | 2 x MGD, 4 x SF4, 1 x H2S, 1 x W | HHblits | 0.28 |
| ``` target    MPTANKADEVIILRPGTDAAFFLGVARELIEKGLYDRAAVIERTDLPLLVRLDTGERLDARDVIPGYELAALTNYVTLKP 6sdr.1    --------------------------------------------------------------------------------  target    DAEIKGNPPPPPFTAGGQVVPTELRDAWGDFVWWDRATGRPRPVSRDEVGARFDGDPALLGEFEVELVDGSTVPVRPAFD 6sdr.1    --------------------------------------------------------------------------------  target    LLKQYLDESFDLRTASEVCRVPPQAIQSIARQLAANKRETLLAAGMGPNHYFQNDLFGRVQFLVAALTDNIGHLGGNVGS 6sdr.1    --------------------------------------------------------------------------------  target    YAGNYRGSVFQAMGQWIAEDPFAIEPDLTKPATVKRYYKAESAHYWNYGERPLRAVAKDDEGDLTKGEVLTGKSHMPTPT 6sdr.1    --------------------------------------------------------------------------------  target    KLIWFGNSNSLLGNAKWSFDVVKNTLPRQDAVFCNEWHWTSSCEYADLVFPADSWAEFKLPDATASCTNPFLLAFPTTPL 6sdr.1    --------------------------------------------------------------------------------  target    KRLYDTRSDYEALALTAKALGELIDEPRMEQYWRGILDGDPTPYLQRIFSGSNATRGITYDELHESSKRGVPLLMNMRTY 6sdr.1    --------------------------------------------------------------------------------  target    PRSGGWEQRQEDKPWYTATGRLEFYRPEPEFQAAGESLPVWREPVDATFYEPNAILSNAAHPSIAPRAPEDYGVPESQLD 6sdr.1    --------------------------------------------------------------------------------  target    VETRQYRNVVRTWAELQQTLHPLQERDPAFRFVFQTPKYRWG--AHSTAVDADWISMLFGPFGDPYRRDPRMPWTGEAYL 6sdr.1    --------------------------DPRYPFIGTTYRVTEHWQTGLMTRRCAWLVEAE----------------PQIFC  target    EINPKDAAELGLADGDYAWVDADPEDRPYRGWKEDDPYYEVARAMMRVRIYTGMSRGVIRTWFNMYAATPATVANQKATP 6sdr.1    EISKELAKLRGIGNGDTVKVSSL-----------------RGALEAVAIVTERIRPFKI---------------------  target    GNPARNEQTRYVALFRYGSHQSGTRAWLRPTQQTDSLVRKGYFGQVIGTGFEADVHSVSGAPKEAFVKIEKAEDGGIGAE 6sdr.1    --------------------------------------------------------------------------------  target    RLWRPLTLGLRPEAPSAALTAYLAGDYSGTKGS 6sdr.1    --------------------------------- ``` | | | | | | | | | | | | | | | | | | | | | | | | | | | | | | | | | | | | | | | | | | | | | | | | | |
|  | 2e7z.1.A | Acetylene hydratase Ahy  *Acetylene Hydratase from Pelobacter acetylenicus* | 0.01 |  | 21.05 | 0.07 | 320-376 | X-ray | 1.26 | monomer | 1 x SF4, 2 x MGD, 1 x W | HHblits | 0.28 |
| ``` target    MPTANKADEVIILRPGTDAAFFLGVARELIEKGLYDRAAVIERTDLPLLVRLDTGERLDARDVIPGYELAALTNYVTLKP 2e7z.1    --------------------------------------------------------------------------------  target    DAEIKGNPPPPPFTAGGQVVPTELRDAWGDFVWWDRATGRPRPVSRDEVGARFDGDPALLGEFEVELVDGSTVPVRPAFD 2e7z.1    --------------------------------------------------------------------------------  target    LLKQYLDESFDLRTASEVCRVPPQAIQSIARQLAANKRETLLAAGMGPNHYFQNDLFGRVQFLVAALTDNIGHLGGNVGS 2e7z.1    --------------------------------------------------------------------------------  target    YAGNYRGSVFQAMGQWIAEDPFAIEPDLTKPATVKRYYKAESAHYWNYGERPLRAVAKDDEGDLTKGEVLTGKSHMPTPT 2e7z.1    -------------------------------------------------------------------------------S  target    KLIWFGNSNSLLGNAKWSFDVVKNTLPRQDAVFCNEWHWTSSCEYADLVFPADSWAEFKLPDATASCTNPFLLAFPTTPL 2e7z.1    NCLLFIGKNLSNHNWVSQFNDLKAALKRGCKLIVLDPRRTKVAEMADIWLPLRYGT------------------------  target    KRLYDTRSDYEALALTAKALGELIDEPRMEQYWRGILDGDPTPYLQRIFSGSNATRGITYDELHESSKRGVPLLMNMRTY 2e7z.1    --------------------------------------------------------------------------------  target    PRSGGWEQRQEDKPWYTATGRLEFYRPEPEFQAAGESLPVWREPVDATFYEPNAILSNAAHPSIAPRAPEDYGVPESQLD 2e7z.1    --------------------------------------------------------------------------------  target    VETRQYRNVVRTWAELQQTLHPLQERDPAFRFVFQTPKYRWGAHSTAVDADWISMLFGPFGDPYRRDPRMPWTGEAYLEI 2e7z.1    --------------------------------------------------------------------------------  target    NPKDAAELGLADGDYAWVDADPEDRPYRGWKEDDPYYEVARAMMRVRIYTGMSRGVIRTWFNMYAATPATVANQKATPGN 2e7z.1    --------------------------------------------------------------------------------  target    PARNEQTRYVALFRYGSHQSGTRAWLRPTQQTDSLVRKGYFGQVIGTGFEADVHSVSGAPKEAFVKIEKAEDGGIGAERL 2e7z.1    --------------------------------------------------------------------------------  target    WRPLTLGLRPEAPSAALTAYLAGDYSGTKGS 2e7z.1    ------------------------------- ``` | | | | | | | | | | | | | | | | | | | | | | | | | | | | | | | | | | | | | | | | | | | | | | | | | |
|  | 6sdv.1.A | Formate dehydrogenase, alpha subunit, selenocysteine-containing,Formate dehydrogenase, alpha subunit, selenocysteine-containing,W-formate dehydrogenase - alpha subunit  *W-formate dehydrogenase from Desulfovibrio vulgaris - Formate reduced form* | 0.01 |  | 16.36 | 0.07 | 319-374 | X-ray | 1.90 | hetero-1-1-mer | 2 x MGD, 4 x SF4, 1 x W, 1 x H2S | HHblits | 0.27 |
| ``` target    MPTANKADEVIILRPGTDAAFFLGVARELIEKGLYDRAAVIERTDLPLLVRLDTGERLDARDVIPGYELAALTNYVTLKP 6sdv.1    --------------------------------------------------------------------------------  target    DAEIKGNPPPPPFTAGGQVVPTELRDAWGDFVWWDRATGRPRPVSRDEVGARFDGDPALLGEFEVELVDGSTVPVRPAFD 6sdv.1    --------------------------------------------------------------------------------  target    LLKQYLDESFDLRTASEVCRVPPQAIQSIARQLAANKRETLLAAGMGPNHYFQNDLFGRVQFLVAALTDNIGHLGGNVGS 6sdv.1    --------------------------------------------------------------------------------  target    YAGNYRGSVFQAMGQWIAEDPFAIEPDLTKPATVKRYYKAESAHYWNYGERPLRAVAKDDEGDLTKGEVLTGKSHMPTPT 6sdv.1    ------------------------------------------------------------------------------NS  target    KLIWFGNSNSLLGNAKWSFDVVKNTLPRQDAVFCNEWHWTSSCEYADLVFPADSWAEFKLPDATASCTNPFLLAFPTTPL 6sdv.1    DCILIMGSNAAENHPIA-FKWVLRAKDKGATLIHVDPRFTRTSARCDVYAPIRS--------------------------  target    KRLYDTRSDYEALALTAKALGELIDEPRMEQYWRGILDGDPTPYLQRIFSGSNATRGITYDELHESSKRGVPLLMNMRTY 6sdv.1    --------------------------------------------------------------------------------  target    PRSGGWEQRQEDKPWYTATGRLEFYRPEPEFQAAGESLPVWREPVDATFYEPNAILSNAAHPSIAPRAPEDYGVPESQLD 6sdv.1    --------------------------------------------------------------------------------  target    VETRQYRNVVRTWAELQQTLHPLQERDPAFRFVFQTPKYRWGAHSTAVDADWISMLFGPFGDPYRRDPRMPWTGEAYLEI 6sdv.1    --------------------------------------------------------------------------------  target    NPKDAAELGLADGDYAWVDADPEDRPYRGWKEDDPYYEVARAMMRVRIYTGMSRGVIRTWFNMYAATPATVANQKATPGN 6sdv.1    --------------------------------------------------------------------------------  target    PARNEQTRYVALFRYGSHQSGTRAWLRPTQQTDSLVRKGYFGQVIGTGFEADVHSVSGAPKEAFVKIEKAEDGGIGAERL 6sdv.1    --------------------------------------------------------------------------------  target    WRPLTLGLRPEAPSAALTAYLAGDYSGTKGS 6sdv.1    ------------------------------- ``` | | | | | | | | | | | | | | | | | | | | | | | | | | | | | | | | | | | | | | | | | | | | | | | | | |
|  | 7b04.1.B | Nitrite oxidoreductase subunit A  *Structure of Nitrite oxidoreductase (Nxr) from the anammox bacterium Kuenenia stuttgartiensis.* | 0.01 |  | 16.67 | 0.06 | 319-373 | X-ray | 2.97 | hetero-1-1-1-mer | 4 x SF4, 1 x F3S, 2 x MD1, 1 x MO, 1 x HEM, 2 x CA | HHblits | 0.27 |
| ``` target    MPTANKADEVIILRPGTDAAFFLGVARELIEKGLYDRAAVIERTDLPLLVRLDTGERLDARDVIPGYELAALTNYVTLKP 7b04.1    --------------------------------------------------------------------------------  target    DAEIKGNPPPPPFTAGGQVVPTELRDAWGDFVWWDRATGRPRPVSRDEVGARFDGDPALLGEFEVELVDGSTVPVRPAFD 7b04.1    --------------------------------------------------------------------------------  target    LLKQYLDESFDLRTASEVCRVPPQAIQSIARQLAANKRETLLAAGMGPNHYFQNDLFGRVQFLVAALTDNIGHLGGNVGS 7b04.1    --------------------------------------------------------------------------------  target    YAGNYRGSVFQAMGQWIAEDPFAIEPDLTKPATVKRYYKAESAHYWNYGERPLRAVAKDDEGDLTKGEVLTGKSHMPTPT 7b04.1    ------------------------------------------------------------------------------FS  target    KLIWFGNSNSLLGNAKWSFDVVKNTLPRQDAVFCNEWHWTSSCEYADLVFPADSWAEFKLPDATASCTNPFLLAFPTTPL 7b04.1    KLLIQTGKNLIENKMPE-AHWVTEVMERGGKIVVITPEYSPSAQKADYWIPIR---------------------------  target    KRLYDTRSDYEALALTAKALGELIDEPRMEQYWRGILDGDPTPYLQRIFSGSNATRGITYDELHESSKRGVPLLMNMRTY 7b04.1    --------------------------------------------------------------------------------  target    PRSGGWEQRQEDKPWYTATGRLEFYRPEPEFQAAGESLPVWREPVDATFYEPNAILSNAAHPSIAPRAPEDYGVPESQLD 7b04.1    --------------------------------------------------------------------------------  target    VETRQYRNVVRTWAELQQTLHPLQERDPAFRFVFQTPKYRWGAHSTAVDADWISMLFGPFGDPYRRDPRMPWTGEAYLEI 7b04.1    --------------------------------------------------------------------------------  target    NPKDAAELGLADGDYAWVDADPEDRPYRGWKEDDPYYEVARAMMRVRIYTGMSRGVIRTWFNMYAATPATVANQKATPGN 7b04.1    --------------------------------------------------------------------------------  target    PARNEQTRYVALFRYGSHQSGTRAWLRPTQQTDSLVRKGYFGQVIGTGFEADVHSVSGAPKEAFVKIEKAEDGGIGAERL 7b04.1    --------------------------------------------------------------------------------  target    WRPLTLGLRPEAPSAALTAYLAGDYSGTKGS 7b04.1    ------------------------------- ``` | | | | | | | | | | | | | | | | | | | | | | | | | | | | | | | | | | | | | | | | | | | | | | | | | |
|  | 7b04.2.B | Nitrite oxidoreductase subunit A  *Structure of Nitrite oxidoreductase (Nxr) from the anammox bacterium Kuenenia stuttgartiensis.* | 0.01 |  | 16.67 | 0.06 | 319-373 | X-ray | 2.97 | hetero-1-1-1-mer | 4 x SF4, 1 x F3S, 2 x MD1, 1 x MO, 1 x HEM, 2 x CA | HHblits | 0.27 |
| ``` target    MPTANKADEVIILRPGTDAAFFLGVARELIEKGLYDRAAVIERTDLPLLVRLDTGERLDARDVIPGYELAALTNYVTLKP 7b04.2    --------------------------------------------------------------------------------  target    DAEIKGNPPPPPFTAGGQVVPTELRDAWGDFVWWDRATGRPRPVSRDEVGARFDGDPALLGEFEVELVDGSTVPVRPAFD 7b04.2    --------------------------------------------------------------------------------  target    LLKQYLDESFDLRTASEVCRVPPQAIQSIARQLAANKRETLLAAGMGPNHYFQNDLFGRVQFLVAALTDNIGHLGGNVGS 7b04.2    --------------------------------------------------------------------------------  target    YAGNYRGSVFQAMGQWIAEDPFAIEPDLTKPATVKRYYKAESAHYWNYGERPLRAVAKDDEGDLTKGEVLTGKSHMPTPT 7b04.2    ------------------------------------------------------------------------------FS  target    KLIWFGNSNSLLGNAKWSFDVVKNTLPRQDAVFCNEWHWTSSCEYADLVFPADSWAEFKLPDATASCTNPFLLAFPTTPL 7b04.2    KLLIQTGKNLIENKMPE-AHWVTEVMERGGKIVVITPEYSPSAQKADYWIPIR---------------------------  target    KRLYDTRSDYEALALTAKALGELIDEPRMEQYWRGILDGDPTPYLQRIFSGSNATRGITYDELHESSKRGVPLLMNMRTY 7b04.2    --------------------------------------------------------------------------------  target    PRSGGWEQRQEDKPWYTATGRLEFYRPEPEFQAAGESLPVWREPVDATFYEPNAILSNAAHPSIAPRAPEDYGVPESQLD 7b04.2    --------------------------------------------------------------------------------  target    VETRQYRNVVRTWAELQQTLHPLQERDPAFRFVFQTPKYRWGAHSTAVDADWISMLFGPFGDPYRRDPRMPWTGEAYLEI 7b04.2    --------------------------------------------------------------------------------  target    NPKDAAELGLADGDYAWVDADPEDRPYRGWKEDDPYYEVARAMMRVRIYTGMSRGVIRTWFNMYAATPATVANQKATPGN 7b04.2    --------------------------------------------------------------------------------  target    PARNEQTRYVALFRYGSHQSGTRAWLRPTQQTDSLVRKGYFGQVIGTGFEADVHSVSGAPKEAFVKIEKAEDGGIGAERL 7b04.2    --------------------------------------------------------------------------------  target    WRPLTLGLRPEAPSAALTAYLAGDYSGTKGS 7b04.2    ------------------------------- ``` | | | | | | | | | | | | | | | | | | | | | | | | | | | | | | | | | | | | | | | | | | | | | | | | | |
|  | 7qv7.1.L | Hydrogen dependent carbon dioxide reductase subunit FdhF  *Cryo-EM structure of Hydrogen-dependent CO2 reductase.* | 0.01 |  | 14.55 | 0.07 | 320-375 | EM | 0.00 | hetero-2-6-6-2-mer | 52 x SF4, 6 x 402 | HHblits | 0.26 |
| ``` target    MPTANKADEVIILRPGTDAAFFLGVARELIEKGLYDRAAVIERTDLPLLVRLDTGERLDARDVIPGYELAALTNYVTLKP 7qv7.1    --------------------------------------------------------------------------------  target    DAEIKGNPPPPPFTAGGQVVPTELRDAWGDFVWWDRATGRPRPVSRDEVGARFDGDPALLGEFEVELVDGSTVPVRPAFD 7qv7.1    --------------------------------------------------------------------------------  target    LLKQYLDESFDLRTASEVCRVPPQAIQSIARQLAANKRETLLAAGMGPNHYFQNDLFGRVQFLVAALTDNIGHLGGNVGS 7qv7.1    --------------------------------------------------------------------------------  target    YAGNYRGSVFQAMGQWIAEDPFAIEPDLTKPATVKRYYKAESAHYWNYGERPLRAVAKDDEGDLTKGEVLTGKSHMPTPT 7qv7.1    -------------------------------------------------------------------------------S  target    KLIWFGNSNSLLGNAKWSFDVVKNTLPRQDAVFCNEWHWTSSCEYADLVFPADSWAEFKLPDATASCTNPFLLAFPTTPL 7qv7.1    DVIFIIGSNTAECHPLI-AAHVIKAKERGAKLIVADPRMNAMVHKADIWLRVPSG-------------------------  target    KRLYDTRSDYEALALTAKALGELIDEPRMEQYWRGILDGDPTPYLQRIFSGSNATRGITYDELHESSKRGVPLLMNMRTY 7qv7.1    --------------------------------------------------------------------------------  target    PRSGGWEQRQEDKPWYTATGRLEFYRPEPEFQAAGESLPVWREPVDATFYEPNAILSNAAHPSIAPRAPEDYGVPESQLD 7qv7.1    --------------------------------------------------------------------------------  target    VETRQYRNVVRTWAELQQTLHPLQERDPAFRFVFQTPKYRWGAHSTAVDADWISMLFGPFGDPYRRDPRMPWTGEAYLEI 7qv7.1    --------------------------------------------------------------------------------  target    NPKDAAELGLADGDYAWVDADPEDRPYRGWKEDDPYYEVARAMMRVRIYTGMSRGVIRTWFNMYAATPATVANQKATPGN 7qv7.1    --------------------------------------------------------------------------------  target    PARNEQTRYVALFRYGSHQSGTRAWLRPTQQTDSLVRKGYFGQVIGTGFEADVHSVSGAPKEAFVKIEKAEDGGIGAERL 7qv7.1    --------------------------------------------------------------------------------  target    WRPLTLGLRPEAPSAALTAYLAGDYSGTKGS 7qv7.1    ------------------------------- ``` | | | | | | | | | | | | | | | | | | | | | | | | | | | | | | | | | | | | | | | | | | | | | | | | | |
|  | 7qv7.1.O | Hydrogen dependent carbon dioxide reductase subunit FdhF  *Cryo-EM structure of Hydrogen-dependent CO2 reductase.* | 0.01 |  | 14.55 | 0.07 | 320-375 | EM | 0.00 | hetero-2-6-6-2-mer | 52 x SF4, 6 x 402 | HHblits | 0.26 |
| ``` target    MPTANKADEVIILRPGTDAAFFLGVARELIEKGLYDRAAVIERTDLPLLVRLDTGERLDARDVIPGYELAALTNYVTLKP 7qv7.1    --------------------------------------------------------------------------------  target    DAEIKGNPPPPPFTAGGQVVPTELRDAWGDFVWWDRATGRPRPVSRDEVGARFDGDPALLGEFEVELVDGSTVPVRPAFD 7qv7.1    --------------------------------------------------------------------------------  target    LLKQYLDESFDLRTASEVCRVPPQAIQSIARQLAANKRETLLAAGMGPNHYFQNDLFGRVQFLVAALTDNIGHLGGNVGS 7qv7.1    --------------------------------------------------------------------------------  target    YAGNYRGSVFQAMGQWIAEDPFAIEPDLTKPATVKRYYKAESAHYWNYGERPLRAVAKDDEGDLTKGEVLTGKSHMPTPT 7qv7.1    -------------------------------------------------------------------------------S  target    KLIWFGNSNSLLGNAKWSFDVVKNTLPRQDAVFCNEWHWTSSCEYADLVFPADSWAEFKLPDATASCTNPFLLAFPTTPL 7qv7.1    DVIFIIGSNTAECHPLI-AAHVIKAKERGAKLIVADPRMNAMVHKADIWLRVPSG-------------------------  target    KRLYDTRSDYEALALTAKALGELIDEPRMEQYWRGILDGDPTPYLQRIFSGSNATRGITYDELHESSKRGVPLLMNMRTY 7qv7.1    --------------------------------------------------------------------------------  target    PRSGGWEQRQEDKPWYTATGRLEFYRPEPEFQAAGESLPVWREPVDATFYEPNAILSNAAHPSIAPRAPEDYGVPESQLD 7qv7.1    --------------------------------------------------------------------------------  target    VETRQYRNVVRTWAELQQTLHPLQERDPAFRFVFQTPKYRWGAHSTAVDADWISMLFGPFGDPYRRDPRMPWTGEAYLEI 7qv7.1    --------------------------------------------------------------------------------  target    NPKDAAELGLADGDYAWVDADPEDRPYRGWKEDDPYYEVARAMMRVRIYTGMSRGVIRTWFNMYAATPATVANQKATPGN 7qv7.1    --------------------------------------------------------------------------------  target    PARNEQTRYVALFRYGSHQSGTRAWLRPTQQTDSLVRKGYFGQVIGTGFEADVHSVSGAPKEAFVKIEKAEDGGIGAERL 7qv7.1    --------------------------------------------------------------------------------  target    WRPLTLGLRPEAPSAALTAYLAGDYSGTKGS 7qv7.1    ------------------------------- ``` | | | | | | | | | | | | | | | | | | | | | | | | | | | | | | | | | | | | | | | | | | | | | | | | | |
|  | 4ga5.1.A | Putative thymidine phosphorylase  *Crystal structure of AMP phosphorylase C-terminal deletion mutant in the apo-form* | 0.01 |  | 21.15 | 0.06 | 634-702 | X-ray | 3.25 | homo-dimer |  | HHblits | 0.30 |
| ``` target    MPTANKADEVIILRPGTDAAFFLGVARELIEKGLYDRAAVIERTDLPLLVRLDTGERLDARDVIPGYELAALTNYVTLKP 4ga5.1    --------------------------------------------------------------------------------  target    DAEIKGNPPPPPFTAGGQVVPTELRDAWGDFVWWDRATGRPRPVSRDEVGARFDGDPALLGEFEVELVDGSTVPVRPAFD 4ga5.1    --------------------------------------------------------------------------------  target    LLKQYLDESFDLRTASEVCRVPPQAIQSIARQLAANKRETLLAAGMGPNHYFQNDLFGRVQFLVAALTDNIGHLGGNVGS 4ga5.1    --------------------------------------------------------------------------------  target    YAGNYRGSVFQAMGQWIAEDPFAIEPDLTKPATVKRYYKAESAHYWNYGERPLRAVAKDDEGDLTKGEVLTGKSHMPTPT 4ga5.1    --------------------------------------------------------------------------------  target    KLIWFGNSNSLLGNAKWSFDVVKNTLPRQDAVFCNEWHWTSSCEYADLVFPADSWAEFKLPDATASCTNPFLLAFPTTPL 4ga5.1    --------------------------------------------------------------------------------  target    KRLYDTRSDYEALALTAKALGELIDEPRMEQYWRGILDGDPTPYLQRIFSGSNATRGITYDELHESSKRGVPLLMNMRTY 4ga5.1    --------------------------------------------------------------------------------  target    PRSGGWEQRQEDKPWYTATGRLEFYRPEPEFQAAGESLPVWREPVDATFYEPNAILSNAAHPSIAPRAPEDYGVPESQLD 4ga5.1    --------------------------------------------------------------------------------  target    VETRQYRNVVRTWAELQQTLHPLQERDPAFRFVFQTPKYRWGAHSTAVDADWISMLFGPFGDPYRRDPRMPWTGEAYLEI 4ga5.1    -------------------------------------------------------------------------GRYTVLI  target    NPKDAAELGLADGDYAWVDADPEDRPYRGWKEDDPYYEVARAMMRVRIYTGMSRGVIRTWFNMYAATPATVANQKATPGN 4ga5.1    NEEDAKEAKLHPDDLVKIEAGK-----------------KAVYGSVALSNLVGKGEVGISRD------------------  target    PARNEQTRYVALFRYGSHQSGTRAWLRPTQQTDSLVRKGYFGQVIGTGFEADVHSVSGAPKEAFVKIEKAEDGGIGAERL 4ga5.1    --------------------------------------------------------------------------------  target    WRPLTLGLRPEAPSAALTAYLAGDYSGTKGS 4ga5.1    ------------------------------- ``` | | | | | | | | | | | | | | | | | | | | | | | | | | | | | | | | | | | | | | | | | | | | | | | | | |
|  | 4ga6.1.A | Putative thymidine phosphorylase  *Crystal structure of AMP phosphorylase C-terminal deletion mutant in complex with substrates* | 0.02 |  | 21.15 | 0.06 | 634-702 | X-ray | 2.21 | homo-dimer | 2 x AMP | HHblits | 0.30 |
| ``` target    MPTANKADEVIILRPGTDAAFFLGVARELIEKGLYDRAAVIERTDLPLLVRLDTGERLDARDVIPGYELAALTNYVTLKP 4ga6.1    --------------------------------------------------------------------------------  target    DAEIKGNPPPPPFTAGGQVVPTELRDAWGDFVWWDRATGRPRPVSRDEVGARFDGDPALLGEFEVELVDGSTVPVRPAFD 4ga6.1    --------------------------------------------------------------------------------  target    LLKQYLDESFDLRTASEVCRVPPQAIQSIARQLAANKRETLLAAGMGPNHYFQNDLFGRVQFLVAALTDNIGHLGGNVGS 4ga6.1    --------------------------------------------------------------------------------  target    YAGNYRGSVFQAMGQWIAEDPFAIEPDLTKPATVKRYYKAESAHYWNYGERPLRAVAKDDEGDLTKGEVLTGKSHMPTPT 4ga6.1    --------------------------------------------------------------------------------  target    KLIWFGNSNSLLGNAKWSFDVVKNTLPRQDAVFCNEWHWTSSCEYADLVFPADSWAEFKLPDATASCTNPFLLAFPTTPL 4ga6.1    --------------------------------------------------------------------------------  target    KRLYDTRSDYEALALTAKALGELIDEPRMEQYWRGILDGDPTPYLQRIFSGSNATRGITYDELHESSKRGVPLLMNMRTY 4ga6.1    --------------------------------------------------------------------------------  target    PRSGGWEQRQEDKPWYTATGRLEFYRPEPEFQAAGESLPVWREPVDATFYEPNAILSNAAHPSIAPRAPEDYGVPESQLD 4ga6.1    --------------------------------------------------------------------------------  target    VETRQYRNVVRTWAELQQTLHPLQERDPAFRFVFQTPKYRWGAHSTAVDADWISMLFGPFGDPYRRDPRMPWTGEAYLEI 4ga6.1    -------------------------------------------------------------------------GRYTVLI  target    NPKDAAELGLADGDYAWVDADPEDRPYRGWKEDDPYYEVARAMMRVRIYTGMSRGVIRTWFNMYAATPATVANQKATPGN 4ga6.1    NEEDAKEAKLHPDDLVKIEAGK-----------------KAVYGSVALSNLVGKGEVGISRD------------------  target    PARNEQTRYVALFRYGSHQSGTRAWLRPTQQTDSLVRKGYFGQVIGTGFEADVHSVSGAPKEAFVKIEKAEDGGIGAERL 4ga6.1    --------------------------------------------------------------------------------  target    WRPLTLGLRPEAPSAALTAYLAGDYSGTKGS 4ga6.1    ------------------------------- ``` | | | | | | | | | | | | | | | | | | | | | | | | | | | | | | | | | | | | | | | | | | | | | | | | | |
|  | 2ivf.1.A | ETHYLBENZENE DEHYDROGENASE ALPHA-SUBUNIT  *ETHYLBENZENE DEHYDROGENASE FROM AROMATOLEUM AROMATICUM* | 0.01 |  | 16.67 | 0.06 | 320-374 | X-ray | 1.88 | hetero-oligomer | 1 x MES, 4 x SF4, 1 x MO, 1 x MGD, 1 x MD1, 1 x F3S, 1 x HEM | HHblits | 0.26 |
| ``` target    MPTANKADEVIILRPGTDAAFFLGVARELIEKGLYDRAAVIERTDLPLLVRLDTGERLDARDVIPGYELAALTNYVTLKP 2ivf.1    --------------------------------------------------------------------------------  target    DAEIKGNPPPPPFTAGGQVVPTELRDAWGDFVWWDRATGRPRPVSRDEVGARFDGDPALLGEFEVELVDGSTVPVRPAFD 2ivf.1    --------------------------------------------------------------------------------  target    LLKQYLDESFDLRTASEVCRVPPQAIQSIARQLAANKRETLLAAGMGPNHYFQNDLFGRVQFLVAALTDNIGHLGGNVGS 2ivf.1    --------------------------------------------------------------------------------  target    YAGNYRGSVFQAMGQWIAEDPFAIEPDLTKPATVKRYYKAESAHYWNYGERPLRAVAKDDEGDLTKGEVLTGKSHMPTPT 2ivf.1    -------------------------------------------------------------------------------A  target    KLIWFGNSNSLLGNAKWSFDVVKNTLPRQDAVFCNEWHWTSSCEYADLVFPADSWAEFKLPDATASCTNPFLLAFPTTPL 2ivf.1    ELIFMTCSNWSYTYPSS-YHFLSEARYKGAEVVVIAPDFNPTTPAADLHVPVRV--------------------------  target    KRLYDTRSDYEALALTAKALGELIDEPRMEQYWRGILDGDPTPYLQRIFSGSNATRGITYDELHESSKRGVPLLMNMRTY 2ivf.1    --------------------------------------------------------------------------------  target    PRSGGWEQRQEDKPWYTATGRLEFYRPEPEFQAAGESLPVWREPVDATFYEPNAILSNAAHPSIAPRAPEDYGVPESQLD 2ivf.1    --------------------------------------------------------------------------------  target    VETRQYRNVVRTWAELQQTLHPLQERDPAFRFVFQTPKYRWGAHSTAVDADWISMLFGPFGDPYRRDPRMPWTGEAYLEI 2ivf.1    --------------------------------------------------------------------------------  target    NPKDAAELGLADGDYAWVDADPEDRPYRGWKEDDPYYEVARAMMRVRIYTGMSRGVIRTWFNMYAATPATVANQKATPGN 2ivf.1    --------------------------------------------------------------------------------  target    PARNEQTRYVALFRYGSHQSGTRAWLRPTQQTDSLVRKGYFGQVIGTGFEADVHSVSGAPKEAFVKIEKAEDGGIGAERL 2ivf.1    --------------------------------------------------------------------------------  target    WRPLTLGLRPEAPSAALTAYLAGDYSGTKGS 2ivf.1    ------------------------------- ``` | | | | | | | | | | | | | | | | | | | | | | | | | | | | | | | | | | | | | | | | | | | | | | | | | |
|  | 5e7o.1.A | DMSO reductase family type II enzyme, molybdopterin subunit  *Crystal structure of the perchlorate reductase PcrAB mutant W461E of PcrA from Azospira suillum PS* | 0.01 |  | 12.73 | 0.07 | 320-375 | X-ray | 2.40 | hetero-oligomer | 4 x SF4, 1 x MO, 1 x MGD, 1 x MD1, 1 x F3S | HHblits | 0.25 |
| ``` target    MPTANKADEVIILRPGTDAAFFLGVARELIEKGLYDRAAVIERTDLPLLVRLDTGERLDARDVIPGYELAALTNYVTLKP 5e7o.1    --------------------------------------------------------------------------------  target    DAEIKGNPPPPPFTAGGQVVPTELRDAWGDFVWWDRATGRPRPVSRDEVGARFDGDPALLGEFEVELVDGSTVPVRPAFD 5e7o.1    --------------------------------------------------------------------------------  target    LLKQYLDESFDLRTASEVCRVPPQAIQSIARQLAANKRETLLAAGMGPNHYFQNDLFGRVQFLVAALTDNIGHLGGNVGS 5e7o.1    --------------------------------------------------------------------------------  target    YAGNYRGSVFQAMGQWIAEDPFAIEPDLTKPATVKRYYKAESAHYWNYGERPLRAVAKDDEGDLTKGEVLTGKSHMPTPT 5e7o.1    -------------------------------------------------------------------------------S  target    KLIWFGNSNSLLGNAKWSFDVVKNTLPRQDAVFCNEWHWTSSCEYADLVFPADSWAEFKLPDATASCTNPFLLAFPTTPL 5e7o.1    KYIILWGSNPTQTRIP-DAHFLSEAQLNGAKIVSISPDYNSSTIKVDKWIHPQPG-------------------------  target    KRLYDTRSDYEALALTAKALGELIDEPRMEQYWRGILDGDPTPYLQRIFSGSNATRGITYDELHESSKRGVPLLMNMRTY 5e7o.1    --------------------------------------------------------------------------------  target    PRSGGWEQRQEDKPWYTATGRLEFYRPEPEFQAAGESLPVWREPVDATFYEPNAILSNAAHPSIAPRAPEDYGVPESQLD 5e7o.1    --------------------------------------------------------------------------------  target    VETRQYRNVVRTWAELQQTLHPLQERDPAFRFVFQTPKYRWGAHSTAVDADWISMLFGPFGDPYRRDPRMPWTGEAYLEI 5e7o.1    --------------------------------------------------------------------------------  target    NPKDAAELGLADGDYAWVDADPEDRPYRGWKEDDPYYEVARAMMRVRIYTGMSRGVIRTWFNMYAATPATVANQKATPGN 5e7o.1    --------------------------------------------------------------------------------  target    PARNEQTRYVALFRYGSHQSGTRAWLRPTQQTDSLVRKGYFGQVIGTGFEADVHSVSGAPKEAFVKIEKAEDGGIGAERL 5e7o.1    --------------------------------------------------------------------------------  target    WRPLTLGLRPEAPSAALTAYLAGDYSGTKGS 5e7o.1    ------------------------------- ``` | | | | | | | | | | | | | | | | | | | | | | | | | | | | | | | | | | | | | | | | | | | | | | | | | |
|  | 2vpz.1.A | THIOSULFATE REDUCTASE  *POLYSULFIDE REDUCTASE NATIVE STRUCTURE* | 0.01 |  | 8.93 | 0.07 | 320-375 | X-ray | 2.40 | hetero-oligomer | 10 x SF4, 4 x MGD, 2 x MO | HHblits | 0.23 |
| ``` target    MPTANKADEVIILRPGTDAAFFLGVARELIEKGLYDRAAVIERTDLPLLVRLDTGERLDARDVIPGYELAALTNYVTLKP 2vpz.1    --------------------------------------------------------------------------------  target    DAEIKGNPPPPPFTAGGQVVPTELRDAWGDFVWWDRATGRPRPVSRDEVGARFDGDPALLGEFEVELVDGSTVPVRPAFD 2vpz.1    --------------------------------------------------------------------------------  target    LLKQYLDESFDLRTASEVCRVPPQAIQSIARQLAANKRETLLAAGMGPNHYFQNDLFGRVQFLVAALTDNIGHLGGNVGS 2vpz.1    --------------------------------------------------------------------------------  target    YAGNYRGSVFQAMGQWIAEDPFAIEPDLTKPATVKRYYKAESAHYWNYGERPLRAVAKDDEGDLTKGEVLTGKSHMPTPT 2vpz.1    -------------------------------------------------------------------------------A  target    KLIWFGNSNSLLGNAKWSFDVVKNTLPRQDAVFCNEWHWTSSCEYADLVFPADSWAEFKLPDATASCTNPFLLAFPTTPL 2vpz.1    RYIVLIGHHIGEDTHNTQLQDFALALKNGAKVVVVDPRFSTAAAKAHRWLPIKPG-------------------------  target    KRLYDTRSDYEALALTAKALGELIDEPRMEQYWRGILDGDPTPYLQRIFSGSNATRGITYDELHESSKRGVPLLMNMRTY 2vpz.1    --------------------------------------------------------------------------------  target    PRSGGWEQRQEDKPWYTATGRLEFYRPEPEFQAAGESLPVWREPVDATFYEPNAILSNAAHPSIAPRAPEDYGVPESQLD 2vpz.1    --------------------------------------------------------------------------------  target    VETRQYRNVVRTWAELQQTLHPLQERDPAFRFVFQTPKYRWGAHSTAVDADWISMLFGPFGDPYRRDPRMPWTGEAYLEI 2vpz.1    --------------------------------------------------------------------------------  target    NPKDAAELGLADGDYAWVDADPEDRPYRGWKEDDPYYEVARAMMRVRIYTGMSRGVIRTWFNMYAATPATVANQKATPGN 2vpz.1    --------------------------------------------------------------------------------  target    PARNEQTRYVALFRYGSHQSGTRAWLRPTQQTDSLVRKGYFGQVIGTGFEADVHSVSGAPKEAFVKIEKAEDGGIGAERL 2vpz.1    --------------------------------------------------------------------------------  target    WRPLTLGLRPEAPSAALTAYLAGDYSGTKGS 2vpz.1    ------------------------------- ``` | | | | | | | | | | | | | | | | | | | | | | | | | | | | | | | | | | | | | | | | | | | | | | | | | |
|  | 2vpx.1.D | THIOSULFATE REDUCTASE  *POLYSULFIDE REDUCTASE WITH BOUND QUINONE (UQ1)* | 0.01 |  | 8.93 | 0.07 | 320-375 | X-ray | 3.10 | hetero-oligomer | 10 x SF4, 4 x MGD, 2 x MO, 2 x UQ1 | HHblits | 0.23 |
| ``` target    MPTANKADEVIILRPGTDAAFFLGVARELIEKGLYDRAAVIERTDLPLLVRLDTGERLDARDVIPGYELAALTNYVTLKP 2vpx.1    --------------------------------------------------------------------------------  target    DAEIKGNPPPPPFTAGGQVVPTELRDAWGDFVWWDRATGRPRPVSRDEVGARFDGDPALLGEFEVELVDGSTVPVRPAFD 2vpx.1    --------------------------------------------------------------------------------  target    LLKQYLDESFDLRTASEVCRVPPQAIQSIARQLAANKRETLLAAGMGPNHYFQNDLFGRVQFLVAALTDNIGHLGGNVGS 2vpx.1    --------------------------------------------------------------------------------  target    YAGNYRGSVFQAMGQWIAEDPFAIEPDLTKPATVKRYYKAESAHYWNYGERPLRAVAKDDEGDLTKGEVLTGKSHMPTPT 2vpx.1    -------------------------------------------------------------------------------A  target    KLIWFGNSNSLLGNAKWSFDVVKNTLPRQDAVFCNEWHWTSSCEYADLVFPADSWAEFKLPDATASCTNPFLLAFPTTPL 2vpx.1    RYIVLIGHHIGEDTHNTQLQDFALALKNGAKVVVVDPRFSTAAAKAHRWLPIKPG-------------------------  target    KRLYDTRSDYEALALTAKALGELIDEPRMEQYWRGILDGDPTPYLQRIFSGSNATRGITYDELHESSKRGVPLLMNMRTY 2vpx.1    --------------------------------------------------------------------------------  target    PRSGGWEQRQEDKPWYTATGRLEFYRPEPEFQAAGESLPVWREPVDATFYEPNAILSNAAHPSIAPRAPEDYGVPESQLD 2vpx.1    --------------------------------------------------------------------------------  target    VETRQYRNVVRTWAELQQTLHPLQERDPAFRFVFQTPKYRWGAHSTAVDADWISMLFGPFGDPYRRDPRMPWTGEAYLEI 2vpx.1    --------------------------------------------------------------------------------  target    NPKDAAELGLADGDYAWVDADPEDRPYRGWKEDDPYYEVARAMMRVRIYTGMSRGVIRTWFNMYAATPATVANQKATPGN 2vpx.1    --------------------------------------------------------------------------------  target    PARNEQTRYVALFRYGSHQSGTRAWLRPTQQTDSLVRKGYFGQVIGTGFEADVHSVSGAPKEAFVKIEKAEDGGIGAERL 2vpx.1    --------------------------------------------------------------------------------  target    WRPLTLGLRPEAPSAALTAYLAGDYSGTKGS 2vpx.1    ------------------------------- ``` | | | | | | | | | | | | | | | | | | | | | | | | | | | | | | | | | | | | | | | | | | | | | | | | | |
|  | 1kqf.1.A | FORMATE DEHYDROGENASE, NITRATE-INDUCIBLE, MAJOR SUBUNIT  *FORMATE DEHYDROGENASE N FROM E. COLI* | 0.01 |  | 11.32 | 0.06 | 320-373 | X-ray | 1.60 | hetero-oligomer | 3 x 6MO, 15 x SF4, 6 x MGD, 6 x HEM, 3 x CDL | HHblits | 0.27 |
| ``` target    MPTANKADEVIILRPGTDAAFFLGVARELIEKGLYDRAAVIERTDLPLLVRLDTGERLDARDVIPGYELAALTNYVTLKP 1kqf.1    --------------------------------------------------------------------------------  target    DAEIKGNPPPPPFTAGGQVVPTELRDAWGDFVWWDRATGRPRPVSRDEVGARFDGDPALLGEFEVELVDGSTVPVRPAFD 1kqf.1    --------------------------------------------------------------------------------  target    LLKQYLDESFDLRTASEVCRVPPQAIQSIARQLAANKRETLLAAGMGPNHYFQNDLFGRVQFLVAALTDNIGHLGGNVGS 1kqf.1    --------------------------------------------------------------------------------  target    YAGNYRGSVFQAMGQWIAEDPFAIEPDLTKPATVKRYYKAESAHYWNYGERPLRAVAKDDEGDLTKGEVLTGKSHMPTPT 1kqf.1    -------------------------------------------------------------------------------A  target    KLIWFGNSNSLLGNAKWSFDVVKNTLP-RQDAVFCNEWHWTSSCEYADLVFPADSWAEFKLPDATASCTNPFLLAFPTTP 1kqf.1    NVVMVMGGNAAEAHPVG-FRWAMEAKNNNDATLIVVDPRFTRTASVADIYAPIR--------------------------  target    LKRLYDTRSDYEALALTAKALGELIDEPRMEQYWRGILDGDPTPYLQRIFSGSNATRGITYDELHESSKRGVPLLMNMRT 1kqf.1    --------------------------------------------------------------------------------  target    YPRSGGWEQRQEDKPWYTATGRLEFYRPEPEFQAAGESLPVWREPVDATFYEPNAILSNAAHPSIAPRAPEDYGVPESQL 1kqf.1    --------------------------------------------------------------------------------  target    DVETRQYRNVVRTWAELQQTLHPLQERDPAFRFVFQTPKYRWGAHSTAVDADWISMLFGPFGDPYRRDPRMPWTGEAYLE 1kqf.1    --------------------------------------------------------------------------------  target    INPKDAAELGLADGDYAWVDADPEDRPYRGWKEDDPYYEVARAMMRVRIYTGMSRGVIRTWFNMYAATPATVANQKATPG 1kqf.1    --------------------------------------------------------------------------------  target    NPARNEQTRYVALFRYGSHQSGTRAWLRPTQQTDSLVRKGYFGQVIGTGFEADVHSVSGAPKEAFVKIEKAEDGGIGAER 1kqf.1    --------------------------------------------------------------------------------  target    LWRPLTLGLRPEAPSAALTAYLAGDYSGTKGS 1kqf.1    -------------------------------- ``` | | | | | | | | | | | | | | | | | | | | | | | | | | | | | | | | | | | | | | | | | | | | | | | | | |
|  | 4ydd.1.A | DMSO reductase family type II enzyme, molybdopterin subunit  *Crystal structure of the perchlorate reductase PcrAB from Azospira suillum PS* | 0.01 |  | 12.96 | 0.06 | 319-373 | X-ray | 1.86 | hetero-oligomer | 4 x SF4, 1 x MO, 1 x MGD, 1 x MD1, 1 x F3S | HHblits | 0.25 |
| ``` target    MPTANKADEVIILRPGTDAAFFLGVARELIEKGLYDRAAVIERTDLPLLVRLDTGERLDARDVIPGYELAALTNYVTLKP 4ydd.1    --------------------------------------------------------------------------------  target    DAEIKGNPPPPPFTAGGQVVPTELRDAWGDFVWWDRATGRPRPVSRDEVGARFDGDPALLGEFEVELVDGSTVPVRPAFD 4ydd.1    --------------------------------------------------------------------------------  target    LLKQYLDESFDLRTASEVCRVPPQAIQSIARQLAANKRETLLAAGMGPNHYFQNDLFGRVQFLVAALTDNIGHLGGNVGS 4ydd.1    --------------------------------------------------------------------------------  target    YAGNYRGSVFQAMGQWIAEDPFAIEPDLTKPATVKRYYKAESAHYWNYGERPLRAVAKDDEGDLTKGEVLTGKSHMPTPT 4ydd.1    ------------------------------------------------------------------------------NS  target    KLIWFGNSNSLLGNAKWSFDVVKNTLPRQDAVFCNEWHWTSSCEYADLVFPADSWAEFKLPDATASCTNPFLLAFPTTPL 4ydd.1    KYIILWGSNPTQTRIP-DAHFLSEAQLNGAKIVSISPDYNSSTIKVDKWIHPQ---------------------------  target    KRLYDTRSDYEALALTAKALGELIDEPRMEQYWRGILDGDPTPYLQRIFSGSNATRGITYDELHESSKRGVPLLMNMRTY 4ydd.1    --------------------------------------------------------------------------------  target    PRSGGWEQRQEDKPWYTATGRLEFYRPEPEFQAAGESLPVWREPVDATFYEPNAILSNAAHPSIAPRAPEDYGVPESQLD 4ydd.1    --------------------------------------------------------------------------------  target    VETRQYRNVVRTWAELQQTLHPLQERDPAFRFVFQTPKYRWGAHSTAVDADWISMLFGPFGDPYRRDPRMPWTGEAYLEI 4ydd.1    --------------------------------------------------------------------------------  target    NPKDAAELGLADGDYAWVDADPEDRPYRGWKEDDPYYEVARAMMRVRIYTGMSRGVIRTWFNMYAATPATVANQKATPGN 4ydd.1    --------------------------------------------------------------------------------  target    PARNEQTRYVALFRYGSHQSGTRAWLRPTQQTDSLVRKGYFGQVIGTGFEADVHSVSGAPKEAFVKIEKAEDGGIGAERL 4ydd.1    --------------------------------------------------------------------------------  target    WRPLTLGLRPEAPSAALTAYLAGDYSGTKGS 4ydd.1    ------------------------------- ``` | | | | | | | | | | | | | | | | | | | | | | | | | | | | | | | | | | | | | | | | | | | | | | | | | |
|  | 3ir7.1.A | Respiratory nitrate reductase 1 alpha chain  *Crystal structure of NarGHI mutant NarG-R94S* | 0.01 |  | 9.26 | 0.06 | 319-373 | X-ray | 2.50 | hetero-1-1-1-mer | 2 x MD1, 4 x SF4, 1 x 6MO, 1 x AGA, 1 x F3S, 2 x HEM | HHblits | 0.23 |
| ``` target    MPTANKADEVIILRPGTDAAFFLGVARELIEKGLYDRAAVIERTDLPLLVRLDTGERLDARDVIPGYELAALTNYVTLKP 3ir7.1    --------------------------------------------------------------------------------  target    DAEIKGNPPPPPFTAGGQVVPTELRDAWGDFVWWDRATGRPRPVSRDEVGARFDGDPALLGEFEVELVDGSTVPVRPAFD 3ir7.1    --------------------------------------------------------------------------------  target    LLKQYLDESFDLRTASEVCRVPPQAIQSIARQLAANKRETLLAAGMGPNHYFQNDLFGRVQFLVAALTDNIGHLGGNVGS 3ir7.1    --------------------------------------------------------------------------------  target    YAGNYRGSVFQAMGQWIAEDPFAIEPDLTKPATVKRYYKAESAHYWNYGERPLRAVAKDDEGDLTKGEVLTGKSHMPTPT 3ir7.1    ------------------------------------------------------------------------------NS  target    KLIWFGNSNSLLGNAKWSFDVVKNTLPRQDAVFCNEWHWTSSCEYADLVFPADSWAEFKLPDATASCTNPFLLAFPTTPL 3ir7.1    SYIIAWGSNVPQTRTPD-AHFFTEVRYKGTKTVAVTPDYAEIAKLCDLWLAPK---------------------------  target    KRLYDTRSDYEALALTAKALGELIDEPRMEQYWRGILDGDPTPYLQRIFSGSNATRGITYDELHESSKRGVPLLMNMRTY 3ir7.1    --------------------------------------------------------------------------------  target    PRSGGWEQRQEDKPWYTATGRLEFYRPEPEFQAAGESLPVWREPVDATFYEPNAILSNAAHPSIAPRAPEDYGVPESQLD 3ir7.1    --------------------------------------------------------------------------------  target    VETRQYRNVVRTWAELQQTLHPLQERDPAFRFVFQTPKYRWGAHSTAVDADWISMLFGPFGDPYRRDPRMPWTGEAYLEI 3ir7.1    --------------------------------------------------------------------------------  target    NPKDAAELGLADGDYAWVDADPEDRPYRGWKEDDPYYEVARAMMRVRIYTGMSRGVIRTWFNMYAATPATVANQKATPGN 3ir7.1    --------------------------------------------------------------------------------  target    PARNEQTRYVALFRYGSHQSGTRAWLRPTQQTDSLVRKGYFGQVIGTGFEADVHSVSGAPKEAFVKIEKAEDGGIGAERL 3ir7.1    --------------------------------------------------------------------------------  target    WRPLTLGLRPEAPSAALTAYLAGDYSGTKGS 3ir7.1    ------------------------------- ``` | | | | | | | | | | | | | | | | | | | | | | | | | | | | | | | | | | | | | | | | | | | | | | | | | |
|  | 3egw.1.A | Respiratory nitrate reductase 1 alpha chain  *The crystal structure of the NarGHI mutant NarH - C16A* | 0.01 |  | 9.26 | 0.06 | 319-373 | X-ray | 1.90 | hetero-2-2-2-mer | 2 x MD1, 2 x MGD, 2 x 6MO, 6 x SF4, 4 x F3S, 2 x 3PH, 4 x HEM, 2 x AGA | HHblits | 0.23 |
| ``` target    MPTANKADEVIILRPGTDAAFFLGVARELIEKGLYDRAAVIERTDLPLLVRLDTGERLDARDVIPGYELAALTNYVTLKP 3egw.1    --------------------------------------------------------------------------------  target    DAEIKGNPPPPPFTAGGQVVPTELRDAWGDFVWWDRATGRPRPVSRDEVGARFDGDPALLGEFEVELVDGSTVPVRPAFD 3egw.1    --------------------------------------------------------------------------------  target    LLKQYLDESFDLRTASEVCRVPPQAIQSIARQLAANKRETLLAAGMGPNHYFQNDLFGRVQFLVAALTDNIGHLGGNVGS 3egw.1    --------------------------------------------------------------------------------  target    YAGNYRGSVFQAMGQWIAEDPFAIEPDLTKPATVKRYYKAESAHYWNYGERPLRAVAKDDEGDLTKGEVLTGKSHMPTPT 3egw.1    ------------------------------------------------------------------------------NS  target    KLIWFGNSNSLLGNAKWSFDVVKNTLPRQDAVFCNEWHWTSSCEYADLVFPADSWAEFKLPDATASCTNPFLLAFPTTPL 3egw.1    SYIIAWGSNVPQTRTPD-AHFFTEVRYKGTKTVAVTPDYAEIAKLCDLWLAPK---------------------------  target    KRLYDTRSDYEALALTAKALGELIDEPRMEQYWRGILDGDPTPYLQRIFSGSNATRGITYDELHESSKRGVPLLMNMRTY 3egw.1    --------------------------------------------------------------------------------  target    PRSGGWEQRQEDKPWYTATGRLEFYRPEPEFQAAGESLPVWREPVDATFYEPNAILSNAAHPSIAPRAPEDYGVPESQLD 3egw.1    --------------------------------------------------------------------------------  target    VETRQYRNVVRTWAELQQTLHPLQERDPAFRFVFQTPKYRWGAHSTAVDADWISMLFGPFGDPYRRDPRMPWTGEAYLEI 3egw.1    --------------------------------------------------------------------------------  target    NPKDAAELGLADGDYAWVDADPEDRPYRGWKEDDPYYEVARAMMRVRIYTGMSRGVIRTWFNMYAATPATVANQKATPGN 3egw.1    --------------------------------------------------------------------------------  target    PARNEQTRYVALFRYGSHQSGTRAWLRPTQQTDSLVRKGYFGQVIGTGFEADVHSVSGAPKEAFVKIEKAEDGGIGAERL 3egw.1    --------------------------------------------------------------------------------  target    WRPLTLGLRPEAPSAALTAYLAGDYSGTKGS 3egw.1    ------------------------------- ``` | | | | | | | | | | | | | | | | | | | | | | | | | | | | | | | | | | | | | | | | | | | | | | | | | |
|  | 1r27.4.A | Respiratory nitrate reductase 1 alpha chain  *Crystal Structure of NarGH complex* | 0.01 |  | 9.26 | 0.06 | 319-373 | X-ray | 2.00 | hetero-4-4-mer | 4 x MO, 16 x SF4, 8 x MGD, 4 x F3S | HHblits | 0.23 |
| ``` target    MPTANKADEVIILRPGTDAAFFLGVARELIEKGLYDRAAVIERTDLPLLVRLDTGERLDARDVIPGYELAALTNYVTLKP 1r27.4    --------------------------------------------------------------------------------  target    DAEIKGNPPPPPFTAGGQVVPTELRDAWGDFVWWDRATGRPRPVSRDEVGARFDGDPALLGEFEVELVDGSTVPVRPAFD 1r27.4    --------------------------------------------------------------------------------  target    LLKQYLDESFDLRTASEVCRVPPQAIQSIARQLAANKRETLLAAGMGPNHYFQNDLFGRVQFLVAALTDNIGHLGGNVGS 1r27.4    --------------------------------------------------------------------------------  target    YAGNYRGSVFQAMGQWIAEDPFAIEPDLTKPATVKRYYKAESAHYWNYGERPLRAVAKDDEGDLTKGEVLTGKSHMPTPT 1r27.4    ------------------------------------------------------------------------------NS  target    KLIWFGNSNSLLGNAKWSFDVVKNTLPRQDAVFCNEWHWTSSCEYADLVFPADSWAEFKLPDATASCTNPFLLAFPTTPL 1r27.4    SYIIAWGSNVPQTRTPD-AHFFTEVRYKGTKTVAVTPDYAEIAKLCDLWLAPK---------------------------  target    KRLYDTRSDYEALALTAKALGELIDEPRMEQYWRGILDGDPTPYLQRIFSGSNATRGITYDELHESSKRGVPLLMNMRTY 1r27.4    --------------------------------------------------------------------------------  target    PRSGGWEQRQEDKPWYTATGRLEFYRPEPEFQAAGESLPVWREPVDATFYEPNAILSNAAHPSIAPRAPEDYGVPESQLD 1r27.4    --------------------------------------------------------------------------------  target    VETRQYRNVVRTWAELQQTLHPLQERDPAFRFVFQTPKYRWGAHSTAVDADWISMLFGPFGDPYRRDPRMPWTGEAYLEI 1r27.4    --------------------------------------------------------------------------------  target    NPKDAAELGLADGDYAWVDADPEDRPYRGWKEDDPYYEVARAMMRVRIYTGMSRGVIRTWFNMYAATPATVANQKATPGN 1r27.4    --------------------------------------------------------------------------------  target    PARNEQTRYVALFRYGSHQSGTRAWLRPTQQTDSLVRKGYFGQVIGTGFEADVHSVSGAPKEAFVKIEKAEDGGIGAERL 1r27.4    --------------------------------------------------------------------------------  target    WRPLTLGLRPEAPSAALTAYLAGDYSGTKGS 1r27.4    ------------------------------- ``` | | | | | | | | | | | | | | | | | | | | | | | | | | | | | | | | | | | | | | | | | | | | | | | | | |
|  | 1q16.1.A | Respiratory nitrate reductase 1 alpha chain  *Crystal structure of Nitrate Reductase A, NarGHI, from Escherichia coli* | 0.01 |  | 9.26 | 0.06 | 319-373 | X-ray | 1.90 | hetero-oligomer | 2 x MD1, 1 x 6MO, 2 x HEM, 4 x SF4, 1 x F3S, 1 x AGA, 1 x 3PH | HHblits | 0.23 |
| ``` target    MPTANKADEVIILRPGTDAAFFLGVARELIEKGLYDRAAVIERTDLPLLVRLDTGERLDARDVIPGYELAALTNYVTLKP 1q16.1    --------------------------------------------------------------------------------  target    DAEIKGNPPPPPFTAGGQVVPTELRDAWGDFVWWDRATGRPRPVSRDEVGARFDGDPALLGEFEVELVDGSTVPVRPAFD 1q16.1    --------------------------------------------------------------------------------  target    LLKQYLDESFDLRTASEVCRVPPQAIQSIARQLAANKRETLLAAGMGPNHYFQNDLFGRVQFLVAALTDNIGHLGGNVGS 1q16.1    --------------------------------------------------------------------------------  target    YAGNYRGSVFQAMGQWIAEDPFAIEPDLTKPATVKRYYKAESAHYWNYGERPLRAVAKDDEGDLTKGEVLTGKSHMPTPT 1q16.1    ------------------------------------------------------------------------------NS  target    KLIWFGNSNSLLGNAKWSFDVVKNTLPRQDAVFCNEWHWTSSCEYADLVFPADSWAEFKLPDATASCTNPFLLAFPTTPL 1q16.1    SYIIAWGSNVPQTRTPD-AHFFTEVRYKGTKTVAVTPDYAEIAKLCDLWLAPK---------------------------  target    KRLYDTRSDYEALALTAKALGELIDEPRMEQYWRGILDGDPTPYLQRIFSGSNATRGITYDELHESSKRGVPLLMNMRTY 1q16.1    --------------------------------------------------------------------------------  target    PRSGGWEQRQEDKPWYTATGRLEFYRPEPEFQAAGESLPVWREPVDATFYEPNAILSNAAHPSIAPRAPEDYGVPESQLD 1q16.1    --------------------------------------------------------------------------------  target    VETRQYRNVVRTWAELQQTLHPLQERDPAFRFVFQTPKYRWGAHSTAVDADWISMLFGPFGDPYRRDPRMPWTGEAYLEI 1q16.1    --------------------------------------------------------------------------------  target    NPKDAAELGLADGDYAWVDADPEDRPYRGWKEDDPYYEVARAMMRVRIYTGMSRGVIRTWFNMYAATPATVANQKATPGN 1q16.1    --------------------------------------------------------------------------------  target    PARNEQTRYVALFRYGSHQSGTRAWLRPTQQTDSLVRKGYFGQVIGTGFEADVHSVSGAPKEAFVKIEKAEDGGIGAERL 1q16.1    --------------------------------------------------------------------------------  target    WRPLTLGLRPEAPSAALTAYLAGDYSGTKGS 1q16.1    ------------------------------- ``` | | | | | | | | | | | | | | | | | | | | | | | | | | | | | | | | | | | | | | | | | | | | | | | | | |
|  | 3ir6.1.A | Respiratory nitrate reductase 1 alpha chain  *Crystal structure of NarGHI mutant NarG-H49S* | 0.01 |  | 9.26 | 0.06 | 319-373 | X-ray | 2.80 | hetero-1-1-1-mer | 2 x GDP, 1 x AGA, 3 x SF4, 1 x F3S, 2 x HEM | HHblits | 0.23 |
| ``` target    MPTANKADEVIILRPGTDAAFFLGVARELIEKGLYDRAAVIERTDLPLLVRLDTGERLDARDVIPGYELAALTNYVTLKP 3ir6.1    --------------------------------------------------------------------------------  target    DAEIKGNPPPPPFTAGGQVVPTELRDAWGDFVWWDRATGRPRPVSRDEVGARFDGDPALLGEFEVELVDGSTVPVRPAFD 3ir6.1    --------------------------------------------------------------------------------  target    LLKQYLDESFDLRTASEVCRVPPQAIQSIARQLAANKRETLLAAGMGPNHYFQNDLFGRVQFLVAALTDNIGHLGGNVGS 3ir6.1    --------------------------------------------------------------------------------  target    YAGNYRGSVFQAMGQWIAEDPFAIEPDLTKPATVKRYYKAESAHYWNYGERPLRAVAKDDEGDLTKGEVLTGKSHMPTPT 3ir6.1    ------------------------------------------------------------------------------NS  target    KLIWFGNSNSLLGNAKWSFDVVKNTLPRQDAVFCNEWHWTSSCEYADLVFPADSWAEFKLPDATASCTNPFLLAFPTTPL 3ir6.1    SYIIAWGSNVPQTRTPD-AHFFTEVRYKGTKTVAVTPDYAEIAKLCDLWLAPK---------------------------  target    KRLYDTRSDYEALALTAKALGELIDEPRMEQYWRGILDGDPTPYLQRIFSGSNATRGITYDELHESSKRGVPLLMNMRTY 3ir6.1    --------------------------------------------------------------------------------  target    PRSGGWEQRQEDKPWYTATGRLEFYRPEPEFQAAGESLPVWREPVDATFYEPNAILSNAAHPSIAPRAPEDYGVPESQLD 3ir6.1    --------------------------------------------------------------------------------  target    VETRQYRNVVRTWAELQQTLHPLQERDPAFRFVFQTPKYRWGAHSTAVDADWISMLFGPFGDPYRRDPRMPWTGEAYLEI 3ir6.1    --------------------------------------------------------------------------------  target    NPKDAAELGLADGDYAWVDADPEDRPYRGWKEDDPYYEVARAMMRVRIYTGMSRGVIRTWFNMYAATPATVANQKATPGN 3ir6.1    --------------------------------------------------------------------------------  target    PARNEQTRYVALFRYGSHQSGTRAWLRPTQQTDSLVRKGYFGQVIGTGFEADVHSVSGAPKEAFVKIEKAEDGGIGAERL 3ir6.1    --------------------------------------------------------------------------------  target    WRPLTLGLRPEAPSAALTAYLAGDYSGTKGS 3ir6.1    ------------------------------- ``` | | | | | | | | | | | | | | | | | | | | | | | | | | | | | | | | | | | | | | | | | | | | | | | | | |
|  | 3ir5.1.A | Respiratory nitrate reductase 1 alpha chain  *Crystal structure of NarGHI mutant NarG-H49C* | 0.01 |  | 9.43 | 0.06 | 320-373 | X-ray | 2.30 | hetero-1-1-1-mer | 2 x MD1, 1 x 6MO, 4 x SF4, 1 x AGA, 1 x F3S, 2 x HEM | HHblits | 0.23 |
| ``` target    MPTANKADEVIILRPGTDAAFFLGVARELIEKGLYDRAAVIERTDLPLLVRLDTGERLDARDVIPGYELAALTNYVTLKP 3ir5.1    --------------------------------------------------------------------------------  target    DAEIKGNPPPPPFTAGGQVVPTELRDAWGDFVWWDRATGRPRPVSRDEVGARFDGDPALLGEFEVELVDGSTVPVRPAFD 3ir5.1    --------------------------------------------------------------------------------  target    LLKQYLDESFDLRTASEVCRVPPQAIQSIARQLAANKRETLLAAGMGPNHYFQNDLFGRVQFLVAALTDNIGHLGGNVGS 3ir5.1    --------------------------------------------------------------------------------  target    YAGNYRGSVFQAMGQWIAEDPFAIEPDLTKPATVKRYYKAESAHYWNYGERPLRAVAKDDEGDLTKGEVLTGKSHMPTPT 3ir5.1    -------------------------------------------------------------------------------S  target    KLIWFGNSNSLLGNAKWSFDVVKNTLPRQDAVFCNEWHWTSSCEYADLVFPADSWAEFKLPDATASCTNPFLLAFPTTPL 3ir5.1    SYIIAWGSNVPQTRTPD-AHFFTEVRYKGTKTVAVTPDYAEIAKLCDLWLAPK---------------------------  target    KRLYDTRSDYEALALTAKALGELIDEPRMEQYWRGILDGDPTPYLQRIFSGSNATRGITYDELHESSKRGVPLLMNMRTY 3ir5.1    --------------------------------------------------------------------------------  target    PRSGGWEQRQEDKPWYTATGRLEFYRPEPEFQAAGESLPVWREPVDATFYEPNAILSNAAHPSIAPRAPEDYGVPESQLD 3ir5.1    --------------------------------------------------------------------------------  target    VETRQYRNVVRTWAELQQTLHPLQERDPAFRFVFQTPKYRWGAHSTAVDADWISMLFGPFGDPYRRDPRMPWTGEAYLEI 3ir5.1    --------------------------------------------------------------------------------  target    NPKDAAELGLADGDYAWVDADPEDRPYRGWKEDDPYYEVARAMMRVRIYTGMSRGVIRTWFNMYAATPATVANQKATPGN 3ir5.1    --------------------------------------------------------------------------------  target    PARNEQTRYVALFRYGSHQSGTRAWLRPTQQTDSLVRKGYFGQVIGTGFEADVHSVSGAPKEAFVKIEKAEDGGIGAERL 3ir5.1    --------------------------------------------------------------------------------  target    WRPLTLGLRPEAPSAALTAYLAGDYSGTKGS 3ir5.1    ------------------------------- ``` | | | | | | | | | | | | | | | | | | | | | | | | | | | | | | | | | | | | | | | | | | | | | | | | | |
|  | 4nvs.1.A | Putative enzyme, glyoxalase family  *Crystal Structure of the Q18CP6\_CLOD6 protein from glyoxalase family. Northeast Structural Genomics Consortium Target CfR3* | 0.00 |  | 17.02 | 0.06 | 145-191 | X-ray | 2.38 | homo-dimer |  | HHblits | 0.30 |
| ``` target    MPTANKADEVIILRPGTDAAFFLGVARELIEKGLYDRAAVIERTDLPLLVRLDTGERLDARDVIPGYELAALTNYVTLKP 4nvs.1    --------------------------------------------------------------------------------  target    DAEIKGNPPPPPFTAGGQVVPTELRDAWGDFVWWDRATGRPRPVSRDEVGARFDGDPALLGEFEVELVDGSTVPVRPAFD 4nvs.1    ----------------------------------------------------------------FYDLDGHIIEVGETMS  target    LLKQ-YLDESFDLRTASEVCRVPPQAIQSIARQLAANKRETLLAAGMGPNHYFQNDLFGRVQFLVAALTDNIGHLGGNVG 4nvs.1    SVCRRFLDSGLSIDEVAKRMDVTVEYIESVLE------------------------------------------------  target    SYAGNYRGSVFQAMGQWIAEDPFAIEPDLTKPATVKRYYKAESAHYWNYGERPLRAVAKDDEGDLTKGEVLTGKSHMPTP 4nvs.1    --------------------------------------------------------------------------------  target    TKLIWFGNSNSLLGNAKWSFDVVKNTLPRQDAVFCNEWHWTSSCEYADLVFPADSWAEFKLPDATASCTNPFLLAFPTTP 4nvs.1    --------------------------------------------------------------------------------  target    LKRLYDTRSDYEALALTAKALGELIDEPRMEQYWRGILDGDPTPYLQRIFSGSNATRGITYDELHESSKRGVPLLMNMRT 4nvs.1    --------------------------------------------------------------------------------  target    YPRSGGWEQRQEDKPWYTATGRLEFYRPEPEFQAAGESLPVWREPVDATFYEPNAILSNAAHPSIAPRAPEDYGVPESQL 4nvs.1    --------------------------------------------------------------------------------  target    DVETRQYRNVVRTWAELQQTLHPLQERDPAFRFVFQTPKYRWGAHSTAVDADWISMLFGPFGDPYRRDPRMPWTGEAYLE 4nvs.1    --------------------------------------------------------------------------------  target    INPKDAAELGLADGDYAWVDADPEDRPYRGWKEDDPYYEVARAMMRVRIYTGMSRGVIRTWFNMYAATPATVANQKATPG 4nvs.1    --------------------------------------------------------------------------------  target    NPARNEQTRYVALFRYGSHQSGTRAWLRPTQQTDSLVRKGYFGQVIGTGFEADVHSVSGAPKEAFVKIEKAEDGGIGAER 4nvs.1    --------------------------------------------------------------------------------  target    LWRPLTLGLRPEAPSAALTAYLAGDYSGTKGS 4nvs.1    -------------------------------- ``` | | | | | | | | | | | | | | | | | | | | | | | | | | | | | | | | | | | | | | | | | | | | | | | | | |
|  | 5cup.1.A | Phosphate propanoyltransferase  *Structure of Rhodopseudomonas palustris PduL - phosphate bound form* | 0.00 |  | 37.04 | 0.03 | 634-660 | X-ray | 2.10 | homo-dimer | 4 x ZN | HHblits | 0.40 |
| ``` target    MPTANKADEVIILRPGTDAAFFLGVARELIEKGLYDRAAVIERTDLPLLVRLDTGERLDARDVIPGYELAALTNYVTLKP 5cup.1    --------------------------------------------------------------------------------  target    DAEIKGNPPPPPFTAGGQVVPTELRDAWGDFVWWDRATGRPRPVSRDEVGARFDGDPALLGEFEVELVDGSTVPVRPAFD 5cup.1    --------------------------------------------------------------------------------  target    LLKQYLDESFDLRTASEVCRVPPQAIQSIARQLAANKRETLLAAGMGPNHYFQNDLFGRVQFLVAALTDNIGHLGGNVGS 5cup.1    --------------------------------------------------------------------------------  target    YAGNYRGSVFQAMGQWIAEDPFAIEPDLTKPATVKRYYKAESAHYWNYGERPLRAVAKDDEGDLTKGEVLTGKSHMPTPT 5cup.1    --------------------------------------------------------------------------------  target    KLIWFGNSNSLLGNAKWSFDVVKNTLPRQDAVFCNEWHWTSSCEYADLVFPADSWAEFKLPDATASCTNPFLLAFPTTPL 5cup.1    --------------------------------------------------------------------------------  target    KRLYDTRSDYEALALTAKALGELIDEPRMEQYWRGILDGDPTPYLQRIFSGSNATRGITYDELHESSKRGVPLLMNMRTY 5cup.1    --------------------------------------------------------------------------------  target    PRSGGWEQRQEDKPWYTATGRLEFYRPEPEFQAAGESLPVWREPVDATFYEPNAILSNAAHPSIAPRAPEDYGVPESQLD 5cup.1    --------------------------------------------------------------------------------  target    VETRQYRNVVRTWAELQQTLHPLQERDPAFRFVFQTPKYRWGAHSTAVDADWISMLFGPFGDPYRRDPRMPWTGEAYLEI 5cup.1    -------------------------------------------------------------------------AQRHIHM  target    NPKDAAELGLADGDYAWVDADPEDRPYRGWKEDDPYYEVARAMMRVRIYTGMSRGVIRTWFNMYAATPATVANQKATPGN 5cup.1    HPSTAAKLGLRNGDEVDVEA------------------------------------------------------------  target    PARNEQTRYVALFRYGSHQSGTRAWLRPTQQTDSLVRKGYFGQVIGTGFEADVHSVSGAPKEAFVKIEKAEDGGIGAERL 5cup.1    --------------------------------------------------------------------------------  target    WRPLTLGLRPEAPSAALTAYLAGDYSGTKGS 5cup.1    ------------------------------- ``` | | | | | | | | | | | | | | | | | | | | | | | | | | | | | | | | | | | | | | | | | | | | | | | | | |
|  | 5cuo.1.A | Phosphate propanoyltransferase  *Structure of Rhodopseudomonas palustris PduL - CoA bound form* | 0.00 |  | 37.04 | 0.03 | 634-660 | X-ray | 1.54 | homo-dimer | 2 x COA, 4 x ZN | HHblits | 0.40 |
| ``` target    MPTANKADEVIILRPGTDAAFFLGVARELIEKGLYDRAAVIERTDLPLLVRLDTGERLDARDVIPGYELAALTNYVTLKP 5cuo.1    --------------------------------------------------------------------------------  target    DAEIKGNPPPPPFTAGGQVVPTELRDAWGDFVWWDRATGRPRPVSRDEVGARFDGDPALLGEFEVELVDGSTVPVRPAFD 5cuo.1    --------------------------------------------------------------------------------  target    LLKQYLDESFDLRTASEVCRVPPQAIQSIARQLAANKRETLLAAGMGPNHYFQNDLFGRVQFLVAALTDNIGHLGGNVGS 5cuo.1    --------------------------------------------------------------------------------  target    YAGNYRGSVFQAMGQWIAEDPFAIEPDLTKPATVKRYYKAESAHYWNYGERPLRAVAKDDEGDLTKGEVLTGKSHMPTPT 5cuo.1    --------------------------------------------------------------------------------  target    KLIWFGNSNSLLGNAKWSFDVVKNTLPRQDAVFCNEWHWTSSCEYADLVFPADSWAEFKLPDATASCTNPFLLAFPTTPL 5cuo.1    --------------------------------------------------------------------------------  target    KRLYDTRSDYEALALTAKALGELIDEPRMEQYWRGILDGDPTPYLQRIFSGSNATRGITYDELHESSKRGVPLLMNMRTY 5cuo.1    --------------------------------------------------------------------------------  target    PRSGGWEQRQEDKPWYTATGRLEFYRPEPEFQAAGESLPVWREPVDATFYEPNAILSNAAHPSIAPRAPEDYGVPESQLD 5cuo.1    --------------------------------------------------------------------------------  target    VETRQYRNVVRTWAELQQTLHPLQERDPAFRFVFQTPKYRWGAHSTAVDADWISMLFGPFGDPYRRDPRMPWTGEAYLEI 5cuo.1    -------------------------------------------------------------------------AQRHIHM  target    NPKDAAELGLADGDYAWVDADPEDRPYRGWKEDDPYYEVARAMMRVRIYTGMSRGVIRTWFNMYAATPATVANQKATPGN 5cuo.1    HPSTAAKLGLRNGDEVDVEA------------------------------------------------------------  target    PARNEQTRYVALFRYGSHQSGTRAWLRPTQQTDSLVRKGYFGQVIGTGFEADVHSVSGAPKEAFVKIEKAEDGGIGAERL 5cuo.1    --------------------------------------------------------------------------------  target    WRPLTLGLRPEAPSAALTAYLAGDYSGTKGS 5cuo.1    ------------------------------- ``` | | | | | | | | | | | | | | | | | | | | | | | | | | | | | | | | | | | | | | | | | | | | | | | | | |
|  | 1wlf.1.A | Peroxisome biogenesis factor 1  *Structure of the N-terminal domain of PEX1 AAA-ATPase: Characterization of a putative adaptor-binding domain* | 0.00 |  | 33.33 | 0.03 | 635-661 | X-ray | 2.05 | monomer |  | HHblits | 0.35 |
| ``` target    MPTANKADEVIILRPGTDAAFFLGVARELIEKGLYDRAAVIERTDLPLLVRLDTGERLDARDVIPGYELAALTNYVTLKP 1wlf.1    --------------------------------------------------------------------------------  target    DAEIKGNPPPPPFTAGGQVVPTELRDAWGDFVWWDRATGRPRPVSRDEVGARFDGDPALLGEFEVELVDGSTVPVRPAFD 1wlf.1    --------------------------------------------------------------------------------  target    LLKQYLDESFDLRTASEVCRVPPQAIQSIARQLAANKRETLLAAGMGPNHYFQNDLFGRVQFLVAALTDNIGHLGGNVGS 1wlf.1    --------------------------------------------------------------------------------  target    YAGNYRGSVFQAMGQWIAEDPFAIEPDLTKPATVKRYYKAESAHYWNYGERPLRAVAKDDEGDLTKGEVLTGKSHMPTPT 1wlf.1    --------------------------------------------------------------------------------  target    KLIWFGNSNSLLGNAKWSFDVVKNTLPRQDAVFCNEWHWTSSCEYADLVFPADSWAEFKLPDATASCTNPFLLAFPTTPL 1wlf.1    --------------------------------------------------------------------------------  target    KRLYDTRSDYEALALTAKALGELIDEPRMEQYWRGILDGDPTPYLQRIFSGSNATRGITYDELHESSKRGVPLLMNMRTY 1wlf.1    --------------------------------------------------------------------------------  target    PRSGGWEQRQEDKPWYTATGRLEFYRPEPEFQAAGESLPVWREPVDATFYEPNAILSNAAHPSIAPRAPEDYGVPESQLD 1wlf.1    --------------------------------------------------------------------------------  target    VETRQYRNVVRTWAELQQTLHPLQERDPAFRFVFQTPKYRWGAHSTAVDADWISMLFGPFGDPYRRDPRMPWTGEAYLEI 1wlf.1    --------------------------------------------------------------------------ENVAEI  target    NPKDAAELGLADGDYAWVDADPEDRPYRGWKEDDPYYEVARAMMRVRIYTGMSRGVIRTWFNMYAATPATVANQKATPGN 1wlf.1    NRQVGQKLGLSSGDQVFLRPC-----------------------------------------------------------  target    PARNEQTRYVALFRYGSHQSGTRAWLRPTQQTDSLVRKGYFGQVIGTGFEADVHSVSGAPKEAFVKIEKAEDGGIGAERL 1wlf.1    --------------------------------------------------------------------------------  target    WRPLTLGLRPEAPSAALTAYLAGDYSGTKGS 1wlf.1    ------------------------------- ``` | | | | | | | | | | | | | | | | | | | | | | | | | | | | | | | | | | | | | | | | | | | | | | | | | |
|  | 7dg9.1.A | Cell division control protein 48, AAA family  *DPBB domain of VCP-like ATPase from Aeropyrum pernix* | 0.00 |  | 29.63 | 0.03 | 635-661 | X-ray | 1.60 | monomer | 4 x ZN | HHblits | 0.34 |
| ``` target    MPTANKADEVIILRPGTDAAFFLGVARELIEKGLYDRAAVIERTDLPLLVRLDTGERLDARDVIPGYELAALTNYVTLKP 7dg9.1    --------------------------------------------------------------------------------  target    DAEIKGNPPPPPFTAGGQVVPTELRDAWGDFVWWDRATGRPRPVSRDEVGARFDGDPALLGEFEVELVDGSTVPVRPAFD 7dg9.1    --------------------------------------------------------------------------------  target    LLKQYLDESFDLRTASEVCRVPPQAIQSIARQLAANKRETLLAAGMGPNHYFQNDLFGRVQFLVAALTDNIGHLGGNVGS 7dg9.1    --------------------------------------------------------------------------------  target    YAGNYRGSVFQAMGQWIAEDPFAIEPDLTKPATVKRYYKAESAHYWNYGERPLRAVAKDDEGDLTKGEVLTGKSHMPTPT 7dg9.1    --------------------------------------------------------------------------------  target    KLIWFGNSNSLLGNAKWSFDVVKNTLPRQDAVFCNEWHWTSSCEYADLVFPADSWAEFKLPDATASCTNPFLLAFPTTPL 7dg9.1    --------------------------------------------------------------------------------  target    KRLYDTRSDYEALALTAKALGELIDEPRMEQYWRGILDGDPTPYLQRIFSGSNATRGITYDELHESSKRGVPLLMNMRTY 7dg9.1    --------------------------------------------------------------------------------  target    PRSGGWEQRQEDKPWYTATGRLEFYRPEPEFQAAGESLPVWREPVDATFYEPNAILSNAAHPSIAPRAPEDYGVPESQLD 7dg9.1    --------------------------------------------------------------------------------  target    VETRQYRNVVRTWAELQQTLHPLQERDPAFRFVFQTPKYRWGAHSTAVDADWISMLFGPFGDPYRRDPRMPWTGEAYLEI 7dg9.1    --------------------------------------------------------------------------RKIVRI  target    NPKDAAELGLADGDYAWVDADPEDRPYRGWKEDDPYYEVARAMMRVRIYTGMSRGVIRTWFNMYAATPATVANQKATPGN 7dg9.1    DRQTAARLGVEVGDFVKVSKG-----------------------------------------------------------  target    PARNEQTRYVALFRYGSHQSGTRAWLRPTQQTDSLVRKGYFGQVIGTGFEADVHSVSGAPKEAFVKIEKAEDGGIGAERL 7dg9.1    --------------------------------------------------------------------------------  target    WRPLTLGLRPEAPSAALTAYLAGDYSGTKGS 7dg9.1    ------------------------------- ``` | | | | | | | | | | | | | | | | | | | | | | | | | | | | | | | | | | | | | | | | | | | | | | | | | |
|  | 5e7p.1.A | Cell division control protein Cdc48  *Crystal Structure of MSMEG\_0858 (Uniprot A0QQS4), a AAA ATPase.* | 0.00 |  | 17.86 | 0.03 | 634-661 | X-ray | 2.51 | monomer | 2 x ADP | HHblits | 0.31 |
| ``` target    MPTANKADEVIILRPGTDAAFFLGVARELIEKGLYDRAAVIERTDLPLLVRLDTGERLDARDVIPGYELAALTNYVTLKP 5e7p.1    --------------------------------------------------------------------------------  target    DAEIKGNPPPPPFTAGGQVVPTELRDAWGDFVWWDRATGRPRPVSRDEVGARFDGDPALLGEFEVELVDGSTVPVRPAFD 5e7p.1    --------------------------------------------------------------------------------  target    LLKQYLDESFDLRTASEVCRVPPQAIQSIARQLAANKRETLLAAGMGPNHYFQNDLFGRVQFLVAALTDNIGHLGGNVGS 5e7p.1    --------------------------------------------------------------------------------  target    YAGNYRGSVFQAMGQWIAEDPFAIEPDLTKPATVKRYYKAESAHYWNYGERPLRAVAKDDEGDLTKGEVLTGKSHMPTPT 5e7p.1    --------------------------------------------------------------------------------  target    KLIWFGNSNSLLGNAKWSFDVVKNTLPRQDAVFCNEWHWTSSCEYADLVFPADSWAEFKLPDATASCTNPFLLAFPTTPL 5e7p.1    --------------------------------------------------------------------------------  target    KRLYDTRSDYEALALTAKALGELIDEPRMEQYWRGILDGDPTPYLQRIFSGSNATRGITYDELHESSKRGVPLLMNMRTY 5e7p.1    --------------------------------------------------------------------------------  target    PRSGGWEQRQEDKPWYTATGRLEFYRPEPEFQAAGESLPVWREPVDATFYEPNAILSNAAHPSIAPRAPEDYGVPESQLD 5e7p.1    --------------------------------------------------------------------------------  target    VETRQYRNVVRTWAELQQTLHPLQERDPAFRFVFQTPKYRWGAHSTAVDADWISMLFGPFGDPYRRDPRMPWTGEAYLEI 5e7p.1    -------------------------------------------------------------------------RRGVVRL  target    NPKDAAELGLADGDYAWVDADPEDRPYRGWKEDDPYYEVARAMMRVRIYTGMSRGVIRTWFNMYAATPATVANQKATPGN 5e7p.1    HPEVLAALGIREWDAVALTGT-----------------------------------------------------------  target    PARNEQTRYVALFRYGSHQSGTRAWLRPTQQTDSLVRKGYFGQVIGTGFEADVHSVSGAPKEAFVKIEKAEDGGIGAERL 5e7p.1    --------------------------------------------------------------------------------  target    WRPLTLGLRPEAPSAALTAYLAGDYSGTKGS 5e7p.1    ------------------------------- ``` | | | | | | | | | | | | | | | | | | | | | | | | | | | | | | | | | | | | | | | | | | | | | | | | | |
|  | 7du7.1.A | mkDPBB\_sym1 protein  *Crystal structure of the rationally designed mkDPBB\_sym1 protein* | 0.00 |  | 25.93 | 0.03 | 635-661 | X-ray | 1.20 | monomer |  | HHblits | 0.34 |
| ``` target    MPTANKADEVIILRPGTDAAFFLGVARELIEKGLYDRAAVIERTDLPLLVRLDTGERLDARDVIPGYELAALTNYVTLKP 7du7.1    --------------------------------------------------------------------------------  target    DAEIKGNPPPPPFTAGGQVVPTELRDAWGDFVWWDRATGRPRPVSRDEVGARFDGDPALLGEFEVELVDGSTVPVRPAFD 7du7.1    --------------------------------------------------------------------------------  target    LLKQYLDESFDLRTASEVCRVPPQAIQSIARQLAANKRETLLAAGMGPNHYFQNDLFGRVQFLVAALTDNIGHLGGNVGS 7du7.1    --------------------------------------------------------------------------------  target    YAGNYRGSVFQAMGQWIAEDPFAIEPDLTKPATVKRYYKAESAHYWNYGERPLRAVAKDDEGDLTKGEVLTGKSHMPTPT 7du7.1    --------------------------------------------------------------------------------  target    KLIWFGNSNSLLGNAKWSFDVVKNTLPRQDAVFCNEWHWTSSCEYADLVFPADSWAEFKLPDATASCTNPFLLAFPTTPL 7du7.1    --------------------------------------------------------------------------------  target    KRLYDTRSDYEALALTAKALGELIDEPRMEQYWRGILDGDPTPYLQRIFSGSNATRGITYDELHESSKRGVPLLMNMRTY 7du7.1    --------------------------------------------------------------------------------  target    PRSGGWEQRQEDKPWYTATGRLEFYRPEPEFQAAGESLPVWREPVDATFYEPNAILSNAAHPSIAPRAPEDYGVPESQLD 7du7.1    --------------------------------------------------------------------------------  target    VETRQYRNVVRTWAELQQTLHPLQERDPAFRFVFQTPKYRWGAHSTAVDADWISMLFGPFGDPYRRDPRMPWTGEAYLEI 7du7.1    --------------------------------------------------------------------------KGIVRM  target    NPKDAAELGLADGDYAWVDADPEDRPYRGWKEDDPYYEVARAMMRVRIYTGMSRGVIRTWFNMYAATPATVANQKATPGN 7du7.1    DKASRAKLGVSVGDYVEVKKV-----------------------------------------------------------  target    PARNEQTRYVALFRYGSHQSGTRAWLRPTQQTDSLVRKGYFGQVIGTGFEADVHSVSGAPKEAFVKIEKAEDGGIGAERL 7du7.1    --------------------------------------------------------------------------------  target    WRPLTLGLRPEAPSAALTAYLAGDYSGTKGS 7du7.1    ------------------------------- ``` | | | | | | | | | | | | | | | | | | | | | | | | | | | | | | | | | | | | | | | | | | | | | | | | | |
|  | 7du6.1.A | mkDPBB\_sym2 protein  *Crystal structure of the rationally designed mkDPBB\_sym2 protein* | 0.00 |  | 25.93 | 0.03 | 635-661 | X-ray | 1.60 | monomer |  | HHblits | 0.34 |
| ``` target    MPTANKADEVIILRPGTDAAFFLGVARELIEKGLYDRAAVIERTDLPLLVRLDTGERLDARDVIPGYELAALTNYVTLKP 7du6.1    --------------------------------------------------------------------------------  target    DAEIKGNPPPPPFTAGGQVVPTELRDAWGDFVWWDRATGRPRPVSRDEVGARFDGDPALLGEFEVELVDGSTVPVRPAFD 7du6.1    --------------------------------------------------------------------------------  target    LLKQYLDESFDLRTASEVCRVPPQAIQSIARQLAANKRETLLAAGMGPNHYFQNDLFGRVQFLVAALTDNIGHLGGNVGS 7du6.1    --------------------------------------------------------------------------------  target    YAGNYRGSVFQAMGQWIAEDPFAIEPDLTKPATVKRYYKAESAHYWNYGERPLRAVAKDDEGDLTKGEVLTGKSHMPTPT 7du6.1    --------------------------------------------------------------------------------  target    KLIWFGNSNSLLGNAKWSFDVVKNTLPRQDAVFCNEWHWTSSCEYADLVFPADSWAEFKLPDATASCTNPFLLAFPTTPL 7du6.1    --------------------------------------------------------------------------------  target    KRLYDTRSDYEALALTAKALGELIDEPRMEQYWRGILDGDPTPYLQRIFSGSNATRGITYDELHESSKRGVPLLMNMRTY 7du6.1    --------------------------------------------------------------------------------  target    PRSGGWEQRQEDKPWYTATGRLEFYRPEPEFQAAGESLPVWREPVDATFYEPNAILSNAAHPSIAPRAPEDYGVPESQLD 7du6.1    --------------------------------------------------------------------------------  target    VETRQYRNVVRTWAELQQTLHPLQERDPAFRFVFQTPKYRWGAHSTAVDADWISMLFGPFGDPYRRDPRMPWTGEAYLEI 7du6.1    --------------------------------------------------------------------------KRIVRM  target    NPKDAAELGLADGDYAWVDADPEDRPYRGWKEDDPYYEVARAMMRVRIYTGMSRGVIRTWFNMYAATPATVANQKATPGN 7du6.1    DKYERAKLGVSVGDYVEVKKV-----------------------------------------------------------  target    PARNEQTRYVALFRYGSHQSGTRAWLRPTQQTDSLVRKGYFGQVIGTGFEADVHSVSGAPKEAFVKIEKAEDGGIGAERL 7du6.1    --------------------------------------------------------------------------------  target    WRPLTLGLRPEAPSAALTAYLAGDYSGTKGS 7du6.1    ------------------------------- ``` | | | | | | | | | | | | | | | | | | | | | | | | | | | | | | | | | | | | | | | | | | | | | | | | | |
|  | 7dxs.1.A | ap1h protein  *Crystal structure of the ap1h peptide homodimer.* | 0.00 |  | 30.77 | 0.03 | 634-659 | X-ray | 2.10 | homo-dimer |  | HHblits | 0.37 |
| ``` target    MPTANKADEVIILRPGTDAAFFLGVARELIEKGLYDRAAVIERTDLPLLVRLDTGERLDARDVIPGYELAALTNYVTLKP 7dxs.1    --------------------------------------------------------------------------------  target    DAEIKGNPPPPPFTAGGQVVPTELRDAWGDFVWWDRATGRPRPVSRDEVGARFDGDPALLGEFEVELVDGSTVPVRPAFD 7dxs.1    --------------------------------------------------------------------------------  target    LLKQYLDESFDLRTASEVCRVPPQAIQSIARQLAANKRETLLAAGMGPNHYFQNDLFGRVQFLVAALTDNIGHLGGNVGS 7dxs.1    --------------------------------------------------------------------------------  target    YAGNYRGSVFQAMGQWIAEDPFAIEPDLTKPATVKRYYKAESAHYWNYGERPLRAVAKDDEGDLTKGEVLTGKSHMPTPT 7dxs.1    --------------------------------------------------------------------------------  target    KLIWFGNSNSLLGNAKWSFDVVKNTLPRQDAVFCNEWHWTSSCEYADLVFPADSWAEFKLPDATASCTNPFLLAFPTTPL 7dxs.1    --------------------------------------------------------------------------------  target    KRLYDTRSDYEALALTAKALGELIDEPRMEQYWRGILDGDPTPYLQRIFSGSNATRGITYDELHESSKRGVPLLMNMRTY 7dxs.1    --------------------------------------------------------------------------------  target    PRSGGWEQRQEDKPWYTATGRLEFYRPEPEFQAAGESLPVWREPVDATFYEPNAILSNAAHPSIAPRAPEDYGVPESQLD 7dxs.1    --------------------------------------------------------------------------------  target    VETRQYRNVVRTWAELQQTLHPLQERDPAFRFVFQTPKYRWGAHSTAVDADWISMLFGPFGDPYRRDPRMPWTGEAYLEI 7dxs.1    -------------------------------------------------------------------------GRGIVRM  target    NPKDAAELGLADGDYAWVDADPEDRPYRGWKEDDPYYEVARAMMRVRIYTGMSRGVIRTWFNMYAATPATVANQKATPGN 7dxs.1    DKQTRAKLGVSVGDYVEVK-------------------------------------------------------------  target    PARNEQTRYVALFRYGSHQSGTRAWLRPTQQTDSLVRKGYFGQVIGTGFEADVHSVSGAPKEAFVKIEKAEDGGIGAERL 7dxs.1    --------------------------------------------------------------------------------  target    WRPLTLGLRPEAPSAALTAYLAGDYSGTKGS 7dxs.1    ------------------------------- ``` | | | | | | | | | | | | | | | | | | | | | | | | | | | | | | | | | | | | | | | | | | | | | | | | | |
|  | 7dxs.1.B | ap1h protein  *Crystal structure of the ap1h peptide homodimer.* | 0.00 |  | 30.77 | 0.03 | 634-659 | X-ray | 2.10 | homo-dimer |  | HHblits | 0.37 |
| ``` target    MPTANKADEVIILRPGTDAAFFLGVARELIEKGLYDRAAVIERTDLPLLVRLDTGERLDARDVIPGYELAALTNYVTLKP 7dxs.1    --------------------------------------------------------------------------------  target    DAEIKGNPPPPPFTAGGQVVPTELRDAWGDFVWWDRATGRPRPVSRDEVGARFDGDPALLGEFEVELVDGSTVPVRPAFD 7dxs.1    --------------------------------------------------------------------------------  target    LLKQYLDESFDLRTASEVCRVPPQAIQSIARQLAANKRETLLAAGMGPNHYFQNDLFGRVQFLVAALTDNIGHLGGNVGS 7dxs.1    --------------------------------------------------------------------------------  target    YAGNYRGSVFQAMGQWIAEDPFAIEPDLTKPATVKRYYKAESAHYWNYGERPLRAVAKDDEGDLTKGEVLTGKSHMPTPT 7dxs.1    --------------------------------------------------------------------------------  target    KLIWFGNSNSLLGNAKWSFDVVKNTLPRQDAVFCNEWHWTSSCEYADLVFPADSWAEFKLPDATASCTNPFLLAFPTTPL 7dxs.1    --------------------------------------------------------------------------------  target    KRLYDTRSDYEALALTAKALGELIDEPRMEQYWRGILDGDPTPYLQRIFSGSNATRGITYDELHESSKRGVPLLMNMRTY 7dxs.1    --------------------------------------------------------------------------------  target    PRSGGWEQRQEDKPWYTATGRLEFYRPEPEFQAAGESLPVWREPVDATFYEPNAILSNAAHPSIAPRAPEDYGVPESQLD 7dxs.1    --------------------------------------------------------------------------------  target    VETRQYRNVVRTWAELQQTLHPLQERDPAFRFVFQTPKYRWGAHSTAVDADWISMLFGPFGDPYRRDPRMPWTGEAYLEI 7dxs.1    -------------------------------------------------------------------------GRGIVRM  target    NPKDAAELGLADGDYAWVDADPEDRPYRGWKEDDPYYEVARAMMRVRIYTGMSRGVIRTWFNMYAATPATVANQKATPGN 7dxs.1    DKQTRAKLGVSVGDYVEVK-------------------------------------------------------------  target    PARNEQTRYVALFRYGSHQSGTRAWLRPTQQTDSLVRKGYFGQVIGTGFEADVHSVSGAPKEAFVKIEKAEDGGIGAERL 7dxs.1    --------------------------------------------------------------------------------  target    WRPLTLGLRPEAPSAALTAYLAGDYSGTKGS 7dxs.1    ------------------------------- ``` | | | | | | | | | | | | | | | | | | | | | | | | | | | | | | | | | | | | | | | | | | | | | | | | | |
|  | 7dxs.2.A | ap1h protein  *Crystal structure of the ap1h peptide homodimer.* | 0.00 |  | 30.77 | 0.03 | 634-659 | X-ray | 2.10 | homo-dimer |  | HHblits | 0.37 |
| ``` target    MPTANKADEVIILRPGTDAAFFLGVARELIEKGLYDRAAVIERTDLPLLVRLDTGERLDARDVIPGYELAALTNYVTLKP 7dxs.2    --------------------------------------------------------------------------------  target    DAEIKGNPPPPPFTAGGQVVPTELRDAWGDFVWWDRATGRPRPVSRDEVGARFDGDPALLGEFEVELVDGSTVPVRPAFD 7dxs.2    --------------------------------------------------------------------------------  target    LLKQYLDESFDLRTASEVCRVPPQAIQSIARQLAANKRETLLAAGMGPNHYFQNDLFGRVQFLVAALTDNIGHLGGNVGS 7dxs.2    --------------------------------------------------------------------------------  target    YAGNYRGSVFQAMGQWIAEDPFAIEPDLTKPATVKRYYKAESAHYWNYGERPLRAVAKDDEGDLTKGEVLTGKSHMPTPT 7dxs.2    --------------------------------------------------------------------------------  target    KLIWFGNSNSLLGNAKWSFDVVKNTLPRQDAVFCNEWHWTSSCEYADLVFPADSWAEFKLPDATASCTNPFLLAFPTTPL 7dxs.2    --------------------------------------------------------------------------------  target    KRLYDTRSDYEALALTAKALGELIDEPRMEQYWRGILDGDPTPYLQRIFSGSNATRGITYDELHESSKRGVPLLMNMRTY 7dxs.2    --------------------------------------------------------------------------------  target    PRSGGWEQRQEDKPWYTATGRLEFYRPEPEFQAAGESLPVWREPVDATFYEPNAILSNAAHPSIAPRAPEDYGVPESQLD 7dxs.2    --------------------------------------------------------------------------------  target    VETRQYRNVVRTWAELQQTLHPLQERDPAFRFVFQTPKYRWGAHSTAVDADWISMLFGPFGDPYRRDPRMPWTGEAYLEI 7dxs.2    -------------------------------------------------------------------------GRGIVRM  target    NPKDAAELGLADGDYAWVDADPEDRPYRGWKEDDPYYEVARAMMRVRIYTGMSRGVIRTWFNMYAATPATVANQKATPGN 7dxs.2    DKQTRAKLGVSVGDYVEVK-------------------------------------------------------------  target    PARNEQTRYVALFRYGSHQSGTRAWLRPTQQTDSLVRKGYFGQVIGTGFEADVHSVSGAPKEAFVKIEKAEDGGIGAERL 7dxs.2    --------------------------------------------------------------------------------  target    WRPLTLGLRPEAPSAALTAYLAGDYSGTKGS 7dxs.2    ------------------------------- ``` | | | | | | | | | | | | | | | | | | | | | | | | | | | | | | | | | | | | | | | | | | | | | | | | | |
|  | 7dxs.2.B | ap1h protein  *Crystal structure of the ap1h peptide homodimer.* | 0.00 |  | 30.77 | 0.03 | 634-659 | X-ray | 2.10 | homo-dimer |  | HHblits | 0.37 |
| ``` target    MPTANKADEVIILRPGTDAAFFLGVARELIEKGLYDRAAVIERTDLPLLVRLDTGERLDARDVIPGYELAALTNYVTLKP 7dxs.2    --------------------------------------------------------------------------------  target    DAEIKGNPPPPPFTAGGQVVPTELRDAWGDFVWWDRATGRPRPVSRDEVGARFDGDPALLGEFEVELVDGSTVPVRPAFD 7dxs.2    --------------------------------------------------------------------------------  target    LLKQYLDESFDLRTASEVCRVPPQAIQSIARQLAANKRETLLAAGMGPNHYFQNDLFGRVQFLVAALTDNIGHLGGNVGS 7dxs.2    --------------------------------------------------------------------------------  target    YAGNYRGSVFQAMGQWIAEDPFAIEPDLTKPATVKRYYKAESAHYWNYGERPLRAVAKDDEGDLTKGEVLTGKSHMPTPT 7dxs.2    --------------------------------------------------------------------------------  target    KLIWFGNSNSLLGNAKWSFDVVKNTLPRQDAVFCNEWHWTSSCEYADLVFPADSWAEFKLPDATASCTNPFLLAFPTTPL 7dxs.2    --------------------------------------------------------------------------------  target    KRLYDTRSDYEALALTAKALGELIDEPRMEQYWRGILDGDPTPYLQRIFSGSNATRGITYDELHESSKRGVPLLMNMRTY 7dxs.2    --------------------------------------------------------------------------------  target    PRSGGWEQRQEDKPWYTATGRLEFYRPEPEFQAAGESLPVWREPVDATFYEPNAILSNAAHPSIAPRAPEDYGVPESQLD 7dxs.2    --------------------------------------------------------------------------------  target    VETRQYRNVVRTWAELQQTLHPLQERDPAFRFVFQTPKYRWGAHSTAVDADWISMLFGPFGDPYRRDPRMPWTGEAYLEI 7dxs.2    -------------------------------------------------------------------------GRGIVRM  target    NPKDAAELGLADGDYAWVDADPEDRPYRGWKEDDPYYEVARAMMRVRIYTGMSRGVIRTWFNMYAATPATVANQKATPGN 7dxs.2    DKQTRAKLGVSVGDYVEVK-------------------------------------------------------------  target    PARNEQTRYVALFRYGSHQSGTRAWLRPTQQTDSLVRKGYFGQVIGTGFEADVHSVSGAPKEAFVKIEKAEDGGIGAERL 7dxs.2    --------------------------------------------------------------------------------  target    WRPLTLGLRPEAPSAALTAYLAGDYSGTKGS 7dxs.2    ------------------------------- ``` | | | | | | | | | | | | | | | | | | | | | | | | | | | | | | | | | | | | | | | | | | | | | | | | | |
|  | 5g4f.1.A | VCP-LIKE ATPASE  *Structure of the ADP-bound VAT complex* | 0.00 |  | 13.79 | 0.03 | 634-662 | EM | 7.00 | homo-hexamer |  | HHblits | 0.28 |
| ``` target    MPTANKADEVIILRPGTDAAFFLGVARELIEKGLYDRAAVIERTDLPLLVRLDTGERLDARDVIPGYELAALTNYVTLKP 5g4f.1    --------------------------------------------------------------------------------  target    DAEIKGNPPPPPFTAGGQVVPTELRDAWGDFVWWDRATGRPRPVSRDEVGARFDGDPALLGEFEVELVDGSTVPVRPAFD 5g4f.1    --------------------------------------------------------------------------------  target    LLKQYLDESFDLRTASEVCRVPPQAIQSIARQLAANKRETLLAAGMGPNHYFQNDLFGRVQFLVAALTDNIGHLGGNVGS 5g4f.1    --------------------------------------------------------------------------------  target    YAGNYRGSVFQAMGQWIAEDPFAIEPDLTKPATVKRYYKAESAHYWNYGERPLRAVAKDDEGDLTKGEVLTGKSHMPTPT 5g4f.1    --------------------------------------------------------------------------------  target    KLIWFGNSNSLLGNAKWSFDVVKNTLPRQDAVFCNEWHWTSSCEYADLVFPADSWAEFKLPDATASCTNPFLLAFPTTPL 5g4f.1    --------------------------------------------------------------------------------  target    KRLYDTRSDYEALALTAKALGELIDEPRMEQYWRGILDGDPTPYLQRIFSGSNATRGITYDELHESSKRGVPLLMNMRTY 5g4f.1    --------------------------------------------------------------------------------  target    PRSGGWEQRQEDKPWYTATGRLEFYRPEPEFQAAGESLPVWREPVDATFYEPNAILSNAAHPSIAPRAPEDYGVPESQLD 5g4f.1    --------------------------------------------------------------------------------  target    VETRQYRNVVRTWAELQQTLHPLQERDPAFRFVFQTPKYRWGAHSTAVDADWISMLFGPFGDPYRRDPRMPWTGEAYLEI 5g4f.1    -------------------------------------------------------------------------GMSRVRL  target    NPKDAAELGLADGDYAWVDADPEDRPYRGWKEDDPYYEVARAMMRVRIYTGMSRGVIRTWFNMYAATPATVANQKATPGN 5g4f.1    DESSRRLLDAEIGDVVEIEKVR----------------------------------------------------------  target    PARNEQTRYVALFRYGSHQSGTRAWLRPTQQTDSLVRKGYFGQVIGTGFEADVHSVSGAPKEAFVKIEKAEDGGIGAERL 5g4f.1    --------------------------------------------------------------------------------  target    WRPLTLGLRPEAPSAALTAYLAGDYSGTKGS 5g4f.1    ------------------------------- ``` | | | | | | | | | | | | | | | | | | | | | | | | | | | | | | | | | | | | | | | | | | | | | | | | | |
|  | 5g4f.1.B | VCP-LIKE ATPASE  *Structure of the ADP-bound VAT complex* | 0.00 |  | 13.79 | 0.03 | 634-662 | EM | 7.00 | homo-hexamer |  | HHblits | 0.28 |
| ``` target    MPTANKADEVIILRPGTDAAFFLGVARELIEKGLYDRAAVIERTDLPLLVRLDTGERLDARDVIPGYELAALTNYVTLKP 5g4f.1    --------------------------------------------------------------------------------  target    DAEIKGNPPPPPFTAGGQVVPTELRDAWGDFVWWDRATGRPRPVSRDEVGARFDGDPALLGEFEVELVDGSTVPVRPAFD 5g4f.1    --------------------------------------------------------------------------------  target    LLKQYLDESFDLRTASEVCRVPPQAIQSIARQLAANKRETLLAAGMGPNHYFQNDLFGRVQFLVAALTDNIGHLGGNVGS 5g4f.1    --------------------------------------------------------------------------------  target    YAGNYRGSVFQAMGQWIAEDPFAIEPDLTKPATVKRYYKAESAHYWNYGERPLRAVAKDDEGDLTKGEVLTGKSHMPTPT 5g4f.1    --------------------------------------------------------------------------------  target    KLIWFGNSNSLLGNAKWSFDVVKNTLPRQDAVFCNEWHWTSSCEYADLVFPADSWAEFKLPDATASCTNPFLLAFPTTPL 5g4f.1    --------------------------------------------------------------------------------  target    KRLYDTRSDYEALALTAKALGELIDEPRMEQYWRGILDGDPTPYLQRIFSGSNATRGITYDELHESSKRGVPLLMNMRTY 5g4f.1    --------------------------------------------------------------------------------  target    PRSGGWEQRQEDKPWYTATGRLEFYRPEPEFQAAGESLPVWREPVDATFYEPNAILSNAAHPSIAPRAPEDYGVPESQLD 5g4f.1    --------------------------------------------------------------------------------  target    VETRQYRNVVRTWAELQQTLHPLQERDPAFRFVFQTPKYRWGAHSTAVDADWISMLFGPFGDPYRRDPRMPWTGEAYLEI 5g4f.1    -------------------------------------------------------------------------GMSRVRL  target    NPKDAAELGLADGDYAWVDADPEDRPYRGWKEDDPYYEVARAMMRVRIYTGMSRGVIRTWFNMYAATPATVANQKATPGN 5g4f.1    DESSRRLLDAEIGDVVEIEKVR----------------------------------------------------------  target    PARNEQTRYVALFRYGSHQSGTRAWLRPTQQTDSLVRKGYFGQVIGTGFEADVHSVSGAPKEAFVKIEKAEDGGIGAERL 5g4f.1    --------------------------------------------------------------------------------  target    WRPLTLGLRPEAPSAALTAYLAGDYSGTKGS 5g4f.1    ------------------------------- ``` | | | | | | | | | | | | | | | | | | | | | | | | | | | | | | | | | | | | | | | | | | | | | | | | | |
|  | 5g4f.1.C | VCP-LIKE ATPASE  *Structure of the ADP-bound VAT complex* | 0.00 |  | 13.79 | 0.03 | 634-662 | EM | 7.00 | homo-hexamer |  | HHblits | 0.28 |
| ``` target    MPTANKADEVIILRPGTDAAFFLGVARELIEKGLYDRAAVIERTDLPLLVRLDTGERLDARDVIPGYELAALTNYVTLKP 5g4f.1    --------------------------------------------------------------------------------  target    DAEIKGNPPPPPFTAGGQVVPTELRDAWGDFVWWDRATGRPRPVSRDEVGARFDGDPALLGEFEVELVDGSTVPVRPAFD 5g4f.1    --------------------------------------------------------------------------------  target    LLKQYLDESFDLRTASEVCRVPPQAIQSIARQLAANKRETLLAAGMGPNHYFQNDLFGRVQFLVAALTDNIGHLGGNVGS 5g4f.1    --------------------------------------------------------------------------------  target    YAGNYRGSVFQAMGQWIAEDPFAIEPDLTKPATVKRYYKAESAHYWNYGERPLRAVAKDDEGDLTKGEVLTGKSHMPTPT 5g4f.1    --------------------------------------------------------------------------------  target    KLIWFGNSNSLLGNAKWSFDVVKNTLPRQDAVFCNEWHWTSSCEYADLVFPADSWAEFKLPDATASCTNPFLLAFPTTPL 5g4f.1    --------------------------------------------------------------------------------  target    KRLYDTRSDYEALALTAKALGELIDEPRMEQYWRGILDGDPTPYLQRIFSGSNATRGITYDELHESSKRGVPLLMNMRTY 5g4f.1    --------------------------------------------------------------------------------  target    PRSGGWEQRQEDKPWYTATGRLEFYRPEPEFQAAGESLPVWREPVDATFYEPNAILSNAAHPSIAPRAPEDYGVPESQLD 5g4f.1    --------------------------------------------------------------------------------  target    VETRQYRNVVRTWAELQQTLHPLQERDPAFRFVFQTPKYRWGAHSTAVDADWISMLFGPFGDPYRRDPRMPWTGEAYLEI 5g4f.1    -------------------------------------------------------------------------GMSRVRL  target    NPKDAAELGLADGDYAWVDADPEDRPYRGWKEDDPYYEVARAMMRVRIYTGMSRGVIRTWFNMYAATPATVANQKATPGN 5g4f.1    DESSRRLLDAEIGDVVEIEKVR----------------------------------------------------------  target    PARNEQTRYVALFRYGSHQSGTRAWLRPTQQTDSLVRKGYFGQVIGTGFEADVHSVSGAPKEAFVKIEKAEDGGIGAERL 5g4f.1    --------------------------------------------------------------------------------  target    WRPLTLGLRPEAPSAALTAYLAGDYSGTKGS 5g4f.1    ------------------------------- ``` | | | | | | | | | | | | | | | | | | | | | | | | | | | | | | | | | | | | | | | | | | | | | | | | | |
|  | 5g4f.1.D | VCP-LIKE ATPASE  *Structure of the ADP-bound VAT complex* | 0.00 |  | 13.79 | 0.03 | 634-662 | EM | 7.00 | homo-hexamer |  | HHblits | 0.28 |
| ``` target    MPTANKADEVIILRPGTDAAFFLGVARELIEKGLYDRAAVIERTDLPLLVRLDTGERLDARDVIPGYELAALTNYVTLKP 5g4f.1    --------------------------------------------------------------------------------  target    DAEIKGNPPPPPFTAGGQVVPTELRDAWGDFVWWDRATGRPRPVSRDEVGARFDGDPALLGEFEVELVDGSTVPVRPAFD 5g4f.1    --------------------------------------------------------------------------------  target    LLKQYLDESFDLRTASEVCRVPPQAIQSIARQLAANKRETLLAAGMGPNHYFQNDLFGRVQFLVAALTDNIGHLGGNVGS 5g4f.1    --------------------------------------------------------------------------------  target    YAGNYRGSVFQAMGQWIAEDPFAIEPDLTKPATVKRYYKAESAHYWNYGERPLRAVAKDDEGDLTKGEVLTGKSHMPTPT 5g4f.1    --------------------------------------------------------------------------------  target    KLIWFGNSNSLLGNAKWSFDVVKNTLPRQDAVFCNEWHWTSSCEYADLVFPADSWAEFKLPDATASCTNPFLLAFPTTPL 5g4f.1    --------------------------------------------------------------------------------  target    KRLYDTRSDYEALALTAKALGELIDEPRMEQYWRGILDGDPTPYLQRIFSGSNATRGITYDELHESSKRGVPLLMNMRTY 5g4f.1    --------------------------------------------------------------------------------  target    PRSGGWEQRQEDKPWYTATGRLEFYRPEPEFQAAGESLPVWREPVDATFYEPNAILSNAAHPSIAPRAPEDYGVPESQLD 5g4f.1    --------------------------------------------------------------------------------  target    VETRQYRNVVRTWAELQQTLHPLQERDPAFRFVFQTPKYRWGAHSTAVDADWISMLFGPFGDPYRRDPRMPWTGEAYLEI 5g4f.1    -------------------------------------------------------------------------GMSRVRL  target    NPKDAAELGLADGDYAWVDADPEDRPYRGWKEDDPYYEVARAMMRVRIYTGMSRGVIRTWFNMYAATPATVANQKATPGN 5g4f.1    DESSRRLLDAEIGDVVEIEKVR----------------------------------------------------------  target    PARNEQTRYVALFRYGSHQSGTRAWLRPTQQTDSLVRKGYFGQVIGTGFEADVHSVSGAPKEAFVKIEKAEDGGIGAERL 5g4f.1    --------------------------------------------------------------------------------  target    WRPLTLGLRPEAPSAALTAYLAGDYSGTKGS 5g4f.1    ------------------------------- ``` | | | | | | | | | | | | | | | | | | | | | | | | | | | | | | | | | | | | | | | | | | | | | | | | | |
|  | 5g4f.1.E | VCP-LIKE ATPASE  *Structure of the ADP-bound VAT complex* | 0.00 |  | 13.79 | 0.03 | 634-662 | EM | 7.00 | homo-hexamer |  | HHblits | 0.28 |
| ``` target    MPTANKADEVIILRPGTDAAFFLGVARELIEKGLYDRAAVIERTDLPLLVRLDTGERLDARDVIPGYELAALTNYVTLKP 5g4f.1    --------------------------------------------------------------------------------  target    DAEIKGNPPPPPFTAGGQVVPTELRDAWGDFVWWDRATGRPRPVSRDEVGARFDGDPALLGEFEVELVDGSTVPVRPAFD 5g4f.1    --------------------------------------------------------------------------------  target    LLKQYLDESFDLRTASEVCRVPPQAIQSIARQLAANKRETLLAAGMGPNHYFQNDLFGRVQFLVAALTDNIGHLGGNVGS 5g4f.1    --------------------------------------------------------------------------------  target    YAGNYRGSVFQAMGQWIAEDPFAIEPDLTKPATVKRYYKAESAHYWNYGERPLRAVAKDDEGDLTKGEVLTGKSHMPTPT 5g4f.1    --------------------------------------------------------------------------------  target    KLIWFGNSNSLLGNAKWSFDVVKNTLPRQDAVFCNEWHWTSSCEYADLVFPADSWAEFKLPDATASCTNPFLLAFPTTPL 5g4f.1    --------------------------------------------------------------------------------  target    KRLYDTRSDYEALALTAKALGELIDEPRMEQYWRGILDGDPTPYLQRIFSGSNATRGITYDELHESSKRGVPLLMNMRTY 5g4f.1    --------------------------------------------------------------------------------  target    PRSGGWEQRQEDKPWYTATGRLEFYRPEPEFQAAGESLPVWREPVDATFYEPNAILSNAAHPSIAPRAPEDYGVPESQLD 5g4f.1    --------------------------------------------------------------------------------  target    VETRQYRNVVRTWAELQQTLHPLQERDPAFRFVFQTPKYRWGAHSTAVDADWISMLFGPFGDPYRRDPRMPWTGEAYLEI 5g4f.1    -------------------------------------------------------------------------GMSRVRL  target    NPKDAAELGLADGDYAWVDADPEDRPYRGWKEDDPYYEVARAMMRVRIYTGMSRGVIRTWFNMYAATPATVANQKATPGN 5g4f.1    DESSRRLLDAEIGDVVEIEKVR----------------------------------------------------------  target    PARNEQTRYVALFRYGSHQSGTRAWLRPTQQTDSLVRKGYFGQVIGTGFEADVHSVSGAPKEAFVKIEKAEDGGIGAERL 5g4f.1    --------------------------------------------------------------------------------  target    WRPLTLGLRPEAPSAALTAYLAGDYSGTKGS 5g4f.1    ------------------------------- ``` | | | | | | | | | | | | | | | | | | | | | | | | | | | | | | | | | | | | | | | | | | | | | | | | | |
|  | 5g4f.1.F | VCP-LIKE ATPASE  *Structure of the ADP-bound VAT complex* | 0.00 |  | 13.79 | 0.03 | 634-662 | EM | 7.00 | homo-hexamer |  | HHblits | 0.28 |
| ``` target    MPTANKADEVIILRPGTDAAFFLGVARELIEKGLYDRAAVIERTDLPLLVRLDTGERLDARDVIPGYELAALTNYVTLKP 5g4f.1    --------------------------------------------------------------------------------  target    DAEIKGNPPPPPFTAGGQVVPTELRDAWGDFVWWDRATGRPRPVSRDEVGARFDGDPALLGEFEVELVDGSTVPVRPAFD 5g4f.1    --------------------------------------------------------------------------------  target    LLKQYLDESFDLRTASEVCRVPPQAIQSIARQLAANKRETLLAAGMGPNHYFQNDLFGRVQFLVAALTDNIGHLGGNVGS 5g4f.1    --------------------------------------------------------------------------------  target    YAGNYRGSVFQAMGQWIAEDPFAIEPDLTKPATVKRYYKAESAHYWNYGERPLRAVAKDDEGDLTKGEVLTGKSHMPTPT 5g4f.1    --------------------------------------------------------------------------------  target    KLIWFGNSNSLLGNAKWSFDVVKNTLPRQDAVFCNEWHWTSSCEYADLVFPADSWAEFKLPDATASCTNPFLLAFPTTPL 5g4f.1    --------------------------------------------------------------------------------  target    KRLYDTRSDYEALALTAKALGELIDEPRMEQYWRGILDGDPTPYLQRIFSGSNATRGITYDELHESSKRGVPLLMNMRTY 5g4f.1    --------------------------------------------------------------------------------  target    PRSGGWEQRQEDKPWYTATGRLEFYRPEPEFQAAGESLPVWREPVDATFYEPNAILSNAAHPSIAPRAPEDYGVPESQLD 5g4f.1    --------------------------------------------------------------------------------  target    VETRQYRNVVRTWAELQQTLHPLQERDPAFRFVFQTPKYRWGAHSTAVDADWISMLFGPFGDPYRRDPRMPWTGEAYLEI 5g4f.1    -------------------------------------------------------------------------GMSRVRL  target    NPKDAAELGLADGDYAWVDADPEDRPYRGWKEDDPYYEVARAMMRVRIYTGMSRGVIRTWFNMYAATPATVANQKATPGN 5g4f.1    DESSRRLLDAEIGDVVEIEKVR----------------------------------------------------------  target    PARNEQTRYVALFRYGSHQSGTRAWLRPTQQTDSLVRKGYFGQVIGTGFEADVHSVSGAPKEAFVKIEKAEDGGIGAERL 5g4f.1    --------------------------------------------------------------------------------  target    WRPLTLGLRPEAPSAALTAYLAGDYSGTKGS 5g4f.1    ------------------------------- ``` | | | | | | | | | | | | | | | | | | | | | | | | | | | | | | | | | | | | | | | | | | | | | | | | | |
|  | 7di1.1.A | mkDPBB\_sym\_86 protein  *Crystal structure of the rationally designed mkDPBB\_sym\_86 protein* | 0.00 |  | 25.93 | 0.03 | 635-661 | X-ray | 2.10 | monomer |  | HHblits | 0.34 |
| ``` target    MPTANKADEVIILRPGTDAAFFLGVARELIEKGLYDRAAVIERTDLPLLVRLDTGERLDARDVIPGYELAALTNYVTLKP 7di1.1    --------------------------------------------------------------------------------  target    DAEIKGNPPPPPFTAGGQVVPTELRDAWGDFVWWDRATGRPRPVSRDEVGARFDGDPALLGEFEVELVDGSTVPVRPAFD 7di1.1    --------------------------------------------------------------------------------  target    LLKQYLDESFDLRTASEVCRVPPQAIQSIARQLAANKRETLLAAGMGPNHYFQNDLFGRVQFLVAALTDNIGHLGGNVGS 7di1.1    --------------------------------------------------------------------------------  target    YAGNYRGSVFQAMGQWIAEDPFAIEPDLTKPATVKRYYKAESAHYWNYGERPLRAVAKDDEGDLTKGEVLTGKSHMPTPT 7di1.1    --------------------------------------------------------------------------------  target    KLIWFGNSNSLLGNAKWSFDVVKNTLPRQDAVFCNEWHWTSSCEYADLVFPADSWAEFKLPDATASCTNPFLLAFPTTPL 7di1.1    --------------------------------------------------------------------------------  target    KRLYDTRSDYEALALTAKALGELIDEPRMEQYWRGILDGDPTPYLQRIFSGSNATRGITYDELHESSKRGVPLLMNMRTY 7di1.1    --------------------------------------------------------------------------------  target    PRSGGWEQRQEDKPWYTATGRLEFYRPEPEFQAAGESLPVWREPVDATFYEPNAILSNAAHPSIAPRAPEDYGVPESQLD 7di1.1    --------------------------------------------------------------------------------  target    VETRQYRNVVRTWAELQQTLHPLQERDPAFRFVFQTPKYRWGAHSTAVDADWISMLFGPFGDPYRRDPRMPWTGEAYLEI 7di1.1    --------------------------------------------------------------------------KRIVRM  target    NPKDAAELGLADGDYAWVDADPEDRPYRGWKEDDPYYEVARAMMRVRIYTGMSRGVIRTWFNMYAATPATVANQKATPGN 7di1.1    DKASRAKLGVSVGDYVEVKKV-----------------------------------------------------------  target    PARNEQTRYVALFRYGSHQSGTRAWLRPTQQTDSLVRKGYFGQVIGTGFEADVHSVSGAPKEAFVKIEKAEDGGIGAERL 7di1.1    --------------------------------------------------------------------------------  target    WRPLTLGLRPEAPSAALTAYLAGDYSGTKGS 7di1.1    ------------------------------- ``` | | | | | | | | | | | | | | | | | | | | | | | | | | | | | | | | | | | | | | | | | | | | | | | | | |
|  | 6hd3.1.A | Cell division control protein 48 homolog A  *Common mode of remodeling AAA ATPases p97/CDC48 by their disassembly cofactors ASPL/PUX1* | 0.00 |  | 17.86 | 0.03 | 635-662 | X-ray | 2.80 | homo-24-mer | 24 x ADP | HHblits | 0.31 |
| ``` target    MPTANKADEVIILRPGTDAAFFLGVARELIEKGLYDRAAVIERTDLPLLVRLDTGERLDARDVIPGYELAALTNYVTLKP 6hd3.1    --------------------------------------------------------------------------------  target    DAEIKGNPPPPPFTAGGQVVPTELRDAWGDFVWWDRATGRPRPVSRDEVGARFDGDPALLGEFEVELVDGSTVPVRPAFD 6hd3.1    --------------------------------------------------------------------------------  target    LLKQYLDESFDLRTASEVCRVPPQAIQSIARQLAANKRETLLAAGMGPNHYFQNDLFGRVQFLVAALTDNIGHLGGNVGS 6hd3.1    --------------------------------------------------------------------------------  target    YAGNYRGSVFQAMGQWIAEDPFAIEPDLTKPATVKRYYKAESAHYWNYGERPLRAVAKDDEGDLTKGEVLTGKSHMPTPT 6hd3.1    --------------------------------------------------------------------------------  target    KLIWFGNSNSLLGNAKWSFDVVKNTLPRQDAVFCNEWHWTSSCEYADLVFPADSWAEFKLPDATASCTNPFLLAFPTTPL 6hd3.1    --------------------------------------------------------------------------------  target    KRLYDTRSDYEALALTAKALGELIDEPRMEQYWRGILDGDPTPYLQRIFSGSNATRGITYDELHESSKRGVPLLMNMRTY 6hd3.1    --------------------------------------------------------------------------------  target    PRSGGWEQRQEDKPWYTATGRLEFYRPEPEFQAAGESLPVWREPVDATFYEPNAILSNAAHPSIAPRAPEDYGVPESQLD 6hd3.1    --------------------------------------------------------------------------------  target    VETRQYRNVVRTWAELQQTLHPLQERDPAFRFVFQTPKYRWGAHSTAVDADWISMLFGPFGDPYRRDPRMPWTGEAYLEI 6hd3.1    --------------------------------------------------------------------------NSVVSL  target    NPKDAAELGLADGDYAWVDADPEDRPYRGWKEDDPYYEVARAMMRVRIYTGMSRGVIRTWFNMYAATPATVANQKATPGN 6hd3.1    HPATMEKLQLFRGDTILIKGKK----------------------------------------------------------  target    PARNEQTRYVALFRYGSHQSGTRAWLRPTQQTDSLVRKGYFGQVIGTGFEADVHSVSGAPKEAFVKIEKAEDGGIGAERL 6hd3.1    --------------------------------------------------------------------------------  target    WRPLTLGLRPEAPSAALTAYLAGDYSGTKGS 6hd3.1    ------------------------------- ``` | | | | | | | | | | | | | | | | | | | | | | | | | | | | | | | | | | | | | | | | | | | | | | | | | |
|  | 7di0.1.A | apDPBB\_sym\_79 protein  *Crystal structure of the rationally designed apDPBB\_sym\_79 protein* | 0.00 |  | 25.93 | 0.03 | 635-661 | X-ray | 1.60 | monomer |  | HHblits | 0.33 |
| ``` target    MPTANKADEVIILRPGTDAAFFLGVARELIEKGLYDRAAVIERTDLPLLVRLDTGERLDARDVIPGYELAALTNYVTLKP 7di0.1    --------------------------------------------------------------------------------  target    DAEIKGNPPPPPFTAGGQVVPTELRDAWGDFVWWDRATGRPRPVSRDEVGARFDGDPALLGEFEVELVDGSTVPVRPAFD 7di0.1    --------------------------------------------------------------------------------  target    LLKQYLDESFDLRTASEVCRVPPQAIQSIARQLAANKRETLLAAGMGPNHYFQNDLFGRVQFLVAALTDNIGHLGGNVGS 7di0.1    --------------------------------------------------------------------------------  target    YAGNYRGSVFQAMGQWIAEDPFAIEPDLTKPATVKRYYKAESAHYWNYGERPLRAVAKDDEGDLTKGEVLTGKSHMPTPT 7di0.1    --------------------------------------------------------------------------------  target    KLIWFGNSNSLLGNAKWSFDVVKNTLPRQDAVFCNEWHWTSSCEYADLVFPADSWAEFKLPDATASCTNPFLLAFPTTPL 7di0.1    --------------------------------------------------------------------------------  target    KRLYDTRSDYEALALTAKALGELIDEPRMEQYWRGILDGDPTPYLQRIFSGSNATRGITYDELHESSKRGVPLLMNMRTY 7di0.1    --------------------------------------------------------------------------------  target    PRSGGWEQRQEDKPWYTATGRLEFYRPEPEFQAAGESLPVWREPVDATFYEPNAILSNAAHPSIAPRAPEDYGVPESQLD 7di0.1    --------------------------------------------------------------------------------  target    VETRQYRNVVRTWAELQQTLHPLQERDPAFRFVFQTPKYRWGAHSTAVDADWISMLFGPFGDPYRRDPRMPWTGEAYLEI 7di0.1    --------------------------------------------------------------------------RKIVRM  target    NPKDAAELGLADGDYAWVDADPEDRPYRGWKEDDPYYEVARAMMRVRIYTGMSRGVIRTWFNMYAATPATVANQKATPGN 7di0.1    DKQTRARLGVSVGDYVEVKKV-----------------------------------------------------------  target    PARNEQTRYVALFRYGSHQSGTRAWLRPTQQTDSLVRKGYFGQVIGTGFEADVHSVSGAPKEAFVKIEKAEDGGIGAERL 7di0.1    --------------------------------------------------------------------------------  target    WRPLTLGLRPEAPSAALTAYLAGDYSGTKGS 7di0.1    ------------------------------- ``` | | | | | | | | | | | | | | | | | | | | | | | | | | | | | | | | | | | | | | | | | | | | | | | | | |
|  | 7di0.2.A | apDPBB\_sym\_79 protein  *Crystal structure of the rationally designed apDPBB\_sym\_79 protein* | 0.00 |  | 25.93 | 0.03 | 635-661 | X-ray | 1.60 | monomer |  | HHblits | 0.33 |
| ``` target    MPTANKADEVIILRPGTDAAFFLGVARELIEKGLYDRAAVIERTDLPLLVRLDTGERLDARDVIPGYELAALTNYVTLKP 7di0.2    --------------------------------------------------------------------------------  target    DAEIKGNPPPPPFTAGGQVVPTELRDAWGDFVWWDRATGRPRPVSRDEVGARFDGDPALLGEFEVELVDGSTVPVRPAFD 7di0.2    --------------------------------------------------------------------------------  target    LLKQYLDESFDLRTASEVCRVPPQAIQSIARQLAANKRETLLAAGMGPNHYFQNDLFGRVQFLVAALTDNIGHLGGNVGS 7di0.2    --------------------------------------------------------------------------------  target    YAGNYRGSVFQAMGQWIAEDPFAIEPDLTKPATVKRYYKAESAHYWNYGERPLRAVAKDDEGDLTKGEVLTGKSHMPTPT 7di0.2    --------------------------------------------------------------------------------  target    KLIWFGNSNSLLGNAKWSFDVVKNTLPRQDAVFCNEWHWTSSCEYADLVFPADSWAEFKLPDATASCTNPFLLAFPTTPL 7di0.2    --------------------------------------------------------------------------------  target    KRLYDTRSDYEALALTAKALGELIDEPRMEQYWRGILDGDPTPYLQRIFSGSNATRGITYDELHESSKRGVPLLMNMRTY 7di0.2    --------------------------------------------------------------------------------  target    PRSGGWEQRQEDKPWYTATGRLEFYRPEPEFQAAGESLPVWREPVDATFYEPNAILSNAAHPSIAPRAPEDYGVPESQLD 7di0.2    --------------------------------------------------------------------------------  target    VETRQYRNVVRTWAELQQTLHPLQERDPAFRFVFQTPKYRWGAHSTAVDADWISMLFGPFGDPYRRDPRMPWTGEAYLEI 7di0.2    --------------------------------------------------------------------------RKIVRM  target    NPKDAAELGLADGDYAWVDADPEDRPYRGWKEDDPYYEVARAMMRVRIYTGMSRGVIRTWFNMYAATPATVANQKATPGN 7di0.2    DKQTRARLGVSVGDYVEVKKV-----------------------------------------------------------  target    PARNEQTRYVALFRYGSHQSGTRAWLRPTQQTDSLVRKGYFGQVIGTGFEADVHSVSGAPKEAFVKIEKAEDGGIGAERL 7di0.2    --------------------------------------------------------------------------------  target    WRPLTLGLRPEAPSAALTAYLAGDYSGTKGS 7di0.2    ------------------------------- ``` | | | | | | | | | | | | | | | | | | | | | | | | | | | | | | | | | | | | | | | | | | | | | | | | | |
|  | 7di0.3.A | apDPBB\_sym\_79 protein  *Crystal structure of the rationally designed apDPBB\_sym\_79 protein* | 0.00 |  | 25.93 | 0.03 | 635-661 | X-ray | 1.60 | monomer |  | HHblits | 0.33 |
| ``` target    MPTANKADEVIILRPGTDAAFFLGVARELIEKGLYDRAAVIERTDLPLLVRLDTGERLDARDVIPGYELAALTNYVTLKP 7di0.3    --------------------------------------------------------------------------------  target    DAEIKGNPPPPPFTAGGQVVPTELRDAWGDFVWWDRATGRPRPVSRDEVGARFDGDPALLGEFEVELVDGSTVPVRPAFD 7di0.3    --------------------------------------------------------------------------------  target    LLKQYLDESFDLRTASEVCRVPPQAIQSIARQLAANKRETLLAAGMGPNHYFQNDLFGRVQFLVAALTDNIGHLGGNVGS 7di0.3    --------------------------------------------------------------------------------  target    YAGNYRGSVFQAMGQWIAEDPFAIEPDLTKPATVKRYYKAESAHYWNYGERPLRAVAKDDEGDLTKGEVLTGKSHMPTPT 7di0.3    --------------------------------------------------------------------------------  target    KLIWFGNSNSLLGNAKWSFDVVKNTLPRQDAVFCNEWHWTSSCEYADLVFPADSWAEFKLPDATASCTNPFLLAFPTTPL 7di0.3    --------------------------------------------------------------------------------  target    KRLYDTRSDYEALALTAKALGELIDEPRMEQYWRGILDGDPTPYLQRIFSGSNATRGITYDELHESSKRGVPLLMNMRTY 7di0.3    --------------------------------------------------------------------------------  target    PRSGGWEQRQEDKPWYTATGRLEFYRPEPEFQAAGESLPVWREPVDATFYEPNAILSNAAHPSIAPRAPEDYGVPESQLD 7di0.3    --------------------------------------------------------------------------------  target    VETRQYRNVVRTWAELQQTLHPLQERDPAFRFVFQTPKYRWGAHSTAVDADWISMLFGPFGDPYRRDPRMPWTGEAYLEI 7di0.3    --------------------------------------------------------------------------RKIVRM  target    NPKDAAELGLADGDYAWVDADPEDRPYRGWKEDDPYYEVARAMMRVRIYTGMSRGVIRTWFNMYAATPATVANQKATPGN 7di0.3    DKQTRARLGVSVGDYVEVKKV-----------------------------------------------------------  target    PARNEQTRYVALFRYGSHQSGTRAWLRPTQQTDSLVRKGYFGQVIGTGFEADVHSVSGAPKEAFVKIEKAEDGGIGAERL 7di0.3    --------------------------------------------------------------------------------  target    WRPLTLGLRPEAPSAALTAYLAGDYSGTKGS 7di0.3    ------------------------------- ``` | | | | | | | | | | | | | | | | | | | | | | | | | | | | | | | | | | | | | | | | | | | | | | | | | |
|  | 7dxy.1.A | mk2h\_deltaMILPS  *Crystal structure of the chemically synthesized mk2h\_deltaMILPS peptide homodimer* | 0.00 |  | 26.92 | 0.03 | 634-659 | X-ray | 1.40 | homo-dimer |  | HHblits | 0.36 |
| ``` target    MPTANKADEVIILRPGTDAAFFLGVARELIEKGLYDRAAVIERTDLPLLVRLDTGERLDARDVIPGYELAALTNYVTLKP 7dxy.1    --------------------------------------------------------------------------------  target    DAEIKGNPPPPPFTAGGQVVPTELRDAWGDFVWWDRATGRPRPVSRDEVGARFDGDPALLGEFEVELVDGSTVPVRPAFD 7dxy.1    --------------------------------------------------------------------------------  target    LLKQYLDESFDLRTASEVCRVPPQAIQSIARQLAANKRETLLAAGMGPNHYFQNDLFGRVQFLVAALTDNIGHLGGNVGS 7dxy.1    --------------------------------------------------------------------------------  target    YAGNYRGSVFQAMGQWIAEDPFAIEPDLTKPATVKRYYKAESAHYWNYGERPLRAVAKDDEGDLTKGEVLTGKSHMPTPT 7dxy.1    --------------------------------------------------------------------------------  target    KLIWFGNSNSLLGNAKWSFDVVKNTLPRQDAVFCNEWHWTSSCEYADLVFPADSWAEFKLPDATASCTNPFLLAFPTTPL 7dxy.1    --------------------------------------------------------------------------------  target    KRLYDTRSDYEALALTAKALGELIDEPRMEQYWRGILDGDPTPYLQRIFSGSNATRGITYDELHESSKRGVPLLMNMRTY 7dxy.1    --------------------------------------------------------------------------------  target    PRSGGWEQRQEDKPWYTATGRLEFYRPEPEFQAAGESLPVWREPVDATFYEPNAILSNAAHPSIAPRAPEDYGVPESQLD 7dxy.1    --------------------------------------------------------------------------------  target    VETRQYRNVVRTWAELQQTLHPLQERDPAFRFVFQTPKYRWGAHSTAVDADWISMLFGPFGDPYRRDPRMPWTGEAYLEI 7dxy.1    -------------------------------------------------------------------------GKRVVRV  target    NPKDAAELGLADGDYAWVDADPEDRPYRGWKEDDPYYEVARAMMRVRIYTGMSRGVIRTWFNMYAATPATVANQKATPGN 7dxy.1    DKYERAKVGVKVGDYVEVK-------------------------------------------------------------  target    PARNEQTRYVALFRYGSHQSGTRAWLRPTQQTDSLVRKGYFGQVIGTGFEADVHSVSGAPKEAFVKIEKAEDGGIGAERL 7dxy.1    --------------------------------------------------------------------------------  target    WRPLTLGLRPEAPSAALTAYLAGDYSGTKGS 7dxy.1    ------------------------------- ``` | | | | | | | | | | | | | | | | | | | | | | | | | | | | | | | | | | | | | | | | | | | | | | | | | |
|  | 7dvh.2.A | reDPBB\_sym4 protein  *Crystal structure of the computationally designed reDPBB\_sym4 protein* | 0.00 |  | 22.22 | 0.03 | 635-661 | X-ray | 1.70 | monomer |  | HHblits | 0.33 |
| ``` target    MPTANKADEVIILRPGTDAAFFLGVARELIEKGLYDRAAVIERTDLPLLVRLDTGERLDARDVIPGYELAALTNYVTLKP 7dvh.2    --------------------------------------------------------------------------------  target    DAEIKGNPPPPPFTAGGQVVPTELRDAWGDFVWWDRATGRPRPVSRDEVGARFDGDPALLGEFEVELVDGSTVPVRPAFD 7dvh.2    --------------------------------------------------------------------------------  target    LLKQYLDESFDLRTASEVCRVPPQAIQSIARQLAANKRETLLAAGMGPNHYFQNDLFGRVQFLVAALTDNIGHLGGNVGS 7dvh.2    --------------------------------------------------------------------------------  target    YAGNYRGSVFQAMGQWIAEDPFAIEPDLTKPATVKRYYKAESAHYWNYGERPLRAVAKDDEGDLTKGEVLTGKSHMPTPT 7dvh.2    --------------------------------------------------------------------------------  target    KLIWFGNSNSLLGNAKWSFDVVKNTLPRQDAVFCNEWHWTSSCEYADLVFPADSWAEFKLPDATASCTNPFLLAFPTTPL 7dvh.2    --------------------------------------------------------------------------------  target    KRLYDTRSDYEALALTAKALGELIDEPRMEQYWRGILDGDPTPYLQRIFSGSNATRGITYDELHESSKRGVPLLMNMRTY 7dvh.2    --------------------------------------------------------------------------------  target    PRSGGWEQRQEDKPWYTATGRLEFYRPEPEFQAAGESLPVWREPVDATFYEPNAILSNAAHPSIAPRAPEDYGVPESQLD 7dvh.2    --------------------------------------------------------------------------------  target    VETRQYRNVVRTWAELQQTLHPLQERDPAFRFVFQTPKYRWGAHSTAVDADWISMLFGPFGDPYRRDPRMPWTGEAYLEI 7dvh.2    --------------------------------------------------------------------------KGIVRM  target    NPKDAAELGLADGDYAWVDADPEDRPYRGWKEDDPYYEVARAMMRVRIYTGMSRGVIRTWFNMYAATPATVANQKATPGN 7dvh.2    DKYERQNLGVSVGDYVEVKKA-----------------------------------------------------------  target    PARNEQTRYVALFRYGSHQSGTRAWLRPTQQTDSLVRKGYFGQVIGTGFEADVHSVSGAPKEAFVKIEKAEDGGIGAERL 7dvh.2    --------------------------------------------------------------------------------  target    WRPLTLGLRPEAPSAALTAYLAGDYSGTKGS 7dvh.2    ------------------------------- ``` | | | | | | | | | | | | | | | | | | | | | | | | | | | | | | | | | | | | | | | | | | | | | | | | | |
|  | 7dvh.1.A | reDPBB\_sym4 protein  *Crystal structure of the computationally designed reDPBB\_sym4 protein* | 0.00 |  | 22.22 | 0.03 | 635-661 | X-ray | 1.70 | monomer |  | HHblits | 0.33 |
| ``` target    MPTANKADEVIILRPGTDAAFFLGVARELIEKGLYDRAAVIERTDLPLLVRLDTGERLDARDVIPGYELAALTNYVTLKP 7dvh.1    --------------------------------------------------------------------------------  target    DAEIKGNPPPPPFTAGGQVVPTELRDAWGDFVWWDRATGRPRPVSRDEVGARFDGDPALLGEFEVELVDGSTVPVRPAFD 7dvh.1    --------------------------------------------------------------------------------  target    LLKQYLDESFDLRTASEVCRVPPQAIQSIARQLAANKRETLLAAGMGPNHYFQNDLFGRVQFLVAALTDNIGHLGGNVGS 7dvh.1    --------------------------------------------------------------------------------  target    YAGNYRGSVFQAMGQWIAEDPFAIEPDLTKPATVKRYYKAESAHYWNYGERPLRAVAKDDEGDLTKGEVLTGKSHMPTPT 7dvh.1    --------------------------------------------------------------------------------  target    KLIWFGNSNSLLGNAKWSFDVVKNTLPRQDAVFCNEWHWTSSCEYADLVFPADSWAEFKLPDATASCTNPFLLAFPTTPL 7dvh.1    --------------------------------------------------------------------------------  target    KRLYDTRSDYEALALTAKALGELIDEPRMEQYWRGILDGDPTPYLQRIFSGSNATRGITYDELHESSKRGVPLLMNMRTY 7dvh.1    --------------------------------------------------------------------------------  target    PRSGGWEQRQEDKPWYTATGRLEFYRPEPEFQAAGESLPVWREPVDATFYEPNAILSNAAHPSIAPRAPEDYGVPESQLD 7dvh.1    --------------------------------------------------------------------------------  target    VETRQYRNVVRTWAELQQTLHPLQERDPAFRFVFQTPKYRWGAHSTAVDADWISMLFGPFGDPYRRDPRMPWTGEAYLEI 7dvh.1    --------------------------------------------------------------------------KGIVRM  target    NPKDAAELGLADGDYAWVDADPEDRPYRGWKEDDPYYEVARAMMRVRIYTGMSRGVIRTWFNMYAATPATVANQKATPGN 7dvh.1    DKYERQNLGVSVGDYVEVKKA-----------------------------------------------------------  target    PARNEQTRYVALFRYGSHQSGTRAWLRPTQQTDSLVRKGYFGQVIGTGFEADVHSVSGAPKEAFVKIEKAEDGGIGAERL 7dvh.1    --------------------------------------------------------------------------------  target    WRPLTLGLRPEAPSAALTAYLAGDYSGTKGS 7dvh.1    ------------------------------- ``` | | | | | | | | | | | | | | | | | | | | | | | | | | | | | | | | | | | | | | | | | | | | | | | | | |
|  | 7dvh.4.A | reDPBB\_sym4 protein  *Crystal structure of the computationally designed reDPBB\_sym4 protein* | 0.00 |  | 22.22 | 0.03 | 635-661 | X-ray | 1.70 | monomer |  | HHblits | 0.33 |
| ``` target    MPTANKADEVIILRPGTDAAFFLGVARELIEKGLYDRAAVIERTDLPLLVRLDTGERLDARDVIPGYELAALTNYVTLKP 7dvh.4    --------------------------------------------------------------------------------  target    DAEIKGNPPPPPFTAGGQVVPTELRDAWGDFVWWDRATGRPRPVSRDEVGARFDGDPALLGEFEVELVDGSTVPVRPAFD 7dvh.4    --------------------------------------------------------------------------------  target    LLKQYLDESFDLRTASEVCRVPPQAIQSIARQLAANKRETLLAAGMGPNHYFQNDLFGRVQFLVAALTDNIGHLGGNVGS 7dvh.4    --------------------------------------------------------------------------------  target    YAGNYRGSVFQAMGQWIAEDPFAIEPDLTKPATVKRYYKAESAHYWNYGERPLRAVAKDDEGDLTKGEVLTGKSHMPTPT 7dvh.4    --------------------------------------------------------------------------------  target    KLIWFGNSNSLLGNAKWSFDVVKNTLPRQDAVFCNEWHWTSSCEYADLVFPADSWAEFKLPDATASCTNPFLLAFPTTPL 7dvh.4    --------------------------------------------------------------------------------  target    KRLYDTRSDYEALALTAKALGELIDEPRMEQYWRGILDGDPTPYLQRIFSGSNATRGITYDELHESSKRGVPLLMNMRTY 7dvh.4    --------------------------------------------------------------------------------  target    PRSGGWEQRQEDKPWYTATGRLEFYRPEPEFQAAGESLPVWREPVDATFYEPNAILSNAAHPSIAPRAPEDYGVPESQLD 7dvh.4    --------------------------------------------------------------------------------  target    VETRQYRNVVRTWAELQQTLHPLQERDPAFRFVFQTPKYRWGAHSTAVDADWISMLFGPFGDPYRRDPRMPWTGEAYLEI 7dvh.4    --------------------------------------------------------------------------KGIVRM  target    NPKDAAELGLADGDYAWVDADPEDRPYRGWKEDDPYYEVARAMMRVRIYTGMSRGVIRTWFNMYAATPATVANQKATPGN 7dvh.4    DKYERQNLGVSVGDYVEVKKA-----------------------------------------------------------  target    PARNEQTRYVALFRYGSHQSGTRAWLRPTQQTDSLVRKGYFGQVIGTGFEADVHSVSGAPKEAFVKIEKAEDGGIGAERL 7dvh.4    --------------------------------------------------------------------------------  target    WRPLTLGLRPEAPSAALTAYLAGDYSGTKGS 7dvh.4    ------------------------------- ``` | | | | | | | | | | | | | | | | | | | | | | | | | | | | | | | | | | | | | | | | | | | | | | | | | |
|  | 3hu1.1.A | Transitional endoplasmic reticulum ATPase  *Structure of p97 N-D1 R95G mutant in complex with ATPgS* | 0.00 |  | 17.86 | 0.03 | 635-662 | X-ray | 2.81 | homo-hexamer | 6 x AGS, 6 x MG | HHblits | 0.29 |
| ``` target    MPTANKADEVIILRPGTDAAFFLGVARELIEKGLYDRAAVIERTDLPLLVRLDTGERLDARDVIPGYELAALTNYVTLKP 3hu1.1    --------------------------------------------------------------------------------  target    DAEIKGNPPPPPFTAGGQVVPTELRDAWGDFVWWDRATGRPRPVSRDEVGARFDGDPALLGEFEVELVDGSTVPVRPAFD 3hu1.1    --------------------------------------------------------------------------------  target    LLKQYLDESFDLRTASEVCRVPPQAIQSIARQLAANKRETLLAAGMGPNHYFQNDLFGRVQFLVAALTDNIGHLGGNVGS 3hu1.1    --------------------------------------------------------------------------------  target    YAGNYRGSVFQAMGQWIAEDPFAIEPDLTKPATVKRYYKAESAHYWNYGERPLRAVAKDDEGDLTKGEVLTGKSHMPTPT 3hu1.1    --------------------------------------------------------------------------------  target    KLIWFGNSNSLLGNAKWSFDVVKNTLPRQDAVFCNEWHWTSSCEYADLVFPADSWAEFKLPDATASCTNPFLLAFPTTPL 3hu1.1    --------------------------------------------------------------------------------  target    KRLYDTRSDYEALALTAKALGELIDEPRMEQYWRGILDGDPTPYLQRIFSGSNATRGITYDELHESSKRGVPLLMNMRTY 3hu1.1    --------------------------------------------------------------------------------  target    PRSGGWEQRQEDKPWYTATGRLEFYRPEPEFQAAGESLPVWREPVDATFYEPNAILSNAAHPSIAPRAPEDYGVPESQLD 3hu1.1    --------------------------------------------------------------------------------  target    VETRQYRNVVRTWAELQQTLHPLQERDPAFRFVFQTPKYRWGAHSTAVDADWISMLFGPFGDPYRRDPRMPWTGEAYLEI 3hu1.1    --------------------------------------------------------------------------NSVVSL  target    NPKDAAELGLADGDYAWVDADPEDRPYRGWKEDDPYYEVARAMMRVRIYTGMSRGVIRTWFNMYAATPATVANQKATPGN 3hu1.1    SQPKMDELQLFRGDTVLLKGKK----------------------------------------------------------  target    PARNEQTRYVALFRYGSHQSGTRAWLRPTQQTDSLVRKGYFGQVIGTGFEADVHSVSGAPKEAFVKIEKAEDGGIGAERL 3hu1.1    --------------------------------------------------------------------------------  target    WRPLTLGLRPEAPSAALTAYLAGDYSGTKGS 3hu1.1    ------------------------------- ``` | | | | | | | | | | | | | | | | | | | | | | | | | | | | | | | | | | | | | | | | | | | | | | | | | |
|  | 7dxv.1.A | mk2h\_dY protein  *Crystal structure of the mk2h\_deltaY peptide homodimer* | 0.00 |  | 26.92 | 0.03 | 634-659 | X-ray | 2.30 | homo-dimer |  | HHblits | 0.35 |
| ``` target    MPTANKADEVIILRPGTDAAFFLGVARELIEKGLYDRAAVIERTDLPLLVRLDTGERLDARDVIPGYELAALTNYVTLKP 7dxv.1    --------------------------------------------------------------------------------  target    DAEIKGNPPPPPFTAGGQVVPTELRDAWGDFVWWDRATGRPRPVSRDEVGARFDGDPALLGEFEVELVDGSTVPVRPAFD 7dxv.1    --------------------------------------------------------------------------------  target    LLKQYLDESFDLRTASEVCRVPPQAIQSIARQLAANKRETLLAAGMGPNHYFQNDLFGRVQFLVAALTDNIGHLGGNVGS 7dxv.1    --------------------------------------------------------------------------------  target    YAGNYRGSVFQAMGQWIAEDPFAIEPDLTKPATVKRYYKAESAHYWNYGERPLRAVAKDDEGDLTKGEVLTGKSHMPTPT 7dxv.1    --------------------------------------------------------------------------------  target    KLIWFGNSNSLLGNAKWSFDVVKNTLPRQDAVFCNEWHWTSSCEYADLVFPADSWAEFKLPDATASCTNPFLLAFPTTPL 7dxv.1    --------------------------------------------------------------------------------  target    KRLYDTRSDYEALALTAKALGELIDEPRMEQYWRGILDGDPTPYLQRIFSGSNATRGITYDELHESSKRGVPLLMNMRTY 7dxv.1    --------------------------------------------------------------------------------  target    PRSGGWEQRQEDKPWYTATGRLEFYRPEPEFQAAGESLPVWREPVDATFYEPNAILSNAAHPSIAPRAPEDYGVPESQLD 7dxv.1    --------------------------------------------------------------------------------  target    VETRQYRNVVRTWAELQQTLHPLQERDPAFRFVFQTPKYRWGAHSTAVDADWISMLFGPFGDPYRRDPRMPWTGEAYLEI 7dxv.1    -------------------------------------------------------------------------GKRIVRM  target    NPKDAAELGLADGDYAWVDADPEDRPYRGWKEDDPYYEVARAMMRVRIYTGMSRGVIRTWFNMYAATPATVANQKATPGN 7dxv.1    DKAERAKLGVSVGDVVEVK-------------------------------------------------------------  target    PARNEQTRYVALFRYGSHQSGTRAWLRPTQQTDSLVRKGYFGQVIGTGFEADVHSVSGAPKEAFVKIEKAEDGGIGAERL 7dxv.1    --------------------------------------------------------------------------------  target    WRPLTLGLRPEAPSAALTAYLAGDYSGTKGS 7dxv.1    ------------------------------- ``` | | | | | | | | | | | | | | | | | | | | | | | | | | | | | | | | | | | | | | | | | | | | | | | | | |
|  | 7dxv.1.B | mk2h\_dY protein  *Crystal structure of the mk2h\_deltaY peptide homodimer* | 0.00 |  | 26.92 | 0.03 | 634-659 | X-ray | 2.30 | homo-dimer |  | HHblits | 0.35 |
| ``` target    MPTANKADEVIILRPGTDAAFFLGVARELIEKGLYDRAAVIERTDLPLLVRLDTGERLDARDVIPGYELAALTNYVTLKP 7dxv.1    --------------------------------------------------------------------------------  target    DAEIKGNPPPPPFTAGGQVVPTELRDAWGDFVWWDRATGRPRPVSRDEVGARFDGDPALLGEFEVELVDGSTVPVRPAFD 7dxv.1    --------------------------------------------------------------------------------  target    LLKQYLDESFDLRTASEVCRVPPQAIQSIARQLAANKRETLLAAGMGPNHYFQNDLFGRVQFLVAALTDNIGHLGGNVGS 7dxv.1    --------------------------------------------------------------------------------  target    YAGNYRGSVFQAMGQWIAEDPFAIEPDLTKPATVKRYYKAESAHYWNYGERPLRAVAKDDEGDLTKGEVLTGKSHMPTPT 7dxv.1    --------------------------------------------------------------------------------  target    KLIWFGNSNSLLGNAKWSFDVVKNTLPRQDAVFCNEWHWTSSCEYADLVFPADSWAEFKLPDATASCTNPFLLAFPTTPL 7dxv.1    --------------------------------------------------------------------------------  target    KRLYDTRSDYEALALTAKALGELIDEPRMEQYWRGILDGDPTPYLQRIFSGSNATRGITYDELHESSKRGVPLLMNMRTY 7dxv.1    --------------------------------------------------------------------------------  target    PRSGGWEQRQEDKPWYTATGRLEFYRPEPEFQAAGESLPVWREPVDATFYEPNAILSNAAHPSIAPRAPEDYGVPESQLD 7dxv.1    --------------------------------------------------------------------------------  target    VETRQYRNVVRTWAELQQTLHPLQERDPAFRFVFQTPKYRWGAHSTAVDADWISMLFGPFGDPYRRDPRMPWTGEAYLEI 7dxv.1    -------------------------------------------------------------------------GKRIVRM  target    NPKDAAELGLADGDYAWVDADPEDRPYRGWKEDDPYYEVARAMMRVRIYTGMSRGVIRTWFNMYAATPATVANQKATPGN 7dxv.1    DKAERAKLGVSVGDVVEVK-------------------------------------------------------------  target    PARNEQTRYVALFRYGSHQSGTRAWLRPTQQTDSLVRKGYFGQVIGTGFEADVHSVSGAPKEAFVKIEKAEDGGIGAERL 7dxv.1    --------------------------------------------------------------------------------  target    WRPLTLGLRPEAPSAALTAYLAGDYSGTKGS 7dxv.1    ------------------------------- ``` | | | | | | | | | | | | | | | | | | | | | | | | | | | | | | | | | | | | | | | | | | | | | | | | | |
|  | 7dvf.1.A | reDPBB\_sym2 protein  *Crystal structure of the computationally designed reDPBB\_sym2 protein* | 0.00 |  | 14.81 | 0.03 | 635-661 | X-ray | 1.21 | monomer |  | HHblits | 0.32 |
| ``` target    MPTANKADEVIILRPGTDAAFFLGVARELIEKGLYDRAAVIERTDLPLLVRLDTGERLDARDVIPGYELAALTNYVTLKP 7dvf.1    --------------------------------------------------------------------------------  target    DAEIKGNPPPPPFTAGGQVVPTELRDAWGDFVWWDRATGRPRPVSRDEVGARFDGDPALLGEFEVELVDGSTVPVRPAFD 7dvf.1    --------------------------------------------------------------------------------  target    LLKQYLDESFDLRTASEVCRVPPQAIQSIARQLAANKRETLLAAGMGPNHYFQNDLFGRVQFLVAALTDNIGHLGGNVGS 7dvf.1    --------------------------------------------------------------------------------  target    YAGNYRGSVFQAMGQWIAEDPFAIEPDLTKPATVKRYYKAESAHYWNYGERPLRAVAKDDEGDLTKGEVLTGKSHMPTPT 7dvf.1    --------------------------------------------------------------------------------  target    KLIWFGNSNSLLGNAKWSFDVVKNTLPRQDAVFCNEWHWTSSCEYADLVFPADSWAEFKLPDATASCTNPFLLAFPTTPL 7dvf.1    --------------------------------------------------------------------------------  target    KRLYDTRSDYEALALTAKALGELIDEPRMEQYWRGILDGDPTPYLQRIFSGSNATRGITYDELHESSKRGVPLLMNMRTY 7dvf.1    --------------------------------------------------------------------------------  target    PRSGGWEQRQEDKPWYTATGRLEFYRPEPEFQAAGESLPVWREPVDATFYEPNAILSNAAHPSIAPRAPEDYGVPESQLD 7dvf.1    --------------------------------------------------------------------------------  target    VETRQYRNVVRTWAELQQTLHPLQERDPAFRFVFQTPKYRWGAHSTAVDADWISMLFGPFGDPYRRDPRMPWTGEAYLEI 7dvf.1    --------------------------------------------------------------------------KGIVRM  target    NPKDAAELGLADGDYAWVDADPEDRPYRGWKEDDPYYEVARAMMRVRIYTGMSRGVIRTWFNMYAATPATVANQKATPGN 7dvf.1    DKASREKLGVSAGDLVEIKGS-----------------------------------------------------------  target    PARNEQTRYVALFRYGSHQSGTRAWLRPTQQTDSLVRKGYFGQVIGTGFEADVHSVSGAPKEAFVKIEKAEDGGIGAERL 7dvf.1    --------------------------------------------------------------------------------  target    WRPLTLGLRPEAPSAALTAYLAGDYSGTKGS 7dvf.1    ------------------------------- ``` | | | | | | | | | | | | | | | | | | | | | | | | | | | | | | | | | | | | | | | | | | | | | | | | | |
|  | 7dvc.1.A | reDPBB\_sym1 protein  *Crystal structure of the computationally designed reDPBB\_sym1 protein* | 0.00 |  | 14.81 | 0.03 | 635-661 | X-ray | 1.71 | monomer |  | HHblits | 0.31 |
| ``` target    MPTANKADEVIILRPGTDAAFFLGVARELIEKGLYDRAAVIERTDLPLLVRLDTGERLDARDVIPGYELAALTNYVTLKP 7dvc.1    --------------------------------------------------------------------------------  target    DAEIKGNPPPPPFTAGGQVVPTELRDAWGDFVWWDRATGRPRPVSRDEVGARFDGDPALLGEFEVELVDGSTVPVRPAFD 7dvc.1    --------------------------------------------------------------------------------  target    LLKQYLDESFDLRTASEVCRVPPQAIQSIARQLAANKRETLLAAGMGPNHYFQNDLFGRVQFLVAALTDNIGHLGGNVGS 7dvc.1    --------------------------------------------------------------------------------  target    YAGNYRGSVFQAMGQWIAEDPFAIEPDLTKPATVKRYYKAESAHYWNYGERPLRAVAKDDEGDLTKGEVLTGKSHMPTPT 7dvc.1    --------------------------------------------------------------------------------  target    KLIWFGNSNSLLGNAKWSFDVVKNTLPRQDAVFCNEWHWTSSCEYADLVFPADSWAEFKLPDATASCTNPFLLAFPTTPL 7dvc.1    --------------------------------------------------------------------------------  target    KRLYDTRSDYEALALTAKALGELIDEPRMEQYWRGILDGDPTPYLQRIFSGSNATRGITYDELHESSKRGVPLLMNMRTY 7dvc.1    --------------------------------------------------------------------------------  target    PRSGGWEQRQEDKPWYTATGRLEFYRPEPEFQAAGESLPVWREPVDATFYEPNAILSNAAHPSIAPRAPEDYGVPESQLD 7dvc.1    --------------------------------------------------------------------------------  target    VETRQYRNVVRTWAELQQTLHPLQERDPAFRFVFQTPKYRWGAHSTAVDADWISMLFGPFGDPYRRDPRMPWTGEAYLEI 7dvc.1    --------------------------------------------------------------------------KGIVRM  target    NPKDAAELGLADGDYAWVDADPEDRPYRGWKEDDPYYEVARAMMRVRIYTGMSRGVIRTWFNMYAATPATVANQKATPGN 7dvc.1    DKASRDKLGVSAGDLVEIKGS-----------------------------------------------------------  target    PARNEQTRYVALFRYGSHQSGTRAWLRPTQQTDSLVRKGYFGQVIGTGFEADVHSVSGAPKEAFVKIEKAEDGGIGAERL 7dvc.1    --------------------------------------------------------------------------------  target    WRPLTLGLRPEAPSAALTAYLAGDYSGTKGS 7dvc.1    ------------------------------- ``` | | | | | | | | | | | | | | | | | | | | | | | | | | | | | | | | | | | | | | | | | | | | | | | | | |
|  | 7dvc.5.A | reDPBB\_sym1 protein  *Crystal structure of the computationally designed reDPBB\_sym1 protein* | 0.00 |  | 14.81 | 0.03 | 635-661 | X-ray | 1.71 | monomer |  | HHblits | 0.31 |
| ``` target    MPTANKADEVIILRPGTDAAFFLGVARELIEKGLYDRAAVIERTDLPLLVRLDTGERLDARDVIPGYELAALTNYVTLKP 7dvc.5    --------------------------------------------------------------------------------  target    DAEIKGNPPPPPFTAGGQVVPTELRDAWGDFVWWDRATGRPRPVSRDEVGARFDGDPALLGEFEVELVDGSTVPVRPAFD 7dvc.5    --------------------------------------------------------------------------------  target    LLKQYLDESFDLRTASEVCRVPPQAIQSIARQLAANKRETLLAAGMGPNHYFQNDLFGRVQFLVAALTDNIGHLGGNVGS 7dvc.5    --------------------------------------------------------------------------------  target    YAGNYRGSVFQAMGQWIAEDPFAIEPDLTKPATVKRYYKAESAHYWNYGERPLRAVAKDDEGDLTKGEVLTGKSHMPTPT 7dvc.5    --------------------------------------------------------------------------------  target    KLIWFGNSNSLLGNAKWSFDVVKNTLPRQDAVFCNEWHWTSSCEYADLVFPADSWAEFKLPDATASCTNPFLLAFPTTPL 7dvc.5    --------------------------------------------------------------------------------  target    KRLYDTRSDYEALALTAKALGELIDEPRMEQYWRGILDGDPTPYLQRIFSGSNATRGITYDELHESSKRGVPLLMNMRTY 7dvc.5    --------------------------------------------------------------------------------  target    PRSGGWEQRQEDKPWYTATGRLEFYRPEPEFQAAGESLPVWREPVDATFYEPNAILSNAAHPSIAPRAPEDYGVPESQLD 7dvc.5    --------------------------------------------------------------------------------  target    VETRQYRNVVRTWAELQQTLHPLQERDPAFRFVFQTPKYRWGAHSTAVDADWISMLFGPFGDPYRRDPRMPWTGEAYLEI 7dvc.5    --------------------------------------------------------------------------KGIVRM  target    NPKDAAELGLADGDYAWVDADPEDRPYRGWKEDDPYYEVARAMMRVRIYTGMSRGVIRTWFNMYAATPATVANQKATPGN 7dvc.5    DKASRDKLGVSAGDLVEIKGS-----------------------------------------------------------  target    PARNEQTRYVALFRYGSHQSGTRAWLRPTQQTDSLVRKGYFGQVIGTGFEADVHSVSGAPKEAFVKIEKAEDGGIGAERL 7dvc.5    --------------------------------------------------------------------------------  target    WRPLTLGLRPEAPSAALTAYLAGDYSGTKGS 7dvc.5    ------------------------------- ``` | | | | | | | | | | | | | | | | | | | | | | | | | | | | | | | | | | | | | | | | | | | | | | | | | |
|  | 7dxz.1.A | mk2h\_deltaMILPYS protein  *Crystal structure of the chemically synthesized mk2h\_deltaMILPYS peptide homodimer in complex with malonate* | 0.00 |  | 23.08 | 0.03 | 634-659 | X-ray | 1.90 | homo-dimer | 4 x MLA | HHblits | 0.34 |
| ``` target    MPTANKADEVIILRPGTDAAFFLGVARELIEKGLYDRAAVIERTDLPLLVRLDTGERLDARDVIPGYELAALTNYVTLKP 7dxz.1    --------------------------------------------------------------------------------  target    DAEIKGNPPPPPFTAGGQVVPTELRDAWGDFVWWDRATGRPRPVSRDEVGARFDGDPALLGEFEVELVDGSTVPVRPAFD 7dxz.1    --------------------------------------------------------------------------------  target    LLKQYLDESFDLRTASEVCRVPPQAIQSIARQLAANKRETLLAAGMGPNHYFQNDLFGRVQFLVAALTDNIGHLGGNVGS 7dxz.1    --------------------------------------------------------------------------------  target    YAGNYRGSVFQAMGQWIAEDPFAIEPDLTKPATVKRYYKAESAHYWNYGERPLRAVAKDDEGDLTKGEVLTGKSHMPTPT 7dxz.1    --------------------------------------------------------------------------------  target    KLIWFGNSNSLLGNAKWSFDVVKNTLPRQDAVFCNEWHWTSSCEYADLVFPADSWAEFKLPDATASCTNPFLLAFPTTPL 7dxz.1    --------------------------------------------------------------------------------  target    KRLYDTRSDYEALALTAKALGELIDEPRMEQYWRGILDGDPTPYLQRIFSGSNATRGITYDELHESSKRGVPLLMNMRTY 7dxz.1    --------------------------------------------------------------------------------  target    PRSGGWEQRQEDKPWYTATGRLEFYRPEPEFQAAGESLPVWREPVDATFYEPNAILSNAAHPSIAPRAPEDYGVPESQLD 7dxz.1    --------------------------------------------------------------------------------  target    VETRQYRNVVRTWAELQQTLHPLQERDPAFRFVFQTPKYRWGAHSTAVDADWISMLFGPFGDPYRRDPRMPWTGEAYLEI 7dxz.1    -------------------------------------------------------------------------GKRVVRV  target    NPKDAAELGLADGDYAWVDADPEDRPYRGWKEDDPYYEVARAMMRVRIYTGMSRGVIRTWFNMYAATPATVANQKATPGN 7dxz.1    DKAERAKVGVKVGDVVEVK-------------------------------------------------------------  target    PARNEQTRYVALFRYGSHQSGTRAWLRPTQQTDSLVRKGYFGQVIGTGFEADVHSVSGAPKEAFVKIEKAEDGGIGAERL 7dxz.1    --------------------------------------------------------------------------------  target    WRPLTLGLRPEAPSAALTAYLAGDYSGTKGS 7dxz.1    ------------------------------- ``` | | | | | | | | | | | | | | | | | | | | | | | | | | | | | | | | | | | | | | | | | | | | | | | | | |
|  | 7dxz.2.A | mk2h\_deltaMILPYS protein  *Crystal structure of the chemically synthesized mk2h\_deltaMILPYS peptide homodimer in complex with malonate* | 0.00 |  | 23.08 | 0.03 | 634-659 | X-ray | 1.90 | homo-dimer | 3 x MLA | HHblits | 0.34 |
| ``` target    MPTANKADEVIILRPGTDAAFFLGVARELIEKGLYDRAAVIERTDLPLLVRLDTGERLDARDVIPGYELAALTNYVTLKP 7dxz.2    --------------------------------------------------------------------------------  target    DAEIKGNPPPPPFTAGGQVVPTELRDAWGDFVWWDRATGRPRPVSRDEVGARFDGDPALLGEFEVELVDGSTVPVRPAFD 7dxz.2    --------------------------------------------------------------------------------  target    LLKQYLDESFDLRTASEVCRVPPQAIQSIARQLAANKRETLLAAGMGPNHYFQNDLFGRVQFLVAALTDNIGHLGGNVGS 7dxz.2    --------------------------------------------------------------------------------  target    YAGNYRGSVFQAMGQWIAEDPFAIEPDLTKPATVKRYYKAESAHYWNYGERPLRAVAKDDEGDLTKGEVLTGKSHMPTPT 7dxz.2    --------------------------------------------------------------------------------  target    KLIWFGNSNSLLGNAKWSFDVVKNTLPRQDAVFCNEWHWTSSCEYADLVFPADSWAEFKLPDATASCTNPFLLAFPTTPL 7dxz.2    --------------------------------------------------------------------------------  target    KRLYDTRSDYEALALTAKALGELIDEPRMEQYWRGILDGDPTPYLQRIFSGSNATRGITYDELHESSKRGVPLLMNMRTY 7dxz.2    --------------------------------------------------------------------------------  target    PRSGGWEQRQEDKPWYTATGRLEFYRPEPEFQAAGESLPVWREPVDATFYEPNAILSNAAHPSIAPRAPEDYGVPESQLD 7dxz.2    --------------------------------------------------------------------------------  target    VETRQYRNVVRTWAELQQTLHPLQERDPAFRFVFQTPKYRWGAHSTAVDADWISMLFGPFGDPYRRDPRMPWTGEAYLEI 7dxz.2    -------------------------------------------------------------------------GKRVVRV  target    NPKDAAELGLADGDYAWVDADPEDRPYRGWKEDDPYYEVARAMMRVRIYTGMSRGVIRTWFNMYAATPATVANQKATPGN 7dxz.2    DKAERAKVGVKVGDVVEVK-------------------------------------------------------------  target    PARNEQTRYVALFRYGSHQSGTRAWLRPTQQTDSLVRKGYFGQVIGTGFEADVHSVSGAPKEAFVKIEKAEDGGIGAERL 7dxz.2    --------------------------------------------------------------------------------  target    WRPLTLGLRPEAPSAALTAYLAGDYSGTKGS 7dxz.2    ------------------------------- ``` | | | | | | | | | | | | | | | | | | | | | | | | | | | | | | | | | | | | | | | | | | | | | | | | | |
|  | 7dxz.2.B | mk2h\_deltaMILPYS protein  *Crystal structure of the chemically synthesized mk2h\_deltaMILPYS peptide homodimer in complex with malonate* | 0.00 |  | 23.08 | 0.03 | 634-659 | X-ray | 1.90 | homo-dimer | 3 x MLA | HHblits | 0.34 |
| ``` target    MPTANKADEVIILRPGTDAAFFLGVARELIEKGLYDRAAVIERTDLPLLVRLDTGERLDARDVIPGYELAALTNYVTLKP 7dxz.2    --------------------------------------------------------------------------------  target    DAEIKGNPPPPPFTAGGQVVPTELRDAWGDFVWWDRATGRPRPVSRDEVGARFDGDPALLGEFEVELVDGSTVPVRPAFD 7dxz.2    --------------------------------------------------------------------------------  target    LLKQYLDESFDLRTASEVCRVPPQAIQSIARQLAANKRETLLAAGMGPNHYFQNDLFGRVQFLVAALTDNIGHLGGNVGS 7dxz.2    --------------------------------------------------------------------------------  target    YAGNYRGSVFQAMGQWIAEDPFAIEPDLTKPATVKRYYKAESAHYWNYGERPLRAVAKDDEGDLTKGEVLTGKSHMPTPT 7dxz.2    --------------------------------------------------------------------------------  target    KLIWFGNSNSLLGNAKWSFDVVKNTLPRQDAVFCNEWHWTSSCEYADLVFPADSWAEFKLPDATASCTNPFLLAFPTTPL 7dxz.2    --------------------------------------------------------------------------------  target    KRLYDTRSDYEALALTAKALGELIDEPRMEQYWRGILDGDPTPYLQRIFSGSNATRGITYDELHESSKRGVPLLMNMRTY 7dxz.2    --------------------------------------------------------------------------------  target    PRSGGWEQRQEDKPWYTATGRLEFYRPEPEFQAAGESLPVWREPVDATFYEPNAILSNAAHPSIAPRAPEDYGVPESQLD 7dxz.2    --------------------------------------------------------------------------------  target    VETRQYRNVVRTWAELQQTLHPLQERDPAFRFVFQTPKYRWGAHSTAVDADWISMLFGPFGDPYRRDPRMPWTGEAYLEI 7dxz.2    -------------------------------------------------------------------------GKRVVRV  target    NPKDAAELGLADGDYAWVDADPEDRPYRGWKEDDPYYEVARAMMRVRIYTGMSRGVIRTWFNMYAATPATVANQKATPGN 7dxz.2    DKAERAKVGVKVGDVVEVK-------------------------------------------------------------  target    PARNEQTRYVALFRYGSHQSGTRAWLRPTQQTDSLVRKGYFGQVIGTGFEADVHSVSGAPKEAFVKIEKAEDGGIGAERL 7dxz.2    --------------------------------------------------------------------------------  target    WRPLTLGLRPEAPSAALTAYLAGDYSGTKGS 7dxz.2    ------------------------------- ``` | | | | | | | | | | | | | | | | | | | | | | | | | | | | | | | | | | | | | | | | | | | | | | | | | |
|  | 7dxz.3.A | mk2h\_deltaMILPYS protein  *Crystal structure of the chemically synthesized mk2h\_deltaMILPYS peptide homodimer in complex with malonate* | 0.00 |  | 23.08 | 0.03 | 634-659 | X-ray | 1.90 | homo-dimer | 4 x MLA | HHblits | 0.34 |
| ``` target    MPTANKADEVIILRPGTDAAFFLGVARELIEKGLYDRAAVIERTDLPLLVRLDTGERLDARDVIPGYELAALTNYVTLKP 7dxz.3    --------------------------------------------------------------------------------  target    DAEIKGNPPPPPFTAGGQVVPTELRDAWGDFVWWDRATGRPRPVSRDEVGARFDGDPALLGEFEVELVDGSTVPVRPAFD 7dxz.3    --------------------------------------------------------------------------------  target    LLKQYLDESFDLRTASEVCRVPPQAIQSIARQLAANKRETLLAAGMGPNHYFQNDLFGRVQFLVAALTDNIGHLGGNVGS 7dxz.3    --------------------------------------------------------------------------------  target    YAGNYRGSVFQAMGQWIAEDPFAIEPDLTKPATVKRYYKAESAHYWNYGERPLRAVAKDDEGDLTKGEVLTGKSHMPTPT 7dxz.3    --------------------------------------------------------------------------------  target    KLIWFGNSNSLLGNAKWSFDVVKNTLPRQDAVFCNEWHWTSSCEYADLVFPADSWAEFKLPDATASCTNPFLLAFPTTPL 7dxz.3    --------------------------------------------------------------------------------  target    KRLYDTRSDYEALALTAKALGELIDEPRMEQYWRGILDGDPTPYLQRIFSGSNATRGITYDELHESSKRGVPLLMNMRTY 7dxz.3    --------------------------------------------------------------------------------  target    PRSGGWEQRQEDKPWYTATGRLEFYRPEPEFQAAGESLPVWREPVDATFYEPNAILSNAAHPSIAPRAPEDYGVPESQLD 7dxz.3    --------------------------------------------------------------------------------  target    VETRQYRNVVRTWAELQQTLHPLQERDPAFRFVFQTPKYRWGAHSTAVDADWISMLFGPFGDPYRRDPRMPWTGEAYLEI 7dxz.3    -------------------------------------------------------------------------GKRVVRV  target    NPKDAAELGLADGDYAWVDADPEDRPYRGWKEDDPYYEVARAMMRVRIYTGMSRGVIRTWFNMYAATPATVANQKATPGN 7dxz.3    DKAERAKVGVKVGDVVEVK-------------------------------------------------------------  target    PARNEQTRYVALFRYGSHQSGTRAWLRPTQQTDSLVRKGYFGQVIGTGFEADVHSVSGAPKEAFVKIEKAEDGGIGAERL 7dxz.3    --------------------------------------------------------------------------------  target    WRPLTLGLRPEAPSAALTAYLAGDYSGTKGS 7dxz.3    ------------------------------- ``` | | | | | | | | | | | | | | | | | | | | | | | | | | | | | | | | | | | | | | | | | | | | | | | | | |
|  | 7dyc.1.A | mk2h\_deltaMILPYS protein  *Crystal structure of the chemically synthesized mk2h\_deltaMILPYS peptide homodimer in complex with malate* | 0.00 |  | 23.08 | 0.03 | 634-659 | X-ray | 2.30 | homo-dimer | 2 x MLT | HHblits | 0.34 |
| ``` target    MPTANKADEVIILRPGTDAAFFLGVARELIEKGLYDRAAVIERTDLPLLVRLDTGERLDARDVIPGYELAALTNYVTLKP 7dyc.1    --------------------------------------------------------------------------------  target    DAEIKGNPPPPPFTAGGQVVPTELRDAWGDFVWWDRATGRPRPVSRDEVGARFDGDPALLGEFEVELVDGSTVPVRPAFD 7dyc.1    --------------------------------------------------------------------------------  target    LLKQYLDESFDLRTASEVCRVPPQAIQSIARQLAANKRETLLAAGMGPNHYFQNDLFGRVQFLVAALTDNIGHLGGNVGS 7dyc.1    --------------------------------------------------------------------------------  target    YAGNYRGSVFQAMGQWIAEDPFAIEPDLTKPATVKRYYKAESAHYWNYGERPLRAVAKDDEGDLTKGEVLTGKSHMPTPT 7dyc.1    --------------------------------------------------------------------------------  target    KLIWFGNSNSLLGNAKWSFDVVKNTLPRQDAVFCNEWHWTSSCEYADLVFPADSWAEFKLPDATASCTNPFLLAFPTTPL 7dyc.1    --------------------------------------------------------------------------------  target    KRLYDTRSDYEALALTAKALGELIDEPRMEQYWRGILDGDPTPYLQRIFSGSNATRGITYDELHESSKRGVPLLMNMRTY 7dyc.1    --------------------------------------------------------------------------------  target    PRSGGWEQRQEDKPWYTATGRLEFYRPEPEFQAAGESLPVWREPVDATFYEPNAILSNAAHPSIAPRAPEDYGVPESQLD 7dyc.1    --------------------------------------------------------------------------------  target    VETRQYRNVVRTWAELQQTLHPLQERDPAFRFVFQTPKYRWGAHSTAVDADWISMLFGPFGDPYRRDPRMPWTGEAYLEI 7dyc.1    -------------------------------------------------------------------------GKRVVRV  target    NPKDAAELGLADGDYAWVDADPEDRPYRGWKEDDPYYEVARAMMRVRIYTGMSRGVIRTWFNMYAATPATVANQKATPGN 7dyc.1    DKAERAKVGVKVGDVVEVK-------------------------------------------------------------  target    PARNEQTRYVALFRYGSHQSGTRAWLRPTQQTDSLVRKGYFGQVIGTGFEADVHSVSGAPKEAFVKIEKAEDGGIGAERL 7dyc.1    --------------------------------------------------------------------------------  target    WRPLTLGLRPEAPSAALTAYLAGDYSGTKGS 7dyc.1    ------------------------------- ``` | | | | | | | | | | | | | | | | | | | | | | | | | | | | | | | | | | | | | | | | | | | | | | | | | |
|  | 7dyc.2.A | mk2h\_deltaMILPYS protein  *Crystal structure of the chemically synthesized mk2h\_deltaMILPYS peptide homodimer in complex with malate* | 0.00 |  | 23.08 | 0.03 | 634-659 | X-ray | 2.30 | homo-dimer |  | HHblits | 0.34 |
| ``` target    MPTANKADEVIILRPGTDAAFFLGVARELIEKGLYDRAAVIERTDLPLLVRLDTGERLDARDVIPGYELAALTNYVTLKP 7dyc.2    --------------------------------------------------------------------------------  target    DAEIKGNPPPPPFTAGGQVVPTELRDAWGDFVWWDRATGRPRPVSRDEVGARFDGDPALLGEFEVELVDGSTVPVRPAFD 7dyc.2    --------------------------------------------------------------------------------  target    LLKQYLDESFDLRTASEVCRVPPQAIQSIARQLAANKRETLLAAGMGPNHYFQNDLFGRVQFLVAALTDNIGHLGGNVGS 7dyc.2    --------------------------------------------------------------------------------  target    YAGNYRGSVFQAMGQWIAEDPFAIEPDLTKPATVKRYYKAESAHYWNYGERPLRAVAKDDEGDLTKGEVLTGKSHMPTPT 7dyc.2    --------------------------------------------------------------------------------  target    KLIWFGNSNSLLGNAKWSFDVVKNTLPRQDAVFCNEWHWTSSCEYADLVFPADSWAEFKLPDATASCTNPFLLAFPTTPL 7dyc.2    --------------------------------------------------------------------------------  target    KRLYDTRSDYEALALTAKALGELIDEPRMEQYWRGILDGDPTPYLQRIFSGSNATRGITYDELHESSKRGVPLLMNMRTY 7dyc.2    --------------------------------------------------------------------------------  target    PRSGGWEQRQEDKPWYTATGRLEFYRPEPEFQAAGESLPVWREPVDATFYEPNAILSNAAHPSIAPRAPEDYGVPESQLD 7dyc.2    --------------------------------------------------------------------------------  target    VETRQYRNVVRTWAELQQTLHPLQERDPAFRFVFQTPKYRWGAHSTAVDADWISMLFGPFGDPYRRDPRMPWTGEAYLEI 7dyc.2    -------------------------------------------------------------------------GKRVVRV  target    NPKDAAELGLADGDYAWVDADPEDRPYRGWKEDDPYYEVARAMMRVRIYTGMSRGVIRTWFNMYAATPATVANQKATPGN 7dyc.2    DKAERAKVGVKVGDVVEVK-------------------------------------------------------------  target    PARNEQTRYVALFRYGSHQSGTRAWLRPTQQTDSLVRKGYFGQVIGTGFEADVHSVSGAPKEAFVKIEKAEDGGIGAERL 7dyc.2    --------------------------------------------------------------------------------  target    WRPLTLGLRPEAPSAALTAYLAGDYSGTKGS 7dyc.2    ------------------------------- ``` | | | | | | | | | | | | | | | | | | | | | | | | | | | | | | | | | | | | | | | | | | | | | | | | | |
|  | 7dyc.3.A | mk2h\_deltaMILPYS protein  *Crystal structure of the chemically synthesized mk2h\_deltaMILPYS peptide homodimer in complex with malate* | 0.00 |  | 23.08 | 0.03 | 634-659 | X-ray | 2.30 | homo-dimer | 2 x LMR | HHblits | 0.34 |
| ``` target    MPTANKADEVIILRPGTDAAFFLGVARELIEKGLYDRAAVIERTDLPLLVRLDTGERLDARDVIPGYELAALTNYVTLKP 7dyc.3    --------------------------------------------------------------------------------  target    DAEIKGNPPPPPFTAGGQVVPTELRDAWGDFVWWDRATGRPRPVSRDEVGARFDGDPALLGEFEVELVDGSTVPVRPAFD 7dyc.3    --------------------------------------------------------------------------------  target    LLKQYLDESFDLRTASEVCRVPPQAIQSIARQLAANKRETLLAAGMGPNHYFQNDLFGRVQFLVAALTDNIGHLGGNVGS 7dyc.3    --------------------------------------------------------------------------------  target    YAGNYRGSVFQAMGQWIAEDPFAIEPDLTKPATVKRYYKAESAHYWNYGERPLRAVAKDDEGDLTKGEVLTGKSHMPTPT 7dyc.3    --------------------------------------------------------------------------------  target    KLIWFGNSNSLLGNAKWSFDVVKNTLPRQDAVFCNEWHWTSSCEYADLVFPADSWAEFKLPDATASCTNPFLLAFPTTPL 7dyc.3    --------------------------------------------------------------------------------  target    KRLYDTRSDYEALALTAKALGELIDEPRMEQYWRGILDGDPTPYLQRIFSGSNATRGITYDELHESSKRGVPLLMNMRTY 7dyc.3    --------------------------------------------------------------------------------  target    PRSGGWEQRQEDKPWYTATGRLEFYRPEPEFQAAGESLPVWREPVDATFYEPNAILSNAAHPSIAPRAPEDYGVPESQLD 7dyc.3    --------------------------------------------------------------------------------  target    VETRQYRNVVRTWAELQQTLHPLQERDPAFRFVFQTPKYRWGAHSTAVDADWISMLFGPFGDPYRRDPRMPWTGEAYLEI 7dyc.3    -------------------------------------------------------------------------GKRVVRV  target    NPKDAAELGLADGDYAWVDADPEDRPYRGWKEDDPYYEVARAMMRVRIYTGMSRGVIRTWFNMYAATPATVANQKATPGN 7dyc.3    DKAERAKVGVKVGDVVEVK-------------------------------------------------------------  target    PARNEQTRYVALFRYGSHQSGTRAWLRPTQQTDSLVRKGYFGQVIGTGFEADVHSVSGAPKEAFVKIEKAEDGGIGAERL 7dyc.3    --------------------------------------------------------------------------------  target    WRPLTLGLRPEAPSAALTAYLAGDYSGTKGS 7dyc.3    ------------------------------- ``` | | | | | | | | | | | | | | | | | | | | | | | | | | | | | | | | | | | | | | | | | | | | | | | | | |
|  | 1pt1.1.A | Aspartate 1-decarboxylase  *Unprocessed Pyruvoyl Dependent Aspartate Decarboxylase with Histidine 11 Mutated to Alanine* | 0.00 |  | 10.34 | 0.03 | 635-663 | X-ray | 1.90 | homo-tetramer |  | HHblits | 0.25 |
| ``` target    MPTANKADEVIILRPGTDAAFFLGVARELIEKGLYDRAAVIERTDLPLLVRLDTGERLDARDVIPGYELAALTNYVTLKP 1pt1.1    --------------------------------------------------------------------------------  target    DAEIKGNPPPPPFTAGGQVVPTELRDAWGDFVWWDRATGRPRPVSRDEVGARFDGDPALLGEFEVELVDGSTVPVRPAFD 1pt1.1    --------------------------------------------------------------------------------  target    LLKQYLDESFDLRTASEVCRVPPQAIQSIARQLAANKRETLLAAGMGPNHYFQNDLFGRVQFLVAALTDNIGHLGGNVGS 1pt1.1    --------------------------------------------------------------------------------  target    YAGNYRGSVFQAMGQWIAEDPFAIEPDLTKPATVKRYYKAESAHYWNYGERPLRAVAKDDEGDLTKGEVLTGKSHMPTPT 1pt1.1    --------------------------------------------------------------------------------  target    KLIWFGNSNSLLGNAKWSFDVVKNTLPRQDAVFCNEWHWTSSCEYADLVFPADSWAEFKLPDATASCTNPFLLAFPTTPL 1pt1.1    --------------------------------------------------------------------------------  target    KRLYDTRSDYEALALTAKALGELIDEPRMEQYWRGILDGDPTPYLQRIFSGSNATRGITYDELHESSKRGVPLLMNMRTY 1pt1.1    --------------------------------------------------------------------------------  target    PRSGGWEQRQEDKPWYTATGRLEFYRPEPEFQAAGESLPVWREPVDATFYEPNAILSNAAHPSIAPRAPEDYGVPESQLD 1pt1.1    --------------------------------------------------------------------------------  target    VETRQYRNVVRTWAELQQTLHPLQERDPAFRFVFQTPKYRWGAHSTAVDADWISMLFGPFGDPYRRDPRMPWTGEAYLEI 1pt1.1    --------------------------------------------------------------------------EGSCAI  target    NPKDAAELGLADGDYAWVDADPEDRPYRGWKEDDPYYEVARAMMRVRIYTGMSRGVIRTWFNMYAATPATVANQKATPGN 1pt1.1    DQDFLDAAGILENEAIDIWNVTN---------------------------------------------------------  target    PARNEQTRYVALFRYGSHQSGTRAWLRPTQQTDSLVRKGYFGQVIGTGFEADVHSVSGAPKEAFVKIEKAEDGGIGAERL 1pt1.1    --------------------------------------------------------------------------------  target    WRPLTLGLRPEAPSAALTAYLAGDYSGTKGS 1pt1.1    ------------------------------- ``` | | | | | | | | | | | | | | | | | | | | | | | | | | | | | | | | | | | | | | | | | | | | | | | | | |
|  | 7di1.1.A | mkDPBB\_sym\_86 protein  *Crystal structure of the rationally designed mkDPBB\_sym\_86 protein* | 0.00 |  | 28.00 | 0.03 | 635-659 | X-ray | 2.10 | monomer |  | HHblits | 0.36 |
| ``` target    MPTANKADEVIILRPGTDAAFFLGVARELIEKGLYDRAAVIERTDLPLLVRLDTGERLDARDVIPGYELAALTNYVTLKP 7di1.1    --------------------------------------------------------------------------------  target    DAEIKGNPPPPPFTAGGQVVPTELRDAWGDFVWWDRATGRPRPVSRDEVGARFDGDPALLGEFEVELVDGSTVPVRPAFD 7di1.1    --------------------------------------------------------------------------------  target    LLKQYLDESFDLRTASEVCRVPPQAIQSIARQLAANKRETLLAAGMGPNHYFQNDLFGRVQFLVAALTDNIGHLGGNVGS 7di1.1    --------------------------------------------------------------------------------  target    YAGNYRGSVFQAMGQWIAEDPFAIEPDLTKPATVKRYYKAESAHYWNYGERPLRAVAKDDEGDLTKGEVLTGKSHMPTPT 7di1.1    --------------------------------------------------------------------------------  target    KLIWFGNSNSLLGNAKWSFDVVKNTLPRQDAVFCNEWHWTSSCEYADLVFPADSWAEFKLPDATASCTNPFLLAFPTTPL 7di1.1    --------------------------------------------------------------------------------  target    KRLYDTRSDYEALALTAKALGELIDEPRMEQYWRGILDGDPTPYLQRIFSGSNATRGITYDELHESSKRGVPLLMNMRTY 7di1.1    --------------------------------------------------------------------------------  target    PRSGGWEQRQEDKPWYTATGRLEFYRPEPEFQAAGESLPVWREPVDATFYEPNAILSNAAHPSIAPRAPEDYGVPESQLD 7di1.1    --------------------------------------------------------------------------------  target    VETRQYRNVVRTWAELQQTLHPLQERDPAFRFVFQTPKYRWGAHSTAVDADWISMLFGPFGDPYRRDPRMPWTGEAYLEI 7di1.1    --------------------------------------------------------------------------KGIVRM  target    NPKDAAELGLADGDYAWVDADPEDRPYRGWKEDDPYYEVARAMMRVRIYTGMSRGVIRTWFNMYAATPATVANQKATPGN 7di1.1    DKYERAKLGVSVGDYVEVK-------------------------------------------------------------  target    PARNEQTRYVALFRYGSHQSGTRAWLRPTQQTDSLVRKGYFGQVIGTGFEADVHSVSGAPKEAFVKIEKAEDGGIGAERL 7di1.1    --------------------------------------------------------------------------------  target    WRPLTLGLRPEAPSAALTAYLAGDYSGTKGS 7di1.1    ------------------------------- ``` | | | | | | | | | | | | | | | | | | | | | | | | | | | | | | | | | | | | | | | | | | | | | | | | | |
|  | 3qc8.1.A | Transitional endoplasmic reticulum ATPase  *Crystal Structure of FAF1 UBX Domain In Complex with p97/VCP N Domain Reveals The Conserved FcisP Touch-Turn Motif of UBX Domain Suffering Conformational Change* | 0.00 |  | 18.52 | 0.03 | 635-661 | X-ray | 2.20 | hetero-oligomer |  | HHblits | 0.30 |
[truncated: 171,114 more chars]
