# Supplementary material for: Metabolic capacity is maintained despite shifts in microbial diversity in estuary sediments
Source: ISME Commun. 2025 Oct 11;5(1):ycaf182. doi: 10.1093/ismeco/ycaf182 (PMC12687941; doi:10.1093/ismeco/ycaf182)
Supplement: Supplementary_Data_1_ycaf182 [file supplementary_data_1_ycaf182.zip › SWISS-MODEL/4_1_Jan_SF_Bin61_scaffold_1987_c132501_1/report.html]

4\_1\_Jan\_SF\_Bin61\_scaffold\_1987\_c1:3-2501\_1 | Report


|  |  |  |
| --- | --- | --- |
|  |  | SWISS-MODEL Homology Modelling Report |

## Model Building Report

This document lists the results for the homology modelling project "4\_1\_Jan\_SF\_Bin61\_scaffold\_1987\_c1:3-2501\_1" submitted to SWISS-MODEL workspace
on March 29, 2023, 6:30 p.m..The submitted primary amino acid sequence is given in Table T1.

If you use any results in your research, please cite the relevant publications:

- Waterhouse, A., Bertoni, M., Bienert, S., Studer, G., Tauriello, G., Gumienny, R.,
  Heer, F.T., de Beer, T.A.P., Rempfer, C., Bordoli, L., Lepore, R., Schwede, T.
  SWISS-MODEL: homology modelling of protein structures and complexes.
  Nucleic Acids Res. 46(W1), W296-W303 (2018).
- Bienert, S., Waterhouse, A., de Beer, T.A.P., Tauriello, G., Studer,
  G., Bordoli, L., Schwede, T. The SWISS-MODEL Repository - new features and
  functionality. Nucleic Acids Res. 45, D313-D319 (2017).
- Studer, G., Tauriello, G., Bienert, S.,
  Biasini, M., Johner, N., Schwede, T. ProMod3 - A versatile homology
  modelling toolbox. PLOS Comp. Biol. 17(1), e1008667 (2021).
- Studer, G., Rempfer, C., Waterhouse, A.M.,
  Gumienny, G., Haas, J., Schwede, T. QMEANDisCo - distance constraints
  applied on model quality estimation. Bioinformatics 36, 1765-1771 (2020).
- Bertoni, M., Kiefer, F., Biasini, M., Bordoli, L.,
  Schwede, T. Modeling protein quaternary structure of homo- and
  hetero-oligomers beyond binary interactions by homology. Scientific
  Reports 7 (2017).

## Results

The SWISS-MODEL template library (SMTL version 2023-03-23, PDB release 2023-03-17) was searched with
for evolutionary related structures matching the target sequence in Table T1. For details on the template search, see Materials and Methods. Overall 249 templates were found (Table T2).

## Models

The following models were built (see Materials and Methods "Model Building"):

| Model #02 | File | Built with | Oligo-State | Ligands | GMQE | QMEANDisCo Global |
| --- | --- | --- | --- | --- | --- | --- |
|  | PDB | ProMod3 3.2.1 | monomer | None | 0.70 | 0.68 ± 0.05 |

|  |  |  |
| --- | --- | --- |
|  |  |  |

| Template | Seq Identity | Oligo-state | QSQE | Found by | Method | Resolution | Seq Similarity | Range | Coverage | Description |
| --- | --- | --- | --- | --- | --- | --- | --- | --- | --- | --- |
| 7b04.1.B | 37.28 | monomer | 0.00 | HHblits | X-ray | 2.97Å | 0.39 | 1 - 829 | 0.96 | Nitrite oxidoreductase subunit A |

  

### Excluded ligands

| Ligand Name.Number | Reason for Exclusion | Description |
| --- | --- | --- |
| CA.10 | Binding site not conserved. | CALCIUM ION |
| CA.11 | Binding site not conserved. | CALCIUM ION |
| F3S.4 | Binding site not conserved. | FE3-S4 CLUSTER |
| HEM.9 | Binding site not conserved. | PROTOPORPHYRIN IX CONTAINING FE |
| MD1.5 | Binding site not conserved. | PHOSPHORIC ACID 4-(2-AMINO-4-OXO-3,4,5,6,-TETRAHYDRO-PTERIDIN-6-YL)-2-HYDROXY-3,4-DIMERCAPTO-BUT-3-EN-YL ESTER GUANYLATE ESTER |
| MD1.6 | Binding site not conserved. | PHOSPHORIC ACID 4-(2-AMINO-4-OXO-3,4,5,6,-TETRAHYDRO-PTERIDIN-6-YL)-2-HYDROXY-3,4-DIMERCAPTO-BUT-3-EN-YL ESTER GUANYLATE ESTER |
| MO.7 | Binding site not conserved. | MOLYBDENUM ATOM |
| SF4.1 | Binding site not conserved. | IRON/SULFUR CLUSTER |
| SF4.2 | Binding site not conserved. | IRON/SULFUR CLUSTER |
| SF4.3 | Binding site not conserved. | IRON/SULFUR CLUSTER |
| SF4.8 | Binding site not conserved. | IRON/SULFUR CLUSTER |

  

```
Target    MPTANKADEVIILRPGTDAAFFLGVARELIEKGLYDRAAVIERTDLPLLVRLDTGERLDARDVIPGYELAALTNYVTLKP  
7b04.1.B  SPSAQKADYWIPIRNNTDTALFLGITKILIDNKWYDADYVKKFTDFPLLIRTDTLKRVSPKDIIPNYKLQDI--------  
  
Target    DAEIKGNPPPPPFTAGGQVVPTELRDAWGDFVWWDRATGRPRPVSRDEV---GARFDGDPALLGEFEVELVDGSTVPVRP  
7b04.1.B  --S---DG-PSYHIQG---LKDEQREIIGDFVVWDAKSKGPKAITRDDVGETLVKKGIDPVLEGSFKLKTIDGKEIEVMT  
  
Target    AFDLLKQYLDESFDLRTASEVCRVPPQAIQSIARQLAANKRETLLAAGMGPNHYFQNDLFGRVQFLVAALTDNIGHLGGN  
7b04.1.B  LLEMYKIHLR-DYDIDSVVSMTNSPKDLIERLAKDIATIKPVAI-HYGEGVNHYFHATLMNRSYYLPVMLTGNVGYFGSG  
  
Target    VGSYAGNYRGSVFQA---MG-QWIAEDPFA-IEPDLTKPAT------VKRYYKAESAHYWNYGERPLRAVAKDDEGDLTK  
7b04.1.B  SHTWAGNYKAGNFQASKWSGPGFYGWVAEDVFKPNL-DPYASAKDLNIKGRALDEEVAYWNHSERPLI-VNT---P-KYG  
  
Target    GEVLTGKSHMPTPTKLIWFGNSNSLLGNAKWSFDVVKNTLPRQDAVFCNEWHWTSSCEYADLVFPADSWAEFKLPDATAS  
7b04.1.B  RKVFTGKTHMPSPTKVLWFTNVNLINNAKHV-YQMLKNVNPNIEQIMSTDIEITGSIEYADFAFPANSWVEFQEFEITNS  
  
Target    CTNPFLLAFPTTPLKRLYDTRSDYEALALTAKALGELIDEPRMEQYWRGILDGDPTPYLQRIFSGSNATRGITYDELHES  
7b04.1.B  CSNPFIQIWGKTGITPVYESKDDVKILAGMASKLGELLRDKRFEDNWKFAIEGRASVYINRLLDGSTTMKGYTCEDILNG  
  
Target    SKR--GVPLLMNMRTYPRSGGWEQRQEDKPWYTATGRLEFYRPEPEFQAAGESLPVWREPVDATFYEPNAILSNAAHPSI  
7b04.1.B  KYGEPGVAM-LLFRTYPRHPFWEQVHESLPFYTPTGRLQAYNDEPEIIEYGENFIVHREGPEATPYLPNAIVST--NPYI  
  
Target    APRAPEDYGVPESQLDVETRQYRNVVRTWAELQQTLHPLQERDPAFRFVFQTPKYRWGAHSTAVDADWISMLFGPFGDPY  
7b04.1.B  ---RPDDYGIPENAEYWEDRTVRNIKKSWEETKKTKNFL--WEKGYHFYCVTPKSRHTVHSQWAVTDWNFIWNNNFGDPY  
  
Target    RRDPRMPWTGEAYLEINPKDAAELGLADGDYAWVDADPEDRPYRGWKEDDPYYEVARAMMRVRIYTGMSRGVIRTWFNMY  
7b04.1.B  RMDKRMPGVGEHQIHIHPQAARDLGIEDGDYVYVDANPADRPYEGWKPNDSFYKVSRLMLRAKYNPAYPYNCTMMKHSAW  
  
Target    AATPATVANQKATPGNPARNEQTRYVALFRYGSHQSGTRAWLRPTQQTDSLVRKGYFGQVIGTGFEADVHSVSGAPKEAF  
7b04.1.B  ISSDKTVQAHETRPDGRALSP-SGYQSSFRYGSQQSITRDWSMPMHQLDSLFHKAKIGMKFIFGFEADNHCINTVPKETL  
  
Target    VKIEKAEDGGIGAERLWRPLTLGLRPEAPSAALTAYLAGDYSGTKGS  
7b04.1.B  VKITKAENGGMGGKGVWDPVKTGYTAGNENDFMKKFLNGELIKVD--
```

  


---

  

| Model #01 | File | Built with | Oligo-State | Ligands | GMQE | QMEANDisCo Global |
| --- | --- | --- | --- | --- | --- | --- |
|  | PDB | ProMod3 3.2.1 | monomer | None | 0.69 | 0.67 ± 0.05 |

|  |  |  |
| --- | --- | --- |
|  |  |  |

| Template | Seq Identity | Oligo-state | QSQE | Found by | Method | Resolution | Seq Similarity | Range | Coverage | Description |
| --- | --- | --- | --- | --- | --- | --- | --- | --- | --- | --- |
| 7b04.1.B | 39.26 | monomer | 0.00 | BLAST | X-ray | 2.97Å | 0.40 | 2 - 824 | 0.95 | Nitrite oxidoreductase subunit A |

  

### Excluded ligands

| Ligand Name.Number | Reason for Exclusion | Description |
| --- | --- | --- |
| CA.10 | Binding site not conserved. | CALCIUM ION |
| CA.11 | Binding site not conserved. | CALCIUM ION |
| F3S.4 | Binding site not conserved. | FE3-S4 CLUSTER |
| HEM.9 | Binding site not conserved. | PROTOPORPHYRIN IX CONTAINING FE |
| MD1.5 | Binding site not conserved. | PHOSPHORIC ACID 4-(2-AMINO-4-OXO-3,4,5,6,-TETRAHYDRO-PTERIDIN-6-YL)-2-HYDROXY-3,4-DIMERCAPTO-BUT-3-EN-YL ESTER GUANYLATE ESTER |
| MD1.6 | Binding site not conserved. | PHOSPHORIC ACID 4-(2-AMINO-4-OXO-3,4,5,6,-TETRAHYDRO-PTERIDIN-6-YL)-2-HYDROXY-3,4-DIMERCAPTO-BUT-3-EN-YL ESTER GUANYLATE ESTER |
| MO.7 | Binding site not conserved. | MOLYBDENUM ATOM |
| SF4.1 | Binding site not conserved. | IRON/SULFUR CLUSTER |
| SF4.2 | Binding site not conserved. | IRON/SULFUR CLUSTER |
| SF4.3 | Binding site not conserved. | IRON/SULFUR CLUSTER |
| SF4.8 | Binding site not conserved. | IRON/SULFUR CLUSTER |

  

```
Target    MPTANKADEVIILRPGTDAAFFLGVARELIEKGLYDRAAVIERTDLPLLVRLDTGERLDARDVIPGYELAALTNYVTLKP  
7b04.1.B  -PSAQKADYWIPIRNNTDTALFLGITKILIDNKWYDADYVKKFTDFPLLIRTDTLKRVSPKDIIPNYKLQDISD----GP  
  
Target    DAEIKGNPPPPPFTAGGQVVPTELRDAWGDFVWWDRATGRPRPVSRDEVG---ARFDGDPALLGEFEVELVDGSTVPVRP  
7b04.1.B  SYHIQG-------------LKDEQREIIGDFVVWDAKSKGPKAITRDDVGETLVKKGIDPVLEGSFKLKTIDGKEIEVMT  
  
Target    AFDLLKQYLDESFDLRTASEVCRVPPQAIQSIARQLAANKRETLLAAGMGPNHYFQNDLFGRVQFLVAALTDNIGHLGGN  
7b04.1.B  LLEMYKIHLRD-YDIDSVVSMTNSPKDLIERLAKDIATIK-PVAIHYGEGVNHYFHATLMNRSYYLPVMLTGNVGYFGSG  
  
Target    VGSYAGNYRGSVFQA-------MGQWIAEDPFAIEPDL-----TKPATVKRYYKAESAHYWNYGERPLRAVAKDDEGDLT  
7b04.1.B  SHTWAGNYKAGNFQASKWSGPGFYGWVAEDVF--KPNLDPYASAKDLNIKGRALDEEVAYWNHSERPL-IVNTPKYGR--  
  
Target    KGEVLTGKSHMPTPTKLIWFGNSNSLLGNAKWSFDVVKNTLPRQDAVFCNEWHWTSSCEYADLVFPADSWAEFKLPDATA  
7b04.1.B  --KVFTGKTHMPSPTKVLWFTNVN-LINNAKHVYQMLKNVNPNIEQIMSTDIEITGSIEYADFAFPANSWVEFQEFEITN  
  
Target    SCTNPFLLAFPTTPLKRLYDTRSDYEALALTAKALGELIDEPRMEQYWRGILDGDPTPYLQRIFSGSNATRGITYDELHE  
7b04.1.B  SCSNPFIQIWGKTGITPVYESKDDVKILAGMASKLGELLRDKRFEDNWKFAIEGRASVYINRLLDGSTTMKGYTCEDILN  
  
Target    SS--KRGVPLLMNMRTYPRSGGWEQRQEDKPWYTATGRLEFYRPEPEFQAAGESLPVWREPVDATFYEPNAILSNAAHPS  
7b04.1.B  GKYGEPGVAMLL-FRTYPRHPFWEQVHESLPFYTPTGRLQAYNDEPEIIEYGENFIVHREGPEATPYLPNAIVS--TNPY  
  
Target    IAPRAPEDYGVPESQLDVETRQYRNVVRTWAELQQTLHPLQERDPAFRFVFQTPKYRWGAHSTAVDADWISMLFGPFGDP  
7b04.1.B  IRP---DDYGIPENAEYWEDRTVRNIKKSWEETKKTKNFLWEK--GYHFYCVTPKSRHTVHSQWAVTDWNFIWNNNFGDP  
  
Target    YRRDPRMPWTGEAYLEINPKDAAELGLADGDYAWVDADPEDRPYRGWKEDDPYYEVARAMMRVRIYTGMSRGVIRTWFNM  
7b04.1.B  YRMDKRMPGVGEHQIHIHPQAARDLGIEDGDYVYVDANPADRPYEGWKPNDSFYKVSRLMLRAKYNPAYPYNCTMMKHSA  
  
Target    YAATPATVANQKATPGNPARNEQTRYVALFRYGSHQSGTRAWLRPTQQTDSLVRKGYFGQVIGTGFEADVHSVSGAPKEA  
7b04.1.B  WISSDKTVQAHETRPDGRALSP-SGYQSSFRYGSQQSITRDWSMPMHQLDSLFHKAKIGMKFIFGFEADNHCINTVPKET  
  
Target    FVKIEKAEDGGIGAERLWRPLTLGLRPEAPSAALTAYLAGDYSGTKGS  
7b04.1.B  LVKITKAENGGMGGKGVWDPVKTGYTAGNENDFMKKFLNGE-------
```

  


---

  

| Model #03 | File | Built with | Oligo-State | Ligands | GMQE | QMEANDisCo Global |
| --- | --- | --- | --- | --- | --- | --- |
|  | PDB | ProMod3 3.2.1 | monomer | None | 0.38 | 0.43 ± 0.05 |

|  |  |  |
| --- | --- | --- |
|  |  |  |

| Template | Seq Identity | Oligo-state | QSQE | Found by | Method | Resolution | Seq Similarity | Range | Coverage | Description |
| --- | --- | --- | --- | --- | --- | --- | --- | --- | --- | --- |
| 3ir5.1.A | 23.01 | monomer | 0.00 | HHblits | X-ray | 2.30Å | 0.31 | 1 - 795 | 0.78 | Respiratory nitrate reductase 1 alpha chain |

  

### Excluded ligands

| Ligand Name.Number | Reason for Exclusion | Description |
| --- | --- | --- |
| 6MO.3 | Binding site not conserved. | MOLYBDENUM(VI) ION |
| AGA.5 | Binding site not conserved. | (1S)-2-{[{[(2S)-2,3-DIHYDROXYPROPYL]OXY}(HYDROXY)PHOSPHORYL]OXY}-1-[(PENTANOYLOXY)METHYL]ETHYL OCTANOATE |
| F3S.9 | Binding site not conserved. | FE3-S4 CLUSTER |
| HEM.10 | Binding site not conserved. | PROTOPORPHYRIN IX CONTAINING FE |
| HEM.11 | Binding site not conserved. | PROTOPORPHYRIN IX CONTAINING FE |
| MD1.1 | Binding site not conserved. | PHOSPHORIC ACID 4-(2-AMINO-4-OXO-3,4,5,6,-TETRAHYDRO-PTERIDIN-6-YL)-2-HYDROXY-3,4-DIMERCAPTO-BUT-3-EN-YL ESTER GUANYLATE ESTER |
| MD1.2 | Binding site not conserved. | PHOSPHORIC ACID 4-(2-AMINO-4-OXO-3,4,5,6,-TETRAHYDRO-PTERIDIN-6-YL)-2-HYDROXY-3,4-DIMERCAPTO-BUT-3-EN-YL ESTER GUANYLATE ESTER |
| SF4.4 | Binding site not conserved. | IRON/SULFUR CLUSTER |
| SF4.6 | Binding site not conserved. | IRON/SULFUR CLUSTER |
| SF4.7 | Binding site not conserved. | IRON/SULFUR CLUSTER |
| SF4.8 | Binding site not conserved. | IRON/SULFUR CLUSTER |

  

```
Target    MPTANKADEVIILRPGTDAAFFLGVARELIEKGL------YDRAAVIERTDLPLLVRLD-------TGERLDARDVIPGY  
3ir5.1.A  AEIAKLCDLWLAPKQGTDAAMALAMGHVMLREFHLDNPSQYFTDYVRRYTDMPMLVMLEERDGYYAAGRMLRAADLVDAL  
  
Target    ELAALTNYVTLKPDAEIKGNPPPPPFTAGGQVVPTELRDAWGDFVWWDRATGRPRPVSRDEV------------------  
3ir5.1.A  GQ---------EN-------------------------NPEWKTVAFNT-NGEMVAPNGSIGFRWGEKGKWNLEQRDGKT  
  
Target    ---------------------GARF-----------DGDPALLG---EFEVELVDGSTVPVRPAFDLLK-----------  
3ir5.1.A  GEETELQLSLLGSQDEIAEVGFPYFGGDGTEHFNKVELENVLLHKLPVKRLQLADGSTALVTTVYDLTLANYGLERGLND  
  
Target    -------QYLDESFDLRTASEVCRVPPQAIQSIARQLAAN-----KRETLLAAGMGPNHYFQNDLFGRVQFLVAALTDNI  
3ir5.1.A  VNCATSYDDVK-AYTPAWAEQITGVSRSQIIRIAREFADNADKTHGRS-MIIVGAGLNHWYHLDMNYRGLINMLIFCGCV  
  
Target    GHLGGNVGSYAGNYRGSVFQAMGQWIAEDPFAI-----------E--------P-----DLTKPA-TVKRYYKA------  
3ir5.1.A  GQSGGGWAHYVGQEKLRPQTGWQPLAFALDWQRPARHMNSTSYFYNHSSQWRYETVTAEELLSPMADKSRYTGHLIDFNV  
  
Target    --ESAHYW----NYGERPLRAVAKD-----DEGDLTKGEVLTGKS--------HMPTPTKLIWFGNSNSLLGNAKWSFDV  
3ir5.1.A  RAERMGWLPSAPQLGTNPLTIAGEAEKAGMNPVDYTVKSLKEGSIRFAAEQPENGKNHPRNLFIWRSNLLGSSGKGHEFM  
  
Target    V------------------------------KNTLPRQDAVFCNEWHWTSSCEYADLVFPADSWAEFKLPDATASCTNPF  
3ir5.1.A  LKYLLGTEHGIQGKDLGQQGGVKPEEVDWQDNGLEGKLDLVVTLDFRLSSTCLYSDIILPTATWYEK--DDMNTSDMHPF  
  
Target    LLAFPTTPLKRLYDTRSDYEALALTAKALGELIDE---------------PR---------MEQYWRGILDG--------  
3ir5.1.A  IHP-LSAAVDPAWEAKSDWEIYKAIAKKFSEVCVGHLGKETDIVTLPIQHDSAAELAQPLDVKDWKKGECDLIPGKTAPH  
  
Target    ------D-------------------------------PTPYLQRI-------------------------FSGSNATRG  
3ir5.1.A  IMVVERDYPATYERFTSIGPLMEKIGNGGKGIAWNTQSEMDLLRKLNYTKAEGPAKGQPMLNTAIDAAEMILTLAPETNG  
  
Target    I----TYDELHESSK-----------RGVPLLMNMR------------------TYPRSGGWEQRQEDKPWYTATGRLEF  
3ir5.1.A  QVAVKAWAALSEFTGRDHTHLALNKEDEKIRFRDIQAQPRKIISSPTWSGLEDEHVSYNAGYTNVHELIPWRTLSGRQQL  
  
Target    YRPEPEFQAAGESLPVWREPVDATFYEPNAILSNAAHPSIAPRAPEDYGVPESQLDVETRQYRNVVRTWAELQQTLHPLQ  
3ir5.1.A  YQDHQWMRDFGESLLVYRPPIDTRSV------------------KEVI----------------------G------QKS  
  
Target    ERDPAFRFVFQTPKYRWGAHSTAVDADWISMLFGPFGDPYRRDPRMPWTGEAYLEINPKDAAELGLADGDYAWVDADPED  
3ir5.1.A  NGNQEKALNFLTPHQKWGIHSTYSDNLLMLTLG---------------RGGPVVWLSEADAKDLGIADNDWIEVFNS---  
  
Target    RPYRGWKEDDPYYEVARAMMRVRIYTGMSRGVIRTWFNMYAATPATVANQKATPGNPARNEQTRYVALFRYGSHQSGTRA  
3ir5.1.A  --------------NGALTARAVVSQRVPAGMTMMYHAQERIVN--------LPGSEIT--------QQRGGIHNSVTRI  
  
Target    WLRPTQQTDSLVRKGYFGQVIGTGFEADVHSVSGAPKEAFVKIEKAEDGGIGAERLWRPLTLGLRPEAPSAALTAYLAGD  
3ir5.1.A  TPKPTHMI------GGYAHL-AYGFN--YYGTVGSNRDEFVVVRKMKNIDW-----------------------------  
  
Target    YSGTKGS  
3ir5.1.A  -------
```

  


---

  

| Model #04 | File | Built with | Oligo-State | Ligands | GMQE | QMEANDisCo Global |
| --- | --- | --- | --- | --- | --- | --- |
|  | PDB | ProMod3 3.2.1 | monomer | None | 0.12 | 0.35 ± 0.05 |

|  |  |  |
| --- | --- | --- |
|  |  |  |

| Template | Seq Identity | Oligo-state | QSQE | Found by | Method | Resolution | Seq Similarity | Range | Coverage | Description |
| --- | --- | --- | --- | --- | --- | --- | --- | --- | --- | --- |
| 2fug.2.C | 20.00 | monomer | 0.00 | HHblits | X-ray | 3.30Å | 0.28 | 342 - 706 | 0.23 | NADH-quinone oxidoreductase chain 3 |

  

### Excluded ligands

| Ligand Name.Number | Reason for Exclusion | Description |
| --- | --- | --- |
| FES.2 | Binding site not conserved. | FE2/S2 (INORGANIC) CLUSTER |
| FES.6 | Binding site not conserved. | FE2/S2 (INORGANIC) CLUSTER |
| FMN.10 | Binding site not conserved. | FLAVIN MONONUCLEOTIDE |
| SF4.1 | Binding site not conserved. | IRON/SULFUR CLUSTER |
| SF4.3 | Binding site not conserved. | IRON/SULFUR CLUSTER |
| SF4.4 | Binding site not conserved. | IRON/SULFUR CLUSTER |
| SF4.5 | Binding site not conserved. | IRON/SULFUR CLUSTER |
| SF4.7 | Binding site not conserved. | IRON/SULFUR CLUSTER |
| SF4.8 | Binding site not conserved. | IRON/SULFUR CLUSTER |
| SF4.9 | Binding site not conserved. | IRON/SULFUR CLUSTER |

  

```
Target    MPTANKADEVIILRPGTDAAFFLGVARELIEKGLYDRAAVIERTDLPLLVRLDTGERLDARDVIPGYELAALTNYVTLKP  
2fug.2.C  --------------------------------------------------------------------------------  
  
Target    DAEIKGNPPPPPFTAGGQVVPTELRDAWGDFVWWDRATGRPRPVSRDEVGARFDGDPALLGEFEVELVDGSTVPVRPAFD  
2fug.2.C  --------------------------------------------------------------------------------  
  
Target    LLKQYLDESFDLRTASEVCRVPPQAIQSIARQLAANKRETLLAAGMGPNHYFQNDLFGRVQFLVAALTDNIGHLGGNVGS  
2fug.2.C  --------------------------------------------------------------------------------  
  
Target    YAGNYRGSVFQAMGQWIAEDPFAIEPDLTKPATVKRYYKAESAHYWNYGERPLRAVAKDDEGDLTKGEVLTGKSHMPTPT  
2fug.2.C  --------------------------------------------------------------------------------  
  
Target    KLIWFGNSNSLLGNAKWSFDVVKNTLPRQDAVFCNEWHWTSSC-EYADLVFPADSWAEFKLPDATASCTNPFLLAFPTTP  
2fug.2.C  ---------------------PEEALKGKRFVVMHLSHLHPLAERYAHVVLPAPTFYEKRG---HLVNLEGRVLPL-SPA  
  
Target    LKRLYDTRSDYEALALTAKALGELIDEPRMEQYWRGILDGDPTPYLQRIFSGSNATRGITYDELHESSKRGVPLLMNMRT  
2fug.2.C  PIENGEAEGALQVLALLAEALGVRPPFRL------------HLEA---------------QKALK---------------  
  
Target    YPRSGGWEQRQEDKPWYTATGRLEFYRPEPEFQAAGESLPVWREPVDATFYEPNAILSNAAHPSIAPRAPEDYGVPESQL  
2fug.2.C  ------------ARKVPEAMGRLSFRLKELR-------------P-----------------------------------  
  
Target    DVETRQYRNVVRTWAELQQTLHPLQERDPAFRFVFQTPKYRWGAHSTAVDADWISMLFGPFGDPYRRDPRMPWTGEAYLE  
2fug.2.C  --------------------------KERKGAFYLRPTMWKAHQ-----AVGKAQEA-----------------ARAELW  
  
Target    INPKDAAELGLADGDYAWVDADPEDRPYRGWKEDDPYYEVARAMMRVRIYTGMSRGVIRTWFNMYAATPATVANQKATPG  
2fug.2.C  AHPETARAEALPEGAQVAVETP-----------------FGRVEARVVHREDVPKGHLYLSALGPAAG------------  
  
Target    NPARNEQTRYVALFRYGSHQSGTRAWLRPTQQTDSLVRKGYFGQVIGTGFEADVHSVSGAPKEAFVKIEKAEDGGIGAER  
2fug.2.C  --------------------------------------------------------------------------------  
  
Target    LWRPLTLGLRPEAPSAALTAYLAGDYSGTKGS  
2fug.2.C  --------------------------------
```

  


---

  

## Materials and Methods

## Template Search

Template search with
has been performed against the SWISS-MODEL template library (SMTL, last update: 2023-03-23, last included PDB release: 2023-03-17).

## Template Selection

For each identified template, the template's quality has been predicted from features of the target-template alignment.
The templates with the highest quality have then been selected for model building.

## Model Building

Models are built based on the target-template alignment using ProMod3 (Studer et al.). Coordinates which are conserved between the target and the template are copied from the template to the model. Insertions and deletions are remodelled using a fragment library. Side chains are then rebuilt. Finally, the geometry of the resulting model is regularized by using a force field.

## Model Quality Estimation

The global and per-residue model quality has been assessed using the QMEAN scoring function (Studer et al.).

## Ligand Modelling

Ligands present in the template structure are transferred by homology to the model when the following criteria are met: (a) The ligands are annotated as biologically relevant in the template library, (b) the ligand is in contact with the model, (c) the ligand is not clashing with the protein, (d) the residues in contact with the ligand are conserved between the target and the template. If any of these four criteria is not satisfied, a certain ligand will not be included in the model. The model summary includes information on why and which ligand has not been included.

## Oligomeric State Conservation

The quaternary structure annotation of the template is used to model the target sequence in its oligomeric form. The method (Bertoni et al.) is based on a supervised machine learning algorithm, Support Vector Machines (SVM), which combines interface conservation, structural clustering, and other template features to provide a quaternary structure quality estimate (QSQE). The QSQE score is a number between 0 and 1, reflecting the expected accuracy of the interchain contacts for a model built based a given alignment and template. Higher numbers indicate higher reliability. This complements the GMQE score which estimates the accuracy of the tertiary structure of the resulting model.

## References

- **BLAST**  
  Camacho, C., Coulouris, G., Avagyan, V., Ma, N., Papadopoulos, J.,
  Bealer, K., Madden, T.L. BLAST+: architecture and applications. BMC
  Bioinformatics 10, 421-430 (2009).
- **HHblits**  
  Steinegger, M., Meier, M., Mirdita, M., Vöhringer, H., Haunsberger,
  S. J., Söding, J. HH-suite3 for fast remote homology detection and
  deep protein annotation. BMC Bioinformatics 20, 473 (2019).

## Table T1:

Primary amino acid sequence for which templates were searched and models were built.

MPTANKADEVIILRPGTDAAFFLGVARELIEKGLYDRAAVIERTDLPLLVRLDTGERLDARDVIPGYELAALTNYVTLKPDAEIKGNPPPPPFTAGGQVV  
PTELRDAWGDFVWWDRATGRPRPVSRDEVGARFDGDPALLGEFEVELVDGSTVPVRPAFDLLKQYLDESFDLRTASEVCRVPPQAIQSIARQLAANKRET  
LLAAGMGPNHYFQNDLFGRVQFLVAALTDNIGHLGGNVGSYAGNYRGSVFQAMGQWIAEDPFAIEPDLTKPATVKRYYKAESAHYWNYGERPLRAVAKDD  
EGDLTKGEVLTGKSHMPTPTKLIWFGNSNSLLGNAKWSFDVVKNTLPRQDAVFCNEWHWTSSCEYADLVFPADSWAEFKLPDATASCTNPFLLAFPTTPL  
KRLYDTRSDYEALALTAKALGELIDEPRMEQYWRGILDGDPTPYLQRIFSGSNATRGITYDELHESSKRGVPLLMNMRTYPRSGGWEQRQEDKPWYTATG  
RLEFYRPEPEFQAAGESLPVWREPVDATFYEPNAILSNAAHPSIAPRAPEDYGVPESQLDVETRQYRNVVRTWAELQQTLHPLQERDPAFRFVFQTPKYR  
WGAHSTAVDADWISMLFGPFGDPYRRDPRMPWTGEAYLEINPKDAAELGLADGDYAWVDADPEDRPYRGWKEDDPYYEVARAMMRVRIYTGMSRGVIRTW  
FNMYAATPATVANQKATPGNPARNEQTRYVALFRYGSHQSGTRAWLRPTQQTDSLVRKGYFGQVIGTGFEADVHSVSGAPKEAFVKIEKAEDGGIGAERL  
WRPLTLGLRPEAPSAALTAYLAGDYSGTKGS

## Table T2:

| Template | Seq Identity | Oligo-state | QSQE | Found by | Method | Resolution | Seq Similarity | Coverage | Description |
| --- | --- | --- | --- | --- | --- | --- | --- | --- | --- |
| 7b04.1.B | 39.26 | monomer | - | BLAST | X-ray | 2.97Å | 0.40 | 0.95 | Nitrite oxidoreductase subunit A |
| 7b04.1.B | 37.28 | monomer | - | HHblits | X-ray | 2.97Å | 0.39 | 0.96 | Nitrite oxidoreductase subunit A |
| 7b04.2.B | 39.26 | monomer | - | BLAST | X-ray | 2.97Å | 0.40 | 0.95 | Nitrite oxidoreductase subunit A |
| 7b04.2.B | 37.28 | monomer | - | HHblits | X-ray | 2.97Å | 0.39 | 0.96 | Nitrite oxidoreductase subunit A |
| 5e7o.1.A | 26.27 | monomer | - | HHblits | X-ray | 2.40Å | 0.33 | 0.73 | DMSO reductase family type II enzyme, molybdopterin subunit |
| 4ydd.1.A | 26.07 | monomer | - | HHblits | X-ray | 1.86Å | 0.33 | 0.73 | DMSO reductase family type II enzyme, molybdopterin subunit |
| 2ivf.1.A | 26.18 | monomer | - | HHblits | X-ray | 1.88Å | 0.32 | 0.74 | ETHYLBENZENE DEHYDROGENASE ALPHA-SUBUNIT |
| 3ir5.1.A | 23.01 | monomer | - | HHblits | X-ray | 2.30Å | 0.31 | 0.78 | Respiratory nitrate reductase 1 alpha chain |
| 3ir7.1.A | 23.62 | monomer | - | HHblits | X-ray | 2.50Å | 0.31 | 0.78 | Respiratory nitrate reductase 1 alpha chain |
| 3egw.1.A | 23.27 | homo-dimer | 0.06 | HHblits | X-ray | 1.90Å | 0.32 | 0.78 | Respiratory nitrate reductase 1 alpha chain |
| 1r27.4.A | 23.16 | homo-dimer | 0.05 | HHblits | X-ray | 2.00Å | 0.31 | 0.78 | Respiratory nitrate reductase 1 alpha chain |
| 1q16.1.A | 23.38 | monomer | - | HHblits | X-ray | 1.90Å | 0.31 | 0.78 | Respiratory nitrate reductase 1 alpha chain |
| 3ir6.1.A | 23.46 | monomer | - | HHblits | X-ray | 2.80Å | 0.32 | 0.78 | Respiratory nitrate reductase 1 alpha chain |
| 1aa6.1.A | 19.87 | monomer | - | HHblits | X-ray | 2.30Å | 0.31 | 0.54 | FORMATE DEHYDROGENASE H |
| 2iv2.1.A | 19.87 | monomer | - | HHblits | X-ray | 2.27Å | 0.31 | 0.54 | Formate dehydrogenase H |
| 1fdo.1.A | 19.87 | monomer | - | HHblits | X-ray | 2.80Å | 0.31 | 0.54 | FORMATE DEHYDROGENASE H |
| 1dms.1.A | 19.83 | monomer | - | HHblits | X-ray | 1.88Å | 0.30 | 0.58 | DMSO REDUCTASE |
| 1e5v.2.A | 20.29 | monomer | - | HHblits | X-ray | 2.40Å | 0.30 | 0.58 | Dimethyl sulfoxide/trimethylamine N-oxide reductase |
| 7l5i.1.A | 19.58 | monomer | - | HHblits | X-ray | 1.73Å | 0.30 | 0.58 | Trimethylamine-N-oxide reductase |
| 1e60.1.A | 20.21 | monomer | - | HHblits | X-ray | 2.00Å | 0.30 | 0.58 | Dimethyl sulfoxide/trimethylamine N-oxide reductase |
| 7l5s.1.A | 19.58 | monomer | - | HHblits | X-ray | 2.09Å | 0.30 | 0.58 | Trimethylamine-N-oxide reductase |
| 4dmr.1.A | 20.08 | monomer | - | HHblits | X-ray | 1.90Å | 0.30 | 0.58 | DMSO REDUCTASE |
| 7z0t.1.G | 19.87 | monomer | - | HHblits | EM | NA | 0.31 | 0.54 | Formate dehydrogenase H |
| 1eu1.1.A | 20.81 | monomer | - | HHblits | X-ray | 1.30Å | 0.31 | 0.57 | DIMETHYL SULFOXIDE REDUCTASE |
| 1e18.1.A | 20.08 | monomer | - | HHblits | X-ray | 2.00Å | 0.30 | 0.58 | DMSO REDUCTASE. |
| 4ydd.1.A | 31.43 | monomer | - | BLAST | X-ray | 1.86Å | 0.36 | 0.57 | DMSO reductase family type II enzyme, molybdopterin subunit |
| 5e7o.1.A | 31.43 | monomer | - | BLAST | X-ray | 2.40Å | 0.36 | 0.57 | DMSO reductase family type II enzyme, molybdopterin subunit |
| 6tg9.1.A | 20.05 | monomer | - | HHblits | EM | 3.24Å | 0.30 | 0.53 | Formate dehydrogenase subunit alpha |
| 2ivf.1.A | 40.10 | monomer | - | BLAST | X-ray | 1.88Å | 0.39 | 0.24 | ETHYLBENZENE DEHYDROGENASE ALPHA-SUBUNIT |
| 3m9s.1.C | 20.00 | monomer | - | HHblits | X-ray | 4.50Å | 0.28 | 0.23 | NADH-quinone oxidoreductase subunit 3 |
| 6zjl.1.C | 20.00 | monomer | - | HHblits | EM | NA | 0.28 | 0.23 | NADH-quinone oxidoreductase subunit 3 |
| 6ziy.1.C | 20.00 | monomer | - | HHblits | EM | NA | 0.28 | 0.23 | NADH-quinone oxidoreductase subunit 3 |
| 6zjn.1.C | 20.00 | monomer | - | HHblits | EM | NA | 0.28 | 0.23 | NADH-quinone oxidoreductase subunit 3 |
| 6q8o.1.C | 20.00 | monomer | - | HHblits | X-ray | 3.61Å | 0.28 | 0.23 | NADH-quinone oxidoreductase subunit 3 |
| 6zjy.1.C | 20.00 | monomer | - | HHblits | EM | NA | 0.28 | 0.23 | NADH-quinone oxidoreductase subunit 3 |
| 2fug.2.C | 20.00 | monomer | - | HHblits | X-ray | 3.30Å | 0.28 | 0.23 | NADH-quinone oxidoreductase chain 3 |
| 6zk9.1.C | 13.14 | monomer | - | HHblits | EM | NA | 0.27 | 0.21 | NADH:ubiquinone oxidoreductase core subunit S1 |
| 7zd6.1.4 | 13.14 | monomer | - | HHblits | EM | NA | 0.27 | 0.21 | NADH-ubiquinone oxidoreductase 75 kDa subunit, mitochondrial |
| 6g72.1.G | 11.93 | monomer | - | HHblits | EM | NA | 0.26 | 0.21 | NADH-ubiquinone oxidoreductase 75 kDa subunit, mitochondrial |
| 6zr2.1.G | 11.93 | monomer | - | HHblits | EM | 3.10Å | 0.26 | 0.21 | NADH-ubiquinone oxidoreductase 75 kDa subunit, mitochondrial |
| 7ak6.1.G | 11.93 | monomer | - | HHblits | EM | NA | 0.26 | 0.21 | NADH-ubiquinone oxidoreductase 75 kDa subunit, mitochondrial |
| 7ak5.1.G | 11.93 | monomer | - | HHblits | EM | NA | 0.26 | 0.21 | NADH-ubiquinone oxidoreductase 75 kDa subunit, mitochondrial |
| 7dgr.10.A | 12.00 | monomer | - | HHblits | EM | NA | 0.26 | 0.21 | NADH-ubiquinone oxidoreductase 75 kDa subunit, mitochondrial |
| 6qc5.1.C | 13.71 | monomer | - | HHblits | EM | NA | 0.27 | 0.21 | NADH:ubiquinone oxidoreductase core subunit S1 |
| 6qcf.1.C | 13.71 | monomer | - | HHblits | EM | NA | 0.27 | 0.21 | NADH:ubiquinone oxidoreductase core subunit S1 |
| 5o31.1.8 | 12.00 | monomer | - | HHblits | EM | 4.13Å | 0.26 | 0.21 | NADH-ubiquinone oxidoreductase 75 kDa subunit, mitochondrial |
| 7zm7.1.I | 18.48 | monomer | - | HHblits | EM | NA | 0.30 | 0.11 | NADH-ubiquinone oxidoreductase-like protein |
| 7v2c.1.L | 15.96 | monomer | - | HHblits | EM | NA | 0.29 | 0.11 | NADH-ubiquinone oxidoreductase 75 kDa subunit, mitochondrial |
| 5t5i.1.D | 17.50 | homo-dimer | - | HHblits | X-ray | 1.90Å | 0.31 | 0.10 | Tungsten formylmethanofuran dehydrogenase subunit fwdD |
| 1h0h.1.A | 16.67 | monomer | - | HHblits | X-ray | 1.80Å | 0.30 | 0.09 | FORMATE DEHYDROGENASE SUBUNIT ALPHA |

  
The table above shows the top 50 filtered templates. A further 175 templates were found which were considered to be less suitable for modelling than the filtered list.  
1cz4.1.A, 1cz5.1.A, 1g8j.1.A, 1g8k.1.A, 1h0h.1.A, 1kqf.1.A, 1ogy.1.A, 1pt1.1.A, 1q16.1.A, 1r27.4.A, 1tmo.1.A, 1uhd.1.B, 1uhe.1.B, 1wlf.1.A, 1z0r.1.B, 2d9r.1.A, 2e7z.1.A, 2fy9.1.A, 2ivf.1.A, 2k1n.1.C, 2k1n.1.D, 2k1n.1.E, 2k1n.1.F, 2ki8.1.A, 2mrn.1.A, 2mru.1.A, 2mru.1.B, 2nya.1.A, 2pjh.1.B, 2ro3.1.A, 2ro4.1.A, 2v3v.1.A, 2v45.1.A, 2vpx.1.D, 2vpz.1.A, 2x48.1.A, 2x48.2.A, 2x48.3.A, 3egw.1.A, 3hu1.1.A, 3ir5.1.A, 3ir6.1.A, 3ir7.1.A, 3o27.1.A, 3o27.1.B, 3o5a.1.A, 3qc8.1.A, 3qq7.1.A, 3qq8.1.A, 3qwz.1.A, 3tiw.1.A, 3tiw.2.A, 4aay.1.A, 4ga5.1.A, 4ga6.1.A, 4kdi.1.A, 4kdi.2.A, 4kdl.1.A, 4nvs.1.A, 4rv0.1.A, 4v4c.1.A, 4ydd.1.A, 5b6c.1.A, 5cuo.1.A, 5cup.1.A, 5e7o.1.A, 5e7p.1.A, 5epp.1.A, 5g4f.1.A, 5g4f.1.B, 5g4f.1.C, 5g4f.1.D, 5g4f.1.E, 5g4f.1.F, 5glf.1.A, 5glf.2.A, 5glf.3.A, 5glf.4.A, 5gpn.24.A, 5nqd.1.A, 5t5i.1.B, 5x4l.1.A, 5x4l.2.A, 5xtb.1.L, 6btm.1.B, 6cz7.1.A, 6f0k.1.B, 6gcs.1.A, 6hd3.1.A, 6lod.1.B, 6rfq.1.A, 6rfs.1.A, 6s6y.1.B, 6sdr.1.A, 6sdv.1.A, 6x89.1.H, 6yj4.1.G, 7a23.1.O, 7a8y.1.B, 7a8y.1.D, 7aqr.1.F, 7ar7.1.G, 7ar8.1.G, 7arc.1.F, 7b04.1.B, 7b04.2.B, 7bkb.1.F, 7bkb.1.J, 7bkb.1.L, 7dbo.1.A, 7dbo.2.A, 7dg7.1.A, 7dg9.1.A, 7di0.1.A, 7di0.2.A, 7di0.3.A, 7di1.1.A, 7du6.1.A, 7du7.1.A, 7dvc.1.A, 7dvc.5.A, 7dvf.1.A, 7dvh.1.A, 7dvh.2.A, 7dvh.4.A, 7dww.1.A, 7dww.2.A, 7dxr.1.A, 7dxr.1.B, 7dxr.2.B, 7dxs.1.A, 7dxs.1.B, 7dxs.2.A, 7dxs.2.B, 7dxt.1.A, 7dxu.1.A, 7dxu.1.B, 7dxu.2.B, 7dxv.1.A, 7dxv.1.B, 7dxw.1.A, 7dxx.1.A, 7dxx.1.B, 7dxy.1.A, 7dxz.1.A, 7dxz.2.A, 7dxz.2.B, 7dxz.3.A, 7dyc.1.A, 7dyc.2.A, 7dyc.3.A, 7e5z.1.A, 7nz1.1.E, 7p61.1.C, 7p63.1.C, 7q5y.1.A, 7qsd.1.G, 7qv7.1.L, 7qv7.1.O, 7t2r.1.A, 7t30.1.A, 7tgh.58.A, 7vw6.1.A, 7vxu.1.L, 7wbb.1.A, 7wbb.1.B, 7wbb.1.C, 7wbb.1.D, 7wbb.1.E, 7wbb.1.G, 8b9z.1.G, 8ba0.1.G, 8bqg.1.A, 8e73.55.A, 8e9g.1.G

Swiss Institute of Bioinformatics
Contact Us
