# Supplementary figures and images for: Metabolic capacity is maintained despite shifts in microbial diversity in estuary sediments
Source: ISME Commun. 2025 Oct 11;5(1):ycaf182. doi: 10.1093/ismeco/ycaf182 (PMC12687941; doi:10.1093/ismeco/ycaf182)

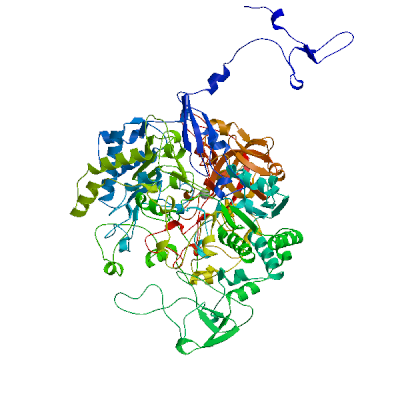

Supplement: Supplementary_Data_1_ycaf182 [file supplementary_data_1_ycaf182.zip › SWISS-MODEL/8_1_May_SF_Bin5_scaffold_55982_c1/models/03/03.png]

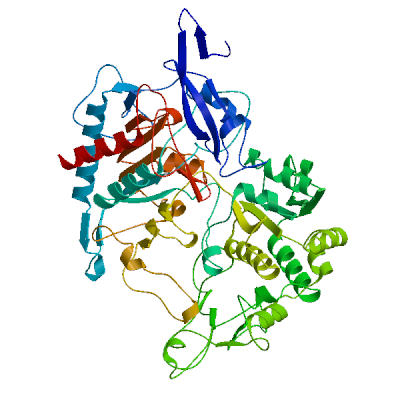

Supplement: Supplementary_Data_1_ycaf182 [file supplementary_data_1_ycaf182.zip › SWISS-MODEL/8_1_May_SF_Bin5_scaffold_55982_c1/models/04/04.png]

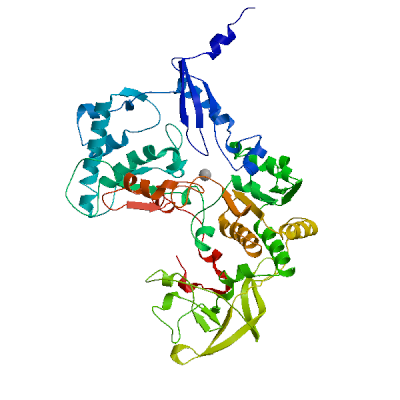

Supplement: Supplementary_Data_1_ycaf182 [file supplementary_data_1_ycaf182.zip › SWISS-MODEL/8_1_May_SF_Bin5_scaffold_55982_c1/models/05/05.png]

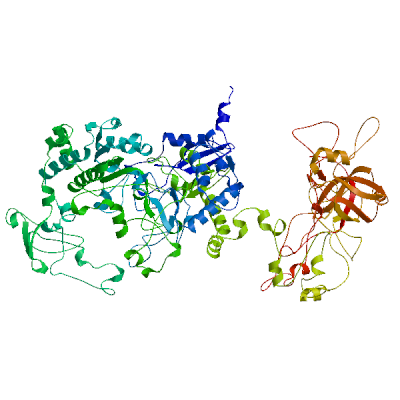

Supplement: Supplementary_Data_1_ycaf182 [file supplementary_data_1_ycaf182.zip › SWISS-MODEL/8_1_May_SF_Bin5_scaffold_55982_c1/models/02/02.png]

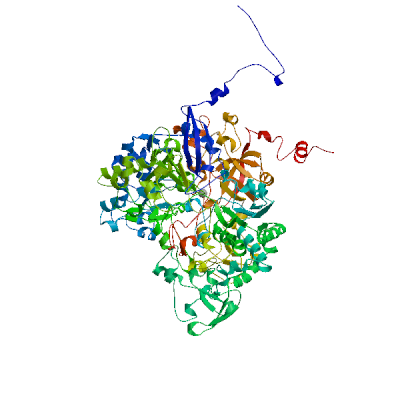

Supplement: Supplementary_Data_1_ycaf182 [file supplementary_data_1_ycaf182.zip › SWISS-MODEL/8_1_May_SF_Bin5_scaffold_55982_c1/models/01/01.png]

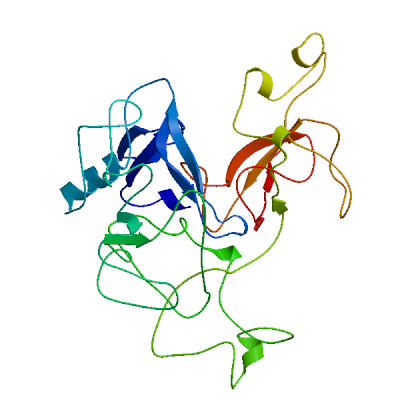

Supplement: Supplementary_Data_1_ycaf182 [file supplementary_data_1_ycaf182.zip › SWISS-MODEL/13_July_SF_Bin2_scaffold_9994_c1_22545_1/models/03/03.png]

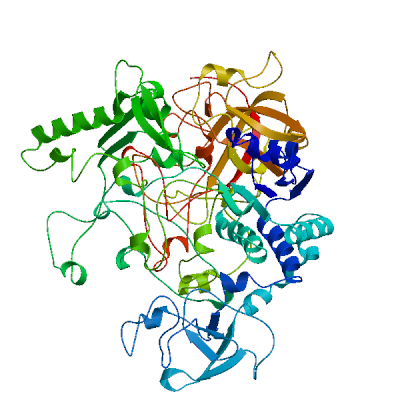

Supplement: Supplementary_Data_1_ycaf182 [file supplementary_data_1_ycaf182.zip › SWISS-MODEL/13_July_SF_Bin2_scaffold_9994_c1_22545_1/models/02/02.png]

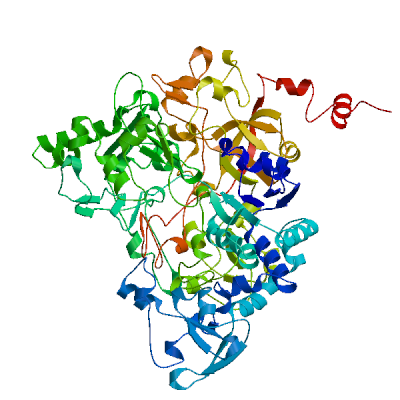

Supplement: Supplementary_Data_1_ycaf182 [file supplementary_data_1_ycaf182.zip › SWISS-MODEL/13_July_SF_Bin2_scaffold_9994_c1_22545_1/models/01/01.png]

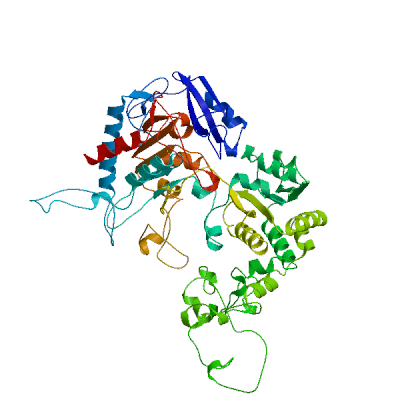

Supplement: Supplementary_Data_1_ycaf182 [file supplementary_data_1_ycaf182.zip › SWISS-MODEL/4_1_Jan_SF_Bin18_scaffold_700_c1_62709776_1/models/03/03.png]

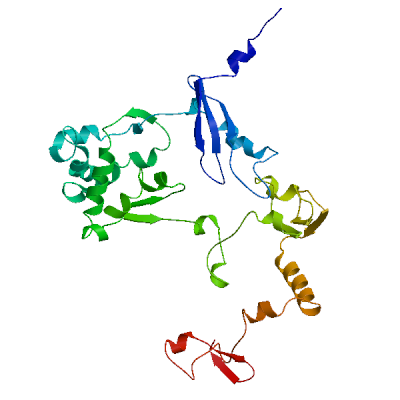

Supplement: Supplementary_Data_1_ycaf182 [file supplementary_data_1_ycaf182.zip › SWISS-MODEL/4_1_Jan_SF_Bin18_scaffold_700_c1_62709776_1/models/04/04.png]

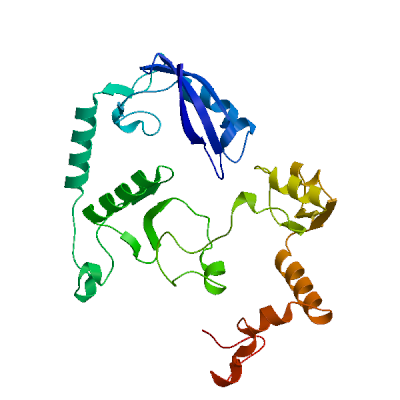

Supplement: Supplementary_Data_1_ycaf182 [file supplementary_data_1_ycaf182.zip › SWISS-MODEL/4_1_Jan_SF_Bin18_scaffold_700_c1_62709776_1/models/05/05.png]

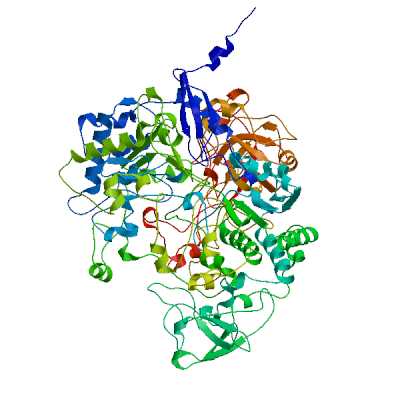

Supplement: Supplementary_Data_1_ycaf182 [file supplementary_data_1_ycaf182.zip › SWISS-MODEL/4_1_Jan_SF_Bin18_scaffold_700_c1_62709776_1/models/02/02.png]

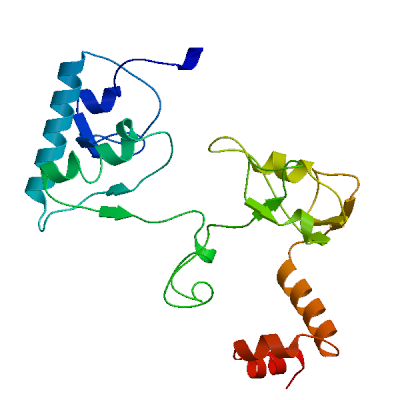

Supplement: Supplementary_Data_1_ycaf182 [file supplementary_data_1_ycaf182.zip › SWISS-MODEL/4_1_Jan_SF_Bin18_scaffold_700_c1_62709776_1/models/07/07.png]

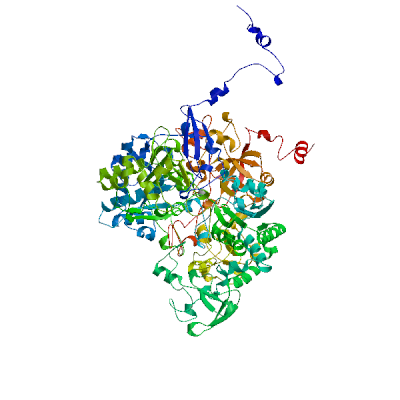

Supplement: Supplementary_Data_1_ycaf182 [file supplementary_data_1_ycaf182.zip › SWISS-MODEL/4_1_Jan_SF_Bin18_scaffold_700_c1_62709776_1/models/01/01.png]

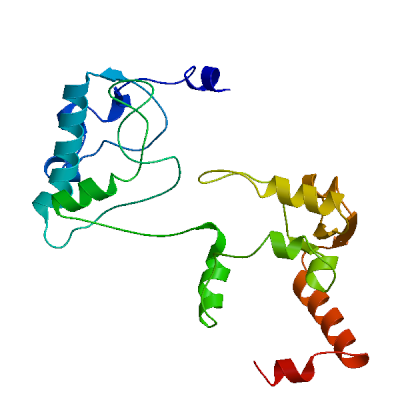

Supplement: Supplementary_Data_1_ycaf182 [file supplementary_data_1_ycaf182.zip › SWISS-MODEL/4_1_Jan_SF_Bin18_scaffold_700_c1_62709776_1/models/06/06.png]

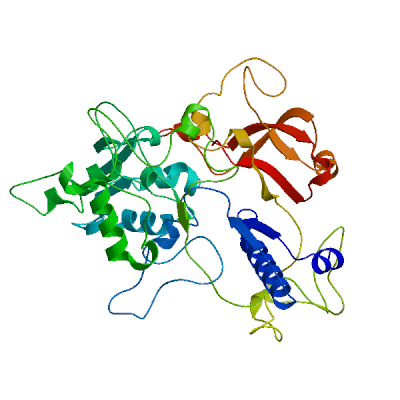

Supplement: Supplementary_Data_1_ycaf182 [file supplementary_data_1_ycaf182.zip › SWISS-MODEL/13_July_SF_Bin2_scaffold_20759_c1_11143024_1/models/03/03.png]

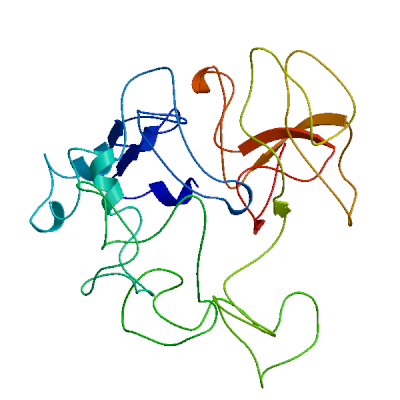

Supplement: Supplementary_Data_1_ycaf182 [file supplementary_data_1_ycaf182.zip › SWISS-MODEL/13_July_SF_Bin2_scaffold_20759_c1_11143024_1/models/04/04.png]

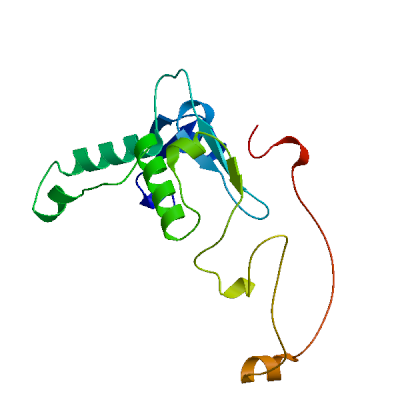

Supplement: Supplementary_Data_1_ycaf182 [file supplementary_data_1_ycaf182.zip › SWISS-MODEL/13_July_SF_Bin2_scaffold_20759_c1_11143024_1/models/05/05.png]

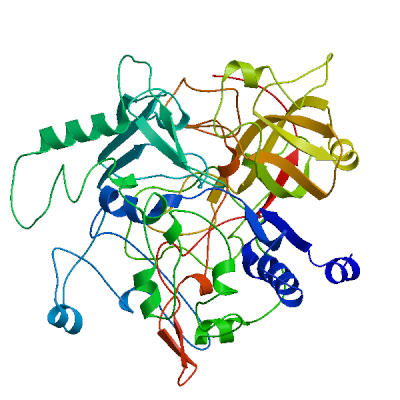

Supplement: Supplementary_Data_1_ycaf182 [file supplementary_data_1_ycaf182.zip › SWISS-MODEL/13_July_SF_Bin2_scaffold_20759_c1_11143024_1/models/02/02.png]

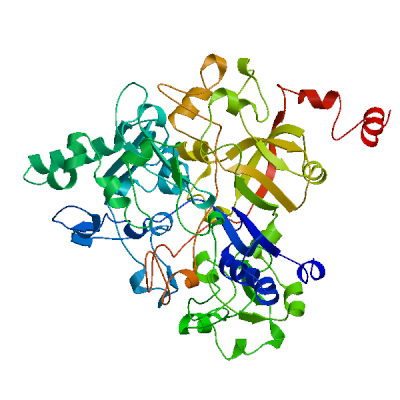

Supplement: Supplementary_Data_1_ycaf182 [file supplementary_data_1_ycaf182.zip › SWISS-MODEL/13_July_SF_Bin2_scaffold_20759_c1_11143024_1/models/01/01.png]

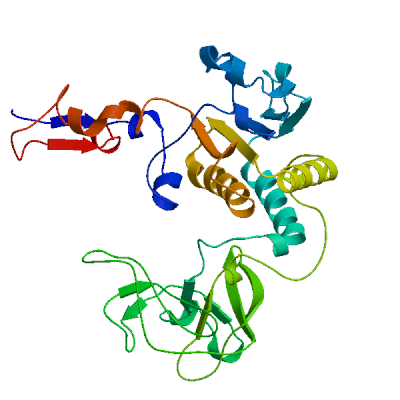

Supplement: Supplementary_Data_1_ycaf182 [file supplementary_data_1_ycaf182.zip › SWISS-MODEL/4_1_Oct_SF_Bin32_scaffold_7496_c1_22440_1/models/03/03.png]

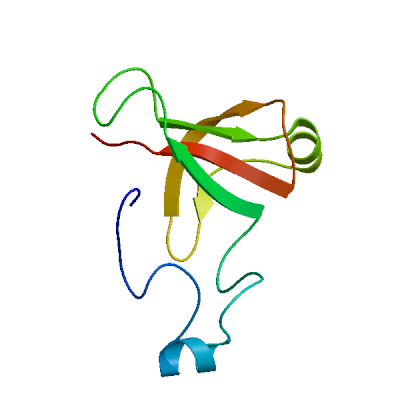

Supplement: Supplementary_Data_1_ycaf182 [file supplementary_data_1_ycaf182.zip › SWISS-MODEL/4_1_Oct_SF_Bin32_scaffold_7496_c1_22440_1/models/04/04.png]
