# Supplementary material for: Metabolic capacity is maintained despite shifts in microbial diversity in estuary sediments
Source: ISME Commun. 2025 Oct 11;5(1):ycaf182. doi: 10.1093/ismeco/ycaf182 (PMC12687941; doi:10.1093/ismeco/ycaf182)
Supplement: Supplementary_Information_ycaf182 [file supplementary_information_ycaf182.docx]

Metabolic capacity is maintained despite shifts in microbial diversity in estuary sediments

Marguerite V. Langwig^1†^, Sunny Lyn Sneed^2⦁^, Anna Rasmussen^3^, Kiley W. Seitz^1°^, Jessica A. Lee^3‡^, Karthik Anantharaman^4^, Valerie De Anda^1,5††*^, Christopher A. Francis^3*^, and Brett J. Baker^1,5*^

^1^Department of Marine Science, University of Texas at Austin, Marine Science Institute, Port Aransas, TX, USA; ^2^Department of Biological Sciences, Rensselaer Polytechnic Institute, Troy, NY, USA; ^3^Department of Earth System Science, Stanford University, Stanford, California, USA; ^4^Department of Bacteriology, University of Wisconsin-Madison, Wisconsin, USA; ^5^Department of Integrative Biology, University of Texas at Austin, Texas, USA

*Corresponding authors: valdeanda@gmal.com, caf@stanford.edu, and ﻿acidophile@gmail.com

^†^ Current address: Department of Freshwater and Marine Sciences & Department of Bacteriology, University of Wisconsin-Madison, Madison, Wisconsin, USA

^††^ Current address: Fort Lauderdale Research and Education Center, University of Florida, Fort Lauderdale, FL (FLREC), Department of Microbiology and Cell Sciences, USA

**^⦁^** Department of Microbiology and Immunology, Brody School of Medicine, East Carolina University, Greenville, NC 27834, USA

^°^ Current Address: EMBL Heidelberg, Meyerhofstraße 1, 69117 Heidelberg, Germany

^‡^ Current Address: NASA Ames Research Center, Moffett Field, CA

**Supplementary Text**

**Metabolic handoffs and functional redundancy in denitrification and DNRA**

In addition to *nxrAB* sequences from relatives of known NOB and comammox bacteria, we identified 17 genes with homology to *nxrA* from 11 SFB MAGs in phyla not known to oxidize nitrite, including Acidobacteriota, Actinomycetota, Desulfobacterota_B (Binatia), and Gemmatimonadota (Fig. 4, Supplementary Table 8 and 9). Due to the difficulties associated with distinguishing NarG from NxrA, we analyzed these sequences from a phylogenetic, protein structure, sequence alignment, and genomic perspective. Most of the *narG* and *nxrA* (14 sequences) appear to be part of a new clade that was proposed after the characterization of *nxrA* from *Nitrotoga fabula*, a cultured nitrite oxidizer [1]. Three of these are phylogenetically related to *Candidatus* Brocadiaceae anammox bacteria, as well as Nitrospirota and Nitrospinota NOB. Representatives from all 4 phyla have conserved residues present in NxrA and NarG, including nitrite/nitrate binding residues (Fig. 4) [2]. Protein modeling using SWISS-PROT characterized all 17 genes as NxrA (Supplementary Data 1). Protein modeling with FoldSeek provided the strongest support for NxrA in Gemmatimonadota (8_1_May_SF_Bin5), which has the highest quality match to a protein structure of NxrA in Nitrospirota (e-value 4.57e-56; Supplementary Data 2). Nitrospinota, and Nitrospirota NxrA (13_July_SF_Bin2 and 21_Jan_SF_Bin67), have top protein structure hits to putative NxrA in Rokubacteria, though these organisms are uncultured and have not been experimentally verified. All other sequences have highest quality structural hits to molybdopterin oxidoreductases from uncultured organisms, and a mix of NarG and NxrA structures from cultured and uncultured organisms in the top 20 alignments, making it difficult to discern whether they are oxidative or reductive.

Detailed characterization of these *nar/nxr*-encoding SFB MAGs indicate that most also contain the beta subunit of nitrate/nitrite reductase (10/11; Supplementary Table 9). Of the 11 SFB MAGs, two Actinobacteria in class Thermoleophilia (4_1_Jan_SF_Bin18 and 4_1_May_SF_Bin55) have the most complete genomic context to support potential nitrite oxidation. These organisms encode the nitrate/nitrite reductase gamma subunit that can act as an electron shuttle in the electron transport chain (ETC) and also code *nirK* and chlorite dismutase (*cld*) which are present in most NOB [3, 4]. The ETC appears to be complete, including NADH dehydrogenase (complex I), succinate dehydrogenase (complex II), cytochrome *bc_1_*, cytochrome *c* oxidase (complex IV), and an F-type ATPase (complex V). These results suggest Thermoleophilia have a fully functional membrane-bound Nar/Nxr complex. NOB in *Nitrococcus*, *Nitrobacter, Nitrolancea,* and *Nitrotoga* utilize the Calvin-Benson-Bassham (CBB) cycle for carbon fixation [1] while *Nitrospira* and *Nitrospina* assimilate CO_2_ via the reverse TCA cycle (rTCA) [5, 6]. Thermoleophilia MAGs do not have core genes for CBB, and code 2-oxoglutarate ferredoxin oxidoreductase (OGOR) but lack ATP citrate lyase (ACL) for rTCA. However, these MAGs have a highly complete reductive glycine pathway (rGlyP), a recently proposed seventh CO_2_ fixation pathway [7]. The two Thermoleophilia MAGs have top structural hits to molybdopterin oxidoreductases, as well as putative NxrA from uncultured organisms and NxrA from Nitrotoga fabula in the top 15 hits.

**The role of rare taxa in sulfur and nitrogen cycling**

To verify the presence of *nirK* in the Heimdallarchaeota MAGs, we examined the conserved active site residues and sequence coverage. The MAG 8_1_May_SF_Bin13 nirK has both active site residues Asp and His required for nitrite reducing activity [8]. The two *nirK* sequences in 8_1_Jan_SF_Bin58 are partial (198 bp and 101 bp), and thus one has the Asp active site, while the other has the His. The coverage values of the *nirK*-encoding Heimdall contigs (8_1_May_SF_Bin13: 9.8; 8_1_Jan_SF_Bin58: 20.78 & 17.9465) are within the standard deviation of the mean value of coverage across all contigs in the Heimdallarchaeota MAGs (8_1_May_SF_Bin13: 8.6 ± 1.9; 8_1_Jan_SF_Bin58: 21.06 ± 3.6).

**References**

1. Kitzinger K, Koch H, Lücker S, Sedlacek CJ, Herbold C, Schwarz J. Characterization of the First ‘ Nitrotoga’ Isolate Reveals Metabolic Versatility and Separate Evolution of Widespread Nitrite-Oxidizing Bacteria. *MBio* 2018; **9**.

2. Boddicker AM, Mosier AC. Genomic profiling of four cultivated Candidatus Nitrotoga spp. predicts broad metabolic potential and environmental distribution. *ISME J* 2018; **12**: 2864–2882.

3. Mundinger AB, Lawson CE, Jetten MSM, Koch H, Lücker S. Cultivation and transcriptional analysis of a canonical Nitrospira under stable growth conditions. *Front Microbiol* 2019; **10**: 1325.

4. Park S-J, Andrei A-Ş, Bulzu P-A, Kavagutti VS, Ghai R, Mosier AC. Expanded diversity and metabolic versatility of marine nitrite-oxidizing bacteria revealed by cultivation- and genomics-based approaches. *Appl Environ Microbiol* 2020; **86**.

5. Luecker S, Nowka B, Rattei T, Spieck E, Daims H. The Genome of Nitrospina gracilis Illuminates the Metabolism and Evolution of the Major Marine Nitrite Oxidizer. *Front Microbiol* 2013; **4**.

6. Palomo A, Pedersen AG, Fowler SJ, Dechesne A, Sicheritz-Pontén T, Smets BF. Comparative genomics sheds light on niche differentiation and the evolutionary history of comammox Nitrospira. *ISME J* 2018; **12**: 1779–1793.

7. Figueroa IA, Barnum TP, Somasekhar PY, Carlström CI, Engelbrektson AL, Coates JD. Metagenomics-guided analysis of microbial chemolithoautotrophic phosphite oxidation yields evidence of a seventh natural CO2 fixation pathway. *Proc Natl Acad Sci U S A* 2018; **115**: E92–E101.

8. Helen D, Kim H, Tytgat B. Highly diverse nirK genes comprise two major clades that harbour ammonium-producing denitrifiers. *BMC Genomics* 2016; **17**.

**Supplementary Figures**

**
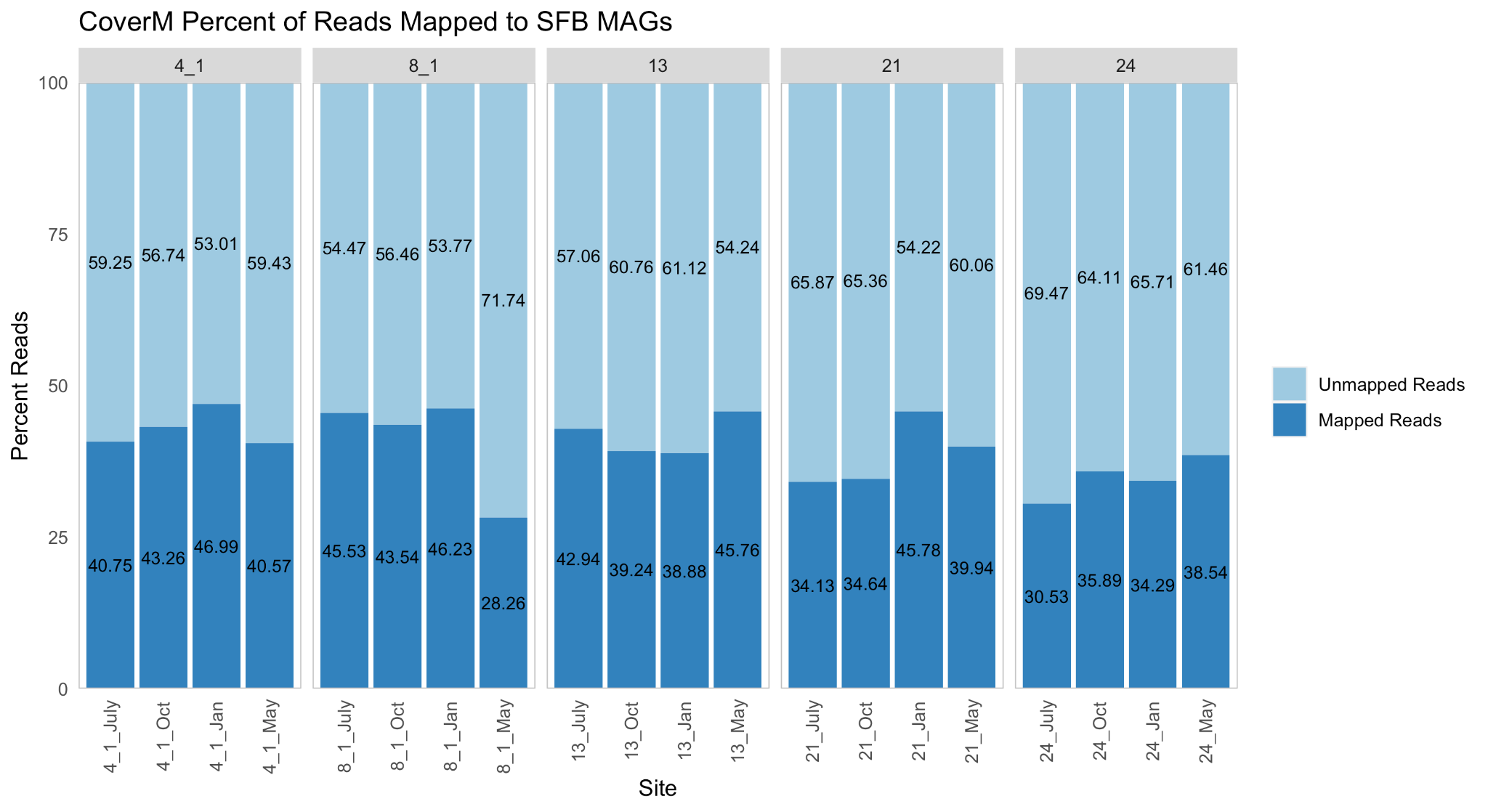
**

**Supplementary Figure 1.** Percent of San Francisco Bay (SFB) reads mapped to 639 SFB MAGs, calculated using CoverM (v0.2.0-alpha7). Sites and months are shown on the x axis and the percent of reads is shown on the y axis. Dark blue signifies reads mapped to the 639 MAGs and light blue shows reads that were not mapped to the MAGs. The numbers inside the bars show the value associated with the light or dark blue bar. The script used to process the CoverM data and generate this figure was written in R (Reads_Barplot.R).


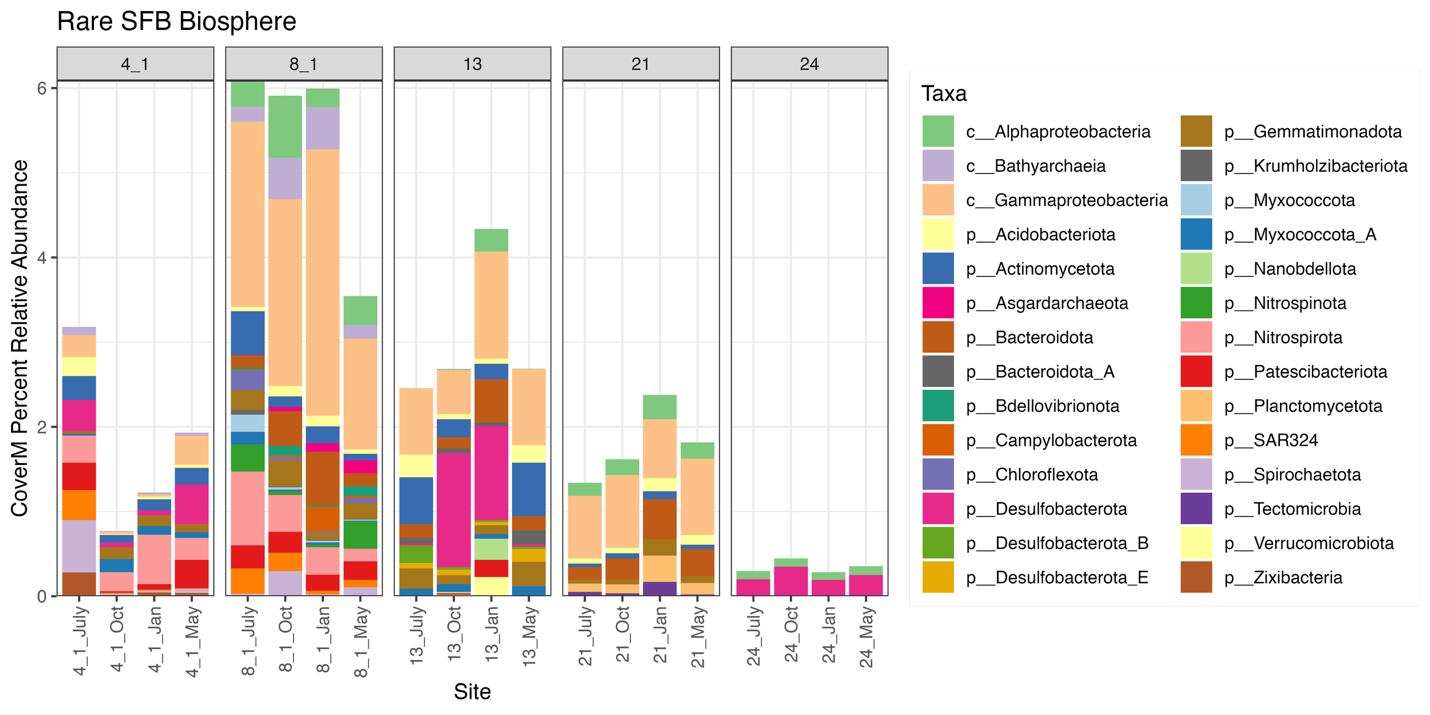


**Supplementary Figure 2.** SFB rare biosphere relative abundance calculated using CoverM v0.2.0-alpha7. Sites and months are shown on the x axis and relative abundance is shown on the y axis, calculated using CoverM v0.2.0-alpha7. Taxa are shown in the legend with distinct colors and correspond to the same color in the stacked barplot (GTDB-Tk r226). Rare biosphere MAGs were designated as taxa below 0.1% relative abundance at the site where they were reconstructed (AbundanceBarPlot2.R).

**
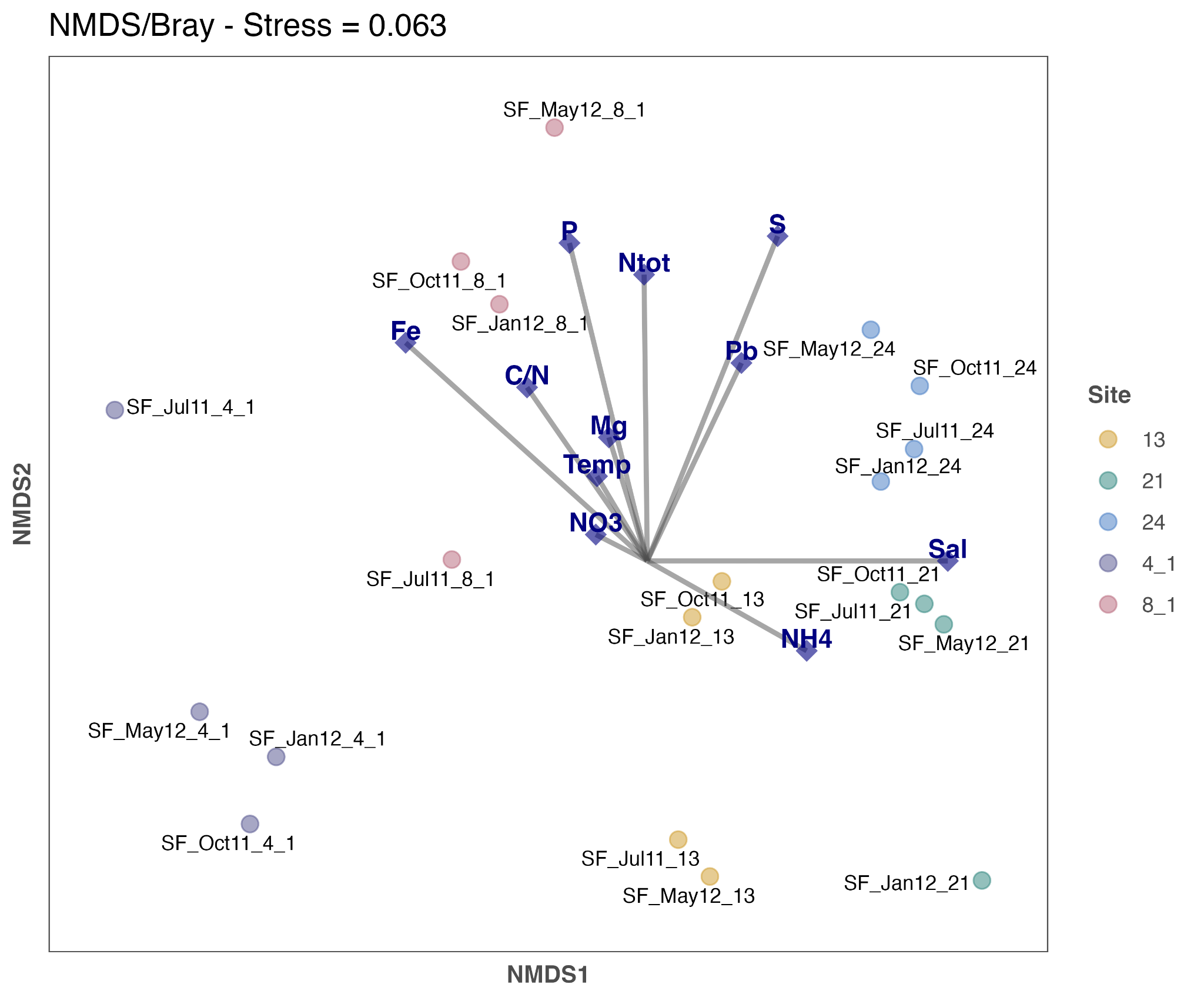
**

**Supplementary Figure 3.** Non-metric multidimensional scaling (NMDS) ordination of microbial relative abundance. Relative abundance was calculated using the relative_abundance method with the software CoverM. The NMDS was generated in R using the Bray Curtis distance for the dissimilarity index. Salinity, Fe, phosphorus, sulfur, and total sediment nitrogen content were statistically significantly correlated with the NMDS axes (all p-values <0.03). Salinity and Fe were fit separately to the NMDS due to their collinearity, though both are shown here. This plot was generated in R (NMDS.R).

**
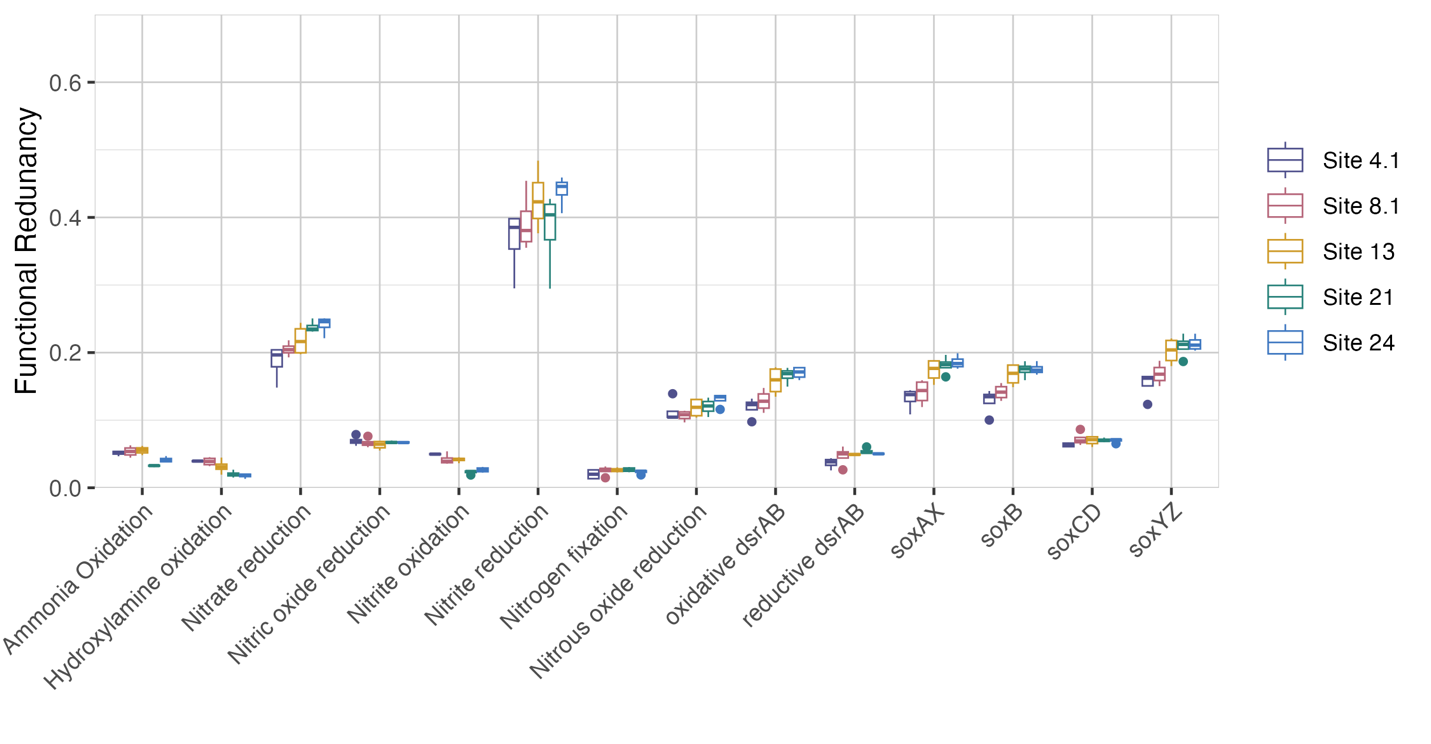
**

**Supplementary Figure 4.** Boxplot of functional redundancy scores calculated for nitrogen and sulfur cycling pathways. Functional redundancy was calculated using the Contribution Evenness metric.

**
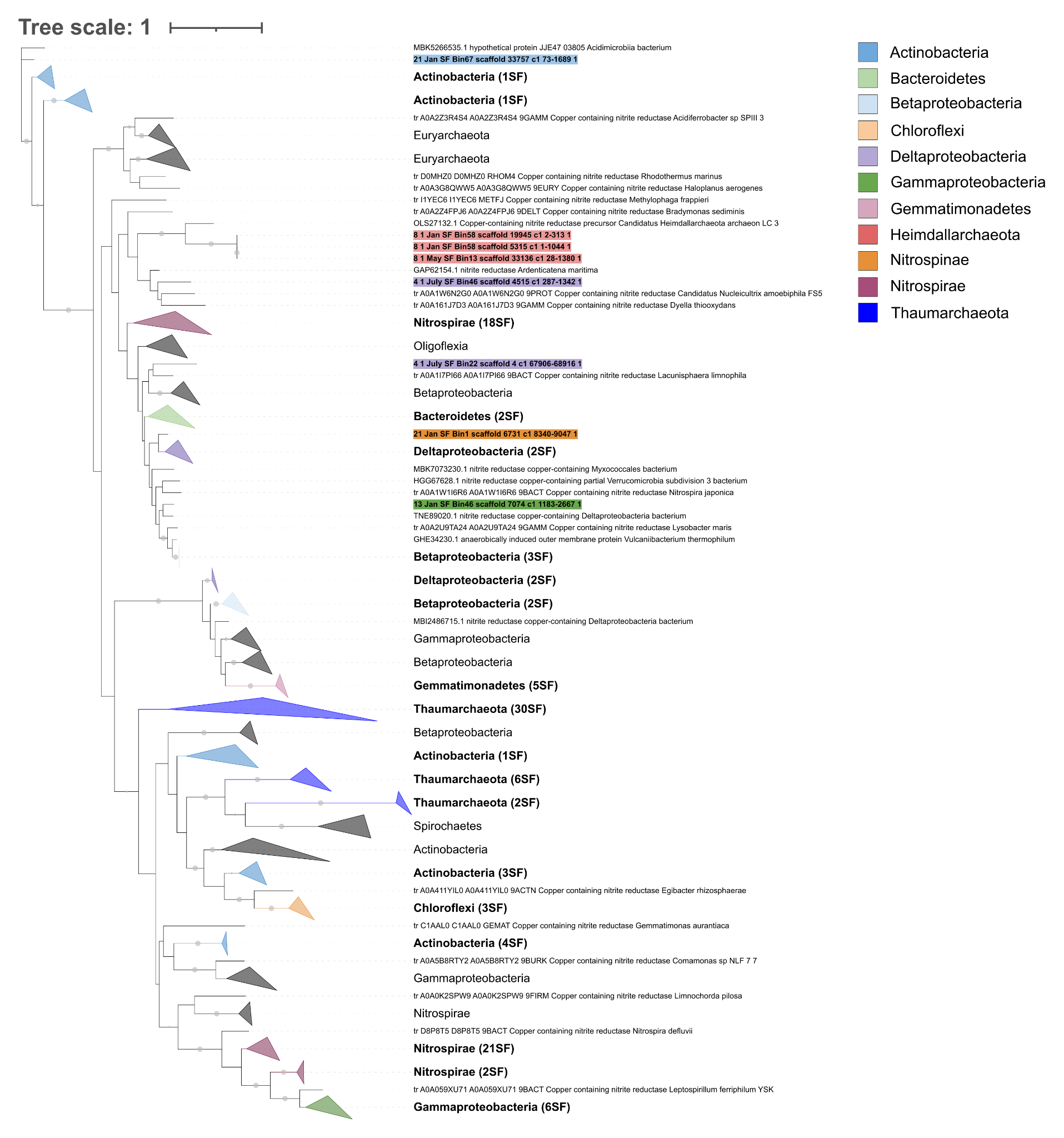
**

**Supplementary Figure 5.** Phylogeny of 122 NirK present in SFB MAGs and 229 reference NirK. Colors highlight different taxa, which are designated in the legend. The phylogeny was constructed using IQ-TREE under the LG+F+R7 model with ultrafast bootstrapping option -bb 1000 and -bnni to reduce the impact of severe model violations.

**A**

**
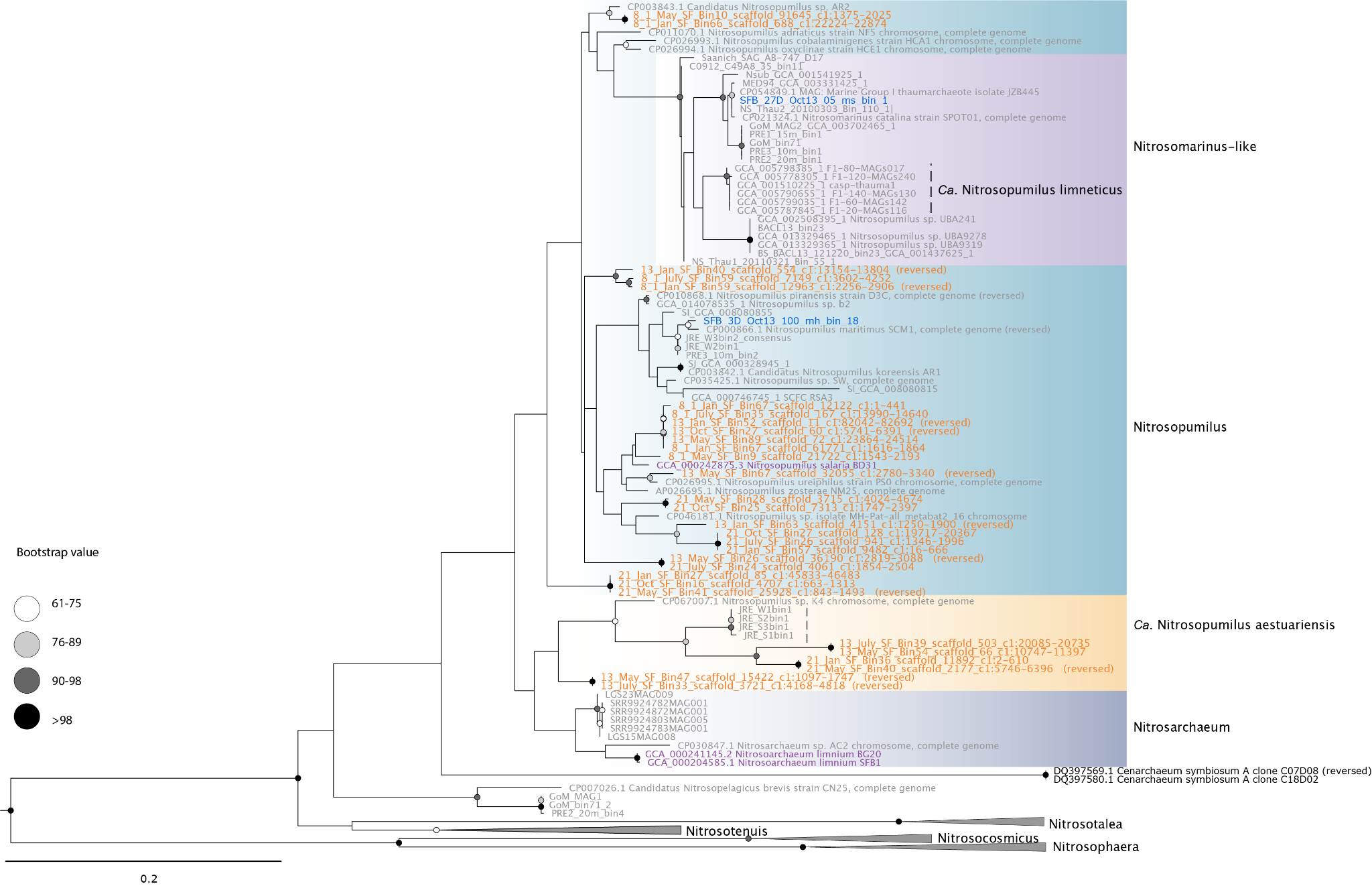
**

**B**


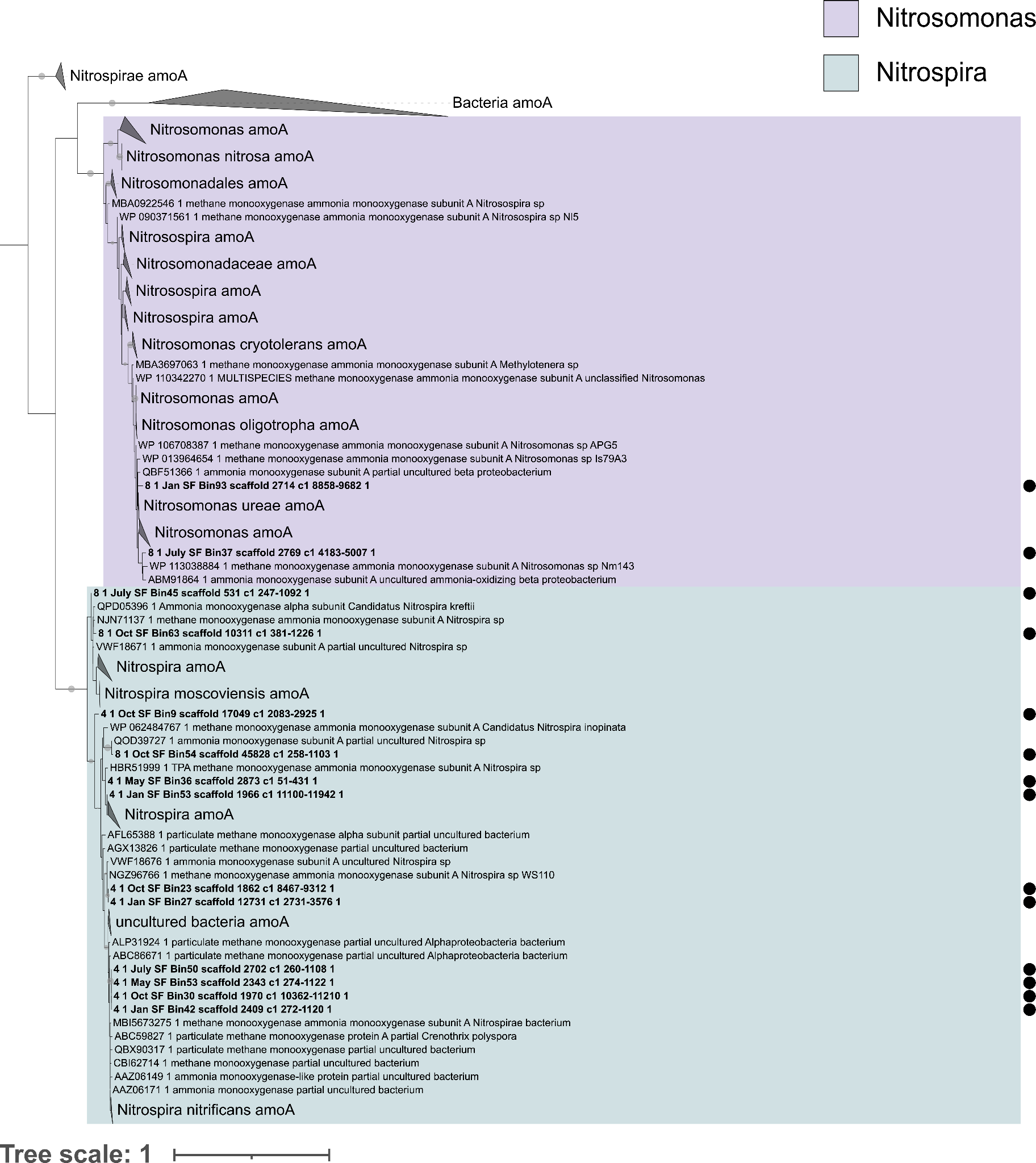


**Supplementary Figure 6. A.** 30 methane/ammonia monooxygenase subunit A (AmoA) identified in SFB archaea, as well as 78 reference AmoA sequences. AmoA sequences identified in this study are highlighted in orange. Reference amoA sequences in blue were reconstructed from the SFB water column and those in purple were reconstructed from SFB sediment. This phylogeny was constructed using RAxML under the GTR Gamma model with 1,000 bootstraps. **B.** 14 methane/ammonia monooxygenase subunit A (AmoA) identified in SFB bacteria, as well as 357 reference AmoA sequences. AmoA sequences identified in this study are highlighted in bold and with a black circle. This phylogeny was constructed using IQ-TREE under the LG+F+R6 model with ultrafast bootstrapping option –bb 1000 and –bnni to reduce the impact of severe model violations.

**A**

**
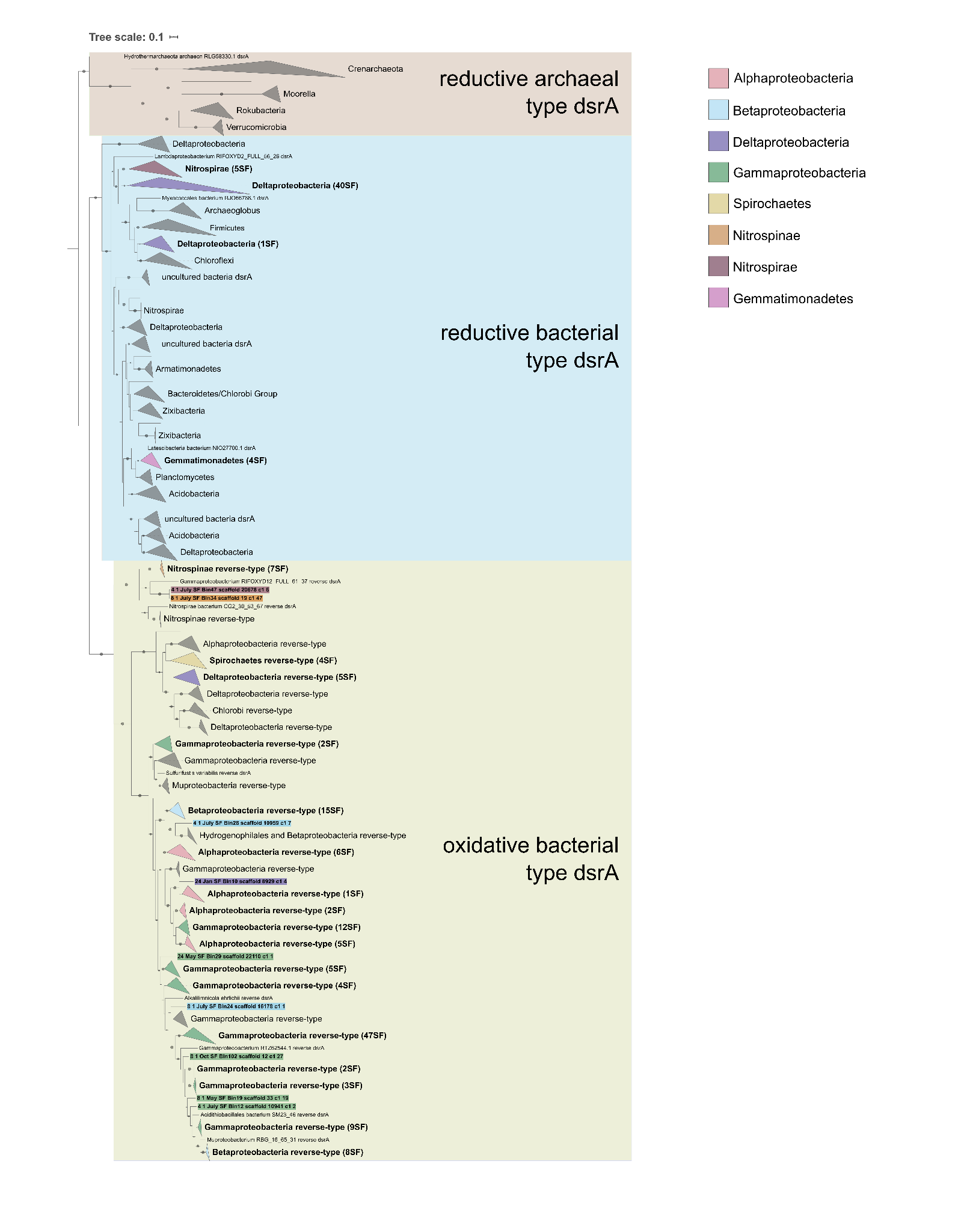
**

**B**

**
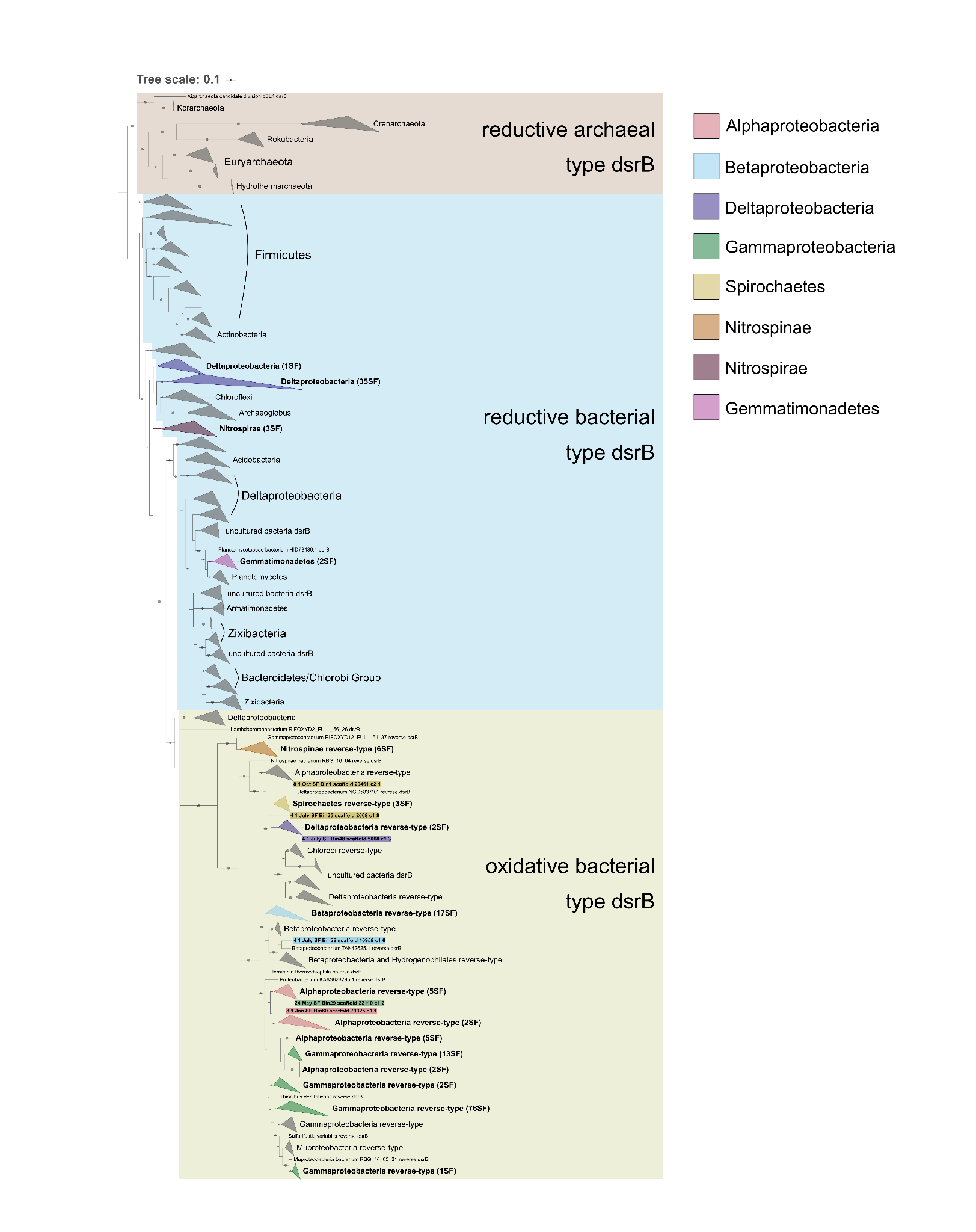
**

**Supplementary Figure 7. A.** 197 newly identified dissimilatory sulfite reductase subunit A (DsrA) sequences (indicated in bold and number of sequences in parentheses or with a colored background). The phylogeny also contains 1,306 reference dsrA sequences. This tree was constructed using IQ-TREE under the LG+R10 model with ultrafast bootstrapping option -bb 1000 and -bnni to reduce the impact of severe model violations. **B.** 181 newly identified dissimilatory sulfite reductase subunit B (DsrB) sequences (indicated in bold and number of sequences in parentheses or with a colored background). The phylogeny also contains 1,513 reference dsrB sequences. This tree was constructed using IQ-TREE under the LG+R10 model with ultrafast bootstrapping option -bb 1000 and -bnni to reduce the impact of severe model violations.


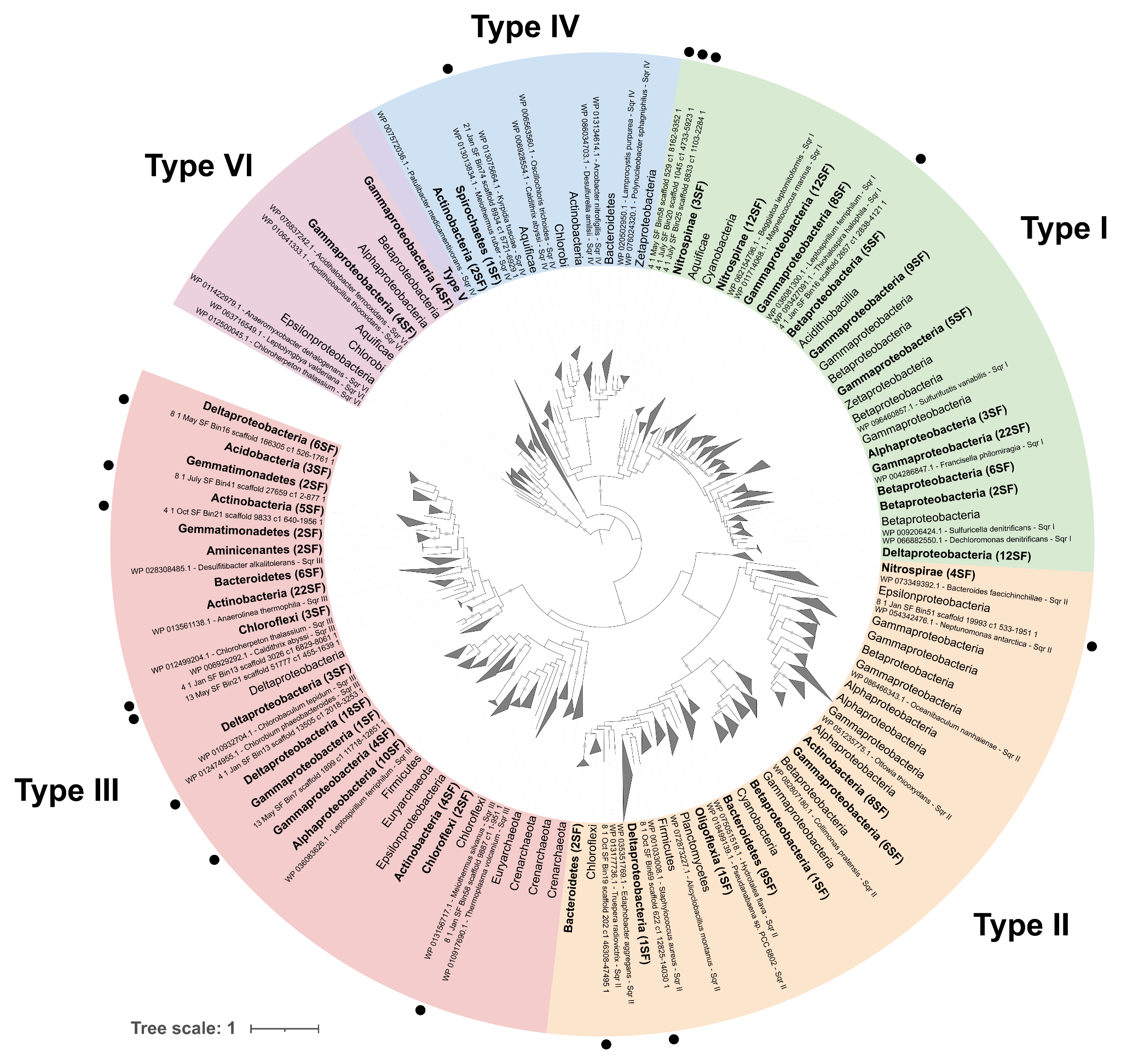


**Supplementary Figure 8.** A phylogenetic tree of 251 SFB MAG SQRs and 333 references. SQRs identified in this study are labeled with a black circle or have bolded font, if multiple sequences are collapsed. Different background colors signify the distinct SQR types, I-V. This phylogeny was constructed using IQ-TREE under the LG+R10 model with ultrafast bootstrapping option –bb 1000 and –bnni to reduce the impact of severe model violations.


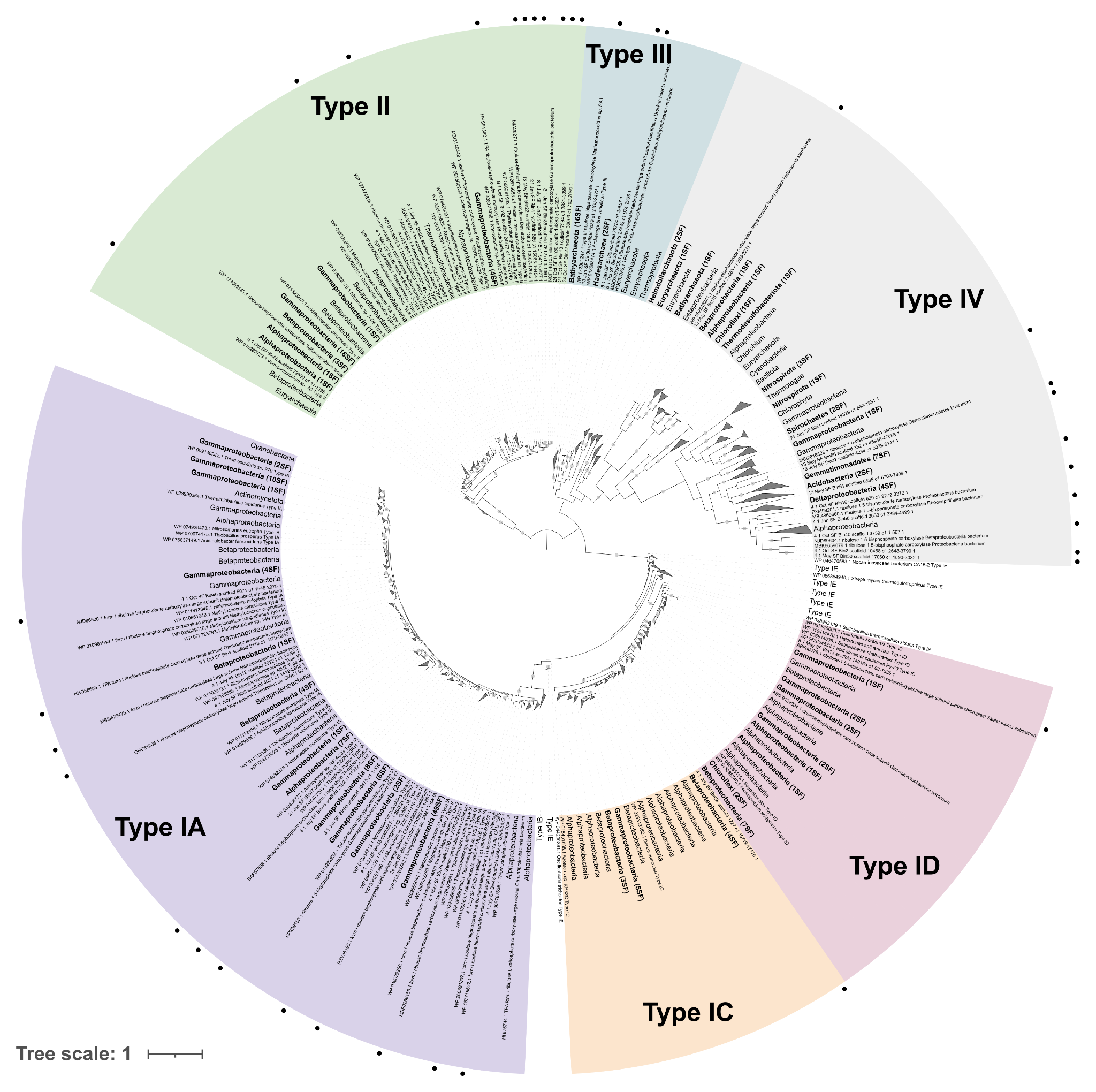


**Supplementary Figure 9.** A Rubisco phylogeny of 237 Rubisco genes identified in SFB MAGs and 885 reference Rubiscos. Rubiscos identified in this study are labeled with a black circle or have bolded font, if multiple sequences are collapsed. Different background colors signify the distinct Rubisco types, IA-IV. This phylogeny was constructed using IQ-TREE under the LG+F+R10 model with ultrafast bootstrapping option –bb 1000 and –bnni to reduce the impact of severe model violations.


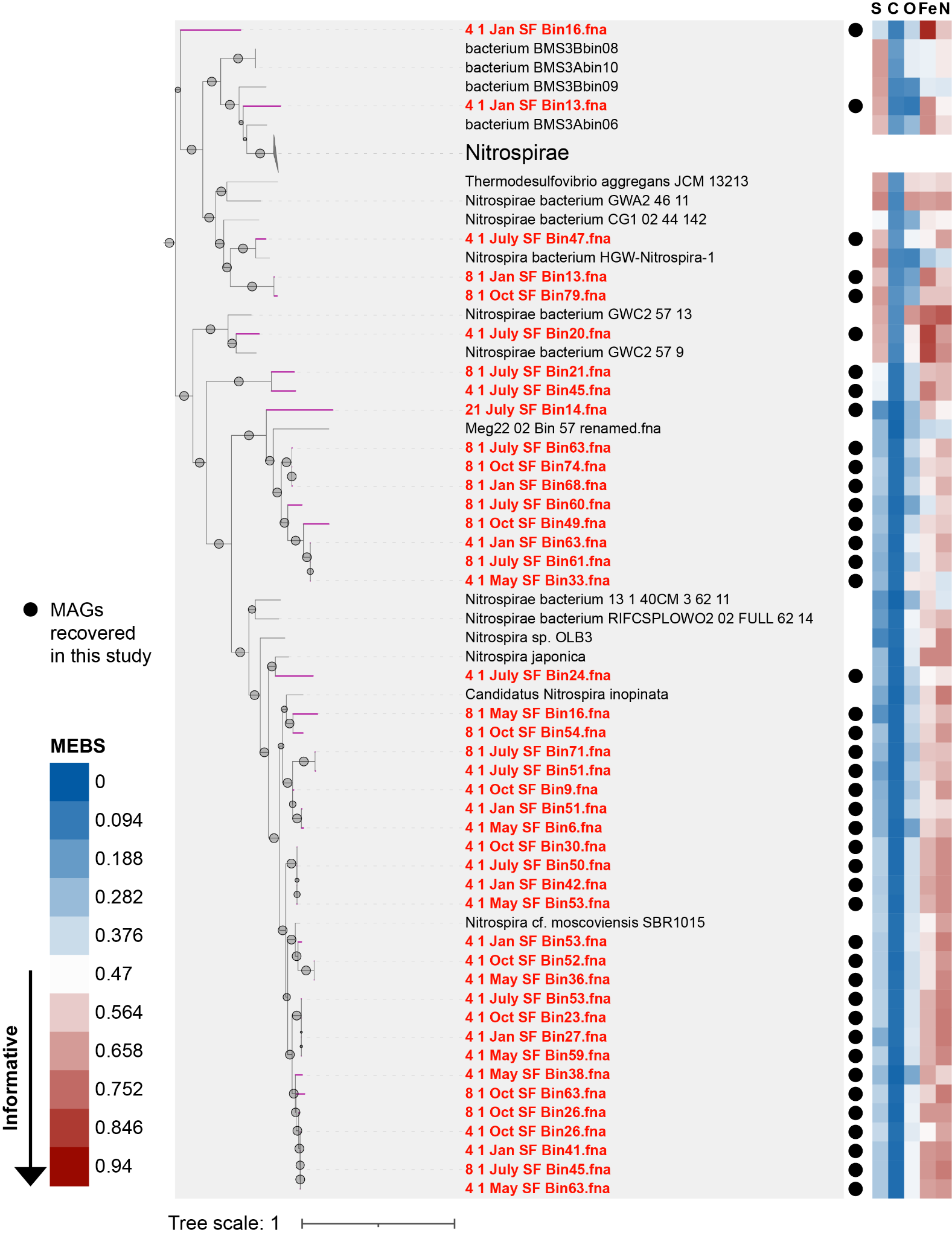


**Supplementary Figure 10.** Nitrospirota SFB MAG diversity based on 37 marker genes. This is a zoom in of Nitrospirota MAGs from Figure 1B. Black circles and red font signify MAGs reconstructed in this study. The heatmap shows entropy scores calculated with MEBS for nitrogen (the outermost heatmap ring), iron, oxygen, carbon, and sulfur (the innermost heatmap ring). White and red heatmap colors indicate a higher number of proteins present in each genome for a cycle. This phylogeny was constructed with RAxML version 8.2.11 with the -f a option for rapid bootstrap analysis.
